# Immunoinformatic design of a putative multi-epitope vaccine candidate against *Trypanosoma brucei gambiense*

**DOI:** 10.1016/j.csbj.2022.10.002

**Published:** 2022-10-07

**Authors:** Ammar Usman Danazumi, Salahuddin Iliyasu Gital, Salisu Idris, Lamin BS Dibba, Emmanuel Oluwadare Balogun, Maria Wiktoria Górna

**Affiliations:** a Biological and Chemical Research Centre, Department of Chemistry, University of Warsaw, Warsaw, Poland; b Faculty of Chemistry, Warsaw University of Technology, Warsaw, Poland; c Groningen Research Institute of Pharmacy, University of Groningen, the Netherlands; d Department of Biochemistry, Ahmadu Bello University, Zaria, Nigeria; e Africa Centre of Excellence for Neglected Tropical Diseases and Forensic Biotechnology, Ahmadu Bello University, Zaria, Nigeria; f Department of Physical and Natural Sciences, School of Arts and Sciences, University of the Gambia, Brikama Campus. P.O Box 3530, Serrekunda, the Gambia; g Center for Discovery and Innovation in Parasitic Diseases, Skaggs School of Pharmacy and Pharmaceutical Sciences, University of California San Diego, 9500 Gilman Drive, La Jolla, CA 92093, USA; h Department of Biomedical Chemistry, Graduate School of Medicine, The University of Tokyo, Tokyo 113-0033, Japan; i Department of Medical Laboratory Science, Kazaure School of Health Technology, Jigawa, Nigeria

**Keywords:** Trypanosomiasis, Immunoinformatics, Multi-epitope vaccine, Chimeric antigen, Bioinformatics, Transmembrane proteins

## Abstract

Human African trypanosomiasis (HAT) is a neglected tropical disease that is caused by flagellated parasites of the genus *Trypanosoma*. HAT imposes a significant socio-economic burden on many countries in sub-Saharan Africa and its control is hampered by several drawbacks ranging from the ineffectiveness of drugs, complex dosing regimens, drug resistance, and lack of a vaccine. Despite more than a century of research and investigations, the development of a vaccine to tackle HAT is still challenging due to the complex biology of the pathogens. Advancements in computational modeling coupled with the availability of an unprecedented amount of omics data from different organisms have allowed the design of new generation vaccines that offer better antigenicity and safety profile. One of such new generation approaches is a multi-epitope vaccine (MEV) designed from a collection of antigenic peptides. A MEV can stimulate both cellular and humoral immune responses as well as avoiding possible allergenic reactions. Herein, we take advantage of this approach to design a MEV from conserved hypothetical plasma membrane proteins of *Trypanosoma brucei gambiense*, the trypanosome subspecies that is responsible for the west and central African forms of HAT. The designed MEV is 402 amino acids long (41.5 kDa). It is predicted to be antigenic, non-toxic, to assume a stable 3D conformation, and to interact with a key immune receptor. In addition, immune simulation foresaw adequate immune stimulation by the putative antigen and a lasting memory. Therefore, the designed chimeric vaccine represents a potential candidate that could be used to target HAT.

## Graphical abstract

**Figure:**
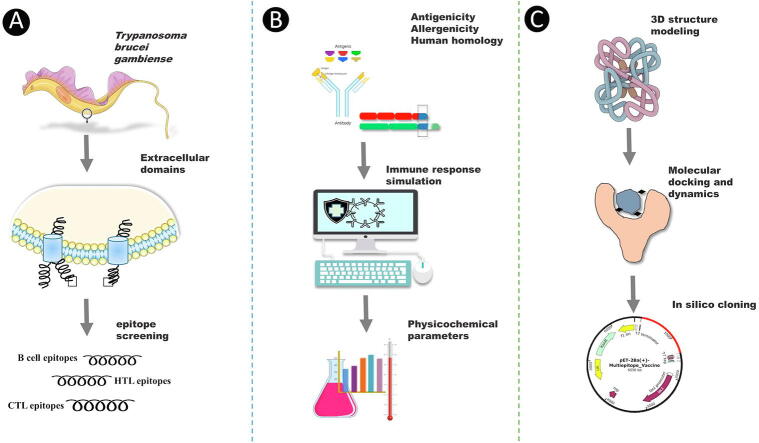

## 1 Introduction

African trypanosomiasis is a vector-borne parasitic disease transmitted mainly by the tsetse fly of the Glossina species. The disease can assume two forms: human infection or sleeping sickness, caused by *Trypanosoma brucei gambiense* and *T. b. rhodesiense,* and the animal infection for which *T. b. brucei, T. congolense,* and *T. vivax* are the prominent causative agents. The disease is widely spread across 36 countries in sub-Saharan Africa and has proven to be debilitating to this region when neglected [1]. While the recorded annual cases of sleeping sickness have significantly reduced (fewer than 1000) since 2018, the disease is still of concern considering the economic state of the affected population, which has limited access to healthcare. As such, the reported numbers may not reflect the true situation of the endemic countries. Considering the evidence that domestic animals serve as reservoirs to human-infective trypanosomes [2] and the ubiquity of the tsetse vector in affected areas, up to 65 million people are at risk of contracting the disease [1]. The COVID-19 pandemic has well demonstrated the possible implication of the zoonotic nature of infectious diseases; therefore, it is crucial to take necessary actions in the light of “One health” policy to prevent the resurgence of a trypanosomiasis pandemic. Very few trypanocides such as nifurtimox, and eflornithine, or the newly FDA-approved fexinidazole, are currently available to treat Human African trypanosomiasis (HAT). However, typically these drugs bind to multiple pharmacological targets, which results in non-specificity-associated toxicities in the mammalian host. Besides, these drugs often require an inconvenient dosing regimen, are effective at a particular stage of disease, and resistance to these drugs is rapidly spreading [3], [4].

Since prevention is preferred over difficult treatment regimes, the ideal approach to eradicating HAT is the design of a safe and effective vaccine. Yet, the parasite biology has proven to be quite deceptive and undermines all efforts towards vaccine development [5], [6], [7]). For example, essential targets such as the variable surface glycoprotein (VSG) [7], the invariant surface glycoproteins, and subcellular proteins of the cytoskeleton, *i.e.*, actin and tubulin [8], have all been utilized as vaccine candidates. Still, the responses generated by these molecules are generally unsatisfactory. Nevertheless, the genome sequencing of many organisms and the advancement in bioinformatics has restored hope towards developing an effective vaccine candidate against many infectious parasites. The so-called reverse vaccinology approach capitalizes on omics data from a given organism to screen for potential antigenic molecules that would otherwise not be identified or at least take time to be discovered using classical approaches. This approach is cost-effective, less time-consuming, provides a better safety profile than traditional vaccine development methods, and has driven the development of multi-epitope or chimeric vaccines [9], [10].

**Fig. 1:**
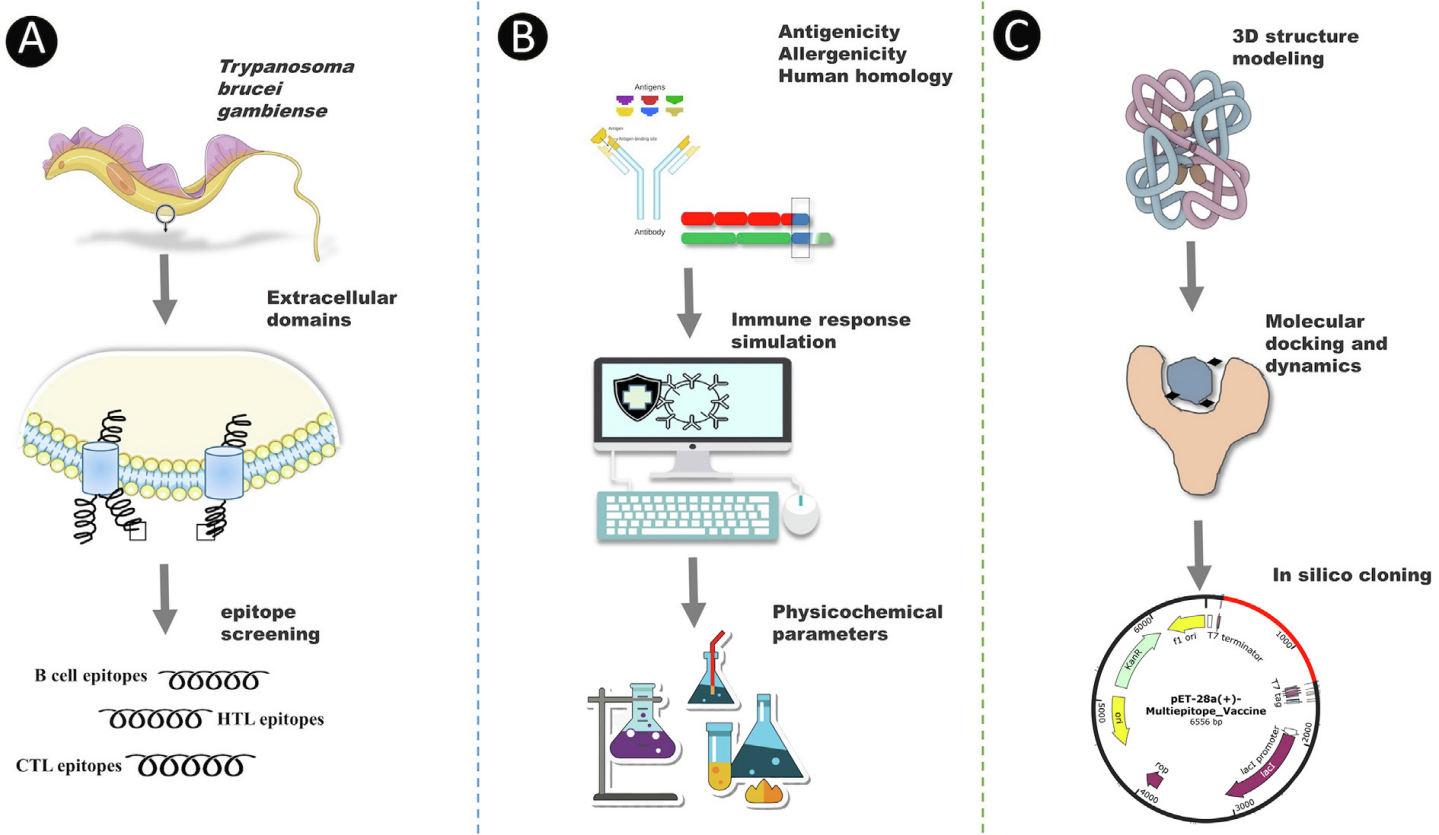
Schematic representation of immunoinformatics design of the multi-epitope vaccine against *T. b. gambiense*. (**A**) Selection of epitopes derived from extracellular domains of membrane proteins. (**B**) In silico testing of immunological responses to the epitopes and MEV. (**C**) In silico prediction and validation of the MEV structure and interactions.

A multi-epitope vaccine is an ensemble of short immunogenic epitopes designed from one or multiple pathogenic proteins, which can elicit high immune responses and avoid allergenic reactions. An ideal MEV should be designed to include epitopes that can elicit cytotoxic, helper T cell and B cells responses as well as induce effective immunity against a targeted tumor, virus, or parasite [9]. On this account, several research groups have reported the design of chimeric vaccines against different pathogens. Diseases that have been targeted using this approach include leishmaniasis, schistosomiasis, brucellosis, cholera, toxoplasmosis, dengue fever, onchocerciasis, and related filarial disease, COVID-19, and even cancer [11], [12], [13], [14], [15], [16], [17], [18], [19]. Indeed, some of these multi-epitope vaccine candidates have recorded remarkable success; a promising example is EMD640744, which underwent phase 1 clinical trial for patients with solid tumors [20]. Most of the reported multi-epitope vaccines are designed from surface proteins as they are more likely to interact with the host immune system. Consequently, we utilize such an immunoinformatics approach to identify all hypothetical plasma membrane proteins from *T. b. gambiense*, from which we predicted epitopes to construct a multi-epitope-based antigen as a vaccine candidate against HAT (Fig. 1). Worthy of mention, African trypanosomes are exclusively extracellular parasites and the targeted proteins for the design of the MEV are the parasite surface membrane proteins. We hypothesize that cytotoxic T cell responses can be induced to neutralize the trypanosomes. Moreover, it has been shown that CD4^+^ T lymphocytes mediate the effective killing of African trypanosomes [21].

## 2 Materials and methods

### 2.1 Prediction of transmembrane helices

*Trypanosoma brucei gambiense* proteome was retrieved on 28 May 2021 from the UniProt database (ID: UP000002316) and used on 28 May 2021 to predict proteins harboring transmembrane helices using DeepTMHMM [22]. DeepTMHMM is a deep neural networks-based algorithm that predicts protein topology from primary sequence using pre-trained protein language model. Proteins containing the desirable helices were further submitted to DeepLoc [23] for prediction of subcellular localization using Uniprot-trained Neural Networks algorithm. “Profiles” was selected for most accurate prediction and only proteins located on the plasma membrane were selected for further analysis. Finally, extracellular helices from these plasma membrane proteins that are at least 30 amino acids long were selected for epitopes prediction.

### 2.2 Prediction of cytotoxic T lymphocyte (CTL) and helper T lymphocyte (HTL) epitopes

The predicted extracellular helices were submitted to NetMHC [24] for the prediction of the CTL epitopes. NetMHC is an artificial neural networks algorithm pre-trained with enormous quantitative datasets from Immune Epitope Database and Analysis Resource (IEDB), covering 43 Human and 12 non-human alleles. For our prediction, Human super-type representative alleles were chosen for maximum population coverage, and a threshold of ≤0.5 percentile was considered to select strong binders. Similarly, the extracellular helices were submitted to NetMHC-II 2.3 [25] to predict HTL epitopes, employing 27 Human super-type representative alleles with a threshold of ≤2.0 percentile rank were selected and they adequately represented the human population. The representative alleles were curated as “HLA supertype representative” in the MetMHC server that was used for this analysis.

### 2.3 Prediction of continuous B-Cell epitopes

The predicted transmembrane helices were submitted to the BepiPred-2.0 standalone package [26] to predict linear B-Cell epitopes. BepiPred-2.0 performs prediction based on a random forest algorithm trained with epitopes data from known antibody-antigen structures.

### 2.4 Selection of overlapping epitopes

CTL and B-Cell epitopes were aligned, and overlapping epitopes were selected. Similarly, HTL and B-Cell epitopes were aligned to select overlapping epitopes. All alignments were done using MULTALIN [27] with server’s default parameters.

### 2.5 IFN-γ inducing epitopes

The final selected HTL epitopes were submitted to the IFNepitope server [28] to assess their ability to induce the production of Interferon-gamma (IFN-γ) [29]. The algorithm was trained on epitopes datasets from IEDB and our prediction was performed with the Motif and Support-Vector-Machine (SVM) hybrid algorithm.

### 2.6 Construction of the final vaccine candidate

B-Cell epitopes were connected by KK linker, HTL epitopes by GPGPG linker, and CTL epitopes by AAY linker. TLR-4 agonist RS-09 [30] was also added to the construct as an adjuvant. This order of sequence arrangement was chosen because the construct will result in high yield of highly stable and cheap vaccine structure.

### 2.7 Antigenicity, allergenicity and homology analysis

The whole multi-epitope vaccine sequences were submitted once again to VaxiJen 2.0 server [31] for antigenicity analysis using a threshold of ≥0.5 for parasites. VaxiJen 2.0 is an alignment-independent algorithm that predicts the antigenicity of proteins based on the physicochemical properties of the given protein. The chimeric vaccine candidate was further submitted to the AllerTOP server [32] for allergenicity analysis. AllerTOP is also an alignment-independent server that predicts allergens-based on physicochemical properties of a protein. Similarly, the designed vaccine candidate was aligned against the human proteome using the NCBI P-BLAST.

### 2.8 Immune response simulation

The final vaccine construct was submitted to the C-IMMSIM server [33] for immune simulation. C-IMMSIM is a machine learning-based algorithm that uses position-specific scoring matrices to simulate immune interactions. The simulation was conducted for the 1098 steps corresponding to 366 days (1 simulation step is 8 h). Three shots each containing 1000 molecules of antigen were administered to human at the interval of four weeks, each corresponding to 1, 84, and 168 simulation steps, where step 1 is injection at day 0.

### 2.9 Physicochemical analysis

Expasy ProtParam [34] was used to evaluate physicochemical parameters of the designed vaccine such as molecular weight, isoelectric point, half-life, instability index, aliphatic index.

### 2.10 Tertiary structure prediction and model refinement

The 3D structures of all proteins that harbor the selected overlapping epitopes and the structure of the vaccine construct were predicted from the primary sequences using RoseTTAFold algorithm [35]. The predicted 3D structure model of the multi-epitope vaccine was refined using galaxyrefine [36] and the quality of the model was evaluated using PROCHECK from SAVES online server [37].

### 2.11 Prediction of discontinuous B-Cell epitopes

We submitted our refined vaccine model to the ElliPro server [38] for the prediction of conformational B-Cell epitopes. ElliPro is a structure-based B cell epitopes predicting algorithm that capitalizes on geometrical properties of protein structure to make predictions. The server’s default threshold of 0.5 was adopted for the prediction.

### 2.12 Molecular docking with TLR4

The vaccine candidate was docked to TLR4 using ClusPro [39] protein–protein docking server. The 3D structure of human TLR4 was retrieved from Protein Data Bank ((PDB ID: 2Z63), and co-crystallized substrates and water molecules were removed using PyMOL 2.5 [40] prior to docking studies. The docking was performed using server’s default settings and the protein–protein interactions were studied using PDBsum package [41], [42] post-docking.

### 2.13 Molecular dynamics simulation

Multiepitope vaccine-TLR4 complex obtained from docking studies was used for molecular dynamics simulation. A coarse-graining approach was chosen to enhance phase space sampling, and the simulation was conducted in Martini 3.0 forcefield [43]. Briefly, hydrogens were added to the complex at a pH of 6.5 using the H++ server [44]. The complex was transformed into coarse-grain beads, neutralized with the appropriate number of ions, and solvated with Martini water using the martinize2.py [45] python script. The elastic network bias was equally introduced to preserve the secondary structures in the system [43]. The system was then energy minimized and equilibrated (NPT ensemble) to a temperature of 300 K and a pressure of 1 atm with position restraints on heavy atoms. The final simulation was conducted for 1 μs without restraints, and trajectories generated were used to calculate the Root-mean-square deviation of the protein backbone atoms. The first 200 ns was omitted for the calculation of Root-mean square fluctuations in the system. GROMACS-2021 [46] was used as the software for all simulations.

### 2.14 Codon adaptation and In-Silico cloning

Finally, to present the designed chimeric vaccine in a suitable expression vector, the putative vaccine sequences were submitted to JAVA Codon Adaptation Tool [47]. The *Escherichia coli* (strain K12) codon usage was adapted. The adapted nucleotide sequences were flanked with *Eco*RI and *Not*I recognition sequences, at 5′ and 3′, respectively. The final sequence was then cloned into the pET-28a(+) vector in frame with the *N*-terminal His-thrombin-tag sequence and C-terminal His-tag followed by vector-encoded stop codon, employing the same restriction enzymes already present at the vector's multiple cloning sites (MCS). Cloning was performed using SnapGene 5.3 [48].

## 3 Results

### 3.1 Prediction of transmembrane helices

We analyzed the *T. b. gambiense* proteome using DeepTMHMM which predicted 2334 proteins that harbor transmembrane helices (Supplementary Data Sheet 2). Out of these, only 450 proteins were predicted by DeepLoc to be localized to the plasma membrane (Supplementary Data Sheet 3). As trypanosomes are well known for immune evasion by switching the expression of variable surface glycoprotein (VSG), unannotated proteins and VSGs were eliminated from further analyses. Finally, a total of 186 extracellular helices were compiled from the plasma membrane proteins and considered for the vaccine design (Supplementary Data Sheet 2).

### 3.2 Prediction of CTL, HTL, and B-Cell epitopes

From the 186 extracellular helices, NetMHC and NetMHC-II predicted a total of 288,537 Cytotoxic T lymphocyte (CTL) epitopes (Supplementary Data Sheet 4) and 251,226 Helper T Lymphocyte (HTL) epitopes (Supplementary Data Sheet 5 & Supplementary Data Sheet 6), respectively. Based on the threshold of ≤ 0.5 and ≤ 2.0 percentile rank for CTL and HTL respectively, 1176 CTL and 1818 HTL epitopes were selected as strong binders (Supplementary Data Sheet 3). Similarly, a total of 725 linear B-Cell epitopes were returned by BepiPred-2.0 (Supplementary Data Sheet 3).

### 3.3 Selection of overlapping epitopes

All predicted CTL, HTL, and B-Cell epitopes were initially subjected to antigenicity and allergenicity analysis using VaxiJen 2.0 and Allertop servers, respectively. The outcome was 377, 236, 157 antigenic and non-allergenic CTL, HTL, and B-Cell epitopes (Supplementary Data Sheet 3), respectively. However, it is not practical to design a vaccine candidate from all these epitopes. Therefore, we chose to present the vaccine candidate as a set of overlapping B and T cells epitopes. In addition to achieving a practical number of epitopes, this approach allows the selection of epitopes that can elicit both cellular and humoral immune responses. Multiple sequence alignment revealed 9 CTL epitopes overlapping with 9 B-Cell epitopes, while 13 HTL epitopes were overlapping with 13 B-Cell epitopes (Table 1). All 44 epitopes were subsequently submitted to VaxiJen 2.0 and Allertop servers for antigenicity and allergenicity prediction, respectively. Finally, 10 B-Cell epitopes, 2 CTL epitopes, and 6 HTL epitopes were found to be antigenic and non-allergenic and were selected for vaccine construction (Table 1).

**Table 1:** Selected overlapping B-Cell and T-Cell epitopes. Underlined in bold are epitopes used for vaccine construction (Key: *CTL* Cytotoxic T Lymphocytes, *HTL* Helper T Lymphocytes).

Protein UniProt ID	B-Cell Epitopes	CTL epitopes	HTL epitopes
D0A254	FLPFLSTRNSVV	FLSTRNSVV	
C9ZXD7	AKMLPHPILASDDDRQY **ASSEGDLSTTGPSAGAAVPQVTRGHNTKNG**	KMLPHPILA	GPSAGAAVPQVTRGH
D0A0U3	**WREALAFAASPFV**	**ALAFAASPFV**	
C9ZP82	GFLTKGPHEINLPQLQR GHRGEVIVDDPTAFEFDSNCIS	FLTKGPHEI	GHRGEVIVDDPTAFE
C9ZL71	RGAGRNLFERPAHAFERAETYLLDAVQQRMHEPLY	YLLDAVQQRMHEPL	
C9ZZ34	**PSMDENNPEIQSTL**	SMDENNPEI	
D0A1S7	AQQRRAYINFDLVPTVNS	AYINFDLVPTV	
D0A1Y1	TDDDSFFSSGSAVLEEDTRRLQNDPLRG		DDSFFSSGSAVLEED
C9ZXR1	**MVSMGTEIAEEDIEN**		**MVSMGTEIAEEDIEN**
C9ZRE0	**KRFSFVLPQPLRKSPIDV**		**KRFSFVLPQPLRKSP**
D0A7R4	SMGTGSGNTIEQYFRDHNMEYTQE		**GNTIEQYFRDHNMEY**
C9ZMP2	**QNFQTCFYPDGDTFLVGDRGIRLSGG**		GDTFLVGDRGIRLSG
D0A939	LFPSLAALANVGSVSAASAEAQGPGMDGGT		**ANVGSVSAASAEAQG**
C9ZUV6	GAAAGALLPGSTEDEAQRRR		**GAAAGALLPGSTEDE**
C9ZJU1	**QSSPRAATASPSTCVWQCRQDYLQ**		**QSSPRAATASPSTCV**
C9ZN41	**RSYLSANPGVTVFMARITSTTTTWMGG**		TVFMARITSTTTTWM
C9ZUC9	**SDRLRRDPACATNNDGAAAGPTSSAGGGELQ**		NDGAAAGPTSSAGGG
D0AAR0	**HETHGRGGTEAQTVGGGAGFPMRSTPSG**		
D0A513	YLSHPFLGVPMKDEEK	YLSHPFLGV	
C9ZN46	VAVLDDNATLVGRLPN	**VLDDNATLV**	QTVGGGAGFPMRSTP

### 3.4 Protein structure prediction confirms the surface exposure of selected epitopes

Although we have confirmed the extracellular localization of the selected epitopes, these epitopes could be buried within their respective surface domains. Hence, we predicted the structures of all the proteins that harbor these epitopes. The results show that all epitopes are surface exposed in all proteins (Supplementary Fig. 1).

### 3.5 Prediction of IFN-γ inducing epitopes

The cytokine Interferon-gamma (IFN-γ) plays a significant role in maintaining the action of both CTL and HTL during infection [29]. As HTL is responsible for IFN-γ release, the six selected HTL epitopes were evaluated for their ability to induce the release of IFN-γ by CD4 + cells. Two (2) of these epitopes returned a positive score from the IFNepitope server (Supplementary Table 2) and hence could potentially stimulate the production of IFN-γ cytokine.

### 3.6 Construction of the final vaccine candidate

**Fig. 2:**
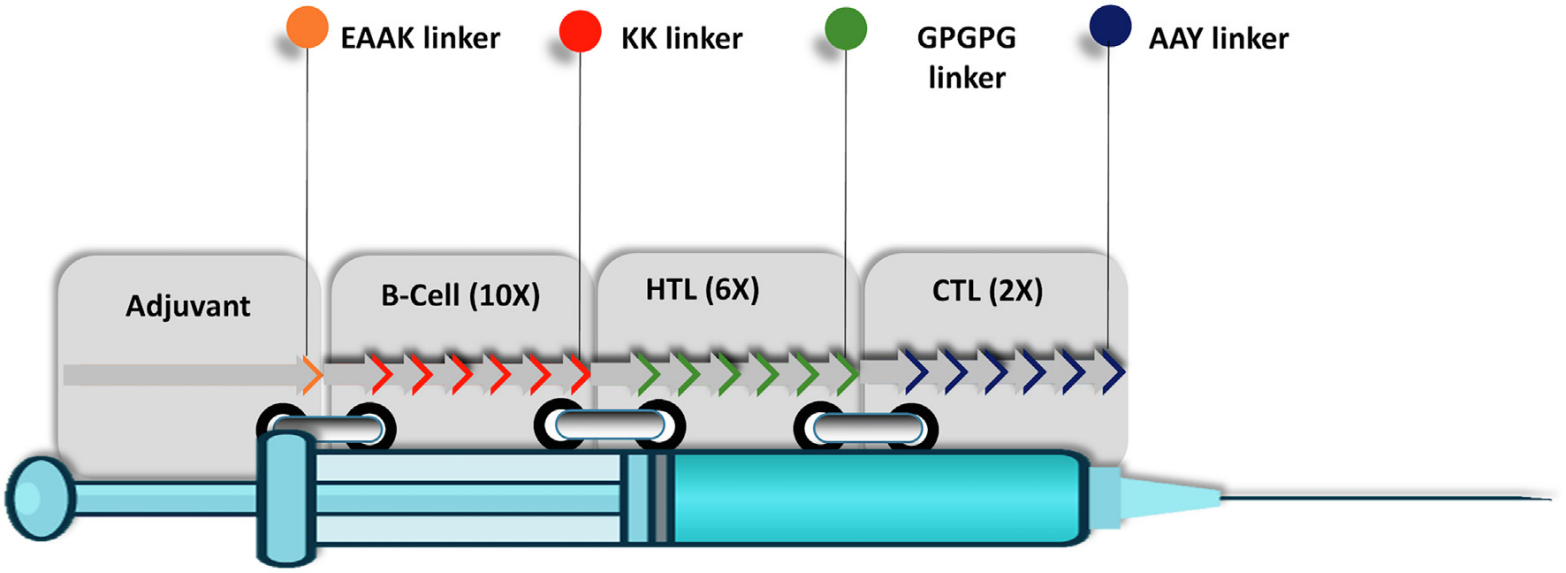
Arrangements of epitopes in the multi-epitope vaccine construct. *B-Cell* B lymphocyte epitopes, *HTL* Helper T lymphocyte epitopes, *CTL* Cytotoxic T lymphocyte epitopes.

The final antigenic and non-allergenic epitopes were arranged in the multi-epitope vaccine candidate starting from B-Cell epitopes, followed by HTL and CTL epitopes (Fig. 2). This fusion order was chosen since other arrangements yielded less favourable predicted tertiary structure at the later stages of our evaluation of the vaccine candidates. As toll-like receptors are essential for inducing innate and shaping adaptive immunity and the role of TLR-4 in recognition of Trypanosome’s pathogen-associated molecular patterns (PAMPs) have been described [49], TLR-4 agonist RS-09 [30] was attached using EAAAK linker [50] to the *N*-terminus of the vaccine construct as adjuvant. RS-09 was meant to stimulate TLR4 by mimicking the receptor interaction with LPS, and its inclusion in the fusion protein facilitates establishment of a ready formulation without relying on a separate adjuvant. Starting from the *N*-terminus, B-cell epitopes were connected via KK linker, followed by HTL epitopes connected by the GPGPG linker, and finally CTL epitopes which were connected by AAY linkers, as described before [18].

### 3.7 The multi-epitope vaccine is predicted to be antigenic, safe, and can induce the desired immune response

**Fig. 3:**
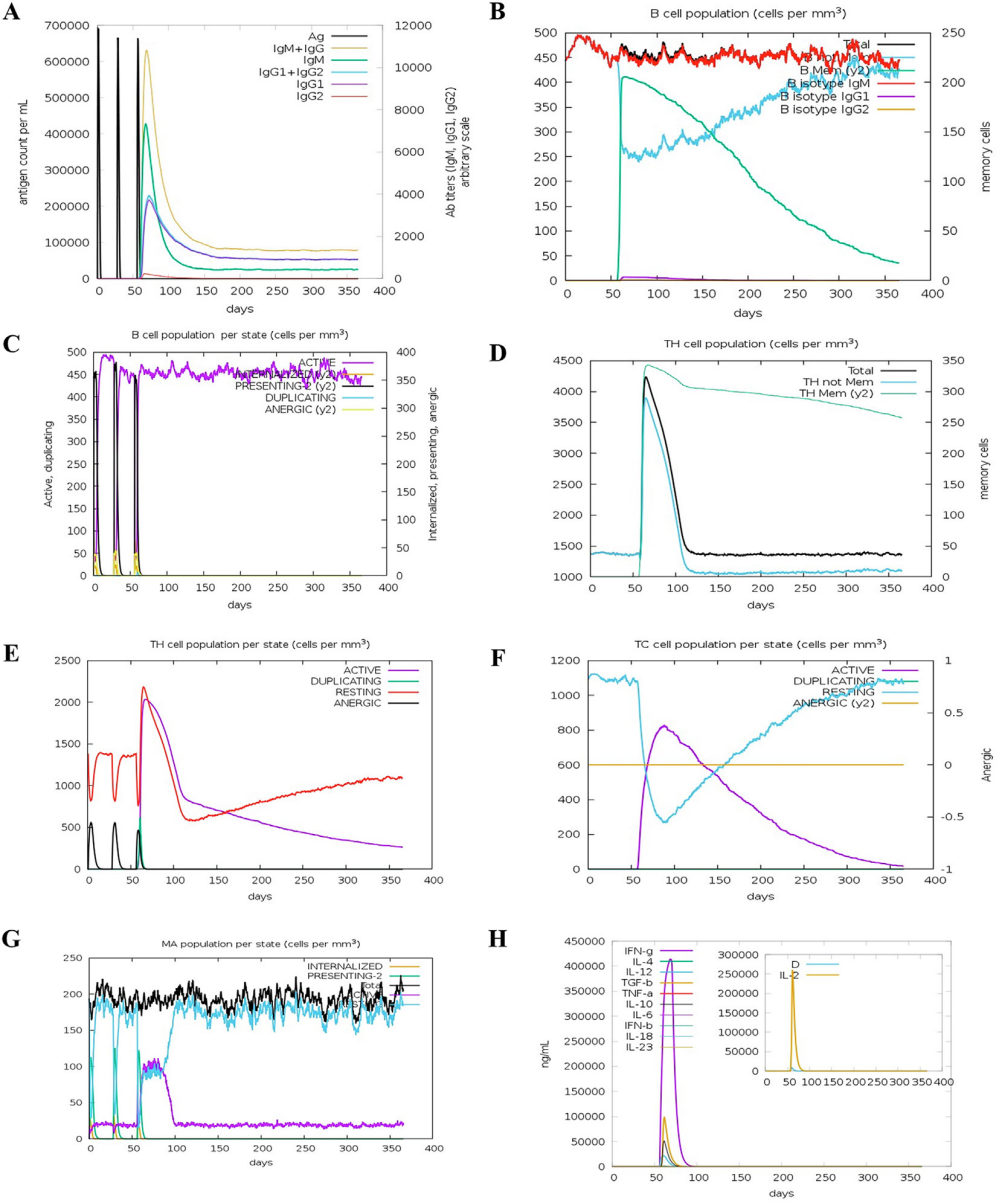
Simulation of the immune response against the multi-epitope vaccine. **(A)** Antigen and immunoglobulins, **(B)** B-cell population, **(C)** B-cell population per state, **(D)** T helper cell population, **(E)** T helper cell population per state, **(F)** T cytotoxic cell population per state, **(G)** Macrophage population per state, and **(H)** production of cytokine and interleukins.

The putative vaccine candidate scored 0.7340 on the VaxiJen server during antigenicity analysis. This value was significantly higher than the parasite’s threshold of 0.5, suggesting that the designed MEV would be immunogenic and a potential vaccine candidate. We further evaluated the vaccine’s safety through allergenicity, toxicity, and homology analysis. AllertTOP 2.0 server classified the chimeric vaccine as a non-allergen, and the ToxinPred server revealed no toxic peptide within the vaccine sequence (Supplementary Table 3). In addition, while a vaccine candidate needs to be antigenic and non-allergenic, it must be non-homologous to human proteins to avoid an auto-immune reaction/toxicity. Sequence alignment of the putative vaccine with the human proteome showed that the vaccine candidate shares 30.1 % identity with human OGFR (UniProt, Q6PK21). However, we did not observe more than three residues stretch in all the homologous regions (Supplementary Fig. 2), thereby restating the putative vaccine’s safety. Accordingly, the ability of the hypothetical antigen to induce immune response was simulated *in silico*. Upon injection of the first and second dose, little or no production of immunoglobulins (IgG1 + IgG2, IgM, and IgG + IgM) was predicted (Fig. 3A). However, a rapid rise in the immunoglobulin levels was predicted after the third dose, and the level fell with decreasing antigen concentration (Fig. 3A). This data indicated that at least three doses of the putative antigen would be required for adequate immune stimulation, which scenario is although not ideal but also not unusual in clinical vaccines. Active and memory B-cells were also predicted to be stimulated, and their concentrations maintained a high level throughout the simulation period (Fig. 3B, 3C).

Similarly, high proliferation of both helper and cytotoxic *T*-cells was predicted, and the effect persisted throughout the simulation (Fig. 3D-3F). Moreover, increased macrophages activity was predicted starting from the first dose (Fig. 3G), and cytokines such as IFN-γ and IL-2, were adequately produced (Fig. 3H). Therefore, this simulation illuminates the potential of the multi-epitope vaccine in stimulating both humoral and cellular immune responses.

### 3.8 Physicochemical properties of the multi-epitope vaccine

Prediction of physicochemical properties revealed that the vaccine is 402 amino acids long and has a molecular weight of 41.5 kDa. The isoelectric point was predicted to be 8.96, and the total numbers of negatively and positively charged residues were reported to be 37 and 43, respectively. Although the vaccine’s instability index (54.72) is classified as relatively unstable, the estimated half-lives in mammalian reticulocytes, Yeast, and *E. coli* were 30 h, >20 h, and > 10 h, respectively. Therefore, this implied that the vaccine candidate would be stable enough to be expressed in *E. coli* or yeast cells and stable enough to exert its function in the mammalian system. Precipitation of insoluble proteins is a major challenge in the production of recombinant proteins. Our hypothetical antigen has a predicted grand average of hydropathicity (GRAVY) of −0.535, classifying it as hydrophilic. Thus, it could be speculated that the chimeric vaccine could interact well enough with surrounding water molecules and hence be soluble in biological fluids. Overall, the vaccine candidate met the prerequisites of being an effective putative antigen.

### 3.9 The multi-epitope vaccine adopts a favorable 3D conformation

**Fig. 4:**
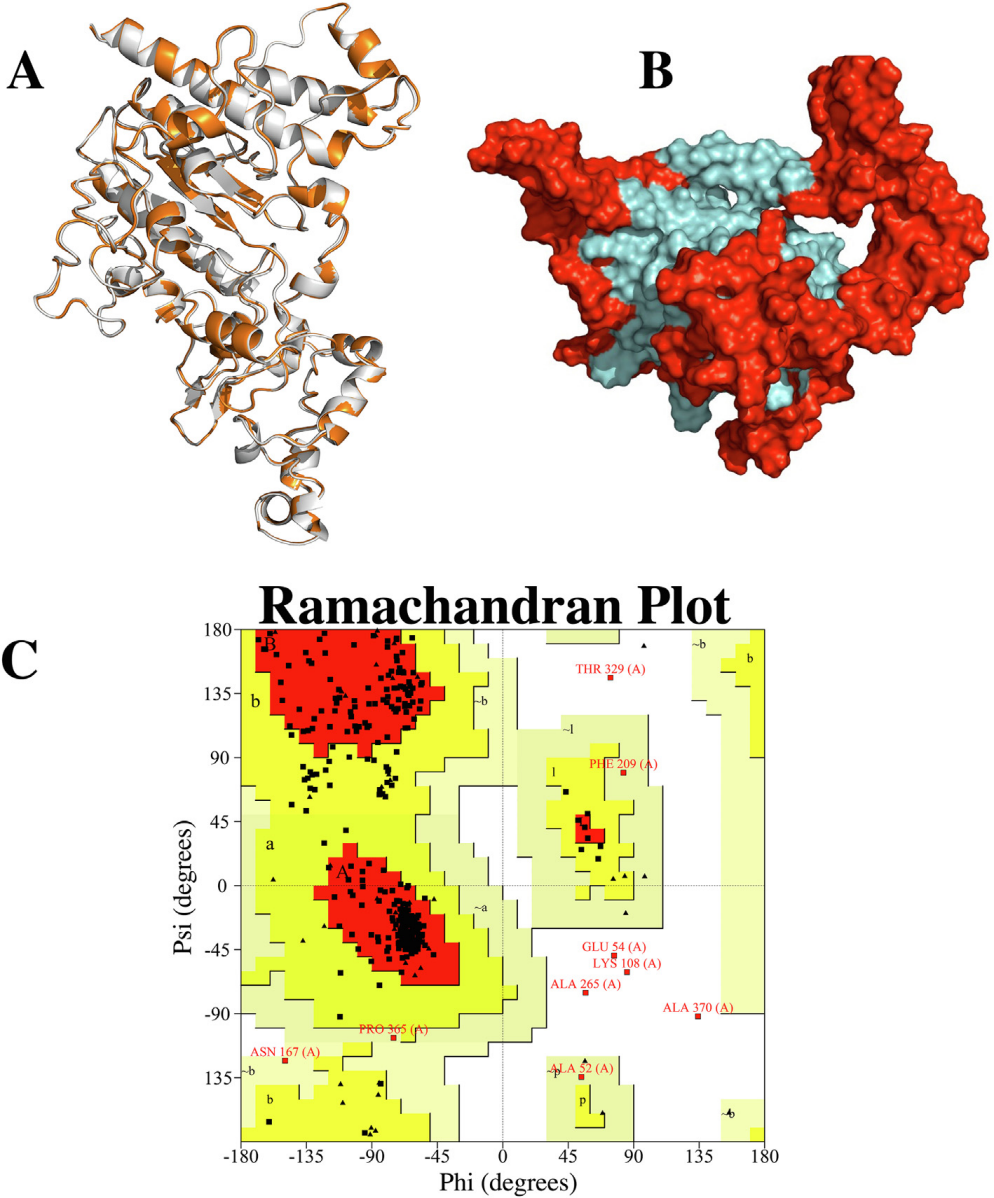
Multi-epitope vaccine 3D structure modeling and refinement. **(A)** Superimposed structural model of MEV predicted by RoseTTAFold (Gray) and Galaxyrefine-refined model of the multi-epitope vaccine (Orange). **(B)** Refined model of designed multi-epitope vaccine candidate, showing discontinuous B-Cell epitopes in red color. **(C)** Ramachandran plot of the refined 3D model of multi-epitope vaccine showing 93.5% of residues in most favored regions, with 0 poor rotamers.

To predict the structure of the multi-epitope vaccine, we chose the RoseTTAFold prediction method because the algorithm belongs to the newest generation of methods employing a three-track neural network and has the best contact area difference (CAD-score) in continuous automated model evaluation (CAMEO) [51] CAD-score represents a consistent framework for evaluating the accuracy and completeness of the structural model. As expected, RoseTTAFold predicted our putative vaccine to be comprised of solely α-helices connected by variable linker loops (Fig. 4A). Ramachandran plot analysis of the initial model showed 90.5 % of residues in the most favored regions and returned a poor rotamers score of 0.3 (Supplementary Table 3). Therefore, the model was refined (Fig. 4A) and resubjected to another round of Ramachandran plot analysis. The refined model showed a 0.0 poor rotamers score (Supplementary Table 4) and an increase in the number of residues in the most favored region from 90.5 % to 93.5 % (Fig. 4C). Hence, the final model could be considered of reasonable quality.

### 3.10 The multi-epitope vaccine conformation favors the formation of discontinuous B-Cell epitopes

Considering that most B-Cell epitopes are discontinuous, we verified if the vaccine 3D model folded into a conformation that allowed the formation of discontinuous B-Cell epitopes. ElliPro server predicted eight discontinuous B-Cell epitopes that passed the threshold of 0.5 (Supplementary Table 5). These epitopes covered 231 residues of the entire protein sequence and were well exposed on the protein surface (Fig. 4B). In addition, the server also reported nine linear B-Cell epitopes, which is in agreement with the number of B-Cell epitopes used for the design of the vaccine.

### 3.11 The putative vaccine candidate interacts with TLR4

**Fig. 5:**
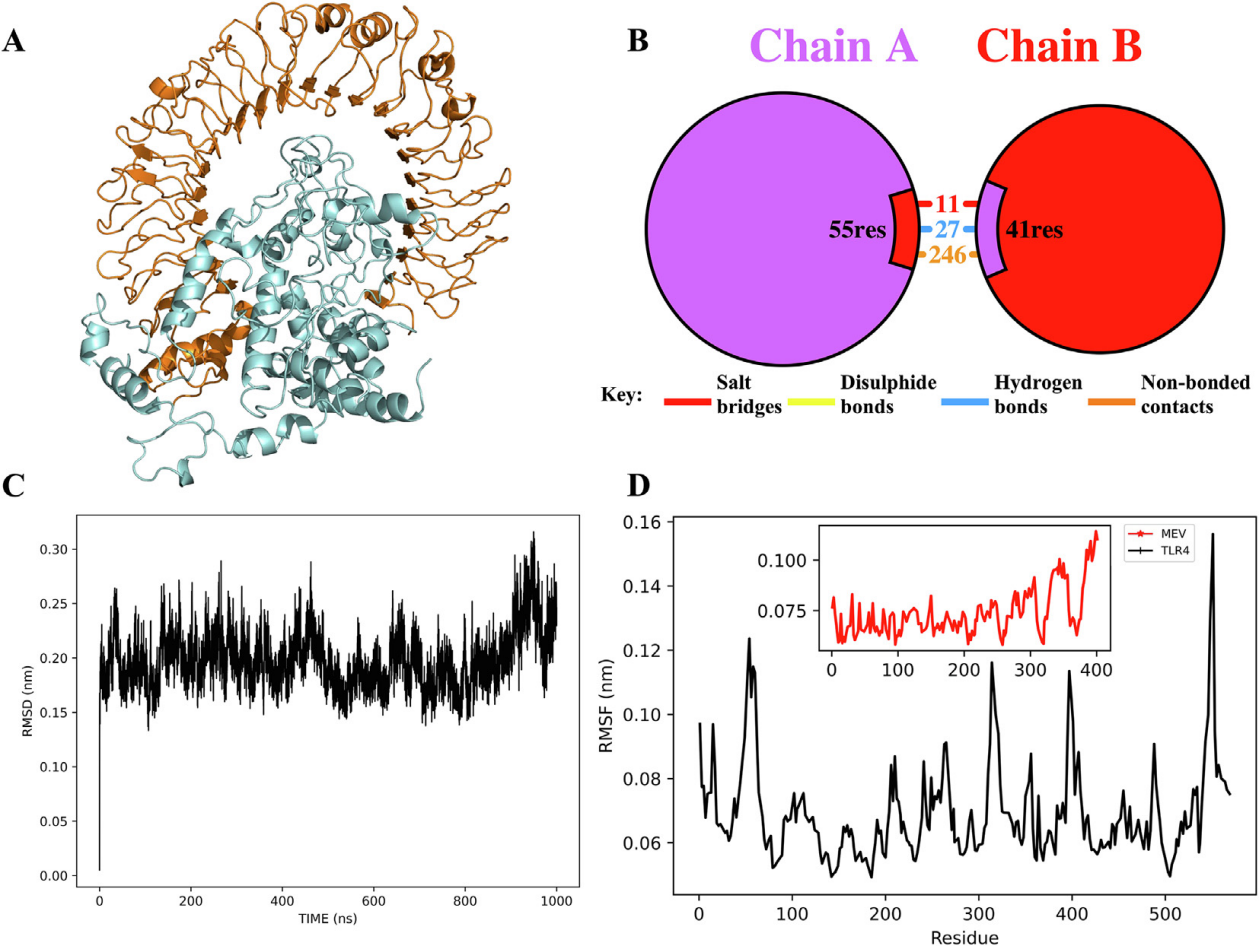
Molecular interactions of the MEV candidate with TLR4. (**A**) Ligand (multi-epitope vaccine, in cyan) bound to TLR4 (in orange). **(B**) Representation of the interaction of MEV with TLR4 receptor. Chain A represents TLR4, while Chain B represent the MEV. (**C**) Root-mean-square deviation of the ligand-receptor complex from 1 μs molecular dynamic simulation. (**D**) Root-mean-square fluctuation of ligand and receptor residues (*MEV* multi-epitope vaccine, *TLR4* Toll-like receptor 4).

Activation of toll-like receptors is essential for activating an innate immune response against *T. brucei* [52]. For this reason, an unbiased blind molecular docking of the multi-epitope vaccine against TLR4 was performed to assess the ability of the former to trigger the latter. ClusPro server predicted ten different binding models of the ligand to the target. However, as described by ClusPro (ClusPro 2.0: protein–protein docking.), the server’s docking score cannot be used for the ranking best model. We chose the model that fitted best into the receptor cavity (Fig. 5A). In this model, about 284 interactions were formed between 55 residues of TLR4 and 41 of the MEV (Fig. 5B). These interactions included hydrogen bonds and several other hydrophobic interactions (Supplementary Fig. 4). Hence, the result suggested that the multi-epitope vaccine could have the potential of binding and stimulating TLR4 in vivo.

### 3.12 Molecular dynamics confirms stable vaccine interaction with TLR4

We employed a coarse-grain molecular dynamics (MD) simulation approach to study the dynamics of the interaction of our putative vaccine with TLR4. The approach relies on mapping 4–2 atoms into a single chemically representative bead or interaction site. This allows the simulation of complex systems on a long timescale that is otherwise not feasible with contemporary computational resources. Although the technique suffers from loss of interaction details due to the reduction in the atomistic degree of freedom, the local energy minima from coarse-grain MD are comparable with that from atomistic simulation. Hence, we ran a coarse-grain MD simulation of our system with Martini 3.0 forcefield. Trajectories generated from the final 1 μs simulation were used to calculate the RMSD of the backbone atoms. The RMSD rapidly equilibrated and was maintained around 0.20–0.25 nm throughout the simulation, indicating that the conformation of the models included in the initial complex remained stable (Fig. 5C). Based on the RMSD, we considered the first 200 ns as an extension of the equilibration phase of our system. Thus, trajectories from the last 800 ns were used for other downstream analyses. The RMSF of residues in both the receptor (TLR4) and ligand (multi-epitope vaccine) showed little overall flexibility, indicating the complex’s stability during simulation (Fig. 5D). Considering the relatively flexible residues in the disordered regions (*i.e.*, the loops) in both the vaccine 3D model (Fig. 5A) and the receptor’s structure, a degree of flexibility was expected. Among the criteria of hydrogen bond formation, it is expected that the donor and acceptor will maintain a distance of ≤ 0.35 nm. To evaluate if the hydrogen bonds predicted by molecular docking could be retained during the MD simulation, we calculated the distances between the residues as a function of simulation time. Both Gly150(B) - Gln505(A) and Gly116(B) - Lys533(A) interaction pairs maintained a distance well below 0.35 nm throughout the simulation (Supplementary Fig. 3). This reinforced the plausibility of the interaction predicted from our docking studies. Overall, the molecular dynamics simulation supported a stable receptor-ligand interaction.

### 3.13 Codon adaptation and *in silico* cloning

To adapt the putative vaccine sequence for prokaryotic expression, the sequence was optimized for *E. coli* K12 strain codon usage using the Jcat server. The adapted nucleotide sequence had a codon adaptation index value of 1.0, indicating a high possibility of protein expression and a GC content of 54.97 %, which fell within the optimal range of 30 % to 70 %. The nucleotide sequence was therefore cloned (*in silico*) into a pET-28a(+) vector (Supplementary Fig. 5), exploiting the *Not*I and *Eco*RI restriction sites within the MCS of this popular expression vector.

## 4 Discussion

Efforts towards the eradication of African trypanosomiasis should be intensified since the pathogens continue to undermine the available control strategies. Although efforts towards the development of new drug candidates are ongoing [53], [54], [55], vaccine development remains the most preferred eradication strategy. Yet, not a single vaccine has been registered to prevent trypanosomiasis. This is mainly due to the ability of the parasite to manipulate its host immune system and destruction of the B cell compartment [56], [57], [58]. For example, in mice models, the most evident change during trypanosome infection is the induced destruction of the host’s lymphoid tissue, which results in splenomegaly [59]. The host’s immunosuppression caused by trypanosomes results from the summation of diverse local manipulations by the parasites. Starting with innate immunity, phagocytosed trypanosomes release factors such as adenylate cyclase (AdC) and kinesin heavy chain (TbKHC-1) that prevent the release of cytokines, which can otherwise activate macrophages [60], [61]. This ensures early trypanosome infection progression. Adaptive immunity is equally compromised during trypanosomiasis, majorly due to the infamous antigenic variation mechanism, formed by the repertoire of about 1000 VSG and over 10,000 pseudo-VSG genes that the parasite bears in its genome [62], [63], [64]. Trypanosomes use VSG to shield the invariant parasite’s proteins from attack by host neutralizing-antibodies while rapidly switching the type of VSG to prevent an attack on VSG itself. The trypanosome’s immune evasion mechanism is not the subject of our work, and detailed reports on different immune evasion strategies by trypanosomes can be found elsewhere [65], [66], [67]. The VSG-induced immune evasion mechanism is not the only means of compromising the host’s adaptive immunity. There are reports showing that trypanosome infection leads to damage to B cell compartments leading to loss of memory recovery of the host immune system [58]. This is the reason for the loss of efficacy of non-anti-trypanosome veterinary vaccines during trypanosome infection [65], [68]. Combination of all these mechanisms by the parasite undoubtedly led to the failure of several vaccination approaches, such as the use of purified flagellar pockets, recombinant proteins, and even DNA vaccines [6], [69], [70], [71], [72], [73], [74]. Despite these challenges in the development of an anti-trypanosome vaccine, the need for vaccine is further compelled by the reported ability of non-HAT trypanosomes to infect humans [75].

The immunoinformatics approach to vaccine design, on the other hand, has proven to be superior to other vaccine design approaches in several ways. The method is time-saving, cost-effective, and allows screening potential immunogenic epitopes from the entire pathogenic organism’s genome. The solution to trypanosome immune evasion and destruction of host’s adaptive immunity [56], [58], might lie in challenging the parasite with a cocktail of epitopes. The immunoinformatics approach offers such a solution and has been proven to be an efficient tool for the design of antigens that are capable of stimulating both cellular and humoral immune responses [13], [20], [76], [77], [78], [79], [80], [81]. Multi-epitope vaccines have not been effective only in the lab, but several of them have made it to the clinical trial [20], [82], [83]. Even so, the recent breakthrough in anti *T. vivax* vaccine reaffirms that stimulation of immunological memory against trypanosomes is very much possible [84]. Consequently, we employed such immunoinformatics approach in this study to mine potential immunogenic peptides from the whole proteome of *T. b. gambiense*, a subspecies that accounts for most of the reported HAT cases [1]. We predicted all hypothetical plasma membrane proteins from the organism’s proteome and collected overlapping B-Cell and *T*-Cell (CTL and HTL) epitopes from transmembrane helices of these proteins. We hypothesized that inducing CTL activity could be effective in subverting *T. b. gambiense* infection as evidence suggested that CTL can directly target and destroy extracellular parasites ([85]. Moreover, CD8 + constitutes a major source of IFN-γ [86], which is equally essential for resisting *T. brucei gambiense* infection [87]. Several groups have previously used this approach [14], [19], [88], [89] to assemble antigenic epitopes from different pathogens. Extracellular domain helices from plasma membrane proteins were selected because of their high potential to interact with host receptors. We also chose helices that are long enough (≥30) to be exposed and also to increase the chance of avoiding VSG’s immune evasion effect. We additionally confirmed their surface exposure through structure prediction of proteins hosting these epitopes. In addition to being antigenic, the predicted CTL, HTL, and linear B-Cell epitopes must pass some safety filters. These include being non-toxic, non-allergenic, and non-homologous to proteins from the human proteome. As such, we selected the epitopes that passed the criteria above and connected them using suitable linkers to construct the vaccine candidate. Considering that protein expression profile of trypanosomes varies significantly at different stages of the parasites’ life cycle and that the proteomic data used for our study represent procyclic *T. b. gambiense*, our hypothetical vaccine could be ineffective if the epitopes are not expressed in the infective metacyclic forms. To this end, we confirmed that metacyclic expression was reported for all proteins harboring the selected epitopes [90].

Linkers play a vital role in ensuring proper protein folding and flexibility and allow the generation of functional domains, such as discontinuous B-Cell epitopes, as well as improving antigen processing [11]. For our vaccine candidate, an EAAAK linker was used to append the TLR-4 agonist RS-09 [30] at the *N*-terminus as an adjuvant. The introduction of RS-09 was aimed to increase the stimulation of immune receptors and consequently to elicit a higher immune response. B-cell epitopes were connected by KK linkers, HTL epitopes by GPGPG linkers, and CTL epitopes by AAY linkers. The choice of linkers to join the vaccine’s different epitopes was based on previous studies that reported the design of stable antigens using these linkers [16], [18], [91]. The designed vaccine as a whole was predicted to be antigenic, non-allergenic, and non-toxic. In addition, the size of the putative vaccine (41.5 kDa) was large enough to be an antigen and was predicted to be soluble in a polar environment. The predicted half-lives in *E. coli* (>10 hrs) and mammalian reticulocytes (>30 hrs) reveal that the multi-epitope vaccine is stable enough to be overexpressed in the prokaryotic system and exert its function in the mammalian system. The yield of recombinant proteins from *E. coli* expression system is often orders of magnitude higher than from the yeast. Furthermore, *E. coli* is more economical to handle than the yeast system. However, it is recommendable to investigate both expression systems. These predicted physicochemical properties of our vaccine candidate agree with the properties of most of the multi-epitope vaccines reported [18], [19], [81]. In fact, a vaccine candidate that shares some of these properties was shown to be stable enough to trigger both cellular and humoral immune responses against *Toxoplasma gondii* [17]. The whole construct was further made ready for adoption in vaccine trials by codon optimization for bacterial expression and a proposed sequence that can be inserted into a popular expression vector pET-28a (+). This way, the vaccine could be expressed as a His-tagged recombinant protein to allow large-scale purification.

The ability of the chimeric vaccine to induce an immune response in the human body was also simulated. Macrophages, B-Cells, helper, and cytotoxic *T*-cells were predicted to be adequately stimulated, and their population persisted throughout the simulation period (1 year). Antibody production was predicted to be triggered only after the third dose, indicating that at least three doses of the antigen would be required to provoke a sufficient response. In agreement with the predicted IFN-γ-inducing epitopes in our construct (Supplementary Table 2), IFN-γ and other immune-cells proliferating cytokines were predicted to be sufficiently released, and their effect was evident in the population of macrophages and *T*-Cells (Fig. 3). A study by [87] confirmed that IFN-γ release is associated with resistance to *T. b. gambiense* infection. Taken together, our simulation predicted that our putative antigen would be able to elicit protective immunity against *T. b. gambiense*. Recognition of pathogen-associated molecular patterns (PAMPs) by pattern-recognition receptors (PRRs) such as toll-like receptors (TLR) is essential in the initiation of innate immune response [92]. When PAMPs come in contact with PRR, a set of adaptors that bears the Toll-IL-1 receptor (TIR) domain of that PRR are recruited. This event leads to the activation of some signaling cascades that eventually result in the secretion of inflammatory cytokines [49]. The cytokines, in turn, facilitate the activation and recruitment of effector cells like neutrophils and macrophages. As a result, the infected pathogen is neutralized. TLR4 is one of those PPRs that is central in recognition of the *T. brucei* species [49]. Thus, docking was performed to study the possible interaction of the multi-epitope vaccine and human TLR-4. The vaccine protein fitted very well in the receptor’s cavity, and about 30 interactions that include both hydrogen bonds and hydrophobic contacts were predicted in the receptor-ligand complex. Molecular dynamics simulation confirmed that the predicted interaction was stable, and binding of the multi-epitope vaccine to TLR4 did not lead to structural instability in either receptor or ligand structures. This strong interaction might, in part, be promoted by the TLR4 agonist RS-09 conjugated to the vaccine construct [30].

In conclusion, we have herein presented the first report of a putative subunit vaccine candidate designed from a cocktail of *T. b. gambiense* plasma membrane proteins. Transmembrane proteins are generally rich in antigenic motifs, since their higher exposure to host effector cells makes them ideal candidates for antigen design. Indeed, transmembrane proteins have allowed the prediction of highly immunogenic epitopes in different infectious agents such as *S. mansoni*, *P. aeruginosa*, *L. infantum,* and even melanoma [15], [19], [93]. Similarly, the computational simulations employed in this study indicated that our putative vaccine candidate met the prerequisites to trigger the desired immune response in humans. Interestingly, all the epitopes used in the vaccine construction are conserved in all the subspecies of *T. brucei* and in *T. congolense*, suggesting that it could be applicable to both Human African trypanosomiasis and nagana. In contrast, post-translational modification of proteins bearing the vaccine’s epitopes could present a major stumbling block in stimulating immune response. Moreover, information on post-translational modification of these proteins is currently lacking. Nevertheless, the potentials of the designed multi-epitope vaccine needs to be evaluated *in vitro* and in vivo and these studies are currently underway. Finally, our vaccine construct has shown promising results *in silico* and could serve as a ready-for-use recipe for such future vaccine production and trials. Due to the unavailability of disease simulation platform for trypanosomiasis, we were unable to simulate the effect of the construct on the disease’s pathology for its potential use for immunotherapy of trypanosomiasis. We hope that the present report will stimulate efforts towards the development of such an important computational tool.

## Author contributions

AUD, EOB, and MWG contributed to the conception and design of the study. AUD performed all major experiments, data analysis, and writing of the first draft. MWG provided overall supervision of the study, interpreted the data and reviewed the manuscript critically. EOB provided supervision and critical review of the manuscript. SIG and SI conducted part of the analyses, data visualization and wrote sections of the first draft. LBD contributed to data interpretation, data visualization and revision of the first draft. All authors contributed to the manuscript revision, read and approved the submitted version.

## Funding

This work was supported by EMBO Installation Grant 3315 to MWG. AUD is a beneficiary of The Ignacy Łukasiewicz Scholarship Programme from The Polish National Agency for Academic Exchange (NAWA). MWG is the recipient of L’Oréal-UNESCO For Women in Science scholarship from L’Oréal Poland and the Ministry of Education and Science, Poland. EOB is a recipient of the Emerging Global Leader (K43) Award and supported by the Fogarty International Center of the National Institutes of Health under Award Number K43TW012015. The content of this manuscript is solely the responsibility of the authors and does not necessarily represent the official views of the National Institutes of Health.

## Declaration of Competing Interest

The authors declare that they have no known competing financial interests or personal relationships that could have appeared to influence the work reported in this paper.
