# Supplementary material for: Immunoinformatic design of a putative multi-epitope vaccine candidate against Trypanosoma brucei gambiense
Source: Comput Struct Biotechnol J. 2022 Oct 7;20:5574–85. doi: 10.1016/j.csbj.2022.10.002 (PMC9576565; doi:10.1016/j.csbj.2022.10.002)
Supplement: Supplementary data 1 [file mmc1.pdf]

Supplementary Material

Supplementary Data Sheet 1

Supplementary Data

Multi-epitope vaccine protein sequence (linker residues are highlighted in bold):

MAPPHALS**EAAAK**WREALAFAASPFV**KK**PSMDENNPEIQSTL**KK**MVSMGTEIAEEDIEN**KK**KRFS
FVLPQPLRKSPIDV**KK**QNFQTCFYPDGDTFLVGDRGIRLSGG**KK**HETHGRGGTEAQTVGGGAGFP
MRSTPSG**KK**ASSEGDLSTTGPSAGAAVPQVTRGHNTKNG**KK**QSSPRAATASPSTCVWQCRQDYL
Q**KK**RSYLSANPGVTVFMARITSTTTTWMGG**KK**SDRLRRDPACATNNDGAAAGPTSSAGGGELQ**G**
PGPGMVSMGTEIAEEDIEN**GPGPG**KRFSFVLPQPLRKSP**GPGPG**GNTIEQYFRDHNMEY**GPGPG**A
NVGSVSAASAEAQG**GPGPG**GAAAGALLPGSTEDE**GPGPG**QSSPRAATASPSTCV**AAY**VLDDNAT
LV**AAY**ALAFAASPFV

Codon-adapted sequence (the first and the last codon encoding the multi-epitope vaccine protein are underlined, EcoRI and NotI restriction sites are in italic):

*GAATTC*ATGGCTCCGCCGCACGCTCTGTCTGAAGCTGCTGCTAAATGGCGTGAAGCTCTGGCTT
TCGCTGCTTCTCCGTTCGTTAAAAAACCGTCTATGGACGAAAACAACCCGGAAATCCAGTCTAC
CCTGAAAAAAATGGTTTCTATGGGTACCGAAATCGCTGAAGAAGACATCGAAAACAAAAAAA
AACGTTTCTCTTTCGTTCTGCCGCAGCCGCTGCGTAAATCTCCGATCGACGTTAAAAAACAGAA
CTTCCAGACCTGCTTCTACCCGGACGGTGACACCTTCCTGGTTGGTGACCGTGGTATCCGTCTG
TCTGGTGGTAAAAAACACGAAACCCACGGTCGTGGTGGTACCGAAGCTCAGACCGTTGGTGGT
GGTGCTGGTTTCCCGATGCGTTCTACCCCGTCTGGTAAAAAAGCTTCTTCTGAAGGTGACCTGT
CTACCACCGGTCCGTCTGCTGGTGCTGCTGTTCCGCAGGTTACCCGTGGTCACAACACCAAAAA
CGGTAAAAAACAGTCTTCTCCGCGTGCTGCTACCGCTTCTCCGTCTACCTGCGTTTGGCAGTGC
CGTCAGGACTACCTGCAGAAAAAACGTTCTTACCTGTCTGCTAACCCGGGTGTTACCGTTTTCA
TGGCTCGTATCACCTCTACCACCACCACCTGGATGGGTGGTAAAAAATCTGACCGTCTGCGTCG
TGACCCGGCTTGCGCTACCAACAACGACGGTGCTGCTGCTGGTCCGACCTCTTCTGCTGGTGGT
GGTGAACTGCAGGGTCCGGGTCCGGGTATGGTTTCTATGGGTACCGAAATCGCTGAAGAAGAC
ATCGAAAACGGTCCGGGTCCGGGTAAACGTTTCTCTTTCGTTCTGCCGCAGCCGCTGCGTAAAT
CTCCGGGTCCGGGTCCGGGTGGTAACACCATCGAACAGTACTTCCGTGACCACAACATGGAAT
ACGGTCCGGGTCCGGGTGCTAACGTTGGTTCTGTTTCTGCTGCTTCTGCTGAAGCTCAGGGTGG
TCCGGGTCCGGGTGGTGCTGCTGCTGGTGCTCTGCTGCCGGGTTCTACCGAAGACGAAGGTCCG
GGTCCGGGTCAGTCTTCTCCGCGTGCTGCTACCGCTTCTCCGTCTACCTGCGTTGCTGCTTACGT
TCTGGACGACAACGCTACCCTGGTTGCTGCTTACGCTCTGGCTTTCGCTGCTTCTCCGTTCGTTT
GCGGCCGC

Supplementary Figures

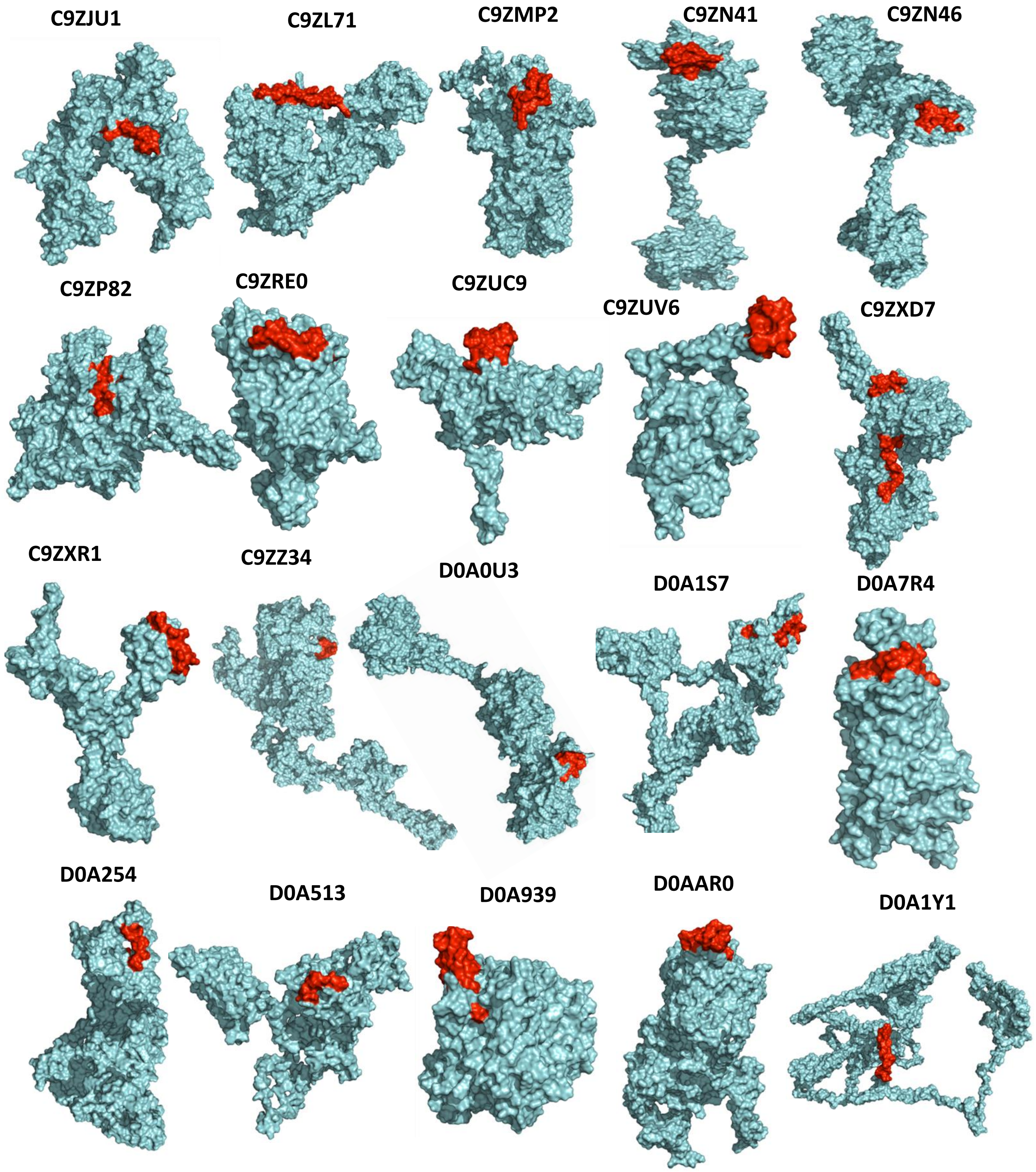

Supplementary Figure 1: 3D structures of proteins harboring selected epitopes for multi-epitope vaccine design. Epitopes are highlighted in red color.

```
EMBOSS_001             1 -----------------------------------------------MAP      3
                                                                        .|.
Q6PK21_HUMAN           1 MPSPSRSGRSRCLKAPRRSRSGLSGPTSSCWASTGIRLEDRGTGTVGRAQ     50

EMBOSS_001             4 PHALSEAAAKWREALAFAASPFVKKPSMDENNPEIQSTLKKMVSMGTE--     51
                         .:........||                ..||..|...||.:..:|.|
Q6PK21_HUMAN          51 NYQKRFQNLNWR----------------SHNNLRITRILKSLGELGLEHF     84

EMBOSS_001            52 -------IAEEDI----------------------------------ENK     60
                                ..||.:                                  |:.
Q6PK21_HUMAN          85 QAPLVRFFLEETLVRRELPGVRQSALDYFMFAVRCRHQRRQLVHFAWEHF    134

EMBOSS_001            61 KKRFSFV--------------LPQPL---RK-----SPIDVKKQNFQTCF     88
                         :.|..||              ||.||   ||     ||.|
Q6PK21_HUMAN         135 RPRCKFVWGPQDKLRRFKPSSLPHPLEGSRKVEEEGSPGD----------    174

EMBOSS_001            89 YPDGDTFLVGDRGIRLSGGKKHETHGRGG-----------TEAQTVG---    124
                          ||.:....|    |..|.:    |.:||           .|.|..|
Q6PK21_HUMAN         175 -PDHEASTQG----RTCGPE----HSKGGGRVDEGPQPRSVEPQDAGPLE    215

EMBOSS_001           125 -------GGAGFPMRSTPSGKKASSEGDLSTT---------GPSAGAAVP    158
                                ||.| ..|..|...|.|.:..|..:         ||.:.:.|.
Q6PK21_HUMAN         216 RSQGDEAGGHG-EDRPEPLSPKESKKRKLELSRREQPPTEPGPQSASEVE    264

EMBOSS_001           159 QVTRGHNTKN-GKKQSSPRAAT-----ASPSTCVWQCRQ-------DYLQ    195
                         ::..  |.:. ...|.|.|..|     ..|...|..|||       |.::
Q6PK21_HUMAN         265 KIAL--NLEGCALSQGSLRTGTQEVGGQDPGEAVQPCRQPLGARVADKVR    312

EMBOSS_001           196 KKRSYLSANPGVTVFMARITSTTTTWMGGKKSDRLRRDPA------CATN    239
                         |:|. :....|.:..:|         .||.::..|...||      ...:
Q6PK21_HUMAN         313 KRRK-VDEGAGDSAAVA---------SGGAQTLALAGSPAPSGHPKAGHS    352

EMBOSS_001           240 NDG------AAAGPTSSAGGGELQGPGPGMVS-MGTEIAEEDIEN-GPGP    281
                         .:|      ...||.....|...:.|||.... .|.|.||...|. ||.|
Q6PK21_HUMAN         353 ENGVEEDTEGRTGPKEGTPGSPSETPGPSPAGPAGDEPAESPSETPGPRP    402

EMBOSS_001           282 GKRFSFVLPQPLRKSPGPGPGGNTIEQYFRDHNMEYGPGPGANVGSVSAA    331
                         .........:...::|||.|.|.|.::.....:...||.|....|...|.
Q6PK21_HUMAN         403 AGPAGDEPAESPSETPGPSPAGPTRDEPAESPSETPGPRPAGPAGDEPAE    452

EMBOSS_001           332 SAEAQGGPGPGGAAAGALLPGSTEDEGPGPGQSSPRAATASPSTCVAAYV    381
                         |.....||.|.|.|...  |..:..|.|||..:.|  ....|:....|..
Q6PK21_HUMAN         453 SPSETPGPRPAGPAGDE--PAESPSETPGPSPAGP--TRDEPAKAGEAAE    498

EMBOSS_001           382 LDDNATLVAAYALAFAASPFV    402
                         |.|    ....:.|.:..|
Q6PK21_HUMAN         499 LQD----AEVESSAKSGKP--    513
```

Supplementary Figure 2: Alignment of the multi-epitope vaccine with human OGFR protein (Q6PK21).

I

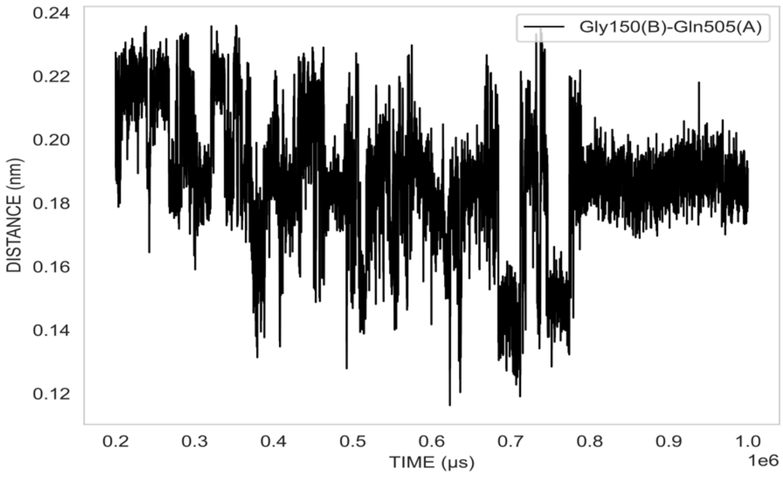

II

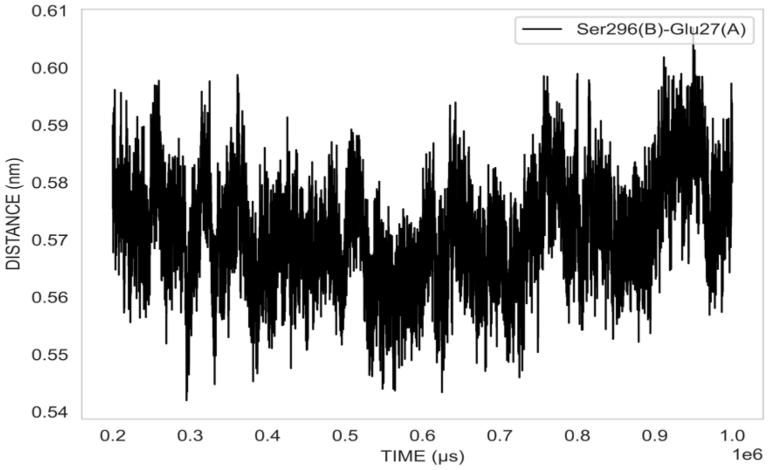

III

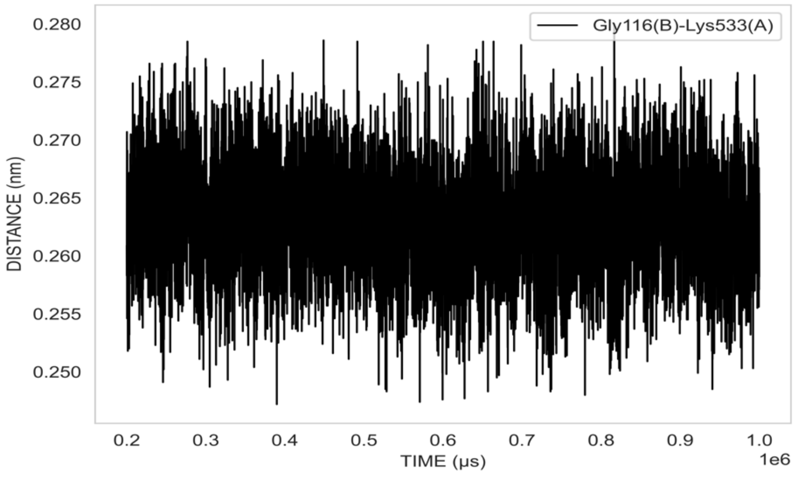

Supplementary Figure 3: Distance between predicted interacting residues of multi-epitope vaccine (Chain B) and toll-like receptor 4 (Chain A) during md simulation. **(I)** Gly150(B)-Gln505(A). **(II)** Ser296(B)-Glu27(A). **(III)** Gly116(B)-Lys533(A).

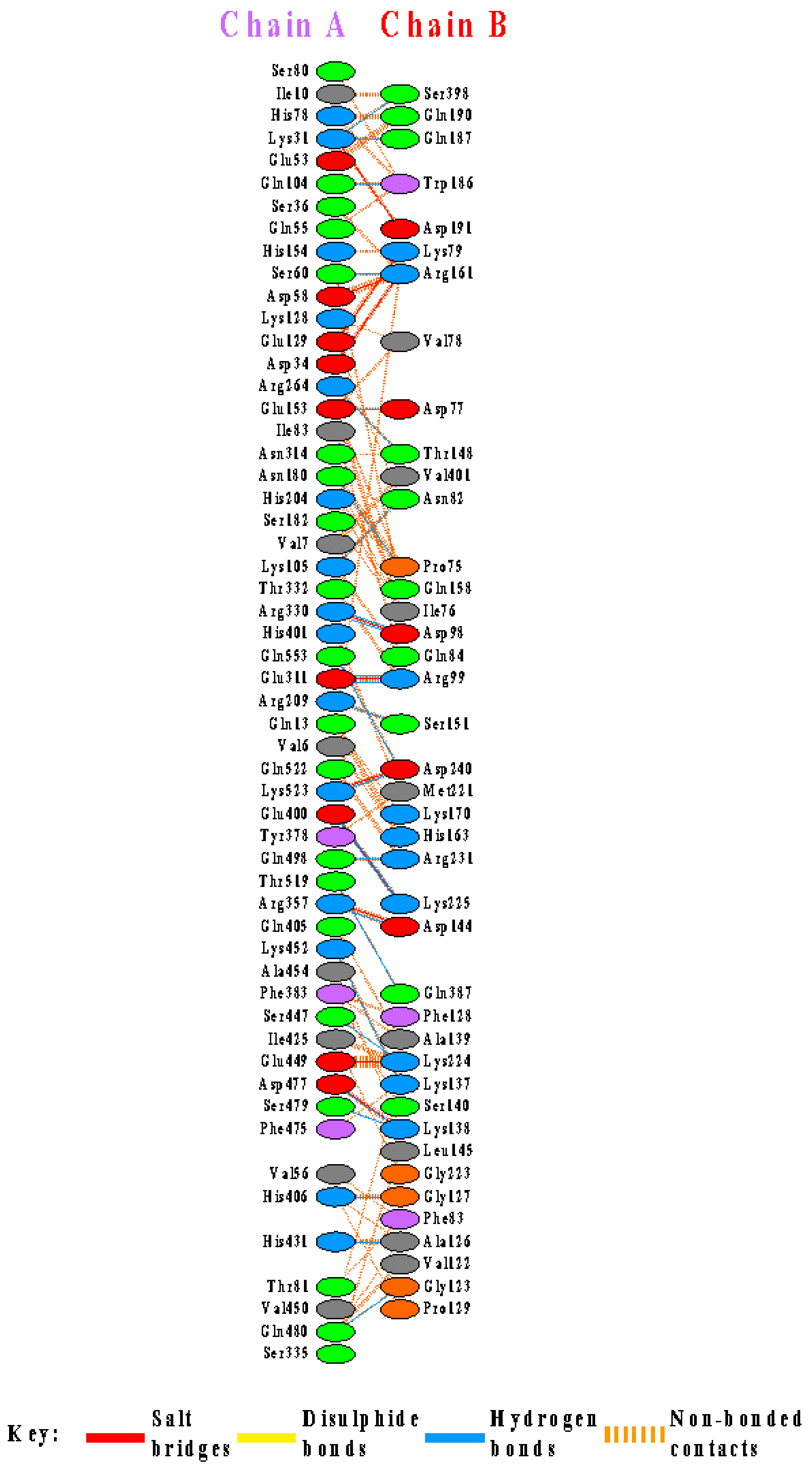

Supplementary Figure 4: 2D interaction map of the MEV with TLR4 receptor. Chain A represents TLR4, while Chain B represent the MEV.

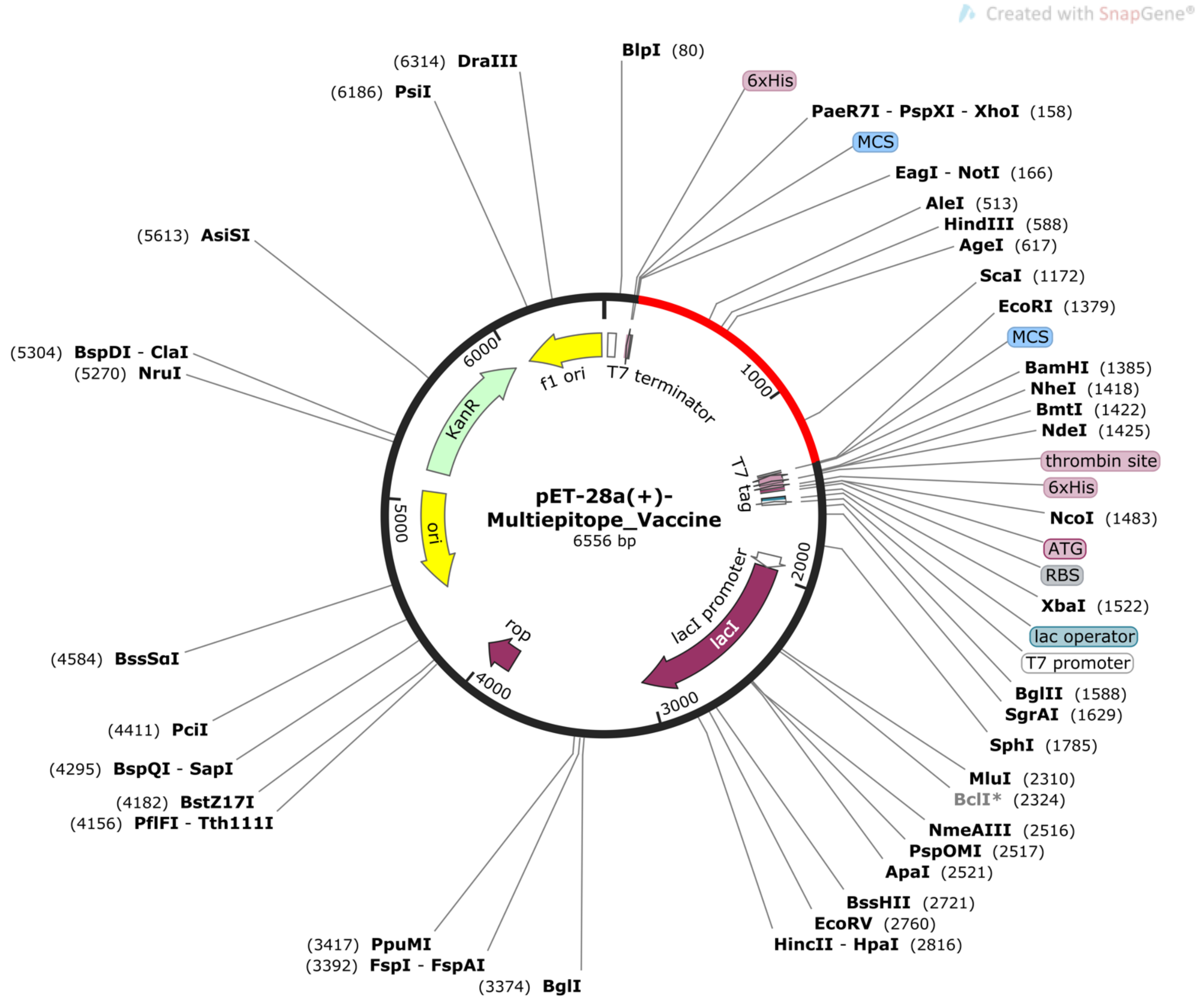

Supplementary Figure 5: *In silico* cloning of multi-epitope vaccine into multiple cloning sites of pET-28a(+) plasmid. The vaccine insert is shown in red, and the sequences are inserted between EcoRI and NotI restrictions sites.

Supplementary Tables

Supplementary Table 1. Selected epitopes for vaccine construction.

S/N	B-Cell Epitopes	CTL Epitopes	HTL Epitopes
1	WREALAFAASPFV	VLDDNATLV	MVSMGTEIAEEDIEN
2	PSMDENNPEIQSTL	ALAFAASPFV	KRFSFVLPQPLRKSP
3	MVSMGTEIAEEDIEN		GNTIEQYFRDHNMEY
4	KRFSFVLPQPLRKSPIDV		ANVGSVSAASAEAQG
5	QNFQTCFYPDGDTFLVGDRGIRLSGG		GAAAGALLPGSTEDE
6	HETHGRGGTEAQTVGGGAGFPMRSTPSG		QSSPRAATASPSTCV
7	ASSEGDLSTTGPSAGAAVPQVTRGHNTKNG		
8	QSSPRAATASPSTCVWQCRQDYLQ		
9	RSYLSANPGVTVFMARITSTTTTWMGG		
10	SDRLRRDPACATNNDGAAAGPTSSAGGGELQ		

Key: ***CTL*** **Cytotoxic T Lymphocytes,** ***HTL*** **Helper T Lymphocytes**

Supplementary Table 2. Predicted interferon-gamma inducing epitopes.

Serial No.	Epitope	Prediction Method	Result	Score
1	MVSMGTEIAEEDIEN	SVM	NEGATIVE	-0.47379613
2	KRFSFVLPQPLRKSP	SVM	NEGATIVE	-0.91347497
3	GNTIEQYFRDHNMEY	SVM	NEGATIVE	-0.25438988
4	ANVGSVSAASAEAQG	SVM	POSITIVE	0.49034067
5	GAAAGALLPGSTEDE	SVM	NEGATIVE	-0.025256707
6	QSSPRAATASPSTCV	SVM	POSITIVE	0.26807514

Supplementary Table 3. Prediction of toxic peptides from multi-epitope vaccine .

Peptide Sequence	SVM score	Prediction
APPHALSEAA	-0.93	Non-Toxin
PPHALSEAAA	-1.13	Non-Toxin
PHALSEAAAK	-1.17	Non-Toxin
HALSEAAAKW	-0.91	Non-Toxin
ALSEAAAKWR	-0.89	Non-Toxin
LSEAAAKWRE	-0.61	Non-Toxin
SEAAAKWREA	-0.70	Non-Toxin
EAAAKWREAL	-0.70	Non-Toxin
AAAKWREALA	-0.62	Non-Toxin
AAKWREALAF	-0.47	Non-Toxin
AKWREALAFA	-0.46	Non-Toxin
KWREALAFAA	-0.85	Non-Toxin
WREALAFAAS	-0.91	Non-Toxin
REALAFAASP	-0.91	Non-Toxin
EALAFAASPF	-1.31	Non-Toxin
ALAFAASPFV	-1.21	Non-Toxin
LAFAASPFVK	-1.29	Non-Toxin
AFAASPFVKK	-1.23	Non-Toxin
FAASPFVKKP	-1.13	Non-Toxin
AASPFVKKPS	-1.20	Non-Toxin
ASPFVKKPSM	-0.97	Non-Toxin
SPFVKKPSMD	-1.11	Non-Toxin
PFVKKPSMDE	-1.05	Non-Toxin
FVKKPSMDEN	-0.96	Non-Toxin
VKKPSMDENN	-0.88	Non-Toxin
KKPSMDENNP	-0.74	Non-Toxin
KPSMDENNPE	-0.78	Non-Toxin
PSMDENNPEI	-0.75	Non-Toxin
SMDENNPEIQ	-0.82	Non-Toxin
MDENNPEIQS	-0.83	Non-Toxin
DENNPEIQST	-0.64	Non-Toxin
ENNPEIQSTL	-0.90	Non-Toxin
NNPEIQSTLK	-1.02	Non-Toxin
NPEIQSTLKK	-0.95	Non-Toxin
PEIQSTLKKM	-0.95	Non-Toxin
EIQSTLKKMV	-0.84	Non-Toxin
IQSTLKKMVS	-0.90	Non-Toxin
QSTLKKMVSM	-0.63	Non-Toxin
STLKKMVSMG	-0.81	Non-Toxin
TLKKMVSMGT	-0.88	Non-Toxin
LKKMVSMGTE	-0.77	Non-Toxin
KKMVSMGTEI	-0.70	Non-Toxin
KMVSMGTEIA	-0.62	Non-Toxin
MVSMGTEIAE	-0.85	Non-Toxin
VSMGTEIAEE	-0.78	Non-Toxin
SMGTEIAEED	-0.71	Non-Toxin
MGTEIAEEDI	-0.89	Non-Toxin
GTEIAEEDIE	-0.88	Non-Toxin
TEIAEEDIEN	-0.90	Non-Toxin
EIAEEDIENK	-0.61	Non-Toxin

Supplementary Table 4. Multi-epitope vaccine 3D structure model refinement

				Clash	Poor	Rama

Model	GDT-HA	RMSD	MolProbity	score	rotamers	favored
Initial MODEL	1.0000	0.000	1.751	4.0	0.3	89.2
MODEL 1	0.9876	0.298	2.325	18.0	1.3	92.2
MODEL 2	0.9832	0.295	2.393	15.7	2.0	92..5
MODEL 3	0.9888	0.282	2.255	18.7	0.7	92.0
MODEL 4	0.9882	0.283	2.258	16.8	0.0	93.2
MODEL 5	0.9876	0.283	2.283	16.7	0.7	92.5

Supplementary Table 5. Prediction of Discontinuous B-Cell epitopes

Serial No.	Residues	Number of Residues	ElliPro Score
1	A:K81, A:Q82, A:N83, A:F84, A:Q85, A:T86, A:C87, A:F88, A:Y89, A:P90, A:D91, A:G92, A:D93, A:T94, A:F95, A:L96, A:V97, A:G98, A:D99, A:R100, A:G101, A:I102, A:R103, A:L104, A:S105, A:G106, A:G107, A:K108, A:K109, A:H110, A:E111, A:T112, A:H113, A:G114, A:R115, A:G116, A:G117, A:T118, A:E119, A:A120, A:Q121, A:T122, A:V123, A:G124, A:G125, A:G126, A:A127, A:G128, A:F129, A:P130, A:M131, A:R132, A:S133, A:T134, A:P135, A:S136, A:G137, A:K138, A:K139, A:A140	60	0.812
2	A:V46, A:M48, A:G49, A:T50, A:E51, A:I52, A:A53, A:E54, A:E55, A:D56, A:I57, A:E58, A:N59, A:K60, A:K61, A:F64, A:V67, A:K295, A:S296, A:P297, A:G298, A:P299, A:G300, A:V402	24	0.699
3	A:D32, A:E33, A:N34, A:N35	4	0.695
4	A:M1, A:A2, A:P3, A:P4, A:H5, A:A6, A:L7, A:S8, A:E9, A:A10, A:A11, A:K13, A:N204, A:P205, A:G206, A:V207, A:T208, A:L382, A:D383, A:D384, A:N385, A:A386, A:T387, A:L388, A:A390	25	0.674
5	A:A156, A:V157, A:P158, A:Q159, A:V160, A:T161, A:R162, A:G163, A:H164, A:N165, A:T166, A:K167, A:N168, A:G169, A:K170, A:K171, A:Q172, A:S173, A:S174, A:P175, A:M222, A:G223, A:G224, A:K225, A:K226, A:S227, A:D228, A:R229, A:R231, A:R232, A:C236, A:A237, A:T238, A:N239, A:N240, A:D241, A:G242, A:A243, A:A244, A:A245, A:G246, A:P247, A:T248, A:S249, A:S250, A:A251, A:G252, A:G253, A:G254, A:E255, A:L256, A:Q257, A:G258, A:P259, A:G267, A:T268, A:E269, A:G320, A:P321, A:G322, A:A333, A:E334, A:A335, A:Q336, A:G337, A:G338, A:P339, A:G340, A:P341, A:G342, A:G343, A:A344, A:A345, A:L350, A:P351, A:G352, A:S353, A:T354, A:E355, A:D356, A:E357, A:G358, A:P359, A:G360, A:P361, A:G362, A:Q363, A:S364, A:S365, A:P366	90	0.633
6	A:V26, A:K62, A:R63, A:S65, A:F66, A:L68, A:P69, A:Q70, A:P71, A:K74	10	0.609
7	A:S75, A:P76, A:I77, A:D78, A:V79, A:K80	6	0.580
8	A:I275, A:N277, A:G278, A:P279, A:G280, A:P281, A:G282, A:R284	8	0.537
9	A:K28, A:P29, A:S30, A:M31	4	0.525
