# Supplementary material for: Immunoinformatic design of a putative multi-epitope vaccine candidate against Trypanosoma brucei gambiense
Source: Comput Struct Biotechnol J. 2022 Oct 7;20:5574–85. doi: 10.1016/j.csbj.2022.10.002 (PMC9576565; doi:10.1016/j.csbj.2022.10.002)
Supplement: Supplementary data 2 [file mmc2.pdf]

Supplementary_Data_Sheet_2_ DeepTMHMM

DeepTMHMM predicted transmembrane proteins

>tr|C9ZVJ7|C9ZVJ7_TRYB9 | DeepTMHMM Topology Prediction - Predicted
Type: TM
MHRITVRLMPTDTCTVIPLTPSITLCGINCRKKHTMAVSASSVGRAFAFLGTALAGGAIGMVVERGGWFGV
DKCHPSPVPYQQMTERTSPEVLHVQCPQPAYPKERCNEDGGETTPFVLRLTSKGLPSNDHLRYYKGFVSSL
NYERRIPNWVLEYIPGTTTAADSVVTADNDLVNASAAEAQRDGMRFFADMTVPQLFRVQPGDYIGGGRGQH
SRGLSRGHLAAAQFHKSSTVELAQTFNMNANTVPQDMTMNAVDWLRLENLTRKLRRYYERGLWVVTGPVFH
PRLVDGDVRTWRWAEPSQCPSPVKPVSSGVLASGEHCHCGNDKAAVHLRKIVCYELVGKRDVAVPTHLFKV
ILGERADGAHEAAAFLMPNEPIAVERPLTAYQVPVVEIERLTGLEFFRNVAAAGSDARFWGRELDALPNIC
RRVVCEARTTGMFRSYRDVARLRAAGSLPELQRVYSILLAEQQQKCGNTAGITVALDGVVAQEYRERLREL
MAVSTNDTG
>tr|D0A1M5|D0A1M5_TRYB9 | DeepTMHMM Topology Prediction - Predicted
Type: TM
MSRKSYLTAVVTRYARSISSSSALDRSFNRNGFKKSAKHETTVPPRSFLSSCLKFTPVAWLIRNAMGIAFY
QLVLEGILTAVCSLMFLGHYTTTAGIRSRLESCHYPFTSFVDWDGGQHDQSFTVGQYHLTAEVLTAAHTAH
NIASGLLPLQLVVLALTFPAARRVWVGVSTRSKALWPHKVEEQISNNPYGKRRNYRR
>tr|C9ZMH8|C9ZMH8_TRYB9 | DeepTMHMM Topology Prediction - Predicted
Type: TM
MYMTRKKQTNKQTVMQNRTKADRFFFFIKKEKEEKEKKRVRIRNKEKNVFGKLILFILSEDDIRKYTWQVL
YLSVFFFFAFVRVCVFVCLFFFVIRKKRMEKQKKKKKKGETRLSSL
>tr|C9ZNJ2|C9ZNJ2_TRYB9 | DeepTMHMM Topology Prediction - Predicted
Type: TM
MYEGCAGQILSPFFFSPLACATACVCVCVTVCTYLYVFLKPVISIFNNTRTYVHLCVCVCVCVCVCVCVCG
CLSKQCLTASLFVLFSFFFLFDFTRSYFPSLSFSVDDSV
>tr|C9ZN09|C9ZN09_TRYB9 | DeepTMHMM Topology Prediction - Predicted
Type: TM
MEKEFTKALQSCDKCSTFHDLCDCFDATVRPLVENCLCSALLKPVRDHVSPNECNPQVSQLRDVLCNIRGG
SDDVWFFLYRTAGSLLSRFTKKRMRNDKCNVSTLAVNVFVLFYGRALPDLSKLCLRWDFLRDEKKNYESDE
AYVHSNAAERRKLLMSSVLSVFSERAHRHYFTALWMPCLQHAAEAALHVHILHRMGDVILPYLTNPLVVAD
YLSGCFASGGLIAVLALHGIFILMLDHGLEYPNYYQQLYTLLTPDSFASRHRYDLFRLLDLSLTSLRVPAY
IAAAFAKKVARVATLSPAPVLYFVLPFIRKVLQRHQNCLALIHRSTKEAFVPANDDHEVAHSDAGDRAREA
GRLATTLFDGNDPFVPDAPLEESHALHSTLWELTALERHFIPTVPLMVSVFSSPAEDQAPLRYEKTYARLF
TSEVTRPISKSQLPTVAYREPGSEDGALLFV
>tr|C9ZVG0|C9ZVG0_TRYB9 | DeepTMHMM Topology Prediction - Predicted
Type: TM
MSDLQPMSGKNSFQLALMIMGIVGAAASGTMTLYKVMWYTVENIDAARVFIARLYVTFFAVIMPSAELGMM
EHNEMVKFLLSYVGRAFLYLFMGGLLLGEEVASCIIGVFMIVLSIMNIVGQVIANKS
>tr|D0A5E2|D0A5E2_TRYB9 | DeepTMHMM Topology Prediction - Predicted
Type: TM
MDVDSDRRDEWAKLLESGDAVSLITVFRETSSCSVSCENTLLSISEFLESCLFQSALKVDMHSLFSATESP
TEVTMEKPEEGTLDSREVLTELLSIGSIKKGRALVAYLRVTRATLRQLFLDTMRRRRRSCGSISEQLRMTR
TVLGAWFPEVEKNVCYCAASLTLRDATPELVRTTNTLDGRKEGKDIANSDESTEEDDHSECCVSDYRFEAL
EALSYLVLDIVYLVGDTRKTVPLLLLLRATVEAAQPYTENSTFLHRSVGNLKDWSFKVCPATGSKSRDVPI
TSGAEGVAAPVDLADVKAIREAITLALLQLIPEGELVTFLSEHVGDMFHGDLMESLSYDDSERGLDPRSNR
LVATVHDLKRERESIYILSDEDDEGEPTFNGEGVAPVNSYVSLQRVGAMFLLCDLMATGDSCSCLLFSNSP
QTLLFLAGPMILEAMKNSSLTVVMTGLTFLSIILTFVTPYSMRISGEDTAAPPSVADTANKDEMGGSLKFS
TRKFGLVFELLKSLISLSTTCPSEGPRVMARAVFTLMIQRCSFDLRLRTYSSFLVLTPFASVCSLVLHALR
NEWSSDDWRADQSSGTNFLRDKLPYFLLQAQQSWLRRLQVSEASFVDPMVQSVNFVRCVIAEDFRQSSNYA
LFRHSGSEGKIRIPEAATSVERCSSWEQYLSYFFKNVASHLRKLAADTSDDSTPRMGVAQMCSLSPLDRFS
LNIALDGVEERVEVFVPERSGG
>tr|D0A8Z0|D0A8Z0_TRYB9 | DeepTMHMM Topology Prediction - Predicted
Type: TM
MCMDTYRPNVSVCFRFRISIFISFRCCRILCVCPVFPLFLYCFQLSHCYYYCYSYCYYHFYLTLIWTYLLR
LRSLGRSRTSFPLFRFQRAGYLLPSTITVCMMSTESLECCECSLPVGNEGKMRAVLLVPCQHVLHAGCSEF

IRKRRRIQQRLSACGDGFDAEPEACAPVEPSDGRLVACPACLCPIQRLVPLFLNSSNTPGVGDTSGYGGAG
NIRHDGDEGDVIGQDISQRVVGDGRSGDTMLQFKQVFYRQQAFIEKMSELCKQRENVAQLTRSCAMLHARR
TDLESEIERLSSIIPNVMASANTPRLNGDKPPVEHMTATELELYITQSSAELSNLERELRDQRSATEKKIK
KAKELQTKYRRMRATVLADVKKDSYVGIHCEESERKTPVEEGSLLLTCSSDVENEVVRYSERPHALSAPAS
DTEEAVMRKHALIISSGSSCCSTSDVEEVKHVERGAEEISLDDGDGDDDVIIIDDSPQPRSSRLTRGSWGR
VGSEYNRGRGSAGQQGLANDRAELLDEDDVMWHPQAQRTRPMFSQTASTPRASQFLPRRVDRLFQSSLEGF
R
>tr|D0A8I2|D0A8I2_TRYB9 | DeepTMHMM Topology Prediction - Predicted
Type: TM
MEFSKFWFRRAITSLLDWLFSCFVDFCFPSSLCFRLFSSRFCTFTLLRLLASLLVGSLLPPPCNGVLHKAG
TSPQYFFAAFASGGARRAPPLGRSAALAHAQHLLSSELKTLPPLASLLFPSIFLWPFLQALPASCIYDLVF
PFARQHFLASFCRFATSHIQRCGNSPFTPFRIAFSLLHLQ
>tr|C9ZM23|C9ZM23_TRYB9 | DeepTMHMM Topology Prediction - Predicted
Type: TM
MCACAPIYIYIYMYICMFFFSFRLGDYLPCCSRRFFFVFNFPDSGFFLFVLRNRSSAPNGRFTYNVVRKYR
TDLLKSFLIFLFLLRIVFCQHVTLFPPPFILPCLWCIKICVLRLFFLLSNFVAWVTAPSYGLTLLLLLVLV
PFILTVRTPFFF
>tr|D0A9Q1|D0A9Q1_TRYB9 | DeepTMHMM Topology Prediction - Predicted
Type: TM
MSWPKGVWRGTRTHTLVCEAGTRIQGKICAFGAYTRRQTYFILAFVPASGPILGDFGIPFIFFWQLWYALT
FLPFYIAVLCACLLLASLCFVVIALGNSHVLLPQFFFFFVFTWVGAW
>tr|D0A3U3|D0A3U3_TRYB9 | DeepTMHMM Topology Prediction - Predicted
Type: TM
MTERRDNVSHAPDAIEGPNDGAHAEDTSPGFFSLENLGVAQVQVVGGTLNGFSIGFVAVYILLYEVATNCS
LFKTTEACKAVGSYGCEWKDTEVCSWKKECDSDSDGVNPCESLIGYSSLYSGIFASAMIVGSMVGSIIAGK
CITMFGLKKSFIIVGVMSVVASALNHISVATNEFWVLCAGRVLMGIGLGVVCVICPMYVNENAHPKLSKVD
GVLFQVFITFGIMLAAMLGLILDKTVNYDNDPDMAGRFHGFCAVSSVLSVAMFLVGMFLRESTATFSQDDD
GKADGGMDPNEYGWGQMLWPLFMGAVTAGTLQLTGINAVMNYAPKITENLGMDPSLGNFLVMAWNFVTSLV
AIPLASRFTMRQMFITCSFVASCMCLFLCGIPVFPGVAEEKVKNGVATTGIALFIAAFEFGVGSCFFVLAQ
DLFPPSFRPKGSSFVVMMQFIFNILINLLYPITTEAISGGPTGDQDKGQAVVFILFGLIGLICFVLQFFYL
YPYDANQDHENDHGAEPVERIASPVDVPTPRN
>tr|C9ZXN9|C9ZXN9_TRYB9 | DeepTMHMM Topology Prediction - Predicted
Type: TM
MPHILPERWPGHNLFNLQRMSCPVLFHSQQYIPSQPLPTSVVFNPMCGLGKRTVVLMYGGFMTQIMNHKLC
FILVAPTNVRRLVLATLVKIIRVFFPFLSARAL
>tr|C9ZNH3|C9ZNH3_TRYB9 | DeepTMHMM Topology Prediction - Predicted
Type: TM
MFLLSRTASRPESSGANSKAVVDATSPATRHSMRLIVLFLLSLLMVAPVVCVADNVTVKVYSLLYDPFLPD
AYNNGVNAGLHASFAVRQWSTASNVNVEVIHPLSYEVPPPELLRSIIEENKNEFFVVVGPLSDSDTLSVLP
SLEEEDLVAFAPFTGSDAVRGWSPNAYFLHVSPAAELLALLRYAVSQLHLLRIGFMYLQNVHFGDSEYELA
VELMSQMGRSLCGVFTLESSFDGEADDAEFTATWELFAGTHPQGVMIFAPQVSDAMRFLMKLVTDNRTSSA
YILSPTPHVTNIEIAWTLAGTTSGVKMFPGQVVLSGVLPLVSDNGFRATRRLRADIKAYALSGTGAVEFDP
LAFDGDAAYGGQVMFGWIVGEVLARALECSEFLKSRMTFMDSLYNQRRYVIDDIVIGDFGGECEPLAAAYG
ATCYCNEGGRMIYMMILGDDYLPRPAADGLITFDACDESGVQMLAPLYTLLFSSVNDPFARSVNGAIHRGA
LFVSGNGHLGKSERLFIHSVPSTSGNVMSDLRKMLDTRAVTSVLGVVDDATLSTPDVVFIDPITLNPRLRH
PGRNVIYLSPTLEQQLFVIAGYLASENASTLHAVMRDDATRLIETVVNRTLLSFNRSLNSVVSLDRDAPLN
AALPTDGSLLLIGLTASDVGGIAAHLSANRNVRVFIPFFDVALFYDDFIRAFRGLKSAERLIFATNLPHWA
DHRPSSETIRRFHANRPNASDRTPLALLGFTSASFLDAVAQHIVGVDPQKVLTTIYTHSVISVDDMQYGAF
ADEDCSRIVGNHTDAVDGCLVNYGAMHISLWPLARALNAAVPPLTKPETPSMHYHNLEEDDHPKSVLAGLV
AGVLSVVVMLAVLLLVLFQLRGGARDNVNAPKELTGPVTLIFTDIESSTAQWAAHPEMMPDAVATHHRLIR
ALIMHYRCYEVKTVGDSFMIACHNPFNACQLACCLQRCFLQHTWGTAAFDESYRQIECQRALENEEYAPPT
GYLELSVYQKLWCGLRVRVGIHTGLCDIRCDEVTKGYDYYGRTSTIAAQTESIANGGQVLLTHATYMSLKT
AEREQLNVTSLGPVPLCGVPEPVVMYQLEAVPGRAFAELRIGHVSGTAGACGTASETGSLVVELSDEAQVV
ATSLESVLSTFAPAQRRKALMTFCERWRVSLPRNAKEVWDDAFCCDVIRVIAAKACRIAEYEPRSTSNSVT
QTPGHSFALPSRVFGSVSLGSQLPLAPPRVCDAVNQAPCPDATSSSNCDNSSESYIIVVEFPEGFGENTPR
NGE
>tr|C9ZI28|C9ZI28_TRYB9 | DeepTMHMM Topology Prediction - Predicted
Type: TM

MFVFICRDFVSFFPFYLFLSLFIPFFLFLLFHFECPYVSKCFLLIEDCPWQFLSYHFIFLSLVRFILYHFS
FRFILYHFSFRFIYYFILLLFLFLFPLLTYTHTPCSSSCMTSLHTNCSGAPFAVC
>tr|D0A0E9|D0A0E9_TRYB9 | DeepTMHMM Topology Prediction - Predicted Type: TM
MYIYLFFKKYHLYICICMYICIHARIVFFVGLRSNATPHTRSHERCSPQARRMCENAMARHLSISETLYSL
FCFLPQFFISFSLSFFLHSSLQYHMKFKRKRLKTGPQKKVSRYFIIIIIE
>tr|D0A212|D0A212_TRYB9 | DeepTMHMM Topology Prediction - Predicted Type: TM
MGIVRVQRSLLWLLGAVFLLWVLRRQRRCSRFFASLKTSILKKLTCKSLGRKQPRVAVRKVDLFVEKVLAQ
GGLQDVDKIVGIFPRRFELVGHVVVVKLNRGIARDVFAPYARALAESFFPRVIDVVLLDTMGIVGELREPH
LEVLWSSATSHFSVNDSLLKVTKERVRKASTFTSEDAELLGSCAEAVTFTTHVENGVRYSFDACKVMFCSG
NVTERMHFASIMAKDEVVVDMFAGIGYFTLPLAINGGVKIVHALEKNKYSALYLAFNAVQNKVSDLIVIHC
GDNRDMGSELCGRCDRVIMGYIPSCESFLPRAISFLRRSTRGEPMGVVHYHLLSEKDQVINTVTHHVRSTL
DEATTSLMRIVNFRMVKSYAPKRFHFVVDMHFSSLQEPE

>tr|C9ZNV8|C9ZNV8_TRYB9 | DeepTMHMM Topology Prediction - Predicted Type: TM

MQLLFIHFFSVLHIYIYIYIYSVCVCMGYVLLNRILLFFVLKGGSGIRIMRWRVRVDGCKWCLEGEEKNSV
SIICLYYLYLLSFLLRLVGCVGSRLLPTIP

>tr|C9ZJ87|C9ZJ87_TRYB9 | DeepTMHMM Topology Prediction - Predicted Type: TM

MSENPAQSYPSGGRETEMALVVGEVDISKPGQGYPQPPKSTYVPPGQGFGDGASGAPGSENEWETGMITAP
CKDCCFCLGSCADTIFCAYAQREALLLSDFQQYTCFGGMCGQCSCCVCHGFEKCCMGTEVFCCPWCAVFAN
RFMVLQHYGLQESLVDTVVIAAACFLPLLLLLWDPRFVPGAWLALQKMILGCLLTQQQHQMRVQGYPRRVE
MV

>tr|C9ZZ87|C9ZZ87_TRYB9 | DeepTMHMM Topology Prediction - Predicted Type: TM

MTDLDATKISIISVKKANPKLLAEVGDEANELPVTVKEKRAHEKRCNFCCMSIGLLFSAFMVVMVASIGTG
MAVYTEKRARYTVNGVMRQVGWISLPGLLVGTTLHYFLSEAMWSGRRNSWGQAWMKAFATNTAIWSVAIGT
GTLLWRKGLLINAAGRRLYYRYPIPSDPLEYRLVRSEAQFFTGMGWSYWLSGVVSGQVGLVSCVGFCVWND
RPYFMMNPHGGYARRCMPNWRREQLARQANVSLSE

>tr|C9ZTT9|C9ZTT9_TRYB9 | DeepTMHMM Topology Prediction - Predicted Type: TM

MKKERKIRGMEYKIVIIIIKKKVNRRKNDNDNSNYNNYNNCKAKKKKSKPKNGFIFLLQHELKFASLLSHL
MITIEDERMKGYDRKEEGRGEEKGVLICNVTCARQTVAVYCVWIERLWIYIYIF

>tr|C9ZI79|C9ZI79_TRYB9 | DeepTMHMM Topology Prediction - Predicted Type: TM

MCVCVFYISSLCLGLYDGDAGVILNWRSEICLSFKVVFTGERNEEIQSRGEGKCADICVECVCLCAASLCI
CPQ

>tr|C9ZV92|C9ZV92_TRYB9 | DeepTMHMM Topology Prediction - Predicted Type: TM

MAYVSPARVKWATFVFWNFLDPTFRLHFRYYQRKLAVDRYLERLGVVANVGIGVTFGLMFYNLLIARFLLP
RPVSSGHSMEENANEVLRVLKYDTTKELPAFLLMRAKREVISKLHVAADKAEVKRQREEVRRLLDSIDQQR
LPK

>tr|D0A3D4|D0A3D4_TRYB9 | DeepTMHMM Topology Prediction - Predicted Type: TM

MVNMANGTDCTRVFLSYHRRRFPHEKYKSLQVSPSDVLDGAVTDEPDTTNFDKYLDLCSLIRPVIAPTGGF
APWHYFIKALFWCSLVFFLDIYAIFAFRPFYLTIIQSLAMAMVGLNVQHDANHGAISRYPLINTIFGLTQD
ILGGSRISWIIHHDFIHHVYTNEPHHDLDLDIPLLRLHRFVPKRLCYAFQHIYFLLLEAVFGPVHVISSMK
FVWQGPTTKQRFLQREWTMSRILTLIPPLRLALNILHAPTLLHALISTFLQYAIGGMYLAFFFLISHNFHG
VRKDGTEVGECFVKAQVETSSSVGGWWLAQLNGGLNYQIEHHLFPRVHHSYYHYLAPIVRDYCTKLGISYT
RFDTVLDNVVSTSKHLAQFGKGIEDKRL

>tr|C9ZWQ8|C9ZWQ8_TRYB9 | DeepTMHMM Topology Prediction - Predicted Type: TM

MTSINAQPPNSATYPRDDHGSAEAGQANAEVERPQTKKQKDGGGCFARLSLFMATIIPPGGIAASAFNIGS
TTIGAGIFGLPAAANSSGLVMAMIYLIIITAMTIFSIYALGVAAERTNIRTYEGVARALLGPWGAFCTAAA
RTFFCFSACVAYVISVGDILSATLKGTNAPDFLKQKSGNRLLTSLMWLCFMLPLVIPRHIDSLRYVSTIAF
SLMIYVVVVVVVHSCMNGLPENIKNVSVGKDDNAEIILFNSGNRAIEGLGVFIFSYLFHITAYEVYMDMTN
RSVGKFVLVVTIAMGMCLPIYALTAFFGYMDFGRNVTGSVLLQYDPVNYPAIMVGFVGVLVKLCVSYALLG
LACRNALYDVIGWDFREVAFWKHCIAVVTLSVVMLLCGLFIPKITTVFGFAGSISGGLLGFILPALFFMYS
GGFTWQKVGPFYYISTYVLLITGVIAAVFGTGATIWAVTVG

>tr|D0A6R2|D0A6R2_TRYB9 | DeepTMHMM Topology Prediction - Predicted Type: TM

MYPSLRMLFCFVFLCIYYVKKAMSVGLYWSARSVRYFTNRYVGFGITLLLLVSCSLVMLCHLHLAVLTNKP
TRFDVCLTVLFLSLIFLAFWSFGFCSFVNPGLAPAWLAESRVEGFVRRMLSDLQKMEEVGTDWLNTTDPQE
DSSSRIAMKKALAVLQERARRDSCGSSGSVANYDTVGCSERDSLLGNQQQHEVPLRPFRSDPDQTAPRRRE
SPESHSPMRGYSRQFLRVVSEAIGALDSTPENFEQEMSFLLGGIRWCRYCLLYKLDETHHCSTCRSCVYHM
NNHCPWIGQCIGRGNHKCLLLFVGYVSLAALVVVIHAIIAVRSGKVKLLSLDASISFSFFFLVCVFTVLVM
VPFLVTELRWLARGEGALRAMRRLIRQRHNPLHGGESSSGAGASADSLSHRSKLQNLRLVFGTDDVFPYWF
LPTFPQWPPREEQEAAFWDKVGDTIVKQLVSAMVDELEDGSVGVI

>tr|D0A0Q0|D0A0Q0_TRYB9 | DeepTMHMM Topology Prediction - Predicted Type: TM

MLFLEPEVDYFRMQLHHLAIPTSICIPSVPKTSRNPETRANGDQECETGGENAKVDGTVPFITQEEQNKVI
QRALRVALYQRFPYESYDTLENVYILAVTHLALPLVGDTGLSYEDLATLEICEHSLPPELRHRTFPSHLNA
LIAQRNPPDSGSVIQDRMLDHSATQGSSEEGTGKQRFNDSYFILIPDLTDEQAMVYCKGRQYAGSMLGSGK
ASVGPASDAARFPRSTLWDDARHSRANALEGPSEDLLSFHVVEGDMIHYVETPQGGLSEPAPRSLRFAAFK
VALSQFSFEEQQYYTKKREEVRSHGSTTTLWGSTLHSSEANEVHINKTVRTILASEVPGRWRAEEEEAMKK
FFTKCNSVLRAYSTPSSQMDDASKGDGGVLAPKQPGAPVSDEHKTRNVAESLGQTERLSHLYGVRRLVYSF
AACDCYHLVAEDNNPLYYAKSSRATYEPHTVNVTLISDLTVSPRLTPAFSASPQRCLGARIGTTKTVEGLV
VSNQPSRAPSPDIKPCAVGEKFSVGSKRHSPSPGVSPPTSGTHGSDSHAPLQRLPSNTEGYLRLTTSLVCN
NVSGNLKHRKENLLEKGLSFGRLNPCHEQATEGNVKCSYEKAVCAGDVVNLFRLLATSSIAQHQLRHNVIR
PVISAAVLDSSGNAGTRGALHRHYQRIKCRALLDHTWYSIPIQPVPSTQDEVQWIPIVFGSAECISDVGYR
TAVHIGFFPNAEVTDDSVRGNPLRPKGTAFATDVSKQSASPGTATRSAMMPLDTNHATLQTSSSDGSKGLC
FSAQLKTAVSSSNEEVPAGVSARRGGVAMSFNTGNEEVPTGVSARRGGVAMSFNTGNEEVPTGVSARRGGV
AMSFNTGNEEVPTGVSARRGGVAMSFNTGNEEVPTGVSARRGGVAMSFNTGNEEVPTGVSARRGGVAMSFN
TGNEEVPAGVSARRGGVAMSFNTGNEEVPTGVSARRGGVAMSFNTGNEEVPAGVSARRGGVAMSFNTGNEE
VPAGVSARRGGVAMSFNTGNEEVPAGVSARRGGVAMSFNTGNEEVPAGVSARRGGVAMSFNTGNEEVPTGV
SARRGGVAMSFNTGNEEVPAGVSARRGGVAMSFNTGNLTGVGSGNVACSSEREIALRRSAMKGGSGWEQRC
SSALFSPRPYRPTSLRVWEPGADDQSNSNRFEQGTRVTCAGPPAGFSATSTPWWRLPSQGPRRGDGVNAGR
GECSTGGFCNDWQQCDTCGGRFPQGSTSDPRFCEHLQSRPTSVLNTYGKFSRSHSPHPSYNAMQQRQTQHY
STMGRDGRLRPPHTDRWDSNDVNGDVSPRTATDPLRGTFWKSPTLNPSNYRNGADPLLSPLSSPSCSGVLT
PNAGRRPTIGEFAATNTRSSAFNCLSSFKHHGAPTEICNRTASGLENNYHGPSFCKNRAGVPAKRSVVPTF
GRMDTLYDNSASGIPIYNEYGYPQGDSRVRPRGMQVMDMMYDRQWQRTQPPARMPVGAGPTNRGSLSTPWT
NTTKNSVCGMRRACLPGKPPLASTRPNGASNTLNNGIAPTAVRRLTLRERIQLQRRLQQREANISAFETNI
KENYCVGNDVKRLRTDVGDLSSANKTTASNGGERTTTETNSACGARTAAALAAVTFGGDPLSKGSLHSIGK
DIMRLKSLTSPFAEADGVNVSFCLRDLTNAFLRMPQSEIKEYDLSKAAWLEDGVSCFVSAKPTKFNGEFEN
YVRHFNFHQQPFYLRERAFLFVLRVPTQPQLRLIIVVHNMHDFLSVLRQRPLRPLESWRGPHSMDEEMGGL
SERENASQLPADECNSGRTRTFLQRVCKFVKNCISGGNGGGTQQMGWAPITWGQRLALLRIQASAAVHGQA
WSECTTPLLTDPVVLYSRMSFLSNRTASFAVLRCQLDTLKAFASTWIKSKRTTADENQVRMIQNRISKVES
IQLGCDAPTAGIGEPKTSSSSEVQAAGRVQQRLRSLPLRKRMGAPFAWKKADTTSNSVEKKGETQRQPQLN
STISSYSSSASLCRSKPEVGGANSSTPPGGVPASDAAGAATPLADADGTLIRIAEKLTSSTDIVSGNNPFP
PMRTILKLVTLWEQNAQRQRAFNDCPAEPAPYSYGGSDTVLVSNLGARAELRDHQDDYDISVSHIQQLLQW
QWEYHQNSYEALSLPVFRGALVASRGCRYGSDACSSVDPKVMGVNPLSRSLLLALVKWGWLVPIDYKALHK
RAVRHTAVGDNMLSRVQFGFGVRRRTLFVGVLGFTCYRGWALSLLILYAAYRCFKLAAVGLLSTGAPWPAP
LHSGTSIVYSRSEAAVEAMLEERQHFLSQLLATRVLQTQHYVHNVASRLDMLLRGYSPILSLIIGIELLFV
WIFFACWSGAARVSATFGVNIDLLHQYMSELISPLPLYDNVMWCLTYMKPNVAGVETFSLVAPLFVVLCLM
TQSPLRWVLYKTWEMFLHDDALVRRPVLSF

>tr|D0A924|D0A924_TRYB9 | DeepTMHMM Topology Prediction - Predicted Type: TM

MSEFQRFVKLLEQTDGRDKILKAFSGVFKALGSLDTCQSRSSAFGAVGKSIGDARCLLRMAKWVGDVPKMQ
NAIQDCRAKGKVNMKEVLKFLRVLCNFLYVLGDNVAFVARYNLLALRHKSIHLKAKTAQFWGFFLAAVLDV
VALYGALQKRASDPATSKKEMKAALISFVKDASDTLVTMAFVGYLREVWRPSATTSGALTAVAGGVATYLN
WNKIK

>tr|D0A514|D0A514_TRYB9 | DeepTMHMM Topology Prediction - Predicted Type: TM

MDGGLKSLFFNCIPFLSFFFLLYYFLPRTNLCLCNVFYFSLSTHSSGMLMSSLFLDVLVVLPFLLLLLLFP
FPQISPFLFCSLAYEFRTGKREFRDGRRHVTGKDSTNGMDHVTVRTPIWGNPKRAHRVRLHVPLLFSFFLF
LLFFLHFNSISPINTHFTPSPVSFSLLQHLFPQYMQVFPLPPPHTFPSYRWRRRRKFCLSATKLFLWFPCF
GIPPPPPSHNSKPIFLFHFFFHIAVSLPLALSFHSVTSPPTKKSPFICQFTFSSHFC

>tr|D0A6V5|D0A6V5_TRYB9 | DeepTMHMM Topology Prediction - Predicted Type: TM

MPSVLVFLRDALAHYESGELCTARQAAEEALHAAQPHEHEAFADSLLLLGNISAAEGDFITAERYAATCSC
YVEEHLGPQHNGAAVVRLNRAIFLLESLRVQSVTTVAVVEEAHALLLEAEQLLVNVCHVGRLVLAEVLHNI
GVCCALLGRYVAALTAYMRSMQIRVRFKDAARVTDLKLALTMEQVALLYRLMDEPKRQEAALLMEVVASTR
RQLLGPHHPLLAAAVLAQGVIAAELGQRCRAQSFLRNALEMFLSLYGKESFQLQCAERLLAEVS

>tr|D0A966|D0A966_TRYB9 | DeepTMHMM Topology Prediction - Predicted Type: TM

MVCDGLSFALRHENWGGKHARDARKSPGGAVLPVFLLSLRALFLCAFPNVQTIPFYLVTSFIAFCMVLLPC
RCAGLRLFCIPCSMLLCRSVASNLGCCWHFFACLWHSGLWERASYLNSSIRRGRKK

>tr|C9ZR76|C9ZR76_TRYB9 | DeepTMHMM Topology Prediction - Predicted Type: TM

MSGIRYRPVGSDVMEVPIEPDEKFPRGTSKFCAWLCPHDPMGAERRVGDLYWNGWSSFVLGFALSGVGLTL
LTLAILCFYFTWDVPRSIALIVVSAICIIPGLFSLVVIWFYISGKEGYDYEQLMPN

>tr|D0A9K3|D0A9K3_TRYB9 | DeepTMHMM Topology Prediction - Predicted Type: TM

MARGGVVRLPSFIDVPPSTETDREGGRSCFGKYGRTAYGGNPIVQQTLPSTFFYIAPQYVIPLFFAVSFVF
IPISITLFITCNGHYEVRGNYQHIHKYQYIPSDPKVNINEGIRSFYVGNEVHRQGTRTRVSFKLEKPMKKP
VYLYYTLGNFHQNFRAFHEGRSLDMLRGHRSIIGSYPECQPYERPGTINKAEKTEVKVVVDGGNVTLKYEE
FLYNPCGIAPWSMFNDTFVLYRSRDVSSAQNDSVKLDEGAELICNTSDFGPTGEPLYQSKTPNKCKKKGIS
WPADEKIRFRPLERDKKLWSLRYPHKNDNVYLTNGWYADEPGHRLTDPEDYDMQVWIRAAFLSNFSKLFRI
IDEDLREGNYFLDIEEFFDVTTFHGTKGYLLRTSSMFGRSGTLFAATFLIVGSVAFVVGVAFAIQYCMAKK
GLGNSLPQPKASWYTFNRTGLDIQNYFQLRTKRYEICPQSSDDE

>tr|D0A1N2|D0A1N2_TRYB9 | DeepTMHMM Topology Prediction - Predicted Type: TM

MCTGITGSHCRYRFSTLLFPCVCLPLWIQGRVFVKSLTGRGEGAYRCVRIIGVLTFLLFVRFPRFVSGMKT
NNLERRRQIRQQLRAFYGDNLTAKDEPVTRESGEPDHSSASMNTRVNSIPPGLDMDSDSFDVGEYTTELFR
VKSLKGVVETDAWLARTASRLETELRELVYRNYSKFISATDTIREIRSDVTEMGGRLHALSGNVENIDDVS
KDVSGKLQEHRSRIEKVITKHRMLRKAQFLVGLADTMRLHMERKEHSECVRKWVMGDSFIAKHANIPKMAI
VHKECKELAMHLYGVLREEVLSVSMENPDASEVIRRIVGELRLLRATSIFGEDKEITPHAGTDGALAPFEE
EILSVLKINVREAFVVAVRSFNATIEAALLIPGLSEMHLEERAGVLMRPRLQEALAQLKSSLAIFHSGSEQ
LCSLFNQRDESAFSKYIIEEVEPVLTDTIVAITTPLGNLLVAIVDAIAKDGSSLLGSPATVQTDLSTVFTT
LAKHLMHYSTTMKGLGVTYLNSAMNQHSEKYAEMVDKSVLGVLLKFVNYLETHVTETNDAQKTKLGDDNAS
DPQKQQEVCFNMCLSRFVLANAAVAADLKSVSAIIESVTSRRSELIDLQKKNARLTRALLRRGIVLSGQFF
LNSALPAFSPATAVDPSASTASEHGGIAVCLRNTVVRLGELYNFLKQGVVPSVDDGNTKCASPPAEKHRGN
DSASGSTAGGSQLLYSAPGSGRNAGATRATRLAVCFTRKKEDALQASVERIFSKQSQNNAVLQTMPLEFRA
ASVVAAIAVYLVKGIVEYVRRVTFTRCGFQCVQTSCTFLLHAFTPTALTSSSPAGTSQSLKVTVSLEEWLS
DCGDERLLKGLPFLLNECCTCAYERCGEKVPLTAVVVERLVRSVLEEMEGVSVSAEA

>tr|C9ZQ80|C9ZQ80_TRYB9 | DeepTMHMM Topology Prediction - Predicted Type: TM

MLLVGLTGGIACGKSTVSLLLKESHHIVVVDSDLVVRELQRPFMPCTRKIARRWPNCVDPQSGEVNRGALG
SIIFSDPSARRALARIMNFPIFRATMKMVIGLWWRSLRQQLRGQGPLLVVLDVPLLYESNIYTWLVDRVVV
VSCSEEQQVERMAKRNGLTREQALQRINAQMPISEKCKRADRVIHNEESLSELEHSVADTVAWMQQQSGGR
VAFALSGALAVGVGLGAVVVYCCLRIVF

>tr|C9ZZQ4|C9ZZQ4_TRYB9 | DeepTMHMM Topology Prediction - Predicted Type: TM

MSCLRSISLSVPPHALSTFSRTGMLYVMYVLLLLMPYPLQVHGAPANINVKVLLCTWNTRVPKIFTTAVNA
GFNASMESRNWTIADRVKVQVVQSSKSHKTPEEFIKDELSKETDKSGITIVFGPVGDDTTLDSISELQKHE
VVAFGPMTGSGEVRRWVRELYFLRPSPTIETMVLIRHALGHLGVLRLGFMYLQGHHYGEKEYEVALRVMEE
MGYKLCGVFVVLNVNDKPAPDKEFNAVFERFAATKPQAVLLFGAPKSDTGRFLRKLVADRRTSGAYVLAPS
GAQVFLELMWKRVLLDSQVSPFPGKLLIAGTNPLAKNEQYVAIKRFQEVMREYLKTHTSETGITDPNYFLK
HDTDGEMMVYGWAAGEVLSQALSVPEWLKDRTTFMDSLYNQRRYVIADFVIGDFGGDCEGEAAKQGAVCYC
NQGGNVVYVREVEDDFSMQTPKDGTVGLAALRCNADSVTLHSPLNGLVIFFGDNTIAVKATASWLVGALRH
DTGSLEYSGQLFLHAFGTTTSDSAKALKEEKKKRTVTAVFGIVTKALMKMTNTLFIDPITIEPQLNKFRRH
VIHLSPTVEQQIYVLTRYLSNNSGKEANAIVCAAEAHGIMKVIRKSLEKWGGSLNISLVRRRGAALTGHLP
TSGAVFVFGISASDVDVIERHLAAHSELRVLVLFSEVALLYEKFVTAFNGSAAAPRLVFATNLPHWYDNET
SSPTIRKFHATVESREKWTPMTLMGFVTGTLMSLALLRVERVNMSTLANFFFVESSVNVDDMRYGVFSDSE
CGSGETGYGDMCRTNYGATRISVWSMARVFNASIELLAEPVTPSLKFLLEWDGLTRAQLVGIIVGSTLFVM
LLAALGVFMHITLRDARDNVSAPMDPTDPVTLIFTDIENSTSQWASHPNVMADAVAAHHSLIRTLIGNYDC
YEVKTIGDSFMIASKSATAAVRLARDLQRCFLNYRWGTDSIDNFYRAEEKQVAELNSKAEPPSAQLDPEVY
RKLWNGLRIRAGIHTGLCDIRYDEVTKGYDYYGQTANMAARTESIANGGQVLLTRATYFSLSTAEREQLDV
TALGSVPLRGVPEPVEIYQLDAVPGRTFAPLRLDHEAYVADESSDVSHTTFNDCMSISSQLGTGGESVSNV
LHALLGTFTIVQRQKELTAICERWRVPLPPWKEAVWNDEYYQEAIHRLAVKVGHIVDVAAVERVGRPTESS
DSSSVILISNPAVDQSTEDLEGDAFECGWEEKGKQ

>tr|D0A0S8|D0A0S8_TRYB9 | DeepTMHMM Topology Prediction - Predicted Type: TM

MIESNLLSQVNTASPLPTFMEVELVNSINTTVGKAFQFAHVFLAEKCDYLAALLPYNSEIWLVLHALLEHR
LLFHADTSFAEMMFSLCRGTIISPSRPLPSQGRLSWLLRGPPPVPPLEARMMDTPAATDSRAVGEAMVGMK
AGEIAAADRAPYGHLKFRPLTNRQKYITLFLLTVKPYLQQRLASWYEANKDAQVAGESQSGSALSRQTLGA
RLKQLALQLYPALHAGWEGLNLAFKILFLLELTPYTAPLHRIFSIVLRRPTGDDLIAASNPRAQAALMLGR
VLIVVLLLGFRLMEFSGNTGGASPSHANSDDLTIPRPPEWGVDVVVPPGTPDPQPGVCPVCERPVTNAAVC
TVSGVVGCYPCLTQFAREKNACPVTRAPMSLECVRRIYEC

>tr|C9ZYM5|C9ZYM5_TRYB9 | DeepTMHMM Topology Prediction - Predicted Type: TM

MHMSNVTDERMMATEFRRELLPEVRFQNPNQNTESQPRAALTTLTLLGVMYTACISGGYGLEESVSAGGPL
LTIIFLCLIPIFWGIPVSLCVAELSCAIPSNAGPIMWVNVTFKPWLCFSTILWTAMLNFVDNSLYPTILAD
YCATLLGISAFSKSLVKLGFLWFCAFINILGVHVVGKMSVLVMALTLIPFVLIFFIQIPEGFDWTRIRTVP
QSIDWPLFIPVVAWNFSGFESAGNVIEEVTNPQKTFARALVLMIFAALATYIPPVLVGASAEGVRDIPFDQ
WGVGFWVRVAHAVGGYKMAVIMMVGGAASTFGLMATQLTTTSRSLAGMGTLNAFPFVSSWLSRYNRNLGTP
INAIVTNTVITSILSVCLTFTVLVQIDQVLYSLRLISILFAFLKLRLKRPTLERPYRVPGGLWGEAICGIV
PIAFSVTLIVASMCASLKIALVTVIIVWGTILVSIIWTHFFRRDGFEGSIVEILEDADMQAYESLK

>tr|D0A9D5|D0A9D5_TRYB9 | DeepTMHMM Topology Prediction - Predicted Type: TM

MAQSFDDWLQSLNPVTKGVFAAAVLLTAAISMHIAPYTYFILDTSAIMGLQLWRPFTAALFFGKFSFPWLI
AMAMFVSYLKYNEEYDYQGKTADFAWMIILVVIGLTAGGLLLGLPVVSGALLMALCWVFCKRHPQLRMKLY
SFEFDAKTFPWVLALFHFILGQNILEDALGIVVGHLFFFLNDLIPLKHGTNPIATPSWFVRLTGLENGGVR
FGGVHAGGQAFAARFARQPPPAAAGGRPHHWGPGHRLGTT

>tr|D0AA26|D0AA26_TRYB9 | DeepTMHMM Topology Prediction - Predicted Type: TM

MEIDAENVVTIVTSPGNVTEHNRFSTASKRPRDSTEDSSTFRLRLKEFGQRAAALRDEVAAGADKPSEALQ
GKMWNLVLDLRPYVVGFASPNVAQLPGEVRELMLGNPQRVARYSVLTLRMYATVTRIVVAAPAILIHCILT
LFSLFPILGIPSKTCAIGDILFKVLHHAFDVLYNCYQPLLLQLFKVIVQEEVIGAFVFTTVPGRRSGYGRC
TIQVLRLPEVLGFKGDTRQLFRTTIVAESLYEVVSVAQCFCTVINMLLTGNFPLLPTWRERKSILRLACSL
LSVNVAAGLSTSMYESWKETVGNGERRNGEEGVNLAFALHTTAASLLHAMLMSDLLVKELHAPMILYTLVQ
HVYEMSRAHVKKGWSKELALLRWLVDQKGERQCEVRQELFPPHVGKSFTQQNTGQTRKVGQRESPFCQSEW
LTNSLFNTNMCDDTVSAITCCSTGQVDAEKREAALLLLALLKRALSCKQDLLPRFEETFYRIGENVLDALV
TRRVTPESIYFHDMSVEASVVHQLLCFFPRDAVSSLLRKILQRRRAVMGNNDYLLAKANASAILCEEAATF
RCVWKQLVTEYDSSILDAAIIILRFGCEKETMQSVVLDSLRDLTSGKVTVRIGDSPLRYSLLYAELVRRLP
ITDFSYTNNIARLIVEVINVRAGEQDVAMWRISGVLRGWREHVTRNYQQSGNDVTPSPLDGPLDTARVSAT
VIASQKLLDALGWSTSFAVSLAKSGATYGNDALLHFLNQLLGITTELFAIFERMRATQADYCEVEPSTDET
ATTGVEVVSSSSTNDDVVEVKPYDGEKPKAQCSELLNEHEVNPFVLKQPVAAVMELTGRTDTVVYDLACAV
QQLVPDKCATNNGAVQDAFISLMCLCNVLLSARKSSSTDFVLAHAFGHRNEVIRLGYAALSLRVLLERSGN
VDVAAVLKDTISKHSNADERTGPSLLLRQLETLRAAFTEEWEGFVQQGPRSGTLRDFSPSSLSLTTISSLR
DHCNTKVCRDVINVCRAICELDGDMYATKATALEVAHGVMSRFSWSMDYLFSCDPDIIHTVFFRAMCYLLG
KNVREVVMRGLDDILPRSLLADDGAEQLLRINELLGEPLSLMSKFPMIMAYILLCDAANGASERLDNVKKV
LQFLSKRLSDLPDMVNRSLGSIIASLMYLHGGREVLSRDRGSLTEQKRVAVGNNIEAEKREDDVGGLSEFD
LAVEMALCIGECGPSVTDIKLVLEMQVTNDTVDGGSLTKHVFSVLDTVSQCVGINNRVTAETVDVSVHTVQ
RWLYGLISLIRCLGSHTTVIASKLPAILDFCSFRPQLVRVVCSVWRELLSRCTPRYLSEHSPSIVVDLLSM
EALTEPGDIEALLHLDEAMRVVYERSKSEPFWELYFKIMSRSNILIRRLRSNGSSAKCQTYESGNAPLSAK
GSADVIVTGLFSVVQSSSTKCKKVFVRALYQYLSTTDVAGRMELTCAARQHPQLIPTLLNCVFELHDEYVQ
YVLACVGMIGAVGHSNGSMLRHGCQHTGSNNLSPVSLHLSGRVSSSTRKLLLSHPTEVLHWREFAVELLSV
HCPRALANTADPTMHDRAAFAVQELLRVFANAERESKCYPTLQNDEALHIEELQRYSWWMELEPSCRQMIE
GYTTTKYTACVLQRTELRSPVYYVSGMEINTWLNVWFCDLVLRCRGVFGQMMKALRNMAKGEQTLISYLLPH
MIIHIISKGDEEDTDAIVTEVNLLLEAASRTGVQESCKAASLHSQNLMMEVNTHMANVTSLEEHVQQIFNV
LEGVEHLLFELRRTPRKSRTGHEGGIDNEKADLVVKVIRDFFERVPWTSKVEAAIGIGSSMRALRCLEGQR
YLPKVQDVVGKGISLQRIFAALNDRDSSRSLHRMQDHNPEDAAFSHENNGEWVQALQACELVLQQRPQSIN
HQFTALRCMQQLGQLHLMSRYSQALLKDSPRPSTLQSATWCRQTQLAALQNYANEAAWRLGEWDSVQARQD
LPVSLALPMVALNKMLKRRGTLHDVFAACLFQRMKIAPIIRAACRESYAQVYPHVVFLHALTDIESAAVAV
ATSMAQVSPDPSDPVVSFRATGLRDTKKMQELGPTLQRRASLTETTLDTQELLLSLHRSIFRAFSMEEEVS
KTWMSHVKLLRNEGFLEPALSAVKQASLNNKFTSPSYWTTPAKLLYEMNMSKQAIEFAEDAASDESIPPEI
RAKLRVLLTRWKQDISYQTPQEVVDSYELALVMHPSEKAHHHLALFYETVYHSVHSSLLQSSSDAAKSTRE
TFERKEVEAVETYVLLAIKHFGMALQLGCKTVLISLPRMLNLWLNCSSSLASSAAEAYPDSNIVAPSLQKM
ADMIERFLLVEETKLSPQLLITALPQLLSRIGHESKHVVNIITKTVVNLMKTFPQQCLWQVLPIDRSKQAS
RGEVARRGILEPYSRLSANEKKLVDNMIIVFKSLIDLCNCSVGELTNGNRSSEPSLAGRPFIQRMEKIFTT
TKVLLPTTANLSPNVLHIPSKSGVFAGSATFQEFIPKVNIMSSLQKPKRITVVSSEGEPVSFLCKSRDEPR
KDMRMMEIATLMNTFFLSDPEARRKRFALRRYAVSALNDDCAIIEWVNNLVPFRKGVEECYAIDGTGVCIS
NVRAWKAKVDSGIMKKLDMFERFIFPRAPPVFHIWFYSNFRSHQDWYHARTIYTQATALWSIAGHIVGLGD
RHGENLMIDVRTGELMHVDFACMFDKGETLEVPERVRFRLTQNVVDGMGVLGVDGPFRACCQAALRCQMKN
KTAVMSVVETLLHDPLVEWMREQSKRQRSFDPKQLIGRVSRRLDGFLDLYNPSKEKDTLALGCEGQVSRLI
SHSSAIENISEMYIWWMAWL

>tr|C9ZM27|C9ZM27_TRYB9 | DeepTMHMM Topology Prediction - Predicted Type: TM

MGVEVADCSSGASAQNITGSEAMTVGHEKAKEQHMHVKRDSYTTAATFVAGGVAGACSRTLTAPLDRIKII
VQEGHLVSGTGKKSLLRPAQLIDVFHLIRNDGGWSAFWRGNGVNCLKAGPEFALVFTLRRYFLSLYEDSLD
EETARVTEWEAAMKATGSELIAYDLSAVSVLPAPLNRWFTLTSIPRILLNFLIGAWAGFGAQLTLYPLEVI
KTRMAVSRRSEYPGGMRQVIYDTYKNSGISGFYRGLTPNMVGIFIYRGLEVGIYSTAQQMIMYRMNNYGM
SRHDSSLSSIETAAVSMFASMFAQTVSYPLNVVRTRLQTQGTNGRAVKYKGMTDCFVKMVRTKGVGSLFSG
ISANYLKAVPASASMFVVFEKVQSILVGDD

>tr|D0A0D8|D0A0D8_TRYB9 | DeepTMHMM Topology Prediction - Predicted Type: TM

MTDKKREPAPKLGFLEEFMIGGVAAGLSKTAAAPIERVKLLVQNQGEMMKQGRLDKPYNGVVDCFRRTIST
EGVYPLWRGNLSNVLRYFPTQALNFAFKDKFKRMFNYKKEKDGYGKWFMGNMASGGLAGAASLCFVYSLDY
VRTRLANDTKSVKGGGERQFNGIVDCYVKTWKSDGIAGLYRGFVVSCIGIVVYRGFYFGLYDTLQPMLPVD
TFIVNFFLGWAVTIVAGLLSYPLDTVRRRMMMTSGAAVKYKNSMDCMLQVIKQEGAASLMRGAGANILRGI
AGAGVLSGVDALKPIYVEWRRSN

>tr|C9ZML3|C9ZML3_TRYB9 | DeepTMHMM Topology Prediction - Predicted Type: TM

MIYELALLISTFLGLSIFVIPFSLLRTWTRRQASSRFDSSSACQRHPTKLDEQVNAKRPLKVVFLHPDLGI
GGAERLVVDAAIALQRYQKVTPVQVIIVTNHHDPQRAFAETVDGTVTVQVFGSWLPASIKGRAKVFAATLR
MCWAAWVTCWMHPDADCFMVDQVAAVLPLLSFVAPQIPRLFYCHFPDQCCDGNRDENQQYKKKPSIFRLLY
RKLFDEVEVFAMNYASSIVSNSKFSRAATLKVFPKLSNRIDAEADIFYPPVSLAVREGAKHNGDTKVFDTE
ELDKLRDAIQGRSVVLSINRYERKKNLVLAIEAFARLLNSGKTTCSGAPLLVLAGGYDTRLEENVAHLNEL
QKVADTYKLMDSQILFLKNITELEKRYLLSQCCCLLYTPTSEHFGIVPTEAMISAKPVVAVNRGGPCESVG
EGGTLCDPTPEAFAEAILLYLNDDELRRRVGEAGRKRASDVFTIERFGEKLATRFVKLWTETNAAMGRAAF
WGKWLEGGKKAD

>tr|C9ZJC6|C9ZJC6_TRYB9 | DeepTMHMM Topology Prediction - Predicted Type: TM

MRRLETISSHVGFRRFCHLQRRGDAVVLILHHELLNALTVDMRAALLHFFNEADNDSSVKCIIIAGEGGAF
SCGIDINDFAASLVDTTKENGVRIPSLPSLTTRIEQSGKVVIAATSGITYSGGLELALAAHYRVASPTSVF
CMPEVKLGIVPCGGATQRLPRLIGVRAALDIISTGRKVSAKEALRLGLIDHLTSSPHEERRDQDVNGVSAQ
GPFCCTDPNGNLESAIEAALQLSARWTKPRRISCDTRKLGIMLYNSILFRRVGSEITKKAPKEVSAPLQCL
QAIRAATNSASFKEGLAEETRIFKQTLHSPEAHAMQHLLRSSYTVLSDTLPTLPVRAGTGLQQQRLRKLAV
VGCGVVGIGIVIMALRAGSQVVLLGEDDNECEYALHVIKSELPNDPLGYNISADCVNMYLNNLKVLPYHDD
LQTVLQDVDVMAECIVGDLDTKRQVFTMLTDLCPPHCVLATCCSSLELREFVKVSRRPEKVVGMYFAPPVH
NVPFLEVTRGYRTDHTTLQRAIHVGRLFHKATILTRDVGFSIISRIFFAILYQALSMLEQGAFPVDIDRAM
RHFGFRLGIFAMEDLAGLQVVSQILNSLKVREGCSGVRSPCPPADVFTIHRRLVEMGHFGQKTGRGWYTYE
EGDLFSSLAAWRQRLIGSASNSTKNTYGVWSKGGASLMRRPYQDRDVELLILDVCREKKIMRRDISRKEMI
ERILFAAVNEAAMLLREEAVSSSSAIDIATTFGHGFPAWRGGLCYYADKFGLPNVVHRMRIYNKTFGDALF
PLPCEELRQMASSQQTFRSTWP

>tr|C9ZUM8|C9ZUM8_TRYB9 | DeepTMHMM Topology Prediction - Predicted Type: TM

MSEDSAAGGCTLRQTAPFLLKFVYATGIQLAAQNSLLPVAVGSAVVCTAFFTYICSSSPRWKGAPGLFGHA
NRNAPRRVKLGATQINVATGESTTTSTLWDIDSTDEGTRCAGGSGVVTATSSAVLLPRGTTPLCVEVSPSS
SLFSSAHIPILYMHAPICRGGRRNGMVRHKRRKNASQGLMAHAVAQGMNLLSDVLWPRAVAIMSLVVDAVP
RFVRCEIPNLNFLRNRWTFSFPSSQLLLLRSRLISSEADGMQTRCGTQQQPVAQADVKVMDETSDTHCELV
VRVGDNSAAGITGALHADAVAQHLLRSSYSTNQCTTVPPLNPLSFSCGHPSVVKQTDDVEAKAGTAHPPSG
PWIQGATVRHGEMRIGRFLGRGRWGKVFQCCNPETGEVLAVKQLIFDDNDPKVRQRVGELRLELDVLRLAN
RCRVPWIVGFRGVERRGSSVLLFMEYCARGSLLDYLTQHVAAARRLTSRCGAEIVSERGGSPLPHRRDDLY
RQSSCVEKGAGGTSNHRPVEGSLREETDGGILPPLPQVSPYSPDCRQRANAERPWSNPLALPIREIQRFMR
QTVEALHFLHSNGYAHLDVKTANVLVTADGDVRLADFGCCGRLRQGPTSLHTCVGHSHAVWPSGLLDGTPH
AAAFSGSIAPSDPTRHNLSTPPVASMSESGDATAGAAGIHGSGSCNCVSGGDSVMYPTLVDDELTAEPRGT
ALYVAPEVIRLDKSRIGAPSDVWAAGCVAMELATGEVPWQHVAEEQLCVMFRVASTKEDLPLPPRIVEAVR
DARWWLACYSNDAEANGRDVHDHHSAAKLLAHEEEEERRYDAATVRRCDRSENAANHFKCDIESLVNMQLL
VSLEDFLHSCLHIRPEERPNCEEMLRHPFLVTR

>tr|C9ZZ11|C9ZZ11_TRYB9 | DeepTMHMM Topology Prediction - Predicted Type: TM

MDATLHDKSTRQNTAPTCLSKAETKPSIHAAAGLLGASISTAMFYPLDALRTQMHVCKGGDVNQLSSLRQV
IRQKGLRRLYAGFAVSVTSYGIGWGAYMAVFKSVQQNLSAYVSSNQIGGGSGSAKSGSVTAGCNVLSGCAA
AITTGTVVTPLCVIRTRQQLFDGSNGAKPQNCWQGFKAIVENEGCGALMRGMIPQILIMGNTIIQMAIYEE
LRHYIVEQKIQPTSFDVALISSVSKAVASALFNPIEVVRTRLQDKRNCTSPEYRSMTVGLRTIWRTEGIRG
LYRGVWVNLCRVVPTTSVSFILYEKFLAILSHHNARRAACLPLVAD

>tr|C9ZRF6|C9ZRF6_TRYB9 | DeepTMHMM Topology Prediction - Predicted Type: TM

MHERWEMMMKGWGFCCVPPPPPLSSFSSLKIFFKFTYLFRSTFFMLLPRRYLWFHHILVSSLFSSIFNFNF
FKIKFTTIVSVTAVLVLLCLRKCFCLMVQRIRVLHCGVDSYGCGHCGAYFCDKSDVISRNFNGKFGKAYLV
SRCFNFYFGPAEEKELMTGKHIVRDAFCSNCDSYFGWTYDFAYEDKERYKVSRFVVERQLLQAIAAGPAAG
S

>tr|D0A3X3|D0A3X3_TRYB9 | DeepTMHMM Topology Prediction - Predicted Type: TM

MCCRICVVRRFYTVQRSLCVHFSAACLPHARGEHPLGIINHTLHRVDAAFPSAYGHVSPFLCFSCPTHIFI
SAVSCFLQHIYLRPCVWLRELRPVSCSMLAVIWYAPFCHFYLSIFLSFYFSIFTFNFLTCAVFFCFCSSFF
FLFFFLIFHVRSHQFWR

>tr|C9ZID7|C9ZID7_TRYB9 | DeepTMHMM Topology Prediction - Predicted Type: TM

MDSFGNQKHPMRQQQQQQSPGYPDQSSWGGTQQNAMLEIGMQYGQNVLQEKSQGFMSYISVVTGFRRYFR
VDNQYVKRKLTMLLFPFLFSMKKTEGYSNNDYEERRYPSGFGGDSPPPMQGGSWSPTSTQSTEQVPPPTED
VHAFDLYVPLMGAITYVILSGFLYGLHHNSVPNEQLVGPAWSLLFWLQVEVFILKLVCHLLRTTPASTILE
LTALCSYKYITICLAVLLREVLRLEGETVYTWAILLYVVLANATFAAKTLMRSHQREQRVPYNAKVFAYVT
ALLQAPMTLWLAIRPFQ

>tr|C9ZSL0|C9ZSL0_TRYB9 | DeepTMHMM Topology Prediction - Predicted Type: TM

MLRTNGKILNGKTPSNVVAKPSGVKKKIVKIKSSSAPKPVESALTGNYGRVLFQMAICIAGIYVCFGFWSI
KQERIMTKPYDAVVVVGGNTTVVSTKLSTVFVLGLVQVVMAVVVSCVLLLAERLYAGKSQKAEKEETQKPK
LEKKKKGTVKPKNGEMVNRREDKKVVVGRTASNKPKRKTEEDSTALSTGADKELEANKAFRNAVLIGFTNG
FASMLGFAAMRRLPYPVVLATKMSKMVPVILVGFFWHGTRYSLSKCLACALITGGSFCFYMLGEAGDESQA
LKSKTRNRSEVVSLFGFVLLFVNLLADGFTNSTQDKLVKVHGWTSNKLMFVTNLSTALWIGAVLLLMECLQ
PFATAYLSISEPVTFSPSFAAFHPLLHRLDESFRWFLRDVAPFNDFSKTMDFFNRHPEALYDVTVMSVLNA
VGQMFIFRTISLFGSLTLTALTLLRKSSSVVLSIIVHGHSVTLEQWFSLAVVFAGAVWEGLIHARKEPVSS
K

>tr|D0A9H6|D0A9H6_TRYB9 | DeepTMHMM Topology Prediction - Predicted Type: TM

MADNSNVGVENADHARSFVEVNILRGDLNAARRFPSNYVRTSKYTLATCIPKSLLNQFRCVSNIYFLFVTI
ITMIPVVSPVNPLSTLLPLCIVVGVGMWKDLWEDGKRRKSDKLVNSVGVQVLRGSDFVSVPSRDVRAGDVI
LCGLGDVVPADAVVLNTSLVDGVTYIETSNLDGETNAKTRRAKPETIKALGTVEDIIEGCLPDAATCAHFL
NNGSLVGWKRCDSRGGDNGKGAISQNVLTENGRTSGGTSGQRARASSAFRDVEVTEVEDGTPQSVELPPPT
NVTQSFAEQTSKKGTEGGHGRTSTATPTPLETRGSVCNTLELSSKERTITECDDRVPSSTVETSDIRDRVR
GGPSGLCSSSNPFAAGTASNIYPRAGAPGSAVALANDGNGVLNDARGDGDFKGVLLRGATPCPDLHSWIGQ
LRLRCGSVVSLSIDQFLPRGCIIRNTEWVLCAVVYTGKNTKMLLNLKSKGEKSSLTSRRINLINIILLFVH
QTALITLCTMSVHWRAERLNGLEGAGSGHTTWYIQWALSRYGGSKYFALMYLTNFILLSFLIPISLYVTME
LNKVLQLYLIANDRRMASYDEFKGVLRYSRPKTSCLNSQLAYVRYVFTDKTGTLTENVMTYVGGCTATERH
DEKERPGALGEAFLRLVEARRLSVPPAVGEPIMTTDALERRQVPQQGRFDFDEEAMEKEPLFRYLRNLSLC
HSVVCFDRPEVESAVAAAVEAAAASGHSVLGGSLPPGSQHAFTRRPSAADATLPNNNRIMGCSGTTDAVEM
VCHRRVSSITPGSGSVFGASPSTGVGRFLHGHTGSASWRMTCHNDALMHERSLTMSRKVREFRDESKIYEG
QSLDEVALVCAARDNLFALQGRTSKHVFVKVVQKVMCYEVVAELQFTSQRKLMSVLLSRCPDMDNASTGTQ
DNVRISYHRKVQETQTSPSFARAWETPTRSSGQHKTMKVLGDQKGDPTHPVTVEEDRKDRPVDSNARGKKL
PFLLLVKGADSSMMSIMNKQNPRNIDLKDLFEVEIDSVAKKGLRTLVLGQRWVSEEEARDWLVKFNEAQCR
LNDRDEALHEVYALLEKDVDLIGTTAVSDELQEDVPETVKFLMQADIVVWMLTGDKRETAVTIACTSGIIE
SGCEDMVHHLDVCSQLSGTTDLQTELKSERIREVLRSQLSAASNKCDSAEEQYGKDTHKMVLVVDGLTLDA
IFCDADLTREFFSIGMRCRSAVCCRMTPLQKAKIVKLFQENTGGVALAIGDGANDVSMIQESSVGIGIMGL
EGSQAELASDYAIPKFRFLKRLLMVHGRFSLYRDAHCLVYSLHKNAFLTSAIVVYTISSGFSGMVLIDSWL
ITFFNLVYCSLQPVLMGVYDKDVEDELAESLPSLYPPLSRENMFFRWGYFIKWFVDGVLLGVLLFVLTYYV
LGDDDALHPYRSGSVEDYGTLYFILLLFLVNLRAASAIVCYNLITVAVLALCFIAIPFLTLFYSALPNVFG
SNRCVYVAIELVGNIKLWLMLLLCFGIYVMYIMGSNAYIELFKPWLNGERAMRAAWESPYKGEHLAKVKLL
RERSRTR

>tr|C9ZSW8|C9ZSW8_TRYB9 | DeepTMHMM Topology Prediction - Predicted Type: TM

MQQKYLGRDEVGGVEVFMRQMLATILPYGIVFGSVLLKLPQIVKILRNHSADGISIISLVVELMSCVISSS
WGIARSLMFKDYGESTLIMIEMFLLLLIVGCMQRKLLITVLVFIVAVFLLVFMSAGYAPRNIHEGMLRLQI
FFALGSRIPQIVINYQNKSTGQLSALTFFLAMSGGISRLLTTFHNIPSDKGRDIMLTQFGVVVFLNFVIVM
QCILYKAADRRQVGTTEAKAVVKKNN

>tr|D0A2I4|D0A2I4_TRYB9 | DeepTMHMM Topology Prediction - Predicted Type: TM

MAVKYSIIVPAYKECGNLEPLTKQVFDALADDGFSKNEVEMVIVDDNSRDGSVEVVEKVRNEGYGVRIEVR
TNDRGLSSAVIHGISVSKGSFILVMDADLQHPPKTVPCLLRALEKPGVEFVCGTRYGAGVEIDKDWPLHRR
FISWGARLLARPLTPLSDPMSGFFGLRVDVFQRGREVVNPIGYKIALELFVKCAVRKYEEVGFNFAARTVG
ESKLTGKVIINYLEHLKLLYFYVYGTALTVLLVLLPLIFYCFYILL

>tr|D0AAA1|D0AAA1_TRYB9 | DeepTMHMM Topology Prediction - Predicted Type: TM

MEDSQTTIPLQPSFGDQAGSPSQLRRLFDTAGSYIMSALIGRRDLGSSEPFNVAQVPHDSTSNDEQWDDRA
VHDPFDPARPPSLCNDNSLCSSDDVGVDLREVILQYRRTPNDSYMGIIQASLHAENFRINGDILQAAVGDR
HLTKTLLKSGRVDVNDVGVQQIIQEEIDSLMVGDENFGGRINPGNIDYLKVLCNAPSATFTKEQALSCRYA
GLEFVKMLYDNPHLLRNLPPPNWLLGFFLHICFVLDMALTWVGLIAVFLHLACVGWVIYHWRVVGHIECSS
WTIVCYVVGYVVSIVAVMTSEEGRIRDYEDRVWDYPDNTTKVVPCVPVFEVWLVYVTLRYELLKNKAKYFV
IRYDLYNGLTNVLLLHTCLFSIPQLLLQYYLAAFYAYGDSFIGHTILAITNYTSYGVSTYLFLRNAICNFS
CNRFGFAVVATKQVSLRPRSVTNRILIATTSCYLECCLFAVLLTFPVIGQCHTETVVFVSTASFATVVGFV
LLVVVLVFDAHRVIWAACWPAAAVQGVFTIVYTYSGKLVDDPLGCSFFSLTSSSVPILTYVTFGFLCLSIL
AGIGVFIYDKVTGGREVMWDSYYFWSMRR

>tr|D0AAE3|D0AAE3_TRYB9 | DeepTMHMM Topology Prediction - Predicted Type: TM

MHRCVGVRLHRLFCLFIPTTFSFCPSVSFQLIFLIAVLFLPFPSLTSLHFLSLSLFFLLHPRVHALVFIPS
TVVLIYPRLCTLLLFFSLSLLYVRDVSSTSTRLRMRFWYSWHCVVSLPAFFYFSFIVFFF

>tr|C9ZSR5|C9ZSR5_TRYB9 | DeepTMHMM Topology Prediction - Predicted Type: TM

MMVLSELPAEKEEGNGGGIEGMCCQSAVPDATPPPPSIIVLYFSYGLPFLHFYFVFSKRSRRKEERSATKL
VPGDHCGNKALCTQGRRLALIGHPGTHRRTHLYIYLYIY

>tr|D0A310|D0A310_TRYB9 | DeepTMHMM Topology Prediction - Predicted Type: TM

MCEKKKSFFSFMFVNFLSSLRLLTFLFIYTEDTSPSLSTTTFFGPPRNSYLLSLFPFISYFFLYFLFPSLI
YFLKLFANANKVMLRSTFLSFHSRVLLLRPSKTKKISKKRRKKRVSSHSQIIVSLLRIPQTPQIYKYSQIG
EE

>tr|C9ZPQ8|C9ZPQ8_TRYB9 | DeepTMHMM Topology Prediction - Predicted Type: TM

MKAESPHSNYQPLNSEEGRWFHRLRNFIMGINVFLMAFAVSGLVVGFIELDEIDSAIREICSPCQHAHVIY
MSSFGALLLLSFLGFVALHTRKRCLRILNTTCLVLVFIPLVFGSVLYVLMSTEHINMQYGWNLVVAERSDD
TCKLELQWKCSGWNKLCATHSTIGLIDLKPLEGTEKENIINNLSVVTNTTSYCSGADEICACPICTEDDQK
YIDKFDQTCEMVVMGALRSHLIVFLFVSLCVVVLTGAGIVVSVAYPHAEW

>tr|D0A7H6|D0A7H6_TRYB9 | DeepTMHMM Topology Prediction - Predicted Type: TM

MLMKLKCIRSYTFISFSSFFFAFSPSFLSLPFSFFAFIHSFCLFIYLVDVFFCFSFLFCVCVRCVCLCRWF
QTLFFITRLQKQWREVNVNERNEKKRRK

>tr|D0A3N3|D0A3N3_TRYB9 | DeepTMHMM Topology Prediction - Predicted Type: TM

MLGNSQGDTAEFGSCPGRNFSSLLRYPPLALYVVSIGLVAYLIGWGPGEGTVVNAVSGSAVACFVVARTLR
GVLHFLWRERGRITFGRVTRCTRSRAPTGLKSTGAIHMSLADAALLPLCTLVSTFHKNSVVSYLADVLYPS
GIKYTLAYHFAWVFGMVLTAGCWNVLLPLEEGRLMPLEKIKSFLYHGAFTLSAWLLVPTLTFLSSALPCSG
SKMTLLFNERCLSSRHRRYVAEGYLGLVLHALVVDGIIGSLSTLGGWCVNLQLCDEYLLQLTMAHRLIYVY
GAVFIGGPFWSGFCALFSASLLFKMRGSFFSSCRLLQYVAEASLELCVLSSLCCMIEPLTPYAIFIWLGVF
FGGWTVIFASLLHRYGLTFCENEEEMLFDL

>tr|C9ZS85|C9ZS85_TRYB9 | DeepTMHMM Topology Prediction - Predicted Type: TM

MNETIQKKKNMLVRFVHRSPLHLISYINSRRESLRMLSPNTAVPFFDYNTPSPTSHNPTAHTADTPPEIFK
EGFPSSSSSSSFLLSMYALYNRKYLCVCVCVCVCVCVCVCVCVCVCVCVCVCVCVCVCVCVCVASHLVP
LLQSI

>tr|D0A009|D0A009_TRYB9 | DeepTMHMM Topology Prediction - Predicted Type: TM

MFSRCQRRAMNRGHLAPTPSKYTDQPGMWGPGTGPVKDTLARRSMFFRMLVMNKIGRLTKPFRTNRVRWIW
RKTQLQLWKTFVMTMVFFSAVMFGNIWLSFVYHAYTVGPTTPVLERKVREHRVSKQVLQMVRERENELLAE
EELAAAKEAIAAQKAL

>tr|D0A3A1|D0A3A1_TRYB9 | DeepTMHMM Topology Prediction - Predicted Type: TM

MEPLEKLQSEIDRLKREIAVLSCAEDEEALRDIYDEFRMDIKEHYSNSIGVYVLKCFTVPVIVGICVFLFW
LSYENIRSWWMRKSVTKGLNNISIVADEISERKLTQAAWRSTRRRVCFFVVIKCFIAVTCIFFFVGMRFLM
EASVRVWLRNVLVPSMEENVTAYDSPVGTSNDSNPMSGIRYEVHNFLASLLGLDWVRDLQSVYLFYLFGPG
LLAFGLGVMRSAAIDMMALGEKFSLPLDTLKMYRTKKKEVYDLQMQKRVEEVMTKLYALADPSIPLLEEDK
RVDDEEEMPGE

>tr|D0A6Z0|D0A6Z0_TRYB9 | DeepTMHMM Topology Prediction - Predicted Type: TM

MNVTSAHMQPRRCHGGHCTSASIHKHKYTQNRLKKKKRSRIRMKENGKKKLHGWKECETTDTNQPRININK
LEITRLNIIREEVKSPPMQSLPTHSCTRTQEQQKRGNLFNHLFSSFVSFVFLLNLSLFFFCFSSLSLFRPL
LFFPVVFLFLFLCLNSLFYFFLLCVYFFF

>tr|D0A087|D0A087_TRYB9 | DeepTMHMM Topology Prediction - Predicted Type: TM

MVISFFYKCTRTPSFLCALSGLGFPKCVCVSGGGAKVYTYFLCRHRSCCRHSGGNHCFFCFVLSCYGKPLR
AFVFFLPLSWRLNSNIDDSDDAWELWREGLYVSVGINKGMFSTCEFF

>tr|C9ZM24|C9ZM24_TRYB9 | DeepTMHMM Topology Prediction - Predicted Type: TM

MPENPQRRDGAGNEVEPVPPLEGNVETSAPDAAEECQTSSTPTEAVIMLDEPCRNLPKLHWQDFKLIGRCI
SFLGESVVNDETLFLHYEGYVSMVNKNTITLLNVNRFTSDDFLKRRERLEQEKTKRNARREVKAQEGSSKE
DEKDIVTRENIWGENNGSTEEVDESGVASGAPADENNRRSMFLSLFYVNSRRGKGDAETQSERQKLICGAG
SIGPIPYVTFARSAIHDVVVGNEQRSSFMSIFRHSSKKFFDMQCLRMYVRRFLVSASRENKEQSLTLRSFV
ESKCNYKGIDDNVLVSVAKEELAALANIDKNIAKCSSRPTDARVGIQLQLFRAPPGLFSGTGILFLTRLPM
QTFWLAVVQSLMTLMLLTVAANTFISDRNGIVNGYMFRYISPILIGCFHCLFASAVTAFHAIRMRMPVRFS
PHAMLRAAAVGIAIGFCSLALLAVAEAISFPRIQSYIRERAEDPIALCAYYKSNECRGYNENCDVDRDSPT
CLWGVCKGEWKKAPCTRKLNRTIVMGFVPFGVLSILLFGMFVIDGFLHYRLFRVSRLIAAITSR

>tr|D0A5L4|D0A5L4_TRYB9 | DeepTMHMM Topology Prediction - Predicted Type: TM

MPYFYCFASRYDGDSPLGVDFEVAGLHLQCTDGVGCVKMRLLPLCQHAAALCLFFLLFPPVWFPYTAFSVV
FFLLPSSIFFLTFVLFLVCRATFPPPRMLQCLKPMCRQKAHFCGANNQNK

>tr|D0A123|D0A123_TRYB9 | DeepTMHMM Topology Prediction - Predicted Type: TM

MFASLMHLCFSLSFFLIYLFLLPNITHVFDILLPFYELFIYFCVCVFMFMCVPAATSCPYYSSLFFVFIFC
LIRFFSPPFLLCLVMSCLSFFFFFFFGFYCCFFLLFVYLHGNLFPPFVFFFLLYIRNV

>tr|C9ZZX5|C9ZZX5_TRYB9 | DeepTMHMM Topology Prediction - Predicted Type: TM

MMLFLPPLLFAATFYYSVLRPSLLFFHFVYFDAFVLKGESFVKSLLRWEFLDALLPISFVCYRIYYTPSPL
SDVVATKRVFEHLTSADKYIYIYIYYQAAVMNVNLDDLFDTGSRGRQNQTGGYVPPPPPSDPPPPPVMQQH
DPFGVNTLLPSAPPPPFESVASAPPSFTCHGATQQHRHVQGYAPGIYQATAANPTIPVTSTLGGAAYSPPP
LQVQPPPAPLPPQVAPYSSPPITAPHGSIVPEALAFSAVVPHTANALQAQIPVVAGTGAVGPAVSANDLAL
AQEEEDRKQLEKIIKLRKEIEKEREKERRKREELETWGCPKCTYRNPLTVNTCEMCEAGRPGCNLPVNAGP
PSGNHGAPAPHRHNNVPLAASVAGPTAWLCSMCFAPNEAHHTNCKVCHSYQKNGTPVTTASIVGGPTASSH

VVANAWLCGICGKNNKVTNPRCEKCQSYQSNGTPIVDKSSTQLRTDVGTAGDLTWVCSACTLENPVSEALC
TACQSGQRPRHLAPSKKDHNEEQNLGSPKWWSCPSCTYQNTWALEACEMCSAKQPAHMRAAQVGGGGLKQE
EVVEAVQWQSDDAAKECNRCQQPFTFKRRRHHCRACGYVFCATCSPFQLPLRGDVPERVCVTCFEARK

>tr|D0A0T5|D0A0T5_TRYB9 | DeepTMHMM Topology Prediction - Predicted Type: TM

MTELILLRYFPASVIITMPQKRSRVVHTNTPTIRLPFSYLFLFLFSLFVIIVKYIRLHRHYLFIFLKRFVR
PLFYLFSFHRLYGYFDPLLAYKLLSPFFFSFCGIHYAPTPCIYSVFLFSFIFLFFKKNINKVYRIQRLSEK
KTSTQSNKPFK

>tr|C9ZMX6|C9ZMX6_TRYB9 | DeepTMHMM Topology Prediction - Predicted Type: TM

MFRIPRSAHLSFAAAALQTSKCFCATGSNSDGKADAPKEGEKATVAGGVSSKSRSNNNTSNTNTSDSNAAV
GRAPGLSSEKRYPKGFEAFYSVSLLRRLPFSRPSSESVNRAYAMSSDERMLIAIISAKARKARRVRMLVIF
SCLFGVTMAVRFYFLHSRAEGDISGYTTVEVDLAGHRAVFRGQKGEALGVRNFVDYRAFEASCDSNKDILV
GIQTYYPWVPMLLLCLLPLLALTHSLFNTSARLASLTAEKSSYSFKRELSVATRLSDVAGLTEAKHEVVEV
IDFLKNPSRYQALGAKLPKGVLLDGPPGVGKTLLAKAIAGEAMVPFLSCSGSEFEEVYVGVGAQRARELFK
QAHECKPCVVFVDEIDAFGRKRKSDSGGSLRGTLNAFLSEMDGFKDSTGIMVLAATNRADILDNALTRSGR
FDRKITLEKPSHKDRVAIAEVHLAPLKLEPSGTIRGFAETVAALTPGCSGADIFNICNEAAIQAAREGKEY
VSTRHFHQAVDRVLVGLEKSAVKLSDAERERISFHEAGKVVLHWFQEKTDPVIKTTILPRGQHRSGVTQKL
PQTAFISTQEQLMQGMVAQLGGYVAEEYFFKDVSTSAADDVQHATNRARQVVCTYGMDPENIGHFGYNLDQ
EDSIQKPFGPLKEDIVDEAVHKLITEALNRARSILKQYLREVRALAGLLARQETLTAHELWLLLGDRPVMT
KEFRTYLES

>tr|D0A3H2|D0A3H2_TRYB9 | DeepTMHMM Topology Prediction - Predicted Type: TM

MTPLQVLFILLGVAVVAQWNLLLRLPQTVVAAVWLAVMTAVISASWSTMLLVERVCGLCEANAHGQLVNPS
SSGTPLRKTVFMALFYPLLLTFSAGVSVTLAWRDSLLLGGVASGCVAFLEQQLEMWPRRRDLKFRVVSCTL
CGFALFCTALLWSFHVTSPREESSMGSLAQFAVIMWTAALTTGLLKLLLFATLHAWHTSNVLTGRAGSVDF
DPMEEVGLSIRICCSLILLVVSGEHLPNFTGILVHVVVLAPFLDVLYAASILFRFRHVVDSFPEVGTAADC
VICFDPVTDSARGRQLRCGHIFHSRCLRRWLMRAARCPTCRQYVFHQENALFPVELFYDFRANIRHRGVGD
EDAQRGGGRGSCHMPGGVLACKHLFTKAYSLSGVMHHPALWPFLQVLQWRPLWATVLHLPMRSLSRRACRL
FGLQRRRLGRR

>tr|D0A2B4|D0A2B4_TRYB9 | DeepTMHMM Topology Prediction - Predicted Type: TM

MCVCAHRTRQLILVCYFPPFPFFSSSREMTKYLKNFLNISLKISRRSGIATFFFFFFCYARLYPPLSVSNL
KVVPFFFFKKKNLHGKLHEIVSGKNNNNDDDQKRKTKGNNRYTRTEKKRKN

>tr|C9ZZT3|C9ZZT3_TRYB9 | DeepTMHMM Topology Prediction - Predicted Type: TM

MSPTRAQRLEGLERIACCSDVKLFFLVSGSRMFLLLLMLFLRVALPHIYGTELLWDSGTLLYDDMNSTWSL
LNFPRSWDGVHFFHVAKHGYSHENVCAFFPLVPFIVRTVSWLNDVLLPEFLTAVPVTFQVALLNVAMGGAA
AICLRRITILTLLSGSHGVDYTNKKGKVMSMTSGTWLDSLPLPPPYYRHEVTVKEHLNARLWKEVGATVLM
WMLSPTAVFSVVLYTESVFSFATFLGVYMLIVSPQGKWRTAAAEAGAVLCFAVAGCARSNATTYIGFVLYP
VFLQVFFFDTYRKRYAKRYGVTSPIRRWPSLARGVIVLIEVVIIITPYLGLNYLCYSRFVPLWDDMSRAAI
GGRFWAFYGWMQKRYWNVGFLQSYRWGNAPNVLISAPIMFFTVRGFVLFIVQPALARSMETESNISKEYQG
PRSDCREDRCGNKKQRLFSIYKLMENLVQSSNIICLLVILAFGATMAHVNVVNRLVMSSPALYWLWGRQFV
CDPWGGNTVVMVRIFLLWNCIGALFVPNGLPWT

>tr|C9ZUS5|C9ZUS5_TRYB9 | DeepTMHMM Topology Prediction - Predicted Type: TM

MSTPSVVGSEQQQDRNRVAYGMRREWKFSLTFAHQAARIILHLSPYWESTFIDPRELEQLNRLDRDVKHCP
MRPALRYNDLGTTPTRMEEVWTENLKKVTILYSHIPPDTTLVGYCVRSVQHVLSDPQVVDVDGCTDFSRLQ
VGNKLRCRHKLGGRRSFHFRYTTSSKTKTKSTVYYVIGTIINQRGYLCTVESTGGEDLDSYVKKHFLPHYTD
KDKFMIDQDLVYSDVSSTILKEQQELFGELRYSDTDAAVAFTIPMHPMSIRPDYSPTQTVGVGSIACMTLE
LNVYQRPVESDEIDIAGVINFKVNQALLFLDVEDMVRMGYPRIMSTEQYSNMKINRLLDIFSDAKVVGTPG
TSYLGVRTGRSRTLTFTYEPFNAVVKVMMTSAIVCGLGVTAVFITKLGGGIFDAHLYLYQQLLRGIKFMES

DIPRGCLSRFNSTCVRLVEQNITRCYAAAPADGRHTMEQHLKLLRESCDTRVEGGISVNVPVKEGGLGKLP
GEGQRCSSASDGNIRTATPTEELASEQEKVSCHEGDATEDTPTSPVGSSTGGSHSSFHSTLSISVTGIRTK
QEVVGGDGTPTYGRPHPQRASILASATPDVTDLNVEPPASPTVTVLLPSPQPPTSAAAIPSPAQAVAAASG
SPRTSSEQVSGGADMSRQLSVAVPPSSSVESNVRVPSPARSPADGVEANVAGSPSSVVFPQMPKDELEEDQ
DSKVQAVLKADDADAYFGLSMYDAYVHSCDMLKCKPNSYLLKKFPTNPRFSNLIHEVDISSNYLGHGGFVA
VLNILPNFPNVHTLHFNDMSLDNEDVEHLCEVLKDNKSVREVHLRDNDKITLPSTRFFSRLLKVNPNIVTL
SLEGTRLGPSVIGRLQEDVKKNAEKKGDAEAASVSDSK

>tr|C9ZW87|C9ZW87_TRYB9 | DeepTMHMM Topology Prediction - Predicted Type: TM

MGCLDVNVCVCVYGKDKNSSEHGIKGEENDCFSQLGKGSRNKRLRAYHKKGSPFYRAKVRAPPIACTHGYG
RKNVTSPVLSLPQKQVSVIPGESCTTASVKRLFCFVVCCISCFIIFFFFSLLFCVIFSFVCILVRLFSCA

>tr|D0A2D0|D0A2D0_TRYB9 | DeepTMHMM Topology Prediction - Predicted Type: TM

MFLRPIALPLWVLFVTVATFFVLRALDPVDVGAHREKVYGLPPQVVWLFPLVPVCVWLCYEAIMLMAPAGS
KVALKVYD

>tr|D0A1Y7|D0A1Y7_TRYB9 | DeepTMHMM Topology Prediction - Predicted Type: TM

MYIYIYIFQIFLSFPFFDQICFNTLWYCLFSFSFFFVHDFDGQNCGICSRFSFPLLPFVPPSNYIYIYIYM
PWMKSWKARVPVELLLLLLLLTEMHRCTQC

>tr|C9ZM59|C9ZM59_TRYB9 | DeepTMHMM Topology Prediction - Predicted Type: TM

MGGHHGTWWTYAKLTATAVIGAVALNDSVNNHTLPSVPTATAAAAGVASEVEASWWFLIVADSILLCFAAI
FAGLTLAIMGLDTLSLEIIADSGPEPDKGHAAKILPIRRLGNQLLCTLLLGNVMVNTLIAQITDSHLSGWG
ATVVATALTTIGGEVLPQAIMSAHALRVGAKSVYLVKFFVLLFYPVCKPLSMVLDRFIGMDPGQIYERNEL
KKLMFMHAARGAESGLGEREADLMVGAMELHEKTVMDVLTPIWETFMLEASQPLNEETIQLICERGHSRIP
VYQGNRNNIVGALFTRDLLMVNPDEETPVLVLVKFYNRSCHIVHSETKLSCMLECFQTGRSHIAVVQEVQQ
RPCGDPYYEIKGLVTLEDVIEELIHSKIFDEYDTVPKRLVGNSSYTQRRQVGLSSRCSRRVHMGKNQLKAV
ALFLSRSLPEFHDCSQGECAHLLRAVEMYAAFYKTRAPHDARGLSKDDKANIWLYRAGVPSDVFTLVLSGK
VEIFAGGEEIRFEQSCWSVIATRALTDERFVPTFSCRVVQDSTFVQITREAYGMILSMLSNSSL

>tr|C9ZQA8|C9ZQA8_TRYB9 | DeepTMHMM Topology Prediction - Predicted Type: TM

MVLGGVVGGRITANRKSVLQRLNTVTTTHLLCNRLCSGCAVTSLSGSDSEKPKSQRLPRRTKRTNYADREQ
LMFPDLFIASSEGEAVSCGSWESLDEGVMQPPQPHAGASSLRLSAFVLGALAMPLVGPITYGILNAMAYVG
YTMGRVSFYTRYSVAPPLSTSGGNSTRRHYRHQEATQHRQHPSPVLLRTSLSSPMDVEASIQQLIAAGAYA
QAAALSTTTVHQAECANSRESLTASDNSGTVSAGNIFLENLNGTPYFRGLLQGTTLDCIRVGSSPIHSRGL
FTTKALPRSTRVVVAPQRTYMDAAQLVLLLGDTHTRLPDTFHYTHPTGSLMELVTQPLPHHLMNHSCEPNC
CCGLSKEFWPAAAATGEYSECKEVLSRIENFPYFGDANSFFTTRDVPAGSELTISYSHRVAPLFYGENALK
KYFVVCRCGSSNCRHFVYKQTDEVSQYFAGRKHCKDKNGILSRFICNGGNNVRVNGNRNIGDDSIKEVGKL
LRMGYDDETVFLSLLSSRKPLLRYMQAHLAASRRSATKRELLMCYRHVFKFLNEASPVD

>tr|C9ZTG5|C9ZTG5_TRYB9 | DeepTMHMM Topology Prediction - Predicted Type: TM

MTQPSADEIYGSRAKVNSVIKHSLCSGVCAVPQWIVLSMAGLLTGALFFLPFSTATLHIVFVAMSIALGLL
FALFSWILQRHQCKRPPRDSELINRSATEVEGTQPRRLSGGSEGREFNAASSEGELEAHCFFESGSHWCLE
ALAIVELLVLGALVLLAPLGVRMDVEGKSLVPSDAREDPSELTYPVEAAWKVFLLHLWFTFAVSMKCAASF
SVGLIHFIVLTASFKKPGTIPVLLLAASFIIPVILFLVITARTALQLRKLPKHTHHVGCDDVAGKELSRGG
DEFNRSSNATTGLSTGETETLPSQSSCGIFRPPPGHRSAEFGEPGSSGDSSPCHCEVVAKEELGRQNQPKW
SPCGLQCSWNIPRGLLDDTRIPFVALDTTTGCIECASAGFAENVGMPVARLTGREFGGLLSELHVENAATV
LDVVRTVASSTVSKPRAIHTEGNGKEVSGVRRTVFGKFGIRPLFSKQRQVGPLSALVADADKSSMEIEMNF
PHIAKDSGGRRVLVRGCRFQDEMETHSSKSLAAVVDEAKTRGTGTSSSGIERERPMVSEPNSVTGIKPSHF
FSLSLDVYLYQHHDTCNSRYLVLRQPSLHYALDCVPLPCFLVHPQTGHVLHWNEAAERETGLSAYDMVGWP
VCLTSVVDPTGSLGVFKDAQKKHSLMPFNSAPSAVLHPPLASPPLLVSLHLVAGGGVEAEQGPLLDQIDCL
CWPGRHLRDVGECRGYAETDGSVLKDTSACFSSDGDSEEAGGMLPPLNPASVNLEPLDDYISRKALFLSPA

WMPESLTESKANTVTWDALHVPLLFLIHGEAPGVRECSPLADCCQPKKQRENNGGVEGLSQSFVDIVEEFK
ESLSCALVKECGISSLPLRDGKETLPNPDITQYLGELRELFTCLAQKVDSFRECRGVPMSGRGDICGDFSD
AEDGDTSSAVRGGRRPVTAFGSSLDLGRGVTTARYVGDGSRGIKEGNDIFGEEGAPKGIKEQQQRTRYDAP
AAGVLALAHKNTASHPVSSFAVSQDALVTPPARGAALVDLGNLTRPSGEERFVERGPGSGASKRCDTHRIP
PMQPHSKPLGYSNPAAEIPSRSVTPTSPLRLDHCESPGDLPVWAMLKSNDEATVPSCFIRVPFGEVFRFGR
SSKCHATTSDVFVSSVQFTVSRWVPAQLCAIRSSTPPHQDNGSSCGSCSPTPSGYGPCNSSALADWRVELC
DCSINGTYVNVKRIGKGRTCTLRNNDLITFQLRARRFFLGFRFVLTDERGVPLKECSSPTCSSFRASGISG
RGTPRLWQRTLSGSGSTFALNASQTESAKLNCSDSASQAVRSASGVRHGARTPNARRIHTSQRGSGRHQRG
TIEWKIGEEMLGKGGNAEVYLGMNLTNGKLIAVKRVPLPKEAGGGGNGKGLLKRYMSLQEEIKVLSKAVHQ
NIVQYYGSSQNKDYFNILLEFVPGGSLRHLLENFGALSPGVICSYLAQTLEGLRYLHENDIVHSDVKAANI
LVTDKGRVKLSDFGTAKHLLWQQGQSIDLANISGAAERTADDAACSTHHVAGTLRWMAPELIRASIGPTKA
SDIWSVGCALIEMLSGDAPWNEYEIESEEEIINLLKYTTEPPDVPECQVLPELALIAKKCLALSPRDRPTC
EELLQLVEEAREKLGLQEDETQSRCDDSTSLRLSSVLGNAAVEGN

>tr|C9ZZ34|C9ZZ34_TRYB9 | DeepTMHMM Topology Prediction - Predicted Type: TM

MCALRDIASTSPLGDAIRDRVKGGIIVTTGEGAGSSPASTINSSPLGMGRGSDDSAHSPRSGNPLGWEGLE
GQSLPFFGMRSRDGTDTCIHSHSYDQPHVCPISMERNLRPSSSVSWLCDETLSEGLAGSPKSSFSPLVASP
RFVNLRDSVVAASPLDYSEEHSPRFASGPSERGGSFCSQFSDNIALGKQLQGALANPTLWCSKEESPRSTA
PSPHSPRVGYSGNCLESPDLKVASASQPGPAAPSHDPWKKVLRCLTITTTLQMLLFVVVFATHYFVTTWVL
VQIQAKCTNSSLAPARCHAFIGDRRPHRLGFITTENFTVVGTLSGFVATALGVMILRYLLWHTLRSFSMGT
VSLICRVAPLLQQEKDAIVYQLQEATSFSRKEASDAGNKERSETSQPNDKKTGYAPNRLCDHKGGSTNFVL
DSSCNIPKVNTRYENFHELAGPDFAGGRVPLRAVRSHEIIPVSSSPLLKNLDRSNKFSTPTAVRHNYWSVR
EARASDSTSLSTQQSDACIDAFALPRPRELKVCCDVEGHPLKTNLHKHADPDKFDSATTNFSQEVTGRKED
AQESSSCKTKSDFSTSNSDQLLLETFGSKNAMLSNSSFLAQQVTFLVCRLFLPALDLEMEGVVTTKRLRAV
QAMSQQFTEVVLRVAREEFGVPFDIRLDSVVVTFHTPSGPNSVNLVRPRDCAFRLVSELQKLESQWARTSS
LPFVWGIAMHLSQLLVGVIRTPSGRTSSLYGEEVRLAYRVTELCRILDCPLLMLQPCYDVFRVCVTAVPVD
VIHRRTCGGDVRIYLYDPKAPKERECVGSSREQYAPLMAAFGLMCEKRFSQASEQLEKVLELDHNAPRLHR
LCKYLAQEHEGEKMNSSLSDITRYVREGPQWCAVDREAKKFIRKKIEKAGMSIDMNATSLSVSQYPFNDVV
EPGCAQEISEEYRLLHRVANSCAMMDLSRFGQCECHVSPQYYAAPGSFQPNEIQSEYALVTLRQNTLMNSS
ITRDVCIVTHQRSSSACGVMPECNYKSDRTIAVVAPPHGENTIQNGDCYNKAERLEFDIYGPVGAGSFGRV
YRGLHPDGNIVAIKEYPVPSMDENNPEIQSTLSEIRMLSASHHKNIVRYVDRCFQNGCLYIITEFVSGGSL
AALVETFHGLPCDIIRRYTGDILRGLQYLHDRQIVHRDISPNNVLVSIDGVCKLSDFGGAVECAMQPPTSE
DNVTLTSDEKSPTRKRTMVLTTESDYCSTGSTTLKTCFGTPVCMSPEACMGVVDPRNDIWGLGITLCFCVA
CSYPWSQEDTADVRSFISKLRRGCISPEPPFDLMDVHFADFVRQCLKRDAKDRPSASELLFHDFMVN

>tr|D0A561|D0A561_TRYB9 | DeepTMHMM Topology Prediction - Predicted Type: TM

MLDAAPAVCLVLGGCQANMFLLELIVAKNRDTMYAMTFAQYVAVALLSLPYVCTFRKVPGKAHLLPLQMRP
GRLSTLHKLAVGVTAWVMGVATNLAFNMHVSVPVHSTFRSLPLLLNMLVGFFFLNKRYTFLQVACVSMITV
GLVLLTVEKSRRSSRSTTNGSSGTQDTSEYFWCLCGMLILLFTTVLSTALSLMQEHMYKTAERREKALETE
SKKTDNPKMPRSVEEATETSPAPMWAEALFFSHAVGIPLFLSQPSRLFTEFASVSSENYVYFLLNAVTQYM
CVMGVYYVLNNKTSAFTLVLILTLRKLGTFTLSVIYFGHYRHFNTTEWIAMFGALGASVLYPLLPKA

>tr|C9ZM90|C9ZM90_TRYB9 | DeepTMHMM Topology Prediction - Predicted Type: TM

MSSNSGGGTGADSAEGSSSDIFTPIPNWRPPKAFERTPSGSSPYSPRGNELLIARRRSATPPPNPTTSLSQ
MLLHGPLAERFNRGASDCHKQMQPSVTLPLMHVRFVSSAKLPQCPVTLDPVVAGPKPIDGPDQQQQQPQRT
FVPFKDRLRSTNFSRGEKSDRSATLCGSSVTSQQLSKDAGSLSVSFSTACWRLASSASVRSGDGSPMNNDD
GGGGCRTQGQPLRLPTYTGTQQGTSQTTARKQTSKLSFKDRQRSSVSNNHSVSAGAEGSGRDAEPPCNESN
TYTPANALTSNDGGQQKHYQRKFKARVQTGALAPPLPPGAPPLLLPGTASPSANYVNYCCHSEQWKDGEAE
GTELDNTNNSSERRRDKMLPRASSTRRGSAPRASAETLPPVVDASIPMTDEGGGRLNPSCGGGGGGGGGKH
YGDRVGAGGNEGEARDVFSFLPISPRIAAEFPAGEEGSALAATRGNRRRSGFSSLFFSMLKPLKVEYQLPF
LVQPSIPLDEETVDEPFGLGSSDCSLTDKGLESLTHSLTSGVGVVETEEHVFIPHYELLKEGRSRAEVILS
TVRAVCSGLNLSVLSAPPDTIASCRKCDEGGADETKAADPSPCGRSGRADRRHTREGGVGLSQSSSTNEDT
KSVSNVGTIIASEEDDCFTFGVVSTSYPLFPTEDQDKSDCKVAARLLFNAEKGVMLISAEDEVKKYMNKPL
KAGTPNGRAEGGKGARCSFERSNIGVSRDSAVQERPQQAQQKGIEKRQEEEEGEETEGLLWRYGGNQESGT
LDATLVLPSHGTEKDSTRDRNAFYEEDGSVSRCTCTQQLITKDARGVDYSRYHAHTLYHHCKCCDRRPASF

LCLHCLTALCPSHVTRHYNDSIPAVGTSKRGASSVCETMDGEETGRNAVGACSLFINILDIMTSFDRVYWC
EPCQSFTWRYTEVYDPFVDQLAATRGTYLEDPVRDIACVGYEVQIKCYGEAQPSFCGRTSAHSSVLRDRSL
VNRNALQTDIALFYPPNAGQDVTPTEGGSAAISGTIECGLGSPRLVGSASPLPRCSAMEDTLHVGVTAPKL
ISLGAKVQGWRATQEDAEAAFVISIPCLSDTPDTGADDDKAEETIAARESNDAVAMAVFCVFDGHGGDAVA
KLAASRFESHLRKAVCSVRHDEAEASAVLRRVDQESTTPQYSVSEASPLRPRESGCFPQLSSNDVNSGAAL
GDVGVRSVSNVTVEMVRQLTEGSADKSVARGSAADHSETSDTCHGLSASPTVSRREMEALRLYFAGIMEEA
LLSLDDELRSSDEGRRGDYNCTGCTACVVGITTNFILCANIGDSGAAFYTPQDIIPISITHRTTDPGERAR
INAAGYTLVEHRIEGLLAVSRALGDYDFKQCGGRGPREQAVTAVPDVTIMPVPPAAAVGRWGVVVACDGVW
DTLTPHQVHHAIMNTPNDLEVASSATEVVIRAREMAYASQKEGDESHINTEKYRCGADGERENGGSKTVDN
VCGGKCCHGGAPGASIDALLLTSAAGIFAQCVAPVDNEEGVGMDNCSLFLIEQR

>tr|D0A6F9|D0A6F9_TRYB9 | DeepTMHMM Topology Prediction - Predicted Type: TM

MAEDAAPDTSHQSSNTSDEGGAKSEVIVHMNNREANEEYGYPNNFIKTSHYTALSFLPLGLIAQFMRVSNF
YFLVCMCLTLIPGLSPVNPVTAILPLLFVIGVSLAKEGVEEFRRHTADRLANSVEVDVLINGVMQRVPSRD
IRVGDIVRVSNGEEVRADLLCLSTSDEEDQVYIDMCNLDGETSLKNRKPHEHTASLRTPEQLQEVQVKIVT
TQPDAELHSWSGCIESNGEAFAVDIGNFLCRGSVLRKTDWVWGVVVYAGVDTKMFRNLKGHPMKMSDLDRR
LNVMIVTLLLFKCVVLATLAFLLVWWNRSNKEHIWYLHWYMNQYGSTVLLLRSFVTIFLLLSYFIPISLFV
TIEVCKVIQAYWMVADGKMTDVVNGRLCRCRPNTSNLNEQLAMVRFIFTDKTGTLTENVMKFKQGDFQGFC
LDSACGTKPTDLLDRCNPAREAAYEYFLSIALCNTVQPTEDPNAEGGISYDGTSPDEVALVSMAAEHGFRL
KKRTTREMVLDIEGVEHEYRILATLEFTPERKMMSIIVRDNVSHHIVLFTKGADSSLLPRTCTNRQAQNYV
QKLRGTLQDMSVCGLRTLVIGRRFLLPEEYKNWEDSYKTASRTLIDRSAALDDVCMRIEGDLWPVGATAIE
DKLQQEVPETISFFLEAGVVIWMLTGDKRETAVTVAATAKLCDPQKDFIIHIDIGSFDPKSAEAIRKVDSD
LSKVRRTLESGGENGSKCTIVIDGLALGVAMSEHFLTFLELSMRVNSAVCCRLTPLQKAEVVRMFQGSTGL
TAIAIGDGANDVSMIQEGRVGVGIIGLEGSQAALSADYAIPRFRHLRRLCAVHGRYSLVRNSGCIMISFYK
NAVLGMMMILFCFHSAFSGGTLFDGWLLTFFNILLTSIPPFFLGVFDKDLPEDALLRRPHLFTQLSHGLYF
DVMTTVRWFGEALIHGTLIFYLFYLTIRNLDWSTHNISMIELGTMQIFIVVLVVLVRCGLAVRCWRSLQLL
GLLASLAITLALTLTYSSFKSVGGSSIYWQMFDLALGPKFWLYMLLVLGSLIMINLSVLYFQKRRYPTLRD
TADEENRVMCRGSLLDENVSQGDKAGYVTFGVESSQSSDREDKSIDIQDMRRREIEVGDTLHG

>tr|D0A6F9|D0A6F9_TRYB9 | DeepTMHMM Topology Prediction - Predicted Type: TM

MAEDAAPDTSHQSSNTSDEGGAKSEVIVHMNNREANEEYGYPNNFIKTSHYTALSFLPLGLIAQFMRVSNF
YFLVCMCLTLIPGLSPVNPVTAILPLLFVIGVSLAKEGVEEFRRHTADRLANSVEVDVLINGVMQRVPSRD
IRVGDIVRVSNGEEVRADLLCLSTSDEEDQVYIDMCNLDGETSLKNRKPHEHTASLRTPEQLQEVQVKIVT
TQPDAELHSWSGCIESNGEAFAVDIGNFLCRGSVLRKTDWVWGVVVYAGVDTKMFRNLKGHPMKMSDLDRR
LNVMIVTLLLFKCVVLATLAFLLVWWNRSNKEHIWYLHWYMNQYGSTVLLLRSFVTIFLLLSYFIPISLFV
TIEVCKVIQAYWMVADGKMTDVVNGRLCRCRPNTSNLNEQLAMVRFIFTDKTGTLTENVMKFKQGDFQGFC
LDSACGTKPTDLLDRCNPAREAAYEYFLSIALCNTVQPTEDPNAEGGISYDGTSPDEVALVSMAAEHGFRL
KKRTTREMVLDIEGVEHEYRILATLEFTPERKMMSIIVRDNVSHHIVLFTKGADSSLLPRTCTNRQAQNYV
QKLRGTLQDMSVCGLRTLVIGRRFLLPEEYKNWEDSYKTASRTLIDRSAALDDVCMRIEGDLWPVGATAIE
DKLQQEVPETISFFLEAGVVIWMLTGDKRETAVTVAATAKLCDPQKDFIIHIDIGSFDPKSAEAIRKVDSD
LSKVRRTLESGGENGSKCTIVIDGLALGVAMSEHFLTFLELSMRVNSAVCCRLTPLQKAEVVRMFQGSTGL
TAIAIGDGANDVSMIQEGRVGVGIIGLEGSQAALSADYAIPRFRHLRRLCAVHGRYSLVRNSGCIMISFYK
NAVLGMMMILFCFHSAFSGGTLFDGWLLTFFNILLTSIPPFFLGVFDKDLPEDALLRRPHLFTQLSHGLYF
DVMTTVRWFGEALIHGTLIFYLFYLTIRNLDWSTHNISMIELGTMQIFIVVLVVLVRCGLAVRCWRSLQLL
GLLASLAITLALTLTYSSFKSVGGSSIYWQMFDLALGPKFWLYMLLVLGSLIMINLSVLYFQKRRYPTLRD
TADEENRVMCRGSLLDENVSQGDKAGYVTFGVESSQSSDREDKSIDIQDMRRREIEVGDTLHG

>tr|C9ZUI4|C9ZUI4_TRYB9 | DeepTMHMM Topology Prediction - Predicted Type: TM

MITKNSIIKEIKLLRHRHWSAWPIIWPFVPLYAVIFTLHLNPEIAWDRTTYLVHGTYINFFHAVCIPIVIF
FHGLLSLFTIWSVRFRSLVQFHCVPPKEVDTATHVYVCTKEFKGESEIVPLVHASSDHPCCFVFQQRKWKL
DAVTEQFVKPRFPTKDNLSMYFAWEGLATTADRSKQLDMFGRNETEVVIPDFQTLLVDHALAPFFVFQMFC
VLLWCLDQYWYYSLFTAVMLVAMECTIVMQRIRNMKTLRSMAEVPVRQVTVLRAGREVSVKTTELLPMDLM
VVDNNAPCPADVILVRGTCIVNEAMLTGESTPQLKEAIDAANLPLEMKKHARHLLYSGTQLLLSNGPHGQS
DTDRGRALAVVLKTGFETKQGKLLRTILHSQERASENNGEAFGFIGLLLVFALMASGYLLKRGLEDPNRDR
WKLFLACIQIITAVVPPELPMELTLAVNTALLGLVKQNVFCTEPFRIPYAGKVDTCCFDKTGTLTTDEMLF

SGVDMADGKGLLNTLKTVPPKAELVLVTCHSLLQLEGTDTVAGDAMEKASLGALGYRVNIDDTVVYDPPAQ
KEDIKGKSTTGSSKNETSGSSKSQNRKNNSGFEKQYKILVRFPFLANLRRMPCIVSAPDGKYVVAKGSPEA
IAQLCESIPPDFHSVANAHAIKGYRVIALAYRPLKEEERSKEAIHNMDREDCEKNLIFAGLAVFQCPLKKD
AKDTIEMLQSGSHRCVIITGDSVQTAISVGRDVTILKCRQQLVASSMKKKGNGEDEVDDCIVWTDAATGKE
VNLDRRSILAKTFVQTRRHKVPSDDEWDLCVNAESIPTTTLATLIAQYSEHIAVWARCAPTHKEDIVTDLK
QREHMVLMAGDGTNDVGALKQAHAGIAVLNATSMDASQNVGGSGEHNSNEPHNEPDVPKDHKIPPGFKLTV
VPPAPSADAPFMEQVRHKMAQARRKAEIVQIARWNKQLEESKKSRETAEAGKVVSQPEMNAPASDFLMESI
FNADDADMGGAPQVKLGDASIAAPFTCRSRALTSVCDIVRLGRSTLVTTLQMYKILALNCLTSAYSMSVLQ
MDGVKHGESQMILSGIILTVCFLCMSKSQPMPTLCPQRPITKVFHPYMMCTIFMQFGLHLYSMVETVRLVE
EADAEGVATMRQAGAEGEFKPTLLNSAMFLLTTLIGGVTFAVNYRGEPFMQSIRKNRPMFYSLVVLALAVF
YFASEMDPTLNQSFEIVAFPSKEFRERFLQILLMDALGCFVIEGLSLKLLTEYD

>tr|D0A3G8|D0A3G8_TRYB9 | DeepTMHMM Topology Prediction - Predicted Type: TM

MEGNGSPPCFTQFTDAEEEETSFLVKGPNQEDPASLGPEKPVALTFRNITYTVQGDKGRPKEILRGISGYV
RGGELLALLGPSGAGKSTLLDIMAQRQKSGTIGGEVLLQGRPIHLGSFRRISAYVQQEDLLWPYLTVKESI
SYAAQLRTPPSFTRSVLETHIQRVMRLLGIDHVQNSRIGSQMVRGISGGEKKRCAIAMELVSQPSILFLDE
PTTGLDTFTAQHLLTVLKEIAAGGVAVVFSIHQPRKSIFQLFDKLLLLTGSGEQAYFGPASAVMRFLEGVG
VAPPQLDNPADVLLDAVAVPPSEEFFKSGVGQNCALAMSRTCSGIVTAFHTSLLAVVEREIGAINERCSYG
YDGLPGGDPSPYYRGITKQIYVVAWRAVLSKLRDSSAAVARIVAAAFFGTVIGSVYFQLGNDQLSIRNRMG
SLFFVTMNTSLSCLATLNLFIEERAIFVREHRAGMYCVLAYYIGKIVQDVPITVVTNLLFDIIVYFTMGLQ
QGVGKFLLFSCTCTFVVLNSYFLCLFLSSLSRNIQVANIVAPLVLVLLLLPSGGVLMGTQSLPFFWRWLKY
VSFVRYGLAGLVVNEFDGLKFVCEPKVPFCFPDGTTYARLQGYFPEELPEYIVACAASVAVYMVLAFFALL
ARAKYLQ

>tr|C9ZJB0|C9ZJB0_TRYB9 | DeepTMHMM Topology Prediction - Predicted Type: TM

MRKKISGEKSHPSPAFFLYSQLCGISLDLMIAFVEYLVVNFLLLCLALLFCFLFSSYFSQQLQRYIFFLCC
DPTFTICWLCSFEMHVPVFSDITVVFVGMCLYLCGSSLFNLIYFSAALRFERLSRCFLYFFRYRHHH

>tr|C9ZJ59|C9ZJ59_TRYB9 | DeepTMHMM Topology Prediction - Predicted Type: TM

MTYNADKILHKVELHTHLNKLFCAFLFSPKHHPYEIPVLFLFLSFSFSFSFSFPFFFFAAVLFCINFSTEM
FLSSSPISHANIFLVSFEGFVYSGRFNTAVLSIPFKTHIITICRPSYYCYYYFYYYYYYYYYYYYYCCC

>tr|D0A952|D0A952_TRYB9 | DeepTMHMM Topology Prediction - Predicted Type: TM

MGMGTMMCGARAGWESTLPSTSTLSFGYSLLPRVSFLFVCFIIIFIVPRSRFVFRSACLLGLLIDSGGSFG
VVRWVSLLAYRGSVKQTNKDNSEWIFLLSAREKR

>tr|C9ZWQ1|C9ZWQ1_TRYB9 | DeepTMHMM Topology Prediction - Predicted Type: TM

MRKLGARSSRPYRSVQWYRIPATVRDVQAPSAKLQRWEAPGINVTEPNAVLGLHGPLLLQPLALFRKQSSD
VVSKHMTVRSCVYFTKLLRCLGVWTDDLRSQEALHQLQLFRTVSTSGTHVPTPAALVGLISLVLLCLCMYV
CLCEEIIVKKYIFSRWLEARRHEDFTTYVWEERVERCRRAIVHQVPLQAAWRIAREGPFLLFTGESSTTNA
AFWAFHAQLTKASVDILPGRTAQTICR

>tr|C9ZU97|C9ZU97_TRYB9 | DeepTMHMM Topology Prediction - Predicted Type: TM

MCINVLWRRQPHTTVVFLDFSFPFITSLLSSFPLCASFHTDLSFAHWVGGVAVVVVFLLHSWMLIIFLSAH
LNHFPLYAEFLTKGLPTFFPLFFCAFVCVCVGAYGLEVLQGKGK

>tr|D0A5W1|D0A5W1_TRYB9 | DeepTMHMM Topology Prediction - Predicted Type: TM

MGQLTQQHNALLLLFSPALFPRCFFVSLAKYRSHADLFAVFKAMASPLCAPLWRRVRCIQHNSFFFLRGSL
LAASSCSTLISIFFSRVLPPLCGFYLCLCCCVLP

>tr|D0A451|D0A451_TRYB9 | DeepTMHMM Topology Prediction - Predicted Type: TM

MISSRFRGENVSSASTTITTNGGDQSEKTCSSSSSMRAIEAAVPYNSNPDLPLYTVDQVPPHLAENLYIHT
GYRMNYSAGMCFRSVLALHNETFNVWTHLIGFVIFLIVSFVFSIGVLIPRLTDRPGDGSETGGSSFPDTVS
LLGFHWPTLSIFAVYSVSCLMCMLCSAAFHTLIPHNHPVIYRIAHTLDYFGITFLVVGSFLPMCYFCFACK
PHLRYIYLIMVSLFGVGGLVGPCFRRWTDPAYMCAKITFYVCMVGSGLLPVCHIYLTLPSSATASVVQGLL
LMMGLYGVGVMIYALKVPESLYPGEFDIYLSSHQLWHVFVLCAAVVHFFNCASMYINFDKMALNC

>tr|D0A886|D0A886_TRYB9 | DeepTMHMM Topology Prediction - Predicted Type: TM

MANVNNETTECGALLSNITSLQTTLTSMGCQVPALHHHTHDHGHHHHHHHHDDHTHGADDHEGHGHSHGGC
ESGHGTYSIGLHVVAIFVVLIASFLGTLIPIIGKYVPALRLPPFVFVLGKCIAAGVLLSVSTIHMINEAVA
QLQEDCVPESFRESYEAYAFLFAVAGALLLHMVDVIVDARVTNKSDSSTNKPEGQPDAEEAQAAPAALDAY
DGHHCHYAVGMPQSRTRRLVSAMFMEFAVTVHSVFIGLAVGIARDAETKTLLVALAFHQMLEGLALGARLV
DAELSLKLEMLFALLFSVSAPLGTAIAVGTIAIWNVSMVGTAFVITQAVASAVCGGMLLYLAFCLMLSDFP
SDMQKHAGKDKVRRFFRCFGMFAALWLGAALMAVLGKWV

>tr|D0A550|D0A550_TRYB9 | DeepTMHMM Topology Prediction - Predicted Type: TM

MSTKLGTDWSAEGPRSFSFLFLDIIIFIVFYSFMSLRVTLQVSERSWATPLDIQNFCSVLDSPVCPFSSYT
VLVWYPLWLCTGFIFVVLIAYCRGSVKIQIRSSVAGMEERQRRHPRVLTSEVYNDAEATFNLLENKEKLVS
LLDCVTLLRAMGMNPTQDDIDYLRERMAEPVLRLEEWRRNEEIRRDKEQRREEMKDRKRIGTVAMKKNTKL
AGDRSAEGTVQDGQNPQQPVKITPAEEVKNIDWNIFISCVEEIYRDAVTEQKEVLGALRILDTEGDDSITI
DELVELVTTNGESVLNPAEVQQLRSLLPKECSLPELASRLQGTYVPPTKEELERAAFEEIERRRQQEAAAK
AAADDAMPLV

>tr|D0A7R0|D0A7R0_TRYB9 | DeepTMHMM Topology Prediction - Predicted Type: TM

MRQPFPTTWKVYLPGLFVMAIYTFVIYFNNTRGNLVKAGHDFTPIYSEEGKLEGFTHPVLVRAGDNYRSRE
NAKADSS

>tr|D0A2H1|D0A2H1_TRYB9 | DeepTMHMM Topology Prediction - Predicted Type: TM

MFIHPGHHWSALFSPCALPASSYTRNLRLCECGVFVYLSIFVSFDVFPYFFFLRRAFLTTRCCQIAETLWM
DCHFRQQLECAAQCIKEGRHNDAESIVRVLQSIDNEQAAECVVTVLIDSAGAGVSDDMIYVHFTSLRLLRV
YLTRCGLTVSKRGKGLVDSLAAYAKVIGDRLLSPPWRPVLSETALDVAVILKLGCANAEGAFDATACEKMV
SEMLDQLSNCESELLVMLNGSVVIHLIEEFGLYRPPSRGRGMSLKFHRDCRSAFECDGLARFLNVLLCAAS
SGSVLSHRVLQTIMAGLNTALSWSHHIFFEEDIPEGDPCNQPLRVSGALWHELLVAGVPMQGARRRGIEEM
LQAWYLEGRICDVPFEQRPVVELIREFCNIVVDAWDLNDRLRYGRKFLLLVTAVARDLLARVEQDEEASTA
LSLILSGSSSILENLSDIFSLSEIMEPFLSELSFIANRLIELDRRYPDNDGIMAALDEVLSCLFRTTQMTS
SAEASVAAMALSVFDAFLSSKLTYAHSSDDQEHFTEAFTTSHITLVAHMGRQDPEKTTVLLCNALDHLQRW
RAGLGSRVGSEWEQVQESMWVVLKLTAAFIADPCDGEHISTPVCFVPYPLSASHPALRLTTSAMNALQDLL
QNVSLASPAVMSALLDVLGNFITVYMHDPLGDNLALASACVTIIRCALYSLRVFAHDEDVGLASCKVLDAS
TRSVSIIAALQESPEVLREAEEMVHNNQQVQGTARGCIAAFCIACLPLDATPTDLLRILCNFDTQVTDFTS
VDVDMCLERCGSLGGFLSSLKDGDRLVSCLEIIMGICNRVLSVSATRFYEKELAVRSIKMVSQLFLAYSPL
LMDEPLLWLMEKVTATLHAAMKVLREDPTWCSENAEEEKQSMLKLMSSLLRDVAQWSMMESSLPPDVTHSL
GLCVVSALATFLSFFDERCLKLPELKGSVLQAFQLCADAFTAEFVSSPDFQAFLIVLMFTLNSDAIDVQRI
GTSVAETVVTFLHRTGADNSELFSSLLSALTKSFLSGKLSMALSPQVARCLVALCGCLPLDYMEEIIQTTT
STLSGSNPHAAAILRRMFTTAQQCSVFNDRQRIISLRSLSEIISESLCGIRGILLV

>tr|C9ZRV4|C9ZRV4_TRYB9 | DeepTMHMM Topology Prediction - Predicted Type: TM

MSSGLTARRLILQQLTAVTQHIPLQARRTSGKGWWLSSFVRPEGDRRVFTLITEEEVAFLKRRCEEEGDEG
RVILSTRPTRNIVRAVGGAALNITLSFLVSPLLALSVSLEAIRLRGLRPAALVIAPGVGLFWGAVFMAIAW
YAAVQQMLLCGVHTLRAPIFYLFCPGCCWNELSCRYEYPSGVVGNSHPLLSLQAPPEKLFERAMKRETRRK
RRGKRDVGYERKGGTHPSEGDDYYAILGVTRDATPQQIKEAYNRLALEIHPDRNPSQSAASQFDVLTKAYR
VLGNAEKRRKYDMGGRSGVEDIGKKKRGAVRALFGGDALYAIVGDVKTGSFSQRVIDGLDWTQEELAIFRQ
RTLERCRDELLSVYLQPLRSEKDSKGAPALQELKGRLQRLLNTGLAREVLHAVGHEYMRVVLYSKASGPRE
RMTLYLNEAGPHRMRRRLDKWRHLCRIRQHTLRDSATMVDLAWYTSVEELESTARWVATSLLLDHQVPEEE
RRQRLEALQALAEIFITYGQSYKGANKQTMDALMNSLRDYYQQQKRGEGG

>tr|C9ZLM9|C9ZLM9_TRYB9 | DeepTMHMM Topology Prediction - Predicted Type: TM

MCVNVCARVGFIPPFSFLSFFFPFFFSLFSLLSFFFLIVLLLLCWVNFPHGGELPYTLGEGKYNDGNTTTT
TTTTTTTTTTTQHQQQQHNNNNLSLFTPFLTVHPQHCCYIFLIFLFPPSYWLHINPHTHTHFLSIFVCLF
VLLLLLLFFSSGGGCVYCHLSMGYQLFRLPFPSPIFFLKPILLFSSLKPPHGK

>tr|D0A3U9|D0A3U9_TRYB9 | DeepTMHMM Topology Prediction - Predicted Type: TM

MNRTPASGGGCSGGKVPVTFCVVIIFPPSFHCTLSTNVFFPRASRFTILCARRLFCGCMCVPVCASVGATP
LCLLMKMCSSFTFFFFFFPSWVVKQHHHQNEISAEEGQLDQ

>tr|C9ZY69|C9ZY69_TRYB9 | DeepTMHMM Topology Prediction - Predicted Type: TM

MLGRKCSGSLYTLMEVNENATPQEIKRAYHRLALILHPDKTDGTTTEQFTRLQEAYSILSDEQQRRTYNTF
GREGVATLNKLNEMNVPVNPSAFRFLCFLVMMLILLLLLQLELVVVRLDYELSWKWPVVFIPLWIALAPPT
LLALGLLAAGIKQLDFLCVCVGVEILLFVGSIATFVAGLCGALSWPVALSPAIAFYAVRVLHIIPHLFPSR
FVQNSEDGSGEVTFSRKKYCLFVLMVLFEEGCAIAFFTLLMLRAMQKSEESKDEPLSFWIVFSPLIACLGL
KALLHLCFCMICSKVEQPNQQASQKSTSAFLFVVYSSMIYVACMIAAKANVEINKWTDGFNPSAAVAVIPI
QVIFISLFLASVIMFCTASRVYEAMLFGVANEEDDAERGATTPLSPRNTGGSNSFRCETVPGEHQQRKQGA
DEVV

>tr|D0A4F6|D0A4F6_TRYB9 | DeepTMHMM Topology Prediction - Predicted Type: TM

MRGSGMHEPTLRWCQHCRVASDTPPAGLNRSRIYIYIYIYIYDIRFFHLQHVLSVAHQGALPRLLYGIRLL
SMPIAYPLFDLLSLSPASLANIRVPKELVVATVVGLYNSEILYLLT

>tr|C9ZR86|C9ZR86_TRYB9 | DeepTMHMM Topology Prediction - Predicted Type: TM

MLCRSALLIADGFLSSGYSFHTLFVCLVPYSLKRWFPRHALRKSDHRKDSIATAMSRLGRDEPLQPSQENS
REPSLELNGAGTLSHIKRQFISSLVGSSAENGAIAWSPSVSSEGSETDDEEPLAETLQGNDKADLCEDVPP
RMSSELVVFDGEGVRPSQPDRPEGGSDRVKPILLCTLAGGVAASIMRHRLGAVCRFTLSGLAMTQLLSILG
YATVEWRSLFDDVIGLWTSDGTDGFICNAVVALLGSVMRRCAFLTGVIGGFFVL

>tr|D0AAG8|D0AAG8_TRYB9 | DeepTMHMM Topology Prediction - Predicted Type: TM

MSDKGKSATDVENTTSPVTQQTFPAEHPGVSKVSEPVYADDKNKGVDARGDAPPSVWQSFSAKMKHIAGTV
VPYGGLASSVFNLCSVCIGAGFLGLPAAANRSGLVMAMLYLVIMALMTVFSLHILSLVMEKTGLRTFEHTA
RGVMGGRFVYFVIAIRLLNSFGTSTSYIITIGHVLRPIIENSCGAPEFLRTPGGIRLLTALTWMVFMLPLI
LPKRVNSLRYVSGFAIIFVLYFALTIVIHGAQSGLPKLTSDEEDGVKLFNSGNSAIASVGVFMFAYVCQIN
IYEVYWEMKKRSCARFTLYAAISMAFCGTFYGFVAFFGYGEFGGTVTNSILLMYNPITEVMMLIGSIGVVV
KICISYALQTMAIRNSIYHVLGWELETLPYWKHFSFVIPLSLTVLLAGLFIPNINTVFGIVGAICGGFLSA
IFPSLFYMYSGKWTRRNVGNFHFFGTYALFILGVVGLVFGTVTVVTNTIMSFIHGGGGAAAIKPPPVC
>tr|C9ZSL4|C9ZSL4_TRYB9 | DeepTMHMM Topology Prediction - Predicted Type: TM
MDARSITRSSAPTVLRTVFHAILCDAFFSFEGIALYWLVSLRVEIKQLFCWRLKRECWDSSFLPCYALFLY
CCTLLWRHPVHNADNVSVSDFRPFLPFLIRCFQRCIAACVLHLSFIHRFFVCFLFRSFHPFHSPPPSCERA
QMRGSVFRSQWNDGNRV
>tr|D0AAQ0|D0AAQ0_TRYB9 | DeepTMHMM Topology Prediction - Predicted Type: TM
MTIITKRVKFFFKKKVTNTRRESEEKGNHETNTRPTGFPQYHAPPSFQKIGFPSGAISSFFLRFFFNYGKI
DVYTHRFFPFHSFLFGSPLLLLFFRTTLRSARIHTQKKTCTTAQ
>tr|C9ZUE5|C9ZUE5_TRYB9 | DeepTMHMM Topology Prediction - Predicted Type: TM
MATAVADFPFRRCPCCAYMKALWCTLRTAGGCLLGCAVSMTSLLPFSPGTRLTRWRLNVVTVGNAIEPEYI
RGTSVNHSLALSPNPITFHWFLARVFHKGASADVGNTRN
>tr|C9ZS45|C9ZS45_TRYB9 | DeepTMHMM Topology Prediction - Predicted Type: TM

MLMHTAPWLHMRLSRLFRQSPLSLPSTKLNPSPDHYAVWGKAIMAENNRRVGPEHMFRTAIRAQQQLQGLA
DKWTPDAKVYCCGSMVTYGQMEWGSDLDLACMFDDPYPSHEVQAKRTDKLWTVIKRYVPHYLRNNLLGLTE
ARTPVVKLRFANDEKVARARYTPLSEEEDRKARTALLDVRNQCVGDNDVEYIAEKMGRDNVEGIWVDRTTY
GCRIAIQCTSKEQMIEAIGFFPDGKIMTRGMREDYTRDVLDVRFVPEMFMYRWDISFVGYGVKNSYLIRHY
LHNGPVAARHTAMAVKAWGKATNVGAGSAAMLTSYAVTVMFIYYLLVTRQVLWVDPWSLPHPAHLPRYPDF
SPLYDCDPTELGRLLHGFFIFYAHHFDYEREVVSLNRNRRSYRSDIGWNFPQNKKGTFSYNFCIEDPYEDV
GTGGLNLGRHLHPAKFQLVKQEFLRAAQCMERFLPTNAPEKSILGVKRADLRHFERDRDCE
>tr|C9ZNQ2|C9ZNQ2_TRYB9 | DeepTMHMM Topology Prediction - Predicted
Type: TM
MLFDCTLLSLLGAMVAAIGLYRVVQFVHLTFFSTGHDLKRRYSKAGDWAVVTGGTEGIGRAVALDLANRGF
NVCVISRTQSKLDEVVAEIEKCGTRGHSIAFDFATAGEAEYKMLFAKLDSLAVGLLVNNVGVNYTYANYFD
EADLVDDLRIIKVNCEATTRMTKFFAPRMKARRAGGIVLLGSFSAVTPAPLLATYAGTKAFNVSFGDALFY
ELKKFGVDVLVVTPNLVVSRMTQGASTRAPKETFLTVGAAAMARQTLNQLGVVNRTAGHRNHIIIEAIARL
LPESLRGEKMLAMHESIKKRAERKAKQ
>tr|C9ZVJ3|C9ZVJ3_TRYB9 | DeepTMHMM Topology Prediction - Predicted
Type: TM
MPLWKQTNCEVETMNVREVVGTVHLGYVSQMLLLVATVETIVVRSGAAPIELKRHVTTVAGKYGHIGDKDG
FPGMSELSSPHAMCRGRNSDEILLGTVDRFRAFSRKNRETTTITAWETDEDQNSGSRSVKVDKPRACVQWT
VGDSTFVYFVESMGEVKYFKDSGVFSHDVVRNGSLTGVALYGNHLYLTEQNTNTVWTCEVGSDGDPIACHS
HVALSAKCSIYGPIGIAATQQGIFVVARGPAKQGTICWFDLQGHKIAEVDGEYVDITSTRSGDLLAATQNE
LHRVSTDGNKLTTKRFAGGSTNSCLPNTEGDDTLLCEITRLLVVTEYEMYVTSEKKSVLRSVTLPPVYVQG
VFPGRPLPVGYPDKDIMEWIVGNLTEDINTALGTTESIVASSSVHVDSTTWLTNFTAGVQQPDFDDEKTEQ
ALHKSNYEHTKEAADEYYNLTDEQVYMDSTMVPYCNRLSLDALRRKLAKEAGEVLNFTLIYADMPLKAESS
DAGNITTVKLLMPASFNNTVTHDLLSDANLTETAHSFIKYLRSSDTHVDVTFSNPPFNFSSLTPDEEQEVR
WYIHDEVMNQIKKCEERSTGRSMARREEVGDYSRTTIATALDSNVTGVCQSTITNRTVSLFYQPPYVEMSL
YEVFIPGNYTFDVSECVGEIDWQDLNDHLNNDTVRPTTEKAPKCGRVCLIIIAVVCALIVAVLIVLAVVFT
SKRRRLAAVVAPARPKFVSTLDEDEQDYASAYGNKERVEQ
>tr|C9ZJG5|C9ZJG5_TRYB9 | DeepTMHMM Topology Prediction - Predicted
Type: TM
MPYPDVYERDYSSLSQPIGFGAFHLVTVVMTGILFCFALFSFFCAMNLISDLAEEFPTRAGKVLRVLVVAV
MGAHVFIMVVDRMNFLCSLVSCITNGLYLRALRHFPLLPLLQPFTWFMGFFVLLESVLWLHLLVSLDAPSE
VVGYGNMFVFMIMLWLVPTGLILSVEVGPCGISDGLQMLRPPQGTKLPADQSGKRATIVSSLRKYFFSKS
>tr|D0A4L1|D0A4L1_TRYB9 | DeepTMHMM Topology Prediction - Predicted
Type: TM
MKLYNYQYFFLHFKCCDDYVYRLCGFVFSRHFLSSSLFGCFFHFCVCSLPPAAPLSRSVSALPVCDCAYHS
VRCCKLMGLRDLDRVRVTPSTATGTSSGVAGPATPSTTKTSGVATPPVVDDETPLSTEGGSSTGREESFVV
GMGILCMGAFTIYSAWRSSRRGSPQLLWGGEGRRLLNGGENTTLGATGAKTNFKPKLVLVNFPRKLRGQLQ
HCLSPLVSTDEVELVDAPVVFYHEATELTAGNVASVVRREVLAAFDELERTPCVVYHSAIAVDGGALDPFW
SARRAVDASRGRAFHECHCIGYTVDGSRVEVLEHDVHGKVASTLSEEAFACPGTAHGLSGVSEALRHHFSV
TPDTFSTQRTECLDHGKHFKPCTSHDPNGGSCSSAVRTLWLSAAPLFSMFAAALRFSRSEFDGWHYLSERR
TGSQLLPWLRNVLKWGKPKGERPVEGGHEVEGVQEKGSLMLEITISQDVSTLRGAGCDALREVLVGAYGNK
DSGQCKGVRIGISPQMKGGGAVVSCYFPSSLSVFQALRESWRVVSFVAHATFDVVSFRLLAPVIIDSSGAG
VPVVDTSETSVPTPMFPRSRQDLEACPSFFSSTQYFCTLSGETFLDSEEAFRRMREGNARDLHYVIVEYST
NDLVV
>tr|C9ZSI5|C9ZSI5_TRYB9 | DeepTMHMM Topology Prediction - Predicted
Type: TM
MDERKRWENCSYFISVCCVSYILYLQGGGGMFEDADSPSLCRYLVHFQLFALSGWKDVGNPISSVMFWCGL
FFLFSTRLVIVPNPCFFVGCVRTFYTLLLYPFIITCLFPRLVA
>tr|C9ZPK5|C9ZPK5_TRYB9 | DeepTMHMM Topology Prediction - Predicted
Type: TM
MKRGKFIHDVNYFFVPLVAPSFVFNGGGGGFICFSVVHLIPISLPFILLFLLFYKLLPAIGFSPLYRLFRR
HVRHRGCDQEICGSPRFPVSRIIAFFFLKHIPLTIYNPFFILFPLFLFTRCVFSRLRK
>tr|C9ZSL9|C9ZSL9_TRYB9 | DeepTMHMM Topology Prediction - Predicted
Type: TM
MSTLSSWLTGGKTGGGKESEKEDAAAIAARNELLNVIEGAPPLLVSQQVDQFAFLRSNARHKIADVVLALT
AYTFSGFIHMYGQSTALTTLSMWAAGTTFFLNMQVRKVFELEDETRRKQAVIDMGLQEKNVFQLEAVASWV
WMLSSLQQFKMHKRLKYCGYSSWTALCCCTYFTFRHMYQMLLEA

>tr|C9ZJT8|C9ZJT8_TRYB9 | DeepTMHMM Topology Prediction - Predicted Type: TM
MGLNIVFLLLLLHFLEELTNLCMLLLRALKPHCRGKLCVCTRPCACAFVGWVRGIPLFLSCIFVPFLFFLS
LFFFSLVRIRKHFAMLTFPGFVPLGEKQSVQAACEARRRYLAAHLVPLYANTNPTELWNNIVKYREHSGTD
SNDCVETYEELRISYKGYKSMVYNLVFHMTIEEDKAAPASERESRKRLYFSHPFLSPATFLSFPRAEDGTI
SAVPMYAFAAKRLLLYRLRIKLELTARVVPMVLQDPVSKVAGQLRCPPSASSPLSNGLTQDDIENFLVELV
PNLRLVRDIPPWMQPYYLCHASRKFMFMCDTRRTGAIAIDTMMKSDVFSELLRMYESDAQDAITTFPEGCT
VDVAASHLVADTGVDDTVAALVISYEGEGNHPDDMYTVKALEEETVLRVRRSQLYWNPGSTEFLTQDVLSM
DNWFSLPLMGRIYEHYTSLDLDGDGVLSIDELARYCDSSFTSLVVERVFECHVPHSGKHHVMDYKTYLDFV
IATEHAATLPAMKYIWSILDLEGTKSYVTVDTLRGFCKEVASELIANGLMTDISAQSILSEVIDMINPKWH
EWVEFDDIVRSGHQATVLPILLSYRNFYAYDCREQTAAEANDEYA
>tr|D0A2U3|D0A2U3_TRYB9 | DeepTMHMM Topology Prediction - Predicted Type: TM
MKARRRGGSLHAELPFTYTLFSTIDVYGDDLLDFTGDDKPERHAEVTEFLRRRGSGACRDAPLGPSSYLHM
REPFSYIAEGSISENTSDAGGSFARVEDAMQHVWFISQRDGISTPFALNFSTSAQFLKYAERESLQFPTLR
CGSSTMASEPIAAHEGPAKWLDIQTKNKTLVTDILSHFPVSADTVEHCCYPDDMDRVIAHSTLGYFFCNLM
CTPIALGEVARGRHQVKSVIQQRLEAAMSVGPRSSTPSAVAVSVIVFFDWVVTVHEEPFHEFDDLLRFILI
NCGNRAPASNNRRLMQVMTAPFIFASFFQIVVGHLLDSESLTMIVDEVSDLVFLSKSTEGHNEEVVQRITN
ARRCFAEYAAELTRREYIASVLLHSHMMDSFLTKDKVCRTQIESAQAYLYRMSDEVGDCRDTVALTNWYQN
VKDTWHHLQYGNKALRQTVLFTELINIMYPLITLQTLFSLNVRIPFAYYEDQMEKDLKPFFIMMGIVLFFL
LLCGRAMYALWHKKHWSTRLLAT
>tr|C9ZSD9|C9ZSD9_TRYB9 | DeepTMHMM Topology Prediction - Predicted Type: TM
MERTNRARVTFGDVTVVPYAGTRPGPAGNKRASQADESSSYSYSDYSDDYSYYGSYTQSSRTNSNESSSYS
QTIESSGTKTQPPPAREQQQQQQQQQSYTQSVQLQPQLQVQPPAPQHPPRAPVAPQQPVPSKSVQGPRGNN
AARVSTESYTGSYSSYSYSYSDSTDEGHNVVASARSGHVAGAAQLDSEKKRAAAGTTTNISAPKAPIGSMQ
MPTITQQTSQTSGHQHPPMAQPHLQMGVQQVSPWAQQVPVGNQQPPAVHSQPPANIQQWHGDDPLMSARSR
QQPPTGSRRPSLRTPEIPKEDQQQTHTSVQQRPANAKYQQAVVGHQHPPMAQPHLQMGVQQVSPWAQQVPV
GNQQPPAVHSQPPANIQQPHVGSQQPVGMAHKYQAARTQQMPSKTRLGRAPATSTEGLKIDNFSGSGSDYY
SYSPYGSHSGSYTGSYSSYSYTQRTPGQSYSSGGDYTTTVKAAAPARPVEPPKPETHSHTTPPAASPIVRG
RVQDDDSYSYSYSYSYGSVSSPEVKHPLPPSHVVKAPQPEQDANVVSVGSSELGYTGSSYDSEYDYSYDYS
ESATKTQKAVEEPKGAEQHSKSTVETTTSLIKPTTSGYTGSESYSASKSGDYSIELSGEGGVTSSESTEIP
SPARIRKTILLAAPSEAEPRFHSLPRGCIVPNSSMEDTLSSFLDQLAELQVETMERNRVLKVLNTEVEAAK
DLKERKVSDSQRIASDEANSPADIVEAPISVIPSRNLTRKSLMFALRAVGVEKAKERYKILEDVDTVVPRE
KARTILEYYGSRCLTEKVMSMFVAYDEFYSGEVPLSIAFEILEYCGLVTQTVGKFKIFLRPQVSQGQILLC
VADQPQEATENIVDDVEGEKRWIQPQTYETTVRCCGEGWKYGDHKVYWNGESGDVFVGSFIVAKGITERKV
AVQLLRCIEAFLYVCHAIMVVTVGTGMKLRVLYTPLVEKLLNPTVKGVTDATSFLYELAFGSVFNPKKYVK
LLANNSNLRDRYLCEGDDAVLHDTIRPKHLVPKAPFMINGDKDDELNTLLLSTANSYTTVSANIWTQKGVE
GCYLEIGVEKLRITEKVPENARCYCLVSVRKSKDSEWMTAEVLQAVSVGKANKGGFKWKFGKSSSDRLFFT
GAIEDSLYVEACYDIVDASGETTTYCVGHVLVPCGKLKSGKLPVRPGSCYTYTPLDEALKQPVEASGGFCA
PRRKTAGPPAKSLTVTIKRLRSKDVPDLKTLPSRCLSLHRHVSLMSLMCAAVISGRDGGSHPLRWLRQQQV
QCCLMVAADSELMDILQGLWKRVMKGKLGPKPQNLGQKYKRILILATKVVAAHNNITGGDQGIITDIVAGK
KGIPFTENMNRPLRPVCV
>tr|D0AAS1|D0AAS1_TRYB9 | DeepTMHMM Topology Prediction - Predicted Type: TM
MLMLMLMFVFFFLFICSSLSHSLVTSFFFVSPSARRSHSHSHFLFLPAASTSSFFVCVFFKIFLLYCLTSY
FGFFPSVCIIFFAHCVFPLFCFTYEILPRHSEKGVHN
>tr|C9ZIR4|C9ZIR4_TRYB9 | DeepTMHMM Topology Prediction - Predicted Type: TM
MHFGLYIIYVYIYIYFTYTLQYLNGIHFLFCFTLFHFLFVSAMIMIIEKKKKKKKKSKENERVKRKKKRER
NEAKARAPAVVIVIIIIIIIIIVFFSFFLCSRFLQIFFLFCVCVCVFVFFFFSTQAEEERVQEKEKKKKGR
EKKKERQNDDI
>tr|C9ZYF6|C9ZYF6_TRYB9 | DeepTMHMM Topology Prediction - Predicted Type: TM
HRHCVGSGEPELPCNIPGVSRFFLSVWLCKENCRIELRNIHTIAWIRFITSYALLLLFRSAVIVMTSLPAP
DDLCQDPPKIENPVKNVILTVLTAGGGSIHCGDLMYSGHTVILTLHLMFHWIYGAMVHWSFRPVVTVVAIF
NCYCIVASRFHYTDDVLVAIYLTIATFIAVGHNADGAPWQLQLFIRWWPCCGANSREVTEDSQPVMVAFKS
EAAGQSSRKVVDERNH

>tr|D0A2V8|D0A2V8_TRYB9 | DeepTMHMM Topology Prediction - Predicted Type: TM
MRGLIKSFSGRSAPTISTVTLRASICHPAGPLAVPSRLQSTSPMEQLIINGAAWESSNYYHRLGFQEAVRD
SSRIKEHYHILAKHFHPDNPNAPPNATAAFQNIKEAYDHLMEEVKDEVPKSHPHTGHSYRFTDHERRQQQM
RFLGEGIGLFMAMTLVFIFIVSRHNSNRVDGRYLGHFAIIFLTIQVFPRLLAAAILYACHSNYLVSIAERT
EQAAVSLIVEQRESHLAIRAEGIDKRVRDRVVMQVTYPHNDEPRRHTEEGSKDNENNNKKEADTRKGNLET
SPRVLTTVTFDSGVTQVIVPATRADREPAECRVKVVDEARGFVIVDRTFEI
>tr|C9ZSU8|C9ZSU8_TRYB9 | DeepTMHMM Topology Prediction - Predicted Type: TM
MQKRRECLERFVLKSFLLFLFRFFPFFLSCFFSPLLLFPLEIPSLSPSFLQELELLPPLLPLLLSSSSLLL
LLLCYHYHHPPFLYKKLFIFSFFYHFFIIFLSFSFLVCLCLCVCVCFFFLKLRATYHYWTVDCARVTLQEI
QRSTVISFFFFPFL
>tr|C9ZU99|C9ZU99_TRYB9 | DeepTMHMM Topology Prediction - Predicted Type: TM
MELLSVTPGDLLVGCWVLTFVGIIFFRWFMHRRRSVEDQPPEAAQTTAGAQIHNGKSIIPNRRDAGEGSSC
RCCSSNDDVITSENVETCGKQQKRKKILVAYATQSNTSYKLSNKLISMLNLALHENSSAYGTCGGSLVLDA
ASGVSCCCSRDPKAGVAPEIKLLQLKEEATQCSTEHLLESGAYGLVVFFVSTYTGGTAPEPSHSFASMLKD
AYLDFRVPRDVFAGVNFAIFGLGDIAYGPEKFNRFAKDLYTWCKGLGGKFVIPPVYASESNTTTLFHVFST
ALVKWIGRAAFTADGVTVMKKKSMNGAQSLNRRLNNKNNEEEEQSVGGECVKDDEAVASGSGNHNSQNGDP
SEGCGSDDGDSDSENSDNNDDKNNNNNNSDDGDAEDVEDIVGEGDDYFSSTTNGEPPELLYPRLRENLQRQ
GYHLVGSHSGVKLCRWTKAMLRGRGGCYKHTFYNIASYQCMEMTPSLACANKCVFCWRHHTNPVGRSFRWK
VDPPRELIEGGLAGHRRMVKQMRGVPGVTPQRLEEALNVRHCALSLVGEPIMYPEINTFVDLLHEQNISSF
MVTNAQFPEQLRDLKPVTQLYLSIDAPTPEELQRVDRPLFEDYWERCLACVRELRRKPQRTVFRLTLVNKY
NTENVSAYADLVRLGWPDFIEVKGVTYCGTSSTSTLTMKDNVPRHTEVVEFCEALCQQLATSNEPKEKRMW
LGRDIVEEQQGAVAAAESGAETHVEGGMTTSSLCGPYRIACEHEHSCCVLISLRRFFIDGVWHTWIDYDKF
SELARSGRRDFTAAEYAAPTPPWAVFQSEERGFDPTQVRVIRKSGKSEVITSGC
>tr|C9ZT53|C9ZT53_TRYB9 | DeepTMHMM Topology Prediction - Predicted Type: TM
MLLILHIHCPQPLRKKKKKKKIIYIYMPRVWSHMNEETILRDLPPFSLFSSLFIDQTVKHKNTITTKHVSS
QYSLSKPFAVHPFVIHYRDIRWGPIPFFFSFFFLLFIYLFFASLIHYLHI
>tr|D0A3I1|D0A3I1_TRYB9 | DeepTMHMM Topology Prediction - Predicted Type: TM
MHRKPLFETIWNEMLDSNALMAITVFVGAVSFYMFLTRNDHQDRRRESPPPTPKKHGASFLRRIHLGEFSG
RKICISWETLVEKHEWRDCAKETLLALSSNMVVYLMCHISNANEKGKVLSMVKDIPQLARHNILFCETEKG
YEAFSRQIKPAILVTHDSAQAEFLSLVLPNVVLVGSSATGSAMTSISSLSELLF
>tr|C9ZU04|C9ZU04_TRYB9 | DeepTMHMM Topology Prediction - Predicted Type: TM
MEKKNECGKKKETNNMKSGTYRHKYEHKHSPTTAFPFHFFFSDVLLSAYSMSLATSLFCCFSSFLFLFLFL
FLLILIMISTTFPSPSYTLPLRLCSAHGVRTHNKKKK
>tr|C9ZR51|C9ZR51_TRYB9 | DeepTMHMM Topology Prediction - Predicted Type: TM
MVNSVFFSFSQHKHVQLVISLNACLFFSAFYICISLALFGNIHWILEAHGVALVRMFGPVDDAERVANSGS
QQHGRQASISSPVPSGYAQPSDRREQFTPVSFGGSQANIGSLGTPNMFRKMRRPSSSTSSRRSTLRSTPAC
TVSHEQAIDTTNVVLVATRPLLKTLARTCRPHPPWCIGMSVPDTVTEEVRLFSSFLRPTALELYCYEAVVT
SVTAMLRELWPTAELHPMSTTAAGLPPLKDMTLHFYAKNTVVTPDAKQYTQMHLQSITNNAGFQLEFCYDY
RNVMCLLLTDSRSGLRMNVRYGAEACRVEVASRLFASAVASNEACRQTFLLLDALLRQNKILDDTGTNPEA
LNSEAVATMLVAIFNSYDANDTPDTGRLLMDFFLTYGFCANFDMFSHSVTARGLREPTSKVHPNAQISVLD
PSNEGHNLTYRLEKAAHLQAVFNYCYTAISQFAQVEERQLRAQSVLSTIIGGESYWSRVLQLYHEGISPYF
EVVNEKKIFLIRAL
>tr|C9ZX83|C9ZX83_TRYB9 | DeepTMHMM Topology Prediction - Predicted Type: TM
MEFQSDDGRKSCATMSSAGAGCGLQTRRRVGLAAVRFGLCGVFNVVVVLCVYPCYIIFSSFPFFSNLTTPL
NDALWVVYSILLFFFRCLLVNNVMPCSVFLLFLP
>tr|C9ZPN8|C9ZPN8_TRYB9 | DeepTMHMM Topology Prediction - Predicted Type: TM
MRCRLCGRVYLPLICFFFFENKMKWKDETKKRILQTDDKQQNNNGSCSQTQKNTINKRMFKCVRVCERERG
GEMEKVNVRRKNVNGVMSKGIMNASMRGFIIIIIIYLLIYFGCND

>tr|D0AAC2|D0AAC2_TRYB9 | DeepTMHMM Topology Prediction - Predicted Type: TM
MPPRSRKGTKSRSASPAPRTPGRTKKSPVTPRTKTPVTNKNRSYEWGGPLGALGMVVLLPLTVIGLNVLCS
ETSCSVHNVWDLPAMMLLALDVGVPRLLLALGVELLWMAFHALLYITPVGKQVKGVKLSDGTCLTYNINAL
HVFTLVHAILGGMHYADVIRLAWLADMFMPLMVAAIIISVFMSIVLYVASFRSSQVSFSPGGNTGNYLYDF
WVGRELNPRTGSLDWKFMCELRPGLIGWSVLNWAFVAKAMEVGTCSPSIIVVALLESFYVLDGLLYEEGNL
TMMDIVHDGFGFMLCFGDLAWVPFTYTLKAKFLAYHASQLSYMHVGICAAVALVGYAVFRGSNNQKSRFRQ
NPKDPANAALKVMHTSSGKSLIVSGYWGVCRHPNYVGDWLMTLSWSALTGFTEPLPYFQPVYFALLLIHRQ
LRDEEQMREKYGAEDMHKFHRIVRYRLIPYVY
>tr|C9ZPE8|C9ZPE8_TRYB9 | DeepTMHMM Topology Prediction - Predicted Type: TM
MYSFCLPCCISFICIIPFLPNFVIIITLYDTILFCVHMLVLVQRTQIRSRCSGWHQRNNSEVVGRNILDKT
LVRKNKNAVRRLGLKHLGTIKKKGKGQHGNKDAK
>tr|C9ZTQ7|C9ZTQ7_TRYB9 | DeepTMHMM Topology Prediction - Predicted Type: TM
MERPADKPQSFLFFFVCAHLILLKNHRVRETSKKKNSSNPNRRFINGPHVLLFSSPYYNFLCCRCCCFFLY
ALGKRVISGIVCVFFCLFAVSFFFSHINTNLLVILCACFVGLSHIHARKGDIIFLNLKYGCFIVVFFFNHM
LTPHRRHVLPLYLLSIFFSFLKNHAKYLLTMFV
>tr|C9ZI33|C9ZI33_TRYB9 | DeepTMHMM Topology Prediction - Predicted Type: TM
MNSSEENRRPCTSNYSFIYLFFPFHTIISLFLLRREQKKKRKKSFPLKSLIIRILIIIIMEPALSVLH
>tr|C9ZUG8|C9ZUG8_TRYB9 | DeepTMHMM Topology Prediction - Predicted Type: TM
MTNAKCSTCLCPTRVGDDDEYARWLRGPYWVENQDLAGAAGRISRLLLLSPAFVWESVTEDILALTVSYNN
LTNSMSYARPLSTLLRLCLVFVSTLDGNALVMRSIIRFSVLTVESFPYQAPHDHDSGMFTNFHPLLLIFIF
CNHHVAKWI
>tr|C9ZQ44|C9ZQ44_TRYB9 | DeepTMHMM Topology Prediction - Predicted Type: TM
MGTEISMTACGFARMVVLFTALLQCLSGSSHLFAFGKVYPTAYVKEYILFPNRKFWHGGDYLVDYSNSVHI
CREAGGTLATDHSQATNECLTAYHLSVADGPFIYSYLGGDATYSASAAQDAGSRCTAGDYDTSLNCVYRWN
SGLFEASGIEGNGVAFWRGSYTYKSGSGALGGYPSFFAQSYPQDGQLHVMGWYTNVTKWFGWFDGGEVSGN
KPGGKAPGYKSLTYFAALCEVTVPQPSNPVPGNGNSPANEDEVSGNGNSTANEELAVINESLHSDEESTNS
QDQPSATPKPKGEAETTLTSLSHITICVGGELCLLIVFFSLAIVC
>tr|D0A3H9|D0A3H9_TRYB9 | DeepTMHMM Topology Prediction - Predicted Type: TM
MATLNVRCPTCLNGVVYAEELWLVCCCIAHCCEQDGGVAPSTVFFCKLSWVRPLPVRKCSLLPFELSSTRS
CGLCTQLDALFCGVIILPLKVVMHNAICQEFGVYITALFSEFERSSVFFSSEHGVFCGEICVSGCVVSVGA
>tr|C9ZXY2|C9ZXY2_TRYB9 | DeepTMHMM Topology Prediction - Predicted Type: TM
MAVDRIGNEEGTTKRKGHGLLCLPAGVIKEVLSFLPATITVRVMTFVAPRFAYITRSDIHWMSVWNAYINE
CTSRYSQSGPLSHRKGRLEMLCANGKSYSGPIILLASVPKRALPNTRVTSRGSGKIALVEWRDDPNSEKEG
EKCDCVVHLILPIRSLPSLSGSPVIASTERMAALRISRVEDREGNEVDHPNDGEHNSLGVGVCDTMLLRKA
LCFLHGNNSNMLFIRPMIDGLHAVRLLFFSSKQSSLDRREGTTEEQEATIVEFINAVAYALECCTSRKALA
PAGTARLATLYLETGSESVFTIGNDAATVAENAIAVEAAETASGGSNDGIFFSLFDSQPSEKPEAGANGLR
YSSVSTQVYLFLLQHVSFSDVHVHVLENERKCAVCSRVLSPTGRAVEFRFFYSLFLGGYPRFFAKMVFAEA
DLPRTRIQQVFERNCSRYVPTSAADYSSNEEDENEEETLPMLCNSFCGGYGRVEVDIKPTISRDGFERLRE
ALGLSAAVLPMPMLWNVVMLATGVGGVVLREQQSCFASYYRTSFAAAFEQEFESS
>tr|C9ZVM4|C9ZVM4_TRYB9 | DeepTMHMM Topology Prediction - Predicted Type: TM
MKYFICHYYYYYYYTLSFVTQLPRATRSTTLHCIELLFSTLFSFFLSFLFLAFSYCSNIIYVIDTVTSIIS
VGDTLLLRGEIMFYYYYCCCCCSFPFSYFILFFFSGSTSGCYEKAGENTECRDVEGAFEHRFFFLKKKV
>tr|C9ZRN2|C9ZRN2_TRYB9 | DeepTMHMM Topology Prediction - Predicted Type: TM
MIEWCKEKCSPSIRLFIYVRLSFFFLKGFFSVFPPDRIRSEVLDRFVFSFYGKKQKKGGRENEAERNGTSR
PQEMASMQIIVSLPFFSVAVFVLRRGKFFFFFFFNCLWLFRPLYSFLSFKTTVLHHFFFYSPFSFRAKVSP
GVVLSSSCVSLYIRGQNEGNYVFSLRH
>tr|C9ZTC8|C9ZTC8_TRYB9 | DeepTMHMM Topology Prediction - Predicted Type: TM

MVLLLVRFLTVIVVNSHAGVTLLLSPQRQSLGLLWDMVVVSLLMLAVQTLVYHYSSADILSWMRPCWSFPH
AFYSLVTYYSLVNKYLYIGVHPTVFVLNCSCLVTIKYVMLN
>tr|D0A8Y8|D0A8Y8_TRYB9 | DeepTMHMM Topology Prediction - Predicted
Type: TM
METGMMRCRWLKWMCPIPVDSFFCMGDFVQLLLAEDGPLLTKGFTSLRSRPIYVMVLFVTHFNEEVEAVLM
KPFSQRFFMGGGVWVPVLLLHTCMFSASGCVCGFISCYFYPVFLFAVSTCVCVCVDTTSDIP
>tr|C9ZZZ3|C9ZZZ3_TRYB9 | DeepTMHMM Topology Prediction - Predicted
Type: TM
MQSQPDNVAYPMELQAVNKDGTVEVRVQGNDDSSNRKHEVAEAQEEVPGGINFWAPRELRLNYRDYVAEFL
GNFVLIYIAKGAVITSLLVPDLGLLGLTIGIGVAVTMALYVSLGISGGHLNSAVTVGNAVFGDFPWRKVPG
YIAAQMLGAFLGAACAYGVFADLLKAHGGGELTAFGEKGTAWVFAMYPRDSNGIFSCIFGEFICTAMLLFC
VCGIFDPNNSPAKGHEPLAVGALVFAMVNNFGLASPLAMNPSLDFGPRVFGAILLGGEVFSHANYYFWVPL
VVPFFGAILGLFLYKYFLPH
>tr|D0A6J8|D0A6J8_TRYB9 | DeepTMHMM Topology Prediction - Predicted
Type: TM
MYKQRKIRDKKESVRGLKKKKENTSLTSLNSTTLSFLFPSSSLIFFSILTQQHHHVVPKTIITISISIVPT
IINNNIAFITHIICLLSRPSHFIPFSSSSLLVFFPFFFLFSISFPPPPA
>tr|C9ZKV9|C9ZKV9_TRYB9 | DeepTMHMM Topology Prediction - Predicted
Type: TM
MAERRQPRVLGAQRTVSEVLGPKPVDISDIRLLIDRCVGGAVDSTPTLGELERVLSYFARHNSGYIRAGVD
DLHILASEILNNNTRAVMVFGEFGSSFGDVEMYLISVVVQYNMSLEAITINAVNVSDDAVSMLCEALTKSR
VSFIDLSNTPLESEAGHSLAALAHVNPYVRTIILDDTLVAEEVLDEIDVACQFNQSNFEANGGVVDPGGTA
EVARIRHRIRNIIRAKHKKIIYCVPHVLGTCPDGDMCMFSHTPMTSGAPDTNANLHERIVDVFAVGGGLNK
LPAPPKAGASWKNPEELGEHTLRLNLEKCRPGKASRKEVSDDGGMLRKSLMITVIPVALVAASVCALRLWK
R
>tr|D0A238|D0A238_TRYB9 | DeepTMHMM Topology Prediction - Predicted
Type: TM
MECFCNHNPWTAALSSVTKKVARFSTNSCFVFFFSVLFFLFHLKNEYITVFNPHILLSMCPSLFAYTKLAV
LVHLVKLCVTLTWRFCHFFFSPFPFNYGTCHLRVRFIKVQHTFARRNKRDFIMRSLRSICASEPRCSRFNT
HSFRLRAAGTSPGAGVSASRTTDCVTALFDVGVGSVEEIFSLPAPQLQKNCGDVVGSVFRKELKNARVGNQ
DFSEVLWMATTRGELLSDIRKLFRHAGVEEAYADPNVTTHPSGAPIGIRDEHNGWYSQQPHYSPPVFPATA
AVSPRSPWAAGMDVFSYAFAYGFWQQRRESLQLAHHQMQKLSSDIQYVKEALDGDHRNRLRLSLRLWCMFL
FPFSLLVLLLLSTDQILYVAELKRVSLCDYDAWQRRYPRGR
>tr|D0A6M0|D0A6M0_TRYB9 | DeepTMHMM Topology Prediction - Predicted
Type: TM
MDTVLMLSAYSQLSLCRTKAMNLSNYEILGAGINTMVYIMSYRGYNIEDAKVYTTAVRSIVAMGGVLFFVS
MRWNWKDFPTVSMYDIILPTD
>tr|D0A7W8|D0A7W8_TRYB9 | DeepTMHMM Topology Prediction - Predicted
Type: TM
MATEQHQQQRQKMLNSKDLENALDYIATFGRPHKQDETLPLFNYIFDLKDKLIGRMSSHEDVRMFVESDAG
RRMSEGLVKALDADGDGHVTPRDLQTIYESKLKGAIRNNSDTLDTVIPWAGQWLVGIGSGYLTGRVIRRVY
ARKYFITLAGATVYTGLQFLAQRNFIEKQLLEAAFKRKVKELADANGDGVVNADDLSYLVENRMRHVSTKL
GFGGVAPGVLGYLALAVGMRRGLRRV
>tr|C9ZJ23|C9ZJ23_TRYB9 | DeepTMHMM Topology Prediction - Predicted
Type: TM
MIPTCIAIRLTPVWICLLVHFADGKWPTPFQCLARVQDYGFSVSAFKRNPLVLVTHMFVHASDEHFVGNVM
LLLCTLLELGEDCFQIGVADRGMWKSIKEAAGATAVLIIGGVSGVAGQLLFDYAQLKWKRWKGMFSVKSLI
GFFHEEEEGIFSTLKSQFDSWVGSVTYSMGKHRVDTSFMCGASAGGYAIHGFGAAYRGTWITVWMAAALEV
FKLATATLGGKPTSGSSGTGWGWWWGPGETVGHAAHLGGLGAGVLMGLGWRWWREHRRRHRFRRTLYT
>tr|C9ZRE7|C9ZRE7_TRYB9 | DeepTMHMM Topology Prediction - Predicted
Type: TM
MPSSHFFSSSHYKTGWSLLVFAFFPISFSFLIFCCSRGTSSILMIVSTCVNAPDDLLRVLVYVRCLTFPIP
AHMQTHVCFSVKYLFHFMISLFIVWAFIQFSISFHKDMFFFSLWGYIFIFIFICFAFFFPSSCTFENVLFS
LFPFFFLLLSRLTFSFTPPMRVLLSVIPFPHLCLAAQCHYK
>tr|C9ZX18|C9ZX18_TRYB9 | DeepTMHMM Topology Prediction - Predicted
Type: TM
MHCTTQHTLSPVSAAFLYLFPFIGVEFFSLPLFSTLLLHFFICGHKTYDNFACHNDNVCEYVKCHPHNPLI
FVLNYFLSSVRLFLYLVVIIVPLPSFRNHTK

>tr|C9ZZT9|C9ZZT9_TRYB9 | DeepTMHMM Topology Prediction - Predicted Type: TM
MFSYDDLVWTISFPTFCFLAIWFSITVSLVVRAESGRKKWKQAREHIAEIESRGDKATGAQHRNSKAVLRR
VVVVVGGDFARSPRMQYHALSLAKCGMFQEVVLVGFDMGNRLSEELKLREKKKNIDGSYDDSSVEKFECVV
ETAYLIPPITPPLWFRILFPHPKLHWLASTFYRACACAVVFTWVLVRASMMFVNSRGQLMLVDLILMQSPP
AVPFVPVVKYIVQPCAFVANAVTYYCIILTGSWLSTALKEITEGIKLQRQRQLPVEKNPMKKDPTNSNSCP
KFVLSPALVVDWHNFGYTVLRNDGRPAVAVWLYRLLECNLCFGDRNITVSKAMRRALLDVSKQSKVANRVD
DVWVMYDSAPSFFGLVPRSRFVQEVIRPVMSAHSQDGEEVIGCSLPPDWVLQSTAATDSRGIFIVASTSWT
PDDDYTMVVEALKQVDEKLQECSQGKDSKPTAAKSVWLLVTGKGVARKRFEMAVAEAHLSSLVVVTTMYMQ
SYKHYAMALGAADVGLCMHNSSSGLDLPMKAVDMLGSGLPVVALRYKSLHELLDDKRGWFFSNAEELGQVM
WKQLILTNGPLLEKRRQVAQNGPGTWDENWGEVLMPLLTNLL
>tr|D0A788|D0A788_TRYB9 | DeepTMHMM Topology Prediction - Predicted Type: TM
MLRRLNLRVTSCIALKHEAAVVPILMTGTQLFHKGQLQLCTEVKETTPFCQQQRCFHTQPVRHMRVAGSIK
PQTSPMNFDGSSGNSGNTADSLTPYVREHLFKVYGLLAAGCVAAGFGSVLMFATPLCKTVPFWLPMAAGFV
PLLWLSFAPPQNPNLKMGLFFAFTVLEGMALAPLIASSMAKGVLGTAIVLTGAVFCGFSAGAYLAPRASLL
ALQGPLFGMLLGMVAISVLNLFYPTAFVHSIILYGGLALFSVMVSVDTQAMIERARCGAGDVVQDALQMFM
NVVNIFVRIAQILGSGDR
>tr|C9ZZC9|C9ZZC9_TRYB9 | DeepTMHMM Topology Prediction - Predicted Type: TM
MLASSLIGKLSAAKNKKTTPIVTWSCVAFCCTSILLFMLPDNVVFSPFVTSFILSFAFLRFFFIHFFLFVV
LPLRILKKDKEKKEGENTHCLCGAYYSLSDNSVFCVLRSLSPPLICFSNICLCHRLCYSHTYIYSVAGSAV
CSARLEERKHTVRSSSETVSCKCKQ
>tr|D0A124|D0A124_TRYB9 | DeepTMHMM Topology Prediction - Predicted Type: TM
MSADGSGELPPVMLHRYPVTLGLPTADPYALAIECMLRIVGAHYKKRDGGLPVTLEVPVDGRNKPAMKRHE
GLTSCLQLIESATHQDAEVITSALEPEALCVRLTAEVVLIPAFVYITHSDAGILRNAIQKAVEPKVASMWQ
RFRGSYSRHVVQQTSFCRSYKNMGEAIENVERCLRAIEAICSINTAGNGMFILGTERPCSADALIYAAASS
FFHADFASRQASGDIIAMQQRLREGCPTLLTYVERLRGLYFEDYSAFYHLRAPTCGMEGEATQKAAEETFV
RGRWKVLAFTGVFSITYFLVANIDVFSAVLLHYLEDDGEEEFLEDEE
>tr|C9ZTE4|C9ZTE4_TRYB9 | DeepTMHMM Topology Prediction - Predicted Type: TM

MELMYNMICTLCVYAVGCGMNCLGRYLALCSGSISVVVPTGSYVLFTVFSTVTKFLPRTLEV
HPRRFVEYASTPLIISVYLPSFLYIFLYGMTIRIRYQKE

>tr|D0A2N3|D0A2N3_TRYB9 | DeepTMHMM Topology Prediction - Predicted Type: TM
MPWYLSTFRVSFCAIYSYFAYAQMRWIVDAEERYRRSLTWKPAVGTIYDHKIVMKRGGSSHVQYRFDVNGK
EYLGDRFRSGGVHKEEMVSNPALLGAGTQLVVYYNPSDPSESAIKLQTDRAAESAFLVGITISLLVAYRSV
RCETILPNMFYSFLAVNRRLGGITGLREARTHSKQKMKYGKGTGAI
>tr|C9ZVS3|C9ZVS3_TRYB9 | DeepTMHMM Topology Prediction - Predicted Type: TM
MQVAMPVSLVDSCLGMQELLVLVYSCVWQSLLPLYELARFFSFPFQSSSYQAAHTHTHTHTHVHTQQTHLL
NMLSPTEPLGSGKAHTEVVTDEGEGYGAMSAAEEKSHHKNGDTPTTDSKFMQCINAIIPHGGALSTTFNLG
SATLGAGVISLAIAFQMSGVIPSILILITVTVLTIYSVGLMMQAVEMTGYNSYADLSRNLFGPGWDYFTIS
VSWLFTFGTCVSYVIATGYLVDSVLSGSSALEFFQGKTGNRVITSIIWFVGMFSLSLPKEINSLRYASAIA
VLFVFYFVICIVVHSAKNGLKDGKLPEDVEMFKSGNRAIEGLSIFMFSYLCHMNCFSIYSEMRKPSARRMT
LHTTYSMSMCCVVYIIAGFFGYTDVGNKSVETVFEIYDVKGDVMMAIAFAGMLLKICVGFSLCMQPARDCC
YYIIGWDLNTLETWKNCLFCGCMALCALLLGLFIPDLNTVFGLLGSFCGGVLGFCIPALYRMYCGNWGISQ
VGVVNYVCTYLLLISGVIAVVFGTAASIYNVAV
>tr|C9ZW89|C9ZW89_TRYB9 | DeepTMHMM Topology Prediction - Predicted Type: TM
MKATSAMCYSTFLPTRGTQTWSERKNDGYITFSTQRCISSIVVVFLFLLLFDILRCFVFPSFTYSHLLLLL
LLLLFLFSRRFYFNFCRNITTSHNWSWLG
>tr|D0A148|D0A148_TRYB9 | DeepTMHMM Topology Prediction - Predicted Type: TM

MTRTSRSTHEAVWYFTYIMVQRGGIVVLTMGLSLVVFCSVTTVRSVIPMLVEPGTLGFMILYGLCCILSFG
ILFNYVAVACLCGPNIDPKETKRLTEQAEALPPQERCRLLDAPRRFCHICERLKAPREYHCRICGRCIARR
DHHCPWINNCVQAENNRYFLMMVLYLLLSTGFVSSMLCAIYTHSVWHGSTVAARDVKRSTSHQFCKSGVYS
SPILLLFGVCTITFFILLFLFGAATLAALRNETAVESFIVAQKQSAFQGSLVPFRNPYDLGRQRNLLALFE
TKGDPLITHLRRGGRISNIWIGVWLLLPTLRPSAFDGMHYVVFDDVVHAV
>tr|C9ZWQ0|C9ZWQ0_TRYB9 | DeepTMHMM Topology Prediction - Predicted
Type: TM
MVPNGGEAAGASREHTQGQNAGSCALNVATNGKWEPYGYNDREREQSHLNSSRGAPLQVRVVVQLPNGTVR
EWKDFAQLQALPQILREVYTTTPDQGRSSAGSLSPLKFFWIDMSGMPQREDLSALFELLHVCKHTERRWRA
MAAAPPGVGLGGFPLNNGTCEEGAERSHYDEDDYYDDATELDYLRAFVAEQYVQISLTVLESPNVTVYGLP
MEKDTEREMGTEKHQRQREGSRPNLLDACESGTLASVQVLCFVNGVVTWRPQPAVQGWEYIPHGLERRLSN
STNIGDGRAVGDGNEEPRGALTTSLLVLTILDELYMAFLPDTTIVLSEVDTIDSMLPLVRQRMSDQADILR
RIQMLRRSLSVHRRVLMSKVSVLELLSRPTVRVLLPFMNPITDAVNEKEREHCREPFDPRVYQPRHRHGEL
HYEQSEARARRAPSSGQQSDTYADIACRILHVLSKLEDARRILTNSIIIHSSGVAAVNNYNSNQSDTLSIT
LGYVALMSLPPTIVASQWGMNVYVPWVDMNSTAPFWGIVGAVTAYAALVLSYPIFCWIRGRPDKLVF
>tr|D0A3E0|D0A3E0_TRYB9 | DeepTMHMM Topology Prediction - Predicted
Type: TM
MSVFYPPLIPSEHYFQETIKDKTGAEAMPYACLHGGSLCLLISLLSCLGAQAVQTLHLVQLVHRHGARSPK
VKHNQSQICGEVPCGYLNAAGKMMLINAGEFLRNHYNSNASEPFFPEESYNSCVTYSRSTDVPRTLQSAGC
LLRGMFPNASEFFPAIHTADVSTDWLLRYDVIPQAYAFSHLDEHWWRNVCNPKLDTLIDTNTLLSVSREVF
SEGFCADPQNRCHCAATLFDIGVAMQSDGRIDKHPLLRENLGRLRDIKFFEDSHRFVYNASDRTHAKMGSL
GQHLAQEILKNAENHMNGLTSYKLYHYSAHDTTIAPLAATLGDSTTTGITPPYGQLYAFELLYDHEVKGYI
IRIRRGAPGQKPEEGYAFSWGEFQMKCMNENHTVYTVEDNKCPYHDFRRFVKSTKPHDPAGLCYLNRRYRE
LFHCPGNVGKPPNKQCKVFRRVCPAWSCEEGYTLNSVTLECVCSSRKCMESRDLSISTDTLGRNLFTIFIF
VLLALLIPIFSVFVCIGKKLRARTRRTRAKG
>tr|D0A1S7|D0A1S7_TRYB9 | DeepTMHMM Topology Prediction - Predicted
Type: TM
MFLRSDQHRGVRERVAGGAKTTGPECPKLSPGRNALENNHEFSAFVNRMKAINYSFATKKKRGTEVEIITI
PNIVCDGEASEEELAEHFEAESRWLDCCLREFTHRRGRYGSVSFLLVRIIIVLTFLLPALWYFWPLWEVLI
DVESELHYSILGIPQGSDARAIKGAYREAVRRWHPDRNPNCDSCRVHMMKIQHAHDVLLAKGSERYELVDR
YGEELAQLRSLVFFRLYNIAFYAAQDIYYLIQAFNGDAHINGSKEFSWFLQVMCRTLTMGVFTVYDTLFIS
GFRVIVLLQVLFYCVSCAKSSAEEWEIIGMVKRSYVDLYREAMFFAGGPLILHCLQMYQSGRILCWADAFE
FFLQLAFGVIYVLSHLYHMTPNLLDNIFMKKCSIPLAYIKLPTRRLSYLNFICTEFGLLLDDLFAFSCRVP
SVYRLTVIVVHTVFLCELLWFPWEPPILSVPDRKDKGKNVHREEEGKHTDMAPHREVTGEPPRAISSEEMT
LLKGVDNEAVNWFDVVSTRFAKQMRSAIHRCAQQRRAYINFDLVPTVNSQEVAFVAVTREHPRAPSKVDVL
FRVRDEFANRMLTTLRPPLEHILGVRLTDATHSEIVSRHARLWEENKRKNPSDTWRRRCTDSEVKVSFDLP
MLSATVISTIVLLYHFL
>tr|C9ZN88|C9ZN88_TRYB9 | DeepTMHMM Topology Prediction - Predicted
Type: TM
MLGFDSANEFIVYVTFLFFGMSVVVVTNSIFSMPFFFIEYYKYAQGKPDAKPEDPKFWKHMFTYYSIAAFL
VELVLASLMLTPIGRRISVTVRLGVGLVIPIVLVFSVMMVTIVTTTETGAKVTIMLIAIANGVAMTLCDAG
NAALIAPFPTKFYSSVVWGIAVCGVVTSFFSIVIKASMGGGYHNMLIQSRIYFGLVMFMQVISCALLVLLR
KNPYAQKYAAEFRYAARKGIDDKGADGDEGNGAAKGPADQDDDPHGGDDTDKGNVMTATVDPDTMKDMDQV
ENITTSQQMLMARVWNVFWRVWPMLFACFMVFFTTFLVYPAVYFAIKADTGDGWYLTIAAALFNLGDFLSR
LCLQFKALHVSPRWVLIGTFARMLLIIPLVLCVRSIITGPWLPYILVHAWGFTYGYYGGISQIYAPRTGSL
TTAGERSLAANWTIISLLGGIFVGAMFALAVNEGLSK
>tr|D0A339|D0A339_TRYB9 | DeepTMHMM Topology Prediction - Predicted
Type: TM
MPPVMVLARDCLSVFFLFLHLSFHPSFFVHFYLYSFPFSLFVRCCNFILFHIPLSIGVASVHLYSFLRMCR
LHCSLFLLAKQPFCAAFKMGPHPSKPDRRMGSGDMGAPVEMPARL
>tr|C9ZRM9|C9ZRM9_TRYB9 | DeepTMHMM Topology Prediction - Predicted
Type: TM
MMHFGVNVGDFIFPVVSSYLISLFFFFLHLLLISLYNVLRHSQAANAEGYKAQVAHINTGHRQEYHQRGLV
RGDLFLFGPLYFLLSLERGTSAVEGERKRGEKKRSPIVKTKASG
>tr|D0A167|D0A167_TRYB9 | DeepTMHMM Topology Prediction - Predicted
Type: TM
MRVHICTRLKQVCDERQGKSEKKKNYVNVLHTTLFSPSFLTKKKLENEGRETEPIPNLRCICNFISLYLFS
CFFPVDFPPFFTFSKYIYIYIYIFFFSNLRVDRCCSFVCVHVCPKLLIVCWISVVTRVRYVITVIVFRFVG

FFYLTVAVGGRLNTNLCNQPHFETFPALLCSLFLVVLLCFVLVAMVLRFVWFAFSVSDGLSWCLLQVTLCI
YV
>tr|C9ZNR4|C9ZNR4_TRYB9 | DeepTMHMM Topology Prediction - Predicted
Type: TM
MCWFTYSHPPTCTHDEVVRVKVFVWSLFLTIPHHNPSPFYSLFFLSRSWPMCVFMLCVIYKYICGSSPQRG
HRVQPIEMMRRVLLRCPRPRRNSPLAFQSTSSPVTKCPLSPKLQRPLQTPTVVGESRGCPMIGTAPHLSCP
IEKEQLGQSVQVRDVVTQLGKLKLSAFVTATALGGYVICGGTSPLVMVAVTVGTLLQCCSANTANQIIEVE
YDRMMKRTCRRPLPMGLISRRSATILCAVELLSGSCILGSVSPAASALGAFNWLLYVAAYTPLKRVSAINT
WVGSIVGGIPPLMGGIAATGTITGPAYLLGSLLLVWQIPHFMGLSFHCRRDYEAAGYKMLAFYNPWRASFY
AVLLSVMMAFITLAGPALINMAAEGWYYPVVAAANAVMIYKALLFHSDPKRHCRGCFVFSYMYLSVVLAVL
MLNHLEPVKRTTTLFQHFTAVAL
>tr|D0A3B1|D0A3B1_TRYB9 | DeepTMHMM Topology Prediction - Predicted
Type: TM
MMFVFALTVWHCRTGQANVYVLCFCLYVHIRFVYLTLYICLCSLLYTCLFAACTILLLQERTTVAASVITP
TALPHFSVLCVVSKLAKVLAVRVINNESVAIATTVSFRIDTRRKVKGRVLH
>tr|D0A287|D0A287_TRYB9 | DeepTMHMM Topology Prediction - Predicted
Type: TM
MSGSIVESGKVPPCIKGGSLLFLFFGGGHMELYHNLSLFPRPPPPCVPANPTGLFGITVLVNFPLCAFTFM
QMSKKKNEGAHLFYVHVYISLYKRRCAYVVLCALIYTHVYIY
>tr|C9ZVK6|C9ZVK6_TRYB9 | DeepTMHMM Topology Prediction - Predicted
Type: TM
MQKHGFVNHCSNLAFYRPISVITDLHARLNIIIIIMQNLHNNSAEQKKKEAWIALLNSKMSTHMCKTIFLF
ACVCHMSLYSLLSVIFSFFHLHGRRGCGKLSCTSLVVLACKGRHRKLEVGTLAKPKGKLIYRDFLQRVGAQ
RKTTDILP
>tr|D0A831|D0A831_TRYB9 | DeepTMHMM Topology Prediction - Predicted
Type: TM
MYKCINIYIYIYIYICINNIYLVSTPFLKGTMELSLIHYYLCRKTTIHARAYIYIFNLVCWITLPTQNLDK
YPNCPMKMRKPSCFPSHIFQSYCLLSLF
>tr|C9ZZK9|C9ZZK9_TRYB9 | DeepTMHMM Topology Prediction - Predicted
Type: TM
MRPLRAWFAMCLVAILLFTVVDVSALYVRGKATRAIFIAVIASSVWICYVLLLFIFLYRPLRRCTALYRTG
CCCRNGSSAANNGVRLWFKEQHSLDVCVEELRNGMVDIEGLKRT
>tr|D0A889|D0A889_TRYB9 | DeepTMHMM Topology Prediction - Predicted
Type: TM
MANVNNETTECGALLSNITSLQTTLTSMGCQVPALHHHHHDDHTHGVVDDGIDGDTHGGCESGHGTYSIGL
HVVAIFVVLIASFLGTLIPIIGKYVPALRLPPFALVLGKCIAAGVLLSVSTIHMINESILQLQEDCVPESF
RESYEAYAFLFAVAGALLLHMVDVLSGFCVRRDEMVYSDTSAVVPHEDIDAEEAQAAPAALDAYDGHHCHY
AVGMPQSRTKRLFSAMFMEFAVTVHSVFVGWQSVSQEMRRPKLYLALVFHQMLEGLALGARLVDAELSLKL
EMLFALLFSVSAPLGTAIAVGTIAIWNVSMVGTAFVITQAVTSAVCGGMLLYLAFCLMLSDFPSDMQKHAG
KDKVRRFFRCFGMFAALWLGAALMACIGKWI
>tr|D0A3Z8|D0A3Z8_TRYB9 | DeepTMHMM Topology Prediction - Predicted
Type: TM
MHFSLFPSKHSTMQTYCYFSVFPFHFIVSCKPRCFCFCFVLFCFVFFSFLLLSFVGRMISVPSSPTLLIFR
ILFFFFFSLSSFDCSHGSYLYILRIAVQMNIEG
>tr|D0A3U7|D0A3U7_TRYB9 | DeepTMHMM Topology Prediction - Predicted
Type: TM
MIGCIPLLPGAQNRIVHAACGAPLEGKSGNDVSNIGSLLSPLLLYSCASAYRETYLHSFFCGLIVAFPFHS
SWPSASCAVLSPHMHVGLAYNGVFDPIFFCF
>tr|C9ZJK5|C9ZJK5_TRYB9 | DeepTMHMM Topology Prediction - Predicted
Type: TM
MISMNLKELLTFLNICFFEVYGRRRRFLFVDSSGISVKAAWFSYVVKYSMMQSGDSCNQLFGALLLSRASG
LNEVYFYEESSRLLVGFLVCRLFGAVDGVVGGVSCCYRKRMYLHQAVKLLKEWANEAYFGFFWSERCKSTF
SHCCLHGVESILLSCGVLAKGDCAFLAQLISNVVIQKKNIYFLTSDAHMLEVSQCLLSSVETQLSEAYVED
GVFISVNLPIESRMLLEGDCREGGILLFSCSLEMRGAVLEDFLTLVGSIGVSFLGSQRLMCCELKELLFDM
GIVSLERLSLCVCDAIQHATGATPFHTEHEFCAWAEYAGSAKRGFFKVGMVRRATLSREGGLWIYGKNSVA
TIVIPVGDPITKLALLEVCEKAAQYAYEVYSGSMYTNGGVVGICCLFLAEYIWRRLPRHGSQNAYLRHFAG
AIHAVLVSHAICLNPSLVEPPATYVQEVEKIAEDHAKLMSSMSYANSVVGQYRSCRSYVSSGHMVDCEEFR
RSVRAVELALDCVSLISKLKL

>tr|C9ZU85|C9ZU85_TRYB9 | DeepTMHMM Topology Prediction - Predicted
Type: TM
MILSGSRTSPFFLFFCFFFSFLSRLSVCLFFHYEFSLFFSSSSPPPCVFFFTFLKLFTRDTYVPGDLFNVA
PQPFLLLLIRTRRHTLLFPPIFSLIFPLYHTRGVHLHHLLLLSLPLLLL
>tr|C9ZTY7|C9ZTY7_TRYB9 | DeepTMHMM Topology Prediction - Predicted
Type: TM
MLAIIFIFDEKESSTLSHAYPIFTSCLHFNTLLFAITLNITIAAISAVQSIHFRVKNWHHQLLMLILSSTR
TVRYQNSIISHRILFCLVGFSITIWADNVASSFILYNELCFFLMLPTIISLCMLVPVVMFVTVNVNITYPF
LPLSSLNNIHFALCPSITYTYSFTHSHSSIVICCTRPLF
>tr|C9ZTB3|C9ZTB3_TRYB9 | DeepTMHMM Topology Prediction - Predicted
Type: TM
MSLLCNISIYILMVLPIILLSRGYNMRPQRLLPLVAVIALSLIAQSTIITSLTGSGLFLNCLQQMFKVLAV
PVMDAALIDFALNDPKARKSLQVMGGGDDASAAVAVAWTAVDVALYRWFNWYHVMGQPGFDEENLRSAVEA
FVNIVSLLIAARLIEGRVQGNEHNSNKVNSTHNVSSGVWIWVAVVRVMGNGVAHFYGNNLIGSALNVVALL
CIRCFLGSKTGAKI
>tr|D0A2C5|D0A2C5_TRYB9 | DeepTMHMM Topology Prediction - Predicted
Type: TM
MLLPYIKYINIFASIFHMPCIRSRFFDNPRRWKQNNRVQRKKKWSRNGAVLKQPVPHFVSRVRSGILLFSD
EVPGGGGGKGWEAFFWWRFFLFFLVCVWNGEWG
>tr|C9ZTI4|C9ZTI4_TRYB9 | DeepTMHMM Topology Prediction - Predicted
Type: TM
MLRVIVVIINSFRFVCFLVLLMVLIYKLLIIINFFFRGFVFSLCLFSRLPSFYFLSVSTRLNVTFTFSTFC
VFFCFLFLLLTKKKQKMSNNNTHKKKEK
>tr|C9ZYX7|C9ZYX7_TRYB9 | DeepTMHMM Topology Prediction - Predicted
Type: TM
MTTLKGGGDKTYRENCPPASGDNFVIDDIGSSPARDLPPAHNSVGTKLGNDSFGLPLYVPLQDSDRPGKNS
SLTVPWSLRKSDGRKEVRVLEFLRSPQFWRQVEFALRVTLIAVFPSVGLVVGFIPLNILGTSTSILSAIVL
ASKVTVGEMIAFIFTWLRAGCIWLPFATCGVALGLGNHIGVWCAYYTLVLFVIATFTENMVRRVCLLLFNI
CMIGLLVKPDSSLVYPSRVMADWCIGTLLCALAVFVPYPIFSKTRAQKALCEIANCTGAAFTGMTSCFWSP
SNVERNMSMTKVRMLIATVDEALQTFYLEQDHSFYEFLFDSGDARRARWFKAQLFERLRTNLIALSQVLDM
VEGRPWVIDESERSLAFGQHLSPHIKDVAASVDKLMDALTSAHTIKAVSDLDELFTDVSAATRKLQHEFNA
ARLDLFYQHRPETLEEFVPLMTYFMFTIINSHDTISQFGCDMSKVEVSRMSSAKVVVAKVVWEPFKEEIEY
VLKLFRTFRRREIQRLIEAAKVSAAMIATVGFSLLIGVDKESLSGPSIIAFVSGSNPVEAVQASVVRLTAC
ILGTVIGFFAGTYSSTPTDKIISVCVLMFIGTFFRTDKDYGILVVYAMFVLIPLNTMESTTTEDTLSRMNQ
ITFGILIYIIISAAVFPLSPSLILRKKRINILIRFGEAVTKLCGLFSKPLTTDLLADGSNDPTSNAPVGND
SVLRSNISASRAFTLSFSERLIVSTDSCMEEIDALLNETSRRLKRTVPFEGFARKERGLLFVHYPTKACER
TAFTLNRMMGLLRSMWCSWSILRSQKAYTPEMRHILRTLQPIALDASSSFNRFVDLMCYALRNPTTALQTE
LMQAVLDFMQSVEELCLRKNHIMIAVITKAVNAEYGMNNLTHGNGNRSHRSETLTPPCDATGLAISECARS
SGIKSSALPLLRKKTFNSIDSMGISGQNIVSLSDNFVMPISGEDSEGLHALSLSLSMFSNDAKLLLMSLEE
MLDHMRKTA
>tr|C9ZX87|C9ZX87_TRYB9 | DeepTMHMM Topology Prediction - Predicted
Type: TM
MIRGSHLVHLGINCSVSCNFLQNRGTISVAFRHRCGSSVSFAPAIRAASPQPVSHQVDVITISMMRRHSTG
GTSGGKDIDVVDKEKPIHIRVAEQRDSQIFTFQEYVEREEEAERRKLSERLSEVRDSPAWMLWGLLFLSLG
VITVVISIRVRREQMRFDPKLRAVKAFDSPEGPSIGGPFSLIGVDGRRYTEKDFLGKWLYIYFGFTNCPDV
CPEEMAKLSRVVQHLDKKVGRDYWQPIFISLDPHRDTPEKIRDYLADFNPRILGLVGTQEEVESVARQYRV
YFALPDETVLSEDDYLVDHSIIMYLMNPEGKFCDYTTKEFQWFESYSKLLRRMMDYERDKVIQRREAGASD
DNGQAAINIKVANVATMLDENVASVQKPSEI
>tr|C9ZN64|C9ZN64_TRYB9 | DeepTMHMM Topology Prediction - Predicted
Type: TM
MHTRLFSTVLANWFFFLFFLLLVFVVSLFWFAWIIPPIPPSKIDALSYWHDKWSHSPLQQRQHPHFRRLSK
LFFLITAVVNQLPSRVFLFVLIMCGTPHTAVTVMRVICAFICVWIYIYICVCVCVCTCIS
>tr|C9ZZE5|C9ZZE5_TRYB9 | DeepTMHMM Topology Prediction - Predicted
Type: TM
MYVCVCVSACFTFISPTIMYFDLSLETKKTNKNKIDRTDRQTCSSSSNSSSRENGKKNKRKKIRKGPNREI
TAVTARHPRIKHAHTHTHTHIFDYLKKKNIK
>tr|C9ZKM7|C9ZKM7_TRYB9 | DeepTMHMM Topology Prediction - Predicted
Type: TM

MFVRKFQGEILKKEGHEPPKGDKAQGSHSMSLTTSFVDPENAKRERPVVDVSTPYNPLDETHQIRKLGVQK
LAIGFLSLNGFTGAHEEVPYTDSMSTYECVRYGMIGLNVWFANFFLVRAGNSGSPVGHPRTRFAWNIAFLM
GIIRVNLAGIVGIGGYFYSYEWLYTHGPAMFRIKDPSPNAYKRAWDEGQSCYLSRAAACIFPPLGYTIFMG
RWKKTAFWFVFTLAMSMQYEYARHYILPGMRLFYSFQANRETQRQANWGSLAPVLEHRVDPDTNRNASVAA
SRYMRITQGPLQDTIWANATHENLPFHRYGMKLPNPYYNWQKAPQGYNSKPYRFKNDLWDLPNVLNTSKAN
FRI
>tr|C9ZSD5|C9ZSD5_TRYB9 | DeepTMHMM Topology Prediction - Predicted
Type: TM
MCDVIVAELPTELTTNFVFFIPFSAANHLRHTNFHTFLYKLLLEIYIYIYIYIYIYILFLFSFMFSSSYLS
CIILFIGYVFILNSFHHYYYYCFCFFRSSTCRLSLPLVFHLFLPVHFASTCLLCFVPRPLTK
>tr|C9ZN43|C9ZN43_TRYB9 | DeepTMHMM Topology Prediction - Predicted
Type: TM
MYDVAYYDPAYVPPALSEDVMTVRVYSLLYNPLIPDNLCDAINAGLNASLASRKWTVAPDVKVELIPPTSY
KIKPVEVLQVALERHKGEFFVVLGPVGDGQALASLPLLERENLVGFAPSTGSNAMRGWNSHVYFLTASPNA
ELLTLVRYAVSQLRLLRIGFMYLRGISFGDTEYKMTTSLMSSVGRELCGVFTVDGLMRGRVNDDDFNTAWE
QFAETRPQGVIAFAPPAKDLIKFVTRLLSDSRTHDAYLLSSSSLEFALDTWREALEADGVEFFLGQLMLTR
TGPLARDTNYRAIRRFQDHMRSYLSANPGVTVFNGTDNFDHDDVDGELMVYGWIAGEVLSQALSSREWLTS
RKAFMESLYNQRRYVIDDLVIGDFGGDCKGGAGERGAACNCNQGGNVLYINVVGNDERFHTVQGGTTVFEP
SRCLAESVRLYSPLNTLMFLILDRELAQVSSEALYYGANVLTGNGRFGQSDRLFISMIPSPSNATYLALQS
ELDTRSVTAVFGVVDDAMLSIAEVAFVDPVMLTPRLHHRGRNVIQLSPTLEQQLFVVVGYVTNTSASAPMS
AIVRGADATIIEVALRKIVWMHGGTLQTVAVLDDNATLVGRLPNRGNAFVIGLAPGDPSLLAAHLDRNPDV
RVLIPFFDVALMYDELVSAFNGNPNAERVQFATSLPHWADANTSSEIVREFHTALPDSSAWKPLPLLGYAA
ARFAQAVLPRMEYVTPKTLLDTIYMQSIITADEMRYGPFEEEEEKECFTANDPVPEQGEVCVVNYGATRIS
MWSLARALNASVPPLTSPVTPLIRYADPNAIKLSSAQLAGVIVGSLVALALFAAPLVVVLYVLRRGARDND
SAPKEPMEPVTLIFTDIESSTAQWAAHPELMPDAVSTHHRLIRSLIVQYGCYEVKTVGDSFMIACKKPFAA
AQLASDLQRCFLRHEWGTTAFDDSYREFERQRADDDNEYKAPSARLDPEVYRQLWNGLRVRVGVHTGLCDI
RRDEVTKGYDYYGRTSNMAARTESVANGGQVLLTRAAYLSLSNSERGQLDVTALGSMSLRGVPEPVEMYQL
NAVVGRSFAALRLDHEVVEDGDLSSTSFSDTGSLRGSINASAQKFSISIKAVFGAFAPAHQQQLLMPLCER
WQVSLPPSSKATWNEEYCEGVIRRIAVKVGRVADHCAASGSEHSVSTLGSASLIIISNHGLERELHGN
>tr|C9ZVJ2|C9ZVJ2_TRYB9 | DeepTMHMM Topology Prediction - Predicted
Type: TM
MMGRIRVCPQPTYPSVLHLFCIGGVTALTACGGALLLYYRGVWVPERRRLLQQQTCLPPRRTRQFIAGSGS
YQQQVAVFQYHPHVLREILFCGLVVLRTILLVVHMVPVIWFTFLTYGLHFCSEPVFFGKVRDMLLAMGPSY
IKLGQWIATRPDIFSPVMCSALEKLYDNTEPHSWSHTEKTLRNTFRESPQGSNDEGRKGDTGTRQNVLYYL
REIEKVPVNSGSIAQVHRAVLREDVDGIPTGTTVAIKILHPLTREIICADLFVMKCFVNVVTTLFRDTVYL
DLRRALGEFSSLLCSQLSLDLECDNLKQFAFNFRDFPGVIFPKPLPSFCTPDVLVETFEEGRPLQEIQPCD
EFRDAAQRGCHMFLKMLFEDNFVHSDLHPGNILIRANPGAPLNVPPTTFFSPTTVERYPDGKPKLKYELII
LDAGLTTSLSPEERNNFISLFAAVACGDGSLGADLMIDRLPPDVRPPYPEAKREKFRMDMTNIFNTVAPGA
SDGFTLRHVRIGSTLCKIMNTLRENKTPIDGNFASLVLTVVVGEGLGRKLIPDFNLFAEAVPYLMVLLEDN
ELSYLANKLRSTYGAGTLLRDSLSLVELQRTPTYIEAGLRKASKTVDRILQQLTRQSAVTCASQEEKA
>tr|C9ZMC7|C9ZMC7_TRYB9 | DeepTMHMM Topology Prediction - Predicted
Type: TM
MCNSLNFLLAPSQCTGSIALCYISFNHISILSYLFIFHMHVCICKYVLPLIPYTFSPFTLLYYYYYCYYYY
YYRFSSIPFHSTPPPRLDTHTHTHTYTKPQKPVHQPNSSSFWSRVLQTSGGSYPSPTESTGEACILRFVNT
PPIPFLLVRGS
>tr|C9ZJ95|C9ZJ95_TRYB9 | DeepTMHMM Topology Prediction - Predicted
Type: TM
MEREPAVTSKTNRLSSNEKDPHDVPETNDGAINGSFNNRKRGIKRLCSIAQAIFAWTFGVLVLLFISFIAF
GVLYSGIRYPEGSGKVEKHCLNPVDAILGAHEGVFSYSNCGATENTTTYNNVTVAGTSYQSGLKWQCVEYA
RRYWMLRGTPQPATFGSVDGAADIWDLKDIQLLNGQKRKPLLKYHNGNATSANSKPRVGDLLIYPRQPNGF
PCGHVAVVVGVTGDRMFVAEQNWENAAWPGPYHNYSRVLNLSCNPNGTACTVREKDNVTVQGWVRYE
>tr|D0A5L5|D0A5L5_TRYB9 | DeepTMHMM Topology Prediction - Predicted
Type: TM
MRNITQEHFPLCTTYHSQLLDRTIMRALFFVNTAAIFLSGWVMAKGAFPAHTFAAVTWAIVLTIHILREQT
LTSREREGKVEKQMRKENYGTVGRLNKKK
>tr|C9ZPP9|C9ZPP9_TRYB9 | DeepTMHMM Topology Prediction - Predicted
Type: TM

MAAEITVLTFNLWGIFNSKHRAERMAHFASKVEDYDIILLQEQFSESDFDIIIQNMPEEVRRTRYFKRYPT
AFYGSGIAVISRFPVKSGVLFTFPLQGFPEQVLHGDYYANKGAAMLCISVPCNNDVEASGGSVMRRDVLVY
STHLVAVYQVPSQLRDWRDEVYLAVRLSQAISFANFIIATSNPTDHIIIGGDFNSELTSMEIRTMLILLRG
HGYCLRSVLPVAKRPEEGMSEHECIENLARMTFSYENKFTCTDGKIITHGAYSPVQIDHIFISFNTLQLCS
YEDCPGADAKYPFKQIMDGQELPAGVVVFRRNDEVFLGSQSRPGSSPRGTAGVRPVPLGEQEAKEMSCPLS
DHYGVAARLRLLTEEEKNGSSGKVSSRATASKLTEEDEASLCEAVSFLEHSVKRLKRESRKYIMLSIVFAV
IMMLCIIYMVARPYLQMHPTRSTLKNIISLCHHNEHPNLKGIGDTSQQRGGTVRRIEQFLKDTFSLNLEGS
RGKTQAVELREQDYEAVASVLHGAGLHNIFFPLVAVVASIMSFCCLVIALLNRKSYAKIISDQITELGNSF
LRHEMHKGNGCK
>tr|D0A564|D0A564_TRYB9 | DeepTMHMM Topology Prediction - Predicted
Type: TM
MGDTGPKGVPGTNDAGEVHKPQKPQRRQSVLSKAISEHREGDDGSVPLLPPSKGLTSAEAEELLLKYGRNE
LPEKKTPSWLIFLRNLWGPMPIVLWIVIIIQFALQHFADGAVLLGIQLANALIGWYETIKAGDAVAALKNS
LKPIATAYRDGTWQQIDAALLVPGDLVKLGSGSAVPADCTINEGVIDVDEAALTGESLPVTMGTEHMPKMG
SNVVRGEVDATVQYTGQSTFFGKTATLLQSVEADIGSIRIILMRVMVILSSFSFVLCLICFIYLMVNFKQK
FRDALQFAVVVLVVSIPIALEIVVTTTLAVGSKKLSKHKIIVTRLTAIETMSGVNMLCSDKTGTLTLNKME
IQEQCFTFEKGHDLRSLLVLSALAAKWREPPRDALDTMVLGAADLDECDNYEQLEFVPFDPTTKRTAATLV
DKRSGEKFSVTKGAPHVIIEMVHNQDEINDSVVDIIDKLASRGIRCLSVAKTDSAGRWHLCGILTFLDPPR
PDTKETIRRSRQYGVDVKMITGDHVLIAKEMCRMLDLDPNILTAEKLPKVDVNDMPSDLGEKYGDMMLSVG
GFAQVFPEHKFLIVEALRQRGYTCAMTGDGVNDAPALKRADVGIAVHGATDAARAAADMVLTDPGLSVVVD
AMFVSRQVFQRMLSFLTYRISATMQLVCFFFIACFSLTPHDYGIENPEFQVFYLPVMMFMLITLLNDGCLM
TIGYDRVVPSKLPQRWNIPVVFTSAIIMSVVACASSLLLLWMALDAYDEKRYPNSWFGKLNIPSLKEGKIV
TLLYLKISISDFLTLFSSRTGGRFFFSMAPGTILLVGAVVSLVISTIAASVWKKSSSDGVPVEGLARGETV
ADRLYPLWVWIYCILWWIVQDVVKVLTHMLMEAFDIFGCVSRASGGKEIVYNAKSSKEPI
>tr|D0A8B2|D0A8B2_TRYB9 | DeepTMHMM Topology Prediction - Predicted
Type: TM
MAVGLSSSKGVLALLRESDSDVLQFALKRLRPLVDTYWCEISDDLPLIESLSESSDLAEETRALAALIASH
VYFHLGAYNDSVHCALAAGPAFSYVERSLFTDTILSCCIDKFVEYQQKPEGAREELDPRLESLFTALTSKW
IGQEGMPVKELVGFTIRARRLDFLEKVLRQHIKSTKSAAILNFTFEVASVLLRDIAFRREILKLLSVLYAQ
GGLTTVDYFSQAQCLLFLGDSTGTADLIEDLIRSGDKATAYQLAFDLHEYGNQEFLSAVAGLIEKRATLSI
PAPSQVQQVETATEDKSGSGADHSTSSQAAAGQTTQEGDKKDISEVHQKLLSALTGELTASLYLQFLSGQC
KADSNVLNRIKQTTDQRKAVPHNALTIAHALMYCGTTIHTFLRNDMEWFGGATYWGKFIATASIGALHYGH
VNEALKIMDSYLPKDNIGVLPHSEAGSLYALGLIHAPVGVGRNQEIILYLKEMLRKFSASEHIIHGASLGI
GLAAMGLKDEKLHDSLFTCVGGCDAVASEGAALGIGLLMMGSGNLGAIGNLRALASEDNQKEKTIRGISMA
MALMMLGREDECWSTANELLEDSDPWVRLGGCFMLGLGYAGAENTKVLERLLSVTVKDTSDDVRRNAATMI
GFLTIKDPALCVELTRVLVDSYNPHIRYGVGMALAVAAAGTGMPEVIDILWGLKDDIVDFVRQGAYIALAL
VMVQVTEVENPKVKELRDIFTKIIAGRKEDMCSKFGCIIATGLLDAGGRNCTFALHRQRHRLDKAVVGMFV
FLQHWYWFPYLLMITLAMRPTCFIGLNSDLEVPEYVFKSNAPASRFALPKSVLAEKKEAKAAAVKAVILST
TKKEEEFVRLKRSAVAGKNQGTGGTGGAANAAASQSGATKEQETEKKREPEANFVILSNPARITARQFAVI
THDVDKRFVPLKENPSSICLLRDTNPAEGKPKVVTDLGWNSSDDAPPPEPFTWP
>tr|C9ZZF3|C9ZZF3_TRYB9 | DeepTMHMM Topology Prediction - Predicted
Type: TM
MGVGLGFVPAGYHSELGALCTACAAAASFAFLPNQFLVVAVPVWLVLQRLVASGVRNRARPRRRGDEVSCC
KGTGSSVSLDKPTSSVVDESRSVFVMTRDMKDEERPVAIVTGTNSGIGFWTAVGLAVEGYRVICTCRDASL
SEITAGRIREKAEQRRLKDTKGQEYREVPSTVIVDGRFCIECDDFSSVRRFVDRFRASYDRLDVLVNNAGM
MRRRLEFSRFNPQLEFHTAVNFLGPLLLTELLIPVLKQSRGRVVYVSSAAHRYPQLVLHEGGFLRLLFSRG
AADCKLNGRLLEALKALNSGEANALGALTSSTLLYAFARYGTSKLLNIYHAHHIARHHGIAVCSLHPGCVG
TNFSRDLVFSGFVACAYQLVCLLFLKSPEDGAQTTLHCAMCDAEELRPVEPHGGDPNAFVSPYFAECMNQT
HPWLLRYAWDVKEGDLIVEWGKDIVGLPLNAKEDHSA
>tr|C9ZI51|C9ZI51_TRYB9 | DeepTMHMM Topology Prediction - Predicted
Type: TM
MCVCVCVQPLLSFILTVECPPYNGLCKCLFTVSFFFLIFGRREEGEGGRGDKAFFKRFFFKIFIFTDGEWN
LLKYCGRH
>tr|C9ZS36|C9ZS36_TRYB9 | DeepTMHMM Topology Prediction - Predicted
Type: TM
MDVVLEVLRTSNNNDERKKADAQLRQMQESDPQAFLMLTWDGISSTAVDTTTRFFLASTVVQFVEQSWQHS
VPKDLQVVMINRYLHLLLCSEPLPSVTLARKVALLLGLMLKRCNARGEPGALPPPLEHAGKMIGESVVHAV
NNLSPTLIGLATQYLLVLHVVLKEMEGKRVGGVFPRLCASLVPVMSSVFVSSATWDYVTCYMPLLYMMKCV

LRVFGSGVFDAGFYPHLLDITWRLAHSVSVTGQPTERVERGQRLMEYAIKVQLKMLSVFPSKMDGLPPAFF
ISQGNGDMEDRSLLALLVAIIESPIGTVVTEKLVCRALLTFKALLTVEDSQPFVANCVIALANAPQLLSRV
IDRITYFLADATGDAVLRAWDLSPEHHAAELERLIDDTEHVSSCAEELLLALTGSTHCSESSLRLTWEVAN
ALLDRGGVEEVTAALHVIGICCYTMADGPYSASFMNFLAAKLLPIITAAATSTVGDQIYPSPFVLRRVVWV
VGMWCESVKGSVARSEVHRALTSLLGAPTHVVLQLCALQAIGHFILDLNFSIEDVPAECVQGTLCAIQNLL
PQLSAPATIEQLAGLVQGLIARKLLHVGSGELLLDMMLPAVYRAISGAQRTPDGEADHSGGNDDGDEESDD
DVSLQGRCIGLLLECVHSCVTIAAPELEERVWSLFRAIVLPCTEPGSRLSLWADEQAWELLLSMCCAARSW
LPETNDALLFCLDNMTREISSRHIVVRCVYSILLLCPEPAKHISVALVDHAMAELTQTCSSDVASAYFALL
AVMLRRGEVTLRCHLLSLALHKFLSIKSVHSESFSEQLALLLAWGFLPYGDCENWEVILLDTVAKVLLHHP
NANSFVEWIVLLFDVSPGPFVTEQLVRLLQHSLSAQHTVLLQLIGEDDRVMARKAVEGTLLPPSSMQERGA
DTDEVGELVRDPTEMLMELFGDTALIGASPHVIRLCRLFDVCAIS
>tr|C9ZWC0|C9ZWC0_TRYB9 | DeepTMHMM Topology Prediction - Predicted
Type: TM
MHLVRKGRVAVGASIVGTLRFQADKVSIFRHHSTTTPSPRTTQPKMHTLSENKADASAYAFGDDLANYSSL
RHNFYLLLHGRRMWLPLVMLGIYNVLAPLYLTTWITNALYWSGCILLQRYSVRGRMNRLRKDFTDHVCVVT
GGTSGIGLYTAMQLWEMGAHVVVASRPGKETETREFIRKNCRLPKESEEDTPLERLTFVSVDLSDQLEVMA
AAARIKGMFDNRVDLLVNSAAVWREVPNATRQGLEEHIATNFLGPFHLTEALLPSLRRSRRGGRIVYVTCA
SHNGVRRADVVRERMTLKPSEDTQQLTARCYSASKLGNIYHVQSLAARRYEGIPLNTPSADLRPVDVCAVD
PGFCATGHTWRESPPFLGTGLLGRALRSLWMKDGYEGSQTVVNCCVREDLESGGFYAACMCMPSGLSRRAH
DSKSCREVMQWAMAKAIARYYTVRPRADNKGDTSNSVSNNLSKVSSAN
>tr|C9ZW39|C9ZW39_TRYB9 | DeepTMHMM Topology Prediction - Predicted
Type: TM
MISPSTLLRSASSRGPFPAALRRLFSQYPRNGGEFLGNLLVGHNVFIADQPRKYDVCHARHFSLLESLNIV
PLFTLTVVHYFSTFLLFPSRRNMIPVLMTELTNKSKMEQEWLEALAAKSPADAVAWRAAMLLSHLVLFPMF
LILSAIAPQLVHATLERTNEILYQKYASISTGAPTFVKKCMEDARDASTYHSMQLNISTDYVAALIIVVLV
LYLNS
>tr|D0A526|D0A526_TRYB9 | DeepTMHMM Topology Prediction - Predicted
Type: TM
MRSATEYTDGLLGLPTSPQLATHKERSGGTQVGLCPCFRIAAGEFMHIYAILKEERLSWRDDFFDVSQLSV
PRSTSEVLERLNLNLPFYAANYMTVCIVVTSLLLFLNPYFLILLCSLVLLLRGLFLHGKYQGRPNHCIYIG
GVTVSYYRLLIVTLFVLFLFPVVWSGILNLLFLLLVGTALVLPHAVSRRPVYFHDEELEKRRPKALQYVVV
FLLHFLSYMESPNK
>tr|D0A5X4|D0A5X4_TRYB9 | DeepTMHMM Topology Prediction - Predicted
Type: TM
MFFRSFTFFLCCRSAPSLFSMCRVCLCGIESSLDERYCYCAMLLLHYLFFRRFSLFFYSLSFRSTPPSGAH
VPSRMRRIISDACDLFPFGNKGCPEAAEEGA
>tr|D0A332|D0A332_TRYB9 | DeepTMHMM Topology Prediction - Predicted
Type: TM
MARATVVAGNAITYGGYCSLWLDIYIYYIMLYYLRFLHQQLSPLLCTRGGGALLPGIGWSNELGSAIPLGY
LEVPRHYVIEGVLWCCGPLPLLVERMNPSSGALRSLLFLPSGVISSDIYHTVIMVSACVKKAYRFYCYVCL
RFCFTALRAV
>tr|C9ZY87|C9ZY87_TRYB9 | DeepTMHMM Topology Prediction - Predicted
Type: TM
MFIVGLFFRGYITGLSCGQSNDSHSVFFSLLVFIPRFLSPFSVCCVASLWVSLLRGGSAWGRFWSPLPRGA
SRLEEVMNSTKFHCMWVWLNLYHAARESGNGGHVLVSDIEFGRSVLQMQLVWRNSLKHISYCLVSFERLTL
IDHLVTLVALLIFKGLPCFVIQKGQSIYKDSNCYLKVQDLKHPLPGST
>tr|C9ZXQ7|C9ZXQ7_TRYB9 | DeepTMHMM Topology Prediction - Predicted
Type: TM
MAFSCLQAPQMFFFFTCVLPSSYLLIIYSVFLSTLLVFITPKPSLLFFVFFLSVSSTWFSLAYTPFFFRII
HTHLTVLPPQRKQKKKGTDTAHKKQREKESCKRSF
>tr|D0A508|D0A508_TRYB9 | DeepTMHMM Topology Prediction - Predicted
Type: TM
MFECTYLFVSFVVSKNTPQYFTSTLASLPFLFSFSLFHAISPRRSSKQMSSLPICLFSFPFLFPCSLFHSI
QFYYTPYLYCHFSSFSFFFLRSSFVLVRFRFVILFI
>tr|D0A0M4|D0A0M4_TRYB9 | DeepTMHMM Topology Prediction - Predicted
Type: TM
MKKNNSKGNSSANKASAGDVSQVQFWNYLKKGDDEGLEKTLLQRVENSETGEVTVVVNSAVANQLVNSPNA
KKILPLSYAISQGMSERGLHALLLAGAKIDAMDGTSERATALHAACWGENDSAVMLLLRCGANPLAVDSQG

RTPLHVLASLNAISLFSLVLETVTKSGVDQNTCRGDDTEQRLEGHLSLEGHSPVIVPAERLLDSRDNAGLT
VLHTAVSDISSGSDGVISKLLSYLEEMAKTSADKVSRLVNMTTDSESTALHLILSWPNCDEGVMMHTVERL
LKLGASASAVDKYGQTAVTVAVTTHVGITVANVVRSLLRSVEEGEDNEARLKNVFMQCDNEKGYALIHHAV
AANNIEVVKVLVNFLGEFDGASGSQYIRHWLGGLLTDNNETVVQLIVEYGCDEIADLLIGFQAINKIDYEK
YKEEHEKIREDESVGNLEDGDIDSNEEGGHEIGDEVGLRGRQFAGRSTAAGASSGGSRIQLARRARARAQA
QSAAQRKKDAPRQDSEVADRQRNAAPLWVMLCAVLIFIASAALVGFTFRNTIVSLFG
>tr|C9ZJ97|C9ZJ97_TRYB9 | DeepTMHMM Topology Prediction - Predicted
Type: TM
MYKHSRGFIYLLNSFCISPSMRANIFKNIFQLKKYSFSESTPPSPTFGFGFGLVFLFFFFVAFMYLHIPFT
RTLDLAFFFFTFYSTSPFKFFANQYVASSSLFVSSSFFSVISLSML
>tr|D0A352|D0A352_TRYB9 | DeepTMHMM Topology Prediction - Predicted
Type: TM
MGAPEGPSTEGTPVAAAFAGDPPAMMLSMHFASVGCDTVWHGGGGGGRRCIGMGCAPLVSGLRASVQKISE
QGARGCSKGSCRTRLRSTGPCCVESFFFFWTVWGVGGSCCDSRLAAIVSLLPLQWQVCGLTCFLSGVIICR
RGGTGYITTRGLIVCWRHTRVYQHVVGWCGSNLTQPLKVLSHHARQRVLNRSRCIPAHMGGDVVFVLLLRE
ATCSCIKKRKKTYKGRFQ
>tr|C9ZZR6|C9ZZR6_TRYB9 | DeepTMHMM Topology Prediction - Predicted
Type: TM
MQCLRFVNGKKCSVNTVGASLSAVACVVAFIAFIAEINSEGWQYRGKVWLDAPIERQVPFRNPKVVFVVVQ
TRPSPGWCRMLMTAVVTNVSVISIGMGGNYSHTIRANWLLNFLDDEGLHDDDVLVMFDGADTFFTDEINRK
EMLDPFIKMSPPLPTFFNQTAIYRGDAWPPMLHMAEPDCYAPQLNITYNPQNESHWDRCARFYAMGLSEAK
TFGAERLLGLPPPVRGHLNSGGIVGRVWAYKEAFNVYLKFRETSSKWWCDQTMWTILFIWSAGNATGVDPK
YIIRRGIISLDYDKRYFYYPTAAYNTRAMIGHFTGNPHQWLRYLPKYFTRLPWYRNLAGNNTYRQSVVEAL
RNTTVITYKYTREKVLKSYEDVCNLEEMTDPDFVVDPLDK
>tr|C9ZIN9|C9ZIN9_TRYB9 | DeepTMHMM Topology Prediction - Predicted
Type: TM
MAKGKGIAGTLSRKLAPRVNYQHHQVYSNVPEDKLIGTVSHGRKLPHLLLLYSLLLLQLALPVSPYFIILG
SPSLSLCHHLFNCLANLISHSMCTNWTCIFSVLFSPTKPHTHVVR
>tr|C9ZVM9|C9ZVM9_TRYB9 | DeepTMHMM Topology Prediction - Predicted
Type: TM
MASVGNCFRRFMSVITAVLLALSTIIYAVCLSVVVFREVKRDRFTVFHMLSVAFCSLSLFCYAALHCISLK
QRYLLYIITFVLLITFGFLTHSTGLTLLVVLDCRDLGILNHSEFFDALNNPAELTSMLNESGTIDNETAVI
GKSGRAVHSCSLHSMVVFAASVTALFQMLALFDVQKVLVEKTKKKMYNVDVYGVPCVK
>tr|C9ZYF5|C9ZYF5_TRYB9 | DeepTMHMM Topology Prediction - Predicted
Type: TM
MAVPPVEMYSGSFWNRMRKPLPLRTQVIRFTVVFVIVSFILAVALQITHERMPDPKVTKPLPDLGFELLTK
VPGMYVLADCCIGFLNILSVFTAFKLYLLHRHCVGSGEPELPCNIPGVSRFFLSVWLCKENCRIELRNIHT
IAWIRFITSYALLLLFRSAVIVMTSLPAPDDLCQDPPKIENPVKNVILTVLTAGGGSIHCGDLMYSGHTVI
LTLHLMFHWIYGAMVHWSFRPVVTVVAIFSYYCIVASRFHYTDDVLVAIYLTIATFIAVGHNADGAPWQLQ
LFIRWLPCCGANSREVTEDSQPVMVAFKSEELDEMNGVLEGRQKKHGGVGDGESLMFKCGAYV
>tr|C9ZTT3|C9ZTT3_TRYB9 | DeepTMHMM Topology Prediction - Predicted
Type: TM
MAFCAVIKPYRNVPFHSLNITACNAFHILSLVLLPSDPFCPCLILSGGFYSEHYGIYGIRTLMRCCVNGVK
RWWINTVRREEVAVCGIRFSKLCLCFLFPNPDFGGVLILVAVAFCVSFPCDPGCHCVASLSVYVIRLFMMG
TISFLCVKHNIPLPTHSLLHCFPCVLFFCFIAVKR
>tr|D0A7R5|D0A7R5_TRYB9 | DeepTMHMM Topology Prediction - Predicted
Type: TM
MCTYVNIHVCYILTFLYFDFLLGQQCVPMPSRAVLRKKARRESKRRKDETEGKTTAFKLQAMEQLGKSHKA
DATKHVASDRKVQRNKRPRGETVKEKDTDVCEVLEGASRKERKRFEAKRRFERQLGALNRSLAAVTSNTDG
TSGDAAPSAEEAHQRHDPKYKNGTFWRDRKERRRRTVFLGNVPAKLTEQDVTSLISDTLRKGWTPPEEDGI
ADVDVVTEEEVVESVDFIKSMPRAKRRHMYVTMCSIKAAESATKLLDGKMMEGIALRCNFAADKVQRGEAI
QRRSASGSEH
>tr|C9ZYX9|C9ZYX9_TRYB9 | DeepTMHMM Topology Prediction - Predicted
Type: TM
MIGTMEDDGVPVILPQASSDDVSTLTLVKRFVLVGTPLMVTTLAQFTLNAVMMAIIGKHFGVKELGGVSLA
LGMLNATAFAFSAGLCGALETVLSQTYGVFQSRGGEGTMYLYGTYTQRMAVMLLVISIPIGIAVIYIDVLL
KSLGERPEVVYYTGRFCCIAALGIPALQFSQLISRYLSCQHQTAPLSAVAVGSAILNPILQHLFIRMFGFN
GSPMAWVLLYVVTDVLLVAYTYYTKTYVTTWGGWDSNAVKNLRPLVNLAAPSLAMSLVEWVVLEVIMACAG

FAPPTDLAAFSITVQVFSACWGVASGTMLIVSVFIGNAIGEGKPLLAKRIANIAIVMVGVTTMLDILLCWK
FEDRIPLLFSDDKEVGHVYRKLMRFVFPYHAVDTFQSTVMGILRGCGLQKIGAVIIGVTLCVVGAPLAFFL
FFYVGVGVEALWIGPLCSVTFVGVPLYIYLLYWYIDWSKLQPQQESLDLAVEPFISVNNAPLEEDMYGAVE
SLGSKD
>tr|C9ZWW6|C9ZWW6_TRYB9 | DeepTMHMM Topology Prediction - Predicted
Type: TM
MQGQLIQTGRRKTQQRKKKNISLNRENVCESSMMKRKQNYSNDIKFKKEDTSVASLTLVQVGPSIIKSKRK
LSSHARRHAGAMTSYLQGQQSKRAFIYIYIYIYIYMHNSVYLCWCISRRGRRTLFFSF
>tr|C9ZWF7|C9ZWF7_TRYB9 | DeepTMHMM Topology Prediction - Predicted
Type: TM
MSSVTTGSSSYAAVLLVLLLTVTQCGNSKFPNLGCQKLWTTRSAQNDPLTCIKDKTRILSHWEDILLPVLA
GLLLALIVVIFPLTFFSTCLCSSCCKPSSKDRGRKQRCCLWMWIAFAFIWAFGVAVFVLFGAQQLKITLDD
LLSHRVNRPLNSLRCTADKILGLAYDWRTGEPLREGIGRETIDSTVDMARSYIAMAKGYYDQYVHWVPTVS
FCIGAFAVLVMVPMFMFAYFRCCSKWLPRLLSCVYWLFAILFTALGLVILLLAYAFGLVCGEITLHYDRSP
GLIQLYALPMCEEKFNFANLNKMILDAQKDVSQKACEEILKYCDNDTSQLGDITGVGGVAALLGRNLPPGL
DPKFLPPGVKAEDLEDEKGLDSLLESHIPGVNDKEKIPRRYEGSVPPDGSLPGNMRDVIGARTTQFPQLPF
GPSNKMLTCGQGVGSKEECSTFGFTAAVISGTKVKGGYMVCPDKGRPCSLQVCADRCRLPEAKDIAHKLAV
AAQLAVNVSIGLSIGRPLLECNFIFDTLLTALPNCDGLYWGTMMLGLGFFLGGMMFALSIYILLRGSCVWS
DLKRWDEDEEKDSENKRESDKRPARK
>tr|C9ZRW4|C9ZRW4_TRYB9 | DeepTMHMM Topology Prediction - Predicted
Type: TM
MTSFTGTTFHAQETQVNGGGSTFMVSPMSASSTENMVLEKDELISLVCAALMSEVVFSAPDPENAASLDRS
RDAARIATLVHSISAEDGEFVLKLALYVRRDLSIRLTAAFLVALCAYERRCQPFLACYMKRIILLPSDWLN
IANIAYSKPHLYLLARDGGESVTATTGTAECCCAQAIKGIPNALRDALAVTFTMFDEFSLAKYNTERATKR
SGRNQTTSCDEYVSRSEDGHTEARQVTIPSRLTFKHLIRHLHLSHPVYAINCLLGKRYPNTVDEFVQRGLD
EGGIRAFNSALCGQRMRLPTPETWERRTSREGNTAAVWDDLVARKCLPFMAAMRNLRNIVLCGCNPTTHDS
LLQLFSSEEHVFNSRQFPHRFMSAYEALDFDPEDALDKFSGKAMISVKPPGSPLPKIIKKRVGKRIGKLPS
LEEVEKVKKMYRGAMQQAAEISARLNIIPISGRSLVILNITRYDLPARIQDLKNGVQLAVSFYYACEDCSI
ILLCRGEFRIVDSEIRRENGILSCVENVCDICRTLLQQRDLEVMAELSRDLEDSHRSRIFNFPYLYLDELI
EKRVNLQALIVMDCVHSCYSGENHTPSLGDLPVYLERLRRTCNENLLFVALKVSGSKNADRRTGLRYQHKN
DFLLTGFSAAALRVVAEGVSCGPRRYVERIDQVYDVNVTTVRAGRCKFESDLRVLREVQGIEKERSNKSTE
GLVKGASGEFSGSTPTDSAPGATLLSSAAEKVDSVCSDYSIPPRSLQSRYKNFRFFLSSTFLDMENERNTL
VLDVFPQLRRWAAENNLHINIIEVDLRWGITEDSTRANLSPSVCLNEVSRCAPFFLGVLGSRYGYRPPTLF
HTVDDDVDSTDFAWIQTLQQEGNTNCMSVTEMEMRHAIFAANRRTKGNKLHTMAFLVRDNAALVKSLPPKH
KRAYAPDTPTAAASIERLTKYLEEQGAPVIPYTATYKQASLGDRFSHSSLTAPRTAIGNISIGSSGMLSSS
IEECPLDMADFSRKAFVALKSIILRHMNLPLNFSGDVCEKGGERKQCRGDGENNCLSERGSYHSNTLYSRE
CMNQLSFSSSLLKKLVPPQELMGVLVRFACTGRFSSGGEHESIVPPAITEMSAWGGSPLANETRDRSNVLV
IQAQEGDGTSTVAAALAMYLRKMDLTSFLVTHFACQAGDGSLQHLAYYISFSLIYGLGLQEDFRVQETDSV
TTLIQLLPRVYEAAGKKRDVCVILDGMNRSSHAPEMLQSLSWIIPPEPIGGIRFVVTLTTGNNPFSNAFAL
RVPPPFCVTLPHLTVAEGAELVRKHLASSGKRLQESFHSNQLRLLLRKADASHASYLTYAIMYLRLFSTFD
TLTNDITQLPSTLAQLQVATYERLEERFGKDTCRAVLVSLYIASDVGGLNEFSLYRLVSNVASASRLVVLL
SGTCLRIRHRRITVTSTSFASSIAKRYLSRASDVVDACLNMLVSELYYKSLSLQASRYELRCSIQSIVDGI
KRDSGGELAFDPRRYTAGELLTILHLAKRAKEYDVLIALLLYVPLLENLVVSAGYLQRLLSILSQSTLLAT
PHARKLDPVVDFIQRKYHVLVCRPFHLRQCIRNNARSTMIFYGLSHSVCDGSKGCGGSKGSAWVKWINNTR
HIEEGRLVMFPSIDPVKCFSINSDGEFMAGGGDDMNTYVMPHTSLDQITASLKHSDTVTAVTFLTNRPHIL
VTGSGTGVVRVWNSEDGRMLQQSENFHRRRVSSLSCHPVSNVVCSGSNDCACAFWPVVGSTAEASTRSSLV
PTELLQHHKAPISSVAYHISGAILATGSWEGKVYFIDVQQRHDEATVSEEGIVVKSSNREQSSGKGVRTKR
YTTPPYSHCVLETWSPVRALAFVPSMVVTCAAALCNGDICLYDYASATCGARFSLHAGVPITALAFSPDAK
WMASADERGSVLITYAGIRGTVLCSLNGHRRAVTTIHFHPQNPLNLYTVSLDGTVRSWSVGEREEQSAYLD
HHNQTGAGAATLNGSHAVTVTACAVASDGSFFVTAAADGVALVFTDRRDDRSLFSCLESSDNEEVVFEPQF
TLLHDQHRISYICIGLQNTRIMCGTAKGEVFVWDSTPGLNRREGRLLQRIRVAEEGAHPVVYIGCESCRNT
LGQEDETNKPLVTYLPCARITAITTSGMVVSWVAQGGDSFVSCLDETQFTNSCTLLPEGELDCQCADGRQP
AVAGKKLQRSVLSWVGKPEADSSSKEEQSTSSCFLSSTNSDTEGVPSNGAEEIVCAVPLLRATRSPSRETH
EESQQPKAVDTIIGLGESHSFSGDTTTGSCDEYYIVVGRRQCHFLSARLCSVIQLPMFTSQSQYNDCSDDA
FSSVACLPAGGVFLCVSNALCVKETRGGGGEYSALFAVSTSCCMIWLLEVTFSPPVADRGKELTAEEEKDA
EVLMREYSENTRLSVLHSVAIYGPQGKPTPVDSLNLAVVSPTTVDFSCDHAPEEVDHSDVSSVLLLTAGCR
DGSVRIFVVRPFQNPSKGTEGEVDASQYENAGNTGGKSDSIAGKAEGRCVNDNMVWQERGVFFASSSVTAV
VTMRKPIYLPTVGPNSCLTVPVTALPKKDEADWGFETNLSSVVHLAGDCLGNVYQLQLKREETKTASKDSR

GKHRFKTALHCPLVSADITTVGTPVHGDVMDCSTCKYLPSPSPLTKHYCECANEYGIATDAGNDEEAWRSE
SRRIMAMFAPSAIPSDKTSQVCEGENKLPTAVSDHELSGETKVTVELPPFERILQKAPSSLSVEEQRAWVK
GQQSLLVEAIRVQRCAVKAREVLAEYSQNVLSQVGLQ
>tr|C9ZYI9|C9ZYI9_TRYB9 | DeepTMHMM Topology Prediction - Predicted
Type: TM
MSLQTTLIQSIKEVNPCIVLFASVVQVAFAALWYKLIVKTIVDYHLAADKGVRRVEHILQRYPPYVHWIVT
LLSACFRIVFIATIVGICKGNSLHDYQNAALLVAGISCVSQHLSIQHQRPLPLLAADVGYEVIAALLASLT
YYAIMTVNVF
>tr|D0A159|D0A159_TRYB9 | DeepTMHMM Topology Prediction - Predicted
Type: TM
MQGGTKIYGGVVVRLTDRLLLCKAPGFTTSDFSIPELMWAELVSKCDAPNMRTSSTMMANNATSSGSAAPQ
LSYHICTDNELAFAILSDMGLTRHKAHATLDEASKTFRKMFVESVASFTPKAVEVFVKPYRDLLLRSSDGG
SSDDKVNKVKKAVDEVKEIALDNVERVMQRGQRIDDIVRSTEDLQLQAQGFQRSSRDLRQQMWWSSVKGKL
LIAGVALFFILIVVFVFVGGKKEEKK
>tr|C9ZWC5|C9ZWC5_TRYB9 | DeepTMHMM Topology Prediction - Predicted
Type: TM
MEWNGGTMTKKRCFDCCCHSRRVIVLIPIALSSSTPFFLFAVLLFFFFFFYKKDKKTSASSSLSFSFQLQM
PLGGRRWYRKSNTERNTDLKSTVCGWLCG
>tr|D0A5H5|D0A5H5_TRYB9 | DeepTMHMM Topology Prediction - Predicted
Type: TM
MPPPFPYSSLLGTAAPFLFLSASVRKRCLLTSFFVFIWYKFVFVFLFFCVHHERTFLLFVCCFVLSKSHYM
WLKQISFTFCFVCVFAFLFSLSMFYFFFFLIHNTSEWLELLKSAISCPLFVHSFHFISLYSLAFIFLRAYF
LAIISCVVCWANLGGQFLVVFVFVFSTFFFCSFVCLFVLVVVVHMLFLLFSPPSYFHMWVSFVPVDRCCLL
SLFPFSLFSFFLFFSFSFLLLLCTILPIPPFRKVLASSRLNFLSFFVLFFFFNVLLCLEVSLIFSLTVCVC
V
>tr|C9ZST6|C9ZST6_TRYB9 | DeepTMHMM Topology Prediction - Predicted
Type: TM
MQGITGRNGRMLPTKKVNNVGLEGTSILFTFFANVSSLMRIVSSAVICRKALTCRGEANRGGAHCFAGNPA
RVCVCVFVCLFVLRQPQHQEAWRMEKEREKKVVVEVA
>tr|C9ZZ73|C9ZZ73_TRYB9 | DeepTMHMM Topology Prediction - Predicted
Type: TM
MKEVGKKKRKKDKRVSEKFNFRVSHAATYVLLLVSLFHFCRFCIDHPFIKHSLFKKVYQIETLPPLEFPFL
PLMVATSLYLHSLLPLSLPTLPCYYKYRSMYIYI
>tr|C9ZRH7|C9ZRH7_TRYB9 | DeepTMHMM Topology Prediction - Predicted
Type: TM
MAVESDVLPPLTRLPPDEALSFIWKITRSILILVFVIFAVIPALCGTVYPPFSALWDVIAAISPYTWGSVG
IGIGISLSIGGAAWGILTTASSLSGAAIRAPQIRSKNLISIIFCEAVAIYGVILSIIMMGKMEADGKAVDS
SGIFTYRAAAAGFTLFAAGLAVGLGNMCCGIAVGVVGSSCAIADAHSSSLFVKILVIEIFASALGIFSVIV
GILMAQKAVMV
>tr|C9ZYH5|C9ZYH5_TRYB9 | DeepTMHMM Topology Prediction - Predicted
Type: TM
MTTATELKQKGNEAYAAKRFEEALKYYEEGLALDGGNIDLLNNAAAASFSLGRCEAAISFARRSLGVRDNF
KAHKRVGEAFWKMGNLREAAAEYEKALALEPTDSSSRENLQKLRQEMNRGQAQFGGSPVVMPQITPHGTVG
LLVDTLVLVLSFAALASSLIGVNGSSAWFLLLIAAAVRHAVIAHSRHMLVPDLSILKSWFDVRCTLDLILC
LVALISGVRAQLPLVVVQGLYSALSLATNFSRVQQVAPAAYQAGLRYAQSITSNAQRLIMHAVSLEAIMLL
TVIFSGGAIFTLFYIQYAKNLYRIDGNVRLAFYGIRENLTRLARKGFMPAFVDSTLQRVCDTLYQFSQQPF
>tr|D0A3W2|D0A3W2_TRYB9 | DeepTMHMM Topology Prediction - Predicted
Type: TM
MDRASELMDEFFKKDPDIRRMLSPCLDELHGLDDAIRRVGHKHSLDPNNQDVTFEYATVLISHTRNSYVES
GVRLMESLAFALWQRRWGGPTHAQLQQACKIEEPLQADGSGAPASPNLQQLVSSEEGREGQQMNGDVVSRN
GKYGQRGCENPTRCSKDTLESDLVIFHYYLAVGWIKLKKYDNALSSLNRMLELKPGHPQGIALKQYVEAVS
RQTITVAGLAGIAAISVAATVLMAFRRS
>tr|D0A6R9|D0A6R9_TRYB9 | DeepTMHMM Topology Prediction - Predicted
Type: TM
MFSLILYLGHFTPISTSPRRFRREGYVCLVGCIDIAVVLFIVLFLSFSFPLVVQLRFAPSLSRVYMCVYEG
FVKHRSSDKCICRGPRNQSAFSCGNIYTK
>tr|C9ZQF3|C9ZQF3_TRYB9 | DeepTMHMM Topology Prediction - Predicted
Type: TM

MEEKKKPNKFSASRAEILMAATNPHLRTLIVPPPSPAAPSIRNGAENYATLRTCGSLSPLFFFAIHNFAPV
SSTTILSFRFFTCHSSITSSSSSFLALGGPLLFFLFSLTSGKGK
>tr|C9ZM60|C9ZM60_TRYB9 | DeepTMHMM Topology Prediction - Predicted
Type: TM
MYIYIIIIIILCASTPLQVLLCPNLFVRMRNHMKLWQHLPAGALRKVRKMLSPSGASAAELDEALNQYISA
IHPTVFPHLTPRHLLRVIPAQPLPVRTVWRRAISMETVPSDMSFEAFKSALMPLVPLFLSLSSTTLGEDGD
EGPLVRWQPWVSDGWRNEQERTREQCGDGVISELSGVVPTYFVSYALLKEQTRLGQLSVATLHTLERKELV
GGSVPPVLEWSEDHSVVRLSNEVIASSSSTRTANSFYSLQSQPEVLRRAVNILALLLPTFKASVGEVRAVY
KLNFITPGGLRHSAITHEFAIRFPDALALVDHPDVQFVIDRDSEGCVMLHSTFLHTSRGAANVLYMCTSVG
HPLSISAHGVDTNEGAGCLDRYYLRVDLPYAPRCGDEVVLQAICSATVTGLYCVQQPDKDRFSLEIATAQG
AVIVFDSTISLLCRLSTAGAPCCGFPAEIVTLLASPNIVKLIHAAPTVCTANQGVYGNVLDGLSLLYAHLK
TDFQCVCTFHQLALFVGIPTTETERPGQCSLLSNRLITCHGGVALPYVRPALLAAVCASRLIALHLADGHE
FDRALLQQLLRDAVEGSNALVMRNLQLRLKRYGMEPHGTCTKHSCEALLQEFLVVYGPEVCFADKFDDFLS
RAASSTEIARTVGKNRSEEDDGEYIRELLLDQPTSTVERDEEDMDVEHDVQGELSKCCKQNVESGDSEVTK
HSEGEQDNMEEAYNFLFDCSGAGATDKGSNIPPSHRSSNHMNCSRGNEQSERSTVFVLPLPNLKGHHMTSR
NVRWTLPPSFSRTNVRGTITTKAEQSVKTIHGASTRSLLPEQRDPTNWHIDKSDEHTEGIASEGALIERLT
QEVLCATSSSKTNKRRLPACSSDRLISE
>tr|C9ZP89|C9ZP89_TRYB9 | DeepTMHMM Topology Prediction - Predicted
Type: TM
MNRKGYKALPREYDEGFSEERVGHALNKDAPSKTYEEFIVRRLQEIMETAQSDSKKPATFGRAAAADVFLR
QDAPITVTFAMLATSCAMHPTTDVVTFSVLFAALYTSCMALQVLLLWLRITEKRQSAISVMQKVLTRWKKR
LVEGPPRDKLDRRTVFRLFSSFLPASAFRIVAVVDTTTGRLKRTLKCLVMKGALRLHRTTLKVSRHTEPIE
LFKLVDIVRMILKGEAVMLRSLPDMAPPPRHPNVTGDANINGTGVEGNDEDISTDESDDEYDTRCEANSVH
ALSVPQQMAFLVYRFVLLAWLLGIVACTFSGYIYHVLTDIPLSDGVFLNPAVALLGLLPVNAKLLNSILVM
YANVNLECLFRHLVSKTTHSVDDRIPRLPFRATMQAFRRATSATCSRPPLCFSSSLVETLGTTTVVALLDT
TGVVTDMVPLPTRLLMLKSYTAPETPSSTSSTTTSDSDNDEFNGAAAFTGLDHSNDPDPFLRAANFDPEYR
LALKRQVRQKKTQKRRFLELQLAPGTDDDLAVQFADCKERQKWEKNVDSLSLCLLIHAMAPEPTKFETWQE
PLRFCDQTPRWGPSIHWVPRASEFDDSLAKSFRIVARIFHINTVSWPGRSRECPEQTCTLLVVASDGLLHA
FTVGTVCMVTHSCPFHFNGYEIEGFDEELREEVVNTGTVVWKDGRGLETVAASHTMLPDKFRSCAESLPRE
DGSYAEFLFYNGVQITADNCLAELKLSPSGEPSTNGCEGTNEEVPRECFLGVPTNTNGSTPATSEADVIGA
INSSERDAINEGNSFCCSKGFTDIHGNSHLSFVPTSVELLGDLLHILINGSHAFLGLVGLQDSIRPNVQNA
MSVLDEAGIRCMYFCSDGERRTKSLGNRLGLETDWNCCISLDEGAKDLDAHSIRAQLPVGIKSIRRHIVHV
DSIPLQVNMFSHAHSASTRAMLSILQDNHEVVSAIGSTFNHSNVRSYIQADLSVGVLPQRRGAVEGKEGLC
LENQKRISEPSDIRSMKSETGDQLLYRDVVDLIGCACTLSDTSTTSMLPIVTTLIRQARFRLSGIGNCISF
VMHANFFVTFLNVIPLIIGGPLLIQPAAAVFELNVIIPILALSCTYTAYADKDPMKSIQSRHNHFVRLKIL
RHSAVVWCLRYIPSLAALLALGITCGLQKCDKGSLHELALSSSDKCMAATRGYIGLTLNYWLMIHSWTHMS
RYDRLSPAFLFKPRYGGQYMYLFTSARWVAANALTVFFSVAFVVAETFWDGILEDAFFPSRTHFYISAFFP
VLLVLLDIPIKSWRLKRATLMQKFRQLSFGTRLGMHSPRGDYEPEVGSATGCSGGANEGGGGVQGSTSDAN
VRNELPLRRRLQDAFYRFTTMRRGKLELNCVCCDHIGGNYATYHINANDM
>tr|C9ZME5|C9ZME5_TRYB9 | DeepTMHMM Topology Prediction - Predicted
Type: TM
MEEASSEHQRLLPCSSGPHSNYNAAAVEVGTPAAPSPWGGLEDADQQSLNSTEVRRRHESKVLLAALMFCF
VFMLVELMFGVVAHSLALLTDASHLLIDVGAYALSIMSLRAASRTSCGKYSYGWHRAEVIGTLVSVFSIWA
LVVWIVMEGLDRSWNVVKCSRIHAMLATTAQQYKRNNSTSYYGFGNISQRPTVDKDGALTEATHMEMCTSI
DSPIMVVVGVLGMVVNVVCAAILYFGGSHGHSHFGGSHHHSHSGNGEEEDSLCEENTGHNHSHDHGHGHGH
SGSEGEGHDHSHSHSGRGFAVHAALLHALGDCVQSLGVILAGIFIYVANRYSYGVPSYRYSIYNLADPLCS
LLFAVITLNMTRPLLRDLLGILMESTPPGINYSELLSALRSIKGVEGVHDLHVWSIASDYAALSVHLEADD
KDAALQEAQEVCKRFGITHTTIQVDTVENGAGLCHSLCASAQTIA
>tr|C9ZX19|C9ZX19_TRYB9 | DeepTMHMM Topology Prediction - Predicted
Type: TM
MPKAKTTTPLQVCLFYPNLIGYTRVILSLISFCLLGHFPIAFLFCYIIGFVLDAVDGMVARRFGQCTQFGA
ILDMLTDRASTAGLIVVVVQVLQPLPHWGATSLACLVFLDISSHFCVMYVSLYAGRTSHKDVSSSIFSLLR
LYYTNRPFMCALCVGQELFYLNLYMYGVYGIAEPAFLFFMALTAALSAFKQVVNVQQLLDSMYHLAVLDAA
GSRQ
>tr|C9ZYC0|C9ZYC0_TRYB9 | DeepTMHMM Topology Prediction - Predicted
Type: TM
MVAWGGMTFYGTALIGLNALALWDLYLQRRQRKAYANAKMPEHLVGVVEEKEFRTTQEYEREKLSFSILLH
VKDIVISNVSLLAKLPAKLYGSLGQLLPGATGSFSHCYVYAVATDVLTTLISLPFEYYSTFVIEEKHGFNK

MTRKEFFLDVAKYFLLRLTLLHVLTSGLILKVVELFGEDFPFYFFLGATGLITIFTFVYPTFIQPLFNTYT
PIPKDGELGKKIYALAEKHKFPLKKLYEVDGSRRSGHSNAYFYGFWSKHIVLYDTIVEQTKGDHDALLAVL
CHELGHWKNSHDKFLFGFMVAQTWCISYGAKAVIFNTDLYKQFGFSDANPLIGFELFSQVFLEPINTLLGY
LVSLVTRQFEFQADRYAVSSGYGEPLIRGLMVIHKENKNLLTPDPLFAALHYSHPPLAQRLDAIKEENKKR
K
>tr|D0A7J0|D0A7J0_TRYB9 | DeepTMHMM Topology Prediction - Predicted
Type: TM
MAGDGFVDEHQYFEHPRYHIFRQRQETASPIESSADVEGQTNDGEQQFILDKGAPKRSLSVPMLMGLMYAY
TTSGAYAIEETVLGGGPLLGIISIVLVPLLMAAPTTIVVAELATAIPSNAAFLMWYNVSFHRVVYFAMVLL
TFLLIFIDNALYTVLISEYVCTAVPCSDTISKLLRLGMVLVTYTLNMVGVQAVGKLSIALSIVTVAPFLTL
FSMHMIKRNFYLNWPAISYIPPSIDWATFITTTSWNLCGLEQAATVIEQTKAPRRTFIRALAPLLGLAYLT
YIPPILTGASIREGLPDLSQWVTGFWSDVAFSVGGVPLRVFMVVASALSAHALLLSSFCTTTQIIAGVAYT
EAFPGPINRALYKRNKRFGTYHWTLTLNAVLSALFGVFLEFGPLVKVDQVLYGLRVLMIFIAFLVIRHRHP
HLKRPFRAPFEGKLLYLLIIPMILFAGLIVLGMVESTQSVIVNLSVLGVVMVISLVYCQLVRKEDFYGRIV
TGTLSEDEKQ
>tr|C9ZR01|C9ZR01_TRYB9 | DeepTMHMM Topology Prediction - Predicted
Type: TM
MELQSLIDTVSLQKLLLLGALLRLILIAYAFFHDQWFRVKYTDIDYMIVVDGARHMWNGGSPFDRTTFRYT
PLLAALVMPSIWIANPMGKLIFASSDLGAAWYCYGVLKSFAKERSAKWMVSLFILFNPIVLSVSTRGNSDM
LVTFMSLMVLSKFARRKCYQAAAVLGFAVHFKIYPIIYALPLTLGVWEQSVAASTNTWRRVVKTAVVVSIC
ALMAAISFAVPTVLCYMKYGQQYLNEAFIYHVYREDHRHNFSPYWLLMYLNMARRHLGQGVDFSPRLVAFV
PQAVVLSFVSYKLRRNTAHACCVQTVLFVAFNKVCTVQYFVWFIPFLAFLFCEPKEVEDDESDGSGAFKFF
SWVKALGVVLMWAATIPLWVTTAVPLEFHGYSDFAKLWIVSCLFFLAMVVLASMLARIAYRVQCTKCSAKS
IKVA
>tr|D0A2B6|D0A2B6_TRYB9 | DeepTMHMM Topology Prediction - Predicted
Type: TM
MKRWRRRRKKKREKEVVFFSFFFLCVCVRECVYVCVCVFFFLKRGKKKKGRRKHTKRRKTTTKEEEISERE
REKKKKEGNNEARKKKRRKEKKERKGNEKPHKHAKKKRNGNRNEIRQQEQEQEE
>tr|D0AAL7|D0AAL7_TRYB9 | DeepTMHMM Topology Prediction - Predicted
Type: TM
MTIKMNDFVNLRIRFIFLICCSFLCLFSSFFSFLFVPSRLLTLLLSNIDVFMGCEVGLSPPSFFRSFVSVC
PCFVCCLVFGHSNDIAVMIITIKMKNMIIIMMIIIHV
>tr|C9ZKU9|C9ZKU9_TRYB9 | DeepTMHMM Topology Prediction - Predicted
Type: TM
MLRRSVPSLYSTIVAPIGKGRVVPLNFSGPKPVYQPKSFVTQFNVLGMWTISNMIPNFIFGALLIAGAHGG
LAGAWPPDPHSLHP
>tr|C9ZIV2|C9ZIV2_TRYB9 | DeepTMHMM Topology Prediction - Predicted
Type: TM
MIDGGAAPRPDNHHIQCESPTQLLRTTQKAHSIRWHLSKISAYNRITGGYESSDFESLGFLVTVVTSACSA
LAVLVNELALKRRGCCGMHIAPPQVPTTQIR
>tr|C9ZL31|C9ZL31_TRYB9 | DeepTMHMM Topology Prediction - Predicted
Type: TM
MSHSAFLHNRLWRKQAGGLTIKSFNRFDDLGPLTVPVSPPTSDLSDAGITVRRSAYSVLRMRNSQPGVCAQ
NLLSCSGKSIEPEMHKCCCSALLYRSPHRGEASTTLSSDEELERDDFVFSLKLAEVEQMLQELVGVVRYEE
LVGPALADARWIGMCQSMPEAVEGAGGWIARVLLTPEEEHRIKWLREDGVQLSDKLACIPSFKALYVAFHT
VGGEGLGLSHFEVIEKRMREGSVKWDMSDAAVACFFAMMLLCNAPRESRPFCCHVVRDVLLETGSQGGMVA
ATAPEPTLRHVLGATLLLRMVGVLLLEAQGTSGAGLDPPMYLFAAITEFLAFVKSLPVGRFTCVLREIAVE
CRARVASLASFYTVPVGSSCLPDFTLWLLREVPNSASCHLHFRHMTLRSHTEAITLLEKSFLLSPQCLDLH
VVVDVLFPLLDSSEVDDFYFIVFNEVLTRLGERLHRNVQSFTEDDFRLITALGVRLFEGQGSGLGQSSSQT
AGNCPFDAKKLEQLEQLQVMGGSPWSFIVLSYDYLKEVMRSLVRDGRQSKYQRQYGRSPISSVAEAAALGG
GGGIQRKSLFPKAVERGKNNIPCIITTTGTECPDASEGGLESDGAMTFRKGYPLLSPSSLTSLRERFVVFV
EELVKTLVDERGIHHRSSSVVSDDLFTFVSKHMSLPNIHDEYRRKIYLLVNIMTKETSTRIGDEGRSIATS
EDDIPPELRYLVTSLSLMLSPWAAVMILRDVLSESSGMNPRYTYVLNAAIDLQLPTSTTRSRMATTLNMWR
FISSQSRKMTFAEHMAMKRRCAVNIPLSYILSSYWSLLTWLTLLTIVGLNVAGLDLESQYAATCLFRDFAP
PEDVIVPLADESCLNTVHGAVSSYFEGSGLNGAKGLTGKSGRREVTWDRISQEGLFSSAPYIFSRAADRRL
PMTVVSVGSEGSVSAVDQVSREFACASTFLHELAGRSIFPFYLSIDQMLSADLIVARVAERIRRVSFDNAW
FVSRAVFRTFPLGRAHTEHSLVTHTCDQASSKRRRITFLLYVQRNIPIPPQLLLRLSDSVLAQHGDLVLVT

HDVSLKVGGCTPKVLEQLAEAHRIRCIPCAPTGGVDGGESMCLSRFWELWAHGNELTRRCGAFAKLLWSAD
HVVCSSRHLRNPSAAALHPTKRVLTDFRCYSL
>tr|C9ZZ32|C9ZZ32_TRYB9 | DeepTMHMM Topology Prediction - Predicted
Type: TM
MYADEEESRFTGNIHTMPSMTANGKNKESATETTDLLRNGGSKDDENVGYTSAFTQTIVVMRHGERRDGSV
DAEPEADPPLTEQGLANVSNAATELRNILGSRTTQNLQILTSPFLRTMQTAERLQKCGIGVRRQRIVDNSL
CEVYGPLRIKSKEPPRLPDDVVVSGRGSLPLWGESLESATKRFADALQMNSRTYSEANLLFVTHGDALGAI
VSALYPMRMVYEAEYLSFIVLRKNKIENSLASGCREFELVASKGVQWMITGPEDCDPQESGHAASEDSALG
KKSDPFGQPRGDSPLHISRRPFGAEDGASPINSHNLSNEEGRGCLLDFWLLFVFRVLAVASQAPLFFFLET
NLKDAGTHITVVLLLELIFFYASTTDERYFGFYQALVAVENFPAVCRRSYRSLSGGHGASTPRPNVLLVLC
AASLKCFAIFAIASGAAAFLSLFPGIHFTMFDSYKMIFNSFWSALLVLFLLLFNFVRVYCYVSVALAS
>tr|C9ZMC0|C9ZMC0_TRYB9 | DeepTMHMM Topology Prediction - Predicted
Type: TM
MARQPLGDMYAMTPERAREYGFVSYYNHTGSEASDKLANNITLTSEEWAQLLTSPVQFILQSGGWGGAGFD
VTVLPQLLLCLPWTLAFLIFRIFAQRQLSRFGLWLQVVVPKDGSKATLNNAQRRKLRKFQNQVWLTAYYII
SAVFGYAVQCTKPWFGLPVSESNRIALLTPHPYKPDGGLMCYYQSGLGFYFSEMLALPVENDIRRSDFVEY
FVHHIVTCALIVFSHCSYEHRFGVYVLFIHDASDIMLAAGKVINYVVSAERKRAQRLKSNGGGDKQTKAKP
SLLYRVIFNETTVNVCFVLFTLFFVFFRLVCLPYLALANIVYGVKIRMFTGSYCLLIILLQGVLQGLHVYW
FTLIMKIVINSLTGRRVDDIRSEDDDDGDGVEGQAVPKNSKPHRE
>tr|D0A325|D0A325_TRYB9 | DeepTMHMM Topology Prediction - Predicted
Type: TM
MLRRVAGTELSRTVARLSVPSRRYTNYDHLHYATDTKAVLCILGYFVALWFVMLRMGSLFSCRAKYKDDYL
RVWPRKLGPGYQWSDAWGPQIDKFFRNVPDRAV
>tr|D0A3I7|D0A3I7_TRYB9 | DeepTMHMM Topology Prediction - Predicted
Type: TM
MDALRRVGCPNSSEEHRKGNEYLGDAAVRYARRGFPDQMCFGECCVDARQVLQHLQFLYQVNGGVEYCKHD
YSGEGSTAFPALLSFFFLMIFFVLCFLPVWVSRRSDDRKHRAFSIWMQGKVFQFSTFGERKGCFAPLQWKL
SKRRRLN
>tr|C9ZKM2|C9ZKM2_TRYB9 | DeepTMHMM Topology Prediction - Predicted
Type: TM
MCMSYVHVCELGAREDKLQHLPMFFERKKILLILLFCFTALATHIFIFIYAHFFFVSFSLVTVSVFSSKYG
YVCLCLCIFSREEEGKRGKCNTDDCVKFLLHVKVYLRFFLFFLCSPLFSFLFFLFFYFPLFLLILIFPFFL
HSFRKKKLRKSNGWW
>tr|C9ZZ01|C9ZZ01_TRYB9 | DeepTMHMM Topology Prediction - Predicted
Type: TM
MVCRCLALSSHYFNAAVNVLCHFHFSCWCCGERILCVALPFIFIPCTIKLFFYFFFLLSARSSIRMWVRFT
VTNIVCFCSSQYLPLLHFFPFPVKVVLIKHYFTCTGCY
>tr|D0A072|D0A072_TRYB9 | DeepTMHMM Topology Prediction - Predicted
Type: TM
MTLCIPFFPLFYSFMHRTFFYFFLFLFFSLRYPYFFFLVLSSLLFPSFFLLCFFFQLNPYTYHPVAFHEYF
PTYQLLQGKIMFFFFFCFISKRKRKKTNKQKQTKKDATWLSPLTPSPSKARAHVGTFINTN
>tr|C9ZI45|C9ZI45_TRYB9 | DeepTMHMM Topology Prediction - Predicted
Type: TM
MKRYFFCNLSSAFMNEVMKPWKAENTKTSAAFSCLHAPEVLLFYIVYTPLPNCLFVVVNFYDCHSSCLFLL
VI
>tr|D0A9Z4|D0A9Z4_TRYB9 | DeepTMHMM Topology Prediction - Predicted
Type: TM
MSYENFAAHAHDRALASGNIKPPPPASYLLIWSLPLLASFFVFGIARPLVADREVTPSLTHIGSGRSKRWW
VVRLHLCVSTWLLFYCNFSVCAYLLLPTRVLLRLRMLPLSNNTTYGWRMRELRRALSFGIHLYDWDDKMYT
GRISVVKSVESLASSDGPVRDELAPVVPLTNSEMWKYRSVVLAALVVTLFTLYFLASMIFRLSLGLPPCGE
HRSNLALPQGSEEEEHDEDDDKNDEFDISFFSVDNEWWNMGNPDDACVDSPEAQAEEERLRSREQKRQNRQ
LPEKKTQSVRSVVWECWPLFASVCYSALYAISGGVAPFMWWVFPSSVVIVIVGSVLV
>tr|C9ZL63|C9ZL63_TRYB9 | DeepTMHMM Topology Prediction - Predicted
Type: TM
MSSGVPVSSQWPPVRGGLIILRAAFSGARQSLDVVKFLSYVGSSAEVSRNLRSCTLVNFLFFFGIWISSIV
FSPLWSLAVTLMRGRLTLAGDPDAITNSASLSNVNRVVGGATGVVTILWTCVFYFVWVFPMYGITLLFGIR
WYNALYAAANSEKRRRALLMAAARSPGRPVVPVRGAAAGAGPKPTASSLSFSDVVVSLTETLFKVVATSLC
TVVMTVVGAILPVPLGACVDVAMRSWLHAFYVFDYRVSSQYVTDRVTQRRNYVGLSSAIEFFEANWAYFLG

FGASRCLVTGVLDLTGIVTSWFAKEAAVSVLFGAHVVLSVEAKLPPRAPFSIPMFTPFFILCDKIIRAVVH
VAQ
>tr|D0A6S1|D0A6S1_TRYB9 | DeepTMHMM Topology Prediction - Predicted
Type: TM
MKLILPFSLLNPRFLCDFALLTCLPTFLFEDVDLWEGDHFSLKMVYFIILFSDINEKENYNNKKPKAIRSD
KGLRCITSVKSLWFPASVETLAFVSTVTFFRFLSVTHLLSLSTTLYSPVHFLPFP
>tr|C9ZP86|C9ZP86_TRYB9 | DeepTMHMM Topology Prediction - Predicted
Type: TM
MLYNNGNCQVEIMAKVKNEKEIIKKKKKKKGSLEKKKKNSQNETKRVRNTIKNNNSNKNNNNIKTIDFFFC
MIKFKEKRNVRVEVFVCLYSSQFPSRVVYFGSRGNQSEHFKTHSFISFFFSSFSLFLFLFLSFFFF
>tr|D0A6G4|D0A6G4_TRYB9 | DeepTMHMM Topology Prediction - Predicted
Type: TM
MDGLSTQYLQMANGPQSQMHDPVSVVNFVDQMQLAFERSTAPRKNFLLDSTTPRGDAIITEVSAMKPGNGW
VTLQVTEGIRRGVHEWGVTIENQGETTDGSGLMLGIVPKSFSKYDSFISQGGGWCLSRAGKFYGHWRRHEA
NASVTFGTGDRVIFILDYEAARMTVRVGDKYVVGEISNIAPEVFPAVSLHYRHQFVRFEYRKVHDRQSKKL
NWIERLAFPHATVFLPLTRQQLEDVPLGSYVFSPLFAEGQGCAPPRKRQGEHGPATCESLDTEERGAKLHC
DYEAHSAAKARLTVVLRAIQAVRRYCTNGVVAAHTFLDRNVTAEMLRSQRHALATRGSDRRGGGDAAVDCG
ALILIHMFSHSSMTVTGVTMLPVLRSLQEYLRSVDLFSLSEASVGCTNAGTVVSPDVVRMAADNVAGLIDR
ARRNECDSSNDTAVSGMGSTDAGISLVPGLTELLVLVALQCGTVCEVLRAVRVLLRAPTSDVSPEMLTWLR
LNKAALTPKCYLPKLHEAYDAIEEDMEHVTPFAPTIEVKVVSVGYHRGAIYVHTADTLRRWGVFVGAYTPT
STTTEPRNCCLNCTSSSIAFMKDEGVIFHTDRMAGAGIAVVVYSSSLEIQQTVMLASVPQWPTTSNYVRMC
SGPGHHILLVYDVPHSDTSSALRGTAAPVPSTTAGALTDDASKASATGIEVQVLNGKKFPEVKWRTKLQFP
PFDNTTRSCVTLRKGSVIDFGNPETCLNTTGGHITVEVWIKMLDKNDVSVFYQHGDRSTSGEVFLETARLE
GAWRIRGGYRHDYRGMCVVSAPFNPASESRFVHVALVFDGNWRLHFDGEEVSRTKQCLQVALENPRQRWTA
GNDCVCQLAGLRVWKGGRTFREIARDSSRVLGGDEPGLVCQYLFNEHNGNVVYNHVRGEAAAGRHAIVRGA
FSRTVCNDHPLLASAHSEPALGTSTVRVTAPTYDLPKLEHAHVFYDGVHLGILEQKERAFHGFAEMKCLHQ
AVFFDIETGRGVYGSFMLRHRYKLGLLGCDENGRYWELFQAGGQPNVSLFGQQQQQQAQLPATVSAGTLSV
SPSAVLEEDHIAWHDNLEFLSFTSSVKSETPGDAPKGSIYGDMGTSSPTDNDTNTQHMSFGALAIWLLNLL
SSFSESNDTASTNSLFSPLLDDVSVRCVQEIRQLLHEHCRPVKSISSRRSLSLKDSTSTIAIGTAMRILLR
LVRRVRDFRIHPDTIGLSEAASVGDLRGVFRELNAIQRRRSTDWSKAGDSFLTTPSQAATMAKAALSVPPK
DIFDGISPTYIPHVGLGLLGILLDIINESGLSLPTELGSIACIIVNEGVTLFFPTAVVRANLLREILGKEE
DSMTRSPALSVLLHAIVRSFTDVTSAAPLLQITEPDNGIPRRNDGIEPNRGKNDDTAAAYGKLAAVKSTLS
TLVVECTQQVMRQAPNNLRQSQLSLLSTMAEAIGVLQMLLFSHCDEFGTVDVHEKMNKGIENMHSLHGVVG
LGATARDYYTELFDNAGLIFETFAQRLEVMSLGEEKETSGRVSFVLDVLGNSFVGSPLHTAITALPLMCSQ
EESGWLLGRLNHLSLRYRALLCSLRASVTTGTTLGSHRPLHVLDTALSLASSWVASFMSLGQISAPTIREE
SASPSHSTVSTPPHDNATDAPSCELSTTLKEICDHPLLAAGIKKDDDKNDKKMDVLMKLQQRQAEWQSIAR
RDVVLASKTPVEIEEVISMAAIAVVHLSSARIEALTMNECIELLAKTLVRLKGLRSGLLDKRSKSPGDFLA
VQQNAKECCHFLLRVARVSDSTVIQMPSLYLARTYADRAQGDPKRPGNVNVDNWKRAVLIMRMKRLYHRAL
VYGWSLIQIPAAVALVERIILSPEIKASDMREAFEERERGALRRLSGLQHMYQLFQNNARLGCKVASLLFN
GRVGGGRHFELGISASGDSTRCSYRQMMYDIIRRLCEITRPITNHKGFPEATTDGSASPASSPRGVVEPCR
SATLSEVLNRQWLPSDFCFFREVRVVEVMFLSFCSIFQPNASETLRHERALELSHRGAVKGNRTHPVAGIG
GSNNGTTFPSSTTAMSGGRTSGSGRPLAVVAPGGVGDEGNEQTLQRTPSEWALLESLNALKSLGLQCAAAL
GDENAAPNEVRGCVNFLDLVFEVLDEELRKCANYASQPYHHSQDRVLEHIGLLCTLISTFARALPAGFICT
EVVCPKAAFLAFRLWLVADVMVLASAMNVGACPLAEVCVHTSILLLRHCSPAAVNPLFTKESIMSVVRKLL
PAVTADCAAVGYFFSFAIRCVSGDSSTGGSGASGSALGNFGDIGISCVASLHALLCSHPWDETFSAMRGPF
LADLADSANGGEAMRPNGVRLLMWIYLIGGPPSASLGPGRKVFMCVDETLAATEEAYIVDCNAKAGTATVL
PIQVGAFTEEHEVPLDNLINASQDEVLLPRSEVLFQFLLPALEQCFSPRLRPHSSPLPWAVIASLLRITLA
VLKKDPSASYVLLERGIIAQVDDFAFRTPAKTPLPLRYLYEMWPLLLPQTMNCQQRALFDAVTSSSKDNDA
KALSSGYVYPVRTPCHDIQGPPSDPSAHTANSLFSVAAAARYSVLPRLDVECSNADVDDYNGSGNMGGSPA
NISGSSVGCTGLPPLFSRLQQAHSSLCLTPSVLCFSGARGDPAGTLIMKGVSKDSCIPPWADGITIEASVL
LYDRLFPELGEAFVIPASWGRPPISFSLFSLYEKKGDGRRSFLRVVLDEARIDCIIETAAVSAVVISTKLL
QEDWDHWIHLAVVIDSKSITLYKDGAGVAAALPKHLGIYLENLFREGSVDRLVIGNISSDGKGTGRGVVSV
DETDSDEASSSGGGRSYGKSSNGVVVALDSVRLWGCARSMKTQRSAADFVAREQTLPVYGVNDGSNVTFRF
SEATGNETYAEKCEGVGVLSGSVRWAPFPVFSSNVNLDKVQRTGVAPPTALPLFIPRREFESFFATLDHSL
ALRLGGEYMQSICAHLSRQCVVAAIQQAMSPQYRVVHFEHCRTKGCGDVPASAIVDPRNIIASTELISHLI
NLLRYSDTGYLEEKAVLAVSQFVKVYFSSMTSEKRLLKGFREAAVAITKALHDETEPFCVELPTVSYVATD
ANLVPIATINGPGGVVSFDANSRGIEQMTLLRDRKQRVLLAKYPDAQSGWPELEVPSDSLWFYAKPSIGTR
SVAPSFTVSARSLKPQVACAIFAALREVLSSDARFHCGLGSFLNAPFISLLTVRTGTTKCAAVPSCRIFTS

VLELWREFPQFSQQDQCPLKITLANLNAPLMSVLQRGHASVASSPIGRHNVRVQSVFELLVAAIRTEAAWD
ILTHNQRTQIRWRRLCSLWERSIGLCAPAITIGVNSSALPASATTALGNSNRNHKVSNVNAGERGLSSSEH
KHGYVHLVLLPFSGSTLQHQPKASIFQRGSGVWNAFCDRGFLSARSNVGFKHGRFYFEVRIPANGDPISVG
AVTERAQQNSFLSPRGLGHDGDSWGFESAQMCRFYHGFRHEFTMRTKWKGLDVIGIMLDLETDTLACLHEE
RQVSVFDNFRASLGAGVPLSFFPAVSFGAGGVDVNFGAAPFVYRVPAGFVPVDPSNYMASPTSTPWMLISA
IDVSECLARSGKKGTGTDASDGVAVAISSSKETRQLPPFLARANASAATYYEGRSGPLCVNLLPCGPKNKM
TSVQGSEVRAEEDPRFFRGSVCVRSGRWYYEVGLRGDALISVGWATSTTATDWSRSRGLGDDAESWVLEGN
RATARHNKHTRSVGGQLWKHGDIVGCLLDCEAGTIAYTLNGVPLQEAQNTDGEGILFRSVNHANGLMPVIG
VDPKNTAVVCFCEEELSHLPKRCRALGTTSPLRKAVENHFCGVDTSGVAERGVSGVCSTQFSPSLARRLLL
TLSSLTELNFHNGAAYCLEDTLTKTDKAVLCDKFLGHKNAMQLLVTLQNLTSLAEAVIPYAGVPSIPMECD
VLLSGPIRRALDHLHDYMLPAVGLRFLHGFFSQTSSAGENLKLTLNRRKALSLVKDTTATLPERLSGSIFG
QVYQLLHDKNVSLFCTSRKLWSVNFVGEGADDIGGPYRESITQLCSELMGPSLPLFIPSPNQASEIGESRE
VFVVRPEMEAPVRLNMYHFFGRFLGGCLRSSEPVPIYLSSRVWKTILGAPVGVSDLGRVDVATVQSYRYIQ
QLSSLGGGEAGATDGEVEELCPGGFTVVDDIGVERELFPGGSAISVGRHNVDLFLELAMDYRLHVMGEAQI
KAIADGFHQVVPVSAVSLLKWYELERMVCGLPDYDADELLDAARYEDLNPDSVIVQYLRQVLRQFSRHERA
LFMRFVSGRERLPSGVRLKVMLDTSAQQTRDERGNNASGADNAENSNMGGDTFDDSRLPHASTCFYWLSLP
RYSSVEVMRERLLFAIQHCLDIDADFMVRDTGSMEDEGEPMLAVIVEDDDEFEDFSHLR
>tr|D0A8D6|D0A8D6_TRYB9 | DeepTMHMM Topology Prediction - Predicted
Type: TM
MYAFTTQIHIYIYIYIYIYIYICVCVCISVKVKMNKQKVWTRKSEMNDALHPALGVPVHHLVTRQKGKKQI
SFISHITSPTPSVYVFLLQAFPFLPMRMCAHPRTCAVTHLANLSYAPCGKIRKKELRPLFASSPPPSRQCY
TLWRQRRRHGTTAATALQNNSNKTYKQKEE
>tr|C9ZPN5|C9ZPN5_TRYB9 | DeepTMHMM Topology Prediction - Predicted
Type: TM
MSVHWIESQCASITLGSYSFTSRHVKTASGCFGTCSRSNGKPNDDNGERLVHFFFCYSCMCVILIVCQCKW
SPPFPFLTHVFFFLFSSAAASREAYLLNHSCLQLS
>tr|C9ZYS9|C9ZYS9_TRYB9 | DeepTMHMM Topology Prediction - Predicted
Type: TM
MTFFFFFMCICTGRHLEGVGSLLSLSFFSLFFFLSFCYCFSIGLSFLNFFFHFTFSHSISPTLLPNYSLHA
FVLLLKGYMRRRLLFFLFCSICCVLCNFATAVKRRKGVRAVGNFFVFFVFVFLSVERIDFSWGDIQSEKGS
KGG
>tr|C9ZRR8|C9ZRR8_TRYB9 | DeepTMHMM Topology Prediction - Predicted
Type: TM
MRPSRRCSAAEPIKGDAYGKKLQESTSTRSAGPISGTDAEKPVLHVWKNLGDDSGTITSFWKTIKSPNCRY
SAMLALRITLVAALPLMLISHFYKEKLPMYAVLGLLGVANSQDTVGEQVSFTITLVQVGVWSTIWGSFLSL
FDIPKNEALWWSLAFLGCFAVGLTGNLRCRRQAVLFTIIVMEVQRSKEEWSALTALLVGRDIIMSAFFAQL
QAILLPVSMNRRVDREMAKACGYLAAIVRDAAKVCWSKDPLEVQLTLPKLSSDPLQDCFITIPGQLTYVSY
EVWVSTRQLELRRERLKVLQDVMPMLHGLTAVARIMSAEGAYRKHRHRSTEKMGEDAIMSTALERLNDSVN
SFCDALSNTMEQLSTVLEPRDVIERVPFDEFATATSNLQSALDKLHYDTMAHDFVPEDTVWYMHLVFAHHM
MILLGEEIHKYAENLRNFDPSRFKSGSRRFIEFFFLDVFHGFVKMIKNRFTLATPHDVRVFKNAFKIACAY
IVGCAYSLYIDKDNVYYFGMTILMGVGLPTAGESIMSGVQRVAGLLFSTSLAYAVDRNYHHGWESFVCVLF
GVFIALFARVAPAYASCALHCAITLPPSLHVVSDFRLTLSRLVSNAFSVMSYYAIVVFIFPVDPIKVLHNT
EVCVVKALSENFSELMGLVHPPVGEGDDEGNEIMSLLLRIKGRREAVWETLKSVRVCMGVAATEPILHGIP
YPARGQEEFVPVLRRLAASVDIILLGLLHTHGQRPGGMDHEIQQVFATIQPVVREIDKYSVHVMQDFVDAV
HKPMEWSYDLAAEHYSKLRELTVLLRAAFKSVYEDIIAGLRRNAVQIKLGKFPNHNFLDMSCTFDGASFAH
DANEMSLSFGDTLRLDEQLDCEELTAPRNASFLLAPKDFTINHDMNMSIAILVGINLFCTQLKKTMEATSQ
LNAFERSRIGS
>tr|C9ZMJ0|C9ZMJ0_TRYB9 | DeepTMHMM Topology Prediction - Predicted
Type: TM
MLCVDRQVPGTLRRSHYLVRLCCFPPLFPLLRLFRWLIGVVPLIFIFLAGIHTLPSVSVFVRPQRQPTYFP
CNEEEGGSEHNRAFRRCTCCLRGATARQKGIMRREWETKVENEEKKRDTNGKEERERSIVLSR
>tr|D0A7I3|D0A7I3_TRYB9 | DeepTMHMM Topology Prediction - Predicted
Type: TM
MFVVRFVSRLLPSFFNLNHGCRVADDNYQPHHWVVVGLLLNFLTVVLSPLVFFVLNVFLLVYMSASPHIDF
FFSPFLCIVTFYVLTPFSSRKFVHLFPFFPKKRRNALKREQGVTCLCDVANFHILFCYIDMHAHMRANNKF
QLPLYGTKVNEEECVGIVRDQACPGAGRRRKLCFFLFLSIRFYPF
>tr|C9ZL76|C9ZL76_TRYB9 | DeepTMHMM Topology Prediction - Predicted
Type: TM

MDQMPLIPLQSYVQNFLTSLTTSTQTLPLHSLPYPHRVPQPHYSSFSHSFSSTPPIFKVFMYMCLCVRVYV
SVSIFWSFSSTSPLFSISLITPFWMVTAVTNISKRKKKEHLTLLIPVTQKTWGRNERNKTVNEEGE
>tr|C9ZQX9|C9ZQX9_TRYB9 | DeepTMHMM Topology Prediction - Predicted
Type: TM
MPVTSFNNRHSIVDEATAAFMLCATEVKRMWGEQSFIAKLCAILMKHWRTRRCMLFLVAWIAFMGCCYGLR
SRLKRLLLFSTDQTDEEKDFPEEGTISEGSCSVCENQCIEVGENSFSDVLYGSEWPRFEGAIPNEKVKSLL
GDDFDPTDQFDATFNHFLASQSGRCDGDDTEECGSKRESGE
>tr|D0A2A7|D0A2A7_TRYB9 | DeepTMHMM Topology Prediction - Predicted
Type: TM
MMWCDSIAVITISFIAFVGAGCFRERLLLQSLIMFLIYRLLYLFTSSFFFFFSALFSYYFLRCAISLHPLF
VGKYGVEFYHMQLNNIIKWSSQPIAHSRLRREKCLSLFVCQAVASSLDTALAIF
>tr|C9ZJS9|C9ZJS9_TRYB9 | DeepTMHMM Topology Prediction - Predicted
Type: TM
MSLSTLLYKAEVRQGLVSRNAGVWLNMCWCTTQEVAEVLSISRLRGDVSESARKTADERFMQLHNGSMNIG
DILTEVAVNRGVCGGAPSDSLQLAAALALEPLVTKGRWCEGNGFNDKVRVLDMLLGFLSDEAVMSSGNATH
TGRVRVSLCLCLCQIITFESPTPYWNGVLSDCLASIVSDWSGSQYEMIDNDSEHIFKSFRHIVVLQLSELC
ASNTALWWYMVSTLSRCLVEGSSSFPVGDVRRHFSFSVVLRLMRRRPRVSDTHEGMRPADSFIFGPAFRQL
IDGSLQQVVALAGSDRWSHVAVQQECRLLLDISSHLISNAEFAAQLFRCSVDLLVLAEPTAYDTIAASTLV
AAVAARVDSALECMSGVLQAFPEVSLRFDAGEVTSKLLNCMAGAAAAARGDECDDDDDKEAELLGMLREDY
VVPLGCAGGDTTSTAGAVLELLVEANPQQLSTIVDALCNCCSSAEGLLSLLRSAHWRAWAAALRCCCRVCE
EGETSLIDRMPHVEDKLMLPLAQLLHESTESMVSVELIDLLSLCLVRTTQGALGCSFLALLDGVFHHCNSV
FGLATGGTGIAARGGGSSQVALLSVSVYAVHRLLSRCGESVTSTLVAACDVQLWLERSLSVLASGGLLAVY
CGAYAVKTFLSFAPRTASHYPALAAAAVDSACRHCGIPDASSLFAGLLTCLCDHKEGECALAGELLSVVAH
HCQLATPHRSVLLRSTSDYVLRASKLCLEEKSRLQDGVESNTCQQCKVLQLVRCFPLITHFTVSDAPHDEA
TTRLLSSCFAASSSVLFVLDAIDDDVLADMGSSLIALFHASTGANRSCTTLSTVCTAMSVVGLVNPSFFND
GAVLSAVLTLFKKEVEVAPRKGMDYIGCCLPVALSCVRHPQQLSVIAGGDLTPAAARGVPVASAWLVMFGV
WSSISPFADHFTSLYFLAAWHRLLLYAVSVRASDANRLDMLATSAPCCCVYALPSLHVKSLPARRLAGCSI
AQCIAAGLSLVVVRGQRAAKSQMLDAEIRKPLYIARRYKALSGLTMHESGADIFLSMSVPEGASWLLQQMC
DAGYEAEVNMAMLFAADC
>tr|D0A7A2|D0A7A2_TRYB9 | DeepTMHMM Topology Prediction - Predicted
Type: TM
MLRCSYFNSLSFSLFFYCDRILLAQFFSQAILKFVMFSKSVALVFAIAFVMEVVRAAAPDPAPGGTTEKEF
PLVWVCLGITVVGIGIALYVAHRRPDELHIPGASDEETEEMTRADGPENNENPGANETAAADDGAKKEQAT
V
>tr|D0A5K3|D0A5K3_TRYB9 | DeepTMHMM Topology Prediction - Predicted
Type: TM
MFGRGFGANEIREEYNFGPSNADDQAPSGNHDSRTHQSQRQGARGSHDTPTNMEEMNVFSESDESLHRSRR
SEDVPAASRYRDAHSSSFYPEGSALSTTEIPSHPPPSAVASNQGVSGGDGDGLNVTAGGVAAQLPEVSKAD
IVKEVTGRSNTNTNGMTVDGKEHALLVERWKNAMAEEERLNILQRQVEYQLKATEGANLEANFPPKFLCIK
PLVNHTIGMVPEPRRKYVKFNYVIWMVNCVLLVANASVAITVAFSPYENEFEGPKASNKAQATALSIAYLL
GVPLSFFVWHWPIYKACITGLSTRHLVSLCGLLVALAFAIFMLVGLPDTGACGAILAMEVYEGKSNYLLIP
LGVVISLWCFETAYFFYCMVVQWKFYRLDIMAQREARSHIDNVIGV
>tr|C9ZVB3|C9ZVB3_TRYB9 | DeepTMHMM Topology Prediction - Predicted
Type: TM
MEVAFDVYASLLPVDRNSLRDQMFVYWLLLTTGGIVMYLLCASFSTYLFFFEFAETYFPRTIAKGGLHDQI
IHEIFIAVTSVPFMAILMTPAAVLSHRGYSKLYYNVDDYGWPYLFFSAALFFVFTDFMVYWFHRGLHHPTL
YRHLHKLHHTYKYTTPFSSHAFNPCDGFGQGVPYYAFIFIFPVHHYLFVLLFMAVNLWTVSIHDQVDFGGH
IINTTGHHTIHHEKFSYDYGQYFTFWDRIGGTYKAAKQTHSLSLFGRGGRVSAQKQE
>tr|C9ZTB9|C9ZTB9_TRYB9 | DeepTMHMM Topology Prediction - Predicted
Type: TM
MQFNIYIYIYIYIYIFVHLSPSLRSLHAFHYSFSNSFLFFFLSLSLHVLLRNPLCPLLFPSAKERKRKVKE
KYHALIPLAPHALTHKKKKKKMETQTQTHKHSGRERERNIVWK
>tr|D0A8R5|D0A8R5_TRYB9 | DeepTMHMM Topology Prediction - Predicted
Type: TM
MLHLQFNAIFVSLSFHLSFFCFSSSVFSPTTFYFFNLFSLSFFSESYLTLPFHIGSHGRGELERQKTISQE
KKKRIDAVRHVYSEKPSHRNLLRLFTTVAQMIP
>tr|D0A3C5|D0A3C5_TRYB9 | DeepTMHMM Topology Prediction - Predicted
Type: TM

MNGRSALISLGGTKQAKKKIHDNHKKRACNQKKTTTNEIGKGTTGTKCITPPFLPQLLHLINSKCAPHIPK
ADAHLHIFFLPLSFFYTPTKKMIAARRWCQSALYLFFFGGGVGVCL
>tr|D0A5D1|D0A5D1_TRYB9 | DeepTMHMM Topology Prediction - Predicted
Type: TM
MNRVSKNMRERRRLPPFIYTISESALREVSLWEHYPVVSRRVPYFLRSSLVWTDGVIVTTYAPSDLTARRV
TQVPDYTPHVCRVVVAFTSPFIFMLCSPLTHRCLPHGIFCGFVGR
>tr|D0A7M1|D0A7M1_TRYB9 | DeepTMHMM Topology Prediction - Predicted
Type: TM
MENQKMGWLMLQQRCVIIDSVCLCLCVCVCVGGKSDEVKLPAARQPPSLYRLPFLFFLYSLPKFKSFIIYC
YFYFFLLLHFLHASYFVSGVFFLLLESLAPRPVTHLLRRGKEKWGLLTARQLATEVSFIILFYFFHKHPSF
FTFCNMQVWAHIYVFPLCSVACVITSL
>tr|C9ZJ94|C9ZJ94_TRYB9 | DeepTMHMM Topology Prediction - Predicted
Type: TM
MAARKAGTARPLLQREGEARNFDSYKLYAGASFAVGVVIILCLMKVPRAENSKDTVSTNTGSLSVGCQQNC
SAPFGNVLGIYNGVPAMSNCNSDSCTAELWNTVKVEDIRIPAGRVDPHAVPPIYGMQWQCVEYARRYWMLR
GTPQPATFGSVDGAADIWDLKDIQLLNGQKRKPLLKYHNGNATSANSKPRVGDLLIYPRQPNGFPYGHVAV
VAGVTGDRMFVAEQNWENAAWPGPYHNYSRVLNLSCNPNGTACTVREKDNVTVQGWVRYE
>tr|C9ZWX6|C9ZWX6_TRYB9 | DeepTMHMM Topology Prediction - Predicted
Type: TM
MTNQSSGGNDRGRRDTDPFAHAGDVKLSSDAIALDVTQKGQDDVVKEVKPSLFAVLLEKFIPHGGLWSCAL
NLASATLGAGICSLPTGFNLSGIVMSCIYLVCVAVGTVYSLNLLAKVAVKTGSRNYGEAARMVMGPLTGYY
AAALMIAMCFGGNVAYIIIIGIILKAVLNRDGVPEYLKSESGNRLMTSMVWLVIILPMCIPKQVNSLRHLS
FVGVMFIVYFSCVVIGHSINKIINEGVADDIVYMRTGNSALDGLSLFLFSFICQSNAFEIFREMKHRSPQR
FTIYGTVGMSICAVLYFLVGLFGYLEFGGKSVDTVLSLYDPGENVAVAIAYIGVAIKITVAHALHAIPIRD
GLYHCVGWHVDTVPYWKHVVVVVTINFTSLIIGLFIPKASTVFGLVGAFCGGHIGLVLPPLFYMYSGGFTR
EKVGNIDFFGTYLLLFVGVVAVVFGTVSTIYNTVP
>tr|D0A0U3|D0A0U3_TRYB9 | DeepTMHMM Topology Prediction - Predicted
Type: TM
MCVGTRAAGFGGSAADTSIVVGPARRRSYCGRALVGAAPLPVLLLLLLSVMCVKADIEIEVFSLLHHQKIE
KRFVEAVNAGFNASMTSRRWKTAPSVHVKVMHPFTPSASPISGFQQAVERNRGKLFVVVGPLGDFGTVSSL
ITLLAEQDVVAFAPLTGSSGGRGWNPNLYFTRASPSAELLALIRYALGRLRTIRLGFMYLHNVFFGVEEYD
VAQRILRRMGYGFCGVFTLSSSLTGVASTRHFDSAWNTFVESRPQAVIVFAPPVGDTARFIRNLVADSRTR
SAYVLVPSMLQFAIENMWREALAFAASPFVDGQVVVTGTNPLARDTQYHAIRRFQRDVRSYLKSNPGVTVF
NASSNFDHDDIDGQLLVYGWITGEVLAQALSVPERLTDRKTFMQSLYDQRRYVIDDLVIGDYGGECAGWAA
GQGAMCWCNQGGNTVHVRVIGRGYRLLDAPDGIMMFDSSHCYPSEVEMQAPFNGVSVLIPDRPTALHAAVE
MEEGASLLEVREHNQDSRLFFDTIVSPFSGAAEELQREWSTKITTAVLGVVDEAMLKTPGVAFIDPVPLAP
RLNKMERNVIHLSPTLEQQFYVCTSYLSRNDKKRLHIVIRSTDAAAIEDVLKKTLATFSVEPQSVVVLDGN
ATVEGHLPDSGDVYVIGLTAADPVVIAAHLNSHDSARVFVPFFDVVLLHEEFFNAFEGVSSADRVLFATNL
PHWADEITASEVVQKFYTAERNASRRTPLSLLGFATAYFMRVIISPMKAMNATALVDSIFAQSVVNVGEMR
YGPFADDGCFLKGVPRLTGCAVNYGATQVSVWSMARALNASIPPLTNPMTPSMRYLDPDAGKLSRGQLAGV
IASSILVALLLVALTTVLLHVSRHNTRDNNSAPKEPTDPVTIVFTDIESSTAQWAAHPDVMADAVATHHKL
IRALISQYECYEVKTVGDSFMIASRSAFMAVQLVRDLQRAFLRHNWGASVFDECYRRLEQDRALESEGYVP
PTARLDPDVYRKLWNGLRVRVGVHTGLCDIRHDEVTKGYDYYGHTSNMAARTESAANGGQILLTRATYLSL
STAEREQLDVTALSAVPLRGVPDPVEMYQVDAVVGRSFAALRLDGEVDLVAESELAVCHASDTSSATTALD
ESSQIITSCFETLLGAFTPSKRRDLLLPICERWRVTLPQKTKSVWDDNDCQEVMRRIATKVGRVVDFHVVD
DGGCSTETVSSASVIIISNRVEDFTAHQT
>tr|C9ZKV3|C9ZKV3_TRYB9 | DeepTMHMM Topology Prediction - Predicted
Type: TM
MRVNSKKKRAVNIYMYICMRVCVSVPLGRMIRRQKVGGHIIILLRLLGYCYVRLTHFHVVVVFFCFRYLLS
TIFLCSFVCLFLSCIFLFHMIFLLLSMLTIDFPLASSLLLRPPSSPPHQFTVSLSSVIYTLPFRVKKTFFP
SFTFSVFHYYEKHRFIDPVTHAPVCLFPKIQKRKRRIGSFSLLFPFPSFFVVVILLLFTSPPKEKHQKMNL
LFYLVYLLLLA
>tr|C9ZWX3|C9ZWX3_TRYB9 | DeepTMHMM Topology Prediction - Predicted
Type: TM
MTNQSSGGNDRGRRDTDPFAHAGDVKLSSDAIALDVTQKGQDDVVKEVKPSLFAVLLEKFIPHGGLWSCAL
NLASATLGAGICSLPTGFNLSGIVMSCIYLVCVAVGTVYSLNLLAKVAVKTGSRNYGEAAKAVMGPLTGYY
AAALMIAMCFGGNVAYIIIIGIILRALFSRDGVPEYLKSESGNRLMTSMVWLVIILPMCIPKQVNSLRHLS
FVGVMFIVYFSCVVIGHSINKIINEGVADDIVYMRTGNSALDGLSLFLFSFICQSNAFEIFREMKHRSPQR

FTIYGTVGMSMCAVLYFLVGLFGYLEFGGDAIDTVLSLYDPGENVAVAIAYIGVAAKVCVAFALHIIPMRD
ALYHCTGWHVDTVPYWKHSLIVTSITLAALLMGLFIPKASTVFGLVGAFCGGHIGLVLPPLFYMYSGGFTR
EKVGNIDFFGTYLLLFVGVVAVVFGTVSTIYNTVP
>tr|C9ZS50|C9ZS50_TRYB9 | DeepTMHMM Topology Prediction - Predicted
Type: TM
MSYNTSSSSDSLLCEIDVNIQQQLDNSNFFLPVPKHLWNVQSKKDKHRCQGGGTSPKNTAQSNQPSTEMTC
VMLQKSMRNALCNLELMYTNDTERLKQRLSEEFHRLRESAAERTRQLIDLSTKAQPLSYRVLFVLNLRRWM
LCRGFEFALQDLKDWLRLALLLRETDTLHFLRECTKKLGPYARQRVEDALILHGSDIDSVCQVVMDIPVPT
DETTTFGGPYYNLTLNDLIAMGDEDTHSVHVVNAQHTMPSHVREGLVEWMLLVNVELNLQLETFFLAVSIL
DRYLLRATIQPDREYLTAYAILLLASKVEEKCLFPLRDSVRLCGKTYKVGVLIATTNRIFEVLDCNVVYAT
LSNVGFGFLWQQEPAACEKQYSFLTYILTTLAIRTQYRQYRLSALAAAAVYVSRLRFNIPTGRPCDEVVVL
LPVIKDAISCNISARSGGVYDLFKRSCFHEASRFPLPDLLYGL
>tr|C9ZUN1|C9ZUN1_TRYB9 | DeepTMHMM Topology Prediction - Predicted
Type: TM
MSGDGVGVYGSKRDGATGGVAKTKSTEQMMREANENLLRALRTNTSTNEAGYATMQELSRQKETITNSVEH
VGETRGELREARRIIRDIRLRVYKEWVIKGLVLTLLFVLDVYLFYRKFLGKV
>tr|D0A006|D0A006_TRYB9 | DeepTMHMM Topology Prediction - Predicted
Type: TM
MLLFSKAIFVLFLSFILPFFLSAFLSSFFFPAYFIHPVLPFPFSFSFSVICFDRTCCFSNCCCNYHYNHKQ
LPSNGQTEGMMMKENEGINNNNNSNSNKVNQRKELFLFVCLFVLFFFPLPFLLFLFLCGVLLIVFLSFFFL
YYYSTTSLLLLLVLLLLSLILLFFFEFFMIKVKFFCFVL
>tr|C9ZTH5|C9ZTH5_TRYB9 | DeepTMHMM Topology Prediction - Predicted
Type: TM
MSAVATFSLFFPSGKGGEIFVAGPRDCGAVMSVFSWGLRSILKCISTTGQVCNKLFFIPIFYFLFLLLFIC
IVVATFFPFQHSFEKRKSALSVVPSLCFSRLLLAGSH
>tr|C9ZQ31|C9ZQ31_TRYB9 | DeepTMHMM Topology Prediction - Predicted
Type: TM
MYEYYNSSNDNSKIGESCECEYTNIIIRAIIIIIMLMILIGECCECVYTNIIIRAVIIIIIMIGECCECVY
TNIIIRAVIIIIIMIIILRECYECVYTNIIIRTVII
>tr|D0A7Y0|D0A7Y0_TRYB9 | DeepTMHMM Topology Prediction - Predicted
Type: TM
MCVRTWQKCPGLVSVKHRCIGGTLTNNGLRTEQYSHAPGPVRYFFLCLYDALLAHQQRLDQYWLNPLYQLN
VHSFPFVYLYIVHFNTKIIFSFSFKANKNFFPLRLTTGKNCRKTKSTCRQPPSPNIDDRSSL
>tr|D0A3Q7|D0A3Q7_TRYB9 | DeepTMHMM Topology Prediction - Predicted
Type: TM
MRRIQCCIPGGISHSRRWSQRFHSVEGVPRLSQEELRRLVDILESDPKILGDIIGRLDAASRRRLIVAGGA
IEWFGKDNAVTEMKRAGVDKDRFISPKDFDHWLEGALRRRQEEEEDEEVTDRLQKQAMDEADELVPYSTLL
WVALVAGLPFVGFGFLDNAMMILAGDAIDNSFGLYFNCSVLTCAAMGNIFSGVLGMQLHGVIEKAVQRFNF
RVPTLTENQMKGKRVFFAGHIGGTVGIMTGLLLGMIPLLLLDSDDAKNERTAGESLAVAKTN
>tr|C9ZRZ1|C9ZRZ1_TRYB9 | DeepTMHMM Topology Prediction - Predicted
Type: TM
MLVFVPPTSTLRGRTRRKKRYNSSKNTQITLQCELRCKWVTNYCRTGLWNNFVYVYASEIIMSTSVNLCSD
YLKTLLSGRLCGNAFLAVFFCFCFYQFLELCGAFLPGPHHVTKGKNRRKEEEGFKKKTDNSARMTRRQIR
>tr|C9ZYA7|C9ZYA7_TRYB9 | DeepTMHMM Topology Prediction - Predicted
Type: TM
MGRTAIEAANRGRRYREVEGLIITTKIFVQLLTNINPTPPSRLRVPLYFFVLIYINIYKVIHMHIYIYICI
TLPASFRGWKVEGGKRQRKTVRTIPLSKKKNTNKRKKERRVKKKNYNKVRERTKCHISPSFISYSPSSFSF
FT
>tr|C9ZWX5|C9ZWX5_TRYB9 | DeepTMHMM Topology Prediction - Predicted
Type: TM
MRATKKHPNDGKGATTDPFVDGSNPIPSEVAAFDPSQQEHVDVVKEVKPSLFTVLLEKFIPHGGLWSCALN
LASATLGAGICSLPTGFNLSGIVMSCIYLVCVAVGTVYSLNLLAKVAVKTGSRNYGEAAKAVMGPLTGYYA
AALMIVMCFGGSVAYIIIIGIILKAVLSRDGVPEYLKSESGNRLMTSMVWLVIILPMCIPKQVNSLRHLSF
VGVMFIVYFSCVVIGHSINKIINEGVADDIVYMRTGNSALDGLSLFLFSFICQSNAFEIFREMKHRSPQRF
TIYGTVGMSICAVLYFLVGLFGYLEFGGKSVDTVLSLYDPGENVAVAIAYIGVAIKITVAHALHAIPIRDA
LYHCVGWHVDTVPYWKHVVVVVTINFTSLIIGLFIPKASTVFGLVGAFCGGHIGLVLPPLFYMYSGGFTRE
KVGNIDFFGTYLLLFVGVVAVVFGTVSTIYNTVP

>tr|C9ZU07|C9ZU07_TRYB9 | DeepTMHMM Topology Prediction - Predicted
Type: TM
MMYLYVCVFNAGHLKVMKKKGENWYHETGCLGAWECARKPVKRKIKERTVDIYIMYIHIFIHVYVYIYIHV
YARMPMYCCILWVSLSFYDVVCVCVCVLECRYESAHVHALTILSQLLLAFHGVSFFPDSFVCYTSP
>tr|C9ZP21|C9ZP21_TRYB9 | DeepTMHMM Topology Prediction - Predicted
Type: TM
MFRCTVRRRIGTTSTILANLALFAANTLSIHDVFGVLLVGGVVFRFISLAASIYGDRCVARAACALPELQE
AHEQYKTIVDHPRAIFWEKKVAAQKLKSDRNRIFRSHHVDNVRLVLPHAVAVLMTWYSFCSPAQQLSDHLA
VVSVTSPLSFRLMGTVVDPTLCFAATLTLISIRDHLQRRMGFSDGLDAWIRRGKGYVTGAWGVFTVITLSG
QFVTSFAGFLPPHVAPTWLGISITSLCKSILVNHTAPMRALFRIQDYPPSHGKHGATCTAEVHEYRLAFTG
VDVEERRHMWQTQKKALDYECNVRLHRLLNRTGLFDSVEEAEHEAERLKKKLNIARTRRKERELRERGDGE
SDGVAPPATLAFKEERGPSADEVAERHFDELQLRENDRRRKRRGHDGG
>tr|C9ZMD5|C9ZMD5_TRYB9 | DeepTMHMM Topology Prediction - Predicted
Type: TM
MFLLPRLLFNSLKSSSEISNWLSESEWGCGRINVKEEVARYRLTVSLFLFSFVYVCVCVCLSCFPSRLRRS
GDVFMLWTVLARVTYTYSFFFYTHIHPPY
>tr|C9ZPL1|C9ZPL1_TRYB9 | DeepTMHMM Topology Prediction - Predicted
Type: TM
MLPENLPTDPAAMTPAAVAAALRVDTKVGLSSNEVEERRQAFGINELPSEPPTPFWKLVLAQFEDTLVRIL
LLAATVSFAMAVVENNAADFVEPFIILLILILNATVGVWQENRAEGAIEALKSFVPKTAVVLRDGDIKTVN
AEELVPGDVVEVAVGNRVPADMRVVELHSTTLRADQSILNGESMEAMKQIEAVKGRQERFPACMVYSGTAI
VYGKALCVVVRTGASTEIGTIERDVREQEEVKTPLQVKLDEFGVLLSKVIGYICLVVFAVNLVRWYATHKP
TKNETFFTRYIQPSVHCLKVAVALAVAAIPEGLPAVVTTCLALGTRRMAQHNALVRDLPSVETLGRCTVIC
SDKTGTLTTNMMSVLHAFTLKGDGSIKEYELKDSRFNIVSNSVTCEGRQVSSPLEQDGALTKLANIAVLCN
DASLHHNAATGQVEKIGEATEAALLVMSEKFANIKGDSAVNAFRTLCEGKWKKNATLEFTRKRKSMSVHVT
STVTGSPASSTNNLFVKGAPEEVLRRSTHVMQDNGAVVQLNATHRKRIIEQLDKISGGANALRCIGFAFKP
TKAVQQLRLNDPATFEDVESDLTFVGACGMLDPPREEVRDAIVKCRTAGIRVVVITGDRKETAEAICCKLG
LLSSTADTTGLSYTGEELDAMTPAQKREAVLTAVLFSRTDPSHKMQLVQLLKDERLICAMTGDGVNDAPAL
KKADIGIAMGSGTEVAKSASKMVLADDNFATVVKAVQEGRAIYNNTKQFIRYLISSNIGEVVCILVTGLFG
LPEALSPVQLLWVNLVTDGLPATALGFNAPDRDIMEQRPRRMEEPIVNGWLFMRYMVIGVYVGLATVGGFL
WWFLRHGFSWHDLTTYTACSDMTNGTCLLLANPQTARAIALSILVVVEMLNALNALSENASLIVSRPSSNV
WLLFAIFSSLSLHLIIMYVPFFAKLFNIVPLGVDPHVVQQAQPWSILTPTNFDDWKAVIVFSVPVIFLDEL
LKFITRRMEKAQEKKKD
>tr|D0A2P7|D0A2P7_TRYB9 | DeepTMHMM Topology Prediction - Predicted
Type: TM
MLFSYFHQLRGGWRGVVGLVLLLLLLLFWQFAFIQFHFICSFFFFPPCVCVCACLIIIIIIIIINIPVLFL
FIPARYIYIYMFVSFYIDVHVFYFPFLFFFATVMMTDGASNYYYYYYYYYYFYH
>tr|C9ZUZ0|C9ZUZ0_TRYB9 | DeepTMHMM Topology Prediction - Predicted
Type: TM
MSLFIISCAITFAYFGFYQMRLPRTSFFLLFFFRYFVGAVDLIFVCLCFSPSPLSLFLLNDAMCFVSLLQP
ITLLSFNILVPIYGHGTFVPLLFHSLSKKKKRQNNKNVQLPVPVLVELWDSSCYSFKVLRFFKLRGATLPS
FASVPYPPPLFHVLRLSWSNEAQIFLLRMCLRYVSLCFSFLSVFFFSFFSLFFFPMRVSHGFVALKER
>tr|C9ZRW7|C9ZRW7_TRYB9 | DeepTMHMM Topology Prediction - Predicted
Type: TM
MRIVMGPSHFFFYFPSLGYLYACFCRYMCGRRLNSGTYCIFFFWKLIVRVTFFSTLVVCIAQDFGTRNLTG
FPFTCFQNGGGDACRLKGACLHLSFTCFVISHLVDSTSNN
>tr|C9ZYT9|C9ZYT9_TRYB9 | DeepTMHMM Topology Prediction - Predicted
Type: TM
MLHEGLVNDIMSYYFIAVNPPTPCLGGSSLLHVYFKSPLLILSFLIRLFIFCASPFARTAGFRVVEFVAVV
ILSMGVDKKISSLEDFFKRVSFPLQPLHYETKLVLDTFLDKGGFLNICDMTFGSPFHDGRRSGAGELSWMD
VANSNPNGKTALALFRFLAPGGYFCYLLEALSLPQHDFAVKLKKSWKGDAETEIETGSYLRYFDFPFSTFH
PTFHELFEEAPEANLFDLPRSRCPPLMLVPFWAPPLVTRLEPSSRRVLTSLRCSPISYFILRMLLYGVRRT
EECSSDVVRLPRINAMGGKINVFFKSVVRLFKGSSITGLPLYHRLLTAYIQYFVSSTCIQKLSSKRSFGDI
QWSVSDVTAALLLSAPSHLWARSVSKVDREKHRLSCRDVASAALVFRLVPFLTECLFALCQKDRGRIYTSC
WEGDNAVRKDFCDSHITPAVWDVLNPHISFYRNTLSVLRQALISFENYDDCRREHFNNALELWYAVVSPTG
RVDDVSESYVVHHYEAYSFILFDVILMFLRSSFLEAIDVTGATIWSRCVEVFTSTAVQNVFREVSNATTNA
RCGIIETICRYFTLNWMGEDGIMRIMKPHAEETLHFLAEVYLATEVRLGEDGISDDTKCSLRLSLDYLAKS
FDGIIRVVDERRSSRVSRLTAATSASPRPSGEGEYTDRVGDSKDLYFRGARLSCNFSGPVDVRFNAENEGI

PFYAQSSNVGFSDEFPRLVTLTEWMETALVFVFELYYKSWIPQCSNGHKLWLLSSDKHCCVNHPQQSAIWE
CFLCEEVYGSCCRSKPRCPRGGSLTRTEISQSISFPYCNQCFVMFKLGSVAYNKPQSDTLFCSNCASRPFK
RISCRFFASHIVVVLCIIVSLMVFLFFAI
>tr|C9ZK67|C9ZK67_TRYB9 | DeepTMHMM Topology Prediction - Predicted
Type: TM
MKVTVISGSSSEVVELPSNAGLTDLKKVYKPRVDIHRKSFKILRSGGDKNDKSAYITLDAKRALTEQGVKD
GSEVVYKDLGPQVGYRTVFVVEYAGPLAIMLAYAARPSFIYGSSIVKEYCYTQKLYIALFCAHFIKRELET
FFVHKFSHPTMPRRNIIKNCVYYWTFALGIGYALCSPYYTEPASPTLVNASAVAMVIFELLNFAVHVQLSG
MRKGDGDATRPVPKGILFSLVSCPNYLFEILSWVAFSLGTSMLTSWGFTFAGLVQMAEWAVKKHHKNYIKTD
PSVRNKKAMLPFLL
>tr|D0A437|D0A437_TRYB9 | DeepTMHMM Topology Prediction - Predicted
Type: TM
MTHYVDSVLYWFPTLVLVVCMIVLIVTAVGLSLGKFWRGLSSWSWRRIFSLTRMSMRQTTFRASMNAEEAV
ETALRKAGWKHVFLRRRVFVPRLQHNREIDVVAVGPVVLVVEVKHWRGSVWCNGQRWFQQVHKTARALEFE
DIREDNVVKAAALRRHIENDKRVPLPDFNLLSDCLQESEGDGTWYEDRRLHKQCGTVIIPVVVFTNPLVRL
DPSTVKQKDDVFDLPGLERYARNLLHNTRGASVFDYYKQRVDEFLRPVFIGRSITPDTYCLSFFTEEKVAR
VVERMRTWDMVYLLNGRLVHGDIVSFGLPSTNRHFVRQQISDVEITWFDGLIGFARTMWMNSGGMVKITLS
SPRKRKVFSFPLVPRHLKSIYKNDRLVLKLAGTTNLEEIPLADIATLRLSQHRETDGS
>tr|D0A807|D0A807_TRYB9 | DeepTMHMM Topology Prediction - Predicted
Type: TM
MEPRRWRPRFFNPFTESRRRSSHLPVYRGFFRVMPLHRWSLNNIQAVLGYAAANPSLFGFPEQPTCIVNTA
YFLPSHRSRQRGIWVFGTERGCKSIVVPSSCNGGQFDAMDAKLMEPLFRWNPGVEYIYLSQNCQAMASQLV
PYLQRCTQLRTLTLEGWNDAVAIHRVVIACKHVDTLDAFSFDDPVRQWKNELSVQALNTLIEMHPKLICVK
ATRVYLTDWYEATQFSRCKHNIALVPFTCDFVLLHYGFFLLLVSVVAYAVYRETWWLLRDTCSRAYLPFWS
ALAAVGTFTVIVALDVAGWRSYGRAWVHMHKYVIIARRWWDMIRGRHHKSLMLV
>tr|C9ZXH9|C9ZXH9_TRYB9 | DeepTMHMM Topology Prediction - Predicted
Type: TM
MSESSATEKHVYGYELYSKNVHQYPSITDVKTVKGAIAGNIHDYLNHLGAFEGNARRERGEVLAYSLLAGA
LSLACVIYFAKDVLLPYKLLTLSLIGVTGAATLCAYVCDCRQKSDNIAFLATCSASTEAKDYLRTLKGHRV
CIRLGEVPNSSALTMEAQVVGPPCFFGAPEVHSSSSKVVPYGRYFTKSGYFFPPPLMNDVDNLLSSLKTST
SKKKRQS
>tr|C9ZPJ6|C9ZPJ6_TRYB9 | DeepTMHMM Topology Prediction - Predicted
Type: TM
MLRRAVAISPSASVVASHTSSRHSSTGSKDLSSFGGNRPHPPAIQHNEPITPMDFNPHIEWFEYPKEVFDP
KYPYTREESIRMTEESWESTMDEMASRYGMRLNMSTKPTFWMAWSLMFIYSWYTLWGCYRAMGTEPGWTHF
RSVVLDQPNVPTLEEDDIYLDQWIRPEIREKHAKYERFWNWKPLTKRDGAQTSWKIPMQYQDESEWRHMTS
AN
>tr|C9ZJW1|C9ZJW1_TRYB9 | DeepTMHMM Topology Prediction - Predicted
Type: TM
MDFLDETVIHPMTAFARNSRMLVRKCQKPNYSEFNASAMATLVGFVVMGLLGFFVKVVFIPINNVVLGA
>tr|D0AAJ4|D0AAJ4_TRYB9 | DeepTMHMM Topology Prediction - Predicted
Type: TM
MSAAGVYLLLVGPSWRQPFTYFPLFGLVSVFFGARIGRWEEKIDVLYYKVGVRSLGGYTRTYMGQKKGQRE
IPPSRGEHQLGKVSMLSHFRSYRGREGPSDEAVKVFGGISAVHFIAECFFFFFGGGRHSAIYIYSPVCKYA
RWGPPASEILGYT
>tr|C9ZU80|C9ZU80_TRYB9 | DeepTMHMM Topology Prediction - Predicted
Type: TM
MNLFPTVPSVCFVLFCLPFRFFFVSFLKKIKKKKCYFSCVTMRCFIPLAAMCSRGEPFSLPLFSTFSCWHR
FKDVTELNNKEKKKKCIWGLLYGLFTCAALVCFILSTTCCLFFFCFNSQFHPVTLFCCSLWEVQPERKKTS
VQHPLFFFPSPCFCYRYQS
>tr|C9ZN59|C9ZN59_TRYB9 | DeepTMHMM Topology Prediction - Predicted
Type: TM
MLFLFHPFVPETLWVKEVPKSANRLQDAMSNSKQQQEVQTPNNLSNEPVQTEDNVKKGRNTFFTKVSLCVA
TVLPPGGIAASAFNMASTTIGAGIFGMPPAANSTGLIMGMFYLIFISSVTVFTMHNLSVAADRSGAPTFER
ATRALLGRGAAYVLAGIRALLGFSGCVAYVISVGDILSAILKGTNAPDFLKEKSGNRLLMAVVWACFMLPL
TIPRHIDSLRYVSTFAVTFMVYFVIVIVVHSCMNGLSENIKNVSVTKSEDAEIILFNSGFQAIEGMGVFMF
AFISQITAYEVYIDMKDRSVRKFVIAAIIANTLCCIMYIITAFFGYMDFGKTATSSILLMYDPVKEPAVMV

GMIGVVIKLCVSYALVAMACRNALYDVVGKTADSLPFWKHCVSVITLSFLVLLLSIFIPKITTVFGIAGSV
CGGSLGFVFPALLIMYSGGFTWQKVGPLYYITTYVVLLCGVFLIVFGTGSTILDTARG
>tr|D0A8X9|D0A8X9_TRYB9 | DeepTMHMM Topology Prediction - Predicted
Type: TM
MKFGKRLIEEEVEQWKDFYVNYKRLKKFIHSSPLTGVEFSEELFRIIGEELERAGGLFKELLEELHRCHDE
LLEQDPQLPAVPSKLPWMFNRKRVHREKLNSPREGLSVAFESSTENSMMNEIVVEQREGFVSKFKKCFLKL
IGDSQKQEIEEDTPRARFLEWHSSAHMLQHFAELNLEAIRKSAKKLRKYRRNEGDFTISIEAEISRSQIGR
SLPRLHVFMTDVDADYERKFGEALVQFKNITINQTYHAKWRFVFLSAALFWILMFFPILESHPSAHNCVAL
FGVIISLWITEAIPFFCTAMLIPIIAVPLGIITDPATHRVASTTVASRIILSRMMDHVQILVLGGLTIGKA
LGRTHLEVYAAGALYRLTAHRPSMYLLGVMLCSSVLCAFVSNIAAPLLVLGVIRPTLWEFPEDTEAPHAIL
LGLAFACDIGGMLSPIASPQNAVALSVLSFHDVSFLSWVTVALPLVLVGVVASWAIILLVWKPFKNVSSIP
LQVVNTEGNKEVRLADQVVVVMVSLITVVLWVLPPNLLFGDTGVVALIPIVVFFGIGILAKEDFNTLSWHL
MFLLAGGNMLGLCARDSKMLDIIADSLHDTLVSQPPYTTLVSLILVVGVVTTFVSHTVAAMILLPMILKIG
LVLPKEEGVFAVSPFTLVFLSALMCSGAMAFPISSFPNVNSLLAEDSRGRPYLKAKDFLFCGTIITFLLFI
CFVTWMVPFTYVVFEGKP
>tr|C9ZJ02|C9ZJ02_TRYB9 | DeepTMHMM Topology Prediction - Predicted
Type: TM
MNCELLVLVLFSSSSSSSEPHLWKKRVPVEVNYFIHYYYYIYIFLFICVCIFFFIFYRISLFYFFFLSLFL
FGICFCFHLLKCILQRRGKGHHVAQGHRGAEPPDGRGVK
>tr|C9ZIR7|C9ZIR7_TRYB9 | DeepTMHMM Topology Prediction - Predicted
Type: TM
MTCWCCGNGGSKNFVFARESIDYSTFAFPTFLFVFFFPLREVSFRLRLFVHQQERVALVLVYVLSPLFLPF
PFLFLALKAEQLHITCTPFLFRLLDGRIGRGGDVVSMRINA
>tr|D0A7W9|D0A7W9_TRYB9 | DeepTMHMM Topology Prediction - Predicted
Type: TM
MGFDIHFCSVFFFYFSLLFVCLFLPFIILRSTLNEFIELWGCSCSLPLVPVTPPSNALHSSFCFSFSFLKK
KNNKFPPLPHITKKTQTHVCRKKKNPSASFLHDP
>tr|C9ZNB9|C9ZNB9_TRYB9 | DeepTMHMM Topology Prediction - Predicted
Type: TM
MNVAREIFQKVSEHGDMKAALGQKMLDQMRGVQRVANRTKRSVEETWEKAKRAAANSSKVLKELLKWHCIN
KEAIRDSFDHMANANCDPSAYKHDYHRNFGHDDARAYAIYCEYKSIPAAKTDVTFSNMEGAVEAWNRAKPK
ADARDAVECSSGHSSRSASSSDKPCTLLESWRWDYDAARYAILKLETLVRNSLGVTHYAQRFQQIGRESLA
QYLEWKKAAEARAAEEEAKRQAAEKAAEEARKALEEAEARRVAAEEQAEARRLEAEKAEKAKEAGQPVSEE
KKKMLLEAVEKAEATEKAAEKQAKDSRKAFEEAEEERIKATEDAEVARWDKEGAEESEEKLKKDVEKLAEE
LKEESKESGEEDDVNADHDDEGSEAKSGWIGTTKVLIFLIPLLLLLLGLLVFFVIRGRRKAEVKDDISIGE
ANAKSKNTKTAAGLDSDI
>tr|D0A9X1|D0A9X1_TRYB9 | DeepTMHMM Topology Prediction - Predicted
Type: TM
MKRKGLHFPIIKYKRVFLFFFSFFLFFFFCFVSCFVLKVLSLFFSSLFFLFLFSFFLSIFCCFCFHLLLHC
HLPFFFLFLPGKYKIIPFLFLSVLPTRFHGNTMTEREEF
>tr|D0A1N3|D0A1N3_TRYB9 | DeepTMHMM Topology Prediction - Predicted
Type: TM
MVGTVLRVYTHRTRCIGELCSVAPLCVILEQRRPGPSFGIRSQSREDNSVHFLPCEGAVDLSLRALSTIEG
LRNYREHRLRPQLLVKRAELRQADDVKTRCDALGLRYADRCIAIAVALILIRNVVLFYWVYFLFDWNLVEP
ITYLLERGAVCLGFLWYGATSSDARFESFRQFLVSRRTQRLYAQHNFNAGHWKELVVEVEGLEEQLRGLES
V
>tr|D0A9C7|D0A9C7_TRYB9 | DeepTMHMM Topology Prediction - Predicted
Type: TM
MEKYRKFGDAATGINPFISMKTPTAFSTLFAAVLFPVRCVIAVVIMSLLFLVDTFNYLFYLLPGLSSLSHL
LFGGLQRGLLRGLLFSLGNVSIARVPTSGSLSPSAGDVVVANLQSVWDMCVIEVAEQIPLFVAAFYGGKAG
SSPEKSKKDEDGTLFVMEPSPLQRWRVWWHIYNTGTSTFLSTVADDSSNAHSGEALPIDITLLQRRCHRLG
VPLVLFAEGSCTNGKGMLNISPIRVGVTPARLLVSALSYDTAALHTVVRPHSVLSYLFSMSASLYGSRDPA
WYSPQFPTATARLASVGTDPPAEAVEGVVVVEGTKVRQVLCGISRPRCALNVGLREKRRFVEVFMAQ
>tr|C9ZU78|C9ZU78_TRYB9 | DeepTMHMM Topology Prediction - Predicted
Type: TM
MKTSLLIQFNGISSFLVVFFVFPQRNMCFVTRARTKRRVKKKNIRGGESGRHTRSKREREGKRVSFVWRTV
CIPPLTLQKTVKHKPKGDTHEGGLYYHNVFR

>tr|D0A122|D0A122_TRYB9 | DeepTMHMM Topology Prediction - Predicted Type: TM
MGPFKKLMRKKKEKKMKQMFYCSKGNSRCFSLFLFPFDPIRLHPVKFLYFFPPFPRAFVYIFYRFFFCVFV
FFSRCVTASVYINIYIIYFPFYVFYLITTSRNHHYHTCAVVTASFFFFFFLTFLHSNMYFCFHVSE
>tr|C9ZXB0|C9ZXB0_TRYB9 | DeepTMHMM Topology Prediction - Predicted Type: TM
MEVCAVMCCCVSCFPIVTLSFSATVFLCDVFKHLSLYRNIFVFNVFIIFVKCFCLHGATTVTLLK
>tr|C9ZM22|C9ZM22_TRYB9 | DeepTMHMM Topology Prediction - Predicted Type: TM
MTARDGAVEVMASIVVYSIFSTVMTITNKLLVANYALNYPMGIIFVESGTALLFAVMGKMMGWVYYPNFCS
RVARKWLPLTLFFVAMLWSSIKSLETMSVAMHTIMKNIAVVLTAIGDSQLYGTRVTPVMYLAFFFMSAGSY
LCAMGDQWVTTWGMIWTTLNIMATVGYTLYMKRLLGDVSKTIGRYGPVFYNNLLSMLVAFVIALPSMGSMI
HTIRSISLPPLLALTVAGTGPLLTFATFWCMEQTTPTTFSVVGVVNKVPMSVAGMVVFNQFPTKTGYVGIT
LGLVGGVIYGCASRERDSGRVGPLLHVAGFSVWGRNTPLLTSSGKYEGRSRSEETI
>tr|C9ZQ36|C9ZQ36_TRYB9 | DeepTMHMM Topology Prediction - Predicted Type: TM
MQTLSASFAAAAGVSHESLPRHVPTLTLLKRILVLTVKLSIAQVAQFSFGITLLTVVGKIGVGELGGASLA
NGLVNATVFAFGAGFSGALETKLSHTFSRNPKDKMYGVYTLRMLIMLLITFVLLSPAILFLDRVLVAMGQD
PAVIDFTGEFCRLSIWGSFFAMLLELLRRYFACQHLSTSFSVSLVIGAVVYPFLLIGLVKVMGFSGVAVGW
SLLMICTTTGLVLYVVVTKKYLATWGGIEDAIYRNWGPLLKLGLHQMAMMLSEWVALEINSICAGFGTKEE
LAAFGITYQMSGICWAITSGTFIAASVLVGGAIGEERPMFARRLAILCLGTSVAISLCNVAILLATRNLYP
RIFTDDEKVVEIVDSLMNYVFVYHIFDVFQSCMMGVLRGCGMQKQGAIVIFFVYSVVGVPLGLLLFFFTGF
GIQALWLGPLVGAAVVGFPTYLYMMMRYIKWDTLKPSVEVYDSDLEEESEESGVLEGVAKVNEPIGTEGDV
ITTLSSNAPTRVTTNSTEQEDVSRRHQ
>tr|C9ZLQ1|C9ZLQ1_TRYB9 | DeepTMHMM Topology Prediction - Predicted Type: TM
MVSAEKDISDVCVDVAGETLKQLGASVSSTNRRSDVVELHIAVGSEKCPPIAAALFSGFVKAACGSEAGTT
KVGLTDVNIDARENFTVLRFAEAVRVRDLDFNELPGDSPPITSVVEVTYMRYLIKHSELQVFQVSDFPKCL
TPRGGQRLDTVVAGYVLFSLQFSEEDDTKVLLYMKSLRRVLGGMCTSSFAETVLSQAWGNMDSVEPSHVTD
MLEDAQMAITDFWNDSDQDTAIRTKVVFLMKTVGSNLREYFAKKTLDAGGVFSGSKNITEVALGCCDEWVN
MCKRLTTVDWGSSWGTAFEDVQLITVRDRLSVVVSIRDLVDEIVELLTAADKLQSLRTETLWETFDSLDIF
ATTPAVEQLWTNCLDAFYRRLQPVEHRCASALSDFFGERGNLAPQTILNEVVKFRQLIRRPAVSKELVNER
DALLAKLNERLQSIRMEFERRSESVEDDVALDEEDRRCQAGRFMPGVVNNMIWLRQLRGRTSDMISMCKSL
LNDLQNAGEFLRSANTLMEEIDDYVIETFKQWTMDVEGSKHVLMLDANAPLMDIAGDGSVRVNYPERLVQL
IREVRIFRSLGFRISTAIQEMVDQGIRFYRNGVALKQVASTYNSMKENIIPCTQAMLLEPALAFENIITAS
GDRKLTWRNTDDAERFIGKLRRASQALTDANRRLHKLHREIESIVIELFSIDLLRLRERWTGKVRLIREKM
ETSGFNNMESWKQFWDMQIYKALEHQYQLGLESLHEVVAEIKADITYDPDTGLATLRPNIDVIRGQYYQRI
KDFITFPLRFRGCGNSDIFGAMPGNNERGIFAVMKHAAQLFKKVQQELKRFHPLLVIGRCGKGGNPSLEDI
VGHTLKEVQHWEQSVRLLKQKGKEINAEELFIKCGCITICTAGVKGTVEDHLHKLSQVLHVTLRDSAQKNL
SRIEEYIAEVTTALGARLTKLEEIGAANLQHAKLMEKRPAVEVEFHHLHNKNVLLQSMANKSDFDFRGVKQ
RWDEVTRRLDAFEREMEKQVDSLKMSVEDTVSAWMKNLERFSNQWHEVKPKTVDTPNAMKIVTERREQFKA
LKAEGEECIQQCKYFQLDEPDIEELEDLERDIEEYVQMWDILVKFQEELDVLRAEPWIIMRAKCYRFEDFG
RHWEEQLRTFSSSPITVHIRTLLDSWMRCLPVLKYLRGDGFTPSHWGEMFSLIGVRGVTQDTLKFGDVIDQ
HETLLRREADLKKLHARAQGEAQIREALDDVRSWGNNAKFTLIPHPDREGVKLITEWKDTMSALSDNQALL
MSMKESPYFGIFANDANKWEERLACLDEYLRNMSQIQRKWVYLEPIFRRGALPQEQERFSRIDKEYLQVMK
VVAADSRLVSLAGHTEFKDVLYTVLEQLDRCQRALNQFLEAKRDSFPRFYFISDDDLLEILAHSRNPSVIQ
SHLKKLFMGVHSVRFDDAKEHILQINSLEGEVVPIEESVLITEEVEEWLGRLDLAVKDTLKLHLVRCVDKV
NIGAYSSQILSTAGLITFTKQTEDAIRDAKSGGLKKHRANLQAQLRELTTYAGGNSDVVIGLKVKALIMDL
IHNIEVVDALIDAGIEKDTDWLWRKQLRFYMDYSDSCVLRMVGAEFRYSYEYQGNAPKLVHTPLTDRCYLT
LTQGMQLGYGGNPYGPAGTGKTESVKALGNAMGRQVLVFNCDEGIDFKSMGRIFTGLVKCGAWGCFDEFNR
LKVDQLSAVSQMIQVIQEAIKNGEPRCHLMGKEINVDSNAGIFVTLNPAGKRYGGRSKLPDNLKQLFRSVA
MSVPDNELITRTILFSEGFENAVDLARKTVEVFKLSRDLLSFQTHYDWGLRSMKAVLRLGGTLIHEYLTER
ACGKVNPSPEEIFEKESEIIIKSLRVNTLSKLTFDDALLYNNLIADIFPGAPIKEIDYAELRPAIEESVKE
LKLQLVEAQIQKVLQLYEALRQRMGVVLVGPGGSGKSTLLLILRRALQRLGKVIPQYIMNPKALPRTQLLG
YMDNDTREWFDGVLTEAARKVVKEETSVLSWIVCDGDIDPEWVESLNSVLDDNKLLTMPNGVRIQFGDNVN
FIFETHSLEFASPATVSRTGIIYLSEEDVDPKMMVTSWLVEQPEDVRDKLERWINDYFYKAIDALLATGKL
IVDTTRTGLVASGLSQLQGCTGKAQFALALVYGLGSYLTEEYRKDYAKEIHSMMSERLPDPKNPLDVYYDE
SSNCYRTFDVEPCTDLSVEDLYRDPMVATVDCQRNVKILQAWMKPVRPGIYRPFILVGPEGCGKKMLLTNL

FGKTPGTRVTAVNCSAQTEATHVIQKLKQMCQVYNTNQGRVLRPKEVERLVLLLKDMNLPKPDKYGTVQLH
SLLQQIVLYNGFYDTDLEWISLERVQIVGSMNPPGSMGRHPVAPRFLAMVSVLAMSYPSREAMQNIYTEFF
NIMIQSGRLQLNLPGKGAVDIARIMTTVYEAVASRYTVNVASHYMFNPRDVTSWVLNLLNYNPEDVTNAIG
YEGRRIFVDRLVTTEERSKISKVIHDNLIFLVGHKSGLSEKETTSFVSWMDTSPIGKKKLTPIANEELKKP
AEDFVLGYSREFADLDVQLIPEVCVWMARVDRVLSQERGNLLLVGRSGVCAAGIVRLAAYGLRMELVTLGI
TREYSMKQFNAELKTIMMKAGVEGQHVVLLLEDHNFTVNSSFLETINSLLASGEVPGLFAQEELDAMTAPL
KEDALGEGMSAYAYFVDRIARMLHVCVVMDPTNPNYEPQCRSNPALFTRCNVYWVGTWHTDSFKLIPRLLM
RDVFKSIDSRDNKKDFSLTTEIVHVHKEYINTFSPQHFKGLCLTYESIFKEKSRMISEGITRLQTGVSKLD
EAQENVDQIATDVVEKRKLMEVKQKEADDALQEIKTNMEAASDQKKNIQKIRKDLEKEQKAIEERKSVIEE
RLSGIQPTLDAALSAVRSIRSEHLSELKSLKQPPPAVQDVMEAVIITIGGGGGGDTNWASIRKILAGDIKE
QIINFNIAGFTEATRLRVTQFMQNHENSFKREVIGRASKAAAPMAEWIKAVLEYSSVLQTMGPMQAELKEY
EVSLASRSEKKRKYEEKLTKLEERVEGLKRNFGERTAEAERLKDHAEQAERLYACAHDLLAKLTSEHDRWV
SQIKVIRENQVLLPKRCLLAAAFILYLGNATEDGRRTALNVWKERLKDVDNFEFFTFMRPESMQLHYKSEG
LPGDELSMDNAVIIQEQVTTPLIIDSSGQALSWLVNHLKGKGLSVEVCSVSEERFVSSLELALRFGKSFIL
TDVDGIEAFMYPLFRKELRTEGTKRVIQFGDRRTVDYADGFQLYLVTNSTDLWIPPDVLSYLTPVNFSITQ
NGLEGQFLGATIQHEQPELEKEKLAVLQKEERLKMQLSDLEESLLKDLAESKGSLLDNKTLIDSLNEIKTQ
AAEISVALETSKKVQEEIDTKRNVYRPFAKTASEIFFIVKSLKALSHMYQFSFSFFMGIFNDTLHHHEEDR
TDIDTKIDALTKTFVRSVVSSVCTSLFKEHRTVFGIHLARSLYPNDCTAAEWDFFLDRAIAPEAKKSEVRV
PTWVLPDSRDLYRSFAVLFEDLTPKLSLHEADVWLQWMRSSNPEVSYPKFISQLSRFQRLLVVKTLRPDRL
MAAVRSTACDMLHVKSYGDNNTLAQLLTRTEANTPILLITTTGADPSQELQAIAHQKVGRDHFHQLAMGGG
QAEEAVRLLRVCAESGDWLFLKNLHLVIPWVTALQKELNVLKPDSKFRLFLTSEAHDDFPSIFLSQCLKIT
FEAPPGVQQNLLHTYQDWESGPYDAKGTLHTQLLFITASFHAILQERRSYIPQGWTKAYEMTSADLKSASD
IVLQQAKGETDWRAIRGLMEDAIYGSRLENEYDMRVLREYTDQFFNPNVLSSAKQQMNLFAKVKVPASGKH
GDCLKVISELPENDIPFVFSLPPNADRVVQLSKVRALTDDLQLLIEARGESSMSREQWANKLRPILQVWEE
LMRPNPDLLQQTVSIARDASPILGFMGAEMTSSLRLVSIVDESMKDLGKVLEGQALLREDRRAEASTMIAG
EVPAQWDNYYQGSPRILPWLQSLLHRATSIAKLFELATNGNLLKSSLNISTMFRPHTFLHALRQETAHSLH
EPLVALRLVTSISTPPEGKTVPVCLEGLMLQGAVLDESNVLQSIEAADEAALFPMPKTYVGWMTTMPDSVT
TVGVPLYTNGTKEMFLAELHLPCASELAAKAFILAGVALVLEP
>tr|D0A3A6|D0A3A6_TRYB9 | DeepTMHMM Topology Prediction - Predicted
Type: TM
MIFLLFISFIGWVLWSLYSLLSYRRVSVAGKTVVITGGSVGIGKHLALHFLRLGATVHVWDDNKEKLSQLS
KEALLIPSPRPTKSANCDTEGLTETSANELEGNDCLKTVVVDLSNRFHLHRLVKQVGTVHIVVNAALNVSS
KAFLDHADNAIERILHVNALCPLILARAFLPAMLERREGYFVTITDANGLLGNASQPDFAASQWAAVGAHE
SIQMLIRENGCCGKVRTTLLCPYNVVSSQPTLLRTPSPFSSSRVMNTADAANTAEGQSVNTIRRSRILGYF
LSIFRRPVTPEEAAEACVWAITHGVERLYIPYSLLFLTLFRFLPVPWFMWVISPSTQSTNVETSKGNCPSE
SDSSRSSSEK
>tr|C9ZW54|C9ZW54_TRYB9 | DeepTMHMM Topology Prediction - Predicted
Type: TM
MHALVHFGNEGWLVSTCTWMTEEYVHTVVAGTISGAAGVLLEYPLDTIKVRLQMGGGRYTGYFNCASRMIQ
EEGTLSLYSGVSTRIVGSAFEHAVVFSSYKWTLRAVGTDEQHPRVWQIALGGVGGGAMSTVLLTPLELVKC
RMQVANVQPGAKWRNGSVADCAASIVREGGLTALYKGGLAMLAREIPGTAAYCGTYDKLKEFLTPEGGSTA
NLSIWSLMFAGGCSGVAFWTIFFPADVAKTRMQVDPAFAKVGFLEALRRIYIEGGMRLLYRGWTGTAVRAF
PSNALIFAVFDLTMYALTRD
>tr|C9ZX23|C9ZX23_TRYB9 | DeepTMHMM Topology Prediction - Predicted
Type: TM
MSTRLCEPSLRDDGAQRGTPASHGKWCDTGAFKLDETPSPVVANVSSTSDASFQFNRTPSPGKHQNHAADR
SMWSLDDFVIIRKLDEGRFGKVYLAREKQSKCAVVLKCISKDMIRFHSLAHQLQREVELQEYAGRYHKNVL
RLFAYFWDDVRIVLVLEYADSGTLQDLLDCYREKEQRSSLPDDKVRTILLQLLSALAFLHERDIIHRDVKP
DNILFHGEKLLLADFSWAVRVNNSDPRYCRRHTVCGTLDYLAPEQVLKRGCTTKADLWAVGVVAYRMLCGF
LPFEMLDARDVCSCIASGAIRYPSQLSPTARHFLQGLLRVDEALRFSCQEALNHPFMRGDCNCRISNRYIE
SKYRARAEEPVLQHQGSFDTTKNGTRWIQQKKESWCCSRAPTLNVPEPVHHTGTNCVVNHSHNHKDMDKST
TLSCPSASDIVRVRTTTERVRCPVDDYSGTSASNITISPLRSTECMQSLSNVSTPSIVSQTHSEWLHSASP
ISHTPVVAAAATSKCSRDDVGTSSYGVSYSGIRLHNSASDGSGTTTGQHHVQLGTSTHSSFAFAPPTSARY
RRDIRGYHRINGRGSSPSDHEEYEVASCALRLCFDDEDDADNNCSGAPHAAASNRPKTLKSGVMMNGTAPP
SHHKGLSYDVR
>tr|C9ZR39|C9ZR39_TRYB9 | DeepTMHMM Topology Prediction - Predicted
Type: TM

MLEGNVLGDTGTSIQTLLSCLAGFFLGFILPGVVAQLGVWQMKTRKQKPLPPRIDRASEALKELLKRERAA
TLSKLGQQSADESQEQCLHSDNSDSAESSEGDEEETSYSSGSSGFERMEELGLKMVLVIRCDGKKTPASEV
ATHAAGAAMNLAQTIQSDGACVQWKRWYLWWNMVGCAKIALKGPDVETLRRVLVSAQERGLPCCSRSRGDA
PGVPVGNPPSAVRDIEEDLVVVAVGPVPSSEVNPITGNLKLFP
>tr|C9ZWY7|C9ZWY7_TRYB9 | DeepTMHMM Topology Prediction - Predicted
Type: TM
MASVGQSAGFGGFAGVITGRHITTYSTGGTMRRRPPAITFFLIPLLLVLPHIVRPLAAKKSIKVKVYNLLF
TNRAPRRSIETMGVGLNASFAARHWATAENVTVEIIPPPPRSVSTLKLLETAADQNKGEFFVVVGPMGDTR
TLAVLPLLRQEDLVAFAPLTGSSAVRGWDPHFYFLTPAPNAELIALLRYAINRLRLLRVGFMYLQGVHFGD
REYQETLKLMTRMGRRLCGVFTKMHAATVMRADEEFDSLWEEFVMTHPQGVIVFTPPIREALKFVRRVAMD
ARTRGVYVLAPSAMQFVIGATWRTAVEEAAVPYIPDRVIVAAPNPLASETKYHAVRRFQEDARSYLRYHPG
VTAFNASDDFDHDDADGALMVSGWVIGEVLSQALSSRTWLESREAFIKSLYNQRRYVVDDLVFGNFGGECH
GMAGERGASCLCNQGGNVVYMNSLGDDHRMIPLRDGITVFNIDRCSTIGVKVPTPLIVVVVLVVDDSAAVS
AFFSLVVGITNADAIRSITNPDRVAFHSVVATSRNTYGRMQGELDTRSVTAVFGVVDLAMLSVGRVAFIEP
ITLSPKLSHPGRNVIHFSPTIEQQLFLLVGYIARDKKTTSLHMVTRGGDVPGIGSVFQKTIESLGGSMNGI
VVPDNDTTLNEDLSPHGDTLVFGLTEADITTVAEHLDNHRGVRVFVSFFDVALLYSEFVKVFKKHPQAAER
LLFATSLPHWADNNTTSETVKRFHDEMKDDESKWTPLALLGYATARAMESVVSRMGRVNSEELINGIFSQS
VIVADDMWYGPFEDSCVPTTGFAAEGCAVNYGATHISVWSMARVLNPSVPPVTKAATPSMRYANRNRGSLS
GKQLAGVIVGALSALVLFFVLLLVCTCLLGKGARDNENAPKEMVIPVTLIFTDIESSTAQWAAHPELMPDA
VATHHRLIRTLISKYGCYEVKTVGDSFMIACKSPFAAAQLACDLQRCFLEHDWKTDVFDTSYREFERQRAE
DDGDYVPPTGHLDPDVYSRLWNGLRVRVGIHTGLCDIRHDEVTKGYDYYGRTSNMAARTESIANGGQVLLS
RSAYHALSTSEREQLNVTALGDVPLRGVPKPVKMYQLNAVPGRTFAALRLDNEAADDDGSSASFSDAGSVC
GPLTGSSLMIASSLQAIFGTVCAAQREKLLNPVCERWRVSVPRKGNNGWEGVEFEDVIRRLSVKVGRVVDH
CVSNSGEHSESTLRSASLIIISRHGGENSTNEA
>tr|D0A3B3|D0A3B3_TRYB9 | DeepTMHMM Topology Prediction - Predicted
Type: TM
MDFGGDRYARERICNPLLLFVFVFVLFYRTHGESGMAAFYTPRCVKTICRPEQLCGAKSCSSPMTVGRRTH
GCKSVTRFVYRGGVDGRTTAACLRPFASLCMLGWA
>tr|D0A6L4|D0A6L4_TRYB9 | DeepTMHMM Topology Prediction - Predicted
Type: TM
MIKCLFFEVKAKKTTTTTTVGLQVRESFGWCICRSSFPIYIFWHPTNSHVMCCHCYPLSFSYGVGRRVELE
SELISRVGSDCKTGMIVIGNADAEMNENNYDVVCMCRHMSIYSYIYIYIYMNVPQRAALALLQLLLWCL
>tr|C9ZQL2|C9ZQL2_TRYB9 | DeepTMHMM Topology Prediction - Predicted
Type: TM
MIPVWYTIMVVTYVMGWVVLPISQSYTEVGGFTVRRKLVSSIKVNAKFYAIYTCIFAVLFAYVVVLKGAYT
SLTSIGNLATALANAWGLLLLVLFMSTGIVGVPKVLWRKSNPIRMLREVYYSAVEIQEDLDIAVLDLTEVR
TELMAISSAVPEEHRPYWTRMIELIDESDGRSSQFPMPTSRTNAVGKRTEIDVSLEHLEQLHERVKGSIKI
AQRMTYRWDATVRDAKFYEMLARGSKATNNGFKKVWFPMRGIILKLLALLCGAITLLILWSEVTLPFRPLT
EKSISVVAIMANSGWELPASVIFLFYMAYCSYWAIFQLKVFDIYVILPEISDNSSLCFGATFLSRLIMPLC
FNFLLMADMANGAVDVMYGHVYRDNMDASYILGDWLNRFLPAIIVLVSLLVFVNIAQCILKLIGLEVHSPN
NINSEEVRQRIEDGYRLVSNALGRPLTAIDARVLGHAASEVERTEVSTPRHNRGPPAERGDRYREYLAQRM
ATEGARRS
>tr|D0A598|D0A598_TRYB9 | DeepTMHMM Topology Prediction - Predicted
Type: TM
MIGTVCYYLLLLLLLIFFNLFIWIFLLFSHRCLRYLERLFISYSSSLACCLRFTSTRLSLILSSLPLQLRQ
INKKKKKRKKKRKKKKNATKPFAHLLSLSLLFF
>tr|C9ZMK2|C9ZMK2_TRYB9 | DeepTMHMM Topology Prediction - Predicted
Type: TM
MQARITTFDITAFTHGWDMPQKTEACRSAGVLYLILGACFSAYALYLRYATRAEQLRRYEMELAGMPEGTP
LLRGPVTNGSFPQSKQNQGATGPRSKESYGADSSN
>tr|D0A4J7|D0A4J7_TRYB9 | DeepTMHMM Topology Prediction - Predicted
Type: TM
MQRLLNQPALTINATLWCYFFFCLSSGRMVVKLKLSLYSCSTTFLTLVHPLLMLSKVYLLAYNGVLLAGWS
TILMKIVQHLSTGGRFADVYSLIAPLLVVSQSAAVLEVLHALFGLVRSPVGTTLLQVLSRLLVLYGALEIG
PTAARMSPFATQMIVAWSLAEVIRYTFYASNLAGVKLRPVTWLRYSAFTVLYPMGITGEIACFISALPYIR
EEKPWTVELPNRLNFSFSWYYTVLLLLAVVYPAGSYVMYTYMLQQRRKALKATDDASQTDVSNKKKAS
>tr|D0A0I1|D0A0I1_TRYB9 | DeepTMHMM Topology Prediction - Predicted
Type: TM

MASKGCFIAVLIARTHDHVPLCSFTDENYANWNQIRQQEQRILERMESPASVPDSAGTRGSYYQSFDHKDC
IYFAFQDAGTGLTIITVLNKLLLRNSSDVTSTNHLACSLLDMIFSEFTQAYSTSEISARTVRPFQFIKFES
TLEKLIHRVQRERQNDVVSDSHGGQRRQVNTQYDAIKQELTDVHFVMRKNLEDLMVRGEKLETMNQFSAQL
VDESSRFYKKTARMNRMRLLKLYGPPAVVISILALFFYLYLF
>tr|C9ZQ30|C9ZQ30_TRYB9 | DeepTMHMM Topology Prediction - Predicted Type: TM
MTHKRLAVMETTNVIHNATLLRNHVGSSSVATFLRAMIANFIAVDAPSWVSNFSRESSGAYNNQWMVLNMG
AVESEAMFKNMAPNTFWVLEQLPGTAPPLGITSKDMTSVLNTTGYWASYNRPYFPMCTIFQVRMQEEYGDF
YSYKNYSRARIFERDQGSVVDIESMKRLMRYNNYTKDPFSLIPNCTGAVGMDDDGNVTNVCKPPYSAMLSI
AARGDLNPPGNATEYGPLVRSVGHVNSGAIDAKIATWTGMVKNPESYTAHVVCGPTTDNQPPFQWVDGMFD
PMPPTYGLLKLYNFSFVVMETPFFKSDVVDVVWWIISGIGIGATILLLSIVLYYNTECSVGVDEDELLPEE
AEGLIDPQN
>tr|C9ZL99|C9ZL99_TRYB9 | DeepTMHMM Topology Prediction - Predicted Type: TM
MVYLVDDLARVRMLMAGVYAGLVVSSTYGFSLFSVYMRKQYRLSQSEITTVSTVGNCIGYCSFIGGALFDY
AGPMVVLPIAGFLGFLGFLLFGLTFDGKIANPSVGLFSTFNAILYLGTPWLDVSTIMPLMLQFPLDRGYVV
LVSKTISGLGTGVLMAYFNGWFKDTTSDDLSNNNYSGFAYFIAIQLVVVSLIALSVTRMPMYFPCAWRKQR
LSEEEWTKRQQTLQLYMNQPAPPRRMKIAVGLVLSLLLFLTTQSLIGGYVKLPPAAYLAFSIIAVLMMASF
CVVALPFQWLGRYTPVRPTDMDTIGEALEDVGTESAVATTKNEVKPLPQYSGSFWQHLLTVDLWCMWLTCF
GMWGTGTVMQMNAAQIYESKSYGEKKSSTLTLYITMMSVGSAVGRMSMGYLDMVLTRRQREGLKTFPTTIA
LPFCPLMLCIAFLLFALLPANALILPFFLGALGNGAGWGSGVLAFRIMYSQDLGKHYNFGFSSGVAATIAL
NLFMFGGMYDAEAERLDTKPECKNPSCVKNQMLILMGANIVAVIAAAIVHFRFSRFINAEQNRCNEPADEM
SGVAAPVTEDAGQPNEGGSHSGGAAQQ
>tr|D0A1R9|D0A1R9_TRYB9 | DeepTMHMM Topology Prediction - Predicted Type: TM
MQPMRTHRPLGVSLCIAAAGHCFLTYTHSLAAAVSGNVNSKIRQKREVAKLLYFRLAVFKKTFVIMPPSIF
FSRGCLCGISPSIYKSFGGLLFRTGVVNNDPRFRCFLFLRFHIEEKISPTPLFDALNLVK
>tr|C9ZL72|C9ZL72_TRYB9 | DeepTMHMM Topology Prediction - Predicted Type: TM
MLRCGCVAGALCLAACLHYCLFMFIWRWRCLVVRAFFPPFVRLHQSCTASLFISLRGLLFHWVVNPIRVCV
VPTVVTSFLLYFLKVYCKWSNAHVDVPYFILERAFMASSFIILSSVS
>tr|C9ZQH0|C9ZQH0_TRYB9 | DeepTMHMM Topology Prediction - Predicted Type: TM
MYIYIYIYIDIPTCLRVLVFFIIASVVSVIGNKKKGLEAMPKEQRSVPMGVISAEPLQAAARKLKFTTKVT
TLGKPEIRKATKKSAPTGLKTGGSQSGMVFNKGFGQHILKNPLVIAAIVEKAAVKPTDIVLEIGPGTGNLT
EKLLQAAKKVIAFEVDPRMVAELNKRFQNSPLAPKLQVIRGNCLDHEFPYFDKCVANVPYAISSALVFKLL
KRPNFKCAVLMFQREFALRVCAQPGSEAYCRLSVNSQLLARCSHLMKISKNSFNPPPKVESSVIRLDPKHP
APSVDFEEWDGLVKHIFNRKNKKVSSIFRTKNTVRTLYDKYCSYQRMEGVNDVKTFEEFRQHLESILQEPI
FDKRARVLDQESIMELLCCFTKNDIHFV
>tr|D0A971|D0A971_TRYB9 | DeepTMHMM Topology Prediction - Predicted Type: TM
MIRLRYRAREVAFGWSRGPALWSTRRLESMGSTSWRVPSTNKAVAQWLYGSAAVVGCVVVVGGVTRLTESG
LSIVDWKPVTGVRPPTTQDEWEEEFAKYQNFPEFKQRNTMTLEEFKFIFFWEWAHRVLARSVGIVYGVPLL
YFVCRGRFRAQPALLATLSGILALGGAQGAMGWYMVRSGLDPQLLDERRKARVSTYRLAAHLVLAFTIYAS
LMRVGFGLKMPRMAHFPGRTRIQACARCCFAAVLCTVISGAFVAGLGGGLMYNDELPWMGGGFIPPRDHLM
VVEPWWRNALENPAAAQTWHRLMAAVSTTAIMAMNATCFRYRVAIPLSVWKSVAAVNAMLIAQVSLGVWTI
ASYVDLPVAVSHQLGSLLLLTTTIRVCAVLGSRGLVLV
>tr|D0A027|D0A027_TRYB9 | DeepTMHMM Topology Prediction - Predicted Type: TM
MEKANELMQRFGELNNPSHIFLAVVIAWLVLRCIYSIGLVLLVEVALVVGAVSYLIRKEGKRTAFNILHAH
RLMRDKEFMKTVLERDLPEWLINPSANNVQWLNSLINEMWKPISEATATTVKNCLEPLLETYKPSFIYSMN
LKQCTMGSQPFVITGIQYHPSREKESILDVTMTWDSDMDIVIHLDMPGPDMNVHVRRLQLSMQTRVVLFPY
ASVWPCFGNMSVSIMKLWMLNFDISAGGVALDAVPAVGSFLDNFFRKTLVGMMQYPKRWTFPIVQGYEMDT
SLADSAMGTLRIRFLRANEWYHRYVSDRAKTPYYIKLLMSGEDPKKRLLKSNIYSGLDTTFSDVFSFILYD
TELTLHFWMYFDVPGYDVLIGECVVPVKSLVESKGREYTCMMSKTSGSRTTVRSKLLIMPEFLPYNTGGTT
TTGSAPQQAPSRAVSESFANSLKSTSDAIVPPSTRSTVPNDDGVENHGGGTLFVTVQRCRNLKNKETIGVS
DPYVKLQLRKQTRKSPYISSTLNPDFNFEAALEVYDIRSDVLHISILDKNDLVKDRLMGTLRIMLSQVAAA
PGDIIRGDMNLDPEGQISLELKLLRH

>tr|C9ZSK2|C9ZSK2_TRYB9 | DeepTMHMM Topology Prediction - Predicted Type: TM
MAAPTRSKGRDQKVYNKILLVNTCLLFACYKPVFTKEKKSLNLAILISLSTPLHFEETTNHYDFFIYIVDN
CSLIQVLCMIYNFLRSLPHFTEGSHHEGEHVYAYRFTAALSNTFCVFVLAPKLFPYLYPSLLPQCHFKQQR
LTKQEY
>tr|D0A837|D0A837_TRYB9 | DeepTMHMM Topology Prediction - Predicted Type: TM
MIRLSEVVRRVLLHASGGRSCRSFRNGCGVENDHENGNAGLLSLRSTLPNQNDFFFSRSSASNCCRLCGEV
SQTILSHTGSQAHVTVEALMWLLFQRARQYKDGAVGVSPTAARNFFVEEARRWEGILQCSLVNQRSSSLHA
ITEDGSPCLEESAVYHGDPATLWKEVAEKSALRLQSQIRVLGKLGVLNVVDSLFTTASSGGLQRDAAFQRM
ECIGDHNWGHSVCRRIVLLFPEVNWRANSNVLVMDALRTVLECNQHLVHVFTLLQLSDKLGGRDVKEKSVK
FKADIVEAIIGELHVALWSLQSSTNDGFTSIPSIHGAPYTIPLVSMVEECLDGIVGLVILALMARYAASLV
PAVVDLVRREKYLLDVSCPFYTLRKRRERFRQDEHAMQWLLPPHPPLQQQRQELLKGARSSVDTCVNEWNL
SNTNPVGDVLVNASGGENMALTNAMEKRTPTGVVCWTKNIFLSEMADFYGLLRLPGGQSP
>tr|C9ZS21|C9ZS21_TRYB9 | DeepTMHMM Topology Prediction - Predicted Type: TM
MFRYRCRNAKNLLLMRNIRRWYTSAGNDENLPSAQEPPLAGVSSPTLWRNGGVLGEGMLGNPRVAATTPQE
VEETFRCSVLARTVSNLKLRRYLQKLEPKDHTLVLAALRGAQAGGLRLDTKTNEVALSKLMDGGQLQASME
IYRKMIQNRMNPTANTYATLMHMCIERDMPEACQKLFEEMVKRGQSPNTRNYEMYIGSLAMDNPPKWKKAI
EVFDRMSRERHGKHLTAATYESLMRVYLNMTPFDWRVVYNCYYEMRSRRPQIQLRWETYHLVAEALRRGQA
GYTRRLITYLDAWFCITYFRSWEFFMGFMFYICLMFLIKGLISWVVVWYYGRAVNASRGASESVIL
>tr|C9ZM99|C9ZM99_TRYB9 | DeepTMHMM Topology Prediction - Predicted Type: TM
MVEEPSGIVLYGDVVERTGIVEGGTSKNSSSGNGNIEKGRSILRPIQAVVGRAFPHIDPNRFYGNNATEMQ
CEGDPNDGRMPMKTTKSATFSSITTSGFTAHVQPGFSGNIEAASISADRGFGAIDCMSRQRQWDPKDFENL
ECGYYTVPLNTCNAAPAGTCVSHPEVPSGELYVELLLQFLRSTVRKHRECAMEALRARLLVDRPLRELFTT
GTECAQLLHLIINELASAGHALPCRVAAECLVLLLFDPTMEVEEAIGEIGCPPTDLAEISTNECNDMTFAE
LSECMRSGEDKGNALERLGMTKAVLDSLNLIPCTLASRLLLGSGVLLLPNVAIRVASDPRFVRFVRERLRS
VLLGSETVSGVVEELLVLRHVLRLQQTCATLLPALHETLMLFIRFVCSIPVEQARSPHDVVGCLAAFLVVR
AAVRQGFVGLFNDTAEGLLSATVLGSGIPAEMWLLLASSSEGDGVGRVFPTMEVFAADLARAAVRTIQAHI
TVAETGVANGEETGHLVSLLAALSSMHYLATYFTLYAGSDDLLFLLGTEHETQSRLKEFMAAKVLTSKGLQ
YAFLRLSSKRHTPLPLDLLNTRNVKTTVGGGEDGKICLNKIELVGACWASLTHARVRLAAAFTGLVHMTEW
EVPVGYATALATSFEEKCLGLKDVMGRLLPDELCTMVEVVQLMRRGVHGTSAVGGGSTVSQQQQRDSCAQN
SSSRARTQKLHIAETFLLHSIFLTKHGQDVAPGLIDAILGLQDVTIADEAAVIDPFVEPTVTLAESLRRAV
AQTRWPWFLFPLYDEDLREKGVWCGWVHRTLGMHQRMKDVLQWDLILCHTLRWAISSCHSFDKWEEAEVRT
DEEVKRLLHELCTGVLPRAGRLIATSASDVLKALASTIPLHEEVSMEYCLFVALAITCTACEPTAAVSIAR
LLSNTLLEGSTYCLTSSSDFEVTEAPIPHHSGSNSTAARSNPAVSSAHGYDNKLLNQWAASGSLLCDNTQV
SATAQWDLGNFTEVLHSVEFYGPGGPAHGSNAIKKGILQNLVEGIARLYLRQRPLTAFECQALRQDLQQLQ
WCPPCLREELDKCNN
>tr|D0A117|D0A117_TRYB9 | DeepTMHMM Topology Prediction - Predicted Type: TM
MPCRGRDCFLRVRLYMRWSHVSSSCVIPYMWSRLLTVTHAKTRDILLVLFFFHLCWLLAYRGPVVGNDKRF
GCFLICDCTRISIYIYIYIYLHMCDLNYHIHFIFCLYTPVLLGYYNG
>tr|C9ZZ63|C9ZZ63_TRYB9 | DeepTMHMM Topology Prediction - Predicted Type: TM
MHRKIFACDMAVSIHGGSPDPRAICLRYVFSPLSILLYSSFPPTKGFAASTAAIYITKFISYTLNIHLYLR
LGFLKKEKKSYRIEEGYREGNKAMQKNAPKFSVSKWNILPANRRSKFFWEKLNEYLEPLLSADFPLMDEEF
RGLCNVANQPPGTHPVLSETLIATVKNLRSYCEGVTLWNPLSLAAHLDIPPTDVIEQLLLAKEAGLVSMVF
VVPCQSCGCDMLRANSVGEICFRATYHKSNVIDCPMCTDRNEVDNLARVAVYFQDATPLPLLSRKFHRLFY
SQEVVRRCLETVFCPQDCAFMFNMRLPEGTFLIIAPGLGVIVQLEVETGAQFLERNRPYHDVRLLLSKFVT
ENNSDSAETANVAQDRNIIKVKHGKLSFRVFNDTQHATFLDICIAFDVRLEFLSPKKPVVTTVPMLLHHST
RGLRSPLFYLFTPAPPGVRTSGVYVLHSFEMPNQDMEEVILSGSDSTVAVIREVHRYSLEDHSGLLLSVGV
GGATFESSFMSYTAALASSLCFAQRLVTRLGNDIAGALSCSITAGELYMASFKGQYDDDENERSAYPSVQI
IGPAVYAGHHPPPAVDPPCTNELMQKYTRNVRFEMRVVSDDNTGVCLKSLAADEMLDPFLTYMQENFAGVE
IKEEPQALVVVVPLDTLNAKVKLASLVDSTTYTKEIHGPFG
>tr|C9ZKM9|C9ZKM9_TRYB9 | DeepTMHMM Topology Prediction - Predicted Type: TM

MLRLINRGNKRCDEPWSPLFYPLQPCLIVIRVGRAAISSVASRKLCVALSPRLLGEGIASNVSLSSRVQRE
AFYYVVSYLSLRRPVLIAWPVEKVLWIIGRNASGSVSGTTTNIMSRLAGQPLVRQVTAVALHGGIASTAAA
VVVQIGGRSFVLLRRAITDSSFSKLSLQQYCHDVVTILASAGVSVAGGGVGAVVGGFILPGIGTVIGCIVG
SFGGGLVPYALRDEGPEKKQQQMAQHCYNPTRPLKILEEDDGWLYITDCTGDSQYMFYDQMGGGETKSILE
EDGAVSGVKVENCDVDGSDDSVGSEWEDVKDADS
>tr|C9ZL71|C9ZL71_TRYB9 | DeepTMHMM Topology Prediction - Predicted
Type: TM
MERRQAGGNLLDSMNSASVHVNDLPAEFPRFPPQTISTGVASQSHRKKSPASGPPMNGSHSFSRHTNTGTV
PAIPRAPRRCRLTNNAPFPDPACVDCQRHSCSRHSGGSTLAGGVELQIEGLTRFVWRCICFLWLAALVAGI
VLQSLPSITWRVLDICGRDAPDMWKYGTWNSNCATIELEDRHTDTYRVQWNGRPYSVDFGDPLLRFRSLVI
NLASPDDWEVFHPDERRFTLRVAIPFMEENSNWSTAPLPIVCRRGDDRCVYINLPPGAVLNDTGGGRVSLS
LSDVPGSIARNINNSAVGILFQGRAYALTVLIWRYIALTITVLHTIRFVVNLKYNNSLYEQWWVLVLLLAS
VLYLNPLTALAISPDRLPLPLEFLELHVPWWFVAVVVSYMFSVITASMPRAANPTKQEGAKNLGCLSRMKA
CFCRSRSIYDPPLWTKVVGVLFIIIAIGLDIAVAWRCCSATLHGRGSGKKIYIYLLCSLFGFGSLVCCIML
YRLRRSMSKKSYLDSRPQQLACRVFMFMFFTAIIFSITQFALFYVLDFRIPGMLAWQPLIQLPALLVWPVL
VNVMTLIYTTRHRPETVPIHPRDTRWKESVWPDDWYRWLARHGGSMYIFHTEKEEASFNWKQLEYRVRRYL
VRLKRRGGVHNLSLLLQSIPNDSSSPPVEGPSDPSINYTIMCSVHSRPQDIEFWKGLRESICNRGAAEIVN
MDMTSTCFLPYVEAGRENDLGPERPSGASQVMRQNVGREGGGSRAPLLENDGGALNGSVALRLSSTTGDAP
PGDGERRLGSVDDHSGGNGDDGGSTNNPKSVWQALRGVISASVRGAGRNLFERPAHAFERAETYLLDAVQQ
RMHEPLYLPFFNLETAIDCFNIAFESYNWKGQSHARAQERKVGTSGCCRGSSNYRSNARESAAYPRETNPD
ACTTSQGDVEMRKFCPSTEQQSVAATPDHNGTPTIDVKQYGYKPIAVFEALDVAAVCAVMDTEFLHHRGKA
PRIVIAFRGTANMSNVREDIKMRRRAWDEMKNDRDNASLNSSCCWEPTVHSGFLEIWEAHQTSIEEKLGGF
LKENSSTVYRVFCTGHSMGGAVACLCAYSVRRMLREIEYPLDEVTVYTFGQPPMGNAAFQTAYDKAIPRTF
RVVNESDEIATFRLYGTQVGTEVDINRHGNYICKPTYMEQRCHPMKNKAFGIEGHQVKSYARSLNALALDT
SCKIRASGDPEASYVAEPGREPDAM
>tr|C9ZW17|C9ZW17_TRYB9 | DeepTMHMM Topology Prediction - Predicted
Type: TM
MRNKVGLSWHVPTGNVLLEIFSATAVSSRGYISMRRHHAATRFPLAVTGTYNPEADGAVAIGCDFRRAITA
PLMAITVWWWLVERCFYHSCIWLFSLLYRHLPVLLWVYL
>tr|C9ZMG3|C9ZMG3_TRYB9 | DeepTMHMM Topology Prediction - Predicted
Type: TM
MPVSVHFLSSTLSYILLQLLYVKECAREQRTCQEGRCATLKRNKKGQSHSEFVCCSELLPNATLLACTRIQ
EVIPFVRLLQELACNVCMYEFQFKLFVKVYFERLIHWCPAVVCWGIA
>tr|C9ZQ66|C9ZQ66_TRYB9 | DeepTMHMM Topology Prediction - Predicted
Type: TM
MKRKVCVFDVPFFFFNFTPLPHFSPVCLCVSIAYTSSIRPPLLFFCNFVCLFFFFGALFHCVTFRKKKSVT
PQVCCIFFFSQFYIFSKLYAFLLCRKWHTILTHMYVY
>tr|C9ZQG3|C9ZQG3_TRYB9 | DeepTMHMM Topology Prediction - Predicted
Type: TM
MDIDLSYGDLEAHSLDTVVPNHEIVRTFNVSGNNIPPDVFVDMVREHIVRMKKLEELDVKNNRIGPPGAQC
LCKALIRHCPKLRYLDISENSILDESLVDVAYLLDQGCIETLLLVNSHITPRGVPTLCDGLLSSKCITNLS
LAFNMLGDAGASLLARALGAHPTLRSLDISDNRIGDQGAIDIADYLFLSPYSRLESVFLSVNVIGDTGFSA
IGEALSRTSNTRLSHLDLGCNANVGPEGRCAFIHYVEHMRHLYSLDLCSSNLSDEDMQALVHAVLSPTCGI
GLIEWYNNPDVRLSTEIELDAALKTKRSDREIRDMRTRRFCVAAASVFSVVALTYLGSVLLKRRRVAAR
>tr|D0A2Z2|D0A2Z2_TRYB9 | DeepTMHMM Topology Prediction - Predicted
Type: TM
MRTKHTSNSLFCFVFLCYNIFLSLSRFC
>tr|D0A5H6|D0A5H6_TRYB9 | DeepTMHMM Topology Prediction - Predicted
Type: TM
MRLTMQSISLQLVSLCFSFLFFFIFFFHFFFLRCTSPNSLRPLMSSCVFFSAFLFMLLFHHHHFHHNNLLS
SAAHALPQLSVCMFLYPLLFFFEFSLLFILLIVRSFVLLCIHSTVLSSHIQRNVTCGELTDISTRQVNAPN
HHEDLSSISSCHIYIYIYILMKSILPLRSSSLSSLFSYYFFIFLFSLLFFLFLKKKVSLALFCPHTSTHTK
TNTHAKSLSLLFFPFF
>tr|D0A4U2|D0A4U2_TRYB9 | DeepTMHMM Topology Prediction - Predicted
Type: TM
MHIYIYIYMHTKRRPSIPSSMRTPDGLKRKGTCEFNQRKERRAKPKAAEDVKKWKMRYGHRFKFFPTPLHY
FEPYASLVHPLLYVCACVFAFFFTIPQCRLFHFSSPSR

>tr|D0A4W2|D0A4W2_TRYB9 | DeepTMHMM Topology Prediction - Predicted Type: TM
MFLFIRVHSSARFNTYIQSFFSFATTSAYRYSLYCSFSYSFPLVFLISFSPALMLYIIFHALAPLISFCFF
FVSLLFVFVYLLFRRAKRKKRKKEDIFLLRIDSFL
>tr|D0A744|D0A744_TRYB9 | DeepTMHMM Topology Prediction - Predicted Type: TM
MPMRPGESSYCITLLCFICFLFICVKSPKARGWGCRTKLYLPSHNFSLSIVLTRTFTFVCPLLSFQCQTYC
LGAVGRQYIRGSTLSNYVSAHLLTYPSPKEERRLIRHFRF
>tr|D0A2T6|D0A2T6_TRYB9 | DeepTMHMM Topology Prediction - Predicted Type: TM
MISYKRRNMFLADGRRVLVGNFIMSISTIIVTFYLTQGHLQVGQRRLLDDISPRLSEAARKIGMLEVELPY
PFNHVSAVVASPTGIADAGHKVMRFFGTNLRSNIITEKVNGEAANLLATGFSSESTILAHLLSPKADGPLG
IMGILHLCSATSCMAPYFTQQYATVTVRPSGNYRLLVGALVYGDVFLGSVVENDLAALDVVLPRASRGREA
TGAMPLHGVVGGSYAGKSVTVKCSVSINSCQYEFRDDDTDASHPSVTVVHLDDVKLGEAELPVMGFRVVHI
DLNSEEQLAIVTYLTGLLHVSARPEHIIVTLYPSPFLEETITAVDVLRVSVPYNVFIEGGRCRLTTNSSTV
PSAEVLRLVLRRAGTSASSALNTRSYAAEPLCVLVLSKSITSLSQLLEHVAPTKQEDVMKTFLGTSLDQGS
HRGVPCRGKHSKPNAFKWILLITVPLLVAGLLACGMLYGRRRVPLLWEDKRP
>tr|D0A0H1|D0A0H1_TRYB9 | DeepTMHMM Topology Prediction - Predicted Type: TM
MYVYAFHYLVVVRCGCRKSEYIHSPVQKVKEGRWIVFFFFFFLLSFFPFFPFLLSFFSPFFSYFRTPHFTP
ILWVCFFFSVWMESGDSPPCEVKVLRCRQPYHIIFHLIIFLFHGLPREPVFHVLCFFFVSLLFISLNSLLP
IYFLITHTGLLFAVVVVDYSRAATQFFFFFFRRRISI
>tr|C9ZM76|C9ZM76_TRYB9 | DeepTMHMM Topology Prediction - Predicted Type: TM
MEDVNRQNHNDAQKRHEYPGRSGENEPSHNQSPLREGESGYRKALCRISLQQMITRPLLQNYNRREVSGNI
YVYTYIYIYIYIYIYIYIYIIQVARGDVLRGASQGAQLLQHGNRHINTSLCTYVCLLSPSLFTWNVFHHVR
LFLQPLQYLNNILCVVTYLFSSFSFSPFLTMVAFFASMSVVAPNETIIGLR
>tr|C9ZL05|C9ZL05_TRYB9 | DeepTMHMM Topology Prediction - Predicted Type: TM
MDAGKLFHLLAAIVFMKLLFGCIFARVPLEARANDHPPGESFQHAGSMGEVSVGVPRRSTRNSRMDTDRSV
RNWGAGHGVGGALPGLFGLIPGIQELMIAFSCLTWPDILWRYFLFLLLLLTFLYFTENVRDLLYVRPRMLR
LGDSYLTEKPPRVGRDVALSYDTPWCLYEVMKTTFFAVTGLLAVRLFIFVLFSFMTLVVMSMAGRRGRNRN
DNPLWFSFFTLLAYITFCVASVGIGFYNFRVFGRIADRSECKILISNHSCVFEVVLLFALAQFPSFITRKE
TKLPLFESIVRLSDSILVDRSAAESRRRAAEAIAKRAKDRDPLVPQLLVFPEGTTTNQRTLFMFRKGAMEP
GEPIQMICVGFPYKHFNPCWNGRCCGGNSFGVLILRLCSQFVNRVEVRPLPIYVPTESEREDPILYANRCQ
EMMANVLGCGVSECTYADYVALLNEKSASAPVPPKRFTKG
>tr|D0A864|D0A864_TRYB9 | DeepTMHMM Topology Prediction - Predicted Type: TM
MINQTGFASALASRTALRHSALRMIPVRRDCFTLFSCRRCFSFSRARAPRVNLEEVRFDKQSARYAGTVFG
VPTDAVVFYAKVGAAVVGVLIVFCIFVKGYGILARLNLGTVARLGFTSGFLSCMVLNAVFIGIARRFRISP
NAVYNQSIAIVMQNEKVVQHLGPHPRTGDFKAYCQTGGFRLPLIRRIRSGSYELSDLLGLKPQKLQMMFVL
RNQANGGEGFVTCEVRRETTGFLSSANTFRSLAVTLTDPSRSSARTIVLIGKPEDVVYRGLMNL
>tr|C9ZHY9|C9ZHY9_TRYB9 | DeepTMHMM Topology Prediction - Predicted Type: TM
MQIYVKHRGKFIFLALWSGFTCIAMFWLMIRRSGFGTSYSRCCYLVMWGEYKQLLEPGTFPSNGFLPPHLR
LDSRSYLDLPETEIALPPLIDVSIAERDQVRNLESMLPSTQRSDLKVQFGPIRTQTIVMTSGANERYVRRR
LGLSEKDRKRLQEEEEQQKAVEVNK
>tr|C9ZWY0|C9ZWY0_TRYB9 | DeepTMHMM Topology Prediction - Predicted Type: TM
MSNVRGNITPAVVYASNEPNALNASDDGIKNYEAYEDIQSNNSESKFAPFSKLSKKIAAVIPPGGIFASAF
NIAATTLGAGIFGLPSTANGSGLVMGILYLVIINCMTIYSMYNLALAAERSKALTYEGVTFVVLGRWAAYA
IAAVRAFDGFTSCIAYVISVGDIFSSILKGTDAPEFWKGNTGNRLLTALLWLCCMLPLVIPRHVDSLRHVS
TCAVTFMVYFVIVIVVHSCLNGLPENIKDVSVGKSDTAAIILFNSGNAAVEGLGVFMFSYTCQDTAYEIYM
DMKDRSVRKFVISSAIAMCMCTVLYILTVFFGYMDFGRDVTGSILLMYDPVNEPAVMVGMIGVLVKLIASY
ALLAMACRNALYSIAGKNADVLPFWKHCASVVTLSVAALVLGLFIPKVNTVLGFAGSITGGSLGYIFPSLL
LMYSGGFTWQRVGPFHYLVAYGLLISGVVGVVFGTGATIWGTIVG
>tr|C9ZKA4|C9ZKA4_TRYB9 | DeepTMHMM Topology Prediction - Predicted Type: TM

MSAIYSLLFDSSSTLGVIIVSVVPWFVVLLYYLYYRIKRIGEVPPEVVAPLAHFLEVLTDGATAGYDKELM
NLFHPELLTVRVERGVVRAMVRWVCDRLGKVTNIMRDAVLVKDDGDEAHIIALVDFEKVQQVKCRMSWKWR
HTTGAAPRNQSSAKTEVSKRFFVTAFRFEPHTEVKSDVLQFLKTDDFIPFAEKFVERLFERPPKTAVEMMV
PSLKDKYISNLDKLQGDVRGVCGPLLGGSVDVNCSLIDATVLRGPLSTAKEEGQTEEGVRGIEMSFFVSGV
GCRNVNVNLLLVFADLRCYVGRYEVRVVPDTRTQVIVDRDTGEKTFIG
>tr|D0AAU2|D0AAU2_TRYB9 | DeepTMHMM Topology Prediction - Predicted
Type: TM
MVWYIPFVYQAFFFSTFNAVGSLHAWYMTQRHMMLVSGSLNSSLGAVAVYTHSFDPTLSNACTSIASISAF
GHFGLHAFRTKALLRPSAMSFLHFCWCISLLAFGIHRGRWAYTLRHD
>tr|C9ZK48|C9ZK48_TRYB9 | DeepTMHMM Topology Prediction - Predicted
Type: TM
MCARVGEREMKKNKKEDEGSVESVHSYVCTVRMQTRATQKETGRGGYVHFTYNLLFNLLIRNIRIYIRTCT
CKRFLYRVGMFCVMFLSCCCCLQSHAEKKKTT
>tr|C9ZVF9|C9ZVF9_TRYB9 | DeepTMHMM Topology Prediction - Predicted
Type: TM
MVCLVGFVFSPPRPYAQPTCDCHYGVVERENPRCQCVCFGDYLLPNCLYTAIDVVDVELWLTTKSGKKRGN
AYPLSSGDIERGLDRLFGHKNSSGLFKFRRGTSSFSSEGLVNSSIRSGKFYVAAVVSMPGWAAQHLLAYVA
QKAGKGGEMTNSASGVSYTLYAAYDMAKGPPLPVMYYYESMAFFTGIDSNIFITAADCGWMFGAIVLVLLM
TRIEQLWMYSIVAGLGAISRRSRGDEKGSLFLNEDGAGSSNVSRHDSLRRSSSKGSSSMKSSSKDKKKNKK
ADKEKKRNNSKRSRASGKRAASKSERRAPDSEAPQLGERRSKSPAGENPGGAQWRNPLTI
>tr|C9ZSC2|C9ZSC2_TRYB9 | DeepTMHMM Topology Prediction - Predicted
Type: TM
MNVSIYKCFCYCWEPLEGSIREKKERETLTGTSIHPSIKVEYCNGERCLHNHRKSQQRNIISPHSFLSANC
EEMWLLKTVPLTVVKLFHALPYLYIIIIFLLF
>tr|D0A6V2|D0A6V2_TRYB9 | DeepTMHMM Topology Prediction - Predicted
Type: TM
>tr|C9ZSH1|C9ZSH1_TRYB9 | DeepTMHMM Topology Prediction - Predicted
Type: TM
MQTHTRICIYRKVIFKKIIKGHLLQWSHIQSLCFFPFSAVILFLASSQLVLLFFLSVFLFGRLLSPSIWAS
CFYWYIFLNFFDCGCGAFSVYYYSLSLTLHSPLPHPP
>tr|C9ZWS0|C9ZWS0_TRYB9 | DeepTMHMM Topology Prediction - Predicted
Type: TM
MSLPLASAILPLLTSLFVEHASIVPPVTVMKSFKSLLIVALSILFLRAVMYVRDCDCSIQTMCVLFSPFHV
VIPHKVSLRQLGYTTPSVYRRTFHVLLVRYRYLQCQLDIG
>tr|C9ZSH2|C9ZSH2_TRYB9 | DeepTMHMM Topology Prediction - Predicted
Type: TM
MKGNEQKMELNRTGESGAKQKRKCSKSKDAYFIIFFFSLLHPSAIFIPLFTAQSTVAYFPHIKVTHFHLML
PTPPHYFIVNFPFHLSCMFLSTTFTHAAGPHPHTHTHVPLLFFFCLILCSLFIVTPLFTI
>tr|C9ZSU3|C9ZSU3_TRYB9 | DeepTMHMM Topology Prediction - Predicted
Type: TM
MFRCNKATRSLPLMTLNKPIMMANYGSCGNDDIMMTVVIMIPKKKKITMSRIHTRLKFLPFSTSPGTFVQL
LLFILLFLSNIYMYISLPFIFLSFFLSCTVLFLPPPPAPRLFPYIPTTFLCFLKRRIKI
>tr|C9ZNA9|C9ZNA9_TRYB9 | DeepTMHMM Topology Prediction - Predicted
Type: TM
MLSAKYRRAPVMAAPTAARWGGCSLFTRDLRCMRPFDGYVDPSTRVVPFLFSFLPLLNASAHLLTYKPKVL
PLLVHIYDNTHGKFGETSVYLRSCHVMSCLMWWWWCVCVCG
>tr|C9ZTM8|C9ZTM8_TRYB9 | DeepTMHMM Topology Prediction - Predicted
Type: TM
MTIVVCVDTKEVYAPVLFFFFGFALVWGFALLTGFSLLPSCLPPCRYTCLFYRIFFFRNYLCTLSCRVFFS
SHSFPLRHLGLLEVSPDHLYHLVYQRIPVSFFS
>tr|D0A402|D0A402_TRYB9 | DeepTMHMM Topology Prediction - Predicted
Type: TM
MFCFVVSFFFFLNDYRLISPSFLSMCFYGGFEWREGGCVSVSVPQTSRTFIYAYVYIYIYIYISMQLCVCI
STCIFVSESGLITSSPIKMGLCCCQVAVSSSALTLIFHIFTLIASLFGLFVLLFFFLSFLFLFSSLSFFFR
IICCSCCG
>tr|D0A128|D0A128_TRYB9 | DeepTMHMM Topology Prediction - Predicted
Type: TM

MTLRTGERDGFSLPTLLPYIYIYLHFFVYFFFSVILSFFLNIWFSFLYKYVRICYSPFRIVHEHLLVCRGA
HSGAMNVYGKDQRIHNGSRTKAESWVMHSFTAITGLQASCIAVCLFRMRGRLDNVLQNSYFITSPLQLNPV
YHSQSCALEVNEDIAMFRNVTYTITGENVEYFFSRGAFNTRILFFLVAMTTFIALVNHTLLWLDISKIRYH
FVVIRKDPLAVFQVSAAVAGIFQNYIVSREHKYMNDYLNTCNAPDRPSLHSVTPFIELYLLFGGTLVTFLI
NFLIAVILRCQKNPKEELIRENELMQMEKSMTGELPFSGDQLMLSQRSRGDANVSNSSIPNQRLAHSRGPS
EGKEFRHVDPLASPTSQLSFARRDSVPASRVSVAREGRLSRGESARRALPSQASAWPPVSGRRSFTGPVSA
PAFDDIIGDSTHR
>tr|D0A7G8|D0A7G8_TRYB9 | DeepTMHMM Topology Prediction - Predicted
Type: TM
MAEAYVPTDTPSVLVRCSSFVYRSYFYFFFFFRILSFFGLCTCFHRIVTKVEFMQLRHELKFAGYGADKST
VLLGDNTSVTHCLQPIWKGEEVKVCVDGVNGNDGSSAESDFVESECSESYVSSASSCSYRGGSPYESNFSG
CTSSPRIGRSNLQGCREDDAVAGVGTHDGQDMTSVSFPVHGTLLSIRDPALTGGLLFQLHLADKEHTPYAE
ACRTAVVLGYSEQGEVFLLPVPSPCMTKKPSYYQSTPLLQLSLSELREYYSWGDNQLLEPTCDDVQSWELP
HLGGFMVRPAFEHMWLATVFAVASDRLYYLSAWSAFVQFVGVAWGLPWVRKFPVNIPEGDPIVSFVAPLVG
CNCGRSLREVYGLRELRRREQQGSTDVGTVPVPPGPVANADSSGWREFLRELQEVVGDAVADWSQLPAAAE
HCDSKETTPPTSAAVTAAERLREDDGQLTSSYVVGKRSGDGGLLSEASVGVVPAVESMLRSFGTEVQQDIG
RNISKDTGLPSPFLFTLFLKAVAYWKSNLLTEEQQSSQPLAFIDYYGTNPHERFVEAIDLLRVHQSKFLSE
TRPNARSSGNYCDQCVGPWTVQRIVRMLAEPAVTN
>tr|C9ZM40|C9ZM40_TRYB9 | DeepTMHMM Topology Prediction - Predicted
Type: TM
MLTLFNLFRLINRTLTFPPFLSLFSLSVFITFTLLSHIIARIALGTSEHTNMSHCKFEHPRHGHLGFLPRK
RSRQIRGRARSFPKDDPSQKPHLTSFMVYKAGMTHIVRDVDRPGSKVNKKEVVEPVTILEAPPMVVVGIVG
YRQTPVGHKTIGTVWAHHTSVEFRRRFYKNWKQSAQLAFTKRKQFARTTEGRLAEARTLKAFEKKADIIRV
VAHTQLRKLRNNRVGVKKAHVSEIQINGGTIAQKIELAKSLLEKEVRIDSVFQQSEACDVCAVTKGHGFTG
VVKRWGVACLPRKTHRGLRKVACIGAWHPSRVMYTVARAGQHGYHHRTHLNKKIYQLGRAVSMEPNQATTA
YDLTAKTITPMGGFVGYGTVRNDYVMLKGSVAGPRRRVITLRRPMAPQTSRKLTEKITLKFIDTSSKIGHG
RFQTKKEKSQWYGPLKKDRIRREERLRKERAARAAERKAKGGATTTAAPRKSKK
>tr|C9ZKK2|C9ZKK2_TRYB9 | DeepTMHMM Topology Prediction - Predicted
Type: TM
MVSEGTSAAGRLEGESPLALRVDTGEIVAGCLAGFVEHFFMFPFDTLKTRVQSGDSTNVILAAKRISRNER
LAHLYRGFAPIIVSAVPAHGAYYSTYEAAKRVFGEDSTVSITVSASCAVAAHDTISTPFDVIKQRMQMDGS
RKFASSLQCGQCAVAEGGVRCLLLSLPTTILMNIPHFSAYWLVYEGFLAYLGGERRNRETEVAGDYITGGL
LAGSVASIVSSPLDVVKTQLQLGLRKNIPDAVRYVLVNRGTKGFFAGVTARVMCTAPAGALSMITYETAKK
FMEER
>tr|C9ZWR6|C9ZWR6_TRYB9 | DeepTMHMM Topology Prediction - Predicted
Type: TM
MARHPLGNMYAMTAERAREYGFVSHYNHTGSEASDKLANNITLTSEEWVQLLTGPVQFITQSGGWGGAGFD
ATVLPQLLLCFPWVIAFLMFRVFTQRQLIRVGLWLQVVVPKGGTAATLSNSQRRKLKKFQNQVWLATYYIV
STIFGYAVQIGKPWFGLPVSKANRVALLTPHPYKPGNGLLCYYQYGLGFYIAEMLALLTEYDIKRSDFVEY
FIHHIVTVALIVVSHCSYEHRFGVYVLLIHDASDIMLALSKILNYVLGAQAKRMRQRKAGKKVDVVEAKSS
FLYRMIFCETTMNIVFVAFTAVFVFFRLVCLPYLALSNIVYGVKIRMFTWSYCLLIFLLQVALQGLHIYWF
TIIVKVLINTALGSRVDDIRSEDDEDDGNVREKASFKHSKTS
>tr|C9ZXD7|C9ZXD7_TRYB9 | DeepTMHMM Topology Prediction - Predicted
Type: TM
MGPTCDRIAKMLPHPILASDDDRQYVQESILSLPDMVENFFTSLERVYTDGKCEEAKQAFATYHSIQTIVS
VLCRVPASAYRTNDEKRSIDWEIYERIKRMGHLFLGDIVVVGPQSLNWRVPVMRLAEEVAQRMRCAYVEFP
FMSVLLREAVSYSSVAFILVIAIFAASVVFTLVVLVTLPTLSPSTTVGLLVGLTAFLLVSVVSCGFLYLFV
TSAKALKVLHTWILEERMYTVLLSSASSEGDLSTTGPSAGAAVPQVTRGHNTKNGYYTIPYGNALGYIEGK
HVDAQVVMIGFDDKYRITRWNMAAEVVTGFLESGCVGKPLSDLVITPTGDIERDLAPLQVFSGEVLKLKLR
AFATVPVTLFTVAVPILNQEGSLIGRILICANAKDNLGEYRTYIRDYVVSEVNLSLSEILEGKAVTPRGLS
VIGPLQSFLEYGYGKQVEELARGMLAEWEWTSSEQLLGQMLRLSPLDHKTSVDSLFPGTLCLHPCIPEAVA
TLLNSLRVPCHLRLQILNSSRNTFALQIVATPVAQAAPSTAQISELREKLLPHLRSTCGSIREDDGTVVFR
FPCQITAALEDIDDATFRPADALNERYVIDQTRAIVNCTVNVLTLITNLVDQHNISMILLRTMFVSLTSVR
ERCELEKRLQSSPSDIDVIVCDRGWLNSVQDLVHTLSYDIIVVPISEPGVRLALEGFQYVINTPISSTEVR
KVLMAIGTAVSLRKNAATAQEERERILTLRQDSPWTKGVLLGRGSFGAVYEATSDLTGGKMAVKMFYFTED
LEESINALLNEIKIMCSLNHPNIVHYFHCERKENSVNLFMELCCCSLGDIIYGRSQKPPDLTVIKVLRQLL
TALTYLHARGVAHRDVKPQNILIKGDVVKITDFGTARQGVGSEDVQGTLRYMAPEVYRGEDHCSPCDIWSV

GCVAAELFDCPPLFMETPHMLADMTDVDYYVDGLTSNPVLCDFLRMCFCLQPEGRATAADLLLHRLFSSSC
SAEVESLPDIFSREKQYVSSLTITTQPHS
>tr|C9ZKG2|C9ZKG2_TRYB9 | DeepTMHMM Topology Prediction - Predicted
Type: TM

MHLCGDEREDDCSAYTGNGPVSGYCFDKGLCDEHMPSQEDEELSEIGAPSSGVNPAATSLPQ
RKKNLVVLFTLFSLAAALLYALSVVRLGLQIRDYANNNRFRVFPDSVAWHPLSYRRLFLQNP
KWGDERSNRGSSPPYSFFEVSCPDCYSTYAPTQEKPPEVIIFFAHGNAGSYMQAHRLGILFL
QEQKIKGRLYTFDFSEQANAHRGALLHHQADFVADTIITLYEDRKNETHNPPFIWLIGHSMG
GVVVRMALSTLSDSQVFPLIAGVITLNSPNRQMPVFLDLPMWRLYKTLWDPVQKNVLANDRN
GYRNPRILSLTSGPLDLMVQSKHTRLSDSIGCNNGSCIDVTTEHPKVCGKTLMTIMRDYCVV
EFWTSAVIRNSLVRGDGISPESRNLKLRNWSLPEGINDPAPPPHSFSDAFSWWAEASYTHVG
LTVLVAFSYGSFVLSAIHPLLLRLLLNLRVLSHCCADWSWKSLFTSSHTAVVMVTPIMGIVG
HLLLVQIPCCLLPAMEGCKSPWIADTLVCKPTFDPLMLVWCAFAASGPALKGIWMGIIAYRL
LQLLLRGSKFGWTKFWLVTHRACIYLRLPRWVRRSPTRSDDNCDEPWNRIIPVTVFGVVVFL
CGVLLPLRICDRALVWLCLTIVLLPLSASYQTATFDSSDGRREEAYMLVSAYALLHLIHLHP
FFAWRNALRSATFDDATVVDFTSRAVEVLLLVLILSYVMWPLYHEQRLCEQLHRDKLSSLSK
ALASPWIQRLAPHLCILLVLIPLVWMISVPVASFRIVWVLLYAFPCFLLGPALVSGGSGGTL
LAPGTSN

>tr|C9ZYR4|C9ZYR4_TRYB9 | DeepTMHMM Topology Prediction - Predicted
Type: TM
MLRASFFFGGGELCKYLRIVYGVFTLWEIGCLFHVVLQPNIVNMSVCVYGQAKTHLPAVANYNSFFR
>tr|D0A7Q9|D0A7Q9_TRYB9 | DeepTMHMM Topology Prediction - Predicted
Type: TM
MWWSFIFSFYFDPSPPCYRYALHESSLIGDDRKGWVPRLSLNGDNQVMLPDTFNECCIPIATLYLFIVYLI
RGEVSTGKWFVNFRGIYSVGVDSFSWWFVTADLQCFVAFSVVAAIFL
>tr|C9ZLF8|C9ZLF8_TRYB9 | DeepTMHMM Topology Prediction - Predicted
Type: TM
MCVLRFIRVMFPMYSCCFFLVFFRFFFVATVVTFEPCGSGITFRYLALCCVQACARIFDSHFRVAFVFCVS
FHPRVELFSFFFFLKCCGGVGHWSGGNLSFFVTFPRFSPFIVFITIITNDVGVRDSSTEHFSRFFFFSCCW
FRFRSLYSFFLTAVVTSHALLSWNPNRAMWEGENKREYV
>tr|D0A487|D0A487_TRYB9 | DeepTMHMM Topology Prediction - Predicted
Type: TM
MQDERETRGDRVRLCRGKHMISVYSFHPSYHRQKHMWMKISLREVQAEHLIVFLSYCMCVILCLHFVRATL
PPFRSLSPLPVRSHHMLHIFLPCRTRMWRWLCGKEEEGVAVGRVMYIYIYIYI
>tr|C9ZU12|C9ZU12_TRYB9 | DeepTMHMM Topology Prediction - Predicted
Type: TM
MWRVALRRPRVAMPCGCIGHIYRRGSGVPYGSVPADIESSRHSRKPREEDVLKNISGRSGSDQANEETPAY
EAEGYDPWRVLGLKPGASTHMVRLRYHELMREVHPDLEPNRVGDISRLNQINKAYEIITKSPTLDRRYRNL
VSDTQYFYYKFLPEWMARNVDEMPRYWSWVRWRTPGAFQVFLLCCGCYMVGRFYAAFPVLTTAFLLSLSFD
ILFHTMTAPATCSMLFLYAIMSSQSYDMAWLTSPKSFLKRELSY
>tr|D0AAM7|D0AAM7_TRYB9 | DeepTMHMM Topology Prediction - Predicted
Type: TM
MDAFLESLKEAHEEVMNATGWGAFFSGEGQKWSMSPIGAFQQFRKEVAAFISAVNWWEPYFRYLALFHLTV
AVTVVLTTWRASIDRIFVVTVLLLLVVWGSSYLNEWGSSHASQIFVEKGANYFDPSGLFIMVTLDMPLFLL
VLWLQARAFLQLLRLMVVVKRNQIKREMEKFPCGRKGERPAASSKKKT
>tr|C9ZST0|C9ZST0_TRYB9 | DeepTMHMM Topology Prediction - Predicted
Type: TM
MRSKKLAVICGMAHRAIITTTVIFSFVYTRIVALSNAPLIYHCDVLIYIHCLLPSAIIILLLFYCAVYTIH
TLNSLCLCTLRDERGRKKEGVNTKRKHHR
>tr|C9ZPY9|C9ZPY9_TRYB9 | DeepTMHMM Topology Prediction - Predicted
Type: TM
MMDCRDASACQGVGSLCFFPYLNPLVSWHALVLGHLLYLFVVFVMRSIMRGRRALNMSRVLVVYNVLQICL
SAAMAINLSPPLKNGVFNLSGKFCPDIEFWMFVHYCSKYIDMLDTVFILCKKKEDQLSFLHVYHHCTIGLI
WGILLRNGLANGTAFFGTWINSFVHFLMYSHYLWTSLGYRNPFKFLLTKIQMLQFSLCILHAILVTLLDTQ
FTLGWNMLQLLYNASLLVLFINFYMNSRGKGCAIERKPQ

>tr|C9ZWW5|C9ZWW5_TRYB9 | DeepTMHMM Topology Prediction - Predicted
Type: TM
MSDSESIQFNVMSELESPVVGREGRNASRGESRKPSVKDLKRPPLHGAPSPTSRKSLRRSSEGSSLQFVVC
GEAGGDDSDNTDTIQFNVMSELESPVVGREGRNASRGESRKPSVKDLKRPPLHGAPSPTSRKSLRRSSGGS
SLQFVVCGEAGGDDSDNTDTIQFKTEGGSRNISLKAASPTSIAGNRRNDSNGRSLRRSSTESGDESVVFVI
EDTQSTGGKKPLSRREASNDDTISFVVGDSGGGDEEGADGSSMQKSLSFTVVGSGELPVAPPVFTGKQGSS
SPQLMSPLEAPSKPGVGSRDMQRPPKAVSRPKEANNLKGKYRSSSRANTGDGGMTRSDSYDVVEEGGTLKI
CSTASKHTRAGRLDIPHTTTRGKDVLPPTASEAGKLSPGETRTSTALAPSASALVPLPFPHPSQKKFSLKA
ADIEEMFLQAKVYTEHWRMFNDIQREEVARLAQRIVEIRDGSSNTAGKMKGKSGEEGKDKPLCFADLFRVK
SFLKETDLPWNTERLGNQKKRLPPGRRPAAPARLVEGGKLPAGRGMSRRDDSGANRRQRPSPQGNKGTRVP
KGAKLGVPHFENVAEEMECDAVAQFAEIYGVAWPPSSKNELFFLTGVRLTQKQCEEFYDSMRLYVVASSKV
NANMNLLNAHERDLSPSRLSDAKRKVRVTRTTLLRKAFTVMDVEKNGVINAALLPSIQRLLEEERRHLQSV
LGGDVTPETFLSRSRGEKAKLNGNVETVVSPKVPQNSTAVNAVGTLRIYSLVLDVLLPLLCASGLLTFDFT
TVGLLVFGTLSLSTAGASPSFLKWREAAQRCFESLLKNPV
>tr|C9ZY09|C9ZY09_TRYB9 | DeepTMHMM Topology Prediction - Predicted
Type: TM
MASNGLAESSEGSLPDDLWLIIGLGGSGALLILLIPVVFLARHFYDRRHFISAAPLPSQNHLPSSKIRLRS
NSAPPASLAALCGQSGSYAGFGIGATSAGGGVYNEAVPHNGPTNTNCFNSGTLGAAYNPSTPSGWEAEAGR
SFTTLSIGLSAPQPTWSDTTGERQHPPATPVGDTSQLGYNPLAVTKEAHLFQFHPNVPQECGGPPQESADP
AQPSPQCTTEDAEPQPCLRRRNSRVSFVGEFRA
>tr|C9ZJ51|C9ZJ51_TRYB9 | DeepTMHMM Topology Prediction - Predicted
Type: TM
MSSEHAAVVGSRKTSSRATAAGIAGVMEIGLFHPFDTVAKRLMSHHGPILCRPISASFCTVNEIIFRAKAH
ASAWEKLLYLYPGSTYAVGYKVLQRVYKLAGQPIVRDHLKCHYDRGFDNVFGRHSRLAMEAVAGSIVGGGE
VLLLPLDRLKVLSQTNESALGRGVVELVRREGVRGMYAGTLVTAMRNVPGTFCLFGGAAFTKESIFRLEDF
SKASFFQNMCASGVGACLAIAVTNPMDVIKTRVQNKDPGSTLTGLVVFRKLLREEGLTALAKGITPKIIAS
APKLVFAYSMTEFLLQLLEGEKRGVVRH
>tr|C9ZX94|C9ZX94_TRYB9 | DeepTMHMM Topology Prediction - Predicted
Type: TM
MQTSSVSEVDCLAEQMYSSPDPSIRQRAQASLEQLTKAEADQSIICAILQQSNNQYALLFMSQCLVLWFKA
VRKWISEEEKQNVIVVHCGGCVKRALENGAPKHVVSALLSAYAKLTKLAFEADPLLEGAVNYPIELLRHEA
DGTNMQLLGLMMLNALVVEFSKYDSSRSKTYLGFVAHRHCSGNFNEKCLLNILVEALKLLEKLTVNTPHIT
EIVKLVENCFSFDFRAIMVDDTEDLPFVHFPCAWKPTILSDQTLQTLWGQHAALPHPHCASLLSAISNICG
TYRSFFETVEERLQYIEFTLTKLIQVTMLQDGRLKIPRYIETLAEAFRRVVLSLGYRELRQVAVFEQWVTA
FQSISVDVLSITFGQEGSFSTATAVMAFWVALTTSKRRSYSEQCPQDIEVAVLPVLRAFLVARIHGADASG
GDSFSLEDADGGLAEAVLAQSDAYANVCLLDPATYLGDFANYLNQQVGMSIFTSPLSTGWLFYIAGGVAWL
VLFSMESSGIEPCSHVFAYARGCADHRRNHSGNPALFSSFVERGMLHFLTNMQSVITGARHGNLSAVVTNV
FQDRGQLFQFVLDNVGHNLLRGPDSLDAMDIIRSSADVIVEACREAPPQLLRELSLELPPTSDLPLAQSEQ
TYKLRTNITKALWFVRSTGSYTRERMESYLSNVDFSMQRTVNNEVNSPSFIAGWVRDLRGACQALKEDQLS
SLDFIDWFLSRYSVFVTIVDTAGDSPIVVTALMRFLCELVTPGKYGRLHISSSSNSAVGLMLFKHLCDLVE
KVEKRTFSIDHLMALSSLSGSYNKVLKPWMLAMDIMKRCMEGSFVPFGAMLYYNDDTFERTTVDLLRKLAL
VGTNVFKEHGKFTVVAVDLLRLLVEENLYFCLRGLTGDELVGLINAVITVCEDVDTQSGVLVHGLGFLTFI
SGLVQEVRAIALSPSLRPTDTHGPDQSPAPFTQPFGSSRLSSPMPPVQQSIVSRPPRLATEVREHLARLLA
PHDSVWQRLLSTAMNIIVFQDRAVNSSCAVVHHIFEAHPPFWFNYVEQLIMSFPEGKHATLREAFSVLTNA
AETREKFFSEVFTLRQVLRRLGT
>tr|D0A225|D0A225_TRYB9 | DeepTMHMM Topology Prediction - Predicted
Type: TM
MEGALFLEGRNVVFFFVPFKNLFVVAVKRDGVEEQMRCVLVWEDEKGLFNVITADGGGKWYRLMGRGRIHV
MFRVVATVARRDFFPPLIVHKFKRPSIFPFPFFSWELPPSFFLPTRPYFGPQRCGGVSYPFYPFPFPFSFL
FVLFTDLYFKGIGVLEYNKDAMYLLLRCFFPLFSFMHVICLPFSYGGGG
>tr|C9ZLV5|C9ZLV5_TRYB9 | DeepTMHMM Topology Prediction - Predicted
Type: TM
MVGFKTLSSTAPLAFSEFSHLLLEALLAAALAVALALHFIRRRNTSKIVEPELPPLEEQQRRIAAFRSQMF
RADAKPNANTQLPNRTGMSEVEARNSCYITLRDESDSSSREYLDLVTEDFHSFSTHPTVVDVAKKIVVAYG
VGSCGPRGFYGTIKPHMVLEEDIAKFLGVEDSLIFSFSFATISTLIPCHASRGDYLIVDDGVSLPVHEGCT
LSRANLLKYRHNDMAHLEEILREVQIKEMKEKKLSRRFVVTEGVFKNLGDVCKLPQVLELCEKYKFRIILD
DSCGFGCMGPTGRGTHEHYGIPTTRIDLYVGSLSQAMGGVGGFCAGEHAIIEYQRLTAAAYVFSASLAPYI

TAGVSAVLKLLDEDHSFPEKLQRNAGLFRGAIRSAGMNPEKITLVECAGDVSPIVILRPTDAYVKCHHQKV
EEELQQVVEAARRKGVLLTRHLFSKEEACSNFSALRILVKGTATEDELLRAAKVITEAVKSVFV
>tr|C9ZMP2|C9ZMP2_TRYB9 | DeepTMHMM Topology Prediction - Predicted
Type: TM
MTTKNRPIEEAAAVYILRIIHTILLWSGVTLGSVLVVCSQPRFVPIRTAISSFVYLLASLCVIFVSILELK
GHISTISGVTHWMDRVGCSYWFLLLFIRCIKPSHAVEWVASALLPLLVLTEWAVVTTYILISSTYHPVITV
DLFLTVLHTVVFTAFVGLDRLMISDITVIFSAARAAQDADAVEACGGNMGMSRSGPRSFRSDDMLELSHEL
QSSASSYSSALCRGNSRCYILRVVARRWGGTYAVLVFVRLLYEFGGLLPAHLLRILVDNLSVVENKHGKKH
GSDGLCMASVMIVVIIAYNLICTFLRAHYRVGLQKIVLYIRGLLTSELFRCTLCRRKHLFDGRTQGDIMNH
LSLDVARVADAASSFNDLWALPLQLSLALYLLYIQISFAFVAGVVVSVALIPINMWLTKRIQGVMSELMRE
NDERVLRITEIVRNIIYVKMCGWSQLVQQWVKESRDRYLVHLTWLKYLDAFCVFFWATTPTLVSLVTFITF
ILMGGELTPGKAVTALALFNSLTMPLNAYPCVINGLVESYVSWCRLSPFLVMRPDACHGDLYGFASGESDS
GPKMFTEITEEYQDMVVSSEEMPCASSLNEIQPLLQAKGCVGEMRRQRLAGIHGLPHPFRRNHDLLIDIRS
GVIRLNTQGGAGLPRPSFMLHIPTFQATSSQLIAVVGVSGSGKSTFLDGLAGEHLIDPNDATGHAAVSRFS
TAYVEQQPFLMSGTLRENILMGLMYDSVRYEAVINAVALTQNFQTCFYPDGDTFLVGDRGIRLSGGQRARV
ALARALYAGKELYLIDDILGCLDPTVARHIVINALVGSAKSGSCVIVATHNQELVERADVVYRCVEGQLLF
SQRLVKTAATTVAEPSSPAAKPHATQQHVALANKEKGHGKVGNVLGDVPWPSSDSSPARAPAATPESGSIE
AAEVLETSKHGTLAWETLACYIGRVGWKLSAFIIISAALMQISRNAGDQYVVTWAKSGDGNTTRFIWSLAI
LAGVNSVLALARGFSFAFGGLRAAKRLHNELLGHVMSATFTFFSDTPPGRIINRLCGDTYTIDDSLPFIMN
ILLAQTFLLCGAVVVILLNSSSLLIVTLIPVSVLYHRIQCPYRASSRELRRLEEAANAPLLDTMRDALDGG
VVIRSLGGRVVMSHLRRASRNTDLLLRVKFNSMLLGAWFTIRLELVGLLPLIFVGGTATYYHGSASAPFIG
LALAYVQPLTSYVGGLLGAFASTETELISVERVRQYFSLASEEPRTLGMFFSPPRSNWPSGGQVVLSNVSM
RYDPSGPEVLRSLSFHVNAGEKVAIVGRTGAGKSSIFSALLRLVEIESGSIHIDGYDTRRLPLEVLRTRLS
VLPQQPFIFSGSLRQNIDPFGLHSEDAVRDVATSVKLDGVALDYNVTDSSRVSGGQRHLIALARVILQRSP
LLLLDEPTAQSSAEAEAALWSSLAEHLRSTTVLCITHKLSHIDFFDRVIVIENGHVAGDGTVAQLRAASVW
PFSSPNADASLT
>tr|D0A6R6|D0A6R6_TRYB9 | DeepTMHMM Topology Prediction - Predicted
Type: TM
MVHEGATFVVSMESLLSSPSPPWTCQASFRCRMPFRGVYAFWRVSLIVFVLLHFIFFRAYHRSKRKIIRNQ
MNPLFAGAVGLRVRCTAKLFVIRRSLSTYLEPRAYCSTGVGGCTTGACGGTNKDCLSFVLEDNLTEKSLPL
FHSYRAAGTGTASKIAVITDVAAFNNVLQGVFKSDGEGVNGVLCDLEGAWRHQLDEIAQKVIAKHRVDDYI
DNLYLPLLGYLIVILLIVQFIVLFRWVFVVFDWNLVEPMTYFIAYTAVWVGLVFHCYSVRGLSLETLLAER
RRQVLYKRARIDGAAIDRQMKLLGGMEKLLARYGKV
>tr|D0A1Y1|D0A1Y1_TRYB9 | DeepTMHMM Topology Prediction - Predicted
Type: TM
MAEPPPPQPLRKGFFRADPHGLRPPESPRDAEEPENHDIVEDPPLPASRNLPPPRLRPMGGGPPLAGEEAA
ATSHSNNRPSPGEVPPLPRPGTTTAVTSAQYNSLTPRQELFSSPQTAVEENIPAGYDNQMNVTSNDFSHSQ
DTGIKRIGDGATGSSAKTRSTIAHAPSGRFSEVVQERPPIESVEQLCQLQTLAHPVERFFVEYQPMATRGC
RSTQVPVEEDEALFNHLAEYTDDDSFFSSGSAVLEEDTRRLQNDPLRGRAIRSVFETLSLRSLTKGRIPYE
LEDQERSSAPWLKQVHQRAIALHHSSFFIFPAGMKARVIAYNILHHWLTEMLLMLIIILVYSMMTAAWSRDT
WPTLEKPSFMFFADVFFLCIYAIEFVARLFTSGAVSHSRAYFRSPWHCLDAAVLLLMILNCTNLQSMWNFS
AFRLIRVLKSSTYVPIPINMKLLAKSLLRSTSNVVKVSTILFYVLLFFSLVGLQLFSGVLQYRCVSPTTKN
VTNQLCRFNHSEKNESYYHGATCPSPHLCVADTYGNPHHGYRSFDSVGHSFLSAFQIMTFQGWTSLLQETS
DTTSVAAILYFFLAILICAWIIPSLYLGVFIEKIEKTRRLFVQKQLQLFDGMLLEQRQRLNEAIKLRDFVE
RDESGKLRRHPIELIRSASRRIQRSKLSNSQTSIATESESGEPAVVVKKPIGDTTKGRSRWTDEQRVQLHL
SLTRQRDIASGGERRRRVVIKNDGEISGGTAGLHSQRKGSESIGERAMMVTGDFALGGRVGVVQHHPLTHT
IGAAHGTDLPLAVRLDNEEEQLRFLKDYQNPIDNDIMRRTNTFEDTNGNLHPSFVLTSTQRRGGSFDEASN
TIRTTTEAAGVIPSRHSATLNGHNTSRMDSNGSLDEPTSKTQTVSKRGSPMPRLSSGQVPEVIIHDPEGGD
FRFAETRSQKWGIVRNILHMFTEGYPRIITQYIREHRRMQRRFGLTPLNYVNKYEDDVLRKLRQRRVLQVK
EPGAPSQTSRASGDEELVEVNGNKVILTDSDDIGDLSPIQMATNIVRNVPVTPFGAVMLVIVIVNGIFNAT
RYFQQPEYWETALFVLGIIFTSFFVLEIVVRVIGLGLVSFLLDFNNLLDVTVTILGFVELAYARSNVVTVL
NWVRLLRLFRTLPFAPMRRVSRVLLLGFADMLYALFFFSIYMFMWILIGMSFFGGPNGMVDHTFQDYYTRG
NFDTFSGASFAVSQAFSYTREEWVYLTWNGMQSRGEYTVLYFMAVVGVAFIARYFFVAVFAWAWQSEEEEE
ENYAAIAKGGSGGRREVTRLHWFDFTVWRSFKHIHGGFERRDVAPDEVFHLNEDMRKQLRIAEAKERFTKE
ALAQTDLAMSQRRMGSPMASPSATMGYNTDGCAPAAQVGTAPRYVNVGGQLQRHINPSVDFVDAQVPPLNA
PISQFQAENAQLRFARRYSTVPASVYTPVIQDDGIDRPSPSARSSPSDAGERSGELGGESQQNGQEEGDGQ
YSPRGVSPNGTGGRATSTGLQRKSSVLGRFPRLGYDRAAGKKDGGSRSVSASAMQQTGDGNELRGDYVNEG
EANGGSMVYEHILYPGPRLRYKHVMRNQYVRVFERCLDCNTYQQMPLRAPPNVQQRTPEELHAEHCHMAAV

RSSRQLVLNAIMGYVRLQKDINQPPTRDAVETVLGQAWSCGMLLFETIEYLSCSDIEQREYRTWDRTLEAL
QLQQWLIGLHVGEEQVGRATLAYTLAHRKREKLAVEHKSFELSWRQRSFFFISPSNPVRRLSTRIIQSRWF
DIFILTVIFIASFCLCFHIPGKANDPETGFVVLRAFDGIFTCIFLVEMIMKWISMGVILFRPEAYFWHWWN
VFDFVIVIVSLIGLADQHSALRSIKVLRCFRILIPMRVSNFNRSLSKISSALLDCLPTVANILLLFFINYF
VWAVLAVRLLNGLTHSCSDPSFVDITACEDAKHEWLPKVRNFDSFFQSLLTMIEVSVGSKWLDVIYTGVNG
RTSEHAPMDDHYLARGFFFIVYYYVSHLILFSLFTASMIYSYLLTKNAAEGVLGITFEHQLWIRMQRMTLQ
LKPRVKLVPLCNQVSQFLHNVVIRPIFEVVGASVLLLNILTMALHWYGETKSKASVLAAFQYVWMFYFTVE
AAMKIGAHGMRAFSRWAFSFDFFVLLLSFIGLIVDAASSEGMPFNVNVLRMLRLGRFFSAAKVFKPMRKQF
SLLHEVLIRSAVSLANVTLILFLGVFVFTVLGLHLVGGVPVGEGGYFDDRYTNFNNFGNSLMMTFRLTTLE
NWSPSLREGMNVTRKCTEDDCSVNYGSAFYCLLLLVFLGLIVLSFYMAVIVDHYVTAARMNTSITRIEDLR
RFRDLWSEFDPNGALVLHTHELPKLLESLRPPLGLTSRHNRVELLRLLREYDIPNHRGKVHYHEVLLPLAR
RVLAMAFSRDTMDYRTTFDTLWRHSEKSLRALPTVLGKRSHATAAQHFAASYVQAVCRRKKACREVQRVRS
ELWHEGRAVCDELGLPYADYGFGNLLLEGPDPMRDLVPRSASAASSGGKGTQKGASEAENAASSPLWRAGQ
AESPSSPRSEGETIDGRAPAARLPGAYQPAIEEREKRFGPDVPNALRRHETRSEKLRRKDEERMLQSTPDD
AVSSPVSNVRSNSQRVNVGEYQPPLGTDPTSWLGSNVNRGSTVGGPTTESRTSSVMPAPQGPTAPE
>tr|D0AAL6|D0AAL6_TRYB9 | DeepTMHMM Topology Prediction - Predicted
Type: TM
MGILQHQADVWHALRYPSKHSWGERKRAYLFVFAYCLAAIALFASVMHFIGAWIACGLLQVIMLIFAMLFA
LNIADCRDKCLNVLECERAINPVMEVYIGLRFIQFLHATFLLTNIPMGVSILVALLYSLWRMWCGTYFVDA
TSLWREVGRLERDSYIHICIEIALIVIYLIAIVFAMVDKYS
>tr|D0AAT7|D0AAT7_TRYB9 | DeepTMHMM Topology Prediction - Predicted
Type: TM
MYVFTTMRIANRLLSFPNSARALCVIFLFICFFRGGKPQLRYLFCPLAGSFGVIVLLLFFHSCLPRHMYTQ
IYASLFKLYRIAFKTDDDDDDGGNAIFRRTVEEMEEGNNGRSNVNATRQLQCGDCFNNHE
>tr|C9ZKD4|C9ZKD4_TRYB9 | DeepTMHMM Topology Prediction - Predicted
Type: TM
MEKNAHGLLADADSPKAHRHIRNTLKVNKLSSALMFMAVVTPVMVLVSFYSAKLEAVREKWVDPYELPPGF
NPKTGRFMSDSKPQGVTEPPAPLLLRGAVPGATRQ
>tr|C9ZUV4|C9ZUV4_TRYB9 | DeepTMHMM Topology Prediction - Predicted
Type: TM
MSLCAEINRTGFLGIIGFDQCGWNGTAGFVWEFWRLAPCCGAPDFANALLCIFNCLFCSPCILCKTYASSL
GDVCSVWPHCLMVLLCPCARWFTRYNLRKRTGTSGNIIGDFFCVFCCCAPCACCQEFRSINIGSWRIVPDA
SRMQFFTPGCRLLR
>tr|C9ZRU4|C9ZRU4_TRYB9 | DeepTMHMM Topology Prediction - Predicted
Type: TM
MRRRKKQMNAKKKKKKTSRHTEKNKMRKERNIAKGGRRGSVAEQHHPSKSNEGTSKLRQEAEVPLPFPFDF
LFHFISSHFILFFSFSLLPPSNFRFLTSPLHLFMYLICFCFLFDFVPPSPISHIFLIAISPASYLHHLSTF
HFFFFFFYCASSS
>tr|D0A6D7|D0A6D7_TRYB9 | DeepTMHMM Topology Prediction - Predicted
Type: TM
MSSEKVAGYVQCVVGIIFGVVTGGASIYVTIAMELLQVAVPYVLVTLGLQRRPPKGGVDKAMAEAESVEAE
VMKKPSKQQRSFRALFIGIDYKGTPAELRGCQADAVMMAGTMEKIGIPITERCVLMDTDDPRFNAIKPTRA
NILQHMAWLVKDAKPGDALFLHYSGYGAQVRAEEDKEEEFDQCIVPCDYEENGCILDNELHEIISTLPRGV
RLTAVFDCSHAGTLLDLPFSLICSSNDCSAVGEMKRIRTGGDVNAHVLMFSACGDDEAAADLPNAGDFVEG
ASGSGGAATQCFISMLLNKTPGTIYSLLSTTRDKLREKGFKQSPQMSASRCLSLTEKFTLTELFSVAEPCP
NILTGEPRWKTTPLGGK
>tr|C9ZRU1|C9ZRU1_TRYB9 | DeepTMHMM Topology Prediction - Predicted
Type: TM
MGWWRRSSSSSNGMQRVVETHFWSLTRYRIGRRFLGLDYADNVIFFPAYRSKRRGGWAAVTLGVTSSSNYF
TNDTSAVNVLEDGYPLHWRANSRLKEFVAEEKKLWQARVKEEVAQLAAEKVSASRTGNPNGGASKEPGDLM
GDINCEHRFRDTLAIKCTSPGFAQRHLRLSRLWTSLYKFLYFYLWGIGISLVVQAYLLFRSWLNPPARQGL
KNIEAHVLHIPKLIFGFCVSCVVWAAHHLQPVVSPVLEALEGVFPQVNWSAASPEALATKAQQLAGHDVSG
GKHGSGQLDQLNVKKTTETMAGNWAVFFSGNFTSIMQGLVFLLAVLLFLL
>tr|D0AAF6|D0AAF6_TRYB9 | DeepTMHMM Topology Prediction - Predicted
Type: TM
MSPRATEPIINEDEHPTSIPSLSQGNVLELPPDSDKSTAAADGEHRGCLNTVFDPIKGIVPYGGMASNVFN
LESATLGAGIVMLPSGFLNSGIIIATLMLVYICFTTVYSIRILVITRDKTGFRSYEEMACGLLGRGADYFT
AFLMFVFCFGTCVGYVISVGDLLSPLLNQPSTTGFLRTSMGKNVIVGVVWLVAMLPLSLPKEINSLRYASA

VGVFFIVFFVICMIVHAAMNGLKDGIGSDIRLVGDGWGILNGFTLFVFAFICQVNCFEVYEEMKGPTPRRM
TRDSSVAMSMVGLLYFLSGIFGYLDFGSDLEGSVLKLYKPQDDVMMAIGYVGIAIKICVGFAICIQPSRDA
IYYVLGWGKTSDVDSWKNLVVSGVLATLALVLGLVLPSIEVVFNFLGSFCGGFLAFILPALYYMYAGNFSL
KEVGWFNYAVTYQLLIVGVFAVVFGTALTIYDEVKK
>tr|C9ZP01|C9ZP01_TRYB9 | DeepTMHMM Topology Prediction - Predicted
Type: TM
MCILTLCASFIYLFFKKNVYFGSSLGPSVTYSDCLQLLLLLLLLLLSSHPCASVSSLLLSFRQTYAYPKG
SSRVVVRICRWMLFFFVCVLLLVSFGRVIMKRCVWGKGILKKEEKKTI
>tr|D0A187|D0A187_TRYB9 | DeepTMHMM Topology Prediction - Predicted
Type: TM
MNTKIHKHIRGSSLRIIASQKHIEGGKKNFAYGNECCVGMGCYEISHGVVNHAGDEQRIVFRRGMTATASI
LLSIAYLFTLLLLLLLLSILCFFFFLKETAAKHHVAQPMACSNCITTPNFT
>tr|D0A4M5|D0A4M5_TRYB9 | DeepTMHMM Topology Prediction - Predicted
Type: TM
MSMLVCRIFGSILPVKPCSPIHLGERVISFLLGVCNYSGVLRYRFWGCRGRRRILCYGSAKGVSVELQSPS
EPGNEVFLCFLKVAHYYSTVKIFATRVLMKELLKGKSLATKSVHSIQAAFRTKFRKRASFILNRRFCFCFP
EST
>tr|C9ZX94|C9ZX94_TRYB9 | DeepTMHMM Topology Prediction - Predicted
Type: TM
MQTSSVSEVDCLAEQMYSSPDPSIRQRAQASLEQLTKAEADQSIICAILQQSNNQYALLFMSQCLVLWFKA
VRKWISEEEKQNVIVVHCGGCVKRALENGAPKHVVSALLSAYAKLTKLAFEADPLLEGAVNYPIELLRHEA
DGTNMQLLGLMMLNALVVEFSKYDSSRSKTYLGFVAHRHCSGNFNEKCLLNILVEALKLLEKLTVNTPHIT
EIVKLVENCFSFDFRAIMVDDTEDLPFVHFPCAWKPTILSDQTLQTLWGQHAALPHPHCASLLSAISNICG
TYRSFFETVEERLQYIEFTLTKLIQVTMLQDGRLKIPRYIETLAEAFRRVVLSLGYRELRQVAVFEQWVTA
FQSISVDVLSITFGQEGSFSTATAVMAFWVALTTSKRRSYSEQCPQDIEVAVLPVLRAFLVARIHGADASG
GDSFSLEDADGGLAEAVLAQSDAYANVCLLDPATYLGDFANYLNQQVGMSIFTSPLSTGWLFYIAGGVAWL
VLFSMESSGIEPCSHVFAYARGCADHRRNHSGNPALFSSFVERGMLHFLTNMQSVITGARHGNLSAVVTNV
FQDRGQLFQFVLDNVGHNLLRGPDSLDAMDIIRSSADVIVEACREAPPQLLRELSLELPPTSDLPLAQSEQ
TYKLRTNITKALWFVRSTGSYTRERMESYLSNVDFSMQRTVNNEVNSPSFIAGWVRDLRGACQALKEDQLS
SLDFIDWFLSRYSVFVTIVDTAGDSPIVVTALMRFLCELVTPGKYGRLHISSSSNSAVGLMLFKHLCDLVE
KVEKRTFSIDHLMALSSLSGSYNKVLKPWMLAMDIMKRCMEGSFVPFGAMLYYNDDTFERTTVDLLRKLAL
VGTNVFKEHGKFTVVAVDLLRLLVEENLYFCLRGLTGDELVGLINAVITVCEDVDTQSGVLVHGLGFLTFI
SGLVQEVRAIALSPSLRPTDTHGPDQSPAPFTQPFGSSRLSSPMPPVQQSIVSRPPRLATEVREHLARLLA
PHDSVWQRLLSTAMNIIVFQDRAVNSSCAVVHHIFEAHPPFWFNYVEQLIMSFPEGKHATLREAFSVLTNA
AETREKFFSEVFTLRQVLRRLGT
>tr|D0A225|D0A225_TRYB9 | DeepTMHMM Topology Prediction - Predicted
Type: TM
MEGALFLEGRNVVFFFVPFKNLFVVAVKRDGVEEQMRCVLVWEDEKGLFNVITADGGGKWYRLMGRGRIHV
MFRVVATVARRDFFPPLIVHKFKRPSIFPFPFFSWELPPSFFLPTRPYFGPQRCGGVSYPFYPFPFPFSFL
FVLFTDLYFKGIGVLEYNKDAMYLLLRCFFPLFSFMHVICLPFSYGGGG
>tr|C9ZLV5|C9ZLV5_TRYB9 | DeepTMHMM Topology Prediction - Predicted
Type: TM
MVGFKTLSSTAPLAFSEFSHLLLEALLAAALAVALALHFIRRRNTSKIVEPELPPLEEQQRRIAAFRSQMF
RADAKPNANTQLPNRTGMSEVEARNSCYITLRDESDSSSREYLDLVTEDFHSFSTHPTVVDVAKKIVVAYG
VGSCGPRGFYGTIKPHMVLEEDIAKFLGVEDSLIFSFSFATISTLIPCHASRGDYLIVDDGVSLPVHEGCT
LSRANLLKYRHNDMAHLEEILREVQIKEMKEKKLSRRFVVTEGVFKNLGDVCKLPQVLELCEKYKFRIILD
DSCGFGCMGPTGRGTHEHYGIPTTRIDLYVGSLSQAMGGVGGFCAGEHAIIEYQRLTAAAYVFSASLAPYI
TAGVSAVLKLLDEDHSFPEKLQRNAGLFRGAIRSAGMNPEKITLVECAGDVSPIVILRPTDAYVKCHHQKV
EEELQQVVEAARRKGVLLTRHLFSKEEACSNFSALRILVKGTATEDELLRAAKVITEAVKSVFV
>tr|C9ZMP2|C9ZMP2_TRYB9 | DeepTMHMM Topology Prediction - Predicted
Type: TM
MTTKNRPIEEAAAVYILRIIHTILLWSGVTLGSVLVVCSQPRFVPIRTAISSFVYLLASLCVIFVSILELK
GHISTISGVTHWMDRVGCSYWFLLLFIRCIKPSHAVEWVASALLPLLVLTEWAVVTTYILISSTYHPVITV
DLFLTVLHTVVFTAFVGLDRLMISDITVIFSAARAAQDADAVEACGGNMGMSRSGPRSFRSDDMLELSHEL
QSSASSYSSALCRGNSRCYILRVVARRWGGTYAVLVFVRLLYEFGGLLPAHLLRILVDNLSVVENKHGKKH
GSDGLCMASVMIVVIIAYNLICTFLRAHYRVGLQKIVLYIRGLLTSELFRCTLCRRKHLFDGRTQGDIMNH
LSLDVARVADAASSFNDLWALPLQLSLALYLLYIQISFAFVAGVVVSVALIPINMWLTKRIQGVMSELMRE
NDERVLRITEIVRNIIYVKMCGWSQLVQQWVKESRDRYLVHLTWLKYLDAFCVFFWATTPTLVSLVTFITF

ILMGGELTPGKAVTALALFNSLTMPLNAYPCVINGLVESYVSWCRLSPFLVMRPDACHGDLYGFASGESDS
GPKMFTEITEEYQDMVVSSEEMPCASSLNEIQPLLQAKGCVGEMRRQRLAGIHGLPHPFRRNHDLLIDIRS
GVIRLNTQGGAGLPRPSFMLHIPTFQATSSQLIAVVGVSGSGKSTFLDGLAGEHLIDPNDATGHAAVSRFS
TAYVEQQPFLMSGTLRENILMGLMYDSVRYEAVINAVALTQNFQTCFYPDGDTFLVGDRGIRLSGGQRARV
ALARALYAGKELYLIDDILGCLDPTVARHIVINALVGSAKSGSCVIVATHNQELVERADVVYRCVEGQLLF
SQRLVKTAATTVAEPSSPAAKPHATQQHVALANKEKGHGKVGNVLGDVPWPSSDSSPARAPAATPESGSIE
AAEVLETSKHGTLAWETLACYIGRVGWKLSAFIIISAALMQISRNAGDQYVVTWAKSGDGNTTRFIWSLAI
LAGVNSVLALARGFSFAFGGLRAAKRLHNELLGHVMSATFTFFSDTPPGRIINRLCGDTYTIDDSLPFIMN
ILLAQTFLLCGAVVVILLNSSSLLIVTLIPVSVLYHRIQCPYRASSRELRRLEEAANAPLLDTMRDALDGG
VVIRSLGGRVVMSHLRRASRNTDLLLRVKFNSMLLGAWFTIRLELVGLLPLIFVGGTATYYHGSASAPFIG
LALAYVQPLTSYVGGLLGAFASTETELISVERVRQYFSLASEEPRTLGMFFSPPRSNWPSGGQVVLSNVSM
RYDPSGPEVLRSLSFHVNAGEKVAIVGRTGAGKSSIFSALLRLVEIESGSIHIDGYDTRRLPLEVLRTRLS
VLPQQPFIFSGSLRQNIDPFGLHSEDAVRDVATSVKLDGVALDYNVTDSSRVSGGQRHLIALARVILQRSP
LLLLDEPTAQSSAEAEAALWSSLAEHLRSTTVLCITHKLSHIDFFDRVIVIENGHVAGDGTVAQLRAASVW
PFSSPNADASLT
>tr|D0A6R6|D0A6R6_TRYB9 | DeepTMHMM Topology Prediction - Predicted
Type: TM
MVHEGATFVVSMESLLSSPSPPWTCQASFRCRMPFRGVYAFWRVSLIVFVLLHFIFFRAYHRSKRKIIRNQ
MNPLFAGAVGLRVRCTAKLFVIRRSLSTYLEPRAYCSTGVGGCTTGACGGTNKDCLSFVLEDNLTEKSLPL
FHSYRAAGTGTASKIAVITDVAAFNNVLQGVFKSDGEGVNGVLCDLEGAWRHQLDEIAQKVIAKHRVDDYI
DNLYLPLLGYLIVILLIVQFIVLFRWVFVVFDWNLVEPMTYFIAYTAVWVGLVFHCYSVRGLSLETLLAER
RRQVLYKRARIDGAAIDRQMKLLGGMEKLLARYGKV
>tr|D0A1Y1|D0A1Y1_TRYB9 | DeepTMHMM Topology Prediction - Predicted
Type: TM
MAEPPPPQPLRKGFFRADPHGLRPPESPRDAEEPENHDIVEDPPLPASRNLPPPRLRPMGGGPPLAGEEAA
ATSHSNNRPSPGEVPPLPRPGTTTAVTSAQYNSLTPRQELFSSPQTAVEENIPAGYDNQMNVTSNDFSHSQ
DTGIKRIGDGATGSSAKTRSTIAHAPSGRFSEVVQERPPIESVEQLCQLQTLAHPVERFFVEYQPMATRGC
RSTQVPVEEDEALFNHLAEYTDDDSFFSSGSAVLEEDTRRLQNDPLRGRAIRSVFETLSLRSLTKGRIPYE
LEDQERSSAPWLKQVHQRAIALHHSSFFIFPAGMKARVIAYNILHHWLTEMLLMLIIILVYSMMTAAWSRDT
WPTLEKPSFMFFADVFFLCIYAIEFVARLFTSGAVSHSRAYFRSPWHCLDAAVLLLMILNCTNLQSMWNFS
AFRLIRVLKSSTYVPIPINMKLLAKSLLRSTSNVVKVSTILFYVLLFFSLVGLQLFSGVLQYRCVSPTTKN
VTNQLCRFNHSEKNESYYHGATCPSPHLCVADTYGNPHHGYRSFDSVGHSFLSAFQIMTFQGWTSLLQETS
DTTSVAAILYFFLAILICAWIIPSLYLGVFIEKIEKTRRLFVQKQLQLFDGMLLEQRQRLNEAIKLRDFVE
RDESGKLRRHPIELIRSASRRIQRSKLSNSQTSIATESESGEPAVVVKKPIGDTTKGRSRWTDEQRVQLHL
SLTRQRDIASGGERRRRVVIKNDGEISGGTAGLHSQRKGSESIGERAMMVTGDFALGGRVGVVQHHPLTHT
IGAAHGTDLPLAVRLDNEEEQLRFLKDYQNPIDNDIMRRTNTFEDTNGNLHPSFVLTSTQRRGGSFDEASN
TIRTTTEAAGVIPSRHSATLNGHNTSRMDSNGSLDEPTSKTQTVSKRGSPMPRLSSGQVPEVIIHDPEGGD
FRFAETRSQKWGIVRNILHMFTEGYPRIITQYIREHRRMQRRFGLTPLNYVNKYEDDVLRKLRQRRVLQVK
EPGAPSQTSRASGDEELVEVNGNKVILTDSDDIGDLSPIQMATNIVRNVPVTPFGAVMLVIVIVNGIFNAT
RYFQQPEYWETALFVLGIIFTSFFVLEIVVRVIGLGLVSFLLDFNNLLDVTVTILGFVELAYARSNVVTVL
NWVRLLRLFRTLPFAPMRRVSRVLLLGFADMLYALFFFSIYMFMWILIGMSFFGGPNGMVDHTFQDYYTRG
NFDTFSGASFAVSQAFSYTREEWVYLTWNGMQSRGEYTVLYFMAVVGVAFIARYFFVAVFAWAWQSEEEEE
ENYAAIAKGGSGGRREVTRLHWFDFTVWRSFKHIHGGFERRDVAPDEVFHLNEDMRKQLRIAEAKERFTKE
ALAQTDLAMSQRRMGSPMASPSATMGYNTDGCAPAAQVGTAPRYVNVGGQLQRHINPSVDFVDAQVPPLNA
PISQFQAENAQLRFARRYSTVPASVYTPVIQDDGIDRPSPSARSSPSDAGERSGELGGESQQNGQEEGDGQ
YSPRGVSPNGTGGRATSTGLQRKSSVLGRFPRLGYDRAAGKKDGGSRSVSASAMQQTGDGNELRGDYVNEG
EANGGSMVYEHILYPGPRLRYKHVMRNQYVRVFERCLDCNTYQQMPLRAPPNVQQRTPEELHAEHCHMAAV
RSSRQLVLNAIMGYVRLQKDINQPPTRDAVETVLGQAWSCGMLLFETIEYLSCSDIEQREYRTWDRTLEAL
QLQQWLIGLHVGEEQVGRATLAYTLAHRKREKLAVEHKSFELSWRQRSFFFISPSNPVRRLSTRIIQSRWF
DIFILTVIFIASFCLCFHIPGKANDPETGFVVLRAFDGIFTCIFLVEMIMKWISMGVILFRPEAYFWHWWN
VFDFVIVIVSLIGLADQHSALRSIKVLRCFRILIPMRVSNFNRSLSKISSALLDCLPTVANILLLFFINYF
VWAVLAVRLLNGLTHSCSDPSFVDITACEDAKHEWLPKVRNFDSFFQSLLTMIEVSVGSKWLDVIYTGVNG
RTSEHAPMDDHYLARGFFFIVYYYVSHLILFSLFTASMIYSYLLTKNAAEGVLGITFEHQLWIRMQRMTLQ
LKPRVKLVPLCNQVSQFLHNVVIRPIFEVVGASVLLLNILTMALHWYGETKSKASVLAAFQYVWMFYFTVE
AAMKIGAHGMRAFSRWAFSFDFFVLLLSFIGLIVDAASSEGMPFNVNVLRMLRLGRFFSAAKVFKPMRKQF
SLLHEVLIRSAVSLANVTLILFLGVFVFTVLGLHLVGGVPVGEGGYFDDRYTNFNNFGNSLMMTFRLTTLE
NWSPSLREGMNVTRKCTEDDCSVNYGSAFYCLLLLVFLGLIVLSFYMAVIVDHYVTAARMNTSITRIEDLR
RFRDLWSEFDPNGALVLHTHELPKLLESLRPPLGLTSRHNRVELLRLLREYDIPNHRGKVHYHEVLLPLAR

RVLAMAFSRDTMDYRTTFDTLWRHSEKSLRALPTVLGKRSHATAAQHFAASYVQAVCRRKKACREVQRVRS
ELWHEGRAVCDELGLPYADYGFGNLLLEGPDPMRDLVPRSASAASSGGKGTQKGASEAENAASSPLWRAGQ
AESPSSPRSEGETIDGRAPAARLPGAYQPAIEEREKRFGPDVPNALRRHETRSEKLRRKDEERMLQSTPDD
AVSSPVSNVRSNSQRVNVGEYQPPLGTDPTSWLGSNVNRGSTVGGPTTESRTSSVMPAPQGPTAPE
>tr|D0AAL6|D0AAL6_TRYB9 | DeepTMHMM Topology Prediction - Predicted
Type: TM
MGILQHQADVWHALRYPSKHSWGERKRAYLFVFAYCLAAIALFASVMHFIGAWIACGLLQVIMLIFAMLFA
LNIADCRDKCLNVLECERAINPVMEVYIGLRFIQFLHATFLLTNIPMGVSILVALLYSLWRMWCGTYFVDA
TSLWREVGRLERDSYIHICIEIALIVIYLIAIVFAMVDKYS
>tr|D0AAT7|D0AAT7_TRYB9 | DeepTMHMM Topology Prediction - Predicted
Type: TM
MYVFTTMRIANRLLSFPNSARALCVIFLFICFFRGGKPQLRYLFCPLAGSFGVIVLLLFFHSCLPRHMYTQ
IYASLFKLYRIAFKTDDDDDDGGNAIFRRTVEEMEEGNNGRSNVNATRQLQCGDCFNNHE
>tr|C9ZKD4|C9ZKD4_TRYB9 | DeepTMHMM Topology Prediction - Predicted
Type: TM
MEKNAHGLLADADSPKAHRHIRNTLKVNKLSSALMFMAVVTPVMVLVSFYSAKLEAVREKWVDPYELPPGF
NPKTGRFMSDSKPQGVTEPPAPLLLRGAVPGATRQ
>tr|C9ZUV4|C9ZUV4_TRYB9 | DeepTMHMM Topology Prediction - Predicted
Type: TM
MSLCAEINRTGFLGIIGFDQCGWNGTAGFVWEFWRLAPCCGAPDFANALLCIFNCLFCSPCILCKTYASSL
GDVCSVWPHCLMVLLCPCARWFTRYNLRKRTGTSGNIIGDFFCVFCCCAPCACCQEFRSINIGSWRIVPDA
SRMQFFTPGCRLLR
>tr|C9ZRU4|C9ZRU4_TRYB9 | DeepTMHMM Topology Prediction - Predicted
Type: TM
MRRRKKQMNAKKKKKKTSRHTEKNKMRKERNIAKGGRRGSVAEQHHPSKSNEGTSKLRQEAEVPLPFPFDF
LFHFISSHFILFFSFSLLPPSNFRFLTSPLHLFMYLICFCFLFDFVPPSPISHIFLIAISPASYLHHLSTF
HFFFFFFYCASSS
>tr|D0A6D7|D0A6D7_TRYB9 | DeepTMHMM Topology Prediction - Predicted
Type: TM
MSSEKVAGYVQCVVGIIFGVVTGGASIYVTIAMELLQVAVPYVLVTLGLQRRPPKGGVDKAMAEAESVEAE
VMKKPSKQQRSFRALFIGIDYKGTPAELRGCQADAVMMAGTMEKIGIPITERCVLMDTDDPRFNAIKPTRA
NILQHMAWLVKDAKPGDALFLHYSGYGAQVRAEEDKEEEFDQCIVPCDYEENGCILDNELHEIISTLPRGV
RLTAVFDCSHAGTLLDLPFSLICSSNDCSAVGEMKRIRTGGDVNAHVLMFSACGDDEAAADLPNAGDFVEG
ASGSGGAATQCFISMLLNKTPGTIYSLLSTTRDKLREKGFKQSPQMSASRCLSLTEKFTLTELFSVAEPCP
NILTGEPRWKTTPLGGK
>tr|C9ZRU1|C9ZRU1_TRYB9 | DeepTMHMM Topology Prediction - Predicted
Type: TM
MGWWRRSSSSSNGMQRVVETHFWSLTRYRIGRRFLGLDYADNVIFFPAYRSKRRGGWAAVTLGVTSSSNYF
TNDTSAVNVLEDGYPLHWRANSRLKEFVAEEKKLWQARVKEEVAQLAAEKVSASRTGNPNGGASKEPGDLM
GDINCEHRFRDTLAIKCTSPGFAQRHLRLSRLWTSLYKFLYFYLWGIGISLVVQAYLLFRSWLNPPARQGL
KNIEAHVLHIPKLIFGFCVSCVVWAAHHLQPVVSPVLEALEGVFPQVNWSAASPEALATKAQQLAGHDVSG
GKHGSGQLDQLNVKKTTETMAGNWAVFFSGNFTSIMQGLVFLLAVLLFLL
>tr|D0AAF6|D0AAF6_TRYB9 | DeepTMHMM Topology Prediction - Predicted
Type: TM
MSPRATEPIINEDEHPTSIPSLSQGNVLELPPDSDKSTAAADGEHRGCLNTVFDPIKGIVPYGGMASNVFN
LESATLGAGIVMLPSGFLNSGIIIATLMLVYICFTTVYSIRILVITRDKTGFRSYEEMACGLLGRGADYFT
AFLMFVFCFGTCVGYVISVGDLLSPLLNQPSTTGFLRTSMGKNVIVGVVWLVAMLPLSLPKEINSLRYASA
VGVFFIVFFVICMIVHAAMNGLKDGIGSDIRLVGDGWGILNGFTLFVFAFICQVNCFEVYEEMKGPTPRRM
TRDSSVAMSMVGLLYFLSGIFGYLDFGSDLEGSVLKLYKPQDDVMMAIGYVGIAIKICVGFAICIQPSRDA
IYYVLGWGKTSDVDSWKNLVVSGVLATLALVLGLVLPSIEVVFNFLGSFCGGFLAFILPALYYMYAGNFSL
KEVGWFNYAVTYQLLIVGVFAVVFGTALTIYDEVKK
>tr|C9ZP01|C9ZP01_TRYB9 | DeepTMHMM Topology Prediction - Predicted
Type: TM
MCILTLCASFIYLFFKKNVYFGSSLGPSVTYSDCLQLLLLLLLLLLLSSHPCASVSSLLLSFRQTYAYPKG
SSRVVVRICRWMLFFFVCVLLLVSFGRVIMKRCVWGKGILKKEEKKTI
>tr|D0A187|D0A187_TRYB9 | DeepTMHMM Topology Prediction - Predicted
Type: TM

MNTKIHKHIRGSSLRIIASQKHIEGGKKNFAYGNECCVGMGCYEISHGVVNHAGDEQRIVFRRGMTATASI
LLSIAYLFTLLLLLLLLSILCFFFFLKETAAKHHVAQPMACSNCITTPNFT
>tr|D0A4M5|D0A4M5_TRYB9 | DeepTMHMM Topology Prediction - Predicted
Type: TM
MSMLVCRIFGSILPVKPCSPIHLGERVISFLLGVCNYSGVLRYRFWGCRGRRRILCYGSAKGVSVELQSPS
EPGNEVFLCFLKVAHYYSTVKIFATRVLMKELLKGKSLATKSVHSIQAAFRTKFRKRASFILNRRFCFCFP
EST
>tr|C9ZT52|C9ZT52_TRYB9 | DeepTMHMM Topology Prediction - Predicted
Type: TM
MYAEMGYTSLRHSVNVAHSPLTREESCLFCFVLFFLQIISLFITTVAASFAFIEPPPQCFNIFSFHPFFPH
FLNVFSFAFCFICVVLCVCVCVCVKNKQTNRQTNSSFQLYIYIYIYIYIYIYIYLFIYLYFLSHPLYA
>tr|D0A988|D0A988_TRYB9 | DeepTMHMM Topology Prediction - Predicted
Type: TM
MSSSYSDYFHYYYYYYYLVDFGEHIRHYILPFSFFSYYLFFLEYFLSLFFCCLHFETTRKICALIFPHCYL
EKKKVKKMNESKNLKKILGLFFKKKKRKKCS
>tr|D0A0P2|D0A0P2_TRYB9 | DeepTMHMM Topology Prediction - Predicted
Type: TM
MQYIRKEYLRGWIPCCEAPCALMPACLLYSTAIGSAVVVTIVWLCFYFPSTSLWQLQRGEGVGRFSEFIVL
FFNTYIYIYIYIYMCVCVCVCVCVFVGFLPASTMTRVYFGGGGGVFNPLWGSSEKKLKNLFPTSERKKETR
VNSTPLIWE
>tr|C9ZQA9|C9ZQA9_TRYB9 | DeepTMHMM Topology Prediction - Predicted
Type: TM
MHRFVSVLREDHRTLLRRIVLYQINRPYSTKIGSCDNFTRVDNLTEATEPVENPVVNSPNCTEPTTDLEGR
CDVNERTISSKNGAHGMSKLLETISKRKKPKAGPTVGVFPKPVGTMLAEIFTSTAKSNTPLEARGRVVPKP
RAFVAQGKPVVSSSLISKLRENVLRKQMDWQCVAQSYPSLQRLGVWQKRSEWTNLFLANSAVPLPPASAKR
LKNNGGNEPAAILTDFPQPVTDVFVCNAPTGSGKSSLVPLFLLDSHWQRMLQRIDISHKAKIVSSAASSSV
IDSKEGVMRSVETFLPPSPFPVNVSTREYATVFGASSRLCIIVSQPTRLACVELAKFTAAILASSSSTPAA
SNVGDRVGYAVGGDSCFSAASEIIYATPGYILNALQHDHSLLSHTTLIIDEAHCRDMETDLLLAWAKQQLL
RAYQREHGKDREQSWGMRHLILMSATLSAEQMVSYLIGAQFSAPPSEATRNLSAFCKPYVLSLGNEAAQPP
DEVSDVAVRSGLAGSGPYRLEEYFIDDLPSALESSQLLGADEKSADNGSKGVSSLNGRLCPLLLPPARRVM
ASLVHFFSYIRFSPEIHSPQARALAQFVLFTLQSIASLASCRSKPHTTEGSGAVQEKGGITQDQNNEQPES
ILVFLPGFAEMSLVLQSLEVLCRKTLVNDEGTNDTQRHAEPAGATTKISGDSTMNYAYDNAGSGNVTFLEY
EGCSFSVALLHATAVGSPQRQLRETTSPFPYRIILSTNVAESSVTIPNVRCVVDSCLERRFFSDPLTGVTL
RSTAVVSVSSSRQRAGRAGRTCDSFVIRLAPRRMFTLQKEVVGTHYGNCGQEELVTSDEKGDDGKVKDNRG
EDNESGKAFFLTSHQRSLSGLVPAAPAHTIPESAAANVASLLLRVKYLFPHQASSLLLLLPDAPKPKAIAQ
GIRQLLDLGLLQCSKSPKQRSGDDVHCIGDSCLELSKDMPRLIDLYNDVCLTAKGEFVSYFPIPHEQALML
YYALQFACIEDAILIACAMTVPTLLLYPRVSVQVGDSKSCEGAPAAQYLQSLLIQRSTASKIGERGKSTSG
HTLSEPLMLLSILYEWYACTSSADVVAFFRKFRVNGRALRTVDSSVAQCALRLWRLVTQGSDSHETVNKEK
AVTSGVGGGDKDDVFALYGEHDRVERRPSASLIKQLVLSTFPESYRVLLANSLLRLHRCALEHSAHRPSSQ
HRTMTQWYTQDEQLIRKLQPQLPRAPKQNQRSKKPLLTFGHVQDRLCAAFVAAFARNTMRGEDASHRAHQR
RMLRNLGALQEDADRTCSFSVELHSDQRHQQSLLERLTPEVLRDVITPYLADTNVQQIELFSTRRSGVVRF
GSEVNGSVDGDRRGGNSGKEEDRLLSAVALPCDPAGLTEAEKGRNLAAVVLAPIGISLLVSIQNVLTRLVA
MLPPTSPPASSAVRLPGEQDRNDGETESIRRAEAHEKVASLDRSLPVPGIRTVNRTFSLRFTETEGDSSAA
SDTCVTWHDSYGREHSLLLSDARRMGYEEFFPQPIAAAAADADHEDCSICRSTGNGVILSGEPVPAPGVDE
DVSSSGVNSDGSDEQHALLSIHRVTFTGAVQWKVTLPTVRPSQHEYHSCEKEEGETAPCNKDYYCYVCHNL
WFREKSFHHHCRSVTHLTRLSFAVQFGMRREWVGSFCSGTLSDLLLRKRGTNSVSSIGCSDVAVELRSCNV
SPLSFLNALQWRPRGEGGTAMLPMAVAGTLIGNAPSYTVTRAAPIKDSGMTLQALHVWVLDTERPGASTEP
AGSELPPSLFVLAGYLAAASTGFDAALLTNTAHTRAHAVMLHDWGVWRFPTPLSVQKLKSVMRVYAGKPWR
GVPVDADDECEAGGTATDQSSESGNRCRKDDAGGACEEESLHWCVVSDVCPQCTYSGSGGKGRQTRRGILK
VVLDDDAPSVDEVVQSTLLIVLHEYQNSLRNVVSFADYIQRVVAKLNAWSNAQQPQLRPPAAAAAVGHCPG
AELLARLVASRRGQSIENLLRDIGCQFLSPPSTLVAMGDVDGTIIPTKGVPRVNTGGTNEKLFLIPTVLPS
YLPSPNLAELLRRHRVDIRNFHAHAACDFNATSQVGGAAEPEKGTLSRSLQVTKGVNTKQGHGPDVLF
>tr|C9ZYN3|C9ZYN3_TRYB9 | DeepTMHMM Topology Prediction - Predicted
Type: TM
MLIIIILSSERTPFSVPQFVAYYTSSASRNYPGIRGKRCKKWGRHSLRVLSATFALDRRTHHQAMLCRTFVR
RVHLFTALVPTNVVQLPHVLKDAQLDAAKKAAIDASPFLGVAPLVPALDLLSDLSNFHQKRDARNLLDECI
TQCRAELYKPTVSDPFHRLQLHEAIMAAGFYLSTAGSTALKGEATRFVLHHYNFDVRRDTIITRTVHNTLL
EVRTSTPESDRLLSDLLLLERRLFGTCRFAPTSGRRWFALGLPLEDIKTEEDLKRVLDIPAVKEKGHFELV

VEDTEKMWKKLIVRPMPEETHSLIEQGEFVVSHTEKDLRFECRVQKPPEPIDFWDKLKDTLLRYWVIWFSI
WVTFFMVDEEIITITALIFLKWRQTKILEEEAEKTGGKIYIAASTGRALNK
>tr|D0A7P8|D0A7P8_TRYB9 | DeepTMHMM Topology Prediction - Predicted
Type: TM
MIGLRKRSSESDDEPVPSSFLLRKVAAVDLFTKPKEDYCRSQTRAGAIISIITVFAVGLLASWEVMSYTLG
WNAYKTELSVDTSPEKNITFNIDITFMQEPCHDLFLDVSDVSGTFSINVTENLLKTPVDVGGNLAYLGTRR
FFTDPRSPLYTRRNDPNSPDFCGRCFTGNKAIAGGKNCCNTCEEVMAEHDRKGLPRPNKNVVEQCIGELSL
ENPGCNYRGALNVRKVSGVIFFTPKVIKNTIKMEDLLKFDASHVINKFSIGDESVRRHSRRGVLNPLEKQR
FNGSGRFMKVRYYLNIVPTTYGSGASSGLHPPTYEYSANWNSREVAIGYGGFPSVEFSFDFFPMQVNNNFK
REPIYHFLVQLCGIVGGLFVVLGLVDSVVARLTRLV
>tr|D0A8A7|D0A8A7_TRYB9 | DeepTMHMM Topology Prediction - Predicted
Type: TM
MVLYHFLLRLPHHLFRFLVPVCCCMYEKKTAKSTTVKQKEKKTTTFRGSTSSVDDTMNLCSVKVLCVCVCV
CVCVCKQTFPLSFPQPTYPFVYFPRLFNGMQRWTGETTYNTHAIKEKKETVEYIHTYLFVQEGEEGLRRMD
>tr|C9ZPD7|C9ZPD7_TRYB9 | DeepTMHMM Topology Prediction - Predicted
Type: TM
MKEFRRRMFGAHLLGNSLRPPSTPVLTAAKMPCAHLSAAVRQKLASEKVPPPPSKCVGALNTSNSAATLVS
TTVAYKDPGVIVGNAPGSVDSSRPAVSPPLLVPQSGVPERCDSNDAGNNSGYQRVEEESRVADAVEQLHSL
LSSWCSREGGGEGSTVQPPPLEITAENREVSQKQREELLSALVCVLENDPSFGPRLLSGLEDDTLRMLLLF
GTAQEYFGFEEVEKQLRKVDTEKEDCVSAEEYNAWVEFALRECADARNEARQSGVKKQQQSRSSEASKASQ
STITNDHIAWNLWLRIAYSASVPFMAFGILDNSIFVTAGDAIDRRCAEAFGLTSMTAAALGGVVSGVAGVQ
MHGVAERFTQRVRLARVPVLTAAQQKSKARGSATRVGNTAGMLVGLLLGMIPLLFVHPSNSRDGVKGETEA
ECQIHK
>tr|C9ZP00|C9ZP00_TRYB9 | DeepTMHMM Topology Prediction - Predicted
Type: TM
MEGVQPLNVRIVREAGDRRKVKLFLHNTSGTEREGYPVMFSIRCCEPDFYIPVGIETTDGILLSGECRGVL
LQLRDPKERAQSWGMEREGKFVKCGVINEQQMRVSCGTNVRASVEAASRFGNGSLDALHSFRDSDSSFLTT
GNRYDFGVESSGGVVRGSPLQRSSVKSWNAVVPSVLPSDGGSAKHLRLSASEESHTPGSLEDELVHSSVRS
SRSPLCTDSSRCSSSLFVHYTRLGGEKRSIAAAKEWLEAQQGKYNRWLEKVHRLQGKDTAGVNSPPTTVAS
APAPTAPHFSALCEPVRWVKRPQKAPANLKLSAFWRKPFSKGSAKLFKVGKESNLCIRVSCELDDPNRSAT
DIKHSGGPLSDGLLRLNTAPSHIGFLRREQPPVTGEVQRYEHSEDVRSSNGNRRKIPEDTTDRSPTALFRL
ERHENQVATGRIPFGKLDPPARGDVDVGCSASTRSDFAATLNGKQSLHAEPHVVHKEVTNCPTPNGSASPL
DDRADKPSMSEKLHLMRWDKTMSILRNLSAPQMGSAICEFVKRCSPHLESLGTVTLNTVVRGLHSVGNTIE
GSINTLHVVVLSVLFVATICLLFAIIRGDSYSNELDIAMSEIG
>tr|D0AA32|D0AA32_TRYB9 | DeepTMHMM Topology Prediction - Predicted
Type: TM
MQGEPRGAPGGQSLFEQMHQQHFAQPQGFSPQQHQIPQPAHYVPPQTSFTQPHGGQTYNQPNNSYYPPAAP
LQPPPFISPQQDSDYYKGFYDGVVKPSGPVVGNIVSWVAPLIVNIAIFVGPLWYVRRKYTQAMAQGAAGGA
GKKPGMGNLMEMMNPMKPKNFRVDVKGTTFADIIGIPEAKEDLKQYVDFIKDPKKFTRLGARLPKGCLLTG
SPGTGKTLLARAVAGEANAPFLSCSGADFIEIFGGSGPKRVRELFAQAREAAPCIVFIDEIDAIGSRNQSG
RSMGGGGSEENRTINQLLAELDGLTSKEAIVVIAATNYPEVIDKALLREGRFDRKVNVPMPDRSARVELFE
YYLKRIITGDAECKPKIQRFRTRKESEDGDNIKTESTASPKANDGKEKVASLTAEEKAPEVKVIPGVSNKE
YAIALADRTPGVSPAQIATIVNEAALTSAVAECEVVPLKTLQESIDDVLIGKKHRQRMSDSSLERTAYHEV
GHAIMAWTSPLQKDVLKISIIPRGRAGGYTQQMQDEAMEPRTDAFLFSQLCVLMGGRVAERIFMKDISTGA
MDDLQRATRIAMEKLLLYGMSKSIGQLAFKANERNEGRGWMNFSEELHAKVEEEARHLVASAYKHTEKVLL
EKRELHEKLTKLLLEKKELTKADIEQILGPRPIVTDTS
>tr|D0A0W7|D0A0W7_TRYB9 | DeepTMHMM Topology Prediction - Predicted
Type: TM
MSMLHLSDRNASLAPSGGEHSLPTGGAVCRVAMDTLPVILRAPVALLLLLIVLPQLSVGAEANATVKVLSA
TWNSYMPQEYVNAINAGFNASLESRQWTVAGSVKVEVVYPRNLDLMPQDFIKEQLELETDQNKIVIVYGPL
GDIVTYLALPILMKHNVVAFSPMTGSTFIRQWNPYLYFLRADPAAETLALIRYALCQLRVLRLGFMYLQGV
HYGDEEYALTVNVMSQMGYELHGVFTVMSSDGEPAPDDEFREVFERFAAALPQAIIVFGAPEKDTAKFLMM
MVAEERIARSYILGPSSVQASLAQMWLHALATAGTSFAPGQLLFTGTNPLAKDSQYIAIKRFQGVMSEYLK
AHVSETNITEADYFLTHGTEGELMVYGWICGEVLSQALSSLEWLKDRTTFVRSLYSQRRYVINDIVIGDYG
GTCEGEAAKHGATCECNQGSKAVYVKEMLENGQTTTMESGFTVVKTSQCYTESSELQGPLNGLAVFMEDDD
TASKAAALWQKGASHLVGKGDLGHSDRFFLHAFNTTIAEAANDLRYEQGERIVTAVFGPVTEAMLDTPNIT
FIDPLELKPRLNKFRRHVIHLSPTLEQQLYVLSSYLAGDGVGTVDAVICSNEADGIADFLRRSLTEFGVSL
RSAVIREDGEGVGKYLPISGTVFVIGLSVPNVREIARKLEERNDLRVIVLFGEFSLLYDLFTTALNNTAGA

ARLVFATSLPHWGDTETSSKTAQLFHDVEKDSRLWTPLSLLAFATGRLMRVILLHVEEMSPETLVNFFYAD
SSIISDDMRYGVFDDTKCDTAEKLSKDDCASNYGATQVSVWSMARALNASIPPLANPMTPSMSFRDPSEGK
LSGASLVGVIIGATFALFLMVALGVVPYFVLRSTRDNNSAPKEPTDPVTIVFTDIESSTAQWAAHPDVMAD
AVATHHKLIRALISQYECYEVKTVGDSFMIASRSAFMAVQLVRDLQRAFLRHNWGASVFDECYRRLEQDRA
LESEGYVPPTARLDPDVYRKLWNGLRVRVGVHTGLCDIRHDEVTKGYDYYGHTSNMAARTESAANGGQILL
TRATYLSLSTAEREQLDVTALGAVPLRGVPDPVEMYQVDAVVGRSFAALRLDGEVDLIADSGIINASTSDC
SSFCELGQSAQAIVAVMRALFGTFTASQRKKLLVPFCERWRVTLPPKTKSVWDDNECQEVMRRIATKVGHV
VDFTAGNTVEPSVDTRRRSSLAFLRLQGLWPGWEGAPRNSPTSSERE
>tr|D0A6Y9|D0A6Y9_TRYB9 | DeepTMHMM Topology Prediction - Predicted
Type: TM
MCLFIYFPSPDLPHFFLIISLAAVFTIPHPLRPCSSSSSSSSSFHLQFPHAAYYLTRPLNSSHHTQGHTKK
ERHIRIYIYRYIYIYIYIYTHICDSSLFLFYSLFGYCPFFFFFLPHALHTHMCMYICIYTTIFTIKYIKIY
ISTCKKYSDLGVP
>tr|C9ZW49|C9ZW49_TRYB9 | DeepTMHMM Topology Prediction - Predicted
Type: TM
MNTPLHAAATVGDIRALVRISRVSSTNVNAVDEEGRTPLHIAAEKGNMEFVRMLFRKFNGVDTTILDNSSK
TAVQRAPVEQQQQLTLLLSVEEANTIARAKDNLNVKREKEEEENVYKDHDVEGSDMVNPQVLRKIVICLLV
PFVYPLLNMGTIFVLQYAALTFVFYFVVVGYFLSEFTIKPPWYHHHPKSNELTARGCPDYWNGCVNDPFKD
LGLQFDNVSFSSTDQYVLRGWHVPPPSDKPRGMGVVLVHGGGRDRRAWLRHVPFLHNAGYGCLLFDFREHG
LSDGNMRGFTYGMKERFDVVAACHFMRSECGYNRICAMGTSVGASSAIMAAAIDKTIDIIVAENAILTCAA
LQDQQIVNIIGGYFARRVYSTFFFNLLRRTASFWLNYRIGNKPSKHCQALHCIAKVSPRPILLMHGTADEL
VPCRHSQKLFEEASEPKELYLAEGAFHCGLHNTHKEEYEARVLGFLQRYGGG
>tr|C9ZP04|C9ZP04_TRYB9 | DeepTMHMM Topology Prediction - Predicted
Type: TM
MDGCKFMEVRGGMEGSFLFFFEKKERISMVICTDYFTILKHHTNRQDIIVCCFFLLFNVRQMCVVCLCVWK
KKKSGGRGKIFYLFFFLKKKKDEEAGGKRGGGTKE
>tr|D0A9F8|D0A9F8_TRYB9 | DeepTMHMM Topology Prediction - Predicted
Type: TM
MPPKSHKRSRKEGEVEEPLLTENPDRYVIFPIKYPDIWQKYKEAESSIWTVEEIDLGNDMTDWEKLDDGER
HFIKHVLAFFAASDGIVLENLAERFMCEVQVPEVRCFYGFQIAMENIHSETYSVLIDTYVVDPDEKQRLLH
AIRTIPCIEKKAKWAIEWIGSQTSFPTRLVAFAAVEGIFFSGSFCAIFWLKKRGLMPGLTFSNELISRDEG
LHTDFACLLYEKYIVNKLPRDRVLEIICNAVSIEREFICDALPVRLIGMNSQLMTQYIEFVADRLLVSLGY
DRHYNSKNPFDFMDMISLQGKTNFFEKKVGEYQKAGVMSSERSSKVFSLDADF
>tr|C9ZNV9|C9ZNV9_TRYB9 | DeepTMHMM Topology Prediction - Predicted
Type: TM
MSLITSPGDAGFGVLLLSFVCASTVLIFLKLAIRRRQHLKEAAPPTIFIPRGYTVEELSEYDGVKSPLAFV
GVRGIVYNGATSFYGNNAPYNAFAGRDSSRHFAKMDVGRQEANMDWTTLSPSHMKTLIEWEALLRSKYEVV
GWIVPSDSFFKKDENLA
>tr|C9ZMW8|C9ZMW8_TRYB9 | DeepTMHMM Topology Prediction - Predicted
Type: TM
MPAPPAASGVRRLQKELRDITLDPPPYCNAAPSSESIFTWYFTLDGLPQTPYEGGRYVGELRFPPEYPMKA
PKIIMLTPSGRFVINSPICLTITDFHPEEWSPMWGVRTIITGLLSFMVSEESGLGGMTATAESRRALAAES
HRYNVERVPVYKELFNSEYQKDLKKLNEEAEALRKNCGDVSENLGNKTTNASRAQLRGIVASIVVLVALAV
GLLVLRN
>tr|C9ZKE4|C9ZKE4_TRYB9 | DeepTMHMM Topology Prediction - Predicted
Type: TM
MLELIKRNFRLAVESDVDAFLHPPGGGSYIFSDKNVDPLKVLKTSFFIFLSIFFSICYFCYCYCCCCFSPI
IGACLASFHHSHHILCCFHLFPHTYFTSLSSFFFILLFFSPSACVWFALRVGKKIK
>tr|C9ZMD1|C9ZMD1_TRYB9 | DeepTMHMM Topology Prediction - Predicted
Type: TM
MHVSAQARQTLCYHCCWMASAVVPTTNKEQVNKADVTSPFGSCEVISGEDEGLKGKNALPANEGEGEQSSM
KCFTSMIPPGGLVSTAFSLASICIGAGILGLPAAANSTGLVMTFVYPIIIYFLCVYSLYCLGAQMERHGFR
SYEGMARALLGQPCLYFTGVLRVVNAFGAAVAQIIATGDIVSTILKGADAPNFLKEKWGNRLLTFIMWLCF
MLPLAIPREVNSLRYVSTISVIFVFYLMAVIVVHSCMNGLPENIKNVHVTGAPGDEGIHLFGTSNRAVEGP
GVFTFAFLCHISVFEIYFGMAKPSAHRFTAYSSIAMGICLVLCVMTAFFGYLDFGRDVTGSVLLMYDPVKE
PAILVGFVGLLVKLFASYALLAMTCRNELCGIIGLDTEKLSFFKHCTIIGTISIIMLLCGLFIPNINTVFG
FVGSVCGGFLGFILPSLFMMYGGNWSLSTVGWLHYIATYAVLFAGVALSVFGTGATIYGVAVGW

>tr|D0A6T7|D0A6T7_TRYB9 | DeepTMHMM Topology Prediction - Predicted Type: TM
MEETLPLHEGNSIYLLLNFPMRGVQPLCAVDVIRMMSLLALQKLILSFWPVADLVITVGCGLWRVNGYIFL
VFVCGTAVGTVDRNVNFPLLLSFCFVAMEFTR
>tr|D0A6W3|D0A6W3_TRYB9 | DeepTMHMM Topology Prediction - Predicted Type: TM
MRPDYMGFMFLTSGVMSFNAGIWQLHRRRQKRRLMENHKNIRKPPVYELPPGDATIDEFEFLPAAFEGTFD
NEGSMLVGPRALPTYKGASSNEESNGGFLVVTPFEIAHTGQFIMVNRGWVPIDAGKHRTLLMQYVGEGFTP
GSVRGIFRREEYMSASLIWGPNKDNEGPVAADLSWLVMRPWNMAVHYYKRRWGPDRAEESVEKHGARHYYL
EMIEDYSGDDQRMVRGHPWPWRRSVDEITYVHLPPSVHTMYIFFWFSVTLGSLYGMGKCYKRQKELFALRR
QMTAQTTLLERKRQEEAKMYMEAMKEVERLKKLGTVAAPRIGSQPTEQQEQQKSDEKSPAAGEKS
>tr|C9ZN63|C9ZN63_TRYB9 | DeepTMHMM Topology Prediction - Predicted Type: TM
MTVFELCKEYIASRSRNHELCACMLTGGVAAILAASAGLRSNKPLPLKAERSKPASSVNGKEAQAQDKVKV
QSFAAAVRRFLALLEIAIPSIRSRESGMLMIISLLLISRTFLSLRITSVAAAVDKSAISGNPATVVRVVGL
LLCWFAPVALANTTLRYCVGMLGLRLQSNLAHHLHRIYLNNDVFFAVASSHSVKNIDERITRHVASWSRNV
AGFFTSILEPLINIVAFSYKVGSTSGSRTLAMVIGYYALFVAIAQKFSPDMEDLVAEQLSREGTLITAHNR
LLKYAEELVMSKGQLFHRNLMNQYLESIVQHRSRAAFVQGRYGLMENLFLKYGSRILSNFVCLGVVLSRNT
EATSGQDLLALFAETSYVFMKLSQGIGGLVRNCRGFFVLRSLSDEIYELQESIQHAAEVQRTSRRALDDAP
KGSAGEIVRGDYIAFENVPIILPTNEMLCRGLTLHVKPGMNLLIVGANGCGKSSLLRLLAGLWPLHGGRIV
KPRMDQIYYVPQRPYVPCGTLRSQITYPKQLSELEVSESMLYECLEMAKLEDILSRPHITWDTVFSWSDGL
LSLGEMQKLAIARLFYHRPHFAVLDECSSNMDIEIEERLYSMCKQLGISLISITHRRTVWRHHNWVLWFND
LGSFMFSPLSFNEHGTAVLTRVVAATDSSMIGRQVTLDVSDGKQ
>tr|D0A801|D0A801_TRYB9 | DeepTMHMM Topology Prediction - Predicted Type: TM
MQPIERVEELLAHEREKIGLFRMPFRTLQLSCAYCVKTVTSYVVKCIKSSAFMFVLPVILLVVGVSTFLID
SPAGRAFRFLDADGDGYVSVAEVEGYFRDKLNRKLGAGRGANSIFPSGTARLDKSKFTSWWVEGYGDAVRQ
NAFFNQGPWREAEYMLADALWWLGLGILSSIGLGTGMHSGLLFLFPYIYQLCAAVDSCGNTNFWTYPVNPI
YGPRDRVFACLNPQKKDVPTSVLTRVLMLLPACFIWGVGTAIGEIPPYLLSYTAARQGKRNSELDEASRYD
ILNKMKAWMLEKIQRYGFVAVLLLAAWPNMAFDLCGMACGQFLMPFWTFFGATLIGKAFCKITLQAVFFVH
LFSGDNVERLIHRVGDVIAAVVVIPSSVYSGGTQGLVKKAVEAVVRARQSIALRARGEATVGEGMQSASLL
ATIFGWVVVAAIAFFAKSVVETFAQNEQQQYDKIVLEYIGKALARGRSKKSVTDEELLQLIEESQRLCVEP
EHLMSFNRETLVTYVCVVVAGFVMVHYSAVAGLSLLFHMFVCMVHNNESLHPVTLWLLRLLFASAALYTLC
GLETL
>tr|C9ZYN4|C9ZYN4_TRYB9 | DeepTMHMM Topology Prediction - Predicted Type: TM
MLRGTVLRYGVHCANATALVIISTVINWSHLPHSSAVASFTYSCLFFSCVCVCKFFPACDPSIPRPARSTR
RICLLPTRLNSNLELEIPAFLVRNDMHYVWTMRFPSPCVWGINAVMYRTNTRDATRNIYTYKYIYIY
>tr|C9ZMP7|C9ZMP7_TRYB9 | DeepTMHMM Topology Prediction - Predicted Type: TM
MSLWPFRTGNDERFQVTSTEETIYDAIKNEMPAYPYGTEVDRYLEWRWRRGLVQSIFPATCYGAFVGFAVG
YRQARVEGRYIGRYKVVWRYSSTFAAVGLLTTAFHHVLVVRNNYHDRFYYPMMAGASGAVVLTVASQMGTL
GQGMLAGSLVGVLYTLSCYGMTYYHRRRLKMFLRQQQTQQVPVHKVSPELQRMYRAYLYDNRPLEEKDVMV
RRAVVLSMSEDDTCLDAKRVLQNMTPEIYDWVNFPDWWPLKFPQQTEEERMIYERQRDEEVERRKRAFLET
DDGALIKRVNRAKKYRDL
>tr|C9ZXA0|C9ZXA0_TRYB9 | DeepTMHMM Topology Prediction - Predicted Type: TM
MGSIMSCLEVGSDSRCFGLPTPVMAALVAGAGGLLFLLYKVYRCQTRKVCRRTPVLQSVNIAVNQPELAVK
KAAEHIMKGEITTVGQLVFFFRGGEEKAIRINRKLFECLQGAFPVGTADQPPADGANGAEGHVCHMMQQLC
RCEEQAARLEAERATAFDDGNPQHIALLERLWVAAGKPKSAFARRSSEWNDLGFQGMDPVTDLRGGGVLAL
RQFLHFAEAYNDHLKGMMEFNKRALADEKNHWYLLAVVSIQFTAQLLLQRDYKVFLPQLEVLYDTISRGHK
PGILTGSLRSAAAMSEVGAQSIGALSQNSVDCSEGEETSDFEVGYFALHHQLLLSFKECWHRDLPHVMEYN
KYLSKFLESFFSPE
>tr|C9ZVM5|C9ZVM5_TRYB9 | DeepTMHMM Topology Prediction - Predicted Type: TM
MEATKTLPIFLSSQPIEQTDKRKHGRKRDIKKTKKQKNSFNIHLRMQTIQAPQVFLHVVSSSLPFYFIFIF
FFCNINSLPLFLLSMYPKQTNTQINKHVYGINNIYIYIHVH

>tr|C9ZQK4|C9ZQK4_TRYB9 | DeepTMHMM Topology Prediction - Predicted Type: TM
MKISAETSPNSSFLSISTFLLFQPAGCNTENITYNIGTCEHPLKHTKALGPPKPKQQKKKHSLDGRGGASL
CERLFSVFPPFHFTVHIVCFVLALLITLCFFLRISVIFMSGRKAPCHWRK
>tr|C9ZUR4|C9ZUR4_TRYB9 | DeepTMHMM Topology Prediction - Predicted Type: TM
MASQNAATNGDGVTAYRTESASLMNDDKRRNDKQTEDQRPPFTVGSYALGIVMILCVAVIWTYASVLIQYI
YEAEEYEKPYFMTYFNTNTFGVNNIGFLLLSSWRRLPWKNGERTSSLVIYDDALKRTFGSGASAPDNEPAD
GADVSRGGPQSEAERIRPYSKFRVFKCAAFFCPIWFLANSLFNASLAATSVSSVTVLSNTSAIWTFLLSLI
FFNQKATWPCLLAMTMTIIGACLVGFSDAENTENETVGGDIYALLAAIFYAVYTSIIRWHASDDDRYSILM
LFGFVGALNTILFWPFLLIFHFTDFETFQTPGGIQFALLLVNALVGTNLSEVLWARAVLLTSPTAATLGLT
LTTPLAMTSDLLIKQKSFNAMYIIGAVLLTLGFICFNLEQQLMKLLKGSRSS
>tr|D0A070|D0A070_TRYB9 | DeepTMHMM Topology Prediction - Predicted Type: TM
MMASEDKTQRNDPHETNEHEEVQTTPEEVDPTAEEPVSRWGRLMTPRIVLSIFTLLNFVTYYDRGAIAGCL
VVIKGDPTIAGSSNVLTDTKAGLLFSGFMIGFMVACPLFAGLGGVVQSKWIIAVGIIVWVASLVGTGLARS
YEFLLACRIFDGVGEAAFVGFTVTVIDAIAPPESRTSWIGTFYSMIPVGTAVGMAAGGVMGAYGSVGGLEG
WRVTFLSLAIAAAPILLPIVFLPKRYNMRQKRDNEYLPIHKAALQLFTNVAYILVVFGYAMYCFVIGGLSV
WSIPFLVEGPMELTNMTASMIMGGVTALTGIIGSIVGGVVVDKLGGSLGSSGTMKCQLFCVVMIAVSVPVG
LAALFMEVTWLFTSLLVVSVFTLFAVTAPINSAILTVVPWDQRAYAVSYSVLLIHLLGDFPSPTLAGYLSD
NAFSRGCPAHGNNTQCRNDIDNLCKWINKTGNSTDGHCVSKYQLRNALLVIFSFLGLAIPCWLAVYCIMLR
EASAPHSNADSEGSETGGNKKTEEAEKANS
>tr|C9ZI10|C9ZI10_TRYB9 | DeepTMHMM Topology Prediction - Predicted Type: TM
MDTLFYFPIGCLIGAVCVVICYLFGMECPVLVGSTVKGAACFMVQRLTNCDVSLLRSSTLFIPSDSSSFSF
VPNHGIGSAARRSLYGRNDNEIKNPLMTQRCQSPHSAGSTLSPERLTVMCQLAMCHEDEEDYVVRNCHLVM
SESFMMLYEVLSSHSVGNHERAITNERFIGRIQMDRVVSRAIYVSSADKRSLRTGGLHGHMLMLTTHSGDD
GLFFEAEDGECGIEEEDPSLGVTVRGSKWWDASSKTLHEVMLGRRRSSGLVGVTKRDESVEKLSNSASLRA
SVGGHGENSCSDHVGGGSPWDEDNGSEEPDPDAKGTSQMYIFLKFRYAREQERVHNLLVGMHEAVHWYEYL
QTIPTPNAFNVFLSRVIFQSLRSTALANFIREKIQKSLDIMAVKKFPRGLEGRILLDDVALNSEVPVISNV
TDPLILAKGEMMFDLDILYRGGASLLFRSCLTYRGVRIPHVTLYVKLVRAEARLRVSVGPPPSKVFWVGCL
APPDVQLEVSQGMESGHGLLHRLLTSLPNLSGIVTKLLKLYLLHEMMLPLMDDFPLPNVEDTPPVTPRDDG
KKTWNVPFCRREAMRYIQEHMNIHWFQEAGDH
>tr|C9ZRP7|C9ZRP7_TRYB9 | DeepTMHMM Topology Prediction - Predicted Type: TM
MGSASCSKVVADVLGVSIVTVRGCSLLCPFLLPLSQMVYLLCRDLILRHIIFSVFFLSLFSKMQKQRITFD
AAYKFTLWYSLLCEAQRDRAKSVERARHGSHVRDVASVLVRASLPRRAYKSCCLAGVCDRDSLTHKAVHFG
RPEVHITHGERDDVYHCLAHSTACIGRSVDLCVKNYCSAAPLSGNCYGGLRCIIQHLQLRSLTFSSITSLH
DAQQVVNTLSAAVRCGLRWLTRLSIGGIPLYADLEPLLYSLRETDAITELRLEECGIENVDGLGHYLKSSK
KLQKLSLRGNQGICGDMKSLAVAVSLCSSLQSFDAGLCRLNDTHLSEFLLGLDNFLKRSCFSLDMSSNVLT
KTCFLCFLEAPLAFRREVTQLDISGHNFNGAGDGAAAMLLELPALRGVVFDRCDIGSRDVQRLVRVFTRKD
RTWSCLSFRGSALTLNDMKRLVMTTFAERSRLCIGGNNIGGGIQKLCLNIVLPFLCELDLSLCDIGDTGVL
QLANSIEKAQPVPLRSLRLDNNGIGGEGVRGRGGLQFLGRALRSSYSPCLEVLSLAHNKLSLKPLLTLVEQ
VSSTLRELCISYTPIASDSCEMADLVGRIMRRQKGSHAFDELDVWALRSTDFDSSCGSEATSWLERQRAVR
VIMEARV
>tr|D0A4C6|D0A4C6_TRYB9 | DeepTMHMM Topology Prediction - Predicted Type: TM
MTERTDTIRELFHKSSSYRPGGDDFAELTALKSHTFYETFQVVTSTAKVAVRNTRASIKAALRAMEQFSPA
VSDQESALLLQEAELLAASDAIKELIASLKNACVQSEKSGSRWGAAVSRKGDEAQDAKTGKPSNQMLLHLE
TALEVVGLCTGRWRLLLALCKEELILYKQEMFSFGRIGVLSCQDPVKEKCGDAESSISRREPDVERPRQLD
LPIGCSVEKPKDATQAVMSVVDRVMKEAARRVTVAPLPLNSGVWSKIRGNKSNCRNVWNADISEVPVPQFE
YSLEEQQQLRVENAMLEKHQRLVSAEDAKAVEVSVRELSQLTSLISERVIQQNEQFSILLKNTEAAQNNMQ
RGIGEVKQTLTHFWNPTRQLIAILWTSIIVLLLANWIIR
>tr|D0A071|D0A071_TRYB9 | DeepTMHMM Topology Prediction - Predicted Type: TM
MTCLKQRQIFPRPQAQVADETGCVSVKCLFARSSRLFMFGYFLVPSIFPVLLFFFCFALFGCLLLLCFVAS
LKFPPSDSFYHLCRMKIPSSSVISLSFFLFLSFAFFK

```
>tr|C9ZY45|C9ZY45_TRYB9 | DeepTMHMM Topology Prediction - Predicted
Type: TM
MWVFRDRMMERGGQSFPLAHYLQHEEQNDVPLPEPHMLLLCGRTFHESLGGDWLFPSVVTFLLGGADVNAR
DKDGATALHVAVTQGNDIATVCTLGREPFLGECSNEMIIRFLIDNGADINARNASGETPLMVAAAKGNITA
MRLLLERGAVITQRDDAGYTVLHHASRSPFSLQLLQCFVDSLLHQVVSEDLLHFVCQKGGKGANFVILFLV
EQLGMDVNAREGECVKASTGEELKPEETSVMSAVAVRSSYTPLHRAVLGGDVALVCALLSCGADVNQTDVI
GLTALQLAANNAGVSAVGGSWLQRFVNIRSLWCPTAAERRVRSKEVYNLLKAYCKETSLSGREALLQRFAC
GNSPSQNLLSFEGALVFADGLIVVHALMVLCATLTNEMILIRGAALFSLFVSFRLSSRNFHTTEAKSLYPA
GCFVGYTIALAGCCVYQLHYVSRQPTPSGLYCIFTLLCGVGSLLCGAIVARRDPLVVNSTPAQRAAIHKTV
FCAKGVLPDETKTTFDTQCMVRKPLRAQRCQYTNRIVLRYDHYCPWLANSIGAGNHRLYVTVLVVHAIFLV
SMWLLVGWSSSRISPGGTTLQRLSEAYMMPVLPVVLIGVTIMLLRQLWYISRGVTMYDVQHPSQCLWCFQL
GARTYSLFDAGTVENLRGFFMLREDFAKCEYLVPGISSRLKDIVKKHQEMQLPCGDHCEGHLPTDAANSQH
QPSMPSSYVDANLLRFEQLTRSISVNKAAQEADALPSSSVVNAPSVSASGIDDAAMRLFQRMVQSNSADVT
VTDIKADITPSEWQTVESRAREMFSFFVESMKGSTVPSA
>tr|C9ZPJ5|C9ZPJ5_TRYB9 | DeepTMHMM Topology Prediction - Predicted
Type: TM
MVQRSTVPFHTSTGERRLNYVSPTGKGCRGDATREKTTCNLFFCTPTHRHIYLYKHILVCPCSERMFVYVR
AFFSSPTADEKSLYVMPLSSAVTIVCFSHLIRSLFPRSRNRRQTYLGE
>tr|C9ZNG7|C9ZNG7_TRYB9 | DeepTMHMM Topology Prediction - Predicted
Type: TM
MEHQTEMTVSLKKMCRFDAVFAFASVSFVGKVYNKNNANEINRVLYNREHGLLFNRHLVRSYGKRFDLCNR
TSTGPFLELEPRKFSRLFFPLSCYAQLCFFLPFTFPIWLAIA
>tr|D0A2D6|D0A2D6_TRYB9 | DeepTMHMM Topology Prediction - Predicted
Type: TM
MSVFFDIEAAAYVLLLIICTATYLRQYSPTLFHRDHTELHRKFLYKCSVVGDRLSLWVATGCIVVAVRMLF
VY
>tr|C9ZLT6|C9ZLT6_TRYB9 | DeepTMHMM Topology Prediction - Predicted
Type: TM
MGHTEHRAAFNRREVVELQQRLTSFLLIFTAVAIAFVVLVASMQTRVATCQTQASMAIHSMYGRFGSNNST
VLDTIVASLSISGVRVVPVEVAASHQAIKLDVFDTSQATLQSAGFRLARQSGLASVVLWKLLYIDHSLCDP
ERTISMEALPNVDYEKASYRVKAITVDNTRLFLYETNVATKDKDRITTFSQLQSVFPGFNSKVGTPHMILI
KSLSIEPEFVAELYRGSTSLSVELRVEKIQQVDNHTSIWRILVVARSPQAEQNFHRVHKVVANALAGVGAL
CNKEGCGTSIVDEFLL
>tr|C9ZXP4|C9ZXP4_TRYB9 | DeepTMHMM Topology Prediction - Predicted
Type: TM
MRRSRVPADGVSHILGKRAVALLVPVLITLMLVTWSVLNLSSLIGERQNSLVIVEPHGNESSSLQVFEASV
INALAVVALIVVFTFIMLALYKFGFEIVLYVWLGVSVGSILFITMWVFLDLVLTRFQIPYDFITMFLVLWN
TGVVGLVSIFYYSHPTVAQVYLIAISVIVAWSATSLPAWTTWCLLVAVAIYDVVAVLCPYGPLRMLVEAAD
ERNRPIPALVYDSDARIIVATSFPGEDAQMVEIAARARKQEGGRQRPRKRPPSPLDSLLGDTPFKLGLGDF
IFYSLLCGIAASYSFIPWLMSVIAVLFGLVGTLLFLVLFKEKLTALPALPISIALGVITYFSSRYLVVPLD
WFATLSVLAL
>tr|C9ZLA1|C9ZLA1_TRYB9 | DeepTMHMM Topology Prediction - Predicted
Type: TM
MTYIIDDLARVRMLASGVYTMASTAAPYCFTLMAVLLRKKYRLSQSEIATISTVGNCIGYCSFPIGALFDY
AGPMVLLPLGGFLGSLGFLLFGLTFDGKIANPTLTLFCVFDAIVYSGIPTLDVATIMPAILQFPLDRGYVV
LVLKTISGLGTGVLMAYFNGWFKDTTSDDVEKNNYSGFMYFIAARLLIVSLIVLSVTRMPMYFPCAWRKQR
LSEEEWTKRQQTLQLYMNQPAPPRRMKTAVGLVLSLLLFLTTQSLIGGYVKLPPAAYLAFSIIAVLMMASF
CVVALPFQWLGRYTPVRPTDMDTIGEALEDVVTESAVATTKNEVKPLPQYSGSFWQHLLTVDLWCMWLTCF
GVWGTGTVMQMNAAQIYESKSYGEKKSSTLTLYITMMSVGSAVGRMSMGFTDMVLTRRQREGLKTFPTTIA
LPFGPLMLCIAFLLFALLPANALILPFFLGALGNGAGWGSGVLAFRIMYSQDLGKHYNFGFSSGVAATIAL
NLFMFGGMYDAEAERLDTKPECKNPSCVKNQMLILMGANIVAVIAAAIVHFRFSRFINAEQNRCNEPADEM
SGVAAPVTEDAGQPNEGGSHSGGAAQQ
>tr|C9ZIM8|C9ZIM8_TRYB9 | DeepTMHMM Topology Prediction - Predicted
Type: TM
MRKKQVSHAHTPLHFVCFSIFGLPASTPPPTHTHTQARHLGLAYSQTLASSTYAYIHIYLYIYDTCLCLMW
FTSLHSSSFVHPSLITLF
>tr|C9ZTL2|C9ZTL2_TRYB9 | DeepTMHMM Topology Prediction - Predicted
Type: TM
```

MTPLGFDFTSFVLSFGTGVFVALFLMRWGCPFQFSVRSKMRQANAFACCGEPLKMTLVVRRDLKMGTGKIA
AQCAHAAVAVMEEVQSRRSDNGANRNDSCSTYGYSGNSDHYSPAASQMCVGSGADRWVSWYDAWRFAGSKK
VALQCENEEQLMEAYRAARREGIPHAVIRDAGRTQIAAGSKTVLAVGPAPDSLVDRVTGHFKLL
>tr|C9ZR23|C9ZR23_TRYB9 | DeepTMHMM Topology Prediction - Predicted
Type: TM
MRIVAPLDGSTAAAFCDNKVTNSRYTVWNFLFLNFYEQFRRPVNFYFLLVASLKFISIVAPVNPLSTLLPL
ALTFSLTAIKAARDDIKRHKQDAIYNKKERKVLNREAMTWETRTNHSIRVGDVILLREGEDIPCDVVVLAA
TNPIVYIRTDNLDGELDLKPRDVVAPQLSSDHTGGDDVPNAIAHQLLSVDDSCASIVGKLGQMRVTCSDPS
PMINCFDGVAEFFFSRSPAAETVAANNSAPMRVSLSENNILPQSCVLKNTKTAICLAVYTGEDTKCCLNKR
NPKVKWAQIDRDISKYAIFVFIFQISCGFLFGAVGYLMNNNVEKTYWYLPMTTGEDGLAFGIYTLRFFLLT
TVFIPISFKFVTDMSKYYFALVIENDVAMHHDGEWCNVRNSSIVEDLGQVDYVLSDKTGTLTQNVMEFLFA
TINGERRCLAPVEAEEVQGSCGEHVLHFGRVLSLCNTVEVVYDDVSQEMTQSGLSTGCGAVGTMRYQAASP
DEVALCNGCEKLNVRLVARDATTAAVEVNGIKEEWFVHYVFAFASEFKTMGVIVEEKSTNAIYYFVKGADD
RILEMALDENSSTGGPQWGKGERMSSKAAILAEVEHYAVFGLRTLLVAEKRLTRNELDEFLEKVREAELSM
NNRKEEIYKLRLEMENSVTILGVTAIEDKLQDHVPETIRSFLQAGIKVWMLTGDKVQTAEQIALTCSLCSP
GDCVLRVLADKLDAFESWEGYMESLLQFSKGVMADVQYGDAAFPTGSSSDPAVGEKACAMGVMQKRNGSVT
TETNESADVNPLSTGSSYVLVIEGGQVLERILTTPSLLKLLTELSENCVSVICARTTPKQKAAVTRLVRSR
GFITLAVGDGGNDVAMIQEAQVGVGITGREGKQAARAADFSISRFSDLRSLVFVHGQLAYNRTAFVIKYSF
YKSVLIGIIQLVHNIFHTHYSGGSFWDGFGLTLWNGLYSLPQTMLYCLDRKVPRRVLEQTPALYKVTRSGV
DLGVCQFFGSFIFRGVFQSILAYFLVLSVHGTGFASPNDAGQSAKDVAFTLTYAILILLQVVTVLMESHTV
TALNAIFIFGMPVVYVAANMIYSSLESFYYYGVWKKTTDIVSFLTCIAVVSALVVPVLGVLTLIKIWRPDP
RDVMRSAELRRQANDPLAVEKRASVSRLSRCLWCVPEEPSTYVTVVLADDELTIRNANSV
>tr|D0A539|D0A539_TRYB9 | DeepTMHMM Topology Prediction - Predicted
Type: TM
MVGQILSRRGLILVCLTFVAILLIFSTRSKRIVREASQASFSAPPPLPEFDYTEKDRESLQYVPPDVVRAW
RERNFLVMLGVLSPDNAVRRRRRHLQRLTCWQYQGVARRSNNFTGDLLVTFVLARHPDHNYTHSPQLVEEG
LRWQDIIALPIKEGRVSTNKVIGQDGRYWGPDAEIGMSRKMYKWFSLMLRLLPNVNYVAKSDDDMFVHTPQ
YLADLRALPRRRLYWGNIVNARFMPLLFATGGLYTASREVVEQFLTYEPLKKLVDVPYSKEREDEFTSLYM
QHEDLLMGRVLYEMGYQPLFVGEPMCRFHNLHGKGWVGPLSHSSLMIHNVKEEEYAQLREYFGEKKQYRPS
RFKVRRGRLYATCGKR
>tr|C9ZWK8|C9ZWK8_TRYB9 | DeepTMHMM Topology Prediction - Predicted
Type: TM
MTQRKVYPPYSWRKPRASVLVLTTTFTMALIIIMWDIYHISEVHSNSSYDLLGTFENDYYLTLTPPSAVSI
WKEREFLVIVGIPSVDRDEWQKRRNLQRRTCWQYAGVATLENNFTGELLPLYLLAPHQLNGYEISESLRDE
ASRTNDVVMLPTNDVCSFSRRKIGEGGSWGVESELVMSRKTFLWLQFAVTAFPNVSYIVKGDDDVFVRVPQ
YLADLRVMPRNGLYMGRVYGATFFWRSGGIPFAAGYFTTFSRDVAEAVASYRPLERLLKAPYSIWRMRQYL
SMSVLHEDVMTALVLQDKIRYKGLIIANAAGCHFHNKAKEDIREAVSNRSVVVHHIREEDYEVLMDHFSNI
SERPQPYGVRWLRNDRAMVLC
>tr|C9ZYS3|C9ZYS3_TRYB9 | DeepTMHMM Topology Prediction - Predicted
Type: TM
MYLRFSLCFFSFALSFRLSCTCLLSSAFGSPLFFGYKRYHFLQFCIFCVPFLLVAFFFFDLYLVGIPAMAM
SGVSSSEWKDMKGVTLRPATMSYLTECAGFRYMAPVQARTIPLLLGNYDVVVEAITGSGKTLAYLIPCLEM
LQYDRVVEVCKDRKDAVVSVIVLPSRELAQQVHQLAKKMLHYVSYDYLGGKNGLPKYSCQCYIGGRDIKLD
VDMFSRTGGNVLIGTPGRLYELLVSSKHSGLFNLTSFELLILDEADRLLEFGFKAKLDAILKRLPKQRRTG
LFSATQTKELAELARAGMRNPVSVAVRVNSLNSAMTNAAKPQIPELLLNYYTFTRASEKLDRLLEFLSKRR
EQKVIVYVMTCASVDWLYACLVGVLLKDDADNVFALHGQMKLEKRQRVHRAVTKRNRCVLVCTDVAARGLD
IPEVGVVVQYDPPVDPATFIHRIGRTARMGRQGETLVFLMPHELEYVAFMKLQNVSLLPYNEEKDDIGEAQ
KVVEEMNVRRTLTSSLQEKRKSLHRAQKEQRMSRKERRAMLQEERNAKSATTQRTRKEVHGDLCESPAILE
LRRAVRQQENKKILDLAARAFVSFIRAYKEHECRYIFQLQLIDLTDLTHSFALFKVPNCGEIKHMRILKIP
LQEELTDIMEIINQQTREKRERETQEREEKRRRVEGDEDEHGEASAKRHRTERNEKLEALKLAKMSRGERS
RTMKQVEIDELLKDSYYVKKERRGEVSGRTVDAIMGVDAIENALMSSRERQEAKRVRRAVK
>tr|D0A9Z0|D0A9Z0_TRYB9 | DeepTMHMM Topology Prediction - Predicted
Type: TM
MAQSTARHVMNTPQPQQGQNLTPAETDNKPRQKSPSSHILASQLASAVSTTMFYPMDTLRIRYMSQDGTIQ
RQHNGQTYRSIYRAVGVIWKEEGLRALFRGCHVAVLGAVVAWGVYMFVYHALCDLYIPTSNKRAGDDFLFR
TVLSSIASCSCAVVGNPIWLLKTRMQIEEIASREAAVAGASIFRNSKNYTSFFGGLRYAIQTDGVLSLWRG
VSAQVLLGLPNALNFPAYEALKSFWLQRSDRETLYSYEACICSTASKTAVSIIGYPLHVVKTRMQDQRSRR
GDLQYVSFLQSAWLVLQTHGFAGLYRGMVPSLLHSVPRLALTFVLYEKLMQQNFF

>tr|C9ZYK2|C9ZYK2_TRYB9 | DeepTMHMM Topology Prediction - Predicted Type: TM
MCIHVFVVVVVVRSLFFLFVSPMAVAHPHQCCAFLHSYRSESHQRLEKRGESMSKLEQRKLGKTPRKPQRS
RARNPKEAERAFKIQQRKKERLQRVRELSTKLRDEINNEEKRARESRKANLDRKAENEKKNMVVQKIKNDK
AIRKLSPKHRKKARIYMMHEL
>tr|D0A921|D0A921_TRYB9 | DeepTMHMM Topology Prediction - Predicted Type: TM
MWGKKNFLLVDAYRMLIAHRWKSQGRWGGASRVTSTASRFLVYTLVNTLSVLFPPESAPVSTVVTAICRLL
FLISCDYWCFQPGATSLLIFFFARRLFSRGEG
>tr|C9ZLY8|C9ZLY8_TRYB9 | DeepTMHMM Topology Prediction - Predicted Type: TM
MMESVKQFVGPSLMVAVIIFLYVTSIASATQLVIGTAAACLLGLIPANAYFPPVWFRLNLFVCLYNLYCSE
LVGYRWLRFLIHKRYSVPHSDYRHGVRCLSGNIITRCPFPPHVLKGSEFHTRGGGTVDLKHLGEKCREGCY
DGVFVMPVPVLLDNYAYFILSCKTKRCAVVDPADPTLVLNMLEVVRNLTQIDFMITDILTTHKHWDHAGGN
MEMRALSQSSSERCRALLSPELNIYGSEVDRPHACNKLIKGSDELIVAGGGAKVVVLSTPGHTSGSVMFLV
GDALPKEDLPQRLALFTGDCVFCGGCGALFEATSVDETLETVDIFNNNRTWVHPATGDIIHTDDVLIYVGH
EYTERSLDMILSVQGTANQTGEQSAIATSNYCDTLAQARNGARRLRSCVVADDVYMKVTSMETKAPLHLRA
CTVPSTVTIEKKVNALLSLKRCVLEEFQGKPYASMAVQRAIYTSTKRNDTG
>tr|D0A5D5|D0A5D5_TRYB9 | DeepTMHMM Topology Prediction - Predicted Type: TM
MCYRDAITTMEAVTFHSPPVPTNRSVPRMANNRRHAHIRKLLFAALVTTVVLMRTVRASDKVTVKVLNLMY
NMKADVEIVNSLNAGLSASLATNSVEKASDFEISLQTPSSYNQTIEETFNDAVKNSKEQLLVIVGPLGNQN
VLWIRDKLEEHDLVAFAPLSYSDEVRGWNPHFYYPSVEPKAELLALIRYVVVFLRLRRVGFMYLKGTNFGD
SSYTFTEQVMSMMGYKLCGTFVIDGNVNERIGDDVFDREWEKFVKTRPQAVLLFGSPHDTTKKFIQYLVRE
DHHTADAFVLTTSSSQTFLLNTWKEALEETNTELKPGQLIITGTGPLGSDTRYKIIQRFREEMTKYLNANE
NWGGFAKPEHFDTNHNSGELMVLGWLAGEMLMRALVDSISLTNRTLFRLSLYNQRRYLIDDMVIGDFGGEC
GSAAQLQGAVCRCNQGGSVVYMKEVVEKYQFASLRRGFFLQKGSECGTLSVPLYAPMSSSIIMMADMENAH
RANLRYYRGIFATFLRIFGEGRDRLYLHSLNSSYSGAAKDLIDTLDERIVSAVFGVVTNEVLTTPGVIFFD
PMVVFPQVSTFKRNVIHFFPTLTQELYVLAQYFSRLTNHIAHTIIRSDEAAEVAEVLSMSLMTFGASLGSR
VLLNEHVSDIHMEHLKHGGDVFILGLTTPDVKTISKYLDTHPQARVFITFTDLLLYYEELKLEFSVTANAK
AQRLLFATSLPHWADNSSTSETIIAYHEAILDPSQWSPMSLRGFAIARVMQTLLLPMKKVSSSLLSDEIFW
QTSFVVDDMRYGPFSDADCIANGVQLSGNCVWNYGATDISVWSFGRVLDPTLPVMQEPVTPSIVYKKIRVQ
LSSSQVVGIIIGLVFALLLFVTLGVALYCTISNKRDNELAPRQPTDPVTLIFTDIESSTAQWATYPDIMTE
AVAAHHRMIRQLVLKHDCYEVKTIGDSFMIACQDPFKAVQLAADLQLMFLHNDWGTDALDNFYREFEETNA
KEDNEYTSPTACLDSEVYCRLWSGLRVRIGIHTGLCDIRHDEVTKGFDYYGPTTNMAARTESVANGGQVLL
THATYMSLSESQRHQLDVTALGPVQLRGVPQPVQLYQLNAVPGRTFVALRLDHDYYFEDGNETTNSTSENS
SSHVELSESAQMIMSSLQMLLATFKGQQREKLLMPYCERWRVPLPRTNTEEWNDEYCREVIHRIAAKVGRV
ADHYAGTHSNHSITTLSSTSVVIISDNKILLDED
>tr|C9ZM06|C9ZM06_TRYB9 | DeepTMHMM Topology Prediction - Predicted Type: TM
MPEVENDSCCSMSTMEEVTPCPVPPIKKRSQRMVIKRWLNMETACEGEPRTIPLGCAPHVWTAAGYPRNAV
NNRRYSIITFLPLSLFHQFRPFFNSFYLFLTLTQFVDALKVGFLFTYISPLALVVILSLIKDAVDDIQRYQ
RDKTINEEKVEKLLLNGEVSVITAAEVQVGDILILHHGQRIPADCVLLRTSEACGTCFIRTDQMDGETDWK
LRYALKGTQTLNNVSLSQLRANIRCEPLHKDIYKFVGAFDIPGKESEAISLQNTLWAHCVVASGTLAVAVL
HTGVDTRSAMNRSKHSTKVGLIEHELNYLGVLCLSVLVLISFLLVVQQHFEGSWLTMFFRFLILLSSIIPI
SMRVNVDVGRIWYAYVIGKDDKIPGTVARNTNIPEELGRLQYLFADKTGTLTKNIMNFRFIQVGSDTVLNY
HEVDRFQSSIAAYFGENYVPRGTNTVDSGKINSHSTQRNFTRDVTSVGEAIVSLVLCHNVTPVVEDGCLQY
QASSPDEVAFVNFCRSLGVTLTHRDVTCMQFTTPGGRLVHYDIIKTFPFTSERKCMGIILREKVDSESVSC
GANSDGVYKYFMKGADFKMTSVVRSSSWLEESCQEFAQVGLRTLVFAQRQLTKEQVDTFLARYDEANADLS
EARDSLLDAAMALIERDMKLIGVTGVEDELQDDVTTSLETLGMGGIKVWVLTGDKVETAITIGRATRLIPR
HGKIEVMSCRTEEETQRYLDSLYLQYYNSMNDAPLDVPWTLVLDGGRLSMCLTKATSKTFVRVARLAYSVI
VSRCSPTQKAEVVRTMKKFTSRNIRTAAIGDGGNDVGMILAADVGIGVEGVEGKQASMAADFSIAKFSHCV
RLIMWHGRSSYRRTCNMSQFVIHRGMAYSVVQAFYSLLFAGTSMSVFNGYLLMGYSTIFTMAPVFALVLDE
DVKEGDVREFPQLYKELLKSRSMNTRSYLQWLWISVFQGGTMIYLALALFGKEMFQITTVAYTTLLLTELV
IVAGTLHLRILWTQRRKHLYYFVAAECFSIVTFLLAVLLLPDMIDQDYLFSWSCAWQVFVISLAIVIPIYL
IRLLDTYILSNARMK
>tr|C9ZSY0|C9ZSY0_TRYB9 | DeepTMHMM Topology Prediction - Predicted Type: TM

MDHDQLYDSPQSAKDSLLDMTALLIVTFAQRAVMAPSYRVTLLATVEGELVREGRLPPKGFGGVFGCIKRL
YVKEGVRSFFRGLLTDAVLSLPATVVENISSTLVSFALQVAIPVRLVESMNPWTYLTLSLSSTSAAVLLAT
PATGLHSTIVTNYVADIVAPVPSEKSKNPDKNDGENNKVIKEGEKGGEESYRYATATEAAASIFRRWGFSG
FYRCIGADAIAVFLYRGTYYYGLQLLPSTLHNRFPYGISRCLAVVAGFLTQPFEVVSRRMQLTASSTTGRR
YKGILHCARTIVAEEGYTALWAGMQARLLVTCVGVAVLELHRHFWAV
>tr|C9ZQS8|C9ZQS8_TRYB9 | DeepTMHMM Topology Prediction - Predicted
Type: TM
MRKQLNMQEEGDASTARTHRRLNDLRMQPLSSLPMTIFMMWMVGNDVSIFSIVFVGMAVTNPLQSMLGAAK
VFEEFNEEAEKDPHVRSAVGHSKLIYIACCFAALAVALIKLNWMGLMPVNAMDWLDSTPPQYKEQSMGTFF
S
>tr|C9ZPP6|C9ZPP6_TRYB9 | DeepTMHMM Topology Prediction - Predicted
Type: TM
MLLKVSHLYTFTNCIHILSLFLKTSVHELTVIPLIAFLRVKEPLFPSGAPQIIAQRRQQPAAREVSFSCIF
YWLGLLHISRVPFRYTLWSVYGYRVFMAPPISLPSLPSVDRSSTKVLVAGHISAVNAVLDVFQTDYSESVR
MYRVCRRGKNGFAKIFPFLRPVYANIALLILVVGCLVGSQAGLRGVLPNIPQSWVSWSKWTGSGTAEHVVM
WQLSCFHIFILGCLFLLILQFVKSLFFVHVDEVTAIRGLGLQLNSYNALGRLCFQRFVDIRLIRSLVIHDA
FFRTQVLFFLSATVENEASRLVLFEETLPRLDVLQPVLCGLRHVLFGEPEDVVTEDSFECQTATQNVLN
>tr|C9ZK27|C9ZK27_TRYB9 | DeepTMHMM Topology Prediction - Predicted
Type: TM
MSDVGICALLCGGFVGTIFLWRKERTFALLCMKDIEKAWECGPEHLDRNDPSTFRRRLISFVGSTAVSWLL
LEYLLFKEARDKTSVGPSAVIHFLFCQEGNVLRALHVTVSTALATLALFSGTLLEKGIALFQQDADEIFLR
DNVICPTGEEIFFRALLLQILLQRRSVTSSIVISSVLFAVSHTHHIFPSVAWEYRYIMGNNEPQPRKKLVC
WRRAAKKLPGLYLCTFACGLLTGYCYVVVGKRNLPSTIVTHALCNFIGPPTFDFLQEQTWLKRLVYGGAYC
GGIAGWYIITSTVARRST
>tr|C9ZRP1|C9ZRP1_TRYB9 | DeepTMHMM Topology Prediction - Predicted
Type: TM
MCSCLVSSWGLLIGSFLSLVSCFILLLTYQLVPTTFWRASVRLYYFVPKNSTHLSKGLMTSRLFADIVDAS
KCAKFFPKTPSAVQEVAAEAKRRALEKLERIYSLSSASRTFLNTASVADVAAAEIGVSASLLSVAMNVSPD
EATRQEAKQQMVDLQSFSIDHFESNKRLFESLKAVSQSNAYRDEYVNGGRDREYSYWLDEELKGYKRKGME
LPDSELEKVVSLQKELSSLCTTFSRNISEDKSEIVVSAEELAGVPENGISGLSKTKDGLFVLKMDYPTLFS
VMKNCEVASTRREMSRAASNKAYPENLSVLREIVTKRQKLAELLKFSSFSELNLDDKMVKSPEAARAFIDD
LVPRLQEKWKSELELILRNLHSSCALTEDGRLRDYDVPFMINQVKKSKFNVSETDLQEYFPLDTTVKGLFD
IYERFFDVTFTRVDNGDELWHKDAFTLHVLNNKTKDTLGHVVLDLFPREGKFSHACCISVVPPVLLEGSES
KFSPALSVVLANFPAPSAERPSLFLHDDVVTFFHEFGHAIHSLFGRSKMATFAGTRVKRDFVELPSQMLEE
WVWEVDILRNISCHYKTKEKLPTELVESKVNTRNTFSGRDSLRQLEFASYSLELFSAPFARVKDIKEMDTS
GLMADIRSRINPHIKYDDETHFECSFGHLTGYGAGYYGYMWSKVFALDVYDFIKNNGGLLNPAVGERYVSE
IIGVGGGKDPSEMLRSFLGREPRSDAFFKSLGA
>tr|D0A8M9|D0A8M9_TRYB9 | DeepTMHMM Topology Prediction - Predicted
Type: TM
MTGDKVGKVCLSYWCAPRSLRRSCECTYIYIFLFKKNSFIFFVCAELCEVKSKLLNPCTQDVKVIKQKKKN
RIPIHQRNVHRSGKPLQTTVSAQLIFQEAHRQDTVNQQSTAAPTERFWKTIKLSPTHFVFSLHRGLPPLSH
AHTHTHTHTNRNANHITLFKAHYVRIHMLLNIPALSLSSLPSLLLSLTFINKYIYIYMLPYLFHQKEKRKK
RK
>tr|D0A904|D0A904_TRYB9 | DeepTMHMM Topology Prediction - Predicted
Type: TM
MGLACFISTLVADFCRQARNICAGVLTAANKLNNVLLYWTALLFANCKLRTNKGTFPSRISMLFFSANSQS
LRSMDTLCSHVVFVHALKAILLASVSNATTIGS
>tr|C9ZNZ3|C9ZNZ3_TRYB9 | DeepTMHMM Topology Prediction - Predicted
Type: TM
MRSSDSHGDPRVADMSSEGPGSSGGSLHFSGVTDMLLGGLQRKETSAPLHPTDIDAAQPCSVGAAFDAVMQ
SASDESRSADINSERTRKVKSSQLVQDSVCPGFVQSNGVTSSAHYLGVKYQGGLVQELQQSGRSQEEMDLS
SCGPKGMEGQSLSMVIEVSKQRGVWGEFAEITSCSDATPRRASPAAQTSSIQPTHSSIFYPYGLEHLPPVL
LHYVTLGGWRTVAGGWDYFSHRRLGDGEVSGHTVRWLHNARPRDRFAFIISPVERRDKTSRDTLRNKLRKY
FFGTPPAFADCGRQSTFRCVPRADCEESMENSSALPPLPLRGLFQKKIGGAMSGSCASSKAEDHSTVGVEV
VEVKHEDVTAMDYNPRLVSSRFAIEAKVVNVYFFPTLEGALEYYNCPGDQYLMDQQEQRGITNVLPCVGVR
RHKYAGCTESCTGLSRARSHASVSTAAAPPEMSAGEGRWIHATRQEPSMRYAEHSAALDDCGRCDCRQQQN
TTNDFPRDDGWVGTSLTPQCSFGTRQTSQQTVVTSVQTDPTVSTGEGNVLHESAEKFHFLPQIAPTIRSRL
MPELTAVHFDTIVTLYSEDPNFNAVLREVLMPEPGTHPYTASLREQRRMLAMYELGMPTWTVFLASTGLPY

RRALRLTFVGLVNIWPIISLFVGLYDLYKHLPQMKRFFSSTFEPLLIWLEEHVTVRVSMMITYTVSVCVTV
ASALVSFLTQFYPLEIASYPLSIIVFLLKHPSRLLLDTLLAFVSIVASLVKLLWITVKILFAGPFLLVSNF
ASLEIGCFGVGGGGGAVFPAAVEGTSLTLKWWRAWQEFWVTVAAPVKNLAKAWYDSIVHVCVSATRREASI
RRWYTAKLHGITLLTEEVHEAVEPSLVTLWGRTRVFVIPLVCLVCLLYWFFLPYVSTLVREEISALTNLMS
GNIGSTQMEYGEGTVSIPGRAEEKAPLEVDKREWSSMFELSQLYNGDIYHLAVECLTESNAGWFLLWAIVE
TLVYKW
>tr|C9ZTN0|C9ZTN0_TRYB9 | DeepTMHMM Topology Prediction - Predicted
Type: TM
MAVEQQRRKTHVESGANGFGWKQWQRSHLLMGEKKECCSGVEIFIQLMRIYRLLRFRTSSSSIYLWVCHAV
CLCWSVYFWKDHTHCPCLYSSLLLLLLTPLVTRYISPTRV
>tr|C9ZTD2|C9ZTD2_TRYB9 | DeepTMHMM Topology Prediction - Predicted
Type: TM
MCFSWYTTDAIFFSKRRKFRSSCGNNRLNHAKQFVFRVFSTSGTFFTGASESFVCRWWHIFDLGCGCRSFY
QRLAVYHWWKGWSPFWGVHRVRKLAAAFFLLPTVWVHDLSIF

>tr|D0A419|D0A419_TRYB9 | DeepTMHMM Topology Prediction - Predicted
Type: TM

MASLCFTDVGTLCRDVEPMRSFPASPTCIEVLSGVHLIASVNARANQCIVELLVGDKVADTFSRAGSEEPG
NSGSRLSTPTGAGKTPNFTVPTVDTAVKEWLDALRASFRTAKVLDVLKEDEGVVTTIEFVRDVTRASSGGE
AEQEGVNRSVKRPYVHLFIGTSRGTVVICNALRGTIMAIAMFQDCNRFVRQGSRRSSLAQGASVTSAQCTE
SVVRFAAHMSTLDTFLNPNVPQNTSAVHSGHLNTVYIVMSSGRVVALGRGAIDVFLETAEGHLDGARPHLV
LEWVENSTFSSFPVAGPAARFASHVRCILSMSEVDPNTGVRLRAPRSTAATTDRSIKDAAFYLSCKCDTTD
VLTDGPENREYLLLCGRAPALASYACKKTEKGFSARQAIGAVASMLGGVKRLLWAGGSEGEGDESSFTAKG
LSVSSQSARNVFPEGDMTFDVVQVDPTLQWVACYSDSAGRIYVYDLMGDAVCQVVKGCRSVEFQWHVATIG
GKRMLLLVIHRILRHSVEMYSLRLGRRVAARYVPSGSILLRSGSSVLIPRVLMLVPDGVVTAIEVKLDRAH
GALPSSSSCISGVTMGRGRSDTVVTGIDVHGGVTCVRGGFPTPYDVLVAALSLPLPNPVTEGDSFDVYCLR
ISKLEDVIKRQFSPGVFDDCCLPVGAGTEEADIPPDVSAAQCLNYVQLRLVCVASYRRALFNGANTKTAQT
SLKEFAMRSAAGPVFTAQGELHPILKGMVGDVTAVLLAKKDETCFSHVAGILPSLEKLLMRRSTASVPEGD
PTGPVVPMPLGDFLKLFYCGGHKLAFLRESVGYANGCVDSFDEWAEIGSVFFGGGVGCFPQQVDVFNSLGF
RTVDVVMMALCWLCRSFARTLSPSCLGTVAELLFFLRNSGEEAFLTGVEAIPCLIGRKHLERRRSEAHVSL
LTLLVLLRQREKPFGSAYHARYELSLKRLLGLHLMIIRHETPLEDGDGWESQTTEAPLYLSLCDLLPQEGG
DALNILLLRQFPPPLTLKLCSEMPGSNVVKEALAGVDHLHWVKQLFMMNAAPPPWAVDSPWYREGTWDHVV
FLSEVIQPTIKFFKLVTQGTLPTLVWVSAASSKQLAGLSVLLVVGLAVLERELTPLRCERLAFLTDGMLPD
SNLFEGNETEFSDYSRHPLGSRSTRDYFNSCREFLHLNIQMVRQLLGGDDYIHGKKVADSVVEFLSVDDGA
IFPLVPVELRRLLYFFTVHICGNLRGLLQRLREACCFMDTVAFFAFKTAGSLCLPSLQLPAISWVTMFEDR
STVLCFLKGTCADNQEGKSLPTMVERMKARSELVRMITFADVLDTRSALSNSFVEDLSEALGLNHVAADFH
LLVLLDVCSRLLLPSEEVCRFLNNVASRHLAALIAVFNCKLLIKCCFGYYADAKKTIPSCNRQEKQRLALK
VQGLDDAMSEDFRCWVHSNELEGDEAIGSSETVDSNSNSQLEYRLFWGSAWCEVHYAGVVKCKVMSRLDQS
QDSEKLRDFHRLLFVLARTAAEGASTLPLGLRRIACELPSVAARVTALI
>tr|C9ZUC7|C9ZUC7_TRYB9 | DeepTMHMM Topology Prediction - Predicted
Type: TM
MYPQQQMSPPGNMQQWNAANSGYSMPYARPYSGYSNGTPPQQPQQPQQQMHTQMLPDAQMQQIPQQQMQQQ
RQQQMQMQQAPPQQFQQQQMQGDSNGQQPMWGYPPPNYQGYRGNPNMYDRRYSEAAAVPGCCNYERPGAAS
SGCCCTATEGTNPYCCGPPTEPGGPLNCCASMCDRTFVAMVFFFLLSTSAFATIMSLGDKDNILVKFKVPN
TRALQAKINSNIVSSSGGCTDGFFTDSRLCMGDGIDAFALTVQLKTWSTQRNAMYFLAMIYSLFTLLYAYL
INFHRRSNRGANDENDPLASSQHQHGGHQGSAVGYPSVKGQLDATINDSQQGSQNTQMQPGLQMSPRTGAA
DPIQVYGTPFYETMRCAWFVMGFAVVTWTYTLMASYMAQSQQYRDRASGELQQFLTAFHQRFIATVVCVTI
YLAWPISNILIEIALWFVLIIPWLIYRTMCNPGLGNLRPDVPLDEMPGCIRLDMFFMDFADTRRLGFSDSQ
WRLLTGTGKPFFASCVDESTIDRDPSMPSLRYASNWAAGAQPGSYPQVPMSPGQMQPHGQQLQQQQQQHVQ
QQLQQQQRMMQSSMTGDEAEGGEVHDRNPRRRRDHSRRRGDRTHSPGGAENGKNMEPPGFGNFPGALWLRG
DKT
>tr|C9ZS39|C9ZS39_TRYB9 | DeepTMHMM Topology Prediction - Predicted
Type: TM
MRPCAAGVALQLVVADPWQRCARRRTSTSANTKTCKSFTSRIPFKLRSDQVNESNIPSAREVSEMIATHEE
LQEQLPSAKFLESRYIKRPLHTESCGSCPATVCGPPLWPDCDTPEEIMDEERYGGPEAMDRVPAPSAEHML
LAWRALMWGTLYAFVGVTLVVAVAIYVSGVNGISSVLQHLRSRSERELHRLSAEGQEVHHFVLDLTNPVAF
GRQLQEAWQLVQDIANKQEEGEESIAKEIKGEL

>tr|D0AAF8|D0AAF8_TRYB9 | DeepTMHMM Topology Prediction - Predicted Type: TM
MSPRATEPIINEDEHPTSIPSLSQGNVLELPPDSDKSTAAADGEHRGCLNTVFDPIKGVVPSGGMASNVFN
LESATLGAGIVMLPSGFLNSGIIIATLMLVYICFTTVYSIRILVITRDKTGFRSYEEMACGLLGRGADYFT
AFLMFVFCFGTCVGYVISVGDLLSPLLNQPSTTGFLRTSMGKNVIVGVVWLVAMLPLSLPKEINSLRYASA
VGVFFIVFFVICMIVHAAMNGLKDGIGSDIRLVGDGWGILNGFTLFVFAFICQVNCFEVYEEMKGPTPRRM
TRDSSVAMSMVGLLYFLSGIFGYLDFGNDLEGSVLKLYKPQDDVMMAIGYVGIAIKICGGFAICIQPSRDA
IYYVLGWGKTSDVDSWKNLVVSGVLATLALVLGLVLPSIEVVFNFLGSFCGGFLAFILPALYYMYAGNFSL
KEVGWFNYAVTYQLLIVGVFAVVFGTALTIYDEVKK
>tr|C9ZUS1|C9ZUS1_TRYB9 | DeepTMHMM Topology Prediction - Predicted Type: TM
MSLPAVDLKFLSNVSRRLQERALHVYMPGDSRSSVAIIFRFGDDRQQHAVRRLLAGAKEVQQVFQRHLSAG
IGMDLPSTLQVLLVRRLDLVKDRWSGSVTYPGGRRDRGDADDYSALCARVNEMLGIPLTSSEFLLLGRLRD
YPIRSRRLQLGGMVQSRFVFLHIGELAPTLRFASHMVEAARWIPFSALEANQRCARSTVSHPLRSFVHPSD
AETRLLLTELFPHACLSFPAVQVPDQQWKVWGLALRSASELMALDDRSTADWPLVSSNNALLQHFVIDPLH
GYYELLYTYHRVRGQWGRKGNELRNLSRCDVIPPTCHPILWAVSDEANPRHVFFLLLCAAFVTMMAYAIAT
VVHGACTVVRLAFGCNEGDGFERTLGYCTASNNDGEV
>tr|D0A3X2|D0A3X2_TRYB9 | DeepTMHMM Topology Prediction - Predicted Type: TM
MLRRIWSHKVLTTFVTLPVASVGGIAIYVEYRQRTRPVLPVFAPVVDGNGSLLLDGKTVPKPSRWVVARRV
VELFLIFFPVAVLYVVMRLRRDWYLLWLQLLLRAVERAGPAFVKAGQWSCTRQDVFSPEFRSVFKKLYDEV
DTHPYEVSLQILREELQQDPAEIFSTIEEKTVGSGSIGQVHSATLRHTGEHVVVKVMHPNVVETIVKDFCI
INTLASFLHHRVPALEKYDLPALAIAWTTHLAAQLDFRLEARNLTLFRKNFRNEPYVRFPKPLLSTQRVLV
ETFCKGEPASVEFLAAQEEHARDKIANMGLNTWCKMLLHDKFLHGDMHPGNVIIDASDPHEPCVWLIDAGL
CQQISEDEGVITHNLMEAFVHWKADLCCDALLSMGKRQKYADENKFRSDMNWLFNHWRPLHSKDAVVTNIL
QAIFECVRVNQVHMDPPYVSLLFAVLVLESFIMNLNPEFNMVRHAAPWLVADGHLTRGLMKNIVMTRLDLI
KREMGVLRGRLRDGAHNDLAKNENVHLNANAW

>tr|D0A4W6|D0A4W6_TRYB9 | DeepTMHMM Topology Prediction - Predicted Type: TM

MKGQSSQCRVPLLGAGSSDDCDGDNNANEPVAAVRRCTNRFSTNMPLLEERHDLSLRQKLQATIPTRHPRF
YSSEERRKMERYESVDTFEPATTVYKDHLAGRSQEPRWFIWVFVVVIGIAVSFTAIVVVALLHVFASVRYE
LLGYGLNNFSFYNPNRYPERSPDHAELDFTAMFEGVGVGLYGISPRSYMKGYLMWVSFSFVTSLISSVICI
CVPGSVGAGMPEVIAYLNGVDYPMLGSFGVLLAKIASVVFSVASGVCTGHCGTLMLTGAMVGAQTLQRRRW
IQIEHVNIIECFRNPRDRRTIVVIGAAAGIASAFNVSIGGLMVVVELISTTIPVRFALYVFAASLVSSLFI
QVYFSHFLYFAGRDRTGYSSGELISELVQVFASTIPFEQIVRMHILYFIPTVVIGFICGALSGVYVRLSWF
ALVVRRHLEQRFKVRAFRALLPVAFTVAYVSLHYWMVVAFGSAGWSPPSSALNPTDNTTAGGLTTLRWGNS
SAVSPHFVDAQSGPCVAVPQTLSDSRDVSVISFYGANGFFCAAPNNTVIPVTATVHEQQVWIVLHSYASLA
FANADSALQTLLSLRTETMLSLPVLMIFLLIYYTSSAIFLGISPCGDTVFPTLVVGATVGRIVGLVVFLIV
SPGGRSSWADPGIFALIGAGSFVGGTTGLAFSICTILMESTGQFQHMLPLMVGIMIAKKTAEIFTHNINAV
LLEARCVPMLNYMNVVEKYPMFDARHVMSSKVVVLETVTPIGRVVDVLENTRHNAFPIESVRDQTYKGVVM
RRQLEIVLWHLYYSHHLSTCSYECGKRVEASLYRDNLHGVLPPLEKWLGAQLDLSPYIDYSGFCVLATATL
PRTYQMFLTLGLRHLTVVDSGNRIVGIITRKDLMADRIIESIIRTDRRRRRIQGSRKHSTNTGSSDVADVE
VAGGEESSEVASGRSAGGWEAALKYEFYRSGEWCVSDEEDRVSENNSIREEPGGTWKPDATF

>tr|D0A400|D0A400_TRYB9 | DeepTMHMM Topology Prediction - Predicted Type: TM
MNAPHRAPGSNSAGNTGPGSHRSRCISLDPRVHEHIPPHFLPNLAKYKYSGSDSGIISNYVMQPYWNFIVS
LVPMTVAPNAITVTGFVMCLSSALLVMFFYYFGNAEYPCWVWLYAVICLFAYQTLDAIDGKQARRTNTGSP
LGELFDHGCDVILTPFVQMMICCALNTPPCVTFVYITLSSCAVFGAIWEQFATGTLDLGYVNGPTDGILLA
CGIFLITAIMSPAVWDTQVAGPYEVPLPSWLGSCGGSFVIGSVRSMLFTFYVVSGTVTLLTNILHVLKRPN
IQKPGMAVTTALPVVCLLVLHVWMYLVYRPIHEKYPYALELSFHVLMSYTATRMTVSRLCAMPFNLFSGLF
IITLLFTVAPLFIHTYLPLAEEKYVLPSLGSATVALAVLGLWQYFHMILSVITQMAYFLRISVFSITSRHD
ANTKWE
>tr|D0A1T0|D0A1T0_TRYB9 | DeepTMHMM Topology Prediction - Predicted Type: TM

MQSCMMHMELRIAGIDSLSLFDAESFVRMHQLSQNSPQVLYANNSLVRLPRGGVNASDHFKPQVKVQFVFE
NLVRIESSTVEAVSDRRQHEEQIKSKWDASEIRTKRFHCNYSQAIIASLPRIVVERYVKIRVLQLAPLLSK
EETIAVGATFPHYFNNDEPSLCVGVAKISLRDLIATSGGVSVTLQPKRSMIAAVESLNLNAVATNAMFGKD
AGGSGVLTFQVVSVVFNPKTLSSMLCCPDYNDNATPTSVLENGRRMDNASDGAALSTIGPELVTDDLMALH
QQMNSGAVRFVLVPYMVLADAVLRFHDAFSWRNPLKTLLLLCIICVVISVEVVDMGLVFAIVSEALTMVRT
MVFFYRVPPRTSMGPNAVQFLAERKGTFQAVLYGSHNHVINSLIRARLFFTQGLQPDCYFELVYLFYRLKK
LKRGVSIFIAALVSGSILFSFETFVLFGMLFAFLVYPLLLRFSHSRLRKTWRRQLTSGTLWKTISLSRSMP
VARVVQVIGPHERCVPTQNFVASTSRFSTRRDVDELKISKDHSTLLGIERPAVLAKDDEEKVLDPQGDVQG
PKLPVNYNPAENSFSCTGGRNAGVFGCNSLQPLACRQKLAPRKSEMIKHEPQQPLHLTFVAIVIGSEVAPA
CHSRTTSINGTTTNITLVQRLWRVFQRAGALGRPPPSHLLNSQYQSYFNSVLTFLQFVGRQCTLDAYMSVD
ASSTLAELAGGTVKLDQVAPLCNLLEVGKDDGITSTLLAHSGQQIVLSKLQLPNSAESSARALALLAAYLL
QGSRLSVYSEPGGKVCSVIIPLKMDDSDIETATEPHPCPAPLQKALVGFWKGLAGGDCSIDISPDVVLTVI
TTYKDTWNGDNATTNPVHEYNHISGQNDDNSTNREVSKRLMQTLNPTQMNKRYSMASAELPPLDTEDKALF
FRHAGGPVRPDDSAGISPRNYHRATRTWA
>tr|C9ZXI5|C9ZXI5_TRYB9 | DeepTMHMM Topology Prediction - Predicted
Type: TM
MAKDTAKKNMRGNAVRMQVFSLITLTVNVICFLVSFWRQSSFPGFGSLAALAFWAGQEYASLALLKHFARP
VFNAEGELLDCPDASDPKELGYYSLAQDVLWVCWVVQVLCFLFHWGFMVLYLPVPVMGLYKLWGIIGPLLT
PAQPAAGEGCGFAPGGLSRAERRKMELQQRKKRV
>tr|C9ZPF9|C9ZPF9_TRYB9 | DeepTMHMM Topology Prediction - Predicted
Type: TM
MLRRTRFICVGAKQFFRTDGQPYQPYTIKSLFLFGFIASTAFLSVFTVKETKKAGPLELPDELEDERRRRN
DPRRPPWPLLHQRSVLLREGKVPHDDLLLLWEQTRHYYPADWLVPLEITQVLKYTSGAYLQNYVPDPDQLR
KEVLMQLLNIQYGRVRDPNGGRLNRDVKEIIAMAIEDLENMDLSPGTEPALVPTHT
>tr|C9ZXI0|C9ZXI0_TRYB9 | DeepTMHMM Topology Prediction - Predicted
Type: TM
MVSATAVRRFGYVGAAANWLIPLAAIVNLPTRKPSEIDPLMTGVLGTYSAVFVRWSIAISPPNYPLFLCHA
TNCVVQAATLVRKAV
>tr|C9ZZU6|C9ZZU6_TRYB9 | DeepTMHMM Topology Prediction - Predicted
Type: TM
METNFQLASVHGMLYTGGNVVFSPDGRQLYSPVNNYISSIKLQQEGHHSLPCSNSNIQCFDISSDGDLIVA
IGRRGVGFFYSLSAAVVLDTISFPPDCEVSCVKFSPCGRYVALALDTTLQVYTAPAQRIVSYHGCHRVENL
HNALTLPIMCIEWTPDSEHILLSGQDARMKICPRQCRVQQKGMALQHNALVGHRAAVCGAWFVDSACNRVV
SVSVDNVVMTWKQTRTTRQEVMRAIASAKLNARVGAQSEEDEYTHAEDDEGAAIPKSFLEKQRLEQLKIEG
VRVSVADDTFLPDILRYAFEVDKKHMLTHKGNVSVTAFHRPRGLLAIGYNSGIFAIHTLGGSGEAEFPLVH
LLSISAQSLTAAAFNASGDFIAFGSAHLKQILVWDWKSEAYVLKEQSHYYDINRTAITADASSIISGGDDG
KVKVWRASTGQCYVTFTEHTAPVTGIATSAATNAFFTCSRDGTARGYDLVRYRHFRVYSAPEQAQLSCVAV
DPSGEVLAVGSGQTDRIYLFAVQTGKLIDQLQGHEAPIACVAFHPSGTALVSGSLDHNLAVWDLFAHGDGG
DRLKGTVEVTNVGSEVLSLSFSNSGRYLAVLTMKQEVTVYETLVPTEPIVIKTFQTSFDAAGGWSKSVGPR
SANYNARFTTIAFSPEGEKIVAGGDSKWIVLYHAKQGYMLKKWPITTNLDVQGAEEQYQWRQMTEAGHIDD
IDVDDTDIHLRRGKLLEMPGSKHRHFATGKRQTALSARTMSVAFASTGTEFVAATSVGLLVFSTHVARPRF
QPLQLTANITTQQVRDQLARGESVMALIGALNLGDRMLGIECMRRMPQSAIPVAVSSVPSVLFSLLLKWVS
SEVEESRGLERALLWAQSILLHSSECAGSFSCDRSLIIPPLKVLQRSLQRHRALGDLARENYFSLQYIIDM
TKMQRDVLKPAAEVTE
>tr|C9ZKN6|C9ZKN6_TRYB9 | DeepTMHMM Topology Prediction - Predicted
Type: TM
MEKHTKQDILKKKSKKEEEPHFRSSLQTKPPSPLPFLPFFFSVNLVATPFPSPPIFNRLINYLTNSYFIFS
FTHNEKRNKKKTHTDTHKGGKSGRATIHSFFILFEIFIFLYIPSLNHIFFSYKYIYK
>tr|C9ZNH8|C9ZNH8_TRYB9 | DeepTMHMM Topology Prediction - Predicted
Type: TM
MPTRAILRHAYSILTTDTPKLCCFDHRVTQPIRCRGVFLIYSKPLLALPMGSLAKKLCLIPVVLFFVCPRE
FILSMNSIYPKLLCDIPHLNDIVPNRVLSILALGFQPYGNYPIHLVRWDALCGDSVP
>tr|D0A1E4|D0A1E4_TRYB9 | DeepTMHMM Topology Prediction - Predicted
Type: TM
MLCSQSPYCTETDVWNKFLFAPCISLLSSSLFFFLQRVTSQITVVGRSHGSKVFLFLSPFFLEGGTVFSQF
TIPFPPPVYVVGRTTGRRGGKEERGIIQTKRSECKKNKRGEGEKNTPAEGIFH
>tr|D0A254|D0A254_TRYB9 | DeepTMHMM Topology Prediction - Predicted
Type: TM

MLSPIVDMKSVGTDDGFDIYLPGSDAFYFKRQLHYQPYTPCAFPVEWLGHQGKMHKTSVHMRISPSNLEEA
CASGSLGYVIMFWAQGQNIKGHESVVGGGDTLLMTAAYKGHIHVVRFLVDAGADINATNSAGFSALHMAAS
AINYHIVRFLLDHGADPTKKNHQGYSAYCVAVQNASLPLLLLLAEHRPIDIHERDEMGHALLHWAAYNNSV
AICQYLVEVCKMDINVVGKDGRTALVWAAREGYADVVEYLLCAGADVDVVDTDGYTALNYAKHRNHPEVQY
VLENYPLAQRGATGMAAGDGLLGNQSRVNGGYVTAHNMRCRGTFSMLYHEPFFAAMSVLGAVHVLLAHYSL
MLLPPLFSYAPFGMFFFRNTLWMAVFGYPVQGGKPELTILQQSGIGRSFSDTIRGTWRFRDRDAASLFALT
TFMLLQLRAWALMGFEPFLPFLSTRNSVVKLNTSLNTTDKDLHDGGSRAVNFMQPVMCFLTLFLLVSVVLC
KLLSMRSVYKPEFGSLVTSPVWNIIKHRAYNWLHPRIIFMERHLVLPLRAFYCYEKDVVMERYDGYSAVMD
CPVAASNHAWFFIAVFCFMVLQWLIFISGWRQARVMLKCPRSDSLGEYISSSAWNLLVHALPCRDPMTIDV
TGKSYFWVFMFRYIIPTTTNRLGVWLLHYSLIAAIISTYCFYRQLVAAAIGATRMELANRTATSGNGGLVS
IFPPSFVPFKDTNSAAAPWTPPEGHCEGMLTIVDDLAAGNVAHRKVGSHCIYADSRFSILNIIYFLLGISG
RRWRGAVAVSSFNSPISSVSLLEGPLA
>tr|D0A731|D0A731_TRYB9 | DeepTMHMM Topology Prediction - Predicted
Type: TM
MRLYVCVWKCSAMRFSMPFMRMDRRPFPNILVVQPPVMSSHVLEVTLNFFLLSLFFSFSFSFFVMGKVQLQ
NKEKRNKRYCLLIAGGGRKWNYGYFFPLFLTVEEEVRWRKDELKMPSEGMYVFDI
>tr|C9ZK83|C9ZK83_TRYB9 | DeepTMHMM Topology Prediction - Predicted
Type: TM
MFRPVLLMAQRSAGVNLLSPLAKLGQSRDLAADIKAGRHRNNKEVIASFEKIQTFDEIHYRKSNRPWELVP
RYAKKRVSDLCGLLSSEDRMEIEQAIDKMQSLCEVDMYVVLVPTVGYTTPRAFANSILFDWGIGEPRGNGL
LLLIAQSEASVQLVTSPAIEEYFAEHFLQAAVKEIFQPLVREGKASYAVVQLVYAIARQAQEMHTLWNRGF
LALPTRNKVRFAAKTAAYGVTSVPYLLIGIIFFGLCSTVLVNQIIDTMCPTCYGSMHRVRDDATLQSIMTR
GQYLEFSNGCAHYRVWKCPRCTDSSRVTLTSRDLHQSTRCLQCMDCNYYTCTLTKEVQKLPTKEEDGLKQL
LYTCENCRIGREVLLPLLRPIDTTSETNWYDFLIDRASTHKKTGIKL
>tr|C9ZZ74|C9ZZ74_TRYB9 | DeepTMHMM Topology Prediction - Predicted
Type: TM
MSVVEMEKDSQPEQPMRSKNFLHRVGTTILFVGTSFALSVCTPRFLPSAVKDFAPTIMWGFTCFCVRRAFK
MMMTLVLITHLLASGASLWSCGFSNRVANRALIFPSFASLAALLATWRLPTMPLERVIGVSLVQTWSISCG
LILFAVFPATLISGSNWDQAKRDARFDQVIERATDQRVERFTVQSTGGVQLDAVAVMPEDPTEHWVAYFGG
NAEIMETSTEDMAFSNHLVHANWLFHNPRGVGRSSGYVCWANDLVDDAEAVVWEAVRRYNINPKNLILWGH
SIGGGVAAALAGRYSDFPFPVLLDRTFSRLSDAAVSFSPFPATVTRGAIKVTTGDLNVVKSCRSLKGRNVL
VIFHRSDEIIRFNISSIARPTAVKKSGLNKSSFLELQSTFVPSPHNTPLDYFPEVTQIQRRIIQLYE
>tr|C9ZJU1|C9ZJU1_TRYB9 | DeepTMHMM Topology Prediction - Predicted
Type: TM
MHARINIRNAVFVPLGISICAAAVVIAARALLRERARPAVLLDCERKAYEILEMLTRATIKTDGHDSKQTE
CKDDASIAIFLNCNGVESIHVRTLVFNHNIMQLPSVFAVHCGLMKNSHPDHLLVCDIWNCLMALVNCSIRD
ILLDDGVALCILDDVKAEEVLALCMLLRGKYSIARYPPAAQADVVVQEFPEELYLGLITAYNRKRSLQSGI
TSFWKLMCSSCSIPQLVTLAATVLFSPEVILKCILGMHAFVHWRPIYAAEIGKSLNGDVSPKLSPFRFIIK
QLMGLPATFVSMAVASTFAFESLYCIRGFVIQSILSDIITSIHIDAYLALATADYDPSDDWLLSRCVSQAL
WQASGSVMDELQSRVPALLEVMHGCRVVFSFPVVSLVMWLGTQWSNFLYFLNESLYMESQFTKSQLASVTN
IATSTYAGNGEENQVSPTCSFPVKNSLTSSPLTLLRKQKDDGIKCMLSSTDNKMLFQQTFSHPSRHDKSSV
QYGLVLFSMVFVESGRPLGYMWDLLTQHVIWPLVPSVFSGCVVDALKRKLQAGGHTCAVSVRRLGWIKDVI
QSSPRAATASPSTCVWQCRQDYLQGFAALRLSGWELMAATAAAAFATASERNDRYEQCGRDVLHGLFQMAD
TLPLLLVSAVALRLSPNFFTQSALDQTEIVNQLRSIQHWCPLYHAFLGSFYDECGMSEAQVDEAHLRRHLA
FSGSSLGCYQVLEGLQSVQRKIDRPVRGVAPPSLDGPWSVEFREVSFRYSDRHPYVLRRVSFVVNKGEFLG
IAGYSGSGKTTLLRLLNRTYAPTEGDIFVNGVHISQYPARMLRRRIANVWQEENNLRFIEDLSIGGNIALG
NLWDCSDENICAALSASRALNFVQKRSSGIHAPLNVREFSGGEVERLCIARAFVKAGSAVGLCIFDEPTSA
IDTITEGEIFDTLRLREGNNLLRDSTIIIVAHRLATLKHADKIVVLNEGMVAEVGSWETLSRTGPHSCFSR
MRQSQQLPHFVKK
>tr|C9ZY95|C9ZY95_TRYB9 | DeepTMHMM Topology Prediction - Predicted
Type: TM
MKGGETAAGVAEAGSKQSKEGVVDSKGDAWSLVFLILSIIAMALVISSSLVTLVVRNNTVADTFLELYMLV
FAFLGLSAELRRFEGIRRGVYLWLRYFYFLTTYSGRGLFYIFLGAIAVGGTPLRYTSCVITTVLGFLMFVV
NCFVNLPVYKDQ
>tr|C9ZVV4|C9ZVV4_TRYB9 | DeepTMHMM Topology Prediction - Predicted
Type: TM

MSQQRQVAAKLGEVAFDSGEKASAEFFAITYGALVQQMITDLPQADDVEVVNQQLDTMGRRIGSRLVEEYS
ARSGAPACRTFAQAADAVAIVGIKMFLNVNATVQPAEESGGGTFTLTFADNPLALFVELPEGPVRQRLWYS
NVICGVIVGALSMVGFIAEATFVRDTLRGDATNEILLSFKGREKETFKVEQ
>tr|C9ZTU1|C9ZTU1_TRYB9 | DeepTMHMM Topology Prediction - Predicted
Type: TM
MKPIPVAMNRSVRRAALMTLQPLVCSGTSARCITTGENHSFRPGRVTLFLRSLFVALTPQRLRSRMATGMQ
RGMERVQILEQEQLWLQRHGSPLRILGLPDHAELPEVRARYRELVLETHPDSQQRVEAVSGNGTERYDMIQ
TAYAMATNPTSLWHQNGAAPTLRRGLASGNSASLHVTLFAVMSYVMMGLVAVIFSLVVVRHLLEGALRLLD
PKFYAFMTQQEEEERRRQLAGEVVDTDPKRLAPTVVKRLLFPGRFVHGGGSGDDFGYNNISESTGGNSPAD
>tr|C9ZI08|C9ZI08_TRYB9 | DeepTMHMM Topology Prediction - Predicted
Type: TM
MYICFCLCLCLCLCVYMCNHRPIFFFVHPCCSFHLSPPFALLRFRFRFLSFIFYFSVFAYYSFNFNFNFVL
RPYFHWFSFFFFLFCVHFLVTLFAYTF
>tr|C9ZMQ8|C9ZMQ8_TRYB9 | DeepTMHMM Topology Prediction - Predicted
Type: TM
MGALKRQAAVTASMIIIYYYYLHHHHHHRNYSHQFSLRNCTTSNNTHTHIYIYIYLYIYIHLYKYISIGVT
LCIPEIFLSLTFNLCRDYSFSLPSIRLSILFQNIFLFSLKKKNHLPFNCLR
>tr|D0A9W5|D0A9W5_TRYB9 | DeepTMHMM Topology Prediction - Predicted
Type: TM
MKTGASCVEHPSSMPDKAFAAQLQSICEVLNTSGENSGKWGGRLEALRQLEVFSRADMSNRRDFMMLMNTF
VKVPLQKHIEEDRAALSNEACRVVTSLAKHCSNRRAWQAVSEWFIQPLLRITVRKKKVFVDAAVETLCNLA
RTNSFGPRAFAELLRGCTAAHAATRSYTFSVLQLFIQQCKNNEGEFALSPYLGDVCRVLRSGLSDADAQTR
RCARDCYWNFRSAEAEAAETLYNKLEAPVKRMLGQDRNRLTIEKMVPKIDRNPTTAATPASKLACGTDRDL
STPPEEPGAVEAAAPAENSKTKGEPEAGGKATEECGPDALLESLSSSAWEERLRGVRRVVEDFPYLRHKEE
CVKLLVVRLNDSNGQVALAALEVLPLVFRLAPLLFKALLPELITSLLINVSERKGFSGVSRRLLVSTIRAN
SIDDVMDAIYCELRRIVSPSLKVHALEYAKYLYIDNAVHFAQLTPMRRAVEELLPIIQTEGPHNLVRKSAA
SALTALYVTSKGTFIRVLSHHLSPSDCESIVDELQLAIPHLGQECRRTLAGQKPLPHPVPVVRCAFADILT
KEDRTPPKDANAKELTVSKSTDPGNATHSTIQRPSTACAKGVHDRPPERRSNKRYLGERKVASPAPMDRRR
SPMGRTSNGDGVSPLRGKGATHHVPMTLPYALPIHGGGNDYSPADLLEWLDRTNVSTDTRDILDAIAFAIA
KNPSSWGEVFNRLLVLLERLIPDSCEPSHTVRFLALKVLHTVVGSGVLRQAVNRSLKRILLLVRAGIDDVF
PEVHMDAAAVLHLIINSGLYSTDHLLNAVAMTLDTWLRSNKVGYSTRGWLTMLEAIKHIFFQLGCSAVLVP
CAFKEYAELKDTTPGVVSEPVLHRVCSTVVSAVQHSVPEVRLTAVVACVAIWMTFDTAALPYFVDLTASQR
KLISMYFTKLADVPVDDLKITNTERDMTSDMRAAGLPYATHL
>tr|D0A678|D0A678_TRYB9 | DeepTMHMM Topology Prediction - Predicted
Type: TM
MHKCIHMTFEIYVCLIFRTFFFFFLLPVRLNLHLILIHLNTFFQLILTVTVCIYIIIIFILRISAAFSVSS
FLCFFLCFAFFLLFFCLFLSFFFSSFHFTHTHTKQLTVKSLISFTQSSFIGFDIFRCAIKAPSFHYLLILS
PFFFFFWFLKVIVFVVCNIHFHLVDLSPDFLLPSRKFDLIFFFKIPHLTNLPLFPRFNLFI
>tr|D0A129|D0A129_TRYB9 | DeepTMHMM Topology Prediction - Predicted
Type: TM
MRSRMSFVVHFSAIFYQFQEIHCPIYVETSIFYYYYYYYADDSVGWGTVVATVIVLTTGTFHTLFFFVCLI
VCSFVSGVGEVRTYNTKRKQEAVGNREGRPKEKKGGCSHVVSRRKCSMGKHDIIVY
>tr|D0A9V0|D0A9V0_TRYB9 | DeepTMHMM Topology Prediction - Predicted
Type: TM
MTKEDHGEEMVTHTPQVLNVRTTGESVSANPLRGVGLKDCGVFSPTSSTFCDTSRLMGNTGRSVENSTVTF
VTGAPGKEEADRRHCRVGTIAAVALLMLTGVFTLSFTSLLSAHYIGSSVVIGTTGLIEKEAVRSVVKIMRS
TVLQLPDFAHIVNAKYIRNQTSLYTVNRFPKDPRALLYSLMSLLSTFKDTIAYFKFVHEGGSYAFAVNYDV
GDGENNSVVLGAYNNDSSRASFVVYDAIRLKRLHEYSNRNWDISHDLSRTLSPFGAVARQWHEGGSNKRWI
PSEIEHTGEYFNYVIPFHVGGALGACIVGLSTDRLLLESRPLVEALRSHGRAMLFDDSRDIVILNSWGQPQ
SWDENPSDGELVFKHLTLEGINDSIVVDLVREMEKHRDPTTDRLSDSVELTFPNSGSHVDGRLGRITDESG
LDVTLAVVTMRSDFLEGVVRTRNVTIVSISVIILLSAVFSLVSTYYLVKPLMLLVSALKSASNLEIPDNAD
IFLGRSRIVEVRKIQGHYAKLRRQLAVLLKFIPEAILARVESKNEAGGHIRANGSATETPGESSTAFVRIM
GASISVTWNAGDQQRDVALNCGIFKEKPNVFNRRHCTVIAIQVYHPDVWKSSTKYHAALIEVATEHSGCVE
ALDPDRALISFGAHAALPMHCRRCCQFAFDLFAKLPLDHRDSVTMLVDTNEFLVGTCGAFSRNARVLFGAD
HLFQLSYAVRDGPCRIAATSGGASHMQGFLAFPVDCVLLPCSTLPIVLFELRPGESSSEVSLSISKQFRLG
FAAMRQGRYGEAMAHYLPMVKMDMQAKRLMRLCRQRCRRNDTVPYVRVLDNHYCVNSSDCKPEEQDESDIK
DRVPETIDSPRRPDGEGSESDSSDQGSESLFKMYAPSSSSSLDGSGCAEGANAADVVPLFLTDSNRNVWTR
SLKKISEGAFSTIFLGVAEDGAQVAIKCIPRLRRDVVAESLEKEMEVASKLHHPNIVRYVSCSITPSHLSI

IMEYVPGGSLHSVIKNFGCVSPYVARRFTVDILQGLNYLHNLGIVHCDVKPHNVLLGTDGVCKLSDFGSTI
SEASVMARTLADGVMLRGTALYMAPEVAAGGRCTPQSDIFSFGISLLEMLLGRPPWQWSSTAPAGSDATRL
RSLFNRDLLFVQSLALGHLEPVIPASVDHEAAAFVRACCHMNPSMRPSASSLLSYAFLL
>tr|C9ZYL6|C9ZYL6_TRYB9 | DeepTMHMM Topology Prediction - Predicted
Type: TM
MEGDVIPSVRSHLQDFTPLIPTDAYGTKHMKGFRVGLGKLDFSSEFVFLHPISSELLDQLKRLTKEEKDGE
RGMSVGSGVTNPPLVEALKCSDIQRCALRSNNVIVATSFGLWGTVDHCGDIIDAVKARFLPGADERRMSKK
VKLQLKHFFKFLNEMQVDVVVLSPDRTRVAVYSAVRSRSSFTPRTELSAAEATRRPGDGGRTLLALEFDHG
DGSWCPLRLAPELEREKEEVGDNGEGNASPLPFIPEDTLLLDGCNASITHGASLTGALGYDSPADSRANS
RTVYKPQRVSDWKGYKDEVTESHEGSDTPEGKSPFEGMRYPNIRPLDIQFFDILLRGLNPRFRLNRIRLLV
AFPVAILGAGFGVILTVYLRSAMGSCCRDASSNTCSGSADHNISDEYCEQTFCDDNNNTALAQVHTAFLFG
NAPVPGHFVVGATCCMLLASVATLLVGYFTARSLAIKSRPLFHMVVMCVQILLAAAAAVLSAYTIFILSHS
RHKIDCSFFGDGDALSCMMAQRFCPNYIIEVVRPGPWPVEFVLSSVLFLLCVVELVAAFLPPMPSKEVVES
CVKAIPETGTFYPSVYAPDGITSREKATMRCTMKKNLRMQLKTRASQKDILLTRNVTIGELIAANRDQSLK
EKTERLSVPTHNSTTSHNNREVEQIHYMVYDAAD
>tr|C9ZYQ6|C9ZYQ6_TRYB9 | DeepTMHMM Topology Prediction - Predicted
Type: TM
MNVTENPVWAFLFFFSPSPNLSLINLYLSIFLSYVHEGIIAVSAYANHRATAYAFPPFLPRSNVPSIFRLS
PLKINALFPPPFFKKKKENSRFFSPSFRTDAHFPACRANSEGSFSFSFCFYTFSSHLLPLPLTVTTFPN
>tr|D0AAU0|D0AAU0_TRYB9 | DeepTMHMM Topology Prediction - Predicted
Type: TM
MPLRAWVHWKIGLAESSNWATFSVFLFNDVCTDVLVYSALLPTVKLWWGLCYSFWFSFGTFLVSKSSCSHK
FICWYPQEPCCMASVVLVSPFLFIFFSRHLPIIYIYTYIYIYLGDSADSFCRHAVSN
>tr|C9ZVP8|C9ZVP8_TRYB9 | DeepTMHMM Topology Prediction - Predicted
Type: TM
MIDLTKQMTKIKRKSAAVMWTNLTCTVLSFPAIVVLVVAWITSMVKTEWGFVHGFMCIVALLFVVASAALS
ILLHCKKPSTKIFFIQQLISLIAFALTAITLGMGHVTLYVCRQSEENEPPDCTLQSVEVAVEVIVCFFMAL
NYLFTQQCVAWYACKQIVEGVHRCGAYGVPLAGKV
>tr|C9ZQ00|C9ZQ00_TRYB9 | DeepTMHMM Topology Prediction - Predicted
Type: TM
MCFIFGVEMSNLAKRPMSLRKLPQLFLLIMIGIAFVAVECIGAPVKLPRRVDTVAGQFGFDGTTDGSSNVS
MLSSPYALCRGRTNDEILVGSSNSFRNYSRKTKETGTFLRGGPTGGLVSADAKISKPRSCVRRGSGNHTII
YFVDDQNGLKYINDNEIQHVTVGNGLSLNAVAIHEKDLYVTDQNNKSVWRCNVGGAGKPQNCEEKKFTGVT
FTAKPEGIAVTSKGIFVTARDSSNKGALLWLDMNGGGRKGNVSGGFVDVFSTESGVLYAATEKELYTVTAT
DTSLSVTLFAGKNTSQCYFPTNGEDIVLCDNSRLLVIEEYEMYVTSKAKHTMRALTLPPVNLTAIFRGRPA
PVGYPNTTIMEQFVASLTEDVNKALGTNDSYVDPDSVRVDPDTWETNYTVFVQQTRFDNTTEEKLRSLTYT
QTDKTVDEYYGLTDEYVYIDTVLVPFCDDASLVTIQRALAREAGRALNFSLIYADKPITFGSDVAENVTAV
KLLMPHSFKNATTPKQLSAANLTDFAHNLVKDLRASDTRVDITFPDPPFNFSAVVPEREQEVRWFVHGKVM
KQLEICERLGSQGDAAVIAAAADATARGKANVTLNTSGVKANDTGVGPNTTNTAGGANTTANVATNGTANV
IVNPSTNATPTGTSNASATNTTERAVPVVAPTQPSNGYAECRSAITNRTETQNMEPPYDRKHRYEVFLPKK
YDFNVSWCVDIIDWRDLDEMLNNRTDEVVEKSLSWCGHGCIIAFAVVGSLIAACLVVLAVVLTSKRRRLAA
VVAPPRPKFVSTVEDDEEDRVSNIGVPLTDGKGTTAP
>tr|C9ZYA1|C9ZYA1_TRYB9 | DeepTMHMM Topology Prediction - Predicted
Type: TM
MRDSFFFLSSSRCYPIVVFSKKDDFLFAVAVYVFFLFTAFEFFLFVCVCVSLFFVFFFALFLPIPVRFPFP
PLIFFFFVFFVLCAGVLAQIENLDESLETN
>tr|D0A842|D0A842_TRYB9 | DeepTMHMM Topology Prediction - Predicted
Type: TM
MHGGANSAVASVHVDPTAQLQYNRQRGTEAYAECEYSDGSADENNRYPQRGQPPQTHETAAVSITDNRQQS
NMTPRAHAGGTGGGGGGSTMRRNVINRFDYGIISPSDARSLFLGASLLNIFFAILGTSLSQLDEVGGACFT
FWGYKTDCDSVSYTIRTQLLPCAPIRGRLQTGAAFSIISICLLASIIYLYIRSALAKYMSQYNAEGQTPAR
RMESRSGILPAEDSIVASNKWTIVAVGGVVVLCEVVSWAMTISVYVSRFCEDASLDRNKVYGPGFALLIVG
TLLFLMALVVFALRA
>tr|C9ZRK9|C9ZRK9_TRYB9 | DeepTMHMM Topology Prediction - Predicted
Type: TM
MYLWVQSKRRYMWQPLPFLFFFLIVVVVGGAKDFVWKEGKKKGLSRFSFFFFFNFLCFIFTLIITSHIFTH
LYFCVYIYIYLYIHICTYIFMFTCMHACASARVTRLHRNGRNKKCKKQKEKENEGSKGILY

>tr|C9ZS60|C9ZS60_TRYB9 | DeepTMHMM Topology Prediction - Predicted
Type: TM
MEEEVNTTSSNLIVTWRSRRCVAPVVEVLQSRPHLLDCLTMFPNGTKALHSPTTCKKLFVLFPGNPGIVNF
YEQFVELLSLKDVDVLVMGYSGHSICDRNNGKVYGLRDQIDNARDFFDSIQERALSYYGNDIYIGGHSIGA
FIAAKILVVFPCIRRCFSLCGVLSRIQETPNGRHMLFVGGNVIFYYVFVYLVVAVLVLLPRFVSSWFIRRQ
APTLSPVLVSQFIEHYNTGALRNCFSMCREEFQVVREPDQQFLGLVGKRMVFYCVQDDGWAPLSHVQEAKK
LCGDCAGFVVESDPSVIHAWCLSNNETVIEKGILPFM
>tr|D0A319|D0A319_TRYB9 | DeepTMHMM Topology Prediction - Predicted
Type: TM
MFKFLDTFVVGRSMAPCRDTKRKDEHEAEYRVFLNHSAASEVKDELERLVSSGSKSSFPYPLSSDTGLELS
VLRLIQSNGACRQLPAKMLSLMLKRLEYACTKLGDTAKINFLRDSIPVLEAALLVNPLKVGLLMLELGFDR
HMLHILQFLRQALSDEKMGGSSVSHQVSDSAAGNVSTPLDPSDDIPLEYSEGGGDEDVVTATRTCFDSCEP
PRATEIRYRELLTILRDTLRVLKVVYVRCGPSKRPSPGEAGVYSDMGNATGISNSQLAKGWVRLRPDSLYA
EMHRRSCPAEERKGSTSGSTLRTHTKRDGILLECSRLCLHCSEVAIGSVERGGEEERTISEALCNLPLVVL
SLLRNPSELVVRNSLISIGAELTHFVAVERTGDSELQTLRQASCSMLLLSRLLRESPLLVVKAMKSVNFSA
NVVRLLQVISVRYHCVHCDGCRIAGQEECARELRTLADAGNDGLHLDSGCVVSLKRPAFNRLADNAVVDEF
LHCYLGLLETQREVRRTAEVHRDLEAARHFMQIEFSLLEALLHSGDALAPTSCLYDALMKYAINSLAPMET
PVQERRQVNTAGSLVDADFYRVVFLNPVFGVFQKDPRLQHAVLYLLHHRLNSLPPKAESSDGGAAIENEVG
VILDLLCSIKVGEQSTESVEGEAAGAGDDNEKQNRGGVVARQEELADVLLKLCVIFHSSLCGTNSDAVATA
VIRCGGISKLVGLLRECYTSGRLSGDGISMLVLHLLLRLLQRLDVQCSVLMGEVDIFFSLLSVKEIRPNAK
RMLLLLYTHSCPDAEYTQRMDQLVKCTWDALDKCARLESGCEADNEDEEMFLSVLNCMSASLSALRSDIFQ
WGSMRQLQNALCGGKENAMDAFLRLFHRTAAPWRVVDSSYGLSCIMNTVVMLVKENPPLRAVLIHTIGADQ
LVESFKTAWFTSHNGSWLQFIRCMLLLVYEDDEAHVSAAVGSVGPSCGFDNGLDGRRSVVGGEEGTLTVRN
PELILPLLRLFLDLPEFKEDHRDALQCLVTRLSSDVMVSQSSLWMVANAGVFDSLTALIPVVAGTALIDSV
LSLVMAIAGHHVTVRETKQFLMSIAHAKSEEERRFLVPIVIEVLSAASRHILNCQATQQNYIAFRQHNGPT
GLKAILSDFPLDAYTLCLWIRLETHGGRRTRQCIYSLQSIEKRTILELVVKSNGQLAVSYDVEHNKATEVD
LGCQLAALVWTHLTVVHSQSKFPFSASEFVVFVNGVEVCAQPSVRYPQLQGLFFHVGTRGEDIEQRTSSNN
FVGQVAAVHFFTCALSPKSVFELFLEKSGGESVRRGFFSGIAVYVDPRFGERGQLHNLAALLRGKPTERPL
ITYEGTVACNTKSIMDSVCVLGALQTVVIPLLVLLVNPQLPFYCRVPPGRRRPASEATRKAMNELLKFVEA
LLLSNIVRADVLEVGLFPMISHAVQQFTVYDCPELPRRLYDICVALVSDEALFDAAYHSLFLSGDLLHVCS
EPTQLLLLQVQYLLCHNNAPARRHLRGLDLPHFVVGQIIQTYNGTSEHHREMREGFFLLMEAVMEGGLTMN
DADAIQRLVSVMLRKQDLHRDVLIEILVRTRVLVANREPNLASFLGKRNFVKDLLPALNNSPREVRNEVVL
FFILLASRSRKTQELLNPTLLASREAVHVTRDISLSWLRDKLNDCAVDVSLYTTLLAALIGRFDVSLQQDV
TITADDKICFAPVLTPLLLLMKRTPNKVMKEKVMTDIATLVQQDSVAWRSILSVQGWYASIAELYLSDAES
LEARRGEQSLFMATTAFIFSRTIFQALIQESYGASELELLITYLFRRRANVFLNAVLLGVVKEYTSYLTNR
RDDNTGGCLSLGSQLALANFTAFLSVVEDVLFYSTTAYINCDATRPADAHRKRGYSEEEELVVFTDEGMLK
LEFCEGYPEGSEYRRDEMLQNGVSFLSGDSMLLRTAPDGVWLHAALAVRTMQLLTTSGAILNSTGTWNNSF
SSSSGNGGSAPGSDAVPQGLRPRKGGFIRLFVRLFRVVCGFTLRDAAQLDDILSLALRWVDVVDNDYSPFA
LLMWQWSEVKEHSPLSSSMTLILSLHELLNRRLRFSLLGPSLRFPDANQEILRRMKAICILHKRDLNQMQF
FSRTVPSSVGEQTVKQCTLEWLCGRSGEDSMKEFVEVASREDYEAFISQCTLAMERDQLTDKSMARTIEQY
HSVTMARLHGILTDFSISRRTMLEVLEQHLQDGTEGEGDDAAGGVESSAERFATLAVREVATVFFNTVWAR
FLSRCRGTIWDVGPGEQRNMKYVRLQDVEQKLLIRRKFEFDPCGTDHANITAAGAPVSDTKVFGKQLPRAR
GGEQMLLTSDSSVDCDEILDKQVSHEDTNSITGTNTAQTPKPIVHFSSSCEVPSMMHCWSAMLIIRDCELC
VFFDEENKAYNQRVADEASSLVVKPRDIIYPTGHIAQLAPGRRFRMRRSAVEMWFRDGRSVLLNFASVKEM
RTAVNRIRMSVERHKVPYHPFYLFHETPRKEPLLMCRTNQWRERKISNFEYLLWLNFFGGRTVNDLTQYPV
FPWVIADYKSDRLDLEKSSTFRDLSLPIGICNGPQSREYVETRYEEMRQTGDVPAHYFTHYSSPAVVLYYM
IRVEPFTTLQVILQDGHFDCADRMFHSLASCWNGVLTNSQDVRELIPELFYLPEMCVNTNGIRFGCRQDGR
PTDSLELPPWAHGDPYEFVYRMREALESDYVSLNLHHWIDLIFGYKQRGKEAIAALNVFNWHSYEDLDRSQ
TDDVDERLLIDSLDNIGQTPIQLFTRPHVERRAMEYADPISCVLGMRAVDIRHLCTRVARVVVLDNDRVLV
VCGNGAALLYRISVSPVMRRMQQLPSPAVTPTLTTPRVGPVADGNVKSHFSNQTPAPSVAAAVAHTSSYLL
GIVSGAPVAPVPQGSVDGSGFRRPIVDVAEDFERRIPPLPPGMIPNIGQKAGGPCETGNVAVLLLDNEVFV
ALGGVFDNTIVIRNFLTTPAFQEERLHAHCGRVVLVAASADSRYLVSGAEDTTFIVWSCHFQRNRLKLRVD
LMFTVYGHEDDPTAVSVCPILDVVATASRDGMLMLHSLANNRLERSLRHPAKLPIDRVLIQPNCYLPNILF
TSTIDNVIHQISINGVLLRSVSAPGRITGWCTTPKQTVLVSTAPLTNTSASEDSRGNLHFMHAFHLNVLKS
VQCPLPATGDTISSCACHPSNPQAVVCGSTHGYLSLLCLGSKVDAPP
>tr|C9ZJ33|C9ZJ33_TRYB9 | DeepTMHMM Topology Prediction - Predicted
Type: TM

MVRSLAKEHRFAVNYAQFTPLRRGWRLPAPVAVGLVPFVVLKIVCVSGGRLRCFLICSRCGATGPFGCSIC
DISSSPRSASLFDGYSVACALLSVGIGALPCHGGWPPSLHML
>tr|C9ZVC2|C9ZVC2_TRYB9 | DeepTMHMM Topology Prediction - Predicted
Type: TM
MWRFGASSTGLKAGSPLVGSASRRCGSTATPSRRRSGFFKMGDMYKPGRYTSKDLPKGPSGSNQPWDPVYA
NYAFQLVTGPLKRKQNTTPDDHLTVDELDKWMDAKGAAKVLGIKEEELPTLTAPAVEEYWAKAYGQRNNAH
QQETVIAAEVLLEYIDSTVHRKKSRQYYRRYMDNARQAIDHETEMRRSEHRQKFIHLFGVAMFTGSAIVVI
IAFFRGYFTRQDVEKIGTDAAAYLNMVFSQPPNLEPPPDYSTRYRDTPPAMELAKQQGVYGVVDPARLSNL
SKDVKQYRADEEMEMLRMLNDENEKARQRRKEDQVRESRVVVYRAGDFDDAGELKDLAKEKEAVKPLSAFS
QQSFRDFAALMASQFGGGSRFQRITQDSVRRAEEMERMQKRMNEIEE
>tr|C9ZLQ7|C9ZLQ7_TRYB9 | DeepTMHMM Topology Prediction - Predicted
Type: TM
MAYVSNAVKQQLRASRSSYTAEAVHAAYKMLQEFEESSEGDPSLERNVSSELYILCTEAAIEKKEWALAEA
CLKKYTGRRAQTKALEARALYCDALVALSGVPHSLRGRAKLAAHIRCATKIVSGIKIVLEEWPKEPETLIA
GIDYLWLSIQDVCGRIISNSMLDILVLVVTLHEKLSLGGIHTQLQWIVRYAVCLRATGRVQDALNQIVSGA
ELVAKTGSERLQLQFYRIQVAFTASTSSNRTKQDNTRPVFQALAAVYSFFCGATDDSSAKQELIQATEKLL
GDPEKKEVKQKGRKAAEAEAAFRDPAIISDVLSEVALALAMCGADDTCNTILPKLLNGDNARARLFTEYAK
AIMRARDCGSLAASKALDAGYLTKNMAEKLFACIGHVEDVIDAARTSVDVSESMYAIEVGCVVLWNLCLPL
LQPQTRGRLRLTLSRIVMLLRLCNSRLNRLFVRAAYESALAEFEEENMNATIEQIDKALAVEQLSGSADNT
SAYPMEFALMWLRRRAVVRKAHEGTSFISDGDHIVHLIEQARLASANRVPLLQAAADRLPPITRTESVAET
RTKNVSVNGEKNKGGKSRKQDVVRETVDERVPFTFEDFSTPNVYYLLLKEASTTNDPAVDEIIIKAAEGLA
RIAVPDNGFARDLLAMKAEAHLVMSELLEKRGQTSPRALGDATHEDGMSHISKAAQLGARLSAAGYGGGWI
VLNACTALINQNTEMFKKGLFLPAAATLRDLSLALGQVNADPMGEYKLFDSIGFGYVMSLVQTYISAKGEV
NGELHCESLAETMRKTYLYPICEAHNSVLKQAAEACHQIMSKWSLPSRRKNFSLILPCINRLLDRTHETST
HPQEQLLFLLGRLNGPIELDERRHLVNTECLDMLRADPSVELCARLASHSVQIPQNERVTLEICRTADRLY
HEGKLGWGTKSLITVNSGRDKSLASTVVSTQQPSPSVMYLGNLKGSVVANSNGGMQPKPSDMDWYWYADIL
LHEATLFLHLGVGADRTHRLEFEKRVLVAITNSAVAASRSIPEAQITQITKAYRALCRVATSISRASYRVL
QSGLRILLSPPILRIVNKFLRSRTNGRCDAEFDDLCTLTSTLGGYLFTSLLEDENYDEGIKQMHELLRVLP
SRYHGSLCAYEAQLRTKLRLSTSELHEKARTVDAEVEAAVCVAVAKNAHDISEGMRAWNQALKILEGKPVQ
RADLVLSMAEWLAGRNAVTKSELISLLMNGLSDLERVDSMLLSAWLNKSAGRRNTDVSPALRHALTGTMVG
RTFAIAQSLTCTNVVPVDKQKDLPSLLETLTALRLLYFLFVVAPITPCASRPFVYKRCCASTLLYYVLSCW
KVVAVLLQIHRGEDIRGVKVDPLHLPETLYEWRGFHANPQHAIIIRDLCKDGLALNTHRLWGMLLDVCDYL
LEDGMELYVFLILSWVEFCIYWRYDESDPRGRAAVRAVRLKGFISAARCGWAEVCDAYNVEPPDAECWSSV
NEVLLSTETPALSVPWPSSGPLDFSNFVGSTHRFILSEAEDLFTLGRVQEALLWKAKVKQCASRTGDRGAL
ARCHLLEARASILRGRWDPPTSEEASAEDSTEEDTKTEDMGDGEGTGLPTSLTVAAWTELQLVHIDGFLSC
DKVAEAVAFVRQLLTRLLWRLEEVGEGDIEKDTTEMCHKVLLQRCAPRFVAFLRRPFDTLFPGGPREGDVK
GLLDEVCSQLDQYADWKSRLVSLRVRQILRPCVDDVLATGKGDVDKALPERLFNEYSEILKVADMLQSEVN
AVVPVLALGGDVTTCPSAAAAYDEVLVMCARNCLERKLLKNFISQRFSALSVADLGIPTGGPVELEGYVAS
YMRDPALYEQSRMLNDSANAALPSQEGICGHDNEVASSLSHFGIPLNYMVNTSTPHIEALLYRAMGGVGTV
SSVQMEASIIAALSEFRRRLTAATRAMECPEASVNTTFSGDVNERENAFLTAQWSKITISVPHPSVPRRMS
VAERRGAKLASIHVEDPNRLAANTWIEINSNLQMLLLAAVRQARHMFEYKQMAKCYIYLAHNFMLSGNFNA
AGTAIEYAETAAMYGFVSDLVTSLTHTTPEAELIRLVNKTKMDAPHLVNTKAFDSMRNNLMEISSMWRRFD
LVGAWPAEPNKADPLLSDICTLSAVREGTTSYFLVAFRRQDGEVCSRRAELDMGALRECTSQIEELKRLKK
VEVAAGPSAGERSPDAKWGSVFHSYLEKVRTVVFPLLGDFSDLFSSQRAASCSLYLCLDPILQPLPFEQLP
ELSSFMAVHRELSVLNLRQKTALRTGKPQSNGVAYIIDPFGENEEALQYIFGGGRTKTSNAEIVTFVKDGQ
GSSSRPSLAYVHRIFANKAYANVLVSACGSFSDIIKPSVIAELSLDHLQTVLIAMATNDASYCREQGCDTC
RAYMDFFYEKNYVVSILLLARGVHHVLSNIFTLTASECNVFSKRCLSFVGGGGGKALAEAVKGSNGDAAGP
STQLHISYGVIHHNVKAK
>tr|D0A1C5|D0A1C5_TRYB9 | DeepTMHMM Topology Prediction - Predicted
Type: TM
MGMRKKETIKKAHKPGVAKGLSYRRPWATFVPTLICFLLLNYLAFGTTVNEEGTDLVVPSGLGDNNTSSLS
KLQLLFEDRLMRSLFRVGLFMFREMKVIQLVAVLAFVIHCGEAGLAAGICIRCKADRRTFGLYTVLTLLGG
ATQLGPLFEAEKDYLKDTTNITTKDDVSKKA
>tr|C9ZVJ8|C9ZVJ8_TRYB9 | DeepTMHMM Topology Prediction - Predicted
Type: TM
FVESIGEVKYFRDSGVFSHDVVRNGSLTGVALYGNHLYLTEQNTNTVWTCEVGSDGDPIACHSHVALSAKC
SIYGPIGIAATQQGIFVVARGPAKQGTICWFDLQGHKIAEVDGEYVDITSTRSGDLLAATQNELHRVSTDG
NKLTTKRFAGGSTNSCLPNTEGDDTLLCEITRLLVVTEYEMYVTSEKKSVLRSVTLPPVYVQGVFPGRPLP

VGYPDKDIMEWIVGNLTEDINTALGTTESIVASSSVHVDSTTWLTNFTAGVQQPDFDDEKTEQALHKSNYE
HTKEAADEYYNLTDEQVYMDSTMVPYCNRLSLDALRRKLAKEAGEVLNFTLIYADMPLKAESSDAGNITTV
KLLMPASFNNTVTHDLLSDANLTETAHSFIKYLRSSDTHVDVTFSNPPFNFSSLTPDEEQEVRWYIHDEVM
NQIKKCEERSTGRSMARREEVGDYSRTTIATALDSNVTGVCQSTITNRTVSLFYQPPYVEMSLYEVFIPGN
YTFDVSECVGEIDWQDLNDHLNNDTVRPTTEKAPKCGRVCLIIIAVVCALIVAVLIVLAVVFTSKRRRLAA
VVAPARPKFVSTLDEDEQDYASAYGNKERVEQ
>tr|C9ZLJ9|C9ZLJ9_TRYB9 | DeepTMHMM Topology Prediction - Predicted
Type: TM
MPSPSETIQLSIALAYLMASAVTIVYMGSQRLLRETIKMKKRSGNQSEVMKTGDAMMMPLMGSVVLFSVYV
VLRFVPREYFNSIVSFYLSIFGVFSLGSFVKTYTRPNVLTGCFCCVAGGLYYITGNWVVNNILATGIAVSA
ISSIHLGSFKSSFVLLLGLFFYDIFWVFGSDVMLMVASGVDGPIKMVFPRDIFGGCKSMSLLGLGDLIIPG
FFIGQTLVFSSQYVKKGSLYFNVALTAYGLSLVNTMAVMVIFDHGQPALLFIVPWLLVSFSITAVIQGDYK
AAWEYTSDAVTEPDNSSTDKVKSEEGDQRGTGDEMGLGDFLVKQMKGLFVWDGEEEEEEVCEGTKKND
>tr|C9ZTA6|C9ZTA6_TRYB9 | DeepTMHMM Topology Prediction - Predicted
Type: TM
MYLCRAYPSPSSYHVLLWTVCVTADSVPYVINLFEFTDVFPCFHCLFFSFFRKFKVELETCRGLAFPLEKE
RMCSASGLGVCVVAKSRVIHAQILLCRYFSSWKMG
>tr|D0A7J2|D0A7J2_TRYB9 | DeepTMHMM Topology Prediction - Predicted
Type: TM
MVSRSGLRFSGFDVKALYPHGVPKRTFPYREAPKQISTAPTAGGFYLTKHALGWPFQIPFEWLFYRAPIFT
FVACVVYDLFFGIPLPLMKEVPPGTPKHFFYNNSGGTPHHFWQHQEGWKVPNMSGARRWVD
>tr|C9ZMA7|C9ZMA7_TRYB9 | DeepTMHMM Topology Prediction - Predicted
Type: TM
MIRRTAPAVSFTTSHRALMLRTNRPLLSADMHSLERFKVAWDEMPVHLIGASRKQSFEWYWKCMYQLGIRS
TYRMTKSRVVMNWCAVFVFMYLTYISVCFSSFYHIYYQDWPEEFKRENARAYAQSKGSDVWAADGKFIKPY
FHINPPMLTMTTEDL
>tr|D0A362|D0A362_TRYB9 | DeepTMHMM Topology Prediction - Predicted
Type: TM
MRFTEPLFSRVIPHGGTLSNTFTFASATLGGSIVALPWAFHAVGIVMGTVYLFLMTLVTAYTVTIIGFVMK
KSRFSGFEQMSLVVLGRGAAYLMSVVMGASCLGAAVAYVIAVRTLLEPLLQQSPLTSNFFGTTAGVRLTTF
FVWLLGMLPLVLPKQINSLRYFSAIGVVCVVYFSVAIIAHATMAGVPKRGDVSMFQGGNIAVEGFGIFIFP
YLCHCVAFQTYYEMRIPSVRKLFISTTIAMIFCSVLYWFAGVFAYLEFGPEVKDSVLYMYDPISDPMMFVA
YVGLVAKLCVSYAMNMLPLRNTVYFLLQWEIQQLPYWKHTAVVTVMSILVLICGIFFPKISTVFGFVGSVC
GGLIGFIYPALFYMYSGDWSLATVGWFHYIMTYCVLMLGVASVVFGTAATIYAIIN
>tr|C9ZJX2|C9ZJX2_TRYB9 | DeepTMHMM Topology Prediction - Predicted
Type: TM
MWWGLLGSMPARVQTPSEFDNALVGAQGVRGLVACDASPEKHKARPAFGVVGGKVTCPCRRSAPFTLARSV
RSTLGGGGLCVGTGALLVLGCVPPCGMLPNLSRPSSNAMHFARYLRAIAPICQ
>tr|D0A0F5|D0A0F5_TRYB9 | DeepTMHMM Topology Prediction - Predicted
Type: TM
MRKMKRRRENTRVSFAVVTYASHTLPPYSLSPSICLNCTAVCLLLFFSHLSERHGYCYDILIFLSPYCFLF
SFFFFYFLLLCIKVYVYITVKRESMRCYHCSSFTSLIRKTP
>tr|C9ZT36|C9ZT36_TRYB9 | DeepTMHMM Topology Prediction - Predicted
Type: TM
MPQIFSDESVEFLERFQYITRRKEGAVPTDSFSLLDICLHTNSVFVQLLITRLLPVTRETEARSVVNEELN
TSANATISGMGGVQSVEDGRGRNPTNGIVHGTTTLVADDLDVFNTRLSEDVLAPVLFSFLDFYVDRRLERR
AIRQGLCAYFESTPYLREAEPSDAGLSMYSTVAFLFLSLTRALLQCEFAKDTFAETMGYKRNCSLPTLPWL
KSRSEYTIGEPTWYWHGTQRLQTIQKIVAQAEINEDVDETVQESGETRGRTSSDGYAINIVYDDFQTMETR
KRKRDASNIPSGHSRVHYKSVGISTLQRSFSTLPYELQEYVLNYLHPADLPVARTVCKAWQALISHSARMQ
FYILAGRAVARIFFAYMKEKWGRAWVRVDKLTTAHRLFHQERQLKGYLSHLSHVLGRGAIASVMRSLLLKT
DKYRIEWDMLRGIWSAIEMEVKQFPFVAHPAVSLMQCMQRDAGNLVPLFALIYEGMEREVIILQLARNCVL
YDFIVLEGSELILGIIITQMMYFIKGERKIKHNRRDV
>tr|C9ZXP3|C9ZXP3_TRYB9 | DeepTMHMM Topology Prediction - Predicted
Type: TM
MTVSEFGRSRVLYRRNGVSDVIEVGNTTELNDVIALLRSFSGVPKSYTTLVTPSLAELCEDAVGVERLWTS
SAERTEPKDFAWLEVETESDEVMRQVLSAFPIHSKTSELVRSSENRVEVVEIFPSCGYVWVSIAAKGAASE
SSVPEDDEPVVVSLIAYEQFLITMHRKPLSGFEDMKAHMEMLVKSPASYGAPVPTAVCSLISGFVKEYQKE
LLSLLVDVDNVNELVLEIQPSECDQLDLLRRIDDLRHSLSRVQASYFAKERVLQRLLLPVVKRTFISSAVG

VAARYQRMLSGLILSIERLRKGRDVLNMSSMGLVSGVSMRLLQRCYWMDYLNNVMTMMTLVSMPISIIPGL
FTMNVRVPFEDSESFVPFYVIVAVTAGIFFLGMSYPVYLYLTFKSPGALVPTSH
>tr|C9ZPZ2|C9ZPZ2_TRYB9 | DeepTMHMM Topology Prediction - Predicted
Type: TM
MWFSPFTSMFFSWYTTNAIFLSRRKFRSSCGNNRLRRVKRFEFRVFSTSGTFFTGASESFVCRWWRIFAVG
RYPCSFYQHLAVCEQHRIDNKPMTSIKADYSQQPSSITRCSDEPLLDC
>tr|C9ZYY8|C9ZYY8_TRYB9 | DeepTMHMM Topology Prediction - Predicted
Type: TM
MCVCVCVGDGGTCAGGNYTTRETSHDFTIGGNVGVADCDAFVCLFVRLSVLILPLTLELCLIRCFFLFIRY
VLLYLVMGPSNSQKTRQKQQQQGKKKWNVKKEEIKLNESHDEGMHVKGEGKMKLYLYKRYHL
>tr|C9ZMB7|C9ZMB7_TRYB9 | DeepTMHMM Topology Prediction - Predicted
Type: TM
MTSSWGPQQHEALVQFLRNVTSSDMNVQRDNILQLQRFAFSGGAAIFLLEVLCSGNESYEASIRHSAGLIL
KQIVQINPTIIDCLNADIVGPPLLRALADTIPSVNVAASLVVASIVRGEGLLQRWPQLLETLKNESSLTFL
RCQQATDMEGFFTVSRTVQGLATCTRIVCEECNEELRELIEADNSACECLGELFSYMLQAVAFMLRSGGDV
ASAMQLVLSAATSCFVEKVFFLSDNPDESFDDSTPEASLDVSTAPDHTRHYAKQLHEQVCALVAELCTAPL
NQHVLVAATEYLSEALRWDTESSASTINATVLFLPHVLYRIRSHESEEESIVIRCLTLLGDIAETHATVLS
GIAREVAETLFYNAVHLTDIFPNTSEEETTSAPDDEAAVRPVVLNRRNVEAGDAEDHSDENKSDMNSCGAT
DRSDEQESDAECATTKKQAFTHILDVMSCTCPGDLLAHLIPLITSALTREANSVESLKEQQAALFILAEVV
DELFDDLHDDFLGHVSANCWGALGPNSTAPHHLRYQAVRCVARMSTGWAASARRPAHLQGLISPPPVLHLL
IGVTASEVSKQVQLEAINSITKVTLYSLDDCRIGGETRAVASALSTVIRGLVSALPSMQYAARTATYRCLS
EVLSVASGNEQRQQEPLIDDITTMHTITALGKQGQQLASLLHHGENKKKSGTNAVELISLMNAMVEVVSAA
SKGTLENVVSPLTEFALVVLNCSADTEGNSSSILEVYGSDITTLVFDMLDGCCSALMNEEELLCMIDSSLR
TAVLLKNVMVGYGTGAANNVVSVCAQILEERQRRQQFGEVELARSCVAFLSTAMHFHRDPHMVATICRLCL
TEVACEMPAVKAISNVFLCLGLIFYNLLFDHNADPVPILHVVVSSQSLDNLTETLRSYLTSSTAQDRGYVK
MNAFICAVALAALHQVGRLHLDVAEPLLFAVIHASPSFIPRVKNHIGEAYRLLLMLSLLLAADQSNWAPVV
PTIVKAIDSFTSTAPTVLSAEVRRRYLPLVGANGQ
>tr|C9ZX32|C9ZX32_TRYB9 | DeepTMHMM Topology Prediction - Predicted
Type: TM
MITAQQTGELPEQTQGRFVCHRLTSGEVIDFPIADMMSIPLDRLCQLAWPLLQFGLDASLRARPLKVPCVS
QSDNDDMQLARVIYDTVAEIVRTVRPEFCWDQVTCHWLRASLFQKALQSIRERAAVVPQEQCLENPTLPSS
QLEVQSSSVCNERRAPGSGEAMNQKTQRRLSESTFICEDVPLGEAMVSPERRGFAPLSWVVPYLKGRQDER
WLQGRIREMVDVVASPQNDGQKQLVSTAASLSGQALHHKRSAVAFLGILREVLDRWNESSKTPGENDIFGF
NVVLPILHITRFTKLDEADTAAVAHILERVRGPPRPCGSNRGSLSTATFVTKYVLSLEVGPRNESTSVSPS
NTQLGEGRLPVGLDEPSTIRTSAAETELPKVQAGSLELNPPASELAKTTEVFLVAPLARKVTVHVRKALNC
DDVQIALIPHASLGGGSTGDDAGETGRSTNVVWFEPVEEATHELIGGCSCVLRYRRGYEGARVPMLEAEVR
LEMCVEVNGRWAEEFRAALSSAQLEIATHLLQQIKCPPVATNLREESIMLFSNGLQPRMETRELPPLLKEI
AKMDDGALRRRVSCRQSDVRGVRAALCMIGSIIAEREPDQVTWGSWVWQDVVKRVSLASSVIGGNPDATDV
AAFASEHASSCKLSTTQIVEGTVALLSSTATVAQLRDYLSTQSERARTCIRALLLLLRAEESFVPQDMFRV
VSLLRDLMMYESDHYLMRFSGCGEVLTEQIRVLVHRLFTAVFAALQHVMSMPEGEQRRLGPHRFLWDTDKF
GPFALISLSLLATPLDERDLDFIWHKIISQVEGLLPSFTDFARTASFKVDEVEENSVIESVRALIGDCDAH
RHGGQTGDRVQCDGGNDGGTFVGLTMVGASERGLAVWVPSGREPACSTSTPLYFPSIQLSSLSSTTVSISS
TLPSSVLSSPRQPPLVSSSNAASSMLYAADINLTAMPSKELIVALASGQHPGVTSNIVEGDPLGFYFEPHS
GKLRHANRVFSLPPLSRGDRVTFCFPFSPKTSTRAVCLLVNGVCFASFPAPPRALYLLVGVTDSLSLEGKA
VRISFRSDELSVLGSIDATVAASLVGKGDASICSDCLPTRHFISMFATLVFAYLISLCTRRFAQAHRSVSG
VSSPPTSSSLQVKNRTASGRRHIHSEEAWRGFIDGCCCRLRQNVESLIDSIKHLSLLDDVNDATERVKRSA
ASFVYHRFLMDYITIMRTAIYCSSHPADILTTLTKVVCCEEAGERARCAALTTIHVVLQEPGRNFDITTYN
PIPLWDCCCQLSRQVTAPVYKPFFTTTSAATDLRVVSGGRHVKSLTAEECSSRGVQNTTSFASQGIPLDGS
LGDVVSFSVRVRRGFEFDSLGRLYYVGVACHDSLPTSTELEQHPGTRQDMKHVYAITDYFADIEAPSARVE
LFRHSKHWMLKKDGIIFGSGDIITVTVDTKARCISFGRNELPLGTLYTNIPSAVKVVFPFVEMYNKDATSS
WMYMPCETGIRARLVMRAMLSCWDSVLVPLLSQMLRQDEIVALQVLGADGDEMSFTYTGPPRGEIKGSFDV
RLVRQKGSLAEIVLGEGGSGSITVPAVAIEPNYESVVSGDLPLGLLANEIMGVLSRSISFTTDEHNNEIVH
IHSTKTFICALRLLSELDMGEVTLWDKETQRLLLSQFIRILAMPDTVHPNGSQILLEAWNELMLLPDNDKL
CMTISNKKDIDDNVYSECGEGGSDDGNARADEHAGEAEKIVYLRCPVCDEEWGHCAGEGHAAPYSLCVTLH
HVMQRLGLPSPFVGFACEWLLVEEGVCVTLRVTAGNEVEGEGADTHGPFSFSGKYVSSHHLRGRCVYKCME
RPNNVNRAEEEWTCAVCTFINLSDSTRCAMCTTARPGATWTCMLCSYAFNSNHNKVCTTCGNMRLGLGSTD
ASTVGDNKQAFCTQCGKMREYISFIQFESRYLCEQCQKETLWLPEEKHVGVAEAKLVGAGDRMSWFVSFGC
EKQSYTDMKSTAYSFRKMLDAVLAVSPQAGDLTRYVPPLMHSSDDDGEAESGCLSLKVNPIQEQKQDGRGY

RAVIPAIIYFCSRIVCSWGPNLAPKGLAKFSVLCRLRVLEDTWLTQLRKVTTDAAQSIFTSCLQILLSGKR
SEQMVWSLSSIARVLMNCNTPFQKQRHYLLYALSLNAVRGGGDRTFREVCYAALNLLLEEASGQKLNVQCL
REVITMVPVVANQRQLMVHASLRGVNASISADVTLTSWLIEVGERMERSQRLPALFDEPSPDTFPYVVELP
DTRMGSGGELIVGQVRGSVGVLGNKGGRYYYEVVLPPNFDERGKTIVMGWGTIQHEVVSSGQHVGSDIHSW
GFNCQDRLRIMTGEQALVTPRPIVGGDIIGTLLDLDTMMMCWSVNGEELTWIAVSTQGKGEAIYPYVSAAM
DPHGVLVRLSYTQFKPEGYKDFSPGCGEEGGRLESSVKPQCFDFYVQLCDLVNDVVNCGFTVDSLAETDTW
MEGGREALRNYPLLSSEVGGGSLYKLKPYLQHLRSINSLAVSVAKSHNIFRNSQFLMRNYEKVRRLLFFAA
RWAIVERQIDRRLLRNNVKRHRHVLIDLKEAKGVLEHGETLDFITLFDKSVTGQLFHQTHDADIYRDAVMF
TTRLSGEVADDAGGVTRSVVSMMCDELQYREDEGGRRMEPLLPFFKLSSHSTMVTLVPNIDFYRSNPNHRQ
LFLQFFTWFGKLIGNITLSGYVMLSITLPRLVWMFLTFDEATVKDYYADIDDSVRGALEDDDFLLNDEFYY
SIPVIEWGLVCAASDTVGALRSKRSLAPAFPKGCFSEGAVVTSQGLCKSIEVERRRTEVGRALVHQYDELL
SAMRLGVTSVVPSHSLQLIRWDDLQQRVCGSPCATAEDVMSSLDVSLLTKAVCDMLVEVVRGMSNRQRAMF
LLFCSGQRRVPLPEKVQVSCGDDPAAVPTAHTCSPISLLLQPYSSAATMREKLEVSLHHMYEFGFV
>tr|D0A5D6|D0A5D6_TRYB9 | DeepTMHMM Topology Prediction - Predicted
Type: TM
MRSTVFGGQRLEKQSDDLSAEKRAQFEEDLVKENEALLNALGNTAAKVKTSADTLRQEVEEQNQMLSFVGS
AFSRASSGVNRSVGRIGEVVQRYGFRHTLLIGVVVFFVLIFFWCVLLR
>tr|D0A9P3|D0A9P3_TRYB9 | DeepTMHMM Topology Prediction - Predicted
Type: TM
MTHQTHAQGANSPDNAADFCGADDITSPASSNHHGAEHAKRTPVNREHNRAARYRDCSPNYNFEWGSMTEE
MTMVGGTEFTERRGEIVVQDDEESIAFRFTNTRKDVLKFRACISYKTHRSTKNAVLPSQVGPFNDEILSVI
FLYVSLDMREILNAGQVCRYWRFFANLAPHWTYYNRLDLGRRLMQLPRYLQKMVGRPRIVTRDEYFRERKK
AAEFDERATEKSSTQHVRWCIAIALLSAAACAGNFFFSYYVGVRFAERYSDAVLGSIAFLILLGLVIVQVV
FIIIPLGTGSTPGEKQNSMRLLAWAAFLLLTGSVLGTITTLTTTRVESTRQLLGAPVVELVPGKGCELVDL
SREPAFAILPAPLSDVRWRPITTDKEEKTFLPHCASPGAGEKEVCFVLLYFDELYNSSVFSNHTQLVISKH
IGTRYALSFDPFDANSSSRWCETGKRPQTIAVTMPVYVRLVEERNRNYFDDSYLDPLRRPGRISSISYQCS
VKYPRIVTEDPPGASEIWYKSQVPWRRHHIPLVTDVENTPLRYLEEHNHFLHYSYACYIAAALLWLFLFVL
QCFLRNHALKVLGLATTCTLLVMNPVALLVSGVLCITLSDYYFICNETTGGAMIGTGISLTAFALAAYVCF
W
>tr|C9ZIJ1|C9ZIJ1_TRYB9 | DeepTMHMM Topology Prediction - Predicted
Type: TM
MYMYSFFEVQVESRAAAVQKMCWVFVLFGFIVVVVVVSVVFLFCFVFIFVYSFLSSLFASFIHFFFDCRAL
VINLSNKYQQKKTHLYLYTFLFIFLHVYYVCAVRCETFSFFFFSFYPLRSLPFTVCKKKKKKYHDKIHSCL
HIFTNICLFVCLFVCLLEVVINM
>tr|C9ZU74|C9ZU74_TRYB9 | DeepTMHMM Topology Prediction - Predicted
Type: TM
MISFTSNCYYYYYFAPTAMSVICPSLVSFRFPLLLLLSIHTHTHMHTFILFNPKVSFKVSRVGMWSTWMTL
LSLSTHFVHSFIPMSFFYLFFWLIPSLRMCATCTCSRYAGWCSFIDTVKCY
>tr|D0A664|D0A664_TRYB9 | DeepTMHMM Topology Prediction - Predicted
Type: TM
MTSFKDNISSERLLRGAQLASWVVVGGYGVALAYRFGFLQRIFPKSIAASLECAVEKVKSWTEPVGDSVRY
AVVRRHLTQVYSIASVGMLAGALGGALFFSFPKVPIAIPVSLTVAPAALLLLLPREVMFPACRVACFLTSS
FAVGCSFAPIGWVAWDSLSILMMLTASTMSGLCIPLFLTRGMASYVLSSQLLSCAATIIALTTTPPLLTST
GLNNGGATEVVRVLQRTDVNVLLVMQLMMNWGINLLHTLPTIARFVKWRGLEDELLLTVDPVKEALCICSG
GAYVFWRCSQWACRRLVEGTLGAGVGGEAERRGHHATSLYDQLKSTMRSRRVVDAGATVMLVLCYVRAVST
LQKGETVKALETLRVCCARVSPINLVLTRV
>tr|C9ZXB8|C9ZXB8_TRYB9 | DeepTMHMM Topology Prediction - Predicted
Type: TM
MIRTVSDDSYIISYVKCQFFCSSICICIYRADVAYYVLICCIWINQAGVTASMCLFSNMFLLILIVRECGD
TMLLSCCCVIMLRVTVPIPSLPVYK
>tr|D0A978|D0A978_TRYB9 | DeepTMHMM Topology Prediction - Predicted
Type: TM
MVQFTVLYTMLMCLLKRCVQVYYVMSLLSCRIIFLPIYLFCPLSNSEGIKQTKMASCRTGRLPMPDDPLFNP
IHMANFRSTARIAGVGLLGVIWIMYQKLHHSHHYITYKGPENPFARIRHRRFPGGTFAFGWGNNGLNRDCG
LKEWECWAGYTGKEYTY
>tr|D0A4C5|D0A4C5_TRYB9 | DeepTMHMM Topology Prediction - Predicted
Type: TM

MRVLTHHRHAVNRGSATLGGLTMVNTAYSFLFAFPVAVVSLVLRWHKKLTLNQRALCVTCLVVWTLVDVLR
LHLGYSGNRKQYVPSLIGFFVMTLLPQLPLAIAYNLAWPHLSSLDYATSTVMIMLLVAEIVFSLRVIVRLI
RNNRIDYYVYGPYHVRDLY
>tr|D0A164|D0A164_TRYB9 | DeepTMHMM Topology Prediction - Predicted
Type: TM
MYHYTIHLFSLLFLSYTFFFLFRLYSASHLLHCVPSSLSLFVFSFLCSVVSFIFPHNEECLLLLLFPFFVP
SYYSRSHSATSHIIFFCPQPHATIVCNELKNKRNGMKTGEKTFKKKKEKRRKDKIREEKREGKKKEWEGKR
EGNVDKKTGWVWSE
>tr|D0AA93|D0AA93_TRYB9 | DeepTMHMM Topology Prediction - Predicted
Type: TM
MSDGFSILAGGSSRPLYPATRSHSAACGVTSLMVVVSLCTTTLFFFLTKKKRKINASGGECQWCCKYSKMQ
ATKQFSAGFTCHGNPAFSLFSSISSILCARVASYLDYIRDTHH
>tr|D0A6S4|D0A6S4_TRYB9 | DeepTMHMM Topology Prediction - Predicted
Type: TM
MTGLLILWDTGYVTALTFSLFTHSHFVVHGERWGWTTATADDMEMWDGVVVGFEGSASDREAVSEGRGDVT
CLAISTCVCTFLCLIFFYFLVICITLLLIIQHSLLPPRLKLLFY
>tr|D0A4C8|D0A4C8_TRYB9 | DeepTMHMM Topology Prediction - Predicted
Type: TM
MMANKRLCRHASSLLSPYTMVPTPIAATGSSCLSQRTSVPCSRAALLQAKCFSSERLARLFALELNRARAE
YRRTPTKAQQEACLRTIASVPLQQVRGLDGKTVAQCLTTLVTLDAPADMPIIHDCAVWISVHPDALSPHSL
AQSLYSLVCLEYSDSASVVCAVRQQLLSAMEDMAASSICMVLAAVLTSYATPARGQLAAGAGKGSEEGVSG
TLQLPMDMSQLAERALPFMDESLGLTEKAMVAVALGKYLSQTATATWTEGLIADRIMAQLSAVVWGCIPGA
ELSVLSMADIVGIVSVLHVVPLPIRDSVATAALQELKTRVCNQSLESSRSVVQVARVLSCLCNVNRECVYY
SASQDVLEKVVSALETYVASDAITLDAAAKLLEEFSKITGSSIATPPALTRYLVRAINGSKEKWDAQSVRT
VLSVFSKDSSLQADAQALATRRVELIKSKCTAGASVDASDVAALLVEGIVDPHAELLAETVTNNISRWSVS
QVMTFLQAAARVNGKPTNAIMRDVASKLVPCMEKATAAQVAAVMSSYGRARVRNDAFCAAVTSRAALLGPE
LTLQQISTILGGLAAVEYRDTKLFLDVAPTVIAQSATADATQTANLLAAYAKMLVWNFKVIWSLAERASVI
HEQFTLTQVFAVVASLNRMDVQHDVLVQALLRKAIRCASDDPNLPLPDVVMILSAFSRSGVWDATLFADLG
KRVVQQQQSLTPEELAETLMAFARVGLTQYNIFDELTLRALAVAPTCSLMALANVSVAYATIGCKHEELFS
IIADRFVNQKLDVPAVTIASVLSAFAAIGIRNDRLFIEAIPRVRHVGQYGTPKDITNVVYAYSQVGLWHYK
LFVRLADRAVQLRGEFRCDQLARLLEAYARVDMRYEKLFVEFSPRVQTVAHLLTAGEISTVVNAYAKVRVL
DTAVFKACVDRAQEVLNSFEQSEAALLVGALRKAKFDHEGLAVSLGKMFPGMMASLQQVEDEEREVGTTPA
GGNEGDETILVNSNDLGSSVVEAADGGASA
>tr|D0AAF9|D0AAF9_TRYB9 | DeepTMHMM Topology Prediction - Predicted
Type: TM
MSGEIRYASGASAQALFDKQQYTMGISRNMFSSHDKVKEKKCMIPPALMKGVTVAKLWDAIFSDKAEFLQR
YHGSRKETNLELSKWEYAPDMASGFRTLTFQCTVDLPRGPVDTTLNQAHRFAYTSSTSGGITLVYHVSSQT
PNVPMGTTFRTEALLEITAPSEDADITLAVYGGCKKMSMGFAAIQYMANPRAIKEMTRAYQQMLEMISKDL
TGDVLCVQTSDVSEGTCSAKETSVSEPSASEEVGQSSTLFQGLLLVLALVVAVSLLWHLAGLQNIGRMTSL
LSARVNDQTMNSAPHPSSLTDALNMGSSADTKTARPSLTQDKALHHAARDVHIQSLRQRWLEQRDRIVALE
VSLDKLWSFFFMQLLIIMFIVVKLFMSPS
>tr|C9ZJE0|C9ZJE0_TRYB9 | DeepTMHMM Topology Prediction - Predicted
Type: TM
MFASGRAFSLLSLFPLLFFVLVLITYTSIVPPLFTLSFSSCSSFTFVLCNCLLSFYRMIVVVMCSTFFSFF
FQNWYCMTPSSFCCCCCSFFFLFFRRNTCCVGHSTEEIQGKGTKT
>tr|D0A7R7|D0A7R7_TRYB9 | DeepTMHMM Topology Prediction - Predicted
Type: TM
MSTQLVREVIFSSVVWTAGDFLAQFLDVHIDAARRRAAGEPKSGHPSGKQMIMMVDQQRLGFAAMFGAIVA
PGMIHFRGILARVVGSAHGNTLAAFSILTAQQLFATPLMLLFYHNSATMVRGGFTDPSFLSAHETSMIARL
RGRYDAMAVERRIAIDILPQTLLASWCVFLPQVLHSYMRGRSLRSRYAACLHIPWLAYVSYVQSTMLL
>tr|C9ZM77|C9ZM77_TRYB9 | DeepTMHMM Topology Prediction - Predicted
Type: TM
MCFYVGIAVCAHVYVPPSIGVCYVTSLSCIFDYFISATASRDFFSTAWEARHIVNDVFTRPFNRLNPVLEK
IKRRDSCGIVKGCWSGATFFFCGELMQLIFPSSLTYSFSPSPYKQKKNIYIYAPAGDNRLS
>tr|D0A736|D0A736_TRYB9 | DeepTMHMM Topology Prediction - Predicted
Type: TM
MLRTKSTTAAGRQDKMLKHQRASARGGETGSGTRTPEGLSPSREVLRRSEPLATYRRYSDSIGEDGYSDSG
KIYCEQYQAAWTRVTTGCMSGIAFVLVVAVLYALYVLCSSYIYSVGCAVVLSIALHPNRCGGESRNWKGRY

IERMRSTREKWTGRSRVFGLLGSLLSLTHFFRYALGRGVAFSGVDKLLGRAGKRGRKFVKICDPSAGSNSG
KDSKLQQKPANVLPAGDANSLKWQEESTLSCILLVVLICFVAHLMFGILLFLGIHSILIALFIITVPFMTP
DRFMSVMWRVWLLAITVFFFVGFSYNVALDVISISDVVKQTTSVVVEAGEKWTGGVKEAIVQMSGGKVDGW
GEGSNRTNVAASFSNGTAASSSSFAEFVNMTRSKLNKMFLQELAYAMNHTNATELALTVQHAVSPLMSSLS
SDISVRSLLKNGGNIRGNFAQLKKLYEQVVWTDVAQKLMERWQELFVYANQLLMKLGSNVLNLFDSVYAVM
LLFVVMHHLLQLEHTVLYYILAKLLKVLDPQCGEYHARKIETEITNSFHTLLQSFWHKACFRFCITFCLFK
CWSFPTPLLFGVVSAVIALLPLAPKWISPVALTFMYKLFCAAYGDGFAAALADPCLWCCFLAFCATYMDER
LLCVSQGFLDDRSSVASAPGQWKLPTAVISTALVLGFFQYGVCGIFLGPMTVVLAKVLYDNWDTAME
>tr|D0A7B3|D0A7B3_TRYB9 | DeepTMHMM Topology Prediction - Predicted
Type: TM
MERGLRKRTTSLKRVRKPTIRRKNSSEDNQVVPWETRSIALLLCAAGCADIVAMAALSPLIVMFTQTLSIT
VFETIIAQLLYLVPQIFSTFVAERFAIRFGGIVLYSLALFATSVSAFLSTAALHCKSVPMYLVSRTVAGLF
RHSATVSAVVGHQYPEFSRCCDVKHFAPYFLGVAVILGGVLGDWWPNIAAISGVMALVEFITAVAVGVVAF
IFARTPKTVPTAQRQSTLREWLLLQPGSKIAHLLPVALLGFCASIVQSLYPSVDRRVFNFSYLAVGAHMMV
DTFMQIFVAPLVLRRYKGTPRVLVCLCSLLFFLSMWASSRFAKHGMGFYFVVSTLLTDLPAAVLQSAFTAY
TTNSFKEAERELALKMQVCARKIMKQWQPVLFMVIQMLLPDEKNVNQLVGVPLALGVMMFVLSNRVDASMA
AVAVGCAAFIIFSPIDDEEMMENLSSMLRWEFPTFLMG
>tr|C9ZK77|C9ZK77_TRYB9 | DeepTMHMM Topology Prediction - Predicted
Type: TM
MFVSKNGLEWSGVEGSVCVFVCCHLFSFFFCAILNLVWRSSFLFLRHCWRPEKMLHFCFFLNFALCSPRIF
VGMSALFPFLSFLLSAASPVVFAFLIFIFMHSFTFLLYFFCLLYFTIKIVFMFLNVHRAEISVPHVIVLCT
HC
>tr|C9ZYY1|C9ZYY1_TRYB9 | DeepTMHMM Topology Prediction - Predicted
Type: TM
MVTMEGTLAAVSGISNVGRHPPITVLLMELFKIAMPMSISQIAQFSFMVVMLICAGHIGVHELGAVSIALG
ILNATGFAFGSGLCGALETLLSQSYGQNPRSTMYGVYAQRMFFILMVFVVPLSVFLLFVEGMLNALGEPPD
VAVRAGHFCHIAIFGLPFFMVLELLRRYYASQHQSNPVFVTLLAAALVNPIVEGVLVYIFGYTGIALGWVF
VMLGMDIALVCFLKWSGLHTRTWGGWSSAAFRNWYPMLKLAIPSLGMAFSEWTAMEVNSLCAGLLSTEELG
AYAVTSQIANLCWSVVSGLFIAATVLVGNSLGAGNPDLGKQYALLSSVLVLVVSLLNAAVVYKFRDRIPRI
FTHEDGISKHFSDMVPYFLTFHVLDAVQSNFLAILRGCGLQLVGVAIVFVCLTIIGTPLGIYLAFTRHYGV
VGLWVGPVASCAAFGIPAYLYVLFCRIDWASLRPHLEECEMKKFVEVTLVDEDELT
>tr|C9ZPJ3|C9ZPJ3_TRYB9 | DeepTMHMM Topology Prediction - Predicted
Type: TM
MRLRLRVFCVLVRVECENLYGVQFIARYALIVCLIIFITYDVHCFFFYFLQRNYFVCSFGIVSLCIFITTF
FLLLSQLKCTIFLHLSRSIVGGSEGQNGG
>tr|C9ZQ56|C9ZQ56_TRYB9 | DeepTMHMM Topology Prediction - Predicted
Type: TM
MFRKSSELEGKCSSSPFVRRDSHTEASTKQAATLLLTFWFILFPYFSVGVFLLPPLGIGHSHMKALASAAK
GGSVWYFLWRHLSSFPFRTAFCPGRRAFGIVLPDDLCPVRSIMNNSYKSVCRYGGGYFPNRFGCFYYFAGR
FKVGNSVHFQKNCDSYIWFYMEGGRAENVYVLAWMSRKINSSFRVFSLHYVIYGLAFRFV
>tr|D0A2M3|D0A2M3_TRYB9 | DeepTMHMM Topology Prediction - Predicted
Type: TM
MEDMGDLAKGNLASSKWLGTFSLLYAEVVVSFASAIVLLVLTVVQLLYRTSWRARKLWRVGNEVRFDGERY
LAKPEGNIGRKILERGIKSHENVYFERPSSTLPPIVANDCDARVNCLSMIAETRKILYKQYGEPARLMSMR
CCLSCIENVLPSSQAEDFLRIYESVLYGQYRADGVPEVISDEEIKFLHVFYHNSILKEIQW
>tr|C9ZK44|C9ZK44_TRYB9 | DeepTMHMM Topology Prediction - Predicted
Type: TM
MQIRITHSLRNTEFFLRIAFFFFKYKIQVSSFIFFSTLPPSHSLFFLSYLCNNLLPRQPFAIFKAYCILLF
YYIYIYFFPLSSSSFVSSNTEFNSSSSHNSPPTAPRCFPHVSFWIIFCFIFILAIITASNRAQQTAGIYVH
IHMGGGEK
>tr|D0A7V5|D0A7V5_TRYB9 | DeepTMHMM Topology Prediction - Predicted
Type: TM
MYRYFSPPLVSVLPEGNEKEIYIYIYKAPSADVMQRNKKRNTRQERKEREAKARQLQMVGIKCSVRLSLVP
FLFLLLFDFLLIFLLLCPTSFPLFPTFHFRHGTAAVPYMPRVWITKLLVPSSFIIHH
>tr|C9ZWR8|C9ZWR8_TRYB9 | DeepTMHMM Topology Prediction - Predicted
Type: TM
MKFYTKTRILAQHAQRCMFVHKMCTIAYFHVKYAFIRVIVQKQCCNATCNNVFWCKTRILAQHAQRCMFVH
KMCTIAYFHVKYAFIRVIVQKQCCNATCNNVFWCKTRILAQHAQRCMFVHKMCTIAYFHVKYAFIRVIVQK

QCCNATCNNVFWCKTRILAQHAQRCMFVHKMCTIAYFHVKYAFIRVIVQKQCCNATCNNVFWCKTRILAQH
AQRCMFVHKMCTIAYFHVKYAFIRVIVQKQCCNATCNNVFWCKTRILAQHAQRCMFVHKMCTIAYFHVKYA
FIRVIVQKQCCNATCNNVFWFKTRILAQHAQRCMFVHKMCTIAYFHVKYAFIRVIVQKQCCNATCNNVFWF
KTRILAQHAQRCMFVHKMCTIAYFHVKYAFIRVIVQKPVLQCNM
>tr|D0A7C1|D0A7C1_TRYB9 | DeepTMHMM Topology Prediction - Predicted
Type: TM
MAPPFVKEFPPSISFWRLMCFRMWGRGKRKQRVGICPFAIFFFCFRGAVLWSALHFAIKKKIPFIRCLVNF
FLFNRCNFHHTRSFTTFEHSLGHHLQYFFSTD
>tr|D0A3D1|D0A3D1_TRYB9 | DeepTMHMM Topology Prediction - Predicted
Type: TM
MRIRLYEVTVISYSFLFSLFLFLFLFNDSLMHTIKNTKRNKPKCACVCVCVCVWKKKTMFVQPFTLLLSLF
SLSLSSFYYSQGTAFIVAPPQGKASPHNTT
>tr|D0A1I9|D0A1I9_TRYB9 | DeepTMHMM Topology Prediction - Predicted
Type: TM
MQGTVRYVSPQKGFSSLNLCVSYLLRPSRTWWHSFVYRSVPHKRCAVYSSPPSLLSLRILARYILLLACTN
RCSNARVCVCVCVCVCVCVCVCVCVWMAGWLKPPYAEIMFRCF
>tr|D0A0V0|D0A0V0_TRYB9 | DeepTMHMM Topology Prediction - Predicted
Type: TM
MSDGLPWPSSTVSTLQRIPFRERLKNFYFLIFSAKTNRCILATSIVLLLISVVRSSCVTTLWYHTAEAIVT
LLFVCEVTIRLMVMRGNFWDSSYNVAEALSCVVCITIFFVLHYTRQMSSSVEHQFLIALRYLGQVLRLAGL
VAVDTVLLGSGEATLHLSFVGTRPCVVSGRENLCDVL
>tr|D0A776|D0A776_TRYB9 | DeepTMHMM Topology Prediction - Predicted
Type: TM
MADRLWLHAHLCWRSTGAKVIFFFPAVQFSERFIMTEESFHHVTCWRKCHLKISKKKKKEIDIKKKVGSFF
LRNGVMRLLLRNRRGSLSLCAVFRTEKNIPIIQFFFFCSTLKIVFFSLMILKPTIAAY
>tr|C9ZRD4|C9ZRD4_TRYB9 | DeepTMHMM Topology Prediction - Predicted
Type: TM
MYVGDKSKGWIAITRCWGIFASIFMSLVLVFCNGTTHFFIFLRMTKKNVIRFTDLGLSTPVPQVLSTSKGE
KKKKRLEVKSERNLRTKEAEKQLSLAREKVEKHKEYVKSEACAKQKEKAEKRKALNDMRVSLHERRENEEK
LQKQRVVSSQQEYISKLVANIAEEEGRTRQVREAIDRDTLDLAKFAPQSVCIHVKRHPHIELTRKELPVLR
EEQAIVEAINSTSRTCVLICGETGSGKTTQIPQFLWECGYGDPKGSPFGREGCILVTEPRRVAAISMARRV
AEELNVPFGEDVCYQVRYDNNLSDGFKIKFATEGIVLKEIQSDFLLRKYSVIIVDEAHERSVTGDILIGML
SRIMPTRNDLYLEELRKNGGLPQMTTLKPLKLVIMSATMRVADFRDNRKLFPVPPPFICVEARRFPVTNHF
SKRTELFNYVDEAFRKVCQIHKKLPPGGILVFLSTQYEIGLLCDRLLLHYAKTKIEYCETSYSKHALLTSE
LPTTPSESDESESDIERDEFGLATEDYALDKDDTELENCNIGRKRCRNAAGSPEKAEGFADESEVNGELNT
LHVLPLYALMNFSKQQEVFQQPPAGKRLCVVATNVAETSITIPNIRYVVDSGRVKTKTVDESTCASCFRIE
WTSQASAEQRSGRAGRVAPGHCYRLYSTAVYSNLMPKHSAPEILRTSLESVVLLMKHFGINHVGTFPFPSP
PKEADLKRALTHLGLIGALNSDDEFRITATGRRLVAYPIPPRFSRVIVEGIDRKLPRFLITLITLIASIFS
TTTSVFTDEGHRIKWKSKDISDDEKERKQRLQALLHPGSDLLTSLNALLVYMNNSSAVNCDRYCLVQKSLS
EAKQLGDQLLVLASRDTAEEPVEGASDADVVGPEQLFEERALAHLSKNQEIVIRKLFIIGLVDQVARRATV
QECRSHGVEYKSDKTTKTPYITVANHIIVYVHPSSSIARTYPPPEYVTFVTLQKNVRSETKESLTLMLGLT
IVTKEWLHECAVTVE
>tr|C9ZM26|C9ZM26_TRYB9 | DeepTMHMM Topology Prediction - Predicted
Type: TM
MYRDVVIRTAFVGSRRDVNTGFVFHPFLLLLLSLLFSLGVFFSLSVASYRRVHLSPLTSLQDHLLPFRLFS
LTPHPLSVLVTDADSLFATCTHTLDHVAARRSRCVFFFFLLFSFSHLPFYYSQVLLPPPPPSGLSRPRSLC
VDVFVFCFFFVLMAVLLMLMKLIFLSLLT
>tr|C9ZPB8|C9ZPB8_TRYB9 | DeepTMHMM Topology Prediction - Predicted
Type: TM
MDGMMLCVTSSLISFCFPHFHHCFLSLFLYLTHTIFRPKYFAVVVFFCFSFSSSNTTFKISGMDELSDIYD
SIVARRIADALLQRTMNFCIRKRRSFESLHSLRWRLLTDLLPTDVQLNNFSEKWSSCTQKCMTRWRTMNED
LNNRAPDDSKKGEVVKRKARFEESDSSSEEVDDVTIENPLLPKNGSFYALRYRLNKLCSIIALDVDRIHWD
IPLFELRTTRDALLKILSVYCVQHSCEYKQGMHEVAAFVFYLTHNDATILEHLRNERGWGHASLPNIFAPI
CPVEGVVAVAYHIFDAIMTESGVNLSFLYFSASYGQTDGITAATHNVQGRLLLKLDAQLHKQINTVYKITP
TLYLMRWLRLLFLREFTLEQCADLWDVFLSERFVTPAEDYHLENSVVTLFAAVMLLNIKANIMKGYNEAIE
KVMRYPSVGQISFLIQEAVLQLERVKHCLGRYFVVEDAVHVEEKQLFGVDSDRHVSSVITESSVRGMQQNI
GALEFVVTMDEQCGTSTKSSLGKAVNTVNSNGYYDVIADGAYPK

>tr|D0A4X0|D0A4X0_TRYB9 | DeepTMHMM Topology Prediction - Predicted Type: TM
MLDISSPLFFFTFPCRSRMTQNLLHVLGTKPLLPYWVTLCPPIIQCTREHIIYIYIYIYICYFLFGCLVTS
SGGMYGAFWLIYLLNLCDMSTRRVSISPHFTPISVSFCFRVALVDGEGVQAISSFLWLHSPLPTPVTVEVR
TRGLMFSC
>tr|C9ZRN9|C9ZRN9_TRYB9 | DeepTMHMM Topology Prediction - Predicted Type: TM
MLQQHLSNLTSPTALSRFSKSKKGRASHAAAPGIHSSLRESTLNIANVAALVERQVCPPYSPQDKKGDMTS
NTTIGYLQWYRCKHDKLNFRSNIRPSEGASPHQMVGSGRCLKASYCAGVLCVAPFLAL
>tr|C9ZTT2|C9ZTT2_TRYB9 | DeepTMHMM Topology Prediction - Predicted Type: TM
MEVELEPFASWLVCVLSVVSHATLVPTVYHFLRKRYAYESVLGVFGLTASLMYHICEAFDAKFFMSSLAWH
RVDNILVISLLGAWSVYMCALHDPFVERFTKYSLMMLCLIFQTRGPWEAVNSVFPAAVSFSIPIGVYVYRR
RIPALEIGRLVGFILIMAVAVIFFIKGLDDKRDHFRMYHSLWHFFCGIASYLMWTLLKVPGVTGVMGKSIH
V
>tr|D0A3S2|D0A3S2_TRYB9 | DeepTMHMM Topology Prediction - Predicted Type: TM
MQGKVVSVHGADAQWYEPTQIPQDKYLKIVQGRNLFLVTFITGSAVIWGLSLIPQWCMYRSVSWMYRKYCR
GRVLDLTPKVADARDVRYYEMSNAVRVEFIVKERVDDDEYKTDLSLSEEEQTERRRALTLGFMLKNDYNWA
GSSITFDTVERRETEMPDFRKYDTVVIRNELLNMGREGARKMLNSAAAYVKEGGYVLLMDFGKPRWPLLAS
LTRWFGAATRSSMNLTHDYGRWVAEDNCYSIIAERRCLMGVHYAFVLQRKP
>tr|C9ZTT6|C9ZTT6_TRYB9 | DeepTMHMM Topology Prediction - Predicted Type: TM
MEVELEPFASWLVCLFSVISHVTFLPTVHHFFKQRYVYEAAMGIFGMTASLMYHICQVLNAEIILDEAGWH
RVDNILVVSFLGAWSVYMCAFRDLFTERCSKYCLLMMCVLFQARGAWKAVNTIVPVFICIGFPICVYAYRW
RLPSVFPNRLFGFALVMAVAVIFFIKGLDDENDPYKMYHSLWHFFCGIASYLMWTLLKVPGVTGVMGKSIH
V
>tr|D0A330|D0A330_TRYB9 | DeepTMHMM Topology Prediction - Predicted Type: TM
MAHSGAKRTSRSGTSTPLRTPTRRRLELHNAAVDEAFEEEDGEGQREPHFERTPKTSRQSVSASLGDGLGS
GGTNHGSTSSGVQHPEGALAMSDAGERPVVSPQVAKAASARSVEGIIDYSPSLCSEQLEVHGFSHLRESIS
FGLPQTRGSVPDVRSSCGVLEVGTTLVPHSTSRSSALSSHTSSKQSHFHTRQSSDGRRPKHGGSRSDLSFL
STRSAIAYLKSTAPAAGVGSPKTSGRSSQATPSSNSFSRSTLGPLELSPIPKENSNPHGRRRNGYLRSRHQ
PPPSWPPVDVPVAVANCSSSTVSVTEVGRIDSEMRREFRLFLAYCIRIVIMFIVTCSAASFALFLAAPLVV
QRSGSDGLFVRQYVESVAELQLIYGAPSSSLTSSEAKQLYLRMVSESLERLDQATLNARPKPSEGPKVHEQ
YYSRMTRAYQAVRYRVALYARSRHRSWFYRNVLYPLHDVWKYGVVRHGAQVTREECQEILLYSLWVRIVDV
AACLRQDENMPCPTLNFLKGEVRGSTGPGGPVLPVASGYPSEGNGVQGEVSSKALFAFVAKNFRHNNREYN
HLYFEGGMGSVFF
>tr|D0A1P4|D0A1P4_TRYB9 | DeepTMHMM Topology Prediction - Predicted Type: TM
MSIDVVPATETAAAAATTATYMDNIISVRKANAKKLAGGYTQTKRIAEICYFSLAVCLWMRNVAIVVLYLL
HSSESSSNQLLWSPLMILSAMVLSDFISGVAHWSFDTWGTPETFLFGNFIRSFREHHVNQMAMTKHDFIET
NADTTLPLIPLLLIQLYFLGCNSKNNSTFRHNIDNDNAGFHVFFLTFALFIGMTNELHKWAHLPAPHPFAR
LLMSCRIVLTRKSHRRHHRGSYDRSYCITTGWLNAPLDRVDFWRNAEAVVTALTGAVPRANDRELLGVRHK
EETDGEGCNEEFGGL
>tr|C9ZS44|C9ZS44_TRYB9 | DeepTMHMM Topology Prediction - Predicted Type: TM
MHEKGYADVDVSPSDKGESADCKIAVVSLAEKIASKAWLFAGCFVVLAMATSFLWEISDSDQTIGYHLFVS
PKFVTDGTTFYGTTAEGKLVRVRLRSVEVPTLRQPYGRETRDHLKSILLLSVGQSTGVSCFVASVDDVGGV
IAEVFFNNVRAFASCGKEIDSCAASSLPSYFTPLSGGFTADSIVNVGDEMVRSGWAWVIDNGWTRNTKLQA
AMDEAKVAKRGLWGGNVSKFPYRRSPHSRKIGKKLTEQRQLISRRRLFASR
>tr|C9ZJA4|C9ZJA4_TRYB9 | DeepTMHMM Topology Prediction - Predicted Type: TM
MDNGCDVDLSTAQKFFKSAASVLEVADSRRREQEMKSSHDKTSSSGTVSSAAGISMEERNGWKEAATLLWR
IMRENKLQHLSQVSGDACCLLYRLCEGLLNYASQRLTNDGSSSSSATLLERVTSLGNDHVLSFIGAFCTPS
RCTKLMNGDSDAGNDLCIHMHDILINQQHFRWMTAVFQDFCSAAQRAYAKQTGLASSLVCTVREILGILEE
CSMTSADCARALMHTEPKPLVVNLIHGLRIRIAHNSLIRPHVYDDKPLQDDNGMLTSSVSIAPTDPSLALP
VIFQNTESLERLGKGELVDLLTRGRGNVYDELNRKALEVALIVVEMAAGYESPTVITALCLIIELSARSLM

PERKDGLPVNAPPEVRIAMYENVISAWRGLCKLLHSTKGCSSSTTLGARRDAFLRHQLKHDMAGSLKQLLE
NCAELLSADPTLFRVVTLQCTPSVFWAKSYGDACDDSLGGLLGNSGAEESLQNSQLGSELDSTWWYNNKPF
ITITSSITQVLKGALETAIMSTSLTEHIVESLIQITMRSKDAFVAVEGHYVVQCILSLSLKCVRRSQSTST
PMEAEERLVLLTLLHRVTAALLHLYGKLRVQPRPCDVADIIELPPLLLKHLDTMESNEERELSRRLMSQAT
ALWYTVLALNVDEANIKRSQEVLPELLRNILDDGETSIIQQTLVREHRSGADDGGVSASSSGKDELVSLSG
NQQLEESDDIVVHLRGVLALLLQRNPRFLSARELEMLLNLVSAKGELLTRLGSASLSCSLSHSEVYVVLEE
LIRGASPALLLDLLSFLSSQLTSDGLAAAAKYERQVWLFKFNCVDAVIVVMERVLKISNQLKQFKQLIRQL
FCFLSVAEPVYGLGAHLSNTNLAQVFSSSLASIKSVEHIVFIVQAVLDAAMSTYDSAGNASSKLNTYGQLS
LPHPNCCLRKPVFMELLPPLLRHMHVHASELENDVLRAVRDVLRATAVVRCDALLDWAIEMGHASLVPYLE
LDATSAEQRLPLVSSGDAEDLKAFWTPILSNASERSEMRFSWSGGVVITVGEWPKKGFSMASCFRFEEIYP
RVNIFQFVGIDGTLRSPTCVFLLGGESVHIEYAGKTVQLSEPGMLKGLAPREWVHFHIVMSVAHTVSVYFN
AVKIGTCSLPYFHSGSQALIHIGLVNTVVPNALYSIGNISLWEEELITPQVEAHLAGRSHEAGVCKLLPCE
VPREISGSRSVSPVGDCVAHFSPCEVDDGQVLLNTLFRSAEGCSMITAKAIGGCVQPPRCWIDYKSFFSNR
GGLMHLLRWIGKSKDSAELERNTSLLCTTLRCTANASATDFRTYAMLHYYLRRSAHLITPAVCDSLVHLAT
AEVRAGNEFHPFIINRLVFDHLLGDMELLAAMPLESAMHVTERVGKMFDVFSCRFADHNVQYIRPFRFVDG
ILNSLSYSRLSLPFTVLNRVISNLKHIIIACGFETNLVSSFMTAAAVLTPAEVDAQSKQKQRLKLPRARFT
QIQASHKTAYNVSLAILRCLIECSRVSDAFLSVFSRLVDLNWFVVCVSRFAETTCVVYATHLFLGALRLNE
ELRAEVMQHPATVATALEPHSFSEDLLLLLLGHSVGADGFLDLLHSSKSLHLQLDSALGSSLHDSDTVVTP
IFFRLLMLHLSASLTRPLLFRPGPISQVGGRRLLRSFRLFKYLHLAVVCSRLMLGIYVKRLSHRPTGAVPD
ACSSLWWNPTLSRDSSNTHLPEAAGSASEHWAQAKDSSLPVVTRSLWHRPSFIVVGIATYIWRRVQRSRYA
RLLRNDIPFLLIDGGGARGRAQKEEMCGTLFILKTLLQISMQPNAFALMTRSPFQVATLAFFGSFLRKEDV
MAKSGELASAMHDFGCREIMRLRGAVETSALQKQEVDTDGKKGSAADSIFGILDLKTPLYADSEDNAYEDD
AYEESVEGDDCQTQNGKCCRSSSSMESGIRKVPPTPQENVLSGIGEVENVALPCHMSPSLPLTNLPELSES
THTHDDVPQSGTVVSTLERSDAALPTLISDPFESVRSLDIRKTRFSPFARGFAGFEERDGCELEEEEGNED
LLLAETIGSVVRPDVLQSHSLASLLEPAVDILSNIVASSLQAMPARTAPANPYSYGACGYLLFQLILITGT
MANEEEGASVLINFFMHQVLELVATRKSPKTQSALQLPPETGGRMKSSLPLSRSTESLADNGQRVDECAAS
VPVENEMNGGIAYAGIISNIFAFNVCAFNDLLVDLLSFHVVELSTIAPYFVRLLLYARVSWNLSDLLEIRL
QIVKICVAAINRKPVSEMSLEEMGLIHTMLTHVLDSPWPMKPLMACLIGSLMRVYDDPSSKDTMNPDSRKR
KEVITLCLRRIALVYQGSKDLKKAVTVNSLASRVSIYEGFVSALLSQDETNAIVAFDVYISSNTSKLKAVM
SGRLKMKVELAVNHFLKKRSFYVNQMRLFNESYNSTAKLSNGYSVAVLVQAYNGRFSSRVGSIARLQPAQL
HWLAVPRSSLSRGDSHLSEAFRIDSRHKRSTILTASTFFANAESDDMKGVSAEDHITILNTSSCEPQVCHI
SALIPPVAVRCRPYVDCGSVPFIDPRCKVTSSAAVLLQNLLEPNEVLRYISNGFRVNGIHVAPCLILVTNV
AVKVFCFSRITEKGDVFLYDYEGNGGNNLGGDCDEETDEVNASSRGGEGGVQKGQKQRSHNTTRGFAVSMT
NKLQRFLTDGGTKGRRRREHEEHEDGTKVAQSVRQATGHPCKSLYWCYPVRNIRSIRVGLYMHQDTAIFLD
VMYSDGLMLSLVDPRQSMNTRARDEFLEVLQEVIGTQRCTIHDHGKRISNMRNMLAGWGGRSVSTFEYLFF
LNRAAGRTILDYNQYPIFPWVIADYRSSTLDLGSDSTYRDLSRPMGAQTKERRRSVEDLYQQMTEVAQQLG
DGGVAAMELTQPFHHGTHYSTSGGVLYYLIRMEPFTTFARIFQGGDFDVASRLFDTIDGSFQSCVNGPADC
KELTPEFFLDGSFLVNMNRCNFGTKSDGTAVDDVKLPPWAKDSAQVFTAVMRYILESDRVAHSIHHWIDLV
FGVCRRGKLAVDCHNVFQRMTYGEEVVKALKESQNSRDIDVIVAEVDNFGQTPMQLFQEHHPPQSDLHLLV
SGLSESNPTASLSSGIFTVVSTSSTAKKGDHRYKNSTNTAGSNSSFACNNSIASTSSFALRTVTSKLHGAR
DPSPKVHLMMEFVAAGRQSRFTLKDLTPGSLMTLPVAAVAFCNTQREPVVGFATTRSGEIACCYRYLIPVD
DEDYLICFDLGADTMIYIDLKGGDLLSTMRYSTLLEPSANISCTCVCCRETLVAVGSTTGTIYCLRPSMDS
GVLTLSSTLCHHMHAIAGLTIDTKYGRMVSFTVSGNDAPIVWCVQQERVVMLHRLNVVHALGHLFVDEDDS
RVVASVIDPLTSNTIVVTRRHLLIFDSNGDRYGVGSLSATEGTKTPMQGNGKLHGAVHSFDGSCTAAITAV
TPYNTLEWAYGTQLLLTGHEDGSISLWRAVRLPPDGVTHGNIVSVTHHAVIVDGSCQANLGSVTAMQQQQW
GEPAFLIGYGSGKVKALSFATPYGGKA
>tr|D0A408|D0A408_TRYB9 | DeepTMHMM Topology Prediction - Predicted
Type: TM
MLAGRYCFTTHDAPTRHIEWLPLYFHSCHYLLSFFFFLFFSSSFPVLCVCQTEKGNKAPAMLRVSRLSLAI
GTYSLFMKEQKNNPALKGLPVAKRGQATAKLYRELSVTEREELAKRAKAAPSATRKKSVPKTKGKEKVTGG
GGKRKASEYTEFVKSNISKYSNLPQRERMTAVAKLWKQQKQMRK
>tr|C9ZYF8|C9ZYF8_TRYB9 | DeepTMHMM Topology Prediction - Predicted
Type: TM
MAVPPVEMYSGSFWNRMRKPLPLRTQVIRFTVVFVIVSFILAVALQITHERMPDPKVTKPLPDLGFELLTK
VPGMYVLADCCIGFLNILSVFTAFKLYLLHRHCVGSGEPELPCNIPGVSRFFLSVWLCKENCRIELRNIHT
IAWIRFITSYALLLLFRSVVIVMTSFPAPDDLCQNPPKIENPVKNVILTVLTAGGGSIHCGDLMYSGHTVI
LTLHLMFHWIYGAMVHWSFRPVVTVVAIFSYYCIVASRFHYTDDVLVAIYLTIATFIAVGHNADGAPWQLQ
LFIRWWPCCGANSREVTEDSQPVMVAFKSEAAGQSSRKVVDERNH

>tr|C9ZQG4|C9ZQG4_TRYB9 | DeepTMHMM Topology Prediction - Predicted Type: TM
MHCELFNCARLQLQESIRFCQVFASSIIPRLLLHLSTVLCWFFRTAAPLLVYFLFFLKRQKGSARRSVVRM
LGTVCLWTSYNSLIPFRSQSSRKCVCMVCCR
>tr|D0A9F9|D0A9F9_TRYB9 | DeepTMHMM Topology Prediction - Predicted Type: TM
MPPKSHKRSRKEGEVEEPLLTENPDRYVIFPIKYPDIWQKYKKAESSIWTVENIYLGNDMTDWEKLDDGER
HFIKHVLAFFAASDGIVLENLAERFMCEVQVPEVRCFYGFQIAMENIHSETYSVLIDTYVVDPDEKQRLLH
AIRTIPCIEKKAKWAIEWIGSQTSFPTRLVAFAAVEGIFFSGSFCAIFWLKKRGLMPGLTFSNELISRDEG
LHTDFACLLYEKYIVNKLPRDRVLEIICNAVSIEREFICDALPVRLIGMNSQLMTQYIEFVADRLLVSLGY
DRHYNSKNPFDFMDMISLQGKTNFFEKKVGEYQKAGVMSSERSSKVFSLDADF
>tr|C9ZXS2|C9ZXS2_TRYB9 | DeepTMHMM Topology Prediction - Predicted Type: TM
MMETDAVLPPQEEELQVLFFFLFFLFCLSAPFPRYFCLLFLRFFFVNQQRKTLKCIFGAILPLSHYVCVLF
FKKMISLPECRLSFACSALLPSVSFNKKRGRKVKLFLVYCVDVYIYICPLFVSPFFFSFYCLSVSLRPFFL
NSSAVSFPLSFPLTFLLSSFLTLRCVFGMCCELSRWRGCDQNVCVCAYTEMLW
>tr|C9ZZN8|C9ZZN8_TRYB9 | DeepTMHMM Topology Prediction - Predicted Type: TM
MNAVDLIYNGEMYTTPYFFCFFLNLNTVVRCGKLSVTYAPHICFTCLFLKLRFPFPPPFFLDTNALFIVVV
LDIYIYIYIYCVSSFVCFFFILFFRVRYMILHYSAA
>tr|D0A3F7|D0A3F7_TRYB9 | DeepTMHMM Topology Prediction - Predicted Type: TM
MFFVFPFPTSPRQRVERVMRVLYQLGAAIFVISYVVLLTEADVRLQAGMHGSFSAPGANLSDSLEMVMSNC
EYEGIMSIYGVGVVVGVNAKSLPFAIISMLQHMRAVLVVQFLAALNGVAGGYCYSTNIRHMHLFGLHVPVH
LILNVLAYVLTVTIIALVAVSGPVRSYYSAILEFCAAKLAEEDNEKLLESGFDGFHLFGTRLEWAMGAAIV
NLIIYTIGLVTRVYKSHDPAIALFSMSDVPWEKTGIRLSVDKAALSIHTAARERIISEARDAIGRGERVRI
VRSYALMTEADYNEQVEEMRRLFATRAQEEQFKNMHSLLEDEPHAGGSAMHWGSSGLQKAEPLRPDGTPVV
QGVGDTVPPFGSGMMGRGADNLADVFNVDNLSDLSLNRAGLAPGAVGGFDASVGDNQAATVQQWQRPGSIG
YPDVFGGERQNESTAFGDGGTAFTPDALQGNDDFSHAFTNISDTRRVVSRRRTATGAVAGTRANGSHEPHN
LSGEELWDVSNLGDDNALSPRIDSPDAL
>tr|C9ZI23|C9ZI23_TRYB9 | DeepTMHMM Topology Prediction - Predicted Type: TM
MRGSGIEVKTLTHKSKGRVRVCVREDWETEKGNEEAIEGSEMFRELQKFKTVAAPQKCFVKSHICFSPLLF
LLLYPSVCWKVFIPLFYFSLLLLLSLHWRI
>tr|D0A8L5|D0A8L5_TRYB9 | DeepTMHMM Topology Prediction - Predicted Type: TM
MLRRTSLRAIKPYLHAFSPGARASEKGMLYRNSNMNAHARVASIQQAQRTHREGKIFLMLLVPDQVFLSVV
LVGVSLVAVLLYFRQQPFSFNTHKQRWILGAINEHEFYQRNREMSNVLAAHREAIEGTRDVIGDCPKVFEP
PVGSHYISRSGDFAPRSD
>tr|C9ZL25|C9ZL25_TRYB9 | DeepTMHMM Topology Prediction - Predicted Type: TM
MANLFRACKGGRRGVSEPGTRNTCLYIYIYIIDGILVCNPQQAPKKKSCMGVHGEKEKKKRKSLVSFSTRP
VGDSALQHSSQYVRRSPSSIYMRFCPPFFLLVLVPSLCFPYV
>tr|D0A0L0|D0A0L0_TRYB9 | DeepTMHMM Topology Prediction - Predicted Type: TM
MVVFSHCCFNRSLHIPLFFSLFLFIIYHFLFSRMLMLLLLLLPHIYPVAVFFFFPSSSLHLFFCHLLSSVT
NKSEKMMKIRVIPLHTCVFVFVFVFVCVCVCVCVFECVLVCMFVGFFFFFILSLSPSVCFQRNIETGK
>tr|D0A249|D0A249_TRYB9 | DeepTMHMM Topology Prediction - Predicted Type: TM
MCICNKQINIYIYIYIYIYIFVRQNFFCVRFVGSPFSFLHVSEDLSAGISINTRFWRFNAVPPRCLSLFFF
LIFFAQLVPSAKFHLPEVLKLKLKGKLQLGSREEFPIPLKYRCVCVCL
>tr|D0A405|D0A405_TRYB9 | DeepTMHMM Topology Prediction - Predicted Type: TM
MFPSDSSCPTGLSLPPFFSFIFFPSTPSVIPSLLFLPSFQVVVQNLEIYSLRVRMVLLIQIILPLFVCLVW
IEPLRFPAIMFSKITPFYPTVQMIVLSQVRCWLFCTCFFLLLFFFLVRLSLSFTLRVSPTPNVYIYIYICI
R
>tr|D0A5I0|D0A5I0_TRYB9 | DeepTMHMM Topology Prediction - Predicted Type: TM

MRCYVTHGVKETPPPPPPLSSSCHKKNTQHLHLIPFLTSIHNSHPIKTRQLIHINFLPFLLFFFFFLFFFV
TLFVCLFVCFCFCFCLTFPPHPHIHIHNCISYM
>tr|D0AA63|D0AA63_TRYB9 | DeepTMHMM Topology Prediction - Predicted
Type: TM
MKVYMANTLSVSPFVAALVVFYSFVLPFVHFLPHFLLCVMRSVRGTEGGVCLFHWFHAIVTIPFIFFFSFL
FLFTRFLLLPRVFIYLLTSIFFNFFPSHFSLLFLILIIIIIIILLSFSTMHFFSFHCCFFVMTLSHK
>tr|D0A6J4|D0A6J4_TRYB9 | DeepTMHMM Topology Prediction - Predicted
Type: TM
MALGFSSAGEVYMYATCILLGVSLLMPLNALVSAPRFMVDYYKYVSGKEDAEPNLPFFWKNIFTFYNVVSL
ASQVIAGPTVLTRAARRLSLSVRFALSITLMMSEVFVVLMMPVIKVPQTVAIVLLCLVTIFAGIGKSYHEA
TCYVLVASMPSKFMSAVMFGVSLCGVITSTLQCIIKASMEDTYESVLTQSYIYFSLGLLIMAGTLAMALCL
RYNSYAQEHVAEYRMLELQEQGVDAESQNDENEPVAEGKGEGEGKSEGAMTTAEQLTATAVMPVARIIRMM
LVTVFCGFFLTLFIFPSLIIPIDRDHNWFATIAILLYNCGDAIGRFSTSFKCVWPPRRALLYATFARFIFV
LPFMLCIYQYIPGHVGPYIFSFLLGLTNCVGAMSMVYGPITPGLETAGQKLMAGQLMGISLLSGIAAASVL
AMIVVVFLP
>tr|D0A867|D0A867_TRYB9 | DeepTMHMM Topology Prediction - Predicted
Type: TM
MAKGSDYDAVAYEPMPREGFDAGDEGPLVGNHDPGFLFDDQADGAHSTQLTYIAPSVKKPVYALGITLLFL
LVAVVLTVILQPTFLYGETCVTPFGTIIGVHNGVFAYSNCRRDHISTQKNSIKGEGSMETGMEWQCFEYVK
RYWIMRGVPQPVILPTARKSSELWSFTQATFKNGSKVQLERHDNGGSQPLVGDLLVYREQPALLPVGHVAV
IVRVGKTHVWVAEQNWFNKQWHPPFHNFSRTIKMHHNAESQTYELEDMAGTTIKGWMRYKT
>tr|C9ZRG8|C9ZRG8_TRYB9 | DeepTMHMM Topology Prediction - Predicted
Type: TM
MRLVADLTLDTTRVIPSHYVFATITALDSTVIHFASLFFLLLLLSFCSLAIAMSFTANSGNSPMSQMSPLS
QPYTSPQMLPQSSLRPTAPPFTLSGTEDQPATGAPIRKGGTDPTRYKTTICRNWEMGSCSFKGCTFAHGEE
ELRMPPRVERYKSSGFDTRRSLTAETPPLYPLNPLPHSGVGRIEHLLNMLYSEVLRERNRFVAHEEANQAL
EALLKKEQMQREETEVQLEAARWKLEQLRTTVHEASVEINTLLASSPANEGLRDRVAMVTQKIASVFSSED
TAVGGGREDEERVMKLLSSLQNCQSTEDM
>tr|D0A4M1|D0A4M1_TRYB9 | DeepTMHMM Topology Prediction - Predicted
Type: TM
MVSFPVCPAVLPPYVCAYAFLNKKKTVYEVGGGVSCCSLKFCHIPIYFCGRVLHGEWRCIVCETLPRVAAN
MFFQLRTASMDFLSNELPASTSDVLWAHLDTSIFYSRSVSRIRLAAGFVLHAACINLLLAIAPPM
>tr|D0A7L5|D0A7L5_TRYB9 | DeepTMHMM Topology Prediction - Predicted
Type: TM
MGGSKLSELNRAVKTHQHTRTQHTRRAAMSVIGTSQHVVEELAKVIAMVHQQRKQMTEGALPTSFVTLVAQ
LESHRAAICRALPAAAAAVEASGEESAAEKVNALRSLMHTRSILNAELHKVQGAVKELAGSSESLEMLHGA
LQNVNATVEIAQKMVGKLLSIKTVDDIVLRVSLFCFVLVVAYIITQRVFGFFPSVVRR
>tr|C9ZYD5|C9ZYD5_TRYB9 | DeepTMHMM Topology Prediction - Predicted
Type: TM
MAKDSEKSPMSLHTGDVLLMDRNCWEMRHPLGIAICLLSKTESRYDHVAMVVKLNDGEVERGRERGIINPK
DPSSPSGTYVAEANLSGFSLRPLENRVARSSSKHIAVRPLSMGSDMHKFEEYVQSHLRDFHSRPYKRDLLM
FPPMVLSPPDKMDRIKAAHKLNLLKGETNDIDKLLAGKLSESDKEALLRIKVVYHDAAQFLIETYFAHLDR
VDGESFPSVDYGGSHFTVDGVNAEEEVVCTELIIQLWQRCGVVDLFPPASSFRSFDFLDNTRFNFKDARTA
FGDVFTLKGNDAPETPIKRATRKKTPTVEGCFDVYRSTSANGDPHNPDVDSMYMWLIQSNTNKVVNSDLGL
NIASVGALFALCGLVIAPLRLRWIEYQLGVVLRRGSVWSLSAGFFARDMLCVLTQVITTSIALKSLLYRQS
DTGPLGPPLVHTHLFDTRHPYYYVCIVWLLANAVAHVTTTPLLNSVIAHHFGPVLPGPLSLRKLMRGSFAL
LPLGALLPFQAAWITWYETMGAAIIPTSSSVLRRRADLLDTDEWRHFRFEALTGAFAATTALDFIAYIFQR
RCWRSFLVQLYRPAATPSCGRRRCAGYGYRFLGNTITMLTTSLSLSFLGVL
>tr|C9ZV67|C9ZV67_TRYB9 | DeepTMHMM Topology Prediction - Predicted
Type: TM
MPISTGTQPLWPVTARTPRASTLRYSACPFLTHQRDRASQTDSAFAGLVRQQAHTRHNPDAGNTPVRGVAT
VSWCLKQHVRVELVSWGARIKCVSAAAVINITFSFHALLDGVPLVWAPTVYRFTSFFTFSFLGWGTQPPRT
MLQG
>tr|C9ZIL0|C9ZIL0_TRYB9 | DeepTMHMM Topology Prediction - Predicted
Type: TM
MSDLRMFPLVLRPRVLNPNISLSPGLRVSGAGSWFPSLSLSPSDHSSSMLICYAQRRSIGNTVQCSKEVAN
FSFYIRFLNCLVTGVIFLYLDPILLFPNSQFPVRGLALATYMAQKYSCSVPSSQACSYRKHSPAELLFSIA
EKGSTFFPVQIPA

>tr|C9ZYP8|C9ZYP8_TRYB9 | DeepTMHMM Topology Prediction - Predicted Type: TM
MSLGDFFGNIVIDGESASFVPNDDSYPRLIWNLERQCFVLPSNVAADDAVSHVSKDSEKAVCDVVDDGVMS
DASSSGFEDFTPVRCIPCYALFGVLRIGSASILLYVSERERVTTITIGETHDVFAVKQLSWLRLPETASDL
SVNKSGDGDDGGSGEKDEASASQGSSQSHAREEMVLEYCRVVDSFCAQSEQHCGASYFYYSPTANLTLEPG
DVVKGMKEVLTPSLNGSSVKGGGAFSLAISSEGQISQRVVFQWNSPLLGAFDEVVASLNCDYHVYVPAFIR
GIVEATTSPEEGVQMLLINRLSYRWAGTRYNRRGLDNAGSGIAANFSASTLWVFPLASGNEGDKAANEKQR
VAAFTILRGSVPRCWSQPANLTFIPTITISSPSSGVDELVLHLNALLALFDGMTSIHCLDTTSLSKAELPI
SKAFEAAALKLREGNGALSRPVDVYYTKYNVKERRAKNAPYNLMRMEVDTLLNRKNEGGSQFVDFWKFSNE
GHPSPSSGSSPPSVPSTADADGATCELVLVHQQKHYVRVNCLDCLDRTNLVQSMIAINILPQMIRYVLGHG
EEMNLSDDEDEYEDEDDGSDGARGNGEFHCSVERCKHMWVELGISLSRLYAGSDPHFVDFLLTGEWGPATF
DVVRIALRRWWQQNFFDGQKQDAVSLLTCQHDPALFHTQFESPFSRNFSGLNRMVLGGMAAAVLATLVSFS
MLFVPRYWMRGEILFFVIFWMSYIALTISRILKDGVSYTNYPLLK
>tr|C9ZKV5|C9ZKV5_TRYB9 | DeepTMHMM Topology Prediction - Predicted Type: TM
MRLRGQQQFLFSPAVGGQCSAALRCVHVTFSPKVPLVTSPLSTAARFFARVSPIQGTIDSPNDGFVTKDVK
IDINPNIGGAYARGVFARREIGYGREIMNIPAFVMYISDSSNQSLRDQVLVVTKQIFSKLVLGTPEEQHYI
KHRVATLMSGGYSYFTRERDVFEFAEEVRVPGPEGVLKNGASYLLSGEFSSYDLQKLPLIVEFNRYDVEYR
GRRGICLFPEAQYFNHQCQPNVEVTITYNNLKSNFYLSARTIRPVREGEELFIDYMPGNTMPLSRLALAMK
KRWGFECTCVRCKSRGIGAVMFLFVVVLIPMVAYLRSVNVRRVQNKQRGV
>tr|C9ZW32|C9ZW32_TRYB9 | DeepTMHMM Topology Prediction - Predicted Type: TM
MLSLLPDRGYTMEGISFNFLLSSDHLSICHPQIPFPPLLFCFNLSLLVRLFLYCMSVPQYLKKNLSVFFLP
VFPPPALHICNALPPVAPTGMRVAFAFFFSVPFSFFN
>tr|C9ZNH0|C9ZNH0_TRYB9 | DeepTMHMM Topology Prediction - Predicted Type: TM
MLVSKQISPFLHAACNESAALPLIPALFFFVSALPVKPTRKYMLKCALAVIWTMGMRLGEKVWKHGSHCDQ
VAVMINVIHLVAGRWVRVFVGWPRSMQVRLCGSAGASVGVSAAVPCGAKSTVFFVLVVRATRRIGQLCRTF
S
>tr|D0A007|D0A007_TRYB9 | DeepTMHMM Topology Prediction - Predicted Type: TM
MCLDMPVVHIHAYMLTMLIIYIYIYTYIYIYACVRLYVLFSSLPFFLKKILTDFVFVLFIFIYPSSNRVLL
SATLFFLFVFFLFVLFVFLFAMGRKEVTEDLHPPLLNLTYIRRVMFVLLLILLLLVLLLLLL
>tr|D0A699|D0A699_TRYB9 | DeepTMHMM Topology Prediction - Predicted Type: TM
MSTFTVCKCLYIYIHIYDFLCLHVQLFFFLSCVCLYVFFFPVVFLSIYFSICFVVFVCVCVCVCVCVWVNF
PLFFPCTKKKNTFSLLYLRSLASSIRLQDVFFLYSGFDQYFLLSFPFLFFLSFYFILFCCVVLCCVSFFPP
FSTHVCK
>tr|D0A5P1|D0A5P1_TRYB9 | DeepTMHMM Topology Prediction - Predicted Type: TM
MRRFVSQQDGKATALWTNLPHARVSSAFFSSLTHESRVVQSVTCVRRVLAPPTPSIYPAFRNAFKSTKRWQ
GSTTSAGGDYYKLLGVKPDASQDEIKAAYKKLALEFHPDRNHDPGAEEMFKNISEAYNIIGNKTRRKEYDM
QRRAETSNAGSARSSYHPGGAAYHSPPGYQHISKEEADKIFRDLFGGMRVDQIFRDFEEMQRGTRSGGRSG
FPREFGAADCSFRPSFRSESTRVYTDGLGNRMEERTFTDSHGNTYTVHTTTSEQPNASMNQRAEDYYSGHA
SNSGGGRFRMGNSSFRVTPRDAANDFGANYFGIRTHGRHPAVAMMILVAWSIVLGTLLFATLAFFVSHPFF
TFAVLFLILLKRMRLF
>tr|C9ZYQ7|C9ZYQ7_TRYB9 | DeepTMHMM Topology Prediction - Predicted Type: TM
MEREKMCVHSTISCTSHSLLIPLSPLSVPILSYVQPLFFFYFGYFSSFSLPFFVPFISLTYTTYLPISPSL
SFPLNGESHRSSHCFIIFFFLLFFSWSLPISHTVLKRMCVCKSAVK
>tr|C9ZMG7|C9ZMG7_TRYB9 | DeepTMHMM Topology Prediction - Predicted Type: TM
MELSFAFLLSTVVLIVFYAVDFFFARLLNVLKQDHLELDECSPEESPDLDPVLVVSSLQQTAVEMLEYQRR
RFRMDEGKGLSVTPEIDPAPPTTVDVGCLSIAASPRRSENVVHVVVIICGLVGPFATVCQALFPNAFGWCR
FNFPYFVQYIGAFSGVASVSVYTGRVWHCSSYSEVSRKQWLELMWFRLDFMCLTAVAAFLGAGSWVVTICL
LAVGFYLAHRVMRIEKRIEALQLETQLVRGEIDVDRVFAPGTAEAARALGTSDRHGYDALA
>tr|C9ZTZ5|C9ZTZ5_TRYB9 | DeepTMHMM Topology Prediction - Predicted Type: TM

MGRLDRNSPRAYGTAVALVRASAWWNFECHVGHILGGIGSDCSNDTDAGHITTYHATFSSLFLHLYLILFS
VHNILRRKETSCVVSSGRTNFSIRDLGSGMMYRNRFK
>tr|C9ZLI7|C9ZLI7_TRYB9 | DeepTMHMM Topology Prediction - Predicted
Type: TM
MRGHTFARVRACRYCAAIFCTPFYLMEFYVNQPVRFAFFFFSSGQECFGVGNLVGDVLSFRGRGGRQLLSI
KTPGWVILPLCIHRTLHKELCASAGGAHLV
>tr|D0A4S0|D0A4S0_TRYB9 | DeepTMHMM Topology Prediction - Predicted
Type: TM
MVDPSVLLAYCPTHDFQRIEAVVREWPGVEAVGSLRTKEGKDNTTSVFINFSDKEAARNATSRIDAVPGLV
RKIEEPKAKQRMRKMGKNMLIDPAMEIFSFGATDKWDVTFNERKREGNRRRHPSVIGPGSGSERTSTKGNN
AIRHHPFTPPVRASVALVDNVPFNMTNDQVARLFSPFGELIDLSRYETMAMVFYRSPDSVLKCIQQLNGKT
VKGKIITVSSGAITIPGLLAAVVGVQVHN
>tr|D0A5I5|D0A5I5_TRYB9 | DeepTMHMM Topology Prediction - Predicted
Type: TM
MYILTVLRFFFLFFLLRHMTARCITKMVRHAHLLWYPHHPETLLSVKRFASSVRQRNHYGEASPTQRPSRS
FSPLYVVEPSTRKGSFWRYLRTVRQEAVLIGAAVVGVGFYSLATLAIPATFGKLIDFAGNGELPLGTSMQL
LGWFTLAGVANFARLACIGYTGERVIARLRGQLYRAIFRQPAAFFDVAENSAGSLAQRLSMDCNLIGASLT
DAVTQGSKNILQTFGSIGIMLYYSPTLTCVVCGMIPPLAVFAGVYGKFVRKLQRQMQDALAVSGSVASERL
NNIRTVKAFAMESKESKWYEKKVDVVFQISKRMLFFNASYVSSIQFVGYGALYCIIWAGSMLVAANQISSG
VLFSFVLYTVYCGLGLMGLTNLATEINKGFGASIRVYDILDTADDIQKLQEQTKGVVPLECHWNIKLTDVS
FAYPTRPEVAVYEKLSLEIKPSRCTCIVGSSGSGKSSLAMLLMKLYEHSDGTITLDGTDLKSIDTRWLRSK
VGYVGQEPVLFGGTIAQNIAYGAEGHDWDDAVDRWLYSSVVESATKANAHQFVTALPEGYNTYVGEGGRSL
SGGQKQRIAIARALMRSPGILILDEATSALDSESEIVVHEAVSRLIEDAKKGSEKRTVLMFAHKLSMIRKA
DHIVVLERGRAVAQGSFDEVRMHPLFCQLVGLPLPRSVREQQSDTLEAGAEV
>tr|C9ZU98|C9ZU98_TRYB9 | DeepTMHMM Topology Prediction - Predicted
Type: TM
MNGIHLNSTTPMPEPSLGTIERCTSVGADLLEKSHFLYRPHSVPGSSSFLEETPAPDNYTPRLSKPAFCSL
PSNLSSLGGRLSLHVAPEVEQLPDKTGVTDFMKSDQTMYGGAAVNGLNTQVTPKTEAASLLVGAKNTPAVF
TMKRGTINVSVRVRPALRGSVKPTCCRLHKEDGLIEYVPPPKEFSVQPKAQGFASGSPFDSNSQQREIEER
GSFFAFDAVLGGDTTQNEIMDRVGRPILQNVLEGFNGTILCYGQSGSGKTHTMLGPAGGLPERLADPDEAG
ILPRMLQELFARLDNKGLHQTSSATTATEQERSQMRKTEAMSGGEASQPAREWGYAVEVSAMEIYKEDMYD
LLQFILKPKTSKKGCDTTNETGAGGQSIDAKGVVCQNRVAVGTASRRAKSTASSATLKIRDVANVGVVVEG
LSWHPVRDAEEALHVLKWAAVQRHVGSTAVNQRSGRSHLFFYIALRQREAPVVNKGQGALGLAPQPPLVIT
EIRSLATLVDLAGSERVADTKANGKRLEEARHINLSLTLLGNVIRKLTTSPKGGDAVHIPYRDSKLTRLLQ
ESIGGNAVTALLCTISPDLKDATETLSTLQFAKCAKRVQNRPVVGRTETKAELLAKVRRLTVRNKWLEKQL
STMSSRLGSFGATSYSPELSDTPARDPRIVGLCSSRLSTLHHERESDDTFLGGFQDSLCSSPPVSLSRIVP
HLCLRRKDGVNEGQVGRTPASEPRMRARARRIYRVTVFTSLSFAFWAFAARLLRMFVPSVRCLPAPVEEVF
IPVADCPACMTVPLRRSRATQFESCETSL
>tr|C9ZYH0|C9ZYH0_TRYB9 | DeepTMHMM Topology Prediction - Predicted
Type: TM
MLDHTIAIGAALFALALVYFVFRSIARTDVTTISHMKQSKLVSRPSKTKKPKERKDRREDERYQREMDALI
AREVARENVSMRSDTHKPQPKLLEEVQKDTSRRPKTPTSLSTAAVEKQAKIDKESGFQPVVNQRKERQQQQ
HQQQQTSAPKPMLVNEALERKLNHFFSNLNRKEKLTRLSQPEEKPTASKGATIIVRKDIANARSWQQQQQ
>tr|C9ZKN1|C9ZKN1_TRYB9 | DeepTMHMM Topology Prediction - Predicted
Type: TM
MRFRFSSFSFSFSVFFFLCVRCAGMRDNNVWGGGWEGCRNYYRRKVRVNSKAKGKTKGKGKESFKDYKREMGE
KRQDIYIYILYIIYNINLVVCARIYDLIRSYINHCFFLSPTSFMT
>tr|C9ZHU0|C9ZHU0_TRYB9 | DeepTMHMM Topology Prediction - Predicted
Type: TM
MAEVAQVCCPAFPHIFTATHPCTVRTIRDRPDYFLPPPSPFLFFPFFFLCFIIPLSCLCSFNLSVLNHTRT
AFAPSAAWIPRTLWSHVARRVALSGGVEGCRVTDVRECLKQNIMLEKVAGSSSSSR
>tr|C9ZQ03|C9ZQ03_TRYB9 | DeepTMHMM Topology Prediction - Predicted
Type: TM
MLYGATCKSDQLFVPAGSSTFESPQPPYNFILSFLIPVATNHSLPSATINNKVPVCCVVILLANCILTIKR
RQGLTYSNSKLCFLEKSQDLLGIGATCSHVGCVQMHFTM
>tr|C9ZJB3|C9ZJB3_TRYB9 | DeepTMHMM Topology Prediction - Predicted
Type:
TM

MYDCVCVCVCVCVCVCVCVCVCVCVCACVCVCVCVRVCVCVCVCVCVCACVCVRVCVCVRVCVCVCVCVCV
CVCVCVCVRVCVCVCVCVCVCVCVCVCVCSVCVCVCVESVCV
>tr|C9ZWQ9|C9ZWQ9_TRYB9 | DeepTMHMM Topology Prediction - Predicted
Type: TM
MTSINAQPPNSATYPQDDHGSAEVVNLNAEVERPQPEERKDGGGCFARVSLFMATIIPPGGIAASAFNIGS
TTVGAGIFGLPAAANSSGLVMAMIYLIIITAMTIFSIYALGVAAERTNIRTYEGVARALLGPWGAYYTAAT
RAFFSFSACVAYVISVGDILSATLKGTNAPDFLKQKSGNRLLTSLMWLCFMLPLVIPRHIDSLRHISTIAF
ILMIYTVVVVVVHSCMNGLPENIKNVSVGKNDNAEIILFNSGNRAIEGLGVIMFAYLFQVVALEVYENMTN
RSVGRFVIASAIALGICFTLYVMTSFFGYLDFGRAVTGSVLLMYDPVNEPAIMVGFVGVLVKLCVSYALLG
LACRNALYDVIGWDFREVAFWKHCIAVVTLSVVMLLCGLFIPKINTVLGFAGSISGGSLGFILPSLLVMYS
GGFTWQKVGPFYYLTTYAVLLTGVIAIVFGTGCAIWGTFTG
>tr|C9ZWK3|C9ZWK3_TRYB9 | DeepTMHMM Topology Prediction - Predicted
Type: TM
MLSTVTVITLVVFFCFLSHFHSTPDVPYFTFCCFCCCFVFFFLLFLKKIYLTLSYLILSYLFFSLIDLSYL
CLFAATVSCLFCFAFFFPPLYSSLAGVVYYFIWSHF
>tr|D0A4E0|D0A4E0_TRYB9 | DeepTMHMM Topology Prediction - Predicted
Type: TM
MPCDSPVSFSIFCFLFFFSSSRGIAVEKGYQQSDWRVDCSFFAALRFTSVDISSACAYICILLATKYMGAI
VSLFKILQAFMHTHTHAFPTDFSPPPPSNHQQKKYPTINRVYFRIAQYARRSNEREN
>tr|D0A137|D0A137_TRYB9 | DeepTMHMM Topology Prediction - Predicted
Type: TM
MHVYKYIYIYIFIYIYSFSHLQVHYPYVNTMTDSSFQLPVTHSTTFGFLSSPALPHLTFPMMDLHPFFSFI
SSPPPPPIFTLLSTLLPQITPLPSPLHGADMSLNLFFFLLFLVFFLFF
>tr|D0AAR2|D0AAR2_TRYB9 | DeepTMHMM Topology Prediction - Predicted
Type: TM
MQSVQSPVVLCPCTSICTGVYMFHSYEYLRMHGYSCPLCICTYTTRLMHWILSKSVLLMRLPLFLFVYYYY
YFEEKRGGRKSYTTTCKYSTVWACVCGIEYQKKKKGGGEKSFVVIVSHRVTVMGVRMFCDFLKLRRWERHV
LAQRERQESALAAAFRCIPWSGVAMFFFVMIFFGIDLGAGIKHTARQIQEKKEEAAQSVEVQKMQARQWSV
EQVKNFREGEIPKPSWAEGGPRGSNNPL
>tr|C9ZI02|C9ZI02_TRYB9 | DeepTMHMM Topology Prediction - Predicted
Type: TM
MEKVATTASTAALRRGINKKEKEKGEVVDGGSARRKKGVNAVEGGQSLGDSEARQRQLLLSRKDAVTPIPE
VALRMFNIAMEDLCNKYLQDYYTAAGNLFLVHSRRLSPQTNGPRSSQQVQSQRHPEKVIQKISSVPVKTAS
SERPSQAPSRGCSTSFRSSRLSAGTAGAATITRSSQSIPLSIRRPTSLLSSSKVVQSRKDRSTCQGGTGTT
EDINHTSASRNKASRTEFMDARTTAVSDERQRSRLFTMCSGTTSPSAGNSSRRWTAAGAEANQTHPSGASK
TSANSPASPVPRIPISKVWKASVSSRTADKQLESPGRLTEPLRTRLYATDAEVSPEHRRQTSPSLLSRKHR
ESRASATPAKPEPPLNESSRHPRASSATTPRMSRVSASSTPQFSRRSLVTTPRVPHRASVRRAGESPHTSV
SRLTTPRQLRPCTHCRGRGIEGGIIGLVSSTRSADLVKESVDCSTEYRLEWSCTNSCAQSYTQQTSSQRRS
TALVGPNRSTLTGVFEWPVGRHTPLSRPYSQTAVPRGSITRDGGCPTTDVTPPSPLVRSLTLISESVNETP
AEYISRHFSSCVINSVLECVRVKMGGRKSDEADPRADELNGSDSSDDTDKIGNGDATNDISLGLQKGDLLL
ECNGQPLSGAASLQEIIRKALLCDNNTVLLRVLRGADSIAVRETLLKDSSAVRXXXXXXXXXXXXXXXXXX
XXXXXXXXXXXXXXXXSTQAVIDGCILFFVAFFVSLFTQPVVFFFTQLLLYVLEMTICV
>tr|D0A0N4|D0A0N4_TRYB9 | DeepTMHMM Topology Prediction - Predicted
Type: TM
MNLGQVPRPRDEPEPFNDFEPRRKCSICCGLRCAAVTGGALFLIVLPCNLIMIRGRIFDGRGFIGFADRHL
LVPLPFAAITFTHQIIQSEALWSKNRKTLWQVNTQSAGLNILLWSILTVACTAVSRRQGPRWSRSYRLLLW
DYRRTRRCCANRYLPTAFGRISADLDWYNVLWTVSVYHVLWGMFAVMVERGFGAHYAMFYRDWPYSRWCSP
RWREWRELEAMKYINTEQVVAPSRWGSFITNDRWRSSSV
>tr|C9ZSU0|C9ZSU0_TRYB9 | DeepTMHMM Topology Prediction - Predicted
Type: TM
MEEPVFSLVASSWRKSSFSLFFPLLLHLLYNYLYIYIYIRIVSHTCVEGRYRMNREGACTFFRWGKCLIPM
SSFCMFCPSEKRGDYNRLWNCASHSSLTF
>tr|C9ZMG0|C9ZMG0_TRYB9 | DeepTMHMM Topology Prediction - Predicted
Type: TM
MKGLSRLDVFPKFDERFERDARQRTALGGVLSMASILIITFLVVGEVRYFFSSVEQHEMYVDPHIGGIMHM
KVNITFPRVPCDLMTADAIDAFGEHVENVLTDTARVRVNPDTLVPLGEARPLMDMKKQPADGNGAEHGKCP
SCYGAESNPGDCCHTCDDVRRAFAERQWEFHEDDASIVQCVHERLKMAAASASTEGCNLHASFSVPRVTGN
IHFIPGRMFNFFGQHLHSFKGETIQKLNLSHIVHSLEFGERFPGQSNPMDGMANVRGATDPSEPLIGRFSY

FVKVVPTVYRIESLVGGGRVVESNQYSVTHHFTPSWETPKGGENNNAKHDPSVVPGVFISYDLSPIRVSVK
RTHPYPSIVHLVLQLCAVGGGVYTVTGLIDSLFFHSIRRMQIKMNRGKQF
>tr|D0A018|D0A018_TRYB9 | DeepTMHMM Topology Prediction - Predicted Type: TM
MSKWLAAVCISPIRFLHRTAVGAGLWGPVSCIFHTIVHMKCTYHLKLLLSQSPVAAVFVVCLSFVRLQYIY
IYIYIYIYIYICHLLFYRELFLLQIFVFSILVYHFTVCVAPILIHLKKMQ
>tr|C9ZIU3|C9ZIU3_TRYB9 | DeepTMHMM Topology Prediction - Predicted Type: TM
MNRLFGSVAACSYCYCYLFVVLIASLPVRLSSAWLGSPPKQQNARGRGFSTFFFSWRIILLCLPRECCLPH
HISLLSPSFAHCLIAFHGPSN
>tr|C9ZYN2|C9ZYN2_TRYB9 | DeepTMHMM Topology Prediction - Predicted Type: TM
MVGKRGRRQSPVVPLPVGLLSGSTVVGCFRAWSRKELLVLLLSASFTLFFAFSAVRWLTTTVRTDLQAILV
EAEPTFVEVPFDPPTGKFYKLWRYRDNKTDSFRKLQPQAFPNVPVIYIHGNAGCYQDMRFFGRVVGESVVR
LRRYNAIHYGERVRNKIFQLYKNEGSELPRAGIQIPKDIQRRAENMVVADTPMLGVELFAPDFLEESNAHS
AIVMAREAMYLNHSVHELFRRFLDHYHDVLKQPPGDLRHAAGGNRSEIPVVASVENEYVDSACGTWSEYSP
DPSACSTLKKEVAMFSSTERIRQEVQRVEREGIWLWTESIGGVLGVFAALLAPELYAGLVMAGPPLRYSPL
LFDLPAVYFQKTIQDAVTVPYANISNTQRNWSKILAGSSTYELLKNLNSVPHADIAGRLERVSLVSVHGGA
LEDIVPPRSSHIMRTVSRRSTSPEHQPLLATKPPYAGRRDVCTEELRGCGISLSHRGLVYAVQLLDSAGYY
TVVASLTGEAGRLVGVESTLPVGRERMFPMVLETLSRNSDAYRAEKFRFTTALHYDSENDKYHMTDGRHFM
NGLTRLCADGQSTLNLQDLPVSEDEDTESWSPLHIFVGATTYEAEEVILPELTLVADEENEEPLSQSNLQV
RLATKLHLPYRRKDSNVTEGTVLRTALSFQVLRRRREQSSLRLRPRFCFFVRNEKVNVSHFALVQHDVIDP
LRELSSPEVHDATLYSQLSGDISVEKYGRFSLIRNMRVSQMETSLSLYVKNRTVYPVTICGSLRSLRVTFR
GGEKVALDEPEAQNQYFFGPYGENVSSFTYSWRPFHTTPFNVTNVYVVVYVLTPDIQPKMDFEDTKMHQSPE
WIKPSYWLWRLNYDSLRWMAIGTTYSVAIQLISCYVLFFFVIIVVFHVPRLARGGGEPEKGVRGFFEVSPT
ILAIAVGLGIELASLHVVRQTLSVCLDEEPPRVSGGNITDMMSLSEALTLLFLCALPPRLDTCRHSWIRMV
AAVPADVTLEHMVHLVFSYCLTVLSVSLMQVQYYLLWPLFKVIRPLLIRRPSGRLAVWPFAFLWLAPMVLH
ITVYWMHVYVTTNISCIFALASLWCIPEEMFTNTGRRYQQLCLLVLFPIQLATHFEGSGLLIRNYFMLPTE
TLTDSERFESLPEQIIALTVAQGCLAVVYGTVYLALLHQDGITDRGSAKKMQYVGGTNCSGGEKRREGKKR
KQRERLESQEVLGERTIEHLEYSMGDLGRRYPKLKLLAKVGSFTFMFVACWMGVVALRRPLEGTVFLFGLS
VVAARVAFALLSFW
>tr|D0A994|D0A994_TRYB9 | DeepTMHMM Topology Prediction - Predicted Type: TM
MSSHFSGREEAVHRSSLSYLLSPNRPRSAGEQRFIRHAVFRATAFIGFIFYFIYCNPEYSYTYSYLQKEYG
LGLGEPWLPKLLKLQKRPPVE
>tr|C9ZZ64|C9ZZ64_TRYB9 | DeepTMHMM Topology Prediction - Predicted Type: TM
MPVENVIDRDAVLRTKSLNNDLKDIYTCTMKHKKIRCANERKVALVPFCFFSLHYFSYLNFLFFKLFYLKK
MVKQFFHFLCHCCCCLWLRRLSCNLKNHFSSKKKK
>tr|D0A0J0|D0A0J0_TRYB9 | DeepTMHMM Topology Prediction - Predicted Type: TM
MYRLMVPYKTAKWASASAPRAFRNAFRRCSTQEGEAAAASRTGKSTVGDTSAGCQQTGPQRKDWRTGKEVV
YVKGVPVKGPLRKLPRKEQILVCVIFSITGSAAVYFVRPVIRNCVTNGFLGLPEDSSWSNGPWLYRLLYVS
IMYPSYSFLLFVIGSLFGRRVWFSFMIHKMWSRFLPKKAAKRLEHMLDLQHY
>tr|D0A9V6|D0A9V6_TRYB9 | DeepTMHMM Topology Prediction - Predicted Type: TM
MRLNDALRGAPHWFEEKSKHKRRCICDSSFFLFKRLGGSTRCLLGFISMTHNKEMQNVNIYVSLLRRRRQR
ESLTEISTNCMRCYYGCVACNICIIVLGHFCPILPFL
>tr|C9ZJ06|C9ZJ06_TRYB9 | DeepTMHMM Topology Prediction - Predicted Type: TM
MCGWVSCVFLLLPLFIQLSPSITLYWPQPLFFSFLLLLMLMTMFSYFMVVTGILLTCFFSFFFVYVSQLCS
PHDFYQHSSKLLEFLLFFSFFSTIHFFHILYLILSSCCFFFFFLYSLVSHLSYLLFALLCELEIIIIFFIN
VKRNGEVFSLRKKREKEKKREVK
>tr|D0AAQ5|D0AAQ5_TRYB9 | DeepTMHMM Topology Prediction - Predicted Type: TM
MSARESESRDGVSCVSSVVEEETTSPWRHNEIERYLPYTIGRETAYVALASPDTVSPETSFSGHCSTFARA
TSEADGRHRVISDVYCGDGGSGSGRTGGCDVERISYVNDCDGAEYSGVKTSSVTPVADSVRSMEYGVGYGS
PLLHDTQELRVSWCYQRDDRAAGGDFTPAFTVSPVITPWQHPGNGRKERDVVKSAPLPPNCCWLWPDIREE

KDGSSSYDVGWRGLKVTEGVPRVNYTDGVPDYLEPLLFRDHLPKNSLKGTEGPAEGPTPRTPGKKVKRVRF
SETVVGSVSFVETHNEIQVIVESHYPWLALAGLVIAVTFFVLHWGVIAVIAGPREGIERITAVSLVDFVSS
GLTSLIMLLFLALLWRPGREEVDVISSCSGISRVMCVAFCGALATLSLVCMFMFTNSAVGFVCFCCCPFVG
THLYEVLKLHNVPLLSTMASILIVWAFAALVIGCFVEGDAVTFRCLWPVAIAISGGAAMCVYLFQFRSVGR
EVSGLFLSFVTLVTVTLMLALASYASGGFSLPTQSTQSSLFVLVMTDFSSVLFSVFFLVLYHLIYHCSSPF
FSRLCVPGSFVFGGAISLLISRLLLESVVFWPLEATGSILMMAGSALIFVAEYHQWQSAGAIAGVE
>tr|C9ZSX5|C9ZSX5_TRYB9 | DeepTMHMM Topology Prediction - Predicted
Type: TM
MASVIEKGLASICCVTTSSKPWAEGEADGDVKYEDLRGPGGRNTSGSVSDKRICVPQKIDPKTFFANERTF
LKWMSISVMIGMMSLTLLNFGDTSSNASELAGLVLLPVSILFMIHSLFVFKDRANKIYMREPMRYDDTKGP
TILVLVLGVSLGIAAIFSVQKQYYRTASSSFNDGPRFS
>tr|D0A9X0|D0A9X0_TRYB9 | DeepTMHMM Topology Prediction - Predicted
Type: TM
MSNNNNNNSNNNSNNKISDFLIITLFFFFIQSIYIYIYILSSILFSFFLFFHITSLTRSFSHLLKCEGNVK
KKKTQQRKKNGKRKEKKKGIAKEKERGKKKMKASFLFFYFIFIFLFSFNSPFFWSYGIRQREEVIRSTKII
>tr|C9ZLS6|C9ZLS6_TRYB9 | DeepTMHMM Topology Prediction - Predicted
Type: TM
MMMVMMMIIIITIITVWCVFYACGSDNVVLSMNGLLQSGLCVCVCVFLLPIVSNVGFFLLGMLWRVVLRKF
YIVCCSIFASRCIFPSFPPLLSLILLLIFGRVNTSTALTFFFLLFFFKNKNNLSDSRQ
>tr|C9ZKS8|C9ZKS8_TRYB9 | DeepTMHMM Topology Prediction - Predicted
Type: TM
MKPLSTLMVLHDFFFTLSDNSFPSEFDPVEPECALFVTNSSFPERGNRIVVPVESQLPEFCREFGCSNCYH
YEHKNLCIVLQEKQKNLKRRNNLGAAFWSLWLMWDFFGLISLLQSRIPRVSFHCMCIKPILGLAI
>tr|C9ZLI9|C9ZLI9_TRYB9 | DeepTMHMM Topology Prediction - Predicted
Type: TM
MGKRKEREPEVGKSSRSPFPTVHVKISVQACTGCGNTKKIQTILVGIRIQRQINVTKHMKVSSIKGGGKEK
RSGTGPVRNSSPKQIDPRHASYFSFAFIFFSSTFPTFLNTRDLTGRYVNTYICVYENMRRRRSVVLALKRK
YNTKGKKKERWGGGAGGGSYVAQNHFLFFFCRRNPVWCYQIMKEINRQTTNH
>tr|C9ZNK1|C9ZNK1_TRYB9 | DeepTMHMM Topology Prediction - Predicted
Type: TM
METSEEGNKKVWGIAPLKSKKRKKKRTKHHLPSGCFSSLLLVFLSLLLLLFPSFLFSLLTSLIIFFSDKLC
ANANHTYIHIYTYTYVYKYYFMCICAASVQMIVYSHLLYDFFLFCFPFLFLFVCLLLLLLLLLLLFLQLFL
FSLILFPFLFFFFLIPSFLLFSFDFLFFFCYFFFFFLSFVKLSWPFALHPQLQSIQTENTHTEKRKEKKKK
QEEERDWHKNRHKIKTQKNGRKMEEKGKKKVK
>tr|C9ZZ78|C9ZZ78_TRYB9 | DeepTMHMM Topology Prediction - Predicted
Type: TM
MATRDRTTEFLQYRSAKTRQTDSQGLLQEDRGASTFSTFVAPLWMQKMDEVRELQRKIRKHMESLEKLWRN
NLKIEFSSSRDEGREEMDIERLRVSIDNLFKQSEKVVNELEVAYMRELPDEGTDAELSILRNVKMCLVNEL
SNIGKLYRESERRYVMDLKKQQSVAKRWGNSERQRVIEQELETDAVMNRCLQKGMSQEQVEAMLLNQQLAD
ERVKEFEHIYTSIKSMHEMFSDMKTLVIEQGAVLDRIDYNMSITHERVQSGRAELEKAAEYQEAGLFKTCF
LFLVVTIFVLLFILLFQKMLS
>tr|C9ZQQ5|C9ZQQ5_TRYB9 | DeepTMHMM Topology Prediction - Predicted
Type: TM
MHGNFVFTGKSRPNRLTLSNDRQPSKNVILNNARAQREQRQKEKRECQAATCIQRNTRGLLARMALAELAR
AALKEIKETQDAAADGGTSSEADEPLERLCWLYEFVRRGIVGLLVCSVQLRECSQILLNRLSAVLRGGALE
EQLLGFPHAIMLLMKLTLEAYLTPSCDEEAVVERSREHLQPLASLFAAAKKLGNGADILICALDLVIRHNA
TPDEEIISALYGLYATLSPAAAASSVQSTASRTLLAYAVGSTSNVSYYLSPTTCALSVLLGTVRRDCYPRV
SEETNAIFLEAIVDVTLKILESTASSPPLQILGKLVRVLPYFHHGADEDAQKQVLRRKWLICLSRATLLCS
SEDELIRETLSDFHHSHTTAHQEHAAHKHLSTYLFSTGYGLRLLEEGLRTSLSAHSTDSFSDVNLLCCVFI
WPLYKFATASARQRLEATTIISKFVYSSGLLRCLWDVYRNCHSSTFESTAAMSAAELLECVATGKLLDLPS
PVAPPTELLSKLSLPRSTHGLFYDPYPAVSVTLFCLMSYFVDATGFLEGLDRCAVFDRADTLKLIFALKGI
LYQSFFHGVLPYSKCEVVVQGALTLFLKLHVVGEAQSFVPHPSVWIICHDHLLLKTFKSIECGTWSAIVSN
EDDEDCTEDDNEEREDGHWAPSLREGNHESYVSLPWWNGSLSWPQEERCIHVLQRIPFTVPFGARVTLLTS
FLSSHVERSRPTTWGHFVVRRGCTFADAFDRFADNPGSSDMYYVRFRAANDLVEEGYGDGVYREFLLSLCK
EGFAAEHGLFCLTDAGYVYPNPFSWEVTGDRDHLKRIKFLGAMVGRGLRDGVLQDIPFALHFRNAILGRSN
SINNLRSFDSQLYRHLVSLMSLSEEEIENLELNFTYTVEALDRVHEVELLHGGRNIAVTRRNCLNYIHLIA
DFKLNRETAKQTRAFRSGLESIVHRSWLRLFDSNEVMKLFGGDVECNIDVEDWKQHTQYHVPDDATSKPVQ

VFWEVVQSLPLEQQRKLLKFSTSMNRPPLLGFKFLNPPFKIHVLWNEAEERLPSASTCFCTLKLPPYQTFG
VAQQKITAAIEETDDFGLS
>tr|C9ZL95|C9ZL95_TRYB9 | DeepTMHMM Topology Prediction - Predicted
Type: TM
MFSSNAGCCLATCTTGFLVPRGLLRSRGGGSAAAFAGSSPSAPLICSKCAFIEHNTCPKKWRNPEKVEVSR
RHGGVKLGSEAVRIREQAAVEQQLVDADKFTNWNIVWGFAAAAALLLVALNILLEAVEPVPSPEYTPYVPG
ATTITAPAERLKNEKFCSSVGSG
>tr|D0A1K0|D0A1K0_TRYB9 | DeepTMHMM Topology Prediction - Predicted
Type: TM
MTDNQGSYITTTKLEEVRLLAVAGDRSAAVYAAHRRMEDNLAKLPAVLADTARYHPTSVDPWVAVFAAVDS
LCKDVELCLQLFTIGAQVERSKGLPTVAVLYEQQKKPAARLQSFAATVQSAFTCKGVTIPVPASLMLRFAE
AIRPFDSSQATKWLQCSFEEMFNRETTGEGAGNNYVASGPTAVAVTPAHATQEPKGLVVAMLTLLIKWRAE
DGEDVLWAQRKRKELSQLVEENVLESLIRFEVESVAAVARRKQRLQKKGERQATKRQVKPTPATEALPGSA
VEATVPAQEVSTLGRLRRYVNDLLATNWSQNPSRAACVIIAIILLCILARKALCTVSLFGGFAGGTPRGGR
KLIDL
>tr|C9ZZW2|C9ZZW2_TRYB9 | DeepTMHMM Topology Prediction - Predicted
Type: TM
MSVVTSERLTLHKCVHLKSPRISFSIPKRVRKAQGKDLYFGYFQLFVCRSCFRRFIASVASRLWAGSCGWA
PNTFFLQLQCLHLGYNYFPFPGFLRNHSFRGGICSYIYIYIYMCVCVCVCVCVCAQLLSFSPLVNPPCYVV
PYFLCFTYTPVSSILCSVSGRE
>tr|C9ZNT5|C9ZNT5_TRYB9 | DeepTMHMM Topology Prediction - Predicted
Type: TM
MMEVVITALFYFIFLFLFIYFFPHHLNFHFHASHLYILLHLPKGRGGSRVLIPTISSPLLFFFFLSSSLLR
SGYVALNLLLFYFIYLFSLRKDTYVFDTMFCFFPRTPTCLLFFLSFMVVPLVPLIFSSPSLSFIFFFSHPT
PPLSLLFFPLISLTNISLRLPRCSTSFRFYLFSKKKKMAPPVLLLLFCAFARFHCTVASCKNENNNNNNNN
KSRNRNSCSCG
>tr|C9ZJQ7|C9ZJQ7_TRYB9 | DeepTMHMM Topology Prediction - Predicted
Type: TM
MTIVAAVLPNTEFYFSRNFASVVLPVILLFLPLLYCTSPAVFFSLLHYPLSFCPVICVDSGSAVGMLMGDP
LCARIICLFFRWELHNYPQCVVFDYLFLHPRGSVGDPLGCILHA
>tr|D0A882|D0A882_TRYB9 | DeepTMHMM Topology Prediction - Predicted
Type: TM
MSAFEFLRGKLGGKVPVRLDCVPPWAQCSSGGKVEEADWQSALDALSAGQLFLLRGITSEWRSGNLVQVNR
YLQGCTAYFDPSKPHLLAYFVGLGGIDLFVTLLCDVNVPNGAADSSPQAPFRADSRDATPFHLVGAIDLML
RVLTELMVCHNELGWYYYDRYPGLFFRLLELANVPELRLVSLMMLEHLLLCVGPVLEISKVPALQKLIRVG
DDVVLAVICRVVSLLIVPGVVLDQRESVPHRLLFPETLLPLQRIQRVIDSNVLWLIGEKGLVQRLVALCEV
TEPNSFRMALGSENLSNSLAGYSFPVLLPNEARAATPITTRGLDVNGLTEYGLGDPNVLQPFVADGNGVDR
GEGGRVRDYASSVGANPVSPTATLPVEVVDTLRAFLQPSLGVSAALYQDDDGASEISVETTGSGIDFSWFV
GCVDAKQRWRLRDLTLCEELDDNRVLLCGFGPPSDKKAKESFEEKNRAFWNFLSPVMRVPLTLHNRYAIVG
AQSEVIFVLNVMLSTFFVGDVWRTMKECKWIEVASKFYDRAFQPRGDSQVLSPYLEQWQHMQELRLLPRFL
IPENGAGEARRAPKQRTIKRGYAAEKYHRNEDGVDDNSNSSDSEADSDDEREEGHLLSAMRPGEDKEAYRR
PNSGHMNADTEEGIDEKTHHHEAETIRKLELLRGVLEFLNTQDRFECSMLQQGDILSQAAPLALKVAKMLA
NRSEDSCVETTACHALEGYLRCFSFGCVSRSSPNEPQTAIGDILMRSILEHRVYNATFVPGLSDSLTPSKR
IESVFSLLGELVRYHYDNLMLLQEYVIGNVELSHLNDPAVTVSSHSRQLHVGSGQVEQLIRLPPLDREEHE
PFVRVLLRRLRGYGCDTNLFMRSLLLSLTPGLRSKINYMWKPVTEATVDAGESLSSSSRIGDIVTGYPHRF
SYITACSRRFVVVISEMQRMEKALGKAVDAPTSTPDPTRALENARSSSFGHIAHLTLWDMLQLLLRSPHRF
SLEERPFPVFFDDTDRAIVMGEIDLPLVGPPPHLVQPLFEKRDHYKSLSELEKTLLREPHELVYGMLGPLN
AERIHGTGRLCVVTTCILVFARVACLGGAGAVRGVLEKLKPLARVGYEKWKKEREALGRGVKKRRRGDRWG
PGRCFNDDRAASDGQCHCKAEDGRLRCSAFLLHGHCKPLGAVELYYRCYGGCFFRNMFRLLCFWVGHYGAC
QRYVETLFYCTEVPFAELKSVMLYLFRLLPDYFLPDMA
>tr|D0A4X7|D0A4X7_TRYB9 | DeepTMHMM Topology Prediction - Predicted
Type: TM
MYVYLFLFFLYKIQEGAKKKKTTTTIIPSQLFNIIFFSFLLSSFNIIFFLHVLVLLFLYSFSPFFSFFSFF
FSFFPLFPSITIPSPLPLSSPFPSNAFKCTHTCNPSLHQNMLKYNIFSS
>tr|C9ZQI9|C9ZQI9_TRYB9 | DeepTMHMM Topology Prediction - Predicted
Type: TM
MYIGLCLCLRKSLKTDQGATCNSPNTVWSPILFCLVFFFPLLMLLYLTFCISTDSTHGNISIYVCIYVYMY
SPVFVPIRIFRLTPFALPFFFFSSTLCFPLFFTLFQLTFAYNFCLSTTFGLVHYKYMYTNIRSSA

>tr|D0A4B3|D0A4B3_TRYB9 | DeepTMHMM Topology Prediction - Predicted Type: TM
MHSVHESLLGDDAAARNNGRQSTINRISMLVAQRLSVVGMEEEVRHGSVAGASVNTLCNVIGAGVLSLPLA
MHEASIVGGFTLMLFMALLGGLAAFMVIMGCEATQRFSFAEVVAHALFPSYTFEDFCTKVGKLALHDLGCV
GGGAMETEELHRKHKEREAVSRRKRRAVIVLLELLVFINNYGTLIIYSRVIGDSIPPVVSSLLHTSGIIAT
RTFWLVTSGVIFFLLSCVRHMDELKWTSFLGFITILYIVVIVVVRYVTSLQNPPYPDVDPAALEGINWCKI
SVNILRSVSTYSIAFSYHSNIPYFYRELRNRKPHTMLKSVYIAFPIVTVCYATTGFFGYLTFGTLVASPAA
GGDIVRNYPADDLLVNIGRFGLFLHFACVYPILSVCARRGLHRVIMHALTWNRLSTPDEEDETPPANETTK
YYSTDHRYKMSGGSDELPLSDDADRDVGSPEDTTTLAIVLEALFIVCTSVVLAAYISGISVVIDFLGTLLG
TVMMFTVPGFTGWCILSRASPLGGSTVVSHTRLFMVLTFLLVVMGIACTSLGLLFLIKQYVLPAL
>tr|C9ZIY4|C9ZIY4_TRYB9 | DeepTMHMM Topology Prediction - Predicted Type: TM
MRPRVLLFFMEGQALRAERAIVKVTNEDSLGGADLYQRVAQALRIFRTHWSQGGVLYVGISWVWMCCTTLH
HRMWWYMHMEKTLKGSVYWKEGNSASLVQNF
>tr|D0A423|D0A423_TRYB9 | DeepTMHMM Topology Prediction - Predicted Type: TM
MSFAPTRSLPNEVNEPLPTLQSEESGEATYVHPDARALFNKVPCLRRLPMFGTAAEGYGPKAVMSISLSYF
LCKGLSDYLVRDSLLAMFTQRFGVDVSVYQRLSNVANMGWSVKPLTAVVSDIFPLFGYSKRWYMFISCLIG
PLLSLGFGLLPGKESSAAIGAALVFLCCFTKANVDILAEGHYSRMMRRVPAPGPALVSWIWWFIIAGSFVS
SSVVGPLGDAKIPQVASFIASVVQILVLPFFIFNWFGELPNREERYGDALLLYKKKREEREKKCDPFSDSL
TQPDRAINSESNPLSTEGPDAAGLVPYEGGAPTGDGDLSSFHFQEPGSCCCGVFEYNKEVVERNGRATVYS
ILIGLFVLVVAVTSVLGSTTYLTVVAIVVSLLHCSFNFYAFPLIIAKANLFGYLQRATSVSFPGALSNFFI
AEADCLADGPHFSFAFYQTVGGIISCAASIVGIMLFNYIFSKRTYRMTYLSTLSLGIFSSIFDLVLVMRWN
RPHVSDHALYIMGDAIISPVISMLNWMPQIVLLSRLCPRGSESTVYSILAASSNLGASMGSVVGSVVMEYA
LPVSSKLPCNFENLKWLVVIAGFLAPLLQIPMVFTLLPRARMCDDLDVDNATTKKKSEIQKEVAEVQESGS
T
>tr|C9ZYP1|C9ZYP1_TRYB9 | DeepTMHMM Topology Prediction - Predicted Type: TM
MILWCLSVDEYIEGRVKVCHEIRLLILLLLLFLIYPFFVGFKRRSRKRNIDGVFFFFGYWFTLIGLDVTFM
PYLLRRSTYYHSYKHAYVCLCVRLRVCVCVFMHL
>tr|C9ZNF4|C9ZNF4_TRYB9 | DeepTMHMM Topology Prediction - Predicted Type: TM
MPYFPGDLSCGFSEKFSSCRALSDPCRCVSSTAHPVGTVGRRKGQFNLFPFFALHRFLRQVSFSLLKNRLG
GYPRHVAACVPIVIAFVQTFYHRYMAAVVPSVRCLCFAPCLGCFPCSFPFPLMRFYR
>tr|C9ZJG2|C9ZJG2_TRYB9 | DeepTMHMM Topology Prediction - Predicted Type: TM
MGERRRKKSKSNNSRNKSLFIGLYVCYVVFFAQLYCAHVGSPFMCFLHSVLLYYSTLISTFFVFVVWERWK
EICCGAPRVLFYFFLVIFSQPSISLAPLCCVCLI
>tr|C9ZKF8|C9ZKF8_TRYB9 | DeepTMHMM Topology Prediction - Predicted Type: TM
MPSVAVMVLGISSVPKTSRSVASHSSSIPHFTSSYWNVLPVSELGSLSPHEVVLPREGKHSIVSWRIRAKR
SLMIYMYVYIFPLLLVVALNLVTTAPQLWLKNGMNKTMGRAGTGLREVSHCQ
>tr|D0AAR7|D0AAR7_TRYB9 | DeepTMHMM Topology Prediction - Predicted Type: TM
MSSGDSSGGGGGVRELGTVTAGMAMMNFDEKTKPYTRADLIKFLQNYEENLTPPEKQLVWQGMLGTLVGMP
LAFFVGYKVSSRFAWHRVRRALTPIGGKGREEKPSWLIRNISTVGQVMFGLAASTIPYIVVQQWFISRVLA
ADEHEGNLSFHVRRLMITQRSSMMFTRTATREVTREEQERLMGEAAQHSAENRSGRQGGAPLGVTDVNLRL
GQQAMTPVAQTGYKPMPGQSS
>tr|C9ZI58|C9ZI58_TRYB9 | DeepTMHMM Topology Prediction - Predicted Type:
TM
MNVTNPSTVRYADAFMSYLRFLEKDVWWTIVYVIFLAWMYLFHAPFRVHNVWRTDWYAQVTKVLQVWIGVV
VFLFHTGSGFFQPQRSLQAEICNADDDTVATIFFPIFSTTTLIILYIHSKKRVWGLGVTYGSSTIVVVLNS
LLLSLFSTVHFAYGMRIQDFEHSSLLKGAVGVLFPSPKQTLFPTLLLLSVINVLFITFDYQYKKRCGFDPL
RGLLWSEVCRSRKAHEVVPTTIRDGCDWRKLQPKPKRISKVEQPWIVSPEGGKRLSFALSSLEIKQLSVQY
PVNRPGMVPWFSTFIAGTALQSVLGNSLNFLTFDQRAVESQMNPKVFKLSFTKHRSKISKSYSLSRVSECS
PSTSVDAVCCDPYGDDDYENVWFDFVADVGDGFNSTYEMARLMAQPFLRLASSDSVQTRSGLVNSVSVRDG
GGRRGLMTPPLTAGDGAFLAVEGNGQQNRCGRTLSMCDYEPQENFSLPRASFVVVGGDLAYPNPTNETYRT

RLLEPYNNALRCCAPLCKLVKKWYNRLVVPEEDNKDVARIHMLSASKVSEMTQRRDIADMCLGEDEVVHST
PLLFAIPGNHDWLDGLVTFKKFIIDESWMGGWFMPQKSSFFVINLPYNWFLLCVDTGSVTDIDPGQRNYFL
NYIEEHLDVSSCVILISHEPGWIYEAMNTNLTSTMQPELHRVVDALGTRLRMRLCGDIHHYSRHTPTDALS
EAPVLVVSGGGGAFLHGARNNTVIYQGTEYKREAAFPRDNHVTSFLTRLVGFRLINWKFDIIAGFMCFGLI
TSSLPLNMEDKKLEEIVDIHVLFCSTVTRTAELCLYTFDKGIISLFVAVCFFIGFFSVGSGRKSVCFRIAY
SLCWTALVVFASSGMLAVVQTTMAYMMNHGLILSTKEQWSSMLESQVRTAADDTSNHLIEWLGDEHALSRG
IESLRSAAHGSVLVGAACTLLRSMDMIENLAYLSYHVGTNVTGTLSHTTNRVQVVLYYLHILLIYWLLATP
LLSFIIGVFLFVSVHYFDLTYDASYSSFQIEDYKHFLRFCLDGRTRCLHAYVVAEKFVPKVWICDGRHVTE
CRDERTKHLPPHLKKHPSRWVPHGVSGTDYTPTVLEHFVVRPHRVTQPDPLRE
>tr|C9ZS14|C9ZS14_TRYB9 | DeepTMHMM Topology Prediction - Predicted
Type: TM
MMESMNLEQPPRLNADPWRDMEPKVDAMLGDLLMISERFRRIVNPTVSDADELIEAINSVGGEVETMRVAL
DTTIERPELFSIDAEELKRRTEKTRSWERDVAKAIELRSKILSNREGQIQDPLESDVMTPNTWVDLDTTHI
HPSAQSSHMLMNSCEATTDTSVGVVESQALMPVGIPIREQTVKASEYSTASVEVHTVDDVDDSRSSNSAKK
TVLIIFFVSSLLVAILVIFF
>tr|C9ZXW1|C9ZXW1_TRYB9 | DeepTMHMM Topology Prediction - Predicted
Type: TM
MPDSPETGRAPINLEGELCMERVVNYTWENLAFQVPAKDSDGRKIKKTLIHKMSGTALGGRVLAIMGPSGA
GKTTLMSAITGKLHGTEKNLEGCCFLNNAIFTDRYRAVVSFVAQDDIVMAMDTPYEATYFACRVRLGLGPR
ESEVLVNEVINRLHLVECQDTVLGIPGLVKGVSGGERKRANVATALVANPCVIVLDEPTTGLDSVNALRVG
QMLQDLAKKEKRTVIATVHSPSSELVETFDDLLLLSEGHVVYHGPREEATAYFASIGHQVPPRTNPCEYFM
ELLQLPKEELEVLWRAWELYLTTPAASTNPCLLKVSGPITERDPYLEDHLKKKGSSWIVQLVELTKRSYRM
YPRHPSAVFVRLVQTLFVGILMALFYFRITLDQNGVKDRLGVLHMVLINGMFSSAMYGAAAYPPERAVYLQ
EQSTDSYNALTYVLAKFIAETAFQVAFPTAFALITYFTIGFYASFSAFMVHWFLLVQLALTAYAFGLAFAT
FFKSINTTYALLPVVFLPLIIVTGLYANTERLHPYWSWLIVISFPRHAYLGVVINEFSRLEKICDPPDLHC
RYPDGQSVIEFFNFDSWSWWKSIVVLLAYQAVLIIITFVSLLIQGKRSRGRLVFRKNLDSRSDPAQTAACA
GETEEGK
>tr|D0A9L7|D0A9L7_TRYB9 | DeepTMHMM Topology Prediction - Predicted
Type: TM
MRIVRIQFLLPSEADALIELQVTVASLVLAVIVFVCMRYIRCLWTSHTPKASAAPSVEEGNADDNGNGIGL
LMLFPRLPPLHEVTTTLERRLPAILQPKLGGEGGTIGPVFFREHIVMREMFQNQSYRASSPRSTQSSHRGE
QSPLSRREPSRTVSVPSAVSESFARLPMSRNASPTVQEDGRNSSRKRVGQRNADTAEATTVVDAPAVRRDD
NRRIFGSLGRVPVKCSDVAPTQDTVRNGSDWKWLAQQKAAQLMNAPSTLSVVHGVCDVRYIKVYDNGDEGQ
SGGDGNHCGGILLRVRSRAITASALLQLVVKCGLIQYTTTSPPVFSSNINNNGSDSSSYNEPSTAVRSKDD
SHECTDHVGEGDTDPRKHEHTSACVFLFDSHTLTGRLLRGLFGFTAVVLHALHTVFALLIKYVARGSGEDI
NSDSAGGQVRLFISPFCSKILLQSAADCMNEWIELRAAGSPEVCVSDIAIALLLGAMRRQRVKHRRRLTLR
QKAVLTGNRGALPEALSRTLLRCCRFLERCCAISCYLAGSPRLVGERIRHPLHPRPHERVAETMSTAVVEV
ETMSLPEYGDTNPPNPKDWVERVLKCTECRLLTAGTCRQPLRTSMDIVTYSGMRDRATSCHFCCEQMEALY
CIEPLYHFPACLPVVESSPPVQCHAITECGDALYQTVWLPVKECPGDEFLEDLAEELLVMTKNILKMCTDD
VLEVLRLTHGR
>tr|D0A288|D0A288_TRYB9 | DeepTMHMM Topology Prediction - Predicted
Type: TM
MSSTGKFVDANHASMRIDRECLHDATFNESAEEQLEKLTPGGFLHYDYRTPMAKEKRLPDQFHVVQWNIER
GIQWDRVKDTFRALKADILILQELDINCRRSGYRNVAKELAKALEMEVYFLCEFEELDSTIRSPKNAVGPL
SQPTLHLDSRSGNSGAGGISGQRRVQRHFHGNAIFSSVATLSNVTAIPHYVGLDWEKHGVKLREPRCGFRY
FMRCCIKSCERENVSMPDLYLYSCHFEIFCGVLGRARQLVDVLRDANALLKESEGMDQQPAFVLGGDLNTV
VYGVVRLSKTVANDRMRFLSIGEKESCWLQRKILSRGMQWVDSAEPASQLSNIVSTLRRLYNRVINSDTMY
RLLYGFSPEELEAMDNKSLCFYDPSDKVRSVTLDNPDFYGFVKGKLDWTLLSNVRVLPPELPEDIVHRLQE
SGSITSSSAESLRSGQPVPDGYVLFNEDFSASDHKGLLIHLMQNPGKSTETYPGDTTFRITPVSNVMFLSF
LWITLWLVVAVFARSIINRNL
>tr|D0A1X9|D0A1X9_TRYB9 | DeepTMHMM Topology Prediction - Predicted
Type: TM
MLCAAVEVLTVLSFMVTLSSVCCYTFAYLVTINVSFTSKWMCCNQMKGTGACGTNRDNYQYIFTTRVMTLL
SLLYIVLCLHLLISVLEFPLRFFCFNCVSCTATIRPELTLTCAAQSLRE
>tr|C9ZL38|C9ZL38_TRYB9 | DeepTMHMM Topology Prediction - Predicted
Type: TM
MRAYVCIINSLSLLIFPVASLFSLYVTGFFILEVGLFLSFSLDFFYRPLPWPVEDVEISNNSGELVACVCL
NGGHRYLTHTENNCAVVAVLLVTLK

>tr|C9ZZI6|C9ZZI6_TRYB9 | DeepTMHMM Topology Prediction - Predicted Type: TM
>tr|D0A4L3|D0A4L3_TRYB9 | DeepTMHMM Topology Prediction - Predicted Type: TM
MRGLVGRLSFHESEKCSCSLFVLFILSRCPSFSHGSSLVYNSGGKALGVGSLSMLEKDAYNTHFLYILCTS
LTFIITLLFYVTCALVQHHCRFCLVFPLPCSPSDELYYPVGCVSEAFHAVV
>tr|D0A6Y0|D0A6Y0_TRYB9 | DeepTMHMM Topology Prediction - Predicted Type: TM
MSGRIYKLFTGREQPQWMWDLALETRIPALFMAYAVYVNTRCYYDALSSYKANSKWGITRGKLLELRRLDY
RYISVPRYALRYEYEVDGKKYVSTRATTGSPYRNWMERFYNDTITESEYLQAIPVLRVGENCTVFYSKKHP
GVHSALAHDANSFEISILCFLAVFPLLMGYNMKAQWWMIKRAYRPNKMLRIRFPPNCRTPPPPEAPSNPCH
ISAIPKS
>tr|C9ZNE0|C9ZNE0_TRYB9 | DeepTMHMM Topology Prediction - Predicted Type: TM
MEFELTGGCSVLCHTFDFFFFLPLPSFLLRFYSLLGATSHAQDVLPSAFLSSSYGANNAHATFLFWYLSPV
PSVCDLLRFRICRATIHTRRMTATVVRKHIIVYLKLHRCICLAIFLFAYQRPVLSLASASSTVISLFVPRR
KHSVFSL
>tr|D0A392|D0A392_TRYB9 | DeepTMHMM Topology Prediction - Predicted Type: TM
MTQKGYVGVPEEPYRSKFTPEVGPYYGFEGEGTHAVPLQGIPLYQGEGNNQEGLKRFVVQGYKDVWAAILY
ILCILATIGLGIYNICQQGLRSLISADVVNIFWACNDDSCIDSVCLFFLLAFAISVIVCLSSLSLMKRFAS
GIIKGCNVILLIFYVVAFLFSVWALNNAFAIPWFCMLVVHLLWFFFAGRRIPLAAELLKSSSTVVCNYKAL
CAVNAVLFGAHVFFSVMWASVMKPLYAQLAKMELNPSAGGARNEEDLTLNILFLSLLALLFVMFWATQVTT
NLMHVTTAGLTATWYFAGKENMPKNPTLASFKRGTTTSFGSICFGSLLVAIIRLIRWLVSTAEDSEHEILR
CIFLCIIGCLESLMEYFNTYAFVHVAIYGCGYTEAAKMTWELCKRCSCAALFNDRLIDVTLFVASFIGSLL
VGVIVGLVSASGGGFAFGFTVSILVHLFIFSPVGSAVTTLFVCYAEVPEGLEHSSPDLYAALQRTDQNGTS
NGAAPPRV
>tr|D0A8K8|D0A8K8_TRYB9 | DeepTMHMM Topology Prediction - Predicted Type: TM
MMRRTPLVARVVVSSFLREPSERKSFKETLAECDANGIDMASSLTSDFALLQSKLFKYRVVRLGDELQYVC
RHPLGSLPSLPALVVHIILLTTVFFLFRNLGRFSSTALVKPPVALPGEGDS
>tr|C9ZWP9|C9ZWP9_TRYB9 | DeepTMHMM Topology Prediction - Predicted Type: TM
MPRLFGQWFLPPPPLPRVYLFIFVLILKVSEEMRSPAAILFIFNVFLNCIIELYCTVLPLVCKQNSKCRRI
RAHCHEILRHA
>tr|D0A189|D0A189_TRYB9 | DeepTMHMM Topology Prediction - Predicted Type: TM
MSRSFFFYVRVGEDGGEMVDVGRFEWELNHNTIFSVCFFFFFSSFYCSRCKTKEQIAYRHIARFFIIIYYF
PPPSLCGVSLSPIAFSFLSFMFVHLLTVRIEEQLNNGNNKGQLHTSPSNMRNGIFEL
>tr|C9ZNJ3|C9ZNJ3_TRYB9 | DeepTMHMM Topology Prediction - Predicted Type: TM
MILAISLLFFTFLLFDRLSFVFVSLPLHQQSRASYNSNAHPLLHIFFLAALSSFCFFASPLHTKKKNKYKK
EKNTEAVRLKEVNVSTQNHACAACLFVFVCITSICTPPVFLQHIRHITPTTKYKSIKQKKKRKAIRNTHKK
KKK
>tr|C9ZKG4|C9ZKG4_TRYB9 | DeepTMHMM Topology Prediction - Predicted Type: TM
MWCKRVRMIMQWGARGSTKWCSFIFLEMSWSIFPLSTGQTCLLSFPYGYLTNQEKEKERGYQDEKTLLLQY
CAFIFVYFDLRYRFSQRDRKTTRPARSDSQKNYCCSATRHLCHSVFFPLCHSIIIVCFASAASTLPRSPL
>tr|D0A1U6|D0A1U6_TRYB9 | DeepTMHMM Topology Prediction - Predicted Type: TM
MGRRSRFFHFLIVSADYYIFFLLILKHSPSAFLLTDWLETRREKSVNLLCSNIFPCSFSFTVLRLMCSLHP
SNSLNPCVLCARLLRVPLNCFFYICLCDPFPLDGYRWS
>tr|C9ZX99|C9ZX99_TRYB9 | DeepTMHMM Topology Prediction - Predicted Type: TM
MTSQLNANAKEWVPTGDFSAFSLPDSAVAEGEGLEQKSGVGTNGSMNTVPGSPPQEQYDTLQWNDMVSDPP
PYFPSFMLAGQSISAPNATEVGEFTLEGIDWKKEFDNVMALTRELLERQLNGEQDTPGRQEAAEHEAENAV
SNPKGLRATPKTTPGAASILDPAAYPDLPGCDVVRPLQSGKWLKAASAMKGKAQTVLTNREGREHNGDRVH
GTSRIVGGHGVASSVGDDDVDEFGRPLPPLTANGNRRWKKPTKGQQKREAAQKNAFEAFANALLHSVSPFM

APLRERCKTSLPHIKVDQRFGKVGQPHTAVAQFVVAPLVTNYRPRHFHDLTPGDLMEFHYDFAQVLKQLKS
ADEVLFGYGPVWKRYAVPFCYVNCKEYRDEVLSLCPDEVPNMRVEAIYEDDVVKDITDVVLSVEELEPLQN
MSFLQAMGRRVNLAPLFDAFDTVFQPIENYRIIIKELHSAPSTYLIQTSQHEVNQKPQPLVLYNDPKLWYD
LPVVSRIKVESTVGRGASERGGAAASGQPGVMSASSLGGPASEKEGQGRNAASGGTTGRKAKDISTDYQNG
GGYRFNRAQIYGVLIPLVGSAAFLTVTGFILWRRRKTK
>tr|C9ZVB5|C9ZVB5_TRYB9 | DeepTMHMM Topology Prediction - Predicted
Type: TM
MFTHDNTIQLDVAYVFCYQEKEKGKKGIQTVGSMNQKVGQFNHSHLPPVFTPLNFTRGGAAAAVACAHRYC
IVVHSWVFSFALMFFFFTSSPFHLNAGASAVGG
>tr|C9ZV88|C9ZV88_TRYB9 | DeepTMHMM Topology Prediction - Predicted
Type: TM
MFSKYGGRVSIVHSLPFFYLSYDHCVLPSFHPNAQLGAKEEEETLARISPLRCLLYIYIYIYIYICLFACF
FVPSSNFCFPHHAHVFFFPFSLFLAICPLTVLEAVRTSNEEKFSAGGKTKASTTQQYVSVYVFSRICVLTY
CFTPIQLMHLFPVFLYSLCCIAVKFTLTMPAVMIFFFFSPLLSSSSSPFLSLVFAVASFLTAMRITILYRP
HGHALTLPPLLLQMSPADTPVSP
>tr|C9ZT56|C9ZT56_TRYB9 | DeepTMHMM Topology Prediction - Predicted
Type: TM
MKEIKEKRRKKRRSLSNIIQYLFLVPPFSVSAKFFLILPHIKYGNIYIYICNLPSLSRYPPRPRPLLLYFL
FCPPFLILFVLHLPSFLHSSVPPFLFFFNTFRLRPSVSK
>tr|C9ZTW3|C9ZTW3_TRYB9 | DeepTMHMM Topology Prediction - Predicted
Type: TM
MTLSFFPFSCVFPPFIHIYTYIYKYIYICIPPQCLYIYLFFCLPFFFLQDVAGISAFCFVAGREIDTIKLR
EGVLITAPLFVPSRSYFSSLLSLYHLLLSLLSLPLPLPLLLLLLLLLFPLCLR
>tr|C9ZQ90|C9ZQ90_TRYB9 | DeepTMHMM Topology Prediction - Predicted
Type: TM
FVKKIAQDNRTVDMYLLAPSSFQHFLIKTWSDALVSLNRTFTPGQLITTGTVPLASDNRSSMVRHFQRDMD
NYLDTNSDWKGFAKPEHYLKDDKLGEMMVFGWLAGEVLFEALNNAPQLTNRTSFMESLYKQRRYVIDDFVV
GDFGGECDEGAALQGAMCNCNQGGSMAHMRVVDDSLSLKPMKKGSVTWSVSECSSANVQVSAPLIGLYVVL
TDDKVAQRASMRWSLGARSIEEADDVDKRIFFHSLKVNLKNLTQSLEQVRDTKAVAAVLGVVTADILSVPN
MTFIGTLPLSPRLNKFWRNVIHLQPLLAQQLYVLAVYLSNTSSTGVKALVRGGEASEVVDTLDKSLVTFGV
SLDSSKTLGDDDPISSYLSGNGDVFCIGLTPPDVAAVARHLQTHRRARVFVPFNDILLFYQEFVAAFNASK
ESIASSERLLFATSFPHWAEKNTKSDMVARFHRHVNESHWDPLTFLGFATTRLLQVVISNMRKVNAELLAD
RIYTESNIRVDDVRFGPFSDAECVSGTSVSANECASNFGATNISVWSMARVLNSSLPRTQVGMTPSMDYVI
PQEGQLTQSQIAGIISGCVSALLLFIALGVFLHISLRNARNNNRAPKEPTDPVTLIFTDIESSTALWAAHP
DLMPDAVAAHHRMVRSLIGRYKCYEVKTVGDSFMIASKSPFAAVQLAQELQLCFLHHDWGTNALDDSYREF
EEQRAEGECEYTPPTAHMDPEVYSRLWNGLRVRVGIHTGLCDIRHDEVTKGYDYYGRTPNMAARTESVANG
GQVLMTHAAYMSLSAEDRKQIDVTALGAVALRGVSDPVKMYQLNTVPSRNFAALRLDREYFFDEGEDGTTT
STSDHSSSRAELSESAQIIATALQSLLSTFKTAHREKLLLPYCERWRVPLPRKAASEWDDAYCEEVVRRIA
VKVGRVADHGAHSGSESSSTQGSSSIIIVPFYDMHLQEY
>tr|C9ZVH5|C9ZVH5_TRYB9 | DeepTMHMM Topology Prediction - Predicted
Type: TM
MSVYVIHVAFSCFPALPFYRWPPIVRYPYPVYSAKYFHSVVCLLLFLFPFIIFLNSGAPKESMCAGRDCSS
ALNVLAGPPTDNGKQPASLPPDAGWKTPPTAMDDQFLYGVYVPPAAYGKLHNRGNLDESAVGLLSVPYGGA
QDWRWSNEWETSMLGAPCSEPLFCLGACCCPWCCAFLQRKKLLENDWSRYLCCAGMFCGRRCCVCEGCEPC
CLCLESCFCLACAAHGNRFIIRKHYNLQNDCCDSVIIWCAYLCSCLACLLNDESLKTIADVFFYIVIACML
AQHQHQMKKQGYPWCRIMK
>tr|C9ZN31|C9ZN31_TRYB9 | DeepTMHMM Topology Prediction - Predicted
Type: TM
MFRNRFFIRLQSNKGVGHHKFPRRATHHAVSSAKLGKERFDLMESLRRDREEAERNRMLPWRSRLKKLQEY
PWKFFIAFMVFWSWLGTYAVPYLKNMRPGELPTVWEGRPIPKELKERATPMPHFRRLSKGIDES
>tr|D0A5A2|D0A5A2_TRYB9 | DeepTMHMM Topology Prediction - Predicted
Type: TM
MVSHYLSDHSFFFTPLERDRVAHADTSVETRPVSFVTLVLHSLWVFLDSTFVPEAVAPNTITLVGLMSSVQ
SYQILSEYYDQTPQSHTAAATGPILMSSLLCVVAIMCGALDGVHARRCRSATPLGDIFSRVCSSVLRIFFA
LTLMKAFNIVDISTQWYALMVLQLIEFNTVLGRISAENLRGGKAKTVVYHLTYCFRDSELSFLILCALIAR
VVFPDMNFYSPVYPNFLRDAFIFLVMVSFTNLLLLKMEKKHKAAIAVCLATRVVPLFNIFSFTNNNVFSLI
SGSLAVGLLSTEVHVSNVSGRRVHAAVICICVGSVFNDILSIGASILYVIGMMVDLSYSARIPLFAPVRNV
FCDGVFDLCHAGHKNFLQNALHYGNRLIVGVCGDDECEAYKRRPIMTVDERVNEVKMCKFVSQVIRNSPVT

GLTEEMIKRYNIHVVVCGDEYNRPDDTYYAVPRRMGILRTAPRTEGISTSLLIARIRDATEVELSRRDNAS
SRSTVMEGS
>tr|D0A2Z5|D0A2Z5_TRYB9 | DeepTMHMM Topology Prediction - Predicted
Type: TM
MTFSKIYSAEEGECFRAILCRKLYCCSKIEICCVRVCIRICWVTRTFKYSLVRLLNQSVKPYITRQALLLS
YIKPKRKIQRQKYASNSLFCFVFLCCNIFLSLSRFC
>tr|C9ZQ47|C9ZQ47_TRYB9 | DeepTMHMM Topology Prediction - Predicted
Type: TM
MCGSVVAGYSTLLLFFLLRVNTLLCTVNVPPTTEGFFNSDNADIRKVPSFSLCYKGSCVFHSCSLILFSST
PGIICVIYFNSVRVIPIYLFWLFPIRWSATMLTFR
>tr|C9ZU20|C9ZU20_TRYB9 | DeepTMHMM Topology Prediction - Predicted
Type: TM
MRLIRDATTFSYCPLLTLYSPRISPEPSLDSNFTFPPRDRPQLSAAFLFYVLLFLFFSPPPPLSSLAVLMP
SKSYTQIYIYIYIYICVCVSVFIYFFYFFLFMPQLMRNKLSLSLAITQPLRRKETCGVVYDAGGDGL
>tr|C9ZK20|C9ZK20_TRYB9 | DeepTMHMM Topology Prediction - Predicted
Type: TM
MIAAENILEGPAHLVSKVEGTALSLLIVTVAILNFLPVSDLMVTGKVLSLSLLHWEVSKRGCPSCSRHRSG
MREGGNKMWGEVSNFSQRACALFALVVHDAWKVKKSGTSRLGALLDHLRCGVCNTEI
>tr|D0A4E5|D0A4E5_TRYB9 | DeepTMHMM Topology Prediction - Predicted
Type: TM
MPLRCTKPLGATPATPTAPPTGRKGHQVAINGFITWILVTMSLVVYFLWAFIPTSFLDMVLASYYPDKYWA
VAIPAILVMTMVYYLTVHFLLMLYRTDPLTDGFCVAQTNGAVRHESIENLVDVEDGVPPITDIPVSVASRL
LFQPWN
>tr|D0AA08|D0AA08_TRYB9 | DeepTMHMM Topology Prediction - Predicted
Type: TM
MENLVDVGKSNGTTNGAATRRGKTQCTEWRGKCKRQKVSKQLSDGASIGFLPRLTNEHVVPPSNAAKGGKE
PNSTALTESANHGSVPKDVPCFQQSNDSLSCGLSSSRITFVLPCSASDENKVVTPVYLHIYELSHGILHRH
SKKVTGKYVKGVYHSAVVCYGMEFFFEGGITVTCAGRTRFGEKFDKVEIGHTNKPLSSFLDWAGQRSRSTY
IFHSYHPTEHNCHTFTSDAISFLMGPEASLPSYLTETIDTIVKTPVGTGVVELLVHFLGGMKYVAAENQRR
RMSERKACLTRARRCIANNVINTTPPASVTLFRVSDPSSCHRTLAAVEPYGRRLLEHKAIEPPAMNFLRCV
QGLCAGVEFIDPDYVTEYVNLATYALIHTTPTLRGPILNSLRVAVLHKMVLCTCAFHPLLLCALSETVRNF
MNLTAEGRLALLRLLCNMGGSIHGAVALSSAEYSQMWVAAAGQAVMDYRNPAIVYTGAALIANLAVAFVLT
ENFVSKTVEYPHISDQCHNLITVTLFYLGSWPRERVPEAATTLMLFALFILLSSSFDASRAAKLHPFQLNF
DCLIREVRTLESSTLIRMLQDILRCPHA
>tr|C9ZRE0|C9ZRE0_TRYB9 | DeepTMHMM Topology Prediction - Predicted
Type: TM
MVSWSRPEFAEAFFGKLVRRRCIYATKAALDSSPFVRVLGLLSLVSLGVGAVVGAGIFVITGQAAAQYAGP
GLTISFVLCMFPCFLTALCYGELAAMLPVAGSAYTHTSVALGEFASWTVAVCMTLECLVSGCAVSVSWSAY
VQAFLKRFSFVLPQPLRKSPIDVVGGRFVLTGSVVNFPAVVITVVCFVVLCLGVEQTASMNSFFVVVKLAA
LVCFVFYGIYYSLGNWAEVNANLTPFVPPNDGHFGHFGVSGILRGASVVFFANVGFDTICASAQECRSPQR
DIPRGIILTLLLCSTLYVMVTVSLTGLVKYTELGTDAPVIAALEKVKAPSFLRLFIEVGTVAALSSVCFVS
FYAMPRLIMAVAKDGLLPALLTHVHEQFRTPINATIFCGIPATFICAVFPLGMLGELISFGTLIALACVCV
SMWKIRIDHPEFHRPFVAPLFPYVPILGALLNAAQLFFLPLTTWRNYFVVMATTSLWYIVYGIRHSTVGED
GITRRPDSLLGTVEPPLCEALEGVQGAGGSLSIELTERYVHN
>tr|C9ZP59|C9ZP59_TRYB9 | DeepTMHMM Topology Prediction - Predicted
Type: TM
MANTWTMTLRVPNPVGSNKKNSSKCCAVETNIEIQMHFRRKYEGCNTFGFTTRPLLTFHVVPCFLFLFLVD
LKIWALASPFIAYFSNYHTCTHTLSSKYETLLSNFKSDVLLAGSIPVPRGELSRYVSISTTSTKTYRI
>tr|C9ZQT7|C9ZQT7_TRYB9 | DeepTMHMM Topology Prediction - Predicted
Type: TM
MLSHKLIFVIHTTSGKNQTYKFLSSTFPPLFTIWVSPSHHSYLIIKRLQELVLNAALDGLRRVLLSTLSHM
FLPFLLLLLSLPHRRRVLCQFFFFLVIRVW
>tr|C9ZKF6|C9ZKF6_TRYB9 | DeepTMHMM Topology Prediction - Predicted
Type: TM
MLPIFLDGLATVMYCFVGVGYFPFVIVSTDLLHDVFCNALNVFLLRCLSICYQQTVLSNVFVIISLLCICF
CLGFFFCVDLDLYRWYYNNIYLRKLVTACVFMLLNHRCPLLALDKCESPVAWLLLLIKILKDVTSYFISSH
TDKYCGR

>tr|C9ZU53|C9ZU53_TRYB9 | DeepTMHMM Topology Prediction - Predicted Type: TM
MNIYIYIYIWNTLYMYTIFSPIPSLFAFCFYGHPVLGLVSGLEAFSREPPCGSVGALALRQTPETRGTAWE
FLSYYPILPSRCLRDPFTILQTHIHVYNSII
>tr|C9ZWK4|C9ZWK4_TRYB9 | DeepTMHMM Topology Prediction - Predicted Type: TM
MNGIVPVCSGMSEKINKKRMNATDVHSHSLFKGAHSQCCGFVCALPVPRNSPLFQHLSFSSTLVCLLCVCL
VLVLFFFCASTCFCDALRHNRRGRKEDTQAEKQHGGTTRGVCSSCPENLLHFVEVRRHKRKKRRK
>tr|C9ZMX0|C9ZMX0_TRYB9 | DeepTMHMM Topology Prediction - Predicted Type: TM
MYFSPVVRKFYSLLSSISVLTSCDLVILFPLLFHSVRITSVQLSRTKLSLPPLPHSYTQRFIYSTHTHTHK
KILKGGYKRKVYIHTTCVFIFFHNLNTMRSFVATVPSLEKDRGYENTFCV
>tr|C9ZTU9|C9ZTU9_TRYB9 | DeepTMHMM Topology Prediction - Predicted Type: TM
MHTESYICFLEPTPLTFVFFFLHFVPRSGGTRSARSSLFRLFGGGTCVQICVWCVAEKVFVGHLVLNSPFP
TFINMFLSSGQELREAPWKGGGKGRLESNLFNLFLFFL
>tr|C9ZL43|C9ZL43_TRYB9 | DeepTMHMM Topology Prediction - Predicted Type: TM
MVAESHVNMGKGLTIPFAYFPPSPPEKKKTFSILIWARWRQEASCGFSAAQEQSCVHGPSICCVFLLVAPS
LVVVTVWFTHLFSPGVLAVQPAVGVNYHFNQWGTKEE
>tr|C9ZJX6|C9ZJX6_TRYB9 | DeepTMHMM Topology Prediction - Predicted Type: TM
MAQEGLKEVTDSPYFLKKWLAFIDYAANDLFGGPRPLKAAHVINLQKGGTLFFCLWLMKKSGNYSATAITY
TALHGGYGLCWLLKELVFPDPKWQKHITFAGALTIFASVLGPYWYIVYNAIMRKAERSEGALCAATLVYLI
GLVMMMCSDCQKYFVLKKKKGLITDGFFSRIRHPNYLGEMMIYGTFGFISNHVGSFGVLAWVWVGLFLPFM
IQKEASMSRYSEWRAYKSRTGFLLPSVLPPKQKTTTVE
>tr|D0A6G9|D0A6G9_TRYB9 | DeepTMHMM Topology Prediction - Predicted Type: TM
MRSGTFLKALFSVDALVTLKRERVRLHRCTRSASPSIFYFISSPHPCDHSLCQCYCLVLHSQQVPLYKVKG
KTNFRSHCGLLRQGGIYSEHTCQFRCCVLLFSFFSTIFFLAYWLRFVLLTSEERRGGEVTRKVASLFFPFV
KFLLGEGLHTSSVNVGGGKKVVRWESKMLEPLTKKEGKYAGVHLSPPYFIFFHPIIS
>tr|D0A7X1|D0A7X1_TRYB9 | DeepTMHMM Topology Prediction - Predicted Type: TM
MKLSKLGPNVEILRDVLRDKLLITACIFFCFLFFFLFVLVSFRTLVYTTSPVSFFYLLHESGSLFVSFFFF
FFGCCCCKWCVPVGRRRRNNGPFGVPLVFSFFFFLFLKFTLMFFFCSSLVFGVFFFLLTFFFFRFLLF
>tr|D0A8R2|D0A8R2_TRYB9 | DeepTMHMM Topology Prediction - Predicted Type: TM
MYMRISKSPQAAFFHTYIHRREGEQGGKDILGVGKMRKGACGTSSLLSLSTSTPKRREKWYAQNRQKKKKK
TNTCNSVLLSLKLLCGTAPLFLKNLLPLFLQMLFLIYLLFFFFLLLLF
>tr|C9ZQV7|C9ZQV7_TRYB9 | DeepTMHMM Topology Prediction - Predicted Type: TM
MRIITIHQSLYEVFSVSVKSRLYLFVIILFHFFFLKASTAIFFFTSFSASFFSISFVAAWLLAKFVALSLA
TSPFFLLLEETVFMCILFLIRSTYVSFVLFLFSH
>tr|C9ZJI2|C9ZJI2_TRYB9 | DeepTMHMM Topology Prediction - Predicted Type: TM
MCHGAVTYTTAPSYRCCACVTCIFHLKCSVDDGFPIEGCTRQELTFAVAPTLLIVTCVILCRAPLIGLSRC
LAGCAYGEGRENEVVMMVELVAHGVRRRWKVKWYIPTIRGGSDVLVSHRVH
>tr|D0A0N3|D0A0N3_TRYB9 | DeepTMHMM Topology Prediction - Predicted Type: TM
MHGVEVSAENQLRQVKSDIALLNHYLTQAEKGHEMPCEELNERIERTQRILRSLSDGRVDYQLDSVSGGVG
APSLLQGTVPHTRVVGKQGVSNLQRRTAQQLLSELSLIESSLQRLNHKAEKRNMYLSEVDQLMGSLRNNGT
HDDVSALQHAERERASLQYARARVQAMINESNSVMKALQDQGRSLESTDSRVADILESLGVSNSTTLQILR
RNKVDAWLVYGGIALTLLFIYLIW
>tr|D0A3N1|D0A3N1_TRYB9 | DeepTMHMM Topology Prediction - Predicted Type: TM
MNKQTNKQTTNKQINKGKTKTEVVLYYTRPKREKKVEDTTSRRTNKQKHTFTPALAQQFICGFACAQFACI
LLSCIPFVRLLPTQHCSSPCRSSKLRIATSSLYISLPFLLSSLSLFFFPPPLSFACLHTHFPPSFIYARHR
FLFLLRSRFRSCFFFFFALHRR

>tr|C9ZXZ0|C9ZXZ0_TRYB9 | DeepTMHMM Topology Prediction - Predicted Type: TM
MREMRGSELRGCVAVFRQMYVTRVYACMYVCMYVCMYVCVCVCVYVCVYVCVCVYVCVCVCVCVCVCVCVC
ICVCICVCVCVCVCVCVCVCVYMCVCVCICVCICVCVYIYMCVCVCVCVCVCVCVCVCVCVCHWFVELKGF
SKFCLEIFF
>tr|C9ZKW1|C9ZKW1_TRYB9 | DeepTMHMM Topology Prediction - Predicted Type: TM
MSESEKIDVSGARNYKSISAARKAFETGDIEMSRMEHQKHIYKEVHNPSASDYVKSVVFGGLDGIITSFTV
VSAAVGSNSSVASVLIFGFSNVIADGFAMGFGEYVSGEAERDNALSERRREEWEVENAFDMEVDEMVQIYE
MKGLSHEDATTIVNIISKDPKLFVDFMMTEELGIIIDTEDTHGPKKQGLVMFLSFMFFGAVPLLAYLPGKG
KGIDGVFALSCFLATCALIVLGMLRGYLSGVSMLRSAALMVFNGVVSGLFSFTVGSLVEHALRSSIEV
>tr|C9ZSU7|C9ZSU7_TRYB9 | DeepTMHMM Topology Prediction - Predicted Type: TM
MGNRLAKQKVQYIYVYVYIYAYTHLLLLVGFRCCFVFFHFWSHFFLLLFLLFFVSFSSMLPYCMPSTPPVC
SPLRYFFFFLIIIAVLTLQLVSYIILPPLFLDGTFFFFCNIYIYLYFIGERNNPCN
>tr|D0A0Y2|D0A0Y2_TRYB9 | DeepTMHMM Topology Prediction - Predicted Type: TM
MSSSVGFLCENPRYCNLFWQCRNGNTTVTASDYDGRCISLATCDELPLVGLWWGISLLLVKFLAAKCSTVA
TSKTHGGNPPDPFTQNKQMLATSLLGCRQPPISSVRPNPHPEIQLVPRFHPHLPQHWVVPTTQKGRRICCH
QRSSGGGAHYKTY
>tr|D0A3Y9|D0A3Y9_TRYB9 | DeepTMHMM Topology Prediction - Predicted Type: TM
MMWGGEIRVSMYSQCENNVRWYLFFLSSSLFYRSSFGGRTYGDGVFVRLSLSFFFFVNPLVLFIFYARTPQ
RCVSLCCLLCVIVGVNSFSYVFRRTRGIIIVIIIIIIICLHI
>tr|C9ZLN2|C9ZLN2_TRYB9 | DeepTMHMM Topology Prediction - Predicted Type: TM
MCFCMCVWMSFHNLNAMLFPLHPLIMLFTTVVIFFSFYITLSFLPLYVLSLFLHLFVLFVPLVFMIFLFFL
FYLILSHVMSLSLFSFLFFLFFSFCLLPKLPLEKIVRAYEALAIY
>tr|C9ZV76|C9ZV76_TRYB9 | DeepTMHMM Topology Prediction - Predicted Type: TM
MLPYPAAPFRLLCRRINVCVCSSPCVPHRGSLIISDFLLTFVRGMLCALVPQGGSAGWFRCVLKVRMMCDM
SVGATLCVRVPGCDSYGFHCVADQNVSSV
>tr|C9ZQ14|C9ZQ14_TRYB9 | DeepTMHMM Topology Prediction - Predicted Type: TM
MPWHVVVSRYLSWFLAVIFNASFRFSVGCRTSFRRPSGKNVSFIVKYFRTSSNLSVGELAGALSSSSTMHF
FNTFCIRLSVGAALSAQSTVVQTLFQYVC
>tr|C9ZJ30|C9ZJ30_TRYB9 | DeepTMHMM Topology Prediction - Predicted Type: TM
MQCDTGSMGGGKMCRCWGKVLAGGNHQKGCRASLCKYKYGVKCSFVYPPPLLYPCVACTCWRTSVLPFFSL
TPFYFRVVGVFFFPFVINRVPFYFLSLRSLYYFFPHPAPLVVDCIPVRRPLFLQINGLFPPSLTRCATVTV
T
>tr|C9ZI30|C9ZI30_TRYB9 | DeepTMHMM Topology Prediction - Predicted Type: TM
MFVFICRDFVSFFPLLSLFIPFFLFLLFHFECPYVSKCFLLIEDCPWQFLSYHFIFLSLVRFILYHFSFRF
IYYFILLLFLFLFPLLTYTHTPCSSSCMTSLHTNCSGAPFAVC
>tr|D0A785|D0A785_TRYB9 | DeepTMHMM Topology Prediction - Predicted Type: TM
MDEEFAGKFSLISLTIISISSLLLHPPPLFFRFRFQLGQYLFSCCASFPTATSSHCRNCFPLQCCSPLDGT
YLLHYVFFCLGLRVGLVSCGITLRPSTGKVVK
>tr|C9ZWR2|C9ZWR2_TRYB9 | DeepTMHMM Topology Prediction - Predicted Type: TM
MGGVKFAVLALLVFALLYVVTRVLLTRHRSNPRNDGRQQHTEPSWDITNRANIPVVQPMESAPPLSEPGVL
LAIPLAWHREIPLSSRPSGSDTGNRPVNPLERAPDGPEVVYSHAKYIPRASSAS
>tr|C9ZL54|C9ZL54_TRYB9 | DeepTMHMM Topology Prediction - Predicted Type: TM
MASAWCIQFRAFLCKVLLQLKSALFTTIFMLLLPTLVMLVLVAGYRSTAIIHVPEMQYDENPLINSSQIFE
NHFCTNRDAPFSFRRRLPVPMCYPPSEEFECLPSVRGGSLCVLDATLAREALAQLFVAAGPVVIPTLDAYL
GLSAFVTHELRRENPSLFARSAKEGMGHYGKLLLVDTKSANHGAAARFRTFCSNVSVLCNEVLGDLPIFAS

MVDARRYAMENDGEVWAIADITDDHRGVGNFGGRGNHFTISMNYSATPWTTEAVIPTTKAMEEGNNLLYIT
SGFLTLQNAIQQFYVRERLGGGLNSSAIFQYTNLYGPTTVAMPSPPQFKSTFYKWGHYMPLLAIIAALLSG
MVFARLIVNEKARMIRGCMEVMGLRWSAMALGWLIIAFSMDFIAVCVPVLLVGFTFFHYVNLAVLFVLYWS
FLCQIRALCLFLSTFFTKPRYVKLAICVVTVCCIMSYYRTPESYHAPKVKFMSLLPCVGYLASFDQLIQHA
STSQKFHWRDTVEGCNSVALLVGMTWVSTFIMLVLWSYLDQVLPTSSCCRKHPLFFLQAFRRFLCHPRDEN
ADVISFPWRELGKDEIPPSMKSVLDQHPECRDTENQTIAAVLYGLCKQKKRQNWFCWKRGSSAGISFGTSG
SAAVDDVSCALEFGKVNMLIGPSGCGKSTLIGMAVGAVRPDAGAVYICGHSTVTEPEKCRRNIGYCPQSDV
LWEDLTVEQHLTFYARLKCGGVWDVREIVNDIIDTLNLEAQWLTKARNLSRGQRRRLCVGIALIGDPAVLF
LDDPTAGMDVKGRRAVCEALSKGREGRAVLIATHEIDDAERIGDYIHVMQGCTVRDSGSPLVLKSKASAGY
VLKCVVSPGLTIEEEDDCINRLVDFVRAIAPGDYGSRPARESHGTVCGVGLLGVERRGRQVSFRFPLALLS
SEGVSVVGEIEARRSEFHLQSIGLSIATLQDVLDYLTVQHQPTAGANGDSGLCGLSLDETNTPRRAGGNGS
TVSVEFTPSGTIRDDEKFSATSGLGRADTNATRNGGLQWTFFSHFAALFVKRLHSAKWDAQLMFYYIVMPL
AFALLSLPVGKVKPTVQPALTLDSSMYRAGQKSPNSTLVWTYSSVLDDAFGVAKSDLRNVFGPYYTPVMVE
CHRAGCTEALSDVLYQYLNGNNSHADVAIALTGAMYGVATSVTMHNLSSPHAAAQSLNMLYDVVNNQLFGE
GSFVTARNEPMPMGPHEEEMFAAFHRIIAALFVILAFILIPANVVGRIVKEVQCGAYHLQCLAGANAFSFW
LSAMLFDFLCYVVAEILVLIVLFASGCDELVGDGHTILAALALFTMFGLCHIPFSYVLSFLFKSSRRAQST
VLVGSLVLGIMWILIEPLAVKNNRVAGIVMGVTNFLRVTPCLAFSEALMAMVCTRLANIRKPLRERPSLFS
PLGYSRGGLTGGTGTGLLYMVGTFFVCLLLFALLELLRRRGAVCCLGPCSEGDGDGKLNTAAHRGNRKRMK
RRGKGTPEDWCANEEMTSYRNGELEEGRGAAAVGLSLQHVTKRYAGVSTLALRDISISVYKGETLTVLGLN
DSGKSTILSILAGRVPPTTGFAGVGETIVRPNAAQSKVGYCPQKDALMDNLTPYDHLLLFSRLRGSPEEQI
HSEVPRLLCVLSLEDVKNCLVRTLTPAQKRRLSLAVAFVGGTTYLLLDEPTADMDFMSRRQVFAAVQGLGR
AKSVILASRHLEEMEVLVDRAAFIEYGRLRYIGTPQELMSHFTCDVMYTVRVAFGNTVAPQLDPLGETVRN
LCKCFDKAQGSRGCRIKSVVGRTVTLAVTCDLLFVCQQSAAISEGAIPGLSPVVQVSATQPQLDDILLDF
>tr|C9ZVJ9|C9ZVJ9_TRYB9 | DeepTMHMM Topology Prediction - Predicted
Type: TM
MHRITVRLMPTDTCTVIPLTPSITLCGINCRKKHTMAVSASSVGRAFAFLGTALAGGAIGMVVERGGWFGV
DKCHPSPVPYQQMTERTSPEVLHVQCPQPAYPKE
>tr|D0AAP0|D0AAP0_TRYB9 | DeepTMHMM Topology Prediction - Predicted
Type: TM
METELKELRKQLSDVADSKSSLEKELDELIVETKTKKSSGPECQLKGESSDSIKKQLGGMNDSKALMENEL
KELRKQLSGVADSKSSLEKELKELRKQLSDVTGSKSSLEKELKELRKQLSDVTGSKSSIEKELKELRKQLS
DVTGSKSSLEKELKELRKQPSDVVGSKSSLEKEMKELRKQLSDVNDAKALMETELKELRKQPSDVVGSKSS
LEKELRKQLSDVAGSKSSLEKELKELRKQLSDVTGSKSSIEKELKGLRKQLSDVNGSKAALEKELKKQLAE
EHYSKEAPENLNEMEGGAKFSDPFHTELGKLKEENAVLMRQLNEINAGRDISKNVVKLTCSPNEKSCSADV
DFAQAKLANFSEGEGSCSLEDALDSSYDTSVVGAESGLAEDYFESDCAVHGHSELGTVLHDRPSQRRRRSK
RAVDGEFCHTTNSDEGAGENSESYNISALQSLGAPIVLLLRKIKAIERDIQRCGKVVSQIAPCIKEARHFK
RVMQLAERHSDSGDNYGARTELQAAASDYKRRVMRAEGTWKRQDENLRLLQARIDAVGEELDCLMVSPRHE
HCEGSGESRVLSSGVSAPNDLLQQMQDRVRLMLFNVRKLLDDMHKQRQNRHEAMLAALQRCSPAVAAAYMQ
EGGISEWKEAAAFSASAVGMLVLAVGSIMFGRYELR
>tr|C9ZQN1|C9ZQN1_TRYB9 | DeepTMHMM Topology Prediction - Predicted
Type: TM
MQENFSFPFSICGVCYSYIVTFSIALYHALFYIGSSRALTFFFATELVTKRNMELSAVLESNAQRFLETEQ
PLNAVPLTAMLREVNPTSVKFSLLHATTLLAARDYDQAFRIAADVAKDFEGNMQAVAIAMKSAYELGDVRG
CSAYAERLKDSPSMNVVALCYLGRCAELSGDTKRAVHNYCAALDIDPFCGEPMNALIERRLLGVNELRDTI
ESLRLPPEAEALRASYYARLPGEFVPKEFDKYIPRTTLLLQAARTEYERNDLQQALSLTTSLLKISPFNRE
CVCLHLSILVDMKATSKLFDVAHLLCSSKPHAELAVYAVGCFHFSLSNYERAGRFFTRATELDASFAEAWI
AYGHCYAKLEEGEQALIVYRRAMNFFPGLPCCSTFVGMQYGRAHQWRLASHFLEEAKKAMPNDPLVLNEIG
VLYMRTQRVDKAREMLEEAYKSLVNPENASEHRDCIIFNLATVYRKLQCYKQAIAFYTLYVKCRPSASHGH
CALAFTHHLMGDMKMAIAHYHTALSIKADSFCRDMLDRALATEFGEASHGFAKRIEESLCSPSPDDISFLA
ASRTLRSDPTATSSKDHSHHPSVGRSLFFSA
>tr|C9ZRI1|C9ZRI1_TRYB9 | DeepTMHMM Topology Prediction - Predicted
Type: TM
MDHEEVHAGARTGAGGGEEDEITENMESGTYDGTQQQANYDPNEQLQYYYDPVDGNYYYYAPQYANEADPT
GDVNENVGRSDKRVTAQELENWEPVRYNWFSDTVLSIFMSLGYKLYLCMLLYCAIILQLMLSLNWTLQVLY
RFYAPQSEGDPSTWSPVCFFTVFILLSFFVVTALCTSCDMLHGAWRAKREDIYFWGATTWAGKSPPVWLHF
FVILLTTGFPFLWATVEAAVAKGSFTYFLCIYAFIAVVTVEFMIVGCYVWFYFLGIRGKITAMRLYRGRDD
FETWERASKYSYNKQLKKRWYHASTLLEEYGLDEKTLRSNALSFTIGYVPLFAVYTAQAFSTNVDYPEMTW
AAICTVALCCVYFLAWFTVFRKRSHWSVYFAIFLIVTLVLTGIIAAITCNLPGALGLIIVLFVLSQCMLAR

KREHTLTRVEQRTLFGMTDTTEQEDPIPNRRVDMHLCCCGDVLANCFRCLGAREAIGYRHPDVVRVEEQYN
RENVSLRTDQRLLMWWWIFVMAVVASIIGIGNHMAYDYRTEIAVANGVPIEGNNTGLFLCQIRYNKNGSAP
LGLYDLSLLSALAYTVGEPGERDFATWFSFFPNFVRMYPRQLPPNSTYATDGIEIPFSHYVDMTSDYHVFT
LNSNSRGLSFMRDVDEWGISITLQLAKVFSPFVGMWPEGFQRSFVQRAAFLQSWFPGVDVLGSISKSISQL
IDDGKKERILLVGDGFNGGYVKLLSTIHGVPFVALNAPGIGQKAALGTEGTQVLSPRSLLSYVDSIEDTTH
TIYIPCDSRLSLVRCSKIEATVNTLRGLCGDVHGRMLH
>tr|D0A4X4|D0A4X4_TRYB9 | DeepTMHMM Topology Prediction - Predicted Type: TM
MLVSTPYAWMKGGKKLHPSFYCVFACSSCMTPVKNKCKVSRVGYTMSGLYLVFSFAYVTFCILPLSFFPLS
LVVSEGGCLLNRQEGNVVRGVLPRCVGF
>tr|D0A7T4|D0A7T4_TRYB9 | DeepTMHMM Topology Prediction - Predicted Type: TM
MGSDDSELEVLQDPSSYTKSIQKDNGARWVRGLTPIETNDSVKLTTAPSSTRSWVLRCMRHWLLVPVILGV
CCLLALTMDMADMWMTHGTTYLERRRHPLDFPLNEDELRGFTALQGESLMHHLSRVRRIWCVSVLSTLFSL
AISWRIGIAARDFLALCGVGVSILSLLATGGQWILLWTACDTKPKRGNIECKAPFVLYWCLTVARMGGPLL
AVWFTASVFDDVRGYRFFWKIFLALPVFAYVSGAALLANCCKRGGGSPSCDGEHAVYRWCSVATLLSWTQL
VVSCWAQHRFNVVLMVKPHNE
>tr|C9ZTX8|C9ZTX8_TRYB9 | DeepTMHMM Topology Prediction - Predicted Type: TM
MSNAEAYDKKLKMLEVKAQGLLRDLKTTADPIRRLRCHTEAQNTLNEIQQNYQLLKTEIQLLEGDEGKLYK
NAEQEHAHELSNLKRMLQLEKAPVPVVPGDQSSSMFGALPATGDRREEARRIAQSVVTIQGTTLGSLAQAE
RALHDTEEVSVNATTRLIAQTEQIRNIRDRVEDLDSEVTRARKELNEFIHRMATDKIIICFYTLIMIGIII
FATLKFLQK
>tr|C9ZIT5|C9ZIT5_TRYB9 | DeepTMHMM Topology Prediction - Predicted Type: TM
MRSGRKAGMRISAKDAEVMGIEVQPYHVIIGTAAFVVCVLLLHFYGKLSSISA
>tr|C9ZZM4|C9ZZM4_TRYB9 | DeepTMHMM Topology Prediction - Predicted Type: TM
MHTKTRTSEKKRGKKKYACFHSYDQFVTTVILIFIYTFDFFFPVSFEGICACMYMCAFLPFFPFISIFFSS
ENFIILPLCCTFSSNKITPNRFIVFYFIHTCAHTFNYLLI
>tr|C9ZPC1|C9ZPC1_TRYB9 | DeepTMHMM Topology Prediction - Predicted Type: TM
MNVIYRAFLPRRRHPIPLRGVILVICILRIPELCLSRSIFISHSPSGESPSQRGWFVASPFYAALPLCFVF
GNMADGCSRIYNAPGLVLGPLIFEFGGVGV
>tr|C9ZTS0|C9ZTS0_TRYB9 | DeepTMHMM Topology Prediction - Predicted Type: TM
MLFCFLLISCWLSFSFTYLNIYIYIYIYVYSCVPILHYNEKCNGVTYITHSQQNIFGTYNLFKENKTFKNK
STKTICGDPTAHQRNYSIDNILSTATLHPRKHHVILFFLRWERGECRTTQAPQHR
>tr|C9ZLB1|C9ZLB1_TRYB9 | DeepTMHMM Topology Prediction - Predicted Type: TM
MAIPLGASLSDPRFVSVQILHVVSAFFLIMAMVRVALGVLLLLFSHSENISYLKLLSRCFHIPLRSLFMVH
VEDMADGSASRFFFMHVMTALVVSYPLAHTIQRRKFALDFAFTTYAIYFFFCCLVGWRVTGGGFAWWLSVV
SGFGITCGMTAVICRRCELQDIILASSPVTSRAVGGNGAYRARENVGNPAKTSVERSALLNSGQTDSVPTR
AAWRNDLV
>tr|C9ZRN3|C9ZRN3_TRYB9 | DeepTMHMM Topology Prediction - Predicted Type: TM
MTTPLRCFCHSYFSPLLLYLPPIYVYVYVYIYLFIFQQLLYFSTFLSFSFNFLHFFNVTFLWLFLHPFLLF
FFSSSPFHLSLSCRYTSDSSFLKETRIPFHLSFLLYHLVFLILYLWKTLYIRTEVGEKN
>tr|C9ZIZ3|C9ZIZ3_TRYB9 | DeepTMHMM Topology Prediction - Predicted Type: TM
MCARMCIDFKFPSLLPYFFLFSPFLFLFNSWQPTCLRRIKFLFSFFFLRTNVWWRLREPKKKETKLKKFPQ
QHTSKEVRNATKIITIIVVKKKQQENKRKKKRGGECMRISVCVCVGGGGGLGCFFKEKLFSLCIF
>tr|C9ZMF0|C9ZMF0_TRYB9 | DeepTMHMM Topology Prediction - Predicted Type: TM
MGFQSAKWVMQFRGATTSLDFDLSKNGGNAQFCLLLLLWSFSRFRCPSIYRLVLCCFFLPGVWWQAALHQK
MAALESVGCREWGNENWMAFMHGNIYLSVSSGRTTVRETRYKRSGRVAMVRRKDL
>tr|D0A888|D0A888_TRYB9 | DeepTMHMM Topology Prediction - Predicted Type: TM

MANVNNETTECGALLSNITSLQTTLTSMGCQVPALHHHTHDMTRPPSHHHHHDDHTHGVVDDGIDGDTHGG
CESGHGTYSIGLHVVAIFVVLIASFLGTLIPIIGKYVPALRLPPFALVLGKCIAAGVLLSVSTIHMINESI
LQLQEDCVPESFRESYEAYAFLFAVAGALLMQMVDVIVDKYVTNKSDSSTNKPEGQPDAEEAQAAPAALDA
YDGHHCHYAVGMPQSRTKRLVAAMFMEFAVTVHSVFVGLAVGIARDAETKTLLVALVFHQMLEGLALGARL
VDAELSLKLEMLFALLFSVSAPLGTAIAVGTIAIWNVSMVGTAFVITQAVTSAVCGGMLLYLAFCLMLSDF
PSDMQKHAGKDKVRRFFRCFGMFAALWFGAALMAFIGKWI
>tr|C9ZMF9|C9ZMF9_TRYB9 | DeepTMHMM Topology Prediction - Predicted
Type: TM
MYSFWASPSESNGKEPRQFLPPREALSPSSLLVHVSSSASPRQCIRASGSAILGYPTSICCKRPSFHRRVS
FLLNLLDQLASLTFSRSMGHWWVSLYTFHFLIKASGNPSERAIGFSMHFSCVCLKCAPLLILWSPTCPVNI
KSLLGTFYSNPTLFSLSSPSLTVTSTSSLFLFWFQHRRGYG
>tr|C9ZRW5|C9ZRW5_TRYB9 | DeepTMHMM Topology Prediction - Predicted
Type: TM
MLRRSSAALIRRTPVRHSGGELFVRPKLEEIPPADQCRGFFGPLNDSLKFLRLLDIKWMMNRAVAMRREYL
IATPTLFTFIWMFTWKGAVIYFWGDRAPPRRMDWNTEETGRLPLGFKPTPAPL
>tr|D0A9G1|D0A9G1_TRYB9 | DeepTMHMM Topology Prediction - Predicted
Type: TM
MTANDEYHISFHQKLRYFALQQDAATQSSDIRRNRVFLEQLLDSRKRNIAMGGAFCSPTGSIRSRAACSIA
SFNRSNASRRYGGGPPSLSPQAASQTSPAAKGEGRKSFIVAGLHADRRDRHPAFDVTVQYAEECIFAAYFL
VSRGLYDDAMNICLKSIEKDVNTTLRDACAVAGTVSAISKSARKTTAFGRLLQTEGETPTAGRRSLRFVSK
EVDQERRAKFTDAWRVVVQYELLTQACQFFRLFTGASSFRCRPGVLQQATSCLENVVHRLVSACDVEADLN
YFVLFNATVMIYEMCLHLMRFASGSTAVVMPVIARSTAYCIEVCESGTLKLSTSRYLLWRVRLYELLCNCY
ERQGMYEEALHQAQRTLVKVRELVELEFVDGVGPSEETREILLVALYNVHLTVLRYTWYVNGSGAERYSPP
QPSVGGAGDGAHGTKQVPSVGDSTVRDGSEAYNGDLPCSGLDVLDKAWETLMRVELPTASEVSAALVGGAG
YRLNKAKQQQKSRDRRNMDALKKTRLVERARGPLMRFLLAVAITSIPPTTRPRSRTDQLGDAAFDPSTFMG
KLHLWATSALEFLARHAVRNYLKENPTIAERCRLEGFDSSETTKAANNAFAVDAPATKSTGKKSAAPRRKG
REGNVVPQEFSVVEDDVLLGSITLEALLCEIGYPKPPGPFLFHGNEEQRDGRSISRLTETEEESIATALLL
HCVLYDAPDVDLYGRLCICSTSLLWRKLGESDEGLASRPVSPSPESGKVGGRHSRSPVWALANVFTASLHI
LRHMRRVRGCGAAESGADRHSLEDIYSIFPVAEHLRRLLEQCKGPLLISVSNSNNNDSAIAVAFGAPVDGL
SSASPSMTSSSVLYPPLLSSTTAVSESVRVVCQQLLERGAEFLLHRMARYSPSKLPVVTRSAPLLRDGNGV
SVASGHQLDKDPVELYHEIICTYLDVKASSGLTSAVDFSELVLRYAKEVVAEVEVVKEKVVGAQGAGDDND
GNAVSGEAAAGSASPTSHQGGEVGGSVDGLPKGVDSACVFRKKHHHAMLGISAINIALRWLSSATHLLDMH
NSAQVAGVDTGARLGLGSHVSALVASGEQEVRDIRTDLMRCRIELCWCASLYQQSFAAIERHREDVTRIKE
RQAKANIYGAVTLKEKNILKSLIQEEPQFLDTNERERKRLVAWARESSDALLLALVLLCLASHQPRRKVQR
EMLEEAFHLLCRPEWSGSNGSFGVDKLAPREGSAYSPLMIWCYAVTICGKLGFTEWMEKSRDVLHAAFLCD
KSNAPCLRDSAASRSVGTITREAATVTAEGSRRSPSPTGDGNTIISPLEFQPSEEILSASSPLQLRALVSA
ILWMSVGDMKGHAYKNFCTTGLMPFTEHSMLVEVPFPKRYKLQETQVVYMRCAYRIRFVMELAFRVKDYGR
MMRCAFELFNALLPTLVDDNYCPVVLRPLGTLCKILLLHPTSTSDDPDVQLLAVRVLGALLLTIRRMASMP
CSAPCANLHADTNSKDQIVGQQAGATTPAGAPVVSPERCSEDLSDLPSTIAGWYELLLQAFERVWNDVYNA
PNLRQRRHRHRIRCSARRVQRVVNSKGSAPEGTQGTPAPGNRGENASRGTSSRLRRGSRVGSAAPSQTTGA
PAECAAGVSTFPGSSYVMLDDIVDDCVPIEYVELLGEILFKVPAQVAGSLRKVFSSCDKALDSVLAATPNC
VVLEGIPPWLLEVVRAVHSRSAQLAIERLHQVCDHPMYARTAAYVVESLMEQGDVVNARRLALDALKRLKD
IRTLINEHQNGLIESMRSWLRREGYLHIQLKGTSSVDASTTDNPTALESAKELAAEDARAQVAPINELGQH
IVSNTAEGEWSPVEKETLRQLTRGFAWVWRRQIARWLRARIMQFCVPFTAKLYFFLEKMSLLQLEQCQVLQ
EKATLDTSGKVSRRASKVKGGVDESVSALTEDMPKGKRAVQGTTLDEGDEEERFLSHAMRSARLFNRCGLP
AQAFQVVLLAVDGIRTFVCDDPADVQPLNDAQQTLAEDAGGRGSPSCGGTYSSDAADGFIDPRLYRFVGTL
SATEQRASVMRTGPRSREKLVALGPYICPLAQTMQRTLLLLWEGYVDYRRDLGIVQPNAVKVRRDVLLDEQ
LPFQGRVPSLFSLFSPAGQYEEAYLEHARDVEQRKRGHSLLYQTHEVCARESFERTRIQWQSSLQLSSLME
MWVDVIPDVGYFLRELEVGCSLLEQEAEERKCAYDTAVVQRQQWEARVSKKRRKKTEEAGTVRCITKPISF
EDPNGMELAAKRLGYTIPMGTAIGKLVFLLKCAQFSCQLASLQIAEEVQKLTSGSFSRHLLPFVLNVQCMI
GEPIAKETVDKLLEVYRDESRQKSLSRNARWTWRRYTKTEAYSRAMQRLGRTLLPSWGTQGDSKMSVLSMP
NASFNFSADECSLVSDVLVETSRPRATVRADDSVVCGTNPPNADDTNGASRPTSLHDVTGSYDNLISYLRQ
RRLFGPLAEELYELGRIYILHRQRDEAERCWLDSVDAALGVPESLRNSTAVDTWSEFQAASVGCPRILLAI
LSLTSLAMYTYREQQKRAVDACLLSARVLERLFEQSSGSGLPQCLRDFCGFALEDIIVLPHLREPLTTLMP
QIIHHLLFLGWELLQFKFPVYSAMVACLAEYLARTYTRHVPLTVEIRLLQAKAAAYSGNFRASMGILRDVC
QGKRVPCVALESFDLCASGSEALKTTKGLKGADTGAQSRRQAEPAAEAPNNQALQQQHQEESSQSGLYND
TELPTSPGNVACIQVFITQCFTSPGSDDRPAADAAGASVGQTVSSLMSGASGLPEPVAAYYGKRLSQRVEL
TLAECLVVLGGKEAAYVWGSLGGQSQPPASGTTTGADRRAMRSRGSQRPMNLTYNNSACREALNAAEQILQ

VILARLQKVQQQRQGERQQHPRQSGNSTSTVGDGNAFVLRGRKSVPRDSAPPSSRLPDGGNAKRWREVEDT
YTRCTSERLLSRIYTVRGESMKALGLLKGLVRSFENGGSFVCNIPHVPSFLWTVGTHNFWCEVYELMTQNH
VRLLEYSVAQKVVDHALALCEQCGDSYSARVFSLYRATISMRTGTASDAEETLNGLLNVSRSLVCDSRMDL
FHPWTILALEALRREKQCKESQKGSTSSIDLLEGAVLNLQEYSQVHSLLPLTFCFNERKQDDSREVTKREN
RREVLWKQLAPLPWSTDAVYVHRAVNTLAEEHIRVGLLDQAERLLQDVILSVASRYDTAAHPTALIESHFM
LARLLCLRNPSLITARQEENPLQQQRKGGETMGGAPVTVMAHEMSSEEEMIASLEPYRQNPVRLLMSVVKQ
VVSVGIHDYNILRVALLLLSALFSHAGRAFCIVAANCAVLAKLVADMKFHVFSGTSVFSLYGDDAISLGTD
VVFAESVTAYIHYQQRNVGNVMEKEGPGWDNAAGGGGSLPPSGPSHPRTRDDRERAAQRNNVSLPAVVSAF
AALHRERSLDCLLPPQESLDLELALQHVRSFLQTRTAPLSCSYLWHSEGALESILKQQASSATRPGGAFRT
QRTGASTATVIGDDNASPAGFLPPVIIDALPSLLSPQTLQQLHVPRANTVICNTFQYESASTDPVAYNENM
SIRAASPRCGQGANGGGAACKQAEPVSTLKFVLVVSPANDPAATQPPSVAEKPYHPTKKTARATNKGMTHS
LAASTGEGPRWVDAGSIFNMWNSTQCVVFDCPVAEMRQLQAQAAHTLLLMQSPAPSLPLAKAPPGVEGAVD
EEDIAAGAALTQQLNEVLSVLFTKLAPPTTGRKAMRAPMTVEGLLSGASNLNATPFGNNSGAVGASGNHGN
PWGGRAAIVGAGATDKVASGTAPGYKEKGADDGCDESDEVSPVDEAKSKLVADFIQMIVASIIPRAVVDDA
LMERNVQMLMPRCAVTVDVVRFLMAVTSNDGHGMSSFNPGLHDWFSRIAAFGSGQVVR
>tr|D0A9B4|D0A9B4_TRYB9 | DeepTMHMM Topology Prediction - Predicted
Type: TM
MIHATGDFCIGALFVGLWLAASVLPFSRVGAMLEDRDHPFAAPLHMMKAILLVVLGAWAICYALIGELGLL
FPANVWRFTPEFCFYALGRHTLRQVAILLYCIIISTPGAVLSSKGRRRCETRECDGADIELDELHSGREAV
VPILNCIHGAVEMGRSAGSDSFREPHISFNGSRSHLPSRTNRDDNSRLHSAAAPAEWNNTHTYSPCENGVG
PGYLHEGGVVMPSDIHIQLKPSLSVSDTDNFMDGPIPSSSVHFRDSGANPASQFPRGSRVNMNSSSFPPSR
VQNNEECGIRPGAASLREAESYTVPLNQQTLVSVHISGGASSATRKPPNSFKRKLCFVRNWLTGGPLPTFG
TDDEVTRATDKCGPLVHRWKRLVLWDAVLKLRLVLLMWIITFTLVVYLAVAASFEDVQDCHEPISETAICW
VNRSATPAQRLHPPRSFTTIFLFSDVILLLVWMICGVRCLQNIGNITAKQKFRRAYLMGASLIMLSITYSC
FSIFDPLRYNGIVTVVLLIFRNVCDLLLILLLVVQVENSSGPKPSWLIWLRGTVVYGK
>tr|D0A0T4|D0A0T4_TRYB9 | DeepTMHMM Topology Prediction - Predicted
Type: TM
MSDDEHKRHPHAQRSRHSTASPGMIGHDGNFMESFKDMLGDIYAPLKTGLKLEHVIYNACYMLLMLYGFFD
VADASKQWAAEFYIEPPRWSILTITSLGSTGYNGTGDAQWTSIDRGFGLLLSALALFVIGARGVRSVFHGE
NGVKALQRYYLFAGFLFTAFLHGPMFWLPITIIALNYVFIIILLKIKMPHWVHMAVMWTAVVSLMLSVGYY
GGRLIIGPRTLGFWGGMASWVPTFNMSILRMISFNTDLYEAIHASAPARATTTRKHDNGCLDCARLRDKHP
EKEVTAVRCYKFRSEYPRNANEYNLLSYMAYMLYPPLYIGGPMSSFNAFASHCQYSTVAMTRSQLIVYGIF
IIILYVTQVSMLHFVYLSALRQRGDLVMKLSTTQAAFMLYYSLAFLWLKFSLVWKTGRLAAVADGVDVPED
MRRAYSNTLSVRDFWRDWHASFNVWVVRYMYIPMGGNRRKYFSILPIFFFIAVWHDLELHLIEWAVWIIAF
FLVELFVGYLWGLPLFAPVRHSKYERLLRSLAGMVSVFGLTITNMIGFATVAAPHGGSLTAQIILHILGTL
NLTLFFFFLLFFFLLSATGVLLRDEEANQIKQLKERYGIVR
>tr|D0AAP1|D0AAP1_TRYB9 | DeepTMHMM Topology Prediction - Predicted
Type: TM
MITIIIIYIITMPQQQKKKQNHGNSSLLSLSLPPTRLLFLFELYCRCFIYFFLLIRIYLYKRKTIASSDSH
TKQPLKQNKRALTEKVRRQRKKEKTSQAHKYVIKYKFININLHHNAKDKYQDYIYK
>tr|C9ZI88|C9ZI88_TRYB9 | DeepTMHMM Topology Prediction - Predicted
Type: TM
MPRWQTHTFRLHENTAARPPGKLYVTFFFLFVKFSTFRTALLSFSLTFRSCSLFVFLTLILFPLPQSTVDW
LCFIFYFLFSPLPLFYLINLLHSYFLFFCFRCCFCCCSFDPPFLSFFF
>tr|C9ZJ58|C9ZJ58_TRYB9 | DeepTMHMM Topology Prediction - Predicted
Type: TM
MREGDRITRICVTLTPKVFSLFFFLHICPCVPVFFFFSSSILFFVFVDVKFWGREGRRGTGRGREKGVRKL
TGVCVCVIIAVSFNSFFCVLSFFLSFFVFFFQFSLPPFFYSALFFIRCITAMIAVWGRGE
>tr|C9ZTI5|C9ZTI5_TRYB9 | DeepTMHMM Topology Prediction - Predicted
Type: TM
MRVEGPASGRVRRICCFLFFFGNNENIKITIIIIMVMIIIIIMIIIIIMVMIIIIIMIIIIIMVMIIMTVS
AKKEKKEKEKKRTLRHGGSGNGGQKERERGV
>tr|C9ZUS2|C9ZUS2_TRYB9 | DeepTMHMM Topology Prediction - Predicted
Type: TM
MLLKLGAVLLLLVLYHVGSLFSRLVFVYTIQQGTTKFCFRFVFALSTYAFILLLLDASQLSERIKSNFSIQ
TLRCVLAVDLVLIALFCPFLVVRSLLPNVRLYYFFLVLLACGLVKMLFGASVAAWSWTLSSTQGIPTLFSW
DVVSVAVSTTGVVVVGLLSGYAAVTTPLAFVEPLFEHGSGDQARLAVGVLAKRQKHLLELWMLKRQQIARA
YGAVSRADGGRDGSNRGSIAAGRRMWNWVANSIYSSVRASGADIAAMETESDGIQAVSMAVFLQMSEMDTL

VRSAGSGETWRRRFNALFGLILFTHALVKFLSTVISLLRWGVLAAENAAPHREDTATKVMNFLEAYGLATP
HGDGAEQRVVWVSIALNAWMIASAIRGFFLLVFRLMTHIAFISLDTTVTILTAGMGAFFVGQLVLLRLTPS
LERESVLYTALREQLPQHGAYCHLNDLVFVVAAALVAMMRRCMSSSSATALCAAAD
>tr|C9ZI92|C9ZI92_TRYB9 | DeepTMHMM Topology Prediction - Predicted
Type: TM
MCLVTSVLVYVYKHIYIYIYMYSLYYPHSSTSRRAKRKRNKRRRNVTLCFGFYLPPLPVSSLCLSSPAYTC
AIVLRAKMVSFVCLFICSVLKSFHCFIICSCGGAFFFLFLVCAYIYIYIDVVYFCSVRCRVTAVVPNFWVD
ITKTHFYFVVAIVVVFFPKKNICLLLLFFNNIFQQLKKKNNYYNNIYYNLIIYWV
>tr|C9ZZ84|C9ZZ84_TRYB9 | DeepTMHMM Topology Prediction - Predicted
Type: TM
MHLSVYVRVLFLPFPLSNTPFFSLLPLTFLYCTGSSTYFHPTIHLFIFLFFASALFNFPLTSSTSFTLQHT
YTYTYVNTCTNVACVLLLLLLLLLLFRQCVPQLLCHTGSDIKFVQTFGQGKIVDTEVEKR
>tr|C9ZRD0|C9ZRD0_TRYB9 | DeepTMHMM Topology Prediction - Predicted
Type: TM
MHMFAFLCTTGPASMLFNINCCCRYWFYLFDHLHLLSFPPARGLLYISVSVGAVVGDKLLYLATGCLLSPH
HNFIILYVNVSVLVVTVVGFAYLWLGLLLRINSAPRLSGNIWIINTYLGCLEASFCTLAWSVWPFVK
>tr|D0A8N3|D0A8N3_TRYB9 | DeepTMHMM Topology Prediction - Predicted
Type: TM
MDSKQRIWDSLRNDARQADNLIERKLAALEDIARRVDDETSGVSFGAKGTASTTGFNQYNNPSSSSSTTFG
NTAVVHFPSESHVRSVQLEFEHSQSEVEVVLQRFETLLETMAETARELPLESAAMTHMERFQQLAAEKRRT
LFRVAADFKRRCERVELLPNISRELDVHREDVGTQLLLKEQESLRHTQRMLNNIIDRGEQAHLQLREQRDT
FSSVSDRLLEITQRVPFVKNVLNRIDSRRRREAVIVGALIGLCMTIFVLFLF
>tr|C9ZYZ8|C9ZYZ8_TRYB9 | DeepTMHMM Topology Prediction - Predicted
Type: TM
MVSKISVVLLVAQNRENNRYNKKKKMTHFRKRERGKGQIQKFSIFFSRVYAPARFSTRFMITFCLLFLLFT
SPFYSFALIFSLSLSFLPGGHADTPYMIATSKRN
>tr|C9ZQ18|C9ZQ18_TRYB9 | DeepTMHMM Topology Prediction - Predicted
Type: TM
TLETRAHSNNGYSKLSDADIKKVKEIYEKAKGKVSEQLPKAKEFGEEAGKRHQEVTEAAKRARGWGLDDEG
QNSSGLHQLLEWYCGTKEDNANNQKCDGVKVKEHYLGRERNPIDCKGTGSTVPFYLDVTSGTMKEALENWE
RKKPKSDGEPVNNNWKANYDSAVKKMEELEESHEKGKKTVNDVSGFYNAAYALHSGLSAGKPLSEVLVEAK
EASRKGAKFTNPGEAAPETTQRGIGTSTGESGATETTGGSTTISTGTGTTSGTEPEVVGADADFGDLLETS
DRSALSSKIKESKVILMAVLIPVAILAIITAVVLVFVRRRRGNAEDVIDEKGEAVSSPDKKGGATSPCYRK
E
>tr|D0A138|D0A138_TRYB9 | DeepTMHMM Topology Prediction - Predicted
Type: TM
MEKVQHPIQISQMLFLFVPTLILHFSFHFLFFFSLVKKKSNNKTKQNGAAGRGAHPYHSSSRKKQQNISFL
NRSPALLLPKFIRPFVYSFFPSFSSLSRAFIQQEEKKKRKIVLHLCEMK
>tr|D0A0Z8|D0A0Z8_TRYB9 | DeepTMHMM Topology Prediction - Predicted
Type: TM
MEHVVARVAGVQKPVDMPLRLLKKQQWVRCALDCSHKRVLKKEAFIELCTSNPYTPLCERMSKSEGIKFLQ
ALHNARQVTVVQDYVYINPADVVDAVHVRLELPNVARRAPVTSRNCSSGQPKDSEVTLRSDMERRRFFWAI
VSLLSSAQMSILAYLTFSVYGWSVMEPICYFVTTSTSLCAYAYGLCYKRNCSYEAIDSQLASSDTLVEGKS
PSTVCAQPFFMEAVELLRAVQAESDNSGLAEDSSSDTKDKA
>tr|C9ZIF9|C9ZIF9_TRYB9 | DeepTMHMM Topology Prediction - Predicted
Type: TM
MLWDHLYYIMLYVVSLFCLFVVSFRCAWLLLFVFVKYILFFTSPCLSPPSHPPPPPFFCFCFLVFCFFVFV
LLFFHLCLPAPFAMVQRLHRQIIIINNVVIMY
>tr|C9ZRP8|C9ZRP8_TRYB9 | DeepTMHMM Topology Prediction - Predicted
Type: TM
MTERSPALVPAVSLLREELQLLNGVVEGQARSRVYRHHIFFHKASFLRSALRNALPQFARRAADPTDLRTV
AGRLLTLTIRCAESATLELSASHIDTVSVALLLLAITSRIGCVLCVVLGRKPETFGKLHFIGSKFGVKYKR
SRSEAELAEETIGVATPIRRRTIGGVIEAVIK
>tr|C9ZVR2|C9ZVR2_TRYB9 | DeepTMHMM Topology Prediction - Predicted
Type: TM
MLAVCDLVRYPSQHRQCVALRSLSPVLKGKCYAEGHRIFPPFLFDSFYSCLLLAFCYTSLLSILLFYFSFG
RRFRTPLNTVVLSLVTRLHRLRCSVAYTAVPFFSILGLPLYNICACTPHSLHICTYIHICIPLYIVTSICV
FTRVCVLLPTFT

>tr|C9ZU93|C9ZU93_TRYB9 | DeepTMHMM Topology Prediction - Predicted Type: TM
MGRTGGKIWCETGMHFVMYTCPFYSFPSIIINLFLKYIYVILCKLVCCSLFFFLPSHHAYTHTPLFLFFLC
VCGCVADVILSVWLISWLVFTLPLIFIC
>tr|C9ZTD5|C9ZTD5_TRYB9 | DeepTMHMM Topology Prediction - Predicted Type: TM
MPGCLSALASHVESASVVSTKSKRKRKSKKKALYDQYLADSGSEDSSELSKNVNGTPSKYEYEEFEPGMDI
NILIQQTIQNVVQSVCLEDCDGLSQFGDNDLICFSAAVKHNSSILSLQIRYLDVSDVSLVPLCRALECHPS
IRALDLSGTRGGRPSVKAVFQLVCTNPNILFVRLDDTMVSPHDAEDIRVATLYNALACPDPTNNPFYLGLL
RKISDIEEEKQKYKEQLSEQLWLFSSRPQNNLNSGKEKKVGFSEKVSESRIGADVCAQFMSGRCAYGSRCR
YIHPDKTTALRNAIALSKYKMAQMIDDDAKSVKSSATSLGQTGRGRLQSRLRPTNFTMNKVHCAVATVHEG
SVEQCSAGSNEEAADVALRLSIWTFALVTAVCSVVLIVVY
>tr|C9ZVF1|C9ZVF1_TRYB9 | DeepTMHMM Topology Prediction - Predicted Type: TM
MTTSPNACQDQPPQHSAPQAHEAECTTHKLSAEETMDARPVHPDARALFRKLPCVWSIPVLGTAVEAFGPK
FVFALGFCELFGKGIADNIIRSSLFPMFTYTFGADAKLYQRMSSLVTFGYAVKPFAAMFSDLFALFGYTKR
WYLALSCVVGSTLAIVYGSLPGELSYVPVAGILVFVTSFTKANLDILTQGHYSRLIRRVPLAGPSLVSWVW
WCVLTGSLVASSIVGPLTDKRLQRVAVFISAGMQLVPTIFFILNWYGERRNREERAYDLKIIREKQLEHEA
DAVRLQGSEATSGSLDNPSDTEEVGEGGARILPCCCGAFEVNREVFARNKKVVFYCMLLTLGAIGMVLVTV
LGTRLQLLITSVVASFTLCGLGFVALPLVIAKANMFTFISRVAYIQLPGAIDNVFMATPDCFPGGPNFSYF
YYSTVGNMIGAMGGVIGVTLFRYVFSKRSYRLTFIVTTLIEIVSSIFDIIIVERWNRPYVSDHVVFVLGDQ
IIHQVCYMMHFMPTVMLISRLCPRGSESMVYALLAGFANFGRSLSNTLGWLLMEYVWNVQSDITVGPCDFS
NVKWLLLLGHFGTPLINIPLVFLLIPAARICDVLDENGKAITKKAEDVHAPSNDSPRRREPTAN
>tr|D0A863|D0A863_TRYB9 | DeepTMHMM Topology Prediction - Predicted Type: TM
MDRLLQFHSQSGVGFMRGSPPPGSSGGLSRLTAVVPVAAPGSVEAETVTTLETFFEVVATVTESIDRVNVL
MCEMSKKHEQAMDTVNVAKCDAIRKEVGDLDDEINNTIQKACKGVEDMENLTKKLKETPEMEGRFAGVIRL
EENQRRFVLQKLSETMEGLQKRQLVAEKNYLSQTERRIKIAYSNPDGGEMDDETAHQLAMQVMEKGATTAI
FQQSKEVLAQMLETRSDIYRIEMSMRSLNRVFSDLAILVEEQGDLMNVIIRNIDSTNLYMEKAHRELQQAR
AYQRASRSKLMCLLMIGVIIIALFVAAGLLGSLL
>tr|D0A673|D0A673_TRYB9 | DeepTMHMM Topology Prediction - Predicted Type: TM
MVLFLCAAIVTEDGKYAAIRKEWMRGEIRNKMKEKVHVVTLGNAAYTPSLPLKRELTLCVFGASAAITITG
SFCRCLHPVVRNFFLFYSIRCFPFTDAVKSINAAKEWKFFSHLLCIVWFDPGLRFSDFASLLPVSLLRLRV
LSLNLFE
>tr|D0AAK2|D0AAK2_TRYB9 | DeepTMHMM Topology Prediction - Predicted Type: TM
MSSREERSCRMCHSSAGKCVSPCCCDGSIKYVHSKCLARWVRHRKSLICEVCGTPCRVAKLSSYSISATNY
RWVTLIWIFSRVWIRENARILSISLLVPIFSWLTFVLEGSLKGAGSIYSPVKVTLTHTHTLSEWGPTEALS
YTVWVLTKLTAASMLLKLVREHWPAIEPFVREAPNADEQGRPVLEGRAVNQLQNWRIDAVEEGKEGEYDAS
GEGGDVEGEPMVSATSGLIVEVAVHPRDDGVVLQAIEAAHSDAGKSKTLRGKLKEELLHLVLPFFFLNVFC
TVLCVLAPEVVRIKMWELWVAVDGNSRAWEVADTLLATLSPSENVARMRGAMVKPLIEALGSSDLLHAPLY
FARLTFVIWIVVFVMKGVRCVPLRTFVLEALSLLRCIMAALVLPTFFIRTTTLLVLFLSMDFFDDDRNVGI
AEDKKRAWHVLTAPVSVRHEDERPTTLLVLKSLKTVLGSERREVCFDFGAARVLRSGINATVDQGVILDGE
EAEDPVLFFLFALSVIVSVVDMPSMWPVSDVILAVVISQFFFIVGFATRLPGRWFRWTMLHKVASVTGLRQ
LCALLDLWALLRVCAEINFIVAVICGGFLPYAFGAYQFFSRNDKPLVLLVTRNLLSSFRWAKGVFMLVDVS
LNIAWYVDSRLGDKMGELFDVRDLALGDGISITGFLSKALRCTAFVACSSICSCIWMGICLALFDCLVVRS
SCFVDTFFYGCRCIITVYMINDNEAWSRMLSIVELPFKGVRQFTEWKCRWLFSSSFGIWAGPQYVDGVNVA
QRELTLITGMPTWYTHLCCSRIMVALDELQRNATADDAEVIQRVAIEITPLIRREEQACTLTEEQMMILFR
CLKFPGMGRLLTLLLVAALLLLALPFMVGACVVSSVLALFLSPSCVVASGFVCGFLWVVGVVATGTLIILT
DKLKSDLGPVRLYFSCGCHHMPLFEALAFAVMPIFMTTTTFIVFPFALAVMAWPHVRQVDSFGAFFMRFDI
ATLCLIFRLLLRCWTRLSPAVFAFFGRVQQVRVRNNGENSRVVSAPQEVAAPAAAKPGASAVLMVREATAN
MWKGCVDVGFECCAKLCYVRNHILRRLVETAESESIVGVAFIDRPT>tr|C9ZVI3|C9ZVI3_TRYB9 | DeepTMHMM Topology Prediction - Predicted Type: TM
MRGSTDPANVDQYDSRTYTRKGLATRVVVREKPYVKSRSQRLVDFLRPSMRTGYNVVQGFELAKVMFCVIF
PVFMLIYWKSVQRKLPDQWESQLSGLQHRQLKEEAVPEHDTDYFSIIETFQERREKALQKKQREVVGTS
>tr|D0A1S6|D0A1S6_TRYB9 | DeepTMHMM Topology Prediction - Predicted Type: TM

MYCGEKLTRDTSSLWIKVNIHVSTHTFFSCICPCRFPFYWVIIGNSFGWTLCRCVYVLLIMLLERCSRVCW
FTFALHVGFTEKSGLWGIQHLAFYSFALPTFQGNVVLLLDHLL
>tr|D0A665|D0A665_TRYB9 | DeepTMHMM Topology Prediction - Predicted
Type: TM
MVVCFTFVDVNVYVCVSGRLFVKVLMFVFLNYFETWSSIFAAASPICACGRALQTLLLVNECLEGTTCERL
FLLFAPSSSLLLCESLESCSTFRKWHTLLVRVEPFVHFSFQFLFVRC
>tr|C9ZQ43|C9ZQ43_TRYB9 | DeepTMHMM Topology Prediction - Predicted
Type: TM
MYSAWSIDFTWFFPRSVPDPATHNGTSGYFQSVKTVTLPVIRASNIPVFAPALHSTVQTIPVCCCFPYITA
PCSRAVKRTTIRAKPHAVMLISVPIVSITCLSVYGPRVKENRLGRVSTIQGEQGR
>tr|C9ZYP9|C9ZYP9_TRYB9 | DeepTMHMM Topology Prediction - Predicted
Type: TM
MEQQQQQPEEKKEGGEWYKYLITIAVYIGLTALWIVGCLIHKRSVARRQAAGVAQSAKGRTESNDNGNTTN
EHKKEEAANNNPSNNQQTSQYPTYDFSSNQNREYGTDAYYSNSYDTNNNASNENRRDYRM
>tr|C9ZK71|C9ZK71_TRYB9 | DeepTMHMM Topology Prediction - Predicted
Type: TM
MVIVIIPKQMRLQPSSNFPVPYSIVFFCYYRFSSVLMFVLLQQYACWVSTESALVCVWVCVCVKGSMRKKR
LFGGRHDPSFGREHRGQLLFLFFLVYMLICLVIIIVINIFFIRFFSLTSLSSHLFFSLQCHGERNLFTVKW
KEMSLLFAYTYLCMCILAVISENSMNTWSHCQ
>tr|D0AAN5|D0AAN5_TRYB9 | DeepTMHMM Topology Prediction - Predicted
Type: TM
MAPPKASLPARVIAGDFLGINWNKVTWMAPYSNFYPITVCGGFGGRMWSSFIQYGHFHNRATRIVLRNAVL
SIPSALAAFVMISNVDWFQALQCAYYFPGWQVPRWASDKYAEEMVLLSRNKPGAMAKHHYAGPVS
>tr|C9ZNC8|C9ZNC8_TRYB9 | DeepTMHMM Topology Prediction - Predicted
Type: TM
MRGCGRGNAERNVERVRACPRWVATKISYGRHKFSIFSPVFCWCYRYDRCCDSVFLFLFLSFFAFSFSVGY
DYVLIHRFSKKNPECTLHHFFSLLFWGLEKKRGKSVM
>tr|C9ZNJ9|C9ZNJ9_TRYB9 | DeepTMHMM Topology Prediction - Predicted
Type: TM
MRGKLEQCVTVVIVVTFNSLICSCCCCFLVSVFSLLFFYSLLFSFFFLFFVSCLVNFLLSLSNAPFFSSFP
TPRSFVRSFIFFVFVISTSAILFFLLSLFRLSASFKPTHKPPPPQTLAIRKEKKEKKTQTNTQTHTHTKKK
TQSVL
>tr|C9ZNQ0|C9ZNQ0_TRYB9 | DeepTMHMM Topology Prediction - Predicted
Type: TM
MFCSTCVSLVGVISALMLLSYLGFVIIYVPVYRYLAITVLLLLLSYRLSVWRFLPTERAHVRRLAEKIVER
RRAYALPTSEEPRSDVGANALYVPAAPYL
>tr|C9ZZF5|C9ZZF5_TRYB9 | DeepTMHMM Topology Prediction - Predicted
Type: TM
MFFFSFFFFSAVFFSFCWIFICGHMWLDPALSLSLSFFYNFLLVRFFFDVCSVWLLSFVLFPLRPLGLLII
SFHSFAAVLRLICCYCCYVTVIIIIEKLYTSYVSLHFFKKKGEPD
>tr|C9ZNV3|C9ZNV3_TRYB9 | DeepTMHMM Topology Prediction - Predicted
Type: TM
MVNTSDSKQHLPKKNYTSYGAIIICRQVLFITVIIIINVIFCESCGWLYLFYFVVTFPYSAVTETCFVDYV
IHNFVITYVFELIDVDKRCHSIMHFYWFISLPEEDASIFLFFRNLNGFI
>tr|C9ZPL0|C9ZPL0_TRYB9 | DeepTMHMM Topology Prediction - Predicted
Type: TM
MLTRGSPLPLPGLLTVTSSVTLVIYIYIFVTDGCARFLATSINPCHLAVFVIMVCENPWLIFCLCCLRIIL
LFPNRRGLRYPVLPSFPPSPLAVTAPPFNFLFRFINAFRITDVMFGVVLYSFPSTCR
>tr|D0A440|D0A440_TRYB9 | DeepTMHMM Topology Prediction - Predicted
Type: TM
MEVVAFFFFSFCLLHVMMALYPPSSYLYCSLRSDCCCFPGAIIGLLCADSVLVMFVWPSAAQTIFFFFLST
FLFHPLPPPKKKKGRPADDMWEDVYELMQNWQR
>tr|D0A0G9|D0A0G9_TRYB9 | DeepTMHMM Topology Prediction - Predicted
Type: TM
MCEGDLKKKRVKGVKGENEVIAAVAVRRNDNSFEWNALNSATLSTFLCLSALLRLTGPLPSNKNSPSYISF
SIFPSPPFCSVLFCSVVLRCVVLCYCVFCVHVCMCACVHVCMCVFLFLRLALILLTVPFCYYCCCWYKSSV
VRWGKKMNNQDGQSAKVNRECCGEGGGGGRNCFFFILKEEWLKRRVFVFFF

>tr|C9ZNR6|C9ZNR6_TRYB9 | DeepTMHMM Topology Prediction - Predicted Type: TM
MPSSAPMKTIPPKSRVPPDWIHPALHRQWQRRDKLRTPHELRLEELDVQRTEMEEASRRIMSIVSEKKSAL
EKLDRQREKAKADIDLDEAAAGEITGVLDRLTSQITVHKTLPRCDARLLDTSDSENFASGAAHCVLSVECG
STWGAVASCFPCDNEIKRSFFSQYAPLFNVTSDDSSAAQKVGGAAFFDEGCLLSVDQVSCFNPACPYWHKE
QLTHLKLTARKLVSRAANFIRGNRNNCDVAALFHRFRMSMEASTALHDAVRIERDMMNCIATLGWAAIFLQ
KDEVAENKQGRYRPPPSRITWDAPIQSKPHMPLMQKLYSLLRNPQECSAWESLIGSSPSTLIADAVSLFQR
HVDVLSWRCLMRVAGDTPERLLWLASRGIEIFPTSPSIRLSHLYALLHSGNTASECVDVCLESVRILSVQA
SKCTLSSSGKVEWSESVARYIAYMIAMTCVRVAPMDAQAAMRLLTYAVKVPGRVCLLPLAQQNLTLMLIAI
CQTGKLEGMNDLPLASISDVTFALSEHFPGRPQDACAGLLSRQLNMNAGCAAAGIDAELMDRMQSAVHLSL
MRAFSSNAVLVERILTKVKMGSVTAMAELWCDYLRVVRQHDGTEALVSLIRSLLEECKSPLLILHFVKILD
FNAEDTTAVASSAVQRFAEENGITVENIASLASSETVTFPVSDWVPFILLHARSLAPQRRVELIFSIPPVL
YCEVPELVFLLWFEIIPPTLLLKDDELFRRCAEYGLVLLREPLLNHFSPIDCNFDEMIAIPHIASLALYRA
VPVLLGAAHHLTAHYRKIVLDVSTELHVIHPYLYAT
>tr|C9ZJF0|C9ZJF0_TRYB9 | DeepTMHMM Topology Prediction - Predicted Type: TM
MSHSSRDDGDCNQLAPPQNGREVSVWNLQTHEGFSLESVSGPNHEDEEQEVSLASPRLEGTLSSLPARDPT
SPLAFVHQRWNSIRGSPRVTSSALGSTTTSYISVSGRSRVTPASARPRDSRDNFRAVAATLVFTHNSSEHV
RRSPPLDDTLPLPAVRQADGEVSICRPNMNNSSASSLSAASDEMPRPATDLQSSVKFYDGCSGAPTQEAEH
LNLCFTRSSLLASRRFNVMREGSTPFSDVADAMCGAQSSEKVFGIGAHRGVDSEHAFRGGAIAAPGRELLL
DCFRPGYNSSTRDVATGVVEYKYIEVQPIVGVRSEGDNLVEDEDNGCCCCRFPSCCTFFCSSCCCCSRSSH
RKLVKGTDARVCLGQNPRTAELTGGGCIVKTWNFINVFSGDISLMSRVFCLLNFTAALSTIVAGGLHLLFT
FDEQRSRVSDDSVCNGIVLLESFSPDMCFAFICFAMNSLVAVYLALHAVRCENAGSFFCHFLTVVLMVGCF
VNYLFSRSSSTSSHSIPAWAIVVFSVNIGLLSTACCLYVPVSRTFAHYLCAKGTVREELLRRRRQWMCIMS
CLQADVVTTLNSGVAALCLASGPIQVIGGAFLVVISTTASLLFIPMLKRRAQWFIVVFVFVAVATTGFNCY
VASHGVYEYFITNALNVYEVSPCYTNLLQYCLSGDFTQLRHSHGLLRYGGGGTSYGHTNEIPFTTGLSREK
YGPFNISTECCLDYGRCRLLDSARFYASGVIALLVVLVAIVRLVLVKLWIHATVEDGSDVYMIPLAWARND
TGKELGQPLRDAVK
>tr|C9ZNW5|C9ZNW5_TRYB9 | DeepTMHMM Topology Prediction - Predicted Type: TM
MKKGKCMRNKMISPRRSSPLHSHSHSRTTHTHTHTHTHIIIIIIIVIVIVITLQATQSTQAHKMKNMGKPM
GRMRKKHTFQSSPQQSATCSKKKKLKNREHQTIPKKQEHTKKKTQ
>tr|C9ZUC6|C9ZUC6_TRYB9 | DeepTMHMM Topology Prediction - Predicted Type: TM
MQPGLQMSPRTGAADPIQVYGTPFYETMRCAWFVMGFAVVTWTYTLMASYMAQSQQYRDRASGELQQFLTA
FHQRFIATVVCVTIYLAWPISNILIEIALWFVLIIPWLIYRTMCNPGLGNLRPDVPLDEMPGCIRLDMFFM
DFVDTRRLGFSDSQWRLLTGTGKPFFASCVDESTIDRDPSMPSLRYASNWAAGAQPGSYPQVPMSPGQMQP
HGQQLQQQQQQHVQQQLQQQQRMMQSSMTGDEAEGGEVHERSRRRRRDHSRRRGDRSHSRGGAETGETMEP
SGFGTSPGRSVTGEDEADIQADRGRRSRSRRRGHRDGSRNARSGDFNDWDDQSGGEGGRRHSRRGRSETNN
STMNSTGFLNPQDIDRMLNEV
>tr|D0A3Z9|D0A3Z9_TRYB9 | DeepTMHMM Topology Prediction - Predicted Type: TM
MHHSFSTPITLLRKHISLFLSFPLLRAASILLLFFLFCILLFSFFFVCVRVNFSESYFASLPSLTYIYSCI
FVHVFPFCHSSLFASLLLNPQHPFPSNKYVYIYIYIYI
>tr|D0A5A6|D0A5A6_TRYB9 | DeepTMHMM Topology Prediction - Predicted Type: TM
MSKETKAPANAPLPKVYTIGIAGFSGMFAWLFTHPYEMWKNTVMTAPKGTSQKECLVKVWERGPFRGLSTG
ILRQAVYAPARLGCYPIFRDAIMSLKGDADGMPTVAERALAGALAGVFSSILTSPVEVCLVLQMTGASKQS
LTRAAITVYSTNGITGYWRGVSALASRAALVGVAQVAVHDQVLSALRRRNVSYSQLHGTQPYGDNIVVNAA
SILTALFYSVITMPVEFARVRMSADTTKAKYKSVTQTIGRVVREEGALAVYDSFAPYFFRCATHTVVCFFT
IEYITRKVKGWRAAKLQAKQ
>tr|D0A0A4|D0A0A4_TRYB9 | DeepTMHMM Topology Prediction - Predicted Type: TM
MAAFSGETAVKGIHLINDDGELLPEADINDFLISALAGKGSSDILYRTGVNYHVVGVFGGQSSGKSTLLNS
LFQTEFMTMDEAHHRGQTTKGAFMTRAILDAQTHRKEREEGEGADLLKREKPQPLFVLDFEGTDGIERGED
QNFERQLSLFALSVADILIINMWAVDVGRFNAANMNLLRTVFEVNLQLFSHGSYVKEEKPTLLVVLRDFTE
NDPAPSFETVRKSFDKIWGNIQKPESFTDATIDVVFDLRYRVLPHYKLQRPEFDSAVSEFREWFVSPKNSN
FLFSNCSMFRGVPADGMPSYLSNCWNAICSSKDLDIPTQRDMLARHRCADAKHAAIEEFKDVCEEYTKKIQ

RGDVIPQFTRALEETIERLLKNFSDQTKLYKVSVVHETAEALEEELGDMELHLLKQYAKSIAVTVLVALDG
VIGSSVDEAARWLQNEARSVLLLEGKDNKGDRIDGGGLAQGVLDTAEGLVDNKRCRLFVEEFWKRICSSLQ
GAFDMLNGRSKSHQAALSSLYGKFATAIMDDQAVREGVAHAAMEGAQHKLRNRFVAMAENAAETVHQVFEQ
ALTSKTDGTVRFFRTTDGLLGAEKQARQAGLVLLGCLLYYRLKLVPVEVDAGEVEGEGTTRALQRLVRDRC
RFQVRDNRTEKNFFLHFTNISDVPRYPLDAPTSVVDSGDTTADTVNADNVLLSHNALQRAFHLYKQKSDFT
LQMQLRNIESGKQSLPPWVLPVMLLLGWNELYYLLTSPILLIAIIVIAVLFFKTFLKSQLEVLEEKCPVWL
VVSVKALLQQAQALQNAYAPTEAVRGGGGGAQFRDPTQPTSVSGASAGVSSESSSAASPRRRVCRESRDKG
ED
>tr|C9ZZF1|C9ZZF1_TRYB9 | DeepTMHMM Topology Prediction - Predicted
Type: TM
MLHVESSEMQAAITMCQFLLIWAFTHLFGWFWRVNMSGVSTTLLMISSCLICRRKAKGRSCRWPANTGKNS
RVALVTGASSGIGFAVTQQLVEHGWRVVMAGRSEERLLEARKKIMVRNPSGCAIVVGVLDLSDLSSVRDFA
EVVTGQKDQLSLSLLVNAAGVLRRRLHRCDGTGMEEMIATNVVGPMLLTELLLPLLDETALRTGASSRVVN
IASSCHTFLGVAPQQGPLEMLKELHSRAPLSEDAGPRDFTLWNFVGYYGLSKLCMIWWTNILAQRVSSLYL
PTTGGAQPSQPLPRVSVACCHPGIITTHLYRDLFPTFVLDYLIYYPSLLIGKTWTDGAQVVLRMAVEEERL
VQGGYYLCDGEYGEKSSNCCLSAYAKDMKAAEEFCAWANMQIELQKDTPRVPKLRTKKVDLPRRLIAVKLL
>tr|C9ZYQ0|C9ZYQ0_TRYB9 | DeepTMHMM Topology Prediction - Predicted
Type: TM
MSEADDERPQPQGGADTLRTLQEEEHREREYIRKELYSSLLNGDNAESGDEPSTKETLRMAMEAREELNRA
AFRPLKDKAAGFRLFAQNMRMPHNQRVASVFLLFSVLMLTVPILILLIGMHIVAPFADVDPGTCGGLMAVF
STIVIMTVYVVYSLREAPLAGEAEAINPDKKQD
>tr|C9ZRM1|C9ZRM1_TRYB9 | DeepTMHMM Topology Prediction - Predicted
Type: TM
MSSKDIIGQKSAARKFTDNIKMTWSRLLDQTVPHRPLRWVMFVFMLSLYILRVYFCGGFYVISYVLGIHLL
FLLVQVITPLADEDLGSEGQLPHTAASPDEEFRPFVPRMQEFVVWCSMMKSVLVCTFLTLFRILDIPVFWP
VLLLYFIFLTIIQVGERIRHMIRHRYVPWSAGKPKFVPKS
>tr|C9ZQ68|C9ZQ68_TRYB9 | DeepTMHMM Topology Prediction - Predicted
Type: TM
MRVNSEAIFLGVCYTSIRSCTAVYAYKLFGASLGCALVWRDIFGSCGSFDVNFLSLSYGRHLHLISYLLLF
HLLMAHCASLICLSFSPSLQSSSIFIGIVFVFAYLFRSH
>tr|D0A5Y9|D0A5Y9_TRYB9 | DeepTMHMM Topology Prediction - Predicted
Type: TM
MYKCFPFPALAHYLSDSSVLYRGNRFVQFTFSVSVTFLLQALGRSGRRCASYHHVLCFFSDPVMQFFALVL
ICVCCRRAGNVFRMLFLFCALVVGISYIT
>tr|C9ZQ02|C9ZQ02_TRYB9 | DeepTMHMM Topology Prediction - Predicted
Type: TM
MKFTSLFSKNTRNSPFSNFNIPPACDVKFLSDEPFPIENGVCSLLYTKAFVYVKAFVHSTSRMVCGVAVGI
SVKLTFFFSKKRKSSLLYTDFIYERKKHNKSFSKV
>tr|C9ZT14|C9ZT14_TRYB9 | DeepTMHMM Topology Prediction - Predicted
Type: TM
MGSLVEDGEEHARVSAHVLMCFDNVEGKKKKRKMRRVMGGVRRRHDLWRVPALGSISCIGFISFSLFLFIK
KCTRTQSHAQLFASVACYSPTILFFLCVFCSCNFHLPLLSL
>tr|C9ZIN0|C9ZIN0_TRYB9 | DeepTMHMM Topology Prediction - Predicted
Type: TM
MRRTQVGNSRLHDIKDSPSNEAEVQAVLAAAKASEGDAWNAVRLLAGCARCTLQAGRGHLALPLIVASFEL
SPEPFRQLVGLQQLTVDGSQQQILQEIAENDAFTKNERGANGIMSTVLIVWHGVVVSVAQNGSCPKDFLRF
SHCIAHLLSLLSGADGVVHEDLSACWLYNIIMFAHKCKCTRAVAHYLLEGMLADVSELPEDLRDASTLQSL
PFAFRKWGAAKAFVHAGVRKHPVATMFKRLCLALYTEGCDDLRTVQREIDSRALAVTNVLQQTFGSETSHY
MVDFHRPFRMEAQHIALLCQHAMDEDQLCTPRGQFGCIRLACLLKRAANKIMAPTMNECKLVSLSSVVALA
LRHQYIDIAAEILQTGVETVDPSDESLLLWGKDLELVLAQNLSAHPPAAAQCPVKPVERARDEASSGVDDK
VGVGTVGAPEPTQLLHEVVQGSLSDAEAVRVLALADDLTQLLPIGLTLLRGRGTLQQRLLADKIAVPFVIG
ALARLVQLLVNEGDMPLVRCFLPFIAHLCVGVPSRAHLLFTLQAIAAFAKGFPGDTGEWASIASALSQFLP
LRSIDAAPSYCHTKATTTGRTFASRRRVFDALRVCGKTSKTMMHSYCQVQVSLLGEGGGVRLLRTTHAGTT
ADTRWEKVLQIEYLLLQLVEEMKIIERRNRDHLRSTDQGESPLCEDLPVSSLRSDISVGPVCCSVGGAQDA
RKAREEWWNARRALDRSIGAVVQSMQSPEGFGCWRAALCGELPDSCQVAVWDATKELLSGLGLPAQHEGDV
SLVLAALPFVGDRHPEDGDLLFNPSHVGPTNPCGCCDETLRRLSTALEQELITHLDAKLVNEPTACRKACL
HVLTAMHAVITDKKCNQPVEHGGEPEKRLTDGCYHVNLYEIPRTPVYLVLDNELHCLPFEGIDVLRHGSVS
RVPTVSFVSTFTSTLGQQNDSCHSSGDYIEKGEKNAAGCAGTVCCVIDPAGVMSKTLRRLLPLCGRKGWVV

KSHNSPPSARLLREMYRAGVRLYVYVGHGKGEQIIQRGELYERVPDPANFPSVFLMGCSSAYMDGGLTYDC
YGMPYAFLHAGAPLFVGCLWHVTDGEIDRLTKRLLSFVSYGGGSGDDFGVCRTMAAGEALRLARKSCKLPY
LTGCATVLYGMNLPLGGTPGGAM
>tr|C9ZWS6|C9ZWS6_TRYB9 | DeepTMHMM Topology Prediction - Predicted
Type: TM
MNASSASEAGEISKQRTRAVARVVEYNMHTAFTLDVASFCGNNMAMNENFLVTFVVLEFINIITAIVVVLR
VNLIVIRPFLLLLLFFLYTENPFSVPREARKGREYFKDLVEKGNKKQQQKQQKRDKSNC
>tr|D0A6S6|D0A6S6_TRYB9 | DeepTMHMM Topology Prediction - Predicted
Type: TM
MEQPSQMSVSAPPAPSIKRPAAPKLTYEEVRRAARLSVLEDFTSQRDHRQRVARASALGTDVHVIPAAAGG
PLSKEGSTRLLKEHTSASWSVYLYQWSIFGLTGVSSISTAMGFYLTIYHNRMFLPVGPMMAVVTWRLWSSL
ESAWEEQRFIDNAASIRESRKGNPMNLVVKRVVVNDEEAEEPAEGMV
>tr|C9ZWM5|C9ZWM5_TRYB9 | DeepTMHMM Topology Prediction - Predicted
Type: TM
MKGLSRLDVFPKFDTRFEQDARQRTALGGVLSMASILIITFLVVGEIRYFLSTVEQHEMYVDPHIGGIMHM
KVNITFPRVPCDLMTADAIDAFGEYVENVVTDTAKVRVDSSTLKPLGKARQLVDLKKQPTNGNETGNENCP
TCYGAEKNPGECCHTCDDVRRAFAERQWEFHEDDVSIAQCAHERLKVAADSASAEGCNLHASFSVPRVTGN
IHFVPGRMFNFFGQHLHSFKGETIRKLNLSHIVHALEFGERFPGQNNPMDGMVNARGVKDPSEPLIGRFTY
FVKVVPTLYQVVSMANTGNLVESNQYSVTHHFTPSWAAPKEGETDNPNSDPLVVPGVFISYDISPIRVSVT
RTHPYPSIVHLVLQLCAVGGGVYTVTGLIDSLFFHGIKRVQEKINRGKQF
>tr|C9ZZQ8|C9ZZQ8_TRYB9 | DeepTMHMM Topology Prediction - Predicted
Type: TM
MKAFVSLLAFSHPAIKCACLCVRLSCYSLITFHGFPSLFSYSAVLSASVPSLIRHTKQFFFAITMQIHNMP
FPTNFPALLSLCSKFEICFPNSFFYVFHLASFSPVNPLLVISVFFQSNTSFSYLH
>tr|D0A7B1|D0A7B1_TRYB9 | DeepTMHMM Topology Prediction - Predicted
Type: TM
MHLEQAGERTYALGCTLERRWFLQFFVSILICLNNGACFCFGIFTPFMKGGAFMFNQSQVNVLSTIGVIFS
YFSLPTGFLYDAKGPKVTLMVGTVLNVVGWLGMMLIFLKPEDPLMGTSLWVMSLFYAISQFSASFYETGSL
LTNLDAFICYQGRVILVQKTFMGLGSSLIVQIYIAFFEIHFDGIWPFFLFLVLYSFTVGVLGTLFVRLPTE
KTQCLGLSIPDKGVVASGGGESSLFNVPFNVGTGILFVAIMYTFIVTMVENYREISVSDRHIIGITTIILC
VSFLFMILATPSYSNNVGGYHSRSANSSWSSHLVDEMTTTVPGQNNCNSQRAPTEAVDNETARRCDVNAPD
AEGIGRGRGVSKDDTNGLNEREPTEPQNGDNLLNPNEEGRRAAERSNHERTVNNSEVVAELQGIKLNGDSL
LTNILRREMWVMWYSCLAAWSSATLVSTNSTQIYKALNFDNYSSTVNVAYVSIYGVASAVGRVIVGSIHPM
LVSRKIPISIFLCGAPVLNIIGLPLFIFIPKSALFLPFFIIGLATGVSWGSTILVIKSLFAPTSCGKHYAV
LFTAGIVSPIIFNVGLFGPIYDHYSKKQGLWDVRECVGTVCVWIPLVVCAIVNVLALPLAVYFFLRIKKRG
GFIY
>tr|C9ZZ24|C9ZZ24_TRYB9 | DeepTMHMM Topology Prediction - Predicted
Type: TM
MMAEKEVNGVWLRGRITFFTKLCPLPFLPHGIFYLFIYLCFTKEMIGVCRLQQCKSFYFPLVISFFFVFTH
RNHWLCSHRLDAKARNLPLYRFHHHWILVD
>tr|C9ZVL5|C9ZVL5_TRYB9 | DeepTMHMM Topology Prediction - Predicted
Type: TM
MAFFVFLDKYDASSASDVEVYLYPSLPLSLVSFFSICVVAGNVTCSLPTRDALLQALISCRCVRIQDCICC
GVVDIQRGWLWLLPLLSFLSYRLLFFFLFGSSSLSLFRCRLHSFVSSAYTDTHTHTHTHTHTHTHLAPP
>tr|D0A0K5|D0A0K5_TRYB9 | DeepTMHMM Topology Prediction - Predicted
Type: TM
MFTSSPHSIVVVVLIIVFFLFFPQISSQFLPLTYIVTRVCRCVTIYDIFQFFFPRVLVLSLLFGWIIYFSS
HLRINKLSFSFCSFGSLVCYIIIVLCCYCRCDAPSLFPPLFFFYSFPLFARALWFLCYSRCYPCLASTLAI
GLL
>tr|C9ZJQ5|C9ZJQ5_TRYB9 | DeepTMHMM Topology Prediction - Predicted
Type: TM
MTAACCNYVAYGELCSYIKRCLSRVEANPLNQSLYNQLPIVRELSGLTIAPDVSAVHLVMSTAFLSEPFPF
AISINTLLLLLLLFILPLFSSPLYVRIPMYWFL
>tr|C9ZTU4|C9ZTU4_TRYB9 | DeepTMHMM Topology Prediction - Predicted
Type: TM
MLLLLLKLFVYYVFCTIVIIYYFYYYYWALVIVVVMVMGRGIKGVIHNFCFFCFHWWGKRMITITITIIII
IRGIIMDVCVCVCLYIYIYVYIYAAMKEDQYLKRNG

>tr|C9ZMD3|C9ZMD3_TRYB9 | DeepTMHMM Topology Prediction - Predicted Type: TM
MHVSAQARQTLCYHCCWMASAVVPTTNREQVNKADVTSPFGSCEVISGEDEGLKGKNALPANEGEGEQSSM
KCFTSMIPPGGLVSTAFSLASICIGAGILGLPAAANSTGLVMTFVYPIIIYFLCVYSLYCLGAQMERHGFR
SYEGMARALLGPYGAHLTGVLRVVNAFGACVAYIISVGDIVSTILKGTDAPNFLKEKWGNRLLTFIMWLCF
MLPLTIPREVNSLRYVSTFAVVFIFYLMGVIVVHSCMNGLPENIKNVHVTGAPGDEGIHLFGTSNRAVEGP
GVFTFAFVCQCYAFEIYFGMAKPSAHRFTAYSAIAMGICLVLCVMTAFFGYLDFGGKVTGSVLLMYDPVKE
PAILVGFVGVLTKLFASYALLAMTCRNGLCGIVEWDAEKLSFFKHCTIIGILSIIMLLCGLFIPNINTVFG
FVGSVCGGFLGFILPSLFMMYGGNWSLSTVGWLHYIATYAVLFAGVALSVFGTGATIYGVAVGW
>tr|D0A7S6|D0A7S6_TRYB9 | DeepTMHMM Topology Prediction - Predicted Type: TM
MIFFKVVVVAVDDIAVLKVTTNIMFVCVSAVVISHNSLRVYGFCGSLSLEGVATFLMLRSCLPTVMVPASR
IVEEDCRVDGETEVHLTKSRKGEKEVSSNVCYFFPPSL
>tr|C9ZWH2|C9ZWH2_TRYB9 | DeepTMHMM Topology Prediction - Predicted Type: TM
MQRLFSPSMPHLLCLYVRHGCTCSHNSRNHVCFLSTQMPAFPLPCFRPLFVAVYAVTFLLLFLVLLYASCV
DKGRSYPHQMGTSGSTIDVARALRTMTSDVPISDGVLQLLLDTPLSHEELQRALPFHTLRTMRHCYTRNFA
LLLLKCVEVLANTAASCRSSNSKGQVVGVQPFLNALRVMRRILPIAMEDGGTPCEEVDGSSAEVTGSSDNS
KADGGDAVPMQASSKALRDKRKRTAFTEMFVQSFFARGCVCNDEQPEETFPPLPGQSEPLGKFLVRLLLDC
CFLEGLGAGVGTTPTEASGCTHSDVSLLWYSGVAGQRVAAESKAVTATTHAVRHELLGTLTVLLSYPLLLP
PNTPDVFFTEVLLSPENASLLKPLVASTLNALLSYVPYGLLPYTSYWVGEEEDVVLMSARFLSSVICYPSA
PVGNTGASCECDQLEDGSAASGVAVSQGARDFFRCLTREEATHIVHNLQNIVGLRLYAKRTYLPDSQRRFA
ASNESMMLLWRLIDLSPACSQAFGCEEVTLKYILPLVDYALDARRSPRLSSRLQLVLFILMRLTGSSSFCL
QCNTPFRESIPFSFDTFVGTYNDLIVITLCYFLLMPHHSIRLLSPMCSAVISNMAPFVTTVSPVTAEKLSL
VFTSVATRCLAYESVLSASSAAVENNVVVADEVTMVNVVETVCDVVQRRATGAAGLLSAFVPKRGLIENVA
EVFGTRPNAEEEDGQQQHPSFHFTLTSPFMVNTLLAAVATANEAAHRAECEGGDRLAAIQGTVLEDHTPRC
QRTAVKRLDPSWDMEIWSFVNHWYSMYQYSAPGSYGDSKSIKMLRFR
>tr|C9ZPD2|C9ZPD2_TRYB9 | DeepTMHMM Topology Prediction - Predicted Type: TM
MTVFRFISEAVCSIFYSFQQEVAHMYHLLSFSAHYLILFLRCCDNMIYEFAFVLFSPSSLCTIFVVSKGRR
VQQFAVHLLAALVRSHPFFFRLIDAPKLDHLLSCRLLFTPNK
>tr|C9ZN60|C9ZN60_TRYB9 | DeepTMHMM Topology Prediction - Predicted Type: TM
MCIARENTSNTVPHNCFEPIGHTVVNNNVGNCETSQNVQSNEPQSGKQPPGEQSKQFAAFIPPGGTIASAF
NIAATTLGAGIFGLPSSAGSSGLIMGMFYLFFISCMTIYSMRNLALAADRSKAPTYESVTFVLMGRRVAYA
IAVLRALDGFTSCVAYVISVGDILSAILKGTNAPDFLKEKSGNRLLMAVVWACFMLPLTIPRHIDSLRYVS
TFAVTFMVYFVIVIVVHSCMNGLSENIKNVSVGKSDTAAIILFNSGFQAIEGMGVFMFSYTCQDTAYEVYI
DMKDRSVRKFVIAAIIAMSLCTALYIITAFFGYMDFGRAVSGSILLMYDPVKEPAVMVGMIGVLVKLVASY
ALLAMACRNALYDVVGKTADSLPFWKHCVSVIVLSTAALLLGLFIPNVNTVLGFSGSITGGSLGFVFPALL
IMYSGGFTWQKVGSLHYLATYVLLICGVVGIVFGTGASIWGTITG
>tr|C9ZMD2|C9ZMD2_TRYB9 | DeepTMHMM Topology Prediction - Predicted Type: TM
MSNEGADSIVTDSCSREVPTAMEVRNEPIGCCDAAVDPKSQEQREGTGFLARMSTFVATAIPPGGIAASAF
NIASSTVGAGIVGLPSAANSSGLVMAIVYLIIITVMTIFSIYALGVAADKTKTHDFEGVAKVLFGAKGSYL
VAATRAFHGFSGCVAYIISVGDILSAILKGTDAPNFLKEKWGNRLLTFIMWLCFMLPLAIPREVNSLRYVS
TFAVSFIVYLVIVIVIVVHSCMNGLPENIKNVSVGRNDVAAIVLFNSGNKAIEGLGVFIFAYVSQITAYEVYV
GMTNRSVGKFVMASTIAMAVCFTMYVLTAFFGYLDFGRDVTGSVLLMYDPVKEPAIMVGFVGLLVKLFASY
ALIGMACRNALYSIIGWDAEKVIFWKHCVAVVTLSVIMLLCGLFIPNINTVLGLAGSISGGLLGFIFPALL
LLYAGGFTWQKVGPFHYIATYTLLLSGVLAIVFGTGASIWGAINI
>tr|C9ZWX2|C9ZWX2_TRYB9 | DeepTMHMM Topology Prediction - Predicted Type: TM
MRATKKHPNDGKGATTDPFVDGSNPIPSEVAAFDPSQQEHVDVVKEVKPSLFAVLLEKFIPHGGLWSCALN
LASATLGAGICSLPAGFNLSGIVMSCIYLVCVAVGTVYSLNLLAKVAVKTGSRNYGEAAKAVMGPLTGYYA
AALMIVMCFGGSVAYIIIIGIILKAVLNRDGVPEYLKSESGNRLMTSMVWLVMILPMCIPKQVNSLRHLSF
VGVMFIVYFSCVVIGHSINKIINEGVADDIVYMRTGNSALDGLSLFLFSFICQPNAFEIFREMKHRSPQRF
TIYGTVGMSMCAVLYFLVGLFGYLEFGGDAIDTVLSLYDPGENVAVAIAYIGVAAKVCVAFALHIIPMRDA
LYHCTGWHVDTVPYWKHSLIVTSITLAAAL LMGLFIPKASTVFGLVGAFCGGHIGLVLPPLFYMYSGGFTRE
KVGNIDFFGTYLLLFVGVVAVVFGTVSTIYNTVP

>tr|C9ZN78|C9ZN78_TRYB9 | DeepTMHMM Topology Prediction - Predicted
Type: TM
MSDTESIQFNEVTDNEEPNVSVGSRTMPQGVDKTGSAQQFNRPPLHGVLSPTARKGQMYSSSASTPKFVVL
NDEGVGDSDHTESLQFKELTDVDSPSARGEAKALSRSISNRPVTQESKRPPLHSAVPQASRRSHGQSSEGT
SLHFVVHGDAEGDDDFDDSDSIQFHTEGASRNVSFKVQSTTSMAETKRGASAVRGQQLANMETADDSLVFF
VEDTQSSIGKKINSQRENSHDDTISFVVEDPVTGGGEGTSSAEKSISFVIASADEASEKSPPVPLAAGGKR
ISTSPSPKSPSEKVSKRAAGSGNAQRKSKRTSVSKKAQGPKEVGTGSARANSRGSTPQHDPNGGAGEGATF
NVYKVDGKHTLNKAGATALRPVAEGGLAPRTTNLAGGRALNERQAADDNETLASLVPNAATLLPLPFPQTP
GRKVSLKAPDVEEMVLQAKVHTEYWRMFNDVQREEVAQLTERIVRTREGGTNVWEGKAEKEKPSQTSRPLC
FSDLFKKTRKDSNRSGNRGRTNAGGAHTSPGAMSTGCGRLTDENKPSANHLGGCYDETGIGTLRIPSPNKR
RWPLTRKARRPRPPKTEKTGNSRFENISKEVRDDALAKFAELSGVAWPPSSRDELLFLTGVRLTPKQREQF
YESMQLYTVASWKVNMNVYAACEQETAPSNAREVKRKVRVTRTTLLRKAFAVMDIEKTGVISVALLPSVRQ
LLEEERRNLQDALSGRKTQEAFLTRSRNEKAKLSGRVNTTVSQKKTEGDDDVKAVSALRIYRLVLDVLLPL
LGASGLLTFDFTTVGLLVFGTLGLSTAGASPSFLKWREATLRCFDSLLKHPV
>tr|C9ZXQ9|C9ZXQ9_TRYB9 | DeepTMHMM Topology Prediction - Predicted
Type: TM
MLSLYAYIFLSLVVTIGFVVDYATVYTDFFSLVVALTDSPIFRLLCVNSFIALSWVLWLVARFTFFGTLSR
TESDAVRSVTPVYAMEFIVCPLYFGISTLSSAGVVSLVTVVVAVLHRLAQERVSTLQVMEDRVLRTPMLVR
LLIFLYLFSMIDLYVVFDMIGNTAESYGDQLSMQYCIALLYVQFLISILKSFTQLFFTVATKESTYNSLAF
YMEMFFSLSNNVVFFISFLYICTSSYVPFPLMRMFLQNMIMCGKNVRLVARYRKLTALLREIPNATEEILS
RDPHCAICYDDMSADQTCKQLPCGHCYHEACLLHWFEKMSTCPYCRSDIAQRTSAMAAYAKRVRVPADATT
TPSEQTGSDASATPSPEDDTTSMPMPSEEEMRRSYERYLAEMASRQQAQGAAISAEADASRLGEEVSAAST
DRLSVVEEINKSLTQSNAVRSVDVPAVSTKEAQRLAAYEEHAAAVRAAQEKLQQRLKSIDAS
>tr|C9ZSM1|C9ZSM1_TRYB9 | DeepTMHMM Topology Prediction - Predicted
Type: TM
MSYKAAIVFFFFVPHCDAKNVLFAVVFPFFFSESAGSRGAQWWVAFEILEYETVNPRLRYFRTYTCTYVYM
STYEKHRVTLNYSHVSLVSLGVRITAPRHRVVTPVICSVMLPFLV
>tr|D0A2Y4|D0A2Y4_TRYB9 | DeepTMHMM Topology Prediction - Predicted
Type: TM
MEKYIFIYTSLFYISIFMYIFYACTFQFYFYYFFIFLDFICRSFLPLSFVFLLQHHAAIDNPKSGVRFLDT
PTTTRTKTKTKLFPLPSLSLLLLRTSLHHPKSQFRFANFPDGVESETVKKFHKILVKEGKR
>tr|D0AA69|D0AA69_TRYB9 | DeepTMHMM Topology Prediction - Predicted
Type: TM
MIGEAVRLFKKGDDLEMRKLRCVATVFFFLWVVDTLIGTFSGCRILQVNAFITFNTCAAVASGVVSLNYCR
IVQAPSFRVKEQITAQPNPQPHSLLLGGQEHRNQQNGAISLEGIQGYYFGAKRLHTLISFGASIFVLFGSL
TVMLESVHDIAHTRQPSPKLLFLTGVAHALLITVFGGEIDAHDRISGTLSSQREPRGLPSIHVTIRQVLRR
PLLLFSRAFSQQYRIIRIVLRSFSAFTCILVSLLVRLGGSSMWETIGVMLLAFYVLAVTLNQSTSMAWLLL
NNATCQPNVAAKCERAIRSVQLIPGVMQIKSSCFWEVSEGELMALVQLILLTSADPLAVTQEAHKILASVA
AYVFVEVRKPDEEDNFDPGESSSYQCHNHSHGHDHGHSHGHNHDYGHHHHHGGGERHAFSSEGCKPVVTPE
EPEPTPSANVALSSSLYTVPPATNNIAGHSGVHANVSTLLGGTRGSVTAV
>tr|D0A033|D0A033_TRYB9 | DeepTMHMM Topology Prediction - Predicted
Type: TM
MRIVPTNAFPLPSPLFFLCLYHSGAFLLHRLIMVFPFLFWLATTFCSVFNCSCCETIFHSLPFFSPFSILL
ACLLLFFRSNSSPWLIPPHVCLCMCFKLPM
>tr|C9ZSM5|C9ZSM5_TRYB9 | DeepTMHMM Topology Prediction - Predicted
Type: TM
MDEVEKRKEVRRRRVPPSVLIRRGLAVALGVICAARCYCYYIFSPPPHTHTHSISTLAPFATLVVFYDKKS
AGGGWGGVGGACDWREGGAVLTHNGVSYLYLCYGLVAASLGELLTLCVSTLPSALPFVHFFS
>tr|C9ZJX3|C9ZJX3_TRYB9 | DeepTMHMM Topology Prediction - Predicted
Type: TM
MPFGLDFHARFSILTAHAMGGCGITNKHKKCDSATSCCAAFHTAKYGELSSFSETKEVRACVSCHLCYIYV
WIHSQKSGKGGLNCARIMDQYKIFILSLPLLITPFLLVRVIIVCVLIQMR
>tr|C9ZKL9|C9ZKL9_TRYB9 | DeepTMHMM Topology Prediction - Predicted
Type: TM
MEGVCEASFPPGFLHFPPVSILICICFVSSLFFFFANYKYIWLCSSLCFFKSVINNNNNNYYYYYCVTYSA
YRNMSPHNKRSMEQKKRNKDRIAAYHGQRVNSHGGHRNSHSHSGHRHRGGHTVRRNRIYDPGNPFDRQLFD
YLIVVDVEATCEYKNDNYPHEIIELPGVLVDVRRGVVDKERSFRSYVRPQRNPLLTPFCKALTGITQEDVD
SAPTLQEVVKLFEQWYTETIPRGAKVALATDGPWDLKNFVHEHSILRDHISFPTIFYEYLDIRTTFAHFFN

RGTPLKLVPMLERLQLTFEGREHCGFDDAVNIARLAVSMMRAGCVFNYLVAIPLTDEFHYDMPNTALYRRK
EGSGYLDPDVVDDIAKKCFGVDYFTFGERHMAEVMEHRRRYPQNFNQRNQLWKQTKMKNRGRRLYGVRFRM
VAVAVLALLGLVLFLLLIYRKVMTLL
>tr|C9ZSY6|C9ZSY6_TRYB9 | DeepTMHMM Topology Prediction - Predicted
Type: TM
MFLLLLLLLLFFSNFLILIAYIIIILYYIITAFIIIIIVIIFLSFCFWDPHIPSFFSLFLFFFAIFYCCFS
VSESGCGVEDLSSFFFFFENEKTNCICKFTKGNERVKKKKRKERERKKIIFNNFKKKKITIIEGRDSEALN
H
>tr|D0A1M2|D0A1M2_TRYB9 | DeepTMHMM Topology Prediction - Predicted
Type: TM
MTSGFGSPVGGDRTCVRFVVWPTLQLHALTYTYATFIQAFIRLDRLSTHPGYTCPLCSCEWPLMSAKLIAC
RWSRALFPLVEIREVPPPTHPHLCRAPSKPLLDSYVLYIFLCVFFFS
>tr|D0A6B6|D0A6B6_TRYB9 | DeepTMHMM Topology Prediction - Predicted
Type: TM
MHGRNNAGSGVSPSNISTDVSGAFEYLDRYCSSGVETGFGSRVSSRGLSPLPDSPVNNVAAPPPGRWVPIA
PPAEMLSSPPAKDDAAEGVATRLWRYIKGGMDRVMENMLEPNRSSRPPTNASGSEPILEVVRLSEPFPQGA
GSAVASRGLVYDDNSYLYHEEDEEDELDMEDLDVRVDLSSALRAYRKSPNECFGHLVAAALAGRSVENPVT
AEELKEARLNPYLVRMLLESNKLNLDDYNVQQELQRCIDALISTEVDRVPQAVFEYLLPLFKPRFVRLDAE
QYSVVESSGFEFVQLLYECPHLFTGSKHFIRMNNACCGWHLAVFVMVISALAEVLCNVGIALVSVHWLTSA
PESYYALYTAIVYGAGYASHLIAMILLMRARERNRVFGNVIFPFPSPHLHVLPVVPLYNIVSIVTYVRYCF
VNRGGLFVDIIHDITAAQVLSTVCFCLCLAVPQFMCQMYLVSTVDGELAFNKRYPFRMLSTAVCATYLLAL
LRLCWIFTTQTSINNFGFACYSFHSKRIIQRYSALLRMVYAASLFLLELNVFIFVVTVTSAKQCHNYEKMV
LSAMSIVSFVAIFVFLVLRGHTVFRIAWALIPLTGLQIALVVHKGKDYPPECPKNRETPEYGRHFQILVCF
VPLLFLVLWLLQLCYVFVVKKRICSLF
>tr|C9ZTS1|C9ZTS1_TRYB9 | DeepTMHMM Topology Prediction - Predicted
Type: TM
MSAPVDNVVVERLSTANQKPINEPRRFATLVLGVFCCMCTSFMYAFNLISGAMQERYDLTQRDLSTITTVG
IVVGYFLLPYGFIYDYLGPRPVFVISMTVFCLGTLLLALTFQEVIEGSVVRLSVYNGLMVLGCMLFDLGAV
VTVLSVFPSNRGAVMATMKTTTGLGSAILGCIRLAFLSRNTSAYFYFLMSFAFAAGILAIAFLRLPPFHLT
GYQEKHLDEEEKAQLRVTKGVYLKQKAPMWRFIYGFAILVTLIVFLPLQGSLSAYLKLGSNFKVGFALVVI
ALIVIFPFMAFPLTTFDGKRPHDDSDSKAKEHVGAGDEISAAEDKVVETDVDYIAPQFQETFIEGLKTARL
WCLLWSVFCCVGVHYVVIYNARFIYTALTGEAPEDALNTLLTVLNGVGSAVGRLCMSYFEIWSQKRRAEDR
VPITIALFIPSVCIITMLTLFLTLPKAALPLPYFIAAFSNGFTAAIIALVTRTIFAKDPAKHYNFCYLASV
LSAIFLNRLLYGEWYTQQADKLGQDVCTERVCVVMPLAFMLGLAFPAFATSTYLHLQYRRLCMLALDERRR
IREEERVLNELPSNPIEPTGNAGEPVHQLK
>tr|D0A2F6|D0A2F6_TRYB9 | DeepTMHMM Topology Prediction - Predicted
Type: TM
MSRAPPPPDFSRIAAEIRKRLGNFGDIAGLTALVGFGGLVCAGLYKSIYFVDGGCCAVKFNAITGLKNRTY
GEGANFAIPFLETPVVFDIRNKPTEVLTATGSRDLQTVNLAVRVLYQPHVSALPDIYRNVGMEYAETVLPS
LVNEIIRAVIAQFNASDLLVKRPEVSNRIGVMLAERAKRFHIDITDVSITQMSFGKEYTSAVEAKQVAQQM
AERAKWRVEQAEQEKEGAILLAKGEAEAAKLIGMAVQKNPAFITLRSLEASRTIADLMRQKGSGSFYIDSD
TLSLNTQTIGH
>tr|C9ZTZ0|C9ZTZ0_TRYB9 | DeepTMHMM Topology Prediction - Predicted
Type: TM
MALVVLCQLVLGAIVRIEVAVRAHGLLFLLRVLGLFGFAFLCMPLCSEDLLFSVNEDLFLCPPPPIFIVCG
KLLSLMYSVASRPKLTFGILTLICHLNSLFSASMW
>tr|D0A3U1|D0A3U1_TRYB9 | DeepTMHMM Topology Prediction - Predicted
Type: TM
MTERRDNVSHAPDAIEGPNDGAHAEDTSPGFFSFENLGVAQVQVVGGTLNGYVIGYVAVYLLLYLTATECK
FTTEGACGGAKIYGCKWSGTTCKFENPKCSEGSDPSDSCKNEVAYTSVYSGIFACAMIVGSMVGSIIAGKC
ITTFGLKKSFIIVSITCTIACVVVQVAIEYNNYYALCTGRVLIGLGVGILCSVCPMYVNENAHPKLCKMDG
VLFQVFTTLGIMLAAMLGLILDKTGASKEEANMAGRLHVFSAVPLGLSVAMFLVGMFLRESTATFSQDDDG
KADGGMDPNEYGWGQMLWPLFMGAVTAGTLQLTGINAVMNYAPKITENLGMDPSLGNFLVMAWNFVTSLVA
IPLASRFTMRQMFITCSFVASCMCLFLCGIPVFPGVAGKEVKNGVATTGIALFIAAFEFGVGSCFFVLAQD
LFPPSFRPKGGSFVVMMQFIFNILINLLYPITTEAISGGPTANQDKGQAVAFILFGLIGLICSVLQFFYLY
PYDANQDHENDHGGEPVEQKTYPVEASPRN
>tr|D0A6R8|D0A6R8_TRYB9 | DeepTMHMM Topology Prediction - Predicted
Type: TM

MEKVTCDAPKIMRSSEYLGVLVVISFIIFATSFSFFCSSVVASFPNRHAATWCRAVGRLWCSCARLLLAYP
RLLTLFISSYSSLCAFNSRFFCVCGVTHSKKMTRCA
>tr|D0A314|D0A314_TRYB9 | DeepTMHMM Topology Prediction - Predicted
Type: TM
MYKRACCAAFFYFPVVFVCILLFRYTKLFLPLSQSTAFFRINFSPPYCSALEAVAAATSAWALRWQMQPVR
TSVRNFIASGFPSKKLRYARLVDVEECASRPQFQKLLQSYLSDFFAKSHENGNTGSSPWFTAVPPSLRNSV
EETVNYLGMLVAIDFRHWGEDPPDATPTGSRVENICGFYAELPGGSAETSDSVSYGGKRLIRGSMAMVHLL
CRAVEVYHLNWHCPQFLQQFATTEDAMEALERCFLGYREDGHTSMWMPAARERVELLLSLSRSLVEKDTSF
FKMLCLSEGYLYHPIFPHLGFVEMLVELHPRYYDVCVLRGEVGGGKDSDQGDEIVIPMLKLAQLTVMAIEE
AVSAMDTVAANTPNVTEAPFPFSGAQGVFKDKHHLTVCCDYQLPKALRSLGLVEYDSYLASLVDTGVLLAA
GGVEECCIRVAALVASDLLLDYLRSSCACTTGREWSPESTSRVWDAPALDCMLWWIGRHYVDSAVKHHLCR
TIMY
>tr|D0A0D5|D0A0D5_TRYB9 | DeepTMHMM Topology Prediction - Predicted
Type: TM
MRWKGSECFAALIGTFSHAVVCTPLFNIRLGKGSASLSCDLRYVRLPFTLCKHSLNLISSLLHNTSLPHMK
WLEGAVLSDNLAFFVFFFLVVVVEEGECNFHDK
>tr|C9ZNT2|C9ZNT2_TRYB9 | DeepTMHMM Topology Prediction - Predicted
Type: TM
MWKNSLRDCPHFHKLPGLLSGTTGKKMKIYRPTLLSFIRAKRRLLAPRSIEICYQVSAHIYTHKHANIYLP
SPHRYMHMYYIYIYIYIYIFFFLIIKKKKVSLPLLPKRHLVV
>tr|C9ZT58|C9ZT58_TRYB9 | DeepTMHMM Topology Prediction - Predicted
Type: TM
MQTRKKKLTTKRFKSFVRRAGKRKKKEGFRNLYREWRVEVVGCYYREGDYMCLCLYLWVCFLFVFLFSLLL
LLLLFCAPASRCVAECLSSFLFYSSALLFFSFFHYPHFFFFILF
>tr|D0A5P2|D0A5P2_TRYB9 | DeepTMHMM Topology Prediction - Predicted
Type: TM
MHDTTCGYGVPLSICCCCCCSNVLVSVCCVCWIKFLPPKAFQHGRCFLFLRTWLGTSLRSITTYRVRHSSL
TIRAFPVWLLVFSSPSLCLSFVYFVCMCVCLLQGQEGVSFLCPYSFFSFSNFSNFDTQT
>tr|C9ZLU0|C9ZLU0_TRYB9 | DeepTMHMM Topology Prediction - Predicted
Type: TM
MHFLCSRPRHALSKEHWRCGSPVCLLISAAGSNGVLTLYRAQRNSNTCGGAFAYRLRQRVLVFFVLFHAGC
WHDLCHFRMHMRIFCRVGGGSLAHRCGQVGVLVSDAYDHRVVMCCAFVEVTYVTIYHAMSFNCRCCSFRFY
VC
>tr|C9ZXI3|C9ZXI3_TRYB9 | DeepTMHMM Topology Prediction - Predicted
Type: TM
MSDVKGALTRLERLHEACGGAGLSTVGALPADGGANMSVNEVGSYEKGQYHVACLMKRARESMTILAETGE
SMDIARRAEISNSIRRDMSAVKKECTALNRVAMKEGKRGDYMQLLSFVNKTEQFQRRLHNGPVLGEASGAI
GDGSTHVGGTTPGVPEANAVTTDSENREVGSFISASEVEGFLQFFEETRKRDAEIDQVLERISAGVTRLQE
NALTLRSELCTQQRLLDDTEEKVDGIHAKLDSLNIKLRRTLEQVDKDRMSVYILCCLLLLGIYGAIYNVSR
>tr|C9ZTG6|C9ZTG6_TRYB9 | DeepTMHMM Topology Prediction - Predicted
Type: TM
MSFSVPFSISDIGPGVGIGARMYTYIYIHIYIYIYVAMEDKLWTLNCFHFAPAFIYHRRLQFHLYFSIRSF
LIANRRVCARPHVPVAHFLPFRAHVSNPVAKHLRLRHCDTFVLFCFVLCQTFFTLCILMPSFLLLSFSFRS
FMI
>tr|D0A5M0|D0A5M0_TRYB9 | DeepTMHMM Topology Prediction - Predicted
Type: TM
MELAGDTLLTSLSVSFLFCIPHRDFFCFFFCSTVPTRFTCLPDLVHDLFADAACAIVCTYFHMLLCLKFGV
LFFSIISFFFLVVVAFFFFFSCVVSTGWGPYAPVLVELSDIRYLVWKGEGNTCPAVPAF
>tr|C9ZJL9|C9ZJL9_TRYB9 | DeepTMHMM Topology Prediction - Predicted
Type: TM
MHSNYLNPDFFFVICFYSTFVSLLCRHCWCKTIHKRMRLSVSGRSSARSGMSSDTRLPAAVLERETKVINY
SHSTSRAMAAQLENCVRLIRGYQASLQYCNEEIEGALLSLGVATTHSLISSITSVMEGLDTLSGVCRAMHE
GYNEEVRHREDLHSSLTGAYISLKELHEATENELREVVAERDRKVAAFGKAVAYVEAVVAERMLWQEANGY
NCAGLPVVEMSFSESCHEALRKFDDTVSEAQGEGHSNAESLLASTDVPQTLAKRWDNVENLRHTNGLPSAR
VLYVPPKDEVELLRNVLEMGTGDRALMWDVQQRERALQDSLTNIRVFGHSCLKALHSMREELAEFRQWFLT
IDRKCEPIVVAMRKLGNGVCGLSSASDKNLPH
>tr|C9ZSZ3|C9ZSZ3_TRYB9 | DeepTMHMM Topology Prediction - Predicted
Type: TM

MGVGRKKFDALMVEEEELPTFSFLPLLFPLFFPFSFASATLCLTSFLNSVASSHDAFFFFTPPRGRLPVPS
PPSFLTISVPIPTKMTMTINKSLSNYFPFSYYSLI
>tr|C9ZL37|C9ZL37_TRYB9 | DeepTMHMM Topology Prediction - Predicted
Type: TM
MGVYVCVCVRNEGKSTIKQKERFSNSSGTQIDIDLYYFRMRKLSIPRCRFVPAAMSTLFTVLLPGFLPLLA
PFVMGCAKGFHAGGIRFPTIPKLSAVFTIGKGPFVPVKALAEGKRRTGNMLDEAEAEGEEETVEGSVNPAP
PHIGTDAGAETTQGLIFTSAGSGHSALGAEKLPRSAI
>tr|C9ZIN7|C9ZIN7_TRYB9 | DeepTMHMM Topology Prediction - Predicted
Type: TM
MLLFHVPCTFYTLSPCLVTLSFPPFIFIFIFYNCLLVGPLISTGNERNKRRRVTPLVLSLLEDPLEVLYSV
TRYKANFLQKVPLLTSLLPRLLTFCEGSL
>tr|D0A582|D0A582_TRYB9 | DeepTMHMM Topology Prediction - Predicted
Type: TM
MALVPAFNCLYYVFVCVCGYDCRCSCCSVSVWRKRKKGKEITKESSYVFRLLLLLPLLLLLVFFFLFLLVS
FICDCVNKLNPWSMSTIATCSRKITKEKKL
>tr|C9ZRL9|C9ZRL9_TRYB9 | DeepTMHMM Topology Prediction - Predicted
Type: TM
MCSQFAPCNLNCMEIGEGKRELTNGEGSFFFLRRSHRDILYCSAPSLGGFINGIFEVPSVLSHLCSLYRNV
LTVLGETEQVYYLYHLFYIPLCIFFIFQLVAFFHRFCRIHKLLPPRQFLAFFVCPKGSLRLCDHVTGNGGI
PVSRIFVDGCCKWR
>tr|D0A4C2|D0A4C2_TRYB9 | DeepTMHMM Topology Prediction - Predicted
Type: TM
MVVQHYLNVFLVVCGCFALLSPLSVWKYCRRGWLRSDYIISTITLFFPLHGVCFIFFLIPGRRSILNSSGS
SQLLRSLTELDLRSGKSNIRGAEYNKIRGERAELPFTVCSFFTSPSTHNLQPYT
>tr|D0A9Q4|D0A9Q4_TRYB9 | DeepTMHMM Topology Prediction - Predicted
Type: TM
MEGFVSLKELQKHAAEGDLWISIDEKVYDVTKYVSQHPGGVDTLLGVAGKDGTDDFNSVGHSDIAKEELKK
YCVGRLSPEDVKILKASSETSTTSAFSLELIAVTSSIVAIIIYFLFSS
>tr|C9ZUY7|C9ZUY7_TRYB9 | DeepTMHMM Topology Prediction - Predicted
Type: TM
MDDVKRKSAMLMTKGIIELRQSPPALVCTIRRFKHPMSGKEVTLYPVPNIAAPHYFRRVLDAHHLTNNFDK
VLCEDGRLPFQAGTALARRHEVFKRLLPFLSLRPVVVNGDKFDGIVERDPLESRMAYQMLLDGADPPVDPR
ARRAIERIEGYADATKTVCPWGVYHLVYMTYRLRTLGYTVESEEELEVVGMKEVMVLGCFMGITTFWMMYA
LYRMLFGF
>tr|C9ZJS0|C9ZJS0_TRYB9 | DeepTMHMM Topology Prediction - Predicted
Type: TM
MPPVYLHTSSYFPLSLPTTHTYARGKKKKTLQKKKRAKNRRNIYIFRTEKKNGSVPHIPSRPREGAHTTTA
NNVSAVCVDKTTNLGRIKAFIIFFFIASLFLSFVLFLNFFFPAYRSLAIRHLLKPVANAQWSQDCKAVAGK
RKKKKNIEGKKNEEKVRFFNIKGGSKTLSFCGKKKRI
>tr|C9ZU61|C9ZU61_TRYB9 | DeepTMHMM Topology Prediction - Predicted
Type: TM
MHLHAGMCTFFEFYRMYIAFGLHLLLFGHSISVSAFTIAAVVVFLIIFPKILPVPSLLSVLNGTTHTHTHK
LKHATAPGNVKRHRRHFDKKVVRDNKFEYLI
>tr|C9ZTP6|C9ZTP6_TRYB9 | DeepTMHMM Topology Prediction - Predicted
Type: TM
MLCFPLFFSVYVHFFFPSLLCLLRWLVLFFVLFLFLFLFLFLFYTTHSYNFFCFCRSFVSLFPFFFFWYFSVW
LRVGVCVCGGGASRLYFIFWFHALFFFFSLLFRHFAFAFVFFYVLLLIFLFMFFINFFFMKGRKVRGDVNR
NK
>tr|D0A0I4|D0A0I4_TRYB9 | DeepTMHMM Topology Prediction - Predicted
Type: TM
MLAVKYLLKLYETPPENGHISAFDTYGNTVFAGTDSGVLMRFVVEGATSPEIVPTEGSLSGDARDRHISGG
NSARSAEAGGVGEEQQAHEEMHADDFHAVSQLEKIYTTLVHHVVVSETQRRVERIQHSRTHKILFVLCEHR
LLVLNSITFEHIYTVSDYVGTFFVSDSRQTSSQRVGRHVICTTETHGRELRVYEFDIAQNKHVRPMAPHKV
MLHEQAQTLVTYGNMVCVGMRRGGYRLLSLPDGNTCSVLPLSGDMQPLLAVGDGEVFMRYDHSIFSVSMRS
MPSGRVLGRTIQLEDEVRHMIARHPFLFAFTESYCDVYSLYDDDVSERLPMSGCLFGSQLGGGDFLYAASA
TKIWMAGLHPLRHQLADLVERFKVEEAFHLLSTQRSRNSLDWQAIELELHVMVGFAYLHRCRPKEAMLHFN
DHIDPRDLLLLLPECIPPGPDEYSSELRGLLKGDCVGETRTSSDGVDFASKDEDVVNVPVGGNNVEPLLAK
CGPDIGCWDGGFWEEWSGCCPYNTYIGELEKAWLETFETFPTVPRSTDQADGVIHRQVMEWGCITAEGFLE

RSWEALKDELVVYFRSRLDQASPVHARPMEYALLVLALEARDHREAYQIVVKSSSLSVEDCYDLLCSLHEY
RLLACLLYCRGYTQDADRLLRQRVCVSSLLPPWVAGRCNNSKQSPAYMNVPTALHSQLTRLLTPLPKNECL
VVADLSSPRGAHSPNDVDETHTINLPLSLSLPMYLVSHLNIPALQELLAEDPDAAVITDEEGCTLLHVLFS
LFISVRDLTEGEAMTKGSALVGLVLSCAVLLLDHGADVAAPNVHGITCLDVLAIAAGGVFFDIVVSALLAD
RDVRKAAAFTNKDDNTLVNGFIPIA
>tr|C9ZXJ5|C9ZXJ5_TRYB9 | DeepTMHMM Topology Prediction - Predicted
Type: TM
MGTNKIREKKGKKQTNKQTNKQRDERMQRKRRGKSVPVVAFFLSTILFSFFLSYVYYFPSDSLSFFVFLFF
FNGYLFFVHSQEKNHFLVFFFDVVAVVIVIVIVIVVLFCFRDNPYGELSHLFFFFSVSFFSCFYNLNY
>tr|C9ZPM9|C9ZPM9_TRYB9 | DeepTMHMM Topology Prediction - Predicted
Type: TM
MPIKYSCVNEGTTILAEHPQGELPKLAELTQKVIATVPGNEYRRKTVEDKDGGVNYHYISNGEGRTVACVT
TNDMRMRTVFAFLEAVESVVRSSAGQSGGELRNGKKLLQQKMEFYNNPQNDRITALNDDINQVVDVMMDNM
DKVLARGDRIDTLHERSATLSEQAQQFQRRSTQLKRNMCLKNLKLTIMIVLTVVVIIFIIIIFICKPNFSN
CKS
>tr|C9ZR91|C9ZR91_TRYB9 | DeepTMHMM Topology Prediction - Predicted
Type: TM
MCLKRKAPHLFLFLSLVHFPQLPVLLFSFLCDHAPTTFFSDNSDLHFPFIQIANYYLFTFSLFCFCVCLRY
SLLWVTELTLNAQLVLFLLYHCAQTQRGPLKEGEMPICPGLCGELAAVPFRVFLGTLPTLAVEERFLRQLQ
PVFAWYSSRKRVKEQANEFIEIDLASCDAELLLRYSHIYYVRRQLFDELIERQMTLLDSGKAPKMAEPSLL
QCLAGCNMTIADRLQLEIRQLGAAKRAASVPGRRELDPVARLEVYDYACMMRLVEEDAGAVGDAEMKARAY
LPREVIESKLGHLTQLLLGSDARAALDKKDVKLLNRMIPPDYTRVGCVEKLRPFDVTAYFRFYGERINNVK
VENYFKRALWGHVYRRFATTPSFLSGVSTYWARHSGLDASFTTTTMPQEVAVAVCDQQIQFPAIKFRAQYV
YTSPETARQLWRTDAAVPLMRLFPLMGSRTAEDLAAGVLTDAFWMHLGLSEEENLLQDSLLLKVRRFVDEV
GDMYETNIDSVLKRVDDNFKQVVPQLKAEDLQVDAPLQDGEGEDTVRETVAA
>tr|C9ZJ37|C9ZJ37_TRYB9 | DeepTMHMM Topology Prediction - Predicted
Type: TM
MPFAANVVQNTNDHSHTGDKGNEGTIVKVGEGVSLQSSTLDIVKSVGEGSAAGEAPGTSFRTIAAGAMGSR
AKGAWEVTSTSGKPTTSSEKGDTAGFAESPGNNPNFLRSPLQRVLVPKTSSRGTNRLDGRNPLGEGAVRKD
GDKSSGSCVKPSVNGDNKCDTIGSLARRAPPGLTMGRSELGSARDEWPVQSPRGVWPHPALSGKSSPNFYG
SPVNTSTRAPFFSPLPAASLPPNNTVPGTLKRQLYNVDTANEEMMMPNNSFASTIVTSGSRCDNRDPAFSD
CGEAGIFTPKRVGRDSRSATPTRWYNTSFHGNYAGGPMRRTSTAERGKLSAANEQNGERREVQSATVSPGL
GLTTVEVYDTSISSRTGNAMKEQRSLSPNVQPACFYPINWSSCPSRDNDDSNAGDRISVPGAVLVPVDSDA
TNRDPQEGAPTNSCQLHNHCLNRTNSEWWFKDPTIFLLNVNLIKLLELREEMERLNEQRSVDRPHSAYERR
GREASLHSSHSIMLAGMLEKLESIQFQIAYSILDIKNVLLKHHFAKWSNTGVRDCVSNVVSAWDAHGPASK
EFKEASYILLDLVSAEERGSITYTNVHRMWNALGTSRTASCAATAFCFVSLLCFVVVAAAGTLPGWVCAAV
ASIGALLMVSVVTITSVQLHYSSVSARVWGFSHNEMLKRVEAVQQNLYDGGEGATGVPVGYSDLVAVQKAP
GSSSTCFQLGKTSSQGHMLPQQDAPRDDCARDCGPLSSRNSFYSRSQLRIPNDTTGILRKVPSMSNVKCSS
GASNGAISSGRSEKQAGSLRDSSPCLRRADRQENDWWNQPAGGRWNDDSFNRCSPMERVPVGHADNPLVAG
GSGREWQEALSEQKRECDTAVRKQIGRRQSVDGDRAAANVLAVPSEFNHGEERPRQFRGAQRPQSRGVDSE
VERMLRDAASKAALNSAYVAPMNIFSDEASPGSSLLLTSATGMQGNLLGTGSDGLPITAFVYCLDDVVAST
LLANLWNRSIYVMQRNGLEDIDMTYKSCAVQTKVILVHAPDVDGAHLDVVLSWMKGGERLVFFFASSSEFI
PSCIPKASQLVLPLTSHDIGRLFSSSLTDEMASKSLFGLSRNLQIPSYTLGRRLGGGAFGAVFEATMDDLN
GRCAVKVMCLRGNKRDNGARNNGKGVRMPEVVREIEVMRMLNHPNLVRYLFCNWDGKCVSIFMELCPGGTL
SDAITNGDIQGADHIISILRDVINGVVYLHDHHITHRDLKPENILFRDGRAKVSDFGTAVQRDSGLKNTRG
TLAYMAPEVLLGEPYGKACDVWSIGCIVAEALGISTNCSNKCAAQDAQQQKQQRPVTDQQFSPTNQGLAEL
CERYRTMDERETRVFDCDDPTVCDFLQHCLHRNPEKRPSPKELLEHPLLNARCGSAVWEWVESVIARQRAM
PLRRRTSTVILTSVAVNKGAGIGRDDDTDGADGMGGGEGGGLHTTYISRVASMSAASLQSISEEGQNSASW
GTSFGDQGVV
>tr|C9ZXR7|C9ZXR7_TRYB9 | DeepTMHMM Topology Prediction - Predicted
Type: TM
MLRTLLQFAPRQLFSFCVRECGITTEHIFWYFPVHISALYLRTFPCELAPWGWCCWTSTPLLFTLSELRNS
LVQFFSISPVFIFDHCFRLLPVLGRAAGKWMGLTDHFPVNVLSFHRAAAAIQFSESIMHHDKGIIL
>tr|D0A5Z8|D0A5Z8_TRYB9 | DeepTMHMM Topology Prediction - Predicted
Type: TM
MRVFRRWWWQQYPKKLYTYESAAAGVFGELNTRLLRVLCHSFIYAGTGNRGEGVFRSASRDFFFHFLLITS
FIAVFVVLSPTFPPSWIRREKEKNSPVNAGPSSGASCLPCE

>tr|C9ZJ89|C9ZJ89_TRYB9 | DeepTMHMM Topology Prediction - Predicted
Type: TM
MHVGDGTQHHEEKKNQNHSGAQTGSEAGNKWSHGEKESRGVDEATPSCARSARLSGYSQPLEERLFGSNAL
METPNDFFTANRRSNEDDCVRIDDSLVYSSVHRSGQSFCDYHRMGNYRVERYSHSEDHRSLETQFDQNRSE
PADFIAGLYRNSGGPLSLYSSRLDIALNSVSEQPPIADRPTASHDLSPVSIPDRADRKGMACASPNDLTLR
VNTSRVWSSGTDFAQASLHSVLTNTPGEALEMPVFPSFSELGSPYRNTASVDEMQQAQKLQPTLSGIVMYD
GQLRGRKPLYQAYGFQSNPDGDRHDPGLHHSSSVVFILYGILLASVINVLFLVFNDLHHEENKLCNAEGRC
PCLLTDVLKYVVTYGLHVYVPFALFPTALFFLTINSITEDRERKRRLARMHCYVTSDDISQHAHDNVAGDD
FEVSEPYGTRNESAPTRALRWDLPPALSTLASNIIFWVVVLIRFGSFSVLDTRVVRPDKLAFIYDAGLVLA
LSAPLAAVAKYHGCFRLVVPFMLLDAFPLLSPPIIAPIKVDKGMEHIFHPLLLVVIERMLWYLSAAVMPET
TPVGVKITLSSSFAAIYTLFVLTSAVSLPYDWWLVVSTVMAVEIFIFEMLFNTLLLEFTALRLFTVAVACV
KRQPPQVFSIKVSDPVNISTQVRWPSLALALCGVSPVLWLPRWDRVLPQSCCDGLIARELVPFVVPFILAV
TAFTLSYLLTAMIRMRYDRLRAPLIARDWFLALMWGWYVTCTVPFALSALM
>tr|D0A9V2|D0A9V2_TRYB9 | DeepTMHMM Topology Prediction - Predicted
Type: TM
MTGQIKKKNQRQKKNKEMHLNNTTAKCHTAHRFYHCTKQLSLHIPLNLYRNIRVKAGDFILFFFKLFIYYF
DLHCHSFFLILYICLSFSSLHLKQSERRGKEIIQSHLTTSPRFTSFCFILFFFPPTNTPSSLVRLLYFDFS
HIYMRELINLLF
>tr|D0AAL1|D0AAL1_TRYB9 | DeepTMHMM Topology Prediction - Predicted
Type: TM
MNSQRSEYRSLAGGGEGGRRPPIPRRSAPPEETDDVKQSLLRYVDLGGMRVEEQIQREKLVEAVAIKEGVE
DLRAVAEDFALLVEQQQEPLDAVRVNVTAALDSVAAGREQIAEASERNASSRKLFCFFPIFLTIVAIGSLV
ALFFKGKIYKDT
>tr|D0A584|D0A584_TRYB9 | DeepTMHMM Topology Prediction - Predicted
Type: TM
MNMPCCSSFLFFFKRALNRISLFFVLVISVLVSLWLVLRWTRLAHPAHTHSDIFGSDVCLFVCVCMYVCVR
ALVCLFFFSGVLACVTNARTTFHAFRSFLKK
>tr|C9ZY52|C9ZY52_TRYB9 | DeepTMHMM Topology Prediction - Predicted
Type: TM
MGNQKAVAGRQDATLSSAAGTKHSAKLPPLLAPLTRVITLEMIWYLLCHIVCLFTLIFGAGDELFTSRKRW
FALGWTKQPQRREYSAGGVYKMFATKATIENKPMLPPHRLNSDLVLSLLFVLTFNGFFLALSRSVSRLIAE
GDERRDFREGTFDIKSGTDDKVSKGKGVPFRLINCREIIYKVTLGATLVFCVLITTCVILQPPSEVQGAAP
SLVLLSSVVPIYYGDRPNGARALERFYSAYQSVIITVLVGLVLDANFLAKA
>tr|C9ZSF0|C9ZSF0_TRYB9 | DeepTMHMM Topology Prediction - Predicted
Type: TM
MTTQDEKNRDSFLLTAGPALISGAAQAVLFNPFDRALYVRVMYRRHHFLDRRNFEHPFQGFVNAAVYRTLV
AASYLFWQDSTRIFIDRYMPACFHASNSPGVNAFLIGAVAGTLNGSLLNGMQVVKYRMWSVEEKFSFFHVT
RNVYGEKGLSIFFRGIVATILRDSVFGIVYEMCRNSRNVHCFFTFLGQSITQKAQYLGLASPACSHATDNN
SPSPFVAASHNSGEALSTHRAEKPSVDSAVFASNLFAAMLASVFSSPFNYVRSVVYGVPSGSGPVRYIQLL
QFLYLQTLFVYRSGESYTSVHATHGGSERVAMNQQDPLRTRLMERSTQRHRYPMAALRWMNSRLNIGWGSA
RVGLGMAISQSIFVFVQSCWKAV
>tr|D0A0N1|D0A0N1_TRYB9 | DeepTMHMM Topology Prediction - Predicted
Type: TM
MAIWACIARSGLVVSKIIACCGLSYFISHFFSWTEKSVGGFADVSLMIFLPSLTFVSITKFENAERAYMFL
WAAIFACVPRILALASATLLRCAYPTRWHGLVMLSCVLQNSFTFGLGTLFMLKGIPWFTNEVGEEAIAFFL
CYSTVNFLVCWLTAELIVRPYAKAPVAVLSACAQEKAEGCEREMSCRGEKNRCGIVKDTATSDNRLRTTRE
TGATPPLYAPKGEPTRVSANEAGEQCAPTSGGGSDDVIHNSASSSCFSVKTLKLVLGVIRKPLIVTTVIAI
IVSMTPIRRVLHVPVLGTTLVGGMKLVAYGTLPLHFLLLGYEAGRTWKVHATTASERADAAQGDSRDSHGE
VSGWETSAGRRDSDDTQIKTFVLAFALAFNAHVVVPLLCFLIIILAFKTYDLIPTSKSFLLAIFVGSCAPSA
IDPFLICSNNALLPLAYSKIMHVMVLSGGLTTFAWLSVYLCVLEE
>tr|D0A6D4|D0A6D4_TRYB9 | DeepTMHMM Topology Prediction - Predicted
Type: TM
MGDFTREDEYQLDTLRAARDCGNLSPGEFESLQYLERKYDAFIEDGIRKLERMAPQSARREHMLPFVFISW
PALWSLLTLLLVTFYLLTYGQQGILVQTLLRWQVCLAALMLLASTPLTFTRCRDRRFVEVGVLVAFMMFSG
VFMLTSLWVIHHLRSLSDSEWDINTCVIVGVANSSLMLLSGLLLMKALEM
>tr|C9ZIZ2|C9ZIZ2_TRYB9 | DeepTMHMM Topology Prediction - Predicted
Type: TM

MSYVENVMARATKRKGDPFLRDGTRSLRFIRYYRTLPYPIRMSGIFALGFALGSIVEIFACKTHLYESVMA
NKDARRHDFDEFVVEFRENVENWQQQDAMRRADAQGK
>tr|D0AAV3|D0AAV3_TRYB9 | DeepTMHMM Topology Prediction - Predicted
Type: TM
MNMLRFDDRNASPAPSGGKHSLPTGGAVCRVAMDTLLVILRAPVALLLLLIVLPQLSVGAEANATVKVLSA
AWNSYMPQEYVTAINAGFSASLESRQWTVAGSVKVEVVYPERYETLPEDFIKEQLELETDQNKIVIVYGPL
GDKSVIYSIPYLVNHSVVALGLMTGSGEVRRWNPYLYFLRADPAAETLALIRYALCQLRVLRLGFMYLQGV
HYGDEEYALTVNVMSQMGYELHGVFTVMSPDGEPAPDGEFKEVFERFAAALPQAIIVFGAPEKDTAKFLMM
MVAEERVARSYILGPSSVQVSLAEMWRLALEAAGASFAPGQLLFTGTNPLAKDSQYIAIKRFQGVMSEYLK
THVSETNITEADYFLTHDPEGELMVYGWIAGEVLSQALSSVEWLKDRATFVRSLYNQRRYVINDIVIGDYG
GTCEGDAAKHGATCECNQGSKAVYVKEVLEDGRTTSVRSGFTVLKASQCYAESSELHGPLNGLAVFMEDDD
TASKAAALWHKGASHLVGKGDLGHSDRFFLHAFNTTIAEAANDLRREQGERIVTAVFGPVTGAMLDTPNIT
FIDPLELKPRLNKFRRHVIHLSPTLEQQLYVLSSYLAADGVGTVDAVICSKEVDGIADFLRRSLTEFGVSL
RSAVIREDGEGVGKYLPISGIVFVIGLSVPDVREIARKLEERNDLRVIVLFAEFSLLYDLFTSTLNNTAGA
ARLVFATSLPHWGDTETSSKTTQLFHDVEKDSRLWTPLSLLAFATGRLMRAILLRVEEMSPETLVNFFYAD
SSIISDDMRYGVFDDTKCDGAENISEGDCASNYGATQISVWSMARALNASILPLTNPMTPSMSFRDPSEGK
LSGAPLVGVIIGATFALFLVVALGVVPYFVLRNTRDNNSAPKEPTDPVTIIFTDIESSTAQWAAHPDVMAD
AVAAHHKLIRALISQYECYEVKTVGDSFMIASRSAFMAVQLVRDLQRAFLRHNWGASVFDEYYCRLEQDRA
LESEGYVPPTARLDPDVYRKLWNGLRVRVGVHTGLCDIRHDEVTKGYDYYGHTSNMSARTESAANGGQILL
TRATYLSLSTAEREQLDVTALGAVPLRGVPDPVEMYQVDAVVGRSFAALRLDREVDLAADSGVTNLSTSDC
ASLCELGQSAQTIVAVMRALFGTFTASQREKLLVPFCERWRVTLPPKTKSVWDDNYCQEVVRRIAAKVGHV
VDFTAYNIAEPPLTTSSSSSVIFISDAAVGLCAVGERNVSTPKEEN
>tr|D0A4V8|D0A4V8_TRYB9 | DeepTMHMM Topology Prediction - Predicted
Type: TM
MRGESSAVSFQYESPDVTGASPSFTNSSLARIIGQKELPRFGIEIGGHEGLLKLLGVRYDGEPTWRGGGGL
RGGILSDSVHQRRCRFGANRLPRPRDKSVGALVKESIEEDKILQLLIGAALFSILLGHLTSYHQKEGGNMC
PSWVEGAAILFSVAVVVTLGALNNYNKQKQFSHVLLQEDGTRQSIVVWRYDTLDDRAMVRELCLAAREVPS
EDLVVGDVVQISSGMELSFDAILFGGNYVVCDECCVSGESEEVVKSLEADPFLISGSSVLEASSEAIAVVC
AVGEKSFSGEIAMAVRDTEKKVTPLQEHLSVMADHIGKFGLAVAVLTFVVLFLKEVYEVVAMGKPFFVMSF
VENLTTSIAIIVVAVPEGLPLSVTISLAYSMRYMLRDGNLVRHLAACETMGSATVLCTDKTGTLTSPHATL
SRVLFEGKVYTANDSGGDGSSCVEGGRWNKSGTGLFVVASSQATAGLLMECVVSNALDPVRGRPVNRTAEA
LLQLAQHLYVSCGDDFSSPFVYDMGRLAQQMCDGSRCVRFPFTSVQKKSVTILKLPTGELRQYVVGAPEAI
LSNCRNFITTAGALVEINTESREFLQSIIQEFGCRGLRSLCCAYAVVYPIEGRIMPLEVSSSPLNFLAAVA
LEEEVRPEVPAAVRASICAGIRVIMVTGDGLLTSINIAYRCGLLNPVGGETSNCFSPPPISTLINDGYAMD
GPAFRACSDTDLLVNYIPKLCVLARATPLDKKRVLQLLKMHDPLAVIAVTGDGTNDAPALKLSDVGFAMNS
GSEVAKRASDIILLHDNFAGMVKATMWGRNVRDNVRKFLQFQLTVNCVACVFAFCGALINESNILPLKPVQ
LLWLNLIMDTLASLALATELPLEKRLFDRAPEPRDTPIILPGMLFQVAVQGGYQFVVQIYMLLAGHRLFGD
SSTGEVERRSRPPIDYLCPKHLSIVFNVFVLMQVMNFFNARLLHEEDSFFENWGSSRLLLLIVAVIAVLQV
CIVQYGGRFMSTVPLSTEEWLYCTLYASGSLAVGAASRFCWRWMRRRGAHSTGSCDSYVLLSYLPRWLRAL
IDGARGGSGRRRQRGSYCKAAVKGKGACKTAPAYV
>tr|C9ZZU8|C9ZZU8_TRYB9 | DeepTMHMM Topology Prediction - Predicted
Type: TM
MGVVDLLTSTRCEDLSSPVVPQLSYLFEYVLSPVYKYAASLYPTTWTPNKVTLTGIFATVVSSLLLLTAMP
LNTFFEPPFATFVPGSYFLIKSPKWMDAPGPSPLYPSMLQPYFNSVFTPTSMLLLCGFLNLIYCVADNTDG
CLARRLKKTSNIGEYLDHGLDCVTSLMSTCVSMSVLGFSFSNVAVTTALVALPTILSHTLHYEKNIFIWGN
RFVSVDEAMLFFFLTSWISLMFPNVGKATFSPALLNAVLPESWARQLIPLRCIDAGLVVCWISQCFVLVNI
GAKSKSMFLRLPTIMLVLNLALLLAIIPCHAVHIEKGGYGTYTLGPFSYVALWIITMACTCSSIVHIPIYA
HCAKLPQTDPLPLAGVILVCLIFVSCPPTAAVLAVVCHVGQIWWNVRNLEGSVRKVE
>tr|D0AAH1|D0AAH1_TRYB9 | DeepTMHMM Topology Prediction - Predicted
Type: TM
MAEVRRITTAAVDWASFDAGKVLTVSTGRRGYKSVGNSSGSWVSEARVFSDTGVLLAQAKLPDVPDGGIDA
STNSSDVLVCCATHEPDKGQRTTVTFVSGPQVKVHELPRMKRKPKVRRHSVVCIRNGFCDVALYILGTGEL
FSCTRGQGGCHVQILYDFGNTCPVRILSGRTTAELIVLLDDGCVAVAELYESSDRSILCRITHLLRVSSSA
TPTHAAFAGGSALWVICDDYSLCAYWFPPNVRSGAMMAVDEEVADCSVTPVRLLDEQLSVPAGSEDVGRIF
LAGESRAYGIHLAVQTPVSVNFSSLSLHSSGGPGTLPTVGGWSHSVFVWSGHIHDLAFRQRAYELLVQNEL
GSSSLNIISLRQNASVLIFGDLNNKQLCHQVVCAAPTQLAGLREECESLVRELETEAAVVRDQHSVESAIR
KLLDLQHCMVATLHRERELNRDLMSYQSISMHLIRRLHIVYIRLSVYQFLHTVGAQECLDVPLREALGLDG
IRQQVCTLQTELRKQFQQEFDLLEPASSMEALKLWGSEGSAPSMCIDAILSHVNCTDGAPLRSIVQQVASL

KPEIALLVLYCTFTGPHCYHDYEKRSARVQFLSTFCLPASVDSWAYLAYAADHCICPTLPAREIGSPPFLD
IIPGILSGLTHSGAYESVFHLIGSTLAITTVGTLPSSVAVKLLYLAYKRGSTAVLETLFRRSDGTPWHCVA
TRVLAWAALQTADIKIFSGLIKPQSPEEEIVETVLRRFPDPAAADVLRLDFYILMQRYADALQVCERITAN
CSVDAQKLQVVASHLRSLMPNGNVSYSHRPSMEGRCDEMLSAVGGQAGSVVLSTKAAFAPPHTAALSICPL
QNEEQQLEEDVARAASRISALRHEGRPLDIIVFGNSQSRSSTNAVPASSVSVAETQETNVVSHGQERAPEC
LSPVSGAQLGNDSISTLDGSLLTQAPATSGSAAVAETVTFSVGSATQREQLYCEAILKRSKKPCGRVRPCE
YHDRVRK
>tr|C9ZYQ5|C9ZYQ5_TRYB9 | DeepTMHMM Topology Prediction - Predicted
Type: TM
MVNWKEGGSRSHFLANLPPNPHFPLFPLSLLLLTFFCYFTVLLLFFFVCFSFSSFRSTLMFLSSYHFLHLF
FLSLRLLQHTYCINNNNNRGSILALSPNPTKDRSHKCTSTHTHKRI
>tr|C9ZX60|C9ZX60_TRYB9 | DeepTMHMM Topology Prediction - Predicted
Type: TM
MQPQPNGLYFPQGNQGEVNGANNSNYYQQPPTFAPWPNQSAGARPEDFFYNGGDANHMNPIRQQQQQQQH
YQHQYQYPQQQMQAQPPKVASATPYFSRPASCASPKAFRTGSDRNSFQSSPSHFVSGTQSPKPTAAHPFIQ
MPVATTVPLMNPAAPVFHDYSPKGQQLPLQGTSWHQRTSSTGPPPTPPPTSYSPTQPGFFGNLLQTLALRN
MMTAFNNERGVDTSQGQVPLHQQRFGYPEDDLPLLDELGIFPHEIRANALAVLNPFREMGENVSDSMDLAG
PIVFAVLLAILLSLRGSMRFSTIYGQFVIGVIFMRVLLSLMTENAVSLQFVISALGYGLIPNVFLAASQSL
MYWLFGYVGKTMLVPALLAVLWSAWCATSMLVRGFHMEKQRYLIMYPLSLFYAVFATLTIF
>tr|C9ZVZ0|C9ZVZ0_TRYB9 | DeepTMHMM Topology Prediction - Predicted
Type: TM
MPCFSPLLPCWSLASSNTNRSQRIVLLIGLNIYIYIYIYYPLCFCFSVFPSVVEDVTCGVSGCGYGERIAS
SFLLPLSFLFFFLYIFGINALEWSVFTATPSVAVGKMLRCARVALRADPLNGGSSMTLGSKGSKLSPEPHR
RRMPWTAAKEYVPGVVLNARDKMVLDGVQLLDIESIDRASQLDPLEVLRAVVATREYNISTGKNIFQLASQ
ATYNGRGQRFYRKEWQEGTYDKYVTLSAIDFDRDGNKGTAYGYITFHGETTTRPVQVDFADVPGWYMDFVE
ERAVPFTGIVPPPPSIGTDVPVDPHSYRLKAYPYYDAPNPPEFVERLLKDRGVLPDTPTETADVDKDPTTS
DGSVHYDGK
>tr|C9ZKD8|C9ZKD8_TRYB9 | DeepTMHMM Topology Prediction - Predicted
Type: TM
MAGAKEVQSVHPLTRWIAGLSSPMSLTDQRAAATRISHLMLTTVFVATVCGYFIGNVHTAFFAVAAACGVS
LIVWGPNWYQNEDPDQRWCDENEVKKYYETLEQLRDEAKQKIIDELESKGEKKRN
>tr|C9ZMH7|C9ZMH7_TRYB9 | DeepTMHMM Topology Prediction - Predicted
Type: TM
MGSDTSVSLYIKVPRRCGGDMLTSVHTFHKCFFFFSIGPFIQSSIRKRGKDEDEDEERKKKRKEKKEKRKK
NRGRWEEKRQQTGEWRVTSFKQIKFSFLILFCVFVFVFVFVFVFKTVINTFSLLYSSFLLYFILVLLPTIFTT
VKRRTHTHKQKKKRKEKETRGGIYFPLSLQLRTGGANKAKKK
>tr|D0A9L3|D0A9L3_TRYB9 | DeepTMHMM Topology Prediction - Predicted
Type: TM
MTTLLDPQPQIDPSQKAMMVLKDSTITPAAMNVVGGYVMGFGFSLFGAMISAESTTQRMGTADFFRYSLKT
AHRLGFSFAYFGFLFGGIEVALEKRRGRKDVWNATLSGGLLGGVYGCRYYKAPGLVGGTLGGAAVSLMLER
VMDALGLAQR
>tr|C9ZLV4|C9ZLV4_TRYB9 | DeepTMHMM Topology Prediction - Predicted
Type: TM
MRSSDLFYLKSFFFFSTFLKPCASFQYFLLQHANKFTCCRPPPPQLLFIILFPPFPYCYVLLLFVSAPSLS
HVVSTLNILTSLLFSFFLKTF
>tr|D0A2R1|D0A2R1_TRYB9 | DeepTMHMM Topology Prediction - Predicted
Type: TM
MQWTSFFLFSPFPFPFKLHHSSRFHIKLYHIIFISSSPSRKTRTSFPFFFFVFTFSLSFFSLFFLFFLSLL
LSFFVASPPPPPHFFFPLVLLHCRHTRTHTYS
>tr|D0A869|D0A869_TRYB9 | DeepTMHMM Topology Prediction - Predicted
Type: TM
MAFSALAAVFFTGFCVLFATILAFSPRQTHDRKKVLARLRAKFPYSVSKIAHRGGSILGPENTLYAFHRAV
KEGAADMLELDVRETMDYRIVVCHDEWLERLCGSAYKHVTVKDITVGDDPNTNLPQLQRNIPLHFVSSEKT
MYCATDSVPVDGTTRLCLLEEVFEAFPTIPIHIDIKYASSDFTDRIFDLIKKYGREPVTFVGSSNWRNEIY
ITRYMKRLSSQKDKCKFHTFAGPIDYVLVHVAHYIGVLPLIPLNFDIFSVPLFTKRKKQEIPFFLRPIAQL
LNSPSLWMHLQQRGILVVGWVLNDVDEFEEASRWPINGVMTDDPISFNGFLISHDVSNTMNLLN
>tr|C9ZTS7|C9ZTS7_TRYB9 | DeepTMHMM Topology Prediction - Predicted
Type: TM

MLKIITCKGASKLSDVWRRVCVVLCFPLDSDYTMPCILLFSKLACISSFGILHTGAMLSSLPFAFVCIAVA
RTLWNKVAFNCFSSQFYFTPLLTKVSASAA
>tr|D0A2E6|D0A2E6_TRYB9 | DeepTMHMM Topology Prediction - Predicted
Type: TM
MDSCIVDGALGYMASVTTTIILFPYRDYVKAFDARAVRRVDPVSFCAARYRGMLFQPSQPLLIALPSGLLY
TGFLLGNGSVSGAFCGGALHGLGKVGVRTLAYRWNLSHRPKEVSYKSVLKCLQQNVKHYGALSFFSGASAT
IVISTAWHGTTLVALQRCGERGFFESWWDAFRTHSFLTFVTSPLRNTFRSALFSRERSSGIHNASTFLAGE
AAILKEAKGVFSNMLRTEGVRFFVGGVLRSTFKTSLPFGLTFATFSLIGGSLPRGGEGRGNDHRRHHVPHR
RFI
>tr|D0A5H8|D0A5H8_TRYB9 | DeepTMHMM Topology Prediction - Predicted
Type: TM
MYTRIPKCIYLNTFVEIISLPPCFLLSLTFPLSFVSPSSFYPLANFPDYRFFSLALPTPPSFFSFFLSFFF
FLFSHLFLPSLICVKLPLPNIFFWFLFHFIFLLVYPL
>tr|C9ZUQ6|C9ZUQ6_TRYB9 | DeepTMHMM Topology Prediction - Predicted
Type: TM
MCGSAFSVAYVLGFVLPFDWESRCLECAAVLWTVGILLSRGLTKKKKNRTVAQSLPTTVDLCGHYRAKGFW
FGCSVSPLCARRKPTKRCHFSATQRVSQRTQETLLIISTVFPRSFVYLRGRVTVEFVDVVRNLMNLSKMLS
GAVLGFETGGQVRF
>tr|D0AAC3|D0AAC3_TRYB9 | DeepTMHMM Topology Prediction - Predicted
Type: TM
MSKQSRKEGTVGGRRYLLYSLLAGTFGALSAVVGKLAFAHDNSGSATSLSGVVSMFFALVGIDARSNSLAA
ALVLGLRAVSLAANGYCTAQMWRWYVRALSCGSTPVCQVVNTGANFGVSAILGFFVFHEVVTITWLAGALL
VVVGLMLVVSDTDVSHE
>tr|D0A3Z3|D0A3Z3_TRYB9 | DeepTMHMM Topology Prediction - Predicted
Type: TM
MTTILENDRAFRIESNRAMTVSIHLIAVVLAFLTLILAPTEVEGVPAIQYKTSTRRTMNCDRFTKNIDECD
TDKYHTLLLLQALPYILVAVSLLFFIPIYFVCKYVFDCCGGRRQSVNFCHPDKRSATVYNRMDLLRPRIFA
VVAAVVCLAAAILGCLAMLVIQQCMRDVRSRVRNIVDMAVGYEKLHMSAMKVALYNSETDSEYPFLLSSLE
SNGPQLNTLFRSRVETVKGIYDRTVLSAVNAGRRAGFWMLGLFLAPTVLTLVGLPVAFCNYRRYVSMFLFL
FIGVFGIIVWTTAGAFAALNFFITDSCFEVEEFAEGRSNILTALSECDETSLTRPTAIIESLFKSQAGKTC
EILKPYCYNDGQDSTSSATSGSVFSCPKDMSCDNVTDLQMAAWVDSTIVIAQGIVNNSGALQEAKNKGHFC
SSVRDGGMCDLRKCASDCKLGNSLSNVGRVAKSVLVGVTAVSRARAMHETVSSALGSCESVLTTLASAMLS
PCKTATKSLFVVEECLGLLGLGCILAMFVYAIGAKRFISLKKAYVPQND
>tr|C9ZNT7|C9ZNT7_TRYB9 | DeepTMHMM Topology Prediction - Predicted
Type: TM
MLREKLFRKLSCVRVCSSFLLYIIIIIIKLAFFSFCLSTTLLLFFFSYVVVIIVLVRTFDSIQRSWKREEK
QGRKIINDHNKKELAVQVWRMGLLSIRFNDMLLFRLTGNVFCCFYKGKQKSDDVEKESFF
>tr|C9ZZ15|C9ZZ15_TRYB9 | DeepTMHMM Topology Prediction - Predicted
Type: TM
MKVFVYLYIFIYIYILSKKKTMDEFLYCSPCLHMGLGWMVVYLREFDMRGIFLSLVMRTCAIFFLLLLLLL
FPLLFFSFVPSISSSFFSFLVLLLQPFHNSAF
>tr|D0A1J0|D0A1J0_TRYB9 | DeepTMHMM Topology Prediction - Predicted
Type: TM
MLGLKHRCWKMQRSVIPLALHSNRLVGRLLGGSQLFIISSALFCYISGKVNKVCFLALLYYLTVAVKLTFG
ETFPPSVADLLVGGAAVLPIFSFFVFLGEFRMLFLNEYVSLRSHFQCIDDLDNLKLH
>tr|C9ZXL2|C9ZXL2_TRYB9 | DeepTMHMM Topology Prediction - Predicted
Type: TM
MLVATASVTSHGCATDLSTKTRSASVTMEGESVRSDERHHVNVRGDESEGVSVSLLSTRHTVHALNKQSSS
AWVTTKLVEQCVTGYRDALLVIREYGHRVEGEGLHLRLQLGEAHRELQSKGGLGASVTHIVEKQLSEHLMQ
NGNANISPRSESLRHRQILRDMKHLAHGLLIPLDIFRHQHNWLLVQEDVQLSLADRLRHYRVVVRQPPPPA
LVERLWFDLWALLGAAWRQRGRLLGVYAEGVLPVNVDGGTAVVPPSVLSPPSHREAYREGQREYRSKRMEG
SPASGVPADADDCYAFGPITPHRVGIMPSRHYTVRWPLMSILRSLRVRVAGKKPGSESGIRIAMSAGSLGE
PKDDHQEESDKCAQTSRGSDFFSPRVSCGSVGGSSQEYPAAFFDLSSEDIETCRVPYLSPECFTQRFRSAG
CHSSEPVEVSVSHRFSDDVWNIAVLTIEFMLTNFPLEQSCCHHASYQGFRTSFIFDDIIELLVVYHNIPLR
AAQNFVYAALHELSLLVSGVEQVGVDSGEGSVLTPRCLAQQWKDYLSETCSNSTVLREVVTNGLLWLRRER
WEVFQTVHSLDDYKEGECGNGSNGCIGGDDNEKNTSSRYGDGAAKRVLDTLVSPYLPLNPALCSAVLREGG
EASQGSLSITGSIQQWCEKEDAFWHTQFDDLQSTVQKWRKCIERYTVLGGMSGKGNEARQTVLFKRLVRVV
RSVLQLQLEKVKAGDQTKTILLSSDRESRRMQRGEVLSHAVQQELLRGLTERGALFMHILDLVPNQFVPTN

STVAGDDLTRVVPFCGVFMSISHTLSQLEKDMAQLTELSPASTAPFRALDTILGVEYNAARVTHLSACQLN
EEADEGKLSMLENVRSVIRQLDLDLKMQVKLVKDMRCLMCTSVPVETRASLVRQYLLRTQAAHGTVEVPIP
ATLRGEIWAVLLLVSPEPEERASRYFALDTARPTPCNRQLSVDIPRCHQYHPLLASSDGHERMWRIIKAWL
LLNPELSYWQGLDSVCAVLLAASFHDEPLVLAQLQELTHNYIPHDLASRETDLPQSMAGKFQLFAVLLRYC
DPQLATHLLDTVECGPELFAVGWFLALFAHGLPMAKVFLLWDFLFVYAAVFPHCLAVLCLAVLLQHREQLM
SHDFSVCVGALSRARDIEVRIVLYNASLLLRSTPPLVGGPCAPTGQPHSSSNSVPRMRVRTLLQAFRRVPF
DETPYGEEWTRNGLFLVDLRETRGGTASLELEGEEQMWSTQRETEERVVGALLFPLVSCQQDGAEIDVMQQ
ELQGRLAVQLASELLSQTRNIALAAVPPVPSSGSRPSAGMRQCEENPAAGTQPEVIARHSECPHIVLFTHS
SNLCEVATSELLARELMRCGAPHVSILLGGFVQLKREAADLIVEMVPT
>tr|C9ZYS5|C9ZYS5_TRYB9 | DeepTMHMM Topology Prediction - Predicted
Type: TM
MEYRLVERKEENKKKKRQKIKIKIKEARRVGGICVAFLQRGRGGANINVYIFFASFCVCVCVCIWNVGNAT
QTLVMPFRKQVTFFLFNLILSFFFLFQLHHIVPQGGFFFFELWGSYFLKVKSQSPGCLRCELLKVFLLVVP
SISACCLQSPILYEEYRTASLRGTVEFTPRCFTVRCVQRV
>tr|C9ZXW4|C9ZXW4_TRYB9 | DeepTMHMM Topology Prediction - Predicted
Type: TM
MHTLVSYVRSHFLPMSSTDNDIVEEPRSFRKELENSNVRGVLLFVFIDGAASSIWSSQPYQVMVSRLAGDT
AVGWVSAAAGVAQIVGALIAGGVRNVPRQVICRLSAFCGLVAVIMSVYGITTQDIMIYYFASALWGMYGGM
AFTGTEALFADSVESGKRGFVYNLKWINETTSSCVGSLILLIMTLYLGNDWDTNVLKILMYTGLAVHPTAF
FALLGMKDKNILHVDDGCEDCEQFASEASQRRLTDGDSSTKPLLGGTKDIIEEDGWDEESEEEERNCCDYL
CTGVTSSWQWLFTVSALPFLLALGNLLISVGSGVTMQYISLYFIKERHVTPIMFFITNIAVNICIALSSTM
VRYLSEHHLDRVSATILVRTLSASLLLTMGVIELPLVALLPIYVCRNALMNSTAGVTRSIIMDCARRENRA
MWAAFECFVSFMWSASSVMGGYIASAKGYKYTFVITALIHFIAMFVLVPAISGVRQLDRRGKVLR
>tr|C9ZP49|C9ZP49_TRYB9 | DeepTMHMM Topology Prediction - Predicted
Type: TM
MYRNVWLSDRDTIFCRTKIWDISSLHYHEQIIDVYPTRWARATPSSIPCYNLFAALGLVMLSDCACVWGFT
PSCHAFRGWCDSICVCFMCFVSLTTLCGEVCYSYDECALCCVRV
>tr|D0A1G3|D0A1G3_TRYB9 | DeepTMHMM Topology Prediction - Predicted
Type: TM
MMRRLALQSSIRRATPFATPLVASTKALNPMCSAITIREASTVAISVQGLHYVGTGLAAIALAGVGLGIGT
IFGNLLVACARQPNLTKMLFNYAILGFALTEAIGLFALMLAFLMLFS
>tr|C9ZR20|C9ZR20_TRYB9 | DeepTMHMM Topology Prediction - Predicted
Type: TM
MNQKEDHEDSQKLNPHPPSSVFTYDPCNSQSVLWHEDPVRDDHSHGDEPPLILRARRGSSEVSSITGIMAT
DYAIAIDQPFQIQGGTGLSRRMSLRSPGGTPAPSDEVQEGEPPARHQLYQTPNMSQTTRYFLADPHGLRSE
SDTQCLNSLTGKHWEYYYQQPRHCDTDTTVDEDHLLHQPSLLEPTVKHVTRSRGDRSGSSSPASIEMRSTS
LRSYTAVMEPRPEVHAESSEEKLVSGTVVRQSDAATDGNSAVAERMKYESPEDPRNYIINIIANNEPGRVT
LQLIHEELNWEERFAEANGTVLEYLQGYQSIFAVSPLDDRVTMRKPLKAAKGRRRLRGHSGYHAMSNRIGS
VSCSYVARKFDLELLSSLYKCRGYRAAIIHDVLHVSSFDTFDLFLFPGGVVVWWGMNRCDHWLVEDDFLSA
DPSFVNEAIQERHTQKSIDELFPMWHSYELDENYDATTQLGRRQALDRFSTNLCFDHYLIPRSNPLRSQVM
LTVSYALGRISVVDFFDNMTHKFHKEVLQIPSEIRGFFDYFSAQQQITRLEGELHIANMAITEFFDTPDFL
WEMGWLHDYHEIAERQNSSEKIFSWFIAKSDALLQQLANIKGRRHRLFILGSDVFLILLLVADVIFLMTSF
ILRLYFPRAED
>tr|C9ZK37|C9ZK37_TRYB9 | DeepTMHMM Topology Prediction - Predicted
Type: TM
MRCHPYMPHKYVYIYIYIYTYKYIKVYTCVNIRFKEEVKQKKDNGGCSGNRVNETVRHEMTRKDEFVISLA
FSLYTILPIICFLFHLCDSPKQHRFYFLSLANIYAWVEVALHLFIFALLYLFARLYACVCLCVFSLLLFVS
VLLRIYDAFLLR
>tr|C9ZYS8|C9ZYS8_TRYB9 | DeepTMHMM Topology Prediction - Predicted
Type: TM
MDVICSALAGVMARAVCHPLDTAKTVTFTGFFGDSSSSLHVNSKGSLRHVLSSIWRREGPCAFYRGAGVAI
VGSAPGTALYLTTYTWSRDFLQGYVSASHSSSFLSTIPSSFIHLICGLFAESVSCIFWVPIDVTKERLQAQ
SSFVEGRYKGNWDAIRTVARYEGVRGLYKGYWSTLASFGPYSAVYFGCYEVFENVLSEHMSLGTFSSSLCA
GGMGNIVACVVTNPLELVKTRLQVQRAVLSVSGKPTAVYGFPFRYKGLLDGLCAIVKSEGVCALWKGLPIR
VTFAAPNAALTMGFYSYLKGNMA
>tr|C9ZRR7|C9ZRR7_TRYB9 | DeepTMHMM Topology Prediction - Predicted
Type: TM

MHVFVLFICRKGTAPSFPSSYFAFSCVSSPSSSHCDCDCDCVFCLHCCNNTYVALPLVCLLLLLLLLLLFFA
NSLVTGRRSAQHRKGRKIFFFLSAAVPLPHL
>tr|C9ZV59|C9ZV59_TRYB9 | DeepTMHMM Topology Prediction - Predicted
Type: TM
MEYAAPIEMSRQSTHEPGTDPKSMNAAFATHHPRHSDKTKREATTQYLLVYDPFILFFVVCWVVRLFFRGG
DNSPAVRHHESAVFAVNFEWERKRPSFSSFRASFCMGRSVGKLLTAWRCLVSSSFSLFVYVGWSILVLKTG
GLATPNPHVLVRVWAEYGFQKGRIKKRG
>tr|D0A9S5|D0A9S5_TRYB9 | DeepTMHMM Topology Prediction - Predicted
Type: TM
MWRFRAVSAIAPRGCFRCVFQSKAFAAATKTLPCTPEGKNPYDVLEVTVTNSTTLNDISKQFRELVVRNHP
DQPCGSHEKMSELNAAYKIVKEHHEGVLRRLKEYEADTKGNGAFQQHRRSRAQDDRDLGRTGGVFRRNARA
AEQASASKKARSLQEITSSWSRYREDTEHAVTSMCNRYELAVEKGCFFRKTSMLNEITVRERWLRKSYIKA
VWEEVHELRGELLRRGARSSQQSQLAEEMVSFASATQRKLNEDFQRLTQLSVQSQTRLFLQRILVALVTVV
VFIKLWQSLLSGMFRNSLTVRFRQGILSH
>tr|C9ZRG6|C9ZRG6_TRYB9 | DeepTMHMM Topology Prediction - Predicted
Type: TM
MRPRCGYANAVVTFSSLPCLLTTFCFGAETCGAFTAAIVCPQRWCAALGERRLSRRRYSAANTKGRCKHVY
NIINSLKLVPKAVLASEFDKLHPRDKLDVKRALVRRRSRRSSCLPVFKSALFNRKYFFREADLTEDSTLGR
GPGGQATNRRKQTAIVKHVPTGITVKFSKFPSYWLNRRAARDVLNLQLEERMLGSKSELGRIRDLRERRRL
WRLRTTCKLVERASKIAAKRSQRHEFHSVLTNQQPLSRVAVLQLDLDQSKQPMYLSDLFDRECGQWWPLLS
KAFVRINAESSNEKSAKVPDILFYTFPSVRRHGESVTSVEQYEMNQVKKCAADEVCLANVKRALKCFVELF
GLRLYEKPTTTAKNCSVLVLGRDGLNWMEFRGRMVDSSGLMTPLALACFAHVTLSLAQLRCAREVNAIRSF
FRREAKAGVPGKWAVQGQEGITEVLRCCELHEEHHPTGVDGVV
>tr|C9ZYZ0|C9ZYZ0_TRYB9 | DeepTMHMM Topology Prediction - Predicted
Type: TM
MFFLIKKKHFPSFNMPFYWYLTDFSFLSLGGGSGERRVCCLCVYVCLYIYIYIGGAEACKNNILARGHVDF
FNVLICDSYCACAHFSSYLFFLVSFPILSFFFFYSLFL
>tr|C9ZJ42|C9ZJ42_TRYB9 | DeepTMHMM Topology Prediction - Predicted
Type: TM
MARLQAHIPFFNFDTSPLCMFVCSFNVVFLCVCVCVAMESSTLCLFLHFISFPFLSIGMEVCICTCTCANG
CFFFFGGGGVCVFYSVVCVGSSMREDVFLCLCLCVCVCVCVYGGREGAPFPNLNLHLLHCGARVFCFVSFPFP
LIFLQPAFFFFFQNNC
>tr|C9ZTB7|C9ZTB7_TRYB9 | DeepTMHMM Topology Prediction - Predicted
Type: TM
MSVFFLKIILMCTCVFSSCDYDIYLSIIIIIIIYFFSFPLSPLVLFLFLFFLIRRKHEERVLVKVFVGALV
VFGVELAAAATCGVCVCVCVCGVSGGSKWILLLSSFFWGGVFSLKSLARPPLLIREERHRKAKRK
>tr|D0A2I7|D0A2I7_TRYB9 | DeepTMHMM Topology Prediction - Predicted
Type: TM
MASNPDSKLHIPDEMFNFFNSLNTAVDTGDISSFHNLYENIFPSHLSKYYAAEQGEFRPLPVLQRMEVAEC
FGNDTAGKLYSFLCFKHLFTDRDVTAEDAKVSWRTFCDLFVSLPGSCDIPNWFLWDIFDEFLFQMTVVYQK
RFAEGAEWSVTEAVQMMEKVISESGIEEVMESDKADDITKSGENHVRWMSGFFGIITVAKINVLLGDYNSA
LSVLKPLDIYGRGKKILAEVAPANVSLMYYVGFSYLMLRRYADASRVFRQSLSAKVSSRKFSERVRLDCAF
MHVVSCILCGTQPDNLSWLMDSRKLQVLEDDKELLATGDEERFRDVFDRCSPKFLAVPPVPPTVCKGMEGK
ELQARLFLRAVKQQQDTIKLRVYLGVYQTTTTELVKTVLDVNDGLVPLFAMKLTSRQLVHDGVSADLHSGT
YVVRAALDCTVEGDNICVVQKSSYRTIESKYFKRMTQRRPRHRHFDNPRQGRRPAPDAHNTN
>tr|D0A4S1|D0A4S1_TRYB9 | DeepTMHMM Topology Prediction - Predicted
Type: TM
MTCYTQSNGFTPRVAKVCLRGWFNLWVILVFFLIWSHGEVLVFLWFLDHVRLPLIINQTLYRKNITFPVLF
QRFTFPADDPCISWSRPAQYISNVLERTPTPFSIFFPPKRGLARPLFSPAGFIYPTQLRRWLCSFHGKKRD
RVGECFLRVYTGTHLFIFLMFGHILNSVLIKYTTLSPVHFPATQCSCFVYPRGKGCFPPLVHTRQDQVLQR
GDVTHVLPSSFNNQIRILLNISAVHSEGVEHIVP
>tr|D0A9H5|D0A9H5_TRYB9 | DeepTMHMM Topology Prediction - Predicted
Type: TM
MMRIRISTPSRTYHSSAREEDGIRMHILFLLLLFLVAIVLPPFCIRVFECSDFPPFLYPQIVLCSADLLCN
DSPRILASFPLESYVFVFIVHFPVATNSGLCWHVTFAFAQTHIDSITRSSVVKVFARFTRAILTGPAGPRR
V
>tr|C9ZST8|C9ZST8_TRYB9 | DeepTMHMM Topology Prediction - Predicted
Type: TM

MLDQLSTCEAFDFVGVGNFILYFPSFVPSFICSLHTFLFLNFFWIFFCFYSFTGEITGCLPGPEAWRCAGR
KWRAEILLGRVGNSKQKPHRDTGVYFPGLKEELSLFGSYHYQYYCKYY
>tr|C9ZJU2|C9ZJU2_TRYB9 | DeepTMHMM Topology Prediction - Predicted Type: TM
MHATQYLRKVYFLHLNVNFFFCKGQTHRVFSDRASSNFSPSRRIMQLQRVICFLLLLRLLMICVYACCFLF
YIVSNRRQKRGYYILHILFCMLAWLHNLCCCRGVCYGL
>tr|C9ZML2|C9ZML2_TRYB9 | DeepTMHMM Topology Prediction - Predicted Type: TM
MTISFASFRTLFFFCFLFFSFFFTYFLGLFLRYISLFFFSSTTLPYLPKKTHIIFSTLPSFHSRIHLFRHP
SCITKWFPFVFYCYCCVIYLFFPLQIPPPRRLICYPSKSSPVIPPPLCITP
>tr|D0A996|D0A996_TRYB9 | DeepTMHMM Topology Prediction - Predicted Type: TM
MPFSKAWRSAVYPDFREQGAYINYKATKDTLHRMKEDIANPATPDELYNSLLMQKATVYKWCENKVKELQM
MAEALMKASDYLSEEETPTNMSMVFSMVGSSEAKYLPPSDARRVADAITYELLRFVECRNLNTDTIEHIIA
RMYRYAVLGPTGDRWKNINKEYDYHALSIDEIFFMLSKVYEHVNEVESMRRDGRSSIPCGTVGSQVFDRRS
VKYWVHMQDLPFVIARIIPHLPLSTFQDTYAMSKERGVPFTLGSPISSVYYDNDKFLLYHRRLERLDGATL
IRMRWYGRPLDSDWNKLESKDSVFMEIKVHHEAWSGERSNKRRFALKEKDVDAYIRGDLSLKPALEKLRSK
NASEAEQEKFMSLATEILTKIHAYDLKPVLRTQCQRAAFQCGLDQSIRISIDTDLRVVAEDFGLSYHWRYN
GADAPLSHFPYAVVEVKLQCAENERIAPWIEELMNCRYMESVPKFSKYAHGIATLYGHTPFIKMVPYWMPQ
LDIDIRASTKPEYNQWDPTIGIASGCWERTTDRVIFGTGHAQTQTVGASEARFLPRTDCLRTYQRVLKAIK
RGAHMNSVAPTMSPTDRPPSDEKKLKEQQELAPVVQYDTDRRHKAYTAFHLYPYCEDGVESLCFTSTGGKH
VAAEVFSGLIPWQTGKRIRVPQKYDPKTLLTSERFMVKWAEQATRVGVVGLAVIRFGNSMSLPNDMVAVHS
FWRANFHIVLGSLMVVVAECVLVYAYVTFKSRSRRVYARRKIRYDDRRGPVALTFVILAVILITVMMHVMV
RYGPMLTGSDTF
>tr|C9ZW01|C9ZW01_TRYB9 | DeepTMHMM Topology Prediction - Predicted Type: TM
MQIPKPGAKLLFVAVGLPSQQPFPRHRGSRSGPRAGANFPMPKSAKNMFLLCSLFPGAVHYFVWYFTLLLP
MLVSLSVTDFGSERTLVLARGTFSVGVVLFLVASNPQRYFQNEEKAPEFPPLHLRQLCRQVKMSHFPLRAR
GASVTVPLTGRSNFDPSRSNVDRIALSLSRRREPTRWRAGQTKRTSGRMLFGY
>tr|D0A1E8|D0A1E8_TRYB9 | DeepTMHMM Topology Prediction - Predicted Type: TM
MWIYARTYATVSSKGYIYVSFFSLVLFCYLTISFHFISFHFSFFFSFSPLSPFRAIRYFPPSLFSRSLTPF
PRYNLQTQTHSHSHSHLHACTHFYFSFLLFSFLFFVLFFLLIIRECSGRCFCLFVCFFYFFFFFLGGGGEG
EVSLFIWSRYSSIFCFVFCFYIGASSASFFSFSFSFLSFLKNKKPFKSSLFLHPHLQTRVCVWGGGLFFFLPS
CFVLPTVLLYYYFFFFFLLRSDFSFFFCCCFNSFAFFF
>tr|C9ZPH6|C9ZPH6_TRYB9 | DeepTMHMM Topology Prediction - Predicted Type: TM
MFVCFSFSFDFLICSFSCTFITFAVFFSPSLAFVDSSHTFPRIHASFHMTLVRKTLRDTHRNMSSNEKVST
TGQSTSPMVGITWKRERSPTAEMQASALYHCTVQKQLELLHRLPSLTAGPCFRNAEHYISGGNSSSSSNSK
VTGNDQSHALEALGLWVTEVPTGDCKNSAERQTPIDYYGEYARSVLRESVIRQGFCHNALYSIKKEQGKES
TNGKQGSSKVARTIHNSLPDFWGIARQSQ
>tr|C9ZKL4|C9ZKL4_TRYB9 | DeepTMHMM Topology Prediction - Predicted Type: TM
MPSAETLLKDLFGSHTVELLRQDGKMVPATTALEGKKYLLVYFSASWCPPCRVFTPQLATFHELFSAKHNF
DVIFVSRDKDESSMSAYFYNPKYSTLSVSGGECSHGDWLALPFTQAQTVGKEIMSRYGLNTIPNILLFDLS
TEELVTSEARQLIGSNCRSAEGFPWRGASAPVISFQGLATVFVVFLMLYQFWQSWS
>tr|C9ZTV9|C9ZTV9_TRYB9 | DeepTMHMM Topology Prediction - Predicted Type: TM
MGCGSSVPDVVNPGFAMRVQRQPSTVSQHQSSENFLNARRLRRKSSGIRRSSSGARIDEEDMLSDISSAGS
SGRSLLDVHSMLARTSFALEDLEHQRRQLPDLIDENLFVYDDVVVAKDEVNSRQWKAEVNRQPQEMRRSRS
GSRRLSMGPRKGKNNVFCFADFKVWVDEMETGANEGMVKVGKQRLSILSRSSEEDEALKGARVSFTCSTRP
NAFAVLRARMIAQQEFSKLKLAELGVPRNIRDFRIRTLIGHSARVRCLSLSPNEKALVSCSNEETYATLRN
LMADEERGSFSGHRDVVMCMAFSGDGKYLATGSKDKTLTLWDAAITKVLTVFKHEKVVICCCFSPDSKRIV
AGCQDRVCRVWDVMRGAQVVTYSRHCGIIASVCYSPDGGNICSGSSDKTLRVWGAERGGTRTVLSGHVGVV
LSCSYSTDGEHIFSNDEAFLCMWNPKDGVCTMRLSVAEFVRKCGGIPKVRRLGWTLCCSAPGAFTHYVVAA
CANRFVYLIDVRTGEEYASTFCKAPVYALTRGRCSTVAFGDTFGNIYIQELM
>tr|C9ZWL8|C9ZWL8_TRYB9 | DeepTMHMM Topology Prediction - Predicted Type: TM

MELSFPFLLSTVVLIVFYTVDFFFARLLNVLKQDHLELDECSPEESPDLDPAFVVGSLKQVAVEMLEYQRR
RFHMGETGVPSTAADSDPVPSATVDFGNLPTAASSHPSENVVHVVVIICGLVGPFATVCQALFPNAFGWCR
FNFPYFVQYIGAFSGVASVSVYTGRVWHCTSYSEVSRKQWLELMWFRLDFMCLISVAAFFGAGSWVVTICL
LAIGFYLAYRVMRIEKRMQSLQVETQLVRGEVDVDRVYVPGTVEAAKALGTSEKQGYNAVV
>tr|C9ZMZ8|C9ZMZ8_TRYB9 | DeepTMHMM Topology Prediction - Predicted
Type: TM
MLCCGCLTILMTLLGASTSVCTIIFPVFRMKNETKEAIQTLWYYMEKTASTESKTSVRESECYEYSVYFQV
SMGAVAAAAAVGLISLLFLCGVVACGKKMKHLKVVSLSLVFFSLLGAITCIGLVTLGYLRGYCQGDPKLRD
KYAPFKERGFKIDVGSYLLCAAVALYLLTTITHCCL
>tr|D0A0F6|D0A0F6_TRYB9 | DeepTMHMM Topology Prediction - Predicted
Type: TM
MFICSNFDDLLYQVERFDCAAEGGGVQCAARTCNREFCCLFVITILFPPSPPQQMIPPTLFRVLLLLLRLR
PLVKGIVSCFYFYFSHAVISLAHTHMHIYVYIVFFYIFIPLATLNST
>tr|C9ZN21|C9ZN21_TRYB9 | DeepTMHMM Topology Prediction - Predicted
Type: TM
MLCKNACFTPKHLTCCVAPLFLRNHTHKHVFDVKIRNSAQFVNKHATLCRLCKNACFTQKHLTCCIATLFL
HNHTHKHVFDVKIRNSAQFVNKHATLCRLCKNACFTPKHLTCCIATLFLHNHTHKHVFDVKIRNSAQFVNK
HATLCRLCKNACFTPKHLTCCIATLFLHNHTHKHVFDVKIRNSAQFVNKHATLCRLCKNACFTPKHLTCCI
ATLFLHNHTHKHVFDVKIRNSAQFVNKHATLCMLCKNACFTPKHLTCCIATLFLHNHTHKHVFDVKIRNSA
QFAHKHATLCMLCKNACFTPKHLTCCIATLFLHNHTHKHVFDVKIRNSAQFVNKHATLCRLCKNACFTPKH
LTCCIATLFLHNHTHKHVFDVKIRNSAQFVNKHAILCRLCKNACFTPKHLTCCIATLFLHNHTHLSLVSIC
FVFVDCVFFNSCFFSSCTVLFTADKCFFSLFCIFWFFFFC
>tr|C9ZMQ9|C9ZMQ9_TRYB9 | DeepTMHMM Topology Prediction - Predicted
Type: TM
MFIARTLPVLRQAMPRQRSFLSSVLKGRRNTPTYYSGRGDMAGNPDILAEAACCGRPDAEWAWLRLLCFVC
VSSTVGVWLWQVYFYKQAMFFKDEPWSPFKN
>tr|C9ZJD7|C9ZJD7_TRYB9 | DeepTMHMM Topology Prediction - Predicted
Type: TM
MSVMLLHFRHALHYYFTLFTYIYLCIYVSCRFFFVQFPTHDENLSPRFILLGICMAVVSLTNDCYYMFFLF
FEQKQNQQQKPFPLPSCGCCGGVTGDSTGSGFCYLPTSYAVVPHFKQLYYFLFPHVYLCVVELHFLLRLHL
MCFCFLLTSSSLRPTIDYYYYYYFVCLKHKLQLLGVSLQEQ
>tr|C9ZXJ7|C9ZXJ7_TRYB9 | DeepTMHMM Topology Prediction - Predicted
Type: TM
MFRFTLLLFPRTSFLSFLFFFSLPSLPSLLLWVYFRFRFFFSFSHARSCYFPSFFLFTSFFFVYLCNLRCV
CEDVSLSVDFLSPSFLFFSFFLFCYTVVSFFFPFFRLFCCSFFAFCVLFFIFSCFYVVRHMTVAVGRTLKK
RKTYRQ
>tr|C9ZXB3|C9ZXB3_TRYB9 | DeepTMHMM Topology Prediction - Predicted
Type: TM
MEVCAVMCCCVSCFPIVTLSFSATVFLCDVFKHLSLYRNIFVFNVFIIFVKCFCLHEATTVTLLK
>tr|C9ZQT4|C9ZQT4_TRYB9 | DeepTMHMM Topology Prediction - Predicted
Type: TM
MGETVRRCCPHSASAELTGHLISSSGDIFISSTITLDFTNAVSREVANASKELATPPVVMRRVAESHPMLL
LTLSPLHRIRQARVLISKVCEGSDTPGANATCDSDIVSEWHELQLNEQTLYATDRVSAESKHHVAQPSWTP
LSTEEYPMGGELELDIGGDSDDTVEPECDDKFEKLFILELVHSAVSGVSAEDSSTFCVLKLCVDIVVESLE
RLQNVSYPRFTVTSKPTVGQRIANACFAERGNGTQDESDDNYILLVGQHQYEYYICPRIEYANGHDTGSGF
AAVDVNVVSISPLQEGDDSGWEVLLLSPLPSQPADLLCAFVRSVGEDTAQPSVEVYNGAKWLIQQYPAYTH
MKAEEDAGHLSESLASSIYFFHCDHAIYQYLMGLLQIADFAWTAAVNRATASTSNTWVVARSDGKNALLSL
RRGFLCTRHAVLLSDETLYGTVGGNPAVSPAVALRGKTAVISAALELKWKATHKDNASFPELLRANKETSF
LSVGSALLRTWSMCLLSDVPGWTEFLHQERQIALYSLCVMPLPPNSWCLPQQYVREVYVSLLLLSSAFVVT
VLRQNLCGPWELLRKGVSSSFMRSIGVTDILPTTGMHLDLVRLLILRDGWGKCRLFDATSLSSVCFVGTLS
YEEKLRQGKSTKDEGSSYHDIHLHITVPPTKRDVQVSHILDQQLAEDAMWLVDLPFTVLYFVAEYRAQTYS
TPSGFNAEAPSSRCRVAHMLPCYWRVSALDWARGLSQHASFTLLRPSGDDGVGATEGSISAEGCGDKEGNS
EGQDGVPPLLIPFLVLNPKVVPQALFLHVDHSQFFTDLSEKTGFIMRQSVLQNIMEESVREGEEDAGEVDM
LFTPLLNQLLLSILDATPSHSPVATAELLPIQLFEESLEAVSGGAEAVWGRVYADTRVRLMRRVGYHRKGG
VNSHSLKRSRDNGTAGYGGGCHESLRLLGNIQSHIQTIAKYQSTLPQLQLRSQRRRPLVLRLYERTGSSV
GVQPKGKEQHESNIPRSVSDLAQLVSRVMCASATRLEEGESTTARRAVDSLLFPLWLLLLDLRECGVTTCG
GQVGTEVVRRICEVSFKGLTTNGVVLPVDTFLLEAYQFLMQDA

>tr|D0A0U2|D0A0U2_TRYB9 | DeepTMHMM Topology Prediction - Predicted
Type: TM
PARLIGGASVAFDSNTPQLIRKGGAVTTIREDGEGVGKYLPISGTVFVIGLSVPDVKEIARKLEERNDLRV
IVLFAEFSFLYDLFATALNNTAGAARLVFATSLPHWGDTETSSKTAQLFHDVEKDSRLWTPLSLLAFATGR
LMRVILLHVEEMSPDTLVNFFYADSSIISDDMRYGVFDDTKCDTAEKLPKDDCASNYGATQVSVWSMARAL
NASIPPLANPMTPSMSLRDPSEGKLSGASLVGVIIGATFALFLMVALGVVPYFVLRSTRDNNSAPKEPTDP
VTIVFTDIE
>tr|C9ZP82|C9ZP82_TRYB9 | DeepTMHMM Topology Prediction - Predicted
Type: TM
MGVPKFASWLRNKYPAIVTKNCPSSVHGLYIDLNGIIHPCCHNDSDTAIALLPEEEKLECICSELELLVQT
VQPKEVMYIAVDGVAPRAKMNQQRARRYMGRAAPAQTGEVASTYMPNKSGMAIVEEVVREFTSTEMAQAND
DLDEVRQTLLEDPLYSGALYNEGEEEAGDALNDGDGVNSFCVRSTDDDYESTHENRYVESNQVVDDVFGHR
GEVIVDDPTAFEFDSNCISPGTDFMAKVSDAVLNMLNEKMSGNDPTWTRLCVIFSGSNTPGEGEHKIIDFL
RTQSSLAQFGGKANHVIVGLDADLIFLALSLHITQVFILRDTTRNPYKRRAVARQQQRERRSRRKRNFIDD
TCIISLNPSYIDEEESDGVIGVPVCESSENSSSDTDNESPIESREAHVTTVPNTGFEYFDISVVGGSLISE
VSALCDIKKFTPATESAFDKKTLVSSAGYYFFGQPNHIEDGTLPSSTECGSPKGRRSKRVSKKAEAERSWK
NFRPCSSPANSKIIDDIIVLSILLGNDFLPHAPSAFSGESALDNLLELYVSEVLPYGFLTKGPHEINLPQL
QRLFEAYAKIEAVKFRRFLLFKSTNKENSNSGAEFRKGSNNDNVTVADVAVALHSTIDDRWRDTYLKSTGL
SNSVQEACEKYVEGVCFVWRYYTTTTTDSDWSWYYPFYHAPPVIDLAIYLKEANIKRMPLPVVKSTPPDTL
VQLLSILPPTSHALLPMVLGEVMVRSPQQAELESTFPLQWNVDYHDSNVVHLATVMLPFADMDVLQQVVDA
AWPQLTEEERWRTQKLDFHLVITSNKTSFVPKGDLRKLSDALDTILRRVPSREGVEIRQLYDVPLERYRPR
TYSCTVEVPRLSYGADGQVIKRQRRHRHSGQLKGNGEGVIEGKKSNSRVTVVNKGPCFATFLLFLACASVF
TAVVLLPCSLASFMQVLSINICAILISFVLGISVHDPSSGCGMSRNNIRQTFVDWLCTECLSLNFSSRTRC
FVCRAPFDNRRCLAVFSCTQSADQSLYDPDHSPHIEAVKLQI
>tr|C9ZPI1|C9ZPI1_TRYB9 | DeepTMHMM Topology Prediction - Predicted
Type: TM
MVCDIFFFGLISLISTSSYVSLSLCVCVCVCMSVPLCLFCLLVIDLVVLLLLLLLPFFQQKKKEIKFAFSP
TSSCLSTTRRSHIRIVINLCRTAEIIRIKKGRKEEKKNVTSSWVHF
>tr|C9ZWB0|C9ZWB0_TRYB9 | DeepTMHMM Topology Prediction - Predicted
Type: TM
MAAPLAALVLLGGAYYIFRLAPRITQRVSMAQGLTCAANRQLRPYRRYEGGFEKSMTKREALLLLGFTEDV
ASGGFLSLPSDEEIKTRYYGLMKQLHSDVDGSPYIAAKLNEARDILGKK
>tr|C9ZIS7|C9ZIS7_TRYB9 | DeepTMHMM Topology Prediction - Predicted
Type: TM
MRAIVNLFASSSPVMLAFLLSPVTNSVSFRCPSIYALFPLFKFVLRFKSTSRLRFCAPGVEVSCFRDISNF
FRMLPWEFCKME
>tr|D0A119|D0A119_TRYB9 | DeepTMHMM Topology Prediction - Predicted
Type: TM
MYIFHFLFTSVVVLSLFYSSLFSFFQRGMRGCPFLYSVIFRLPPHDGEDCFFPLFFIYLTSLPSAKTALGN
DYLEYRTSHIRRSCEPPTVRLRVLFSLSFFSQSRFKEIKIPLFAVFCLLGWWYCTF
>tr|C9ZL48|C9ZL48_TRYB9 | DeepTMHMM Topology Prediction - Predicted
Type: TM
MPPVTGRPIGRAPAPHPVIRRTRSWRGIMQVRFNDTRADRNRILKHAFLWAPVRIFGICFVTTTIIYFTIG
HDRFMHTLFGYESEMHYEARVNPDASILIGDTLLDKDRAWKSPLRNLEKPLHPRREFDVLRDPKST
>tr|C9ZVE8|C9ZVE8_TRYB9 | DeepTMHMM Topology Prediction - Predicted
Type: TM
MTTSPNACQDQPPQHSAPQAHEAECTTHKLSAEETMDARPVHPDARALFRKLPCVWSIPVLGTAVEAFGPK
FVFALGFCELFGKGIADNIIRSSLFPMFTYTFGADAKLYQLMGGMSSLGYAVKPFAAMFSDLFALFGYTKR
WYLALSCVVGSTLAIVYGSLPGELSYVPVAGFLVFVITFTVANLDILTQGHYSRLIRRVPLAGPSLVSWVW
WCLLVGSLVASSIVGPLTDKRLQRVAVFISAGMQLVPTIFFILNWYGERRNREERAYDLKIIREKQLEHEA
DAVRLQGSEATSGSLDNPSDTEEVGEGGARILPCCCGAFEVNREVFARNKKVVFYCMLLTLGAIGMVLVTV
LGTRLQLLITSVVASFTLCGLGFVALPLVIAKANMFTFISRVAYIQLRGAMDNVFMATPDCFPGGPNFSYF
YYNTVGNVIGTMGGVIGVTLFRYVFSKRSYRLTFIVTTLIEIVSSIFDIIIVERWNRPYVSDHVVFVLGDQ
IIQQVCYMMHFMPTVMLISRLCPRGSESMVYAVLAGCAHFGRSVSNTLGWLLMEYVWNVQSDITVGPCDFS
NVKWLLLLGHFGTPLISIPLVFLLIPAARICDVLDENGKAITKKAEDVHAPSNDSPRRREPTAN
>tr|D0A436|D0A436_TRYB9 | DeepTMHMM Topology Prediction - Predicted
Type: TM

MNRCYSLLCSVLNSGKRAQTSFPSTRCTVIHSRVLPSESITSSFSPPETTTASKDKSRGETASKTTMTGPL
QNFGDELNFPKMEEEVLKHWEDIDAFKECLKQSEGKKPYTFYDGPPFATGLPHYGHLLAGTIKDIVCRYAH
QTGHHVDRRFGWDCHGLPIEFEIDKEYGIKSSHDVKKMGIENYNNACRAIVMRFSEEWRKTVTRMGRWIDF
DNDYKTMYLSYMESVWWVFKSLWDKGLVYRGFKVMPFSTACTTPLSNFEANLNYKDVSDPSLMVTFRTKDD
PNTFLIAWTTTPWTLPSNLALCVHPNIDYVKVLDSKTKRHYIFGEPRLGEVYPKKKGDGKKGTETSPYTIV
SRMKGKELVGTKYEPLFPYFEEKYGATAYRVLCDAYVATDSGTCVVHQAPGFGEEDNRICIDSGVITKEDM
LCPVDENGSFTPDVVDFQGRYVKEADSDIIKYLESKGLVHSKGSIVHSYPFCWRSEAPLIYKAVDTWFVKV
ESLREQLLSANEETEWVPDFVKVRRFSNWLADAKDWNVSRNRYWGTPLPIWHSEDWEEVVCVGSVAELEEL
SGTKGITDIHRHFVDQLTIPSKRPGMPPLRRVEVVFDCWFESGSMPYAQIHYPFAAKDSFVGEKFPADFVA
EGLDQTRGWFYTMLVLGVALFGRVPFKNVVVNGLILAEDGKKMSKRLKNYPEPGIIINTHGADALRMYMIN
SPVVRAEPLRFREQGVKGIVRDVMLPLFNAAKFFIANANYCVELGGNVATDVVSSNEMDRWILASTQTLQQ
YVKREMEKYHLYNVVPGVLRFVVDLSNWYVRMNRRRMKDTVDLEDRSKALSTMLNVLFAVSRIIAPIAPFV
AEMLYLRIKPLLPQEQQLKSVHFHMFPEDDISKHDEVLERAMTRMVTIVELARVLRDRMVIPMKRPVRQVV
VVHPDEAYLDDVRKVVTYIKEEVSAFEVVLSSSDEYVTTQLDASMENLGKLYRNEAPQIRKAIQALGPAAV
TEFLKTGEVEVLGKKISRDDVKVIRKVKDGITDFESNTDNDVVVLLDKRDDQTLVDSWRAREFVNRVQQLR
KKAKLIVKDIIEVYFETEDPELTTSILNCSEQVNKTIRGKWETMDKLPKGAKLIAEEDNSISGVGIRIVFT
QPSAGES
>tr|D0A4K0|D0A4K0_TRYB9 | DeepTMHMM Topology Prediction - Predicted
Type: TM
MPRAYYKPFTVYEEVVGFHGARRNAYICCAILFGGLLFKILVLSQYSTVTDYSGVDGLQSNPDYQAMLAQR
EALAQSLSSREEIHRAIASRRAPPA
>tr|D0A6H1|D0A6H1_TRYB9 | DeepTMHMM Topology Prediction - Predicted
Type: TM
MLQCKFYITLFLHIICSRRHIPSVIPCCCFFLLLSFGYKRCMWTRRNVFPLMSLRDAARAPVVISPQAKNG
GPSPLSPTAKEECKKSDSIVSMNYTLIPGIMPEEAVMILSWALCIDDPMAVFGKEGIEAFRQVEYFFAGTS
GGRLFVYPLVPPAMRPFLPSDNWEWLQGEGVKQKLSVPWNAGPRRLFHQHRYDEPVTAIAILGRLVVSCST
DMYVHMSLFHPTPHHILTIRHPSPLRCVLLWEGAVFASRLLDSQRRAQVASAECCVVYLITGDDGGVARIW
RSNVEAGEYFLVAVLAVVTSTCYVGFATPLHYIAREDADAEHSNRRPTAETTRQTKAIYSLAVDDDRRLIA
GVEGGVVVWSLADLPYKQKEKDHLLCWDEERMVMEPQATSTLRIRGQRLMNMTVWVKSAAFTEECLSSCRI
DQRGRSSYERGESTNNHAGESVMGDADGSRKPPFHTDARWSSWKPKYGTHVAGGLDVGVVSNVVCMPSLNG
DNLDERGSAESLLRAACAPIIFPSLKNIVHFPLWVVEPVFAPMRILSLTGSICTALLVLVPSGRVVTGGSD
GVVTLWLWDVAEATYVRAIVSERPQSHSGLCRCLTALREPDIFTSCGYEDGIIKEWHVYDEPELLIRLERS
FALQSESSGKSSTWPAEQKQHPFGVGVSCAVSFPAFCALFVVGVDECHINTFGLLEVLGCKPPPDYIFDGY
KTVRVVPSSHDTHGIDKWGK
>tr|C9ZZH3|C9ZZH3_TRYB9 | DeepTMHMM Topology Prediction - Predicted
Type: TM
MMRSLSKEDLLFLARQAVSGVRRAMSFPQLGVQSQQKILFSTLETLEDCFRRQASIDLRTVDLWVTSFSEE
LVSQLNCCSGTTAEHAEYGGNGSHPAHRQGDAPYILSTPSAPGTRGDKQRDVGESALIGCARLLSELLQRI
GEGAAKVFSQRGIVTHMMKFFVHVPLSKKMRQAFSTALADVMGYNAALLGEGVSASRSLLLKCSEEGRVFS
VVPQQVHLVSVLAAAISRGEYGARQEEVSLFQRDGDAFLSDLICTCSALAGDATGEQDIRGIRLTGCLAYF
DLLKVVLRSCAESKAMCRSRHREKFMAAWVRTASSLHNLRLEMEMPTSNPGGSAGPIIGSEETWVFVLVDV
LVEVAATNSFSRSRETFFGSLKCHGTIADSVIYPKTSSLAATFSSLAPSMSYLAVVRPTGRAVESFPSFWE
TLMTGESSGVWQLASLLFDCVLTGDGCSLVAEALLCVNSGGDITDCIYRYVIFTLALLCTAAPQNAKVLAE
GPVIDALLACIASNEESVFSCAESPSVLSCTIHPLSARSIEASVALLNLLTSLHANERVMKRLMDGVGKMC
SQRVLAADVNIVESFLHVLGCTSFPRGALHFSGASCIRCSLDWDRFGSLFSTYTCVSWLHPKCVWREGSPL
FCCEYAQCGVSVTLVIVANGRLCGLVVRFRIFEEVTDVKVPGVSFQADTWSHVVFTQHPAGFTVCVNGCRA
EVRFPHDTYEAVSKEKVVKISLGGVLGVPSFFGFAASMELLDRALTMEEVMKLYDLGPKSLSEAAASCYLP
LNVLCGVSFGEDVPIIKVVDEIRQRPQNLSVSNLTTFLPPNMGEIFAHHDVAGWAVRTVAGAGAAAESFPV
VARLCVRFLCTTMKLSTIDGGIDRIIDGGVIERLRGALLSWRHMPVDVPALLISCTIPRGGKIMRNHDTTQ
SILSLMLDLVDGGGLGPHDASCLLRELSDTLLFPENVAIFRLVPGRFERLLNMSVSLPLECVGNLIVLVER
LCKEPREIEQVLKFLLIEAVSKTHERVKAVVLHMLFDIARTDTTVCDLIETAFDNTGASFLILLAGGKSHG
SEVIRVLALRILSLMLHSREDSHKKFINSRGYEVLAAVMAGPEAACVPIGMATFNCLFQMAFGAFLPAASG
GLERARQIRTASLGSRRDNLPHEVAGVSTTEKRLHVVGSQGYLPCLIHQPLRMARRGYGFGSFCEGSGGDC
SQYQLCEVHELSRVRAYSEGVLREPQVIYPLLRLLERLLQNMNNHMEESCEDILDAAVVTGEFTFAVSGQG
NVQRGSSFTGIPSSSSVCGERESPAVVALRVLTYIEKIVGCPKSSEMLLTFPWLTLLWDSIQHVMASPTSR
SSYTGEGLLASQRPRIFAGIEKRIRRAATRLAIRDLGCNSSAGVVRDIVQGRHPTIFRRIILEDIASHFAL
NHHGFSNRAEAMNIVQNLDLLFRSIEDILSPPPVPLVLSIVNCINAIAVRNDSWVRMRMREYSRLFETRDH
LSYMLLTTTEAFGKLKPVALRQVLEANENRPRTIRILLFHLGNAVTRGDTVEVGVLLLMIWHLNKVDKSNI

HALHSIVGEEYARFVDNVCLRMDTTLSRAPVVAEGASAYPCVMREATTNVYSDDAAMALVREVSASVVEWH
REDSDRWKVVQQRLVAAPECVGSGSVGSEEDAFGCSSDITGVNCAVQSSSNWKIEILGHLERIKREVNTRV
TESC
>tr|C9ZN46|C9ZN46_TRYB9 | DeepTMHMM Topology Prediction - Predicted
Type: TM
MIYHSETDGPHTPAASKSAGSAVWHCRLSTVMALLLFSNVLPAHSEGNIKVKVYSFLSLQRLPLRLTEAIN
AGLNASFAARQWTVAPNVTVQVVPPPPNNVSFMETLHDTINQNKGKFIIIIGPMGDVETLHALPLLEREDL
VAFAPITGSDSVRGWNPNIYFIRASPTAELIALVRYAVSQLRLLRIGFMYLQDISFGDSEYKHAVELFSHM
GRELCGVFTVKSSMEAFADDGAFEAAWEAFAKTRPQGVILFAPPAARDTVKFITKMVADKRTRDAYVLAPS
ALEFVVEVTWRFALAAAGKQLKPGQVILTGTNPLAADIRYQAIRRFQDHMRSYLSANPGVTVFNGTDNFHH
DDVDGELMVYGWIAGEVLSQALSSREWLTSRKAFMESLYNQRRYVIDDLVIGDFGGDCKGGAGERGAACNC
NQGGSVVYVKQFAENYKLLPAKNPVKVIQHGMCDADGIILYAPLNGLFILSNESHRKKKINREIHKSASAT
NRNADMTQFHRLFFHSMASSSAESARTLQHQLDTRSVTAVFGVVDDAMLSIAEVAFVDPVMLTPRLHHRGK
NVIQLSPTLEQQLFVVVGYVTNTSASAPMSAIVRGTDATIIEVALRKIVWMHGGTLQTVAVLDDNATLVGR
LPNRGNAFVIGLAPGDPSLLAAHLDRNPDVRVLIPFFDVALMYDELVSAFNGNPNAERVQFATSLPHWADA
NTSSEIVREFHTALPDSSAWKPLPLLGYAAARFAQAVLPRMEYVTPKTLLDTIYMQSIITADEMRYGPFEE
EEEKECFTANDPVPEQGEVCVVNYGATRISMWSLARALNASVPPLTSPVTPLIRYADPNAIKLSSAQLAGV
IVGSLVALALFAAPLVVVLYVLRRGARDNDSAPKEPMEPVTLIFTDIESSTAQWAAHPELMPDAVSTHHRL
IRSLIVQYGCYEVKTVGDSFMIACKKPFAAAQLASDLQRCFLRHEWGTTAFDDSYREFERQRADDDNEYKA
PSARLDPEVYRQLWNGLRVRVGVHTGLCDIRRDEVTKGYDYYGRTSNMAARTESVANGGQVLLTRAAYLSL
SNSERGQLDVTALGSMSLRGVPEPVEMYQLNAVVGRSFAALRLDHEVVEDGDLSSTSFSDTGSLRGSINAS
AQKFSISIKAVFGAFAPAHQQQLLMPLCERWQVSLPPSSKATWNEEYCEGVIRRIAVKVGRVADHCAASGS
EHSVSTLGSASLIIISNHGLERELHGN
>tr|C9ZNS1|C9ZNS1_TRYB9 | DeepTMHMM Topology Prediction - Predicted
Type: TM
MSVVLADKVVKVKEERKAGRKTEVNTQSQMSTYIYIYIYIYASMCMRVQKYILKKKDVFSFRSCRLNLHPL
VANYFLFPFRLLFLSASATQLLPTPIPNDDSTHMRDVGEMKQ
>tr|D0AAA3|D0AAA3_TRYB9 | DeepTMHMM Topology Prediction - Predicted
Type: TM
MGGSSSREMMNPYAVNTPLMGICLASIMFNSVQGRTLRSSNVFNNLILIYALGFSTGLSTVMQQPIWGAKV
GIAAALGFTFGPNLRLIYLQRLFPDYVRYGIGSVYIAYHSLQWYSEVHAWEDAMEDEVAE
>tr|C9ZNK0|C9ZNK0_TRYB9 | DeepTMHMM Topology Prediction - Predicted
Type: TM
MTRHIYGAKFFLKKRRGAHSSLFFLCFVLLFFFPVLRRHRPPPPLTPLLSFFSFFQKKKGNFPLFSSHHHN
FTPNLTFSFLLSSPPFCFSPLFLSLHKYINMLTQVHPL
>tr|C9ZN69|C9ZN69_TRYB9 | DeepTMHMM Topology Prediction - Predicted
Type: TM
MTPAASRLRRALREARRLLEEVKLSEDTAVGQCNQRQVTELLKYIKRIGFLLSLVKQEMRANTDNSDKRET
ATTEVSLSGDDVGNAPTVVDAAEAFLSQFLDSYFPHLHVPVACVGLVARHRAPKQPFAVCEPFLIHEDSNA
PCVRASIQLEPSPTVKSSTTPAKMGQSGGGDIAITNMAARSTNASSLITSSAFSSAPQDADDAAQKQVINE
INDAIREIKEGALRVSDIMNQEKGRLEENAALLQRGVDGTTSQCKKMDRVGSAFGGGPVLPHFLRALPGVE
VFWDSILAPLWRIIAQAVFICAIVAVTGGTVMLMLVTPKSYVYER
>tr|D0A235|D0A235_TRYB9 | DeepTMHMM Topology Prediction - Predicted
Type: TM
MCALSREPSVLLPPHAHTHTRTHTSYKHKHHVVTVSITSPTHSAAMEEGKEHSYLKKQTNKPMITVVSSYH
LTPLRMPSTSHWRLWLYLYEVCVCVCICVPVVPAFSHFCNYLRYGLLKKDGGAQQPDKSNITIPLIISLST
SFRSHAHTHRRIHTRPSATLKVAVNIWVTGRTKREDKSSY
>tr|D0A204|D0A204_TRYB9 | DeepTMHMM Topology Prediction - Predicted
Type: TM
MWLNTRCYLLNSPLDFVRAAPIGAAVVERRFLLLFSPFPLRIEYFLVFLLLYYRFLDGACCKESGKVPTTP
ELTQRYQGLKNCKTSPLPTNTFAAASHVFCSLKEQWAVGAVETPVFNDIRQLK
>tr|C9ZWI5|C9ZWI5_TRYB9 | DeepTMHMM Topology Prediction - Predicted
Type: TM
MNVAEEGNSQLLQQDEWCEPLRLKHHRDSWTQGGFSSCAVLYFFLSPTLHSLVLVVITLLGMLRYNVFHHG
DFKKVLVANRGEIACRVFRTCREMNIRTVAVCCEGEPNAKHVLEADEAFVLGPPPASTSYLRGDRIICAAK
KLQADAVHPGYGFLSENAEFASAVLAAGLKFVGPPPAAMLSMGSKSESKRIMEAAGVPIVPGYYGEDQNPD
RLLHEAKTIGFPVLIKAVSGGGGKGMKIVMEETEFHLMLESAKREAINFFKDDRVILERYVMHPRHIECQI
FFDSFDNGVFFFERDCSVQRRHQKVIEEAPAPGLSVDMRRRIGDVALTAARAVGYVGAGTVEFIFDTEKDE

FFFMEMNTRLQVEHPVTEQVCQVRGRPLDLVRLQLQTAMGLPLGFRQEDISMSGASVEARIYAESPRNGFL
PVGGRLRYLKEPPQGNRGTVKVRLDTGFRAGDDVLVHYDPMIAKLVVWGDNRAAALEGLRTALASYHIVGV
ETNIDFLQRCLSNPGFVEGGVTTRFIEDNSVNLLQPREIPNNVLALAAVSYLCSQRGASTLFWPNRQISQG
VCFTVGGNPVVVRVTVSTKMCFTCDFDSSSVTVYVESTSNMPDSSTFIRVTVDGETRFGFTSFVTDSEVAV
ALPQGFYTLALQPLATDFGSTSAQANGSASVLSPMPGKVTKLLVADGTLVRQGQAILILEAMKMEHVVKAS
CDGEVKFCVHADGIVGGSTLLAHIASAAV
>tr|C9ZRQ8|C9ZRQ8_TRYB9 | DeepTMHMM Topology Prediction - Predicted
Type: TM
MLCTCGRFFFYFVQFLSLSIQNTFANEGGEWEAVLRHIIPSSSNERDRGGATARWAVFIIIFFFLGGEGLG
VAAAWRNKKASRVLANFHYKQLFADGTKTRFFFFMALATRVVVDIYLHVATGRL
>tr|D0A0Y0|D0A0Y0_TRYB9 | DeepTMHMM Topology Prediction - Predicted
Type: TM
MILLSTFVVSVFVYGLSVCGVCDAVNVHPWGRHSIRGTIHIHILRGPRSQFPFFLVCSSSVSRVGIYNNGA
VNNSFNKVRSFALSRLLAVLKCTFLHVLTAVMFNTLLSVMKEEQHFLPMNCFLVLSNPLQMQWHHWCFQLP
STRVFTYLQSSGTQCFGEFGQSTIFFMSLDTATLSIIQL
>tr|D0A8N6|D0A8N6_TRYB9 | DeepTMHMM Topology Prediction - Predicted
Type: TM
MLPISLGRVHSHVVSVCVYWWWCFTARGDSSLPLLVHPFPFFCVFIRSSIQVLSFFVLFFSFLLQQLTTTF
FHFSPSSFYPSPVIPFLCSFTSVCFTFCVIFLFFVFVLDCSCFFVPSLLHSSLITFFSFSFPFIFHVSFYH
ISLSVVAAPLNSKQSGGDYFSRLFVVRATPHTLPKGEKTVIVGVGQCGRKRRKVETPEVRRNSNRDIYIYI
YIFGRGEELR
>tr|C9ZRY1|C9ZRY1_TRYB9 | DeepTMHMM Topology Prediction - Predicted
Type: TM
MYGELGIHQKEGEKSSKEVNECMCGSMEGRWKKKKGEINIRMSVPLLQEVRFLNTEEIYFPPPLFYFVVPQ
RISAGYRPVFVACFTAASSLFFFLYFIRCIGLSLLSFVPRLESLAITVSPL
>tr|C9ZRP4|C9ZRP4_TRYB9 | DeepTMHMM Topology Prediction - Predicted
Type: TM
MKNKSSKQVLLPLQGNISPSAESPRPSSLMTTEGRTRNYKDLINRTVFSLIMAYFFLGLVSIGAEAIIFLL
IIILCLMFHEVSRINQRERKNKQLPSVFIMKVWFLCTTMFSMTAYSIRDPLVATYPGAMRYYRNAWMIAFG
FPLVGMVGFVLSLRKGMYRYQFMQLAAIVMTLLYVTAQGYAQISNVMRGMLWFVLPISCVINNDTWAYIFG
KLFGRTKLLALSPKKTVEGFVGAFVFTIIWSFWFAGFLSYFPHMYCAKTDFHSAFHCEKDPLFVKRDVPMP
AFVQALTFNRLTTIRCARVQQHALVFAAFASLIAPFGGFFASGLKRAFKMKDFGDLIPGHGGITDRMDCQG
IMGFFTWVYLQSYVYRDENCPSWHTISSCALQLPEEQRRSLLSTLNRSLTE
>tr|C9ZZL0|C9ZZL0_TRYB9 | DeepTMHMM Topology Prediction - Predicted
Type: TM
MERIASGHRMSMLLLGVGLLCLYGSLLVNGFAFDLPANQKRCFSEEVPSGTELRISYAALPGYAQFVDAYV
VGPAGKMYMTTVGQDRGSMVEYITKGGEFTLCLLSRVATGVKQSEGMARSVSVDFRLGSGKNDYANLTGKE
KLRPIEVELRVLEDAVRSLHTECLYYREKEAEMRNANESVIAKVAFCAAAIITFFIIFSLWEMWHLKRYFR
KKRLID
>tr|C9ZNU5|C9ZNU5_TRYB9 | DeepTMHMM Topology Prediction - Predicted
Type: TM
MALPTSHVVQTPKRQEYLASCLSGCVAGVCSTCVINPLDTVRVRLSVSRSATGKAHRSLLYTVRDLFEGGI
VHAFSRGLSANLMASLPSNGIYLPTYRCIKDQLSSAGVNQNVQPAIAACGAVCVTNTILGPIFLVRTRVQV
NEKLTVRQTFRDVLKHEGFSGFYRGTMTNIVGRFVEEGLFWSIYELLKRLSSEASFKGSSNFFLTSVAVAS
LSAVAKIAATTVSYPYNVVMNHMRSVSYVTGKPEYERIMPTIRHIYYQDGIPGFYKGLAPQLLRSTLSKAV
QIYSFELAMFIYFSTVQRPVVSCAPA
>tr|D0A051|D0A051_TRYB9 | DeepTMHMM Topology Prediction - Predicted
Type: TM
MVTGGRSSFAVYLYYAKVLRLLDYLVAFCFGATGFLLGRLIRPYCRTFSWDDLSISNSFAKKETFPDWSLV
VMALLSCLFIFIIEKMRERYSPVGEWYMDEQLPEPSSNSITSSTNVLSEERAANRGGASVRERNGLENLPP
APSFERKVQRKFIVDQVINLWILSVVFAFFFSLGIVDVLKIYAGRLRPDFLDRLKSEGYNATSSLVGVCDH
AREGRLSFPSGHSSCAFAAFTPLTMYFLGLSRAFNSGPVWRIILSMFPIYLAICVAASRTRDNRHHFSDIL
GGSVIGLVIGAFSVNIFFRVRSGEGYLVPRRLEPPRRRSGSPNDNDTV
>tr|D0A575|D0A575_TRYB9 | DeepTMHMM Topology Prediction - Predicted
Type: TM
MSVYGNGGDHEWTPSFSPPLYVQRAQKVQTLLRENGCSSFIDAGCSRGGLLRHILTSQLQEHSFSRALAID
LDKVALHEAKEAITALGFSSPVALLHPMHVEFVQGDLTKPPVFTPFQEDKEREREIGKEATQLGEAVPRTQ
LSHQYDAVISIEVLEHINVRDVPLFTEVLFAHLAAACGARVVVITTPNRDRNGIGNAKSSVTGQFGGSIQP

RLNGAPHSLPGLPYNVRHEDHKFEMTAAQFRRYCDYVIEAYHPRWVSYTLFGVGEMFTQGSIFHAGPDRVA
VRRQKVLPSLPVTLDALKETRFPLSQLSGVPPLTPSDAGGSEINWTHDMLARGRLFPWEEVFGELSTRPDE
DMLCCIRTTSPYCSLPPVEMPYKPLWNRMGEAVRGAFAAAAIEDEAVQEHDSYLSFADVSVNYHYRFFAPF
NASLCALISCMLRKVHRRNGIWNPNDRPARPLVRCSESASGYVRLVLAWILYDCWGVDALKRGVGDDSYGS
ARRTLSSDEMKVLLFLSSLGFFPGSVHHLRSVLHRGRLLTGSGRRRGVGGNRRAASATSVCSSEIDKANRE
KIRWLSYALFQHGVSARHIASCWNNVA
>tr|D0A2P9|D0A2P9_TRYB9 | DeepTMHMM Topology Prediction - Predicted
Type: TM
MFGEGIFQWWVPKEPKEKKKRKRGTILPFPAAGGVFFSIVIYLHFCSLICHSFACTHLRVSLSRCTVKQAE
RDSELRRGFCVVYSFLDPSSSSSEGFCLFVFNVLHGMWIPVADVATCFPSTSSLILSPPRPCVSL
>tr|C9ZTB8|C9ZTB8_TRYB9 | DeepTMHMM Topology Prediction - Predicted
Type: TM
MYCDDMRCVCVCVCVCVFVCVHVFFLNFNIKRMNVWVWVSKKKKEERINKKKKGKRKEKKEGDSKTKETND
GSGETNVTIENKKNDFFLPPFPFFFCFVFNILFLSHAVLHVAAAGMQGEAGGGRRE
>tr|C9ZNU3|C9ZNU3_TRYB9 | DeepTMHMM Topology Prediction - Predicted
Type: TM
MLLIPTCTGVPFPVVVETYCDCSLRLRKSTASPFFSPLFPPCLLSVFPLQVLTRFTKEEDIFFPISLWRCR
VRRISRHRRKKKIVTVIMQSCAVIGCGAAGMAASMALRRSGLLVTCFELAPDPGGVWNSDTRSSFSTRGLI
SPLHPTLRCVLPKDLLAFSDMRFDYTVPQFPHHSSVRRYLDRYAASKGVGGLVRFNTKVQSVRYDATSAVW
HIITVNVVNGDVFEWAFDKVCVCTGQAHEPRYPEGLKELLVPYVESGGELHHAFHVKDFRQFKNKRVVVVG
DDVTAWDYCWDLKRCGADVYHSGCSFPDEGHFSDSCDESGLIKGSYGTNMRDIVSAAVQLLSRTPGWRKDV
ARGNKVVSNWLRFRNGAILGRLSNLGQPIGCEGKGILFSNECLGKVEVLKEMRACSMAGKALGYSDLPQSV
FMDNVDAVICATGYHRRYPFLHEDIREVLEKPTCFLPQKGNKCADDTSANATKAVDHRSLYLGTLFAPNPS
IGFVGMQKELLPPFLLFEAQSKFVAYAFTHRLNLPNDAAGLLARQEELMRRYPLLANLYAPYGLGLYSALY
FNVLQEELQVGARDTYTSAIMERQKWILLTGLLRLVHKARSLAPLKRKQQHILFSNTV
>tr|C9ZYT4|C9ZYT4_TRYB9 | DeepTMHMM Topology Prediction - Predicted
Type: TM
MHLITKKRPTRLGVKNVSGRPSLCLGLTCFSGILIDFSFSFLFAFFFCIIKVITRWVKATSRANMTTTPTG
DGQLFTKPDTSGWKLSDFEMGDTLGTGSFGRVRIAKLKSRGEYYAIKCLKKREILKMKQVQHLNQEKQILM
ELSHPFIVNMMCSFQDENRVYFVLEFVVGGEVFTHLRSAGRFPNDVAKFYHAELVLAFEYLHSKDIIYRDL
KPENLLLDGKGHVKVTDFGFAKKVTDRTYTLCGTPEYLAPEVIQSKGHGKAVDWWTMGVLLYEFIAGHPPF
FDETPIRTYEKILAGRLKFPNWFDERARDLVKGLLQTDHTKRLGTLKDGVADVKNHPFFRGANWEKLYGRH
YNAPIAVKVKSPGDTSNFESYPESGDKGSPPLTPSQQLEFRGF
>tr|C9ZKI1|C9ZKI1_TRYB9 | DeepTMHMM Topology Prediction - Predicted
Type: TM
MYVFSPAPQQKDPGGGVEVKVKSSDTGNGGQCSDAQWKVNFLSCLRGPAQTCRLLTCLSNTIVDVASGQTK
VGKTANSEQKCISSSATDPVVQESYLTELCRYLAPEASSSDTGASASAKNETSLLSVPKGEKGRMLYSTFL
RMAEIPYWRQTLLGPLLRCWCREGCDELGGTFLLEAIVNAIERELCSLNMLEQRHPIPEDASGGPELWDLS
KYDKAEAQQYEVLINELEGVVRGAGSFRGICIVMDCIIRNSIQRLVEGVHYVVCGMSARGGALPTGGVRMN
EEVVRVVDSVVALVWREIGSFFQRCEVDVEAALSETQWLFLMLLITGYKGAHALLAAPPSVSSSSASSNTD
TSTTTTITDRIHLRARRMFFEKLQSNLRDYLSMRLLAGGDPASNSFASVHTVTCHQPFFWRCTLEAESAPG
HLPPALSVNIVIKEWVKKEAATAWGNAVRVVRCFVHGQMAVEDVPLTAVPAVSESVISSIQRCHRQLVEQH
IEHLSASQKYFFSAGKRPREDEGKHAMDGGSTWLSYMVEFFFCVYHPSCFSILSPVKQGEFVSLLRQLHDM
ILYKGHDALEGSIASLLPGEKASEVKLRDELMQWHTQYVHTRVVNALRENSFLVMCLLHGVFALFSRAGRT
NIQGHEVLRCTLLPILSILCSPQDEGEGGEEDEEDVTAVEENATTDETDAFFVDRQERQQCMILLVTSGFM
SIPWNLRGLRIRLLLFGILGTRLRDSFGSEGAASELYQLLSEVVSGQRTPEDIASEIFGDCTGDPFLSLLV
SEREDTDGGTVVDAMEDILRRDVPVQFPAPCGTLLLMSVFRTLSQELLRLRTEWEQQIKLLSESESDYLGR
QATFCTTEDIVVFRILVRVIHHMVFGFQVQPLGLLRLWISFLVHLFAFIVPPLTEDDELILLAPHVLERRL
HKQQEDNDRKLNDSAGAISSSNFRRSANESSQGELRAALGELLADLVLMPLLDGEVAKQCPAAIPSHPGSR
MWVKQNIRRSADILLLQRVLPAQVLEELCLALGVNVSAMNDKGPNGVRATRQVGGGVGKSFLGVLGVFAFC
QRLDHFVVPGSVSDDGVALHLQALWHAVEVGYASSAV
>tr|C9ZKX2|C9ZKX2_TRYB9 | DeepTMHMM Topology Prediction - Predicted
Type: TM
MGASFSLSRARLYFFFFSFEFHLYLQGNFFFFSFTARGLRLLGFFFSLLDFTFAFSFFLFYLFCFCFVHQQ
TFLCLYINTKILFLFSFFFIFSLFFLFFFFESSK
>tr|C9ZIS3|C9ZIS3_TRYB9 | DeepTMHMM Topology Prediction - Predicted
Type: TM

MNSGSIACWISHRFVAKYLHVNSPSSRLFLFAKSMSYLISPLHLNAFTTGESARLTKGAFYLRTWCRWLPF
TYHFSCFSYVNIGCNMFLFQPAPSLPLSGHVCKSSKALSREQPPHAPSTSLAWRDPFTSVTPPFLFTTDNQ
PLIIFILIAHFSTVARFVGTFFAFRCTVYTYIYIYIYIYIYIYIAQYPMYVDFFEFSSYG
>tr|D0A8F4|D0A8F4_TRYB9 | DeepTMHMM Topology Prediction - Predicted
Type: TM
MDGLYFTAKAQFHQLATHISLYHEDASPTYRTLGEACLQLAGLRPDRFTFWNVPNMSGYFNKALPLDIHGG
YVLVDEAAVKAAAGTYGVLRYAYLAAAVRARAGGRWRYDFTTMNAALCMGVASGFAVLSVGRRRWPLMRRR
PVGAIAVGVTTCFVAVVATRLLLRAMGAGITHARNSNRRALEKLRCVDCYDDVARYTEQRKEEVEAQRVPQ
PQPGMPPLPEASLRQFERLSALQVQLLESNLCEIRLAKRRANSQLCDVHRGLRDDEQYAVSAGLPIQYADV
ALARERARQLPSGG
>tr|C9ZY48|C9ZY48_TRYB9 | DeepTMHMM Topology Prediction - Predicted
Type: TM
MGEVCDPSFSPLCGGNVLCLRFGICTNVLDPSAQLFLIFPYFVIPSVGLLVIRVVFCISYLLSPFQCVQFP
FTYILLSLYARGMFFKKLCVIFFRFWLFSPIPWSVTAIITSIGCSHWKKVEVKY
>tr|C9ZLG2|C9ZLG2_TRYB9 | DeepTMHMM Topology Prediction - Predicted
Type: TM
MTLVADSDYPRVISALHGLETRQIRVGKTVTAAFHQITDAILASHPYDRIVVESGTYFENITIKHPLELCG
AKDAEPPTIVSVGPCLVVDVDGPIILHGLNITAKGNRASENQAVVVKRGNPRIEECDMTSLYVKNDSKPHV
SHCRIHSSRHGVGISVIGNGSGIYEHNHIFGHEGESLYFDTAGKPIVRHNRISETKGRSGVVVHMSGRLSG
TTEVLFHDNIITGGGEAASNSQVDQVPTHSLQVLLKRVAPEGAALVRVVHNAHPVMVSNMLSNGVTGFRIN
DCRLSPECFCGNRIGNCSAWGIVVGGDSTVTVDGNDVQGCGAGIYARTTHVREESGGGDGEDAIGPCVSIC
RCNLQWNSYYGFVIDRSAVKVNECNVAESSTGMAFVGDCTGSVVTQNVLSRNSVTGFSICNHGSVVIDDNL
VSGFNESMYGIFIQDNSNVVIRGVKVSRMATAITMSGGSRLVLENSSIKDVSVHHIVIEESSQATLSRNTL
KGSGGASVVVTGRSNCHLVGNSLLISQKEGVLVEEGSRATIEKNNLGSMREVIYVKKQSTAHVRGNDVAIS
RYGVVVAGPGSECTVVGNNFRGLTGPGVYAFEMGTATISSNHMRDCSATCIQAGTGAEALIEGCTFTDCRS
GVVNADGAETHCRVVKCHMNRVGHGIQYTGAAKGVVEENSINSCRDFGVSCESHTPVVVRKNTISDSAVGL
NLCAEGTLSRLIISGCKTGVLAKFGASDKVNDIQVLNCSIGITFTRGTMVSLDTVKVNACQKYGIVVAGPS
PNATVVQATVEKCGVAGVAILENGACSLEQCVINRNGKGVLLRQPDNVSFKECFIAQQHQGVVAIPGEGER
IRETSFSRSVREGSALFLSCTIGGDECKEGVVAGDCDTTLYFEKCRIGAGIAEKGVGVVVKSGAVVRMNRC
DITKCSRAGFLASTAARLLAERVTVEKCAIGVLFASPADEETMAIQELDSEAQALVEECPTETHTSGGSNK
IIDVELGGSILRWATFRDLTISRCENAGVWYMSQSVGEITKSTVQECTTGIVAEHGSRTILQAVQVSGSRG
RGVLLPARLSKEAEVTFITIQNSESHGVEITADEAGDESDDVALVKVKNCEIFDNKGSGIVLQSSVTLESC
CISGNECSGILCSGNDYQGNLRPHISLCQLENNKEANVVAAGGCIPELHSCVLEGAGVGVRVESAVSMVKC
ILRYMGVAAMFTHPESSPSDSGFIDSHLSRCVLKDNGIGLSCHWNAPASDESRILVENCMFDNSTKASVEV
GQGPLVHIVATTFETSRKAICVEEYGRVDAEGCVFIGNLQGVIITSPQYVEVHRSRFLRDGNCGITVGGDR
GEVQIRDNCFKDSSKKSVHISTENVLTCVQNNIFEQATSGIVMEESPRVYVYNNIFRKCGTAVTMRSPGCC
GVVAGNILEENTCGCLCETEAKTKVWRNEFSGNKKCGIMVTTGAHPVVVENIFKQQDSSNSRAVSVCDGGI
GHFASNRFIKNVCGVFLESTGSVVIVNKNTFDGNDVGVQIGKETTAHVVTCLFLNSGTADVCASQQIARGQ
CILAYNCFCSEAGTAVTLGEQAAVIIYRSLFIGSKGRGVALEGVSQSMVCESYFSGLSLGLFAGERAGGRI
VNCIFLRCLRAVEACQHAKTAFHDCFFFARSSAQPGMVTLNKHAEPVFSHCEVIGTDKSSSPLLCSVGNGV
VEECLFSSGNTSVSLGSECGTKLSGNKFLRGIYGVVLLAECAPTMDGNTFDSHDKAAVKIMSHAGGVMREN
AFVQPIENGGILAGANNVVIENSKHIEATSIGEKSGKRVGNHAAVEERFLSTLAKWLRAAPAWTRNDLRGM
PSAATLPPVIRESLNTVEDDEKEGDQAEGGEQGGVQPQMSRANRGTDEKTPGGEGAQADVKQKRAPVVGRG
AGRRRKIPPYEFTPDNDISRTPEDIVNELQSWLEGSNITLIVEEENRPTTGSAASQTYNSNMLLGVDEGIS
QMKKLQSDKKESHQLTSDREEDQGTWMSNSHVTSVVGSSERVRDPLDKHQDTASGWGPGPDPSADLGASKS
EDETMLQIPLEEHLEAVTEDPQLSARHVEKGLERAPRRPFPPLYSTNVEVQQRGPPRRGPPETFVRTMGLS
TVARRNREADLRRRSSVAMQSKKRRRTRRKSLAAINVGRSPVPGGKGKEEGTTPAGSERPRYPTPSGGFMG
IEGDSCSAPSFDPSHLSHYNGDAEELSGTTLGNDIYLTEFGVESASTRRGMPEFNGSCESQGERITSGSRP
DRDLLKNVTLNFSLLGQLSFSRNSNRNPSPLPCGEFLGADDHRFDGKPNKNVARSAPVDLAKNDSKGALGT
GSYQKGPKEHVGLRAMMMDQSGDMKKQLDSTRPRAVRESETKRQLLPADSAGEAMGARTAPSGRTGREGAP
RCSVDQFVVHSGAAQQTKRISKKGRAEKKFVKKKVYPVSGTARRGRKDNNYSAEETQTFNKISRREGGKEN
ENMLSEKTAEISATVGDEGAPKGGSTTIAEGPPTRASTSRPALGEKQLHANASGQPSKVSLLGITKRPTEP
GVEIPHDGLSGWTSTAASESHTCGLSVGSREGPRTGASGRAVGNTTCAGAADGSLLLNRMGHAMSSRAVTR
EGEGKGGLSPSSDRAKDHLVKKSRTIPFSGEKGSFASPNERSQDCARGNKNVTVPQLSIGVLRDKERSTPS
RREADAPSATYPKANREQVTHDAAEPLKLKQAGKLRNQTCGEGKGDPLYPPGDILSRSGESQGWESGATFW
KPVGQLSENGRAMLGALQNEVFAGEANVFDPPWGDVTGPKETEVFSLATPTTARTETQAGEPCGSSSEAGE
LEASVEDVIALLRWLADMRQRKLMTREGFSRAVDCMKETSAVPFFFCENSAAGGPTVMVRLPKTADQVVVV
PLLKPNQKIEIEVVRNTHILPPCMVKPSTVASPKQEEKVNVVRINTCVALPSLVKKIEETPRGSSPPHPVS

ANMSSLSTGYPRQSPPNSPGASVGHFATLESSHSCLGWRPNVESEEKGVKSARTAYTASKQQVTASSGFGR
RRFAAAIPKDANRTLVPFNPADPKGTSPFINAGKVATFITAGTPNVIL
>tr|C9ZPZ8|C9ZPZ8_TRYB9 | DeepTMHMM Topology Prediction - Predicted
Type: TM
MSGSNRKGNVSGGFVDVFSTESGMLYAATEKELYTVTATDTSLSVTLFAGKNTSSCYSHANGEDIVLCDNS
RLLVIEEYEMYVTSKEKHTMRALTLPPVNLTAIFRGRPAPVGYPNTTIMEQFVASLTEDVNKALGTNDSYV
DPDSVRVDPDTWETNYTVFVQQTRFDNTTEEKLRSLTYTQTDKTVDEYYGLTDEYVYIDTVLVPFCDDASL
VTIQRALAREAGRALNFSLIYADKPITFGSDVAENVTAVKLLMPHSFKNATTPKQLSAANLTDFAHNLVKD
LRASDTRVDITFPDPPFNFSAVVPEREQEVRWFVHGKVMKQLEICERLGSQGDAAVIAAAADATARGKANV
TLNTSGVKANDTGVGPNTTNTAGGANTTANVATNGTANVIVNPSTNATPTGTSNASATNTTERAVPVVAPT
QPSNGYAECRSAITNRTETQNMEPPYDRKHRYEVFLPKKYDFNVSWCVDIIDWRDLDEMLNNRTDEVVEKS
LSWCGHGCIIAFAVVGSLIAACLVVLAVVLTSKRRRLAAVVAPPRPKFVSTVEDDEEDRVSNIGVPLTDGK
GTTAP
>tr|C9ZV86|C9ZV86_TRYB9 | DeepTMHMM Topology Prediction - Predicted
Type: TM
MYDPAPLPFDVATVHLFLSSGHVFVLSYLKYRWEKPFIVFFVSAVLLCLCSCFCAFACDCGAGVFSFFLSL
QSIHSCAITSFSVDFFLFRGFVFLLFPLPVRMRAWNIHK
>tr|D0A1C0|D0A1C0_TRYB9 | DeepTMHMM Topology Prediction - Predicted
Type: TM
MAEERCLEGPMEYFFAALERQFPKQTALIGGAKSLTQAILRALDVLYPFVEALFVRFMDYAKSLSSHELNQ
ILPLIAGFSICFFGGCFFTLFSTVEIVYLTSWDRIKKSAEVIHRNYVAAMEASRKDDAPGYGYTVTTGDES
LSKNELLSRKLRIFLQSVDPVSVKEELAAVALAFMAVVAALRDRFAYYVTLGCILADTGPRFFPLKAILEE
SLPPELQKLAGPFSNFLFGALGTTIAVLASQYTITLHCATRGSKMLVDSALELAKTRGIVVQDVKVDDSWV
KAFAFGLAVIGFTWQAANGFSLPFPLNLLLFPVTVLEWIVNVIFRASGSAVWATTLSV
>tr|C9ZQ64|C9ZQ64_TRYB9 | DeepTMHMM Topology Prediction - Predicted
Type: TM
MIKLVSGVVNSFTQLDNALLEELMLKLTEDRSTRVVDCLPGILKELSDHVRDPVQECQEQHKQLCSQQELQ
QQQQQRFERRENTPHASEVTRLLNLAMDSGPGAVKWTAVESELSNFLRSLMAGRRQVGLKESASQTVQALG
PLLNVLTSTADTDGSEVEKDALMPLSFYRGFYARAVSLLLHLHLSGFDGSDRIAWLKRCGWKVELYDILLP
FIQKEVEPLEKELVYHFQEGKNTARREEPQHFFLFLQQIYTRLVNDARAHGWAPEGIGEQDGVNTLIALGS
AALAATAVSVFRSAYGWHEESGYLSVKDFTIHGVNCLVDFLTKSGGIVHLGAQAGIVERLISPDIFCTYAD
AARSFVCKAFASGHQVLWRLEFLTNSPTLTDQTATLKAVRGTYHMLRCLEAILRRLAVVFALLPACVIITW
NSVVVPSLVMFMQHLEDAKEEAASLCETRAELIITSFHLLDSVQAVCSAAEEWRERFCEHCGEKDVSTAQL
GKMTRWREKLRREVTESTSQFLAHLFAVEEVSPRGLHAWDGILQGLLHGPSTGSNIVQEVMRTSIPCLIPK
EKRKHLKEYCEATGANALANLLSEDVL
>tr|D0A5V0|D0A5V0_TRYB9 | DeepTMHMM Topology Prediction - Predicted
Type: TM
MNNGSAMEDGNMLLLQALFPYAGKKYFAAALKFNKDVEDAVRFVHSKVDLAPHCGKYLIQSVEDTQPVRRC
NEQEETLSAEAAAKIEKMKNKPLSNVSQHDAYNEVAIAFPVDTSSWDVKFIPPFVAPHPAASQPLLLPEED
DEMLRRGGPTRTPTLPWANITALREAKREEEGSTIDSKPQQTTNTSGNAQRLKAMPKIGVARIGVHRVGIE
RNCATASEDGTKGLRFDGKEIHESCNISGSSVGCMDLLEFTVAKERECVNLKNMMRQWADVFSRSSGCFVL
REIVLKCNNMAPILPDLITSCFSLMEQELCGRELVAEPTHFVSCFLRREYEVKLGPIKMKTVELSHNYASL
EEGGKYLRICLNLRRIVLETLSYSYRNAKNKSADFTHGTVDLKVENCALKVDLIILLAGGGFVTIYRRCDN
LKIGSIGADFSGFIPNLIFFIIKPIIRRSLRNQIMAVMNERTFGKT
>tr|C9ZN44|C9ZN44_TRYB9 | DeepTMHMM Topology Prediction - Predicted
Type: TM
MIYHSETDGPHTPAASKSAGSAVWHCRLSTVMALLLFSNVLPAHSEGNIKVKVYSFIYSPYVEYRQVEAIN
AGLNASFAARQWTVAPNVTVQVVPPPPNNVEVVDALQRVATTEKGLFVVFGPLTDIETLHALPLLKREDLV
AFAPSTGSSIVRGWNPNIYFIRASPTAELIALVRHAVSQLRLLRIGFMYLQNASFGDSEYKHAVELFSHMG
RELCGVFTKNIDDEGLYGGHDFDFLWNDFALTEPQGVILFAPPGKDSIQFMKKLVADKRTRDAYVLAPSAL
EFSIVGAWREALEAAGAPLKFGQVILTGTNPLAADIRYQAIRRFQDHMRSYLSANPGVTVFNGTDNFDHDD
VDGRLMVYGWIAGEVLSQALSSREWLTSRKAFMESLYNQRRYVIDDLVIGDFGGDCKGGAAKQGAACNCNQ
GGSVVYINVIGSGYRLFPVNGGVTIFDSKKCYINKPRIPSPMSILSLTLFDTALPVDTYASMSEVLYASTR
GRESALSRQLFFHSMASSSAESARTLQHQLDTRSVTAVFGVVDDAMLSIAEVAFVDPVMLTPRLHHRGKNV
IQLSPTLEQQLFVVVGYVTNTSASAPMSAIVRGTDATIIEVALRKIVWMHGGTLQTVAVLDDNATLVGRLP
NRGNAFVIGLAPGDPSLLAAHLDRNPDVRVLIPFFDVALMYDELVSAFNGNPNAERVQFATSLPHWADANT
SSEIVREFHTALPDSSAWKPLPLLGYAAARFAQAVLPRMEYVTPKTLLDTIYMQSIITADEMRYGPFEEEE
EKECFTANDPVPEQGEVCVVNYGATRISMWSLARALNASVPPLTSPVTPLIRYADPNAIKLSSAQLAGVFV

GSLVALALFAAPLVVVLYVLRRGARDNDSAPKEPVEPVTLIFTDIESSTAQWAAHPELMPDAVSTHHRLIR
SLIVQYGCYEVKTVGDSFMIACKKPFAAAQLASDLQRCFLRHEWGTTAFDDSYREFERQRADDDNEYKAPS
ARLDPEVYRQLWNGLRVRVGVHTGLCDIRHDEVTKGYDYYGRTSNMAARTESVANGGQVLLTRAAYLSLSN
SERGQLDVTALGSMSLRGVPEPVEMYQLNAVVGRSFAALRLDHEVVEDGDLSSTSFSDTGSLRGVLSGTSQ
MIDSCLHAVFGTVPLSQRQKLLLPLCERWQVSLPPSSKATWNEEYCEGVIRRIAVKVGRVADHCAASGSEH
SVSTLGSASLIIISNHGLERELHGN
>tr|D0A3K0|D0A3K0_TRYB9 | DeepTMHMM Topology Prediction - Predicted
Type: TM
MVESNEKCSSVSGFFVQRNRLYYSYNLNPISILISQDPFVASMQSQKVIMKVIRTSIAYLDRRELYIYGRN
LPVCLRLCVQVLFGFVLCLYFRCVKILLVANIYL
>tr|D0A0L9|D0A0L9_TRYB9 | DeepTMHMM Topology Prediction - Predicted
Type: TM
MYVEVTKPTKVIADDLHYASLDIFAVCHLCFGRFFCFFSRSHRRNRRNYFRYSVKSYLEQGSKGNLYKICQ
VGSRGTWSESMISTNTAPSVREVKAKSENKYFCISGRVASPRYGSKDFGSFLL
>tr|C9ZPM7|C9ZPM7_TRYB9 | DeepTMHMM Topology Prediction - Predicted
Type: TM
MFVSLLVVIALPQSVVVYFSLMSIFYTYMGTPRWKCQPLKNMVRCCDRSFNHLYLLNRSIFVLCVCVLVYL
LPSNRLSVFYSVPVRDYRARSTTWGDRGFQHLFLYIFPKGLSHLFTYCTV
>tr|C9ZYS0|C9ZYS0_TRYB9 | DeepTMHMM Topology Prediction - Predicted
Type: TM
MSKTDISPKTTPIPLLPEPIFLYDLQESVNIILRKKKRKKVLVLNMKKRKRAATTTNEKTFKICLPCFTVC
HISFIFFFISSYTRKTKTNENKKKQTHSKKKRMRKQTNIHISL
>tr|D0A707|D0A707_TRYB9 | DeepTMHMM Topology Prediction - Predicted
Type: TM
MEMVVNDNDKKKKKISKTHVCTCTRVYIYVYAYAFHYLFFIFYFLLSHIHTHTHTQINKDVKTTSSCPVTN
HKYPLCTPKNYNHLTTSFHITTRLNPDFAAAAAAAVVVVVVVFAAISPHPLYHVSLSLISFPFPPSSAAHP
SAV
>tr|C9ZNN5|C9ZNN5_TRYB9 | DeepTMHMM Topology Prediction - Predicted
Type: TM
MVLSKIEVNNKSNGQTNKRINKTNIRAGKYTFRFKHTPYFRFFFCCCLPSYLASFLSPLLFFFKFPSLSHH
LDSWEKQQQQQFDTIYNYMRRPTVKMPPFFFAAA
>tr|D0A8Z8|D0A8Z8_TRYB9 | DeepTMHMM Topology Prediction - Predicted
Type: TM
MQSMSAGSDFTLGTSVTPFLELLPLLVILLIFAFGIQAVVLLSKSRWEDQQGNGEGEVVRTITGTFYRSSF
YHRFYENSKCEHTGILLSSTLLNIARHVQNGSKQLVCTTVYPRDARHGPDKQSVLFLFDPLKSDSELVLGP
SSFESTGFFYAGVALRGFNLRDQLPRYAVASLPTCGMWITVDDGIEVTYQRCRKDEDFAQRVDHVLYLKAR
GPGAAQKLERFVDQSLKEYISNLPRTAGGQQYHFQLTGGEKTLQFTKQLLSTSKTFDTLFFSQKDELLSRL
EQFVEKKGRFAIEGFPYKLGFLVYGESGVGKTALVSAIAAYTGRHVISLHLPIISTDQQLSDIFLTRTLRC
TKDFQPFVYELDDVIFLFEDVDASDDLVRCRQASTGQGPEGGNQCGYGLGGVASQQARCDGRYKVPGLSGF
LNVLDGVVDMHSLIVVMTTRDPNDIDSALLRPGRFGYKIHMENMRTDELVGLVGLHFGTEQRHTAEVEGKT
EGERSVNAGRNLYRLSRADEKTVREYIVSRCGEEFSINGRVAEAMCMGSDTLDEFLARLAKWYGEK
>tr|D0A6F5|D0A6F5_TRYB9 | DeepTMHMM Topology Prediction - Predicted
Type: TM
MGEWKVYIGAICDGLCENITSFLAGALRGQMFFFLFPWASSHQLQVVLDAAVEILARMLTCRIPPIAQYVD
LLLSSIRQVCLFVFPCSRFPHLSFLFLYCFSYPKLYLNFCWLIL
>tr|C9ZUP8|C9ZUP8_TRYB9 | DeepTMHMM Topology Prediction - Predicted
Type: TM
MVGGDGEEPGLQRQAKRALGDPLPLDNGMESEAPNDFSNDPSDNISVQSLQVACVQKCCLCTIVAPLDRIK
FVMQCQKELQRTGVLDRTFRSSWHCFRCIHSIEGIRSFWRGNLVQVGSLLPVTAAHMLLGGPMQMWVYNNF
PRAFPFGHSAATYASILCGALAVSAVSYPLEFARFRLAVDLRRSPCDLYDYRHSLAFFAQSVFSEAPHLLY
AGFGLYVTGSIIYGVMYTGLTQQVLSRLPSEPEGYTATVVQVGAGVGVSAVSTLGLHPLDTMRRRMMIAVT
MDGLRYASARHCFHHILSTEGIAGFYRGAAFTMVRMVYISSLYMWFLPAA
>tr|D0A9Y8|D0A9Y8_TRYB9 | DeepTMHMM Topology Prediction - Predicted
Type: TM
MTTALRDECSPLQMYSELNPPVAIMTAVPAADMQPAETDNKPRQKSPSSHILASQLASAVSTTMFYPMDTL
RIRYMSQDGTIQRQHNGQTYRSIYRAVGVIWKEEGLRALFRGCHVAVLGAVVAWGVYMFVYRKLCEVYNVS
SFASRTGMSVVASVTSALLTTPIWLLKTRMQIECRAMGTCGSYSGFFKGFHHVLVTTGVRSLWRGASAQLL

LVLPNSLGFPIYDSLKDCINQNIIGISAESCSWVHGRDLTVIEACLCSVVAKVFVVTLSQPFTVLKVRLQD
QRWNQGEVRYMTIKQSIPLIIRREGVYGFTRGLASSLLYSVPRGVAYYVLYEKSLQFFSRRV
>tr|C9ZYA5|C9ZYA5_TRYB9 | DeepTMHMM Topology Prediction - Predicted
Type: TM
MRAGCNVFFFFSGRGLDVSVSNWLLCGSFSSLLLFGGFICKRKFWAAKLIPCRGTETSRKVLTFSVPSSGI
RCYRCTALPFFPSSNSLRALCKRSPMSVLRFWQVGRRRRFISPFTRLKAWWMGIEDASLLARYGEDGPFRS
VWIKWRGTIIVATCTAVMFGRMGETSRSNDILDNIELNRQRYYKREFAPEYVPNAPEAVYDGPKGYSYRDE
VSGIMVNADGKLTSDLTREERRARLEQSEISSGMLEAARRLRESPRYQRNDS
>tr|C9ZVD1|C9ZVD1_TRYB9 | DeepTMHMM Topology Prediction - Predicted
Type: TM
MQQNIHSFTPTNGKDRCKLCLSYCGPIYLVVVILSFLGAYAAFFIGLCLRYPTSWKLAVCISCLFTGAVLG
GILIFTLLRAVFTPAGYVPQSPWQYPPRYIGECPGFVPPSGEGVGENPNTVRQLDRHNQLRYCTACKQFKP
DRAYHCESCERCTFDFDHHCPVLNNCIGRGNYKMFVLFLCYVPIVGCVLGGLMFVGFFLVDEAEPAVAWMV
FAMIMMIFCAVIGIFGCVHLCWLCRGESTMGRHVSSFNKQRGRSKEERAREREEHCNAVCGVRRVWWRLVM
PVLPLREEAASLV
>tr|C9ZNM8|C9ZNM8_TRYB9 | DeepTMHMM Topology Prediction - Predicted
Type: TM
MAAAPFPSSLFTYMSSTAVPAAGTESEGDVFVIFVYFICGYHFHRVVIALFFFCSPLPKCTCGSANKPPIF
QKHVSLSLSLSIYIYIYIYVAQREKQDSLWRHALLEVFKKLLTHHLPFSLIVTYVGRRRGN
>tr|C9ZVS2|C9ZVS2_TRYB9 | DeepTMHMM Topology Prediction - Predicted
Type: TM
MPLQITWCFFRYASSYAREFSGFLSGHARTLGTCVFMCLAVFTSVVRIGSLLFFPFSVIKLSSSTHTHTHV
HTQQTHLLNMLSPTEPLGSGKAHTEVVTDEGEGYGAMSAAEEKSHHKNGDTPTTDSKFMQCINAIIPHGGS
LSTTFNLGSATLGAGVISLAIAFQMSGVIPSILILITVTVLTIYSVGLMMQAVEMTGYNSYADLSRNLFGP
GWDYFTISVSWLFTFGTCVSYVIATGYLVDSVLSGSSALEFFQGKTGNRVITSIIWFVGMFSLSLPKEINS
LRYASAIAVLFVFYFVICIVVHSAKNGLKDGKLPEDVEMFKSGNRAIEGLSIFMFSYLCHMNCFSIYSEMR
KPSARRMTLHTTYSMSMCCVVYIIAGFFGYTDVGNKSVETVFEIYDVKGDVMMAIAFAGMLLKICVGFSLC
MQPARDCCYYIIGWDLNTLETWKNCLFCGCMALCALLLGLFIPDLNTVFGLLGSFCGGVLGFCIPALYRMY
CGNWGISQVGVVNYVCTYLLLISGVIAVVFGTAASIYNVAV

>tr|D0A990|D0A990_TRYB9 | DeepTMHMM Topology Prediction - Predicted
Type: TM
MRRSPCADYFTSLMATAGFVRCRGLSSSGQTNTTQTVHVLHSLYRRLRKEGEERKMLEDALSVNQLETNIR
IGLRLLHCHTQLVDSNRQLKELPFWRLVRRMSLAVARRYYAWRIFSIRLRLRSTTAISNALVYTLFLVVCF
MLYEIYCVCRIGVTRAEDRYKSLAVPIVQTFEALEEAARRRKDNLLKEMEGDIVRQRN
>tr|C9ZV14|C9ZV14_TRYB9 | DeepTMHMM Topology Prediction - Predicted
Type: TM
MMQGSEKEEKMCARCYACNFFFAIISLFCFCFSFHVSVMLNFEVELSPRVTEVLTLNPFKRNNREGRGGTE
FACAPAVVIDSFFFSSVHPLFSMCEKPSTSLSKNYCF
>tr|C9ZZ06|C9ZZ06_TRYB9 | DeepTMHMM Topology Prediction - Predicted
Type: TM
MASAPRLGRKGGKITASPIIPTHALTPGSSTSPVSLHTPGEGTTIIGDPNAAHPFGSDMLTVTKKQTSAEA
NVLQFDVGAIQLLGKGVVVGRAKQARTRTYQPQSDATEVKQRLHGVFSARRSKVPHRAASAGLDALQESEV
VATRKSQDTLSLVAALLSADRMANILVVVSKYYELSHVRSSVEAILREEGEDTQSVGVLFGQTFEGSKSTR
AWVMTADMALLYMTQLGSPAPFTHVVVPCCVDTTSSLSCFLKMLGGWMMSNTPRSQSVRLVVTTDCSNDGH
VARTIGVPRVKILDDKVIRLHEFSYNEVCALLGKQTMEMDKDAAGKFPSPPKRLVHYTADVAAELVRYVVT
HTPTAQIFSIFTADVREVLTALQGAKIEDCTVYSTLKSAADKVETKHRVHVINHVSHALDTENEFTMVLDM
GTIRRSSVQHKSESFIAASTTEWESKAEQAERKSILGENTTCCYFALFQDDVGASFQDEAQFLPDIFNVEN
AFVQCARLNLSVCEVGRLLPSVPRDVVDQVMQKVAEKCMISTPDSLDITFLGEIKSRLPVEIDVAYLIMGG
CSLGLGEATLVVSAVIALPFRSTAPPTYTVNRWTEATQESRKRCAGDIALSSDLLADAFVFLEWLRLRTTS
AATAAFLEAFLVQEFKFEKIEGLMNHMRDQLMNYAFLDRLDDVDTVNKVAESLRENASTMLMLLSMALSRR
AAFIRDAGHINEKDRHASMVFVRTSKQLVVHPFIPSGTRWETGGIVIPVILKNSTTILGGMFSLVDTSLFF
ASLLLLYPQIEYSRPVTTERGRVVYFGVACNWQMKRFVVSIDDATQILDFRENINTAIGCMRALRMLPHPI
SKTRFAIALKEHDRFFDMERLHRETQRRLHSLAAALNVQEHQGSFETFAKHYTAPKEIIPFNDVAATDVLL
LRRFADGTLWDEQRPSPQSAPAIGSSPQKSAVLTPFDTAIPAPYDDDDDDVQIIQNSYFMLHGPLIEDDDD
D

>tr|D0A7M8|D0A7M8_TRYB9 | DeepTMHMM Topology Prediction - Predicted Type: TM
MQMSFRYLFRFLCFFFSIFPHALQCLQFFSCTLFSGVKNQQWNRVMNKGEVRRIVHTTQKAIRPSHGNKLR
VAVCALSLWFLDQQVGTPEILSLIWVIMLPFPSLSFFLLFSKQNSCHLPTCFLHTKAYVYR
>tr|C9ZU73|C9ZU73_TRYB9 | DeepTMHMM Topology Prediction - Predicted Type: TM
MVASFLFFFQKSVGAGSRNRTKVSTDVARPTSASASTAAKKNERENMNVQKAIQRLGREKEQLDKSRVREF
YAAPLEDNIFEWHFTLLGPADSPYAEGLYHGVLRFSNDYPFSPPDITFLTTSGRFEVGKSICSSVSSYHPE
LWQPRYDIALVLVALRAFMAQDDEEGIGALARQYVSADEKRRLAREARKFSCAVCGMKSAESLWHEEMEGH
PPVGAEVEASVPQLPKRKKPEANERGDNDKKAKACADAGNNNSTSTSDNCNNATTGSSGGGEEEAAATPET
TEDTAEKAEEKLLDIAERSVQTEERSEPVAPGVRLRLGQRLLINLSYSMLDNVIWVCSAICFAILVKKALW
G
>tr|C9ZK96|C9ZK96_TRYB9 | DeepTMHMM Topology Prediction - Predicted Type: TM
MHTKEFKRWMLFDRGHCACVSACAVRFTFPSLRVGREMRQPESGLKRAQRENICLSRGKGVKKKKRTCGGS
VASCCVNRAHGVTHRSLPFFVSSFHFLPFSIPFPWFLFSLPFIVITSCLFTYLFLFTPTPRVSAFTSFSSV
SMRAFR
>tr|C9ZL34|C9ZL34_TRYB9 | DeepTMHMM Topology Prediction - Predicted Type: TM
MATSNGFESPRESVAAAAACSSQLREAAERARKDPSKDIGLTTEELQERKRCAAEPHRFVSKSTSRPFEVK
SSSRAQQIMTKGVVERQVTARQKEVLRKVSSLRKLQVGLFFVGVGFAYWVGVEFLLPHYAAVQERNRILRL
RYEMAQRKREEHLRVQGSSQ
>tr|C9ZPP7|C9ZPP7_TRYB9 | DeepTMHMM Topology Prediction - Predicted Type: TM
MFVRLPTPGETCVSRLLISRRFASAKQHVTRRNISSHNGGATTGQKSVGGKNFAKRPEDVAPHSSKGSAAG
PTMRRSRFYRIALDHGFGFAVYFYILGESMTLSVLYALHSNALGTGDTFAWMNAVGAERFVNLDRWAHAGP
TIVGVTLSFRLLLNYLAANAIMYPMYGMQMRFCVATFGVLGKGLNPLRRLRTLGRNVTSSGAKRTVPRAPS
ATAPKNKVNRLP
>tr|D0A553|D0A553_TRYB9 | DeepTMHMM Topology Prediction - Predicted Type: TM
MDSLYSWNDSTKIGIALTSLGVFFNFIGIVMFLDSVLLTMGNVLFVAGIALVMGPSRFKSFFLFRRRASCC
FFIGMLLIMLGRSLIGLMIQGFGTLNLFGNFFPMVARVLESVPLLGPVMLSPPVQKLLSLLGLQARGNRNV
>tr|C9ZWF5|C9ZWF5_TRYB9 | DeepTMHMM Topology Prediction - Predicted Type: TM
MSSVTTGSSSYAAVLLVLLLTVTQCGDSKFPNLHCDNVWDGPSAQNDPLTCIKDKKRILSHWEDIFVPALA
ALLLVAVLVAFPISWFFTCLCSSRCKPSSKDGGKEQRCCLWMWIMFALIWAFGVAAFVFFGVKQLWATSNH
FLDVTLMNPLNVVNCTAEKVIDFASNWTSGNREPYADGVDVSFFYDISENAVRVVEMLRGRAGDYIKLLPV
VSYAVGSVCFALMAPMVILACCRRGPLIVPECFACAYFVFGLVFSVGGAVLFLLSYASSSVCGEIALHRER
KPGIIQWYGIPLCNSKFRPDAINKKVTDAEIGICREACNYLLDNCDNLDMRSPSMSRFSGSSVSYDGYVPS
GYLKDRNGKPNTRSSDISPDALASFIASGFVSHAAARNVGGTSPVKVLTCGKNITSSDECPNFGITATVLE
DTRVKAFVGSCPTPGNSCTVVECAANCTEGRAKNVSIEVVRVAARSRNVSVALSIGRPLLECNFMLDIALT
AMPDCEDITPGVFMLSVGFLLGSLMFAVGIYVMLRGSCVWGSAKTSPEAS
>tr|C9ZYG5|C9ZYG5_TRYB9 | DeepTMHMM Topology Prediction - Predicted Type: TM
MSAAVPVSEGNGGVATQNPLPSGELPSTTSLAQQPLSPVGTSPPSNALVTAPGTAGTSQNAMPVNTVDQTN
RYNNNNEYANSYNTGMGYGGLGMGYGGLGMGYGGLGMGYGGLGMPGMYGGLGMGGLYGGLGMPGMYGMGMS
EDFQRSQMTFMLVGRLLEMCGMFAGVIQMTFGSALQFMGNYIGMSQQYNKLKSGMYMDEAGRWVELPKGTD
SSRESAVSGTRRRPSRHRKQEKQSHPIFGVLRRLLFLLLAVMLAKRLTR
>tr|D0A709|D0A709_TRYB9 | DeepTMHMM Topology Prediction - Predicted Type: TM
MAVFTFGRPHLPNGAITDTFKKRLVHAVRIALLTGAPWIIVPCSRGERTNSEAFLRNMGSPGDLLFNDAVV
EDLPPELQELAKQLRDSMRFAASLDEWIQSGNPIEHFARVANVEGTGVSIASPRLLRHGQSQWIIPEFVWV
DRNESWVVNTKEDIKHGLLVLLRRVLQTDFYGENSESQSKQDADAAWLERGIFDIIVVGSMHNEGFLYSAT
SRELSFLSRAHRQSTVLQRQGRENLLVSWLSKNINVRITSAEDSVPEITVPEPWIVDAQPEPRALRKAWWL
FRMLAPLSHTIHLNYCRWSACIRCSLTSVRIINFVFEFFAAIEAILTEKVNMLGYF
>tr|D0A7H4|D0A7H4_TRYB9 | DeepTMHMM Topology Prediction - Predicted Type: TM

MGFPLFLFRFFLFSAQNFLLSPLRPAAAVAPFSFKLIVTKSNIPLLFAMICVVGIQIRYIFLSYTHLLNCK
LVPIIIVLFCFCFVLFFFLPSFLPYILPSFLFPLHLSLSSSTSFFSVSIIITFHYILLK
>tr|C9ZNY5|C9ZNY5_TRYB9 | DeepTMHMM Topology Prediction - Predicted
Type: TM
MVNLHELGGKQGCGGPQFTPVGVSLTPLLQYFSLLVHVVGKLPGFAAGFVTVWLVYIMFIGRDVLLNTCVP
VAGFHITALLLLRELLTPWQEGIAGAAEVRRRVAAQRSFVRDVQLQWGAKLD
>tr|D0A832|D0A832_TRYB9 | DeepTMHMM Topology Prediction - Predicted
Type: TM
MCPGPVLIATWLDLLFLFSFVFFPHFLIIFFFHPFLCQIVDVLMTSFCRCVVLPFFFCFFFSMYHPFLCLA
LFPLPPPSAPYFTSSLLALPFRVLPSGKRSYELSVISKNCEQKKKRILRPFHQVKRKEKCNMYLYLYIRRG
AKANVGVFFFHFVSAFFLCVCFG

MAAAPFPSSLFTYMSSTAVPAAGTESEGDVFVIFVYFICGYHFHRVVIALFFFCSPLPKCTCGSANKPPIF
QKHVSLSLSLSIYIYIYIYVAQREKQDSLWRHALLEVFKKLLTHHLPFSLIVTYVGRRRGN
>tr|C9ZLP1|C9ZLP1_TRYB9 | DeepTMHMM Topology Prediction - Predicted
Type: TM
MRTSIDSFRVAYRLMVTSITTTPRNSHKTTRTVGLCLSFSISTDMPPLIVLYLFFLFAGVLSWMWGTIIKN
LGSSSQKVPVVLFTTMRCQWCMLVRAFLFLSKRKKI
>tr|C9ZYF9|C9ZYF9_TRYB9 | DeepTMHMM Topology Prediction - Predicted
Type: TM
MAVPPVEMYSGSFWNRMRKPLPLRTQVIRFTVVFVIVSFILAVALQITHERMPDPKVTKPLPDLGFELLTK
VPGMYVLADCCIGFLNILSVFTAFKLYLLHRHCVGSGEPELPCNIPGVSRFFLSVWLCKENCRIELRNIHT
IAWIRFITSYALLLLFRSAVIVMTSLPAPDDLCQDPPKIENPVKNVILTVLTAGGGSIHCGDLMYSGHTVI
LTLHLMFHWIYGAMVHWSFRPVVTVVAIFSYYCIVASRFHYTDDVLVAIYLTIATFIAVGHNADGAPWQLQ
LFIRWWPCCGANSREVTEDSQPVMVAFKSEAAGQSSRKVVDERNH
>tr|C9ZMS0|C9ZMS0_TRYB9 | DeepTMHMM Topology Prediction - Predicted
Type: TM
MEEGDHMEFSAREIEELENDTINPTNCNHKLDKEHVEKLRQATLQEIEEKDKLVEKGMRLFFMNKVKDAED
IMNNNAPLDPLHALGACCLAALKSLLSMGVNDTKYALEKADFSIAFSTEVATIPKGFFGSVTNLFWGTAGR
EFQPGMFRAKTIRAQSFAVRGILLISQQGDMMALLRGGMALKNSYKLLQSLKRELEDLKKKKDANSYEGLG
ADRNSVYGLLLMVGVTHVVISLLPSRVLSILGFLGLKYDRKHGMKFISMTWESRTLFAPFAALFLMVITSF
VPGFCPLHVPGWLPAAKAIAEHTVDQPPMGESLLHLWILGRIKRLEQDVEGSMSALSKCLKAAEEGQVAKW
MPQLRDFAIYDQGWNFIITMQWREAIELYKRLEDHSAWSKLFYGYAQACCFDMLALEAEQAGNKEGVAKYQ
QEASEALWRTAHYRINIMGGRAVSVEEFVSVRLGETFKHCGIEHPNKGNKRIVEPTPFPLEGLKLRNPVRV
GVIELLALFDITHQMPQSNTEKFLDIIDRVSKKCSDSPQIGSTDDGNGACGNEAPAEKGDEKNIETYTTIV
CAAVKANLLCRIESRREEASACLDRAVAISSKYKREKCSVSWVRPQMIYEQAVLASVEGDEGRMNKLLDKV
KESNHKRLFQNVMDVKLHLTEYGKEAPVVSHII
>tr|C9ZXX8|C9ZXX8_TRYB9 | DeepTMHMM Topology Prediction - Predicted
Type: TM
MNVCVSLSLSCGQPHGGTARGRKKKRRGKEERNKTNRKENEMKEFLVPITALWVDSSLAIVLCTLFLKKKI
FLSSFSSLFIRGYYCSSFHTYANKYIYIYIYIIPSFHFTKQFRDSICHQCELARTTFSFLKKKTNCRGTLC
LFVPGYPFLFVNYAQICALLTPYKHYYYYYHAVIFSLVYKEEEAHVRGFFFNFFFVVVCLYLTSLSWMHAS
YRLGGHKTQGRK
>tr|D0A7H7|D0A7H7_TRYB9 | DeepTMHMM Topology Prediction - Predicted
Type: TM
MSPLVVLIKPLQSSAHSIIFFSFLFFRFLFNLLLHIFFLFCDAPLVSPKPSRKHKKKSINKRKTHKKTKKK
GKEKTIQQEKQDNQLDEKRKRKREGEKHRSNTTPKKKKKEEEEGCGEGVLIYSGAKYFSLLRNRHHRHRHH
NFHILLRVTSVRGMNLAKRRKNINDKKKK
>tr|C9ZWQ7|C9ZWQ7_TRYB9 | DeepTMHMM Topology Prediction - Predicted
Type: TM
MTSINAQPPNSATYPQDDHGSAEVVNLNAEVERPQPEERKDGGGCFAKVSLFMATIIPPGGIAASAFNIAS
STIGAGIVGLPSAANSSGLVMAMIYLIIITAMSVFTMHNLAVAADKSSARTFEEITGKLLGRGASYCLAGV
RAFHGFSGCVAYVISVGDILSATLKGTNAPDFLKQKSGNHLLTSLMWLCFMLPLVIPRHIDSLRHVSTIAV
SFIIYLVIVIVVHSCMNGLPENIKNVSVGKDDNAEIILFNSGNRAIEGLGVIMFAYVCQVVAVEIYMDMTD
RSPRRFVLASAIALGICFTLYVMTSFFGYMDFGRAVTGSVLLMYDPVNEPAIMVGFVGVLVKLCASYALLG
MACRNGLYSIVGWDADKVAFWKHCIAVVTLSVVMLLCGLFIPNINTVLGFAGSISGGSLGFLFPALLVMYS
GGFTWQKVGPFYYLTTYAVLLTGVIAIVFGTGATIWGTATG

>tr|C9ZP80|C9ZP80_TRYB9 | DeepTMHMM Topology Prediction - Predicted
Type: TM
MQTLSASFAAAAGVSHESLPRHVPTLTLLKRILVLTVKLSIAQVAQFSFGITLLTVVGKIGVGELGGASLA
NGLVNATVFAFGAGFSGALETKLSHTFSRNPKDKMYGVYTLRMLIMLLITFVLLSPAILFLDRVLVAMGQD
PAVIDFTGEFCRLSIWGSFFAMLLELLRRYFACQHLSTSFSVSLVIGAVVYPFLLIGLVKVMGFSGVAVGW
SLLMICTTTGLVLYVVVTKKYLATWGGIEDAIYRNWGPLLKLGLSSMAMMLSEWVALEINSICAGFGTKEE
LAAFGITYQMSGICWAITSGTFIAASVLVGGAIGEERPMFARRLAILCLGTSVAISLCNVAILLATRNLYP
RIFTDDEKVVEIVGSLMNYVFVYHNFDAFQSCMMGVLRGCGMQKQGAIVICLVYSVVGVPLGLLLFFFTGF
GIQALWLGPLVGAAVVGFPTYLYMMMRYIKWDTLKPSVEVYDGDLERRVRRAVFWRVWQR
>tr|C9ZKV0|C9ZKV0_TRYB9 | DeepTMHMM Topology Prediction - Predicted
Type: TM
MYIYIYIYIYMCLSVCLCFGLHISCILFSLFYFFSPSNFSYLYIYKFVYEFARISTIVFFFLPPILWEMLL
YFIVVAVCAVAGVANAAFQPSLALFDAKLNETLSITTWRNVDLGLRSTIYAGPNPARLFALHAIVNFSNYG
TEEKRVLKVKKFLFLIPFCTPFNSSSDAAPKVDSCSLLHFGGTIMLRFRGGWESHFVSLLRKDSVLGPTNK
EAVRRFISVTGGSGGNNSNLTFPFPIEGTPSSNLPFFGGGPRHDAVQSCNELIDECVQASSFRYTDSYKNI
PLESFSGRIIHHPHNPLSWNTPWLLRISAALPPDSACEVDILVVLRTPADAFHFRMWLTGSVTGSLFLLGG
CLCIFFSFVIGTKELGVRLWRAVPLEGRQRTVLFEEVEGDDLPCFPILRILAALRRASVEVWTELLLPTLR
TLLERIRRLFRISREDEGVILISADEETLGAGNTREGGTRGTLAQGTDGNNNNNNNNSVDDDDDDGESGDL
CRICRCTDPVEDLFSPCACDGTSKYVHRQCLEKWRNTTTNVEHRRVCAECKTPYTLVLECVPLSPYGSARH
PVCVPTCCILLSYVMRLFLVLVVFCLGGYYLKVCMYIATGFDGGILWSFHHFYHWVLGLYFVIAFCVNLIG
LEYVIRDFPKAWQQLLILLISVGVVEIPLNYVGQFFVLMLCSTDVQLEVSYGVGILTASLFYSQVLPSVYE
GMESLLGVREVVAPRNADIV
>tr|C9ZWX7|C9ZWX7_TRYB9 | DeepTMHMM Topology Prediction - Predicted
Type: TM
MTNQSSGGNDRGRRDTDPFAHAGDVKLSSDAIALDVTQKGQDDVVKEVKPSLFAVLLEKFIPHGGLWSCAL
NLASATLGAGICSLPTGFNLSGIVMSCIYLVCVAVGTVYSLNLLAKVAVKTGSRNYGEAARMVMGPLTGYY
AAALMIAMCFGGNVAYIIIIGIILRALFSRDGVPEYLKSESGNRLMTSMVWLVIILPMCIPKQVNSLRHLS
FVGVMFIVYFSCVVIGHSINKIINEGVADDIVYMRTGNSALDGLSLFLFSFICQSNAFEIFREMKHRSPQR
FTIYGTVGMSMCAVLYFLVGLFGYLEFGGDAIDTVLSLYDPGENVAVAIAYIGVAAKVCVAFALHIIPMRD
ALYHCTGWHVDTVPYWKHVVVVVTINFTSLIIGLFIPKASTVFGLVGAFCGGHIGLVLPPLFYMYSGGFTR
EKVGNIDFFGTYLLLFVGVVAVVLGTGSAVYYSV
>tr|C9ZJ13|C9ZJ13_TRYB9 | DeepTMHMM Topology Prediction - Predicted
Type: TM

MHYASRGAKRQNQLRLPPAQKPGRLRVLARSFRGVNDAVGDFTQRNNESPLLYALLFVVGLLILLFIVLFV
VLLCILLRSDGWSTIDAFSSKTHIVEDPDKSALLEIRRLYDTMMRTGSDREATALLEFLSTYQRSMEARVS
KSTHGDDENAGDNGNVGDSGVPQGKSKYASEKPRVSNGTSLSQRSFAEVLADVRETIQDIMTLDPDVGLDR
AAFFVKEKFGASNDDRSQRTDSTGSHDIERGEVEAFEPFDDILYLQLLSFFAKEEPTRSISSADRLWWRRK
RISAPSVMEGRFYYPLRRFHRMQSFSPDTISTVAAWFDTMALDYYGEEMEPFESSLQQSYEGAQVSNHSGS
SPENPEIKEHAVSFSPSHTASIFVSVASFRDVECQSTLQQVVHRATNMFRTYVGIAEQHNKSDPPCLSYDL
FQPTLCPSAAIASDTASARAVFSDVLCFPMDNIRLRHIAPDAARGPTYGRYMTMLLYRGEDYVLILDSHTR
FVYGWDSRVVAMHMYLRHPRIVLSHYPEGFEKELSNFTYERTTTVYLCRASFIESDGYVRLGGILVNEENV
NKFSHQGRVLRYAAKGFGRKPDADVSRPLPQPWAAGGFLFARGSIMREVPLDPHLPNTFDGEEVLYSVRLW
THGYDIHSPNRTICYHVYTRNDQPKVWNNNPLWSSLRLRSRERIQCLLQTREKGQTVPKVPVNTTDPAVTI
DVDRYGMGRVRTVENWYRFAGLDPVRYTFDGRWCGKGKI

>tr|D0A596|D0A596_TRYB9 | DeepTMHMM Topology Prediction - Predicted
Type: TM
MCFCYIYVKNMYQTICKIPYGVGYPTQCSCTSCKHRAYSRRLNFLVLFRFFVCLLFCFLVFFFFFIFIYCF
SSLISLYFFLSMMMMVIIIIKWIYVGENLFFLLLVYFFYYYQLFLI
>tr|C9ZQV9|C9ZQV9_TRYB9 | DeepTMHMM Topology Prediction - Predicted
Type: TM
MYLYIQTKYVYVYAFTCFSKKNKTPYAQLVEKFLSSLFFSFSLAFFPSQMFTYNLHLLFSLFPLYPFFFNF
FQSFHCFVLPPHVGNYDPKVRQPPSPPPREGTSIKPHTSHIFS
>tr|C9ZLH3|C9ZLH3_TRYB9 | DeepTMHMM Topology Prediction - Predicted
Type: TM
MNERMGSLMTASNYMLRNFGIEMKNEGCRRTKKKKRGECLPKITGREKEIFFIVLLLLFLLLLLLLLLCWI
QTDGQKKKERSNTWRGMCWTCFFLFFRISVFCYLSFMK

>tr|C9ZVR4|C9ZVR4_TRYB9 | DeepTMHMM Topology Prediction - Predicted Type: TM
MILGLGDGTFAILFFIILGIVGTLFGSYVCPRLVLPIGLACAAMPFIAYGCIISSPHDPVPAPLPQTSYNR
ERPFPTNPDEAVVVDYLFPVRVVLMVLTCGSLLAAAGYYVVRVVVLEPPFKAPRVQCLREQLEEEHPTWYR
>tr|D0A0C2|D0A0C2_TRYB9 | DeepTMHMM Topology Prediction - Predicted Type: TM
MSNTKWSIRFSGRGVLERSCCCFLRGGGIDSGRFEERDSNELSHHALCRIKIKKNWKIAQFHAMMDFFFSV
SLGEVLSFMRRFMDFKSLTDTGIAFCLLVFYFPLPLFLILFLRLGFCGSNNMLVSTWRLYRGSEGGQCFAP
VHVCVVITLWPHICTVVSELPLCIVCSSPLHTCRCR
>tr|C9ZTP3|C9ZTP3_TRYB9 | DeepTMHMM Topology Prediction - Predicted Type: TM
MWYRFDVLITVSLIFPFAFVSALTSFCFCFCILVFTGIWFVTACVKGEWGGSWRRSNAEEKKTEKREEKLL
FVFFFPSTDLVLFFFNIFRWFFLFLLRKGAREEGSAAFGYLRVEF
>tr|C9ZYF1|C9ZYF1_TRYB9 | DeepTMHMM Topology Prediction - Predicted Type: TM
MFFFSVRLGSRAVHCIPLPLFYVSFSLLILSLHFFFFFFVHMPYISFVSLFSSFFFSSSFFSSPPFFCNFK
LTFRFLKEFTPEGRKLVARHVVVVSVLPITILPFYFILSFFFF
>tr|C9ZZN1|C9ZZN1_TRYB9 | DeepTMHMM Topology Prediction - Predicted Type: TM
MPPSKDSATPLDAAILCHIRTPYLSGSGLVGTVQTRLSATLADVRRLLESLVSLARQGNDRNVGNAIGSSE
APVNVSKEEMRKIMSHALHPFDLSADFSFVRGGCGAMVPREREKSTRLVDFFPLLPQLFSVNKKNYHIGWA
MEPPAPLVRILARLEDVEVCRNAVAVLFVSMQTPDEMLKDRLTLYQSAANVGFSSKSAQLNSFKKSLNLWN
CNVRDCFGRTVLHESVYHGNKEVVAHLLSLPFIHTNEQDAQGLTPLHIAVRKGDERIVSQLLMRGVDVLLQ
DASGDTALHTALRLRDSRLVELLCQCLREAGIGAEKLSQYRNKRGLSPSDVFKLRWPTFFQLCAVGDVQAI
HTLYNHYLFKCDLAHAPDNLLHQSVVHVAAAAGHADVVRYLLDEVGFGRGRAECFINSRLQTPLHVAAERG
EFATVKFLYERYPKWLAARDITGATPLVAVLRNRRRWGTAIAEYFITVASSETIAPNTCDNGGNGALHLLC
ELGLFHLARLLVESHGADVNLAHTGTVNTHHFLRVTRRSKFLMEQMKWKLKHQKTGKQVKMTPILCAIRGG
RCGIDIIEMLLSHGAATREDEIVELLFYLVSNEHYEVAERVLARENVKPPGHNELLCRFCREKHVVGISWC
SKQGFRQLNVMQESHHPLAVSSALGDAAAVAILLEHGANANIGKSVLHTPLALAINGGHDDVVRCLVRAGA
CLLSADGSWSALRAAVKRGEEFIMAGLLAEPSLPPSEVVHAMVFALQDARGRGVEIRERLCVRLARALNLA
ECGGVHPTELLHLAASRSCFAVVRVLVDKLLALPREELENILKDAPAAPSLAYALIEPKAVPLPKGVPSRL
YALHPSPAPFKRLITLRTWQEGLHHRLHLRDAFSYCASAREESLLETLLFDVGLKPWEGPDFRGWNAADYA
VASGLYNSVRILLVSGLAPLRHHHVRSGTWLGILCRSMATPAARPSETNLLYFTLKRLVCAGETLLIRHIL
WDVCRRCDVSDMRDASVWLTDVLLCCARSRSLEILHILTREFRVTWGRKFFPTAEPLLMAVLNSDAEMALF
LMTHGAVPTAFGPIPKMHDNVKASFLREERRAVSPLWLAARLGETAILQQLLQSTDLLCHEATLDNSTQCG
DILQALIDGATRRVTKERDAGMARTIFMLKRAGYLALTPDVVRCAARKGLVQVVNALVDCYGPDPFVEDLK
CGGLCAIHFMVANQELCGTLRSLLVAGRSTATETVGGSVSLASAMLATPFRVNPVDYALRHGCAEGALLLL
CLGLYGSGTVFAKRARTLSVVIRLAVQRRCTTGWSGYTALHAAIEMQCHSLAKTMACEMAAIHGPNSGPYA
EMTIKNDDAPSLHAFMAFHLREAHFGTLLSSGVPPVVDISSPCDIPYIVWSPHASEGRFREAVRESATRQG
LEQPLDLMKESLRVEVRQHMLSYNENIFFRSRSFAVTALTPMACAVAAGKLSWVQLFSSCGVSLTNDSSII
AAKYSAVSTQGRIKAPPVYKLVDMRDSRKRKAQKSVRRKHVCSKDTGAWLLTAYVHNRCHISPIMLSMAIV
VECVRSGAVEQRLLQQLQIIRYLLGSDECPLREEVNLLAIVAAELQLWGLLEDTVVTLERCGAVFDPISEG
DIPPFMHNVVGSAHHVMHKVARVAPREIILLVARNSSFVHVEELCDAKGRTALYHALYHPTPAAVDTLLSL
NVSVSKRCCSGTGRTPLMVASKLGKLGHAERLMRKEVLDLEDNYGNTALLLAAEGGHKQVVEYLLAQGSSP
STKNAKGMTAVMVAALAGHDSLAVPMVERFSGISDLFTPHTTILHCAAVGGAWGVATTVVSCLAQADPLAV
DHFGYTALFLAHAFGNARVLRTLLGAVLHKGITVPSSFVRERQIISRSSELVPRGWLKGTLYLAEAVLGNG
RKGSSSSLSSSHYGPMRSSETLYGHRTSIRRNDVPLLLWCVRNNNTLGVRVLGEINVGDSCYALHEAARSG
NVEMVKLLLKLEVSSPDVLNESGMLPFEVAAVHKHVECASLILLHTKLDPVRLRVHIDHGDDPDSTANSTD
SNTHNPMQLLASSENAEVFADVVDSVQRMSGDTWPSVAQQLFDTLSTPGPDGMTALELQLALGRAAGALRL
VKILQRLSLDVAGNCAFAVSSSILHHLTNVSPAVRVLLHDMFGLTDVAKGYGMRRRRFGRLSFADVRLIGI
SALQSDAYCAAATSSIFTFGNEVGATSSLLERLPFEVRFVPRPFERRSAAERAKLIRWLGSSLILSSYKQL
RTCAQFDAVEVEIVSHPAEEFAELVGDHLHHSIYVDGDCQLVVPDLNIILGFATRREKQRLLDEVEHLCGV
LTDTMQSLPHPALSKGEVKVDWRNCNIDDVEQEAIECLIENGLKKLRVFLEGNLRDCLCGVNMTDILSVKC
TAEPVAEKVHITFQYTREALKKFKPSATELGSTVDGLLCVVRFSDAGMGGLDFVMRSVLYPATLSDVNLVG
VSRIRDAWVADVSRRVGQRMGGETIAFKLQLEGSELHELPLHLLKRVMADVTDAIAMLMSRPSAKKHRFLV
SHIVGESLASSLRAVFVLFSTSRQPTARRAQGNLLICFNATMTPTMSDIYQCLRRSALKDEAERLKGMLMS
IVSTMGLQLSVALPAVPLLMDVLGSLRHQDTENVVSFLSVLCHNDGSLVLKPLIEGVSIGWKTELGRVVRR
HVRQISVALEVSGGSSCELKENGTFVYKCPLNCAHAGSYAAHSKGLLSAQQIASLLLIQIDTMDPTMRSLI

STTKAMACWSRACGTCTRLLDAGSQRCSIVIKRRNILDQPVGQSASDAFSFRGGWSGVKVKGSTGVVRFTA
PTKAGYYLQHILLNDQPLFNSPLRIRVRPLGPHLPNTKILSTFNAVVVKRPFHIQLLLHDRYLNRVAHICP
LRIEAVDGGAVHVAGWKRLHVDTVEVEVVVKEVCEQCSVRFRLLASGGAGNFVVDYTVASVTPDTYRQQFG
PVKSRLMAKGLQGKTGRPQRPPPPPVGIMSKRQKNESRTPRYIFPPLRAVAKGEKNTKRKSKPPST
>tr|C9ZKP6|C9ZKP6_TRYB9 | DeepTMHMM Topology Prediction - Predicted
Type: TM
MPARASQRTPRGTTSKSERQVTNKENPQEQATTVPNDSTRTDIPDVVAEVQKVEAILARRRETMGAGSNGF
TCGTWVALVVLEVLAFVWLSWAMDVYAMYRGARP
>tr|C9ZKN7|C9ZKN7_TRYB9 | DeepTMHMM Topology Prediction - Predicted
Type: TM
MSFFLFFSLSFICRKSGVNKVKGKKRKKERRKKRKKSRKKGNGRVKGLLLLLLLLLLLMQLREEVLDGIYL
YIYIYIYIYIYIYLLLHLFIFVYVRVFLCVDVLQQEREESNKRTYRERGKKTDK
>tr|C9ZUT6|C9ZUT6_TRYB9 | DeepTMHMM Topology Prediction - Predicted
Type: TM
MNCAADHVVERLPTARQQPAGEPHRFGLLAVAAFSCICVSLTYGFNLISGAMQELYGLTQRDLSTISTVGI
AVGYFGLPYSFIYDHFGPKPIYFLGLLCYLLGTVMFALTFQGVIEGTVLRLSIYNASVTLGCSMFDMGALV
TLLSVFPSNRGAVVAMLKTLNGLGAAIVGSVRLAFFSENTSAYFYFLMTLVIVIGTLATAYVRLPSYHLTG
YEENHLSEEEKVKRLMRKAVYLRQKAPTWRFVHGFVILIALIVFLPTQGALLAYLKLGSDYKVGFAVVTII
LTLLLPLMAIPTTKFDGNNQRIGIDDKSPTEGAVGSNDGDSSDSNVVETNVDYIAPQFQESFLAGLRTLRL
WCLMFTIFFCAGSLFVVMFNARYIYTAMVGEAPDEALNTLLTVLGGAGSATGRLGMSFFEVWSQKRKPEQR
VPITVVLFIPTTFVIIMLTMFLTVPRSVLPLSYFIGALANGCNSATIILVSRTIFAKDPAKHYYFCYIGSL
LSAIFLNRLLYGEWYTHEAEKRGEVVCTDKVCVMMPMMLLLVLSLLAFVSSSYVHVQYRRLCTKALEERRR
IREEEAAARRQLSGATATAELPN
>tr|D0A8K5|D0A8K5_TRYB9 | DeepTMHMM Topology Prediction - Predicted
Type: TM
MQLLSWSSAAKALFGLTVARLLLAFLYRRLYRHLYYHPLVPGSVALITGGGSGMGLEFARYFARAGCHVVL
VGRDADALRSALASCVELGSPSAEMVVADLNTIEGTNLVCFGLRDIIEQKRLHGQFRYLVLNAGLGAILPF
SSGVHFYETCESVMQINYFANVRLLQGLLSLLEETHSAANPSRVIVVSSLAGVLPSVLRSAYTASKHAIQG
FANALRGETEVAITLFCPGYVDTDFHSKATLIKGDGAPVTSHRRGMAPDVAVGKCMEGVLSGRSEVLTPFV
GKLGYILRPLFTRLVDSRAKKMSHRSLQK
>tr|C9ZQ45|C9ZQ45_TRYB9 | DeepTMHMM Topology Prediction - Predicted
Type: TM
MRSYGELWREGASTSLQCVTSTEHLAYLLHASLYIIFFLRVFAPYSQYLLPLSPSNYVFPCSVLFRVADQY
LHHSPRISTGRCFYDAGQLRCDHLPCSPCIVLTLPNLFSFTRGP
>tr|C9ZUA4|C9ZUA4_TRYB9 | DeepTMHMM Topology Prediction - Predicted
Type: TM
MINISFFFFLLSFITFFRLLVRVREVKKEGIAMNIMITIIMKGGRARTVKNVLVNPFLMIISFLPAISFLP
FVPFAGILILPFMLINIYLNIFEWHQCHSFFFVYTQLHLF
>tr|C9ZZ17|C9ZZ17_TRYB9 | DeepTMHMM Topology Prediction - Predicted
Type: TM
MKRKKKRRNANRHVIVINTECSAETQGDSTISSSSFLSDTFVMKIGVVVDSLKQAVVFTTAVTVVIICRKG
VACETKNKMINGMKRKEEIIPAISILFFSFFNLFFFL
>tr|D0A1K2|D0A1K2_TRYB9 | DeepTMHMM Topology Prediction - Predicted
Type: TM
MTTPGTSGTAKHDVKVFRSSIDTSSVSKTIFSQPTLYMGIVNLILMYATQRFPEEFMTPKYMTMSYNGVRA
RAMLTIPFCCIGSAYFAVASIITKSSSPLAGHLLGYGVSLGVGLTMMTFRRVSWYYPLLGLMYLSFGGFHH
YRRMMVYGDNAPIYNWGDLTEIWSARRQRKLEGRKKKHEAKEVAATEGRN
>tr|D0A100|D0A100_TRYB9 | DeepTMHMM Topology Prediction - Predicted
Type: TM
MVNFSPSPPPFIVILTISPFIYIYIYVCVCFFLIFDSSLSHRPTFHSIFTIFNNNNNNNKPNISPSTRKAA
TCSGMGEKRREREKTGWQKEYTRRKWKLALKKKKQQTSTTTRNRQPITVKTLQKHAGLRRWEVKWYKL
>tr|C9ZNN7|C9ZNN7_TRYB9 | DeepTMHMM Topology Prediction - Predicted
Type: TM
MQGRTNAGYEVQGDGWSKGLEKRRVGSRFVYIYIYIYKFYLCVRDNKLLAGDLPFSFLFFSFLFFDRAYIH
IYVFGTSYPLFWPVFYWPLASLFHLTTVNQFIALLLRLDSCHLSLTFPLMEAPNCSVWHGAKA
>tr|C9ZLH5|C9ZLH5_TRYB9 | DeepTMHMM Topology Prediction - Predicted
Type: TM

MNGVFVSSGAGRCAQESDSEEVMIKCHFDVLLVIRFAIIYIYIYIYVCVCVCVHLSGWCDHYCLCICSVEA
YSLSLSHGPHILLFPLETPAAYFVISRAFFSFSFFVVWSLHNRSLGTRAFRQLASRAESSVNEVLVVVVII
IVVIIVAIFFF
>tr|C9ZZ38|C9ZZ38_TRYB9 | DeepTMHMM Topology Prediction - Predicted
Type: TM
MCYCVQQNDLGCIFFSFSSRSRSHPLSFCYLDFSFTLTRMHSRCNVVDSCICGHPLPRRCIGVAVFLLFFS
LFSYLRYVLLLLLSRKLLCSCPPPSSPPPSCRSLALSEILRST
>tr|D0A390|D0A390_TRYB9 | DeepTMHMM Topology Prediction - Predicted
Type: TM
MMQGFPSLGEKDPAAKPPAEGKPTSASGEKQPSEAVQGIPLYQGEGNNQEGLKRFVVQGYKDVWAAILYIL
CIFAIAGIGVHAMWSRGSSEEKQEVKTTTAENEPSNSKSLWSVYFLVGVAAAASIGAASVSFLLMRMFPRK
TILFANIAAIIINIISALLAILSGHMVSGILMMTLCALHILWFYFARHRIPFAAELLKASIDVLSGYKAVY
LFNVMLCVGCTGLLMLWANAFMWTYQRSQAQDSSFSGIIYLFLMLFLFWTSQVTTNLMHVTTAGLTATWYF
AGKENMPKNPTLASFKRGTTTSFGSICFGSLLVAIIRLIRWLVSTAEDSEHEILRCIFLCIIGCLERLMEY
FNTYAFVHVAIYGCGYIEAAKMTWELCKQCVFSALFNDTLIDVTLGLLATCGSLLVGSVFGFLLKSWAAFA
LGFAVSILVHLLIFSPVGSAVTTLFVCYAEVPEGLEHSSPDLYAALQRTDQNGTSNGAAPPRV
>tr|C9ZNP5|C9ZNP5_TRYB9 | DeepTMHMM Topology Prediction - Predicted
Type: TM
MFLSPPSFLWFGRGSRAFSVLCLSSAPFLLLLHASASGRHVLLILLLSSSHCCDYYYHYFSRTFSVFVFYF
QAFAFFNLSLYMLERPDLLLKVRFSYPFLRLCDSFTNTC
>tr|C9ZUT9|C9ZUT9_TRYB9 | DeepTMHMM Topology Prediction - Predicted
Type: TM
MVKFLFPVPCLVEFSFFFLDTRRVLRGLATTVAGISGVAGFGVYVCICAFVPSYLPFGSLSFLCEDVFVHV
WISAPFHLSFPNRRGLRWRFAFRYAFATFPTVSVCLFCVCVCVCLSPSPLFFIIFSIYSLIPIGFFFSPS
>tr|D0A3X7|D0A3X7_TRYB9 | DeepTMHMM Topology Prediction - Predicted
Type: TM
MTGCGCIYFVCFSFPLHLFVAYLHGGSWCTVNGSVGSVSIITVPVISSLKITMFTVRSGVVRCARLWVLFF
SIRTPAIAHSASWNDKRANKQIGQLGGFWRRGAMHIHETIPVAIVDPAEPNAAPIRRHIEDRTLFAYRNMG
FALTHYHSSLTGANRHPIVIENMNQLRVYHRREYTYNLLLRRTYNVKTFQTVLGIIADSREPFNNVNQLQN
TKLYAYRNTSADQEVVHRFSKEAVGGSYTPPQYMRFDALITGPHASAFITSRDSITEEFVINFVRAGHYTY
YLHQHLSNPRYLPAWEKIYGPRGSRPAKPVLFYIGVGPQEFPESSMSAGMQRSLHAMVERCVDCLETPPPG
IAADLIPFSVRETARKYGVRLLQRWRMRYFALRC
>tr|C9ZSV2|C9ZSV2_TRYB9 | DeepTMHMM Topology Prediction - Predicted
Type: TM
MRLTATHLLLFIHATFLFHYCAVVYSSCISPHIFYLSYVFLFFFFRPCFSSFFPCFKSFLKKKLLLLLLFP
LTLFSPLFLTNATAKKTTIITRATTIIIIITPQIHEVFKYQNAINIVGKVQNPLTKENKKSPLRINLNMQM
YTYIIAFLFFLILFIHSSSSPPFFPSVVQHIKQKQKKQKKTKNKNKTEIKVAVESANVKLSKLYYNGYESK
IK
>tr|C9ZTE8|C9ZTE8_TRYB9 | DeepTMHMM Topology Prediction - Predicted
Type: TM
MVFEGRIYTISYFFACARVFLFTLIFFPLLSIFSLYHFSHTRSTISPYALPIVSVFFEGTTPFINIFAAFV
LYYFSFPLSLTHAHTRPTGTRLFCLPLLVVYVCLLLRECFTSLVLTTSFRNGEGEGRGGSKVNRYNV
>tr|C9ZR46|C9ZR46_TRYB9 | DeepTMHMM Topology Prediction - Predicted
Type: TM
MLIVKTEGDIPYIRLPRGKETKYDEPARRQEVAGYLTFKRIIRTTICATVFAVPFCVSAPSSPLLKLSQTQ
DTKKTLIPRMMSDFFYVNFIASWLSYYYIIEPGVINEKAMHGFMPWLGPISAQVPGLSCLLASHLFYPGMW
ALFNERTWGERIREFVRLNTKCALAYSPIHLPAAVAIGSIIGIVFYPFKFFKKRSCGRAPLDKEHR
>tr|D0AAR0|D0AAR0_TRYB9 | DeepTMHMM Topology Prediction - Predicted
Type: TM
MSNKLLPSQPPQEADAGGWNRRNVLESLSTNAKDRATSAWFQELARTEEEGDAAFARARPPLFRRAQRTHV
RSDEERRRMDNYESIDYSEPQSTVYKKRMAQWKKEPRWLKWVMFIAVGICVGLWSVLLFQTLEYLERRKRG
MLRTYLHETHGRGGTEAQTVGGGAGFPMRSTPSGVSWAVAGKCYIIYILWCAGFALLSSLCCLVMPTAAGS
GVPEVMAYLNGVMFPRVFNIRNLIVKTLSCIFVVSAGVPVGAEGPIIHIGSLIGAGLPTGRSRTLNCGATS
LLSTFRNPRDMRSFISAGAACGVTSAFSAPIGGLLFVMEEVATFFSVRLACMVFVSCLACMCVIQIVNSYM
SGWHLLAQSPMTHGEFLPSAIAMFIVNNVPGNHVPLNVYTFIPTVVGSLALGLLAVLYTVSSVRFLRWRRE
RLFPNTFLRVLEPCLFSLAYNTVCYVLPLAFGCIEIPPYVKDHKAEMKVELFTEFCADRENTFNPLATLAL
MGPYNSIRVLFSRHTTGLIPWYACLLQLMLYTFSSSYAGGMFVSCGTVIPSLFIGAMGGRLVGTLFNNEVW
ADPGVLSLIGAASYFSGISRLSFSLIVIMMEMTADLTHITCLMVGVVFARALADRFCHSLYHSLLDLKSVP

FLEAQTGVHKFDMFCAKDVMTSPAVTLNTVESIAQVVEVLQSTQHNTFPVVAMAKMTYKGVISRSQLELLL
WFMYFRETDGSDVIDSGRLSMLASNVPCGGKNGESVGRVQTKATGIEVCESEGGSACGPFTDCNESIITQA
GRSMDTIVPPETNVTDGPPRGPRSHATYADLNKVRECIFWRRLPPMPPVELLSKSTMRCHVDLSPYVDLST
YYVRDVMCISRTYYIFRHLGLRLLPVVDRRHRVIGVITRTNLFGDRLQERLRDAEEAGRLAAVPRAWE
>tr|C9ZZI7|C9ZZI7_TRYB9 | DeepTMHMM Topology Prediction - Predicted
Type: TM
MWFIAPTPFALKHRCPAIVLGGFSLTAAVCCEVVALKQRRYIVKILKKGNSGAFSCSCILIGTLRWWPMAL
LVRGRYHEVTKKILKRQKGEKGKIFCHCATADAMILHESPHHYCKWAARGGSEDWDYSNSFVVVCAVLLEN
IATNEREGKCHLTFHAATSMHHDYMLVALRGKVVKAKVSFRFREV
>tr|D0A2X2|D0A2X2_TRYB9 | DeepTMHMM Topology Prediction - Predicted
Type: TM
MTSREGIHPPYEVPVDAIFQPEGEGADISRSGTLISGAASARTVASPSQTQPADSRLSLSPPLKSPKITAT
PREVFHGERFSGESLDQPLTPGCAKERVGPSSSRGSDLASVGDGFTRFTYGSVQDCDELDRHSLGPQLTDA
LECSFRSGTFVPSSGQSYTSLGRAAFHIFKGNVGTGVFLLPAYYRDAGYALGGVVVVLMGWLIIDCVLALI
RAKQIIGHTGARTYPAVVKYVLGKLWMHFAKFSLLFTQFGFCVVYIQYASSLFAEFFTGHDLYKLFVFISI
VVVTFMTFVSHRLGFLAYMSMIAAVFVMVVLAGATAEEVCSLSTTGVAPEVWAIVPTMRIFLFISGHVFSL
EGIGVVLPVENSISPEDYPKFEKVVKYVNASIVALYVFFGVLGYLAYGEALESSVVLAMPASTMKVLMQVL
LGLSLIFGYPIQFVPAIQLLDRALGIELHREKSMFVMVRVTFNIFVGAIAASIGAETVSLFAGFLGAFTGI
HLMVTLPALLAIFTERVENARSSTDVDGVSAELSFCDYMKIFCTFPDNPFDCRWYAYIIFSLFVWVAGIYF
TFAPMLSK
>tr|C9ZVD6|C9ZVD6_TRYB9 | DeepTMHMM Topology Prediction - Predicted
Type: TM
MNVYTIAMRRCTGTYAHGGEVSYWLAKPPFSLLFFGGRDGAVEFCIYFYRPHSFTIGIFFNISISFYPASA
PTTECVKNKMLGCHILYVYTRRPFFIWTPPPFLKGHSMMVSSAVIIIF
>tr|C9ZTG7|C9ZTG7_TRYB9 | DeepTMHMM Topology Prediction - Predicted
Type: TM
MMLYQRRGRGEMHRPGIRTIVGSGNSTPSSLCAPFPFDSVIPPPPRGLLERFLRRLTSTISWVVSFLLVVC
SIPFLLLIHALLVYCDGYNREVVARHRRKWLHQTVPHVRPVRVAPSPNFPLEGWHMRCEDGRQRWHYGRLL
NVEEGNELGKAQAEGCPYTPYGEAGNANDDFEDTGATRTGPRGGPDMRAVKEERCRFVERYQLGLTGTAHV
KPRGSVEEAMRAGAEFLLRLQHPYSGHWPNDYSGPLFLTPGVIFVRYIVARGNIRNMFPPHADHQHEGDEP
CLCGEAERLELIRYIRNYMNEDGGFGQHTEGHSTMLGTVLNYVALRLMGVSDDDSDAARARQWIRGEGGAV
SIPTWGKVWLCVLGLYEWDGVNPIPPELSLLPNWIPFSPSRLWCHSRVVSIAFSYLYGLRWRQPDNLLLQC
LRYEIYLEPYGNIKWAKHRSNICAKDCYTPLSSVYKVFAAVMSLYEKRPIKFLRRRALEVAWTHIAYDDES
THFICLGPVNKAFDMLITWIREGETSGRYLNHLNRLDDYFFMGPEGLRMSGYNGSQLWDTSFAVQALCACN
MELLYPEEMALAHHYVDVAQVQENPVAATQFYRHRTKGAWNFSTRPQAWQVSDCTAEGLRVLLLLRHKPFP
HQRIYDAVDQILSLRNRGGGWASYEPTCAPHYVELLNCSDVFKDVMTDYVYTECTSSCVHTLSLFREHFPD
YRREDVDRAIRDGVKCMLANQRTDGSYYGSWAVCFTYAAWLCASALRISGEIYSMERHPTCVRLVNFLLSH
QNTDGGWGEDVSACARGVWVDNPSGSQVVNTAWAVMAIMAASGEASSTELRRQLRILKAVSAGIHFIVSRQ
LSTGDWAQERISGVFNGNNPIHYPGYKNTMPVWALGVYRRWSKTYGQHFPRSMD
>tr|C9ZUN6|C9ZUN6_TRYB9 | DeepTMHMM Topology Prediction - Predicted
Type: TM
MHPLESPQEHKTQVSPENNGNETKSVLQKLFTCNEDPKPLYEELGGVEGIAERLGTSITDGIDSFSVENRR
AVYGRNELPEEAPLTFWKIFKAAWSDRMIILLTLAACVSLILGLTVPEPGHEKVDYKTGWIEGTAILMAVI
AVTSASSIQDYRKELKFRALVEENSAQPISVIRDGHKVTVDVTEIVVGDLVSLSPGLVIPVDGLYVRGLSV
VVDESSVTGENDLKKKGAEHPILLSGTVVSTAEDAYILACAVGESSFGGKLLMESRLDGEPRATPLQERLD
ELAAFIGRVAIISAVLLFIVLCIIEIERIATNKQQFYPKKFLNFLLLCVTIVVVAVPEGLPLAVTIALAYS
QNQMQKDNNQVRRLCACETMGNATQICSDKTGTLTQNRMTVVQGYIGMRRFRVTNPGDPSSTVNLEGVSSD
AQSLLMLGLALNSSSEKELLPGNVGAESDLLSRWTWRTDKGNKTDQAILDFVDRVLISVPGSCNDKELPHQ
KLRMTNRSRGFAIFPFTSERKFMTAVVAGADGVVMQHVKGGSDRVLGMCNRYLSSEGREEPLTEEVTEMIT
AQIRSIAGDANRTIGVAYGRIGTDGAVPEEEPEGPFVWLALLGIQDPLRPEVVDAVRMCQRAGVTVRMCTG
DNLDTAVAISRQCGIYNRLRGDLALTGKDFRNLVYDTYGDEANMEKLWPVLDRMMVMGRSQPLDKQLLVLM
LMLRGEVVAVTGDGTNDAPALRLANVGFVMRSGTDIAVKSGDIVLLDDNFRSVQRAVVWGRTVNDNIRKFL
QLQLSINIASIVVVFVGSFLSAHDMSPLTTVQLLWVNLLMDTLAALALATEQPTEDCLNRGPSSPRAPLVS
RRMWLTILTATVVQVVSVLLLTQYGGKWLKAKGKELPTVVFNVFIFFTIFNMFNARKVYDEVNVFEGLFIR
SKSFLVIVVCCVGFQVLAVEVLKEFMSCVPLRAEQWIASILIASLTLVFVSVSRLIPVSEPSFEKGAELED
MEPGARRIAVKLAEDVEHHSSASNNVGSYMRFGQRLVARAQWQRVREHVTMRGVSQFWWSRHSHPRERGAR
METYGRLVRGTY

>tr|C9ZQF9|C9ZQF9_TRYB9 | DeepTMHMM Topology Prediction - Predicted
Type: TM
MSDEKINVHQYPSEADVRGLKARNGGACEVPFEENNEPIPNRSANPQEKNENELVGDNADNEAHDAVDVNY
WAPRQLRLDYRNYMGEFLGTFVLLFMGNGVVATTILDKDLGFLSIAFGWGIAVTMGLYISLGISCGHLNPA
VTLANAVFGCFPWRRVPGYIAAQMLGAFVGAACAYGVYADLLKQHSGGLVGFGDKGFAGMFSTYPREGNRL
FYCIFSEFICTAILLFCVGGIFDPNNSPAKGHEPLAVGALVFAIGNNIGYASGYAINPARDFGPRVFSAIL
FGSEVFTTGNYYFWVPLFIPFLGGIFGLFLYKYFVPY
>tr|C9ZKD7|C9ZKD7_TRYB9 | DeepTMHMM Topology Prediction - Predicted
Type: TM
MQPATAPYLLRSLYKDDHIISTHLNRQITNIVTALFGAHTTNCYDAQLCYLAKGIYVAFALLRGQTLGQEF
CDLLPVTGSNPPRLVGMRRKLLLATFLALEPAVVFQFAVRLFPRLPPHDVVSNVSKCTLMMLMLLETYGTL
AHRFLRVRYLSLVPSGALQNGEGAPRTYLKLGFVLMLELLIRLWRAVAEWRGNRGAGEQNEEGGAAGRGED
DSDTADEHASVPGKCMLCLGNRKQPTATLCGHIFCWRCLSEWIKSNTQGAICPFCRRQITVNSLVPLYFYV
AKEPPVADGDSGAS
>tr|C9ZP87|C9ZP87_TRYB9 | DeepTMHMM Topology Prediction - Predicted
Type: TM
MSSEPTVDESSKQHCNQLAVPEAAVAPTEPQHTTNSHLNDGKEVSVQLGELDETPEHLHPLVHQINTLNEC
DLLHIAEITEYVVTRDVYSSLPCRGGSWFLPEVRNVEDNEPQRCLSCIGRGTVCVTICGRRIMFPILRMCL
CAMIIALVWFTLWNALPHSLLEPGGYIWDTATIVIVSSFLGGLVCRVLQLPALVGVLWVAIAWNNIPYEGY
LTSGIGLGIKDISSKFGLTVIMVRAGFSAYLSGIALHWKHTLMLSFIPFALETVAHALIASAVFPYYDFNW
AVVQGSICSIVSPAVVVPGVLYLQNMGYGRGSGPLSLMVSSVGIEVAVGVWLASTFLERIFFQQSILTFGL
VGIAQLFGGAGLGIILGVAFFYFVELFKGDVQRLPNGKLQKKYLFPVLDLVTFVFLTVAALLVFFGYAVSL
AGGGCIACVLFASTMTHMCLRKGNPELEAQRKYIGKRLAQVWDNLMMPILFAMMGAKLSTASVFSRDFFPK
VLACFFGSTAVRLAAIFIVQTRSGLSLREKLLVCVGYCGKASAQASLGPVATALVATKMASLSDGEEPSVE
ILKMKKFAENVQQASATYVMFLAVVASVGLMRGGLALFQKQERRLREGSEQDSGTREEEMEQKV
>tr|C9ZV78|C9ZV78_TRYB9 | DeepTMHMM Topology Prediction - Predicted
Type: TM
MGGDRRHMGSIPGKSDVGEPGADLPPAPPVDLCDDQLRMASTIKSFHYKLQQRMTTAGDRACTQETEEERW
FRLIAEESEQMKSYTAAMHGIATQYWDPQEDKLRDGGQTKSCVTCPPITTRVIPSNNAICVTKRVRDTETV
VVGSACERQRAACLRHNDRIKYSLQCVTEYFLGICRVPPLIASKDTNRSTCSEVADVLSCSAVAGAVYYEK
LPLLASSSVKRWRRTYYDTHGRQAYPYEIDAEAYRVLLASERTVGDGNTAAPVCPLSSSGLVTTGTAGGTA
ANYMTMSRTEACRRINLDCPHPRLLGQQRVPPLLVLDIGSCYGPFGGRSVTNGVLQVPLAVTALDLSPYEG
SGVIKADWLAIRFHDSDRATGRCSCDDEGCISGTTFDDHGHADCGVSLVRWCDEESGKYEGTGSARRILSL
ARGAFDVVFFCLLLSFLPHPRLRYRACLHAYLALKDGGLLVIVSTRTQGARRSLWVDEWIKCIEGIGFKRV
HKNVMKQIVGLSFSKQPASEVSGDDSVLCGAEVWVERMMNRADALGGLRTIGDESPW
>tr|C9ZVV6|C9ZVV6_TRYB9 | DeepTMHMM Topology Prediction - Predicted
Type: TM
MFRLSLIQVIKLTLYIYIYIYIYIMLFIISFAVWLITGSTVIGAEQKKKIKKICRVFATIIERKGESEKDA
CKHGEDNFLSTPVMRGQEGKKWVMCRASAEADGTKGTRGLHNSHHPPLLLLFESNITFI
>tr|C9ZP12|C9ZP12_TRYB9 | DeepTMHMM Topology Prediction - Predicted
Type: TM
MDLFVCGESVARNFVLLLHLSSTPSPLWKALQCRMRESLQLSTFWHTPHCTSPFPGTSTFPSLARLRGNCT
TTTVLSSVTMLLCLNFLCSVREERVPKLASHRSH
>tr|C9ZQC7|C9ZQC7_TRYB9 | DeepTMHMM Topology Prediction - Predicted
Type: TM
MKRKDVQQTSKSSSRRIPLMCFSPLISACHFSTQCHTPINTLQLLDVSRHRCHYCYRSFTQFEHLSELLSV
SSSLRLVSYCCYHYLIYSIQLLLSLVSFVPLLFLVAPSPFLSILFFSSFSTTMVQLSPSLLPLAALFTARH
THTYEHKHASTRTHTHEDEKTDEGVQTIEFIYLIHAQHTCMCILYKDEYMENKCCLIDVPNIGKK
>tr|D0A2G3|D0A2G3_TRYB9 | DeepTMHMM Topology Prediction - Predicted
Type: TM
MFVTYLIFSSNGSRLYGYCLFMSFCFVVKSFSVFFFSFHFSEKGWFMLKGPWVGGDARKRLYVMAAVMLLI
VLMFMGVMDFKKREAKRWVKNSSLNDHSPGVYYVVVQTKPSPGWCRMLLSSVLTNVRVATVGAGAVYVHAW
RWAWIRKYMLWKRMQDNDVMVIFDGGDTFFSEAARREEAIEYFMNTTPSTRELFSEEDTLHGKVAPPLLFA
AEKNCHAPQTYIMTGVDTRKVKPRDMCMNLYEGALAVSTKEGTQALLRETPSGESHLNGGGMIARVWALKE
ALEVFFALKRRSFKWWCDQSMWTMVFIWSVTRPKHANRKLLLRRGIMSLDYETRYFHYPSGVPVKNGMILH
FPGPPAARSEKMLQFINETSWYRALRDSSTQREAYKYLLERYTTEIHTVWGSRKYVKFSTVCNVSNAANPR
WLIGPLNKK

>tr|D0A9C6|D0A9C6_TRYB9 | DeepTMHMM Topology Prediction - Predicted Type: TM
MSSRMSQKLRGHSLAPKRQKPLVNPELLLASPTDSTRGGSKQFVRAFNDEGCLSTREVGVRWFNFALLSIF
YTLRSQPWSLLITYTILLYVGILFVFSAAYVVWARGCGAQDGSTWVSALYFTVVSFAANGGYVGEQQDTML
DPHHVCFTGRTVIVTLLSFGNIIFVGLVAALVVGKAAYGEELGHRIVFSDFCSLAMAPGTKDRDCWDLTFR
MANSSSSKALAHGQLRLFIVTSEPTDNSRHQRKRRKRSVDRTETRRHATSSHHHIDDKSKRSGQLSKHLEH
HISDGTVQTETHPQRREGELDDVTRMDRESREWADAESHIVEVLAEEHLKRKRGREHTSKRQVLSSPATSS
CSSDADGGASFGGATGSGCSYVPINSPESALKQVSIQVEELRWTCSGEKHLDGRDGRLLLWFPVDITHTIN
RYSPLYRYVKHNMTNSLCTLNSLQVASPAIPQEAISEEERGPAATAAFPCSFQLVVTFDATEMESGRHISA
RHTYAADDIIKHYRFSNKVVRMSPEKREVLVDYHYFNEMLSDVVSPLHRTERVRTR
>tr|C9ZM03|C9ZM03_TRYB9 | DeepTMHMM Topology Prediction - Predicted Type: TM
MSDGGISTGSHQHLGSIYRSLEYLPRYFQIFALFGAFFLASIRENPVIAWLAVTMHSSFGTEIVWSVEYQR
RLELMTLNERLCLTLYGVALSALAMFTYVKTGRDVLYHLVQESGVDSNSRRWPVAIMCLMLACEGMVILKE
EAACELSSSDSIAHFVLTGFRGFEQTGFDPRLKYANPHFLIQVQILKAYAMSFVKRMLPAVILAFVYRSGR
DARRSAHAALRWYIMTIMIFRSLCEAYTMMYGDGLRQYSSWSLLLTSVVWYAMVNYTFGPSKRAGWTASTS
SASSVRETTNGISKCIVLFFMVYLSVLALVVSVHVVELLLIWLMFFLLTVWIPAIIDSCAVSYIICITTAV
SFVVYKMNHTTKWGSLRLIPLFSLWWLDICILYSLVANVTHSRFGGTLAVLVALGFHCYHSLEDLEHGVRT
SGSYVEKVVTMLREVVEETVTMDEEVSAGPRTEKACYKTLVVVACAVMGFLISVAALLEYSQSSDVRVHLL
NQNIILVTPRVLFRKFVRTFILVISLLVFVIAGLVMYRLVPQLAMFVPDFIALGVAVGVACAILGRLIDLQ
DPLYDALMRLIGAMEPPALTEDSVLDEGYGRWKHA
>tr|D0A251|D0A251_TRYB9 | DeepTMHMM Topology Prediction - Predicted Type: TM
MRKCIAHHHNHHLFFSLSFLLLYTMVINFLQMPHLMRSLTFTVHAQHLRKKLKKSTCITVPPTKCCIKSND
KKKERKTHLYLPHTTILNVFLQFPYHLTTIIYFFLFVFIRFIFYWVSTVTVFYLRRLSRHFCGHPLLKKKT
REWDERKKNGSCLGNTYTKCVEEGEEKDMKGRKQTKKTSTRKKRRKGNGKCVFASH
>tr|D0A236|D0A236_TRYB9 | DeepTMHMM Topology Prediction - Predicted Type: TM
MLRRVRPLRAWNNRQHSDRERDRIARERQRHILYDNAGNVKLYGILFLLWEEFRVPIIAVATGFFVLLAYN
KIVLYYSARQLAGERELDQKSESEARLSGKLKGDRYLIKPWRQVEDPDFLNIPSYAGKGVYSSKLFEDDVA
STDPLFSERRRN
>tr|C9ZK42|C9ZK42_TRYB9 | DeepTMHMM Topology Prediction - Predicted Type: TM
MKSFDGFALFYFVQLFKVISFYMSCFLSLFFFCPAFLIFLFSHFRIPFFSPPPASHILQKKKEKEGNIHLH
IEANPIKISFICICIYRFIFTCIFKTRGAQSERNKIKIKKTTKE
>tr|D0A472|D0A472_TRYB9 | DeepTMHMM Topology Prediction - Predicted Type: TM
MYEYTVCGNPCFISFLEKMKGAKKFYGKRRKRTCVIGAALLLIFLLCRMLFIRTEEVEDTTFEEGCWEREM
RSEGVGDVKVRYVVIQTKPSPGWCRMLVSSLVVGIDVITIGLDGVYHHTSRPHWFLNYIESAGLSDDDVIV
TFDGADTVFVNKHNLQCAISKFISTTPSKPENFDEEKILKGVQKSPLLFTAERGCFASQLSVLFSIRGRKH
EKRCERFYRGEISKAKATGAERVMRMPKSGRAYLNAGGVIGRVWAFKEAIGGFSKLREKSDRWWCDQTIWT
ILFAWSVNQQDTGGKAVHLRKGLISLDYDARYFLIPSYTSPIRSMILHFSGLIRDWKWWFPGVVRRLVWIQ
RMRDDVYQRDSRSLLTKTSLTIYGAKGEKYIRKFADVCNVDEAVDYTWLSTVRSKH
>tr|D0A782|D0A782_TRYB9 | DeepTMHMM Topology Prediction - Predicted Type: TM
MFESLYELFSTSPGRTPSNVVCNNEMPPALIVVMLKADEQRSHTSQSEGEGRRLLNGLMAAYPSSNYMFVI
DAAESTSEVFAAVTGEYNLLRELSPKFCYVVFAALRDPEVVVPCTSVFNPAWVGELRRMLDGLRNPRHMVA
ELLLRKELCMQLEGLLRFTAECRWIGDESLSAKLSRLVDSRVTDLSSSKNYSAPSCGGLQVSCISMLKEGE
EAGSPPLVLQTGIRIRGVVPDLTRSLRLSDCNEREYSPPLLLVVLHAGESIRENIVDGSVGRDSSVQELDC
LLQQLSSSDEVFVSLGGYVVVPLCSAAAARKQWLDTKEVLTRRVTYDGADSHALIREMLTQALLPLRKWHR
KHHTPAEDEKSNSPRSRVAILFYTPQEPCSVHQCVHSIVRQQLAFLQVPLFCITTPVGVDTAYKLLDISST
SGERCICTLEASCTANVNHDSECGGKGQACYGVLGSSTGERVAQAALTAIRSVTHLGVSVTVHSIDNSTED
TRFEVGDLRQGLDTTVLISRPVQNPDRGACAKHQLTILAYSVDKSRYDIVRFMPSETVKEVPSSEHSWWSQ
VEIAGRLLKSMPSKAPPSVSQLGNTLMQHSEEVPSLGGLAEQQHKLVEWLLSNYGTQELEGTPYTLVIGWC
PSPYMGNGSTEEEDGWPTSSGYLSASPCIADPLVELRWTNFPFCDGAPVTTISAIISRTFPFEALVESTAS
NSLALETLRNDSAEIRLASMKHIPELREARARIVAFLSVDTSTGAMSLQQLVPAKTEDDVKGGMLHTFRSL
RLHQMYCVVHLALRCSLDSSFFMQNDVIIADGITFITPRASVHDVRVTTVTHCRADVEWKGTATSVRIECK
PMKWISTQKNSERSPGGISDVTDSEVVGADGATTIVEYSCDGFQAAVAGLQPFTLYHLRVTPIGDSTHGAM

IREKLEEGEAYFATCVDAGVIDTLSVESIVPDSETLKLTVAHPVLCKTAAISRTRSLVLTITPTMSMSNSL
DKEKPLQMRRRKFSEGESVVTTFTLQKVERVQVCIEFNMKLCFGHVYLEETSSTCSHTCGSDDNDAQYIDD
SGQVRGVCVWFARGVSSWTKEVALCGNSSPRSLSAPPMCSSPTQLLDGYRISRSDTPFAEMRSDFKASSDL
KEHSTEENVVVTEGKEGCGEMEWSESSIVSVSHEKRLSKYVVDDSDPSTTGEATRSLYCVFSKDPTFRGVR
FLTDMSSQVLLASFAVGGNSSGYISAVIMPLNVSVDNLDGDNTSLKVSYAHVDRERPVTVKLVQHLEQRYQ
RVGRKTMHLYAVTDVCCRSREEVLGVNGVKTKKMLLSWTCGAQQEGSSLFVLIGSKRYKPKPNNTLLIDCN
DTLVAAPVCFCGSNIIKETCSIYLVPPPPPLSSQYCTVRAVKDEALHIFITEKYCVLLYRCLACVSDLIMV
LLHVGGDNATELPCSQQKVSGGETGVHLQVPLDAVDPEKNTVELCVEVRVRNSACCIGDNPSVSDWRDRTG
FGGSGSSSSLISTNILKGRRQQPILLIPWSASESFDVPVASSTLIKNLQVTELHSTRAKLSWTTVPYKETA
DEKLGVFLTVQCAAAEGHVKKYKEYVVCSARSFLLRGLLPATPYHVELSLDGGCPPGRVVFVTPQDMEDNV
LRKLIPSITAVWASRRGRNWQHVMRAACGGTDNAEQQRFVRLQLDIPNVESKHWTVSPCSAQIIKEEISGC
GTLRLAVTAKVQVLLVAKSGKLLQMWSTVSGTQSQTVSFAFPVCGGNPAASWPHIELRMDSFAQFSSAATE
DETGDEVSPSVDQDSWSFSEKDKISDLRAGHLVSRKHLGDRTNCPFANSHVTVHLVGCPQLIKVDENCCVV
EWSGSCSGYAIHWRVGPSGVVNTEHIEAERKQKPSITVDVSALDTVVLLFSLHGADCASNCVLFAVLVPPK
VSKQIGCCCETTRGLSVPTSPRCSNKGLIKVDYTRGGQPGSTLTDGESLGQNGVSGVSGGLCCTASVVDTS
SLEWREGFKGLLKDEVCCMLLPFPMEVVWLNPVVTAFYSRRT
>tr|C9ZR66|C9ZR66_TRYB9 | DeepTMHMM Topology Prediction - Predicted
Type: TM
MLSLFGFFEIKRLHIAQVLTISAAFCGYVVFNNFSLLANTVSVYQTSKILCTPLIVLIEYAAYNKQETKET
LLAIFITCLGSGITVCADTRLTVEGTIWALLAILANSLYTIWGNTKQKDLGVNAAQLLIYQAPVSSLMLLF
AVPIDGLTELRSYEVTPTSVWTIALSCILAFGVNLSFFLLVGQTSPLTTNIVGYLKTVLVFIGGFVFISSE
ADTKTLLGVTVTLVGLGCYTATKVRALSSPSSAKESRLT
>tr|C9ZXB6|C9ZXB6_TRYB9 | DeepTMHMM Topology Prediction - Predicted
Type: TM
MIRTVSDDSYIISYVKCQFFCSSICICIYRAEVAYYVLICCIWINQAGVTASMCLFSNMFLLILIIREYGD
TMLLSCCCVIMLRVTVPIPSLPVYK
>tr|D0AAS2|D0AAS2_TRYB9 | DeepTMHMM Topology Prediction - Predicted
Type: TM
MSFFFLFPFCVYARMCAYVYLHVYMSACAVIITITITIIIAIIIIIIIIIFLFGLLFRLKHDASDLQKYVC
VYVLVFVFVFVCTSSRMLVRFHFIFTFFIIFFSYSLLSGASAPFCVVFSYFFLFLIIVIINKFRCLSRNNL
YLLFCVVIFLPPLPSLPFPSLPSFFSFSIFTSIAHIHRWFFSAGVDLTALLCAPHFFLFFSFATICTGGSF
VPLLLLFGFNLFCLCVSVCLFFSFFC
>tr|C9ZVS4|C9ZVS4_TRYB9 | DeepTMHMM Topology Prediction - Predicted
Type: TM
MLPPQLNCVATFSMGIGNVVQAPFWVRYSNGRKNLDYRHVLYSVTRSDNKKKDYREFRHHQNFQWSENEAY
HSLAGIKRPRKTSHSTLIGAAVREARLPCIGILCIPTFIITFHFQINAIIFIFLFQTRGSMCGSGKVQVDY
>tr|C9ZMW4|C9ZMW4_TRYB9 | DeepTMHMM Topology Prediction - Predicted
Type: TM
MIPPYYYYCYHFFFSFAIIHPIVVVFISCLACFSLSLFLYRSVCMFRFPQPVSFSFLLFPSFLFVFPLSVC
VCVCVCVCVCVCVCVCVCVCVCVCVCVCVCVCVCVCVCVCVCVCVCVCVCVCVCVCVCVCVCCIFGLVILL
SFCFHIVHGV
>tr|C9ZLK5|C9ZLK5_TRYB9 | DeepTMHMM Topology Prediction - Predicted
Type: TM
MIVLPFPPFLSFFIVLSLFLSPFLVPLFVVFVNSLVPFVYFTFPIHSSTLPCLSSIITARTFIRWQMICDG
REPLSFGVGRLLFMMCLFVYTTNVEGDSQRWRPLF
>tr|D0A490|D0A490_TRYB9 | DeepTMHMM Topology Prediction - Predicted
Type: TM
MCLCCISYFNVSLTTIIYTLVWFLFSSVTLWGTLPHFVTHFFPFHCAIICIYCLLTSIFFFLFVGGDAHQY
HGIFHSYVLVPVLNVSILMFFPPPFSSYFAT
>tr|C9ZZD1|C9ZZD1_TRYB9 | DeepTMHMM Topology Prediction - Predicted
Type: TM
MRKDEYRNRQQGSFPLSNPYSRQGRGMSGNQHVSLTVRKDNYTDDGDIRGGAYSSTVGDNRRLQHHRSDVD
PYLHHDTQHTRDKRGWCDEQRSTKSRPCDYPQMVKSSEGAIRRSSSQQRWITQQQRCDDSVREVHRWDKET
RPSDEWNQQRRHSEDALGSPPYPRSHGHPHGMNAGRQPPHSHLFPIPSYDRRAPRGAPPGYSGEEHRNSSE
GHGQFADSSSHLMNAQRSIACEHDTQQQPQPEKDVRLHITRGYNSEFDVLQHLLQLQPKGETNTTHPRPLP
NYRDAEGAGHHEPQSRCPKHQQEAWGESQRMEGRNWDTYQDRDAAMMDRDPRRGMDRDAAMMDRDSRRGMD
RDAAMMDRDSRRGMDRDAAMMDRDSRRGMDRDAAMMDRDPRRGMDRDAAMMDRDSRRGMDRDAAMMDRDSR
RGMDRDAAMMDRDPRRGMDRDAAMMDRDPRRGMDRDAAMMDRMIDGGAVGAGRMIDRRGGNRRGGRPSGSN

VMPPSSLPPDKEALEKFKRLYALQEDQNETADHLRKQASSTTGVVGTLDVSQVLDLVAAHDVTIVVTDTGT
GKSTLIPKAILDDDPGAKIVNTQPRRTPAIKLAERVSVFYGEKVGSRVGYWVRGEHAGEVGQTPIMYVTNY
TLFLYLLHTTPDCIGMTHVIFDEFHERSVEVEVSLLLMKLVMKRNPGRIKLILCSATAEASKWAGFFDGLT
VGEYSKANAMYPVHDYYLEDVSRLIGVACVAPDIESSGIMSSIQLHTIIFYMKKLLEFLATAAKPGDSILM
FVPGRTVVEQLTLWIRDNLGEELDAIPWYRDIELSYVQEAIQRKSGTKKKVYVATDIAEVSITLPDVVFVI
DSGTVKRPYITESNPNSVAFPPLELMWESAVNLKQRRGRAGRVQQGFFFTMVLKEQVPQLPPCDCRLSNAV
IHEIVLHCLYLTSAPYILFSMCPEKPRNVSVQLSLNTLCDGGYIVPEEDHTSLIERIDNPEHLSIKKVWSE
LVSEAYESRSETKQGGLEGVDSRQLATKPPANLRYHVTLRGLIVGRLPFAVNAGAVVFHGLLTGLTTLSIV
AASCIACNSPFYVPYDVTDRVERLKVKNCVQETMLQFKGRLRNDVVSSVGAVLEYMQMQQEGLSEEGQNTW
CEKRYLSRMRIVDILLLVQQAKDQLGALIPFEDIDDVGDLRRQYDTHAQMLSILCSAAFMQRGIFVLHDAE
TAQKERFAGSGVFVNINCSRDMSVPTACPWTRNTVCVPYTLHTAYDRLVGSFSSQLSQEVYNVMLLVFSST
ILFEEIVDEAAPVTFEVWQCGSRVFITCDCQTALQLLQLRRLMCARLCILHMLLQREASITDVETMSDLLI
ANGSNFGIPCNMEARPDLIPAMITSNVIRIIEGIEEAATKVRVGGNEGSAVPHCKPREPPPGFNPTRQSAL
VFSSNGCAPYHRPEPIP

>tr|C9ZPV0|C9ZPV0_TRYB9 | DeepTMHMM Topology Prediction - Predicted
Type: TM
MLPFVALRVVAALVVTLFGVSGVVSPLLCLSNKCTTLFGGGTHTTFPQTLSLANCFAAGMLITIAVSHFFL
HALEDAAARNADPSLVSLIMLSGILLPTVLDRIVDKKDGEESERNRSNGCCHGHGALLAQDDIHQGGRCGV
PLVLLLMFFHAAMEGAVLGLEPDDPSLLTIVVPLCVHRILDGVAIGVAISKKLFLATSCSSEELLLLHADG
IGECTHNAGRLHGGVASTEMCLVRKFDRKLRDELWRWPVLLWLAITPAVALISAVLCSSSSDAAGGHCVGK
RGGRYCKGHRPIPHNDTRELFESEHTRGIGSLNSLVGGAGGGLFLFAGLMTILREEVHGLAACVSLLLGVL
VTLLLGRIEI

>tr|D0A6F2|D0A6F2_TRYB9 | DeepTMHMM Topology Prediction - Predicted
Type: TM
MGNEMEELMCVCVCLCVTCSCNLKFKFQPYGHCGTDLGFYPRPPSSHCVNPLHIYVSIYLFICLFIFIDLI
CCYFHPDIILDLSPIVVSPKKINILILIKEKGRFLTSTKLKGKTSQHVEKIKRNK

>tr|D0A540|D0A540_TRYB9 | DeepTMHMM Topology Prediction - Predicted
Type: TM
MANFVRKGAMGRINAERLYAFNKTLKKDTLRERGYWEVSKDSHPINRWSYRRWKQRKEELSVFEDKLEKVP
VDQKFKIKKLLTVANISVLGPIMILLYFAFCYLRYRLWGITPIDGAGAVVRGVQNLPRPPGY

>tr|C9ZLA6|C9ZLA6_TRYB9 | DeepTMHMM Topology Prediction - Predicted
Type: TM
MSAAGGVLTHEQKEKKKKRNIAKNNNNVRSYIPCTAQPVILSPPFPFFFLLCFKRDQKVYVRTNINSPPFS
VYRFFFLLLRGRWGYGTRNVLCVTFTTYIFINKYINIPVYVHL

>tr|C9ZNG0|C9ZNG0_TRYB9 | DeepTMHMM Topology Prediction - Predicted
Type: TM
MLHACSQIVHYCVFSRQIRIHAYDCAKTVLHNHTHLSLVSICFVFVDCVFFNSYFFSSCTVLFTADKCFFS
LFCIFGLFPSADVNIFRALFCLLGMRLLKGEEAMLDVRLADMPTFPLLCLLLSFFCLFSC

>tr|C9ZT71|C9ZT71_TRYB9 | DeepTMHMM Topology Prediction - Predicted
Type: TM
MSQSRFYSSNKDYLGLTGCGASFFFFSFYIVLFPFCRICPIPFLPSLTLLFSSFPFVVCSFDGDISKYLLF
LYGMPFGTVRYPWVFIHFGSHNGFMVLLCYVL

>tr|C9ZZI1|C9ZZI1_TRYB9 | DeepTMHMM Topology Prediction - Predicted
Type: TM
MSALVRRVPHIGPVRALHGTTLGRVVVVKLPLQHQRARICGSARWLQGSGCGDRSAGVGPIVIHDDGGNAV
TRVKRLPLTLIEEVPLPRRVLTDLELVMMAWCEEHARQYAIIMTVIRLGIFFLLLIILYVFYRTQLSSERM
LRGVDHMPADLRIGSVVYFDITENGMDIGRIVIGLLNENCPHYCEYFHRRCTGSGGNGESFRGMQLSAIVP
RHCLIFGDGREMTHDVPGFSPHYLPTEYLGTGSWRGALSSIAYGTNKESPNFAIHVSAGDYAPQIFALVIG
GFDVIERINSAGSKHGNSPKKEFIVVECGELCTLAKSHIVPMPWKLYKSISHGYDEEKFGRKISYNFLESS
DKGTSAEEAREAGSFNSGSNPPTPWWRFL

>tr|D0A2E7|D0A2E7_TRYB9 | DeepTMHMM Topology Prediction - Predicted
Type: TM
MMRSSRFLLVVCNKAPARPGPMLYGWGRTEQKRRLEYESVESKYHKREFNKNWDLAGVEQRYTDFMVVRTY
FSIGSRWGTWVYNMLQFYVLAFLPIFAFMHTLHKNVEWYDERIRHAAWW

>tr|C9ZPS6|C9ZPS6_TRYB9 | DeepTMHMM Topology Prediction - Predicted
Type: TM
MTGKNHSVDRSSGFSLLIDGNEQENAELTSAAASSPSSFTVRRRMSMREHLSLFAMSFSYGLCFNTLNNIV
IPKTVARITHGKESLWVGVLMGIGALCQITSPLFGACSDRLGNRTMFLTNGAMLTVVGLVLFTFVEITNSM

LVLCTAHFVSSVGLSVAYSMVVALLNDYVAKEETGKGSSAMALLAIIGSGVGYTMLAVGVSTVFCLGTYAL
ATIFCLVITLNSIPSESLRQPPQALHFSDTILNSFTIPSFRIFPDFGFACVGRGLFNCGLAVQVYIIFFLR
DIVQLASPAEVTSALSVAALLGGLLGAGLSGPVSDRVGRKSLIYLAAVTCSLSLLMLLEVRSLEFLYIIGF
VHGMGSASFLSVDYAIGVETLPRKDGMPIDTAKDLGIFGVSATIGTFAGQLLYGMLLHMYVSKGEGNTQRY
SSIGFVAVYSVSCMAFICSGITLAFINTK
>tr|C9ZHV6|C9ZHV6_TRYB9 | DeepTMHMM Topology Prediction - Predicted
Type: TM
MSHVFLCIIAILSPLLFSFPNPSVLLRLSRHTFFCLHNLTFLFQVMHRPLYISFSFFFSFPAILYLLYLAF
TALFFTVVRFEEKEREISG
>tr|C9ZSP1|C9ZSP1_TRYB9 | DeepTMHMM Topology Prediction - Predicted
Type: TM
MILTYLYIVSHSWGALCLHLHTGLRARGWVFVCVGASESRGEPLSCVLPLPLPAFSLFFPLSLAPLAFRLP
RSSRLGASPSCNILMWAFRVRVLVAFEQPIAPDFDHLSLPLLC
>tr|D0A835|D0A835_TRYB9 | DeepTMHMM Topology Prediction - Predicted
Type: TM
METKAIMLLVVSAIGVVMLTWLFVSICTRKSKADDMHNEPLLSKHANSKLMGVDGRVLVLYGSQTGTAEMF
ARNLTREGSRRGFPLKVLDIEKYQASNLVKEKRVIIVCATYGDGEPTDPMVEFHDWLMSDSRVMGEELSGV
RYTVFGLGDRQYVNFCREGITVDRRMSELGAQRFYPLGRGDYSDDIEEDFDKWRSGLWPALSTELALDVKS
GEEGPVAPECCMKALESSDEAPLPFPKADPGQEPTQRLPSWVPVKVNKELLSNATGRSTRLIEFDTSETVI
SYQAGDHLGVLPSNPSEMVNTYLRVLGVSEQESSQVISLENKNTGECVFPCRASIRTALTWYIDLAGPPKK
STLRAFAHHCTDPVEKDTLLKLLSTEPESVEAYGKLVLELRTVLGFLQRFKSMSPPLSFFLEMMPRIAPRY
FSISSDSLTHPTSVAITVAVVEGGLCTNLLQQAAVGQNIPVFVRKSNFHLPLRAKDRPIIMIGPGTGVAPF
IGFLHRRSAWLEKGNKVGDALLFFGCRRREEDHIYADFMEKCLSNGALSVRDVAYSREQADKVYVQHRLAA
RGKEVWEIISRGGNVYVCGDAKNMARDVEKQLLDIAQKYGAMKEDEATALLEKLATDERYLKDVWTA
>tr|D0A7Q5|D0A7Q5_TRYB9 | DeepTMHMM Topology Prediction - Predicted
Type: TM
MPVLPPWRRRTSIFHRSEGRVQSVGATRISLVAAAGLVASQSATFTVLYLFLPEGLAGLTHLMVAAVLFNA
FMLWNWGSDPGFVTSSEEPSTARERAIRRWCSVCRLWQPLRTKHCDKCGRCVRKYDHHCYCIGGCVGEFNH
LRFVLTLASAVPYFVLLPPALLKCFSLGDITDLDRVISRNIVPFIFVAYTTIQLVLVLSLLGLHCTLLLNN
RTTWELSSRGRITYLDSRAANPFNKGIVQNVYFLFRRKPINWYSVLEEDECALV
>tr|D0A2F5|D0A2F5_TRYB9 | DeepTMHMM Topology Prediction - Predicted
Type: TM
MNCVPLSCIKIRSEGRITLLAKRLHLCRVTLVLMAGCLCVVVHPVPLAIPAVCKKKYFTLRRISWRRFHNA
DLSCALDSRLATHSLFILSTLSVILFATSVLLTSLVNKAEVKSGKIVT
>tr|D0A5Y6|D0A5Y6_TRYB9 | DeepTMHMM Topology Prediction - Predicted
Type: TM
MLPGFPYKGNDISVANAISQLFSSRRERNLAMFLLLTTGVMVLELVYGIAVNSLGLISDAFHMMLDSASIA
IGLCAAVVASFPSDERRYPFGYARYEVLGGFVNAVLLLFIAWYVTLESIERIIKPPEIEAGYLIQVSLIGL
IVNILGIIFFHGMHGHSHAHGGCSGSVDHNIRGVYLHILADLLGSISVMTSSIIITLTGARISDPICSILC
SFFIAASAFPLLEETGKVLLLSNQPYGELSFFRTLISEICSVVGVKRVLCLCAWTHSTSPRDSSLCAVKLL
KHDSVDQSSVRGLVKGSIRSFITSATGVRNTGIIVHVE
>tr|C9ZR93|C9ZR93_TRYB9 | DeepTMHMM Topology Prediction - Predicted
Type: TM
MALRKRCGVTCCFYGSWPPTVGFANLTLTSICLFILDGLCFAFARVKVCVMPVMLPADWACVDILVCVCVF
GKLMSVAKIEEGAIKRKPLLPMCVCVWEKIARKKHGTSPALFRMPCYTVVRRFGGPQNSCSFPLCLSGGIR
TVWLCFICFVRYATHLPAFWGGCGSCYDAVQVNCFSI
>tr|C9ZSK7|C9ZSK7_TRYB9 | DeepTMHMM Topology Prediction - Predicted
Type: TM
MKGMPCSFFPSLFHLSPIINSLLSHNSSTRHIVPQNLKKKLPRDFTTHTRSIHKYVIFFYATTTCFTLVSP
CPVCAHYYFHVHPLKLLSKKKKKKHKNRLPITRHPHGKSTARG
>tr|C9ZMR2|C9ZMR2_TRYB9 | DeepTMHMM Topology Prediction - Predicted
Type: TM
MHSFVFPLFFFILFKERNKKNLFQTRLNHFSFGVGLKSRSAERISLLGELLCCISTTLFPSLNSTFPTIIL
IYIYLYLHINKSVVVAICGGVFSPPPPSFLLKLL
>tr|C9ZYW1|C9ZYW1_TRYB9 | DeepTMHMM Topology Prediction - Predicted
Type: TM
MGYLLQLSLVFVDIYPSHSLSFYFLDLLLYFVQYLLRHQPEPLFTCKERKECENTQRGGEEVGNVGGRGIN
SLRAWLCMDADLNGLRKRLVEAHERVGRMTFLMLRAVLQHIAQRRPRAAVSSVNGLNTSAELSRVKSSATR

DNNSRGHTAEPATKRGRAVAVDQAKLSRDFYRAALEAARQRAVEEHQGASTAMSDGQEGDEYTPSVALLLL
QTARERAIKLAVFLRFRLAAVRGQQLNEECELRFSYYLLRRVVRAWNTYTLGSFYRRRQMITYVVGHWCAV
TWRNRRMREHLQIFRDGLLVRRQAFNFILLRRLRRYFVQWLQRLETRRVIRDLEERAADMRRKHTFVTVAY
PNGRPSAFVVKNRVFAHWKNKTEYRLDGKLAELISNKSLMKRAWSNFVRRYNALVNIDLGAPCLPREQQLL
VVHVEPQVARLMLLKIQCARQIARECLKRFVFSKWRSLYARRVADSFFVYQRRLGAMKMWLEALRRRRLDT
FVMVECWRQWRHRFLCRVRSVHADYWRRCRYMRRPFVFWRCSAAAIRFHHLHIRRWCMQHWWQRVARRVIH
RRMCASTERRIFMFWRSKAAVVKSNRMMLCVADSLRELVVLLGCFRRWRQRHEDSRRVHLSESILSELRRE
KQRASLFNRWKRLTFWPRPAVGFGGELVD
>tr|C9ZLN6|C9ZLN6_TRYB9 | DeepTMHMM Topology Prediction - Predicted
Type: TM
MVSSLRRKKIIRNKCVYLSKNCMMQKGIESNEHIKEKKDCREMSGKGVRVGTKKKKQNHRGETDRRKNATR
ESHTELWHPAIPQTTQTRLISSFSFSSSLLLSFSSFLCVFFLL
>tr|C9ZKW0|C9ZKW0_TRYB9 | DeepTMHMM Topology Prediction - Predicted
Type: TM
MIILLFPVCLVFFARWPLHIFVEWVLVSPYVLGIWSYRFGCLPISLSILYGEGEEALTAYGVRRKLHPLDP
RLNVLVFPCLTFLYIFVSISHLLISLPVSNWKD
>tr|C9ZIU9|C9ZIU9_TRYB9 | DeepTMHMM Topology Prediction - Predicted
Type: TM
MYLIYFTFHLFQFSFSLTFLYFSLTLLYFSLISPLSYTSTALPLSRLHVFSFLTVSLVALFHSLFFFFASL
FTLSHTSILFLSCIYSLISSSFSSSFCICISTLSHTR
>tr|D0A628|D0A628_TRYB9 | DeepTMHMM Topology Prediction - Predicted
Type: TM
MSVCVWGGGGGGGVCTTTQITSSTSRAVDKEILTAAAYSSEFVLSPCFIILYQTFVDTKWNKIIKQGKERK
NTRGTMFRMFLFSSFLFLRCCWPFSLCNFPPYSALFLHSAAP
>tr|C9ZZS3|C9ZZS3_TRYB9 | DeepTMHMM Topology Prediction - Predicted
Type: TM
MADGFLFSPPPLHISTPHAQTFPSLICRHGATFPQSLRAVVLIHNVVPILTLFRFQHFDAIFFSAWSNDPF
LAAPCISVTQFPCFHAVAVCATTASEFSV
>tr|D0A4I3|D0A4I3_TRYB9 | DeepTMHMM Topology Prediction - Predicted
Type: TM
MLGRFDFLGKFRLYFVVVSRLFSFEGDYVAYRLFFVYFVFFTLSFLFVWSRRVVVEFSAFYFFCCFLGGVI
SGLAHTSMTPVDLVKCGVQVGLYNSMTDGFRSLWRNCGGCWFRSISVFTRGWVPTFFGYSSQGGLKFLLYE
LLKFWFCSRLEGSAASPMVLSYVSKLGIFVVSSGVAEIFADVALAPWEAVKIIIQTSNVAHTELSYFFPLV
YSSEGIYGFYKGLPALWCRQVPYTVVKFLSFEVIVRLAYRYLLTSPSDPAPKYVQLLVSVISGVLAGFLCA
AVSHPADTVVSKLNQRVEGSPAADKRKVVQIVRELGWSGLWKGVELRMMMTGALTALQWLLYDSFKVSVGL
SATGGNGVRISNHVDSDGRPPGDNK
>tr|C9ZLS7|C9ZLS7_TRYB9 | DeepTMHMM Topology Prediction - Predicted
Type: TM
MYALDYRNQPLKCFLLGWPSLITAWAGVLWFVLCMWECLNRPIERSERSVMDPMWYRALIPPLVPPVIVVA
AYFSWLGWKIFTHN
>tr|C9ZKD6|C9ZKD6_TRYB9 | DeepTMHMM Topology Prediction - Predicted
Type: TM
MLSFLSPKRAGAIRVWRNGAFCVGPNYIFAASCFLSIIAVSTASIALNNMLMLRILCGTSAFLSLGFMWLC
STSDPGICPWRTREEMERDTKNGVSKGKDAELVTFINKNGEEESALLRCKWCYTCNQFRPLRAVHCSYCGV
CILRRDHHCPWVGTCVGERNYRFYWFFLWSVTCLSLTVLVSGVWGIAIRVARLCGTVFCTEKSMFVSAFGE
THYIEPTISLVALISCAFVAPLAVYHAMLVTKNMTTGEELNCDGVSVHYFSRGGCVANVKASLCSPIPPSI
FQEGVSSLTPLATAVVMVEEAEV
>tr|D0A2U1|D0A2U1_TRYB9 | DeepTMHMM Topology Prediction - Predicted
Type: TM
MHVCQIFLSVILPLCLFSDHPAQSFFPFVLYVVIFVIKPVIILVASPHFTPLLEAHTTARHFHNYNRRHKD
RKAKKKLIETTVEKKGKKLRNINKLHVKVNFSPPQTQHPFLLVLHNNPFFYHSFLQKKKQILFRKGGKKKK
REREQREIIKYVFS
>tr|C9ZNJ1|C9ZNJ1_TRYB9 | DeepTMHMM Topology Prediction - Predicted
Type: TM
MCLVIYLFLCVEAVVLAGRVGEEERKACVCVERCCWFKGVKKKKKTEKKNKVKWMKRERKRKRKGKGRNVS
GISFSFSTTLLWRLPFSFVIIFLLLPFNYLFLSTISLFPTFSSFVPPFCCFNGLTFIASFTHVTVVEELSF
EERLTVSINILFFFSLVLSVTTPEFEEGKVKKKKSNTN

>tr|D0A6K4|D0A6K4_TRYB9 | DeepTMHMM Topology Prediction - Predicted Type: TM
MITSAAHFAWCYSNCFSSLRHCSRSKLKIDMTDSESDAGFIQLRNRLLELLYSSNSSFSVDQIARILAVDE
SKIVNELKHVAHNDVAYDEAKGIVQVRADMWKPRSMKAGDVVIDTAATTTPAAVADLAPGSVAVEAYGGGT
SGGGVERRNINAGGPLTPSPVLGSPLNPMFKAYPKYGSHNYYPNRGMRVLAPKGALRKTKYRRTGDAETLG
KGGGMRDEGTSPTGRVGYVCVADEFRLSEMESYYCAQGYYAKFAFDVLHIRFSDREMKAKDPRGDGLGAGG
SGCQDGSGNNLDNSSTNPAGGEKVTDRTTSSAFTKLSRSAGFDLFVFGYGAVVWWGFDQRFFKIVENDFML
SSSPISNLMVNRYATHLVNANYPVWCTYNLARKESLEPDEHFREQLRFDHFLIPCGRGDFSTGNVCMLCVS
HALAQSAKIDYLELKVQELAERCSPLPRELRENGRVTIAERRLLQLRGEVLSYRLMLKSGSNLMDEPDFFW
ENAYLKPVFQATKEYFEIAERVEALDNKLDAANEILSMIAEEFSQRHGARLEWIVIWLVFVEVILGVLELI
INIKPWVERGK
>tr|D0A2A3|D0A2A3_TRYB9 | DeepTMHMM Topology Prediction - Predicted Type: TM
MFWVITGSNVATALEATTKFGEGPAAEAQSSATDRRRSQYATGAPTLEPTFRKPRPALPREADNPFYYSEA
ASNEPLQRLKEITYATILVPLRVLYITLLIVLYWLLEMIIQKRGCHTAANEVDNLSKVSCLFRYIAGTLPR
WSILGLGYLRVNRRNKCNYGRRADGSRVVGPVIVANHVTIQDGLLLLFECDASLVTGNLAEANFTSMLLRG
RTYNGEDRRIVKSLDLKHRQKTQEEIDSTEASTNNNPQTEMSADMSDCNETEVREGGKESWPMSYSLNRAA
RMIVLMMRFSTDAFATGLPVQPVVLRHSHKYFNTSWCGAASPTSILLGTAAELFNQVEVIYLPVCEPSKEE
KLNPSLYAERVRRAMASTLNVPATWHSEADVHLALVAARLSLPVDAVNVETAHPHFAGMPYDRVVRMLYRF
SALLQRSDGGPQPQMNITPKGYMSPTALGRFLSPIGLYPTLNERLLLHLSRRASVRGCYISFRDFLSALYT
EPIIVGSSDSHGTSDKTGESSVPSNAHMVFIDELQKGEADDEVALDFILRRTFAMMLLAGEDLLRQQEKYR
SHIKDTFVTVGEVQGAGQENVEAGDANVISCAESPLPTGTCPSSYTTVYGCKVRFLFRPCQNINRCAPVAA
SLAATALSVEERRLGRAEFDALLDMLFVPHHSTLWRGPATKSQVQLAEEDSLFSYIRSGATEEWEATVGKK
TVQIEEVGRGTNRSGEYITFDAFLRFCFRHRAMAEYFHACCEHFLLCDDLA
>tr|C9ZNX1|C9ZNX1_TRYB9 | DeepTMHMM Topology Prediction - Predicted Type: TM
MYIGTHMYKCIYIYIFIYLFIFIPFYPPLTVKAGNKGKPAKQAQRNKVTGRKTFLLASPLYLPLPPFLFCK
LFPFFFFFQPPPFSRITHAQTQKKNKFFLPRHHRHIHK
>tr|C9ZUQ2|C9ZUQ2_TRYB9 | DeepTMHMM Topology Prediction - Predicted Type: TM
MQKLWHDLYEAETLSDARRAVLSTVPQWVPWLGGGVCLIAYAAVLRTRLFLARWRRAARKRAERKFSGFRR
WKGCDAPPPCVVITRIEEEVDGDEDGDGGSDAEGSRCGDGGAATMLSVGDIAEFRCSVGLRSPRDDDLLIM
IEEMLLVDDVPDGWVLCRTTSGVVRFMNLNTQELCLFPPGRQGRGHYIRKELRRRNKLEIESGFSLSYNND
EVFNESWGGGGGHTGKFPNNDSLGKTSETDSSLQRNHSRGTDRGTRDDAHFVSDEDTLSFSSDEADLYGEG
EQSAFQRVFRYFLEREKRRIERDVEMQFWSCDTNGDGTGKDDNVASAKVENCGLRLLVSNGSPGQNMRDIV
SRPNGRD
>tr|C9ZIB7|C9ZIB7_TRYB9 | DeepTMHMM Topology Prediction - Predicted Type: TM
MVWLHWTTEQIMERKGHARVKFVCFPNFILLLLFPFLLFVFTLLGDLFFLFYCYVQSLTPSPLISNFFSRF
FSFCCLISCFILLNGIFFCTRLLFFFLFSFPLLKLLFVCPTYVFFFTLVDCEEQMRQFIFYLPYFSSITFL
SLK
>tr|C9ZQT0|C9ZQT0_TRYB9 | DeepTMHMM Topology Prediction - Predicted Type: TM
MRRDCVRCSSSVDRFMDVIDICMQRVNIGYLLKGYFPFYSLGVAFAACIISLSLLILPAQPLYPGAVPLVL
HAFSILILTGGLFNAAGGKIKQRKHQGELITESVKDVLSELLRCATSSSAVYVLLALEVFVQMSTIFSEAG
CICCLCWVWFGWLLCGKREVLLTCWREGGHLTSDS
>tr|C9ZVQ3|C9ZVQ3_TRYB9 | DeepTMHMM Topology Prediction - Predicted Type: TM
MLSSFPFDMFLACFFFLHQRLSSPFVFLHIFHHFRHVLTQTYSPLEGSEVNEEKRKDKIRKKKKERKKPSE
EKGKNLTNGGEKKKEEKCGKHLYIFFLFIY
>tr|C9ZL52|C9ZL52_TRYB9 | DeepTMHMM Topology Prediction - Predicted Type: TM
MQPQSSLFRARAQAHGEARPNGTGALTENAVAENDQIMSALLSDVRAVKKNFTSMGVEVRRQNSFLDSLQD
TFGRTRARLNRTMRYLNLPELTSAKHMWVLFVFVFVVLVLIYIMLKSR
>tr|D0A031|D0A031_TRYB9 | DeepTMHMM Topology Prediction - Predicted Type: TM
MFAVFVFLITHYRHRSRMCTEGKGEKTGFDLPLCICAFHFTSSKQKQQQQQQQTPPIIFNIIIITIVEWMS
SLALPFPPFFFCYYSSSLPSLVTVLFHRVL

>tr|C9ZIF3|C9ZIF3_TRYB9 | DeepTMHMM Topology Prediction - Predicted Type: TM
MSFWTVPSVDDIPPNVSSGTDLEPRTAVEVEAVERRRWLRLVQEAWLRRGRRVPLSEREPPVGGIAAEAAV
VPAVTTAVVVSININPLLWGEEGDGNQEENSIQALDAPYVFGWRSLGLLPGDSFNILGELGKQLERRYQAL
SKRSLMVKHCHNTEVSRLHRGLMQCRERAGGRGKVIFHYGGYGVPRPQSGLIYLMEPSGSAVKCRTRTLFE
EVGLPLVVVADCPNASELLRNFLRYQSSPKPEHNTGSDAEGDTSFAQGPPDADRPTLRVPHEGTAGVSSFD
AGSEETCATSSVLSDFFFLGATFSGELPQHPKLPSDILTSCLMTPLQMALLWFMVENDSLTDIHPLLPFLI
PGALDDKKTPLGQLHWATMAIMECIAWSSLPYKTFVHLFREDVVVAPLFRGFLLAERIITALGGEVCVYPP
LPHMGNHPQWDSLDCIIGRTFVSLKRAVQPAPPTSLTTLEFREWLDWNVTKWRCEQRSMSLPSLGNRPVTV
PDFLEEELRCLMAVAERVTEQSLFHCGGIAWHQCRRPGHIGPHRTGPRKASIYRGMGEEAAVDPLLACSTG
SNDLSGCGCSGGAFMRPFPSVAGLPMLLQGLLVVAHRDEAMKVLCRFIDAGLSAAAACAKVGIFDMALARF
WSRSDLQHLLPSMLFVYAKACYADPSLAGLAWAQRSAVTTSCLKALERPFSLPSDPPAGPGLWQCKELGYW
LESEGQRVLSSAILSMTSFSFDQDRKYLLESGALNLCSELLRDASVRVPLLQRGSAEVYINQSRGSRVQHH
FVATTDAYAARLTTSITSLLALFVALTWKASALEPGAARPIDTGTEGALTNGSGGEQKLGLEELASPLKTL
HLLSWASSSIIRGAALKAISMTMSSSCTDEAASLRCIHIIVECANVLVAPREGNMSNRIDLVDIVFLSIRW
LVRYLSAHMPMETVRASVQCASEKIYRASEAGEGGSNCGGEEDILPGLVSGTRSLICDIYFPKGCPLESCP
SKCDNDRAMEVGCPIVFLARLVCWTGAATHDPCPYVSSRAEEALSCLPPLSAGGFFTQPTTPKESGAVEKS
TKVPSDAKSRRFLSMLRSSVIRVLKRGMRGTGNRSDGAASRGKAAGVERDRRLSNESGGAADVQTLACDAV
LPRRIVSTGSGAAVASFVFTLLGFLDELLLVPMDDDDPRNTFNLRRDFCMREYVHKVRNELRLSAPPAGYS
GDSCNLPAVPSPPLPTWSPPVSHVEGVKPLVDEEREPSGMLSPTGNQLPLINLSITSDTSLGASAWSRPDS
NVSFEHDGHIGVMAFHMAERHLVTGTSHGTVQVWSWSAEEHLTAMAGRSSTALSAACSLTAATASASTSPA
TSVGVGSTSVHVKATCRWISDICVPAVTSWVDPVAFARTTTLYNQQQQSGPRKKMTSGIIAKSSDGWVGLP
WNIHSAWQCPEEDSPVISEHCATNSTKSVTGLHFVDAAYRTLLCVVGSSGSVQLFTDYAGGTAVKRVTSFA
TAACGRRCGGGRMPCLSSYHVPATLLHVSGPDGLIDSWDLVCEHKVLEGIGSQQSSFIMPSVITPSSTDDS
TVAVGGGSVYLFDLRKPARATCIFPSSAESEDDAAKRPVGGAENSKGTCLHISFPYCYPHVIVTGYGGAKG
VVTLWDKRFPRDPLRQVVVAEPVAPASSALRNPVCRMDVQPYHQTLSTVSATADAIFISDVMEESAVNGQA
RASYTRVKELPGAVAFHPILPICAVATGGPPRIYGRSTTCVYD
>tr|D0A8C1|D0A8C1_TRYB9 | DeepTMHMM Topology Prediction - Predicted Type: TM
MCGRQKTENAYREPCWRLSFHNTMGTGGRRNHPPRQPLHVCNEDRLPHCHADHQKRYWLGPLMWMEASAGE
YNKPRSGHFAWIALKQQPQWLQNLPYYRCYTLQQTGALVCFSCYRSLLFPCAVLYYYSQMVGDKYRSHKVC
SIGKNNISGVGSIGGVNYTCRCFQWGYAYAYASANIYIYLFIFTSAWLCNHGKCDVRFTERFRPFLSFPSV
SWLQQELSLSSRGGFTF
>tr|D0A5F9|D0A5F9_TRYB9 | DeepTMHMM Topology Prediction - Predicted Type: TM
MSGARRVCAVCFSFLSTSLTVFAFVVIYFFFLCEDITLMTLSSGADAADERSSVDGQLPPTERCATGHGRE
PEEGTFLGLTADERRAGFIVISFLLFQEVDSNVFRGLLACSIAAYTVKQYVGWYRSCQP
>tr|C9ZTR2|C9ZTR2_TRYB9 | DeepTMHMM Topology Prediction - Predicted Type: TM
MTVVIGLFFFVLFSPSVLQLDFSCCSLHLHYFSFVLLLLTSFILCVTGAPLSVQHNKRMKFISPAVVWTIL
WGCFFACLVLCSYIFICQQSCHIINFVEFTLERALYPFASLCCTSFSIVIAYQLI
>tr|C9ZZF8|C9ZZF8_TRYB9 | DeepTMHMM Topology Prediction - Predicted Type: TM
MHKYKHLACNATGNLCFFYLFFFVLPLLAFFLFFFLFSLRIYHAPLFIIDSLTAKLHKNTKPKRPVSRVVS
RDGYSYCSKFESQCRGVGTKRKTERKKKQSQMNKQIKKRRNEL
>tr|C9ZTJ9|C9ZTJ9_TRYB9 | DeepTMHMM Topology Prediction - Predicted Type: TM
MYYFCRFVTAAPIFYLFVPYYGVSSFNFTAVMALWPRQSFKTLDSLFLKSLLWFFVVFFPNLNYLYYRFIF
FYEVGVFSALFVEMQPYFVTTNECGSYLFRLQSSRIRMPCVHCAAIPPCMSS
>tr|C9ZZ72|C9ZZ72_TRYB9 | DeepTMHMM Topology Prediction - Predicted Type: TM
MQQKSDAVTPLPLKQMFPLALVLLNESLCSSILLPYVGYLVSFFEKCPPEEAGYMSGVVLGSFMLGQFTSG
KMWGWMSDYYGRKPTLALGLIIGGLMVLCFGFSGNIWVCIIFRFFHGLSNGNLLVAKTVLADILDRTNEAQ
GFAMVSFTYGFGILIGPAMGGLLYDPANSNMFRWAGFRKDGVFARYPGLMPAVACFFYAIFALVICLVFLA
ETNPHARPLPGWILALLPSSLRLRATHEVVPNNENIEAVVVEPEPDYFFNGLPDESDILGENHGTATWEKS
QQLSFRKDGTNEEAKQHKEAAPNGTNGRVDSHLEVDREASAPALRGAGDEISVATSQEKEPFGYRDSFVNP
NTRCVLVTYMLICCGDVAFSEIFSLWAIAGTSHGGLGYQASAVGTLLLTNSFPCLLSNVTLHLACRVITNK
LVLWRISVMIISVVVGLMPFVTYAVGGAQIPLLLMCTFARQWFSSWAFGLITIFTARVAPPLHTGTMYGIA

QSCGSIVRCVIPFAITPLFAWSISGHKPIPFNSVLTFLISSVMFISACVGSLGLKVEDENELQNEQQLETN
YVVVMDCEFDDEDARGGIFTGIKG
>tr|C9ZUN0|C9ZUN0_TRYB9 | DeepTMHMM Topology Prediction - Predicted
Type: TM
MPVVCVCLTAVFPFFFLSVFHTIATATLMCVCVCPSVCTPSTHSYNAQMTSPLSLYVIPAGIYLKATHGTP
LSPRPFPFVFLGVSHFVANRCAFCSQTNKCGRRCLANLKQQ
>tr|D0A0R1|D0A0R1_TRYB9 | DeepTMHMM Topology Prediction - Predicted
Type: TM
MHHQLITSTCIHAHMSYPASFSFSHSLCVCVCVCTPASLYTCLWPCQWTLNYSIFFFLVGGFEAECASLIR
EQQARPRCLLVCNECCGLETLSCMMCSTGITI
>tr|C9ZT02|C9ZT02_TRYB9 | DeepTMHMM Topology Prediction - Predicted
Type: TM
MRISQNHVFPLVLVLATTLRAYVFSFTIFFFLRPHVCSFFLYSRYQPVSFVVPVRAFLRRFAPLSGSVSSG
DNGCVAMPNLNYYYYLLFCASPSLLPLFCPFIYLFI
>tr|C9ZVY3|C9ZVY3_TRYB9 | DeepTMHMM Topology Prediction - Predicted
Type: TM
MVVSVVGHWLRNKIRGLLRRSILFRFVFLWATALYVMCTTTAVLFCTPVFLIFYFLRLQHSWSFWKLLPFP
FVSTYFAPNDFLTLQKYHIFFFKRKRLRPTDTIICV
>tr|C9ZP07|C9ZP07_TRYB9 | DeepTMHMM Topology Prediction - Predicted
Type: TM
MCLHGKGKESRNKYKPHLFTLFTLGHTNYQKTRTNGSRHSLQKKKTKTKNKSDEGKKPLHFIPFPPHVRDA
IRSFSTFLLTYTFWVYAHKQHKLPFVTLILYLLFLNLSFPLADGAHIEANILTGS
>tr|C9ZP79|C9ZP79_TRYB9 | DeepTMHMM Topology Prediction - Predicted
Type: TM
MQTLSASFAAAAGVSHESLPRHVPTLTLLKRILVLTVKLSIAQVAQFSLGITLLAVVGKIGVRELGGASLA
NGLVNATVFAFGAGFSGALETKLSHTFSRNPKDKMYGVYTLRMLIMLLITFVLLSPTILFLDRVLVAMGQD
PAVIDFTGEFCRLSIWGSFFAMLLELLRRYFACQHLSTSFSVSLVIGAVVYPFLLIGLVKVMGFSGVAVGW
SLLMICTTTGLVLYVVVTKKYLATWGGIEDAIYRNWGPLLKLGLSSMAMMLSEWVALEINSICAGFGTKEE
LAAFGITYQMSGICWAITSGTFIAASVLVGGAIGEERPMFARRLAILCLGTSVAISLCNVAILLATRNLYP
RIFTDDEKVVEIVDSLMNYVFVYHIFDVFQSCMMGVLRGCGMQKQGAIVIFFVYSVVGVPLGLLIFFFTGF
GIQALWLGPLVGAAVVGFPTYLYMMMRYIKWDTLKPSVEVYDSDLEEESEESGVLEGVAKVNEPIGTEGDV
ITTLSSNAPTRVTTNSTEQEDVSRRHQ
>tr|C9ZV36|C9ZV36_TRYB9 | DeepTMHMM Topology Prediction - Predicted
Type: TM
MSSIYTKRALLFASHGFQMLALVPSVRESTETSSWHLFIEIFQVLGCLNESYDDVGSVFRRTKGFLICHRY
WWMGLSTGYFFFLVLKPLNIIFIVTLLD
>tr|C9ZKC2|C9ZKC2_TRYB9 | DeepTMHMM Topology Prediction - Predicted
Type: TM
MPLISEKRGASLETFPNNRQPQDDVHNIRLLSESVIIMKRAGQITLPVESPPWKPPRVPASLQRRPVLRLL
SFFSWLAAAILVAFSHTLNGGNASAMAKRPDTHNSV
>tr|C9ZPB6|C9ZPB6_TRYB9 | DeepTMHMM Topology Prediction - Predicted
Type: TM
MAAGSEKWMLVRASHVLPLLLTNFKCHSSLNPLMASISVYLGRRRVAFKCNIFHFRWGFLNRLMHYISLTQ
LVPLFLMKHVQKKTLRDIIRQHFHLFTSCFFSFLCGVIKA
>tr|D0A016|D0A016_TRYB9 | DeepTMHMM Topology Prediction - Predicted
Type: TM
MNCMFRGNEEVYHLHLGVKGLEDIKKQFPKLLCVGWTLPPGTLCVPRTLPWLRIVFVHCNALVSLFAWVAS
ELSARYDITVYGNFPLLFMNTCSGEGVLR
>tr|D0A2A5|D0A2A5_TRYB9 | DeepTMHMM Topology Prediction - Predicted
Type: TM
MNPLPSHPLPLQFVIGSISVSNHHQYLWLPHVYLILSLKIQFVSTPPRFFPLGVFMCTCNCLPFFFLFPFL
SPYILYSTTHSLNQLLHFITRPLSKLCFPLFPVYISSTSSSFLMCKQKSNTQIKV
>tr|D0A767|D0A767_TRYB9 | DeepTMHMM Topology Prediction - Predicted
Type: TM
MHLLQLEAFMLFSYIFYDRLKHADIYIYIYIYIYKPFSVFSFPLLLSVGAHKETWNNTKFNQFTRSGLHYL
TVTFTSSLFVYDAFLLFVCLSFSFYLVCFHGGCPKPTGTP
>tr|D0A9R9|D0A9R9_TRYB9 | DeepTMHMM Topology Prediction - Predicted
Type: TM

MSTSGIVRRLDEGMFTVYPYHSGWIVAPILLLLSAAFVPVGVLIIKESDSLLEVSVSYGGVNKYTYRVDAE
DRYPHKFSFNGSNYSTGATTVISFKINETVRQPVYMQYRVTGFFQNYRRYRSSQDYNQLLYNPRSVSQDCE
PFRYPGEVHKAAETGNVYFPCGSIAWSLFNDSFKLYKGNATSTLNDSELICDGSAFDADGKSSVGHSCRKN
GIASNGDIKLFRSAKEPEDEGIWSSKGKSSSDDPYRKEGYYYGEPGHRIPSVRDEDFIVWASLGYTSEVTK
MYRIIEKDLEQGDYKVEIVENFDVYSFKGEKYVVLTTRSWFGEKNHEMGITFLVVGCISFVLGLGVIIQQW
VL
>tr|C9ZJ48|C9ZJ48_TRYB9 | DeepTMHMM Topology Prediction - Predicted
Type: TM
MTAHISEHMSKRGPRESLLILSLGVLFFHLLSSYVEETVFYLPGFHHTGLLSFLQVFIMAVVSYVRLCRTL
PMEERGSARVGLWRRLLNSRKVPLRTYVIISFLYISSAYLTNEGSRLLSYSTQVVLKSAKLLVVWPVRLIV
IELPLRWGDATTSEGVSRNGDVICVADVVNEKECESTCVLVSAEECCDTPLDPAASVAQSSDQSSPTSNDV
FSTSINAPHDGLDMTVNSTAACVANSTQHSREGLWSILKESFPCFVIVFGVVLFMHATNASTTAAVEAVGD
KMENHEHRVREICGVVAIIVALLCDAGVCVAEEKYCFMAHGASNEEVMFYIFSISSCNGFISLLLSGRLAD
CLHFMQGQPYFFPLVLLASICNYCGAYFIVSITSSYGSSTSTMVTSVRKVTTVLFSYAVYLRPIGAAHVVG
LLLVTSGVWQFERIRRRNDERA
>tr|C9ZJQ1|C9ZJQ1_TRYB9 | DeepTMHMM Topology Prediction - Predicted
Type: TM
MVAYASAASSYHAGVLVTSVVLSFGLGLTVWWLASGYRGRMRLRSRVVLVRFVKQVVDDWEEFCLREVEHW
LAVRSPEGNIGEDSLFSNHGAKERGEVQDALPFHGPMRSMAYAPGVDLSRWLQLWNVGRRLCAALPEDMVL
LERLGYIIKYTAFRRLATMAEARYYAAAWRSAVHGSDTKNGKAQIRRPRIPPWLVGHRPFWIVPLPGPGVG
QFLCLPLSYFPVEAVLTSGAEETWVKDLADVLPLIMGQLSFKFLVDGLRTRQARRVAHATIRRRYPPSWKR
LWREIDRLFNTFAAPLAKSQLERGRMARQRFCREKLYEGVELVSGDGPDMEEGMDGIFSVQSSEKVEGVMG
RDVLLRDANLTRKLTLLLRFRTLSHAVRIAINLWAPRYNVFAGWMLPYVLSSSGCLEGRGAVLMDFARSVL
WGSISAAVGAAVRSFDDVFRWKILVMLRDEVLHEVNVKIAVADEAFLRRCSSEGVGRSGGNPLARSFEYAE
NTAARMLGYFDCEQKKYISLIIGVVTAMLRQEVDVAAFAAAASWLNFYEISRAIGRWCGLLLDKSVEHEIA
RMGEEPLPAEPRYGLQLLLEVLAEENDGTTGTNAPVEGDAPVQNGSRASHSRYFLSLIACGGTFHVDWPVG
PLPTHKKAQTDEVAYLRRLQKLFDTVVSGERACALSCTSFICSPNDGALSIARGLRENAACVREFVLKERV
QHALEGDTVQTVPANSSEKLHAVNGGAKAALDAEEAALLARPQAMNRIPSTLGFDDVFSRPPRFILRQLGL
DTVFAQRAALSVRRAESFTITMDLSDPISRPFHNVFTQLLLHLTEFIETLLFYSLLSYRSNLTSIWGVWPT
WFTTRWACDGGATIAAEWSAASLLDRVSAYRALMRGNERVELDHPFRLTQQVARYMPTFVHDASPHNYSAV
FKARRGLWRNKIKHVEGIQFDDAGRISEGFHLRKGIDFRGVWFVYPELHHCATDGSLQPSLANVTVHFPAT
QITAILGRTGSGKSTLLSLLKRMYDPVAVVSLKEERVWEESEGLITVLQRCLGTQLPQANEGNCENIVTID
DIPLTCFSTTFLRQAVGMLEQTPFTFKGLSFLQNISLFTPRVSRDECVAAATLCRCKEFIESRPLGYDDKS
KELSTGEKQRLALARAILVGRHGLGVCLLDEPTSHLDGHTAAYVEEAIGNLTTIQQPEVTVLLVSHRLSTI
RYATHVVILDGGQLEYQGPADERKLRSNSFLRSAAEGQDLQIVRSAARPEGRTQGNVKSAVYADGLLKVSS
VDA
>tr|D0A3D0|D0A3D0_TRYB9 | DeepTMHMM Topology Prediction - Predicted
Type: TM
MFRNHASRITAAAAPWVLRTACRQKSDAKTPVWGHTQLNRLSFLETVPVVPLRVSDESSEDRPTWSLPDIE
NVAITHKKPNGLVDTLAYRSVRTCRWLFDTFSLYRFGSITESKVISRCLFLETVAGVPGMVGGMLRHLSSL
RYMTRDKGWINTLLVEAENERMHLMTFIELRQPGLPLRVSIIITQAIMYLFLLVAYVISPRFVHRFVGYLE
EEAVITYTGVMRAIDEGRLRPTKNDVPEVARVYWNLSKNATFRDLINVIRADEAEHRVVNHTFADMHEKRL
QNSVNPFVVLKKNPEEMYSNQPSGKTRTDFGSEGAKTASNVNKHV
>tr|C9ZMW5|C9ZMW5_TRYB9 | DeepTMHMM Topology Prediction - Predicted
Type: TM
MQGDTKTMGKKKTVSSLNETRERRHLDIGKVRDVWNTFQTAMWHVCLPNELVMCFDPLLRCIFALYYLPPV
ALVVAITHSALARIVWVLVTPVAQEMEKQPYDASRIVTLALGLYQIIICMLPFTTLSIAVATLGHKALNCF
MESMVDSWCTYEQRDYPLLAARLRFMMEDRWRCCTFLVVLFLLGTLVLSGFHTDLLVAWPDREGNVALNVW
LIIFIDLTFVVVSAFTCSPPGLRLLNSKKDTADGKDGSFLEYFFDTWWRKRVFYIRYATGYMVIALGVFTT
YHDGPLRALSMLLETLMYLLIPHVMVHAVLSISIVAVNFHRSVRWLSHHRAAASHTIAFILPYEGVLLYSM
YYFRHHWMTVCLLVGVSVLLIYRSIDIAREFEVTEAGSVLWKREDDGTVAPPDLAKVLEEAHENKSRHLNT
VSLGVSGDMERMRLISDGKEIPLQRVVIERIGESRSLSKKAVFLAYAFPRVLHAQKPNYLGGKHFRTGRLL
LRTITSILLTFFALLVAGVILQAAFPELRKWPVRLRISEGGEILTIDHIVVRMHLLSRNASANPLSPPVTS
AMTPAHAAFQWGNTTAFNTDWYASLCAREFHGASVWEVSLLALATYLSTEEEVRQMLHFMNTHMETDWIMR
ERHGMDCVAVDSSTKPTEWNGYFDFYSAKHDLSVVAIRGTDMTSAIDFLVDFNMFFEVVLYHLLSNFVPGA
GILPSHLIADLIGLASLRSDGNFQYGTWESLIAESKADDKNNKLQCVSNNYRRDFFADVYNHIRYIGSRSK
RPKHVILTGHSLGGAVASIVGAKMGIQAVGFGAPGITLARKKFNVDLRSINKHVGNIISSHDIFPMIGGNV
GEQHRIECLATTRELCHAMEFLVGALWRSCGSIRSRFPSMGSVL

>tr|D0A252|D0A252_TRYB9 | DeepTMHMM Topology Prediction - Predicted Type: TM
MPTNDAPEYTIYAERYSLISPSINKTNKTHTKKNSIVLISFFKKKEIRRANLCGNTHSRGEERRWEYIYIY
IYIYICEFKVVYYYYYYYHHHHFICAFRNFQRNV
>tr|C9ZN85|C9ZN85_TRYB9 | DeepTMHMM Topology Prediction - Predicted Type: TM
MSRVLDPRVPVLSRGVTPSLEYAVMDGSSLSPSQLAGIIVGSVFFVALAIALCVLLCLFVFNSRDNNRAPR
EPTDPVTLIFTDIESSTAQWSTHPELMPDAVLAHHTMIRSLIMQYGCYEVKTVGDSFMIACRSASTAVELA
SDIQRSFLQHCWGTTVFDDFYRNSEMQKAEDDDHYIPPSARLDPEVYRQLWNGLRVRIGIHTGLCDIRHDE
VTKGYDYYGRTPNMAARTESVTNGGQVLLT
>tr|C9ZM16|C9ZM16_TRYB9 | DeepTMHMM Topology Prediction - Predicted Type: TM
MKENGVLGGVLVPRTRRFLVGGILFSLFVILLNLLIAYAVRRYYPRSRAKKERRSRRRSGSGSGNGKNSGR
GKHKERKRSDPKVILLVGMPGSGKSTWLKQYKGRCDDSCRIVDEDELRVEITGKFDDFSKEDELCAAMINA
IVRHISEKHNVVVESNRYVLDEKFRKQIISTVPSCRLLVKEFDIKAHFAQARLAKDAEEGKRHHTYTETEL
EDWEVRQLEAKELFKKEGWSQMH
>tr|D0A8C8|D0A8C8_TRYB9 | DeepTMHMM Topology Prediction - Predicted Type: TM
MLRRTIFLGRVTRSWTKGSIVKASAHFPDSVRADPAERRNEDSSPKESRTWSLHDGPCSEPLHVPKHIQWA
TQKVRVWGQLMRVDKAAAINLLLQPCYWGASLAVTRAIVWEGADPVVLFAPFIPVHLIVLFGAGAFLARGA
GCIINDMCDRKFDRMVERTKTRPLASGVVSMKEASGLLITVAGFALVIALNLSPVALASAVALAPIAAVYP
LMKRITYMPQLVLGLCFNGGIFVGYAAVLNRIDLAVTLPIYCSAVVWTVLYDTIYAYQDRADDLKCGVKSS
AILIGDRKHILTFMILPIGMGILISGLMVSQSLPFYIGVLCCVWYLQGVVDNVNIYDAWSCGIGFRRNVRF
AILVTFSLCLGNVLWALASEHQPEKDAASNAISEKSALMKFLLLNKEAEPMSYSVQDIRWVDRFAHPAFVA
AQKAQHEGEEKTVVVPAWMRREYLGENVGTIMRFFGVDEEAIQTWQKWWYAQLDHYNMFSSIAV
>tr|C9ZUJ0|C9ZUJ0_TRYB9 | DeepTMHMM Topology Prediction - Predicted Type: TM
MWDGPLRSRQNLRTVAKLRSSGCPLTQSAIHPTTTSPSEGETASEILQGQASAGYIEEQQLPATSSQLEMS
GDSNAASDNNVVIMTKRQRDDLSQASQAEVLSRLRTEDAQGPAENNGAQSDLALHSCDMEERVDSSKLNLN
HVTVNKHSPPASKSAGISQQNDDGGCGASENVNNTTTAASKQRGKLLLDSSSSNCTPKQQAQQAVTQVPEW
ELSHEQERIFDIVVNHRRSVFLTGGAGTGKSHLLRAIIAALPLSTTFVTATTGLAALNLGGTTLHSFSGCG
FVDQHTSTHQMVYRNVLGRKKARANWRKCRVLVVDEVSMLDAWFFDMLEYVARHIRGCRKPFGGIQLVLSG
DFLQLPPVNKHSPKQETRLCFEAKSWPRVNPLVCTLSHQFRQKDKEFFSLLNEVRVGALTAPSLGLLSSLS
VITTVSFVDEEKLKLKREVGAEAVDIITDSKGRTRRQRQDGFTILRARRSEVDAINTEKFGELDTEIYSYK
GAHRGEGHFPSDLPSTVSVRAGCRVMLLANLDLSAGLANGSIGTVESFVSSKLHQTANPSTKDDLQHLADH
MMLPVVRFDHKGKQGPGDGGGAAAGRLVVIEPHRWTMRQGDSDVSCSIQIPLQLAYAITIHKSQGMSLSHV
NVDFAGIFEEGQAYVALSRCTDVANLVIENFDAQRVNPNIKALAYYRALEFVGTEHREAEKKLIDNGNKMN
PWGPYDVEDFEASDDDNGGAVKKEVVENLTYDAENISCMVEQFRQRYMPQYIMFSTLRRRVLSNTEDAARV
KGALLVMDTTSLLALTNMTGPTSLYQTIFTERGNMMRVPRVVKEELLFLASTDVKEVSSVTTPTLHSFCST
CSSTPCSTGFSYDFVEVVSCALSIMENAKCDFLLDEQREGEANSLPPVIQEWRSLSPLLMLNSSPDTGEKD
APSVIGFGERSREQHHSTLMFASFLVSRYSGNGAVYVCTETVELAARALAIGLRVCSIAYLCNRPRRVN
>tr|D0A812|D0A812_TRYB9 | DeepTMHMM Topology Prediction - Predicted Type: TM
MLQHFLRPEVLGTTGKDEENGTKDHQPGQQRPTTSRLDSQSLTATDTVSERPKTAEPLDLVSISATYKETA
VLFPDAHQAAFEPKVQVPFEFQRGRIPREIEIERRRRLYESKDVSRLVQVAGLTLKLLAHKSSQELPLQVF
DDTSYDSRIPAEWMEIAAQNENPAGRYLPAEGIYEFMNSDFRMRPCRVIGWDVARNEVKLLWGAKPVPDET
PVVVPRFHVRLLAEDPVVYVERLVNAQKQRVKAMAWIRYRLCCDAMPTDGLPGLDSNLSDRLLRLGTGIPN
LNKAVFPDVDERAQRLIAELTLEWQRSHNRILLQDLMQRDESTLRMVANTTQMSLQELVRGPNVEIRRTIK
SDPSSVIPIGDFDFAERERSFTFSTYYTQPEVVTALTGVRSECMKVLEGSLFNLPKARQMQLSEFQKLQRD
HMAAVEKYLKGEWTENICNVIRNSFVSAGKGWLNVHESKQEIYEISKLKKFFTTVKFMMEDTLFDLVYTSL
QDFTVFFEEVSEFTVNVIDMNNVENKWPGSDADDCVEKQPLFTIRLAEQDGSFTYSISFKDFEEAIIDLFF
SAIQCTDAIPQVEKFVMSQYFWRREGEGPFLDSVKQQEERVCLLRDRVRHALQNSMKPLRDYLQTYDDLLP
LVRLDKKVFITEYAAQEHTMEEMKEEIRAHLKAKKVVAQKLPAFITVGNYVVDCQSFGQIMASKEHELAKL
VMNLICKIAKTKTSYIREEFTKIVRVVEKQPQTPEKLYELKAIIVNTPERISELSAEIEEMRQYYNVLDGF
QYELSDEESRQKWEAISWPRQLTLRIQETNKQLEKVEEELHARLQKEAEEFSKKVDALQRVVATFSKYTDA
TEAEKVAAEVKVNSIEIRKCIEQARSINSDQRLFGDKLTDYRSVFELEKEFKPYSDLWLTTYQWQDCYRRW
HADPFDSLDHEEIDTVVTNAFKTMTQLSKTFKDKNATLKIVSEIRGKVEAFKKWVPIVTSLRQPGMKERHW
KGLSEKLNLPLVPGETILLMEDLEPLLGFKDVIVPHCEVAAKEAQIEKALKDMRAKWESRVFIIEPYKESN

TYIIKDSSEIVELLDEHLNLTQQLQFSPFKAYYAEAITDWERSLNLISDIIEQWLECQRAWRYLEPIFNAK
DIALQLPRLTKLFDRVDKTWRRVMGTVHHQPNVLDFCIGTSKLLESLRESNRILEEVQRGLNDYLAEKRQS
FPRFYFLSDEELLEILSQSKEVRRIDAHISKLFEFIQRLSWTENNEINGFFSGEGEHVPSVNVVYPEGNVE
MWLGSVETMMKEAVAEQLRQSFYAYSNTPRAKWVLEWAAQCVIAVSQIFWTNGCEEGLVAEKSVENYFRVL
EHQLFELVDVVQSPLNARERINMGALITVEVHAKDTVEAMTRHKVDSIQSFEWIKQLRFYFDTDDRMCHIK
QVDAHFVYGGEYLGNTGRLVVTPLTDRIYLTLTGALALCLGGAPAGPAGTGKTETTKDLAKALAKQCVVFN
CQEGMTCLSMAKFFKGLAWAGAWACFDEFNRIDVEVLSVVAQQVTDLQQACVMKQYRIVFEGSEVVVDPTH
AVFITMNPGYAGRTELPDNLKVLFRPVACMVPDYAMIGEIRLFSYGYKKARSLAQKMVMTFKLSSEQLSSQ
DHYDFGMRAVNTVISAAGLNKRENPNEDEDLLLLRALRDSNAPKFLKDDIILFEGIISDLFPGTKLSPTEY
GVVVDSLRQVVTSSQLQPVPGFIEKCLQLYDVTTLRHGLMLVGPAGSGKTMAYTSLQKALSGCSVMQSKGQ
DVGARDYMKVFTHICNPKAVTMDQLYGAYDENGEWKDGVLCVLFRRAAKYGDEGNQIGKHWVMFDGPVDAL
WIESMNTVLDENKKLCLVSGEIIQMSRDMTMMFEVEDLAVASPATVSRCGMIYMEPTACVPTQALTKSWKE
RLPKYVAPQADYLEQLVELYVDELIEYVRANLREYVPSTNVILVHSFFRMMDGYIESFGGLPGQRGPPTLS
PERLEIMAKCITPLFFMAITWSIGATCDEVGREKFADMLREMATRNNHADSLPESGSVYDYCFVYYPSPDD
DEEARWTHWDELRATCDIARTTKFEDVLVPTIDNTRQKYVLTHLLERKVNVVAVGPTGTGKSVAAGGLVMN
GISDRLLGLAFSFTPQTKAGVLQDSLMSKFDKRRSHVYGAPVGKHFLVFIDDANLPQKERYGAQPPLELLR
QLLGHGGLYSFVGGIKWNLVIDTSFVMAMGPPGGSRTQVSNRLMRYFNYVSFPEMSEASKRTILNTILKGG
LHQRGVKEEVVDFITNLVDGTLNVFKRCRKAFVPTPSHVHYSFNMRDVMRVFPMIYINDTNSLPNRDVLLK
QWVHEMQRVFCDRLICNEDREEFLSFIDDEIIQIGYEGGYKSLLPDGRLIFGDFMSTGERSYQQITDMDAL
ATFFNEQLLAYNNANENPMGLVLFLDAIEHVCRITRVLSMPNGHCLLLGIGGSGRKSLTRLACFLIPEMDV
FTIEFTKNFGVKEWREALARLLLDCGKDGKKRTFLFSDTQIINQTLMEDVAALLTAGDVPNLFEDQDIEII
NERFKGVCMSENLPTTKVSMYARFIKEVRSNLHIVLAFSPIGEVFRTRLRMFPALITCCTIDWFAEWPGEA
LLSVARAQLQSAKGDLGDDEGDRLSRCFKSLHLSAAETTERFFVETHRRSYITPTSYLSLLNTYISLVESK
RKFGREQASRLENGLEKLYDTEVRVVELEGQLKAQQPVLEMKKLEIRGIMEKLRVDRKDAAEKEASARTEE
VAATTKAEECARMRRECASRLAEAEPALQEAVKVLSKIKAAEISELNKYQNPPKGVQYVMEAVALLLTFGN
CPKEFYSGPPGGKKTPDWWLCAKSYMKNANQLLDTLVQPPGKGGFDREAMDMPLIEKVRTYYENDEFQPEK
VKSVSVPCMAMCQWVRAMYKWFFVNREIQPLRERLADAERELKRVNRALAETRRKLDAVVEAVAKLEKEFE
DAMATQTALENEVEQTSEKLQRAARLIAGLGGEKVRWKELVEQYKVKDTCVSGDMVIAAASIAYFGPLTGP
YRKHLLQTWSASLAELGIKTSENSDLLSTTGDAVQIHDWQLCGLPKDPLSTENAIILSNARTWPLLIDPQG
QANSWIRNLHKDDNLQVCKASDDKFMKTVEGAIRLGLPCLLENVGESLDPALEPVLHRNVFLIGCTPHIRV
GDSAIPYNEKFRLYMTTKLPNPSYTPETIVIVSLLNFFITRSGLEDQILARTVEKERNDLEQEKQRLTRDC
AEKNRELKELQENILRMLEEAEGDILDQEELIDALEKSKLKSTEISEDLVRARATEVTIDETRNKYRPHAY
RGALLFFCVSELSTVDPMYQFSLQWYINLVLLAIENTEAAVDIEERVEKLIEFFTYSFYTNVCRSLFERHK
LTFSFFLCTSILQQQDELDGNEYHYLLTGPTGSGGEEPNPAPDWLTENSWNEIQFVSSNLPNFAGFAEHVT
QCINYYKELFDSLNAHTYPLAAEWQGRETPLQRLVVVRCFRRDKVASAIQEFVKHYMGERFIIVPQFDLMD
AYKDSTCLTPLIFIISPGSDPMNDLLRFAEHMRMSKKLDKVSLGQGQGRKAEELLSNGRERGQWVLLQNCH
LATSWMPTLEAIVESFTLETVRKEFRLWLTSMPSDSFPVAVLQISVKMTNEPPMGLRANVTRSYYGLTDDD
LEHPTKPNQFKKMVFAFCLFHAVIQERRKFGSLGFNIAYEFNDSDRNVCLLQLRKFISLYEDVPFDVLTFL
TGEINYGGRVTDDWDRRCMMALIKDFITPGVLEEGYSFSPSGTYHTVEACSRAFYLDYLGTWPLNPEPEVF
GLSDNADITCAQSESASILATILSLVSRESSGSSHQSREEMLIKTAQHIMEKLPPTFNVQEFHAKYPTKYE
ESMNTVLVQEAVRYNRLLRFVQKSLSEFSKAVRGEVDMSAELEAVGSSFFINAVPASWAALAYPSLKPLSS
WVEDLLRRVQFVQSWYDRGMPNALWMGGFFFPQAFLTGTLQNYARRKDVAIDSVSFNFSFLQDETPTTVAA
PEQGAIVYGLYLEGARWDGAGRTLAESRPKELYVDVPLLHLDPVVDRVADPNDYICPVYKTLTRAGTLSTT
GHSTNFVLSITIPTVAPPEHWIKRGVACVISLNF
>tr|C9ZZR9|C9ZZR9_TRYB9 | DeepTMHMM Topology Prediction - Predicted
Type: TM
MLTNFGKRIPITLRLYIGLVFPIILVFSVMMVTIGKTTETGARVTIILIGLINGASTALCSSGAVALAGPF
PTKFLSAYVWGVSVCGVITSTFAIVIKASTESNFKRTEDRVASRLTQSRIYFGLVMIMQSISCGLLLLLRK
NPYAMKYTADFRYAARKGNAVEGDDAGDDNEPSSLGKGPADQDDDLKADCKAGKSNVMTSTVDPDTMRDTD
QVENITNSQQMLKASALSVFRRVWPMLAVCFIAFFTAFLIYPGVFFAVKLGPDDNGWYMVIIPMMFNLGDF
VARLFVQFKTLHASPLFVVIGTFARLLLVIPIVLCAYSVIKGTTFPYILCFLWSLTYGYVGGLAGVYAPRT
GSLTTAGERSLAANWAVSSLLFGIFAGCMCALGVNSALPKDESQ
>tr|C9ZJF9|C9ZJF9_TRYB9 | DeepTMHMM Topology Prediction - Predicted
Type: TM
MKVVHVYMHIYIYVCVCVCTCFLFCIQLHKERERGIFWVCYPFYVSQSRCSMVGFFFFPLSSFPPFPFFFL
TVTGNERCFTFFFRCCSLCVLMLCAGLFPCLHAYHNHHQLYFLSRTP
>tr|C9ZJZ0|C9ZJZ0_TRYB9 | DeepTMHMM Topology Prediction - Predicted
Type: TM

MYTELFELLLYFGIATLFPICQFNFSIPLSFCFSPAKMYLVQKHRKRKGYWSTNRGHWASSFFFNIDSLAS
TDTLLCYDPSLLLFLFSQNHSKGTLSLFVIVVTTTPVVCNAAERERIRRPAAPHSAWGVPFFLKKQLLFVL
RSSGPGVAMSLHVGNGLQQMEKKQCVSSCLLLTSLHVRGPLPLPSARSLVASVCVGTSSITTDVLQPVMSD
EVSGSSVPLSPVFQRASSTHQRLASASSVIGLEPLRIMFPLIDDDACHGDGGSGSAQPHKYEFCIGLREAD
SNALLGVAICTRSGAWFPFKPCGGSARITFRYCEQMERKNRILRHLQEAQLYPTSSDTKMQSDASCKLEAS
SSFLAPTASSVLSASTSFVDGGGPGKGSRPTHSGLSATDALAVARPHSACSSPNFPLLPATDTKVKPENEH
SSHIMSVPNVTTEGPRHLASSNVATSQTCKKEPRRNYHCGLNPISVNGFFTTSDPCPPGGLREREGQPRSL
VFSNIHVKGFFPLSLTGSWRLEFLIPHPCIPVTKHTVTVPATFDTDGSSALRDTLTVDLPLFLRGEVQTRL
RATWVDSNNLDDITEVPTVVEFDPIVIKVGGSEVCQQERRYHKLVRHSAEKFTLSELGVQLLVALKYFAPH
REGQNGRPTSAHSDARLWRSAMAWREGEKDADAVKTKGNGLGKGGDGADMDVRLPELQTAATPDGKNMSKN
SIFTEEAMSAKALSFGDNASQSNAHRSPPSKEVPPPRRASKEEVRNPGMGHFSTLKPATNAQLVASMDDLV
PCYTEEQRRAVFALYDTNNRGYMTQEQILHFCRAHVALFDGHQNDANIVRLLQSLFPRMPRRRMNATEAAA
KATSSYLLQSRKPCAPALEQRLKNRGLQTPSGRPTSGQHTAGGSPQGALGLPRRCITYDIFEVIALRLAGM
>tr|D0A2K0|D0A2K0_TRYB9 | DeepTMHMM Topology Prediction - Predicted
Type: TM
MTQNILLDPSIRDWVLFPLIALVIFVGILKHYASILMKTSASPKMETMCCANTVNCARHLLSEGRKLPSEA
FQQRVKALREGPLKKKIEVNPMEIMNDPTVLGDMMKGNVLSMLPSMGMMMLVSYFFSGFVVAKFPFVLASR
FRGMMQRGVEIDDLDCNYVTSLSMYFLIMFGSNSVLQLLLGEGGIPDENAMMMNSMSGGGPQQPVDYNKVF
KSLSDELEYAQDKHRWVYGDAPRLLLEGK
>tr|D0A728|D0A728_TRYB9 | DeepTMHMM Topology Prediction - Predicted
Type: TM
MQGNKGEYKNSLHCAKRIVMEGGPFALYKGVIAPMTGTGVVMALYFVAYDATETLIRKLKGVDSLTPLSMG
EIMLCGGSTGVLGSLVLGPAELLKVRQQTALSSGARGSLRDVILNIYRKEGPLGFTRGIGATMLRDVPGSM
AWFGAYEYTKLLLCKNPKDPSVGEALFAGGMGGIAVWSFSLPLDCIKTRVQASSVPLTPVVAFRAILSEHG
IKGFYRGIGPALLRAFPANAACFAARDKTKSTLNNLCGM
>tr|C9ZRS2|C9ZRS2_TRYB9 | DeepTMHMM Topology Prediction - Predicted
Type: TM
MIIIKHNKKKTILTQNVFAFTITKTALFSSLLFLLSSHYCKRREIKRIKIRVGELHSPPYEPADINKNIYI
CMNIYIYIYIYIFINMCVNIQTSYISNATRPKAMQRRQNVQRCR
>tr|D0A8F5|D0A8F5_TRYB9 | DeepTMHMM Topology Prediction - Predicted
Type: TM
MQVTTIFIKCSVMGEEVLSALIVVVSVVVVVTGTVFVAGAGFLRISFGALIVSLSFSLVFSTLSSILLEAS
VKLPQESFPVALLRAILSAAGKQTVSGIALLALLFVVAAAAARACGLLEDIQILLDAHKAFTDEGDAEIVQ
R
>tr|C9ZP56|C9ZP56_TRYB9 | DeepTMHMM Topology Prediction - Predicted
Type: TM
MMEQCFGGTENWLRDRRDIYCVFFFPVTVSVWPRTFIFTFLFFLCIFTSACFWTKAKAAELVPRKRNFTLL
LFYLFIFFFTCGTFVIRSIFFAFLKIKIILKISPSPLLTVCHISFI
>tr|C9ZRK6|C9ZRK6_TRYB9 | DeepTMHMM Topology Prediction - Predicted
Type: TM
MRQLGSAVLACRNFNTPLKSLRSITLPTTSSLKRLLRRSQLGANCSTNRMCFATTTTVMMLNKFGLKSSGD
ESASDGYNGIYRMESGSNKSGQHALESVEEDLNSLKRRVQQRYSDELRFLSHDSNRQPMVIVLGNHSAGKS
TMINRLLGIELQRSGVSPTDDGFTVIQSGEDDITEDGPTAVSDPRYSFQELRKFGIHFVNKFKVKTRKLPA
TSLLPPGLMIVDTPDMIDTPIHLNDRTSVEGQLRGYDLFAVTRWFASRCDLIILMFDPANPGTTGETLDVL
TKSLAGVEHKLLIVLNKSDMYDKAADFARVYGVLCWNLSKVLQMKDIPHIYTTYFLPRGDEFDDTRDAAGF
AAFNKSSSTEETAVGGRKTASGSPQKESTSIIARDELLRQRSEVVSEILQAPLRRYDNLITELEEGVKRVL
LAGRVCTEIVSTYRKMKVVATLAPPAIFCVSGLLLAVGGFTEVAALLASATAIAAVFAALKTRKSLVDFEN
DVLTNIDVIFDRLYVRHEKTMDTQLRWKDTVKPEILEFLNSSSVAGRQGIASLPTLSKKSQDSIISILKND
IPQLRVRVANYKVKNFLRAGERQPLSDEARVK
>tr|D0A8Q6|D0A8Q6_TRYB9 | DeepTMHMM Topology Prediction - Predicted
Type: TM
MFGPLSFKNQFCHYCFFSLSIHTRTALGIHLIFFCVNINSLLTRACAYVRLRKSRFSRCMFSESLYSFHTV
SADKNQSNNQKGLLEDEPCQYSNGNNEPGNKAKGNRDHELVGRWGSPRHGGGVGVRISTSVSGKFDKRCPK
PSEGYVPPHYVGLPKYWLPNETFHPEINPRSQRITKWENYVGNSERYHAICELVKELWLANAPVNGNEMGI
DGVNGALRQLGIEVWSPLAENFFSELSLNETNGQRLVGYSAFKRVFVRELQALLTPSSGTNRLDGEGVPRG
DTCGVRFCDPNTICSSIQTLRRHQSTQGASSKNTVSSAAASSSFRRTAAGEPSVGWPGISDICSALSGHDL
LGVTAADVYLSPDSRRRSSILGSSSTGTDALQRVMGRGNQGLYPLPIGANGKTLDGVSVVSRMSIANSCEG
DSCTPVARSRRSSSHRSALAVVTSPNKCEGRKLRRADEGSPEAAGAQRRGRSTTKTKGGKQKTRQKSRGSS

ATPRRPSRKSSSKRRNRSGSKRRNRSGSKRRNRSGSKRRNRSSSSRGRLPQECEGRDDSSTSLSSVRIVKN
PTGTQRDGSVVEDESHYGPCDAAGRGSYRSRVMTLHRWRRTSPPVLCVDVRMPDDKRGTFDVYRGDTVEEL
VGDFASAYNLSDRLSKRLERLLARKLEYYKWKL
>tr|D0A0P4|D0A0P4_TRYB9 | DeepTMHMM Topology Prediction - Predicted
Type: TM
MQERQEKRGGGGKPSRPPNKRQKNNNHSTVRASEAPFWCFTIFSASQLLFSSIPPSFPTSQSTNHSNSNIY
IYMLPFLFSFLCTHFFLFFSFHTLRSPLTHPRKKKTAEVKNKKEPLYFYFTYPFF
>tr|D0A5V9|D0A5V9_TRYB9 | DeepTMHMM Topology Prediction - Predicted
Type: TM
MSPLYSCVVCLAPHFSGACAKGKVRCGLLLLAHLCYHCFKISYSCNGGLSYGSVVHMPFGCFLYFLQFCAL
QVLNSDDSVAPSVLGVTTFFFFFLSVWLSHKVDCKRC
>tr|D0A0L1|D0A0L1_TRYB9 | DeepTMHMM Topology Prediction - Predicted
Type: TM
MLFRKQLKHILHLYLWMQISVRCMLTVLLCSQRISNWRLNSEASVTKGKKRKERKEKRRRRGSGSISLKLL
HILFLFLFLFLFLFLLLLLLLLLLSLPLILVSFCFGSSSFFSSSFLFACMFTSCA
>tr|C9ZL41|C9ZL41_TRYB9 | DeepTMHMM Topology Prediction - Predicted
Type: TM
MRRWRSLSRRHVSQYDANSLMLVLLSLGSCACIAHSVCGCACVCVCVCVCVCGVIEGGEKGCRMFPCVYLS
PFSHFEVYFRSFFLNTSSLPIFSYQLCIRLFLLKCLPFVCMRKNNLVTQAYILVYICVCAHVSCYFPVASL
QRNGVRAHFFFSFSFSLSFFACLCLLYRVIPLLRILHY
>tr|D0A5N9|D0A5N9_TRYB9 | DeepTMHMM Topology Prediction - Predicted
Type: TM
MFFTHGPSFSLRYQNSGRIGDVHVFMKNLYRFWVYKGLWFSCLASLVDFLNSVVVFCLVVFLTTVFDWPVA
TACDEVSCGTVTLTHHMRLPTERGASHVLLCLILLVSSAASATYEFLKFLETCSLQMETERMVRVIVDTGY
TSPFHRLAHIWRRMRVQSDGHEELPDHGLPFIGNMAWGDFLDEVCARIQRDRSFGVIEHKEFDSLRAVQAL
MVYENYFITFHQHGILERGALKYADENVIRMLIRSLFDEFSTLVRGKDHLTLLRWKVVKYLVVYALFYPFV
VAHFLLRVLVKNAAMVRTDWGGYMTKDWNKRALWTFSLYNEVPHVLSARIASGRQIVMEMICRLKPHSSGK
RFVCRIASGIILVLLSLSLVNTSLLVSGNLCGIPLLWWLTGSIAAFSVCNETPPVEREYNYLPDLERLVKQ
LHYSQEEWNHSGESFYSAVTWDFLESRFSVIISDIARTLFMPCILLGLLFDGSLPALMECVQSESVRVDGL
GSVAREAAFDFQLLGDGNDYPLAYHLSEKLLKSIASFSAIYPKWMLKHMPDAVPPADQSGGSATSGALDIL
VTKLRNRVDAETEMQVLGDPSVSALGVVNSEAEITRSSALLGKGTAHEREQLFVSHMMQSIYASKSFGIDS
RTLRTAKEGREGCGT
>tr|C9ZVS2|C9ZVS2_TRYB9 | DeepTMHMM Topology Prediction - Predicted
Type: TM
MPLQITWCFFRYASSYAREFSGFLSGHARTLGTCVFMCLAVFTSVVRIGSLLFFPFSVIKLSSSTHTHTHV
HTQQTHLLNMLSPTEPLGSGKAHTEVVTDEGEGYGAMSAAEEKSHHKNGDTPTTDSKFMQCINAIIPHGGS
LSTTFNLGSATLGAGVISLAIAFQMSGVIPSILILITVTVLTIYSVGLMMQAVEMTGYNSYADLSRNLFGP
GWDYFTISVSWLFTFGTCVSYVIATGYLVDSVLSGSSALEFFQGKTGNRVITSIIWFVGMFSLSLPKEINS
LRYASAIAVLFVFYFVICIVVHSAKNGLKDGKLPEDVEMFKSGNRAIEGLSIFMFSYLCHMNCFSIYSEMR
KPSARRMTLHTTYSMSMCCVVYIIAGFFGYTDVGNKSVETVFEIYDVKGDVMMAIAFAGMLLKICVGFSLC
MQPARDCCYYIIGWDLNTLETWKNCLFCGCMALCALLLGLFIPDLNTVFGLLGSFCGGVLGFCIPALYRMY
CGNWGISQVGVVNYVCTYLLLISGVIAVVFGTAASIYNVAV
>tr|D0A990|D0A990_TRYB9 | DeepTMHMM Topology Prediction - Predicted
Type: TM
MRRSPCADYFTSLMATAGFVRCRGLSSSGQTNTTQTVHVLHSLYRRLRKEGEERKMLEDALSVNQLETNIR
IGLRLLHCHTQLVDSNRQLKELPFWRLVRRMSLAVARRYYAWRIFSIRLRLRSTTAISNALVYTLFLVVCF
MLYEIYCVCRIGVTRAEDRYKSLAVPIVQTFEALEEAARRRKDNLLKEMEGDIVRQRN
>tr|C9ZV14|C9ZV14_TRYB9 | DeepTMHMM Topology Prediction - Predicted
Type: TM
MMQGSEKEEKMCARCYACNFFFAIISLFCFCFSFHVSVMLNFEVELSPRVTEVLTLNPFKRNNREGRGGTE
FACAPAVVIDSFFFSSVHPLFSMCEKPSTSLSKNYCF
>tr|C9ZZ06|C9ZZ06_TRYB9 | DeepTMHMM Topology Prediction - Predicted
Type: TM
MASAPRLGRKGGKITASPIIPTHALTPGSSTSPVSLHTPGEGTTIIGDPNAAHPFGSDMLTVTKKQTSAEA
NVLQFDVGAIQLLGKGVVVGRAKQARTRTYQPQSDATEVKQRLHGVFSARRSKVPHRAASAGLDALQESEV
VATRKSQDTLSLVAALLSADRMANILVVVSKYYELSHVRSSVEAILREEGEDTQSVGVLFGQTFEGSKSTR
AWVMTADMALLYMTQLGSPAPFTHVVVPCCVDTTSSLSCFLKMLGGWMMSNTPRSQSVRLVVTTDCSNDGH
VARTIGVPRVKILDDKVIRLHEFSYNEVCALLGKQTMEMDKDAAGKFPSPPKRLVHYTADVAAELVRYVVT

HTPTAQIFSIFTADVREVLTALQGAKIEDCTVYSTLKSAADKVETKHRVHVINHVSHALDTENEFTMVLDM
GTIRRSSVQHKSESFIAASTTEWESKAEQAERKSILGENTTCCYFALFQDDVGASFQDEAQFLPDIFNVEN
AFVQCARLNLSVCEVGRLLPSVPRDVVDQVMQKVAEKCMISTPDSLDITFLGEIKSRLPVEIDVAYLIMGG
CSLGLGEATLVVSAVIALPFRSTAPPTYTVNRWTEATQESRKRCAGDIALSSDLLADAFVFLEWLRLRTTS
AATAAFLEAFLVQEFKFEKIEGLMNHMRDQLMNYAFLDRLDDVDTVNKVAESLRENASTMLMLLSMALSRR
AAFIRDAGHINEKDRHASMVFVRTSKQLVVHPFIPSGTRWETGGIVIPVILKNSTTILGGMFSLVDTSLFF
ASLLLLYPQIEYSRPVTTERGRVVYFGVACNWQMKRFVVSIDDATQILDFRENINTAIGCMRALRMLPHPI
SKTRFAIALKEHDRFFDMERLHRETQRRLHSLAAALNVQEHQGSFETFAKHYTAPKEIIPFNDVAATDVLL
LRRFADGTLWDEQRPSPQSAPAIGSSPQKSAVLTPFDTAIPAPYDDDDDDVQIIQNSYFMLHGPLIEDDDD
D
>tr|D0A7M8|D0A7M8_TRYB9 | DeepTMHMM Topology Prediction - Predicted
Type: TM
MQMSFRYLFRFLCFFFSIFPHALQCLQFFSCTLFSGVKNQQWNRVMNKGEVRRIVHTTQKAIRPSHGNKLR
VAVCALSLWFLDQQVGTPEILSLIWVIMLPFPSLSFFLLFSKQNSCHLPTCFLHTKAYVYR
>tr|C9ZU73|C9ZU73_TRYB9 | DeepTMHMM Topology Prediction - Predicted
Type: TM
MVASFLFFFQKSVGAGSRNRTKVSTDVARPTSASASTAAKKNERENMNVQKAIQRLGREKEQLDKSRVREF
YAAPLEDNIFEWHFTLLGPADSPYAEGLYHGVLRFSNDYPFSPPDITFLTTSGRFEVGKSICSSVSSYHPE
LWQPRYDIALVLVALRAFMAQDDEEGIGALARQYVSADEKRRLAREARKFSCAVCGMKSAESLWHEEMEGH
PPVGAEVEASVPQLPKRKKPEANERGDNDKKAKACADAGNNNSTSTSDNCNNATTGSSGGGEEEAAATPET
TEDTAEKAEEKLLDIAERSVQTEERSEPVAPGVRLRLGQRLLINLSYSMLDNVIWVCSAICFAILVKKALW
G
>tr|C9ZK96|C9ZK96_TRYB9 | DeepTMHMM Topology Prediction - Predicted
Type: TM
MHTKEFKRWMLFDRGHCACVSACAVRFTFPSLRVGREMRQPESGLKRAQRENICLSRGKGVKKKKRTCGGS
VASCCVNRAHGVTHRSLPFFVSSFHFLPFSIPFPWFLFSLPFIVITSCLFTYLFLFTPTPRVSAFTSFSSV
SMRAFR
>tr|C9ZL34|C9ZL34_TRYB9 | DeepTMHMM Topology Prediction - Predicted
Type: TM
MATSNGFESPRESVAAAAACSSQLREAAERARKDPSKDIGLTTEELQERKRCAAEPHRFVSKSTSRPFEVK
SSSRAQQIMTKGVVERQVTARQKEVLRKVSSLRKLQVGLFFVGVGFAYWVGVEFLLPHYAAVQERNRILRL
RYEMAQRKREEHLRVQGSSQ
>tr|C9ZPP7|C9ZPP7_TRYB9 | DeepTMHMM Topology Prediction - Predicted
Type: TM
MFVRLPTPGETCVSRLLISRRFASAKQHVTRRNISSHNGGATTGQKSVGGKNFAKRPEDVAPHSSKGSAAG
PTMRRSRFYRIALDHGFGFAVYFYILGESMTLSVLYALHSNALGTGDTFAWMNAVGAERFVNLDRWAHAGP
TIVGVTLSFRLLLNYLAANAIMYPMYGMQMRFCVATFGVLGKGLNPLRRLRTLGRNVTSSGAKRTVPRAPS
ATAPKNKVNRLP
>tr|D0A553|D0A553_TRYB9 | DeepTMHMM Topology Prediction - Predicted
Type: TM
MDSLYSWNDSTKIGIALTSLGVFFNFIGIVMFLDSVLLTMGNVLFVAGIALVMGPSRFKSFFLFRRRASCC
FFIGMLLIMLGRSLIGLMIQGFGTLNLFGNFFPMVARVLESVPLLGPVMLSPPVQKLLSLLGLQARGNRNV
>tr|C9ZWF5|C9ZWF5_TRYB9 | DeepTMHMM Topology Prediction - Predicted
Type: TM
MSSVTTGSSSYAAVLLVLLLTVTQCGDSKFPNLHCDNVWDGPSAQNDPLTCIKDKKRILSHWEDIFVPALA
ALLLVAVLVAFPISWFFTCLCSSRCKPSSKDGGKEQRCCLWMWIMFALIWAFGVAAFVFFGVKQLWATSNH
FLDVTLMNPLNVVNCTAEKVIDFASNWTSGNREPYADGVDVSFFYDISENAVRVVEMLRGRAGDYIKLLPV
VSYAVGSVCFALMAPMVILACCRRGPLIVPECFACAYFVFGLVFSVGGAVLFLLSYASSSVCGEIALHRER
KPGIIQWYGIPLCNSKFRPDAINKKVTDAEIGICREACNYLLDNCDNLDMRSPSMSRFSGSSVSYDGYVPS
GYLKDRNGKPNTRSSDISPDALASFIASGFVSHAAARNVGGTSPVKVLTCGKNITSSDECPNFGITATVLE
DTRVKAFVGSCPTPGNSCTVVECAANCTEGRAKNVSIEVVRVAARSRNVSVALSIGRPLLECNFMLDIALT
AMPDCEDITPGVFMLSVGFLLGSLMFAVGIYVMLRGSCVWGSAKTSPEAS
>tr|C9ZYG5|C9ZYG5_TRYB9 | DeepTMHMM Topology Prediction - Predicted
Type: TM
MSAAVPVSEGNGGVATQNPLPSGELPSTTSLAQQPLSPVGTSPPSNALVTAPGTAGTSQNAMPVNTVDQTN
RYNNNNEYANSYNTGMGYGGLGMGYGGLGMGYGGLGMGYGGLGMPGMYGGLGMGGLYGGLGMPGMYGMGMS
EDFQRSQMTFMLVGRLLEMCGMFAGVIQMTFGSALQFMGNYIGMSQQYNKLKSGMYMDEAGRWVELPKGTD
SSRESAVSGTRRRPSRHRKQEKQSHPIFGVLRRLLFLLLAVMLAKRLTR

>tr|D0A709|D0A709_TRYB9 | DeepTMHMM Topology Prediction - Predicted Type: TM
MAVFTFGRPHLPNGAITDTFKKRLVHAVRIALLTGAPWIIVPCSRGERTNSEAFLRNMGSPGDLLFNDAVV
EDLPPELQELAKQLRDSMRFAASLDEWIQSGNPIEHFARVANVEGTGVSIASPRLLRHGQSQWIIPEFVWV
DRNESWVVNTKEDIKHGLLVLLRRVLQTDFYGENSESQSKQDADAAWLERGIFDIIVVGSMHNEGFLYSAT
SRELSFLSRAHRQSTVLQRQGRENLLVSWLSKNINVRITSAEDSVPEITVPEPWIVDAQPEPRALRKAWWL
FRMLAPLSHTIHLNYCRWSACIRCSLTSVRIINFVFEFFAAIEAILTEKVNMLGYF
>tr|D0A7H4|D0A7H4_TRYB9 | DeepTMHMM Topology Prediction - Predicted Type: TM
MGFPLFLFRFFLFSAQNFLLSPLRPAAAVAPFSFKLIVTKSNIPLLFAMICVVGIQIRYIFLSYTHLLNCK
LVPIIIVLFCFCFVLFFFLPSFLPYILPSFLFPLHLSLSSSTSFFSVSIIITFHYILLK
>tr|C9ZNY5|C9ZNY5_TRYB9 | DeepTMHMM Topology Prediction - Predicted Type: TM
MVNLHELGGKQGCGGPQFTPVGVSLTPLLQYFSLLVHVVGKLPGFAAGFVTVWLVYIMFIGRDVLLNTCVP
VAGFHITALLLLRELLTPWQEGIAGAAEVRRRVAAQRSFVRDVQLQWGAKLD
>tr|D0A832|D0A832_TRYB9 | DeepTMHMM Topology Prediction - Predicted Type: TM
MCPGPVLIATWLDLLFLFSFVFFPHFLIIFFFHPFLCQIVDVLMTSFCRCVVLPFFFCFFFSMYHPFLCLA
LFPLPPPSAPYFTSSLLALPFRVLPSGKRSYELSVISKNCEQKKKRILRPFHQVKRKEKCNMYLYLYIRRG
AKANVGVFFFHFVSAFFLCVCFG
>tr|C9ZKF5|C9ZKF5_TRYB9 | DeepTMHMM Topology Prediction - Predicted Type: TM
MFKHERKRVNCSAKKFCFYCYVSKTLMTRPVCVCVLTLFAGLVQIVATYGKGWGTRVGVVDMCTDLLKIEV
PAAVAEENRLVKYAEANCTPIRSVPSGATTDGNCTVCGGGTLLCPFNYRCVTAKVSEFLGLSTPAVASPVV
EAGGDSNRDEASNKGEAPPSSEYGPVLSEEPVKNGSAVSATPKKEENGPPSKDDGHLQSESKGTVSSSFGS
ESNGASSVRPPEQQTSKSQPLQPAIYQVFSSVLLLFL
>tr|D0A6F7|D0A6F7_TRYB9 | DeepTMHMM Topology Prediction - Predicted Type: TM
MCGVSGVGGHDVPLPSLVTSSDKWLVYGCATDALVVCALKDGLASSCSSNAGSTGGESDFVQNTSVVSLPA
GEELSVVCIHPVKDLLCVGTTLNGVYLTTAANPNFSAGAKLSLRFSGIVSHCACFVNGVEEGHDILVMSCL
LEEALVKCHKLLLWDICSKALLWRGCTEAMVSITAVPGVVGFASCSRRHLLMWSFRNGASSASSLTSTANS
AVGADEGSGGTITVFSKTFAAVEELHDVEYVCVVTPASSAEKSLTVLTTKGFLVSFNLHSGEPLKWMDCKI
SSVTSAYRCGGDIVVCGGLVRFFSEDWVFRGKIRPPEHSPHPGTPHAPHSTGLTCIGAAPCGGNAIAFFFT
GGGIVRCRIGRLGVEAKMCDSVSTCSKLTFHCVYEYVPISPHDAPIQLLALTPDVICLHSRHQLRLYGTPL
LFSRGNMSFESTCCAYHRGLGVIAMYESSTHQIVAVTPGLERVLCRLGLEVPLTDLITLEDNQFVGASTAN
TIIYLEGQWGEDMQHFSLRLLRIQRFAELKTPFTHLAYSAGVLHVASSREVVNLHTGHITSFNDDIIALIP
ASGGALLVAHTHSCIFLSPGGVTPVGFPFLEADLRGAASSLGRRYVALYNEGNVHVVELSSGKRVKHISAQ
RGASGASPTTIRCVGFTADDSSIVVCDSRGVLEVRGFEGTGLISPGIYGDATSADDTNTNRFSRESSCSSS
RRAKQLDGPTSTELQNRFKDLHGFYEASRRVATERKAYKLPERHSKFASSTGANSSSGRSTPSPPTSANRS
SGKNAFRVQDGDEGLLPTPKKSPYPARRWKAADVPASLLNVGAVGNEILVSASLVEVSALTSIDSKHNDLS
SVLEGGAATLSWAVNDIPRDSGKCRVPDSPAYVLQSPTRKGEPLLSPMKVPEDDVVNVDSFDRVDPRRIER
LHSQTAGHDDPTALRCDRTNSSGGIASLRARSLDIRDGLKELADAYERLGEEDGDDVDETTMEEMFSTVSR
LYTRLQSRQSRRSNSSCSFASDQSSASINAMLANLQLLQCQNSRIEAQNREILSKLSSANGR
>tr|D0A7D4|D0A7D4_TRYB9 | DeepTMHMM Topology Prediction - Predicted Type: TM
MTDILMKMSPILAFLPEVATPLRVVPIRERIMWTFVALFIFLVCCQVPVFGARPGQASDPFYWMRVVLASN
KGTLMELGISPIVTASLVMELLVGVRIISYDINNKRERAVYEGVQKIVALFITIVEATAYVSSGMYGDVRE
IGVFMCGLIVLQLTFATMVCILLDELLQNGWGLGAGTSLFIATNICDTIIWKCFSPSTINTGRGSEFEGAI
IAFFHLLVTRTDKVRALKEAFYRPQLPNLTNVFATVLLFAVVVFLQGFRVPLMTKSRNAAADRQPYIIKLF
YTSNMPIILQTSVVSNINFFSQILSRRFGQFNFLINLLGRWESRAYSQSGQMYPVGGLAYYLTAPSTFYDM
INDPVHAVLYIVFILFSCATFSKLWVAISHTGPRDVAKRLVSEGRWLAQARESEEDMARLLEKYIPVAASF
GGLCVGALTLFADFLGAIGSGTGVLLSVTMINQYYDILREEGEDLGYNFIKRKVA
>tr|C9ZXI4|C9ZXI4_TRYB9 | DeepTMHMM Topology Prediction - Predicted Type: TM
MGVCCCALQGRRMKSVRYDPRFVSLFFIPIPLTFSSSFAPLRWRLPTSGHRLRTHTQKKTVVPSKGGSEGL
HPSVPTRRMQRRVQLRISLTAASSFFFWQHRSYYDKVRREREKSDDTPCAMFDEFPKNPHASSVPNQFRTP
NADAVKDAELLQRGFLLSLVVMLFALFYSGFLDPFNEGYRRPDGYGMPAVEKSV

>tr|D0A0E8|D0A0E8_TRYB9 | DeepTMHMM Topology Prediction - Predicted Type: TM
MVCFPFFFTAFLLFFFGYFLFPTSSPVTRHYSGSRGEVFENSHGFFSPFPLSSLHHHIYIYIYIYIYALSI
VCIRMHAKLYTHAPFFFHLIAFSRPWGYLQRCAYYRFYYDYLFNLLNSVNTGLWWASPPCLPILIMFSVIF
TFVILSLLLLTVSRI
>tr|C9ZN40|C9ZN40_TRYB9 | DeepTMHMM Topology Prediction - Predicted Type: TM
MMLLTTIPHMSTPHTFLPFAFSMPGTKMVRLASNLICGALSNFFSFFGEGVKFIMFCSVNFYIRAIYGCVI
FNFVVMFVCDVNALEEWFIFAGCFFLIYLLFLVIYCFIIVLFLLVHCAFELFILFF
>tr|C9ZID4|C9ZID4_TRYB9 | DeepTMHMM Topology Prediction - Predicted Type: TM
MELRMGRTNIPLRSIRCFFRGEVVVGNEQHKKKREKEKERKGKHKGEAYPFFNFFFSFGVWFLCFFSFFVS
FSFLAFKYYIFHLYLQ
>tr|C9ZI77|C9ZI77_TRYB9 | DeepTMHMM Topology Prediction - Predicted Type: TM
MTEIVSDNYFPPNDKLGETPSVPNEATPPLESPSSTTENATSLKREENTSALHSNSLLDTSSSICAFEFNF
VIEQDETVRGAIETINALMQAGDINGAVAVAEASQLTIDMLALNGTQLAIAMNMTLKYAVRPICGAFLFLF
DTAFGVWAMVPSLEGILRKTAHKVGNNNTTATSYVATREKPFLWGITGGMDVTNRVYAEVTMLVVCVILCL
LIFSIRRLFRHYSASIDTLEAWKQKSWHVQANGAYSPAPLLPLAVNHTHVETLAWEDGLQFANKSKSLHNI
SPTSSGANLVEDNMSPISLSRTM
>tr|D0A5R6|D0A5R6_TRYB9 | DeepTMHMM Topology Prediction - Predicted Type: TM
MEENNATTGQKRRRRKNKRGREAQRHTDSDELQPLVSYGSLAQTPLLAEEQSVGAVSLSAQTVAAPSPVVV
PEFTEAAKSSPSSVQLLGGSTVALVDKTVESHFSLHGLPCASCASHIESHIGEMGGVAQVTVNFASSHAVV
VHNPHVVGASRITEEMEAMGYTAVLISAHSDDAEQLDNLGVREFELLIGGMTCGSCVSRVQSALLEIDVVK
SCTINFSTGTCKLTMTGGRDSLNCVQEEVKKLGYTATPLEERGSGNAVDVMKEALERTKEIQAHKRAFVLS
TVLATPLAVFMILMTVTKYFEDITTMVIVNTIQLYLATPIVFYCGSGFFSRAWVNLKHRTFTMDTLVALGA
GCSYLYSVAGLITMFLMKRHVTTYFDTAGMLVAFMLLGRYLEAYAKRHTNDALIKLMNLVPPTSLVVTPAG
DVSMPSSTVKKGMRVRVLAGDRVPVDGVVVEGNSDVDEQMVTGESVPKPVSAGGTVVGGTTNLTAMIVIEA
TKVGEEAVLSQILRVVREAQNSKPAIQRIADKVAARFVPAVIIFSLVVLAVWLLLGAFNLYPSEWRGNNES
TTVFAFDFFIATIVAACPCALGLATPTAIMVGTGVGAKHGILVKCGMTLEAMRRTRCVVFDKTGTITNGTL
CVVFQRRWGNGADAAVDAVGAVEQRSNHPISRAIAVCVASDPKSQVAAYEVVTSTVHSGLGVKATVQHKDS
GHQLNVFIGNMDMMLSSGIDVSGEVAKVVRWQMGLGRTVTLAAVDGSVRFLAALADEPKAEAAGVIRFLKR
QNIRVFMVTGDNPGVAAAVAQAVGISQEDVYAGALPVTKAAVVKAFQEEYRDVVFVGDGINDGPALAQASV
GVALGAGTEIAIEAADAVLVRNSLVDLLNLRALSITTVRHVYGNFMWAFGYNVLILPLASGMLYPFLHVRV
PPVVAGVAMILSSLSVLMSSLSIRCFSPYKREQFIDII
>tr|D0A2L7|D0A2L7_TRYB9 | DeepTMHMM Topology Prediction - Predicted Type: TM
MLAKDVDAGRSTTSSTTGVPYIPTDVIVKKVAPLIFLPPEHIRELVQCFDHEGKGGLSEAQWSRFCEEHHK
RFTSLGQYEIDFERFQYYGEFSATEEPSSAARVVQGVVRFLEGFAAGGIAGAVSKTVIAPADKVKIIFQVD
SQRRFSLYNACKLGMATVRKHGIAGLWIGNGATMIRVVPYAAVTFVTFDYYREGFQYLLIADRTSTSKNEG
TMVIIRFLSGSLSGATATACTYPLDLMRARLAVHNFDKGVIPSYCRAYRSLVADHGWRSLYSGLVPTVIGI
MPYAGCSFAVFETLKSYIVRWRELSSEKSISVHERIVAGGFAGLVAQSATYPLDIVRRRMQVTPGRYRGVF
HALRVIYKEEGFLQGWYKGLSMNWIKGPIAVSTVFTVNDIVKRRMREYDEEVVKYSRRGNLVSLPEGLVCG
MMAACVAQTCTAALLQLKILFQVCLGRLYSRTTGRHGPLSNGLLCWRGVAAHGGDVTMMRVISYGALTYSL
FDICQTASERLLFSLTPTPATNFVAGAVATAAATALLYPIAHVGARAVKHTTPRHFHSHYWLLHDIAKAQS
PRSFREWSTFAAMGVGPVGGVGFATYEFLKEHCHCTSFGHRLFAGVLASFVGHVTTYFINVGRRRGQVEQL
TSSGVVDAKSVCLKPGFYASFRRCMPRRWPVSATTFGISLAVNDMCRDLVIQERKEILHDIFFTR
>tr|C9ZP51|C9ZP51_TRYB9 | DeepTMHMM Topology Prediction - Predicted Type: TM
MASDNESTVEVREARDTEEYDAQAAARGLCDKACDIKSLLHGHVESTRALERVVGEFCTRDIAEEDAFRKI
SLDVMHAEMRALRDLHYVAFSQRWAEVEKGSAAEEVTSLPSADRSVGSCVSHLLASLCEGKPDATFSLSEY
FGVADVLMEWRSLTPASQEQLDILLLSITRSVEDISSCVLLLNGVLYTPSDSFDCCELTRRELTAIIGSGG
PASAEEALVGWLNFIVRRSLLNQTLLQRLMSSTIEFRRSDLCLQGDGADVLVVVDHRRLLNSAVFRGFFFS
GELRGVEAIGAQVNLMSVLFAGNGSGDQLDIEKCIADIFRALWEQQELLSCKWKNVTVTLAVQARKDVGAG
AQTKLMDFVVLDLRPLSPNIPFQWHFTWGEVCALGQLSTNDGDRARPLDIPVFKTTRVAVASLGPYDKLSR
IMFPSINEYLRLTRKEPPSVAPVAYAVVVAALVVGGFIVGSCILGSRRRR

>tr|C9ZKX3|C9ZKX3_TRYB9 | DeepTMHMM Topology Prediction - Predicted Type: TM
MLPLMLLLLFDIKSLSFPSPSLIICSSLFFFLYFIISCRICSFIYNLPLIHTLPPSTFPPLSHEEIRVYIY
IYIYIYIYVYIYTRAQNFLSTFLSFFLTKKKKKSFSLLSVMPFSFSPSRVCCGAYIFPHPSILLFLSFLKK
HTISYTTDSHYCTSVCQSTFCFILCCY
>tr|C9ZSS7|C9ZSS7_TRYB9 | DeepTMHMM Topology Prediction - Predicted Type: TM
MLRHCFRHHRKVPFSPFFFFNCRVFLLSFEITQTTALYLSIYLLFLCSSTQLYHQSNCFRVETKAKKKWKL
TPLPPSEREQNTDNNKRCVCVCVFLRGGDMRSKNF
>tr|C9ZZ52|C9ZZ52_TRYB9 | DeepTMHMM Topology Prediction - Predicted Type: TM
MLGAGTWFIGFGIPAVLAGGFVASRKRLAYQWREELMDPDGGAYPPLTDADMEFIRHVTPPEWPLPEGQLP
TAKKVTGLFVSPDVLSGDEGGALLDEVKRWIALYGQKVDLRKVAFAESQAQSEGVDIGFYSDIAIISDHAE
DIQLMKAPWETGDRIKHHKMPSSLRYMVNKMQRKFEGLGRLRHVYVEYSPSGRFYHEPKPTKAFDGHDYVV
IPLRRDQNATVVTFAPLLRSRCSFVKEVLMRSWTSHDVDVIIPPGSALRVYGSARYEWGWGIRPGNVWFGN
YRNSIRHIDAIRTPFYLHNILSSMACRILRPAPSVKESDAALVVLHFDGPRDFDKPRSLLLQPESLIFGRP
PTVETYEKWVEEKPTTESVRNEGVFLFMVKNYVEMLRVT
>tr|C9ZL70|C9ZL70_TRYB9 | DeepTMHMM Topology Prediction - Predicted Type: TM
MMVCLTIFLDISFRICLPVFNAHYSKRRKGGSGMPIPTVTMGPDPISEPLLHDADRDEIPFCVTRNIGREM
RRGSNSDSGNRWRQVEPVLELQIETLSTTSAGWFLNSWYGALVVALVLQSLVSFQWGMVNICDSSSVSIRN
LDSWRSSCVERYEYQSAELNCSGTKVLVQACDPPPHSSHRAMLPSLLGCGAVPLSAPSYAEDSPGPNHDGM
SMRSGGYIPKQRQQRLFNLRWVDRNVIDIPASKANRFPRVVFSLASPQDAISGDIPFKTYQLQVVLEKFPV
HAPSEGDINVNSGTSRPATTGVYRYNTTTTCFRTTPRCSSVVLPQDVVVVGGNSRITLTIIGAVEELASVA
SMSSVGIAFQRSFYTIFTIACRYTLIVISGVHLIFFLYRIRRTQTIYEHYWVTALNVALILYLDPFFAAGV
YDENGVELYRFFEYNVPNYFIAFLNCFIFALVGASINASGEVGETKRRLKGETVPGCDAPVFEEPSPRPGR
PPATVPPDMPPSSPGGDPRDLQRKEKNSWTSEAHDSSVLLASPDGDRAALVRRRGMPLWVTVSITCYFVIL
VGLDVGRAFVENWDWGTVADCATFCCRYLASMFYTMLLLLVFASNVLLLWLKRNLGRQPYLETRPRQLACR
VFIFVFASAMVYFVVQAVLIDLLYPHIVRIVVYQPFSQLAPVMVSSCFVSHITFVYTPTTASRRVPIRPNN
PMWRRVVWSRQWYQWLQFHGGILYIFHNEEQERYFNFLQNKQRLAKVLHRRSGRVPVATEHEGVGPICSTA
ECVWNGDGPLGTYDAESSDSSDSSGGESSFSYNSSHNEDDDVASSGRGSRRRNWSRIRRGVSTLFERAECR
FVEKSALLLDSLEGAILDPLQSLQLGGNRRVPFFNLEAAIDCLNLSWEAYAPLCSDVDASRRDADGGGNGT
SSYCADCTPCAFPPETRGEGDSDKSTLDERAFLEGAVDSSAVTTVDNRPIDGDLQSFTVAREGSSTSVGPA
MCTKQYGYKPIAVFEALDVVAVCAVMDTEFLHHRGKAPRIVIAFRGTANMSNARENIRVRQRPWREVDGVR
QWWGLTKRARVHSGFLNIWISLKPAVLHTLHRFLKENSSTVYRVFCTGHSMGGAVACLCAYSVRRMLREIE
YPLDEVTVYTFGQPPMGNAAFQTAYDKAIPRTFRVVNESDAVSLFSLFGGTHVGTEVDVNRHGNYICKPMF
IEMLFRPTGGKGFALKNHTLAAYAQSLNAVADRNSGRECKVRCLQPYVRDVVDPSLSALSSAAANVSVEHQ
AANV
>tr|C9ZLB8|C9ZLB8_TRYB9 | DeepTMHMM Topology Prediction - Predicted Type: TM
MTFFLCMCGGGAHMTCDGRWGTSGAEASKCKRPLMFAPVFFFFLSQKCDICLFPIPSLPSLLLFFLRFCPL
THFCDHLQYQTDCRTDKMYQFGWCSPQASQYGIISCLFFFRLLFSFYYYYYYYYFSCVRILLIFSPPLSLS
SVCHYRHRSCSSIRFFFKVGSASLFPLTRSVLFVSLQAPFFFSLFPNPARREIVTPNWLCVSVFFLNQ
>tr|D0AA67|D0AA67_TRYB9 | DeepTMHMM Topology Prediction - Predicted Type: TM
MPNEIPYSFFILPFSSRHRFSSFFACVLVHFFFFVMSLKVKKLIDNKPDDERKPVKVILLGDSAVGKSKLV
ERFLMQRYVPVQMSTYALTLFHYDFVTEDDEAIDVDIWDTAGQERFSTMHPAYYHEAHACILVFDVTRKAT
YKNLEKWLGELRNYREHIPCIVACNKIDTDPSVVNKAFAFVEKHNLSLFYVSAADGTNVVQLLESAISEAV
KYKKSPKKDDLMSQVLGFIKE
>tr|C9ZRZ0|C9ZRZ0_TRYB9 | DeepTMHMM Topology Prediction - Predicted Type: TM
MFLISLLTVCFTINFLSAFTPFCNGVVVAAVCCRFPTLSLEVAVICFHPYLFVLFSTFLLVASTQMSGEKT
HTLKQVQTFGKKKTAIAVATVTKAPQCNIRINGVPISQILPETLRAKIMEAVKVVGARYFSRLRVDVRVRG
SGQVAQAYAVRQAIAKGIIAYYQKYHNEIEKAALKDKYLEYDKFLLIADPRRCEPKKWGRHSARTRFTKSY
R
>tr|C9ZNI9|C9ZNI9_TRYB9 | DeepTMHMM Topology Prediction - Predicted Type: TM

MFVVRVVVRDGSYAARGPYKNRSSCMAFPHVSYRVYSSSTSGALRPSCRVLRPQTIKPNPAHTPSGKFDRS
FEKLSSEIENRMPFRQQQILFGSKYDRNFGEESQDELKKAAQDDLFGREPERAAARQARSYFTGDDLSPTA
LHVKSPRLKVSQLQHRQNYDKNILKTSKEADDYYYRRETPSERGARRRVLYSVASVVGIGACWGTYCLCQY
MLE
>tr|C9ZVD0|C9ZVD0_TRYB9 | DeepTMHMM Topology Prediction - Predicted
Type: TM
MVKMPSNLSDLFPLPKCSVASAVELTLLIGAVAASTYLAVANSVWRVYCGLTLVLFAVYCLRLWRLLPGEK
EYLEMEAHKLADHMMVKQREATATQQTLVGQRGHATNPAQTPALQHSHFQGVNPILGSHQLQQLASSLALS
QSQQFMSSMMLPHHQQLMSSVALPPLQISNSLPIPLPLQQLANSMALSQMHQPQFHAVNHMVAPQQHQREL
ITPRITVASPLPTPSVLRQFPPTTLRENDGEEPPQKSQMPYGDGHLG
>tr|C9ZV11|C9ZV11_TRYB9 | DeepTMHMM Topology Prediction - Predicted
Type: TM
MLPGKLFKSTVKETDMSISCSLAVFLVVYALGRWADGTQTRDRPFMYADSMPALAPGEVRRNEAMRFPLTL
ETEVRQRYVLTNMQRDVYYIAHLSYLGSPSIMYNLHLTRLPRATVGELQQKNQAGPGQRHLSDVNRMYVKA
RKDVLLFDFLGGEGEVDNGDHDPANDDSFVPVLEVRGYRNGFPHEPEKWKDFRYNIRLDVVGRADGLTMAI
MWNVITVIVMGVFVIGFVAPWVISTAAGDTTNQRSRRDFLIFFW
>tr|D0A9M6|D0A9M6_TRYB9 | DeepTMHMM Topology Prediction - Predicted
Type: TM
MRRRHADAFTYAHEAEKVVCVYTPGTVSGSVPEGGKWNIMLHGFFSNEKKKLSIWWNRKWYGIVSHLPEVG
DDGGDVDLIATESSTEYTLLLLFGCNTYFLYSDISLLFFSTVSFPPPKKKKTLKHLFLQYCLPTHNSVHIF
LLHGDAATPIVPMSQNRQLLYPREEMVSLVRSLDRPQENGLFSQDVLLQYPELAESYTKVCPNRCDLATAA
DRAAKGAYGYDVQLTTLKEDIRLMVNNCILFNGAEGAYADAARTFEKFAMGKIDAYISQKVGGRRLSSFRV
ASVSVPEKHSTGKRGRDGESRERGPEAVDNDSSRRQPGRGNGIATSEDDSNRRSAELIKLIDSLNRREDDG
AFAVDVAEAYPELKSAYEAMCPLKMNLIIMKERAQNGYYLGQQHRRRRTGDDPTLYMGSSIAESLTSLRDD
VELMVRNCVNFNAGVVEWERRAASFHHFAHKKIDDFVLRIDPSLRGTRTGVEVYVQEAKKMQQSQLKQKIE
GSERSNRGDVGAPESVAQSISPPSHTTATPMNPPRKATIPGTARIPTLSAHVNVVNTVTPIVQPTALQPVF
NTPSTLRRRLISDHLHRETLHARLIHRLENDKSGTNGVPEIDRSGDAPQMYEPALSCRAVLDAFITSVREF
HKAQRESQDFVNPFMYAQQEENLYCDYVTLIKQQLERLFLHIVLYNREKAEMYDWAAAKAAQMAVADASLL
PTAPAVSSHVSCCWLDEAHLCYLVRFLQHLPQLLGLACAEVDTAKSDRTGNAQLYLTTVEQGVVGKIAKIT
EELLSFIARYEEKITASHQNSESLKEQAEAPAL
>tr|D0A8I4|D0A8I4_TRYB9 | DeepTMHMM Topology Prediction - Predicted
Type: TM
MASRFQFIGQSPCFFFFFGHPARQPGSGCGGGYWHLSVGQMSLAETVWVGWDAACSSLLLFVVPVAFHCLV
RGATFASRGKAFGKEDGVLARTVATFLPLVSVVKVGRKGRWGR
>tr|C9ZSI4|C9ZSI4_TRYB9 | DeepTMHMM Topology Prediction - Predicted
Type: TM
MVSNCSQLCVAGFASLITVLCFWCARYADQEARRRRWWQFFLGLITPLKVRGQSRCNNCVLRKNGVRRRLT
LAAASIPLPGRQGVASLLPVFFFLKGMTFLFCLFFFFSDLAAMRCAALGWFAAFTAFRAVTEFRLRLGCAR
VPNSPLPLRRRCLASSFPFRFCLRK
>tr|D0A9R0|D0A9R0_TRYB9 | DeepTMHMM Topology Prediction - Predicted
Type: TM
MLWEVMAGDEYLHAANEYTMFLRVEVSRASFVTVVYVLPPRCMLPCDWQSLPLFVLPLSRSTMGHLQSTQK
QEPNEISSEGRVATEHESTNIDDLIRSGRYKVAKKETLEMMSAIQDERDRGKLYPDSEVKDLKQYIIEQEG
LNVGDAKRRALSQQWRFFLSCEPMQRGLDAGIIVGSFAAAVYAFRSPKNRIPAKVGLVFACGCCLGLITVP
VLVVAAESYNNKRIKKLEKELFAKQRAEFLNKER
>tr|C9ZTE5|C9ZTE5_TRYB9 | DeepTMHMM Topology Prediction - Predicted
Type: TM
MTGGELDVVNRIHREQRNRNAFCMWWIGMINNFHYCLVLSGSLSLAEGYGMRQYVALITWANVFFGIVARI
INALVLSLLSFNIRVTVTYFMGLLAIFLVSFAYDIGGHNNVAAFVVLLIGVVFIGTASSYGESVFLGYMER
LPSKQVGAWSSGTGLSGVAASLIYLGLTHAGFSNSSIFLMSTPFLAMYWAFYFFGLKLPVETREGGYKAMV
NWKGTPWRSITPVPHNKMPECLKEMRQEQCLATEGYEDGVYSEQDGDDSHRSQTFNEYTWPILKSMHKFTL
WNNFNLASVYIAEYAVQFMAPFCFPCKQLKESKSFWIKNAFVLTQFCYQFGVLISRSSLVCVRIRQVWILT
VIQVINAVAWFVEAKVHFLEDPDDEKRQLAFTFILFAWMVFVGLLGGASYVNVFYNILEETKEAQNAEIAE
FVAWRGRKRAPGLSSERVFNDTEFGEPLGSEEEEATIIRDCVAHITAEWKTKRDMAMNIGALYVTVGITLG
SLLDLFFTVVVLRGGGCS
>tr|D0A1L4|D0A1L4_TRYB9 | DeepTMHMM Topology Prediction - Predicted
Type: TM

MLAVTARRATMLVGCYSRHWCLPYVRVVTTNSFCFVLFFYKFFFFVFCRMTAGYPVLVVDNGGYMLKALYI
TSDGKSSFLRGKIAVIPNCVGAASYVGRGIVGEQLFKLPHFHGFMVRRPVDRGFIVDAGLQSYIWEYLLQH
LAVADESEVELVMTVPFGAPKQVGELLHYLVAQRFRFRSVTFVSSSFLTLVSSVSRDWLYGKAPGNGKSNK
KVREKSNFGSDGVGSESVTGCGMVVDFGFSSTTVVPYVNFIPLQDSIVRIDVGGKLLSNRLKELISFTQVN
VTEDGWLVNHIMEQACYVALDPLVSLRKAEYAKRFDKGNGAFAGLRYYLPTVPPLMPLGCREEQLTQIIGK
DVVLQEKNSLQHIVFRHEAFLIPELIFNPVDVGICQMGVVEAIVHGTCCRGSLQNMRTLHGAMMRRIIAFG
GITKFKNLPERLSTELRKHLKEEGASRVSVPPVKGSAPLQEFVQWSRIGRVRIDLQYNGVGYTPSDCELQP
LFGALALITCPVLEPQLKLVQSRSRVELLPGSTAKGSQNRQQASAGPRTLSAVLSALQNLL
>tr|C9ZXC5|C9ZXC5_TRYB9 | DeepTMHMM Topology Prediction - Predicted
Type: TM
MTIVLLCTLLSQLSSFVLRCIPLYRCFLFRFPHLRLVAHMGFKRYPTTVLSICFLSRCASTSLSLCICAHT
STGKRCTLFVPGAFIIFSSPVVLYLAQLQFSCVYLCWLQNVAAVSLRTHEGVVYFSASKQFALWMREEGN
>tr|C9ZK55|C9ZK55_TRYB9 | DeepTMHMM Topology Prediction - Predicted
Type: TM
MFHSHNNWRSCIALQQLLLFIHYIKNSSKVTQTTAAQRSTAMRRRKERRGILSQSECRAHMDAENRTIRSG
CFSLLFDFCFVLHFFLISAFRHTLYPSS
>tr|C9ZQX4|C9ZQX4_TRYB9 | DeepTMHMM Topology Prediction - Predicted
Type: TM
MSWKYSNQEKLFLLRKWPELLEDAINVDGELSLPKDEVGGAGRQADAAKNSFNFSPTFRLPFKAWEAIFTL
LDNIAPTDLPSQLCEELHSVYGVNPLIALMVHSCCWFDKGIDDKVFSDQLSSLSGVTKELQLQSQNYSMPS
DQKGALDAATVATGILIDVLSLRKRWDEGSEGTERGVEEAINSLIEWSAPKRPVIELARALIVGVVAYAYV
KPQFAAAATNLLRKAQTSVNESMVECTRSRSGRSTEAVSPYNITDWRVVKCLETELVLRQKHQDISDFLSA
TNRWWLTLPETNPFKEKKGSRTVSAEERWHAVRSRYVGQQHVWSSLIEHFLSIGVVEAVKPTVIVLFGPSG
YGKSEMARLIACALHKCTPSEAETEGHLVHIHLPSFCTRDSIYSLVDPPAAHIGEGILLSALRRNKEAVVV
LDEFEKGSAEAIQNLWLSAFQKHGTLRSLKDAARSISTERVTFVLTCNIAADVIASDEERYLKASNEKERA
AMRAEWTKICMDVCRKTMHDPFVNRVDYFFPFVPYTVEEKQQFVKLQLSRIIEDQRVKGVHMYIAPQLVRV
LADQLQTFHSSNIEGILRPLLMKIYQKKWKKAVITVEERINCHSYVVIPAADSEGGEIPWTSMPGGASSLE
HYDGPERTGNHSAERQQRRTVEQEAINSIESSRRSSKIASNSRGNSRSANHTRTSQVAQSADCAMLLETDV
ERELRLELEKATELLLEKDKEIECLKHKVLLLERIVAVLLATTLCFMLLLSMIIGTKMVLILAVALTGFMS
LLVGMPLKLLIGALRTLYSVLGPMGSAIAFGIMSLWLSNAVRSVATC
>tr|C9ZJG7|C9ZJG7_TRYB9 | DeepTMHMM Topology Prediction - Predicted
Type: TM
MESNKRVTGRNDAVDMEPRENARENRKQHQRRERQKYDEESSDVVPSAVAVPHLLGTEPIQLTDGQRRCRR
EAEQVALRKERKYYRVNRFDRFIILLFFILIPSLFLRMLSFLTSEWIVTARPKNHRAVGIITTCFLSIVNV
CVEHGYSNVHHKLIDALSGRVVCEMSPTDVARLTTSMWALSGLQLCLNLLALAVMLRISCRPTRSCLHAVV
MGLVVIGTLLSPCIVGLFYLRTKCEKKGCVAHHLAPPQCTVAYGWGFGLYVAAFILDFVAVLTCICLISYN
TGLQNRVTQRHNGTVQSKAPASSAEAPEASEALNDQYLTAASLGIKIEGADDWVYDSKSDFFYSFKLDAFW
DPVQRRYYHRKLKSWLATPDGRVELPEAIADV
>tr|C9ZXC3|C9ZXC3_TRYB9 | DeepTMHMM Topology Prediction - Predicted
Type: TM
MLGVFLRVVYLRSNTVEPLLVFFLSLSPPPTLFIPVLAVLHDRVYKEAATRCLQAPYYSFYLTRGLPSRKV
VGNWKGKKVKTGGGSRGRVETTTAAAEWFLWCFLSVVVDRHVYVLLLFVVCGLWGQ
>tr|C9ZK85|C9ZK85_TRYB9 | DeepTMHMM Topology Prediction - Predicted
Type: TM
EKSTLIVVKRNCGDFFFLRWGVKIVGQIMAALLPLVLSASLVVVAVVLSILACTVVAGSNVLPLFSLLLSF
ITPLPFLFFWRSESTFDDDGEINGFVVFLSGALAVSAPSLSIVLYHTGHSSLGAFLLSLGSQVTLLGAAAF
LQSGERQDEEGNYDL
>tr|D0A8D8|D0A8D8_TRYB9 | DeepTMHMM Topology Prediction - Predicted
Type: TM
MDILQNIALIFLWPYGIISCCMCFYVFFSSGEGIHWFTTFRNCFVILGAVVATAALPFFRVPSLSLFIAFS
AWSVYAALVAAHYWRFRLWRRHLIPVNMPLVVMFLWEAYRVAEQMWN
>tr|C9ZRC8|C9ZRC8_TRYB9 | DeepTMHMM Topology Prediction - Predicted
Type: TM
MLRLCRRLSYRYQEFEMSKGAKYPFNYAREAPNTSVFPQYQMNFWMFWGWGRQELWIDRDTRKYETTWEML
RRLLMWFFPCYFIFPFGIPKWVAANYYGEYGEKPFLDYTMHYPMDNHYIEWGGSVLDAEWLRHIHTKESY
>tr|C9ZX73|C9ZX73_TRYB9 | DeepTMHMM Topology Prediction - Predicted
Type: TM

MMQLNISEGVGAGCFCYSLHLLFTFYFIVCFMFMEGYIYIYIYIYIANLLIVRETIVIITVTVMKNKRLTS
LIVRCTFNLKGFPFLK
>tr|C9ZRX9|C9ZRX9_TRYB9 | DeepTMHMM Topology Prediction - Predicted
Type: TM
MGPLLCFSLPNNAVYPSAYLGVRASSLVPLPSLFSVSVALHFLHPFSPCFPFAFMPFLFWGTPGVHDDALF
FFSPPLVTHFSPFSLIFSLSTMHVYICLQCAVSCMTCYPMTR
>tr|C9ZLV1|C9ZLV1_TRYB9 | DeepTMHMM Topology Prediction - Predicted
Type: TM
MDFFFFSCFFLQVCHLSLVVVSSFIYSFFFPLLIVPSFRTSHLVVHSATSPHTPFVFPELLLLLLLLLYYY
CCYYYYFTFFFFLNYFRFLTISWLFILLHFISFHFYLFCTLYHTFSFLSFCTLTISPLLIPFFKPRQRKT
>tr|C9ZRD6|C9ZRD6_TRYB9 | DeepTMHMM Topology Prediction - Predicted
Type: TM
MYMYYPLYYVDLCALVSLYMFLISFSFMQLYDHTSPSFQFFSSEVNNVMAAQGLLKERDLAGTAAFEVALQ
HIVVRQDRTYHFVQLLIALSFLVLIVEVVLIVVTYAVLPILSIEPEGYVCHILSFASPPCLILPVVCLGLL
RTATQRFAIESGSQMVHRLNATVMEPCLGMHFKYLSGKISSDGYCYADRDLLSKPDYTSQT
>tr|C9ZKM0|C9ZKM0_TRYB9 | DeepTMHMM Topology Prediction - Predicted
Type: TM
MKITCFVVFFLLLHVLVLHMFFFIMSWLCYLSGEIPSFSWLRMHIFPCRLFSGFPLWGLFVCFLVVHYYRH
TLTSSFHPLILSFLFILFILFFFKKNALLRFKPPQQRALTWRRTVTASTGRFHHLSGVCIRGV
>tr|C9ZRN0|C9ZRN0_TRYB9 | DeepTMHMM Topology Prediction - Predicted
Type: TM
MQRLLHAKPPLLSSSSQLSQRFQQTSNSVVDVFVFGDSEILFLVSSDKSGTGSRDQVFFPDKVSRSHDGGS
AAHKDRGSTLLCELQRVAAVEVKKEQQAAKKCLICSLCGGYCGIAAASTVRVEGLHVVVFFVLECDCLVFA
SFTLNTATPGPGLEVRSLGKASLLAARWFRNGAGGLACSADAFRQNYVPISSVIDCCDDAAVTGAAATAAS
VPTFPPSSSSSTLQRQNRPLSMGPHSNSSRGHLAVLRIAFVRVVGWVEYVELVWWEKGTADDRDGSSQQWR
ELRGFSQVAAGALFMQAAGWTNDSFAAVREARAVHWLPCSYDYEQQYPLLAVLHQHMHVKGDALVDRLSIF
ALSMRRQSVDQSESDLRGQCSWNGLREKVSGFCMNGPWRLEALTLAHPCFLLHVSDAATSHAPPTRFLSYS
KACAGDVLGVFLRPAHVMYPRSSFLREDSAYATWEPVAVRAMEVCEKGGGFDFVLYGECGEVARCPVGGFI
ESAVSCAVEPFEERRVSGAASGVQRIVAAVAAIRSGGALLFLEVISSASPLGNKGKRPSGDVPIVFSILAR
VVTLCEPRGCNPFQHLSQLVAVRWDGGKGSVMTSVVCDEPDDKTVEGTYSRDVRHMYCSLTHGELLAKDKG
ALQLKYGETGQRVSGKQQCNSGCLWPRVGIRPTLQFSSDTDIYYSVGAQRSVDFDVLSHCILPPAKAATCG
YVLDDGEEHVGGEEIAVFRSGDIYLCRHFSSEPASELFPAVSRSEKNYKWLSKVHWPSADELDMSAALTLL
FSSATVDICMAAMRNRKRVGGGGVDVKAEGNTFDSLDEINLLFVCWSTAIIVTAGGKVLWVGDLRASPSET
IGAFPSLVACMLRPFATYDSSFFFLLSASTTPYVHGDVGSTGVNSVFLLLQVPWLLSESSEGSEHVIAVCV
TDVCFLTTDGHPLFAFMPSVCPQQHVTLNHLEKQVAGHTVCRGEMMYCSSGALRAVTSALLFVTSATGLDL
VARFLPSCEDRVDHASRDNTKVVLDLRNVIADECRADVIPLFTETTLIQCVRLPAGDCQLYAIVISFLSGL
TLVAVSHSFHCGWWDLQLVAIPPVASTGVSEVGNKNMSIVAPFVRLQRAPATVVADRGILFCAQDACGGKW
AFCFRLTDGASQECGKQREVGGESLFARLNFLHNQTQRSLGESHTSGETGERVVVTRSAGGPFTFFLIE
>tr|C9ZMJ5|C9ZMJ5_TRYB9 | DeepTMHMM Topology Prediction - Predicted
Type: TM
MLLPHPSFVGRFFQCRSKEMAGDRLWEDRNNRFITIFVSEWGGVTALSTSTAVIWCLHSIYGLRALCRCYP
FRTANKTRVNTNICFIPHVYKCFAISARCLYVSPFWMCCFSAPDCQSIGPLCGVDFVVTCTAEVPRLGRRS
WALIVFVFGRARPRSPGSLLQLGGGGGAPVFDLVIAAGLWVEQCLPV
>tr|D0AAF1|D0AAF1_TRYB9 | DeepTMHMM Topology Prediction - Predicted
Type: TM
MRVKGRKEVRTVKRKGKGFSALTVVKTRLKAKGVAKICVSHLKESNLSSLYMVNTRFYIVSSLFTESLLLL
LLYPYFIYMLSWLFTYFPVLLNTGRVCSRTVGGIPCAKRSAPPILRDSGEGCQKKLCAS
>tr|D0A393|D0A393_TRYB9 | DeepTMHMM Topology Prediction - Predicted
Type: TM
MTQKGYVGVPEEPYRSKFTPEVGPYYGFEGEGTHAVPLQGIPLYQGEGNNQEGLKRFVVQGYKDVWAAILY
ILCILVTIGFGTYNTISQNFSDFISDSVVHLFWRCSDECCSVFLSVLFFVSSVSSVILSLFSLSLMKRFPH
DMIITANLLFFAVCIVGFLLSILWGNLYLLLLCLGLFIVHWLCFTELRNRVPFAAELLKSSSTVVCNYKAL
CAVNAVLFGMFTLFLVLWCSAFSALSNVLSKQPDPSSGVSGKRGNSFLIISVFLLHLMLLFVMFWATQVTT
NLMHVTTAGLTATWYFAGKENMPKNPTLASFKRGTTTSFGSICFGSLLVAIIRLIRWLVSTAEDSEHEILR
CIFLCIIGCLESLMEYFNTYAFVHVAIYGCGYTEAAKMTWELCKRCSCAALFSCFFVDAMLCLFAVLSALL
VCAVVCTAYGLVFDLSFGILHITAEIFVFSFGVCMLVHLFVFSSVTSAVTTLFVCYAEVPEGLEHSSPDLY
AALQRTDQNGTSNGAAPPRV

>tr|D0A2X5|D0A2X5_TRYB9 | DeepTMHMM Topology Prediction - Predicted
Type: TM
MVPDDAVTRDHGVDARLAAEKKIIRKGMEWKTRVTSPLCAPKLNRLLLLIGVTHSSFFFRREPFFSPFLRF
FSSFSPLFFFFLFFLYFSICSSCFPLKILLRSTNHIIK
>tr|C9ZZZ8|C9ZZZ8_TRYB9 | DeepTMHMM Topology Prediction - Predicted
Type: TM
MVVERDRSNELHSIFNGMKHGEVLLHNGVRHIDPSRGELLQSNSGLRSSNETQIFNRFAQAFAADLAKVSE
SIMRLTQLTQRQTVFEDRSSEVTALTQVVKTSLQRLHADLNTLDELKARALDAEKVVLARTRASSGSEAHS
LWGGRADVDSLVQSQTKHSDTIVETLRTRLARTGQTFRSTLQQQTKEMKSNAQRRHMFTTGDRPQTFESAL
FHDQEMQQQQQMQLASRGENVQYYKQRSEAVREIEAAVVEVGEMFNDFTRLVHEQNEIVLRIDTNVETSLR
HVNAGSNELLRYLANLTSNRGLIIKIFAVLFFFLLFFGFLVVR
>tr|C9ZT98|C9ZT98_TRYB9 | DeepTMHMM Topology Prediction - Predicted
Type: TM
MRLSYADQLIELDRQRRNIVYGRETKRFHLKIIAAISGVLYAALYVMAWLYPRGVTHVWIDDVLVEVLEDS
MIEQVGGVSGCIFVASLVLLLCMSFLKESSDKDRHGKKHRSPFAGACGPYRKAPGLSVPFSPATVQSTTTA
GATEVLTEPIGVKGHHDLEGEYAIRTRAELDKFLSAKELPKRMEGSLPTAHNSVNAAGTVSGSMATAPAAA
TSISAPLAAGGGFGSVSSEGIRVQYGSGGERASAAASADRPAETMWNSLGIVDPERSQVKVRKWLSDLCQT
LVEEVDSTNRWFVERQLRHFDCGHSLDETIVMPPPSSAPRVGFGVPAQPVSSPPSVRKMDALMDERNKIAT
QGQNVQNIDVTMHIDQRLQLEMKLDTTATFTPSSPASVAEQQARRTYVVGRIRTFASQKSLASYHHNCGDI
STWRDDFPTDAHLLTHILRTCVPGFTNYVRFPHQPLNAQQHLALVVGDTG
>tr|D0A5P9|D0A5P9_TRYB9 | DeepTMHMM Topology Prediction - Predicted
Type: TM
MTTHWVRDYGAIVTSAATFFLCASYQRVLLRHSDALIPSEKKEKGGATSSFFREFMQVARIALPTVWCKEA
LGTFVFVLLFFVLAVIRALTSEANGRVLKSMTEGTGGTRLRNFTHTLLLRTALHLLSSVCSSCVEHLRTWL
IGCYRVRLSKHFQERYYSQLVFYRAAVIDKRLEAVDSVVTTYCAEFAEHFTELPYYFVLPALGSATSMVAL
IRRVGAGASAAACGLVLASVLLMKKFSPPFGKIYASLLSKEDAYRRMLSNSLSNVENIALHRGGEHTRKKL
DTQLGVLKGYLDHFALSRGHFNLLELSFATTLRNIASIIVFGDALRKENKSTSDIYVELLYLRDLSKSVTD
VVSNFREISHLSTYTLKLAEFDRILKDIEGDTCDTFPCVASIPDVGTVVPTVVSPSVPIAGALSFPLFTFT
NVKLKTPTGHVLFENLNLTIQNDQDWVITGNNGSGKTSLLRLISGLWRACEGDITMSSCVKLLFAPQESYV
VPHCTLVEQILYPKIITSLNEGDIACIKEAISLAGAESVVEVLGGFESPAVGCDLSNVDETYDWSSLSGGQ
KQRVNMARVFYQVLQADRSREIPVVLLDEATSMMDDTEEKVMLNLRKLNARMISVTHREQVVKHHTHVLRA
LPGGRWMTTKVTQYISSDDVVTQGISSVGGSSNAEGVGVDLHRGF
>tr|C9ZSV8|C9ZSV8_TRYB9 | DeepTMHMM Topology Prediction - Predicted
Type: TM
MAGKLNGATGSFSSPKGTGASRKGIDSRKGSVSSHSAGLKKVKKLNSLPISREINPKQQSEETCIANGGTE
DAMYTPRTLSVLIVLLAVFLFMVRYCHYLDMDVVSSVKLGLAASGVSFIAFGATHLPDSMLLRPHPSFWRA
VLAVGVLYLALLSFLLFQSLDTIRAILLLHDPSLKSLPEERQYAEDCRIFTSDDPFLFVRTTFDIFIVAHT
LGYFAKTIIVRDWRASTCISVVFEIVEVTFQHALPNFKECWWDHLLLDVLICNGGGTLLGILALRIFHARR
YNWVLSDNVKSKCGKARRFISQLVPQSLVPYEWNVFLSPKRFVQFLLLLSLMTLQELNTFTVKHILHIPPK
HHLVVLRLVMWLFLAIPAVCEYYFYISGLDPTNKLGPSVWVSVVNLLFEVVLAGKLAVEGNYFQEPMPGYI
AIPWITSLISLCIWFAIFFGVLTLKQRMEKRCLLYAISSVFFYFGCGCVLAMFAMGMPDLQIGREAFQRYV
YPYERYIIFWR
>tr|D0A2Y2|D0A2Y2_TRYB9 | DeepTMHMM Topology Prediction - Predicted
Type: TM
MDLCHLVGMHMCCWRHLLYLFTFLPSPTSFRQTQTLFPFGRSLDDCCVFVFIFVFVDGSVFFFCEWDVGDS
YPTAAAVYGKSSFYTLMYYLFHHVIAIACHAYLFGFLFCFFFFLPPVLP
>tr|D0A9Q2|D0A9Q2_TRYB9 | DeepTMHMM Topology Prediction - Predicted
Type: TM
MHFQELKKIIIIKAPPCYSYLLNPFLQYASDILCGELQMCTSMNTSFIQVLFLPFCAYILFSFGVVAVLSH
TWVGIFIVSVCAMCIFFSVFISDSCCLLFSFLQFWFFSFFPREKGEKNIIHQTQYEMRKGASPAKGLSPAS
FSTSFSFFFCIMFCFYFSLKNRSPGTRRVRLYNQRRK
>tr|C9ZSX8|C9ZSX8_TRYB9 | DeepTMHMM Topology Prediction - Predicted
Type: TM
MPLWFICGLFSVYLVLEMTSPRHLLFCAKQGNIRRTWTFPPLCAPRYGYSRPVMMFFSLCRRWAGVLGLMG
NEKPVEGRSVGVVGSAIPNYVGVEPPPFKRSVRALSAGPLTIHMQRRPYRVTPTPEEPYSLPARVRELTRS
NQWQDAIHLIYTLTHLSSSEHCSEAQECSGKKTGVSDVFEMFLFEFLRSGHVESAFAVWRKCSTNLKFDPG
SELLMKFATFSLVVDGKTQESLDLALMARQSQREGDAPGEHFVEYGMSWILLCTKLSGTAKIGASDVTEVQ
NLAEKLFVRCWCQPQQSSGGGVNQFKTRKLRFMLHGMVALVSLEGGLEVLRRLSTRYHGGASDSVFQEFVS

LAEIFESDNHKNKAAHTLTAAPLPSMLIHLFPLPLPPSGLVVFRYLFCDDDDAPSRREPPQPRLLSHRTVG
TTFLERLVENGSVDSFLRSAVEHDISESCPSLLTRVMQSQLGANARVLPTVFGRQLLPSVQRALRSRPAAG
TLHLLLLRFLSRVLGPFLGTRAGKGSHMEFPAGGGSIASSAVFYIELLGCVAALVPTSGIRAWHRFVQRVT
RAIIRECGSVSGAAIPSLLQVQLHTQWFLPELLTYVDTTRERDMYFACQHPLMQSRERNWDRILCALIWEC
HGKPVSAYEGTGSVSWHRHAGGLVRYAGEEDRIRAFEGAPPFFDVKRSFVQRVVRWQWLLESLWQLVGHKN
GDRNHCSWEELLCACSGILSNCRKYHRGDIVRDFVDVLFLPAASFVSPRGSECCRRTNVLTLLSTCADSGC
GTTESVTLARWYFLNELSLWFTTLHVSGRFRALGDVLQPGPDRTAQRFALLRNYVSSVSANISRSGEVCKN
KRQQLQDLLTYYNEVCKSDNSLRTPQLLSPLASALRRADLLDSLEELIKVSLSELSGGLRGRSGTRAALKK
LLFFHDFHRGSAVCAESGCTSHVTDENYHGAAVAVANIAEVYILMLLEHGHLETVEKILLRPPASHSLQDP
ATEVASPTHGGADGFLSPLQFDITLLMPSTNREAMQLLFFLYLAQEKNVSCWRVLDKLPKYGASAAGVVAV
VGRLVSQEKHLFGQFSRTAIRLADGSLHPKTAGTIIYGSNRGCIQNKPQMVATVIGGKKRASNLKDLNHLI
NNGDWVLALRSIPFVLTDPLHITRKALLVCEAVPHGAAWEGAVRVFVRANHICTDISHGVHCMKEHTCSFP
VMGIQEIGRLLTLLASARRWQESLQVFESVGSHAVDGYMFAETCFTLRSSGHPGLAVDLWAMWRAAVGDAV
APTPRMCGQFLACGVVGDVDVADAACVMVREATIPKYGTWSSAKPELPASDEKCSSSPCVVTIPGTELALS
FEREEDTVATLLRDRWNGSWQDALQMALASGRSRIIQEVARKSPRNHSIYKAVVGWAAKEQRQLSVAERCA
IAGHLITNPVSDGGEYDRVGRVLEELLGSEDD
>tr|D0A7Z5|D0A7Z5_TRYB9 | DeepTMHMM Topology Prediction - Predicted
Type: TM
MEKQVVSRARVISYFLLLFIYLFLEDGYLGFVTLVELMVGHYPTELCCRSLWSILFFFLLRHTGVPIFFCE
NEKTVTDFYGVIGSMFQFGLIWFMAFVDVCY
>tr|D0A2U6|D0A2U6_TRYB9 | DeepTMHMM Topology Prediction - Predicted
Type: TM
MKRAPCLSRSPAVGSAAVILSDVASDSIKPYMPWWLISLTFIYRLFLCATIRTVEAPDEWWQSTEVAYNMV
FGKGHLPWEWRYGLRSVFFPAVVALPFYLLKLLGRDTTWAVWFAPRVLQALVLTLIDVSVFCMGATLDELL
AKRELELAEETRQSKTKGFSYFCEVSVSRSRRGICNSISYTALLLSLSNWYMAYCGVRLYGNVIEALLVLL
TLQQRRYVPFLLLTGLASAIRVTSAVVLSPLVFRHLANATREHGFIRGLFRIVLTGLIVLVAVLGGVMVLD
YCFYGRWVLTPLAFFRFNVLHNLSRFFGEHPWYFYVGPVLVGIVGPHVLFTIAAPLVLWRDTASRAVSRPV
LGMLGIGAWTLGFYSLIDHKEMRFVFVVIPLSLITAAFVLVRWSRTSAVVVKMNRLFVLFNIVMIYLMGYV
YRRGPLDVMAEVRDGPRINRLDVIATCYTVPGYSYMHKKVNHLGFVDCSIDLDEKTGLPKVTEDIMFRRYP
KEYVLWRYDGKHSFNMSDLEESRKASELQSVVMPKSAPHPDAMVMTRAVAKEIEEPFLKRHGYRLYRTFLH
SPLTLAPYEDIYIQMWVKVTK
>tr|C9ZWE2|C9ZWE2_TRYB9 | DeepTMHMM Topology Prediction - Predicted
Type: TM
MRKFMQAFSFQCFIYFFLPHLQVYCFYLFITLFFVFFFHLVLLYFFIFILCVCVSICPCIRGGGKKKGGLL
KCFFFISVFLYYFFFFGRCCSFMFFFFFFFV
>tr|D0AAH6|D0AAH6_TRYB9 | DeepTMHMM Topology Prediction - Predicted
Type: TM
MLMQVSHLIVCIVDAAQSTHHVLVARAGTPPCAYNMSFFFFHCQRKENCASSGWYLCCLIIASPVLFRCVA
VKQQSKGICSCGASSLRSHLPSSFASARFIFGSCVFCFVFQCASSLFIFFFNPNCCCCIRCGGVRVHKASN
QAREQTRLEASKKERKGKKEDSAEKRWIEIGQRGKG
>tr|D0A819|D0A819_TRYB9 | DeepTMHMM Topology Prediction - Predicted
Type: TM
MKYIFLHCAYMYTSPYIPYLSPFLSTQFHCYINDYTNTRVIYIYIYIYIYIYVDHFCYVFSFFFCVSKFFV
THIFFLVLTPVLIPSSHFYSKKDEGKGV
>tr|D0A0F2|D0A0F2_TRYB9 | DeepTMHMM Topology Prediction - Predicted
Type: TM
MSLLGGIFAFATSVRKGGKIGERVCLPLCNVLILAKVLIFRFCLPTLFSFLFFLERRSPFMTQPVDDSWYD
AELLDALLGKEDVDPSHVPLSSLAAAAPVTTPKGSCRALEITDLGHPRLFMIGGSLFSRYCRPSPALQNHR
ARFHWSRLSWLDYSAIYCPFTKQFLSPRSPHWSHLVSAGFGTPRRLLFGRSIVLVKPQKLQALAAHIQQSK
EGNAVSGKNPTAMWSPPLQQTLGLLLETLPYHDETSLARRVQEVLSAGDTGAATGRRIPLGLYNHVPDVKR
VMPPPRSSQEGEEEEELAQNDVDAVEVVALGESYLRRVLPWRTAVLQHRPKMKLNMKVGHKEMVGLSSEGA
DLHPAHSKADVLVERYSLLCSTPCCVPKTQMAYLSLRYVPDVPVMQVVVDALLRTEETEYVFYCPIEHGVD
CHTVVDIVAYRVRRTGDGDVRAAERFPVLHLGLHANPTDSSVAPVIVVIGNAHLLSQRAISRLLDQWTRNG
AAAVAVRRRNGGSGDNGNSDVGIDADEAVGRSWRGRELLLRIGDPLRAIACGVPLFGNVRAVFVSYSAFDC
APSDCSAHSPGLNNSVSCVRSANATVHVLKPLREGVFTLEEPVVAALRRVAPTFQRRMDVSDRRLFIKEVC
DVISSTHVSTLSNRGADRHGWTTSPDDSYHEEDVLLHLYEAVEAFLECPVKSGDVSLLSCAHLMTKYPELA
TARIRHELIREFPLYVEMNKSELTVCSAVSAPAPDSVVECAAGGSGSCECAAGFNIPLVSSRRLARKKAEE
EHDLLKKTYVLNATLDRVVTSDASGCGSSFTSVIMRGSEHQRYECPIEHSYQPFFEPPLLLGRWSQTLLFC

APVVADTVALRCVLLHAVEASVRDDDTINVVDVDIFCVDYVKYPPERRCRLFGVRLRFATPDGFTKGTVKD
CSARERATEMLEHALAKCAVSAELFPLWTSSELRKQRTCRRVTQGSGVAGTFVPHFRFEALDECVRAAQPN
VVEQRLFALGFVQCGPLCLTVGSHVIALRDISSNIKRGTLCRVARFAPMMVAHEFGAPVDRMVQCFFEQQR
CKGGGMGALPVVRPVLQPNNLQVTPFGEAEESERETRDEEALVIPAVSLVGGYRSLHYYALPALQLPLLVP
SQAAAASTLFHPLFAHQDSLLEFASAETHSKAATMIMSLPPSLGARRSVSCAFLYDAVFDNGESGAEVHEE
GGQFPPHGSKGSASPSHTVCCDVLSALSLFPLRV
>tr|C9ZXL8|C9ZXL8_TRYB9 | DeepTMHMM Topology Prediction - Predicted
Type: TM
MQVPVFTQTSRPSHIHIELRVLVRSSTRVILADESNIVHILCIYACTYALIFTCFSFLFLALLNPLTRFLF
LLLLSPFFPVTHILSHKVVPNLLFFKKKYNTHRSADHYQARLPSLARTALVGQLRAFCSFMLLFTSR
>tr|C9ZXX1|C9ZXX1_TRYB9 | DeepTMHMM Topology Prediction - Predicted
Type: TM
MVSRVFSAYAEWVRRNADNVASMERFTHMLTIILMSPNNMLKHEIGNTIVNIQNFSNHAIISSAGRKLSVA
EQISIVGQIIRKAECLLELLLRRYRGHRTAWNFLLILQLLKCFLNLVAHQQMCLLPSIWQALRRQLRRLIT
FPKKLLGPSGVSSGNSGAALSNLPQSSQAAERTTPLVIPRVVATRMFRDRSSETVGEDADFSEAGKGSNSG
GGNPDVLPCTAYDVLGAVVDFLLLLRPLFLLYCARSAFPTTAMDACSQIASEVEDTTLDDGPRDSNVTDKK
SDYGSFFSVKSLIADGLKESLLGNWSVWTLFLGLDVIFAALARYIFHYRKPIVCINRDARNYSGVGSNDGR
QIAHDGEVPSPELSGVNSAAEPDTVVSRDSLRVQQTMHNLVCCFLRDPFFGSVLRRFIYDNFIIGRINGIP
LVGSLLALHVASFLCRQHYSFMYSIGE
>tr|D0A075|D0A075_TRYB9 | DeepTMHMM Topology Prediction - Predicted
Type: TM
MDSFVAGAAAGLVVDFTLYPIDTIKTRLQSRDGFRCAGGFVGVYRGLSAVAIGSVPSGAAFFVGYDLTKRA
LLGEDDGQSDVTYAGRKQWQLASQATAAVVGETTASCIRVPIEMLKQRLQAGQHRNLRSALVHITHGVTPG
VATDTAPTSMRVRGIPNLLSGIPVMLLRDVPFAVIQMLCYEALKVALHTDRRPHYLPLCGALGGATAAFIT
TPLDLLKTRIMLGQVSNPRAGRPKKLSVVCGALQELLHEVPRPTDRWGPMQRFFRGAVPRVTWISIGGSVF
FTTYEVVRRYCSCYRFHEK
>tr|C9ZZS9|C9ZZS9_TRYB9 | DeepTMHMM Topology Prediction - Predicted
Type: TM
MSITNIAAVDDITSGSSSKVEYPSSAPSPTSLSRNAVNRSFSDLHKDTWFVLVSFNAMLRTLLLHPLYLAI
SRKRITREAKPPSVFSIIASAYRGEVGGGNNSSKKVRGLRAIYRGVGAAMIGNLIGELVYLHTMEWTKEAL
DVAFADANVAPERGRDFRTNSFSAAVGGMAGELASLLLVTPIVVVCNRQMTAGYGMSSSNTYGSLRDTLRE
VSNLYKQPGVGTSWGTARYKLRGLYAGLLPGIMTLPASGVWWALYSRSKAILYTMAEPTLSRWEREMSLVE
EKKSPWQQNWLLSPTDNPALNAFAGVVASCVTSFLFNPVDVLHTRMQALPTVSGAVKGAQYTSLRTVVCDL
LSAEGWRGLLKGTVANVGASVVGGVVFSSVFELTKLGSDRELWKQI
>tr|C9ZXN5|C9ZXN5_TRYB9 | DeepTMHMM Topology Prediction - Predicted
Type: TM
MSKSRAVVKFGGDQSKETVSFRNFFDVLNQRHSNVSVSGLAVTLLFELLSISIYVEASFYAHPTSIYDFNW
KTGRFITVAIINAIFFSEWIVMLWVEEQKVRYCLSLLSIVNALTCLPMVIVGIGAIVKPTWQSVWVPLFLR
VWWLRKCILVLLDYPQVAKWMMDIRRDICRFLITMLAVLSTCVGIQQFVETLAGNYMDPFSSLYCMVTTFG
TIGYGDVSPQTAPGRFLMIGFLVVALSYFLPLFQRLAQIGRDHLNYNECHSCWGRRPHVIFSGIFTGLGAE
IILMNFYAGWRKYLGVRVVLLSPVDFPPEVRLLADIPWLRNRVVLMIGDSAKQVDLIRADAANAEAIFLFG
DTGSAAYHADYQVIQQSLAIRQFDPELPQHLYLRSERHTRHVASYAASVVEVERLLHHLLGLGAAVPGAVP
LIMNLLRTYEPLKVKGTASRPWIEEYEWSLQNDIHCLEMQQTLRGYSFHSLARLLLQHNVTPIGIIDENGE
VQLNPHRISSSAVKLVVVAKALRSARSALEAAEEAHSQTSFGEHTGEVEGKEPCIYGMDREVAAGAYYTSD
FPGREAKSGVEALQLVDDAYDFENHFVVIDLSMAKAKAPETEGAREGSLSSAAMDVFHVMRSIRQSYPQND
IVLLTKDTSFSAYFGRYWNSVPGAIPVKYIDGCGLNANDLRRCNLKRSAGIIIFFSGDIGGASTGGLSLLV
FLSVASILPSSHNIPVVVELDSTQYLSLFPPYADDPYLCSRAESDFVFEPNYVIGNALSRHMFFPSVHRTY
FMDEFVDIIDMMVSGVDERTPSLGRLPLLFTTDSLHIYQDVVEYCLKLCYLPIGLHRCISDPETPYINGQR
FVLTNPPGDLIVDQKCDAVFYLLPAS
>tr|D0A365|D0A365_TRYB9 | DeepTMHMM Topology Prediction - Predicted
Type: TM
MEYRWLNRVLTVELCMSIIAFHLVHFTGIDWNAYMQEVKGFLDGELDYMKLKGDTGPLVYPGGFVWSHSAL
YFMTKGGVDVEMAQWLYLGIYVMVLVLVAHLYYNSGLRGRLFIRLLLSKRIRSLFMFRLFNDCWAMFLVYL
SVICFARGRRWTVGCLLYSMAVSVKMNIFLFAPGLLLILCKSLPFTGVVRCLAVCALWQVVAGLPFLLHNP
RSYIVRSFDLGRVFTYRWTVNFKRISEEIFFSSKFSRSLLVMLAVSWLLVCFRRWSKRAYRRGRYEKREGT
VVLVGASDEEVFHNVTLTLMESNMIGVIFARSLHYQFFLWFFYFVPFVLSATRLPLVVKVVAFLAIQYGFE
VYPSTTASSSVLLSGFLCVWLGMLLFPSEYSETTGKETTVTLTAVKRGK

>tr|D0A0U4|D0A0U4_TRYB9 | DeepTMHMM Topology Prediction - Predicted Type: TM
MFPEASVSWQRMMDKFVSCCVVKNQHSKGQAPSGTKLLRSPHALPAAVLKIRIHSPGGLCVWGSLFSLIFY
FFRSIGSLRLLVSKIPTKLYASYALVAVIPRCCAVGSFCSTVRRAPACRFMRLLRNWPSCAG
>tr|D0A708|D0A708_TRYB9 | DeepTMHMM Topology Prediction - Predicted Type: TM
MLVSVAVCFAYSHVLFYCFFHLFSLATLQNLFTLFVSLWQRISREKGVESNLYCIITTPASIVEISGGNMI
SESQMFSPWRNSARDQAQLRSCALAAPSAAASRYIHRLEEEVSALTQHYNTACHLLHRAILVQDLSSVIID
ELEGRSHIVEEESTLRSNIMWLCMSMTSVLRRTVPQHSTAVRAGTASQVDSDAARDLNGLPERIINAVRHT
VEQGSERVSELVSAVPRRVCMALSTEDIGVNRSKHNASVMQNEEYRSGFSTMQRQLDRVLSRVEDILSEFN
TAQHALVNQRRDDEYSKGYVVSLQTAAKNVEDLSTQVAELLRQLQQMRRDQLQHQQTSVSSSSQPQLSPAK
VTFTETTQTTGPLPSCDAVTQTLPQPEPAASKVESPATQGVKIVQASSVTAQTQTAGPPPTATTATQTKTP
PSSPTDVTRENRTTQRLILTAPLPTVATGTQTDTPKPSAAEPAPSTDKCLRDSRAAEEWKLLMEFAVNAIN
VLSSNVRDYVSTFEKIQRLSEQEIDFLQQVGDSRKTRRWESLLAEKSAEIVRLNEEVTKLDSTIKAAEKPR
TRSSPSPARSEDNNTPSLAQLYAAYSAPFIPPKLRTTQKVKLLPTTELLKAALRAGARSATEGDSTGARSG
TVTPKSVSRSPGKSGEGSPVSRQGLTPDAKAAKKVAISEPNKMPFQSTPNAAVRGGDISPLTESRRSTVAA
PPAATKHVEKRTHRVFDDDTPVATPRVSTVTKAPDTSDKPSLPSSKIRKSDAAAAKSSPRAASPPPTSAVS
IGGDVVTVRKFKRSLSSSSSSSGKADTIAPLRAKKSEGGNKESYEKGEDGKPKDEPKKLEMRQLKKKSFSS
DSLSVSLSDVPVTRPKVEKVADKPKSAAANNSFDESSDDEPKRALHRVSVPAKNNERNEQTPSNKCQDKPR
EKRWSETSSSLDDKEGGNEKAAAKKGVSKNASKSTDTSLTLTL
>tr|C9ZKE7|C9ZKE7_TRYB9 | DeepTMHMM Topology Prediction - Predicted Type: TM
MLRCIQCNAAVYRIVQPENEVVEKCGTCGRRCDRYYEFSNCQKWISITLLEKPAWIHVLFNKKDIRATLFC
TALLSRLIEAYVVRTSLVYGALRMLRSKGPVSNVSSVATLQLFRNVNPKIEPLMAYQDTLPNIFICACGEY
LLCLLVTVIFALHSWRRGGSALWDVVLTWMTCVNLAYSAKLCFVVFLIWRIPIALVSLVDLISLLWAARGF
SLVENRYPPLFTSVVVLICTAATRYLFRSVTQWSPQLLV
>tr|D0AAU8|D0AAU8_TRYB9 | DeepTMHMM Topology Prediction - Predicted Type: TM
MSLGKTIYVDKELGENVGCVSRIRIMSALVAGVGAGLLGLTNLAGALFFVVSALFTSFAILCFGCEGGPER
YFPSGKKELFSFGSLFTGGMTYILAWTVAYDAIYIF
>tr|D0A0A8|D0A0A8_TRYB9 | DeepTMHMM Topology Prediction - Predicted Type: TM
MPFLAFPSFLFGVFFLLSSFFFFPPFFLLSFSCFSLNFSFSSIFIVVVAFPLFCSSISFIPSLVLMLRFFV
LFSPSMLLPSPKSCSVRVQMRSHTHTHKKKKRFGLLPLPL
>tr|C9ZPZ6|C9ZPZ6_TRYB9 | DeepTMHMM Topology Prediction - Predicted Type: TM
MISIHQSILFGRDVSRVRMENSPVPCINQFRCRLTMSSLSLLLVFLVVVMPPVEALDNITVKVYSLLYHPF
VGRRLIDSMNAGFNASMAARQWTVAPGINVEVIHPASYRIPGPRFLQRAINDNKDEFFVVVGPMGDGQLAA
SRPLLQKENLVAFAPSTGASSVRGWSPNIYFLRVSPTVELIALMRFAVTHLRLLRIGFMYLQGLTFGDSEY
EVAIKLMSHLGRELCGVFTVASSHGKGAADSDFDAVWDKFVVTRPQGVIMFAPPAKDVVKFVVKMLNDSRT
QDAYFLASSVLELTIASWSSDIEAANTALDLGQIVLSRTNPLATDTQYQAIRRFQDDARSYLSANPGVTIF
SGTDDFEHHYVDGKLMVYGWIVGEVLSQALRSRAWIKDRETFKKSLYSQRRHVVDELVFGDFGGECEGTAG
ERGAVCNCNQGGNVVYINVARRDNVLEVIHDGQVVVDSSLCYHDEVRLHSPVNGLLVFMQDDPVAQNAAEE
IYDGAIPLTGDGRLGQTDRFFLHMLTSETAEASSELESELDTRAVTAVFGVAGDAMLSLQTTAFIDPISLL
PGLPHRGRKVIYLSPTLEQQLFALVKYFVGSGSTVVHAVVCRGDVVSIEGLLYLMLMTFGGHMGTVVGPDS
STNLEGSMPETGDVLVIGLSKDDVAVVASHLDRNPGVRVAVLFFDIALLYTEFVKTFKSSSSGGRLLFATS
LPHWAEANSTSVTVQKFHAAVPDSPRWTPLSLLGFATGRFIQTLLFHMDKVTPDSIINAIYTLSVVNSDDM
RYGPFVEERCPSTKDAPEGGDHFCGKNYGARRLSVWSMDRALNASVAPLTSGATPSLVYIDPYSKVLSGGR
LAGVIVGVLFILLLLVALLLVLLCLRRSARDNDSAPKEPADPVTLIFTDVESSTAQWAAHPELMPDAVAAH
HRLIRSLITHYRCYEVKTVGDSFMIACKSAFAASSLAQGLQQRFLSADWGTSAFDESYREFEQQRADDDNE
YKPPSARLDPEVYRSLWKGLRVRIGIHTGLCEIRQDEVTKGYDYYGKVTDMAARTESVANGGQVVLTQATY
FALSTAEREQFDVVSLGRIPLAGAPQPMEVYQLNAVPGRTFAALRLDREFGDDFEDRMSTSTGDSSSLRSG
MNGTTQMIASCLQAVLGTFTAAQRQKLLVPLCERWRVTLPRTAQLTWDEGFCEDVIRRISVKVGRVVDLCA
NSGGERTVSTLRSASVIIMSNRLREFEAEATQSPEA
>tr|C9ZJU7|C9ZJU7_TRYB9 | DeepTMHMM Topology Prediction - Predicted Type: TM
MAMLGFESTAEFFVYLTFIFFGMSVMNVTNAIYSNYNFFSEYYKFVKKQEKAEPENESFWKHMFTYYNVVV
FTMQVVLEAFMLTPLGRRIPISWRLIFGLTIPMVEIIVILVIPAVGGSENGAIATMMMVAFVGGISKTLCD

SSNAALAGPFPTKFYGAIVWGLAISGLMTSFLAIVIQASMDSSFTSKNTQSQIYFGLVMLLQVVACVLLVL
LRKNPYAIKYAAEFRYAARKDGVTGDGADGEFDAKGTGPADENRYPDEKENKNVLNADIDPDDMKDTDQVE
GTTNAQQMLDASVMVVVKRIWPMLVACFFVFFATLLVFPGVFIAAKTGDTSGWYFTVVVAMFNLGDFLSRL
VLQFKQLHVSPRMVMIGSFARALLIIPLSLCAAGTVTGVWLPYIVSLLWGLTNGYFGGLSMIYGPRTGSLT
TAGQRSLAAICINVALLMGLFVGAMFALAVKEGLPK
>tr|C9ZLB3|C9ZLB3_TRYB9 | DeepTMHMM Topology Prediction - Predicted
Type: TM
MDAPLQLRTLGSNGASSPQGAVEARGISDGADVEVAHEDACGASPHRDSVEMVDSKERNSTQITRGIEPVG
PSGSQNDSTTSVVVDARECWICREASDTPENRLTSGLCRCRGSIGLVHTGCLNYWVFSQRRVRCPSCNATY
NVISVSSEDFPKGFLHEAVLLVRHLYLPLFCKCASILLGLVINGFAIAFAVGCAFYHEEVFADAGDQAPKS
FGNVGAAADLGGSTATTRANAPNGGVGSSSGVWLWVGVMLFGWCSTALWRSLWVSWGQWRAEFINDAVDDA
PKPPPYTLVECLHYYFDIVVSMTGCTRQMLWLRSMELCALTAIAYIISFTYGRVLVFTLIFLGAVLMRLLF
QRKKINDNMRRFDEAQERRHNATYSDLVKWFITYITEMALFSFALTIIGGLVIHYALSPHILTFPTSIPAL
NESITFLRLLLYWASGTLSSILLMCIETTVIVNIFAPGVDLFFVRSVDLNVDSDSAYWSFILAQIFDSDPL
QVLFDFPRLALIEVVTLFAFLALPLQAMFFLNDLLATKVFGSAGIKLAWVLHNSGEYLMGNSPANGVIPPF
DGSFESGWGSLQELLTEPLEVPSLVVSGLLANTLPLVKSLIHFLSSTSSINILLGAGTGVIVLCLKVFPIK
RTQLRVMRAIAVWLAAHVVYMEDFLFDKERLQTLDNWLQAGGEGDVPTQRVPLAFVFLRRERVLPPEQKRP
AWLKVRLIIFSAMFFIASTGVFWALPVLLAALLLLVMPCNAALLCCTFNASFLLLDYKLYLKAVGEFVFIS
AVLLVGLPLQLLHALRLAVNFGYPRKRLVKETFEYCLNINRTIGKYCGEPRDEQQEDVKAELGEVDSNSSD
DIIAVNDDDDEDFE
>tr|C9ZQ74|C9ZQ74_TRYB9 | DeepTMHMM Topology Prediction - Predicted
Type: TM
MLATRQIVPSTVAHIVCPLSLIGGGVVLLAAVGTSIHVYKPVWRADSDDDIERCYFSATGDEMHLGNTPIS
MVACAHQCFAVVTQDHRLVVARYQTNLPVDPLSWVAKDPLHHNNAWIVLHRASLSTDFSGRCSQPVHTPQL
ACSSDGLVVQCAATGGGRYCVSINFPSTSQGAEKLAQTKWVFDVPGSVAQQTFPTAAFKRDVLEQSDEELL
ERYTIQELVEQKQKQERPEPCIRCGMPPVSVSATSHVCDSNDVSVALLSVASPTTVKSECVEKRDSFAFAS
VDSDCLDLCMVNRPKFDQIFDDKYPMPSCSPYLVLSLHKTLLVLARLGNTHKLTVFSVDEGPVMKICKVVR
LAQSAVQPIAGVVHQRRQPFQVLERVDGTGESEVSNTDGDVLWLLLSNGNLTEFPLGSVARATGPVVVQDA
PHLLVGDCEGCDWPLAHISGLPSGFRPSAAVPLPHESTVTFEPHNTTRRYIMLSDGVADSHVIDLETRCTV
GALLSNGAMTSACGGPSADIIVSYAKGVLQRLSPGIGAVLRVRATFAGSQRMFLLSTAVGNRLQDKEPEHT
LLEFYVVITTAINTVLLRGSGSQLEKVNDVQHLILDEPTLAVYCAPDAQELSPPSSHFFAQCTPTRINFSG
KWIRLPNVLPEFTSASHACFGDDKWLAVAYGRKFAVFSTANVNDTYVVLLETLPSDVSHVTTWKASESNSG
CCNSTLWCVAACLWSHEVWVWMLDCGVRGELRTRYHIFHLDAVALSSFLITKRTAESCDGGVHEVGMGLVL
LDQGVVVLQCSIEGPPSLTALKDVDGAPFVADLCLSIATHGAAAAGGPHFDFASLRAGSVELVSLDGMSSS
GEQLAVALAPHPGDNSANTDGGMLLPQLHCGLVVYLPAHSFYLLLLADFDGISLRSITELVPPPQPSHLAL
ALGVATNGEAAPPISNPPCDLRRRGVLLRGPVVYQLTHSMRLPSVTSPSHSLTKAVYLHQCNIIVAMLDRG
EQASFISTVDVDTFRVVDAMAMKQDEVAMCMEPLMSLGGGEVSGDFIVGTVILSSDAPNAVDKEAATAGRG
ANTVLGRLIVVQARPLRISTAADIVGPQLKGNGGVVDLSVQSLGDVHLIAVAALDMVLIFRLVGSTLSLMC
STSCTPACTTVALQYPFLSCSLYSWGTRYMRLVSKSSKSNSDKSPNRIHCPEPVAALGDVILTQCLQDDLS
LRHCALEPTPFANIHSQTTFADGFVRVDGNRNVVILSLKNPKVNGQNSTAVAAGIVGAEGGLRSSITRCVR
LPSCIQRVSVQRERSHHHLHPNGKNNNNNNIHDNGGSRGSFRPSELTPWRYRQVPFVCWHGRPTALRVVGP
SLLLPCADGALHCAREIPQAFVGVLLRLEQRATELYDTTFSLSRRGSHVALGESPSCGLQRTYHTVSYEAE
TALQSPTRVLMKQSFVSVDAVGELVLLRRLVEIPDAALTSEERLLVERKASLLDERLEHVWAEYGGELKDM
AVAELLYLW
>tr|D0A0M2|D0A0M2_TRYB9 | DeepTMHMM Topology Prediction - Predicted
Type: TM
MIIIVIILPFPFIVEPHYQSQFFFLHTLLQRAMRPKGKPIRVYTVNYMCGEEAFRFRSYSSHIFLFLLPRP
SACMPPSTFLFHSPILFSLNFSSSLHKIG
>tr|D0A359|D0A359_TRYB9 | DeepTMHMM Topology Prediction - Predicted
Type: TM
MHLKECPPASSVGWLRPSLTIYMFGSKSLALSSAVFVLDCFTMGRKQRLLSAVVNVPLTLRLMLYFSTSYK
GEEPRLPPSTCVLGSSQGNNFLLLPGAFAESGCTTFFPGREGLMSELQDELWLQLPSRLLILARLDLANFL
FAVVGRLPNSGINLFQQRPPSSLAVLPKRCMGMTLVALTTL
>tr|C9ZS47|C9ZS47_TRYB9 | DeepTMHMM Topology Prediction - Predicted
Type: TM
MVCAFVFLLLFLFSFLHFVFSDLYLFLPVFYLCFGKLDGTRRTFSFFLNGTHACSTGRGIGGGAEGPNNSE
GGSPLTHIFLRYKSTSVAPNVWKEEGRGTKVPLITHTHTYIYIYIYIYGHYTYIYCS

>tr|D0AA37|D0AA37_TRYB9 | DeepTMHMM Topology Prediction - Predicted Type: TM
MFSFFYCFFLCVCCYLWITFSFSISGTRMDSVPLFIYIFCRVWLVVLYCRCDCATDFFLFCYLSHTGSRKE
DRKEKWKSKNKKKDMISFVMRRDGLDLVLFPNLYTGANPLCSLMRHRNISV
>tr|D0A8Z1|D0A8Z1_TRYB9 | DeepTMHMM Topology Prediction - Predicted Type: TM
MQHIYADVYIVRSSRLCEHSLPTFFLAKVYLFYQLLWLELVPYCSCECIYCVVVDLLVVLFLSPRPLPLWG
YPCHSIPWFPCFFSLSKNFIYPQSIWTPTFFFFFSNAAAWLMNPFILPPLERPATGGPWTSHVRLPPESEK
ILETMPKRLLPAIEKSYLSASVKKLSIWKPEPRFEDMIEALERQQRKLRATIGGREHECRYRLLWQHQEEK
VIIECRTEAASVSELMRQRARYSQKVRDFEARGHVLHVETLTRGHIYKSEEDARKHIESLEAMMSLKGLRH
KASRDVESAESILHGLCDVQEFERCVAQGSFHPSKIFKPVTSLIMTRNTGTGHYQGRTDLLMDYTVCDEEC
RARARLLFGERLALMDLYCEHNAVVTIAVEESSARAAMWRAFRWGRCQARLEALLIIQRWWRMLRVKPWLV
RRKQGISECFRKKRWKMTASDYHALLSRMRRSRALVGGPSSLTDIRQSMAAVERAAELSHQWLYDYFVYTL
MNRAAQLHRLIQHDLGIRSVEEYAERSTEEEDEATSSLPLSLLRMPYRVFVRMQAKQPYPSWRINRWVEGS
ISLLRLQEGLMAEESRLRDVLHGREARETDALKLFQFVLNKGDWPWRSFCTAMHFIVHDESIGRDLVAESE
RNARSTLSVEEAAEASQYFTLESERLYILQEEALRREAIEKEESTVFGDATEGEWRWENLCKTRMRFAASC
GCVLHWERERDRGLGSKHVSLDSVARVITRFFRRVQRRRRLAAARSKDASEETRVREIASAMKRLLEGIIE
EDKQLIPHYIAQFGAAATAVLSPVSTSRATELCSQAIGTQEDASYIDTSASLFSEWFDAQASEPMERNYKE
IVARFIAAHKDLYRDMQHLFYIVRRVREGVEESELSERIALQQSEAFLQVATYQVSVCEEKERLKLVEEIE
SAHLDMCEQLEMLGALAWCTTPSTRPKPFVFPLTKARKAKPEVTNALLPLPIYMTNTNRLLCREEVTRVRI
AVEELREYKAYLENAHNLLHDDVVREEATQRLLLCAKVDNYYPSNPPVAQLAALVEEETHTRIPTEEEALG
TYGSPMLRSVSEYAEHVLFKRFLQGFLKFSSEAQYFDRHVTVLLLQRVSFLGSKLRELLAMESCARGRLEF
YEERYRDALREAYYEK
>tr|D0A576|D0A576_TRYB9 | DeepTMHMM Topology Prediction - Predicted Type: TM
MARCIIGARRVTFWSMEQFACLLRSFHRNGQVVVNKNESSQRFASLRPFLIDLPYSAAMKLLIRLFMLSFH
FYQFVWCVALVGYFIYIYIFVKVIGCAP
>tr|D0A5M8|D0A5M8_TRYB9 | DeepTMHMM Topology Prediction - Predicted Type: TM
MYGPSYFSYGEVRYIRIYYVPPPEISLFVPTLITMIIITIIISYGHVCLFVFCGSGGCGKCASMHFSASFP
EREDCLHHIFFSVLDLSATHTCPLLQLFSTSLRYALRNVEEPLFFCFLRSISF
>tr|C9ZJY2|C9ZJY2_TRYB9 | DeepTMHMM Topology Prediction - Predicted Type: TM
MKTRVEWEPLQPRYPTENPPHHATGKYMRAPHLLPLFLSTFCPYTRRLQRHFISFHLSFFFSFVPVLFVLW
CSPLPSASILTVPSLLQEARYCCTQVHILCARGAAVNEEEEAQQAYHSIIRRRYWSGGKGL
>tr|C9ZI98|C9ZI98_TRYB9 | DeepTMHMM Topology Prediction - Predicted Type: TM
MSLRRHVITRSISVHNRFISSSGEQKTGGSRGGFFSVLRYGPRNRAELFGLCLGCAMCPLSIYLINWCEGC
SNGNNNTQPELVLGNETFVTHGVAAPDKGSKYPLGGPFRLRESRTGNYITDKELFQDHWTLLYFGFSKCAE
VCPSTLRFITDVMKACDEKLAGDKNLSTEAARLQAVFLSVDSRRDTPEVLEGFVSKYDPRVRGLTGTSKEI
EQAARAWRVYYSSIDETDEEKSAREAKGVPMVGADDDTYQLDHSSAIYLVGVDGKLKDFFFKEMGVADAVG
RLEVHLQDVYGFKDTRG
>tr|C9ZKB1|C9ZKB1_TRYB9 | DeepTMHMM Topology Prediction - Predicted Type: TM
MDARKGVITHSHAKKRHIKVSERGGGACHVHVLSLHILLIPDRNMLLWVSLCAEGGDNTLSLFSPFACQWL
VCSVCLFFFSIQVYAWAYGHKCGNDPLRKVGSEDVCVYVCVEEGQADANNRKRSDCL
>tr|C9ZVQ2|C9ZVQ2_TRYB9 | DeepTMHMM Topology Prediction - Predicted Type: TM
MEVFGSNGVSVATCSNEREWMEKEVKEVTGITHHIMGFFSSFFSSLLLFYLLSHVVCFFFSFLFFSFPFIP
LSFLHWFHDASCAVQYSCVKNGRKEERNGKKK
>tr|D0A3P0|D0A3P0_TRYB9 | DeepTMHMM Topology Prediction - Predicted Type: TM
MAIHVARQGRRKMYYWVGTFICVVGILLLLVIPFKGVVREKLGMPSFIGGYCAVFATLLSFFQILEHLTCF
SDPECQTKVVRILFMVPLYAMISWVCLLAPGAAEYLNILRDAYESYAIYAFFQLMLALMGGVDTLYRALML
EEWPPVPHIFPLCWLEPMKVSPTFVRNCRLAIFQFMVVKPLGAIVIIILKAKHELGGILDVSKGHFWTALI
CNFSITTAFTALVYFYVGLKEFMEGTDALLKFICIKAVIFLSFWQGILIQLLAAMDWLPNFGYWTKEEAPQ
GLQDLLICIEMMFVAFAHRYCFGSDVYDPANLVSIEESQGNLDPYAAGRTIPPIRYSVRENLKYTLRNEDI
FNDIKAIVRNQ

>tr|C9ZIP7|C9ZIP7_TRYB9 | DeepTMHMM Topology Prediction - Predicted Type: TM
MHFQTSKSFCCAVKRLCYPMHVLARALLFVLGNRLPGKHVKYIYIYINTHITSPPTSANGKKKCITRCFFT
LFIIFFPLSAQSIYYTSTFCACAIFSFLCVCVCARAWCFTFWVHCPYARHMNEHTNTPLSIRVAQWRPLGT
NFFLLIYHFFSSSYPPSFVTRMYYSLG
>tr|C9ZJ03|C9ZJ03_TRYB9 | DeepTMHMM Topology Prediction - Predicted Type: TM
MKRSHVILTKATQTQKRAATATKAVVNSFFFLFGATRRKKTRGRKKEKGINKTKQNQNTHTHTHTHTHTHK
HTSASAFLCHFLSFVTCEYRYTQTLSLFFFLTPTHTHVLFLFLFFPFHTTVISL
>tr|C9ZZZ5|C9ZZZ5_TRYB9 | DeepTMHMM Topology Prediction - Predicted Type: TM
MQSQPDNVAYPMELQAVNKDGTVEVRVQGNVDNSSNERWDADVQKHEVAEAQEKPVGGINFWAPRELRLNY
RDYVAEFLGNFVLIYIAKGAVITSLLVPDFGLLGLTIGIGVAVTMALYVSLGISGGHLNSAVTVGNAVFGD
FPWRKVPGYIAAQMLGTFLGAACAYGVFADLLKAHGGGELIAFGEKGIAWVFAMYPAEGNGIFYPIFAELI
STAVLLLCVCGIFDPNNSPAKGYETVAIGALVFVMVNNFGLASPLAMNPSLDFGPRVFGAILLGGEVFSHA
NYYFWVPLVVPFFGAILGLFLYKYFLPH
>tr|C9ZXE1|C9ZXE1_TRYB9 | DeepTMHMM Topology Prediction - Predicted Type: TM
MRGGVGVLSPCGVCWRCASIGCHRCGSTTPSQVFLLRVVATQGLPCRGELPEHAPTCSFPPSYPMGSECGC
CCCSCCGVWCGTNSFETAAEGRRILCLFIFGPSTLFSFCGCCLFLFEKPRRGCTAIF
>tr|C9ZHU1|C9ZHU1_TRYB9 | DeepTMHMM Topology Prediction - Predicted Type: TM
MVHPYLWVIIVGGIVSFLTGCGVGMNDLANSFGTTYGSRVLNLWQIVILASICEFVGAVSLGSEVTSTISG
GIANPMTFANEPYVLMYGMMCALSATFIWLLFATMMSLPVSSTHSIAGAIIGFALVYGGFGAVSFAKKIDE
FPYVTGVAPIIASWFISPVFAGAVAASLYALLRLVVLRPANSVNRALFALPLIVGVTFFLESFFVLFKGAD
SHLHWGPAKASWVAALIGLGAASTSAACIPLLRRRVRLITERAERERAETGMNTAPEISGDAGAISENAAG
VGAAVEGPVDTANRIVPPSSEPTSDSPTTEYQSKNMSRLSMTGVVDEALKFDVQIYDERVEYVFRYLQVFT
AACASFAHGANDVSNAIAPFSAMYSIYINQQVVEENDVPLWILVLGGAGLVVGLATLGVRIMRLLGERITK
ITPSRGFSAELSAALVVSLCSAFGIPVSSTHCITGAVVAISIMDCGFRKVRWMMVGKMYLGWIFTLLITAA
ISALLFAQGIYAPSLTSQ
>tr|D0A1A4|D0A1A4_TRYB9 | DeepTMHMM Topology Prediction - Predicted Type: TM
MQIKSTDEIVNLIYFFFLSSEETICPKRVDRTGTHVHSYSRLYAKRQIYIIYITYIRVCMCAGKFIPFLTA
EHGNLAPTHILNQYYDRHFLYAFYIKQGEGVIYLVSVID
>tr|D0A126|D0A126_TRYB9 | DeepTMHMM Topology Prediction - Predicted Type: TM
MGNEPDEARVGGVPEMPKVYEKKRTCTDIVFLCLFFVMLLAFAALGIIAFVDGEAKDSLNGRDRTGRYCGS
GSPPDGFADSIPEGAPFQSKVWGENKYLWYPFPLKQTAMSLNPLLYLSLGLCVQQCPMANERLLERLLKNP
SSVTEEEKNAVKVYSYGGSSVVESSVQTVNHAIPVYHTGPILGRCLPTITQPPSVRELLMTSEYSQRVYAF
VLKGVLEVKASWKVFLCAAAACTLLCFIFLFLVRFLISFVVWASIIACFLLLLSGGCICIQLYLNDGHFMN
LHLDVTKYSVLLLCVAIGLWIGALVYVFVIIGACPRIRLVCALAKIASRVVDNAPSTVLLPILMNVAILCL
LVWSILVALGLYNARRKTGKISFVPVTDAIGINDGSFKGIGDTKFYQGVVHGVEHRYALVYLLFGELFAFL
WCVSFLNAVSFTSISFVSTFWYFSNLNGGKKRVPFFGVLRAFVWTVFYHAGTLALGSLLIAILQIVRILLV
YAAEKAEEAVDRHDLIQCLHCYLQYALLCFENFVSSINKNIYVVVCLTSRSFYTSACTGLSVLKGHSSDLF
LISWMVLCVKILGKLFVTVGTVVTSYLLFKHTTLEPGVDSMIMPLILIGIGAYFLSGPFFDVFDSSTLALL
ICYCYDHHINQSVGIFYVPAELEHQLSDYSQKKKLRRLKNQQAAAHRRG
>tr|D0A9X3|D0A9X3_TRYB9 | DeepTMHMM Topology Prediction - Predicted Type: TM
MNVFTLNSNIYCTFSFFVCPFFSLSPSSPLPFLKFIFPRALIWQILPLPSPLQLPPLPFCIICSHFFFPLF
SIFRVFAFPFVITLLFFFYFFPLILNIISYAHNHFFVLLLFFFVPPFPLLSRLNMHLLRVPVMYLLFVLR
>tr|D0A047|D0A047_TRYB9 | DeepTMHMM Topology Prediction - Predicted Type: TM
MPSVKWLSAASISLVVLMLQNSLLVVLTRYSRINIPPEKRYHTSTLVLNQEILKMVVCLFLLSQEGGKCST
ALPNAACMNTPSGGGFLTVLWGVCFCKEARELLVPAFLFVSQNYLIFLSLANLEASAFQVLSQTKLPFTAL
LSKYMLGRHLSSMQWLSLLLLSIGVLLTQAQGSNPRHTATTATQRPVVGTLACLISALSSSYASVYFEKLA
KTTKPSLATRNIQLSRFGILFAALAMLIFDVLPSYGSNAGQGREPFRFWKGYDQWLTIALVCLNALGGLLV
SAAMKYADNILKTFATGGAVILSGIASYFIWETPMTLLFIVGATLITLSAVLYNKYDSHAHHTAAGQTSKR
EEHH

>tr|C9ZUP6|C9ZUP6_TRYB9 | DeepTMHMM Topology Prediction - Predicted Type: TM
MSPSSLTLRYVLPCHSCTSCDEATVADRVSLLDTPSVPVITWTTPSEFLTAVKATVPVDSAKHNGSLAVGE
DGLGLHLLSADSAGAAAGDGEAGPRVIQLTLDWVLALPCVSDDAEGNSSVELHVLRTNRLDFLVQTWSAVT
SHIVRLTTWPRMEVSFFPSGFPCEIVSGMINGSEPSYYEVRARIPIRLVLSPTPEGTKERKNDDRGSVSEM
LEEFASTKRMYAHQLRALIHDAAFSCISSNPTSFCGFDEAPSSGRGTDTASPGESVKEEEAVSSASTNQQS
GGGVSAGKEPKGPQQVLPRVFHSIPPPPVPVALNICLTHHGEDGEVPTEGDDQRERDVVLFVHTVVRAVRP
RSGASPRLRPRRIALDALVNLFPERFAVHGVSDVAVAEVNVEDLSCMWVPFNDTTIEGDVLALSAEEYFSF
HGRQDSEVSGQASPRGNGSKMQHNPYDRMRVSPADVDPHGRKTAPQPCFGWGRGMCFNGGGPGRPFTFNPA
LCGTFQCFPWTPSFALRKGRTASAGAAETSEVRGVVRSSWTGVSSWKNVTPGSSCSWRGESSGSRRGGKSG
SLRSGASEAPWAPWAAETPDDVSVKVPPQAEETSAVSASPQDDNEVMDLSDSRQMILEWADSIGLSRRRIA
NLAQQLERLGEQFASISLQHCVTTDGADKRKKGRGGRNGQKAVDAGAPEEDSDAVHLVEGLIGASMRPHGS
ISEEVLRFAARLLGGAKQCEEDYGSVDECTKEAFDMIQKLDQDLRATALEVQRTQKDIRKTLSGILTPKGE
RKAKVETPNLFYPAIKALNPEQSHRTELFGIGAPSGLSRSGDILTLLRFKKGDYEVAKQSVMTVSDKLRKA
EVWCAAQLEQMQLVVKLYKDILVPCLDARVATRRDEERKRSKLIVDWAVSIAIVLESQRSAESQESNGVER
HEDEHLSHKGPRGAASTEKPLRLSNGKPLLNGGSNGRAHEGQAEPLKTHDATTRSEGKGLEASSERPAKVE
RPVKAERPAKTERPMKADRVAPPVTETTRDLRRSTSPKRKRKAPAVVAQPWSLMTVTPAPVVTTQRRGSRY
RRFLWRLARFSQVDRLRRNAYLLALLASVAVAYLVVVFLYG
>tr|C9ZY31|C9ZY31_TRYB9 | DeepTMHMM Topology Prediction - Predicted Type: TM
MLGFESFSEFVVYVTFIFFGMSVMMVTNAIYSIPAFFTEYYKYAQGSSDAQTENENFWNNILTYYNAAVFS
AQVLLETFMLTNVGRRIPIRIRLIFGLTIPLVELIALILITVCHTSEAGAKATIIIIALVGGVSKTLCDSS
NAALVGPFPTRFYGAIVWGLGVSGLITSLMSIIIKASMDDSFDSMLTQSRIYFGIVIFIQVIACVLLALLT
KNPYAIKYAAEFRHAAAKESAVESNEPVQETITDQEANAGEEGERVEKSTSKMNVLNVSEDPDKMKDTDQV
DGTTNAQQMLDANLWFVVKRIWPMLVSCFFVFFATLLVFPGVFFAVEVKDGWYITLTAAMFNFGDFLSRLV
LQFKQLRPSPIVVLIGTFARLLIIPLLVLCVRGIIPGSALPYILCLLWGLTNGYFGGMSMIYAPRTGSLTT
AGQRSLAAICGNLALLLGLFAGSMLALAVMTALPES
>tr|C9ZKF9|C9ZKF9_TRYB9 | DeepTMHMM Topology Prediction - Predicted Type: TM
MGVVRNSSPQRWVSKALCFQPCTNIIFVSCINTCYFLFLHQYSKSSLFYFCFCLTVHDIVHGTPQRLGAVR
HPRGRYQCDIYRGKEEERKEVQRFPYWPLEWLKECSSMLKSVICNRLHSRDW
>tr|D0A5M2|D0A5M2_TRYB9 | DeepTMHMM Topology Prediction - Predicted Type: TM
MVDHISLCVLFALPLSFPLSNLFLFIFAFYSRELSSWKRRRVMAKHPGEGEGEGEQQRLSMGAKRSTHAQSPS
QPPDATAAPIEILGASKPLPGDGSDCKPGSSETIIVFPPPSIFKRVGSTQRPLRGDTKPHASGTAVSFDEK
TTSREPISHQTKVNPPPSINVHRVPTGGQPVPAGPRQVNEEPPKMDDDAADTASTPEPDDEHLRSLTVDVM
SPIISEEGEAKGTVTCPVDAICEQVDNLSSDSDKADIAFVLYSQYLKDYERKPSFTADTWDEIKYVGDSGR
RFGVLYDEERGAVQLILFGKCYRIFILSFLLRFICLILFWAVMYMLASRSKFSPGGLYFDTLCTILFAGVV
GSIISRVTRVPSVACAVLAAALYNNIPPTGSLTAGTSLDMRGVISLFGLTVGMIRGGLALNLRALKANFFR
YLCFSVVPMVAEAFAHGFLAKILFRYPTTTWALLHGFIVTANAPGIIIPALIELQRKGYGTRGGPGVMILI
SVSVEATFCVWTIQLLLAIQFNTMGLLLAGLLGPIQIIVGLVVGVGLGYAFYFVVFDILYKEGQRVPLRES
TILYATQRHLSHVRLTSCFIVMLVSMVCTSVGRMVSCIGGAAVIIVTMLALFSHWCDVASKMEHLTAKGDV
LVFFRVLWDYVAMPALFAMAGASVNFSEVFSGDFFAPGIACVLAGLVVRCLSSMITPLLLRMPFTWRELVF
CGIGSLGKGPMQAAFGAVPLMFLQTMGANSGNSTATADDFSADDDIKHAQTLKNSAVLSMLVACPLCSILL
GVLAQKLLRRDAPSVPAAAK
>tr|C9ZJQ9|C9ZJQ9_TRYB9 | DeepTMHMM Topology Prediction - Predicted Type: TM
MSDVEGSISFSNVESHILASEAVMQHQEVVDTFSDGWNSPTGVHVTGNNDRGSIVCNTAEWATGSDGLDDG
DLGRRRSSIYLDAKDGDGPVSVAQGRSTIDNVALTVDTRPSDLVATLEGENYPYYDLDYVCSYAFQSEHAD
LNSHDYGLDAFIHGMRLGPWPLQSTVTTSPPRVLDGQSNRLAQFGGLQSSLTVASESPLRNDVGVLREANS
LQGKTSPNGTSERWLRWSFRAFDGKCNLPQGTTRGCDTLGVGESSLYDVDMVLGLLCTDNGIPGVLSSGSC
GEGHTQKASEICMGVMRRGDSGSNNELMLFPRHDDRGTGALTNGTSGAVHQRCSEDIIGAPTGVVNAQPLR
TEEGKASGNVELTMVGDVLPKTFSCPSAASDFVGAEGPLGDVKEGTGLSLLSKALPISAILTDRARGRVGT
PSCNSLVDSLIGGMEYPYFSGLKGDSNLIISSPLPSRQSICTAEEGEGAILSAEGVSVGMERECEQYLQSH
ISCSVGVHLRRGTELRSINPCIARCGDSRPGSTCRPVSCSLDKNQVVGQGVCMGGSERLSIQKRSREKAVQ
GPGRMNGSSISLVAGAESITASQSFVRPPAAVVSRKRCHPCSELSTGGVSGSHTVKVAEDGMCLVEGRSSP
IIRASRERQSVPSNRVRLIVLNHKTGTGQNDVQTAVNLRGESTLEVCPPQGLPHFFSVDEYLECTDDNDMC
SVTLAELQGKFLRGMNVAMFVAGSESVPLSGWCAVFEVMNHVFRDINDECELFMSVVLIRQESARDLLSES

EGGVRTLSSSVMAPWVAFENVTHMKLKSAHHFITLLSRGLEVADLKHNCVGSRVVLSVMLKQIDVNDVILS
SMLVTGGATPNDLDVLLREGDQSARKLFHEALGGPYFTVVVSSLCERDRDLLQLLSAQHKFTSVVNQPCTV
GSVSRLIDNAEAEVAGCSTDDQIVLERKQFLLERIALAKHIMYNPKGLSLVVDVEESVRLEKRVHCGVKFD
KQPLPGPQSSSVSACGGDSHNAASALCVEEGQSSKQRQEPCPKFFSEAQWLKPSSKGDTRVTLMGGAPPSS
LPQRNHYGEGKTPSCLPCVTAAGMPTPRDPRLERQVRPSSRRLSIPVEPDQASYATTCSMSDPQRGEGLPS
LGPLATTCGMELAPSTQRRAHRCFPSPASSDHTTCAYSQRLFSLTPARGTSHDAEVSCKIDTPGVPDAEKV
RTSTTPDAFSCPGISTKVKYDPGLPLPSLKAARGPPCSMTSCFPFGPILTGPPVVVEPVEHCGISQGPALC
RQREQPRLPDASVACSGGSPSLTCKRVAHASQNSVHTDIKKKQISDGGSDSSARSAEGSEDDVLHSAMSAP
SKVRTLVVVDQGCGHVIGAGNDDGGPFLRTGGSHEGYEVDEVVKWGDGCGDVPSKVLGELFDVFVLGCNAA
ILTAESGSSTVSSTVLRGLVRNVLSEVVDASGLQNSKRRGLLSLSIVKLSGERMLDLLDDSSEPERLVIAM
SPIFGPCVHNARRFPVSSCSGFDSLLASALRRAKNAGVDDGLVFVSVVLKQKLKADGDVLVSSLAVSLAGE
NVDLYTSVLNRVSRAPRALFHYALGGPCYTVALLSIDVATTQTENMLRVQKCIGEVVNRPIHRGSIVKFIS
GIRDDLVPSLREKFQGSNKCEPSDNILYRLEEMVRDAELLLENFESNDPRAYLSDKETRSPKGCVSLLETK
ARTGDEGRVHSLLFFEQRLLSDGTAGVQGNSVFTRAGAITRRYGVDEVVLRGPDSDSNATLCSRLLDELVR
KFMSGHCTAVLAADSHNSASTPLILRKIVYLILQSTKAGVGCGPVGGDLFLSMALVKNGVTVDLLAAEESE
AVHRFGVEISPLFDRHVRGVSHRVVADTEAFDRFLVGAVEHVVPTLQAEDPGLMVVSLKLTHHVEEPVHDV
FVSSLMVTTVFDHVSHYSDILSNRSSELTDLFKLALGGPCFTIVALGLCDEDDDAEALLSVQAKLSQVRNL
PSHRTSALRRMDELTKTIQVLGVQLQEEESEEEKAQLRVRIVRARRFLLEVESLLKNPPREMGCVRAYKSV
H
>tr|C9ZXK9|C9ZXK9_TRYB9 | DeepTMHMM Topology Prediction - Predicted
Type: TM
MCMRSGGVINLSILSTGNESVRVHFFLSFPSVCVLFSFFLFFLKKRKERGKHVECSMAYYFSLPVWSVHLK
GLQAKFVIAFVCLCGCVFMCVQQIFCFSFFFYFCSFIGYGKTKVLDRR
>tr|C9ZIC5|C9ZIC5_TRYB9 | DeepTMHMM Topology Prediction - Predicted
Type: TM
MSLTLFSFTVAQFCCFFLYSLTNFPSFFLFPCSHVFRFVLFFFTSFVACVLFFFRLLQVLYFLLSLLPFAR
SHMSAAKIPSPLLHSGLTFSCHPFHCLFDVSPHTHTCYRSAFHLFIYYKNI
>tr|C9ZQN2|C9ZQN2_TRYB9 | DeepTMHMM Topology Prediction - Predicted
Type: TM
MPGVHYAYVYHAQVRQPADSWLSPSVIRGTGLGRANEEDGNGATLPMWGGFSFFRVLPSSSGARLEVVLRA
LGCLSGVFCLVELAAACDVHTCLVQVRMRGGGEGSEGVTGVLLALLCQRCVLDVGGFVRFRTVNPVSPFLV
SLPFL
>tr|D0AAT9|D0AAT9_TRYB9 | DeepTMHMM Topology Prediction - Predicted
Type: TM
MRRAAFCHLVHCVRVRSSSVSVLNGHHFCGIAFFCRIHGLTLPITTAVPVQRTAVRAFHHPNRPMFGPSPS
VGTDRKGKRNAKPTQRKDQEQWEPSKKPDIAGGSVVTDEDSNKIPIYRVMGHVLHHLWPAGKPGYRALVVA
SVTCVVLAKVLKVAVPFWFKTIIDVLTNSTEVGACVTTVTEALHLGVFGLVVAYGISRLLSSFTEEMKSAL
FAPVGCHASTTIAMELFAKLHSLDLQYHLGRETGVLSKDLDRGSRAFWSLAHALLFMVIPTAFEVVLVCTV
MKSCAGTPFILTAFSAVVLYIGWTYVVSNWRAEYRARFNKGDSRVGGLTVDSLLNYETVKYFGRESYEESR
LRRETADMNHQLMRLDQSMSLLNFGQHAIFVLAALVSLYLSTCGVLAGSMTVGDLVLVDALLMQLYTPLSF
LGMIYRDIQASTQNMQAMIALLDVQNSVKQKANAKALELVSGTIELRDVCFAFKTGGDDRFVLRNLSLAIP
GGSTVAFVGPSGSGKSTIFRLIYRFYDPLSGSVLIDGQPLTDLQIPSFREAIGVIPQDTVLFNESLRYNIC
YGRPDATEEEMIRAAKVACIHDSIMNMSDKYDTVVGERGLKLSGGEKQRIAIARVVLSNPPILLADEATAA
LDSVTEMHVMQQLRDAGGKRRTLILIAHRLTSIMSADKIFVLDGKGSLSESGTHEELLQRGGLYSELWSKQ
LHDAHRAEE
>tr|C9ZZI2|C9ZZI2_TRYB9 | DeepTMHMM Topology Prediction - Predicted
Type: TM
MNRRRKARRRAGLKLSLASFVSWGILIIIILIFEVLCCSFFFFFRGAKGYWLIKMSVSSQLNTDGAHRYARA
NVEKPREYWDYESARIQWSSPERYEIIEKIGRGKYSDVFLGWDTKVRRQVVIKVLKPVKKKKILRELKVLQ
NLQGGPNIVELYDVVRDPCSKTPSFIFEYVEASDFRTVFPTFSDYDVRYYIFGVLQALEYAHSMGIMHRDV
KPNNIAIDHKKRDLILIDWGLAEFFHPLTPYNARVASRYFKGPELLVELPMYDYRLDMWSLGCMLAGMIFM
REPFFHGKDNNDQLVRIAKVLGTDELFEYLHKYNLTLPQHLETAVGRHGKKPWTMFVTQENQHLCPKEALD
FLDQLLQYDHVKRAQALEAMDHPYFDPVRAECTARIREKKRGNVVTE
>tr|C9ZZN4|C9ZZN4_TRYB9 | DeepTMHMM Topology Prediction - Predicted
Type: TM
MTGSGGREPIDVATGTAPAVDKGETIPTKYEPHNKWWSCDIPTTLSLVQLEDPSVGVPSSDVSRRAAELGH
NHIPLKGGPSALWILAQQFLNSITLILTIVMVISAVFEDWAEFGVVLFLILFNALLGFYQEYSAEKSLESL
KAMTAGSAKVLRDGTVQVIFIDEVVVGDVIIIEQGSSVPADCRIIESSGLEVDEALLTGEALPVVKHTNEI

PDPGNSCALGDRKNMVYRNTTVTQGRARAVVCAAGVDTEMGKLAARLADGTGSGRTELMRKLDLMMYILFA
VCLGLALVVFAANRFKFKRSTLSYATAVAVAILPESLVAVVTVSMTFSVRNMARQRCIVRKLAVMEVLGNV
TDICSDKTGTLTENKMVVKKALIGIDNELVVTGAPHERYGTFIREEGEEHVNMLEVYETNKLAVEFMRCAA
LCSTTVLHPDSEDADLLKGSGNPTEVAIQVMTWKADAPRNKLENQGWECIAEYPFDSKIKRMSTAWYNSET
KSLYLCTKGAPERVIDLCTTKMLESGKLAPITDDDRQELTKKITSLASHGLRTICFSMNASTVNEFPIPDD
DTFQVTHPRETIEQSLTFLGIVGIYDPPRPESRPSVIACQHAGIVVRMLTGDHAITARSIATILNIITAKD
GEDPLKVLTGPEFDRIDMATIEEWPDLPLVVGRCSPESKVKMIECLHKRGRTVAMTGDGFNDSPSIKFADV
GCAMGSGTDVTKGVADLIITDDNFATIVKAVAEGRRISQNIRKFVLHLLSGNVSEVIALICGLPIRYMGEA
LFVLSPVEILWLNLCTSAPPATGLSLDPASADVLLVPPHTAGLFTFELVTDFLVYGFWLGGLALSAFVWII
YGINDGPKGENCNERTAVGCEMVWQARGTAFGILYFGLLVHSYTVRHPRQSVIFMKWFDNPWIYGSVIVCS
LLFFPIVYVPAIARNIFQHHSLTWHWGMMVPAVIIFVGLCELHKIFKNCLFPVKRPVIDRSDEEEYRRFAV
PGPDDDRDVESIAEDQLRQSFVSAAGSIASGGRRRKGRRFGGKGGKM
>tr|C9ZLR7|C9ZLR7_TRYB9 | DeepTMHMM Topology Prediction - Predicted
Type: TM
MIRRQTYVWSIFTGASAAYRWFPPRRECFRFAGAEANSAGGGAGASSGVHQTVAVNAAPEGFWRCLFSSWQ
QGLVASIGAVTGIGAVGFFLYVSMKDDTVHHTAAVASEALGDARLRERATNLSKEVVESVLKDQKSVELVV
GLVVRVLQEDDSMIAVSSFLRSLFEDHYTQEVTKKFVLMTLLDPWIQEQVRLIAKDLVKGLLRDPEVKKAL
VEFLSDSAATSLHDERLHYNMAHAVRSVAKRAVSPFT
>tr|D0A942|D0A942_TRYB9 | DeepTMHMM Topology Prediction - Predicted
Type: TM
MGSSVPTAVRPVSPLIRMSLWMHICVRVYLSDFCNSFMFLRIFFVEGEGKEGEGRGTGRSVRKADGVRTGT
CRFFFPLLRDWYELHSLCHAAHSTLFLFFLFFSSLTLNYWFHRIKSVNGMDVVLLRNVETSG
>tr|C9ZVN3|C9ZVN3_TRYB9 | DeepTMHMM Topology Prediction - Predicted
Type: TM
MADSNNSGCDAAVAGAHNKNNNNNNEEEFLPDTIAGSSAIKAPVPHKRVVKIGTSSQLLQEELGFPKESSK
RKDDAADPAGDGSPEGFLDSGTVDPFMTVIYPNTGTEKDEDGLDDDEEADIANRVVSRRDGDNFDRTQVRV
GGFLQGADDNMAFLIPNDLPRSTFLYLLTASDGRDRLFKCMQCSLHLLICLLMRPSILPQGSEETADYWAE
RFRKNINTIRHGRSLFKLGRWIVNAFYLQEVYERLALKYGRTMREVKGEFKAFLCRYLSLPPGSVMRRLIG
WDNNGVGGGSTGQFVNREVLVPGDTYEHVARVEFPRQFVSELHSIDAKLVTTGPSLTGIQQKEVGVSGAIK
ASDMALQSEEAKEDGERKVAGFSTLSEGKGLMVRDPLRTEALTSGSLAPSATPPSLYPAATEMYAVGIGNT
TRASLSKPATLLRPLRCTGADDESSNSNNTSIAHNNNTAPSSICGGSLNYSLMHEGKPKGDTYNEGFSTSR
NPDDSHQSYFKMYQTVPYEPKLGDALRLPHQGEGDSMNKMSKNQLSRAKFARCGSAVDNGDDIGAEENMFS
LGAAFDEGDNHLESNGTVSPSDDREEERERGLAAEMGKPASRRKGILQFSTPLILLQAVRSIATIARRLLR
DLLLLSSEHFLNLSPVEKNRALLQRHCHWLWLIVASIDVLLNTIRLLNPGWYKYTNVRDNPRFRCGCKDPD
TRGSVMLFRELVIRRQANLYFPDVDLDFGVPICSSPGYFEAADPENIAPACRVCGKLFVEMTSPDEQGEKL
EQGGDDTTAEEKGVTDTEEVETNGNAAVRQKASKSITGRAEVVFLFIPWLMRRLFNYIWLVRSHSNWSATI
WLQVSYLAEFYLAVMYCFGGYDTGKRDAPLKAMIHPTGAIVGFVGAIIGVYRVIQSAPK
>tr|C9ZP68|C9ZP68_TRYB9 | DeepTMHMM Topology Prediction - Predicted
Type: TM
MGASGIKQNKEDVFSLGEKRRCSRVFVLICLFPPFPLPFFFTVLPFNLFPPSFFGIYIYIIPIISERNDFP
SSFLLLSFLCYSLSLASVTFLLFLLFPCMIYFHRFYLHH
>tr|C9ZS37|C9ZS37_TRYB9 | DeepTMHMM Topology Prediction - Predicted
Type: TM
MMRRLAIQSSVRRTTVAITPIAVPMKVASPMCSAATRQASTVAISVQGLHYVGTGLAAIALAGVGLGIGTI
FGNLLVACARQPNLTKMLFNYAILGFALTEAIGLFALMLAFLMLFS
>tr|C9ZVW6|C9ZVW6_TRYB9 | DeepTMHMM Topology Prediction - Predicted
Type: TM
MTRETPHQILSSILLLRSLLFGHHRTVIHCPPYASLYTSITLLVTAYPHNRIIKHIYIYIYIYIYIYIQIN
YTRHYETATTRNFFLSSTSTRVISIPLHDSTSRSANITHVFTARSTALTTSLFSCS
>tr|C9ZZS5|C9ZZS5_TRYB9 | DeepTMHMM Topology Prediction - Predicted
Type: TM
MILFCAKRVFFIFTYAFFFFFFFCVVTSFHPPSFLPLFVSRALPQEVVWAPASSRDCRFHGAASFSIVFFL
LNFTSRVLSFGSSINWGYHLVKSSDTCGTLECSVRRFCVKAKKYVMNIGEVFEDCKNLWCEVSSHCSTERR
HFPLPVFLISDSPIRRLPHRCPREGPPKGKKKL
>tr|C9ZUF4|C9ZUF4_TRYB9 | DeepTMHMM Topology Prediction - Predicted
Type: TM

MRPDGSYHKVLPRFPIGGTFHEHRPRFPSGCCHLDERGRWDCGLLIDEEVGFIQCHRPRKKSNVCLKLTTP
LGGVREGVTGCCVTSRHRWGVPCISDPTIGMLLSRTAHNLQAYSSCIYIYTLCVLLLPFLLHKHMSCSSCL
ALQGREGN
>tr|C9ZUM1|C9ZUM1_TRYB9 | DeepTMHMM Topology Prediction - Predicted
Type: TM
MRTEGLRRALISDVAARWYVFQRRFLSTSTAHLVEEIRQALRVLSLSDSASDAEVRDRFQSLAKSNHPDVL
QGGTEESSVAEDKMRRGVEAYKLLRRFTEAERHALLKQRVQQEEETERGRRVYQGVVGRKGGGSRATFEFN
KFEQRRREMRNEAPPWKVDNHPPRNQRRSFLFYVLGGAATDARVSAAEILREYRRTGHLAGGRFRDCCAGS
FGSRAPSGLFGANADEWARTRQRAERAQVVSAAFGRHIVAGVFAVTVFLLICMIFVAVKKQYAARGLYTSS
ESFAKRELR
>tr|D0A2K5|D0A2K5_TRYB9 | DeepTMHMM Topology Prediction - Predicted
Type: TM
MCEGNKSCLIIFCVCLCFCVFVFVREVHVQKFMCYIILILPPWVSTPQPRHVPRVLCCICYTLLLLPRRIY
VISFFLSPFCCRCVRALNMGSSGYDTSYKSSLSSAPSSVGSRTSRQISEGATSVFSGETSSVSGHDPVFRN
VKRNPNLQFLKPDDSVIHVSPSARNVVRYIASNYDSFPDGHYRVVDYFRVAVEMFLQQVRLEYEKQAVQAV
KNGVKKPRYTWGNKSGLAISYATSCDMLKKLLYDVISEENRPKWDEVVKQFIYFLISESGIPGGVFSEQTYH
TKFLDWRKGGTRYQNLSIASNVRFPSASHRRQGVENYLRSGSWLSMKTTEVTVLANTDGTSRGESMGGVTD
STPSCSNSHASSMCYPPATRIQQRINMEHPVVPAMAKQVDDFSPRRTRWREKLTQIMGSPSARGLGEDRSN
MVLTFVRLCQVCDSAEQAESLTNIIVESDESIQRSFEGNGGIAVLRRFVGEPKWGCQFKFLFGLVEKLLRM
RLMSWGTESRQSWLRDLNGRGRRNLDWLRNIEVIPKGEREKWTNLVRELEGRYVLMPSHRDLEQPARVKRP
PTGREGLEQKVASSDGHSVKPQLCVSNSRCRRLPTEFLTLPDPEAEGDVNNFMDRKAKYCEDGQAEWRKAL
RKAAGVDLGGVSVPIWYDPYQLLDTSQCSSA
>tr|C9ZWE1|C9ZWE1_TRYB9 | DeepTMHMM Topology Prediction - Predicted
Type: TM
MGSFVVRKCRLLALIFSIFSLALTGLTFLTPIFEAQGERPGIITLWKYDMEDINESADGGEEENINNSNSI
NKNGTSRATRADDSVGKRKKDQPHENEAGDTDDIILYEDYFTCNYGRFHIQVVEGLSIVAASLNFMNFFMC
IFFFGTHSFLRVPLVTYFLLAACCSGVVFGLLLDWYKNEWCDSQPCLSCLEEDGWQLGYGWMFLVASSSFS
FVGAVTAIISS
>tr|C9ZJ52|C9ZJ52_TRYB9 | DeepTMHMM Topology Prediction - Predicted
Type: TM
MAVSDVKLVMSRQMRKLRLYFIYIFIFFPRPPLPMDNNIDTHSTPIRLTCFKKTNIFLADYGTAPPTTNST
QHSPAQKYQPSFHAIQINVPPPSPFFKFFDFFCCCCCSFVLFCF
>tr|D0A8H7|D0A8H7_TRYB9 | DeepTMHMM Topology Prediction - Predicted
Type: TM
MERLMEGSENVKEALLQCIAKSDTSAADGVLPLLRTFSRPVSKTPFFDVQVETEHAWPSSNLLNGGGCCKV
HYRNGAHLVSSGNRLLVVRQDNHFYFPPWGSLIQDAYIQRCGADKRSLIIVGLVNGIHAALLQEESPPVVL
DDIFIETGRSVEKIVSFENGKLTLCYGNAQVESCRLVFRGDKVSEMVLSSNQRPRRIYDAVISLWDTKRYQ
DSAYDPYRGNLFVLSNTDLAVWKRTLTGEFVVAGSVAVLRNTVTVLASFETSHHAVLIAADGGRQPVLLDE
APAAGGGTACVNVRLASSRPLPSGLHVEDIRFACRDTNGTTLLYDALTHLLILITTNCAVYEGVYDEMELV
STFHLDGPITGIGYVSESVRFGTSFVVYGGSGIQCRIGALPIGLVTANLLKLGNPHDEIFSSLLKLGGRRG
MEALVGANLAGVPAETLAPLLGQFLHPFPQSNNMRVAPGAEGLLYLVHRQIVVAERLWTSPFSWDLALRLE
GAVKLMEEWRGSIESLLRLNGWLDYPVQHMGLVWQGFVVSSPQAFTTRTAINTQALFLYTLLKGLGDACII
CKLYCMLLTACGEEALSMASSIGNQMGLEYIVWRCDTDAIISEFCTTALACNGANTLAQLEAMKHALPVKA
RHAISVHSLIMKGNADEALTYACDNIVSLRDEQMVSYVEERLNVAFPDHMVPIRLLLCWLRHDKSSIGELL
SLLEQNTADGSPEQVKRCLRLVMQAAARSPALSRDVVRWIVGHALEDDRLMCFAEIIEEYIDDLGEPQTIP
ALFYSCWKDRLRCPQVAARGFGDIAKSKQRVLLSTRLRCINLALEYGPTDSDRLTYVLLLLQDELVKVIER
TGQSFNYNSSSKLPRQETQSHVDELRHFYVVESRLFELAGVYRRNGGAKVQMDILKMHPETPERVTVQVLH
DLLASLVHEGGQGASEAVRCIVREYYGGYAAGLPLLPFVTFLVHNREDPGTIVDTLRSSGVPAAVVFDTFL
HCLDDRCDDTVLTKSGVITALTAAVTNMVGEGRSVYATYLLERIHKLLENEHQIALLRRSDVEQLQNAEAA
MMRFVSSSQSVS
>tr|C9ZS63|C9ZS63_TRYB9 | DeepTMHMM Topology Prediction - Predicted
Type: TM
MLWGNVRVAPRSCFTSFSLSLSLLRWVRPSLAVMCLWQLALLPFCIALRTFLFLFGTSVASIAHIERAENS
SSTRNISIPGTTSPLFFRRSGKERQYMRYSVVRTRPSRGRRLRHCVLISFLALLLMMLSLSISFYNTRRSV
DSLKMKLREDAAGNPLLREGAAQTAWRLLNTENGSLWGYVGGIDSLIEKRVDRAYWGPTMFRSNEGTVNES
TVTNVVSERFRSLNMSEAEMKRRYIRHQLVKEVTGCDSLSVPFNPLHDSRCINFMTNSSNWLDVVPIGQNV
DQRTIKFRLLFKPLRINASYSVEYPLETFVKVPQKYFVLEAASEVVAFNVDRLLLVNRVPPTGLGCLPLNT
LRGSVNKYKHNTSTFKKFLQDSKAENYEQWIEKDLFHFLRRAKHHLRKNGNNQTCVLVSIQLKIADVVHFL

ETPMRIPYRVFSDTWFDYFDLRANVGELEDGLPTFAHERHYPGVLHLAALAMFDYVIGNMDRSPFKNNFVV
GGVANQLVSDNTTLLHPNHPTFVYLDHGSSFRLRRPERNPIAKSHIGFENGKEDTFCLFRGPLLRRIQELT
GPSGEVTHGNKTKKEAYGAAHNHRSTETLFTHMLRERVPPEAYSVIDSSNLDLVVVRMKELLAMAGRCLSD
ERIRRTVLFP
>tr|C9ZLF0|C9ZLF0_TRYB9 | DeepTMHMM Topology Prediction - Predicted
Type: TM
MVISIYKYINRQIPFAVSLSLSLFSFLNAPFHICEGKRGRRLCMWSISHVGMGLFSLFSFFLFFSLIIHHI
LPSMHLSFCFLYCYMIHFALSATNSALVRYFFFFFLFLFFLQGVLRVHFITILRLGTATLVSRPHASFFLL
PQPLTHSHFSCHTQKQTVSKENKEKKKDGTKQNIKDNIKDNTTTSRVEK
>tr|D0A469|D0A469_TRYB9 | DeepTMHMM Topology Prediction - Predicted
Type: TM
MNKTSGDAFSNRWKKKLILNTSPFPLLFRSVGIRIKLCLFILTVLFTTEMYDIKCTIFNVTINISASFPPS
FCSWCCFRGPSRPLALTSIAFWKLYHLRSVHTQFRSSPAHRC
>tr|C9ZTK6|C9ZTK6_TRYB9 | DeepTMHMM Topology Prediction - Predicted
Type: TM
MVTSTSPRSGNLPFLYVISFIKWDCEVCGIVENNNISGDLVGWCANVCLRFCFSFFFFFDCYVNLVDPFYR
MCFQHAYTYRFLSHPLFLLVCCDGYFVLISFCE
>tr|C9ZV47|C9ZV47_TRYB9 | DeepTMHMM Topology Prediction - Predicted
Type: TM
MSQSRVPIRYGAFIHLSCDEGYVTAGGLGNEGLFIRNKNELSDDAEPLPLFGFETSVFQILPPTVAKVAAE
EGLSKGNRAPNSNPLSDGVSNIYSTQQVTFGQLFVLVHAVSRLHVAALPSEPSERDPDCARLVLAPPGEIE
QTFCQFIFTPRYTTHGEGDVVCRGDEVLVQLASIPIFLQTTVVSPTRPVKHETFGSASFPYEPTDGCGRGN
SWSAGTRMALSAVLGAPEVNLSEAKALVFVVERYDIDRDKAEHQRILHRIPRPCVSAGVPVIFYHLEHKRV
LATSVAMPPCQGGGKIGVSGVAPRGNEGGGERDHLLKTRQTHSVMGNVTVICHASGRFAAAGSSKSQGSDS
HSAGATSLDSDPCSTKGKAPDDRKRVNGVGGAALPLFAVDKGVADGTFSEEGALSGLQCSCTALWILENEQ
PTVGGAVNMNSGVYRLRQACSNLYVAVEGSAVDTILEGDGSPEGSVVSQRSCCNLTGDIVNEEGGDVVRPT
TLSMIPPPRTPKDLQRTLFRLSPMFNTDCGYLIENDCLLLQNVATDMYLCTSEGSETLSLSWKPSNIDLIV
VRRAATDVQDSVLFLWSQCETLSGYRDAFQVLTTEGTATHQQQTEADGEHGQGRYPSAEGSTSPTLMEGRM
AIVSDYDHIPESFVRPSKTCPKGSNVGTGYASLLPVICACQRTLAELIIFCSISPERNVLRRDGIPIPNHQ
HMLVELCVHRLVIDVILAPFSKFGVRADRSVGKHAQCCGQWGCSSWLPPLPLSGGVVDVNDLLLKMHREIH
IVCRLGFRLLRQMVRQAPELKAGFENYIPYFLAFDGYKLEVVDSLTRLFSENPAVRNSSLELVVNHYIAGL
HLTRSGRYLQLLCSMCSVGTHGVTERQRLVCQKLLVENANALYSFVLDSGGEWAVKTDKDEPPIPCNILFS
GQQQDGGEEEGTKLREYVQSELELLGALCLDGCPPLCREEVAKVFPSPVLLRALRNFSPKWPEVSDRSRVC
DVVRSHLIRLAMHCYILPYIDDPAVQLREGAVLLGSSKLHLKVDEKTAFSGKPDSELTQAVKEGTLHVIRS
NTHFVRSDTGRSILIRAALAAWLRFVSAHQVSATETACLVPLLLELLDSRDDEAHDGSSKIAEYTWTRLEV
SEAGLLVVRAREMICQILLQILETATYRAANEINLLLHGILVTDHGASLAHQHDYCSLLRRADVSSTGDPF
VTSPTERDALLWHKGKANDGNGNRCFGLNRRKGPSDYQSTVAGSAVTTQLSGSTKDVAAFLDYVKGICSCI
VRPLRVDQLVPRLVDLAHHDGSQLAPYAMELLVRICTVRRSVARLVLQVHPFPSSEVIQCFDNMYFAAVQV
RSSYIRGSVEEAIDVALQGIDGLSTQTQNEVTGTTNEGRVDDGGTDEYYNDLTEDDEEIEEIGICEEQEVE
ASSSSAPEDQHEVGGENKARRLWLKAAGAARIVVYRNAIIARRRSVGLSETSRVPLRVVVRAETVRHWQVH
ITMLEMYPFIGPSSPAFSKWMRFFYVFTLSQSNAESLKAYIDVFMGAFNLSSNCVVMGLHIVLSILATIKD
PTPHLTDAFLRESARYIDGEIAALHPDGEFATKLGLHVFTKTTVGGIPRRRMLQLLRDYDAFRCLPSPGVT
EKHGRGRFTACIVEMVCRICGTSMGAVALGRSALPVTHLLEITLSYGTSYTPLVEVPRPRALWESNSFHLL
GAYLLALVSLYIAAGDAGGDGGRRQRQMEWMANRDWWSVVLLLSRQLKELTRLMQSRTETVLWRGRRILQR
YRRLWLVNLPLALLTFMTECFNEAGFYRYRDVVGATFHEMCMSVAGFSEVLLASADAIRLQAREMVGYRRL
VALLQVQTGNLVGHELLSGTMLTTRRNLRHGVICYYKKINEAEEEARGLHPDALPLAETGPQAENDGIQGG
LIATADVPTHTQNDADDSSPMGLLISGAATKCLDAERLRGALRSLVNRDQLITMEDSTDLGEPAGIMNALL
LSCRERSNVLDFVSTTLGCMRERSFGSITLIGMLNIFSNALHTALREQERERLKHVSAENSSVITDIFTVR
SFETDYAKENAGRLLQTTFSDLGATRAIASLCAVDDQVVAYSAVQLCVGLLEGGNEHAQKALLAYFQEHQE
RFFHNIRDMLHKAVDWVQCTNAEHQIVVLERGGVVPNVSNAHEFTRMLLTNALTTPPSLYSSLKVRVGRGA
AVARRRLSAWDRAGGSLNQRFVCTLFRMLQLFCEGHNLSMQNYIRSQYDNLHSVNAVHEVMNLITEIAAVV
HPATVRMLQSAFALLTELCQGPCHENQEALLGYGVCVIISKLLSRLNLPDVTGTPSTGTGITNWGGGDSNS
TDNKASVDCCTLNETDSEGNAFLRLQGGFLLSKDDAGNLRIALTQCLLSLIEGCRSRDVFRQLLEQIPVEV
IERELTTVDPGAYDSILENEELASDPGVEALFNWLIFLKTVRPYAEADYLKRIDAMLQHTNKLCTRLGFIE
IQRADGMLEKVLFHIPHVWRGLMRRNRKQMLAGINCSSRAAKLGDFMYHSDNVIFEVERSYAFQCWVERRT
RWRLDNRSSGWRGKECDAGKVDTSVDAGKPRWTPKWKQCSDAPKYFWNHFIAPVLFCTHLGFYEYSSLLVA
VVLNIALINGEGRHRNLEESQLWANIISGLCVLQLVLSLIAITVDTIVFFPVSLYVHYRQKQQRFSGRAKF
NETLQGVLRGLSAKEISLLLVTRFSFQYRLLLVVMAVLSIFVSYYFAAAHLTLMVYTFPTLRTFVSAITHN

GRQLLLTALLGVMGLYLFAIAGRIMFPEQFGSNGEVDENSGKKNDENGNCDTLLRCFTFILWQGLRQGGGV
GDVMDEVSWNSSTLVPRVSYDLIFFALVNVVFLNIMFGIIIDTFGELRDDRRERENDLRSTCFICGLDADT
LEKGQVGGFRAHVEDAHNMWMYLYFIHYLRHKDPNEFTGQESYVHEKIQRNDLSFFPEEDCLALQECREGN
GKRTGDDEADSDDELASSVVVGGSAPRGPKPESAHPDTGVKLVLKELAAVREAVSALAREATMEGERTRGL
AQQLELINRSSQSSSLRKFPGGGSAASVAETSTSKGTWLRHSEPEH
>tr|D0A586|D0A586_TRYB9 | DeepTMHMM Topology Prediction - Predicted
Type: TM
MLCAYTPFLRTGTSLFVYNVIILTTVHTITSCFVHFIFVFILLNTGGLGVSGGLVRTEITKAMSCPRVQYR
RRMHYATRGNRMRLVRTPGNRLVMQKRGKRSQGPHTPWVLGHKRLAGTKALRHTKARLAPRHQKTTSRPYG
GVLSHEQVRDRIVRAFLIEEQRIVKRALKAHAKVQKEKKRRAAKRKSKEEKVAAVAKKVAAKVGTKSLIAK
KEAPKRKAGKAPVGAKLKK
>tr|D0A5I1|D0A5I1_TRYB9 | DeepTMHMM Topology Prediction - Predicted
Type: TM
MKKKKKKEIHCRYRCCCYCYCVCVCVCVCIWVFFKKKNHKKRNEMTLFVCLCFISNSYLLYFLPIFHCLSW
ARTLLFLCVFMFVFFVFVRRYFFSSFLLFIFPPIFFHLYLVVIIASVASCGCGKLFVVFFFLCDSLCLCFL
FFPHVPVFIYAVIYVFI
>tr|D0A162|D0A162_TRYB9 | DeepTMHMM Topology Prediction - Predicted
Type: TM
MILKPHRYRPQFALFFLLSFRNNYSVQRFLRSTCKYGFSSTSLSLSLFQYFLLHILSTLIKSWRSEVKAHE
RHYNIEIKGEGEEARGKGNETIKKTIIIIIM
>tr|C9ZWD5|C9ZWD5_TRYB9 | DeepTMHMM Topology Prediction - Predicted
Type: TM
MYQMNDTKEQKQTATISLSTMEEKTTKIDTVIGSALFGANRPKETGTPYTSAGGGEVYFNPSNLLGYVWSY
VKDERARGDSSGMGVSGPDLHMRPPQWMEDKNVSIDGTPLTEEELRAPNFWQTKYKRELDIGEAIQEVKRT
RRTEATHDIVGSISWLGRKLGWSEPLDEQYARKKVEERYRLLGGQDTTGSVVDEATTGQKIGVATPWKVLL
ESVETGRPVAKVANERRLCVDFYGVDHFFDARLPLIWFTTKCGIAVGIIQGSLKAIQAVNVDVQFLKASGV
GILSILNMSVFASVVKWGGNCALFSTAFCFGDSLATSLKYFLLPPHDARQRSILNYSLGFAMSGSTVGILP
WWILSDMSLAFRLSVSGAFVGGILGLAVGTVMRRLVILNTTRLEATNRELRRYEALMRRQRCWVEEERLKA
TNQRLVWW
>tr|D0A4I6|D0A4I6_TRYB9 | DeepTMHMM Topology Prediction - Predicted
Type: TM
MCRCLRSFRVCVCVRVVHALTRRHSHSLLAPLAHFTVCMYRCGRRRYVLLCKAACHGKEVAVSCYPRLLLL
LCGHIIFLSPLTCVRCPRVTCALKCNYFFFHFFSSTLYCGAVTSDLLVCA
>tr|C9ZTP9|C9ZTP9_TRYB9 | DeepTMHMM Topology Prediction - Predicted
Type: TM
MRGGKKYKQIYIVCLYTKGKENTIPQFSTLSSTCLHKTQAHTCRFSQFLFFLLYLFILYFVSLLYPNLYLF
LALTHLFHYSFLFLSIFLFSPFQLLLLLLLFFFIFTFIYLYQNGGG
>tr|D0A203|D0A203_TRYB9 | DeepTMHMM Topology Prediction - Predicted
Type: TM
MLCSTCVVPLVANYLFSFLALFLDTVGLVEFSVVTARKILEKKKELKRKKKRRKGGNTISSARTQNSRTKK
QTKRPARKTTVCNAFAFSGLQKGQCTYISFSSQP
>tr|C9ZW15|C9ZW15_TRYB9 | DeepTMHMM Topology Prediction - Predicted
Type: TM
MREPIQTSTPVSTGETDPAVVAAEHANPSVFSVPVVAHDVAPDAVTASTAVTDVNVEVQSGGTKDITVSSS
PRKEAGVIPENTNIYKTAFHIFKANVGTAVFLLPVFYQDAGYILGPTIAVLIGVCVIDASQLLLGTKLTID
RPRVDTYGRICKFIFGPPLQWVLFVCLLLSQFGFCLLYMQLTVDTMNTMVQFKGDTYVWSFVMFFIEFGFT
CFSSNFSTLAIISISASVAVTFTLVATFVGTCMEINKNGRVHPTVNAFGNNIPIGWFNNMASNLMGLEGIA
IVLPAHTGCNQKTRFKFTLSLVLTLTVSIYLLYGITGYLAYGTSINTSIIDGLPESQLSTAVRAMLVINLV
CTYPVQFQSAIQAVDQVVGCSAFSVKGILLRLFINLVIVSIELAVGPKAVHAVVSLIGALPAAVMVFILPA
LLTMQVDHAVMNPEEDRVTLKYWGSMFTAAPVFSWTRIRCYFYIIFGLIVMVMGTYSVIAEL
>tr|C9ZV69|C9ZV69_TRYB9 | DeepTMHMM Topology Prediction - Predicted
Type: TM
MECGALYAATVLSITDSEGTRELRWSTLYRLFSTQSLSAPSWILTRVLRFPTPEGWEHRKWKSNCNRARLR
RFSLQDAVFFFLLCRWLNFFFSCCALKMRSEWKEERCSGRGAKGVGCHEEVISRRMIARILAYINDQQ
>tr|D0A2S4|D0A2S4_TRYB9 | DeepTMHMM Topology Prediction - Predicted
Type: TM
MSRVSCVTMIGQSDGEWHMLLLCNMCAGEVIIPCVCLCVIYLLIGIIYVMLWCVTCSSGCMNKMRLRQNIS
FLRFLMLLSYTTPQCSQVTNLLMCPHLLFPILTAISRQQHALHVNTQRFVQLCSLWNSASCFRG

>tr|D0A6C4|D0A6C4_TRYB9 | DeepTMHMM Topology Prediction - Predicted Type: TM
MTALSNGSFIFTAGRGRELHLLEKGSDQKSYIVYGGEVLAFACCEEDHVIAIGLRSQSGPDQCVIRIYQFN
GSEVGDMLAEASCLKVTQLMWIESRQLMACGPAGCSLFVWKKNLSVSFKDSAVGCFMQIGRVALLYSEFQT
IKYWSFDTKKMLAAVKLPQRDHRVLTVASAGYFCFALYEDGTVQAFYVTRSTVKKLEVFKKLFPMLSTNGA
VGGTSAQLLLCALSSRKVLVGLRGGGRVHKLEYKNDKVLVDSLQEDLPTPFALMGISLDGRRCLVLDTKEG
RHCTLVLSFGGTKELSPGTEARSVQKKLPPAEKTRRTGGDRGPGKVKWPGSYRVSSVLVATGVVVVLSALV
IRRFSSR
>tr|C9ZZX7|C9ZZX7_TRYB9 | DeepTMHMM Topology Prediction - Predicted Type: TM
MHMFLFLCSCVYSVRERRHSPLCATFASIGMFFLLLHSFLYTSLVQTSKFEPFFLNACAGCYHRCCCRRML
PSFSFLRYHSVSHICGTKSIKVRVHLSLSMC
>tr|D0A337|D0A337_TRYB9 | DeepTMHMM Topology Prediction - Predicted Type: TM
MVQLYFECKKKFCQSPIGSDMHRCVGTCAPFLFFLFFTCQIYIYIYIIYVSRSYWFVATLSYFFDKPAITC
ECHRVVRVDHLLKEEKKKKIVHSSVSRQGIGEV
>tr|C9ZUK0|C9ZUK0_TRYB9 | DeepTMHMM Topology Prediction - Predicted Type: TM
MQSDERSSAEVSLTVGDATDVSKKRNLQEQWLLPYLDCCTHPRLREAIEREDRMAIEKGMLLLLAFGTPEE
VAGRINSNNVDEQRGEHGEKGSSCARFGVADGTGTPVPLVPICSSPRNEYSDWSDAEDELVCRCTYFRRTF
TNKNFSYNLPRDVQRNVVIFLYTWIVNWDFMIRNVSAWSSVVSCFCSVVRGRRKYALKHPGFSLPWKPLVD
IVHSLVFESSRLSRIRFYTHVKAEVAEILANLCWCASLHFDGESLNGLWEICAPYFSQDSEQAPQALWFLY
ILLPIHATYDRVTGRALPMTERIVRFLLVDARYWQQRSGSWLMVSLDIVFKICRERAGIMDLDEYAETLFS
MILSVMRPPISSNDKPERLVSSAWSFGGRKVLEVSQGRFMKIVGRISEALPCCTTSPLWNQLRRFVSSTSV
FLTPCAVDSRALGHLLSFYNTFLETAYLRVKHKHSWLLTGEKDPGNGFVINGYRWKRETVDSLVEIVAPAI
IPALYHGHHSIINAVGLLTLLSPKIMQPPTFKYVEAGIRGCLESPLQCSVAFKLFTKTLLPFSECSETRES
CRAFLSEILPLVPQWINCSSIQLSNSVREFLFAVFTVLNLDELLGSEVAECEFAVSVVEQLFHSAKRGDTE
DSDADVLASIIDSLSHNVSDEAYSDCLKKALKEAETQVKNSQVPSVGCLLEPLARRSPERVMKWAIARLLP
PLRSDVNTCNDEEVLWCSSLLSSCVCGAGLACFPYRYELLDCIQAQLFSITNRKRRCCAALLYTAVFFAFS
ETRCVGVRAVDSSLVRGDHMMEEESCAAEGDSEQKQDKSSMGLYRYFDVEPVWQEPSEEHVSFLHEVYQLF
LTDLTDIIQNIEHIRVPSRKEAARGLRAFLPPHMSPSSHHDRVNEGLRENGGCVDDSVRQLVSHQAPSAPD
AEGSHDVVTPHNVLCGAVCWLDTVWAVNCELRVQCATDGETNMTPWHYKDPKTPWPLHEVSPSLVEKSTRL
TESIHSLLLDYVLRRVTGGVADEAVIATVLRRDQSLLVRGKLPLDVAHTKGVDVDTLCYVLTLFFREMGLD
TRTNIPNDYTIPFDVEFKHFSAIIVESERHRFYFPALFWRLRSEYLLHCRRQQNPMVVYPQYLGDTLSIAY
SLLFSPYPDVRKHCSCILRTYLPYLGFDGCRQFLLRHFDVLEGIVDFVRQEGDVCSGNKNSICDKEGHTGP
GEEILQIENCGSEGNDSCVEGDCGSDAAKICDVFFVEGCTGAETSQSKSDDHGKGSALANASDDIATSGVG
EKGSLVQLRKALSGAIMQTVIGFSLDVYQRDASFIRRMFEVGLKIPDQLRRTGSACSLYGDRAAEVCTPFL
IEPRAIACLTDELLPLAMKCSHTAPTFAVQMLRTLSCNHIQLHYELLSPASIAALFKLTVDICAETRRSAF
SVLRVVLYSLKEKTPKVNILMRKGSMEGEDTTSFFSNRYRFLQENHTSFSLHGKMGLVFPPKSIRVDRTCA
PPDISGDGEVFTVPDNEVPGLRLRMQSFTACHEEKDLAERRMVRQGLKNLISKLAGILDDSENTSKGAQEQ
ASDTDDLVGKVLVNEAAIEKRIRGTWVWNVLLANRDQTSFSVGLASMWRSLGEAAGVEETVKRYIQVAYLW
FSDYMKLVQSESNRSKEYPQELPCLVDVIVAAICLSKRNPKLRREALDCYVSMLLQACSNILLSHTVLMSF
ISGLGALYGKVKTHDVWSMYEPFFDMLTPLDDGSGSPVNVTSGVRDSDSVEPNIKVAFTGVKRGILRALHV
ITKLLDVFAADVNIVLLPKLCRCILDRKKIFLFSDSNEIRHYAATSLRYLLRLSLYQSKYIPSNNSAQSVV
LDFLRQLNCIVRLQVCYPPTVLNSGFPFQVATTCITAVNTNEDMLVVNKHPVASDNKNHGNTSDVSTTAEQ
AGLKTLTSMWHSPPPELFSVMWMEAITTATTALDGASLDNDDFETVALSTLSSFALSRQRKDVIQSVVKFL
CDVLEGNYPFGKTRRAKVVLTRAFYQMLLMNLHRIGKYETMRSVFSACVGCMSHGDGDLRANTRSLLAVLS
RVASIEQINSLVQQCAKELQEYSISSDRDTGGVAEEPLPAVIERENNRKRRRAALVRCLCAFLSSDVGPIT
PHVKFLMRKLAPLEHDAMAEVQREVKVAFEQWWKAHANGWELYHRESFTPKEIELIMPLMKMPKYLV
>tr|C9ZRT9|C9ZRT9_TRYB9 | DeepTMHMM Topology Prediction - Predicted Type: TM
MRRLFCFLWYSATACIRLIMYAWIFTFIHIYTPFPTFYFCLPFIPSPLTNGRFCLPMLTQFIIKKEKGPSL
PMGEDSHASPCFGEGGLCLTVVKCFVVCYTWNQRKLPSTKGTPLK
>tr|D0A8W1|D0A8W1_TRYB9 | DeepTMHMM Topology Prediction - Predicted Type: TM
MSKYVNLDSLMGKTTSVPMTNFEISGMVQMEQEGVMEDNSLFPSLSFKERVSGYIIAFIVSFLLSTMSWIA
LPHSLRKYAALNTMANIVSVGGTMFLCGPSAQLKRMFDETRRGATTVYLTSLLMTLISALILKFVLLTVLL
MLAQYLAMIWYTLSYVPFGRSAVLKVLSRFT

>tr|C9ZRB0|C9ZRB0_TRYB9 | DeepTMHMM Topology Prediction - Predicted Type: TM
MWTCACTCVWLSLLFCFVCLFHSLMFVINTDPLSSFFLFPVDHISVSNLVVSFLMAFVLPQIHINEQSSWG
PPEVQGGKIETTVGSLYHKREAVEPFDWLRVLEPVEGGRQREFTIVEDEKRNKILKSTRVKERRQVPDWSA
PRRHQNSRRNFQNKPKRNITLPPDTVKVPSDAVILEQFRQAELAKMPNLTSLPTVSDISQHNRPPVYKNEM
DKASCKAPIPLNEKETKVDFTRSDSFTDNVLRGILKSEPPGTYPIVVATDEVLALLMTCSRSVYSWHLHFY
RVGRFYFISKVDGCNVEKQWVDETADVSRVPSETEVVETDRTSSLEAESSKVNNFFVAQSCTAARYQMDCE
KSPFPGKHPRLYRYRRFVMHADTKDRYDLIVRCEVDAMQGDKHIRLFGLLEHCIKGEENDWRKMLASQTAT
CISEEYRRNAQKMARWIALCHLSGAHMKIGFISRCRKGAGVFDPLRHEVLATFTNDPSPLAAQLGIKVANM
WTVADTIITAFVQSDFSEAALVKRSGDTSILLVEKCEEEFYEEEEDEEDDDEEDDDDEGEEDG
>tr|C9ZVI8|C9ZVI8_TRYB9 | DeepTMHMM Topology Prediction - Predicted Type: TM
MEHWKKLLAKHSCFILLKYPSYFFFSYTFPLADSFLSWGGGRFFSSPYLNMTRVAVCVKQPGYICVCGGIT
LYLDLILILREVFHQHLPLAVASVMRFFVVAVSTTLGLASPSRWVLRQQALTAVLTPARRYVNALYRVPPQ
NLIPTHSPIGEEVDTELFVRQVKDLFLALKQTQLVDQVHEMRACPYYDSLFAADILFQHYDIVLPMPKYGR
EHRSSEISETLRYLRACGSTGDFNHVLQDVKGGSADGGDFVSTTHGILMGYGTPRTNKLCMMAITDASAPN
EAAAAQQRALNIVPIEMSPIAPPLADVLAFAGKRTLIVQDTEHGRSAGEAAASAIRKVPWQILRIEPNCSI
ISHLCGVNSVFDVLVDQDFPTSMERIGEAGLNPFPVEWTEPRKLGLSMRRVCLIARFARGTMSAGGYADSD
GHRAASFTYHSRNIASNSRLIHNGHRKHGDSGAPLEAQLRSGELPAPVYQRPPRYAPAIHRTGGLATTATI
RDKRERE
>tr|C9ZKD9|C9ZKD9_TRYB9 | DeepTMHMM Topology Prediction - Predicted Type: TM
MKRCNSESETEFDSLKSENPLLHSPSLKSWNIMKNTPVVPPFSSAMTMAMKEHCGASTATSAPNERQYKEL
NVDFNDKYFGEVTRSGSIEVPHGNGRLFSGEGKHFYEGGFVNRCRDGYGVLNTERYALWCKWKMNRPDLTS
QSRVDYRDGTRYNGFLSAHQNNSAAANIPSSLRCRLSKFSIWVHSLTLVRERWGEIVHSGGDRYFGQWDND
MPSGFGCYVTKYGDRYIGLFQRGRFHDTGTLFVHACRWGNRASKIPDGVGFLYQQEQVENQHVEAAEAAKQ
RIEDRFKNTFTESCGCSGTDYEISGHKRQGPPRGWDGVIFDGVWEMGRFLGEGHVTLPCGSRITAEWKNLF
SPTQGRVFMASSNDNKASGRQDMDTRGWFQCFHWESLLSGLTDESKEQKYAACASFRERLGLATTEEEEQV
VLADFCSNEDAIRNALKVFQRCFYFLHGSCGSSSEIGSGWGSNPLGWCYVRNSYGGCIHNIKGRRITACDV
DLALTDIISFVRSTERWVVEMLGDSSLSSPSSHLFVMRKLLDLTLRSVYNVLFNLYVHAYDVEDIALTQAL
ERVREHTTLDDLGVSFARQQSSEELFDPYADAVNRIERLARGVWTYGSKLKILAQWSMEIDLSTRLARVTL
DDGPLALLPKRNAQPASGSADDLIPIHQFVLMKAKVDHLYVHTKLLVDLSSEDVFMEFTSQENFFVITFQA
CTMILSKFHPLLRDESHVLAPPSLFEERLRSGVHSIRRLAETFIAQLLKSHGVFEGEESRELSAEEFGAVA
LGYIKAWLAEAVDMAATHGGTAVASGSDQILPVAELLQMEETRELALLSEPNFPRVLALFCWLAVANVLSV
LHIHLGVMAANGDDLLNVDTDPAKLLDDYVAWRNVCGGDVSSLVLLLGKGAPRPLFLRRAAAFIASIL
>tr|C9ZNW8|C9ZNW8_TRYB9 | DeepTMHMM Topology Prediction - Predicted Type: TM
MVKYGVGVVAEYVCPIHVPFLLPLCAYFCYIYIYIYNLLVHIRVDTVCLCVCVCVGVYMLCTGLNSFIHYL
DLHVIARVPSTQELRCYSCNNRHFFFWFTTPGIFVTPPILLSFFLFSLGSFFFVFFFLFRPPLL
>tr|D0A887|D0A887_TRYB9 | DeepTMHMM Topology Prediction - Predicted Type: TM
MANVNNETTECGALLSNITSLQTTLTSMGCQVPALHHHTHDHGHHHHHHHHDDHTHGADDHEGHGHSHGGC
ESGHGTYSIGLHVVAIFVVLIASFLGTLIPIIGKYVPALRLPPFVFVLGKCIAAGVLLSVSTIHMINEAVA
QLQEDCVPESFRESYEAYAFLFAVAGALLLHMVDVIVDARVTNKSDSSTNKPEGQPDAEEAQAAPAALDAY
DGHHCHYAVGMPQSRTRRLVSAMFMEFAVTVHSVFIGLAVGIARDAETKTLLVALAFHQMLEGLALGARLV
DAELSLKLEMLFALLFSVSAPLGTAIAVGTIAIWNVSMVGTAFVIAQAVASAVCGGMLLYLAFCLMLSDFP
SDMQKHAGKDKVRRFFRCFGMFAALWLGAALMAVLGKWV
>tr|C9ZRX5|C9ZRX5_TRYB9 | DeepTMHMM Topology Prediction - Predicted Type: TM
MLVLVVKFSSTFVQVAERYFTLLIFLFWLVVAMSRRRPNVMLTIPKNTLQVFDMGLDFRSYLSRVDVNGLP
VLGNVVSYEASKLTEVFTPTVGLLVTILHMLAHCTSSCSAVAWLKAYDAIQKMYIENSEEENTEAVRYAVE
ARAAIKDYLTGDAVPGTNLLCEKLLTLLDQQSGIHGSQAPSQRKVPREPKGAFCNLHLSDQKCYQDFACNQ
LHLQEVKTVRHRKIFYMRLIEVLKAEKRSRQQIRHRIKDSVFFARYGNKVTVQCQGKWIPFFKTVYTDGRL
EQALRTQHFACGDSACADENCLGIHPQDRRVTTSSKLMDLNNPDVVLQQIEEEAERHLSMEGAFNDHEAEH
ISAAASSSSMPSPTSEMWRNTVREYVRQTGIPQVEADESLLRTLQDANVLATPTASTESLTPLLDELDMRT
FSSSSPVREHPNAEIG
>tr|C9ZK17|C9ZK17_TRYB9 | DeepTMHMM Topology Prediction - Predicted Type: TM

MPCATKGEASPPVTTPVGRDGCLCVVTWQTAGRMSLQTCALESAADALVFLRNNLPGIALCGLWVDMQKCS
EEEHRQVLQLLYPNMQPSHVETVLANDMYDVVELQPTEGEYVVGCLSCSPSHGGPFPAATAMSRAEFGCEE
GVLCSFVCSERVLLTLHTAPFAGLAELFRHVLRDNSQWPMSMSAILCTLVCYTCELSLPDPTALIGEVDCI
DEMMLLVAPGKRDQTDMLRRVALLRRHLSALNRKLQEKAKLISEMTGPAMRTTFVSRELHLGYMYREALEG
LSQVLSRLECAQDTLDHANLNFMYAITMRMSQTSTGCDRQVMIVNKIATICLPAILVASLFGMNCKVQWVA
DDCDSLYPFWTIVALMIVWMAALLFQPVRDLIREKGG
>tr|C9ZVP0|C9ZVP0_TRYB9 | DeepTMHMM Topology Prediction - Predicted
Type: TM
MSDVPWAQKPEENQSNIEKHTAGDAPTEFDGGTATAELGPHEHTNSLNRSYSDLDSMWFVTYAVILCWLRT
AAQQPLNLALARKQTSSVANKMTTRQIIRLVYNTEGGLLGLTQGMAALALGCALSEAIYLWLFEYSRENIP
LANKPTRDAVSGYIADALCRLVHLPLSIVALRQMTANCEGVQRGRAVKPGCMIHTFASMYREGGLRTIYAG
YGTTLVIGCQWTAVWWAMYGVSKGYLYAAADGFLLDGREDPVTEPVFVYSPWLSWKRWLLARDDNVALNTV
ASVITSAVTAVIFNPYLVVRANLQVAPKARLWGVTREVYRTRGILGFYGGLALNIGTCLIDGVLASTSYEY
AKLWADRAHQSG
>tr|C9ZUG5|C9ZUG5_TRYB9 | DeepTMHMM Topology Prediction - Predicted
Type: TM
MADDGKTGFAKFVYYIKVFHALDYILLVTFALAVVYGMRTVRPPCRTFSWNDTDISHKKGKSTFPSGTLFS
MEFIPIGLYLLFEALRARLARKGEWYLDVKETDQLIDGATTRVEMLEPEGKRSEGAHSAKAGRYPDESPEM
NRENEPKRLMTCLEATNYWVLAHGFSIILAVCLVEILKVYAGRLRPDFLDRLKKANVTEGTDPKDLCDIAK
ARDGRLSFPSGHSSCAFSVCTPMALYFLSVLHAFSGASVWRTLVGLSPIYLACACAISRTRDNRHHFADIV
AGSLIGMGTGLLAVAVFFRFGKEIAVLVPRRMEFVRSRGGRVLADVAE
>tr|C9ZJJ9|C9ZJJ9_TRYB9 | DeepTMHMM Topology Prediction - Predicted
Type: TM
MMCKGLFSKLPPNIVYRFLYANTGLKSCCWKAPLTAQRRTFHRSPQLHFELNPSQQMYSMEHAFEEPTFRF
ICEGIDIDYKEEVRRALMEVLPRNPSNFVVRDDLPQSQGSSVQRTVQLLRAIAKKRLLSFAQVNSTPHLFC
TFYEVIAMCCTPLALLAADHFTFAAMIALYGSKEMKALLLDHVDSCHIVGAIAHRELIAEGVPLNTEARYN
RGENVFVLRGSGKFAVVGAVCADWAIVTATLTLNKDDNMGIHAFAVQLREGGVLKKGVSARPINGENEAMT
AAGVGVLHFENVTIPVTSLLLPSEIVDGKLVFAGGAATLTSVQALTRQMRLASGALYVGTLKRSLTNIVRY
VAQKWMVGPDGRRNYPVFGVQHVQSPLVKLLCTSYVYMTLWRRVLPAFTKPEAQPADYEDEMKLAGTLYFL
TDNLSTLKSFADDFMDVHSSLRSTGTCDVLLTVQMRRDGLDHSSLIREVAFKSVVKNVGTTHWGWWLSSCF
QSFAALDRFVKNPFYSPRIADLGRHRIFFSHKHYREKKRLRQSREIERRKGGSEHQWYDWVMFRHEQVVHC
GEAYMEMFALEVMMDETQACTDPRARKILRDMGWIFALSRQQDRLDYFLTQQMMSPSKSCILSSHLDNIVT
VMAPQCVNLVDAFQVPRALRAPCAAEDMESYWTIPGTDTGIQRGDKVLLFGDRKPFGNTKAEQEKMEESRE
DFDLFHGLAGKQSFAQEK
>tr|D0A855|D0A855_TRYB9 | DeepTMHMM Topology Prediction - Predicted
Type: TM
MTTFPPVVYCTADATVSLSPAVCLLAAGCRRPCCSWRIVLKVNFSQTPIAQCFSYNLLSFALCLRLCVAMR
LRTQVVGIGQLRTSFTSRICMGRRSNSVFFHLWLSFASLCLGNSDSSEGSPPVSNGTSPVVLSDQSVANME
ANDSQWKPIRIVVSAKDLDDSLKYCVVAGVPRPDFMGGTLRCKTGDVLTNEKRLILTEHALPSAIHLHAER
LLVGMEIDNIVVPEFDSPACKSFTVPIHHRSVGVPQADIILYVASGPAPHDGPAYATTCASLLSGRPIAGA
INFSPSAITESYLYIRTVAHEIAHVLGFNFEAMKQLNMVGTKNIRGKSSVKVVKTPNVVKVARQYYGCGKI
TGMELEDNGDDSVRNSHWKRRIARDDLMTAIMGVSHYSELTLAFFLDTGFYRVNWEKGERMRWGHGAGCSF
IEGKCMENNETNFPDMFCNDSAETLSCTHDRQALGRCTVHSYAVPIEESVRYFTMSWVGGSDNNLMDYCPV
VEPYTDSYCRDGRRELLPGSRIGESSRCVKGEGLVAFTTHVGDICVEIHCGKRRGVSIRYSGDDSWHVCPE
GGHVTPDKTFSEGRIVCPKWEEVCDDNAMTLSCLAAVFAIFVSALSLVV
>tr|C9ZJT0|C9ZJT0_TRYB9 | DeepTMHMM Topology Prediction - Predicted
Type: TM
MCICMFFFFFLQALFVCRYRNFFFFFSFLSSIFFCFILFNGPSESDKAMVRMHGNGHGKAASALPYRRTPP
TWLKSSSRDVIDAVCKLAKKGLSPSRIGMQLRDSMGIAQVKNVTGRKILRILKHKGMAPEIPEDLYCLIKR
ATQMRKHLERNTKDKDTKFRLILVESRIHRLARYYRRVKQLPPTWKYESSTASAMVA
MHLQVYLVDGKRQYTLKKMDPDGKPTLSAHPARFSPDDKYSRHRVTIKRRFKALASEKRPKPL
IIIIIIIIIIIIIIIIIIIIIIIIIIIIIIIIIIIIIIIIIIIIIIIIIIIIIIIIIIIIIII
>tr|C9ZMM0|C9ZMM0_TRYB9 | DeepTMHMM Topology Prediction - Predicted
Type: TM
MIEGSRNSSAVKKRLVAKPLEECAPLNLLGAEDVMGALHTSSVSFFTWRILQTVIVHCFAGALFGYGIGFV
GPYYTLYKMGSDCMTVTTQSTCPMFIPEKCTWKDNACKFVVDNCSGQLKEACGASSSSSVSQTCYWNHKTN
VCQPMAGFTALQNGIFAGAFIFGGCIGSLISAPLLSVSGHRITFLLCGFVGTVGGALTHFATYTDSYMLLV
AGRFLTGVSAGVLCVSSPAYVEETAPIQHRQLVGVFFQIACTFGILSASAMGLGLAQRSFSTQHDMQQRQQ

MFCLPGTIIALLMMFISGPMRESVVWMEQRHLSMPGEQDDLVTFAEGRISYGTVISSDPKPGWGGVKGQLL
TALVLCIAQQLTGMNAIMNYAPIITDRMGLEPLFGNFVVMAWNFITVLTSIPISSRAHADRAYVVALLLAS
LSCLLVAVAVLPFFGFSSNLKVKLSATGILLFITFFELGMGSFFWTLSQGIFPPNFRHRGSSFTVLVHFLI
NIVINVGFPLAVEWLSGGPSGNQDVGMGIAFLFFGIVGLLSTAYLHRHLRLWQSS
>tr|D0A4S5|D0A4S5_TRYB9 | DeepTMHMM Topology Prediction - Predicted Type: TM
MIGRLGWIEPFPFVGKTLLLRGGGGGGGGGQQTWEAIRDLNKDTRNNLKRENMSVIIICRSGTPTLFMLFL
FLCSFCFGGNKNANTRLSISVLFYQLVDVEYFLVFANFCYGCHYASTTEKERMLDIVLAEEGKYGSDERQP
YYRCWAFSFSLEAWMRWLTWRVPTVEGKVRFDAS
>tr|D0A112|D0A112_TRYB9 | DeepTMHMM Topology Prediction - Predicted Type: TM
MTSVGFPCDMNTSSSLTSLANFYFHTRNCVDKNVRTTDRSRMEFWVQRLPTVILQEIFMEVIRSDGFCFAW
VGILSSVCRSWYCACQHPVMWAFMAKKVFVAYPLVLRAQAVPEDKTNLRMRSVSDPIIDDADDFGTVSNSR
DAMELPTLMTNQSLRRAIEDVRLFDERRSYHTYVRQIRVLVLGMMLSGTLLLFSMFLFITVCVLEGIKLGG
VFTTYAALSFLWATYWGIFTIIIANIVMEAHFEPAPLFPRLRKNKPLILTSTAILLIGLFTLVLPTLLVHI
NLTREEKFSWMWCGATPLLFLLSWQLYVVLSCFSPSKWGELLRSPHVVLRPMKVVMSLILNIPHAFPFCCA
VALYSLLQYIQYGGRTYLLVAVFPLLASLPILSTLLLLDFYMKRQMRDLLTGVSLLLASIFPLSLFWTDFR
GFSLLPLAAASLLLFITHFRQVAQQSMLELVEEIQRNRRPARWRCSHSVRM
>tr|D0A3A7|D0A3A7_TRYB9 | DeepTMHMM Topology Prediction - Predicted Type: TM
MWQSKCSFTFDLHGLFSSTNFFFFYINVGDHMEKDGDDERLRSYPALVVIQFTTGFMASACTLMRLVVAWE
LFTLGPMNLLIAVRGSTYRLLRSFERICAVLLKRMDMCLI
>tr|D0A269|D0A269_TRYB9 | DeepTMHMM Topology Prediction - Predicted Type: TM
MFPPIYIYIYIRHVFNNNISPSPISFLFFLNTVILLSFFFTYSLYFSRLSCFSTTPPHLIPLTFFFSLCYH
CSIGMMGEEAGNGRNILLTTFFHERKYIKKFFLREESALREYV
>tr|D0A026|D0A026_TRYB9 | DeepTMHMM Topology Prediction - Predicted Type: TM
MRQVMGDGRKHDGEPIVVLHQPFPFYLYDLPPLPKFFSSYQTKGEKKLVYSTFRYVDVRPYCEVFVPGWQI
DVRLLIVFLLLSYSFRFFFFFFVVVCVAYDFLVLRTTKKKNVELFYPLASARVSHILLSYSARLFVCCCRW
GRRTVEGQM
>tr|D0A528|D0A528_TRYB9 | DeepTMHMM Topology Prediction - Predicted Type: TM
MTDKDGGTEMSFVDGATEGQTAGFADAPTSNPMGNAAMIDPQSFVSKAWGFVRFPAEAVRQQLNRVRPWSQ
FFDREQFASPEGFGDAVSRLRCNVVHFYHNYFVVALLGSLIVLIVNPMFSICMFLMLLMWAYTHKKQMEAA
ETNVNHLLIGNYEISFSKAYILISIFGIISFFLFNGSSVMFWMFFASLGVATVHAVLRKPHSENELAHFV
>tr|C9ZMF7|C9ZMF7_TRYB9 | DeepTMHMM Topology Prediction - Predicted Type: TM
MVSLLALEVNALIVPAFIVFVLFIIPIPLLSRAMSRAMGYAERVNFYGVSVLTIVTVTTFIGFVLQVIDWR
RKYSGGKPSFAEMTMEIDWEGRKWRLERNMYIHALATVLSAAVMKFARLHNALEKKER
>tr|C9ZVS1|C9ZVS1_TRYB9 | DeepTMHMM Topology Prediction - Predicted Type: TM
MQVAMPVSLVDSCLGMQELLVLVYSCVWQSLLPLYELARFFSFPFQSSSYQAAHTHVHTQQTHLLNMLSPT
EPLGSGKAHTEVVTDEGEGYGAMSAAEEKSHHKNGDTPTTDSKFMQCINAIIPHGGSLSTTFNLGSATLGA
GVISLAIAFQMSGVIPSILILITVTVLTIYSVGLMMQAVEMTGYNSYADLSRNLFGPGWDYFTISVSWLFT
FGTCVSYVIATGYLVDSVLSGSSALEFFQGKTGNRVITSIIWFVGMFSLSLPKEINSLRYASAIAVLFVFY
FVICIVVHSAKNGLKDGKLPEDVEMFKSGNRAIEGLSIFMFSYLCHMNCFSIYSEMRKPSARRMTLHTTYS
MSMCCVVYIIAGFFGYTDVGNKSVETVFEIYDVKGDVMMAIAFAGMLLKICVGFSLCMQPARDCCYYIIGW
DLNTLETWKNCLFCGCMALCALLLGLFIPDLNTVFGLLGSFCGGVLGFCIPALYRMYCGNWGISQVGVVNY
VCTYLLLISGVIAVVFGTAASIYNVAV
>tr|C9ZNW3|C9ZNW3_TRYB9 | DeepTMHMM Topology Prediction - Predicted Type: TM
MSLSTFFFLFFLSLSLPCSLSFVICLHFICMTTSFQHLLDIVWLFPNTHKQTNIFIYLFIYLGSTHCNPFH
RHREGLLFFLFFSLSLFIYFPFCVSILFPQFIFFVSSDVSFCHFPCWLRLLSFSSFFFSSFSIILLLLLRLL
GWSMTSAFRV
>tr|C9ZPJ1|C9ZPJ1_TRYB9 | DeepTMHMM Topology Prediction - Predicted Type: TM

MSMILYLLCMYCFLFFVFTPVLLEFRCSVAYPSYHTRCEERTKFSHGAFTEISHSFVPLYSIFPFYFSSTA
LMNIFVVTLVVNQLSDYHLLQYVRHLCRSVF
>tr|C9ZTR9|C9ZTR9_TRYB9 | DeepTMHMM Topology Prediction - Predicted
Type: TM
MSAPVDNVVVERLSTANQKPINEPRRFSLLVLGAFCSMCTSFLYAFNLVSGAMQARYNLTQRDLSTITTVG
IAVGYFLLPYSFIYDYLGPRPIFMLSVTVFCLGTLLLALTFQEVIEGSVVRLSVYNGLMTLGCMLFDLGGV
VTVLSVFPSNRGAIVAIMKSFAGLGSAILGSIQLAFFSDRPDIYFFSIMSFALTVGILGIVFMRLPPFHLT
GYQEKHLDEEEKAQRLARKGVYLKQKAPMWRFIYGFVLLIILIFFLPLQGALVAYLKLGSNFKVGFAVTVI
VLTAIFPFMAFPLTTFDGKRPHDDSDGEVDDREEMSEEPFPVEDKVVETDVDYIAPQFQETFFESLKTARL
WCLLWSIFCCVGAEFVIIFNARFVYTALAGEVPDDALNTLLTVLNGVGSAVGRLCMSYFEIWSQKRRAEDR
VPITIALFIPSVCIITMLTLFLTLPKAALPLPYFIAATANGFMATTIALVARTIFAKDPAKHYDFCFLGSM
LSAIFLNRLLYGEWYTQQADKLGQDVCTERVCVVMPLAFLLGLSFLAFITSTYVHLQYRNLCLKALEERRR
IREGEEALNDESLSHTE
>tr|C9ZPR1|C9ZPR1_TRYB9 | DeepTMHMM Topology Prediction - Predicted
Type: TM
MVPQINSMGAGIEWSFSRYCLPHSPFCQLCLHKFILSRFHSCTFSVFWFHFSFLVMPFLHPFYFVWPLSFF
PCPSPPPSPSFLFFSLYYGQVEVSERREGKGQGRDVSVTRVLNSKRKQRLKNKHTQTFVDNAIAGIFLLRN
QTRERERK
>tr|C9ZPW8|C9ZPW8_TRYB9 | DeepTMHMM Topology Prediction - Predicted
Type: TM
MSLMIGGGVDHRCSRFVYSKCFFLFFLFYFLFTSPSFFFLMRMCAIMDRCHSKSRSMTLCSVCVLLLYPDS
YESIIMCHFFFFVCFSFPFFFLPFFIILTLVCGCVIFRMAALF
>tr|C9ZSS5|C9ZSS5_TRYB9 | DeepTMHMM Topology Prediction - Predicted
Type: TM
MAAPAQAVVKEKPPIHVYPQAKIGTKIFTHHGIHSMRYNGVCALTGSTVLMASGRFVMFVDVHKGTIESMQ
GPENGGVGAVAVHPSRQYYVVCERKPSDPAIRAYSWPSRTEVGEFVKGATKGFSACAFNKDGSMMATVGMY
PDFFLTVWDWESRGMVLRSKCHNTDVYTVLFSPFDSGLLVSGGAGHIKFWTMANTFTGLKLQGLLGKFGRL
EISNVSGFVVLSDGKVISGSESGLIILWEGDLIRCCFAREVDREDDDGTAATFMARSYDYTPCHEGAINVV
ELMEGGRVLMTAGDDGYFRFWRVSELEVAEGEGAPPLYVPECLGEILVHAGAFIRSVTYCKDVDEWVVLDS
AGVLWRVPYVHPDDILNNAVTKPKEQAVPALEFNGGSITSAALSPIDHTVVTGGEDGTIRLVDYVTPRELY
KMCLPQPNVVIGLRFFQKDPEKKKFLACCKSGAVLLVKRGSTAFTLLGQWRPHNDGLALFAVDAAEHRLCT
IAHGTVFFFTILDDFSSLEPIGFCKIPLPGATCVAWDDASSCCLIGFECGKLLAIRAPTRDMVDQSVSYEF
TCNYALVGIRQRKKVEKKQANVSAGEREGFVEEEEEEEYLGPWSVRLICPMADGDFAIGAGGVELLYKYGL
HVRYEGQKELPPLPPTGIEPPDYVEEPLMNLCYRDYTPEASSMSYSGRYLVVICEGSQMLLRQLDEMGRVR
LEPILVASAHDRLDGPIAAACTSFDDKMLVSVGSDGLVVAQLLDGCIAPQPPSPVAQLQPLRAEEIVEPQL
APFSITEQKDLDDRRRAEDEKRRELNLFLDKLKDVHQKYARLLRENQSLALTHRLSKEEITIHPQIYRELQ
EEMRQRVEESRKPTALELARENIRTRKMRNRFVDNLAHDRFLVRSFSKEFSVASFRTPYVDGSIKLFQQQI
DELLGSERCSSLACDGDRGIVSVASGEASQQSPRNLSLSTAAGVVRWLNSEERKRNEGEQQKMNAAHREAE
ILTTTMRQYLNKMDERREERHWRKKGYEMLLAHKPDPAVEEANLNEELRRETRRRGECILRTDPSYHSAPS
AVIKLQQLIRLEEIIFNMRNNFSNELLKLRDEKERLCGTLNVSLQRIRAINEKLKDKSFHADDVKLTPEEM
PGKRFEISRDGLVAFMKQRQEEKLREQTAKKAQRGFGADLATGEPATNTDTSGADTPATRRSEGEDSRKVT
ISANRDSFGTAAARTRSVRTGTLAASKSGRPFSGGGFAAGAARERMNHELRVKHENIKLTEMEEEELQIER
NRLLAERQRLHTQVQAMMDEFDVRLWSMYEERSRVDANLCLAHTHSLLLFREYNILLVFRQKDFELQSSYD
EARNSRDRCLREMEELQRLVQDQTASIEKLQEANKVFRREVEIFISNSFPAEHVPYITKVFLRQIKRRKHH
SDMSGNDDDITSDDDDDDDMGEDEAWEEICPPNCSEERWCEVIEKREVRLDYVDAITEERRQLEATEQRIE
EHKALADKNNAAVSTCLKAIGDFQGEKRKQLNMLETLVAMRCGQVRCLDEEGRCPDTFRRNDLVVVSDKVI
TGLHDRIRALAEEKHDRRGKLKSMVAEQQALQRERSEKQALHTQWEEKICEAMLLKFGQIVNLEVLESSCG
SREVEQLKERLRLEELSWEKELRKRDKKIAVLREKLHESLEYNTSLLQTIGDQESDRQSVERSLAQSTQKV
VSKMYDSINVATEEDRSNLRLLIAAQQEEIDALRTEVALLRTKGGHVYAAAMAAGR
>tr|C9ZI76|C9ZI76_TRYB9 | DeepTMHMM Topology Prediction - Predicted
Type: TM
MFECRICVHVFLATDENSGDIRGCKKKKKKKREKEKKKRGFVGIFISKAIKRRTQMSLCGNIGESTAVVLM
ICFNFFSPFTFGFSINNSSLFYTLFPLSLSLSFFSFIILCFASF
>tr|C9ZSL6|C9ZSL6_TRYB9 | DeepTMHMM Topology Prediction - Predicted
Type: TM
MAKRREVADDSDVYYVLEFQKNYSDTTRQSLIVSDEVPDSTLLWRNMVMFTLLKIIGSYDSGAFSAAVGAEN
GIADEWGLTNLEQGALSASVFLGCMVGCPLAGHLFSQYSAKIVLIRVLVLHIFFTFCFATVTVYVISMVSR
FLIGVTLSFIFVYVPVWVDDFAPRDRQSVWMALHNAGVPVGVLGGYLCGAILPSYTRISWEWAFYSKCIFT

VPVIVYFLRVDHRSVDRNSSRKSNVQGSLGIGHGGNGLPTNGTESAVRRGTENVFDRSSGARNLVSSACDA
VLHIWKTAAVLLGNIEYTCSVLAMCSLYFVVSGLQNFMTQYLHAEPFNASMKTIMVGFGTAIVASPIGGVI
TGGVLLDRLGGYQQNTRRVMIFTTAWGAGAAFFSVLCIFAGSTSALLVLMSLMLFCGGAVVPSGSGRVMAS
LPDTQRPAGAALAQMVYNLVGNFSGPLVCGSIAQWMGDLKYGIRAVFCCSVIGLVPMVILLFAADRHPSGV
CAMSSCGPVSTVVEERLEVKSSGVVVENGNDDTVDAK
>tr|C9ZHX6|C9ZHX6_TRYB9 | DeepTMHMM Topology Prediction - Predicted
Type: TM
MHFIILMCSRLLFTHLPLRVSLHVSACVCVFFFIILFCFFLFSNAFFARTSQRIVCTRFRVAPPPPPLSLF
PSLLPSLRFYFNSLFVFTPIFVYFPLPIFFSFFFFSLFYIVGPLPVATVTVGPSPPLSSSSHFFFLFILLC
ICTSKRASRIFFEHVPSLRVLSVCLYEFFFLSGLEV
>tr|D0A450|D0A450_TRYB9 | DeepTMHMM Topology Prediction - Predicted
Type: TM
MESTVTGEPCHACEKQETTDEHSRCGRGTLPLYHIHSTKVPEYMKDNPYIYTGYRAQYTTMMCLRSFLAVH
NESLNVWTHAFGFLVFVLLSILLFTNVVLAREYVWHCVVYGGFSFACLMCMLCSTVYHLFMCHENEAVSLF
VELVDYHGISVLIVASYIPLLYIGFACKPYYRAIYMVSIIMFGTLSVVFSSLPSLRDAKYRWIRTTVYVLM
AVGGIVPLLHFYAFTPHNTESMMPLKGVALMFELYGAGVLFYTSRIPERWFPGRFDIYLSSHQIWHVFVLA
AACVHFFSCTALYQQWLVSRHIC
>tr|D0A675|D0A675_TRYB9 | DeepTMHMM Topology Prediction - Predicted
Type: TM
MFPFYGSPSRNLFNAHTHNTLPSTFFTVWFSNGTNCFSVFFAFFFLFSLHVCHSSARSPSITRLIPLLCIF
VIVFSFFFCDSFNRFLCCQNFGAHVFNFLLPSFSFLFFLVLDFLSTELSPE
>tr|C9ZK84|C9ZK84_TRYB9 | DeepTMHMM Topology Prediction - Predicted
Type: TM
MERTRINFSPLRVSSSPARRILSDIAFFSGGRGGSAVFRPVLLMAQRSAGVNLLSPLAKLGQSRDLAADIK
AGRHRNNKEVIASFEKIQTFDEIHYRKSNRPWELVPRYAKKRVSDLCGLLSSEDRMEIEQAIDKMQSLCEV
DMYVVLVPTVGYTTPRAFANSILFDWGIGEPRGNGLLLLIAQSEASVQLVTSPAIEEYFAEHFLQAAVKEI
FQPLVREGKASYAVVQLVYAIARQAQEMHTLWNRGFLALPTRNKVRFAAKTAAYGVTSVPYLLIGIIFFGL
CSTVLVNQIIDTMCPTCYGSMHRVRDDATLQSIMTRGQYLEFSNGCAHYRVWKCPRCTDSSRVTLTSRDLH
QSTXCLQCMDC
>tr|D0A1V4|D0A1V4_TRYB9 | DeepTMHMM Topology Prediction - Predicted
Type: TM
MEHGFNHSSLPSSLSAPVASLNSFFHRVCSNPQCLTSAIHGSVSCGSVNRTPPLRKVSIDNRLKYYSTTLL
ATVTLAFVFIRGCAFVVPVICSFSSLLLSFPFTAPLGFWFFSFHDFVYRPFPPREEPFWIRCKH
>tr|D0A3T7|D0A3T7_TRYB9 | DeepTMHMM Topology Prediction - Predicted
Type: TM
MDRSETFKVFCERVEAFLGKGVGIDLVLKCMLGLTRLRMVNASDEATKEVYAGFAMAVINGRMLGNHFRYP
SSFSLALRTFKAKEGPLFHWFLFGLSFLLRTFEQMTGDLNYYQMIIMRHWSRSRLSHTYWFFKSLSLTCLL
LDEVLLLRLELRSPQWREKTSEERSSFLRRKVISIMRCLLDMCMYYQWVPWYNPYKTLQYCCVTASGFLGV
YSGWGDVCDSLAASKARRVDSIKAD
>tr|C9ZJK9|C9ZJK9_TRYB9 | DeepTMHMM Topology Prediction - Predicted
Type: TM
MLISASFVSYQRVIVAEHRVTATMKGVVDVMLEQLPRYDTKAVYQYDESVFHFLVENELVYGCVTGVDHTK
RVVFEFLSRIRDFFKKEFAGSDRRYPRPSAISFSACSKFGAVLSDNMRSFNETNSTDKLGEINKLVDDTKH
TMLGNIDVLIDRGERIELLCHKTEVLNTGSRAFQSSARSLKWKVCMGKMRFVIGTFLLFLFFATISFIFIC
GTNLKCKK
>tr|C9ZIS9|C9ZIS9_TRYB9 | DeepTMHMM Topology Prediction - Predicted
Type: TM
MGCFAVAHVWLPRRSDVFLFVLRYPFLPRWVDPCTRGGRGGFALSGVHRMRLATLLGPGNHMLSEPVVPFF
FWVVAYSLLSVWRVPLYSPSRAFASIPTFLRKLYR
>tr|C9ZSY8|C9ZSY8_TRYB9 | DeepTMHMM Topology Prediction - Predicted
Type: TM
MIIAYEEVNEKMHVFFSKTKRKEEEENENEKGKRKRRRRRKRKERKKKKNLTYASARENAHNLLQCFTTMH
VFFLFYTSTNFPLKFFFSEFLNCFCLCLPTSYFV
>tr|D0A2Q9|D0A2Q9_TRYB9 | DeepTMHMM Topology Prediction - Predicted
Type: TM
MFTRVHLLTMSRWFNRVEGFTTLFRSSSFSLPSVPVAICVRNSLHILIIFILIVAVAIVVSYFITYICTCT
LTHVFVFSLSLSLFLLVSFFLFPILCLAAASLIVATGCRVDERS

>tr|D0A1N6|D0A1N6_TRYB9 | DeepTMHMM Topology Prediction - Predicted Type: TM
MSCGGAWEALIDRAVMEVSKFLSATKDAFHQDSPTAEWMLNLLDDNALRDVVMERNFNALCGMVGCAAMPP
AAAGRVEGQLQQEAIGTGKNILNRNDEEDTVSDGEDDGSDAADAFRRYEKYREQICAGREAATALSGVDMT
RCFCSPDCAEQFAAMLAKVPRTLVYSREGLVNSVGGLFPNMQFSVLQQLAGAEATVVPDIREKEVVEGQEK
KLDLPIPSVGNEENKDHKVKLRELLESIQAVQSVWSRGVQEVRPFNTRKGTMQKMPIPLMVYDWLGTVSTD
KTKSIFASVCYSNTRKSDRFPCVGGGRSTNLFVRCMQPVLDACIRKGKELSEGRAADEGCAENPCVDPALQ
HQRLTLFVRHVFSGETSATLSRLLMYDQATLERAWGVWNSTGLLASLSFPSAVPGDFTCGGGSPARIYLAV
VLTAAAALCVPAIWVEWLQEDNGLAEVLEALGCTSDDFIACVRALVIE
>tr|D0A6E7|D0A6E7_TRYB9 | DeepTMHMM Topology Prediction - Predicted Type: TM
MDTHGKPKTKKGERRVAVHAAKRIHRGVCVCVCMEKRANKFVLLFDVVGFIIFVLPFLFSCQHLTCCLFYY
IYIYIFFYYFLFIFFDFVHCGNFCDTESMSTHPYPKQEWQRPI
>tr|C9ZWR4|C9ZWR4_TRYB9 | DeepTMHMM Topology Prediction - Predicted Type: TM
MATNKGITFQVIVGIAALLSAFIIIMICIIVIERRRLRNARARQNVQLDPAALNLTGEQLRHRLIRHAVDL
LLADNYVCEGITLTSTVGVTEVLADRELRSTRGRPYFITTANGRRATISQVVQQTRASGQAPDSPPGPALM
ELLEKLQHARRAEPVPNEAHCKQVEELMERLIKLEEANQRKDMKTDADEVYGKAAQYATNPANKCVRHVMN
AVGVGRSLVASR
>tr|D0A306|D0A306_TRYB9 | DeepTMHMM Topology Prediction - Predicted Type: TM
MTPAILHHPVPSLLHPFKCPFTCAQIGKRKCLKTNNKKKRGEKGCSSSNSFPCRPSRPFPFRFFPLPPYLW
GGLSLFCNYFHLMGMYPLLIFLFNPFILFTFFFIIFSKKIYPTLTSFQCCFCSFSSILSNSASICYGCVVA
SFAFPFISFLLPSLPSP
>tr|D0A7I5|D0A7I5_TRYB9 | DeepTMHMM Topology Prediction - Predicted Type: TM
MVKPAVFSPVGGGNPEPVDGVGAGFRGPETVMPLLTAGAGRVRERRFQRQLGFWFVALAVAVPWWVLRSRA
GNRQSTGDDVRDLFASTCATTGGVGYILYAGGSSVLII
>tr|D0A885|D0A885_TRYB9 | DeepTMHMM Topology Prediction - Predicted Type: TM
MLGANLGINFHTDDDHGDHGDHEGHEGHDMGGCAPAAGSYSMGLHIAAIFILLIASFLGTILPIAGNYVPR
FKLPPFLIVVSKCISTGVVMSVAVLTLLNHSLHSFMEKCIPHGLSMEVYSAFGLLFMLISALLMHSFDSAM
DLLLEGWAVRKEEEKLADGAPQVADSVPTAAALPPTQCGMKRCTAQPGVSCETNGCCQSSPGPAYGATGCC
GSRGEAAALLTGARRVMALALMEFGLVVHSIFLGLSVGIASDSRTKVLLVALSFHQFFEGLALGARLAEAS
LKAKLELFLAILFSISVPVGTAIGAVTMRDGGKSITGSSYATMSAIVNAIGAGILLYIGFVLLLVDFPTDL
RIYAGVGTPNRFVRRIAMFVALWVGFGVMALLSKWH
>tr|C9ZNL6|C9ZNL6_TRYB9 | DeepTMHMM Topology Prediction - Predicted Type: TM
MRPLDVLLNGLRGLCTAIKETPLFIWVYLSILGVVARKLTYRFGLTTKSKKIGKHVIRVTDAEETLSEDVM
DVEEINATFDDVGGLEDVKKALIEHVKWPFTRPELFEGNTLRSHPKGILLYGPPGTGKTLIARALARELGC
AFINVRTESLFSKWVGDTEKNAAAVFTLAAKLSPCVIFVDEIDALLGLRNSVDAAPHNNAKTIFMTHWDGV
VQKKSKIVVIGATNRPLAIDEAIRRRLPLQLEVPPPDITGRRKILNILMEHDVADESNRSRLVDYVASKTF
GYTGSDLTELCKAAALMPIREIGCDNELPCLECRHFDEALKRVRPSMASSV
>tr|D0A7H1|D0A7H1_TRYB9 | DeepTMHMM Topology Prediction - Predicted Type: TM
MTTISTVGLVLSYFGLPYAFVYDYFGVFPVLVMGFVMMATGLLFMALTFGGTITASVVLLCVFNGIFNFAS
GLYDLACVVTTLTQFPTAKGWIVAVMKTFIGLGSALLGAIQLAFFEDDPTNYFYFLLAFGAVVGIVVMLVM
RSAPYIITDYMLKHLTEEEITRREATKAVYLRQEPPTLRFAIGLLIITVLIIVLPLQSALIAYTDVSPFNR
KASTIVFVVIWLLYPIVCLPAKCLDKSWRFWRKESQVSAGSIEDQRRDDGSSEGEIDELDYIPPQYQTRFI
DSVKTLRLWALFWSLFCTLGAEFVVLINTRFLFAALAGKEIDDSLNTLLTVLNGTGSAAGRLIMSYLEIWS
QKRKAEDRIPITVTLFIPTAAITIMLVLFLTVSNEYVLPFAFVVGAIGNGIIASVTILVVNTIYAKDLGLH
YNYCFVATACSTILYNRLLYGEWYTYEANKLGVEVCLERVCVQMPLLVMLGLNLTAFGANTYVHCEYLKLV
RSALEGRRVAAESLPEKPEGANDINSIRLELPAETSAAPYGSRDRRGTE
>tr|D0A5R4|D0A5R4_TRYB9 | DeepTMHMM Topology Prediction - Predicted Type: TM
MSHTMTNASTLMSITVCDPNMKGTGLEMECLRPSMHIPELVLIPPIYVLIISGFLCTLVNAFFNYVSFGRS
SITTAMEKHTRVKMLIHHSLIVRDMEGHTSVSRRDNIYNETDHLLTHSNVSVDHNDSAVFFQGILNSDALG
FESPRVPASVADEALYGSLECIRRVGQQIVFSHNPKYMIISITKSLVMLLVLQYHFDYNGGWDRFVVCTAA

NLMSGLLTPVLEYGAEFLLQLDPDSNLKVAQEHDELDDVVLISRILTRIGEFKLEDLETVLVQGCTFIVFG
AVLLPPVFVFCLVGAAMFLWLFGPLWFAYGYFRHLYYKPRQSKGLSSPNARGWLRITPFRELARIVLLKAV
TLFLLQWTVQCSFLLGLMLLQGESYLKALHLEVKHQLWRWNNPLKMGGFRLLCLVSQLLF
>tr|C9ZIP0|C9ZIP0_TRYB9 | DeepTMHMM Topology Prediction - Predicted
Type: TM
MVLSAAIEKRQHPTEQGGEEMQWMGSSPRCPSSVEACRRICIGEGLANNDVRISGWLLLCVSNTKEATTSP
LSKFDSPANKGCGTKLNNDVDDGDSSGSNRAEVGGNGNASSMKSDLHDSVSRSEAPAGTTNTSSSSLSETH
GDSAVVSKRKEKPVDEDVDAKDHVEEGYGDSAQPVDALEESSTISDSWGADLPANCHARVIVADVKRSLWK
LYPNETVREKKRKMLCNILAQILSNNPERHYYQGLHEMVGFVMYVMEGAREADIVAVCSRLLVQQWRSFSC
KQLERSQSMMYAMHTIVVKEEKILAEELEACSVGPESHYAMPWLITWYTHVCDDVEALSRLFDFLVASEDE
NTVIFFTAALMLHEREQIIGIIKEVKSSCCDCDDSDGTATAEEINKTLVMAQVYSRLVRLPKEVLRRDRAR
DLESIIRRATVLHEKYLSYANDVRGRFMRNIVSVSTTSDSSGKWHQCKRWLRFSLERRWVSAGFICIIAIF
AFWYRESTSRGVTLLGNWITDLYRVSCSIRSSVFTV
>tr|D0A9K7|D0A9K7_TRYB9 | DeepTMHMM Topology Prediction - Predicted
Type: TM
MRSKTAAPSVIVTPDRLHMTAKRKEECLVQITNISFEKVLFRMLTTTPERYLVKPTKGVIEPSASASVLIT
LSPTTARGEDVSDVNATDDFRLEYCLQEPEDCIEPRCTNVPALIKEKKQQDRRLVHSKTVRCTVDLTAVNG
KWGEVRLRDDGNGTGRGSKGKNVISAVLNSRKREEVPGQVKASLAGQKQAGGGNSLMWIIGGAATLFCWWW
FAY
>tr|C9ZIU2|C9ZIU2_TRYB9 | DeepTMHMM Topology Prediction - Predicted
Type: TM
MFRCSVAGGCAADKGENFCVCTCFDLCRLNFLVTHSDCLCSGRRDVFFFFLQQSLGWLGPITHFRCPSFLF
LVLVGWFIHMHATC
>tr|D0A0T8|D0A0T8_TRYB9 | DeepTMHMM Topology Prediction - Predicted
Type: TM
MRRRLHNVVTSITSRVGKPHDDGRGARHLSHGHSHSHGHGHSHTLEDTVQGKQLRLCQIATAVGGATNIFF
STTKIWFGLSGGSVALVADGFHALVDLLADVVSYMALTVSTKRLPRCRFPFGIGRTETAGAVIVASMLLFG
AVTLLFTSMQECTRELLKLINPNGESSAASTGAHSHNEHIPNHHQHHGNAMGGHPHEHAHHSHSHYQVAQT
DEMGRVTIMWTMVALAAASVVCKEALFRWTKRVGERAGSRVVVANAYHHRADAWSGAIALVGVAGQCIGMP
GIDGLAGLFVSASICQIGYALMRDSVLEFFDFQRAEEVAAVRRVLQDYNKLHLVNVFLIRHGHSYALHVTL
LTEMDTAAMVLARTSNELTKLAQRSVRVADTFTTIAPCDRGSEESLSNILRLVEEFHGLQSIPFDWGTRRI
SLPQTIDEECMRDVKSIAAFFELEIDIVAGENDFKRTAHPSVGCC
>tr|D0A870|D0A870_TRYB9 | DeepTMHMM Topology Prediction - Predicted
Type: TM
MRALYQFVAGALFSASLFVFADGLIVATQNALPYNFLMWLPSLLMFCGMFVLCYVDAGAISNRYDLMGDGD
SSRDRAFFFASVLFMVSGFAVSLWKAIDPYTNAGVPWPGASLVIQSALLIASFALLFWQKTQDSNSFL
>tr|C9ZRL4|C9ZRL4_TRYB9 | DeepTMHMM Topology Prediction - Predicted
Type: TM
MFCFCRCRFRRLWGWDFVFPFLYFHAKFILQFYECSIVRLLNATMYIVYLKAFFFCVWTISPSQSLPEIFP
PIVTSILTPWRAGGEKNGEQRRCAMCEKDDEWGRNKISRSSLNVAQLLIMWEPKRGKRGPNMKGNFQASEP
K
>tr|D0AAP4|D0AAP4_TRYB9 | DeepTMHMM Topology Prediction - Predicted
Type: TM
MFPLRGVSSSQFSSQANSYSLFHYVLLVLLTVQNATIVILMSYTQQRQSSKDPSNRFNTSHVVMMTEMVKF
LMSLAWCAWDVFSTIAPRGSRVLDDSGTNASETPLGHRGSDVMRDVKTLKQDQKLLCYEDSHEDNSNGAKL
ATDMESAPADLSSDAQSIEVEINRESFCGLFLLQLLHRSAVPTAVPAIIYAFQNYVMFVALANMEPTLFQV
TYQTKILGTALLLWIFLGRTFSSQQWMALFLLMAGVVLAQLGSKHSNRKPEEKTNSVEISGSYVVGVVATT
MAVLCSSAGAVMSEWLFKSKDASLSSHTSTKNVHLSAYSVVCYIVAQLLAGSGSNTQGQAQVNATPDDVNA
GTSFFQEYFRGFDSLVWLMIFVQAVGGLLVALVIKHTDNIMKAFAAGCSIVLSGILSLLIYSFVPGILFVI
GSMLCIVALIIYSRG
>tr|C9ZVX4|C9ZVX4_TRYB9 | DeepTMHMM Topology Prediction - Predicted
Type: TM
MCFQFSFFFSFFHCERRRGSERLTARHFSTVSIIQTLMCGSYSFALHHWDSFNLFLPPFKVFLLCFRLLFD
NSYYFYYSTPSSQRVHGRWRRRSLVTRLTA
>tr|D0AAP2|D0AAP2_TRYB9 | DeepTMHMM Topology Prediction - Predicted
Type: TM
MCICGGSYKSMYVVLFFSLCVSFTILLFYFVFVFSFLFFLSFFRSRICFVTCGSSDILVSLSFLPLLFFCS
FLCVCVRLCLCLYIYVCGFLLTSLRVVIYCYCCWSTRRIFGTQQQQKKKGQ

>tr|C9ZQK5|C9ZQK5_TRYB9 | DeepTMHMM Topology Prediction - Predicted Type: TM
MVPLTNLLHMYCGMRGNLACTRKGKSVFGGGGDNKQTCQQKYKKWMSCTSIRGVERSFVTCMCSTKLVRLQ
DDSVTKLCLRSCSSPSFLFPSSSLIFFLFFLKKKRDLFTSYIIGGTSNLRDHLFTEYCSTFPFLLFLSVKR
EPSSPQESL
>tr|D0A5W3|D0A5W3_TRYB9 | DeepTMHMM Topology Prediction - Predicted Type: TM
MTTMEKPRLPTVATAWLLLTAPVVLLDAVFVLNRPKTPDTPHPLGELVLFQPWTVYATYDRRYAPNEDAFV
VAQSWMNLLEVTLGLLAPLLSFSGYWKSAIKLAAVVSVMTFSKTLLYFVMDFVEGGEYTRHIGYLDKLLMV
LLPSSVWIIVPFFTAIRCLQSLSQAGSPGSGDNSPKKRKNK
>tr|C9ZZY6|C9ZZY6_TRYB9 | DeepTMHMM Topology Prediction - Predicted Type: TM
MMASEDKTQRNDPHETNEHEEVQTTPEEVDPTAEEPVSRWGRLMTPRIVLSIFTLLNFVTYYDRGAIAGCL
VVIKGDPTIAGSSNVLTDTKAGLLFSGFMIGFMVACPLFAGLGGVVQSKWIIAVGIIAWAAALVGTGLARS
YEFLLACRIFDGVGEAAFVGFTVTVIDAIAPPESRTSWIGTFYSMIPVGTAVGMAAGGVMGAYGSVGGLEG
WRVTFLSLAIAAIPILLLIVFLPKRYNMRQKRDNEYLPIHKATFHIFTNARYLLVVFGYAMYCFVIGGLSV
WSIPFLVDGPMELTNMTASMIMGGTTALTGIIGSIVGGVVVDKLGGSLGSSGTMKCQLFCVVMIAVSVPAG
LAALFMEVTWLFTSLLVVSVFTLFAVTAPINSAILTVVPWDQRAYAVSYSVLLIHLLGDFPSPTLAGYLSD
NAFSRGCPAHGNNTQCGNDIDNLCKWINKTGNSTDGHCVSKYQLRNALLVIFSFLGLAIPCWLAVYYILSR
EASTPHSRVASEDRRLVGDQTPAERSKDTL
>tr|D0A267|D0A267_TRYB9 | DeepTMHMM Topology Prediction - Predicted Type: TM
MTCMLQCEEKVPFAHVNTSLHDTRFLCIRACVTTVVSPTHLFRKFAALLILCCISPLWFLLFSSFIPHNQM
FRRTSRCLVEVGDTCVPNNFVHATDLAFYFSRFQPRSHLHPHGIPELRDAVELLQPPEGKPCSRWSAPRLF
SPAALTAPAAVAEWKKKREKAMMLLSRGEEIMENVSGVRSAAVADVVRPLYATKFELLNGLEHLPRSEFAT
RIDVHRFIVKHAGRLLYFNDSWDKSDVKHLDTTCVILAHFIRSFCALHQRPFHVKQANVSDSKADRNPSSW
TMAVVGGESGDAMISLQTVMDRLDELLALSEKITERQMGEMPEIRWNKPKLLLLKGTLTIPLTGNLTHAQE
LVNQAAKTVYEFHQRKKPIVDGLVARKQEHELGLFMLVQAEMAARVFDWTINTGEVDMEVVEMFEAAAKFY
SSPCNTPLDADGIMSAELLDKRRFEVDAYTTCMLSFGNFLLGAPRPAAKTRRDAPVFLPKQLFTISPIATV
ASSSDLIYADVQRRAPMTVEVARKRAGEALERGLKLNRELYPDQKQNPKAGWTLLAMASLYADMRDYLYAT
GLFASAEKTVIENYGGVSLERLLVSKLRYEFLAGVGSEEEAKASAHEIVQLLKQMDIMPHG
>tr|D0A1F5|D0A1F5_TRYB9 | DeepTMHMM Topology Prediction - Predicted Type: TM
MGGGACDHRCVTVRCVTRGRQGKKSYYYYYYYLLLLKFQYQCTCFGLRGGRVDCTVFCHVGLHPRHFFLRK
SFVLFCSGYNLLLSLLIAFLHIASFVSTSRLVSFLFFFPFFLLHKLFFFFLYTLCKP
>tr|C9ZV06|C9ZV06_TRYB9 | DeepTMHMM Topology Prediction - Predicted Type: TM
MKKRVAVSCCVAGVLLFAAAVAIHRTAPTTIEQKKLAAWIYTGFRSATERTKATVTALVPEPMKPHIKQLL
AGATSVTLVGQGFRRVPGIATAISASAQTTKQQNVLEIIMAKPVDKLLYAVGAIFAQAFLLRLLVKPPGYW
GPVRDQKVWRNFFTTQAGWFLLVVCVALPNCVVDAGFIITLLGRLPSETYLPALVVGKLLQPYVATALFCS
PTFVRWLPQVWKVMDAKGSEDDQSGAKVVFWALLTTAVLLAGTNGVTMLWRRDGSNDEEEELVLEDAEAEN
>tr|D0A1L6|D0A1L6_TRYB9 | DeepTMHMM Topology Prediction - Predicted Type: TM
MCGCIYTLCLLLESPAKPCIILFTFCPLRFVSVHFPIIISFPCSFFFSLYLFSPPPVTHPPHLFIISLSIL
PVVTFPSFISISISHCFSFFSFFPFVLPKNITRVGEKGKYGVVTLNE
>tr|C9ZTI9|C9ZTI9_TRYB9 | DeepTMHMM Topology Prediction - Predicted Type: TM
MSSLLKVHQLDQRMQFNPNITTGYRPHMPVKEAFHTFFHLHNESFNMWSHAVAACFILYLMVFPPVEDLLE
VNGKVVLRQRVVPAEEANFLLSASPTVVFRSTCLMAFVAFVCSVAYHILIPCGASRSVWVRLLSCDAIGTV
LTTTGTAWSFLYRGNACASLHTSHRVAVLLLLLSAAALTAVLRCGSCSSPSARGKVMGFFSLPYLALVLWM
EVPKAYTQGHCTAVNYHTLSWVFIALGTFVNASRFPEVQVCRITRCTARKPEKRSDMKPKWWRWLICCLLS
SDEIDYAWNSHNIWHYCVILSVIAKLLGCRYDMVEFELARCVT
>tr|D0A679|D0A679_TRYB9 | DeepTMHMM Topology Prediction - Predicted Type: TM
MFVTFILVSVIALSHTGVFFCFFLFVGSYDSRFFLLFLSSIKLLFLHCFFFSVSGFFFFVRRPRLFSLLDV
FTLFHPSFVFSPFSINSSRVWFRYGVPRSHIFIYIDVHSLSPSGLFVQKEKKGNFSFFFGPLSFLCYIYLF
L

>tr|C9ZSY7|C9ZSY7_TRYB9 | DeepTMHMM Topology Prediction - Predicted Type: TM
MASCTHTHIYIYIYVGALASRYISFLFVWLCFIPFCLFKRPLFFLIILFYCFCLVLSKSFFFFFASATVWS
STCDMREQKDIYAYISSRPHITGLDEKGTKLNATGTSD
>tr|C9ZKY3|C9ZKY3_TRYB9 | DeepTMHMM Topology Prediction - Predicted Type: TM
MCHGGRTNHFFTDSESCFMGDDMRLYQCFCPIATCTQTAVGPACRLTTSFLTFCGLLLLFWFLVAAAYVYI
TGLMSEHQRHHLPESYSALPGGYYYEKLFGDGTDSNQNTAVPVADAEKSVSLIPNRRVER
>tr|C9ZMW9|C9ZMW9_TRYB9 | DeepTMHMM Topology Prediction - Predicted Type: TM
MVTRSLHFLPHIVRVVPLWLNTSTLNENNSHFILTLFRFFAISCMVVAYTRLMGTLTCTHFVGRCESWTVF
GTVFRKALLRIFVFAIFLPVVVYIYFISPIL
>tr|D0A5Y3|D0A5Y3_TRYB9 | DeepTMHMM Topology Prediction - Predicted Type: TM
MRSRLLSGGLLAGNLLGGNLFGDNLLGGNLFGDNLLGDSLFGDNLLGDSLFGSSFLATAFFGAAFLAATFL
ATAFLGTAVVFAIFYFPFYFPMKCFG
>tr|C9ZIN3|C9ZIN3_TRYB9 | DeepTMHMM Topology Prediction - Predicted Type: TM
MHVVSEIQRRPNKARLKSEKYIVHFYSLCTLVQLVCLIVFVTQFDMASNRTFTSSLWVENPFELAPSLVWL
SKRCSHSVQNERVFAFTGVSVNISDKVVGVMFAALATEMHATFFLAVTALLVGAINRYAVKANFFEFKWRN
FNVRKDCFFATEIVLISALLHSVLLAEDTHRMLHDYLDHCNTRSRGFLPYCSTVPMIIFITFAFATYFFGF
LVYMWNALPKYGIMSDEEVVEYREWLRRREESVAEVKRMEEEVRRANTRLQLMLENEKNMKLGKSTYQSRR
PTIRREAYGSKQGDDAQQWGT
>tr|D0A7I4|D0A7I4_TRYB9 | DeepTMHMM Topology Prediction - Predicted Type: TM
MCVCVCGKPTFPGICCTILFWPGAGTALPPAIILVSRYMFPKLCLFANRRNKGSTSSNLVELTRQPLLFIC
RATCKLWDASLVYRRFETVQVLYQTVRVEVNNVLTRHDGFFFLNESVIADGNVRCVVSTLPALCLLV
>tr|D0A096|D0A096_TRYB9 | DeepTMHMM Topology Prediction - Predicted Type: TM
MRAHTHTHRMSTEPHNEHAPHVLGRKLKARKVERLQSLRLRRRPVVSGALPAATEFCDAAGVSEVTGNKSA
AQVDTSTSVCKERERSFPSTGDGCGAPRAYLNSTTSKLTKQAHFSSPYMDTRIPIACSEEYHWAPLHTSFA
HELVAEHNTLVEWYKEHFIKCQQQSTERQPPFSPEENYTEVLPVWLAVARSRHRELSRFVQYHRTYFNGID
MEELQLTEECLRRLEQCLVSAGCQELASCSDCSATLSADCYGEAAGHSPEKHPGCTALGIPRQCTNVSVEV
VDDVGKGSQLSLHSFPVADSHAVQLCLGETNWRTAARVSGKDCRVVCNRPPPPVPGDLGCEGMTDDVITER
DVYLQPFLTYFSFSPQRRQSRRVKLFLAVVALSLIFFCVAVALVSQKFR
TM
MCVCIYVFLYLFGGKTRERRRCVRRNRERKQISIRPLFAFHSVKCSSIFFFVIFPRYPSYIHVCCTRRLVC
SHRRPVASTGSVGEQVAARCF
>tr|D0A5L1|D0A5L1_TRYB9 | DeepTMHMM Topology Prediction - Predicted Type: TM
MKMAPVGKSSNRYWRYAEANNKMISFIYTGVASCLTTRSTRGCSSSNLINSRKCDSTYLCGQGQCACMLAV
CHTWSSRYARLYVFRQRSTLYMYVSRKGASIYIYIYIYIYAYIYHLFTFKED
>tr|C9ZSG8|C9ZSG8_TRYB9 | DeepTMHMM Topology Prediction - Predicted Type: TM
MYLWRFKCFTLCLFIIVICAALSPLSLHYLFPSSSSFLIIHEMSCAMGCVGVGCWIPNPLEFYLSWLPPFP
PFNCHTSCSHFTSSLVLLLLSLLLSLSPSRCD
>tr|D0A217|D0A217_TRYB9 | DeepTMHMM Topology Prediction - Predicted Type: TM
MLRSPWTMLCGVGPPGLAPKRKPKIRIESGYERENQKYYEERGHFFSAFASISILSLGCTFLFVPLYRMYC
APTGRGADPKFYTPQAQRDREQLNEMYPVPKKLLKVRFLSDVGNTMPIAFVPLQKEVEVLIGEPALAFYSA
YNRSNRTLLGVSSYTIAPPEATNYLNKIQCFCFEEQRFKPHELVEMPVFFYIDRDFLNDPMVNWLDEVIVN
YTFFNLEKTKDIIFRSNLA
>tr|C9ZXG7|C9ZXG7_TRYB9 | DeepTMHMM Topology Prediction - Predicted Type: TM
MPQFVLPVCVCVLLFACITLLPFSCLRSAPSLDIIPMTPAVVSMREKNNGLLLSFAVTAMGVACLVKLYKR
CFVRVPPHCVTVVYETRRNSVLASSMEEANNELPQVSHFRPLMYIVSLLLYRSKKLVIVPPTSFFSTFTLP
CSVLDESGDGEVVDCTVEDIHVTDGSVRMVMTIRYCIPVDELERYLAAVGPTAPNERIALAAAGAARARGA
ELSVGLIINKSKRDTAFLEPFRAHLSSKLMSESCVKVLDVVVEAAEIADRSLGG

>tr|C9ZS24|C9ZS24_TRYB9 | DeepTMHMM Topology Prediction - Predicted
Type: TM
MREPPGFDLLSLRRTGSLRNDGLSGTIAWVGWGVLVYIRMCICCGRGGVLLIVRVGCGIPGLVLLCATVPS
PLGAFLASIGDGAFVGVEGLLRGHWIMSERYGCFRFALRGYGWCAYADYLCVCLCFHFALLLRTGSCQQG
>tr|C9ZLK3|C9ZLK3_TRYB9 | DeepTMHMM Topology Prediction - Predicted
Type: TM
MRRALYRGVTESGPEIRYFAVHAGCMRRWCSAASGSSRGDRPTDYDVPPPRPAFYVPPEERASDDASLQFL
RKDSGVRSGLMKGCGDATGEVKLEPGQLFAPEPPVRPAESPSYINEKAARLSSTSVRAGASLLTGDIGAEV
SRLAVVKNSTSTRNRSEKTDASPPLTYGGSKGSIFESRDVADGVPEGKKIRQHEGTVGRGEEGGETTGGNP
TGDELPLLQQMQVDLRRLEYYQGTPQYPEMLKEFRAKYLSDGGSEGEGDGRLEDLRSYTQEELARGLESQP
IDYLRASSKLKVELTSGPRAYDPVLVMQQMGVMRFQGYAFPPTTELGRLCDSDGKLPDSSEGAHRFAKYVA
QTAPTAIKDQLKGNEDRHILYRTMGLDVVQRRQVKAMLSDFDHGDRHTSYHVMMSYPYADWLHVFYMVLVG
VAIYEMQVRFCAYDFYDEYLGLDLRQVPKLKKPFLVTVTVVVMVVALFHPLLLVSIATTRLYRIAMKRPVG
PP
>tr|D0AAQ2|D0AAQ2_TRYB9 | DeepTMHMM Topology Prediction - Predicted
Type: TM
MACSLRQCLLSYGVAESAMLREQVEEDVTTPVSEHEEEEEAAPTTHLLSPRGTVRLIDGYESTGHRDTHCL
SPDSRERGSWIYHRPPIYGLQALSEPKRFWQLVVGALCCFVVSSSFTFNLYSGQLQAKFNFTQNDITSIFT
GSDVAGILMLPLGAVYDKYGARPVFILALLTQPVGAILQALTYDDFIKGNLYLFIFYSALQAVGTWLLDTA
AVMTLLSIFPSDKGPVVALSKVITGIGYGVIGAIHSAFFYGGEAKDTRNFFIFLASIGVVATVLGYMYLED
PPYVVKGSEVDTITRKERVTRRRLRRIYLRQRPSGLRFAIGFGIVAVLMVYLPVQAIVSMYFNLGHRYRVS
FACTTVAILALYPVMALPLQCLERSQSLILPMTSTRCSERSSCVSRAASESVGSVACVDDLDYMAPQFHIK
LADNIKTLRFWALMWTMFSLSGAEVLVLANMRFLLAAFDGGLLADTYVGYLLVLTSVGSGFGRIILSLFEM
VSQNRSAEERIPITAALFVPAVVQVVALSLFFVLPAPLLAIPCFLVSFAGGCSAAASVIVVRTIFASDVGK
YYNCITVATVVSSLLINRGLYGEVYTHEAMKEGKTICIGRQCVILPIIVVLVACVSSLLAVGYIHRDYSQH
CEHMFELHKQRHASNEAPVKADHIGFNFKRSGKQSPAGGSAANI
>tr|C9ZVY0|C9ZVY0_TRYB9 | DeepTMHMM Topology Prediction - Predicted
Type: TM
MMPPFTPPPPIYQQRLLACSWFTFLKQFIPFPTSFFLVNIVIAPAHAPTGAEPYCVSFIFFFLQPVADGCH
NFCICIYILKLLLFTCYIITSLIEHQAFWW
>tr|D0A0L3|D0A0L3_TRYB9 | DeepTMHMM Topology Prediction - Predicted
Type: TM
MFEKYFIWFCCCLEDGKLEAGMIFVFFFSCVCVYIYILFVFVRSFSFFLFLFLFPPPFPFLPLIFLSFFLL
FSPSLLLFFFFFLLLLLHMPSFLPLVVERKERGGKK
>tr|C9ZSS0|C9ZSS0_TRYB9 | DeepTMHMM Topology Prediction - Predicted
Type: TM
MYCYLCSLLLPFGTTCWICCVVVASITGFMLLHVCSLPLDSFLPLLIYSKSGSSLKRSGESNVTPTADICG
SDLTLCAAEGMDKASVNDSLGRYCIYMCLCLSMVFKKYDDFT
>tr|C9ZYW2|C9ZYW2_TRYB9 | DeepTMHMM Topology Prediction - Predicted
Type: TM
MQSLVSMNLVHIFESIPHRIYCWAWLGTLVQECFHVYVCVHSSTFQGSVSAARDLESAKFRHFVDRRARVF
LHIFFVLPLSHGRFSLPCVVCTLCTFYCLCLCYHILSPSLFCRPVQLEIF
>tr|D0A734|D0A734_TRYB9 | DeepTMHMM Topology Prediction - Predicted
Type: TM
MKWRVKIFAALGGFLFGYDTSVINGALFQMKEHFDFPAHSWISGLIVSIAIAGAFVGAFASGFISVRWGRR
SCIALADIFFTLGSIMMAFAPNVEVIFVGRAIVGLGIGICSATIPVYLAEITSASNRGSSIVFNNVCLTGA
QFIASVVTALLVQFTGTNFGWRVALGLGAVPSVIQFVGLIFFLPESPRWYLATGRVEKALKTSEMYDIDIV
DCAEGGGLVIDYRALFSTVMRRRLLIGCMLHILQQTSGINTIMYYSSVILYDAGFKDPKTPVLLSIPLAAI
NTLFSLFGVFTVDRWGRRLLLQISACGCFVVTVGMTVVGFMLDKQIPYEIGGWIFLSLLGFYLVFFAPGLG
AMPWVVMGEIFPNTLRTSAASVATMCNWGSNALVSQVFPMVLGSIGVGGTFSLLCACIIAAVLFIQFFVVE
TKGLTLEEIEEMFDPRARHRGSDGSQCSESCSKVERENEGEGTDHGLNQAETARAPI
>tr|D0A4L6|D0A4L6_TRYB9 | DeepTMHMM Topology Prediction - Predicted
Type: TM
MCCGRRISAETFILPALFLLTSYSIFSFCVFIFPRLLDALGQAFLQPFIRGIRQPTSATPLHVEHLLSISA
GSWGLFGVSAVLFSFTVWAYVAAATTDPGRVPYAYHKGAPKSATLALKVSGAQHHCPVCAHYKPQRAHHCS
RCRRCVLKYDHHCPWIGRCVGFFNYKLYLLVIFYTFLCTLWVVLLLLLACSSFAVQHYEAVNGQVRHRRVD
MPAWDSRKCWAEAEALDRPHLRAKDLVVSAQAVAQLSLFEEFGVCPPFLGVYVCFLEAFIFLILSGSLLRK
HWGLARRNLTTLDLVIQQTQIEQGICVRPPVNPFDIGVKGNLHQVFGDGDADGEHIHANFIARWFCRLLPV

AAYPEQEQLRYASSVSSERSALVSTDPALWAGGAQASIPNYGTLQKGDLSGGVGQVTVIEGRGVCNVYHLS
QVSSHVGSLLGQTFPTAVPLTSPTPV
>tr|D0A588|D0A588_TRYB9 | DeepTMHMM Topology Prediction - Predicted
Type: TM
MEGSHSTHNYPPPHTHTHTVKRGPTNNTIRTSHTQICIRFCRKGIYTYEYYIVPLTLHNFFVFTTSLYSFY
RFHSFSPSRYFVRLFSLLPPSPTSYRPKSEQVKQELPPPPTFASFCPFKVVFLFPPILSSQLPSSI
>tr|D0A261|D0A261_TRYB9 | DeepTMHMM Topology Prediction - Predicted
Type: TM
MDPLDDWDVVEADDFTPRVSSGDKNEDPSTKKDADDSLLNHEENAVSSKDSVNVYNTPSFLKSKCRIPSGP
QVVRNDSTPSNNYNIVASPGNEGEPSVQRPPAHREITGASVLQARGILHESFDNSNAQEVVHRCQYVKHTV
EPQQNSVNAVSNSRLYNLIGKLLCAGQLLGLATPISDGEEQNCWALSVKLWSHTSCIIKQMGSAGQLMCFM
VFLYFVSAAPVGRNKTEHCSPSINCCSLFKDGVRYFCYLLFLVGPLLRIAEFAVQKRSFGKALHGCEVGGR
AHKIVCNAVTWFGKCILTTALIVPQEPNGGAAVSYVTAKETVGSCKTKVSDTTECFARVNSASEKAVLGVN
RLLFVVVCISLVSFMRDAEERCTPSSR
>tr|C9ZI83|C9ZI83_TRYB9 | DeepTMHMM Topology Prediction - Predicted
Type: TM
MFQPLCFFFTFFFFSSVVKFVCWTRGKNKVRYLSVRLFPSGFVFSHCHFLPCFHYCSCKHIIFYTHTKRKR
KMEKRETKQKREAAATYSSVYPCAVPPPLNLNHLLHWRVNGM
>tr|C9ZJU3|C9ZJU3_TRYB9 | DeepTMHMM Topology Prediction - Predicted
Type: TM
MAMLGFESTAEFFVYLTFIFFGMSVMNVTNAIYSNYDYFSEYYKFAQRNADAISSNPSFWKHMFTYYNVVV
FTMQVLLEAFMLTPLGRRIPISWRLIFGLTIPMVEIIVILVIPEVGGSEDGAIATMMIVAFVGGISKTLCD
SSNAALAGPFPTKFYGAIVWGLAVSGLMTSFMSIVIKASMDSSFESKRVQSQIYFGLVMLLQVVACVLLFL
LRKNPYAIKYAAEFRYAARKDGKTDDGEDENDAKGTGPADEDGYPDEKENKNVLNADIDPDKMKDTDQVEG
TTNAQQMLDASVMVVVKRIWPMLLSCFFVFFATLLVFPGVFFAVKGSMDLNNFWYFPVAIAMFNLGDFLSR
LVLQFKRLHVSPRMVLIGSFARALLIIPLSLCVSGAIPGVGVPFTVSLLWGLTNGYFGGLSMIYGPRTGSL
TTAGQRSLAAICINVALLMGLFTGAMFALAVKEGLPE
>tr|C9ZQG2|C9ZQG2_TRYB9 | DeepTMHMM Topology Prediction - Predicted
Type: TM
MAASRSDEPFDPFKEFGGSPAPVAAPPTAATPFQPAMAQPTTASYGQPAQPYAPMAQPSPQPYAQPNQPFA
GVTGTYGGQPSGPQMSPSPTGMVPPAGVPGGTYGSQSPPPPLQQQQGGEIFAQPSMKIWTIEFYQQFFDVT
TEVVLNRMRDSLIPTMTPDYMKNHTWVAGTGSLADVATDGQDTANAVKPDLYGPFWICTTLWMLLGIVSNI
MSRIEYGRNPNHDKKWTYDFTMASIASLVIYLYCFGFSCILWGVMRFKSLPLSLTDTLCLYGYSMFVFIPI
TILCAIPISFVQWFLVLMGGGLSTAYLLTNFGKLWKAMLPAQWHLGLSGLVAVLHLLITLSFKFYFLNYSF
>tr|D0A1K6|D0A1K6_TRYB9 | DeepTMHMM Topology Prediction - Predicted
Type: TM
MKQEFVRHVDAYDPLSAAQPPVLETTASTLVSDKPPNDSFDVDVNNRPNVERCFSPNLLGQPCPVVSNGDS
SRRQATSGFGACGVEIHLAEPSVCSTRDSQVFAETHGSRFMRGIELLDRLPHEESRDDCQEEMASVFGAPR
NHTKPQPEQQQRLNHEELRSPLSVHSVCVWEVDAPRDNRIVITEETEPGWEQPDSQRDAALQQLLCDASKA
RGIKLTWLLCGLLLPFMLVLISMVVGTELLLNVKVAEPRKDVIKLSTRLTLSRLKDMYYDIRRMPQVSEVA
RASSKVAEKRAALDSVCSFLRGAPVVLATYSGVNASLTWTHNCSAGDQTDIALPSTMRLSDPTSPTPRISR
YRSDLLIVTEKYGPEADVFVVILRRDITGLMLLGHSLVDDFTTGISPLVAILLPSFSSSRLTVSLYNNAQW
LEDANPQNNPHDDEVLLLFEEWCSSGDTHTWHSVGRLLPGGLANTTVGEDVAHFPAPVPRLAGGGFFSVTP
PLVMCSTFCVDGTSNTCGKDNPSSVWLVTYGSSSYRGLDGLLTTAMVVGYTGAVLFMVMLCFAYISIDAPV
SYLKSLIFSAAGGTERRKEWDHTVHGWRKIWLGDLRALVNTFQILALCFRLNKKYVPQHILEKQVKNLLDV
KDKICSADTDGEAAVEVAEGRHNDDDDAVDDKANVGAFVCAATVADVKSSISKRMCQGSQSVTSLNRIQPL
CEVGDLQATDIAVESGRAAALSGAADGREPVNRGMILVENATILTVHLPAVETAYFTDFGLAVEQHRHIMA
LLLRSVRQYRGELFQRSGECISAAWNAFDGCADHAIRAAACALRILDRLEAYRRAGFRVGIVLHQGPFVCG
VVEDRAEAFTTVFGSVPRQAIVFSELAASLTVFDVLISEPVKESLSSHYECIMVDVIKYHEDDPPITLYEL
SKERQLPMTKGMPRGPSAFAEEHARVFFNFRNHEFGLALAGIEKMKRSFSKTELRLLWRIEQLCKYYMHHE
KDLPLPYYRRFPTWRIYEVTESVEGSNDFLALSSRGGTVLCGDIPPSVMAHKSSFDCDAMKFRQELHDNVL
ASRRTGSKESGLAFSAVKEADMGTGSPSPAREADGVNRRMSSMCLLTDAHSGNLESGLERSLGRPTTSLRD
TLGKGRPSTVPGGEFGTPEAEGASALNVAGRNAGSFDAMKSTNPQTKTCADEENYGDVELGVGDERRRFSF
TNCRPSIIAEGRRRSYNRCAESCVSLTSDSMEDAHSFSVVDPGGSLMATYTLPKKIVAKNGITYLRSSRIL
GKGSFGCVYLGMDVNSGRMTAIKFLPMPSGEEEVSKVETEVVAMQKVKSGHVVQFISYAFQSNLIIIIMEC
MMAGSLKGMLDAFGSIPPATACLFIRDVLRGLHKLHSNGIIHRDVKPQNVLLTLGGTCKISDFGASAFLSE
VVRREMEGNGLQIQGTPVYLAPEAARGKPVEQSDIWSCGIMFLQLLTGGLPYADHFLRMPPQVLVYHIGSA
SAKPIIPDDLDEFCLEFVQICLKSDPNERLSAQQLLALPVFSL

>tr|C9ZI15|C9ZI15_TRYB9 | DeepTMHMM Topology Prediction - Predicted
Type: TM
MTTTQGEVISTTFFSSKALLKSLQDVSRSLHISGNQFLPPNTVLLCAVGLGFLVDGVYTAAVWFRNRRALN
VARAVNHHNGPVKFVVTDELEDRDEEAKACLVQQYSDELCKAILGAPRTSPGSWARSPPADAVAEYETLPE
PIRLLWEDRVRYTLDAREVVGLLRRRLEPHLAGSRYGPSAALMALLWRLQLLRTVHESIVIAGDVGRCNAE
LTELGKHTGALYREFISTLHAPVRVPFDSCWECLSAEDDRLRGQARALQLRNLWSLTRYVFAGGLDKLCTY
GMVGLLTAFAARSASAICTIRVDVEQLLPNLFGTAASINTGGTTGDGAIVSWTAMHIVVARLLVSEWMLHM
LRLGITRVTQDYTHASAAWRRDTVKHRLYDALTRTPLSYFDKTERYAVEEIVYYVNDLEGVDVFVHDFFTR
LAQSGISLCVALRVLDMRSAVTVMGAVAGASLLNASLSFLKKTYCALSYCNGIEFEAVDSPSDAEEEENRK
DTTDHLMFCGMDIIEHIPELRPYGADTKLMEWWNNYTAHYRRRSCGLRRVFQLTYNSRYLWAIDALKPVAR
WLLPAIVAANAAGTCQIQVFLLEAIRASQDVLERVVDVQRVVDVVGYNAYKAGVLERILDSKNWEDGDTAE
RICEHTDKGNSSDTTALTCGMNGGTYKEHKEGGELRLKGNDVEVLAVRANGLQFRYPTAPTVDAFLKPASF
QFELRNRTTGLGRLVCITGVSGCGKTTLLRLLLGLYAPENPNTLLLEFRLRRHHVSESNGGGTGNSGNKEQ
WAPVELIPRAQLRSTFFSYVPQAPTIFPGATIAQNVSLRNRVSITDTLVLERVRVCTEAAGCGHFIKRLPN
GIMTPLCVNVGWATPDAVRLSCGQGQRLMLARALFHCSSVLLLDEPTAGLDSETKKAVMSQWRELLSSGLV
GGIICVSHDADVLQMADETVTL
>tr|C9ZK51|C9ZK51_TRYB9 | DeepTMHMM Topology Prediction - Predicted
Type: TM
MLQKISAESASGYNAEFDPTPPATVEEMHRQLDLIIEACKRLEDLKTSGVRLTHREVLKLRKAYWLAVGET
QKLRRDNSSRTGSLDGLETSETSIVDRPLSSGSSVQVNCSDDMGLKRQDAKALSCDEELVKLEKCVEERPE
AHTFDVVHDLAEAMNTGWRIFGIPLERRLQTLAVSVFNFFAYVSLSLMLIVVMMMNQVTMVFVILYVLYIF
TIGRPKHPLKKKDALMSLGIWHHFTNYFPVRLVVPQKVRCQFDSSKNYLFIYHPHGINSFGALSCFMLDTM
NLRTILPGIRIHLQTLKLNFYIPFWRELAVAGGCGDASAQCIRDTLRKGPGECVALVVGGAKESLLARPKR
NEVALQDRKGFVRIALQEGTSLVPVYGFGENDVYRIPRFAESNAWRRVEGLVRKYTRFAIPLVKGRGWFNY
GFGLSPHRCPITVVFGEPIEVPRIPEPTAEEVQIWHAKYVEALQRLFSENHTVFAADSKGLVIR
>tr|C9ZLG5|C9ZLG5_TRYB9 | DeepTMHMM Topology Prediction - Predicted
Type: TM
MWVIGCGFPFPSLFSFLLICFVFFLLFRLACWLMCYANHLLYCNTKAEVGKKKRKCGKVKESVFFLFFFLF
LLPSSVAKKNFFDSEREKIKRKYKIKLYVIVFFFLNLEDPLSLSLSLLLLLFSMCVKVFFVIDFTFPLSHI
HASPLSFSTPFTCLFLFFFIPFILSLHYFCNSCFKCRNVVCSSLHKHGISHSFNLSFFFLFSRRVVQSSLS
TYLFISLFLFFFLFVVSLPALYYFSCGW
>tr|C9ZSU1|C9ZSU1_TRYB9 | DeepTMHMM Topology Prediction - Predicted
Type: TM
MSAFDYEKAKRRQNHLMQQTFPFHTQNHPIIHSYTYIHIYIYTYIHIYIYTYIHIHIHMYPSTRRLSHLLF
SLFCSFFSLILLYSLFRVFVLLWMTAKVEG
>tr|D0A4Z7|D0A4Z7_TRYB9 | DeepTMHMM Topology Prediction - Predicted
Type: TM
MKGDSPCTSIIILKPVGCRGLIFFILHKSTDIYNFYKCLTLILRFFVVLYPSCTPATCHILHRISCALPYT
CPFSLCMCFAFVYVHCRSCYYYCSYSLMRFLFYRQRYLEFCCCCCRKYVIFPFPEIARL
>tr|D0A625|D0A625_TRYB9 | DeepTMHMM Topology Prediction - Predicted
Type: TM
MGAEKKKKTVCICARVRSVFKKHLSWKVTHNIILVLLSDVVVNKLLHHVCFFFFGGGGGGGGGRFAFFFSP
FPSLFLCHRCCCVSACLCKWCGHSNVEQMYPRHFFLFCSSRYLRLFLSLYVYNGLTFQLNRIVNSILSLRL
RKVFIYFCK
>tr|C9ZV97|C9ZV97_TRYB9 | DeepTMHMM Topology Prediction - Predicted
Type: TM
MNSVPHNIIPSLLTPPAIPQSLLSPFFSLLFPNVSTKGTDSFSPFFHIDITPFSFMVSLPRLTLLLFASKY
NLPLIVFYPPYVPFPPPVVSYLLFFSCLLVHFYLFIYFQLPISF
>tr|C9ZT74|C9ZT74_TRYB9 | DeepTMHMM Topology Prediction - Predicted
Type: TM
MFQLRSCVSYSLCYVQVNQRRTMMGLFEEQAPPGSGENKVSTFARSLDQLEAKNTRIVKKACGIPFHIGEA
EALDAVCRHHGATVRVHSVERYMMPFWLAATAAGGSFRAEILQRDPAYLTQQHCLVWVEGPNYQFSYPFGE
HHPSNQVSASYVHPLSLVERCVVGTHVPSMLISRFELLKELEEMEVHPKIIPFAMSTATALTILDSRLTRD
TVLRRIDQELVKFHGSFVRSNVTLTGIYKESISIRPVFLPMLRFTVTTGNSSTQCPTFVCGATGKVAGPVL
HLTKRGKSGVALLFSAVTLIASATVVEPGVATTASIFAAALSLRVQQFLMAMCFLREQTNQMAELKTAGML
HFTSDQQGYRWSPEDEEREEYEYREELRRQARKKESFEQRVKEEAARDEARRGRHVNPKERRRTDLENVDP
LGYYKLLGLSGREFSATSKDVAVAFREAARRHHPDVSNTGENDEISRSRMQKIILAYKILRDPITKKAYDS
GKLTSIQYDEGG

>tr|C9ZNV7|C9ZNV7_TRYB9 | DeepTMHMM Topology Prediction - Predicted
Type: TM
MENNDTTPSNANGTGGDGGVERAMTWEEYRKKYVEEQRRRFRLHQQGINRNDGEGGAISETNASAQASSLP
LQQPTPQPNAPEQQQQQQREEEEEGAGQPVPQLVRAALRRGLTHFLNEETVIQGLRVVGRAALASVLVFR
SFSVYNVFVMLGIYMGWCLLKFMFSSVRVEGSSERKRNQGRESVQPEGINPEGTKPQAAQAVQRVTPVRKL
LYVITRCATSFVLSISPTYSVEQLEAELVADGIVDPHLHVD
>tr|D0A623|D0A623_TRYB9 | DeepTMHMM Topology Prediction - Predicted
Type: TM
MAGTQAAVDYTKVLVKNTVRFILLVLFLISLVVSLLLCMLADIFQAYAPVFPLWPMVFASVALNVACAVST
SLYHRVTCKASRSYKSSGNNHAYLAMQGFGYGLLFICPAVLFLYIELLASPVAVYRYGLITALGGSCAVAN
GLLIASLRYSEKTQRRVKEGGLGGGERPQRDSGLWRRRLLLLPDGETAIVVLLCIIALTCSIIAEYFTHLR
RALTIVGMLLLVVSAAITRVGTGYRSATGEHPFSISRCSFRTGIVQGLGWFLGGATLCFDLVLCNSGAGAP
PSWHIISGLSSMVSVAALLFARFHRFTEPTFKQGLQLSSDGPYVMTATSLFQLTFVWCLTLLLSTEYCELG
MQLRQGLITCQTLTAVSMFALPLCTHFLGRVVFGKEYGIWISLDCRLEFAILQRLAWFCYSIAIFFAVLHM
TESNHFRFVVIQALLVGVSQCLVHASLWAFGGGTLGRYSSDVEDQESLSRTTSTKSTAQEEDSEGEASVSY
EGGGSALPLVLNAEMVTAIAVCVCGIALRLVADVESMDGAFVGALLGVPRKSLLNLARVMATVAVPLAHIS
SRDRVPLWQPFVGCGGYVSMQAVGWSVYAINTLIEAANYFHEERRSLASPVFGTQSMPLEHTVDGVCATVP
FICIFLGSLFETWAQQSEKIHQRKMQQKIVELNSLLQHVIADPEDRERTKSLLKFVIGPRWKDLRSSGSSD
EDERAEEVETRKEGMRNIVAILCVCVMMLFATSAFSATHQPAFTLIFGISGMMMTSVSCFSLQLFYGSIVH
GATGTYSYFMPFSGGQKFVAFQTAGWSCYAAALLLILISCLEGRGSPTAFVCMGFFSVAAQFLILNSIPHF
DSTPRPASLLEQNAEAALAVFALVGSFTFGVVWNAYTGGGIPGQAPSPLPIIVTAIAASCAAPLGIVSLKR
HMERSAMFAVADGLESSSEASDGEEEIRSDGHTDAIAHVSNGSGHQFPSSLIQGQVNTGVDSERGTNDSFD
TQTSRSFPGSTGSFGSGKRCRSVQHMEVIPGTLYTICLILALLATLIWVIFVPIVLFYMFYSYAYSGLAVF
HMTFTTFHIVLFLLSIAVVTPVLVQIIYDRRCKGLRGKFWSPLVAFTVYSLPTIVVSTVLVVWTQVDTMGA
EVFALNMVFMSCLSFLSYAYLVASLFNTCFLGYVLHFYLYTCLVQGLAPLVVWKCVTDVGFTVFWLWYLRG
YGKLPHITGCMCGAKSRDLFRTYLSPAIVDYFSARLIVDGRGKAGRGKTVIYSNSEGEEENAGPDHNDPSN
KYIYSFHPHGVFPGTALWLPMSPQWEELIGRNEETIVTTHGADVIFAVPFMRDALMSVGTMSVSRKGIENC
LKQNNSPIIVTGGMAEMVYQKGSDTEMHIVMHHSGFVRMALQHGVPIVPILCFAEQNVMMNVPFPRLQRLT
SRKLGFPFPTMPYGRWFLPLPHARPLTVVVGKPILPDPAMCNADDPDHVVHYRLRYFGELQRLFFKYRDEA
GYPNMVLHLHCHNETHIVTEFRGSESVKDDQNQ
>tr|C9ZL27|C9ZL27_TRYB9 | DeepTMHMM Topology Prediction - Predicted
Type: TM
MLPSAQEVVEVRSPETVKLDLVPDDATAGPEIAGAEHHHSEDSGAAMSHSEGDHREDTNGAMVSDTKSSAL
DLMEKPEGDGSEEYRKPPAGTDSDTCDTAAEHLFSEEDGEEEGSQQEERDEGSYEDRSEDEEEVEGEPDGE
GSDEEQDVMEQEEDYGFVLPPETSKDAVAKVESFVINGKKYTASSFVHNAGFRVLRELKGKKARDLVICAS
RKPHNILSCKNLHLTNRAVCVKDCYLRLNTLVNNPGKEALLVDPTSAATALFCERTDADHDQSACTALHLM
PGIVYVGFPPLRVGYFEGELHKNIALTRLQEGSDTCGTWCAGRRSHIVVTCSFVHYNRGTVYKEAPSPPAL
PTALFPGMQPREHVGRGRGRNPLGRGRGARGRGGLGRGRGCGAATEGHDRSNNARGRGRGASKELSAGRGR
VRPVNVVAAQPPLSSAVVCEEVGGVAPPPEVAITEGGMRAHGVQFLNFVLVLLSVIIAVLVYMLM
>tr|C9ZNR7|C9ZNR7_TRYB9 | DeepTMHMM Topology Prediction - Predicted
Type: TM
MPLQPSYLMRPPKACSVYPTCVFIFWEAPRAVTQVDEVENITGQLRPSLVQWSYVVEWTTNNSPKTEVAEM
FTASSHAVLELLYPQETLQVRIVARGSCDGSEITRVSTSCTVEPGLAVALPWLHPRSMRCLCIFDALFFSY
SGIHCVDAIPSSWKHVRAKMESVRLLESRFSCAIVKAVYEEPPYPITSTTVYYFVMCPKALVNPRDKLRHC
DIKYLLERDQEQKPCAVFCGIGETGSSAALAAHLFISAIPPSSNELCSRVFCIAYGSPRRLLVEDYLVLAS
SPPFAGNFLHYTALLAPKDPLFEFTGDVLNSDASVGVSGAAAGGGSPKEVHAVQHFNMPLGLRCGPVFDGT
SGNVHLEARCSGVETLGREECNELLDVSTHLSMLNQLILQCYHSGDTGDILVPMINNVQHAMEDGVTVFLT
IEGESLHFGPRVICTSSRNQAMAAVSVTAVTPTRLTATFSLMDMLTTKFFARHRRPPQSLLVEISLFTDHG
YVSFNEYRIVIAEDISLLLFHSSHSDGPHQWIFGPPGDLVDGAISVEPLLTSLVAGGPNTSTFVPFQSTSL
LSDIAAVSELVGKHAVKKPETGLYNFIFRGAAQSTKPISIGSIPMHEAVLLKDALKRHAASETRDPNALRS
ELDPWKRRLLARLPSLDSGNYREKLLSLLLMLGGPKPAADTTAVALETLLLSKVLHNLRRSQNAIHLNYTN
SLSTCMGFDVFYERMLPLALAWVKHSEEAELEALHNIATLWIACLLYHLRGAYLFTHAVVVAGCAGSGCNT
LCHAIVRERAARQHHFVGTQRERVIVVRRAKLGSLADVKEALARGLGITAIVCGELSDVAGKEFFELWCSL
RRMLCRRMAQRLFAFLSKVDELLGRNQLIKPLEEACIGAEGGAVAFQSLQRQAALAARTEWDVGSEKRGED
ASLAGFVAVSFAPSVLLLRNSPFVHSSGMSAEEFAQKLMSISSYQLRRVLAGIVEGEFVNAR
>tr|C9ZMC4|C9ZMC4_TRYB9 | DeepTMHMM Topology Prediction - Predicted
Type: TM

MICVVHHTPPAYHSSSVTLSCLTFPCFFVTLYPSRRSHMETRFFLFLSFAVRLLVHRTLPALVRYTFICFK
IGFWKEKNEGKERKERGGHLKITCVYLGKYIYLYMHVSRHAYTFIYQTHEMKEKEKKGNRSVEFVCLLVSF
SFLKKK
>tr|C9ZHY8|C9ZHY8_TRYB9 | DeepTMHMM Topology Prediction - Predicted
Type: TM
METPFGEVIDKPYTVFIFLLHFIFCCIFAFVLVDGSLGRLVIVRCRAFIYITPVNKYMRIFTVSVVALLSS
FSCPQNPVANIGEDKILFCFLRFFFYFTALLIVFSSF
>tr|C9ZT06|C9ZT06_TRYB9 | DeepTMHMM Topology Prediction - Predicted
Type: TM
MNVCVRVGNKRYIVMNECMSAVARVIVSVGLLAHLMKHGESLSEAEWRRMRRIRLVILISLLFLPYFKKEK
KLLASKLALEILVLVILRAMACRGNGFDVTWWSNCGFCYLSRW
>tr|C9ZSR0|C9ZSR0_TRYB9 | DeepTMHMM Topology Prediction - Predicted
Type: TM
MPITSWCGGSGRIHFSTQHQHLCESSTKAPPGEGASCRPSLANPKLTNLISGHRPQIPTVGSFPYPREYVG
SAAHHNVTKWPSPNRTETPAFSSTRRTSESSVLSAQFPRKRGTVHHNTSTCHHPRLRPRKIPVMRAFCVCV
CVCVCVCVCVCVCVCVCVCVCVCVCVCVCVCVCVCVCGFVEVCCGQRKMAGFCSYSE
>tr|C9ZSA9|C9ZSA9_TRYB9 | DeepTMHMM Topology Prediction - Predicted
Type: TM
MLLMTSFPCECWCISIFSLFMIRYYKLFLYLFIKCYLILVNTIPKYLCFLSLLLSISGVVVHTSLPRLNHT
TINSINIYHLPCHVTRKGIPIGKIPCNVCHKFL
>tr|D0A2G6|D0A2G6_TRYB9 | DeepTMHMM Topology Prediction - Predicted
Type: TM
MQGMIQNKKNKEGKEGSFFFVFFSFLFFPHWRGGGDDGGGRLAKYACGATRWTAGFGRLPYFYTHSLLYFF
FFCFLLAFFFLRFTCVMLFSLLLLLLLLSFLFGCLSLSYLLNDTSLFDLCVVHLFCVCFVFVFIRINILLP
TFFLLFRFAVWLRGDLVFVFFVCFSFVFSFLFCLFFFSPFPTVGIDACGFCVRASTAGLCESAFSLFALLT
YICASPSPPLPSFFFFFSFSFPSFCLCFVCT
>tr|C9ZR37|C9ZR37_TRYB9 | DeepTMHMM Topology Prediction - Predicted
Type: TM
MCTLLHFSSTFHSDLRTATLISFSLCVAQVCCRTSLSISNFQFHCFLLFFLLFDVLKTQKDSTWQCQFLVL
CIDVMEHLPTIDSRVETLRLAFGSFLRLIIYSVTYYTGRGLFLQYVLRRRGSVSQSKQSMISYVASQGLEI
GVSVIICPVRYLAAVTTPRFLFDYTLTGWSEILRTLDLFSPGRFASYAVYAFSSTSEWDLDFFVWQMPSVA
LTLAKLWYRRRHLGTKKCCTIRVLLMLPLQILLRAYLSSFSVMIPECGEELLEAIVSAGLEAGVSAYIIRN
TSPFVEENNKLRLVGTLKHHSIGSQAGEREDANKKQE
>tr|C9ZYN7|C9ZYN7_TRYB9 | DeepTMHMM Topology Prediction - Predicted
Type: TM
MHTLLVTTNIFALCILSVFCSVVLFVLWTSATSRTVNSLPQELLLGLGNVDDILTAITLKDSLRNMNMANI
ICFALLILLVILLSLVEGVLIRMKSDLEECAKLPPVTLVQHMNNSYRAGLENSGLAYREDAKG
>tr|C9ZKR2|C9ZKR2_TRYB9 | DeepTMHMM Topology Prediction - Predicted
Type: TM
MPPPALPAIRHPSGLQKPPLPAIRKPTMDDGVARMLETGNLSADVDLEEFFNKEGVFKAVKAPVHPVKERQ
LPRVMTKTSSYARYNGVLLDIEDIRKIPPKLNLQDLREWENGVDDDDDASDVTSSLYGTVLKNAGEIMTHL
QAPQRNDTRTYVELLDEYSMHEFIIRKGMTLRNTPEFASFKRRYNSCWGNIEHIVVKLENFLKMYNVELAF
VDGRKVAELAAFQVDDILKREDLLQCFANHEEVEEMTANVSQLYAAADSGRKLAVEKIQATWRMYQQRIAY
RHLLDGKKAATTVQRAWTLYRLHCTTRKTIKALRETRLLRWKHTMEEFIRKWPKIRDGPRRIIHVPSLSYP
RFHAKHVPFYLLFQMGQIIRVSDLKDPNVEIILIVPMRPEPEILEYYYSMLASAGVPDVKSRLTLLVPEEA
KRLPGVLSLTRLMLLSSRLMKCLASASKGRDTYIVPGVVGAEELSLATELNVPMLSADPNIAQAFGTKSGG
RRLMDLAAVPAPPGAHGLNDRNDLLSALALLMVNYREVSRWLVKLDTESGSRGHAYFDVNRIHVMAGVDES
KKTYENVLAELKQHAGKRARLLNPLSYSDWEAYLNMFDVVGGCVEAVPNRIKTSLTANLFVEPSGVVAVNS
VVEPMLAPAFTVIGCQYPFSKVVPYAAVRDAALALAKAAYHKGIMGYMSVDFVVTDVSENSLPRLYAVDVD
LCLTNNAAAHSFACLVAGCEWNAEEGTCLFRGTANSLSYAYSGLIYSPLLASVRHKSFFTLCHRKGLTFDS
QLQSGIVYHLLSIITCGCVGVLSLGTQRNVVARKMKEFQSLLNLELPKQGEHTGESNIMYFYSATHQLVRS
VQVSEMK
>tr|C9ZMP4|C9ZMP4_TRYB9 | DeepTMHMM Topology Prediction - Predicted
Type: TM
MGFGAVVSYFGLIFLLTVFIASGVQIMMYPTSGAMMLAKSNFPKLLEMAGIQYRLSVAEYTMVVQGIGASC
IGFSLLMLLGVGRSFFAFLLAVLVSLLTLAFHIDLRAPTKMSETNAFQVLKNAAIFGGLMFVAGSSGRSRA
APRAAANAAGKEKKKN

>tr|C9ZIU0|C9ZIU0_TRYB9 | DeepTMHMM Topology Prediction - Predicted
Type: TM
MFIFHLPSFPVGVCLSLYVFVPRKRGAAFFFFSAAHRGVREERIRGNRTVW
>tr|C9ZII9|C9ZII9_TRYB9 | DeepTMHMM Topology Prediction - Predicted
Type: TM
MSLLLRLCVSLKELRQGFHAAVFHAKINAPTRLFIYIFFSLPSPLFMSRTHFTPNVIIIIIVVVIVDVATI
FSVPHRCALLCC
>tr|D0A471|D0A471_TRYB9 | DeepTMHMM Topology Prediction - Predicted
Type: TM
MAKGGKRVVARRRRVRLYLIVIALLIVVGVMVTMDIRSSHTETGHGLRKENFHSNYAPRVSYVVVQTRRSP
GWCRMLLSSILTNVSVTTVGMGAVYIHAWRWTWIYNYMLRERMHEDDVIVIFDGGDTFFTETHLREDAMKY
FLATTPSTPEKFNETEILQGAMTPPMLFTAEKGCYAPQMYIMTGVDPSKIPQRERCLNVYEEAFEASTRAG
TQAILRKHESGRWHLNGGGVIARVWALREALDVFFALKRQSYKWWCDQSMWSLILAWSVSRPKHVQPVLLL
RRGIMSLDYETRYFHYPHTTPIRTGVILHFPMPIVLWKNKMAKYVGETTWFRALRDSEGRQKTVADYLKTV
YVDIRFVFGFKKWRKFSSVCSISDVVNPNWLSGVLRK
>tr|C9ZHZ5|C9ZHZ5_TRYB9 | DeepTMHMM Topology Prediction - Predicted
Type: TM
MRKRNVGIKARPQGAVILSNQRLCLVDATKKGGKHLWAFITVSVLRAALFHIFFFLFSH
>tr|D0A7K9|D0A7K9_TRYB9 | DeepTMHMM Topology Prediction - Predicted
Type: TM
MWIGIADFFFSFTTPFLCYFRTQRHAPTRTHICIYIYIYKYIHKHIQAHARVIDPFIQCSIAGTVIPPVCY
ICSPLFRVSLVFLQFFLLFFSFFSPLSVSDKRTCTHANLHTHQIFPRAFSRFFLSFLLGR
>tr|D0A7A0|D0A7A0_TRYB9 | DeepTMHMM Topology Prediction - Predicted
Type: TM
MNDYRLCGGCGAVLNCRPASRVVRAPPLVASNLPRPFTMAVALLALLSVGVRVAAGAEVTVNILYLMYNPK
FPKVSVDALSTGFEASLAARRGDIPNGVKVSVIRPSSHDQPIEELFESAVEASKGKLLIAVGPLGNDNVLW
SLEHLKNNDVVAFSPLTYSDEARGWNRHLYFTTAEPDAELLTLIRYFAVTLRLSRLGFMYLSDSFFGKESH
AFTLKILSGMGYELSGTFSLEGTGGLEVVSTAFDAEWEQFVNTRPLAVLLLGSPNLVTREFVRRMATDDRT
TGVYVLAPSSAQVFLINTWRDALEESGRELIPGQLIISGVNPLPNHARFAVVKRFREEVDEYLSSSDEENR
FAMRQELLEDDTSGELMLTGWITGEVVSRALRSTLSLTNSTAFIDSLYEQRRYLIDDLVVGDFGGDCDAFA
AWQGAVCQCNQGGSVVYMKEVVDGFRLQPVVTGFLTWGASECSSAGVVLRAPLNGLIVQLMDNVVVYRASL
RYLNGASALLGNGRIGERDRFFLHPLEATGDEAVEQLEQMRDVKVIPALFGVVNKEIMATENLAFIDPITS
NPRMNRFNRNVIYVSPTLAQELYVLVQYISEHPGGNVRVVIRTHDADMILEVLVATLRTYGIFMQSKVIVD
NEGSLKPHLPSSGDVFVIGFTALEVYDLAEHLDAKRGLRIFAIYPDVTLMYEELRAAFSRTPPTTTNRFVF
ATNQPHWAEENSSSLTVQAYHKAVPEPAMRTPMSLRGFSTARLMVSVLDHMEKVDAKLLADYFYSESTINV
DDMRYGPFSDVDCIVNGVALASNCLSNYGGTNISVWSMTRLLDPATPPLQNGVTPTLVYVDENKLTQGQVI
AIAIGSTLVALLLAALALVLYFTLRNARDNELAPREPTEPVTLVFTDIESSTAQWAGFPELMPDAVASHHK
IIRSLMVEYNCYEVKTIGDSFMIACRSPFAAVQLVSELQRHFLSHKWETEVFDRFYHAFEEQRACDDKEYT
PPTARLAPDVYGSLWNGLRVRVGVHTGLCDIRYDEVTKGYDYYGGTTNMAARTESVANGGQVLLTRATYLA
LSEDERKEIDVVPLGLVALRGAPQPVELYQLNAVPGRTFAALRLDREYYFDEGGDGTTITTSDHSSSLADL
SESAQMIASSLEALLSTFKVPQREKLLRPYCERWRVPLPNQANPVWDDVYCGDVIRRIAAKVGRVVDHSAN
GNNELTTSTHSTSVIYISNRCSQLDECFSADQPFVSV
>tr|D0A1S4|D0A1S4_TRYB9 | DeepTMHMM Topology Prediction - Predicted
Type: TM
MENWNPERFPTSQGGARPVAKGRIEKWGTPCHFKGISSGGSWWVLPLTRIASAAVEAVRPVWSGGFFFVVL
PPVPFGMCPPSVEKSAWARSVLFPGLELLRCQRVILGSSAARPLLYLYDVANSSVPLCCATCFLELRCG
>tr|D0A5V7|D0A5V7_TRYB9 | DeepTMHMM Topology Prediction - Predicted
Type: TM
MLRCSSFWAALPKVERRRRKGQQMTRSRMPDLPKSAGGYFFRQLRPYLPPAWQYGWRWKLLQGIWLGSALS
FASYLVYVMYFRDSDSDIAQRAAQYTYVRNEHGQVVDIGYKPLIDAAHRARRRAQRLLEEECEE
>tr|C9ZKU2|C9ZKU2_TRYB9 | DeepTMHMM Topology Prediction - Predicted
Type: TM
MSISPAENDWFSFLLDQSWVAFVILGVFLVGIKIYNMRQREEGRKLLTEAVKLELLCQQIRRSKAQSERGK
KDA
>tr|C9ZRL1|C9ZRL1_TRYB9 | DeepTMHMM Topology Prediction - Predicted
Type: TM

MVTCCQLFLCYYYYHYYLFICLFVLAGRTFVHALASVNDCVARNSVGCAKGKKKRCANNSRCCFFFMLSNL
SQILWEFMCRSIFSPRCSPIEMYRNAFYMSLLLKSSSFFSFFLLFSFVSSLACLLLFSFFLFWFSCLCDLS
PLRNCFLLVLTVIYPVPSTKKNTTNKYIYIYIYIYINND
>tr|C9ZXZ3|C9ZXZ3_TRYB9 | DeepTMHMM Topology Prediction - Predicted
Type: TM
MRPQNFLLLFLFPIFSTVGLVDFCGHFLPVTVSGNAISDFDCWLCLCPWFNLHFPSFSSTTLITTVWYLQG
LMGPRMAGFLLGSVAAASVAAFLVQYDVLRRKDITEREIDDMETQAALVKDRFYRVQSRLLECQGGDE
>tr|D0A599|D0A599_TRYB9 | DeepTMHMM Topology Prediction - Predicted
Type: TM
MQRNEWNGLRHDFPIHFTQSCFPYLSPNFLPLHVPLLYGRGNNKKIIINNVRMLRNPNRKGRKREEEEKKR
TTQLFAQSIYVFFFFFLPFFFLFLFVSFPSPVCYFPPPLPFFFFRIILQ
>tr|C9ZK31|C9ZK31_TRYB9 | DeepTMHMM Topology Prediction - Predicted
Type: TM
MYDWPLYSLCGVYFRTFYCILCKYGEFISRLGGKRHAFTHILSIFFLFFFYIPFQTHVLLLLLLVRRIFCS
FVSSSFFFFLLFLFKYLRSLSFPERFSLIYFFVFLVYQNHHIYIYIYIYIYIYIPS
>tr|D0A8Q5|D0A8Q5_TRYB9 | DeepTMHMM Topology Prediction - Predicted
Type: TM
MWCTVEGEETWFMREQCGKGIEVSLNTATCLASPRRKMHLLVVPRIHTFSAPLCMRTFDPVAPNVEKKKGR
KEGRKEESRKDRAGAVSCVKGRNICLAFAVSFIWFRLRYPFFTLRGLVVGATISIFPSQWMESFSPFRVVG
SVCGELPVSSTMLYGAPQLMVATGRTFQLFRGKELTMLRGGPHFEKRVRAVTQAGKYRFVAEGPRIHAFVH
HKPLWNCMHNDVTQSRVDHILAVDDILFCVGNDKRVVVREIKSGNMLTEFLVDGSEETRAMVTPAGYNNKL
LLATAQGSLQLYNFKSGVCLWREKREPGAQITALAASNFKDIIAYGTSNGRVVVLNMATNEDIMSFDQEER
GAVTALAFRTDKDVLVAGSSSGEVAMWDLENGCLDGLLTRGKQVKTETEVLESPHTNTVHSIVVLPTETAT
IVTAGADNALMQFRFDTVDGLGVLVRERRGHMGSCTEAKFYNSDLLLTAGTDRSLRVTHLFSDRASWELSQ
GQLGRRGREKQMGREAMKMPPAVAIASCTARNYQWASIVSIHESSSKMCGWRMDTRSLDCKLSGIKTSMHI
ARAVAMSDCGNFAVIGYSSGDVSVVSIQNKGVRQLLDSSLKPDDRAHCGSVECVEVACGNNIVVTAGLDSM
IKLWSLFTCRLRTVVKTDCPLHKSCIHQPSSLFIAAQHFSIRVYHCNPDIGLTTQELRVPVRTFSGHTSPI
TTLALAPDSYRYVVSASGDAALLVWDLASSACVGQYRLASPAISLAFHPDALFMVSTHAGERGAFLWSNNL
RYGFVPEVITDPRARSVEELPQLHFPTAHGAAEDAEDDTVDNAVKCEKGLPGSGNEVKEEGESDGNDDGML
GDIAGAGGNAEQEVELFDAKKDTALMQIEREKVKWAELDEIISGGMRLAGVPRSMWFDLTLLEQIREKNQP
LLPPKKRDVPFFLPTTQELRPTFLVAASNSKDKETLVSRVVSAPIAQLTPVQQMLVRNEHDAFLSYILSLG
SPQAVDLEIKRAVDYVEGCSYTEKELERIKDCLHGLLSFVAEWLKRRENVDLVQGILAAVIHSHGPLITKC
GAELINVLEELAELQNSIRYSVDHLVGYPSCLAGTFSGSHF
>tr|C9ZYT7|C9ZYT7_TRYB9 | DeepTMHMM Topology Prediction - Predicted
Type: TM
MAQGGFTLSSRWVSRLPEFARRAFQYDQMELDSALAQMYSLCVKPSLISKMSKARKMTKNHYHRDDPAFIV
LQIFSLVLTVAAYGLALRGGLLQILYNTLYSVLLGYFVAGGAIATVTWLFANHFLAASSQPHESGWEVDWR
YSFDVHCNGYFPYFIWTKVIQFVLLPIVLHNSCLPRAIGNCLHTVGLVMYAYVVFLGYLELPMLAQQQRLM
YPVPLVVVFMLLVTLFTSWNVSYWSLCQTWCS
>tr|C9ZK41|C9ZK41_TRYB9 | DeepTMHMM Topology Prediction - Predicted
Type: TM
MSTPRLQYPPPTGRKVKQKAEIKTKKKKKKKGKQRQTKEQEKDIKESKNKKKKEINIKPHYPIKTCDRNFS
DTYLCVGLSVLICVRVFCRWFKRTIATRQGAKYNTNEYTERGRKIRNQTERYTYIYVYIYLFSYLVINTNN
TVNINVLKIPVVSNQVVEATIVPKILEKKKTQKLLKIWHLLPFFSRIKKKMTSFTVTSVTHCSFSISSLAS
LFLPETFYV
>tr|D0A9K5|D0A9K5_TRYB9 | DeepTMHMM Topology Prediction - Predicted
Type: TM
MTYKHMTNHRAAKNNKNGKEKSHKDEPATVGTADASPVSPHMGRSDGVEESPMRHTSQSNNTDNNMRKDVT
RSPNGCDCDEGSEIAWKCQSQVERALAMFLFKGTARSSPADDVPVLWDVGCDALVAAKRPVMYLIDFFTRR
RVMFVFLVIAVLLGYHNFQEEISSLTDSFVVADADRPGVRFLQNHTMRRKHPVMIIPGFISTALEVWQDVV
ECTTSQAYSSRFRQRMFGPSMLFLLATDPACYMKLFSLDKGTGFDPPGVKIRPDMGFGAADFFMPGYWVWA
KIFVNLADIGYDPQSMGISSYDWRLSPRGIHRRDGYYYHLKNYLMYLYHKNEERVVIVSHSYGSLVVVDFL
RWADEHEAGWTNKHVANWINIGGTTMGVSKTVSALLSGEAKDTLALPGTARAILENYFSRNLRTETFRTWS
CQAAMLPSGCEGVHPQILRLHNGTVLPPKEAIRLLTRRLNESGHVAVVKQAREVLGRFGKRPNLPKAPNTT
VFCLYGVDRKTEIGYVLGEDEAVDDTYNEGEHIVNGVINGDGDGTVPLLSLGYMCRAKNGWKRDVGRVITR
EHKHSSGSSMNLRGGSSSGDHVDILGNHELVWTILKVVSGNAEEGELSDRIYSNIDEKIEQSGDCLLMEGQ
>tr|D0A6X6|D0A6X6_TRYB9 | DeepTMHMM Topology Prediction - Predicted
Type: TM

MFTVSSSMRCGTAYRTAADRCQVSRYHQQLVDSRYWNCSTPSQALSTLSVLARSRTVVDALDVAALGALEA
MAPSMTQGERTLLQKFSTFLKFKNTTCLPPASPHESNASPLTEDPNANVVALYTKLMGEKPSVGVEEIGSI
SFSSDSILQLSTVGALMLFEMMVSTTFQPCASWRIEDNIVTLLNYLRNTVIRGDTVDSRTLGWIMSKLNSA
GSPIACRPSECGRLFKACVKRLNDILPQMSVNECLQILPLIDTTAYERPFIVCVEIVKRLDACSEIELSDV
RTSTLLSALRCEDVTLKTFMKICRVISKEFRIVELSKGESLLFLTILVARLNSSASAEDVGIIGSNGKVWE
VLFAQLYVDTGDMSVVECIEALMCLEVLYFSPLITAVPGGLVEKLKKRVFFVIRKAMKQRHVTAQEVELFL
KSLQRLGELLKRCLLFPLLEKDTAIITELQAELALRLVQQSVNTA
>tr|D0A7D2|D0A7D2_TRYB9 | DeepTMHMM Topology Prediction - Predicted
Type: TM
MGQCIEESSYDEGCSPTCGARNPETTQEGACASRTAEDEANAWARITARMRDAGENEGPTKLSALDSPLAR
GTEKANSAAEEAALSPLNHRISSHKARGGRKGGLSPSSHHSRATALGSDRDTGRTFPSSMASVVPAPLPPI
EGPLQLAWPPNDRMKGSTVSSFCEQHSVVRPPGGSDRRNHGEVTFMNNQLNAQHGAFSSQKINTSDRGYSE
TTTNSSDGSSESHAGCSADGIAVPQWSNPNQTPSDGALRASRDVNSLCSDSPADRVRNHPPCASCCVDRSN
PDSWRYNKPRRHAFQWPLHSLQIMAICTIVTFTALFVSSVVPGYVLLYRDEGCSECLWEVIVSSTLVLTSI
VCTSGLMLVIAFRENGDINDEGEPCSFCERRTLLDSRHCKACNKCIEGFDHHCKWLNMCIGSKNYRLFIAF
VTSALCSMVLGLIAAVVFLAKWWNRLLPYSVYFRAGPLLFCALVLLTCVPLIHLLGFHIMLNRANMTTYEY
IMSKRQVSQPRQGNVLPAAAAKELERL
>tr|D0AA58|D0AA58_TRYB9 | DeepTMHMM Topology Prediction - Predicted
Type: TM
MSFVFQISSCPFHPRLQFFFYLLYDNRNIIAWGTVYNLSGGLHSLPSFLMIVQLFPRKGPCPLVVVSLGLL
LCSSHIFDVFTCLYFAAIFVDMWATASCICLSSCCSYSAFPLF
>tr|C9ZPA2|C9ZPA2_TRYB9 | DeepTMHMM Topology Prediction - Predicted
Type: TM
MEKGEATELNEKKNKCSFNSLMAVVQLVTQMWRCCCALRLQLLCYLFSNTVVVFSTNLVIIYLYVCIRLCF
YKLERRIMRVPLRKRERKKEKDKCEGKFKKKNIYI
>tr|C9ZSX4|C9ZSX4_TRYB9 | DeepTMHMM Topology Prediction - Predicted
Type: TM
MVLRGHSYSILLALSLPISHTRVHYLRVMQLHISLYRRPRYTACHPWLVRIRKLVQPPIIRFCRRVSLTYC
SRSYSLIKGTVISADSVGSPVISIIFVGFLLLMKPQCVYVCLGARKAKVEACLFEGL
>tr|C9ZTR5|C9ZTR5_TRYB9 | DeepTMHMM Topology Prediction - Predicted
Type: TM
MSAPVDNVVVERLSTANQKPINEPRRFALLVLGTFCCICTSFMYAFNLISGAMQARYDLTQRDLSTITTVG
IAVGYFLLPYSFIFDYLGPKPIFVIAMTVFCLGALLFALTFQEVIEGSVVRLSVYNGFLTLGCMLFDLGSV
VTVLSVFPSNRGAVMAIVKTFTGLGSAIVGSIQLAFFSKSVANYFFFLMSFSLVVGTLAVVFMNLPPFHLT
GYQKTHLDEEEKAQRIARKGVYLKQKAPMWRFVHGFAILVTLIVFLPLQGALVAYLKLGSNFKVGFAVTVI
VLTVIFPFMAFPLTTFDGKRPHDDSEGETCCRREVAEEVSASDDTGVETDVDYIAPQFQETFIEGLKTARL
WCLLWSIFCCVGVHYVIIYNARFIYTALAGEVPDDALNALLTVLNGVGSAVGRLCMSYFEVWSQKRRAEDR
VPITLSMFVPSVCIITMLTLFLTLPKAALPLPYFIAALSNGFTAAIIALVTRTIFAKDPAKHYNFCFLGSV
LSAIFLNRLLYGEWYTQQADKLDQDVCTERVCVVMPLAFMLGLAFPAFATSTYLHLQYRNLCMLALDERRR
IREGERGPKEPSSCPKEPTRDAREAAPQ
>tr|C9ZVN0|C9ZVN0_TRYB9 | DeepTMHMM Topology Prediction - Predicted
Type: TM
MLYDVVELLANYVQYINLTVLIAPSVFPPAYSSFLRFVGRAMNAISHGGGVAVTVNGTSKHNNTTRMFEGS
TLNLPKWMPPDLRRVFALTNIVAPLSLVVLFAPVLGPPQFIAFAYALCTAVFFLAGCTLLLHAQHSLEVLE
SGSALLKLVRAIPSDTKVAITAASAVSVGLLAAVGLTLRLIWGKRRQAQLLEQLRHLEQAPHSVEERVAES
LMNVAASHNGCVWRDGEGGMERPVEEREREETRLRCAKAELAYQEMMQRRHGETHAFSVWWALLKLLVLLG
SGTAAAFLLRKSLWESTESLFCSPLIYFPVSMLLLTTFFLALSLSVGLSEGGRRWLFDAKLWFRHLFLYTL
LLLVSFLYAPLIRSAIGLIPCRDVGDNNAPVACGADNKSTSVLEVDTSKWSTTEARKLMVGSNVSCDSVNF
FLYASALLTSTTYAAFFILLYGLVTRQALLALEHYPLEDSGSCSARSAEVVPAIGGNTAEAFHHSGNFFGR
KWEPAWGTVPREGVSQSGRERMRHKNYYGRVHSSQNEVHFLYAPYTFSWRYFKLVVLAQKTAVVIFSAVMQ
EDDGLASPWTGFIASVILHLGMLVILVVCRPYSGPVEFALSLALQLMLVVLATMGLVSSFKPAVVSRSLWS
FVASCFLLVPCGAILVGGLLASRRQCRLKHGKRTKRRQRGNQVCGFLWGFLTCGLRPKPRHSGSHWFLHHN
QEPRSYQIFVPALYFSLLKTELSANAGPLGAATSDRLRRVGSNHLHLRRNRVGDGSNSRAASPSPPAVDHS
VPSANSYSTCGSPSPSVPSARPVVTSTSRSRERWAHVRSALFSGRLRSSSPAKTLFSIQQTPVTSSTWREE
TSEHGGVTTPQMLVPTLSISASSSPLAEEHQLCQRAMTVLFATVGGACRWGASPCVDVQKSNTRRRLSSVA
VYERGSCPHQLISRRNPSASLEGRHCEAFLERAQAVAARQRLIDFYQNQRVHMLRKQRAVDCYINAQAAQM
VRWLLIFLGISAAAAAALFLRGVLQVGERHVVVGVLPNCGA

>tr|D0A222|D0A222_TRYB9 | DeepTMHMM Topology Prediction - Predicted Type: TM
MIILSFVCCLHWPCRRGGCVPFSMYISCASLMDLPRRAATRGVTQQSKSAAICVLPFPAFFFYTLAFRLDI
HYLGFSLGTSFFSILGNMLFLSTHTAPQTLVVYCLLREKGRR
>tr|D0A618|D0A618_TRYB9 | DeepTMHMM Topology Prediction - Predicted Type: TM
MQSRRNSMMNRVCRKYARVPGQHGDGNVPAFYPIAINPGWWGQPFGKVKDAKGQILFFYGLVLPIALYWIF
DVTFAQRTRVGNVGKRPMYSNFFFRQMDLDDPDHAIKYEKLRQEIAENKLEVRWGGTNFLASYLWEPGDPE
PDIRRREVHAHH
>tr|C9ZV85|C9ZV85_TRYB9 | DeepTMHMM Topology Prediction - Predicted Type: TM
MERYKRVKVKATVKECICGESVLFRVVLLHSFCFIFGLEGAYVFIPFADSNTFLTNLLAIYSWAFLFPHVV
FLLFLFRVVFASIPSTSSLSLYFFRLRSLFPLVFFFFTFCAFIMITIIIIIIVFFSFSLLPTLPKTRERKE
KETCTPLSQMDVGAQFSFPVFVFV
>tr|D0A3D3|D0A3D3_TRYB9 | DeepTMHMM Topology Prediction - Predicted Type: TM
MPFFFFIFLNLRFGSIYLSERLSLRWDAFPTKXXXXXXXXXXXXXXXXXXXXXXXXXXXXXXXXXGKGEK
GKGIRKKWKYFLSFWKAAQNAVVVSKTGGKFFLKFFSFPPVLTPFPLFSTVLNIIINYLLHHPLGLVIFYS
FFIYILQILLLVVVMTMGLVVVVVVVFLFFFVPNFNQP
>tr|D0A2R9|D0A2R9_TRYB9 | DeepTMHMM Topology Prediction - Predicted Type: TM
MCVRLCNWESEMVVEFLYAGSLSLQKKRFFFFATATGVLTCVSPLYLFSSFCIPNITPFLKNSYPYFSLFF
FPSCFIPPIFFPAALRLLIYFFFVLFLNCAFLTAVTTFEFMRVVSCFIFFF
>tr|D0A5I8|D0A5I8_TRYB9 | DeepTMHMM Topology Prediction - Predicted Type: TM
MNRSCSRVTVLRGAPSALYLRAPHTVSVRLRRSAVASLLGPTFLRVQVRTQFAQIPGKRDQEAFLAQFNPL
DVLGLNESCTVDDIDEAFKRMSAKYGPNGPTPNAQMVDRVFRAHEVLKDPASPYYLRAHSSDSDRQRLQFQ
LLPKRQRRLIKAQAGMLVLFLAGIAMLVISMTFRPVKRMLRAATR
>tr|D0A653|D0A653_TRYB9 | DeepTMHMM Topology Prediction - Predicted Type: TM
MYQSFWKHHRIHMTITPAATEHLHKYIKWNEYAYEYKSICLFVYSCLHVCAVTFSRLFFSIPLLPPPPHTH
TQPAHTRTKKKKTATMTIIMKKERKKCVVQLCENGDAAVEWLVQKCNTSVCMQQKKIIEEIIKIYSLIIVM
TINIVTIVKHTH
>tr|C9ZP75|C9ZP75_TRYB9 | DeepTMHMM Topology Prediction - Predicted Type: TM
MYFRSLRISTFSFFLFSFSSFCSLSCDTVSASFTSFHVPSTHVFTHSSFNLFPFVFVCVVLLCRITSFLLF
FWRLSFTFISFLNNLVNRMAAYLYKYIYIYMYRK
>tr|C9ZYP5|C9ZYP5_TRYB9 | DeepTMHMM Topology Prediction - Predicted Type: TM
MDIYIYIYTQQDKTSTTYRIVYRRIASIFLLFFPASAAATVFVVCLFYLFYFASTLVAFDSTRLPLPSAKC
TKKKKEKKRFTTILFTRQIHTYHFPPPSLTTSPFVYISIYLLPRLFPPYVLRVSLHRIHIYIYIPNSFFDP
TLFFSFPYPFFFNNGKELSSQPPGSDEKKKQTKKQKKKITTTPKVSRK
>tr|D0A8F6|D0A8F6_TRYB9 | DeepTMHMM Topology Prediction - Predicted Type: TM
MEAMVTDSMERCEVCMITATKQLSETRSNTARHATREGVTDAASVCLLVWLYYFFIPVVVICFFSLLFQCG
NSARPLHREVTVKRRAVPPLTALLPGSA
>tr|C9ZZB3|C9ZZB3_TRYB9 | DeepTMHMM Topology Prediction - Predicted Type: TM
MPNRLFFFIFCSFFLKKGRKRKYCNSFSIKGLYYSPRMAVKVIFVKLRRSILCAAASLLVFPDFRCATNGE
YVINIYIISLNMWIAHFCDILTLLLLPSRFLMRT
>tr|D0A9X2|D0A9X2_TRYB9 | DeepTMHMM Topology Prediction - Predicted Type: TM
MSVFVRFFFYFTDRSLLPLPSSLFSLLHYFLFLLFYFYYFIVIINIIIIIIITARILQGFERGEKEENSIN
IYKYIYIFIYISEWLCTLGTSSRFRISSHPPPHKKKIMQIYVAAVNFLFLFFFLYSFLISFKLYCNRLTSL
LVSSLLLLLLLLLPLSCCCCCCSLCVYLSMKCYCYYYLMFVDAVSVFFFCFLCFIFFFFSPLFFFLLPAITR
>tr|D0A268|D0A268_TRYB9 | DeepTMHMM Topology Prediction - Predicted Type: TM

MSTNGGAECSNAYITEGVKLSPISSEKAENTQNNDGVTLLQPSWFPREELKEAVNGSTQANPLKGPSPTDQ
PPPPQVLSPAQPQSRNAPASLSESVRRLHERFRNTSWLEMLCIFVIITVVIVGHFIEIISIKYWHSKFPNG
KTPGTLTTLVLPSILLAAFMVTVMLLFIFFTTPSLRFAFCCQSLVMLFRIGCIDAIQSGVGVYSVIKTPNA
LAALVKPAAPLFATLFTKMFLKDKRSYGSPWLLISLAFVIAGILVASIFDLKHGFNKIGKNSWWASIYLVA
VALSSLVNAMQAVYMLKFTYDPKFDELYKIRARGGTAVPSMGSQSTLLSAQNVLALGVGNGNHQLAVEMGN
IPAAQEPEDVARTALKRLRQGRGTSVKIVMLTIMLLFRFAGTLAFLPLDGVKPWGESNSIGDAWNNLIAGE
RCVATCDNNLPPFVMYTSGVLLSYVGSAYLNQYSVTMCSIIKQIAWPLAALILVFVPRWSIDPVVTPWYFS
LVSIFILLLAVLLYMFWECSTGDEKDKNERQLKERMMYHLAK
>tr|D0A8S9|D0A8S9_TRYB9 | DeepTMHMM Topology Prediction - Predicted
Type: TM
MLHNFMFIFFLLQNCLVLEVNSTNATLAPFPFIPCVMYATRAKVLVFRLATFVLVSVVRGQEEVTEHKYNI
VFSRDPVPSGLSEEQYYPMRLSNGSAYLCVLPDITVEEKKTLQAEDSELDVPLSLEHVAVVNRALKNMCYT
MEESWWTYRLCWGSGVEQFHRSAVAGDSKSNAPKQMKEDPHFVLGVAPPADVLDLRYGVNTKGLRYIYTIY
SDGLTCDLTQLPRTTEVQLYCAREGEGNSPTMRVREAEVCRYIVSLTAKEVCLLGLKEIQQRYGVITCHET
KPTNTVDWNNKQQG
>tr|D0A7Q4|D0A7Q4_TRYB9 | DeepTMHMM Topology Prediction - Predicted
Type: TM
MALVGGEHRVTEDIVAVKYLESVRRLYFNPHAKDLERRTVHLLAQLLACPPHSLLVWDSLRRTRLLTTVAR
VLGSEKKLQGLLSVGGRPEDYLRVVELASNSSPCECMNHLTKGEDSHTELEPFFAVLGRLGKLDCDAATYR
ACYDSFARAVSNLLSDARNCARLVRADISPIKILFNTLKGSPSSRIDVSLCVLRVILQILLHTAAFTCIWI
KEFSDLDGPSTCLSVVLTWCGTCEDPSLPLSTESTVVFDLLCLLLSFPGNGRGPSSALYMYLRGCHWEAEN
LEVGPLDDTATIGMFGLNVLVEACSIAMNFRPAIFTHLARLLVRALKLGGDDVVSAGLVSAALVAVLRQYA
KFCDDCDKTILAIVRLCVTVCSCHGAYDAQTEGATQLCTLVVELVCDMRGHISRCTVQAVMCLTSRVNAES
TFTRLFSVHLLERLEGRLAISQDPAEVYLFLQMMNEICGNVPVLRREFMLTLNTLFQLLGTEAKNGRETLM
LCVDLLSTMCRDDEETTFNIYAGLREFITRSDFCSVDSIRLGCWGLQALIDLLAHASSSLTHFADSVDFLV
TCVEKLCVAMCRAFDSQMTVTECFGVPSQILGPQPLEVGDFDVNPGSVVPNVYYFYCQFLVLGLHALCYIQ
ATSTGTFFRDLPAKLERVLARGPFLRCRVYTSAVVRALIAMASSTSSVVFDEVFFANRGDCAQWFAGWGAV
HRHSWRFNTSFGDVSFFHERQRIVHIGYFWCLQRLLLQLSDTSQKSMLGASVLELWFNVFSNSSENIYILM
DSKMTLTLASLCAVSAADDCEYSFERYEILLKTMHEMGVYGMDCASVLYLLRLCLCDRDSSSVLYNDEATV
KVLSTLKTLLRQSLPSNGPVLFYGVSSGCTPQSHFCNNTAALQVRGPRIGGGVDIPVLLGRVKSDRCFSFV
LWFLLEPKSPHTVEEVDIILMSIAFNRNSAFIQLVYTVQGTLHLRVILRSAEDRNQQQQRLHSEYVLFSND
EIPTKRWCMVSMLVSQKRTKSFGRNPKYLLNVQLSLYTGSQADAQPSVVMKLSDIRCHGTCKGGQLLREVN
MVSLVMTDGPPTNYPRRFLLGSLGAFGGVLSRVEVEMLFAMGSDSLFSLHFMDKKFVNNLFPALAYMSERN
RGKHGAQLMRLAVPWLAQGYANAENAILASTFPFAVYQKRERQVVVAGVRNGDLCVESNAEYLQPTNDRPK
VQGSSAEETLPPASARSVKSGTRIPVPIFLGVRSVSDLQNSGVAREDMLALLNAPQFICSTPLYSILDALG
GPPFFLCFSSLQLPHSGAFEEAWECACAAVKWHTTNNLLCPKAQRGDAHLLALVKALLYHRIHVSETTASS
LCDVVGDRLITRMDILPLLLEFTVWARDTGAFRFVVGKLRQYILDDVYGRFNRQILGAGRHVASTEGECNA
LEEFLLHLHIHFSEGIVGSWDTSLIPFATEFLTALCQTATHARRVVKAATALLALDKPLTSAAIEWVIGLL
EAVFYLMNENVILCGNESASLAEDITAIFKSSHEAVRRKALSLFSRTPMSRTQLDAVISQYMVRGVLSSVD
DLLSEEEFLTIVGIIERSAASGDTTRARGLLYFLVTLLSHVPNEIRLGILSLLEEITCERGAFEGGLLSPL
DTGGFPVSLLYGRLLHVLHMVEGNCSSLVESFVRLVIRMCVGMVSVSCERNDMNCGHLLGEVGLASLTCVA
SILKRSGREGSLDACISSALIRAYRYSSEVLRAYSMHLDKGTATGAFLMSYVSFLKSTAIVVILLSWCERD
YRLSSEVKVEDESCVCHAAAEAYLAQFSKPIHFGDHHLSARLCMSFFEGGQRLFFQLQQASTTGLHAKQVK
QISQELLTLQWRVAVTLLNDADTSGEDLMKELALLYVASSGSASYGFKFSTWKERERLVEPPLGSDWKHLQ
FAPGALITELLKVDYERAVRESAPWLIVSRGASSLLSESRLENSSRYNMAVSICRLVRYNSGIWSNVKGGF
TPLSVAELDSNIKTNAMSIGGAALKPLPEGGDGISVHWEVEFSRSGVASLERYINDIHSTALSSLNQCSER
FALPLLSVSAEQALQHREKSGLAVEQYCRSVLRQVASHRKCIEGESKAVLAAYAAYRFSLTPWWGQSVNLY
VASPVPMNQWELDRFTGPEWQRIRFRRFAHAMRVKHVKSKGDMLPYVYRGDISYPDDTQLLEIYSVTAVPQ
LIGVHCDGPQPFVAKCVLVLPMDRVPITLYVSPHTISYVHDESEPSPDATERGVTATQATKGNGGSCFSGL
PCISASADQESDTVACNGDVRRYQRVFAVSSLRAVWPRRNLLQPSALELLFSTGESIFLVFHSQDTMKLVS
DIVTTVACPYLDRALVLTEANLKMWCNWWREGRITNFHYLMYLNFAAGRSYGDMRQYPVFPHVVADFSSLT
LDLTSPSTYRCLKRPIGAQTPEGIQRAAKTYAETSTDVGMVVEASCCGSPYHYGSHYSPLGGALHYLVRVQ
PFSDFFMKMNSKLDDAGRVFDSVGAAYAIATCGKDVKELLPEFYCLPELFVNANRIPFGTKQDGEVVNDVQ
LPPWASTPRKLSQTLRRALEGCYVSENLHSWIDLIFGFRQRGKEAVAALNTFHPLTYEGSVNLPNISDNVL
RSSHETQIDCFGQTPLQLFSQPHKSRRKIADITFRQSPCPTLRCDASNLALCVLLNPGRLIPAGVCKRSCG
TTVVFPRTKLNGIGSSIDEGTERKAQDGQVRPLVLPKGVFPFTVVHEQVRDCLRYKGSCIVLVDGKDASHQ
TRSFHVGYGEITALTVDSPNVYVGMESGAIHMLTMTYDTFLEVELTFRDGKGVRLGGEEVFQRFLQGKLKF
TESRQTACRTLCVLYGHTARVTALCLSSEWGILVSSSEDCNVALWDTERRVLIRTIPNHLMSPAYSKFPMK

SVMNHRLQGSSGSWYFDLITVNAKHGDIILAGGSLAGLHEVRRYSINGEFLGFYSLGETPATAILSVGDIV
FVGRGSVVHILKGNSLEWSCDLIHPGIEDCIESLALSPNGQSLVACDRRENLVTWKVAPQ
>tr|C9ZKD5|C9ZKD5_TRYB9 | DeepTMHMM Topology Prediction - Predicted
Type: TM
MKILRDVYRQTFPKKLRFDKSSRILYAQIYVIARRHKHRKYIPKEDIEDVLNNYTTLPGSPWLQLPIIRHV
LLIRQINGIRGFFGKRSWLLNRAVEEEFNKIVRWMNAQEQLVLRTGAGRCAANLVLREKFVKEVLDVFHVA
TAYQRMVRVGALLIVILLLLLIVMVNVDRFVYVYLVHWSGMCRTEVMEWFREITEQHTVAEVPPAYRSLLP
PPCVMRVAEDGRVKYELKIAELVSKDENIIVLAVPCPQVGTKSFFAALGKTVGLCDAVLMEGVSFEYIDRI
APASLFPLRDDTFPALGVHHRFLDILRDSREPPFLYPAGTELSWSAYLQHLLIPFEVKCIYWPTFSSASKG
EARVGWGRLRELIEKVTVEQERAQLGEDKKELKPYVICLPWTVNQIVNLEASLVKLGFRVRRVFPLEWIDR
DHMGEHFCNYYSLVGE
>tr|C9ZTR6|C9ZTR6_TRYB9 | DeepTMHMM Topology Prediction - Predicted
Type: TM
MSAPVDNVVVERLSTANQKPINEPRRFALLVLGTFCCICTSFMYAFNLISGAMQERYDLTQRDLSTITTVG
IAVGYFLLPYSFIFDYLGPKPIFVIAMTVFCLGALLFALTFQEVIEGSVVRLSVYNGFLTLGCMLFDLGSV
VTVLSVFPSNRGAVMAIVKTFTGLGAAIVGSIQLAFFSKSVANYFFFLMSFSLVVGTLAVVFMNLPPFHLT
GYQKTHLDEEEKAQRLARKGVYLKQKAPMWRFIYGFVVLLILVVFLPLEGALVAYLKLGSNFKVGFAVTVI
VLTAIFPFMAFPLTTFDGKRPHDDSDSKAKEHVEADDEVSAAEDKVVETDVDYIAPQFQETFIEGLKTARL
WCLLWSIFCCVGVHYVIIYNARFIYTALAGEAPDDALNALLTVLNGVGSAVGRLCMGYFEVWSQKRRAEDR
VPITLSMFVPSVCIITMLTLFLTLPKAALPLPYFIAAFSNGFMAATMALVTRTIFAKDPAKHYNFCFLGSV
LSAIFLNRLLYGEWYTQQADKLGQDVCTERVCVVMPLAFMLGLAFPAFATSTYLHLQYRNLCLKALEERRR
IKEAEDNQTNAENVCAEPTCDDNADACLEEKAADSSK
>tr|D0A2C0|D0A2C0_TRYB9 | DeepTMHMM Topology Prediction - Predicted
Type: TM
MYYSLWGCIRDCGRMLCDILVWRLLHFPTLFTFCCRLFVSVCKSFFAVYAFASLTCTLPCRFPAFTPLYIS
AVYNYFSSLLIVDLVMHTIGSKKGEENNSRIINIDAVHQLQGETRRVSEWHFKINSYVCVPARTHGEKLNK
LVGRRDDKKK
>tr|D0A076|D0A076_TRYB9 | DeepTMHMM Topology Prediction - Predicted
Type: TM
MDRQFLCGNWCLTCITTVRVSGMCLYTHTHTHIFVFPDFSLLFFAFSSSWTLFFIFLKYGSFCLPSFVAVN
ISHLIPGLFSPLSPSPREVAGGGGGDLPFV
>tr|C9ZLN1|C9ZLN1_TRYB9 | DeepTMHMM Topology Prediction - Predicted
Type: TM
MRDEQKEYATISGVWTRLILFSTLATAVIYVAVGLFACRRLIRTNWQWILIAVVYFAVGLIHAFFSLSLLC
LAIGCVFWVFGAEPMSLLEILTYTTIMVVHMCFFAMGKKTILHAL
>tr|C9ZY22|C9ZY22_TRYB9 | DeepTMHMM Topology Prediction - Predicted
Type: TM
MPCTSLCFLLLCCYVISPSPHSLLLLFFLFLQRHSNMRKNGLLHSHLSPLFTQLIRLATPSVILFPFIGLY
ARIILFFYCWLVYMFASLCFYIILYIYISA
>tr|C9ZZG6|C9ZZG6_TRYB9 | DeepTMHMM Topology Prediction - Predicted
Type: TM
MFRRFIPCMRSGAAGPLRAVVTTARRFQGTKSGGGSGREEASTEGVAPEVSPSSDKSSSITNVNMGVERRE
GFLEPSDDVVLEYARHALARDGRDKEPEALIWRWDTTYPPILSRGKQNFYDYTDDIPTHVKPFWHHEYYQQ
REYFRLQRAKLPLKERVKTWSAVVFCISLVGGVLTFFRIWVEQPKEVRQLREELLQQTYGRVLELAAGHGQ
NIGAYPYAVHEIVMCDANAQQLQAIRYRIPQTAYPKYDVRRVRSENLEMFADGEFDCVVDMFGLCSLHDPL
KALRQMQRVVKSSGLILLLEHGKSPYLPINWFLDYFEERHSVNTHGCKWNSPIREYLKESRLEIKELRNMH
YGTTYYVVAYPEVLEALSARGGSPDVQKPRE
>tr|C9ZNH2|C9ZNH2_TRYB9 | DeepTMHMM Topology Prediction - Predicted
Type: TM
MTGAPFYFSIVVGPNWTVRSFIQRCAMTRYVPHLGRAKSSFKYVGILGLVVEPLCVRVPYRRVLSCETPFM
ALCVCDFGDALPRSDKGLVRPFSLRCSSFLHTFFYPIICFYFLPKIRINSCVVCSLSPLSVLHEEPVGLFF
L
>tr|D0A284|D0A284_TRYB9 | DeepTMHMM Topology Prediction - Predicted
Type: TM
MRPYFPGANGVLRSHCISCFVSRRQQSAKKDGTSADEGKQTPPYCHTGENYCGSASVPRTEAVNARMMQTS
AAYPRVHVTLHDPQKSKSEEADELSRCGGGKGVNGINDKAETVFDGNRVGDLSENEVRAFLFRKEREIAHH
HQQRNSTIPYPPHPDDVVPQFRRIKRHQQMLVVALDPDYPVYDRRDNVPELPPAQPHPWVKKTPTGPFIVH
GDGQLGVVGTGEVGFEDAFTSDNTTEVAAATMGEVMLPLPRDFRGLQHRSTLHQSLPSCNGKVLQETVIKN

SFALTGRGVFTTRDVSAGETIMIVRNTARNLGVKSEIERLVEMCTDVLSDTYNNFCNGNPQKLDFLHDWVL
TGQPSSLLLHWPRSATKQVLECIGGAEVLHALELHEIHIARLAAIMDMNSFLVESSYAVRKGMAYFPEAGF
LNHSCSPNATYDVIPAHTFCETDYYVDELGDSANAAFCATNYGEYTKSSNNDIVDATSGRNTTSDTKSDRD
ASEDECMEDVHNADGSQIIPSGTVEYLFCCRATADIPAGSEILISYVPPEWSFDNRQYVLHDRYRFWCKCP
KCAPTLDSKYTRTPRLLVAMLIFSIFLQLLVMRQRDMEHATMRERETDEEEEMRHGESGREKPKRRARGLF
ELLEESRQEEMYTPDRGPIPERVAQDPWARPAR
>tr|C9ZTR8|C9ZTR8_TRYB9 | DeepTMHMM Topology Prediction - Predicted
Type: TM
MSAPVDNVVVERLSTANQKPVSEPRRFLMLVIGVSCSMCTSFMYAFNLISGAMQERYDLTQRDLSTITTVG
ICVGYFMLPYGFIYDYLGPRPVFVISMTVFCLGTLLLALTFQEVIEGSVVRLSVYNALMMLGCTLFDLGAL
VTVLSVFPSNRGIVVATMKTTTGLGSAILGSIRLAFFSGNTSAYFYFLMSWALAAGILALTFVRLPPFHLT
GYQEKHLDEEEKAQLRMTKTVYLKQKAPMWRFVHGFAILVTLIVFLPLQGSLVAYLKLGSNFKVGFALVVI
ALIVIFPFMAFPLTTFDGKRPHDDSDSKAKEHVGAGDEVSAAEDKVVETDVDYIAPQFQETFIAGLKTARL
WCLLWSAFCCLGANYVIIYNARFFYTALAGEAPEDALNTLLTVLNGAGSAVGRLCMGYFEIWSQKRPAADR
IPITAALYVPSVCIITMLTLFLTLPKAALPLPYFIVAFSNGFTAATMALVTRTIFAKDPAKHYNFCFIGSI
MSAIFLNRLLYGEWYTQQADKLGQDVCTERVCVVMPLAFMLGLAFLGFLTTTYLHLQYRRLCKLALAERQR
IREEERVVNELPSNPTEPTGNAGEPVHQLK
>tr|C9ZYW8|C9ZYW8_TRYB9 | DeepTMHMM Topology Prediction - Predicted
Type: TM
MFGGVSYVALQVRGAPHVTHFSVMSYRSNAHSICFYFPFLSFPRVVILCLFLYFTSYNKNIDLPLPPPLST
LFFFFCFLPLENTTLCTLSEALALSFSKSCRYKLFCFSLVWFSF
>tr|C9ZUL4|C9ZUL4_TRYB9 | DeepTMHMM Topology Prediction - Predicted
Type: TM
MQMFLRRDSTHCGTGTSHASSLSPRYRHCCKVPFTFWASVLCIVSSTFLCSSGAASLVLRPNKPLVNVPFS
VETTDQSIKADIFVSRMSTCDNTQIIGSKCKISKNAPCNFTVTSNDVGIDPWYDTKVREVYICAGDAFPSL
SLSVQLSLVEAIPRYLKRDVDNTIRFGDAVPVGTFITFHRNENCDNVSMIQGLPPAELGSNREVRVTRSVQ
SVIYLCAKVPTSDGKTFVVPASVLLTVPRYDTNNTDALRHTNETFRLEAVGNLVWATFSQSPICDPILQDP
VGGETLQVVNMEVSVPKGDYFLCNGWPYHKGGRLYSPSENKVTVREYGVQPRTLYSGYGTRIKYTMDAATA
AANDVKLVLDFYMYSNCTGAVRVDDPSGIQYRGPPVKKRRSRSDGGAVALPVSGTYYACLEGSTIAYPKAR
AAVVTVLPPPTVSFDEANVISGLDLTVLLSGHNAGRSGVITVGLSVTPECEELNSKGDVRVGASSVTFRVP
EDALPNMTLCVATPSSVDLQADEEPDEGYTYPVKNIATRRYKLKHRTLFVGVAETIHLDANVTLKAGTTGY
FSLDNCLTPVGETYSMNATALYGVAFSAAGRHVLCVKTPGTEHPVNRPYSNVGNVMVYGPAELSPASIVKG
VSTPVKVSAVPPEAPIVFSESESCTPSISEVNATAEGEASVTVMRNTVGKVYVCVGYHDSDGGAWKVRVSG
MLAVSDVEVFPKTVFVGVSNRVNFIVPDKTSLKGLKVLFKETGDVNCGEIVDDGTAVAIRITEVGPAYVVY
TAVGMKKWKVCLWKGNGYEDVGLIQSQQQLELVPDSPIGVVGLPLSMRFRGEALTALQPTRFFVSESIESC
QSAPGGGIRVYGEGWVDPISGSAEPFVVQKAGELHVCVGWGDTNESYYLYGGKVSAEDFAVDSLYAVRRST
NNFVARPLLKKASLFLVRCVNAASCKVPLNARICEEATERYYTGPTTPLEDVLLGEYALCQQDERRLGVAV
GQKPLKVINPFTASFNVSLVRKHTPLRLTLDGGSLHIGASNLTVYIVPPELNCTEANASYDSFPFASGEHS
KDVTIVKTPAVAKGARVCVGISAYDKLPASTFNIFHYMTPATIIAGRSVTVESSGIQSGRVRISTLEACTD
SILSEYDTVIANSMSTLHVDKCKYRNRDLTDVYYCERGESGGYVMRGTMQLIHLDECPGGKQPAVRPVTAL
PAKQVTDYGIDKAILTSPFLSTRSDCNGVLSQSRATMTPRYNRNLTFYVCTHTIRDPGYVFTTDGPTLSVK
NFRVVNPSIHGRVDSDSEVLGPANLVMNYATRTPDTYLSDCAVCGEKIVAAPGLAETPAEEAVTLIGISGV
KCVCVLGEQPTSPPIPVAEVLIITPPIVKKIDPAAVPTARFHAKLQTPVVSGVKPLYSLVPGEDAENPLAY
LHNSDFRGVYLSENACATTLTGNSVGYVRPSGSVVLGPTFIPGRLPSVSLCVGTPAGNLSVVSEVEVSTDI
IFPSSFVLGTEAEVYIPLSPNSAFRLRRDASCSGEDVVPFFTTDEEARGNISFKNVNAAGLPSAGVWTLCQ
EVFDGSATRPKIVKISANGIAARVASAKQLLPIAQIETFDPTYYNIRGRDVLLGVPGVLYLMDDLYAESLL
PGFSTDRSCLRRNESHGSWALVGDDDAATARRVSVTAQNGTDSIYFCATTPVNRSVVSVPLSSSLRFIPPV
SVFPSIVEPCKVTPLESCRAPDSSHRQTVVRVIKGDCCNPTDKGVTVGEASEGSDGKCKLRMNHNKIRDYP
AGTEFSVCAWDLTDNTYCVTLGNVKASGDICGGSAWGPLGMGAVIAIIVAAVLLFLLLLLLAVCFLRRCCS
KEKEERQLVVADKMQLDNLDMSCISGTSPDPEYLESGNNPLLLGFYSGDGSPNTNTTARTMIDGMTSGVDD
FAWGGDFPGRADWTSVEEQECDDRDQIALQEARDRYNMALVFTDGIERIRVDAKELELQFDYSGEFSMYEG
PTGRGVNVNPIPVTVPADHPDLLNAIYMRRASQRRRLQSQTREEIEVSVHDTMSEMSTDVHDTSRLTSYDM
MSYTTTQHFYEESTFMLESEAGRRVRLVNWEEEEWQAIVDAEFSDYVRLQQAMRSIPLPLPQAVVLPFNST
ATRGRDTINYSGHTHVNGEGVTALPVYDTPPFHNPHAASVEEAQAYPNHDEESNVSLSCDSSHN
>tr|C9ZQF1|C9ZQF1_TRYB9 | DeepTMHMM Topology Prediction - Predicted
Type: TM
MAPYVRCWRRLLPLHLWTRKRGEPARPRRTLLSTSHPLFGQQQATLPTAQSSRRLLPTDHPVFGTKGLFRV
EEPFKGPIRVSGFFLAIDPHIAAGPGKPMNPNAQITVSDKEGNMSRVRYRCLLESRRGLRLEALIRMQVGW

ISPKVTFRSIPHVIHLGKGFDVSIPLLMHFTMVSVLDECGNKTGSFLYLSGTPLLGFSVGFTRITQGKLVL
DPSRSVNPAEEARQFVGGCHVRQLGFLFLKCIAYVLCLMACFPTSKRVLQFMTSQPGPSPERSKNLT
>tr|D0AAD7|D0AAD7_TRYB9 | DeepTMHMM Topology Prediction - Predicted
Type: TM
MPGHKRQSSRSFVVLVRASLSIEGARVVPSRTTTGAGALVRWPSCGTIHLHPVVFFSVFLYCFSMTNLVPK
AHPFPFLFASGLLYPQQRLIFMVYIPAVSILGIWCSQPLAAFACIP
>tr|C9ZSF7|C9ZSF7_TRYB9 | DeepTMHMM Topology Prediction - Predicted
Type: TM
MGSASQVPGASTSSSEQGSKSVNLLYLLLLLLVIPVVGGILFAIRYFRLHQYVSVARGLKLWTSPSAADEY
SHASGAVAGPKSTGVDGQTVGVQVLDMDGEGCTAERGEKEFDPIFDEAEKHHRRWLQRRTVGDDGNSDVVE
EDRGVTTEFEELDESDVEMAVRREAENNKDGDIEVVNEEASPEPEAVEDDVKDDVSGKRFRKCDAVAARAW
LKTLGTEDLAHDDNGAPQSIDAADEGEGEKSPSSVRSPVARGDVVGKFHARSLSREGRGTSSVIVDSAVEK
ENFREIISTLAPLKPLPTGIRPKTTQSSGMGDSGLGSDSGKVEANSSNQTNCMKEAKEEVGDHVEAIREGR
GEQSDVGTEGSKTEESIGVEVEGDGGNEATGEEMDLR
>tr|C9ZJR0|C9ZJR0_TRYB9 | DeepTMHMM Topology Prediction - Predicted
Type: TM
MRYAFFALYRSVVWEWAGLTRLSASTKKRMYLCWFINIYVFFIAFTTHYSHVAPLSCIKAIRVCWRCFFFF
LIVSNARQLQVAILWSHVRFCLHISVLLTSLRHFTASLHSLLKGIPRYI
>tr|C9ZV32|C9ZV32_TRYB9 | DeepTMHMM Topology Prediction - Predicted
Type: TM
MAFFVLFCLVVPVYSLPLKRLYLTRNMWHRADFRFISEATPSLRTSCLTTTARPVIIAPSMLCVFFFFSTA
ACVCVCVRLRRCPCWKLVLVCNRALLTPSPDTKGRKFVLLFNQPLSRANLNLHPPTSGPFMWTTAAAWALA
ANM
>tr|D0A089|D0A089_TRYB9 | DeepTMHMM Topology Prediction - Predicted
Type: TM
MSPSQLSREEFTCAYQGYHGRLIVSFHNLAFNSEQLNVDFPWEYVKHIKVTTKHLKEVPTTMLEIKVSRNK
QQPSQKPQIDGKQYFYGFEDIVVVEREIQKFRNAASTADHANDDSAPTGADNNANTTSAPMSNAKTNATGP
RNGHQVGTSSFVQCDRATNGDAAAASPGSPMEDGDYVHGVSRSRQGAHTAREGEEDRRSNLFAPNATGNPL
FLLTKSRPPDREHNSPGYGQQNQQLQPKHEKRQKCRAVSFLRRLLPLNSAYISDSGLQVIIGFLLVIVPVL
FILVTTMEGRWESPSEGKLEKVIRDLESLQRWRKGQQQQKHGGTVSQTSEAPVHVTLSDLAEGVKELTRRF
VDRQHELTALRLRHLQREVPNDVSPLTGMGGVTLSELNAAVDEGAKEEDVENGNGKTSTVETEQQRQQQTR
RFSLARVKHDLYRVASLVQKILSFAHWIITGGGKVKQNSYDVPRATPWFFGILGGGGNAAHHHKKRKFVRY
VERLPNGQSVTEYVDEEELEERRTCIRLTRSLVASADLLDGVLLQYMGVVMMPQYEAYLNLMQASTGVSFA
SSLDSNGASPSPSSSSRPSAGQGDTVGSEATGEQGPRLLKTIALRRHASLLLEMEPLSSWGHGYNSQGWKG
QPDPSNHGSKNEGYEVPQSVAGNKSISKILNEFLLATQNKLLSGVDTSTPRLMFRNILKEVRYWSRHEEDW
QTLVLWQLNRTNTNITNSNHTHERLEAETLKAMDDMPLFRGQWCFLEKPIASPLDSASGRRNTDRTEDHDT
RSVESAPREAVYSSHNSKGKETQGEDGLACSAEAVASFPRGIHSSFLPNIKAVGCQKRGSGSVENPGEDLK
YGSKGDALGREEETGESHPLDDAADDDRARLRNAEYISREVQLRWKNALELFFSAHNPEKDQWHERDAMQK
WTSAGRGGTDSWWEREGVINDENTDGQDDGMSAEERDMVKIRRRLLQLFVYVGRRYDQYLFVPASSYPWSA
AAFRKCTSEDFGCHFWLRSSLFSFFWPSLWFHTEWLSHPMHEWLRRIKGRDPVIRHLFRELLHIPLGEVED
VSWVLLNTMTTSQELLEVKHNICALMCRLVFVLTAVIVATVAFFRCSH
>tr|D0A020|D0A020_TRYB9 | DeepTMHMM Topology Prediction - Predicted
Type: TM
MPALQWLGRKWRTGTDDFFLSSFVFFSALTTSGTLILSRFGGWFSVDMKGCPGVPPKYERCVYALGIISCV
GASFHLASALLSCRGGPFHVSKRRHVGTLLYLTTATALSCLPLSSVVLKYSLYDGFMKMCDPFSRRSLYAS
LLVNIVVSLLHVISLLLSFDPNGGRKWGDSEEYKMLWGRRCRLLCCCCFRPAEDDDDVFEDAAATLAAFFQ
GYDLVPSDIFAGMILLHDAQRRVLLERVAHVRFPPPDDGCKERVSSQAHYFPLLTSQQRQCVVELRQYSRF
YMAAYGCLLYLHMNLCTGLPKLCCSDPKMCCRKRANSHQGAGCFCDLTAALKVSGLAEEDIILSNWRNALF
RPVFYVALDRETASIVVAIRGTLSFVDCITDVTATPEPLFIPDLANSERACANDYYVHGGIKRSAEYVLRE
LRESGVLEAVLHGGLNSYRLVVLGHSLGAGVAAVLSILLYATEEGVRERLRCLAYSPPGGLMSPALANYSK
DFILACFVGNDVIPRTASHTFDDLRESVLDVLESCNMSKPLIFANRCILGRRNSSAGRCEPLSSEESRAVR
AQLQSKACVLPMDQRKLFPPYTLVHLRKAVVRWTPKSCCCIPCCCCSKRENVFVPTFETPDDVQTVVCSPS
MFSNHFPDYVFDALEETTERLQRGELERFFDCQYYNTTNFAHPTYGATPVNGGMGVV
>tr|D0A1T1|D0A1T1_TRYB9 | DeepTMHMM Topology Prediction - Predicted
Type: TM
MTMGRHIFFNFRGPSPIWRNWGRKHRVGSAMVGGTNMQERVVRAITIIAFCPYVARFDPNIPLHTRYQLSL
FCSPTSFLMLVGCRKLFLCYPFVLLYIPSQSPLKLAPFLSVFASSGP

>tr|C9ZWS5|C9ZWS5_TRYB9 | DeepTMHMM Topology Prediction - Predicted Type: TM
MRMRLQRCTCYQRIPISTLTGVRVYVHDMLFYFFPSPAAYFSLKYIHAHAGVLKSLTDDMRFLPGMTQYPF
FSSAPHVHQFCFSVSFCFVWMCCPLSPLDITFPLAPACVRYSCFCGISPLTSSPPALPFRFPFYIFFSFMA
SPG
>tr|D0A1S1|D0A1S1_TRYB9 | DeepTMHMM Topology Prediction - Predicted Type: TM
MHESTVGRRAQQRRRFSTRPGTEGSVGTGSVGHLRIFPGVMLHLWILLSLCVPCSLAQGGHEVRMTVKVLS
LMFNSAGATVDIINSLNVGFNASLAAQNWTVVAGIDVTVIRPPSYNVSAAEYLENYVKNADDGESLLVVFG
PMGEGNIRKSYKVLKEHNLVAFAPLTELTESRKFLPNLYFLRPEPSAELVALIRYAVNHMRVLRLGFMYTE
SLAGAPSAHSRATELMSQLGYELCCLFTVPNDVEETASGEAFEAEWEKFAQNLPQAAIMFTRINDYTKQIV
GRLVSDQRTATTVLLAPSLLQKSLVAVWRQALEASNVSFVPHRLIQTGTNPLAKTTYFGAIRRFQNEARDY
LTRHPEWSGLSDSNHFLTNDVDGELMVYGWIAGEVLMRALRSNTKLGDRISFINSLYDQRRYVIDDLVIGD
FGGECATGAAAQGAVCDCNRGGKKVFMKEVVKGYHFQHVLPGIFSTSRDHCYSNAIQLHPPLSGVIMRMSD
NLTMLRAALEFYHGISSTASLLNVGELNRLIMLQVGSTTEQSMNDLVELRKNSIITAVFGVVVEEMLTVKN
LTFIDPIVMNPRLNKFRSNVIHLSPTLEQQLYVLVSYLSKNRQGPVHLAVYSREGAEIAEVLTRTLVTFRA
NLSSSKIFRDVGELEKYLPAKGDVFLLGIGSGNIPTVKEYLRTHQDVRIFVQFSEVMLMYDEFVGGFNGSA
GADRVLFATNLPHWGDTDSKSKTVRKFHKVVKPPHRTPLALLGFATERLMQRNIRRMEKVTSQLLVDLFFE
EASITVDDMRYGTYDRESCLISGFAAAANCISNFGATNISVWSMARVMNSSVPVLQDPVTPQMFYVDPNAN
GLTAAQLAGAIAGSVLLFFVLLFIAVPLYFATRSGRDNDNAPKELTAPVTIVFTDIEGSTAQWAAHPEQMP
DAVATHHRLIRSLIVQYRCYEVKTIGDSFMIACRSPLAAVQLACNLQRSFLLHNWRTTLFDQSYRQFEEQR
AEVENDYVPPTAHLADEAYSQMWNGLRVRVGIHTGLCDIRHDEVTKGYDYYGRTTNMAARTESVTNGGQVL
LTRATYLAMSGMEREQFDVTALGALPLRGVPEPVEMYQLNTVAGRQFAALRLDRDVDVLNDGTDGSVLSTS
DHSSSRAELSESSQVIVTSLNALLGTFARAQRQKALLPFCERWRVVLPRKPPAIWDDSYYQEVIRRIAVKV
GHVVDYCASSGADHTFSTLTSASLIVITQPRGSSS
>tr|C9ZLM1|C9ZLM1_TRYB9 | DeepTMHMM Topology Prediction - Predicted Type: TM
MAQSEMAAGGTNHRLVASVEGPFALAVPTSLTDIPPEVLLSEAHVRELPAAQEDSVSTDSYSSVPHHQQML
PETTRAPPRRRRALRPSISREENDERNIKHTVVVPEMYRFSKRQVHGNVWDINSELDPCENINVYPERGIS
LSLDNLVRYSPADLFSRAWPLVPAPVKGASFAPFAQLPELPDILNVHHPDTEEVARFIRFTAVEFARRQRT
LSPLFLRCLLVKAAASSETTQAELVSLTGGTCFDEPVRSAWEDENSCDDDGDDIEDDQSVSRSGVPVSDVD
TVSSFDWAIAVVSDSLHPRVPMQVVRMLHKVIDRCGINSTNETLRSWGMRRVFRILLQQVEQESCSFSEGG
GDSRWLFGAIELLFKLVLASKDIDSLLSIFRWVYRHPDLAERVTLKGIRPWITSIETYARPRMRVLFPTSW
VKCHDVSLAHSLGMEISRSVIGACVVSRNGELHGVVLTTRGIYKVLLVPPYNLVCKNENISLTGCRGVFLE
NNQIAVQSKTVGVVTFYDAETLQVRRVLEACKTNGNSGVYVYSGMDRFVIPHFHPNKSAPFYAKFSASMSV
HGTVGPPTLLIPPEAESLSVQFFLCPVRAEQCTSMGLVHVRCFNKEWLSVKVAINSKTSLLSICFGHDEDL
RVEVHEPLRSEWALWSATLQNTNGVFTWNVYKDAVMIKSCAVRCKVANLSTSTTGSVDLLRGSFSGFISNI
QIWHRAQCINDMLTSAKGRLSPASQCGLLCSFKMNEGSGCCLRSSDGAITWKGVISWGPPPKVPFGCDVTE
KEAIPYTPLGDYYVVTNTFETAIVEDGFCTWMDANGRILEQCTADVHPKELYFLCGFTSRMYSVICDVSSG
LSLRWVDTQAPPAMHVRELESMRDEETVSEKFLEFEGDKGIVTPFSLSNLILHRLNAVLVAEGRCCGGTQY
TPQFFSWNSTSLRTVSRLIDISQDLILRVKQPLGRNSSSLLLLCVCGRLLTQQLTWIKRGCPPHLVAIIVD
MFETLSRLEPEDRGILMETQFVFKELHRVLVMECVSYDYLLERILSTERLTDIKDLLVSDYIPSLVTSVLE
KDLKKPFMAFLNRLKEACLAESQLILTGKGASFRPSCVLLPTLLSILSRKANPQWHLVAIALLNSLCKGTM
RLFNGLFSNVASGHTGGTEELNLLKQTAIGVVVFPVVHYLVDLLLDSSIARDVLTLLNETRNVLYPYADIL
PAQYVNHNLTETHRIFVPAACTHTTSLDLRYARSIEVLHEHPTTRETNKVRVALVTNEGRRTFVTMRDEHL
TIECGGRVEVTVTNTKKEESLITVNARVMLQTELSYWISDICLALGQAILHNTQQLLLKGLPDTVVFPQNS
IFRGGLSEEVLKKHNVPGMSDEVLLDKEDQKGLTDLFEGFGDLLSQWQELYRKKHVPYQDRLEYVMRAFCA
AHAWHLRRTKNVSLLKHVEKSLDFMRKKSYLILEALQQGDEKAMLDRARFLLDTLNPRDRLESAMAEQQQT
EEPVTRVSSPTSVHSVANVRTTLSKSAHVSHERTESWNVSALGRFTSSLSDLSKHCYEEVGSMNRPRIPLL
TSDAISELVATNTESISTLVFKFLLNGYNGVTNEDLVRALVEKARYASNNKAVLRLQEKLLSSHQLDEDMS
WLIVNSHLFYREFIRSRRREYSNEASNNVRTNSGYSEATLEDLTEVHYIEPIIGCGFQRELELQRATCSFF
HCVIHRNFSRIVEAKLQCEASRGMEALSLCAVLCHPWDSVDLSAINPSKVFRVLKTYILAPIADTVVTPLN
GSSTDIAWHRFLQDRGLFNLLYRSVIVPNSLQPAADGATVVEGDTLGVHVEGADICFLARNQWRAFKAEEA
FDMSPAQRDCYAALAGGCVLADMAEVLYFEITLELALNEEVFVCIGVTSSSISSCESVGKDCSAAFCSDGT
VRYSNANVEFSTPWSVGDVIGCGIMAPSSSLFFTRNGEFLGIATECTFSTIIPFVAVQTDGMLVKLIVNFG
ENAPFEFDMASLHSSCRTQTVSPVMISDASFIAVHYLVTVCLKNLQRLGQPGFNANGDSICALLEEASVFL
RDVVVELVTSLKHLYSDESTHLLKRQRLHIGGVLLTRLFRIVNVVIDSFRCSLVSRSAHLEILRICSITLS
EAHDHWVKARAARSLGHMARTLRQDCFAEAVEALRGSMSPDVIVNSLTELARAKIYPECSQRAFIPRWTGG

RTRVTGGGAFYGEKPLPARGTHKVGFRIRRQMQMGHGVGASLGGCYYIGLSHGHTPVTTMASLISRDDVYI
LQDADDGDQVPHLLLRRQTIPRNSQRRVYGCDEVVWVELNADVGEITYYRENMVRIGLAFANIHRVDDLYP
FVFHFNEDAVCDIIADPSQVKESCELLLSSLRRGVAVSVLQQLHVTPYFGPAISDWIYSFFVRSSYDLEKC
IVALAILGGERSGLFCQHKTHGPVVVDSISESASRAVVYLEADPDGRQFSTDVADLKPSFVMPALFSVSEG
ESLRCCGWLTSGLRRILFEASAVVPLMNDEKEQRMDIAENFVKTITDAMNMEYTALQIIRHETTNQKGSVT
LLRRNAPVSATQSTSSSFRMVNGQQPMSRTPVVIDRRFSTKSLFTPSPSIGVTSAAHYSAVRGNVVLEDVF
SFAVSITIAESKGTLMYIGVTAETEEPAEPQAIVRCENIWALCNRDSEGTNNTNCFVEPGMIYSAGQLLFD
SDDVVIIKVNRQEGTASFSRVRGGKCVDFGVLFEKIPEEKKLRPFVLTGPDSVVVFSFLDCHTFPARNTYP
PSNISALTRSTPPVLCASCGSEMLEAWYEGEGGICLCSECFNSWRYPKCMFHFTDHNDNLTDYLLSQHSPK
ELVVGSIVGFMETAAFAWMRDKSVNVQIDGSTCIATDGAAFAVLDDFSTYGDSRVDVTVSHVAGVDGFSFG
GLHSFDLLWCVNNLLQTSVSIKSNMILISPTVIPNSSKVVVSMLFLDEETEPSPVDASAIFVGVTSEECDY
RVMTSAEFERRIAHGNIWGTWSDSKTKAATPRSVYLSVDMTKGSIMLSSSLRGLHLHPTVATCNCPIEEDT
NLRVIVFTRTPCQVTSTGGWVSVNDDALDINSSSVAVGLIPSNCIGPDVGIMGCNNLLFDTLSYTMAQCGT
AWKVAPRIAEEGTGILFSIGDTITVERVDREVAAYRNGLLIATYTIPPETLLHRRRLVAYLSTRGMTATVV
PPLFGKAHLGRVVRTCGSGVGVVKCLCPCQGKREYVVRKRDVRHCALSADAAAIVVGAKVAFKAGSLVQPK
RGLVTSIGGNTVNVCDETDKRVCFSLDKNSIYVLDNEGPHKVLQPPYPALKAPDGSVTRVFVVGSTKFRLL
RNESYNGIMFDVRAEDSVVLTGLTVLTHASGRHRVEVFFKKGSHQMHERTSGSWTKIFSNFVKMRAGRQFS
VTFHGIRIEGGSTFALYINATHNFGVGHYAKEGGCSGAVSSEMDSDGTLTVLMGRTSESSTPFTDVSSNSC
GFCGSIVYYVRSEETQALAVEETDSVVWREWDERNNDGCSDHLPLVSRPQMSTLSERAEFVVTVFDGPVVLR
ELLLPVFVQPYVGHTIGPMSFHVSLYCADVNDSTEGVTSRERNWVWVHHEVLLPKECSHRIVMNKLQLKMK
RGTYVFSAVCERTMGMVRERGRERIRCFFTCSEQGHCVSNTFFSVRGLVGNILAVPANPMSVSEVLSAIST
GGHLSQNNSASYNGIMFDIRSKQDVLLEEIFCISQTTADNINVRVFWKEGSMKGAEKVPSKWKEVATRYLY
LADKQTFSPGPLNLQLRANQMYALYVNTTSSCGVRFYNSSDGHVGDVGDEFESDGVLTIYVGKKSEGIVPF
LEIPSEPRAFRGRVTYRLIVQRRLLGRNMAVFPAVQGFVITRLLTALVAFASGPFSVTQPFEDTGVIESLG
KLVKARPGNVGRQEISEDLLSGVVNGNLTDALGDGVVTLPFGVNGVAYFNSLCKGDLAVIALGSGEFSAGE
LVRLVSDPDIKSQVAVEVGRGGTPVCVTVPVDCLVPVIECGWCHQPFAVESVCQATGTVHFPALREEEHLI
SILARYIAQRWNLSFDAGSACASSLVRHTRSTNFAADRIIKARRHDSISGRALDSGSVEYMLSPELLHEDG
WNEKCCSFACPFVYNENLAAFVSLLFTPLGDSWGVAFVTDLPLRLSSKSRPKLLQVSLHIVFDDDIEQDVL
YEPATYYAAHGGPLHEQLGVFAPSQYCFFVKRRGCDCLRLSICEMTFPPPTPFMSSVSHPWRWAGGYDTVE
LKPSEACELEGVFSTGAWPVNKPRFWKLGAVSATSANVFFLEVLVQLFRPIFKINWEGSRNSVIHRSFSLA
RDEELTLAFTADNDFVVYDEDDNVRCHVSYREHVGSSLKGISPNIAVMNVGPHPTSLYLHSPREVIDRGIS
TRSRSWSEGVVEYTPVWTLCNPSKVVISNKGRTASCNDASGQAVIGSPLPTVGLSGFVVQITRSDRAGGDS
LGSGHFAGIVVSTFDQLEPHFVKLREQVDQVWIVQDVLDGDSLPTQERIPPIDSSGNTMFLAGTKLHFLLD
RDNGTLSLARNNESPRVVFHNIPSNLSVSPFVRLDHANASATLATFYGGALGAGKFRAPPSVVLASHPITP
TILRRIPFRVVVSTYTVLRHVLYSLPSDRVTAIELIWCDHCGNSKLAYRLSNALKQLAEVGAFSREMIRVE
DKKLEEFCGQAAVRRVVDALSSPLDKPHVFIPGTADEGDTFIRLPSAYEVIGRVGGSSMVRLLYFDDNTTS
KRTLHIFSHDDPEITVTSFNVVTKHPCVMYQEDIMQSLDSSKSFGVITHGWFDGTLNSLDMERFSKQQQKS
IVMSLLAALEHWHLHSLIHGNIHPKNVYVKTGRGATVTCAVWNVYTLRRYDHFASPQLQRQGTRDMKGDLW
SCAQLLRMFSTFLICEEGFRSAAGMLEKGNMTISYILDQLREFADDAKKSAGEETIFLQTGRQACIPGNSC
SGIMFDVIAKRNKVNIVKVYFVSDTTSTATVTLFMRNGTFRGVEEQGGEWQVVMRRDMELARGVEAKVEGF
ESITISPGEHVAFFLHTTNHSSIMFYTVSEDGRPGLSNVALEDKYIGITAGRVTTCTLPFSSIQKEKQLLH
GGITYTIPSQSCGYRSYLCQRVNARDLQFNRPVGGPVTVESLGRGLILCESGEQRLIFLGGTRMEWVQWET
VSRVSFDDSPAWRQLEDDLRGVICGRYLSLGSLNPPHMQSSKLQFNRRICNQCAVTDRLTTEGDFFWWVSD
PIRTSCIVSVKEISSVQGSSLFVSSSEGTDGEDLSKVESLDGKKHIFIYVDELGRCVTSLEDSGYRIVKKL
QDDLSQAALFLVYGPVPTCEDIFPLSVFVNARSYNCDFTQFEDDTAVFSYDTSVPLEHNLLFNGFALKCCA
VPHETPKFSVASCVTYRSDVSAVSLPFCDVPQTKEDAERDLSPILERQNHRRRLLLKFQRQQPWVEEEVTF
SASPQHVTISNGCVQHVSSIGPQSCTVTKPVIEGLKLHEIELLVCTSVQFPARVTFQHPPTAWFVDVPIPI
IPVHGESLILPTSSVKCVRLYIRVIPWAKRALVVVNGETVYEVLQGRDCPADMQLSFTLSAPGTSVKVVHW
RVLHKEDSPVQRSAMDTIWTQLMIDRISPNVSQGMGSLGQHSWNTPAIGIQSGGESSLIPQVEEAMVSYAK
KLIGTVIMSCNSSDTLKTLQHLLPYTTLRNDLSSLTALVRAEVRRSSFFLLAAAYACLAAPFAAQSAENVE
VSVNLIYVSLSLDGVIHLLSTSFGSTLMLLLLRTATAHVRAVRNVALRCVLQLLKMSNCPLPSHEALCASL
APLLAVISSMCQRGKMSCTFVQLGILLVSELLQRYQLERPSLPAPGRQSCVPYAIVICTKLAIEAVTSKPP
RPLPEAFTRGQQESHRISFELLTGPTSPEGYCSCYGVFSQTFRCTFGSKKFEVVLNSARCGNVVVGWNMDA
SLPGSLAAGTSDKDQSSASTLLPSCGYLIKPNGKIHLCLSRKKESQPLQVRAKGGDIVLVTCLYHEQAVIF
NLQRESKNETTTRFETFPNEMLALPVFFAERREDATFHGEQLLTEASTGMDVSQLYSRFQEHPHRLDTEVV
AQSFEFYAELSLFCQTFSISEKLLVSPNGDASLTVPSHDLVEYTHLLSFLGADVLGNDVPIERLVPYVKRL
QIFDTLTAVFSVVVDIRRSTELFEMWKKLKSLCSAESSKKIQNETMRPFRNRTGHKGKVTIHTMHARASVR
YGFYPTLMRSVFGQLFAQLQRSPISIFYVSPMFTVKLAGFGSTDVGGPYRDILSQLATEIMSTHPSGKFQQ

NPLFKDCGCDGQLAVMPNATMALEFQLFPMFEFFGKFLAACFITKDLLAVEFPPLFWKLLLSEETSLRDLR
AMDRDIMRQLTPEALAERTAEELEERFPGLQESWLRFCSENSHLRMREELPPVDLRTAGILAEHIASIEVH
KYDVVISHIRHGFGQVIPLYTLNAFRWQQVELIICGTPKLSCQALRDVCEVSLPSNDSQMFDEVIETMCDK
DRMLLLRFTTGQTRLPLKENIKVKRGGTRDSLPTSNTCFFTLRIPAYSSIDVMRDRILYAIRHCEAIDTDG
LAQEHIVLDA
>tr|C9ZPF7|C9ZPF7_TRYB9 | DeepTMHMM Topology Prediction - Predicted
Type: TM
MCLLPHTQFTFPLHTGRPRRRIEPFAFYSSFHCLIACLYAQLVCPTALFNFSFTIMFLQLMVLVLVEVLPC
GVRRNCGCVVYVTVVLVVVLGSGEGVYWEKKEMPRARAVSALEHMHQKADNINIYVHECTNK
>tr|C9ZLX2|C9ZLX2_TRYB9 | DeepTMHMM Topology Prediction - Predicted
Type: TM
MVDVHRKEGGGIGVSWVRAIVFVLLVYVISVTVDFCYNVYYDREAAFQNVLNCSLQVFRSNSVDCWLQNGT
LLGSARLGRLLLWDADLDIGFVQANHTEKLQLLMNELDSKCFGARSDRRRGVRNPLLVFRKCTERICAEFH
ETSISNGIVTTGDGASPQRELFPLRTCTIGDVVAQCPYNSSYYLREAYGSGWLTASLLEFF
>tr|C9ZPW5|C9ZPW5_TRYB9 | DeepTMHMM Topology Prediction - Predicted
Type: TM
MPIRSLFSLPHSVSLNFPLGACTSFISGALPYRWGKLLNLFFSVYLLYLVDLPCCFSYYLLGNALAPVFFF
QEQDSSRKKSVGRRRILVAESHSKKTNKQSGVKGKEKRESEREGPRV
>tr|C9ZZG5|C9ZZG5_TRYB9 | DeepTMHMM Topology Prediction - Predicted
Type: TM
MSEWEVDECCDPQHMILLLEPKNAQREFPPHHTLCASCLVDRFDKLPALHVVQTLQDIMLSLQYTFSPVLV
KHRTNSLHESSFVDVPAQGCLSSPPWDLLLRFAGLLQDLGAKICRCISLHHHMPLYKAGLSVLALVFTLLE
EATESGLFCHTECLGMDGRSYDFTVQGHTTLLSQPPVGLSICLCETVLAGLSEEVKVTADDFSVPLPLLLL
TDLAEASPPFLWCVAEHCVPHMCSLTTALTDAFRVEILLYLLHLAFEACAKHPVWLESLANALPASSWGVR
RSPFLTYLTWALETFRGEEPVVANALYLTRFVLQYGTTRTALFTTTPLPHHDAAAGTAGGKSDSYGLPLSD
ALGLARNLIILLLHLNGDVVVAAAEALRELFVEKPLLGECPDLSDYIIEALRTASADTAPALVGLLNNLPS
GSVPYGPLLCVLFEVSSISGKAFEDAVRGPHFVDAVIDRHTNLNDVAEKIVRSVRDGSSVSFHCFLLECLC
LERACKHENCGGASLLPETISMLTNTLLHVVWELCGEATAGTEAGGNYHADQQLFPSEFRTIPCGALHSLI
CVAAFLLPGCEVLLMQLVVGLLGEALRQFQIRNDGGIPDTRLVFDSSLGDAILKMGSTVIRRLVGKGDDFG
IGDSQLEQKVCHLLRGNLLTLLLSVPSSFSDLRVSVMNDFLLLAPANRDLTGEDGQIYFTFVKGADGLMSP
QLLVNNSLRRHPHWAASFLLALATAGCEQPLGSGQLDSFLFEQLSLVPLFTEGARQVFASGGYSGAYRTGD
DVIPSKDRCSVAEFSALKVSLAIHKWGGEQPQRDISHLNACVSVSGASVVSEELRLSHLQMFRSAEPQWLE
ALGARTWGKALLAALVSSAIIQIESDREQGRIEEAHDYLFTGSQHRDKDQVTLCSLVVNHAAGCGLFLSLL
FNTLRRRTASNDHPLSCRIISFLCRCLHAEESTDTTVIIEFVRLLLEQHLTPLLLYREAAPFLVGHAARLV
ALSLSRLPVSVASCCDHTLFKWALQNVRQPSGQVYMWMTIFLLLRRAHRSEFALRHEVELGAELNHMVKGG
DVSPCIEAARATVCWMVEGEMMRCGVGRHHSAVQIPLNEWSKVAPGNLTLRAFVICSVEHRMKCCWSSAMN
QFLEVAAPLLLTWVSELMFYHCETVAASRVCHELLGKFPHLSCSLEALGLFFLLTGVVVAGTRSEKQGNDP
VHSVGVAEGGWVFAALLNAWPSWAEAGGLREAVAVFLSGLGEESKLCNGAKRPRELLVSGSAATVAQFNRP
SLNVYEAVRAVLEACELQAERCGPFPVNRAKDFYLRWPVEAISIDMERDIVFRAAEGGGELMVAS
>tr|C9ZUS7|C9ZUS7_TRYB9 | DeepTMHMM Topology Prediction - Predicted
Type: TM
MNRKVKHSHVSSWRALKAHALRIMTTKMIKIKKMRKGVYFRNLLHFLFKIFELTVAQLARGKYIFPLQTQL
NTDTHTYTHTHTYIYIYIYVCVCIQQSFCSFLPLVISLPHIPLLLFSQLSTLLTFFHAFNIFLFISGAVLL
CYLSATSTQHILKCLSSIFLTTPPCSSSSLFAPHFICLA

>tr|C9ZKH5|C9ZKH5_TRYB9 | DeepTMHMM Topology Prediction - Predicted
Type: TM
MQKSVPSYVDPFGTCPSSHANTPRAPCRRHQRRLHKTTHGGISTGNVQDVDEFDDGFVGINLFAEPESPEA
VREGCNCDLQRGIPPQRKHVVLESRPNVFVPVVEQNKCGELTGDDVVSDVMREMPQPQSSCIGCRIKTHNS
AGIGARPLHLPEPVDCAEGNRHAIKVLLLYMNHMEVTMSSLTAGASIFSLLWLIIFVAWEKESVEDSLLLY
IAITHSYEGAAFQFLFILLLVASLTPFAWRFANKQWAEEVSDRCPAGINPLPQHLSRREGIIDGARFPISC
NPVTSHVDTCSNAATVVAPGLRCDVRHSNAGVKTQSHLQQHKDISHGGDVAPNAAAAASWEVEVSLKSPWM
LLLKPIVPLYIICTALTVAQIALTEGWDAAKLQKVTAADASSLRIVLLTVISVRAGLMSLAVLCNLFRPSK
EMKVYFLS
>tr|C9ZN56|C9ZN56_TRYB9 | DeepTMHMM Topology Prediction - Predicted
Type: TM

MVMPWICHFVCIHLIILPVVVFAFFVFRLFFFLFVCLLLQLSFILGFVLLSVLLFPPTTLLFALSLLLLLS
LFLLLLLFGSQLPLCRVASKVPVLLFLSHTYVLHFLRVFRCVKEKYNNRQEQEDQYIFVFLYIFICLFIYI
FKMLLICFCNVYSF
>tr|D0A0S1|D0A0S1_TRYB9 | DeepTMHMM Topology Prediction - Predicted
Type: TM
MCMRVAAFLVLCISYSGRVRIPFQCLVALLLLLFLTNSDALYVLTGVRQATMLRVIACFYPLLCIRFLIFV
TLSGEVAREAIQLRKHNVSLVDHNLFHDSDGE
>tr|D0A6C7|D0A6C7_TRYB9 | DeepTMHMM Topology Prediction - Predicted
Type: TM
MVIIIARSCILFIIYVVAEGINRRVCRRCRFLPAPAASTSLRQCTYSATTTITIPRLKDRTWHCNRCCSFN
VYNHPKPIFVDLRRGKLNGTNEMKQTPTQNLNVLIYKNAKKKKTTAR
>tr|C9ZIT6|C9ZIT6_TRYB9 | DeepTMHMM Topology Prediction - Predicted
Type: TM
MASNQQDASIGVVDRQTEPKRRRRSSGTAFSSMELHRRFEEHVAKAQQLFLQGNKDGAEQEATLALRIHPT
DHLFALLAVIAESKGQFNRASDLRLLQAFMANDAVLWEELLHEFMQERLYYKSAVCLHRLAVLETRDKVRY
RTLQLQLADLYIGLGEFKRAAHILISLWNGSRCRDFEVFAMLSSLFFQLGRWNSLHRLIESSLKSTFRPVL
STMEGGEKPPDAPSSRLGREPSTVEGKEELTAPMARRRVPRRVRFFGIDDNDECDNDTDSVVKRAEGIQQA
GSLSQPTPTVDNTCLAEALCEGDDFDFTSEGCDSQSVHGIRSGACATPSAVIHSMYGDSVKFRTATDKKNF
LTLVNVHAELLNEEGNFPDTVRLVEFAAGCLNVSLLELHPDLLVRLGVAYAFLGGMEQQCREVFHHLVDTC
PMAEYGDVLYDAANALQQVGLHTEARMLFQTVRRYHEFRMKGSNGNADDGNNLDDEEKTEVKTVFVAALFA
ESQCEASLGNIEVACDNLCRVLEVDPHHLQSRLALGRLCMYDMNDTERAIEVLTPRESEPPLERIQLAAEL
VRVFARSKKYVEAIALGVSVFELIMSSQYEGDADSVAPGSTKRSTAPLSMPTLSPASSAIIPPSAISDIVN
GPRLSSSADLSSTRLSHALATSIRASSVAFRINLARRGGTPLTASVYGASAAASTVAGWSHGDEETGQKDS
STIFRFNRTLKPVGCLRPVNKGTCNRKCPRVADEGDVDTEDDCRRVRKRRREEEVAQRDVRSFWKEHADSD
PDNCGEPSNQENETEHQMEQGGAHLSTDKKSENEDEGPCIDDGSDDGDATRFELPSLEEVSKQFGDSYMQE
LFMQASSNEAMTSAGHMPASNVGGTIALDNVLGDDTEAGLGQAAAPLRVTMRDALKVLGCAGFIELAVTVV
DCYGAIGKFTEAKEFAFVVLIGCQRTSIFRHLVTMERPLRWAVLRAALASGECEDAYRVGIRLLQEQCSEQ
EKDRILELLFGVLNRTEMGSSILLRLVAGGYQENPSVLVLLGNRYFLRRTYIRALNMYLAAMQRRPNDVLV
CFLVGVCFLLVSHQKRIRARNACVVSAWHYLLRYQGALRDIGPQRQAEATYNCARALQYLGLHHLSTPLYE
QVAYEYSVPEQCSLPLQRAARFNLYFTYRWRTGNSRLALDALQPRF
>tr|C9ZNR3|C9ZNR3_TRYB9 | DeepTMHMM Topology Prediction - Predicted
Type: TM
MSSEAASGLWRSEDMTLLRLTMQRETAHDSVLKLGQLAAFQFIDLNSDVSAFQRDFVQEVRRCDGMERKLR
YLHDEIEKAGLTCVSTEAIGRESLFALEHKIDEYEGELRELNGQYQSLLEESNRTQEHLEVLSREFGSGIR
QSPGLNLLTGVIPKDRIATLERLVYRITRGNSVLHTDEITTPFSEGEKERMVQKCVFGVYFATPRLWESLK
RISEVNGASLYPYAESTERLQYMRDTLNSQLETMKHTLQQSLLRQRHLLTSISHNVCQWRQTVAVEKSVFS
TMNMLKFSGSTAVAKGWAPVRSLDRIRASLQEAEYLSGAQVLTIVEEISTKEKRPTCFFTNKFTVCFQSIV
DSYGMARYKEVNPGVLTIVTFPYLFGIMYGDIGHGVMLTLFAAFLLIKEKDWEGRKLNEIFAMIFDGRYLL
LLMGLFAIYVGFLYNDFFGFSVDTFRSGYQWPPLNGNTQEGDMQPSSPSGVTPARSVIFGIDSAWAETENK
LEFYNSVKMKCSVIIGVVQMVAGVILSLMNHIYFGDRIQIWFRFVPEIVFLLCTFGYMCVLIIIKWCTNWD
QRTSEAPSLLETMTNFFLQPGTVSVPLYKGQEFVQVLLLLIAFAMVPILLCAIPMHEKKEHERKMRLQALA
RRNEDERHEGSEDDYEEDEKFDFSEVVIHQVIHTIEYVLGCVSNTASYLRLWALSLAHSQLSEVFWSFTFL
MALDMDKGSGIFVFFGLCVWMCATVAVLLGMESLSAFLHALRLHWVEFNNKFYAADGYPFTPFNIAEVLKE
LD
>tr|C9ZTV2|C9ZTV2_TRYB9 | DeepTMHMM Topology Prediction - Predicted
Type: TM
MCFYVSFPPLHTCVYRSICACVHLFCPSTAAVITSLLCLSNLCFLLLLLSYQKDHVSYIYIYNSEKGTDKE
GPHNPLQGVLAPTTPLRRRKTPQGGGAT
>tr|D0A3F6|D0A3F6_TRYB9 | DeepTMHMM Topology Prediction - Predicted
Type: TM
MRFVAAPSQYFFYGCGVAIDLTAVFLPSLFLLSQRYLRCPALCAFYARHSRGWLRFFLEYFPLFFCVPLAT
LLFFKRNFPFLLGLFVCYSFVTECWATSFPSFSTCVARYVIGSVVLQHLRHTVAVTLQQ
>tr|C9ZPF8|C9ZPF8_TRYB9 | DeepTMHMM Topology Prediction - Predicted
Type: TM
MANVADGTDGEVISGRGWKWFDRKGGTSEEKSRSFQRKSCHEDHTPLAKVHTKPVSLFRILLFLPFSRYIK
KVSFLLVCFCAMFLCVSLSLPFSDTDLPTAPFSSSLLHMRTLVRVFAHRDRHKGSGTHWSFVTWLLST
>tr|C9ZTW7|C9ZTW7_TRYB9 | DeepTMHMM Topology Prediction - Predicted
Type: TM

MGTTKMIVYPPPAAKKWCASIYFLFFVFSFAAVTAVCFHFWRGAYGAKQFMLLRGARRQNRDDHLERFLHC
TDRVWGERIGEGRRVCKKKRYACDHKSTGVCSMEVFFLGGETAASAHWRFPSTQHPIFLFFFTYITVRTLH
ISILPYGEVPRTVNICGTAPFSTSPFPCCSTHIGVE
>tr|C9ZMN3|C9ZMN3_TRYB9 | DeepTMHMM Topology Prediction - Predicted Type: TM
MTLADTLHPVFVFILIVNAVSCAFPLLSHRLVFYPIVFVALGLLYVTLLVLLMGATVMMRNQSFSDHRVKE
NTLIIIKRDVNMTTWMVISTVVMVAWMYCWARRVWLVYILPMVEEYNAFKSRRWADALDLRGRMKPTVS
>tr|C9ZT16|C9ZT16_TRYB9 | DeepTMHMM Topology Prediction - Predicted Type: TM
MFFTPPQLQKLEQDWNGLAVRDWMIANVDVVLYISFLYLGFVFIGPKLFAKLVGTNPAAAAAAGARSADGT
GSPIVRRSMVVWNLALSIFSIFGTSTVTPVLLRNLANKGFYGATCDFKETEFYTTNVGFWMGIFALSKIPE
LVDTIFLVLQGKQELPFLHWYHHVTVLLFSWHTYCVGSSAYIWVAAMNYSVHSVMYLYFALAALGYKRVVR
PLAPYITIIQILQMVVGCYVTIFALQELHGEGGRGCGVSPANMRIQLVMYASYLYLFSKMFVASYIRPPKR
PTVGGPSSTAGVSNGSVDKKVK
>tr|D0A4H0|D0A4H0_TRYB9 | DeepTMHMM Topology Prediction - Predicted Type: TM
MLRPPLSPSVHATSAPFFSSLIRVSLLGICLVSFFTFFTSRWSPVVCCVIEGHVVVVWNKKFCWLTGFFSV
FCCFFLSFFCVFVFMNTIHSCDCLCCFDYW
>tr|C9ZT01|C9ZT01_TRYB9 | DeepTMHMM Topology Prediction - Predicted Type: TM
MSDITYENGSPTYTGNTVLKCFRENGNGLLFRIVNDEEKKWAFYNDTKDYNMVVKVAFGKDSTVQPLGNTK
MEKDTATGEFKCELEIAPLATEMFIEGVPNGYKINFEANPIPQMSKMRLYMRVQQMIDSRNVVRRIDSPPF
FPFSLLVLLFLGVAEKMRDPKHFILLYQKSSPLYAALALPYVTVRRVVHFFQVPSVFSSFCIISSLLLPFR
ASTIYVQDNLRYFTSSIPNLRKNKRHSNMSDITYENGSPTYTGNTVLKCFRENGNGLLFRIVNDEEKKWAF
YNDTKDYNMVVKVAFGKDSTVQPLGNTKMEKDTATGEFKCEVKIAPLATEMFIEGVPNGYKISYEADPIPQ
WKSVPT
>tr|C9ZHT9|C9ZHT9_TRYB9 | DeepTMHMM Topology Prediction - Predicted Type: TM
MVHPYLWVIIVGGIVSFLTGCGVGMNDLANSFGTTYGSRVLNLWQIVILASICEFVGAVSLGSEVTSTISG
GIANPMTFANEPYVLMYGMMCALSATFIWLLFATMMSLPVSSTHSIAGAIIGFALVYGGFGAVSFAKKIDE
FPYVTGVAPIIASWFISPVFAGAVAASLYALLRLVVLRPANSVNRALFALPLIVGVTFFLESFFVLFKGAD
SHLHWGPAKASWVAALIGLGAASTSAACIPLLRRRVRLITERAERERAETGMNTAPEISGDAGAISENAAG
VGAAVEGPVDTANRIVPPSSEPTSDSPTAKENPANNASGLTTPGVVDEALKFDVQIYDERVEYVFRYLQVF
TAACASFAHGANDVSNAIAPFSAMYSIYINQQVVEENDVPLWILVLGGAGLVVGLATLGVRIMRLLGERIT
KITPSRGFSAELSAALVVSLCSAFGIPVSSTHCITGAVVAISIMDCGFRKVRWMMVGKMYLGWIFTLLITA
AISALLFAQGIYAPSLTSQ
>tr|D0AAD0|D0AAD0_TRYB9 | DeepTMHMM Topology Prediction - Predicted Type: TM
MCVYVFSATWIAPNANCAFFTPPPSLSLSQRTRHEMHLLTLHVIVNTFFFCRTERREMVNRGVNVTCHTEF
TCALYPHTINLRQIYTLRLFFLISVISSFLSVSEAVAVAGEARIFAYQAGLLP
>tr|D0A9G4|D0A9G4_TRYB9 | DeepTMHMM Topology Prediction - Predicted Type: TM
MLMLHHETYNVFRTNPFVDNLLGGKCMMLKLMSFHAIAVSSLLVVFCFRGVWEVPHVVLLVSTAMLVVDDF
RLLYGVLLNVTTSFVVRELVQAASFVGSFASLLLFTCADVGYVAGPPFLALSVCLAWLMSRLVAFGVLPAF
ATTAAAGYTAIIHTVLALVSFSLAIGICLLLPPSMLQPGPCSCGKSHYVTNFLSCLGRLDPSFFIRSVACG
CLVAVVSLLTVDSELVTLPVLDSLHFKAADIPVVDGSGNWPNGIAISPVVVLLLATLALLLLCLVPSMGPF
VRFVELGPATAAMVLIVAFFFDGKQLTATLAAVCAFSFAIAVGGLLRSLGAVFLSRTVVRDNFGKATVRPT
AALFTTVLPMLLVTAVCSSCVALILGLTAWLAAVRGNGNDSGDGTADRRGWAVMFVVLVFSSVALALQLLV
GMWWWLPRLYKRIAGIV
>tr|D0A5H7|D0A5H7_TRYB9 | DeepTMHMM Topology Prediction - Predicted Type: TM
MAPYTKEKHKYMYTRKSGDTYVFLSNDALDVVVLRFSYYNFLYFFNFSRSKIDFIMFSSFLPLPLPLPLLP
PLSVSLSVCFPSFFFLRFFFHLSFVLFNRVWLTSC
>tr|C9ZM39|C9ZM39_TRYB9 | DeepTMHMM Topology Prediction - Predicted Type: TM
MHKLTQLLRYRKTEDELVESPSVDLTLSFSAGGWLQMYHFGGAQAIVDSGLLEKLAAEGKRVRFCGCSAGS
LAATMLVSKMHCFEKVRERVIMYGEHYRSSWIYLFCMINYLKDSLDMFGKHMRDLENNPELNGKLNDGSLE
IYVTKLPYIKGKVITTFKNYDEIVESMLASCCLAPLVGMPFKLRSTGEWVCDGALTTVTPRMGEPSTITVS

PFYYSSATIHPTVFVPVWWGLCPPAEATHRDLFTLGYNDMLKGLMACGHVSKEVGEPLLKPDVSGAFKDMK
RIGIFHHWAGRPLLLFVRLVVCFFIYFELCLACMACIMRSLLRVDREPLRNMRANVVNMLTFRTLRRLLFN
QDVQGSDQHLERTSCIFRVFRPIVL
>tr|C9ZRN6|C9ZRN6_TRYB9 | DeepTMHMM Topology Prediction - Predicted
Type: TM
MTVVTVQAPSIHVHTVSCSCDISMLHVGTVYRILTYFRALLLIDEESIPCFLRRHLPLSLLWLGATTASQG
FGGGQERDATCKAIFFFFCVPPGLSVVTAIIACG
>tr|C9ZWT9|C9ZWT9_TRYB9 | DeepTMHMM Topology Prediction - Predicted
Type: TM
MEGKFHILWRFIVDILVCTKADKSEGRGKDVKRKKRKRRELIIILLHAFLQLHDSVAGITSQIMLREWGAAT
TETVSCRHTFTHLVVTCVRAAVPSFLPFFSFLFFLPHWWSHSFLLFFLIYIYIYIYIYLFIYFFVFVFSSF
YREQFHKQTSRVKFFFCGSTLPVSLRV
>tr|C9ZYK6|C9ZYK6_TRYB9 | DeepTMHMM Topology Prediction - Predicted
Type: TM
MRKLVRSSTVQRAMPVFCTPILTSRCTVSSNERVTYFDYLDRIWNCHWFGVSDVGIAPSQEPAFITNMFVS
FQEGLNLGAPEAILLLGVLCRVATLGFSLYGERASERMRKAICKLKTPHEAYQRVYHSEGATSLDIQLAAT
ALKGERRRVFAEEKTSNAQCLSSILGSPIVLFGMFQAKSLCENPYLEFGTSPFLWCTSLTMPDPYGILPLA
FCGLTLANFELSISKELKTGWMSNVVWGARLGCLCVLPVALQFRSGVCLYFLGMGFVGLLQPILLRSNKFR
SFFNFPTEGKADTNKFSYTDDGFYTRMSVQFPYVSHLFDSVAEENISRPPQPPVAPVWFGSAQSAMGYGKK
RISSPASVASEKRVAFGKGLNFAAPGWKDHRAEFREEDLIPDNSCFGDKSCKSGKDGALKDAGGTRVRS
>tr|C9ZUG7|C9ZUG7_TRYB9 | DeepTMHMM Topology Prediction - Predicted
Type: TM
MRVWRLRNQNHCIIALNHVYSPPQGVSVGTQIVSTIFAALAGSAMESTCKRIGGNCSKESTQIKSVIMESQ
MPNTSENVPFARYDATEEDEDLNQCEEKNRVKEFGYQKEKRKKEEVIVETKVSKVKGEKSKSKICRRKGEN
SDEADDEYYECREGVTNKRALYNLLIIVIFLFF
>tr|C9ZVX1|C9ZVX1_TRYB9 | DeepTMHMM Topology Prediction - Predicted
Type: TM
MAFQTPWIFIAVIGVPLFMVYTKARRMDAKEIHEIQMRVKYRSEFWEKGNEFVRSHREIVLKGLKETNDPM
VGKEFSELETNGVTKTHHSIWKLW
>tr|C9ZMC6|C9ZMC6_TRYB9 | DeepTMHMM Topology Prediction - Predicted
Type: TM
MSNAPTLQAVLVVLLMLGLGVVVIIYTITKTRWRLGASREQQRLRLGSTANNLSGELLRNRLIRQATEVLV
TNKHAVEGIPLASTVGVTEVLADREMRARRGRSHSITTAGGQGTTTTNGAETSRSNRQVPEASAGPTFIEL
LTGLHHHLQRLNRSQPASTDSHCALVEELIERIMQSEEDAERKADIMNDADEVYGMGFEYSLNPANSRIKY
VEKTWEEEPLIFSPR
>tr|C9ZWF0|C9ZWF0_TRYB9 | DeepTMHMM Topology Prediction - Predicted
Type: TM
MPNTNDIMYCGGEQSVSRGDGANGPKGRPTAVQSRQPHTSLSFIRFLLFTYSYSFYPPVLSLSLLLFSFFH
MFSFSVPTTASHFVCINVCRGRGVVSPWSIVYQCLCGWRFRCQKTFMY
>tr|C9ZTR7|C9ZTR7_TRYB9 | DeepTMHMM Topology Prediction - Predicted
Type: TM
MYAFNLISGTMQARYRFSVKDIMNVNAVGLAVGYFMLPYGFIYDYLGPRPIFMLSLTVLCLGALLFALTLR
GDIEGTVVRLSVYNGLMTLGCMLFDLGGVVTVLSVFPSNRGAIVAIMKSFVGLGAAILGSIQLAFFSDRPD
IYFFSIMSFALTVGILGIVFMRLPPFHLTGYQEKHLDEEEKAQRLARKGVYLKQKAPMWRFIYGFVLLLIL
VVFLPLQGALVAYLKLGSNFKVGFAVTVIVLTVIFPFMAFPLTTFDGKRPHDDSDGEVDDKEEMSEEPFPV
EDKVVETDVDYIAPQFQETFIEGLKTARLWCLLSSVFCCVGGSFVVVFNSRFLYTALAGEPPSTNVGILLS
VLGALGSAVGRLLTSGVEIWSQKRRAEDRVPITIALFIPSVCIITMLTLFLTLPKAALPLPHFLAAVADGF
MATTTPLVARTIFAKDPAKHYNFCFLGSVLSAIFLNRLLYGEWYTQQADKLGQDVCTERVCVVMPLAFLLG
LSFLAFITSTYVHLQYRNLCLKALEERQRVKEQHRASKDDSRVSTEPIGVI
>tr|D0A7U1|D0A7U1_TRYB9 | DeepTMHMM Topology Prediction - Predicted
Type: TM
MIFLVISTAIIAVLAWFVAGVFIRGGGGRGKTAAPQVVGVAQYPSQPSSRVDVRVLFGSQTGTAEMFAKTV
TREGLRLGVPMKLADVENYRPSDLAGEKYVIIICATYGEGEPTDTMVGFHEWLVDDSRAVGEELSGVKYTV
FALGDRQYKFFCREGITVDRRMSELGAQRFYPLGYGDCGNSIEEEFDNWCHNLWPVLGRALSLVLKSNSTE
PVAPECRMKLWGPPEEAPLPFPKLASVLEPTQRLPSWAPVKVNKELLSNATGRSTRLIEFDTSETVISYQA
GDHLGVLPSNPSEMVNTYLRVLGVSEQESSQVISLQNRATGKNVFPCRASIRTALTWYIDLAGPPKKSTLR
AFAHHCTDPVEKDTLLKLLSTEPESVEAYGKLVLELRTVLGFLQRFKSMSPPLSFFLEMMPRIAPRYFSIS
SDSLTHPTSVAITVAVVEGGLCTNLLQQAAVGQNIPVFVRKSNFHLPLRAKDRPIIMIGPGTGVAPFIGFL

HRRSAWLEKGNKVGDALLFFGCRRREEDHIYADFMEKCLSNGALSVRDVAYSREQADKVYVQHRLAARGKE
VWEIISRGGNVYVCGDAKNMARDVEKQLLDIAQKYGAMKEDEATALLEKLATDERYLKDVWTA
>tr|C9ZPW0|C9ZPW0_TRYB9 | DeepTMHMM Topology Prediction - Predicted
Type: TM
MIFYHLFFIHYSLLAPPEGVTEPPIHVLEKAIYIYIYIYIYMRVVVTSCSVFVAWHHFSLCFHYPVIPIPT
PHSTIFIVFLFLLLDLLCPSIWVYECSLRICLCPFRVYLWLGIVR
>tr|C9ZN66|C9ZN66_TRYB9 | DeepTMHMM Topology Prediction - Predicted
Type: TM
MASTWPKLMMSNARLQLPANVPTDRFFFNFAFLSLDVARSPLPVHQKLKKIKWSLLIPACSYSSKVRTISH
IRFCFSFYALQVLHCFLSCVISCFSGCFFLTTRRVNTNHPIIPVYFSRLACLIARGFLELLLLLLF
>tr|C9ZPA5|C9ZPA5_TRYB9 | DeepTMHMM Topology Prediction - Predicted
Type: TM
MQKGGDISVWFHPSRWYGIGGEGRGVCVGGSELVLGFFPFFKKNKGCKWFCFVRYLLLFCFIFTLSRVSFL
CMSGSHYRCLWCDRHAPVVLLYHTRKHKKIYCKLCPLVRGVSWCILLFPVS
>tr|C9ZN41|C9ZN41_TRYB9 | DeepTMHMM Topology Prediction - Predicted
Type: TM
MIYHSETDGPHTPAASKSAGSAVWHCRLSTVMALLLFSNVLPAHSEGNIKVKVYSFIYSPYVEYRQVEAIN
AGLNASFAARQWTVAPNVTVQVVPPPPNNVEVVDALQRVATTEKGLFVVFGPLTDIETLHALPLLKREDLV
AFAPSTGSSIVRGWNPNIYFIRASPTAELIALVRYAVSQLRLLRIGFMYLQDISFGDSEYKHAVELFSHMG
RELCGVFTVKSSMEAFADDRAFEAAWEAFAKTRPQGVIAVAPPINDTMRFLNKIVADKRTRDAYVLAHSTL
EFSIVGAWREALEAAGAPLKFGQVILTGTNPLAKNTLYRAIRRFQDHMRSYLSANPGVTVFMARITSTTTT
WMGGLWMVYGWIAGEVLSQALSSREWLTSRKAFMESLYNQRRYVIDDLVIGDFGGDCKGGAAKQGAACNCN
QGGSLVLMNVIGSGYRLFPVNGGVTIFDSKKCYINKPRIPSPMSILSLTLFDTALPVDTYASMSEVLYAST
RGRESALSRRLFFHSMASSSAESARTLQHQLDTRSVTAVFGVVDDAMLSIAEVAFVDPVMLTPRLHHRGKN
VIQLSPTLEQQLFVVVGYVTNTSASAPMSAIVRGTDATIIEVALRKIVWMHGGTLQTVAVLDDNATLVGRL
PNRGNAFVIGLAPGDPSLLAAHLDRNPDVRVLIPFFDVALMYDELVSAFNGNPNAERVQFATSLPHWADAN
TSSEIVREFHTALPDSSAWKPLPLLGYAAARFAQAVLPRMEYVTPKTLLDTIYMQSIITADEMRYGPFEEE
EEKECFTANDPVPEQGEVCVVNYGATRISMWSLARALNASVPPLTSPVTPLIRYADPNAIKLSSAQLAGVI
VGSLVALALFAAPLVVVLYVLRRGARDNDSAPKEPMEPVTLIFTDIESSTAQWAAHPELMPDAVSTHHRLI
RSLIVQYGCYEVKTVGDSFMIACKKPFAAAQLASDLQRCFLRHEWGTTAFDDSYREFERQRADDDNEYKAP
SARLDPEVYRQLWNGLRVRVGVHTGLCDIRHDEVTKGYDYYGRTSNMAARTESVANGGQVLLTRAAYLSLS
NSERGQLDVTALGSMSLRGVPEPVEMYQLNAVVGRSFAALRLDHEVVEDGDLSSTSFSDTGSLRGVLSGTS
QMIDSCLHAVFGTVPLSQRQKLLLPLCERWQVSLPPSSKATWNEEYCEGVIRRIAVKVGRVVDHCAAVGSE
HSVSTLRSASLIIISNHGLERELHGN
>tr|C9ZV27|C9ZV27_TRYB9 | DeepTMHMM Topology Prediction - Predicted
Type: TM
MRTCLHELSFYSLFSCRFELILCVYNGCVCVYIYIYIYNYLSTYKNVQQDFASHYLWSRLQAHAYACGQKG
EESGGIRKNRRDVYQQKVIVYHSQITIIITESKKKKKLCPHV
>tr|D0A619|D0A619_TRYB9 | DeepTMHMM Topology Prediction - Predicted
Type: TM
MMELIGCPAVAVLTCFYWVASHLVLLLRVALPRFSVIVRYGGRCVTPEGICGGFASWIQSSRDYAKRTVGM
WKSERLSRAVFHCTAILEGSILCRIRVSRKLSFCAFYVAGIVSIVLILILMDGGYCSPIVEGMTVYSDGLN
HSAVAFLVSYAGRPLLAFLMHCTVRLLECLFLHRFRGGSDDCVTGFAAVAGCSFYVFASCSSGVILPCTER
SPLVIGGQRVVNGMTEASPFFPTFALAVDALFVLHMVFQATQVYHHWVLAELRRKPDGTSVPKGEHCSVGR
CKTTPKGVSEEGRIGDGSAVLYHFPRIALFKYVQEPHYACEVAMYAVNAISICLIVYNRSLPSGGTGREIG
DEFSVGSEVVPLLCATCLPPLGVLFFSLFNLAITAREHRRFWECVNARRGDGERELIPKWDLFYGVW
>tr|D0A429|D0A429_TRYB9 | DeepTMHMM Topology Prediction - Predicted
Type: TM
MLSDGALCGLLNSGACMRASAFSNSFPSPFFLLFFPALRYTSPSLILFSFVNVVALIRVLPPFRYMLNERR
PQVHLPVTEARAAIVRMIRRHNAVVIVGETGSGKTTQIPQYVWDDIVSKGDGGIVGCTQPRRVAAVSIARH
VAQQRGGKVGGEVAYAVRFDDTCTKNTKIKFMTDGILLREIQTDPDLTHYRCLILDEAHERTLHGDVLFGL
LKDIARRRKNTLSIVIMSATLNEEHFSKFWWDAPVGVIHGRTFPVTIYHTVEPQADYVEAAVSALLQIHQR
EEPGDVLCFLTGREEIEDAKRMLEQRMKLLPNDIGDFVVLPLYSAMPYEQQLVVFDPAPPGKRKIIIATNI
AETSITVEGIKYVVDSGVVKAKHYNSKTGMEVLAEVDVSRAQATQRAGRAGRVAAGKCFRLYTAQAFESLH
ENTVPEIQRCSLISVVLQMKSLNIDRIADFEFMDAPNPHALTKAEETLMLLNALDSEGCVTPLGKRLTDFP
IEPAAAMVLLAGKALGVEREAVIAIAMTSTENLFVTSRDIKERADRCKGAFAKSAGDHATLVSIYQAFKHS
PKDQRKLWCETNAISYRQMLKVQDVITQLMAILAEGDDEELLATLVPKSVRQALSHKATDESCAVVSGSND

DNTVTNGYDHQWEPGANGRSSGLKFRDFELLRRALCCGYFLNVAFYNAKIGNFQTVVGQQVVHIHPSSVLF
TLRRKPALVLFNSVVRTTRRYMQNVSVVQEAWLRETSSFSRMML
>tr|C9ZNL1|C9ZNL1_TRYB9 | DeepTMHMM Topology Prediction - Predicted
Type: TM
MTKGGKVAVTKGSAQSDGAGEGGMSKAKSSTTFVATGGGSLPAWALKAVSTIVSAVILIYSVHRAYDIRLT
SVRLYGELIHEFDPWFNYRATQYLSDNGWRAFFQWYDYMSWYPLGRPVGTTIFPGMQLTGVAIHRVLEMLG
RGMSINNICVYIPAWFGSIATVLAALIAYESSNSLSVMAFTAYFFSIVPAHLMRSMAGEFDNECVAMAAML
LTFYMWVRSLRSSSSWPIGALAGVAYGYMVSTWGGYIFVLNMVAFHASVCVLLDWARGTYSVSLLRAYSLF
FVIGTALAICVPPVEWTPFRSLEQLTALFVFVFMWALHYSEYLRERARAPIHSSKALQIRARIFMGTLSLL
LIVAIYLFSTGYFRSFSSRVRALFVKHTRTGNPLVDSVAEHRPTTAGAFLRHLHVCYNGWIIGFFFMSVSC
FFHCTPGMSFLLLYSILAYYFSLKMSRLLLLSAPVASILTGYVVGSIVDLAADCFAASGTEHADSKEHQGK
ARGKGQKRQITVECGCHNPFYKLWCNSFSSRLVVGKFFVVVVLAICGPTFLGSEFRAHCERFSLSVANPRI
ISSIRHSGKLVLADDYYVSYLWLRNNTPEDARILSWWDYGYQITGIGNRTTLADGNTWNHEHIATIGKMLT
SPVKESHALIRHLADYVLIWSGQDRGDLRKSRHMARIGNSVYRDMCSEDDPLCRQFGFYSGDLSKPTPMMQ
RSLLYNLHRFGTDGGKTQLDKNMFQLAYVSKYGLVKIYKVMNVSEESKAWVADPKNRKCDAPGSWICAGQY
PPAKEIQDMLAKRIDYEQLEDFNRRNRSDAHYRAYMRQMG
>tr|D0A130|D0A130_TRYB9 | DeepTMHMM Topology Prediction - Predicted
Type: TM
MYCIVIIIIIYNRGVIFCVFALPPPRLFCKGRRFSSFFFFANDFVSCKEKVSLPQTSRAGGLGYMATSSNY
SMQMFFFFLWLCALASGCFLSRKHCCVYLYINKYIFMCCALFCLHMYVCAYAT
>tr|C9ZUV6|C9ZUV6_TRYB9 | DeepTMHMM Topology Prediction - Predicted
Type: TM
MAGEGAVSGAVAAVLCGTALLSTFSTVVPRVLGFIPAHTFSARSYPWNLITYVVVETNIVLAVCSAVYMLT
FGVAVESIIGTRALVRLIAASTVSASLTLLILSALLYNVGFTWFLQCYCGVWPAASGILVPWVGVSPRSPA
FPSQLPRQVQRQHVPTALLAITLVIDWLFRGQHRITENDVGGTKVFLGSVFTPALLGLLTTWYLQSALNTP
SVVPLSVLLEPLLKLCGMVSKAPRQQGSGDGSRAGSGSGGIADTVAVPVLQGAAAGALLPGSTEDEAQRRR
NIALAALSSRIQQTTADTAATQHDAAVV
>tr|C9ZP30|C9ZP30_TRYB9 | DeepTMHMM Topology Prediction - Predicted
Type: TM
MLRHFSRMWHTRIAAAHVYMFYFLSPRLFSFSFSAFWLYTTTYGVVYILAALPTNSPLCRSFFFFCVCLPS
FSLTFFVDTWKSGLCSILFCWKLRKQAGMVVADRYKTMSNM
>tr|D0A308|D0A308_TRYB9 | DeepTMHMM Topology Prediction - Predicted
Type: TM
MVWKPWGTVIMSNEEKLEKRSKRKDRTASGRSITKKKTVQGTREWKRQRKKEREKKQDKKGKKEKKMRQHT
KRQKQARAKIRQACGAMLEAYKHSTYGQLKKMGSVFALFRFVSSLYCEKQQQKSYIALFAFEIFFSLICAY
LNLLFSVFSFPTFIFLFDIGSW
>tr|C9ZKU4|C9ZKU4_TRYB9 | DeepTMHMM Topology Prediction - Predicted
Type: TM
MNSRTPYEKRKGDNKTKTKGKGEMMNHQRPEMMNPVGTMVSGVLNIALLFAAFTAVYIVVKSNHGVDLFSM
AWNDFLRPNYDDDL
>tr|D0A513|D0A513_TRYB9 | DeepTMHMM Topology Prediction - Predicted
Type: TM
MRGSCSEGALSGGCLPSRATTNPLGGPSSAGSLEMAEMLGAEAFYGLPILCSLWSERKLPKRRGARGRGQH
WSCASGESGKKRNCNLPPQLLFTIMSYLLPEGMLKVRLVCRTFNDVFLTYSVQETFAVSLPRSFMEPTVGF
QTLSGLIKEREAVLRNKERSWSKWLSAYVVMWLRKEANNDTISKATVEGQALWVQLQRSRVLCRLAMEEWL
GHLSDCRRAQYNIRRNWFNPTNLGCTLRTGVPPVFLPDGTLCINDPPDVVKFYAKRNRGLPLCEDALSHKT
VSRKNDSLKDMWAPFEELAFPAYVCLMYYDPYVDALHISLASGTCTLWRVNSIGIEAAAHRTASEADFESS
SHQLWRKTDLPCRSIPSRIEVVGNYTISSEFLACSVTMTRDGIKLEDYRWSEDEVMSKVRLFGEGANGDTS
RIIVASCGRDVSASLLPRATAGDAHLTREDVAVLNILQNQLSNASNSVNRPRAGPTRGEASANESYRPSAT
NSVDGINGGHNDVESGNNGGNIISIAGGPGNGTFELLVDDIEHEGVGTAASEVTANHLEGGRGDRITDFRP
SSITINSTERIDIKRYVFGVFNEQARLMCTVAITHEVAVFILTQSGCRTLGVNYSGGIHAASVLSSPVFKR
CESPTAEHPCEEGDALFLCASQHNVLICQQKLYRRLEEVEILVIHQNHREAATSRGALRPSEQRYGTWYFL
KGYSYHSPPMILRPLVEVSSLMLQAFVQPRPLLPEHLEWLSKKSNEGTCKVDCQRQREEHLPPYKSSWWCP
QRDGHPYFHKPFVMFISKFVGYLLAGCDCTSGGYQEVSTDDREHFHSVPLLDGIAVKRLHPSAVALSPSHA
FFVLGMENGSILMVSPSCRKCDQAKDGQGGCTTDAQVNGAHLIEQLDTISIPEGGEGYLASREAPQLLVAG
EDLHEEGADEGESSSSSEDEAPHPRSQHPHTSHLYRTTIISNRVRGSARQKRNLRSFTPQQLFGCAFHEDP
YISALQRCSSSILNSMERLEGEQRRHHPTPIHAVWIHAPRDCYRFSRKSGIWSMHLDDWKLTVLNRLYELT
VYDLSMQSFVGKDNVKGSVVFAPLLTLSPYLSHPFLGVPMKDEEKWLCSKIRRRMRAETERTASGWEIVWH

NGVLVVLGVSKGWRYSVFDFHARFSDPDVKNFSDPAPVAYSAKRDEYYESISGSQMLVPLDFGKKGTHKLA
LPRHFPLLRRDENMYLVRLSVRAVTLVFLCVALILVMLRIDGYITAPHWVAAAAYAPYALDVIANSFTDYD
RYYIVRSDVPFYIRLFSDLLLYIVFPVVFTLRRDLYPRFNTPWIVLTIPLCVAIAMKSLPDIYINMHPGQR
HWFAWVTTSRLLKALYEWLIISTIVLLALYFDGPSGRDPLGPKFHIALALTPVMLLILILKVKSAAAFVRT
GNWRLYLTCLFPLLLLALQVMLFVGEFKDYYEYIGKPITTPKPSASQSLFILPFSVIVVAIYKGYTVASVL
CR
>tr|D0A0L2|D0A0L2_TRYB9 | DeepTMHMM Topology Prediction - Predicted
Type: TM
MTIIIIIKMLGEKSLVFLFLFLFWFGFFFTFLCAAATHKFKYCRGGERRGGEKGWRGANKQNELVERFILI
SYWCQFTLCGALRFLFFLFLFPFLFLVITSLRCF
>tr|D0A214|D0A214_TRYB9 | DeepTMHMM Topology Prediction - Predicted
Type: TM
MEGKSVVGGSHKLADGSSSCGTIGANPGYAGTSTPLDASSSLYFGKVVEDAVPVRVEVGETTTRHTDPQGA
SVPLTRKATAKDRIAVEKTSDFVFCDECGQNIPVSEWADHREHPRVVENVTSWVGRLNKLLIRLFIDFFMW
VLMNIYFREVVVVNEKSIPKTGGVVFYGNHQNQFIDAMMIRANCGRPVRFVMAEKSFQRPIIGLFGHMTDA
VPVIRPQDAPLNSGEGRLIRMDGDMIYGEGTKFTMCLSDRDVIIWYREDVKCTAQVLKINSDTVLQLTMPV
AACDAVTKPVGFQVSRRIDHSEMYASVYQTLQSGQCIGIFPEGGSHDRTSLLPLKAGVALFSLGAAVRGID
VKVVPCGLTYFYGHKFRSRAHVEFGEPITPSAEVVALFNTDKRKATGIFLEQLNEELRGFTINVPNMSALN
FLHGFRQLYQPQNCILATRDHLRLTRRLSVIMEEQKGNPEFIDYRSRVENYQDYCNALLVRDSQAATLGKL
GSNEARQLHLMFRRCFTLFLMGVILVPFFVVGLPIGILAKSLSERHRKKALSQSNIKIVGTDVMGSYKLIV
GFISVPVVFSIVSIITYVYTDLRTALTVSVCLPMTMYVSLLILQEAAIEMRAALPLFMSLISKHKQFCKLY
ERRRALVALTKVLVKKWDPELEEELQHYVQESKQSMKLREPSLFSLRHGGLRRLADIKN
>tr|D0A7C0|D0A7C0_TRYB9 | DeepTMHMM Topology Prediction - Predicted
Type: TM
MSKNISMQLNRSKSVRCIRAQIYMYKHINFYKSINIHRYICKTYTYLVNFWLFSFLLFPSFFSLRLPIGEA
TARSVSHYFILFCICSNLRSLLTTLKLNNKPRVCNHSNKQKCGNTSPSSYTAQTHFQRLRTHNYPLLHLLL
ISHHFLSSVPFEEVSFTYMYAYALLFHIIGRRKIKTTNNITYPSLSFFFFFFISLPAPLSLPIPRTPLPPE
ATHYNSLGQYKTL
>tr|C9ZM25|C9ZM25_TRYB9 | DeepTMHMM Topology Prediction - Predicted
Type: TM
MRKKRKKEGSVWRRIIKFYNFYCYYYYYYPPPPCVYGSVFFFFMCVRVCVCLLSHHMYARSSPNETVVDIL
SFHYFPSFCFLFFFFFVFVFPTSRVYCTLAMRLVL
>tr|D0AAN6|D0AAN6_TRYB9 | DeepTMHMM Topology Prediction - Predicted
Type: TM
MNYSTGYSAAHSLGSTESLPAPGPVLHALLNNFEFFLLVLFAVLCQFIVFAVLGHTGGEVPGTLFFPFPLM
RPIVDSIVTPMGRVLGRQQQVWVGVDYEDDAVDYDYLHGTGHWTGESST
>tr|C9ZP37|C9ZP37_TRYB9 | DeepTMHMM Topology Prediction - Predicted
Type: TM
MLVFSSLFLAFSQAYFVLSGTLFSLPVKPQLIEHSVCSRGALVGKEVKERKGLEKRLITKNEKKTTEASLV
LFLFVSRTEQGNTERKTPLFSLVWFFLSSAFFFSYQKFSSIFFLFNLRSGLATGRNKRRGTGASQQRSINQ
GRRKNRKEEGSEEEDPHWHFSVLVFF
>tr|D0A4D7|D0A4D7_TRYB9 | DeepTMHMM Topology Prediction - Predicted
Type: TM
MSPLPLHVPFFTLEAPNNRVYKYMAVSVLGTNECVWTIARIRRGESRTSTPEERQMEDDECMRVFFFFLCV
CVYASLVLSTQPAVITVAAVHKIRLPNSYIVPLLPLLPRLSFAGFYFFSCFLFDSVRSPRSPSSAAPSFTQ
LFLPSSSFVVTAAATISVAVRVDVICYTCLAAPFSVSSTLKLLPLRVLPNNGNTKFSAPHFFFFLLPLRFG
CCGCLLADFSQPIKCNN
>tr|C9ZI18|C9ZI18_TRYB9 | DeepTMHMM Topology Prediction - Predicted
Type: TM
MLVLVLALRVYLFGHMHAPNAKLFFVAVDFFYFVAFPVCSVAYGGEELCTCSDSLTLALSVVLVRQEQVL
>tr|D0A8T7|D0A8T7_TRYB9 | DeepTMHMM Topology Prediction - Predicted
Type: TM
MFKILFVSTGISNQKTRGKKQRKEMAQPDPHFLFLNLYRWFERKWSVVASSGPMRLVQFLSMVPFCSARRA
LSIIFVDTTTRKKRSCYVFLFPLPHTSINIFIVQWLPMLFLFFFY
>tr|C9ZLG6|C9ZLG6_TRYB9 | DeepTMHMM Topology Prediction - Predicted
Type: TM
MDSFRLTEKDEARCSFGDPKRSRDAHDNRMEHFNVDIRKNSALRNRSLVCGCLTGFTCAAGAIGALAGMYR
SSLGSTSQDKRFAPLLIAHAFSGGVGIAFTVCRSLLFERAVYEVELQRELWEIDNHIAGEIQEMVAIYRAQ

GLLEEEAHMITRIFAKHREAFANLMMVEELGYSRLEPPAGWEAVVDAAIPSSIGYTLGWVLPLLPFMGSEL
SSARSELVALCTLAAGIFVVSVGQSEVFFGSYANVWKAVGATVWNLSAAGLTYGATRFMACRSGV
>tr|D0A3E4|D0A3E4_TRYB9 | DeepTMHMM Topology Prediction - Predicted Type: TM
MFPIPPTTFVHQFPLFPATCCSDSTGFFKGLALPVSPLTIASTPTALTTRRDFVVLRQPWRQLVVALSLSF
RTPGMLLSCLFDQPTWGGVAFLRAWRVRCCCFQDCTVSLRV
>tr|C9ZZJ8|C9ZZJ8_TRYB9 | DeepTMHMM Topology Prediction - Predicted Type: TM
MRPSGTLSFSSNLPYRSLSIPYWHFLALSFLIHFSGAFRRTFFINLLLRTPRSWLREGNTFEPTAATSAEA
V
>tr|C9ZYG7|C9ZYG7_TRYB9 | DeepTMHMM Topology Prediction - Predicted Type: TM
MEETKGKKKQTKDGDQKEKSERMNERTNQSIKAPQGRFHKFCMKPTLEQSLPIYVLFISLHPPLFFHHVTF
CLIIHALLSCGRGIITPFLYFTHINTRICVRVRKSI
>tr|C9ZME0|C9ZME0_TRYB9 | DeepTMHMM Topology Prediction - Predicted Type: TM
MGAQSPTASLPTQPFLDFFLPSSLFLFLFRVCLFVCLFTTRREHHRHKTHTHTKHKNKVEEEKKTSSDQVV
MRRNVLSSLNRSSSVLIVNIGTSGVVPAAAVASRLQTTFHGPPGNVPPNFRAPPLRGAAPTQHQMSPQQQQ
FYQQQHMQQQEFHQQQQHMQQQQFHQQQHMQQQQQPQMHSSAVGTEAEDDFEPPRKPSEPPRLLRLDTSDR
GITTVALSRAPVNSLSLELFEEFNSWMLWLGSDESCKAIILTSSIPTVFSAGLDMSEMHNPEPERLRRFWK
SFQETWLILNSFPKPIIGAISGNSPAGGCVLALGCDSRVMVRHPADKPDRPYRIGLNETKLGITTPPWVIP
AYAYVLGSRRAERMLQLGETPTADEALRMGLVDLVVDGEHQLREAAVKEVERFLSVPQQSRWMSRDMLRRE
FLQFIGSEEDREYDTQFFVELMMNPEVQKSLEAFTARLKGKAVRK
>tr|D0A3R2|D0A3R2_TRYB9 | DeepTMHMM Topology Prediction - Predicted Type: TM
MRGSGSSVHSLTWLWDKMPVLVAEKARSNGPWRLRVSAALESTRRFVGERLGYCDISCEKGHQVDGNVWYA
EQYLYPNHWQPGSGLRPKLVDDGESRTWLRAWARAVLVPLSILLFAFFLYISL
>tr|C9ZXS5|C9ZXS5_TRYB9 | DeepTMHMM Topology Prediction - Predicted Type: TM
MLPIFFFLRPFFRVVLFFVVVLAPSLCGSVALFRCAYACVFSCLHVYVYVYVYVCACVLCFFLLFLLILLL
LLFLFFFVSAGNSKSNIPATFHHYYYYCYLFIYSFYLLFSIWSSFSSAVGSVAHASTHNKVHAHRRYTAAY
AYVHLY
>tr|C9ZT60|C9ZT60_TRYB9 | DeepTMHMM Topology Prediction - Predicted Type: TM
MLLFIPVLASQYRCTQATFMFVSTPVCVRICLSLLPSFPPKFLRCFFLYFCNLFYDLCRLHHVLYFWKRNE
FIPSILPSLSSLEGFDTSQNEKNKHCYKD
>tr|D0A101|D0A101_TRYB9 | DeepTMHMM Topology Prediction - Predicted Type: TM
MLLNSSFFVYFVLIAVVFITPHRVWFVYIYIYIYIYIYTYFCIALYIFIYTYVQMLLLFFTVFCFSFTALA
LSFKFLYIIYFFFVGTSSFRSVWAYGMDSADPTASECDKISSIVSRLEMNEKRQHELMLQISACEARIRAI
EAHMSRNSDEVPKKSLDSQVNDRMKTDDRKQAVALTEERVCDYCLIFTQCWSCPACGREWYCSSRCQRLRT
YLHGPFCGCQRPAVA
>tr|C9ZP28|C9ZP28_TRYB9 | DeepTMHMM Topology Prediction - Predicted Type: TM
MRSGERSGAKKWTDERVLLCLSLHWRNWGSRKRQHRMTGEHNYCDAAFVFVVVVMNIFLLLPWPYPHSFDS
RHESPPPPPQTHSCKGGFLFSPLTDCATYIWFLLGPTINTYSL
>tr|D0A9I5|D0A9I5_TRYB9 | DeepTMHMM Topology Prediction - Predicted Type: TM
MEGFCYTAAADAASKAKGGVPQLPHLSAAALGKASSRPLPHPAAAITTVELPSGSVVIAGGGSVISVEPLS
SLLQSCSKEGECEACNDPLLGGVAVHSTVPGEKVTLTISSGLVSCIGTALLPGGTAAMCAVGTSAGSLCLF
VVESRTGGNALALCKVCEASLCEYTDGLISADDIVLDVCVGLDPAGRPEFVAVATATVVVVIDTQCFNESL
RANATEAVLRETVGSWQRTPCPTEKPVNETFYASFTASEVVRLLVPQRYRASFAVDIALLIVFDNGELRYV
ARRVVGGSLQLLTEHHRQRQLRHSTVGLGMLQHNSEGPIDGSHGSLALPHVLYAYSLSSVVHNVCTAAVGV
SAATYKVVNNAALFCDDMSQTMRIIVVGAGTQLAERYGHQLRGTTGWWAITGAAVLARGTLDQRQLAAWGF
DVPCSGALPRDRRGLACVTVLEGGRVLFACGNELYVCTMADATNAVVDGSSGGGGGAYVALRQVHTCGSVV
CGVACTGTRIDGRNVVVIGFGASLAALLF
>tr|D0A587|D0A587_TRYB9 | DeepTMHMM Topology Prediction - Predicted Type: TM

MLRWRIQSHPPAATNAGYSRIPRLPVIFLAWRGHRSAPGRLRLPKREETHVPPTTSPGRTTVAAANVFLNT
TGMPVGEVEEGRRGFLGFGKVAGRAMPLNPLAVLRPQKTDAKEDRSTARFLKAVELLNGKRRSRKEGITMQ
MTAEQQHDIVNRYAQTRWYGFMWYPFRNVTERQFKWWRRIAHLALIIVGLTGVVLALVMYYREVETVLLLS
PEDRSDYQKIVTGMRFSEIYRLSMEVLGKEDPLEALPSPARYHLILEAAREKGWHKIDWELEGRTRYPRSA
VEDLDFIHIIYWAVMYIGSAVTGGGELFSDRFGDLIEVRQAQKLRDAEASFVEQGSEPPPSKK
>tr|D0A1W5|D0A1W5_TRYB9 | DeepTMHMM Topology Prediction - Predicted
Type: TM
MAELAKSAVLVSSCTDDLLGDAKQVVVGPNQEDLHSAEAVLNRYSTVGFQASNLARAFSICEMMLTPQSPS
PSLMPTEGDQTSESPVMVQPTLFVGVTANLFGTGCREAIRFLCTECVPLPNGVEPATPLDDMAGISCDGTG
ALKPSPCDSRALIHVLVVSGGAMEHDIRRACESYKLSREGAEEEGEQFHHPVERDRSRGKGTDCHFGNVRY
NSSGVASRNLFSCVMRCLVKRLAEAQRKEKANREAAPIPEAYYDVCSWAITPSTLWYMAGLWMADIFTEAL
QETGEVTDEKVASEEGLKRAKSTVLYWAARNGVPIFSPSLTDGDIMEFILTAGDTGVPLLQLDLVADIHRL
NRLAMRSRRTGMMILGGGVVKHHVCNANLMRNGADYAVFLNNAQEFDGSDAGARPGEAVSWGKLRLDSTAV
KVYSEVTIVFPLIVVHVFVAWVRMMRSKGKENIRS
>tr|C9ZPW9|C9ZPW9_TRYB9 | DeepTMHMM Topology Prediction - Predicted
Type: TM
MFCPCHGFLLIPLVCCVPTFSYRDQKKKKKMVTLRYLRSKEEAEARRARLEEARRIANQIGVQRARTYAA
QKREQARCIMKDLRTQWLLERQRESDLIDRMLYEAHARQGEGMKGAANLEAHQHEQAINELSAWGAEYALE
RERHKLALEKASVTRALQQEPQRRIIERKKAVRAAEEARAKVIVSKREKRSVPGFTGDDVARPSPLAGAYP
QTMPTRLRKGKTRMEQPTTTVEWPAREDIVEDATAAAAHYLEERQRAIESNHESVERKRREAAERAASLKR
KQEEEAKHAEEEMRRREKNLLALKQHATEGPRTEEVCTRMQDTRRSVALKSKGEAEFEAIFLRDTLGTEAK
PRQLEDSVSFNTFERLLQLSTISSLLPTDADIVAQLPTTSLCVFADEDPEQSGELSPGFHATKRDSGDHVL
RATAATDDVEVESHAVTPTSGGEPPLLHPLKASEEVPPHPTSICDHSACDDDNSSTHTGSTDSTNKIEERS
ERREQFLTNLKLLQARLERAMGEVPPSNNPQGPPSQTNVTVSSMSSISTPPRREGGGGDFPSEDYKNMSSE
GTSDVSFKSRDAGFSSASSAYTRTHPTMTAEQLKAALLRVKLRLHSEL
>tr|C9ZRS6|C9ZRS6_TRYB9 | DeepTMHMM Topology Prediction - Predicted
Type: TM
MSRFDDITFTFFYFSYFVSLYNFFCACADTTCFLPHDFPPLPRTPFFFTSFFSFLPPFIHRIDCRPNGCTC
CNRSKPNKGGRGGGRERTRNANKKPSNCILKKKKSKCHIHIYIYIYIYL
>tr|D0A143|D0A143_TRYB9 | DeepTMHMM Topology Prediction - Predicted
Type: TM
MSYRGHLNSANLQAVLLPWCKPYSKVIKGNFFFVCVGSRCVQVSNSFFLLVVFLLLLFFSSFVLLQPSTDA
ELAKGTDDRTRHFIRSKKERVPHAITTSPIRLLHP
>tr|C9ZI11|C9ZI11_TRYB9 | DeepTMHMM Topology Prediction - Predicted
Type: TM
MLSLLLLLLCGVACGAVATVFSVWFAFIRFDNFLQRMLERNEEAAIRALEDTRTSVTVPASKVEGICEMAC
FDGDVVWKTIPVRAVLFGSTVSVYRLVTREVSDDLNNTVVCGDQLIGKINTDSVISSVVKISKYHRHVNIA
ERCSPVSGQCLLLRHKPGLPLFLVDPVVQLKQRLCRKQREQTSRGTVHHSPLQDDDEDFINAKDTHSDQRN
NSQSNNLSSSALDSSSSVDGSGEDYSKWTAVLFKMCTRRELERWYNLLQGNSQSEEWRNFIKRLTRADALS
LVVARLYFANTDTSSLQDLLTRKIRRKLRRVSRRLPNHMKGEIILDRLELGEEIPLLDSVSDPVVSPNGEI
EFDFYLLYRGGLHFSLRFSITYRGMRVPDIIFNVKVLQLSNRVRLNVGPPPSAKIWLSSPHTPHLQLEFTQ
EVATNDGFLHTLLKLLPDMSAIMTTIVKVKLFEDMLLPSMDDFPLPCLSYSPSSSEASDDGEEDVDAKVEY
SFASPAEEESSPLPLRSFDAVSMGSLIERRPHR
>tr|C9ZL98|C9ZL98_TRYB9 | DeepTMHMM Topology Prediction - Predicted
Type: TM
MVYLVDDLARVRMLMAGVYAGLVVSSTYGFSVFAAHLREKYRLSQSDITTISTVGNCIGYCSFIGGALFDY
AGPMVVLPIAGFLGFLGFLLFGLTFDGKVANPSVGLFSTFNAILYLGTPWLDVSTIMPLMLQFPLDRGYVV
LVSKTISGLGTGVLMAYFNGWFKDTTSDDLSNNNYSGFAYFIAIQLVVVSLIALSVTRMPMYFPCAWRKQR
LSEEEWTKRQQTLQLYMNQPAPPRRMKIAVGLVLSLLLFLTTQSLIGGYVKLPPAAYLAFSIIAVLMMASF
CVVALPFQWLGRYTPVRPTDMDTIGEALEDVGTESAVATTKNEVKPLPQYSGSFWQHLLTVDLWCMWLTCF
GMWGTGTVMQMNAAQIYESKSYGEKKSSTLTLYITMMSVGSAVGRMSMGYLDMVLTRRQREGLKTFPTTIA
LPFCPLMLCIAFLLFALLPANALILPFFLGALGNGAGWGSGVLAFRIMYSQDLGKHYNFGFSSGIVSTIAL
NLFMFGGMYDAEAERLDTKPECKQPSCVKNQMLILMGVNVVAAVAAAIVHFRFSRFINAEQNRCNEPADEM
SGVAAPVTEDSEKES
>tr|C9ZSP6|C9ZSP6_TRYB9 | DeepTMHMM Topology Prediction - Predicted
Type: TM
MCSCCCEPPVGQWIDIYGGPVRPRRSGFECPLDALQVIAWSVIVTLATLHFTLHVPMLDSLLVCIISPISG
FLTFATAALKLALSCSRIEDPIVFATDVPRYAQEELVQEAAPPGTEPCVFCRRFVILGSKHCSVCDKCVPG

FDHHCRWLNTCVGEGNYVMFCCFMGTAWCSIALVFGVGIYVISNAFIHKQDFSDRLKERFGVSSYVTYMVF
LFLTLALSAAGLAALGNLIVFHINLCLTRRTTYEHVLSKRAKRREKLSKQSKPLVATDRSKGPCGCLAIQK
RRDFRRYKKNPAGSSGAVPDIGVHNLGDTATDGAYPSRDTSQPGETCEIRETTSDHKGGVQLERETHDNEP
IA
>tr|C9ZUS8|C9ZUS8_TRYB9 | DeepTMHMM Topology Prediction - Predicted
Type: TM
MFEEEGLEALLISFFNVFVTMYKISSHVCVHVFVYVFVCLFVCALTRERVRSCTYHIIYIYICIHAYIYTY
THTNVYACTYTYACVRLCVCMCMYVCMYVCMLTQIPQSIVLYSVTLIRSSEK
>tr|C9ZYG2|C9ZYG2_TRYB9 | DeepTMHMM Topology Prediction - Predicted
Type: TM
MAVPPVEMYSGSFWNRMRKPLPLRTQVIRFTVVFVIVSFILAVALQITHERMPDPKVTKPLPDLGFELLTK
ISFLSVVTDVLIAFLSLLSFFTLWKLYLLHRHCVGSGEPELPCNIPGVSRFFLSVWLCKENCRIELRNIHT
IAWIRFITSYALLLLFRSLVIVMTSMPTPVDKCQDPPKIENPVKNVILTVLTAGGGSIHCGDLMYSGHTVI
LTLHLMFHWIYGAMVHWSFRPVVTVVAIFSYYCIVASRSHYTDDVLVAIYLTIATFIAVGHNADGAPWQLQ
LFIRWLPCCGANSREVTEDSQPVMVAFKSEAVDELRERDDSAGLSGEVSTNEV
>tr|C9ZQ34|C9ZQ34_TRYB9 | DeepTMHMM Topology Prediction - Predicted
Type: TM
MCHTRVWASMRLHGCLLSPLTSVSMCHPFWLQFFVCSFHLFLDTVARVSFTGLKSLTHRNRLVGVAMFSGP
AEVYYTKSIQNNHRQLRRNVGGAQPGRGIYYSLLAVLLVVTFIKTASATLERVRVGIDMIRSREMAMVESE
NPRVIVVGGGLAGLSAAIEAAGCGAQVVLMEKEAKLGGNSAKATSGINGWGTRAQAKASIVDGGKYFERDT
YKSGIGGNTDPALVKTLSMKSADAIGWLTSLGVPLTVLSQLGGHSRKRTHRAPDKKDGTPLPIGFTIMKTL
EGHVRGNLSGRITIMENCSVTSLLSETKERPDGTKQIRVTGVEFTQAGSGKTTILADAVILATGGFSNDKT
ADSLLREHAPHLVNFPTTNGPWATGDGVKLAQRLGAQLVDMDKVQLHPTGLINPKDPANPTKFLGPEALRG
SGGVLLNKQGKRFVNELDLRSVVSKAIMEQGAEYPGSGGSMFAYCVLNAAAQKLFGVSSHEFYWKKMGLFV
KADTMRDLAALIGCPVESVQQTLEEYERLSISQRSCPITRKSVYPCVLGTKGPYYVAFVTPSIHYTMGGCL
ISPSAEIQMKNTSSRAPLSHSNPILGLFGAGEVTGGVHGGNRLGGNSLLECVVFGRIAGDRASTILQRKSS
ALSFKVWTTVVLREVREGGVYGAGSRVLRFNLPGALQRSGLSLGQFIAIRGDWDGQQLIGYYSPITLPDDL
GMIDILARSDKGTLREWISALEPGDAVEMKACGGLVIERRLSDKHFVFMGHIINKLCLIAGGTGVAPMLQI
IKAAFMKPFIDTLESVHLIYAAEDVTELTYREVLEERRRESRGKFKKTFVLNRPPPLWTDGVGFIDRGILT
NHVQPPSDNLLVAICGPPVMQRIVKATLKTLGYNMNLVRTVDETEPSGSSKI
>tr|C9ZZZ4|C9ZZZ4_TRYB9 | DeepTMHMM Topology Prediction - Predicted
Type: TM
MWRKGIHDGTLVGCHIFHPLSLSSPHPICLRYHATMCLGEAFKLRLAILLFVSLLYAHACACGNSLLMLSI
LLSSLPNIHSCVTQGALLPYKPRSPYSIARC
>tr|C9ZR36|C9ZR36_TRYB9 | DeepTMHMM Topology Prediction - Predicted
Type: TM
MQGEENLKGATKCLAASPSEETVDPLAPPSPSSSTTSNRIHSVSEAERFYRSECARIGINVNNAFVKQLRA
GSTVFNFDNGYLGERGIIPILTTLQRLSVASLSMRGCKLTAEDIAQMLQYLGFHETLQKIDLRDVPLSVSS
GRKLFALALKNNCLTEILLDKDTPKYLSIQRQCFSNANMKLVASRCLVCNRAVIYSPDQRVEGQILLALSK
ALMNRGESYCEVALGVLFRTLLVCCDTNNGVLFICSYECKNKLSDDIVIMIESVLSEHLHGVKGTCPRNTT
LQRCLVDNVARLERKRVNSEKLNAEGSTKVVNPLSSASSGATRCFDSSSADSSDAEEMHRKNHDDADACSV
CGNHTLCLSNGAYRFLSQLHDDVTVDCYLRPSALLHLSRVMIKHQRFKPCSQLCVQHLVRFGLYSYGGVQV
TNSMTGISLLSSLDIPLTQLSNDDFSIINFASAYIDDAAGEDTCCALTVASAMTDIEGVAIDPYMIYAVGR
FLAKIPPTSLGMELRHACEVALMVGCLPVESAPFNRRKERPTRDLYYLWEKWCKVADMETLVRAAFSRRRQ
GLFIVDGPHSNLFDNTRAVLWAFRKMRRSILIVMRFCVEWLALPNGVIPNESPMGRSFHTTLKVVGQTNIH
NTIYVICQSNFGENVGNRGFFYVPRSVFNLYVSSDACIFVDSNVFALSNGEKISLHASRYLPKKVIGIVEN
CQVLHEAFNYMSDICLSTDGGGLEGSRVFSEAIRFPPALLFFAYRTGECHISQSQWKLLRTLRQLCLTEST
RSLVLFYLSEVLGPNAVEWVARLLHDLVNLPPYPARLQMGSGTVTREHVRERGGDVEWNQVMGLLSESTTM
LTLSSSLPVDRVEKKRLTKRVSRPANRKKDAPIITQDPFHEDLLGMWSFMRKEAESQRKSGGRSKPNGGRL
VLPLSAQMEKGITARALAQEPEVVPPSLIGETWHSICLCTKDGNLTEYALFFIKNHVCCFNVENRSMNIPL
QPMSKTLGLNSFPFMGGFDCAFNSPVNPRTAYFFCGEEWLEWDSYRQRCSGGLFKLRCHKQFRSLPEKFLS
GINAAVPVANTPLVFFFCQLEYVVFNITERCLVGGPMRVGDHGKGNAGETIFGPSLSKVFPHGPMATMLMW
EKSGETEESQEQEDNNNNNWASMGSIRSTREESLVGTTAKISANNNNGDGKGSSITHEKEMVVMLIGRCGR
VATVKNFIYSHDAKCEVRFETIPSSVLSRLPMAFRQNTMTALGSICRTVLETECSVHDVILCDAHRSVHRE
CTVTSTLTQSAECAEDLLSVVLPHNFTTVAPLLCESAVFVQTEESLLGEPSQTIEVRYDPMNPCAFGALVI
VLNLSCIEPRVLQVAPVEVLLESSDDGIAYVRHAHFFITSSITPVCWGVSHVGCVWRVRFVSKLPVGTGVV
RLLLYEVMHVVGTIPVDPTVALPPLESTLVTIDAPCLLSPARDILNCLTPGAVFTTSTSAGWFERHCILPL
LPNGDFCLLFCGPHFVELDCSNGVVVGHRAVPIAAHPAFDTLPAPFLRGVNAAFYPNVRTPNIVAFISGSH

VILWDIEKGAFASGGGGVQSAATMFKDLPWKMEEVENIVQIWSQPEEVFVIKSPHVLRWNIVTSELVEGPM
RLSNSTYFRHPAFEGKKILCAASFPKYPTRFYTFCEDLVAALDVETQGITNLTEAKPVSQSEVFFPVTWYL
RWGIRRHDCVINLDFKNYQKLLVGVTMQSSEPCNDNWVVECSDDGVLWQTVGRHHQITSRCRTIWGSERVG
HHSRRFWRFSLECEEVDSRHEYPVLYFHMLLLTVSSVSSRVLPVRVACSGTLNGPMSSLFVDDKVTVAFEN
PVDESGATKVVRQHLIVDYGEREPSDVMEFSCICVGKPRPVTWIISCSSNGEQWEVCGTWHSHDHYFRAAW
HPRGPRRFWSVELEAREQSTIYENLTFFEYTGPSVYLEDSLYLSCLLDPFVLCEVGSSGDTVANVVVPAEV
GSSIVLDSKQDATQVVGVCMICHENKSPYTAFVVECSSDAVEWRGVGVTFNLRGNLAQAAWDVSDCCQYWR
LRVTQRVGTSPLRICSIRFDTPKPTLYHHVDSKGDIVAGSSGKDLNLRGNCTEVVAVEAELPPLSSFVVEK
RSVDGSSWQAVATLRNDDSADSRKVRQGWKPRGMSSHWRLRPVLAEDNLEETCFSSKSGPFISWKKECDIQ
AEWYSFIGKAFVRYDFSKAPDVKMAVEKFDEVRLPIFLSDNNAKTVVLRSRGMETEASVSWFFDSDTSPHF
KQVFVNGRVETNPDAEAELEKLAALEVTSASSPRAPKKTGKKISKASTAQQEEIVKAIEMEEEVLVIVVET
SNNGVDYVPVAERPFSPKGNSLSFFVDTPTSYWRVRFKGIPKASFLELFEVFWSLEKGARTKLSSLLRCPL
SDSMYETWKNAAYSHEDEFCQQCRAGYDQARSVVSGLREPEDIEHHGRIAQYFMRFKSEYIAFQSKVFRNA
SKAKNLPDNKYISTERRSGNDFMYLLHAVAERYSCGVDKLNMLIANPMLVSRDFSIEKESVSWFYPKIVEG
GTLAESFYGFHNARAFVIILWSMSLHVQRNTDLLVTKKINALNEHVASVMVLVEIKEINWLAIEHFPRLPL
LYHVGFSGQPVLIAFTLNTLVFTPRYRLTFPGCSPLPLNHQMQLFPGVNFIRRTSLATCPCPIFDKLLLLY
TLQFLENTKTEIILSAPTMDSPNATVRFTLPGNDINFGLRGLVIGYMVFEIELCMKATNVDPSEMALPVTE
HKRINFVSYDTQFDSGEGTEPIIELTLAGSIGYPDEQILISGMSPSARYDIGDVFPDAFISDLSINFNAVL
GHQPQSFLQPTVNLSGTLVVPACGSFVCRLQRVALNRNFESLSMEVSEMPLASLVTLSDKLVGSLQKGVDV
SWMEGIPMYFSLELQLEYVERRLTGRGTFTLDGIEGDVLVTLSTQGFGATGNFNTVRIGAVCLQGKDERHQ
VLVNVASLANGHLSVRLDGYSYLFAPRPYPTRVEISQRGVVCISTGVFYDVCIEEDSIFPSKKYANVLLSK
DLFSKCLEEELREIPLITALLAKGVEFSFVVDDVFAPQCMLERQLVFITVRGVLMGCYFDVTVPVTQPDNT
VEEAKKISKELLPRVIEQCDMPLWALYHMFVGCVKRCGSAASGGEGGDADDNNSDGTNLSVEGSASRPQRG
DASRKQCDGVFLSRWIQQERGVFEEGL
>tr|D0A3S3|D0A3S3_TRYB9 | DeepTMHMM Topology Prediction - Predicted
Type: TM
MRQSSLPEEDAFLCLLKQIEREGYTPNIGERAVICRTINGVFNPLGVYFALIGASCGYFGLGMAVRASQRL
ASSVVGFLYMAELSWSFQERRPCAGFLNDVRSLDGQLREKTGAQHLLGEFSLMDRSLIGEIFDFVTLDKTI
RWLLCLTSSCASGPHWLPFVKIKWFDGLPLDPLTIWYWRFVLSFYR
>tr|D0A1Q5|D0A1Q5_TRYB9 | DeepTMHMM Topology Prediction - Predicted
Type: TM
MSKQELQLTDAVSEQDRLLGELHNSVLNTRQYALAIGEDLEEQNTMLDELQTDVERATDESRRQNYNVGQL
LWESESRGFWTLFIILSLILVVLLIL
>tr|C9ZPA4|C9ZPA4_TRYB9 | DeepTMHMM Topology Prediction - Predicted
Type: TM
MAFWEKIWCRLFGRVNIPGVVYSDEIDATATYDRQQRESMWRGNLRNVRLDTVPPLLLRTSGAENALLGPE
EEPYTFWERVGVVPVHPSRVRPRQYYHPITDFGLYDYDEVSPEERDLTARWLAKVLEFNAAVPLGILCAAG
CLVLPLHTVYRMPLLVASAATGVMFEITHSYMNAGKERQDLDDFILAKEIWYIKNVEAYQLGIPLIPKGRE
AEYQAFLDGSNGTSQMLPDELAEALH
>tr|C9ZND7|C9ZND7_TRYB9 | DeepTMHMM Topology Prediction - Predicted
Type: TM
MQRSIIRLATATAPSRVRLSSAAVTEPKTSMQPMHAHVRSSHAYEASAKGAEVGARNEAAFMNYELVRKLD
LGGLFAIPHFVNLLTITPLYCAALAVGCWTWGLFYWDLYCRSHYETVLIARPEALK
>tr|C9ZJ62|C9ZJ62_TRYB9 | DeepTMHMM Topology Prediction - Predicted
Type: TM
MEIPINVSESVLVDDDEEQCLKNLSWGARLRWYLLCTALGFFCSAMGYVALSFGVYWKYSVLNTLGSLISL
GGSFILKGPRAQLRYMFDDYRRAASAVYITSLVLSLVVAIYFKSFFLCLLCGIVQYVALIWYSLSFVPYGQ
EAVASCIRRVTGL
>tr|C9ZMZ6|C9ZMZ6_TRYB9 | DeepTMHMM Topology Prediction - Predicted
Type: TM
MFLYMFHFFCCCRYPIFLFRSLSLSLCFDPLLYVTHNTFLFFLSSIWKLCKFLRQASLSVVVSPGIFRMCC
GWLTTLVTLFAVASAVCALVFPVFRMKNETKEAIQTLWYYMEKVDDVVKQTKVHESPCKEYSVYFQVIEAG
VLISVLTGFAALVFIILSNCKIPLCCLRIISTPLSIISFLVSSVSAVLITVGYGKGFCQWDDQLREDFAPF
KERDFSVDAGFYLIVVTAVLFLVSSVAGCCL
>tr|C9ZNG1|C9ZNG1_TRYB9 | DeepTMHMM Topology Prediction - Predicted
Type: TM
MTMPIFVFNVPFRRSHDRGPPLYGDPVGIYIYIYIVHPFELLATFSHALGIKFLRVTLHTCLQLVVLLLSV
SAETGLAQCRYCGLRRQRIIAGPLSFAILHEESLPRTKRSCSKEAV

>tr|C9ZNI8|C9ZNI8_TRYB9 | DeepTMHMM Topology Prediction - Predicted Type: TM
MPSTICHRRTSHKVAALLFAPRHRTQRTQSQFGSTTGPTSGAKTDFSGKDGSNSINLSSDTDEAKGNDWHG
GGVPRENRSFHFDPSKLPDTIPGLERRELAARLEEHQRELAQKYQQSAADDLAKAIDLMRRAMPPEEFKRF
LQDVEKAAAEGSKEAARINAMSPAQLYRYQRRQRRRQRVQLFAKTVMLFVSVFGCVFFLFFFFFFFH
>tr|C9ZZ96|C9ZZ96_TRYB9 | DeepTMHMM Topology Prediction - Predicted Type: TM
MPSKNANRVTPPTPMSQTKERSFADFGSRNDTVQIFSVGARRSWSSGRILACHAGGPGSIPGLRIVFLPHA
EPTIFFSLIWDDRAILLVWLLLFVCHHFATVTIIPHHALSPHLQKLR
>tr|C9ZNN4|C9ZNN4_TRYB9 | DeepTMHMM Topology Prediction - Predicted Type: TM
MVPSEGTFLLCLRACPELHQHHIFPFLPHILRKSDQSPQFSFHASCPFFFVCVCASCPFFFFAIIPLSLIL
TLVTNGTSPYLVTPCFISYTPIHLPFCVPSSPPSPFPSFFFFF
>tr|C9ZZB1|C9ZZB1_TRYB9 | DeepTMHMM Topology Prediction - Predicted Type: TM
MSVVPSLLYCPQFALFCIMSRDSCHTTVRRKKKRIKRVRGRDERIGHVWFTFCYSSEVSPRFTAFSLPSYL
LFIFLSSLLFLFVPPLFMSVSVRDGGHIKGCGSHVRGFSLTNTCTFYGLTSIVKPTTCLSFCVLFYSVFYS
VLLLPMLFSFIICALPSHSSPFSLFFFLCSPLFEIVTCRSPRWGAEPLLLIHSCVHYCYC
>tr|D0A5Q1|D0A5Q1_TRYB9 | DeepTMHMM Topology Prediction - Predicted Type: TM
MACQEVNSEVPDSDVVFHDTIFMRNNKHVSEQWIRIGELYPNGAKESLLPETFAREQFGQGSHYECFMLSA
LATLVKFPDIIRNCFVSRNVRRDGRYTFQFFRDKEWVKVEIDDSVMLEDDEVLFIRSPTSHWWPLLFEKAY
AKFYTSYDNLEGCTLQEAYHDLTGNPVLNIPMDARLAKAAGVDVTGGQYWLDLAQKLQSGQFVGSLLTRET
DLESMGLQSEQQYGILEIFSLTGTSSVSDIVIHLYNPFEDEEFTYKGPLNSKDTEWTPKLREKYNVDDTHS
IFLPLHVALKIVNSVQLCYISPIDGDATYFDDEWKGETAGGNPTSVTWRKNPLYCVRNAGINPVELVVVIK
QKDQRHLWTSEDDELMYKRCGIVVVQSTTVTQIPTYFVTGNNHKAIHKSLFLNSREVSSFVRIPPNSLCYL
VPSCMKKGAESAFTLALYRMKHQNYTEFNVSRLALPKLLWHNPIQEKLQLQMFTKDRRDFYVDIPTDIHIL
MHQEKPFVSEKTGGDAMVQDYVGVYLYDDTDRKIGGVHAATNFRETSVLHRLPRSGRYAISITCPRASGEV
PIALTIVGSPDANVRIVEAPDDASMFDDEDDIAEGDEDATVSNPIDYIPVAGGGQVLTEVPDSTVPFEDKR
FMVDNKIMTNEPWIHIGDLYPEGKTRSLLPERLSREQFEQGEHFECCCLTAFAALVEHHPNVIRDAFVTKA
VRRDGRYTFRFHRYGQWVKVEIDDRIPLLGGKTLFCRSSDGYWWPLLLEKAYAKFYTLYQNIEGCTLRELF
YDLTGLPVLSIPMELKLAKAVLCDVDDVSFWVELSEGLKDCACAAVARPGFDDTLGLSNGQEYAVLDVVSL
TDGEASSISDLVVKLHNPFLETEYKGPMNASDAAWSSELRTRLCPERRDTIYIPVEVFCNAFLTVEKVMVR
GLIVPGWQFNSEWGEGTNGGNPTLVTWRENPIYVVQNTADTPLQIIAMIGQPDQRRVLHLLPEQEVNYVQC
GLSLAKSIKGESIPTYLLTPNSHVLIHKGRFFSYREVADVIVVPGNSLTFLVPSAMFRDKTKFLLSYWYFS
ANDRNSVTIERLNLGVARHLPAIAHLTVKERVREQVDFIVNVPTDVHILLRQEKPCHGDSVSNALTDDFLA
MYLYNSEDDQLSGVVTATNYREMGIVHHLSEGGRYVIYVVCPRAEGEVPLRIEIVAMQSAQVRTVEALPLC
KSILQVEKELKLSTGSLPYSPLLISEGDLKNFSGRLSDNSMRLESSSDPDLSFLGAEVEGIPTLDLTLMSD
SSFSEMARERMQLKRDPIANASRLAEVEDSMVARANAIAKEMHLRERRYLNPEPEGVPLELLPLNEDEVVS
EKEDRLRALNRKERKDEKLLRSLEDVIADRVHEIARELKVSERDLFLDPTPGGFPVAQLSLDEDDVFHSLE
VERLQLRLRQDSLASKERIAELEHMMNCRTTELIDKMKEEIRKFLDPTPLGIPLEELKLDEHDNYVAKEIS
LIGMIRKGKKESQEAISIREQLLQIEYAVGKEHLNNFRIQYLGDDIEGRQPHELNLEEETYMQMERKLIEY
YNSNQRNSEEAQKIRVNLHHKATKASKHLNRSERKNYIKRDRLEISISNIPLDDNEQFTTLEAERIRKKRN
KKNSEVEQIEMELNNIAQQLAKLKASDSRSFLDPMPEGVPLSELGLDKDEKFSTMEEERRKLIAEDREGNA
ARIAELEAAMNEHSHELAKLKASDSRSFLDPMPEGVPLSELGLDKDEKFSTMEEERRKLIAEDREGNAARI
AELEVAMNEHSHELAKLKASDSRSFLDPMPEGVPLSELGLDKDEKFSTMEEERRKLIAEDREGNAARIAEL
EVAMNEHSHELAKLKASDSRSSLI
>tr|C9ZTV3|C9ZTV3_TRYB9 | DeepTMHMM Topology Prediction - Predicted Type: TM
MKGEVEQQRYESKAPLRGGWLRFLRLSQGACVVSTFAVKPFIISSGLFSFSRCRSFFFFCFLRLLSSLICV
SPLGSPRFIFIFRMFPLFSPLYIPLSQFPS
>tr|C9ZV99|C9ZV99_TRYB9 | DeepTMHMM Topology Prediction - Predicted Type: TM
MYFLLLAGIIWGITNPLLKRYSGGMSVDSSSFLEDLRFLASRPKYLAAQLANLSGSVFFFAGLPSADVAVG
SIVANSLAFVITVLVSVLVLREGTLKPRTLVGCSLVVVGTSLCGIASSS
>tr|C9ZQJ4|C9ZQJ4_TRYB9 | DeepTMHMM Topology Prediction - Predicted Type: TM

MHANWITWAASTVVYDAGDTISFVFALSSLLPSIIVIFIAGLASSSTSHQRDAALLLLVGLCQNTALNTFL
KAFIKGPRPISSMYIMVPMSSSSNYGMPSYHSQFMFFFITWLLRKASANHIPVSWGMWLFFLVSATVVACG
RVYNSYHSTDQVIVGAAVGVINAYASTTPTLERVLRWLMFRLSPVRNFFTSWVTYVRL
>tr|D0A9L0|D0A9L0_TRYB9 | DeepTMHMM Topology Prediction - Predicted
Type: TM
MCLATCIIVYLYIYIYICVCVCVRNICMNHYIDIYIYIYSSLLFLISRLYLPFQWQHTHNIYIYTERERER
GTFTYVFFSPYAAESVAAAHSDSVRPTLNRFSQPGVGISMHPYLFICKEGNNNNGSSTNLKSSMSLSDRRH
TPLGRNLSESTELSQSLSSHSGGSSPIPRPNLDDITRRLLEENEGMLFEINKLTRELTDERAKVSGLHETL
AERSAELELMKEKLSDATVVATDDTGKPINYKRECDRLAADNNRLVKEYERVCTELEKRTEEIRREMGDQR
DEAEGKTISVQVEQFNREITALVPTVEHIVNSLKRCVVTGGIAEERILENAQKELHTMRRITDTVRRMNAG
DETCVKGLLTQIQKGEQLTAPGDVAVVIRWMVVRAFEAGLQSAEGNLRTLVDILNNSKGRHTGENATTGSG
EVPASGISVRQMFRLLKM
>tr|C9ZQP9|C9ZQP9_TRYB9 | DeepTMHMM Topology Prediction - Predicted
Type: TM
MLCSGRIILKKKWIPPLGYRPKDFERKLRSPRSSKPWRDRRAASGGQIEVSPSTAAANIPRGDLVFRPPLG
IRDVRPGSPSELLPTFGVSEEHLAAHALPAAGSQVEGGDVLAVAVPRSASHLTLPFGRALNVNVEEIKTYE
EGPVPIHLQQFRRSRREAADSIILPRHINRLFHKIMGWSEDLVKVEMDEAPTPNTGGVPSDTAAVRAKEAQ
ESEVPLLRMKYDNVVDKMKYGVLLDNYEKDKFAIEHQLEIAGEKFERKFLDWEGMHVLDQDPSAAVREVLE
NKASIVMEQPSSFAIPVVNRDCCRGCGALLQDQDENSFGYVRKGDVERYIIEKQQKMRARAEYADRMSELQ
AHWRKHGRRVGEEWLDFMTQEEFDAFYRDRNAPFVCHRCHALENLGMEGRRRIWSAPDFTDKLRALREKKC
VVVLVVDVTDFPGTMVYDLPGLISMNNDVIIAVNKMDCVRNRSFNYRGKDRAVAACLVTERYVRRWVTGIA
VQFGLPRHQIKDVIPLSAKRGWNVEALIAAVEEASNLNLRRPTKPIPTYFVGVANVGKSSVINAIAHKLYV
PLPPHPESRKVYYTKKAPDGSESVFWRWYTPPNVNQAEMIDIPSRHDKKASKLVTTSSLPGTTVDAVAVRV
SLSKGAEKGRAHLFDTPGLLPHWHRHSPLTLLQMRRTLIRKFRNPQCFILVPGNTLFLGGLCAVDVVKGTS
RGMLFMVYTSQKVRNAIINTDRSDEFWREQLGRALDPPGSVEQVGDLRLTESRSYLFECYQRNRKRPKADI
YFCGLGWVAFCVNEPADVVLRVRTLPGVVHGVREPLRYKDLLAFKGWPKLHLYCMHGKMHLMWFGLCISNL
LL
>tr|D0A910|D0A910_TRYB9 | DeepTMHMM Topology Prediction - Predicted
Type: TM
MCVVVSGFGPPATFNGGYVRMIGEGFRFFATIWVMWVVALSAEIQLLFARAGVSKGGYRYGLSTFIILLAQ
VFLGTEWSRTLRIKFLTLYHCCLSKEKNNHHYDLYLFVSRFSEGSRLST
>tr|D0A5N5|D0A5N5_TRYB9 | DeepTMHMM Topology Prediction - Predicted
Type: TM
MRKGCYYLSEGCASFCCYHRTCTPLCHISFVFLSINPFLFSLTTFHFPPFPVSRLTHTCIYKHLLRTQYQT
HCPHMLFFRKKKMRKRKQKCAIFKISFLSFSTHLEPLFPSLIPC
>tr|C9ZTX6|C9ZTX6_TRYB9 | DeepTMHMM Topology Prediction - Predicted
Type: TM
MGVSCFFSSPSLPFRAFLQLIGLSVRICAECCSSVIVLIHILSVHMYVCVHTSCGLDLLSFPRVILEGSGL
LHEYDWRVRKGDCACVTEQRGCVPLLICIGAVRKKKWTVQQWLLLLPAVATSCPRAGTC
>tr|C9ZL89|C9ZL89_TRYB9 | DeepTMHMM Topology Prediction - Predicted
Type: TM
MKRVASIIIFSFSHHHHHSRLCSCCSFLFPSFICCFFLHFLSVCFASLTEIKLLFQFVLPFLCCGNVPFTI
RSYSCSSKRSQRINMIIIIIFKRKKEGKKEKESQIS
>tr|D0A6K5|D0A6K5_TRYB9 | DeepTMHMM Topology Prediction - Predicted
Type: TM
MRRQQNRQRLKASNAASSDEPSATRSRYNRYLSAITLPHVKHRTGMIIYVLVLFPLFDFPLCLMSNNMANS
LTIFVLCLTLPTPLKPQLILHTRQCFSSSCWPATGAISFYFCRQRGGMSIPRKLNVIT
>tr|C9ZXZ9|C9ZXZ9_TRYB9 | DeepTMHMM Topology Prediction - Predicted
Type: TM
MLLLTFFSSCVNNAFKLTSVRLLVAVHFYPFRFWSCWWVLFEQLSGNAPCRNFVVILCAKQAPLNSFNVNM
ASFSYSLCPLAACLFFFCCSVFRYPFDAFHFIEQVAFSVCFHFHFERRTIVFYFSLGTCFLGV
>tr|C9ZT04|C9ZT04_TRYB9 | DeepTMHMM Topology Prediction - Predicted
Type: TM
MKKLKRTRQRCHQIWLLLSQQWKKSVDAIFSFFFYFFPPPFLDSKGMCQNINIRNTRNSLNNGDNNDDAIF
SPLSHRTKTVSEYIYIYIYIYIYIDGNSGNINNNNTRQNTAVNHRTLPNQNEEKQLMKAVGTGGSLQPLFY
YFFCGGLSDIRCVPSVQPRVCPSHCSSLSSMYSVTFILIYLSLISCCFFFDTSFFFASSTADFGARLFS
>tr|C9ZNM9|C9ZNM9_TRYB9 | DeepTMHMM Topology Prediction - Predicted
Type: TM

MRVEMSFGFLQLSLYPHPFTHLCTLSCLVSSFSFIFLCCRFLLFGHPFLSHFDTTSTIPAKMGLFTVKISL
CFPDSHPCYHCLFGHNRNNSKKYCWSLL
>tr|C9ZVC6|C9ZVC6_TRYB9 | DeepTMHMM Topology Prediction - Predicted
Type: TM
MLWGAVTLLFPAVVMSLLFITAGVPDIRRCCIHSCVAAALVPLAVLVAIGISIALDQCILYLHEAARSSIP
QPPPLRHALLQLKPDSLMTLSFLDVQIVAIFLFVSYGLQILLYHLVDIVRYLKISRRRLVM
>tr|D0A9T1|D0A9T1_TRYB9 | DeepTMHMM Topology Prediction - Predicted
Type: TM
MFCILPAVRLHHVVGAAFPPHQHLLRLLRVHCPVLSLCVRFEDYFLAACIVFLIPDLPLFYPLFFSYHCVC
FFLFFFVPRVIPIFLFLYYYYLGVNRGLCTELFH
>tr|D0A0X5|D0A0X5_TRYB9 | DeepTMHMM Topology Prediction - Predicted
Type: TM
MNMLHLGDRNASLAPSGGEHSLPTGGAVCRAVMDTLPVILRAPVALLLLLVVVPQLSVVAEANVTVKVLSA
AWNWYMPRKYVTAINAGFNASLESRQWTVAGSVKVEVVYPSNLHLMPEDFIKEQLQGETNMSSIVIVYGPL
GDQSVMHSIPHLMNHRVVAFGLMTGSTFIRQWNPYLYFLRADPAAETLVLIRYALCQLRVLRLGFMYLQGV
HYGDEEYALTVNVMSRMGYELHGVFTVMSPDGEPAPDGEFKEVFERFAAALPQAIIVFGAPVDDTAKFLMM
MAVDDRIARSYILSPSAVQAKLPQMWLRAVEAAGASFAPGQLLFTGTNPLAKDSQYIAIKRFQEVMSEYLK
AHVSGTNITEPDYFLTHGTEGELMVYGWICGEVLSQALSSVEWLKDRATFVRSLYNQRRYVINDIVIGDYG
GTCEGDAAKHGATCECNQGNKAVYVKEVLEDGRTTSVRSGFTVLKASRCYTDSSELHGPLNGLVVFMKDDD
IASKAAVLWQKGASHLVGKGDLGHSDRFFLHAFNTTIAEAADDLRNEQGDRIVTAVFGPVTEAMLDMPNIT
FIDPLELKPRLNKFRRHVIHLSPTLEQQLYVLSSYLAGAGVGTVDAVICSNEADGIADFLRRSSNEFAVSL
RSAVIREDGEGVGKYLPISGTVFVIGLSVPDVKEIARKLEERNDLRVIVLFAEFSFLYDLFATALNNTAGA
ARLVFATSLPHWGDTETSSKTTQLFHDVEKDSRLWTPLSFLSFATGRLMREILLRVEEISPETLVNFFYAD
SSIVSDDMRYGVFDDTKCVVTAEKLSKNGCASNYGATQISVWSMARALNASIPPLTNPMTPSMTFRNSNAG
RISGVALVGVIIGGALALFLVVALGVVPYFFLRNTRDNNLAPKELTDPVTLIFTDIESSTALWAAHPEVMP
DAVATHHRLIRTLISKYECYEVKTVGDSFMIASKSPFAAVQLAQELQLCFFHHDWGTNAIDESYQQFEQQR
AEDDSDYTPPTARLDPKVYSRLWNGLRVRVGIHTGLCDIRHDEVTKGYDYYGRTPNMAARTESVANGGQVL
MTGSTYLSLSAEERQQIDVTALGDVPLRGVPKPVEMYQLNAVPGRTFSVLRLELELLNDDEDQTTTSCSDH
SSSRTDLSVAAQTIAASLQSLLGTFTPAQRQKALIPFCERWRVPLPQKVGNVWDDDGCQEVVRRVAAKVGR
VMDFGTRKPSSSVTSLERGGSVFSGGGAAAIMLASSSSSSCVDGHCGTVQLIDVENDSATT
>tr|C9ZQH4|C9ZQH4_TRYB9 | DeepTMHMM Topology Prediction - Predicted
Type: TM
MQTSTGTTGYHASFGFRPVGKFCNPAITVRGYTEPQLSHQQHAAVILSASTITAWLRRPIEKLHAFYFLCD
VPGESSRIYASDKRAHFFRTLFGIKWLAGPSNLWTIASYAFQLTAFTKLRDLIPCESNTTCGFTAGCLTGL
LYAFLRHPYEVLLATAEAEKGPMKFVGAWDVFMKAVTEKPNVLLGIYRGVSVAACSQVALLGTTWGVYNAV
RYDGVYHGTPVLFLYCHGGALLGKILQYPFLSIRQQVRIRNQHTRGRPHTFRSYIVEVRRKHGITKIYDGF
FASRPILNSIPAALLLVTYDLCSRHLTEQLHPELRKMHGEVNQPLYSRHAGPYAETLPTYEFKRG
>tr|C9ZZA4|C9ZZA4_TRYB9 | DeepTMHMM Topology Prediction - Predicted
Type: TM
MGFSLAGILQALLLCLNAMAILSERRLLSRYGLASSAVMDSSDIGSETAFSSGDELAGVPRQRPFRESIAA
LLSSVRTLMRWPLIFINTAVIVFTLLFG
>tr|C9ZJ41|C9ZJ41_TRYB9 | DeepTMHMM Topology Prediction - Predicted
Type: TM
MFTSSGCSGTCSGPTNARRLYFMLRVCAALLWVPHCAASWWVASTNQEVLHVLIFHVLAIALTYYLAVYKS
CGDYLLIVSWAIGVLSRSTWVTPKAGGSVVIHHNAAPSVLHLSTQNNNFSAADGSRDVNGDGDVNGLMAAV
SALSSTAEGSRLLWLGVSMLNGIIGGALCKLKDLDEDRYYPTLDLGARLYVVVPFCTGQWFYFLLRNGWRG
ILLENLTIIIVLAIVGYTVLTYKLLKKYTPLAHGDVIGCFCALLTTSFGVVFAAVLVGNIFFTVLVIPFLK
SLFSLNALVSSLGIVMEIIVYEII
>tr|D0A0H0|D0A0H0_TRYB9 | DeepTMHMM Topology Prediction - Predicted
Type: TM
MKFLGFFCFYVFMCVCMWEMFIFQIIPFCFFFLLVVVFSSTRSSSSPPPQVVPFPFLFFSFALSLDFSWCS
VHVPGEISTLSLNHLFLPFLSYAAAPFSFLLFFVFPF
>tr|C9ZSD8|C9ZSD8_TRYB9 | DeepTMHMM Topology Prediction - Predicted
Type: TM
MHFYLSFPLLSSVPHLCQYLYKQTYIHMLLHYCNCARSAGVAGDNCLRPPLPPLQTHTHHLRKIKDLVKRA
VLCNSVFIYRRRLFVCLCVIRTVTLCESSVPNVFPPRNFSHVCVVAACFPFLFFPFLFVSLCVQHFSF
>tr|D0A6N7|D0A6N7_TRYB9 | DeepTMHMM Topology Prediction - Predicted
Type: TM

MPLPTDPREWVKKVRAKTPVLLEVGKPAPKAVLSFGGCGFLVTYSLGVALYLQQEKADLLAQSFLLGAGAG
VIPAVALACGTRAVNIEKVRDAILDNRFMVTDEERRIEVLTKFINLLLPRNAVELVSGRLALTIGFSNRDP
GYMKQTKEQIHFGHHIAQWTDVSDLAQCILASMAPNTAKPMIFRDADNVMRGTMMSLSSELDQYCRHIYIH
GYCGYPYNKHQTRHNIYFGRHGFLANTYFPFWKQALLAFAPTFGGEARREELLEAYDAGYNDARRYERWEE
DPYHFSKADRSPSDDFSFRQLRANLFGGKKAAERFEL
>tr|C9ZTB6|C9ZTB6_TRYB9 | DeepTMHMM Topology Prediction - Predicted
Type: TM
MNPCLSLFTTFACWTSIFPFDSFLDFLSFFFPPFTSSSSSSLSPRCFLYKFCLTHTDNVTQPCLVYFSFPF
TINQTVFPIFFRPVSVWRRLPSPQIIDDIYRYGLCKGVNA
>tr|C9ZMC5|C9ZMC5_TRYB9 | DeepTMHMM Topology Prediction - Predicted
Type: TM
MFEVEFANKQEKGISFFFFFFAPRGAFPPLFLLQKKNEKKEGLCRTAAPIGGAEAMRRKFHARFPSSSFSS
SNFSFFLRMKHVFLLLLLYCCSFFSVFVPILVCFSPPPFFSPSPSLFFFFYVLSPFNWAVPYVLVLGIMLQ
RGRGKECAYVVRRVSSNSHAKNR
>tr|C9ZV44|C9ZV44_TRYB9 | DeepTMHMM Topology Prediction - Predicted
Type: TM
MISPKFFILPILFSLFCRGFTSFCCLTVRSKATKRVVKKNHAHDVFRSVVVIFFPDALRFSLFHPSHTSKS
TPAITVSLTYIRTRVQPSIQVEEKEGAGSVQTPLK
>tr|C9ZWR7|C9ZWR7_TRYB9 | DeepTMHMM Topology Prediction - Predicted
Type: TM
MTSGAAAQAPNKCPTNEAAAEPLRVPNVLTGEPFVSPNSNEGEALEKPERPTNALARCFHFILPRGGALSG
IFNLASVTLGAGIMSIPSAFNTSGMIMAIIYLVLVTVFTVFSIFLIVSAAEKTGYRSFESMARNLLGRRAD
IAVGFLLWLLCFGGASGYVVAIGDVLRGLLSHEKVPAYLKTDRARRLLMAAIWFVFIFPLALPKRVNSLRY
ASAIGVSFILFFAICVVEHSAEKMVTDGGIKQELVMFRSGNDAVAGLSLFIFAYLCHVNSFSIFFEMKKRS
VTRMTRDAAVSCSVCCFVYLLTGFFGYAEFGTTVEGSVLKLYDPYANPVFFVCFVGIIVKLCAGFSLNMLA
CRTALFQVLRWDLDTMSYVRHSIVSVSFAVGSLVLGLFVPDINVIFGLVGAFCGGFIGFIFPALFIMYAGG
WTRQSVGWVQYILTYVLLILGVVAIVFGTSTSIYYTIKKYY
>tr|C9ZQJ1|C9ZQJ1_TRYB9 | DeepTMHMM Topology Prediction - Predicted
Type: TM
MFLVTFVSFFLCLFVSRNCYFCFFACSSGFSLEFFSCFFFFEKKKYHFNILCFLCDHNYPYPFLKYCSLFS
FFFLSSCVICLLFAFRVCTKLNYSIMMNMCSSLIGPRSAELIFSFFFSVFFFFNMSHGGDADRFLLYFSCT
KKFKKKKKNQCEEFSLIVS
>tr|C9ZRB6|C9ZRB6_TRYB9 | DeepTMHMM Topology Prediction - Predicted
Type: TM
MYGGHIYSQSVTVNHTQAKVTSNAHSHMSQSPLTSPSASGFQTRMLRISILKTHRLRYMYLAASPLRHCIT
FSLPIGIISLLLKRYLILFLPVLYLITHVNAPFLQPHTKAEGESRRYFVTIIR
>tr|D0A3U2|D0A3U2_TRYB9 | DeepTMHMM Topology Prediction - Predicted
Type: TM
MTERRDNVSHAPDAIEGPNDGAHAEDTSPGFFSLENLGVAQVQVVGGTLNGYVIGYVAVYLLLYLTATECK
FTTEGACGGAKIYGCKWSGTTCKFENPKCSEGSDPSDSCKNEVAYTSVYSGIFACAMIVGSMVGSIIAGKC
ITTFGLKKSFIIVSITCTIACVVVQVAIEYNNYYALCTGRVLIGLGVGILCSVCPMYVNENAHPKLCKMDG
VLFQVFTTLGIMLAAMLGLILDKTGASKEEANMAGRLHVFSAVPLGLSVAMFLVGMFLRESTATFSQDDDG
KADGGMDPNEYGWGQMLWPLFMGAVTAGTLQLTGINAVMNYAPKITENLGMDPSLGNFLVMAWNFVTSLVA
IPLASRFTMRQMFITCSFVASCMCLFLCGIPVFPGVAGKEVKNGVATTGIALFIAAFEFGVGSCFFVLAQD
LFPPSFRPKGGSFVVMMQFIFNILINLLYPITTEAISGGPTANQDKGQAVAFILFGLIGLICSVLQFFYLY
PYDANQDHENDHGGEPVEQKTYPVEASPRN
>tr|D0AAK5|D0AAK5_TRYB9 | DeepTMHMM Topology Prediction - Predicted
Type: TM
MVPEGRVVVLVGKAPEVRLLAPRTISRRDQMRGCNTVTAAARVHGNVTSALILMYACACPSVGNYMMAVIG
TAGFASLILLSVPQKKYGSRSFTFIHSFYPFLLFIFVPLF
>tr|C9ZY30|C9ZY30_TRYB9 | DeepTMHMM Topology Prediction - Predicted
Type: TM
MVNLNMNETFIFIYLFICFPFPNFSREEPNVHTHTNTHALSLTHPLFFSFQLFLLFIYLFIYFLFFSLASL
QHPSFHPFLSFFLFFFLPYFPFLSFYYFFNYFFILFFSKKKNNNNFLLNLLFCAFKRMPTFKY
>tr|D0A9E1|D0A9E1_TRYB9 | DeepTMHMM Topology Prediction - Predicted
Type: TM
MESFGPLAVVSATILSVIAVFYIGIMGPSRYHRNGVVGWLYRGMVKLPHACVSCCCCRSGTSGGLFSCFMK
TRCGRCFHYLMNERHWGLVILYIILVWPVEIAYLALVAMRLKASLLSKMVSWGLVLFSEVVYFAAVFSDPG

TVTSRSEKDAQKRAFATAGGAKPQMKKGHEATRKGEERMQYRKFLLSPRAEEEQGQRYVVDGILYGKDSTR
GCGVECPTCNVPRPSRSKHCRMCNRCVRRYDHHCPWINNDVAEGNHRWFLLFLLIHIIECIWGLWDLYTMV
VQFLTAQGLWVSAIRFANGYTYRITNIHRLFAIATMHPLVLFLIIFAAPITVVLAVFWLQQMSFVVSNVTI
NDMNKIDTTIDFITTLPTATDVYEEAQNVRSVLENVAARPPRRLRALKRPSAEAIVVGSKKDKAYRKEVSK
MLMSDLKGLFNRGVWNNLKEVMFPYS
>tr|C9ZUE8|C9ZUE8_TRYB9 | DeepTMHMM Topology Prediction - Predicted Type: TM
MRSDVNGKCPYIADETEKVWSACLISLHPSAPSGTRFTLEWEGAAKWRGIFVILTDLACLLFSYSREYTTL
LYKLLCGRKNDVEAKWCGSCTHETVGTRNLPVLRRSFI
>tr|C9ZKR3|C9ZKR3_TRYB9 | DeepTMHMM Topology Prediction - Predicted Type: TM
MSDNRKAVDVTVLSEDPKKKENEKKAREAESLKMSEEDERIKGQVELLVTRVGDSNTELAAVAVDQLIDLL
RTHTSGSVASVPKPLKYVRSMYGQLERVQKETTNPKLAVRLHDVLSFVAMTIEFPDGQRPALEHKLLGTQD
DLAHWGHEYLRFLAGCISTEWKERVSKGESVVHLDGFVQQIVSYMVKHQDEPTAVDLLMEVENIKAIIPFI
DGHNHRRIASYLAAASKYLTRPMDTEALRVVYDIYVKMESYTEALFVALQLGDRAAVENLFKMCEKSSVSL
QMALSCARYRFFLPNDNEEQGILSSANGNMKLSELYRHVATELDAMTPLTPEDIFKTGLDGKQSSSASDSS
FKLACSFASGLVNCGYGKDMYLTGEEATWPSEQTDNRLFATTSMLGLIHLWDHADGLLEIEKYLHSDTVNI
RAGACLATGISLCGVSHPYDPALGLLNDFVCAPHKEVRIGAILGLGYAYAGSMREEIRELLVPILIDGEQT
MEVQCLTALALSMVFVGSTDEGMVETITHILLEMPEKDLKESATCYIILALGCLFLGRQEAADTLLDTLQT
SSPIIRRYAEIVVRSCAYAATGNVVVIQNFFHAIAENDEPEDTEVPAAEGDGNGQQQKKNRQQQSTENAAH
LNYKAAAVLGIALVALGEDIGTEMAKRSIIHTLLVDTVSKGEANISGRHAIPLAYALLSASNPGMQVVETL
NRLSHDSDMLTAINAIVAMGIVAAGSNNARVVSKLRNLALYYQKDRFASYLFSVRLAQGFTMMGRGHLTLS
PLLNDRSLVSPTALMGLLVFLHSALNFEEIILGKYSYMVSSIAPSINPRMVLAVDDQMEVVKDGVQVRVGL
PVDTVAVAGKPKSITGFQTQTTPTLISVTDKVEVASQKYRPVASVVEGTFVVEEKPNVE
>tr|C9ZK26|C9ZK26_TRYB9 | DeepTMHMM Topology Prediction - Predicted Type: TM
MPCATKGEASPPVTTPVGRDGCLCVVTWQTAGRMSLQTCALESAADALVFLRNNLPGIALCGLWVDMQKCS
EEEHRQVLQLLYPNMQPSHVEAVLANDMYDVVELQPTEGEYVVGCLSCSPSHGGPFPAATAMSRAEFGCEE
GVLCSFVCSERVLLTLHTAPFAGLAELFRHVLKCNGSEAERHSRECEGSVVGIVKDPSSHRSSFVPVAMGT
DALCTLVCFTCEASFPAPASLLSEVGDINEMVFLISPGEQDQTDLLRRVALLRRRISSFRTALFLKEKLIH
QLISPAMRLTFVSKSHPSAVAYKEILARVQKVSEKLDDARDMLNHANLNFITGVSMRMSQASAGLDFKMNI
LNSVAAVSLPINLVVSLFGMNLKVPFMTGEGSTLIPFWTICAVFVVWAGICLTPLFRRAQRGLKNDPIAPY
E
>tr|C9ZU21|C9ZU21_TRYB9 | DeepTMHMM Topology Prediction - Predicted Type: TM
MFKWQTRLLINNVNNSFCLFVLSFFFFFVNISRFSRRLGEIFDVSTVSIYSFVNLLFVLRSVLFYLLPLLL
LLPLLLLLLLLLLACFTLSPISFPFLFNF
>tr|D0A811|D0A811_TRYB9 | DeepTMHMM Topology Prediction - Predicted Type: TM
MNNYHIYDEIGKGRHSRVYKGRQRKSIEYYAISSIEKSQRQHVLTNVKFLRSSNHPRIIKFHNWYETNNHL
WVITELCTGGDMRQVLHPESRLSEAAVRLYGGDIAEGLMYIHSRGVVYRDLKPSNVLMDSTMAMRFYDFGL
SCNFPVNRGGGTIGTAMYMAPELFTKEGVPSIASDLWSFGCVLLEMRTGKPPFDADNLEELITQILTEPYH
PHEELSDELNELLGKLLVKNPLERATWEDVVLSPFWQGRLHMPAAPHPPQPAFESLKQKRLASDGGKGDFV
MTLEEAKKAVEWTVEVAKRNFILTQPDSDGSALICLGVYDRIDTRDLTGEGGGVHDPTSGSLIGDGNSCAS
PSSCVARVGCDSNAGRQGDGAAQREGDHNREMVSDIGTAVGGNAAKNSNVKQLAGGGKFSTALIDSLLSHV
SDAHVRPLVMNNHIERFVEQKYDAKALGFEPLTKSQLKAYDEEQKARFVTKVYQRLSSSSLSCEDKLNVLC
YFESICSESSTANFVVSSSIMTLCLNMAGHRKASSSFRATAASIMGILVRHATFIHPDLAKVNILASIVKM
YAEEESSRVRRKLVACYGEFLIYIAVQQERERAVWGVDVPATFNLYRSLLNDPDDVQKHYAIKTIENLASV
GNRQIALDAFVNAETISLLLSIYALPPTPTCGEYMRAAAACTALKLAMLREELIPAVLESPYLKLEAYGGV
LAAPSPKLAQALLTFVNMTLVKGMVGLRHANITKLGKPDATSPFASSRLTGDKAKGVLTALSGVAEAVVRG
LRNGSEHATTAMKGKTLVLFILLGCMDEQLLVRFFTSSRCVAYVDGIAKDKDSYVQRCTQGFAVYLSYFFG
SQLEGLTHGAPSFMMNNVPSALCNLLGTRHLSSMLQLNDKVFSSIGKCLVMVSSSSRYAAAGTNLHELVEL
LAQNRELVLRHRLVISSDIFPPYLSMLAESESERRFLSLRILRALIVPFGSESSRQNEEAQKEGAMLDRVM
QVVAGMLGDLLKEVEPIPVHGIGLLATCGERRPKTLANLATVELIGELVRYMMRSRQSGVSSPLQLVMLAL
QTERGGVLMDYLAEQNFPAEVLLNILVMAVEKDVDNLLEPCCELFEHFLKQAVANPNAAALRQSVMSVAPR
ALETLWLPLCTSPVGTTAKSAAACVLYFTQLSPEVGQELLSDGNVQYIREVLDKCRSPGAVVCVIRTLRLI
LERGTKHDAQKLMPWLLGSLEAAEQDQKCGGIVALEIRGIKELLRP

>tr|C9ZTT5|C9ZTT5_TRYB9 | DeepTMHMM Topology Prediction - Predicted Type: TM
MAFCAVIKPYRNVPFHSLNITACNAFHILSLVLLPSDPFCPCLILSGGFYSEHYGIYGIRTLMRCCVNGVK
RWWINTVRREEVAVCGIRFSKLCLCFLFPNPDFGGVLILVAVAFCVSFPCDPGCHCVASLSVCMLSVYL
>tr|C9ZIQ5|C9ZIQ5_TRYB9 | DeepTMHMM Topology Prediction - Predicted Type: TM
MLMCLCFSACGFALRGPFSAGKCFVHYFSLCCYVCLGFPSPARSIALNHFFCGRSFGCASKITNFLFHVGR
HNLFCARLLSGIVWHMPPNTCTRMHFHAGYAFVSECAGVNLWEVCVCVCAVSLSVLFCRGNGIPRLFGERL
RTPPFEYMCFLLFGWFH
>tr|D0A4C9|D0A4C9_TRYB9 | DeepTMHMM Topology Prediction - Predicted Type: TM
MHVCILLLALPFFMCVGFLFATICSAFLRGPSCGRRLPILVRTLLFSTSVSPWFKVFSTLSETLFTCIPGP
KLIQIINRAMSSRNCSLSQGRLLSRQKRPLVVAQFLNKVPIEDRHAATNALLCLGLRAYKMVHATTPPDMQ
TLRSAVSAEISAVASGSEGLNDQKIDEGTASGRQRGAVDDVDAFLFARRERGQAPSSSPPVDRRLEGNTEE
EEFYTRIESVRNVGEPSYHDRMPQLMEDVDVYSSSKSGADTRALNRLAGDGGESDRAAGKSGCFTDVLDFV
TNCAVGSLSLCTPTRCREAANVSSSPPANCYNAPGSEQPNMGHASPLRASGSQDLPLGGMKKFNMNTVVPL
LDDNGVVYPVEFIHHLMARIKSTRGVRENPILVAAYMPQSLAVYTLQGSFGDLYRSLLVSYHVSMW
>tr|C9ZYS4|C9ZYS4_TRYB9 | DeepTMHMM Topology Prediction - Predicted Type: TM
MSAERVAVTLLMWVLVALLVSTIFLAAVGYRMRESQNHFLYFPNVPAGSTFVCDSPLERGYRNSEQVHIRT
ADGLTLRGFIMWPPMELQERPRQQHYMEERMGYMLRVPPLSAASDRSNPNPPFLERPEVSGAGSAHPQCSI
LYFHGNAGNVGHRIPIAAMLSTKCRCAVLMVDYRGYGQSDSVSPTQEGVMLDAQACLDYLLCHPHIPADRI
FVMGTSLGGAVAIHLAAEPHNAKHIAGVIVENTFTSIGDMASEMARHALNGAQPCFSFLLLSLFEYYVKPL
CLHIKWRSIDAVQKICAPMLFLSGLKDNVVPPLQMKKLYSKTFSTRSRRFVEYPEGDHNTLPLIPGYGETV
NAFIQDVLRHREQLI
>tr|C9ZKD3|C9ZKD3_TRYB9 | DeepTMHMM Topology Prediction - Predicted Type: TM
MNNKAPGTPPRPEGYETAGGAATNENFFHPKRKMTKSIQRAGFRSARRLMVCFSVVIGTIVIAPSYLGPFY
VNYVAGKNWLERASKSIWTRRNEKDYDLYMTQRKNGWWEYLGLKHYNIGGETNVGEIQTYRSK
>tr|C9ZT57|C9ZT57_TRYB9 | DeepTMHMM Topology Prediction - Predicted Type: TM
MTRGQNDTLRDVEQDTTRMHTGVKLSKEDERMLDDRINANTLVYPKQALIIVAAIASFQPVYLSHAVNGLD
WINPINAFLYLVIATFTAYMLSQAYTVMVESEFWRQQRHYSEVSKGDEKLLHKYRLQVAVGYTLFFVNSVF
SLLCTLLHVYILRHSDPRVSFMLSPTLTAALLWLVAQKNEESRQRRMARHK
>tr|D0A6F4|D0A6F4_TRYB9 | DeepTMHMM Topology Prediction - Predicted Type: TM
MDVIQTSFFFFKVWRVWDWRALITSKQCILLSLFFLFLEKKKLVFAVHYSLFFRSNSFFFISNSNACRPCA
CACNECCYCCFYFPFLQMKKKKETHSMGYIDA
>tr|C9ZJC7|C9ZJC7_TRYB9 | DeepTMHMM Topology Prediction - Predicted Type: TM
MMQHGKYKVIVTVEAKKERKREGIGEKHHSPTIPATFDSSIYYWGFRWKELCRRHSVHHFNSLQVHLSLLM
SLGNGVGEVSLLTSLCLFNCCYWCCTLVC
>tr|D0A727|D0A727_TRYB9 | DeepTMHMM Topology Prediction - Predicted Type: TM
MFSRLLHCLPFVESHYVLESHHKTKVFLYSHTKMMRRLALQSSLRRVTPAAVSVMTPAKVTSPIGHAIAIR
QASTVAISVQGLHYVGTGLAAIALAGVGLGIGTIFGNLLVACARQPNLTKMLFNYAILGFALTEAIGLFAL
MLAFLMLFS
>tr|D0A1Y9|D0A1Y9_TRYB9 | DeepTMHMM Topology Prediction - Predicted Type: TM
MLLSFPPPFFSLFIHFTVNYCGGASPHPIHSRMAIPDVSSLSPLHTHTHEKRITTAAFIPFFFAFLIEFPF
FFFTGIIDIHRCVKCASSPREQRSLQKKNTKKKWKKERKKSVVSRGSGAQLKVKPKAISFFFFK
>tr|C9ZP29|C9ZP29_TRYB9 | DeepTMHMM Topology Prediction - Predicted Type: TM
MLWGRNKKCATLGLPYTYIYIYPSLVHAHPSADTHTHTRLFCFPSLCVYIFDIVLYLSATTIPACLRSFQQ
KRMEHNPDFHVSTKKVNENEGKQTQKKKKERHNGELVGSAANI
>tr|C9ZMC8|C9ZMC8_TRYB9 | DeepTMHMM Topology Prediction - Predicted Type: TM

MELTQAINLAFAAFLFACVAVVMTEYMMVKQRRRRAQLARAHRQGRREATGQSSQERARSRLTRQAIEILL
ANNYVHEGIPLASMVGVTEVLADREMRSNTYQGRPPLITMADGRRATISRVVELVRENGPTPDALPGPSVV
ELLESLSQQQRRAGGAEPFGDSGLSVAELMIEEFAKLEDKKKKRNRAEESSVEEVYGKGSEYTHNPANVRT
ENAFNHLCKRGDVPSTTY
>tr|C9ZPH3|C9ZPH3_TRYB9 | DeepTMHMM Topology Prediction - Predicted
Type: TM
MYLPLICTVTVVEKGSGERNHSVPSDFFVAITCNAHTTRTPFLISSLTFPVINGANFPFIYLFICVVIVYS
TTTLLVAVEEGEEQFVVLWRHCHICYPLVPGFLANCIRTFIQTVIKTLG
>tr|C9ZU10|C9ZU10_TRYB9 | DeepTMHMM Topology Prediction - Predicted
Type: TM
MEFVRPFFLLSLFKFSGIPCFSSRRVPCFFFFVVLLWLLLLSFFPLNVTLLVADSAVTALYSLFPRAYQTF
SRNAAPRQKEKCSLFVFFFVFTLLFAAIGKCTCLPFVVVCVCA
>tr|C9ZQ93|C9ZQ93_TRYB9 | DeepTMHMM Topology Prediction - Predicted
Type: TM
MSESPPRWQILFFCYCCFFFFALRASPLLPNTSTPLAFQNAVTSRKILYYRRLQLRAIRTKWAPICCGTNR
FFPLFCPNVRAWWSLSLCVCVCVCVCVCVCVCVCVCVCVCVCVCVCVCVCV
>tr|C9ZKE0|C9ZKE0_TRYB9 | DeepTMHMM Topology Prediction - Predicted
Type: TM
MYIYIMHYTLFVLYAYYYFYYLLRCTNKYEGSKVLTVVVTDGSHTQTHTVALHPCFMFPFFTLLFFSCSAP
GVCRQSTWNEKCRWKGGKKRKQMKEETNQFHSVNKETSIYPFSGSDCKKKIIIINE
>tr|C9ZRX4|C9ZRX4_TRYB9 | DeepTMHMM Topology Prediction - Predicted
Type: TM
MQSKRDSSSEFLETLYQSTERGKTVSQTYLAAQVEKEINRTLDPVQLYEFQKPENFRYNLRHGGVPVANKV
CDASSLSRERELNGPGANGDHMEPESVSGVRPFWRTHLDEQDSEILRMIQRAMRPPNKSSVPPVAPDDAAV
IRKLKTAEDCITFFAANGSSSHLKYVYLCRAPHVLEFRPYDLCVSPMKRGLEQKEHYIMSATGVVHVRPGH
PSEVVSLAEWMRESSLFDVLRKVHFFKTYLVYKAFFRWYKNVRAKDFLNKRRLLSRNLLISKSTFSTPILE
VLNASYDLTTHPLVLYEAQGKHDYTIEDFRTQQAKQRQKTSAAFVACMEKIETGVMKLIEVMKSKADVPDI
NTKEALEQYLLANAIVTGSGSFRSQRKMKSMHDARGEQLQRMRELKRSIVEYSQIDQLIRMVDCVTSETVF
RVILSAIKTLYNTLHIQSSQELRSVGFQVLLQPTEDQMLFNPPERAIRTLCTELITECIEVTGSVTRLCSQ
KRLKGHFTKAPALWVLGGELESDARTLYLQSSILQLVGDDYAQANTKMQAYAVTLPHIHFLEREWSDILAQ
WEEETGERPLTCSTLFKLYDKIQDAQDTLRVLMASFVGNMLWINAAKLKTEIEPRMRIVRDGIDSTLREIT
RTAISSLHSYFKQKIQVLNERPGQLSQFAEYVAHYKEVVSETTETEAAVAQADALCDLMDRQQVEFLDDED
KQLRERTLGSVSTAMSLQILFDEARLHAEAFIEENLPFHTQTLEDSVCNVEDECVTIKQILTNSEFVVLTE
NVEPIIQYLAELNTKLSEIQASETQLTKYAKLFDRPPIDWSTLKEMRSLVFVRREAWTLLDTFMKQREMWF
NSPVRELDTAAMEDEVNQLIRRTGALNRQVQEKEYEDEVGQHLLTELQELRAIMPVIHDCGNKHMTDSHWN
IVLREATGSDNRYHEGLSLRNLQDLNILEYKEVLSEQVGLATGEWKISNDLDKIKQTWNGISFTTKPYRNR
DGVFILDALEDVIQQLDDNQVELQTIMASRFAAPVRDRVEEWIRNLRHVGNVMEEWTNLQKNWMYLEFIFS
SDDIKAQLPEESMQFSSVDTLLCLLTMKAHNDKNVYQICTENGVLEQLQQANLNIDYIQKKLEECLETKRA
AFPRFYFLSNDELLSILSDVRNPKAVQPHLTKCFDSIASLIFSGDTYSEISGMMSGDGEEVEFEKVVYPIG
NVEKWLCQIESMMRASLLMHMRKTVEAYTPEARETWFFNHPAQCIQAVDMVVWTQEVEEAIRAGSLLEYHE
HYQQQIFQTVNLVKGSLTKLQRTLVCTLIVLNVHNRDIVYTLHESSVTSLSDFAWAQQLRYYWEATDSVKS
GMSVGIHHCDAHLWYGYEYLGNQPRLVITPLTDRAFLTCTSALAMNLGAAPQGPAGTGKTESVKDLGKALA
RQVVVFNCSDGINYKTMSRMFAGLAQAGAWACFDEFNRIELEVLSVVAQQMLDITSALAQRLESMSFDGHM
IKLSRNFGVFITMNPGYAGRTELPDNLKALFRPICMMIPDYALIAEIMFYSEGFKDARTLARKMVQLYKLS
SEQLSKQDHYDFGMRAVKSILVLAGGLKRQHPNDDEDMLLIRAMRDANVPKFLRDDTILFMALIKDLFPTV
KIEETQSDLLVRYVREDMEKKGLQVVEGLITKTLQLYDTLVVRHGVMLVGQTFSGKSTIMHVVQGALTQIK
ADGHDPEGNIPLFNTIHIHLLNPKSVTMGELYGQVNEITREWTDGIISNIARTITRVAQELPDRQWIVFDG
PVDAIWIENMNTVLDDNKLLCLFNGERIKLPSTATFMFEVQDLAVASPATVSRCGMVFTEPFYVDGGRGWI
PLAKSLVTKKATMFPRFRADRVSELLERFLPMSLDFIRKECREWIGSVDAQLAVSCIELLAAFMKTIDEED
LLPPLLRTKEAKPQNDDVYLLPETTYSGVKLVPSEPEEFKAPEIAYEQVSADDETLLDMYFLMSFVWSVGG
NLRDDSRERFSAFVKKHFEELLPGKIAADDGNSVYDYVVHKASMRFVTWRHLVPTFCFSPELPYFDLIVPT
AETVSVITLFGVLVANSRHVLLNGVTGTGKSLAVMDFVTGTLRGDDPEGPWEYFDTVLSAQSRARDIEDRL
DGKLYKLRSNAIGPKPGKRAVFIVDDLNMPALERYGASPPLELLRQLITQGGFYDKRKSPATFKEVHDVVF
LTACGVPGGGRNEMTKRLTSRFHLLCQPSFSDHSIRRIFGCILHGFLSAWEQPDVNALSSVLVEAIKECYY
LISTEKLPTPKRSHYTFNLRDFSKVVQGIMQVSPTSAPTREILLNVFVHEVSRVFHDRMIDDGDRRWWWKT
LSGVSQRVLKMNWDPRLENVIFGDYMRRDRSQYAEIQTGEGLIEVLREYQNGYNIESNGTCELVFFKDAIH
HISRICRIFRQPRGHALLVGMGGTGRQSLCKLAAFMCNLAVHEVTITRSFNMAEFRERLKGILMESGCKDN
PSVFFLTDNQIVQEEMLEDVNNLLNTGEVPGLMQNEDIDQIVEAVRPLASAAGKKEARNAIFSHFVTLCRN

NVHVVLALSPVGEPFRRRLRMFPSLVNCCTIDWFDQWPADALHSVAQHIFSELPLDEEMKNGLIKLCVSIH
IDVQNRSEEFFEELHRRNYTTPSSYLELLNCYHQLLSEQEEQTIAQMKRYQAGLDKLQSTQCTVDEMKEQL
VQMQPKLLQAAQDTEEIMTKVKLEQESAQVVRTECSKEEEAAMAIRREADGIRAECQAELDKALPILRAAE
NALAELRPDDIREVKSFQKPAARVVLVLEAVLILLGERDLSWDRAKLVMGRMDFIKDLQNYKKDELTERTI
RSIQKYVNNPEFQPQEVAKSSKACKSLSMWVLAINNYYEVVKVVAPKRERLAEAEAKLSVATATLREAQDR
LKSIEDKINGLKRDMQENIEKKNKLEADIALTKARLNRSEQLMSGLSREQDRWHSSITFLQDEKLGLPGKV
ALAAGCIAYLGPFTGPYRKRMLTAWWENCKEFGVPIGNSVFDLTTLVSPAKVSSWSSAGLPQDPVSTENGV
IVTKSKRWCLCIDPQGQACAWIRAMERENNLRTIRLSDPSYMRTLENAIRVGLPVLIENVEETVDAALDPV
LLRQTYRSQGRLLLKLGDVEIDYDPGFRMYMTSKLPNPHYLPELQIKVTVINFTVTQGGLENQLLTDVVRY
ECAELEAKASNTQKDISDGKTQLKAIEDRILHLLTSSTGNILDNEPLVRTLSEAKDTSEAVTEALNLAEQT
QKDIEVACNRYRPVATRGAVIYTVLSHLARVDHMYQISLDFFKQLFVKSMHRTEKVSDVDERVAILLPAVT
LDSYRTVCRGLFERDKPLFVALIFSEIYRQEGIISDAEWEFLLKGSEGRSLVDIDNDSWPVWMTEAAWNEL
TALSMALPETFGDIKDTIYDNEDDWSTWFESDTAYEWFPSTTQGLTLFQKVLVLKACREDLTSHGLSIVCS
HYLGKVFTESPAFDLEACFADSSPTAPIIFVLTAGIDPTVLFTEFAERKGFAEKKLVLSLGQDQGPKAEEM
IRRGSKDGWWVYLQNCHVYESWMSTLERCVEKLHISTTLHSDFRLWLTTMPTRNFPVLLLQSGVKVVKEPP
KGLKANIRDSFCNEVTPALWDGRPENPTTWRRLLCSLAYFHAVIQERRKFGPLGWNIPYEWNQSDFSASLH
TLQVYVPTGVSESVPWSALRYMVGTINYGGRVTDFLDSRALVNILDAFFNENVLQPGQFNITSDGVYCIPE
DVESLDAVKEYLADLPAFESPELFGMHSNADIACNRTTARNQLAVMLSVQPRTKGTAGRSPEDKVLEMVSE
FQTRLPEAIDKTKAHPDTYQLTEGGVMISLGTVVSQEIDVFNSIIRKLEATLLELKRGIKGEVVMSARLES
MFDACLLGQVPQIWHENSYLSRKPLASWFQDTLLRVEFFRDWNDNGMPMSFWISGFFFPQGFLTGVLQAHS
RENMIPIDDIRFRTNVTRYEVVEDIVNTPKAGVLIHGLFLEGARFTFEGMSLCESNPRELYTSMPLINLEP
QRLKDQDKSKPVYECPVYKTSARAGALSTTGLSTNYVISLDLSPGSSPPEHWIRRGVALLCMLDD
>tr|C9ZUK6|C9ZUK6_TRYB9 | DeepTMHMM Topology Prediction - Predicted
Type: TM
MFGSSVSAWSRSDGVDSTSGSDAMCRPLLHTRRDSKGAEERMNGRDGVRREHSASIDSATTDLLFMGRPLP
HRFIVSWLMLVGILVLSLAYHVPSYTTSYVKRKAVLGHFLADDYLGKESGDIVRLPTVFVHNRKDLFSRIE
DFVAAFYELAAAAEAELKYHYYMSAATGKGTEFLRYILHGETPQRVEEMDRILTKEMNNLADTSEAETVTI
NAVTPVKMDVELFVFGSREHIGPSDTKIFSVTLTEEDPLGPFASEYRDWINGSSKTNDGEEEYSSRYSRAE
TSSGNVLNSPPNIRINTACAPRLSTLSGKYYSPCRRQIKGDAATNADVFFFSLTDNVRSIRLRGTLPQVIA
KLTDGSRSTSGFSMIIYQWTIEVKFFFHRGGYVETTYNIISTAKHMQPRIHPRFLLTPVMFLLALLDMAIR
FRALKSAISFRREAAAEKSREGGYSLPRAREGIGSGRKCHSEGTDVESSPSEIDGADSDRGVTMLIVKSLK
RWVRHHADQWIMHRTSRPGARDSGPVGFMNMYEQWHHRLQEKGGDGWHFVGLVADLLILLYVSLFSVRLFG
VQLSCFFDAVENMTLGVAAFFVCVNLLSQSRYFPEMYFNVQAMLRLISKLFLGMLSILPMFIGFVLFYCMV
FGPHSDGRFTDVNYSFMTLYFMMFGDALVPAIASAGGSSFSLATFLANTVTVVYILLVIIALNLAMSITQY
QWVRQRRKIGGKDTLLTFRTREDVRVEVLEDITDSIEVLLQLEATEVSAREKKTRSDVAASDSSAVSV
>tr|C9ZR54|C9ZR54_TRYB9 | DeepTMHMM Topology Prediction - Predicted
Type: TM
MSCFLCNQLKGLTPWEVLSWHRPTATGSLFAVLLSTILFFWYMKYTVVTFLCRLLQVLFAAMPLMGFMKWG
TYTNDDIQMMVDRFADCFAPYAVLVLQKIYDLVTWRDRKQSGAIAALTVVLAIVGNYFSDMAVLGLIVTLL
FTAPVVYEKNKEVIDNATADIMAMAEEHLGALRTKVDQLTKKNN
>tr|C9ZQZ9|C9ZQZ9_TRYB9 | DeepTMHMM Topology Prediction - Predicted
Type: TM
MQSAYTIKVSERQADGVRDVSVFHHGELHFSTSHQNMKWAVLTRFPAPFTQHGGSLHPAAISLPDEKCDVA
SAGGGNEKQRDPVVVGKSDIDSGSKLMYEYLCVFHCDEPRNRLSHYTAHQPWTICSAYSTADPAAVTHFVL
NNINPSCVVPGLRGVFVHGTPQSVPTVPSPLIQVGVNTFQPAADLWWMVSQLAINPVYVSVGGGNPATCLP
TGDAAANSTRMHIYNCEDNMEYVAPKHALVGSTVLAQFQVSEDRMMVVAVGGNDVRLAMTTVSEHVGIMLW
VWWPQRLHMPNLRVKGASVSLTVSESCRANVQVLFLFDASRGSLHVLRTPDLLTAKGSLDLLFTISTNCPP
VVLPCMCSSMMQPIMVCNRPSVNEVALYYVPELLRTPLSTNYMATMSLSGVLPKNIIVEGVSYHNGSTIVF
EAAVAGSDREPSVAASHQQYAFNFPSLLDEQHPLARFILEALEPVFETECVALLQCELLRRSWKSKRKGQV
WDFGTGCLELIANIVHNSMSPNSDRRGSPSGGGRYAAVGGSGSGRSVSQMCDAAPSGLELQTSSACTFVDK
NFRLCDVVADPILMTADTLQKNAFRFQDRGHHENHGKFSSELDHGNIWTKKQCGLCVFTLHLLYESLKLQE
RFWILLEPLARLNLRLSEIMQWSQYTRYYSTCLCETETEDFAGHTTTGASRTGTSVSFQRALPENLLIEYF
KFHASSSTAAVMSGAPPLLYTILQRVLKGKARTSGGWPAIRGITSSNPLSLANKLFFMLVDCFDAPQVTAR
VPTSWWFTLCKGLLEYDIDPKFVSTELCAGVGQPIERALCIAKDHPDDIWNDDFNTIIGRLDRLQHNLTST
RCVNPAGDVVRTAQERAIGREYRATLNDDDGVIMRPDFPKRWGDSRLDIVQSMFNTAAPITLPSQVDGTDA
IYGSLRNLSRRATALPVGRGMFTLCTQNFRVRDSVPIPPLNLEGRTSDGITITNNFEEAEPVNIIWPLFHN
GCAAGLRFLSLPHFKGHRSAKEEESITRHWVLYQTRNISCPASRSGLLLAAGLLGHLKVLQRTDIYSLLVS
PQSQFSGREAVTIAVLLGLSCSLRGTSNPVVFNCVSMHVQSLTPATEDIEVSLDVQTAASVSLGLLYQGTP

DAFFVEMLLIQMSRLPSDEHFRDREGYALGAGFGLGLLLLGTGSSHGVPNVENRLLKFMEGARREAAPSAC
EGLEVFNEVNPDSGHFLTRAQMARNAKESFRNQSTRVYEGDCFNIAVSGPAAVVALGFIYLQTNDALIADK
IAPPNRLVGLQGVFPELCLLRTMMSSLIMWSKVEPTQEWIFQNVPSCLLRLVHSPKKSGLAPSQIRYLTMN
LGYCIAGAVLAMGMRFAGSMNADAKVTVLAELNGFLRGFIGSTKTGITSIQNSTGAFLPCISACAIALALV
TAGTGDVQCLAVMQKLHKRMNVGYGDHLAISMATGLLFLGGGQLTLSNSLSSVAALIMAFYPVWPETASDN
RMHLQALRHLYCLAVVPRLIETIDVLTNRPVSVPVRVIVNRGRLFQNEPSSVVKEMWTPVPKGRENQAVRM
VTPCLLPELSTVAQIEIRSAQHYNMTICNMDPNVIGDGGIVVRVLEKNVTSTEDGKSGRSPGEELVVSWIQ
RLFHEQMQLRPGPIEAGVIIDNVNLLFTCQERFLADLSHSEREFSLDFIMNVRRTLNRRYSGLLRHNGRLS
TRHPLSQLIMKQKSVYSTASSLVQTLVSDDGHSSDLAPIAEALLMYAEATGMESTGNSEAFHVSFVMQWLS
QALHFYGLVGRTKPLSQLLTKYTDVLQRREQRVYALYRMNQTLLLRPEVLEDLVDCCVELAN
>tr|C9ZNP1|C9ZNP1_TRYB9 | DeepTMHMM Topology Prediction - Predicted
Type: TM
MCGKDSGVEERKLQHPHRVQQFTAGVALSDIATAGVVGFTAAIPISIVDYSIMARVAGVTNSSGRVLWEGM
RTLFLRPHRFFIPCAENKCAPVFGACFTVYSLTFCTSNLTKSYCEAKGYTPERSNLITGLAGGATNTLMTM
WKDSLILRILPPVGGASAGSGRPKPVPWLTRGLFVGRDVFTCLAAFTITPMVATWLTNYFWNAKENAKLTQ
PRPLEGKTHIPLATVDLAQLITPTALQTVTTFMHIFAIRYRQTYPNFTFGDLSTSLRETYLSSLILRIVRI
LPAFGVGGILNRELRSDLQDRVEGPAYH
>tr|C9ZRX7|C9ZRX7_TRYB9 | DeepTMHMM Topology Prediction - Predicted
Type: TM
MNPKLFSTYVIDCVILFLLVLFAFGYQTVRNQERVDAQFELAASLFGAKDKSAGAEKSISSVKDFFTVFKG
IIDAYYHIPAAATGTFMHYTYGVGNFTPLVPVVEITFHDRPQNSLAKVPLVFSFDVQASDVLGPFISPQSY
YYQNGFCGDEVTSRLRYILCQTSSTLDVFDNVDKVKLSFQLYSMRRSDDGRVRPAEWDIEVMFGMGGYGPV
ITMTTSFSSIEERFPQRFPVMMSCAVAPLVLLSFLFRMDRISQLVTYVKVVLTTCRRSRWYGRLRPDNSGL
RGQSFLLLGVFSDIVALTFSITSLVIQFTPKVSGTTEHALTVLLGFAAFMRSTQLISVLKLSPSLCVVVDG
FVTASDQLFMYVVAVFPILLGYSVCGFIVYGSYQSYFKTVPYSIVTLICAAFGDNLIDTFVDMDRGAYVVQ
VLFTRIFFGSFLAFFICNVLNVAYSIIQDSYNQAIRMHGATSSGTTGANGSGTRLTEELREILGKLRG
>tr|D0A3G2|D0A3G2_TRYB9 | DeepTMHMM Topology Prediction - Predicted
Type: TM
MHLIIIYFFCFYSYSAPSTSLFLVLLRFASFPHGQLLRPRIGCWATLVAVRKKEIRHPRLLTYLKLLLDNI
RGCLSSFLELVLPALSHYFQKILFLEKDRL
>tr|D0A7F8|D0A7F8_TRYB9 | DeepTMHMM Topology Prediction - Predicted
Type: TM
MGSSDGTSLSPTVVLRVQYSLYLFVGIVATMLLRGFLTLLSHLPLIKKGCEYAGKGDANFCVGEVLAYRVS
FSLSLFFFLHLLSVSDLTCCIDTESRVEFQRRFFFAKTILLGLLFLATMWVPNTFFAYYAYTCVFASGLFL
LINVVFLIDFSYQWTEEWGERMEQNSKWLWYLLIVAVLSYIAGIAIAAMSFVVFVPNVNCNYNAFAILSVL
ISAVVYTVLSIYLPHGSIVSSGIVFAYTAGVMFVTLRMGDDANCNTIAIPPNEAGSLKQIIIGSIVSGFTL
VYSVVSTGGSSKGFGHVGDDDVEEDPEESGHLSSYMFFYTVMMLGSMYLAMLSTGWHVSGMGEDKMKSSIN
IAYWVRSGTVWSAVLLYLWSLLAPYYCCRDRDFGIAVDDW
>tr|C9ZN50|C9ZN50_TRYB9 | DeepTMHMM Topology Prediction - Predicted
Type: TM
MSSFLQELGPVAVSHRQELEEVKRELDIKRDELDCWIYGFLENKKFDVKETVAKLQRRFAMEVNEMAKYEF
TEYMRTSLRLGIIQVIGEDKCGRTIFYVTVNRDKKAASHRDGKKHTFDLMVSYGTRLRADNKRCQMVLLVN
YEDASMWSNVDMSLQADVALRISKFFPGCISKVYICNMGRMLCSVAKPLFSQLPSAFSDSIMFFSKSDRAS
GRLLEFIDESVLPVQLGGTNDCDNQEHWDRHGDIIEDYYHGMKAAISERGLTVKEWELQCIEQPNVPVRHP
SLASETESMVSLLTFGSSLTATPVEGNLNGMWRHDNDISPPLEEEENEWCLLMKPLPNNLSLFFLEELYRW
RVAVSAEEGEMRCALMDDYAAAWKREFDELPMLDLSNKKWYRFIPESLRELYHGVLIVVNVASSLSFLVAL
LFFMALTSSAAVTVFLAFFIEWNYVYALFVTLILVTFQGTALCSRAVTVLVVIMKHKVVPPLDMLGKTRGS
VAQLILFCSMIFLQMFSFGYSMTHSSLLRSIQVTFATGWLFAVAFLALCHIVLFFDWIPMGEQRRKKNQGS
MLALYLLLDVQESEEKTEAVLTRRLSTTIACAVPITLDFLLGIAFIISGMIPFAVATCIATVAAILTIDYC
SLGRSSMSSRSVLRSTVAFACMLWLYLCFSFRFLQYDGPWIVSVCTVAAVNVVFISLAVFCLRKRSAKKTL
RGLLFLFVILLITSCTASFFLVGSTVGVISLVLLIHSGMGIFCVRRRSRNIGGTFIFYGALAMLALSCVLL
GWLGAKWQYWVPHSLPPVNASGPRDPLEEYHQYPICTLRTAGGFGIVDFALLTEVSGASKENVFHEDFDNW
FGSTDVVYNNTVKVYGERNEQWSISRFDSLARNVTVLTLQNGRVYSSIITMTVWIGYIALAPLQILLPNNW
LKQVAFLLSFLTRAIDFTWYDVKDEVVRYLTEMKNTTSNEIVLTAWGIAGGIASLAGVESHTQTITFGSPG
LMDALHYTNYTEGEYHKYVLAVVSELDVLNAVTRYDPTTYQRIQCSAGSNTCGSMNYISSELVRVCDTTGR
RHVGQQVEESVHEM
>tr|D0AA77|D0AA77_TRYB9 | DeepTMHMM Topology Prediction - Predicted
Type: TM

MRYAPIRLWQLLFVQCCCWAFSFSFLVPHSGPLLTSPAPMYSCSVLTSISFLLLFLIRFIDPKQSVQFDKC
NATAFGMTAMSKIGEGTTRVSSLYSILLSFFFCLESERFFCSVFFIFVVFATTNDTRDSLCCFN
>tr|D0A3W7|D0A3W7_TRYB9 | DeepTMHMM Topology Prediction - Predicted
Type: TM
MCRRVVPVAQRIPLSSFPFSAFATWIVLHSREFLSCGLLLFVAATILTGLCHSLFASPLCNEPILWQGIFA
PLRAINHSFLMYILLLEQFHMCAHVPLPLLLMEQKQGKGVAGVRCRVDLKKKKLFMPPQRQCSHCEKEKAV
LKRPRNGKLLCQRCFFNLFEAEVHETIMKEKLFKPGDIVACGASGGKDSTVLIHLMKLLNEQHEYGIQLLL
LSIDEGIAGYRDDSLKTVHRNAEVYNLSLRVLSYKELYGWTMDEVVKVTGLRSSCTYCGVFRRQALDRGAA
MLGATKVVTGHNADDLAETILMNILRGDLPRLSRCTSAITSGESLLPRVKPLKYAYEKEIVLYAHFKKLDY
FTTECTYSKEAFRSEARTLLKNIEILQPRCILDTIRTGEQLRVKEQECATENPPSACTRCGYVTSQSLCRA
CVLLQGLNMNGPAVCVREQPQPQQQQQQQNVEVEGQ
>tr|D0A8Y4|D0A8Y4_TRYB9 | DeepTMHMM Topology Prediction - Predicted
Type: TM
MGTGADDGKAGRHQPMPQVQKKEIVHRNPNHVARDVFDLRGTSNPLAMQLYSDGISSGPLLGIPNRLVRDL
QPLSTNARKRCHSDAAFSHHSGDGAGHDGRTTKHGTLQLYRPPVCGEEELKEHFAAVALEGKEVRVPTKRH
RSRPGRLDPLKGTLQSSTSGSPSYGENEGNEGREGQKNIGDANNSAPNENVGPIPRFTKLRSNAPNEFTYL
VMRPRGIRSPYNPYDLQVVSHRDVDPSHYYTVSAAGVTKFSGHEAEFIELHTWERDCRVFNQLRELDVFRE
YKKWKSFLLWRGLVRNHAMSNCKTFLTKNLFHVHPQLSAALQTVQRICLNFVNSSRIHPPATETRTLEALC
ATLAAHLNFQRNRLDKMIKNIRDTVERAAKAAMLAAQIERESISALHAEDEKKKKKLIDVRELGTQQKPTY
IEMSQKKAVCQRITAFIRLCDYIIVNCLTKLAATAVEDVDRDFQYPRPDKQQDSVTSPLNKNSWRGKRQEG
HKAEAPKPTGPFTGPILSLNVVYREDSQSIEITPSLAHITECVEDVVEDYIKTLSAVPRLLTMDAFKVFTE
NAAHDSQKEVLSGPAVGEMIVSEEAYKQHMTSVRYGITRAFGDVEEYVNSFQCFRDMYVENSSLDADSIQK
RTQNLEFFRERLALYKSQSDAISTKIPSSKDVALFTVYTEELRDFFTPSPVVCLETFHRLLPVIAHQQNEL
LLADLQSCNTYLAAQPKTVEHYVEYLAYNKALEARFDTLAASYDFVREFFTLLQDEKVEIEEDLEETYRSG
TRPQFEKLRTQMQIVEDAKDSQQRYFIRNVDEQLDELRRSIEDVYNRAGQPVIADANADMDEVIHFITDLQ
AEAERIAAREKKLRQYQIAIGAEETVVETMSDMLNDVNIKARLWVGMREWDEFVESYGPIPFDQLNVQEVQ
ETVQKYVMTVKQVSTKLPGNSAVNKLKSKVEAWRILLPILQALTNPKMKLEHITKVSNAVGPLKDDNGVSK
TIADWNNQFTLGMLHSNEVVKFKDEIVAISAAATEEDKLQQQIDKVNALWNGGGPKPPVEFQFHNHKELKD
VYVLVGSSVEDVMALLDDSVIAMSSIGSSRCCQGVLRAQVDRWENRLRYMQETLDRWVELQRHWIYLENIF
SSAEIRSQWKDDAKRFEKVDRFYRDLMRKAHDMPTAYRGLLINAPVETGEQQNTKTLKYDLEGNIKELEKV
LASLERKLEEKRCAFPRFYFLSNDDMLDIFAKVKSPELIMPHMLKMFDGIKTLSFTETNDITHLVSMEGER
VELVNKSIKARGPVEVWIDMLEREMFSTLRKHANTCLTDYEQRGDRESWMFQHAVQLVLIMEQLLWTRGVE
EALTSENPAEAMSRFKEANYKALESLAGLTARKLSKVQRILLSTLITIDVHGRDLVDEMYESNVLDVKEFG
WTKQLRVYWEQDTDGYGNCFIRQNNSRFVYGYEYLGAQGRLVITPLTDRIYMTVTGALKLQLGAAPAGPAG
TGKTETVKDLAKNLARQCIVYNCSDGVTYKMMEKFFSGLIQTGAWTCLDEFNRINIEVLSVIASQLFEIKL
ALQNGLETFTFQGTPNVRVRPTYGAFVTMNPGYAGRTELPDNLKILFRPVAVMTPDFRMIAEVILYSEGFK
NAKDLSLKITQLYKLSSEQLSPQDHYDFGMRALKSILVMAGDLKRSQPDVEEDLTLIVACNDSNVPKFVAE
DLPLFRGIMQDLFPGVHFPERQYEELLPAMQKSIDGNKLVALDTWVTKGIQFYETLIVRHGVMLVGVTGTG
KTEIRTCISEALTSMSEAESSNPMARPVHQFIMNPKSVMMHELYGLLDVNTNEWRDGVLSVIAKNCVRESE
VNKDHRWIVFDGPVDTLWVESLNSVLDDSKLLCLDSGERIKLPDTIHMLFEVADLAVASPATVSRCGMVYL
DTTDLHWSAVVRHWSESKLAEAGGEPQCREFIVSLFDAHVQKGLDWLAMQKVLISGGMVNVVQSMCDLFTA
LIKCNNVQLMPDPRCEAKLQHDSKVFKERNELCGILFAFSFVWSIGGNVDVASMDLFDTFVRNLLESVVRF
PNSGSVYDYTIDYGTRLFVPWESRMVEFKYDPKIPFFDILVPTVDTMRYSAIANTLIQYGKPLLFNGQTGV
GKTVILMDHLSRHKEELMLSVIVFQFSAQTSSERTQELIESKLKQKRKNILGAPPGRKVVLFIDDLNMPAL
ETFGASPPIELLRQVMGNGGFYDRKIAGFWKHVQDVTVVAACGPPEGGRNPITARLTRLFHLLHIPTLSDE
SMKRVFHSILHGCFQARNFSMEVREMAKPLVAASVDIFNKVRDAMRPKPATPHYTFNLRDLAKVFQGVTQV
TQRVCKTRLSITRLWIHEMLRCFFDRLATKEDRQCFTEELMMETMTRLVPGNHVYADYFENKPLVWGDFLR
IGASERVYEELPDVSKLPSLFEEYQDDYNTFLTGSGIGASSGGGGEEDEGGSGGGASSLNLVFFKDHCEHL
VRIIRILRQPRGNALLVGVGGSGKRSLTRLAAYIAGRQTVEISVGKGYSMNEFHEFLLELYTTTGVNCKPT
VFLLSDNQIVHEGMLEDVNSMLNSGEVPSLFTAEEREKRVNACLEAAQQRGFVDRDDVYNFFISRVRDNTH
IVLCMSPVGDTFRARCRQFPSLTNCCAIDWFDEWPHEALLGVAQQLFDDEEDGISNELLPVLAPLCVDIHT
AVIEVAHEYWEELRRRYYITPTSYLEFIDFYKDLYTTQRKLLEEQLSRVINGKEKMKETDETIAKMRVEIE
TKRPLLEKASKETEEVVADLSVRQARASEVQVQVRAQQDSAADQQRHASKIANEANARLAEAKPIIDRAKA
ALDTIQASDLNELRSFANPPSAVLKTAQACMVMFDPKDFGGAWSGNTDWKGAREFLSYRQLLDMIRSYPTD
NVKPAILQKMQKYVNDEEFTAEICSQKGSQTCGNLCLWVHAVNEYSKVVKEVAPMREAAAEAERHLSETNA
KLLSAQETLRGVEQELVELQKNYEKSLKRKNDLEEGLKVCIRRLENAESLSNSLKSEGARWEENINQLKKR
LEALPLQMFMASACAAYFGVFTPRFRKRLVRIWVEKLAQRGFDAADFSLPNTLGDPMDILTWQINGLPTDE
SSTENAIIASLSTAPRRWPLFIDPQEQGVKWLLQQYATPGTGGGGGGTSPSGVLGSGGAKGLAKPVPSVKV

IKLTEPTWMRTLETQIRLGGIVILDDVGETLDPALDPLLSRRIFASEGGVPQIRLTPNTGPIDYNPNFRFF
ICTKFPNPHYLPDISTRVALLNFTVTIDGLEDQLLGEVVAIEKRELEEEKNHIIQSISLGQKKLKIIEETI
LGKLKSTQGNILDDSDLIAELKSAQSNAKVLSENQKEANEKMTIITSTRDRYRDVAARASVLFFVLADISR
IDPMYQYSLQFFVKLVQNEVRVTGKPDDFSEEDPVVLQAHLEKIVSRLTVATYEQVCRGLFNKDKIILSLL
ICTAIERHGRRLPDDEWQYLVRASAFVPNELPPLPEELSYLTRQQWELANALFVHVHCFSELGKDLLENGE
LWKNFILSESPQGAEIPGKWGQTLNPFRRILLIRCFREEKLFFSVIDYVSKAMGQRFVEPPPFNLEVALAD
SSPTVPIVFILSQGADPMGALQAFAQSQEQKLQYVSLGQGQGENAKRLIASCKKEGDWALLQNCHLSKTFM
PELEQQVALLLQSGDTLHPNFRLWLTSMPTDFFPVFVLQNSVKLTNEPPTGLKANMVRCFGEITEEEFNVF
SEGQTMGEFSKDFAFKKLLYGLCFFHSVVLERRKFGPLGWNVKYEWNDTDFHVSKQWLRLFFEEQDVIPWE
SLEYIIGQINYGGRVTDPLDRGTLQTILRRYICPGIMEADFKFSPSGLYYAPDAVRLEGFMDHIQKMSLVD
EPEVFGMHTNANLRYQLQVSQYLLNTVTSIQPRLVGGGGAHKGDGDGGNALTPEEEVKRKCEEFEATLPEI
LTREEAGPRSFTTLESGLPNSMSTVLTHELVKYNKLIQTMRKSLHDLQKALQGLTVLSTDLDAMHESFLTD
RVPQLWSAVGYVSLKPLGAWYRDFLARVDFIRTWLRKGEPNSFWIGGLFNPSAFMTGVFQAFSRAEGVSVD
KLGFRFEVISREPSEIDEGPLRGCYVHGIHTDSWRWDAASGVMTDSLPGEPYATLPVVHFLPEPHHRTGEG
WQRIPLYRTVIRAGVISSLGASSNYVLSIEVPTDKETDYWQLMGAACVCALAV
>tr|D0A645|D0A645_TRYB9 | DeepTMHMM Topology Prediction - Predicted
Type: TM
MSLSALRSKRTVNDIWPVRYKRFFVLTDPQNIIGETTDGLATLTTISYLLEREAAVVVASSFGDLKGIPLS
FSRGQREEAIEIFRTEGGMGYTNYFNSLPINLKAEVLKVANGCETPVDSVTLSPVEVNGAFAALSIRQKAA
ALHNVFPRVEFSQCSTYAFVRELTNRFPTVPVKFSPDPLNAPISQLKPGEILVLENLKFYQNEISPNHEER
VAMAEVLASYCDYCVNESFATVCAVHASNTELPKILYHGAAGFSMEKELAFFLSFLAHPPRPIVVVVAGRQ
SSKKLRMIRSLVGKVDKILVAGALIMPFLAAKGLTTDKSFNNSERVERRRLNEAAEVEELSVPCVGFAQEI
ITLCEKNGVELVFPVDHVVTRRITKATEDSAAIVESIAVPSDVYAVDCSANTIALFTKCIRPCQCVFWTGT
FGCTRMGYSEGTYAFARALAQQGKLSIVSGNSTACVVRQLGLTSQFSHISSGGSTCLDALQGHHLPGVEML
SDIPVAVDVRSCILADDLLRSLPLFSNCSSHQLKAVAQKFVRRVHACGDYLTYYGDKHACMWVVASGGLVA
RSGDTTLTLPSRYIGRGQTVGMYGFITQSFATDTIQAAEDETVTYQLTFSSLQDLFNEQSDLAAQLLQNVS
ETLRVMAIEEYREQSSIMNVLRRAALCSRFPTFLAIPKDWGFMEDVIQDIISGTVLSGITQICTGQTALSS
PRTFSLGLGSSTSRGHMVHCLCRVVLRDLLYHKLIEYGIMPATCISSIILSPFRLRAMGMMWSDVTYKAML
DEALLLAVEMCSPIAAHGTFLVVQRRIEVLLRRKCATIVKFFLTALVDVGFGFVLFPLTFHRRTSEAISLP
DLFRSSVFKRHQWKVVLLLVLRCVVHVLLNGVRRVHRFWVIKRKSGSGCDLTPQRFPRVTK
>tr|D0A4A4|D0A4A4_TRYB9 | DeepTMHMM Topology Prediction - Predicted
Type: TM
MLLTKLAPMRSAARHMPLLCIPLHTARHTATVGASGASGRVGEDHRHSSLEGTLLHPPFIGSSSRFTYALP
ALDSDDIAALERETLNHHEPNCWNDYCCVSLVKLLRWCADKLFRERYIHRATMLKAIAPAPAMAGAIVANL
RMYLRKKDATYLPSHGNFSSEVRVLMAQMESHAAHVNILLSMCEITTVERVAAVLLYGLHYFIFTLLFFLY
PRMAFRLMGYLNEESVVIWTHMINDVELGKIVERPIPRAALQYWGLHRFHHRQKQQSHTKSYVTEKEEEGR
RVMHNNEASYANPSGDGAAENGGEDGDVSKRGLTLRYMLLLLRSDEMVWRDSCHAAADAIDEMHEVQ
>tr|D0A6B8|D0A6B8_TRYB9 | DeepTMHMM Topology Prediction - Predicted
Type: TM
MLSEISKHSPTHCLFFCFVLFIERVPPYGLYSFCCCVSWRRIHLWSSHPLSTPSTFPSTVVAAEAMFVSVS
ATTGFPSSLLTFRYFYRFPLSAYVLVILCIRTASLLFLASHRLGESKRMGRRLRQCGPQCVSGCGCCRLLR
EAHETQLEAQRVVLEKRHPLDVPKMGEGARQLMTDEQFNVSNPAVRLRMRFLH
>tr|C9ZL78|C9ZL78_TRYB9 | DeepTMHMM Topology Prediction - Predicted
Type: TM
MVPCVVIFFFRALVTFLPICLRLFLLLFRCYTFALYRHEGRRMVIWFSAGDGVGFPTPTPWEIQPPRGLTS
ADGCRGGLRDGGEPAVISRQQRQQKPSRHQQEHHCVRQPKPKVVIQQSDLRDEQFQRLSVLGEGSYSVVVA
ARHLPSRQVVALKELSRQRLRDAKLETQLQWEINLHRTLRHPNIVRLLSYYITPRSVVLVLELCRGGSLLR
RLQATAERRFDEGRATRYIRHVAQALAYLHEHGIVHRDLKPGNILVDARGVARLADFGWSKGLAGSAAAAV
ITNGYDASEVAPRCAAGETDACSETEDGHGRLTVCGTLDYMPPELLGGTPCSYKADMWSLGALLVEILSGQ
PPFYRTSQQETLQAIQDEGPQLDVDGSVLSPLARDLALQLLQKDPNVRPTAAEVLQHPWLRGKRPRV
>tr|C9ZX06|C9ZX06_TRYB9 | DeepTMHMM Topology Prediction - Predicted
Type: TM
MYNWYLWGKWLGKFQPKPQMIITAAHPVAKILCFCFCNSILCCGCCGYGYFVLCLPLILSTNLISFFLTHF
FSFHVCHLWHHLCIVVRLQWVSDVAYLLLV
>tr|D0A982|D0A982_TRYB9 | DeepTMHMM Topology Prediction - Predicted
Type: TM
MCPINASFYTLIVVKLLRESLIHMHGALAFGFVVVFLSFLVLWQRASCVSKIHLVGDVLFVFAHPDDEAMF
FSPLLDYVRRHGLNAHFLCLSNGNYSGLGTVREKELVASAEYFGVNRRSVRVVDHPDLQDGPDNLWNTEIV

QREVLSYLHSVKDIRTVITFDHRGVSSHANHVAVYEGVLLAKKNLPPGILFLSLHTRDLLEKYVGILSTVG
YTVGIHRCGGRRNHVILIPPTSLFTSFSAMRKHKTQLVWFRYLFVWFSSYSYVNEVKELGVA
>tr|C9ZSD3|C9ZSD3_TRYB9 | DeepTMHMM Topology Prediction - Predicted
Type: TM
MRGDGTENVLSSSSCREREREKGRGIKVRKTLWRINGTSSPQTTPSLSALIACSDGPCDFFLCHISYRSPF
VPFPYASPHTLSLFIYAYLPCSLSFISSTRRSLTCRKR
>tr|D0A7D1|D0A7D1_TRYB9 | DeepTMHMM Topology Prediction - Predicted
Type: TM
MLLEVAIFLLTALALYSFYFVKSFNVTRPTDPPVYPVTVPILGHIIQFGKSPLGFMQECKRQLKSGIFTIN
IVGKRVTIVGDPHEHSRFFLPRNEVLSPREVYSFMVPVFGEGVAYAAPYPRMREQLNFLAEELTIAKFQNF
VPAIQHEVRKFMAANWDKDEGEINLLEDCSTMIINTACQCLFGEDLRKRLDARRFAQLLAKMESSLIPAAV
ALPILLTLPLPQSARCHEARTELQKILSEIIIARKEEEVNKDSSTSDLLSGLLSAVYRDGTPMSLHEVCGM
IVAAMFAGQHTSSITTTWSMLHLMHPANVKHLEALRKEIEEFPAQLNYNNVMDEMPFAERCARESIRRDPP
LLMLMRKVMADVKVGSYVVPKGDIIACSPLLSHHDEEAFPEPRRWDPERDEKVEGAFIGFGAGVHKCIGQK
FGLLQVKTILATAFRSYDFQLLRDEVPDPDYHTMVVGPTASQCRVKYIRRKAAAA
>tr|C9ZUB8|C9ZUB8_TRYB9 | DeepTMHMM Topology Prediction - Predicted
Type: TM
MSANPSFSFALVFTSVPSYFPPRTPLLFSVPVTVRTYQRFPFFFAEEGCKMCTAPIKVLDSTQVSTLHTCL
CPILLLGQVSLQRYAVTLLATHGSVFSSTFVSLPVLGCCAHNFFFPFPFHPSLIVHVRIHVLHECTQQSPE
RDW
>tr|C9ZVC9|C9ZVC9_TRYB9 | DeepTMHMM Topology Prediction - Predicted
Type: TM
MNGAVWGKRRENIICFILSGEIWFGGSGIYSPLRPLPHFFCFHPTDLFFFWGGGRSLLTFLVVSDIFPMQA
GWNIFVTLCDNIYIYILGGEKFGTGKGRFLVSLHAY
>tr|C9ZLF6|C9ZLF6_TRYB9 | DeepTMHMM Topology Prediction - Predicted
Type: TM
MYYHLHPLFPMHAHTPQREKKIHAHARFLSFCYYCYYYFLYFTLVPPCLYLNPIYTHTCTRTLVLPADLCM
CVSSHLQALNQWGVEVEMEENRKSSREVGVGVCVVKHNLLLRYCFVLCFSTSFPPPLLLPFCAATTHKYIH
NTQRTHTHTHTHTHTTNQSAKPAIFFQSQSFFLDP
>tr|D0A6C9|D0A6C9_TRYB9 | DeepTMHMM Topology Prediction - Predicted
Type: TM
MMVELSSFSRDVCCGKSLLFPPPTLINTTVFDILKCWSMLLHFLYLCFCMGDRSHKGGDRAKKKKKKYLVN
VRKGVKPLVDEDQNLFAEMEPRRDGSDGL
>tr|C9ZT88|C9ZT88_TRYB9 | DeepTMHMM Topology Prediction - Predicted
Type: TM
MGYACLHMRGIVFNDAYEHFPFHPALSYCFLDVSTSNYRAHLVVAAQVVLTGFGWLQNNVTHRGGRKKKKS
PRGDTGAAALFLSFFLPQFSRPPAADAREIFKCVACCSWPLETPSHTHTHTYAP
>tr|C9ZPS1|C9ZPS1_TRYB9 | DeepTMHMM Topology Prediction - Predicted
Type: TM
MVGSASRSCGTDMVNDAEMYGGSENQPQNSSNAVTDSTPKLSRWPDGTTFEGPLVDGVPEGHGTCVYKSGC
VCVGSFSRGCVEGPAEALLPCGAFFVGNFCRSAAHGHGVLLQNGRLVRGEWRDGVLVRRTLENVNDARDVQ
FYTRVIAKLCHRVEELNGTIERDSSSVFQQTSNSGLCIREPIVLLRDEQQQERDAVQIRRWARVATQSPPY
MRNEPHGAGMPPLAREPDISPRFDRGSFSSLNTHRRQPTALDLNQLTSFARSVLASGASQQAIFASAAPAA
NEFFSPTRYLQCFFIMLFPFISLPQFSFSPIRRVMLKMEREFVVSGAFLFRSFNVPIYTLTFSTIATCCLI
ATVVIVSLKVELGPVTEGKVTLEELFIPCILWVAQSALYAAYNSYVRVAHALERLDRRLTPQLYACAAGIV
NTKAKVCIFTWDDEGRRVVTNVHYRYRWLGESLFVGILFSLAGPLTRVSYKQPMFGYTKYEVSALVLISAS
VLVFAAMVTFNALKLTDMQRQIKEQMRALTHLAYVEGKSVMHPSEHLLQRFNLDEPFNVSDIFNGVSGWYV
IRSVVVYAASCSNHAARSLAMSVFFMLVNSCFLVTALDMICMLSFHYNDLGKRFSCMHAYGVVTCSVWGIL
LLRYLYKCVETVCESERHLYLLDVASLYHRTRYNNAEGCADIITTCRKMVKAYEQLPCVFLFPITPFIITI
VVILYIVALLIATIHVYFAAVTRFADTGGR
>tr|D0A546|D0A546_TRYB9 | DeepTMHMM Topology Prediction - Predicted
Type: TM
MIFISTSPRLVSTPSVFLHFFIFLTTYISSFSPVFRVRSVSQLCVAPFGAKPKSIFAMKSGILVGGYCGDD
ARVPQRYTLPFLHIVLHPLVMFLWCGYSCSVLLCGMYIYIRS
>tr|C9ZRI0|C9ZRI0_TRYB9 | DeepTMHMM Topology Prediction - Predicted
Type: TM
MHCRTASDEEEHLSLHNAVEEAVHLTWNNNYERAKEILSRHKENHPRFSLEFANVFLVQTLMNSTNETREV
LLDQLKEADALASRAKHGDSMFRVGEVAGNNENLRFISKSQRQKRKKEFERRRKAAQKAGEPFDDAWKLEC

DVIYAEALFMRAVGQLMMNAYFRGGINLRKAWGLYYKLIQQVEADTDDRIPEELKMCIKYGTGTFYTFLAF
VPSTVMKVLSVVGFVADRELGERYLVEVFESDGIRSPFAALVLCTFYLFLPTGLGKVEETLVKAKRILDKM
NERYDANTYFYGYSNFYHRKRGEIEQALETIEKAEANAERVGLVPTLVRYLRADTLFMDLRFEEAKQRYTS
VIEHLSVTKQSFAYTGQVVLSLAACCVMLGDDNTAVSWLKKVGSMYNPKSKNDANSPKFASRVLNNNQLLP
LCGVYMLYINRDLAHMNVNQAERVLMELQRVTTGKDMSSPEAMYMHTLFVAVIQKGCGRTDEAEEGMKKIF
ASEKSIPRDSMVLPYAYYEMGELEFRRGRIEEAKGLFTKGQSIPGDGNETLMNRYRIALKQLKQKTNEG
>tr|D0A7K7|D0A7K7_TRYB9 | DeepTMHMM Topology Prediction - Predicted
Type: TM
MDTSEEATDKANGSRRPSAEEQLRDLQEKTNLWKQSVAQQLSEATKKNKQLRDELKQVNANHGSEMGALRV
QLEKEFRERTSLKDEELQNVQTQLMELRKEKRSMVEQHERDLKQAIARVRETCAVEHTMRTDAETQQHEAE
LHKLRDAMELKEKELALSEHEVCVMKTRLERLAEQYRELSALMTQNTTQLESGGDAANKEKVLENALKASD
AAHQQQLETVRSEMSNLEERHAAKLREMQRAHELEQQRCMNEVYQREEQLATLQTLLHQAQHEAEAAQRRI
SNVRLEREQMEQQLTERVAGLSRELASRMEYAQQLAAEVNSLQRELGRQEGLVRSLEEEANMREEAFQTLL
LSEENRALVMGLQEALQKSRDDAEAWARKYEEAVQQTKSTCAVPIGYPCDGNDGDERKFRELAQREKALEE
ENLRLQTKAKLLRAMELRLEELKRSMASQASTILEHHNSGNRGSRMGDVRYGEERKGSRSASPLRRMQLMW
NQHSSVSGRLSRCAFELSRCRLPQCMLLFAFCVASLLIIFVVRSFSL
>tr|D0A6K0|D0A6K0_TRYB9 | DeepTMHMM Topology Prediction - Predicted
Type: TM
MPHRAHTGKVIYIYIYIYLFIYFIRTCLCVCLLVCDWLFVYVECGEIDRLRACVFSNRRVEECVWKAIGKD
EKGIRGFGLLMFYWTREVSFTPTQFIYFPTFFGIWALLRRPLGKMLQHTHPWECSHCIPPFSHLWSVPLHT
VLYPFYLRFLSLRPHICASVTFTFSPHPSVFFLLFTRLSRVLKVREFIFVRAARY
>tr|C9ZUW0|C9ZUW0_TRYB9 | DeepTMHMM Topology Prediction - Predicted
Type: TM
MLGDGPLFRSLLCLLFSFHVFFPLLRCFFSRCVRVSGSTQRFAVRVNIICLFSSKGSTETKYKKKGMLSIW
RAVKVSLPQAGHCHSYSVTAMLWSRLWLREMCQRIVGDSRMGCTALTLCVACIGASSWGAATVLCHPLATP
EYLIPYYLRSIKSRFRHYASVRSADDGAMLMTMEDFVRSVLVLRKDEPLGASSLEDMSDLFASLDADGNVC
LNLTEYTFLMVLLTAKLKDIRMLFTIVDKDRVGTLGLSEFAGVLRGLGCTANEANSLTTGCKNGIVRRLFG
DDGELRCSYDEMEGSINAINEAIWRAEFHLFDPNNVGCIGAEEFGKLLAKQMIGSNVPFYVVENIRRMRGI
NTTVTVDQWIGFHQVMREADTIGEAVRLFMESGLSLGKREFNRVVKAAGVRPFKEEELDLIMALFDRNGDG
ALDFDEFISLMKRKLSYQYSGSSANEPREVKHFPTRLVECVGETLRGSVG
>tr|C9ZJ98|C9ZJ98_TRYB9 | DeepTMHMM Topology Prediction - Predicted
Type: TM
MIRTVVTRREFNCKKKRWKWKIIQYGTKQFSSLFLQLNSLLFFFFSFFFFKHKSYVIITTITLHPIKFLAS
FFPPFCASHFTTSPTVSRYGEILHFHIVSYFIFPLLPHI
>tr|D0AAH3|D0AAH3_TRYB9 | DeepTMHMM Topology Prediction - Predicted
Type: TM
MNPTDLREGAQPLLPAFPPPPGHSSSGGGGIPDSALQFHGGTRPLWATATPAVATAPVSAAGVPPSQVGFN
TGLPSEPQGAAYAGREGYADAAIRASADFWKNEQARRVTMEQATGSGCSSLPGEFRGPARAEFPSEELEFH
TGSPKYVLPVSLLLAVGLFGAIPLMSSFAFAVLLTGCIVTYVVDYAGYRLGGVFAIIATTLCFGFALFLSN
LRASITFLGPLCMILTVQGLLTTTAMAAFLHFHWLQVGYPELVCLMERSVLGVTPMLVLPSLLSSTTAFVG
SRHAPAWFLLSMCAIHCYFYRPLESSFVLRRRVTRGEQADLGQSPKRRGKADDDGNRSSGIAGVGEEAGES
WMQINGKPEAVVFTVLLLVLPATLYVAFQRDLYEAWLSNIVNVVGMVCVQILYLFWKPERSLWFLLSNKQN
SREFFAHDSFGLCVTIYRYRSCMYALGALILPNWCVYRLLNSRYRFLFDGVAPPINGILLSVALYAALLAA
YMTKKLLDADKEGRSVLTDRHMKERVVAIVAAAVSAVFVGFVVGLPGPLMFNLVICLCSFNLFLIDRGNAG
LMFIFAFFSSLLLAWWMSRTFSFVILRLHVFGESRTIPSPALAGNILSSYFVGCMSFPHAFTGFKFLYALT
LFLQSLNVVVVEHILYSQKEDGVYPAVLVLFTSVVGVLLVSRLHRNGVLGAAGASLVASSYIAKLFKFLVE
VTGSYYVEDAEMESSGPLFAVEITTMWWAALLCGFVAMLFELEKVSQMGNKTIVATVVLFAFSAMLMVAST
VRNFQRATYEFLTKSQITEGAAAYVFVGMSCIVFGALTYPFSARHLGVGPIMRHLNSVARGALGLGIIVLA
SQAIHLESAKDEEEFGTEELGYSKQCVLAGMLLVAAGRYLPMASLPMAISVIYWLVATFFLVAGLGTYLLP
TPSVLLLISLGVFVYFTFVTLDVAHYREKRMQYLWMLSTVAVCFAVFSVALMGRGGMEESDTSDVLLIWET
HVVGLRRLLSVVSVTGLFTAILLKFRLGGEALLPGAVPITSELMEHIGVVVNYNVILSVTCLTVLNMWNHD
YEPGLHVVTSLLLLLLVKDDTLFLELDRENYRYFPPLAYALGLLWGCLIHDAYRAGGSRTSSVAAVLREVL
CALPLAPSHISLLSLVYASKKAARVSVRPAVAFVLVGIMTLLFSSQKAVQWMAVVGICGHSARLLEAQLMG
FFSDVR
>tr|C9ZWE6|C9ZWE6_TRYB9 | DeepTMHMM Topology Prediction - Predicted
Type: TM
MALAHAPITGFLIGISVYYSSAAIRGHVVSQPSRTVLGWTATIKRLPILTFPLGTLGISIFDLGCALFIII
QMRKLERRWGSASFLAFVVTTAMISSFIINVCVVRGSSLSLPFEQLQVLSAAGSLMPLASLVTRFIMEVPS

LDAWESFSFPLAIISKPLLTLSMLKLIVCPETELPMRTSRLRGSVIRADAGLYTRLVLTLLGVVFGLASGR
QGPLAWFLGLFSRRVCRPVLRLLKPILNIVGGQSPTVELKPLKRAGNVRAATDGRYTVDNLVEGGYMNDDE
AFGLWSSGGARHRRNARPMPQREGRNPTGNQQTTHRPVGGRVGDVEVDERVAQIMELGMGFSAEDIRHALS
AAGGQVDVAVNVLVGA
>tr|C9ZJW5|C9ZJW5_TRYB9 | DeepTMHMM Topology Prediction - Predicted
Type: TM
MYIYIEREKKHFSLGWVTYTCITLFFFFIASFLYLSLLVYYIYFASLSLISHVLTCKALEMGMSVFSGLMA
TSHYATQSLLTPFCCLLLFFHIVYIYVQKKKREKKRALTRTVW
>tr|D0A815|D0A815_TRYB9 | DeepTMHMM Topology Prediction - Predicted
Type: TM
MCWWFEPRHVCSLRRFYRDVGPPGVFQSYSLTSTRLVLFVGDVFRLMYKKKHSAVLSVGYRCHCRFPHPAL
DAICRKAVKACPMFSFTSCAQLNKFIFVPSFLLLHPPRCLWIVVVVFLCVGVKTSPVCYKSTVVLKSVFYL
FRGCYTTATEVCLLSRKMRAFDPLFVSSFPNLKCQKLLLHFSFFLSLFVIELLCCCLIVSFVPSRSALEVR
>tr|C9ZZK0|C9ZZK0_TRYB9 | DeepTMHMM Topology Prediction - Predicted
Type: TM
MWASGSRLAPALSKATRLQLGNIKTEYGYVSTAAGSQEKWGFLFKGRRYPLISCGYSICLVCIAITVVGTY
QQYRDMIIMTEHISYEDVKDRCLTPMPGWARLRSLQIASPLGPMTYRDPTPLDWLPFELKLGCVKQASY
>tr|C9ZHY1|C9ZHY1_TRYB9 | DeepTMHMM Topology Prediction - Predicted
Type: TM
MEGGRTKGDKVEGVCWYKFRRGDLIGMNERMEVREGVCGESEEAVLKEDMLQPCAYTNKYALTHTHTHACK
YIYIYIYIYTCAHVCVCVCVSAYLFVAPCTRLCQ
>tr|C9ZQH1|C9ZQH1_TRYB9 | DeepTMHMM Topology Prediction - Predicted
Type: TM
MRVKKNETFSFDPPCAVFFFFSLRFALLCFFFLFSVPTVRCCFVLSFLLFFSFFSSSFPFDLPIRHQVDIY
VTAPTGIILTITKAQTIYIYIYIYIYTNI
>tr|D0A8I1|D0A8I1_TRYB9 | DeepTMHMM Topology Prediction - Predicted
Type: TM
MNVGKSGTASAKKLQPWPLVLNDISRASRKLRRRLRGITYRRSQLPRRRCGLKIPAVRASRPPGGCCRGFG
VRTAGLWLTGAACAVALAVTPSPWSGVGRGAWGATGCFCCITA
>tr|D0A434|D0A434_TRYB9 | DeepTMHMM Topology Prediction - Predicted
Type: TM
MSASSDLDATSDEDENHFRRGNGSLVLQLSVLCDASFDQPQRPFGEFFCRPSMPTRETCTTTLLVNLLWFA
RNYYNLVLLASMGVMLFAPPFALAVLSVSITYILRSRNGKSGAPCTSPQNDMLQKKKKGNHPTVMLFGLIR
FLVFIGTCYVCGFLFITVCFVGVVALCLFHAMFTPYTDKAFELYSDVLCCRRLPLRLPCSPTRQFTCVDPS
FDALSRQGSTVMSPRGGSCSKSSSPWMGTRTPEVLHHSFSSSSRPSQGEGIHNGWPLSESRRTVLSTQRGE
QLTGRRQSHLSEWGSRQQVRGRHRSPAALDVSDFQSDVEPHDSLVCAHAGTVRLTRVKESISISPMEQHLE
RKLSRMCSGFSGEAVTQGSVLDLCNDVCSALETAALEDEEGGFEQTTASQPIAPPTEAAPCLM
>tr|D0A551|D0A551_TRYB9 | DeepTMHMM Topology Prediction - Predicted
Type: TM
MKASLTSVGDYPPVSFPFCLFLFLLLLLRTSRRGIFFLVRFPPHHKSLKEIIMTAKPASQCKEGKEGRVQD
RCADGDEGLVTLLIQTLKILIPTDISGILMMASQTITLMFVGNHLGEKGMSEYSAGLLVFNVLAMSIVCGL
GAAIDTISSQAYGHDPHSPLIGETLQRALVIDLVLWALMSIFFITSKPFMVFSFGEGLGEGGALFLAHCPL
YLLAQIVSGVVSKTLLAQRQPSLVALANFVSAVASPFINYYLTPLGVHGAAWALGCTVSVCAIAVSLIAAF
HPAVVIFDAPWPSPALLRKDEWITFLRVGLPSLVAVCAEWWAFELQACFAVTISPLALAVVGVFMNILSLL
FALSLGISVSASVIVGNALGSGRYKFSKRYAKFIIICDIVLGVATAAALVYNGAFVARLYTNVPSVAKAVE
STMPLVALTHMADSLQLCLQGIFRGAGQPKQAAQGVLLTLWFVGIPSIALYVLVFKWGVKGVIGGLLTGMV
FEVILLYCLMSRWDWKKLAQKALELTEGEEKALLSTN
>tr|D0A9A6|D0A9A6_TRYB9 | DeepTMHMM Topology Prediction - Predicted
Type: TM
MMRFTRDGAVGNMQSDKSRRVLSCILFLCFLGLGALIAFATIPFSVLDGRSDKKQSDLVRIAVDETNWWSE
NMRLRVIESAAAARAIEGFVIGEIDNMPPWKGGLEQRALGWSFERFPYVAAAMFRNVRNNTGTVVMLAPGG
VVSQTFPNTESVLEYDFFESGNGIGVAAAERMAERGGFELFGPVLRRVPVPNASWQLLICSAIHNATSGDP
VSVANFWGFTVVVKDLMGLLDVGAFEERMKELEMNYLVYITDDANNTIPVTTSLRNNLTTAEIEKFTGGCY
NRPVLPQVGHLFMCVRSAAMIEKHSRSTVVLLVAGCVFISLIAFVCGVMVVLPCLREFDSRMNAPKTVPFV
MTIVGPCNAERLFELAPSAAFPVLEKYAKLQKAVITNNHGYVGLQVHPYTATFVTRDVDTAIETCFQLLKG
VQKKHLDEPLKKWLGADGELSIAAAIHWCADAYIRVETVNGSIRYEGNDVKYCERMWMFVPPNKVTISQHA
KDNIRSPSTEVCTTQIGSVFFRGVKEKQALFNVTRVDGDELQTFKDSTVPPSRIFGSAEIEQAEYEETPNA
GFIPISIQTNTRGTGGCEMPPSSPNLHKKKFKDRASQYCCSRDQEEHGTHRNPLAFPGGGDEVLPRTVAFA

SDPDNGRVGAEDVEIGLKFIYPRDHHVNPHIPTGSRFRLANDDVTHGYAAASSDKNVSKATSSGNSPVTDF
HTMDELACVSVTSKGDFITEALMRPWIPNALNTHFRVFFEQYSLFLDFSYESVRTVIFYFYIAYKELLKPL
AGPERTNLFNRFVIAFGVPPQSVLEALAVRCALRHIQQLEGIRTMLWHSEQERLQPSQCANAEDTKREGVS
HVVEGRI
>tr|C9ZR19|C9ZR19_TRYB9 | DeepTMHMM Topology Prediction - Predicted
Type: TM
MKRNSAREGDIEDFFCNSSLSAPRGNCAQNVNSRRRRVVREDYGPGIPGNATIFVHTFGCGHNVSDGEYMA
GQLVESGYNVTDEFGQADAYLLNSCTVKNPSEEHFVSMMNRVRDTGKPLIVAGCVPQADPTNKQWGDVSVV
GVRSIDRVSYVVQEALQGNCVRLLGETEDQRQSNDSNELPALDLPKVRRNKYIEIIPISVGCLNNCTYCKT
KQARGDLRSYPVEVIVDRVREVVRDGVKEIRLTSEDSGAYGIDIGTDVVYLLQAVAVELEGTDVMLRVGMS
NPPYLLRHVDGFATVLKHPNVYEFVHIPVQSGSDSILQTMLREYTVEEFFMCIDSIRAAVPKATVATDIIC
AFPGEGESEWQETMELCKRAKFEVINITRFYPRRNTPAAAMKQIPTDVAKHRTTELTNFFNSYRTFDSMVG
EVHNVTLLETAHDKHHLVGHTKNYVQVLVDPAQARMGESVVVVITSATKYSVMGRVLRSRWERLSADAVGF
ATAPFRTRKGRIASALLMATAVGTVLLYWSSTRRRRCK
>tr|D0A602|D0A602_TRYB9 | DeepTMHMM Topology Prediction - Predicted
Type: TM
MGLLTRYYEKLAEQGRLEQLIVFGMYDERVYEVIDRHKLAGSITALLPSSQAAAFLSRGMAYDCVNEEVLR
NFIKICMMLSTYKTVEEGASLAELIGSCGVPEHYRMSLFNFSETYRHMILSKSYFPQRAVLKLLSYIFCDD
VLTDAVAQLQYKSDILNHVNKVYVDTPDFISWRFSVRVLSLAGGYNEDRSDVEYTEALTRLVTRTIPEFIV
YFGATFMYAMIRHYPRRVRGITPRWLARQRAVFSAAYCAFLGVFVNSILEYRVHKHRMLYELRKSQECRRR
RGAEKRGVDYTPNTYFDSLEGVTRHIYSMQLTTYGASASMLLLSLVPLVPVKFPKWMGDVAWRHPFVPRLF
PAILGMHAASRCMVPFVFAPFTIMTALRFNGWSTTLYDRYVELRMRLWHRKVEAVFDTSTDVTDLDVTRES
KKKGGLAGN
>tr|C9ZZH1|C9ZZH1_TRYB9 | DeepTMHMM Topology Prediction - Predicted
Type: TM
MLSVIRSCALFSFFFFWGGDSCGGESGCEGCLMFVVVVVFPLRFAEKQSESSRFVCFCVCVPVVTRWTSAS
SGAVLCRATLPHDTTRPRVYIRICGAAALLLMCSLGFERGLNERECLFMMYIPPFVPLYR
>tr|C9ZRF2|C9ZRF2_TRYB9 | DeepTMHMM Topology Prediction - Predicted
Type: TM
MDVWKQVVAHVYSNTSNRSPPSSPHSPKKKKRRNDKKKEKEKKNISAAATCLFFFLSLSLFHFLPSPSPLP
RFHPFSFSLRASFRLKPNNYSDPFSKPTPTHYSCHKIPLSFSHPPTPILIFFFFFSCKNIIPSSFVKKKKK
KKTQRKK
>tr|C9ZHZ3|C9ZHZ3_TRYB9 | DeepTMHMM Topology Prediction - Predicted
Type: TM
MHSSFFLFSLGVFVIYLPPSLTVLLPIFGLTTVKT
>tr|C9ZJ39|C9ZJ39_TRYB9 | DeepTMHMM Topology Prediction - Predicted
Type: TM
MCVYIFHLPPFIFFHLLSFLHFLSFFSFLSFSLFSLSLALSFSYFLCSLYTYVPFCFAFFSSLFFYFIFFL
ICIMRGSGCNKWFFTFTFDAPFNVHETKQSKIKNKNKNNNDSKNNNNNNRKTAAEIWKENIIAERRENVFKK
FFKKKKKREKRGEREIFRFNHSLSVLFPRFLCFSSFFLSFFFPCHEAFSIQFISVFSFCFFFYFFFLNLFF
L
>tr|C9ZYW5|C9ZYW5_TRYB9 | DeepTMHMM Topology Prediction - Predicted
Type: TM
MSAQAHTYLCDAWNRDKVMAIVQFLPMALEGPARTAGCESLALSLGNLARMGDAYRAVTRLSLLANALSKP
TLTSLSKPTGDMVASRIDQLSHLFHIGFCLNENTAVLAGHGVFPKSLHRLSGVAVLCWMYTLVLGIVRQLY
LFVKLRPRQASRGAGAGDDKKVPAYTYLELKRAFVNLLKLVCYFLFALTCLPEGKPQLLANASGPLVPLHV
MVKALSPNPLHASNTVRGLLGLIASVCEFY
>tr|D0AA82|D0AA82_TRYB9 | DeepTMHMM Topology Prediction - Predicted
Type: TM
MRQTNKRTNKKEIIRHYPHLFFSFPFENFFVYVVVILCSVMKCIYFSPYYLPLLVFFPFVQNPALLYPFFS
FFLFFFKKNVISFGFFLSLSEYFFRTFFHFMNFTRKQKIKSVH
>tr|C9ZWZ1|C9ZWZ1_TRYB9 | DeepTMHMM Topology Prediction - Predicted
Type: TM
MAGPSVFRLGNMLCRTKSVWCGILCVFFITSIGAVTYCWNRGLCKSRPQPYCESGLFGSLCLCAFAVFAFA
SVFCSVLAGWRAWQQQDVRDCFFSFFSCFQCRDYSAPFG
>tr|D0A2E0|D0A2E0_TRYB9 | DeepTMHMM Topology Prediction - Predicted
Type: TM

MKFCTRYAHCHYLSFSTLPLWQAYPCVNFSASKAVPPIRQSFWSFFFFCVVIYLPLTTSSGLHQTVPSSTL
FYLHPASVVITNTSFSLHAVLLLLFSLFIQTTSFFFLLQPRTENASVKPASKKQDNPDTN
>tr|C9ZYU4|C9ZYU4_TRYB9 | DeepTMHMM Topology Prediction - Predicted
Type: TM
MASRLAAFQPDIIEFFLANKASLQTLRKEWRAVEKGFVGYFKDFRDTLKELRGKEQADKQEGKKEELSSGS
RAVNQFYESEELAKRMKWKRITWDEEADYNGFARRLLDNDDLIKSVPAFSFLAIHVAKDSSCADFVRFPPR
EQRFESGTLTNWVLSERTPATVERYKPSKVRSMRSPFEVDWTAGEARVVDTTSIQEVLVFLQTVAPRLQAL
QVRMEEQQNQIAKDIPKLKLRVGAEIRYNKHDTTAWDDPNRCADPDYVTPDDVQQFVEGMMKSALLYRWFL
KDQRVRIMPPGRPYLLNPERKEVQIPANFAAYNWWGAHARFQKVEAFILFTINMWWLWFTLAIVIVGDVEI
L
>tr|C9ZUY4|C9ZUY4_TRYB9 | DeepTMHMM Topology Prediction - Predicted
Type: TM
MLAESPGFAKALSTLTLLGTESFYSTTLEWRKRVTDIIHMTSDEQLPALLMRVYDVLLQVLLDDGRKQPNY
LELASTAVAVMCFVHRLFLHYREERLLLDEYRRWAFLRAVVRTFEFTLSNHFEGSAALSDASRGVIALMFL
PDADLSRLSVGEAEFSAEDASFQRALHLAHSFMPSAKDLGAFLECLVCCIESLLSVRSVLGHPEVETTDFG
VVFSVLFPLQSAMAQRYEHAERLFRSICSIASAIRGPPSATTSSGTAKGVANFFSAVFTPSQVNTIQRFVS
SVGASGSPKRSPITCREAVECVTSFFVCATEEMAIEACARPLFDLLCLDDVEEELVRFVLHSCTENLLLLK
PCQLQRVTQLLLTAVKRGIAISETVSSVAAVLKKESKAVVEVKIFVLEFLSFYSTAVSHASEVETLHSVFK
WTLEEVTSISDWVEPQAVSYLAAVTQFVRSALASAPLVALLARLEWPHEVLLLLLRAHGDGVREVAVTFRN
LFCSMVFNEGQFVDVLISELRAMGGQSPWPSQISILLCVFVYLLQDKRGAWAAIRSNDIYVCLYLLHYLDQ
STCIEEDAHLIGTSLALLLSHLRWSEAGGIVAHYVDAVRTPWMVQLLVDLATGFYTTTKNLIAADAMRVET
VKTMTPGSRLCCEVVGGFSIPLSCRFEHTFPFGVLLRWIVEPGNQSGDWQKNLMELLSGMLHRRCLPLVDD
AFTSTVVKQKLYELLPYVADVSRRDVLALLSSSKEYLTLEDVSNAQAIWERLAYGRIGEGSQHEFHDYITW
YGNGALHGVLQGVQKGCSLNDRFTTALWVYWESCVGTDTGSMIQLWELLWPGAEGSTKVSLAVDTMQRKVI
FAVSPADEAQQVVTLPFIPPGQWMHVVVSFLCNRLFSSTVTAFYDGVRTLQTDITVPGSHTLLPYPGGSSF
MARILSESTYDFTCTVGFSHEGSSEGCVQRFVSFQLFNAALSQLEVMSLFCVDPYSLTGLRAAKRCPLELP
LLRPEALRYLVASAAHGELQELTSSRALNFAPFPAESLILSLHVQNAELVECSQLQSLHNKRWVVPDSAGR
NGVTNFLLHGSHSEPVATGGNITKVLISHNVPCEWLRWLCVISCAYDEVKMPNTKNVLDSADAKMAVLQRI
AATTLRIIAAYYWKTEEVSDGNVAIFINYVTLQVKQRPQVYLATTDGVDAVFGLGAVKESTGEHCVVYSTL
PVEDVFFNWKVTSFLPEQCQQRILFHLTALLQKSNPFRAVNALRLQHSSFFDGFICGLVREYVWPPVLESA
VAVIALYIECIAGDAVALREVLNLCASIVPVERDVLTVAKRLVSKIPLVAFECIVVVVLNHLLKVLCGACV
GPHNEGLMFAEALSHAMPQCWFHILTSEWAHPVSVTMAMHLFTLCYKRSETFRRRFGDVIPLLYENLRHHA
HQSDLLIVLISGFWGSDWDQQKPPYALLNQLYTTSEGTVPSFLQLAMHLLGRSASLLLRPDAAVNYPHITC
ASFYAAMGAAATAEHCRARSQLRCYISVVVFCAWLRRGQKIPDDGANRISFTKRESADHGEMRRTVVAILQ
WVARRYPSDRRVSKAMYVSQKSYNPMDMALLCLFPALPMDKDVGSVGNMPLPLLDVVEGLETTVGHRDIFD
GGYSGTGGFDFDDCDDEREELVCDARGPNTSPATEARDCDFDALLHQPESELYNACRALFVAHIKRYARSA
KCQPFVTGRKSVRSPPKLVLALQRALALCPSGIGLRARYMFCVAVCCFCLEALTDMLREEQPGETGQCVLD
NMVATGKYLADRLVSGAATPTLAVTKFLQHLLKLHLNTEQVKDLCESLSECLLLLMDSISCGRDARSAASV
IDSLHDEHIGVFGYRIPSEELLRLTTHRLLDISEVLLASDVDRHSVNMEKLVGLWRTFLVANREVPFFRLV
WNTEEKAARVFYMLTESTDGGVGDLVAWVMADHGNFKQSLCKDEWTQMTLRKTFLQKRDRRWAACAVEQRD
RECADLMSSCKVKCNAMNEEWSKLTDTSTFVDTALLARRIECCDVLHRHLQVQLLPNYGMEEKPGTLHLHP
SGAVGYPCGVPFDLLHNLTNAHIRYPSLRCRLAASTASDDGSVSSHSRASVPAALLRIVGSHFTNPVLFVG
NVYYLRGDECSISVMSVTPREVVIVGDSQFSASGDFSIGVLNPRDAWGTLSLDNGSPSCDNRNFLPWLSER
GNTEKLEIARHSCCFRTICVESRVPSSPSFTWRFSTGSILKVHQHFFQHRPVALEFQLENGDRCFIALDE
RLCFSRAKQEEIMSAVSRVAPQADMETYSQKESRMSELGERWKQRRISNRMYLLQLNDIAGRTVADMGQYP
VMPWVLSDYESSTIDLRDPGVYRDLAKPIGALNEAKERQLRDRYEQWFDKSQPPFHHGTHYSSSAVVMYYL
IRLQPFTQRSVRYQGGRLDIAGRIFHSVSEAWNSCGGVGDVKELVPEFFCLPEMFMNKSRIDLGVRLDGVQ
LGDVVLPEWCGGDVERFVHLHAEALESDVVSGKLHEWVDLVFGNKQRGRGAVDAINVFLHLSYGQAVGRAI
AEASSEEDCKAIVATAANFGQTPRQLFNRAHARRFDNRYGPTPQQAFINEAGKLSVRWRSVCYPWGNKRCC
TPITSLRIVDSSAIASTRFSLILPTTPMQVCHYSNVTRELSCHEYGKPTLLCVLPNVWHQGYGELTTMCTS
SLGGIICIGTTNGRVVICSRVTSTAPFTIMSILNACRNDDSAPVKLLKMWNSGHLSVVCEDGPRGSLWHVA
HSGVMFCFKFDISDVVDESSTIRDITRDEQSNCYFIATHNHIVQLSSTGRVLGVASVSKASTATTARKGTT
APPANNDAAADGPKQHFCAAEYINFESYSKTNILLAGHEDGTISFWTVRPLLGFDQNTFATEIKMFHSFTV
DGDTRITTITHDGAGFSAGTASGEVHTYSVPDPFADEE
>tr|C9ZMU7|C9ZMU7_TRYB9 | DeepTMHMM Topology Prediction - Predicted
Type: TM

MLRRWSECGRFSTTLSWCAANPVGRQQTRRVSGETTAKDASVDSTGDEDKDKEAQYRERLIQEILSRDKEI
FELKRQHELSMLRVEQNQKRVLKDQEDRGMYYEQNCNVHTFDTVSVGLYTQRNTLYHTMSIERLRNVKLFT
TLLVTVLTCFYLYYRYMINNDWVYVEKPMKLLGSRACALSEIREKMGEQQEEKEHRERRFAV
>tr|D0A391|D0A391_TRYB9 | DeepTMHMM Topology Prediction - Predicted
Type: TM
MNKQYGTETNRYDYDVWQENQRNCRQECSPQAVPLQGIPLYQGDGDDKEELKRFVVQGYKDVWAAILYILC
ILATIGLGIYNIVKPDASGEEEGESVVSVKSSNPFSGVFKSVYIWIAVAGGIAIAVASLSFLFMRMFPRKI
IIVANVVSIIINIGGAAAAIVMGVLPLGIALLIMCAMHILWFYFARHRIPFAAELLKASIDVLSGYKAVYL
FNVMLCVGCAGFTILWGYAAVPSLETATNKGDSGALAVLLLLFLMLVFFWSSQVTGNLMHVTTAGLTATWY
FAGKENMPKNPTLASFKRGTTTSFGSICFGSLLVAIIRLIRWLVSTAEGSEHEILRCIFLCIIGCLERLME
YFNTYAFVHVAIYGCGYIEAAKMTWELCKQCVFSALFNDTLIDVTLNLLVTCGSLLVGSVVGLLFRSWVAF
ALGFAVSILVHLLIFSPVTSAVTTLFVCYAEVPEGLEHSSPDLYAALQRTDQNGTSNGAAPPRV
>tr|C9ZMS5|C9ZMS5_TRYB9 | DeepTMHMM Topology Prediction - Predicted
Type: TM
MSPSELTLLLLLSYCIVVIFFLPTVRLSVCPFVYAWVRKGTALFCLPSPFPKRAIDAGETVETPKIIVGEG
GTVLAFQLLAGDVRKGTFTRIYPHSHHRVIRSSLLSVPPRPCVVARINNYAFLCPSSLFLYIMMDL
>tr|D0AAL3|D0AAL3_TRYB9 | DeepTMHMM Topology Prediction - Predicted
Type: TM
MSNIKGNNFLLQAPSLKDRKLRRASGIKNRHGSHEWKSAWVLPPLSMNVALLLLFCSLSELLFVWLKKQLK
YRFFTVSITLKHLLSSLICLLHIYVRTHFVLFD
>tr|C9ZZD0|C9ZZD0_TRYB9 | DeepTMHMM Topology Prediction - Predicted
Type: TM
MSLVLRSFGTHIHLYIFIVKCLEEIGIVYPSVSLFDLIFMLFCCQRCCFHAPFFIIGCCIWKMQTDEGGPM
LQFFLCFFRLSHYFFFSFLFFSAPSWQVFEVF
>tr|C9ZU70|C9ZU70_TRYB9 | DeepTMHMM Topology Prediction - Predicted
Type: TM
MGTEGNNIGPPITLLPFLISHLIHYVPIFSVVLLLAFQLVAVRLLMIFLSSTFRYFFSLYFKLSALIRKVK
YISCRYVYGPVTITHTHTHTSPLFFTFLFLVLFMI
>tr|D0A7R4|D0A7R4_TRYB9 | DeepTMHMM Topology Prediction - Predicted
Type: TM
MERQSDEDMLVGTNDEPGTCRMVMNEILELRWRALVAICLLTFGSYYVVDFPGSMGTGSGNTIEQYFRDHN
MEYTQEMNQLLYSVYSWPNTVLAFFGGLLIDKYLGIRTAALLFTSLVVCGSLLFWVGLRFTYFPLMVGSRV
ILGIGSESLGVAQSSYVARWFKNTRGVALAFGVTISFSRVGSSFNFIFTPTIAESLGVEVATLAGVAMCGV
SLLACIILVVVDLYAVRTKYIRAEPCDDEESVMKLSDVFRLPFTFWALTFMCTFSYTAIMPFISIARNYFQ
VKYDIDGTQAALYISAYQLSAAIGSPVIGSIVGALGRNTLWLILSSTFIGVFHLVLLLTNIRGDLLMASLG
VVYSFLVSGLWPSIPLAVEENVVGVSYGAMTSLQNIGLAVFPLVVGKILDAYTPDHNSSSIIDLAFDAELL
NGNSNASADGPHPTLEGYEVAELVFIGSAGAALLASMAVLIADKCGRGILSASAKKRGQMKDEKRESLLNH
LPEEERTLVYLHREP
>tr|C9ZTN5|C9ZTN5_TRYB9 | DeepTMHMM Topology Prediction - Predicted
Type: TM
MAAVVPHTFVVVLCMGLVIFFSTDVPPPLAVHRLRRIGAIRVFTHCLSLMIPPLFFAAVDYICLHMKAASD
RNDEFAVVVSPRDPVILCCCVSFSHVVSMIRSHIVSLILF
>tr|D0A857|D0A857_TRYB9 | DeepTMHMM Topology Prediction - Predicted
Type: TM
MGRIGDTPMIIIGLLGFMGVAAAAIGAHWLHPNLPEKDRRAWDYAVQFNLIHTAAMMAIFAVCKSVHPEGS
AAKWLNRSFVLLFAGTAIFCGTVYAICLGVPGKQVGPMAPVGAITMMLGWVSVAIAGF
>tr|D0A231|D0A231_TRYB9 | DeepTMHMM Topology Prediction - Predicted
Type: TM
MRLNVDTSGDEGGFHWMFHPLLFCFVFRLWTKGDSIKILPVCLSFCLSLFFYFDIKPHRQYIFALFFHSMS
RVFLVSDYLLSFGFYLFVSPFVCLCVCASRHFRIIMQFILFRAPALRALQTKQVGG
>tr|C9ZUQ4|C9ZUQ4_TRYB9 | DeepTMHMM Topology Prediction - Predicted
Type: TM
MHFLLSFFLPFFLSVMSSKVGQEFSQYVTEISQSQRHHVADRVEQLARHQSRAWYYLVGCVTFTTSSVMLV
FRLWGPRHIFKNSVYYTRPLPPAISMGVVLYGILYTCRGMLMRSRICTMMEDYEYELKRISAHHCEEGVNQ
LAWLQFVTEQLKQGAEHRFDFNKLRN
>tr|D0A2F2|D0A2F2_TRYB9 | DeepTMHMM Topology Prediction - Predicted
Type: TM

MVCYVIFFVTYSHVTIAIDINTHAYYFFCYIEWSWEFGWVNNICQSCVGSLDGCLHAVGASRDRTDLPLPL
FLYSFLSPPYSFCHFPHLKPHSPVIAYSVVSRFPHSRCGQS
>tr|C9ZVI1|C9ZVI1_TRYB9 | DeepTMHMM Topology Prediction - Predicted
Type: TM
MSSDLLREYENDFNETLKEANDVASRLQESLQRSGVSYQAPPAAGPQSRSQQCHSLQQSLTRLRELITNMS
YESNEVEPASAKEEVKRRLEDYRGKLVALEKQLSRLRQESREADRTDLLGNGREMGDDGGSMEEHARMLGT
TTKLKEGTGALQKAEALLHGTNELGLETLSVVRGQTETMKHIHGVVIDVDDDVTESRRIVHRMHQVARKQK
LIMAGVIGMLVFTFLIIVFWK
>tr|D0A6H2|D0A6H2_TRYB9 | DeepTMHMM Topology Prediction - Predicted
Type: TM
MLSGAEELISDLFRRTSTTSNAVEFILSSNSAAYHDVMQRICNSNQPQLVEGILRTAQSLLPTELFQVFGK
SVVPVLLLFAPAVLRRVGELLAAPKETPLSTCGEWDAFTSFITGIHCKLLASPECRATNVRSLPPPPVAAR
HTPSWSKVFHGEENVSEGVPPPQPSTDCEVNVTAIGTDNYGSLLCTIVCGLSHLLACVATPPCVIGGPGLT
TLCNEVMRLLSSTPVFCYASKDANLQIACVSLLLSVLNVGKAVQGCVASSFMPTMRRWFDLTLYSANAVCI
QLLFPYILHARAHR
>tr|D0A2X1|D0A2X1_TRYB9 | DeepTMHMM Topology Prediction - Predicted
Type: TM
MLIELCALRIIHTERELSVVSCCAANSFRDLSRIYHFQNGSSLSVSLFFSPFLFRLDISFVSTLNYFLFLP
VYNTNLVSFSTVLFRFYHFLPLSTTQSLSLQNLLLNDTEEEEYLKEKK
>tr|C9ZIR2|C9ZIR2_TRYB9 | DeepTMHMM Topology Prediction - Predicted
Type: TM
MKQQNRGDVKFDQAIKQSNKKKEKKRYLRQKMTQRSEPNTFVIIILLFVHFFFLLCFFFLLCFAHVCVSSY
PSVQETAKLISYHSQKTKKNAVICPVIFV
>tr|D0A3K9|D0A3K9_TRYB9 | DeepTMHMM Topology Prediction - Predicted
Type: TM
MRCFRKKVRDEEEFTLTSSVSEELQGDICNDGKPYGNADPQEQVPETGDITQFFGSFVQPNASMVGNSFCT
TPFPVPVSWHNLTYSLQGRVILHNLTGTALPSRCLAIMGASGAGKSTFLHALSDHLATSKDRKLEGKIQLG
DVEYRHQYRKVMGFVGQDDVLSNISTPKRSLRFSVRVRRNPDPETTKQQVSDVMDELGLQHCRDTTVGTPG
LVAGLSGGERKRCSMGVDLICDPKILLLDEPTSGLDHVTSAKVVQLLNTIARKGRTVIYTIHQPSAGVLNH
FDDLMLLVRGRCVYHGTMEDSVAYFESIGYVCPETYTPTDFYMTLMQDSVQAKVLIKQWKRHVKRKRTLHT
RVVELNHQPWTSDTAKFLHGYIQRFKGSAVSQFSELVRRDFTELIRNRTFIITSLFQSLIFSLVAGLIFFG
VSSDMTGIQDREGVLFMVVINRAMGQTYTMMHRFYDFKALYIREQQVGSYPPLLYLLSKTIVETPYRLLFC
LVECAVVYWMVGLYASAGAFFTFYAAIALLSEVAASLGFLLSATCSTSVGATGFAPIVLLPLTLVGGLYAT
TDRMRPYWYFLEKLSFFRHAYILVLRNEMKHIDEIECDEKTNASGLCGYIPANGKEVLELNGLEDGQSENW
IMWLCLVLFYVLLRTGILGALYKAARHKL
>tr|C9ZR89|C9ZR89_TRYB9 | DeepTMHMM Topology Prediction - Predicted
Type: TM
MSAPRVNTKTSAGKKNQGESSSTKNDDNREAFVSRMLSFWPKVTQEIGVSSILGAAVGVTSRRLTSDALYG
TGLAFIVLQFLNFFGYIQINWKKVEMDAGKVLDQNGDNKLNQLDLKALMQRFILYVGQGVGDIGGFVTGFY
FGARYLA
>tr|D0A987|D0A987_TRYB9 | DeepTMHMM Topology Prediction - Predicted
Type: TM
MKVTKKNVRGNANNISTHTRTHTKKTVGDMSVCVCVCVCVCVCVCGGRGEANSDPIASIYDITHFPIIIII
IICICVCAFVHSLVFLLAFVPVLVLAFFFLFSSFLPPPPPHYYYYYRILSSFCMRVCVCMHAPLF
>tr|D0AAL5|D0AAL5_TRYB9 | DeepTMHMM Topology Prediction - Predicted
Type: TM
MILNSHTIATSARSALILSSSLPSFLYWPNSFNYGPAHVPLFNTPIPALLLFLLNFFSFNLLFFIYIYIYK
YIYIFFRFVFQGLYSTSFSSRQSFFVFYLFRFRCNCKYAWLTSTYRPSVAHVQTQTQTKQK
>tr|C9ZRI8|C9ZRI8_TRYB9 | DeepTMHMM Topology Prediction - Predicted
Type: TM
MTLLHQLLLYISVSWCGKHVFAVLTSPFRDIDGIITCKLHSYHTGCARVPIKWSFVDNCFIKQWVLLYTVR
CTTFSSCFWLLGQLQLRGSGELSKEVSPRSFILPPLFTLFSVGDFMLAHRAYRFRTVPFLSSFL
>tr|D0A593|D0A593_TRYB9 | DeepTMHMM Topology Prediction - Predicted
Type: TM
MIIKYPSIIYFYIYPPQSQSIPFVFLLRLFSFSFSFFFFRLTTSNISSSFSCFLFVYYHSSHFISSSASSH
LTHLSFPNPHFEDFLFFLFSFFLRIYMYIYIYVFVFVCLYVLFFVLPPSWLYLSLFPPCNNSIR
>tr|C9ZV08|C9ZV08_TRYB9 | DeepTMHMM Topology Prediction - Predicted
Type: TM

MMTSQSHSPFTEASGFAAAVTLLFTAMGMVILSSHLMPLTGPRVLELSSKIHLTMAAGTFLLLRFALIAPF
GKHSGLSKVRIRVPALLSWSLQECPTLLSIIYYIVVEYPCCVHQCNPLQWLHMILDRPSMEVTTTVASAAP
TLRLGLLFFAAHYFNHSVLYPLRVANHGTSVPLHITLSAMLYCALNGRLQLLANIDSVDASQRLVVDSTWQ
TILTILGAVIFFAGMLVNVTSDCYLIRLKKRPPLGAYKIPYGGLFVFVSCANFFGEIVEWFGYVAVVYGTN
GTVAGLAALSFAAYVVANLLPRAYAHHQWYIQHFGAEYTALQRRAVIPFVY
>tr|C9ZKK4|C9ZKK4_TRYB9 | DeepTMHMM Topology Prediction - Predicted
Type: TM
MRRYMGSSRNEASHRVASVRVCGIEPCDVPFASLVFPPSLPSFFFLHLFYACSRFSFLPSCFLYGYYDSPY
SNSPFFVASSFLFLYVMILCSQKHAKDMRTYMRYLPFIYREFLFYHIFDEGNNTTPKQRRKRKKKRAQVER
RDIIGRRGGGRGS
>tr|D0AAR3|D0AAR3_TRYB9 | DeepTMHMM Topology Prediction - Predicted
Type: TM
MPFTVVQVSSCRMVFVFVFVFQFYFFFHSFILSFASFCSVCLFVCLFAFLFFFISRLFLKLRLTSFFLSFT
FLPILNLSSPAVFVFFSLIMSCNMPPHFMP
>tr|D0A893|D0A893_TRYB9 | DeepTMHMM Topology Prediction - Predicted
Type: TM
MTGFRLPVVGHQYCVLCPVCSNTVSFSCKSANIYGVKCYLCHTHINLQNPSEPLTPDDPGVLPKHSFDDSV
SSRGVCYSMEVRESSGGSASAQVAMLISKMTKEQRHIMSKGPYSEVQEVFSARRIQAISDKIWFLHPLVYC
VEYLFFGDKPVAEVGNVRLFRREFPVVGPFYIPVVKTSTSSAVPLTIMIVFTGVLTYITHVVGEPHLWHNM
KLIFMLFLPLCGSLWATVYSDPGYARPTFHSVPSLAADGPSPREVEGGVQQERESSWEDVNGQSVERRWCS
ACGIHRPLRAAHCYFCGMCVNEQDHHCGVIGVCVGRRNIGMFLLFVLICIATMVLTVTTAALVLRSCVSQN
MGGMDAAAADSKGKGCKAMTPLLYALLVTTLVVAVFLLLGTLPLFVSVIAGVLTATTTRERLKHIYPSGRS
PFDRGVLRNAWDFITRGKPASIIDDRFVSQCLMQAEKADDFIL
>tr|D0AA23|D0AA23_TRYB9 | DeepTMHMM Topology Prediction - Predicted
Type: TM
MNRQDGICYSALCGNIYLPALTSVMMMIIISIFLFLKSTILSSTHTGWIVKGLLVAMMELAEGVRSGSSVF
AGVSPNNVIRARRPSVEAPPMISIQRSPSAGSGALESRSRSESPRAASIGRLPPRGLPVPLRAQQQRAINL
RSMSSSQTQERILLPRSRSSSLMLSSAAVIPADMVASKRFTPPGGRTPLVGPAKSPPIGFVKVMAGPPPPG
MVKIPVSPSAIKSPGLSPHFNRSQTSVSSPMQSVALPSPASPAPALTPGLARKSSDHVSPGERSRRRRRRS
EGKIVDTPQKHRSRTNEMECMEGLAAATEGSLPAEQSRHRRHHRLSRRATARRSSSALAGEKASKDVPPTP
ENPVVNGDNGDEIAFVVEEEGKEGANTEAKQRSGDARKSSSSSSDSSVSFQFTDTNPPEGQCKEEETTPTP
EEKSVVNEKCAKPMDVESPRSASRTQRHNSHRISAPGTPKSKGSGTSSKSRSRFGLPSVEGVQKASVMEVD
EEEKIEEATPAEGKHEPSLPVAENPKNQWNAMMSTLQDQSSNGVSRRTAPSLETSNKLQEASRKHIAEQFL
SILCPQQFYGKVTKLHRIRLINSERSRAPGLKEHATILVASKGVLPNQLFYPQLRLAQCDGVEATSADIPG
QLDTSIVLCEAAPGNMVFAGTEVEAEHIFENDSTVTSCFILEKSAIIFRDPVRYVLPKAMLDVRWIPYSVQ
GSEPLCPVHHKELQLYDSSTRELCCSLCLSKSGVDTSKLIVVPEALEGDSRRRVTETLGEHLKRSIDKTAE
WMGHHQRIISVAKHKKEAVIRQFDMLISAVKSKRDEFLEHCDASFASTLSSVAKEVLLAEEKVALMKAAID
HLRSDALKPLYSLQVATVASALHVGEETPWGTSTDATDISLLNSGLSVNLEGVMAELQLVSVVPYTRPPSS
RRRRMGDDRQEEVTQTDDYESKRRTSQKSVPTKELTLDDHLNLSTHPHKRPLTSVRRRRSVGRGSPTGRRA
APLLPHEVFEDSDILGDDENIKRVRRLGQWITVPGCRGTCIFNAPIHKIIKACREGRNLSTRPIALQWTLR
VDDPGEWVGIGVGVGGNLTTWSENHTPDLGHLWVVPEGARRQHFNLRVTLAPRVGHAKLTVHNTNGKQLDD
GHIPQWRAARSCYPQITFGGRIGDVRLIDGPQLLTT
>tr|C9ZUT1|C9ZUT1_TRYB9 | DeepTMHMM Topology Prediction - Predicted
Type: TM
MCPCEYTCMCLCECVRMFDFYISVKASEVSSFSYAHSSAVAIIAKLRCVCVLVGYFSHQILSLSFVVVLYP
NELPFFSSFTVIFFSSDVFLVITPECLFNCFFRTEGGRGEN
>tr|D0AA52|D0AA52_TRYB9 | DeepTMHMM Topology Prediction - Predicted
Type: TM
MSHPEHFGISYANIVYAFLVRVDGLHINDSINQMFRSNSREKKTLIEFLLKKRGNHNNAVLLDKDSDDTES
RLPLRGTAMAELTRLAGEDLAQWTHIVKGRYCAPSLEETKSADSTHMRWRLTTSDVVAQLTMVRNTFMANI
LDFKRMSGTTGPSSSVPNNEALGTLGNIPVSLLDVLHENVVAAIKDSISKLCTRLRKTCPPGSLTRSMVAV
DSHVTSGASAQWDWSEAHLLASVVELVWALSVSVVLDAHNASAVCAGGLLYSCRLLCSDADASGANENDYG
SQLSLLPPVFFLTHDLAVKLEAHYHAVLQSAGCSIVSPSRVYYSLLSSSCYDLLSQIDEQAVRGHCMSSVR
LCVSLLKEARLSCETWESVRWDDIVSRVRSGIPQANRQVLVQDLECLWHFHRNWSEKTRGGRTSLTNDAQV
GGLPIILSGKLPSFLSTRFQDLATELFSKCSSLKSVEASKGTKAVESMFVTVFFDALYMCHIFHNGPGSVD
SLVVLLVLVKKILPHAKKEKRLRGVVTLLLLGCLFPQPVPDNGNTKIHDQLVGSSGQPISEMLLGELDSWR
KCKYSMWYAISVAAMLGCVSPASLLKKMPSFLKTLRNLFSSVDNIDRSSILYSTLHVGGLLVDLHLRNATA
LAEDVKSCTECFLYYTEKMLVKDLHNEKWREGTGAPACIMAASDLLLFCLQQEHFYLKATGWLLRLLDCRN

KTRGSVKSVKGPTICYGQLIAMRALSALCELKYFSVDSKMSEQKNGTKNAVLACNLNCGYHNTVDELYDDF
TDTKTQIRSKIMGMIFCRGSCYNYVVTTVEGPTALWHEGHLKNLLTFIKFTNNIQTAINDIRKAAFEYLND
AAQESSMNGTSEFKLQLVSVLIVAGFFTPARAMLDRSLTLPKLLVGADSQLKEAVARVVLPLYLKSGNNSN
GSSYGRRSLCSCAFAIYRLAESVIDVLVSEDNGRVEELVKYLTSHLLELRDEMEYITLNDMYVRTTHDDVK
DSPQLGRRWDFFSAGAEVRAFNCAVGSRRDKWNWLLIPMNRNVDQLEGCFSSPESEGWYVVRALEGTTLMD
ALEGLAIILLGHTRRTVRQQALLQLDILASLLSLQRVHCGDQLSTNEDAVGRTHNAPGTSAGMRLGAHGHE
GAQNQRFTASEVLKEVGESFEDMYLSLEESYIPFPASTSWHPKSERHQKGMGYVGERSFRTHIGVVESSRG
TPADVEMHIRVNNSGSPLVSTPLGDRCMMADSEAYLLIFVSALALVLATHCYYPGSALVRVTCALAGEIVQ
RGLKLHMEGKLPLWRSCYVLRFLILSVDNTKSLPDANRVTAGGERNRPGHRHGGNTTIVGNFDDYKEVTWQ
IISRYAEYNKLMDDITVPLITYGVSSTTTFSFFDVLQFLHLGPLEYTWTRDTPLVTFPGVMDSNHPLIKSL
RDILYKCNCSHSAKDAEHKAWACLSVFLPTLVVVGRRFFVKPPIIPSREQEKLAIQRTQMEKIIVGTIDFL
LLGNTRLFEAGENIEEQQELQAETLFQILDEGNGVAARSWLALSRSLPATNNGDELRASFSRNTVGGAMTI
IFLRGLFYDLVTLIFSKETKYLETQLKSILLLKVFYSLTHQVQWFLDINPLQVDDNPEAQLHPNVLVAMEK
GVLALQTVILSMEQLTGGSPSATVASIANSFSENFLKWLQHALPPNNTNLEGDMPCIFNIAVTLLVADASS
WESMRRVLHQEPVDSPVHRSLFSIMVTAFSVLWGRLERTVTSPRVCVKRISYQTRSDASSDARASLLGSGT
SCDSVNKMIVTNSSLIFYALMYSYEESSVGPVFDPIVRSKAKALLQLLCGELLINDTSGEDGFARWFYLLL
NATRMQRDNVDVSGQLLQNFFTMASSIILSAGATVSSTAPIHPVSVKIALRLLSLFVRQVEATEESLRTML
KVTQACRNSISDNYIAEVWGGWIAYEGAGRDRFIAVLVTSMIDIADKNTIAYYIDRFISSEKLSQGVGEGR
VSLDSQIQSTYYNFFLFSIYQMRLLNQRHASSAPSSSTKYDTALNDITSEFSKDDRIAPSYTAMSSVNCIN
FEMSIGRCPNVNVAGKGKMAWIAEENIWHGLQRLSQEAVRGRAFSTVRRERCSGRTCLSPMPLYPDDVETS
LELSFVKPLVSVLFFASWSIERLQEHGQRLWMEIYRDLSALGEFTSVAGSCEVKNERHVVSSSYPSRDVII
SLAKRDATNADEGSCVEDEQETGSVVQQSEASSESDDNTLVQNDGSNFSSSESEEAGAFRASDEKSVIDYG
SVVEHCSTESNGTVVVHGGTHTTFAAERTITVEKDDKQSEERKRRDRLKRKKDIYRVAHRDYLTLHIHFSC
MLLAHLSSGVLHRSDERDEPGSRFYVVDLAFKVLEKLLKGTVTDPTYRAPNSTKYWLELTLTRLRHRDLMM
AQKGNEMQQIWECWDTLLLEESLVFGDSKLNENHAHSPSSTVDIVFAELFMGITGHGRNVFGRRFLQRCLH
DSSKWLTQGFMMENRAWQRRHQVHSHISLLCMKVIRSISTCLSLSTENLLVGCGSRHLWVEKMSMQLAEAN
LLRHLLAVLSDRVGANSTVQSGANPQHIVTNKRYVSCIPVITTLACLLRDITIHEILYSSESQKQGMDRTK
PEVLAIATPFGSNSSFPSSYLVSCFWAGFALLFASDAWSSVCDLLTVFHRHGCLKFLNVIPKPPPCSSGTF
QLTVLPECAAVQLVLLQQGRLTAKQKRDNDELRSTIGRNYKHNTAVVLKYLSEFSQDNSGSAALLAFSELV
LLCDWVSLNLAGFSPEVLKTLHVEESKSRENSTTNLVRSNADLGGPPVYSLWDCLGLFGETWKQSLVVFSG
GSGSSLKNLSLRMKYVLDELFRVCSEVFNVPSVEMLLSRVDLSKQNGVHPGSPFSGSGLDDIDVCSLHSCA
DTADDRCCLCEDNVLSFQQYFEILVVLPFMSLFRSHHTPFPMWSKLLSFVSALLWYHERGHTYITDLLLKF
LPDLETALDDVIMQNALVDVNEVPLVDRLLVSVIAEEVLAKAECLKACRPAKQKNLVYDSRRHPLPLVSLI
DNEQVITDIVKYLVDSFSTSLGSSAVNLHQLSAVSLGSENATACRYHSTSNRTKGTDGEGMVNTDGKSAGE
ERSYLDCENVDRQEPDEQTEEACPIAGQFSEQSSDCYQESESGLGTRSHRFGAFVYETLPHAVGPSHQLHQ
LPIENYCCSVGNTSVDPPKADDEGEQADTNNSGQLLVTYGDDTRTQSACDADTISRTSGRYSASSVSVDVD
ECSG
>tr|C9ZSD0|C9ZSD0_TRYB9 | DeepTMHMM Topology Prediction - Predicted
Type: TM
MKETTFLINSQSQLQKKKRRKDDYCQFKLQKLQISQKMHNGNVTASKRENLFVVFLKPFLFFFNCMFVSLC
VRRRRNDNTLTFTFTWPYMTGTIFFFLFSILFLFYFVLFLFFFIFVSLFRFYKQL
>tr|C9ZM38|C9ZM38_TRYB9 | DeepTMHMM Topology Prediction - Predicted
Type: TM
MFRVSRRMLTGGFEGHKPGMENKSLKDKAKGELIKMLKIQLVLVPLVVGWVMIMYPQPSPEEEKRLRAEYE
KNAGWKT
>tr|C9ZKL0|C9ZKL0_TRYB9 | DeepTMHMM Topology Prediction - Predicted
Type: TM
MWTSKNSLAIRILLVTMLCSALCCVALFDSFFFLNASSEVRVRFADIVDADVIVVMWRYSILFPCIRAQDC
YMFTAQVMLSSFSSPAFAPFFYFYCLTQETSGRKPKAGSGAEKKKSRSFSREILSLTPLSLSLGLSRTYYI
SLRYV
>tr|C9ZRU6|C9ZRU6_TRYB9 | DeepTMHMM Topology Prediction - Predicted
Type: TM
MRRHVSLVWLNAATGWRLHTATTSEGISEETREQLLEEEEFRAMDASQEDSEMLPIHHRLATKADALEAEL
KRLSFVLFEKPQLVVDDCPSTWTRRPANVVKIIRCYGATNACAKGRHQRPVGWVPPVGSSSTIVQTFNRMR
HALLNMRKRKLGQPETALNDQLATLSADLDYLNRMKRLHALNFYGRMNLTPRRHIWLALYTLLWNAVIAIQ
NSLACVFFGTLRGIRDHGVVMGAIRGPATGFLRASQFLAYGLVLSPLIHVPSGLINSMYGVWNALSGKLFF
EAGSGRWHYCSALTALWLHREVSLERRAIRSVGRLEFRRKNMRAENRWKDRLASMGFSFDRINEKFGGNHQ
KAQHTARGRAENIENPYEVLQVKRNATLEQVKAQYKRLAKVFHPDTVQCGSEEERRKAREKFESISQAYQI

LSNPEKRRSYDLGGAQALRLHESKMGRFMARTPEEVVQSVFGGEIFKQKVLGQLLRSHWHLRNEAQVSVSL
HEFEQLQVLRYFELTLELVRIVDVHAMAPVSKGCKHRSGQPQSEVADALIDELSGRKKNQPARERKQTKAQ
HKNQGDTPNNEGTGSPYELTCFTNEFNCFSRDFEDRCDRYTRHLAEACFGRELMYEVGQSYVISSQRFLGI
LPFYAPKLHVYKKIFSGVDRVYAAFREKVDDRAKDNPEWLARKVMTEYFSMEFDSVVADASCVLRFAAQNV
LQDVAITEEQRRRRCYALWYLGDQMMRKGVPWSRTEVKRDDGELMAYIQQAANSAASTSKPGSF
>tr|C9ZS31|C9ZS31_TRYB9 | DeepTMHMM Topology Prediction - Predicted
Type: TM
MGQYSKPKNEIRETEGEGEAKKFSLRIQLLQCSLLLQLFIFIFKHANQYNIQILVLLLMSLLYLSLGTYFR
VVINSGQWGPFKSGAGSTTQREKVIIMTLSFSLKINKLYCIGGVHKHS
>tr|C9ZS30|C9ZS30_TRYB9 | DeepTMHMM Topology Prediction - Predicted
Type: TM
MDAINESTLMSRAAPNAPKGISVLNKSVHESMHFPPAWVSLSVGHLDDAYSLSSKNNDVLCLAASLVMRGD
TERALKMMEALIISSCVVVHERLKGLLSDDCSSLKRMSQVLLSHVRGRAALAAALGASIDPTSEECQQAAM
GACEIVDWDVIHRWFIIALLLHDGDYKASDIVWFHHFFALPPMTTLDGSPKSAASARKRDADTLSAGFLGE
GPTAMWAPMDASQMKDILLSHCGKDEVLLISTICAWKTRRYRSVTTSATEFLALAGGDQVILDDYMSIMVR
FVLCMSLIELGERTLAGKEVAILMQHKEPFVAMVGCSIATFLLPLPECIGRITSFNGPIPRRRYLIALCEY
IHALTLLQLGSMEAAIRVIREALPYSSSHNVGEWLLDILCIACTAIEDSKTISEWPVSVHRQLYLALAPAF
FGGFNMHELKVNMKSPLGRMVYPRRTALCASIKQMLPVYMVRANQKFAKGEYVEAWENVTLAVACAEEIIG
SVEFAFTDCSPMLVYAFGCRVGEHVIGELMSSSTSEEFSTCSVAGDTAALVQKSDMLGESVIQLCLQWARR
IREFHPNARLGALTMSKCTTFAHTDNFLSRAMDVAHRYPGSALAQNCLTLALYGNHNIPEAADSAANALQT
FPHTVEVKALYGYMLKKDGLYYYNYRGLIPIHCSPCNDYKWAKRNITTLILLLINVVVILVTLSLNLPNVF
SLSETAREIAVRVQLPTLIPISYTLVVFTYAITASFTKNNLTHTILQDLFFDNGPLNRFVFAMRGIAFVNA
SNALLITIAGNNFLFTSHWYTFLFYLVLFIVFVPFTSHLWLLPSSDQPREAMWTWLALVSIDVAATAFLLV
PHVILFVIEPLMFALFFLQGSTYRPTQDNVSGNVKKRLVIHTAYRNVMPSRYAVTTGSGFIHLSLLKLLFY
KTHSSLSTKYLIRAQTDEENYRVFPLIEVFEPISPAPIELDEGTKYMLKSYLHYPGAATTKVERKDGTAEG
GQQGLESDSSEEEGYDDPDWGDQAPLTRSFAGGNTGGESPAVGEVIDQEALNSFLNVGVTVLSEFGKRQGK
RKSNHDMPDNPVELSDIRSQRRDSVPGGERRSRRPSAVFRQGFSSPRDGCGDGRPNRSKSERRASFAFGDS
CAASPRGADKHNRGTNDDENRYLGGDCSVAGSGGASAAQTKYSSGNTTSTFRDPVVASSSSIGSSKCTSVA
TGDAKGGNLAEKPSALPKSKDKRDKKSKGNASDAPPEELQLKKSLEQLCEVCRSVSFTSLSVSDKKNLEGA
KTNVHQSLSSLCKGVEVGPIRLKSEGCCEAVCDALFHLIAEGDVTNPLEGVRGPLAETLLQRVELSCLANL
LVAKEIPLQCVLHGRTNILNIVMVPSCVEGMCADRWSTILTALQTDARRERVESLRLLFKGLKYVKGKDCL
QSIKPMLQTAVTAVLIAESAHVAGEKLFALLETIKKEGRIPIDFWEEEDSRNRTLFSHACASGNVELVDAL
WCTGFVKCPNKVQGDGTNGLMQAAINNQRRVVQWFCDHAMDSTDKSFQHIHPVYGDALNIAKEVDADLHAL
IEAKVEELKTYQWASTTQKR
>tr|C9ZKL3|C9ZKL3_TRYB9 | DeepTMHMM Topology Prediction - Predicted
Type: TM
MESAVIVTIIIMMMKIKITITITTSISNSTVTFVYICCYLFSDIIMLPLLSLLLLLIFAITITVSFYQIIF
YYFFPICFDTHYFSFFFILLLLLLVVVFLFFSFFFPSPNCFLTSLPLHPLFCIYKRMIKYIYIYTYINICI
IRI
>tr|C9ZY34|C9ZY34_TRYB9 | DeepTMHMM Topology Prediction - Predicted
Type: TM
MKEKKGGGNLFCFYFYWSVSVFVYLCVCLLVVSSCFVAVVVAMRVRNNDDVAVDVGMVMSLLYFCFTFPSL
NCNSYLFCFFVVSSCCCHNVCSPLLSFFFCCVFVCLCLWVVSFLHSIINNGKYDMN
>tr|D0A379|D0A379_TRYB9 | DeepTMHMM Topology Prediction - Predicted
Type: TM
MSTTHMKRGSTSFLQVSSQKSGRFCQRRSLHSSTTDSHFPVFNEERQAIHLHSKKRSAHFLRYNFFKGYFV
ERYYFPLPVPSIFITYINIYIPTVLTSGTIHGIRSLFIAYYSLTY
>tr|C9ZPF3|C9ZPF3_TRYB9 | DeepTMHMM Topology Prediction - Predicted
Type: TM
MITDRHPVGALLLTLRSLSPRLRFSFFFLCPITPLSCIFLNGKNRVFLGFHRRLLIQGNSLEKQFVQVMLR
CSWFLHSGFRIPTGEEIDGQILVNRFSRRRGKGGWIPRAITATRLQGWVAIAGILVGAAVFIGPWFIDEYR
EMLGYKFVPEPPSSMPRMSTQWWENEWRKGNPLWRAGESTVDFYTGAFDFVRNVTGRTLYDATAFKLLQKS
SQKPLRPRALVPLCGDSPIIKELAVRGFEVDAIDASETAMRSCVERTERALPQDAYDRIHLHWKDVFSPEL
WNGSLKSVKFDFIYERQGMTSLNRDQREDYALLLKQALADDGVMYVEGIFRTGRVKGNKIRGPPYSLSRRE
LQQLFPADDGYVVQCEETNDAMTKLSREDKVLQRVPKELYVTPFHCAIFRLRSLNPGGVST
>tr|D0A7R3|D0A7R3_TRYB9 | DeepTMHMM Topology Prediction - Predicted
Type: TM

MISSSQGGPKPRGGAKRGVAELRGIAVATATNAQLRVLSYNFNILPRGCGGFQRERISSFLETVDQYDVIM
LQEVYAASVLPYFLQNRMCFQKMLVDELVLRGFQHYAISKQPSYTTMLRNNVFSDNGLIIASRFPIGQRGS
YTFRSHERAVQSVRRGCLFAEVKVPLTSGGEESIIFFNVHLRQEDSDDVTSEHVKETRQFAASVIRNMCSN
PEDVAQIPFVLAGDFDVNGINLHNVGQPTKKYEDLLGELQALGSGVREAVFDAQHRHPPTRPTELFFPTQS
KLVRNSFSPQRQDYFFVSHTVAVKNPDIHKFVSDSQQPYTYLSDHFGVSAVLAIPQTSKQRHKRWMPLTES
NAPESEETVNEHSNPIFSIIVEIIVLCTVSWAAFCFSWVALLFSLLVGVLVWYCLSPIHELRSERKFTRVG
NSNAHGGEGCVSDLPREYESLKHANSVGEMWRRAVSLHSAQRCLGQKNDAGVPEWLTFVSVDARAQELASG
LAALGVGPGDVIGVDCDASVDSTVLELACATYGIATLALVGKGSTIRNLIDEYDIKVVFAARNAVGAILTC
RSRSLETLVCMHSSHDSTDCMVARDVCITLISYNEIFSKGRSQPVLLRPVCDDTTLYTMVVDPSTSNGNLK
VVRVTHADALRAIRTLVGTAVLPNTQHKHLLVHYTPFAMLFNRLFVLGLFAHGSAVATTPVAACARAFATI
QPTIVVATPSLFSTSAVQLRRRNERYWPIFSWIFEQIYHLRLFLINTHNRDSLILRTIFFRGTQHLFGGNV
EKIVVCSSEESLSDTLAEHIVVCYTPCLREVFFLPSEGVFCVDGVPAPNIHVHLEPFDEPSKEAKIGSLVL
SCEGRKERTLPIAAMWTGTRTLRLIGPPDGVLLPVRSEYVLAASLERVFSQSRYVNDVFLYAEPSRPIIAI
ISPNRDTVDFEWRQSREGEVAGEDNLLSNWAKFASFASDLLTADFQVIAKRNALHESNVPSYVHIHPHAFK
KHDSFLTPYGGIRRNSLKSYFKVVIEGFYNDAAPTALPTPGQIIESDEEGQAYERDQKPFSLNTPISIDVG
GTFAKLVYVQPPGDFKVPHYVVKEAAVLAEGFNVRMLDLLDNAEGMKRLLNDDPFSTVGTLQFAKMSSKCI
PDFMSYIVESQMLSYYTKEYRNTLRVTGGGAFKYAALAKKMDLNFSVMREMSAVVHGLGVVIGRAPETIFT
VDPATGERHPHRLKSPPGEPFSPYPCLLVNIGSGISIIKCLGPDGSHVRIGGSPMGGATFWGLVRTMTAVT
SWEEITETMRLDGPGDNRNVDLLVGDIYGYNAKDLPPMLSVDTVASTFGKLGTERFYESQGNVDRLYASSS
EDLSGAVSSSPDSNPTLRDAVAPTLASHGKTSEIDIVRSLLNMISSNVTQLAYLHSRVQDVHNIFFAGGFV
RNNPIVWSHISSFMAYWSKGECHAHFLGHDSHLGALGAATNTEEHVS
>tr|D0A939|D0A939_TRYB9 | DeepTMHMM Topology Prediction - Predicted
Type: TM
MDFKRQLASALVLNVVLKVFTFLLTAGVTRQLAPNENGVNFSFQLYFNTVLFLARESVRSVNARHNLREKS
GSGGAALKVMNCAAISLPLGLLVVLVLELLHGFRITLFPSLAALANVGSVSAASAEAQGPGMDGGTLGLPE
VVQVISVIAALSIEPCLAVAQSLDNVRTVVTSEFWALLARLTATISILWWYGSLSGHPWITRMCFSVANLS
DALATVAYFLCLWNAPNQERGRKAAGGGGCEGDEDGEAATSMYQVRVIAARVLWGDTVQTTTSARSYPLRE
CLPWCYLSLHSMHDVLLREFRLFLQFFRESCLRLLLTEGEHFALAAMGSAAAVGQYSVVTNLGSLIVRLVF
RVWETACFARWSRDIAAGRMADASVLLFVMLRVSLYFGAVAILLGPPLAELVLLRLFTRRWATAETVRALQ
LYCYQLPLMGWYGLLDAFVRATASPRVLRLAQQVLVVQAAVYVAFCFAALRLHWVGDPVAGLIVANGISTG
LRCATSLWMIIKTPGRPQQESRRFEVQLRDFSAVFDSRIATVWFLLFGCTRMLLPFISLTTSGVVSVVLFF
PLFAASVLRWDPETRVVVKALVLSSKRSGE
>tr|C9ZT93|C9ZT93_TRYB9 | DeepTMHMM Topology Prediction - Predicted
Type: TM
MKPPISSFSQLHFTSFHISFFISPHRNHFPFHAFNYLFIYFISTPTATRVAIVTITDIIIYQYYLQFPSPM
PSFIGFKARHFLVLPSMATFSSFPFPFLSSCSSSSLSLGFTHLFFIFKHLICHSDSSTLFSTLLPYMLNDD
MSSFQNKTNK
>tr|D0A4E8|D0A4E8_TRYB9 | DeepTMHMM Topology Prediction - Predicted
Type: TM
MNCISVDAVTSLQKEESSWREGHFLHVPLRIFYVCGYAIFFFFFLYLFVHCLYGCNNNSTLCSFSTACYLI
LPLFSSPPHCMLGTRLSFTFVAVLLGAIHSDAQETGR
>tr|D0A1E1|D0A1E1_TRYB9 | DeepTMHMM Topology Prediction - Predicted
Type: TM
MVMITPADSAYVIIFRCDDLVAYIYIYIYVCVCRYKCRCEHGCRGAHLAFKFLLYPFTVRCCSRSGWGKVC
YITLHWLCVSELFCLTHFSPSTFHLSDTHVCVCLRCFTFPVKW
>tr|C9ZUK1|C9ZUK1_TRYB9 | DeepTMHMM Topology Prediction - Predicted
Type: TM
MCVLQLPFFAFLSFPLLFSLSYGLTTFTVRCFFTFLSTSFPILLLFLIAAPFFFFKAFFPSYHCCRYCTTV
ILAITIENLCSSYVPFFSPPSLCAPHPCEC
>tr|C9ZTU5|C9ZTU5_TRYB9 | DeepTMHMM Topology Prediction - Predicted
Type: TM
MSFHSYYYYYYHHQYYSLPFFFLLSFLLSLISNDFTNNKHKHKTHTENKTKKKESSQSHHLSSPRVSSWTF
LNLSGGLMLSHPTFALVPLFFSFSFFIFLSLKKANHTQSAKRTRKLYSASFLPLHHFTSLSPFLPLPLRFK
SC
>tr|D0A0K3|D0A0K3_TRYB9 | DeepTMHMM Topology Prediction - Predicted
Type: TM
MFSRSFLFCFDLLSFVLFCLSWHGSNFLNSLTWLFGWITAVGVVDVVVVVFTTNNNNNNNVVVVFLVYISI
GISTAVPPLVYYSPFLWLFFCFVFFSAGGRKREPTKKL

>tr|C9ZJG0|C9ZJG0_TRYB9 | DeepTMHMM Topology Prediction - Predicted Type: TM
MHPSVRSFIHPCTHSHILTQNVCTNIYIYIYMYKHLHIFTNALIYTYIRRHMHYIIHKCMCTCAHKSESTV
SATKTFVLLFFFNFFFCCSIKAIIITIITIVNAIAIPLLQHNRPVIDTTRTITGVLISHLLSINFFSTVIH
HHLFS
>tr|C9ZSS9|C9ZSS9_TRYB9 | DeepTMHMM Topology Prediction - Predicted Type: TM
MLRRAAPAGGIIARWGLSNCNVSIRHGRHGLTRHDFSNVFSRSLTEEEMKALQQQQEYRPVSAVVPGKLFM
RHWIAGEQATESVVDRVISGFVFVCFMLWGCGFATLGFNGNSCAHTAVLVFIAYWLYLQTHCRLLISALAT
LAVLHLIVN
>tr|C9ZV63|C9ZV63_TRYB9 | DeepTMHMM Topology Prediction - Predicted Type: TM
MRGNVMNLYPPLEAPVLAPCRGVCFGRCCFFFVAGVALHYPSHCSLYSDIPSYVGLEHLRKRYDRIHIASF
LMFYSFRYLVTFKGKNTFDFTDARLTPLLTVGRSNRATGFLTNRGVYPGAVMLQKMVSTRRIIAIQNRSAV
R
>tr|C9ZNA1|C9ZNA1_TRYB9 | DeepTMHMM Topology Prediction - Predicted Type: TM
MASSFLSCGWSTKFSCCSYAISPHLLAVIQDSLKATRSRSSVKVSHTRSSSQRNMGRTCFMVFPPVILHSP
ATNTFALVYALRLVMIVVSLCTVWRCSLPVANLQ
>tr|D0A478|D0A478_TRYB9 | DeepTMHMM Topology Prediction - Predicted Type: TM
MLAQRGYVGRVTHLPFDRPYLVPFTFGWGSHHSTHPALRSEIFLFRNLWLPPSLPYILLPRVGLGILLVVF
SVFWMAAVSQHRPFPVARHSAVLDPSIFSRVTVAPSFSWRGCAPQGTIWPPDGALFWHQRSMAKSVSELPL
LTNCLTNGEWLSLFRFHGCLLLPALRVGRFIRLPFLK
>tr|C9ZMD4|C9ZMD4_TRYB9 | DeepTMHMM Topology Prediction - Predicted Type: TM
MSNEGADSIVTDSCSREVPTAMEVRNEPIGCCDAAVDPKSQEQREGTGFLARMSTFVATAIPPGGIAASAF
NIASSTVGAGIVGLPSAANSSGLVMAIVYLIIITVMTIFSIYALGVAADKTKTHDFEGVAKVLFGAKGSYL
VAATRAFHGFSACVAYVISVGDILSAILKGTDAPDFLKEKWGNRLLTFIMWLCFMLPLAIPREVNSLRYVS
TFAVSFIVYLVIVIVVHSCMNGLPENIKNVSVGRNDVAAIVLFNSGNKAIEGLGVFIFAYVSQITAYEVYM
DMEDRSVRKFIVATSIAMATCSVLYAMTAFFGYLDFGRDVTGSVLLMYDPVKEPAIMVGFIGLLVKLFASY
ALIGMACRNALYSIIGWDAEKVIFWKHCVAVVTLSVIMLLCGLFIPNINTVLGLAGSISGGLLGFIFPALL
LLYAGGFTWQKVGPFHYIATYTLLLSGVLAIVFGTGATIHGVVVGN
>tr|D0A2P6|D0A2P6_TRYB9 | DeepTMHMM Topology Prediction - Predicted Type: TM
MYTFLLIVPKMKTKKHGQVKKKKKRKKRKTYIHLHLYNLSLRPHHRTWCAHQLLCIHLSLPLTYTRTFIFI
FIYSFYLFIYLFFFGFFFCFRLFFFLFTPFFFPSQLLMIMTFFFFFAFSLVFISLPFL
>tr|D0A5C8|D0A5C8_TRYB9 | DeepTMHMM Topology Prediction - Predicted Type: TM
MEIISVAEEYRYISEWLGCKVYSDLLEGLEKSPNELNLSRYVYSSKFFDCVIHFLERRSEIEVLDVSGMQV
PTDQLVRLFTVLRSSSVTRLVLCNVHLDSTSGAALKDLAVACAGLHVVDLSGTGLSSEVEYAIHLQTEINR
MQWEVNQTRWRKAVVPETAKIWRWRMESWNEDCFMGACHCSTEVCDASCRDIMNNAATSGAPFTDNSFPPD
TALKNSDIKWVRVSSLVEDQRDQNECCVGDKPLVASVINDTSNLRAALSVVQQVSYLLRHLCIWKLPCIGA
FAFRFFSGDTPIEVVIDDFIPMIGDRPAGIHHESNSGDYWGCLVEKAFAKLHGGYKNITGVGLGYALSCLT
GGICFELQMKNLKKHVSVADIFRFLKDCVRKRRIVAFHANPEIERAFRCLEEIGIIPFTPYLLTAVEAPNA
DSGYTCVVRFSSLNDKPIEYIGATPHLVKDGIVEFPSNSMHLEHAISYFERIGLLLWPHGDPLYIHRHVIE
YPCCCLGGSDTVSTFASNPSFLLSNNSNSEKEVVLVVRASSRDNEMHDEWTHLHIFKYSADGLGGMRRCDV
CPRNELLSTKKAKGGEVGLVVLLKGNESLQVTVSSSAPFPCTLGATCLDCFDFVPLSLPELKTIKDWWRKG
SELGPSFVIKNKTDITVRNFVVAISQIPNHKRTVGVGLEIRSACFLCPGGDDLLWLTEFRSDILVVFNLDV
TMKPQESYRLVPRARCVHEDVQFSLTLFCVVPLEWE
>tr|D0A6N9|D0A6N9_TRYB9 | DeepTMHMM Topology Prediction - Predicted Type: TM
MNDRPHFYEAPDLMDEHGAPIVFDDVYADLLSVAVEESADDASRLICGLEAVRPVDNEIHSVADEGTESEG
RHALDVLLYEYEHIRNAMKRCVRASHEVLRNLQQKRHRIEQQERTVRRRTELASSSTSFPIFKLNVGGMRF
HVQRETFLQFDVTLFHVLCNEQFAVQKDECGYVFFDRDPWLFRELLFLLRERRQCLVENEVASPLVPPQDQ
QGDMQHHRHQRRPQELYSVGQRRLAELPPIERQRLLEEARYYGLNELVAELLTRQYEWRHCVFGPLPSLDL
AHGCDQVQPPLLASLREFESQRLEEGGHTPLPPPRCCFASSVFLNGSMYLFGGFGSEGEVIGTLYRFQLKP
GSMCEAGAEIEGNDSGNCREPHGANSDAKGRHTRANACVPGRPFVWYDLVEPHREGRRGVPPPRTGHAAAS

WGNCFVLVFYGNNLYHHLRDVWMYHTIQNAWYEVKVCGADVPARSGHTVTVMQGRFYLFGGKDLFQRDARC
FADVYEGLFDAEGMELTWRLASSSGEVQQTDRLRQPLAACDENCGEPCDAPSAAYHSAVEYKGRYIIVYGG
LRDSEGITNGGNPGGLRGNMGVGTAPAAYDTNLRLYVFDTLGGTWQRVQTRCAESKDQMGSDIPLTGHVAV
LCHDDMYVMGSYDCTHHQRLEVFRLSLVTLLWCRVPTSVAVGHIPPCSRALPSIVLLPPTVEAPRSVIMVF
GGYNTNTCQYLNDSYLISL
>tr|D0A953|D0A953_TRYB9 | DeepTMHMM Topology Prediction - Predicted
Type: TM
MVMRGVGIKRYEAREGKLVALIFGYSFPSFLPSLLFSLPPFLFLLSVFSSLILLFYCYVGWGDCIGEKEEK
GGSKLSKMCHNILFFFFLWVQRYPDGSNYGKKERAVAWYPARCFPLMFEVVVSRWREIKA
>tr|C9ZLT3|C9ZLT3_TRYB9 | DeepTMHMM Topology Prediction - Predicted
Type: TM
MVCCHFFTRTSHLLLCFATGCLFSSIFDYFLLVHSLFFLLYLTALPFFLLHLVANYFVSQGDSFMEKENKS
ICWRPKNALIECIVSTKCFEEKQSVEDCVAASECFMERRNWTLCKVNAVNPRYRLRGNPYDVATEDQKKIE
ARNERIKRRELEEEGIVS
>tr|D0A001|D0A001_TRYB9 | DeepTMHMM Topology Prediction - Predicted
Type: TM
MDDPCSAPLPTSTEIVRRECFPRIPPEILLREHAARYHLMGLGLLSLLEALKPQLGQVFPSFGDCDGSAMR
DVVGNRANGLLERKPPLPQTNIKRDKSPSNRLEEKGAGCRLAYGVHTVPSNDAYCSQVNRSGCVFSEVDSS
RLTAVELVRRFVQKRTGKAGKGNPGPLPQRGDNEATQEDREALWGYSTGAVVHVTFYPFASHLRAMRQSAG
ESKRVRRRGNSASETASSNKGGDDDDDDDDGEQRPVEGSEHGSSDRGAEGDADEAELLCLYEQAPRGVSLL
GYHGSCSVTSLVYHPINDLAVSGGVDGSLLVWDVHNRYRQVLRDKKNCDEDNRETRTLQHFTHHVQTRKLV
QTIGHVHNGSVTSLEVYGDLLLSGGMDGTVRVWQNSEKTVFGSASGLPQYLGYQVFHCNGWVRHICYAGDR
VSHGGDIMICSEDGVISYLKSGEVSPRGNVGLFQGSSRWGKPLLEALRNQRAVAARAEVRKAQRVQLVREG
ESCSTRQINSEFSSSLFSGNLAKEGGGKAPISVSHRGDSPSRLQLTRTMRTISEESRLARSIELSGVKGSE
STNSITRMFPMHRHNILAVVGYSPSVRFLDISRLKLTSMVVHPSLTALSKVGPHASGLSTRDKKQHRPSSG
GSTQLKSHKLISSASHAQVRGCGEALRFLDLLYINALDYMILLDNRNTVFVWDNTSNKMVASYKVPDVSDG
GRNNVAFHLLPSGTRYYEGEHPTGENGGQGFSDASESPRSRSTSHYQAASVQRQRQQPGVTRIPFFVACTM
GLELYDLVISVYAELEFKPHSDRVVGIFAVQQPSGTSSRYTLSPSPINTNGIFDDILKKPPSPSDHMELEE
SSISERLMRPHTAAPNCLGKFKFTCSSSDNEPAELFTEEELMCQVHEVDEHYQPRVLTCSGDGIIHVWGSY
FHPICSYNNESLKKDFAELRVSVVNSAQGFEHTTPMVERMSVEPGKRTANSAEVEKLGRYYDTACFYYNLR
WNTAVTGHDDGSIRYWHCGQQLPKCAWFKGLHQNTVSGVVGARISRWCVQQALSSQGMRDDDVARLGCIDV
LATVSYDGHLKLWGGANEAKAVPYLSTKVSNNELLCVDFSEVGQYFVVGDTVGTISTWSAHKLEPRFSIPS
EPPAPWVPRFAKLSEKTVRRFSARKVNREEPEPVIRVKGIYHTDGVTALLVDGNFVFSGGEDGRVFLWDLV
NGILVREYYLNSVTERDVTRQTLLAGGVPSAARNNSGLFLSKEVNNSFGGTKRRNEKGYVVNISLLKERNG
DLLISTSEGWIYHFRQSCSHPLQMYKHSFRIASVCVLFEGYEQENSGFPNGRNSIGFDGSDGSVFELAVGA
DDGHLVVLHETKFSKLGYASD
>tr|D0A1N0|D0A1N0_TRYB9 | DeepTMHMM Topology Prediction - Predicted
Type: TM
MKKIKKLLGAPLFSRYSSQQHSFKSTNPAVLRFSAVIYPQIFYHDALRLLITTPHYVSFPVFVSAYYLPPL
RVVVFWLLLLLLSIHFLFRSYSVLIVIVFSLH
>tr|C9ZX61|C9ZX61_TRYB9 | DeepTMHMM Topology Prediction - Predicted
Type: TM
MMKSIPLNFVSFFFRYFPRYHQQTHAVISNDFVSYKELSHGSGLLPMFSWKDVLSTVIYSTPQFLLRQMSS
VLLGLVAGYCSILLLFGPLPFAYGILQLTATVLIAYALAVATRYGVRWGTCSVLALMDGGVWRVSFRRKNN
TGSARSGETQLD
>tr|C9ZXP8|C9ZXP8_TRYB9 | DeepTMHMM Topology Prediction - Predicted
Type: TM
MLISAADVSSAALREEIQRRKDFFALLNVVAHRMYTPTTVEWRLIDHCLGEINYRFADFSFLVGFLLVMPI
CRHKRTAFSRYVLPFYAGLVAYDNALRLTNPCPAVTFWNSVVLLDGHLGETARQIYAPKCFYKVENGHRQT
GAPVRYATFLHLLWDTVSVTAGSLLLQTLLRYTFNRSSWMHESGGSTVTSLVISTRLFIWNVFALKSEDTS
VGREYTLRLSLPHFIVSPGLVHSYRSRHLILSRSSFGSHMWYLAHFPWVYCLSSKGRSQGE
>tr|D0A9B1|D0A9B1_TRYB9 | DeepTMHMM Topology Prediction - Predicted
Type: TM
MSAAGIARHGGLRCSRALVQPSAPMAVLVRGCCCWYALRFFAYSPYDSIRKPPKGKGGSRASCNPIKVAGM
SVTNSNCGFPDRACGESPTPWKFPETLKSGRYKDERQRRRRYEGPKVRDSAGPDRRFYRRLKDFREVQDDD
PYAGVFNERDSVKKQRVKEWQRQFEEENADVELPYERTNVLARLAPNWFVRYFVNMRDRGGADSLVHVVIL
AVGLFMTLWIIGRLFYTPPSQARPISELR

>tr|D0A529|D0A529_TRYB9 | DeepTMHMM Topology Prediction - Predicted Type: TM
MRDDLLVVFILLGVSCVVVCVQGENKVRLRKVEGMENLTAPGERHEVYHLFSETSREFFLRWLCMCVWVDF
LKCICTPFDCIFQKSMCAGGLHTNRAVVTVCMKRGS
>tr|D0A0H3|D0A0H3_TRYB9 | DeepTMHMM Topology Prediction - Predicted Type: TM
MTSLRFFLYPFQNPPYIYIYIYIYAYILIYIHTYTYYHHHRFRCYYYHYIYIYVCMCFFLFCCSKKKKKVG
CKTHTKTKKKGKKKGKEGRKYHRSEEKKKKRGKRTTTNVKN
>tr|C9ZW58|C9ZW58_TRYB9 | DeepTMHMM Topology Prediction - Predicted Type: TM
MEPGPESTGFDTSKFLQFLFATLFLFVCVMVLIMYQHKLLSFAVQTVLRYCFLPSTSRVHLGGLLLSPPGC
RIFFQNFFYQDENMTLRVTDGYISFVFWDSWFGDPSWAKVRDFMMKDDLKGTQCLAKVKTKWVRGVIVDVE
RSKRKLLVRLESGEPVNRESDPAPLVDDERFNNLDWGSELHEMADVDGASNSVVGHNGDENTSHRSSYNLE
HSRESHRTVPTRQREVNVSCASIPSTSARMFYAPDCRNSDANSKGGGGDGNYSSVTAVHVEPEVGINGEPP
ESVKLLDMSLVRIQKKCGRVRVHLNGMELTFYNATGNYRKSETVERSEQQRKPRHNDSVFKSLWGIFRPLC
KSENDSTAPRDPMEGREQRQHEVLESEEEMSSRVEDTGVEDVFDGARTRATVEQHRRKNTKSIMQRFYDFV
GAVEVELFTSYVNIGGAAAEHPYFLRVGFKRADGLSYLAQEGVPSVDICRLVTEATLEELCVRWFPMKSTE
EQHEPRSVWDKWRDSFFLYCEGVPPEQKPEPLLDEPAVLIKDYGTIIGNKEESSAHLVYYRDFPNVYAGEE
VSCQSDLPKSGIELLIDTPDLCHGPWSNFSIMQLKEYFVPNTYQSVESLKFELGKKRPHGCFEIYIELLRE
TSLIIPFKRKTIAPTPPFGLCDSGRKGMVAVTLGRGSSYVQKLKNPLPHEEKQCTESLLTGLDVLVATNAI
QEESAVCMSMDKIELFVDKLSDKPYNGRKLWNILPVMENAEVWWHMDYISFFGDLCLDWLFVPHMYHGKPF
TTEEYNTYKLFVEEFIPTVRRFECIFAGTFKIHLNGNMHNAIYSENINDKTANVFLTLSTKTGGYMFVTVP
SDSYLLSSVTEVRRPFEISVSGIELFLSVPHSHPLHELVDVSSPFGCVDKLFVKGLHTVNVPNQTVPQDMD
PIDPETGQSKLRNYLDYEFDITGVRGHLMAPHIATFTELFENLFGGSSCVVRPDELFWPLSKIHPKINTPK
NVRSAFHTYLQKDQPPTNSLDFNTTVKFTDIDVTLRTGEAKSPQVNLRFSTLDITVMRQASVTDIGLTISP
IYGRLPAPLTGDLADSTFLCCGDVSLGIVRHFGLPPLRTLFYSASECVVDGISFYMTVEQVALLTELFTTL
RKQFFKSDEGLLAEIEKARTVAAYSADNCVLTTPAQGGRRRVPTSGRPPACFRRVRTSSLLPRPRGEENAR
CKAARLTLIRFLTHGGPFLKIPFSELVPAVVASGFQEFLEQLDEAEDSAKDYAIHTILVSIPSVQGCVAVG
PDNYFEVKFPSGVQLVSTTKNDLNSNVRTTAALRGIELCGYCRSSNHNSQDETSPFAEVFRLETSLQVQRS
VAYPFDKGLEIHRKKQRHFVFEHDTDRFFTSALAHVRRPCMRDESLQDSKGGADDPHLSFLLKGEEEEGDE
RELRSAGVLHKLPVYNQDGKNGGDSVEDCKNEDGPTEATHRQGCEGTAAPPQQLKCSGREAEQSAGNVSTL
TNTRMGVPAGGSSLVESDESDGTSFNKSETNVFATCVSQTDELLSYSDEDDMMKELCESDDVFERGICSTS
HTPDDEFSRRVDHLPDECNDLITNSLDCVSSVSASPRASEGNTTLSRKLITSTHFLRRFSFDCPDSQKDTP
DGCDNTAHNKRVIATDRTHFRAPMPTVGFVPSKRPPTTPTLRMLQRTVDACNGSVGGEGESAFWSAEGAAM
WAKRTADNRKRSQEGTTSKQVHVEFLAPLNCLVAQDFVEKLLFDANTRLQSVFATHNLYSSLDGGETADTA
AAPMLPVRHMRQTSGLKGMRNLIKSSSTPAEKEMKNLFARKQPSRNRAHQKRWYSEVDIWSVCIPSTRISV
VTDFPLPSDNVVHDARARGVYNTQLCARSAQVVMQQTHPPPRATRQDETKTFSLSTTCSSLAVITQIEHEP
LLRSDNPFVRVPGIDYDRKDTLAVLYFTSIRGRMRHQNTQGIQNGIYHLSVDTIAFHAARDFFFYLHSTLA
LWERLNGAWGAQEYPRWYRGRPSPETWSSHNDDNEAREGSHGAPGSSVAHFEHLHSAVPTTRNYGIEGSRV
FQGVGSIRVVRLELLDVSRDGHYLRVDTRCPNVVEISRLQAKFVVRVPMKRAHDPLSVPSRPLVRQNSSVT
LSSSEVCSGFVPSQEWKRRPPQLSTVQIQFIGEVEHAVARCFPSLLHIMGAVKGSEWVADSPIEIISESKC
SKTPNDEGGSPEVDCGFSSQPRADAANDCNQVTFRGSFALGNIDVCMIQSEMNFLQIKGTGLAGFGEARAE
AINSRECVKTHVLTEAFSERIKRQLRAWADRARTAAPKGHYPTPPSVTPQLRMKNSGFFAAETMRLQYVAD
VVAPCTDGFEVIDYEEVSEKTHRQDPRVFVASTKQLRVDMHHAEQEGPRGSAEGCKTDPADTSWVADSSEK
GEQVNVQVDIGSAVLSLPYRPKASEIVQPQLQQWVHEWLGPSVLQTMYTSQTVLAGGYHLGVRPQSKIYAC
CCSLRVRDTLLQSDLPQNMTSNLTIPLASLFLNASSEGRLALKGHLHPTKLSSSSPTSGRHTMSLPNIFIF
YNSDRLRRSGMCLTETVEVTVSPIIVSHLLFIFNRASTVRSTLTKIFKPEAEQVDPQQKVGNLNLQDNQRR
KSNFLLLVEGLRISYLTSMTNMRFSVWRLKGRMYTTEDDDLCRSHMTLKVANVQVALVDRDDYDQLQTFLH
QSEYLQKRAESSSGRSSKEKLMSSSLPNSPDQERKRSISTLNGFIWGLFETSLTLSTGRSDTNELHSALKS
IAATAPVRTSVSLRSLGNVGNTATRWNVAIFSPLLIARVGLAQLLQQSIDEAKELADKMHRAARRESRKYH
RQLAKSAVYQRLTRLERRESQQMLSTQRQQVERLIALTNNVIPGQSGASQRRASIALSVEPSCEDVVEGSI
FQTTSDHKFFVTMTNFLAVVPFGDAPYRTILEGTTDASHRGCPSDVLLNRKDFVPTMAMKVKVEGTTVVCR
ALMSQQRVPLLPTKSVGALRSDGIGAFLGATDSSTGTRQIFTSKFMLSDAHFYCSDGSPLERGTSARSVLS
STHVFGGIGTLTSPGKGEYRSSLSKVSFNSIEIPLHINRCGRSVSIGSVMDMSAPQISVSTQTASILSQFT
NEMSAKSLSSVFRHREGSADAKTKSAEVIAEPSGIHTASGGPERYRRERGRRTRHTDPESPVTSVLQQAEL
TPILYNFDVTARLEAGEMYVYSIQRGTATNTTASSQQLGSTVNKVNRTSKRQVKFKPIAPGGEAEFPGSPR
VPSGLAAKKLMSVLLKIPLPEVTAMVMGSYGGSMADEERLIARVEIRENTIEIDPSIISLAHEIEEWEVIQ
ARDSAEHVANTLKLVKVWERDEEIKHLARYSTVADVALPLPRAFLSVLSNAELQSKKAVTHQKLGRLGNSH

RPKTTSPPSLQDQEKRLQHGHEDAPKNHSDGKRRLLSVQIRVTEFRLVLTTEPASNISFTLSLDDRVGSVD
FFIKRIQTPSTTWIEDVPVYNPPVVLLLLCVRRLRVECQAKMEVKSVEMYLQEVEAQAALQQHDNALVLTN
VYVHLPRDASSSGKMELTVRAPYVSQMFIFQALWQRSLLESLASINRIFDRGAAIFREKMKANPYIRRRGG
ATKQAERSLVVAVTGSHGNVRVDLGSGNAHLVSVGSMSLALVIAQSSIDQAQKVCIDASVRSIMLRSEGVL
SGVARVDTIYLKGFSLSNSGGSTKVVRSPAGRTFRYLLCAQKLHAVFKERQLRDLLEGQVGEIILNFMDST
GEGVATNVRLDVSLSRSNVVITPSTAPTFVRTFTDWCNIVAEQSAAAKMKLAKLGMETGHTSPEEDKGESG
RLAEQEVGFHASGTRAGRGSSGREGRPPLHDNFNITPSFVAIPEEEEQGQDKVIKSKDGTCSSSNSDAEST
SYIPFMGNKLTRIPCGSIEVVLDRSSILLGNASGGVDKAGCIIATFPHATLSFAECPSEEDTVVKRVLEIN
TNNMELYRPGATKVLILGFHGTNRYEFYTRQKVGSSEVGYVMTLHQVHPWTGNPGLQDFQELIQLVRSFKA
TKKTKPFQRFGEIDDMWNAPEGSMPMTPPGADNRAPTSTAVVEEEGERGGVDADIAISSSSVTGLVATSLS
DMSSRKGKKAADKRYMKPLRNVQFAPQLRFGGAVAVNVDVILNWFGITKNMLPHIIHTKACDVLEGVLCFL
EGVA
>tr|C9ZQL8|C9ZQL8_TRYB9 | DeepTMHMM Topology Prediction - Predicted
Type: TM
MFFPVFLYIITIITTVVDCWHGLLLFFTLFFFLFFFDSSLVHSSLLDLGDWIFFFKFKPLSLCFSFSLSFS
LFLAYVGLSSSFQLFCRCFIFFQFYFGDYVGMKKER
>tr|D0AAP3|D0AAP3_TRYB9 | DeepTMHMM Topology Prediction - Predicted
Type: TM
MSKERIAVKKKKKVRKTIHHTRTLVPPLSLHLLFGPLSSVFFIITINYLFIEISLLPSWSLVEIFLHIERS
SGSGERIKHKRRVASGDEAAIRRAQGSPLRTEAEC
>tr|C9ZSH5|C9ZSH5_TRYB9 | DeepTMHMM Topology Prediction - Predicted
Type: TM
MVHSKALPPFLRVCGSATDCLRCPIPLTRFVHFLVIVVFRNLFLHLRGNSACGSVWIVTAPSFSSPAGVVR
DFPVVRNIFLCGRVTWCRVWGHWYPLTCAFYVCIAAFFFF
>tr|C9ZTC5|C9ZTC5_TRYB9 | DeepTMHMM Topology Prediction - Predicted
Type: TM
MGKLNPALSPLQKKTSTNVYPPVPKNCVVTGGTGFVGTRLVEMLVERGAERVVSFDIVPMESAVSAWQHPV
IEYVVGDITNYNDVLVALEGADCVWHLAAAVGPFHPRELYRKVNYGGTMNVIRACFQLGVKKLVMSSSPST
RFKGGLFHRPCVDGLTEDEMPKLPLDSYMQMYAETKAEAELAVTAASCDDLLTVSVAPHQVYGPRDNLFLP
NMLEAAGTGKLRVFGSGKNRICFTHVDNYAHGLIIAERGLYKGSPILGKFYIVTDGSTHPEPDAYCIFWNE
LDKAVVAMGFVSIHKKIRVSFWLIYVVALTGELVGWMFGFVFKLNVFNVFVLTMHRWFRITAAERDLGYKP
IISFTEGWDDTITWFKMNWLPTFEGRSRSLLGLSHRSQQKIDIQARMTR
>tr|C9ZW76|C9ZW76_TRYB9 | DeepTMHMM Topology Prediction - Predicted
Type: TM
MALSEKACLRIRVTVYTIFLISYLVGAAVFVRKFSNFMAASGFVAASIFIGLAIAMHVMGTEGLQSRIQPR
SLKRVVIMGVLVAADVALLTLSIYTLVVGIKRREKWTGESNFCSCTAMVCGLKWCSVCVYLLYRSGTPSNP
PEFDSLRKGEEDGNGKEGPQTSML
>tr|D0AAD2|D0AAD2_TRYB9 | DeepTMHMM Topology Prediction - Predicted
Type: TM
MKHCSACAGGATLLRATRMTGATFIQARCAHLVRGTNRSLGQHQGTNIPHYRLPLRRALRLIDSTGRHNAS
MVETEDCVSEEQLQHHSHDGEGVSQRQEDAARYAAYHCHHMLTSFRVERQRWGENTGTLSVEDLSRIVEYI
FYASVLLHLTALYQTADKYCEDLIGAVISCSIFVSKNVEEILKSLSTASASGTLMAANKRLAYQGSDMWNS
LHWAAVHYSQGVCYWGLLDHTEPLFDMRRLHAPFRGLFSTYVKLVCKILAHAGMPREDKQLLSYVKSPEGC
NRWFGEEVNGGLAVSTLCFPGSAAGTACSGVHDNHCFPQQRRLQLLPLIWVALLSCHCAVSNEENRFRSAR
HAFLHALPLFSKEAKAENVVMLLARLLYIRPSTVLKANSVDVGMLLARAEQCNVFPPSSSVPSVTYAAFLR
LWMCADVAQEKEPEVRGVHKAYAAFRAIPLCALQLDGEPHSPASGGDAFVRHLLLRALLQWSFRLTAFVSF
RGEVEEILKSTQHVVQLLDHARRRPGWGGRKETIVVDLCEAVLRAFAVRVSVICGMDAPLKKEDYALIAEF
DDVVRMMIRSGEDRSSLCTLPTVYECMRPRRGSRSYAREVEEDSFPSSLTPRNSPV
>tr|C9ZRY0|C9ZRY0_TRYB9 | DeepTMHMM Topology Prediction - Predicted
Type: TM
MYMSFYSLPTHYLFPSATSPANLFFFPLNPSILPVVPFFFLVLLVNFRVIHQHHLLSSCNYSFSGTAPRNV
SKDQKWHERWVCTFSVGKGCGWEECRRIYELGSSL
>tr|C9ZLR9|C9ZLR9_TRYB9 | DeepTMHMM Topology Prediction - Predicted
Type: TM
MCLCPAALIIIIIFIVIIFHFPLLPPWPSFACFPLTRRFRAFVNLLFCLCSSLHQRERDNPTVEMLRRLVP
RVMMAPMGGATALCTSRGYNMLVFRDPKRRPQLSEEERAKVVVNQAEWPEEFKDFDPDDPYKNSPEIIKGM
SSWNLFLWGVECAFIYQFYELVFPKSI

>tr|C9ZPP5|C9ZPP5_TRYB9 | DeepTMHMM Topology Prediction - Predicted Type: TM
MRARNLAAGVTDKGPTKLIVLGVSLSSQLLFSTIFLASGLNTFSAVLVRIAVHSSSLFPSRYMSKLLGGSS
LSRDGGVKEVLMWCKRWREMRNYSNYPLSFFSFLSFFFLPSLSGGMNRLLWLL
>tr|C9ZHZ1|C9ZHZ1_TRYB9 | DeepTMHMM Topology Prediction - Predicted Type: TM
MVLIYIYIFTNAFIFTYVATHFAFLFFLVAREISALLLKVK
>tr|C9ZXI6|C9ZXI6_TRYB9 | DeepTMHMM Topology Prediction - Predicted Type: TM
MGGTSTKESLLYFMDVARGVSPPDEKDFSSHLARCRKELCSGAELYRTLLPIMQTLMPANAQASQVQWVVR
FCLKQVEHASCNYSQGVSDIFLTTVRAVQFVVRHACETSRGDASRLLWTIGEDLGESVHGALRVSTTGDSD
TVFMNRWLSTGIGKLCIALEKFIVFVPLTVSTVEAHGEVVSLLLCMCSTALYHSTACDTNFTDPFTRLILA
SGDLHALVLVLLQRLMDWGEGRLPKTPVLYRCAQYSSLWTLYNMFAGGTENESITCGDHLGRHCAQLLSVL
VSYGKGYRPNEALHYIASLGDSSQIQVKALLVTFNRHVLGCPALCILLYTLLHDNPTFIHTVMTAYPDEFL
GVIQGVLRLSYTASSEAWASGFNSSDDDIDGGQCAIPIVEDAITPEAVASIVRRMTAFSYPFISFMTGTLV
LVCSQDQVINKRMSNTIAETTFKVGRLVGRCPVIAVCIVIIVHSIARALNDGNEALAAVFIPCLANFAPFV
HDIDVYTSQRLVGLLLLTLRKVQRTAEHARSGAGDCLGTLEGTTPAGGCSSADTSIQMFLRQLLALTEAVG
GLLQGDCRNNYSFIYELVYQRSHLEEGAKLLSTVSCHSSEAQTALQSLLSVAKFYDEEIANSTATDSYGGA
LAVIRQVSQNKVVPTGPSSRLSMRTGTYAATTSADVEYCTSLEMGEIPFLYEESTCSYDFFGPFLWSTLLS
DAAYPGGILWATDLSTLGIFPH
>tr|C9ZV49|C9ZV49_TRYB9 | DeepTMHMM Topology Prediction - Predicted Type: TM
MLVIRCSLLLCSPLLRRGTVVKVSAGVLLSLAVAVAVVTFAPLQALIPFCCCSFPVYLGVPYEYIRAVKNS
FNSVDFFTAVRIYVHVYAPSRQRTEATQKEEHLRKETKRKEGSK
>tr|C9ZU15|C9ZU15_TRYB9 | DeepTMHMM Topology Prediction - Predicted Type: TM
MHEYTHTHTHTHTRGKKKTTNKKEGFIFCFWFFRLASTPAYTFFFVVVGLIAHFSFSSFRLSTFFFKFFSS
SEIEVTSLDFDPFQAPERRTAGIEGEVTGGVEKVPKEEEGRKQKSKAKGRERRKGERERNSTPTLKCCVHL
TIIIIIVVVVVVFEGGTNIASEVNIEKIRERRFIYVIEVVSLP
>tr|D0A3X4|D0A3X4_TRYB9 | DeepTMHMM Topology Prediction - Predicted Type: TM
MTFVSLSLPAFPTGTFCMLYFHPCLFLFNFFFKKREEKRCFCRFLLANVVCFRTQWFAGCGMWMWRRSVDF
SFLFFICLFKVSGRILMRICTGGFSSWEL
>tr|D0A3Q9|D0A3Q9_TRYB9 | DeepTMHMM Topology Prediction - Predicted Type: TM
MVIHLCGLIIVPTVSSLITIIIITLLYYVHYMMCCSCCFFFFCLLVVAHTCLPSLYASYTHRVGRSYIGGM
SGWFGSPAPTAAPPPIPLNNPIGVTPQPSVLAVPAPQPVSLSVPKAVPIPQISALQQPLSSVDANLLMPLL
STPPLPLSTLPAVANESRLTAELPPTLSPGRSSEPLTVSLEGCGGVNVAAKAAILPVAALGGSEGQSGEQA
AAVVGGAEQISPALGGVVTDGDTLAACRKSLPSYVVFGGARYNRVDVPPGAVKVLKRSNEKLISVSPRNTA
KYMSHQLQLASAILRLDSLMRPPSSSFDAAARFSAVAPQDAGPLECDTFSLKNIWTIAYAGSVKDLHAFIE
LDGSAVSARGFVVYNRRHYGVKRLSEKFVLGLGQKATPLQYAAIAGHVDSVVLLLCMGAEDDSYPYLRDIL
GDELETTVKGVNAKLRSPRKHHCSRRKGKRGPAPTVAPPPPAAEEDCPAGTQP
>tr|C9ZK39|C9ZK39_TRYB9 | DeepTMHMM Topology Prediction - Predicted Type: TM
MEKEERKKSVAPLVMNMRRECSRKFLPLPPLYLLSESSCTIGEGGKTENWPAYASLGHQLSFLGRTFFNCF
RYYVSPLLSFPILFIAVAKVMPLACHQTAQHFTCFLLTHALVSNLFVLTFLLRAVPLLSFRCVFTPLKALT
LYDRTQTDANPT
>tr|C9ZT15|C9ZT15_TRYB9 | DeepTMHMM Topology Prediction - Predicted Type: TM
MFPYVTDYSGFAIRKWMIDNVDVAGFLCLLYLGLVWKGPGVVKSLREKNLINATLLQGVFIMWNLFLSTFS
VIGMIVVVPAAIAHISNKGLVPALCERDVNMIYDSPVGFWVGVFALSKIPELFDTVLLVLQGKQPPFLHWY
HHTTVLIFSWQSYCEGSSTIFVFVAMNLTVHAVMYFYFAMCASGLKAIMRTIAPVITIMQILQMIVGSAVT
MYSAYVLYNPQPDGPQTCNVTKASARMGVVMYLSYLYLFAALFVESYLKPKKRTEKSK
>tr|D0A7R9|D0A7R9_TRYB9 | DeepTMHMM Topology Prediction - Predicted Type: TM
MTVLVVCVVKHMREWSCISAMLCFCFVLLLLFFPCFILPRPVFSFSSIGSGASLLEARFTPFWHYFYRWYR
SSMFISRIGIIPHTNPQLCIRARIQRIVTLLVSGKGGSNK

>tr|C9ZKK1|C9ZKK1_TRYB9 | DeepTMHMM Topology Prediction - Predicted Type: TM
MSDSHALRRLLYAGAEEGSVAPRGHHHGGSYGTLISSVAVEQPAAVQPRSGLRLSSACLAGCFLGCATMRA
ANRERWWLGLFFMGGLYYNAVKAHKVGNGLVGHQSGFFAAGLGAIGCVGRLLVRVGNPKANRRLLCMFAAL
MWYEVGRYHLWSEHATEFRKEVTPEHGYGLLTEYVPPHIDTDLLPYRSVSRRRD
>tr|D0A4L7|D0A4L7_TRYB9 | DeepTMHMM Topology Prediction - Predicted Type: TM
MGTFMRWYHRYARYLSCSLGGGGFKDCVVLSQRPRVHCEWRAVPQGLFPFLSLALCHVVISRLPHWLRCVY
VCAHPLKRNGLWLVGGRERHHLAFIVIANELRGLGAGFVIHYW
>tr|D0A4T9|D0A4T9_TRYB9 | DeepTMHMM Topology Prediction - Predicted Type: TM
MTLSVDTAIGDTAICMERSCDAPTPTYAVKTRAMVLSGGSGCTLSRCRRLGRSARQRSPSNKRFSGRMSAA
SVTPSGGPVMDRSGTGYVDVCRGGECAPMLCRSGGCDHGVSIFAAAATPAAAVPLLDGTVAAECTNGAGGM
PADGSRCVTQLYDAGASLRNESGRRYRNNYSCHYSNSGGSGYCCCSASYRGQKGHKGGQSQVFHNSPLFMG
NGGNLPPNRGVISNYHYHHHPPSGASHHHHQRCAVRSHRRRGFGAAAVEAIRSRFQAGATMSHHAPPLSAV
KCNRQTKQTASNGDQEPRQAFQVPLPRSKAAAGVTQPSATSCDAGVVRPSKGDFRIEMDTPFSATDSMNDP
VNGSSRLSGIAPLFDLAIQQLILECDRRVANASGTACASAHSPSNSCNSQKQSSAHFSVNTHSFDMPTASH
TKTTFAQTPKESSFATFAVEQAPLERGRLESPHRDAEAEVMARDGACTTKLHRVEPRLEERVTARRENHVG
NVVGRLFRDQSEDAVDLGSDCSKLQLLIVSVGLTVSFGESAPLVLIGTLIYLFRIVKRCDSEYSSVTAANW
YRLTAVALLVATKMYVDGSGSWNECFSNATDIPLKELNKLEIDFLFLLDFDALITEEEVEARADWMDSVAS
RHDMMTPLRTFVLGNSCAPSCVATPLSPAADEAFRSVPLGSSSNMPSTPLSLTPSNMTPVNCFSTPFTQSR
PIRNQCVGSLVSGVDASDKQQQQTRPSSMRRLSSSLCPCDRMSPLPLSVSSLSLTERLFSVVHAPAEPETP
PSLRRFARQDDEPVSPLTPPDQERMSPVFFFCNATRGRRCNAMAAVGEKTNKTSPAGSAVQYRVSAGVAGD
AWKGDEMDEEGGTPPHPRRCILSPPTQEAAPLPPLGMTDFHHRLPRK
>tr|C9ZU95|C9ZU95_TRYB9 | DeepTMHMM Topology Prediction - Predicted Type: TM
MNSTKCKYIYAFVYDQTHRYKCIIAVFQLLLSFFFCCCCCCYLFLSFFTLTQTLKITITLIIIIIICVSLV
ITKDHLSKKGLFKKKKKSSTKVKFISKK
>tr|D0A3C4|D0A3C4_TRYB9 | DeepTMHMM Topology Prediction - Predicted Type: TM
MRCFYLADVDRMVGCRRLWTGALSTFRVIVRHATDVVARLSNSAAERCILWNYGLDGKTPPECRFHQQETP
EEPHHRCEVVALVFLGQCLTSFMNGSYQPTLITIYGSVVSSLTPFKRRLEQFAENVQFYRLFLQGSGTLVC
GACDAPLLVWLVMPWEKDGPAIHKFVIRLAQSLRCNALVVAVPLNGSCGQKETVKRLSVLLELGRQRARAQ
RVVLGGSNLAGMFVLLYLQERYSSLRLGPYAAAAPETAHRAVCCSVILVDPFVGWEVAHLFHTNAPQRSAF
QGVSDANRDSVPPNPPQAYSARSPPSTPISVKDRFKHAVMMASGSRVRKWFPPAIAYEAPPMVLLGKESGI
WIEELRDFNAKVDRFCVEENRHRVLFFPYSIRTTAAGMLVLVNCRVELERMLRMVEGFIIRHLSRDGWCAG
VRHSNTPFNWGEPMERQRSPAGEVTIWAALPPSQRGVVETSGRR
>tr|C9ZUD6|C9ZUD6_TRYB9 | DeepTMHMM Topology Prediction - Predicted Type: TM
MKQVTVTGEVSLLSKSHLAVICWVLPNGLGIELIIVSVRWEDKRGERKGKWGGVRWLKGLGNLQKRDASEI
ALYCVRTNFAVPLTECAGARVSMWRYKHSLAHRKHRECMVLCAIRHISLC
>tr|C9ZXR1|C9ZXR1_TRYB9 | DeepTMHMM Topology Prediction - Predicted Type: TM
MSGAILVYIMLHTAYKTISSPTRVVMEKLNLGCRTTCDIRACHCSPVAECMFLPLLSLSTFLSIVCAALCF
LRKMVFALLELPAGVGLFKVDGNKQKLKALLSFKSTADALATTTQVVNGELAKPVRKFLKKNFVEKQITEE
LAVADAKLAKAIKDALAIQCVHGDDTLATFRALRANLDDLLEDVSTEQLNQTALGLAHNLNRYKLKFSPDK
VDMMVVQAVALLEDLDKEINKYAMRAREWYGWHFPELAKIVNDNILYAKIVLAAKTRFNVRDTDFSDFLEE
ELEQKVKDAAMVSMGTEIAEEDIENICRLCSEVVAASKYRESLAAYLSSRMQTIAPNLTTMVGEQIGARLI
QKAGSLLSLAKYPSSTLQILGAEKALFRALKQRQATPKYGILYNAQVVAKAAATHKGAMSRVLAAKASLSA
RIDSFGEGDNSAALEYRGKVEERLRQFEEGVTYGRTGNVRGRGGGMQLQQKRGPPNGNGGGAPFKRQRLDS
GGFRQRE
>tr|D0A8K1|D0A8K1_TRYB9 | DeepTMHMM Topology Prediction - Predicted Type: TM
MQYFHTPLPLLIFILLYFSSRQCVRMANFFLFSLRKIFFLWSVGFPPFYAFICCSHVSVRSERRLIMRGRG
MMWVYQFGSRLPLISFLFLFFFIFIFYVLV
>tr|C9ZVL1|C9ZVL1_TRYB9 | DeepTMHMM Topology Prediction - Predicted Type: TM

MDAIITNERRMYAEGEKKIKTEFRSFLFCFYSFHFLFPFICFVYHPFFLYLFNFKICAFSVLMKCACEFLL
IPLLLTGFCIYFVPPHTHTHTHTHTHTHTHTHTHTHTHTRTHTHTRTHAHTHAPFLKPVIFGLTIYILRHL
PYTAGDITGGKGGKKTQTNKQTNKKKESFKRRKNKMMMT
>tr|C9ZNG8|C9ZNG8_TRYB9 | DeepTMHMM Topology Prediction - Predicted Type: TM
MMLPVCVGLSMSLYTFLYTQHFNTACMFASTRCPLHGRVTFWHLDGLLFLSISTRSAGILCRKLLERDCRV
LSREGEKNNNKRTPLPPYRYVGFLQAARWFVVPEGRHGDLKLANAEYDEPRAK
>tr|C9ZMK9|C9ZMK9_TRYB9 | DeepTMHMM Topology Prediction - Predicted Type: TM
MITCLRCCSPEAGLVRRGELKMWMSEYGLCARNGTVKRRRDIPTFPSFVCLFSCLICSNEPLPPYCFPSLR
AYEIRQRDFVFLCFLLSFPPPCFVKVPFACLFVFTSLRAFVCMCGQKYIYLVLIIVTSFPSLTHSQLFIPP
FGVTLKRRDHHVRGLEQRKAGEKEATKQSYKKKKDESTNSNSNSDINEKQKQIIIEDVRVAQGLFGVAVY
>tr|C9ZZG2|C9ZZG2_TRYB9 | DeepTMHMM Topology Prediction - Predicted Type: TM
MFRRSCISAFQPTAFLRVSLVFKQLEGSNPLTVKDRPVNSWSDEFLKPPVSKEMADKYGRYAKYSDPTLCS
VDTSSEVVLNTYPEGSREGRIEATAGVALKDYDASMWDEEFFRKYILKPKLPNELEDRARVTDYALNSALL
GFVILMVRYAVLPLWYVGQPAMSMVGQMNIEAEVGELEDRECKTVVWRGKPVFVYRRSERQMNDVLGTPLS
ALKHPETDEARFPDHREMAVVIAICTHLGCIPIPNEGLFGGFFCPCHGSHYDASGRIRQGPAPLNLEVPPY
RWVDDKTIYLGKL
>tr|D0A318|D0A318_TRYB9 | DeepTMHMM Topology Prediction - Predicted Type: TM
MRNCQPASLRETSAMEEAAQLRGTIGSVSTETATVRDEGRVWRKVLYTRQPYEDNYVDPQQFLQDLRQNVN
VATYEYNKVVLDTFVVIQQFSFVVFYLFVFSMMLSGRVDECALMWVNATLFTLAFVFCIVVQRQQAVDRGE
IPASFTHYLMGLCRQGIPLVGVLILLSPVLQTLTVAYSNDTIVTLSSLSMFVHVLLTDYNYLNCYSERYQQ
NTAVNAATFGIIIASRIQRVFQSGALIMFGILCFTLSPIPRHNLKHVSLRAHVVLTFTICALAIYFLMEV
PVFALLYCVVVVVISVVIPFFFVKLHGSMKDQINGPWDEAKPTNSAAAVEWANAGMLQ
>tr|D0A5E9|D0A5E9_TRYB9 | DeepTMHMM Topology Prediction - Predicted Type: TM
MHDGNFLKEVTAGSVGGALATIVEYPLDTIKVRLQDDPHRYRGSLSCIIEITRSEGIFGGFFRGLPLPVIG
AAVENATLFATYREAIGPAQDLFYRKRCEPDTEPYLAVFTAAAIGGIVVSHVLTPAELIKCKMQVQNTLPE
EKRIYKNSAHCVTSIYRSSGIRGLFKGHVSMIMREALGCGMYFSTFQLVIRNMLQEGQVFTDASPFVHFLG
GGCAGVVFWTSIYPIDVLKTKVQTNRSEYGNLSITQGLMRLYKIEGMRGLIRGYGVTAIRAFPGNAVLIAA
YEQVNRIFEAQGSTNKLLCVCSGLNG
>tr|C9ZKD0|C9ZKD0_TRYB9 | DeepTMHMM Topology Prediction - Predicted Type: TM
MAWITDAAVSQFPLATVLKQQGALPTSNMFTALPENRVLRIFQLNALTTQEEVVEILRGYLLDIFNIGALQ
SYRYVYQDEFSFLLDVILFFGSTWRCGQSVGDRMQNLVLRNEIKALESGQSDAVQLVHSLVPSRARLLLYG
VLNLFVPYLFRKLQRRALEEGWEDGGAGVVKRKLAKLLKAVSVSWMIISLLHTLHFLATAQYRTPVERMLS
LRLVYGSQDTRRFTNLIYLNQHIFWQIWSSFISVLNIGRYTSRVTRFLQAFTAKPGNPSLTDNACSACHNK
PTLPQRSNCGHLYCYYCIKSRLLGPGSAKSFRCLRCGTVVSTTTPWAQ
>tr|D0A6T2|D0A6T2_TRYB9 | DeepTMHMM Topology Prediction - Predicted Type: TM
MKSNIKDKKTPLTLSLGKHLSLRFFFLGGTLLAAHARLCQADRRESMSHRGSCMQCGSFDEGAEDPRDRQW
YCNTCWAAYNDSRKKGRWENREGCDDRRNNIGANHRRRNPNAGSRGATETAGAKGGSKAVDKQHRYKAEEP
ERKGPAASPSWYHSAQDAVRRLESVLGSFAPFRQDYRHHLMPDATSLLDDLQMLQVAERSSFSNREGEVRR
QHTCSFVGQFMQRHQSITVIAHTGEPLSPRGNRVGGVSRSAYLENNKVSLDLLLSSVDDIVSQVYVRFDED
MTRIIILNALDRVTACETQKQRDAVRVRVIEELLQVLGVEHCELIQCVMHRPRDIFLRLLEEFCTLDGGNDG
GTTGDSAAPDHRGLTVRFVKTAKQRSVLREAEFDRRGSADEQWLAHMTQRYRQLVRESELDEFFKRDLSIN
GSCPSFASGATVLQREDHIRVHVPPPERKVLPEEDRVCIATSLPEWTHAAFLNITHLNTIQTSLFRTAFHT
SQNMLVCAPTGAGKTVCGLLVMLRCIEEQRVDGVLDRNFKIIFIAPMKALAQEMVENFSRRLAPFAMEVRE
LTGDMQLTKRELAQTQVIVTTPEKWDVITRKQANEELTTQVRLIIIDEIHLLNEDRGPVLEAIVARTLRQG
ELASEQKHRTRLVGLSATLPNYKDVANFLRVDLAEGLKVFGAEYRPVPLEQSFIGLRMGLKDKERRMDQLA
YEEVVRNVREGHQVMVFVHSRKQTVALARFFMEEANVRGHEALFKQDETVPKEAQKLGCSLQGRDLSNLFA
AGFGAHHAGLIRYDRTSTENLFRKGFIRVLVCTSTLAWGVNLPAHSVMIRGTHIYDPKRGGLVSMSVLDVM
QIFGRAGRPQYDTSGHGTIISDEKEVGRYLRLLACALPIESKLQEGLCDHLNAEIHAGTISSIVEGSGWLE
YTYLWQRLRVNPLMYGLKVSDVRQDPKLKKIRYEIVSKAAEELADAGMIRYNPQTGAVDTTDLGRIASHYY
ISYKSIATFNTKMRRPDESWIDSLDLGAAMNVVACADEFSQLRVRQEELDELKKLHALLPRQVQHYGIVGE
SADETSVEWKVTTLMKSYISRINVDMHSLASDVNYVVQNAPRISRSLFEIEMQRGHPLTTAVFLSLCKCLE

QRRWEFEHPLLQFGVDMTDAVYRNIEKKRPSMSLLQEMSASDIGSLVQNQRFGDVIARLVASFPTVSLAVD
IQPITCTILRVKVTISATFSWNSRYHGSVEPFWLLVEDQDNHFIFHHELISLKRKEVEAGLPQVVNLSVPI
VAEYDMYSVRLYSDRWLGSQEEYSFSIGHLHLPDDSQKTTPLLPLAPLRREVIPEKYHTIYESFREFNPVQ
TQVFHAMYHTDSSIFLGAPTGSGKTIAAEMSILRLFEKYPVGSKVVYIAPLKALVKERVKDWMARFSRRLG
RRVLELSGDVTPDITALVQADILCTTPEKWDGLSRSWQVRRYVTAVRLVIFDEIHMLGSDRGPILEVIVSR
MRFIGWHRNAPIRLIGLSTAVANPADLTSWLGVSHKWAVFNFDPSVRPVPMRVHIAGYHGRNYCPRMAAMN
KPVYNAICEKSPNKPVIVFVSSRRQTRLTAMALIGFLVMEQNTAKFVRMDVEEVNAYVAKVSDPYVKHCMQ
FGVGVHHAGLLEGDRTVVESAFLKGKLQVLVATSTLAWGVNFPAHMVVVKGTEYYDGKTKTYVDFPITDVL
QMIGRAGRPQFDTEGVAQVLCHEPKKGFYRKFLYDPFPVESALHRQLHTHINAEIVSGTIKTRQDAVDYLT
WTYMFRRIVKNPSYYGLSDRSPASVTIFMSTLVANVLDELEQCGCIEHCQSDGCDADADPNALTYTVLGKL
CSYYYLSHKTVYYFDTNINENSTHVDVLKALCEADEFEELPVRHNEDKLNLTLSQALPLPIKANNADSPHV
KAFLLFQAHFERCNLPISDYYTDQKSTLDNAMRVVQAMVDITANNGHLHTSLRCMSLLQCIVQGLWWHSNT
LLQIPHVTDTMLPIIAEHCAGLHHVSQLVNSPLSVLTKLHETLCDHCGLGDKEVREAMEAVRSFPLIDVHI
RLSRTVVDDGHATEEVMVKRGDGMDDDEEGDGYELTAYLTRLSTPIRHVVAPHFTKSKEEQYWLVIGSEHT
GELIAMKRVGRLMGNATTATTLRFDWDDDWTSFAENGSVPLSLYVVCDSYVGLDQQYNFRVSVPSRE
>tr|D0A6R7|D0A6R7_TRYB9 | DeepTMHMM Topology Prediction - Predicted
Type: TM
MFGLDRLSILFYQIDPRDYPEYMECARSTEQPGTILMESRAMQGVRRVVPIGYLLSVLYFYGYRRPRLSFA
DRELSKLHHYFYPSAGYSTPIGILGGIAYACYYDGFCGCSEERAARAVLWERSKAMVAWRQHQRMQREKEN
AEGQQHHWLWSRLTFSWSGCGHSSGDGKAQTLSYEDFLDPYGVVSVGKQAVEVHDPSFYQIYSKEQVDTLV
SAAMRLRKSPEEERWLRTSGRFGGYGIIGMLLTWNSGGMFFRLFMGLGFGVVCAGAISGAKLDS
>tr|C9ZPR6|C9ZPR6_TRYB9 | DeepTMHMM Topology Prediction - Predicted
Type: TM
MVSLLIICVFTFHKNLHGDVQRINFRFRPYVPLLMCILDSPNNVVHHASPRTSVCTGNQCLVLQLYRLGKE
SWRIIRHTSSYLVYVNMHTRNLLLWYVMFFFFASLGFLHGSANCDLSARITVRK
>tr|C9ZXJ9|C9ZXJ9_TRYB9 | DeepTMHMM Topology Prediction - Predicted
Type: TM
MKEKSGNIRHFTHFMQQLCYAAYVYMSLCRRGNGTNKSGEARLRKLQWRNYCVFLFLFTFFLSLKYNKLLS
LRLYPLAHSRQIGVYKSTHLSLFVCSFVFVFKNTIPT
>tr|C9ZJ45|C9ZJ45_TRYB9 | DeepTMHMM Topology Prediction - Predicted
Type: TM
MFLFFFLKFSSFLSPSTTTFCGMHTTYTQEGILYVFMHCLGIYPSFGFCFCLFHWFYFLVSALFLFLFLFF
FHVCFRRMFVDCSCELTFLVVVVVVTFFFF
>tr|D0A2W6|D0A2W6_TRYB9 | DeepTMHMM Topology Prediction - Predicted
Type: TM
MTRRSAMSDNTTSTSSVTPERYPFCIVWTDIPLVSWLFPFVGHVGICDSTGRIFDFEGSYCIGVDHMLFGN
PVKYWDISAMYVPSSRFPPGGLLSGDVEERRRRETEEYDRALSGVTTRFRKTQTYNFFTNNCHSYVASVLE
EMTNGPRRPWNMFWIAWGLAIHGRYVSPGRFLKAHLPFLVIVALCVTAAALLG
>tr|D0AAG2|D0AAG2_TRYB9 | DeepTMHMM Topology Prediction - Predicted
Type: TM
MPLKPVCILTLYYPCYLPSAVLLFLVISHWISVLAVMKFLKPGKVVIMTSGRYAGKKAVIVQNTDTRNKER
PYGHSLLAGIKKYPRKVVRGMSKRSITRRSQVGVFLRVVNHKHFLPTRYNMDLSRELRGRINVSDASKKAK
SKQLVKRLFQARYNAGNNRWFFQRLRF
>tr|C9ZZQ9|C9ZZQ9_TRYB9 | DeepTMHMM Topology Prediction - Predicted
Type: TM
MLHYIAIKILCTTAAGIGLGERRSHCFHRVEQPFLFASEASYCIAAGVTSSPLVILVCLCGSKTWCADTII
VRGSLNSSQIVTICSGKNATLDPRCLTVFHHLIFFSCLY
>tr|C9ZTB5|C9ZTB5_TRYB9 | DeepTMHMM Topology Prediction - Predicted
Type: TM
MSTGPSTTLAGSGCLTVALAGAAVVVSAVTYISNKRRSALSTIAANRGGFQSVSVQPQTKGNVAGQDASSA
NSRGVPHSTFPQSNTGPTNRSYYYAFAERHVKRHNATTMEWRDDVLGVRMLFSPILFAVETEERQAPLLLV
GLRYLRQPEHRVAITFEYCETEETAENYRELSLERVCDCAKLLSGTGNIRIGSAQLPSAEYCYLDRGNKLR
FALSVFLTSKRLAVTAQYIADTRVKSVLPTAFNELVRSIQFSEPRSSPSYLLCAEPRLGLGFRLPLDFVMD
EHLRELLTIAEETTVTSATPRASGGEGPNGSASHSALRRIDKEGRNSSNGPRAAGNAAPSTPDVLWGFSVP
HQLPDLCAVEHATGGGRYGFLGNGETNGNSDSVSVQFATISSLGPRRIVVVACYEPLPAGNFSWQTFFEHH
LRAVVRRFSVHHSTDGSNAYSVSVVRSNDELQCAKSGKQRNFVVQLQQIMVPTNGDSAGEDDPNPTLQLEG
ALCVQEVLIDPNNVCAAHLRHPQGRRSDGDAVSPTDLMHMHTDKDGPSGEAFISAYLSVFCLRIRSECVSM
CFLFPTACHTLEEVVTFCRRTIDTMSLGNHYGQSTSLIYCNKRHEVLPFSILLNPACAAAAATVVVREPIM

GEPLAAFCVGGMDGLTVHLRVFPIPFVAHTSQVARRRATSRLEKLVRDYLLRLPGRVCVHHWETTMLGASA
ALEVHYEQLCDSDDDDGDSDSFVMDNDMGRFNPFSLLGSIYCHEGHDMEGAECPQEQHTPVIGMSASAVGS
SVLSRSAEHMLLRAPSLDSREESTTLQVAVVVCCEGCAFLFLASLEGYPLAAVRQVVRQFASNLSVSTGVT
A
>tr|C9ZI65|C9ZI65_TRYB9 | DeepTMHMM Topology Prediction - Predicted
Type: TM
MATIYHEGFKVRYVTRGIGSWVLYFLSLAALFILPFVIATSMGGIWLTNSHFTQHPRLKFSGRCLLRYTTV
RGKEKLWTCSDAFNEKLLYGHAELDTLPFFTIYEEDRDSDGSVDCVTIVLGLPVNSLGSRVENSFPPPDEM
DAIERVEFIPEFIYEIIHPSIHLNMTAAPLIVFQRPGGGHASASGTGGAHTGGPVCASTSGDILFHTTEPL
YSWSNVHYAHTYADSPLDAVESIDDVLNVPRFAQKYASRNQSLLFRRYAESAGGASLLPDITYDRVLGEDM
DTVGDFTWKLTLRVQDANVPYVSSIPELIKWAWIQYFTIAYVIQWVLWKLRGMLLKSGMIGSTAIFHRGYA
PR
>tr|D0A1E9|D0A1E9_TRYB9 | DeepTMHMM Topology Prediction - Predicted
Type: TM
MVVAFLSFLVLFCCCFIFLYIYIYIYISFQVKNKKRKKKKKSQQDGPPHSCMSHLFILTPSLLFCFCFCFF
LLLPPPFVLFFSILRGLTALYLLPFIHLKKKKEDVTGTGCGGVKTKTAGAGSSFCYYYFFFFCSFCSLNIN
IILYKNKTKNKKIALSLPPPFLFRFIPFLCMLYHIISLKFCFFFCFFLLFFFFFFSFYFVFGLFCLPLPPS
PHRRVFPLLFPLLFT
>tr|C9ZL64|C9ZL64_TRYB9 | DeepTMHMM Topology Prediction - Predicted
Type: TM
MDEWKILAIRASTNASRRLTLVAYFCMCVCELFKSPRVCLHLYIRVLLIFLECDYFSPLFFTFSFFFFTSC
CPPCKWDERFTLLLSSPFVEVCDFLLMREEGVFYDTVCYISTVGDSICVCVSGWQKENWGVARFAVHCSPA
IFCSCAISLLSCYPFLHPYRFWPPSRLFSLLCCGKNKNKHVNLSGTTRNR
>tr|D0A9I6|D0A9I6_TRYB9 | DeepTMHMM Topology Prediction - Predicted
Type: TM
MLHRRTEDTMTRRTVRGNNLLSTECVGEIMRSSLCFCDKRIYTNTFGNPLTRNRRAHFSPVMLALWIAVRC
CWKCFPFFLLLCCTTYCCVIFLLSVLVVCVLFR
>tr|C9ZNX2|C9ZNX2_TRYB9 | DeepTMHMM Topology Prediction - Predicted
Type: TM
MMIMIIVIAVMRGIIMRIICCNTMTHVRAGRAVVCFIADGRGEVRRQGNKNCVFVFVCLFVCFLFCFSFSV
IVAVICWYVYYHYAEDTFLPIHFSSIFFFLFILCASSALSRVSYWGFPFCSFIIVLIPANLYRLLYCHE
>tr|C9ZPM4|C9ZPM4_TRYB9 | DeepTMHMM Topology Prediction - Predicted
Type: TM
MHGVYPILVVPCRRGGIPYLTPEQARDILGEEERILSLSIFDAYEYKDACKKAGKSFAEFCGLGEFRVILT
VRSPYVGAHASVSASETAVFGVHEKGRISFSNESWAEIVKSVMPNMAITLYDSVPLHEQHSKRRKTASTRS
LKWAKSAECAPDIGCELIKASSVADRENVFVYADELGQNETIVQYASRLCEITKNHYVMSPTPSLGAVLMA
LKVGASFIECALPWTLAERGIALVFDMNPVHGCSPQRYESQIDLNDHCFAVDINPLSQACVCSTCRRHTRA
YVHHLLTVQEMNSCILLVVHNLAFVVQLIGLYRRSTAEARESLLTWVLAQL
>tr|C9ZLE5|C9ZLE5_TRYB9 | DeepTMHMM Topology Prediction - Predicted
Type: TM
MMDHCYSFCKTCTVVSVGGGTVFFCFMAFIFSSHLITEWLLLLLSLTISCRIVLFFFVHGARIPSVLSYVL
MYVCVGVLTDHMLQLSALTLKYKWQKRKEGAAVHQGKGNGLHLAHYFPYVFTSPEYVCVIIVHFHLYIYIY
ILFSLSFYLCPIPLVRRKKGFVHLVLCITGETAAKVYLYCHSVCMRVQSIRTRRRIRFFFLGG
>tr|D0A6D9|D0A6D9_TRYB9 | DeepTMHMM Topology Prediction - Predicted
Type: TM
MLRWSPEEIWDNMRHKLSGYHNSFCVLFFLSCLPLHEHWTTYAFCLTFMILYAFFLFLKCEAGVRYFCFRR
VLRLSLVRIAHTVLDFIGTYICFKIPFLLFICFDSTVDRDGCFPVLLGSPR
>tr|D0A0C4|D0A0C4_TRYB9 | DeepTMHMM Topology Prediction - Predicted
Type: TM
MPNYLPNRDGFMRAMLRETIKRIRRVRERAGRTDEPKEIETTGDPFRDLTLAFIRCVERVKENIKERNEGA
ARHGQDRIAVEQSLAIHKDIRNLETILEEMKQEVNKSGAALEKETRRKKAKPHKIALLEKAHSAKSGQYKD
CLATLELVKESDHQRIAATSGVNVGQELLVGRRAQLRGELGSLWRDKKDGGGHVDPYAGANLEDDTVGGGR
LEDHEDTAEAMKTIAAQDKKIQNSLEVVSKGVSRLHTLALEIGGQIDMQNKHLDNTEQVMNKQTEQLHTLN
VRLKKLVKEMKPMSVFLYVCCILLIMSLVGFFLMQFDVI
>tr|C9ZIW3|C9ZIW3_TRYB9 | DeepTMHMM Topology Prediction - Predicted
Type: TM
MHTYKNKYIYVPSRYTYCPQIHLQRIHFFFSIFSLLFLFFCFKETKKSTKKITFLSFSSAGTPLQPDLSCS
KKKKHTKEKKEKKTPPTWVGNTTKLLKHQNKKRKKKDRKQHKHRLCSFVIIVITFI

>tr|D0AA53|D0AA53_TRYB9 | DeepTMHMM Topology Prediction - Predicted Type: TM
MFTNEGSGPEVLRCKYVLCGLHLAVTAHVTPYTSIYPLLCILFSCFKGRNNVCVCVCCRIISADCAVTHSG
RCIFFVLFFVVIVVVFPFFFLRCSPSCCVQLRTLHVIEHVCVYLIALKVAT
>tr|C9ZKJ8|C9ZKJ8_TRYB9 | DeepTMHMM Topology Prediction - Predicted Type: TM
MCVYVFLAFSSYFFPPFPFSLFLFFLPSFNFTACVYVYLSCGVKARFSVAVTPQNLTLWQMEGEDVWRKKK
TVEVTRCREVDGRREGEWWQQAVFFFFFIFFLILFRCSPPIYIYMFLFTCDLVEGCFIAVTPLHISLSFLF
FP
>tr|D0A0K6|D0A0K6_TRYB9 | DeepTMHMM Topology Prediction - Predicted Type: TM
MNKLTTPLPLPKQTHTKKRNNNNNNNNNSSNNSKYHYSYYCYYYYYMNNSLQLLFFIFIFYFFSFLLFPPAP
PARPFPFPPPPSYFNDISTVLITTLENNIHKI
>tr|C9ZPJ9|C9ZPJ9_TRYB9 | DeepTMHMM Topology Prediction - Predicted Type: TM
MRNVSSLGDSHSFTTEKENNHSKRDGFNVGNAAPRSSRECNGRETAPEIVRNTARSTEHPFSSDAKPLPSS
GLVGAAVGHHGGGVHLLGFGGIGPWSPRPGGGYAMKYDHHSPNTHCNTGRGLQIVTHPANSRSASVVGSME
EEVPRVSNQTTTNPTRGVSLRCWVLFPLIVIMTCVYAISIALAIAPWARASVAVMGSLHDVVEVNYVTLSG
FTPFLRMTRMAKGIGSMYFSNNTFSNPVMDHAMPLKDGMVSSLCVTLRDVDKRRMIASIGAFSLTKKQAAL
CIASKSNPGQYYGHVSEGGIMKDFYYMDPVTMEYQQPLKRYESTTSGLTITEYTERFHFDSVVRIWNESVR
EGREMKPSEYWVRPRFPPTYAAYVYPFFEREDDGTVGVGYIYVGMHTGKISVRWHKASDSGVRVMLVDPNN
ESGQFYVFANNWGQPLANVSDEWKSAFMGEPIRFLSPDDVSDPLMSKAIRYLDLQQAARGKNQRSWFMYGG
LAAVASAHHIVTDSGVEMVVVVVTNPSYYLGPIATYGGIAGLSSFVMYLIVIAACYYFVELCLHRPLRSTE
EKLRAGLVKSSDEEKQKVIALREVCELHGVCSVLRRRLNAVRTYMPDRAFDLSGAAASVSRSKDACASDPE
AASSPRRYGEELKPVACSVAYVYYTPGRTRNPSDAVVELMMQIVVSAAAACGGCFEVQRPDYCIVSFGVQS
MDREFAAEATRAVEFARQVATKLMARSEVSGAYRVIVESGAFPSGIVSGGGRSRYVLLCRNIYRRVGDALQ
SVGVAAAVTEETALLVRGHFRLLPFRSVFLEGEDGVCVTLYEVLSGDAGQPTWNEFEGHYNEAYDMMVRGN
YVSALRLLDKAISVGSLTLSGESPSFMSLQPQRLRDECAARVGRRDRTPFVNQLLLVPDSLNSSATSLISS
PPLLSGTGKTSGGSSSPCDGGSPRTPSVFVGENAASTLNGEIPRFLEDCHGNSWQRSIDPIHEGANSVGVA
YMGMSATGVLALLKLYPLTELGERLTREELDVALDKVLQVGESVSLVQCLSYCHVPPHGVLLVWEYVPGGT
LRDLKVRYGRKLPAATVKRHVTSLLRGLACLHERGLVQGCLCPETVVTCVGGHCRLTGVLLDAAPLLKHQM
TYCVSPEEARGKPQSWMTDMYALGLLVIEMLNDDFPWRWTTNAQISRSRNELLAVLSDHEALMESLREGLL
EPVPPPEDADPTIQLVVSSCLRIDPAQRRDAVFLLQRVGGVVAVDA
>tr|C9ZM09|C9ZM09_TRYB9 | DeepTMHMM Topology Prediction - Predicted Type: TM
MLRVFSGSCLSLEHMETVRDFNLVKQPHVLGPGADYFTEDGQPVRPQPCAHTLHKVFSGSTLDLNAACGEI
LSDEDVALSNGTRGSPHRAGQQGQQQQPKIAPGATVSTILSQLSSETEKERAKSLRSKTVVLIVGGVGYVS
SHVVNKALEAGYSVRITGSGALTPQQQAALSSVGRDHEQRLSIFEADMTNTNSLRDALRGCKYVIHCGCPV
SSTDKDTVEFHLSAVQALFNAIRQAGKSTVKRVVIHGAASSVFHVTDPEPPSGAFDESCWNSVATNDTDPI
PYARIYFEKEAWRLKQMLGVELVVILPSITIGPSRTEEVSDAMKRIQHLATASSYFPYAPNLHWNFVDVRD
VAEAHVRALECQEVKDQRVIVSGGCFSFAEIGKLIRNEYPHLTPPTRTANTLMTLIIGATHSQVSIRFLWR
TLGVRKCLDTRKATTELGMKFTPMQETLRASIEQMISAGELPPADGSVNARSVSRAGLVVTTMVAGLIGVV
AWIVVRRRGKN
>tr|D0A6L0|D0A6L0_TRYB9 | DeepTMHMM Topology Prediction - Predicted Type: TM
MMLAVIVAFAVIFFVTLALAGRGLLPFYRYPPIALNPDVYQSFKLVKKTRVTHDSFIFRFALHASHQCLGL
PTGHHIRFRVASKHNFTGTPQVVQHSYTPISSNDDKGFVDFLVKIYYKGSNPAFPNGGRLSQHLDSLSIGE
AVEMLGPVGKFQYMGNGDYTVEMGKGEVKRQHIAGFAMVAGGTGITPMMQIIHAILKSPEDPTRLWLVYSN
HTEEDILLRDALDEACKDPRVKVWHTLTRSAPPDWAYGRGRVNEEMLRTHLPPPQLEEGSVTVLLCGPPLM
LQDAVKPNLLNIGYSQDNIFTF
>tr|D0A2E9|D0A2E9_TRYB9 | DeepTMHMM Topology Prediction - Predicted Type: TM
MDWVFFLMAALAALMVFGLIIHLVSIFSSEGDNNDGFFGKLIIVVSFTLVSYNLLMLPYSVAALQYAALIP
ESKSNVLLLWTIVAVALVIFGLVICPFVLVYHEVGNHNNSNAWKRVCVSVAMTLLVVVLASSAFYCGWHFA
GYANVGYTAYSTSIQPVTSFESIKEFSDQSQGESLRLQVSLFVYLIALLCAAGWIPLCTFGGIGLVAVPQE
FLLYFRDRPRPITASEYAYRREEVARESQRLIDKGRMIEETSAEHSYGGHARKVLAFRQAVRELEAYHTTL
EISYHQQGGKVLQGYLCLLAGLVFTFLSIRWILYITLSNVSDTHPMYGGMLRQLSDTSLTLCVTVYSCFAF

YLLCCTIKGCIKLGGNLALYHIYPVEVNKTLTTSFLFNAILCIITSSAVLNLCADSFPVYAVNSDVSVLFS
VFVANLAVVKYVVSYTPYFLVVVSCLALMWLIVSPRRYARVSRW
>tr|C9ZLA0|C9ZLA0_TRYB9 | DeepTMHMM Topology Prediction - Predicted
Type: TM
MVYLVDDLARMRMLMAGVYAGLGVSKAYCFSIFTPFLRSKFNMTQGDITTISTVGTCMLYFSFPGGALFDY
AGPMVVLPIAGFLGFLGFLLFGLTFDGKIANPSVGLFCFYNALVNCTFSWLDVSTIMPLMLQFPLDRGYIV
LVSKTIGGLGTGVLMAYFNGWFKDTTSDDVEKNNYSGFAYFVAIQLVVVSLIALSVTRMPMYFPCAWRKQR
LSEEEWTKRQQTLQLYMNQPAPPRRMKIAVGLVLSLLLFLTTQSLIGGYVKLPPAAYLAFSIIAVLMMASF
CVVALPFQWLGRYTPVRPTDMDTIGEALEDVVTESAVATTKNEVKPLPQYSGSFWQHLLTVDLWCMWLTCF
GVWGTAVVMQMNAAQIYESKSYGEKKSSTLTLYITMMSVGSAVGRMSMGYLDMVLTRRQREGLKTFPTTIA
LPFCPLMLCIAFLLFALLPANALVLPFFLGSLGNGAGWGSVVLAFRIMYSQDLGKHYHFGFSSGVAATIAL
NRFMFGGMYDAEAEKLDTKPECKQPSCVKNQMLILMGANIVAVIAAAIVHFRFSRFINAEQNRCNEPADEM
SGVAAPVTSGGHSGKKQ
>tr|D0A6B7|D0A6B7_TRYB9 | DeepTMHMM Topology Prediction - Predicted
Type: TM
MYGLCHAFRTSLSLFLFSLPNLLQCDSCFCPFALYAFFAVTFVLSLPAYPIWFLPLLIINFVYRLDRCDNK
KLGYVRQSPSRAISNMQGQADALTPKAQRVAAGETYFYREHRKPQVETSYRIRVISGTANYALSESVAKYL
KVDLCRTEIKRFANGELNIKVVDDVRGDDCFILQPIAANEHTDINTAMMELLLLVHTLKLSSAKRITAIVP
YFAYSRQDRKTEPRVPISASAVAQLLQCMGVDRVVTVDLHCGQIQGFFRNMPVDNLLMFPEFATYVMRQPW
FDRERTVVVSPDAGGVERANVLADRIGASHIVTILKRRSGPGKVDSMQTVGNVEGYTCVIVDDIVDTAGTL
CKACELLRDMGALRLVACATHGILTDPACERISQCDALTELVVSDSIAQNINSQKCNKLTVLTIAPLLVQA
IYNLHFECSLSSLFRT
>tr|D0A0K7|D0A0K7_TRYB9 | DeepTMHMM Topology Prediction - Predicted
Type: TM
MTQWRERRNFSFFFLLLLFSKCFFPQPFFFLVGWLRPRKYRVSLVFVYARVAVRMFYCHCCCCCLEFVSFF
FFFDFKKKICERKMCACFFFFVCVNMCRVNAFFFYFLFFSFFFHFWEINF
>tr|D0A6V0|D0A6V0_TRYB9 | DeepTMHMM Topology Prediction - Predicted
Type: TM
MGVHCCVCTLHDSSSDCLMALVLPVLPFLCVLALVCYFTAFVIREVHPAVIDLSVSMPGRVQARASHLCAS
QHPFPPPILMTMHIIIGILLFGFSRGALKERVANHRGG
>tr|D0A5R7|D0A5R7_TRYB9 | DeepTMHMM Topology Prediction - Predicted
Type: TM
MTKYELKMQYFDEWMIRWRKFQTESDWEIEKGRQWWRRFNMAVSGALFCGLVLYTSGTATLKRQYGLPHFF
DIGVDGQAKETMLKTLTSRWRYTPQGYGRVLITGVPTYILFVTLEHYRERRRMQQYLQQNTVFGEQMRRLL
STGKIEEYLPVNIKATLPASQQAIYNY
>tr|C9ZP71|C9ZP71_TRYB9 | DeepTMHMM Topology Prediction - Predicted
Type: TM
MSFQVLTTDFIGFCCPFSKIRDWLSKQKKKEKRSDTREKKTINKQVNMKVLTWGWEMRGNSSLFFFSCLKA
FNLSFFFVCVLIFGADCAKLAPICFSFSQKSVDR
>tr|C9ZSM9|C9ZSM9_TRYB9 | DeepTMHMM Topology Prediction - Predicted
Type: TM
MRVCTLACMCPFPFPCLSHFISTLFSKKKRGGVIGSGGKCCESYFCTFRCRRRVLPSRRPHSLRARGGSSA
GCLGASSALCLFSSTFEILPCPLISSLDRTYRLSLWSVNSSHYAYFYLQHVFRFVPLLLFFLFLPLSFAHE
>tr|D0A9R6|D0A9R6_TRYB9 | DeepTMHMM Topology Prediction - Predicted
Type: TM
MCLGTRLCGRMYTFWGQLDFFFFLPSPLCLHLISVYVERIRLFSYTFSKFLFRIVSIYILDDTVYQPRRCI
KFDDRSAGAPKPFSALRIFSYQRMFRGESTM
>tr|D0A0P1|D0A0P1_TRYB9 | DeepTMHMM Topology Prediction - Predicted
Type: TM
MNNSNNSSVGSIGAPPRQRHKFGTCTAEGIAAPVAAGGIITRGGNFQKPTPSSDDSAASTEDLSLLLWCSV
GVFLLLYVISGRFHNLYITPEDNDKAEQNFLQYLSERWSREGDAIITKASNDASFTQHGDGRIYFRVMNQT
FPIKESTPPRVVSKVSFPEVSSDNRWRGDLKNATSEAAMEWRNLTQTMQCAGTDGPLIFHLVAFLSSGVRF
VSTYVPPGKPEIRSVGAHIPCLNAILPLMCKGDKWEIICPPEMAFGSHGFQEVPPSATTIWQVLMLDVTKS
GPRTRAHVQKLLAAATRRHSGELPITRRELYERAMRARGKLLGNGEGTL
>tr|D0A3F0|D0A3F0_TRYB9 | DeepTMHMM Topology Prediction - Predicted
Type: TM
MSARCAAKHTKETIKFVRAYIKFYIYIYIYIYIYIRVCVYAWKFVYYFVIRVFPPSFSCFFVCVSCYRSFG
VTNRLVSQQKVSAAQGRVMSRATYSRSTSPYRSPRGGTPSRSTDASAFIRFLLSENIDQRIADTKRWLRRI

REHMRNNAPRRCSRCFDPSMIFCAIRASSALLTEKPNRHRMSARPRRFRCSLNSFLDGTPFLTPRDLCRYN
TSVAKRLSDDELELMRKYVFDYDEVAFADLLYGDDIIPDDSEEGRDESPRSRRRILAAVRAFNERSRIAME
RLFAAPGGLVDAVMRITLLVPLVSNDMDDSDEG
>tr|C9ZLG0|C9ZLG0_TRYB9 | DeepTMHMM Topology Prediction - Predicted
Type: TM
MPPFFSPHTHTHTPTLFLCYFPPFCFPYIYIYFFFFLSFTVTLDSRVNASCCCFCLLVTRDCRCARFKKFT
TGPIRRGFQQSFHNALFFLFPPVVALVTMATMLTSTPQLEGVKQLRIVTEKKERGKTTKKT
>tr|C9ZNH6|C9ZNH6_TRYB9 | DeepTMHMM Topology Prediction - Predicted
Type: TM
MNAQLIAPSSFVLHILCPSVGPFRTFFPGSPKGGGLMFNVFGFFLSILFVFFLRSIFLVDFSFVFALPFPN
SMLMFFRVVELLFYASATHSKQTWLWYIGRSYL
>tr|D0A0K9|D0A0K9_TRYB9 | DeepTMHMM Topology Prediction - Predicted
Type: TM

MRNCSLDGSIHTKGRREGGGGRERSHAAEASTTFRYCCWSWCFFFFVSINFNLLACLFISSIPFCKPRFGV
STVEYLLSNAGSKTIGEWNKEGKMTRMRVDAKLLLKSEDDFRRLLELLRPFHLRTWKYEDFYFDTPNFLLM
AKDVQLRLRVPFKSSDGKDEKLAGAQGSNMLCHVSLTLKTNSSVDVGGQTAGIFEMFPFTPKDVDDMLQED
SLVSVLKNRSSDEPAKTVLEYLTRASEEYGEELVLTRFASFETTRRQYKFVPHLGFGSTPSSDKDVASEKN
KSDPPRFYVDEVPMGDFKSYEVEMQGVTDPLADVCQDLMDYLNEKGIEFTHSLSGKLNRFMTRTLELEEMK
EESQCVRLRIKGNKGYEEVCRWQNEENEISLDPIPKRGAPPVDNDKPTFGVTQMASSLLGLGPLTSQASRM
THSQSVRLKRIREGNHGDEEYFENYFFDDRPNGTLAAKKYTLRLRCCNPPTAFSLELRKEKWSAGGVKGYE
RRRAYISGDVARLMLRDPNKFLNSLSSQSSLGHLLRRDMGLQKLTIVGYCKTHRITYNGKIIERTMVEDSQ
RTQNGNAGKVAARPPDVEFTLFHGPSSAGCRGEFSIQLNRIMVDTGSDPVKVAKRASEERCCSIFNPRGCS
PFPREPSETESYEVKLAGLPEGLAATAEDWLVSQLHQRQVQWEVVMSAGMGQYHPSLAAA

>tr|C9ZKR4|C9ZKR4_TRYB9 | DeepTMHMM Topology Prediction - Predicted
Type: TM
MEEDKNHWSYDAENEGVLRDTPSPLQIQESFGNDGNTQTEGNEHGDSTTEENISNSEVKCEIEIREKIHPD
AEEEEARLRAEEEARLRAEEEARLRAEEEARLRAEEEARLRAEEEARLRAEEEARLRAEEEARLRAEEEAR
LRAEEEARLRAEEEARLRAEEEARLRAEEEARLRAEEEARLRAEEEARLRAEEEARLRAEEEARLRAEEEA
RLRAEEEARLRAEEEARLRAEEEARLRAEEEARLRAEEEARLRAEEEARLRAEEEARLRAEEEARLRAEEE
ARLRAEEEARLRAEEEARLRAEEEARLRAEEEARLRAEEEAHLESKDSSVKRPIQCDDGCRWI
RADEDAAVSAIEGPTGSALDDCLSYSFVGISSSEVSVGGDNCDAFMGKGDTSSASDNARSDHLTGRSCSKT
VPLKHPLTNNIPCHDEFEICMEEELLRMGYAESDIHVRYLELHHHETAREPGRCLYPPLESGGGVFSPQNI
QIGFFRSKQVVIASHALVSRRVAKRERSAKGGERALSESRFFFENELMSDTTVTTEDRNLPHNSPDEQLVE
EQKLRTNQNNLVAAALSRFTLGDPVQVWEQACGFSLDPVPGIRDGVRQLPTHVTGGTSSYIWAHEIATNKP
VPAMKAFDSDVLLRVECTSIASRLLEVHKDAVDPVIVSVAFYSMGSSTKLKVSETFFFDSCVDIFYPHKER
SELNRESRAMAFIPQEFFASLYLVMRVYRPCCEDQDSYVDLYSRPDRYKSQQVTLMKQNTQLLAMKSDIFE
EIGWYMMPCVKNKSVVETLETTKLYQRGVPDAQLFQLVENERAQKALATLPFFASFSLKHCKGCEVEFPSQ
HDEPLPEENETIIKVCTRSDTEVQEETCRFVPCIIPILNSGFFNSYHNVYYFRLDRVKLVSTGILRTVPST
HRTFVMEINVRDNDTSLSGENLPLLYGNRLSAKTLQTSVWASAVHNSLDFSLSDEFKVQLPLHLGEKFHIF
ITLYACCKKMDNSSEEMQKMHKVGYAAFPIIHNGVVRVKDEWTIKFIAADQVLEGSEKSYLDKFSEAADEA
HLNGGIPVLSVSTQTRTSVHASNAIIASLLKEMPASLESILRNDNLFRVSGNLKDIYRSDDDAIHSSLIRK
MRKLPLAEILAFYPLLSFFILSLISSPSKSVSLPCRTAALEVLLDITIKTQHYDLTTRSRQKQHSARGVSK
TSVTRFLYHHLTNDLLYNGEKQRLYAGFAETWLHLLVACRPHSENTDQGAGDKTAKDDREQDKRNIRKRMA
DLSWFLFGVILRSIYIWALENPSIPRAKLLDPGFYSIIGDLCVEALDVLDGFGIDDVLVRRTALFTRNLFN
FCDRGKVLNITQRIVEFFEKRQDMEGLGTFMSIILDDVDVVTLMIPSSSYTQPVFLTRILVHAFSLLLSSP
SQVVRSSSSDVLYKFICRVTNGVRYPATCLRWMASQLLALVREVSLRWKAYVQMCEKTESVATIEDKRQLC
VSILWIIYYAPPGRLRNWLKGERNSDVISGLLSLVSDAQHLFRYAAGVDKANPHGVKEPTQWMREWDARMS
TFVAAIGAYVCSAVLHAVPDALKSLRTEKVDPIVFPFFHLLEGVLHLGNSTAALQIGSCALLEVAHSIFPE
ILCSTSRMCSGMAVLVMSLMSSCSVHVRSMAEEVLFLMCYACYTGCRSVAKIKAPIFSATVYVAESKKRDL
RLAGGFLELHLSNLVRKAQQSGDNFPPFSQSYVKRCEADSETPGNEVTAGRRHCGESPFTSVERMSPIPSC
LISNGVNGIASLVGNRRTSAIVGSEQGSNNCNEPPSFAEELASVREISMMLFGSVRQLMQTESLRLKEAKA
LQHFKLAVQLLRVGAVHECLRWFQRLHELHKANNNMGEAGIVLLFVAALCFRLTEAFYQVRGKESRGARIP
FIVFSHVFWHDYARLLPEADTLLTGDVLYTVVSELRVLPSDPCLSLSGHVEVLRGAASLLDKDHYNIFSVG
VLSIVERYYRLLNDFKAASIHSAMADGCYAVSREDRQKRENHRYFLLWARMNRTEVELHEMDGNESMQNG
YSLPLKCVFKMPAKTKLEQFLECSKDFVRPLLKDATPLVVTEMAEEQKDMGALEASKLPPENCCLLSVCEV
NPCFSSGRRKLTDSYDRNASLNKFEYMTYTYDNSREYGDAKPDSALLRNRLVVYKYHLERSFPSTTNVIDV

ATTHIDQLDTAATVAHVLGKSIEMLQAPVDNDELVGTLLRALTPGGFARPGAYIKEVITTMSTNSAVMTQV
RSLSKLLKTKLTLCENHDAPLNSTENYALVLKAVMDIECTLITLDDKVGVACSQ
>tr|C9ZY55|C9ZY55_TRYB9 | DeepTMHMM Topology Prediction - Predicted
Type: TM
MTMNFGNMTLGGATATFGGQSNPLCNYTSSLAKKFVYKEINKVYYPLRRHVFRLKVRTAAEIRINDVVKAY
MKDKMTFKKGCYAATITNTVELDHMGSIIPKDEYEVRRLTSYMTSKKMSNDYKRHMQELWTRVLFVCESTN
LVGVTENATHQNSRPGTDEEFMAIIWYSTFASTLFAFVITLLVWWYRYGQAPQYAEMK
>tr|C9ZIM6|C9ZIM6_TRYB9 | DeepTMHMM Topology Prediction - Predicted
Type: TM
MGGKEINKTGREKKCWEYHRRSFSKEKREQKVICVDLHVVVSFLLYLSCSFLPHFNFFTPICSFLSPTPKV
VGRNKYLPLPPPFLFTSHPPPPIPCSLLLLLVIFLRYILCICVTSGGETGVGKERVVLGRLL
>tr|C9ZXS3|C9ZXS3_TRYB9 | DeepTMHMM Topology Prediction - Predicted
Type: TM
MSGSLNVEQGPREASPGSATLLDSPAATGMSTRHAEQVVQSIKVLYELHKSGALSLEEFNAAKQQILFPPH
SLLSVSGRSSEVGHAEYVRSRHIKKRENRGSKRGSSHWSSTSSSSSSISTVSNFMVAPRPRVWLPLVDEGS
GDGVNHDAVMKEELDEGFLSGPVDKAGYYSLRSRTLSQDRGRPCAPPSSNGHGSNLKRYGTFRPPWPVVIG
DTSIPKESVRAPMLPVNQVFVEYFNCRGERGQTFSSVELKEHQLRPPLFLHKRFATGQRERVPEALSTLAH
TQELVPSESAVLHEEYPAAEPPGAPSVSGNSSLELCLNWYWVDMVGRDPSEVMYKNALRHLTKQFDIAESF
LLDREHPLVLPQICSSPEDPSQFLICLRVATAKIALDDDSVKELTNRWILVVDLKRKVVITIHRMDSSYIA
NMRYHWKSLMERSDISFEEFLVRIMHDAVCTYTSHLIAHSDILEKCEAKLFVSSRRGTNNVPGTEYSASKQ
HAEGRIFSLFVDGSSSPFLLKLMDTKNKEPMDKGLMNIFLYHLHRRASVHHRVLNMTRVVLSESFTKLGLC
SKEYADEMCVHCIELIDRALEICDDAKTLLDMHISLQSFRTNELMALLTKFSAFFTPSSFLAAVYGMNFPH
IPELQWAWGYPCYWLACIVACVLIYLYMYRRGLLE
>tr|C9ZTP7|C9ZTP7_TRYB9 | DeepTMHMM Topology Prediction - Predicted
Type: TM
MMLILIPIIRVCRHSYIYIYSYIYIYIMYKYILISSYYHLIITIVIITTYDLTNVVKCGSGGSTNLKEKKQ
KREEKNSNNNNISTEGQQISYYYYYFFKKGTRIGDHHTKSIYAKGNI
>tr|C9ZU59|C9ZU59_TRYB9 | DeepTMHMM Topology Prediction - Predicted
Type: TM
MHPRWVESYGLEGAVGSGRWLLLLSPAVVWQSVTEDILALRVSYNNLTNSMSYATPLSTLLRLCLVFVSTL
DGNALVMRSIIRFSVLTVESFPYQAPHDHDSGMFINFHPLLLIFIFCNHHVAKWI
>tr|D0A632|D0A632_TRYB9 | DeepTMHMM Topology Prediction - Predicted
Type: TM
MYLVLLRLEKGKDGFNECVSSICGWPTCLRRRVDNMLVQSDFGNGNHWKRIDRKANGLCVCVCIHRISPDS
LHDLPQANLHNFLFSPLFISISCLFVYLSVRLCLCLCVRVFGPAALS
>tr|D0A2K6|D0A2K6_TRYB9 | DeepTMHMM Topology Prediction - Predicted
Type: TM
MCQNRGVFFLKILIIVLSFTPWRAASGRVLLPQCHSIYICVQTSVMTVHLVHRIYICLHSTPLRGSGIIFR
YIASFPLSLSLWRTFIFVLYTPPVVICFSCILCVLKFHFVPFHVIE
>tr|C9ZYW6|C9ZYW6_TRYB9 | DeepTMHMM Topology Prediction - Predicted
Type: TM
MSAQAHTYLCDAWNRDKVMAIVQFLPMALEGPARTAGCESLALSLGNLARMGDAYRAVTRLSLLANALSKP
TLTSLSKPAGDMVASRIDQLSHLFHIGFCLNENTAVLAGHGVFPKSLHRLSGVAVLCWMYTLVLGIVRQLY
MLSKMRGHCTAAAASGDDKRKTCPYGGCKRVMVDLLKLVCYFLFALTCLPEGKPQLLANASGPLVPLHVMV
KALSPNPLHASNTVRGLLGLIASVCEFY
>tr|C9ZSR8|C9ZSR8_TRYB9 | DeepTMHMM Topology Prediction - Predicted
Type: TM
MSATTKQAIPKLFPKPLHKYVGNGRVFQCLKSYEKYGVVAYLNSVYNLAPSFKWMLSIVPLYGIFVGNPPV
EKIDVNSSAALCTTGLLWFVYALLIQPQNSGSRSLAAVNVCLAAVHGYNIYRAMSYKRSKALH
>tr|D0A9F7|D0A9F7_TRYB9 | DeepTMHMM Topology Prediction - Predicted
Type: TM
MPPKSHKCSRKEGEVEEPLLTENPDRYVIFPIKYPDIWQKYKEAESSIWTVEEIDLGNDMTDWEKLDDGER
HFIKHVLAFFAASDGIVLENLAERFMCEVQVPEVRCFYGFQIAMENIHSETYSVLIDTYVVDPDEKQRLLH
AIRTIPCIEKKAKWAIEWIGSQTSFPTRLVAFAAVEGIFFSGSFCAIFWLKKRGLMPGLTFSNELISRDEG
LHTDFACLLYEKYIVNKLPRDRVLEIICNAVSIEREFICDALPVRLIGMNSQLMTQYIEFVADRLLVSLGY
DRHYNSKNPFDFMDMISLQGKTNFFEKKVGEYQKAGVMSSERSSKVFSLDADF
>tr|C9ZJQ2|C9ZJQ2_TRYB9 | DeepTMHMM Topology Prediction - Predicted
Type: TM

METDVVRPRIVVGLELVGTASLLLVLAHLVFFGIFSFFIGYSKRCNRHFTYASTEKKGNAKGSPVGGPAKR
TRKRKEHNKEKMSFRRQSFRDGCQTGFVPGDYDISHGPPHEGGTWPHGHKYGSQRSMSCRSCGSGVRLPNN
SFLMSRAGGYGGQRESSFSGMCRASQYGNFATYGMMGSGYRSFGASGFGAIEGGSMYRGYQDSMPVQKLFSS
RRDRGRVPSQVGFFRRPSQLSWSNSSNYVQVSKSLYGIDGGVSKEPARSEVGRKFSVAGGATMSANGLGEA
KPPQALGSRIAEKFAGKSHSLHPREGAPIRKNSPFAVDARQARTKSPASYDLADAEDFHIMEGHASPVCEA
TRAIVETVSDGSSRTIITAESNNVALDIPLSRTVGEVVHPLARVGSAFGEGTEDTQEQTSVASKGETSNTY
GRHRRPFLKRSSSLHRPRGVNAVNISIAEFPEAEEVQDSLEGDILNTRNSTGVPHDTNRPRRNQVSEACDL
LHPTSNEETAGGGEKFASLRPNTNRGSRMQAGESDIRGKLATDGCTGCGPDIARTQSPGSQGELLCDGNTR
AADGATPVTETASIKESQRRTRTNEEQRHPKDLKTNRIKTVVLVEKVPSTSTPLLVEGNNISYRGSKHEAH
EVVIRNPSSTNFYSTTLNGVKDAVCDGYNASVLSVEAPNSDMLFRSPVWPYLTRIVRGVLQRCQTTADSIV
SVTSALGFFHDDKVKDLFDLNGGRFETMQVQPSPIYGPRIPQLRYTKVTSSMAFDESLNKALSRASTDPIL
STMTGGVLVALLLVKQSRIVRTESGESTCDVLLSSLVFASSGSGTHPYDSAISRVKNEYCMLFHLVLGGPS
HTCFLLSLSTADESDEEVNGKEIVENLLELHRKMHSTYNYPLRNGSVTRFVKYVMAANKEGIERLKHEEDP
EKKNRLKRYVQEQTRLLNDATKMLKDAEAELRARANQPR
>tr|C9ZND1|C9ZND1_TRYB9 | DeepTMHMM Topology Prediction - Predicted
Type: TM
MLPSFTRKPADHPIGYLVALSGLLMQLMSYGIDNSYSIFSEDMHNDPSLGFPSITAISLGNSVSLGLSPAF
GVLAGFCVDRLPPRFMMALSTILLFTGLWISSTLAANIYVVTFTYCLFASIGTACMLSPGAAATSSWFNRY
QGLAMGINFAGGGIGSAIIPPLAGKWVVAYGWRKAFQLMSIFCAIGVLATALSARRREPKRDDSSADDETR
EGNKSGNGSSVTRSNEPATVGGGGAANNGHNEGKEDVREMGRKNGSHTNTSKVPPNRRGVGTNQQNGNDGE
GLDVTEQSQRNNTFASAIDVDMDTSMDADEPQVIRSLHTHKLTPWELFLSMFTLPFMGNFLCWFIYSWAFY
SLIYAAVPYISSMGKPGTVYAGVPPIPTDVAATLFTFYGVFQVVGSVLVGWLASLVTAEFAYVFCATVGGI
GCGLLALGRSYVAFALLLCIIGFCMAGMFAVMPTLIATHLYGPNLGFYFGAVFLAGVVGGFVAPPMQATIQ
LRNNGSYAFVCVVMSVSMTLSALVCYATLWRSKRSGIVLAARKTKLVEIM
>tr|C9ZQU2|C9ZQU2_TRYB9 | DeepTMHMM Topology Prediction - Predicted
Type: TM
MAGRLLTAVPRHTADPTNSDWLQACYFVVLEHPLVCIDATLRGRITPLTEQFLRNAEGVSEPSDDTTNLKE
EHVNGTGGSSVKEMRFVVITVDNVSGQLLHTGVPGKDLFMSMREAVSAIPALCPSTPEQYEVFTILEFVAF
AGACLTEDRLYMLFVETGRLLTDDKSCIVTDSGSKPQRVLPVISVERARWYTVPLRVAVPALQQPLGGEDL
QSWVLGPSGGLRDDSAMAMSSSFRQSYLFFTPGIDVSHCSIFEEATRVCGARSDEESGDVGNSSPNGCPGG
LDGSTDSSALAARVMSFLPLPPPSTGREALGRCDCLWNEELIAFCDIFGIADCCCRLALGSFNASTLVVPQ
TAPHVALLLVRLSRLALPGCFNDCSGKSAKTAFQMREYTSAHEAEFQLLLLPDMKLHDSKVDDNLVNSLPS
LIWRRGGDFAGHEDIIQQKQRIDFCTARGMDPLPIGEDYVAAIFSSYQQRYGMGTKIALMHAGCWALIGSA
PPHNTTKGGAAVNTSVNGSRESSKRRTNSQRGLTPASCYSVYASETHHCDAITNELQGRLQSLASHIKSHS
LRPAVFVGAINSWATCLHCVPEKGSAVDALWSRECIRSYNGISCGGTTAGSSGVPGPIIPRICVSNSGEVS
AAASAVVSLILVTVWLFNQFFESQPNAKLLQRHKLLTRKIMDLVVNSAAGLKQVGDSCGHEEMITLLRECL
FQVVGAVPVSCTLTIWETARNMVFLYRANGDRSSPSDFTSTKKGIFRPSVVSRRVILNNPFHSFIMNLFPP
AGGFYQREFARHRTRLLTHVAGQSYIIGAQFMFHTAESDTSSRVVSEANADWGGNERTTFASRQRYLSNPP
LSDDDEATSSYSTNTGGVPTPTLRMLQGLVHPLSQPLLVPLRIAAVELTIALPTVSYITHVAIRVADALQR
EPFAGALTLSLHSSCYVGQEHERLVLDDVPLPLCRGSNASAHAGPHLIFYELPRPSCESVPFSVLNEARVV
ADGATSTFSPIVPVVARFLRYTLRGSGRNALAVFPLLVFGEPLCDIPPLERKTIVGRSCLKQCSLLRELST
AKLHTDSNESSLFQMPLDGAAENCSFRSEESINGKTNIDETTNTVVTPIKGNKSSTASHHDILITEQTLRE
LYVRKLRSACPRGVFALNLPLLFHMESKRLQCKQKRFFRDFCFQKLGLPLWVMKPSSQIFPHSSVCPSRVP
ESFEDLSRVHLLQQSGTCQPPRNDRPIKGANATQRKNVTCAGCDQRFLFGFGLEVCDRCKRHICGECRSKE
PVRLLDVGISSTISSVCRRCLDRVDKLENALVAYNVTTKECEEVDSDTERGAAFEFYIPVTPPSPAEFPIP
YSNYSFYALRGSTYQLTALPFTRVVSAPITEDKSASLFEEVLSFPADVSDSLGGWRCVSNDMPGETQKYVN
ENVVILLPYRCVVSHGLLYYTLAGEIRGSIDLQMHVGETVQTVSRPDAAKSSEGTACARISDCMSGEIGVA
ELRFRHAASDGESASDGSSYSLTVRNPVGILVSLFFAGSSSDLALLHVKHFSVWGCFDSCTRNVRSLFYNC
PPDVCRYESLKLTSLKGVCAPPAAACLPLTIPEKHLIKPGNIIHRLHTDHLLVEYVFSNPVSVCGFTVEGQ
HPASVSSSRGALPTALRLIGILENGFRGNIGYFSLPWPPLAQRVGRCTEREVFGYSYALSSTVHGLVSLLV
EVAEWRLAKGRVRQEVGRGVFCNLSPFYDECESTNKEAEMSPQQPEMHLGKMVFWTSLNPEARHAVSSNAY
NSIFSRAIGL
>tr|C9ZQ70|C9ZQ70_TRYB9 | DeepTMHMM Topology Prediction - Predicted
Type: TM
MQSEIMRHHGDRKHMCRISSGCRHLGRCARPQLLFILFFFLYVTSVCTSHGGHAYIYAPKYEETNKCMCLP
SNSNTFFFSPSIPTFQLSFPLTVIVQLAAEKSSPS
>tr|D0A6Q1|D0A6Q1_TRYB9 | DeepTMHMM Topology Prediction - Predicted
Type: TM

MITGLKNDIVDRKHAGVFPPLAAHIFLHVIPFLTEMLSPRTKPNARRLMRCYMLSVTRPPATRGFQPPLFL
YIYIYMCVCVCVCVCVCMCGKSSKTHTTRMPLHRSICEYHRHYL
>tr|D0A4D8|D0A4D8_TRYB9 | DeepTMHMM Topology Prediction - Predicted
Type: TM
MLLGLKDLFIVRGGAAHRPTLQCDEDGFVVYLMLLVAYCICVCFTTWNYGRTCSPVVLETIILLARVMVGN
SELLFKGDQAAAWAGFGGPHRSPNGETSSHGVGSQQRRKDRVISKPTFAPVPFFSVCAAAFLIVSAFTTLF
CKTDACLLHSTRIAFGLSMVIIPATLTGRKAFMLAGGCYLVYVFWGPFALYVQQFQESGTHTWWHSLYSRY
STALALTVLLMDVRDFTQCQLGRKCVAAFPALEKLVKIIPDSSSHGDDKVWGVSISYVCFVCSITTMTLCL
WASSMMLERSLDVSCTGVFCALVVVLKIADGCLAKGFMKALRESFPLLTISALLVAVVYYCVPVESHPIVA
GSMPCSMVVVYIANRCPCGSYSETIFLLMWLTMFGSVYRGSSFAHVIEGRYVFPNQMLHLVTIGVGFHTLF
VLGFATLTQGSHPCSGPLLPLYKRVTAILRELRLLNKGRCGRKEERNTRSSPPPQTVVERSPLRVETRGAE
KNGNSTEGNLYVVSSELPSLTKLVKVEAELSAEISSTTEQPSQEDDEFFGVVHRGSTGEGVKRLECLVDHG
SAVESTQGVSQHESPTATPILVHEGHFNESHMDAREEEAETTPAFEDIREMECPKKEMEEKVMIILEERQE
SRVRDVADQSSEMGKRKLSANGNSKATYAEAVLEDLQAETKAVKLITAQQKAQQPEESLDKQQLLRESGLS
EPKKNVLRASTSAEGSLKRSGDASERVPVGAVVKMSRQHQATAKSKKESTVPLPKCMVTAQEMSSTPALVA
ADVAVERPSKKEVKHATTPKRNTAEVEAEKQKGTAKGVNQKGGKILQDEPAKKPRAVADSLKQKEKVESRR
RKDEKDPTRCTSDSKPKQFVPSLQNIGAQVGDNCREDGNANDTKTTTATGVAADQPSCTSKNSAVASETRE
CVAISIPQPRSWSSPAQNTSLVLNPIAAEANSRKTTCWNTSSVNNGCETAGFGDQISLLGKTSSPWETPPS
LGESPLAELSQLYLTAGPMCEAPQGETTRSIPLEARNALENFAKAWEPRENVTHLPRESGLEGGSPQKEGK
RRQSVGGVWAGIPLSVATDSKGLQLVNHEAASSPNIFNVSEPPTTMSNSGMAEGLEHQTAPSSVEEHHFAR
LQYPAAQQAQLLMMADGRLAMYPVYSPPYVHQAVVMSPPHGGAAVPHHHVQHQQSLAFYPQVVVPVSQMPS
GTWSPYAQSPGVHQ
>tr|C9ZMC9|C9ZMC9_TRYB9 | DeepTMHMM Topology Prediction - Predicted
Type: TM
MGMHMATLVGSVYYSYFFFLMGVVVIALRFLYFSRRGGVFCLFSVLVCLFGYSWRSVKQWGVKHKTEGSRE
AKESARRLEALAPVFHTALWCGGTSTSSPPCALLLNNI
>tr|D0A0H5|D0A0H5_TRYB9 | DeepTMHMM Topology Prediction - Predicted
Type: TM
MIAINLFVSLFARFVFVTFCCSSSIRVPSFREGGFNLFVNPFCFCVFVCLCCLCCCCFVFFLCHLTSSPSP
FFVFPLCMCTYIAYSFFFPIFLLLFSSRTFCLISPFPRYILFWGLQLPNIYIYICFIYLNGVTFPAASLRA
FKTLLFFFSLSSPLNPSINLLLHVFRRLPASLQWLHLPTPPKKKK
>tr|C9ZNT1|C9ZNT1_TRYB9 | DeepTMHMM Topology Prediction - Predicted
Type: TM
MNDRTSLLGSKWRKRGGEKSVHVKGSANGVGVGLRCEGKDNSRVPRLWVSLFMFSLLMLLPMPLRASVHSG
DNVTVTVLCLMRGLRAPSPIGDAIISGFNSSLAARNWTAARNVHVVPVSWGSYGNQSIDALDKKLSNKSEL
LVLLGPLSDRDVLAVTPLLEKHNVIAFAPVTGSSGLRKWTPHLYFLGADPVAELIALIYYALSQLRPLRLG
LMHLHNTSYGEVQYELTMRLISRMGRDLCGVFALESSDYGSASGEVFNAMWSRFASLHPQGVLVLGPPNTD
TFKFLLAVPSDERTKDMYILAPSALQPAISIIAEELRRSGKVAFASGQVIITGTNPLATDVKHHAVRRFQK
EMRSYLKAQKNESSPGGEDHFLKHATTGKLAVLGWVAGETLLQALSNREWLTSREEFKKSLYNQRRYVIDD
LVIGDYGGECTGSAAAQGATCRCNQGGHVIYMNRLEENGNLSPLADGLITRDVSGCYSDSGGLHAPLIGLF
ILTLDDPIALRAATIMRNAASVSVSRGHHEQSNRLFMHTLPALSDGLTGSLQQELNTRTVTAVFGVVHPTI
MRTPGLAFIDPVPLTPQLNRRMKNVIHLSPTLEQQLFVLAAYISSSSGSGVRAVIRGEDSNAIGDVLTRTL
ATFGVTPHSLVTARVNETIEGALPVYGNVFIIGLTASDVGSVVAHMERNPDVHVLVPFFDVVLLYDEVLKA
FNGSSSANRLLIATNLPHWAEVHSSSEIVQGFHAALPDPAQWTPLALVGFAAARVVRTIIPRIEKVTGETV
INLFYNNIGVSAGDMYYGPFNHGECVNDDVDGLGDGGCAVNYGATRISVWSMARVLNASVPALALPVTPSM
QYHDPNAAGSTGSTLIGIVVGSLFTVFLFVAIVVCQLYFAQRSARDNGNAPKELSDPVTLVFTDIESSTAQ
WSAYPELMPDAVDLHHRLIRSLIVRYNCYEVKTVGDSFMIACKDPFAAVQLAHDLQRTFLHHNWGTTAFDE
LYRDMERRRAEEEEGYTPPSAHLDPNVYDQLWSGLRVRVGIHTGLCEIRNDEVTKGYDYYGHTSNMAARTE
GVADGGQVLMTRATYMSLRAEERRQFDVTALAPVELRGVNAPVEIWQLNAVPGRIFAALDIDKDTCVFGDS
SEGSISSGERGLSGNAITRSAQTIVDSLNALLGTFTAAQRRKALMPFCERWRVALPPEAGFVWSDDYCRQV
IHIVAAKVGKVIDHTVVSQSTGRSTSIATGQLSDIDGFTVTL
>tr|D0A085|D0A085_TRYB9 | DeepTMHMM Topology Prediction - Predicted
Type: TM
MSEEVQRRSSAISTSQGIANAVPVPKKNPYLLRIATCYAHIPMGFREFVLAGPLLLLALGIAYFAPTLAPM
DKLTQGLQPNTAPMSSYRLEPIHNEKGELKGYRRIFIKQDKEAKTSHES
>tr|D0A061|D0A061_TRYB9 | DeepTMHMM Topology Prediction - Predicted
Type: TM

MAEADCSPYGAKRDDRALVVSGLKRTVSTASRNALYAAAGRLAFGVVTGLLKSGLRTGVHKVFLREVLSAD
PLKWAFLSAGLSCFRLLKHIASKLCSHLSIPEKFSYVVAGFISVLPVMVMERGTRVELCLHVLVRALQVVG
TSCVLPLFPKVFRDFEHYDIVVMCLSASQILYGFVFAPYTMPPSYLSFLSKTSMLDERVVRSYAGLTRHQI
SPELVELCVERGRRLPHDPSEHLSVCCSYAHEGLTCNQFCFMLFCKNMVQVGLPLYLPLKVATIVVQYKKL
IRKPLQSLCRAARSVFFSTLFLALYSVCSTRYACLAAQRNIRGGLLFAIASSLAGISTLIEPKGRRADLAF
YCLIYAIRSFVLTQCQFGRLPYPRQSSIFMLYLFSVVLLIFQYDYDPDKLDHRSRYVISRIAGGEILTPTE
QYE
>tr|D0A7X0|D0A7X0_TRYB9 | DeepTMHMM Topology Prediction - Predicted
Type: TM
MCLCMYRRRGGELLTTCYYLFILLKLALPNNSFHSFVSFSTFVQCLMRFLLFLLYRFTALYATPLFSFFFF
FIFSVAYSHRLRVQKLDYTLHARIFELIHHKLLLEVLIQPGNAGGIHPPPFPFPFPFVLFFFSVLRAFFYK
KFALRILLEVFCFVLFCLRKYVIHHTYNMHNKTECYYYYYCYCY
>tr|C9ZYP6|C9ZYP6_TRYB9 | DeepTMHMM Topology Prediction - Predicted
Type: TM
MFFFSCSSLWLCVCTRSGIRGPFAYFRFKAFPNTYQRHAIFLSLNIFLPFLYFECTVRRAATATESVRSYC
IKVTVTIIIIMHTDHIYIYICIYVYVLVFIVRLRLRLCLFIFGSGRLSALAVSNNLCTVSNFVCFLSYIFV
>tr|D0A2C6|D0A2C6_TRYB9 | DeepTMHMM Topology Prediction - Predicted
Type: TM
MPTYVEAENFLDAPRGAVVANKAVTEAESCDEAYNKQGSVRASFAANGATDTICQKESVGTHTQPKRVTHQ
EEEHKSIPFISVVMTYFTFLLLFTFGCIAELRQKLFGAKRKQREGYASLLTFLEYFWQNHFYRRARDCFNH
AIDSRPSRVIGVMERVSTDSNRTFSFTGNIKPCVNLSSYNYLGFADDIPHITREVLNSLDRYGLASCSAAQ
HAGQHGPVSKLERAIADFLKKEDAVVCGMGFGTNFRGLPALFGEDTLVVSDSLNHSSLVNGIRLSGARAKV
FKNADMNSLEKVLREAVVLGQNPKGAYVPWTRIVIVVEGVYSMEGEFVNLPRVVELKKKYGALLFVDEAHS
IGATGRTGRGVTEHFGVDPRDVDILMGTFTKSFGAIGGYLAGDQSVIDHIRTHSSLALHCDTLSPPCAQQA
LSVLDVLNGNDGTDIGPKHIQQLMENSRFFRQGLIDRGFSVLGNDASPVVPVMLYHLGRAIAVWRKCMRRG
LAIVIVGYPATPLLGCRIRFCVSAAHTRADLQFALDVMDQIKKETNIQFLPSTVPPQGEE
>tr|D0A696|D0A696_TRYB9 | DeepTMHMM Topology Prediction - Predicted
Type: TM
MLTTGPLSILRAERLCKIYFYSGFAGLPLLWFTTWLFFRHHAQHSEAIRWYTNNSLRLSVVGGLVVLLWYV
VALIVLPVTSPLFALPPSQKGEWRPGFFTELVDE
>tr|C9ZTQ5|C9ZTQ5_TRYB9 | DeepTMHMM Topology Prediction - Predicted
Type: TM
MYTVGCNTNVSGGAVNKREKEETERMEKEEEKGILFVCLSCMSPCGTTPLLSHTKTQKNIINMSLFCITSC
RSHFIFFCLVVRHPAHMCAGYSKISYSGFNLSFLPSFPLLCSYYYFYFFAISSLHRLPDTSSYRRENSVPS
VGTY
>tr|C9ZU83|C9ZU83_TRYB9 | DeepTMHMM Topology Prediction - Predicted
Type: TM
MEGRRSVRCNYEKKEKEEEKVKNNEIGCLKFSHKVYKHMDINICKYLFTSIRMCVCTCLYILYKTTGKKVG
VVPQIFLKWAIDTRMFVCLLMQTRNLFFFVYIHLKRVETAYVRWDMKGREEMPHIILDHSRWIYTLTYAYS
HPLCGTHFTRSNFLTCTTKETMRCSCLPVFVVFFFRFSAAVV
>tr|C9ZPN6|C9ZPN6_TRYB9 | DeepTMHMM Topology Prediction - Predicted
Type: TM
MTDATKMAGQLLFQNTLAEVVRKLRSSNESEAEVIEQCIADIKSEVTSTVQSVKVTAVLKAVYFSMLGYSA
TYAAFNIIEVMADKMFGYKRIGYMAACLTFTPKTEVLPLLTALLKRDLSSANQYEVGFALYCISTVSSPDL
ARDLVVDVVNLLSHPRNYVRKKAVLSLYRIFFEYPESLRPTYPRLKEKLDSSSERCDNDPAVRGALVCVLC
ELARRNPASFLGLAVPFFSMLSTIQSNWTLIKIIKVFGYFAPLEPRLGKKLVDPIIRIVQTTGAKSVRYEC
ILAVANGMSKTPSLTKIVAEELRVFVEDSDQNLKYLGLDAMSRMVRDNAKLLSGHRDVVLACLDDIDTTIR
RKALEVLSGLVTKRNFVSTINNMMHRCVRLPPDEEWSNRVLATVIEVAQTDDYSYVQDFEWYVKILLDISL
VNLSTYQHGALVQKELVTVLTRVNAVRQFGVNELSQLLSNTNLLKSDPSRSSQWEVLKAAAFLCGEYPYWL
QDKRRTCELLLSERISLLKPEVQVVCVTAVGKIVAYMRKPCQRHLVLVNGEEEIPLPEDSLTFKELRASIL
QTEAITDSNGTAKVVGATGSISNRHDAERKNLLGLQLFRHSVHPDVQERASMILYQLNVDPDIGPLLYEQE
LLPVAIGAQEAVEPPEGLNLDEPFCSHLPAGLSPSDSEEDGGENDEDLYYVDGYDAIVAREQRRREEARRG
EVAPFYIKNDALSRDQLSEELHSAVSAAPAPKTSTSFYVPQKSQVINRYLSRPKNYDAAAHGQRRIQDEDV
DEATKKFRNVDVTRSLAPDERLPEPIPYGRLQQSATTEGTAAAAAAAAALDFMVDESFDPLVLLEEKYLRV
TAFVLSCRVRKAGTQITMAVEISNLASSSSMRNVSLRFQPNKDNYTTERVRLEADKSQEAITGANRPKTGR
GDNKNAVGEGGDGDKEVEESDRSIFVSKCMKGTTTLRTKMLLTFSGSLPPSLTEPLLFSLLYTREKKPTES
LLPMPLSYRYFAKPHVDTTSVEFMQTIMARHLSESPVLTCFVAVAASCVPLVLPAIQQQLRLRPVDILKDA
ASFYSVLQSRKSAANHAHVAVLLLEDEVEGEKGISIAVKSEHTSLAELLAQEIAKLLMTATS

>tr|C9ZVH6|C9ZVH6_TRYB9 | DeepTMHMM Topology Prediction - Predicted Type: TM
MSVYVIHVAFSCFPALPFYRWPPIVRYPYPVYSAKYFHSVVCLLLFLFPFIIFLNSGAPKESMCAGRDCSS
ALNVLAGPPTDNGKQPASLPPDAGWKTPPTAMDDQFLYGVYVPPAAYGKLHNRGNLDESAVGLLSVPYGGA
QDWRWSNEWETSMLEAPCSEPLFCLGACCCPWCCAFSQRKELLENDWSRYLCCAGMFCGRRCCVCEGCEPC
YLCLESCFCLACAVYGNRFIIRKHYNLQNDCCDLLLMSCCPLCCCLAFVIDDERFNLFVGTWGAAFACMLA
QHQHQMKKQGYPWCRIMK
>tr|D0A242|D0A242_TRYB9 | DeepTMHMM Topology Prediction - Predicted Type: TM
MLLIVFVVGFLLLLTAFVALQLRRSQQFPTFIDATPSRVAARRSETRRSQYNDGDYWSNESSDEGSDDDDN
DNCEVGHKREERRMLRGGTSGSAGPLRHRRTRRGAGNAAEANGDGEEDKNGSQTKLSRLQRKKQEKERERQ
ERQQAQEALLENRRVKQEEEQRQQKDREREEREREIAEEAALQELREEKKRQEDEEYAKWVEGIGVVERGE
IGDEERKKHDNLVKYLITMPGNGGERQQQRVIDAENFGEGKKVGDETITRGTESSSRGNILILNDVARMHG
LSVEVTVQVIEKLLEDGVISGVFDDRGKFVMLSEEHYKQIAEFVKLRGRVSMKELARECNRIIML
>tr|C9ZXC9|C9ZXC9_TRYB9 | DeepTMHMM Topology Prediction - Predicted Type: TM
MFQISARFFVGLAAANVCVCVFMFWSYVLFFFLTIVVAKLTGQQSRTSVRFVWLPFFCYLLVNVDTDKSVC
CVCESTCKASRWRTCADCIFSQCQCCFLFSLSPFCWIHLDTFRVLRRGRGKGKVCECIPGIMSFGLEYSEG
QRDYLERIGVGPLLEDFVADAVREKPNDVYEFLRQWATARCAKATAATHEKSARVIQRAFRNYRSRSTATA
>tr|C9ZKE2|C9ZKE2_TRYB9 | DeepTMHMM Topology Prediction - Predicted Type: TM
MFFFPLSLHFTCMALQRTLDFFFVFFPCWRFWRCCFLFFFDGGGGIIIVIIITIIIYSLLFAFLPVRPIEM
LIYLPFDCFHFLFVVDDDDDVFDLFCFVLCPPAPHRQLFTLFRYLFFFCRAGLQ
>tr|C9ZIK0|C9ZIK0_TRYB9 | DeepTMHMM Topology Prediction - Predicted Type: TM
MGRQEDGSFDARGGDAVLVAELHSEESKEATFVHPEARALFNKVPCLRHIPLFGEAAEGYGPKPVLSIGLS
YFLCKGLANGLVGGSIIAMFLNRFTVEGLVYQRLTNIAGMGWSVKPLLAAISDIFPFFGYTKRWYMFVANL
VGPAFSLGFALLPAQPSSAAIASVFLFFSGLSRACTDILSQGLYSRMMRRVPGPGPALVSWVWWFIILAGL
FVSVIVGPLGDAGIPQISPMIGSAVQLLVAPFFLFNWFGELPNREERYIDAHVLHEQKLKEAKDTPPVADD
NDPIDVNGGMVERQVTNSEPVTGPNADGGEAAVKPFVFREPRECCCGVFQMNEEVLERNKRETIYSVLIAF
FVVGVAITSLFGSRLHLLIAAAVVSVGHCSFNFYALSWAVAKVNLFSYLHNASTVSFGGAVTPFFLSGPDC
NPGGPHFNLFFLQTVGGTVGSVTSALGVVLFNYFLSKKTYRMTYFLTLVFLIVSNIFDFIIVMRWNRPYVS
DYLIYFCCDVVISPVIGMMHWMPLTIILSRLCPRGSESTVYAILAASSNFGGTVGSSLGSVIMEYALPVKT
LVPCDFANVKWLVLISGFVAPCIQIPLIFTLLPDARMDEELDGDGKPVRKVAADVSPEGKETVCEGDSDVT
PNAEGNGIAPKRS
>tr|D0A1K5|D0A1K5_TRYB9 | DeepTMHMM Topology Prediction - Predicted Type: TM
MAMRRGITHLDGRANGVRRFFLAPCPYYFLIIWSISSSLFFFLQVITASLFHEEMSIRLLLFVYLIALKLR
SATTSYICLILWLTPVQPSGLVDRGKST
>tr|C9ZLA7|C9ZLA7_TRYB9 | DeepTMHMM Topology Prediction - Predicted Type: TM
MARNQTHSINRFIPPLLERFVCTHLPCAALFSLFSLLITRWYYKFLPLWTLFVHFFHFSPVCQSVNSLPPP
RAILSIVFRRCVTTVGVCQGLPYNFSLFLPPFPFLSFLFLSFLCLNIIPFSRGAVRRARVRATNKPMKLIA
KLGGGGSGNNTKELTISNPEATLNNLRTVIAINFRLPLGSFRLQHATFDLNDDKRLLRNIGIQDGDTITVV
AKHEREESGSGSGKSKENGSAVPPPPTTAGTMEGNSEAVTGVEKSGTEEADGDEESEFDENDGSDGSDGSD
DDDDDDDGQEAMLARLFEVPNLIEMRQRFLADPEGVLRQISADDPVLLELIAKNRQAFLDLVCNDNFFEEL
QRDREEGVMTTEEDEEFDEEMLEEVMSQFLAAASDLDDEAVVEQQDEDGSETQRYLDRVPTEVEEEKIEQL
MQLGFTREQCKVAFFKAKRSIERAANLLFENPPQL
>tr|D0A9K6|D0A9K6_TRYB9 | DeepTMHMM Topology Prediction - Predicted Type: TM
MCASQEKRRAHGNAPSRQHGERAELFLFCIVICIYAYKYIYIYIYIAYMHVTLYVRISVCLCLRVLAYYRC
FLEEQEEKELRRFCVLLRHYAKRHNEYGRNIDYGEEI
>tr|C9ZL26|C9ZL26_TRYB9 | DeepTMHMM Topology Prediction - Predicted Type: TM
MSSCERLSPKQFHSQESRLYTEKCVRELMQTRAFRDYVGHLEYSERMQFSWFVALIGLALASYYHAIFMFP
VNCGTYAGNFDEATPFFLVATLLMALFVAPSVMVAQISGFFEPISREEVSSLLTYITFVNAAVVSFGGSWL
HNPPYIVSEPPVPWLSGTISQLDELATRTILRALPLHGIVVGSWLLYLWQSRGWRRVWKLPLPLLTAPVLL
AVFTWFWFRVTSPICAQLLSSSTDALELVDVFFSASDEMIKSFSSIFSTPLRLGAFISPQDCCRLWCSTLA

FSLLYFAGALAIPVIGGIAILISWVLPTHPTTWGLSFILSIAGLVMQVQRNESSVVALFALLVAIWALGRN
GFSLMN
>tr|D0A6V6|D0A6V6_TRYB9 | DeepTMHMM Topology Prediction - Predicted
Type: TM
MYIYIYIYIHTYFIYLHLHFLFKYVLRGGCWEQEQRWNNSFALFVTYLCVSPSLCVGAACPIHLFVTSFSC
DIYIYIYIFIYVCLCSVLYLLPFFSLVFCAPPFFFTLALCIIQRGGKNENTHKKKEKKGKVKTISREIKYN
TPYYHRYHYCCLTPQKTRKVKKKRLWHLGGFERPTGR
>tr|C9ZIU8|C9ZIU8_TRYB9 | DeepTMHMM Topology Prediction - Predicted
Type: TM
MMQINLIMTRGGGKTRWIENDIYIYIYIYICRMICVCIGGRELQGEQIIRMHVFFFLSVLWRGEKETNKVL
GTRGRGVSSLVSYCFLFFRP
>tr|D0A1F0|D0A1F0_TRYB9 | DeepTMHMM Topology Prediction - Predicted
Type: TM
MLDVYLCDVVHCIPGFVHSFLFFSFFFSFFFSFFFSFPLLPTSPFLSFLLFCFYFFTFFIIFSFFIIFSSY
FFFIVRFPLHTSLIEMERKGRNYHIFQTNKQINNNNNRKRKRERKKKRNEKER
>tr|D0A3Y7|D0A3Y7_TRYB9 | DeepTMHMM Topology Prediction - Predicted
Type: TM
MFAHATHMTSFPHSLSHIYVYMYLSFPIYSNKAVNDTCTRVWQRLQGCIHILFTMVIHIIFSSISSLLFLS
NNKYQQLPHQRSLLRKLSRFRRTFHPTVFFTAPPPSLYICIFYSISMCVQDSVLHPSPLKHNVYT
>tr|C9ZMR4|C9ZMR4_TRYB9 | DeepTMHMM Topology Prediction - Predicted
Type: TM
MHTNRNPYTRCMQYVLFVAERKKKMEGGRDKEERKLMALLPVPFYSVLYLTLDFCDLTFICFILFFHSLSS
VDSFVSLFFSFHLFPFLLSCPSLLFHTHARSICMLMCVIYRVSLEISVVKCV
>tr|C9ZMW3|C9ZMW3_TRYB9 | DeepTMHMM Topology Prediction - Predicted
Type: TM
MKQGNGTTHSIPHNVPFRRIVILSDYGSPWLNAVTAVTCATLPCLSALSNGWCEKRGNVVVASKLDSPRVL
IPAEAGVAPDGTSCSSLSSLSAVCRDLADGSNIASRIFFTADAASNPRAQTFAPSVEDDLFVYVSAWSAEK
DAAHLALRSLSNMFGATSFGIGASGVLTGPCGVPRLIIILADESTRNSVCETARHLSNWCVLSCIVPSFAPP
AFNHQLAASVVRECDVVCGVLECVALGVALGTVKRLPFGFMDPVCVMPLDAAISTALIAFLQRLKGGPAAV
CGGTSATIIIGSPSDSAFVWGMFAQYLMDYYGRVGLLALKESQLRWLFDTSPALQLNADFMNWVNYGTSTM
MPFRCYYDNAAARRRKQLMEHYPHSNVARKVCGTLTNIDTRIHNMVKLTQHSEPTGQHDGERVNKQGKPNT
AGFYSDLLRAVEQDVALRPLYPYISLSCIRWDVFVKVIAQSVLEHAARQIAGHPTVVPPPLPLYHNDIIFY
GPQRAPRNCLLTRFFPESVRWAQRSGLRPNGVLVAAEPGLTEESLACILQRPSIIQVVKKTAKAEGVTEED
VLRRATAILRGMGDNLNQLNVRVFGLMVRKALFRLFDRVSLNASAFERLHAATTQPRTHVMLLPAHRSYID
FIIMTYLLLVMGLTLPHVCAGDDFLRMGQITKLMRGSGAFFIRRTFRDDPLYTALFKEYIHNLVLRKQMIE
FFIEGTRSRTGKTQRPMLGVLKFIADTLVNAQEAIDDACILPVSLSYDELLETKLYAAEQLGLSKPRESVG
NLVRASSVLSRRHGKIHVHVAEPLSLRSFCHNPSQCPEGFEPVTGGVTAAAPNKSSDGEGARSPSHIPKQV
LTNIAWHVTHTLQDNTIITPTALLAAVLGTLSPPAGCMSLREAQAKMLWLRDHIIRRRAHVSEDCVNNNGE
ELSRMALFHLAEFVETRVEAGETWVAMRQDSRAPLGVAICSNQLIHVFVDEGVVALVARASGELSDNKKTA
RVSTDLLKRECELLRTLLAGEFQDYLPWCPYTYASWFKYAVTRLLAHDDIHNDGTVTLEGGGSAESTNKVK
KGDPYAVASEPRLHDSEDIVIHFTRRFRFITGILYPFVESLYVVSVAVAAVAETHVEHLLRRRPLLLACQK
WALELCNEKRLVVHIQSCGSVALKSAFTSIMEFIGLKSRTEEKDLVYILPIPPNGLETQLQPVTEQLKKLL
RPLIKVKEEEEREARNHMVNVYKKIQMSSKRASRM
>tr|C9ZR03|C9ZR03_TRYB9 | DeepTMHMM Topology Prediction - Predicted
Type: TM
MSTEDILIECPLNVEVEEEADGINDVVRLEENQLSTAQLLCRITSLIAIGAFIYLTFWNNSQFWAVVFVYH
PLFMVVAFMGALPELMCTVAGFHSRKFPFSRVIRTHLKWTMVFKALSLLGIVAVEWTKFVKSKNHFKSWHG
IIGAVCEASQFLEALVGLSMYFNAGGRFFSSSQARLLRVAHRLLAVVVILTGLTSMSLGMFTKFAVEVYGA
LAVRVAFAILPIVLALWGYACPFVAW
>tr|C9ZIV8|C9ZIV8_TRYB9 | DeepTMHMM Topology Prediction - Predicted
Type: TM
MRRHRVAVVSDFFYPGFGGVEVHIYSLGQCLMRRGHKVIVITRAYGDTCGVRYLTNGMKVYYLPLMAVKLP
AGSVTLPTMYLTFATMRSIFIRERITVVHGHQNTSNLCHEALFHAGTLGLKTCFTDHSLFGFADVSSIHIN
KVCEWSLRNVDQVICVSNTSRENTVLRAKIDPQRVSVIPNATDCSFFTPPDDMKYKSWASKVENEGLTIVV
IGRLVYRKGSDLFVDVIPEICKRHPNIRWIVGGDGPRRSQFQQMIERHDLMDRVKMLGSLPHSGVRNVLIQ
GQIFLNCSLTEAFCIALIEAASCGLLCVSTRVGGVPEVLPPNMLLLAEPDPSSIITTLEEAIASVPYISPW
ELHDNVRRFYRWDWVAERTERVYDKIMCTKSPSLYERLMNYASVGCVYGVICWLLCIGDWLMLTFLEFWFP
SELIDIAPDFPLELYSRNREKLQVMGSPS

>tr|D0A534|D0A534_TRYB9 | DeepTMHMM Topology Prediction - Predicted Type: TM
MFYIDDLFWTSLNYATSWVGLVSILTYLLVVLLQQLALHVPQNLKKKYAAEWALVTGASSGIGKAIAEKLA
EQKINVVLVALDDPLLSSTFAELQQRYPKQSFRKVGVNLGEEGMRYMQPIVEATKDLHISLLFNNAGYINT
GLFADTTIERLRANMECNAGCAVPITHHFLRQMLQRQQRGLVSFTSSASCYLPGPTATLYSPTKAFLTNFA
TTVAAEVRDAGVDVVVIHPSPVNTNFYKNQGPLLNSLKTAQSAAGSPMNIAEQIFAAAGRLTVWDQGVTCA
AFRIVNKVIDFQLFSELVVRFAWLNGDHKRLVANSNTRGKKQS
>tr|C9ZWC1|C9ZWC1_TRYB9 | DeepTMHMM Topology Prediction - Predicted Type: TM
MPFQLSRRLVVASVRQMRVRIPCSRCYCLYESCRSYRQTPSLFPPRKLSQAKQKPLFPGMYRTVRLFDGEH
TYHYGRHTYTIMRESLTFAIIPLGCVAAYAATYFDPFAAVPLELQGAYLVFLLIGSRLYARGRSCRTVKDL
TGRHAVVTGGTSGIGKETAARLAAAGADVTVLARSSSHAEAAITYIRRSAHCEGQVVQFTPLDLSDFLAVR
DYCRRLRHTNTRLDILVNCAGVMHQRQVMSRCGDDEQLAVNLLGPYLLTEGLLPLIAESGGRIVNVSCSAH
VAVRGNIVQTYLSGRGVWSPRVRGKFDGLEQYGLTKLGCIYHAQELASRSYHSTTKGNASFCPTDTSHPSL
GSSAINKGSNLNLYHYKHNSGNRASNNNDSGDAGGDDEAGAPNYTTCVAVPGGVVTGIYRYVPLAATFRWF
RWIYLLFMRSAREGSQTVVDCCLRDDIVNGGYYQNCRYAPSGLSAAACNIEERKNVLTWVRRKMQPYMQWD
R
>tr|C9ZXQ5|C9ZXQ5_TRYB9 | DeepTMHMM Topology Prediction - Predicted Type: TM
MMLLSAVGTCLSYYKSLCFVLNFRFPQRHFASLLVPQMYTYIYIYIYDFGRRLRLGVRLGPAAKILIHRLS
PPTFRRLSLAFRGLMTTTVDSAPTAAEPMLEVKKDLRLLETGRPVQGYSNTPELLAKHRSVTGGAPYFRFP
PEPNGFLHIGHAKSMNLNFGCAVSHKGKCYLRYDDTNPETEEQLYIDSIHEMVRWMGWEPDWITYSSDYFQ
QLYEFAIQLIKDGKAYVDHSTADEMKRQRENREESPWRNRPVEESLRLFEHMRQGRYAEGEATLRVKIDMK
SDNPNMRDFVAYRVKYNEHPHAKDKWCIYPSYDYTHCLIDSLENIDYSLCTLEFETRRESYFWLLEQLNLW
RPHVWEFSRLNVTGSLLSKRKINVLVKKGIVRGFDDPRLLTLAGLRRRGYTPRAINRFCDLVGITRSMNVI
QITMLEHTLREDLDDSTERRLMIIDPIKVVIDNWDGEMEAECPNHPRKPELGSRKVMFKKTFYIDRSDFRV
EDNDSKFYGLAPGRRAVGLKYSGNIMCTHFEFDEAGRPSLIHVEVDFERTTKPKTNISWVSEAGAVPVEFR
LYDYLLRDDRAAVDSDFLKYINEDSEKVVHGYAEAAIREAKVFESVQAERFGYFVVDPETRPDRLVMNRVL
TLKEDKEKTAISRGNTGVKGKKQ
>tr|D0AAA0|D0AAA0_TRYB9 | DeepTMHMM Topology Prediction - Predicted Type: TM
MLELIHVYISLSIIRVPSLAIFSVFNYFSPVCFFFSPVYYNFVAVCGGSRDLMTQSAEVSVTSYEQPLPGR
PTDGVGADLPRDADDVRDSTQEFDMLAVDVILGDGTHCALDFAESAYLKRLISQKQPVQLEFGSQVAIEEL
KRIVALDLRGSKLSRARRQTRADVKSRMSEADDKVLSSFSTSAAPHREDSQACAFFSETTLSRNGTVSCYN
ACELTRNRELFMEVLELSARLEMEKTFAVLCGEFGRIYKEEVFCDTDIRSLASLNSKEKLCEVLAFREKVM
TAFRIGGGSAEPGSPQPRSLQPTKDVLIATGADKSNHQLPCVAWLKKDRYFYPEFYHVGDLTQSIDPSSTT
MTEASYLGEDFAKKGHTLYQNPVWEGEDSAKTCSECKRSFSSWPALLFAAVRARNCRCCGRRMCEQCTSWM
LDKSLAKLSKPGQPEEGRMRHACKRCYDQAMMINKHTFLCTTFVCAGLSIVDIALLRTVNEQWRAAAELCL
SDYRSSLYENMWNMKIPKRTAQILASSVNLLVGHPEPLIFLFISIDWENNELVEKACAAIEQTAKGAAPPP
MSPHLRDFYWKPPLSHWYMLCTRTCGRMLPPFFGIKILECLHRAPQGHATAEKIRKIVTKHLLRDWITSLD
TCIRECVVLLLLDIFDLESCQPRVVSVLLSLSKLDPQLAIVICQEASARMRVNEFSYRTLQERVIERNADT
FPESHQKFLNTLKFLNLITPEVNLVPVGGDAREYQRALVTQLSNSGLMGGLAETTGESVSGCLSTSSRIAQ
ERSGLCAVSPMLFPFDSSVVITHIDLGGIKIMESSQRPVSIPLVDAEGVTRSILVKRENLEKDKVMCVTSR
FLRWVLYKQIGNVVLPTYRVILLSPSSGLIEVVKDGQTIHRVIGKDKEGRLLQHLVSLEKTQGQLGDSVSE
PQLHERVRGAGHQCGQPNSLIVKGFDSQKSAALARECFMCSAKFFILLNYIFAIGDRHRENVMIHPSGAIF
HIDYGMLLTTRTLAEHVLPSYVRFDSDLEGCIEYFMKEEPAPEVQTEGEQDDSCTRFIIQTADWFLEVRSY
AGIMHQLLSHIVRRNALDGIKHIGELTALMNLTLMRSLAEESCKETFCKRVKDSRGRTWLKDITHDTHKWT
QQAMERGFKWLYKLMSLHPIGVDARNQTK
>tr|C9ZN51|C9ZN51_TRYB9 | DeepTMHMM Topology Prediction - Predicted Type: TM
MTEGLGEDKSAISHEPTAFEQRVHDEALERSRRVAKRRNPDNAFMKALHKIIPYGGIVSSSFNLASTTVGA
GIVALPVAFQMSGIVMSTIYLIVVAIMVVYSFALLTSVGEHTGLRSYEQLTRTLLGRGADYMAALCMWSLC
FGGEVSYVISIRDAIETFVKSSDATSEGLRSESSIRLLTVAIWFFFMLPLCLPKEINSLRVLSASAIVFVL
FFVICIIINSGHFLAVNGMRDDIIYFQSGNASINGLGIFLFVYVSQVNCFEVYEEMYKPSVRRMTVSAMAG
ALMSFCLYFLAGLFGYLQFGSEVNSSILKMYNPLTDVKMGVAYVGIMFKICVGYGLHMFPCRDAVYHVIGI
SVHTVAWWKNALFCCAMAVASLIAGLFIPDIRMVFGLVGGLSGGFIGYVFPSLMFMYAGGFSVAGVGWGHY
LGAYALLFAGVIAITFGTVTAVYDVIV

>tr|D0AAN9|D0AAN9_TRYB9 | DeepTMHMM Topology Prediction - Predicted Type: TM
MESKTWFGSQEVDGSDHGLAASDGASLVEHVRDILMDSDDDVVSVSCVNLFLKTETTSRAVIENEEYLARH
STGRDFRRGATIFSARSSSHSMSRFTTAGAFSSGSKEIEYGVLSMQPSVCVGVDRDVPLKEKVDSFHCGCV
GVVSAVTDVRRSVSGLRSSKLVATGDCCVADCGDQEPLLGAGRGPFGGNGDSSILERTYGTLDGLHSVTAE
RFVDEPVVAAGRWDSYCSEEFPVFVDGDVFRCLFCGSEVHLVQGSPSDVFVPEALVSESNSSFGLRRAVVH
VFAVFIISMLMLHHETYNVFRTNPFVDNLLGGKCMMLKLMSFHAIAVSSLLVVFCFRGVWEVPHVVLLVST
AMLVVDDFRLLYGVLLNVTTSFVVRELVQAASFVGSFASLLLFTCADVGYVAGPPFLALSVCLAWLMSRLV
AFGVLPAFATTAAAGYTAIIHPFCLR
>tr|C9ZZY7|C9ZZY7_TRYB9 | DeepTMHMM Topology Prediction - Predicted Type: TM
MLTQAGEEEAVAETLTKNLSCLYKAFTLGSDRKEQMCVLQHLMSFLSLPVMGITVGTFLGRYGAVGERGVT
ANNSSAFALLLLEYFTVEAWNNSVFYLHINNTEKQGEWARAAWALVDILPPTHRVVSALHALRGITGC
>tr|C9ZWD2|C9ZWD2_TRYB9 | DeepTMHMM Topology Prediction - Predicted Type: TM
MKVICFFFFLLLLLLLFCNTYIVSLYVFDPFTIPLIQTPTTFLRAINPPLKYVTSSFYFCFAHNLLLFWGF
YSFYLLNLFIITSLLFYFCFFLFFFLSFDISFSFLLLLGTRNCFSLYFTHLARAASSYFVLLVLSPSLSLL
GFSATPRDKQETKKKEQNRTEKNILLKTKTKAKKESKRVTLSFCKLLHFDFHL
>tr|D0A7S1|D0A7S1_TRYB9 | DeepTMHMM Topology Prediction - Predicted Type: TM
MSSLPSDSAEGFIISHDSRCNSSPVALGNLSRSRCCRRTFIWSPDAFNQLIPVSITSALVFGVVFTLWNDL
GVAELVFLFFLLSIAYTSAFILSSSDPGVYPRLRLSEVDPLRDRMELVYCRVCNLRRPPRTSHCYECNVCV
REHDHHCGVLGGCVGQRTMRWFVLYLISISGACILGLLWLIRGLLRLGPMVVMPSTNSSTVRNVSDRGAAP
SSAAYKGEDVKAAAIIVMFIILVLITMLVGGLAGYYLYLVATSTTRREAQKRTPRTHTSFTLKGMVSNVVN
VIYPPPSLLIEPAGASDVHMV
>tr|C9ZI16|C9ZI16_TRYB9 | DeepTMHMM Topology Prediction - Predicted Type: TM
MKLTAVSTFVACCYFQFCHLLLSRFIFIFMHMIHFPSVVLCPLFACHNAIWCMHELCGLALEVMGSTLVGM
GGGGKGVTKDFMFRRRSH
>tr|D0A6X0|D0A6X0_TRYB9 | DeepTMHMM Topology Prediction - Predicted Type: TM
MTITVPTLPMRMKRKWDEDDTAVTDVNDNNSANTSNEDEEEDEDEDDTAVTDVNDNNSAHTSNEDEEEDED
EDDTAVTDVNDNNSAHTSNEDEEEDEDEDDTAVTDVNDNNSAHTSNEDEEEDEDEDDTAVTDVNDNNSAHT
SNEDEDDTAVTDVNDNNSAHTSNEDEEEDEDEDDTAVTDVNDNNSAHTSNEDEEEDEDEDDTAVTDVNDNN
SAHTSNEDEDDTAVTDVNDNNSAHTSNEDEEEDEDEDDTAVTDVNDNNSAHTSNEDEEEDEDEDDTAVTDV
NDNNSAHTSNEDEGEEELEFTIAPSPVFLPHPPVSDENSTLLDGNDLSVDRGNYCSLLFSFVFVFLTFVV
>tr|C9ZQ98|C9ZQ98_TRYB9 | DeepTMHMM Topology Prediction - Predicted Type: TM
MSNHTQRLSGGEYTHKKKMHEIKKGKINKQTNKHRSSPLSSLFISPKKKNSEADGKVVQNYININKKKYIY
IYIYIKKKKQRAPSKNNNNNNIINYIKIFVLFISYILLLIFASSFCFFFKRTYTTIACFLNSFARSSTFSL
LFCFYFSPPFKKKQKNKLTNLQNFPSQRKVTSIRTYICQRRIDNNNNNNNSNHNNSHNKRAKNK
>tr|D0A202|D0A202_TRYB9 | DeepTMHMM Topology Prediction - Predicted Type: TM
MFSRSFVGRMGNGTYGGWPWPVKLPLKRGWHHELDRHQSIAEESRRYILFGDALLVSIVLYSVYRLYYLAL
CSDAYRTHLSHLSGAPPAIIANSFDFANLENNRTVPRAVLDEYREAVVDGKVKRAPIESIIFKY
>tr|C9ZP97|C9ZP97_TRYB9 | DeepTMHMM Topology Prediction - Predicted Type: TM
MIAAEVALSSIPSLKLCPVTVLLVFVTSMPTYALYINVRSLWIFFCFLQQQQQNPSISVWFSRFFETFILI
ERAHNFLEYIYISIYARLHERMAVMRCILRIHVTDGISTRYLAYRASFLCVKRAHSNFRTGQEDQYTTKRS
EDESFGGGDSHYPSKDRTTTSPASSQGSSDTHEHTAASSAEEKLSSASRGARDDISHQELEPMRWPQEAPG
SPALTEPIIDEKGQFIVSRIQWPTGELAYATPPPPDTRVAPRFGYNVVQVKKHVSWWKHYQQHPRISVAYI
NIQLLFLLGAAWLMAFLVEEYRRVTDELRTPGAMVGEHRGRGPVEKGKQKISFTNDEMTSLIGRAQDNWMD
ARAEANYIGSKDYTMKKIPRPKEFSVDDFRKR
>tr|D0A1F4|D0A1F4_TRYB9 | DeepTMHMM Topology Prediction - Predicted Type: TM
MWACSCNQHWLTRHPPRMQKCFLYLFLNFPLLLKGLVPFRPTGLFLFLFCPQTQSSFSSFSSRDLHNCATL
AMSSLLFGSLSLFIYSFYSLPTLQASVTPSLNSLSAILWAPRKCGGRNLITTGGPARANKC

>tr|D0A7I2|D0A7I2_TRYB9 | DeepTMHMM Topology Prediction - Predicted Type: TM
MTTSQLPKYTWEEIRKHNHDKDCWVVLYRRVLDVTKFLNEHPGGLDTINDLGGYDITNSFESIGHSSSALA
LSKEFIIGELDPSSAPPPVRVRKLGDDVPLTKVKGGGGVLSVYHIVGLFLLILLLLCYIFAT
>tr|D0A3C0|D0A3C0_TRYB9 | DeepTMHMM Topology Prediction - Predicted Type: TM
MSRGVERYVAVLTLFAWHFVSMMCSVSRNFCIYIYIYISYIIFCKMPSFTFTIHRRRFLFSLEAEWHPALA
KTSNRKESRVLIANCVSAGVCASQNIEKLWKARCRFDMKTVYIVAMCAGGGGLLCILLVITICVLRKRTKT
SNTDDDDSAAQSGCALHYIDMSGNERVLDVNALGARDGITLLATIANHCGVQDSSAIALTYVNDARRCVEV
DPDLLLRSDGELSDRLMQSKFFPLRLGWRVKPPQPEQKRHQKQRVDEDAKACSAEESRIATRIHECPQEMA
NGSYKDVVGSTPLPLILPTNSHITSSNPITITAAQYTVEGSDYVLDVNTVVAMQEALRQHTPRSILLEDGS
VIVVDPAKRTVCLRTPQGNQCAVMEHPAGELKYTIDNKGAGQWYPYRCPIYLPPGRWCVRAEASSADHRRV
RTTSRVFTVDVVEK
>tr|D0A1Q6|D0A1Q6_TRYB9 | DeepTMHMM Topology Prediction - Predicted Type: TM
MSFFCPHTKSVCIRISPSSIFSFSPFLFISFPFFFGAWWWVGVNVAKYKRDDQRRTYETIITNWKKWPQVT
ASATVHTQQKQNHTGKGTTSFNPTTFFLHSSIHYKR
>tr|C9ZSR2|C9ZSR2_TRYB9 | DeepTMHMM Topology Prediction - Predicted Type: TM
MFRPVEAPVPPKLVDPLSRRFGEEFQLEPVPGPHSPVRPQPLPCERNSFAPYSPLIVHLQRNAFAFVLFFF
FCVDLSLHFRQRRAPPPHACALRFQKLLGAGSAAGSVRECAQRPTGRENAVPTRHG
>tr|C9ZSD7|C9ZSD7_TRYB9 | DeepTMHMM Topology Prediction - Predicted Type: TM
MLMSDALTMHSLLFFSFSCSYSLLKFCLSFVVVVVFALTKNSYDNFYCIIIIIVHASLPNFTRIN
>tr|C9ZWS9|C9ZWS9_TRYB9 | DeepTMHMM Topology Prediction - Predicted Type: TM
MTLSGSSARGGFLIPVHSMVGNHFACRTKSRLCFISLLYSTHSTGVALLVLDCAYLHHTLPGNSGLLLMLC
DAGSLPRCFPTPCFDLIASEVMEVAARVSISVHAGAMKGVFLSTDPHAMLPRAMCNPILSPILKKDTGVHA
GKGKTLWFVA
>tr|C9ZPK1|C9ZPK1_TRYB9 | DeepTMHMM Topology Prediction - Predicted Type: TM
MAGRLRDSLSTYFSKHIDCGDWRACLRVLRGCEKARTSLRSLPYKTLFPFLVANGQWEFVLRLSSQFIAVN
EKTTPTTAGTGAVTNDIKKDDEDLCNVLLGAALASEAVISMGSWRAVVMLVRHATEQKLQLEPDIVCSALR
AMDSWQQHGSGQSSNSNEFSTFETDKRREQLMFARNILLANASLTEEHSSVRAVDLCNLGPHTKTRVIQVE
LLLGVRDVFEPSDQADGPGNKSHWLKLQGQMSDCFTSSWENAVYTLLRVCTYHEGTPREVGVEGNGVCDNL
TERCSLNSLISAVANNASLRPQAMEHVLQIALEQMLDGCVIRDLPTYMMSEGKDVKRDDTAPSHFLECNAA
NLLLWCAQLRENWGAAVDTELLKWRAVLNLVILKVEQRRRQFEFRCSPHLLPRKSEEKHLQEDATTLIRLC
ASARLVLLDGGDGEKDCSVCLLDNSELKYAQRVLQLLMSHGALVIAALTHEHEVEMRDAIVGVVVDTVRTI
MTACVNSCRVIDGCRVIRQPTFGDVSKCSADSLSFDANSVRTAMRRNFWTDVFFPNSTLFLRHVQKEAQGD
ELRVCIAWQELLRELFAETSALAMWFGADNYVDRIHRESEAGKTRVVSQSGDESTNKNRRNRKRDPHVNVD
DDIITEDLSPVSDESMKQIQQLVEFLSRRFTVDICGVSLREGGRFYHQHRDDNPTQFRMGSDSSVVVPGLS
EVLKRQESVNDLASMLAHTHMERNSVSVPDITATLGTLVKSIADYRVKGLLEHMLRLTKNKRLQMKTKEKK
ERWQLCRVAVAALVHESGVFPPNEKTGLVVNILEGTEPSEKMWELSLALFAEATISICVQKPCHNPIAGDC
NEVVAVNNSTNKSTTLVYIMDVLVSQTTPPRWREALQVVETASEAALRYRGTTERSVTPTKKLPLSESEHI
DLFETVDSLKQQQQWVDAVSLFTQYEARLLSSHDIAGFSFAFAHAPLSFIRIALLALGHQQSRGIAQAALT
ACARRVRHLEHRNPRHRGQGHHYKERANQGKECNRRGGDDTLCEENKDASTAEDQQRRCFVLHEVRHIILL
ALQVWPVPVVKEVDFTFIWKTVLQGCGGDVEMAYSLLNRSFIYKSPELHSEIAELTHMLDLCHRTHDVKSA
ASAYREFRRRRMGLRVPEESMLKLLELCVMGLNDEQVVEVDGGHATLNEGTSWLLTVLLDVLSMHNSALYL
DSKHNTSGENSVFVRVCVVLFSLRSAATWDSNGGNIRITSGIPFYNDKGYHTTITPIFTCAMKRGMRHRAL
SVLHILLDHLVKSHTRFLRDISSFVGVLLSIPPEVTSVSCAGSETPSEEASFSGLVEVLMVHVEELQQQFG
DVVPIASLFWLKVLHYSLAGCSVGRKFPARKSSNLSPFCPSLFVTENNAAAIEAVDSLLKDVVTKALDETC
HRLHMIHKLGEGVSETSYVHSLRRELLPCSFDAIIYTLCRHFGKHPSLSAFLSGAALMLQQLRELPTCPPD
SADFTASYSTVVYGAVSLQGILSGFYARQSLLQLAVGGHPSGTLSATVEGTFACMAHKAAEYVFSYVKLLS
AWILPLVHTAEVNKIEVGGSSISFIHTEHVRFLIQLVAKATGTCAAWGESSHKEKCMFKLQRLCDSLSRLV
FEGAGTSNFGVTWSTRMVLRVLDSVLRRVTIKDVEFNNLLKEAAVHAVDYQTSNTTDSLQSATLAVALIML
ESAISMPNNFLQDAVLILPFDAKARELLQGDASLLTVLVVAWLIFLGVSPSLIHRVVKSLVPFTPQFIANA
EQLLLYFLPSAMRITMVSELLRMIICSGENLVELLVLGVLVTNRQRDEYTSLHAEEVVFSVINAVGATVPN
RWDLALAAARGCSLVLSQPQRKEVSDANEVLVGKAKLWERLAVAIQSCGPWPRGVALQTLLEVRCGSPCSE

GRRDDNHHSGLQQDYVELLRATSDLNAHFNCSSLVPPQYRGPDSSPLRLLVQRRHQQIFQAASIVLGNARQ
KHDREGRQRVLGTLLSCDSSVQFVACERMASLSHGIGTSPQKQRGRDRDTFDSWIVDCVALWPWEAISNDT
QHFIIWSYATLKVTTALGRRLQSEESQPLLVSPSMALGTPPTTSFSVENSATVPATTVAASSVTLNFPRAL
FGMSWAKELRREQSERVGADGSNGSDFVIPFPYDCKTIMTVLCGDGYITDVTVEDTLLAVSSGFSCLQYVY
ALFSSGGPVKEERELLLFIAGCCRSMQEVAHAVLKRLNGPPPLLDAVISMWNVLCIRRVEFGGLQESTDVE
GVAEAPLDHNTAALFVVLAVGALRRSGDSSLISKNSTQQQRQLIAEMFAHTAIVCGGSRNREWEEKQGHFD
EALFRKRCVDVAGAAIRKLGLQKHWHVQVCESSFFDDHINLAIRLVRDDTTWISLWPPMSLELSRCRRYVD
GVLCSNSVKVDACQVRHPKAICWLMEQCVAASSRFRGEKARLFDVIGLLDILQGYILRGVAYFTLVSPERS
TQVKVEDSTAAPLYVRGTAAGLVVDQFASLAIKRWSREPCHWSGHANIGVYDVIRNLVDAIFPRTSRISEP
PSGAHSFLSGYAPVSTTGVVALTQLLLVREDLEERRTVVVDHVVSLLKLEQRREATRLLVANDVKTSIWSN
FPDNLGVEIGRSRQPCDLKDMLLFHVRALVASVRFVNPLGWHDKRQLTLDTAYTALLDFLLLYDGGQLSLR
EVDIVTGEVESELEKIFGSGVRRVILASRPRRTAVSQDEAVTVEMLLDFLNGKWRRQDTRRRRQEVRLHYK
LHEILASHNGGDVIAGAPEILLLDGEEMKGAEFSALLSAFPDTYTVVQKALWSAAVVPSHEVTFKILDAAV
SGNGDVGAICAALQVFATVAPYMVVTTMMLEKVLVLLIEAQKYRVSKSCAALCRSTLDLILRRFQYVPLNT
PEAVAMMAELYALLSAKSRTISGMSEIEEVELEHLLWRCVVESDRVERAPLEDALSLLEYHSLKSVHRGCR
LFLLYRNNRPHYDPPFLRLGRMLDALLIFRKTPNQSTHVCLTEGLLKSLEKEAYASPMHASSDEIRLLPWR
RSLRFMPDNSDECGVEGLRCRFFIVIMTQLFLLRSRGVNAIRHLWKEALVSITRTAEKNMWCVVSGNEHCR
RATELALRTIHIAQPLDQWVPALNLVQRLPAVSAGSTLQYFRTVDEALSETLRYVLGACSMTSIPIAVLVE
FLRFLAAKPPFVQLISRGCNSSVRHLNCAQMLADLTKKLQKHPYLGWIDGLAVLQSLSFHRPNSSDVTKKT
DPKRDNGDKSYIILREAKAAFVTATMQRMYKRQAYEPLLRFVEAAQQTTDNPRIRGRDTLAMLEHAASVVF
PISADSNGPWVILSLLNGTLIGKTVDDMLREQSSGTLSRKERHLHDLLRNGQTGDAAVVARQAPQLLQSRA
THDACVSLIRRFHHSAVVHFYDELLHGGQDGSECMYAVPTTYIQCYERVAIFRNLPADSLTLAVTAFEVAS
NCPSGLSERSQWTGVFIAVAWLRAQTNDSVCFEPLALTLRWVQRDVPKTVEGPLSVLEATVQEILSTCEAA
EGNQIENDSEGVTSSALRMLCVNRTATRLLVEILSGVFPTTTSRLCGALQREYQKVKLCPPTEEKNEWLDF
LCSTAAYNEALYTNNEQYKLLARTPSDTNQRNTILFWRNREIFRARPGGGSSDWLSALAAYVSARSYIGRP
CVTAAQATQLVNLMMASGSLGVVVTAARDVFLFSPEAVPLGDSVSTADSICTEALSLLFSGLHSMKRQAIN
FVNTETSEKHLPMLLQNTLNELEMLNHFVEDVLERLQEKESVSSTSGIGAFWGNTELGCHMIGCLALTPIG
TASTTRGNISAANIVSSLERWRLLLLCVLNALPPHQRHASVLNAVMVSIRDAFVILNKLQKCPVPAVTGCQ
EALKGLWCDALSLVAGNNELRRCFFSTGKGCNLSQQGMLRQDVVDALFHCASLTGDRGLNAMKALCDSLVR
PHGIRLSEEAMSLLKVACINNMDQRHGLRFLVICESLLGGPPLVAQRQAELLADDMELQELEYLSTVEKKL
SVDCAKLVFLVEKRRAAGVRLQRSHYRKLFLLLEERGKGLKPIEMADAVMFLYFSQLQDGSTPDVRDVERL
LHVLSRAAVPPLLILQYAKAMTLIPYARTGRNTRQKISSLPRSETNAYEVGEMPYFIPSEELLVLITDAAW
KLRFATQEGGCASADETQEGIQMFASFLEWSTQFSRIHCFKPSLVESIVLILCTLPEKASMTPKTMGNESH
AVAVTQQFLGDVRVLLPIALSRTFGGHAFSEDAQSIFQMSMRSSGENIGGDRIQIPELVQTVIPTQESLCE
LRNTALAAAHVIKQQMMLHSSLPISSSLLCRVFAAAVAGCGSTRLSPTLEVLSQIARDDKLITPPLRYLLA
LTVAEQSVLWDLFYKSACPDVEVEGVTAAVVVHYVSKAFDIVRLCFAPKESLTLGEVLLPCSSGEGAEDCN
EFEDKGDWGQGRGLSWEERLGEVNGESTSTRSCDNDLLLWEKSLQLFGVFLLNARPKADLSVPFVTLLEIL
CRCGQWSSACKYVARVGAPGQQPLRRTLQALTNPPKRESGFDTSVNADDIDTEKLLSQHVVVDGFLLERLV
RTVYTAHQLWQRQHVVNTERSNNIPLGFWRLALHLLEEHKLFVKRSLIASDSDARNGKAHSSLSPSTFAVM
MKIRPQ
>tr|C9ZWX4|C9ZWX4_TRYB9 | DeepTMHMM Topology Prediction - Predicted
Type: TM
MTNQSSGGNDRGRRDTDPFAHAGDVKLSSDAIALDVTQKGQDDVVKEVKPSLFAVLLEKFIPHGGLWSCAL
NLASATLGAGICSLPTGFNLSGIVMSCIYLVCVAVGTVYSLNLLAKVAVKTGSRNYGEAARMVMGPLTGYY
AAALMIAMCFGGNVAYIIIIGIILKAVLNRDGVPEYLKSESGNRLMTSMVWLVIILPMCIPKQVNSLRHLS
FVGVMFIVYFSCVVIGHSINKIINEGVADDIVYMRTGNSALDGLSLFLFSFICQSNAFEIFREMKHRSPQR
FTIYGTVGMSMCAVLYFLVGLFGYLEFGGDAIDTVLSLYDPGENVAVAIAYIGVAAKVCVAFALHIIPMRD
ALYHCTGWHVDTVPYWKHSLIVTSITLAALLMGLFIPKASTVFGLVGAFCGGHIGLVLPPLFYMYSGGFTR
EKVGNIDFFGTYLLLFVGVVAVVFGTVTTIAGALS
>tr|C9ZTI7|C9ZTI7_TRYB9 | DeepTMHMM Topology Prediction - Predicted
Type: TM
MMFFHCLFICLLTFLRFEVPSKRCPNTRFSRRVIHTHRFVREVRRKNKTLRNYFFSPFLFSPCLKFLLFRS
VCLLSFQSLLLIFVVLPTFPHPTETQTKK
>tr|D0AAA6|D0AAA6_TRYB9 | DeepTMHMM Topology Prediction - Predicted
Type: TM
MKALKKNITVYSVSLGRLLNEFNNRYFRLKKRSRSEDLKRMPVREEAFLRLSPWHHNVTGSCESGVSELDS
DRVNGDLTRCRFLFGRCQMNVEYQFALSVGTRKDVASSKCTGKGSEGGAVLLRFTKIPNKSGGSSTVTCSI

QVNKEVQYFNREEVVVAMKFCREIYAKLGEANEFLETEKKRVHMGHYGVDEESNSMGEGSRCLVIICAFCL
LMLYIVGSKYSQCRIKNGRGRAYGQKGGQRSKITSNQRFYSIVIIGTASILQVNRIVCWG
>tr|D0A1H1|D0A1H1_TRYB9 | DeepTMHMM Topology Prediction - Predicted Type: TM
MHLFSSYRLDGFLRWCVSSLSLTFLCSVLRQYGRFIFVWLQTFCPTHFGVPLPEGEATDIFVVPVVYGLEV
TSDGGCWRDRGLITSSRGHFCCTFFFVVKKKKKNGVCHHMSSAIRFSVCAFLSALKGKRFPLP
>tr|C9ZV66|C9ZV66_TRYB9 | DeepTMHMM Topology Prediction - Predicted Type: TM
MYSRALPTYIYMLPQQRLRLILTRSVGVLAEISSVQPRKPPFDEQQQAFHSHVQHLPAKDIFSDLLVSASA
LGVGSREDWLRLKKVYLARLPELPCPTVVMLLRQLVKNDACGLVECVSIQDRLAESHFLTTLKTEEQMEAL
SSIVIVGSRLSPKFMPYAQEALRVIISGAQVELLPELSYVVGCLVTSEDNGISAEAQSSILKELLERVRRV
DYTLLTQEKLVYLFWSLSTFCSSIIPVRLVYIEVCEAIVANGIRTGGVESASPRNDLLLLCGLCQIVSVSG
SGDRVVPIQSEGVINNVSWSENDCLGCLSPSFTVKLLAILDSVVLTFVQRVVKGEIQNRFIISTVLESLTY
CGLTKLQKNAQILFHILLRMLMYNGIDFWLRPLGDAAAVTRAEAIGPLVNVYSVAADVAFLDRLMYRLNFC
VFPAVSQCALEKATVKQVRIILEAIAYGNLTVNSRKFYLGECFKIVPGIVRNASRWELPRIINAVMKLSIK
DNFIWCAIRERSRELGYQHGCRGGGYPQTDTKRHEVSGKCSEEDTNSSDVALQICEKALMLSESEGVPLER
LTHSLETLCTRKGMKSSTMTQCAAIVGSLSKLRIPSRGFKPYTAVSTPIFLRLEVLQYPDWGLSLILDGAA
AGVSRTVLLNSLSAVGTRLLQNDLGRNRYESAVFYLIAAHELDARYSVLHPHIVGTIRDQQRLQTLPFHIL
VRLVGALCAFGIDEKCVLERALHRVDSLCVHVVEREKWVEADWRAALVLVNAMWTSAAKTVWKSLIHVTRK
LVIVMIKRIRDGEVGVYPLQAALAIATVVINDLDNSSMGYADGKVNDDIVTAFLNIVAPLFSEDGVSFSLP
RAEALLRSESGALFPPQCNSLTCSLVVSHALQRLIQMNNDSAKRFTPHAIGLWRFCLKHIEKAPLTDRTLM
NTVTFCVRFGGSSQTFDRFMASLVDSGDEVSMLDVCLIAEGLLKNGKRRPIATQFIAHFSQKLPLDNLPAA
ALCSLLLFFARDAEKVALGLSKVEMLLSLVWASLALKMDRLAGSGELLKLVETVPANAANPLLRLICDTKA
IDDTPTVELVRPLNIVLGWKTNTVSPATVLCFLQKVGTSIELREKTDPAECLATEEMKRMIHCYSQ
>tr|D0A7B0|D0A7B0_TRYB9 | DeepTMHMM Topology Prediction - Predicted Type: TM
MVILCEAFSSFLFFPPFYVAACLQLTCVTFCHRYFCFRVFFFSFLRKSVYLFVFFIFNLLTHFYAYRHTRT
RTLTKTQNLNTSFFFFLSVCEVFCSSKSRRGQWHCLSVAVHPKGIGRK
>tr|D0A6I6|D0A6I6_TRYB9 | DeepTMHMM Topology Prediction - Predicted Type: TM
MSTRPGRGGGPAVTYGKSTDGPPSARLNVTGGEIGGGKRYRGQPNLPESHLNELCHHSAICAAKAWGKKRN
IQMSTRPIIMHAYRKLTTTPASAHGGAAFYFFFCVFVCVWRPLVQCESQATRLSPVVTVLGTSTALHV
>tr|D0A7L3|D0A7L3_TRYB9 | DeepTMHMM Topology Prediction - Predicted Type: TM
MTILHHPVPSRSFRSWEVIAGGPQMQLCVQERTCEGMRKNKRIVHRLFYGEVDLKKYRIIRSSNRVDYQSM
TDGSGRNIAEESTGPRMRLVLATSDSRDEIMRDWDELKAKGMPPCGGNWGLGVSQQSSASSIGHQGSELSP
FQRSPVCPNWTGIGGSHLAWENATEYSPLSDLNASPQFTCLPVQGGSSADAYDACLIRIMEMWDWTHPFET
AAYVTVVAAGWWCQVLQWSFIFIISLVMGLLRCRQFMTEKEEREGKARILYVTNFLVMNVSKNVIHFVSGV
VCLHWLVVWLAPQWTLINIFEHVIPPLIIASLLHTGFRRLIGTPLVRRTKPLSSKTESAASCGFNYSLLSS
GSNVCTGAFSGHNSGSPIQREANAAAGGTAFFGERCDRNALANRQPSTPSLRSREWLRGDGLVGDSGAWER
SGTTNTTTIFVEVDVPQGSVNSLQAVLANAVRSERQGNYFFRCNKTLSRQTLVSTKDGSEFTLSTVYMLAG
ARESLRTDTNASCGLDFPVGPQQGEQSAVQSLAGAESSAESPPSADPAIKSMRTPITLPSLLGFAKDEGLL
TWSYDIAGSEREDGSLRLRACYTITSSTNLPESTRHELTKQLSCKADAIVELLGTSGAEIRTAVPKEDRQR
TTSLHVSPVRSAVCTTPPAHVQDGVAENIPKCEENNGALQIAEYVIKEIFLKCKWDFVSQKKGATLWTADT
QWSDKKAVKVVVHIPRATLADVDSVVNDPHLVSVMDNMVEAKVLVRKVSSDVHIYHTKFSSPFWGISARDV
VTRTAVSFYPSEDQRVAMGLEGGKPMFLHTSVDAPSEVPTLEGFVRARVFAFGVLAEVAVGDDGLEGVRVT
RCIAADPEGLLPGYVVNALSVMQLDSILTFAKFVKKRARASVSGAASQCFGRESEERAEATPAV
>tr|D0A4Q5|D0A4Q5_TRYB9 | DeepTMHMM Topology Prediction - Predicted Type: TM
MPLVKAHPLHPSTSHTAVTELSERETDRCRTQLNNPPRGISLVDTAVRRSGEKPSTKTKQTIRGSNSHTGN
SKMISARISYYFLFSFPSFYPACPIPLHLLPFLLYFIFFFIFPFDCFPRPFSSASAYLDMQLLERRSKKKT
EKD
>tr|D0A4K9|D0A4K9_TRYB9 | DeepTMHMM Topology Prediction - Predicted Type: TM
MAGGKLLDQTEPAQGYVRGPDAYAFRNDDYWPSSMYVQVEEEEVESGCCRSCGVYCCCSSCFSFFFILLGA
TLCLPFVVWGLFPTAVNWGDHWSLFKVVLLCTGVFNLLLGVVGIFSVYFKSKMPSMLFSVMLMASAVPVLS
LGLLFLLLLMDILPLDTMLSGVWREAVQEQPELICNIQANYKCSGFEKGRCCFSNATRHIPGIRPRVCYLV

SRDGHVMLDPITLEEITWPKLYCSPVCGESNIYRDTCGPRIVEDTRPALPATVGALMACGFILLLLSAVAL
RRARRPRESPLYFQYEY
>tr|D0A7H5|D0A7H5_TRYB9 | DeepTMHMM Topology Prediction - Predicted
Type: TM
MRVRISEFVVLYSLPFHYFICTQSKMFQQLHMFHLIRIPFLFQFLYSFSYPSTYSFIIDVYILYGASFRIM
EGNTSFPLFFFFLKNKTKQKDINMKALKHVKKGGKKAK
>tr|C9ZJA5|C9ZJA5_TRYB9 | DeepTMHMM Topology Prediction - Predicted
Type: TM
MLRINEYTNQRNNNNNNNNNNNNNNNNNNPYFHFTCALLHFHYSFDHSFGFIICLKPTLILLLSLYFFFFTF
ASQLCAQRKGGTNSHFSPPLSISLFFSPFPSPVVIINKNKW
>tr|C9ZPX3|C9ZPX3_TRYB9 | DeepTMHMM Topology Prediction - Predicted
Type: TM
MNAMKDKQKKKRQSVETAKKEEKKKASESGANKYIKGRREVQNEFKSERQKTTTTTTSKQQHQLKLNPMQT
NAKYLLKKLYGAVGLGRRIRKRKVSLSSVTKSNKPSDVSFGVVTLPSSFSFSCSFSFSFFFLFSPLLKM
>tr|D0A879|D0A879_TRYB9 | DeepTMHMM Topology Prediction - Predicted
Type: TM
MSTGKIIGDHRYLIEGLIAKGTFSNVHRCVDLTTDKVYAVKVISKSVIESQNMSVTVVREVSAMEAAPPSP
YLVRLVDKLVSSRNYYLVMNVVEGCTLLDVIEKKYRPRPPLRWVRSIFRQLLNGLYTLHGANVVHRDIKPE
NLLLNRSYTRLVISDFGFACCAPPGRSLHHSCGTMNYCAPELLVPKPAYDGRKVDVWAAGVTLYVMIFGVH
PFQARKEGCADSLAEVITSGAYTLPHSLQPELEHLLSVMLEPDPALRWSVRKLLRHVWVLGGMVGGVTGRS
SLRLTTPTPGSTGVANRDANTPEDYVVRSRFARALRADDTDPSARQFRSCPDLRREMNNFLDACDSDGEDA
EWDGDFSNGNSTHDGYTKFSSLNVSLTDIGRGRQPSDLDGFSESTAFATNGSYSSLFSADQLLLDPDETPD
TPKGDFLLTVRVIVNFALFCTTLVIVAALRFLFDVPVRDLPLPPFVISIMEYLLVPPHERLAQSFEVRRMR
HRFPCAALRRLVASAERAIQRSSWCRRIMPVRGVDEGTDVPLGTGRRGIVLRWLEKN
>tr|C9ZL97|C9ZL97_TRYB9 | DeepTMHMM Topology Prediction - Predicted
Type: TM
MVYLVDDLARMRMLMSGMYAELGVSKAYCFSIFTPFLKKKLNLTQTEVTTISTVGNCIMYFSFPSGALFDY
AGPMVVLPIAGFLGFLGFLLFGLTFDRKIVSPGVGLLSFFNALVYCAFPILDVSSVMPLMLQFPLDRGYIV
LVSKTIGGLGTGVLMAYFNGWFKDTTSDDVEKNNYSGFAYFVAIQLVVVSLIAFALIRLPMYFPCAWRKQR
LSEEEWTKRQQTLQLYMNQPAPPRRMKTAVGLVLSLLLFLTTQSLIGGYVKLPPAAYLAFSIIAVLMMASF
CVVALPFQWLGRYTPVRPTDMDTIGEALEDVVTESAVATTKNEVKPLPQYSGSFWQHLLTVDLWCMWLTCF
GVWGTAVVMQMNAAQIYESKSYGEKKSSTLTLYITMISVGSAVGRMSMGYLDMVLTRRQREGLKTFPTTIA
LPFCPLMLCIAFLLFALLPANALVLPFFLGSLGNGAGWGSVVLAFRIMYSQDLGKHYNFGFSSGIVSTIAL
NLFMFGGMYDAEAEKLGTKPECKQPSCVKNQMLILMGVNIVAVIAAAIVHFRFSRFINAEQNRCNEPADEM
SGVAAPVTEDAGQPNEGGSHSGGAAQQ
>tr|C9ZZ16|C9ZZ16_TRYB9 | DeepTMHMM Topology Prediction - Predicted
Type: TM
MRTCMKTRVIIATITITNITYIFRYIYLYIYIPQKCRNQQTGREQTKHTHTHTHKHNNKKRQAKERNKQNE
REEHEKANPFKQCKSPHLWGDRLCTLMYWGNGVLLQQRGNYKKKKVKKRKRKRNNFVNLSAYSHPSLSPFN
CLTHIYKTKQKLNPFIFLL
>tr|C9ZNW2|C9ZNW2_TRYB9 | DeepTMHMM Topology Prediction - Predicted
Type: TM
MRAGKGGKKKTALNQPDDTKSLSASDTQRQYKRGFSLKKKKENKRKPTIMEFLSPLLSSPHPSLRLPGNAY
WMGTILFFFSFFLFLTSSHPAAARHFFYFTAGVPHPLFTHQIPKQLPSPVR
>tr|C9ZUL9|C9ZUL9_TRYB9 | DeepTMHMM Topology Prediction - Predicted
Type: TM
MSYGKWAGVCSPHFLMSHVTMCRRVAHILLRGPPCSPQRSRSDTESPFGYVETLVSRPRAYARSTPLMTSA
ALLRSGLTARTGIRFATLPPAWPQVTSRRFASAGQRAPTYDEVRDACSVLGVDVDSDPKHLKKIYRGLVQK
HHPDAGGDAAAMSRITVAYNRLKDLSKLEREQFKAQQATYRGGSYHYSSRAGSSGNGTPSYDTSGRYAAQG
SHDAGTSYDYRGASNADYFKQTANPRRGFYTYKQSDRSFTDNPFSTSNPFSMHAQVRRAWNMPMSSILLRA
IVAYLGICVLFLLAYRRYRDWMHDDGWKMAESFARHERLAELQRLRQEMNDRIQSSREAAAAERLDAQRYS
LARGFGGQSGTDYNVVTTSKEREVRALERARRQRIENNTIARGWPFIPEEKGRLVVRAHHPPGVVFFEPSR
REEQLRQLTSMQRGRAWSDANQSLAEGAAVNPQSVVSDELNAVRAIRTILNGIHDGGQAENRVL
>tr|D0A7U7|D0A7U7_TRYB9 | DeepTMHMM Topology Prediction - Predicted
Type: TM
MLQEVLRLHPLFSASGSSRNLHLYHISSPLLSPLLSLYARTLLTLGCASFAAPMKYSFIGLLSVICVTTVF
ATVGDGNTTRKEACLARMYPQSAGFFGSLGVAVALIFANLGSAYGAAKSGVGLAQLGLLAPEKIMRGIVPV

VMAGILGIYGLIVAVIINNNIKTELYSYSTFSANLHLGAGMAAGLASLAAGLSIGVVGDTTTRAYGKQDQV
FVAMVLMLIFSEALGLYGLIIALLMNNQANRYTGLCDSS
>tr|D0AA81|D0AA81_TRYB9 | DeepTMHMM Topology Prediction - Predicted
Type: TM
MGANYAVRCLATVYLFLLMLIGWIGAWLLQLVVITFTYPFWTSEQRADCCALIFRIANFLSLDALNPFWKS
TILRPFPNVQGKKVLVVMNHLSGADPFLMVRVLLPRDAAWVAKNDLFRVPFGGWAMANGDDLCVQFKNKKA
GLETVKGTVAPMMEAARAKIHRGRMLAVFPEGARNDTPENGLKPFRPGFFTLAKEEGATIVPIAISGTDDC
WPKHSFLMDVGHAYFSCGDPIATNNFNTVEELVDYVWNAVTDLRSTHPSTKENSHKDR
>tr|C9ZQ16|C9ZQ16_TRYB9 | DeepTMHMM Topology Prediction - Predicted
Type: TM
MVTCFDEVVVMASAWLLLKVVPSLPPVTVLMRCLLVFCSVRVQLAPLWLVVASAFPARSLFAMPVGPNWVV
TTSAGSTVGAFAIQPFFSLLSVTHLRFSTHIFLRLDRHVPRLLRFDWPCLRILVSRLQTWRRVRLPLLQLL
PSSFGPLFGLNWLWSCNIQ
>tr|C9ZRU7|C9ZRU7_TRYB9 | DeepTMHMM Topology Prediction - Predicted
Type: TM
MFFPPFRSFSFCFVLIIIIIIILFLFCLGFWFGHLNTTCGPYNPSSNYKLIIFAFFFAFCCFVFVFVCLFV
PSLVSQLHYPLACEKLFPPFYCFVTIIFNFFLLLRCVALN
>tr|C9ZW93|C9ZW93_TRYB9 | DeepTMHMM Topology Prediction - Predicted
Type: TM
MRSSLSLSFFFYSVPLWLFLISSFFFLERLPRASLPPVPPSSFGSVYFPLSLCAKIPKFFPFPLRPQPGGL
SHVGAKTYPFTHIYIYMRICALTLFCTCASFLCSHRCTSLIPLVRVLGMIWEVSCHRVGERNTGCDSPDSL
LSLSLSGGHILDGMSAQKKEKRKKKERTHMYTHHIATGGRSIGDVMCRTEGKEREAKIKV
>tr|D0A2B7|D0A2B7_TRYB9 | DeepTMHMM Topology Prediction - Predicted
Type: TM
MRMRLYTHTKYIYIYINIYINVCVYVNICVYVFSIVISGVLVLVLVLLIECLLFFYSFFFPSFRFLFYLFF
SFSLAIFMYFFLFLFLNIALFQFEFHLFRYLVTETLFFSYFTLSFFFFAC
>tr|C9ZU03|C9ZU03_TRYB9 | DeepTMHMM Topology Prediction - Predicted
Type: TM
MTERTTSHSLPHTHKHTQTYTHTHIYIYIYSFEKGHLHVASAVHPMSSAPFLLFSSDGNAGVKVGIGVASK
ELSYRPVLRTFSVRRLTRLNLQHNSLCFATFLSLFFFTLFTLRQNMKGQNGKRNKYKSS
>tr|C9ZQX1|C9ZQX1_TRYB9 | DeepTMHMM Topology Prediction - Predicted
Type: TM
MLASHLKTAAEKLTEHCGRDDYYWTTDNEMHAVRRRKMLEKYSSEIKKLYGPDPLTWKIATAVVLFQIFLG
SLASSMSWPVFLLTAYAIGGTATHNSFLAVHEITHNLAFRKAVHNELFAIFLNIIVPLPYAMGFKSHHRDH
HNYLGWEQVDPDLPTALEGKLLSSYIGKFFFVTFQVFFYALRPTLVRKMKITRMHVLNIVAQIIFDVIFYM
LFGPWCIIYFFLSLVLGTGWHPLAGHFLTEHYIFQGDGSQETFSYYGPLNWLAWNVGHHVEHHDFPYVPWR
HLHKLREIAPEFYENLEVTPSWPLALYDFVFKVNMNPFSRTVRRKGSYLRPDLRSAEVIGKEEN
>tr|D0A9B6|D0A9B6_TRYB9 | DeepTMHMM Topology Prediction - Predicted
Type: TM
MCAHLRASYIRPTSWLQTLCLLHTCCFKEMKSCLLFLYLMLPSVDPPQVQKLKVSSFFSFRSLSLSLSLPP
SFFLSFRLKLYLNLHLLINLFSFSGYLR
>tr|D0A401|D0A401_TRYB9 | DeepTMHMM Topology Prediction - Predicted
Type: TM
MLRRWREKLSVLFVPLIDCSVFCFCFFIIDVTILSFRKMCVAWLVVVFFFPSFTSRRLHRSSGEQLGKGER
QREKKKERRCSRGSNAEGRNMKQIQAQQEQEMCCISK
>tr|C9ZR87|C9ZR87_TRYB9 | DeepTMHMM Topology Prediction - Predicted
Type: TM
MPVNGKKGRSKEKEVKKSWVVKRYSKTPSLVVRWNQRAERLFFLVLFFVLCCSNVAVSFSPLSVSISLFHL
STLLYREMSTVFPLLRALGKVSHFTEVQTRAVGKAQQILFGSVGSNAQKCTTSHGEVAALRCRYRVCDAFE
VPMSFIGSCQEHGDNMILAHVPGLPGAFPVSTEELHRHKSFFLIDNSRDDWGSATVNNADDMQLSECVQLS
LLCVMEALETEGGGASILDKLLSLVRRRAASLRGSELLLATGSRINEGGLELSFPYHCGNSHASKRYFDIS
QEACTLFGPHVKHGTKMLCRYGTAVVVGVAPEESLGCPVPFWNPLGAPAACLAPVFSGCPAIPVGEVKLEY
NGPTTSSSLLDAEDASRYLNPTVDGRFDVSSWLNEGLFGVKVGQPVEDGCVAHGVCYDLNLCEFVLFVREL
SSGEVRPSSHCLLK
>tr|C9ZZH2|C9ZZH2_TRYB9 | DeepTMHMM Topology Prediction - Predicted
Type: TM
MRVTLEERDLHIAFSVSLATIGLVFLVQHVASTVSRLPSWFINLRWHYISLICLPVCLLVSLSLSSISWFL
VSAGAGLALVSGVRTWERGATTFLFCFLLAGQCLEQLSHTVHVTLPPKARTWTSRYRGQIALWPTGEATGP

EHLLVMYNDVTLTRQPCSGAVAGTIPTQAGAVTRQLGMRRHTLVSFATNTRIGSMESCLGDGANGGFLPLH
QVALLGESGRLEDVTLRCGLPSSARDGNSNGKTFSRCLTLDASTTFENVTVEFYADGNFKAVVSILHADKC
ADALSQLQAVGKSILKDEIDVLLCLPSAVSHRFSKLVGDPTLVPTALRAEVPYFFRCVWYLPMAVRNIPEG
AAFIADRVVIPIAATVVANSLAFVQTIIWTCWNLMPDVLQLLEDVGVFVTGAYCRAVKGTNSTLCAKSLLE
HAEDQRFQLGVRSVDCVFETVRLVLDALELVPGLWRVLLWTLRAEWHLVLYCLNIVQVILTVVVRWPLQTL
YGALQLTLSWMLQTLSLFFLDYDLSRIGDALCSPLAYLYRLQKELLAAEAQAAWGLLWWFFSGVCRMVNVF
FATVCAVCNGLFCVVSLYTGTTFCTHSFVALIQTAIVLIAMRNELHDIATRDGTGYTSFVGRWPGGKYMVM
LTLFAKLHSVVLLRYTVAHTVLIFTLFGLSVVGVLSKLYNLTLYIAFPWVSTTVFLEFFTADPRWSTVAWV
SAAKGLVAILLDRTIGDFTAYLVGEVFFVLGVVFTGAAFVWGCQKELPLLALRVLLRLPLDSSSESSTTGA
KLNTTPQPDVGRAVFLDDATGAEMTKGEIMLPDVVEVVDDTGKEEATECNKDVQ
>tr|C9ZP06|C9ZP06_TRYB9 | DeepTMHMM Topology Prediction - Predicted
Type: TM
MKQQKYCSLPPPTLSFFSNLFLSSSSSNLIFPAKASKRAFHFTASRGSLRTRYASQHTILHSTTKIYKHKN
KINTLPFPSALFCFVLFLLLLSGSFLHSAPLVDFRAVQTSANEAHHLLLKRKKRK
>tr|C9ZQY8|C9ZQY8_TRYB9 | DeepTMHMM Topology Prediction - Predicted
Type: TM
MPLTSASVRGIHWLCTFFFFKLILLCVLSIIVFTVVGVSRLLKKERKCLLFLYIHDFFFHFCSFALFNYCQ
LDMVSVPFPMVLERKRSRNEAMTSSRAAGRLRSATKRTEKIKEQVMKSSTSVSRRTIPPSQRKMLEQTQSV
SQLSEASTGNLPPHKLSQRELVRVHGWEPVVKPLRDTDAVSLPGTATTSVVWRNDITNATLIPNDVGGGVR
TRRAAARLAAGVEPPNYLVYSRDPHVKSKRRGQHNVHVTEDVLSASASGAVLSGVSLTSSKAVRSRWRGIK
SVDNGENKQQDGNEIGEDKPNSVGDDDAKFVGEGNGEKRQDGRVKKSEGPEEIEEENGADSIDAKEEKVGK
VEDMGEMKENVEEQNNNPQSDEEITDQSEDEKESGEEVGNETPAEDAKNREGDGDTPRGRRRRGLASWLSR
RRSRFGCERRKRRRAEAVTSADGQQQQQQQQRIGTLSASQAMLPPPYRRNRRLKRKLDVTEDSPIFASHAE
LRDKASHIFARAPFSTDEDVLRSTIDWRDNPTLLYARLLWELAPREFLGIVTKRQEERTASSVLQGANSTI
SVKKE
>tr|C9ZRU7|C9ZRU7_TRYB9 | DeepTMHMM Topology Prediction - Predicted
Type: TM
MFFPPFRSFSFCFVLIIIIIIILFLFCLGFWFGHLNTTCGPYNPSSNYKLIIFAFFFAFCCFVFVFVCLFV
PSLVSQLHYPLACEKLFPPFYCFVTIIFNFFLLLRCVALN
>tr|C9ZW93|C9ZW93_TRYB9 | DeepTMHMM Topology Prediction - Predicted
Type: TM
MRSSLSLSFFFYSVPLWLFLISSFFFLERLPRASLPPVPPSSFGSVYFPLSLCAKIPKFFPFPLRPQPGGL
SHVGAKTYPFTHIYIYMRICALTLFCTCASFLCSHRCTSLIPLVRVLGMIWEVSCHRVGERNTGCDSPDSL
LSLSLSGGHILDGMSAQKKEKRKKKERTHMYTHHIATGGRSIGDVMCRTEGKEREAKIKV
>tr|D0A2B7|D0A2B7_TRYB9 | DeepTMHMM Topology Prediction - Predicted
Type: TM
MRMRLYTHTKYIYIYINIYINVCVYVNICVYVFSIVISGVLVLVLVLLIECLLFFYSFFFPSFRFLFYLFF
SFSLAIFMYFFLFLFLNIALFQFEFHLFRYLVTETLFFSYFTLSFFFFAC
>tr|C9ZU03|C9ZU03_TRYB9 | DeepTMHMM Topology Prediction - Predicted
Type: TM
MTERTTSHSLPHTHKHTQTYTHTHIYIYIYSFEKGHLHVASAVHPMSSAPFLLFSSDGNAGVKVGIGVASK
ELSYRPVLRTFSVRRLTRLNLQHNSLCFATFLSLFFFTLFTLRQNMKGQNGKRNKYKSS
>tr|C9ZQX1|C9ZQX1_TRYB9 | DeepTMHMM Topology Prediction - Predicted
Type: TM
MLASHLKTAAEKLTEHCGRDDYYWTTDNEMHAVRRRKMLEKYSSEIKKLYGPDPLTWKIATAVVLFQIFLG
SLASSMSWPVFLLTAYAIGGTATHNSFLAVHEITHNLAFRKAVHNELFAIFLNIIVPLPYAMGFKSHHRDH
HNYLGWEQVDPDLPTALEGKLLSSYIGKFFFVTFQVFFYALRPTLVRKMKITRMHVLNIVAQIIFDVIFYM
LFGPWCIIYFFLSLVLGTGWHPLAGHFLTEHYIFQGDGSQETFSYYGPLNWLAWNVGHHVEHHDFPYVPWR
HLHKLREIAPEFYENLEVTPSWPLALYDFVFKVNMNPFSRTVRRKGSYLRPDLRSAEVIGKEEN
>tr|D0A9B6|D0A9B6_TRYB9 | DeepTMHMM Topology Prediction - Predicted
Type: TM
MCAHLRASYIRPTSWLQTLCLLHTCCFKEMKSCLLFLYLMLPSVDPPQVQKLKVSSFFSFRSLSLSLSLPP
SFFLSFRLKLYLNLHLLINLFSFSGYLR
>tr|D0A401|D0A401_TRYB9 | DeepTMHMM Topology Prediction - Predicted
Type: TM
MLRRWREKLSVLFVPLIDCSVFCFCFFIIDVTILSFRKMCVAWLVVVFFFPSFTSRRLHRSSGEQLGKGER
QREKKKERRCSRGSNAEGRNMKQIQAQQEQEMCCISK

>tr|C9ZR87|C9ZR87_TRYB9 | DeepTMHMM Topology Prediction - Predicted Type: TM
MPVNGKKGRSKEKEVKKSWVVKRYSKTPSLVVRWNQRAERLFFLVLFFVLCCSNVAVSFSPLSVSISLFHL
STLLYREMSTVFPLLRALGKVSHFTEVQTRAVGKAQQILFGSVGSNAQKCTTSHGEVAALRCRYRVCDAFE
VPMSFIGSCQEHGDNMILAHVPGLPGAFPVSTEELHRHKSFFLIDNSRDDWGSATVNNADDMQLSECVQLS
LLCVMEALETEGGGASILDKLLSLVRRRAASLRGSELLLATGSRINEGGLELSFPYHCGNSHASKRYFDIS
QEACTLFGPHVKHGTKMLCRYGTAVVVGVAPEESLGCPVPFWNPLGAPAACLAPVFSGCPAIPVGEVKLEY
NGPTTSSSLLDAEDASRYLNPTVDGRFDVSSWLNEGLFGVKVGQPVEDGCVAHGVCYDLNLCEFVLFVREL
SSGEVRPSSHCLLK
>tr|C9ZZH2|C9ZZH2_TRYB9 | DeepTMHMM Topology Prediction - Predicted Type: TM
MRVTLEERDLHIAFSVSLATIGLVFLVQHVASTVSRLPSWFINLRWHYISLICLPVCLLVSLSLSSISWFL
VSAGAGLALVSGVRTWERGATTFLFCFLLAGQCLEQLSHTVHVTLPPKARTWTSRYRGQIALWPTGEATGP
EHLLVMYNDVTLTRQPCSGAVAGTIPTQAGAVTRQLGMRRHTLVSFATNTRIGSMESCLGDGANGGFLPLH
QVALLGESGRLEDVTLRCGLPSSARDGNSNGKTFSRCLTLDASTTFENVTVEFYADGNFKAVVSILHADKC
ADALSQLQAVGKSILKDEIDVLLCLPSAVSHRFSKLVGDPTLVPTALRAEVPYFFRCVWYLPMAVRNIPEG
AAFIADRVVIPIAATVVANSLAFVQTIIWTCWNLMPDVLQLLEDVGVFVTGAYCRAVKGTNSTLCAKSLLE
HAEDQRFQLGVRSVDCVFETVRLVLDALELVPGLWRVLLWTLRAEWHLVLYCLNIVQVILTVVVRWPLQTL
YGALQLTLSWMLQTLSLFFLDYDLSRIGDALCSPLAYLYRLQKELLAAEAQAAWGLLWWFFSGVCRMVNVF
FATVCAVCNGLFCVVSLYTGTTFCTHSFVALIQTAIVLIAMRNELHDIATRDGTGYTSFVGRWPGGKYMVM
LTLFAKLHSVVLLRYTVAHTVLIFTLFGLSVVGVLSKLYNLTLYIAFPWVSTTVFLEFFTADPRWSTVAWV
SAAKGLVAILLDRTIGDFTAYLVGEVFFVLGVVFTGAAFVWGCQKELPLLALRVLLRLPLDSSSESSTTGA
KLNTTPQPDVGRAVFLDDATGAEMTKGEIMLPDVVEVVDDTGKEEATECNKDVQ
>tr|C9ZP06|C9ZP06_TRYB9 | DeepTMHMM Topology Prediction - Predicted Type: TM
MKQQKYCSLPPPTLSFFSNLFLSSSSSNLIFPAKASKRAFHFTASRGSLRTRYASQHTILHSTTKIYKHKN
KINTLPFPSALFCFVLFLLLLSGSFLHSAPLVDFRAVQTSANEAHHLLLKRKKRK
>tr|C9ZQY8|C9ZQY8_TRYB9 | DeepTMHMM Topology Prediction - Predicted Type: TM
MPLTSASVRGIHWLCTFFFFKLILLCVLSIIVFTVVGVSRLLKKERKCLLFLYIHDFFFHFCSFALFNYCQ
LDMVSVPFPMVLERKRSRNEAMTSSRAAGRLRSATKRTEKIKEQVMKSSTSVSRRTIPPSQRKMLEQTQSV
SQLSEASTGNLPPHKLSQRELVRVHGWEPVVKPLRDTDAVSLPGTATTSVVWRNDITNATLIPNDVGGGVR
TRRAAARLAAGVEPPNYLVYSRDPHVKSKRRGQHNVHVTEDVLSASASGAVLSGVSLTSSKAVRSRWRGIK
SVDNGENKQQDGNEIGEDKPNSVGDDDAKFVGEGNGEKRQDGRVKKSEGPEEIEEENGADSIDAKEEKVGK
VEDMGEMKENVEEQNNNPQSDEEITDQSEDEKESGEEVGNETPAEDAKNREGDGDTPRGRRRRGLASWLSR
RRSRFGCERRKRRRAEAVTSADGQQQQQQQRIGTLSASQAMLPPPYRRNRRLKRKLDVTEDSPIFASHAE
LRDKASHIFARAPFSTDEDVLRSTIDWRDNPTLLYARLLWELAPREFLGIVTKRQEERTASSVLQGANSTI
SVKKE
>tr|C9ZKU7|C9ZKU7_TRYB9 | DeepTMHMM Topology Prediction - Predicted Type: TM
MAVIANTSSLNGGFVSGSFMFLNDIFLYYSTSDLLWVGFMSMLMFIIMLWLSNVVVPAIRMDRYLANIPRA
PGGLPVLGHALELLEGSPSSKMASWSLRPWKAKGENTVAVTKAGTNRIVAFTVFSQRVVYINEPALIKRVL
LSNQRNYTKDIASSYKHFMCLLGNGLVTAEGHKWRKGRLMLSHSLRIDILEDMPEMTMRAVGRIMEKLRTV
GSGVPFLDLNEEFRHLTLQVIGETVLSLSAEETDRIFPTLYLPIVHECNRRVWEPWRAFMFFSDGFRERRR
CLKRLNAVICDIIQERWRQRNEGSQKDVMSLCLSQVDALDNNMLLQLRDDVKTLLLAGHETSAALLTWATY
EVICHPEIRDKVVEEAKALFDPTHCDRTMETPEGVWGIPSASAVRSSLRWTPAVLRETLRKHSVVPLVMRV
AVNNDKWPASETGLDKDVVIPAGCSVAVGIEGVHQRPDIWEDPGSFNPERFLDVAIPNDTNSPPTGEKYEK
RIDPYAFIPFINGPRNCLGQHLSMMETQVALAYLFLNWDLQLHGAVSQSDTEINKELQQEVGRPHKFLIPI
VPGNGLKVVGHPRPRY
>tr|C9ZTB2|C9ZTB2_TRYB9 | DeepTMHMM Topology Prediction - Predicted Type: TM
MQASFEAGSGYQCRTGGTLVLTHLTPSVPPTCPFVQLCKHLFPSRFIVVITSVSQYFDLLIFPSLPPPPLL
CHSVVPTFERNILREVVYSSSLSKLHVHLWKIILKRRRNAKRREGVKQERVLEVREQKVNRVIYIYIYIYI
YLYIYREA
>tr|C9ZNR8|C9ZNR8_TRYB9 | DeepTMHMM Topology Prediction - Predicted Type: TM
MCAYARVFACFKVFSVDPCCHPTLGGGGDTVAVIWFLLILVDSLPPLFHHAFIFLAPNFFNITCAACNHPL
SLLPFFFFRFFLEEGGEKMGTTSTDTLELEVLRNPSNVRGWLQFIRSILCSDYPNQVSKANAVNVAYERAL

RANGYSYKLWMGYISYRRENTRELTSPNEWFRSLRDIYDRAVEKLPMMPLLWTSFIEFAMDGSVAPRITLT
RHVITRALEALPFTQHHRIWRLAKLWVSRPHVPMPTATYIWRLYLLYDPSTENQRNYFHMLWEKGNASDFL
VECAAFLLRDSTSHGGLLRDIAFWETVRTALETKGLCFGGDISQVEKIVQMAADYCASPAEFRLSYAVFLA
NQGELSMARETLWAILNDVDNPAVFCRAFAAALAFESQIIDSLAMDSSIHALDEVKYQQLREKLCGDVSDP
LYHLTRLTQQHPMLLNQLQLRADRHCTALWLKRIEILKEMECNGVATSSDVIALYRQAITQCTSGMPNVEA
ATAQLFESYACYLWENNLRTDAVAVADEGAWFVKFSSTTSNVLLMGLVVEFSQLTDPARTLDKLVSRLVKA
TNVSNSIRSKGLARQVAVKNLARDPRAWVLAVDVAFHRLLLKNTGESGGRSNEELKNLISLFCNSSGYTAE
GACYLACRLWQSGDVSAAFQEFERALVAFAAAPLAVLHILQQYLSCLCVSFGTRLPLHRFREFSKLGLDVA
QFTMRSSPVSTVEFLLNCVTLESRLGFSGSAVQIARECLHLALKHQAEYDSLLFGVLDAVLEVTFRLHGSQ
ALRHYCAELLERQKLTPQLIQRLALWWAAVERRTGNADRAHTVMEACCKSQDPSSSHGAVFWSMWESICNT
VKQFEGVHRRKQQAALKYSNGNNSADAAVDGFDGSKNNNVGASGNEAIEVVPVPALT
>tr|D0A2K1|D0A2K1_TRYB9 | DeepTMHMM Topology Prediction - Predicted
Type: TM
MFHFPGNYTPLPLLKAAGGKKKRMAPLKNMPVNHKSIPLCVFTLLALLFFFSPARLFNNVLLCCITDAVWR
LCRRNSKRKVNVYIYVCIYMFSFPFISCLCMYYFFTLFEGI
>tr|C9ZTJ4|C9ZTJ4_TRYB9 | DeepTMHMM Topology Prediction - Predicted
Type: TM
MSPSVRLPVSIYMCTFMCLNKYKYKYIYIYIYVYVCVYGFPSFIYYCYCQMCNLVPIFFLFLFPDGLPLFS
FFFFFFLLHLTLFYLLFYVLLLFFCLFSSFLFLLHPFFFLFYCIFFSNLFYYIKNTFAMSLYFVYYLTLSF
MWLYRNIYCYCYYYSSYYCYCYLLLFSKREFFLFNF
>tr|C9ZTJ3|C9ZTJ3_TRYB9 | DeepTMHMM Topology Prediction - Predicted
Type: TM
MYIYIHKYTHIHIYIATEGLMHCHFFFPGIRDRTRLLIHSTRQLINKNQICRSFLLFFFPCNIYIYIYIYI
YICVYMYVCVCVLVLLLFLSSSISFFLSLFHFFCFLLFFKNKGNPSICCPTPPLYLLPLP
>tr|C9ZTM2|C9ZTM2_TRYB9 | DeepTMHMM Topology Prediction - Predicted
Type: TM
MFLHNRGLAVDRAVLRLNPLIVYVIIPFLLPLLSMCILRWKLEYAWAELFVLNALVSAFTGLVMWLHSERV
ANMFLQYSGHSYGKANEGAAALLGVHRISPVLPACVSLFLMLISFWHVVVMILLAVM
>tr|C9ZWF9|C9ZWF9_TRYB9 | DeepTMHMM Topology Prediction - Predicted
Type: TM
MYKSNKTHVGRVSEWTTQTRRITIHFSHTQAHQRNRRDSRLLFVRQNRDRRKAKGDKIRSQCSGNLLILFF
LFGHFFLFLCIYLSPQPIFTDPFPFAPTSFSHVFYIVLGSFTFPPLASVPPPYALGVLNKG
>tr|D0A2F4|D0A2F4_TRYB9 | DeepTMHMM Topology Prediction - Predicted
Type: TM
MASQEETVSSPICVSNRIASTGLVGLEVGATSPVRDRAHQGGAHVRLDRGLRECEDAGEVPCFNSRIGAKT
TLSAISELLELKTGAGLSLTEAQECLVRRINFIRRHYIYLHAAYIMLIALTGATALCYTEDNLAFVDALFA
AMSAVCCCGLSTVEVAEWKAETHAFLHVMMLAGGAILTSAYQPLLRLWAVSKLCPMLEGDTKDSDGGNALG
GNYLSKSMRLWYASALCVVTTLMYVILVHISLAFFLGMFNRSNLRVPDVVLLAVASFHGAIFTPMEPYVDD
PAVVAVASVGCALGFTMFPVLLRCFLRMEWFFFSGVKRLFSSFRQTSTVRQRSLFPADNGEEAALLGCVDA
PLKKGAKEGEFEGGVKGYSTWDRAFCDILKSKQPASMHTFLFDLRETVYLGVAWVVITVIAAAPFWFQQWS
GDGLLAPYSEPYKVFLALCQAAIVRFGGASFLSCLDYSDSHIAITIMAMYVPPIPVPTYRVYKKWKSVPLA
YVIRPFTSRKFWLFVAVFCILIFEEEWPDGPDAPYYDMITRTMFEVVSAFSGCGLSLPPRWSALSFSGTLG
VFSKLVIVAVMFAGRHYTVDFSIDIGFNSLP
>tr|C9ZKT6|C9ZKT6_TRYB9 | DeepTMHMM Topology Prediction - Predicted
Type: TM
MMLGFESVSEFTVYITFIFFGMSAVVVTTSIFSIPFFFIEYYKYAQGDPNAEAEDQRFWNNVFTYYNATTF
LVEFLLTLFMLTNLGRRIPLAVRLGAGLILSILAVFVVIMVTIIKTTETGAKVTIMLVGVINGVAATLCDT
GNGALISPFPTKFFSAAVWGVAVCGVITSFFSIVIKASMESNYESMLTQSRIFFGLVVLLEVVSCILLVLL
RKNPYAMKYAAEFRYAAKERTNDCENKESGTSNGPAEQDEDPVAIDNNTTKGNVMTVTVDPDTMKDTDQVE
NITNSQQMLKAKVSVVLKRVWPMLAAGFLAFSTTFLVYPGVFFAVKTDVPNGWYMTITAAMFHFGDFLSRL
LLQFKRLQPSPRYVVVGTFARVFLIIPLVFCVRGIIGGTLLPYILSFLWGLTYGYFGGMALIHTPRTGSLT
AAGERSLAANCAVIAILCGLFSGSMLALAVKEGLPQ
>tr|C9ZJ73|C9ZJ73_TRYB9 | DeepTMHMM Topology Prediction - Predicted
Type: TM
MMIGEIPQAFTDFGLVLFKEGENAGESDNCDGVKFSEHHGTHRRRNVIDCGDEKANKYGDASSKTLEDTLK
QWESKKPQATGASGGNDVCKAAASSENYPCTMTEEWQTHYKETVKKLKELEGAHEKGKKAHDAMLGYANTA
YAVNTKVEQEKPLAEVIAAAKEAGKKGAKIIIPAAAPATPTNSTKNEDSAPTEHVDRGIATNETQVEVGID

ADFDGLLEAAEAAEVTHRHQRTAMIILAVLVPAIILVVTAVAFFIMVKRRRNNSHDVDTGKAEGGVSSVKV
VM
>tr|D0AA34|D0AA34_TRYB9 | DeepTMHMM Topology Prediction - Predicted
Type: TM
MFFIIYLNMPLQFGAYPLIMFFFPLPFLLFSKKNYSFSVYFLIYLLLYIYIFASVVLVLLLLPPPTVIMFK
TMCYSIV
>tr|C9ZYI6|C9ZYI6_TRYB9 | DeepTMHMM Topology Prediction - Predicted
Type: TM
MPADSVVGIPADLSLAMVEEKEAELAKRRQEIRRMQGNVASPLGPEPNFPPQFLCIKPLVYHNIKEQVPVP
SQRFMYTLAFMYFALIAIIIYNISIALLSFVFGGSGMHFGLSFVYLVGIPGAFVVWYYNVYSSVVSEANSR
RWLGYVGLALGVIFDIWMAVGVSGLGGCGWIMALGEKNFLVFILAIISASLWTAHCLMLLLLGIKYFRMAS
SAKPEAGAPADTAP
>tr|D0A3Y4|D0A3Y4_TRYB9 | DeepTMHMM Topology Prediction - Predicted
Type: TM
MRGSVTCSITSNSLIIYPFFHCLCCEVPEHASFTRGERSYQCFLLPLHILIGTPPHCTFPGPSFRGNANMC
IRTWCFAVRFECVMFSDGIMGGGTMYQIHCLQESGVCLPSLFLLCACTHLFFKNLSSASHITG
>tr|D0A346|D0A346_TRYB9 | DeepTMHMM Topology Prediction - Predicted
Type: TM
MVLHLCHLVPRDIYILVAFAFLFASCILQLIVVMSRVSGRDVGIIQPQHPGTSGYNAVHVSASFVLYLEGG
KKICEMNDDDDKIFVGCARFVSSFYVVGWILLYSVVTSYLSFFFGMFCVVPFSKTTMNVFALEMSLFWVPL
SLHSAAVGVFWDRLLPSAEKTVQLNLGQNTDLMFVQSYISVLMSVSQGLLTLSFFLVVFRFFVALHRRKAV
KQRAVARGDIDRLMRCHLVNDDWERQKEVLKDYAERLMFEIGIREECKQKSAETPSTSNDPTDRPTEEAHL
NLIEENVSCEPVEC
>tr|D0A052|D0A052_TRYB9 | DeepTMHMM Topology Prediction - Predicted
Type: TM
MCCTFYCIWYCALPPIIIYFSQELQRCARIAGVARERFVELLVKRKRQFINNCYESVTTQTDAVWYVEIRR
PLLCIRNCHTAKGETPPRIVFIPAAALKLQNELEDLSFFKHAVERTVVVRTDDP
>tr|D0A0X6|D0A0X6_TRYB9 | DeepTMHMM Topology Prediction - Predicted
Type: TM
MFNFTAHILYFRLFCHLVLLFSIYFRRICLVNHPYVAYCFGQHCHLCTVGNDSSSHRFFFCRSFSITGFVN
LCCGMCLVLLDGQQILFLQSFLHWSCVAKNTPQLFQLISGKGAAPLRQHRLTS
>tr|C9ZWN8|C9ZWN8_TRYB9 | DeepTMHMM Topology Prediction - Predicted
Type: TM
MVEKPREQHQLLPVRAGRATGYSATAVEVTTSTPHSPMSKSGGGRQAHVEPTEMRRRRESKVLLSALLFCF
VFMSVEFVFGVLAHSLALLTDASHLLIDVGAYAMSVVSLCTASRSSCGKYNYGWHRAEVIGTLVSIFSIWA
LVVWIVMEGLDRSWTVVKCSRIHAMLATTAQQYKRINSISYYGFSNISQRPTVDKDGALREATHMEMCTSI
DSPIMVVVGVLGMVVNVVCAAILYFGGSHGHSHFGGSHHHSHSGNGEEEDSLCEENTEHNHSHDHGHGYGH
SGSEGEGHDHSHSHSGRGFAVHAALLHALGDCVQSLGVILAGIFIYVANRYSYGVPSYRYSIYNLADPLCS
LLFAVITLNMTRPLLRDLLGILMESTPPGINYSELSSALRSIKGVEGVHDLHVWSIASDYAALSVHLEADD
KDAALQKAQEVCKRFGITHTTIQVDTVENGAGLCHSTCGTV
>tr|D0A5Q3|D0A5Q3_TRYB9 | DeepTMHMM Topology Prediction - Predicted
Type: TM
MNYQAITEIPDSNTIFHDTIFMRNNKHVSEQWIRIGELYPNGAKESLLPETFAREQFGQGSHYECFMLSAL
ATLVKFPDIIRNCFVSRNVRRDGRYTFQFFRDKEWVKVEIDDSVMLEDDEVLFIRSPTSHWWPLLFEKAYA
KFYTSYDNLEGCTLQEAYHDLTGNPVLNIPMDARLAKAAGVDVTGGQYWLDLAQKLQSGQFVGSLLTRETD
LESMGLQSEQQYGILEIFSLTGTSSVSDIVIHLYNPFEDEEFTYKGPLNSKDTEWTPKLREKYNVDDTHSI
FLPLHVALKIVNSVQLCYISPIDGDATYFDDEWKGETAGGNPTSVTWRKNPLYCVRNAGINPVELVVVIKQ
KDQRRYMSPEERTMYLQCGIVVVQSTTVTQIPTYFVTGNNHKAIHKSLFLNSREVSSFVRIPPNSLCYLVP
SCMKKGAESAFTLALYRVKLQDYSNILIKKLEVPSMDWGHCAIGKVTLETKVKERVDFFVDCPTHIHILMH
QEKPFVNKVGGDITTRDYVGVYLYDDTDRKIGGVHAATNFRETSVLHRLPRSGRYAISITCPRASGEVPIA
LTIVGSPDANVRIVEAPDDASMFDDEDDIAEGDEDATVSNPIDYIPVAGGGQVFTEVPDSTVPFEDKRFMV
DNKIMTNEPWIHIGDLYPEGKTRSLLPERLSREQFEQGEHFECCCLTAFAALVEHHPNVIRDAFVTKAVRR
DGRYTFRFHRYGQWVKVEIDDRIPLLGGKTLFCRSSDGYWWPLLLEKAYAKFYTLYQNIEGCTLRELFYDL
TGLPVLSIPMELKLAKAVLCDVDDVSFWVELSEGLKDCACAAVARPGFDDTLGLSNGQEYAVLDVVSLTDG
EASSISDLVVKLHNPFLETEYKGPMNASDAAWSSELRTRLCPERRDTIYIPVEVFCNAFLTVEKVMVRGLI
VPGWQFNSEWGEGTNGGNPTLVTWRENPIYVVQNTADTPLQIIAMIGQPDQRRVLHLLPEQEVNYVQCGLV
LSQCTCSNPVPTYLVTSNNHRVVHKGLFVNFRESANIVTIPANSMSYLIPSAMFRDKTKFLLSYWFQKLPD
MKFIKISRINVSVARSLPAIEDLELHNHEKKRVDFLVDVPTDIHILLRQEKPCVTVRTHDAMADDFLGIYL

YNEDEKRIDGVTAATNYREMGLVHRLATAGRYALSITCPRGTGVVPCRVEIVGVEDAKVRITDPPEDAGNL
CEVDLRFLGSEAEGVPLEDLPLDSDADLQAILEELRRLHGDIDANAESIAKHERMMSDRVHKMARQLLAKD
RSKYLPGYDLQTVNSILDAEPHFVVLERGRYHLKCDPRNATKVRNVERSLLTLANEILEKHDDPDLSFLGA
EVEGIPTLDLTLMRDSSFSEMARERMQLKRDPIANASRLAEVEDSMVARANAIAKEMHLRERRYLNPEPEG
VPLELLPLNEDEVVSEKEDRLRALNRKEQKDEKLLRSLEDVIADRVHEIARELKVSERDLFLDPTPGGFPV
AQLPLDEDDVFHSLEVERLQLRLRHDSLANKTSEIKELESRLNRRACELARVKVEEGRAFLDPEPLGFPLI
DLNLDGDSEFVKLESELRKLRWEPRVNSTAMRTVEEDIARVVHRIALRKTCLDRDYLDAEYEGRPVQGLPL
NDSKEVLAMETRRRVLINAKADRDEIGVAENMIRDAVREIARALNVGERLDYLEPCPRGVPVEDLPLDMDT
EFHELEVHRQRLKCDAKKQDTVIMDVERALNDRAEALAREHLSSDRGYLDPASVGVPLRLLPLDSDSVFQE
LEIKRALLKKNPQRNGRAISDLEGGLNERAHVLATEMKHAERSKFMDPNPGGIAIEDVPIDNDEVFCDLEI
RRFGLCEDRHANIAAIRNLEDAMNERVIELADKMRCEIRMFLDPMPLGVSLDELPLDNNETFVAKEAELHK
LRKDLRANGADVALLKSDLNELAKQMAAEMISKDRAFLFPEPEGRLLEELPLDSDKEFRSLEKQLRLLCKE
SPPEAQKIAAIEELLDGRAHQLAKMKNCAERKDYLDSHPCGVPLDDVPLDNDDEFSMLEVKRAALMRDVVG
NKDSIASVEKALNGRAEKLALLKLRSERSFLDPEPHGIPLHCVPLDDDDSFHLLEVERLTLKGLNIRENDH
KLVGVENAMRDYVNDLALEVKRKMREDVLDTSPDGLPLSVLPLDNDLPFIELENGYIKAVFDAKDTLKIQD
LAGAMRRRSEDIARKLRSDVRLSLEQHPLGFTLDELPVDDSELFLGKERELLELKRDGRYNSPTAIEIMEE
LNSIILDIAKEQAIREREFLDQEPEGRHITELNLDSDTTFLGLERQRRLLKKDPFADRSRIVDLEQMLNDR
SHELAKKLNSLDRPKFLPVAIRGVLLSELPLDSDAEFTRLEVERSSLYRDPVRNASTINHVEEALNIRVAA
MADEVVRQDRMFLGEDVEGVCVRHLPLDDDAEFRALETKRATYKSCGSRMGTKAIKDLEDQLGDRAAELAR
TAKKELRNLLGTEPLGVPLSDLPIDVDGRFRALEDNYRDLSRNSANGGKLCEIILQLNERALELAAELHTK
ERVVLSQCPGGIPLSALALNEDEMFKALEMEARKLRHSTAGRGRNAARISELESAMNERAMELANQLRRTY
CDAAPEGIPLELLGLGGVEEFTKLEDALRDATKDPVKNREIISDIEFELNSRAHVAAMGLLEGDRDYLECE
TMEVPLSDLPLNTDSVFRALEVERAVLKLREPHRGARRVRELELKLVERAHRLAEELKEEDLSGLDGEPEG
VSLRALKPHNDSLFSEFVGAMRELKRGPKRNTVAIEELRERMNERAHELAQEKLKDDREFLDQCPEGVPLS
DLPLGTDPLFHKLETERARLKERDAARNITHIHELESRLKDRAVELANEQKGRDLSELEQSPCGVPIYILQ
LHDDPVLQKMLNKMRGLRNRGVTGPEFVALIDEINNCARDAAEELLLRDREYLEPEPLGVPLSLLSLTTDT
VFHGLEVQRAVLKAQNARRNAAKISDFEMRLNERAVQLAEVQRQRELEGLDPEPEGIPISVLNPHGDAEFI
KLVTELRQLCSEGNNEGMCDVLKTTMNDVVHALAKRLKEGDRGYLDPEPEGVPLCVLPLDTDVLFFRLECE
RVTLKARNAHLNARRICELEDALNNRAVELAREQLRQDVDGVDEAPCGIPLQLLRLHEDSTFLVMVDDIRT
LRKNPDANSEAIDAVVMSMNDRAYDVAIAQFDRGFLDPEPEGVPLCILPLDDDELFHGMEVERIKLKLTDA
RRNAQTIGTLEEEMNGRAHELALQQLREELSGIDAAPEGIPLSLLRVMEDPAFALMVLELRDLKKDTQGNA
ERIREVEDAMNNRAYELADGLLEGDRSYLDPIPLGVPVSELPLCSDEPFATMEVERARLKAEDARRNAAGI
GELELKLNNRAKELASEQLLRDLEGFHDEYEGIDLVHLRPHDDKEFASLVPELRRLKRDGDKEAVEQHMRS
MDRRLRELAKELVGGDLWFLDKEPEGVPLTYLPLDGDEILDKLRHERAQLKIQDPRGNADRIAELERKMNE
RIHDLAKCIGDEDFDGVEGEPCGIPLSLLSPRDDEVVAKLINDIRKVKYSTEEKSSNGALNNLQVELSKRV
HQLAEDVLIRDRVKYLNQCPESVDISFLPLSTDEQFRALELERVIINIQSNGDDKRLDGLEDMLNKRAHEL
AKEQLQEDLRGLEQAPHGVPLDVLKPHSDLRFIKEAEQLRNLKRDPLRNKREIDDLVEQMNNTVGEIALNL
LRSDRDYLHLAPEGVPIEVLPLEVDAKFCALEAERAMLKGENNPKNASMILSLEEKLNERARQLAQEQLLE
DLKELSSFQHCVPLSLLSPHTDVQFAADVAEFRRLKKEGEQNTPAIQGVVGRLNARANELAEEYLRTDRAS
YLNPEQLGVPLDLLSLDTDSEFVSLEAERLKLLSYPAVNNEQITRLEAELNACAKKLLVKRLYKERNYLDR
EPLGVSLNALPLDTDSQFHFMELKRLALKAGDPYCNEVAINTLEERLSDRAYELARGQIAEDLSDLISAPR
GIPVSLLHPHNDPEFHEMVVSVRLLKRDSQPDLSKLMRLVDDLNRRVKELADCAVGTCRRKLNPLPEGIPL
QSLPLDEDPLFSQMERDLVSLFLEDEKKNATRITKLEDRLNERAANIAVSVKAKDLEQLEQTPCGIPIELL
RPHEDSTFASLANQYRISKHLGGSDSHGVNVGTLNKRATEIAIELLRGDQSYIDRQPCGVPFSALELEKDP
QFRDLQVQRMRMVLSNPKRNLNRITELEVQLNSYVQRCAQAFLAADRGFVDKEPGGVPLAELDLDNDPLFH
RMEVERAKLKIRGPVINAYRIRNLEEKLNDRAHELAEELLATDLEGIAPEPEGVLLASLHPHDDAECASLI
AAKRRLLKEPSQNMEKIRDVQEKLNNRVFQLAREVISKLRKQLVQEPEGIPLEFLPLDADNTFRSMERDLL
ALQATPRRNDIKVEALRGSLNERACELARGQIQCGRGFLNPQPEGVDLEHLPLDENVVFRSKEVERTKLKR
CPHENAAAITQLESDLNALVHSIAKDIKLSDRAFLNLTSHGIPRELLPLDEDRSFTTMERRRRRLKRDGNR
RATAMMNLEEMMQDRVDDLAFRILRGDRGNYVTLPDGIDAADIPIDTDERFGTLELRRAVVMLQGSEDCKE
QVGKLEHQMSERLQELVRKRVKEDRDFLDPEPEGIPLKDIPLDSDAEFRHLEARRRKIRRESRRRVTNVAD
LEEAMNDRAHVLARCIVAEDVGHLRPDYRRIPTNELHLHNDDTFRSLASKRRELLQVGAVTVDIAAIEDDM
DIRALQVADDVVTEERAFLDPQPGGMFIVDLQLDEDETFFALERERRRMRKDFRFAKRNKEAINDLEVSLN
ERACELAREAFSKQRDFMDQEPEGIPLKELPLDNDLIFKDAEISRYVLLKDPKHAANELIAFENVMNCRAH
ELAKEIIAKDRDFLDLEPDGVPREELPLDTDERFQVLAQEHFRLKRLNGNLKSTEVRVLEEKMNDRLHELA
KEFLQENRSFLDSEPEGVPIADVPFRCDPTFVEIERELLKLSRNPVVPDSAKQIVRDLLNKRLHEIAAAFL
KKERIFLDPEPFGLPLACLPLNYDHILNLLERKRRALKKNGRGEIKTIRSLEEEIQDRVNVIAVEYLEMER
TFLDPRPRGVPLTLLPLNGDWRFRSMEVELRALRKNQTKNRKDIEKIEGDLNLRATELAGAFLQKHRVFID

PEPLGVPIEELPLDSDSIFHNMESMYLQMGRHIPRAAELRRMEEEMNDRVRELATEHLQGERAFLNQKPQG
IPLCDLSLNNDRQFRDMEKRLRALRKHPRSNTEGIKTIQDEMNQRVNALARRKISDDRSYLPLELHGVPVY
DIPLDDDREFRDLEVKRHRAKILSPEDAVLVQRVEEELVSRALDIAKDFVWRERSFLDSNPQGVPIIRLPL
STDEQFRELETRRRHLKKDPRNAGIVALLEQQLRARAHELAEGLLGWQDVQFHEANHSVAEMWPRIGELYP
EGIRNSVFPEITQPGDTSSAPGELGYLAPFIAALSQHPPLIHRLLETKTHPVNGPYSFIFFDPNSNPVRVD
IDDRVPVDAKCEPKFTRVPHRSWYPLLLEKAYAKFVGGYAKLDQCTPHETLRDLTGRPVLHIPFDDRLADA
ANTGDFRSVKFWKGIKRNLAAGDVITCISNHESVDGLHSQCSYALLSVIETVHESNDVSDIVIKLHNCYYD
SPTYDGPLCNDDDNWTDGLKRLCHYNPEEDALYIPVPVFLRNFSSMQRCHINCGDRLTAPGEWTGVSCGGN
PKFTTFRNNPIYLVENKTSRPVTILAELRHHAPAYRDPDDVNHYHQSGLALLKAINTRMPVSPILTSVTHR
FLHRGMMLDAREVCAQMELPPSTTCYLVPYTLGRDNYGKFHISVYPGLAKVNLAPLRSGGLHRDPVRAVLD
VITGCEGGTRTDFTVSAPCDVHILLRQSEVRSTDYAHKSDVIGEEDLTMSVYNDSGIKVASTGCATNCLEQ
ALVFRVPQQGRYSAIVQRLGVSKAPLYRCDLFVYTSRCATACFVPVSVGLKPLKLGKCSALPPCGAEFSGT
SNGVNCGCNSLLPLHSAPYIKNGNQ
>tr|C9ZI47|C9ZI47_TRYB9 | DeepTMHMM Topology Prediction - Predicted Type: TM
MFYDHVVSVLVIQFLSFSSLFLSYFRDALRKYPHSEITMRIVHKKKIIIYNINNKIGESIEVLFFLGGGMA
AFFGSLVKSFILPKPSPTYSADKHPGKLVHIPRVDWDTRKENGTFTYGLLLLDTAAKFIIIYAHTNAVDVA
MVFETMSYVSKRTSTSVLLVEYTGYGIAYGETTERSMNEDVLSAYYYAVRHMRVPADRVVLMGRSIGTGPS
AQVCALLQGEEEVPALLVLQSPFTSLKECANDITPNVGSIVGYLGYDWFRTIDVVAQVRCPIIIHHGQCDD
VVPFEHAQQLKRTIEEATPPGVVELHAEPNRGHNDLPTESANRFIDKKLRSFGQPRCLQPRCRPYHLVNPS
IYEYLFCVKEQPIINMEELLKHWNETLSIGSFAYKREKLYVLLTASVSLFAMRCARAWQHYAGSRKRHYGN
SYIASCNGSVGVGVGVGVVGGSGSGGNGDGSGSGAEELYCAKEDIIKRCLACWGSPLGAYLSVRGPPIRHK
IFGVHLDVCVENVVASDGISNESGFRSCGGNPYFLKRDTGEAYLSVAELEFTHGLTRSVAAAMSTAPTLGG
EEDEMDVFLQKNVVTRIQTQCERVVAFLDSNEWENMLEVLVSFGQRAPSFLSSKALQYYSECSSQARFGDS
CGTGAACCGNDSGPAPRSEVMETIEDVDEWLRPWVVSPDTRMQLGTEVPWDYYLLKARLCVVEYTPLGPDT
SWEEARRITDAWRVVKIIHDLFCSYSRRVLHPSFSAS
>tr|C9ZVX3|C9ZVX3_TRYB9 | DeepTMHMM Topology Prediction - Predicted Type: TM
MLSVAYGVTQGKMAFTKGYECFRCCLYVTDVRREKKILKWMRLFAESRGGFRCMTFYVLHRYALFGSVSFH
HLPFRYRFTGCIIFFSLFFVVFLALLRMFVPLLQGPCRTPPS
>tr|C9ZTU7|C9ZTU7_TRYB9 | DeepTMHMM Topology Prediction - Predicted Type: TM
MPTTSYIYIYLYTYILVYLYTCAYKQIYARSLNRQPTPDTTNAALPDLAFTPLCCVSLVLFLLFLFRPLIS
CLFCRFFFLFWFWLYIPVPSAFTLSVFTPSPFPPLWHYPADGNGDDDITALPIFGNRQPLKRETRGKGKQD
GICTEEGSGTGNVNCTFAQTHAHTHTRARRNFYSLLFYFALFFPFVLFTTLRVCVNVYVFA
>tr|C9ZNK4|C9ZNK4_TRYB9 | DeepTMHMM Topology Prediction - Predicted Type: TM
MLYKDLLHPLSLSWLAYDVLCVVVRSFSPETLVRKRPKRGSKAKERRHRRDHSLLESRDYRSRHNSSGDHE
STDPSPRFTSHNTSLQANASAGSERPPTVPSRAAAGSGSSRPPSRAGSRLNSRSSSRSGSRHHTEVPLNEG
PPQSFFGRLRNWRTINVREPVLLLTHVLRAMPVAVLHCFTQLRTDVATLELLRFSDLSDTAAALLVFNEDA
VFAPDFVWNFTITRCLLGAMQQYVLGRVCLETNFVVNRLRSRRTYRHIIGWAMRLLTRLLTDPISCSILSP
MLLCTVKDFSPVTGPLRYTPSGLCAAALTGFVGVVLEQLVLPTAAICCQRVVVHIYDGLDYVLTRRYATKN
IMRDEAVDSRVNASGIDSATASEDDRESGQRTARKDAESAKRHSHNREDAQQVGKGAQMAVFRAIICRVVG
ALLAQCAVQHPLTVLSRLLYARAVLHGTGLLKSYADFPAKHLVWADFAAFLKQNAHGSSSGVPALEALRGI
GCFVGCEVSLIRSSILRCSAQERAIEELLLQAERRRQGGADSAGMSFTSSVSHASALESMELMTRCIASTS
PLFNMVHLTSFTKLLSFYMDMWVRLRGE
>tr|D0A8E0|D0A8E0_TRYB9 | DeepTMHMM Topology Prediction - Predicted Type: TM
MLFSHPSKGKKEKRAKGKSEGVCVLLHLDLFLYFFRCEFSCDTRPSSWYLCSSAAILFPSYHFFPHSTYSS
FAAFPRHLFLFFFFLHCLRAIACSFPLAGLFDIITPKSTMNELKNPMRYSPGVLVGNWYEDMRVAEDKIKS
YRSKMGHDADILGSQRLEESCVDADTHSMTMGDVIMLGKPLRLLNVATEAVLAVDTAWTHPQRLPHQFLLT
ATGNTAPRQRVEWVLMRAEDENNVGYTKQLKEENVLHYGQHIRIANEAAHSEGFLYLHSSIRDVGQSGAQL
AVASLGTSKDNIFVVAKPGEKRDDIRYGAPVRVGDRFVLYHAATNQPLRCIKKLQRTSFGFEYGMDCSFAG
DNHSRSVAAVTTEPTNLFVVVAANYGVTNTMSVSSLRNLTGRGNVGVLSESYEVDLSAIISLIREGVLYFG
GRLGFRLLSKVLGVACNEQCVTPVRRQDIFHGISLMGVTIHPGELDVIFKKLDRVGNGFVVAQEFLRELRC
ELPQSRLQGVITAFQQLVIEGGGSVDYKDMLNLFVFNACFHPDVEEGIASREEIIFDFINCWPNMNSTSSV
TTDMFVAYYTDVSPAIESDERFFKMLKRCWKIPETDAYKSMKPCRSVTVFRSDNTSSIIYLPDSSVLNIKD
LSSVRRFLTQCGVKDIKDIRLNM

>tr|C9ZW73|C9ZW73_TRYB9 | DeepTMHMM Topology Prediction - Predicted Type: TM
MSEAMESKVCDATAVKKTEYDIPQWSKGNYEINWVAVAVLALPPLMGIGALMAGVPLQTNTLIVGVLFYIF
NGMIGITVGYHRLFSHRSFTAHPILQWICAFAGAGAFEGSAKWWCRNHRIHHRYVDTFKDPYDATRGFFFS
HIGWMIMKQNYAIPARVDVSDFKYNNIIQFQHRHFFKMAIASGIILPTLVCGLGWGDWLGGYFYAAILKIV
FVHHCTFFINSLAHTDLFGAVQTYSDRHTPHDSVVCALLTFGEGYHNFHHEFSQDYRNGIKWYHYDPTKWV
IRLCEFVGLASRLVRTPNDIIKLNVNKLKYKRLLKQAAILEREMDECEPAETKMYTWEDIHSLVEKGRKLI
VVSNRVIDLDKPLNTGTSYTHDNETFQWYEVHPGGRQVLDLYIGKDATKEFGSGMHKHSAGAESLLHHLQV
GYLNE
>tr|C9ZV18|C9ZV18_TRYB9 | DeepTMHMM Topology Prediction - Predicted Type: TM
MCSLKNNPFGVSSFLHSEHAYTDTNIQAYINTIEPHAHPALLSVIYFEAAGVCPCFHPLTFLSLLLFVFLL
FFFIGVFFSLFVVVLYNLYPLLSFCFSFLFRPLNTSTVSSFAILSWVEAPCDRTFVSTPFLQLPPFHNSTK
LSSFCYYYHYYCYY
>tr|C9ZR92|C9ZR92_TRYB9 | DeepTMHMM Topology Prediction - Predicted Type: TM
MEGVSALLDPFSGSGLCCLFFFLFLESKFGLLHALYTSLLHAKDRYYAPHISVPPPQVKFDNQPSTQPLTP
RAGPPGTICCYDKGTNTQIGLVTVNSPAEVQEAVVRARVAQREWAKTSFSTRRQLLYSLMEYILENQALIC
ETTSVECGKTMMDGSLGEILTTLEKLRWTAAHGEEALAEEVRDVGLITFHKRAAVNYVPFGVMGAIVSWNY
PFHNIYGPMISALFAGNAFVGKISEYSSYYASYYLSIVQEGIKELGYSPHLVSFVVGFAETGEALVNSVDK
LTFIGSPAVGKIVMRSAAQTLTPVVLELGGKDPAIVCDDADLEHVVPIIMRGTFQNCGQNCVGLERVIVQD
SIHDRLLTILEKRVRALTQGPASVGLYDLGAMTMGEDAVRKIQKLVDDSVDAGATLVCGGKGDTSFFPPTI
LTNVTPSVPIAREEVFGPVLVMMKFKTDAEAVELVNACEYGLGSSVFSSDIERAKHIADQLVTGMTNVNDF
GINYLCQSLPFGGVKISGFDRFAGVEGLRGNCVVRASTTDRIPGVKTVIPPVLQYPISEASFTFVERLTNV
IYGGWFAAVSSVIEMMRMKSAPRKKDS
>tr|D0A6I1|D0A6I1_TRYB9 | DeepTMHMM Topology Prediction - Predicted Type: TM
MQFFFFPCCYLHSPYTFLHVARGVSVAHVCANIYIHMCIPHVFLLVPSRSSLKSPSPIRSAHAAKYALTEI
MGLPLGFIAGAVPLAPAVEENRTKGLKCSRARE
>tr|D0A3K1|D0A3K1_TRYB9 | DeepTMHMM Topology Prediction - Predicted Type: TM
MEWPSLGTCSMLYSKFAPAKIDWTRASRIAEKPASRLHGTDAQSMLILLVEALESDELAVTGTADDVGDDA
ASPVTSIRWQGLWHLISFLSGRLLVSRNVLAARVILFVVCCWTKRDGSRLLDSTLYLLLGSLATLAQKAFA
KPSSSGPNCYLSFRGKSEKKKLTPDGDNRRHPKV
>tr|C9ZP73|C9ZP73_TRYB9 | DeepTMHMM Topology Prediction - Predicted Type: TM
MFLCLCTVHFIFYVTIAIIIAIVNILFISFSRFLFFIYFSQHFFLFSCCRCCRFSSFICLLGHYLTFLKEI
PVCCSRRSTRSGGEKFNASLPPFKSRRWGTTREKRKKNKVKKVKIKIK
>tr|C9ZTK7|C9ZTK7_TRYB9 | DeepTMHMM Topology Prediction - Predicted Type: TM
MSRPCGVQLAIPARLAAIGLGAAVFTFLLKGTLWGRPTKLAAGRNCERNNTPLGSPKRLRNKEDSLGCVEM
FRRFLKKLGRTSAAEKVVAIRTAKSRDAEIYRLLLLKHQKQLGITFSVAIEEVPELSGELQIVVGPLEDYR
RHNNCSPFSTTAVTERERRVGGSEQLAEVEQLLVCRIKLPERRFCRNIPPFVMRVLAAQANNSLVFSTHAA
EVLYCMPRIEEETREYQNKHLLNTQGEQKQKKCKRSGLTSSSPSLTLGKGAMVLGGSSRPTSNSTFGSLQD
TLQSTPSCGSITIQSQLRVQGHETAVTQNLGENIDVNAVLFSTSLLVCGDSTSSGKVGPAFCCSVPNRVQE
KDEPTHTAVREALLNTLFLVPPKGLNRCPLEKLRQNPLGVWRASRQSVLTTAMLYVIDVASANGDPMVCYS
RVVDEVVAQAVLLHHDGMVLDFIGALSERFGKQYTPHCTVFQQAVESRREDHSRSGNVSFQVDSSNPCENL
QNILEELPYICEFILEHRNDFSNAVLTPGGSNLLEEVFGVPATVFPFLPCARESIAFITQSFNCIVETYGS
LSSEGFCKFRSDVLNESKADVMASSQRCFRELNNSRSGIISFEELCHWMARKLSSDNTLSNPDARLLSIAM
SLGLPMALLLGTRQEWAHLSRAPIFNGEGDQGCRLAHAT
>tr|D0A523|D0A523_TRYB9 | DeepTMHMM Topology Prediction - Predicted Type: TM
MPGSLNSCPNNPNTQHCVLFSTVSHWTGPQSLYYCFPRSRTLLQIARFILDDPDLSTQLGYGALLGSNRST
ELLKQYIYCSAHVPAASSNERYHAASWCIASVGITLILVATVCDYKWRVGTSSVTHEADETGNEGANELNP
RATPTCLPLISAEEVREDSTKLPTTPSPDCSRQCPRAGGLSRFHQIYGVHSPLLTPEEQLTAQSAVDSSSA
SIPSAQEASVFSSLTQQRLGFQGTSMRSDEQFLGIKAGVALASVPTLMQESGDGGNSCVTKPHALNVEKLN
RSLQKDPLRGSDAPVLRSGVAHVVQCLSLVSSFQKWRGYPESESDLNLFNGLRVITFLWVTIFGTFWFTQQ
ISPVNSSVVPSDSMLYAFLQKRVFSACAISTFLVIAGFLTLHRLHIYEEWQMKSPAVRAKWSRGTRRQWLL

KGALSYTRYVVSRFVRVIPVALTVTLLVPNILPATSKGPLWMALSNAPALHKNCEDYWWTNLLLINNMVPT
DGSKCCFPWSYYVALEFQLVATGPLIYRLARSLHTRVYAAGSIACVMIGVALRYSAFMSEKGKHLEKSEAA
PYTFDAGALYEWPHLMFIPFLAGAILHRIYIAVKHRAQTLLMFGPDMFTVMHRCPESTTTEDRLSYFILEK
LRLRSMRFLIMWGGIGIMCLCVFWGWSVLHLSRETTQVSVCRAAYESLILLFWCLGLCMLVFPLLFGYGGA
TRRFLVHPLWCGLSRLVLVGYLLTPIVIGIVNAHWGPTNVGVMLLLMLRWLGCTFLTFVAAFALHMVVERP
FLYISSQGKY
>tr|C9ZY92|C9ZY92_TRYB9 | DeepTMHMM Topology Prediction - Predicted
Type: TM
MYTSMSIQGVVDEFWTAANPYSQCLEPTTEEEAERQARFESIDYYKPYPTMYMNHLRGGSVRARAPSSRNR
YATTDPAHAAAVTVETPLTPGEEHLKNGVESNSVQGACETPPEQTFDAVTTRKSHDRNEALRWVLHVFIAV
TVGVVAIIVSYSVELIQDHREHLLVRLMNKGGFGWCTLALFIDLCTSIWFVMIAAGVVVYFEPGAAGGGIP
DVMAYLNGVHVPKVMTLRTFVVKSISCVCGVAGGLPVGLEAPLIHLGAIVGAGVTQGRSRALRCQTSLFQA
FRNNKDRRDFITAGAACGVSAAFGAPIGGLLFVMEEVSSFWDHSASGQIFLATMVCFSIISIFRSVIEDQR
LLGWVSNAVSVLFEVNLSIPLNVYSIAPSFFLGIVCGLFAALFTKVNIVLIKYRRRCMRQSSIRRFLEPVV
AITLYSLLSYFFAMLSGCAPFQDMEDRGDVLVWGTENTTSLFTATCSENNTFSPLATLVLGSERDTIRHLF
SRQTIWQYHPVQLLVLLLLYTIFACWSNGMAISGGLVVPSLVIGAALGRLFGLFVCFVGMGNIGVERGYMA
SLAWMDPGLFALIGAGAFLAGITRMTMSICVIMMELSSELHYLLPTMVAIIIAKAVAGLIAEPLYHQVLQL
DSVPYLRACLMRPEFEQLTAADVMTSRVISLRQREKTSVVMQALQTTTHHAFPVVEAVRKRGAAVKGGEGV
APLPQLEEEMTELDNDDYTSYKFVGLVTREDLRIYLSLPQLSKLPAAPGESRERNGTSSRQPTAGVVEVNN
VSWREWIAHKSSLFLANGGSNWLEPASPVLESEDECRSGSAFVSSTERSSEYSQINDACFPPVLDISLIVN
RSPWVIPPFFNLEMVYQTFRMMGLRHMVVVDGDTVVGIITRKDLLVRVLDRRFEELQRGPFTNGNCHDRVA
QQHGQATVTAINSCP
>tr|C9ZV51|C9ZV51_TRYB9 | DeepTMHMM Topology Prediction - Predicted
Type: TM
MHIYIYIYIYLFIYLYIYICILCTSLSFLLCFCSTFFLFLLCFLFCLLLVTPYYFYRLYYYYYYYYYYYYS
YYYCYCYSYSYYYCYYYCYFWGYCSAFPFFFFGCSRG
>tr|C9ZQS7|C9ZQS7_TRYB9 | DeepTMHMM Topology Prediction - Predicted
Type: TM
MIRRLGSVSSTSSSSLCRAASVAMIPAAQSRAAVREFHSLYSLAASLVVCSGSVLCFASWANNICNVRKYG
RWRFRNTIDDVIMVSDFKTARYIGGFIGFLFYWFIVGPRKYMYESNLADIPGNKRFGPF
>tr|D0A692|D0A692_TRYB9 | DeepTMHMM Topology Prediction - Predicted
Type: TM
MSPVKAFWHRKCPLRCASSATFRLPLSRVKLGGRGLSDGSNTLSNISSASCSFSGALFVAVRFVQFEQTSA
TLRGINTRKRATAAPWITKSTKDYMSEVNGHVASVNPVKDKLQRESKARRDRFTQVAEETDRIFDTEMDRF
RYDDARRWRKGIDFFKRQGIAFTLFYVSAYLGCLFGLYIGFATGFFKKEAAYEYMYFFLGGYVEKEWFYSR
VEAWGTYINFGFAFVINEMLEFIRLPFMIYTFYTFRPYLTRVNRRVKPSIFRWNAAES
>tr|C9ZKG3|C9ZKG3_TRYB9 | DeepTMHMM Topology Prediction - Predicted
Type: TM
MKAEGQLFFMSYFCFNSLLTLLTECRTPTCVSFVFCLFRLIRASTPFLSPIALEEGWIVRWTHRRKKQMLQ
LSTSYLQRLQRLYAHFKQNLHTLLGGCCTAVELLDEDDLTNWFVQLRYEDENKKEFIVSLRVLFLEELQRM
PLVFVVSPRLFATFIHYGAICSFELMSQQWTVDVEGLSLLFHSLYTTLNPFGCDSRVTVDESRRWLVGNGA
HGGNDDAAWMPQQSAASAYSLEEHDLGIQHIRNSHPHLFRSKVTGQKRIPEANQLATREGLSAAATAVTND
EGQRSSAITSDDGIEGDSGTTRREKVVCSCGELQQMRFSSEVMDIFLDTPRRRALFQSPEHPTKDTVVDDD
MFVCNLPSTSGALRENLSFNILLAPLCAREGMLNPPLDNNANPTVNSPAFGSSAGAGECKTDAAETMENEV
LSRLAEEGRDEVAPVICTCAASEGEAGEESNSVGEGGSGGTGINCAFCNKKKTCECAPIRLVLCGEGGSGV
ESFLTSIRWSSTTEQCVIPPDCTLRLQKLEVYASVITVLGTLEVDSCIVTGNIVVEGQGQVIFSRSKLFVD
VPDGYREGILVLDESKLDLRDGTVMKRYAPPSVDCQDTGFTGVASLNVLHSFIHVSNSARLCARGGCSIAP
SECERVILAEQDASVDISDCDIMAGYINAVSVAGCRAFFAGTRFYGGDQVVFEASDGNRPTGLSVELGGLV
TARHCLAEYVYFGFSVISHSVAHFYSCHAEHVVNGYTVEASRATFDSSSAFTNHVGIFALNGAKCTINNDA
GPPELFEKRCRTLLALRGRENGWYRLPLMNTCGSGGDNESNAVFTVEGGELRYDMGRGWRGVHKSALPLAV
DEVEQENDYVSSIIRAFSKKRRGSGTTKSKGNKANNSNDCEGVATEANTETLLPLSFVGGAFGLEVREATV
VARGLILKDTGITSIFAYDKSSIELTDVIVMSTLPASRKTCAVRIVNSSATLSRCLVTDHSLGFTASQGSV
VLCRECVGVCRSNSFVFDGAECRLYSCGAYAEQVGVLVFNQSKLSIDNSDAPHCILGGIPCIFQCRIRGLD
SRSSHVRCVGVTVRGSLESGFTSHNGGYLCLKKCMVDMSDDPFHGCTASVAPPDLPGEPQSTESPVYAPVI
TCSSVSVDGGCGVCRDLDETATTSPTCLCGGTWSLGVKAWAGSHCELTACEVRHVTFGYAALDADTEMEAW
QCVASDVANGYLVDSAKCCLNHCTTECNHVGVLVLNQGTCIVHHGSYRARVCGIENRNGIVQVEGNVKISG
FSRSGVHVNCGARFDATVDSLLEVSVSGDFGACRLSGPLGPATTCSSLCGQPPSCFVMDKGTAVIHNAVFG
GGAAIAVRCGVEAVAYLYRCKAEFCNVAFNALVGSNVYLSDCLAQNVWNYSLLVSHGGAIRVTASRVFSRF

SSVKNVFSVLQGNVRIFGKCVLDNTVLHLNGDAAAAADMAPQSLELDGVLEQVGCPIRVYEGGQLSMNSCY
VIVQETQAAQLNVLEEDKCDCGHKCPTVRAAIFAKGSNAYVRLRNVLLQCVPTPCLANKAGLSRGTSSGHV
PRATDSFKPGEIGQLHSKVIWHTLVLHSGARGEVCGAADVVPPMHNKNMTFFFHRSTSEGATHSGRLPSRE
ESRRPAAAVAGVPRDDVRYRTSAADIDIGPRQNGGCPLNEESDQQAPKPTKRSDSDTPILPVRLHVTDGSS
LVLENVRVRQLSVAMDSRVAATRCVFAGSEAVSVVSDSSLEATRSVFISAVGGPALQAECAKVSLTGCSGY
CIYKEIIIARHATVTLKNSVLHAIASSTAAHGGIQSPTTSIPWSDLPWCADNQPWDYGNEGKHSRSGPIQG
LDISSFTLAAVQCGHSTQFGSCGSCYEALGGCEVSGCVRLQGGTNHEGELRNFSSGFASPRLMEVKSPVEP
SFMNHNAGQTIRPSLHASEQPALHQRTSVRLEDSHLRLCLRTRIEGAVVADAASHTRVSRPPQPWRVVCLL
RDVSVNISVIISNKLVSAVKFLQWPLKRE
>tr|D0A1S8|D0A1S8_TRYB9 | DeepTMHMM Topology Prediction - Predicted
Type: TM
MPQNMEVRDDARRPNEKPSWKVLLTRGLLVAFIALALKNLGMFGWVDRGSETVPDVPQHAYYSQKGDTYDV
EIMSVEPGLFKRDSFRRIVVGGTEGISRNYTVNLQSCFKRNCSATLKVVMRLYDLVYTQEFPMVRFLPSRR
AAELHNLFTENVPLSRDEEETEANTTWKAYFQPVLTLSPVVDLPSPIPPEIRHLYAREEASGKYLPLLYIN
NIWVLRSHLMELNKTTAEFPLNFTIRISPITSWKLGIQYNFDRSLKQNQDKGLMRSEDAEEIKRIFLETNP
YFLALTLLVSILHMFFEYLAMSNDVMFWRRRKDFRGLSLRTIIMNCYSQTIIFLYLYDNDETSWAILLPSG
IGVLIEYWKLAQTAHFVRGEGGRLRLQFGDGYDKRTRKHDDVAIRYLMYLLTPVLACYTVYSALFNTHRGW
YSFFIGTQVRFIYIFGFAMMTPQIFINYKMKSVAQLPWRTFVYRALNTIIDDLFAFIVTMPLLHRLACLRD
DIVFIILLYQRWIYPVDTSRKEDCEADDDDGEERCVEDKGKKNVGGPTREKGD
>tr|C9ZIF7|C9ZIF7_TRYB9 | DeepTMHMM Topology Prediction - Predicted
Type: TM
MHLYFYFVVVVFHHTYIYIYMSISLSLIFTPYHSSIMSSHVSSCLVSSPFSFSPFSVLYSTAICLFICFGT
FFFSFFFLLNSVNHSKEGFFFLFWKKKKKRKEKEEKKKEGSKRDQICTQWDVGERNIRHVLMHELNKLFNH
YFISVHKKVKREKEGEEMGKRKKKK
>tr|C9ZNH4|C9ZNH4_TRYB9 | DeepTMHMM Topology Prediction - Predicted
Type: TM
MMWYLCRVNCSDGKGRQSNSFFGLTHKHWFVFVRFLNSLKFTFVVIALPYVGFVFAFCARAWNRSASLYTY
FSEKKTSDIIVNKKTYTKMLGMRSYIVFVAVVTLQHICVNSKAHENNIVNGKEFEALCGFINFALDTQTPQ
DLVLKVEEAEDKILSDTFAAGGDAKVSKELQELKQKVETYKEQHSDLWSSSTPTIIQKSLTEALYGNGNKQ
VVVKHVQGNRSDVCGRAGGQTGKKAGETLALDLLCICAASDQDEDDVVTCCNGCNTTHVGVWEPQQNSPAH
WNLLPPKCSNVKREKTRPLQVLSAALRFFVSTLNTTSNSSSGPRNLLGAHDGSVEHGCSGGILDGHGRCVI
YLPRHVSESAPTIPWYLKLQEAATKMEELIQAEEYLVKIQNQVNELKREARPAPNNSSEKEKDESNNTDNG
LEGKKPTTSSKRGCSGFESKVTCLQQKPRCEWNGTECVTSIRRLLTSSGVNRLPAPLKLFFLVF
>tr|D0A0M7|D0A0M7_TRYB9 | DeepTMHMM Topology Prediction - Predicted
Type: TM
MELNNVIFWVCALYNEMLAMVAFFIYYFLPPFTCSSQRCLCIRVLLHKHLPSFGHLFLSFVWVWVCIAALF
IPVERAFLISPLISHLMVAWFLLCVPTRRGEKRKGKEEGAKPQQISG
>tr|D0A6H3|D0A6H3_TRYB9 | DeepTMHMM Topology Prediction - Predicted
Type: TM
MGKQRNVGNIPPASNNVRTSLPLLLAVLLPVCFVRAGEDTNTYKADERVRVYAGQIGPLHNTFETYSFFRT
PGCPPASWSRRSSTLGQALTGRQLQEMGVNVRFGKNVTGGVMCTFTPKQADIKRWRKMIQKKYVYELYVDE
LPIWVLLGEVTPAGPVIYLHRRFHIETNKNQIVHVTLEAEQGTVLTQGRDYTFTYSVIFTESQLSFEDRFK
KYVDQKLFEPRFRWISVINSVILALLLSIINIFIVSRAIHADLKGDEDELGIDSSYGLVEGSGWKQLSADV
YRVPPYPMALCALLGTGAQLLLVFVAMIMSAAFYNSRHKPTYGAVTVVTEAYALTGFVAGYVSASKFVSYT
VYKPALASRWMHCMYLTIAAFPAAILFCGVSTNAIAYLYGSARALHVGGVAYVALILVFLFCPTVVVGTLT
GRYIFWRRFNAVSNRNSLPHVNQIPRLVPRPPYRLLSRPYLILLTGALPFSSVVLELFLVFSCIWMNKLYY
LYYFLLIGFTIFLVIACFTSIAATYLLLNMEDHRWQWMAFGFGASTGIFIFIHATYFYFFQTSMSGMFMLV
FYFAYSALFSLAMSLAGGCVTFLASSQFVQKIYTSVKLE
>tr|C9ZXA7|C9ZXA7_TRYB9 | DeepTMHMM Topology Prediction - Predicted
Type: TM
MIRTVSDDSYIISYVKCQFFCSSICICIYRADVAYYVLICCIWINQAGVTASMCLFSNMFLLILIIREYGD
TMLPSCCCVIMLRVTVPIPSLPVYK
>tr|D0A2J8|D0A2J8_TRYB9 | DeepTMHMM Topology Prediction - Predicted
Type: TM
MKVTINIKASEERRVMSVAHFRHVFVPHFVCMYVRGTRFSVFVNFLFFFFILFCFLCYPLLTLCFFFSLEL
KGDPPDMEFLLSRHKGGKVLFILFFDLILFSQ
>tr|D0A8P3|D0A8P3_TRYB9 | DeepTMHMM Topology Prediction - Predicted
Type: TM

MPAYTGLNGRPIPPGAKRRRHITSALFKTPLLLVPAIFMMILLYYSMRSGRGHRRGLPVPFYDSQETTTSL
DDWKKDRSSSGIPGLPPMSDEGRSVIIVPCTASGSINSKLDGQQQVTTEVQFYRTRCQPVVVGKNDERTVK
EKGGCSDDVRPIVSIIPEGSEKLYFPKEVLLEEMEEEEQGPQDTVGRFPQRMEKVLSEDGTNGFWLSTSSS
GPPIMRCASRALVEPFFSAPLNPQPNWIWGSSGFVVSVLLYTIARDVGKGVVEGSFTFRSREKIHFYGLPL
RKKPSAGWIEADDDELDDEAVTGGTLPSGTVPLVYAFGERSVVGILWLNGSPSHAYTTDTADSKPSGMREQ
TVHFASATGATRVFLLPGPTLEDVLLQYYTLTGFPVFPPLFALGYHHHSVRYRHADDILHVNHMSLSLHLP
IDTVGMNLGAVAADDIFAWPRRRIADSLEVQTRLWRDGGHIIVLAVTPTVVPSSGSAAYIEGKEKGYFIFS
PLKEGAAFVHLVSGIAKVWIDFLNPRARQWYSEMMEFTRFVGSTNLTHFSLVDNEPMLPRVLGGTDGVTLP
SSALHYRGVRHRQARNVYGMLHSMAAYDGQLSRTNNEYRPFVVTQSYFAGSQRYAAVRLRYRHRRNNDLTL
SWARLRETVELCILHSISGLPFVGPDINVPVPNSFWGKKNFDELQVRWYQLSAFLPLFRSDMDVRPRHATI
LEFPKRTIFRIREAVLFRYTLLPYYYTLFWRSHLYGEPILRPVFLPYEKRGPPPEKGVAMSKESFFVGPDL
FVAPVLSAVGEETAWRKEPHHRIRLPPNDLYYDYWTGALQYQGGLVKEPACIRFDKNPPFAEAKHVAPLFL
RVGSILPTFTQTRTMRSSHDPANYTLTIALPQLTSELLWASEPVNSSDGRLLAEGELFVDGGDNYVDYNNH
STIRSDFCALRLECRLFPHSYRMEVRLKATVNSSCGKAVEALKANLVELDDPNVYVIDRLRLLFASPSESR
FLVRGLPAASENGRQEQMQWKVSYGEIDDNVVEVHNFFVQLSDSLSNENTTHVIAALDLRL
>tr|C9ZXX0|C9ZXX0_TRYB9 | DeepTMHMM Topology Prediction - Predicted
Type: TM
MSYQIIHTCLTLTVLCSAGNGGSALCYRCYFPSCDMWLRACTQERNICRIPCASWMLYLLFLLTFMCPLPI
CNQATNLTEELNLLRCSDWFAYAYALRDMLYDRSIARVFIVLATLLYVYYVVWIGVTPFIVSSHFTQNFFP
PREYGIFVAAAVMTLMLGVTVTLASVRTILWAGAVGPEESG
>tr|C9ZJE7|C9ZJE7_TRYB9 | DeepTMHMM Topology Prediction - Predicted
Type: TM
MHKSAVWRVWAFFFWRVGTRCLFVCFTSFSTRLTFHHSFQFFRIMGYSSATKGIVCNYLFIYLFFLWSRLR
FRSFFSLFLFFFLAGLPYEGVCNNIHLHFTTVPSLMGPTLLSNTWFVTLFGWMFQFLGSFVYLFLLFIFFL
ILPFLVIITLTYQGVLSSSLRRCRRVVRKKTHQRKKKSKGKNK
>tr|C9ZVD2|C9ZVD2_TRYB9 | DeepTMHMM Topology Prediction - Predicted
Type: TM
MLNGGEGLGWGEASRLFRNWRVPYAINKKKQTNKLVKELERNDFFLLKWQISMCVNVRVRACTYVYEWLEG
IVRKDKVRSFAFVFLLFFLPHRLPPKVVPDALNIFHGSFKHFSLIPHCSLIIIILPYIRVCMCICMCIALCL
NLLRKFIYRCHYSPPLYFVVYVIIYLPLFRPLLSLFCCGFAVAVVVVFHRFDLREESQRCGRN
>tr|D0A5H4|D0A5H4_TRYB9 | DeepTMHMM Topology Prediction - Predicted
Type: TM
MAPPSDISERRQRASLKSKKRWVRRILWLIVWWFGAVIISRLHQDSLRMYVIVTTFIAIFAALGWRQRRKI
SREGVKSNIHERFGTVASLRRRCVEFNALKNIGTPEAIRVIRDEAFRQNLINSPPDAPSCCCGSGRPFAEC
CRVLQEELRRCGAET
>tr|C9ZJU5|C9ZJU5_TRYB9 | DeepTMHMM Topology Prediction - Predicted
Type: TM
MAMLGFESTAEFFVYLTFIFFGMSVMNVTNAIYSNYYFFWEYYKFAQGNEHAVSANPSFWKHMFTYYNVVV
FTMQVLLEAFMLTPLGRQIPISWRLIFGLTIPMVEIIVILVIPAVGGTSEGGAMATMMIVAFVCGISMTLC
DSSNAALAGPFFPTKFYGAIVWGLAVSGLMTSFLAIVIKASMDSSFESKRVQSQIYFGLVMFLQVVACVLLV
LLRKNPYAIKYAAEFRYAARKKGTVCDFDVKGTGPVSGNRYADEKENKNVLNADIDPDKMKDTDQVEGTTN
AQQILTRVLMVVVKRIWPMLLSCFFVFFATLLVFPGVFIAAKTGDTSGWYFTVVVAMFNLGDFLSRLVLQF
KQLHVSPRMVMIGSFARALLIIPLSLCAAGTIPGVWLPYIVSLLWGLTNGYFGGLSMIYGPRTGSLTTAGQ
RSLAAICINVALLMGLFAGAMFALAVKEGLPE
>tr|D0A8D1|D0A8D1_TRYB9 | DeepTMHMM Topology Prediction - Predicted
Type: TM
MLQLMYSILLSSALLGGFVESCLYFFFLAEHFPPVHASELPCSLFSSFYFLERLRPAIGAARTALCRFPYV
PRFTATSVSPHPYTKSGKTSAETFCRSVHSLFSRTVLLVHCASARHRTYWKNTGHSTPILPHNLSRRTAN
>tr|C9ZU24|C9ZU24_TRYB9 | DeepTMHMM Topology Prediction - Predicted
Type: TM
MRVCLWLFNQPAVWNNAAVQHPLLYFFSIFVNYSRTFAYRRDSGHACIEMNVFFLPNVRVLFLLNIKINQY
NFCFFRVNYFATFPIFFSPPLFYFSFSSFLSFVRV
>tr|D0A2B8|D0A2B8_TRYB9 | DeepTMHMM Topology Prediction - Predicted
Type: TM
MCFVFLFPPVCIYIYIYIYICLCMCMFMFICVFVFVLVCLCFGLRVLVLKLFVSYCFVFFFFPPFPFPALM
LYFVPPSTSFPLYVVIVVSACVFFFFKISV
>tr|D0A810|D0A810_TRYB9 | DeepTMHMM Topology Prediction - Predicted
Type: TM

MMYIRTFGDMLHLLAIFILLGKMLRGRSAAGLSLKTQFLFALVFTTRYLDLFLSFISVYNTMMKIFFLATS
WHICYLMRCKSPWKTTYDHENDTSASVTLIIPSFVLALLFNGHQHGMWVMDVLWAFSQYLESVAILPQIFL
LEYTERYEALTSHYLAAMGAYRLFYLIHWIARYFVHGSVNAVSVCAGVLQTVLYVDFFYHYISQVVWRAKQ
RYDLAR
>tr|D0A866|D0A866_TRYB9 | DeepTMHMM Topology Prediction - Predicted
Type: TM
MGGGCVSQRMHKTFIGLGRAPAWFMSHWSYGRFVCACNWRRKRAPKHLHPPFFFLNPHDCWLLSAVSLPFV
GVTVTQLILIHCMSFLSSTHGTIGFCIVTRRLQLCKGLFIPSSNICQLYCSCASS
>tr|D0A7V9|D0A7V9_TRYB9 | DeepTMHMM Topology Prediction - Predicted
Type: TM
MGRRMMKRKRKGEAAHSHNFFFYLFLLYYYLLSSSVSPLYACNYLVLTLFVICMSYRWKYKLYASKESYGP
AAEGAQTRFCEGGFLKLGVCVCLCLGREAYAHVSIMWRCSQSTFSLLFYLLVNLSAWWVVLN
>tr|C9ZUC9|C9ZUC9_TRYB9 | DeepTMHMM Topology Prediction - Predicted
Type: TM
MALVASCSGLYFPDGESVDNIVTLTWRAPATASAQQPRSVVLFKVKASNPHKFYIVPRYGTILVSDDSGRK
PPQPPAVSITFGLRPQSDDPLEPPPPSLRRDNSDRFAIEYLLVVPDAATHEHLTQELSRPHRRGSNTVETA
ARELWEQVSEGKLKATQGPTVFLQAYTRGVVRALHETQGRRGEGFSDHSHNDGGDEASGTATSNASTTATA
MTISNDNKHCNSGAAVADAYGSAVRNNSDKNRSHRAHNRGISSDEVQRTASSVNSGSHRSSERHEEEQVVV
PPNARLVTSMIHRKLSDRLRRDPACATNNDGAAAGPTSSAGGGELQALKFGIKALRSGPRIHPEEAASISQ
MKTLEADPGMGGGSNNIASPAGTGTEAMNGIPGESSVGCRRTAVADVIMRIPPATSAVAAVKNQKGIPLTA
LLVLMFLSYGAAAFLVERMALI
>tr|C9ZLF3|C9ZLF3_TRYB9 | DeepTMHMM Topology Prediction - Predicted
Type: TM
MMVTNTCIIMWPFRKEQERGGWLIGWNPHGLLVIVVAVVHDPRVENATAALNKLKESEGDIRATAHRKQHK
DVPSATSLLILGWMHSKQATLDKSEYADAWVRSQHSHAELWLELNEGPLLHQLYCCGSPVQPCHLHVLRSD
PTVSYAVTSTAFSAVARHDRGDLVKLVEGKMQPICVEGANSTLEGVLLPSHMVHSAGPRTKMRRMVGSDPL
TQDEEEKQEGAKQRTEDDRVSVGSSGSAEGSTESQSVRGSETPGFGGELHPRLDFPTSTDYGELSSLLRAS
AYGKILRELCENKVSNATDGPAATARLCAYWKNCGNRFTGFLTKILVVFHLVLKWPGNVSYTAALLEFRLR
MVIHWLLLLRGDTRVCCLHPVLPHIQHNDPALRRFCVVDFLVRFAVDTICGMLLSLLLSHGSGLLVTKAVS
WCRWLLYDLHMEYMDWFEGWPAGLKMNGDLSMTLSCIAKAVLEASWASVEDLSNVGLFYKLLIFVAPLGAS
CAFAFVADLCLIASLHLYLVFHSVSLPYRFARFMLRNLFLQFQGKKHNPLRHRTDTYDFPVEQTLVATVIF
TIIVFLLPTLAVYHVYFALICIAVWLLQGALLVAAHLTLYLPLYPLFYWALYRRQWPGGVALTGPKVLSTP
QHSRALDPFARGSLTVEFGVVSKPLELRVLLADFLLVAHVVGRLHPFDMIVAVFKVRRWERTNPGKCLVPP
LPADIMPLTGMTLPKTAVA
>tr|D0A8Q2|D0A8Q2_TRYB9 | DeepTMHMM Topology Prediction - Predicted
Type: TM
MIKYYGGRLLKKVRNGCLHSCKNKLACCQRLDLPNSILYYYRVIIPIILTVNAVSPCAYRNHNKPLLEISS
WHCYHFLINLHWFTQIYISSLFAYMLMNEPLRQFWRPASSGKGS
>tr|C9ZJ92|C9ZJ92_TRYB9 | DeepTMHMM Topology Prediction - Predicted
Type: TM
MNVTGVPGNHHDLFFSISLSLSLFCLLPFYLSFCVCVRTLFYSSDFLFKKPFRCDLCSGVTLAKTSIDGTC
NGLRECGRNANIRKQHKYMNAGKMHIERQPAAELQTNYRRQRIHKTSQHLNISAI
>tr|C9ZX69|C9ZX69_TRYB9 | DeepTMHMM Topology Prediction - Predicted
Type: TM
MTRGKRRTVGTFGKRKKNKPRCEPHFPPCSSYVCWRVFLCMFLNFLDNSLPRIQWIWNCVGAFLCFKFVLE
DPHHCWYLYFLYAYPYLQEKGYLLSQMQRLFWYFSLFCSLSSPPFATTKNTGERSLKKNC
>tr|C9ZIT7|C9ZIT7_TRYB9 | DeepTMHMM Topology Prediction - Predicted
Type: TM
MCVFVQLRNAFLEVEGRLEGFSACWCLFLSLVFGTTKISGGTVLFMY
>tr|D0A6G6|D0A6G6_TRYB9 | DeepTMHMM Topology Prediction - Predicted
Type: TM
MNKAGVYSLRTDPSLQDYYDACAEGSGDEGNGSGDESDQDSGNYWRSTRRSPLRGKARLRLYQRDSVQKLL
RDQGYFASRGPEAEAAASLLSRPLVFAGGSHEANMSCSRQLLPPCTSNDSYVPTTEERLIFDALCALRQSP
STSFAASAGPEGASNLDRPAVWALRRDVMHTASLRGISLSVRSLVDTILTGSNEVARAEWQLQSISTSLSK
EDLNTMEDAFTDYSVTTSGDEALMLASHLVLRRLCGIVRSIMRDVSMLALGFLEDEERQQRQTNSSSFTYH
RRMEIEKGVEEVLLPLRRCIHIIDCACKLEERAESETGEEDDIDSIDRASGLMDYLIVRMSALQDSSTMDL
YRFYMVLLMFFSWPYVQLITSAIFGFVTKIDSDMWRSRVPRIFRLSFSHIRVGEDPRRGQKAAILPTDILS
HVFLCVGHARPEGRRWSGRDEGEGGHQRQRQSLSRAERRAAYGSMMSSRSFILHSFVAFARQRALKGWRRK

RMADHHAISGCVEQDEWELCRFGRLGSSVEGGATDALPLALCTCHTSASTPVGVGATKWGLCVENYKTHHN
IALTDGDRQEKSATGGRSDLWLHPFIPAARWVTVSLLVPIAQVVQRLQERGLTDLLSVAVVSPCVNSSDTA
DFDDRNETDDGAHMVEEGSSMGEFPSLKSINALQVYSSVSDPSESRASFRDYMSLFIDIALCRDTERIVHK
FLYRLFTESHWWYRCGETEYACSGTASSFISNTFAEAIKEHPLGQFVRLSVAPKLEVVDENDSGSTRVDCQ
SEMLRTFAAFELVFTLPPDVDLILVPRYLSVEWDESGDVRETYTSYFWKRRQCSEERTGQPAELTDATEQR
ARDSWSYCFGYLCSLYYAQISLREQRKRLQRQDVDEYNSAAAPGEALFGQRHRAVRVIRGLGSAYFELSFA
VDCLLSFNKNVVTVVACEMEKLTRVGSASSCITLCQSLDALLLRLLIVCFPEGASSSLAGASAAKTSITNS
VTALLVIALDPASLPVRRVMSCTRSAVEALVAVVRTLPASSAVRKHVKPLLVLLTFNRFYGE
>tr|D0A9N9|D0A9N9_TRYB9 | DeepTMHMM Topology Prediction - Predicted
Type: TM
MNPSKVVQGTPNSVEWDPRNPPDTPFFTETTAVERSRIPTEYIRRMEEATRLYAMHREGRHALHTACQIAS
WTVATQCCWLCVGVWLTVRGYRHANPAHSFVSGMTSNKVFCRVFTPIPVLGLTMMGVTAAQLPRDVKVLIT
AREGRMEEENCMKSAFDQCMAALAEGRLAMGKEGILAA
>tr|C9ZHY2|C9ZHY2_TRYB9 | DeepTMHMM Topology Prediction - Predicted
Type: TM
MRFTFPYRSPLVIYIYIYILIYLHISPFPRAYISTHAHTVPPFPFFVPSMNIFCLVGVVFPRSLLFYHYFL
REKQSVCVRVCSVQAMERKKERE
>tr|C9ZNQ8|C9ZNQ8_TRYB9 | DeepTMHMM Topology Prediction - Predicted
Type: TM
MVSCFVRPVDVFGNPKTALGENLEPQHTWLEWIFMPRHKPLFHNFSSNARAGLTVALVNVPLSIALAIGSG
ATPEQGIVSCVWAGTVATIVGSSHFNVVGPTGALSGLLASMVAARGPGVLSPLALQAALWIFLFFLLRVNR
LLRYVSSGVAHGFGCGVAIVIAVGQIPSALGLKNVTPHESLVEKLKEDYRRIDTISIPDTLLFLSTILPLL
ILSKRYNKIPWQIVFTMLGIAMARILPSGSLVLLGSKYPNLSLNFFSFQPLHTLIEADYFDFKTVLYGFGI
AIVALIETLISSIIANNHVDDKSYLNYSGARDTFGLAMANLMSSLVGGIPSTAALARTALMIHSSAFSRVA
GVISCMAVALLCSVLLPLFRDIPMATVAAILMVVAYKLVDLHDLHILHVVDTANLYSTIITCAACVLTDTF
VGLVVGVFISVLLNYSDSQEAEVLFEEEDETFASPHRPEPVMFRVLIVRPQESLLFINAEIMKNTIFERSI
TFCESTPSGLKRRLVLDMALVNRVDFDGTKALGEIISAHREKNWVVDVINAQHLRNSLALCTPFHDLHKLE
YQELY
>tr|C9ZKX8|C9ZKX8_TRYB9 | DeepTMHMM Topology Prediction - Predicted
Type: TM
MRGREGGGFLFSFFVFGNQCDITTISPIGGVEGECHLGAELSSPTGVSVFHFVREKKKRSATKLRVISSGC
LHHSLPLALRFFGNMRANAVICLFSSTIAAACASYFLFIMITFFV
>tr|C9ZI48|C9ZI48_TRYB9 | DeepTMHMM Topology Prediction - Predicted
Type: TM
MQKLLSSMLSLLLLLLFVIVASCCNFCLAVVSGFWSSRAYIFSRRFFAHSICLSLRYFFYLFIYFGLRNCV
CYFYPFF
>tr|C9ZU94|C9ZU94_TRYB9 | DeepTMHMM Topology Prediction - Predicted
Type: TM
MLARTLLRGTQEGSATVAASSSSSSSHAAATVAAITRAAPYTRFSTLRLHGHKEPNFRARPAGWKGWNDPK
LQDPWAPFAQQSPCRHVERQGAGCWFAMGHFALKDFFGVLHWRFWARIAIWGGSGMSIALWTYALRMHRNG
WEWKNRGAVFAME
>tr|D0A345|D0A345_TRYB9 | DeepTMHMM Topology Prediction - Predicted
Type: TM
MSRTDFSCAICYEVASEPVVTRCGHLFCWRCLSRWLHPPRSAVNTECPVCRGRVDENVNGDIIPLYGKGRS
EGASSSFQRSSRWTQGASHGPPPRPAAARVPSSSDGNSFRLRGAFPFLSSTSFFFFSSDPYSLIAISLLWA
MYQLPWREWLTNLSTYLGGVSNTVPTADEGGSNVGAVPSPQRSDDLHSREVGEAITRHVRSAMVLALGLLA
TSFFMV
>tr|C9ZPN9|C9ZPN9_TRYB9 | DeepTMHMM Topology Prediction - Predicted
Type: TM
MFVPVFYPPSPFFFFLFFTSLRFLISFSFLGPLPSVLFTRCCTYVSQHFSSFWDVSPFPFFFILIKKIIKI
SRTPSNGSIDVSFIFIAGRLLLLLLLLLLSLI
>tr|C9ZXJ6|C9ZXJ6_TRYB9 | DeepTMHMM Topology Prediction - Predicted
Type: TM
MYLYVCIFFLILFFFVCFPLSFFFFFFFCLFSVFFFCFFFLFLCLYLRYIFFFPPPLLVILQSFSRHSFIL
SFYCSFVFFFGIPAALVYIFINACAFVVL
>tr|C9ZYJ6|C9ZYJ6_TRYB9 | DeepTMHMM Topology Prediction - Predicted
Type: TM

MGFTTCAIIPIASSILSVAYFSFPNPLASAQLLIVLFESCSRNRSVVTLFGALVHAGSDADTKKCADASRR
VDNCNLFPFGFAAASWGAAALPNEFCGSLFGSHLNSGFKRQTSHYIACAVKPPISRKNCGDN
>tr|C9ZSJ9|C9ZSJ9_TRYB9 | DeepTMHMM Topology Prediction - Predicted
Type: TM
MLTSFLLVLNFFSNFSMVLYNAFQYFGALSFYNEILLMFVLVFFSAAGVVLSEVCRVVNFYFSSGGSVFVE
RRISVYLSTHVVVAVCDRNALLQNAIFYYMYAKFLVSPVGSSCWRNMTSVILLIDPMRSAYEESYDSPFRV
DDDDSDDAGRWESVNWRCQKQLRRFFIIRVPTDGCWLPVGNDGVELTYERKRETVDGSECVVRTIVLRAKG
ATDAATRIDKFVETALDYYIMNLPDVMNEGKVFLELQAPSSCRDEGGLLFKRYPLGCGKTFDTLFFPEKSR
VLKLLDDFMGKGGRFSTEGFPQKLGFLLYGPPGTGKTSFVGALAEYTRRHVVSIHLPFLKSNHSLYDVFLN
PTFRCIGESDPTSLAVEDVIFLLDDVDASSPLVRARVRSGRTICRRHHATLVSDGSVADDFVGVESPVLDS
EVDEEEGEYGGLAPEPENPVDLVNQLLKATFGKRYTGKGGTTRGGAENGTSLLKWLRPSDELNLSGLLNVL
DGAVDTPGRIVVMITNFPERLDPALVRPGRFGTKLRMDYLQLPALLDMLGLHFGAVLRESGESGTDESSDG
NDNAGGGVSQPLRAIRTFERGEADSVNTSAPPKLSAYDVARVRDVVAALNDSRKGREGEDVSGLMISPAEV
ESMCAVSTTLDEFLLRFSSRFGAPQASANVQ
>tr|C9ZL50|C9ZL50_TRYB9 | DeepTMHMM Topology Prediction - Predicted
Type: TM
MSTGHSGVRHHIEIAIVITIIIIMTVDVGITSGKESGLHVPLGYTFNWIIDRIYCCARLQVLHKCRGKYSP
TVCSKMGVVCRFPICSYILIGISLGQFP
>tr|C9ZZG7|C9ZZG7_TRYB9 | DeepTMHMM Topology Prediction - Predicted
Type: TM
MMVRAQGWHGWVKILNSLPVCFSLPLTLYLFPSAIAYTRKGFWSCDHFRLFPISYCFLLPLFPSLALIPHV
VQGFYHERVQYYFFFSFSLCSLPIPLLLSVRGEEEGGLLINKWSTERRRKGRGEK
>tr|D0A139|D0A139_TRYB9 | DeepTMHMM Topology Prediction - Predicted
Type: TM
MQLRCFLRLGVGVIYFLIIIIIIIIIYLAFITCQTRRYVSHFPLAKMSVYFLLHMFPYPLFFCYLTFTHLI
YCYNVAAPSPRLFVYFYFCSSSGCCCSVMSFIFKYFQRGASGFCQRLYMKYNVLYCIERERVVLALMHFTI
AILFHSSHRQSDS
>tr|C9ZN68|C9ZN68_TRYB9 | DeepTMHMM Topology Prediction - Predicted
Type: TM
MTTVVMSVALTATVFLGFSLYNRMVVPLIARVVGGRIPTRAQCLKTLTFKDRLFIALSKIFTVLFVYHSYL
FITDTEVSNMSLNFNDFNVVLCGVAWMPVHLVALFIIYDFFYTLFHWALHWRPIYPLIHKHHHRQVTPFRG
NDDAVNDHPIEYVIGEYNHIFALYLLTRIAPAGQVHVLTAILFVFIGGTLASLNHTRIDVYIPYVFNVRAH
DLHHYQFKYNYGQYIMLWDWVFGTYKCSRVHGS
>tr|D0A273|D0A273_TRYB9 | DeepTMHMM Topology Prediction - Predicted
Type: TM
MEGFRYFTQRVPQCLWNPVAMKHAFIIALVVAPLWAFLLKPLLEKYHQQSEPLHLFLWALLRGVVMQFPVL
TSVKLRAQAEPKPSRGRERPSGHTPELESAKRYATGGKPITAKALDDLFRSPDFEVWYGQHRQSLLEKVRI
RCSQQLWASIATLAVLVFGAFALPLFSFSNEEKTLYAVLRPFLTVPTFKVSIFTSGKDKKEIMYSVIAQLT
ALGHAVVVLVAAFALFTTSFMPVGLKQTAAAALVAFLALVAEATRVEQMAVGAGFVLLFAPVAWRIASVFV
>tr|C9ZSV3|C9ZSV3_TRYB9 | DeepTMHMM Topology Prediction - Predicted
Type: TM
MFSCAVSGDPLSGKGTSGFLHWFIYYVPAAVFCLCADSLSFVALRSSVCLPFSFFFLSILPDFMSFPLCFA
CFVQIPLPPSFCFGWLLLSLFVFPPFKSVFFFSPLSAKLSRAGTHYL
>tr|C9ZTT4|C9ZTT4_TRYB9 | DeepTMHMM Topology Prediction - Predicted
Type: TM
MYSVHWEEDGIASCFASLFSTASYAAYMPTLQYFLKKRYVYESVFAIFGITASLMFHLCNLLGVEIFMDDA
AWFRVGNICLITLIGAWSTYICAFRDSFVERFTKYCFLLICIICHATGPWTPVKLLTKGAYVSFLVPICVY
TYRRQLPAVFTRRLLVLVFAVVIALSFLFISLDDERDPFMFFLSACRSLFGIASYLMWTLLKVPGVTGVMG
KSIHV
>tr|D0AAQ7|D0AAQ7_TRYB9 | DeepTMHMM Topology Prediction - Predicted
Type: TM
MTMINGGTGLKHELNLIEVMVFEGSFFYYYFMFKEFFSFRLARYPSSYLKSSCCSPAGVSSAPPFRVVLYF
NLISCCPFFFYNHLRLILCQVDTFFFFLSYVYHVNYISHCALLTGSPRWSTAGIIDEINDYLNCVWTRCVF
VPLGLPVVVLPNYTTA
>tr|C9ZIR0|C9ZIR0_TRYB9 | DeepTMHMM Topology Prediction - Predicted
Type: TM
MCNCFACESSLPFFPFFLYLQYPVAACIFFFFFVSVRILHPFLCCFLLLFFVSVYCMCIFMHEIYIFGE

>tr|D0A8U4|D0A8U4_TRYB9 | DeepTMHMM Topology Prediction - Predicted Type: TM
MEACVGGCRNEGEVGKQDVLERLRTYWSDERMKQKDVKERLSPDCGQARSTEHALNPFQKMRGGSCAVLPA
VSHPLFSFVVCDKVFSCGGLLLYIYFCVSALSLYGWLVSLQTCDPLLPSVLSLFSVGDAVCPLLCHAGTKD
ATHSVHRYIHIYIYMRIT
>tr|C9ZYT3|C9ZYT3_TRYB9 | DeepTMHMM Topology Prediction - Predicted Type: TM
MGLNNDVWERKKGVEEKERGTREEREEREGGEGRGWGNQCVRVKDLRKGRREWGRRKEVVFSNGGIFLLHV
VTSNLFLSHRYIVDLFFFFSLYVYPFVDVVVIVVVVVVPLSVHTGLYVCLFALFLGGERKE
>tr|D0A6D5|D0A6D5_TRYB9 | DeepTMHMM Topology Prediction - Predicted Type: TM
MENLQGSRLADGTDHFTTLSVDDAANNMSFNVLNELAVRSGEENFEDDIIVSAELVGTLKHIMRRLTKAQK
QIKMLKERLRAQARNTSPESQGETIDTESGDKQTLTLLQEYINLELFLWGQLAKILHDEYKRCTDVYASTM
SLQDRAPFESLIKTVCNNAPFCEYMGEMINFNVERDDLLSRSEGRSIQSEPILYKSVVRTQSIATYTAFLL
VEGSRGNFLDSTPIQDLCKVIIGEKKRQKRLIALSLVFSRLEEDDQAVGEALSQQVPRVLLQRLRHLVHRI
FGYPENNGISPSQRWSRSPFRHYSAVIRSELNSPTLSRKTSPEVAAGSSVGVPCVKEAQQVLPQSKQMSLS
SHSQFAGSEQASSSPMFDRSTIPIKEVEHESTLAEFNQGGGDDLISRPHKKRKFETGEESPRFRKLPDFWQ
PRRGDRVGRGPMWCFNDQGKKCDFGEVVEVTTAEEVVVRWIRSVDCPPHESEGNRFFRYKYKGPNDQVVPW
DHFLNIQTLMPENIDFKMEEFMLCCAVVGALCHQRETVLPALQFQTVESMFYVLSRVDLDAATAAVETEEK
KALGFVLSEDNVEMNADERELLEVRLNWSAELQTTKSHYRKMRELVGRVGWVLNSLFTHKKLALIFLELGG
LKQIIKLTDGRLDPVTMYGCCIVLSQLARAAVFENLLRNHGEYFEPILNFILHQWKVASSHDVQGSAGGFL
FLALSFPYVVTFFDAHQGPQATLGLIERLLQSSEEQFDVICPGVTLAALKCAYVYLISHLMLATRVIFRKH
RFLSVLVTDSAPERSLPRDPATVESVLGYLSAPSATIPDISTETIQSLLTRDRLAAFRFFADNNFHQILLR
CAQFYFTQGRWDLLAASLNVLCVLVVVPFFRPLVADAQYPESGVAHLIMIVSELTSAFQNGNASRESHLIP
CVATSLQILLNLTRLPADKNDDVCVATFNRVCGMIRANDGVRTLLEVLKVRKDPAMSVKLQLFPVVARALQ
LMVTLRRYADTRLLFDALGVGSVARELMTQYGDIQKEYLTMMGPRYVASEVHATGRFMENIKCFLFDETNR
TTPSVSVDPVELEQRQAVIARSHISYSRESLLELICRHLETEGLLNAASALRRDAPLSCDIAMAQHSANAS
PDMALSPIGAPTLDGIVRSYLRQQHEKCTNPITTLPQFDLRKGHVYYPLDAPVDQTRNAFNRRLVQKMGLD
FSLRTRTNENHFTYRNPGYLFDITGSGDELQGDSVAFCDDGETLVVGTSEGGIALFDTFPDDSTDDKLSEQ
HLAFDNGSVVGIYVSDDSALLGLVNAEHKVAVMGRNELPAVKYQVEDSRAAMFSCCNTYLLATCDEEHTCR
LYDLRAQCEVRHFSDPSWVGENVDNVATFDQFSQLVLSDAVLWDVRCGDRPICRFDRFTNSFCNIFHPFNP
LVMIDEKVWDLRTLSILQTVPSFRNSSSFYTSPFRRVIYSFREASALSHTATPVLSVVDSYTFETVFSTEV
RPAFRAFAIEPSDRYCAAILDGDAAAVVRVFSTSSGPLPGQQAFSLPQNDRSNDSGAGLAESDDDDEVGNN
GSWSGGYDEDDDDGEDDSDSYEWASASDSPEYYSNTETTSDSDSAEVDGGGDGSDTWTMGEEETTESQDEA
ASRNGENSDDEASLEST
>tr|C9ZY35|C9ZY35_TRYB9 | DeepTMHMM Topology Prediction - Predicted Type: TM
MLMFQVRDYRTASFNVHIYIYIYMCVCVYFLCVCMVSILFSVMFYVLCSFFFLFLFSYLCRLFSVYPALFL
FLVSEASIKRKNIYTPLLSITTPAPSQEKKKEVFLSLILYFFCCSPYPFFLYFNLKCTCFTFVCLFCFVFS
SD
>tr|C9ZPJ2|C9ZPJ2_TRYB9 | DeepTMHMM Topology Prediction - Predicted Type: TM
MQCELVDYALHQLKRNFYIVLCLLTCTPSCRCFWCFFLLLLFPLKLWCSEQRRHKTTVGYMYRYIYIYIYI
YMILYVCSLQLLFSPTYVCHCSSFVFADDKLFKKKKTVSPRLTSVSIVCVCAPSAFAWEIHELHVGKWKGG
N
>tr|D0A4Z6|D0A4Z6_TRYB9 | DeepTMHMM Topology Prediction - Predicted Type: TM
MTLWSATRMFKRTSTRFSASITQQLYMNCRNRCFSQCSASMRRGSRALAWGRRMLLCAVAVTTSYVFIDYA
TAQSITRSMRALITTARVVYMYKEATPETSEERSNLHRAAALSLLNLCLRNEGLYIKLGQSLTAMNHILPW
EYIDVLTVLLDRAPVVPLDEVRRIIQEETGRSCEELFVRFDPNPIASASIAQVHRALMQPSDPIQSPVEVC
VKVQKPHIRRQVFWDLQTYRFVLHVLGAAFNIPVAWMKETVVEGIRREVDFSIEARNATRIRQDFADRRDV
YVPEVYGDLVTPRLLVMEWVDGVKLVDVVAVREQFDEVKVLQTVFGAFGDMILKSGFVHCDPHGANILVRP
QPYPMEEEASGKSKGLRQPGGRCCNPQVVLLDFGLCCPESERFRLEYALLLKAMIMQDMVTVRKIVHSWGV
DDEKTFSTLQLRRSYASFHRGNCGEMTREEAMHMHNEERERIMNVLKREEQLPCELVLVGRSIDILHGVNR
LYGGLVNHMRVFGRRAVSALGRLNTYEDIQLYLTHLAGLSNGSAGEEAIPLCSSGKLLSLFDPALEHQRRE
EAAAVLKFRSGVTAAFRYRVWEGIISLLMLFQFELALLFLDAYHMFIRWYSKVFEVSIWNWMDARCQRFIK
AGCCGKKENKFYSIR

>tr|D0A7B8|D0A7B8_TRYB9 | DeepTMHMM Topology Prediction - Predicted Type: TM
MCVPFWFFPNTYPPFLSLPLLVVVAVFLGVIFIPWSDCARCFPFIHLFSFCVVASFLFTLFVFDMIAHISS
AALCSTPFVFFLLSSFLSSSHCPLLFSKKKESGSGIWSGIELS
>tr|C9ZTI6|C9ZTI6_TRYB9 | DeepTMHMM Topology Prediction - Predicted Type: TM
MKRNERKKKGEKENGKNIKKKPKQQKKIEKKGKKNEAILSLLLLLLLLLSTFWLHVQGLIAMSCETFDSLF
PFPLLAFFPPSPPFSLVFLPYRPPSQFFSFPILPFRFWFELSS
>tr|C9ZX28|C9ZX28_TRYB9 | DeepTMHMM Topology Prediction - Predicted Type: TM
MRSFPTPRPAAICDLWHLSATGYCFPVTPSCNVHIRYSAWTPFFGLSILFFVSVTVSCVSALVGTGSISTS
HSVAKGRPQLRVVMAAEPVDRGCGPISAAKKVTLLIDDMIRSGKYKILFDALKSFRNGFVYGARIRAPHAL
VLNLVWSSAPYSVIARRVFDATRQHALRLGATAFTFSLLRSLMALVEGRQRPWHSVVAGFIIGCLYWGEQG
AVTVQMSMYILSRILSALFFILMERLAVTTSVHPPPWAFRLYSGVLWMFVMPLFLYHREALQPTMRTSMQY
IYEDCTRYSNWYNLLCFNSDTSF
>tr|C9ZNZ5|C9ZNZ5_TRYB9 | DeepTMHMM Topology Prediction - Predicted Type: TM
MYNDNLYYHPRCRCLYYLLFIYFFLYGGEVLSRFFSPFAMVSSRLFVPMGIPFVSLLHCFVNPFLCVCVCV
CTCHFLLFPPPTFALRLSPFFSLLFYFPAMARVVGGKKGKGQMHEEDPEVAKYKQSVIDVGQRTQLELLQA
EVRHNKECLTALRLENKRLNVILQQAQRGQRRGVEIDPLRREEEQLHNKLCLLKRSLNSVHGKKEELTKEI
ARTVEETGYILQEGKFTTDNSAMGQKIRGLENRLDKCLIKHNEVNAIRRTYEALLERLQQEQAGFDTQLAA
MEKTLQNKEKDLCDLNTVAAEASNGRDAAKAEVLRLKAQLTRERRAQNKDLEERRAFVMTKQKQLDRKAQR
LKDKIERDEERRAGQHMAGGNQQKKVSRVTTQKPERREDVEQQQQLREAYNKLKEVTMSNTVDEVISKLQE
RQDANAQLMQTAEEAEEADAMLKEERSQLMEEWEKLQQQNVVHRSVLKGENQKPPATEERTTGGANSVANV
AEQRALVRRRVLEEFESYLNDRKNELNVAQRVQEGLGQLLMDLGAGVQRLADTVACATINVMSASTHVSMF
DEVCEDELAFCNARGDSIVSVLNSTAAKLENMLELVDVEDVASVATSIYNWRYSLPPTNVCIRLETSATPA
DGGSGELGSDHSDSGGVSGKGRRTSADEMPVGCRRWFGPAPTDDFPENEIHDRHELKQMSIATVERERKKA
RKL
>tr|C9ZT13|C9ZT13_TRYB9 | DeepTMHMM Topology Prediction - Predicted Type: TM
MLMNFGGSYDAYINNFQGTFLAEWMLDHPSVPYIAGVMYLILVLYVPKSIMASQPPLNLRAANIVWNLFLT
LFSMCGAYYTVPYLVKAFMNPEIVMVASGIKLDANTSPIITHSGFYTTTCALADSFYFNGDVGFWVALFAL
SKIPEMIDTTFLVFQKKPVIFLHWYHHLTVMLFCWFAYVQKISSGLWFASMNYSVHSIMYLYYFVCACGHR
RLVRPFAPIITFVQIFQMVVGTIVVCYTYTVKHVLGRSCTVTDFSLHTGLVMYVSYLLLFSQLFYRSYLSP
RDKASIPHVAAEMKKKE
>tr|D0A6C8|D0A6C8_TRYB9 | DeepTMHMM Topology Prediction - Predicted Type: TM
MSSVLDGLKQLLVASPFSHAKYALLVVIALLFQGVHKCVLARRANPSSPRRHAGKASGRHRRPTVRFDSTL
FWRVVGLLRICFPSVFSPESGAMVALTLLLALRTRLTLMLSRVAGNNVKALVQKNFRELLLGIGDIALYAL
PATVVNVSIGYTISSIEWRFRERLQQALHKEYFQGRRVYDLATTGTVDNPGHRVTNDVQCFSRELAVLIPS
ILKPSMDIVTFSSALAEHGGHNESLLIFSYYAFVAVLFRLILPNFATMMAASHAKEGNLRTMHTQLLHHAE
EVAFYRGADVERATADRLLRSYLRLESNIKRLKWWGTLVSSMFVEYGSTCVGLAVCGFDVARRADSMDAAG
MAQLYARNAKLCTSLAKSIARLFSIHLKVSAVCGGAHRVGELQDSLRSLERNERETTLSLVEESSDDKIVF
KNAYILSPSDKMILANYNATFKAGRHVLIMGCNGAGKTALIRVITGVWSLREGSLKRPPPSQMVVLTQRVY
LPPGTLRTQFTYPTSEADKRAGDIEDAKLVEFATRVGLRGLLTRVGGLDAWKEWSEVLSGGERQRVAFVRA
LYHRPTFVFLDECTSAVSQNIEPTLYKLLLDEGMTLITTSHRESLKKFHHDIMMLDGVGGYTETEVGQPRV
TI
>tr|C9ZZU7|C9ZZU7_TRYB9 | DeepTMHMM Topology Prediction - Predicted Type: TM
MDIRGVVAAFQRGSPLIPRAVKVLFNYSHPLTEREVALLNQYVKGVQEQQEAGLRTTEVPENINALAARPL
QRLPAVSVIQCVLGAAKIPSFPIRYQRQLQRQLLPLVVRDTPLTREEWTSILHQTATLPMGYSRTALRCAE
EILKVAAPPLFKGDAVPQAGEEQTLFLNIFATVVLHYAKLHAACRKHSFSSLSLNIPVGNENVLLCGPMRR
LRRILEKSCNHLREKWGALIEKLPPPQALTLSVFPSWSYPISKETVQQTVKSSPMLMLHLLGKKPVPGSLL
VDKAEEVIQLCLYNMGLTNCSLNSTRDYLTVILALPVSDKRGYFMTHGEDSFFQLLMPFVNKGKILTPEVA
QEWETLYCLIFLGIGMEEELLPLFVRAAAAVMQKCCASTEKASIMILTSAVLRASSETSRLRVRQEDIVKI
IERDLVSHSADAVKAAWRLLQVSGVENRVVDEMRAALKSAKGNEEVRWAMALYISPKRAPSDVIEGLNELL
FDSCEEVDAYRPAAFIALRSLVESKAKVSPKLCTVLCRADVVSRIGESRDELDALAETAVALANVFVFSPM
ALLLTHRSGLLRRRLSDSVEDLASDASAAIALWDSVTHVVFGLEEKEKTNILSNSACGALLPVASALLKHT

AKMAVPTALRELVICCGHDHIQGEDPYYFCDNDSLLGRMRTSSKVVRRRLFDSLFSGEHRHLLVLHHDALV
DGLCGYFSESVHLRTAASRGLVLLLGTRTGFASSQIIFNKLLDAVKESVEYMLALDMRDRAIAAASPAAML
QYEEKTLRERQLLKTYPSKPPKGMAEDDFEDMKRRDAAALEKGREELRKEIQKHSRVIEDVLLQRKTALVT
VRTLGTSGDFPLECVAVLFPYLQETLSGNDVPEVLERLLVDAIAGLLSRTAFAHIAEGMSRTVAELEGKAS
LSVDDVSRVSTMATYLRQSMTKMLPPPLFVVLLPFSHVAFKAGRGSQAVRTTIPLATQHQIMGVLIQNIGQ
ANLPQPTETLQLLYTILQRFPSLFKSVQQGINLLMAMIPTSHLVALELGFFNQVDTVKEVTAAAYHRFSHF
STCRRALTLAAVFLHDSSTDVVRSMRGITQNSSHPFTLCPTDWNDLTYFLQAYGQQQKHHATRISASMREL
FLLPNTTDVQQRSWLKDICKIGGLGSVVAIEVLSSSLKGDAFQDVLLYLCTIVESPNTEPFMLVVLSCGRV
VLHDCSLVVLKAMSQTLQVRLSKPPKDITPVHKELYLAISTVWLTIIGCRLKENSLLESIIVQQGSTLNNS
TSAMVHRTVCDSMVEVTKNKDACSLPQLDEFVQKCLKQVLHSGSYIKKKAHAYGVAGVLHGLGLTSLRRYN
ILETMQASMREKQAERSGVMVLLEVLSEVMGPKFEPYALAMSSGLLEGVADKDQKVSECADDASRLMVSSL
TAVGLRQLIPRLVKGLAADQAKMRIPPLNFIGYVAFCSPKQLAATLPEITKHINACLFDVNHNVSAAAMNA
LRRVAGVVSNTEIREHVEVILAALRSPNTETENALDTLLYTRFVNAVDPASLALIIPIISRGLSNQMPHTR
PKAAQIVASMVNLVNDTQSLKPYCQQLVSLLEEAAEDPKTETRTTSAKAIAALAAAIGGTLVDEIVAWCFS
NLHKSHGSSVEKAGAAQVFVEIVESCGDAVLYDSFAVIETGMLDERPPVREGFLHIVVYAPSTLNPTTFQQ
LLPMAFPWVLEGLSHFSDRVRDVALTAGSSIINLYGTRNLALVLEPLMNGVLSEVSTLRHSSLLLTSKLLL
HIVQNIRKKMRVQSVKERGPEDGEKGGKEDEQTNGEPAADDDTGAMEILQVESARSVEKRGISVLGALEEA
LGTEGFVRLLSAIFCGRNEHNLNVRTESNNAWQACVASPCGAVKKIFSGLIDLLIIYAPSENPDCAEMASK
TIEFTSRLSEMIEPFIDTLCDRYKEDDRRSKLGALTCLTCVVGYVDGRRLIGMGGQIVGCVLPGMQEKDPR
VQQCARELFAKVSKIVGPGLIESATEAQLETSVRGVVEVVKVKPNVALEIIFRYLNRQSKYVQHNLELLDT
ILDVEEADDQMRRYIPDTGKILLAFLVQRLDGASESYQKFIAGLSQGYEHIPQEQWQKALRAPATQLGALA
AAEAFGLGISTESVESLSAVFRAAIESLGSDSDEMRALAVSMIPKLFNSIERRIVDSLEEEEQQDLTTSKR
AVGRYLLQYLGVFQETLGVTARAMVTDTEPEFSVLGEGGQARLFDSLMAFYNRGLDYGTSMQKVQAVECIQ
DLLTYAPRRVSAGSTNTVAGRCSKVLFVRNDGGVVLAVVRLCLQLMGYPASGKEAMVEGTMALAMFNAALC
DVGEARVLALRVVIQLLQRSERYADLILGTVVAKKAAVDSPLLRGVMCRFISVVMRYSNLSKTLSHITKLM
DIVKPIWERAETPATAVAAGIAVAALCRSASITDEQFSTLKDTALNMMSTKGTSALGGFAFSYSVIASRVE
RVDASFVNAAMFTVRSAAGFGISDKLSVTWILRATAALVGTGLVPTSELKMEVYAPLLRRVDANDEVLMST
SQYFYDAVSAQFPQTIPSMSDFHREMSTQWCVVGHFDADLDDEVIADTMC
>tr|D0A705|D0A705_TRYB9 | DeepTMHMM Topology Prediction - Predicted
Type: TM
MQPHGGAKNIRPRDVFTYVMRRIWPPDRPMPIFTMLAVIVNGVQLIYSFPVLLSLDCKKWMSQWCAIGTAN
TIGNMLYPIVLMWRFRRKIEEGIPTAESNIHLFVKEPTNIVFLLFLIWEILWMSHSPSLPDSSLSSGQCSH
YVLFLIIFSSVWMFLLLLLVMFTFMTDFGRPPRWREWANERWRQRQEIFSLVHYGVPNPPENTRRKTFSET
FADIMGTHTDLGPAQPDVRRG
>tr|C9ZN45|C9ZN45_TRYB9 | DeepTMHMM Topology Prediction - Predicted
Type: TM
MIYHSETDGPHTPAASKSAGSAVWHCRLSTVMALLLFSNVLPAHSEGNIKVKVYSFIYSPYFEYRQVEAIN
AGLNASFAARQWTVAPNVTVQVVPPPPNNAEVVDALQRVATTEKGLFVVFGPLTDIETLHALPLLKREDLV
AFAPSTGSSIVRGWNPNIYFIRASPTAELIALVRYAVSQLRLLRIGFMYLQDISFGDSEYKHAVELFSHMG
RELCGVFTVKSSMEAFADDRAFEAAWEAFAKTRPQGVIAVAPPINDTMRFLNKIVADKRTRDAYVLAHSTL
EFSIVGAWREALEAAGAPLKFGQVILTGTNPLAKNTLYRAIRRFQDHMRSYLSANPGVTVFNGTDNFDHDD
VDGRLMVYGWIAGEVLSQALSSREWLTSRKAFMESLYNQRRYVIDDLVIGDFGGDCKGGAAKQGAACNCNQ
GGSLVLMNVIGSGYRLFPVNGGVTIFDSKKCYINKPRIPSPMSILSLTLFDTALPVDTYASMSEVLYASTR
GRESALSRRLFFHSMASSSAESARTLQHQLDTRSVTAVFGVVDDAMLSIAEVAFVDPVMLTPRLHHRGKNV
IQLSPTLEQQLFVVVGYVTNTSASAPMSAIVRGTDATIIEVALRKIVWMHGGTLQTVAVLDDNATLVGRLP
NRGNAFVIGLAPGDPSLLAAHLDRNPDVRVLIPFFDVALMYDELVSAFNGNPNAERVQFATSLPHWADANT
SSEIVREFHTALPDSSAWKPLPLLGYAAARFAQAVLPRMEYVTPKTLLDTIYMQSIITADEMRYGPFEEEE
EKECFTANDPVPEQGEVCVVNYGATRISMWSLARALNASVPPLTSPVTPLIRYADPNAIKLSSAQLAGVFV
GSLVALALFAAPLVVVLYVLRRGARDNDSAPKEPVEPVTLIFTDIESSTAQWAAHPELMPDAVSTHHRLIR
SLIVQYGCYEVKTVGDSFMIACKKPFAAAQLASDLQRCFLRHEWGTTAFDDSYREFERQRADDDNEYKAPS
ARLDPEVYRQLWNGLRVRVGVHTGLCDIRHDEVTKGYDYYGRTSNMAARTESVANGGQVLLTRAAYLSLSN
SERGQLDVTALGSMSLRGVPEPVEMYQLNAVVGRSFAALRLDHESGEDGDLSSTSFSDTGSLRGVLSGTSQ
MIDSCLHAVFGTVPLSQRQKLLLPLCERWQVSLPPSSKATWNEEYCEGVIRRIAVKVGRVADHCAASGSEH
SVSTLGSASLIIISNHGLERELHGN
>tr|C9ZJH8|C9ZJH8_TRYB9 | DeepTMHMM Topology Prediction - Predicted
Type: TM

MMGSSWAKSGRKGVRSDTSLAIPVQHLFTFPPISVLIVSRISMNCFRLSTTCSILCADTSFLFLFNSPFLL
IYFVLFFPLSPFPVHVRIIRAYISLWWYVIGKAAATLATHNACLTAGRRHRNLIFKPLLRKKKEEGSNTTF
YIFCFVVEKRQTKRG
>tr|D0A2N7|D0A2N7_TRYB9 | DeepTMHMM Topology Prediction - Predicted
Type: TM
MRKQYGKRWRKAEREVRTATGEEKKNKVESFLVLLFLCCFFFFSFFIVLLSLLLLLLLPLNSLYNEVLIAA
NMVATLCLLPLPLLPFTSSLPLLPFSFFLSFFLFPSLYAHKHAPSLFHYLLPNLHTLMGGETHTHTHTHRP
RHAKSSIK
>tr|C9ZKS6|C9ZKS6_TRYB9 | DeepTMHMM Topology Prediction - Predicted
Type: TM
MIEYACGWLHAPVSSFNSRGETTSSSEGATWGIALGRFGPTAKCSLVRVLAVIVSGYYFFVGLCLVSAAKC
ICTDYWSMIEGLTNVANVSELREVTNLTDQLRYSLIVVSCVTLFVHLAISVVGVRHIRSLFMQLDANVEAL
HRVADALAVPHNGDATGTIDVNPKQSFSHAKSCKGIQNAATPEMANDMLMLLHQLRGLLPDSTFEKELTDV
AVRQCNGAKLQCEKANLPVGSRLEAHSPRVMSQFRVSRRTAAGMVRNREILEHGGTGTTFRKRRVTVVVVC
LSDFAHQVEEDLEHCLHFSRQFLSVVTQIVERYGGIIASVAPNKVVVTWNAFTDSPRHAQNGMQCACDVLV
ALQPFTQGLSVSPSLKLFPTIVATSGFVMAGTIRVSDHEDDVLSVYGNCISLSEELPSLLGALRVRCACVG
ALAHFCPSNFSCIPRDCVTDANNKRHIIYEMQDKALAQGRRHVEAFQAFQRGEHGVAHHLYTRIHEETRSD
WNALRMSQICYYLEESHSVYTQRFPEWQLFPVEREHSCVNELQLGRRAYGFMKCEASPVEDHIRRAILKQT
DTRALPHSYGRNEEESYRTPEVAKSDKCLIVKCNSSSLRYVTEFCDRQGLVFRMSKQVLGTGACGVAFLGL
SQTGALVAIKEIELPLRTRAQNSNLSDLNRRRLRRKGIQVESAMEKTLDGIINEVSLLSRLRHANIMGYIS
SAIWGNKLLIVMELGSGGSLYDLIQKFGSIKESRARRYLRDVLQGLEYLHRKNIVHRDIKPQNVLLLETGL
CKLTDFGTSQNLQKIANSCAPEGTPPYTAPEAARGKAEKASDIWSFGIMMLYVLSGSLPWPNMNQMTSHAF
FYKVGHVESFMPCVDDKISHDAKQIVKRCCQRDPKERATARELLNNSFFNCTDTSTSCLVKDWPSSCGNIS
RSEFHL
>tr|C9ZIS0|C9ZIS0_TRYB9 | DeepTMHMM Topology Prediction - Predicted
Type: TM
MFSGVPSSRWRFLPLVEGWRVWVQTAESRDFPSQRGRAQMKGSGIIRWLSDIVRDFIQVHGCWLFGGAGGW
VCLKGCYVIFSTRSAARLCLVRWRFLCLSIGRARVAVRLWKVIIIIVNSVASRFTRVWC
>tr|C9ZT18|C9ZT18_TRYB9 | DeepTMHMM Topology Prediction - Predicted
Type: TM
MKRNETKTEGIVVFVFVCFCLWFWFWFLILEKIETTSLVLGCKVLTFFLFILFYSFSIFIPRFSASLLHFV
PPIRCGKFIYLCGGGRGRDGVRKLCSFILSTHSTPCFFFFFPLHFFCFFYVFVPCFTNCNVFIVPPYY
>tr|C9ZL06|C9ZL06_TRYB9 | DeepTMHMM Topology Prediction - Predicted
Type: TM
MAGINGKIYLHVYIRWLEIIESRPLSSNVVGTMCSKFFVCVGRENRCDYVYCVTFVHFCRFRIDCSLLHRI
PITVSFAGHFCCCRRRSFLLLLTHFNTCSVFFVLQVGMSEVVPTRCVDWSLNTDSESGKLFLWHNIYNISV
WSNTLGLSEPYDGLDDTSVWEAFLQFQISTVYKLCGVCRKPEGNEDLKVCCYCGQAVHKLCSTKAASEQIE
WKDANKKFVSHMRVCGSCDGVEAPAAFTPPVREAPDARRVVRRALVVRDEYPEEVVRELESLEKKACRPHS
KEEDDRLLGMVCRAVTRFFQPRNSLHLFAKVRQESKGGVGVVALKNIPALTIVGVYPGYCDALSGEHAKLG
RPTAKYALMDLNCADYFNVVFEEFQSTFTPFINEPGEDERSNCGWIQETKNKDGRLSVMTCRGIRKGEELL
IGYGPVYPRSYPYTYDAFTFHQVESKNCAVCFALWHWPTTDAEDAVLECHVGYDSATDSYHLVDIEDDSKR
S
>tr|C9ZN92|C9ZN92_TRYB9 | DeepTMHMM Topology Prediction - Predicted
Type: TM
MSLVPTATVLEKNCATPVLAYNPLINPSKRSDTHTQLPSPKRYQRTRLSSAHCLTFPDGSPVILNWCVCGL
TWYVLTCRLAMITLLVTAPFPNGASASLPHSPLFCVCCTLVTKYKPRTGMDEVALRAKRIIHTRSSDVPIT
AVTAVRRAATGNKKIMKGNISHLARVQTHGKALDWRRSLNFFFLRFI
>tr|D0A4Z3|D0A4Z3_TRYB9 | DeepTMHMM Topology Prediction - Predicted
Type: TM
MFPGGGCNVVSAVNGAPAINVFPAKVGYFGYYFALGSVIAFYGVIFTSKGMSPSHIGLLLSFTPLANTVLF
PTVTYIADRFQCSSHILVMCCIASSFFTAFFLLSNTTVAALISFHLLVVTRSPISPLLDQHTLSIFPKEGR
VKDWGALRSFGALGWGVGSAVSATTVDLTSTWALASFLFAAGQVGVLYCVLRSKPYEVVERTPMQFHEVFL
FVLHHRRLLLFLTASCFMGAGFALVNNFLFVFLETLGGSKVLMGLSLALTVSTEIPIFQNAKYFQELFTDR
QMLSISMATWMLRVVGYSLLQNPWLVLLLEPLHGITFGFTWLPGVHIVNTVFPPNLSNSATGFLYFFVNGI
GPITGSVLGGAIYEWLGPRVMFRSAAFVVFCVLVLFVFLDRYLEKEEAVSATAGDTLACTTDAVVAGEVRL
SGESERC
>tr|C9ZP77|C9ZP77_TRYB9 | DeepTMHMM Topology Prediction - Predicted
Type: TM

MHQYIPQHIISNIYIYIYIYKRSHSSLPLSSCTPLPSVLLTCQVPEEVATSLLAFLGTPPLSFPSVLPFSF
TVLLQLLFLTLFHLFLRTLSGLKEEKRESVMCIHVHVNTKTAKASMYEETP
>tr|C9ZI78|C9ZI78_TRYB9 | DeepTMHMM Topology Prediction - Predicted
Type: TM
MSLLVALVRVIGFVLFFTALLRVKKTRVRHLPSRRTRVSRHQGETCIWLERLTSAVFDIFYSAMREGNEPD
TREQRGQSQNNSCEGDFAANSARREDKSCGSGGGVGGDAWRFVRLIEEHVEALLEDRGIAACATFNIHSLG
DKPPIIRAIRVINRTGMESVPPGSAAPATGSGSPGSGDKDTATPSTVVVPPALNANTSTSINATGVKPVVS
HKTKLRPSQLTLYPTGDGTEKWHNEMHPDEAVAAAPSAANPILSCLPPLPATAIELEAEVEYAGDIDVRLQ
ADICLARGRRLPVFIRVSDVEYIKAHVRLHATLKHENATMDTQRKPYLQCTLWLESEPTFSFKMSTVFSFH
GIRDFFAVPVVAKFLFLRLVNYRMLYPRGAGLSFNIPLPEDVVDGGVYPWFSPMDDVGTVATPSLNSFMMG
RGAL
>tr|D0AAJ7|D0AAJ7_TRYB9 | DeepTMHMM Topology Prediction - Predicted
Type: TM
MIIFWHFATHLLFYSAYRRKQYLHWRMFLALQIHLSTVICLLFFLCIYDSSLCMHYFLFHLIGGGGGTHHL
VLIEEEMFMCISRPPPKKKKACVAYLQCFNFIYRIGSRPVMIGVATEAKWG
>tr|C9ZM52|C9ZM52_TRYB9 | DeepTMHMM Topology Prediction - Predicted
Type: TM
MLLLLLCFRYYFYAFCFMFYRRLPIPVRNTLFFVPVSLFLFGSSLFHFFSHFIFFISCCTFISIRSPDPFF
FSFPLYSFFIYVCVWGLFTHFFFFFFAFPF
>tr|C9ZKE6|C9ZKE6_TRYB9 | DeepTMHMM Topology Prediction - Predicted
Type: TM
MPPKKSSNAKAGKSEAVSEVSKINMDYVQYELPSPDMAVVEKYIETAKAKARASSAQRRREGRKSCWRAAS
PNMSLCKKIRGVFCPNIFQTLKGPVRDFVLGFVVTFFVMYVFFVALYFEKAPSKR
>tr|C9ZZ51|C9ZZ51_TRYB9 | DeepTMHMM Topology Prediction - Predicted
Type: TM
MVTWALKYFVRVVRWSTEAFLIWPTRPLFDYATSLHCVPISGTFITSVLLCFYGFPLLWSTAMVAVGFIIS
GVLFLVSPLPLLKPIGGRYSVGLVHMNGCRSQSIPPVAVFYPTNMVPEKKGLPYVPFGDDRFLRGVAAYAN
VPFFFIRDFSFVRISASRNAVPAALLNQYERVPPIVVFSHGLAGYHLFYSCFALDLAARGAIVICLGHCDN
SASFMRDSSGKESEVPLKDYGWEVPAREAQVAQRVSEVRGTLQRLTEKDFWTTLGYSNSDIDKFLSKPLQV
HLAGHSFGGATVLAAALEENQNPVKGVSVKSVYTFDPWMLPIQNEHFCNPLSDGRKSYTVPTVTVHSDDWV
KDSESWEFFKRMKALVLEQSAYASLNEVEKQALFGIVVTKNTNHLSLVDVSVLSPVMHGNIWATVSPRVQI
MEWCNALLRFAKQNTEVCSTC
>tr|C9ZWR3|C9ZWR3_TRYB9 | DeepTMHMM Topology Prediction - Predicted
Type: TM
METTHIINVVSFSVLFFCLLTITIEYVVSSRRQRRLANARSREREHLEVTRRNTTDHMRRKLTRQAIELLL
ANGYVTEGIALASMVGTTEVPVDRELRPNRPRRRPNLVTVADGSRMSISRVVQRARANGQAPDAPPGPVLM
ELLEKLQQQMQRENESQPFSGPIHTPVEELVVRLVKLEGDAKGLEKPSAADETYGKPVEYTINPANSSIWY
VGNNCW
>tr|C9ZJS8|C9ZJS8_TRYB9 | DeepTMHMM Topology Prediction - Predicted
Type: TM
MINVLFGTFVFVPISVICIYFPSFFLKKFILIPSFVSAVSSTSLQKNVKRERRDGAFVSVGTFYMWRITRL
CCKQLIRDESLVSLPPAVHRLEVYRNAVSVDCEERICAELGLLLQREGHTAFAEGSTPQQKIVKHTYLELH
GAHKDFTEVKSFQRKETRRLPGLLWSPTLVSWMNEVVPRVRVFPVGVMPDTARVVEHNMTGYEMHMEHPTV
GCSFLYLSLLSDTVLTFDDEATGRYGEVFLPQRALMCCSGEIRWGWRFGEQSREVHTFVNASGVRRVVEPD
LRLSVQLWKFTPQLLDARLLQDRVEESMKQREAEESNEVVETIAVDRKKNEEEHKGSASSHALFSALAEKG
ASGTGPLGGEYVEGRTQACKGVEGKSMTMEDIGRDYNTYKQQFQNVHGVLQEMKALQDAGQPINDMWLRKR
ILDGQKDDNHDAQDGFDPNDVEGTWDRVDAKARFYKARLKGMDYDGTANSNSSMPDVTEDAPLDMKKTIQK
IAPLVKDGDKILASLPCGQ
>tr|C9ZLV2|C9ZLV2_TRYB9 | DeepTMHMM Topology Prediction - Predicted
Type: TM
MFQKYIRQCGTYFPFLFFPLSVSSSPLNRHGGAYFNIYIYMRMSCLRLLPHFSEANFPSKKKKILFKIHLF
FFLVPLFFLICFKQLAFFFSPCFRLLFFFCGFSLLMLFYVFICLFAPPFSPFVYLLLANIKQNVFAQWIA
>tr|D0A8E6|D0A8E6_TRYB9 | DeepTMHMM Topology Prediction - Predicted
Type: TM
MITFNPIRRTFGCGIATWDCAIYFVSFFFLRKCPSRSRVASFLESVSRMYLSVVKGGMHDSEECEVFLRGD
VNSRHRLEVTAVMAQGRCCSAVCPCVLQL
>tr|C9ZK36|C9ZK36_TRYB9 | DeepTMHMM Topology Prediction - Predicted
Type: TM

MTFITFGEPPQTLSSYCLVVVGLLFFFFRDIFIIISLVIILLSILLSFPLLMLLFIITSAAVTNPTSCFTI
LLLNPYHFHNLLLQLLVYGGSRANLSLACFFRCCLHLFNWPICCPRVKEVGPLQQLT
>tr|C9ZQ40|C9ZQ40_TRYB9 | DeepTMHMM Topology Prediction - Predicted
Type: TM
MAAMLLTFYMWVRSLRSSSSWPIGALAGVAYGYMVSTWGGYIFVLNMVAFHASVCVLLDWARGTYSVSLLR
AYSLFFVIGTALAICVPPVEWTPFRSLEQLTALFVFVFMWALHYSEYLRERARAPIHSSKALQIRARIFMG
TLSLLLIVAIYLFSTGYFRPFSSRVRALFVKHTRTGNPLVDSVAEHHPASNDDFFGYLHVCYNGWIIGFFF
MSVSCFFHCTPGMSFLLLYSILAYYFSLKMSRLLLLSAPVASILTGYVVGSIVDLAADCFAASGTEHADSK
EHQGKARGKGQKEQITVECGCHNPFYKLWCNSFSSRLVVGKFFVVVVLAICGPTFLGSNFRIYSEQFADSM
SSPQIIMRATVGGRRVILDDYYVSYLWLRNNTPEDARILSWWDYGYQITGIGNRTTLADGNTWNHEHIATI
GKMLTSPVKESHALIRHLADYVLIWAGYDGSDLLKSPHMARIGNSVYRDICSEDDPLCTQFGFYSGDFSKP
TPMMQRSLLYNLHRFGTDGGKTQLDKNMFQLAYVSKYGLVKIYKVMNVSEESKAWVADPKNRKCDAPGSWI
CAGQYPPAKEIQDMLAKRIDYEQLEDFNRRNRSDAYYRAYMRQMG
>tr|D0A1W3|D0A1W3_TRYB9 | DeepTMHMM Topology Prediction - Predicted
Type: TM
MARRGYTCIPPHSTVSRFFCLLRYSSSDSQRPGQPKGQQSDVLYTFQYSDGIHNVCANVSRNAEYTRACDS
VYIYIYVSIYLFSFIYQPDVAMVMALKLRVEEEENGEKGWQFHFSHPLKGKGEKNK
>tr|C9ZUR7|C9ZUR7_TRYB9 | DeepTMHMM Topology Prediction - Predicted
Type: TM
MSLSGILFPQRCHHAAHAFSSRGELWTALVTDHNFLVLRGGTLCASGPLSAPCDGDGAGFEAEAHSDLPEP
PPTPLACRICCSERDDDIRVIVISMSGVYVVDVTGPRMHMTYHSLCVCGGIPGCSRATVHSSPNEAQMPVC
LSEWSHTQKEECARKALGAEFVTPTVLVIAYSDAVELIELSWRGRQWHERRDRNEEERSRGVMRWRVRGSY
SKLAVCRVSMYLSVSQRSSLVEVFPLQPRLSDQEQRTEQSTVSFPPVLHAPREACVDSFRSLSYRTPTPSL
LETAEGGAYVWRGASTRLCNYKITSLEWHRVSGYSLLCVTCHESDRGQPFIVFFIAQLCPVHEGAHVRAGL
ETQHGSGMVGVNSSTVHEVMLMLVPAGKVPLSSISGTPLSVHIVPNYICSHSEVQGSDKLELLCVEKDGSV
FRTSYCYDPQQGNCLVRCSSTECLVPQLLGPIQKHYGDITTVDVLPAWRPRAVAFGPLTPAVAALLEAQRY
RLVLRFRNGMVARVLVDLAAAIVRVEAFLTLGVDAHPPLLATAVVDGVSGPQQMVLGLTQDRLFVIRRTLF
SGGLTEASVSVVAPLALEHLQKLVCGSSVCGDDDAGNEMKGNTCVEESEALRFFMQAKCPEKLHMLPVLLK
ACGGDARKLLRQLTAKYGSPTPAAFSDKTILDEGRSCDGSGDRSKNGRFNSVTPSLLPSALKVGALRLTTG
GIRCCVEMSVKDTDKPHHHQWWYIPATPLASVVGEWDSLIAHGVLPPSTALVQCLDAPLPAELLCERRPSR
DGLACVSWSTSAQSLVVQSRLLPHETPLKFRPFESMAQPYNPVRVDCVKLVNNVIVVCVMGATRDEEIKTV
LMVYVLDALRDVATQPVFEQELVIHNVATFFMCDTGGLSVLRRDSKSIVHWMRVTTNGVESGHCWREYCVR
GGSESQEAITTMAPLYTKSSGMNCHCYEGASCTHFVYVSSEEGLIKVCETSPKKDLRSALKGEIIVEEGGS
CPYTEVGRTGARCAKVELYHPTVITLLLAMHRPRVARAALEGILESAAEASVASQSVDDSGPRRVAEVLCQ
NSTNGPLDALPVPAATEEIAGVAVRTHQLEGGEEENLCDYAAAFGLDIPLELLDQMTEVVMHAKLRGLSGQ
EQLTLLCTVESLRAVRVSGVANDTGASRCLFSHKFLQLQQRRRAAVASAESLPANHTSFLWAALSDCQPQL
LDVLSATDGNVLSWANVEVSGVPFWLDSVHRLRAIAERVAREQYQRSRDVRECALMYCLARKPGVISALCR
TCGNTKLEMFFSRDFNEAKNRSAASANAFAAVSKNLIEYGAAFFVLAAEPRNAAQVLLQREGNVSLALFLI
RIATDDNPDDLSWFIEQRRLEETSCGAMSKSEEACLLWRGGRKHEALSLLTVQAPLHVLEGADRLALLKFA
RRRTGTERGPAQREMLRLLMHTVRLSHAAGMRLPTSLFCQEALSWLLGLSKEDAGDDPTAQGNGSAANRVA
TSGIVADFNSGTLAFNAFDMDGDDGERGEMTASSQSPEVAVASPMSEAVSVPTVRAGAGEIYRIDPAVAYS
LLRELDFIREELQNSVSTQPQQQVQKHQNTAVCDILSCAARLLVAVGGRDPAAGAFSCLQQMLECLPQGQW
GKSCVDRLSESPRYAEGEGNGMGLCTVIIALRVSLMSVATYNKDCALATALLGLPMIDDILLVEGAQTVTV
NTIAVYVRTVCHVLENLRSQTAEWAQEEEKRETSLGSDMHGVDGSESLNFALYNPFEGSGSAGSGVRPNDN
SGRLRNDKLRAMLLVWCTARLHLSALKELLLEASGLRSASQDSQSTQVALQQWLVSYMLCRVTLDFNAAAV
QFVGDVVSVLQLRDPLPADPNGVFIEVEQQLLQIAGVLGHSLPTATSLLGDNGVCESCGQQQRYVDLLKLC
VSHSEVFCQTTTCNTSLSGMTESALTLLSNTVAPRTVENLLHGRLAQGTKEALRFVEATWLQRHLTRHNYR
EQFLSPLREGPSVLRGDESLVLQQCGRAVTSVDYDRSSHDVIVWATAADVSVRGGYCGMLSRPNDTSPVVR
PRADNGGPSFHPTREGGVTGLSPGGSSREGVLFGSPIRSLTKPAASPNSTPGSDRKLLSRRPSLPLLDGSR
VAAHPRLPFFSAPHLDGCIDFYSFTAAECVKTFDCGDSRAVTGVAYSTDGCTFVAGLEDGSVRGWRFDLQP
VDRAVQPLFVYHVLPTGGIRTVMFCGASQSHIAVIGFSYDSLATGQRNPEEEEGVCGLRSEPKPASAKPKS
KKGLLGFPRFGNYERNTTELLLLVDLVLRPGTFAWQELSVVPEHAVYIKGLDSIMCISTNGAVALYDMWSS
RLYTFVRTPNVAVTCVAASYHDDIVAVGLVDGAVLLLDSNSIRGAVQACNGSTSHVASECVAEEALASKES
LLLKASRLQLIPSTSPRCDVHSVVFTPSVMLAGLGDGRVLAVGFPFSTIKFGKA
>tr|C9ZM17|C9ZM17_TRYB9 | DeepTMHMM Topology Prediction - Predicted
Type: TM
MKGICGLTPFVRGAKCDVCTTCVLTHCIPTSLLFILYLSITNALFVLFSYSACEVIRSLCPCGNGYEIVCT
GKILLRSVVTYQSPRVLREAKKGSGGKSKG

>tr|C9ZM74|C9ZM74_TRYB9 | DeepTMHMM Topology Prediction - Predicted Type: TM
MLICPTVSSPICRIFSLFFFQFLLCFLFQLGRSQETNSGNMKNAFTAKSNRRRVKSWMKNHPMEGDDIITS
FYDLLCKRDPKTMKPCRNVFVPDTVVYEHNFPRGWYTTDVRGQEIVRRQGKDLDAATIELGFSREVAENFP
IVATYLSIHEEILDNGVTETTTRVQVLNKDDIGAFVARKNKSNGILQRFVFPKGYHNSVIKVVWSPRIAMI
QRRTNKYPIKDRKRAESDPFVVTVTYEGPEYLSEESGVSAHVAFEVKNVCAEIIKHFFHAEHKYITRMVLY
FKSDGKDRLWFLWCGSLRVAERATQCHMPMNLAPRFGEPMREGSLSEQEMLRRVDKAYYVITRDGPFCDAY
LRGNDSHSDVSSDRRSTRGAKARDCFLSEGKESDSLQSKRFVEEEDLPPELENLLVEMRKLEEMVLETFAD
KFYNAYSYFLGSTYRPFELVVPPNVVNVLTQSSTRELMEFLMLEKKPEAEGKAPARPEGGAAAVSADPEGA
EVTEESRVEMETDTSEQPMVYFIPEKHRLPISLLRERTEKWIKELFSSRYDEVRKDYVGHTEGAHHPEGES
TESDNRANVKNAEKFPSEGAEL
>tr|C9ZNB8|C9ZNB8_TRYB9 | DeepTMHMM Topology Prediction - Predicted Type: TM
MXXXXXXXXXXXXXXXXXXXXXXXCFCFAFFFFEQAPTFVIRIPLSKWPVGGHIKVIFLL
ADIQRTGAALPYKSGGEEYFLSFGFFWKFLSELNHGALRKEASSLSRKKNS
>tr|C9ZMK6|C9ZMK6_TRYB9 | DeepTMHMM Topology Prediction - Predicted Type: TM
MMCYIRLFFRGYLLFPPYLSLIIGAPFFFFHECSASLLVLVVAGRWGKSLPVFPPIFLFLSNHIHTLLSSS
FSYLRNNTPTNDVRARQRFLSFFFGGGVIA
>tr|D0A4X9|D0A4X9_TRYB9 | DeepTMHMM Topology Prediction - Predicted Type: TM
MLSRQLVTRCGMGIRPALINQVSMGCGGVCFTGLRCEKRGVSQIAANLATHALQVPSCFSLSTLLYSPLGT
AMLIVLAYNMVVVGTKQMTYIMEITGKDYVQDQQLHQIMKYGILSCLLLAMEVLFVEV
>tr|C9ZW27|C9ZW27_TRYB9 | DeepTMHMM Topology Prediction - Predicted Type: TM
MIADAVPAVTQVVFAICLCTGVSTFIPNLVETAKDMSVFVSHLLIPSLTFYNIATPLSVELLKKCSVLILF
AALIVVLGVLIAQLASFFLFRVKNTGIPRDLQRSVHFKLLRRRGRRESDDGVSGKSTHILNIVVNGEREEL
PIEPRDVLERLEPPEEEYENEPFYCCAMTLALCVQNAVTIPLSLLQALAETLPWIDLEAGTAYIFMYSIVT
ICVVWGVGPVLVRRAKKTTDKRRIIRELMEQQRRLQNCHEATTQTEVSCTTSRLASPTFPMLSDPPANVDG
PSGEARAGGPVHRSGVAPESSTSDRGAARYSIGEVNQSHDAARGPIVLLTTTPSTCQIFGCDLVETGQIRV
ISRSQFAREEAEANAGVSCLGSSMAWLKRTVAQLLRTPSFTSVILGVFVGIISPLKGLFVGGNLSPVMDIL
SALAKGSIPCSLFLLAANFMNPKATVPAPKPVRMRDAEQNTDFPLDDITVNRSFAETYYRERQDITFDIHA
SFTPAHLPQPGTGSAAGGRADFAPFATSTPATDPRKGFLTTLYDMFSLNGINKNFMFGVIVLRLIVMPAVG
YGLVLAVLYISPSMLGGGSSQNVLLLVLLGQLASPSAINCNILFVAERYMPHVWAKMLFFQYVCCIVTLTG
WYGLSIYIVR
>tr|D0A6J5|D0A6J5_TRYB9 | DeepTMHMM Topology Prediction - Predicted Type: TM
SMEDTYESGLTQSYIYFSLGLLIMAGTLAMALCLRYNSYAQEHVAEYRMLKLQEQGVDAESQNDENEPVAE
GKGEGEGKSEGAMTTAEQLTATAVMPVARIIRMMLVTVFCGFFLTLFIFPSLIIPIDRDHNWFATIAILLY
NCGDAIGRFSTSFKCVWPPRRALLYATFARFIFVLPFMLCIYQYIPGHVGPYIFSFLLGLTNCVGAMSMVY
GPITPGLETAGQKLMAGQLMGISLLSGIAAASVLAMIVVVFLP
>tr|C9ZNX4|C9ZNX4_TRYB9 | DeepTMHMM Topology Prediction - Predicted Type: TM
MHAWTDWCAEVGRDGAWRDGKKKNTRKVRRKGESEVSCKAIRASCRFATMRVWCVVGCGDTLFSFLLFFCF
TTTASAYHHHHSLSTHKLPLYLLLFNNNNNLSLLF
>tr|C9ZPG9|C9ZPG9_TRYB9 | DeepTMHMM Topology Prediction - Predicted Type: TM
MKGKKIRCMLYPYHSIPPSLSLFSLYISCFYSPTRIWLHKYTGVGREIRQNVTTRKKSIRIVQFFFCFKFL
FCLYFSFVLLLFSPFAPLLKYTFIPFYHSLRKSYE
>tr|D0A234|D0A234_TRYB9 | DeepTMHMM Topology Prediction - Predicted Type: TM
MPHMWWRGAHFFFFVESCACPTRGSIFFCFLPPFFLFLYCLHSFRTFTLIFFSLLSVTDRRKVNFLLHSSF
CFPPYLCVASHHSLRVVYDYLFIFIFIYVKKGG
>tr|D0A6P7|D0A6P7_TRYB9 | DeepTMHMM Topology Prediction - Predicted Type: TM
MIGNVEINVSPGMFSHFFFLSHPLLFVFSCYPLFLFIHSFIHPSIHSTSFFLVLLYLSTLRPLYALYFSFM
FLLLMIPSPICSIFPQIRLYSSVKAMLV

>tr|C9ZX80|C9ZX80_TRYB9 | DeepTMHMM Topology Prediction - Predicted Type: TM
MKKHWLPLCMDFVIVLPFLPLPSTHTHTHTHTQRHAVMRRRTCLWDAVQFLCFLFCSHGTLFPLLLLPSVG
EDLLPRHDTSHSLWCFDLRCWGMCVGRGRKVAYKSSMHMHVRLFLFLFSKYCFD
>tr|C9ZUU5|C9ZUU5_TRYB9 | DeepTMHMM Topology Prediction - Predicted Type: TM
MFMETGTYRLQARGISITLNEWYGNAFPRMFDMLPCFPFHIVPFTTSFVLLCLYLTIVIVIVVSLAGIFAC
VPQICHCGPMWWCVFLSLCVYSNILNGCMYVFICTAVQGV
>tr|D0A6X2|D0A6X2_TRYB9 | DeepTMHMM Topology Prediction - Predicted Type: TM
MFVEVTGDAVLYPRRAVLSYIASCEPHGMQFDICPPLLSEKHTCAMDVVGLLRGVICISSLTLGMRGVVGC
MRVSKPHLTRQQSYVFFPFCFIPTSSAPFVAGRYSAVIKEKMIRMHGKHYFCIKVPVLRLAAFQGVQGGGG
GQGVIRGHLSGCGAVSCVLFSSKKAAVMMCEMDSFFKAMSSW
>tr|C9ZWG4|C9ZWG4_TRYB9 | DeepTMHMM Topology Prediction - Predicted Type: TM
MPCQICAWFIFLGPRSCVRWYGKGAGSVLVWMHAQFVSCFRVAFHLFLLCCTGFHPVYPLPHAALIPNATS
FPCESRAMDRYLLGTKHKWVMWSYVEAHHIMQCMAWPHFFTP
>tr|D0AAR9|D0AAR9_TRYB9 | DeepTMHMM Topology Prediction - Predicted Type: TM
MGRSFSPRHITVTITFTLLLFFFFGSTFGIPASLPETSPFYHLPSFGRPYIPHCHHIFMIFSPPPPHAPMP
TIQHLLFVILLPFFVSFAFAINISPRNDVLMEHNTPLIRSLPLLLKNTFFSTMV
>tr|C9ZRK8|C9ZRK8_TRYB9 | DeepTMHMM Topology Prediction - Predicted Type: TM
MRGMDAIHHFHLLNIFPLQNCLSLPPSFPPVEFLYLLFKNYFSSFPLSVFPPLPPLCIHLYVYVCDICLIY
IYIYIVFHIIFLINNIKVTNHNQYYYYYYRSVTLHLLPPPNKAIKIKKKHAISHSSFLQSTHQKTKTKKKK
ERRIITTR
>tr|C9ZS72|C9ZS72_TRYB9 | DeepTMHMM Topology Prediction - Predicted Type: TM
MSSLMSANNSGLFFAMALTTNQTFFLTYHMGSYANNAVMLSSRKPMLMRDVFLTKSSSFFPNPLSCVYVHS
PLDAVLNSLLAWFLVRPIIEIIGWRSGVAIYFGSGFFSSFAYIFSSQLGTGRTNTPFDCADTSNGAFSGFA
TLSLVLPKCYIPTSKRIPTSYLGVPYLIKCFYDEYVAPHYVDKREKGAIELRNWGFVGGVFFVMIYTSLVL
RTKHDMTTMRTFWRNMGITTQKK
>tr|D0A1R7|D0A1R7_TRYB9 | DeepTMHMM Topology Prediction - Predicted Type: TM
MTISTPVGIFEYKFLLFLPNFHLWVPELCFTAVVLHSNATNVHIKMRMCVRKGCTRNKKKTGGTGESYIVT
LLFFKLRIVHLLLFIPPLQFFFFALEDGGCYHRDWLRLVPLDILSYSSSGEVRTTMCSTFSPTFPPSIGFW
RFLMFVRTPSRKTRSLANAAEPLHPLLWIPSVAQPFLG
>tr|C9ZQL0|C9ZQL0_TRYB9 | DeepTMHMM Topology Prediction - Predicted Type: TM
MHMPHPLSSLHQPFVLKQLCYICLSLSLLPFFVCRQEVVEPMALFLWLVLAFSLLVWRFASVLRSVPAPRR
RCGDSPLRVCVVLGSGGHTSEMMRIVETLKTEIWGHHRPFYVVSSTDSHSASLAKQFEERNFGRCCRLHII
PRAREVGQSYFLSIFTTLRALWSCVFLALDEKPDVILVNGPGVCVPVVAGALLVAILIPSSCYCRPAIAFI
ETYSSVSHMSVSGKLLGPISDVCVVQWLKLYENYQHKWWWGCKNIFFVGTRNTAEENGLQQRLSPMLGKFD
GSEEGGGAGSMALVTVGSTQFTPLIEAVDNEEVLRALAKRGITQLLVQKGTSPYVNRISFAHGVSVEVFPY
RPKLHEIIQKAALVISHAGAGTILEVLESKKPMIAVPNRALMLDHQLEFAEALSNERYIYCVQVADLCKQL
QRLDLGALRVYPGADTAELLRLLTPLFSL
>tr|D0A980|D0A980_TRYB9 | DeepTMHMM Topology Prediction - Predicted Type: TM
MKNTNNLRVLGSDPFKDITAKARVRQLLQTHQGRDKLFKVVQYFLRIKLWRDSVAYSTSYLPGGHYTAVER
NLMTIMNTRRLFRVGRFVGELVRMRVTLIKCSELVYIPARGGQWVGFFIQCQMICDLIARLLMCVKSLCED
VAFLAQKGFLHSNVAEQLFNIAFRCSHPVLLVDLFLNTLRLLQGVIDASHQPDEKEVISATYSFSLLSRYD
RVDKLRRGMCESKGDGKSKDDRQNSKIVKARMENNVVLVDSYAKLLWKDFELHWIFVTELKLLLDIFVAFA
SMKRYEAARGVASVAGLFSGILSVYRVWTYGR
>tr|C9ZL59|C9ZL59_TRYB9 | DeepTMHMM Topology Prediction - Predicted Type: TM
MYICMRVHVYVCARNATSKGKGLHFMWLSGGSPIFLLSFLLYFLFVFFSFFLSLYTLYFISLFLFIYLFYF
YFFQPYCPCVQVTFRSVPASLLVLFHECLFLFSSCLVSYTNQIFSFSYFHVSMHIPTYMSAAGGNGWKYRR
QIMY

>tr|C9ZYG0|C9ZYG0_TRYB9 | DeepTMHMM Topology Prediction - Predicted Type: TM
MAVPPVEMYSGSFWNRMRKPLPLRTQVIRFTVVFVIVSFILAVALQITHERMPDPKVTKPLPDLGFELLTK
VPGMYVLADCCIGFLNILSVFTAFKLYLLHRHCVGSGEPELPCNIPGVSRFFLSVWLCKENCRIELRNIHT
IAWIRFITSYALLLLFRSAVIVMTSLPAPDDLCQNPPKIENPVKNVILTVLTAGGGSIHCGDLMYSGHTVI
LTLHLMFHWIYGAMVHWSFRPVVTVVAIFSYYCIVASRFHYTDDVLVAIYLTIATFIAVGHNADGAPWQLQ
LFIRWWPCCGANSREVTEDSQPVMVAFKSEAAGQSSRKVVDERNH
>tr|C9ZQA0|C9ZQA0_TRYB9 | DeepTMHMM Topology Prediction - Predicted Type: TM
MTATTTPSNSSSLKNDCEEGAVGAQLLYNSTEKAASRLLLSAERYVKAGQALLVLAVASAGVVGLLASWQY
RRIHRVWRIRHPRRLAQQRQAMWAFGTFGTATFLLLLSPIGPGGLHEARLEDVKRLDDIAVRALILKRRYE
SAAALAATLRENETTGWWWRTTAQQETEAREMFERCENEWRALMKERIAIDPNV
>tr|D0A0G8|D0A0G8_TRYB9 | DeepTMHMM Topology Prediction - Predicted Type: TM
MVISVYANRETTTTLFSPPSVMRNVSTCTHTTCPPLKLRHFIGIINEFDSTHSCTSFLNPSITGAQHVVTV
TVIGIVIVRSWHQSHSKGLHRLLAMVLKIHTHTQYINIYIYICTLCYTCSFLPPSLLHKHQRKQLLMICST
LPPPLQSFIL
>tr|D0A0N0|D0A0N0_TRYB9 | DeepTMHMM Topology Prediction - Predicted Type: TM
MLPHGSTRRSEVFYCLLPPMRLVSLFVTNIVLPLSLISPFIYTVSVNQSTNRPSCGPTISQVVYEKVFLYA
CIWVSTSASLRQLKENHCFLNPSMCFFFVCSDTTRNK
>tr|D0A5X6|D0A5X6_TRYB9 | DeepTMHMM Topology Prediction - Predicted Type: TM
MGPYYLCASLCFHVAVAVGLMWPLMYATWNGYYSVLFWGSHKIVCTRIGEERFLNFFLDLIPSASVFMLPF
CGCFVLITLCIPLLFWRHRHIDTSFTTGPYCKFFGCFSLWRCGGDMKREQAKY
>tr|D0A9A1|D0A9A1_TRYB9 | DeepTMHMM Topology Prediction - Predicted Type: TM
MVGREIGYEQGKIASYVSLINAFHVIADIPSSLVADCVDLRRLMSFSVFAQAAGCMPVVLLGCTSTSLAAF
CVINGFSTGAFFLARHIYVARRLNPDHCGMVMAFLSGLLRLAHMLGPIFLGVIASLWGDTRYFFFVPMGAS
LLAWCCIQFSPYCLRVGRNAPQGRAHATTATQLEEAKKLDEGSVGGETVPILPGDSLIKGPVLEEGLTRRV
VSYGAVNTLNTDNARSNVGSVGSEVGIACSHECCSLQIEGSKGGTAPATYCSVIMDQWNVIWRLGIYVILF
VALRANRKLLLTFAAMRMGFTDVQLSFLLSLSFSFDAFLFPLGGILLDNCSRRFARLPAVLGLGIAFLLLP
LQHSGQWLYVMAAVFGVVDALGCGLIMTLVADYRHQYFGGLFFGIMRTVQDMGHVISSAAVSLMIHRFDFA
ICSNFWGVLGIFAAVWGWYGVPNPTR
>tr|D0A6U9|D0A6U9_TRYB9 | DeepTMHMM Topology Prediction - Predicted Type: TM
MYQPLLEAPAGSPSAVVGDRLIGEAAGSLFGGSDRAAFEAQGGAYYPPWRPANANVISGGRYRAAGSGIMA
TWQRWPLYRLKTVLRPLTSLFPLLRVVFPFAVPGQDRCPQLCAAAFAERVTAISFHPVRMILAAAVNEGND
CSRVVVYDVAEGREECVLTHAFQRQTCCLVWKPLSRDVLAVGCNGGVLLWSLTFNMSPAGQAVLGNGNLTH
RSVVNGTESSAPYCLFYRCAKNVVVTCIRFSCRDGRYLACGSAKHAALHFHDIRLQPSKSLLLRNVSVEGA
TQDVLFADDDSFAIRLVCGTSVLVLLQFPSCTANVVPTAAPVLGVTKARGLGPNYFFLHCHHVEGVFVAHI
NPFVGVHVIGLISTGIHRGVGGAVRCVASSDRRLFVALETGHLLVMHYGRRGVFTLIPVGTAEMGTECMAV
FDGCTYGSLLAVVEADQSVSFVPAYHA
>tr|D0A0B3|D0A0B3_TRYB9 | DeepTMHMM Topology Prediction - Predicted Type: TM
MSVSFVSFTRFVATVVTLSLHNFFLKIFFHITALKDLMRRIACVRLNVGLCRCLAHDAASGVAPPSNGVKD
QHAVGRGVVLPAVRAAMCVARPCPMFAATITSNEIPHYDDPCVVWREYFSLQYRKPYYHHLVTNVTTFEVP
EGFTTRFPTYHRRQKLFVDEANRVFRNSGSPGVKTNGIVGNTDGFGSSSSSCSGFPPDVNPPPPSGPSPHG
VSGLKHKLAAYGAGGLLLYLIVHNILLAIIFFTLYFLHIDVVAYARSYGFKVGKEDGTSEAPSTDVDSGNG
KGEKKYPSFWTALALSILLNKLLVPLEVAVTVVLAPRLVPRLQPIAARVIPRVKSIIAGCKAGK
>tr|C9ZKA0|C9ZKA0_TRYB9 | DeepTMHMM Topology Prediction - Predicted Type: TM
MSLFAKKKWRVQDIIRTEEKKYLASVLKVTHPFRKQQIVIVPAPRYALDSYYSEWVYQPYAKEHKLYVSND
IFNPTHVYLARILLRRNIFPGYAYFHPMGFPDCIDLNLTRREFISREQPIRTPMLLLLLTPNMYRERFHSW
VGRRVLNIVGERYVTHPSEDNRSLMFILPPAYVPDAVNVLQSLGFNVTDHTTAVVGDTDTVDKLNKWGDMA
QVVALAYIWFMFVLLVVNESHRMEKMFEEYKRELAEKAGRDPTELGL
>tr|C9ZJ57|C9ZJ57_TRYB9 | DeepTMHMM Topology Prediction - Predicted Type: TM

MLPKQQLGGSVCNASIETVNTEATDPSEAKKIVLNAGRSEKVNVYVPPSTLMVRDIQEQIPAEYFQRSMWR
SFSYLSRDMFQLFLTFVIMYNFVLPMLDSSLLNAVPPVAWLSRAAAWMIYWFVQGLNGTALWVLAHECGHQ
AFCNSRRVNNAVGMILHSALLVPYHSWRLTHGTHHKHTNHLTKDLVFVPVQRSAVGEAVEEAPIVMLWNMA
LMFLFGWPMHLLVNVGGQKFDRFTSHFDPNAPFFRRADYNNVMVSNMGVLLTLSILGACSWSFGFAVVVRW
YLIPYLWVNFWLVYITYMQHSDVRLPHYTHDHWTYVRGAVAAVDRDFGPLLNSWLHHINDSHVVHHLFSQM
PHYNAIEVTRKHIRDILGDLYVTDAKPLLKSLVHTWRECRYVVPSEGICIYRS
>tr|D0A1J7|D0A1J7_TRYB9 | DeepTMHMM Topology Prediction - Predicted
Type: TM
MSSLQDPFDESVSDVRELIVKARSIREAIQAKGLIASDAVTELGTVVDSVKEELMLLNDVLRVIEQKNGRV
GEEVLSVNEVVRRQTVVRELEIDLKDISAFREFAESRVTENDVSNLDRGPAEGSHGDTFYLRQERVQREEQ
AQQDVILDRLSHGLQELRETGINVNDELQQQDNLLSIIQVDVEGVQARLRVVNAKVDKMLADMSSRSKICS
LLGLALVALLLFYCVFS
>tr|D0A008|D0A008_TRYB9 | DeepTMHMM Topology Prediction - Predicted
Type: TM
MEIHLFFLFFFSSLVAIRTVFFLLLLSCRDTLHLKKGRCGRSTFRVAQMIDTKQLCSKLDGVLHRFTTHGK
GEDLANCEDMLSDIKIVTSAVEGAAANQSSDSPLPVKVLEELRLLGSACWNVTMRCTDHSREHERRLKVAL
REFATRSFLLGNFVYSEESVRHSYFTHHPREAEQCILMCLKTSRDLSLNHMPEGSKALLAAVETIVPHIPP
GVVQRLPHLKHRNLSWEFEYTKMEVMWNLGHLFESSQSCERLAQMLLRDNTLHRTLLETFFHFVFTVGSAE
VEPPNECFIRDMLMSSINVQNYLKENGGSDAPCRLPMLRGATLEQMALSWLREGNASEAVRWAVEADSALQ
SNTSALLRLKATAAAGMEKEASVQLREYVQRLDVTVDDAVAVCFDFQKLFTTMESGSVESMQLLQNRTKGT
SAAEGVTFRLVQLLLHSKDTESCRSALQIMRNECLDFEDPKYRRYCFKWLWELSDNAEFSHGEAVESLEAA
IRLADCASDSEINALQLHLCTKYVESVEQGQQVNMLAKPKDILLRYTERKPRCVFAHALLFKIFVMEGSET
CMKEEIHQLVACEPSELVTPALCTAINCCLKRNCLNVASLAALQALLSPVPFADVRTELEVLRVYVTTAIN
RSCAHSDDDLYKLTQRIQNILSEGSATLKLTHDEVMWWTQAFLLLGSEFTLEASATSIALFRTAACIAEQD
PSPREAGTQSPLLAAILCMLEDEFRLFSTGNPLIDLADLEKHLNACRDLISTTYTAECRVTFLLSKSEWHL
RGPSIETPQEMDEIVRELSTIPVPYNVYEALAEAATFEASRVPTECSYLREFAMGLFAKASLDLMERVAAS
TEVRAGEEEAVAETLTKNLSCLYKAFTLGSDRKEQMCILQHLMSFLSLPVMGITVGTFLGRYGAVGERGVT
ANNSSAFALLILEYFTVEAWNNSVFYLHINNTEKQGEWVRAAWALVDILPPTHRVVSALHALRGITGC
>tr|C9ZM98|C9ZM98_TRYB9 | DeepTMHMM Topology Prediction - Predicted
Type: TM
MLRGNTSLPELYTQTHTHTHSFSLSLSACFFEWVNEWQREYLVGWLWGLTQPHSDDNLSVCSFSLLIHSRL
WFCVIIFPLIVSCRLRPVPSPCTLVFRNFLFCFQLLLFGPVPQDFACSSFY
>tr|D0A0V6|D0A0V6_TRYB9 | DeepTMHMM Topology Prediction - Predicted
Type: TM
MCAMKRVLSHPVTQHYLSHRYIYEATIIITIIIIIIIIITIIIITFYFCYLKRTGLVIFPFLLSPLWKVPYL
YAFDKRSTTVQSLFSTCLPRLQFLSIASLCSGMH
>tr|D0A182|D0A182_TRYB9 | DeepTMHMM Topology Prediction - Predicted
Type: TM
MLRGVTALLEAIDERAEQHARQAVERNVEINVPSCSTEGHDPAQQGILRCNLPDGSVAIDPAGSGVVHLPT
FPEPPFPLHGFAAPPHHDDAVAPSLNTWSHIEGNGIATTASALAAVGRDSSSPLTGQKEKQIEALTQMVQR
YQAAAEVAKQELLQQSLRVCKLEEALQDTKTELEEHKVKSRLLLEESQRQCEELHARLERRKESVPFYHSD
DIPGQAESHFEGVEGVKTEEGDVGDLRNSVTSLQRQREWLEAQVAAGRRETTEFRQRHSAVLQDLEFLKAD
LKGVQEALDSEVAAHNHSKALLQSRQRELNELRAAVERNGGTFTASGALVALRPDGGKEGDVLLSRQLLEK
HKAMETALRDAAEWRRRCERMTNRLEEERTARVSIEAPYPMGEVGEPHSVTFFGEEYNGVVGQFIGKCGHV
LDSTALHTARLLRRSSPLRIFTMVYIVCLHVLIVVVPLMLSS
>tr|D0A3K8|D0A3K8_TRYB9 | DeepTMHMM Topology Prediction - Predicted
Type: TM
MAYKRHAAVYGEAGGTQSMGHFLLKEPTDLFSGNVTIQYGESSTPLSFTLVACKGLTCFVIFLFLIDSDAK
LIWFPQSPWSTTGGGPFFFFFRSPPQHPVSGVRVSGVHLSGASIRDLCSGCGQ
>tr|C9ZNX7|C9ZNX7_TRYB9 | DeepTMHMM Topology Prediction - Predicted
Type: TM
MERKQKKTRSMYSFVVFFFSKKRKDFTAVLTSFLSFLLFCLLSFVPPTSQDVIKCIFTGEHHKKATEAWGT
SAHINIHKHTHTHTKKKEAMKGKAKEGSERSCIKFAGLLVSVACLISCGME
>tr|C9ZK69|C9ZK69_TRYB9 | DeepTMHMM Topology Prediction - Predicted
Type: TM
MTSLPHIDAHTNTRKDVFTWLTLCFHVFFFQPKKIMFFFYKFWWGINRCCWFISLLSASVSHYLLFFLLNK
RVARNLEGYEWLRAVQTKRKTSAFYDHYFWYYYYCCCSCAPPFFSIFVPHCCKPLPNKHTSFYYLCLFTFL
MLNEVLHSGCASCLQFLRHLLPRESFPAPFLVSFPFFFLIT

>tr|C9ZZ50|C9ZZ50_TRYB9 | DeepTMHMM Topology Prediction - Predicted Type: TM
MHWGYRHALEVALISFYLGVCFTLGVSLMIHSVYYTGSVAEYAVGAYVISSIVLFHMSEFLVAVYFLRHDA
HPGAFMIFHSREYTVAGAAAWLEFFTELFFCSEGWKVSATSRWGWLFRLNYTMVNCAAVLTIFFYLVRVCG
MAHCGCNFSLLIETRRRSNHVLVTDGIYSILRHPAYFGYFWTALFSQLVLANPFCFMAYAIVLIRFFKERI
TYEETVLSSVEFFGESYTKYKAGTWVGIPFIR
>tr|D0A8Q1|D0A8Q1_TRYB9 | DeepTMHMM Topology Prediction - Predicted Type: TM
MHATVTATQHILQSVTTHRYELVAVLISGIVAYFATVRFVQGARIKLIQRRLCGIDINKTTPEQRKKIASK
PYDELDEGEKRLVVPESFGILAGAVYLSAVLITTSVTFGRVSRQLDGSVTSIAVMLLLGFVDDVLDLRWRY
KILLSAIGTIPLVMTYKGSLEVIVPRLLTPYLGSPTLYLGVFYLVGLSLLCIFCTNSINILAGVNGVEVGQ
SIVIAVASIVHCIMQMRLESDPSYGGPATSGQLLAIALLVPFVGVSAALWRFNSYPASIFVGDSYTYFAGT
VLSVAGVTGVYSKTLMLFFVPQLVNFAISLPQLLRIVPCPRHRVPRWDMERDVMQNSGNYTLLNAILLLRG
DMHERDLTRAALKLQVVSCIVAMFARYFLASLLYDHVY
>tr|C9ZJC4|C9ZJC4_TRYB9 | DeepTMHMM Topology Prediction - Predicted Type: TM
MLLIAVTIIVALLVAYYISTLFSAVRGTTRRRSRSVLLVGLTGSGKTTLFAQLVARKRVEVRTSMEPNRGV
MRLASAAENEDPTAGSESSGVTIIDFPGHRRLRESLMRALEEVKKVVFVVDAVTIQDPHEGAEAVAELIVA
VLSSTEFFGVESVLIACTKRDELTSYSAKAVQKLLEKEITHCLSTRHGGVQRLESIVNASGVAVGSGNKSR
RNAATHGCRAHELSLDEARKFSFANFQVPVQFVDISSFVGPEDQVYNVEPVREFATS
>tr|C9ZYY0|C9ZYY0_TRYB9 | DeepTMHMM Topology Prediction - Predicted Type: TM
MIGTMEDDGVPVILPQASSDDVSTLTLVKRFVLVGTPLMVATLAQFSINMVVISMIGVHFGVKELGGVSLA
LGMLNATAFAFSAGLCGALETVLSQTYGVFQSRGGEGTMYLYGTYTQRMAVMLLVISIPIGIAVIYIDVLL
KSLGERPEVVYYTGRFCCIAALGIPALQFSQLISRYLSCQHQTAPLSAVAVGSAILNPILQHLFIRMFGFN
GSPMAWVLLYVVTDVLLVAYTYYTKTYVTTWGGWDSNAVKNLRPLVNLAAPSLAMSMVEWVVLEVIMACAG
FAPPTDLAAFSITVQVFSACWGVASGTMLIVSVFIGNAIGEGKPLLAKRIANIAIVMVGVTTMLDILLCWK
FEDRIPLLFSDDKEVGHVYRKLMRFVFPYHAVDTFQSTVMGILRGCGLQKIGAVIIGVTLCVVGAPLAFFL
FFYVGVGVEALWIGPLCSVTFVGVPLYIYLLYWYIDWSKLQPQQESLDLAVEPFISVNNAPLEEDMYGAVE
SLGSKD
>tr|C9ZJG9|C9ZJG9_TRYB9 | DeepTMHMM Topology Prediction - Predicted Type: TM
MTGKDGKVLVADLPMMYPKDLLRFEKEGGVWGQQYCVCRQQLTLGVGAAAFSTLLGIGYVRGRRNTGFVVF
STFTGFTVLGFCLGATLSPIIYPNVASNRETSMMRRVWWAKECAKHWDYSQMDGNSWHAAHPHDRLPQQLK
DNNEECATA
>tr|C9ZUP5|C9ZUP5_TRYB9 | DeepTMHMM Topology Prediction - Predicted Type: TM
MYVWIDGWMDVSVCIFIRCLRQRKGEGRGAGEGDLVSAGLYPFFLQLFMSFLYIFIRLYLCAFCFLVVVSC
DLVIFLPYDVRRCKVMGISLCLCSFPTFRF
>tr|C9ZQC9|C9ZQC9_TRYB9 | DeepTMHMM Topology Prediction - Predicted Type: TM
MHFIGIVGCFLFLYSPLCLLTAVSLRAYPQLLIIALFGAVSAYVSLLLTGILYTIVQNVRTEDHTNAISYV
VVLIQVCTSILFRVMLFVFLYRLERFARGYGQLIAKSSSRFALTSAAVGCGMGMVSSLRGAGTLLDATRRL
EFYTDGTTLYDFNICPQMPLLQHAVLQAFLLLLCHIAWAVMTGQGVVALLVRRDKRRTLFDPLSDTLLGTD
DYSGDYPNRAADVLVTNYNPDVVDNPQPAGNAVIEGESPAAAGGEGLTSSGTAPAVVSEQEPWGYQLNQPQ
REEEEGTNLLTTSSDKGPKTQEVGDEERISEQDTGVDTQDDGDGPSAQQSTQLLSKSLQEPTVLHRAPTVA
VASLVSAAALQLSFSLFSLLSTGAYNYKTMEEVPFRGCMVTLPAQAAITAASLNAMFGLLRAEGFGNNAVG
LEN
>tr|D0A4K5|D0A4K5_TRYB9 | DeepTMHMM Topology Prediction - Predicted Type: TM
MFCCSPCVSVREEKGLVMFVPVWIERVPSCYFSSNSRWHPLRTGWGGERGGHGSRRKGGDRYPWCRGMMEL
SVFRGDVFFCPAPGGRGTTRNFTTDFRDHKCPRVSVALIVSSNLAAWFSWKGGLTFFFFLVLTPSLSF
>tr|C9ZZ29|C9ZZ29_TRYB9 | DeepTMHMM Topology Prediction - Predicted Type: TM
MEGKKIQRTTEQKGKLKKNERNQTECIQRWETNKNRNKKEKKKKRKHQNGKKFAAFHYSFPHFPPSSSFFF
FSFLLLISLRFIHFPPFPIFPLCGVYFFLLFLFFFFI
>tr|D0A1V6|D0A1V6_TRYB9 | DeepTMHMM Topology Prediction - Predicted Type: TM

MYLPKQPVKVKESPFAVQKSRRFIAATGVAAFVTTAVASYFTLEKVRERQRRPDGVLLQHRNGKRVVIVGA
GAAGCALAASITSACPDVHVTVIEREKKHVFHAVVPLAHVGHRSYDLNTTGGVDFLRSPATWNVTREAALV
RGEVLRVDTTKNEVVVREDQEALTITARAETSEQPSKNVLTWPIGLLSWLLGASQPPVNEHRYPYDVLVLA
CGAARSLGPLQKFLTGEQVDRYRIAVNPGITRDCLVHLYKGTVLHVKVPPTSFAEGATGVRSISTMRYVSR
QHDGTFIGTVNTVWRYLFFFNKQKFCPYMAVTADRGPSDALPIEVNERVMDFWKQRSVNFMPCTYITHLDP
RRSEATLYNYQTQQKEVVNYQLLLLDLPLVAPEFIRKSGLSRSEAAEGFAEVNPHTLQHVNHANIFAIGDC
AALPTVKSYGAVFAQVPVVSHNVQQQLLHGAHGTGEMSNAAVRSRFARYDGYSSFHIVMTTWRAMWPEMTY
GGWRDNLSFPTITDAPLSLTNNHIWDNLAWADVRGFLNAVYYQWFLYEVMFYFVFTRGAWYPPKWFSVPSF
GDDGTML
>tr|D0A8P7|D0A8P7_TRYB9 | DeepTMHMM Topology Prediction - Predicted
Type: TM
MSTPCNIRDALALQFCWVNVLVRRRNGHYTSFARVRAALFASMHACFIVTLLFYFVFEPFSWVLAFLIQLI
LFIISVVHTALISEYEERLNNAMELERQLNPLIIAELTFRCFSLFHLYLLH
>tr|C9ZR21|C9ZR21_TRYB9 | DeepTMHMM Topology Prediction - Predicted
Type: TM
MVLTTVVGGIGTTFERLHKIERQKLAVLRDPLVLVTGSMAGLAGRAVALPFDEGGNKGPKRALQRRAPQFG
ILMWFYVPVSARLLPGTEVNPPYKAFTTFMIGAVAGFVMRLICNPINRVSDECLRTGDSFRKTCQIFRSKT
ILQFFYTTPPLMVNAVYFGTLLTVFEGLRRFCERNRILPLRVERDSSGKEIINFVNHFTVGVGHTVVGGVA
AAVASTVCYPLSAHFYQQTVIHDSAICRGLMPTLRKEVPMMAVSFGVFSLLQSLLARHHGPRAGFGY
>tr|D0A0F0|D0A0F0_TRYB9 | DeepTMHMM Topology Prediction - Predicted
Type: TM
MXXXXXXXXXXXXFTFFVRFSFLLHSVSSSSYQSVYLLLRFPFAYSFVSQLLIHLTHNLIIKKMGEREVIV
VCFRAAHHHSAVFTANRFYLLYKKNIFKHIYIWIYLGYIRR
>tr|C9ZXM6|C9ZXM6_TRYB9 | DeepTMHMM Topology Prediction - Predicted
Type: TM
MSSLRGCQLPDARHAEAHVRRRRRTSRCYFAPPSCASKRFFLFFRGGGWCLSPVLRPFSRWAKLCLADICV
VCIPLNFYFLPTPQPWLCTGTRVGERPFREK
>tr|C9ZUF8|C9ZUF8_TRYB9 | DeepTMHMM Topology Prediction - Predicted
Type: TM
MIARVRLWCPAKLLPRLLWRPAFMPALVCVRSAICVEWWVCGAKHIQAQFCVCVSGSLVVRNCCFFFPPLI
SRNIRPGSFFFLFLHVGRGDFRAQVGKHTEFAESASRLRTIRRGCRPKLMCHHCE
>tr|C9ZWH5|C9ZWH5_TRYB9 | DeepTMHMM Topology Prediction - Predicted
Type: TM
MIRSLGYRLSNPVVTRYARSISLQVEKSCATLADKIVRGRATNVFYTHPSYVMAREKMLYTLWVDSGVFLL
SLRAMLPFFCVALFFKA
>tr|D0A2C3|D0A2C3_TRYB9 | DeepTMHMM Topology Prediction - Predicted
Type: TM
MPYFFKPLGARAWGFIAASVGGTFAIGIFREIALLRQREIPLFNEADVVHSEEHARYPCKTFLGYDVVVTN
HYVREKGQGEQSLLPRGSDAENGNCGHSDRGGVVKKLFACFSNETYTTAGHSDSVAEANELYLVREVVGDA
HAPRGDVSTRSVKGFNGDDDDAVEDTVGNRVGSSMFIASSSWPPGTVKYTSMTIETSHNEVRNDSDALLRR
WSNRVTQQSLVRCDPLAPASMNCLADASYLGAPYLRIMLSALLLLPSPLPRLRVAVLGVGGGSLPSFLQQH
FSHDIMRLDLVDAEQQCFRAAVEDMGMRKKMQGGSVTCHVQDGAAFLQDVVGSSTGTGESNSTFDELVSSR
THSGTSVSFKGGDFPTNGNQHKPLSRYQVQPATCYDVLFVDLFVGSDPPAFMSSLIFLQLCRAALSSIGVA
AFNLPKSDPDFVQMCQRVFGSQNVYQVPVPASANIVVLARCAAGAGVGHTIYEERSHVAHRHFYRRAQQLQ
KSHGLPYDLSSHYPIWWRLW
>tr|C9ZLR0|C9ZLR0_TRYB9 | DeepTMHMM Topology Prediction - Predicted
Type: TM
MRRSAVRLVYEKFEGTIAGDKGYMTYQQACSIFGFEMSDRLEVTEIKKRFNKLVMRFHPDHGGTSEQFQLL
REAHKLLLAHRHDKGESNRAGGTDVNFRRMNYDNMTNTIHRETAGNPEYRSFSLQDFAFFLALVIFVVSFY
AYRAFHTQLQILRSRWSYTESMLHDEGGHKDVRGWHPWRTDRMTRDVMDEIGHLQQSISREMLEEKRALSP
LVHMPWQSGGPFANYTVARQPIGAVLEDADGQ
>tr|D0A775|D0A775_TRYB9 | DeepTMHMM Topology Prediction - Predicted
Type: TM
MMDRNENSGCKALWSNTRLEWCYWCHLIRSFKCSFALFFAVLCVLAIIVGNTILLLSVNFWLDLMVKESTE
GFTPIAVQVIVCLFLVLCFGVLIIVYIAVYGIKPLVRALCDTNRVGSPLYRMLLIFSSGATNGLSSALAIY
AMTYTPEFMQAVLLSVIPFFAQIWTYALVREERDRCYSSMTLIGSLVLCIAGVLLASLSSFFTTDMSTKKA
PWGWAFVYFLSCIVFGLWCVVQRLYFDAIMIKGTKPQEGERELHQPLSHQHPEITNSGRYEGRDGQRLPAE
AAAAGVDGVTQSNEIEEVASSENAENPMLNREWAKQDENDLAAKALVLFLGIIFQAMVSFAFVPIDAIPGF

GNSKDMKESWKNFSATFDFVFASWFNLRFGLLYTLGFLMSFIGCAYLNERSPPLASVVLQLAGPITSLVLI
IIPEWDVFGEHGILSHKASGVIFLIIAGWAYHVWEVAYPNAYKSSTTTAAVMRGTGNV
>tr|D0AAE9|D0AAE9_TRYB9 | DeepTMHMM Topology Prediction - Predicted
Type: TM
MISHILQYWVMFLLVGTNFFFVSCHFLRFRFLLRPDFFFLSFFQRTSRFTHFPSLGRRKGGERFHTKAEVR
TEAIGEYKGKGSMTTSGVESVSTTVNEGLKPQTEGSEERRPTTFKELRPSWLTPFFGANEPEPYKPPERGH
PCKAFSRQVHECLDRNGNNVDFCQSKLALLQSCLKELGL
>tr|C9ZT66|C9ZT66_TRYB9 | DeepTMHMM Topology Prediction - Predicted
Type: TM
MLSFFFCALCCKLDRYGNASVSQYTLYVRPPKILLIVLLTMLSVYICDLICLASEATSSRVFFCSFLAYNL
GGYYTTCNNLLDRPFNRQEIQEEGKQRRFNCYT
>tr|D0A1H0|D0A1H0_TRYB9 | DeepTMHMM Topology Prediction - Predicted
Type: TM
MSSEVPTYRGGGFPFLHFLPSNTKLIFIFKDRNVRYACISVVPQFPVAWCFFPLRNATFTVGAHDRKQFAF
VMGGVLYFLEWVICVVSECQRFVCRTISGKETCLFCCVYED
>tr|C9ZSY5|C9ZSY5_TRYB9 | DeepTMHMM Topology Prediction - Predicted
Type: TM
MKLKRSRKKEKTKKEEKHVFIDILLIEVEKFPHCFRRRRNLLLYTCTISFPCFVLFYFTLKFFCFLAAMDW
MCARIRESPPSFIYLFIFLNKKIKNQVTETRPTNEHYHHFCFDANWKQ
>tr|C9ZSE5|C9ZSE5_TRYB9 | DeepTMHMM Topology Prediction - Predicted
Type: TM
MWSRVNWPLLPLTVLLFCSSVRLHFVSVLVFLFHVQFGQFSIRRRGLVSSSPLAKLFFFFSFISATFTMKF
AVSPSHTPKYQTNICMSTLCLSIRTLLPHVFLKSNCSESSYFTNLFTDSLFALGN
>tr|C9ZNP2|C9ZNP2_TRYB9 | DeepTMHMM Topology Prediction - Predicted
Type: TM
MNDNIITIIMITIIRVGGNVMEKRRETKTKKFSLPRPAAHHASMQNRKTRTYSLTQSFLFVYSRMRHFVLT
ALICYPMLLLLFLSFTSLYFYHPLPLFFS
>tr|C9ZMD7|C9ZMD7_TRYB9 | DeepTMHMM Topology Prediction - Predicted
Type: TM
MTLNKTRVRSAGWGGNIRAKCHQVQHLQRRMNTLLVHCYTVTFRRISSIVNFSVAAGAAVGSWRLFIYTHT
HTHTHTHTHLQAFEAFFCTSLSFLYERRPQQH
>tr|D0A9N8|D0A9N8_TRYB9 | DeepTMHMM Topology Prediction - Predicted
Type: TM
MVESHTVNHSLKVRTDLRRMLSFELGLSRPPRRRGGLCWKLGNGAFSVYTYLFLYVCCVVSSRVAPVSITC
VNWMLWVKECSAHTQADMYCPTSAYVSRPSWVYVSHIVLRVSSFHPFFCLHLEFFRYPICLRVLSCSLKNK
EEYNFQ
>tr|C9ZTQ2|C9ZTQ2_TRYB9 | DeepTMHMM Topology Prediction - Predicted
Type: TM
MNGRTRVLFTAGVPTPPNCAHNVVISGGGIVGAAAMAALQKLRARFLAGSVGAEGSVSHNVHSRCLSRLLL
ADPMARPHYDAANIMHNLRTVSLTPVSSKLLDNLGSWKRLQTKHAYYRIALRHERTNGPVAPDQSSQEKSR
NFFVNGPLGNGTSTAEPLLEFTNLDSPLGFICYNSDINTTLLDVVEEQMKQYQQPLGSDGCGVQEQPTDCI
EFGSSLGSYSLPHRNIVDGPAGRAILRKSVGDQPVEFSLLLGCEGRGGHLRESLGSALVQHDYAQTAFVCT
AQLKRPADGNVCCFQNFFTNGDIIALLPTSEDTSNIVFSTTAEHARELAAAKQSELVEELNRRLHAFAPRD
IPLIISVPEVELGGKTVRAQGMFPVRLSVALQPFGPRCLLLGDAAHGIHPFAGQGLNLGLYDVCALVEVLE
QAVRSGQDIGSVIAVGQPFAAAMMSHTGIMITAMESVFGLLATMPGLSCTGMSVLQKLPLVSSLGKQSIIH
VASGGFFASHHRESFLLA
>tr|C9ZSN0|C9ZSN0_TRYB9 | DeepTMHMM Topology Prediction - Predicted
Type: TM
MAFNRSLIVTLFFTLCDLILLCILPIYSHGLSGHILVALHVGTLVVLASMMSLFTLRTSFLVEGRSSKALF
PQRVTGPLWLLHVVLTPAPLVYKEFIMPWVYTSVESAPRAWADSAYITLSVLNITTYVAFSVSLLYFIFFI
AERRLYKFHCHISRTGTATDNATVALNCNPLE
>tr|D0A6T6|D0A6T6_TRYB9 | DeepTMHMM Topology Prediction - Predicted
Type: TM
MDLIPHITRTGYDVSTLVAARKLLFVGAISVCTAVLGLLLWFKAEMFNNVGPHPSNWAGYDALNFIGLFSF
GVFSTLGVSLLYVGTSTLACIHGGILGLILMGMLGLVFVVLGGLGAVWGSETMQCLAVFCTGSTASTNRGG
RPEAGVAFRSGLGLAAFLSFVPIGVVILFQAARRVFGVDPFWTGHKGRRFVSDRFAPLLRFLFAVAFVSFW
AVVSLYMPNSLESFLPVRIARNARMLSSHNYTLCRTEPHTYTCPDLPWSWIRTRNLVMNDEVVLKVYPSNL
IFYGYLFVILLTTALIRSFRYGRRFLKRRCPVLRDYTYGEVGFLFLTFSMIVLFFLYWIQGHNFKQRYTDV

SNDTVSSERWYRSMGQLAVAFLSLSIFPVSRYSVLHSILGTSWESSIWVHRLLGYGVFLGIVGHAIGWYVR
CFELGAFPQGIFSIPAVDPPGNRDDYTIPLITLTTLFSLVSTIIFALEPLRRRFYELFYYTHIVTFYMLVP
VVLWHAASAWEYFLPGLTIWFLDWLLRIYRRGSTVDLVSAAASGSFVEIRFRHGSLGALPGQFVFVNVPDI
SLLQWHPFSIMCEHEGYYLLYIKSMGEGTWTEKLGKLVCRRGLKFKLNVEGPCGRALDINEHQNILFVAGG
IGITPCASVYSHICDRMALGLRSPTPMLLWSVRDRELLLLMSQLWQGGDSSAVSASSTIEEAPSDRVQVFF
TGGCNVEDGEYTCVVNERMDISDRIPQVIAGKDPRTVLLFVCGPPGLVELARSVAHSLGVDFHQETFLL
>tr|D0A447|D0A447_TRYB9 | DeepTMHMM Topology Prediction - Predicted
Type: TM
MPTCVSKYKNACAYCWFYGVVYMGGCAFIYFCVCVFSLLLLFIVIFFFLLIPLHVSWVACGADTPLHTVLQ
FTLNHLCVSVCLFVLIFHRFMFSFFFFGVVALNLYTLHPSESRRKA
>tr|D0A454|D0A454_TRYB9 | DeepTMHMM Topology Prediction - Predicted
Type: TM
MVLWELCKPPFFSFFFVNTIEASSSFSPPHSTLSGSNTLHRHLPFSPSSPSCIFFSFKHSRYCYHNHQHHH
GHYYIITITIFQPIFLFYSLLRILMTATAIPNCWLRLRLPAFSFDVSGFIRTKGRGKKKMRSFRL
>tr|C9ZLA2|C9ZLA2_TRYB9 | DeepTMHMM Topology Prediction - Predicted
Type: TM
MNADTKVFPSSETMSSEGPPSLSATDVVAASGKAANCMQFPSPVYFTRWLDDRYAVVGAGGGGRRFGMANL
LAIVSVSTLQGPDEDSIKAKREDQLPSRPWSFVTAIDLEGDIPWCASSFLMCNDKTKLSEGVLGYLAISHI
TCFTLIDVWRDPETKGLTLRRQARVSVPADPKNPDKKPIAIVQGAVVVAHDEEGILVYELSSLLRKPLSTG
SGELSGNEGTCRGSTPDYKQSSPGISPSLVESVEPLASWSLPARVNDLHANRFFVPKKSKTKDGAKGCQRY
SDYLIIAVLVQDKTLRLSATKLQIHERLVSTTASELAASIGGGVDWSSRRSESRRVGTRLDDFCVLTGKDC
RIPFSLMKSSMRIVQLFGVENVKPCVASELWRRARRLHCEEGVRGEMLVARIVLVVFNVMSNQSYMLSARV
VSTSSPCDDVAATNASELPCGVVDESCAANDVGRTRRRRLALRVYFSPEPSPVVNDGVTSISPCYYCRESS
DGAAMRGEGVGTAIPHNWLVGTVDGALAAVSYSDNGSFQTRTLRPPKERRGAKWLPALHREPISSVAVSSL
NDVLTADIAQNVVVSALPFHERRDQSTVVSYGNGENSRRSCRGVERASDLSTSSGFGSGVVRCNAEETLAL
PKNKSLLLFPAPQGGMWLMWLKSKPLITIHLVAAIVVVPLMGVLFAFLLRR
>tr|D0A8M7|D0A8M7_TRYB9 | DeepTMHMM Topology Prediction - Predicted
Type: TM
MPRRGTDYLEALSVPKHDVTGVKGEGVPLRKRLQPVQVTGYDSTAKIGRSTRRQELRAALLEEELRGGGLC
GGCRRVALWVSVFALLAVLAVAAMMYFPQGREILLPLLSQLRGYLGV
>tr|C9ZT83|C9ZT83_TRYB9 | DeepTMHMM Topology Prediction - Predicted
Type: TM
MRCGSSSPFSFSLLPFHFCSILFFIRGCAWAYVAKENDVEVTSFPFLVIGSWDHPLPIHARCISAFGHFLS
SSFAASGSRQRSLVQITALVMYWGLSITFLSLKRSFRLVRSHTHFFLF
>tr|D0A7B9|D0A7B9_TRYB9 | DeepTMHMM Topology Prediction - Predicted
Type: TM
MQEPFDANATGGVHEPSTTTLQMDSRSSATTGSVEVSTHDISLHAVRKDSNRIGDGVSGVHNCRASFMDMF
WATVQRLFRQTVRRKSEVVLEVVVPLLFMTVTVILWSIWGTNHIDESPRIDYGALPRTMNPTIYQHFTCSA
KLGGVPGLRVCRNKKEVECIKDEITAPFRGVCVHKRVGATTLVAFFANGFVGRIAPIPPLDTLIMHQWLAR
HANTRVLIPRGLIPNNRLSAIQSSGRLYFVGNASVVRGIVQHLGRVSHYFTNVYGGTFETLPEAQEEVRRQ
TLNWGIVHVRCFEPGSLDVQIYLNGTALPTLRETVANAYPGGFQHNRAEMYALSGYLTLQKEISEHHMGLF
NPGMGLNITPYIMPQGFVEFTETPLLQTARGILPVLFGLAFLFTVTSRVHVNVMEKESKIRETILIMGMRK
SVLNVVWFLKPLLIDLVVCSLITVLLKLTYMPRSGSVSLFVVIFVFALTTIPLSGVIACFFSKTRLALLVS
PIIYFLMTLPYAVERPTNGLLCMISALLSPTAFISIVHGALAMEVSGGFHLTQLRIGGDPVCAEMLLIMLV
ADLVLYTLLMLYLDTVLQNDWGTKHPLFFITNPIRALFWERRKVGTSACPFTDGRADNGVFEDIGGTKEEA
TVVMAGLRKEYQRGGDTFVAVNNFCWSMGRGEISVLLGLNGAGKSTVINMITGMVKPDAGDCYVNGRSVRR
ELPAARQQMGFCPQHNILWPQLTCREHLEFFGKIKGLKGKALDLAVRHVLHETGLSEKSDDLAGHLSGGQK
RMLSVGIAFVGGSPLVLLDEPTAGMDASSRRHAWGLLQRMAAHHTILLTTHFMDEADILGHRIAILNDGRL
QCSGSSMFLKSKLGLGYSLTVVVRSKDNFCFVDDAVKKHVPGAELLSYCGCEVIYRLPLGGVAAFPSLIEK
LEIASDVNLNSYSLAATTLEEVFLRVCGGQQCSAEKPRDCSSLWGRARHHAAGITQLKAIMLKRIFTALRD
RRMHMLQVVCLTSVLLATVLIGSNPAQVGPLPLTFDLYDEKVIVDSANCGLFWGKSPGVPNVHISEISAKD
TRELSIYGMKTWFAHEYPRYAAIFCGDRMLYNPKLRGMPVVMLYNSSALHQVAITMSMFYQLVLQRVSGVQ
ANVSWSVGVLEDEATYVGALQLMLIGAIMMIPLTLISSNPLAWVVKERECGSLHMQRIAGLRLPIYWASNF
LFDITMYFISVSAIVSVLMLFDQEDYVGSETIGAFITALMLYGLTSIVFAYLLSFLFREHSKAQLVVMGFN
FVVGFLSVIVVYVFSLLEITRETSESLRWPFRFIPSFCVGEAIINISHFRFGKAVGNATSAFDMDVTGYPF
IYLAVEFPIFSLLGFLFDHPRRRAWWNRRSYDRTKVFDEAHSGDSDVEEERCRVCLPMVDGPGYPPARVVN
LSKKYPNGKEAVRDLFFLVSSGEIFALLGTNGAGKTTTMSILCQELMPTGGVVETCGCDIVKQGGKALRCI
GYCPQFDTCIGLLSVEEHIRLQAGLYGMVGEEVENVVTDLLYMCDLTKYRKSLAGGLSGGNRRKLSLAVAL

VGGPGVIFLDEPTAGMDPIARRKIWSVIERAACQCAVVLTTHHLEEVEALAHRVAIMKDGTMRCVGRNAHL
KDKYGAGYEMHICVAEGELPALVREFVDRQFAGATLRECKGRQLVYALPRSTSLADAFRTLESSKGLLGIV
DYSVSQATIERVFLQITEQDEWVSKQTTEMDIV
>tr|C9ZMD0|C9ZMD0_TRYB9 | DeepTMHMM Topology Prediction - Predicted
Type: TM
MSETNFTLLLVFTFVFLFLIIRVLLMFRPRRLLQDGEQQEGNDGLSPHQPLHPAPPWNEGNYINIPVAQPT
TVVPPPPEAIILPGIPIARHHEMTTAAQLQQEEPPRSEIPNNPLEQATSHADVVYGHSAYIPRTTGVSLAA
SESKGGEVEKTL
>tr|D0A774|D0A774_TRYB9 | DeepTMHMM Topology Prediction - Predicted
Type: TM
MFFVTSIVRMTVHAPRGIPFQITTGDAVKSVQKKYHKPFFGVSMEYLQLGPPSAEFLPFYFCEGSIKGTFR
GVVSYRDAEGGAKNNAGISSNSGMRQVVTAPQPLQSSFGPHQTQIYAGYKYNLHYVQGVLCSETNPLQLRN
MSSVNVEGATINLFEQSTRTLRVFVEQEVRRQATETARAMISSYHPSASSIVVEFIELNIHIDDVIPVFMP
CYVVKACYDQQEYTLYVNGASGQVTGPFLINSLYAGRTAAVATALVTLCLAPNKGAGFIMGSLFAVPMYYI
AFYAARYFPLLRRDYSRKRRQKLREKHESDDRSGFRPDMSSKRIDEEYSRSSYWDTHAYEQKWSRKQGTVR
DPRGYYAVLGLNGGESVNEIRSAYRKIVLTEHPDTGGSTERMTKVNEAYRVLRDPKKREEYDRSGCYT
>tr|D0A0F9|D0A0F9_TRYB9 | DeepTMHMM Topology Prediction - Predicted
Type: TM
MRFHFYMSFSFSFSLSYLSSFYPTHSSPCYMQSASCQRKLWRGRGRGSFCFFCFSLHFISFHLFVVVVVVL
YSFLFPSPPILFFFSCGRDFVFVFLFVSVCFANPSALFSFPFFSVYLFVRMCVCKGEMWRGKWELLFKKMW
RVYISP
>tr|C9ZJJ0|C9ZJJ0_TRYB9 | DeepTMHMM Topology Prediction - Predicted
Type: TM
MKGKGESFKILMLCNGHELPSIPFEIKNKIFPLCYTLSHIVVIQHYEADCCHCRCHALITATASRRFQRPP
SPFTVLLVYFSPLLVLRLLFFSFCSYPRALCSSRLHNIKGNFGGGGGGDAFL
>tr|C9ZNK5|C9ZNK5_TRYB9 | DeepTMHMM Topology Prediction - Predicted
Type: TM
MIVILFFTSVFLFSSFPPLYLSSHFSVILSCLFTLAALPVFKRPIPVCDTIRQASAGHCSKATRPQVKSIG
SDKERKRNIYTYIYIHTHIRKEPGKGVKNEGPKISGEKEK
>tr|C9ZPI2|C9ZPI2_TRYB9 | DeepTMHMM Topology Prediction - Predicted
Type: TM
MSLLHRADATALRILREQHVESLAMSAQTGPINYIKKTFKDIVDTMPDRRNTFLMLVTLCTFFVGWRTAIA
VTDCESPLVVVLSGSMEPFMFRGDLLVLHNIGEPTMGDVVVFSLPNRTIPIVHRVHRIRLLEDGVTRLYLT
KGDNNEMDDRTLYPRGYHWVEKKDIIGKVAVLVPRVGFITLIAEDHSWAKLVLVPLALIWCWYTGM
>tr|C9ZP22|C9ZP22_TRYB9 | DeepTMHMM Topology Prediction - Predicted
Type: TM
MEKDTVGCTVGRSSHQHESCRFCFQQENTFFFLFFLRRRYKYTIMGHGGMAFCPLLPRACEVSPPPLYRKA
LYTSAYLCSPPNDLLNWRPLRFAALCNMQQQWCSFSILSSVFTAVPLVILHLKLS
>tr|C9ZLI8|C9ZLI8_TRYB9 | DeepTMHMM Topology Prediction - Predicted
Type: TM
MAIVVTILGLLLAAALFMGFWYLLRERRCPVIVMRLWFVSCLVTVVIVLYLLCLPIRIARYNKWVSRQQGA
NIACWLSCLLMGKVLWFLSPHIHIKIMEGSLDPHGIHHSGVMCSCHTSFFDTILFTQLIPLSYMRNVKAFA
KRSLWSLPFMGKVIDTCGHLPVYFTSTGGSFAVDKEKQALVAEEADEFVNAGGNLCVFPEGALNRTPETLK
DFRLGTFAMITKHRSRLYYMVHNGCHEVWPPAMNEIPGFPADVYVCFGEYKYNEDSTAEDISQGLREVMQK
HVNRILLLRQQARNASTEKAT
>tr|C9ZYK3|C9ZYK3_TRYB9 | DeepTMHMM Topology Prediction - Predicted
Type: TM
MVESGIGGSDESFISFCFAIVSVIVTIVYIPWVIRHVRSIIEYYTTPSKRQRMMNNPLEDMWKSIIYVPLV
AAKILTEPSMLPVGARICIKEKHFFFTSLLPRFLRFIIRPKIFILWLWVVLFSSAMYITLTFDAHAILGVS
TTASTGEIKKAYRMLSRRYHPDHNKTEEARLIYVQVRRAYKALVDREAFEEEEAKNTHEFTVGVALPRFLT
SREHDGLVLFGLLGILVAVPVAIWYNFRDQGGISQQLRHIQRGRERLESFLKHLGIPEDQKYVERRDSRRY
LVRLLVSLGMLPPNTDENASLNLPSYPDFITRCVEIDKNITFLRNFFDDSDIEVLHEYLVKNGVRLLDEYD
AAHLGHHQGEGTLQLASPTEYKVISYFLFLHIEEIDKALEELLEKVGSGVPSARKLMNLHEEVRDLLHLVF
EGDNKQVKNHIQKLTTVPQRANDIVDAMGPEIEAVYKKMYKNYLQMQVGSKNEKRLLKASLRRL
>tr|C9ZHW1|C9ZHW1_TRYB9 | DeepTMHMM Topology Prediction - Predicted
Type: TM
MIHHVSVLSLSCAQGYWWSYLRRLLKFVFAVLPSLTPSVVSAAQPFIFIFLTMRRMCVHLCRAAAAAASVG
SSNKSDPIEEKKQKADNIFTIRRSPASLYESTMHSNTLSDHPLTHRARRNELAYRKRRLTERGNYEQAAEA

EAEAERRDREGRGGAETREYYSAICFVVLGYLTAHGVVFKRFYPQDLQLNYSPDRGYSDEVAARRKELAAI
NEMVGVSVLESVYSKEKAKIKSEIDA
>tr|D0A0J4|D0A0J4_TRYB9 | DeepTMHMM Topology Prediction - Predicted
Type: TM
MGPNRERRNERSSQKIVVDFYIDVQGMSFTQANKVIYISILSLPVGYFEGSSPSNAGSLILSAFFLGFFLI
LRLLHAPTNVSVWPPRNENTIIITIIITTLHDQVVKLK
>tr|C9ZK61|C9ZK61_TRYB9 | DeepTMHMM Topology Prediction - Predicted
Type: TM
MCVCVCFIYFSCSRMHKYALYGPVFFHTVLFLFIFIIFIFIFFRSPSSSFLFPLPFPSSPVHMSKRSELRA
LVPFAPCEQAYVRFPTFLSWCSACMIFSFYSGGNEVRRVYLSFFVFFSHQTVAFPSLCPAGMFSLFLVLFF
VPTLVIAFLRPYNSYRRNLSFCVFSMHSFRESR
>tr|C9ZWT7|C9ZWT7_TRYB9 | DeepTMHMM Topology Prediction - Predicted
Type: TM
MQKKRKEKRGVQMEGESNSTPTHHSVPRFIHSLYFIFPTTTPHEIKQNETIHHHILLFDPFPFFFCFFIRM
MLFCFFLFLEKLKKLIRLSPSSHVTPPPRLFSLFYFPPLIFFLFYKTHKHMRAPTRACKKKKKYMSTSTTF
FKKKKEKRA
>tr|C9ZY76|C9ZY76_TRYB9 | DeepTMHMM Topology Prediction - Predicted
Type: TM
MLRYSPMHLIKTLRGPRVTGAPARFLRDNGVSLVGPARSTFLMMYRMQGNQNVRMGAGAYKDSCRTFCSSA
VLRDPKKPSGQLVGTHQVQQRNEGDKGGDEEQKQEDPLGGNAGAGYLPFPPPFHTINVVMLLFLANVITYF
LMNFSGSDDLRDFIVEHFTLSHENAGRIYPFFTNALYQENLLQLLIDCWLLWEFGKTMLGFLGNARMTFFA
SLCTLGGGLIHVARQNFELYYGMDPLEVRGRCYGPNPFILGLVGVEGIIFRHLNFIQQPPVPFLVLTAFVM
VIDVWRIFTTKPEEHGAATGGALVAYVFWALPTRMLGLDKLTAAL
>tr|D0A048|D0A048_TRYB9 | DeepTMHMM Topology Prediction - Predicted
Type: TM
MCGAVAAYLSFIPQIVLAATLHVITSSLTVALALSSLWFPFILLLTTPRLSTALTVDTPTRTSFFFLISLP
LNSTISHFCVHSSSNMRRTFLRLGGGGGDLFPTSKAAAHLEPQWVMERRPPPPPGPGKCYVTVHKIGTKSG
SKWQLLQPHIHTLEMISMDSPPPHKLDVPRPTARAAASFVAGVKSSLEHARKQTTSVSGIAAANIAEKKRC
EARMKTRDYAEGKRPVRERPGWEATTDVVELSEWLHKRVNDHRKIVSDNSNGYEREYTPWEKKPVPPQIKK
>tr|C9ZM88|C9ZM88_TRYB9 | DeepTMHMM Topology Prediction - Predicted
Type: TM
MNADSGEESREPLLRGGHSNDSNNHTDTNDAGTTEVHITADVTPKPTMDILLRSEQQLNTRRHLWQAQIHA
LLGPEPPYALRPEDRAGFLFGRLYHTWTGPLMSLAARGVSLVPEDIPLPTRDVRAFNSGLRLLQTLEEQKF
RRFGWDSYTADGDDAAVVRHRRDRQSVGLLRWVGPVQQLRRPRQMYAGVEWKSAPRHRVREQRRSMRKREK
KGEELDTNGEKNVMPFHNGVIDGEHLFSTTDGAHTATCEAVGDIVFISPFQSQGQERQSNPQGSTRKTYKN
GIFMPITSQKPPKHISVARALFDTFGSSVYILIPIQMLQDACQLAAPVILQKYIEYVQMSDQDWKGGVALV
ATLCFFSLVQSAAGNKLTQMSRRVGLTFHNALLTVLFTKCATVARKGLAHPDMSVGRIVNMVSNDVGSARS
LPTLLPIMVGAPLRLAVGALLLYQLVGLSALAGLGVVLVFLPLQGVLMGRFFGFLNTIARLRDERLKATNE
LLSGIRVAKYMSWEPALVCQIEKKRREELKALRSIQHMYIIVAFLSNAVPSLVVAAVFVLFHVLGNELTPT
VVFPTIALFRLIQMPFIMIPVSVSAFSRFIVSMRRISAFLENDDVESGLLEMQKHERDGRQIRGPDHIRWS
LIGGDSTAVIEFMNAAISTYAPHKLPPCESELKKANKKGKSNGGITGDRVEGGDDADDREGRGDIQTTENN
NGNAGGGRGHGHGQSHAHGHGHGHGAADGTTEYYEVRRKELLHNVTLRIPKGSLTCIVGETGCGKSTLLES
LLPGGYEITSGTLRAPATVAYVPQQPWIMNATLRENILFFSDMDEARFRRALRCAQLDCDLELLANGVETE
IGENGVNLSGGQKARVGLARALYAERDVYLLDDPLSALDVHVGEKVLQDCLLGELSGTTRVLATHQLHVLR
HADLIVVLGSEGTTVFTGNYEEYKTFTGEAAGNSHRGDDDVNDGQSANDNSGCDETHEGISTEMPRERDDT
MGPGERDNGVTTWDPLNNVETTSHGNGGDAAACESPLIDDYCDDSPAETGAASGKHTKLQKLSPLEDGKKE
VSGKLMTDEEVATGSVPFAIYARYAAASGGAKTCVPLLILFVLTEVVMVSPFLWLSFFTMKTFNLPVNTYL
FVYGGLVFASVLCSPLRWATGYGVLRAGSYRLFERLLRSVVAAPMSFFDTTPLGRVINRFSKDMTNIDEII
PDSIVYFVQCALSLTSSVAVMVASQYLVIAAIIPCGFIYYRLMLFYNNANRELRRVTNRVSSPVFSILGEM
LAGRSCMDAFGKTPSFLTEALRRVDVVSACSYVEVVCNCWLAVRIDLLVTVVLTAISGLGVYLVLQFGTVD
VGLLSLSLTMAVNISTILTSMVGQAATVEANMNSVERVLHYAHNIEHEDLMEDMEKAIKKQEERDRQMEKN
ERKKNKKGVASAGKNRMDGAEVNRSTNGMSGPNGHDFEAARGSCVSIRVTGGAESDEENGGTDKPSTAHVP
SRHFHYTTSSAVEFCNVSMRYRQGQPLVLRDLTFRITTGQKVGVIGRTGSGKSSLLLTLLRMVDIEGGNIL
IEGHPIRSYRLRKLRQLFSVIPQDPVLFDGTLRDNLDPLHTSSDEEIFETLRLVGMQDRITSSAEGLRSRV
VDCGANFSVGQRQLLCMARALLRRDSRFVLMDEATANIDPALDRQLQYAIRHTFVTHTVITVAHRLHTLAS
YDLLLLLKDGRVVETGRPRDLVMDENSRFSRMVAAMGENALKNFMEATERDSFPR
>tr|C9ZTX5|C9ZTX5_TRYB9 | DeepTMHMM Topology Prediction - Predicted
Type: TM

MTMEVHAGSSILPLRVLALLDPPPSVMGRFLTVCSRLVCCIMVGSSEEVVGGMLGNLRLSCVRLPNLLRKL
IDLLFPTLFWLLLDTVSNPLGSFSGLHLLLAGPREGDVVYVRVVDRRRQRVAVTGV
>tr|C9ZUV1|C9ZUV1_TRYB9 | DeepTMHMM Topology Prediction - Predicted
Type: TM
MHSVEVMGLFIQRQRRWGIRGLFFHAPRVDSRRFIHLGCSQNALLRPSIAPFATNSFPLTHQKQHQHHSQT
QSQYQPHHYHHKQQHHHSQTQHQTHQHQHQHSKQQQQQQQLGRFSVLRPFRSGWNRFCSQCRGRKCLIVA
AACILGCCSYVVLCLALWWRQQEAVGRLFSPLEEHPLEEWSLVEPTLREGDIILMMGTGEISSKITAAQYV
YSGMRAAALRYSHVAVVVEPAHFDRLRSRRTSFAYDIRNNSDNGSSRSNNNSVDGNIADSVKAEYGGMLLT
RPLSAGIMGEGQSLFERRPRRGAVIMEAIDNIDVNAPDVNGHVRHNCVQLVEASRRLFGRHDEKWCYRRFA
VRRLKGFEWTPQRKRLLREFINENVGRPLDTNSALMLSYIHPRLYEWVGGRPHGNEISCGELIVDLYKHCG
VIRRRERPITNSGVMTSGLLSPTPVTSAVALTTSLEDGVEGNKEREKECVEEYYARPSIQTAPYQFAEGEE
VGVLDFAEGISLGPEVRMCHPYATQQS
>tr|C9ZSR1|C9ZSR1_TRYB9 | DeepTMHMM Topology Prediction - Predicted
Type: TM
MIAGHARHVRYHATQSVTGRHTLFRCTRTYVHECLHGRVGAESAMIAFPGHYRSIQIYLHFLLPPFFAGIP
TMSRYPSSKRLRCKRARVGAHFDILFLQFEVEVPDFARRCLSLQWPPSIFFAVIFSPPLSYALTFSSLIRL
CYRKFGGQLLIYIYFILFFKAPHIPTRHVDKYKATRPAPGNLTRMVEYTFPSDLFLLFDKLKPRKRVKPPE
VLKHQGGGG
>tr|D0A435|D0A435_TRYB9 | DeepTMHMM Topology Prediction - Predicted
Type: TM
MRNIALYELYYPLACMCLFLLRANQRTFGGPGLLAVPLMCVLSAAYPLMLIIFDLSAVMHQLFISFGLVSA
PTMSPTVISHLREPTECLLCASSVVLLLRVTVGLHYLSLLSYLNGSPRYRGDCSHYIRTNAVFTLIAAIPL
TFAFLPTFFLF
>tr|C9ZVZ4|C9ZVZ4_TRYB9 | DeepTMHMM Topology Prediction - Predicted
Type: TM
MGKGKFDPFNSLFGIRFSIFCYLFSSTILRTFSLNVHMRVKYFPLFHPLPSSTATTVSVLWRSHFAGFLSL
FCFFLNILCKVCRNTSGCFSCFAIVSLFLREISLVVSLPYCMLPFGATLTTAFDAGVFFLFSSPRCMSCGS
GGVAAALQCKSSVAMLNT
>tr|D0AAG7|D0AAG7_TRYB9 | DeepTMHMM Topology Prediction - Predicted
Type: TM
MSDKGKSATDVENTTSPVTQQTFPAEPSGIVDASEPIHTDDKNKGVDARGDAPPSVWQSFSAKMKHIAGTV
VPYGGLVSSVFNLCSVCIGAGILGLPAAANRSGLVMAMLYLVVIGGLGVFSLHILSLVMEKTGLRTFEHTA
RGVMGRRFEYFVVVIRWINSFGATVSYVISVGHVLNPIIEKSCGAPEFLRTPGGIRLLTALTWMVFMLPLV
LPKRVNSLRYVSGFAIIFVLYFVFAIVIHGAQSGLPKLTSDEEDGVKLFNTGNSAIASVGVFMFAYVCQIN
CYEVYWEMKKRSCARFTVYAAISMAFCGILYALTILFAYGEFGGAIDNSILLMYNPITEVMMMIGFIGMVV
KLCVAYALQTMALRNTIYHVLGWELETLPYWKHFSFVIPLSLVVLLAGLFIPNINTVFGIVGAICGGFLSA
IFPSLFYMYSGKWTRRNVGNFHFFGTYFLLCAGVVGLVFGTVSVVSENILALIANNVEVVRVRAPETQC
>tr|C9ZQA5|C9ZQA5_TRYB9 | DeepTMHMM Topology Prediction - Predicted
Type: TM
MWCQCCLWWLDHLSVRRCITPLFVFNEIKAAYFFLGEKCERPRLSPSICSHVHTHGVASSPLLVSCTFEGC
VMVYILAHDVVLCLLLDRHGDPTAVQSFVQNKWDSLRETQLPSTMSLVCWLWHSLKAKRGQQSGEMDEDEV
GDGIRVATVPGGNSPIETPYRARNAVEPSHAEFVESPTLATSTSDCYPESTPLTSRVAKLTVKQVLQWVKK
ARPTSDGHESHFAGEADEPSNHTSRERNVTTRCRTAEDLWVLTRLVALVRHAVATLMRQLLPLSCQTNGQR
CDQAEEEEIEEDDHSRFTDFPHECVVLVPLHVCRSIRGLSLRQVIAICTQKQTVLSEWSKSRRSVSFIINP
ALPPTNNPHRAEKNKRLDKIQQAVFAQYYESMAHSSNRRANGAFDPVIPLHQDGAQGLQNRKQEESFSSFL
RGASIGFDMQLMALSGGAVGYYLGYVRGRPANDCIVYATVGLVLMLLVDALLLILMLRRQDESLRRERQNR
WKWLQPIGRDEKKSQRGEDYHAEGRGSVVAAALNAKKNA
>tr|D0A3Y0|D0A3Y0_TRYB9 | DeepTMHMM Topology Prediction - Predicted
Type: TM
MKAAQPVVVDVEETFDSVAEKLWCALPSVAKFHATFEKTPILGAESGNVGVVPQQSSVEDTSEAAAAKLDP
PVTIRDAVVPVDEMQHLAGCVYTACHTNPVDADVEAAVAAILDKGDEENSVEYSWGDFREFLERVESQWME
WADMDQKWLAVTERQHIQVLKDLLESITYDVEPFGLLPRDKVLTMRSTIVHTKQLRTLFKTFVDFVSPPEY
PAVYDKLWGLLIASQNGCEVAEVSPETPLELNSFTKFVLWFCLSIFVNATEAGLVVAAKTLWLRHLNERGV
MLKKQFVDVCRYMCKFYSVGGSDVEYFEKCCARSEEALQGSDELPPFALFQQYMQPTEGRMLVDPNDLYIP
EELERSKWMEELYRMNCSSNRIIVHGRRGVGKSHLAASLAKRLGCVHLDAGELALEAEAAAPADPLGAQLR
ECTDADAPISLATLAALVRKKICSSETRYRGYVFSDIPFFSSENDAEKISFFTDCGLLDELVPTTFVLVDC
ENEFHPERLEATLAARESDHQEELSLLKEEKEEEASYEALAQKIEELKATLSQITTKQEVEGGAEAPEGEE
ANAPELDPEEVEKDLKQLLEDQTVQEERKIEQADSRIARTKKYRELRLRRLVAQTLSEGYESGNEMSLTAL

PCFETALCRARLMGRCLTVDCASVAEEAVTYIVDTLSLQPCVKPWALVDKVERDPEREGSVFDDPDIERLT
EEFATNFGVMTSSRWKRFCPVTFAEHGVLVEGSIAFGCVFRQQLFYLASEEKLSMFRANPCLYLGTLPLSR
EPILLLSLVEPQNDAQLSPTDMQILVRKLHEQLDLTPMAFSEFTTLWDSHRLLKGKRAEVLSNRTKYEVVE
RKQRADRLKKRLAQEKRKKKSQKSPGKGKGKKAKSVVEEVTVEEYKGWEKKAEAPETIATRIAKNLEERLE
RQNTLVPVLVHALDDNPLSGFDQLFSEKVVPRTVVVLQYEKPTKAEDSAVLEPSAAEGVEENSGEVPKVLL
PQEVVLDKLSDLPDGGVDTVAEVSPHDVAIHRIVVNDKDVHALVTEIMQAVCPGMDPVGVGVVDDAVGEDD
EEDAQEFDDDENEEDDVPTAVNPAIKPGKTFLNQFGTTLEFCPVTLCERRLLVRGQSDHCLQYRGYVYTFA
SLEAKTKFEFNPLRYMRSSYSLPPCRMWIVGQSKSGKKTLAQSLHEAYDVPYFQYNRKLFDQCVEVAMTPT
GGVISNIFIPPQTFDNPYLALAAGILKEVQEFDPEQERRMKLREEAERELERREEAANNGDEEEDELDEEA
EARLQEHLAFEPETEHDRQLRLSEAYLKVAGCVTHIEPFASKGYIMVCPPFSDGDIEVLSSVDAIPEVTVN
MEVSDEIYFKRNKEAMSSVSYSEMSSGGIPEVREEPVVNEEAKLLRRLEYEDRRKEREVARWRRRHIGADD
PESDVDEELGDEGAAGGGNDESQEDEANERPFDQDLIVEKEAVGEFAEAVEERSIPMIVLNGDLSRNAVFR
RAVRRLSRFLENRRSLLHAPQIVRYEDATRMLESGEATLSCFGSTDPVTLYDLRHGSRRVCKWRPDGAYLE
EEPLIEPDSSSSDAQGTAVPEGSEGKDESEDTLDSGDRKPEPPQLDTSSTEGDTESGDDEEMSELDSELLD
EITEKFANRRRREWLRTCQRVALLHGRLFFFESDETLLRYMQNPLLFIQQPPPQPPLRSMPVITFYDDDGA
YPPENSGPSRKRCTAEHVAFNLNWIYLSLPKLLSWSAVNASLLSLSRKAIDAVLSGSVDDALVARLLGHRL
NAADAKQNGVVLHNLPRTPDQYRLLLAWGLKVDKIFQFDDNYRDVATLMKSTATIEKQLTTARVSVAGLSE
ICDCIDGFVVNEGRAILSHLTGFPIDIDNSYHTVADIETHLSPYRWFCPYSWCLGENLVDQEKADCRFAAL
YDGQYYFFSSEEYLERFLLCPSQVTLPPGFKALPTPLPVRVQPSTEYAFELEGCCPVLLYDTRENRGLRGV
LEPVARKGDPSCIVEYGGCYYALLDEEAVRRFLMRPWQYVDGAKLPPSRKVPLPEGKTMSTIDEEEFIRRI
LYDPVAHALIAVAEVRPKYYGLSLEESALKYIALHMKCFNPKNSEIQAKQYKKKFEIFSKQSTLYKTITMH
SNSITQNEKFAELCDEWENSKYGREKELSIHCGCETEPV
>tr|C9ZS74|C9ZS74_TRYB9 | DeepTMHMM Topology Prediction - Predicted
Type: TM
MKTWKYCFSVIFPSFVFQLLYAPPKHIVRCYTCACRRTSFYAPFFFFSIIILLCCLLLLFLPYVFFLLFGY
TLVTYLFLISFLTFWCSHVFPLLSCAVGPVAASIHCIANHYPLSRALIMLFSLFYFTERNRIVGDIVEGKT
T
>tr|C9ZJK4|C9ZJK4_TRYB9 | DeepTMHMM Topology Prediction - Predicted
Type: TM
MCGNLVLVFFLNYASVQVKAFILLCIAWSVIAIATVYKFIFISHINFHFLETFVFLGIYICVCCFSIKDMS
GDGSDGWGEMVLPGVFMLRRSAALPRKLIGPVSDGDKMEIIAYKIEEIEKYIQKLKESNANINEFLIDVGR
NSGTRHQTLSSNDISTLNADGDDRVLLEALAENAALISSKTRELEELKALLQNNCCACACRSYSSTDGGSG
VQEPSVTHFTGDEEESSVNDMSIARFSL
>tr|C9ZQ41|C9ZQ41_TRYB9 | DeepTMHMM Topology Prediction - Predicted
Type: TM
MTKGGKVAVTKGSAQSDGAGEGGMSKAKSSTTFVATGGGSLPAWALKAVSTIVSAVILIYSVHRAYDIRLT
SVRLYGELIHEFDPWFNYRATQYLSDNGWRAFFQWYDYMSWYPLGRPVGTTIFPGMQLTGVAIHRVLEMLG
RGMSINNICVYIPAWFGSIATVLAALIAYESSNSLSVMAFTAYFFSIVPAHLMRSMAGEFDNECVAMAAML
LTFYMWVRSLRSSSSWPIGALAGVAYGYMVSTWGGYIFVLNMVAFHASVCVLLDWARGTYSVSLLRAYSLF
FVIGTALAICVPPVEWTPFRSLEQLTALFVFVFMWALHYSEYLRERARPPIHSS
>tr|D0A624|D0A624_TRYB9 | DeepTMHMM Topology Prediction - Predicted
Type: TM
MLFCFIASPRLTSFIFFFHSFLSYFSPPLPPPPFLSTSPNTATVNVCSSPHRNIFLLAFTCPLCFALLLLL
IIVRTVAYSSHVSFFLCCRPLLFCFVMFCLSYLSFCLLFVNSVFFSVLETMRSCRRKIFFASSVFHFSFSV
FFLFFLIVFFL
>tr|D0A3P5|D0A3P5_TRYB9 | DeepTMHMM Topology Prediction - Predicted
Type: TM
MAAEQPTTLAGVLLTDVWRGPLLATSRTSERLGHIDFAMVVPPVRAFIYYMLCPFLCYPVAALHLWGYYRC
LKTLVLPKPYKEERYASHVIYGVQKRVYDSRQKRLHETAVTDAQEIQRALQVADGERNHFVDDPSAPGVAY
YVVEQRIMRAEAIFTGENWAWAFISDSCRYLSPVVLAYLFVPSCRRLKVDLKLWSQGRLRWRQLRHPVLNF
FKSYRDYNDAITRINITKPPPKGKQPWTTNL
>tr|C9ZZS4|C9ZZS4_TRYB9 | DeepTMHMM Topology Prediction - Predicted
Type: TM
MTDKEDASEFIIVDDDESKRREEDPTQENIQLSLTPQNASDHTVSPTNSHESVLALKRGDIVDGAARTGVS
ITTNLVFSATVANPIAAAAVAAYESFRAVSDYRRGALNVLGLPMDRTDLALRIGKEVGTAATGLAIGYAIG
SLIGLGSIPVVGQVIATTVLSVALNFCVVTLLTRHVDRLILKIKLRRQYGYPRNEQGARRRFEELLEPQHC
LLSFETCRIVQHYRDYRVACGWEGLTDISEYRASANIALMPISFQHFAIVQLQRKWGFVNERNECRKVYRA
LLLVHHPDRGGSGELTGVLNHDFEVYAFCQGWHEDCRRLCQENEEAIHTDGARRPRKRKKNAVVSFLQSLF

RTGKNSSQEAQELCDSGFLALEAGTPLELSELEELSVDEPDESIADEEYAVSETKASTTQHSICVVLAAIQ
RCYQLTTEAVPFSWLKQLHRDYEGWSRLKVNVHMFIRMHVFVHIADEVAARDTATTVLPVERSCYDFRWCT
KCEAVAKREDLRRTIVATCFPKHVTGKLIEAFELWKTAQSLAHSFFKQHTGATADVTVVAGTGQTLSTLFD
IQTAIQALCTGDSDSLEESDETEDERTVEFADEVKIIGLIAGFALSGAHAVDTHQRLRETCKEYYRARDSK
MKSVASLRHATHEVEGRTSKSTTKKEKLKSSEQREGDTKESLLFHQVFTEVDAATCELYNIYSVHLPENIG
FFKLLPSSCARCAHQKCEIRRYVKYERERTLVSYKHVVVEPRNVLEPHKPATYQGMVLKDGKEINEIEFAL
LCAEYVDPISGTVTPCWLKRYTFPKYENESSVQTTKVFKTLMERELRLQETCASCRVTSAIEVFGDEHTNQ
IYFHLPRGGTQVRFSSPQDILKRIKQHGVRWLHGALQAIIDIHSCQVTHGDICMSNFTHDDFGNTTLGFFS
NSLKVHKYGTKTAQDDAADFGVTLHAEVLPYLRGTLPQTKSSPHRNASDDRASSGVVTGCGQAEQQIREMF
NVFLEVADRLTGKLEPRWSLLDARGFVRRLIKVNYDCDERQYLFTKEVSYPPYWTAQKNMGPVSLFPDNRV
LMHRSPQGTAVFLNRNIYLWETYWKYRQQLVLARGCSSFSVPVALREQPHFLPCSDDCEVNERFLWYNCKD
EEEAWQICLEGTKEEKCRFAFVPPWMEAKKQTRNDDNPWATWAIAFRVALGAVVSDEDEMETCCVPSAGPD
TDLFVVQRCAMVRGSGPAAGQWELSVPSPGTRSYPEYLVRWFHS
>tr|C9ZZB4|C9ZZB4_TRYB9 | DeepTMHMM Topology Prediction - Predicted
Type: TM
MMCFFVSAFVCAGNEAAEAKEEAKRKTKVRKQREMSEEKVSNKEKKEIVILFFFLPFFLIDYLLVRLLILI
FCFLILFFFLHRIARSYSFFILSFFFIFPRACSLLSFHYCLFFFFFQ
>tr|C9ZVE9|C9ZVE9_TRYB9 | DeepTMHMM Topology Prediction - Predicted
Type: TM
MTTSPNACQDQPPQHSAPQAHEAECTTHKLSAEETMDARPVHPDARALFRKLPCVWSIPVFGTAVEAFGPR
CVLALGLSELFGKGIADNIIRTTLFPMFTYTFGADAKLYQRMSSLVTFGYAVKPFAAMFSDLFALFGYTKR
WYLALSCVVGSTLAIVYGSLPGELSYVPVAGILVFVTSFTKANLDILTQGHYSRLIRRVPLAGPSLVSWVW
WCVLTGSLVASSIVGPLTDKRLQRVAVFISAGMQLVPTIFFILNWYGERRNREERAYDLKIIREEQLEHEA
DAVRLQGSEATSGSLDNPSDTEEVGEGGARILPCCCGAFEVNREVFARNKKVVFYCMLLTLGAIGMVLVTV
LGTRLQLLITSVVASFTLCGLGFVALPLVIAKANMFTFISRVAYIQLPGAIDNVFMATPDCFPGGPNFSYF
YYSTVGNMIGAMGGVIGVTLFRYVFSKRSYRLTFIVTTLIEIVSSIFDIIIVERWNRPYVSDHVVFVLGDQ
IIHQVCYMMHFMPTVMLISRLCPRGSESMVYALLAGFANFGRSLSNTLGWLLMEYVWNVQSDITVGPCDFS
NVKWLLLLGHFGTPLINIPLVFLLIPAARICDVLDENGKAITKKAEDVHAPSNDSPRRREPTAN
>tr|C9ZKQ2|C9ZKQ2_TRYB9 | DeepTMHMM Topology Prediction - Predicted
Type: TM
MLRLSSRCHLYEKLGRTKLGAEVLLKTRNDMRGFGKDSNFIVRFLARRRPIDFLALEVDGRLARELHPSFR
IVKNACTLLLMGPVFVMLVSGMMSPTYYLLVSTNIMTVDRYNGLWEFSHWAVVLCGQLFVFFFLYDLSVYV
RYPFFAYIFVPMYRRLGLQRHQNVTVPLDEVRRRARAKPLGAPLSTVGKKLKNKQTGTRGS
>tr|C9ZRS3|C9ZRS3_TRYB9 | DeepTMHMM Topology Prediction - Predicted
Type: TM
MSKRGTKVYKLSFFFSSSLVSLDCIFVIFVIILSPINFFYSFRISFLFYFCYCLKFTSSFATTRHIESVLR
NHTHTHTHTHTHTSMRTCNLSHSFFFVLNALSFYMRETKQQKGKKRRLKRSFCMIRWKKKGGK
>tr|C9ZU79|C9ZU79_TRYB9 | DeepTMHMM Topology Prediction - Predicted
Type: TM
MYRYASSAAAAGSHRLLFPRHSATTFIHMSATKTTNSNGGIKCTSSECRECPRCNSMRSSRVATIVPLLSC
APSILRHSNCCYGSFSHHHHYGIIANKSCGDVIYGGNAVAFHMQRRGFRRELCEGPLDLKAGERSARKGPP
VAHSLPVYKTAHKLNHSVRWKNANLITNVAGGKDYRLRETQGEGTFDESGMYRDWLYGDERRYANYIGVVL
GVLSISLFLYTMRVMGAETWDIPTPVLAAQRKKRAAALPEGGEGKEENLGAGENNASGTAELQIGEDALAM
LKKPAVVSKAALVTAK
>tr|C9ZYS1|C9ZYS1_TRYB9 | DeepTMHMM Topology Prediction - Predicted
Type: TM
MKKKYIYIYIFCCCCGCLLHPRYILTSCVCASMYSSYACKKIFSFFFFFPSFCLFSFIYFHFFHSYLHLCV
SVSLHSSVFINSSAALNFRCVYVSLKNEKGK
>tr|C9ZZM3|C9ZZM3_TRYB9 | DeepTMHMM Topology Prediction - Predicted
Type: TM
MFISVVFAVCLFIYLSFCFYFCSRVCDFVLFVCLYVYFITLYIYIYIYFIIYENICVLLPSLLQRSILFLS
QLYI
>tr|D0A489|D0A489_TRYB9 | DeepTMHMM Topology Prediction - Predicted
Type: TM
MWLRVRLHFLSLFSLYLLVTFLVLIAEFQPTMRLPHPSNSTWYCRVPFFFFLLPFLLLSIFSSFCKFIYGG
CDPLFLFLFLHFLYSFLSVMLFFFFFFFSVAASSTLSFDCRNICTASPLQFPVFSFTFFLFSFFVTPRVTA
GTAAAFRSSHVFGSFVGGSGGDVTAISGRVRVLCDCGENIWLHTCGEEII

>tr|D0A244|D0A244_TRYB9 | DeepTMHMM Topology Prediction - Predicted Type: TM
MCQTLTTDGLSGEVRPAFTEEEQNPNRCSDDSGDGVNSMETRMKGDPWRFAVAAIFCLLSISNAMQWIAFA
SIFDETRTYFNMTAVQVNYLATTYVIAYVVAVFLSCKLFEVTGLKAGILIAATANAIGASIKLVAVYAWPH
MILLFLAQAFNSVTEILTIATPPLIANRWFPVEERVAANAVMTICLNVGCGLGALIPVFFVSPEKQEQRHF
AALFWFQFGLCAGTFALTFIIPQLPRQSPSYAADRQQKMEAKRLRTLRERQNRQSGAGAPAETQPPLEVVE
DDRLNMYDDDDIESVAEPINVFSTLGDTFRAMRTNSSFVFLSIASAAELGLIWSVATVLPQCLVPFGVTGS
ESGWISFLNLVLGSVIAPIVMHYFGQNWRHKQSLLVISIILVVNVCALCLCYYFGPSGDDHRIYYVVVVFL
LWGGVAGLCQNFMLPIMFDFVVELTFPMRESTSAPVLTWAACLSNLVLTVVFGEVLGENPTRTDSTKVLLG
TVIVCIIGCVALVLVRPLKRREEFEHRMEELDRAALQLPQDASHERVR
>tr|C9ZUP9|C9ZUP9_TRYB9 | DeepTMHMM Topology Prediction - Predicted Type: TM
MTSKVEELVGEVEEALEKLVNASDSIFNASTTLDKKQAELSAKPVMREARSKVEMLNDELRRTEDAAVRSH
YEKVCRAAEEKIRVLDAEIKKHVYAKHTKVALEKTHAERREEELLGPGGADGSGFQDSQQVLEAAVNVQKD
MLVSLQRTEKVMIMTEETGQETLQALQRQTEQMYQVDEGLEELQGQLDRAGRDVRWFFRQLAGDKCFLSLF
GILVVAMAVLMGVMIYKKRQAKKNAEK
>tr|C9ZIL6|C9ZIL6_TRYB9 | DeepTMHMM Topology Prediction - Predicted Type: TM
MAEEAGLPWVARFINHILTPGSALSPVVWCAFNVVMAALLLCWLPLLISMPSNIHLWVFGFLGAGLAFSTN
WFFRELSARPPSAAEATEHEVREKRNQ
>tr|C9ZNY0|C9ZNY0_TRYB9 | DeepTMHMM Topology Prediction - Predicted Type: TM
MRSYVFGQLCWICRVTVELLMAMGTGAKWMFMLTRLVIFAFMSFSYFVPMVYWYFSSPNIIHRVAYRSAKR
SRQRVPRIEPGRALGSKRTVGSTGFENCPKVEEPDDIDTLKSLVTPLLLSRDGRNSEATCSVECAVNDDPK
QGTTSSILSDELRGSCGGSVAFSSVYLGGERNGNGTISERAKETRCDNPLEDVVSSDDDEDRQNLNNRARL
DIYLPVSSDSSSNPTEGEGTPSAGVENKKSPIVICISGGAWIVGCYLWSGLVARLLATRGYAVFCPDYRNF
PQTDMEGMVVDISDAIAWVVHNADRYNGDVSNITLVGQSAGAHLSLMSLLSQAHLHAEEASGGEPPSGAAY
YVKRYNPRTSIRRYIGLSGIYNLQELVPHFDKRGLYSSVLYRIAGGEDKLANFSPTAYFGPKVLGSTEESL
PENIFDFLPRYIYFLHGDADESAPLSESADIAFAMREKQRLLTRRRCGKSVGRCAPFFTCDSSSSIPCQHT
HCSPSHMSSDENGMTSISPCASFASGEPKHQSNGGIRGRGSSELSDEEHRASAVEIRWVKIPDASHTDPFV
EEVLVGHQSSLVEFIVKQDDYLVPGHQWEIEAESSEPDEDECNCATVNTPGDSPAEYSDSVTPVMLPDAIL
PLRVPHESRPLLMRAASYVCPF
>tr|D0A843|D0A843_TRYB9 | DeepTMHMM Topology Prediction - Predicted Type: TM
MESKHPEVRGKTRAEAVFEETVLDEREEKEHPHPRDENSANVVGGQTYKSTNLSDVSLTLDVTALGAGPSA
RDEATPLEPHSEDSEAHSSAAAPASEPYVHSISEQKPVKEKVYLNGNDSLLSVDVGRNSIPRSSSQHSECV
SAEKEKVNVAEETEQHEPVNGSRDNCAEERREEVRAIYAKLPLYRFPCDSNQSQELSQTFVPSPAEATTSA
GCWEMGACATTARPAVTAGKQQCAYVKGQQLRATSDSGLPHSQSSDGTTSVCTRAPSDRATLPQCLHNALV
ICWNTESRWLVVTFCTFLAFLLTLVAHPLSLMDVVGGGCYTFWGYKNDCDKSTYTYRTGLLKCVGLRRVLS
AGVVFNILAIILLAAASGCCILFLKRDVFHKWNLVVFVLLCLTALVQLVSWMLVVTMFLLRFCEDTKQPRR
TAYGVAFGMNVTSWILVFVSLVVMKVFPQG
>tr|D0A255|D0A255_TRYB9 | DeepTMHMM Topology Prediction - Predicted Type: TM
MIKGSDPLHHGVSEGAGGNSRGDENDTVVNSNVEVMCLDMSRSSLLANSTKPPSSSCSDTPSSCSDSNMSG
IDFYCRQVPIFSQGVGVDYFVNGMGGYEGHLHQQPQGGELGFDQRNKQGGKSSSSPSNFHHNVNEECSATV
ATRLAGGLSDDCSGNNTRRRNFNHSMCNSPELRSCGDEYAAEARRNVPGSSRGVMAAVSQTQSLGKRKIAE
SNVQANLPLLRVSDCGTKTVHIIPLGNTNVTSQFKCSCCDHVFELSFVTNHADSSAKEKDQERGRNTEVGL
TCTYGNGLDSNTGEATAVDRSAEFLFRQQPDRESTSLSLLGMKFDKGFRVSAGSVRGNYGSHPMENETNAS
QGAVRHSLEDRRRRYQSGRFSQGTPKEIKANDSLFPSLPTFSPTSFVRAYVGEGNLPWEATLDNVPAVTVS
CCVAALNLIAVQFLRHHVIDTRDHFCLFATGTYMIFASYFVAYYFLGHYTESFRRISRDKQFYIIANLIKA
GILASLVPFATLHLSRIILFDEWDTNTLRNLGCIYTIPDFVSMLIVKRMSWSTWTHHLCVLLFNFFSTMND
YTQENVCRCVVVYAAFSTFAYCVNVLLASRFLGVKPGIARILSHVSVVAYLFFCGLNWVWQTYYIHRLLYG
GNGHWTVYVYIALVGLVMYDDVTLCQWLIHNARSTAFAAAHHKEQQRRQ
>tr|D0A7V4|D0A7V4_TRYB9 | DeepTMHMM Topology Prediction - Predicted Type: TM
MLQNCEVANKRVERGQTTTTDPNDDLAFERHTSAPYGPVKVKRYRIQNIVLEILLVAALCLVLAGSNSSTG
KLEKDNYEVFIDWQNVWVCTDEECLTHDIETTLCKKFVTNVTVFHISALGFTIPCFLAIVFNTTMIFGNAF

VPKTFMLFNLALCTVASAVAVKASVDTVSSPLCYKMTLRSLGFEYGPAVMIFGFSLAFSVLSLVFCTIFLN
TNSDRLSE
>tr|C9ZWY1|C9ZWY1_TRYB9 | DeepTMHMM Topology Prediction - Predicted
Type: TM
MDRSGSQSAASAASELADSRGFVGPISDAHDHGNTDEKNTSKAKDSNGFFSKVSLCIATVLPPGGIAASAF
NMASTTLGGGIIGMPAATNSSGLVMGLFYLMLISSVTVFTMHNLSIAAERTNTHTFEEVTRVLLGRGAAYI
LAAIRAFLGFSACVAFVISLGDIMSSILNGTNAPDFWKEKSGNRVLTVIVWACCMLPLVIPRHVDSLRHVS
TCAVTFMVYFVIVIVVHSCLNGLPENIKSVSVGKSDTAEIILFNTGNKAIEGLGVFMFAFISQVTAYEVYV
DMKDRSVRKFVIAATVANALCFVLYALTAFFGYMDFGRDVTGSILLMYDPVNEPEMMVAMVGILVKLCVSY
ALLAMALRNSLYSIVGVTADKLPFWKHCVTVLVLSGIILLLGLFIPKINTVFGFAGSITGGSLGFIFPALL
VMYSGDFTWQKVGPAYYIATYLLLAGGVFVIVFGTGATIWGAAVG
>tr|C9ZVY6|C9ZVY6_TRYB9 | DeepTMHMM Topology Prediction - Predicted
Type: TM
MDPHPTPLNPDATWDDNGLLFTLLMGVFAFIGLGAVSAFTVYAPSMWLRRVFRLNHTAVVLVEEEPLGGDV
ETLQAPDDKEPRYRRVGCALPYPCSLFVLPSWKCWLSVEEAVEGLENVFKPLVDSESGNPAGIGDNADDRG
SAVGDDDETAPLTRDHISSEYPATVEVDIEGVSSGSPVGNVPSRTNSAPKTPLDPHVAVYLYFLKLFSTVF
ILGSCINLWICVLAGTDDYREKSMVSMDEKHCGAQGSNRTGCMELMPYCHYTDADTCVPVPLHGIYDLTMR
NITPNSWRLWFVALLDMAFCLFFIAAIVYYLRKVDKYVETVMRHQMECAVGHRVAIVGGLKGRVLTEAAFR
RRYLQEDSYFGPNRRGVNTFRYPVAVANALMGGTLSVDDGDTYHQYDCGGLSCIFSSCFFTRYKTTRSNAV
FLQDGSVRRMLFPRDPPPGMYTYMDKTEEAMEGLQEAVADYKVFHKLANHVSYKKRQELHKKLLLVRASFP
FCFSMISKVDYWKKAFIERATKLNRFVDEVPARKPKGIAYVVFDNPLAAYEFVNLFYAQHRGASGTWAAIA
GPPNGIIEMNITTNRQVGWVRRIVVAVIYILMLLFWSVPVGFLSSIDNISKIPGMGWLSRDSSKLPENVRS
AIAALLPVGVLALFNIALPYIVRILAIAMGVINMYEREECALHLQYLFMVLTGVIFQAPLQNGMEELNRLI
TEISRDSIAKLFVTFVTPEGGYWYTKVVMGLCVSTWVAFLNPGTLTLVVLQHKLANVQRVYNELFGPCLFE
WPHLYSFDLSLLAMGLLFHMTVPLLSFFVGIYFVVRYFTQRGMLYDRYRPENHPRQDCTPFGAAGMVIRAA
CWLYCLGAVGGVLFMNELDHLGGLLICSVSFTLSVILLAYTHITTRRWIATLPNARRLLRNRVPRSVQTQY
GTSRSADNVVAPKLPNPKPPARAEEERREADSAVTFQGATLPVVPRKQYSMETLDLAFDGVAGPTHCAPTT
SLLWQSRGMQSSPVGSVCNLQDPDGTEMEGVVEWDAAPDAEDDMLYHRMSLLPHNPNVDSRYNPRHQQLQR
INITHEIERLEETIFHVERYWDAPYSEVNPEECGIIEQR
>tr|C9ZYF2|C9ZYF2_TRYB9 | DeepTMHMM Topology Prediction - Predicted
Type: TM
MKTNQKGTSYNRKDYPFFFFSHSLSRHSPFSCTLEIYIYIYMQIEMKINKKLYKKKRMGHLNNDSNGRDRN
NENADNDYVNADDKPSVLGGRGGETIICTNAKKVVIRKHIYMCVCILYTLCSLCFRPLLLTRITYSHGAIN
KSFFVFFFSF
>tr|D0A5V8|D0A5V8_TRYB9 | DeepTMHMM Topology Prediction - Predicted
Type: TM
MEGLSSTSLLWLYAFTVYHIYLLIFNLPLVTVTVTVRKYFPLWDMSDQMRRLRRNRRQSDRYFHGSSQRGN
GIHERDRGEKSNFTERSRRNGGDDNGLEGSRKRKRAETKQDDDGGEEDTPERLGVIPGITLDDMGGLAREI
PIIKELIELPIRSPHLFSRLGADPPCGVLLHGPPGCGKTKLVHAISGSLQVPLFFVSAPEIVSGISGDSEA
KLRNLFLDAISAAPSIVFIDEVDTIAGRRDQAQRGMESRIVGQLLTCMDQVAQAWRQHNKVVCVMGATNRP
EALDTALRRAGRFDREISLGIPTIDERHSILKIICQKLHLAEDVDFFELANMTPGYVGADLHLLVKEACIL
AIRQKHNELEEKNKLDDPNAEELVSFVVTRDNMKEAVKRVQPSAMREGFTTIPNVTWDDIGALEDVREELI
TSILQPIRSPKLHRRFGLDHPVGVLLYGPPGCGKTLVAKAIANQSGANFISIKGPELLNKFVGESERSVRM
VFARGRASAPCVLFFDELDALAPRRGSDRANPSSERVVNQLLTEMDGVEGRESVYVIGATNRPDMIDPAML
RPGRLDKMLYVPLPSVEQRASILETHARRYPIDASVDLPSIARDERLQGFSGADLAALMREASLHALKNIY
RGATEEMLEQMERDASGEAVANAQLPSVTMEDFEVSMSKVKPSVSAKDRMDYEILHKQLARDAKGPC
>tr|C9ZRQ3|C9ZRQ3_TRYB9 | DeepTMHMM Topology Prediction - Predicted
Type: TM
MVQQRTTSNHNYTHFNNFITHWSRILRILCGTEVRDFAAWNFHHSHILFIFFSLSLVFHIKTRLTPYGFYD
HRKGMCRNQTTEQPTRESACNIFSLPFFFLK
>tr|D0A3D7|D0A3D7_TRYB9 | DeepTMHMM Topology Prediction - Predicted
Type: TM
MGKMCFTRASVTLTGFAGSVMVMKLIIAVRLCSSGEVGGTAHKKENVQGKRGRERAGEREKSHAYPHNAKH
LSFFLLPPFSFSVHERGLSCVPFIRLGRWKGIHTPADI
>tr|D0A1C6|D0A1C6_TRYB9 | DeepTMHMM Topology Prediction - Predicted
Type: TM

MRCGKICSLVYVNFLCNKPLSFDFFLCVTYTSCAFFCRFYRIPHKIGYFTIATFRFHWEKQWFSAMWREKW
DMCCEGISTLWTVGGRLRNVYNSSKSEAERRRDVWSTLLCTATFAMTAVAFAAIDAHGGVPPVVWNVLGGA
KRLLRR
>tr|C9ZU90|C9ZU90_TRYB9 | DeepTMHMM Topology Prediction - Predicted
Type: TM
MSAFVDVVKNAVPTAAAVGSNGSIIEEDETQPICPSLSWKQRLIGCGVCMGIGSLLSLLSFGAILRSDVAT
FAVVYTLGNVASIGGTLFLAGPKTQVQRMFSEGRWVATTVFVIAMGLTLLAALLLSSALLVLLLCLVQFGA
MAWYMMSYIPFARTAVKGCLRGVV
>tr|C9ZYW4|C9ZYW4_TRYB9 | DeepTMHMM Topology Prediction - Predicted
Type: TM
MWLFTRSNDTSAEAGIISSLPNAADAHTVAQKNMKTEGHEDHAVSTSLIDRSKINQLAVTKGDGRVSFFDT
DSIDCIVKHYDKEMTVADQKIKQKQQKKERKMLNLYDYEGDRRYNQWLADQERVRENLKLHWLDFAIDRPM
LCFKYLTRVGTTAGLFYGLGRSVFLYRTMDKMYAKLHGVSFSNIALYEVSLAVIKGTVVSAAGVVGVVVGE
SATNIATTVITGDISAPERTWVNVCVCGTSCGLFSGAAFAALHASTLTSWGMAAAATAMTVTGSIGGFGLG
FYSYKPYAATREKRINDPYWRPWYQRRIADSGGAYMRGRYS
>tr|D0A276|D0A276_TRYB9 | DeepTMHMM Topology Prediction - Predicted
Type: TM
MQLCIPPFCFQCVSVSPVRRRLNWLLQNNYEKETTKTFILWIPFLLFLMSFPESLPTLPPLEKKGKQRYTS
NRSKSLLRIINNSGGEILLFNSIFQSILPHSHHLLLFSLLYYFKRVHMDPEIHKGRATRFQRVCYFFFYCC
YCRCSF
>tr|C9ZXI2|C9ZXI2_TRYB9 | DeepTMHMM Topology Prediction - Predicted
Type: TM
MTCFSASVWAVACVKCLAALFPLCTCCGDMFPTTFLSPAFFFLQITTMIMVATVDAVFPPTALTS
>tr|D0A3X5|D0A3X5_TRYB9 | DeepTMHMM Topology Prediction - Predicted
Type: TM
MEEEFLRVHPEKLSVRERWSKALLRWDGCAHMKKKKLCRVGVPQSQRKTVWPLLLMTYGWQLEKYGDYHLL
MSQQPRDAAMFEIIERDLGRTFPTHRLFNKPGSTGQMGLRSILRAYANLNPETGYVQGMGFLVGTLLIQIG
DEESTFSAFVSIMENPRYSMAKLYAPGFPLLFVRLHQLQKLLGRHCKKLLKRLMEYGIELSTFAANWYLTL
FAYHFNFGLLSRIWDMFLCEGWKIIHRVAIALLLLHKSALDRARDGAELLIALNTAHEGKDEVEVIRKALS
VKFKTADLVRWERKFCQQL
>tr|C9ZP55|C9ZP55_TRYB9 | DeepTMHMM Topology Prediction - Predicted
Type: TM
MALTARGLFFVCALVTTSSTSFFLFACWPVLIVAALRLCTGSAPKRVISLANTYYDFVQQLWIYMMVLLLE
GVLRVRIAYHLVSKGEMGREQKLADFFARPPPGRVKLIILNHRCHLDWLVMFPFLARAGIAKSLRIVLKVG
LSRVPIFGWSMQLFRYLFLTRKWASDRSHVVRMMDYYKNSDGTVVFLFPEGTDLTESSVKKSNAYALRNNL
PQFYQVLNPRSTGMIEMKNMIGAENIDEIVDVTMGYTDFVRGERPNEASLLNGRTPSKIHIVCTRHCFSND
QQDGWEVGDRVKGLRGKGLFSVPADDEALKNWLTDRFAKKELLLSRFYTRNPVGFDEEHIRSVFGQDCDIV
SYDEDEEAARYPEITKFSRIARDLGMWYGVVFVILYWTVPVVLVLLFAGYWVLFWFVLCSILCVSAVRKVG
NLSYYLIP
>tr|D0A7V7|D0A7V7_TRYB9 | DeepTMHMM Topology Prediction - Predicted
Type: TM
MQRTAPSSPLVTTHSNDRPAVGTVNSKKKRSCLNHSDPITFFNILLLLLSFPLFLCFFFDSMRKKKESEEN
RHDNDERKHENKRVDETKKFVTFFPFVERSGC
>tr|C9ZQ95|C9ZQ95_TRYB9 | DeepTMHMM Topology Prediction - Predicted
Type: TM
MREFGMRAFTGHIRSEVASGLEKAPEPLHHPRPEQKEKNTYIFIFVYSYIYIFISYFSLRQHAAIILILFC
LLLLLLLLLLFVCSVFSFFFFFVGVVIIQWCRVFHDVGRRGWMVYV
>tr|C9ZTQ9|C9ZTQ9_TRYB9 | DeepTMHMM Topology Prediction - Predicted
Type: TM
MQPVLVANSEDALSWILIDSRGHGLAKSHLPIRGNVTAITEGFEVITLLFWTIFFFSCLLCLSVPLFYGTL
SGVIHHCNVGFQLLSALTTSNEQHSNQKGGVKKDRFNITTAGNGDNRSGQVHQRSSRAATTPHDHNVKHLA
VIMDGNRRYGQRHSVETVCVKELEDVCEFIFNEDMPLFGVEPIFKRLAFLLKRTKLDGHRVGGEKLLEFVK
DCIDFNISMLTVYAFSTENWSRPALEVKVLMTLFYCYFERMRQTARKQGIFIRFISPAFEMIPSRIRRIMV
DIEEETRRHVPRRIVVNVCVSYSGRDEIINACNGLLQKRNEYSPISTGDLMSQMLRSVTQADHEEEDASVL
FDGGGAEPQILLRTSGEQRLSNFLLFECAYTEFLFVDKTWPEINREDLVKLLQYHDKRDKRYGK
>tr|C9ZM92|C9ZM92_TRYB9 | DeepTMHMM Topology Prediction - Predicted
Type: TM

MIIITPPCHGAAGKTKDERIYKYINNEKYDTEYENSVLKHPIIQIYLIIFNYLYIPFYSARNNSQKKNIGA
KHFTLCYPLFFLSHYVLFLVFSLIYLLI
>tr|C9ZSG2|C9ZSG2_TRYB9 | DeepTMHMM Topology Prediction - Predicted
Type: TM
MATMRRPFGLKKTRNYILCPWYFHSSIFLFFLSPSLGVVNAPDYAKYKTTEGKRITYTLHERKGLSLSIFV
HYFIYPHFSFPSFVSTSSSPPLESEGKGGNG
>tr|C9ZU17|C9ZU17_TRYB9 | DeepTMHMM Topology Prediction - Predicted
Type: TM
MTRFFFFFCLTYIAFFFSFFVSTFHSMHYRACFFFFNFFFLQPFLHIIVCINVFENRVEISMKDAATFVCE
CFKLRNCPTADHSLFAFLLFPSAYNNNNNNNNNNKDCNNY
>tr|C9ZXR5|C9ZXR5_TRYB9 | DeepTMHMM Topology Prediction - Predicted
Type: TM
MMTKTYGSKNPSSNVPLHLAGPPPFAFSDGRAPTRGNMRSFPTPSSAGLSSSVHLPPRSQGSDRRSLVLSE
ISGASDQLLRTLHRELCNLSAHTESGVSRSCRGESTNRDSASPSRANITGHGFDEQAPISQLSSEEVRTAL
SLIIHKQNLDLKSRHQRLRQLAEENKKGDTMGTSSDGGVLSEPCCLVLHRILAEARRRWMRILSSRVQGLK
KELPVREHPQSNDAHTTSGVSNANALDDSISGVDSDEEMEWSVEHLYETLVSGLNSTVTEELIVAEILGGD
TTALHDGIDAFGLLTVFRAPVQAMLVQHQEALAPSLSTLRQYFAALHPSAGCPRAHGSASRAAACSAYSQL
NDFLNPGGSTPKSVAAALRRGASPSMRRLLYARALQLQLVVLEGGSTNNAPGELHGSNCGDVIGRCVSFGT
SRNLESMRRRVHHKYAVKGMEVTVKVLQAVVKVDNMQSVGDSDRYFIFLDETEALGTTLIVDKDVSDVHLK
STLAQLGRPPEQIESYLVYLLHADVPSADAGQQRTQQQHQQTKTQHLLPSGFFPVEKCTLLIAPVCYITGD
TTEQYSLVVALFGQLWCRLQGPTPELAQCCWIFESLVVRFAAPACLHATRALRYPPLRLALRWMMTAFADV
LEPNELLNLWDLVLSYHIEEVFSGHTSLSLPMCACGRSRGSQRLLPAPCALWLLPIVAASIFVYRAPLVER
CGTAEEMLLLFTTGHHLRCRPLLQYLLFMAK
>tr|D0AAF3|D0AAF3_TRYB9 | DeepTMHMM Topology Prediction - Predicted
Type: TM
MMRRCFPLLSAGMVEYHEWHLHEPLLQNYEGWRILDNPKYERKSDAYIYDFTGCVRHLDPIIDDPRLTHKQ
RVCRLYRWALKELQMWLVQLNAHKFNLAYKVVRRRFEMYRYVTDPATCDMMVRQTQKYLRENACFYYLRRN
NASPWSTATLANPMFHPDGAQVYDHWTHPEVLWYDDAKLHRWTGHHPMYGSAGEASDRFGDMDVAPHLRFF
TTVVFGMVFLWAILNFLAFFDRDEDVHFKEWCSAFSDDLKGALYAAERNSRSHTSMLGWDWDRIMGKVQQD
EGFHLYDVDFAAAGSNIARR
>tr|C9ZZC2|C9ZZC2_TRYB9 | DeepTMHMM Topology Prediction - Predicted
Type: TM
MLFPLVCCFRIILLLLYCYGASFSNILFFLFFTHSLHMIYARTGKKKKSQTQIACLYVYTYNTSCSQIDGV
HSRFYTSSFSFSFSFSSLSFSCLRVRVFLQPLLA
>tr|D0A0A7|D0A0A7_TRYB9 | DeepTMHMM Topology Prediction - Predicted
Type: TM
MWIPASTREGSPHEESNKSHFSRFLFSVFSLLFFSFTCKFLNTCSFFFFFCFFVFVFSPLFSIFCFLLLLL
LLFFLVVVVWFVSLCFLYSNKQEGVTILMMMMMIIIIIIRWQWWW
>tr|D0AA25|D0AA25_TRYB9 | DeepTMHMM Topology Prediction - Predicted
Type: TM
MKWLLRLVGRRGKKKTPIPPKPYVDTASSSKHMSDIRMYDPNDLKHVDPEKPHWQDPRFESRESVPFFDVW
GFNRDWSWSLFKLSLLIFVLIFYWEMRSMMYLKDAPPRITSTTTVSPVPDYARDEKATEEELRAAGFAYVG
VKKLDHLGLVAEADAQERKK
>tr|C9ZXK7|C9ZXK7_TRYB9 | DeepTMHMM Topology Prediction - Predicted
Type: TM
MLPCPSFSTTTPRFDMDTYLGRTFYFFSTINPLLCFETSNSLKRHQELLNRVAAGEEGVASDRQLWKARTA
IEICVHPTTKEVIFPPYRMCAFLPVNSFIVPFMMSPTTIASPVLTIFIQWFNQSYNCAVNYANRSSDKQPM
SELSKAYVAAVGVSCAGALGATAMLKKVKGGTLKATAVRAGLPFVAVSAAAIVNLSLMRKNEWIPSGTGLQ
VVDEDGEVRGSSRVAGMQSLMMCSVTRVTWNLISMVLPLLMMRPLLARCAAVRARPVVYETALQIASLGVG
VPLALGAFSTTVSVPANRLEPELRGLKRKDGSPVEIFTYYKGL
>tr|D0A4N4|D0A4N4_TRYB9 | DeepTMHMM Topology Prediction - Predicted
Type: TM
MSKYIYIYIYIYICVYLQLSSPLLLLLLLLLLLLLLLLLLIFISLQMSCVTYECEETRHGGWKNVVRHSKTD
STSRSFPFLPACFISFTETDSFFVCLFELVAR
>tr|C9ZKQ9|C9ZKQ9_TRYB9 | DeepTMHMM Topology Prediction - Predicted
Type: TM
MFSAKRKDVAEALIGFLHAIFTSFYVDVFIGQYVLVGDRDENMGSTHNNGNHIDRTISSRRSDGGAIWYLA
LTQLLYFFLLLKNSVIFRQHASTATFSSFPLRRLSSLPVCGTLLSFCFALMWFPLDLPFLNAATSFTLLTA

AYQALFARCRKFVDALSRSSLRRPEGNIACTLFAAVGVAVACWLHGIPNNPQPMRCFTTFCAVGGAVGFFA
CGRQKSHSNSCIGADITEGDDGDVLQEAVWFVQQTMLRSSMKVALLLWMFQGYNGLIATSFISVFLTACHD
TIPLPLRGLLLFIVFVLPQGTDTVLASFKRVVGKKRLVALLLALLAFIGFLFILVALRDHLFILHMSPTAS
TLSFAVLLVMHRLMIDTLSRVQSWVLRDVVEEDTVIFCRPKPMTANVQLLLDSASQLVQCVALVLTSLHLV
SVSSGKSVKPFKAAWVVGCPTLVMSVVMLLIWVRWYNLEGSHLQFVKMAMRKRADGSDPVV
>tr|D0A6E8|D0A6E8_TRYB9 | DeepTMHMM Topology Prediction - Predicted
Type: TM
MRRVHQIPCKKLFRQLGRRGILSTPPSPVESAEPTTIRSRGYGPSKHLLRGVDATRGVVVEAALSPADVEM
QRRVLSMANPNVAIHAKLEGEPSPVHSGTNSLHGDDQRLADRAGVASSEQLASVATVILDEVKEQLQLNLS
AADASQDSRAATVVNAVAECLRNEVDRLIHANHTQESSITRVLRENTDLICKRVADVICSEQLKTLSFKDV
GQLPQVIKEVGTSLVRLESALKSALDSSFGKDKNLEEVLGDLREHIVSAIEQASRFQQERLLASVSDLLAD
CADAQDSKSRLAQAASLKVIEKNVRDAMSQMVEDTQHKVQSVLRDLRGSASTSDAAGSTMNVSSGNEVAFE
RILASYGERLEELRESTAAAVDLKELMMEIHSMHQTSAGDVSEVKNLVKKLKRELISALQPSNTENTEGST
VERKSSDTIDYNGIFADKLDALADDVISRIDGRLKEQHADVRELSARLLQLEDVIKKTPRAALQVGSVPEL
NDDRLLALAQSISESILSSLPPHSAPAVGGKEQQEPPRPSVSTAVATTAHTFTTTELAEAVSAALTPRLDE
LAAAIQKKVAPTSTASGNEYDNRHMGETQLVTVAQTICEGVREAVRESMALYKPPVASPPTPAPGAVVAPA
VDTSALEQAMVTKVEDLKRCVMDAVQEMDRPQEIDLSPFRTYLDDVLRSMQTTLSKQQQATQDKVDGVVEA
LAKKLQDYQHQLESKLQRQHRDQVEQIQVVRRAQEQDMKADEETRKQRMSRNQEDAEQRTNEQKSAVTGAM
QEVFLQVRQRLKEDVRTLLKGEVAAALIPLDDLYKELRESGQKSRTALEAKLRSVEDTLQSVLDSERHRSD
RLQATLDDVIACIKNNSGEVSGSETEALKSLKRLLEDLAERNTATGMEICSDVKASLERTEELHVQTAEKM
LRGFDTLKYEIHEGRQQLDQLLSSNQQNKDVALAVEAMRTKLDEVRYSLDCIMASVREMQEHPMGQLQEPQ
QQQSLLHETEVKSPIDDKVLEQYEQRQREASDLIASRLETLSATTREVLSQLQRQQPRRSGEEMGSQPAVT
TNLSVESLQAMEGRLGERMQALQAALDELTKVGASVPPTTEVFRKDEVDGVEGNKSFIREVDELMTLVKSS
RSVQAKQMWERLSILRTSLVDVSTKKTGDTNIKQLLGALEENRLKLREEMGEAERELVIKIDAATQKAVEH
FAARIERAVKASADAGLGQLRNDIKESSEILKAHQHQFSSQLQDRMTNIERTNRECAAELKEKLRDTSKEY
HSALGQFVKNDMIAALRSTVDEATTQQMRTFTQQFTWQKEREETNKEAVMRSVQQVSQEVGKLQSMSSEVR
TAVTSSSAGVVQEVRNAQKDLAMLFEKRLAAVLEQQKKQQVVQVLSPVRVGEADSSNGIPSETTDTPSLVT
MVNPQGASHLSWFLLSQSVVTIITIMLCAYFLFAAFLVAFVPIPLTDEFASHMEQAADNDVSEIALKRPLV
SRVADRVIL
>tr|C9ZI17|C9ZI17_TRYB9 | DeepTMHMM Topology Prediction - Predicted
Type: TM
MYTLYPRFDAQPSDPALLGEAAGNELLKEVTDLTSKGHRMDVGEYIPFRRSLRFNTPQSRRPARRRRGLQD
TAPEYESEGSSGFCSDVLATFASQLRCLFPCCGPRRVEDEKLWFGFAMTRGVRNIYLRVVRQHPIVSMIVW
SVLFLCETTMVAIYLWQVSGVAEGATWNSFTEEELGLCFCISSALSILLLSQLIFAYSFTPFTIVVVLVTT
IYQIVLLFMALVFGLTWASRMYVPMFLRCWPMRQYFLFLLDSVAMLSSSSDRLDVVRLAAPSLSLFLTMVF
TTACIFQIQQIFSGTMIGTIDSLYFVMATVSTTGFGDVLPQGNIGRMLTIGIIFVFLAKMPSWIVVVVGMV
KMLRDFPKYGGPPHHFIVYGHVSQEDAVSILDEVFKLYPTRSVCFCNASFPRDVLSLGGHPNYRMRSTFMV
VKVLNKASLHRMRVSEADAVIISPTSDGTAKARDDDVFLSSVTFQRHAPFVQQYLQLRFGTHIKLLEERET
MVDQNMRSIMASALILPGIVPFLVNLVRTASSGGSSPASLWSEEGMDNWKDLYEYSRRATFRTFTVPPYFE
RRPLRDAVYLLKTFNVLVVGVEEGRNRMMILDLDYKLALSDTILVLHESGSDSVDLALRALDPTEGSCGSM
TGCDRSEQWEARMASPAADASAQSGSRGDVPDAEVEELGAVGIDSASLIYPFNPSFEVPTGSNEEVPCLGT
ECDNSPGKTSPVAMQGHKGKANVSLPNSVALPKGTITSVLDMQVERLRRRLLGEDFVVKCFGDVSLSRLPL
GGSLAVLTHLLNWRQAVRSRSEPVESSVERIERQINDILRHVSNEYQCGRSGKAPGENFLFIDHVSSFKQP
LLESMYDHYLNDHASRFELVQMMRCIRGIHSRSRVTLLSWQSLSDSFLRQWDSSFEFPLRHIRGSSTMESH
LNYAIKESGGAANLRGILIYCSQLSHWDFKDVPLVAVENNIRAILDCHATSEKQQQGVGGGSSRGAEVEQQ
VMVELKSFKSCISVTPHHADTEWRQRGEVHFQYSLAFMMGRCFSANMLQTIFIHAHRNRCIMKFLNNVLCL
HRQGSVFDTGGWNSGDKSDTTLFKVCGNQLLHFQTFGDLFVFLLKHRSWVAIGVFRRFPTSEGLPGVPRYF
ITNPPMKMPLRVDDVVYALSGVTGAHNRMGC
>tr|D0A064|D0A064_TRYB9 | DeepTMHMM Topology Prediction - Predicted
Type: TM
MLWGSRKGRRERSIWDLRRWTCSCAARTLLGCVKSAAVLMVLWRHGFAPSTVGPFRTRTMRVWLARVRGAG
AVALGWDNLLLGSGGGRQRKGMDDGPIGVHRLSLCCDPSCNTGMSASCSFPFFCLSACL
>tr|C9ZVZ9|C9ZVZ9_TRYB9 | DeepTMHMM Topology Prediction - Predicted
Type: TM
MARTPQPSIRAYTGSGNRLWAEGSKAATPHSQSRTFHQMWPTPPSFPFRSHSLTDATLPAFLVLFLHSTLF
FSSHLGKFADRKGKAMTHQKEKKGRYRSYV
>tr|D0A2A8|D0A2A8_TRYB9 | DeepTMHMM Topology Prediction - Predicted
Type: TM

MPPFCVCVCCLHIWWYVCISPLCKKMKRSDDVSGFTSFLMYSPTYALTLDVSIVLSCLLAIACRIKLFANK
SACPCAVPSLKKTGNCGTSFVGDYVRHSTVAKDTVSDGRHNSMPEVLQSNDVTMNRSASPSAAKGGCSETH
VSESLTDSKPCGHCSDDALGQLFGMPVNAFAATALINYLSLVSNCLTQYFRPSQVLYATLMSFCDVLLFAM
SDYYGEGTSHCRHDKCVSHSDLCDGWRGLKLRSPSVPHELHDLKGIADHKSQRRGGSDPVYPCGVEASSLH
LETRGNRWWLMILGFMRSNVDDASKPKHADGGVLSKLRVRRYILPILHTISVAALVILRKYQLLFFLGTAA
GYVASCVTLHMVGYGLSALLSVLLFPFIGGGYYGTVRLSTNDERVEMLMGFFSLSGAVHVGGVGVYYVLQQ
TFSGVCVVLYVLMLLEEDDALYSTHVSLMRWYVNPCGQGLLDAQKLFTFLLLLGSMNRHKQRERLLARYRK
NFSSHLL
>tr|C9ZKK8|C9ZKK8_TRYB9 | DeepTMHMM Topology Prediction - Predicted
Type: TM
MITTTNENSDESQQVHTHIYKYINIYIYIYIRENNVAINPPKHKGMTTVKRGEPPQPQSLSAVVIVNRLPS
LHLCFIPSFLFTFFFLFRLSLSSSLFALAP
>tr|D0A3M3|D0A3M3_TRYB9 | DeepTMHMM Topology Prediction - Predicted
Type: TM
MRLTNTKPPPPRNAAFYITKLCTIAVCVYVLKMAKLAEVMLRSRKIIQPVMLLSYASFAIFVALWFYTGLV
VKPKHPNWSETHKGLLHTAAGAWCAGGFLWAVAVWPVFHIWTIPLGLVSLVLFLNLVAITPSFSRKEKE
>tr|C9ZP38|C9ZP38_TRYB9 | DeepTMHMM Topology Prediction - Predicted
Type: TM
MCFPPSRGSAKTSSLCPYWKVLDWFVFGPLMCPLFIVAYIGKKARNLIPQLYLIYIYVDFLLATSQLAFHL
CTILCWFSLLFSLLSLKLILCFLAPFLVCQLNPSL
>tr|C9ZZE6|C9ZZE6_TRYB9 | DeepTMHMM Topology Prediction - Predicted
Type: TM
MIVSFHKMQAFFFKVLFSFYIYSQVTRCSVSGRVEGLSRGECARTHACVNDCICVCSLSTQLLFVVHMHRL
PHLIFEFFFLFVTTSLPFTFTFTLPFLSFSF
>tr|D0A2R2|D0A2R2_TRYB9 | DeepTMHMM Topology Prediction - Predicted
Type: TM
MFVAGSHHIFHFVLFCFVFFFFKKKKSLDFTILPVCLLLLFFFSLLFDSITPEGPGNGAGEGRRGVRVLLE
GGNYENLIYYICAVPVFSFFSFFFFFFFSGRGGRMLPPPRYEVRMRRRGKHGDGDDDDDFFFFNRDFYDNN
DNGGSVGAVMTKRDSSDMKIAVHKCRRSQMFGVCVHIYFVRTCDRVLSSSIFSFNFFSFNFFCFQFTRICE
GQAHVYMYLVK
>tr|C9ZZW1|C9ZZW1_TRYB9 | DeepTMHMM Topology Prediction - Predicted
Type: TM
MLLEKYHYTYWSALVSIVKAEPRVVGLWMLLWVAVVWYGLFLCCVPLVTVPMWRSALSFIIMHLQTVWLCD
VCWLVVVKDLLFSGLVFSFRVCCLPLVLSTFCFSPVSDTSCSSLE
>tr|C9ZQG9|C9ZQG9_TRYB9 | DeepTMHMM Topology Prediction - Predicted
Type: TM
MLRRRITIPAMAYAVPSMRTPQRRFTDKVVLGTCSALSTSTTILAIFHLLVTPVSGTIAAATLSASAVSGA
LVILEGDSSTSGTAFGAVLGVVFGGIGGYYAKSSKEPWRK
>tr|C9ZI22|C9ZI22_TRYB9 | DeepTMHMM Topology Prediction - Predicted
Type: TM
MKGAVPSTWASLASVWKKTVGEFELSTLLMAQAVAMLALYKVTNGFQMPFRSLFFFILVIYVILLVLHLVG
AVPLLFEVYDIIVRNYTSTQEGDSMIHLCRDLHELAETERARVMFVLTLVYVFLQSFCLPGSALINAAIGA
VIGLPLGVPYCVLMGTAGASSCYTISHIIAGRCNGHNNRLVQKLRKQVEERTPSDLFAYFLLLRLTPVVPN
WLLNMASPVAGVPLPTYAAGTLIGIIPQTYISVRFGEAALTTLSGEELMMTPWDMMWIGILCIVVFAGCML
KRRYGKVDCNT
>tr|D0A340|D0A340_TRYB9 | DeepTMHMM Topology Prediction - Predicted
Type: TM
MDSFASYNTRNQVRRRAASVTARSSLSSGADPSLARSRRQMSMSQQPRRLTVRDLWERAPEEDRLLFTQCV
RRVQCRLKESLSSMNFLRELASYYTRTAPLINGFPFCVSLSYATFLFHMQMPHVCIEDIQRYAQLIATAID
IIPDTQRSEHPFVRDVIRGDVFGLSSPSLEGPAHYVVLVPSLQYRAFSLLAVTLVEHGVVPVEIINQWQRK
LRELCRATSSLVSNRALTLILKTAEEVDREEQVEVLMNVIRSSPRRMHVEQIVACYERLKRAAPCISTGPM
YGRSLSIRLTSVFLTLRSPIRKEFVESFLYPSLCSEDVKDTLNQPAVRMHLYRKLLRLCTPGMGTSNPYYL
CICAIIQPFLDDVTEGSLEIVSLVKCLMPHAAYFVATLTLDTRLQAEVLAGIVVSLVGSCARVMKRVLMNS
PVARAVESPATVYSVLFLLREVVRGCSISSSERATLMLQCIGAVANPSVMKTLGKVTKEAFEKFLPSGGDT
CNSLVGGGAFPYIVDPQLLCAEMDMVLHGRHVCEAIDIILQHNWNIGSICALCGETRGQSPLCQASGAAHY
VGTASLGRVLVTITECAGSEVVREKLKSLMCDSSRRLDSAVHLLLFHIFAHAGPHRKELYLEIEPYIRSTL
VEVLNKSRAFGVLDSEERASLLTLHAKLVILLGNAVDRSHVDALLQALSCIQIHSNHDALVLWYLANLLLR
RGGNNVDLLPTAPDENNYCVEYPDCAPTCKEAADNAQLLLKIIHRAHYFSPQMRKLVGCCVCKLIQDFNMQ

ADNIITALLSPFGSVPVSLNSLVEYALPVGANSTFWSFFLRQMKTSAPARTALLASFVKSVTRRFSVASPV
SCMPITGEETTAELFAVMAYETVRRCPPLARVVLHLLTSWVKQTRSTPGRFVSLVYTSLQMVMVIFRRSVG
ADVAEVGAETRQDAHHFGEAVSKAVQEIKMQVGRINDMAREIRDENVVFFRLLVRLLKRTKFVVKETVGES
CDEIADMLGAGDSLDNEEEEGAGVSVEVCDVAQPDGTPYQGGELFSYSLEELQRPSDDNVFDDLTMSHGDF
DAVPAPIPLHLQRRNEQKVEVTLTRRPHANTTFSSQLQDTVTSSADSLSPCSSGGSCGLPPAFDSGTGAAD
ASGRKQGGITPLSKAQERLNESYRQAEGAETILTGRAPTVSKGIQTSPQEGQVKRINPTTSPNGQLSNVAA
STFPEAVLVESNPGRSKRFSEMLKEASTAIGDIGANSTWHEMSVFSEGDEEPIDDTTVPPLQGTNSANCVT
VPSALALQFFATYQTAGDVFDELQRQDERQHYGECAGPPARRQVLATEGERRGDLAVYGFDRGTVSQHNLV
STAAVAPHAAILPVVVEGRANNYDLPHSSRPQVPGRLVVAHVNMVNTVDRKRAQDGGEAEGGACRMTAEKK
NRVEGAGAGNPGQLVTYSPQPEQGQNGGHHDDAKQCYENRGATTTLLPQQETSAALRELEALLGHSGATSV
SPTFGGEVATTTSGGVAMFPQGVVPRFFVEQGTGTTTLELRQAMGARDPNGVSSLTNKERRVRGNGIMGAN
VDGEVGNTWRSDVTAPPVPEYARDPQYSLEIV
>tr|C9ZU89|C9ZU89_TRYB9 | DeepTMHMM Topology Prediction - Predicted
Type: TM
MVGGGVRQQLMIHHGRGSVYFYHHDLIFFSTFILMSLKLHYYIAAIIVIIIIGVVDIGFQADEYRVTYFNS
YASDSPSIITFCQGFPTHTHNLFEVWYYRMETFS
>tr|C9ZVF4|C9ZVF4_TRYB9 | DeepTMHMM Topology Prediction - Predicted
Type: TM
MRSLGLHPPFFLLLARSACFAAAAVVVVVLVRQYPMLCWGVFHFFPLPRFVPANHSACSPENVFVIIIIII
VIVIIVIKIIFPFDEFFFLSLPFAFFLLMFLLLSPQCRLKWPSEGNNSLGALSNSAALFGTTTAFPIPPPS
PPPLLVVQQRVLIFFMHCLFFNLFTCGDPGVWNDFFIPLSVTFSFPFPFFIHLRVGLFSILVIFIYQLIYF
FPFFCCSSHSCLFVYFLGAPTTPFGCYWCLFIYTAWASFFPSKQEAKKKGTKLLVGEREPKP
>tr|C9ZQ07|C9ZQ07_TRYB9 | DeepTMHMM Topology Prediction - Predicted
Type: TM
MKKRVSITYLKRCPYVYFLCLFFSALSSRHIQLNQERILAAKSIISSFSFKCKVIIIIIDSAATRKAVNTQ
RMFFILVVSSTICEIPQVCVDIADLKTYIILSVLFFNIEL
>tr|C9ZN13|C9ZN13_TRYB9 | DeepTMHMM Topology Prediction - Predicted
Type: TM
MRVNRTQPTCLSLLFQVLLYCTCSWCVFWFVTTLSLLIFKGATLYFPPTALFMEIISVFLLLVLGISTLAL
GKRGNLLEEVGSTSLTVFLLLVGIGGAVYYMWLQTYVMMLDFIVSLVMLVLDTLTALCGACTAFGLFRSRR
SKWNGILLVGKAPPIAVVVDIKHGKGD
>tr|C9ZVN2|C9ZVN2_TRYB9 | DeepTMHMM Topology Prediction - Predicted
Type: TM
MTFVDLFDMTVAYVQYLYIMVSLSPNSFPEIILQPLSKGQLALLHISYDYLTGDSQKREQMFTLPNWIPHD
SRLNYTFIVVIIPMVISLGGFLIVSSTGASLCLFAHVVAVFFTVLGAVLVSTPRAEKMEIMTRETQRILVL
AGGVGLIVLFVAGIIYLFMLRRSQKKKRRLEMAADRGAEHREEVSAIQKAVRRVTQNKRATSALLAYEAKK
EVYVQARRWRSILIQLLVCGIAIAAGYFIYVTLPTEDSAEALKIGRYDKAVAITFFVASSVIVIWALLSIS
QKGRQIQVKLTDKLSKSLTSTALIALSIAFTPVTLNMMRLLVCTEVSCNEGEMMIGMKSAVGGSRNFSNSW
DTTEKPCLACNFTEHTQKCSKSLQIKLCGGNIREKRLAYDPRVSCREIDNFYKMSMSLVFIAYFVAYPAFL
ILSIDRATSILVDEYPLEKRICDEFNKKELYYEKTLMSRNVSSSLYVAFKYQFRRSRILFLLQRILLVLIG
VLMRRGPGSVVAWLGISLITCICLVYLTYLMMARPYARPVENWYSISHQLVLTFIGSLGVVGSMSTRELVP
NAVTITAVALMIVAPMFALIVGSVLTFRNDRQRVERLQSRLKRDFETVADTAENQSPDNVVVGESLDNPNC
KGQATACTDPDLPPRGDGTTVGSAQSPGDMANNPQPGAEVNDSSNGRETGLPAQNPQVLASLPAAGGGVKG
DNTRQRSGNRADREGAPASGPSDGADIGTAVVFADEHNAPEETDKKEKAMKDDYFGDKRSFEPQEYFRGYK
EVPLSHDCGEGEGEGEGGGMDDELYEQTAVKRGELKAVLSSLIREGDDYLPDDGYPPGVAVPSNALDGWRH
ETQHGEEYDSAKKEDDATKGRPSCFSVMLRWMRQCGKAFHVHSNTLRYGEVLQSRRTLKMTAGIAERPFWI
TPSESQFLAAKAEGCDVPAIRQSAGGDEGPSGLYEPSQFFAKLVEHKKKCWNPLVGPSSRAVTAAKTAKED
SGKAKGPNSPSSALAVVASNLPVAKEGNVNPYGQTITSDVSDRISAMLSKEQLCAALAELKAADLDRLLPR
DLKDTTVTERRPSICPEFAPHVALEGESEGNWEAFVQRLLEDIPDPLEKYRETGSRESAEAEGSPKDTTTG
RRQSVTLDTALKMAGSPSAKRKSRMDVSSARALVRGAANMNAENMPKLRRHYLLRQQLVDEYNSEAGKLES
LQMAVDHRIAMSIRRYMQIFFVALCLFSTLSLAMCIGGMMYKGDNISLTNAQSAYSVHNQLFGYKSWEEFT
RNCCCSSLSSPAALPPNNVMALERWVCRNRLVKERVRRDVLKGEAVDGFKIRELCGMDFKNGCKLKLHPKG
RVEIENCTTPVTEQEILRW
>tr|C9ZQ51|C9ZQ51_TRYB9 | DeepTMHMM Topology Prediction - Predicted
Type: TM
MSWQEGGGRGCVYPHGNCRRNLTARSPARRYSMYKHSPVITAMSLLHLLPLLLMWMPPVCAENGNVTVNVL
SMMYSLGFTTPEVNAINAGFDASLSAHSWKTGSGATISVIRPSSPNATIEDIFQLGVKQSEGKLLVVFGPL
GTNHVLKNSDELKKHDLVAIAPVAYSSEVRGWNPHLYFISVEPNAELLTLIRYAVVYLRVPRIGMMYEKDN

TASMGAYEFTVRVLAMLGRHLCGVFVVKDSENQNISEDDLNTRWRQFVATRPQAILLFSSLGNTAKWFIKK
VAQDNRTANAYLLSTSLQQHFLIKMWREALVLANRTFTPGQLITTGTVPLANDNQSSLIQHFQRDMNNYLD
TNSDWKGFAKPDHYLEDDGLGEMMVYGWLAGEVLFEALNNAPQLTNRTSFRESLYKQRRYVIDDLVVGDFG
GECNEAVALQGAMCECNQGGSMVYMKSIMDGFRLRPLWEGFLTWGVSECSSANVQVSAPLSGLFVILVDNA
IVFRATMRWFLGAQALDEAYDVDNRIFFHPLTVSSENVTQSLEQVRDNRDVSAVFGIVPAAMLDTPNMMFI
SPMVVGIRQNGFRRNVIHLLPVLAQQLYVLAVYLSNTSSRGVNAFIRGEQAGEISSLLYKSLVTFGVPLDS
SKTLGDGDPISSYLSGNRDVFTIGLTLTDVAAVARHLQTHRRARVFVGFNDLAMYYDEFVAAFNASKESIA
SSERLLFATSFPHWAEKDTKSDVVASFHRIVNESHWDPLTFIGFVAARLLQVILPNMKKVNAELLADRIYT
ESNIKVDDMRFGPFSDVECVSGTSVSANECASNFGSTNISVWSMARVLNSSLPRTQVGMTPSMDYVIPQEG
QLTRSQIAGIAIGCVVGFILFIALGVLLRISLRNARDNNLAPKEPTDPVTLIFTDIESSTALWAAHPELMP
DAVAAHHRMVRSLIGRYDCYEVKTVGDSFMIASKSPFAAVQLAQELQLCFLQHDWGTNAVDNSYRHFEEQF
TEGECEYTPPTARLDPEVYSRLWNGLRVRVGIHTGLCDIRHDEVTKGYDYYGRTPNMAARTESVANGGQVL
MTGSTYMSLSAEDRKQIDVTALGDVALRGVSDPVKMYQLNAVPGRNFAALRLDREYFFDEGEDGTTTSTSD
HSSSRAELSESAQIIATALQSLLSTFKTAQREKLLLPYCERWRVPLPRKAASEWDDAYCEEVVRRIAVKVG
RVADHCAHSGSESSSTQGSSSIIIVPLHDLYCRENYSI
>tr|C9ZJK7|C9ZJK7_TRYB9 | DeepTMHMM Topology Prediction - Predicted
Type: TM
MATRSLSSSVVSLIMSSSHISMGQACFIGVSSTFLCKPNKNILASAFSAASIDKRVPVYLTALLLSSYKLT
MKMGTFSLLGSTLCFAANSQNLLRIDRRCSQVDCVLMTLAMQFDYEAHAAITGSWVASFITITSFTGVFVG
RKTGDAVSLAGSLPFVSSVSSSTIRSLPGKFSVAFLHSLLPTCGSLAP
>tr|C9ZUV2|C9ZUV2_TRYB9 | DeepTMHMM Topology Prediction - Predicted
Type: TM
MVPVCGSCVAKGTSFFLFFSSFSLFPATTTAGGEAKNNLISKVQGGTLLFAFALVFFLSFLFQLLLLLIFF
SVLLKLFVLCHFALICQTEHLFFFPFLCFFVLFMCSSLFFSCFCLSIKYS
>tr|C9ZJL1|C9ZJL1_TRYB9 | DeepTMHMM Topology Prediction - Predicted
Type: TM
MKRVAQSALIARSCGTFLCSRRCSLLEHRTKGSMFMKHLDLYARRDPQLAPYLLREVDIEYKRRCRKVSFC
AWICVFTAVTAVQIRMQSEQLHYMRLYADCVEAEQRAKDEDGIRRRKALVALMNVVKDAFGRNQSWGNEDQ
EKALKEL
>tr|C9ZMC3|C9ZMC3_TRYB9 | DeepTMHMM Topology Prediction - Predicted
Type: TM
MPISILYVMLALLVFVNICVLIALVVVLVRRSAHRMQIRERVANGLDGTAGQTAVVTLAQHYPSLIVGDYR
HQGEAVIFNGGSGVGGITDGNAIPNAADERMARQQIHPLTTDDVSLLHQLAVEGMVVSPTNQMTNSALTET
RHFPSWGLSNRIAVPLPALLLAADEPNAIPFLEGEQQWDEEESSSPAGWTTGTDPQHAGGALSSCFPPTAR
GGSRDESHIVYGRSCYFVGPHWDGPRGEVPLECVLRDGCAGSSSESSSDISSVATEPARCIVANRCAPPTM
LSSFPLSLYPFSFSFPSHMHLSVSSSNSLFASDEEEGEEYAAG
>tr|D0AA54|D0AA54_TRYB9 | DeepTMHMM Topology Prediction - Predicted
Type: TM
MDREKLAWAGLSIVSVVFFLYIQNSSPRRSRGLHRPRGTRASPPRPVPNKYRSGSTPGTAACRSEGSRSVV
SSRPERTNGLTATSNNTFSSKHEPSAHPQESRTNGENTPARHPHWLTRISGSGLNSAARSRRPSTATAPYM
NETPQRRERGTQTTDDAFRVVNENDALVQMVSTDALRCISPIPRQGKETPSPLSQDVDLCLGYSLLGGEKL
PSFLLSPALDGGVARELMELIEAVQTPEVDPKEWLELYNFLTELPRSVLCHILKTRKIPELAPPLLTARSP
QDKRLTHLSRLQQEVFCLLRGERGRYSKIGRFAMDEREAIIIWCPAEGKTTKYENQFHQFTILSSDDDDSE
DKDNADDLNGSPQCVRVAEAVEKILLRRRRAKMPSSATQFPESSSVSGGETMWYTNSSFEEAEVATLLKQL
MKLRHHEAFSVLQRNGTDYSFILNLLSLSTNAQSMLERSILKKKQRARAKVGAPSSSTSSPSITPSGKSGV
KPHEQVGTRSNGMSKHTDVRQYEEPNLNEELSILLDKTPSSSFNDGKEGFMVSKKLFGSERNGERTPRRKG
VHEHYARVSTTSTVPSPLDNIVDHKILPYGRMNTHAHVEMNGDNSISSGEGSNFRQKKSVYDRLYTPPKKT
VKPAATTSSWAKAPFNVYV
>tr|C9ZSS2|C9ZSS2_TRYB9 | DeepTMHMM Topology Prediction - Predicted
Type: TM
MGAGGGGSVSFLQLYLMFLFCFVLFPLLFFCSHHVLAPYVLPCYFVQVSVPVKMCTEVKGVYLVSSIACPP
SPLLSLYFSSSICFFFKLPHPTPHPPSPKPLLLFHFISFPSFWFYI
>tr|D0A4Q7|D0A4Q7_TRYB9 | DeepTMHMM Topology Prediction - Predicted
Type: TM
MWFECTAYYWPPPSVCLAPYFFSLCYFLSLSPLVIPFARVLISKDQASTVVLFVLTTSLRFRHSVRIVSFL
FCAKKLLLPLRRCSRGATEQPIYAIDTEGDEPKDTKYPVGKKPTIRCV
>tr|C9ZI03|C9ZI03_TRYB9 | DeepTMHMM Topology Prediction - Predicted
Type: TM

MDSRVFVCVECITVICALRCIDTAPILSYSILLYHYYYFCMCVFFPLFIYILFIFILPLLSINIHTQGTNI
TKRENCLHINQKQIPKYRIRKKTRDRKGDH
>tr|C9ZN89|C9ZN89_TRYB9 | DeepTMHMM Topology Prediction - Predicted
Type: TM
MCPTESFHPTVVNNSSNKLHRACEAFLFVFDEKEEGKKENNSPISKIFRMSQQINIDTSPHIIINAMPLLA
LLSRRCACRFIYLNIYIYIYICKYKEITTRNHNGAEARQHKKT
>tr|C9ZMB9|C9ZMB9_TRYB9 | DeepTMHMM Topology Prediction - Predicted
Type: TM
MTFEVAAQAPNKCATNEATAEPLRTPNVLADNLLARSDLSEGESPEKSGEAEKPEEQGNALMRCFHFILPR
GGALSGIFNLASVTLGAGIMSIPSAFNTSGMIMAIIYLVLVTVFTVFSIFLIVSAAEKTGYRSFESMARNL
LGPRADIAVGFLLWLLCFGGASGYVVAIGDVLQGLLSHEKVPAYLQSKGGRRLLTSAIWFVFIFPLTLPKR
VNSLRYASAIGVSFILFFAICVVEHSAEKMVADGGIEQELVMFRSGNDAVAGLSLFIFAYLCHVNSFSIFF
EMKKRSVTRMTRDAAVSCSICCCVYLLTGFFGYAEFGPTVEGSVLKLYDPYANPVFFVCFIGIIVKLCAGF
SLNMLACRTALFQVLRWDLDTMSYVRHSIVSVSFAVGSLVLGLFVPDINVIFGLVGAFCGGFIGFIFPALF
IMYAGGWTRQSVGWVQYILTYVLLILGVVAIVFGTSASVYYTIKKYS
>tr|C9ZWR0|C9ZWR0_TRYB9 | DeepTMHMM Topology Prediction - Predicted
Type: TM
MTSINAQPPNSATYPQDDHGSAEVVNPNPEAPLSQNELKNSGGFFARVSLFMATIIPPGGIAASAFNIAAS
SVGAGIIGLPSAANSSGLVMAMIYLIIITAMSVFTMHNLAVVADKTNVYTYEGVARVLLGRWGEYYVAVVR
AFHGFSACVAYVISVGDILSATLKGTNAPDFLKQKSGNRLLTIGMWLCFMLPLVIPRHIDSLRYVSTIAVS
FMVYLVIAIVVHSCMNGLPENIKNVSVGKKDDNAEIILFNSGNRAIEGLGVIMFAYVCQVVALEVYENMTNR
SVGRFVIASAIALGICFTLYVMTAFFGYMDFGRAVTGSVLLMYDLVNEPAIMVGFVGVLVKLCVSYAILAM
ACRNALYDVVGWDADKVAFWKHCIAVVTLSVVMLLCGLFIPKITTVLGFAGSISGGSLGFILPSLLVMYSG
GFTWQKVGPFYYLTTYAVLLTGVIAIVFGTGATIWGTATG
>tr|C9ZSV1|C9ZSV1_TRYB9 | DeepTMHMM Topology Prediction - Predicted
Type: TM
MRGIITIRMTNNKDNKNGNNDYDCGCRDNDDNNNKLRRSHAAFRIVNTDKRLIALLFFFFFSPPSFFFLKL
SPIHSSSPLHFKHTLNIYTGVCTRICIYTYVYAYICTYM
>tr|D0A765|D0A765_TRYB9 | DeepTMHMM Topology Prediction - Predicted
Type: TM
MTEVFRVQLVSLYRSPEVVGCTAEPVLHTELVIQGQGTTHEGHQENERGIIMGSVGLLAKHHYVAIDVVSH
FEEGQPLRIEDSLKSIRARPPPYIHVEGGGRLFLLHPIEHNRYLLSVTVPKTVWRWFGPTALHVLLSTAVR
GYEIGTDVAAVASVPTYYPLHNRISENATAVQDALRTRMQQVVEFTAHLTECISQGTVSSPTISQVSAQVV
ASVNKMCSCSPSALRHSNQFRVMMQKLVWSATNFSEYTSMNGAIASGGLCDGYKRLVVTASAVWHRGEPVF
SCGSCEDFQMYLLPAALTTYMCRCLGEGAQQTITIKCFAMHARTGADCLRVPGYLKQMSQPHHVGATVCLS
FASAGGWCIALQMEVALNDFTGAPMSHIVSGVIKLISTTLEAPQFTKLMANKSAETLHCAPSASLFTAAAS
GVMESVRQIGIYQHYCSNGAYAINILQNTCIYHYCKGDTSHPGETTECRNPWPESLDAAALELVKLTSFSL
RYGSGRASRNGSALCAAPRSSSPFTILRTNGAVVAMLVVPLLFPDAAVALLVLELAPSGSTAPAIRAFASW
LAAKVM
>tr|C9ZZ31|C9ZZ31_TRYB9 | DeepTMHMM Topology Prediction - Predicted
Type: TM
MVLVFQISFHIDKELNCVEMFLLLLLLREFKHFPHITGLHSVIVFINESINDFPVVSYMKLLGITLILAYL
FIYSFVSDFHFSCVMRNRSLFSLFHIYMLISIYIYKCVHLFLKCPIFTVCFS
>tr|D0AAG0|D0AAG0_TRYB9 | DeepTMHMM Topology Prediction - Predicted
Type: TM
MLPFFTLNSITTLSTFHTQMFSTTEKCCTINFPNVLSTGTVPMPLKPVCILTLYYPCYLPSAVLLFLVISH
WISVLAVMKFLKPGKVVIMTSGRYAGKKAVIVQNTDTRNKERPYGHSLLAGIKKYPRKVVRGMSKRSITRR
SQVGVFLRVVNHKHFLPTRYNMDLSRELRGRINVSDASKKAKSKQLVKRLFQARYNAGNNRWFFQRLRF
>tr|C9ZSE9|C9ZSE9_TRYB9 | DeepTMHMM Topology Prediction - Predicted
Type: TM
MKEILKKQKRKRLKLREREHERRSTDPYRQNSTHKQSCSLFLKKKEMMSLLLPLLYFPFEFCSFSPSLLLF
TLFFSFQGAAGRGGGRGIHLQISTFTLNAYKTECEAIKKKRREMEGNDVYAYVNFLRPFLFLRMS
>tr|C9ZS28|C9ZS28_TRYB9 | DeepTMHMM Topology Prediction - Predicted
Type: TM
MNNANDTLDYSGNTSSTYSAAVNEEFFTPTRRTHVSVSTSHVSSSEADFVTGSMVDDEEYYNEYTSGYESR
VYTHASFTYDEFSSGSPNSDDLSSTFMSYYIDNVIGQKTRYFFSVLRKPIVFFSLILPGSNFVGSLGRLVY
AVTVSASRRTAVNYSMGVETLCALPKGYGHMPEHLQRARLALPSFVEYFAKMLLRLSGVPSLADLQKRKWL
ETVRILKSATGEIISQSAHRSSGPMIPLAVFLPVFSLSVITIARGVISRQQTYNEYEEDEDVAYEAAHSGS

YKGVAASRGLDALQLSGLDDSFPNLGSEKSNEVNYSGVETPKGSPPAPELFPSPKPQGERHAPSETQTNPQ
TVPTSRDLSPDSSAVGDEFKLIMSPTSEDPGRAPSAASREAVRHPKQYDLRGDVVIALTGEPSPQEVDVLL
MAAAKYKCRRVILPSLVADTALASVHPLVDVVKVSDVMEHVMSVEESEGRTTVGVFLRPVTDETEELRLFT
HPTSVVYIFVPAAFLEANVIAHLVDRSVYYSASLDEPFPINVCLYDRLTKERQEGA
>tr|D0AA51|D0AA51_TRYB9 | DeepTMHMM Topology Prediction - Predicted
Type: TM
MKGAMPPRQPPVRPCVVPRASIARAFAAFAVSIVASYLVGTKMGPITVIITLALFLFGSFNLLRHALNLVV
VNANEANYNNMLRRADLMQHPSNQKFSDCGENAWVSFGFSCMQGWRRAMEDDHVTLLTCDGGFFGVFDGHS
GANVAKFCGGNIFGFISQTEAYKNGNYSRAIYDGFMTIDKHIYSNFKDEKSGCTAVVLFVKGDNLYCGNAG
DSRSVLCSDGEPVPLSTDHKPFLPTEQTRIERAGGYVWNRRVNGALALSRAIGDFSFKSNTLVPWDQQAVT
SAPEVHRTLLDRTRDEFAVVACDGIWDVLSNEQVVRFVRLRIQRQVPLDKIAEELLDHCLSPHPFGVGCDN
MSVVIVKFKQSPPVSPEEEFNVPAADVAESPEQLPLLSPASDGGGRDFREENRRVGSNVTETNSLLVAEDG
EES
>tr|D0A5S5|D0A5S5_TRYB9 | DeepTMHMM Topology Prediction - Predicted
Type: TM
MWRPYTLFVVPMRFGSPLQLRSLLVSRMPGRFDAFLTRRFVSTATPGGQPTCSDSHNSAACLTAEEFSNIV
PMFLLGQALRQHTSNEENRAGAGEFVITREVFNRYCSESRVEDTDKALQLLCESGVVVSISDGAAVHLRPV
QFLQMHNDAADASDAKVKTPFEDYIRAAQNRLADAEAEEMAMRVALQPAIDNAAKRRRLLWSGALCLMAVQ
LAVISRLTFVDLDWDIMEPVSYFLGSGTSILFLIYLLRNGRVKSYKEYEEKAAASRVRLHAPADFEWRKYD
AVVRRVEVEKNMVEQMRKWFKHH
>tr|C9ZSB6|C9ZSB6_TRYB9 | DeepTMHMM Topology Prediction - Predicted
Type: TM
MIQNNGNMGNTQNVFNERFNRAQGEVQEQGGRQMQNPSVTGKRKSREEELYESLYYAKWSYVMSGYNQEPL
GMKVFFGRPQHIWTEEEVDITPEHCEVDAELEERPTGLEIFVLTSKMGWPSATFQLGINDESGIMDNNVYI
RREMMRVWYIIQQKLNEWWVEKTESTPPTHIVIGIPGTGKSCGVGSFLLHSLLHFHEGMLDVVVYFTDVDA
YLIYNKKGNEEGRVVLYKRKDVTNVIKEMRLKKRGHIIFDTDGPDETPPYETQYLGWGITTLNTPDIYYYE
DWEKDYLSWNVILNCDYVRDIKAFVAWKKLSLFPNYTTLDENVRAKVKEELEDEWRLVERRVDVVGPLPRY
VFSEQSYTNRLQDISDYLRSLENEKKEEYETILEKYFTWKSEKVVHSLVLIVREGSGYGNLESYHCRPLSV
AIGNMILCTLFSTVATLMVEKHSESVYGKVGAYSLETRALVSLLFPGVFCVVTKHLNYLRRLGETEDKRSI
LKDMTPQQFQIFEQKFLPEAGQNAIGNCKYKVLYRSLEESELFVDGFFFVEDCSRRVADMRDGFPQLGVAS
KTIVLIQITDNYRKEASVSELQEFMTNIARYFSDWDTFSRNMAWEMIYVNAIYGGVIKTRQRCVNNNTADA
EQQT
>tr|C9ZIT8|C9ZIT8_TRYB9 | DeepTMHMM Topology Prediction - Predicted
Type: TM
MSDDSATGNLGSPTRLGGSSSPPAFSVSCDNGVSDTRLASSGRCHIDPSRSAAIERFLLGAQTTTAPPLGG
GLATSVASTSAPGGDYIRGPQSAVDNVSFVSVKHPESADAPLSDPKPVASPKNTFTVAKSSGTVVGRSSRL
GEKSLEPSESAEAKIKSPKEIPPEAEPRGRTPPSRGRVKLVLDTPVQLRSPVSERSPTPPVEVRSALAQPR
TATLTKCSSVGRPAAVFYGSPDGTRKCGIKRTKSLGDLSQALLRGDRAADVSEWLAECSSHRRADRSAASV
AGSGVYQRGSGFSEFVSLGPVTARSDRGGKSGGRTQLRAVDRVRRGEDLSRADAHFDPSPGLGRGGRHEWQ
RGGFARGRFAEDRAYGHVGPGSPGLQSHASAPQLPYFGGMRGAERDMDTSIGGRSLGAPSVHGAMSDQFVG
QHIRVRATSVTRAARPESPTTAFTPGTQGSGFASPGVGGKGSQPSPSHVAASNLGQAHPPVGQATHAERMF
MSLIADRSRERRQHFQRTPSTFSLPVNCSTDVSPVRDLSSPGSFQSPSVCHGASGGGVGGGGKGGDGSGGG
GGKGGGGGGDRGGNKKGGRAGKVASSAGDEEENEERQYKERSCTALHSIPWDQRAFLPVGGFGMKRFSIFS
APLFWEKLDWTVRASLFTVLPLMILTLEPKTEHIFPMPSSVAFLAFWISMPTFGSGLREFIIALKGYALSL
LLLLFVVLVDPQSTWLILLLLFIFVLSTAFFAEELKKTCAYCLTVFLMERQAKPAETGIEFVKDYFITLLI
ALAFGLAAFFIPSIRWSSDLAKAKVSFLGNSLSIFVQGTCSSFWTRSPLERELHILRLRQLQFTAKKAADK
AREFLEEADYEPHTGHQMEGIKQRLDFFTNMHGILSSMVQVVELVNDDPGRIETPVCISFGEAIEEDLAII
SSAMDSTILKISDFKNGVNEEDIHLFCEARERFQDRLAEVRQQVILNNEMYETGESDVLLGFFMFSVDELC
EVISSFNPSIEAKSRFLSLSREFLSSFKSPYEALRRLVMTIIHRRTITRRAKEAIKLALCMTLPSIFQVYA
LDNNATSPVAGAAIIAFVYSSTGAQSFTYAVNRVLGTVLGSLCALIAVQVADGRRLVLYVAVAIISFLGAY
VQTSKEYYAAGNAICNSVISVITQYKNSEAAMVRIQQNLFAIIIYFCITMVLWPMRARNKVYMSFDISLRC
FREATCRLLRNLDMPEDVNEVDATTTEALAQWNKKISRQAFFLPGAATEPTLVGASFPEPAWSRLIDVQWK
LWAIVSMMRFAYVTFMASKVDDETELSVHWVVLRRISPYAKDMCDLLYATVDLCLLILNKTAIVPSAHLTR
LRHGMLDAEHSIVETYIQTLAHKIAGDGGSANNSCSDMGSGPGGGSDGVSSHLDMHSPRGVSHPDSGVAQR
RRKPKVTDGYFVYNVTEEEEELMRTFRNRTCNARQLAQTCDNDSHGGRSDNGSDGDGGRKPGHRKGSDSSR
DVRTPDKDDGNDSGNKSFEKAEDIEITHEHGEKGGVRRALRQLLRRRTTGRPSDAETDRRKEASSNAPAVA
ACHGQTDEAADVHNSDDDVEPLVKGPRRDRKKEGGERLYSESEVTLVHSQSDNSQVETREYTMEKSSKAGK

QLEKQSKNETLRPSAATAASPAPAVDICTRSEDGSSSHSVGGDGVFPACSFFDAKEKELVLSNRDIHSLEA
FLFGVRALTVQLGDLQKALLEMVHSNELEKTM
>tr|C9ZYF3|C9ZYF3_TRYB9 | DeepTMHMM Topology Prediction - Predicted
Type: TM
MYLSGGRIPLCKAFACKENQCGAMPFHFSLYIIYIYIYILLLPLRLLLLLSLLLLLFFLPNPFSYLLTYQT
IYILIPHFSCDSPRSPFVLLCCKLLTQVPVSFVGLK
>tr|C9ZX71|C9ZX71_TRYB9 | DeepTMHMM Topology Prediction - Predicted
Type: TM
MFFSFFFFPPSFLPSSNNFVQLLRLRVWTIFVFVFFFDPFRFLLVTSGLLALNRKKKKGGWASEKVGRRGI
CHFSFFFFTFSFWLPLLLFLLFFHPCYGEAIAQNCLGSVVFSFKKKML
>tr|C9ZQE8|C9ZQE8_TRYB9 | DeepTMHMM Topology Prediction - Predicted
Type: TM
MLRRRKERERNALLHAATLKPESAVQQLNQCGSQRPIGLSHFRVEHATLIHPMKYLLLSVVHFQVTLFFSF
PLLCLFSPHLVCCLLFMPKIFKVTEGYTVTAGGTMSKLTPRSLLTPFGVAGFMFGSALMLLLDLIIVARGE
PNAQYRVSLVLLLPALISVVGFVLLTRASPTDIRNNVNSAKGVVLLGWILCVTSATSALVVCHKSFSGLQG
RTRAAPGVALVIFTNLVSLAGCTFLWAKQFAVTGL
>tr|C9ZI29|C9ZI29_TRYB9 | DeepTMHMM Topology Prediction - Predicted
Type: TM
MLWTACPHWLDCSFFPPFFLLLRYVFFFSFSTPFLCRLHFARLH
>tr|C9ZP09|C9ZP09_TRYB9 | DeepTMHMM Topology Prediction - Predicted
Type: TM
MKGFVYTHALFKANETITIVITTTTVIVITTTIIIIVLVMMKITIKIILVVIMMVMAIINFFFKKSKSKAH
LCVFFFFFSSSVSSSKMPNDQVMLSKTKIQTKAKKNYLIETLQLFSFFLSILSFFFFFFFASRCRTSCKGC
FLFFFSSSSSSNPLITCCFVSFSFFLCVCVFVLLFLPSSLPLHFFPLFLLCYLNYF
>tr|C9ZXW5|C9ZXW5_TRYB9 | DeepTMHMM Topology Prediction - Predicted
Type: TM
MHTLVSYVRSHFLPMSSTDNDIVEEPRSFRKELENSNVRGVLLFVFIDGAASSIWSSQPYQVMVSRLAGDT
AVGWVSAAAGVAQIVGALIAGGVRNVPRQVICRLSAFCGLVAVIMSVYGITTQDIMIYYFASALWGMYGGM
AFTGTEALFADSVESGKRGFVYNLKWINETTSSCVGSLILLIMTLYLGNDWDTNVLKILMYTGLAVHPTAF
FALLGMKDKNILHVDDGCEDCEQFASEASQRRLTDGDSSTKPLLGGTKDIIEEDGWDEESEEEERNCCDYL
CTGVTSSWQWLFTVSALPFLLALGNLLISVGSGVTMQYISLYFIKERHVTPIMFFITNIAVNICIALSSTM
VRYLSEHHLDRVSATILVRTLSAFFMLIMGMAELPLVALLPIYVCRNALMNSTAGVTRSIIMDCARRENRA
MWAAFECFVSFMWSASSVMGGYIASAKGYKYTFVITALIHFIAMFVLVPAISGVRQLDRHAKAKSRT
>tr|C9ZYG8|C9ZYG8_TRYB9 | DeepTMHMM Topology Prediction - Predicted
Type: TM
MAEEEMFCVCVCVCVCVTNMKKRGKSNKFDLALSFLRLFFFLFFSFFLSLFNTHFFLFYFVSVHTPHHISS
ATRFFFFCCCYCLVFLSFHSPQFCSSFAFQPPLPSCFLFSFAIFFLVFFLCFIHMCWCVQSCSASGSFPLI
ILLFPSFFFSPSGISCKNAPVIPSFTYEFLPFSFLSFYTSFVFFFRLLAYCMSAGGSFSCFSSFLFLFSFN
FFFRYYYYYYCCYCYCYCYYYYYLLLLLILLFLCLSHAIRLSNGRDSAECGVKR
>tr|D0A4W0|D0A4W0_TRYB9 | DeepTMHMM Topology Prediction - Predicted
Type: TM
MKSGNNNSNGNSTNFKKEKKKKINTCIGVDMAVHLNTIIQENGGNPPHTKKKKPLLSVSLPPKKKKKEKNI
RFKYRYDFFLNYYSKFFSFSLFFSFFDPLLTKGKENV
>tr|C9ZTT7|C9ZTT7_TRYB9 | DeepTMHMM Topology Prediction - Predicted
Type: TM
MREEGKKKKDVTHKRRDLRIYIYILICTFALTDWWIGMKAKGTNEYLTSSAFCIFRKHTYHGIDVFLFHYI
FLHSWLYNVPPPLPKKPHILKRNCALSLSLSLTHTPPSLLLPSFLSFFFSLSFLLSFPLPLPLIPPSPSPP
PSTIPLGMKQNT
>tr|C9ZZ59|C9ZZ59_TRYB9 | DeepTMHMM Topology Prediction - Predicted
Type: TM
MMIYPRMRVEVSYSATLMVLWALLLTFMSWRVNQTSEGMAVLLLTIMSVATPDGESHLFLPLLFLAERLLG
EVMSCRMLPHEFHASTILSFMTTSYSHTLWKRDSRPRIFMCGIRDVIVWLLLIFIAFKDCGVTVGGGVFVV
FGFVFYVTLKRILGESEAILVGGLVGFYAFDAVSGWGFSSVGAFNADAPNKEGLHTVSVMTKAHIVSRTAI
MCAVVMASVLYFASRFCLVAKFDNSKSSEQVMNVPRITFAFWMSLTIIVASAYAVGSMEIGEDMALWLVRY
ITASRFRVLTIAYWSIAVPSMVVVVDVFTKDLSKVVRRKLFHFLAVAAFTPAAIVDPQFLSLSLSVATSLS
ILLELGRYYGVSGASYVNAFVVHHIDSRDSIKGVVRTHIYLIYGLGLSMMLYYRRERNGKTEHHNSLLELS
ISVIPGLVSLGVVDACAGIVGSSFLLSYRRALGRYLSNNVYTERANATITHKTTTGTLGGFVCGVVFWVFV
LFVSNVEQHGQAAMSLVLVVVCSVAECFTDGIDNLQLPLALYGAVTTLFAFSGWGEDSCA

>tr|C9ZTE7|C9ZTE7_TRYB9 | DeepTMHMM Topology Prediction - Predicted Type: TM
MAHGRVIEWFYDSLPSCVGLWDHTGRSDGECDVLQRSSCSLCDSRRHLLRTIPKAYKSYKDLKHGDLTEEP
TDAAERHVTAIRIILQVGYNKYALDVPSGHVLTVSAFLDDLCCQLLLAMAGGRYDMDDDVAVWWEFLEDAV
EALGMHAVLGIGGDGSTPLLRPLITTQCSPHSARGASEHEAGCLFPLRRWIAVVRHICRTVDGVVHLPSCD
AAHSGGPNAGDTRDAGGKGTTGMLLNGANPLDSLLSAVKLLCSCLSNSFFCHVPAVCGLVRGSQAVASGGD
APKTESSAQHLIRLAIRTNIITSCRRHVATALGDSNSPPVTDEALREGRCSTQSVLGQLILLRCTHEEGCV
GSEGEKRGGCFCWLLVASSLVGAAFCVLVDEEGSVPLHTESFLLCFVQVAIEEWKRKLQRYSLGGTEGLEL
LTHLFRRIVYQLQLGRLVDIFGRSAALVPRILSRQYVLASTLVLPNTVGNINDGSGSAAAVTSWLLDHAIR
RWNSHPSLLLPCWCRRGDAVNVVRDAPHDDEGALGDELLIRTVLTGQHGDHYFLRTFVDHCAAARRGRLGS
GGSSPLAEIHYYSVMLNIVRDMMAGDAISFLTKNDSDANSSTTVGNDAFPRYECLRFFNDWMAVIRKELTC
>tr|C9ZVW1|C9ZVW1_TRYB9 | DeepTMHMM Topology Prediction - Predicted Type: TM
MLTLAIICVSSAAVAAACAVWWFLGTIPLAALLVLYACMANQLFRACLMSGPKTSRQVPPTPIAPCQRLSA
VQLSKAYREGVLSCEEVTRTYIEHIKRVNPYINAMVFECFDEAIAAAVQADKVWAKWRANRGNAEPSWLLG
VPCTIKESMSVTGCPNASGLPQRRHIISRMDSPVVKNFRDAGAVILGVTNTSELCMWYESSNYVYGISCNP
YDTRCIVGGSSGGEGASAGAVFSTFSLGSDIGGSIRMPAFFNGVFGHKASPHYISNRGQHPAPVASTNHYM
STGPISRFAEDLEPLCRVAARGGFLEDEKKFPPRPPLRRLPCIDARKSLRVFILEDFGTVLARTSTTQLEI
VREVGQYLEEQFGALVTYVNLHTWKCSGGGEILKVFKPFSKSLRLWLGAMSNDKEEVVFTDLMAEGMTSFS
PLKEIFLWVIGRSQHTLPALSLTVVDAVLQHFPKWGPMGTANDTVREFKKSLEELLNGDGVIISPTFPRAA
PRHHRPIFSPFDFQYTAAFNVLRMPVTCVPIWQKELRGDMRVPTVEEAKELTASVDYHLPKGVQIASREGN
DELSLAVAQVLETAFGGYKYPGWAILE
>tr|C9ZMD8|C9ZMD8_TRYB9 | DeepTMHMM Topology Prediction - Predicted Type: TM
MYSRRLHTSYSKLQVRRYWRVGTHMGHQPCEGDVELGDRGTTFGHSEGINQLKQGTYKLYISEDDSLCNVP
QGSCTCTGGVGEPSHPRCTGVMPPRFHLITQSTDGIIYERRDLTGMGSLTGLLHEAAVSDPPLAFFWIDTS
GVPQPEDLSALFEFVNVNPYTEWRWRALAAASIRNDSGDCPPSNGVDGATNELAQEDADSYYDNATELDCL
RAFVEEQYVQLRITVLQAQSAMPCCLRSAGNGEIGGKVEGPGKHQQQPRNLRANLFDACESGALTSVQAL
CFRNGVVTWRPQHAIEGWEYISHRLIRHLQDASGGEDIKPQNLLSTSLFALTMLDELYMAFLPDITVVNNE
VDEIESMLPLVRQRPSDQADVLRRVQLLRRNLSVHRRILMSKVTVLELLNRPTVRVLLSFMNTSTTSKLDT
VVRYASSYVDIARHILPTLFKLDNARRVLTNSIIIYSSGASATNNCNSNKSDTLNVTLGYVALISIPPTIV
ASQWALEFHVPWRAGDSTTQFWAMVGVMGACMLMVLVYPVYCWIRGRPDKFVFH
>tr|D0A507|D0A507_TRYB9 | DeepTMHMM Topology Prediction - Predicted Type: TM
MSHFPFNFSLGSLLFPLFVFFFFFFTFPCLLYFYLFFFLKPSFFVYTLTFLPTFAFSTASSAYLIIDPLSQ
SRDEKKKGKKNPLTIFHICVWKKKQIVHGRLLSFAL
>tr|C9ZU82|C9ZU82_TRYB9 | DeepTMHMM Topology Prediction - Predicted Type: TM
MYIYMYIYIYIYILGVRAPAHIYPRFPQSSDFPCPAKHIVFMLRHCTAHRRYRTAWRELLHPLPVRARKME
WLKRDAVEENEEILRRPYYTIKSYALPPAVGRQESIHNSNNIRGGMHSSHSLDLIMRQPRRVKTPEQLRAL
RDRLRFIGVTGPMPQATSVSTKSYTDTYGSRLRPRYPESWDTVPPHQPSRELL
>tr|C9ZZF7|C9ZZF7_TRYB9 | DeepTMHMM Topology Prediction - Predicted Type: TM
MHFYQPYTHTHTHKHRQHTLQVLCAHFIYIYYPTHKCTNDKESVTKFAMRKKKKILPLDSLRFLFLLISFP
IIPLSFAYPIRFFFLFVFQLRHYSFLLYLHLSVRFFSFLFLFLFFSLSLYLFSSTSSRNQPLKKY
>tr|C9ZTQ4|C9ZTQ4_TRYB9 | DeepTMHMM Topology Prediction - Predicted Type: TM
MSSSNDIASKIREANRRRAEEGSRIRQLQQERREEMLEREAEKAASKPSGFIPYIIISLIFLSFPLSEPIV
DWVLVACFALLNGAWVLFFRSASWVNWGTLALCDFSLIRISHVIPHIPRLIKETPPHVLAAFIGGNLVAMV
ILYYAYVVRRLPWKTEVGSKKPKRVKRSDGEEVETLEEFARRRERANRIDNMFSGLIVANVVVLVLVGAIP
FRTVFDAVRQIIGFFH
>tr|C9ZNC1|C9ZNC1_TRYB9 | DeepTMHMM Topology Prediction - Predicted Type: TM
MLLEAVEEAEATEKAAEKQAKDSRKAFEEAEEERIKATEDAEAAKEEKKDAEESEEKLKKDVEKLAEELKE
ESKESGEEDDVNADHDDEGSEAKSGWIGTTKVLIFLIPLLLLLLGLLVFFVIRGRRKAEVKDDISIEEGGA
KSKNTKTAAGLDSDI
>tr|C9ZWF6|C9ZWF6_TRYB9 | DeepTMHMM Topology Prediction - Predicted Type: TM

MSSVTTGSSSYAAVLLVLLLTVTQCGNSKFPNLHCDNVWDGPSAQNEIMKCLKDADLMKGQWQKIAFPVAA
AFIIVATLVAFPISCFLTCLCSSRCKPSSKDGGKEQRCCLWMWIMFALIWAFGVAAFVFFGARQLETAATT
LIMKTVENPINFLDCTADKILDLAYDWSAKRSLQNGISRETIDSVVGLARGYVREGRATYEQYVHWVPTVS
FCIGTFAVSLIVPMFVFAYFHCCSKWLPRLLSCVYWLFAILFMALGFLMILLSYAVGSICGEIALHYDRSP
GLIQWYALPMCQEKFNFANLNEMIFNAQKDVSQKACEQILEYCDGDASELGDITGVGGVAALLGRNLPPGL
DPKSLPANMKPEDFQNERELEAKFGPNIPGVAGNPNTWRDFPLVAAGAGGLRYPREGNGDLLRAEPFELYR
ALPHSSEKYLKCGKDLKNATECSSFGFTAAVISDTKVKGKLFVCPEVGRACTLQECATSCGRNEAKEMANK
LVTIAGLATNVSIALSIGRPLLECNFIFDTLLTALPNCADIFGSTMMLAVGFFLGGMMFALSIYILLRGSC
IWTNLKSQDEDEEEYEDEDEEDCGKKNNKNHEETNNYEEYGKKEEQ
>tr|C9ZPT6|C9ZPT6_TRYB9 | DeepTMHMM Topology Prediction - Predicted
Type: TM
MTSFPRSFQHDGLLSAVVTQPQRAFPHLPLSLSKLFPYTSCSTGAAPVDNSFNIKVNSSKGYAKWVGSSPL
LVIPSPKLRTFLSVTFSYISTITFISAVLLFVFLKLILPPNTAPPSKRPAILISKKKNIASSLSYLMTHFN
KRKTFRECESINND
>tr|C9ZN36|C9ZN36_TRYB9 | DeepTMHMM Topology Prediction - Predicted
Type: TM
MNDLRQRALRCRAQLTERLQPYTEKLDGALQTGFDISLRTLKRAFMILTAVGAALLILLIVLLVCLVASVL
GSLFIRWYVLLLAPGTELFTLSFNTMPLETEQWRLHKLNNMVSQLEPTRESPLLHRGDENSTASLVGLGGG
VNANLVSVLQNEAAGLKMSLITKLVRNTVATSTLVIPSRVYRQVFLPHGGVSVEGLFKQRKPMFNARGVYA
AKMQLVFAKEDIGREVSMFLESSMLMAKDPYISQSLGALDVLFKMDSSFTLITGEKPRSWPVELFMWSVSK
FLCVPIWCYQKLAPLLNPDVFPMFDPETEVAVVTPVYSDFEPPLYLQPHLRAINFTLYQSEEGFQPKVRLR
RLHLHTSVELSGLAYYFSNYAVSSFVGAAVLLQVLLGGAALVALVFVFIVGRSWGAELMQLNGDAPDDEGY
>tr|C9ZI09|C9ZI09_TRYB9 | DeepTMHMM Topology Prediction - Predicted
Type: TM
MRDKEKRSESVGHYYYYYYYYYYFFFLDAKLYIPNIMFTAAFISLHLLYSV
>tr|C9ZIT9|C9ZIT9_TRYB9 | DeepTMHMM Topology Prediction - Predicted
Type: TM
MSLGVERRGGSRSADRRQSDATSGVVAQRRGVYTYIYICVSTVECWTTVVSCTSAFTTDCYCCGCRLSLER
GAFFCFGAALSVRGVPLLPPPPTVYICVVTRGGLLRGTGRSCFSLPSLFVASLPSYRCPILLYLRPHAAIN
YELFSLPPFFCCFQLPCAV
>tr|C9ZMZ1|C9ZMZ1_TRYB9 | DeepTMHMM Topology Prediction - Predicted
Type: TM
MLRRLGANVSNMARPMNKYAVTVSPRRHLEPMSTWYLASWAMVWYYAFFFWMPMVWTDIMVPSFVYNKLPV
IHFLQEKRAEQKLRRVLDETYTEWTEELDQAHVTDAITRSLNI
>tr|C9ZK45|C9ZK45_TRYB9 | DeepTMHMM Topology Prediction - Predicted
Type: TM
MRPLQFSLQLPSPRTHNPNFQIYFVPEKKKERERGKKKEKKRSKYKEKKMYNVILVFSSSLPPHVFPLKYY
VLHETFHDILFCLFACLSSFFFTLGLWSFFFVFYWFRRCFFITCFFFSFFSSLFSILVCLFSFFIDCLFLN
YYYYYYFYYYFLFFPSQTSC
>tr|D0A1J6|D0A1J6_TRYB9 | DeepTMHMM Topology Prediction - Predicted
Type: TM
MTHRRRSFCSGCSYYHLFCEKKVCHMVFRQSVNCKALLPYCHASGEMLTEPAKFLHFLPRGCPRFKNWGEK
KMKKLLGGEIGLPYCYAVHPQCLCRHRSQARKVAALNNFTFLVTLGGCKDVFHLFFFFFFGSGK
>tr|C9ZUK7|C9ZUK7_TRYB9 | DeepTMHMM Topology Prediction - Predicted
Type: TM
MYFFFFQVIDREKLAYASNISIILELLCAVLIFVLIGVFLRVAPLKWAHFVAPRRVNVCRAELNIINPKVE
DALESTAVERPKENITNVKRHKEEDSRGNKISPIVEKTTNRAEKTSRVVRDISLEDTLDEFFREEQLLHDT
LLDVETDMNLHRVEIEPTMRLLSPPKVVNQRIKVPASPEKRRENGRGKVVPVSKVHEKSLDEALECFFNDE
ELLEGTLQEVF
>tr|C9ZPK2|C9ZPK2_TRYB9 | DeepTMHMM Topology Prediction - Predicted
Type: TM
MNHSRIKQALYTLFLMTANPRCSVKTFPVPLYGTVSLFWLLQLSLWHIQHYTFSVFALNNEALPFYRYRIA
GISHNYCGKLPISLCSAILFTVSSDYLQKQLLARVHTSKKVRDGDSLDLI
>tr|C9ZQT6|C9ZQT6_TRYB9 | DeepTMHMM Topology Prediction - Predicted
Type: TM
MYRTLTPSCKKFSGFYTTFPRRCNTTILIPFRIFSILLYLTALICVAGSSDDFVLIEFSDNEVSGGGDRVD
LRRSVNVYLVVSFMLLFVTCWGIFTGRTLRSGTVNFMHCCSHTTAAIALIVIWCLKLHLHRMWHVFYAFGA
IPTAMEVCALCGSYYRGLDSYL

>tr|D0A5J6|D0A5J6_TRYB9 | DeepTMHMM Topology Prediction - Predicted Type: TM
MRASAMTFRGRMDVKPWMGVGCYPVTPSFIGGMLSVKPAYNHYKMMRPQRPLRAIGTNVTLPPVLYNRLDQ
IVQIENAIALQRVERQVEMTLSQLALGIRNAEDFLEKHLLHAPVRPSLHMLWFHEDGIRKNQHILSTLGAH
HLVPLFSLLAYDVERGKMSLHMAEELYDELMDCSVAQPKVVQRELTNQMVRAYCLHDEFEKALDVVSEMKA
KGIRRTFVTYAPIFRMIRSKEDVETHLKLEQFMRDAEGGRLQKLCFIDVPRIFYVFGVFIRYNWAAINSIF
TAICTIGALHLFNYGI
>tr|C9ZNP4|C9ZNP4_TRYB9 | DeepTMHMM Topology Prediction - Predicted Type: TM
MLRKKEREIRQFLASSHPRILARFFSSGSCESAEATHQRLPPPPPPSRLSIPFIFSYYLSFFTDVYYCYYY
LYFFLHHSRAHVPSYFPSFSVNSFLFLYFPMHTIHTLHTRIFIYIYIYIYMHAKTNAKAQKRE
>tr|C9ZNQ9|C9ZNQ9_TRYB9 | DeepTMHMM Topology Prediction - Predicted Type: TM
MEGKHYDVVGRCEKIKERKAHEIYDGGSGGGGGGGADYSRVYMGHANLSLIFFFLLYVFANTAILPGRYPL
CCLFVFFPKVIIIIIIAIIIIFCLFFVFVYIYVCVYMCVYLCVRMCVDCL
>tr|C9ZP72|C9ZP72_TRYB9 | DeepTMHMM Topology Prediction - Predicted Type: TM
MRSPNGSVGGRPFGYSHNGPERGEEGEEMENVSERIDAESDYGDSLNNRRFRPPPPPPPPPPPFESREGSPE
STNSNGRQQLQVVSVGVLNVQHDVSGMPLAGRDGTPRSVPQSELTKRKMQGEAQLRYEYYNRPRLTVTSRP
RIVAAPMLDRSDWNQPLFTLCFCLYGNERPEAPLDDEFNSPESSATPRRKHYPHSPSNSGNSSILGHFCWR
MFCLRCSVADQMRLLAIEEDERGYEPLNFCCEGFFGSRMSLPRAFWTMCICDILTGGSPCGCFYHGLGTAL
YGCRLRYLVRCRYRLQGIVLSDFIHMLCCPLLSVDQQGAEMLANGLVEPREFGKFML
>tr|C9ZJ40|C9ZJ40_TRYB9 | DeepTMHMM Topology Prediction - Predicted Type: TM
MKCNAERKWENRKKFSSLTLHIYINIYIYIYIYILYYLFLLLALFISPSIIFFPFPLFSFCIFPLPFPLFF
PPFIFFFFHSMFDVLKKRNNNNQNKKRRSVKEILIMVMVIIIIIIIINIIIIIIIIGVLT
>tr|D0A2G5|D0A2G5_TRYB9 | DeepTMHMM Topology Prediction - Predicted Type: TM
MMDKMGKSLLHSTLSLYSFLSLSSSLRSTFFPFFFLFFSFAKISVAACELVLFCFSFPLMSFFGKFMKKFR
RETPAPNMSEADCSELEEMIKRNPVAATLKDEWALFVHHIIGSPAETRKEIWVEKCKVPQTHSEIDRVGNL
FMKFFKDDLIRRKWRGQFSYAVVGRENEGHLEVEVLLHSLKRKETTREVLWNLKLRYHTNAISSEVTDTRK
CPNLHNIPL
>tr|C9ZVE0|C9ZVE0_TRYB9 | DeepTMHMM Topology Prediction - Predicted Type: TM
MLILFMHWCASASSWYIPPPLSFLSLDVINVTSNYSLKKGIFFFLMDGEGGKFMLIWMRFLGRIFRSHLVS
GVFFLFFTPSSDCCYFIGGVCFFVFLLPFVCVCFVDVVVVLHKYRFSLPFPLFLFLFLFYFLYLYVLISFA
SCFCFCFFLYLFSPNSAFSSSRHIGSGEGSGYLVYLYIFKIFNVVSSFSSSFFFPSCVFLCLCPRLIIHCF
ALSETFNEGFSISLFFFPSPFGCRVCACLQQH
>tr|C9ZYW7|C9ZYW7_TRYB9 | DeepTMHMM Topology Prediction - Predicted Type: TM
MSSLPPDKTLFGSHSQRLVAVAQFCSLVSAGVAGSKHYTLVARSACALAKVLANYLCLSRLKGSYLLLREV
SPSSVRRRLHSSPSWFTGVMRVLTMLAMLLFRITDKIALLANEGVLSNNICFYTSRLIPSLLFYCNLMQTM
TSAVLLKAVRPISFEATDTRNVFRKRYYLQGVLSFLEGVGLMTYAMTLFPRGVPPLAMTLHEKHLLTHWLA
VAASSFPPALSVSTTTQGLIGLAATLPSFFMSP
>tr|C9ZI53|C9ZI53_TRYB9 | DeepTMHMM Topology Prediction - Predicted Type: TM
MYLYTCISFLSVIFVCEVAAHFVVFGVHNFPTFLFFFLLFPKNVGSQFPRIPPLILLCHFFFGDYFPFLFI
ITNYCYYY
>tr|D0A569|D0A569_TRYB9 | DeepTMHMM Topology Prediction - Predicted Type: TM
MATRPGFSVATDYPYLFPGLCGCGGRTGLAAAGGAFAGSSVNGCAFRVLFILFPFALPIPAGVRVDELQQP
PPLPVQTGARFCAFSRSGCLAFPNPGARLLNWMKDCQVPAGSLRMRVLLSFGMPSAPRYGTQGATPGFYSA
ADWRRGGGGLNVQEELTCACPCGGPPLRVYSF
>tr|D0A1B4|D0A1B4_TRYB9 | DeepTMHMM Topology Prediction - Predicted Type: TM
MLCTDVFLDLVSLGCPYRFWTLCFTLCLYCRIDLLCTSMKWHICGSNACVTIECICHCTIEGECSTFWYLT
SAAARNDEFLNFNGRINVARWRLMFLLLLK

>tr|D0A6J2|D0A6J2_TRYB9 | DeepTMHMM Topology Prediction - Predicted Type: TM
MALGFSSAGEVYMYATCILLGVSLLMPLNALVSAPRFMVDYYKYVSGKEDAEPNLPFFWKNIFTFYNVVSL
ASQVIAGPTVLTRAARRLSLSVRFALSITLMMSEVFVVLMMPVIKVPQTVAIVLLCLVTIFAGIGKSYHEA
TCYVLVASMPSKFMSAVMFGVSLCGVITSTLQCIIKASMEDTYESVLTQSYIYFSLGLLIMAGTLAMALCL
RYNSYAQEHVAEYRMLKLQEQGVDAESQNDENEPVAEGKGEGEGKSEGAMTTAEQLTATAVMPVARIIRMM
LVTVFCGFFLTLFIFPSLIIPIDRDHNWFATIAILLYNCGDAIGRFSTSFKCVWPPRRALLYATFARFIFV
LPFMLCIYQYIPGHVGPYIFSFLLGLTNCVGAMSMVYGPITPGLETAGQKLMAGQLMGISLLSGIAAASVL
AMIVVVFLP
>tr|C9ZJH3|C9ZJH3_TRYB9 | DeepTMHMM Topology Prediction - Predicted Type: TM
MKGGGGIVCFSQECLYFPSPLTDASIENIIELSSLLVRTEDPCENVVAFKVLSAVRNRYGVQPSVGLIFAG
ECVRVKFMLDIRRLRRLAARRGGMEEGAVELPDAGTRDEVFVDICVVPREVMAPYESVSGFFGGDCRSKGP
SGPDVAAAFWKQRGRVQARERNAVRCALRCIYGELGVPDSLVMKRSHGKVEGLVEGCETAAVEKAPAAQPQ
LGSAVRNAGGEVSPPSTALRSRHSPKGAGDQTAASASPLVYSPVNCASGTVPSGRCSNGYEAAVGEASRMT
NVIAPDSGREMYSLLSVFLLYKVPFPVCCVLLFLSFLCGIIE
>tr|C9ZHX7|C9ZHX7_TRYB9 | DeepTMHMM Topology Prediction - Predicted Type: TM
MRHFITALVIIIIIINAIVIAHLMPFYCFIMSLHSYHIISLLFSLFSFLSSSTLLLLLFVYMYCINITLGY
SLSVQLHFFF
>tr|C9ZIK3|C9ZIK3_TRYB9 | DeepTMHMM Topology Prediction - Predicted Type: TM
MGRQEDGSFDARGGDAVLVAELHSEESKEATFVHPEARALFNKVPCLRHIPLFGEAAEGYGPKPVLSIGLS
YFLCKGLANGLVGGSIIAMFLNRFTVEGLVYQRLTNIAGMGWSVKPLLAAISDIFPFFGYTKRWYMFVANL
VGPAFSLGFALLPAQPSSAAIASVFLFFSGLSRACTDILSQGLYSRMMRRVPGPGPALVSWVWWFIILAGL
FVSVIVGPLGDAGIPQISPMIGSAVQLLVAPFFLFNWFGELPNREERYIDAHVLHEQKLKEAKDTPPVADD
NDPIDVNGGMVERQVTNSEPVTGPNADGGEATVKPFVFREPRECCCGVFQMNEEVLERNKRETIYSVLIAF
FVVGVAITSLFGSRLHLLIAAAVVSVGHCSFNFYALSWAVAKVNLFSYLNNASTVSFGGAVTPFFLSGPDC
NPGGPHFSLFFLQTVGGTVGSVTSALGVVLFNYFLSKKTYRMTYFLTLVFLIVSNIFDFIIVMRWNRPYVS
DYLIYFCCDVVISPVIGMMNWMPLTIILSRLCPRGSESTVYAILAASSNFGSTVGSSLGSVIMEYALPVKT
LVPCDFANVKWLVLISGFVAPCIQIPLIFTLLPDARMDEELDGDGKPVRKVAADVSPEGKETVCEGDSDVT
PNAEGNGIAPKRS
>tr|C9ZQP6|C9ZQP6_TRYB9 | DeepTMHMM Topology Prediction - Predicted Type: TM
MRAWSNLLASVTMAPFVLLCFVCSCVWPAVATAGSIPLAVVGSVITSEDAYRFIEVQAVDGATGAVVRSVP
LDATLTFTFHGLPQTVSEVRLLPRLPERRFRLDSSASVLTAPLRTKDGDGWLHLSAIVEQQDGSQAGDQLP
GSISAAVMAAMIIALAVIGRYRLLSLFQFPAPKPPKLRRVAVAVNR
>tr|C9ZXB2|C9ZXB2_TRYB9 | DeepTMHMM Topology Prediction - Predicted Type: TM
MIRTVSDDSYIISYVKCQFFCSSICICIYRADVAYYVLICCIWINQAGVTASMCLFSNMFLLILIIKEYGD
TMLPSCCCVIMLRVTVPIPSLPVYK
>tr|C9ZU54|C9ZU54_TRYB9 | DeepTMHMM Topology Prediction - Predicted Type: TM
MFLSVLLRVTLCGTIYIYVRQCSNYFLVHCVSMCVCKCHQLCEPNLSLSLCIYIFIYNTIIFCPSPNESTC
MGWHERHAPRPWGSRGTLRPEALNLDAWNFMLRDF
>tr|C9ZZW0|C9ZZW0_TRYB9 | DeepTMHMM Topology Prediction - Predicted Type: TM
MTTESGTCCSKFVHYCKVFHVLDYFLLIGIAFVVCLWMESLQPYCRGFSWTDATISYKLKSSTFSVLTLLI
MEVAPIGFYFIMEFLRALFASKGEWYLDIHVSNRLPDESRAPVEMLEVSSDGFNNTAAERTGNCPDGSFTT
ENKGSSKRLMTFLETANYWVVAHGFSVVLALCIVEVLKVYAGVLRPDFLARLEREGYNSTSRVKDWCKVAA
EGRRSFPSGHSGCAFSVFTPMAMYFLSVLRAFSGASVWRTLVGLLPIYFACAVAASRLRDNRHHSGDVVAG
SVIGMLSGLLAVAIFFRLGKGKAFLVPRRLDFVRRHGGQVLSDITK
>tr|C9ZZC1|C9ZZC1_TRYB9 | DeepTMHMM Topology Prediction - Predicted Type: TM
MGTCAWWGGGRGEGKVGKRFKSILVYLYVFFKYFFYIFLKIYIYIIFYTIFLRSTKGAWRKINIGTNVYII
IIIIIVVVVAVIYNDGKVKVARYKERKKVK
>tr|C9ZYF7|C9ZYF7_TRYB9 | DeepTMHMM Topology Prediction - Predicted Type: TM

MAVPPVEMYSGSFWNRMRKPLPLRTQVIRFTVVFVIVVFILAVLLQITHERMPDPKVTKPLPDLGFEVLHK
YPFLFSVADCCIGSLNILSVFTAFKLYLLHRHCVGSGEPELPCNIPGVSRFFLSVWLCKENCRIELRNIHT
IAWIRFITSYALLLLSRSIIMVVTSLPNPDDLCQNPPKIENRVKDILLTVLTAGAGSIHCGDLMYSGHTVI
LTLHLMFHWIYGAMVHWSFRPVVTVVAIFSYYCIVASRFHYTDDVLVAIYLTIATFIAVGHNADGAPWQLQ
LFIRWLPCCGANSREVAEDGVPVAIVIKNEEMMNFDGKS
>tr|C9ZND3|C9ZND3_TRYB9 | DeepTMHMM Topology Prediction - Predicted
Type: TM
MFINIYIYIYIYMHICVYMCAVVTSYTWRLLSLPCVAVTYGRLLVTSSIVWHMAQLLFHSSRRGVTHEREE
QISFFWLYPSGRSLPNSFLRYSHRKASFPFSVFFFSLSVGELWRKCNGGDGQ
>tr|D0AA72|D0AA72_TRYB9 | DeepTMHMM Topology Prediction - Predicted
Type: TM
MHTVFFSLVQLSDKPERYVIVRRTRTHPRLHSHFFCVSCSYHQPYFNLSKMANVIASGTLAVLLPLGGYMG
YSKKNSVASLAAGVSAGLISAVSLIYLLSDSNHKVANRVEACMSFLLSAVMAFRYLKSRKPTPLYVSLLTG
MCFLFGFAPYSF
>tr|C9ZYL5|C9ZYL5_TRYB9 | DeepTMHMM Topology Prediction - Predicted
Type: TM
MCVCVCVCVCVCTIHMCVRHHHCYFFNVHSESGSWRETEVFICPVCGEGTSSPLILCGEYLSSLPLTLYTR
AFARRRSVLGRFSVFSYMGERGGGRRCEKKNFQRQQPQVFT
>tr|C9ZVZ2|C9ZVZ2_TRYB9 | DeepTMHMM Topology Prediction - Predicted
Type: TM
MPVRCESKMRRSGDCVARHHAQSSSYVKTDFLKPRVRLFFSAYADVGKKVGLRHLQNFFSAVTHLLASLYF
TLLSFSLLLPLVFLFWHQRLWSAALWFGLKTGELSNSNHSFYDAEGNG
>tr|C9ZWZ0|C9ZWZ0_TRYB9 | DeepTMHMM Topology Prediction - Predicted
Type: TM

MSPPPALIGFVFVFGFWFFCLEAAAFATFSLSEICMLNCGARFLLNCNLLFGCLCTPRLLCCSVMNLEYIL
VRVYWTLEDVHSNSCGKLISVEICCCCSLGCFRGLRELECLSSCAI

>tr|C9ZKR8|C9ZKR8_TRYB9 | DeepTMHMM Topology Prediction - Predicted
Type: TM
MDDPSWQRNCDMLTHALQSLERSTSQIKRSTNKLVTLRDIAQERDKVKKVTSAANAKDVHAVHDALAFLEK
YMRLHPTQLRGQGVKLSTEAQVVLENYKKSCDVFYKKCISLEESLRKNSATTRVRIGTGDGSDVDDFDDEG
ESLLPRGATAGSAQRQAFEDDLHNEIMAERVRETSEIAESVRDINELFNHINSLVAEQGVGLEIIEENVTR
SSAATRNAVGHLQQARNSQQRSGRDKMFIFLIVVLMIMLLLVAYHKV
>tr|D0A5A5|D0A5A5_TRYB9 | DeepTMHMM Topology Prediction - Predicted
Type: TM
MPCVCVCVYWALKMKKRQSDKRPNCGSAVTLLILSFVLYLIAYLLFFFLESFFLCFSHFDFIYTYIYICVC
VFVCTCLNTMIMVPLPVTFQLNARTGGRGGGNLRLCDEYGWMRYEKIIYMGVIFLFPPNLIFFLNSFYSFF
FFREGMR
>tr|C9ZKN5|C9ZKN5_TRYB9 | DeepTMHMM Topology Prediction - Predicted
Type: TM
MFCLGTFSHLMSLVLGRFILVTAVCVGCLVRLISPFFSFSFSCISFFFSFFSLSFLFPLSSSSSCVWPLQF
KRCTNNNFCYYYTASFVCVCVSFPLVVPETLIVAAVFFFPPLSSFVLLCACRGMRKSRNNVT
>tr|C9ZME1|C9ZME1_TRYB9 | DeepTMHMM Topology Prediction - Predicted
Type: TM
MDSSSRSLNHVASHNPNAPKYVSMERVGVAMLLFILFVVILTCIVFIIFVCLIRNNHIVLRLFTNGHRPPD
ALPPPAVLCETRRLRALGGGVNGAVLIGDENRQLLSALDIPLTPVPSLCEDCKELCRSSSNCSTLDIAIND
GCLATRECAKESAVLGAHDCGKCQLGYPKRIRDLSTDRVFELYISQPSSALYGSAQYITIDKEPDASVRCT
RESSKLNRRASESLGSICCARTPTSTTKTQNMAGEVFAEPFSSFR
>tr|D0A0K0|D0A0K0_TRYB9 | DeepTMHMM Topology Prediction - Predicted
Type: TM
MVKYATSTRNSKEGGGKEAMKFVQLLRAPLCPNKCPPVSVSPLTLGISMRICATRERILFFLLLPQFLCLP
NSSPFHTNNRPLFPTIQFSPPPLVLIVLLLIRSAVPFFFL
>tr|C9ZX92|C9ZX92_TRYB9 | DeepTMHMM Topology Prediction - Predicted
Type: TM
MGLYGAYDTYMSPLVRDWPILLPMVAVYGAVQVTAQWIYPKVFGTAFQKLARDDQNNVIVRTVSVFNGLLM
VGSAVCFVTNLRDHGYVLDTDVYREIPYYRFFRVAIVAYFVWDIFVCFAYKWEAQWKVHAFASLVGAYLLS
FPYSDQYGSYFTGMFELSNVPFHISSIIRTLRTTAFSSLATICDVVFAFMFLIIRVVGGSVVTFRWLQLMY
KLLMDTRSDDKVVVHDEASIIVSMVLLSIIQSLQYVWLFEIMKQGYRRFVVADDSNADDQVSVSKRKKKE

>tr|C9ZST5|C9ZST5_TRYB9 | DeepTMHMM Topology Prediction - Predicted Type: TM
MWERKKEINPCFHSSFGCFEQKLGECNNYKHCVYTGVSMKDANFTSSTSNLRHYTIQAREAETFHVVLINL
VYVVPIVPSCVCGMARCQASYHAFLSLCLHLTFYSHSA
>tr|C9ZQH2|C9ZQH2_TRYB9 | DeepTMHMM Topology Prediction - Predicted Type: TM
MYQGVSLLVLFSYIFKYPFQQQIKRMKKKKCWSLPSEKRFGGFLSPSPTTTTTTYTHIFCQGRAYGNRCSY
ILFFFFSPSSFFSFSFLSIFFPSPLMLNVEVCFCFLFLFGYFSFTSHQ
>tr|C9ZN27|C9ZN27_TRYB9 | DeepTMHMM Topology Prediction - Predicted Type: TM
MSWTLESQMQCCFLFLFLFYFIFNIITLRACGRWAFGWAQLPKQGNGGMVRESTVICTISCAYAHVCIYIY
LFIYLFTLYECACVSLTTWCFGPLKQLRCPYFLPLFFDVFKCVCLVVCFVLFSI
>tr|C9ZTW4|C9ZTW4_TRYB9 | DeepTMHMM Topology Prediction - Predicted Type: TM
MIKYMLIYSTVCIFLIFIYFSWGFFPFFCLSALFFSFLFFFFSVLSHVSCFSALNLFLFRFFFNYEINPQQ
KGFKGVFIFLFLRNAAEKQRHLVIPGVSELLYVVVKLVLLWSFFFYMCFFFFSFFCFLFSPLSAFV
>tr|D0A1T8|D0A1T8_TRYB9 | DeepTMHMM Topology Prediction - Predicted Type: TM
MTNTHIHVCTHLTYDVSALSVRSILLFSYFSLSFCRCCGSYAVLCSHSTSSHFVTFFPPPARLSLGSVDEV
PASTCCLISFLFNFSFEISAFVWPTDVTEK
>tr|D0A2F0|D0A2F0_TRYB9 | DeepTMHMM Topology Prediction - Predicted Type: TM
MDFIFFYSSLIFTFCCVYFFSSFSAFRESVGKKFAHIYRCFEAGSSPRFPLRRAASMEYMHHIQGVVVLND
SGNRVFVKYYLNEDMKARGVLTTLEKQRALERVIYDAVSAPKRNWAASKDGDIVLHDVHSILFHVWGSITF
AIVGDIKENEMVMHTVLRCIVDALQRILKTQDITHKGILEKYDALVLAVDEVIDDGIVLETSAQNVADDVA
PFMADAETDTARSALSKVNEYLKENL
>tr|C9ZW29|C9ZW29_TRYB9 | DeepTMHMM Topology Prediction - Predicted Type: TM
MLEKYLSSIIVPYLSQFVENLDSKQLNVDLWNGNVVLKDLMLKKSVLEALIQGDPIGDNFSFEGATKAAPT
PGVNPAPRLPLTVQRGICKRVNLVVPYTQLRSKPVVMEIGELLICVKGNTECRDVVLSKAAKLDAAAARKS
RELEQFEAERRRTRENNANNSAGAAAATSAGAAADTTEKAPGATTGASVTPSATEKGKGKDGYLSRLGELV
VNNIVIKVQSVHVRYEDEATKTVAGVILGDVRLFTVDLFTGNEKFVDPVGMRRMTKRLEFAGLQCYCDDPA
RYELNPHGFFISRVNDMSKWVIAMRERVEEGDVEHSTIVGPVKGHVDVNLILKNFIRDLIWEPYLKVRMRL
DSFQSKFNRAQYLTLIRTISLLSNWTSLTEVLSRRPNAPVTGNACAWWRYAIKAVRSVTAAPKRERLLQRI
SEVCIVDYHVLYRDVVRKTEMSPEKQRAYRFITRFMTVPDMIAGRKYVYAQLANAIQLKRKDTEAKKAEAQ
PAEEGPKKGGWLSWIRRQPNDPEPQEDEEARALEELEREYGLDPGEANGSDTGEENEEEKLPESYCWFDAQ
FELSMHHTTLYLSKTQKLDLVLHDVGLLVKTFNAPNSVQVRIVTDNLTLSNPVEDETLRERLPSLVEGLPF
RCGPSSEDLGVVTERSRSWNSVPLLECSAALNPVEQPIEDTHLDFTMNMRLLPLRVVADPPTIDAIVRFFH
VPKGLDVGYVVGRTKDFALTVGQAASTELRQAMTNTKGYKISLDAAGPHIIFPKTLRGALDEPALAVSLGH
AMFDTQPLTETEKQQRLLAAQAGGQNEEWHYYSSTADFSKFFIELGTLGSVLERQGSGFMLVPEIAYSASV
LQLIDRDMANNREWLIVRMVVPKLCMACSVGQAHVLSNMVEQWVAYLAAAGTEDDNDPVAMPSVDTAVLGA
EKLLAAAGSPGGIGPTVGVGGSTSAGAPSAEGSYFNSCTGNASRPRKAKNSESDSDDALASTAPVVAQRAQ
ADLPYVRLHATIEELGISIFEDDLETRQPLVAPRFTMAFRTALMTLHVRTQKQLLEMVLNDPYCLDAREPD
HLIIAGPSIHCNAEMAADVPMHVDIVMQPPLKFCLNTSCMELLEAVVDITNLIATTSDAIPQLTRSDEDGV
DTVKFERGAKIPFDVMQELQIKQVQHMFRMPDTPVVQMSLRIAGIATVELMERCVERQPSQEQSAGKGERQ
PRDYPIAYATFKEVNLFLRKNSVTMEVNGSVQQVEVSLSEVHDVSPSNRVVLQRRVAPPHASLIKSKVTQP
LETGATVAVAAMTPGSGSGQLASSQENLPPRLQSSESEQRQHLTFTYRTSRPVMPIFQTGADGKRKLTNVS
ELRFSGFAELEFSSSDIHLDIHSVMVLSQYFCTGLFAELSGLSLRTRYDGRVPAGPSLIEPPQLLTSVRVF
AQDLTFNLPVDARVENDIERFRVSVDHVAVQSSLLSAEEKQSMNISLREIRLLHGFPHGVDAERMQSAEEG
KVLMPTTSLDVSLKSPLDQLSDEPLRVELRGDELVLALTEGDIVDLCRLISGNLTRAPPPPPPAPPRLQSL
TGSRRESSEVGDARRPSVAACLPQEITEAQSGAHVVWRAGRLTLSLLDTSSSGARFRLEGNGFTFDQRAPG
GELSLRWDKLELHDVYEETCRATMLLCGCGRVELGNLPAPWCDPCKGANKEGMNDQVDKINATTGDGASSV
DFEDLTPEQLHQLLQEQLNQPQDSASAGGLSRDFVDIQTLVVDFTLERFAVSDQWLAVYDFVCNQAVVEAL
QHVASTDDSAPPRRLCQGFKEPSEGMEGAKPFRCIVTTRAVNVPFLTISREEFLEADITSLHADVIKYLNT
TTVTVRMHDLVVRHSASGERVLFKKHETDDLHLSSFDNTQENLLLTGINSARSPRTPAGGTTTAPAAALTN
PTTTTASPNQPVGTATPDILNLRCHIGGTPENPQQRVRIAVGELATLFSAPLVAQIVEYCAQPDQPIAKIS
NLGVMREQRERLARAADQMVNNGAIHVQVLWQQPCIIFAGNAQELANKSRNIEAHLGLMGSEILFDKRSGS
CTVSTKVVGVSIPEMLEETTVRLRYKLEERNADISVVVDKATALLHPVGVEKLLWVIQWNLMVPPDDSGNS

VANNVTHSGAVAGACEKEGIKEPNVLNDRKEQVVGERSTDLVEEPITQNIHVEASSIVLAMHDVLGLHVHT
TALGGLKIHVNAAGDVDIAVKSVTTVEHFTNQRLMEACGEGALTVHVKSAEKTTSVCMAEVNFRVVPKAVG
SLLNMLLSVQLPPSLPSVSGVPTTVLPSETGWEHRLSVRLNCCRTLALHDGQEVFLAEYKDLNVDLCSFAD
ASSTLTVSIGWLVVQNLYSKKVKGSELMLPMDGEENGSPAVIDFVLTTSPPNNRSVTRDCDCAAAPCYVQQ
LRCRISSFLIVSVPDVTYAAVQVTADILGNISDVNRGKAYDYVAEKTAQQIRERQQLTEVEVAIGRPCIKL
LDAVDSQNVVEVFLGDFVVRNKLRCAAAQGSNVAMCAPHAEVFSLSINRLSLNILGGSAFNHSSSIEVEFS
RLITDDVIPTVASDESVGVPNDEMMSLNVSVPMLSARLTEAQLNTLLDVVGAFSSFGCSPPVWGDSCGAFP
SSHSQGASGHHDGSMSATSFHTSSVASPSGGAAGKRRAKDPLLGLQVQGAAHGCGLPTGDVNVTAATADGT
ITNSESVTNEVSTSMKVSAVMQRLVANIDDKFEITVDHLTVEQVTTSRLSRTANGNDNSNAVPVPPLTNSN
SKLFLEGLVIRHCSWGSFDDNNERASLFASRDVEVASVTVMSVNGNSRETDSSTTVRLGCLHTTITPQILV
DARNILYGPFCMKVARTPLSPIPICRLTDDVYVLKSNLSLDPTHILITGNKSRCSYVLDLNQYKLCLLGPP
APHIVLDDNCELTITNGCIVVPGMYALSSFVSFGGNASVFTTKSCVIEKIAHHDRVVAAVFRPVRRRSRGG
VESTAAPTHKNMAAPSVDPAKQPVTSIQLPQQQPQVGAVSLNSTSVIPQCQSNIVPRQVSGEEVRSTVSVE
CKDVMLRVMAEEAEDIGVCMFMALDFTTTRQLDDGVLKQNCAKVTLRDIRTSTRDEVALQPSTFTVAVAGT
DRVSVTGSLASQGLCLRVSLVRSLVELVKDTMSAFTHEGEVPVYPVNVELEDENLKPLVDVPALGECRICE
MQCAKLATSEEGPGVLCYRCCTGKMALPETNVWFAVDPVDILLLSDSGGMLQLRCVAPFETSYTADKGLHT
HMKLQVRNLNDNAAVWEPLVEHITMTFGGDVRTSAYWIKVEKFDYVLSPMNVKLLRKLSEDFSPTSELQRQ
LRRKFRGNGHSLLQTDDETLDEVSGRGTSAEVAEGVCDDGHKYVAYVEVHNYFSESLEFDGLPIEPRGRSV
ATNVNKNYGTLRRTHSVSSVGVTINWFRSPLYVRYPDMVVRVQTTLSHREGSKRLFRVVSLRCYPVHRTDM
IICLKNNVSTHVEVVSGVPAVGPQESFYFHPDFSLDRKLQLRPVQTVDEEYTEALAVGTGAAPTLSSLLSA
SSTTLHSRGVKRREKLFVFTIHERQEDIWAGTPMFVISIEPHICIENNLPCSVRFRIHKDGPGKQNLFSSV
VKAAERADVIQGETSVDRACFHFEVHHEDKDSNGVIYSTPTPVKLKPRTTHVVLRAQLGDSRELRINVRCK
DNKIVLSTPYSIVNFAPLPIELRECSSSGVEISCAGTNFRFLPKQMKSAYPASPQSADAREFFVNVHIMDY
EARAIPLHVQDSSAIVLGERMDKGSAERGTSNRGPILFHLMCTMQVDMYGSRFVTIVPRWTIVNRTPRDLY
VAQALLDAAPPGSSSSGKDTRTVGGVPDKVSGRCFRCRYDIAQHIPPNSSTPLFVTARCCSELGYAVFVLA
DKGARVYGNPVSLEEVASELVVAHGVCEGGEAKRASVVVETSVVVDGPYTFFTVQDLPVSPYILLNRTPLS
LELHGAHGHRLARVGPGCSTPFVMNPRDKPTRTRITLESGTWDTTATADVGEEPPRWEGEQPTPFVEFDFD
RPMRQEERYNELDIEYVVGFGPFGQQIVELKVATSAGMCTTRGAAGGGVLNPLESDSILNERSLRVYQPPP
PTPLDVVMNLSYVTCSLVTMDQEILLVAIEDVRCHVTRKDTKETVNFTVKNIQIDNQCEEKPVYEVCLVAI
RAANDVQCISAYVEREIVPARALLCVNQVCLNVVPIAVRLSDNVLVAIASFGAQLTVEQPMRAQAPTEQEL
FDNAPVTVGAMNTRVILRCMVVNPLEVRVWFEREAGGHDFIREHVRVRTAALLSMMIQSCEDVHINLPGLN
IERRSGSAGVLWEWLLKVYWDKSYTLMAGLFYQYASSLPLIGAPIKLFSGFGAGALKFFQEPVAGLQESPK
AFARGLAAGSAALLRETAGGGLGAVSNLAKTGASIIDVFGDGSRTSSRSGGAGACVLHGLASGVKGVVNAP
VEGATQGGARGLLFGMGKGALGLVTKPVAGLLADVSRLTGTAARACDSSHLPKVRRRRGLREFHANGAVAE
RNSLLTVFEYERGEHDGSKWTVGSESSWSDDKPQWLPCTKEQMQRKQGGGPRPDMWQVQRYGTNFEGWKYS
KNYHGKYTREQLPRSKVRRRRWVSVIRPMPTSAILRLVHIATKNPTCTSSSDSVSDGVFCSAQLKHGSVSS
ATASSFRGWSGSPADDSFPGTKFKPESPKEAFRTVVLYEYEAKVVFVWSSRVLPQGRSPWEDSEGCRVQRR
SDYNPPHGWSWDSDWFLSRGAAGPDGWEYVGKESHTSTELRRRCWKRRIRKVL
>tr|C9ZW90|C9ZW90_TRYB9 | DeepTMHMM Topology Prediction - Predicted
Type: TM
MSWFGWGRKSGDEKAEREETEKTIAAIFSPSHATVRDAPDRKDSILRESTVRLFKKGEEPAPYTAVGGTFQ
TKHTVETTREDRMSGGEKPVPLDLLPPCPIAGYELRSDGLVDNIEKPPRTLTAQELRSLVAGLSSIEQRAR
YEVDTQLPQQVHNRGRRGLEVVVNPCLHMCGLYLMLWKAPRLYFNASPRGSAFFTRVMTLLRWNMPEIEKE
KLARKHRRLLQATNARVTLAFLTGVFLTAVAVITRPPVDVLDVGPDVEVGKRSVGFQQHSEAALRWLWLVY
YHHPAYKPLAQGTRPPILLESSSR
>tr|C9ZI32|C9ZI32_TRYB9 | DeepTMHMM Topology Prediction - Predicted
Type: TM
MKAEAKHFFASVSFASFSFFSFLMKVYLNIYLYLCIREKQFSKCSESKDEKRKSSKDGRHKIIAIIRHHHT
YANVRMLFLLLYFFFFTSIPRLFFLLFFFS
>tr|C9ZYH9|C9ZYH9_TRYB9 | DeepTMHMM Topology Prediction - Predicted
Type: TM
MRVAERAGVGDANSVVQVFLHVRRRCESSSLVESLVGRFFVYPGKLPTYAWRVFVLFFGCGLLFLLVSKLW
ATFVFFALFFVMCEVCRVVHYLNGRSSTFHSRPLPLFFLLSTVHSFVKENVNTLLYPRSFLSRTCFLYLGT
ESAR
>tr|C9ZYA8|C9ZYA8_TRYB9 | DeepTMHMM Topology Prediction - Predicted
Type: TM

MNSYPVILEFTHASPPFPHPFLPFTYSPYTIVLLIPLLRLSYSLCYEPRTALISYILLIHPICLFIYLSIF
LFFLLSFPSYFPQTHSAHYNMLPCTDSFALVPEMFSHSLLVFVFAFFLFFYMKFLHCSPFPSPHTQTHTLS
FTSFSSLFFFSL
>tr|C9ZUV5|C9ZUV5_TRYB9 | DeepTMHMM Topology Prediction - Predicted
Type: TM
MALLFHPPCRILYVCCRTTFFGSSVYTWLRLERDCSLLPTGFELHSRHMNAQGKVCVIVHGGRCYVSLLLE
SIPFDCITPILADIQVCVLKATCMTVLSLFYSFSLFLRPTLFSQSIRGSRQPVLVTCQMQARLLRSLFGPS
DRERCPDASRHEGSTMCGQQDIGFVKQLRHFVRMPDMFVDIEYFFALTILGMVENLLSLLILPLKLIVSVH
RFERRDAVALVLIVVGVLTYTLLSFKTVELYAYLYHAVRRTSFIKLMMVFNILEVADKLLSALSHEATEVL
TACVGDWRGAAQTCGEQCERFAATWLPVLSAVVAVVSVAAHAVVLLLSVVTLNVAVNSEGNLLLTLIVSSS
LSELKGAVYKKHNRESLYSVAAADAIERVKFLLFVLVMVMQHMHERFCGLDFADTLFVLLSEVAVDFTKHL
FVAKFNGISLSVYRSFTQLTLIDMAAETVLWRLTHIRACCVDSALYDSQDLRKLLRPSDGFFPKYVRRTGF
VPVPYAALLLWSFSPIAHALFRNAPLLLLLVLVAVMLLKVFMSELIHSVSMRFVVRSMLSAESCAGECQYS
KWQNTVGSQLHGVSPLATPPRVRKQRGEEPMHGTAKVLQLTSFLCSLMTLDPFDLQVGKLRR
>tr|C9ZSX1|C9ZSX1_TRYB9 | DeepTMHMM Topology Prediction - Predicted
Type: TM
MLWLKLRVAGGRRRVRRVSSGGSSFPFPLLVLFCHSALALQQRQNQKQRHTNKSRPSILCGRQIWERRALR
LGFLRSIGPVSLMQLLVVAVALLRSGSLAPTKDLSERLGMKESFTVLDFGEPAKAPLFFGVTKEQLWRHVR
TRRCRMCRHRPKVTPDKAPRRQLVFQSPVVLWGYVCLVACLRGGGGVNLVYEWLWGLRGYC
>tr|C9ZYX8|C9ZYX8_TRYB9 | DeepTMHMM Topology Prediction - Predicted
Type: TM
MIGTMEDDSVPVILPQASSDDVSTLTLVKRFVLVGTPLMVTTLAQFTLNAVMMAIIGKHFGVKELGGVSLA
LGMLNATAFAFSAGLCGALETVLSQTYGVFQSRGGEGTMYLYGTYTQRMAVMLLVISIPIGIAVIYIDVLL
KSLGERPEVVYYTGRFCCIAALGIPALQFSQLISRYLSCQHQTAPLSAVAVGSAILNPILQHLFIRMFGFN
GSPMAWVLLYVVTDVLLVAYTYYTKTYVTTWGGWDSNAVKNLRPLVNLAAPSLAMSLVEWVVLEVIMACAG
FAPPTDLAAFSITVQVFSACWGVASGTMLIVSVFIGNAIGEGKPLLAKRIANIAIVMVGVTTMLDILLCWK
FEDRIPLLFSDDKEVGHVYRKLMRFVFPYHAVDTFQSTVMGILRGCGLQKIGAVIIGVTLCVVGAPLAFFL
FFYVGVGVEALWIGPLCSVTFVGVPLYIYLLYWYIDWSKLQPQQESLDLAVEPFISVNNAPLEEDMYGAVE
SLGSKD
>tr|C9ZSP0|C9ZSP0_TRYB9 | DeepTMHMM Topology Prediction - Predicted
Type: TM
MWLIYTNVYIHVCLYTGLYIQGRNKKKKASAQPAVMFYTGCSYRGERTSDDRRFDGQGIFTFANGDTYVGA
HRDGCMHGHGTLFFAKERGGGHYVGVWENGRNVSGAFVFSDGLVYGSGEQTKIPDPSAGGPLTPEVSETGT
ILAAQQSGNTGESDKQITNASVSGEGEEVEAPPVDTSSNSTLARNWLYCRKGDRRLWAEHLREVMPVLPLQ
ALLGGERAPQSRKWAKSLVVAPCVAAEAKTPATFAQGQPRCLSDVPEEFWSDEEQRKSVVSRMELQASPFI
GAQGGAGREETMIDGERLATNLTLRIIKPLESEAMRRAIEAAALASTAARVKSDTFMSPRSRSSSEKSSHC
LNIVEDSL
>tr|D0A6J6|D0A6J6_TRYB9 | DeepTMHMM Topology Prediction - Predicted
Type: TM
MALGFSSAGEVYMYATCILLGVSLLMPLNALVSAPRFMVDYYKYVSGKEDAEPNLPFFWKNIFTFYNVVSL
ASQVIAGPTVLTRAARRLSLSVRFALSITLMMSEVFVVLMMPVIKVPQTVAIVLLCLVTIFAGIGKSYHEA
TCYVLVASMPSKFMSAVMFGVSLCGVITSTLQCIIKASMEDTYESVLTQSYIYFSLGILIMSATLAMVLCL
RYNSYAQEHVAEYRMLKLQEQGVDAESQNDENEPVAEGKGEGEGKSEGAMTTAEQLTATAVMPVVKIIRMM
LVTVFCGFFLTLFIFPSLIIPIDRDHNWFATIAILLYNCGDAIGRFSTSFKCVWPPRRALLYATFARFIFV
LPFMLCIYQYIPGHVGPYIFSFLLGLTNCVGAMSMVYGPITPGLETAGQKLMAGQLMGISLLSGIAAASVL
AMIVVIFLP
>tr|D0AAE2|D0AAE2_TRYB9 | DeepTMHMM Topology Prediction - Predicted
Type: TM
MCKRSDLLRKAKEVKIEEISQQKKQKKLLLRHLLSFLYFILFWVILLFSSGNTHAFCLCWYVHRHFSALYF
SVKKNDNNSIVSVQSGCPFFLLLLNPIRLFPPSPHFLAKSRGKSLCGIR
>tr|D0A0K2|D0A0K2_TRYB9 | DeepTMHMM Topology Prediction - Predicted
Type: TM
MCNTNLNQRSLLLWFVTFRLRRAVKQQQQRTASQTSVKKKKRNQLPFQLHYTPFVKFHFTVFFFLLHACSS
FVVYIFVARWRVGVQTKLLLTVTPTGPLGPLTVE

Domains topology of predicted plasma membrane proteins.

TMHMM result

```
# tr|C9ZN44|C9ZN44_TRYB9 Length: 1232
# tr|C9ZN44|C9ZN44_TRYB9 Number of predicted TMHs:  1
# tr|C9ZN44|C9ZN44_TRYB9 Exp number of AAs
in TMHs: 23.41878# tr|C9ZN44|C9ZN44_TRYB9
Exp number, first 60 AAs: 0.32011#
tr|C9ZN44|C9ZN44_TRYB9 Total prob of N-in:          0.01505
tr|C9ZN44|C9ZN44_TRYB9  TMHMM2.0        outside      1   849
tr|C9ZN44|C9ZN44_TRYB9  TMHMM2.0        TMhelix    850   872
tr|C9ZN44|C9ZN44_TRYB9  TMHMM2.0        inside     873  1232
```

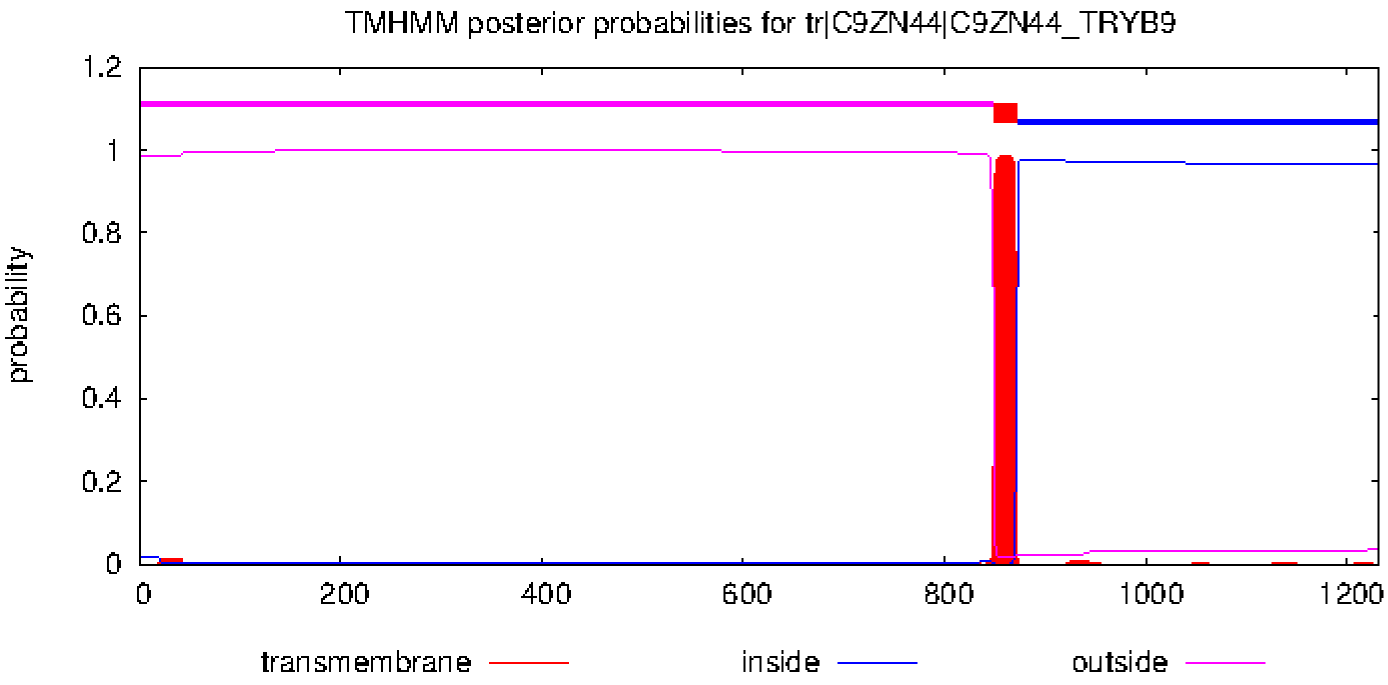

1 # plot in postscript, script for making the plot in gnuplot, data for plot

---

```
# tr|D0A9V0|D0A9V0_TRYB9 Length: 1195
# tr|D0A9V0|D0A9V0_TRYB9 Number of predicted TMHs:  3
# tr|D0A9V0|D0A9V0_TRYB9 Exp number of AAs in TMHs:
63.8735100000004# tr|D0A9V0|D0A9V0_TRYB9 Exp
number, first 60 AAs:                               0.00063
# tr|D0A9V0|D0A9V0_TRYB9 Total prob of N-in:        0.88570
tr|D0A9V0|D0A9V0_TRYB9  TMHMM2.0        inside       1    87
tr|D0A9V0|D0A9V0_TRYB9  TMHMM2.0        TMhelix     88   107
tr|D0A9V0|D0A9V0_TRYB9  TMHMM2.0        outside    108   110
tr|D0A9V0|D0A9V0_TRYB9  TMHMM2.0        TMhelix    111   128
tr|D0A9V0|D0A9V0_TRYB9  TMHMM2.0        inside     129   451
```

tr|D0A9V0|D0A9V0_TRYB9	TMHMM2.0	TMhelix	452	474
tr|D0A9V0|D0A9V0_TRYB9	TMHMM2.0	outside	475	1195

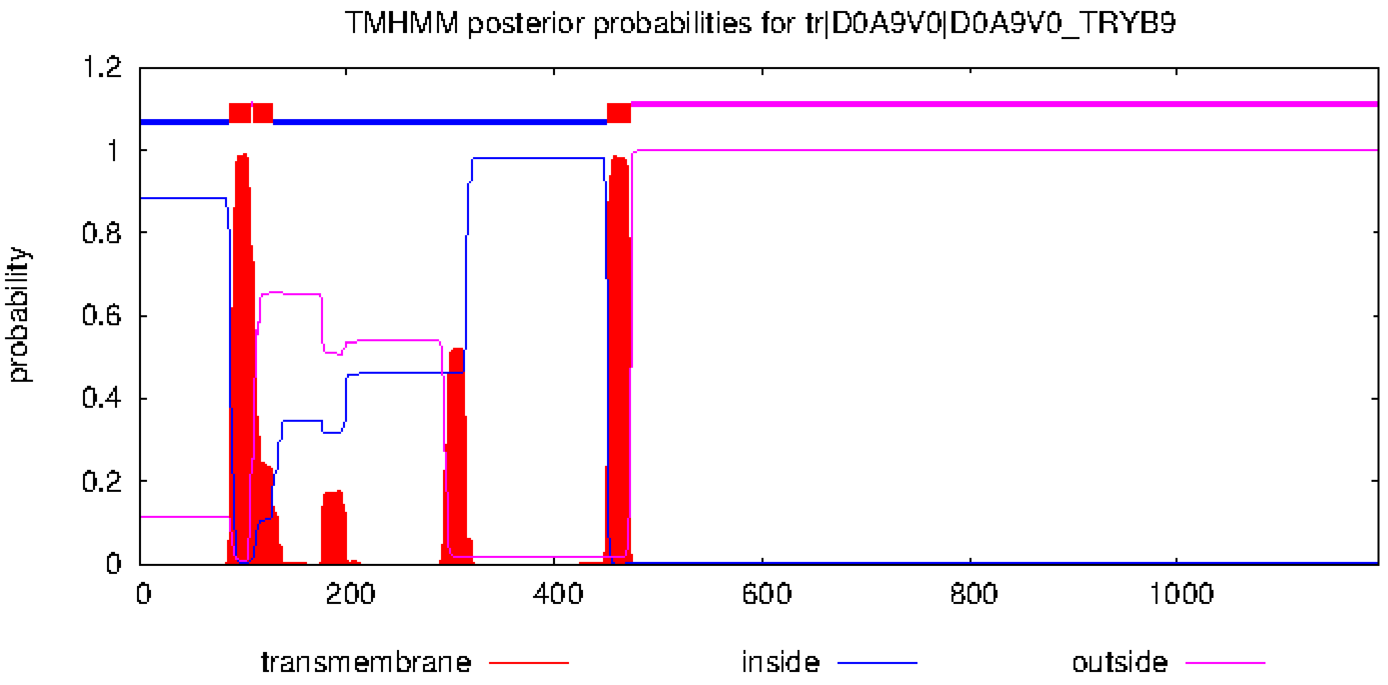

2 # plot in postscript, script for making the plot in gnuplot, data for plot

---

```
# tr|C9ZP82|C9ZP82_TRYB9 Length: 1036
# tr|C9ZP82|C9ZP82_TRYB9 Number of predicted TMHs:  2
# tr|C9ZP82|C9ZP82_TRYB9 Exp number of AAs in TMHs: 43.28301
# tr|C9ZP82|C9ZP82_TRYB9 Exp number, first 60 AAs:  0.00025
# tr|C9ZP82|C9ZP82_TRYB9 Total prob of N-in:        0.00133
tr|C9ZP82|C9ZP82_TRYB9  TMHMM2.0        outside      1   909
tr|C9ZP82|C9ZP82_TRYB9  TMHMM2.0        TMhelix    910   932
tr|C9ZP82|C9ZP82_TRYB9  TMHMM2.0        inside     933   938
tr|C9ZP82|C9ZP82_TRYB9  TMHMM2.0        TMhelix    939   958
tr|C9ZP82|C9ZP82_TRYB9  TMHMM2.0        outside    959  1036
```

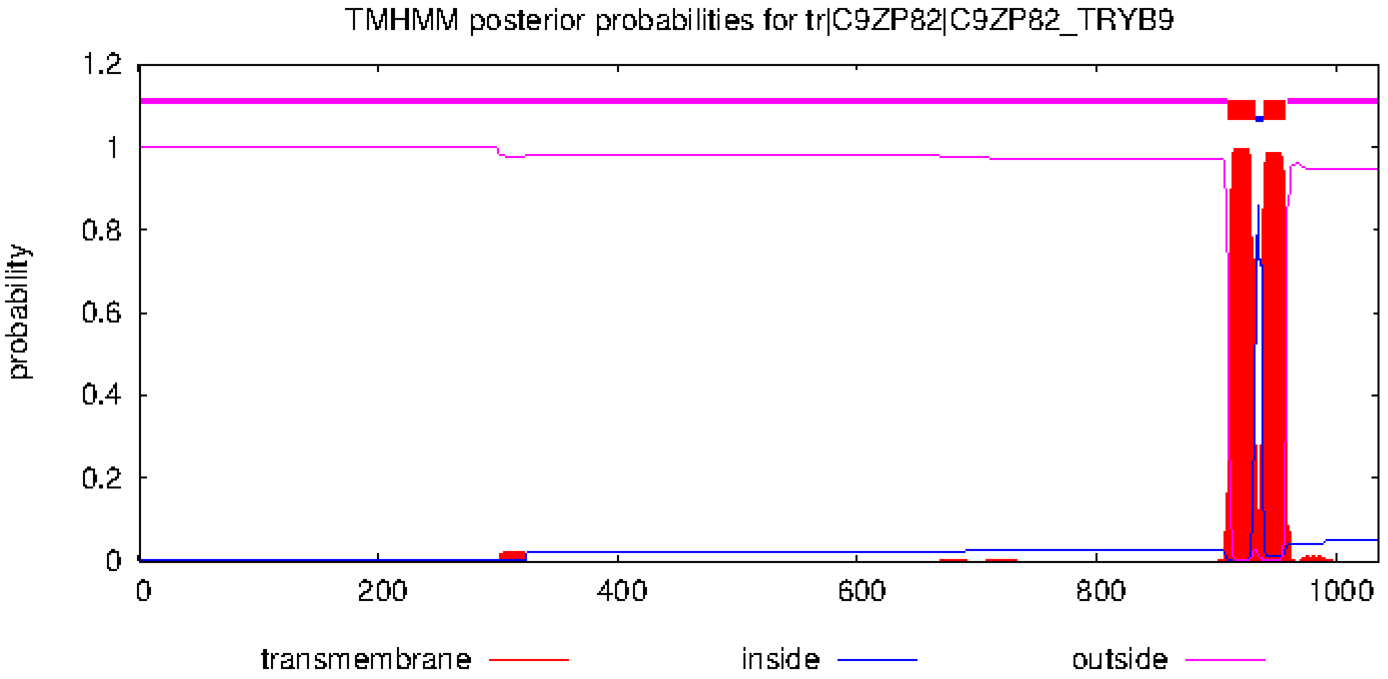

3 # plot in postscript, script for making the plot in gnuplot, data for plot

---

tr|C9ZJU1|C9ZJU1_TRYB9 Length: 1007
tr|C9ZJU1|C9ZJU1_TRYB9 Number of predicted TMHs: 1
tr|C9ZJU1|C9ZJU1_TRYB9 Exp number of AAs in TMHs: 54.4146900000001

```
# tr|C9ZJU1|C9ZJU1_TRYB9 Exp number, first 60 AAs:  21.91732
# tr|C9ZJU1|C9ZJU1_TRYB9 Total prob of N-in:        0.79725
# tr|C9ZJU1|C9ZJU1_TRYB9 POSSIBLE N-term signal sequence
tr|C9ZJU1|C9ZJU1_TRYB9  TMHMM2.0        inside       1     8
tr|C9ZJU1|C9ZJU1_TRYB9  TMHMM2.0        TMhelix      9    31
tr|C9ZJU1|C9ZJU1_TRYB9  TMHMM2.0        outside     32  1007
```

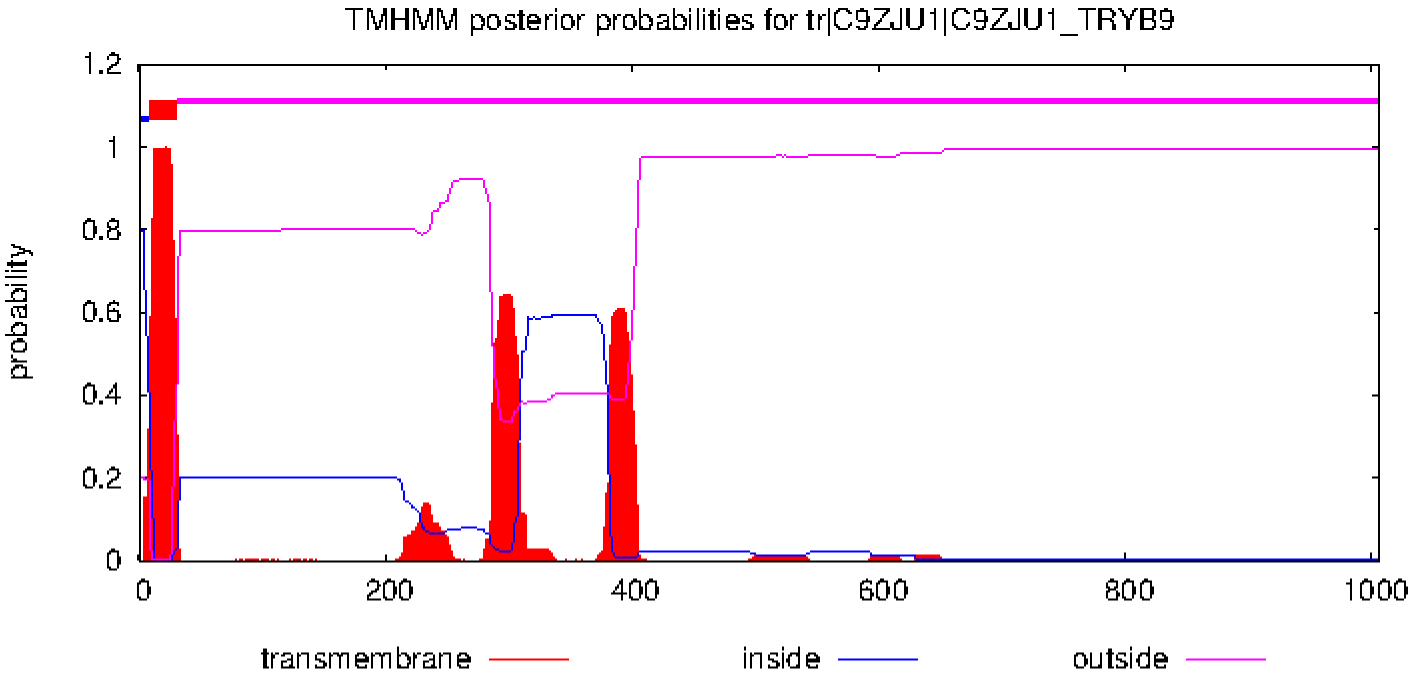

4 # plot in postscript, script for making the plot in gnuplot, data for plot

---

```
# tr|C9ZQ90|C9ZQ90_TRYB9 Length: 962
# tr|C9ZQ90|C9ZQ90_TRYB9 Number of predicted TMHs:  1
# tr|C9ZQ90|C9ZQ90_TRYB9 Exp number of AAs in TMHs: 30.39454
# tr|C9ZQ90|C9ZQ90_TRYB9 Exp number, first 60 AAs:  0.04093
# tr|C9ZQ90|C9ZQ90_TRYB9 Total prob of N-in:        0.28862
tr|C9ZQ90|C9ZQ90_TRYB9  TMHMM2.0        outside      1   579
tr|C9ZQ90|C9ZQ90_TRYB9  TMHMM2.0        TMhelix    580   602
tr|C9ZQ90|C9ZQ90_TRYB9  TMHMM2.0        inside     603   962
```

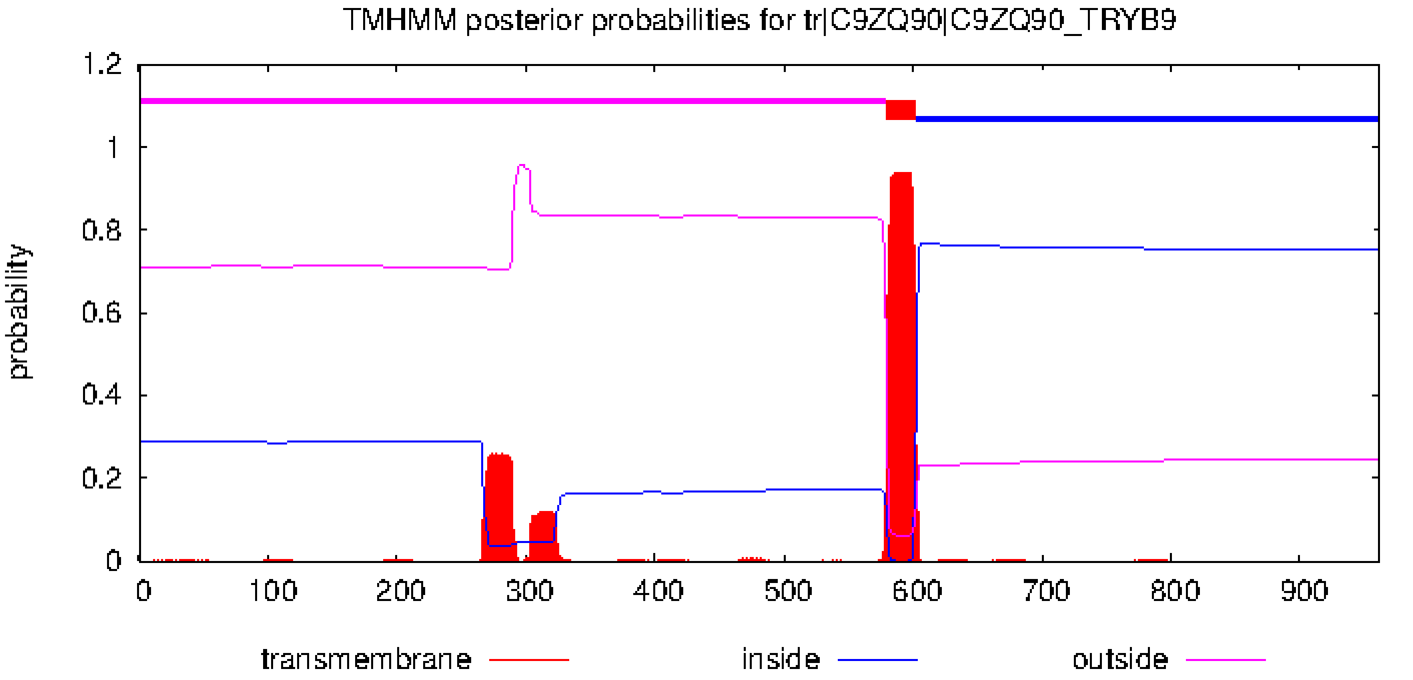

5 # plot in postscript, script for making the plot in gnuplot, data for plot

```
# tr|D0A254|D0A254_TRYB9 Length: 808
# tr|D0A254|D0A254_TRYB9 Number of predicted TMHs:  5
# tr|D0A254|D0A254_TRYB9 Exp number of AAs in TMHs: 131.03922
# tr|D0A254|D0A254_TRYB9 Exp number, first 60 AAs:  0.0123
# tr|D0A254|D0A254_TRYB9 Total prob of N-in:        0.36746
tr|D0A254|D0A254_TRYB9  TMHMM2.0        outside      1   349
tr|D0A254|D0A254_TRYB9  TMHMM2.0        TMhelix    350   372
tr|D0A254|D0A254_TRYB9  TMHMM2.0        inside     373   418
tr|D0A254|D0A254_TRYB9  TMHMM2.0        TMhelix    419   438
tr|D0A254|D0A254_TRYB9  TMHMM2.0        outside    439   474
tr|D0A254|D0A254_TRYB9  TMHMM2.0        TMhelix    475   497
tr|D0A254|D0A254_TRYB9  TMHMM2.0        inside     498   575
tr|D0A254|D0A254_TRYB9  TMHMM2.0        TMhelix    576   598
tr|D0A254|D0A254_TRYB9  TMHMM2.0        outside    599   662
tr|D0A254|D0A254_TRYB9  TMHMM2.0        TMhelix    663   685
tr|D0A254|D0A254_TRYB9  TMHMM2.0        inside     686   808
```

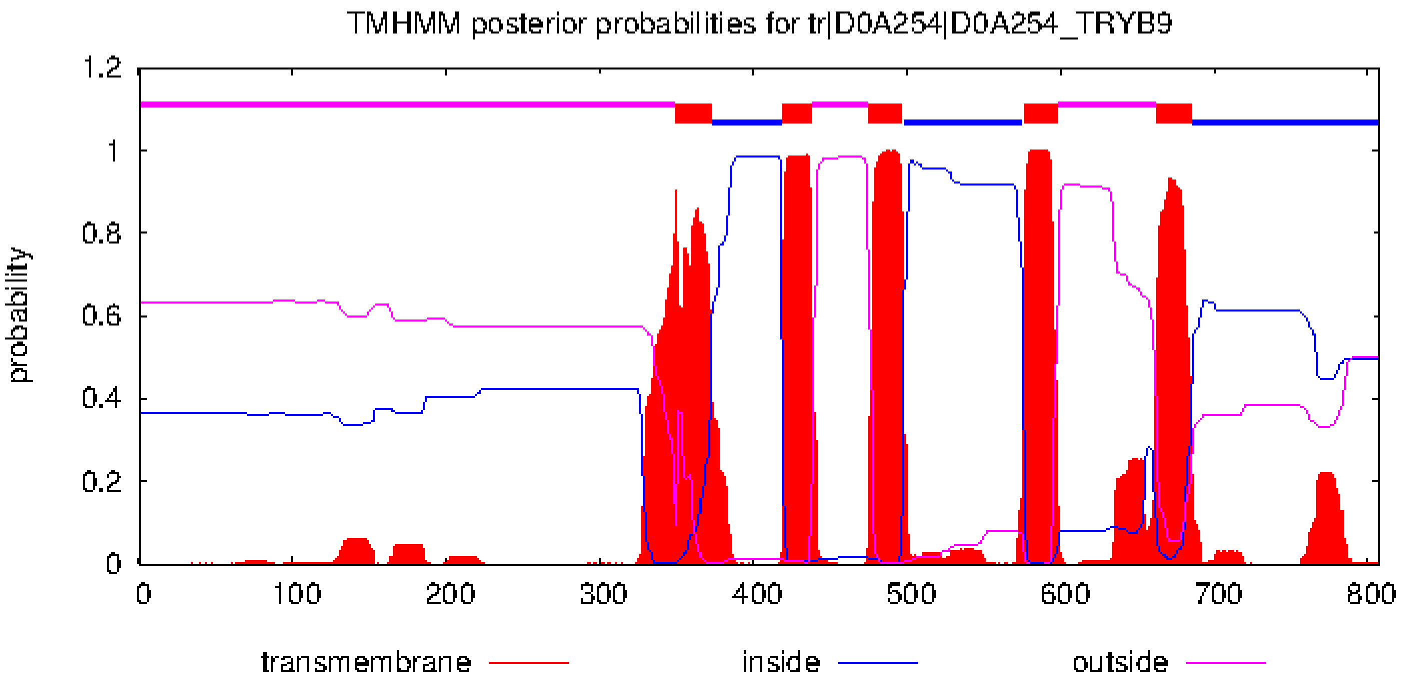

6 # **plot in postscript, script for making the plot in gnuplot, data for plot**

```
# tr|C9ZXW1|C9ZXW1_TRYB9 Length: 646
# tr|C9ZXW1|C9ZXW1_TRYB9 Number of predicted TMHs:  7
# tr|C9ZXW1|C9ZXW1_TRYB9 Exp number of AAs in TMHs: 150.67892
# tr|C9ZXW1|C9ZXW1_TRYB9 Exp number, first 60 AAs:  0.07045
# tr|C9ZXW1|C9ZXW1_TRYB9 Total prob of N-in:        0.04201
tr|C9ZXW1|C9ZXW1_TRYB9  TMHMM2.0        outside      1   362
tr|C9ZXW1|C9ZXW1_TRYB9  TMHMM2.0        TMhelix    363   382
tr|C9ZXW1|C9ZXW1_TRYB9  TMHMM2.0        inside     383   394
tr|C9ZXW1|C9ZXW1_TRYB9  TMHMM2.0        TMhelix    395   417
tr|C9ZXW1|C9ZXW1_TRYB9  TMHMM2.0        outside    418   446
tr|C9ZXW1|C9ZXW1_TRYB9  TMHMM2.0        TMhelix    447   469
tr|C9ZXW1|C9ZXW1_TRYB9  TMHMM2.0        inside     470   475
tr|C9ZXW1|C9ZXW1_TRYB9  TMHMM2.0        TMhelix    476   498
tr|C9ZXW1|C9ZXW1_TRYB9  TMHMM2.0        outside    499   501
tr|C9ZXW1|C9ZXW1_TRYB9  TMHMM2.0        TMhelix    502   524
tr|C9ZXW1|C9ZXW1_TRYB9  TMHMM2.0        inside     525   528
tr|C9ZXW1|C9ZXW1_TRYB9  TMHMM2.0        TMhelix    529   551
tr|C9ZXW1|C9ZXW1_TRYB9  TMHMM2.0        outside    552   590
tr|C9ZXW1|C9ZXW1_TRYB9  TMHMM2.0        TMhelix    591   613
tr|C9ZXW1|C9ZXW1_TRYB9  TMHMM2.0        inside     614   646
```

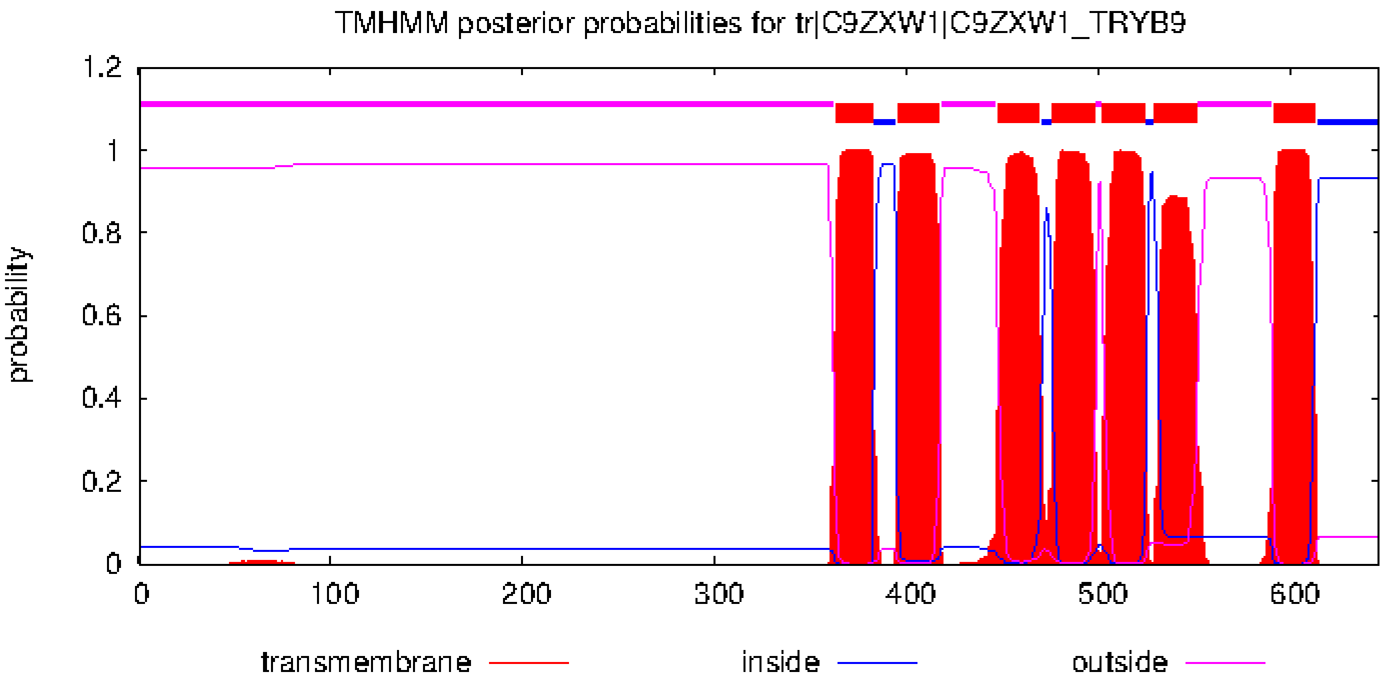

7 **# plot in postscript, script for making the plot in gnuplot, data for plot**

```
# tr|D0A7B1|D0A7B1_TRYB9 Length: 643
# tr|D0A7B1|D0A7B1_TRYB9 Number of predicted TMHs:  14
# tr|D0A7B1|D0A7B1_TRYB9 Exp number of AAs in TMHs: 309.39839
#  tr|D0A7B1|D0A7B1_TRYB9 Exp number, first 60 AAs: 24.91415
# tr|D0A7B1|D0A7B1_TRYB9 Total prob of N-in:        0.99607
# tr|D0A7B1|D0A7B1_TRYB9 POSSIBLE N-term signal sequence
tr|D0A7B1|D0A7B1_TRYB9  TMHMM2.0        inside       1    24
tr|D0A7B1|D0A7B1_TRYB9  TMHMM2.0        TMhelix     25    47
tr|D0A7B1|D0A7B1_TRYB9  TMHMM2.0        outside     48    61
tr|D0A7B1|D0A7B1_TRYB9  TMHMM2.0        TMhelix     62    81
tr|D0A7B1|D0A7B1_TRYB9  TMHMM2.0        inside      82    87
tr|D0A7B1|D0A7B1_TRYB9  TMHMM2.0        TMhelix     88   110
tr|D0A7B1|D0A7B1_TRYB9  TMHMM2.0        outside    111   114
tr|D0A7B1|D0A7B1_TRYB9  TMHMM2.0        TMhelix    115   137
tr|D0A7B1|D0A7B1_TRYB9  TMHMM2.0        inside     138   156
tr|D0A7B1|D0A7B1_TRYB9  TMHMM2.0        TMhelix    157   176
tr|D0A7B1|D0A7B1_TRYB9  TMHMM2.0        outside    177   185
tr|D0A7B1|D0A7B1_TRYB9  TMHMM2.0        TMhelix    186   208
tr|D0A7B1|D0A7B1_TRYB9  TMHMM2.0        inside     209   237
tr|D0A7B1|D0A7B1_TRYB9  TMHMM2.0        TMhelix    238   260
tr|D0A7B1|D0A7B1_TRYB9  TMHMM2.0        outside    261   274
tr|D0A7B1|D0A7B1_TRYB9  TMHMM2.0        TMhelix    275   297
tr|D0A7B1|D0A7B1_TRYB9  TMHMM2.0        inside     298   435
tr|D0A7B1|D0A7B1_TRYB9  TMHMM2.0        TMhelix    436   455
tr|D0A7B1|D0A7B1_TRYB9  TMHMM2.0        outside    456   469
tr|D0A7B1|D0A7B1_TRYB9  TMHMM2.0        TMhelix    470   492
tr|D0A7B1|D0A7B1_TRYB9  TMHMM2.0        inside     493   504
tr|D0A7B1|D0A7B1_TRYB9  TMHMM2.0        TMhelix    505   527
tr|D0A7B1|D0A7B1_TRYB9  TMHMM2.0        outside    528   530
tr|D0A7B1|D0A7B1_TRYB9  TMHMM2.0        TMhelix    531   553
tr|D0A7B1|D0A7B1_TRYB9  TMHMM2.0        inside     554   565
tr|D0A7B1|D0A7B1_TRYB9  TMHMM2.0        TMhelix    566   588
tr|D0A7B1|D0A7B1_TRYB9  TMHMM2.0        outside    589   610
tr|D0A7B1|D0A7B1_TRYB9  TMHMM2.0        TMhelix    611   633
tr|D0A7B1|D0A7B1_TRYB9  TMHMM2.0        inside     634   643
```

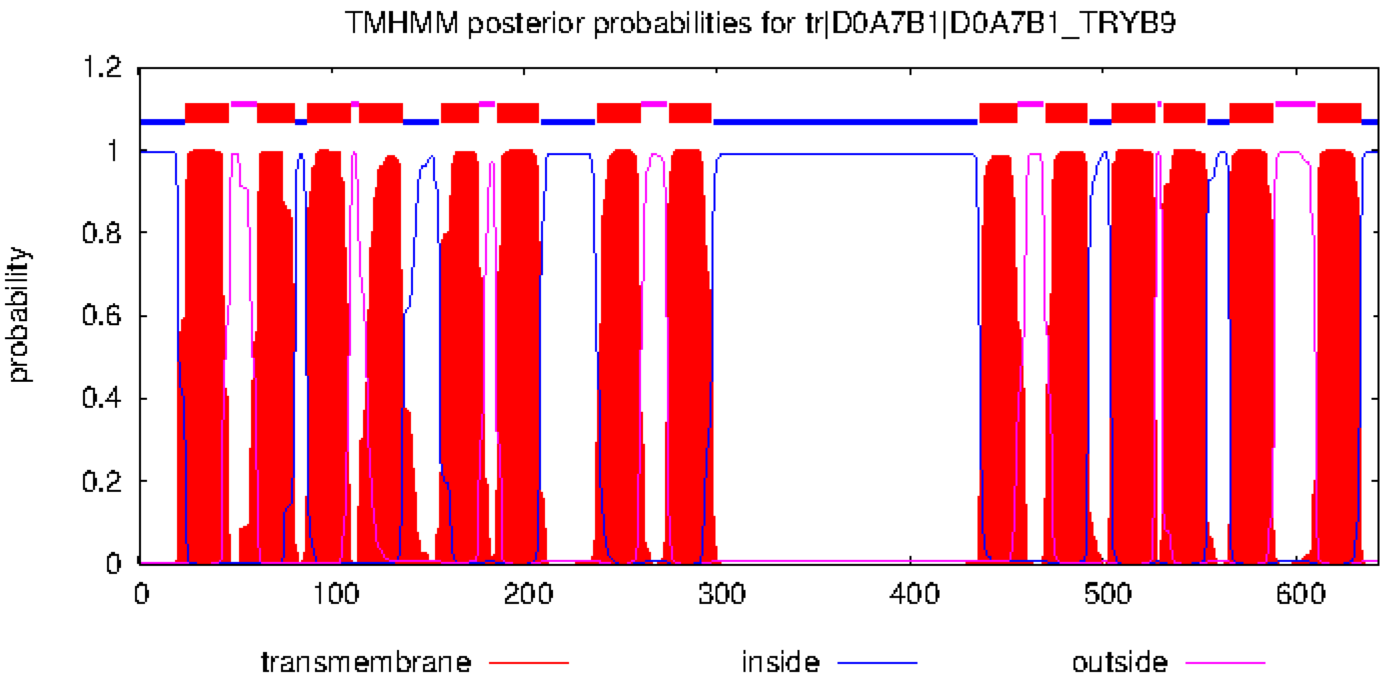

8 **# plot in postscript, script for making the plot in gnuplot, data for plot**

```
# tr|D0A423|D0A423_TRYB9 Length: 640
# tr|D0A423|D0A423_TRYB9 Number of predicted TMHs:  11
# tr|D0A423|D0A423_TRYB9 Exp number of AAs in TMHs: 252.49621
# tr|D0A423|D0A423_TRYB9 Exp number, first 60 AAs:  0.4285
# tr|D0A423|D0A423_TRYB9 Total prob of N-in:        0.41038
tr|D0A423|D0A423_TRYB9  TMHMM2.0        outside      1   132
tr|D0A423|D0A423_TRYB9  TMHMM2.0        TMhelix    133   152
tr|D0A423|D0A423_TRYB9  TMHMM2.0        inside     153   156
tr|D0A423|D0A423_TRYB9  TMHMM2.0        TMhelix    157   179
tr|D0A423|D0A423_TRYB9  TMHMM2.0        outside    180   198
tr|D0A423|D0A423_TRYB9  TMHMM2.0        TMhelix    199   221
tr|D0A423|D0A423_TRYB9  TMHMM2.0        inside     222   227
tr|D0A423|D0A423_TRYB9  TMHMM2.0        TMhelix    228   250
tr|D0A423|D0A423_TRYB9  TMHMM2.0        outside    251   349
tr|D0A423|D0A423_TRYB9  TMHMM2.0        TMhelix    350   372
tr|D0A423|D0A423_TRYB9  TMHMM2.0        inside     373   378
tr|D0A423|D0A423_TRYB9  TMHMM2.0        TMhelix    379   401
tr|D0A423|D0A423_TRYB9  TMHMM2.0        outside    402   444
tr|D0A423|D0A423_TRYB9  TMHMM2.0        TMhelix    445   467
tr|D0A423|D0A423_TRYB9  TMHMM2.0        inside     468   473
tr|D0A423|D0A423_TRYB9  TMHMM2.0        TMhelix    474   493
tr|D0A423|D0A423_TRYB9  TMHMM2.0        outside    494   507
tr|D0A423|D0A423_TRYB9  TMHMM2.0        TMhelix    508   530
tr|D0A423|D0A423_TRYB9  TMHMM2.0        inside     531   541
tr|D0A423|D0A423_TRYB9  TMHMM2.0        TMhelix    542   564
tr|D0A423|D0A423_TRYB9  TMHMM2.0        outside    565   583
tr|D0A423|D0A423_TRYB9  TMHMM2.0        TMhelix    584   606
tr|D0A423|D0A423_TRYB9  TMHMM2.0        inside     607   640
```

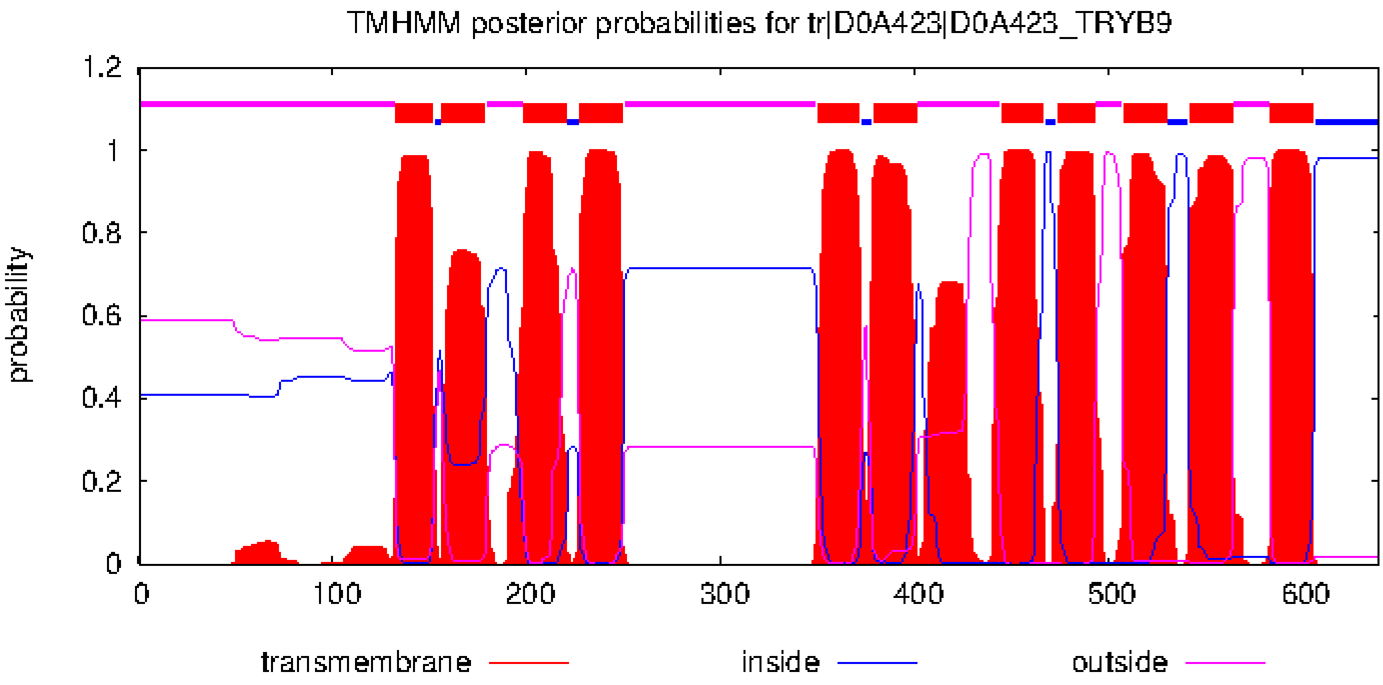

9 # **plot in postscript, script for making the plot in gnuplot, data for plot**

```
# tr|C9ZVE8|C9ZVE8_TRYB9 Length: 632
# tr|C9ZVE8|C9ZVE8_TRYB9 Number of predicted TMHs:  12
# tr|C9ZVE8|C9ZVE8_TRYB9 Exp number of AAs in TMHs: 287.4575
# tr|C9ZVE8|C9ZVE8_TRYB9 Exp number, first 60 AAs: 4.84829
# tr|C9ZVE8|C9ZVE8_TRYB9 Total prob of N-in:        0.83096
tr|C9ZVE8|C9ZVE8_TRYB9  TMHMM2.0        inside       1   142
tr|C9ZVE8|C9ZVE8_TRYB9  TMHMM2.0        TMhelix    143   162
tr|C9ZVE8|C9ZVE8_TRYB9  TMHMM2.0        outside    163   166
tr|C9ZVE8|C9ZVE8_TRYB9  TMHMM2.0        TMhelix    167   189
tr|C9ZVE8|C9ZVE8_TRYB9  TMHMM2.0        inside     190   200
tr|C9ZVE8|C9ZVE8_TRYB9  TMHMM2.0        TMhelix    201   223
tr|C9ZVE8|C9ZVE8_TRYB9  TMHMM2.0        outside    224   237
tr|C9ZVE8|C9ZVE8_TRYB9  TMHMM2.0        TMhelix    238   260
tr|C9ZVE8|C9ZVE8_TRYB9  TMHMM2.0        inside     261   335
tr|C9ZVE8|C9ZVE8_TRYB9  TMHMM2.0        TMhelix    336   358
tr|C9ZVE8|C9ZVE8_TRYB9  TMHMM2.0        outside    359   362
tr|C9ZVE8|C9ZVE8_TRYB9  TMHMM2.0        TMhelix    363   385
tr|C9ZVE8|C9ZVE8_TRYB9  TMHMM2.0        inside     386   391
tr|C9ZVE8|C9ZVE8_TRYB9  TMHMM2.0        TMhelix    392   414
tr|C9ZVE8|C9ZVE8_TRYB9  TMHMM2.0        outside    415   428
tr|C9ZVE8|C9ZVE8_TRYB9  TMHMM2.0        TMhelix    429   451
tr|C9ZVE8|C9ZVE8_TRYB9  TMHMM2.0        inside     452   455
tr|C9ZVE8|C9ZVE8_TRYB9  TMHMM2.0        TMhelix    456   478
tr|C9ZVE8|C9ZVE8_TRYB9  TMHMM2.0        outside    479   497
tr|C9ZVE8|C9ZVE8_TRYB9  TMHMM2.0        TMhelix    498   520
tr|C9ZVE8|C9ZVE8_TRYB9  TMHMM2.0        inside     521   526
tr|C9ZVE8|C9ZVE8_TRYB9  TMHMM2.0        TMhelix    527   549
tr|C9ZVE8|C9ZVE8_TRYB9  TMHMM2.0        outside    550   571
tr|C9ZVE8|C9ZVE8_TRYB9  TMHMM2.0        TMhelix    572   594
tr|C9ZVE8|C9ZVE8_TRYB9  TMHMM2.0        inside     595   632
```

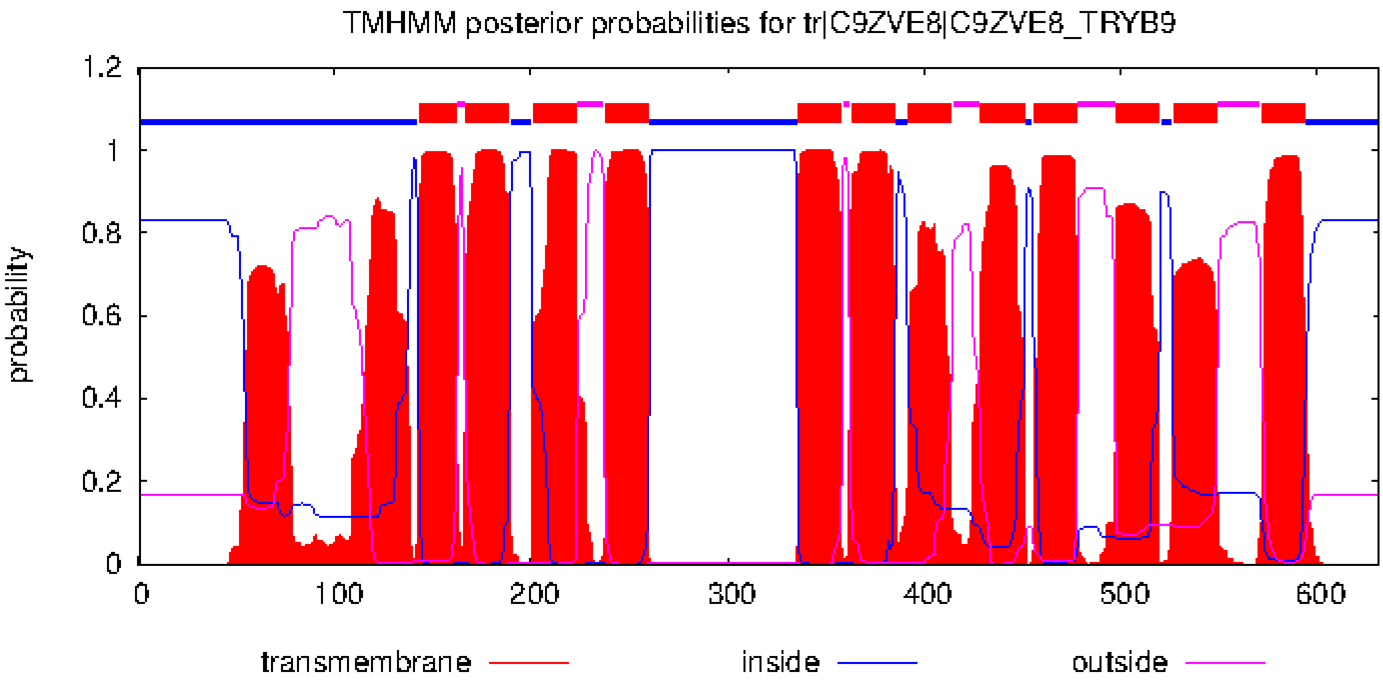

10 # **plot in postscript, script for making the plot in gnuplot, data for plot**

```
# tr|C9ZVF1|C9ZVF1_TRYB9 Length: 632
# tr|C9ZVF1|C9ZVF1_TRYB9 Number of predicted TMHs:  14
# tr|C9ZVF1|C9ZVF1_TRYB9 Exp number of AAs in TMHs: 298.08906
# tr|C9ZVF1|C9ZVF1_TRYB9 Exp number, first 60 AAs:  4.70806
# tr|C9ZVF1|C9ZVF1_TRYB9 Total prob of N-in:        0.82009
tr|C9ZVF1|C9ZVF1_TRYB9  TMHMM2.0        inside       1    53
tr|C9ZVF1|C9ZVF1_TRYB9  TMHMM2.0        TMhelix     54    76
tr|C9ZVF1|C9ZVF1_TRYB9  TMHMM2.0        outside     77   115
tr|C9ZVF1|C9ZVF1_TRYB9  TMHMM2.0        TMhelix    116   138
tr|C9ZVF1|C9ZVF1_TRYB9  TMHMM2.0        inside     139   142
tr|C9ZVF1|C9ZVF1_TRYB9  TMHMM2.0        TMhelix    143   162
tr|C9ZVF1|C9ZVF1_TRYB9  TMHMM2.0        outside    163   166
tr|C9ZVF1|C9ZVF1_TRYB9  TMHMM2.0        TMhelix    167   189
tr|C9ZVF1|C9ZVF1_TRYB9  TMHMM2.0        inside     190   200
tr|C9ZVF1|C9ZVF1_TRYB9  TMHMM2.0        TMhelix    201   223
tr|C9ZVF1|C9ZVF1_TRYB9  TMHMM2.0        outside    224   237
tr|C9ZVF1|C9ZVF1_TRYB9  TMHMM2.0        TMhelix    238   260
tr|C9ZVF1|C9ZVF1_TRYB9  TMHMM2.0        inside     261   335
tr|C9ZVF1|C9ZVF1_TRYB9  TMHMM2.0        TMhelix    336   358
tr|C9ZVF1|C9ZVF1_TRYB9  TMHMM2.0        outside    359   362
tr|C9ZVF1|C9ZVF1_TRYB9  TMHMM2.0        TMhelix    363   385
tr|C9ZVF1|C9ZVF1_TRYB9  TMHMM2.0        inside     386   391
tr|C9ZVF1|C9ZVF1_TRYB9  TMHMM2.0        TMhelix    392   414
tr|C9ZVF1|C9ZVF1_TRYB9  TMHMM2.0        outside    415   428
tr|C9ZVF1|C9ZVF1_TRYB9  TMHMM2.0        TMhelix    429   451
tr|C9ZVF1|C9ZVF1_TRYB9  TMHMM2.0        inside     452   455
tr|C9ZVF1|C9ZVF1_TRYB9  TMHMM2.0        TMhelix    456   478
tr|C9ZVF1|C9ZVF1_TRYB9  TMHMM2.0        outside    479   497
tr|C9ZVF1|C9ZVF1_TRYB9  TMHMM2.0        TMhelix    498   520
tr|C9ZVF1|C9ZVF1_TRYB9  TMHMM2.0        inside     521   526
tr|C9ZVF1|C9ZVF1_TRYB9  TMHMM2.0        TMhelix    527   549
tr|C9ZVF1|C9ZVF1_TRYB9  TMHMM2.0        outside    550   571
tr|C9ZVF1|C9ZVF1_TRYB9  TMHMM2.0        TMhelix    572   594
tr|C9ZVF1|C9ZVF1_TRYB9  TMHMM2.0        inside     595   632
```

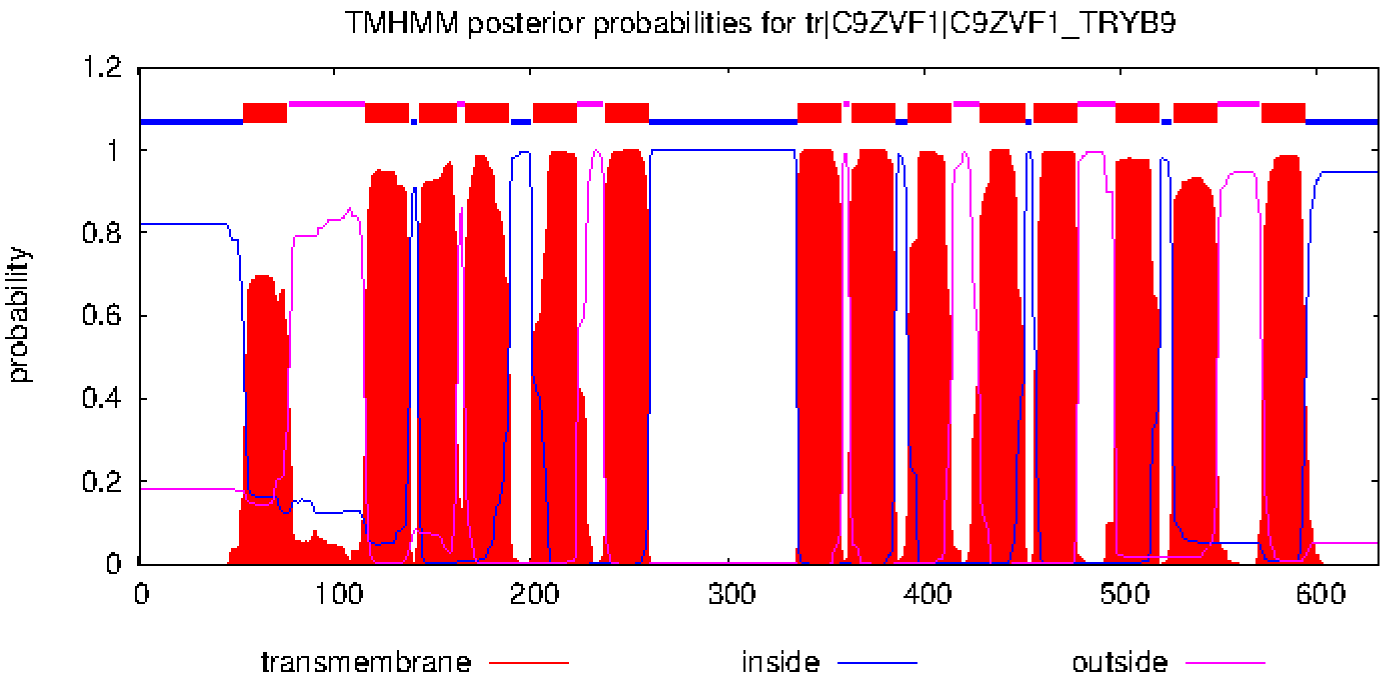

11 # plot in postscript, script for making the plot in gnuplot, data for plot

---

```
# tr|D0A8Z8|D0A8Z8_TRYB9 Length: 563
# tr|D0A8Z8|D0A8Z8_TRYB9 Number of predicted TMHs:  1
# tr|D0A8Z8|D0A8Z8_TRYB9 Exp number of AAs in TMHs: 26.34858
# tr|D0A8Z8|D0A8Z8_TRYB9 Exp number, first 60 AAs:  22.57859
# tr|D0A8Z8|D0A8Z8_TRYB9 Total prob of N-in:        0.71157
# tr|D0A8Z8|D0A8Z8_TRYB9 POSSIBLE N-term signal sequence
tr|D0A8Z8|D0A8Z8_TRYB9  TMHMM2.0        inside       1    18
tr|D0A8Z8|D0A8Z8_TRYB9  TMHMM2.0        TMhelix     19    41
tr|D0A8Z8|D0A8Z8_TRYB9  TMHMM2.0        outside     42   563
```

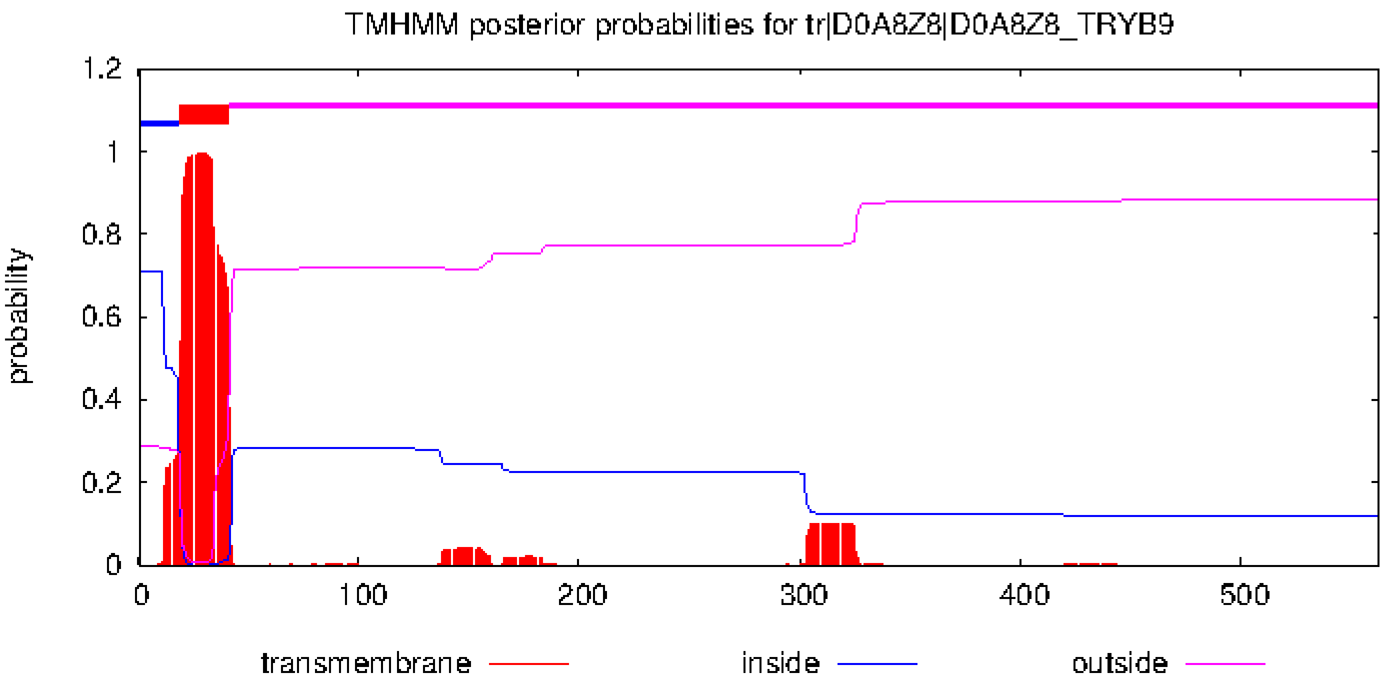

12 # plot in postscript, script for making the plot in gnuplot, data for plot

---

```
# tr|C9ZRE0|C9ZRE0_TRYB9 Length: 539
```

tr|C9ZRE0|C9ZRE0_TRYB9 Number of predicted TMHs: 11
tr|C9ZRE0|C9ZRE0_TRYB9 Exp number of AAs in TMHs: 259.133539999999
tr|C9ZRE0|C9ZRE0_TRYB9 Exp number, first 60 AAs: 22.42813

```
# tr|C9ZRE0|C9ZRE0_TRYB9 Total prob of N-in:        0.31572
# tr|C9ZRE0|C9ZRE0_TRYB9 POSSIBLE N-term signal sequence
tr|C9ZRE0|C9ZRE0_TRYB9  TMHMM2.0        outside      1    37
tr|C9ZRE0|C9ZRE0_TRYB9  TMHMM2.0        TMhelix     38    60
tr|C9ZRE0|C9ZRE0_TRYB9  TMHMM2.0        inside      61    72
tr|C9ZRE0|C9ZRE0_TRYB9  TMHMM2.0        TMhelix     73    95
tr|C9ZRE0|C9ZRE0_TRYB9  TMHMM2.0        outside     96   169
tr|C9ZRE0|C9ZRE0_TRYB9  TMHMM2.0        TMhelix    170   192
tr|C9ZRE0|C9ZRE0_TRYB9  TMHMM2.0        inside     193   204
tr|C9ZRE0|C9ZRE0_TRYB9  TMHMM2.0        TMhelix    205   227
tr|C9ZRE0|C9ZRE0_TRYB9  TMHMM2.0        outside    228   246
tr|C9ZRE0|C9ZRE0_TRYB9  TMHMM2.0        TMhelix    247   269
tr|C9ZRE0|C9ZRE0_TRYB9  TMHMM2.0        inside     270   289
tr|C9ZRE0|C9ZRE0_TRYB9  TMHMM2.0        TMhelix    290   312
tr|C9ZRE0|C9ZRE0_TRYB9  TMHMM2.0        outside    313   334
tr|C9ZRE0|C9ZRE0_TRYB9  TMHMM2.0        TMhelix    335   357
tr|C9ZRE0|C9ZRE0_TRYB9  TMHMM2.0        inside     358   385
tr|C9ZRE0|C9ZRE0_TRYB9  TMHMM2.0        TMhelix    386   408
tr|C9ZRE0|C9ZRE0_TRYB9  TMHMM2.0        outside    409   411
tr|C9ZRE0|C9ZRE0_TRYB9  TMHMM2.0        TMhelix    412   429
tr|C9ZRE0|C9ZRE0_TRYB9  TMHMM2.0        inside     430   441
tr|C9ZRE0|C9ZRE0_TRYB9  TMHMM2.0        TMhelix    442   464
tr|C9ZRE0|C9ZRE0_TRYB9  TMHMM2.0        outside    465   473
tr|C9ZRE0|C9ZRE0_TRYB9  TMHMM2.0        TMhelix    474   491
tr|C9ZRE0|C9ZRE0_TRYB9  TMHMM2.0        inside     492   539
```

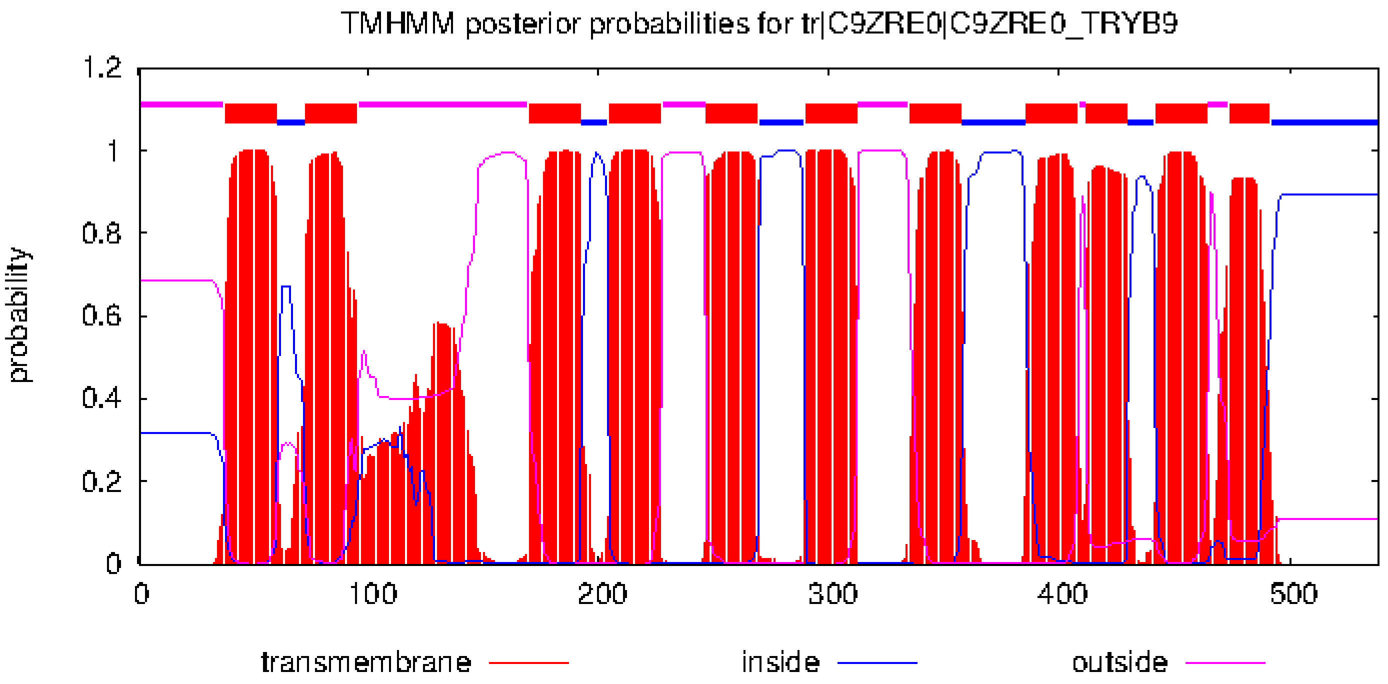

13 **# plot in postscript, script for making the plot in gnuplot, data for plot**

```
# tr|C9ZVS2|C9ZVS2_TRYB9 Length: 537
# tr|C9ZVS2|C9ZVS2_TRYB9 Number of predicted TMHs:  12
# tr|C9ZVS2|C9ZVS2_TRYB9 Exp number of AAs in TMHs: 257.71864
# tr|C9ZVS2|C9ZVS2_TRYB9 Exp number, first 60 AAs:  22.0932
# tr|C9ZVS2|C9ZVS2_TRYB9 Total prob of N-in:        0.14547
# tr|C9ZVS2|C9ZVS2_TRYB9 POSSIBLE N-term signal sequence
tr|C9ZVS2|C9ZVS2_TRYB9  TMHMM2.0        outside      1    31
tr|C9ZVS2|C9ZVS2_TRYB9  TMHMM2.0        TMhelix     32    54
tr|C9ZVS2|C9ZVS2_TRYB9  TMHMM2.0        inside      55   142
tr|C9ZVS2|C9ZVS2_TRYB9  TMHMM2.0        TMhelix    143   165
tr|C9ZVS2|C9ZVS2_TRYB9  TMHMM2.0        outside    166   168
tr|C9ZVS2|C9ZVS2_TRYB9  TMHMM2.0        TMhelix    169   191
tr|C9ZVS2|C9ZVS2_TRYB9  TMHMM2.0        inside     192   218
tr|C9ZVS2|C9ZVS2_TRYB9  TMHMM2.0        TMhelix    219   241
tr|C9ZVS2|C9ZVS2_TRYB9  TMHMM2.0        outside    242   260
```

tr|C9ZVS2|C9ZVS2_TRYB9	TMHMM2.0	TMhelix	261	278

```
tr|C9ZVS2|C9ZVS2_TRYB9  TMHMM2.0        inside     279   284
tr|C9ZVS2|C9ZVS2_TRYB9  TMHMM2.0        TMhelix    285   307
tr|C9ZVS2|C9ZVS2_TRYB9  TMHMM2.0        outside    308   328
tr|C9ZVS2|C9ZVS2_TRYB9  TMHMM2.0        TMhelix    329   351
tr|C9ZVS2|C9ZVS2_TRYB9  TMHMM2.0        inside     352   362
tr|C9ZVS2|C9ZVS2_TRYB9  TMHMM2.0        TMhelix    363   385
tr|C9ZVS2|C9ZVS2_TRYB9  TMHMM2.0        outside    386   404
tr|C9ZVS2|C9ZVS2_TRYB9  TMHMM2.0        TMhelix    405   424
tr|C9ZVS2|C9ZVS2_TRYB9  TMHMM2.0        inside     425   444
tr|C9ZVS2|C9ZVS2_TRYB9  TMHMM2.0        TMhelix    445   467
tr|C9ZVS2|C9ZVS2_TRYB9  TMHMM2.0        outside    468   471
tr|C9ZVS2|C9ZVS2_TRYB9  TMHMM2.0        TMhelix    472   494
tr|C9ZVS2|C9ZVS2_TRYB9  TMHMM2.0        inside     495   506
tr|C9ZVS2|C9ZVS2_TRYB9  TMHMM2.0        TMhelix    507   529
tr|C9ZVS2|C9ZVS2_TRYB9  TMHMM2.0        outside    530   537
```

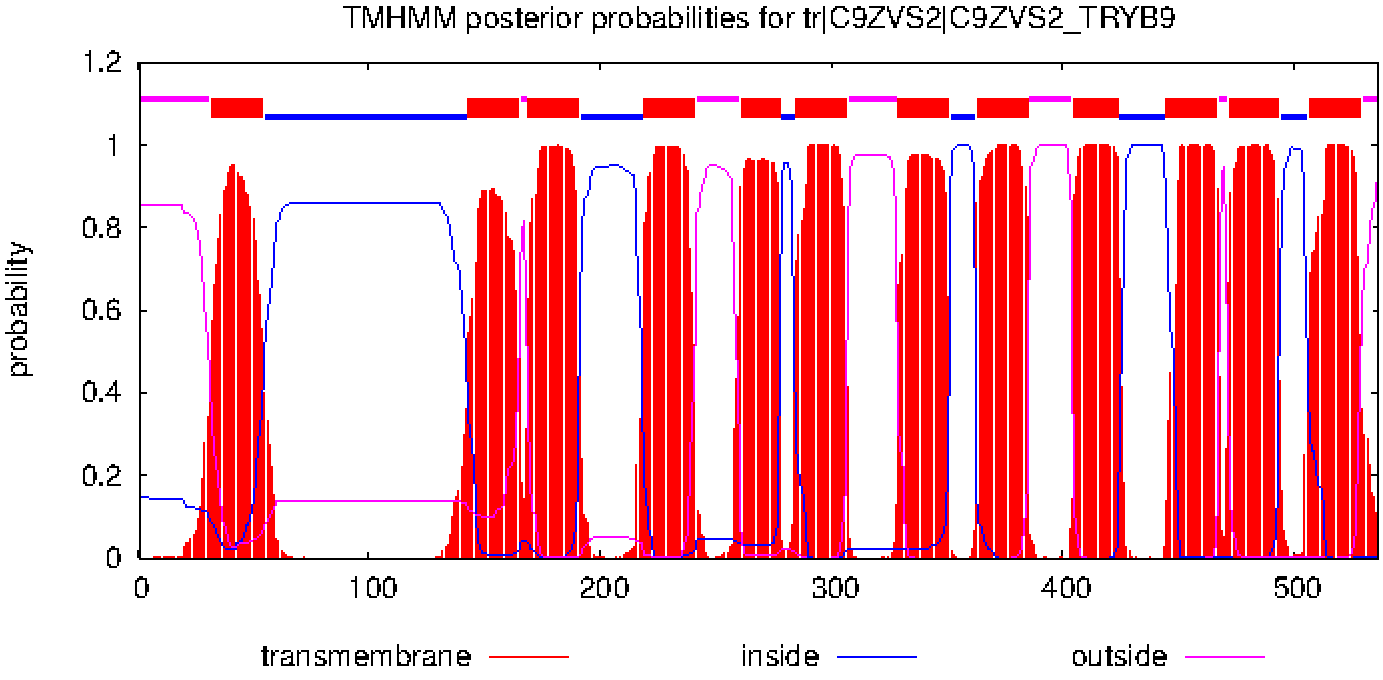

14 # plot in postscript, script for making the plot in gnuplot, data for plot

---

```
# tr|D0A3U1|D0A3U1_TRYB9 Length: 527
# tr|D0A3U1|D0A3U1_TRYB9 Number of predicted TMHs:  12
# tr|D0A3U1|D0A3U1_TRYB9 Exp number of AAs in TMHs: 259.58914
# tr|D0A3U1|D0A3U1_TRYB9 Exp number, first 60 AAs: 16.42563
# tr|D0A3U1|D0A3U1_TRYB9 Total prob of N-in:        0.74151
# tr|D0A3U1|D0A3U1_TRYB9 POSSIBLE N-term signal sequence
tr|D0A3U1|D0A3U1_TRYB9  TMHMM2.0        inside       1    43
tr|D0A3U1|D0A3U1_TRYB9  TMHMM2.0        TMhelix     44    66
tr|D0A3U1|D0A3U1_TRYB9  TMHMM2.0        outside     67   115
tr|D0A3U1|D0A3U1_TRYB9  TMHMM2.0        TMhelix    116   138
tr|D0A3U1|D0A3U1_TRYB9  TMHMM2.0        inside     139   150
tr|D0A3U1|D0A3U1_TRYB9  TMHMM2.0        TMhelix    151   170
tr|D0A3U1|D0A3U1_TRYB9  TMHMM2.0        outside    171   179
tr|D0A3U1|D0A3U1_TRYB9  TMHMM2.0        TMhelix    180   202
tr|D0A3U1|D0A3U1_TRYB9  TMHMM2.0        inside     203   214
tr|D0A3U1|D0A3U1_TRYB9  TMHMM2.0        TMhelix    215   234
tr|D0A3U1|D0A3U1_TRYB9  TMHMM2.0        outside    235   248
tr|D0A3U1|D0A3U1_TRYB9  TMHMM2.0        TMhelix    249   271
tr|D0A3U1|D0A3U1_TRYB9  TMHMM2.0        inside     272   300
tr|D0A3U1|D0A3U1_TRYB9  TMHMM2.0        TMhelix    301   323
tr|D0A3U1|D0A3U1_TRYB9  TMHMM2.0        outside    324   337
tr|D0A3U1|D0A3U1_TRYB9  TMHMM2.0        TMhelix    338   360
tr|D0A3U1|D0A3U1_TRYB9  TMHMM2.0        inside     361   372
tr|D0A3U1|D0A3U1_TRYB9  TMHMM2.0        TMhelix    373   392
```

tr|D0A3U1|D0A3U1_TRYB9	TMHMM2.0	outside	393	401
tr|D0A3U1|D0A3U1_TRYB9	TMHMM2.0	TMhelix	402	424

```
tr|D0A3U1|D0A3U1_TRYB9  TMHMM2.0        inside     425   436
tr|D0A3U1|D0A3U1_TRYB9  TMHMM2.0        TMhelix    437   459
tr|D0A3U1|D0A3U1_TRYB9  TMHMM2.0        outside    460   473
tr|D0A3U1|D0A3U1_TRYB9  TMHMM2.0        TMhelix    474   496
tr|D0A3U1|D0A3U1_TRYB9  TMHMM2.0        inside     497   527
```

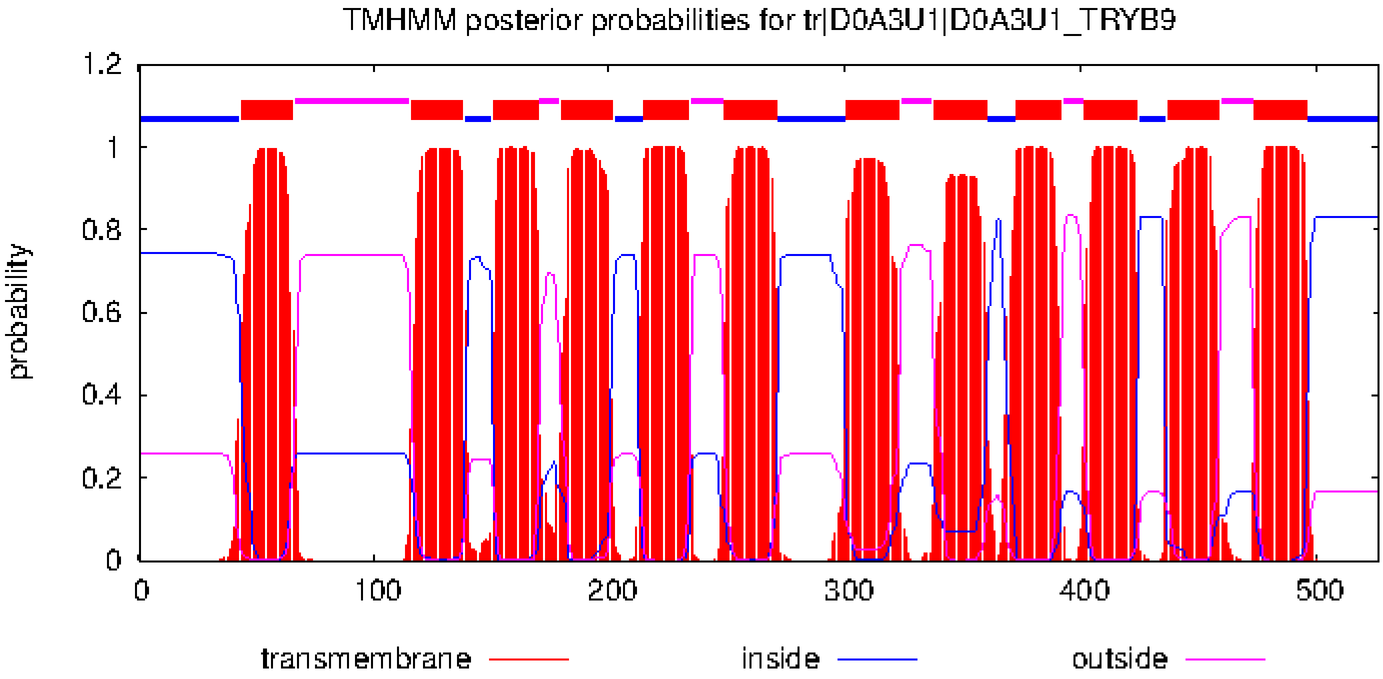

15 # plot in postscript, script for making the plot in gnuplot, data for plot

---

```
# tr|C9ZN60|C9ZN60_TRYB9 Length: 471
# tr|C9ZN60|C9ZN60_TRYB9 Number of predicted TMHs:  11
# tr|C9ZN60|C9ZN60_TRYB9 Exp number of AAs in TMHs: 241.48912
# tr|C9ZN60|C9ZN60_TRYB9 Exp number, first 60 AAs:  1.66247
# tr|C9ZN60|C9ZN60_TRYB9 Total prob of N-in:        0.98701
tr|C9ZN60|C9ZN60_TRYB9  TMHMM2.0        inside       1    62
tr|C9ZN60|C9ZN60_TRYB9  TMHMM2.0        TMhelix     63    85
tr|C9ZN60|C9ZN60_TRYB9  TMHMM2.0        outside     86    94
tr|C9ZN60|C9ZN60_TRYB9  TMHMM2.0        TMhelix     95   117
tr|C9ZN60|C9ZN60_TRYB9  TMHMM2.0        inside     118   147
tr|C9ZN60|C9ZN60_TRYB9  TMHMM2.0        TMhelix    148   170
tr|C9ZN60|C9ZN60_TRYB9  TMHMM2.0        outside    171   184
tr|C9ZN60|C9ZN60_TRYB9  TMHMM2.0        TMhelix    185   202
tr|C9ZN60|C9ZN60_TRYB9  TMHMM2.0        inside     203   208
tr|C9ZN60|C9ZN60_TRYB9  TMHMM2.0        TMhelix    209   231
tr|C9ZN60|C9ZN60_TRYB9  TMHMM2.0        outside    232   250
tr|C9ZN60|C9ZN60_TRYB9  TMHMM2.0        TMhelix    251   273
tr|C9ZN60|C9ZN60_TRYB9  TMHMM2.0        inside     274   293
tr|C9ZN60|C9ZN60_TRYB9  TMHMM2.0        TMhelix    294   316
tr|C9ZN60|C9ZN60_TRYB9  TMHMM2.0        outside    317   338
tr|C9ZN60|C9ZN60_TRYB9  TMHMM2.0        TMhelix    339   361
tr|C9ZN60|C9ZN60_TRYB9  TMHMM2.0        inside     362   381
tr|C9ZN60|C9ZN60_TRYB9  TMHMM2.0        TMhelix    382   403
tr|C9ZN60|C9ZN60_TRYB9  TMHMM2.0        outside    404   412
tr|C9ZN60|C9ZN60_TRYB9  TMHMM2.0        TMhelix    413   435
tr|C9ZN60|C9ZN60_TRYB9  TMHMM2.0        inside     436   446
tr|C9ZN60|C9ZN60_TRYB9  TMHMM2.0        TMhelix    447   469
tr|C9ZN60|C9ZN60_TRYB9  TMHMM2.0        outside    470   471
```

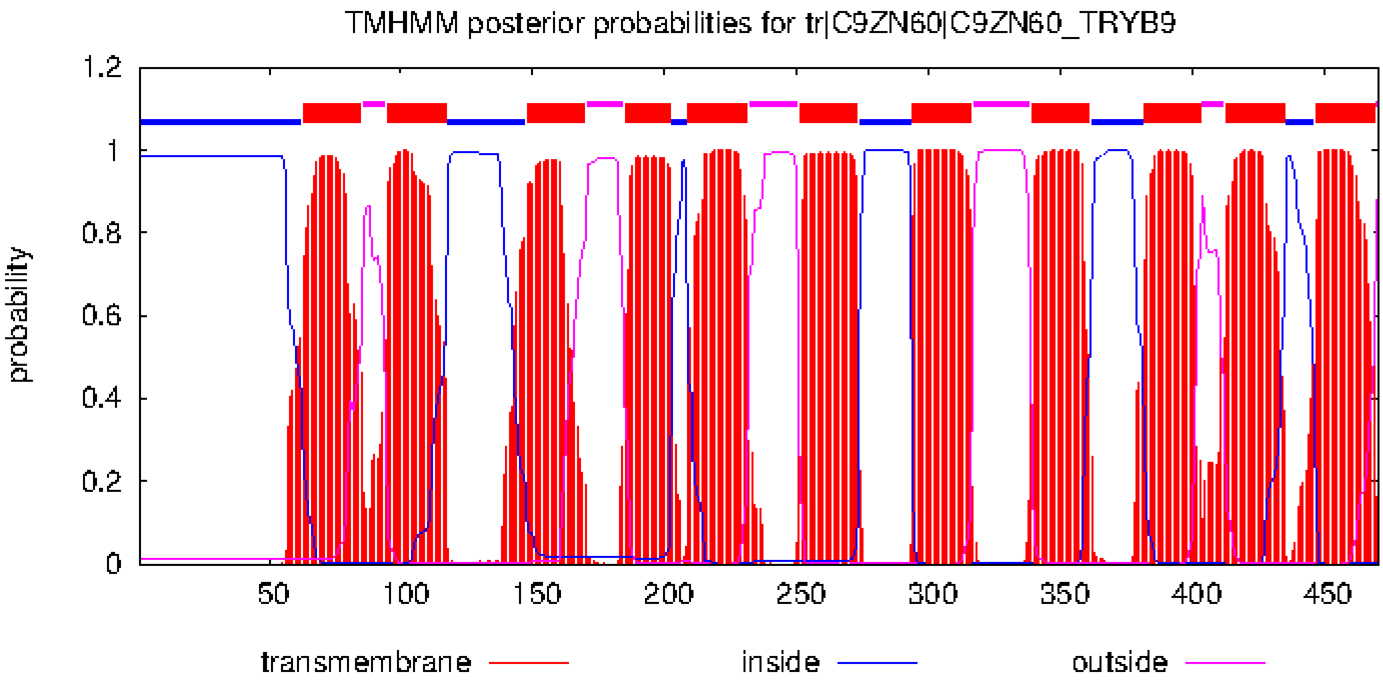

16 # plot in postscript, script for making the plot in gnuplot, data for plot

---

```
# tr|D0A0N1|D0A0N1_TRYB9 Length: 471
# tr|D0A0N1|D0A0N1_TRYB9 Number of predicted TMHs:  8
# tr|D0A0N1|D0A0N1_TRYB9 Exp number of AAs in TMHs: 194.42216
# tr|D0A0N1|D0A0N1_TRYB9 Exp number, first 60 AAs:  32.4738
# tr|D0A0N1|D0A0N1_TRYB9 Total prob of N-in:        0.76355
# tr|D0A0N1|D0A0N1_TRYB9 POSSIBLE N-term signal sequence
tr|D0A0N1|D0A0N1_TRYB9  TMHMM2.0        inside       1    11
tr|D0A0N1|D0A0N1_TRYB9  TMHMM2.0        TMhelix     12    34
tr|D0A0N1|D0A0N1_TRYB9  TMHMM2.0        outside     35    66
tr|D0A0N1|D0A0N1_TRYB9  TMHMM2.0        TMhelix     67    89
tr|D0A0N1|D0A0N1_TRYB9  TMHMM2.0        inside      90   100
tr|D0A0N1|D0A0N1_TRYB9  TMHMM2.0        TMhelix    101   123
tr|D0A0N1|D0A0N1_TRYB9  TMHMM2.0        outside    124   137
tr|D0A0N1|D0A0N1_TRYB9  TMHMM2.0        TMhelix    138   160
tr|D0A0N1|D0A0N1_TRYB9  TMHMM2.0        inside     161   293
tr|D0A0N1|D0A0N1_TRYB9  TMHMM2.0        TMhelix    294   316
tr|D0A0N1|D0A0N1_TRYB9  TMHMM2.0        outside    317   378
tr|D0A0N1|D0A0N1_TRYB9  TMHMM2.0        TMhelix    379   401
tr|D0A0N1|D0A0N1_TRYB9  TMHMM2.0        inside     402   412
tr|D0A0N1|D0A0N1_TRYB9  TMHMM2.0        TMhelix    413   432
tr|D0A0N1|D0A0N1_TRYB9  TMHMM2.0        outside    433   446
tr|D0A0N1|D0A0N1_TRYB9  TMHMM2.0        TMhelix    447   469
tr|D0A0N1|D0A0N1_TRYB9  TMHMM2.0        inside     470   471
```

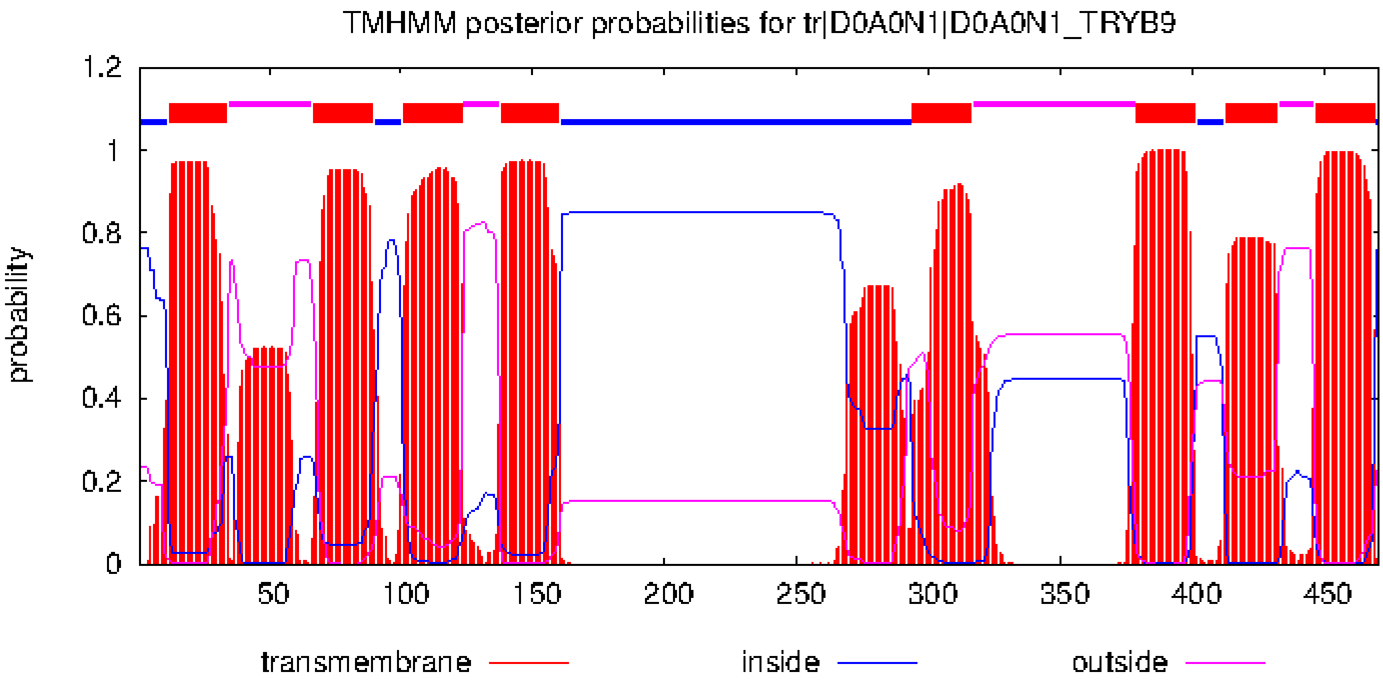

17 **# plot in postscript, script for making the plot in gnuplot, data for plot**

```
# tr|D0A400|D0A400_TRYB9 Length: 432
# tr|D0A400|D0A400_TRYB9 Number of predicted TMHs:  10
# tr|D0A400|D0A400_TRYB9 Exp number of AAs in TMHs: 199.77221
# tr|D0A400|D0A400_TRYB9 Exp number, first 60 AAs:  2.85212
# tr|D0A400|D0A400_TRYB9 Total prob of N-in:        0.66176
tr|D0A400|D0A400_TRYB9  TMHMM2.0        inside       1    55
tr|D0A400|D0A400_TRYB9  TMHMM2.0        TMhelix     56    78
tr|D0A400|D0A400_TRYB9  TMHMM2.0        outside     79    81
tr|D0A400|D0A400_TRYB9  TMHMM2.0        TMhelix     82   104
tr|D0A400|D0A400_TRYB9  TMHMM2.0        inside     105   110
tr|D0A400|D0A400_TRYB9  TMHMM2.0        TMhelix    111   128
tr|D0A400|D0A400_TRYB9  TMHMM2.0        outside    129   157
tr|D0A400|D0A400_TRYB9  TMHMM2.0        TMhelix    158   180
tr|D0A400|D0A400_TRYB9  TMHMM2.0        inside     181   199
tr|D0A400|D0A400_TRYB9  TMHMM2.0        TMhelix    200   222
tr|D0A400|D0A400_TRYB9  TMHMM2.0        outside    223   231
tr|D0A400|D0A400_TRYB9  TMHMM2.0        TMhelix    232   254
tr|D0A400|D0A400_TRYB9  TMHMM2.0        inside     255   260
tr|D0A400|D0A400_TRYB9  TMHMM2.0        TMhelix    261   280
tr|D0A400|D0A400_TRYB9  TMHMM2.0        outside    281   289
tr|D0A400|D0A400_TRYB9  TMHMM2.0        TMhelix    290   312
tr|D0A400|D0A400_TRYB9  TMHMM2.0        inside     313   347
tr|D0A400|D0A400_TRYB9  TMHMM2.0        TMhelix    348   370
tr|D0A400|D0A400_TRYB9  TMHMM2.0        outside    371   384
tr|D0A400|D0A400_TRYB9  TMHMM2.0        TMhelix    385   407
tr|D0A400|D0A400_TRYB9  TMHMM2.0        inside     408   432
```

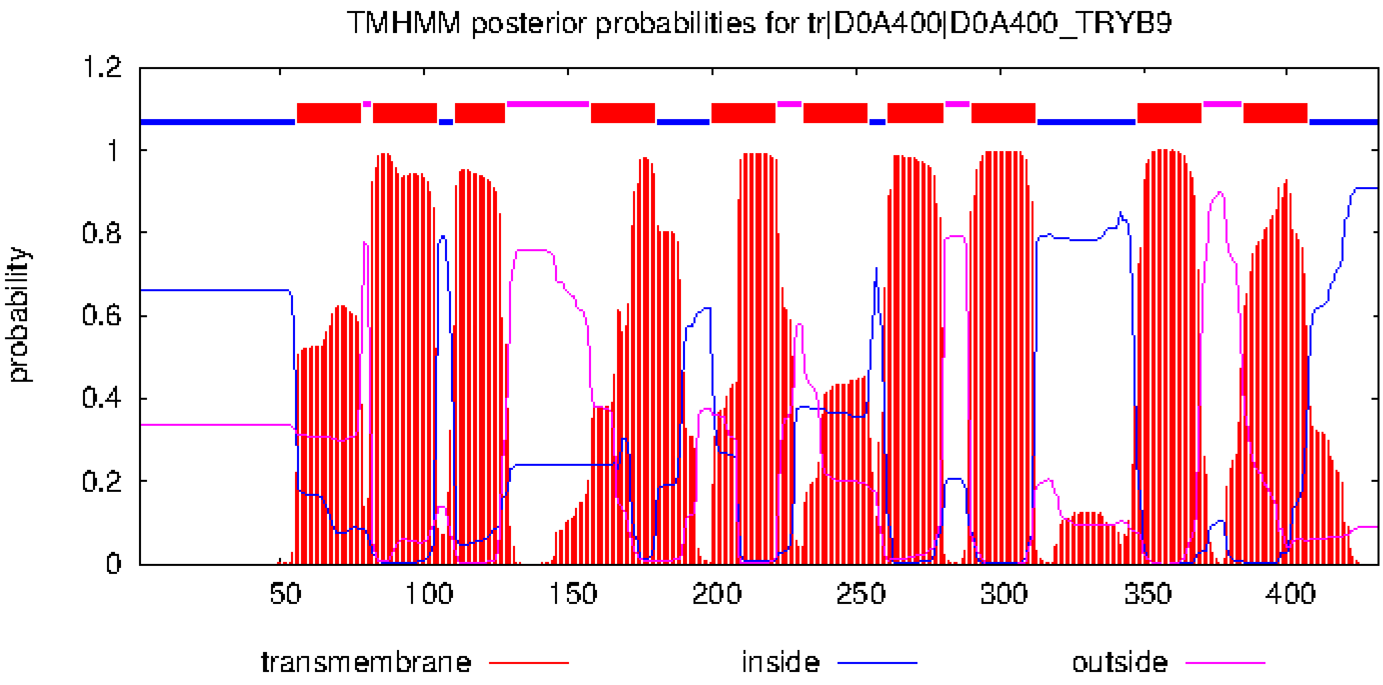

18 **# plot in postscript, script for making the plot in gnuplot, data for plot**

```
# tr|C9ZRP4|C9ZRP4_TRYB9 Length: 406
# tr|C9ZRP4|C9ZRP4_TRYB9 Number of predicted TMHs:  6
# tr|C9ZRP4|C9ZRP4_TRYB9 Exp number of AAs in TMHs: 150.5452
# tr|C9ZRP4|C9ZRP4_TRYB9 Exp number, first 60 AAs:  12.37011
# tr|C9ZRP4|C9ZRP4_TRYB9 Total prob of N-in:        0.42828
# tr|C9ZRP4|C9ZRP4_TRYB9 POSSIBLE N-term signal sequence
tr|C9ZRP4|C9ZRP4_TRYB9  TMHMM2.0        outside      1    52
tr|C9ZRP4|C9ZRP4_TRYB9  TMHMM2.0        TMhelix     53    75
tr|C9ZRP4|C9ZRP4_TRYB9  TMHMM2.0        inside      76    95
tr|C9ZRP4|C9ZRP4_TRYB9  TMHMM2.0        TMhelix     96   118
tr|C9ZRP4|C9ZRP4_TRYB9  TMHMM2.0        outside    119   132
tr|C9ZRP4|C9ZRP4_TRYB9  TMHMM2.0        TMhelix    133   155
tr|C9ZRP4|C9ZRP4_TRYB9  TMHMM2.0        inside     156   192
tr|C9ZRP4|C9ZRP4_TRYB9  TMHMM2.0        TMhelix    193   212
tr|C9ZRP4|C9ZRP4_TRYB9  TMHMM2.0        outside    213   231
tr|C9ZRP4|C9ZRP4_TRYB9  TMHMM2.0        TMhelix    232   254
tr|C9ZRP4|C9ZRP4_TRYB9  TMHMM2.0        inside     255   305
tr|C9ZRP4|C9ZRP4_TRYB9  TMHMM2.0        TMhelix    306   328
tr|C9ZRP4|C9ZRP4_TRYB9  TMHMM2.0        outside    329   406
```

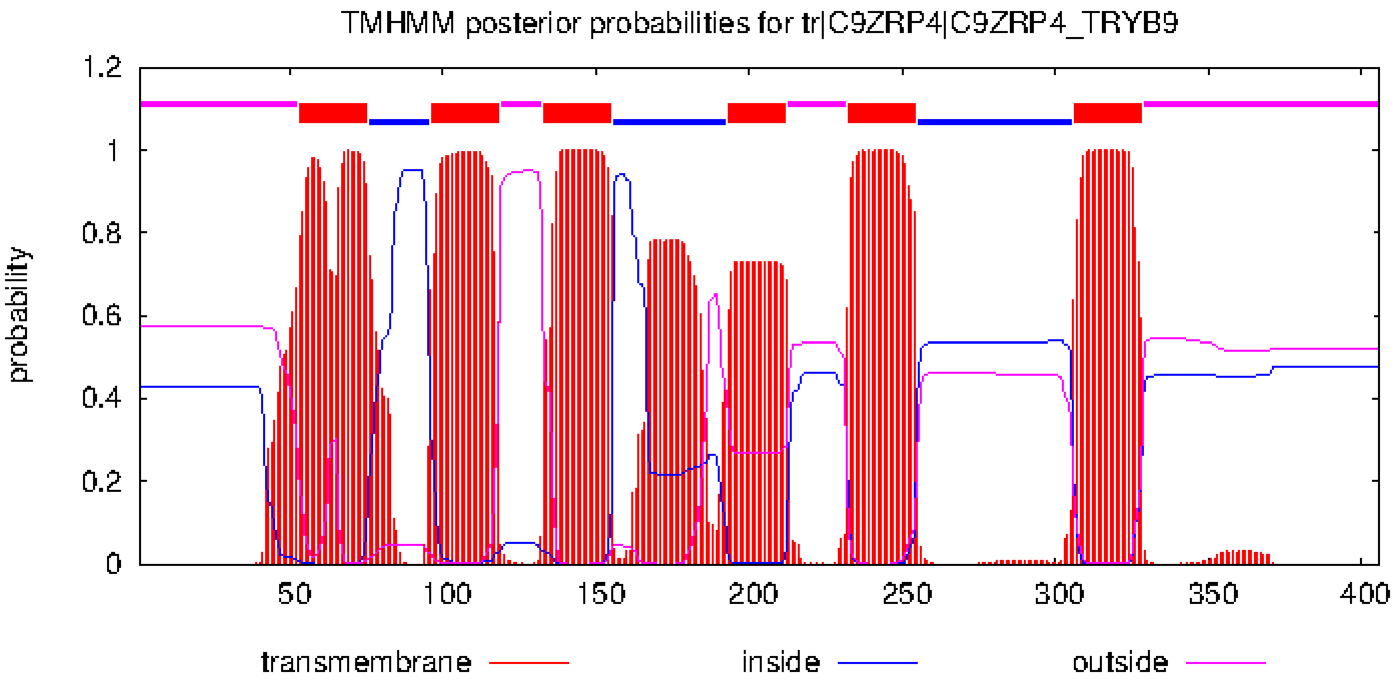

19 # plot in postscript, script for making the plot in gnuplot, data for plot

---

```
# tr|D0A888|D0A888_TRYB9 Length: 395
# tr|D0A888|D0A888_TRYB9 Number of predicted TMHs:  9
# tr|D0A888|D0A888_TRYB9 Exp number of AAs in TMHs: 176.93648
# tr|D0A888|D0A888_TRYB9 Exp number, first 60 AAs:  8.85637
# tr|D0A888|D0A888_TRYB9 Total prob of N-in:        0.77603
tr|D0A888|D0A888_TRYB9  TMHMM2.0        inside       1    12
tr|D0A888|D0A888_TRYB9  TMHMM2.0        TMhelix     13    35
tr|D0A888|D0A888_TRYB9  TMHMM2.0        outside     36    80
tr|D0A888|D0A888_TRYB9  TMHMM2.0        TMhelix     81   103
tr|D0A888|D0A888_TRYB9  TMHMM2.0        inside     104   115
tr|D0A888|D0A888_TRYB9  TMHMM2.0        TMhelix    116   138
tr|D0A888|D0A888_TRYB9  TMHMM2.0        outside    139   157
tr|D0A888|D0A888_TRYB9  TMHMM2.0        TMhelix    158   180
tr|D0A888|D0A888_TRYB9  TMHMM2.0        inside     181   232
tr|D0A888|D0A888_TRYB9  TMHMM2.0        TMhelix    233   255
tr|D0A888|D0A888_TRYB9  TMHMM2.0        outside    256   264
tr|D0A888|D0A888_TRYB9  TMHMM2.0        TMhelix    265   284
tr|D0A888|D0A888_TRYB9  TMHMM2.0        inside     285   295
tr|D0A888|D0A888_TRYB9  TMHMM2.0        TMhelix    296   318
tr|D0A888|D0A888_TRYB9  TMHMM2.0        outside    319   327
tr|D0A888|D0A888_TRYB9  TMHMM2.0        TMhelix    328   350
tr|D0A888|D0A888_TRYB9  TMHMM2.0        inside     351   370
tr|D0A888|D0A888_TRYB9  TMHMM2.0        TMhelix    371   392
tr|D0A888|D0A888_TRYB9  TMHMM2.0        outside    393   395
```

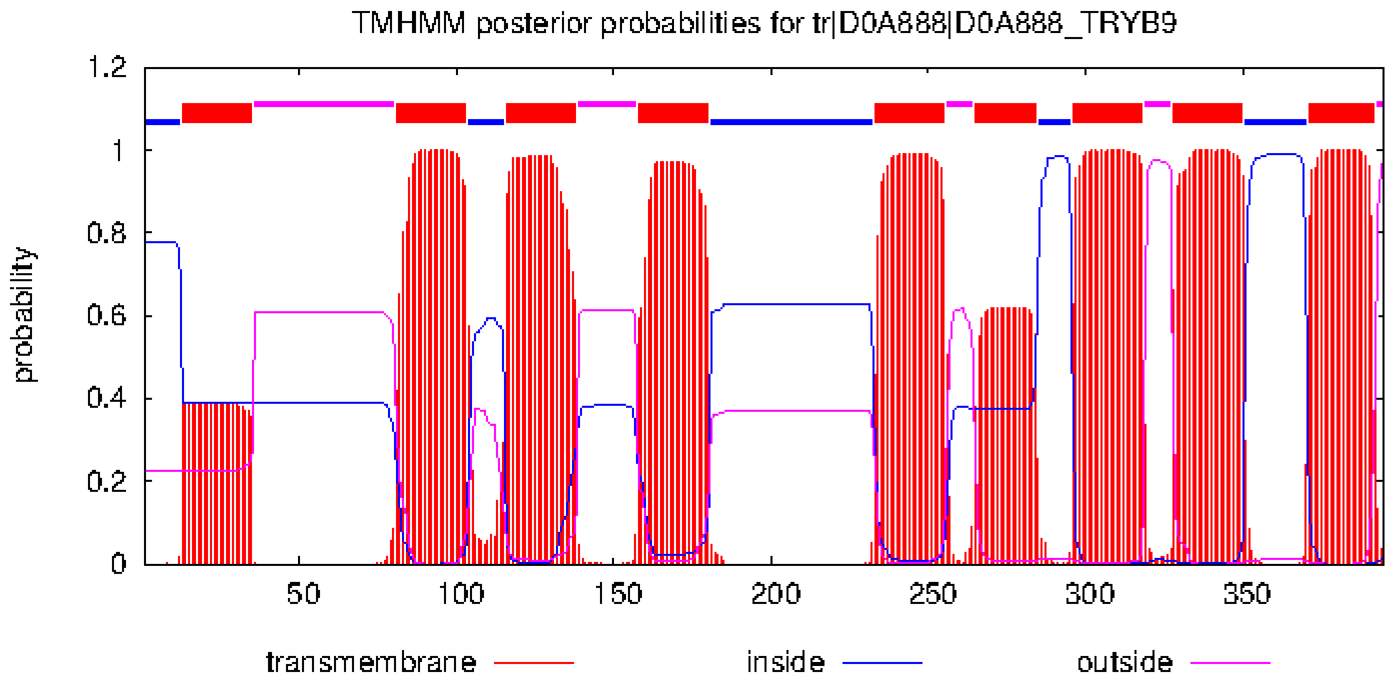

20 # plot in postscript, script for making the plot in gnuplot, data for plot

---

```
# tr|C9ZQ18|C9ZQ18_TRYB9 Length: 356
# tr|C9ZQ18|C9ZQ18_TRYB9 Number of predicted TMHs:  1
# tr|C9ZQ18|C9ZQ18_TRYB9 Exp number of AAs in TMHs: 22.88099
# tr|C9ZQ18|C9ZQ18_TRYB9 Exp number, first 60 AAs:  0
# tr|C9ZQ18|C9ZQ18_TRYB9 Total prob of N-in:        0.02232
tr|C9ZQ18|C9ZQ18_TRYB9  TMHMM2.0        outside      1   297
tr|C9ZQ18|C9ZQ18_TRYB9  TMHMM2.0        TMhelix    298   320
tr|C9ZQ18|C9ZQ18_TRYB9  TMHMM2.0        inside     321   356
```

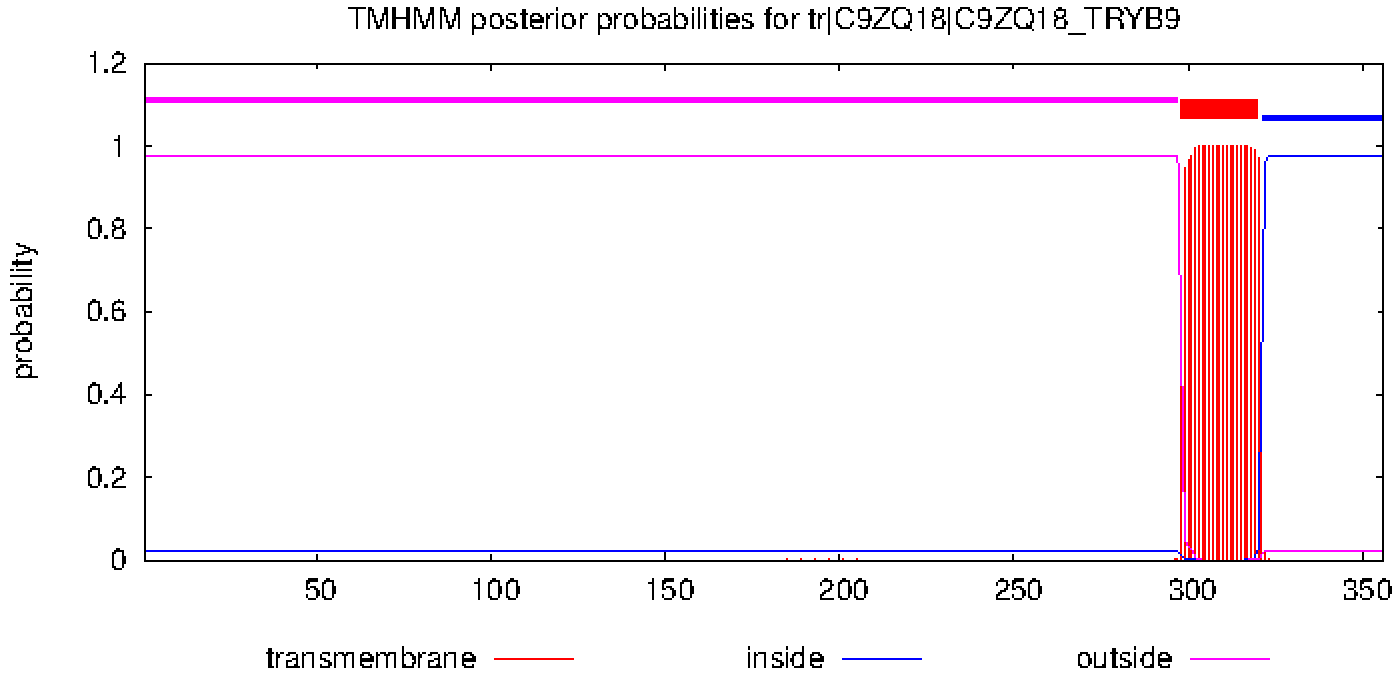

21 # plot in postscript, script for making the plot in gnuplot, data for plot

---

```
# tr|C9ZVD1|C9ZVD1_TRYB9 Length: 297
# tr|C9ZVD1|C9ZVD1_TRYB9 Number of predicted TMHs:  4
```

```
# tr|C9ZVD1|C9ZVD1_TRYB9 Exp number of AAs in TMHs: 92.1344900000001
# tr|C9ZVD1|C9ZVD1_TRYB9 Exp number, first 60 AAs:  27.05003
# tr|C9ZVD1|C9ZVD1_TRYB9 Total prob of N-in:        0.99956
```

```
#  tr|C9ZVD1|C9ZVD1_TRYB9  POSSIBLE N-term signal sequence
tr|C9ZVD1|C9ZVD1_TRYB9  TMHMM2.0        inside       1    19
tr|C9ZVD1|C9ZVD1_TRYB9  TMHMM2.0        TMhelix     20    42
tr|C9ZVD1|C9ZVD1_TRYB9  TMHMM2.0        outside     43    56
tr|C9ZVD1|C9ZVD1_TRYB9  TMHMM2.0        TMhelix     57    79
tr|C9ZVD1|C9ZVD1_TRYB9  TMHMM2.0        inside      80   176
tr|C9ZVD1|C9ZVD1_TRYB9  TMHMM2.0        TMhelix    177   199
tr|C9ZVD1|C9ZVD1_TRYB9  TMHMM2.0        outside    200   213
tr|C9ZVD1|C9ZVD1_TRYB9  TMHMM2.0        TMhelix    214   236
tr|C9ZVD1|C9ZVD1_TRYB9  TMHMM2.0        inside     237   297
```

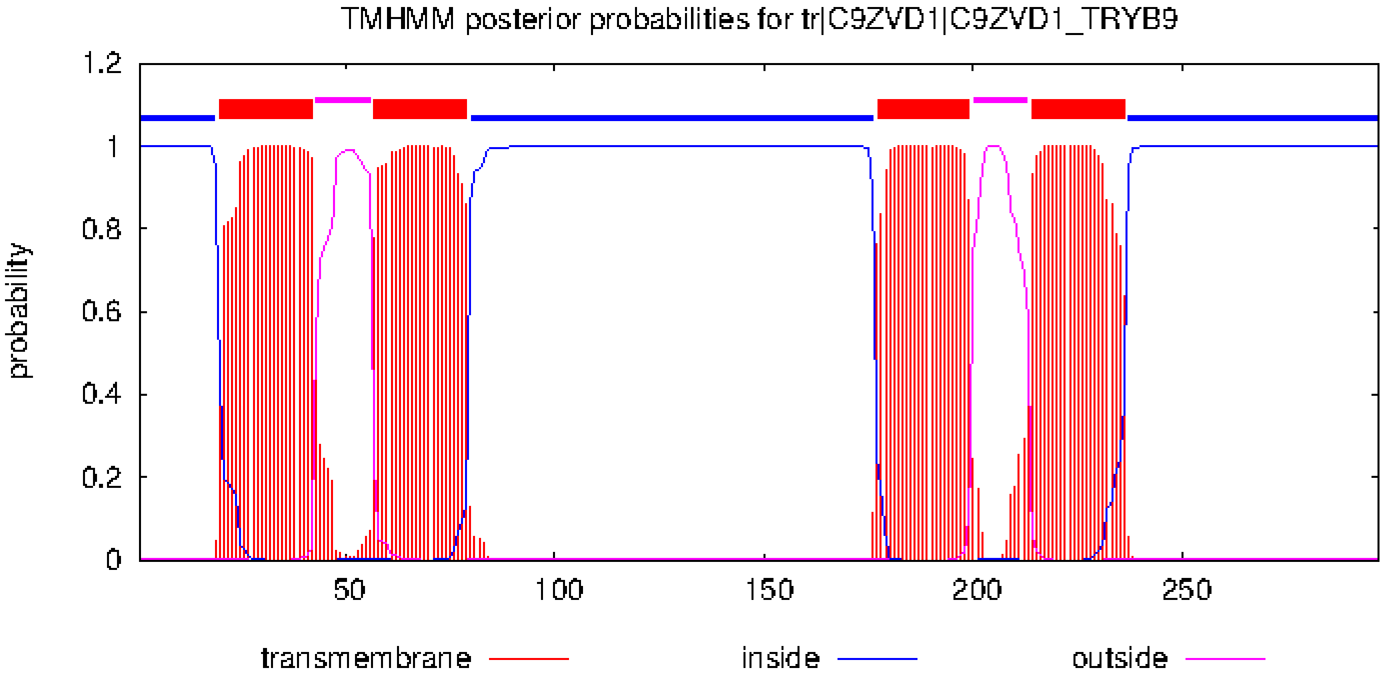

22 **# plot in postscript, script for making the plot in gnuplot, data for plot**

```
# tr|D0A0U2|D0A0U2_TRYB9 Length: 293
# tr|D0A0U2|D0A0U2_TRYB9 Number of predicted TMHs:  1
# tr|D0A0U2|D0A0U2_TRYB9 Exp number of AAs in TMHs: 25.87857
# tr|D0A0U2|D0A0U2_TRYB9 Exp number, first 60 AAs:  0.17622
# tr|D0A0U2|D0A0U2_TRYB9 Total prob of N-in:        0.10775
tr|D0A0U2|D0A0U2_TRYB9  TMHMM2.0        outside      1   245
tr|D0A0U2|D0A0U2_TRYB9  TMHMM2.0        TMhelix    246   268
tr|D0A0U2|D0A0U2_TRYB9  TMHMM2.0        inside     269   293
```

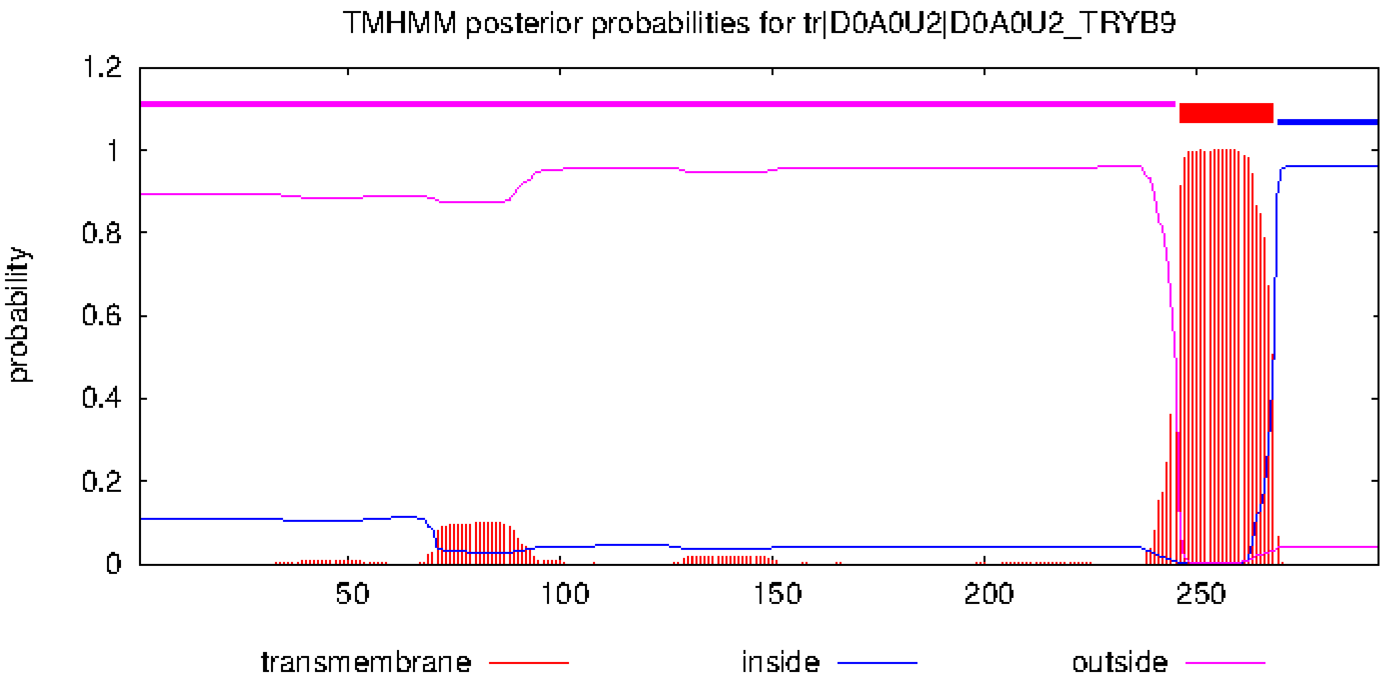

23 # **plot** in postscript, **script** for making the plot in gnuplot, **data** for plot

---

```
# tr|D0A867|D0A867_TRYB9 Length: 274
# tr|D0A867|D0A867_TRYB9 Number of predicted TMHs:  2
# tr|D0A867|D0A867_TRYB9 Exp number of AAs in TMHs: 40.6904500000001
# tr|D0A867|D0A867_TRYB9 Exp number, first 60 AAs:  0.40697
# tr|D0A867|D0A867_TRYB9 Total prob of N-in:        0.97315
tr|D0A867|D0A867_TRYB9  TMHMM2.0        inside       1    60
tr|D0A867|D0A867_TRYB9  TMHMM2.0        TMhelix     61    80
tr|D0A867|D0A867_TRYB9  TMHMM2.0        outside     81    84
tr|D0A867|D0A867_TRYB9  TMHMM2.0        TMhelix     85   107
tr|D0A867|D0A867_TRYB9  TMHMM2.0        inside     108   274
```

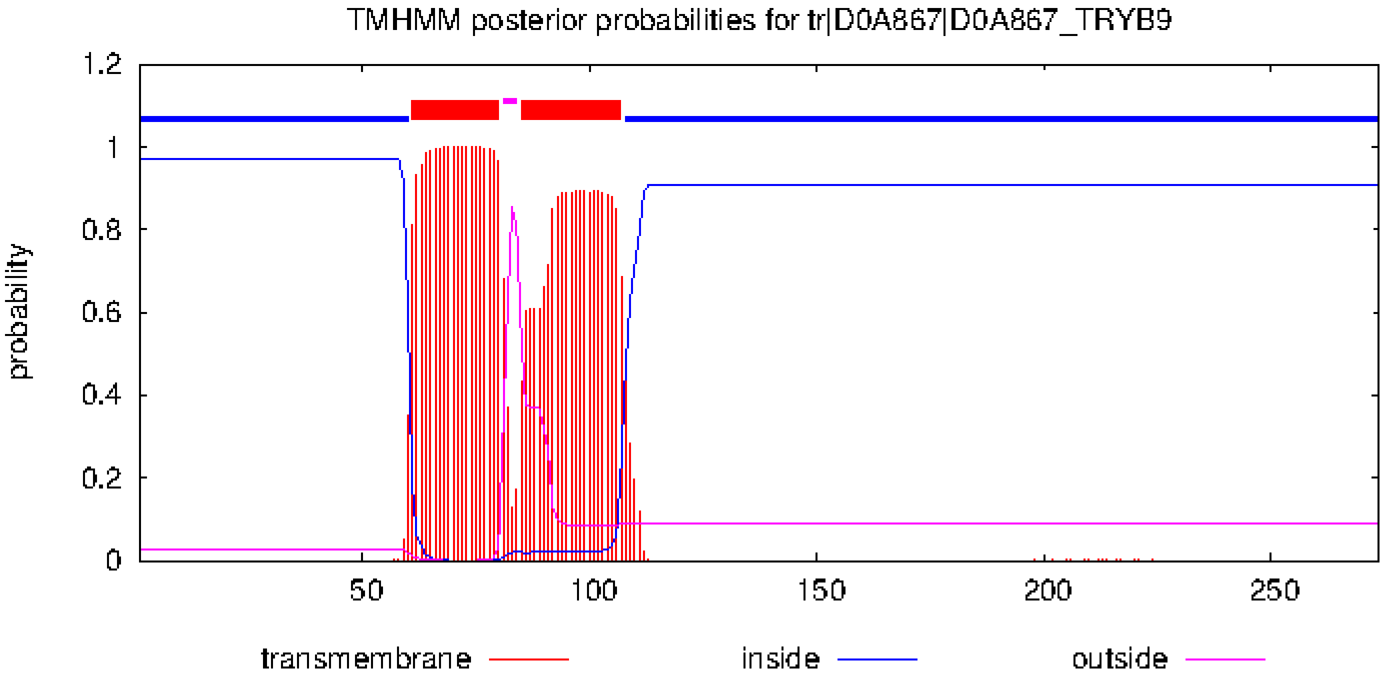

24 # **plot** in postscript, **script** for making the plot in gnuplot, **data** for plot

---

tr|D0A0Z8|D0A0Z8_TRYB9 Length: 254
tr|D0A0Z8|D0A0Z8_TRYB9 Number of predicted TMHs: 2
tr|D0A0Z8|D0A0Z8_TRYB9 Exp number of AAs in TMHs: 43.12932

```
# tr|D0A0Z8|D0A0Z8_TRYB9 Exp number, first 60 AAs:  0.00056
# tr|D0A0Z8|D0A0Z8_TRYB9 Total prob of N-in:        0.99666
tr|D0A0Z8|D0A0Z8_TRYB9  TMHMM2.0        inside       1   137
tr|D0A0Z8|D0A0Z8_TRYB9  TMHMM2.0        TMhelix    138   160
tr|D0A0Z8|D0A0Z8_TRYB9  TMHMM2.0        outside    161   169
tr|D0A0Z8|D0A0Z8_TRYB9  TMHMM2.0        TMhelix    170   189
tr|D0A0Z8|D0A0Z8_TRYB9  TMHMM2.0        inside     190   254
```

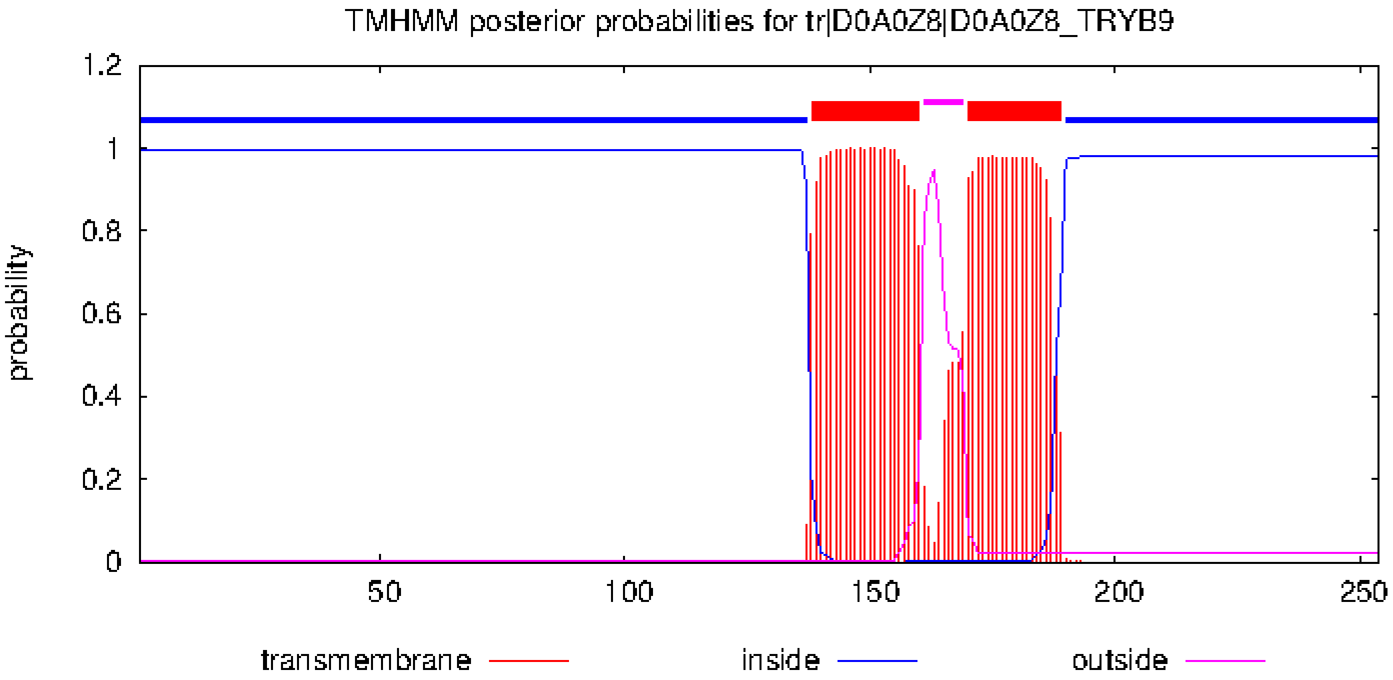

25 **# plot in postscript, script for making the plot in gnuplot, data for plot**

```
# tr|C9ZSX5|C9ZSX5_TRYB9 Length: 180
# tr|C9ZSX5|C9ZSX5_TRYB9 Number of predicted TMHs:  3
# tr|C9ZSX5|C9ZSX5_TRYB9 Exp number of AAs in TMHs: 61.28537
# tr|C9ZSX5|C9ZSX5_TRYB9 Exp number, first 60 AAs:  0.0002
# tr|C9ZSX5|C9ZSX5_TRYB9 Total prob of N-in:        0.96061
tr|C9ZSX5|C9ZSX5_TRYB9  TMHMM2.0        inside       1    69
tr|C9ZSX5|C9ZSX5_TRYB9  TMHMM2.0        TMhelix     70    92
tr|C9ZSX5|C9ZSX5_TRYB9  TMHMM2.0        outside     93   101
tr|C9ZSX5|C9ZSX5_TRYB9  TMHMM2.0        TMhelix    102   121
tr|C9ZSX5|C9ZSX5_TRYB9  TMHMM2.0        inside     122   141
tr|C9ZSX5|C9ZSX5_TRYB9  TMHMM2.0        TMhelix    142   161
tr|C9ZSX5|C9ZSX5_TRYB9  TMHMM2.0        outside    162   180
```

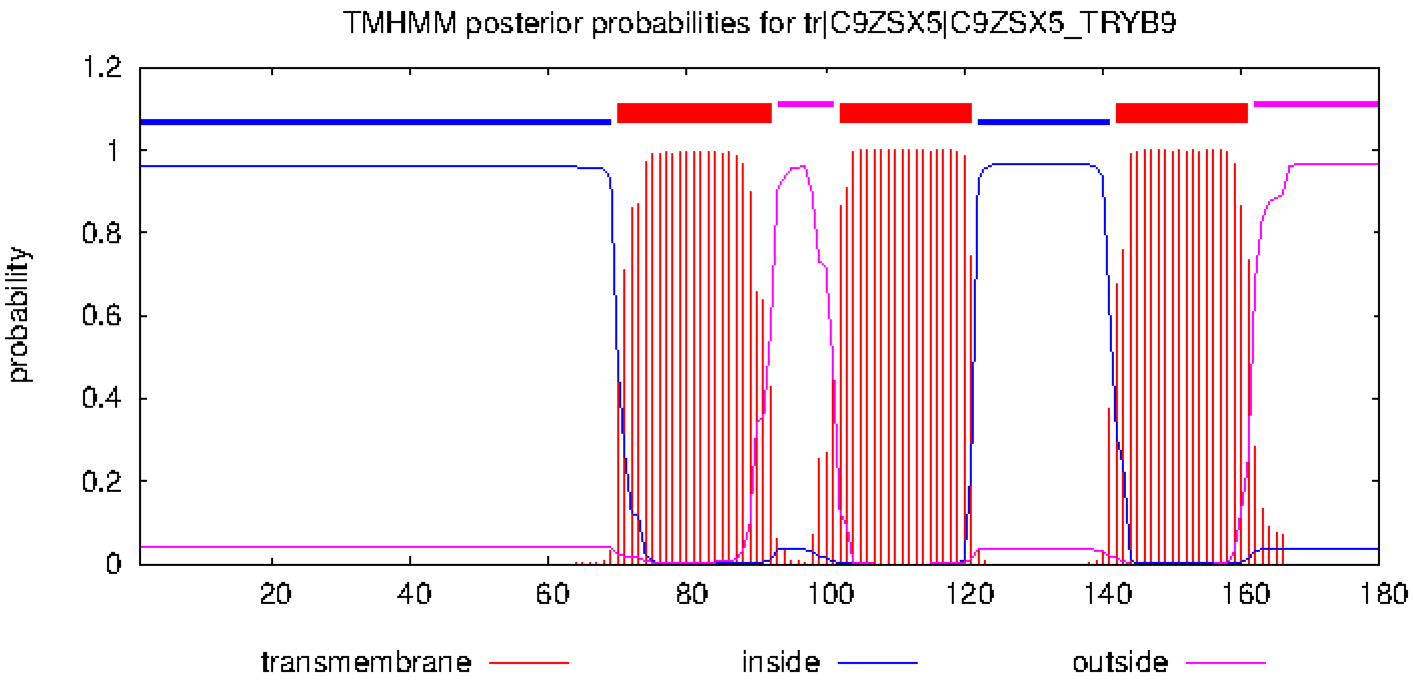

26 # plot in postscript, script for making the plot in gnuplot, data for plot

---

```
# tr|C9ZWK4|C9ZWK4_TRYB9 Length: 136
# tr|C9ZWK4|C9ZWK4_TRYB9 Number of predicted TMHs:  1
# tr|C9ZWK4|C9ZWK4_TRYB9 Exp number of AAs in TMHs: 23.27375
# tr|C9ZWK4|C9ZWK4_TRYB9 Exp number, first 60 AAs: 1.62784
# tr|C9ZWK4|C9ZWK4_TRYB9 Total prob of N-in:        0.01354
tr|C9ZWK4|C9ZWK4_TRYB9  TMHMM2.0        outside      1    62
tr|C9ZWK4|C9ZWK4_TRYB9  TMHMM2.0        TMhelix     63    85
tr|C9ZWK4|C9ZWK4_TRYB9  TMHMM2.0        inside      86   136
```

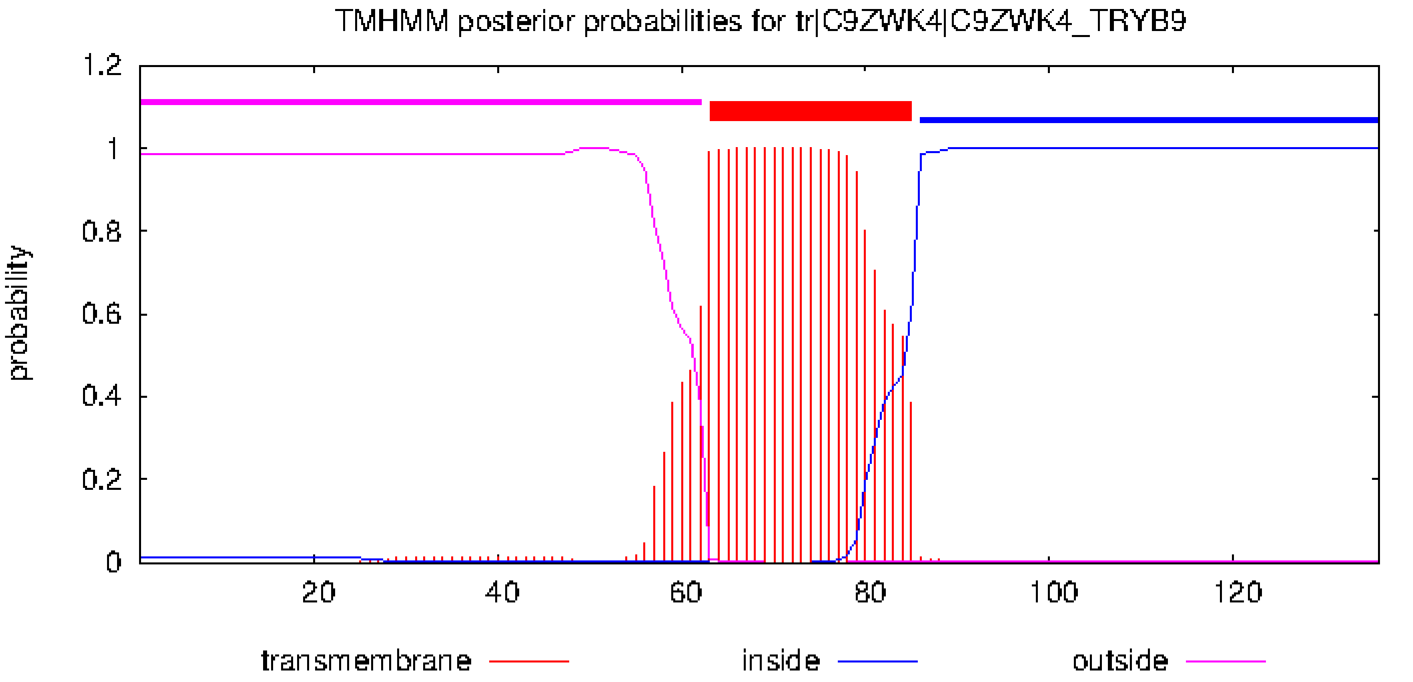

27 # plot in postscript, script for making the plot in gnuplot, data for plot

---

```
# tr|C9ZYP9|C9ZYP9_TRYB9 Length: 131
# tr|C9ZYP9|C9ZYP9_TRYB9 Number of predicted TMHs:  1
```

```
# tr|C9ZYP9|C9ZYP9_TRYB9 Exp number of AAs in TMHs: 20.90662
# tr|C9ZYP9|C9ZYP9_TRYB9 Exp number, first 60 AAs:  20.90662
# tr|C9ZYP9|C9ZYP9_TRYB9 Total prob of N-in:        0.03324
```

```
# tr|C9ZYP9|C9ZYP9_TRYB9  POSSIBLE N-term signal sequence
tr|C9ZYP9|C9ZYP9_TRYB9  TMHMM2.0        outside      1    19
tr|C9ZYP9|C9ZYP9_TRYB9  TMHMM2.0        TMhelix     20    40
tr|C9ZYP9|C9ZYP9_TRYB9  TMHMM2.0        inside      41   131
```

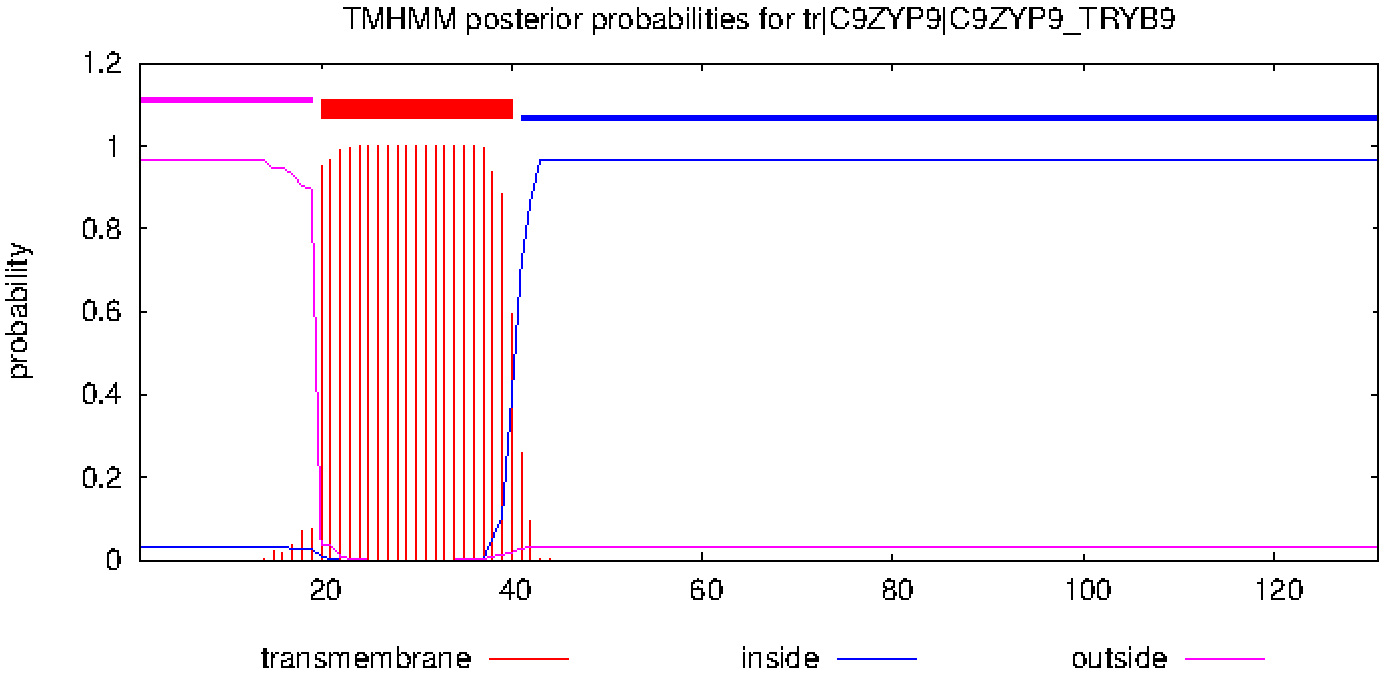

28 # **[plot]** **in postscript,** **[script]** **for making the plot in gnuplot,** **[data]** **for plot**

```
# tr|D0A5M0|D0A5M0_TRYB9 Length: 130
# tr|D0A5M0|D0A5M0_TRYB9 Number of predicted TMHs:  1
# tr|D0A5M0|D0A5M0_TRYB9 Exp number of AAs in TMHs: 43.25574
# tr|D0A5M0|D0A5M0_TRYB9 Exp number, first 60 AAs:  14.41821
# tr|D0A5M0|D0A5M0_TRYB9 Total prob of N-in:        0.34906
# tr|D0A5M0|D0A5M0_TRYB9 POSSIBLE N-term signal sequence
tr|D0A5M0|D0A5M0_TRYB9  TMHMM2.0        inside       1    68
tr|D0A5M0|D0A5M0_TRYB9  TMHMM2.0        TMhelix     69    91
tr|D0A5M0|D0A5M0_TRYB9  TMHMM2.0        outside     92   130
```

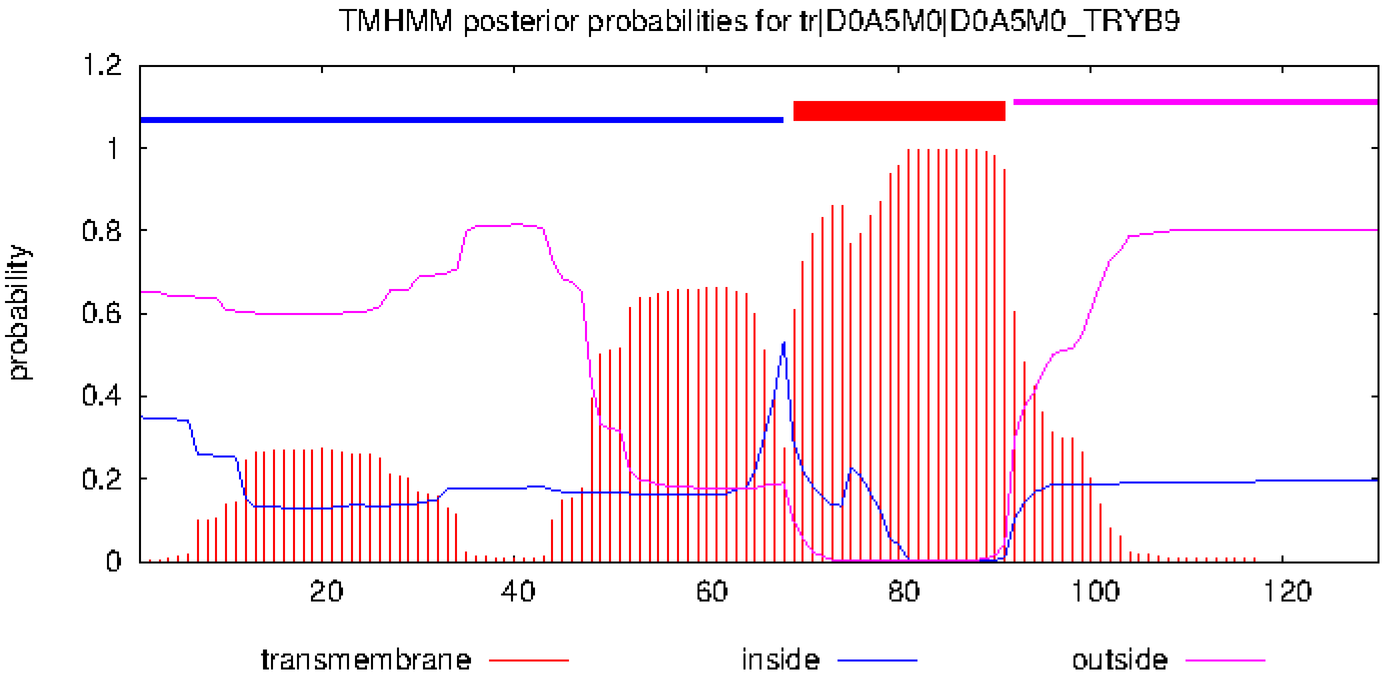

29 # **[plot]** **in postscript,** **[script]** **for making the plot in gnuplot,** **[data]** **for plot**

```
# tr|D0A6S4|D0A6S4_TRYB9 Length: 115
# tr|D0A6S4|D0A6S4_TRYB9 Number of predicted TMHs:  2
# tr|D0A6S4|D0A6S4_TRYB9 Exp number of AAs in TMHs: 43.3955
# tr|D0A6S4|D0A6S4_TRYB9 Exp number, first 60 AAs:  20.16067
# tr|D0A6S4|D0A6S4_TRYB9 Total prob of N-in:        0.09334
# tr|D0A6S4|D0A6S4_TRYB9 POSSIBLE N-term signal sequence
tr|D0A6S4|D0A6S4_TRYB9  TMHMM2.0        outside      1     4
tr|D0A6S4|D0A6S4_TRYB9  TMHMM2.0        TMhelix      5    27
tr|D0A6S4|D0A6S4_TRYB9  TMHMM2.0        inside      28    78
tr|D0A6S4|D0A6S4_TRYB9  TMHMM2.0        TMhelix     79   101
tr|D0A6S4|D0A6S4_TRYB9  TMHMM2.0        outside    102   115
```

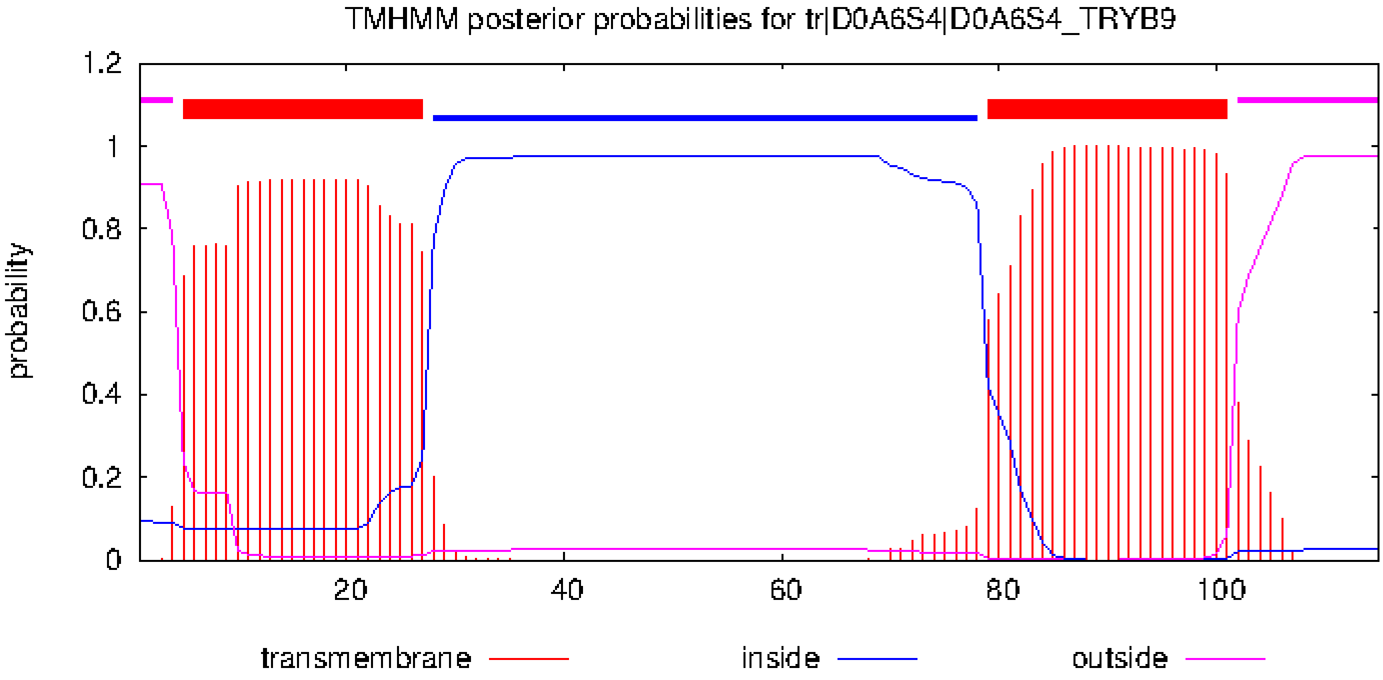

30 **# plot in postscript, script for making the plot in gnuplot, data for plot**

```
# tr|D0AA93|D0AA93_TRYB9 Length: 114
# tr|D0AA93|D0AA93_TRYB9 Number of predicted TMHs:  1
# tr|D0AA93|D0AA93_TRYB9 Exp number of AAs in TMHs: 29.28524
# tr|D0AA93|D0AA93_TRYB9 Exp number, first 60 AAs:  22.56475
# tr|D0AA93|D0AA93_TRYB9 Total prob of N-in:        0.00602
# tr|D0AA93|D0AA93_TRYB9 POSSIBLE N-term signal sequence
tr|D0AA93|D0AA93_TRYB9  TMHMM2.0        outside      1    24
tr|D0AA93|D0AA93_TRYB9  TMHMM2.0        TMhelix     25    47
tr|D0AA93|D0AA93_TRYB9  TMHMM2.0        inside      48   114
```

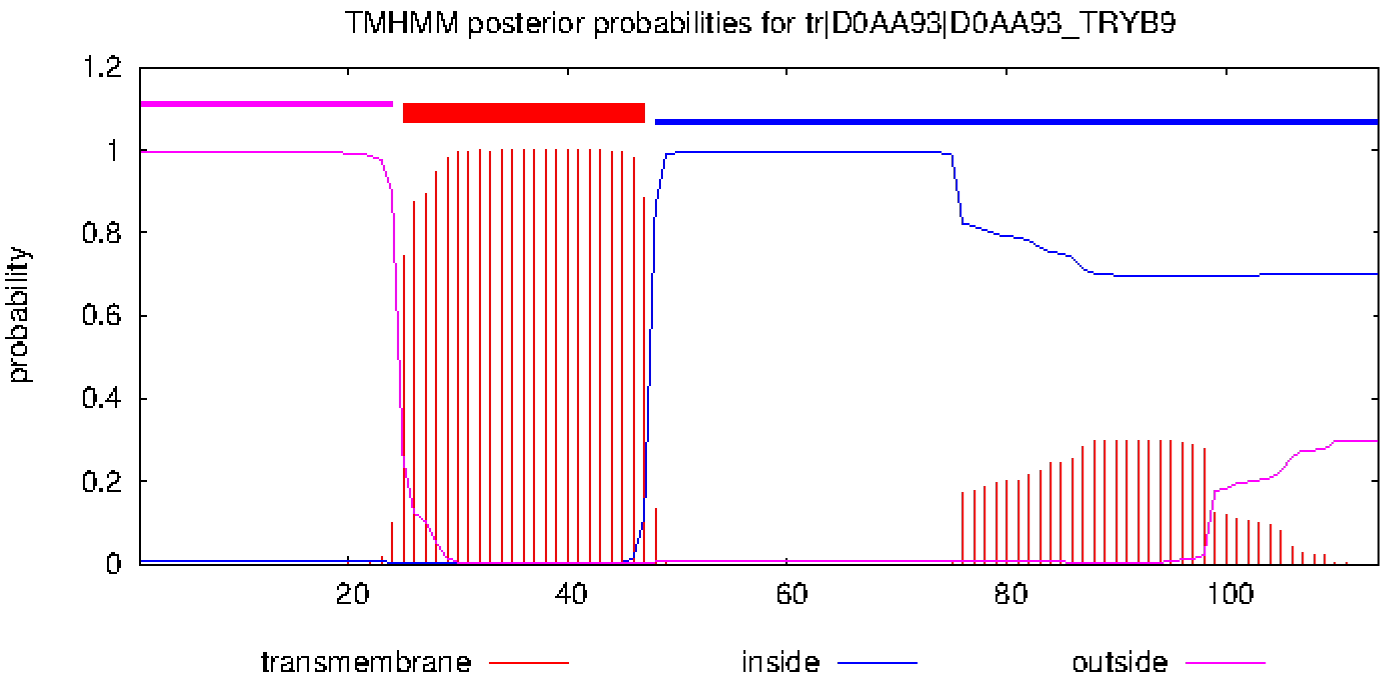

31 # **plot in postscript, script for making the plot in gnuplot, data for plot**

```
# tr|D0A3I7|D0A3I7_TRYB9 Length: 149
# tr|D0A3I7|D0A3I7_TRYB9 Number of predicted TMHs:  1
# tr|D0A3I7|D0A3I7_TRYB9 Exp number of AAs in TMHs: 22.63506
# tr|D0A3I7|D0A3I7_TRYB9 Exp number, first 60 AAs: 0.00182
# tr|D0A3I7|D0A3I7_TRYB9 Total prob of N-in:        0.02242
tr|D0A3I7|D0A3I7_TRYB9  TMHMM2.0        outside      1    79
tr|D0A3I7|D0A3I7_TRYB9  TMHMM2.0        TMhelix     80   102
tr|D0A3I7|D0A3I7_TRYB9  TMHMM2.0        inside     103   149
```

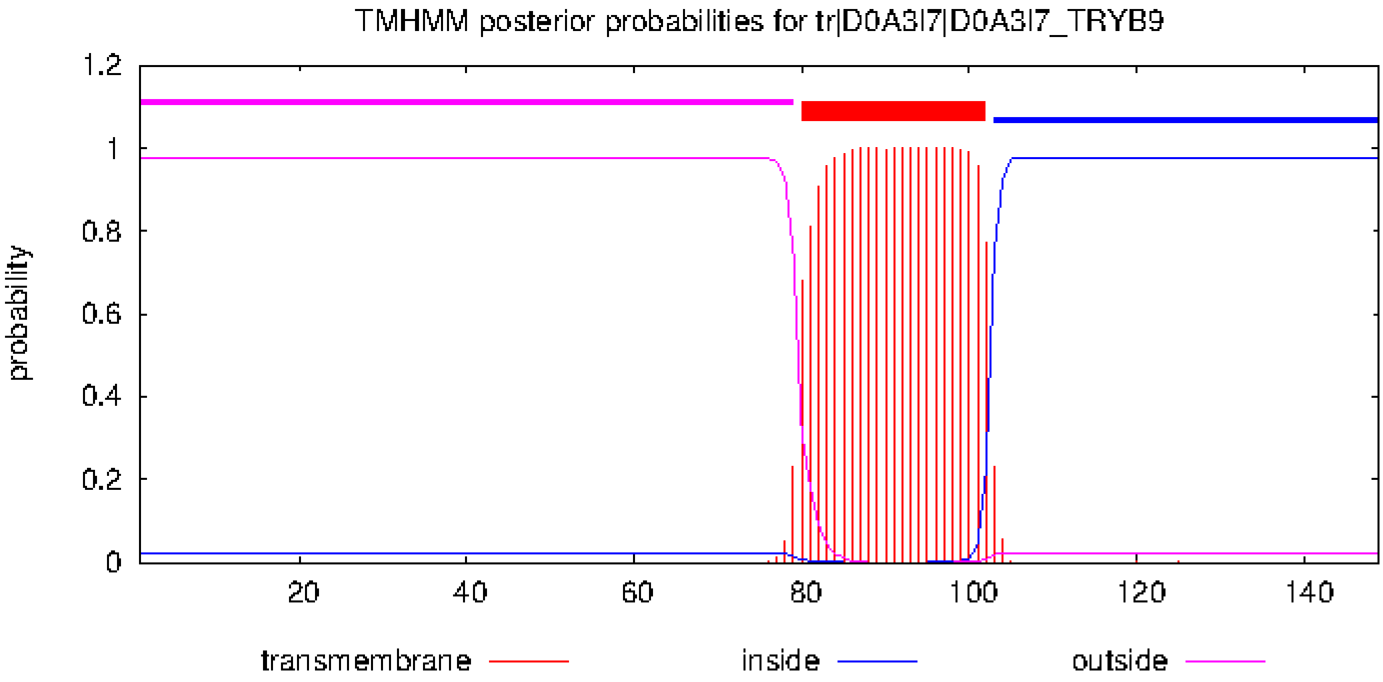

32 # **plot in postscript, script for making the plot in gnuplot, data for plot**

```
# tr|C9ZR39|C9ZR39_TRYB9 Length: 256
# tr|C9ZR39|C9ZR39_TRYB9 Number of predicted TMHs:  1
```

```
# tr|C9ZR39|C9ZR39_TRYB9 Exp number of AAs in TMHs: 22.49183
# tr|C9ZR39|C9ZR39_TRYB9 Exp number, first 60 AAs:  22.46753
# tr|C9ZR39|C9ZR39_TRYB9 Total prob of N-in:        0.31800
```

```
#  tr|C9ZR39|C9ZR39_TRYB9  POSSIBLE N-term signal sequence
tr|C9ZR39|C9ZR39_TRYB9  TMHMM2.0        outside      1    14
tr|C9ZR39|C9ZR39_TRYB9  TMHMM2.0        TMhelix     15    37
tr|C9ZR39|C9ZR39_TRYB9  TMHMM2.0        inside      38   256
```

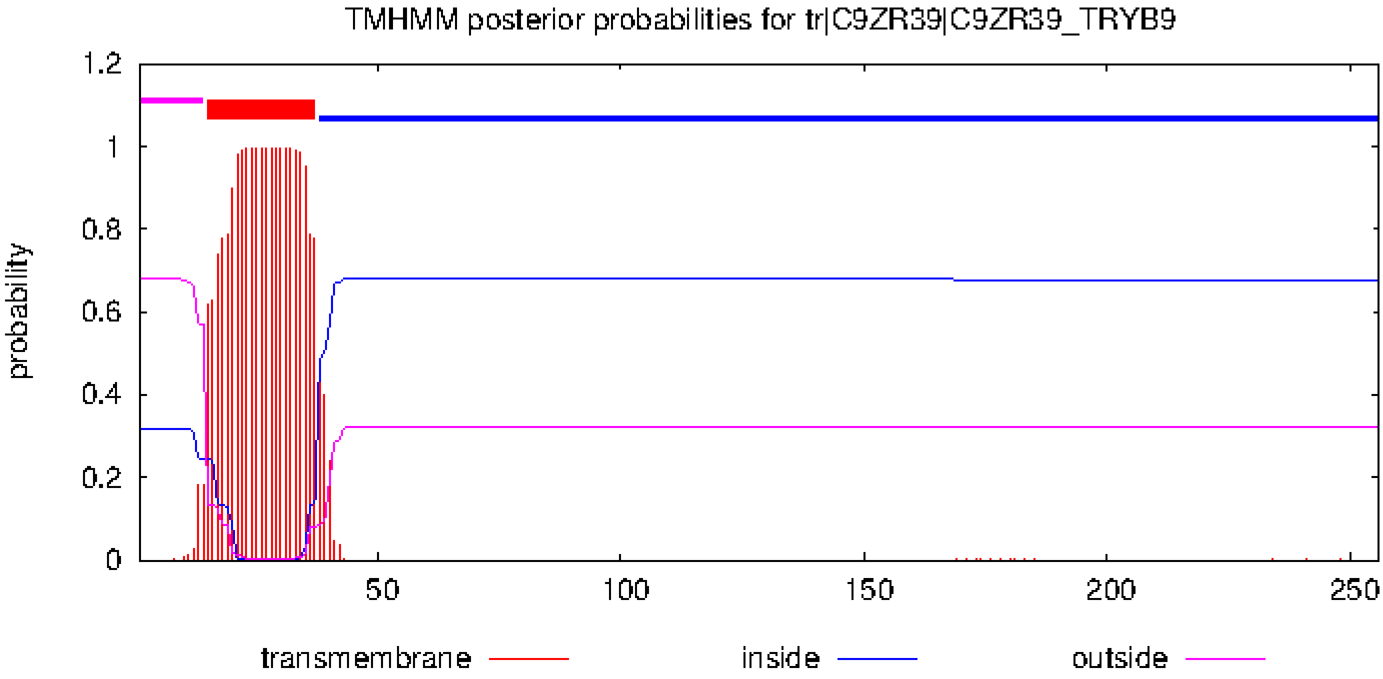

33 # **plot** in postscript, **script** for making the plot in gnuplot, **data** for plot

---

```
# tr|C9ZJ95|C9ZJ95_TRYB9 Length: 280
# tr|C9ZJ95|C9ZJ95_TRYB9 Number of predicted TMHs:  1
# tr|C9ZJ95|C9ZJ95_TRYB9 Exp number of AAs in TMHs: 23.14524
#  tr|C9ZJ95|C9ZJ95_TRYB9 Exp number, first 60 AAs: 9.91185
# tr|C9ZJ95|C9ZJ95_TRYB9 Total prob of N-in:        0.95837
tr|C9ZJ95|C9ZJ95_TRYB9  TMHMM2.0        inside       1    52
tr|C9ZJ95|C9ZJ95_TRYB9  TMHMM2.0        TMhelix     53    75
tr|C9ZJ95|C9ZJ95_TRYB9  TMHMM2.0        outside     76   280
```

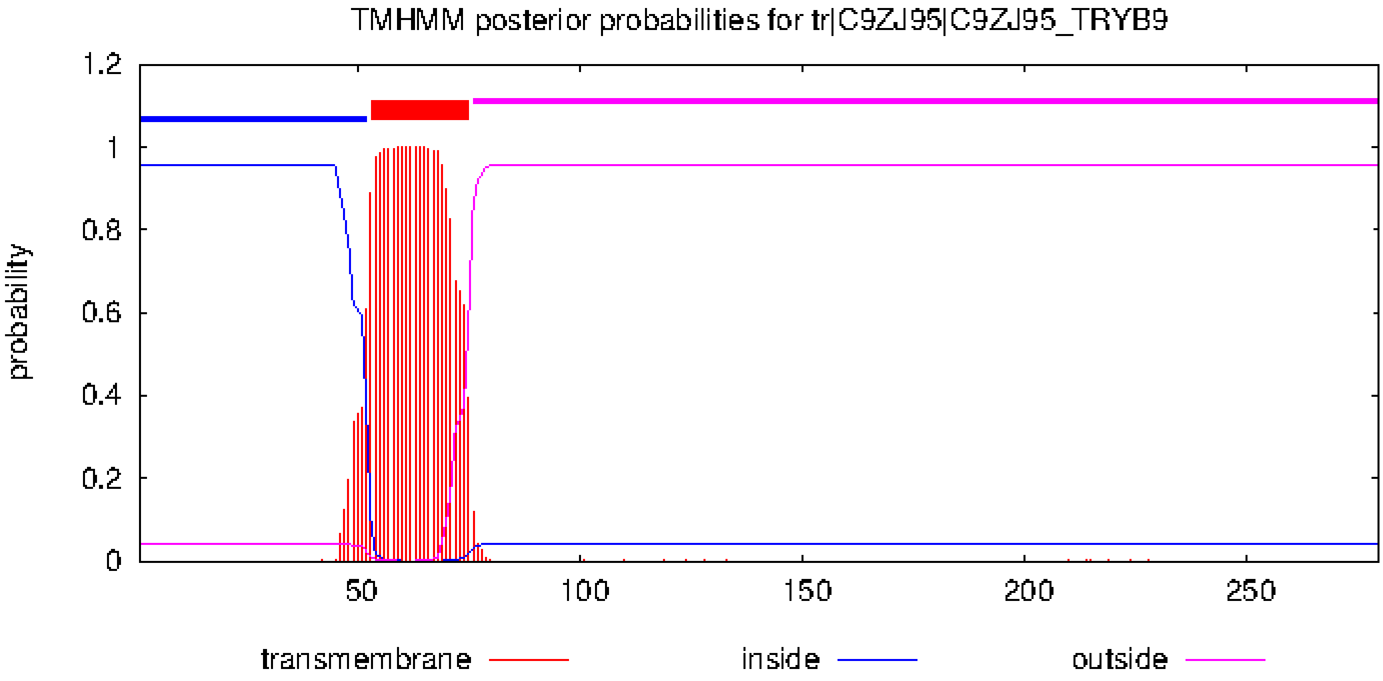

34 # **plot** in postscript, **script** for making the plot in gnuplot, **data** for plot

```
# tr|C9ZZZ3|C9ZZZ3_TRYB9 Length: 304
# tr|C9ZZZ3|C9ZZZ3_TRYB9 Number of predicted TMHs:  6
# tr|C9ZZZ3|C9ZZZ3_TRYB9 Exp number of AAs in TMHs: 134.42323
# tr|C9ZZZ3|C9ZZZ3_TRYB9 Exp number, first 60 AAs:  6e-05
# tr|C9ZZZ3|C9ZZZ3_TRYB9 Total prob of N-in:        0.99407
tr|C9ZZZ3|C9ZZZ3_TRYB9  TMHMM2.0        inside       1    66
tr|C9ZZZ3|C9ZZZ3_TRYB9  TMHMM2.0        TMhelix     67    89
tr|C9ZZZ3|C9ZZZ3_TRYB9  TMHMM2.0        outside     90    98
tr|C9ZZZ3|C9ZZZ3_TRYB9  TMHMM2.0        TMhelix     99   121
tr|C9ZZZ3|C9ZZZ3_TRYB9  TMHMM2.0        inside     122   140
tr|C9ZZZ3|C9ZZZ3_TRYB9  TMHMM2.0        TMhelix    141   163
tr|C9ZZZ3|C9ZZZ3_TRYB9  TMHMM2.0        outside    164   195
tr|C9ZZZ3|C9ZZZ3_TRYB9  TMHMM2.0        TMhelix    196   218
tr|C9ZZZ3|C9ZZZ3_TRYB9  TMHMM2.0        inside     219   230
tr|C9ZZZ3|C9ZZZ3_TRYB9  TMHMM2.0        TMhelix    231   253
tr|C9ZZZ3|C9ZZZ3_TRYB9  TMHMM2.0        outside    254   279
tr|C9ZZZ3|C9ZZZ3_TRYB9  TMHMM2.0        TMhelix    280   302
tr|C9ZZZ3|C9ZZZ3_TRYB9  TMHMM2.0        inside     303   304
```

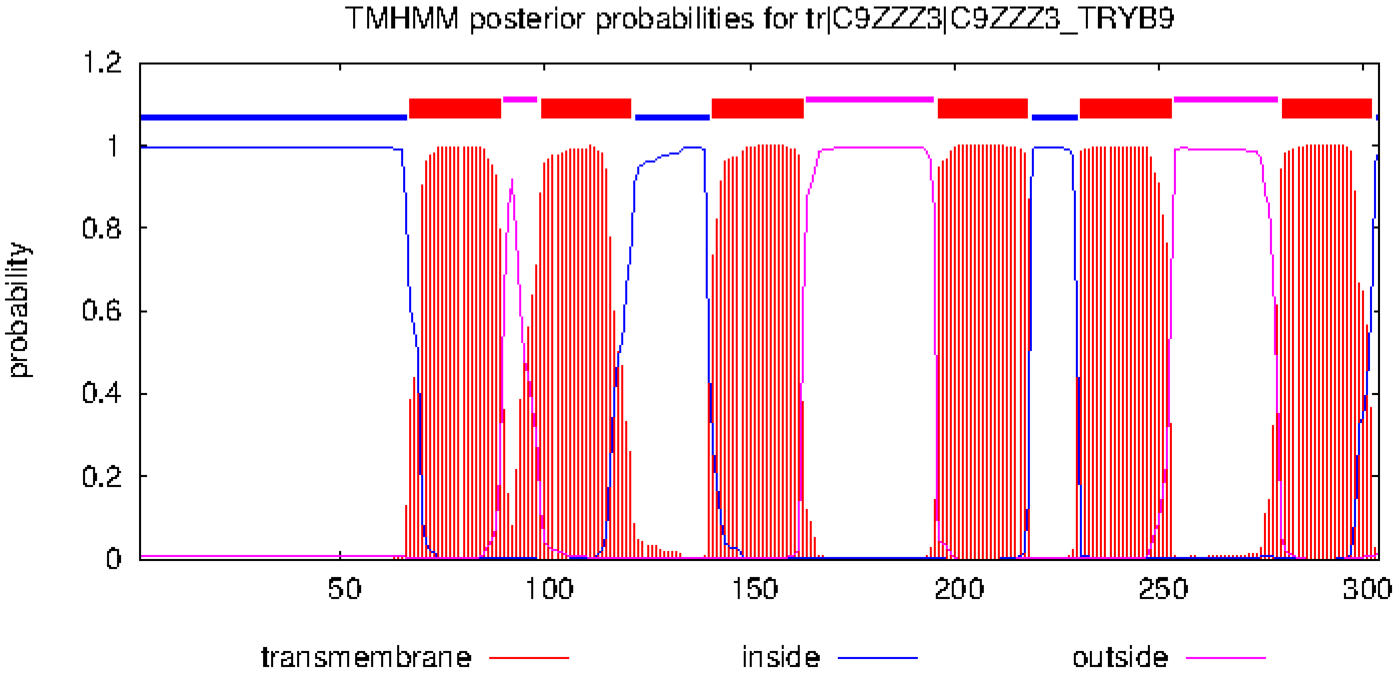

35 # **plot** **in postscript,** **script** **for making the plot in gnuplot,** **data** **for plot**

```
# tr|D0A148|D0A148_TRYB9 Length: 334
# tr|D0A148|D0A148_TRYB9 Number of predicted TMHs:  5
# tr|D0A148|D0A148_TRYB9 Exp number of AAs in TMHs: 105.82117
# tr|D0A148|D0A148_TRYB9 Exp number, first 60 AAs:  26.31529
# tr|D0A148|D0A148_TRYB9 Total prob of N-in:        0.99978
# tr|D0A148|D0A148_TRYB9 POSSIBLE N-term signal sequence
tr|D0A148|D0A148_TRYB9  TMHMM2.0        inside       1    23
tr|D0A148|D0A148_TRYB9  TMHMM2.0        TMhelix     24    43
tr|D0A148|D0A148_TRYB9  TMHMM2.0        outside     44    57
tr|D0A148|D0A148_TRYB9  TMHMM2.0        TMhelix     58    80
tr|D0A148|D0A148_TRYB9  TMHMM2.0        inside      81   159
tr|D0A148|D0A148_TRYB9  TMHMM2.0        TMhelix    160   182
tr|D0A148|D0A148_TRYB9  TMHMM2.0        outside    183   214
tr|D0A148|D0A148_TRYB9  TMHMM2.0        TMhelix    215   237
tr|D0A148|D0A148_TRYB9  TMHMM2.0        inside     238   299
tr|D0A148|D0A148_TRYB9  TMHMM2.0        TMhelix    300   319
tr|D0A148|D0A148_TRYB9  TMHMM2.0        outside    320   334
```

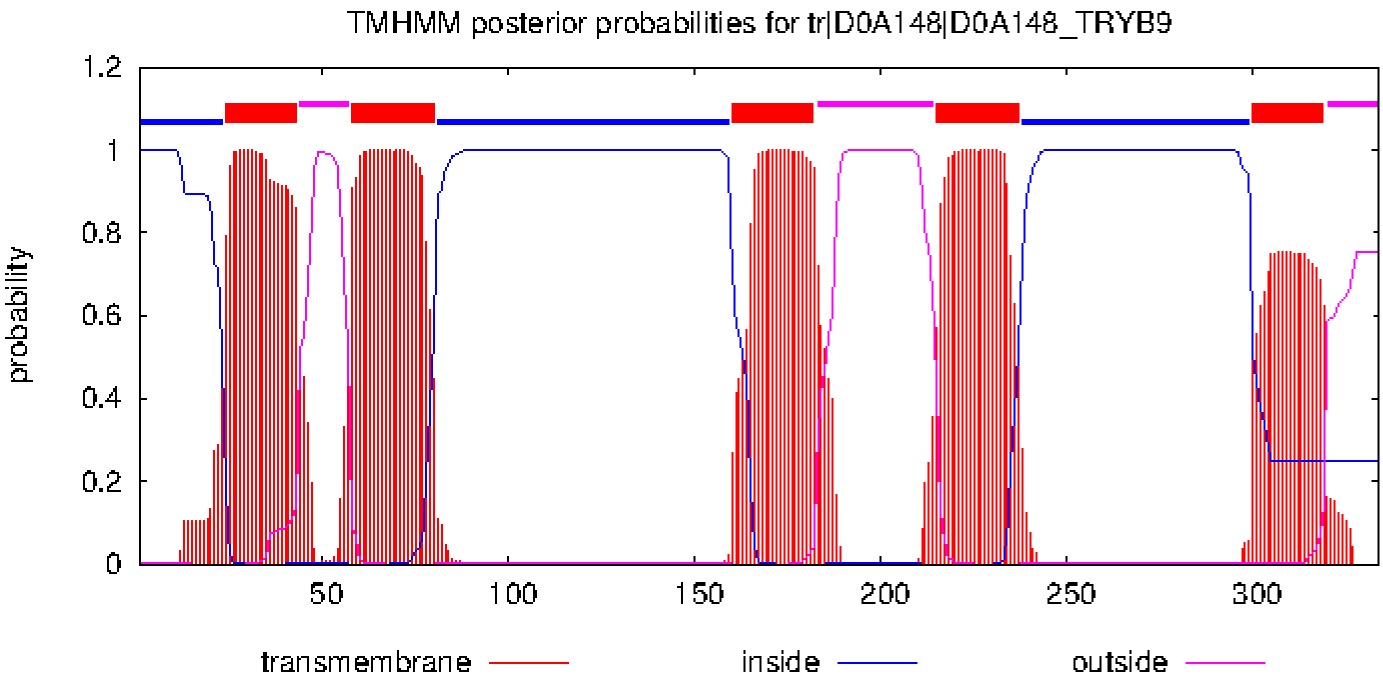

36 # plot in postscript, script for making the plot in gnuplot, data for plot

```
# tr|D0A6D7|D0A6D7_TRYB9 Length: 372
# tr|D0A6D7|D0A6D7_TRYB9 Number of predicted TMHs:  1
# tr|D0A6D7|D0A6D7_TRYB9 Exp number of AAs in TMHs: 22.53082
# tr|D0A6D7|D0A6D7_TRYB9 Exp number, first 60 AAs:  22.47823
# tr|D0A6D7|D0A6D7_TRYB9 Total prob of N-in:        0.99194
# tr|D0A6D7|D0A6D7_TRYB9 POSSIBLE N-term signal sequence
tr|D0A6D7|D0A6D7_TRYB9  TMHMM2.0        inside       1     6
tr|D0A6D7|D0A6D7_TRYB9  TMHMM2.0        TMhelix      7    29
tr|D0A6D7|D0A6D7_TRYB9  TMHMM2.0        outside     30   372
```

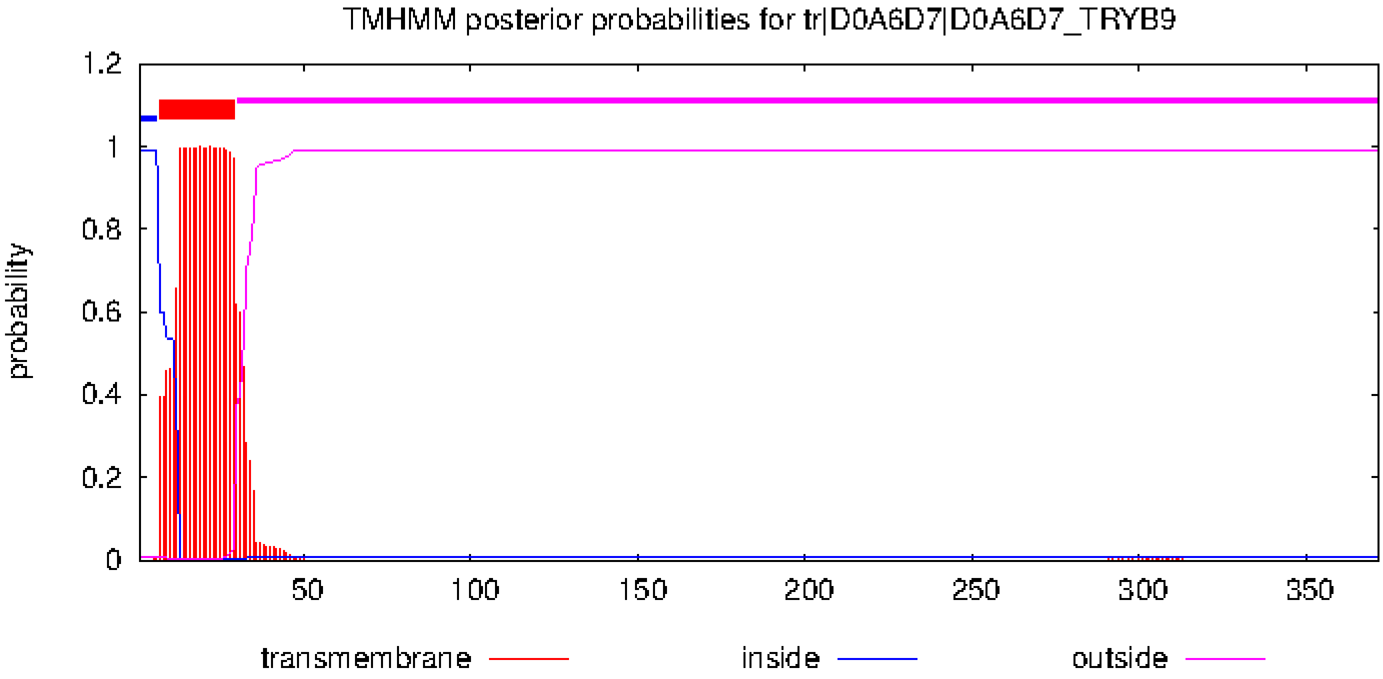

37 # plot in postscript, script for making the plot in gnuplot, data for plot

```
# tr|D0A889|D0A889_TRYB9 Length: 386
```

tr|D0A889|D0A889_TRYB9 Number of predicted TMHs: 7
tr|D0A889|D0A889_TRYB9 Exp number of AAs in TMHs: 181.87552
tr|D0A889|D0A889_TRYB9 Exp number, first 60 AAs: 13.42042

```
# tr|D0A889|D0A889_TRYB9 Total prob of N-in:        0.64904
# tr|D0A889|D0A889_TRYB9 POSSIBLE N-term signal sequence
tr|D0A889|D0A889_TRYB9  TMHMM2.0        inside       1    12
tr|D0A889|D0A889_TRYB9  TMHMM2.0        TMhelix     13    35
tr|D0A889|D0A889_TRYB9  TMHMM2.0        outside     36    68
tr|D0A889|D0A889_TRYB9  TMHMM2.0        TMhelix     69    91
tr|D0A889|D0A889_TRYB9  TMHMM2.0        inside      92   103
tr|D0A889|D0A889_TRYB9  TMHMM2.0        TMhelix    104   126
tr|D0A889|D0A889_TRYB9  TMHMM2.0        outside    127   147
tr|D0A889|D0A889_TRYB9  TMHMM2.0        TMhelix    148   170
tr|D0A889|D0A889_TRYB9  TMHMM2.0        inside     171   286
tr|D0A889|D0A889_TRYB9  TMHMM2.0        TMhelix    287   309
tr|D0A889|D0A889_TRYB9  TMHMM2.0        outside    310   318
tr|D0A889|D0A889_TRYB9  TMHMM2.0        TMhelix    319   341
tr|D0A889|D0A889_TRYB9  TMHMM2.0        inside     342   361
tr|D0A889|D0A889_TRYB9  TMHMM2.0        TMhelix    362   383
tr|D0A889|D0A889_TRYB9  TMHMM2.0        outside    384   386
```

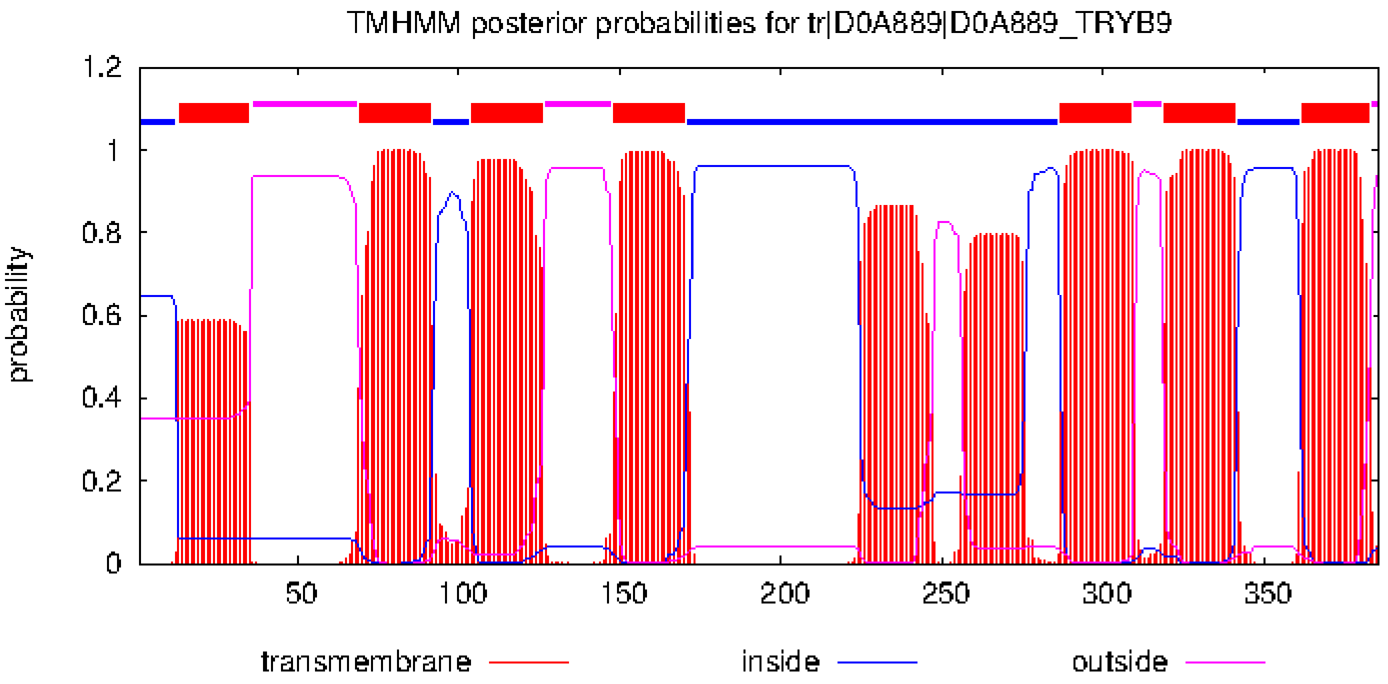

38 **# plot in postscript, script for making the plot in gnuplot, data for plot**

```
# tr|D0A886|D0A886_TRYB9 Length: 394
# tr|D0A886|D0A886_TRYB9 Number of predicted TMHs:  9
# tr|D0A886|D0A886_TRYB9 Exp number of AAs in TMHs: 180.8174
# tr|D0A886|D0A886_TRYB9 Exp number, first 60 AAs:  10.23997
# tr|D0A886|D0A886_TRYB9 Total prob of N-in:        0.68720
# tr|D0A886|D0A886_TRYB9 POSSIBLE N-term signal sequence
tr|D0A886|D0A886_TRYB9  TMHMM2.0        inside       1    12
tr|D0A886|D0A886_TRYB9  TMHMM2.0        TMhelix     13    35
tr|D0A886|D0A886_TRYB9  TMHMM2.0        outside     36    79
tr|D0A886|D0A886_TRYB9  TMHMM2.0        TMhelix     80   102
tr|D0A886|D0A886_TRYB9  TMHMM2.0        inside     103   114
tr|D0A886|D0A886_TRYB9  TMHMM2.0        TMhelix    115   137
tr|D0A886|D0A886_TRYB9  TMHMM2.0        outside    138   156
tr|D0A886|D0A886_TRYB9  TMHMM2.0        TMhelix    157   179
tr|D0A886|D0A886_TRYB9  TMHMM2.0        inside     180   231
tr|D0A886|D0A886_TRYB9  TMHMM2.0        TMhelix    232   254
tr|D0A886|D0A886_TRYB9  TMHMM2.0        outside    255   263
tr|D0A886|D0A886_TRYB9  TMHMM2.0        TMhelix    264   283
tr|D0A886|D0A886_TRYB9  TMHMM2.0        inside     284   294
tr|D0A886|D0A886_TRYB9  TMHMM2.0        TMhelix    295   317
tr|D0A886|D0A886_TRYB9  TMHMM2.0        outside    318   326
tr|D0A886|D0A886_TRYB9  TMHMM2.0        TMhelix    327   349
tr|D0A886|D0A886_TRYB9  TMHMM2.0        inside     350   369
```

```
tr|D0A886|D0A886_TRYB9  TMHMM2.0        TMhelix     370   389
tr|D0A886|D0A886_TRYB9  TMHMM2.0        outside     390   394
```

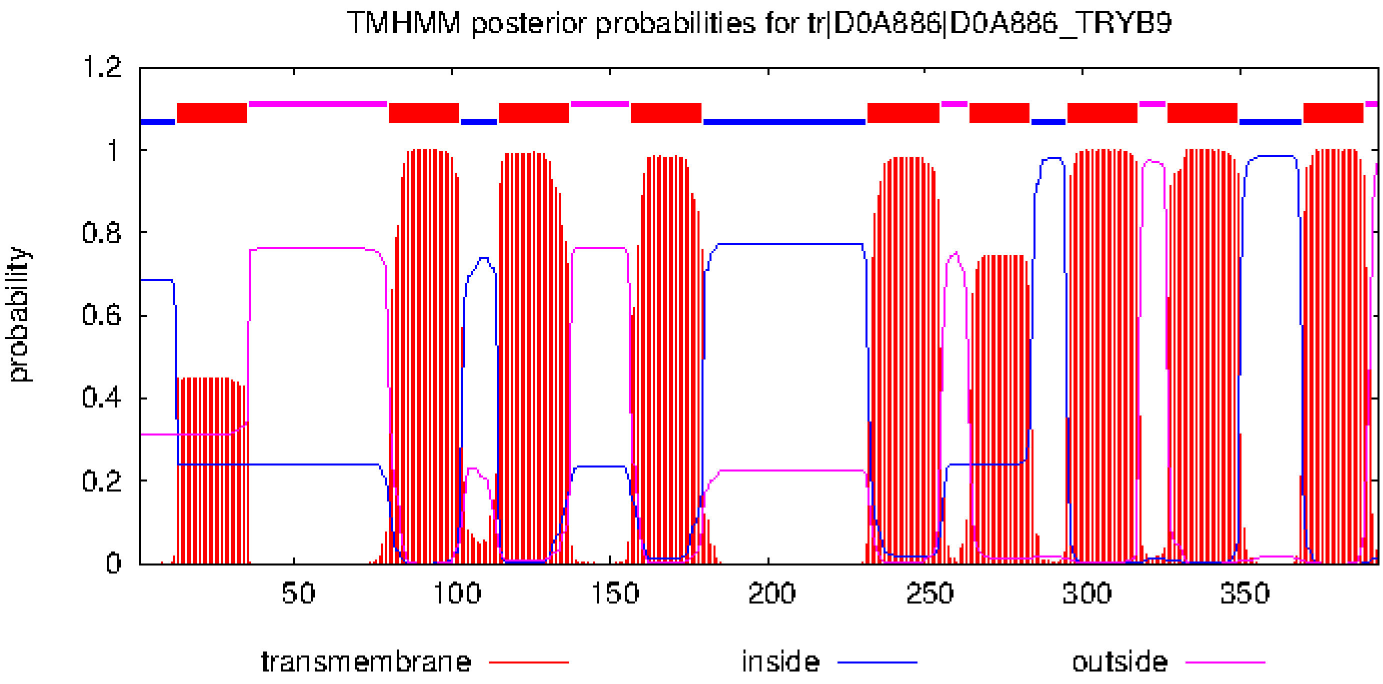

39 # plot in postscript, script for making the plot in gnuplot, data for plot

---

```
# tr|C9ZY69|C9ZY69_TRYB9 Length: 430
# tr|C9ZY69|C9ZY69_TRYB9 Number of predicted TMHs:  8
# tr|C9ZY69|C9ZY69_TRYB9 Exp number of AAs in TMHs: 179.27844
# tr|C9ZY69|C9ZY69_TRYB9 Exp number, first 60 AAs:  0
# tr|C9ZY69|C9ZY69_TRYB9 Total prob of N-in:        0.99661
tr|C9ZY69|C9ZY69_TRYB9  TMHMM2.0        inside        1    91
tr|C9ZY69|C9ZY69_TRYB9  TMHMM2.0        TMhelix      92   114
tr|C9ZY69|C9ZY69_TRYB9  TMHMM2.0        outside     115   128
tr|C9ZY69|C9ZY69_TRYB9  TMHMM2.0        TMhelix     129   151
tr|C9ZY69|C9ZY69_TRYB9  TMHMM2.0        inside      152   157
tr|C9ZY69|C9ZY69_TRYB9  TMHMM2.0        TMhelix     158   180
tr|C9ZY69|C9ZY69_TRYB9  TMHMM2.0        outside     181   189
tr|C9ZY69|C9ZY69_TRYB9  TMHMM2.0        TMhelix     190   212
tr|C9ZY69|C9ZY69_TRYB9  TMHMM2.0        inside      213   232
tr|C9ZY69|C9ZY69_TRYB9  TMHMM2.0        TMhelix     233   255
tr|C9ZY69|C9ZY69_TRYB9  TMHMM2.0        outside     256   269
tr|C9ZY69|C9ZY69_TRYB9  TMHMM2.0        TMhelix     270   292
tr|C9ZY69|C9ZY69_TRYB9  TMHMM2.0        inside      293   312
tr|C9ZY69|C9ZY69_TRYB9  TMHMM2.0        TMhelix     313   335
tr|C9ZY69|C9ZY69_TRYB9  TMHMM2.0        outside     336   349
tr|C9ZY69|C9ZY69_TRYB9  TMHMM2.0        TMhelix     350   372
tr|C9ZY69|C9ZY69_TRYB9  TMHMM2.0        inside      373   430
```

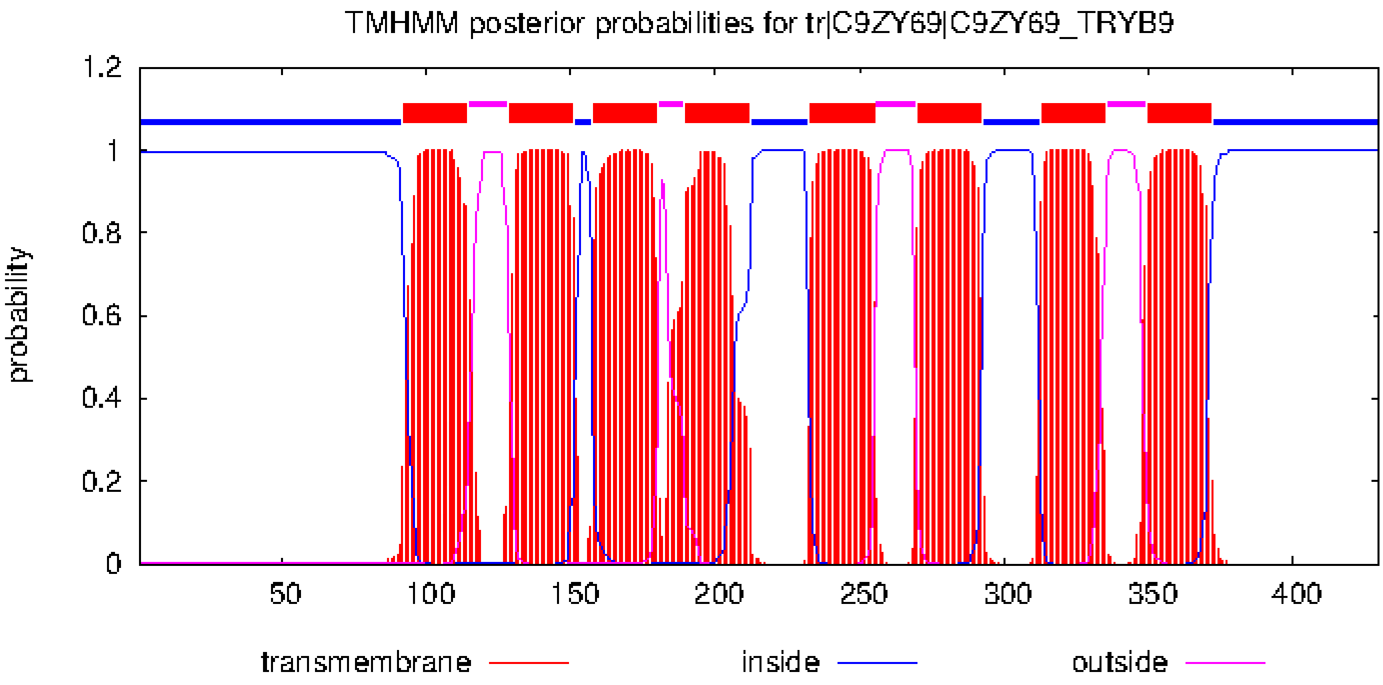

40 **# plot in postscript, script for making the plot in gnuplot, data for plot**

```
# tr|D0AAF8|D0AAF8_TRYB9 Length: 462
# tr|D0AAF8|D0AAF8_TRYB9 Number of predicted TMHs:  10
# tr|D0AAF8|D0AAF8_TRYB9 Exp number of AAs in TMHs: 225.39112
# tr|D0AAF8|D0AAF8_TRYB9 Exp number, first 60 AAs:  0.03171
# tr|D0AAF8|D0AAF8_TRYB9 Total prob of N-in:        0.29502
tr|D0AAF8|D0AAF8_TRYB9  TMHMM2.0        outside      1    93
tr|D0AAF8|D0AAF8_TRYB9  TMHMM2.0        TMhelix     94   116
tr|D0AAF8|D0AAF8_TRYB9  TMHMM2.0        inside     117   136
tr|D0AAF8|D0AAF8_TRYB9  TMHMM2.0        TMhelix    137   159
tr|D0AAF8|D0AAF8_TRYB9  TMHMM2.0        outside    160   178
tr|D0AAF8|D0AAF8_TRYB9  TMHMM2.0        TMhelix    179   201
tr|D0AAF8|D0AAF8_TRYB9  TMHMM2.0        inside     202   207
tr|D0AAF8|D0AAF8_TRYB9  TMHMM2.0        TMhelix    208   230
tr|D0AAF8|D0AAF8_TRYB9  TMHMM2.0        outside    231   244
tr|D0AAF8|D0AAF8_TRYB9  TMHMM2.0        TMhelix    245   267
tr|D0AAF8|D0AAF8_TRYB9  TMHMM2.0        inside     268   287
tr|D0AAF8|D0AAF8_TRYB9  TMHMM2.0        TMhelix    288   310
tr|D0AAF8|D0AAF8_TRYB9  TMHMM2.0        outside    311   329
tr|D0AAF8|D0AAF8_TRYB9  TMHMM2.0        TMhelix    330   349
tr|D0AAF8|D0AAF8_TRYB9  TMHMM2.0        inside     350   373
tr|D0AAF8|D0AAF8_TRYB9  TMHMM2.0        TMhelix    374   396
tr|D0AAF8|D0AAF8_TRYB9  TMHMM2.0        outside    397   399
tr|D0AAF8|D0AAF8_TRYB9  TMHMM2.0        TMhelix    400   422
tr|D0AAF8|D0AAF8_TRYB9  TMHMM2.0        inside     423   434
tr|D0AAF8|D0AAF8_TRYB9  TMHMM2.0        TMhelix    435   457
tr|D0AAF8|D0AAF8_TRYB9  TMHMM2.0        outside    458   462
```

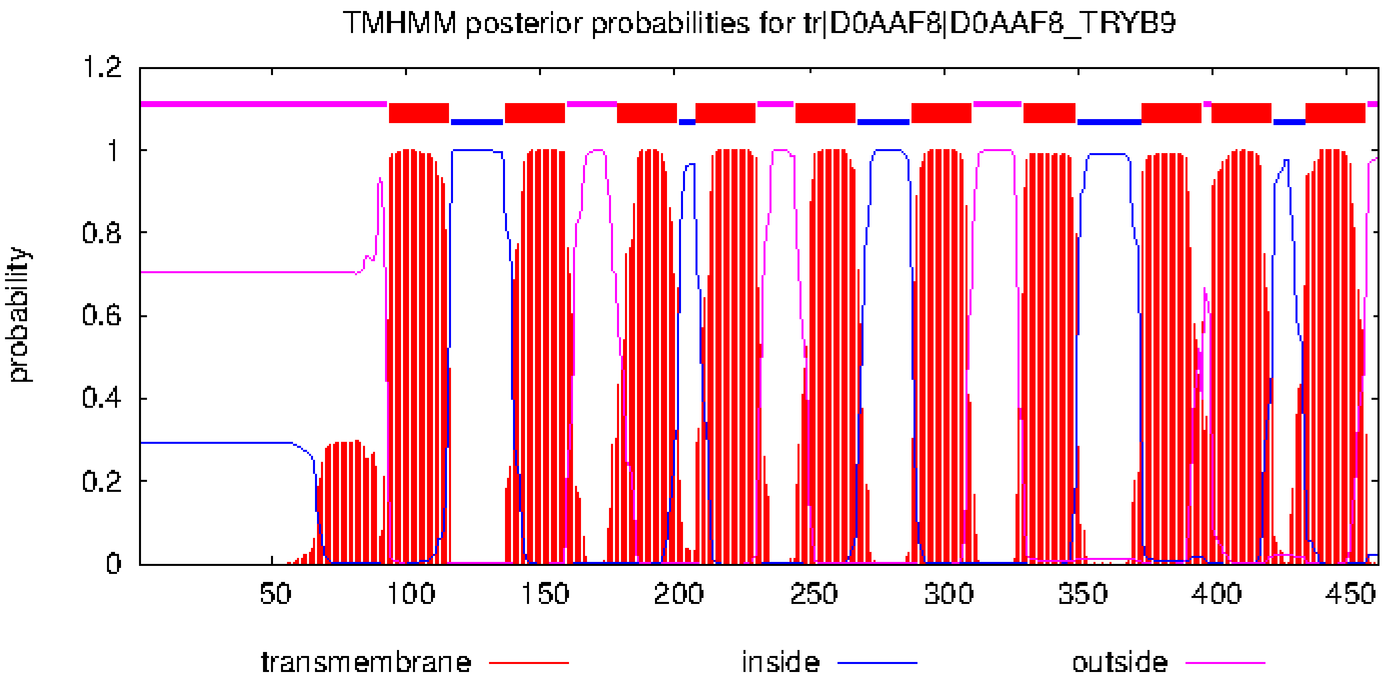

41 # plot in postscript, script for making the plot in gnuplot, data for plot

---

```
# tr|C9ZN88|C9ZN88_TRYB9 Length: 463
# tr|C9ZN88|C9ZN88_TRYB9 Number of predicted TMHs:  11
# tr|C9ZN88|C9ZN88_TRYB9 Exp number of AAs in TMHs: 241.83066
# tr|C9ZN88|C9ZN88_TRYB9 Exp number, first 60 AAs:  23.4618
# tr|C9ZN88|C9ZN88_TRYB9 Total prob of N-in:        0.98811
# tr|C9ZN88|C9ZN88_TRYB9 POSSIBLE N-term signal sequence
tr|C9ZN88|C9ZN88_TRYB9  TMHMM2.0        inside       1    12
tr|C9ZN88|C9ZN88_TRYB9  TMHMM2.0        TMhelix     13    35
tr|C9ZN88|C9ZN88_TRYB9  TMHMM2.0        outside     36    61
tr|C9ZN88|C9ZN88_TRYB9  TMHMM2.0        TMhelix     62    81
tr|C9ZN88|C9ZN88_TRYB9  TMHMM2.0        inside      82    92
tr|C9ZN88|C9ZN88_TRYB9  TMHMM2.0        TMhelix     93   115
tr|C9ZN88|C9ZN88_TRYB9  TMHMM2.0        outside    116   124
tr|C9ZN88|C9ZN88_TRYB9  TMHMM2.0        TMhelix    125   147
tr|C9ZN88|C9ZN88_TRYB9  TMHMM2.0        inside     148   153
tr|C9ZN88|C9ZN88_TRYB9  TMHMM2.0        TMhelix    154   176
tr|C9ZN88|C9ZN88_TRYB9  TMHMM2.0        outside    177   190
tr|C9ZN88|C9ZN88_TRYB9  TMHMM2.0        TMhelix    191   213
tr|C9ZN88|C9ZN88_TRYB9  TMHMM2.0        inside     214   300
tr|C9ZN88|C9ZN88_TRYB9  TMHMM2.0        TMhelix    301   323
tr|C9ZN88|C9ZN88_TRYB9  TMHMM2.0        outside    324   337
tr|C9ZN88|C9ZN88_TRYB9  TMHMM2.0        TMhelix    338   357
tr|C9ZN88|C9ZN88_TRYB9  TMHMM2.0        inside     358   369
tr|C9ZN88|C9ZN88_TRYB9  TMHMM2.0        TMhelix    370   392
tr|C9ZN88|C9ZN88_TRYB9  TMHMM2.0        outside    393   395
tr|C9ZN88|C9ZN88_TRYB9  TMHMM2.0        TMhelix    396   413
tr|C9ZN88|C9ZN88_TRYB9  TMHMM2.0        inside     414   433
tr|C9ZN88|C9ZN88_TRYB9  TMHMM2.0        TMhelix    434   456
tr|C9ZN88|C9ZN88_TRYB9  TMHMM2.0        outside    457   463
```

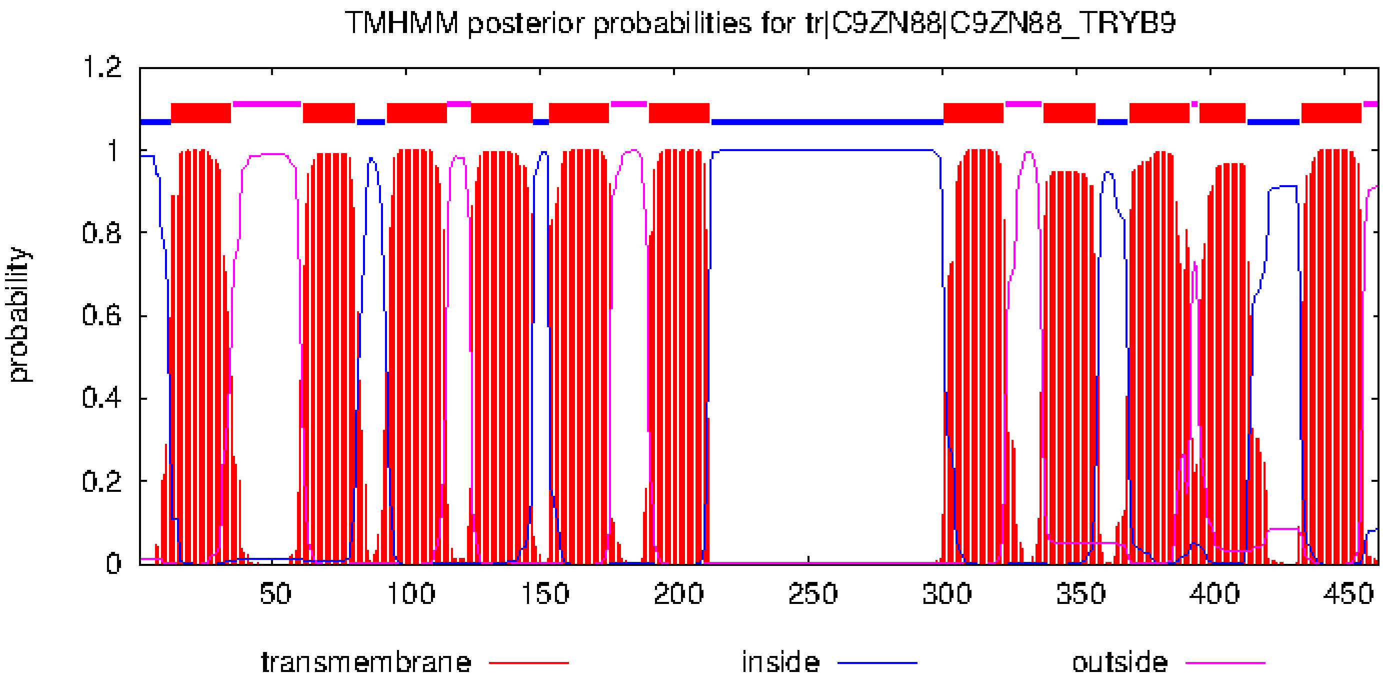

42 # **[plot]** **in postscript,** **[script]** **for making the plot in gnuplot,** **[data]** **for plot**

```
# tr|C9ZL05|C9ZL05_TRYB9 Length: 466
# tr|C9ZL05|C9ZL05_TRYB9 Number of predicted TMHs:  5
# tr|C9ZL05|C9ZL05_TRYB9 Exp number of AAs in TMHs: 124.31334
# tr|C9ZL05|C9ZL05_TRYB9 Exp number, first 60 AAs: 20.84927
# tr|C9ZL05|C9ZL05_TRYB9 Total prob of N-in:        0.17064
# tr|C9ZL05|C9ZL05_TRYB9 POSSIBLE N-term signal sequence
tr|C9ZL05|C9ZL05_TRYB9  TMHMM2.0        inside       1     6
tr|C9ZL05|C9ZL05_TRYB9  TMHMM2.0        TMhelix      7    29
tr|C9ZL05|C9ZL05_TRYB9  TMHMM2.0        outside     30    79
tr|C9ZL05|C9ZL05_TRYB9  TMHMM2.0        TMhelix     80   102
tr|C9ZL05|C9ZL05_TRYB9  TMHMM2.0        inside     103   108
tr|C9ZL05|C9ZL05_TRYB9  TMHMM2.0        TMhelix    109   126
tr|C9ZL05|C9ZL05_TRYB9  TMHMM2.0        outside    127   182
tr|C9ZL05|C9ZL05_TRYB9  TMHMM2.0        TMhelix    183   205
tr|C9ZL05|C9ZL05_TRYB9  TMHMM2.0        inside     206   216
tr|C9ZL05|C9ZL05_TRYB9  TMHMM2.0        TMhelix    217   239
tr|C9ZL05|C9ZL05_TRYB9  TMHMM2.0        outside    240   466
```

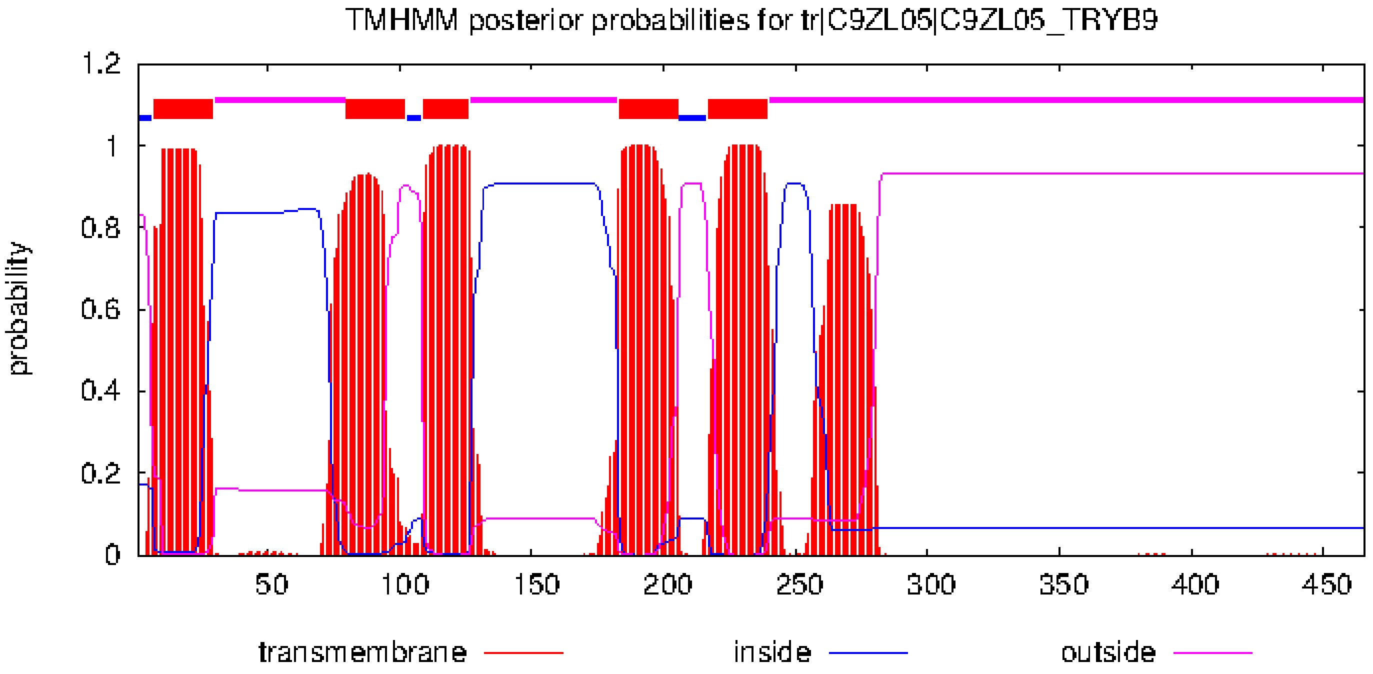

43 # plot in postscript, script for making the plot in gnuplot, data for plot

```
# tr|C9ZWQ8|C9ZWQ8_TRYB9 Length: 467
# tr|C9ZWQ8|C9ZWQ8_TRYB9 Number of predicted TMHs:  11
# tr|C9ZWQ8|C9ZWQ8_TRYB9 Exp number of AAs in TMHs: 244.08186
# tr|C9ZWQ8|C9ZWQ8_TRYB9 Exp number, first 60 AAs: 11.20051
# tr|C9ZWQ8|C9ZWQ8_TRYB9 Total prob of N-in:        0.98859
# tr|C9ZWQ8|C9ZWQ8_TRYB9 POSSIBLE N-term signal sequence
tr|C9ZWQ8|C9ZWQ8_TRYB9  TMHMM2.0        inside       1    48
tr|C9ZWQ8|C9ZWQ8_TRYB9  TMHMM2.0        TMhelix     49    71
tr|C9ZWQ8|C9ZWQ8_TRYB9  TMHMM2.0        outside     72    90
tr|C9ZWQ8|C9ZWQ8_TRYB9  TMHMM2.0        TMhelix     91   113
tr|C9ZWQ8|C9ZWQ8_TRYB9  TMHMM2.0        inside     114   143
tr|C9ZWQ8|C9ZWQ8_TRYB9  TMHMM2.0        TMhelix    144   166
tr|C9ZWQ8|C9ZWQ8_TRYB9  TMHMM2.0        outside    167   180
tr|C9ZWQ8|C9ZWQ8_TRYB9  TMHMM2.0        TMhelix    181   198
tr|C9ZWQ8|C9ZWQ8_TRYB9  TMHMM2.0        inside     199   204
tr|C9ZWQ8|C9ZWQ8_TRYB9  TMHMM2.0        TMhelix    205   227
tr|C9ZWQ8|C9ZWQ8_TRYB9  TMHMM2.0        outside    228   256
tr|C9ZWQ8|C9ZWQ8_TRYB9  TMHMM2.0        TMhelix    257   279
tr|C9ZWQ8|C9ZWQ8_TRYB9  TMHMM2.0        inside     280   290
tr|C9ZWQ8|C9ZWQ8_TRYB9  TMHMM2.0        TMhelix    291   313
tr|C9ZWQ8|C9ZWQ8_TRYB9  TMHMM2.0        outside    314   332
tr|C9ZWQ8|C9ZWQ8_TRYB9  TMHMM2.0        TMhelix    333   355
tr|C9ZWQ8|C9ZWQ8_TRYB9  TMHMM2.0        inside     356   374
tr|C9ZWQ8|C9ZWQ8_TRYB9  TMHMM2.0        TMhelix    375   397
tr|C9ZWQ8|C9ZWQ8_TRYB9  TMHMM2.0        outside    398   406
tr|C9ZWQ8|C9ZWQ8_TRYB9  TMHMM2.0        TMhelix    407   429
tr|C9ZWQ8|C9ZWQ8_TRYB9  TMHMM2.0        inside     430   441
tr|C9ZWQ8|C9ZWQ8_TRYB9  TMHMM2.0        TMhelix    442   464
tr|C9ZWQ8|C9ZWQ8_TRYB9  TMHMM2.0        outside    465   467
```

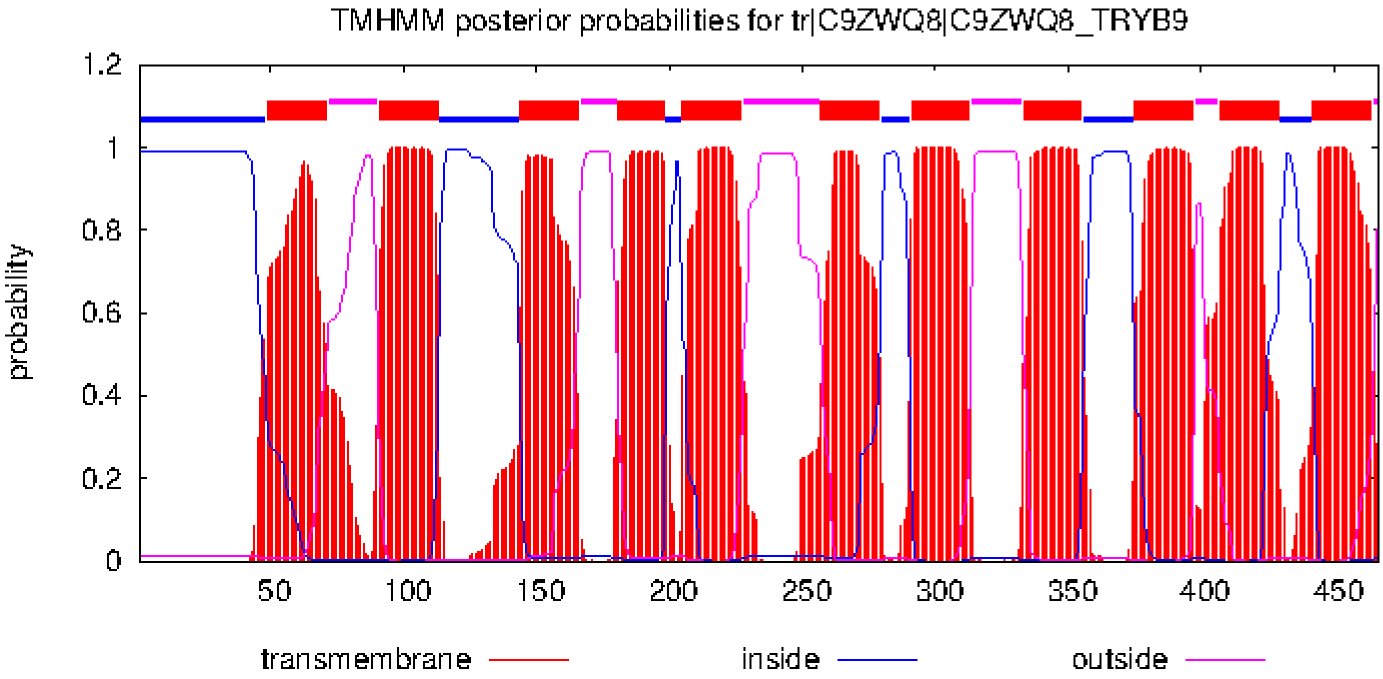

44 # plot in postscript, script for making the plot in gnuplot, data for plot

```
# tr|D0A6R2|D0A6R2_TRYB9 Length: 471
# tr|D0A6R2|D0A6R2_TRYB9 Number of predicted TMHs:  5
# tr|D0A6R2|D0A6R2_TRYB9 Exp number of AAs in TMHs: 111.74886
# tr|D0A6R2|D0A6R2_TRYB9 Exp number, first 60 AAs:  40.12707
# tr|D0A6R2|D0A6R2_TRYB9 Total prob of N-in:        0.24108
# tr|D0A6R2|D0A6R2_TRYB9 POSSIBLE N-term signal sequence
tr|D0A6R2|D0A6R2_TRYB9  TMHMM2.0        outside      1     4
```

tr|D0A6R2|D0A6R2_TRYB9 TMHMM2.0 TMhelix 5 27

```
tr|D0A6R2|D0A6R2_TRYB9  TMHMM2.0        inside       28    39
tr|D0A6R2|D0A6R2_TRYB9  TMHMM2.0        TMhelix      40    62
tr|D0A6R2|D0A6R2_TRYB9  TMHMM2.0        outside      63    76
tr|D0A6R2|D0A6R2_TRYB9  TMHMM2.0        TMhelix      77    99
tr|D0A6R2|D0A6R2_TRYB9  TMHMM2.0        inside      100   301
tr|D0A6R2|D0A6R2_TRYB9  TMHMM2.0        TMhelix     302   324
tr|D0A6R2|D0A6R2_TRYB9  TMHMM2.0        outside     325   338
tr|D0A6R2|D0A6R2_TRYB9  TMHMM2.0        TMhelix     339   361
tr|D0A6R2|D0A6R2_TRYB9  TMHMM2.0        inside      362   471
```

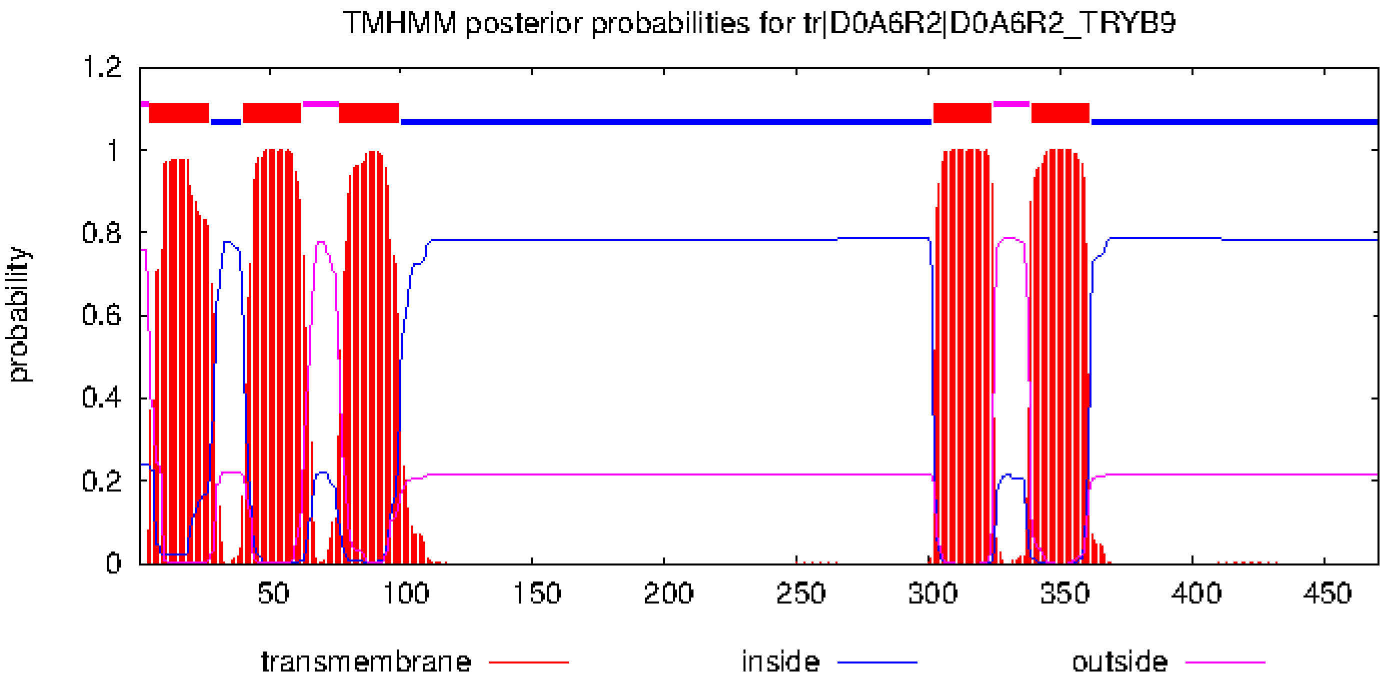

45 **# plot in postscript, script for making the plot in gnuplot, data for plot**

```
# tr|D0AAG8|D0AAG8_TRYB9 Length: 494
# tr|D0AAG8|D0AAG8_TRYB9 Number of predicted TMHs:  11
# tr|D0AAG8|D0AAG8_TRYB9 Exp number of AAs in TMHs: 245.23997
# tr|D0AAG8|D0AAG8_TRYB9 Exp number, first 60 AAs:  0.00011
# tr|D0AAG8|D0AAG8_TRYB9 Total prob of N-in:        0.99793
tr|D0AAG8|D0AAG8_TRYB9  TMHMM2.0        inside        1    70
tr|D0AAG8|D0AAG8_TRYB9  TMHMM2.0        TMhelix      71    93
tr|D0AAG8|D0AAG8_TRYB9  TMHMM2.0        outside      94   107
tr|D0AAG8|D0AAG8_TRYB9  TMHMM2.0        TMhelix     108   130
tr|D0AAG8|D0AAG8_TRYB9  TMHMM2.0        inside      131   150
tr|D0AAG8|D0AAG8_TRYB9  TMHMM2.0        TMhelix     151   173
tr|D0AAG8|D0AAG8_TRYB9  TMHMM2.0        outside     174   194
tr|D0AAG8|D0AAG8_TRYB9  TMHMM2.0        TMhelix     195   214
tr|D0AAG8|D0AAG8_TRYB9  TMHMM2.0        inside      215   220
tr|D0AAG8|D0AAG8_TRYB9  TMHMM2.0        TMhelix     221   243
tr|D0AAG8|D0AAG8_TRYB9  TMHMM2.0        outside     244   267
tr|D0AAG8|D0AAG8_TRYB9  TMHMM2.0        TMhelix     268   290
tr|D0AAG8|D0AAG8_TRYB9  TMHMM2.0        inside      291   301
tr|D0AAG8|D0AAG8_TRYB9  TMHMM2.0        TMhelix     302   324
tr|D0AAG8|D0AAG8_TRYB9  TMHMM2.0        outside     325   343
tr|D0AAG8|D0AAG8_TRYB9  TMHMM2.0        TMhelix     344   363
tr|D0AAG8|D0AAG8_TRYB9  TMHMM2.0        inside      364   383
tr|D0AAG8|D0AAG8_TRYB9  TMHMM2.0        TMhelix     384   406
tr|D0AAG8|D0AAG8_TRYB9  TMHMM2.0        outside     407   410
tr|D0AAG8|D0AAG8_TRYB9  TMHMM2.0        TMhelix     411   433
tr|D0AAG8|D0AAG8_TRYB9  TMHMM2.0        inside      434   445
tr|D0AAG8|D0AAG8_TRYB9  TMHMM2.0        TMhelix     446   468
tr|D0AAG8|D0AAG8_TRYB9  TMHMM2.0        outside     469   494
```

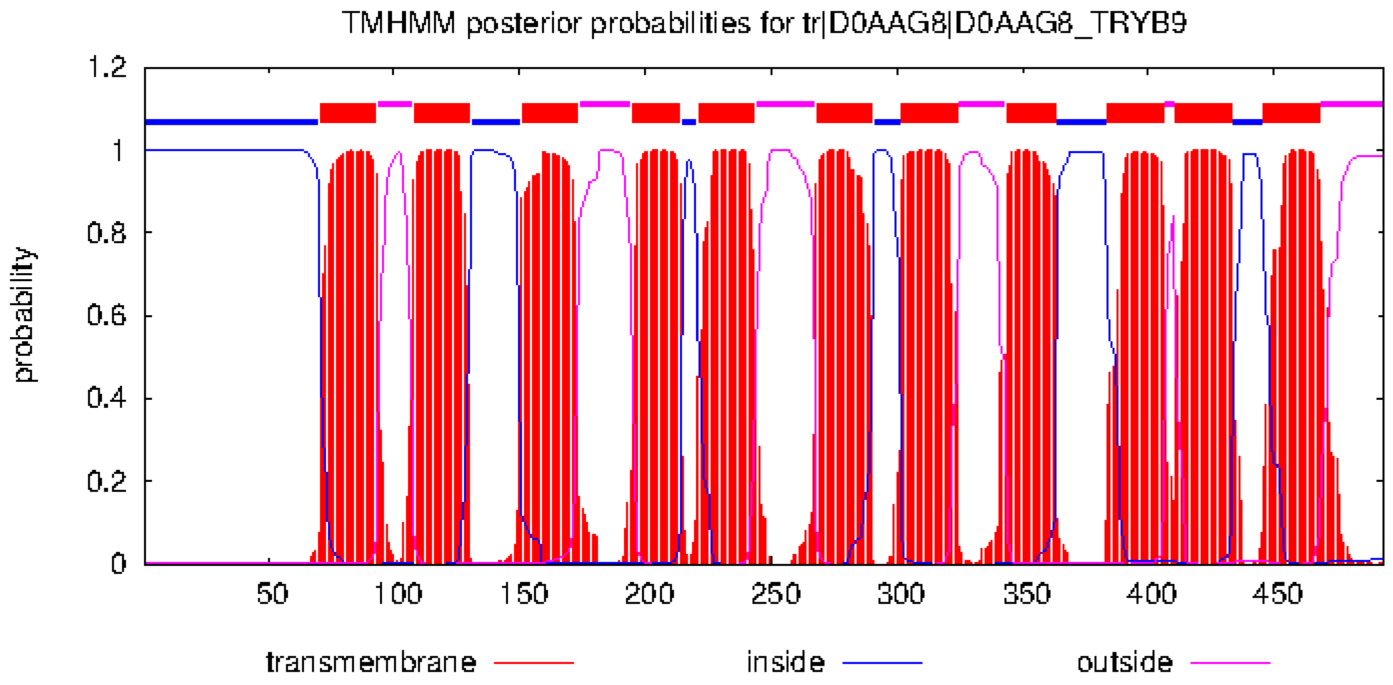

46 # [plot] in postscript, [script] for making the plot in gnuplot, [data] for plot

---

```
# tr|C9ZQ36|C9ZQ36_TRYB9 Length: 524
# tr|C9ZQ36|C9ZQ36_TRYB9 Number of predicted TMHs:  10
# tr|C9ZQ36|C9ZQ36_TRYB9 Exp number of AAs in TMHs: 225.87795
# tr|C9ZQ36|C9ZQ36_TRYB9 Exp number, first 60 AAs: 19.54796
# tr|C9ZQ36|C9ZQ36_TRYB9 Total prob of N-in:        0.76067
# tr|C9ZQ36|C9ZQ36_TRYB9 POSSIBLE N-term signal sequence
tr|C9ZQ36|C9ZQ36_TRYB9  TMHMM2.0        inside       1    38
tr|C9ZQ36|C9ZQ36_TRYB9  TMHMM2.0        TMhelix     39    58
tr|C9ZQ36|C9ZQ36_TRYB9  TMHMM2.0        outside     59    67
tr|C9ZQ36|C9ZQ36_TRYB9  TMHMM2.0        TMhelix     68    90
tr|C9ZQ36|C9ZQ36_TRYB9  TMHMM2.0        inside      91   109
tr|C9ZQ36|C9ZQ36_TRYB9  TMHMM2.0        TMhelix    110   132
tr|C9ZQ36|C9ZQ36_TRYB9  TMHMM2.0        outside    133   151
tr|C9ZQ36|C9ZQ36_TRYB9  TMHMM2.0        TMhelix    152   169
tr|C9ZQ36|C9ZQ36_TRYB9  TMHMM2.0        inside     170   181
tr|C9ZQ36|C9ZQ36_TRYB9  TMHMM2.0        TMhelix    182   204
tr|C9ZQ36|C9ZQ36_TRYB9  TMHMM2.0        outside    205   208
tr|C9ZQ36|C9ZQ36_TRYB9  TMHMM2.0        TMhelix    209   231
tr|C9ZQ36|C9ZQ36_TRYB9  TMHMM2.0        inside     232   294
tr|C9ZQ36|C9ZQ36_TRYB9  TMHMM2.0        TMhelix    295   317
tr|C9ZQ36|C9ZQ36_TRYB9  TMHMM2.0        outside    318   326
tr|C9ZQ36|C9ZQ36_TRYB9  TMHMM2.0        TMhelix    327   349
tr|C9ZQ36|C9ZQ36_TRYB9  TMHMM2.0        inside     350   400
tr|C9ZQ36|C9ZQ36_TRYB9  TMHMM2.0        TMhelix    401   423
tr|C9ZQ36|C9ZQ36_TRYB9  TMHMM2.0        outside    424   427
tr|C9ZQ36|C9ZQ36_TRYB9  TMHMM2.0        TMhelix    428   450
tr|C9ZQ36|C9ZQ36_TRYB9  TMHMM2.0        inside     451   524
```

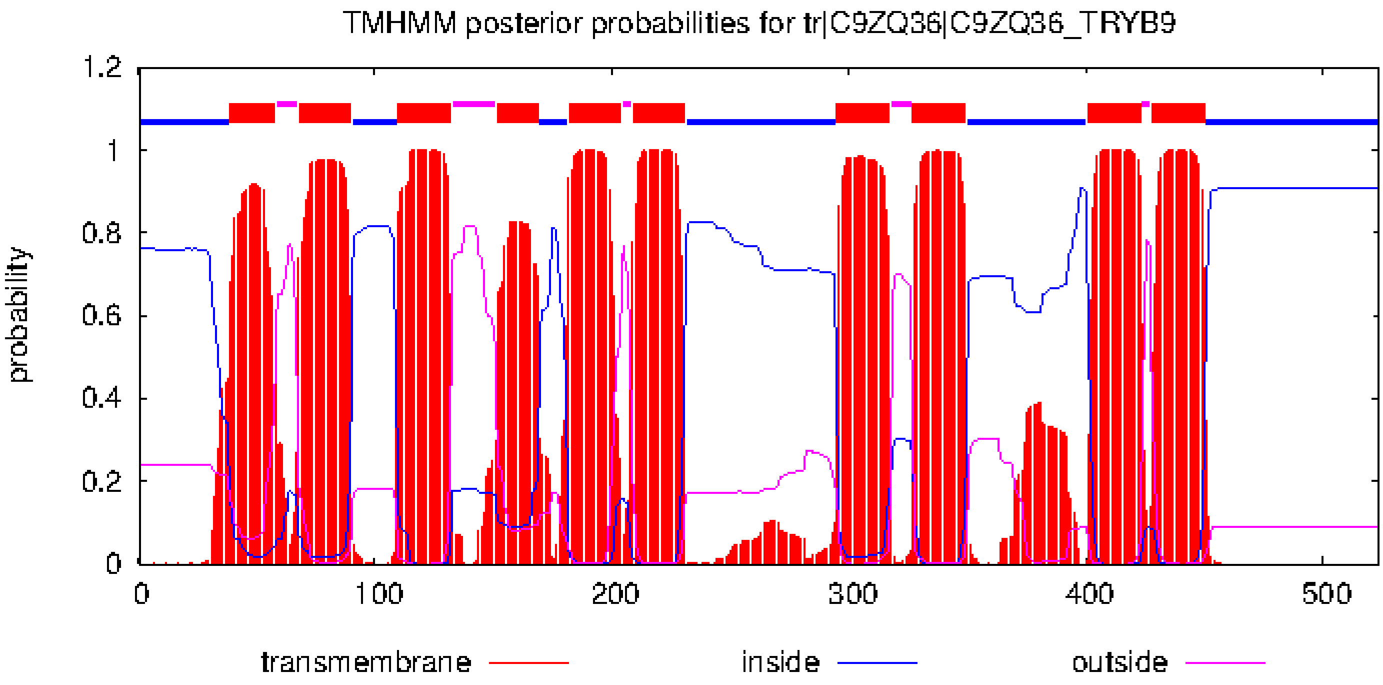

47 # plot in postscript, script for making the plot in gnuplot, data for plot

---

```
# tr|D0A070|D0A070_TRYB9 Length: 527
# tr|D0A070|D0A070_TRYB9 Number of predicted TMHs:  9
# tr|D0A070|D0A070_TRYB9 Exp number of AAs in TMHs: 244.09325
#  tr|D0A070|D0A070_TRYB9 Exp number, first 60 AAs: 10.16173
# tr|D0A070|D0A070_TRYB9 Total prob of N-in:        0.93113
# tr|D0A070|D0A070_TRYB9 POSSIBLE N-term signal sequence
tr|D0A070|D0A070_TRYB9  TMHMM2.0        inside       1    93
tr|D0A070|D0A070_TRYB9  TMHMM2.0        TMhelix     94   116
tr|D0A070|D0A070_TRYB9  TMHMM2.0        outside    117   120
tr|D0A070|D0A070_TRYB9  TMHMM2.0        TMhelix    121   143
tr|D0A070|D0A070_TRYB9  TMHMM2.0        inside     144   180
tr|D0A070|D0A070_TRYB9  TMHMM2.0        TMhelix    181   203
tr|D0A070|D0A070_TRYB9  TMHMM2.0        outside    204   212
tr|D0A070|D0A070_TRYB9  TMHMM2.0        TMhelix    213   235
tr|D0A070|D0A070_TRYB9  TMHMM2.0        inside     236   262
tr|D0A070|D0A070_TRYB9  TMHMM2.0        TMhelix    263   285
tr|D0A070|D0A070_TRYB9  TMHMM2.0        outside    286   299
tr|D0A070|D0A070_TRYB9  TMHMM2.0        TMhelix    300   322
tr|D0A070|D0A070_TRYB9  TMHMM2.0        inside     323   341
tr|D0A070|D0A070_TRYB9  TMHMM2.0        TMhelix    342   361
tr|D0A070|D0A070_TRYB9  TMHMM2.0        outside    362   370
tr|D0A070|D0A070_TRYB9  TMHMM2.0        TMhelix    371   393
tr|D0A070|D0A070_TRYB9  TMHMM2.0        inside     394   473
tr|D0A070|D0A070_TRYB9  TMHMM2.0        TMhelix    474   496
tr|D0A070|D0A070_TRYB9  TMHMM2.0        outside    497   527
```

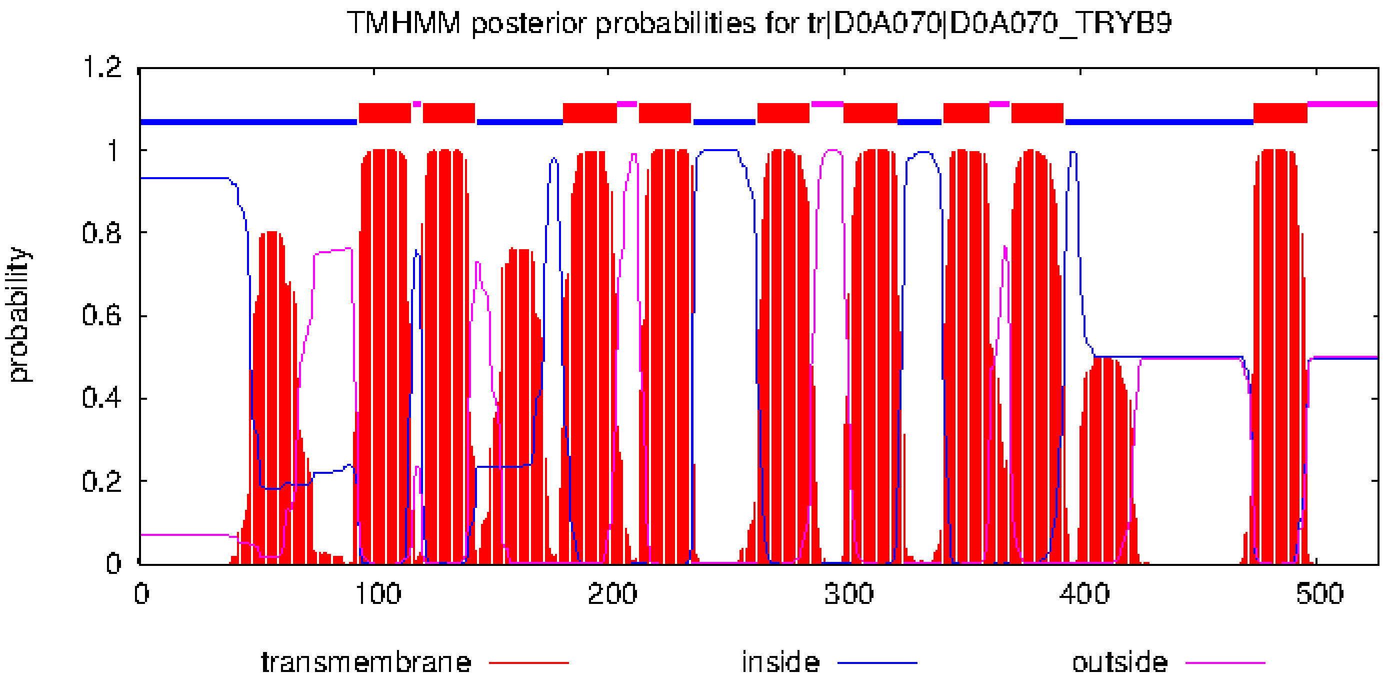

48 # plot in postscript, script for making the plot in gnuplot, data for plot

---

```
# tr|D0A3E0|D0A3E0_TRYB9 Length: 528
# tr|D0A3E0|D0A3E0_TRYB9 Number of predicted TMHs:  1
# tr|D0A3E0|D0A3E0_TRYB9 Exp number of AAs in TMHs: 23.38414
#  tr|D0A3E0|D0A3E0_TRYB9 Exp number, first 60 AAs: 0.68658
# tr|D0A3E0|D0A3E0_TRYB9 Total prob of N-in:        0.03008
tr|D0A3E0|D0A3E0_TRYB9  TMHMM2.0        outside      1   490
tr|D0A3E0|D0A3E0_TRYB9  TMHMM2.0        TMhelix    491   513
tr|D0A3E0|D0A3E0_TRYB9  TMHMM2.0        inside     514   528
```

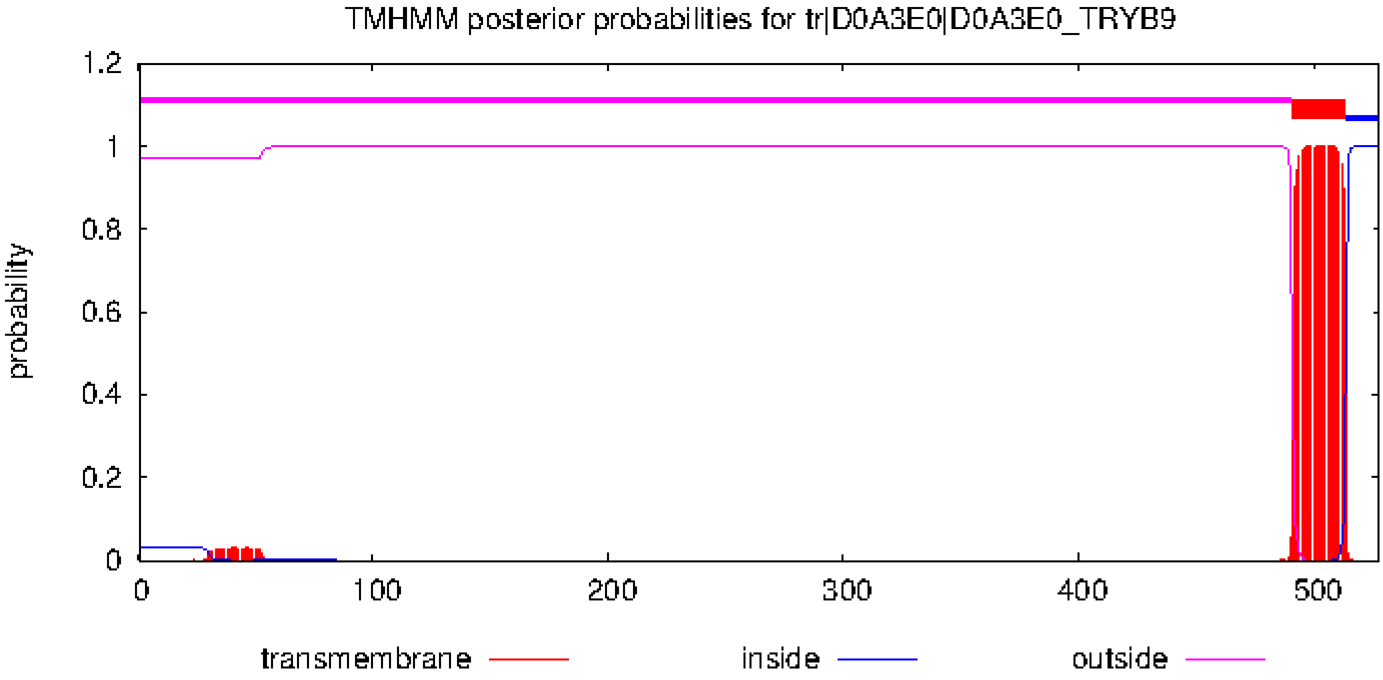

49 # plot in postscript, script for making the plot in gnuplot, data for plot

---

```
# tr|D0A3U3|D0A3U3_TRYB9 Length: 529
# tr|D0A3U3|D0A3U3_TRYB9 Number of predicted TMHs:  12
```

```
# tr|D0A3U3|D0A3U3_TRYB9 Exp number of AAs in TMHs: 261.90344
# tr|D0A3U3|D0A3U3_TRYB9 Exp number, first 60 AAs:  16.74421
# tr|D0A3U3|D0A3U3_TRYB9 Total prob of N-in:        0.91132
```

```
#  tr|D0A3U3|D0A3U3_TRYB9  POSSIBLE N-term signal sequence
tr|D0A3U3|D0A3U3_TRYB9  TMHMM2.0        inside       1    41
tr|D0A3U3|D0A3U3_TRYB9  TMHMM2.0        TMhelix     42    64
tr|D0A3U3|D0A3U3_TRYB9  TMHMM2.0        outside     65   116
tr|D0A3U3|D0A3U3_TRYB9  TMHMM2.0        TMhelix    117   139
tr|D0A3U3|D0A3U3_TRYB9  TMHMM2.0        inside     140   151
tr|D0A3U3|D0A3U3_TRYB9  TMHMM2.0        TMhelix    152   169
tr|D0A3U3|D0A3U3_TRYB9  TMHMM2.0        outside    170   178
tr|D0A3U3|D0A3U3_TRYB9  TMHMM2.0        TMhelix    179   201
tr|D0A3U3|D0A3U3_TRYB9  TMHMM2.0        inside     202   213
tr|D0A3U3|D0A3U3_TRYB9  TMHMM2.0        TMhelix    214   235
tr|D0A3U3|D0A3U3_TRYB9  TMHMM2.0        outside    236   249
tr|D0A3U3|D0A3U3_TRYB9  TMHMM2.0        TMhelix    250   272
tr|D0A3U3|D0A3U3_TRYB9  TMHMM2.0        inside     273   301
tr|D0A3U3|D0A3U3_TRYB9  TMHMM2.0        TMhelix    302   324
tr|D0A3U3|D0A3U3_TRYB9  TMHMM2.0        outside    325   338
tr|D0A3U3|D0A3U3_TRYB9  TMHMM2.0        TMhelix    339   361
tr|D0A3U3|D0A3U3_TRYB9  TMHMM2.0        inside     362   373
tr|D0A3U3|D0A3U3_TRYB9  TMHMM2.0        TMhelix    374   393
tr|D0A3U3|D0A3U3_TRYB9  TMHMM2.0        outside    394   402
tr|D0A3U3|D0A3U3_TRYB9  TMHMM2.0        TMhelix    403   425
tr|D0A3U3|D0A3U3_TRYB9  TMHMM2.0        inside     426   437
tr|D0A3U3|D0A3U3_TRYB9  TMHMM2.0        TMhelix    438   460
tr|D0A3U3|D0A3U3_TRYB9  TMHMM2.0        outside    461   474
tr|D0A3U3|D0A3U3_TRYB9  TMHMM2.0        TMhelix    475   497
tr|D0A3U3|D0A3U3_TRYB9  TMHMM2.0        inside     498   529
```

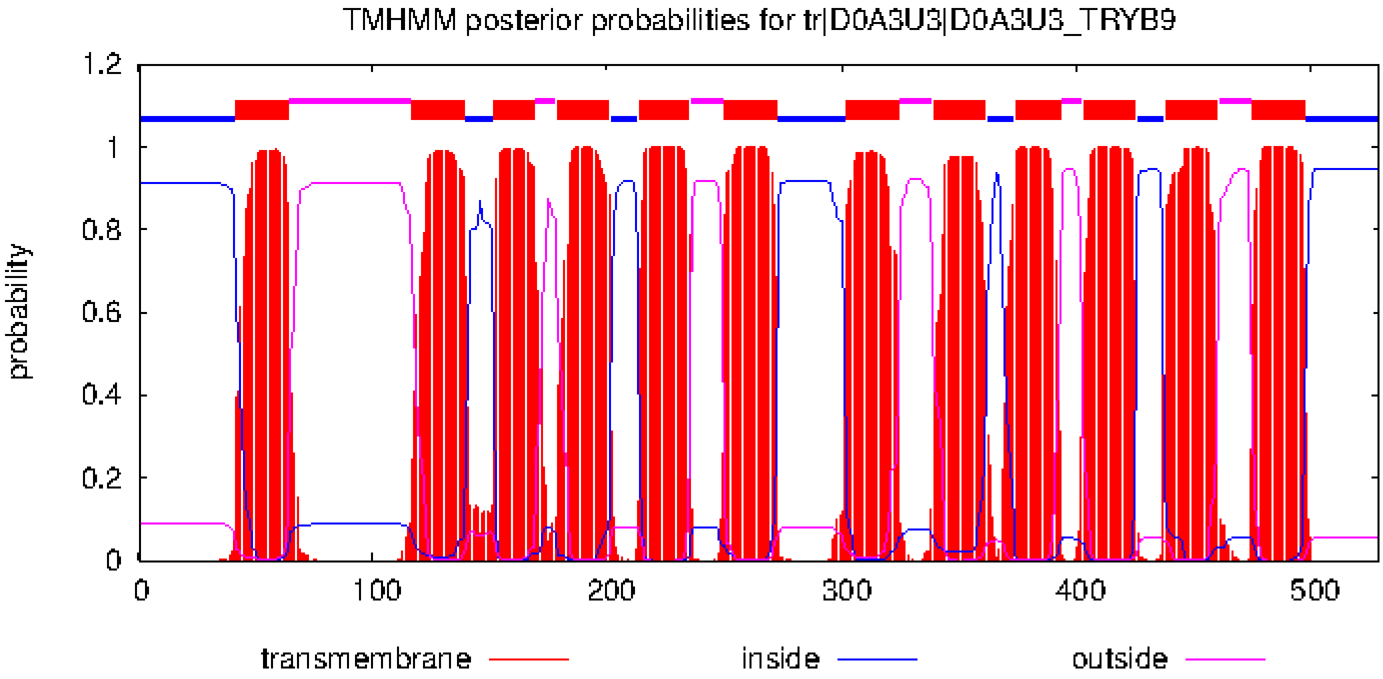

50 **# plot in postscript, script for making the plot in gnuplot, data for plot**

```
# tr|C9ZVS3|C9ZVS3_TRYB9 Length: 530
# tr|C9ZVS3|C9ZVS3_TRYB9 Number of predicted TMHs:  10
# tr|C9ZVS3|C9ZVS3_TRYB9 Exp number of AAs in TMHs: 242.18528
# tr|C9ZVS3|C9ZVS3_TRYB9 Exp number, first 60 AAs:  8.41394
# tr|C9ZVS3|C9ZVS3_TRYB9 Total prob of N-in:        0.48838
tr|C9ZVS3|C9ZVS3_TRYB9  TMHMM2.0        outside      1   159
tr|C9ZVS3|C9ZVS3_TRYB9  TMHMM2.0        TMhelix    160   182
tr|C9ZVS3|C9ZVS3_TRYB9  TMHMM2.0        inside     183   211
tr|C9ZVS3|C9ZVS3_TRYB9  TMHMM2.0        TMhelix    212   234
tr|C9ZVS3|C9ZVS3_TRYB9  TMHMM2.0        outside    235   253
tr|C9ZVS3|C9ZVS3_TRYB9  TMHMM2.0        TMhelix    254   271
tr|C9ZVS3|C9ZVS3_TRYB9  TMHMM2.0        inside     272   277
tr|C9ZVS3|C9ZVS3_TRYB9  TMHMM2.0        TMhelix    278   300
tr|C9ZVS3|C9ZVS3_TRYB9  TMHMM2.0        outside    301   321
```

tr|C9ZVS3|C9ZVS3_TRYB9 TMHMM2.0 TMhelix 322 344

```
tr|C9ZVS3|C9ZVS3_TRYB9  TMHMM2.0        inside     345   355
tr|C9ZVS3|C9ZVS3_TRYB9  TMHMM2.0        TMhelix    356   378
tr|C9ZVS3|C9ZVS3_TRYB9  TMHMM2.0        outside    379   397
tr|C9ZVS3|C9ZVS3_TRYB9  TMHMM2.0        TMhelix    398   417
tr|C9ZVS3|C9ZVS3_TRYB9  TMHMM2.0        inside     418   437
tr|C9ZVS3|C9ZVS3_TRYB9  TMHMM2.0        TMhelix    438   460
tr|C9ZVS3|C9ZVS3_TRYB9  TMHMM2.0        outside    461   464
tr|C9ZVS3|C9ZVS3_TRYB9  TMHMM2.0        TMhelix    465   487
tr|C9ZVS3|C9ZVS3_TRYB9  TMHMM2.0        inside     488   499
tr|C9ZVS3|C9ZVS3_TRYB9  TMHMM2.0        TMhelix    500   522
tr|C9ZVS3|C9ZVS3_TRYB9  TMHMM2.0        outside    523   530
```

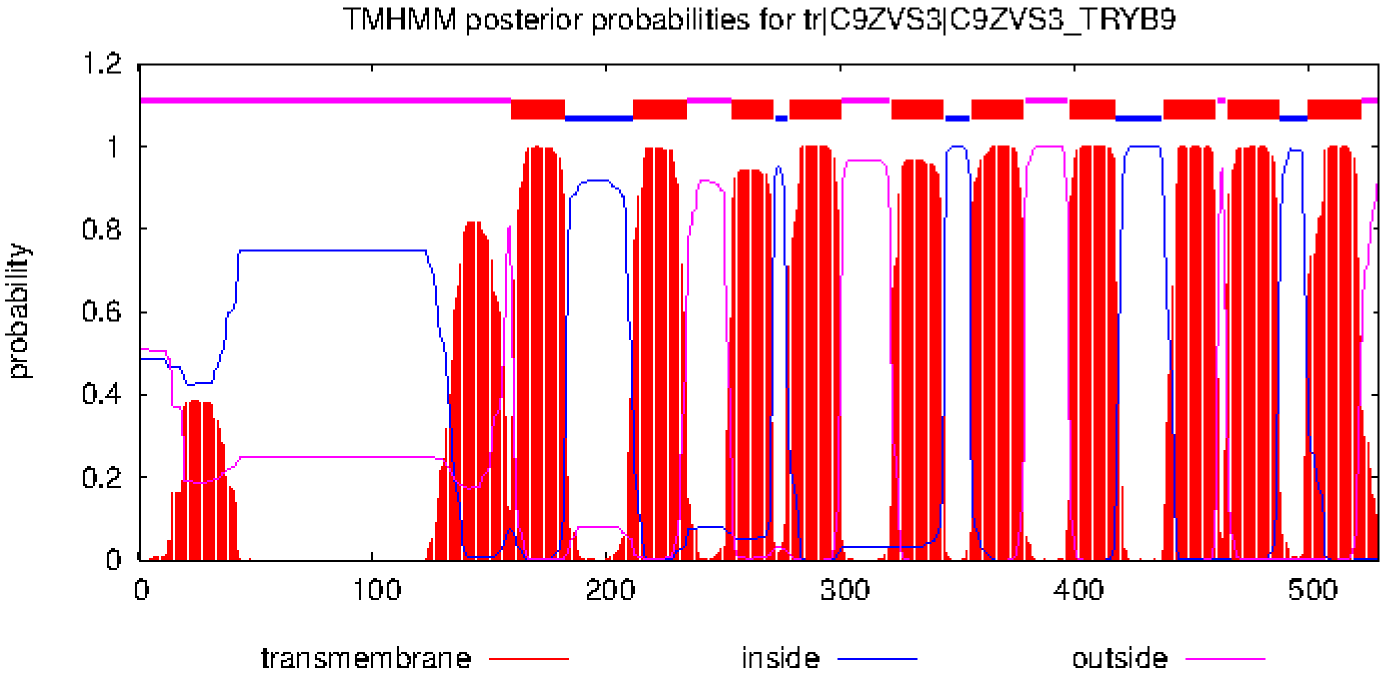

51 **# plot in postscript, script for making the plot in gnuplot, data for plot**

```
# tr|C9ZPP9|C9ZPP9_TRYB9 Length: 580
# tr|C9ZPP9|C9ZPP9_TRYB9 Number of predicted TMHs:  2
# tr|C9ZPP9|C9ZPP9_TRYB9 Exp number of AAs in TMHs: 47.18755
# tr|C9ZPP9|C9ZPP9_TRYB9 Exp number, first 60 AAs:  0.04205
# tr|C9ZPP9|C9ZPP9_TRYB9 Total prob of N-in:        0.90615
tr|C9ZPP9|C9ZPP9_TRYB9  TMHMM2.0        inside       1   416
tr|C9ZPP9|C9ZPP9_TRYB9  TMHMM2.0        TMhelix    417   436
tr|C9ZPP9|C9ZPP9_TRYB9  TMHMM2.0        outside    437   525
tr|C9ZPP9|C9ZPP9_TRYB9  TMHMM2.0        TMhelix    526   548
tr|C9ZPP9|C9ZPP9_TRYB9  TMHMM2.0        inside     549   580
```

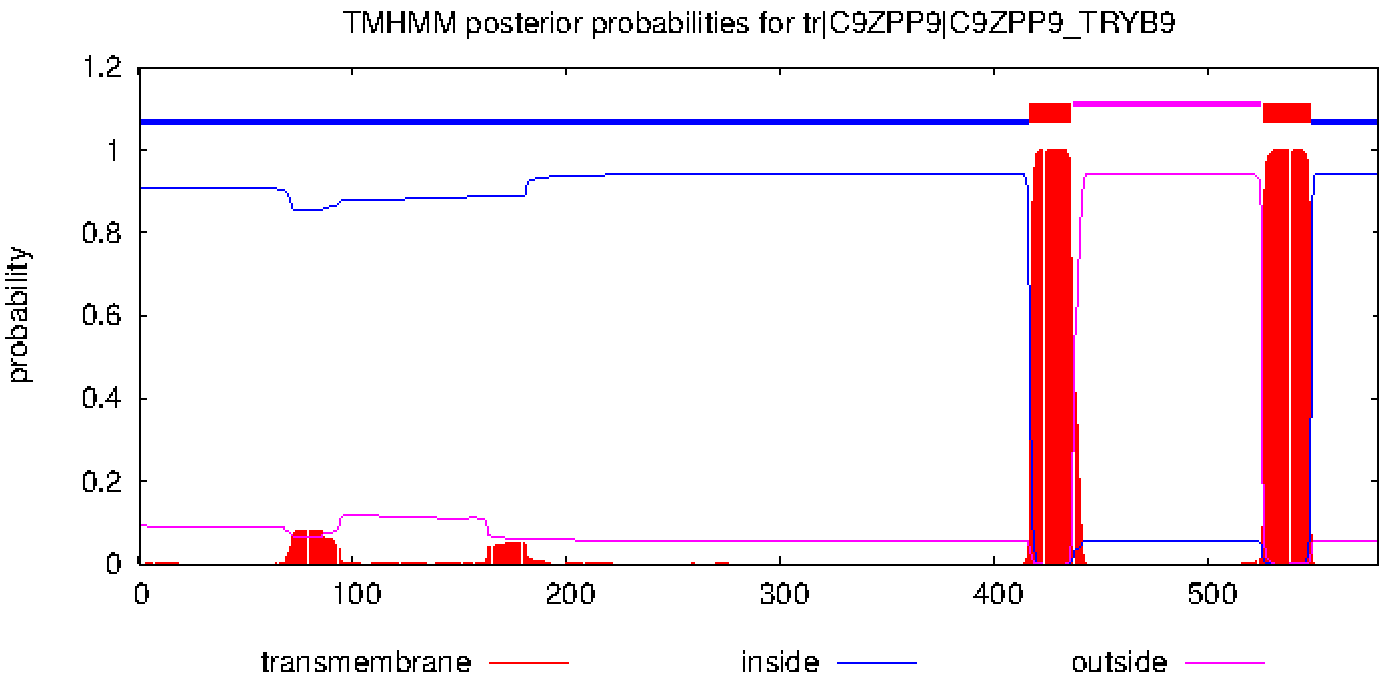

52 # plot in postscript, script for making the plot in gnuplot, data for plot

---

```
# tr|C9ZL99|C9ZL99_TRYB9 Length: 595
# tr|C9ZL99|C9ZL99_TRYB9 Number of predicted TMHs:  13
# tr|C9ZL99|C9ZL99_TRYB9 Exp number of AAs in TMHs: 291.20653
# tr|C9ZL99|C9ZL99_TRYB9 Exp number, first 60 AAs: 32.19672
# tr|C9ZL99|C9ZL99_TRYB9 Total prob of N-in:        0.16874
# tr|C9ZL99|C9ZL99_TRYB9 POSSIBLE N-term signal sequence
tr|C9ZL99|C9ZL99_TRYB9  TMHMM2.0        outside      1    14
tr|C9ZL99|C9ZL99_TRYB9  TMHMM2.0        TMhelix     15    37
tr|C9ZL99|C9ZL99_TRYB9  TMHMM2.0        inside      38    49
tr|C9ZL99|C9ZL99_TRYB9  TMHMM2.0        TMhelix     50    69
tr|C9ZL99|C9ZL99_TRYB9  TMHMM2.0        outside     70    73
tr|C9ZL99|C9ZL99_TRYB9  TMHMM2.0        TMhelix     74    96
tr|C9ZL99|C9ZL99_TRYB9  TMHMM2.0        inside      97   139
tr|C9ZL99|C9ZL99_TRYB9  TMHMM2.0        TMhelix    140   162
tr|C9ZL99|C9ZL99_TRYB9  TMHMM2.0        outside    163   176
tr|C9ZL99|C9ZL99_TRYB9  TMHMM2.0        TMhelix    177   199
tr|C9ZL99|C9ZL99_TRYB9  TMHMM2.0        inside     200   240
tr|C9ZL99|C9ZL99_TRYB9  TMHMM2.0        TMhelix    241   263
tr|C9ZL99|C9ZL99_TRYB9  TMHMM2.0        outside    264   272
tr|C9ZL99|C9ZL99_TRYB9  TMHMM2.0        TMhelix    273   295
tr|C9ZL99|C9ZL99_TRYB9  TMHMM2.0        inside     296   346
tr|C9ZL99|C9ZL99_TRYB9  TMHMM2.0        TMhelix    347   369
tr|C9ZL99|C9ZL99_TRYB9  TMHMM2.0        outside    370   383
tr|C9ZL99|C9ZL99_TRYB9  TMHMM2.0        TMhelix    384   406
tr|C9ZL99|C9ZL99_TRYB9  TMHMM2.0        inside     407   424
tr|C9ZL99|C9ZL99_TRYB9  TMHMM2.0        TMhelix    425   444
tr|C9ZL99|C9ZL99_TRYB9  TMHMM2.0        outside    445   448
tr|C9ZL99|C9ZL99_TRYB9  TMHMM2.0        TMhelix    449   471
tr|C9ZL99|C9ZL99_TRYB9  TMHMM2.0        inside     472   483
tr|C9ZL99|C9ZL99_TRYB9  TMHMM2.0        TMhelix    484   506
tr|C9ZL99|C9ZL99_TRYB9  TMHMM2.0        outside    507   528
tr|C9ZL99|C9ZL99_TRYB9  TMHMM2.0        TMhelix    529   551
tr|C9ZL99|C9ZL99_TRYB9  TMHMM2.0        inside     552   595
```

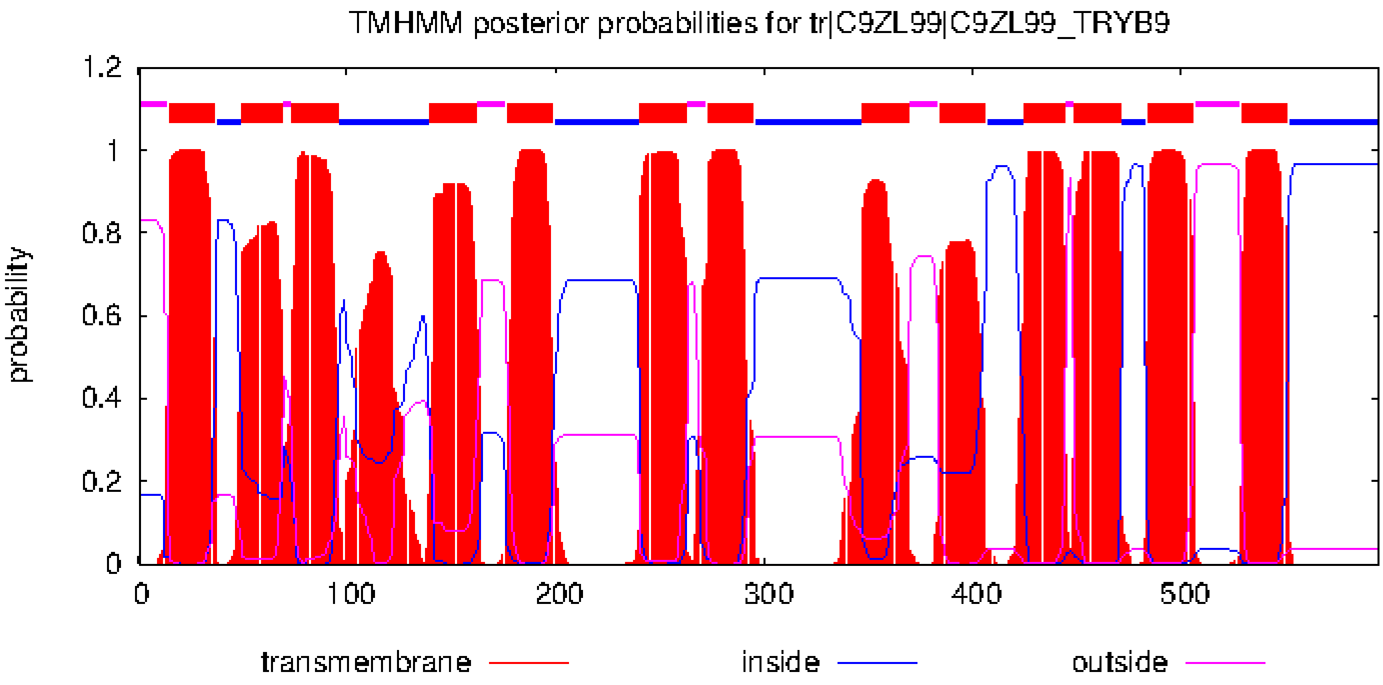

53 # plot in postscript, script for making the plot in gnuplot, data for plot

---

```
# tr|C9ZLA1|C9ZLA1_TRYB9 Length: 595
# tr|C9ZLA1|C9ZLA1_TRYB9 Number of predicted TMHs:  14
# tr|C9ZLA1|C9ZLA1_TRYB9 Exp number of AAs in TMHs: 296.28226
# tr|C9ZLA1|C9ZLA1_TRYB9 Exp number, first 60 AAs: 32.87637
# tr|C9ZLA1|C9ZLA1_TRYB9 Total prob of N-in:        0.58281
# tr|C9ZLA1|C9ZLA1_TRYB9 POSSIBLE N-term signal sequence
tr|C9ZLA1|C9ZLA1_TRYB9  TMHMM2.0        inside       1    12
tr|C9ZLA1|C9ZLA1_TRYB9  TMHMM2.0        TMhelix     13    35
tr|C9ZLA1|C9ZLA1_TRYB9  TMHMM2.0        outside     36    49
tr|C9ZLA1|C9ZLA1_TRYB9  TMHMM2.0        TMhelix     50    69
tr|C9ZLA1|C9ZLA1_TRYB9  TMHMM2.0        inside      70    75
tr|C9ZLA1|C9ZLA1_TRYB9  TMHMM2.0        TMhelix     76    98
tr|C9ZLA1|C9ZLA1_TRYB9  TMHMM2.0        outside     99   112
tr|C9ZLA1|C9ZLA1_TRYB9  TMHMM2.0        TMhelix    113   135
tr|C9ZLA1|C9ZLA1_TRYB9  TMHMM2.0        inside     136   141
tr|C9ZLA1|C9ZLA1_TRYB9  TMHMM2.0        TMhelix    142   164
tr|C9ZLA1|C9ZLA1_TRYB9  TMHMM2.0        outside    165   178
tr|C9ZLA1|C9ZLA1_TRYB9  TMHMM2.0        TMhelix    179   198
tr|C9ZLA1|C9ZLA1_TRYB9  TMHMM2.0        inside     199   240
tr|C9ZLA1|C9ZLA1_TRYB9  TMHMM2.0        TMhelix    241   263
tr|C9ZLA1|C9ZLA1_TRYB9  TMHMM2.0        outside    264   272
tr|C9ZLA1|C9ZLA1_TRYB9  TMHMM2.0        TMhelix    273   295
tr|C9ZLA1|C9ZLA1_TRYB9  TMHMM2.0        inside     296   346
tr|C9ZLA1|C9ZLA1_TRYB9  TMHMM2.0        TMhelix    347   369
tr|C9ZLA1|C9ZLA1_TRYB9  TMHMM2.0        outside    370   383
tr|C9ZLA1|C9ZLA1_TRYB9  TMHMM2.0        TMhelix    384   404
tr|C9ZLA1|C9ZLA1_TRYB9  TMHMM2.0        inside     405   424
tr|C9ZLA1|C9ZLA1_TRYB9  TMHMM2.0        TMhelix    425   444
tr|C9ZLA1|C9ZLA1_TRYB9  TMHMM2.0        outside    445   448
tr|C9ZLA1|C9ZLA1_TRYB9  TMHMM2.0        TMhelix    449   471
tr|C9ZLA1|C9ZLA1_TRYB9  TMHMM2.0        inside     472   483
tr|C9ZLA1|C9ZLA1_TRYB9  TMHMM2.0        TMhelix    484   506
tr|C9ZLA1|C9ZLA1_TRYB9  TMHMM2.0        outside    507   528
tr|C9ZLA1|C9ZLA1_TRYB9  TMHMM2.0        TMhelix    529   551
tr|C9ZLA1|C9ZLA1_TRYB9  TMHMM2.0        inside     552   595
```

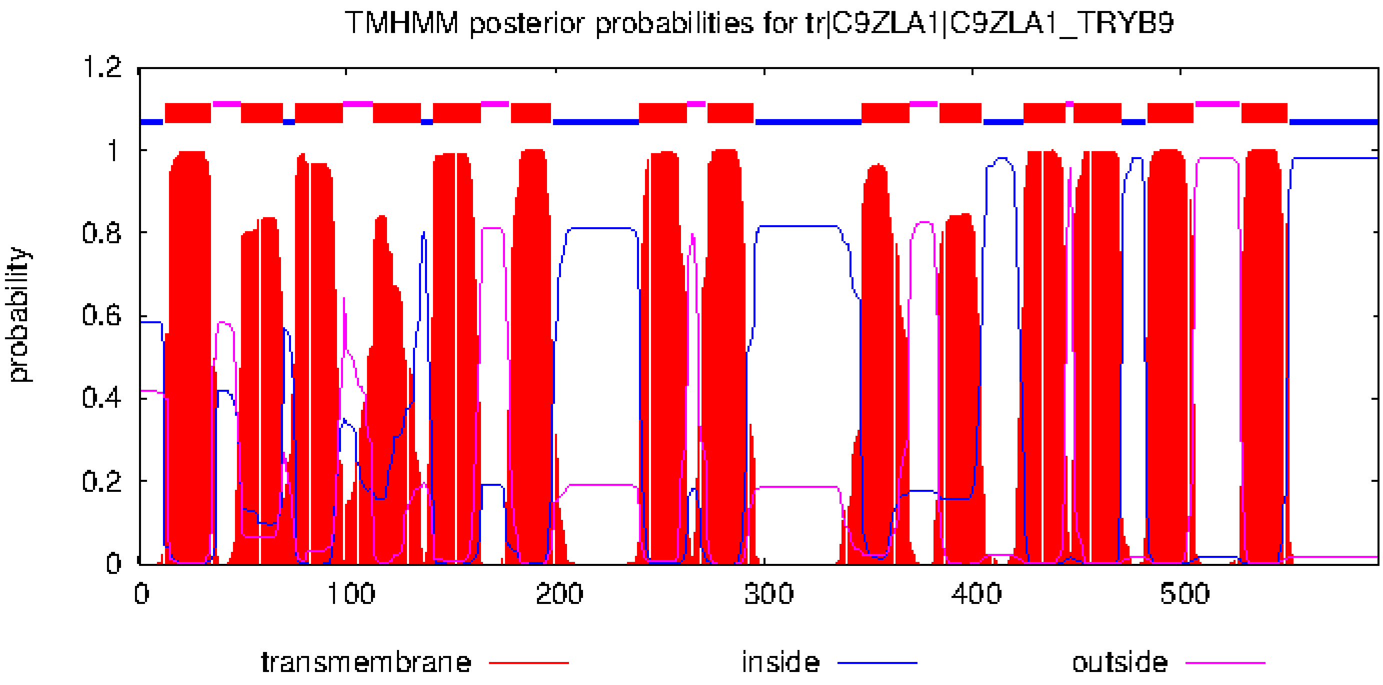

54 # **plot** in postscript, **script** for making the plot in gnuplot, **data** for plot

---

```
# tr|D0A1S7|D0A1S7_TRYB9 Length: 656
# tr|D0A1S7|D0A1S7_TRYB9 Number of predicted TMHs:  1
# tr|D0A1S7|D0A1S7_TRYB9 Exp number of AAs in TMHs: 104.7305
# tr|D0A1S7|D0A1S7_TRYB9 Exp number, first 60 AAs: 0.00018
# tr|D0A1S7|D0A1S7_TRYB9 Total prob of N-in:        0.95483
tr|D0A1S7|D0A1S7_TRYB9  TMHMM2.0        inside       1   112
tr|D0A1S7|D0A1S7_TRYB9  TMHMM2.0        TMhelix    113   135
tr|D0A1S7|D0A1S7_TRYB9  TMHMM2.0        outside    136   656
```

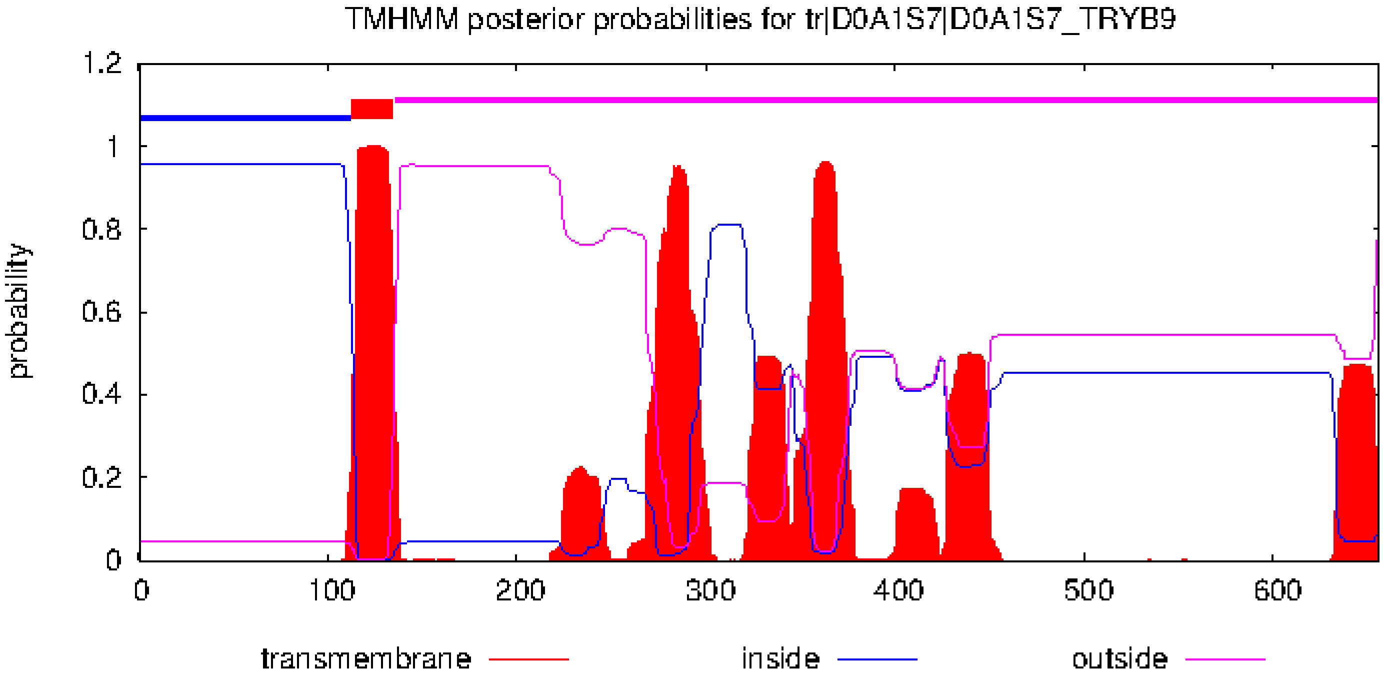

55 # **plot** in postscript, **script** for making the plot in gnuplot, **data** for plot

---

```
# tr|C9ZKG2|C9ZKG2_TRYB9 Length: 813
# tr|C9ZKG2|C9ZKG2_TRYB9 Number of predicted TMHs:  10
```

```
# tr|C9ZKG2|C9ZKG2_TRYB9 Exp number of AAs in TMHs: 218.33185
# tr|C9ZKG2|C9ZKG2_TRYB9 Exp number, first 60 AAs:  0.00072
# tr|C9ZKG2|C9ZKG2_TRYB9 Total prob of N-in:        0.08779
```

```
tr|C9ZKG2|C9ZKG2_TRYB9  TMHMM2.0        outside      1    68
tr|C9ZKG2|C9ZKG2_TRYB9  TMHMM2.0        TMhelix     69    91
tr|C9ZKG2|C9ZKG2_TRYB9  TMHMM2.0        inside      92   433
tr|C9ZKG2|C9ZKG2_TRYB9  TMHMM2.0        TMhelix    434   456
tr|C9ZKG2|C9ZKG2_TRYB9  TMHMM2.0        outside    457   478
tr|C9ZKG2|C9ZKG2_TRYB9  TMHMM2.0        TMhelix    479   501
tr|C9ZKG2|C9ZKG2_TRYB9  TMHMM2.0        inside     502   531
tr|C9ZKG2|C9ZKG2_TRYB9  TMHMM2.0        TMhelix    532   554
tr|C9ZKG2|C9ZKG2_TRYB9  TMHMM2.0        outside    555   607
tr|C9ZKG2|C9ZKG2_TRYB9  TMHMM2.0        TMhelix    608   627
tr|C9ZKG2|C9ZKG2_TRYB9  TMHMM2.0        inside     628   633
tr|C9ZKG2|C9ZKG2_TRYB9  TMHMM2.0        TMhelix    634   653
tr|C9ZKG2|C9ZKG2_TRYB9  TMHMM2.0        outside    654   667
tr|C9ZKG2|C9ZKG2_TRYB9  TMHMM2.0        TMhelix    668   690
tr|C9ZKG2|C9ZKG2_TRYB9  TMHMM2.0        inside     691   702
tr|C9ZKG2|C9ZKG2_TRYB9  TMHMM2.0        TMhelix    703   725
tr|C9ZKG2|C9ZKG2_TRYB9  TMHMM2.0        outside    726   753
tr|C9ZKG2|C9ZKG2_TRYB9  TMHMM2.0        TMhelix    754   773
tr|C9ZKG2|C9ZKG2_TRYB9  TMHMM2.0        inside     774   779
tr|C9ZKG2|C9ZKG2_TRYB9  TMHMM2.0        TMhelix    780   802
tr|C9ZKG2|C9ZKG2_TRYB9  TMHMM2.0        outside    803   813
```

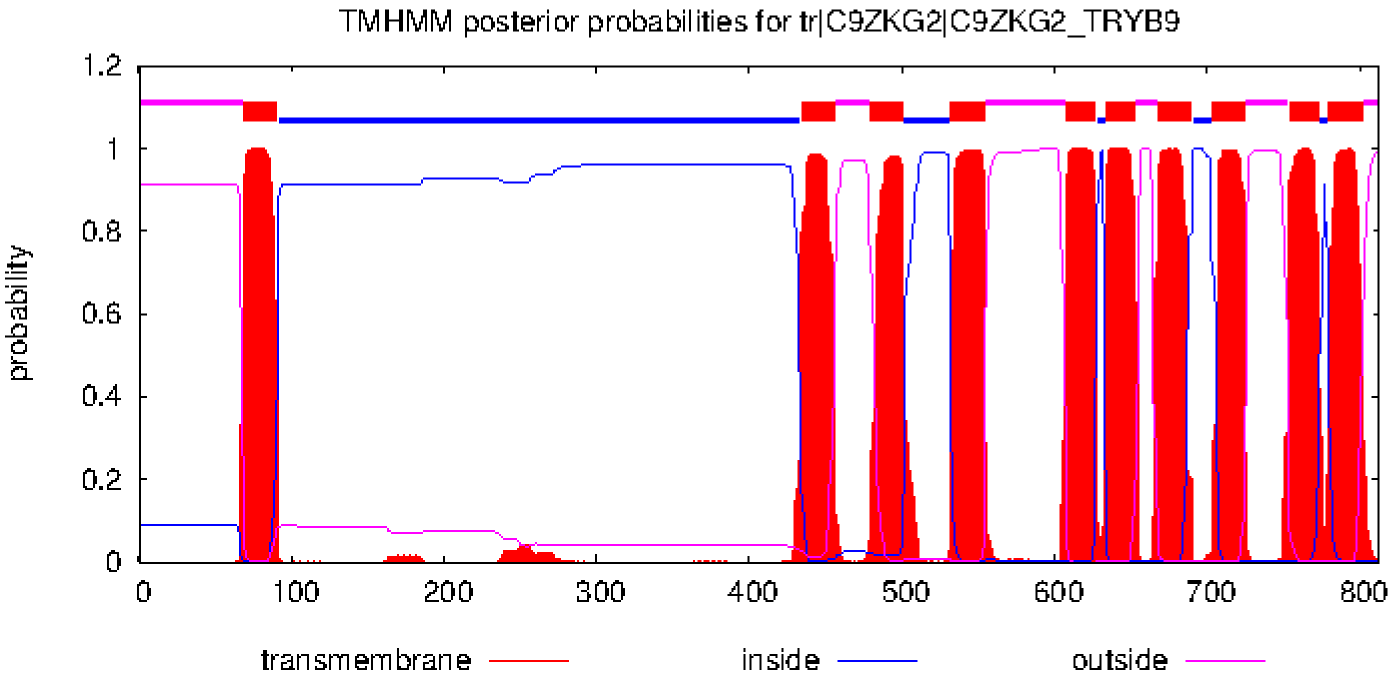

56 # **plot** in postscript, **script** for making the plot in gnuplot, **data** for plot

---

```
# tr|C9ZY45|C9ZY45_TRYB9 Length: 820
# tr|C9ZY45|C9ZY45_TRYB9 Number of predicted TMHs:  5
# tr|C9ZY45|C9ZY45_TRYB9 Exp number of AAs in TMHs: 118.32606
# tr|C9ZY45|C9ZY45_TRYB9 Exp number, first 60 AAs:  0.06933
# tr|C9ZY45|C9ZY45_TRYB9 Total prob of N-in:        0.26344
tr|C9ZY45|C9ZY45_TRYB9  TMHMM2.0        outside      1   392
tr|C9ZY45|C9ZY45_TRYB9  TMHMM2.0        TMhelix    393   410
tr|C9ZY45|C9ZY45_TRYB9  TMHMM2.0        inside     411   421
tr|C9ZY45|C9ZY45_TRYB9  TMHMM2.0        TMhelix    422   444
tr|C9ZY45|C9ZY45_TRYB9  TMHMM2.0        outside    445   453
tr|C9ZY45|C9ZY45_TRYB9  TMHMM2.0        TMhelix    454   476
tr|C9ZY45|C9ZY45_TRYB9  TMHMM2.0        inside     477   554
tr|C9ZY45|C9ZY45_TRYB9  TMHMM2.0        TMhelix    555   574
tr|C9ZY45|C9ZY45_TRYB9  TMHMM2.0        outside    575   593
tr|C9ZY45|C9ZY45_TRYB9  TMHMM2.0        TMhelix    594   616
tr|C9ZY45|C9ZY45_TRYB9  TMHMM2.0        inside     617   820
```

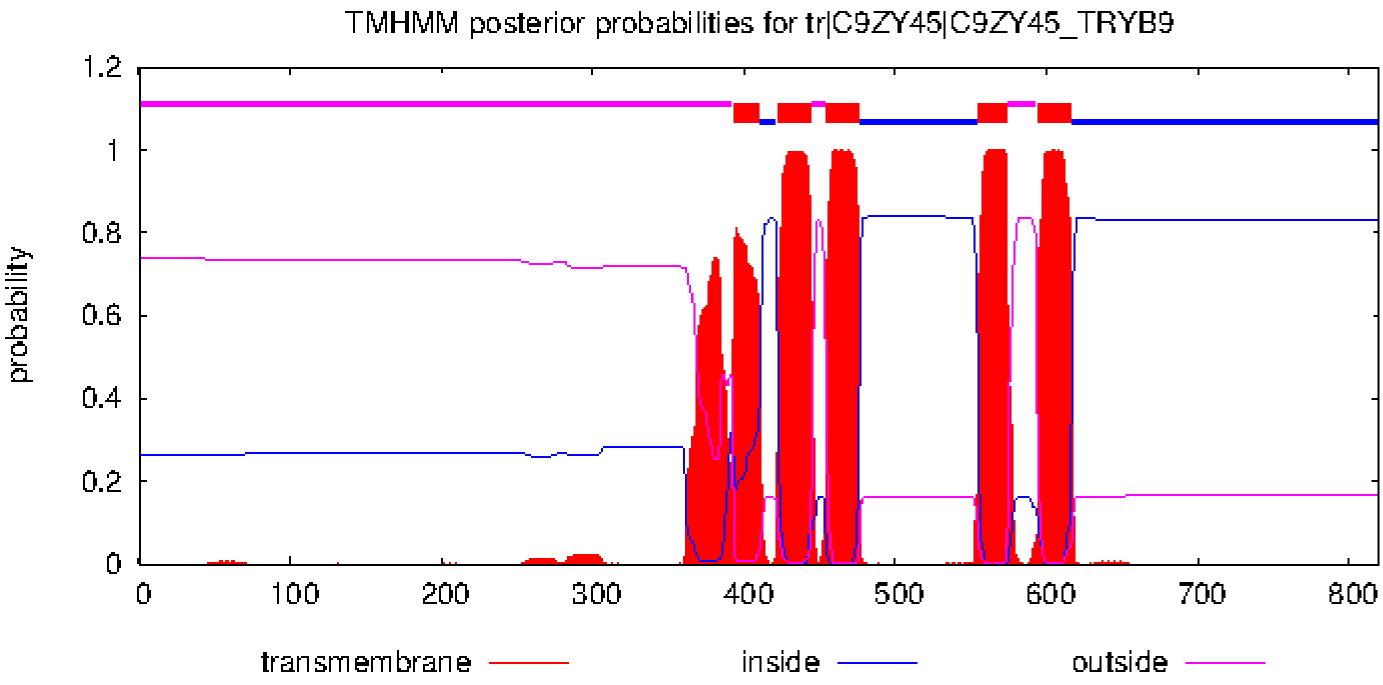

57 # plot in postscript, script for making the plot in gnuplot, data for plot

---

```
# tr|D0A564|D0A564_TRYB9 Length: 912
# tr|D0A564|D0A564_TRYB9 Number of predicted TMHs:  7
# tr|D0A564|D0A564_TRYB9 Exp number of AAs in TMHs: 188.69159
# tr|D0A564|D0A564_TRYB9 Exp number, first 60 AAs:  0.00013
# tr|D0A564|D0A564_TRYB9 Total prob of N-in:        0.61614
tr|D0A564|D0A564_TRYB9  TMHMM2.0        outside      1   257
tr|D0A564|D0A564_TRYB9  TMHMM2.0        TMhelix    258   280
tr|D0A564|D0A564_TRYB9  TMHMM2.0        inside     281   292
tr|D0A564|D0A564_TRYB9  TMHMM2.0        TMhelix    293   315
tr|D0A564|D0A564_TRYB9  TMHMM2.0        outside    316   653
tr|D0A564|D0A564_TRYB9  TMHMM2.0        TMhelix    654   676
tr|D0A564|D0A564_TRYB9  TMHMM2.0        inside     677   687
tr|D0A564|D0A564_TRYB9  TMHMM2.0        TMhelix    688   710
tr|D0A564|D0A564_TRYB9  TMHMM2.0        outside    711   729
tr|D0A564|D0A564_TRYB9  TMHMM2.0        TMhelix    730   752
tr|D0A564|D0A564_TRYB9  TMHMM2.0        inside     753   804
tr|D0A564|D0A564_TRYB9  TMHMM2.0        TMhelix    805   827
tr|D0A564|D0A564_TRYB9  TMHMM2.0        outside    828   855
tr|D0A564|D0A564_TRYB9  TMHMM2.0        TMhelix    856   878
tr|D0A564|D0A564_TRYB9  TMHMM2.0        inside     879   912
```

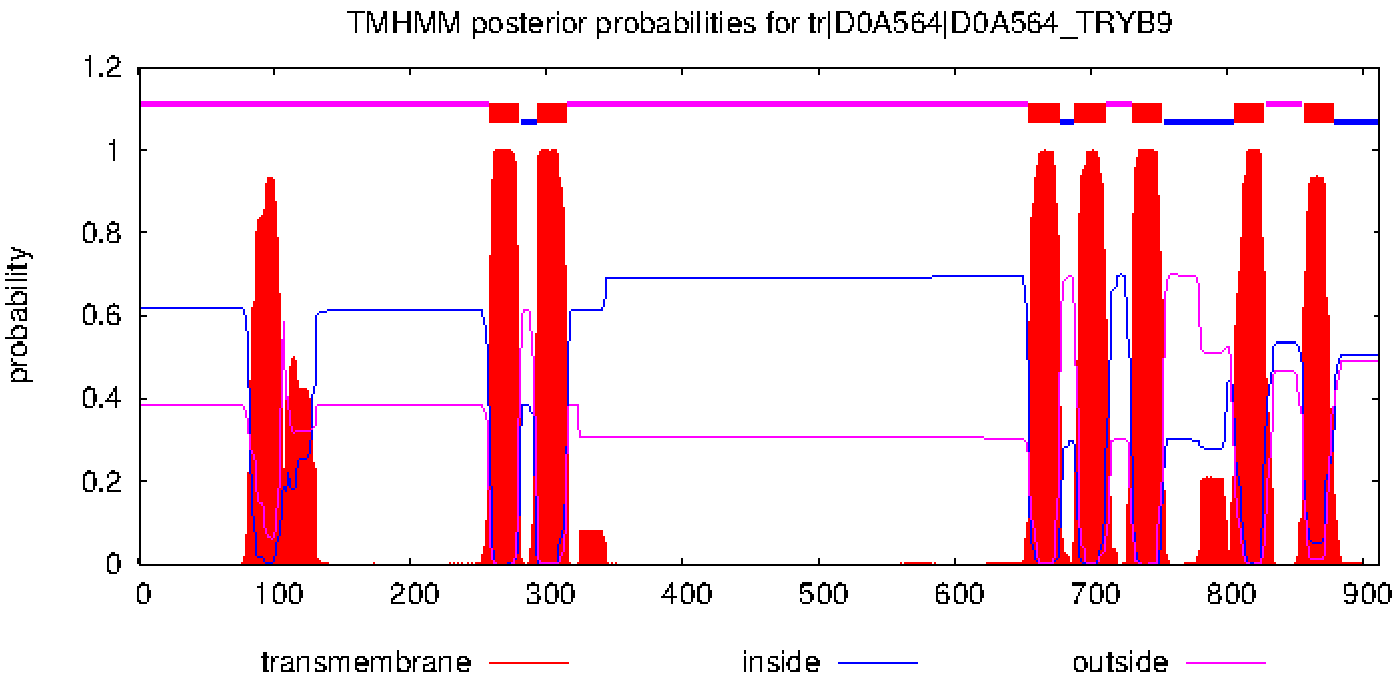

58 **# [plot] in postscript, [script] for making the plot in gnuplot, [data] for plot**

```
# tr|D0A4W6|D0A4W6_TRYB9 Length: 985
# tr|D0A4W6|D0A4W6_TRYB9 Number of predicted TMHs:  11
# tr|D0A4W6|D0A4W6_TRYB9 Exp number of AAs in TMHs: 262.53207
# tr|D0A4W6|D0A4W6_TRYB9 Exp number, first 60 AAs:  0
# tr|D0A4W6|D0A4W6_TRYB9 Total prob of N-in:        0.82960
tr|D0A4W6|D0A4W6_TRYB9  TMHMM2.0        outside      1   108
tr|D0A4W6|D0A4W6_TRYB9  TMHMM2.0        TMhelix    109   131
tr|D0A4W6|D0A4W6_TRYB9  TMHMM2.0        inside     132   199
tr|D0A4W6|D0A4W6_TRYB9  TMHMM2.0        TMhelix    200   222
tr|D0A4W6|D0A4W6_TRYB9  TMHMM2.0        outside    223   236
tr|D0A4W6|D0A4W6_TRYB9  TMHMM2.0        TMhelix    237   259
tr|D0A4W6|D0A4W6_TRYB9  TMHMM2.0        inside     260   305
tr|D0A4W6|D0A4W6_TRYB9  TMHMM2.0        TMhelix    306   328
tr|D0A4W6|D0A4W6_TRYB9  TMHMM2.0        outside    329   342
tr|D0A4W6|D0A4W6_TRYB9  TMHMM2.0        TMhelix    343   365
tr|D0A4W6|D0A4W6_TRYB9  TMHMM2.0        inside     366   377
tr|D0A4W6|D0A4W6_TRYB9  TMHMM2.0        TMhelix    378   395
tr|D0A4W6|D0A4W6_TRYB9  TMHMM2.0        outside    396   398
tr|D0A4W6|D0A4W6_TRYB9  TMHMM2.0        TMhelix    399   421
tr|D0A4W6|D0A4W6_TRYB9  TMHMM2.0        inside     422   441
tr|D0A4W6|D0A4W6_TRYB9  TMHMM2.0        TMhelix    442   464
tr|D0A4W6|D0A4W6_TRYB9  TMHMM2.0        outside    465   587
tr|D0A4W6|D0A4W6_TRYB9  TMHMM2.0        TMhelix    588   610
tr|D0A4W6|D0A4W6_TRYB9  TMHMM2.0        inside     611   616
tr|D0A4W6|D0A4W6_TRYB9  TMHMM2.0        TMhelix    617   639
tr|D0A4W6|D0A4W6_TRYB9  TMHMM2.0        outside    640   653
tr|D0A4W6|D0A4W6_TRYB9  TMHMM2.0        TMhelix    654   676
tr|D0A4W6|D0A4W6_TRYB9  TMHMM2.0        inside     677   985
```

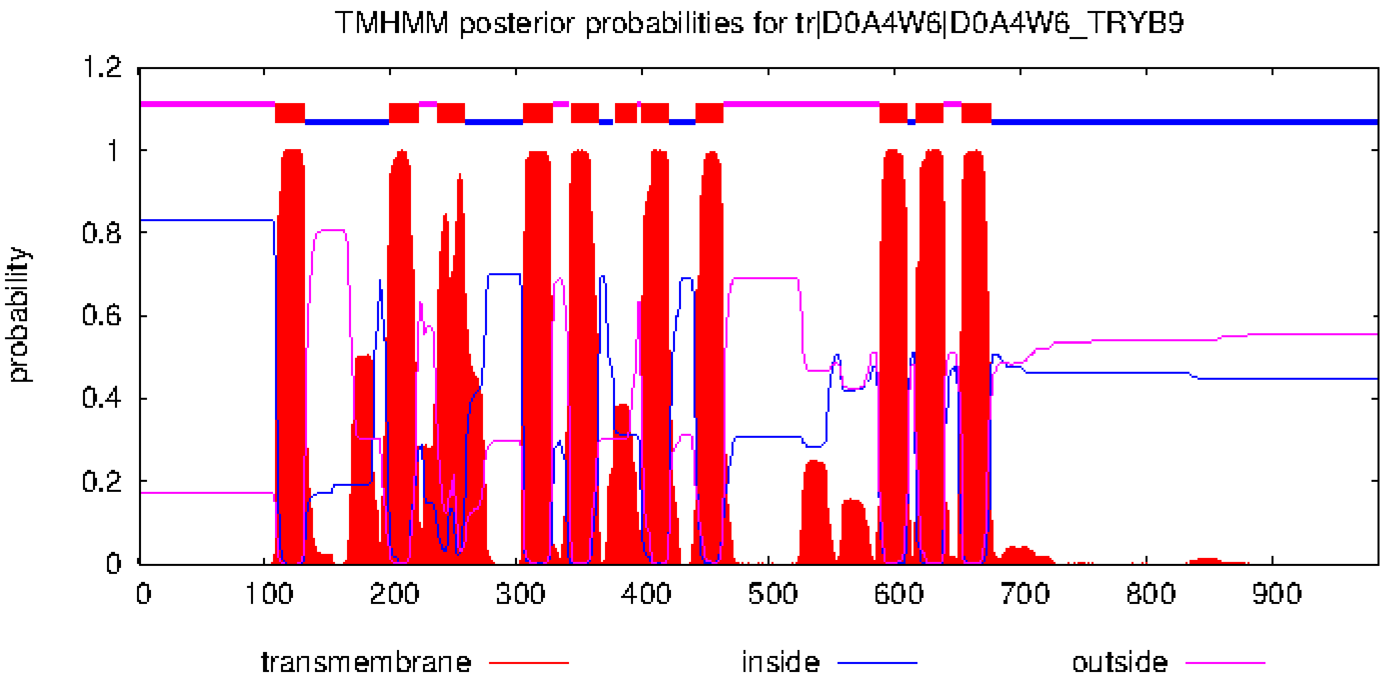

59 # plot in postscript, script for making the plot in gnuplot, data for plot

---

```
# tr|C9ZPL1|C9ZPL1_TRYB9 Length: 1011
# tr|C9ZPL1|C9ZPL1_TRYB9 Number of predicted TMHs:  10
# tr|C9ZPL1|C9ZPL1_TRYB9 Exp number of AAs in TMHs: 200.88955
# tr|C9ZPL1|C9ZPL1_TRYB9 Exp number, first 60 AAs:  0.13019
# tr|C9ZPL1|C9ZPL1_TRYB9 Total prob of N-in:        0.49564
tr|C9ZPL1|C9ZPL1_TRYB9  TMHMM2.0        inside       1    66
tr|C9ZPL1|C9ZPL1_TRYB9  TMHMM2.0        TMhelix     67    84
tr|C9ZPL1|C9ZPL1_TRYB9  TMHMM2.0        outside     85    87
tr|C9ZPL1|C9ZPL1_TRYB9  TMHMM2.0        TMhelix     88   110
tr|C9ZPL1|C9ZPL1_TRYB9  TMHMM2.0        inside     111   257
tr|C9ZPL1|C9ZPL1_TRYB9  TMHMM2.0        TMhelix    258   280
tr|C9ZPL1|C9ZPL1_TRYB9  TMHMM2.0        outside    281   305
tr|C9ZPL1|C9ZPL1_TRYB9  TMHMM2.0        TMhelix    306   328
tr|C9ZPL1|C9ZPL1_TRYB9  TMHMM2.0        inside     329   758
tr|C9ZPL1|C9ZPL1_TRYB9  TMHMM2.0        TMhelix    759   781
tr|C9ZPL1|C9ZPL1_TRYB9  TMHMM2.0        outside    782   790
tr|C9ZPL1|C9ZPL1_TRYB9  TMHMM2.0        TMhelix    791   810
tr|C9ZPL1|C9ZPL1_TRYB9  TMHMM2.0        inside     811   830
tr|C9ZPL1|C9ZPL1_TRYB9  TMHMM2.0        TMhelix    831   853
tr|C9ZPL1|C9ZPL1_TRYB9  TMHMM2.0        outside    854   886
tr|C9ZPL1|C9ZPL1_TRYB9  TMHMM2.0        TMhelix    887   909
tr|C9ZPL1|C9ZPL1_TRYB9  TMHMM2.0        inside     910   921
tr|C9ZPL1|C9ZPL1_TRYB9  TMHMM2.0        TMhelix    922   944
tr|C9ZPL1|C9ZPL1_TRYB9  TMHMM2.0        outside    945   979
tr|C9ZPL1|C9ZPL1_TRYB9  TMHMM2.0        TMhelix    980   999
tr|C9ZPL1|C9ZPL1_TRYB9  TMHMM2.0        inside    1000  1011
```

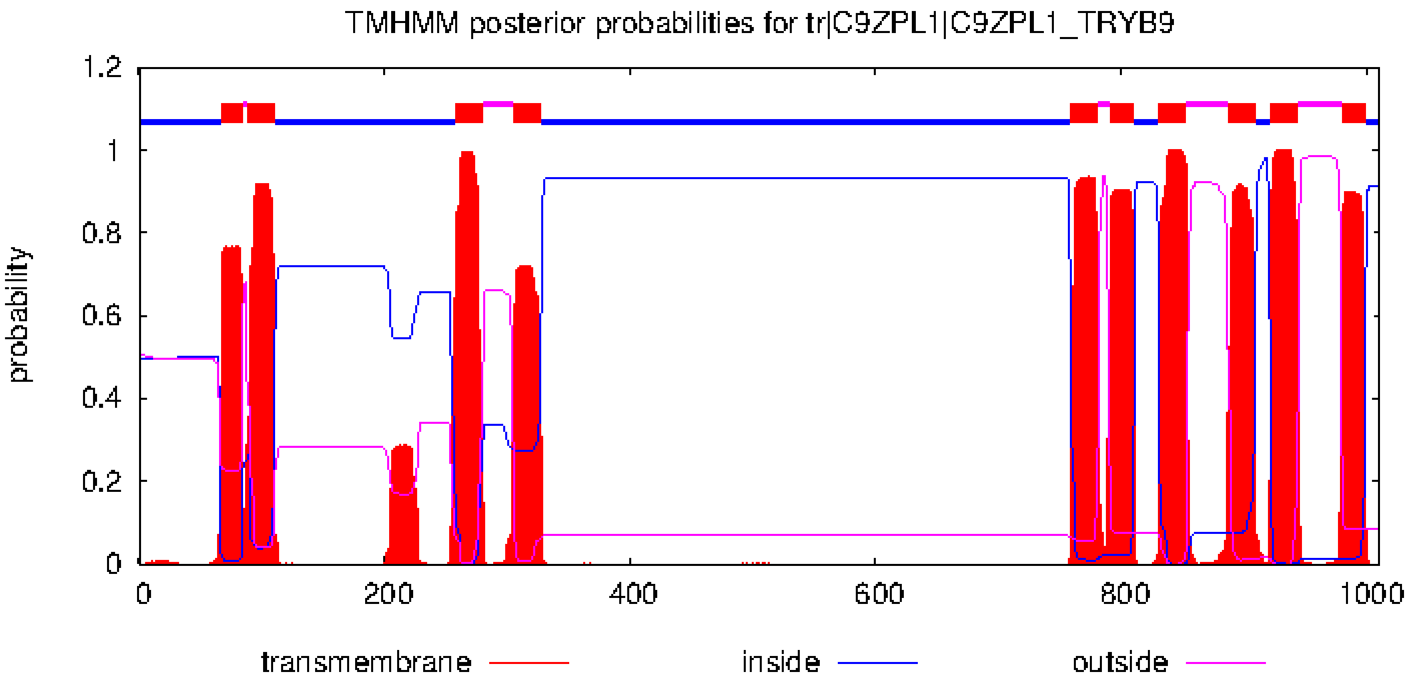

60 # plot in postscript, script for making the plot in gnuplot, data for plot

---

```
# tr|C9ZXD7|C9ZXD7_TRYB9 Length: 1023
# tr|C9ZXD7|C9ZXD7_TRYB9 Number of predicted TMHs:  2
# tr|C9ZXD7|C9ZXD7_TRYB9 Exp number of AAs in TMHs: 60.7062600000002
# tr|C9ZXD7|C9ZXD7_TRYB9 Exp number, first 60 AAs:  0.00539
# tr|C9ZXD7|C9ZXD7_TRYB9 Total prob of N-in:        0.73029
tr|C9ZXD7|C9ZXD7_TRYB9  TMHMM2.0        outside      1   155
tr|C9ZXD7|C9ZXD7_TRYB9  TMHMM2.0        TMhelix    156   178
tr|C9ZXD7|C9ZXD7_TRYB9  TMHMM2.0        inside     179   190
tr|C9ZXD7|C9ZXD7_TRYB9  TMHMM2.0        TMhelix    191   213
tr|C9ZXD7|C9ZXD7_TRYB9  TMHMM2.0        outside    214  1023
```

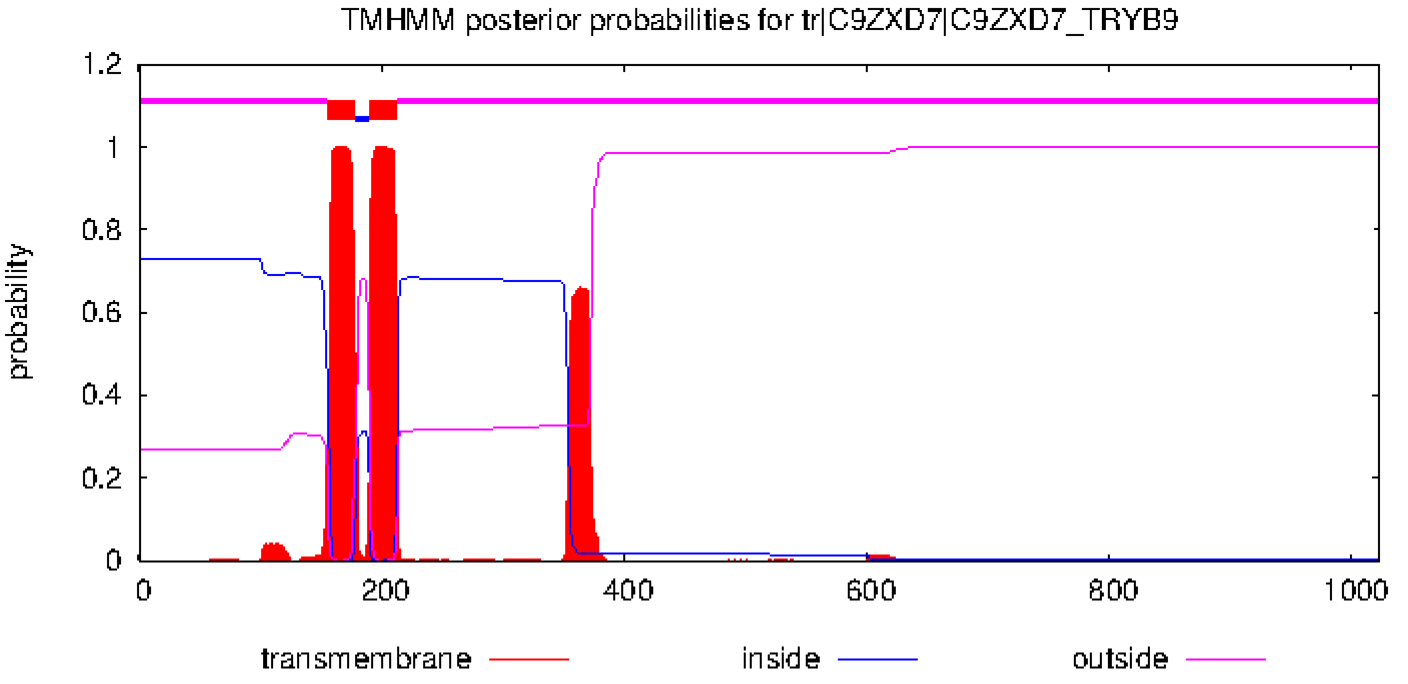

61 # plot in postscript, script for making the plot in gnuplot, data for plot

---

```
# tr|C9ZM06|C9ZM06_TRYB9 Length: 1080
# tr|C9ZM06|C9ZM06_TRYB9 Number of predicted TMHs:  10
# tr|C9ZM06|C9ZM06_TRYB9 Exp number of AAs in TMHs: 216.42076
```

```
# tr|C9ZM06|C9ZM06_TRYB9 Exp number, first 60 AAs:  0.00527
# tr|C9ZM06|C9ZM06_TRYB9 Total prob of N-in:        0.87916
tr|C9ZM06|C9ZM06_TRYB9  TMHMM2.0        inside       1    75
tr|C9ZM06|C9ZM06_TRYB9  TMHMM2.0        TMhelix     76    94
tr|C9ZM06|C9ZM06_TRYB9  TMHMM2.0        outside     95   108
tr|C9ZM06|C9ZM06_TRYB9  TMHMM2.0        TMhelix    109   131
tr|C9ZM06|C9ZM06_TRYB9  TMHMM2.0        inside     132   309
tr|C9ZM06|C9ZM06_TRYB9  TMHMM2.0        TMhelix    310   332
tr|C9ZM06|C9ZM06_TRYB9  TMHMM2.0        outside    333   341
tr|C9ZM06|C9ZM06_TRYB9  TMHMM2.0        TMhelix    342   361
tr|C9ZM06|C9ZM06_TRYB9  TMHMM2.0        inside     362   871
tr|C9ZM06|C9ZM06_TRYB9  TMHMM2.0        TMhelix    872   894
tr|C9ZM06|C9ZM06_TRYB9  TMHMM2.0        outside    895   898
tr|C9ZM06|C9ZM06_TRYB9  TMHMM2.0        TMhelix    899   921
tr|C9ZM06|C9ZM06_TRYB9  TMHMM2.0        inside     922   950
tr|C9ZM06|C9ZM06_TRYB9  TMHMM2.0        TMhelix    951   973
tr|C9ZM06|C9ZM06_TRYB9  TMHMM2.0        outside    974   982
tr|C9ZM06|C9ZM06_TRYB9  TMHMM2.0        TMhelix    983  1005
tr|C9ZM06|C9ZM06_TRYB9  TMHMM2.0        inside    1006  1011
tr|C9ZM06|C9ZM06_TRYB9  TMHMM2.0        TMhelix   1012  1034
tr|C9ZM06|C9ZM06_TRYB9  TMHMM2.0        outside   1035  1048
tr|C9ZM06|C9ZM06_TRYB9  TMHMM2.0        TMhelix   1049  1068
tr|C9ZM06|C9ZM06_TRYB9  TMHMM2.0        inside    1069  1080
```

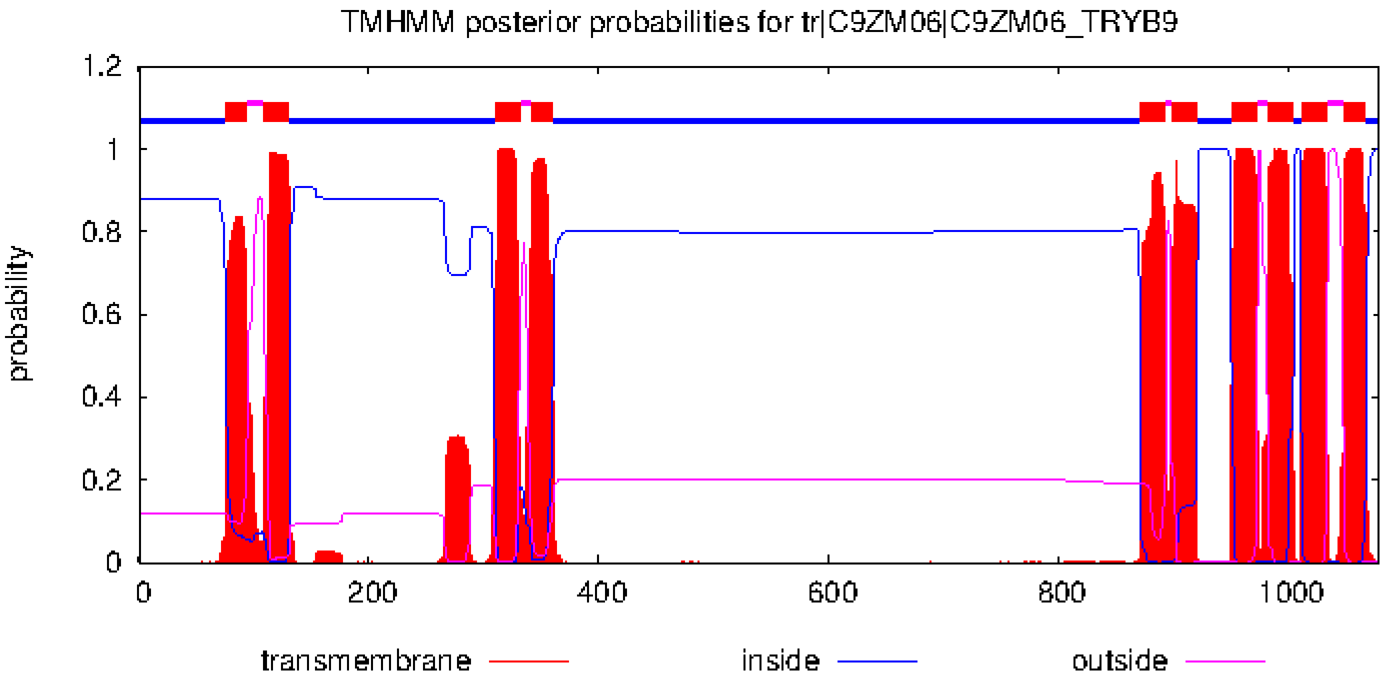

62 # **plot in postscript, script for making the plot in gnuplot, data for plot**

```
# tr|D0A6F9|D0A6F9_TRYB9 Length: 1128
# tr|D0A6F9|D0A6F9_TRYB9 Number of predicted TMHs:  10
# tr|D0A6F9|D0A6F9_TRYB9 Exp number of AAs in TMHs: 213.93793
# tr|D0A6F9|D0A6F9_TRYB9 Exp number, first 60 AAs:  6.46096
# tr|D0A6F9|D0A6F9_TRYB9 Total prob of N-in:        0.73846
tr|D0A6F9|D0A6F9_TRYB9  TMHMM2.0        inside       1    50
tr|D0A6F9|D0A6F9_TRYB9  TMHMM2.0        TMhelix     51    68
tr|D0A6F9|D0A6F9_TRYB9  TMHMM2.0        outside     69    82
tr|D0A6F9|D0A6F9_TRYB9  TMHMM2.0        TMhelix     83   105
tr|D0A6F9|D0A6F9_TRYB9  TMHMM2.0        inside     106   286
tr|D0A6F9|D0A6F9_TRYB9  TMHMM2.0        TMhelix    287   309
tr|D0A6F9|D0A6F9_TRYB9  TMHMM2.0        outside    310   332
tr|D0A6F9|D0A6F9_TRYB9  TMHMM2.0        TMhelix    333   355
tr|D0A6F9|D0A6F9_TRYB9  TMHMM2.0        inside     356   846
tr|D0A6F9|D0A6F9_TRYB9  TMHMM2.0        TMhelix    847   869
tr|D0A6F9|D0A6F9_TRYB9  TMHMM2.0        outside    870   878
tr|D0A6F9|D0A6F9_TRYB9  TMHMM2.0        TMhelix    879   898
```

tr|D0A6F9|D0A6F9_TRYB9 TMHMM2.0 inside 899 930

```
tr|D0A6F9|D0A6F9_TRYB9  TMHMM2.0        TMhelix    931   950
tr|D0A6F9|D0A6F9_TRYB9  TMHMM2.0        outside    951   964
tr|D0A6F9|D0A6F9_TRYB9  TMHMM2.0        TMhelix    965   984
tr|D0A6F9|D0A6F9_TRYB9  TMHMM2.0        inside     985   990
tr|D0A6F9|D0A6F9_TRYB9  TMHMM2.0        TMhelix    991  1013
tr|D0A6F9|D0A6F9_TRYB9  TMHMM2.0        outside   1014  1032
tr|D0A6F9|D0A6F9_TRYB9  TMHMM2.0        TMhelix   1033  1055
tr|D0A6F9|D0A6F9_TRYB9  TMHMM2.0        inside    1056  1128
```

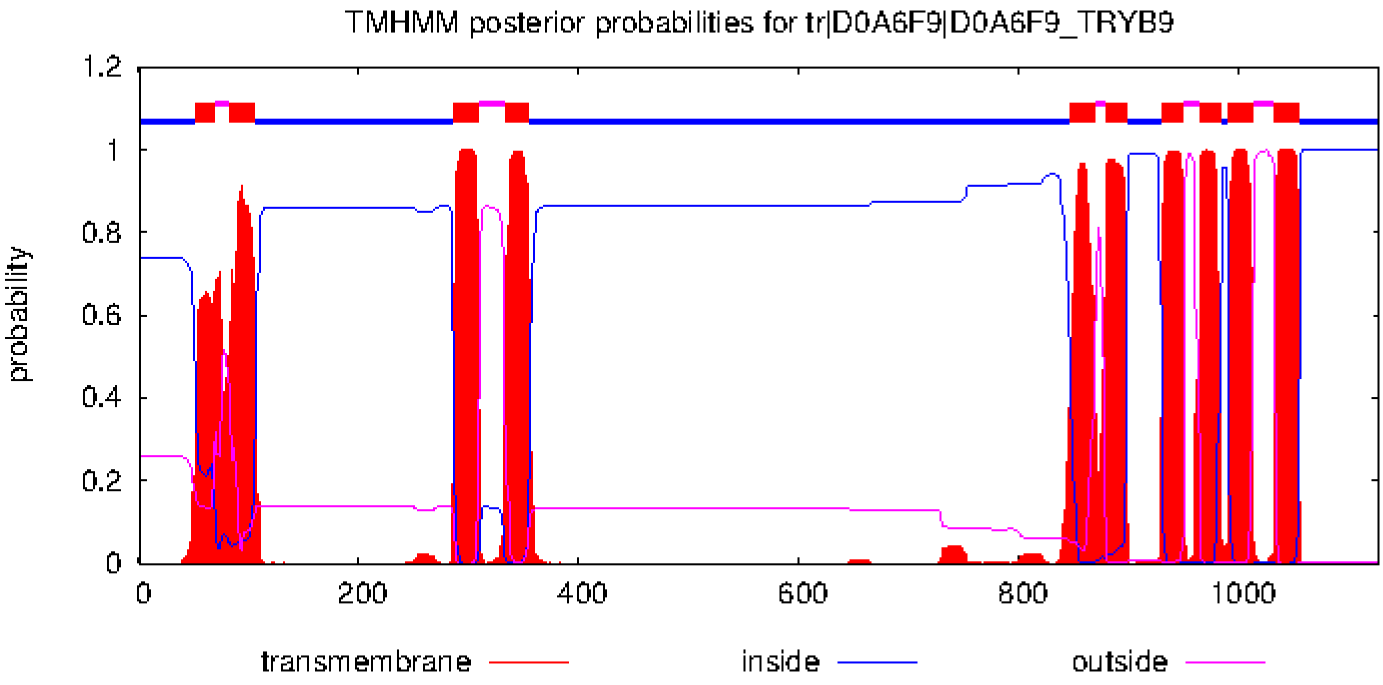

63 **# plot in postscript, script for making the plot in gnuplot, data for plot**

```
# tr|C9ZR23|C9ZR23_TRYB9 Length: 1196
# tr|C9ZR23|C9ZR23_TRYB9 Number of predicted TMHs:  7
# tr|C9ZR23|C9ZR23_TRYB9 Exp number of AAs in TMHs: 193.641900000001
# tr|C9ZR23|C9ZR23_TRYB9 Exp number, first 60 AAs:  10.46037
# tr|C9ZR23|C9ZR23_TRYB9 Total prob of N-in:        0.35662
# tr|C9ZR23|C9ZR23_TRYB9 POSSIBLE N-term signal sequence
tr|C9ZR23|C9ZR23_TRYB9  TMHMM2.0        inside       1    42
tr|C9ZR23|C9ZR23_TRYB9  TMHMM2.0        TMhelix     43    65
tr|C9ZR23|C9ZR23_TRYB9  TMHMM2.0        outside     66   299
tr|C9ZR23|C9ZR23_TRYB9  TMHMM2.0        TMhelix    300   322
tr|C9ZR23|C9ZR23_TRYB9  TMHMM2.0        inside     323   340
tr|C9ZR23|C9ZR23_TRYB9  TMHMM2.0        TMhelix    341   363
tr|C9ZR23|C9ZR23_TRYB9  TMHMM2.0        outside    364  1005
tr|C9ZR23|C9ZR23_TRYB9  TMHMM2.0        TMhelix   1006  1028
tr|C9ZR23|C9ZR23_TRYB9  TMHMM2.0        inside    1029  1040
tr|C9ZR23|C9ZR23_TRYB9  TMHMM2.0        TMhelix   1041  1060
tr|C9ZR23|C9ZR23_TRYB9  TMHMM2.0        outside   1061  1069
tr|C9ZR23|C9ZR23_TRYB9  TMHMM2.0        TMhelix   1070  1092
tr|C9ZR23|C9ZR23_TRYB9  TMHMM2.0        inside    1093  1104
tr|C9ZR23|C9ZR23_TRYB9  TMHMM2.0        TMhelix   1105  1127
tr|C9ZR23|C9ZR23_TRYB9  TMHMM2.0        outside   1128  1196
```

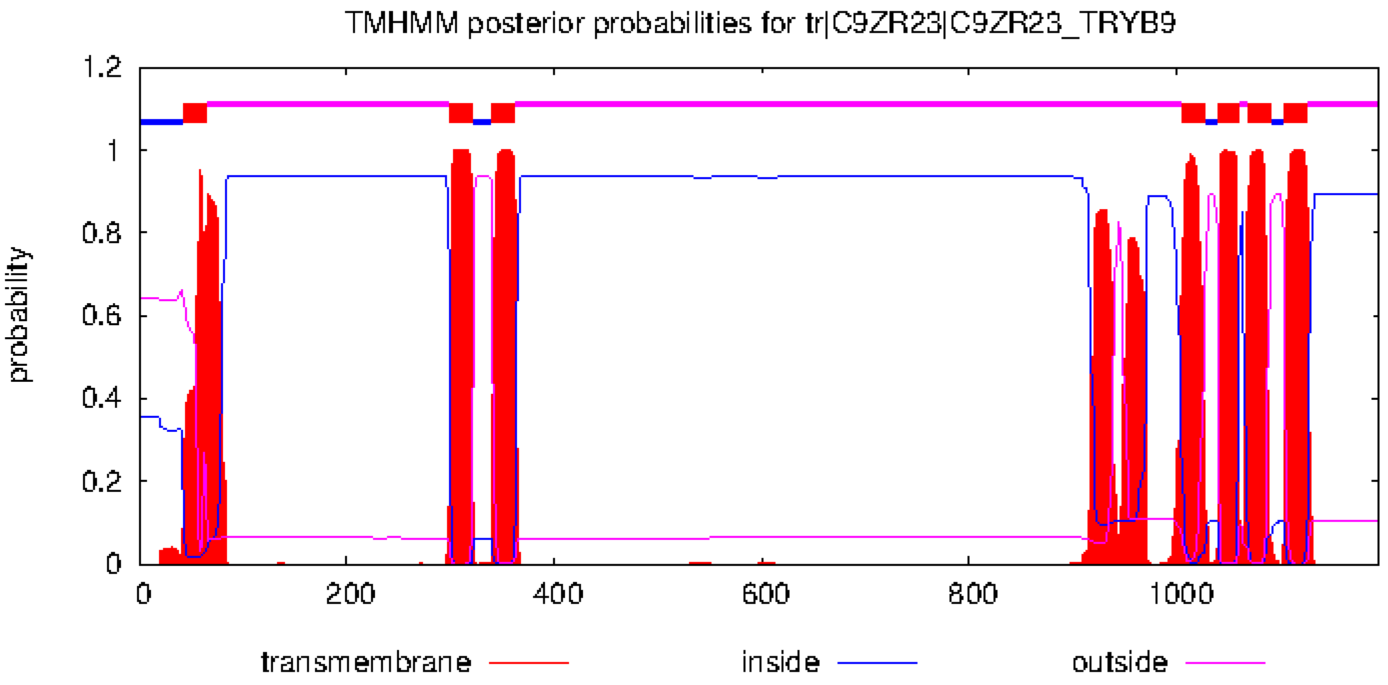

64 # [plot](#) in postscript, [script](#) for making the plot in gnuplot, [data](#) for plot

---

```
# tr|C9ZN43|C9ZN43_TRYB9 Length: 1204
# tr|C9ZN43|C9ZN43_TRYB9 Number of predicted TMHs:  1
# tr|C9ZN43|C9ZN43_TRYB9 Exp number of AAs in TMHs: 23.40836
# tr|C9ZN43|C9ZN43_TRYB9 Exp number, first 60 AAs:  0.00424
# tr|C9ZN43|C9ZN43_TRYB9 Total prob of N-in:        0.01614
tr|C9ZN43|C9ZN43_TRYB9  TMHMM2.0        outside      1   821
tr|C9ZN43|C9ZN43_TRYB9  TMHMM2.0        TMhelix    822   844
tr|C9ZN43|C9ZN43_TRYB9  TMHMM2.0        inside     845  1204
```

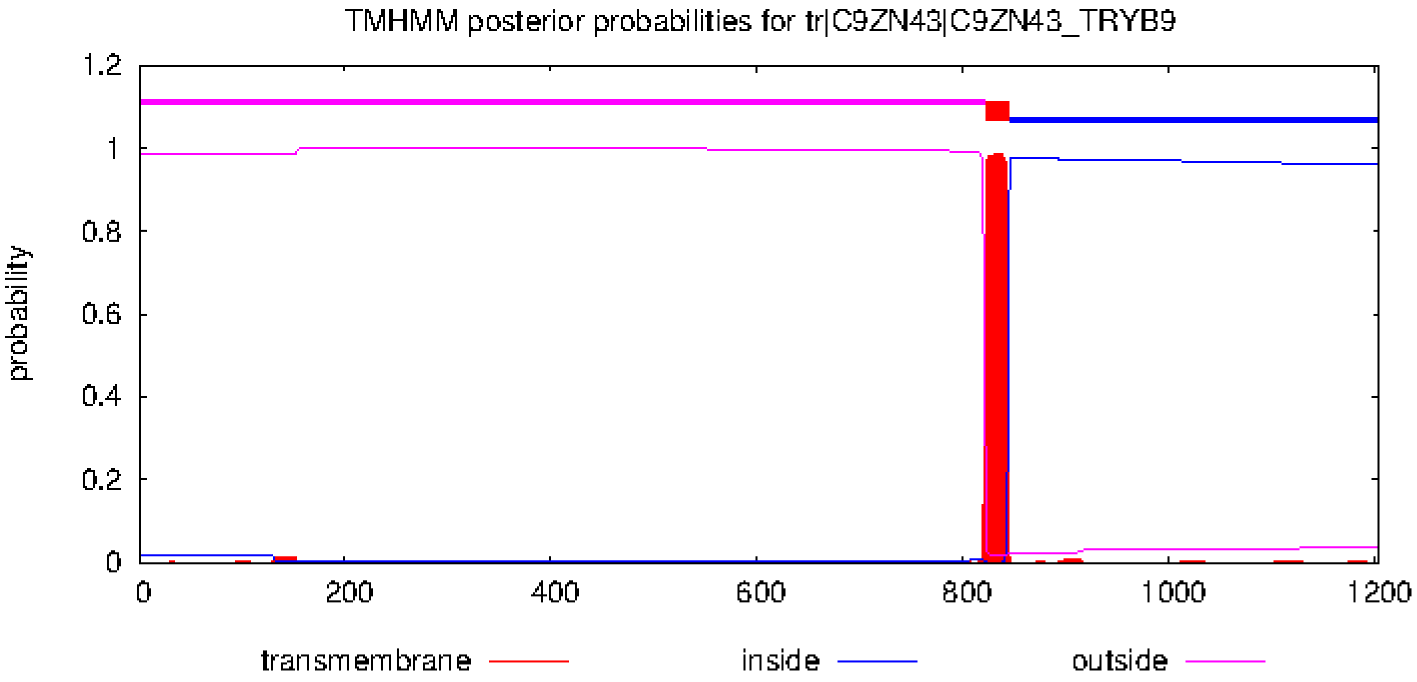

65 # [plot](#) in postscript, [script](#) for making the plot in gnuplot, [data](#) for plot

---

```
# tr|C9ZL71|C9ZL71_TRYB9 Length: 1232
# tr|C9ZL71|C9ZL71_TRYB9 Number of predicted TMHs:  7
```

```
# tr|C9ZL71|C9ZL71_TRYB9 Exp number of AAs in TMHs: 157.13319
# tr|C9ZL71|C9ZL71_TRYB9 Exp number, first 60 AAs:  0
# tr|C9ZL71|C9ZL71_TRYB9 Total prob of N-in:        0.93176
```

```
tr|C9ZL71|C9ZL71_TRYB9  TMHMM2.0        inside       1   124
tr|C9ZL71|C9ZL71_TRYB9  TMHMM2.0        TMhelix    125   147
tr|C9ZL71|C9ZL71_TRYB9  TMHMM2.0        outside    148   314
tr|C9ZL71|C9ZL71_TRYB9  TMHMM2.0        TMhelix    315   334
tr|C9ZL71|C9ZL71_TRYB9  TMHMM2.0        inside     335   345
tr|C9ZL71|C9ZL71_TRYB9  TMHMM2.0        TMhelix    346   368
tr|C9ZL71|C9ZL71_TRYB9  TMHMM2.0        outside    369   382
tr|C9ZL71|C9ZL71_TRYB9  TMHMM2.0        TMhelix    383   402
tr|C9ZL71|C9ZL71_TRYB9  TMHMM2.0        inside     403   438
tr|C9ZL71|C9ZL71_TRYB9  TMHMM2.0        TMhelix    439   461
tr|C9ZL71|C9ZL71_TRYB9  TMHMM2.0        outside    462   475
tr|C9ZL71|C9ZL71_TRYB9  TMHMM2.0        TMhelix    476   498
tr|C9ZL71|C9ZL71_TRYB9  TMHMM2.0        inside     499   517
tr|C9ZL71|C9ZL71_TRYB9  TMHMM2.0        TMhelix    518   540
tr|C9ZL71|C9ZL71_TRYB9  TMHMM2.0        outside    541  1232
```

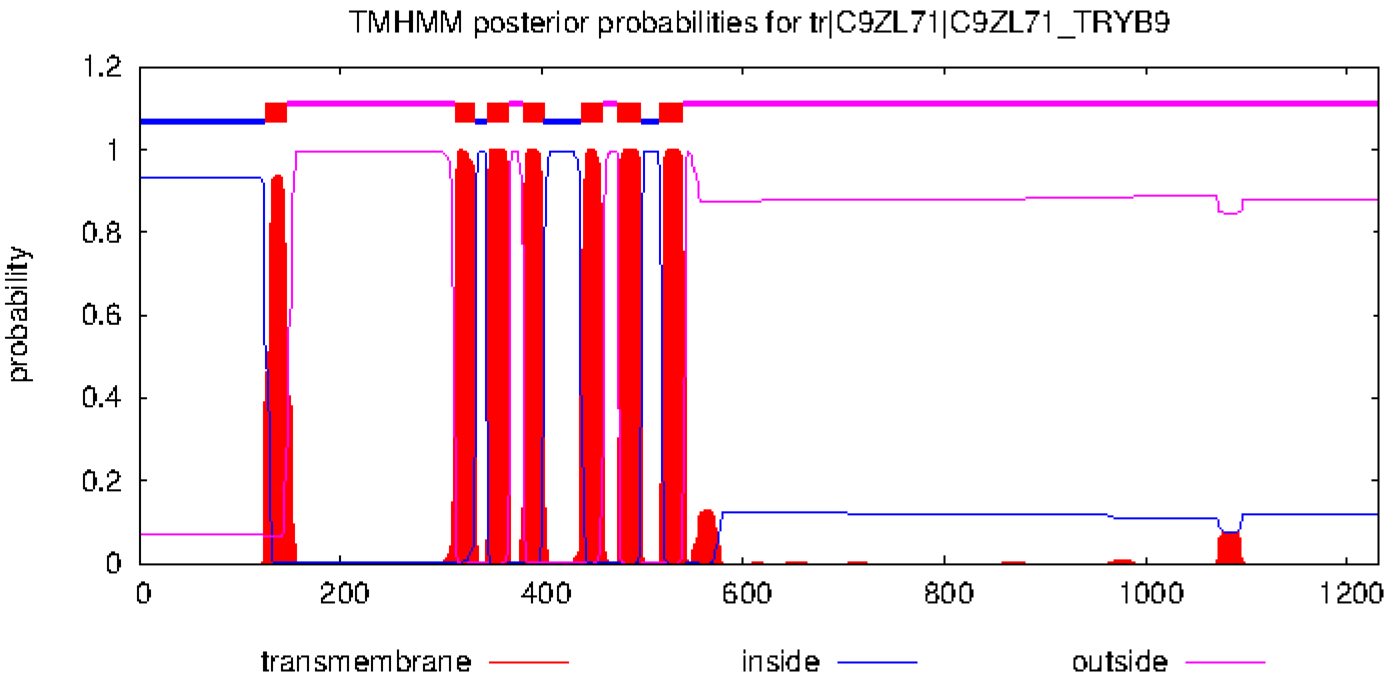

66 # plot in postscript, script for making the plot in gnuplot, data for plot

---

```
# tr|D0A0U3|D0A0U3_TRYB9 Length: 1236
# tr|D0A0U3|D0A0U3_TRYB9 Number of predicted TMHs:  2
# tr|D0A0U3|D0A0U3_TRYB9 Exp number of AAs in TMHs: 48.61435
# tr|D0A0U3|D0A0U3_TRYB9 Exp number, first 60 AAs:  18.42736
# tr|D0A0U3|D0A0U3_TRYB9 Total prob of N-in:        0.81029
# tr|D0A0U3|D0A0U3_TRYB9 POSSIBLE N-term signal sequence
tr|D0A0U3|D0A0U3_TRYB9  TMHMM2.0        inside       1    33
tr|D0A0U3|D0A0U3_TRYB9  TMHMM2.0        TMhelix     34    53
tr|D0A0U3|D0A0U3_TRYB9  TMHMM2.0        outside     54   850
tr|D0A0U3|D0A0U3_TRYB9  TMHMM2.0        TMhelix    851   873
tr|D0A0U3|D0A0U3_TRYB9  TMHMM2.0        inside     874  1236
```

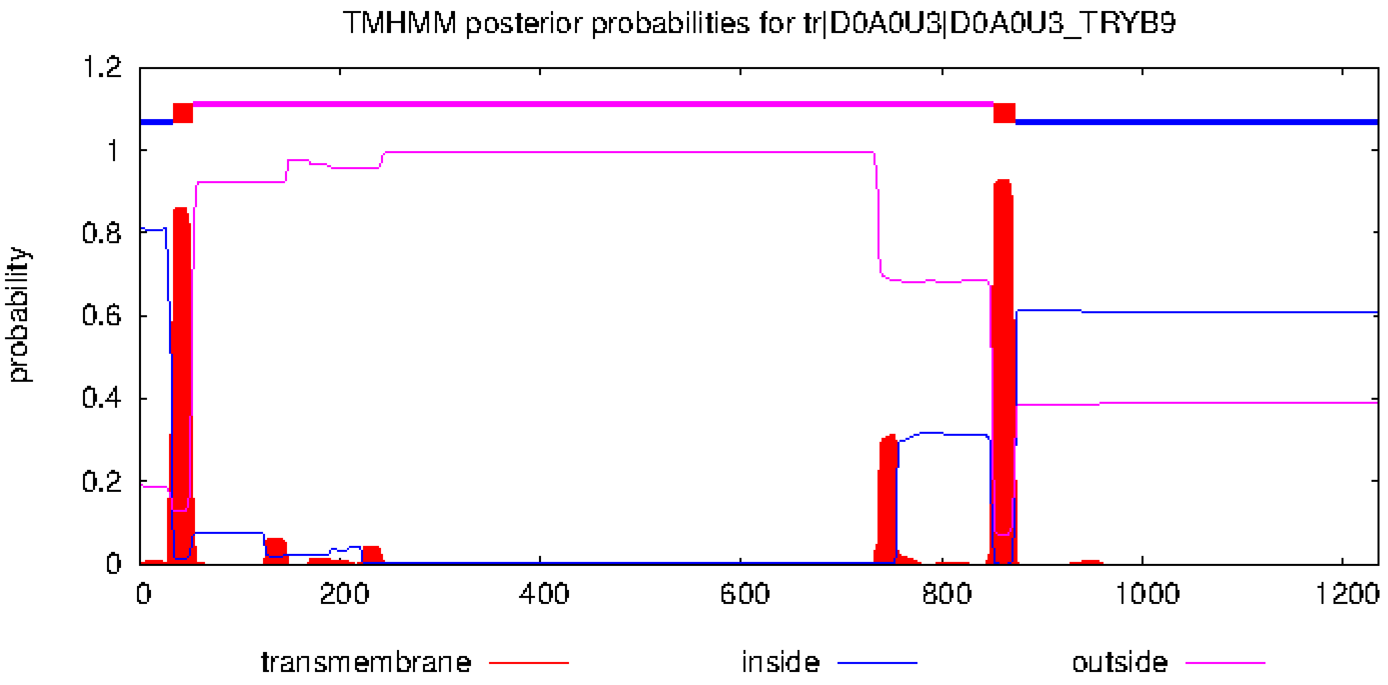

67 # plot in postscript, script for making the plot in gnuplot, data for plot

---

```
# tr|C9ZWY7|C9ZWY7_TRYB9 Length: 1240
# tr|C9ZWY7|C9ZWY7_TRYB9 Number of predicted TMHs:  1
# tr|C9ZWY7|C9ZWY7_TRYB9 Exp number of AAs in TMHs: 66.9811500000002
# tr|C9ZWY7|C9ZWY7_TRYB9 Exp number, first 60 AAs:  26.56532
# tr|C9ZWY7|C9ZWY7_TRYB9 Total prob of N-in:        0.33962
# tr|C9ZWY7|C9ZWY7_TRYB9 POSSIBLE N-term signal sequence
tr|C9ZWY7|C9ZWY7_TRYB9  TMHMM2.0        outside      1   858
tr|C9ZWY7|C9ZWY7_TRYB9  TMHMM2.0        TMhelix    859   881
tr|C9ZWY7|C9ZWY7_TRYB9  TMHMM2.0        inside     882  1240
```

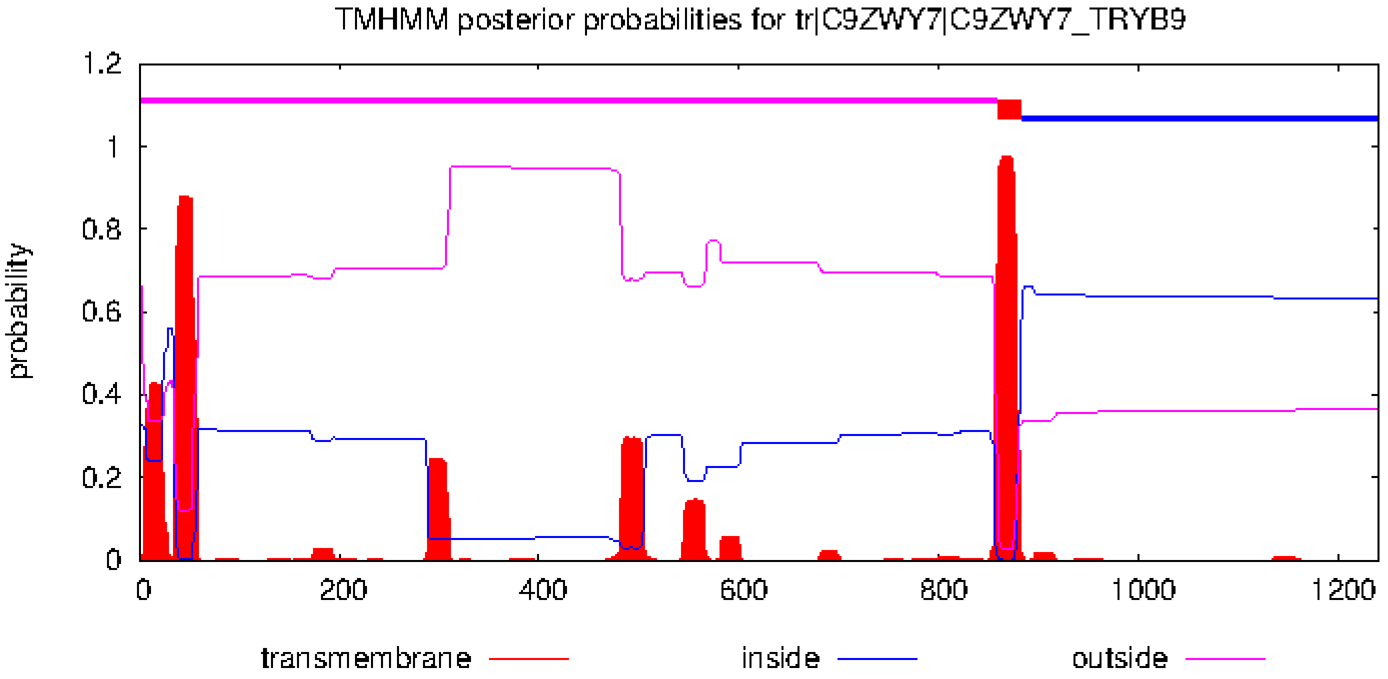

68 # plot in postscript, script for making the plot in gnuplot, data for plot

---

```
# tr|C9ZZQ4|C9ZZQ4_TRYB9 Length: 1242
```

```
# tr|C9ZZQ4|C9ZZQ4_TRYB9 Number of predicted TMHs:  2
# tr|C9ZZQ4|C9ZZQ4_TRYB9 Exp number of AAs in TMHs: 52.35167
# tr|C9ZZQ4|C9ZZQ4_TRYB9 Exp number, first 60 AAs:  9.99187
```

```
# tr|C9ZZQ4|C9ZZQ4_TRYB9 Total prob of N-in:        0.46455
tr|C9ZZQ4|C9ZZQ4_TRYB9  TMHMM2.0        outside      1   727
tr|C9ZZQ4|C9ZZQ4_TRYB9  TMHMM2.0        TMhelix    728   747
tr|C9ZZQ4|C9ZZQ4_TRYB9  TMHMM2.0        inside     748   839
tr|C9ZZQ4|C9ZZQ4_TRYB9  TMHMM2.0        TMhelix    840   862
tr|C9ZZQ4|C9ZZQ4_TRYB9  TMHMM2.0        outside    863  1242
```

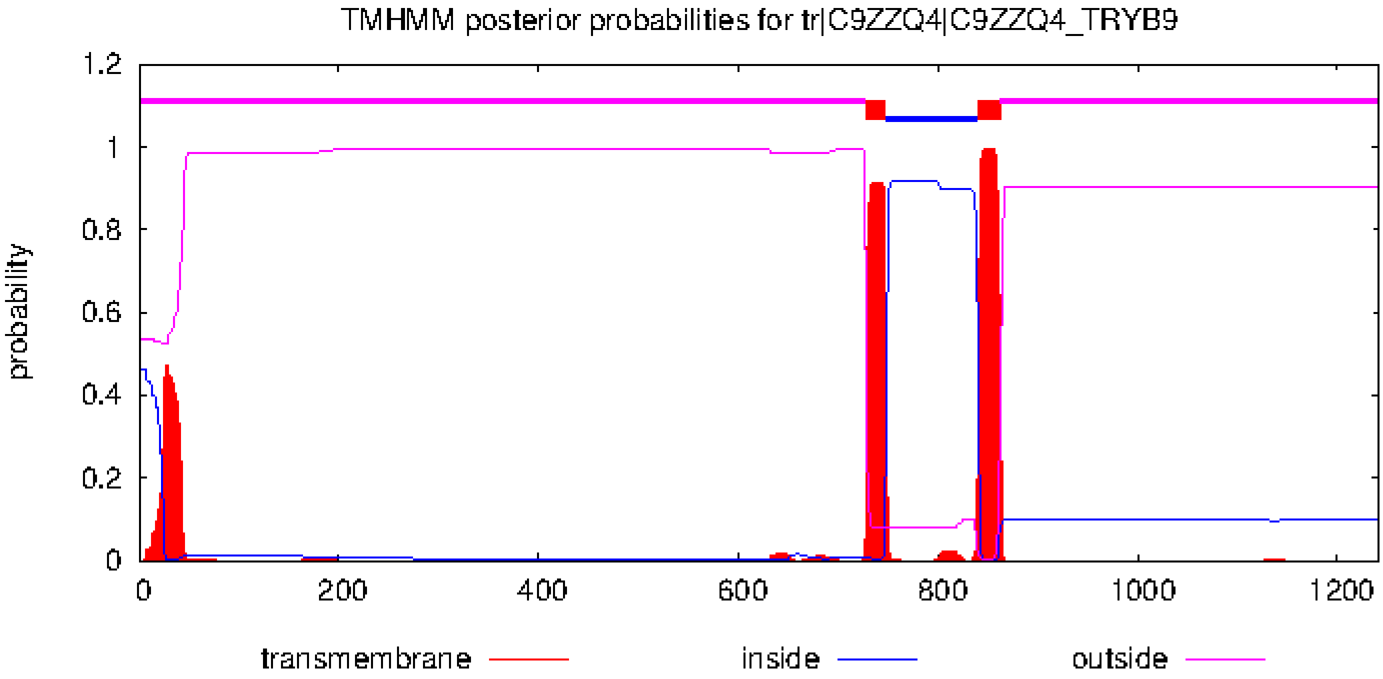

69 # plot in postscript, script for making the plot in gnuplot, data for plot

---

```
# tr|D0A0W7|D0A0W7_TRYB9 Length: 1254
# tr|D0A0W7|D0A0W7_TRYB9 Number of predicted TMHs:  1
# tr|D0A0W7|D0A0W7_TRYB9 Exp number of AAs in TMHs: 42.7728600000002
# tr|D0A0W7|D0A0W7_TRYB9 Exp number, first 60 AAs:  11.71174
# tr|D0A0W7|D0A0W7_TRYB9 Total prob of N-in:        0.36006
# tr|D0A0W7|D0A0W7_TRYB9 POSSIBLE N-term signal sequence
tr|D0A0W7|D0A0W7_TRYB9  TMHMM2.0        outside      1   860
tr|D0A0W7|D0A0W7_TRYB9  TMHMM2.0        TMhelix    861   883
tr|D0A0W7|D0A0W7_TRYB9  TMHMM2.0        inside     884  1254
```

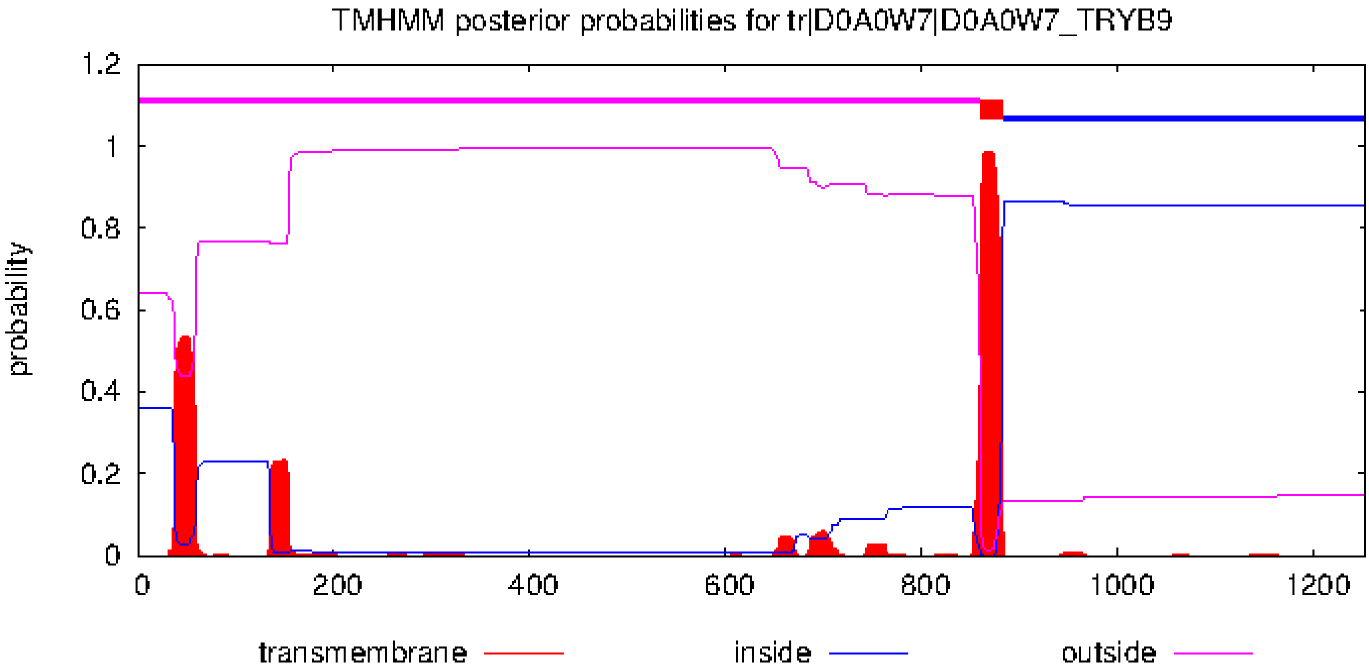

70 # plot in postscript, script for making the plot in gnuplot, data for plot

---

```
# tr|C9ZUI4|C9ZUI4_TRYB9 Length: 1261
# tr|C9ZUI4|C9ZUI4_TRYB9 Number of predicted TMHs:  8
# tr|C9ZUI4|C9ZUI4_TRYB9 Exp number of AAs in TMHs: 179.16826
# tr|C9ZUI4|C9ZUI4_TRYB9 Exp number, first 60 AAs: 22.70603
# tr|C9ZUI4|C9ZUI4_TRYB9 Total prob of N-in:        0.99273
# tr|C9ZUI4|C9ZUI4_TRYB9 POSSIBLE N-term signal sequence
tr|C9ZUI4|C9ZUI4_TRYB9  TMHMM2.0        inside       1    20
tr|C9ZUI4|C9ZUI4_TRYB9  TMHMM2.0        TMhelix     21    40
tr|C9ZUI4|C9ZUI4_TRYB9  TMHMM2.0        outside     41    59
tr|C9ZUI4|C9ZUI4_TRYB9  TMHMM2.0        TMhelix     60    82
tr|C9ZUI4|C9ZUI4_TRYB9  TMHMM2.0        inside      83   196
tr|C9ZUI4|C9ZUI4_TRYB9  TMHMM2.0        TMhelix    197   219
tr|C9ZUI4|C9ZUI4_TRYB9  TMHMM2.0        outside    220   222
tr|C9ZUI4|C9ZUI4_TRYB9  TMHMM2.0        TMhelix    223   245
tr|C9ZUI4|C9ZUI4_TRYB9  TMHMM2.0        inside     246   395
tr|C9ZUI4|C9ZUI4_TRYB9  TMHMM2.0        TMhelix    396   415
tr|C9ZUI4|C9ZUI4_TRYB9  TMHMM2.0        outside    416  1157
tr|C9ZUI4|C9ZUI4_TRYB9  TMHMM2.0        TMhelix   1158  1180
tr|C9ZUI4|C9ZUI4_TRYB9  TMHMM2.0        inside    1181  1192
tr|C9ZUI4|C9ZUI4_TRYB9  TMHMM2.0        TMhelix   1193  1210
tr|C9ZUI4|C9ZUI4_TRYB9  TMHMM2.0        outside   1211  1234
tr|C9ZUI4|C9ZUI4_TRYB9  TMHMM2.0        TMhelix   1235  1257
tr|C9ZUI4|C9ZUI4_TRYB9  TMHMM2.0        inside    1258  1261
```

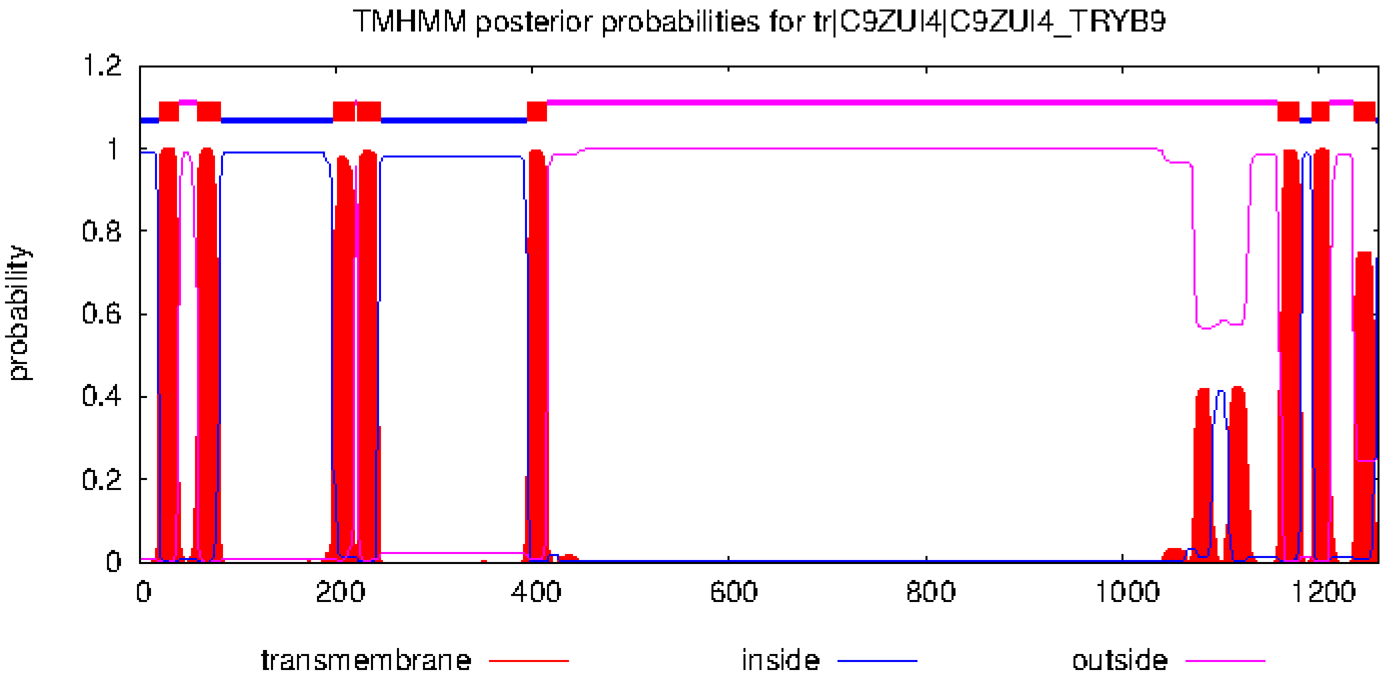

71 # plot in postscript, script for making the plot in gnuplot, data for plot

---

```
# tr|C9ZNH3|C9ZNH3_TRYB9 Length: 1281
# tr|C9ZNH3|C9ZNH3_TRYB9 Number of predicted TMHs:  2
# tr|C9ZNH3|C9ZNH3_TRYB9 Exp number of AAs in TMHs: 48.2827700000001
# tr|C9ZNH3|C9ZNH3_TRYB9 Exp number, first 60 AAs:  21.97196
# tr|C9ZNH3|C9ZNH3_TRYB9 Total prob of N-in:        0.96707
# tr|C9ZNH3|C9ZNH3_TRYB9 POSSIBLE N-term signal sequence
tr|C9ZNH3|C9ZNH3_TRYB9  TMHMM2.0        inside       1    35
tr|C9ZNH3|C9ZNH3_TRYB9  TMHMM2.0        TMhelix     36    58
tr|C9ZNH3|C9ZNH3_TRYB9  TMHMM2.0        outside     59   847
tr|C9ZNH3|C9ZNH3_TRYB9  TMHMM2.0        TMhelix    848   870
tr|C9ZNH3|C9ZNH3_TRYB9  TMHMM2.0        inside     871  1281
```

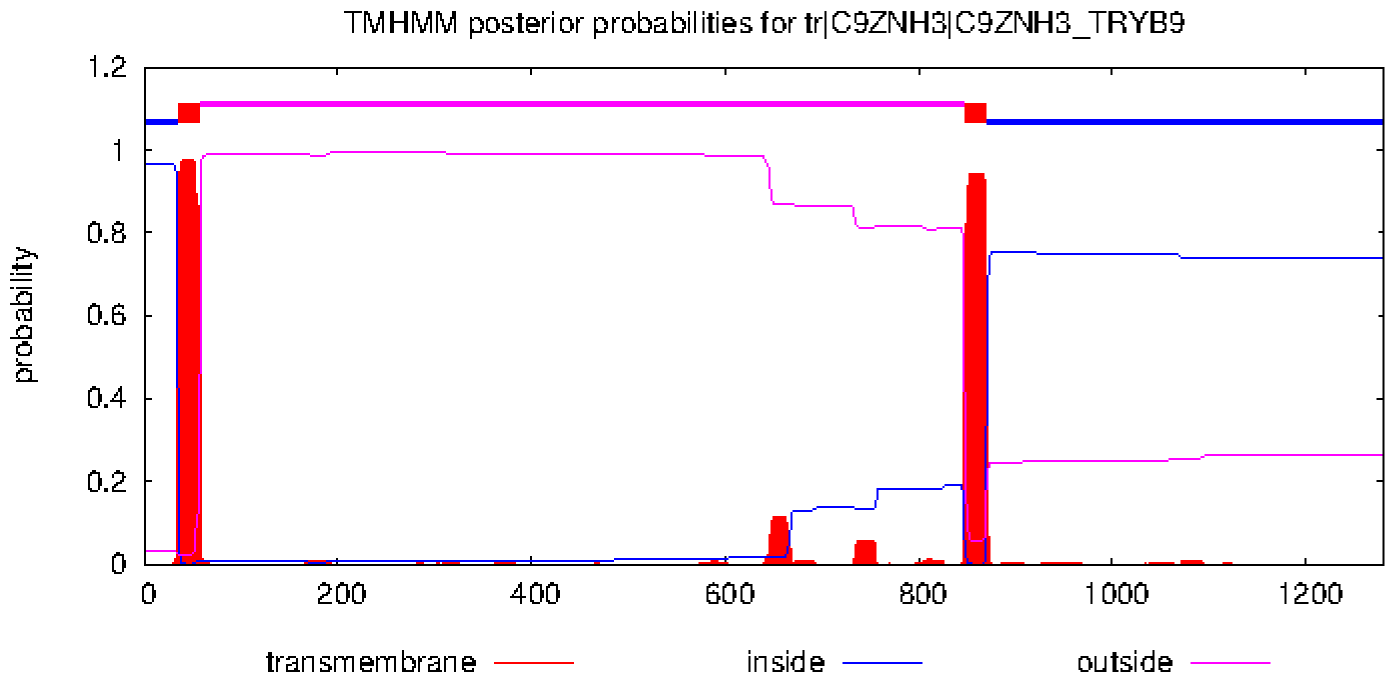

72 # plot in postscript, script for making the plot in gnuplot, data for plot

---

```
# tr|C9ZZ34|C9ZZ34_TRYB9 Length: 1345
# tr|C9ZZ34|C9ZZ34_TRYB9 Number of predicted TMHs:  2
# tr|C9ZZ34|C9ZZ34_TRYB9 Exp number of AAs in TMHs: 45.5945500000001
# tr|C9ZZ34|C9ZZ34_TRYB9 Exp number, first 60 AAs:  0.009
# tr|C9ZZ34|C9ZZ34_TRYB9 Total prob of N-in:        0.01000
tr|C9ZZ34|C9ZZ34_TRYB9  TMHMM2.0        outside      1   262
tr|C9ZZ34|C9ZZ34_TRYB9  TMHMM2.0        TMhelix    263   285
tr|C9ZZ34|C9ZZ34_TRYB9  TMHMM2.0        inside     286   322
tr|C9ZZ34|C9ZZ34_TRYB9  TMHMM2.0        TMhelix    323   345
tr|C9ZZ34|C9ZZ34_TRYB9  TMHMM2.0        outside    346  1345
```

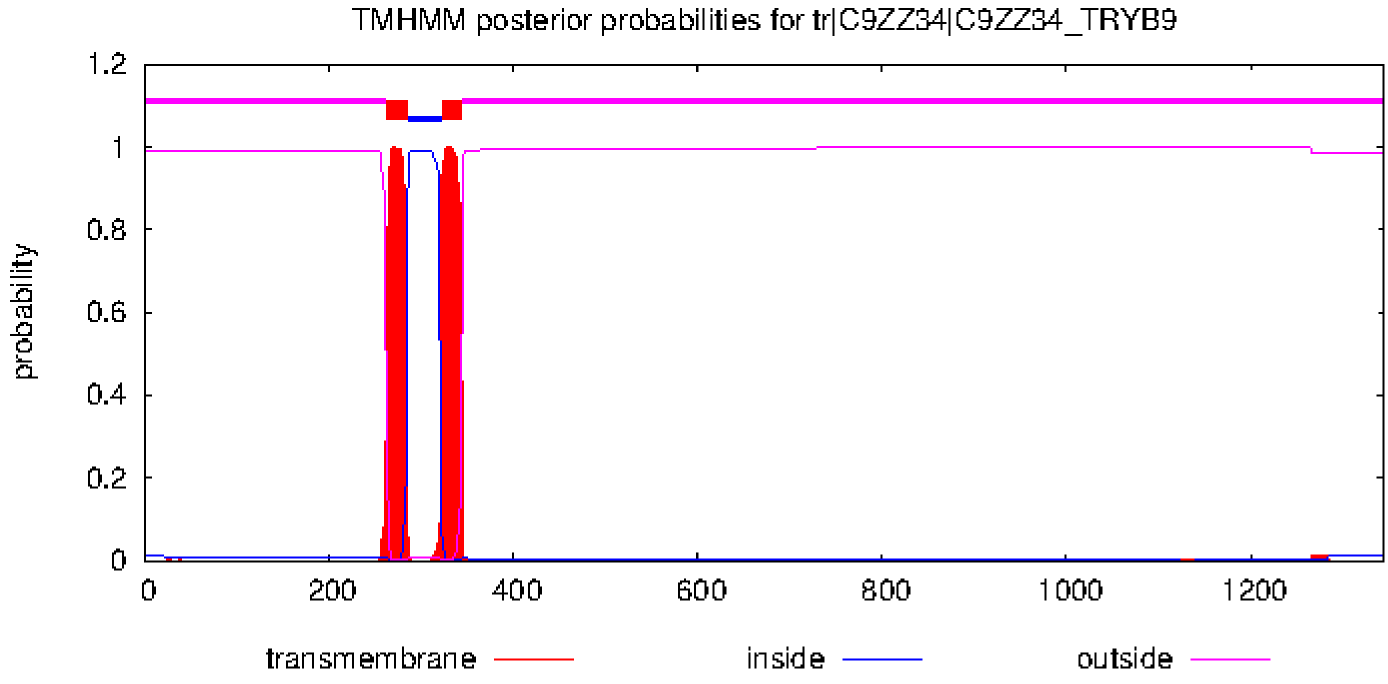

73 # plot in postscript, script for making the plot in gnuplot, data for plot

---

tr|C9ZMP2|C9ZMP2_TRYB9 Length: 1503
tr|C9ZMP2|C9ZMP2_TRYB9 Number of predicted TMHs: 12
tr|C9ZMP2|C9ZMP2_TRYB9 Exp number of AAs in TMHs: 310.599789999999

```
# tr|C9ZMP2|C9ZMP2_TRYB9 Exp number, first 60 AAs:  35.17641
# tr|C9ZMP2|C9ZMP2_TRYB9 Total prob of N-in:        0.11331
# tr|C9ZMP2|C9ZMP2_TRYB9 POSSIBLE N-term signal sequence
tr|C9ZMP2|C9ZMP2_TRYB9  TMHMM2.0        outside      1    14
tr|C9ZMP2|C9ZMP2_TRYB9  TMHMM2.0        TMhelix     15    37
tr|C9ZMP2|C9ZMP2_TRYB9  TMHMM2.0        inside      38    48
tr|C9ZMP2|C9ZMP2_TRYB9  TMHMM2.0        TMhelix     49    68
tr|C9ZMP2|C9ZMP2_TRYB9  TMHMM2.0        outside     69    82
tr|C9ZMP2|C9ZMP2_TRYB9  TMHMM2.0        TMhelix     83   101
tr|C9ZMP2|C9ZMP2_TRYB9  TMHMM2.0        inside     102   109
tr|C9ZMP2|C9ZMP2_TRYB9  TMHMM2.0        TMhelix    110   132
tr|C9ZMP2|C9ZMP2_TRYB9  TMHMM2.0        outside    133   137
tr|C9ZMP2|C9ZMP2_TRYB9  TMHMM2.0        TMhelix    138   160
tr|C9ZMP2|C9ZMP2_TRYB9  TMHMM2.0        inside     161   288
tr|C9ZMP2|C9ZMP2_TRYB9  TMHMM2.0        TMhelix    289   311
tr|C9ZMP2|C9ZMP2_TRYB9  TMHMM2.0        outside    312   383
tr|C9ZMP2|C9ZMP2_TRYB9  TMHMM2.0        TMhelix    384   406
tr|C9ZMP2|C9ZMP2_TRYB9  TMHMM2.0        inside     407   476
tr|C9ZMP2|C9ZMP2_TRYB9  TMHMM2.0        TMhelix    477   499
tr|C9ZMP2|C9ZMP2_TRYB9  TMHMM2.0        outside    500   987
tr|C9ZMP2|C9ZMP2_TRYB9  TMHMM2.0        TMhelix    988  1010
tr|C9ZMP2|C9ZMP2_TRYB9  TMHMM2.0        inside    1011  1071
tr|C9ZMP2|C9ZMP2_TRYB9  TMHMM2.0        TMhelix   1072  1094
tr|C9ZMP2|C9ZMP2_TRYB9  TMHMM2.0        outside   1095  1169
tr|C9ZMP2|C9ZMP2_TRYB9  TMHMM2.0        TMhelix   1170  1192
tr|C9ZMP2|C9ZMP2_TRYB9  TMHMM2.0        inside    1193  1204
tr|C9ZMP2|C9ZMP2_TRYB9  TMHMM2.0        TMhelix   1205  1227
tr|C9ZMP2|C9ZMP2_TRYB9  TMHMM2.0        outside   1228  1503
```

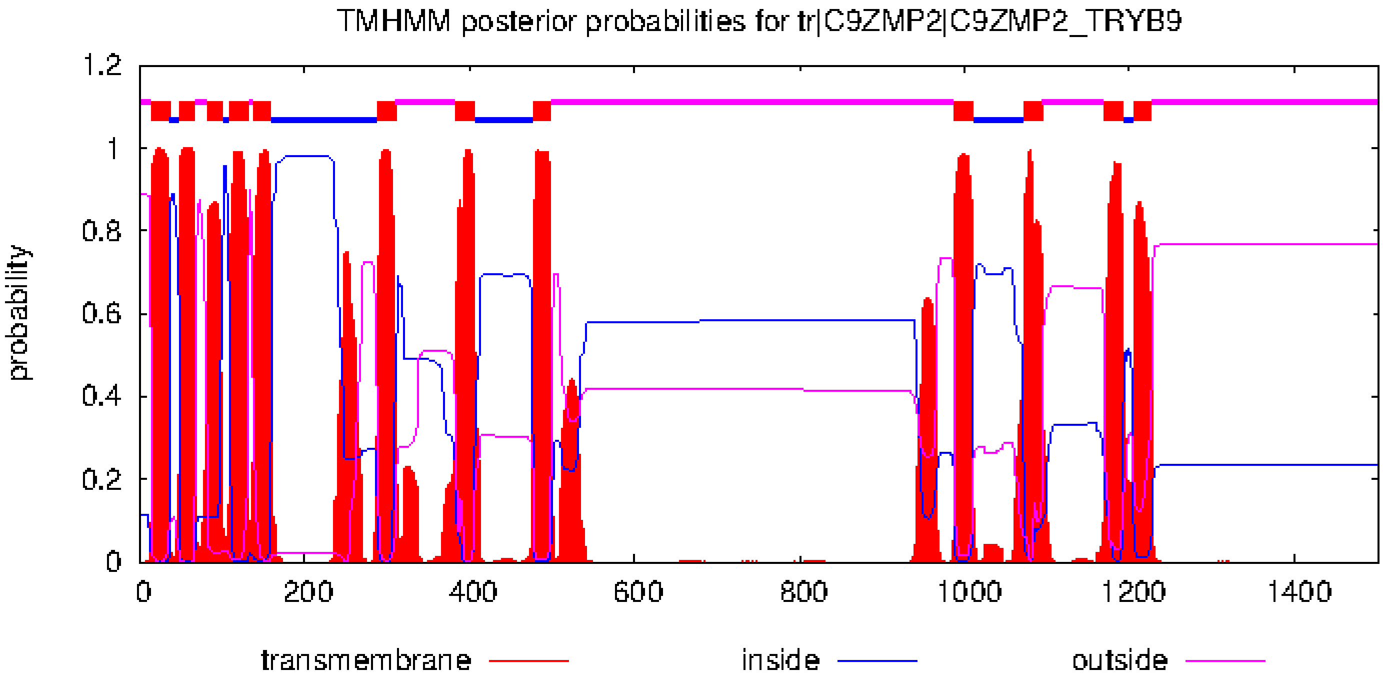

74 # **plot in postscript, script for making the plot in gnuplot, data for plot**

```
# tr|D0A9H6|D0A9H6_TRYB9 Length: 1569
# tr|D0A9H6|D0A9H6_TRYB9 Number of predicted TMHs:  8
# tr|D0A9H6|D0A9H6_TRYB9 Exp number of AAs in TMHs: 177.24968
# tr|D0A9H6|D0A9H6_TRYB9 Exp number, first 60 AAs:  0.47185
# tr|D0A9H6|D0A9H6_TRYB9 Total prob of N-in:        0.94756
tr|D0A9H6|D0A9H6_TRYB9  TMHMM2.0        inside       1    60
tr|D0A9H6|D0A9H6_TRYB9  TMHMM2.0        TMhelix     61    83
tr|D0A9H6|D0A9H6_TRYB9  TMHMM2.0        outside     84   488
tr|D0A9H6|D0A9H6_TRYB9  TMHMM2.0        TMhelix    489   511
tr|D0A9H6|D0A9H6_TRYB9  TMHMM2.0        inside     512   543
tr|D0A9H6|D0A9H6_TRYB9  TMHMM2.0        TMhelix    544   566
tr|D0A9H6|D0A9H6_TRYB9  TMHMM2.0        outside    567  1329
```

tr|D0A9H6|D0A9H6_TRYB9 TMHMM2.0 TMhelix 1330 1352

```
tr|D0A9H6|D0A9H6_TRYB9  TMHMM2.0        inside    1353  1397
tr|D0A9H6|D0A9H6_TRYB9  TMHMM2.0        TMhelix   1398  1420
tr|D0A9H6|D0A9H6_TRYB9  TMHMM2.0        outside   1421  1434
tr|D0A9H6|D0A9H6_TRYB9  TMHMM2.0        TMhelix   1435  1452
tr|D0A9H6|D0A9H6_TRYB9  TMHMM2.0        inside    1453  1458
tr|D0A9H6|D0A9H6_TRYB9  TMHMM2.0        TMhelix   1459  1481
tr|D0A9H6|D0A9H6_TRYB9  TMHMM2.0        outside   1482  1507
tr|D0A9H6|D0A9H6_TRYB9  TMHMM2.0        TMhelix   1508  1530
tr|D0A9H6|D0A9H6_TRYB9  TMHMM2.0        inside    1531  1569
```

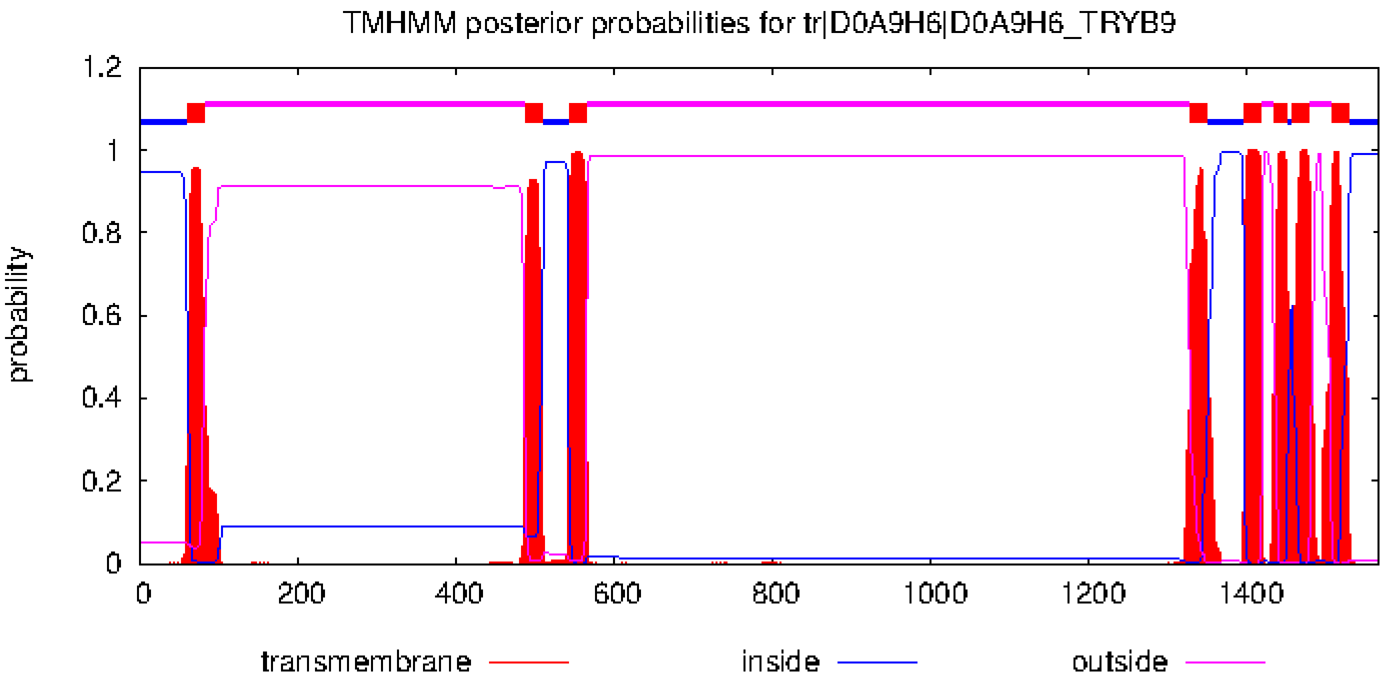

75 **# plot in postscript, script for making the plot in gnuplot, data for plot**

```
# tr|C9ZTG5|C9ZTG5_TRYB9 Length: 1678
# tr|C9ZTG5|C9ZTG5_TRYB9 Number of predicted TMHs:  5
# tr|C9ZTG5|C9ZTG5_TRYB9 Exp number of AAs in TMHs: 111.86556
# tr|C9ZTG5|C9ZTG5_TRYB9 Exp number, first 60 AAs: 25.87872
# tr|C9ZTG5|C9ZTG5_TRYB9 Total prob of N-in:        0.97884
# tr|C9ZTG5|C9ZTG5_TRYB9 POSSIBLE N-term signal sequence
tr|C9ZTG5|C9ZTG5_TRYB9  TMHMM2.0        inside       1    29
tr|C9ZTG5|C9ZTG5_TRYB9  TMHMM2.0        TMhelix     30    52
tr|C9ZTG5|C9ZTG5_TRYB9  TMHMM2.0        outside     53    56
tr|C9ZTG5|C9ZTG5_TRYB9  TMHMM2.0        TMhelix     57    79
tr|C9ZTG5|C9ZTG5_TRYB9  TMHMM2.0        inside      80   140
tr|C9ZTG5|C9ZTG5_TRYB9  TMHMM2.0        TMhelix    141   163
tr|C9ZTG5|C9ZTG5_TRYB9  TMHMM2.0        outside    164   199
tr|C9ZTG5|C9ZTG5_TRYB9  TMHMM2.0        TMhelix    200   222
tr|C9ZTG5|C9ZTG5_TRYB9  TMHMM2.0        inside     223   234
tr|C9ZTG5|C9ZTG5_TRYB9  TMHMM2.0        TMhelix    235   257
tr|C9ZTG5|C9ZTG5_TRYB9  TMHMM2.0        outside    258  1678
```

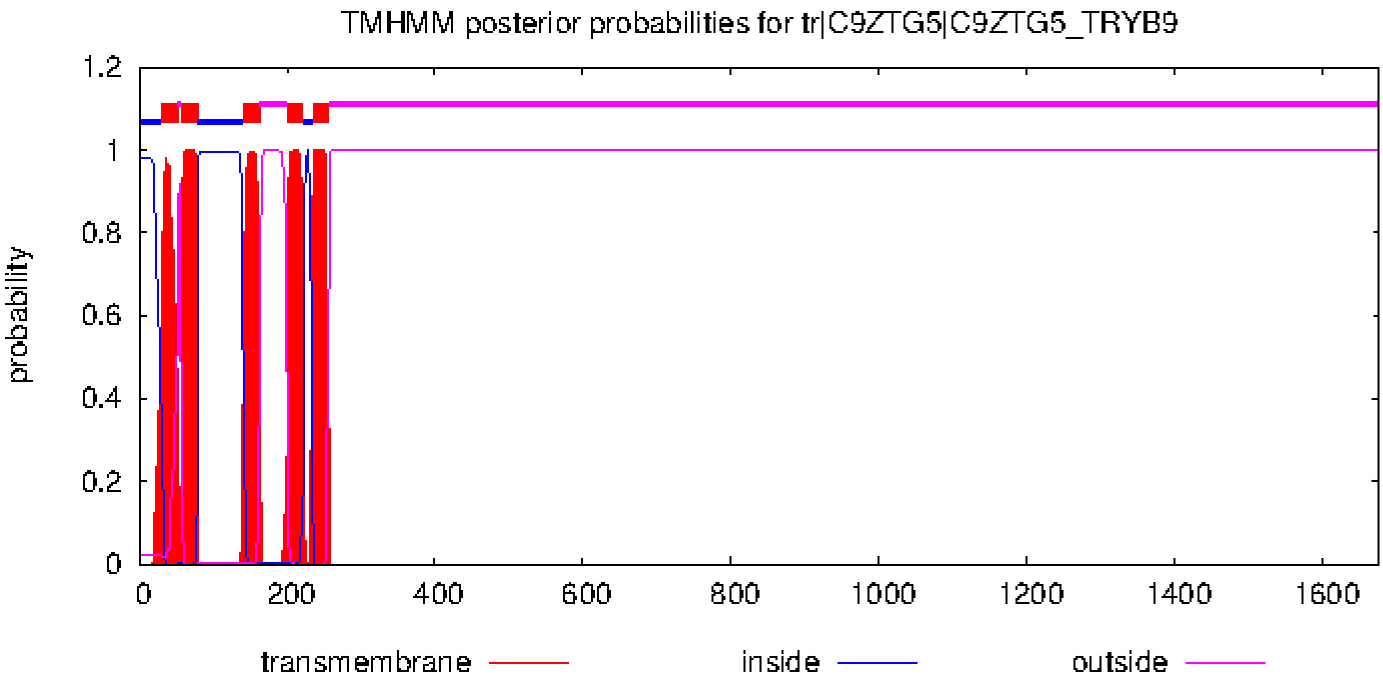

76 **# plot in postscript, script for making the plot in gnuplot, data for plot**

```
# tr|D0A1Y1|D0A1Y1_TRYB9 Length: 2693
# tr|D0A1Y1|D0A1Y1_TRYB9 Number of predicted TMHs:  22
# tr|D0A1Y1|D0A1Y1_TRYB9 Exp number of AAs in TMHs: 464.444649999999
# tr|D0A1Y1|D0A1Y1_TRYB9 Exp number, first 60 AAs:  0
# tr|D0A1Y1|D0A1Y1_TRYB9 Total prob of N-in:        0.11782
tr|D0A1Y1|D0A1Y1_TRYB9  TMHMM2.0        outside      1   321
tr|D0A1Y1|D0A1Y1_TRYB9  TMHMM2.0        TMhelix    322   344
tr|D0A1Y1|D0A1Y1_TRYB9  TMHMM2.0        inside     345   363
tr|D0A1Y1|D0A1Y1_TRYB9  TMHMM2.0        TMhelix    364   386
tr|D0A1Y1|D0A1Y1_TRYB9  TMHMM2.0        outside    387   405
tr|D0A1Y1|D0A1Y1_TRYB9  TMHMM2.0        TMhelix    406   428
tr|D0A1Y1|D0A1Y1_TRYB9  TMHMM2.0        inside     429   459
tr|D0A1Y1|D0A1Y1_TRYB9  TMHMM2.0        TMhelix    460   482
tr|D0A1Y1|D0A1Y1_TRYB9  TMHMM2.0        outside    483   544
tr|D0A1Y1|D0A1Y1_TRYB9  TMHMM2.0        TMhelix    545   564
tr|D0A1Y1|D0A1Y1_TRYB9  TMHMM2.0        inside     565   576
tr|D0A1Y1|D0A1Y1_TRYB9  TMHMM2.0        TMhelix    577   599
tr|D0A1Y1|D0A1Y1_TRYB9  TMHMM2.0        outside    600  1042
tr|D0A1Y1|D0A1Y1_TRYB9  TMHMM2.0        TMhelix   1043  1065
tr|D0A1Y1|D0A1Y1_TRYB9  TMHMM2.0        inside    1066  1077
tr|D0A1Y1|D0A1Y1_TRYB9  TMHMM2.0        TMhelix   1078  1100
tr|D0A1Y1|D0A1Y1_TRYB9  TMHMM2.0        outside   1101  1114
tr|D0A1Y1|D0A1Y1_TRYB9  TMHMM2.0        TMhelix   1115  1137
tr|D0A1Y1|D0A1Y1_TRYB9  TMHMM2.0        inside    1138  1160
tr|D0A1Y1|D0A1Y1_TRYB9  TMHMM2.0        TMhelix   1161  1183
tr|D0A1Y1|D0A1Y1_TRYB9  TMHMM2.0        outside   1184  1202
tr|D0A1Y1|D0A1Y1_TRYB9  TMHMM2.0        TMhelix   1203  1225
tr|D0A1Y1|D0A1Y1_TRYB9  TMHMM2.0        inside    1226  1245
tr|D0A1Y1|D0A1Y1_TRYB9  TMHMM2.0        TMhelix   1246  1268
tr|D0A1Y1|D0A1Y1_TRYB9  TMHMM2.0        outside   1269  1773
tr|D0A1Y1|D0A1Y1_TRYB9  TMHMM2.0        TMhelix   1774  1796
tr|D0A1Y1|D0A1Y1_TRYB9  TMHMM2.0        inside    1797  1804
tr|D0A1Y1|D0A1Y1_TRYB9  TMHMM2.0        TMhelix   1805  1824
tr|D0A1Y1|D0A1Y1_TRYB9  TMHMM2.0        outside   1825  1838
tr|D0A1Y1|D0A1Y1_TRYB9  TMHMM2.0        TMhelix   1839  1861
tr|D0A1Y1|D0A1Y1_TRYB9  TMHMM2.0        inside    1862  1904
tr|D0A1Y1|D0A1Y1_TRYB9  TMHMM2.0        TMhelix   1905  1927
tr|D0A1Y1|D0A1Y1_TRYB9  TMHMM2.0        outside   1928  2005
tr|D0A1Y1|D0A1Y1_TRYB9  TMHMM2.0        TMhelix   2006  2028
tr|D0A1Y1|D0A1Y1_TRYB9  TMHMM2.0        inside    2029  2084
```

tr|D0A1Y1|D0A1Y1_TRYB9	TMHMM2.0	TMhelix	2085	2107
tr|D0A1Y1|D0A1Y1_TRYB9	TMHMM2.0	outside	2108	2116

```
tr|D0A1Y1|D0A1Y1_TRYB9  TMHMM2.0        TMhelix   2117  2139
tr|D0A1Y1|D0A1Y1_TRYB9  TMHMM2.0        inside    2140  2145
tr|D0A1Y1|D0A1Y1_TRYB9  TMHMM2.0        TMhelix   2146  2168
tr|D0A1Y1|D0A1Y1_TRYB9  TMHMM2.0        outside   2169  2219
tr|D0A1Y1|D0A1Y1_TRYB9  TMHMM2.0        TMhelix   2220  2242
tr|D0A1Y1|D0A1Y1_TRYB9  TMHMM2.0        inside    2243  2300
tr|D0A1Y1|D0A1Y1_TRYB9  TMHMM2.0        TMhelix   2301  2323
tr|D0A1Y1|D0A1Y1_TRYB9  TMHMM2.0        outside   2324  2693
```

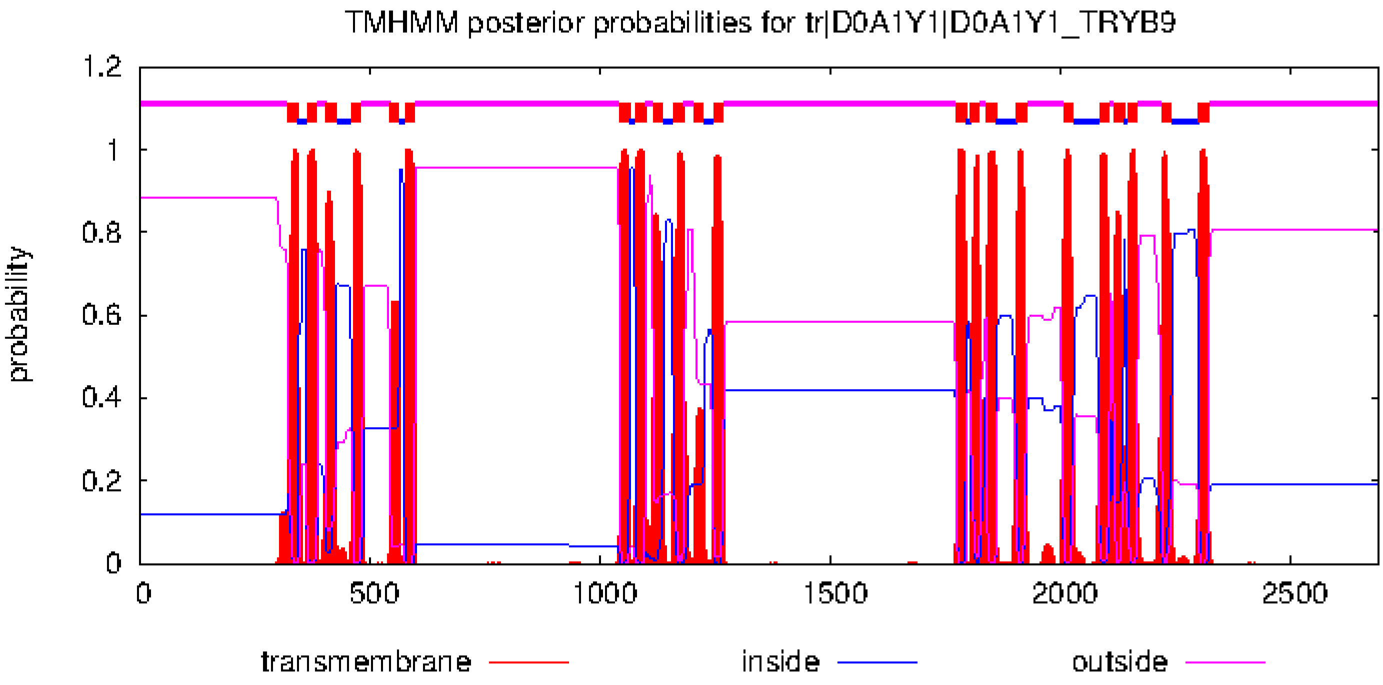

77 **# plot in postscript, script for making the plot in gnuplot, data for plot**

```
# tr|D0A0C2|D0A0C2_TRYB9 Length: 178
# tr|D0A0C2|D0A0C2_TRYB9 Number of predicted TMHs:  2
# tr|D0A0C2|D0A0C2_TRYB9 Exp number of AAs in TMHs: 45.85249
# tr|D0A0C2|D0A0C2_TRYB9 Exp number, first 60 AAs: 2.36328
# tr|D0A0C2|D0A0C2_TRYB9 Total prob of N-in:        0.90155
tr|D0A0C2|D0A0C2_TRYB9  TMHMM2.0        inside       1    57
tr|D0A0C2|D0A0C2_TRYB9  TMHMM2.0        TMhelix     58    80
tr|D0A0C2|D0A0C2_TRYB9  TMHMM2.0        outside     81    94
tr|D0A0C2|D0A0C2_TRYB9  TMHMM2.0        TMhelix     95   117
tr|D0A0C2|D0A0C2_TRYB9  TMHMM2.0        inside     118   178
```

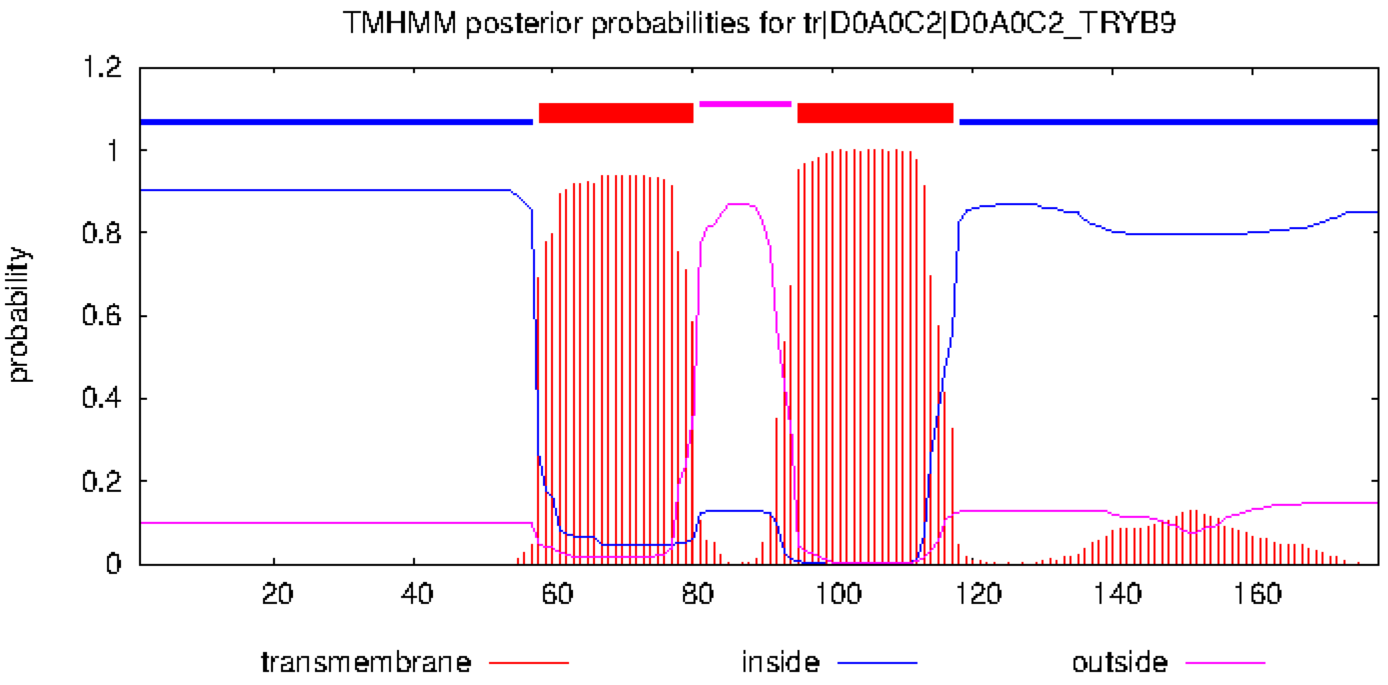

78 # **[plot]** **in postscript,** **[script]** **for making the plot in gnuplot,** **[data]** **for plot**

```
# tr|C9ZN85|C9ZN85_TRYB9 Length: 243
# tr|C9ZN85|C9ZN85_TRYB9 Number of predicted TMHs:  1
# tr|C9ZN85|C9ZN85_TRYB9 Exp number of AAs in TMHs: 23.50554
# tr|C9ZN85|C9ZN85_TRYB9 Exp number, first 60 AAs:  22.88873
# tr|C9ZN85|C9ZN85_TRYB9 Total prob of N-in:        0.00745
# tr|C9ZN85|C9ZN85_TRYB9 POSSIBLE N-term signal sequence
tr|C9ZN85|C9ZN85_TRYB9  TMHMM2.0        outside      1    38
tr|C9ZN85|C9ZN85_TRYB9  TMHMM2.0        TMhelix     39    61
tr|C9ZN85|C9ZN85_TRYB9  TMHMM2.0        inside      62   243
```

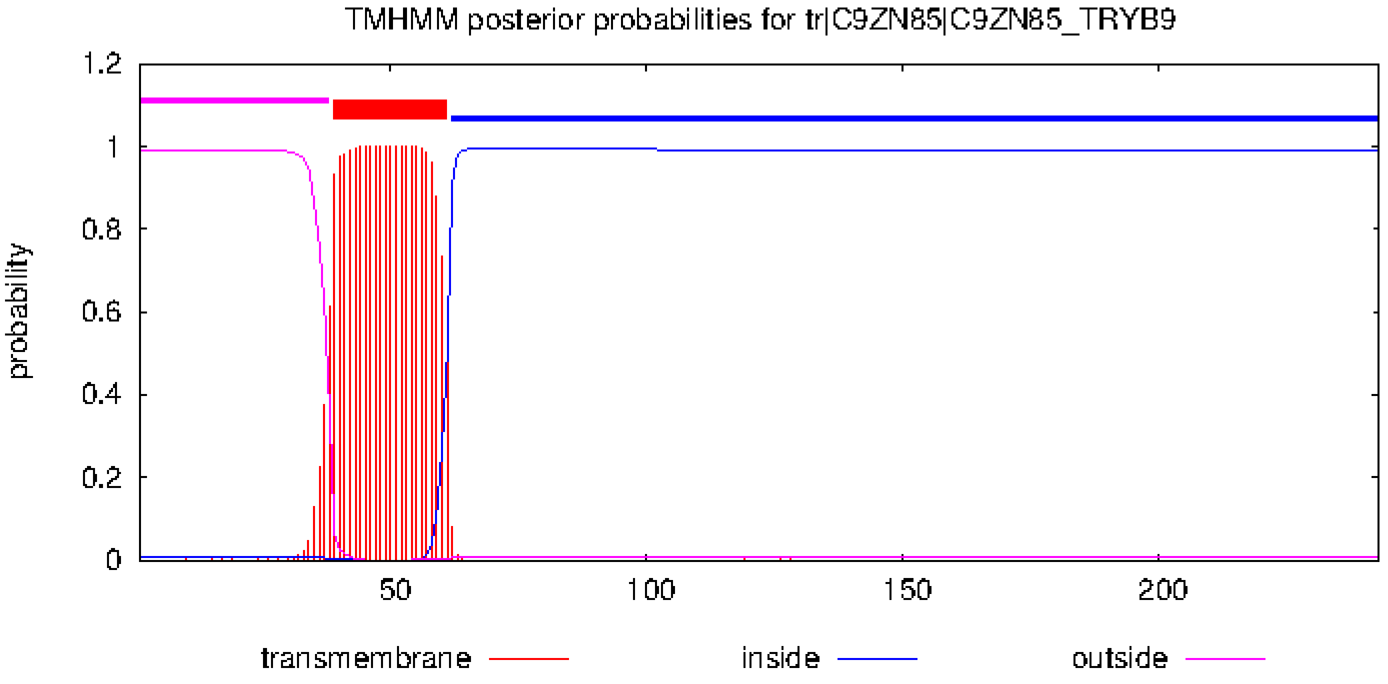

79 # **[plot]** **in postscript,** **[script]** **for making the plot in gnuplot,** **[data]** **for plot**

```
# tr|C9ZKD6|C9ZKD6_TRYB9 Length: 307
```

tr|C9ZKD6|C9ZKD6_TRYB9 Number of predicted TMHs: 4
tr|C9ZKD6|C9ZKD6_TRYB9 Exp number of AAs in TMHs: 88.79818
tr|C9ZKD6|C9ZKD6_TRYB9 Exp number, first 60 AAs: 31.79466

```
# tr|C9ZKD6|C9ZKD6_TRYB9 Total prob of N-in:        0.94600
# tr|C9ZKD6|C9ZKD6_TRYB9 POSSIBLE N-term signal sequence
tr|C9ZKD6|C9ZKD6_TRYB9  TMHMM2.0        inside       1    20
tr|C9ZKD6|C9ZKD6_TRYB9  TMHMM2.0        TMhelix     21    40
tr|C9ZKD6|C9ZKD6_TRYB9  TMHMM2.0        outside     41    49
tr|C9ZKD6|C9ZKD6_TRYB9  TMHMM2.0        TMhelix     50    72
tr|C9ZKD6|C9ZKD6_TRYB9  TMHMM2.0        inside      73   167
tr|C9ZKD6|C9ZKD6_TRYB9  TMHMM2.0        TMhelix    168   190
tr|C9ZKD6|C9ZKD6_TRYB9  TMHMM2.0        outside    191   215
tr|C9ZKD6|C9ZKD6_TRYB9  TMHMM2.0        TMhelix    216   238
tr|C9ZKD6|C9ZKD6_TRYB9  TMHMM2.0        inside     239   307
```

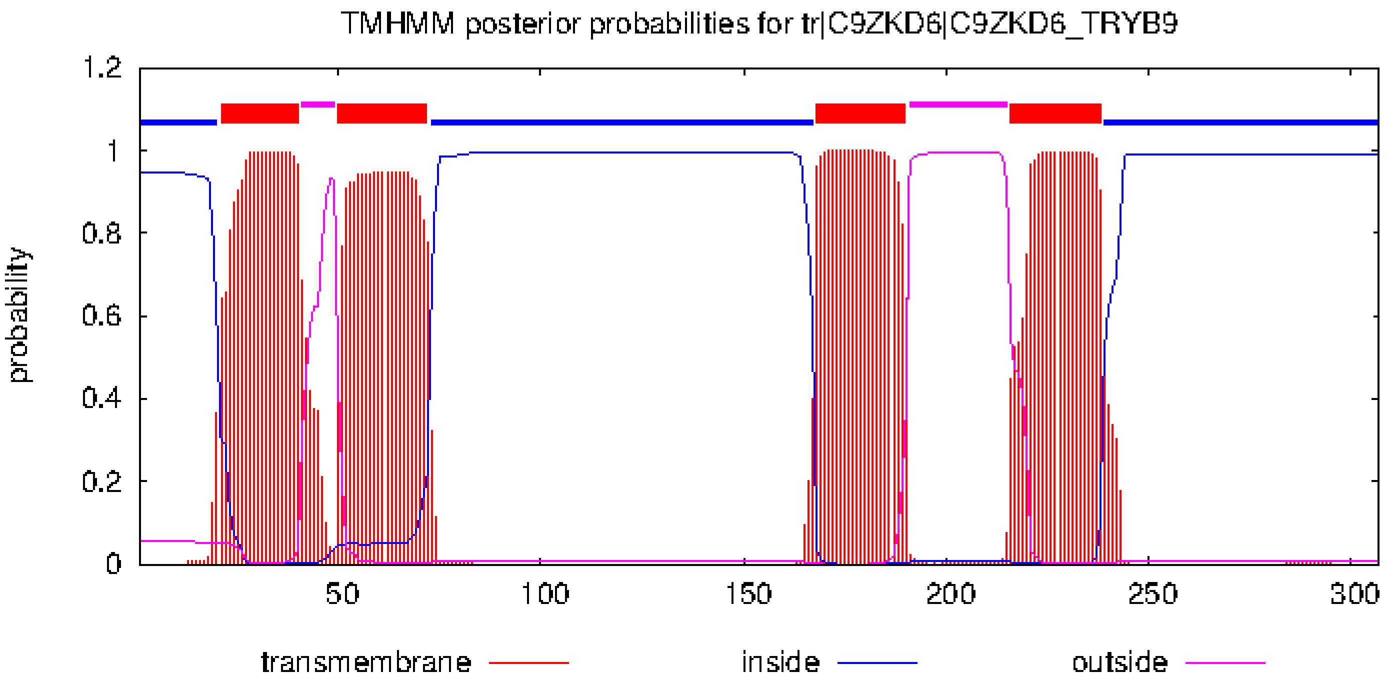

80 # plot in postscript, script for making the plot in gnuplot, data for plot

---

```
# tr|C9ZQF9|C9ZQF9_TRYB9 Length: 321
# tr|C9ZQF9|C9ZQF9_TRYB9 Number of predicted TMHs:  6
# tr|C9ZQF9|C9ZQF9_TRYB9 Exp number of AAs in TMHs: 135.33391
# tr|C9ZQF9|C9ZQF9_TRYB9 Exp number, first 60 AAs:  0
# tr|C9ZQF9|C9ZQF9_TRYB9 Total prob of N-in:        0.97647
tr|C9ZQF9|C9ZQF9_TRYB9  TMHMM2.0        inside       1    84
tr|C9ZQF9|C9ZQF9_TRYB9  TMHMM2.0        TMhelix     85   107
tr|C9ZQF9|C9ZQF9_TRYB9  TMHMM2.0        outside    108   116
tr|C9ZQF9|C9ZQF9_TRYB9  TMHMM2.0        TMhelix    117   139
tr|C9ZQF9|C9ZQF9_TRYB9  TMHMM2.0        inside     140   158
tr|C9ZQF9|C9ZQF9_TRYB9  TMHMM2.0        TMhelix    159   181
tr|C9ZQF9|C9ZQF9_TRYB9  TMHMM2.0        outside    182   212
tr|C9ZQF9|C9ZQF9_TRYB9  TMHMM2.0        TMhelix    213   235
tr|C9ZQF9|C9ZQF9_TRYB9  TMHMM2.0        inside     236   247
tr|C9ZQF9|C9ZQF9_TRYB9  TMHMM2.0        TMhelix    248   270
tr|C9ZQF9|C9ZQF9_TRYB9  TMHMM2.0        outside    271   296
tr|C9ZQF9|C9ZQF9_TRYB9  TMHMM2.0        TMhelix    297   319
tr|C9ZQF9|C9ZQF9_TRYB9  TMHMM2.0        inside     320   321
```

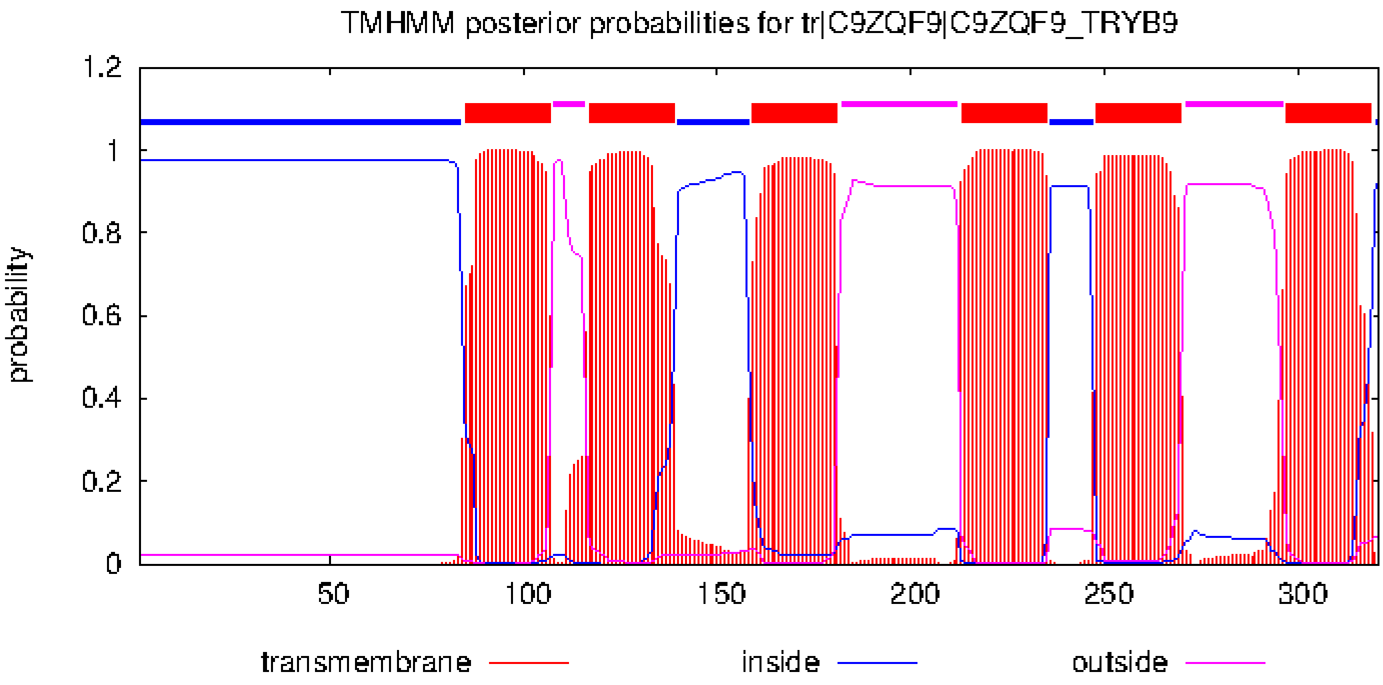

81 **# [plot]() in postscript, [script]() for making the plot in gnuplot, [data]()for plot**

```
# tr|D0A885|D0A885_TRYB9 Length: 391
# tr|D0A885|D0A885_TRYB9 Number of predicted TMHs:  8
# tr|D0A885|D0A885_TRYB9 Exp number of AAs in TMHs: 163.10288
# tr|D0A885|D0A885_TRYB9 Exp number, first 60 AAs: 16.65663
# tr|D0A885|D0A885_TRYB9 Total prob of N-in:        0.24711
# tr|D0A885|D0A885_TRYB9 POSSIBLE N-term signal sequence
tr|D0A885|D0A885_TRYB9  TMHMM2.0        outside      1    41
tr|D0A885|D0A885_TRYB9  TMHMM2.0        TMhelix     42    64
tr|D0A885|D0A885_TRYB9  TMHMM2.0        inside      65    76
tr|D0A885|D0A885_TRYB9  TMHMM2.0        TMhelix     77    99
tr|D0A885|D0A885_TRYB9  TMHMM2.0        outside    100   113
tr|D0A885|D0A885_TRYB9  TMHMM2.0        TMhelix    114   136
tr|D0A885|D0A885_TRYB9  TMHMM2.0        inside     137   228
tr|D0A885|D0A885_TRYB9  TMHMM2.0        TMhelix    229   251
tr|D0A885|D0A885_TRYB9  TMHMM2.0        outside    252   260
tr|D0A885|D0A885_TRYB9  TMHMM2.0        TMhelix    261   283
tr|D0A885|D0A885_TRYB9  TMHMM2.0        inside     284   289
tr|D0A885|D0A885_TRYB9  TMHMM2.0        TMhelix    290   312
tr|D0A885|D0A885_TRYB9  TMHMM2.0        outside    313   326
tr|D0A885|D0A885_TRYB9  TMHMM2.0        TMhelix    327   349
tr|D0A885|D0A885_TRYB9  TMHMM2.0        inside     350   369
tr|D0A885|D0A885_TRYB9  TMHMM2.0        TMhelix    370   387
tr|D0A885|D0A885_TRYB9  TMHMM2.0        outside    388   391
```

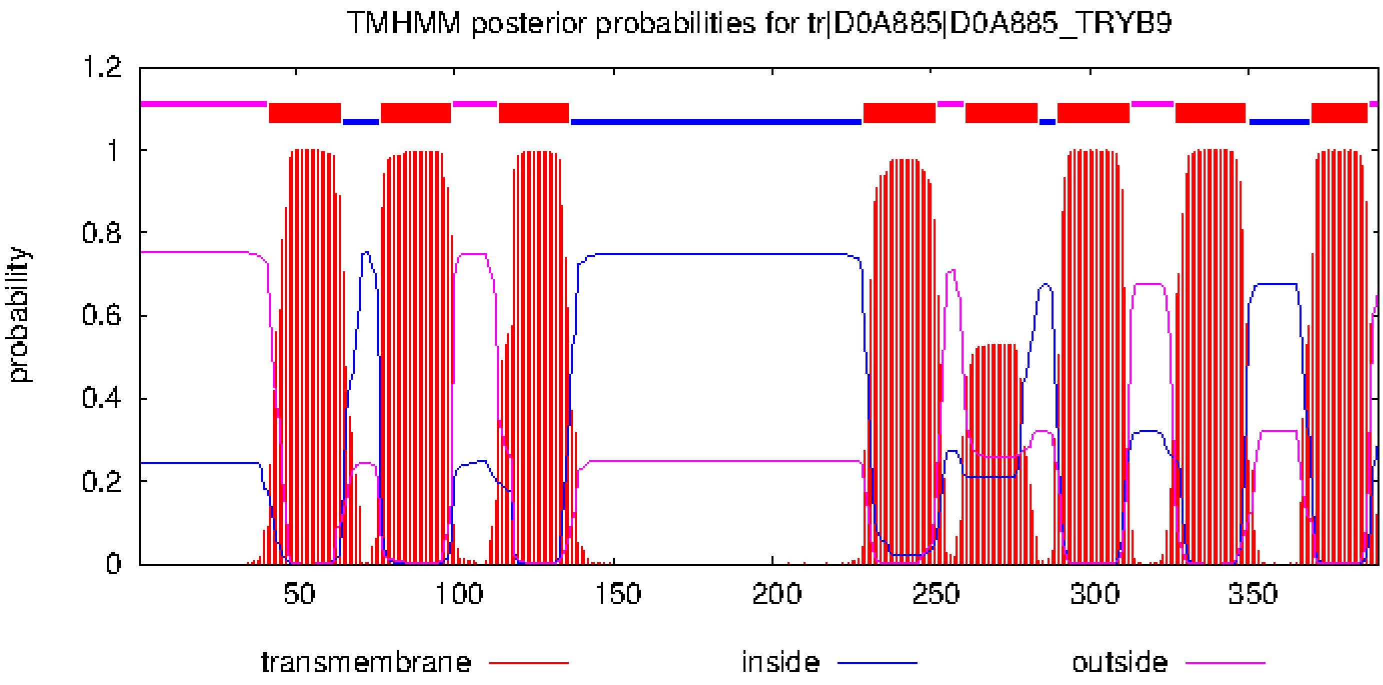

82 # plot in postscript, script for making the plot in gnuplot, data for plot

---

```
# tr|D0A887|D0A887_TRYB9 Length: 394
# tr|D0A887|D0A887_TRYB9 Number of predicted TMHs:  9
# tr|D0A887|D0A887_TRYB9 Exp number of AAs in TMHs: 180.90422
#  tr|D0A887|D0A887_TRYB9 Exp number, first 60 AAs: 10.23564
# tr|D0A887|D0A887_TRYB9 Total prob of N-in:        0.68733
# tr|D0A887|D0A887_TRYB9 POSSIBLE N-term signal sequence
tr|D0A887|D0A887_TRYB9  TMHMM2.0        inside       1    12
tr|D0A887|D0A887_TRYB9  TMHMM2.0        TMhelix     13    35
tr|D0A887|D0A887_TRYB9  TMHMM2.0        outside     36    79
tr|D0A887|D0A887_TRYB9  TMHMM2.0        TMhelix     80   102
tr|D0A887|D0A887_TRYB9  TMHMM2.0        inside     103   114
tr|D0A887|D0A887_TRYB9  TMHMM2.0        TMhelix    115   137
tr|D0A887|D0A887_TRYB9  TMHMM2.0        outside    138   156
tr|D0A887|D0A887_TRYB9  TMHMM2.0        TMhelix    157   179
tr|D0A887|D0A887_TRYB9  TMHMM2.0        inside     180   231
tr|D0A887|D0A887_TRYB9  TMHMM2.0        TMhelix    232   254
tr|D0A887|D0A887_TRYB9  TMHMM2.0        outside    255   263
tr|D0A887|D0A887_TRYB9  TMHMM2.0        TMhelix    264   283
tr|D0A887|D0A887_TRYB9  TMHMM2.0        inside     284   294
tr|D0A887|D0A887_TRYB9  TMHMM2.0        TMhelix    295   317
tr|D0A887|D0A887_TRYB9  TMHMM2.0        outside    318   326
tr|D0A887|D0A887_TRYB9  TMHMM2.0        TMhelix    327   349
tr|D0A887|D0A887_TRYB9  TMHMM2.0        inside     350   369
tr|D0A887|D0A887_TRYB9  TMHMM2.0        TMhelix    370   389
tr|D0A887|D0A887_TRYB9  TMHMM2.0        outside    390   394
```

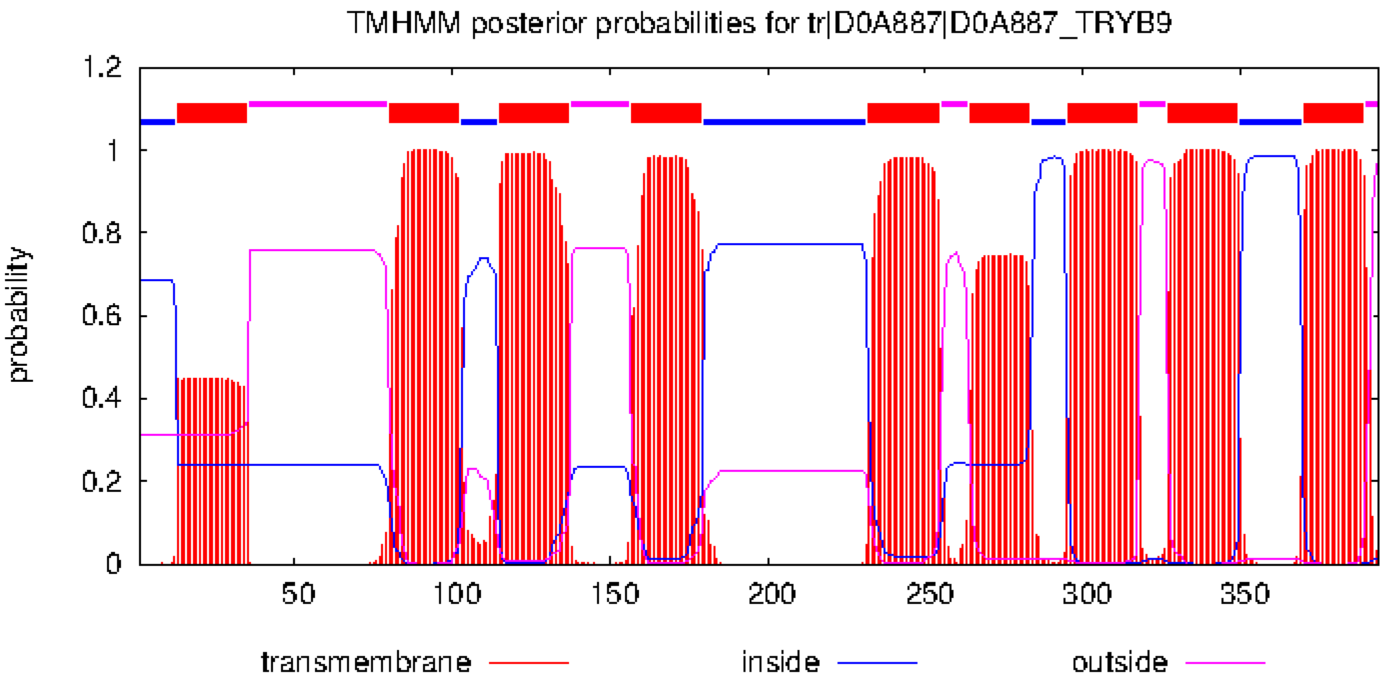

83 **# plot in postscript, script for making the plot in gnuplot, data for plot**

```
# tr|C9ZZR9|C9ZZR9_TRYB9 Length: 399
# tr|C9ZZR9|C9ZZR9_TRYB9 Number of predicted TMHs:  9
# tr|C9ZZR9|C9ZZR9_TRYB9 Exp number of AAs in TMHs: 200.06788
# tr|C9ZZR9|C9ZZR9_TRYB9 Exp number, first 60 AAs: 38.34937
# tr|C9ZZR9|C9ZZR9_TRYB9 Total prob of N-in:        0.88890
# tr|C9ZZR9|C9ZZR9_TRYB9 POSSIBLE N-term signal sequence
tr|C9ZZR9|C9ZZR9_TRYB9  TMHMM2.0        inside       1    12
tr|C9ZZR9|C9ZZR9_TRYB9  TMHMM2.0        TMhelix     13    35
tr|C9ZZR9|C9ZZR9_TRYB9  TMHMM2.0        outside     36    45
tr|C9ZZR9|C9ZZR9_TRYB9  TMHMM2.0        TMhelix     46    68
tr|C9ZZR9|C9ZZR9_TRYB9  TMHMM2.0        inside      69    74
tr|C9ZZR9|C9ZZR9_TRYB9  TMHMM2.0        TMhelix     75    97
tr|C9ZZR9|C9ZZR9_TRYB9  TMHMM2.0        outside     98   120
tr|C9ZZR9|C9ZZR9_TRYB9  TMHMM2.0        TMhelix    121   140
tr|C9ZZR9|C9ZZR9_TRYB9  TMHMM2.0        inside     141   235
tr|C9ZZR9|C9ZZR9_TRYB9  TMHMM2.0        TMhelix    236   258
tr|C9ZZR9|C9ZZR9_TRYB9  TMHMM2.0        outside    259   267
tr|C9ZZR9|C9ZZR9_TRYB9  TMHMM2.0        TMhelix    268   290
tr|C9ZZR9|C9ZZR9_TRYB9  TMHMM2.0        inside     291   301
tr|C9ZZR9|C9ZZR9_TRYB9  TMHMM2.0        TMhelix    302   324
tr|C9ZZR9|C9ZZR9_TRYB9  TMHMM2.0        outside    325   328
tr|C9ZZR9|C9ZZR9_TRYB9  TMHMM2.0        TMhelix    329   351
tr|C9ZZR9|C9ZZR9_TRYB9  TMHMM2.0        inside     352   370
tr|C9ZZR9|C9ZZR9_TRYB9  TMHMM2.0        TMhelix    371   393
tr|C9ZZR9|C9ZZR9_TRYB9  TMHMM2.0        outside    394   399
```

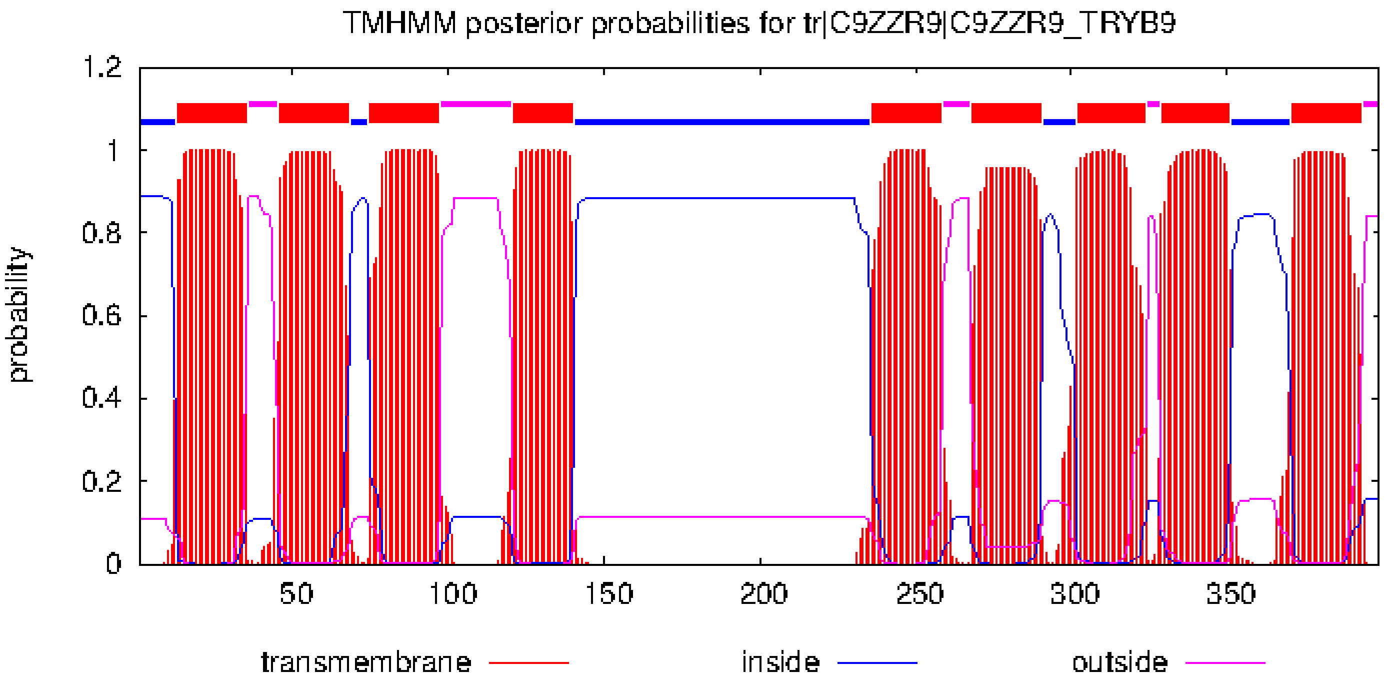

84 # plot in postscript, script for making the plot in gnuplot, data for plot

---

```
# tr|D0A4L6|D0A4L6_TRYB9 Length: 452
# tr|D0A4L6|D0A4L6_TRYB9 Number of predicted TMHs:  4
# tr|D0A4L6|D0A4L6_TRYB9 Exp number of AAs in TMHs: 90.8576900000002
# tr|D0A4L6|D0A4L6_TRYB9 Exp number, first 60 AAs:  22.93162
# tr|D0A4L6|D0A4L6_TRYB9 Total prob of N-in:        0.99995
# tr|D0A4L6|D0A4L6_TRYB9 POSSIBLE N-term signal sequence
tr|D0A4L6|D0A4L6_TRYB9  TMHMM2.0        inside       1    11
tr|D0A4L6|D0A4L6_TRYB9  TMHMM2.0        TMhelix     12    34
tr|D0A4L6|D0A4L6_TRYB9  TMHMM2.0        outside     35    70
tr|D0A4L6|D0A4L6_TRYB9  TMHMM2.0        TMhelix     71    93
tr|D0A4L6|D0A4L6_TRYB9  TMHMM2.0        inside      94   168
tr|D0A4L6|D0A4L6_TRYB9  TMHMM2.0        TMhelix    169   191
tr|D0A4L6|D0A4L6_TRYB9  TMHMM2.0        outside    192   259
tr|D0A4L6|D0A4L6_TRYB9  TMHMM2.0        TMhelix    260   282
tr|D0A4L6|D0A4L6_TRYB9  TMHMM2.0        inside     283   452
```

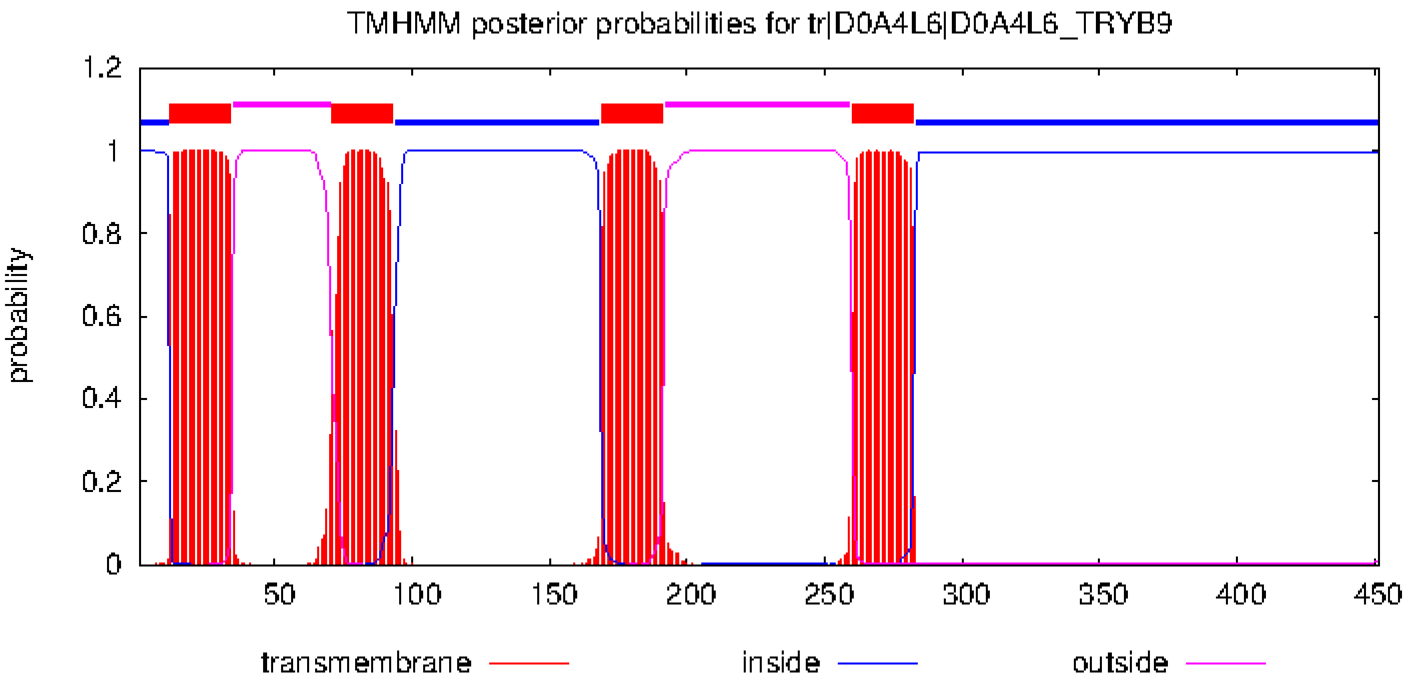

85 # plot in postscript, script for making the plot in gnuplot, data for plot

```
# tr|C9ZY31|C9ZY31_TRYB9 Length: 461
# tr|C9ZY31|C9ZY31_TRYB9 Number of predicted TMHs:  10
# tr|C9ZY31|C9ZY31_TRYB9 Exp number of AAs in TMHs: 225.61554
# tr|C9ZY31|C9ZY31_TRYB9 Exp number, first 60 AAs: 23.26909
# tr|C9ZY31|C9ZY31_TRYB9 Total prob of N-in:        0.16061
# tr|C9ZY31|C9ZY31_TRYB9 POSSIBLE N-term signal sequence
tr|C9ZY31|C9ZY31_TRYB9  TMHMM2.0        outside      1     9
tr|C9ZY31|C9ZY31_TRYB9  TMHMM2.0        TMhelix     10    32
tr|C9ZY31|C9ZY31_TRYB9  TMHMM2.0        inside      33    92
tr|C9ZY31|C9ZY31_TRYB9  TMHMM2.0        TMhelix     93   115
tr|C9ZY31|C9ZY31_TRYB9  TMHMM2.0        outside    116   124
tr|C9ZY31|C9ZY31_TRYB9  TMHMM2.0        TMhelix    125   147
tr|C9ZY31|C9ZY31_TRYB9  TMHMM2.0        inside     148   153
tr|C9ZY31|C9ZY31_TRYB9  TMHMM2.0        TMhelix    154   176
tr|C9ZY31|C9ZY31_TRYB9  TMHMM2.0        outside    177   190
tr|C9ZY31|C9ZY31_TRYB9  TMHMM2.0        TMhelix    191   213
tr|C9ZY31|C9ZY31_TRYB9  TMHMM2.0        inside     214   304
tr|C9ZY31|C9ZY31_TRYB9  TMHMM2.0        TMhelix    305   327
tr|C9ZY31|C9ZY31_TRYB9  TMHMM2.0        outside    328   336
tr|C9ZY31|C9ZY31_TRYB9  TMHMM2.0        TMhelix    337   356
tr|C9ZY31|C9ZY31_TRYB9  TMHMM2.0        inside     357   367
tr|C9ZY31|C9ZY31_TRYB9  TMHMM2.0        TMhelix    368   390
tr|C9ZY31|C9ZY31_TRYB9  TMHMM2.0        outside    391   393
tr|C9ZY31|C9ZY31_TRYB9  TMHMM2.0        TMhelix    394   416
tr|C9ZY31|C9ZY31_TRYB9  TMHMM2.0        inside     417   436
tr|C9ZY31|C9ZY31_TRYB9  TMHMM2.0        TMhelix    437   459
tr|C9ZY31|C9ZY31_TRYB9  TMHMM2.0        outside    460   461
```

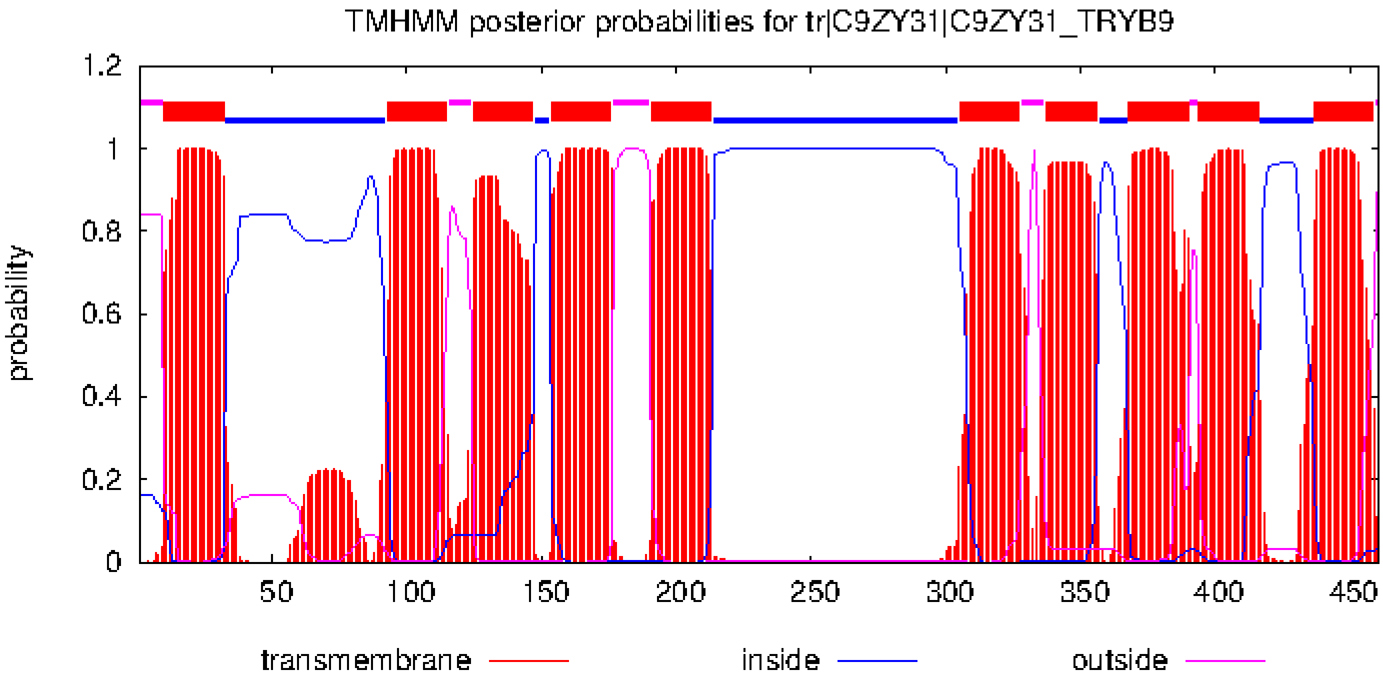

86 # plot in postscript, script for making the plot in gnuplot, data for plot

```
# tr|C9ZJU3|C9ZJU3_TRYB9 Length: 463
# tr|C9ZJU3|C9ZJU3_TRYB9 Number of predicted TMHs:  11
# tr|C9ZJU3|C9ZJU3_TRYB9 Exp number of AAs in TMHs: 240.53942
# tr|C9ZJU3|C9ZJU3_TRYB9 Exp number, first 60 AAs:  22.64748
# tr|C9ZJU3|C9ZJU3_TRYB9 Total prob of N-in:        0.99737
# tr|C9ZJU3|C9ZJU3_TRYB9 POSSIBLE N-term signal sequence
tr|C9ZJU3|C9ZJU3_TRYB9  TMHMM2.0        inside       1    11
tr|C9ZJU3|C9ZJU3_TRYB9  TMHMM2.0        TMhelix     12    34
tr|C9ZJU3|C9ZJU3_TRYB9  TMHMM2.0        outside     35    63
tr|C9ZJU3|C9ZJU3_TRYB9  TMHMM2.0        TMhelix     64    86
tr|C9ZJU3|C9ZJU3_TRYB9  TMHMM2.0        inside      87    90
tr|C9ZJU3|C9ZJU3_TRYB9  TMHMM2.0        TMhelix     91   113
```

```
tr|C9ZJU3|C9ZJU3_TRYB9  TMHMM2.0        outside    114   122
```

```
tr|C9ZJU3|C9ZJU3_TRYB9  TMHMM2.0        TMhelix    123   140
tr|C9ZJU3|C9ZJU3_TRYB9  TMHMM2.0        inside     141   155
tr|C9ZJU3|C9ZJU3_TRYB9  TMHMM2.0        TMhelix    156   178
tr|C9ZJU3|C9ZJU3_TRYB9  TMHMM2.0        outside    179   192
tr|C9ZJU3|C9ZJU3_TRYB9  TMHMM2.0        TMhelix    193   215
tr|C9ZJU3|C9ZJU3_TRYB9  TMHMM2.0        inside     216   303
tr|C9ZJU3|C9ZJU3_TRYB9  TMHMM2.0        TMhelix    304   326
tr|C9ZJU3|C9ZJU3_TRYB9  TMHMM2.0        outside    327   335
tr|C9ZJU3|C9ZJU3_TRYB9  TMHMM2.0        TMhelix    336   358
tr|C9ZJU3|C9ZJU3_TRYB9  TMHMM2.0        inside     359   369
tr|C9ZJU3|C9ZJU3_TRYB9  TMHMM2.0        TMhelix    370   392
tr|C9ZJU3|C9ZJU3_TRYB9  TMHMM2.0        outside    393   401
tr|C9ZJU3|C9ZJU3_TRYB9  TMHMM2.0        TMhelix    402   421
tr|C9ZJU3|C9ZJU3_TRYB9  TMHMM2.0        inside     422   433
tr|C9ZJU3|C9ZJU3_TRYB9  TMHMM2.0        TMhelix    434   456
tr|C9ZJU3|C9ZJU3_TRYB9  TMHMM2.0        outside    457   463
```

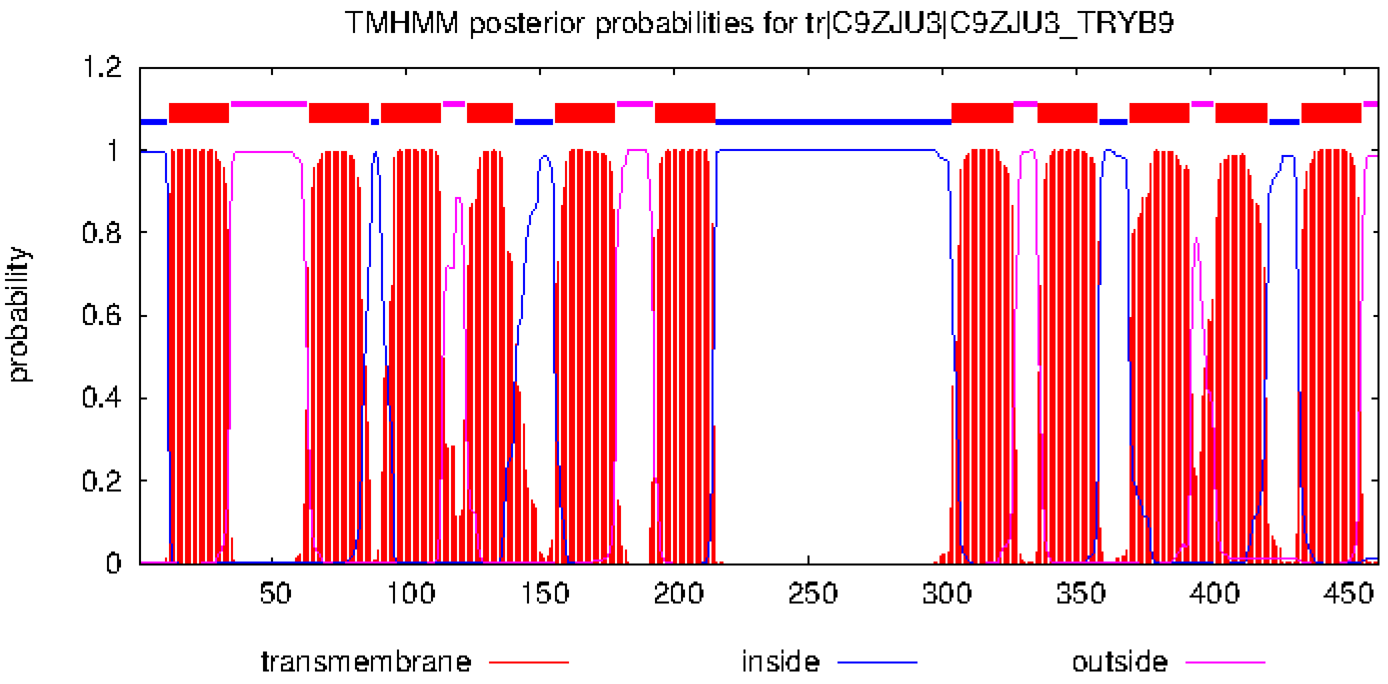

87 # plot in postscript, script for making the plot in gnuplot, data for plot

---

```
# tr|D0A0T8|D0A0T8_TRYB9 Length: 471
# tr|D0A0T8|D0A0T8_TRYB9 Number of predicted TMHs:  3
# tr|D0A0T8|D0A0T8_TRYB9 Exp number of AAs in TMHs: 79.6538700000001
# tr|D0A0T8|D0A0T8_TRYB9 Exp number, first 60 AAs:  1.55427
# tr|D0A0T8|D0A0T8_TRYB9 Total prob of N-in:        0.98136
tr|D0A0T8|D0A0T8_TRYB9  TMHMM2.0        inside       1    58
tr|D0A0T8|D0A0T8_TRYB9  TMHMM2.0        TMhelix     59    81
tr|D0A0T8|D0A0T8_TRYB9  TMHMM2.0        outside     82    85
tr|D0A0T8|D0A0T8_TRYB9  TMHMM2.0        TMhelix     86   108
tr|D0A0T8|D0A0T8_TRYB9  TMHMM2.0        inside     109   128
tr|D0A0T8|D0A0T8_TRYB9  TMHMM2.0        TMhelix    129   151
tr|D0A0T8|D0A0T8_TRYB9  TMHMM2.0        outside    152   471
```

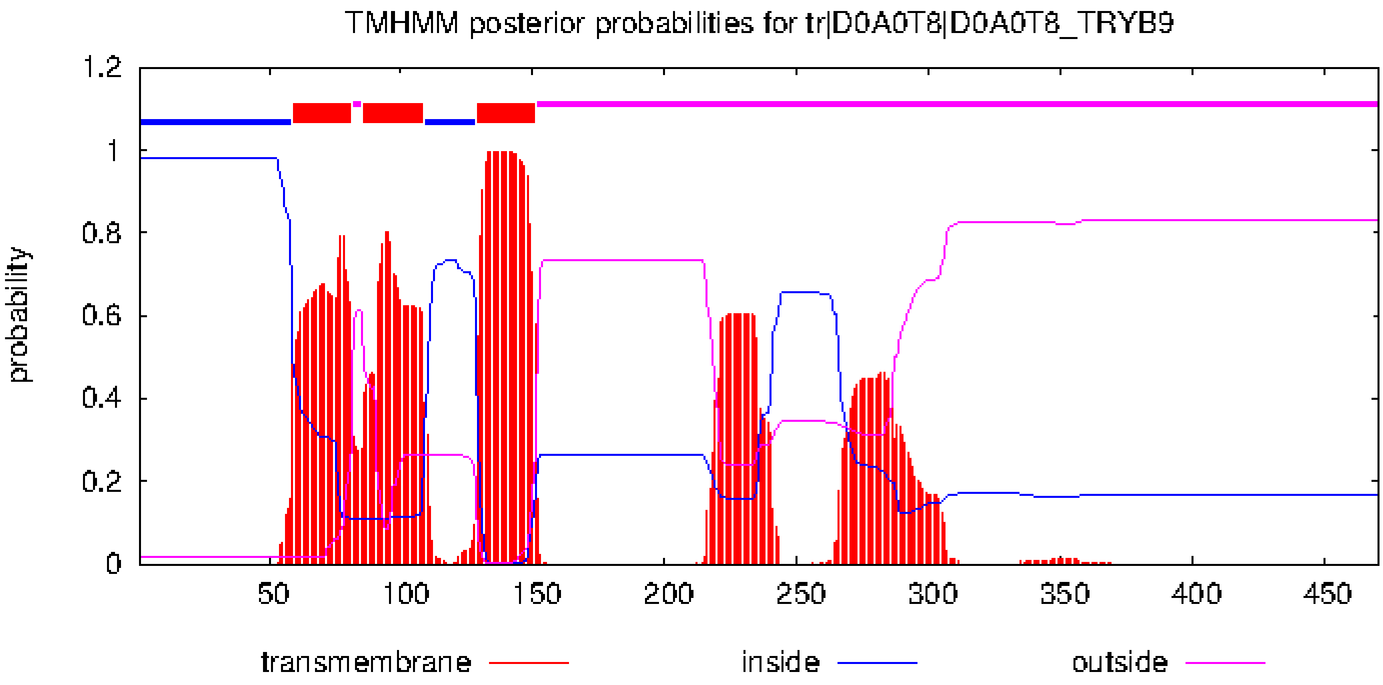

88 **# plot in postscript, script for making the plot in gnuplot, data for plot**

```
# tr|D0A734|D0A734_TRYB9 Length: 483
# tr|D0A734|D0A734_TRYB9 Number of predicted TMHs:  11
# tr|D0A734|D0A734_TRYB9 Exp number of AAs in TMHs: 253.39745
# tr|D0A734|D0A734_TRYB9 Exp number, first 60 AAs: 40.46949
# tr|D0A734|D0A734_TRYB9 Total prob of N-in:        0.99957
# tr|D0A734|D0A734_TRYB9 POSSIBLE N-term signal sequence
tr|D0A734|D0A734_TRYB9  TMHMM2.0        inside       1     6
tr|D0A734|D0A734_TRYB9  TMHMM2.0        TMhelix      7    29
tr|D0A734|D0A734_TRYB9  TMHMM2.0        outside     30    43
tr|D0A734|D0A734_TRYB9  TMHMM2.0        TMhelix     44    66
tr|D0A734|D0A734_TRYB9  TMHMM2.0        inside      67    72
tr|D0A734|D0A734_TRYB9  TMHMM2.0        TMhelix     73    92
tr|D0A734|D0A734_TRYB9  TMHMM2.0        outside     93    96
tr|D0A734|D0A734_TRYB9  TMHMM2.0        TMhelix     97   119
tr|D0A734|D0A734_TRYB9  TMHMM2.0        inside     120   131
tr|D0A734|D0A734_TRYB9  TMHMM2.0        TMhelix    132   154
tr|D0A734|D0A734_TRYB9  TMHMM2.0        outside    155   163
tr|D0A734|D0A734_TRYB9  TMHMM2.0        TMhelix    164   186
tr|D0A734|D0A734_TRYB9  TMHMM2.0        inside     187   236
tr|D0A734|D0A734_TRYB9  TMHMM2.0        TMhelix    237   259
tr|D0A734|D0A734_TRYB9  TMHMM2.0        outside    260   273
tr|D0A734|D0A734_TRYB9  TMHMM2.0        TMhelix    274   296
tr|D0A734|D0A734_TRYB9  TMHMM2.0        inside     297   302
tr|D0A734|D0A734_TRYB9  TMHMM2.0        TMhelix    303   325
tr|D0A734|D0A734_TRYB9  TMHMM2.0        outside    326   339
tr|D0A734|D0A734_TRYB9  TMHMM2.0        TMhelix    340   362
tr|D0A734|D0A734_TRYB9  TMHMM2.0        inside     363   395
tr|D0A734|D0A734_TRYB9  TMHMM2.0        TMhelix    396   418
tr|D0A734|D0A734_TRYB9  TMHMM2.0        outside    419   483
```

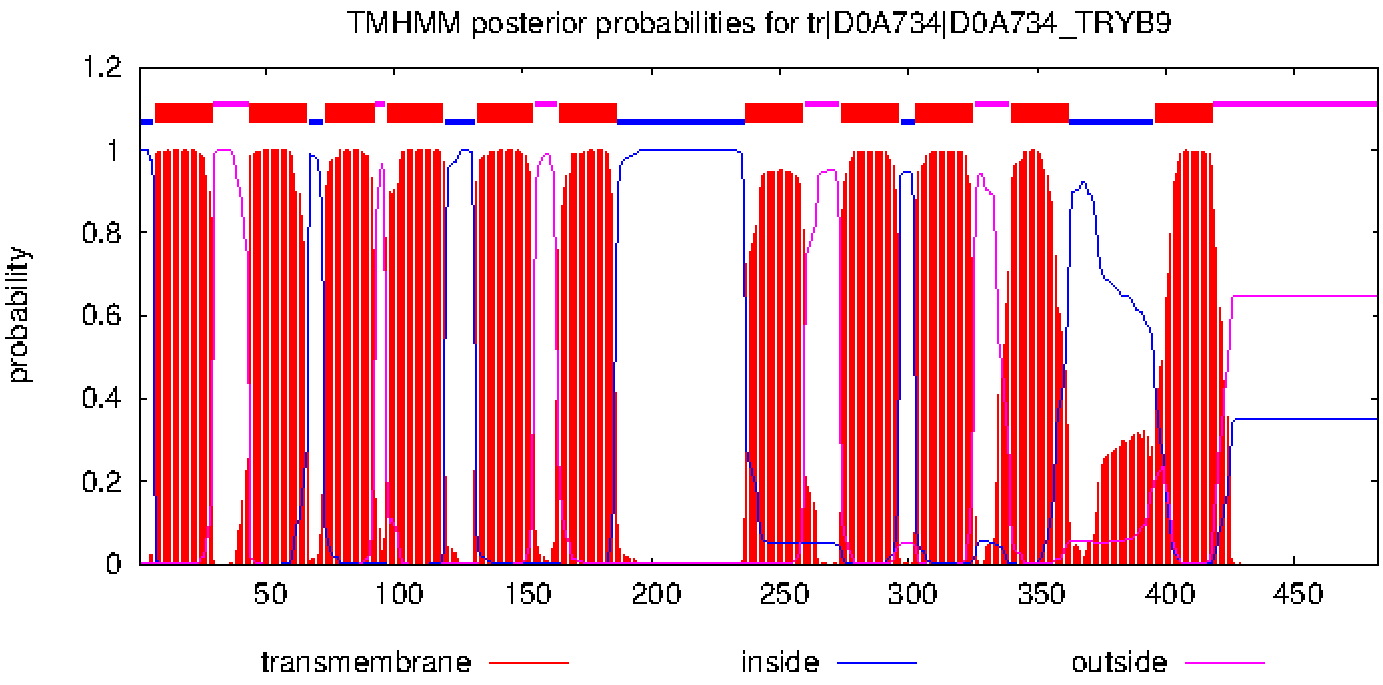

89 **# plot in postscript, script for making the plot in gnuplot, data for plot**

```
# tr|C9ZP80|C9ZP80_TRYB9 Length: 486
# tr|C9ZP80|C9ZP80_TRYB9 Number of predicted TMHs:  10
# tr|C9ZP80|C9ZP80_TRYB9 Exp number of AAs in TMHs: 234.71446
# tr|C9ZP80|C9ZP80_TRYB9 Exp number, first 60 AAs: 19.55171
# tr|C9ZP80|C9ZP80_TRYB9 Total prob of N-in:        0.78689
# tr|C9ZP80|C9ZP80_TRYB9 POSSIBLE N-term signal sequence
tr|C9ZP80|C9ZP80_TRYB9  TMHMM2.0        inside       1    38
tr|C9ZP80|C9ZP80_TRYB9  TMHMM2.0        TMhelix     39    58
tr|C9ZP80|C9ZP80_TRYB9  TMHMM2.0        outside     59    67
tr|C9ZP80|C9ZP80_TRYB9  TMHMM2.0        TMhelix     68    90
tr|C9ZP80|C9ZP80_TRYB9  TMHMM2.0        inside      91   109
tr|C9ZP80|C9ZP80_TRYB9  TMHMM2.0        TMhelix    110   132
tr|C9ZP80|C9ZP80_TRYB9  TMHMM2.0        outside    133   151
tr|C9ZP80|C9ZP80_TRYB9  TMHMM2.0        TMhelix    152   169
tr|C9ZP80|C9ZP80_TRYB9  TMHMM2.0        inside     170   181
tr|C9ZP80|C9ZP80_TRYB9  TMHMM2.0        TMhelix    182   204
tr|C9ZP80|C9ZP80_TRYB9  TMHMM2.0        outside    205   208
tr|C9ZP80|C9ZP80_TRYB9  TMHMM2.0        TMhelix    209   231
tr|C9ZP80|C9ZP80_TRYB9  TMHMM2.0        inside     232   294
tr|C9ZP80|C9ZP80_TRYB9  TMHMM2.0        TMhelix    295   317
tr|C9ZP80|C9ZP80_TRYB9  TMHMM2.0        outside    318   326
tr|C9ZP80|C9ZP80_TRYB9  TMHMM2.0        TMhelix    327   349
tr|C9ZP80|C9ZP80_TRYB9  TMHMM2.0        inside     350   400
tr|C9ZP80|C9ZP80_TRYB9  TMHMM2.0        TMhelix    401   423
tr|C9ZP80|C9ZP80_TRYB9  TMHMM2.0        outside    424   427
tr|C9ZP80|C9ZP80_TRYB9  TMHMM2.0        TMhelix    428   450
tr|C9ZP80|C9ZP80_TRYB9  TMHMM2.0        inside     451   486
```

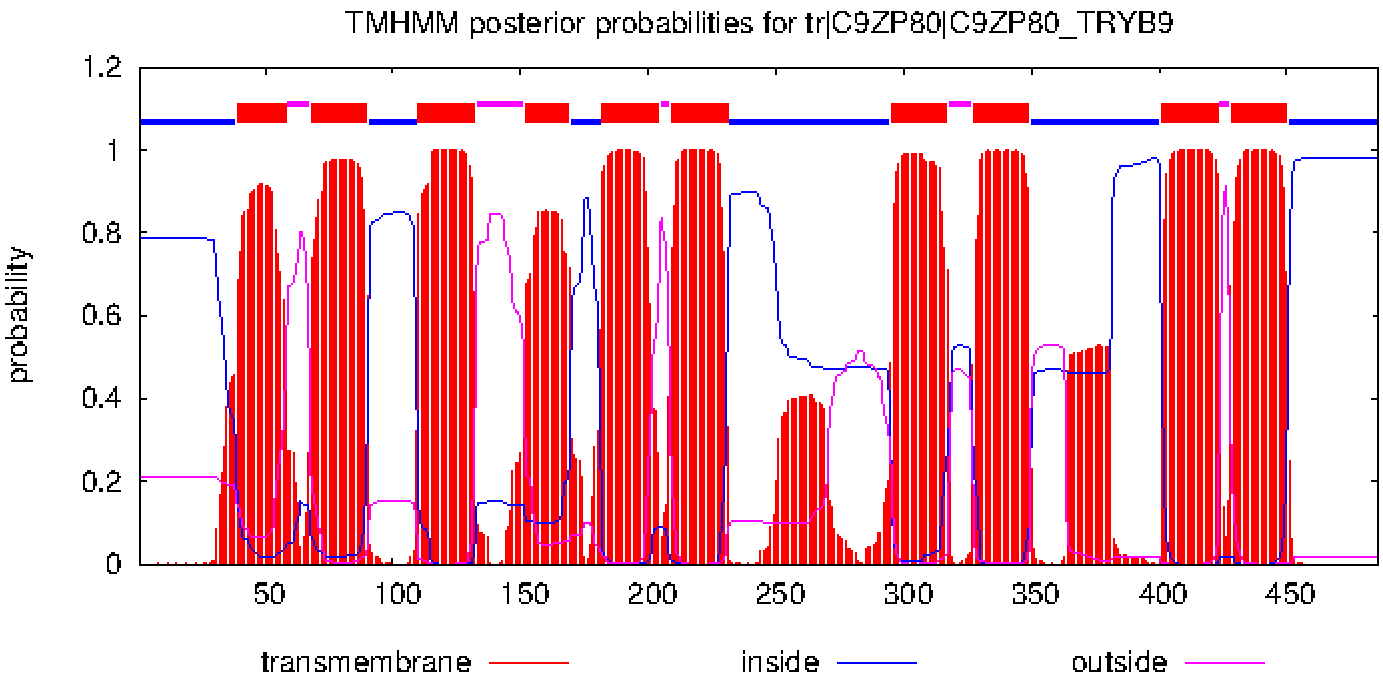

90 # plot in postscript, script for making the plot in gnuplot, data for plot

---

```
# tr|C9ZW15|C9ZW15_TRYB9 Length: 488
# tr|C9ZW15|C9ZW15_TRYB9 Number of predicted TMHs:  11
# tr|C9ZW15|C9ZW15_TRYB9 Exp number of AAs in TMHs: 222.47975
# tr|C9ZW15|C9ZW15_TRYB9 Exp number, first 60 AAs:  0.00224
# tr|C9ZW15|C9ZW15_TRYB9 Total prob of N-in:        0.90060
tr|C9ZW15|C9ZW15_TRYB9  TMHMM2.0        inside       1    88
tr|C9ZW15|C9ZW15_TRYB9  TMHMM2.0        TMhelix     89   108
tr|C9ZW15|C9ZW15_TRYB9  TMHMM2.0        outside    109   112
tr|C9ZW15|C9ZW15_TRYB9  TMHMM2.0        TMhelix    113   135
tr|C9ZW15|C9ZW15_TRYB9  TMHMM2.0        inside     136   160
tr|C9ZW15|C9ZW15_TRYB9  TMHMM2.0        TMhelix    161   183
tr|C9ZW15|C9ZW15_TRYB9  TMHMM2.0        outside    184   197
tr|C9ZW15|C9ZW15_TRYB9  TMHMM2.0        TMhelix    198   215
tr|C9ZW15|C9ZW15_TRYB9  TMHMM2.0        inside     216   221
tr|C9ZW15|C9ZW15_TRYB9  TMHMM2.0        TMhelix    222   244
tr|C9ZW15|C9ZW15_TRYB9  TMHMM2.0        outside    245   266
tr|C9ZW15|C9ZW15_TRYB9  TMHMM2.0        TMhelix    267   289
tr|C9ZW15|C9ZW15_TRYB9  TMHMM2.0        inside     290   300
tr|C9ZW15|C9ZW15_TRYB9  TMHMM2.0        TMhelix    301   323
tr|C9ZW15|C9ZW15_TRYB9  TMHMM2.0        outside    324   342
tr|C9ZW15|C9ZW15_TRYB9  TMHMM2.0        TMhelix    343   362
tr|C9ZW15|C9ZW15_TRYB9  TMHMM2.0        inside     363   381
tr|C9ZW15|C9ZW15_TRYB9  TMHMM2.0        TMhelix    382   401
tr|C9ZW15|C9ZW15_TRYB9  TMHMM2.0        outside    402   405
tr|C9ZW15|C9ZW15_TRYB9  TMHMM2.0        TMhelix    406   428
tr|C9ZW15|C9ZW15_TRYB9  TMHMM2.0        inside     429   463
tr|C9ZW15|C9ZW15_TRYB9  TMHMM2.0        TMhelix    464   486
tr|C9ZW15|C9ZW15_TRYB9  TMHMM2.0        outside    487   488
```

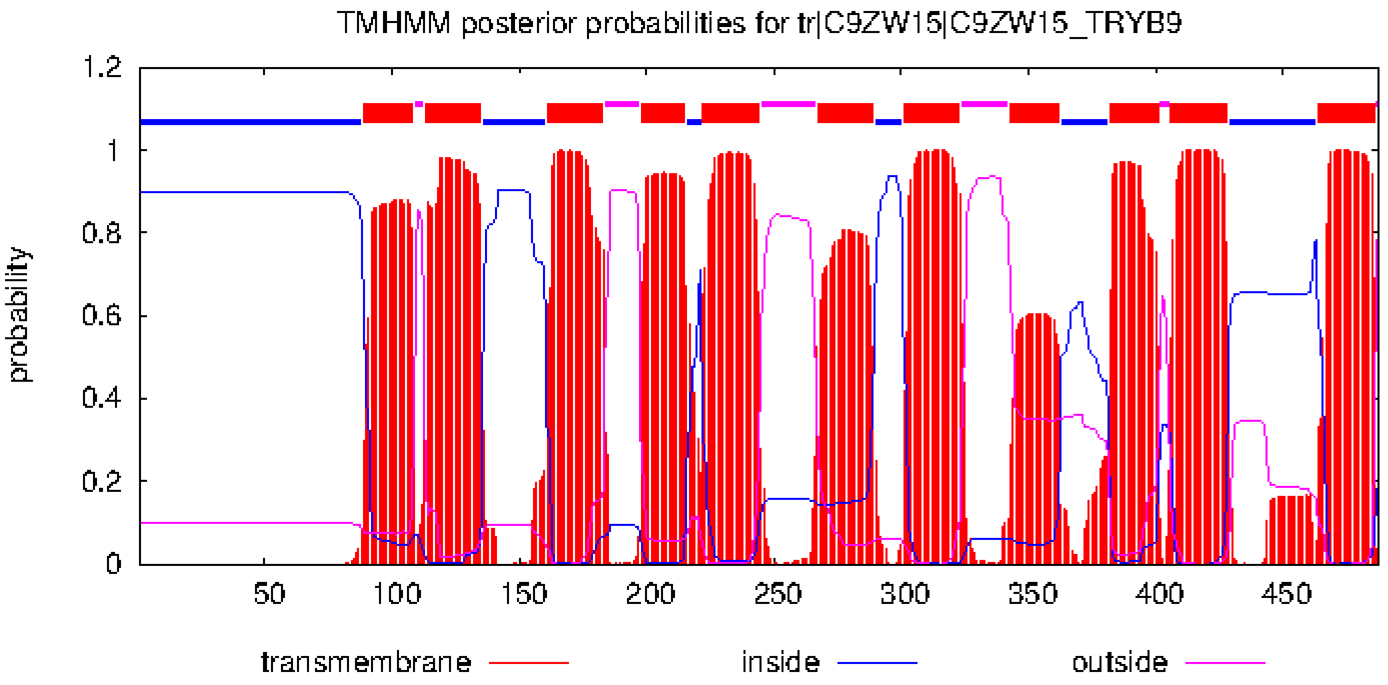

91 # plot in postscript, script for making the plot in gnuplot, data for plot

---

```
# tr|C9ZHU1|C9ZHU1_TRYB9 Length: 515
# tr|C9ZHU1|C9ZHU1_TRYB9 Number of predicted TMHs:  10
# tr|C9ZHU1|C9ZHU1_TRYB9 Exp number of AAs in TMHs: 227.65597
# tr|C9ZHU1|C9ZHU1_TRYB9 Exp number, first 60 AAs: 40.64362
# tr|C9ZHU1|C9ZHU1_TRYB9 Total prob of N-in:        0.04442
# tr|C9ZHU1|C9ZHU1_TRYB9 POSSIBLE N-term signal sequence
tr|C9ZHU1|C9ZHU1_TRYB9  TMHMM2.0        outside      1     4
tr|C9ZHU1|C9ZHU1_TRYB9  TMHMM2.0        TMhelix      5    27
tr|C9ZHU1|C9ZHU1_TRYB9  TMHMM2.0        inside      28    39
tr|C9ZHU1|C9ZHU1_TRYB9  TMHMM2.0        TMhelix     40    62
tr|C9ZHU1|C9ZHU1_TRYB9  TMHMM2.0        outside     63    83
tr|C9ZHU1|C9ZHU1_TRYB9  TMHMM2.0        TMhelix     84   103
tr|C9ZHU1|C9ZHU1_TRYB9  TMHMM2.0        inside     104   114
tr|C9ZHU1|C9ZHU1_TRYB9  TMHMM2.0        TMhelix    115   137
tr|C9ZHU1|C9ZHU1_TRYB9  TMHMM2.0        outside    138   151
tr|C9ZHU1|C9ZHU1_TRYB9  TMHMM2.0        TMhelix    152   174
tr|C9ZHU1|C9ZHU1_TRYB9  TMHMM2.0        inside     175   185
tr|C9ZHU1|C9ZHU1_TRYB9  TMHMM2.0        TMhelix    186   208
tr|C9ZHU1|C9ZHU1_TRYB9  TMHMM2.0        outside    209   222
tr|C9ZHU1|C9ZHU1_TRYB9  TMHMM2.0        TMhelix    223   245
tr|C9ZHU1|C9ZHU1_TRYB9  TMHMM2.0        inside     246   394
tr|C9ZHU1|C9ZHU1_TRYB9  TMHMM2.0        TMhelix    395   417
tr|C9ZHU1|C9ZHU1_TRYB9  TMHMM2.0        outside    418   436
tr|C9ZHU1|C9ZHU1_TRYB9  TMHMM2.0        TMhelix    437   466
tr|C9ZHU1|C9ZHU1_TRYB9  TMHMM2.0        inside     467   485
tr|C9ZHU1|C9ZHU1_TRYB9  TMHMM2.0        TMhelix    486   508
tr|C9ZHU1|C9ZHU1_TRYB9  TMHMM2.0        outside    509   515
```

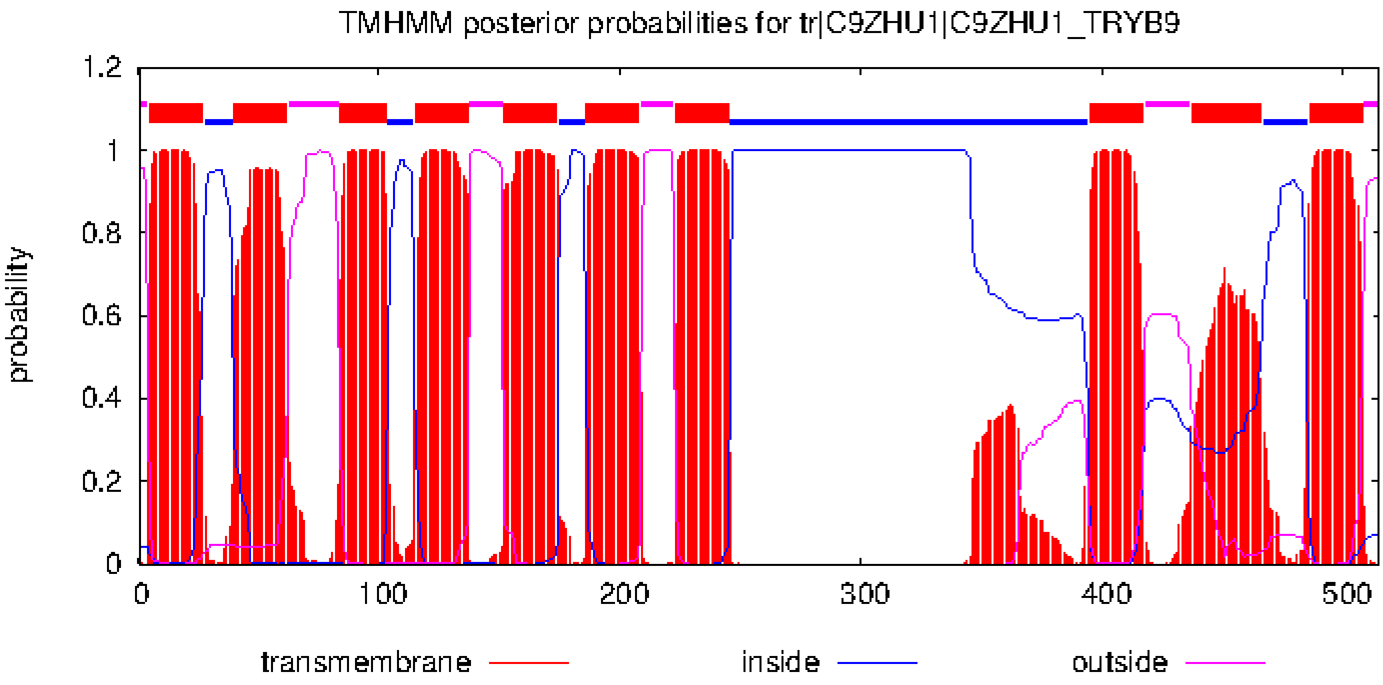

92 # plot in postscript, script for making the plot in gnuplot, data for plot

---

```
# tr|C9ZP79|C9ZP79_TRYB9 Length: 524
# tr|C9ZP79|C9ZP79_TRYB9 Number of predicted TMHs:  10
# tr|C9ZP79|C9ZP79_TRYB9 Exp number of AAs in TMHs: 236.88695
# tr|C9ZP79|C9ZP79_TRYB9 Exp number, first 60 AAs: 19.78493
# tr|C9ZP79|C9ZP79_TRYB9 Total prob of N-in:        0.64827
# tr|C9ZP79|C9ZP79_TRYB9 POSSIBLE N-term signal sequence
tr|C9ZP79|C9ZP79_TRYB9  TMHMM2.0        inside       1    38
tr|C9ZP79|C9ZP79_TRYB9  TMHMM2.0        TMhelix     39    58
tr|C9ZP79|C9ZP79_TRYB9  TMHMM2.0        outside     59    67
tr|C9ZP79|C9ZP79_TRYB9  TMHMM2.0        TMhelix     68    90
tr|C9ZP79|C9ZP79_TRYB9  TMHMM2.0        inside      91   109
tr|C9ZP79|C9ZP79_TRYB9  TMHMM2.0        TMhelix    110   132
tr|C9ZP79|C9ZP79_TRYB9  TMHMM2.0        outside    133   151
tr|C9ZP79|C9ZP79_TRYB9  TMHMM2.0        TMhelix    152   169
tr|C9ZP79|C9ZP79_TRYB9  TMHMM2.0        inside     170   181
tr|C9ZP79|C9ZP79_TRYB9  TMHMM2.0        TMhelix    182   204
tr|C9ZP79|C9ZP79_TRYB9  TMHMM2.0        outside    205   208
tr|C9ZP79|C9ZP79_TRYB9  TMHMM2.0        TMhelix    209   231
tr|C9ZP79|C9ZP79_TRYB9  TMHMM2.0        inside     232   294
tr|C9ZP79|C9ZP79_TRYB9  TMHMM2.0        TMhelix    295   317
tr|C9ZP79|C9ZP79_TRYB9  TMHMM2.0        outside    318   326
tr|C9ZP79|C9ZP79_TRYB9  TMHMM2.0        TMhelix    327   349
tr|C9ZP79|C9ZP79_TRYB9  TMHMM2.0        inside     350   400
tr|C9ZP79|C9ZP79_TRYB9  TMHMM2.0        TMhelix    401   423
tr|C9ZP79|C9ZP79_TRYB9  TMHMM2.0        outside    424   427
tr|C9ZP79|C9ZP79_TRYB9  TMHMM2.0        TMhelix    428   450
tr|C9ZP79|C9ZP79_TRYB9  TMHMM2.0        inside     451   524
```

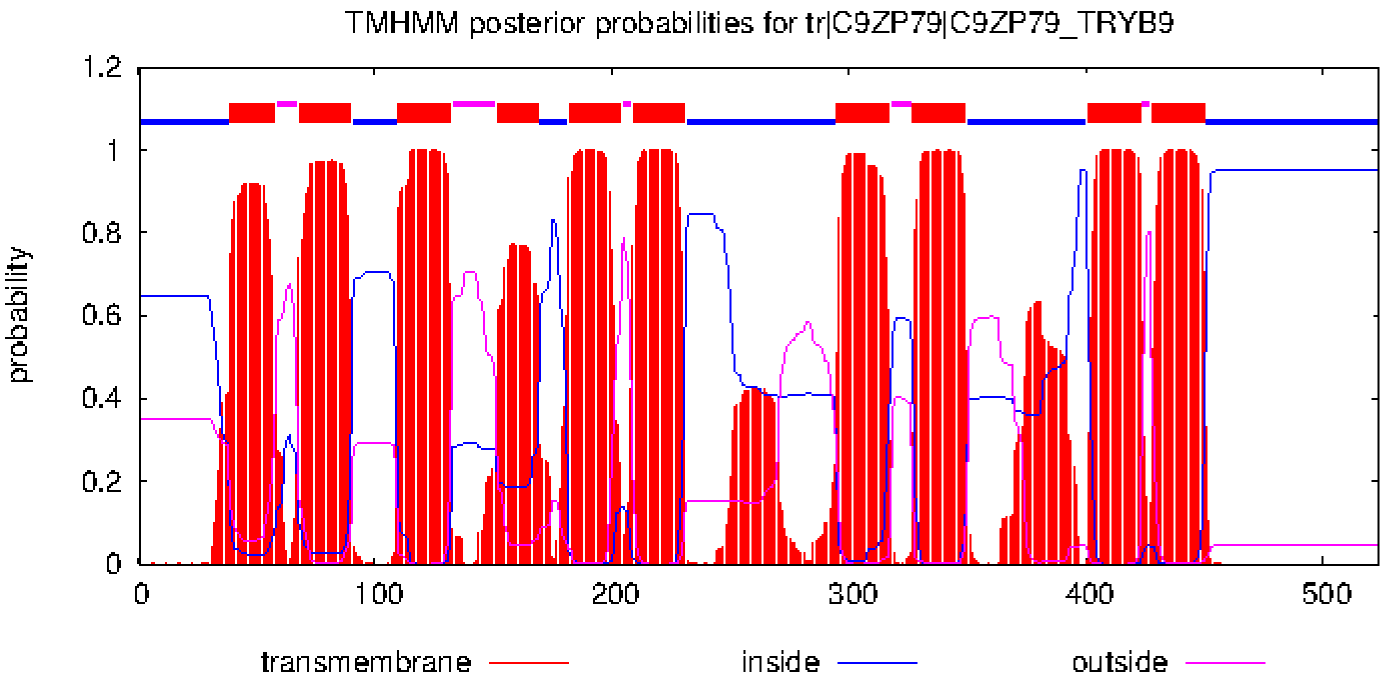

93 # plot in postscript, script for making the plot in gnuplot, data for plot

---

```
# tr|D0A7D2|D0A7D2_TRYB9 Length: 524
# tr|D0A7D2|D0A7D2_TRYB9 Number of predicted TMHs:  4
# tr|D0A7D2|D0A7D2_TRYB9 Exp number of AAs in TMHs: 91.54911
# tr|D0A7D2|D0A7D2_TRYB9 Exp number, first 60 AAs:  0
# tr|D0A7D2|D0A7D2_TRYB9 Total prob of N-in:        0.63436
tr|D0A7D2|D0A7D2_TRYB9  TMHMM2.0        inside       1   306
tr|D0A7D2|D0A7D2_TRYB9  TMHMM2.0        TMhelix    307   329
tr|D0A7D2|D0A7D2_TRYB9  TMHMM2.0        outside    330   343
tr|D0A7D2|D0A7D2_TRYB9  TMHMM2.0        TMhelix    344   366
tr|D0A7D2|D0A7D2_TRYB9  TMHMM2.0        inside     367   423
tr|D0A7D2|D0A7D2_TRYB9  TMHMM2.0        TMhelix    424   446
tr|D0A7D2|D0A7D2_TRYB9  TMHMM2.0        outside    447   460
tr|D0A7D2|D0A7D2_TRYB9  TMHMM2.0        TMhelix    461   483
tr|D0A7D2|D0A7D2_TRYB9  TMHMM2.0        inside     484   524
```

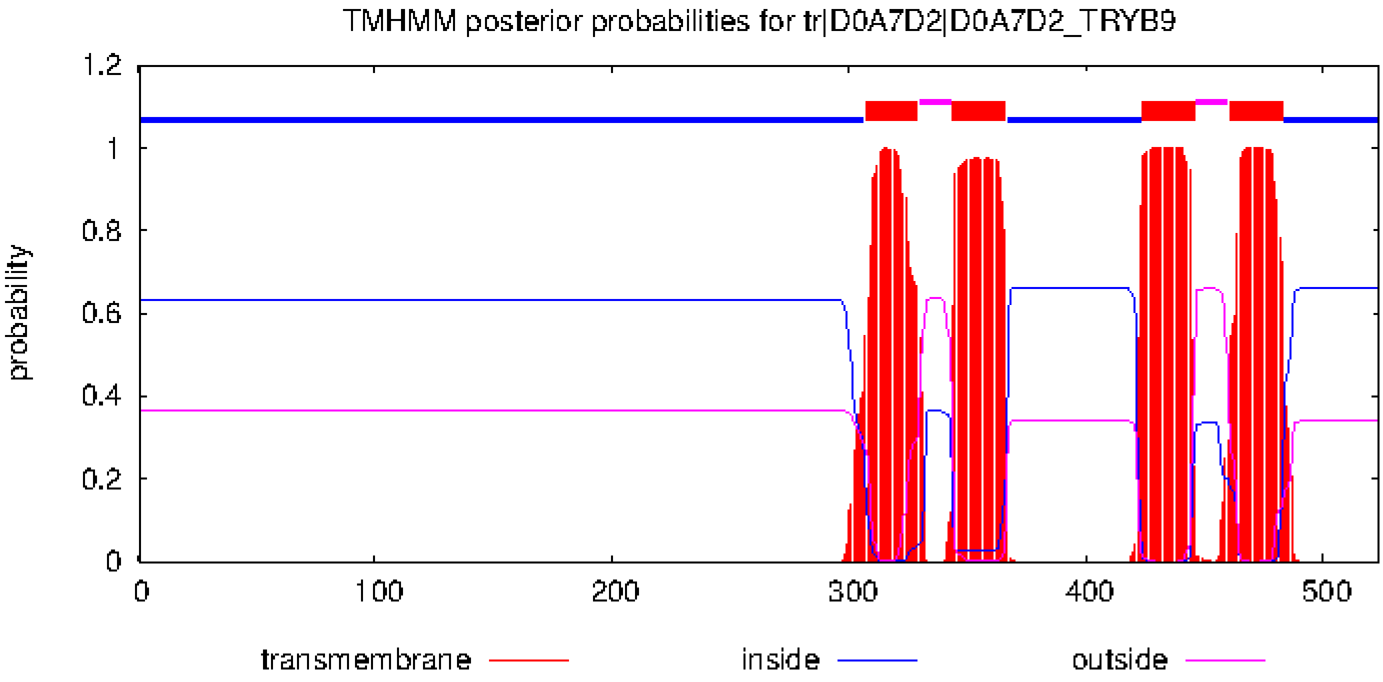

94 # plot in postscript, script for making the plot in gnuplot, data for plot

```
# tr|D0A7H1|D0A7H1_TRYB9 Length: 546
# tr|D0A7H1|D0A7H1_TRYB9 Number of predicted TMHs:  12
# tr|D0A7H1|D0A7H1_TRYB9 Exp number of AAs in TMHs: 268.36211
# tr|D0A7H1|D0A7H1_TRYB9 Exp number, first 60 AAs:  43.24242
# tr|D0A7H1|D0A7H1_TRYB9 Total prob of N-in:        0.69899
# tr|D0A7H1|D0A7H1_TRYB9 POSSIBLE N-term signal sequence
tr|D0A7H1|D0A7H1_TRYB9  TMHMM2.0        inside       1     6
tr|D0A7H1|D0A7H1_TRYB9  TMHMM2.0        TMhelix      7    26
tr|D0A7H1|D0A7H1_TRYB9  TMHMM2.0        outside     27    40
tr|D0A7H1|D0A7H1_TRYB9  TMHMM2.0        TMhelix     41    63
tr|D0A7H1|D0A7H1_TRYB9  TMHMM2.0        inside      64    90
tr|D0A7H1|D0A7H1_TRYB9  TMHMM2.0        TMhelix     91   113
tr|D0A7H1|D0A7H1_TRYB9  TMHMM2.0        outside    114   122
tr|D0A7H1|D0A7H1_TRYB9  TMHMM2.0        TMhelix    123   145
tr|D0A7H1|D0A7H1_TRYB9  TMHMM2.0        inside     146   182
tr|D0A7H1|D0A7H1_TRYB9  TMHMM2.0        TMhelix    183   205
tr|D0A7H1|D0A7H1_TRYB9  TMHMM2.0        outside    206   214
tr|D0A7H1|D0A7H1_TRYB9  TMHMM2.0        TMhelix    215   237
tr|D0A7H1|D0A7H1_TRYB9  TMHMM2.0        inside     238   291
tr|D0A7H1|D0A7H1_TRYB9  TMHMM2.0        TMhelix    292   314
tr|D0A7H1|D0A7H1_TRYB9  TMHMM2.0        outside    315   328
tr|D0A7H1|D0A7H1_TRYB9  TMHMM2.0        TMhelix    329   351
tr|D0A7H1|D0A7H1_TRYB9  TMHMM2.0        inside     352   363
tr|D0A7H1|D0A7H1_TRYB9  TMHMM2.0        TMhelix    364   386
tr|D0A7H1|D0A7H1_TRYB9  TMHMM2.0        outside    387   395
tr|D0A7H1|D0A7H1_TRYB9  TMHMM2.0        TMhelix    396   418
tr|D0A7H1|D0A7H1_TRYB9  TMHMM2.0        inside     419   424
tr|D0A7H1|D0A7H1_TRYB9  TMHMM2.0        TMhelix    425   447
tr|D0A7H1|D0A7H1_TRYB9  TMHMM2.0        outside    448   466
tr|D0A7H1|D0A7H1_TRYB9  TMHMM2.0        TMhelix    467   489
tr|D0A7H1|D0A7H1_TRYB9  TMHMM2.0        inside     490   546
```

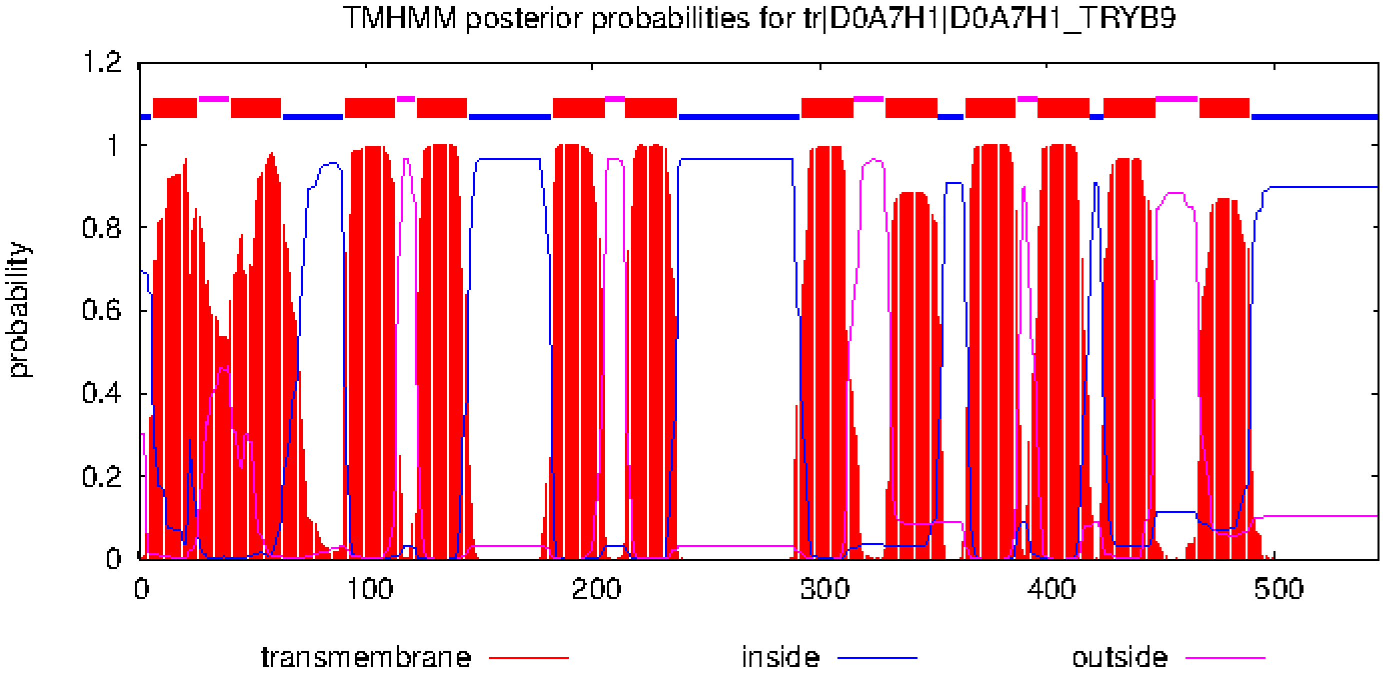

95 **# plot in postscript, script for making the plot in gnuplot, data for plot**

```
# tr|C9ZMM0|C9ZMM0_TRYB9 Length: 552
# tr|C9ZMM0|C9ZMM0_TRYB9 Number of predicted TMHs:  12
# tr|C9ZMM0|C9ZMM0_TRYB9 Exp number of AAs in TMHs: 253.68247
# tr|C9ZMM0|C9ZMM0_TRYB9 Exp number, first 60 AAs:  7.54351
# tr|C9ZMM0|C9ZMM0_TRYB9 Total prob of N-in:        0.72704
tr|C9ZMM0|C9ZMM0_TRYB9  TMHMM2.0        inside       1    53
tr|C9ZMM0|C9ZMM0_TRYB9  TMHMM2.0        TMhelix     54    76
tr|C9ZMM0|C9ZMM0_TRYB9  TMHMM2.0        outside     77   156
tr|C9ZMM0|C9ZMM0_TRYB9  TMHMM2.0        TMhelix    157   179
```

tr|C9ZMM0|C9ZMM0_TRYB9 TMHMM2.0 inside 180 185

```
tr|C9ZMM0|C9ZMM0_TRYB9  TMHMM2.0        TMhelix    186    205
tr|C9ZMM0|C9ZMM0_TRYB9  TMHMM2.0        outside    206    209
tr|C9ZMM0|C9ZMM0_TRYB9  TMHMM2.0        TMhelix    210    232
tr|C9ZMM0|C9ZMM0_TRYB9  TMHMM2.0        inside     233    244
tr|C9ZMM0|C9ZMM0_TRYB9  TMHMM2.0        TMhelix    245    267
tr|C9ZMM0|C9ZMM0_TRYB9  TMHMM2.0        outside    268    281
tr|C9ZMM0|C9ZMM0_TRYB9  TMHMM2.0        TMhelix    282    304
tr|C9ZMM0|C9ZMM0_TRYB9  TMHMM2.0        inside     305    353
tr|C9ZMM0|C9ZMM0_TRYB9  TMHMM2.0        TMhelix    354    373
tr|C9ZMM0|C9ZMM0_TRYB9  TMHMM2.0        outside    374    387
tr|C9ZMM0|C9ZMM0_TRYB9  TMHMM2.0        TMhelix    388    410
tr|C9ZMM0|C9ZMM0_TRYB9  TMHMM2.0        inside     411    416
tr|C9ZMM0|C9ZMM0_TRYB9  TMHMM2.0        TMhelix    417    439
tr|C9ZMM0|C9ZMM0_TRYB9  TMHMM2.0        outside    440    453
tr|C9ZMM0|C9ZMM0_TRYB9  TMHMM2.0        TMhelix    454    473
tr|C9ZMM0|C9ZMM0_TRYB9  TMHMM2.0        inside     474    485
tr|C9ZMM0|C9ZMM0_TRYB9  TMHMM2.0        TMhelix    486    508
tr|C9ZMM0|C9ZMM0_TRYB9  TMHMM2.0        outside    509    522
tr|C9ZMM0|C9ZMM0_TRYB9  TMHMM2.0        TMhelix    523    542
tr|C9ZMM0|C9ZMM0_TRYB9  TMHMM2.0        inside     543    552
```

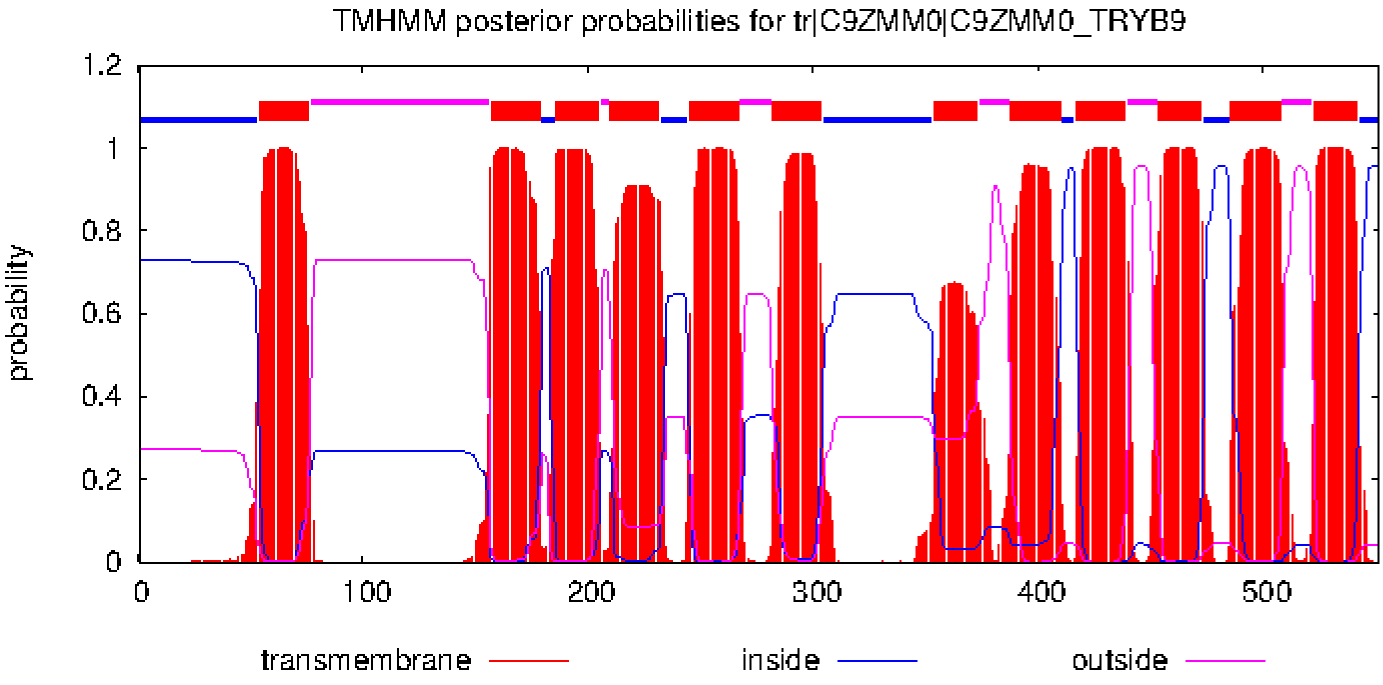

96 # **plot** in postscript, **script** for making the plot in gnuplot, **data** for plot

---

```
# tr|D0A2X2|D0A2X2_TRYB9 Length: 576
# tr|D0A2X2|D0A2X2_TRYB9 Number of predicted TMHs:  10
# tr|D0A2X2|D0A2X2_TRYB9 Exp number of AAs in TMHs: 221.92053
# tr|D0A2X2|D0A2X2_TRYB9 Exp number, first 60 AAs:  0.00019
# tr|D0A2X2|D0A2X2_TRYB9 Total prob of N-in:        0.26055
tr|D0A2X2|D0A2X2_TRYB9  TMHMM2.0        outside      1    190
tr|D0A2X2|D0A2X2_TRYB9  TMHMM2.0        TMhelix    191    213
tr|D0A2X2|D0A2X2_TRYB9  TMHMM2.0        inside     214    237
tr|D0A2X2|D0A2X2_TRYB9  TMHMM2.0        TMhelix    238    260
tr|D0A2X2|D0A2X2_TRYB9  TMHMM2.0        outside    261    274
tr|D0A2X2|D0A2X2_TRYB9  TMHMM2.0        TMhelix    275    293
tr|D0A2X2|D0A2X2_TRYB9  TMHMM2.0        inside     294    297
tr|D0A2X2|D0A2X2_TRYB9  TMHMM2.0        TMhelix    298    320
tr|D0A2X2|D0A2X2_TRYB9  TMHMM2.0        outside    321    339
tr|D0A2X2|D0A2X2_TRYB9  TMHMM2.0        TMhelix    340    362
tr|D0A2X2|D0A2X2_TRYB9  TMHMM2.0        inside     363    381
tr|D0A2X2|D0A2X2_TRYB9  TMHMM2.0        TMhelix    382    401
tr|D0A2X2|D0A2X2_TRYB9  TMHMM2.0        outside    402    420
tr|D0A2X2|D0A2X2_TRYB9  TMHMM2.0        TMhelix    421    443
```

tr|D0A2X2|D0A2X2_TRYB9	TMHMM2.0	inside	444	461
tr|D0A2X2|D0A2X2_TRYB9	TMHMM2.0	TMhelix	462	484

```
tr|D0A2X2|D0A2X2_TRYB9  TMHMM2.0        outside    485    487
tr|D0A2X2|D0A2X2_TRYB9  TMHMM2.0        TMhelix    488    510
tr|D0A2X2|D0A2X2_TRYB9  TMHMM2.0        inside     511    550
tr|D0A2X2|D0A2X2_TRYB9  TMHMM2.0        TMhelix    551    573
tr|D0A2X2|D0A2X2_TRYB9  TMHMM2.0        outside    574    576
```

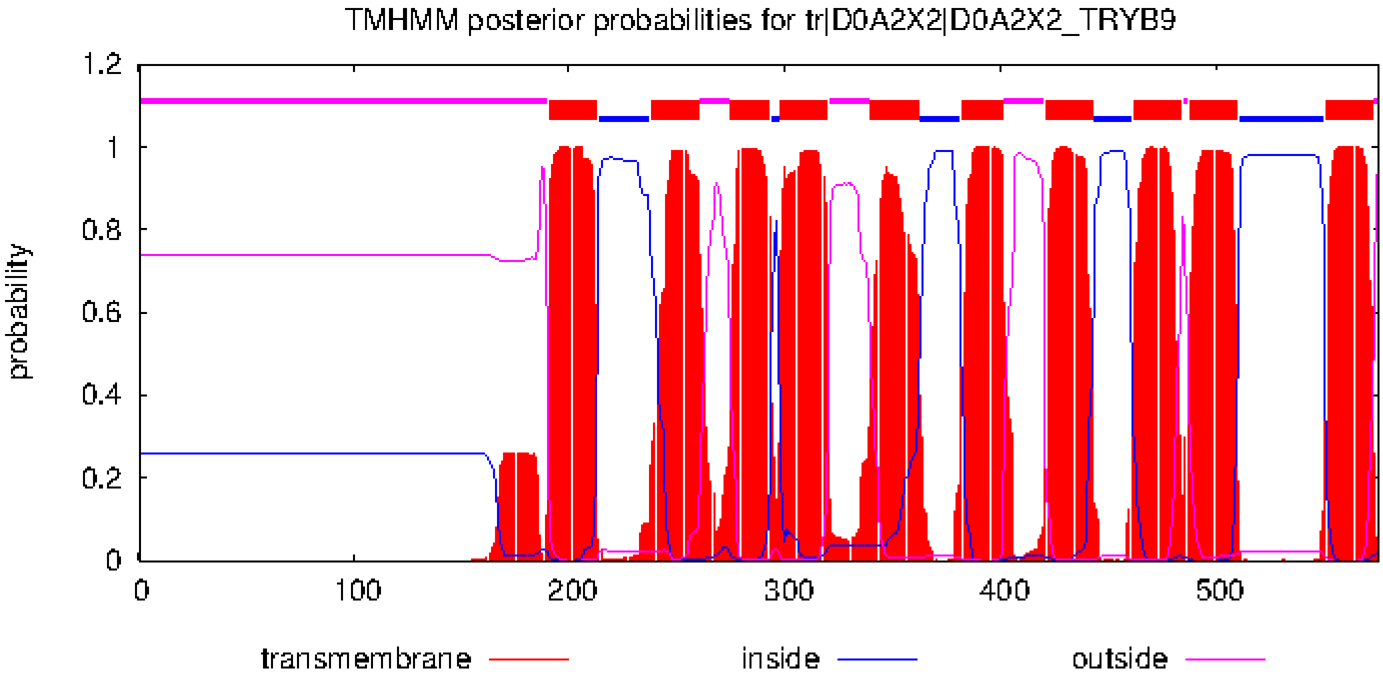

97 **# plot in postscript, script for making the plot in gnuplot, data for plot**

```
# tr|C9ZMW5|C9ZMW5_TRYB9 Length: 896
# tr|C9ZMW5|C9ZMW5_TRYB9 Number of predicted TMHs:  9
# tr|C9ZMW5|C9ZMW5_TRYB9 Exp number of AAs in TMHs: 199.58358
# tr|C9ZMW5|C9ZMW5_TRYB9 Exp number, first 60 AAs:  0.29385
# tr|C9ZMW5|C9ZMW5_TRYB9 Total prob of N-in:        0.99605
tr|C9ZMW5|C9ZMW5_TRYB9  TMHMM2.0        inside       1     60
tr|C9ZMW5|C9ZMW5_TRYB9  TMHMM2.0        TMhelix     61     83
tr|C9ZMW5|C9ZMW5_TRYB9  TMHMM2.0        outside     84    111
tr|C9ZMW5|C9ZMW5_TRYB9  TMHMM2.0        TMhelix    112    134
tr|C9ZMW5|C9ZMW5_TRYB9  TMHMM2.0        inside     135    175
tr|C9ZMW5|C9ZMW5_TRYB9  TMHMM2.0        TMhelix    176    198
tr|C9ZMW5|C9ZMW5_TRYB9  TMHMM2.0        outside    199    212
tr|C9ZMW5|C9ZMW5_TRYB9  TMHMM2.0        TMhelix    213    235
tr|C9ZMW5|C9ZMW5_TRYB9  TMHMM2.0        inside     236    265
tr|C9ZMW5|C9ZMW5_TRYB9  TMHMM2.0        TMhelix    266    285
tr|C9ZMW5|C9ZMW5_TRYB9  TMHMM2.0        outside    286    299
tr|C9ZMW5|C9ZMW5_TRYB9  TMHMM2.0        TMhelix    300    322
tr|C9ZMW5|C9ZMW5_TRYB9  TMHMM2.0        inside     323    334
tr|C9ZMW5|C9ZMW5_TRYB9  TMHMM2.0        TMhelix    335    357
tr|C9ZMW5|C9ZMW5_TRYB9  TMHMM2.0        outside    358    360
tr|C9ZMW5|C9ZMW5_TRYB9  TMHMM2.0        TMhelix    361    380
tr|C9ZMW5|C9ZMW5_TRYB9  TMHMM2.0        inside     381    495
tr|C9ZMW5|C9ZMW5_TRYB9  TMHMM2.0        TMhelix    496    518
tr|C9ZMW5|C9ZMW5_TRYB9  TMHMM2.0        outside    519    896
```

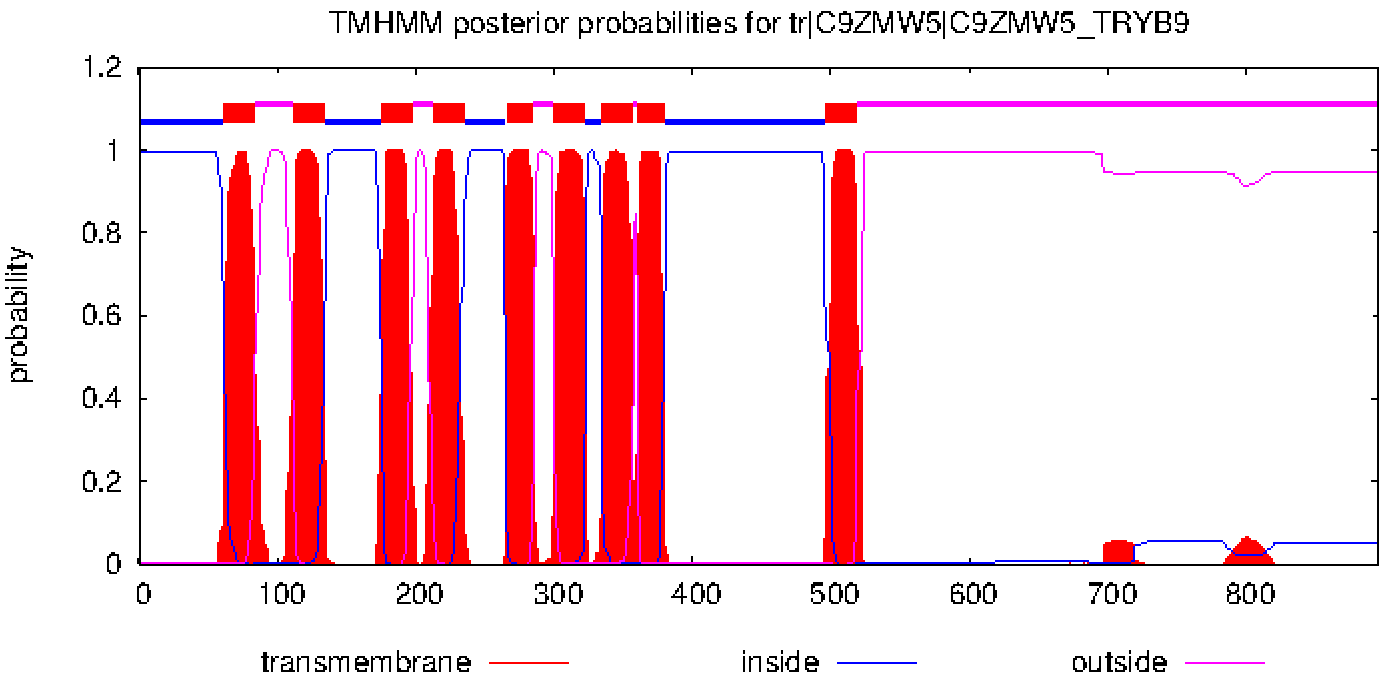

98 # [plot] in postscript, [script] for making the plot in gnuplot, [data] for plot

---

```
# tr|C9ZJZ0|C9ZJZ0_TRYB9 Length: 923
# tr|C9ZJZ0|C9ZJZ0_TRYB9 Number of predicted TMHs:  1
# tr|C9ZJZ0|C9ZJZ0_TRYB9 Exp number of AAs in TMHs: 21.7055799999999
# tr|C9ZJZ0|C9ZJZ0_TRYB9 Exp number, first 60 AAs:  19.88012
# tr|C9ZJZ0|C9ZJZ0_TRYB9 Total prob of N-in:        0.82823
# tr|C9ZJZ0|C9ZJZ0_TRYB9 POSSIBLE N-term signal sequence
tr|C9ZJZ0|C9ZJZ0_TRYB9  TMHMM2.0        inside       1     6
tr|C9ZJZ0|C9ZJZ0_TRYB9  TMHMM2.0        TMhelix      7    29
tr|C9ZJZ0|C9ZJZ0_TRYB9  TMHMM2.0        outside     30   923
```

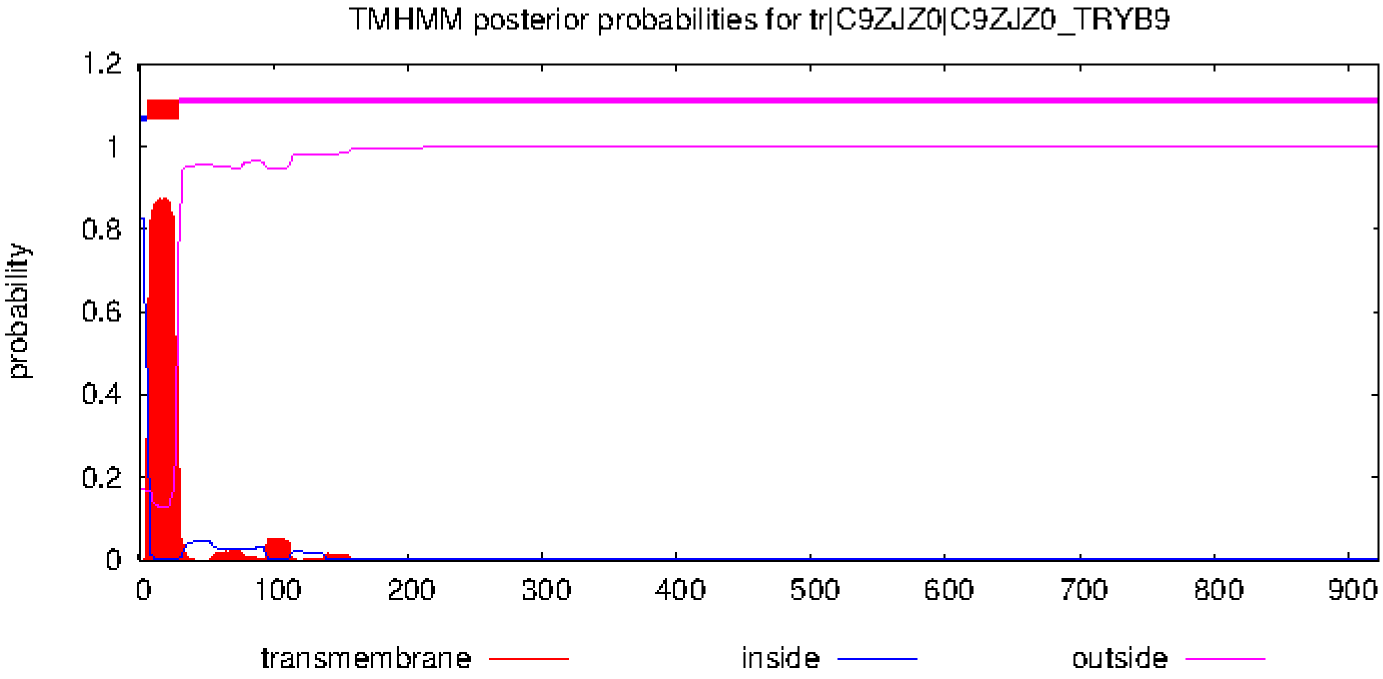

99 # [plot] in postscript, [script] for making the plot in gnuplot, [data] for plot

---

```
# tr|D0A5R6|D0A5R6_TRYB9 Length: 961
```

```
# tr|D0A5R6|D0A5R6_TRYB9 Number of predicted TMHs:  8
# tr|D0A5R6|D0A5R6_TRYB9 Exp number of AAs in TMHs: 178.02289
# tr|D0A5R6|D0A5R6_TRYB9 Exp number, first 60 AAs:  0.00062
```

```
# tr|D0A5R6|D0A5R6_TRYB9 Total prob of N-in:        0.99883
tr|D0A5R6|D0A5R6_TRYB9  TMHMM2.0        inside       1   279
tr|D0A5R6|D0A5R6_TRYB9  TMHMM2.0        TMhelix    280   301
tr|D0A5R6|D0A5R6_TRYB9  TMHMM2.0        outside    302   315
tr|D0A5R6|D0A5R6_TRYB9  TMHMM2.0        TMhelix    316   338
tr|D0A5R6|D0A5R6_TRYB9  TMHMM2.0        inside     339   349
tr|D0A5R6|D0A5R6_TRYB9  TMHMM2.0        TMhelix    350   372
tr|D0A5R6|D0A5R6_TRYB9  TMHMM2.0        outside    373   376
tr|D0A5R6|D0A5R6_TRYB9  TMHMM2.0        TMhelix    377   396
tr|D0A5R6|D0A5R6_TRYB9  TMHMM2.0        inside     397   533
tr|D0A5R6|D0A5R6_TRYB9  TMHMM2.0        TMhelix    534   556
tr|D0A5R6|D0A5R6_TRYB9  TMHMM2.0        outside    557   570
tr|D0A5R6|D0A5R6_TRYB9  TMHMM2.0        TMhelix    571   593
tr|D0A5R6|D0A5R6_TRYB9  TMHMM2.0        inside     594   896
tr|D0A5R6|D0A5R6_TRYB9  TMHMM2.0        TMhelix    897   919
tr|D0A5R6|D0A5R6_TRYB9  TMHMM2.0        outside    920   923
tr|D0A5R6|D0A5R6_TRYB9  TMHMM2.0        TMhelix    924   946
tr|D0A5R6|D0A5R6_TRYB9  TMHMM2.0        inside     947   961
```

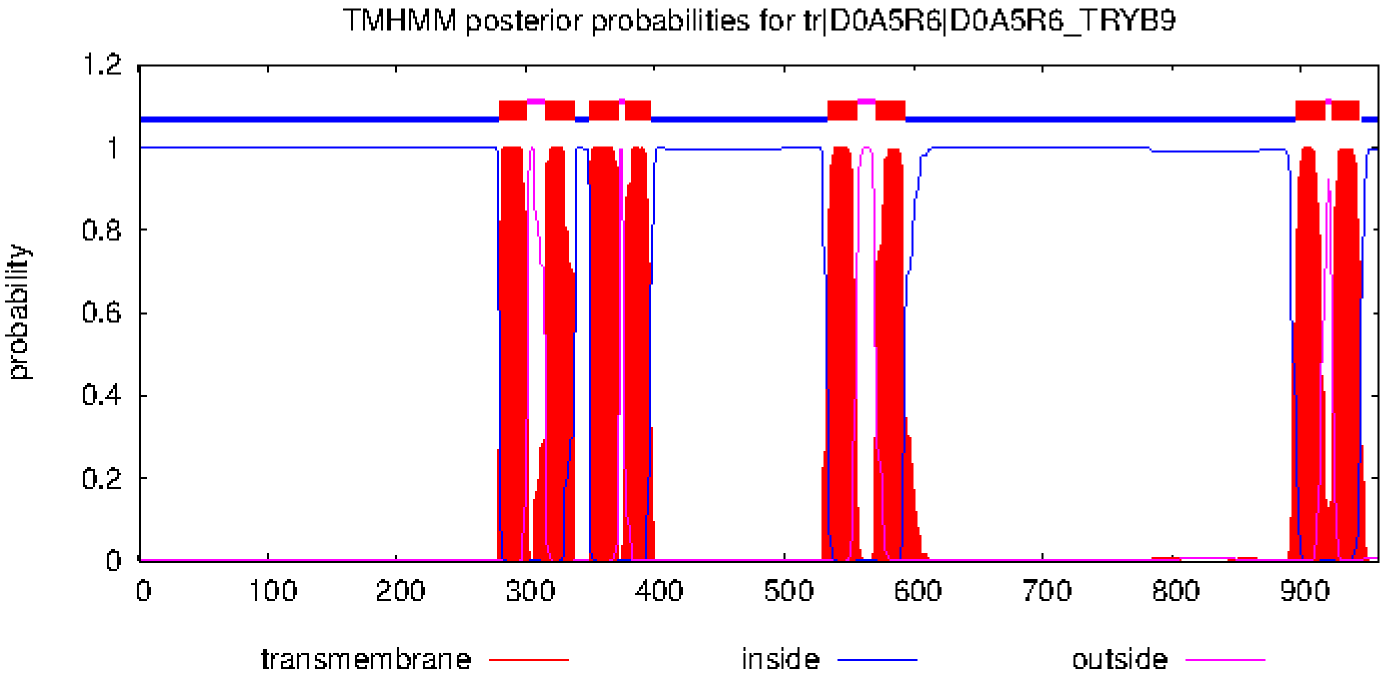

100 # plot in postscript, script for making the plot in gnuplot, data for plot

---

```
# tr|C9ZZN4|C9ZZN4_TRYB9 Length: 1041
# tr|C9ZZN4|C9ZZN4_TRYB9 Number of predicted TMHs:  8
# tr|C9ZZN4|C9ZZN4_TRYB9 Exp number of AAs in TMHs: 186.00409
# tr|C9ZZN4|C9ZZN4_TRYB9 Exp number, first 60 AAs:  0.00012
# tr|C9ZZN4|C9ZZN4_TRYB9 Total prob of N-in:        0.57030
tr|C9ZZN4|C9ZZN4_TRYB9  TMHMM2.0        inside       1    89
tr|C9ZZN4|C9ZZN4_TRYB9  TMHMM2.0        TMhelix     90   107
tr|C9ZZN4|C9ZZN4_TRYB9  TMHMM2.0        outside    108   110
tr|C9ZZN4|C9ZZN4_TRYB9  TMHMM2.0        TMhelix    111   130
tr|C9ZZN4|C9ZZN4_TRYB9  TMHMM2.0        inside     131   276
tr|C9ZZN4|C9ZZN4_TRYB9  TMHMM2.0        TMhelix    277   296
tr|C9ZZN4|C9ZZN4_TRYB9  TMHMM2.0        outside    297   310
tr|C9ZZN4|C9ZZN4_TRYB9  TMHMM2.0        TMhelix    311   333
tr|C9ZZN4|C9ZZN4_TRYB9  TMHMM2.0        inside     334   832
tr|C9ZZN4|C9ZZN4_TRYB9  TMHMM2.0        TMhelix    833   855
tr|C9ZZN4|C9ZZN4_TRYB9  TMHMM2.0        outside    856   874
tr|C9ZZN4|C9ZZN4_TRYB9  TMHMM2.0        TMhelix    875   897
tr|C9ZZN4|C9ZZN4_TRYB9  TMHMM2.0        inside     898   913
tr|C9ZZN4|C9ZZN4_TRYB9  TMHMM2.0        TMhelix    914   936
tr|C9ZZN4|C9ZZN4_TRYB9  TMHMM2.0        outside    937   945
tr|C9ZZN4|C9ZZN4_TRYB9  TMHMM2.0        TMhelix    946   968
tr|C9ZZN4|C9ZZN4_TRYB9  TMHMM2.0        inside     969  1041
```

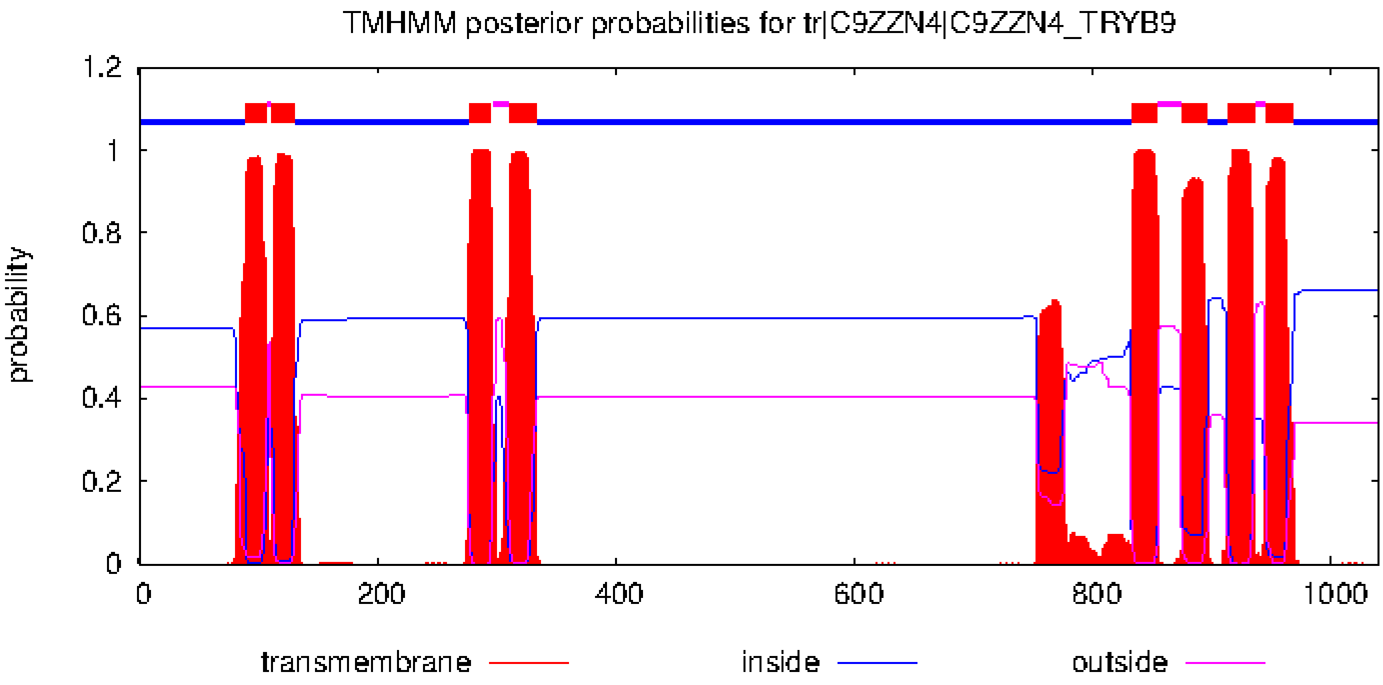

101 # plot in postscript, script for making the plot in gnuplot, data for plot

---

```
# tr|C9ZUN6|C9ZUN6_TRYB9 Length: 1077
# tr|C9ZUN6|C9ZUN6_TRYB9 Number of predicted TMHs:  10
# tr|C9ZUN6|C9ZUN6_TRYB9 Exp number of AAs in TMHs: 207.149380000001
# tr|C9ZUN6|C9ZUN6_TRYB9 Exp number, first 60 AAs:  0.00039
# tr|C9ZUN6|C9ZUN6_TRYB9 Total prob of N-in:        0.81030
tr|C9ZUN6|C9ZUN6_TRYB9  TMHMM2.0        inside       1    98
tr|C9ZUN6|C9ZUN6_TRYB9  TMHMM2.0        TMhelix     99   116
tr|C9ZUN6|C9ZUN6_TRYB9  TMHMM2.0        outside    117   130
tr|C9ZUN6|C9ZUN6_TRYB9  TMHMM2.0        TMhelix    131   150
tr|C9ZUN6|C9ZUN6_TRYB9  TMHMM2.0        inside     151   233
tr|C9ZUN6|C9ZUN6_TRYB9  TMHMM2.0        TMhelix    234   256
tr|C9ZUN6|C9ZUN6_TRYB9  TMHMM2.0        outside    257   285
tr|C9ZUN6|C9ZUN6_TRYB9  TMHMM2.0        TMhelix    286   308
tr|C9ZUN6|C9ZUN6_TRYB9  TMHMM2.0        inside     309   327
tr|C9ZUN6|C9ZUN6_TRYB9  TMHMM2.0        TMhelix    328   350
tr|C9ZUN6|C9ZUN6_TRYB9  TMHMM2.0        outside    351   779
tr|C9ZUN6|C9ZUN6_TRYB9  TMHMM2.0        TMhelix    780   802
tr|C9ZUN6|C9ZUN6_TRYB9  TMHMM2.0        inside     803   855
tr|C9ZUN6|C9ZUN6_TRYB9  TMHMM2.0        TMhelix    856   878
tr|C9ZUN6|C9ZUN6_TRYB9  TMHMM2.0        outside    879   887
tr|C9ZUN6|C9ZUN6_TRYB9  TMHMM2.0        TMhelix    888   907
tr|C9ZUN6|C9ZUN6_TRYB9  TMHMM2.0        inside     908   919
tr|C9ZUN6|C9ZUN6_TRYB9  TMHMM2.0        TMhelix    920   942
tr|C9ZUN6|C9ZUN6_TRYB9  TMHMM2.0        outside    943   956
tr|C9ZUN6|C9ZUN6_TRYB9  TMHMM2.0        TMhelix    957   979
tr|C9ZUN6|C9ZUN6_TRYB9  TMHMM2.0        inside     980  1077
```

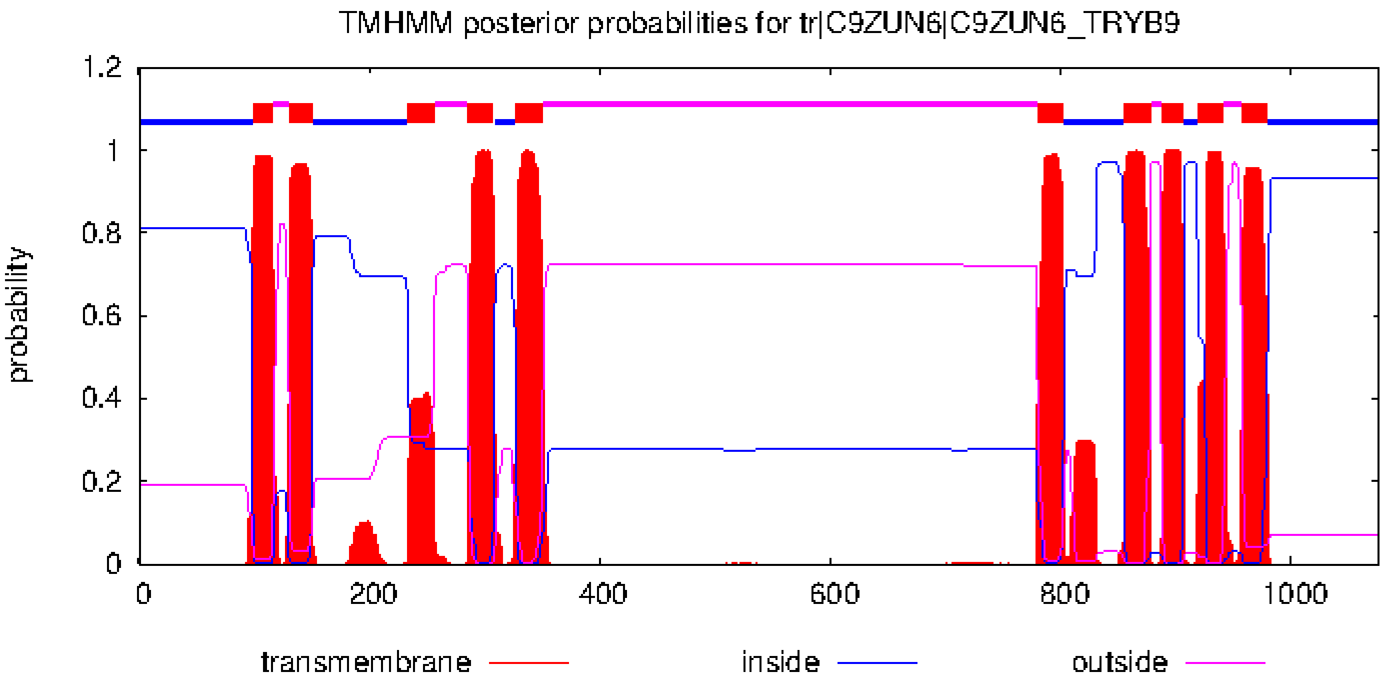

102 # **plot in postscript, script for making the plot in gnuplot, data for plot**

```
# tr|D0A1S1|D0A1S1_TRYB9 Length: 1242
# tr|D0A1S1|D0A1S1_TRYB9 Number of predicted TMHs:  1
# tr|D0A1S1|D0A1S1_TRYB9 Exp number of AAs in TMHs: 41.3050900000001
# tr|D0A1S1|D0A1S1_TRYB9 Exp number, first 60 AAs:  4.819
# tr|D0A1S1|D0A1S1_TRYB9 Total prob of N-in:        0.19375
tr|D0A1S1|D0A1S1_TRYB9  TMHMM2.0        outside      1   859
tr|D0A1S1|D0A1S1_TRYB9  TMHMM2.0        TMhelix    860   882
tr|D0A1S1|D0A1S1_TRYB9  TMHMM2.0        inside     883  1242
```

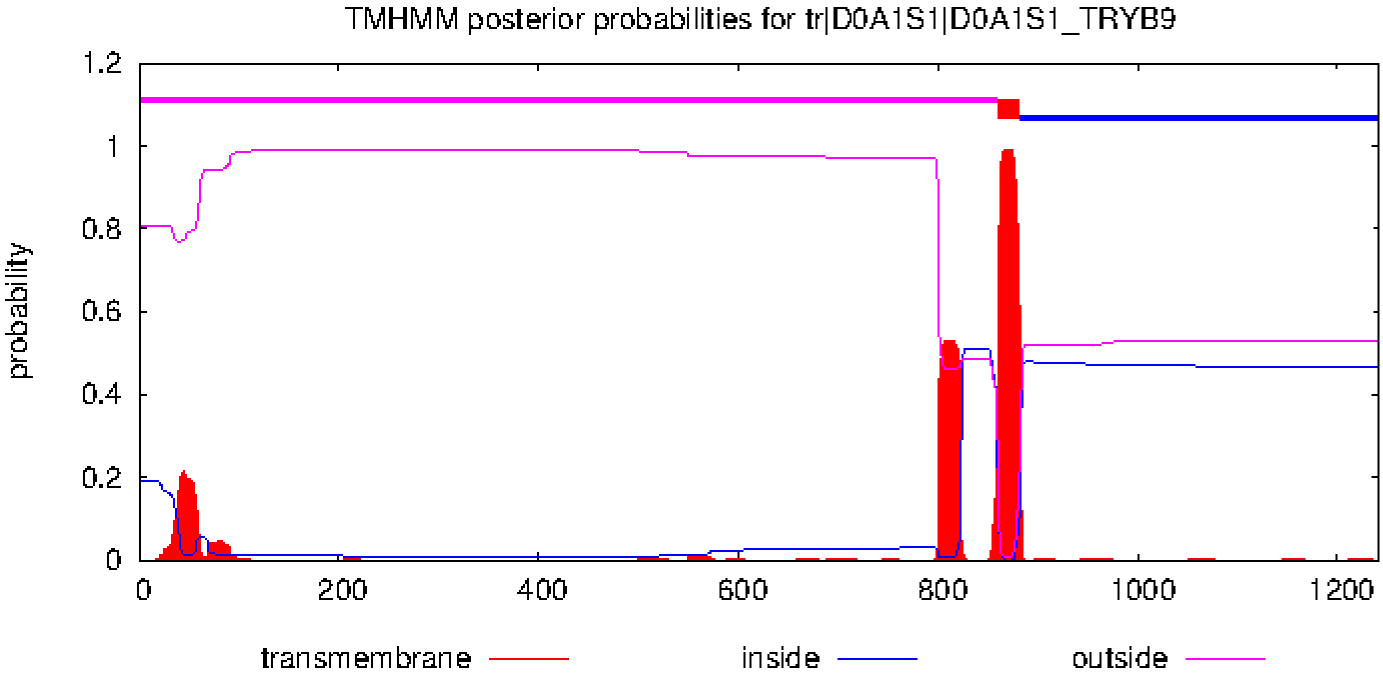

103 # **plot in postscript, script for making the plot in gnuplot, data for plot**

```
# tr|C9ZPZ6|C9ZPZ6_TRYB9 Length: 1243
# tr|C9ZPZ6|C9ZPZ6_TRYB9 Number of predicted TMHs:  1
```

```
# tr|C9ZPZ6|C9ZPZ6_TRYB9 Exp number of AAs in TMHs: 38.1414100000001
# tr|C9ZPZ6|C9ZPZ6_TRYB9 Exp number, first 60 AAs:  9.79887
# tr|C9ZPZ6|C9ZPZ6_TRYB9 Total prob of N-in:        0.46568
```

```
tr|C9ZPZ6|C9ZPZ6_TRYB9  TMHMM2.0        outside      1   852
tr|C9ZPZ6|C9ZPZ6_TRYB9  TMHMM2.0        TMhelix    853   875
tr|C9ZPZ6|C9ZPZ6_TRYB9  TMHMM2.0        inside     876  1243
```

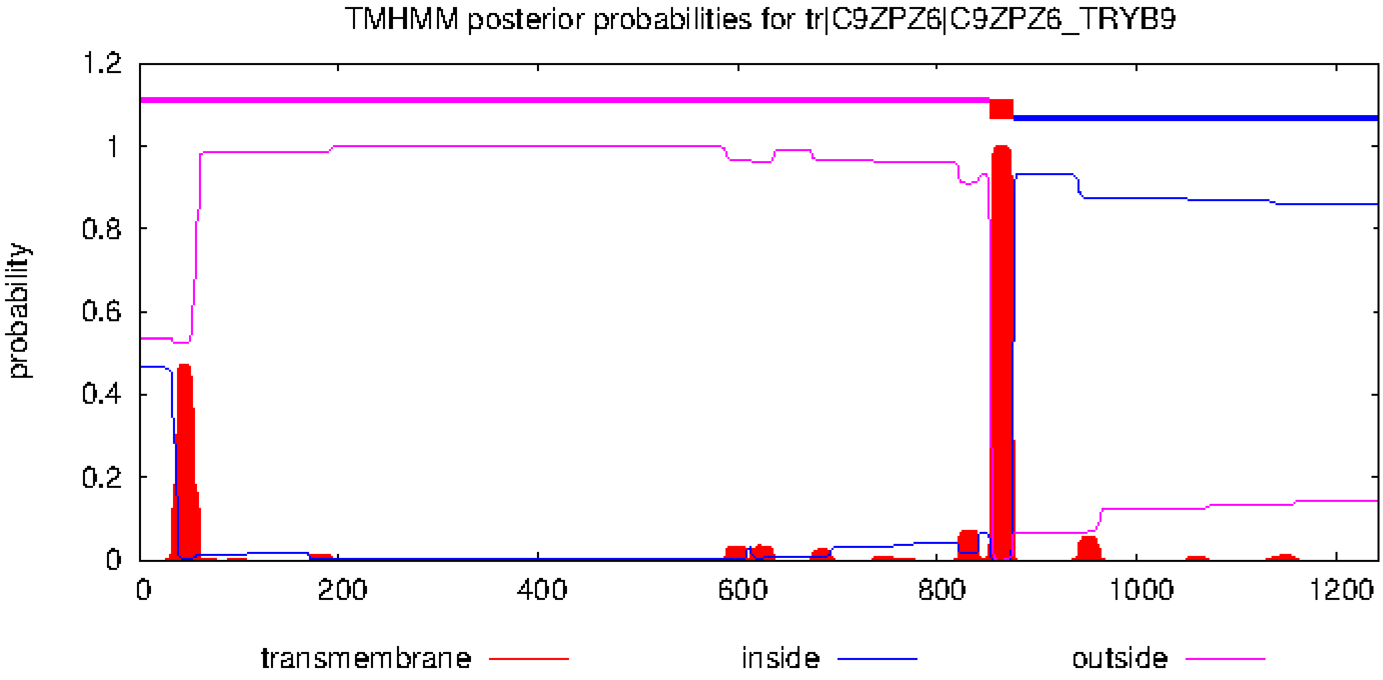

104 # plot in postscript, script for making the plot in gnuplot, data for plot

---

```
# tr|D0A7A0|D0A7A0_TRYB9 Length: 1244
# tr|D0A7A0|D0A7A0_TRYB9 Number of predicted TMHs:  1
# tr|D0A7A0|D0A7A0_TRYB9 Exp number of AAs in TMHs: 33.3612800000002
# tr|D0A7A0|D0A7A0_TRYB9 Exp number, first 60 AAs:  8.35275
# tr|D0A7A0|D0A7A0_TRYB9 Total prob of N-in:        0.41355
tr|D0A7A0|D0A7A0_TRYB9  TMHMM2.0        outside      1   851
tr|D0A7A0|D0A7A0_TRYB9  TMHMM2.0        TMhelix    852   874
tr|D0A7A0|D0A7A0_TRYB9  TMHMM2.0        inside     875  1244
```

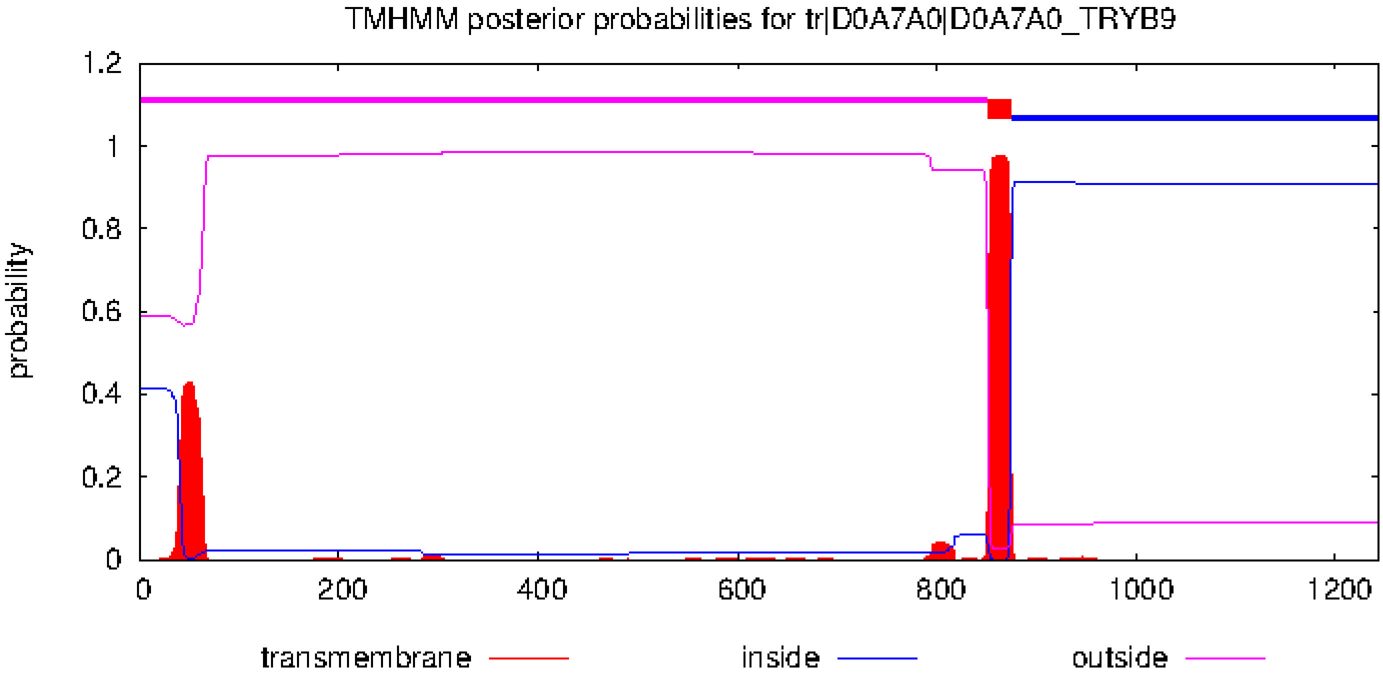

105 # plot in postscript, script for making the plot in gnuplot, data for plot

---

```
# tr|C9ZL70|C9ZL70_TRYB9 Length: 1282
# tr|C9ZL70|C9ZL70_TRYB9 Number of predicted TMHs:  6
# tr|C9ZL70|C9ZL70_TRYB9 Exp number of AAs in TMHs: 146.73297
# tr|C9ZL70|C9ZL70_TRYB9 Exp number, first 60 AAs:  0.778269999999999
# tr|C9ZL70|C9ZL70_TRYB9 Total prob of N-in:        0.14575
tr|C9ZL70|C9ZL70_TRYB9  TMHMM2.0        outside      1   370
tr|C9ZL70|C9ZL70_TRYB9  TMHMM2.0        TMhelix    371   393
tr|C9ZL70|C9ZL70_TRYB9  TMHMM2.0        inside     394   404
tr|C9ZL70|C9ZL70_TRYB9  TMHMM2.0        TMhelix    405   427
tr|C9ZL70|C9ZL70_TRYB9  TMHMM2.0        outside    428   441
tr|C9ZL70|C9ZL70_TRYB9  TMHMM2.0        TMhelix    442   464
tr|C9ZL70|C9ZL70_TRYB9  TMHMM2.0        inside     465   554
tr|C9ZL70|C9ZL70_TRYB9  TMHMM2.0        TMhelix    555   577
tr|C9ZL70|C9ZL70_TRYB9  TMHMM2.0        outside    578   596
tr|C9ZL70|C9ZL70_TRYB9  TMHMM2.0        TMhelix    597   619
tr|C9ZL70|C9ZL70_TRYB9  TMHMM2.0        inside     620   639
tr|C9ZL70|C9ZL70_TRYB9  TMHMM2.0        TMhelix    640   659
tr|C9ZL70|C9ZL70_TRYB9  TMHMM2.0        outside    660  1282
```

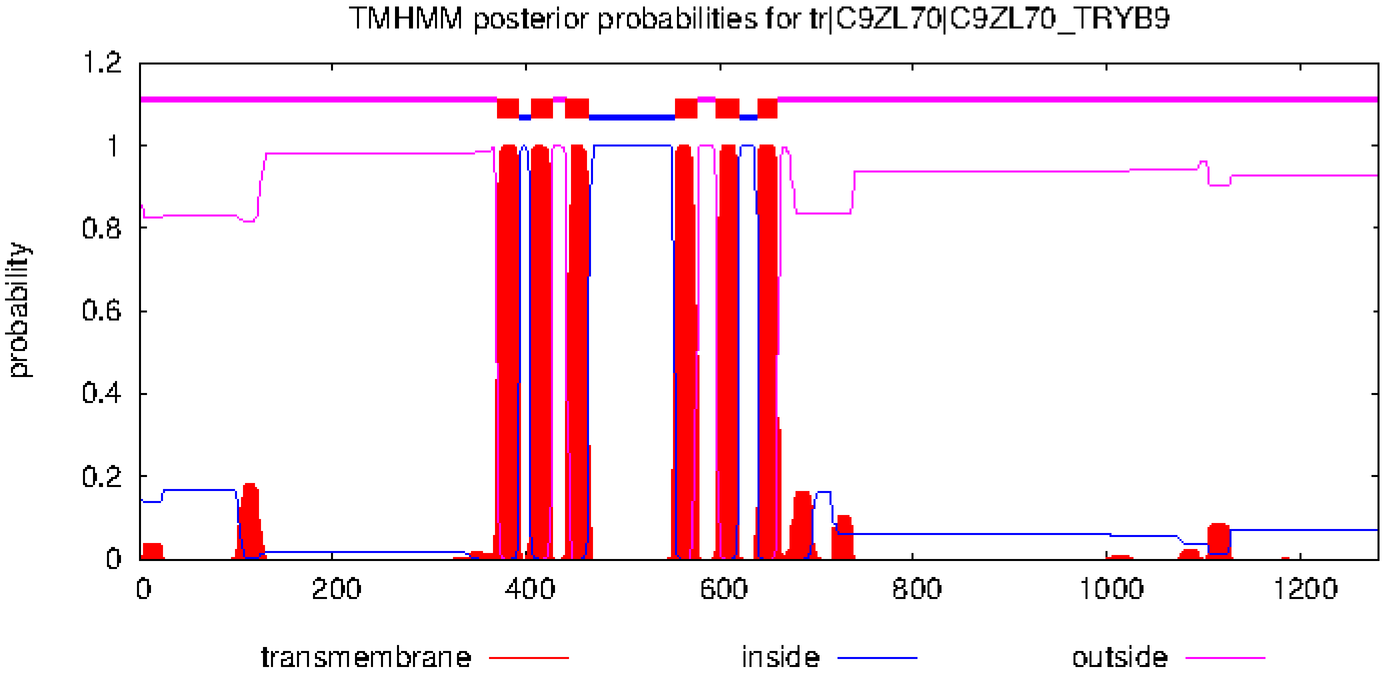

106 # plot in postscript, script for making the plot in gnuplot, data for plot

---

```
# tr|D0A623|D0A623_TRYB9 Length: 1524
# tr|D0A623|D0A623_TRYB9 Number of predicted TMHs:  23
# tr|D0A623|D0A623_TRYB9 Exp number of AAs in TMHs: 551.01872
# tr|D0A623|D0A623_TRYB9 Exp number, first 60 AAs:  30.76388
# tr|D0A623|D0A623_TRYB9 Total prob of N-in:        0.99882
# tr|D0A623|D0A623_TRYB9 POSSIBLE N-term signal sequence
tr|D0A623|D0A623_TRYB9  TMHMM2.0        inside       1    20
tr|D0A623|D0A623_TRYB9  TMHMM2.0        TMhelix     21    43
tr|D0A623|D0A623_TRYB9  TMHMM2.0        outside     44    52
tr|D0A623|D0A623_TRYB9  TMHMM2.0        TMhelix     53    75
tr|D0A623|D0A623_TRYB9  TMHMM2.0        inside      76    93
tr|D0A623|D0A623_TRYB9  TMHMM2.0        TMhelix     94   116
tr|D0A623|D0A623_TRYB9  TMHMM2.0        outside    117   125
tr|D0A623|D0A623_TRYB9  TMHMM2.0        TMhelix    126   148
tr|D0A623|D0A623_TRYB9  TMHMM2.0        inside     149   186
tr|D0A623|D0A623_TRYB9  TMHMM2.0        TMhelix    187   209
tr|D0A623|D0A623_TRYB9  TMHMM2.0        outside    210   213
tr|D0A623|D0A623_TRYB9  TMHMM2.0        TMhelix    214   236
tr|D0A623|D0A623_TRYB9  TMHMM2.0        inside     237   256
tr|D0A623|D0A623_TRYB9  TMHMM2.0        TMhelix    257   276
tr|D0A623|D0A623_TRYB9  TMHMM2.0        outside    277   285
tr|D0A623|D0A623_TRYB9  TMHMM2.0        TMhelix    286   305
```

tr|D0A623|D0A623_TRYB9 TMHMM2.0 inside 306 324

```
tr|D0A623|D0A623_TRYB9  TMHMM2.0        TMhelix    325   347
tr|D0A623|D0A623_TRYB9  TMHMM2.0        outside    348   361
tr|D0A623|D0A623_TRYB9  TMHMM2.0        TMhelix    362   384
tr|D0A623|D0A623_TRYB9  TMHMM2.0        inside     385   404
tr|D0A623|D0A623_TRYB9  TMHMM2.0        TMhelix    405   424
tr|D0A623|D0A623_TRYB9  TMHMM2.0        outside    425   433
tr|D0A623|D0A623_TRYB9  TMHMM2.0        TMhelix    434   456
tr|D0A623|D0A623_TRYB9  TMHMM2.0        inside     457   727
tr|D0A623|D0A623_TRYB9  TMHMM2.0        TMhelix    728   747
tr|D0A623|D0A623_TRYB9  TMHMM2.0        outside    748   756
tr|D0A623|D0A623_TRYB9  TMHMM2.0        TMhelix    757   779
tr|D0A623|D0A623_TRYB9  TMHMM2.0        inside     780   799
tr|D0A623|D0A623_TRYB9  TMHMM2.0        TMhelix    800   822
tr|D0A623|D0A623_TRYB9  TMHMM2.0        outside    823   826
tr|D0A623|D0A623_TRYB9  TMHMM2.0        TMhelix    827   849
tr|D0A623|D0A623_TRYB9  TMHMM2.0        inside     850   869
tr|D0A623|D0A623_TRYB9  TMHMM2.0        TMhelix    870   889
tr|D0A623|D0A623_TRYB9  TMHMM2.0        outside    890   898
tr|D0A623|D0A623_TRYB9  TMHMM2.0        TMhelix    899   921
tr|D0A623|D0A623_TRYB9  TMHMM2.0        inside     922  1031
tr|D0A623|D0A623_TRYB9  TMHMM2.0        TMhelix   1032  1054
tr|D0A623|D0A623_TRYB9  TMHMM2.0        outside   1055  1068
tr|D0A623|D0A623_TRYB9  TMHMM2.0        TMhelix   1069  1091
tr|D0A623|D0A623_TRYB9  TMHMM2.0        inside    1092  1103
tr|D0A623|D0A623_TRYB9  TMHMM2.0        TMhelix   1104  1126
tr|D0A623|D0A623_TRYB9  TMHMM2.0        outside   1127  1153
tr|D0A623|D0A623_TRYB9  TMHMM2.0        TMhelix   1154  1176
tr|D0A623|D0A623_TRYB9  TMHMM2.0        inside    1177  1182
tr|D0A623|D0A623_TRYB9  TMHMM2.0        TMhelix   1183  1205
tr|D0A623|D0A623_TRYB9  TMHMM2.0        outside   1206  1524
```

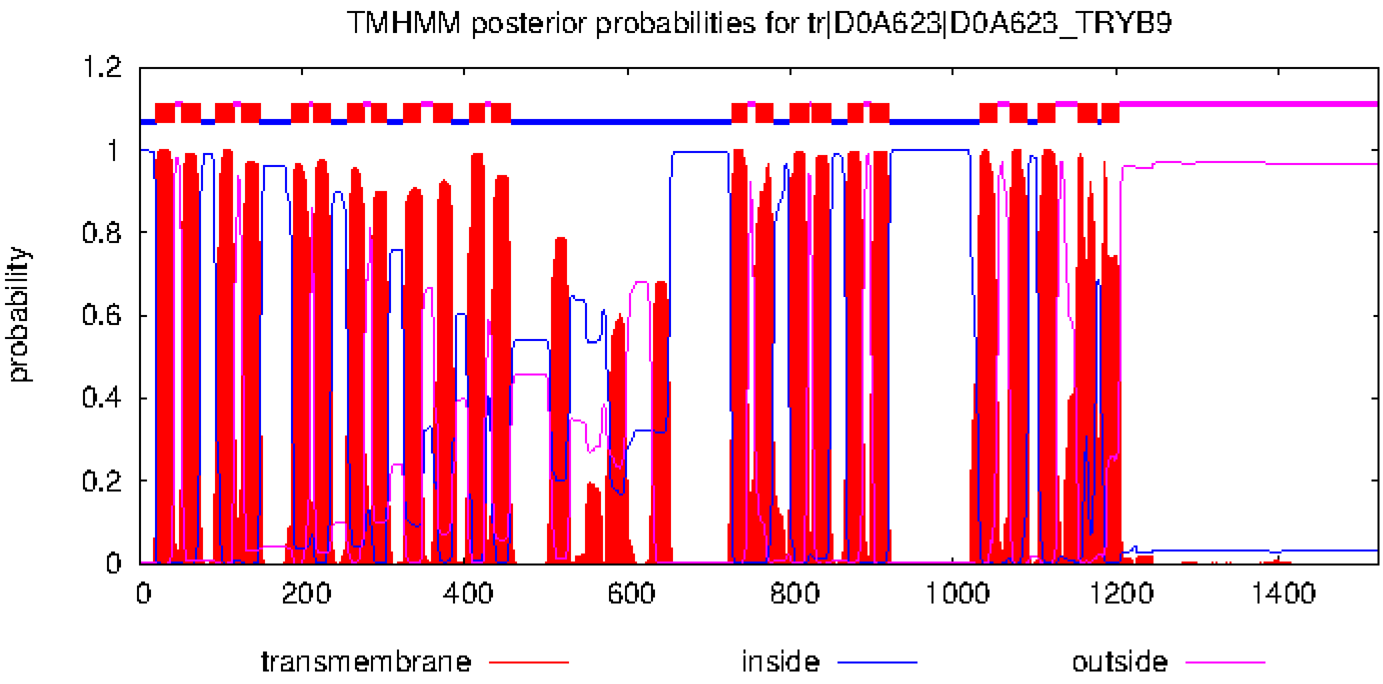

107 **# plot in postscript, script for making the plot in gnuplot, data for plot**

```
# tr|D0A1K6|D0A1K6_TRYB9 Length: 1534
# tr|D0A1K6|D0A1K6_TRYB9 Number of predicted TMHs:  1
# tr|D0A1K6|D0A1K6_TRYB9 Exp number of AAs in TMHs: 54.5257400000001
# tr|D0A1K6|D0A1K6_TRYB9 Exp number, first 60 AAs:  0.00018
# tr|D0A1K6|D0A1K6_TRYB9 Total prob of N-in:        0.64395
tr|D0A1K6|D0A1K6_TRYB9  TMHMM2.0        inside       1   217
tr|D0A1K6|D0A1K6_TRYB9  TMHMM2.0        TMhelix    218   240
tr|D0A1K6|D0A1K6_TRYB9  TMHMM2.0        outside    241  1534
```

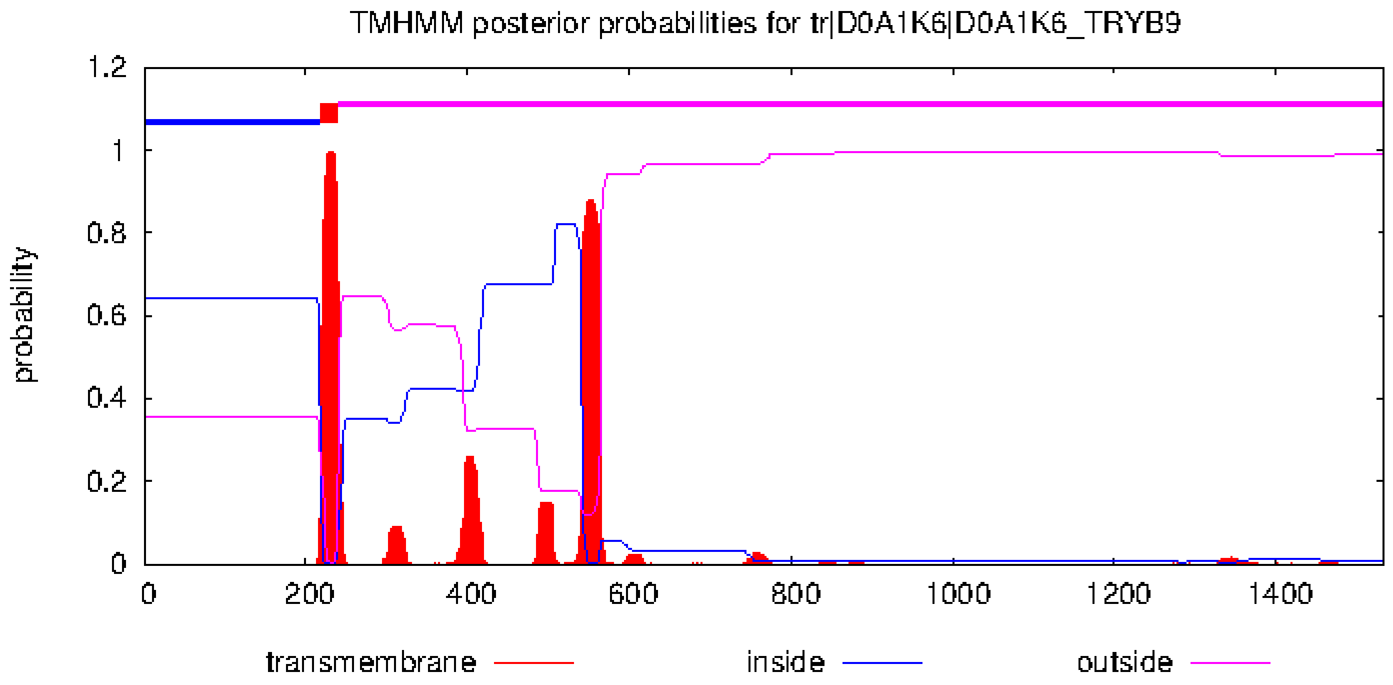

108 # plot in postscript, script for making the plot in gnuplot, data for plot

```
# tr|C9ZW76|C9ZW76_TRYB9 Length: 166
# tr|C9ZW76|C9ZW76_TRYB9 Number of predicted TMHs:  3
# tr|C9ZW76|C9ZW76_TRYB9 Exp number of AAs in TMHs: 72.59239
# tr|C9ZW76|C9ZW76_TRYB9 Exp number, first 60 AAs:  41.90803
# tr|C9ZW76|C9ZW76_TRYB9 Total prob of N-in:        0.35432
# tr|C9ZW76|C9ZW76_TRYB9 POSSIBLE N-term signal sequence
tr|C9ZW76|C9ZW76_TRYB9  TMHMM2.0        outside      1     9
tr|C9ZW76|C9ZW76_TRYB9  TMHMM2.0        TMhelix     10    32
tr|C9ZW76|C9ZW76_TRYB9  TMHMM2.0        inside      33    38
tr|C9ZW76|C9ZW76_TRYB9  TMHMM2.0        TMhelix     39    61
tr|C9ZW76|C9ZW76_TRYB9  TMHMM2.0        outside     62    75
tr|C9ZW76|C9ZW76_TRYB9  TMHMM2.0        TMhelix     76    98
tr|C9ZW76|C9ZW76_TRYB9  TMHMM2.0        inside      99   166
```

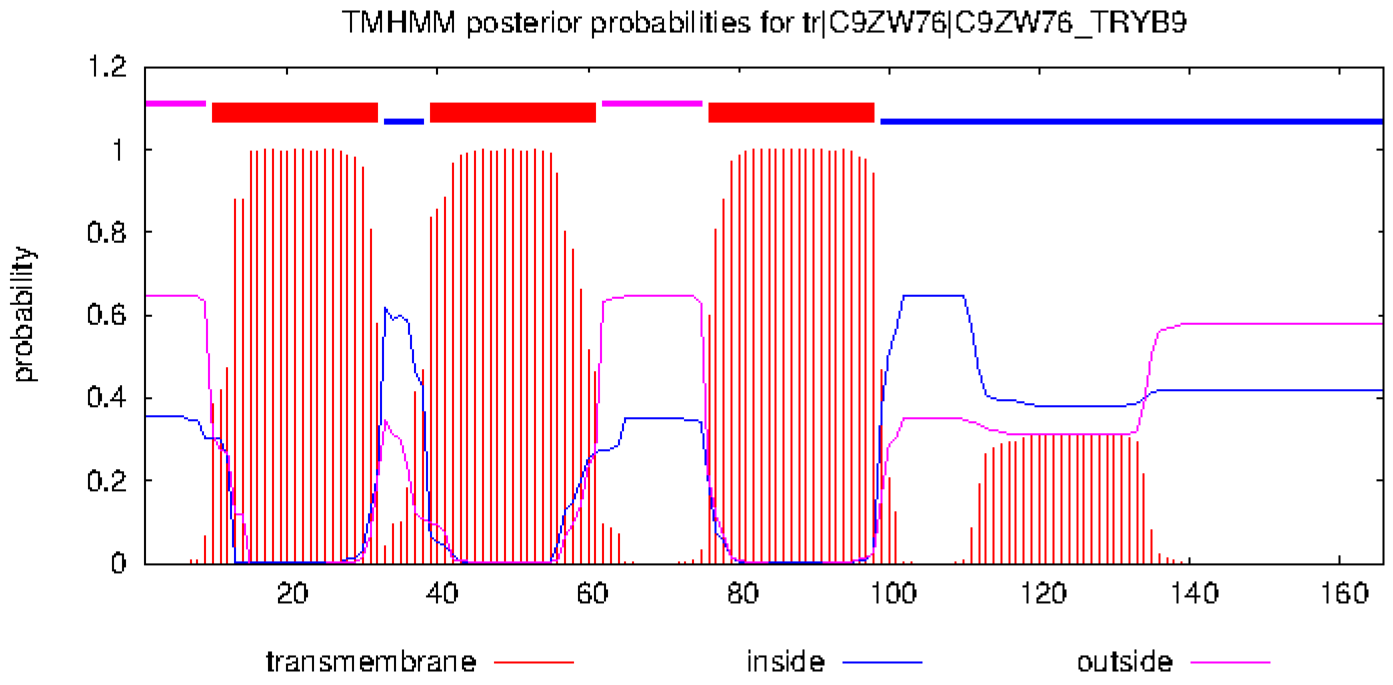

109 # plot in postscript, script for making the plot in gnuplot, data for plot

```
# tr|C9ZXX0|C9ZXX0_TRYB9 Length: 183
# tr|C9ZXX0|C9ZXX0_TRYB9 Number of predicted TMHs:  4
# tr|C9ZXX0|C9ZXX0_TRYB9 Exp number of AAs in TMHs: 79.65172
# tr|C9ZXX0|C9ZXX0_TRYB9 Exp number, first 60 AAs:  25.06969
# tr|C9ZXX0|C9ZXX0_TRYB9 Total prob of N-in:        0.42023
# tr|C9ZXX0|C9ZXX0_TRYB9 POSSIBLE N-term signal sequence
tr|C9ZXX0|C9ZXX0_TRYB9  TMHMM2.0        inside       1     4
tr|C9ZXX0|C9ZXX0_TRYB9  TMHMM2.0        TMhelix      5    27
tr|C9ZXX0|C9ZXX0_TRYB9  TMHMM2.0        outside     28    46
tr|C9ZXX0|C9ZXX0_TRYB9  TMHMM2.0        TMhelix     47    69
tr|C9ZXX0|C9ZXX0_TRYB9  TMHMM2.0        inside      70   108
tr|C9ZXX0|C9ZXX0_TRYB9  TMHMM2.0        TMhelix    109   131
tr|C9ZXX0|C9ZXX0_TRYB9  TMHMM2.0        outside    132   145
tr|C9ZXX0|C9ZXX0_TRYB9  TMHMM2.0        TMhelix    146   168
tr|C9ZXX0|C9ZXX0_TRYB9  TMHMM2.0        inside     169   183
```

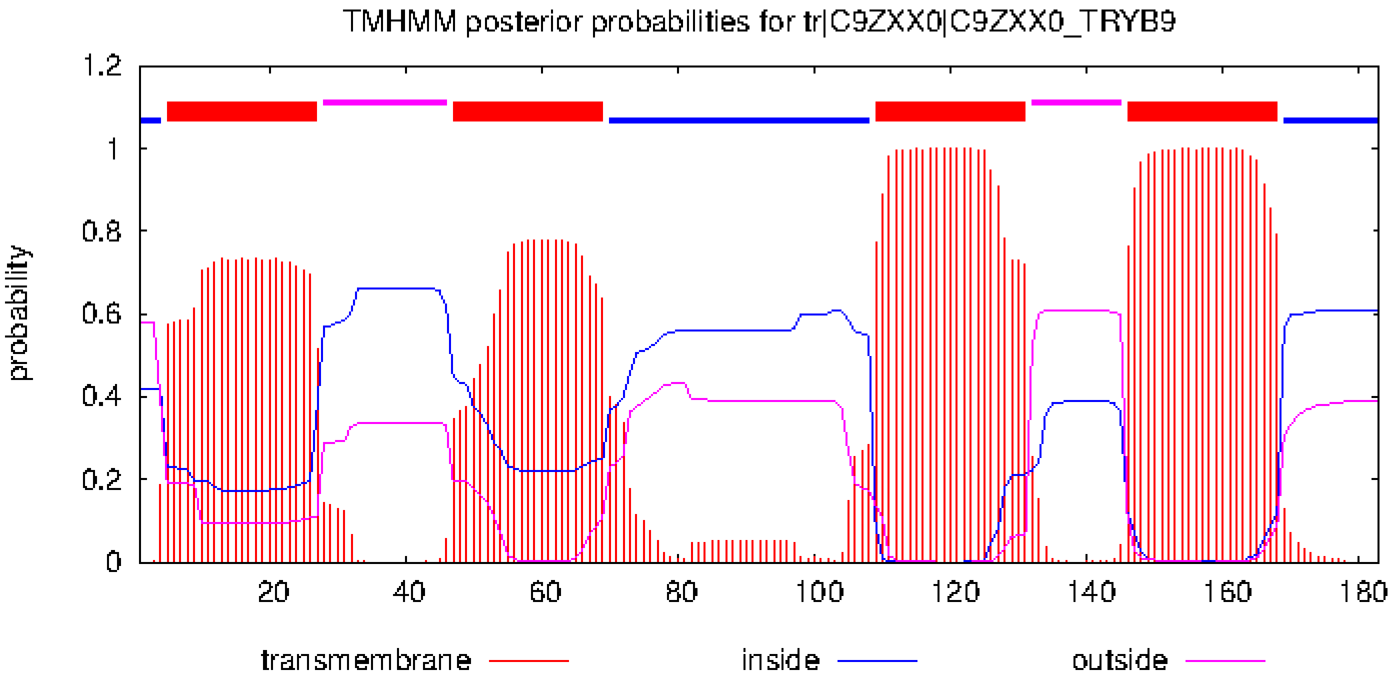

110 # plot in postscript, script for making the plot in gnuplot, data for plot

---

```
# tr|C9ZZB1|C9ZZB1_TRYB9 Length: 202
# tr|C9ZZB1|C9ZZB1_TRYB9 Number of predicted TMHs:  2
# tr|C9ZZB1|C9ZZB1_TRYB9 Exp number of AAs in TMHs: 54.45954
# tr|C9ZZB1|C9ZZB1_TRYB9 Exp number, first 60 AAs:  1.40851
# tr|C9ZZB1|C9ZZB1_TRYB9 Total prob of N-in:        0.13528
tr|C9ZZB1|C9ZZB1_TRYB9  TMHMM2.0        outside      1    61
tr|C9ZZB1|C9ZZB1_TRYB9  TMHMM2.0        TMhelix     62    84
tr|C9ZZB1|C9ZZB1_TRYB9  TMHMM2.0        inside      85   131
tr|C9ZZB1|C9ZZB1_TRYB9  TMHMM2.0        TMhelix    132   154
tr|C9ZZB1|C9ZZB1_TRYB9  TMHMM2.0        outside    155   202
```

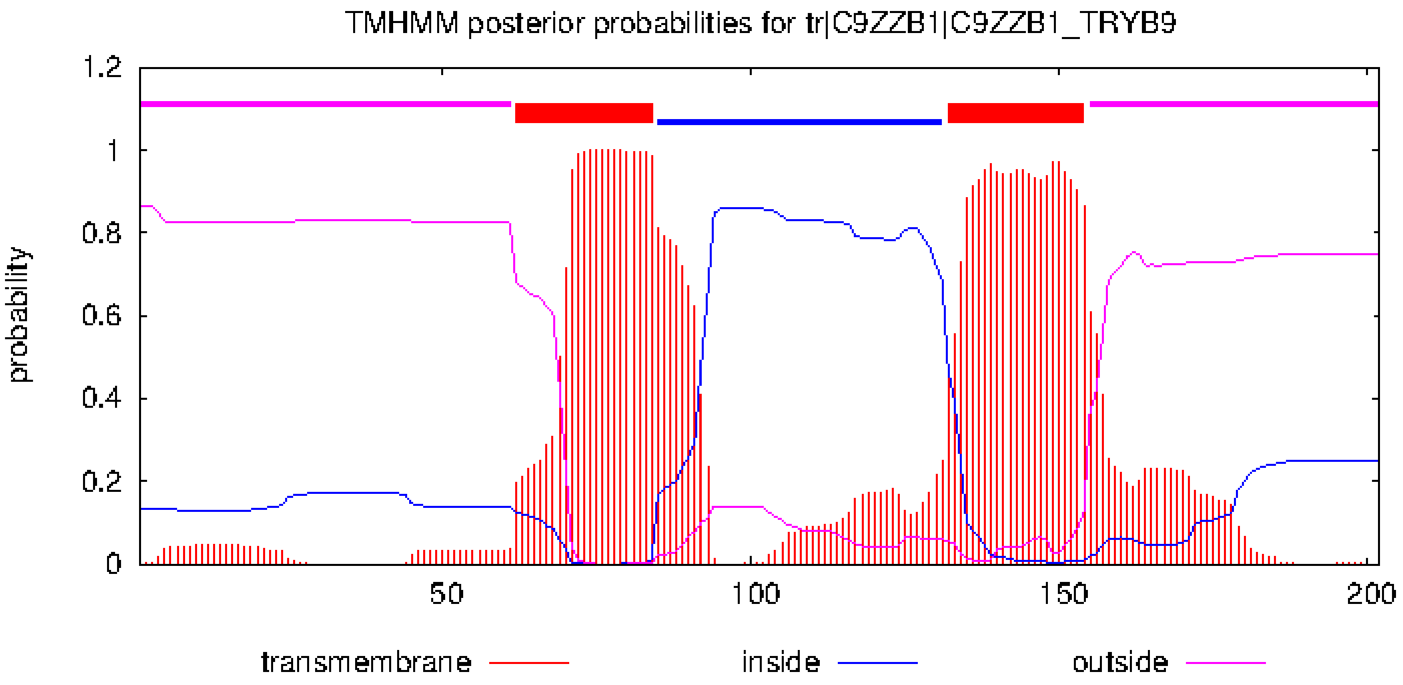

111 # [plot] in postscript, [script] for making the plot in gnuplot, [data]for plot

---

```
# tr|D0AAA6|D0AAA6_TRYB9 Length: 273
# tr|D0AAA6|D0AAA6_TRYB9 Number of predicted TMHs:  1
# tr|D0AAA6|D0AAA6_TRYB9 Exp number of AAs in TMHs: 33.66296
# tr|D0AAA6|D0AAA6_TRYB9 Exp number, first 60 AAs: 0.00625
# tr|D0AAA6|D0AAA6_TRYB9 Total prob of N-in:        0.21051
tr|D0AAA6|D0AAA6_TRYB9  TMHMM2.0        outside      1   203
tr|D0AAA6|D0AAA6_TRYB9  TMHMM2.0        TMhelix    204   221
tr|D0AAA6|D0AAA6_TRYB9  TMHMM2.0        inside     222   273
```

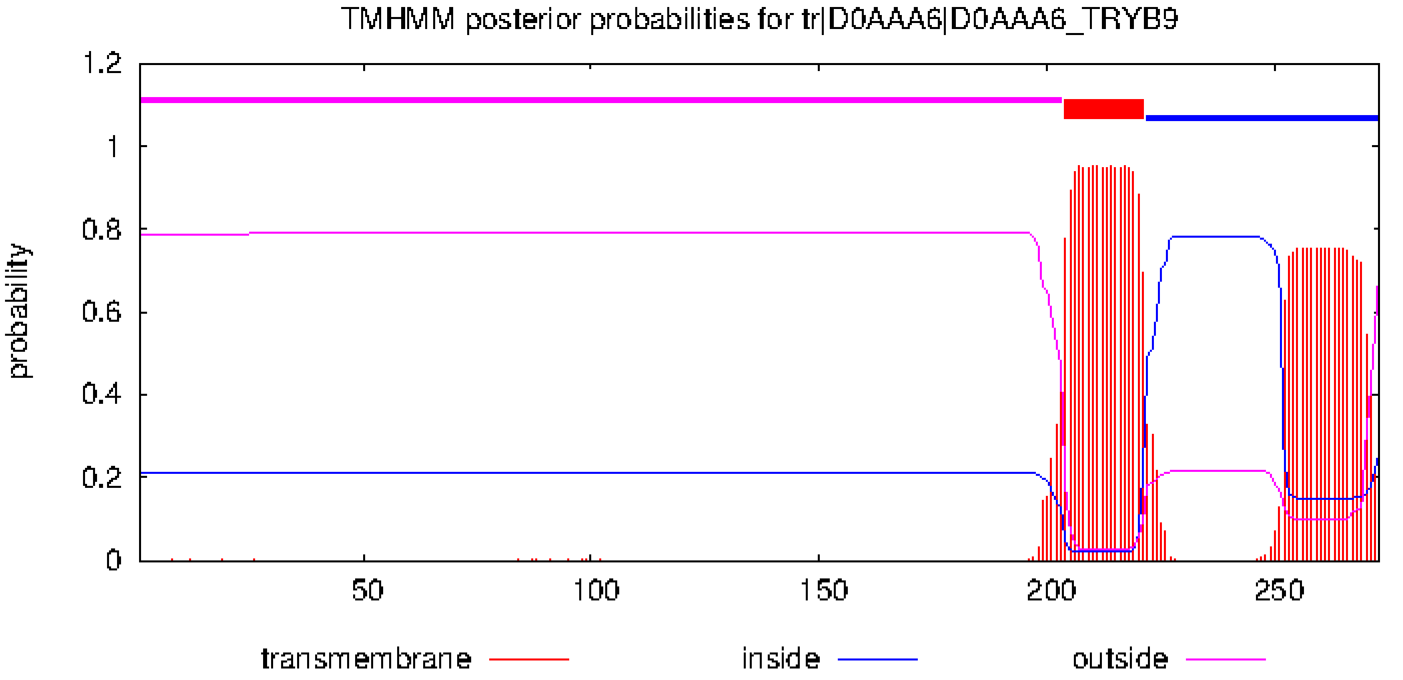

112 # [plot] in postscript, [script] for making the plot in gnuplot, [data]for plot

---

```
# tr|C9ZJ73|C9ZJ73_TRYB9 Length: 286
# tr|C9ZJ73|C9ZJ73_TRYB9 Number of predicted TMHs:  1
```

```
# tr|C9ZJ73|C9ZJ73_TRYB9 Exp number of AAs in TMHs: 22.79466
# tr|C9ZJ73|C9ZJ73_TRYB9 Exp number, first 60 AAs:  0
# tr|C9ZJ73|C9ZJ73_TRYB9 Total prob of N-in:        0.07112
```

```
tr|C9ZJ73|C9ZJ73_TRYB9  TMHMM2.0        outside      1   237
tr|C9ZJ73|C9ZJ73_TRYB9  TMHMM2.0        TMhelix    238   260
tr|C9ZJ73|C9ZJ73_TRYB9  TMHMM2.0        inside     261   286
```

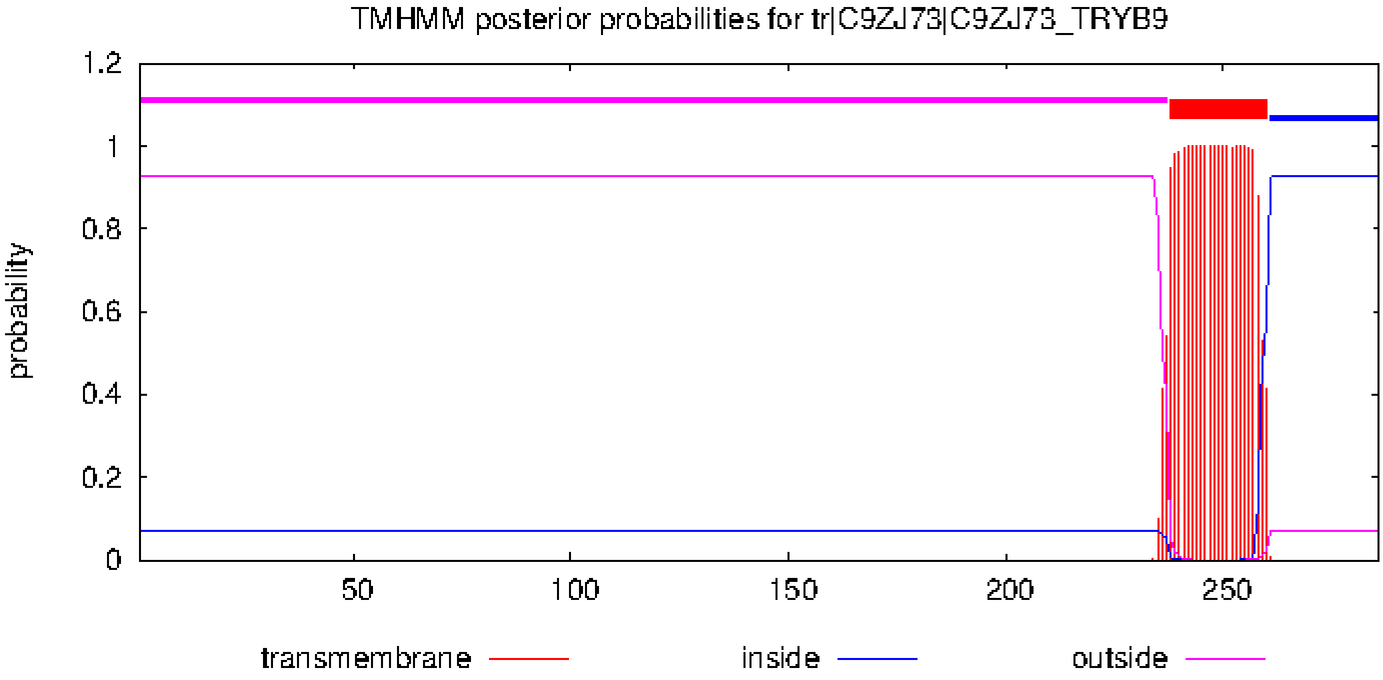

113 # plot in postscript, script for making the plot in gnuplot, data for plot

---

```
# tr|D0A7S1|D0A7S1_TRYB9 Length: 305
# tr|D0A7S1|D0A7S1_TRYB9 Number of predicted TMHs:  4
# tr|D0A7S1|D0A7S1_TRYB9 Exp number of AAs in TMHs: 95.55063
# tr|D0A7S1|D0A7S1_TRYB9 Exp number, first 60 AAs:  14.02823
# tr|D0A7S1|D0A7S1_TRYB9 Total prob of N-in:        0.93527
# tr|D0A7S1|D0A7S1_TRYB9 POSSIBLE N-term signal sequence
tr|D0A7S1|D0A7S1_TRYB9  TMHMM2.0        inside       1    45
tr|D0A7S1|D0A7S1_TRYB9  TMHMM2.0        TMhelix     46    68
tr|D0A7S1|D0A7S1_TRYB9  TMHMM2.0        outside     69    72
tr|D0A7S1|D0A7S1_TRYB9  TMHMM2.0        TMhelix     73    95
tr|D0A7S1|D0A7S1_TRYB9  TMHMM2.0        inside      96   162
tr|D0A7S1|D0A7S1_TRYB9  TMHMM2.0        TMhelix    163   185
tr|D0A7S1|D0A7S1_TRYB9  TMHMM2.0        outside    186   227
tr|D0A7S1|D0A7S1_TRYB9  TMHMM2.0        TMhelix    228   250
tr|D0A7S1|D0A7S1_TRYB9  TMHMM2.0        inside     251   305
```

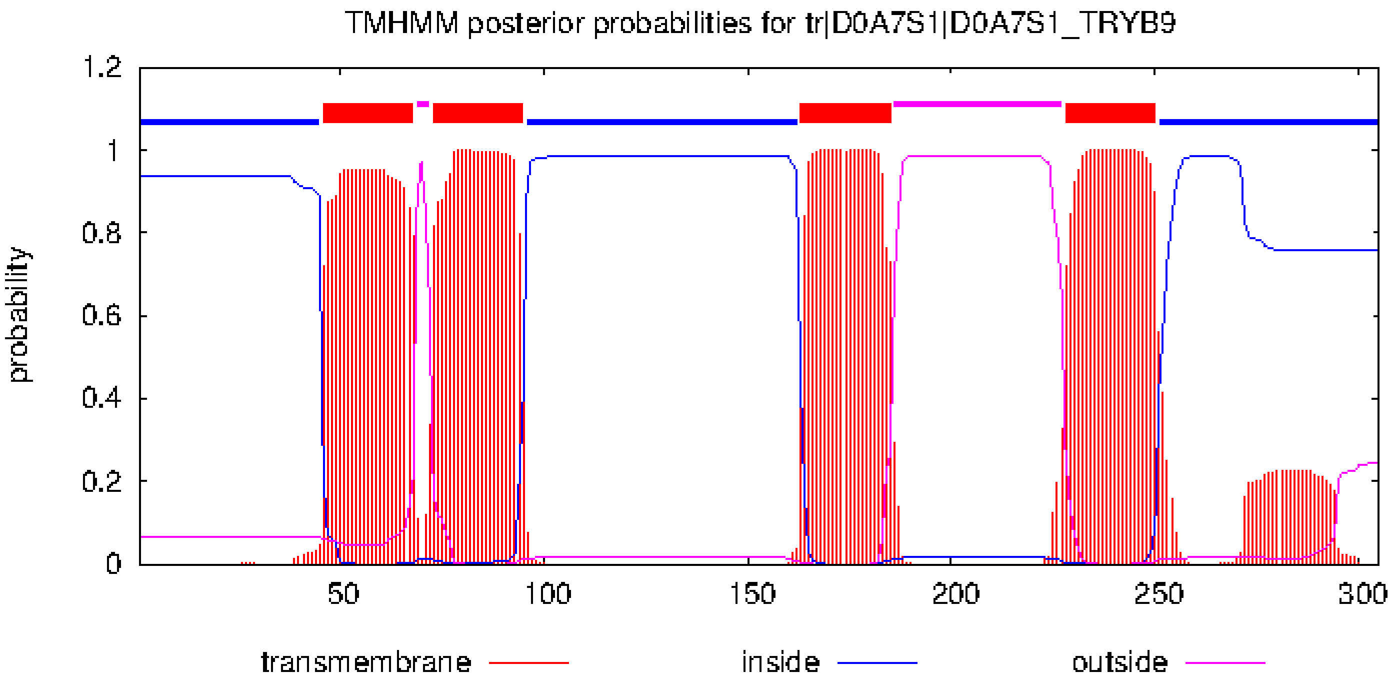

114 # plot in postscript, script for making the plot in gnuplot, data for plot

---

```
# tr|C9ZUV6|C9ZUV6_TRYB9 Length: 312
# tr|C9ZUV6|C9ZUV6_TRYB9 Number of predicted TMHs:  6
# tr|C9ZUV6|C9ZUV6_TRYB9 Exp number of AAs in TMHs: 129.32141
# tr|C9ZUV6|C9ZUV6_TRYB9 Exp number, first 60 AAs:  31.8146
# tr|C9ZUV6|C9ZUV6_TRYB9 Total prob of N-in:        0.34133
# tr|C9ZUV6|C9ZUV6_TRYB9 POSSIBLE N-term signal sequence
tr|C9ZUV6|C9ZUV6_TRYB9  TMHMM2.0        outside      1     9
tr|C9ZUV6|C9ZUV6_TRYB9  TMHMM2.0        TMhelix     10    32
tr|C9ZUV6|C9ZUV6_TRYB9  TMHMM2.0        inside      33    52
tr|C9ZUV6|C9ZUV6_TRYB9  TMHMM2.0        TMhelix     53    75
tr|C9ZUV6|C9ZUV6_TRYB9  TMHMM2.0        outside     76    84
tr|C9ZUV6|C9ZUV6_TRYB9  TMHMM2.0        TMhelix     85   107
tr|C9ZUV6|C9ZUV6_TRYB9  TMHMM2.0        inside     108   113
tr|C9ZUV6|C9ZUV6_TRYB9  TMHMM2.0        TMhelix    114   136
tr|C9ZUV6|C9ZUV6_TRYB9  TMHMM2.0        outside    137   150
tr|C9ZUV6|C9ZUV6_TRYB9  TMHMM2.0        TMhelix    151   173
tr|C9ZUV6|C9ZUV6_TRYB9  TMHMM2.0        inside     174   185
tr|C9ZUV6|C9ZUV6_TRYB9  TMHMM2.0        TMhelix    186   208
tr|C9ZUV6|C9ZUV6_TRYB9  TMHMM2.0        outside    209   312
```

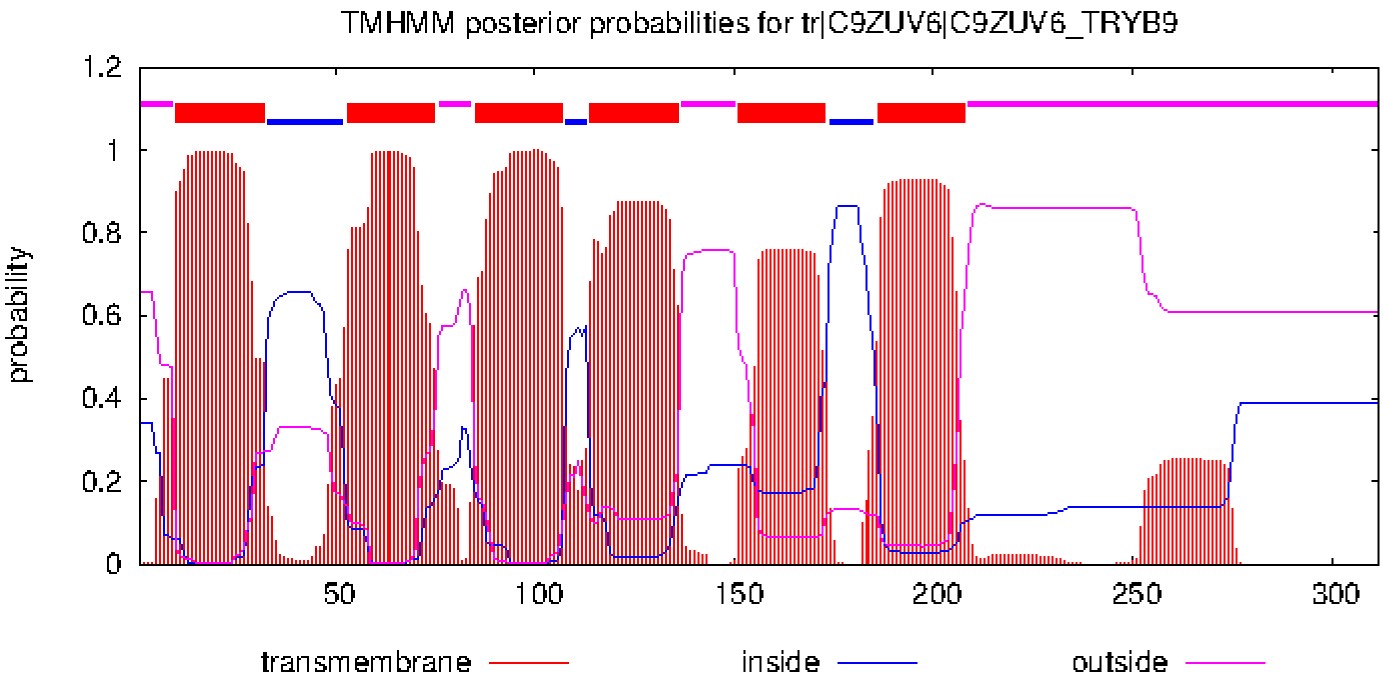

115 # plot in postscript, script for making the plot in gnuplot, data for plot

---

```
# tr|D0A318|D0A318_TRYB9 Length: 342
# tr|D0A318|D0A318_TRYB9 Number of predicted TMHs:  6
# tr|D0A318|D0A318_TRYB9 Exp number of AAs in TMHs: 146.43072
# tr|D0A318|D0A318_TRYB9 Exp number, first 60 AAs:  0
# tr|D0A318|D0A318_TRYB9 Total prob of N-in:        0.99124
tr|D0A318|D0A318_TRYB9  TMHMM2.0        inside       1    80
tr|D0A318|D0A318_TRYB9  TMHMM2.0        TMhelix     81   103
tr|D0A318|D0A318_TRYB9  TMHMM2.0        outside    104   112
tr|D0A318|D0A318_TRYB9  TMHMM2.0        TMhelix    113   132
tr|D0A318|D0A318_TRYB9  TMHMM2.0        inside     133   157
tr|D0A318|D0A318_TRYB9  TMHMM2.0        TMhelix    158   180
tr|D0A318|D0A318_TRYB9  TMHMM2.0        outside    181   230
tr|D0A318|D0A318_TRYB9  TMHMM2.0        TMhelix    231   253
tr|D0A318|D0A318_TRYB9  TMHMM2.0        inside     254   259
tr|D0A318|D0A318_TRYB9  TMHMM2.0        TMhelix    260   282
tr|D0A318|D0A318_TRYB9  TMHMM2.0        outside    283   286
tr|D0A318|D0A318_TRYB9  TMHMM2.0        TMhelix    287   309
tr|D0A318|D0A318_TRYB9  TMHMM2.0        inside     310   342
```

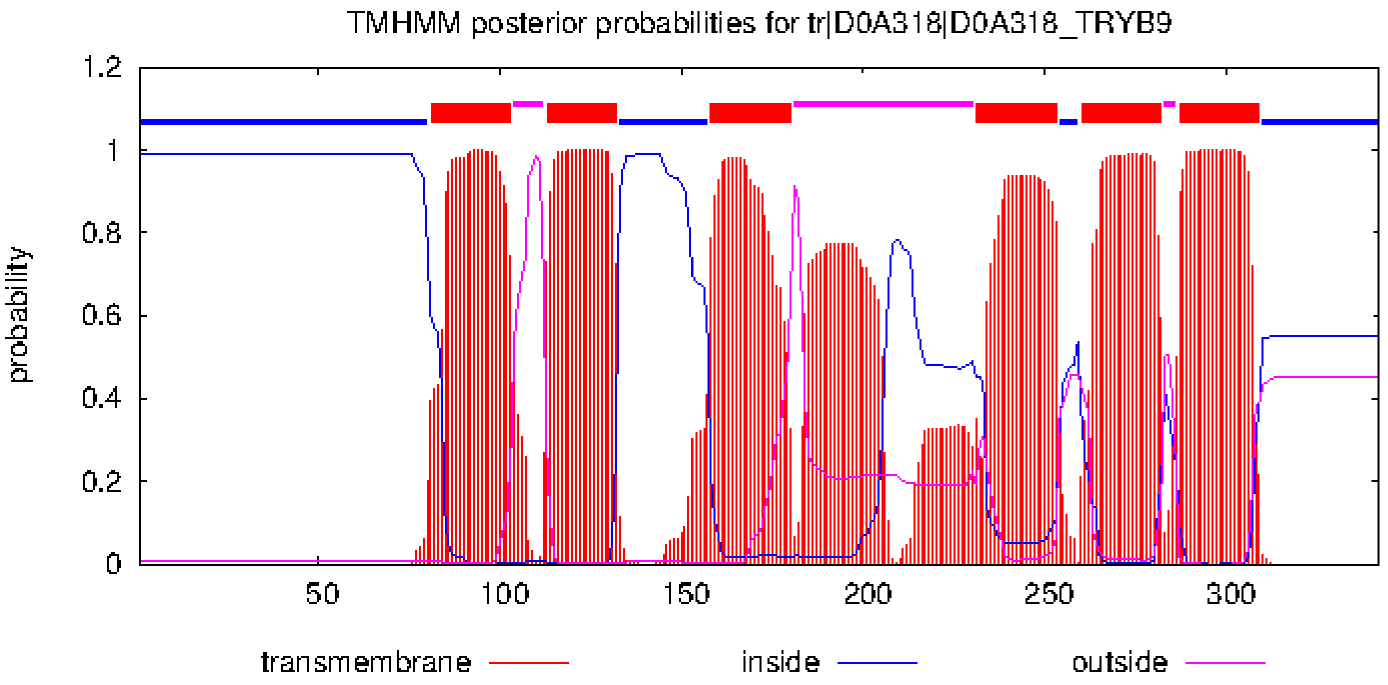

116 # plot in postscript, script for making the plot in gnuplot, data for plot

---

```
# tr|C9ZWE6|C9ZWE6_TRYB9 Length: 371
# tr|C9ZWE6|C9ZWE6_TRYB9 Number of predicted TMHs:  3
# tr|C9ZWE6|C9ZWE6_TRYB9 Exp number of AAs in TMHs: 107.39984
# tr|C9ZWE6|C9ZWE6_TRYB9 Exp number, first 60 AAs: 33.89549
# tr|C9ZWE6|C9ZWE6_TRYB9 Total prob of N-in:        0.85111
# tr|C9ZWE6|C9ZWE6_TRYB9 POSSIBLE N-term signal sequence
tr|C9ZWE6|C9ZWE6_TRYB9  TMHMM2.0        inside       1     6
tr|C9ZWE6|C9ZWE6_TRYB9  TMHMM2.0        TMhelix      7    29
tr|C9ZWE6|C9ZWE6_TRYB9  TMHMM2.0        outside     30    48
tr|C9ZWE6|C9ZWE6_TRYB9  TMHMM2.0        TMhelix     49    71
tr|C9ZWE6|C9ZWE6_TRYB9  TMHMM2.0        inside      72    82
tr|C9ZWE6|C9ZWE6_TRYB9  TMHMM2.0        TMhelix     83   105
tr|C9ZWE6|C9ZWE6_TRYB9  TMHMM2.0        outside    106   371
```

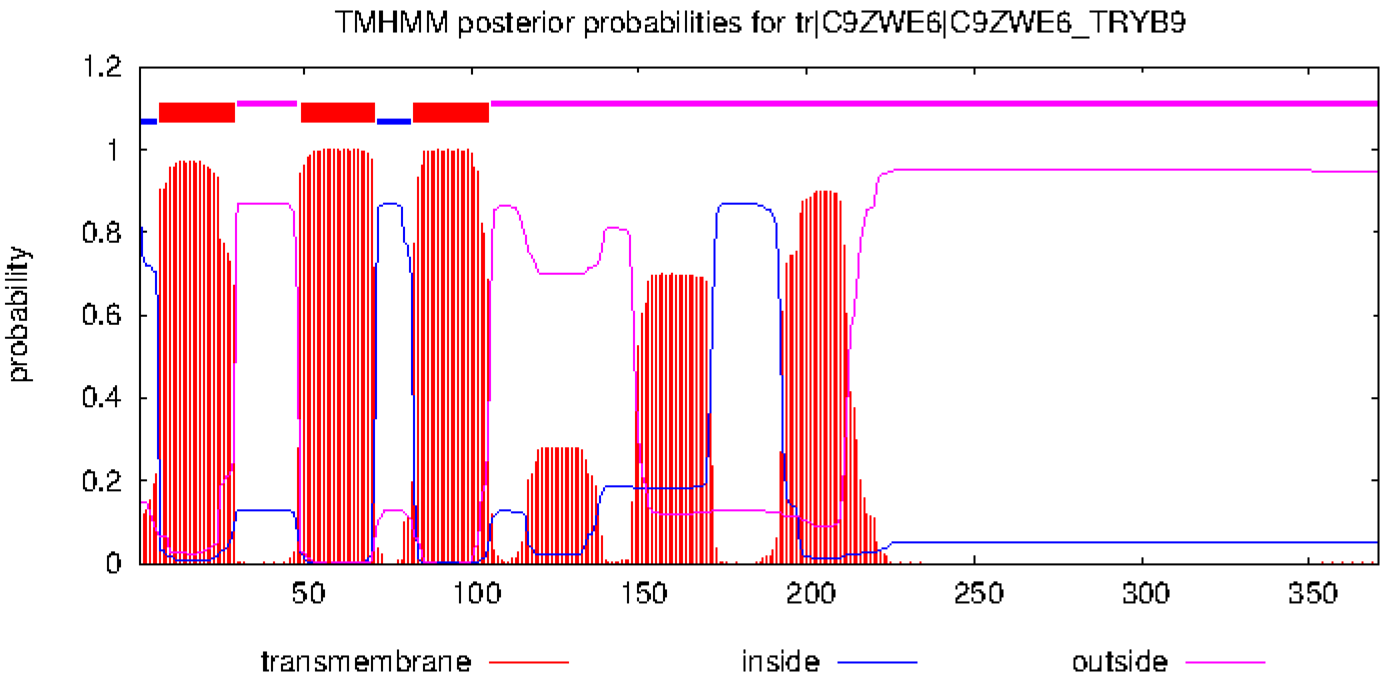

117 # plot in postscript, script for making the plot in gnuplot, data for plot

```
# tr|D0A9E1|D0A9E1_TRYB9 Length: 452
# tr|D0A9E1|D0A9E1_TRYB9 Number of predicted TMHs:  6
# tr|D0A9E1|D0A9E1_TRYB9 Exp number of AAs in TMHs: 125.79977
# tr|D0A9E1|D0A9E1_TRYB9 Exp number, first 60 AAs:  23.82484
# tr|D0A9E1|D0A9E1_TRYB9 Total prob of N-in:        0.17405
# tr|D0A9E1|D0A9E1_TRYB9 POSSIBLE N-term signal sequence
tr|D0A9E1|D0A9E1_TRYB9  TMHMM2.0        outside      1     3
tr|D0A9E1|D0A9E1_TRYB9  TMHMM2.0        TMhelix      4    26
tr|D0A9E1|D0A9E1_TRYB9  TMHMM2.0        inside      27    85
tr|D0A9E1|D0A9E1_TRYB9  TMHMM2.0        TMhelix     86   108
tr|D0A9E1|D0A9E1_TRYB9  TMHMM2.0        outside    109   122
tr|D0A9E1|D0A9E1_TRYB9  TMHMM2.0        TMhelix    123   145
tr|D0A9E1|D0A9E1_TRYB9  TMHMM2.0        inside     146   260
tr|D0A9E1|D0A9E1_TRYB9  TMHMM2.0        TMhelix    261   278
tr|D0A9E1|D0A9E1_TRYB9  TMHMM2.0        outside    279   282
tr|D0A9E1|D0A9E1_TRYB9  TMHMM2.0        TMhelix    283   302
tr|D0A9E1|D0A9E1_TRYB9  TMHMM2.0        inside     303   322
tr|D0A9E1|D0A9E1_TRYB9  TMHMM2.0        TMhelix    323   345
tr|D0A9E1|D0A9E1_TRYB9  TMHMM2.0        outside    346   452
```

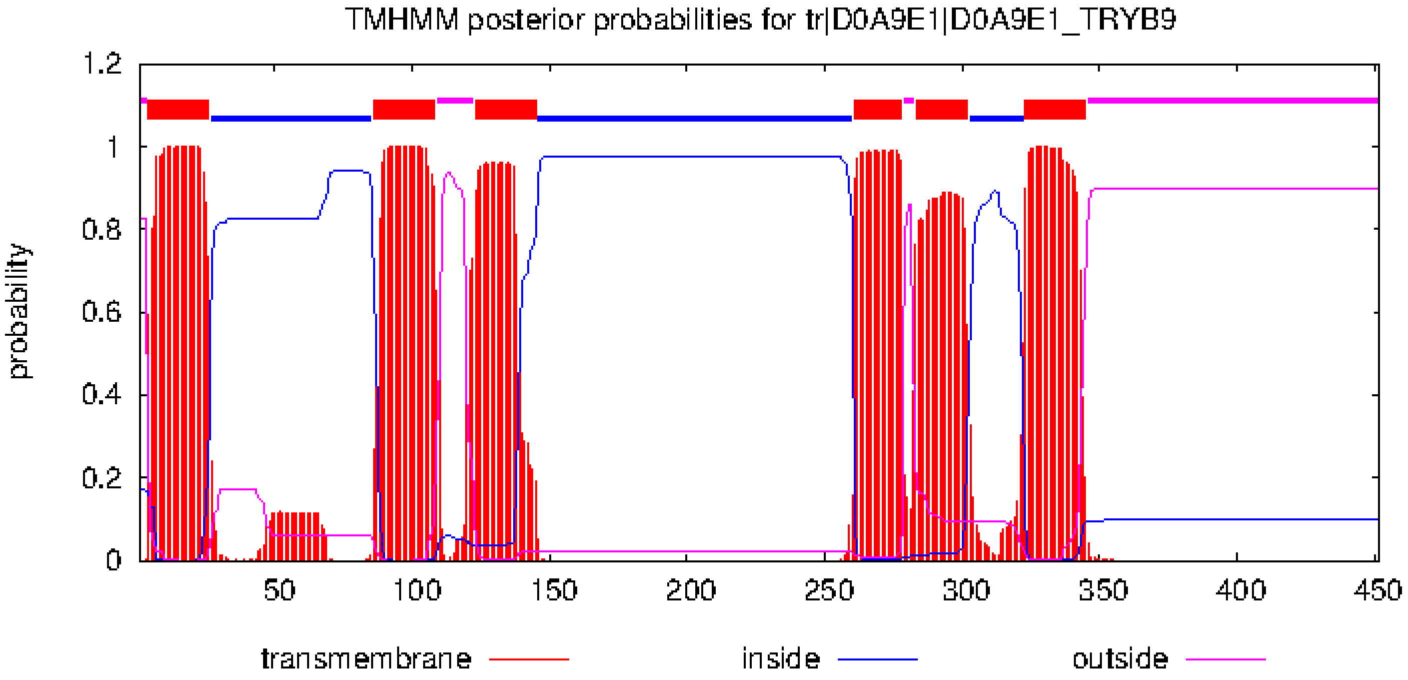

118 # **plot in postscript, script for making the plot in gnuplot, data for plot**

```
# tr|C9ZJU5|C9ZJU5_TRYB9 Length: 458
# tr|C9ZJU5|C9ZJU5_TRYB9 Number of predicted TMHs:  11
# tr|C9ZJU5|C9ZJU5_TRYB9 Exp number of AAs in TMHs: 243.73122
# tr|C9ZJU5|C9ZJU5_TRYB9 Exp number, first 60 AAs:  22.73251
# tr|C9ZJU5|C9ZJU5_TRYB9 Total prob of N-in:        0.99689
# tr|C9ZJU5|C9ZJU5_TRYB9 POSSIBLE N-term signal sequence
tr|C9ZJU5|C9ZJU5_TRYB9  TMHMM2.0        inside       1    11
tr|C9ZJU5|C9ZJU5_TRYB9  TMHMM2.0        TMhelix     12    34
tr|C9ZJU5|C9ZJU5_TRYB9  TMHMM2.0        outside     35    63
tr|C9ZJU5|C9ZJU5_TRYB9  TMHMM2.0        TMhelix     64    83
tr|C9ZJU5|C9ZJU5_TRYB9  TMHMM2.0        inside      84    95
tr|C9ZJU5|C9ZJU5_TRYB9  TMHMM2.0        TMhelix     96   118
tr|C9ZJU5|C9ZJU5_TRYB9  TMHMM2.0        outside    119   127
tr|C9ZJU5|C9ZJU5_TRYB9  TMHMM2.0        TMhelix    128   150
tr|C9ZJU5|C9ZJU5_TRYB9  TMHMM2.0        inside     151   156
tr|C9ZJU5|C9ZJU5_TRYB9  TMHMM2.0        TMhelix    157   179
tr|C9ZJU5|C9ZJU5_TRYB9  TMHMM2.0        outside    180   193
tr|C9ZJU5|C9ZJU5_TRYB9  TMHMM2.0        TMhelix    194   216
tr|C9ZJU5|C9ZJU5_TRYB9  TMHMM2.0        inside     217   300
tr|C9ZJU5|C9ZJU5_TRYB9  TMHMM2.0        TMhelix    301   323
tr|C9ZJU5|C9ZJU5_TRYB9  TMHMM2.0        outside    324   332
```

tr|C9ZJU5|C9ZJU5_TRYB9 TMHMM2.0 TMhelix 333 355

```
tr|C9ZJU5|C9ZJU5_TRYB9  TMHMM2.0        inside     356   367
tr|C9ZJU5|C9ZJU5_TRYB9  TMHMM2.0        TMhelix    368   390
tr|C9ZJU5|C9ZJU5_TRYB9  TMHMM2.0        outside    391   393
tr|C9ZJU5|C9ZJU5_TRYB9  TMHMM2.0        TMhelix    394   416
tr|C9ZJU5|C9ZJU5_TRYB9  TMHMM2.0        inside     417   428
tr|C9ZJU5|C9ZJU5_TRYB9  TMHMM2.0        TMhelix    429   451
tr|C9ZJU5|C9ZJU5_TRYB9  TMHMM2.0        outside    452   458
```

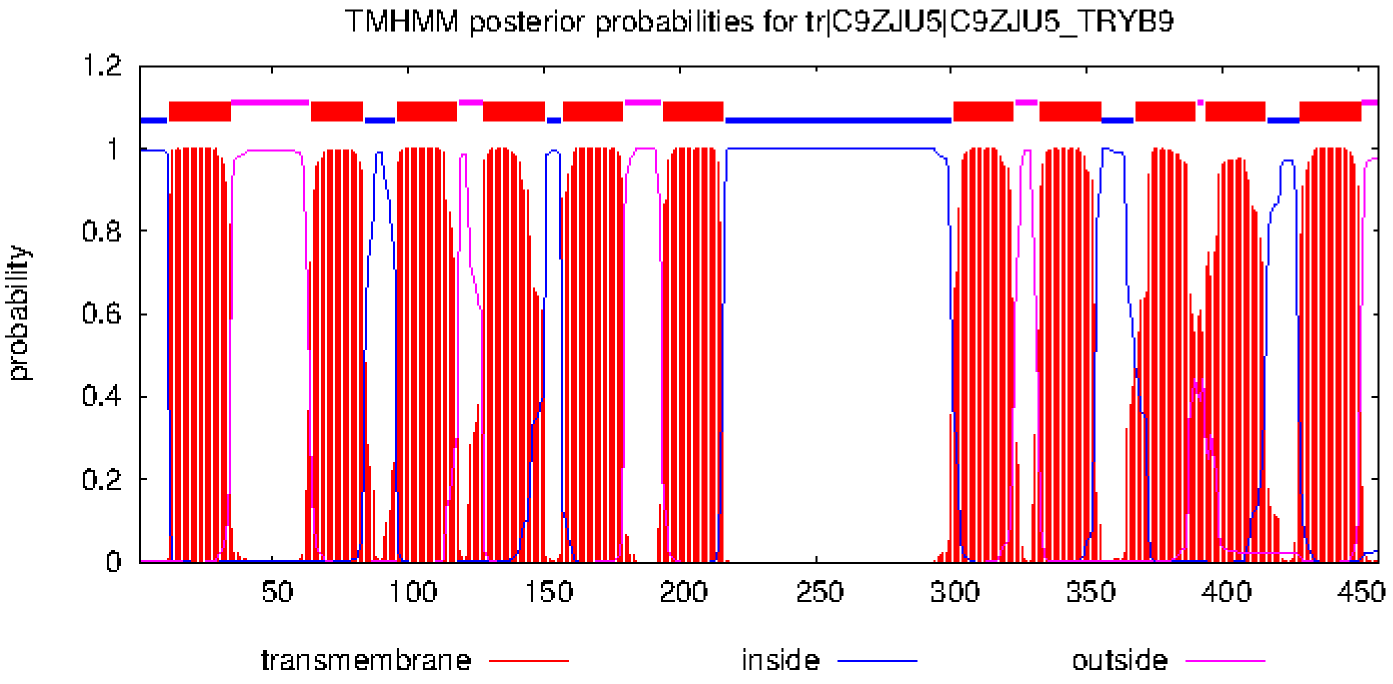

119 # plot in postscript, script for making the plot in gnuplot, data for plot

---

```
# tr|C9ZWR7|C9ZWR7_TRYB9 Length: 467
# tr|C9ZWR7|C9ZWR7_TRYB9 Number of predicted TMHs:  11
# tr|C9ZWR7|C9ZWR7_TRYB9 Exp number of AAs in TMHs: 242.2965
# tr|C9ZWR7|C9ZWR7_TRYB9 Exp number, first 60 AAs:  0.08449
# tr|C9ZWR7|C9ZWR7_TRYB9 Total prob of N-in:        0.98916
tr|C9ZWR7|C9ZWR7_TRYB9  TMHMM2.0        inside       1    65
tr|C9ZWR7|C9ZWR7_TRYB9  TMHMM2.0        TMhelix     66    88
tr|C9ZWR7|C9ZWR7_TRYB9  TMHMM2.0        outside     89    97
tr|C9ZWR7|C9ZWR7_TRYB9  TMHMM2.0        TMhelix     98   120
tr|C9ZWR7|C9ZWR7_TRYB9  TMHMM2.0        inside     121   142
tr|C9ZWR7|C9ZWR7_TRYB9  TMHMM2.0        TMhelix    143   165
tr|C9ZWR7|C9ZWR7_TRYB9  TMHMM2.0        outside    166   184
tr|C9ZWR7|C9ZWR7_TRYB9  TMHMM2.0        TMhelix    185   204
tr|C9ZWR7|C9ZWR7_TRYB9  TMHMM2.0        inside     205   210
tr|C9ZWR7|C9ZWR7_TRYB9  TMHMM2.0        TMhelix    211   230
tr|C9ZWR7|C9ZWR7_TRYB9  TMHMM2.0        outside    231   256
tr|C9ZWR7|C9ZWR7_TRYB9  TMHMM2.0        TMhelix    257   279
tr|C9ZWR7|C9ZWR7_TRYB9  TMHMM2.0        inside     280   291
tr|C9ZWR7|C9ZWR7_TRYB9  TMHMM2.0        TMhelix    292   314
tr|C9ZWR7|C9ZWR7_TRYB9  TMHMM2.0        outside    315   328
tr|C9ZWR7|C9ZWR7_TRYB9  TMHMM2.0        TMhelix    329   351
tr|C9ZWR7|C9ZWR7_TRYB9  TMHMM2.0        inside     352   371
tr|C9ZWR7|C9ZWR7_TRYB9  TMHMM2.0        TMhelix    372   394
tr|C9ZWR7|C9ZWR7_TRYB9  TMHMM2.0        outside    395   398
tr|C9ZWR7|C9ZWR7_TRYB9  TMHMM2.0        TMhelix    399   421
tr|C9ZWR7|C9ZWR7_TRYB9  TMHMM2.0        inside     422   440
tr|C9ZWR7|C9ZWR7_TRYB9  TMHMM2.0        TMhelix    441   463
tr|C9ZWR7|C9ZWR7_TRYB9  TMHMM2.0        outside    464   467
```

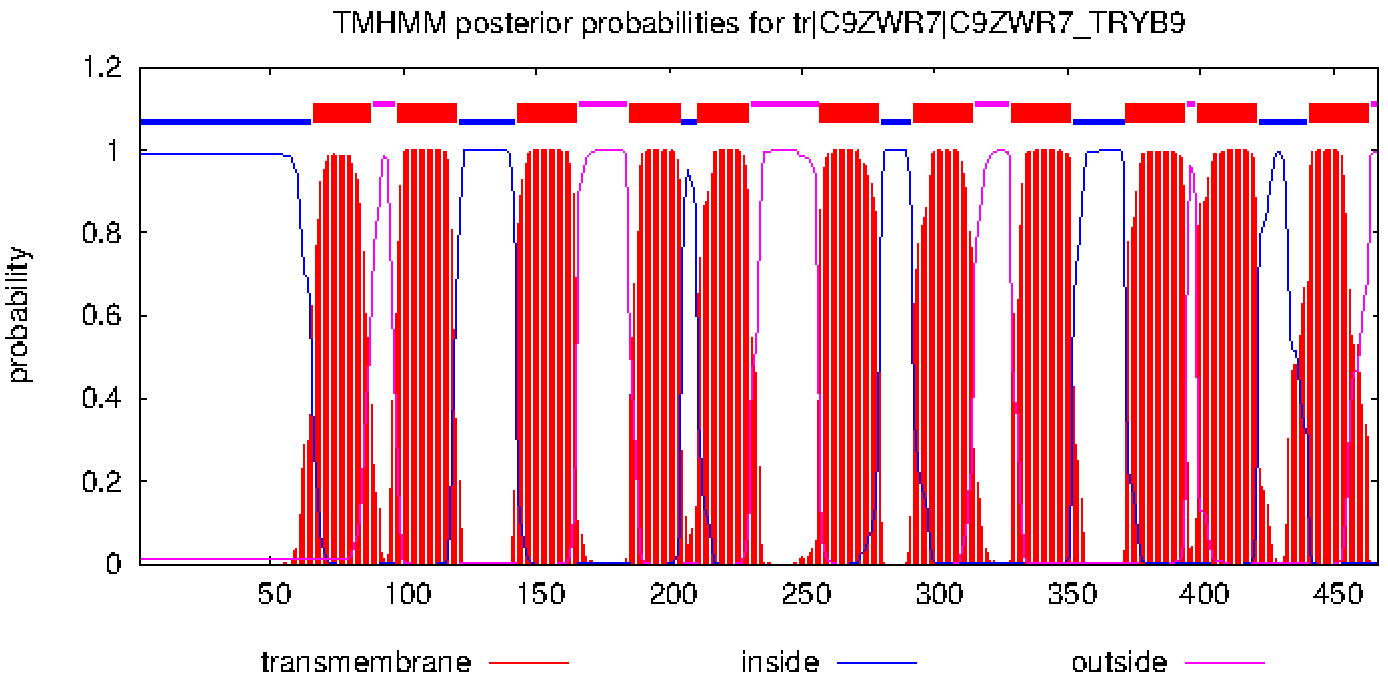

120 # plot in postscript, script for making the plot in gnuplot, data for plot

---

```
# tr|C9ZKT6|C9ZKT6_TRYB9 Length: 462
# tr|C9ZKT6|C9ZKT6_TRYB9 Number of predicted TMHs:  11
# tr|C9ZKT6|C9ZKT6_TRYB9 Exp number of AAs in TMHs: 244.35858
# tr|C9ZKT6|C9ZKT6_TRYB9 Exp number, first 60 AAs: 23.75628
# tr|C9ZKT6|C9ZKT6_TRYB9 Total prob of N-in:        0.94887
# tr|C9ZKT6|C9ZKT6_TRYB9 POSSIBLE N-term signal sequence
tr|C9ZKT6|C9ZKT6_TRYB9  TMHMM2.0        inside       1    12
tr|C9ZKT6|C9ZKT6_TRYB9  TMHMM2.0        TMhelix     13    35
tr|C9ZKT6|C9ZKT6_TRYB9  TMHMM2.0        outside     36    63
tr|C9ZKT6|C9ZKT6_TRYB9  TMHMM2.0        TMhelix     64    86
tr|C9ZKT6|C9ZKT6_TRYB9  TMHMM2.0        inside      87    92
tr|C9ZKT6|C9ZKT6_TRYB9  TMHMM2.0        TMhelix     93   115
tr|C9ZKT6|C9ZKT6_TRYB9  TMHMM2.0        outside    116   125
tr|C9ZKT6|C9ZKT6_TRYB9  TMHMM2.0        TMhelix    126   148
tr|C9ZKT6|C9ZKT6_TRYB9  TMHMM2.0        inside     149   154
tr|C9ZKT6|C9ZKT6_TRYB9  TMHMM2.0        TMhelix    155   177
tr|C9ZKT6|C9ZKT6_TRYB9  TMHMM2.0        outside    178   191
tr|C9ZKT6|C9ZKT6_TRYB9  TMHMM2.0        TMhelix    192   214
tr|C9ZKT6|C9ZKT6_TRYB9  TMHMM2.0        inside     215   303
tr|C9ZKT6|C9ZKT6_TRYB9  TMHMM2.0        TMhelix    304   326
tr|C9ZKT6|C9ZKT6_TRYB9  TMHMM2.0        outside    327   335
tr|C9ZKT6|C9ZKT6_TRYB9  TMHMM2.0        TMhelix    336   357
tr|C9ZKT6|C9ZKT6_TRYB9  TMHMM2.0        inside     358   368
tr|C9ZKT6|C9ZKT6_TRYB9  TMHMM2.0        TMhelix    369   391
tr|C9ZKT6|C9ZKT6_TRYB9  TMHMM2.0        outside    392   394
tr|C9ZKT6|C9ZKT6_TRYB9  TMHMM2.0        TMhelix    395   417
tr|C9ZKT6|C9ZKT6_TRYB9  TMHMM2.0        inside     418   432
tr|C9ZKT6|C9ZKT6_TRYB9  TMHMM2.0        TMhelix    433   455
tr|C9ZKT6|C9ZKT6_TRYB9  TMHMM2.0        outside    456   462
```

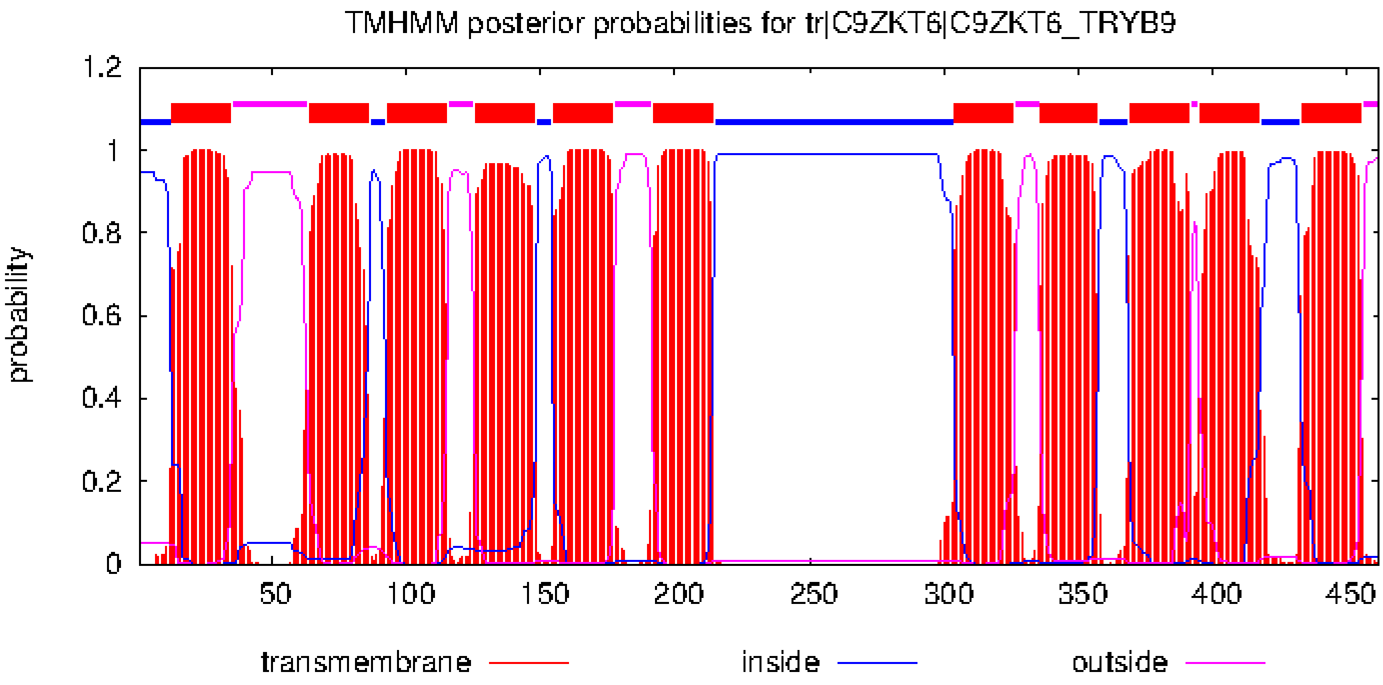

121 # plot in postscript, script for making the plot in gnuplot, data for plot

---

```
# tr|C9ZWN8|C9ZWN8_TRYB9 Length: 467
# tr|C9ZWN8|C9ZWN8_TRYB9 Number of predicted TMHs:  5
# tr|C9ZWN8|C9ZWN8_TRYB9 Exp number of AAs in TMHs: 111.54723
# tr|C9ZWN8|C9ZWN8_TRYB9 Exp number, first 60 AAs:  0.00413
# tr|C9ZWN8|C9ZWN8_TRYB9 Total prob of N-in:        0.99954
tr|C9ZWN8|C9ZWN8_TRYB9  TMHMM2.0        inside       1    61
tr|C9ZWN8|C9ZWN8_TRYB9  TMHMM2.0        TMhelix     62    84
tr|C9ZWN8|C9ZWN8_TRYB9  TMHMM2.0        outside     85   129
tr|C9ZWN8|C9ZWN8_TRYB9  TMHMM2.0        TMhelix    130   152
tr|C9ZWN8|C9ZWN8_TRYB9  TMHMM2.0        inside     153   216
tr|C9ZWN8|C9ZWN8_TRYB9  TMHMM2.0        TMhelix    217   239
tr|C9ZWN8|C9ZWN8_TRYB9  TMHMM2.0        outside    240   308
tr|C9ZWN8|C9ZWN8_TRYB9  TMHMM2.0        TMhelix    309   331
tr|C9ZWN8|C9ZWN8_TRYB9  TMHMM2.0        inside     332   343
tr|C9ZWN8|C9ZWN8_TRYB9  TMHMM2.0        TMhelix    344   366
tr|C9ZWN8|C9ZWN8_TRYB9  TMHMM2.0        outside    367   467
```

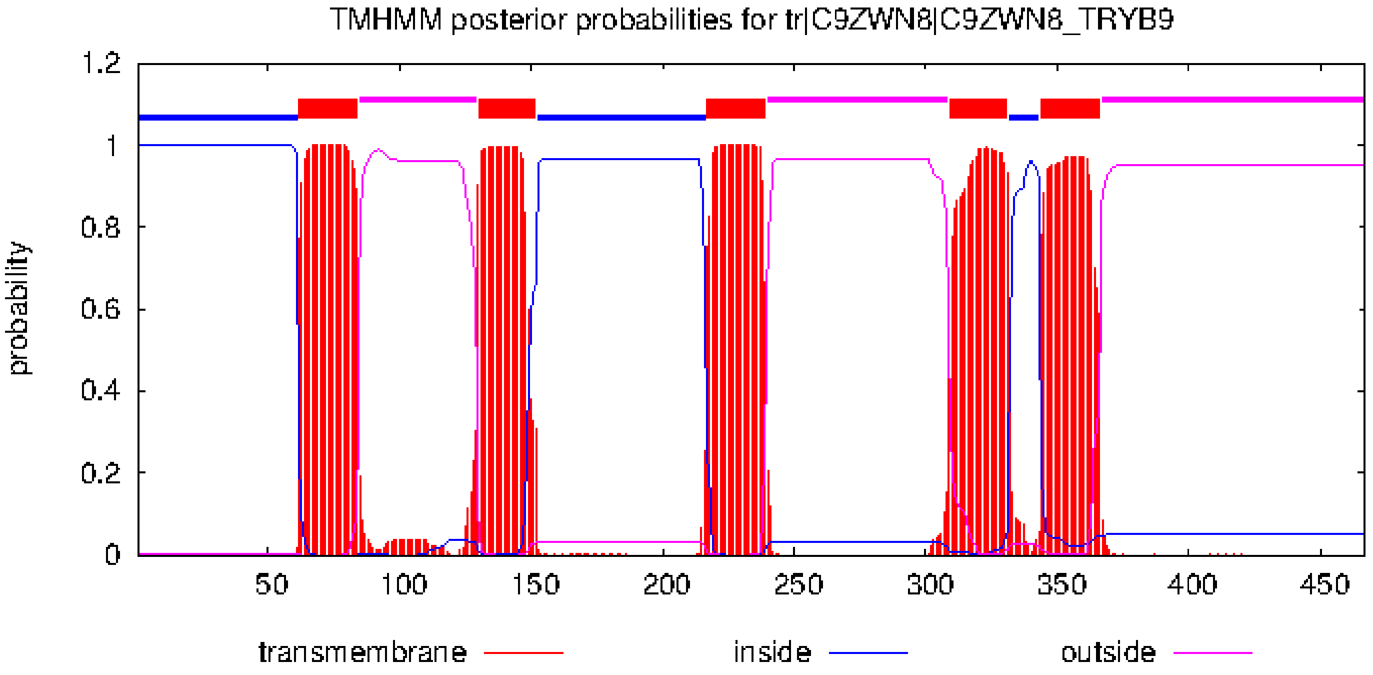

122 # plot in postscript, script for making the plot in gnuplot, data for plot

---

```
# tr|D0A893|D0A893_TRYB9 Length: 469
# tr|D0A893|D0A893_TRYB9 Number of predicted TMHs:  4
# tr|D0A893|D0A893_TRYB9 Exp number of AAs in TMHs: 93.3557700000001
# tr|D0A893|D0A893_TRYB9 Exp number, first 60 AAs:  0.47043
# tr|D0A893|D0A893_TRYB9 Total prob of N-in:        0.89093
tr|D0A893|D0A893_TRYB9  TMHMM2.0        inside       1   182
tr|D0A893|D0A893_TRYB9  TMHMM2.0        TMhelix    183   205
tr|D0A893|D0A893_TRYB9  TMHMM2.0        outside    206   214
tr|D0A893|D0A893_TRYB9  TMHMM2.0        TMhelix    215   232
tr|D0A893|D0A893_TRYB9  TMHMM2.0        inside     233   325
tr|D0A893|D0A893_TRYB9  TMHMM2.0        TMhelix    326   348
tr|D0A893|D0A893_TRYB9  TMHMM2.0        outside    349   385
tr|D0A893|D0A893_TRYB9  TMHMM2.0        TMhelix    386   408
tr|D0A893|D0A893_TRYB9  TMHMM2.0        inside     409   469
```

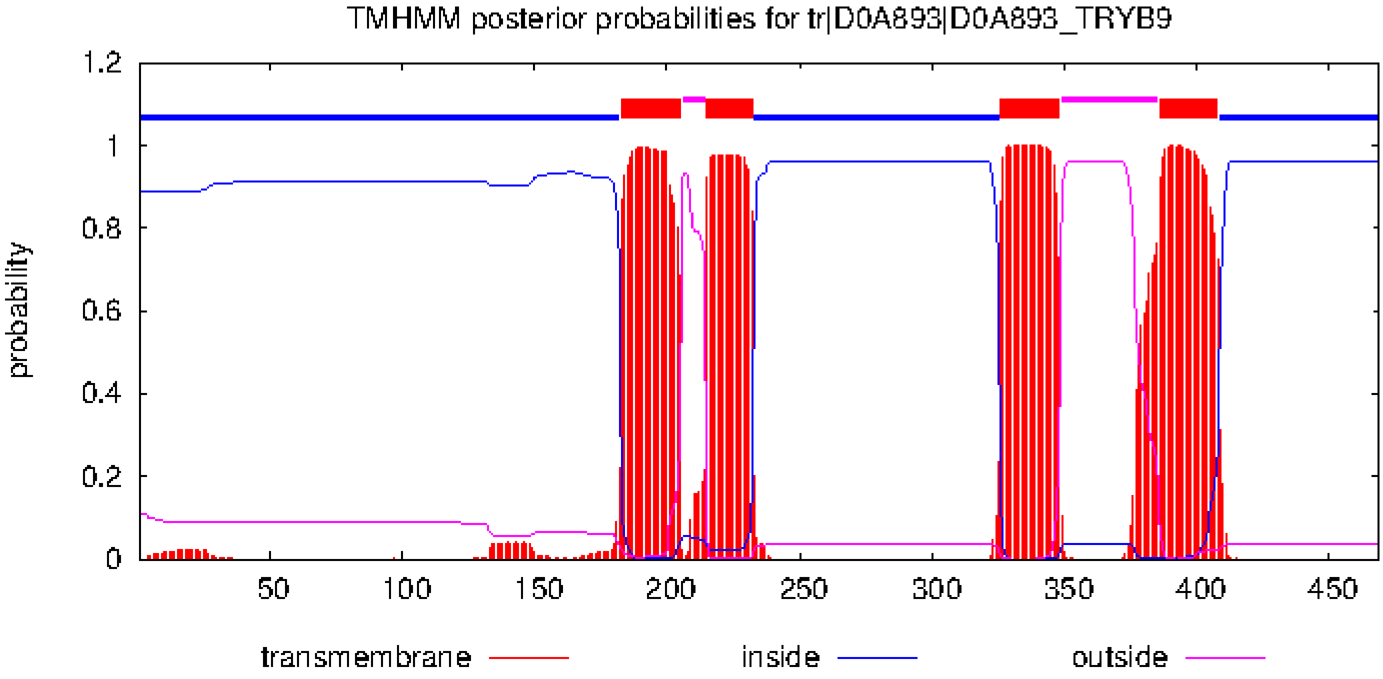

123 # plot in postscript, script for making the plot in gnuplot, data for plot

---

```
# tr|C9ZXR1|C9ZXR1_TRYB9 Length: 504
# tr|C9ZXR1|C9ZXR1_TRYB9 Number of predicted TMHs:  1
# tr|C9ZXR1|C9ZXR1_TRYB9 Exp number of AAs in TMHs: 17.46581
# tr|C9ZXR1|C9ZXR1_TRYB9 Exp number, first 60 AAs:  9.01822
# tr|C9ZXR1|C9ZXR1_TRYB9 Total prob of N-in:        0.63278
tr|C9ZXR1|C9ZXR1_TRYB9  TMHMM2.0        inside       1    49
tr|C9ZXR1|C9ZXR1_TRYB9  TMHMM2.0        TMhelix     50    72
tr|C9ZXR1|C9ZXR1_TRYB9  TMHMM2.0        outside     73   504
```

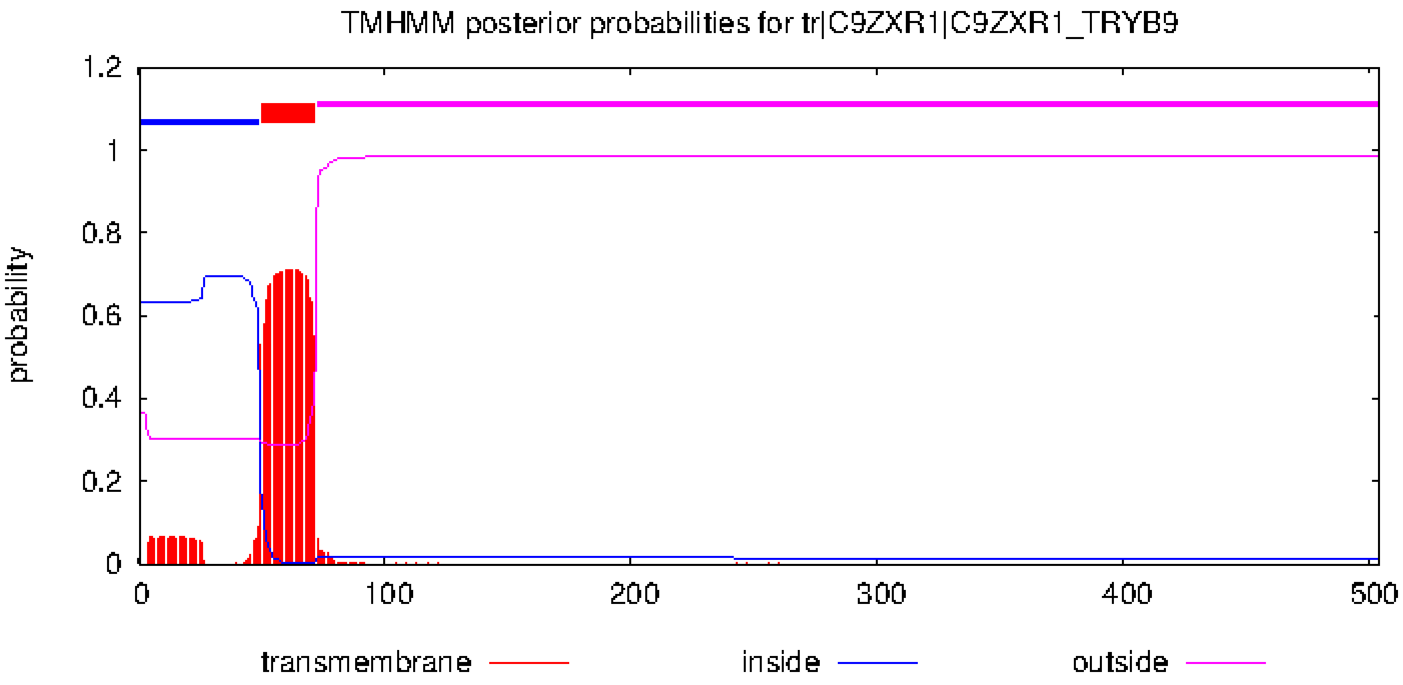

124 **# plot in postscript, script for making the plot in gnuplot, data for plot**

```
# tr|D0A7R4|D0A7R4_TRYB9 Length: 512
# tr|D0A7R4|D0A7R4_TRYB9 Number of predicted TMHs:  10
# tr|D0A7R4|D0A7R4_TRYB9 Exp number of AAs in TMHs: 242.72547
# tr|D0A7R4|D0A7R4_TRYB9 Exp number, first 60 AAs:  19.1591
# tr|D0A7R4|D0A7R4_TRYB9 Total prob of N-in:        0.86185
# tr|D0A7R4|D0A7R4_TRYB9 POSSIBLE N-term signal sequence
tr|D0A7R4|D0A7R4_TRYB9  TMHMM2.0        inside       1    29
tr|D0A7R4|D0A7R4_TRYB9  TMHMM2.0        TMhelix     30    49
tr|D0A7R4|D0A7R4_TRYB9  TMHMM2.0        outside     50   104
tr|D0A7R4|D0A7R4_TRYB9  TMHMM2.0        TMhelix    105   127
tr|D0A7R4|D0A7R4_TRYB9  TMHMM2.0        inside     128   167
tr|D0A7R4|D0A7R4_TRYB9  TMHMM2.0        TMhelix    168   190
tr|D0A7R4|D0A7R4_TRYB9  TMHMM2.0        outside    191   204
tr|D0A7R4|D0A7R4_TRYB9  TMHMM2.0        TMhelix    205   227
tr|D0A7R4|D0A7R4_TRYB9  TMHMM2.0        inside     228   256
tr|D0A7R4|D0A7R4_TRYB9  TMHMM2.0        TMhelix    257   279
tr|D0A7R4|D0A7R4_TRYB9  TMHMM2.0        outside    280   293
tr|D0A7R4|D0A7R4_TRYB9  TMHMM2.0        TMhelix    294   316
tr|D0A7R4|D0A7R4_TRYB9  TMHMM2.0        inside     317   322
tr|D0A7R4|D0A7R4_TRYB9  TMHMM2.0        TMhelix    323   345
tr|D0A7R4|D0A7R4_TRYB9  TMHMM2.0        outside    346   348
tr|D0A7R4|D0A7R4_TRYB9  TMHMM2.0        TMhelix    349   371
tr|D0A7R4|D0A7R4_TRYB9  TMHMM2.0        inside     372   377
tr|D0A7R4|D0A7R4_TRYB9  TMHMM2.0        TMhelix    378   400
tr|D0A7R4|D0A7R4_TRYB9  TMHMM2.0        outside    401   445
tr|D0A7R4|D0A7R4_TRYB9  TMHMM2.0        TMhelix    446   468
tr|D0A7R4|D0A7R4_TRYB9  TMHMM2.0        inside     469   512
```

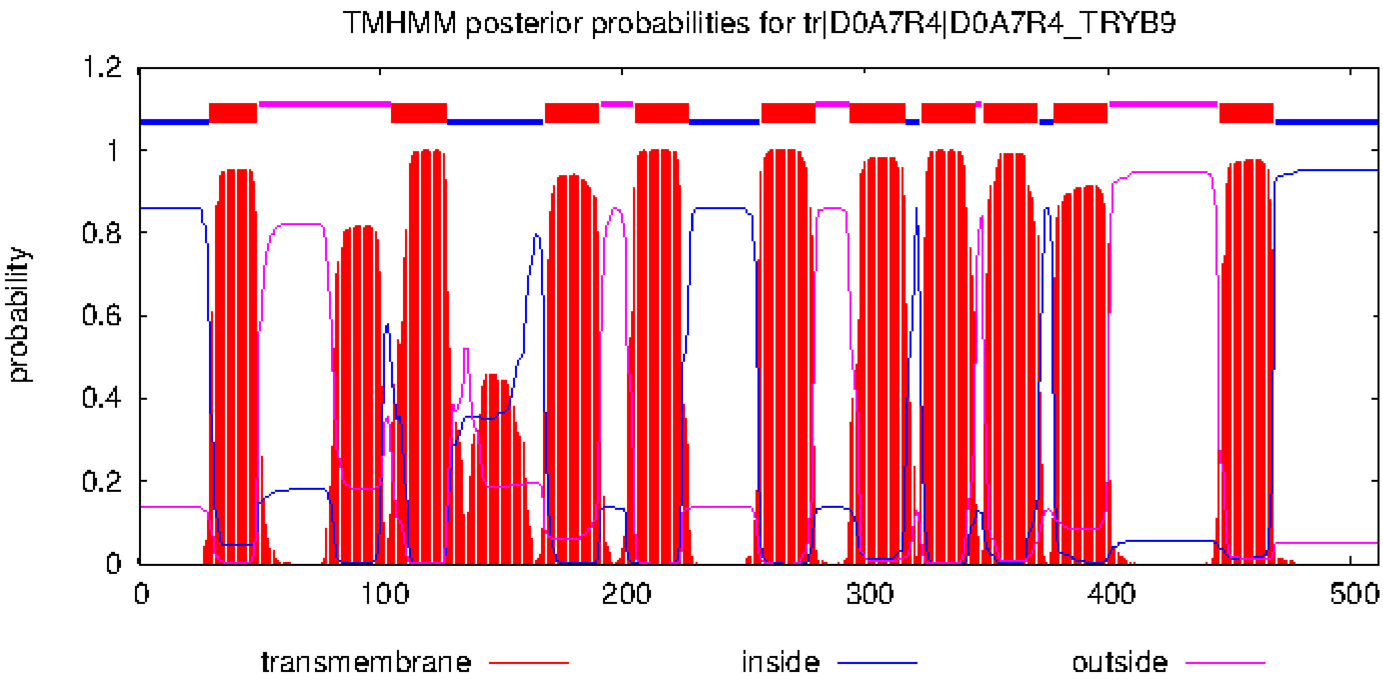

125 # plot in postscript, script for making the plot in gnuplot, data for plot

---

```
# tr|C9ZHT9|C9ZHT9_TRYB9 Length: 516
# tr|C9ZHT9|C9ZHT9_TRYB9 Number of predicted TMHs:  10
# tr|C9ZHT9|C9ZHT9_TRYB9 Exp number of AAs in TMHs: 227.65585
# tr|C9ZHT9|C9ZHT9_TRYB9 Exp number, first 60 AAs: 40.64362
# tr|C9ZHT9|C9ZHT9_TRYB9 Total prob of N-in:        0.04442
# tr|C9ZHT9|C9ZHT9_TRYB9 POSSIBLE N-term signal sequence
tr|C9ZHT9|C9ZHT9_TRYB9  TMHMM2.0        outside      1     4
tr|C9ZHT9|C9ZHT9_TRYB9  TMHMM2.0        TMhelix      5    27
tr|C9ZHT9|C9ZHT9_TRYB9  TMHMM2.0        inside      28    39
tr|C9ZHT9|C9ZHT9_TRYB9  TMHMM2.0        TMhelix     40    62
tr|C9ZHT9|C9ZHT9_TRYB9  TMHMM2.0        outside     63    83
tr|C9ZHT9|C9ZHT9_TRYB9  TMHMM2.0        TMhelix     84   103
tr|C9ZHT9|C9ZHT9_TRYB9  TMHMM2.0        inside     104   114
tr|C9ZHT9|C9ZHT9_TRYB9  TMHMM2.0        TMhelix    115   137
tr|C9ZHT9|C9ZHT9_TRYB9  TMHMM2.0        outside    138   151
tr|C9ZHT9|C9ZHT9_TRYB9  TMHMM2.0        TMhelix    152   174
tr|C9ZHT9|C9ZHT9_TRYB9  TMHMM2.0        inside     175   185
tr|C9ZHT9|C9ZHT9_TRYB9  TMHMM2.0        TMhelix    186   208
tr|C9ZHT9|C9ZHT9_TRYB9  TMHMM2.0        outside    209   222
tr|C9ZHT9|C9ZHT9_TRYB9  TMHMM2.0        TMhelix    223   245
tr|C9ZHT9|C9ZHT9_TRYB9  TMHMM2.0        inside     246   395
tr|C9ZHT9|C9ZHT9_TRYB9  TMHMM2.0        TMhelix    396   418
tr|C9ZHT9|C9ZHT9_TRYB9  TMHMM2.0        outside    419   437
tr|C9ZHT9|C9ZHT9_TRYB9  TMHMM2.0        TMhelix    438   467
tr|C9ZHT9|C9ZHT9_TRYB9  TMHMM2.0        inside     468   486
tr|C9ZHT9|C9ZHT9_TRYB9  TMHMM2.0        TMhelix    487   509
tr|C9ZHT9|C9ZHT9_TRYB9  TMHMM2.0        outside    510   516
```

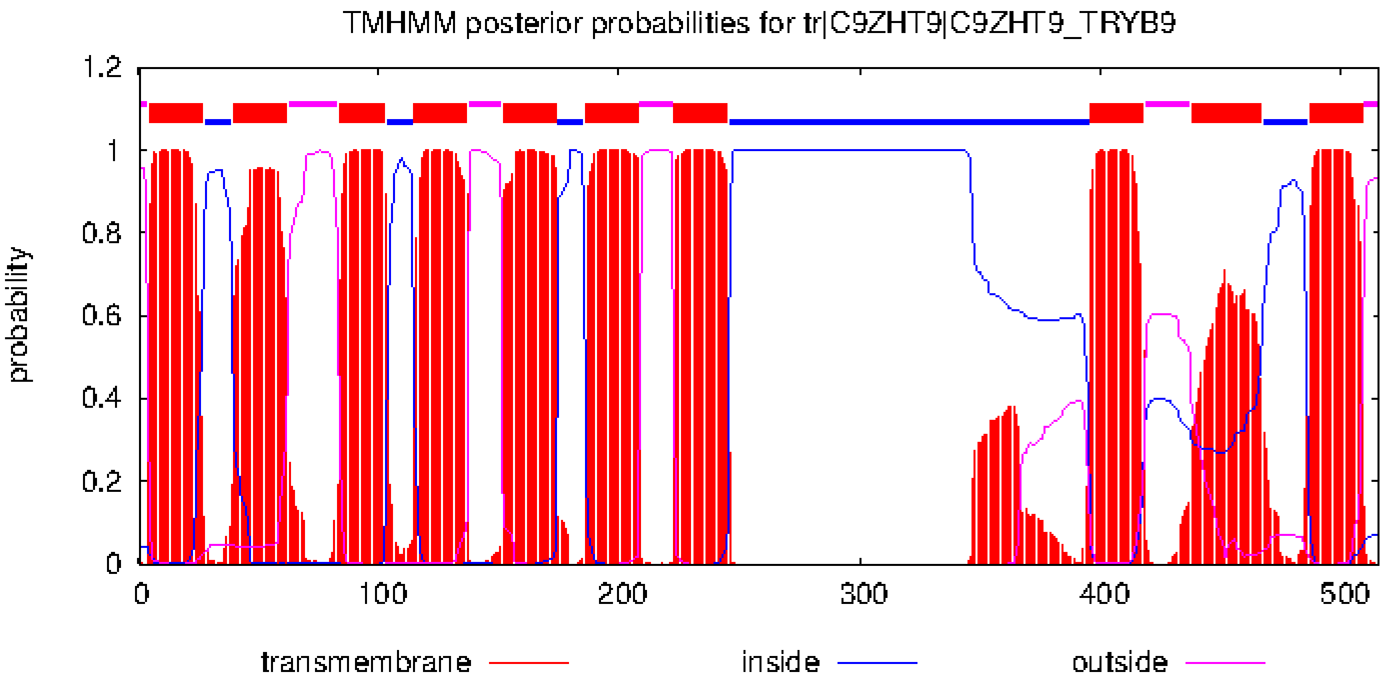

126 # plot in postscript, script for making the plot in gnuplot, data for plot

---

```
# tr|D0A3U2|D0A3U2_TRYB9 Length: 527
# tr|D0A3U2|D0A3U2_TRYB9 Number of predicted TMHs:  12
# tr|D0A3U2|D0A3U2_TRYB9 Exp number of AAs in TMHs: 259.65451
# tr|D0A3U2|D0A3U2_TRYB9 Exp number, first 60 AAs: 16.12081
# tr|D0A3U2|D0A3U2_TRYB9 Total prob of N-in:        0.76092
# tr|D0A3U2|D0A3U2_TRYB9 POSSIBLE N-term signal sequence
tr|D0A3U2|D0A3U2_TRYB9  TMHMM2.0        inside       1    43
tr|D0A3U2|D0A3U2_TRYB9  TMHMM2.0        TMhelix     44    66
tr|D0A3U2|D0A3U2_TRYB9  TMHMM2.0        outside     67   115
tr|D0A3U2|D0A3U2_TRYB9  TMHMM2.0        TMhelix    116   138
tr|D0A3U2|D0A3U2_TRYB9  TMHMM2.0        inside     139   150
tr|D0A3U2|D0A3U2_TRYB9  TMHMM2.0        TMhelix    151   170
tr|D0A3U2|D0A3U2_TRYB9  TMHMM2.0        outside    171   179
tr|D0A3U2|D0A3U2_TRYB9  TMHMM2.0        TMhelix    180   202
tr|D0A3U2|D0A3U2_TRYB9  TMHMM2.0        inside     203   214
tr|D0A3U2|D0A3U2_TRYB9  TMHMM2.0        TMhelix    215   234
tr|D0A3U2|D0A3U2_TRYB9  TMHMM2.0        outside    235   248
tr|D0A3U2|D0A3U2_TRYB9  TMHMM2.0        TMhelix    249   271
tr|D0A3U2|D0A3U2_TRYB9  TMHMM2.0        inside     272   300
tr|D0A3U2|D0A3U2_TRYB9  TMHMM2.0        TMhelix    301   323
tr|D0A3U2|D0A3U2_TRYB9  TMHMM2.0        outside    324   337
tr|D0A3U2|D0A3U2_TRYB9  TMHMM2.0        TMhelix    338   360
tr|D0A3U2|D0A3U2_TRYB9  TMHMM2.0        inside     361   372
tr|D0A3U2|D0A3U2_TRYB9  TMHMM2.0        TMhelix    373   392
tr|D0A3U2|D0A3U2_TRYB9  TMHMM2.0        outside    393   401
tr|D0A3U2|D0A3U2_TRYB9  TMHMM2.0        TMhelix    402   424
tr|D0A3U2|D0A3U2_TRYB9  TMHMM2.0        inside     425   436
tr|D0A3U2|D0A3U2_TRYB9  TMHMM2.0        TMhelix    437   459
tr|D0A3U2|D0A3U2_TRYB9  TMHMM2.0        outside    460   473
tr|D0A3U2|D0A3U2_TRYB9  TMHMM2.0        TMhelix    474   496
tr|D0A3U2|D0A3U2_TRYB9  TMHMM2.0        inside     497   527
```

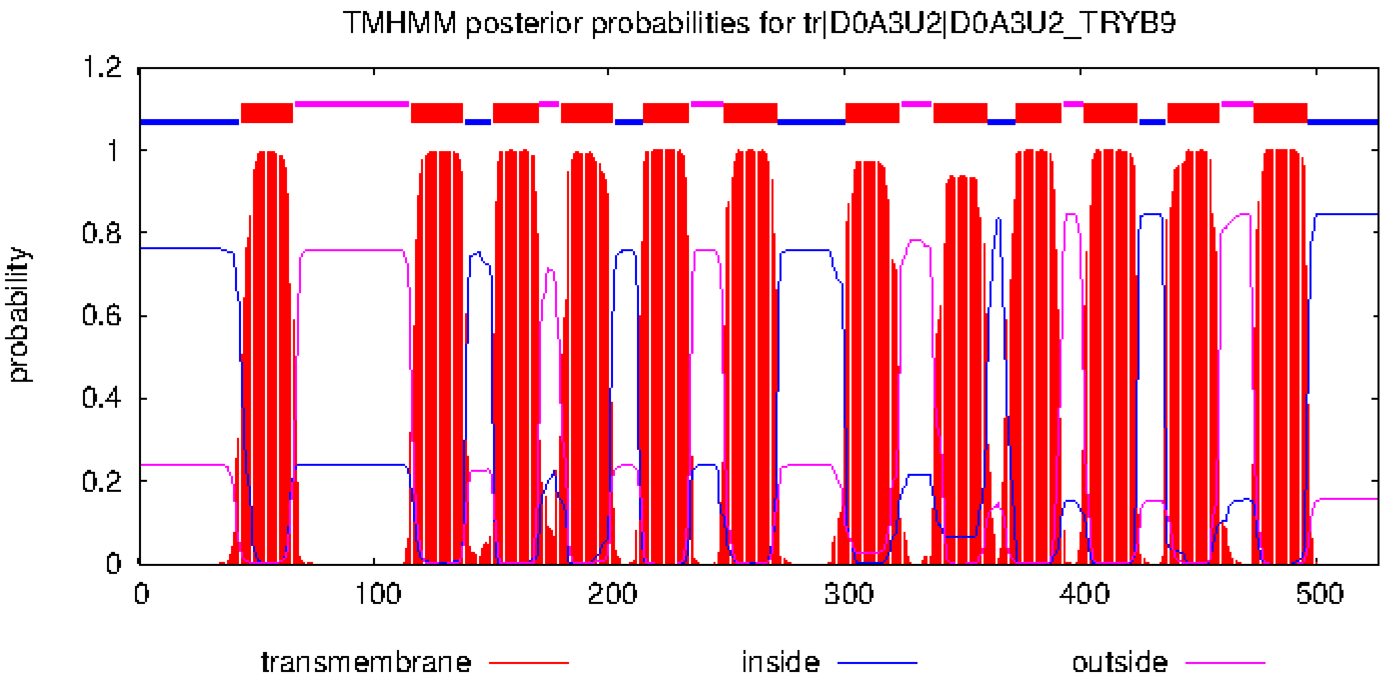

127 # plot in postscript, script for making the plot in gnuplot, data for plot

---

```
# tr|D0A551|D0A551_TRYB9 Length: 534
# tr|D0A551|D0A551_TRYB9 Number of predicted TMHs:  12
# tr|D0A551|D0A551_TRYB9 Exp number of AAs in TMHs: 261.64037
# tr|D0A551|D0A551_TRYB9 Exp number, first 60 AAs: 19.64993
# tr|D0A551|D0A551_TRYB9 Total prob of N-in:        0.17778
# tr|D0A551|D0A551_TRYB9 POSSIBLE N-term signal sequence
tr|D0A551|D0A551_TRYB9  TMHMM2.0        outside      1    10
tr|D0A551|D0A551_TRYB9  TMHMM2.0        TMhelix     11    28
tr|D0A551|D0A551_TRYB9  TMHMM2.0        inside      29   123
tr|D0A551|D0A551_TRYB9  TMHMM2.0        TMhelix    124   146
tr|D0A551|D0A551_TRYB9  TMHMM2.0        outside    147   165
tr|D0A551|D0A551_TRYB9  TMHMM2.0        TMhelix    166   188
tr|D0A551|D0A551_TRYB9  TMHMM2.0        inside     189   199
tr|D0A551|D0A551_TRYB9  TMHMM2.0        TMhelix    200   222
tr|D0A551|D0A551_TRYB9  TMHMM2.0        outside    223   236
tr|D0A551|D0A551_TRYB9  TMHMM2.0        TMhelix    237   256
tr|D0A551|D0A551_TRYB9  TMHMM2.0        inside     257   262
tr|D0A551|D0A551_TRYB9  TMHMM2.0        TMhelix    263   285
tr|D0A551|D0A551_TRYB9  TMHMM2.0        outside    286   304
tr|D0A551|D0A551_TRYB9  TMHMM2.0        TMhelix    305   327
tr|D0A551|D0A551_TRYB9  TMHMM2.0        inside     328   331
tr|D0A551|D0A551_TRYB9  TMHMM2.0        TMhelix    332   349
tr|D0A551|D0A551_TRYB9  TMHMM2.0        outside    350   353
tr|D0A551|D0A551_TRYB9  TMHMM2.0        TMhelix    354   376
tr|D0A551|D0A551_TRYB9  TMHMM2.0        inside     377   387
tr|D0A551|D0A551_TRYB9  TMHMM2.0        TMhelix    388   410
tr|D0A551|D0A551_TRYB9  TMHMM2.0        outside    411   458
tr|D0A551|D0A551_TRYB9  TMHMM2.0        TMhelix    459   481
tr|D0A551|D0A551_TRYB9  TMHMM2.0        inside     482   487
tr|D0A551|D0A551_TRYB9  TMHMM2.0        TMhelix    488   510
tr|D0A551|D0A551_TRYB9  TMHMM2.0        outside    511   534
```

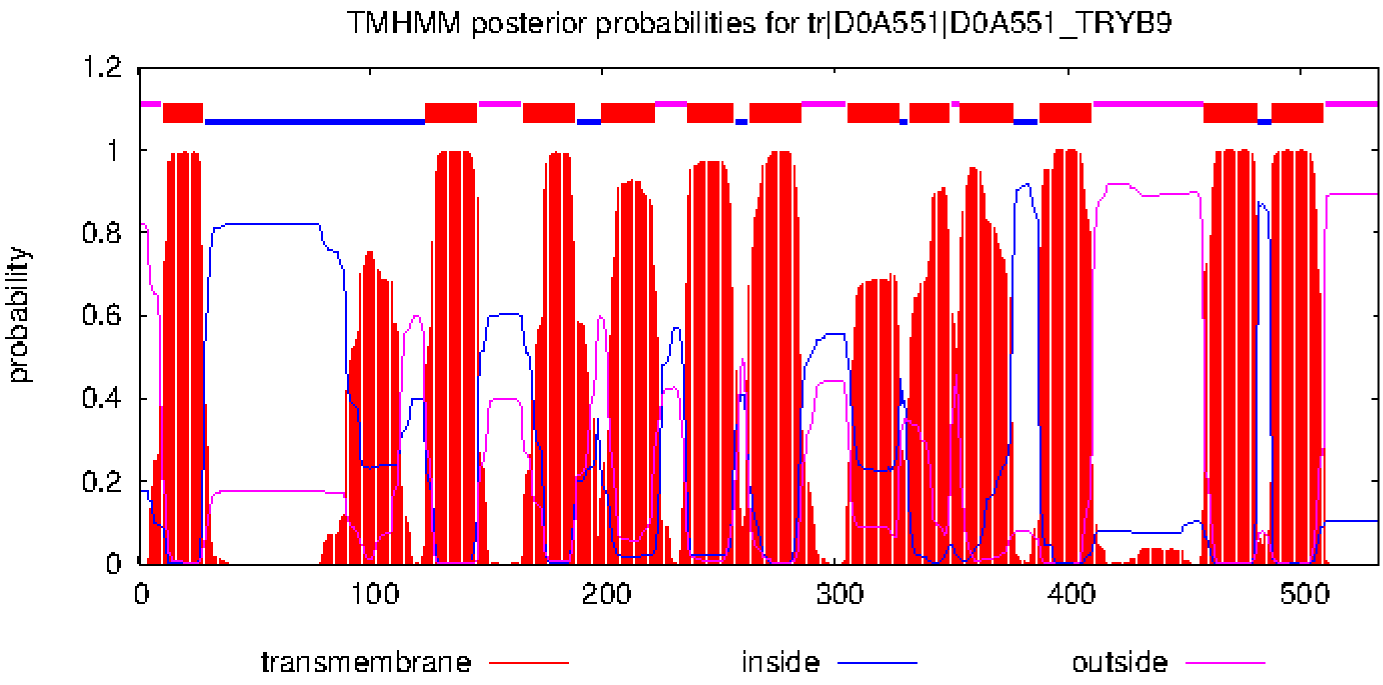

128 # plot in postscript, script for making the plot in gnuplot, data for plot

---

```
# tr|C9ZND1|C9ZND1_TRYB9 Length: 547
# tr|C9ZND1|C9ZND1_TRYB9 Number of predicted TMHs:  12
# tr|C9ZND1|C9ZND1_TRYB9 Exp number of AAs in TMHs: 259.04073
# tr|C9ZND1|C9ZND1_TRYB9 Exp number, first 60 AAs:  25.9644
# tr|C9ZND1|C9ZND1_TRYB9 Total prob of N-in:        0.97015
# tr|C9ZND1|C9ZND1_TRYB9 POSSIBLE N-term signal sequence
tr|C9ZND1|C9ZND1_TRYB9  TMHMM2.0        inside       1    12
tr|C9ZND1|C9ZND1_TRYB9  TMHMM2.0        TMhelix     13    32
tr|C9ZND1|C9ZND1_TRYB9  TMHMM2.0        outside     33    51
tr|C9ZND1|C9ZND1_TRYB9  TMHMM2.0        TMhelix     52    74
tr|C9ZND1|C9ZND1_TRYB9  TMHMM2.0        inside      75    85
tr|C9ZND1|C9ZND1_TRYB9  TMHMM2.0        TMhelix     86   105
tr|C9ZND1|C9ZND1_TRYB9  TMHMM2.0        outside    106   108
tr|C9ZND1|C9ZND1_TRYB9  TMHMM2.0        TMhelix    109   131
tr|C9ZND1|C9ZND1_TRYB9  TMHMM2.0        inside     132   143
tr|C9ZND1|C9ZND1_TRYB9  TMHMM2.0        TMhelix    144   166
tr|C9ZND1|C9ZND1_TRYB9  TMHMM2.0        outside    167   176
tr|C9ZND1|C9ZND1_TRYB9  TMHMM2.0        TMhelix    177   196
tr|C9ZND1|C9ZND1_TRYB9  TMHMM2.0        inside     197   343
tr|C9ZND1|C9ZND1_TRYB9  TMHMM2.0        TMhelix    344   366
tr|C9ZND1|C9ZND1_TRYB9  TMHMM2.0        outside    367   385
tr|C9ZND1|C9ZND1_TRYB9  TMHMM2.0        TMhelix    386   408
tr|C9ZND1|C9ZND1_TRYB9  TMHMM2.0        inside     409   414
tr|C9ZND1|C9ZND1_TRYB9  TMHMM2.0        TMhelix    415   433
tr|C9ZND1|C9ZND1_TRYB9  TMHMM2.0        outside    434   436
tr|C9ZND1|C9ZND1_TRYB9  TMHMM2.0        TMhelix    437   459
tr|C9ZND1|C9ZND1_TRYB9  TMHMM2.0        inside     460   470
tr|C9ZND1|C9ZND1_TRYB9  TMHMM2.0        TMhelix    471   490
tr|C9ZND1|C9ZND1_TRYB9  TMHMM2.0        outside    491   504
tr|C9ZND1|C9ZND1_TRYB9  TMHMM2.0        TMhelix    505   527
tr|C9ZND1|C9ZND1_TRYB9  TMHMM2.0        inside     528   547
```

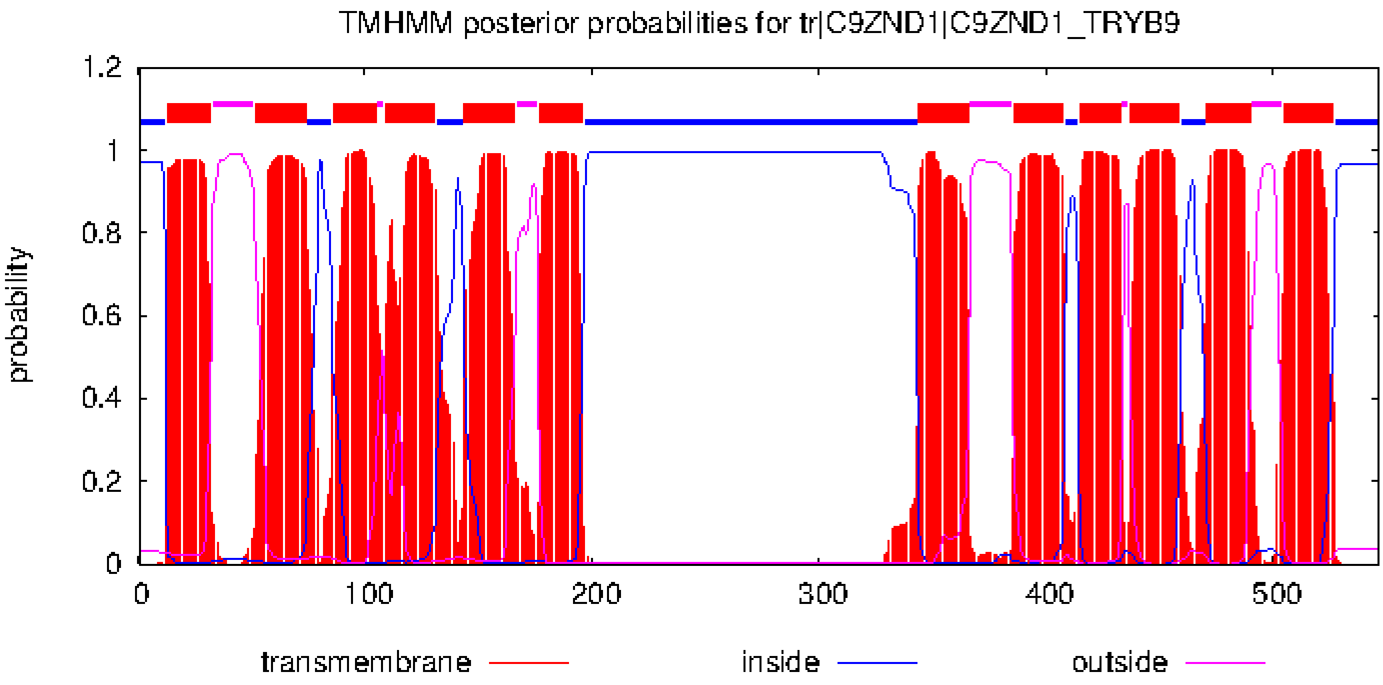

129 # **plot** in postscript, **script** for making the plot in gnuplot, **data** for plot

---

```
# tr|C9ZL98|C9ZL98_TRYB9 Length: 583
# tr|C9ZL98|C9ZL98_TRYB9 Number of predicted TMHs:  14
# tr|C9ZL98|C9ZL98_TRYB9 Exp number of AAs in TMHs: 300.3256
# tr|C9ZL98|C9ZL98_TRYB9 Exp number, first 60 AAs:  33.26319
# tr|C9ZL98|C9ZL98_TRYB9 Total prob of N-in:        0.65552
# tr|C9ZL98|C9ZL98_TRYB9 POSSIBLE N-term signal sequence
tr|C9ZL98|C9ZL98_TRYB9  TMHMM2.0        inside       1    12
tr|C9ZL98|C9ZL98_TRYB9  TMHMM2.0        TMhelix     13    35
tr|C9ZL98|C9ZL98_TRYB9  TMHMM2.0        outside     36    49
tr|C9ZL98|C9ZL98_TRYB9  TMHMM2.0        TMhelix     50    69
tr|C9ZL98|C9ZL98_TRYB9  TMHMM2.0        inside      70    75
tr|C9ZL98|C9ZL98_TRYB9  TMHMM2.0        TMhelix     76    98
tr|C9ZL98|C9ZL98_TRYB9  TMHMM2.0        outside     99   112
tr|C9ZL98|C9ZL98_TRYB9  TMHMM2.0        TMhelix    113   135
tr|C9ZL98|C9ZL98_TRYB9  TMHMM2.0        inside     136   141
tr|C9ZL98|C9ZL98_TRYB9  TMHMM2.0        TMhelix    142   164
tr|C9ZL98|C9ZL98_TRYB9  TMHMM2.0        outside    165   178
tr|C9ZL98|C9ZL98_TRYB9  TMHMM2.0        TMhelix    179   201
tr|C9ZL98|C9ZL98_TRYB9  TMHMM2.0        inside     202   240
tr|C9ZL98|C9ZL98_TRYB9  TMHMM2.0        TMhelix    241   263
tr|C9ZL98|C9ZL98_TRYB9  TMHMM2.0        outside    264   272
tr|C9ZL98|C9ZL98_TRYB9  TMHMM2.0        TMhelix    273   295
tr|C9ZL98|C9ZL98_TRYB9  TMHMM2.0        inside     296   346
tr|C9ZL98|C9ZL98_TRYB9  TMHMM2.0        TMhelix    347   369
tr|C9ZL98|C9ZL98_TRYB9  TMHMM2.0        outside    370   383
tr|C9ZL98|C9ZL98_TRYB9  TMHMM2.0        TMhelix    384   406
tr|C9ZL98|C9ZL98_TRYB9  TMHMM2.0        inside     407   424
tr|C9ZL98|C9ZL98_TRYB9  TMHMM2.0        TMhelix    425   444
tr|C9ZL98|C9ZL98_TRYB9  TMHMM2.0        outside    445   448
tr|C9ZL98|C9ZL98_TRYB9  TMHMM2.0        TMhelix    449   471
tr|C9ZL98|C9ZL98_TRYB9  TMHMM2.0        inside     472   483
tr|C9ZL98|C9ZL98_TRYB9  TMHMM2.0        TMhelix    484   506
tr|C9ZL98|C9ZL98_TRYB9  TMHMM2.0        outside    507   528
tr|C9ZL98|C9ZL98_TRYB9  TMHMM2.0        TMhelix    529   551
tr|C9ZL98|C9ZL98_TRYB9  TMHMM2.0        inside     552   583
```

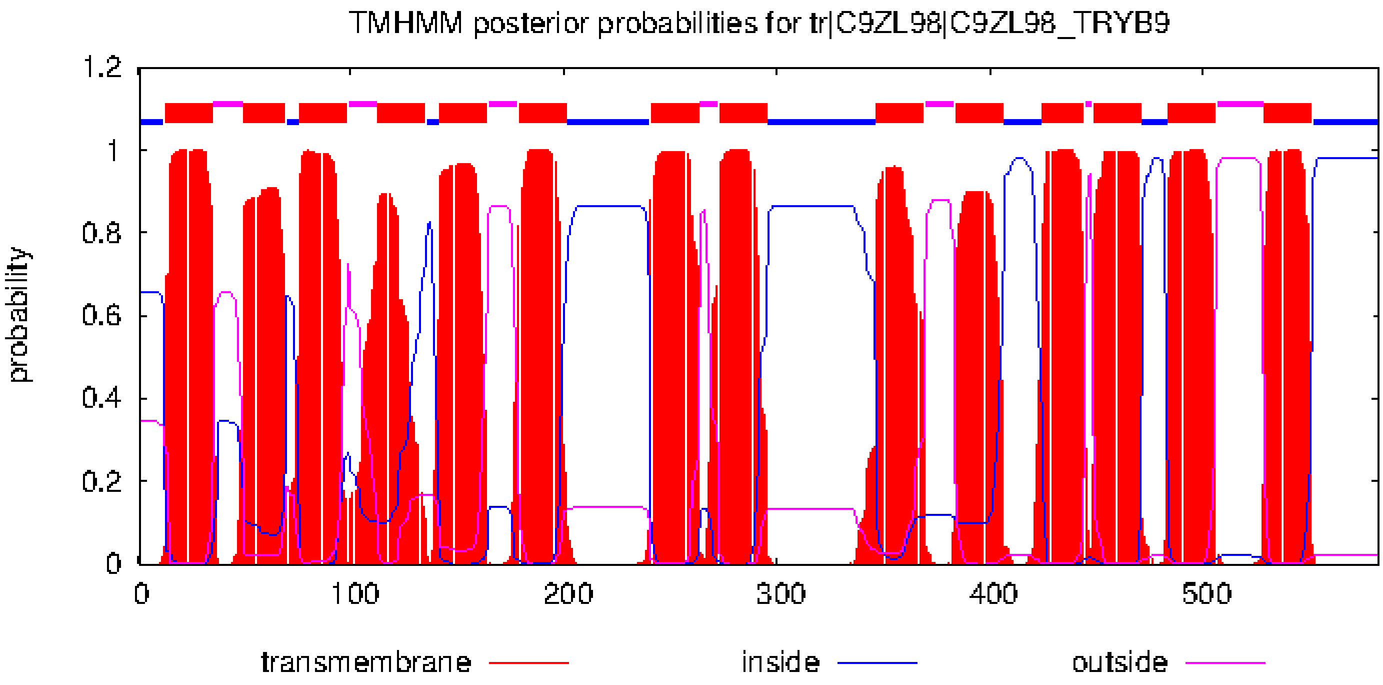

130 # plot in postscript, script for making the plot in gnuplot, data for plot

---

```
# tr|C9ZLA0|C9ZLA0_TRYB9 Length: 585
# tr|C9ZLA0|C9ZLA0_TRYB9 Number of predicted TMHs:  13
# tr|C9ZLA0|C9ZLA0_TRYB9 Exp number of AAs in TMHs: 296.380309999999
# tr|C9ZLA0|C9ZLA0_TRYB9 Exp number, first 60 AAs:  33.64141
# tr|C9ZLA0|C9ZLA0_TRYB9 Total prob of N-in:        0.51391
# tr|C9ZLA0|C9ZLA0_TRYB9 POSSIBLE N-term signal sequence
tr|C9ZLA0|C9ZLA0_TRYB9  TMHMM2.0        outside      1    14
tr|C9ZLA0|C9ZLA0_TRYB9  TMHMM2.0        TMhelix     15    37
tr|C9ZLA0|C9ZLA0_TRYB9  TMHMM2.0        inside      38    49
tr|C9ZLA0|C9ZLA0_TRYB9  TMHMM2.0        TMhelix     50    67
tr|C9ZLA0|C9ZLA0_TRYB9  TMHMM2.0        outside     68    70
tr|C9ZLA0|C9ZLA0_TRYB9  TMHMM2.0        TMhelix     71    93
tr|C9ZLA0|C9ZLA0_TRYB9  TMHMM2.0        inside      94    99
tr|C9ZLA0|C9ZLA0_TRYB9  TMHMM2.0        TMhelix    100   122
tr|C9ZLA0|C9ZLA0_TRYB9  TMHMM2.0        outside    123   141
tr|C9ZLA0|C9ZLA0_TRYB9  TMHMM2.0        TMhelix    142   164
tr|C9ZLA0|C9ZLA0_TRYB9  TMHMM2.0        inside     165   176
tr|C9ZLA0|C9ZLA0_TRYB9  TMHMM2.0        TMhelix    177   199
tr|C9ZLA0|C9ZLA0_TRYB9  TMHMM2.0        outside    200   240
tr|C9ZLA0|C9ZLA0_TRYB9  TMHMM2.0        TMhelix    241   263
tr|C9ZLA0|C9ZLA0_TRYB9  TMHMM2.0        inside     264   269
tr|C9ZLA0|C9ZLA0_TRYB9  TMHMM2.0        TMhelix    270   292
tr|C9ZLA0|C9ZLA0_TRYB9  TMHMM2.0        outside    293   341
tr|C9ZLA0|C9ZLA0_TRYB9  TMHMM2.0        TMhelix    342   364
tr|C9ZLA0|C9ZLA0_TRYB9  TMHMM2.0        inside     365   384
tr|C9ZLA0|C9ZLA0_TRYB9  TMHMM2.0        TMhelix    385   404
tr|C9ZLA0|C9ZLA0_TRYB9  TMHMM2.0        outside    405   423
tr|C9ZLA0|C9ZLA0_TRYB9  TMHMM2.0        TMhelix    424   446
tr|C9ZLA0|C9ZLA0_TRYB9  TMHMM2.0        inside     447   452
tr|C9ZLA0|C9ZLA0_TRYB9  TMHMM2.0        TMhelix    453   475
tr|C9ZLA0|C9ZLA0_TRYB9  TMHMM2.0        outside    476   528
tr|C9ZLA0|C9ZLA0_TRYB9  TMHMM2.0        TMhelix    529   551
tr|C9ZLA0|C9ZLA0_TRYB9  TMHMM2.0        inside     552   585
```

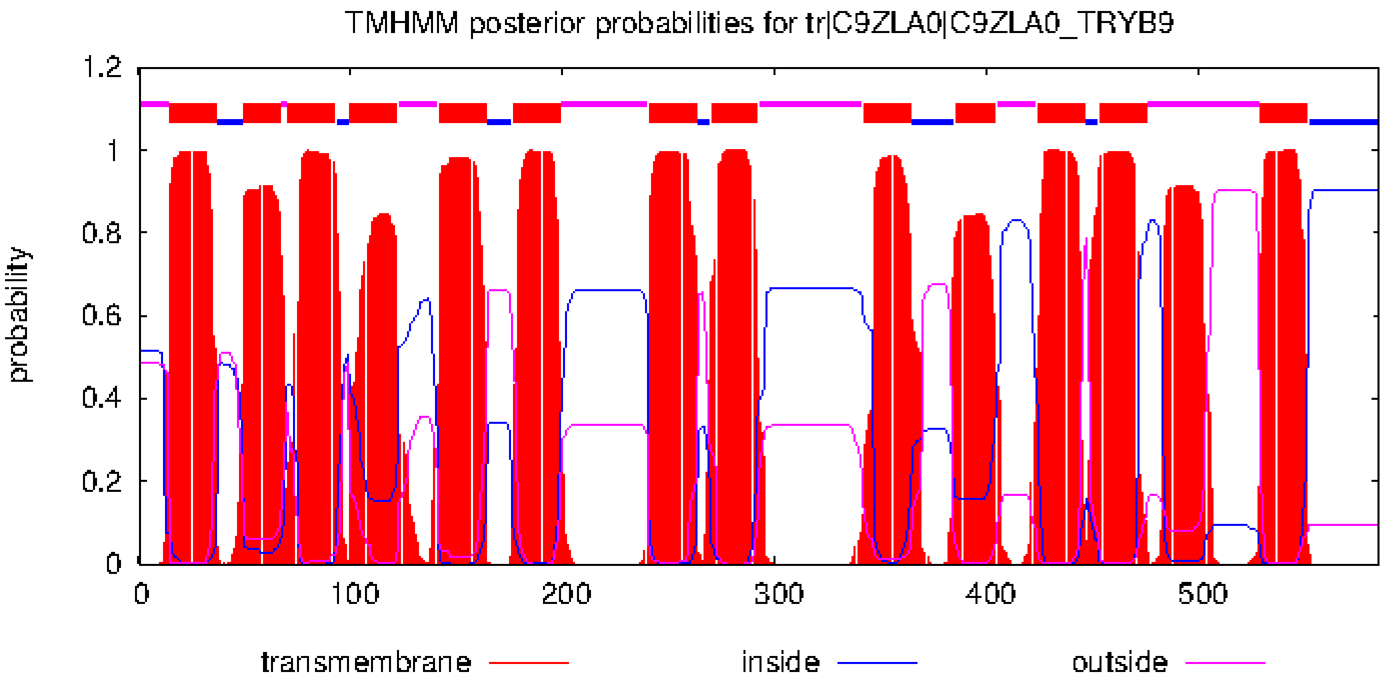

131 # plot in postscript, script for making the plot in gnuplot, data for plot

---

```
# tr|C9ZL97|C9ZL97_TRYB9 Length: 595
# tr|C9ZL97|C9ZL97_TRYB9 Number of predicted TMHs:  12
# tr|C9ZL97|C9ZL97_TRYB9 Exp number of AAs in TMHs: 289.14665
# tr|C9ZL97|C9ZL97_TRYB9 Exp number, first 60 AAs:  26.74978
# tr|C9ZL97|C9ZL97_TRYB9 Total prob of N-in:        0.59405
# tr|C9ZL97|C9ZL97_TRYB9 POSSIBLE N-term signal sequence
tr|C9ZL97|C9ZL97_TRYB9  TMHMM2.0        inside       1    12
tr|C9ZL97|C9ZL97_TRYB9  TMHMM2.0        TMhelix     13    35
tr|C9ZL97|C9ZL97_TRYB9  TMHMM2.0        outside     36    70
tr|C9ZL97|C9ZL97_TRYB9  TMHMM2.0        TMhelix     71    93
tr|C9ZL97|C9ZL97_TRYB9  TMHMM2.0        inside      94    99
tr|C9ZL97|C9ZL97_TRYB9  TMHMM2.0        TMhelix    100   122
tr|C9ZL97|C9ZL97_TRYB9  TMHMM2.0        outside    123   141
tr|C9ZL97|C9ZL97_TRYB9  TMHMM2.0        TMhelix    142   164
tr|C9ZL97|C9ZL97_TRYB9  TMHMM2.0        inside     165   176
tr|C9ZL97|C9ZL97_TRYB9  TMHMM2.0        TMhelix    177   199
tr|C9ZL97|C9ZL97_TRYB9  TMHMM2.0        outside    200   240
tr|C9ZL97|C9ZL97_TRYB9  TMHMM2.0        TMhelix    241   263
tr|C9ZL97|C9ZL97_TRYB9  TMHMM2.0        inside     264   269
tr|C9ZL97|C9ZL97_TRYB9  TMHMM2.0        TMhelix    270   292
tr|C9ZL97|C9ZL97_TRYB9  TMHMM2.0        outside    293   341
tr|C9ZL97|C9ZL97_TRYB9  TMHMM2.0        TMhelix    342   364
tr|C9ZL97|C9ZL97_TRYB9  TMHMM2.0        inside     365   420
tr|C9ZL97|C9ZL97_TRYB9  TMHMM2.0        TMhelix    421   443
tr|C9ZL97|C9ZL97_TRYB9  TMHMM2.0        outside    444   447
tr|C9ZL97|C9ZL97_TRYB9  TMHMM2.0        TMhelix    448   470
tr|C9ZL97|C9ZL97_TRYB9  TMHMM2.0        inside     471   482
tr|C9ZL97|C9ZL97_TRYB9  TMHMM2.0        TMhelix    483   505
tr|C9ZL97|C9ZL97_TRYB9  TMHMM2.0        outside    506   528
tr|C9ZL97|C9ZL97_TRYB9  TMHMM2.0        TMhelix    529   551
tr|C9ZL97|C9ZL97_TRYB9  TMHMM2.0        inside     552   595
```

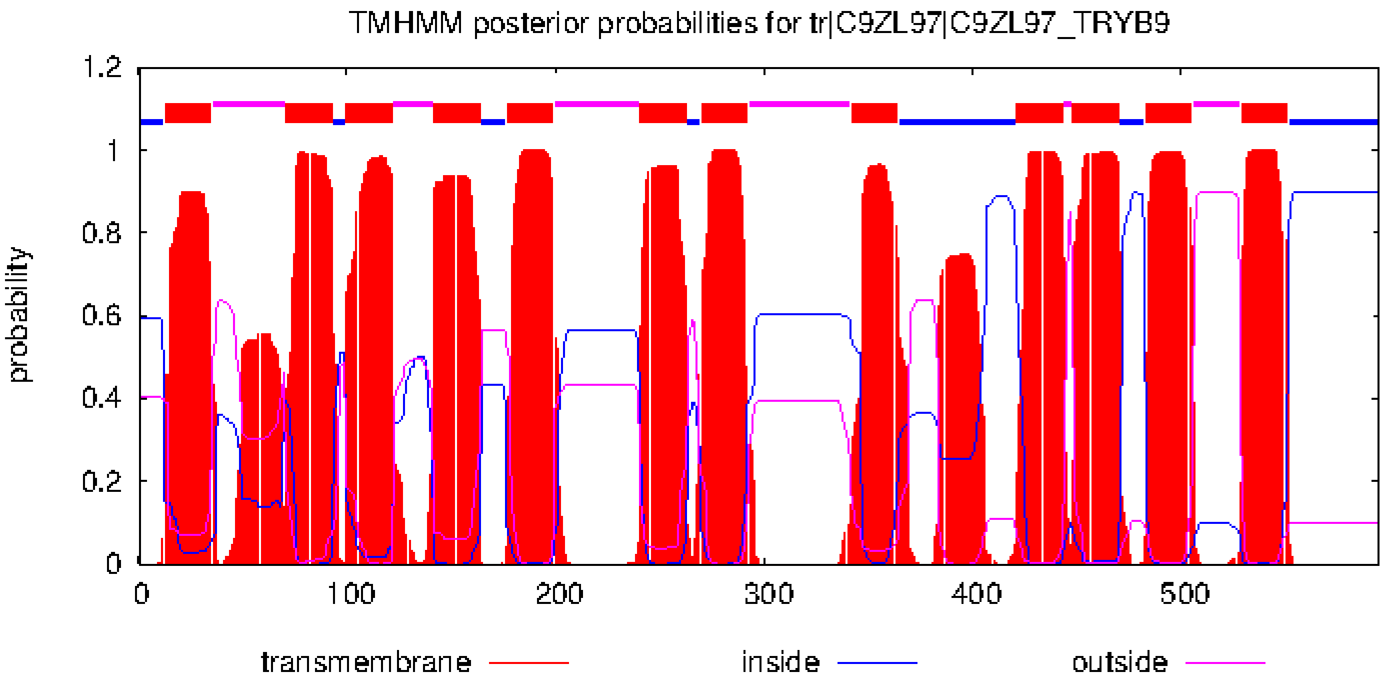

132 # plot in postscript, script for making the plot in gnuplot, data for plot

---

```
# tr|D0A939|D0A939_TRYB9 Length: 598
# tr|D0A939|D0A939_TRYB9 Number of predicted TMHs:  6
# tr|D0A939|D0A939_TRYB9 Exp number of AAs in TMHs: 227.49931
#  tr|D0A939|D0A939_TRYB9 Exp number, first 60 AAs: 30.15866
# tr|D0A939|D0A939_TRYB9 Total prob of N-in:        0.97727
# tr|D0A939|D0A939_TRYB9 POSSIBLE N-term signal sequence
tr|D0A939|D0A939_TRYB9  TMHMM2.0        inside       1     6
tr|D0A939|D0A939_TRYB9  TMHMM2.0        TMhelix      7    29
tr|D0A939|D0A939_TRYB9  TMHMM2.0        outside     30   389
tr|D0A939|D0A939_TRYB9  TMHMM2.0        TMhelix    390   412
tr|D0A939|D0A939_TRYB9  TMHMM2.0        inside     413   423
tr|D0A939|D0A939_TRYB9  TMHMM2.0        TMhelix    424   441
tr|D0A939|D0A939_TRYB9  TMHMM2.0        outside    442   455
tr|D0A939|D0A939_TRYB9  TMHMM2.0        TMhelix    456   478
tr|D0A939|D0A939_TRYB9  TMHMM2.0        inside     479   484
tr|D0A939|D0A939_TRYB9  TMHMM2.0        TMhelix    485   507
tr|D0A939|D0A939_TRYB9  TMHMM2.0        outside    508   548
tr|D0A939|D0A939_TRYB9  TMHMM2.0        TMhelix    549   571
tr|D0A939|D0A939_TRYB9  TMHMM2.0        inside     572   598
```

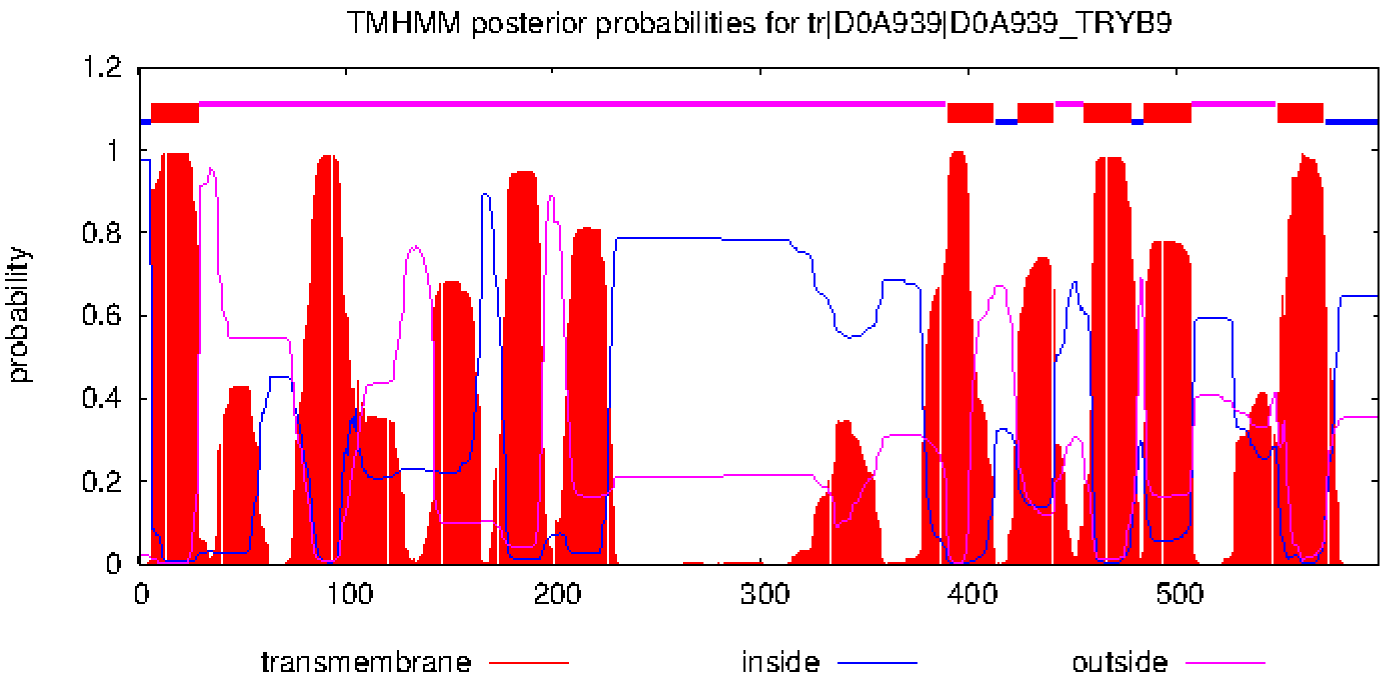

133 # plot in postscript, script for making the plot in gnuplot, data for plot

---

```
# tr|D0A2F4|D0A2F4_TRYB9 Length: 599
# tr|D0A2F4|D0A2F4_TRYB9 Number of predicted TMHs:  10
# tr|D0A2F4|D0A2F4_TRYB9 Exp number of AAs in TMHs: 218.25539
# tr|D0A2F4|D0A2F4_TRYB9 Exp number, first 60 AAs:  0.01835
# tr|D0A2F4|D0A2F4_TRYB9 Total prob of N-in:        0.99350
tr|D0A2F4|D0A2F4_TRYB9  TMHMM2.0        inside       1   107
tr|D0A2F4|D0A2F4_TRYB9  TMHMM2.0        TMhelix    108   130
tr|D0A2F4|D0A2F4_TRYB9  TMHMM2.0        outside    131   133
tr|D0A2F4|D0A2F4_TRYB9  TMHMM2.0        TMhelix    134   153
tr|D0A2F4|D0A2F4_TRYB9  TMHMM2.0        inside     154   164
tr|D0A2F4|D0A2F4_TRYB9  TMHMM2.0        TMhelix    165   187
tr|D0A2F4|D0A2F4_TRYB9  TMHMM2.0        outside    188   223
tr|D0A2F4|D0A2F4_TRYB9  TMHMM2.0        TMhelix    224   246
tr|D0A2F4|D0A2F4_TRYB9  TMHMM2.0        inside     247   258
tr|D0A2F4|D0A2F4_TRYB9  TMHMM2.0        TMhelix    259   278
tr|D0A2F4|D0A2F4_TRYB9  TMHMM2.0        outside    279   287
tr|D0A2F4|D0A2F4_TRYB9  TMHMM2.0        TMhelix    288   310
tr|D0A2F4|D0A2F4_TRYB9  TMHMM2.0        inside     311   402
tr|D0A2F4|D0A2F4_TRYB9  TMHMM2.0        TMhelix    403   425
tr|D0A2F4|D0A2F4_TRYB9  TMHMM2.0        outside    426   465
tr|D0A2F4|D0A2F4_TRYB9  TMHMM2.0        TMhelix    466   488
tr|D0A2F4|D0A2F4_TRYB9  TMHMM2.0        inside     489   507
tr|D0A2F4|D0A2F4_TRYB9  TMHMM2.0        TMhelix    508   525
tr|D0A2F4|D0A2F4_TRYB9  TMHMM2.0        outside    526   559
tr|D0A2F4|D0A2F4_TRYB9  TMHMM2.0        TMhelix    560   582
tr|D0A2F4|D0A2F4_TRYB9  TMHMM2.0        inside     583   599
```

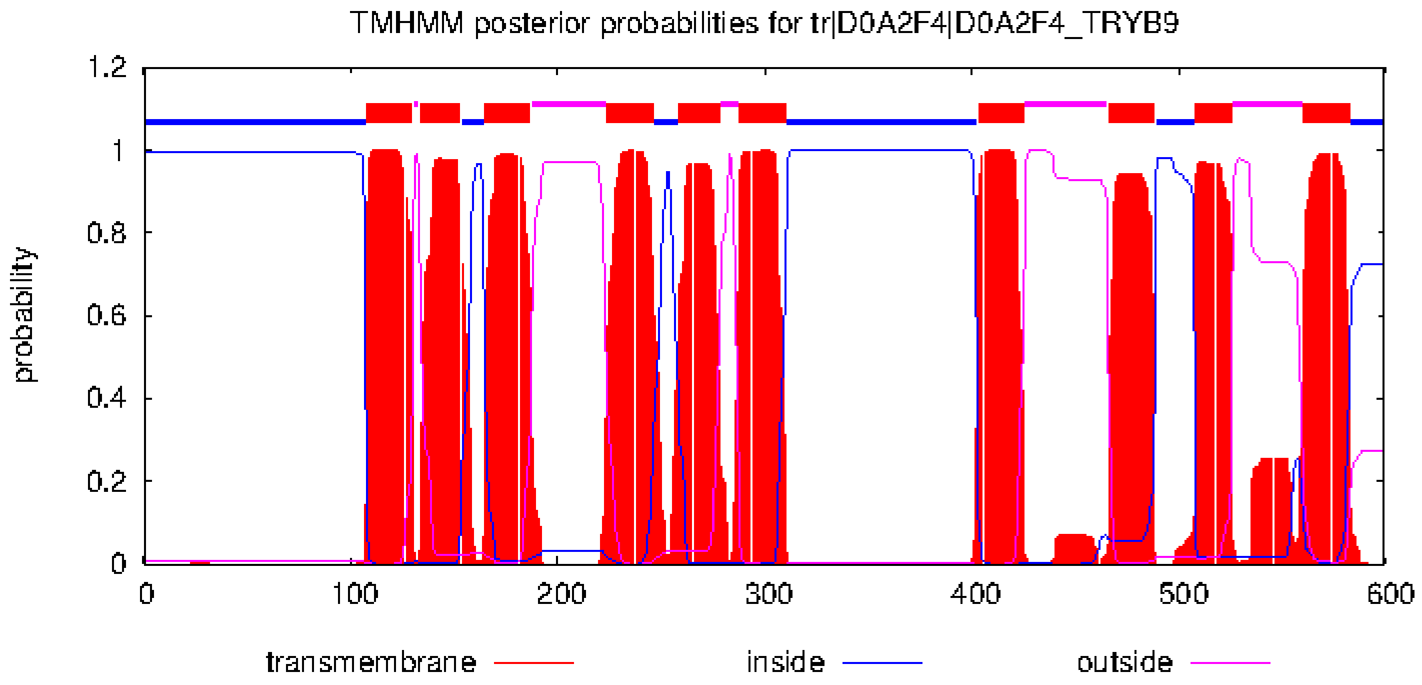

134 # plot in postscript, script for making the plot in gnuplot, data for plot

---

```
# tr|C9ZIK0|C9ZIK0_TRYB9 Length: 652
# tr|C9ZIK0|C9ZIK0_TRYB9 Number of predicted TMHs:  13
# tr|C9ZIK0|C9ZIK0_TRYB9 Exp number of AAs in TMHs: 285.92108
# tr|C9ZIK0|C9ZIK0_TRYB9 Exp number, first 60 AAs:  0.00425
# tr|C9ZIK0|C9ZIK0_TRYB9 Total prob of N-in:        0.37999
tr|C9ZIK0|C9ZIK0_TRYB9  TMHMM2.0        outside      1    69
tr|C9ZIK0|C9ZIK0_TRYB9  TMHMM2.0        TMhelix     70    92
tr|C9ZIK0|C9ZIK0_TRYB9  TMHMM2.0        inside      93   111
tr|C9ZIK0|C9ZIK0_TRYB9  TMHMM2.0        TMhelix    112   131
tr|C9ZIK0|C9ZIK0_TRYB9  TMHMM2.0        outside    132   150
tr|C9ZIK0|C9ZIK0_TRYB9  TMHMM2.0        TMhelix    151   173
tr|C9ZIK0|C9ZIK0_TRYB9  TMHMM2.0        inside     174   197
tr|C9ZIK0|C9ZIK0_TRYB9  TMHMM2.0        TMhelix    198   220
tr|C9ZIK0|C9ZIK0_TRYB9  TMHMM2.0        outside    221   229
tr|C9ZIK0|C9ZIK0_TRYB9  TMHMM2.0        TMhelix    230   252
tr|C9ZIK0|C9ZIK0_TRYB9  TMHMM2.0        inside     253   347
tr|C9ZIK0|C9ZIK0_TRYB9  TMHMM2.0        TMhelix    348   367
tr|C9ZIK0|C9ZIK0_TRYB9  TMHMM2.0        outside    368   371
tr|C9ZIK0|C9ZIK0_TRYB9  TMHMM2.0        TMhelix    372   391
tr|C9ZIK0|C9ZIK0_TRYB9  TMHMM2.0        inside     392   397
tr|C9ZIK0|C9ZIK0_TRYB9  TMHMM2.0        TMhelix    398   420
tr|C9ZIK0|C9ZIK0_TRYB9  TMHMM2.0        outside    421   434
tr|C9ZIK0|C9ZIK0_TRYB9  TMHMM2.0        TMhelix    435   457
tr|C9ZIK0|C9ZIK0_TRYB9  TMHMM2.0        inside     458   469
tr|C9ZIK0|C9ZIK0_TRYB9  TMHMM2.0        TMhelix    470   489
tr|C9ZIK0|C9ZIK0_TRYB9  TMHMM2.0        outside    490   498
tr|C9ZIK0|C9ZIK0_TRYB9  TMHMM2.0        TMhelix    499   521
tr|C9ZIK0|C9ZIK0_TRYB9  TMHMM2.0        inside     522   541
tr|C9ZIK0|C9ZIK0_TRYB9  TMHMM2.0        TMhelix    542   564
tr|C9ZIK0|C9ZIK0_TRYB9  TMHMM2.0        outside    565   578
tr|C9ZIK0|C9ZIK0_TRYB9  TMHMM2.0        TMhelix    579   601
tr|C9ZIK0|C9ZIK0_TRYB9  TMHMM2.0        inside     602   652
```

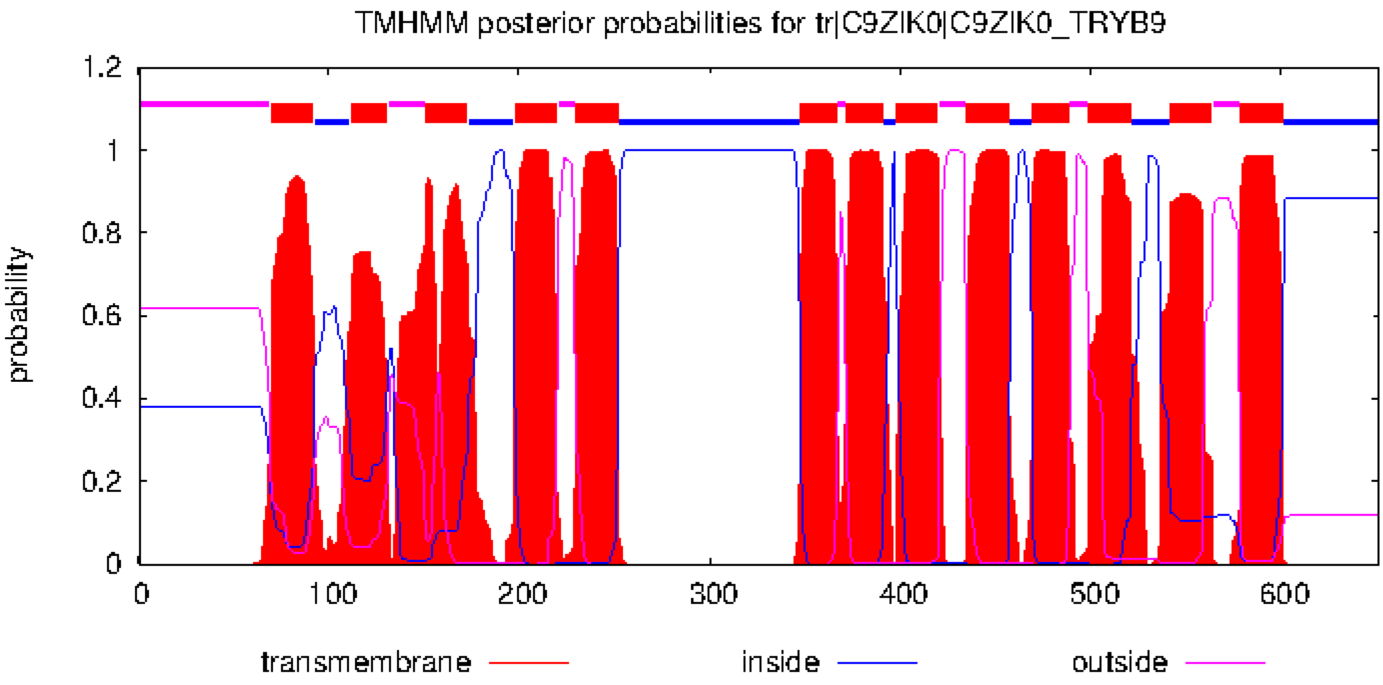

135 # **plot in postscript, script for making the plot in gnuplot, data for plot**

```
# tr|D0A3K9|D0A3K9_TRYB9 Length: 668
# tr|D0A3K9|D0A3K9_TRYB9 Number of predicted TMHs:  5
# tr|D0A3K9|D0A3K9_TRYB9 Exp number of AAs in TMHs: 113.44292
# tr|D0A3K9|D0A3K9_TRYB9 Exp number, first 60 AAs:  0.00171
# tr|D0A3K9|D0A3K9_TRYB9 Total prob of N-in:        0.16657
tr|D0A3K9|D0A3K9_TRYB9  TMHMM2.0        outside      1   404
tr|D0A3K9|D0A3K9_TRYB9  TMHMM2.0        TMhelix    405   427
tr|D0A3K9|D0A3K9_TRYB9  TMHMM2.0        inside     428   493
tr|D0A3K9|D0A3K9_TRYB9  TMHMM2.0        TMhelix    494   513
tr|D0A3K9|D0A3K9_TRYB9  TMHMM2.0        outside    514   516
tr|D0A3K9|D0A3K9_TRYB9  TMHMM2.0        TMhelix    517   539
tr|D0A3K9|D0A3K9_TRYB9  TMHMM2.0        inside     540   545
tr|D0A3K9|D0A3K9_TRYB9  TMHMM2.0        TMhelix    546   568
tr|D0A3K9|D0A3K9_TRYB9  TMHMM2.0        outside    569   638
tr|D0A3K9|D0A3K9_TRYB9  TMHMM2.0        TMhelix    639   661
tr|D0A3K9|D0A3K9_TRYB9  TMHMM2.0        inside     662   668
```

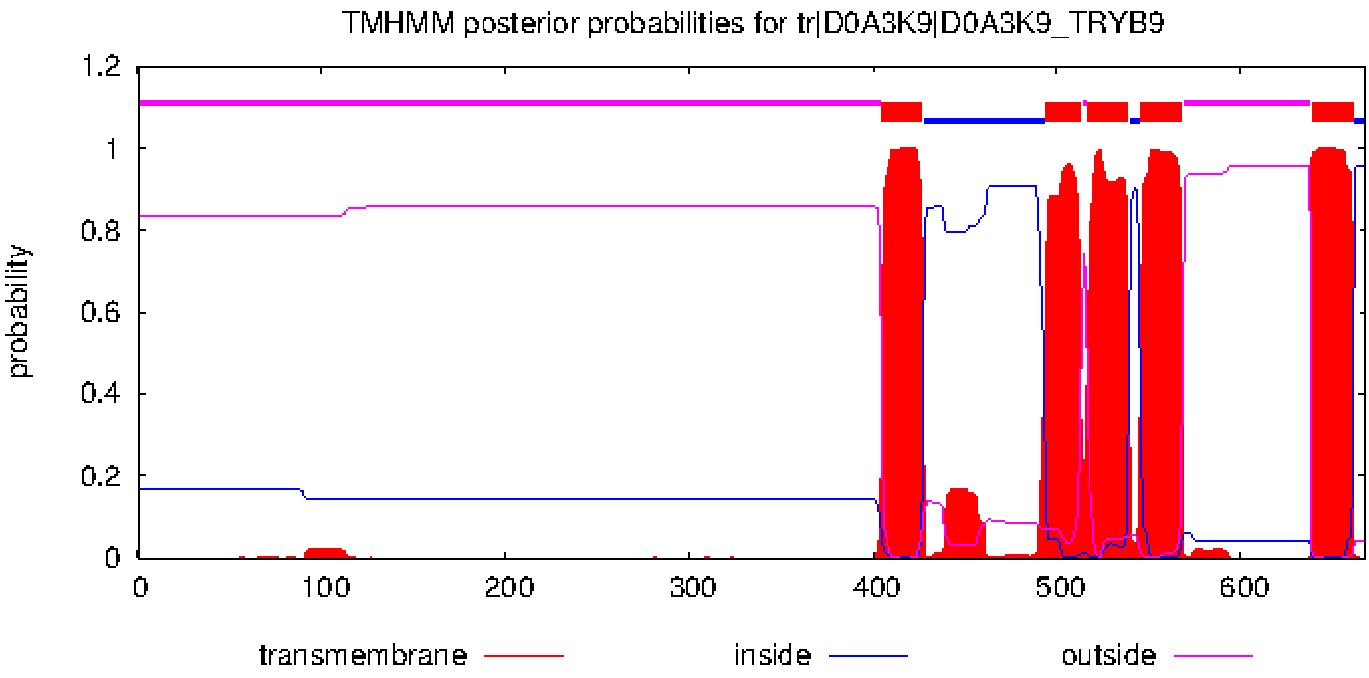

136 # plot in postscript, script for making the plot in gnuplot, data for plot

---

```
# tr|D0A214|D0A214_TRYB9 Length: 698
# tr|D0A214|D0A214_TRYB9 Number of predicted TMHs:  3
# tr|D0A214|D0A214_TRYB9 Exp number of AAs in TMHs: 67.8079000000001
# tr|D0A214|D0A214_TRYB9 Exp number, first 60 AAs:  0.00255
# tr|D0A214|D0A214_TRYB9 Total prob of N-in:        0.07898
tr|D0A214|D0A214_TRYB9  TMHMM2.0        outside      1   513
tr|D0A214|D0A214_TRYB9  TMHMM2.0        TMhelix    514   536
tr|D0A214|D0A214_TRYB9  TMHMM2.0        inside     537   565
tr|D0A214|D0A214_TRYB9  TMHMM2.0        TMhelix    566   588
tr|D0A214|D0A214_TRYB9  TMHMM2.0        outside    589   591
tr|D0A214|D0A214_TRYB9  TMHMM2.0        TMhelix    592   611
tr|D0A214|D0A214_TRYB9  TMHMM2.0        inside     612   698
```

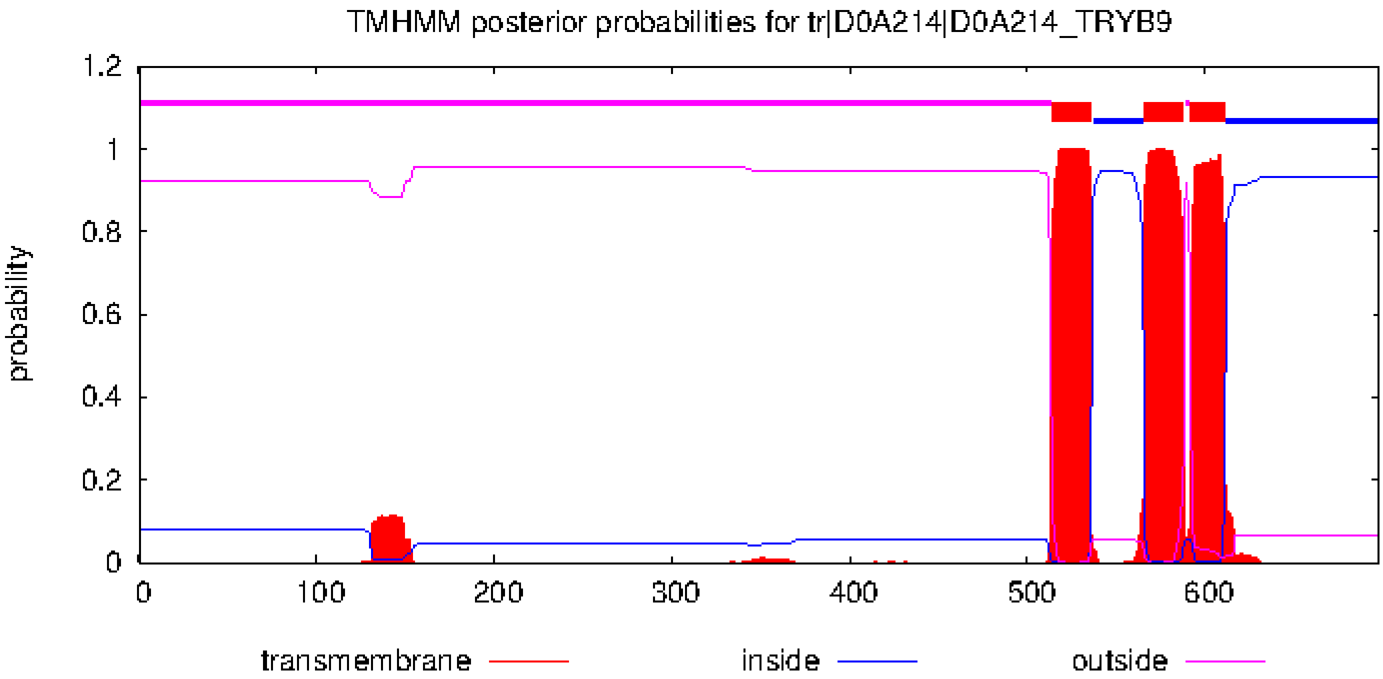

137 # plot in postscript, script for making the plot in gnuplot, data for plot

---

```
# tr|D0A0K9|D0A0K9_TRYB9 Length: 699
# tr|D0A0K9|D0A0K9_TRYB9 Number of predicted TMHs:  1
# tr|D0A0K9|D0A0K9_TRYB9 Exp number of AAs in TMHs: 22.51518
# tr|D0A0K9|D0A0K9_TRYB9 Exp number, first 60 AAs:  20.45118
# tr|D0A0K9|D0A0K9_TRYB9 Total prob of N-in:        0.99531
# tr|D0A0K9|D0A0K9_TRYB9 POSSIBLE N-term signal sequence
tr|D0A0K9|D0A0K9_TRYB9  TMHMM2.0        inside       1    39
tr|D0A0K9|D0A0K9_TRYB9  TMHMM2.0        TMhelix     40    62
tr|D0A0K9|D0A0K9_TRYB9  TMHMM2.0        outside     63   699
```

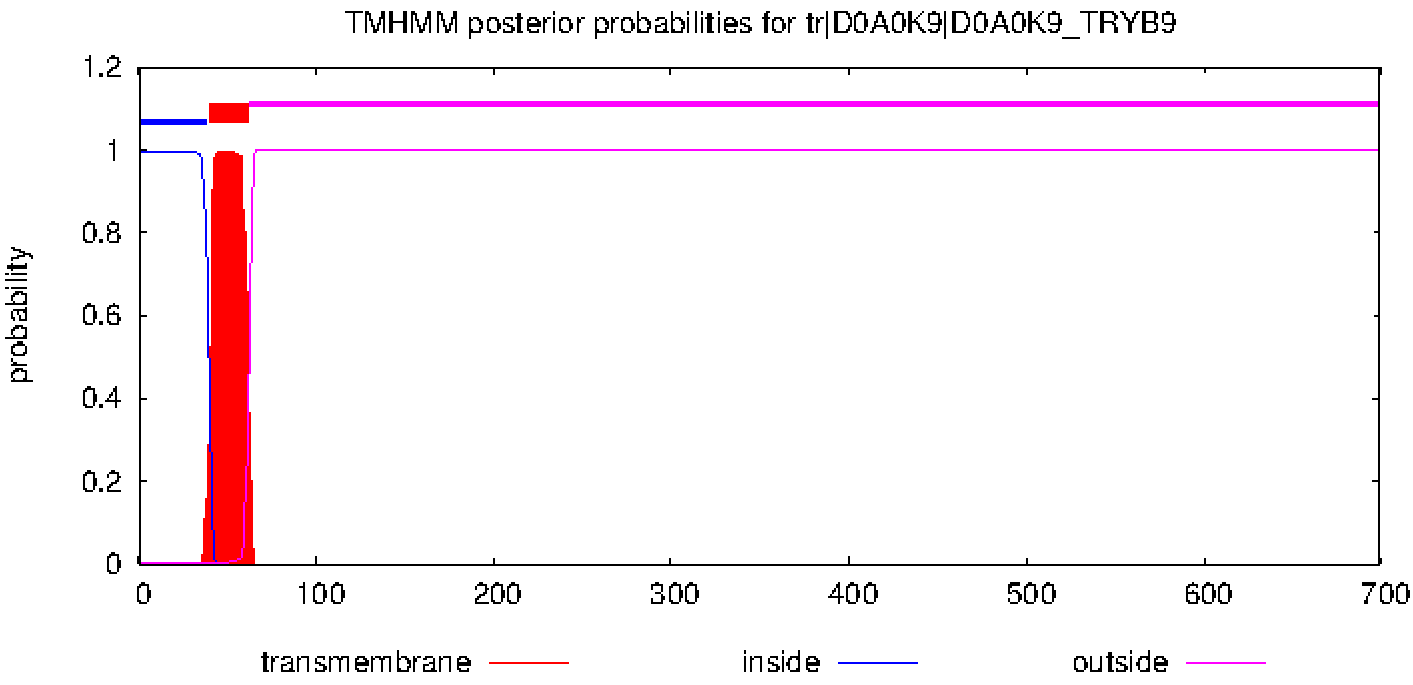

138 # plot in postscript, script for making the plot in gnuplot, data for plot

---

```
# tr|C9ZNL1|C9ZNL1_TRYB9 Length: 821
# tr|C9ZNL1|C9ZNL1_TRYB9 Number of predicted TMHs:  11
# tr|C9ZNL1|C9ZNL1_TRYB9 Exp number of AAs in TMHs: 239.68266
# tr|C9ZNL1|C9ZNL1_TRYB9 Exp number, first 60 AAs: 19.45711
# tr|C9ZNL1|C9ZNL1_TRYB9 Total prob of N-in:        0.11855
# tr|C9ZNL1|C9ZNL1_TRYB9 POSSIBLE N-term signal sequence
tr|C9ZNL1|C9ZNL1_TRYB9  TMHMM2.0        outside      1    39
tr|C9ZNL1|C9ZNL1_TRYB9  TMHMM2.0        TMhelix     40    62
tr|C9ZNL1|C9ZNL1_TRYB9  TMHMM2.0        inside      63   149
tr|C9ZNL1|C9ZNL1_TRYB9  TMHMM2.0        TMhelix    150   172
tr|C9ZNL1|C9ZNL1_TRYB9  TMHMM2.0        outside    173   176
tr|C9ZNL1|C9ZNL1_TRYB9  TMHMM2.0        TMhelix    177   194
tr|C9ZNL1|C9ZNL1_TRYB9  TMHMM2.0        inside     195   205
tr|C9ZNL1|C9ZNL1_TRYB9  TMHMM2.0        TMhelix    206   223
tr|C9ZNL1|C9ZNL1_TRYB9  TMHMM2.0        outside    224   242
tr|C9ZNL1|C9ZNL1_TRYB9  TMHMM2.0        TMhelix    243   265
tr|C9ZNL1|C9ZNL1_TRYB9  TMHMM2.0        inside     266   276
tr|C9ZNL1|C9ZNL1_TRYB9  TMHMM2.0        TMhelix    277   299
tr|C9ZNL1|C9ZNL1_TRYB9  TMHMM2.0        outside    300   308
tr|C9ZNL1|C9ZNL1_TRYB9  TMHMM2.0        TMhelix    309   326
tr|C9ZNL1|C9ZNL1_TRYB9  TMHMM2.0        inside     327   346
tr|C9ZNL1|C9ZNL1_TRYB9  TMHMM2.0        TMhelix    347   369
tr|C9ZNL1|C9ZNL1_TRYB9  TMHMM2.0        outside    370   413
tr|C9ZNL1|C9ZNL1_TRYB9  TMHMM2.0        TMhelix    414   447
tr|C9ZNL1|C9ZNL1_TRYB9  TMHMM2.0        inside     448   453
tr|C9ZNL1|C9ZNL1_TRYB9  TMHMM2.0        TMhelix    454   476
tr|C9ZNL1|C9ZNL1_TRYB9  TMHMM2.0        outside    477   526
tr|C9ZNL1|C9ZNL1_TRYB9  TMHMM2.0        TMhelix    527   549
tr|C9ZNL1|C9ZNL1_TRYB9  TMHMM2.0        inside     550   821
```

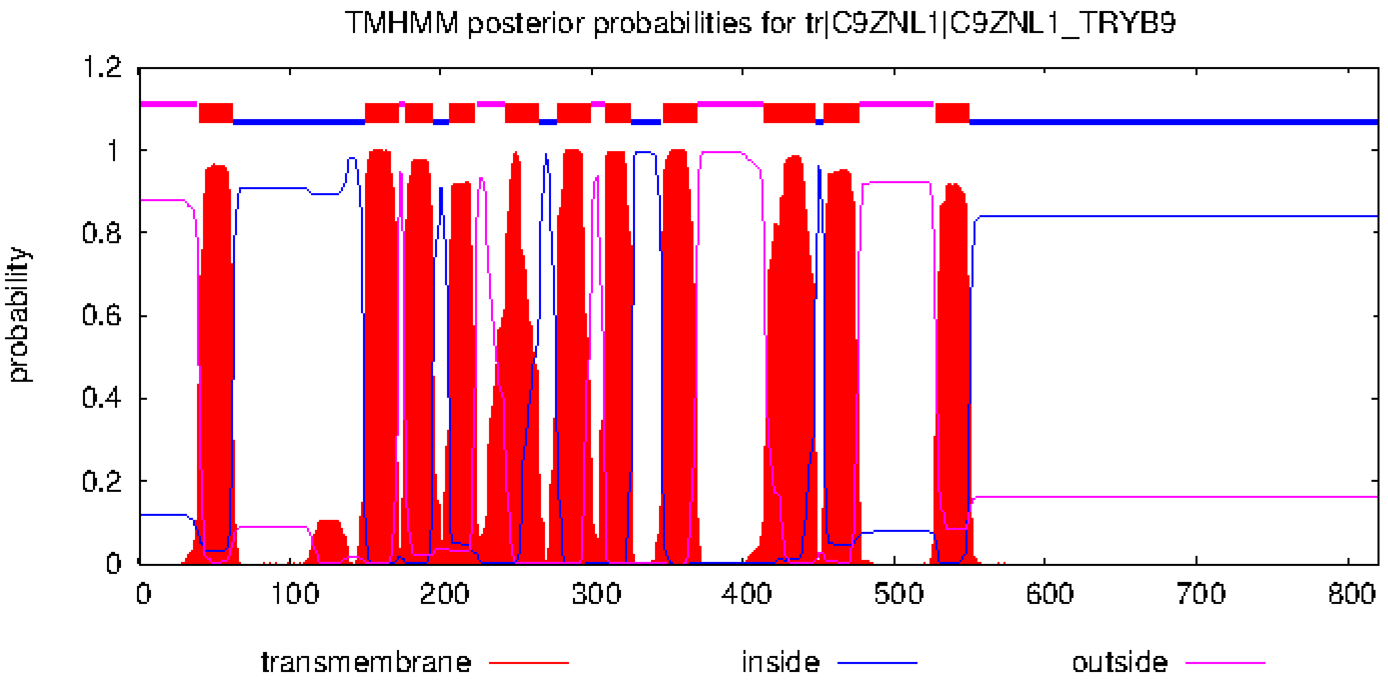

139 **# plot in postscript, script for making the plot in gnuplot, data for plot**

```
# tr|C9ZN50|C9ZN50_TRYB9 Length: 1079
# tr|C9ZN50|C9ZN50_TRYB9 Number of predicted TMHs:  12
# tr|C9ZN50|C9ZN50_TRYB9 Exp number of AAs in TMHs: 284.55175
# tr|C9ZN50|C9ZN50_TRYB9 Exp number, first 60 AAs:  0
# tr|C9ZN50|C9ZN50_TRYB9 Total prob of N-in:        0.02508
tr|C9ZN50|C9ZN50_TRYB9  TMHMM2.0        outside      1   405
tr|C9ZN50|C9ZN50_TRYB9  TMHMM2.0        TMhelix    406   424
tr|C9ZN50|C9ZN50_TRYB9  TMHMM2.0        inside     425   425
tr|C9ZN50|C9ZN50_TRYB9  TMHMM2.0        TMhelix    426   448
tr|C9ZN50|C9ZN50_TRYB9  TMHMM2.0        outside    449   452
tr|C9ZN50|C9ZN50_TRYB9  TMHMM2.0        TMhelix    453   475
tr|C9ZN50|C9ZN50_TRYB9  TMHMM2.0        inside     476   495
tr|C9ZN50|C9ZN50_TRYB9  TMHMM2.0        TMhelix    496   515
tr|C9ZN50|C9ZN50_TRYB9  TMHMM2.0        outside    516   529
tr|C9ZN50|C9ZN50_TRYB9  TMHMM2.0        TMhelix    530   552
tr|C9ZN50|C9ZN50_TRYB9  TMHMM2.0        inside     553   592
tr|C9ZN50|C9ZN50_TRYB9  TMHMM2.0        TMhelix    593   615
tr|C9ZN50|C9ZN50_TRYB9  TMHMM2.0        outside    616   618
tr|C9ZN50|C9ZN50_TRYB9  TMHMM2.0        TMhelix    619   641
tr|C9ZN50|C9ZN50_TRYB9  TMHMM2.0        inside     642   653
tr|C9ZN50|C9ZN50_TRYB9  TMHMM2.0        TMhelix    654   673
tr|C9ZN50|C9ZN50_TRYB9  TMHMM2.0        outside    674   676
tr|C9ZN50|C9ZN50_TRYB9  TMHMM2.0        TMhelix    677   699
tr|C9ZN50|C9ZN50_TRYB9  TMHMM2.0        inside     700   711
tr|C9ZN50|C9ZN50_TRYB9  TMHMM2.0        TMhelix    712   731
tr|C9ZN50|C9ZN50_TRYB9  TMHMM2.0        outside    732   734
tr|C9ZN50|C9ZN50_TRYB9  TMHMM2.0        TMhelix    735   754
tr|C9ZN50|C9ZN50_TRYB9  TMHMM2.0        inside     755   760
tr|C9ZN50|C9ZN50_TRYB9  TMHMM2.0        TMhelix    761   783
tr|C9ZN50|C9ZN50_TRYB9  TMHMM2.0        outside    784  1079
```

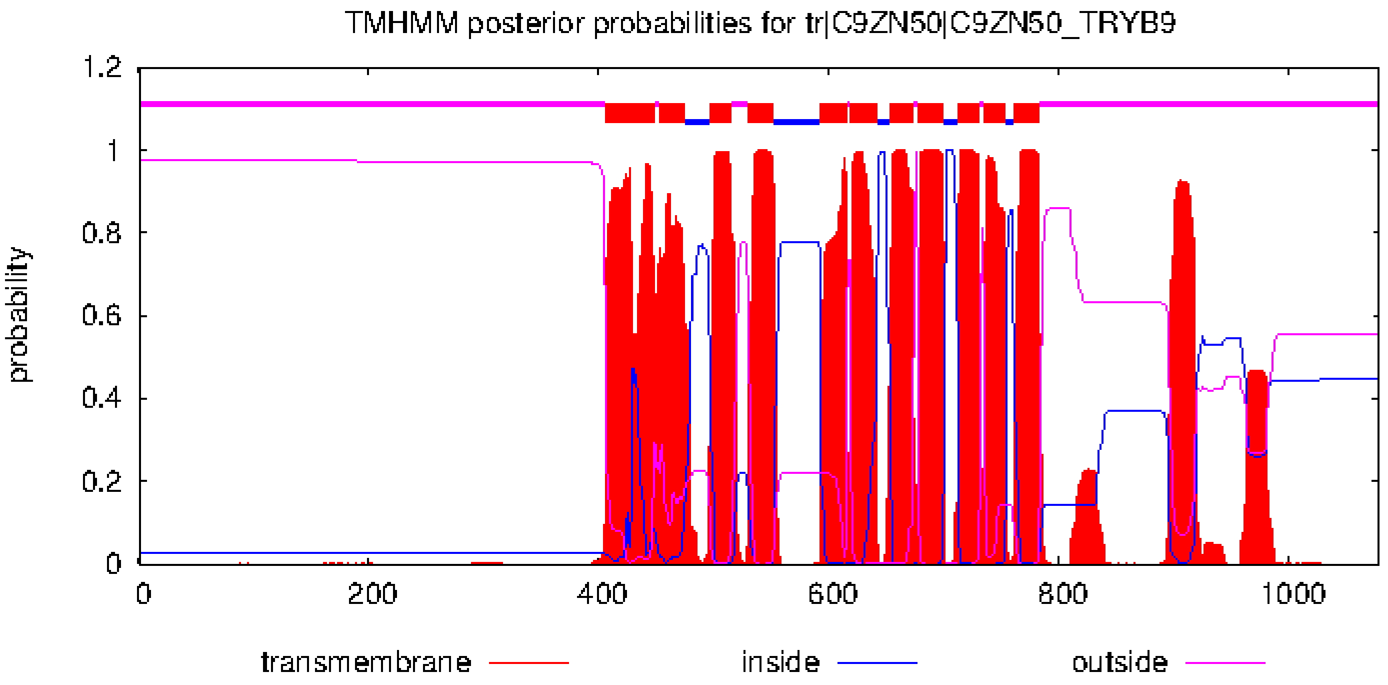

140 # **[plot]** **in postscript,** **[script]** **for making the plot in gnuplot,** **[data]** **for plot**

```
# tr|C9ZN41|C9ZN41_TRYB9 Length: 1233
# tr|C9ZN41|C9ZN41_TRYB9 Number of predicted TMHs:  1
# tr|C9ZN41|C9ZN41_TRYB9 Exp number of AAs in TMHs: 24.12186
# tr|C9ZN41|C9ZN41_TRYB9 Exp number, first 60 AAs: 0.46045
# tr|C9ZN41|C9ZN41_TRYB9 Total prob of N-in:        0.02370
tr|C9ZN41|C9ZN41_TRYB9  TMHMM2.0        outside      1   850
tr|C9ZN41|C9ZN41_TRYB9  TMHMM2.0        TMhelix    851   873
tr|C9ZN41|C9ZN41_TRYB9  TMHMM2.0        inside     874  1233
```

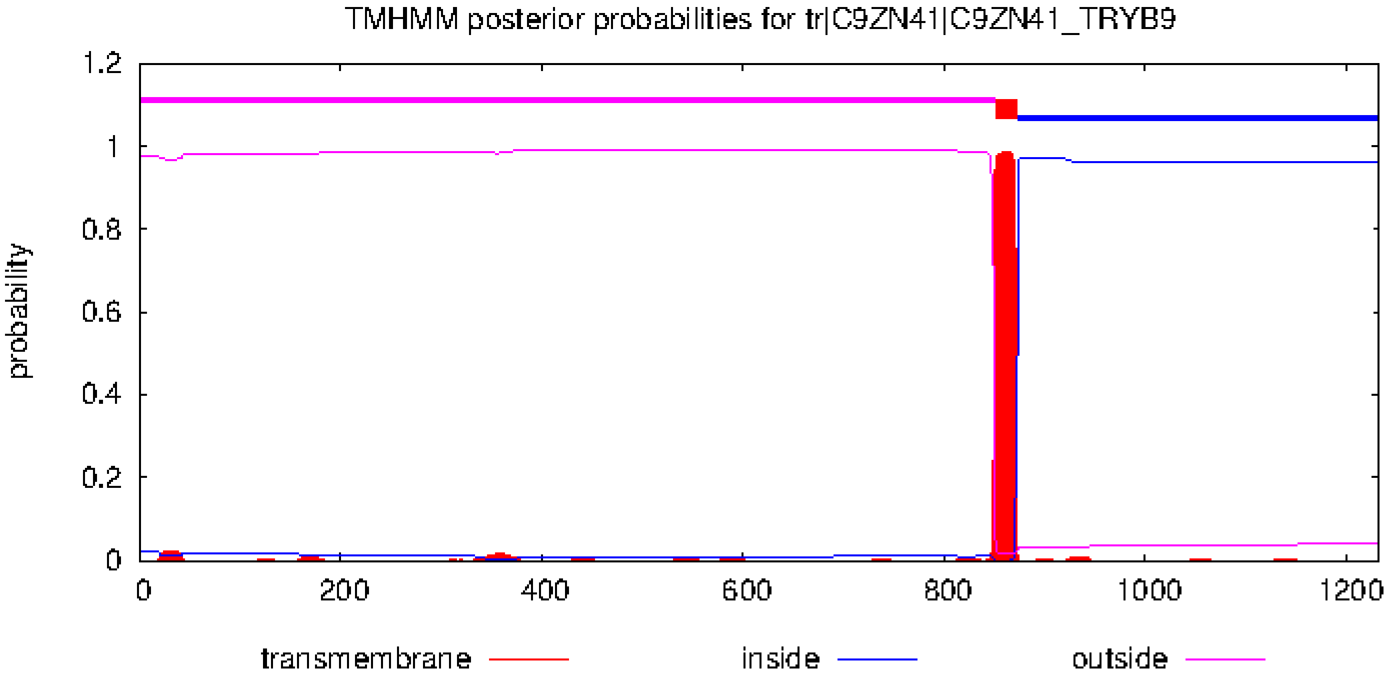

141 # **[plot]** **in postscript,** **[script]** **for making the plot in gnuplot,** **[data]** **for plot**

```
# tr|C9ZNT1|C9ZNT1_TRYB9 Length: 1249
# tr|C9ZNT1|C9ZNT1_TRYB9 Number of predicted TMHs:  1
```

```
# tr|C9ZNT1|C9ZNT1_TRYB9 Exp number of AAs in TMHs: 43.0771000000001
# tr|C9ZNT1|C9ZNT1_TRYB9 Exp number, first 60 AAs:  7.72865
# tr|C9ZNT1|C9ZNT1_TRYB9 Total prob of N-in:        0.43023
```

```
tr|C9ZNT1|C9ZNT1_TRYB9  TMHMM2.0        outside      1   869
tr|C9ZNT1|C9ZNT1_TRYB9  TMHMM2.0        TMhelix    870   892
tr|C9ZNT1|C9ZNT1_TRYB9  TMHMM2.0        inside     893  1249
```

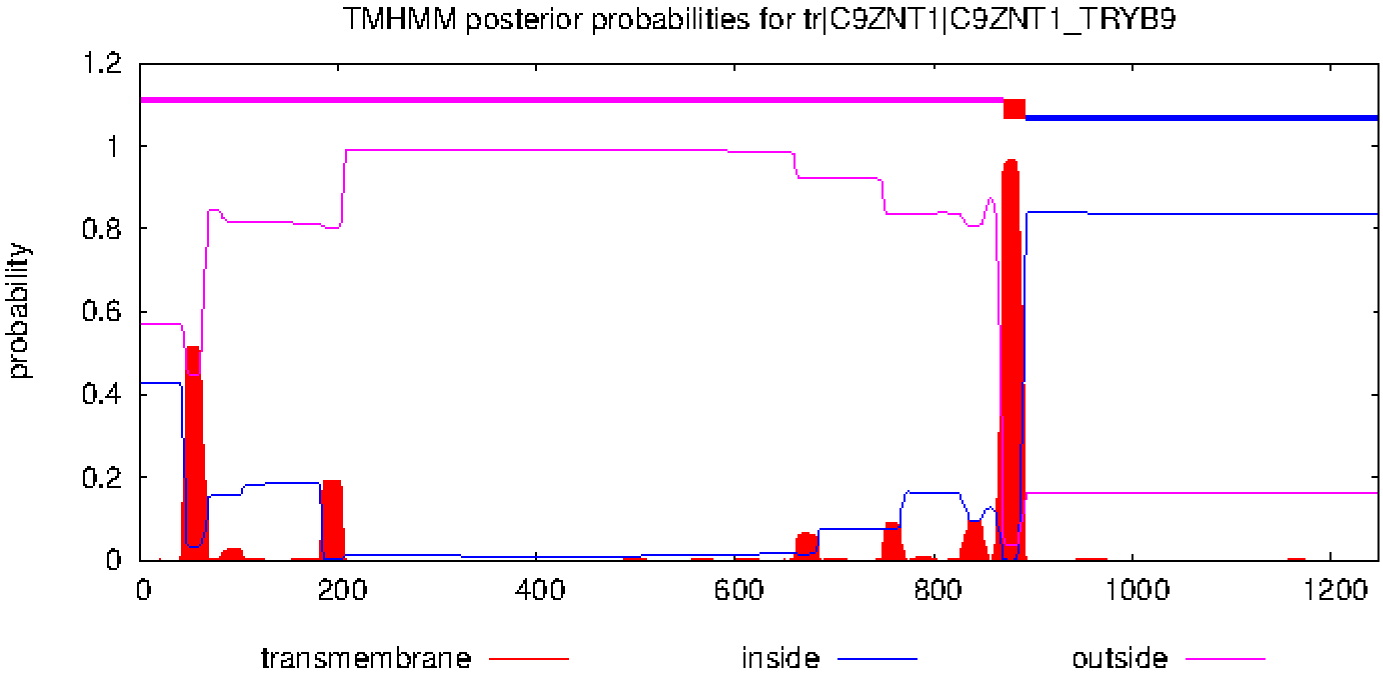

142 # plot in postscript, script for making the plot in gnuplot, data for plot

---

```
# tr|C9ZPJ9|C9ZPJ9_TRYB9 Length: 1253
# tr|C9ZPJ9|C9ZPJ9_TRYB9 Number of predicted TMHs:  1
# tr|C9ZPJ9|C9ZPJ9_TRYB9 Exp number of AAs in TMHs: 78.7766800000006
# tr|C9ZPJ9|C9ZPJ9_TRYB9 Exp number, first 60 AAs:  0
# tr|C9ZPJ9|C9ZPJ9_TRYB9 Total prob of N-in:        0.63665
tr|C9ZPJ9|C9ZPJ9_TRYB9  TMHMM2.0        inside       1   164
tr|C9ZPJ9|C9ZPJ9_TRYB9  TMHMM2.0        TMhelix    165   187
tr|C9ZPJ9|C9ZPJ9_TRYB9  TMHMM2.0        outside    188  1253
```

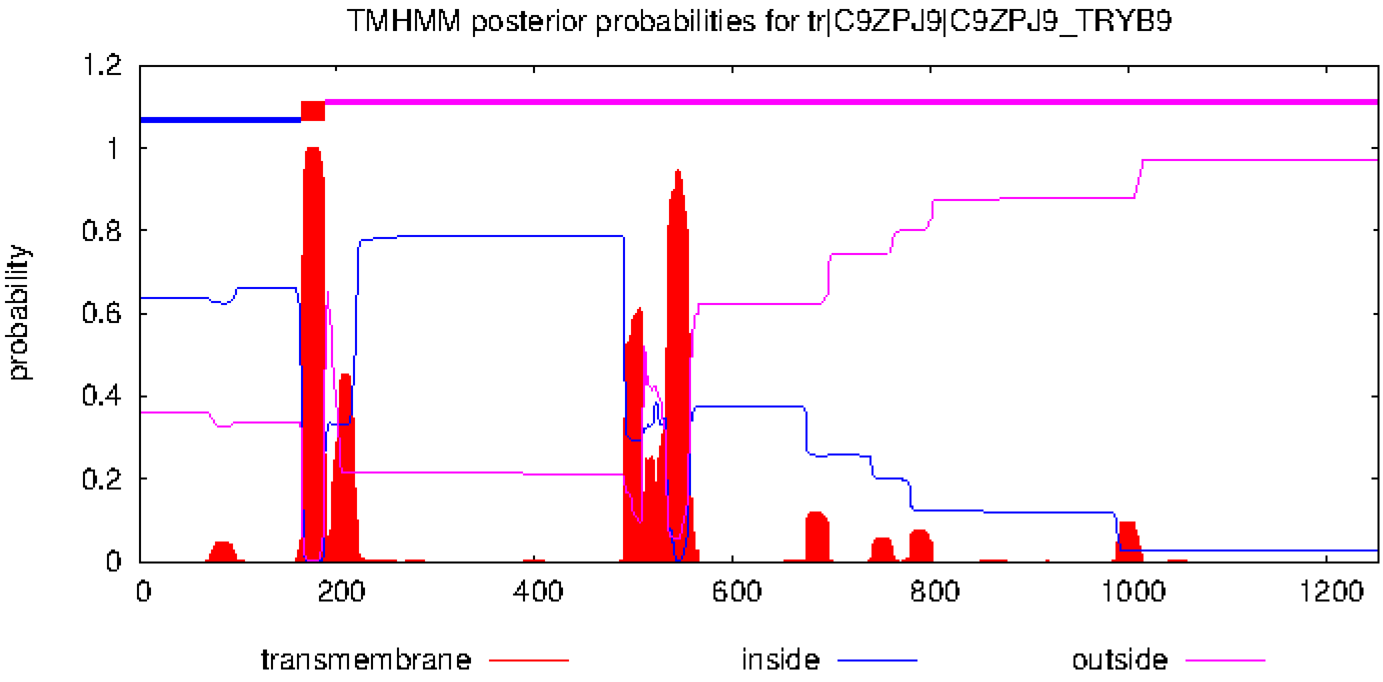

143 # plot in postscript, script for making the plot in gnuplot, data for plot

---

```
# tr|D0A0X5|D0A0X5_TRYB9 Length: 1268
# tr|D0A0X5|D0A0X5_TRYB9 Number of predicted TMHs:  1
# tr|D0A0X5|D0A0X5_TRYB9 Exp number of AAs in TMHs: 51.4627500000001
# tr|D0A0X5|D0A0X5_TRYB9 Exp number, first 60 AAs:  11.18587
# tr|D0A0X5|D0A0X5_TRYB9 Total prob of N-in:        0.47420
# tr|D0A0X5|D0A0X5_TRYB9 POSSIBLE N-term signal sequence
tr|D0A0X5|D0A0X5_TRYB9  TMHMM2.0        outside      1   861
tr|D0A0X5|D0A0X5_TRYB9  TMHMM2.0        TMhelix    862   884
tr|D0A0X5|D0A0X5_TRYB9  TMHMM2.0        inside     885  1268
```

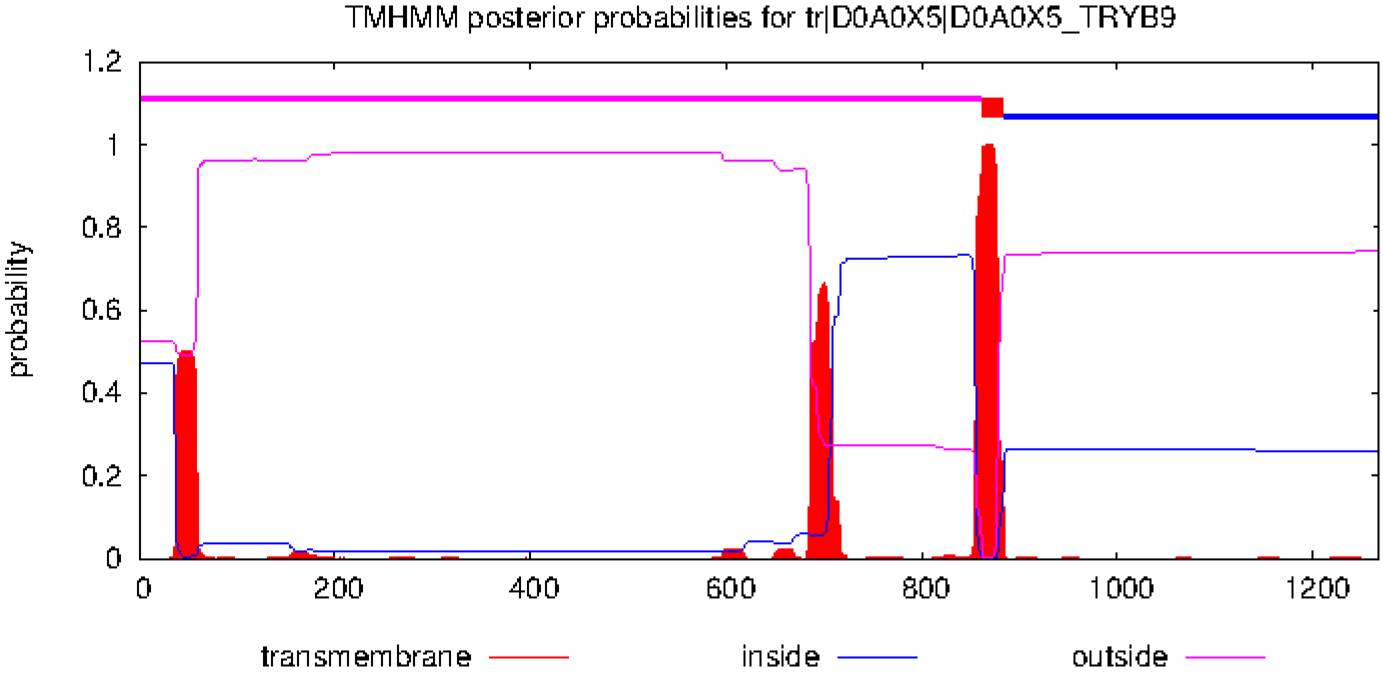

144 # plot in postscript, script for making the plot in gnuplot, data for plot

---

```
# tr|D0A513|D0A513_TRYB9 Length: 1493
# tr|D0A513|D0A513_TRYB9 Number of predicted TMHs:  7
# tr|D0A513|D0A513_TRYB9 Exp number of AAs in TMHs: 163.88024
# tr|D0A513|D0A513_TRYB9 Exp number, first 60 AAs:  0.01165
# tr|D0A513|D0A513_TRYB9 Total prob of N-in:        0.00099
tr|D0A513|D0A513_TRYB9  TMHMM2.0        outside      1  1220
tr|D0A513|D0A513_TRYB9  TMHMM2.0        TMhelix   1221  1243
tr|D0A513|D0A513_TRYB9  TMHMM2.0        inside    1244  1287
tr|D0A513|D0A513_TRYB9  TMHMM2.0        TMhelix   1288  1310
tr|D0A513|D0A513_TRYB9  TMHMM2.0        outside   1311  1319
tr|D0A513|D0A513_TRYB9  TMHMM2.0        TMhelix   1320  1342
tr|D0A513|D0A513_TRYB9  TMHMM2.0        inside    1343  1362
tr|D0A513|D0A513_TRYB9  TMHMM2.0        TMhelix   1363  1380
tr|D0A513|D0A513_TRYB9  TMHMM2.0        outside   1381  1394
tr|D0A513|D0A513_TRYB9  TMHMM2.0        TMhelix   1395  1417
tr|D0A513|D0A513_TRYB9  TMHMM2.0        inside    1418  1421
tr|D0A513|D0A513_TRYB9  TMHMM2.0        TMhelix   1422  1444
tr|D0A513|D0A513_TRYB9  TMHMM2.0        outside   1445  1468
tr|D0A513|D0A513_TRYB9  TMHMM2.0        TMhelix   1469  1491
tr|D0A513|D0A513_TRYB9  TMHMM2.0        inside    1492  1493
```

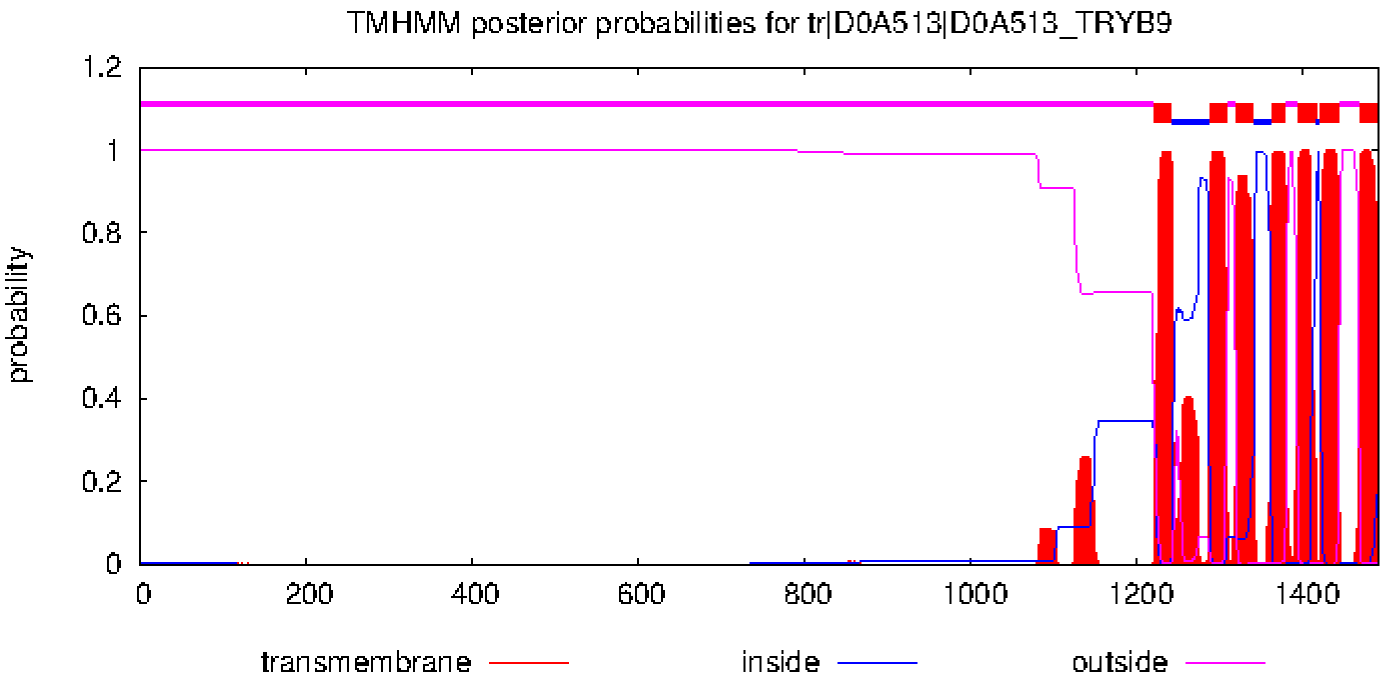

145 **# plot in postscript, script for making the plot in gnuplot, data for plot**

```
# tr|C9ZU90|C9ZU90_TRYB9 Length: 166
# tr|C9ZU90|C9ZU90_TRYB9 Number of predicted TMHs:  4
# tr|C9ZU90|C9ZU90_TRYB9 Exp number of AAs in TMHs: 90.3625899999999
# tr|C9ZU90|C9ZU90_TRYB9 Exp number, first 60 AAs:  17.87654
# tr|C9ZU90|C9ZU90_TRYB9 Total prob of N-in:        0.95990
# tr|C9ZU90|C9ZU90_TRYB9 POSSIBLE N-term signal sequence
tr|C9ZU90|C9ZU90_TRYB9  TMHMM2.0        inside       1    42
tr|C9ZU90|C9ZU90_TRYB9  TMHMM2.0        TMhelix     43    65
tr|C9ZU90|C9ZU90_TRYB9  TMHMM2.0        outside     66    68
tr|C9ZU90|C9ZU90_TRYB9  TMHMM2.0        TMhelix     69    91
tr|C9ZU90|C9ZU90_TRYB9  TMHMM2.0        inside      92   103
tr|C9ZU90|C9ZU90_TRYB9  TMHMM2.0        TMhelix    104   126
tr|C9ZU90|C9ZU90_TRYB9  TMHMM2.0        outside    127   130
tr|C9ZU90|C9ZU90_TRYB9  TMHMM2.0        TMhelix    131   153
tr|C9ZU90|C9ZU90_TRYB9  TMHMM2.0        inside     154   166
```

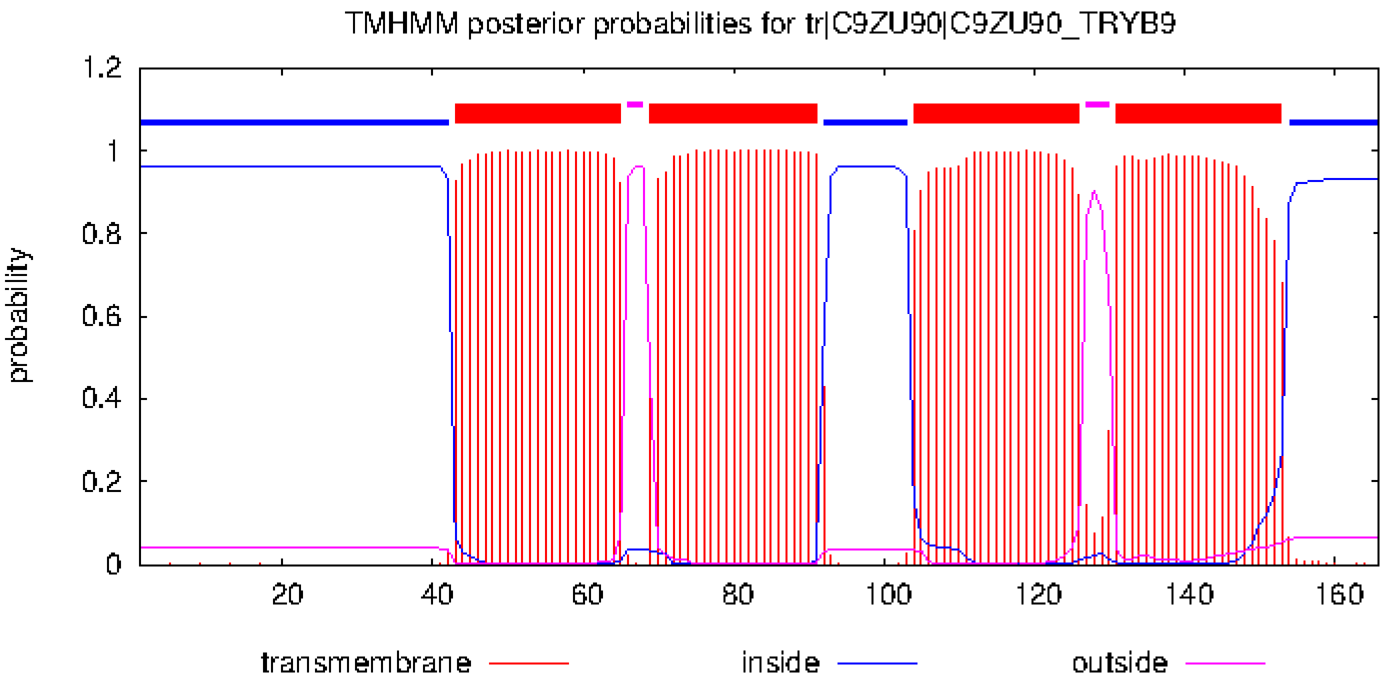

146 # plot in postscript, script for making the plot in gnuplot, data for plot

```
# tr|D0A489|D0A489_TRYB9 Length: 192
# tr|D0A489|D0A489_TRYB9 Number of predicted TMHs:  4
# tr|D0A489|D0A489_TRYB9 Exp number of AAs in TMHs: 88.4896
# tr|D0A489|D0A489_TRYB9 Exp number, first 60 AAs:  36.00579
# tr|D0A489|D0A489_TRYB9 Total prob of N-in:        0.99394
# tr|D0A489|D0A489_TRYB9 POSSIBLE N-term signal sequence
tr|D0A489|D0A489_TRYB9  TMHMM2.0        inside       1     6
tr|D0A489|D0A489_TRYB9  TMHMM2.0        TMhelix      7    28
tr|D0A489|D0A489_TRYB9  TMHMM2.0        outside     29    47
tr|D0A489|D0A489_TRYB9  TMHMM2.0        TMhelix     48    70
tr|D0A489|D0A489_TRYB9  TMHMM2.0        inside      71    76
tr|D0A489|D0A489_TRYB9  TMHMM2.0        TMhelix     77    99
tr|D0A489|D0A489_TRYB9  TMHMM2.0        outside    100   113
tr|D0A489|D0A489_TRYB9  TMHMM2.0        TMhelix    114   136
tr|D0A489|D0A489_TRYB9  TMHMM2.0        inside     137   192
```

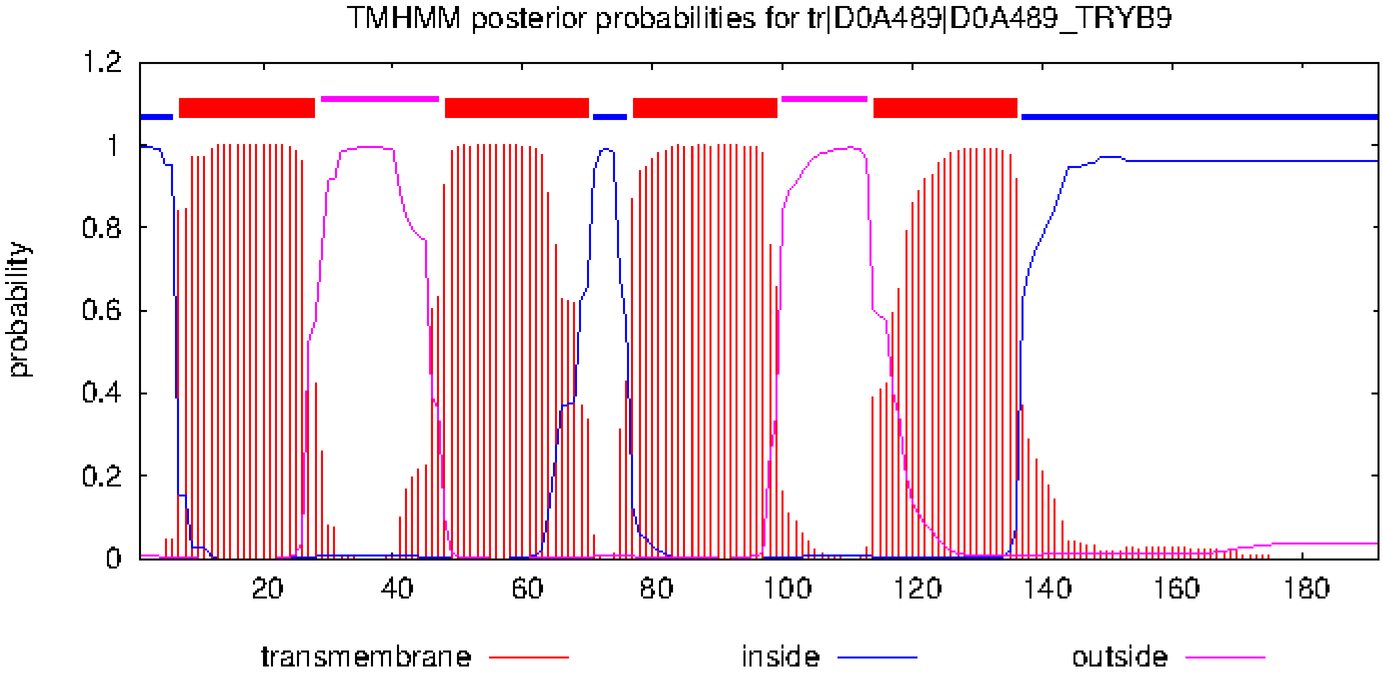

147 **# plot in postscript, script for making the plot in gnuplot, data for plot**

```
# tr|D0A6J5|D0A6J5_TRYB9 Length: 256
# tr|D0A6J5|D0A6J5_TRYB9 Number of predicted TMHs:  5
# tr|D0A6J5|D0A6J5_TRYB9 Exp number of AAs in TMHs: 122.00435
# tr|D0A6J5|D0A6J5_TRYB9 Exp number, first 60 AAs:  21.67922
# tr|D0A6J5|D0A6J5_TRYB9 Total prob of N-in:        0.02060
# tr|D0A6J5|D0A6J5_TRYB9 POSSIBLE N-term signal sequence
tr|D0A6J5|D0A6J5_TRYB9  TMHMM2.0        outside      1    14
tr|D0A6J5|D0A6J5_TRYB9  TMHMM2.0        TMhelix     15    34
tr|D0A6J5|D0A6J5_TRYB9  TMHMM2.0        inside      35   104
tr|D0A6J5|D0A6J5_TRYB9  TMHMM2.0        TMhelix    105   127
tr|D0A6J5|D0A6J5_TRYB9  TMHMM2.0        outside    128   164
tr|D0A6J5|D0A6J5_TRYB9  TMHMM2.0        TMhelix    165   187
tr|D0A6J5|D0A6J5_TRYB9  TMHMM2.0        inside     188   193
tr|D0A6J5|D0A6J5_TRYB9  TMHMM2.0        TMhelix    194   213
tr|D0A6J5|D0A6J5_TRYB9  TMHMM2.0        outside    214   232
tr|D0A6J5|D0A6J5_TRYB9  TMHMM2.0        TMhelix    233   255
tr|D0A6J5|D0A6J5_TRYB9  TMHMM2.0        inside     256   256
```

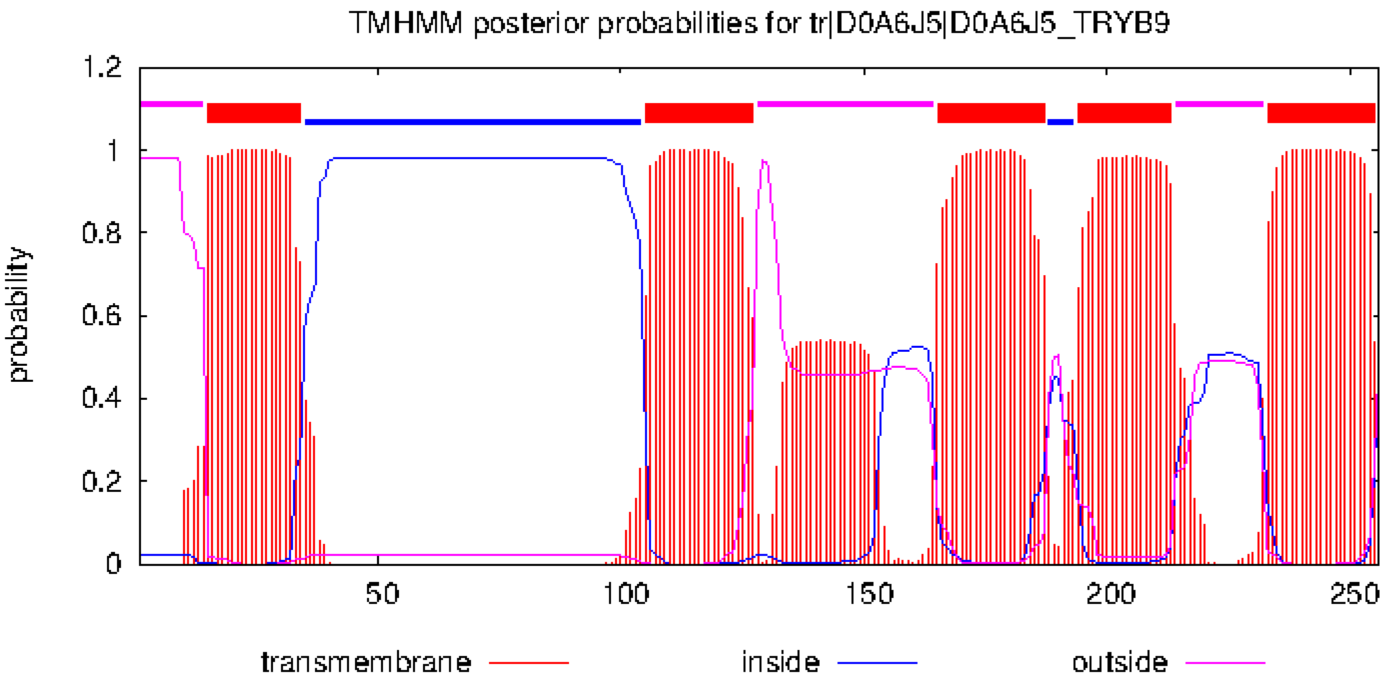

148 # plot in postscript, script for making the plot in gnuplot, data for plot

---

```
# tr|C9ZI22|C9ZI22_TRYB9 Length: 295
# tr|C9ZI22|C9ZI22_TRYB9 Number of predicted TMHs:  6
# tr|C9ZI22|C9ZI22_TRYB9 Exp number of AAs in TMHs: 129.62671
# tr|C9ZI22|C9ZI22_TRYB9 Exp number, first 60 AAs: 23.39165
# tr|C9ZI22|C9ZI22_TRYB9 Total prob of N-in:        0.58489
# tr|C9ZI22|C9ZI22_TRYB9 POSSIBLE N-term signal sequence
tr|C9ZI22|C9ZI22_TRYB9  TMHMM2.0        inside       1    20
tr|C9ZI22|C9ZI22_TRYB9  TMHMM2.0        TMhelix     21    40
tr|C9ZI22|C9ZI22_TRYB9  TMHMM2.0        outside     41    54
tr|C9ZI22|C9ZI22_TRYB9  TMHMM2.0        TMhelix     55    77
tr|C9ZI22|C9ZI22_TRYB9  TMHMM2.0        inside      78   113
tr|C9ZI22|C9ZI22_TRYB9  TMHMM2.0        TMhelix    114   136
tr|C9ZI22|C9ZI22_TRYB9  TMHMM2.0        outside    137   139
tr|C9ZI22|C9ZI22_TRYB9  TMHMM2.0        TMhelix    140   162
tr|C9ZI22|C9ZI22_TRYB9  TMHMM2.0        inside     163   221
tr|C9ZI22|C9ZI22_TRYB9  TMHMM2.0        TMhelix    222   244
tr|C9ZI22|C9ZI22_TRYB9  TMHMM2.0        outside    245   265
tr|C9ZI22|C9ZI22_TRYB9  TMHMM2.0        TMhelix    266   284
tr|C9ZI22|C9ZI22_TRYB9  TMHMM2.0        inside     285   295
```

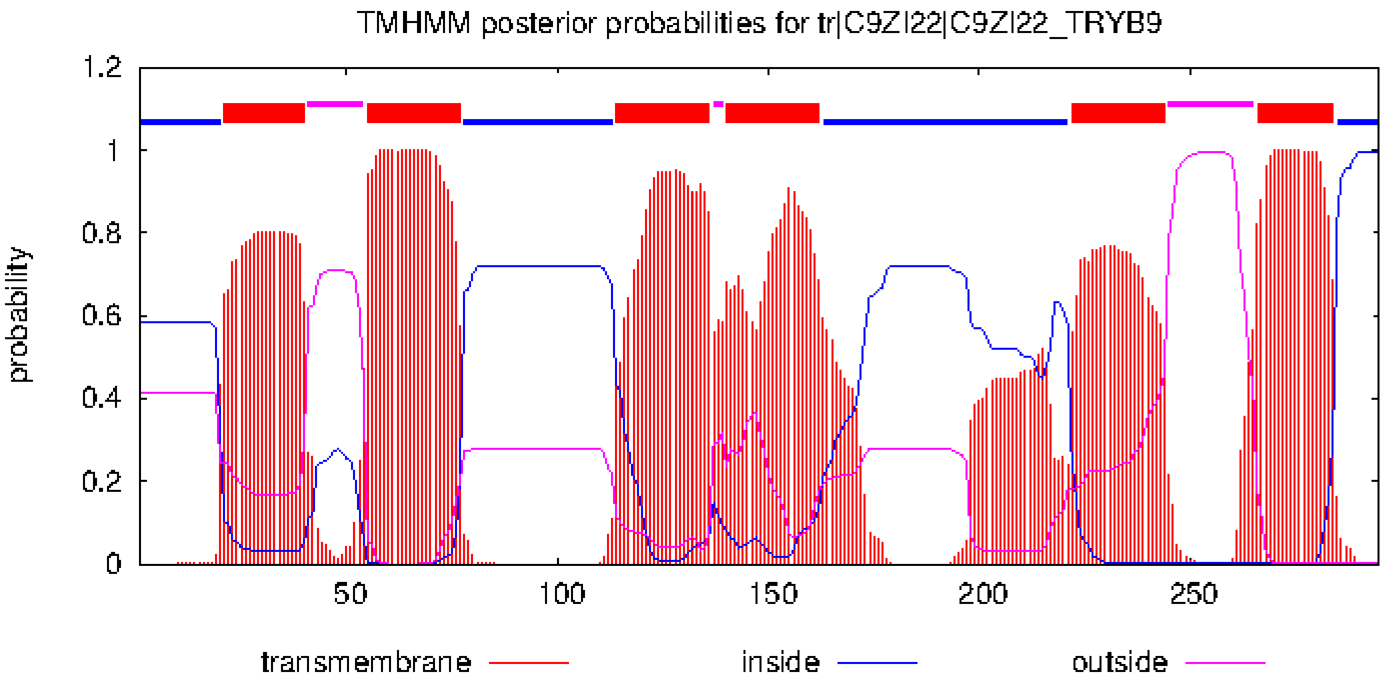

149 **# plot in postscript, script for making the plot in gnuplot, data for plot**

```
# tr|C9ZMC3|C9ZMC3_TRYB9 Length: 327
# tr|C9ZMC3|C9ZMC3_TRYB9 Number of predicted TMHs:  1
# tr|C9ZMC3|C9ZMC3_TRYB9 Exp number of AAs in TMHs: 23.92868
# tr|C9ZMC3|C9ZMC3_TRYB9 Exp number, first 60 AAs:  23.26037
# tr|C9ZMC3|C9ZMC3_TRYB9 Total prob of N-in:        0.52869
# tr|C9ZMC3|C9ZMC3_TRYB9 POSSIBLE N-term signal sequence
tr|C9ZMC3|C9ZMC3_TRYB9  TMHMM2.0        inside       1     6
tr|C9ZMC3|C9ZMC3_TRYB9  TMHMM2.0        TMhelix      7    29
tr|C9ZMC3|C9ZMC3_TRYB9  TMHMM2.0        outside     30   327
```

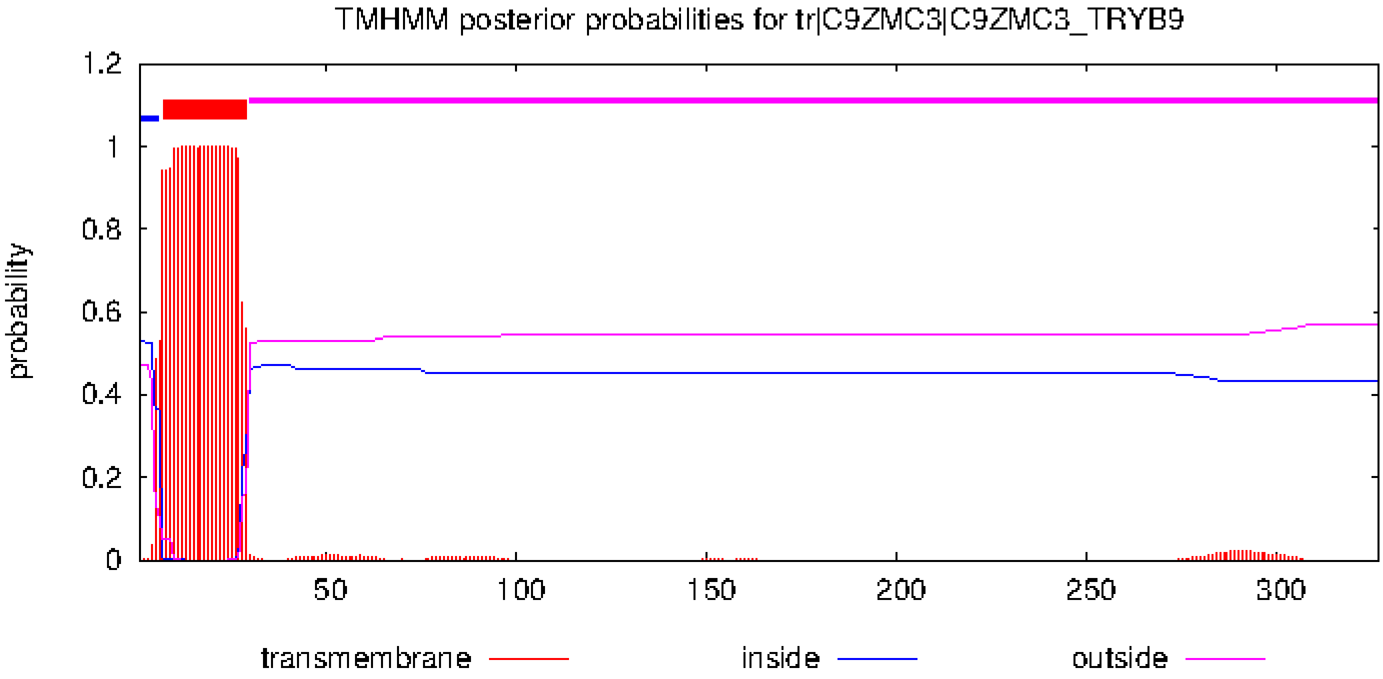

150 **# plot in postscript, script for making the plot in gnuplot, data for plot**

```
# tr|C9ZZW0|C9ZZW0_TRYB9 Length: 330
```

```
# tr|C9ZZW0|C9ZZW0_TRYB9 Number of predicted TMHs:  6
# tr|C9ZZW0|C9ZZW0_TRYB9 Exp number of AAs in TMHs: 129.20217
# tr|C9ZZW0|C9ZZW0_TRYB9 Exp number, first 60 AAs:  21.58542
```

```
# tr|C9ZZW0|C9ZZW0_TRYB9 Total prob of N-in:        0.93367
# tr|C9ZZW0|C9ZZW0_TRYB9 POSSIBLE N-term signal sequence
tr|C9ZZW0|C9ZZW0_TRYB9  TMHMM2.0        inside       1    18
tr|C9ZZW0|C9ZZW0_TRYB9  TMHMM2.0        TMhelix     19    41
tr|C9ZZW0|C9ZZW0_TRYB9  TMHMM2.0        outside     42    60
tr|C9ZZW0|C9ZZW0_TRYB9  TMHMM2.0        TMhelix     61    83
tr|C9ZZW0|C9ZZW0_TRYB9  TMHMM2.0        inside      84   156
tr|C9ZZW0|C9ZZW0_TRYB9  TMHMM2.0        TMhelix    157   179
tr|C9ZZW0|C9ZZW0_TRYB9  TMHMM2.0        outside    180   219
tr|C9ZZW0|C9ZZW0_TRYB9  TMHMM2.0        TMhelix    220   242
tr|C9ZZW0|C9ZZW0_TRYB9  TMHMM2.0        inside     243   248
tr|C9ZZW0|C9ZZW0_TRYB9  TMHMM2.0        TMhelix    249   271
tr|C9ZZW0|C9ZZW0_TRYB9  TMHMM2.0        outside    272   280
tr|C9ZZW0|C9ZZW0_TRYB9  TMHMM2.0        TMhelix    281   303
tr|C9ZZW0|C9ZZW0_TRYB9  TMHMM2.0        inside     304   330
```

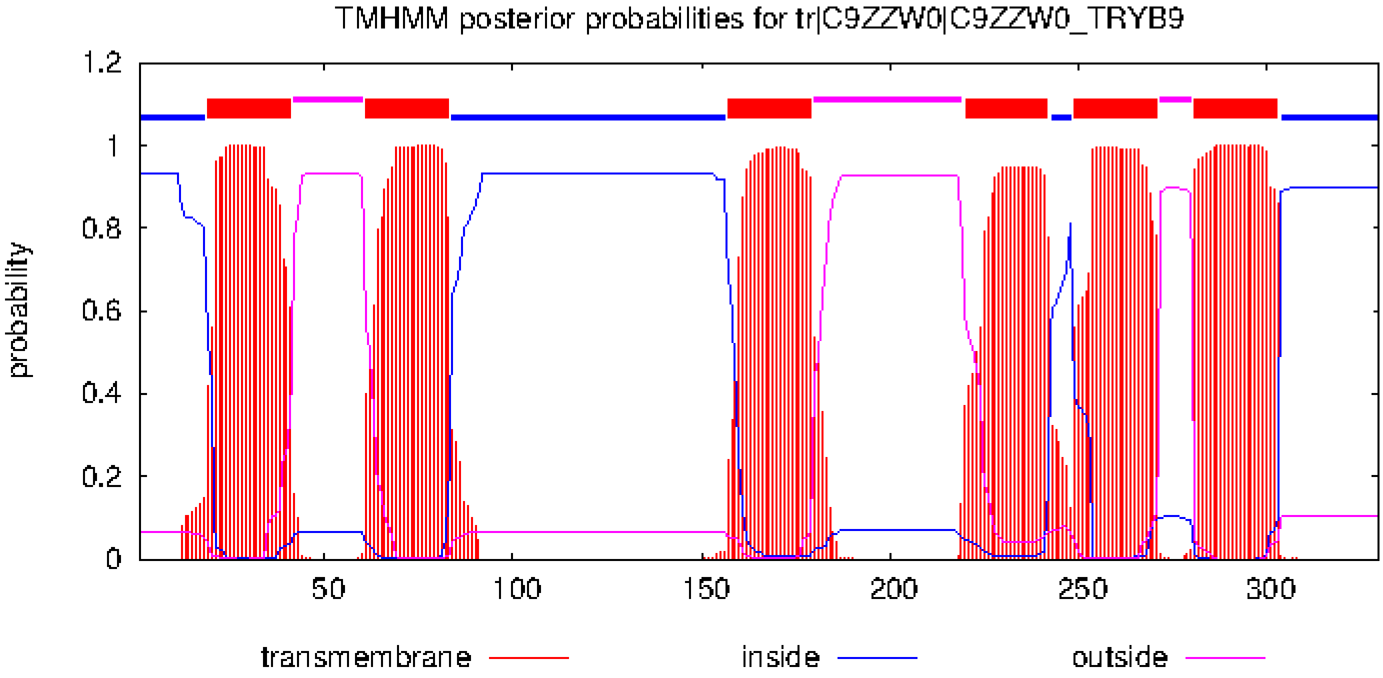

151 **# plot in postscript, script for making the plot in gnuplot, data for plot**

```
# tr|D0A0B3|D0A0B3_TRYB9 Length: 348
# tr|D0A0B3|D0A0B3_TRYB9 Number of predicted TMHs:  3
# tr|D0A0B3|D0A0B3_TRYB9 Exp number of AAs in TMHs: 62.55694
# tr|D0A0B3|D0A0B3_TRYB9 Exp number, first 60 AAs:  21.32117
# tr|D0A0B3|D0A0B3_TRYB9 Total prob of N-in:        0.11475
# tr|D0A0B3|D0A0B3_TRYB9 POSSIBLE N-term signal sequence
tr|D0A0B3|D0A0B3_TRYB9  TMHMM2.0        outside      1     9
tr|D0A0B3|D0A0B3_TRYB9  TMHMM2.0        TMhelix     10    32
tr|D0A0B3|D0A0B3_TRYB9  TMHMM2.0        inside      33   227
tr|D0A0B3|D0A0B3_TRYB9  TMHMM2.0        TMhelix    228   250
tr|D0A0B3|D0A0B3_TRYB9  TMHMM2.0        outside    251   295
tr|D0A0B3|D0A0B3_TRYB9  TMHMM2.0        TMhelix    296   318
tr|D0A0B3|D0A0B3_TRYB9  TMHMM2.0        inside     319   348
```

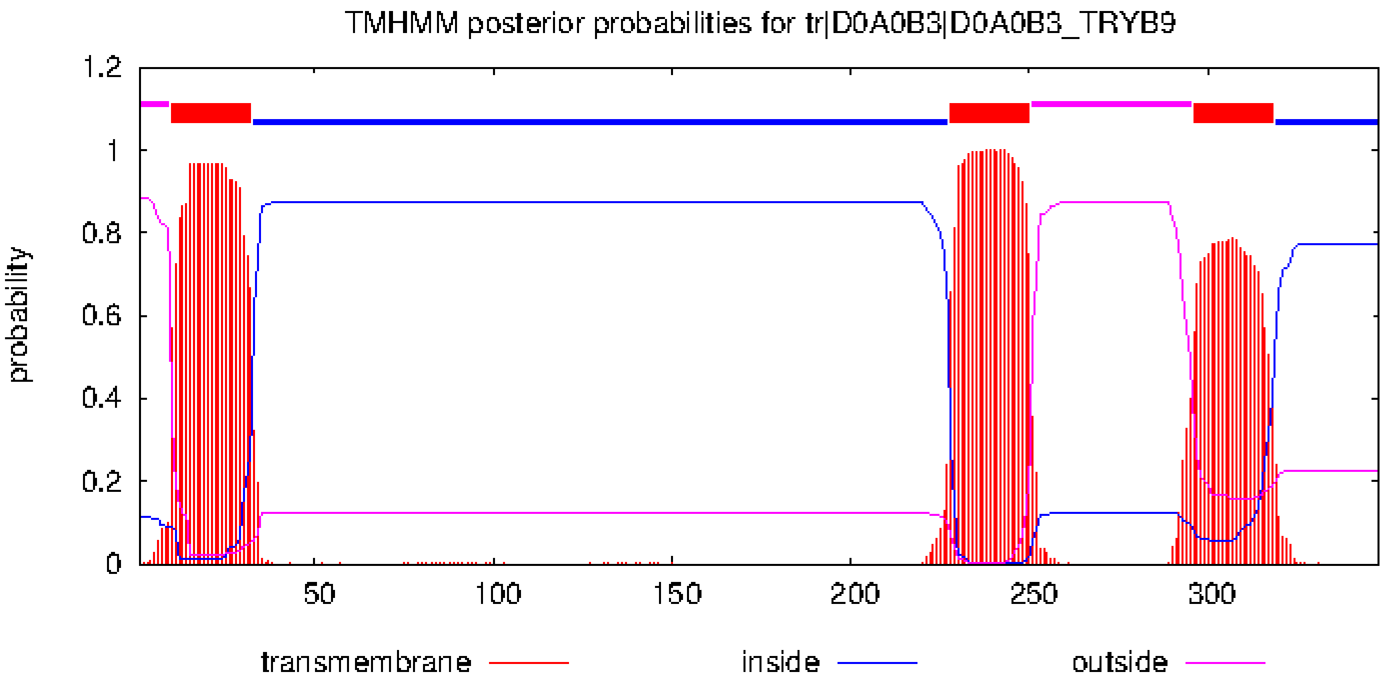

152 # plot in postscript, script for making the plot in gnuplot, data for plot

---

```
# tr|D0A4Z3|D0A4Z3_TRYB9 Length: 433
# tr|D0A4Z3|D0A4Z3_TRYB9 Number of predicted TMHs:  9
# tr|D0A4Z3|D0A4Z3_TRYB9 Exp number of AAs in TMHs: 207.80237
#  tr|D0A4Z3|D0A4Z3_TRYB9 Exp number, first 60 AAs: 26.59563
# tr|D0A4Z3|D0A4Z3_TRYB9 Total prob of N-in:        0.44686
# tr|D0A4Z3|D0A4Z3_TRYB9 POSSIBLE N-term signal sequence
tr|D0A4Z3|D0A4Z3_TRYB9  TMHMM2.0        inside       1    26
tr|D0A4Z3|D0A4Z3_TRYB9  TMHMM2.0        TMhelix     27    46
tr|D0A4Z3|D0A4Z3_TRYB9  TMHMM2.0        outside     47    55
tr|D0A4Z3|D0A4Z3_TRYB9  TMHMM2.0        TMhelix     56    78
tr|D0A4Z3|D0A4Z3_TRYB9  TMHMM2.0        inside      79    97
tr|D0A4Z3|D0A4Z3_TRYB9  TMHMM2.0        TMhelix     98   120
tr|D0A4Z3|D0A4Z3_TRYB9  TMHMM2.0        outside    121   170
tr|D0A4Z3|D0A4Z3_TRYB9  TMHMM2.0        TMhelix    171   193
tr|D0A4Z3|D0A4Z3_TRYB9  TMHMM2.0        inside     194   220
tr|D0A4Z3|D0A4Z3_TRYB9  TMHMM2.0        TMhelix    221   243
tr|D0A4Z3|D0A4Z3_TRYB9  TMHMM2.0        outside    244   284
tr|D0A4Z3|D0A4Z3_TRYB9  TMHMM2.0        TMhelix    285   304
tr|D0A4Z3|D0A4Z3_TRYB9  TMHMM2.0        inside     305   308
tr|D0A4Z3|D0A4Z3_TRYB9  TMHMM2.0        TMhelix    309   331
tr|D0A4Z3|D0A4Z3_TRYB9  TMHMM2.0        outside    332   345
tr|D0A4Z3|D0A4Z3_TRYB9  TMHMM2.0        TMhelix    346   368
tr|D0A4Z3|D0A4Z3_TRYB9  TMHMM2.0        inside     369   374
tr|D0A4Z3|D0A4Z3_TRYB9  TMHMM2.0        TMhelix    375   394
tr|D0A4Z3|D0A4Z3_TRYB9  TMHMM2.0        outside    395   433
```

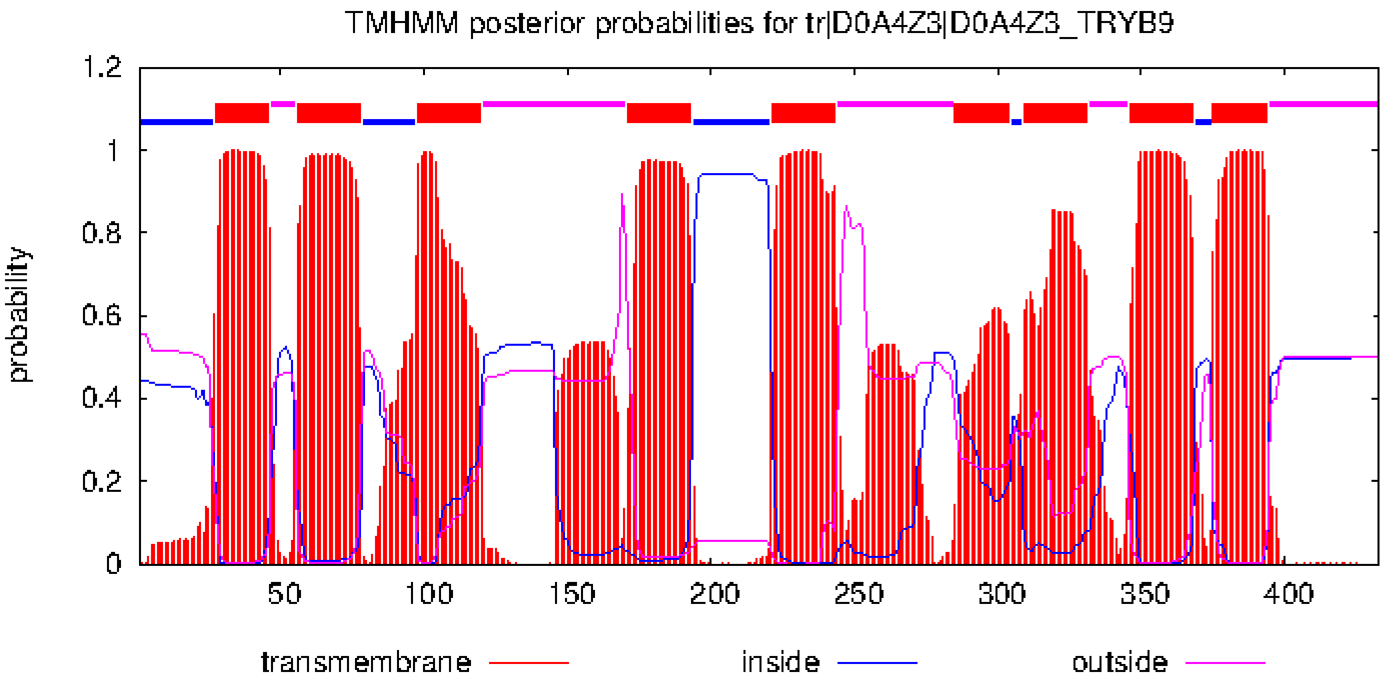

153 # plot in postscript, script for making the plot in gnuplot, data for plot

---

```
# tr|D0A6J6|D0A6J6_TRYB9 Length: 435
# tr|D0A6J6|D0A6J6_TRYB9 Number of predicted TMHs:  9
# tr|D0A6J6|D0A6J6_TRYB9 Exp number of AAs in TMHs: 219.81602
# tr|D0A6J6|D0A6J6_TRYB9 Exp number, first 60 AAs:  23.0292
# tr|D0A6J6|D0A6J6_TRYB9 Total prob of N-in:        0.41186
# tr|D0A6J6|D0A6J6_TRYB9 POSSIBLE N-term signal sequence
tr|D0A6J6|D0A6J6_TRYB9  TMHMM2.0        outside      1     9
tr|D0A6J6|D0A6J6_TRYB9  TMHMM2.0        TMhelix     10    32
tr|D0A6J6|D0A6J6_TRYB9  TMHMM2.0        inside      33    92
tr|D0A6J6|D0A6J6_TRYB9  TMHMM2.0        TMhelix     93   115
tr|D0A6J6|D0A6J6_TRYB9  TMHMM2.0        outside    116   119
tr|D0A6J6|D0A6J6_TRYB9  TMHMM2.0        TMhelix    120   142
tr|D0A6J6|D0A6J6_TRYB9  TMHMM2.0        inside     143   154
tr|D0A6J6|D0A6J6_TRYB9  TMHMM2.0        TMhelix    155   177
tr|D0A6J6|D0A6J6_TRYB9  TMHMM2.0        outside    178   191
tr|D0A6J6|D0A6J6_TRYB9  TMHMM2.0        TMhelix    192   214
tr|D0A6J6|D0A6J6_TRYB9  TMHMM2.0        inside     215   283
tr|D0A6J6|D0A6J6_TRYB9  TMHMM2.0        TMhelix    284   306
tr|D0A6J6|D0A6J6_TRYB9  TMHMM2.0        outside    307   343
tr|D0A6J6|D0A6J6_TRYB9  TMHMM2.0        TMhelix    344   366
tr|D0A6J6|D0A6J6_TRYB9  TMHMM2.0        inside     367   372
tr|D0A6J6|D0A6J6_TRYB9  TMHMM2.0        TMhelix    373   392
tr|D0A6J6|D0A6J6_TRYB9  TMHMM2.0        outside    393   411
tr|D0A6J6|D0A6J6_TRYB9  TMHMM2.0        TMhelix    412   434
tr|D0A6J6|D0A6J6_TRYB9  TMHMM2.0        inside     435   435
```

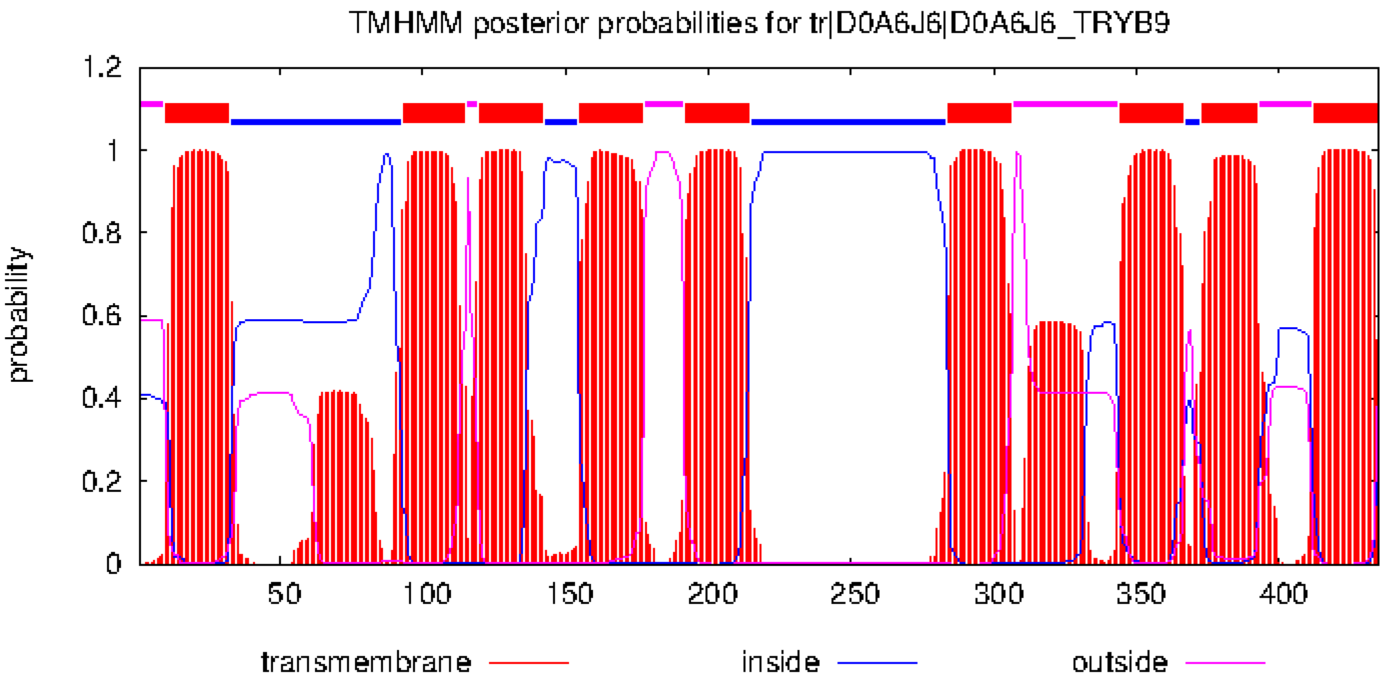

154 **# plot in postscript, script for making the plot in gnuplot, data for plot**

```
# tr|D0A6J2|D0A6J2_TRYB9 Length: 435
# tr|D0A6J2|D0A6J2_TRYB9 Number of predicted TMHs:  9
# tr|D0A6J2|D0A6J2_TRYB9 Exp number of AAs in TMHs: 218.61214
# tr|D0A6J2|D0A6J2_TRYB9 Exp number, first 60 AAs: 23.02883
# tr|D0A6J2|D0A6J2_TRYB9 Total prob of N-in:        0.41194
# tr|D0A6J2|D0A6J2_TRYB9 POSSIBLE N-term signal sequence
tr|D0A6J2|D0A6J2_TRYB9  TMHMM2.0        outside      1     9
tr|D0A6J2|D0A6J2_TRYB9  TMHMM2.0        TMhelix     10    32
tr|D0A6J2|D0A6J2_TRYB9  TMHMM2.0        inside      33    92
tr|D0A6J2|D0A6J2_TRYB9  TMHMM2.0        TMhelix     93   115
tr|D0A6J2|D0A6J2_TRYB9  TMHMM2.0        outside    116   119
tr|D0A6J2|D0A6J2_TRYB9  TMHMM2.0        TMhelix    120   142
tr|D0A6J2|D0A6J2_TRYB9  TMHMM2.0        inside     143   154
tr|D0A6J2|D0A6J2_TRYB9  TMHMM2.0        TMhelix    155   177
tr|D0A6J2|D0A6J2_TRYB9  TMHMM2.0        outside    178   191
tr|D0A6J2|D0A6J2_TRYB9  TMHMM2.0        TMhelix    192   214
tr|D0A6J2|D0A6J2_TRYB9  TMHMM2.0        inside     215   283
tr|D0A6J2|D0A6J2_TRYB9  TMHMM2.0        TMhelix    284   306
tr|D0A6J2|D0A6J2_TRYB9  TMHMM2.0        outside    307   343
tr|D0A6J2|D0A6J2_TRYB9  TMHMM2.0        TMhelix    344   366
tr|D0A6J2|D0A6J2_TRYB9  TMHMM2.0        inside     367   372
tr|D0A6J2|D0A6J2_TRYB9  TMHMM2.0        TMhelix    373   392
tr|D0A6J2|D0A6J2_TRYB9  TMHMM2.0        outside    393   411
tr|D0A6J2|D0A6J2_TRYB9  TMHMM2.0        TMhelix    412   434
tr|D0A6J2|D0A6J2_TRYB9  TMHMM2.0        inside     435   435
```

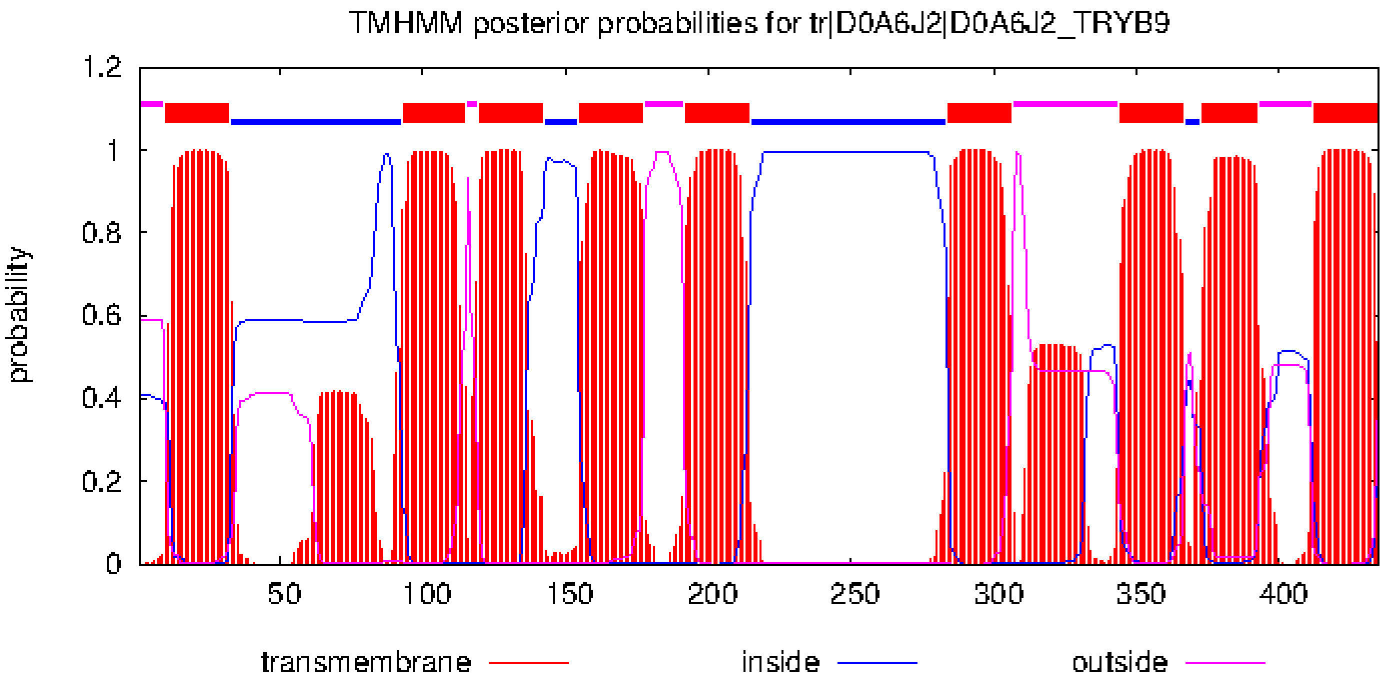

155 # plot in postscript, script for making the plot in gnuplot, data for plot

---

```
# tr|C9ZUC9|C9ZUC9_TRYB9 Length: 448
# tr|C9ZUC9|C9ZUC9_TRYB9 Number of predicted TMHs:  1
# tr|C9ZUC9|C9ZUC9_TRYB9 Exp number of AAs in TMHs: 20.30756
# tr|C9ZUC9|C9ZUC9_TRYB9 Exp number, first 60 AAs: 0.00018
# tr|C9ZUC9|C9ZUC9_TRYB9 Total prob of N-in:        0.01528
tr|C9ZUC9|C9ZUC9_TRYB9  TMHMM2.0        outside      1   420
tr|C9ZUC9|C9ZUC9_TRYB9  TMHMM2.0        TMhelix    421   443
tr|C9ZUC9|C9ZUC9_TRYB9  TMHMM2.0        inside     444   448
```

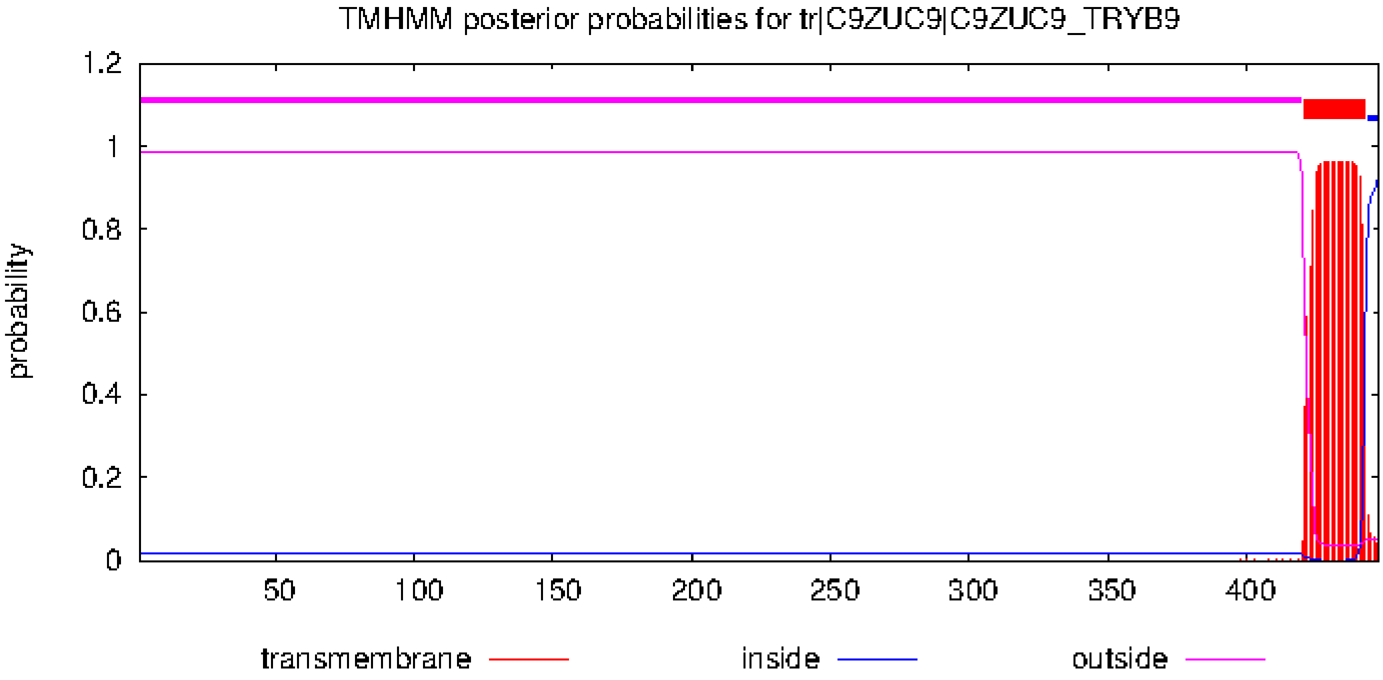

156 # plot in postscript, script for making the plot in gnuplot, data for plot

---

```
# tr|D0A9A1|D0A9A1_TRYB9 Length: 452
# tr|D0A9A1|D0A9A1_TRYB9 Number of predicted TMHs:  9
```

```
# tr|D0A9A1|D0A9A1_TRYB9 Exp number of AAs in TMHs: 199.20204
# tr|D0A9A1|D0A9A1_TRYB9 Exp number, first 60 AAs:  24.05334
# tr|D0A9A1|D0A9A1_TRYB9 Total prob of N-in:        0.88896
```

tr|D0A9A1|D0A9A1_TRYB9 POSSIBLE N-term signal sequence

tr|D0A9A1|D0A9A1_TRYB9	TMHMM2.0	inside	1	12
tr|D0A9A1|D0A9A1_TRYB9	TMHMM2.0	TMhelix	13	35
tr|D0A9A1|D0A9A1_TRYB9	TMHMM2.0	outside	36	66
tr|D0A9A1|D0A9A1_TRYB9	TMHMM2.0	TMhelix	67	89
tr|D0A9A1|D0A9A1_TRYB9	TMHMM2.0	inside	90	101
tr|D0A9A1|D0A9A1_TRYB9	TMHMM2.0	TMhelix	102	124
tr|D0A9A1|D0A9A1_TRYB9	TMHMM2.0	outside	125	133
tr|D0A9A1|D0A9A1_TRYB9	TMHMM2.0	TMhelix	134	156
tr|D0A9A1|D0A9A1_TRYB9	TMHMM2.0	inside	157	271
tr|D0A9A1|D0A9A1_TRYB9	TMHMM2.0	TMhelix	272	289
tr|D0A9A1|D0A9A1_TRYB9	TMHMM2.0	outside	290	308
tr|D0A9A1|D0A9A1_TRYB9	TMHMM2.0	TMhelix	309	331
tr|D0A9A1|D0A9A1_TRYB9	TMHMM2.0	inside	332	337
tr|D0A9A1|D0A9A1_TRYB9	TMHMM2.0	TMhelix	338	357
tr|D0A9A1|D0A9A1_TRYB9	TMHMM2.0	outside	358	361
tr|D0A9A1|D0A9A1_TRYB9	TMHMM2.0	TMhelix	362	384
tr|D0A9A1|D0A9A1_TRYB9	TMHMM2.0	inside	385	424
tr|D0A9A1|D0A9A1_TRYB9	TMHMM2.0	TMhelix	425	447
tr|D0A9A1|D0A9A1_TRYB9	TMHMM2.0	outside	448	452

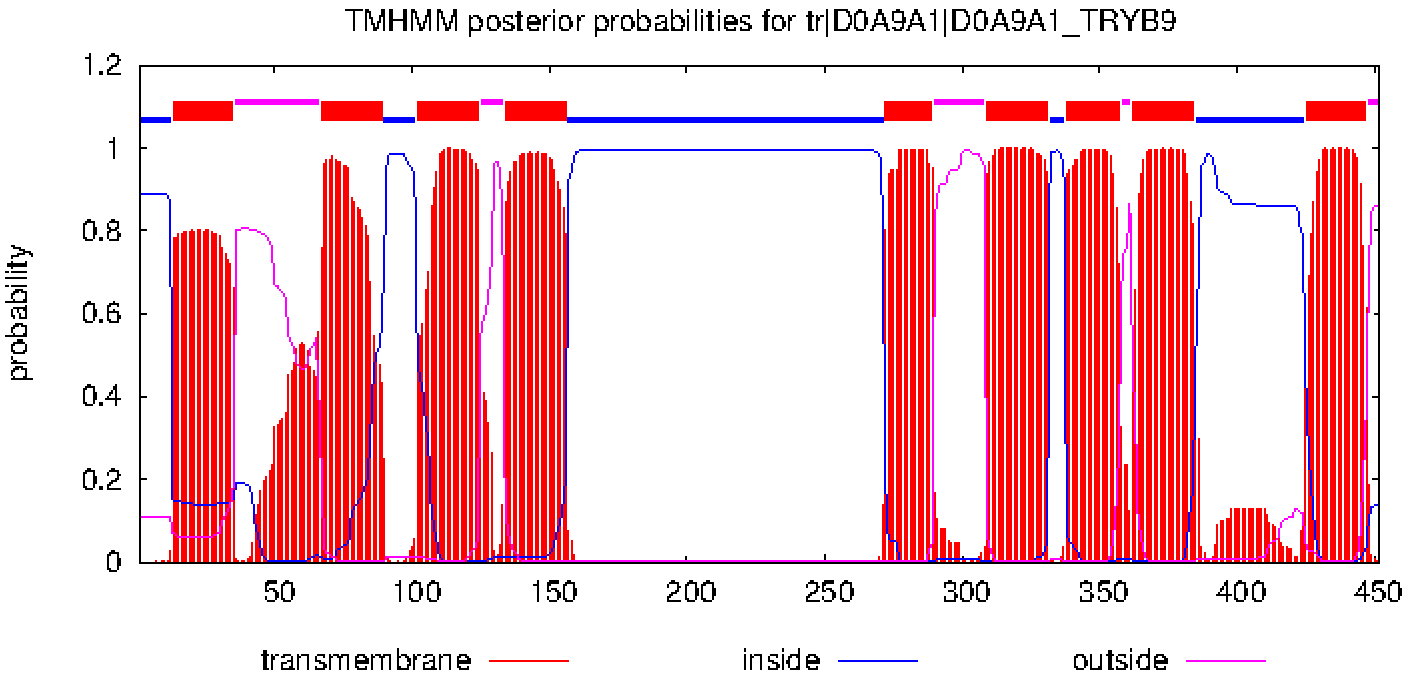

157 **# plot in postscript, script for making the plot in gnuplot, data for plot**

---

tr|D0A843|D0A843_TRYB9 Length: 456
tr|D0A843|D0A843_TRYB9 Number of predicted TMHs: 4
tr|D0A843|D0A843_TRYB9 Exp number of AAs in TMHs: 90.48697
tr|D0A843|D0A843_TRYB9 Exp number, first 60 AAs: 8e-05
tr|D0A843|D0A843_TRYB9 Total prob of N-in: 0.81313

tr|D0A843|D0A843_TRYB9	TMHMM2.0	inside	1	292
tr|D0A843|D0A843_TRYB9	TMHMM2.0	TMhelix	293	315
tr|D0A843|D0A843_TRYB9	TMHMM2.0	outside	316	356
tr|D0A843|D0A843_TRYB9	TMHMM2.0	TMhelix	357	379
tr|D0A843|D0A843_TRYB9	TMHMM2.0	inside	380	390
tr|D0A843|D0A843_TRYB9	TMHMM2.0	TMhelix	391	413
tr|D0A843|D0A843_TRYB9	TMHMM2.0	outside	414	427
tr|D0A843|D0A843_TRYB9	TMHMM2.0	TMhelix	428	450
tr|D0A843|D0A843_TRYB9	TMHMM2.0	inside	451	456

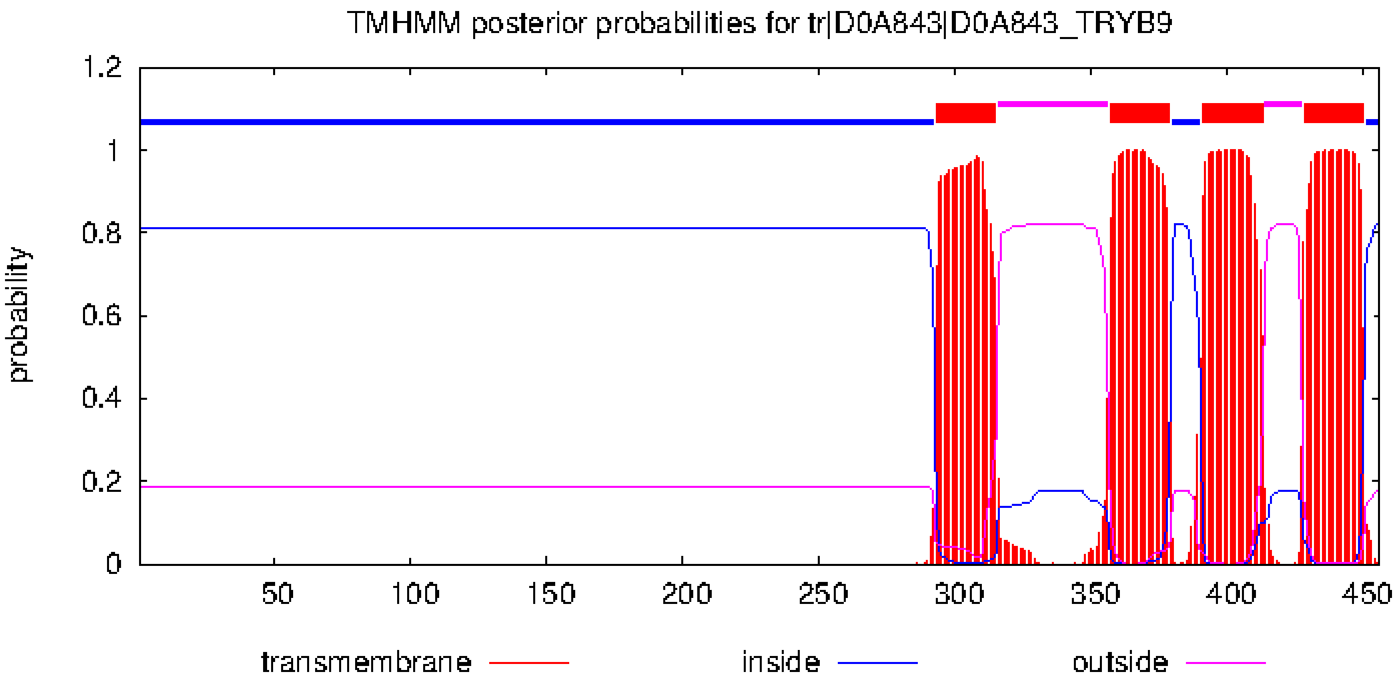

158 **# plot in postscript, script for making the plot in gnuplot, data for plot**

```
# tr|C9ZMB9|C9ZMB9_TRYB9 Length: 473
# tr|C9ZMB9|C9ZMB9_TRYB9 Number of predicted TMHs:  11
# tr|C9ZMB9|C9ZMB9_TRYB9 Exp number of AAs in TMHs: 243.25129
# tr|C9ZMB9|C9ZMB9_TRYB9 Exp number, first 60 AAs:  0.00014
# tr|C9ZMB9|C9ZMB9_TRYB9 Total prob of N-in:        0.99134
tr|C9ZMB9|C9ZMB9_TRYB9  TMHMM2.0        inside       1    71
tr|C9ZMB9|C9ZMB9_TRYB9  TMHMM2.0        TMhelix     72    94
tr|C9ZMB9|C9ZMB9_TRYB9  TMHMM2.0        outside     95   103
tr|C9ZMB9|C9ZMB9_TRYB9  TMHMM2.0        TMhelix    104   126
tr|C9ZMB9|C9ZMB9_TRYB9  TMHMM2.0        inside     127   148
tr|C9ZMB9|C9ZMB9_TRYB9  TMHMM2.0        TMhelix    149   171
tr|C9ZMB9|C9ZMB9_TRYB9  TMHMM2.0        outside    172   190
tr|C9ZMB9|C9ZMB9_TRYB9  TMHMM2.0        TMhelix    191   210
tr|C9ZMB9|C9ZMB9_TRYB9  TMHMM2.0        inside     211   216
tr|C9ZMB9|C9ZMB9_TRYB9  TMHMM2.0        TMhelix    217   236
tr|C9ZMB9|C9ZMB9_TRYB9  TMHMM2.0        outside    237   262
tr|C9ZMB9|C9ZMB9_TRYB9  TMHMM2.0        TMhelix    263   285
tr|C9ZMB9|C9ZMB9_TRYB9  TMHMM2.0        inside     286   297
tr|C9ZMB9|C9ZMB9_TRYB9  TMHMM2.0        TMhelix    298   320
tr|C9ZMB9|C9ZMB9_TRYB9  TMHMM2.0        outside    321   334
tr|C9ZMB9|C9ZMB9_TRYB9  TMHMM2.0        TMhelix    335   357
tr|C9ZMB9|C9ZMB9_TRYB9  TMHMM2.0        inside     358   377
tr|C9ZMB9|C9ZMB9_TRYB9  TMHMM2.0        TMhelix    378   400
tr|C9ZMB9|C9ZMB9_TRYB9  TMHMM2.0        outside    401   404
tr|C9ZMB9|C9ZMB9_TRYB9  TMHMM2.0        TMhelix    405   427
tr|C9ZMB9|C9ZMB9_TRYB9  TMHMM2.0        inside     428   446
tr|C9ZMB9|C9ZMB9_TRYB9  TMHMM2.0        TMhelix    447   469
tr|C9ZMB9|C9ZMB9_TRYB9  TMHMM2.0        outside    470   473
```

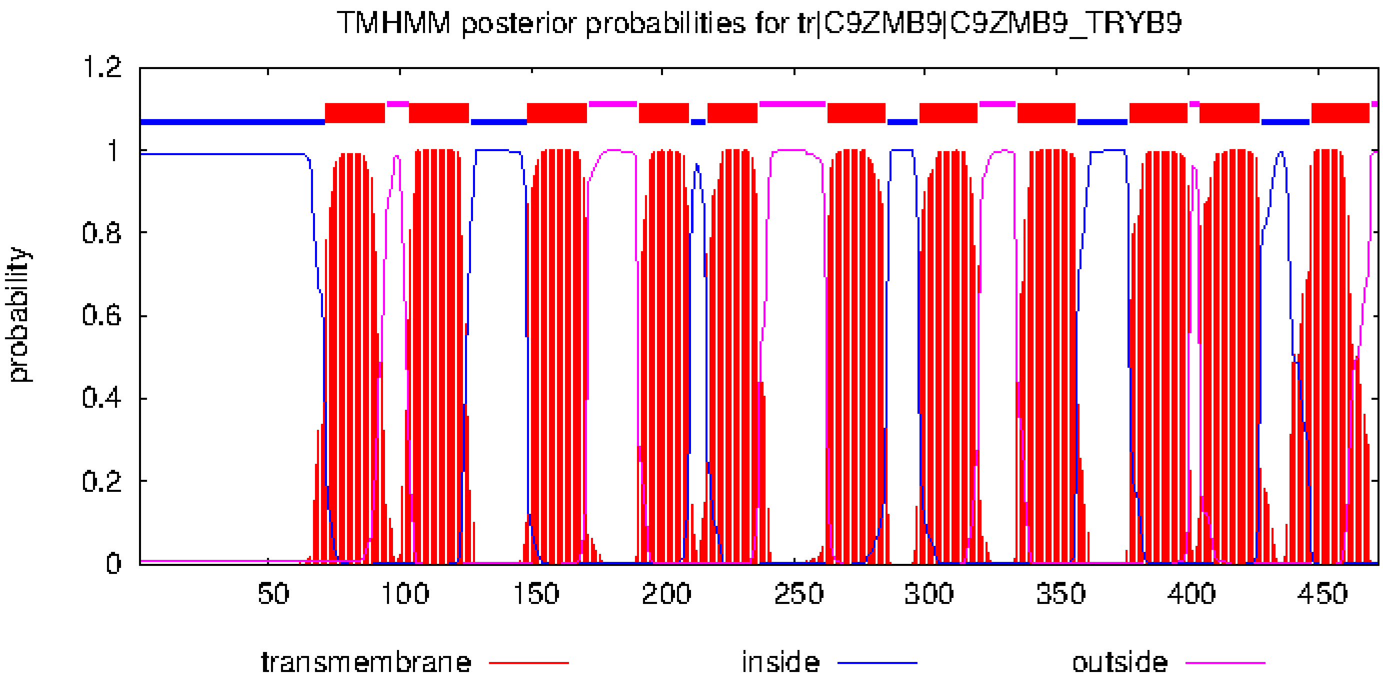

159 **# plot in postscript, script for making the plot in gnuplot, data for plot**

```
# tr|D0AAG7|D0AAG7_TRYB9 Length: 495
# tr|D0AAG7|D0AAG7_TRYB9 Number of predicted TMHs:  11
# tr|D0AAG7|D0AAG7_TRYB9 Exp number of AAs in TMHs: 243.88925
# tr|D0AAG7|D0AAG7_TRYB9 Exp number, first 60 AAs:  0.00011
# tr|D0AAG7|D0AAG7_TRYB9 Total prob of N-in:        0.99866
tr|D0AAG7|D0AAG7_TRYB9  TMHMM2.0        inside       1    73
tr|D0AAG7|D0AAG7_TRYB9  TMHMM2.0        TMhelix     74    96
tr|D0AAG7|D0AAG7_TRYB9  TMHMM2.0        outside     97   105
tr|D0AAG7|D0AAG7_TRYB9  TMHMM2.0        TMhelix    106   128
tr|D0AAG7|D0AAG7_TRYB9  TMHMM2.0        inside     129   154
tr|D0AAG7|D0AAG7_TRYB9  TMHMM2.0        TMhelix    155   177
tr|D0AAG7|D0AAG7_TRYB9  TMHMM2.0        outside    178   196
tr|D0AAG7|D0AAG7_TRYB9  TMHMM2.0        TMhelix    197   214
tr|D0AAG7|D0AAG7_TRYB9  TMHMM2.0        inside     215   220
tr|D0AAG7|D0AAG7_TRYB9  TMHMM2.0        TMhelix    221   243
tr|D0AAG7|D0AAG7_TRYB9  TMHMM2.0        outside    244   267
tr|D0AAG7|D0AAG7_TRYB9  TMHMM2.0        TMhelix    268   290
tr|D0AAG7|D0AAG7_TRYB9  TMHMM2.0        inside     291   301
tr|D0AAG7|D0AAG7_TRYB9  TMHMM2.0        TMhelix    302   324
tr|D0AAG7|D0AAG7_TRYB9  TMHMM2.0        outside    325   343
tr|D0AAG7|D0AAG7_TRYB9  TMHMM2.0        TMhelix    344   363
tr|D0AAG7|D0AAG7_TRYB9  TMHMM2.0        inside     364   383
tr|D0AAG7|D0AAG7_TRYB9  TMHMM2.0        TMhelix    384   406
tr|D0AAG7|D0AAG7_TRYB9  TMHMM2.0        outside    407   410
tr|D0AAG7|D0AAG7_TRYB9  TMHMM2.0        TMhelix    411   433
tr|D0AAG7|D0AAG7_TRYB9  TMHMM2.0        inside     434   445
tr|D0AAG7|D0AAG7_TRYB9  TMHMM2.0        TMhelix    446   468
tr|D0AAG7|D0AAG7_TRYB9  TMHMM2.0        outside    469   495
```

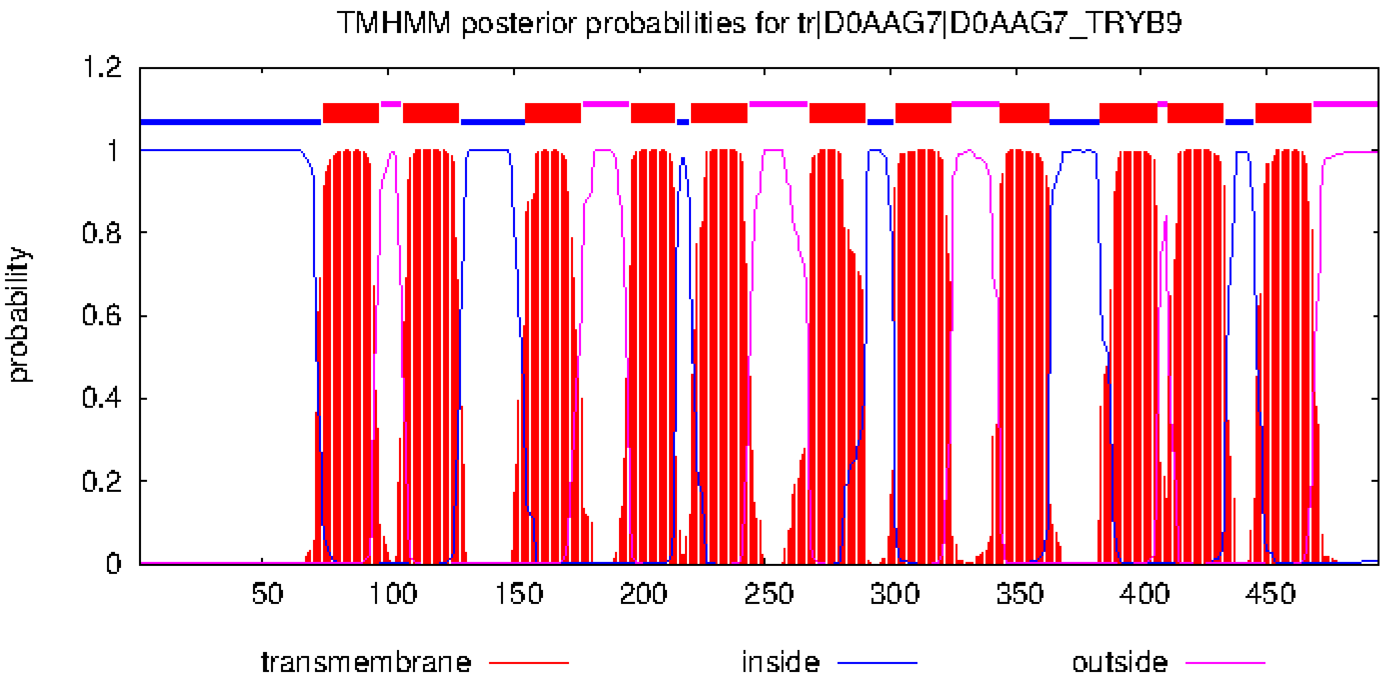

160 # plot in postscript, script for making the plot in gnuplot, data for plot

---

```
# tr|C9ZVE9|C9ZVE9_TRYB9 Length: 632
# tr|C9ZVE9|C9ZVE9_TRYB9 Number of predicted TMHs:  12
# tr|C9ZVE9|C9ZVE9_TRYB9 Exp number of AAs in TMHs: 286.76552
# tr|C9ZVE9|C9ZVE9_TRYB9 Exp number, first 60 AAs:  2.44422
# tr|C9ZVE9|C9ZVE9_TRYB9 Total prob of N-in:        0.64389
tr|C9ZVE9|C9ZVE9_TRYB9  TMHMM2.0        inside       1   142
tr|C9ZVE9|C9ZVE9_TRYB9  TMHMM2.0        TMhelix    143   162
tr|C9ZVE9|C9ZVE9_TRYB9  TMHMM2.0        outside    163   166
tr|C9ZVE9|C9ZVE9_TRYB9  TMHMM2.0        TMhelix    167   189
tr|C9ZVE9|C9ZVE9_TRYB9  TMHMM2.0        inside     190   200
tr|C9ZVE9|C9ZVE9_TRYB9  TMHMM2.0        TMhelix    201   223
tr|C9ZVE9|C9ZVE9_TRYB9  TMHMM2.0        outside    224   237
tr|C9ZVE9|C9ZVE9_TRYB9  TMHMM2.0        TMhelix    238   260
tr|C9ZVE9|C9ZVE9_TRYB9  TMHMM2.0        inside     261   335
tr|C9ZVE9|C9ZVE9_TRYB9  TMHMM2.0        TMhelix    336   358
tr|C9ZVE9|C9ZVE9_TRYB9  TMHMM2.0        outside    359   362
tr|C9ZVE9|C9ZVE9_TRYB9  TMHMM2.0        TMhelix    363   385
tr|C9ZVE9|C9ZVE9_TRYB9  TMHMM2.0        inside     386   391
tr|C9ZVE9|C9ZVE9_TRYB9  TMHMM2.0        TMhelix    392   414
tr|C9ZVE9|C9ZVE9_TRYB9  TMHMM2.0        outside    415   428
tr|C9ZVE9|C9ZVE9_TRYB9  TMHMM2.0        TMhelix    429   451
tr|C9ZVE9|C9ZVE9_TRYB9  TMHMM2.0        inside     452   455
tr|C9ZVE9|C9ZVE9_TRYB9  TMHMM2.0        TMhelix    456   478
tr|C9ZVE9|C9ZVE9_TRYB9  TMHMM2.0        outside    479   497
tr|C9ZVE9|C9ZVE9_TRYB9  TMHMM2.0        TMhelix    498   520
tr|C9ZVE9|C9ZVE9_TRYB9  TMHMM2.0        inside     521   526
tr|C9ZVE9|C9ZVE9_TRYB9  TMHMM2.0        TMhelix    527   549
tr|C9ZVE9|C9ZVE9_TRYB9  TMHMM2.0        outside    550   571
tr|C9ZVE9|C9ZVE9_TRYB9  TMHMM2.0        TMhelix    572   594
tr|C9ZVE9|C9ZVE9_TRYB9  TMHMM2.0        inside     595   632
```

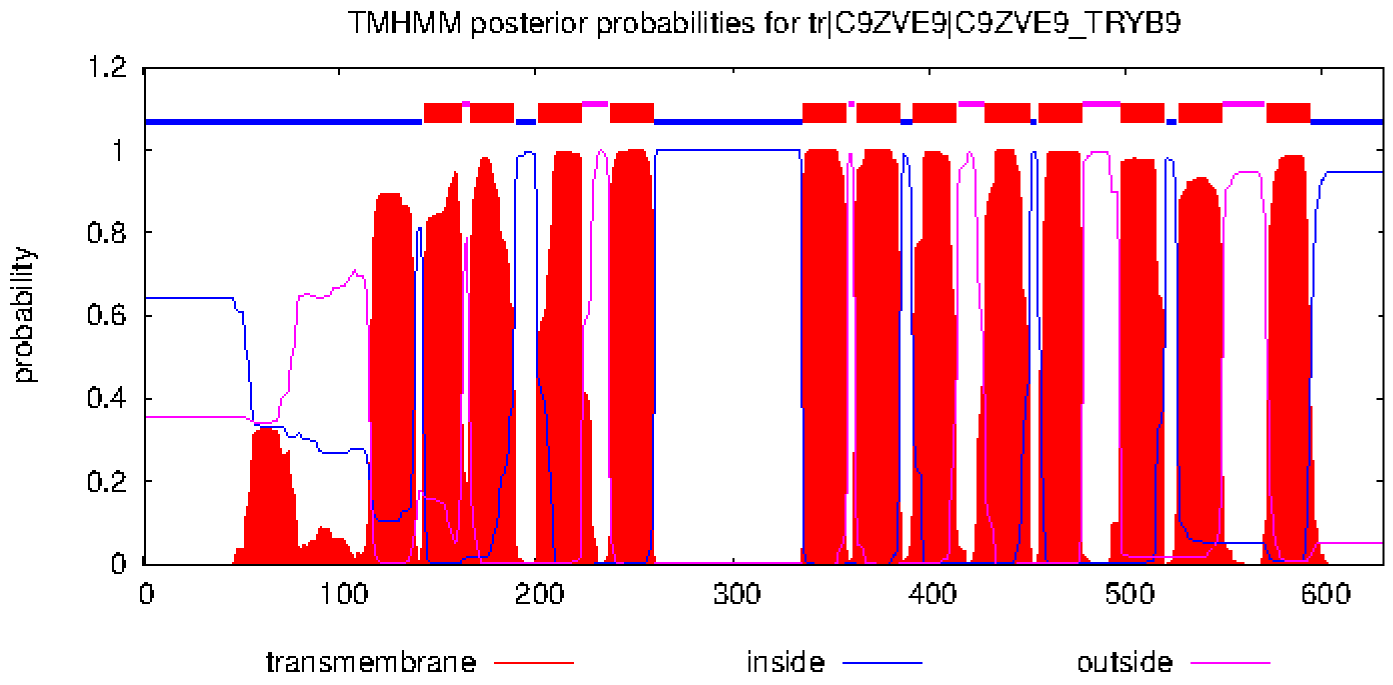

161 # plot in postscript, script for making the plot in gnuplot, data for plot

---

```
# tr|C9ZIK3|C9ZIK3_TRYB9 Length: 652
# tr|C9ZIK3|C9ZIK3_TRYB9 Number of predicted TMHs:  13
# tr|C9ZIK3|C9ZIK3_TRYB9 Exp number of AAs in TMHs: 283.84848
# tr|C9ZIK3|C9ZIK3_TRYB9 Exp number, first 60 AAs:  0.00425
# tr|C9ZIK3|C9ZIK3_TRYB9 Total prob of N-in:        0.37999
tr|C9ZIK3|C9ZIK3_TRYB9  TMHMM2.0        outside      1    69
tr|C9ZIK3|C9ZIK3_TRYB9  TMHMM2.0        TMhelix     70    92
tr|C9ZIK3|C9ZIK3_TRYB9  TMHMM2.0        inside      93   111
tr|C9ZIK3|C9ZIK3_TRYB9  TMHMM2.0        TMhelix    112   131
tr|C9ZIK3|C9ZIK3_TRYB9  TMHMM2.0        outside    132   150
tr|C9ZIK3|C9ZIK3_TRYB9  TMHMM2.0        TMhelix    151   173
tr|C9ZIK3|C9ZIK3_TRYB9  TMHMM2.0        inside     174   197
tr|C9ZIK3|C9ZIK3_TRYB9  TMHMM2.0        TMhelix    198   220
tr|C9ZIK3|C9ZIK3_TRYB9  TMHMM2.0        outside    221   229
tr|C9ZIK3|C9ZIK3_TRYB9  TMHMM2.0        TMhelix    230   252
tr|C9ZIK3|C9ZIK3_TRYB9  TMHMM2.0        inside     253   347
tr|C9ZIK3|C9ZIK3_TRYB9  TMHMM2.0        TMhelix    348   367
tr|C9ZIK3|C9ZIK3_TRYB9  TMHMM2.0        outside    368   371
tr|C9ZIK3|C9ZIK3_TRYB9  TMHMM2.0        TMhelix    372   391
tr|C9ZIK3|C9ZIK3_TRYB9  TMHMM2.0        inside     392   397
tr|C9ZIK3|C9ZIK3_TRYB9  TMHMM2.0        TMhelix    398   420
tr|C9ZIK3|C9ZIK3_TRYB9  TMHMM2.0        outside    421   434
tr|C9ZIK3|C9ZIK3_TRYB9  TMHMM2.0        TMhelix    435   457
tr|C9ZIK3|C9ZIK3_TRYB9  TMHMM2.0        inside     458   469
tr|C9ZIK3|C9ZIK3_TRYB9  TMHMM2.0        TMhelix    470   489
tr|C9ZIK3|C9ZIK3_TRYB9  TMHMM2.0        outside    490   498
tr|C9ZIK3|C9ZIK3_TRYB9  TMHMM2.0        TMhelix    499   521
tr|C9ZIK3|C9ZIK3_TRYB9  TMHMM2.0        inside     522   541
tr|C9ZIK3|C9ZIK3_TRYB9  TMHMM2.0        TMhelix    542   564
tr|C9ZIK3|C9ZIK3_TRYB9  TMHMM2.0        outside    565   578
tr|C9ZIK3|C9ZIK3_TRYB9  TMHMM2.0        TMhelix    579   601
tr|C9ZIK3|C9ZIK3_TRYB9  TMHMM2.0        inside     602   652
```

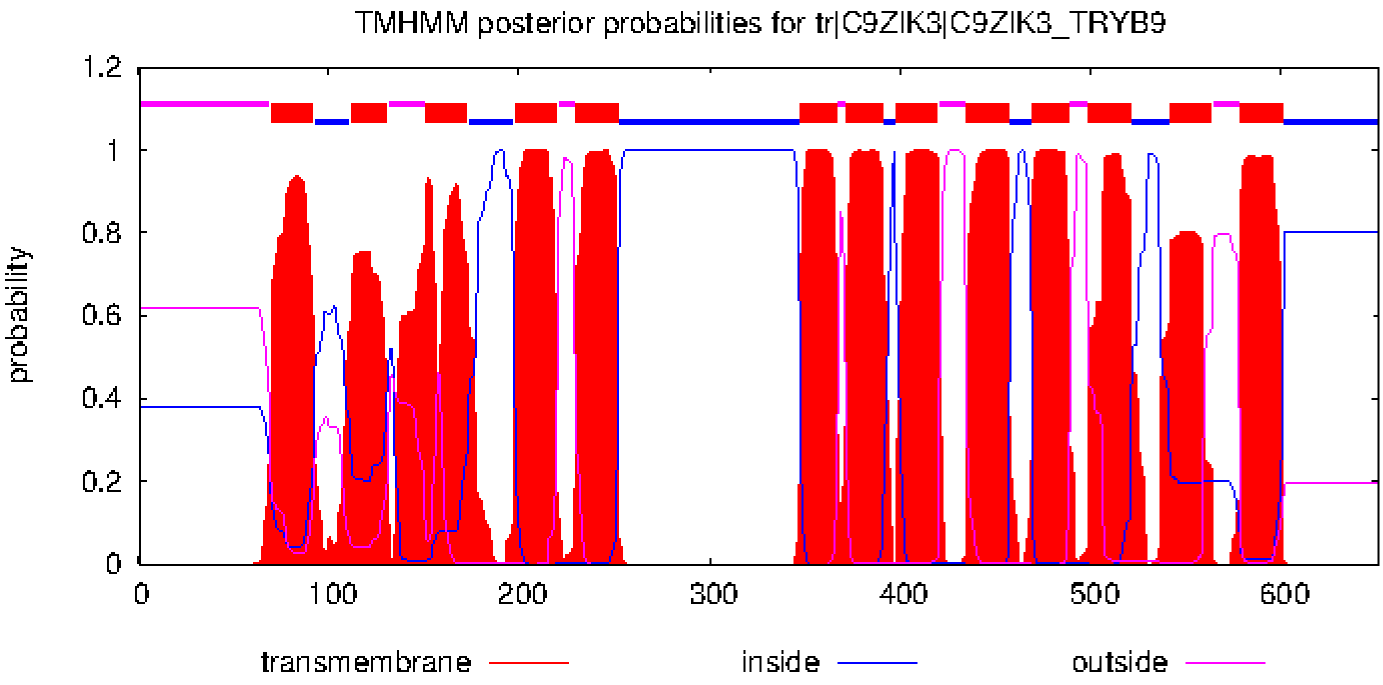

162 # [plot] in postscript, [script] for making the plot in gnuplot, [data] for plot

---

```
# tr|C9ZLF3|C9ZLF3_TRYB9 Length: 729
# tr|C9ZLF3|C9ZLF3_TRYB9 Number of predicted TMHs:  4
# tr|C9ZLF3|C9ZLF3_TRYB9 Exp number of AAs in TMHs: 114.99462
# tr|C9ZLF3|C9ZLF3_TRYB9 Exp number, first 60 AAs:  8.45672
# tr|C9ZLF3|C9ZLF3_TRYB9 Total prob of N-in:        0.53148
tr|C9ZLF3|C9ZLF3_TRYB9  TMHMM2.0        outside      1   398
tr|C9ZLF3|C9ZLF3_TRYB9  TMHMM2.0        TMhelix    399   421
tr|C9ZLF3|C9ZLF3_TRYB9  TMHMM2.0        inside     422   486
tr|C9ZLF3|C9ZLF3_TRYB9  TMHMM2.0        TMhelix    487   509
tr|C9ZLF3|C9ZLF3_TRYB9  TMHMM2.0        outside    510   560
tr|C9ZLF3|C9ZLF3_TRYB9  TMHMM2.0        TMhelix    561   583
tr|C9ZLF3|C9ZLF3_TRYB9  TMHMM2.0        inside     584   589
tr|C9ZLF3|C9ZLF3_TRYB9  TMHMM2.0        TMhelix    590   612
tr|C9ZLF3|C9ZLF3_TRYB9  TMHMM2.0        outside    613   729
```

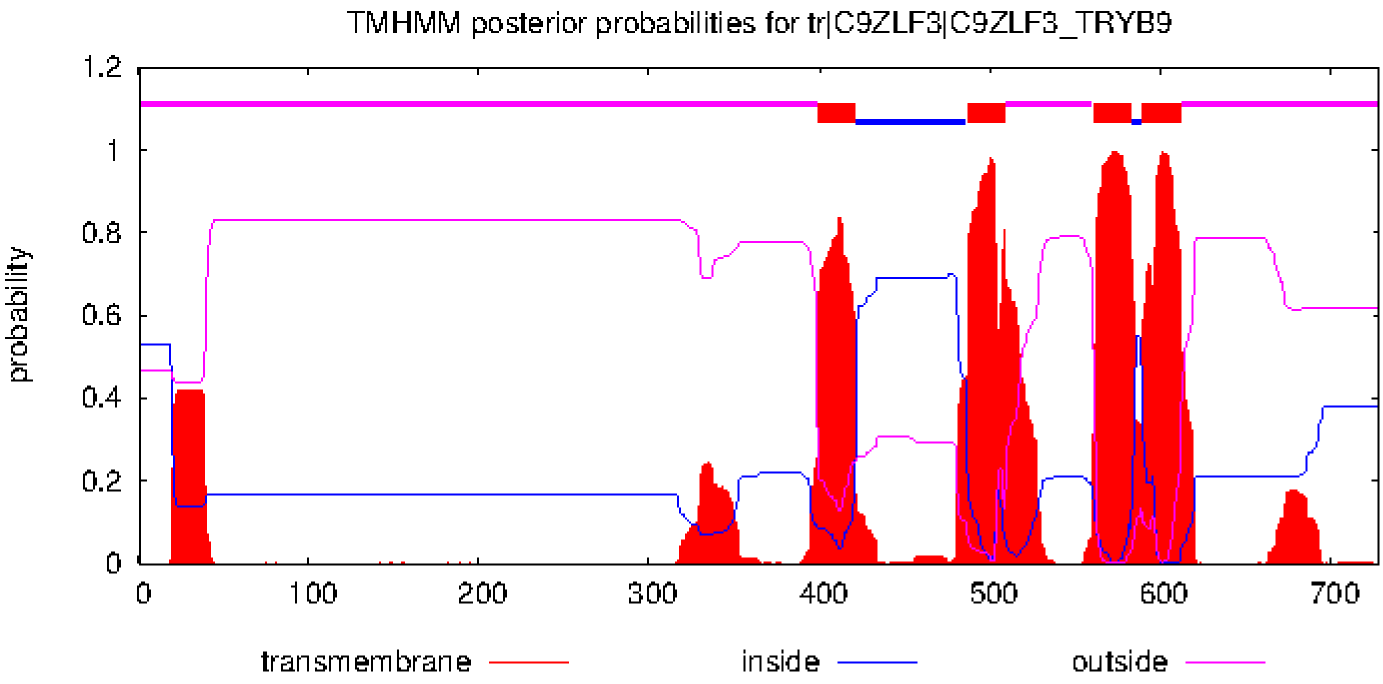

163 # [plot] in postscript, [script] for making the plot in gnuplot, [data] for plot

```
# tr|D0A6T6|D0A6T6_TRYB9 Length: 779
# tr|D0A6T6|D0A6T6_TRYB9 Number of predicted TMHs:  11
# tr|D0A6T6|D0A6T6_TRYB9 Exp number of AAs in TMHs: 257.366799999999
# tr|D0A6T6|D0A6T6_TRYB9 Exp number, first 60 AAs:  20.92997
# tr|D0A6T6|D0A6T6_TRYB9 Total prob of N-in:        0.99980
# tr|D0A6T6|D0A6T6_TRYB9 POSSIBLE N-term signal sequence
tr|D0A6T6|D0A6T6_TRYB9  TMHMM2.0        inside       1    22
tr|D0A6T6|D0A6T6_TRYB9  TMHMM2.0        TMhelix     23    42
tr|D0A6T6|D0A6T6_TRYB9  TMHMM2.0        outside     43    61
tr|D0A6T6|D0A6T6_TRYB9  TMHMM2.0        TMhelix     62    84
tr|D0A6T6|D0A6T6_TRYB9  TMHMM2.0        inside      85    96
tr|D0A6T6|D0A6T6_TRYB9  TMHMM2.0        TMhelix     97   119
tr|D0A6T6|D0A6T6_TRYB9  TMHMM2.0        outside    120   148
tr|D0A6T6|D0A6T6_TRYB9  TMHMM2.0        TMhelix    149   171
tr|D0A6T6|D0A6T6_TRYB9  TMHMM2.0        inside     172   197
tr|D0A6T6|D0A6T6_TRYB9  TMHMM2.0        TMhelix    198   220
tr|D0A6T6|D0A6T6_TRYB9  TMHMM2.0        outside    221   278
tr|D0A6T6|D0A6T6_TRYB9  TMHMM2.0        TMhelix    279   301
tr|D0A6T6|D0A6T6_TRYB9  TMHMM2.0        inside     302   321
tr|D0A6T6|D0A6T6_TRYB9  TMHMM2.0        TMhelix    322   344
tr|D0A6T6|D0A6T6_TRYB9  TMHMM2.0        outside    345   367
tr|D0A6T6|D0A6T6_TRYB9  TMHMM2.0        TMhelix    368   390
tr|D0A6T6|D0A6T6_TRYB9  TMHMM2.0        inside     391   402
tr|D0A6T6|D0A6T6_TRYB9  TMHMM2.0        TMhelix    403   425
tr|D0A6T6|D0A6T6_TRYB9  TMHMM2.0        outside    426   453
tr|D0A6T6|D0A6T6_TRYB9  TMHMM2.0        TMhelix    454   476
tr|D0A6T6|D0A6T6_TRYB9  TMHMM2.0        inside     477   482
tr|D0A6T6|D0A6T6_TRYB9  TMHMM2.0        TMhelix    483   505
tr|D0A6T6|D0A6T6_TRYB9  TMHMM2.0        outside    506   779
```

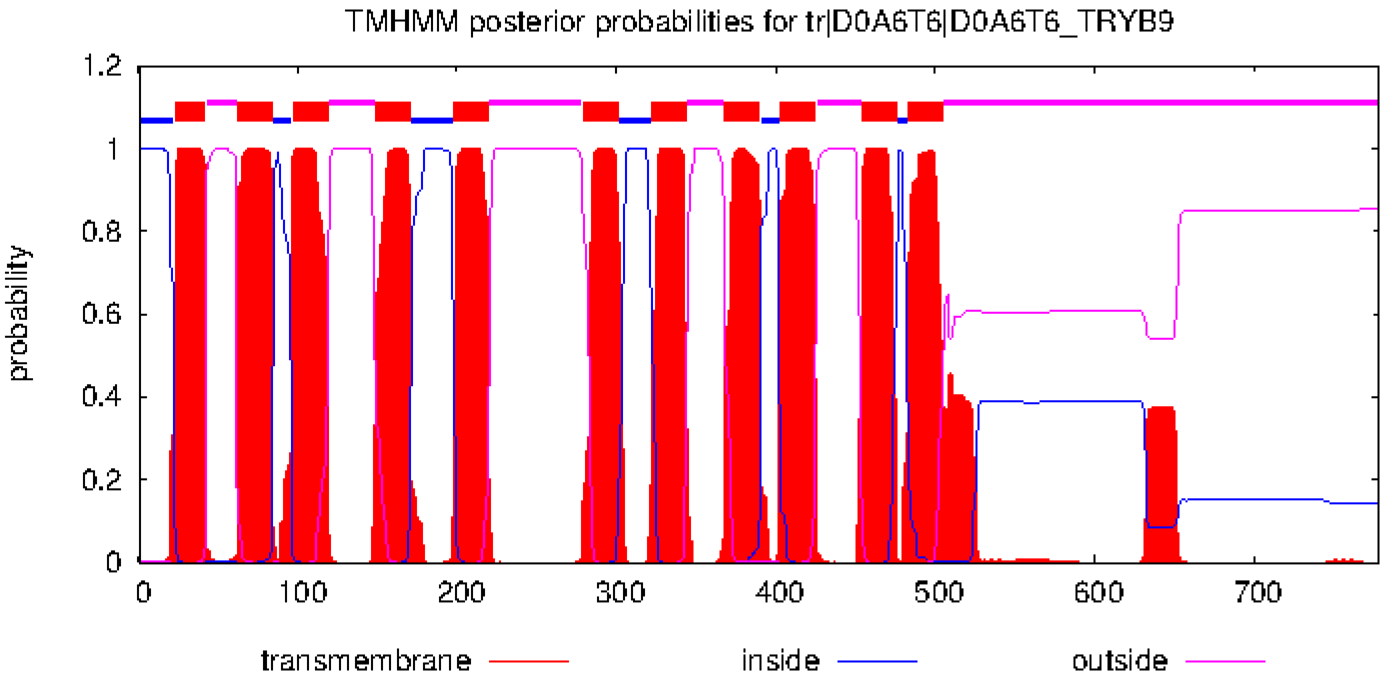

164 # plot in postscript, script for making the plot in gnuplot, data for plot

---

```
# tr|C9ZKS6|C9ZKS6_TRYB9 Length: 929
# tr|C9ZKS6|C9ZKS6_TRYB9 Number of predicted TMHs:  2
# tr|C9ZKS6|C9ZKS6_TRYB9 Exp number of AAs in TMHs: 55.0130100000002
# tr|C9ZKS6|C9ZKS6_TRYB9 Exp number, first 60 AAs:  14.44302
# tr|C9ZKS6|C9ZKS6_TRYB9 Total prob of N-in:        0.00822
# tr|C9ZKS6|C9ZKS6_TRYB9 POSSIBLE N-term signal sequence
tr|C9ZKS6|C9ZKS6_TRYB9  TMHMM2.0        outside      1    46
tr|C9ZKS6|C9ZKS6_TRYB9  TMHMM2.0        TMhelix     47    69
tr|C9ZKS6|C9ZKS6_TRYB9  TMHMM2.0        inside      70   102
tr|C9ZKS6|C9ZKS6_TRYB9  TMHMM2.0        TMhelix    103   125
tr|C9ZKS6|C9ZKS6_TRYB9  TMHMM2.0        outside    126   929
```

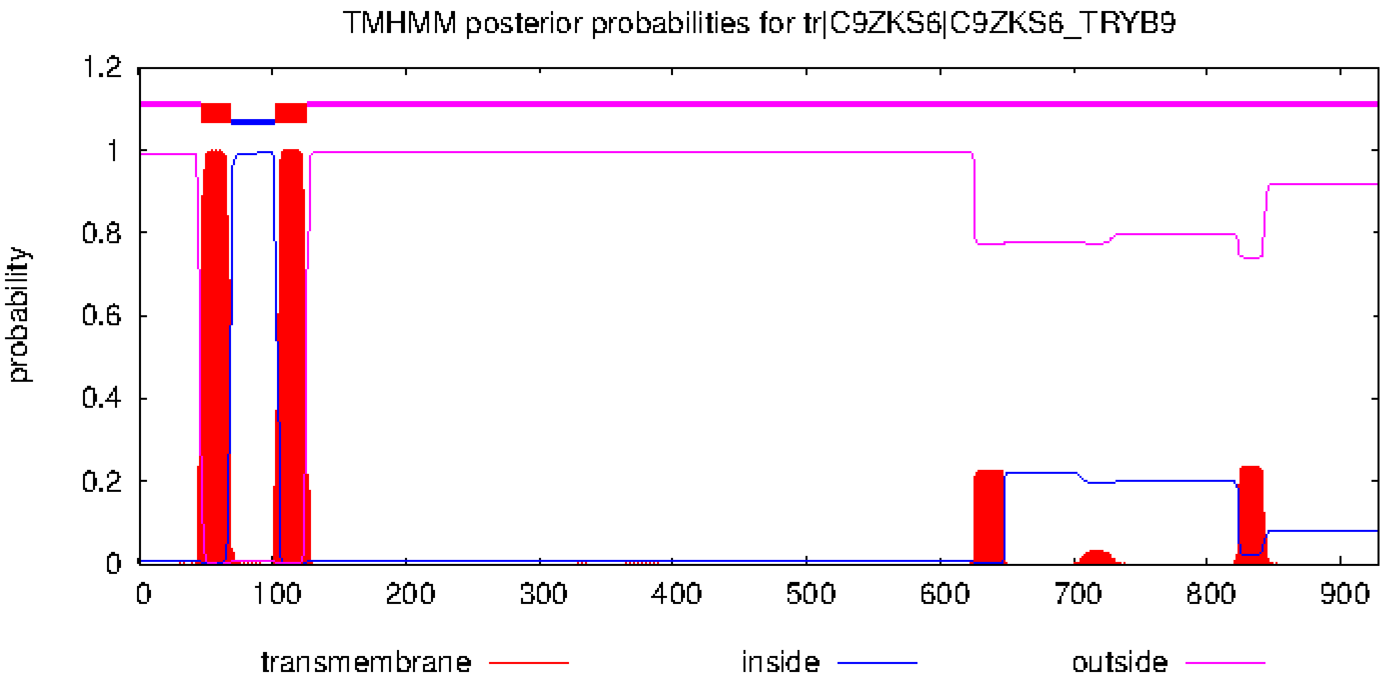

165 # plot in postscript, script for making the plot in gnuplot, data for plot

---

```
# tr|C9ZVY6|C9ZVY6_TRYB9 Length: 1104
# tr|C9ZVY6|C9ZVY6_TRYB9 Number of predicted TMHs:  10
# tr|C9ZVY6|C9ZVY6_TRYB9 Exp number of AAs in TMHs: 216.52238
# tr|C9ZVY6|C9ZVY6_TRYB9 Exp number, first 60 AAs: 22.97654
# tr|C9ZVY6|C9ZVY6_TRYB9 Total prob of N-in:        0.00248
# tr|C9ZVY6|C9ZVY6_TRYB9 POSSIBLE N-term signal sequence
tr|C9ZVY6|C9ZVY6_TRYB9  TMHMM2.0        outside      1    19
tr|C9ZVY6|C9ZVY6_TRYB9  TMHMM2.0        TMhelix     20    42
tr|C9ZVY6|C9ZVY6_TRYB9  TMHMM2.0        inside      43   200
tr|C9ZVY6|C9ZVY6_TRYB9  TMHMM2.0        TMhelix    201   223
tr|C9ZVY6|C9ZVY6_TRYB9  TMHMM2.0        outside    224   292
tr|C9ZVY6|C9ZVY6_TRYB9  TMHMM2.0        TMhelix    293   315
tr|C9ZVY6|C9ZVY6_TRYB9  TMHMM2.0        inside     316   591
tr|C9ZVY6|C9ZVY6_TRYB9  TMHMM2.0        TMhelix    592   614
tr|C9ZVY6|C9ZVY6_TRYB9  TMHMM2.0        outside    615   638
tr|C9ZVY6|C9ZVY6_TRYB9  TMHMM2.0        TMhelix    639   661
tr|C9ZVY6|C9ZVY6_TRYB9  TMHMM2.0        inside     662   680
tr|C9ZVY6|C9ZVY6_TRYB9  TMHMM2.0        TMhelix    681   699
tr|C9ZVY6|C9ZVY6_TRYB9  TMHMM2.0        outside    700   736
tr|C9ZVY6|C9ZVY6_TRYB9  TMHMM2.0        TMhelix    737   759
tr|C9ZVY6|C9ZVY6_TRYB9  TMHMM2.0        inside     760   796
tr|C9ZVY6|C9ZVY6_TRYB9  TMHMM2.0        TMhelix    797   819
tr|C9ZVY6|C9ZVY6_TRYB9  TMHMM2.0        outside    820   843
tr|C9ZVY6|C9ZVY6_TRYB9  TMHMM2.0        TMhelix    844   866
tr|C9ZVY6|C9ZVY6_TRYB9  TMHMM2.0        inside     867   872
tr|C9ZVY6|C9ZVY6_TRYB9  TMHMM2.0        TMhelix    873   895
tr|C9ZVY6|C9ZVY6_TRYB9  TMHMM2.0        outside    896  1104
```

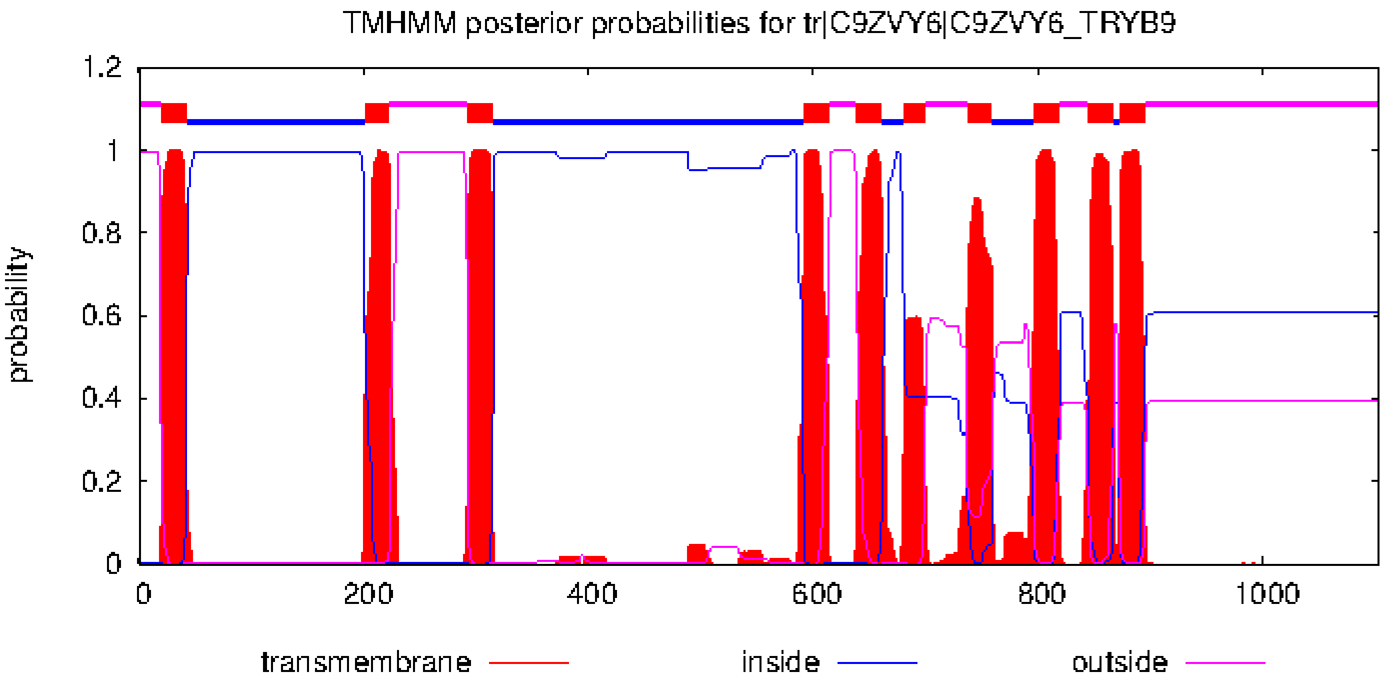

166 # plot in postscript, script for making the plot in gnuplot, data for plot

---

```
# tr|C9ZI17|C9ZI17_TRYB9 Length: 1167
# tr|C9ZI17|C9ZI17_TRYB9 Number of predicted TMHs:  7
# tr|C9ZI17|C9ZI17_TRYB9 Exp number of AAs in TMHs: 156.492680000001
# tr|C9ZI17|C9ZI17_TRYB9 Exp number, first 60 AAs:  0
# tr|C9ZI17|C9ZI17_TRYB9 Total prob of N-in:        0.91830
tr|C9ZI17|C9ZI17_TRYB9  TMHMM2.0        inside       1   139
tr|C9ZI17|C9ZI17_TRYB9  TMHMM2.0        TMhelix    140   162
tr|C9ZI17|C9ZI17_TRYB9  TMHMM2.0        outside    163   176
tr|C9ZI17|C9ZI17_TRYB9  TMHMM2.0        TMhelix    177   199
tr|C9ZI17|C9ZI17_TRYB9  TMHMM2.0        inside     200   205
tr|C9ZI17|C9ZI17_TRYB9  TMHMM2.0        TMhelix    206   228
tr|C9ZI17|C9ZI17_TRYB9  TMHMM2.0        outside    229   267
tr|C9ZI17|C9ZI17_TRYB9  TMHMM2.0        TMhelix    268   290
tr|C9ZI17|C9ZI17_TRYB9  TMHMM2.0        inside     291   294
tr|C9ZI17|C9ZI17_TRYB9  TMHMM2.0        TMhelix    295   317
tr|C9ZI17|C9ZI17_TRYB9  TMHMM2.0        outside    318   331
tr|C9ZI17|C9ZI17_TRYB9  TMHMM2.0        TMhelix    332   354
tr|C9ZI17|C9ZI17_TRYB9  TMHMM2.0        inside     355   504
tr|C9ZI17|C9ZI17_TRYB9  TMHMM2.0        TMhelix    505   527
tr|C9ZI17|C9ZI17_TRYB9  TMHMM2.0        outside    528  1167
```

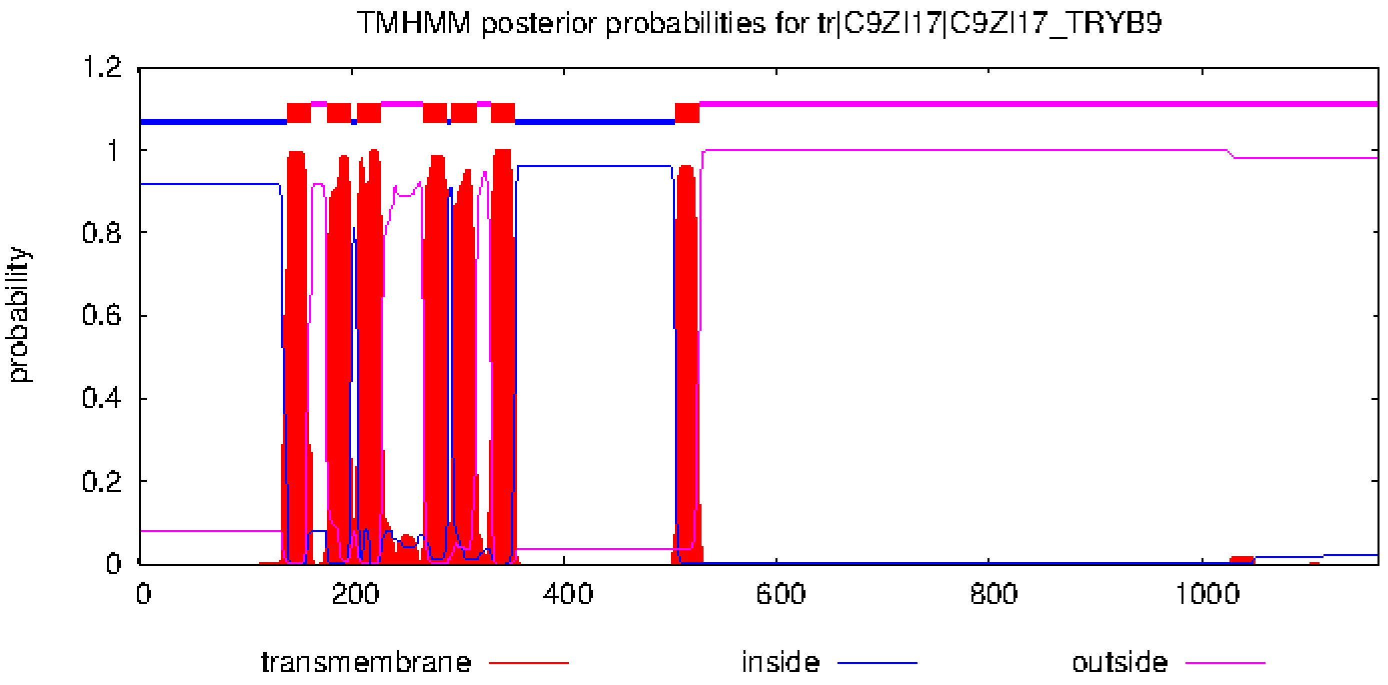

167 # plot in postscript, script for making the plot in gnuplot, data for plot

---

```
# tr|C9ZN45|C9ZN45_TRYB9 Length: 1232
# tr|C9ZN45|C9ZN45_TRYB9 Number of predicted TMHs:  1
# tr|C9ZN45|C9ZN45_TRYB9 Exp number of AAs in TMHs: 23.54054
# tr|C9ZN45|C9ZN45_TRYB9 Exp number, first 60 AAs: 0.35744
# tr|C9ZN45|C9ZN45_TRYB9 Total prob of N-in:        0.01734
tr|C9ZN45|C9ZN45_TRYB9  TMHMM2.0        outside      1   849
tr|C9ZN45|C9ZN45_TRYB9  TMHMM2.0        TMhelix    850   872
tr|C9ZN45|C9ZN45_TRYB9  TMHMM2.0        inside     873  1232
```

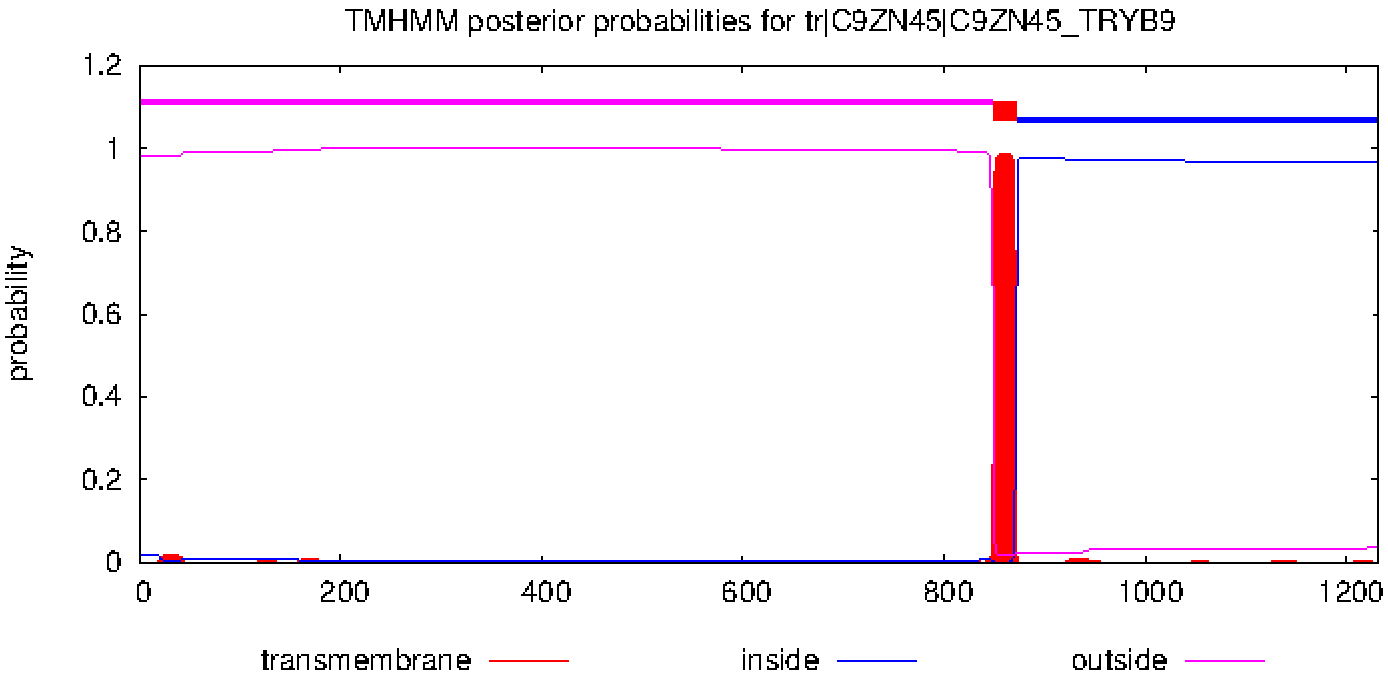

168 # plot in postscript, script for making the plot in gnuplot, data for plot

---

```
# tr|C9ZQ51|C9ZQ51_TRYB9 Length: 1245
# tr|C9ZQ51|C9ZQ51_TRYB9 Number of predicted TMHs:  1
```

```
# tr|C9ZQ51|C9ZQ51_TRYB9 Exp number of AAs in TMHs: 34.9715500000001
# tr|C9ZQ51|C9ZQ51_TRYB9 Exp number, first 60 AAs:  7.6588
# tr|C9ZQ51|C9ZQ51_TRYB9 Total prob of N-in:        0.34878
```

```
tr|C9ZQ51|C9ZQ51_TRYB9  TMHMM2.0        outside      1   857
tr|C9ZQ51|C9ZQ51_TRYB9  TMHMM2.0        TMhelix    858   880
tr|C9ZQ51|C9ZQ51_TRYB9  TMHMM2.0        inside     881  1245
```

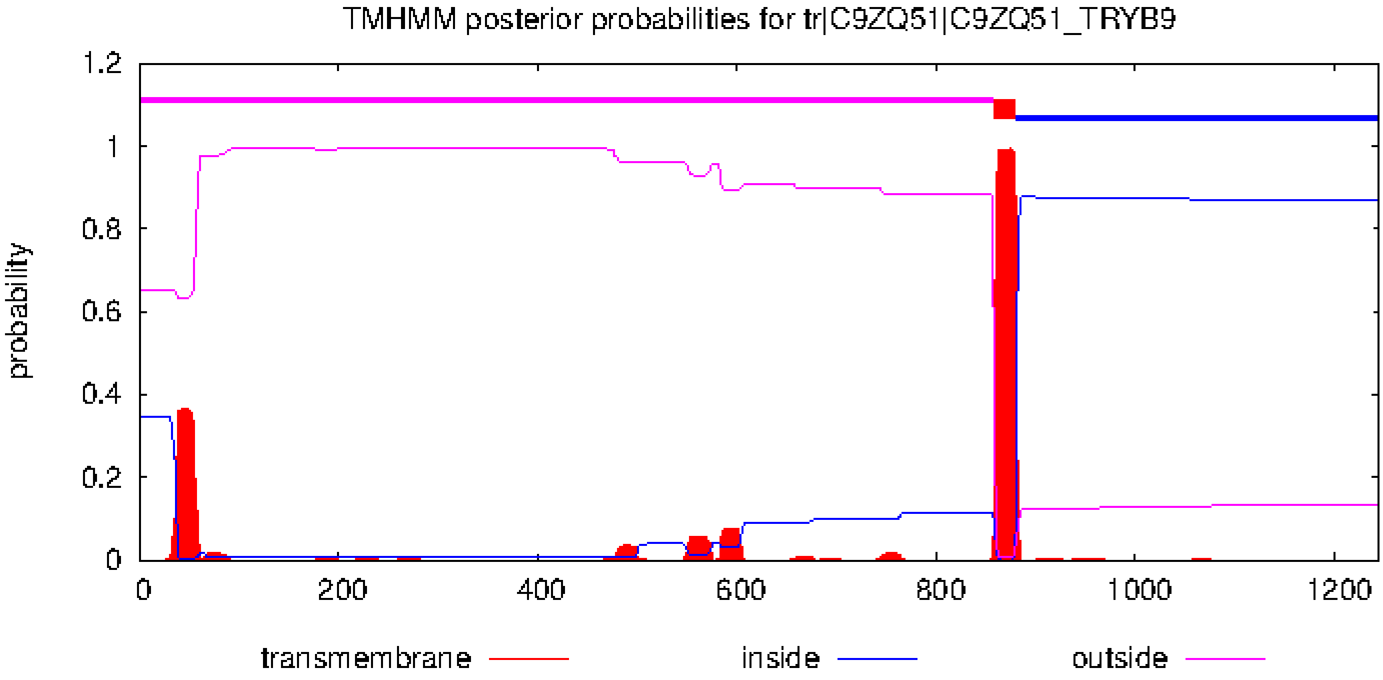

169 **# plot in postscript, script for making the plot in gnuplot, data for plot**

```
# tr|D0A7B9|D0A7B9_TRYB9 Length: 1737
# tr|D0A7B9|D0A7B9_TRYB9 Number of predicted TMHs:  12
# tr|D0A7B9|D0A7B9_TRYB9 Exp number of AAs in TMHs: 319.147449999999
# tr|D0A7B9|D0A7B9_TRYB9 Exp number, first 60 AAs:  0.00219
# tr|D0A7B9|D0A7B9_TRYB9 Total prob of N-in:        0.96289
tr|D0A7B9|D0A7B9_TRYB9  TMHMM2.0        inside       1    88
tr|D0A7B9|D0A7B9_TRYB9  TMHMM2.0        TMhelix     89   111
tr|D0A7B9|D0A7B9_TRYB9  TMHMM2.0        outside    112   384
tr|D0A7B9|D0A7B9_TRYB9  TMHMM2.0        TMhelix    385   407
tr|D0A7B9|D0A7B9_TRYB9  TMHMM2.0        inside     408   427
tr|D0A7B9|D0A7B9_TRYB9  TMHMM2.0        TMhelix    428   450
tr|D0A7B9|D0A7B9_TRYB9  TMHMM2.0        outside    451   464
tr|D0A7B9|D0A7B9_TRYB9  TMHMM2.0        TMhelix    465   487
tr|D0A7B9|D0A7B9_TRYB9  TMHMM2.0        inside     488   491
tr|D0A7B9|D0A7B9_TRYB9  TMHMM2.0        TMhelix    492   510
tr|D0A7B9|D0A7B9_TRYB9  TMHMM2.0        outside    511   519
tr|D0A7B9|D0A7B9_TRYB9  TMHMM2.0        TMhelix    520   542
tr|D0A7B9|D0A7B9_TRYB9  TMHMM2.0        inside     543   562
tr|D0A7B9|D0A7B9_TRYB9  TMHMM2.0        TMhelix    563   585
tr|D0A7B9|D0A7B9_TRYB9  TMHMM2.0        outside    586  1158
tr|D0A7B9|D0A7B9_TRYB9  TMHMM2.0        TMhelix   1159  1181
tr|D0A7B9|D0A7B9_TRYB9  TMHMM2.0        inside    1182  1200
tr|D0A7B9|D0A7B9_TRYB9  TMHMM2.0        TMhelix   1201  1223
tr|D0A7B9|D0A7B9_TRYB9  TMHMM2.0        outside   1224  1237
tr|D0A7B9|D0A7B9_TRYB9  TMHMM2.0        TMhelix   1238  1260
tr|D0A7B9|D0A7B9_TRYB9  TMHMM2.0        inside    1261  1271
tr|D0A7B9|D0A7B9_TRYB9  TMHMM2.0        TMhelix   1272  1294
tr|D0A7B9|D0A7B9_TRYB9  TMHMM2.0        outside   1295  1343
tr|D0A7B9|D0A7B9_TRYB9  TMHMM2.0        TMhelix   1344  1366
tr|D0A7B9|D0A7B9_TRYB9  TMHMM2.0        inside    1367  1737
```

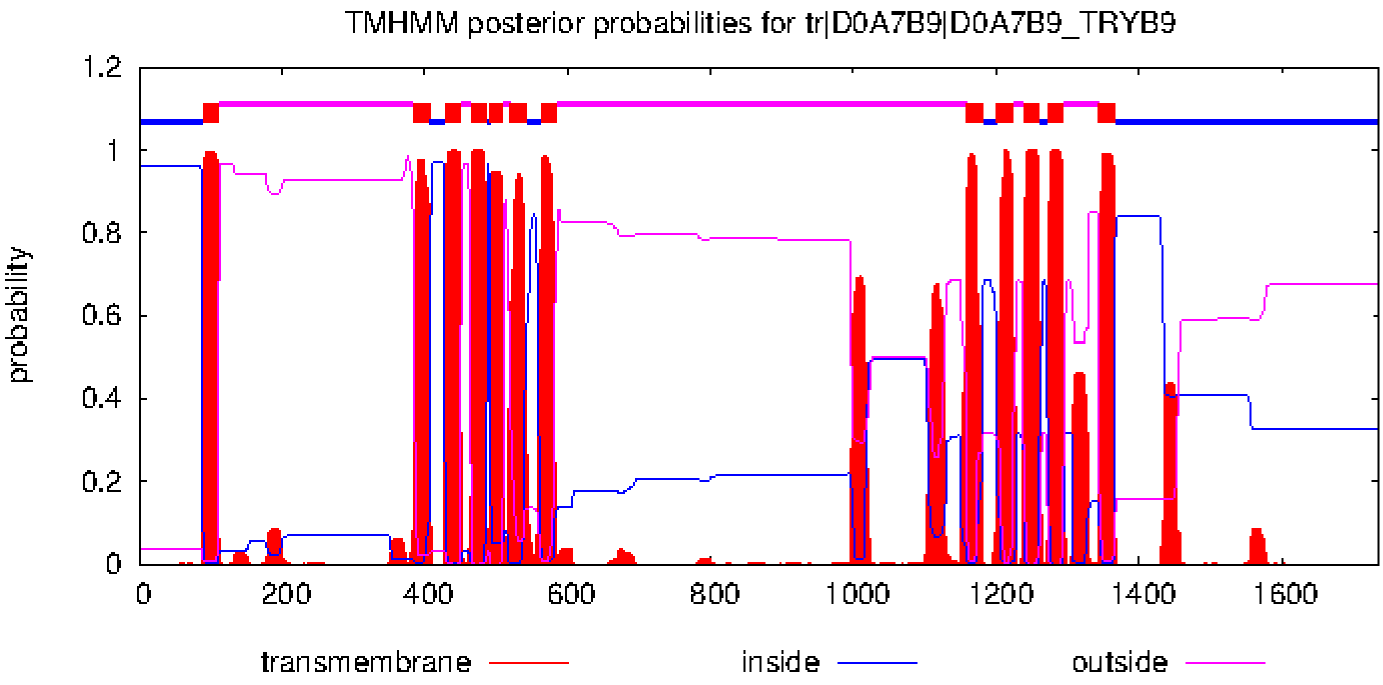

170 # plot in postscript, script for making the plot in gnuplot, data for plot

---

```
# tr|C9ZM88|C9ZM88_TRYB9 Length: 1759
# tr|C9ZM88|C9ZM88_TRYB9 Number of predicted TMHs:  8
# tr|C9ZM88|C9ZM88_TRYB9 Exp number of AAs in TMHs: 213.93401
# tr|C9ZM88|C9ZM88_TRYB9 Exp number, first 60 AAs:  0
# tr|C9ZM88|C9ZM88_TRYB9 Total prob of N-in:        0.25012
tr|C9ZM88|C9ZM88_TRYB9  TMHMM2.0        outside      1   446
tr|C9ZM88|C9ZM88_TRYB9  TMHMM2.0        TMhelix    447   469
tr|C9ZM88|C9ZM88_TRYB9  TMHMM2.0        inside     470   535
tr|C9ZM88|C9ZM88_TRYB9  TMHMM2.0        TMhelix    536   558
tr|C9ZM88|C9ZM88_TRYB9  TMHMM2.0        outside    559   572
tr|C9ZM88|C9ZM88_TRYB9  TMHMM2.0        TMhelix    573   595
tr|C9ZM88|C9ZM88_TRYB9  TMHMM2.0        inside     596  1097
tr|C9ZM88|C9ZM88_TRYB9  TMHMM2.0        TMhelix   1098  1120
tr|C9ZM88|C9ZM88_TRYB9  TMHMM2.0        outside   1121  1134
tr|C9ZM88|C9ZM88_TRYB9  TMHMM2.0        TMhelix   1135  1157
tr|C9ZM88|C9ZM88_TRYB9  TMHMM2.0        inside    1158  1209
tr|C9ZM88|C9ZM88_TRYB9  TMHMM2.0        TMhelix   1210  1229
tr|C9ZM88|C9ZM88_TRYB9  TMHMM2.0        outside   1230  1232
tr|C9ZM88|C9ZM88_TRYB9  TMHMM2.0        TMhelix   1233  1252
tr|C9ZM88|C9ZM88_TRYB9  TMHMM2.0        inside    1253  1324
tr|C9ZM88|C9ZM88_TRYB9  TMHMM2.0        TMhelix   1325  1347
tr|C9ZM88|C9ZM88_TRYB9  TMHMM2.0        outside   1348  1759
```

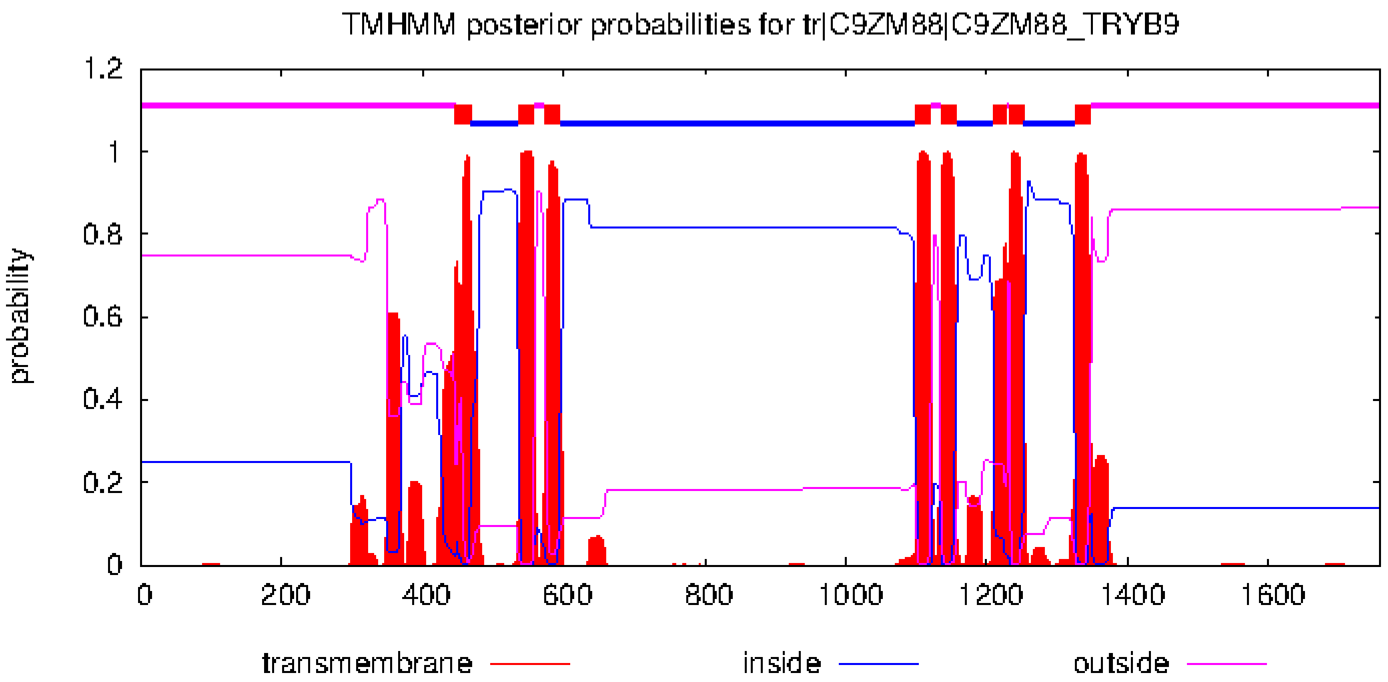

plot in postscript, script for making the plot in gnuplot, data for plot

Predicted extracellular helices from the predicted plasma membrane proteins

>tr|C9ZN44|TITTLTTTRVESTRQLLGAPVVELVPGKGCELVDLSREPAFAILPAPLSDVRWRPI
TTDKEEKTFLPHCASPGAGEKEVCFVLLYFDELYNSSVFSNHTQLVISKHIGTRYALSFDPFD
ANSSSRWCETGKRPQTIAVTMPVYVRLVEERNRNYFDDSYLDPLRRPGRISSISYQCSVKYP
RIVTEDPPGASEIWYKSQVPWRRHHIPLVTDVENTPLRYLEEHN

>tr|C9ZMD3|

VPTTNREQVNKADVTSPFGSCEVISGEDEGLKGKNALPANEGEGEQSSMKCFTSMIPPGG

>tr|D0A7J0|

GASIREGLPDLSQWVTGFWSDVAFSVGGVPLRV

>tr|D0A3G8|

GTQSLPFFWRWLKYVSFVRYGLAGLVVNEFDGLKFVCEPKVPFCFPDGTTYARLQGYFPEE
LPEY

>tr|D0AAR0|

EYLERRKRGMLRTYLHETHGRGGTEAQTVGGGAGFPMRSTPSGVSW

>tr|D0AAR0|

EIPPYVKDHKAEMKVELFTEFCADRENTFNPLATLALMGPYNSIRVLFSRHTTGL

>tr|D0A893|

HVVGEPHLWHNMKLIFMLFLPLCGSLWATVYSDPGYARPTFHSVPSLAADGPSPREVEGGV
QQERESSWEDVNGQSVERRWCSACGIHRPLRAAHCYFCGMCVNEQDHHCGVIGVCVGRRN

>tr|C9ZY92|

YFEPGAAGGGIPDVMAYLNGVHVPKVMTLRTFVVKSI

>tr|C9ZY92|

SGCAPFQDMEDRGDVLVWGTENTTSLFTATCSENNTFSPLATLVLGSERDTIRHLFSRQTIW
QYHPV

>tr|D0A9V0|

KPLMLLVSALKSASNLEIPDNADIFLGRSRIVEVRKIQGHYAKLRRQLAVLLKFIPEAILARVE
SKNEAGGHIRANGSATETPGESSTAFVRIMGASISVTWNAGDQQRDVALNCGIFKEKPNVFN
RRHCTVIAIQVYHPDVWKSSTKYHAALIEVATEHSGCVEALDPDRALISFGAHAALPMHCRR
CCQFAFDLFAKLPLDHRDSVTMLVDTNEFLVGTCGAFSRNARVLFGADHLFQLSYAVRDGP
CRIAATSGGASHMQGFLAFPVDCVLLPCSTLPIVLFELRPGESSSEVSLSISKQFRLGFAAMRQ

GRYGEAMAHYLPMVKMDMQAKRLMRLCRQRCRRNDTVPYVRVLDNHYCVNSSDCKPEE
QDESDIKDRVPETIDSPRRPDGEGSESDSSDQGSESLFKMYAPSSSSSLDGSGCAEGANAADV
VPLFLTDSNRNVWTRSLKKISEGAFSTIFLGVAEDGAQVAIKCIPRLRRDVVAESLEKEMEVA
SKLHHPNIVRYVSCSITPSHLSIIMEYVPGGSLHSVIKNFGCVSPYVARRFTVDILQGLNYLHN
LGIVHCDVKPHNVLLGTDGVCKLSDFGSTISEASVMARTLADGVM
LRGTALYMAPEVAAGGRCTPQSDIFSFGISLLEMLLGRPPWQWSSTAPAGSDATRLRSLFNR
DLLFVQSLALGHLEPVIPASVDHEAAAFVRACCHMNPSMRPSASSLLSYAFLL

>tr|C9ZP82|

MGVPKFASWLRNKYPAIVTKNCPSSVHGLYIDLNGIIHPCCHNDSDTAIALLPEEEKLECICSE
LELLVQTVQPKEVMYIAVDGVAPRAKMNQQRARRYMGRAAPAQTGEVASTYMPNKSGM
AIVEEVVREFTSTEMAQANDDLDEVRQTLLEDPLYSGALYNEGEEEAGDALNDGDGVNSFC
VRSTDDDYESTHENRYVESNQVVDDVFGHRGEVIVDDPTAFEFDSNCISPGTDFMAKVSDA
VLNMLNEKMSGNDPTWTRLCVIFSGSNTPGEGEHKIIDFLRTQSSLAQFGGKANHVIVGLDA
DLIFLALSLHITQVFILRDTTRNPYKRRAVARQQQRERRSRRKRNFIDDTCIISLNPSYIDEEES
DGVIGVPVCESSENSSSDTDNESPIESREAHVTTVPNTGFEYFDISVVGGSLISEVSALCDIKKF
TPATESAFDKKTLVSSAGYYFFGQPNHIEDGTLPSSTECGSPKGRRSKRVSKKAEAERSWKN
FRPCSSPANSKIIDDIIVLSILLGNDFLPHAPSAFSGESALDNLLELYVSEVLPYGFLTKGPHEIN
LPQLQRLFEAYAKIEAVKFRRFLLFKSTNKENSNSGAEFRKGSNNDNVTVADVAVALHSTID
DRWRDTYLKSTGLSNSVQEACEKYVEGVCFVWRYYTTTTTDSDWSWYYPFYHAPPVIDLA
IYLKEANIKRMPLPVVKSTPPDTLVQLLSILPPTSHALLPMVLGEVMVRSPQQAELESTFPLQ
WNVDYHDSNVVHLATVMLPFADMDVLQQVVDAAWPQLTEEERWRTQKLDFHLVITSNKT
SFVPKGDLRKLSDALDTILRRVPSREGVEIRQLYDVPLERYRPRTYSCTVEVPRLSYGADGQ
VIKRQRRHRHSGQLKGNGEGVIEGKKSNSRVTVVNKGPC

>tr|C9ZP82|

DPSSGCGMSRNNIRQTFVDWLCTECLSLNFSSRTRCFVCRAPFDNRRCLAVFSCTQSADQSL
YDPDHSPHIEAVKLQI

>tr|C9ZJU1|

LRERARPAVLLDCERKAYEILEMLTRATIKTDGHDSKQTECKDDASIAIFLNCNGVESIHVRT
LVFNHNIMQLPSVFAVHCGLMKNSHPDHLLVCDIWNCLMALVNCSIRDILLDDGVALCILD
DVKAEEVLALCMLLRGKYSIARYPPAAQADVVVQEFPEELYLGLITAYNRKRSLQSGITSFW
KLMCSSCSIPQLVTLAATVLFSPEVILKCILGMHAFVHWRPIYAAEIGKSLNGDVSPKLSPFRF
IIKQLMGLPATFVSMAVASTFAFESLYCIRGFVIQSILSDIITSIHIDAYLALATADYDPSDDWL
LSRCVSQALWQASGSVMDELQSRVPALLEVMHGCRVVFSFPVVSLVMWLGTQWSNFLYFL
NESLYMESQFTKSQLASVTNIATSTYAGNGEENQVSPTCSFPVKNSLTSSPLTLLRKQKDDGI
KCMLSSTDNKMLFQQTFSHPSRHDKSSVQYGLVLFSMVFVESGRPLGYMWDLLTQHVIWP
LVPSVFSGCVVDALKRKLQAGGHTCAVSVRRLGWIKDVIQSSPRAATASPSTCVWQCRQDY
LQGFAALRLSGWELMAATAAAAFATASERNDRYEQCGRDVLHGLFQMADTLPLLLVSAVA
LRLSPNFFTQSALDQTEIVNQLRSIQHWCPLYHAFLGSFYDECGMSEAQVDEAHLRRHLAFS
GSSLGCYQVLEGLQSVQRKIDRPVRGVAPPSLDGPWSVEFREVSFRYSDRHPYVLRRVSFVV
NKGEFLGIAGYSGSGKTTLLRLLNRTYAPTEGDIFVNGVHISQYPARMLRRRIANVWQEENN
LRFIEDLSIGGNIALGNLWDCSDENICAALSASRALNFVQKRSSGIHAPLNVREFSGGEVERL

CIARAFVKAGSAVGLCIFDEPTSAIDTITEGEIFDTLRLREGNNLLRDSTIIIVAHRLATLKHAD
KIVVLNEGMVAEVGSWETLSRTGPHSCFSRMRQSQQLPHFVKK

>tr|C9ZQ90|

FVKKIAQDNRTVDMYLLAPSSFQHFLIKTWSDALVSLNRTFTPGQLITTGTVPLASDNRSSM
VRHFQRDMDNYLDTNSDWKGFAKPEHYLKDDKLGEMMVFGWLAGEVLFEALNNAPQLT
NRTSFMESLYKQRRYVIDDFVVGDFGGECDEGAALQGAMCNCNQGGSMAHMRVVDDSLS
LKPMKKGSVTWSVSECSSANVQVSAPLIGLYVVLTDDKVAQRASMRWSLGARSIEEADDV
DKRIFFHSLKVNLKNLTQSLEQVRDTKAVAAVLGVVTADILSVPNMTFIGTLPLSPRLNKFW
RNVIHLQPLLAQQLYVLAVYLSNTSSTGVKALVRGGEASEVVDTLDKSLVTFGVSLDSSKTL
GDDDPISSYLSGNGDVFCIGLTPPDVAAVARHLQTHRRARVFVPFNDILLFYQEFVAAFNAS
KESIASSERLLFATSFPHWAEKNTKSDMVARFHRHVNESHWDPLTFLGFATTRLLQVVISNM
RKVNAELLADRIYTESNIRVDDVRFGPFSDAECVSGTSVSANECASNFGATNISVWSMARVL
NSSLPRTQVGMTPSMDYVIPQEGQLTQSQI

>tr|D0A254|

MLSPIVDMKSVGTDDGFDIYLPGSDAFYFKRQLHYQPYTPCAFPVEWLGHQGKMHKTSVH
MRISPSNLEEACASGSLGYVIMFWAQGQNIKGHESVVGGGDTLLMTAAYKGHIHVVRFLVD
AGADINATNSAGFSALHMAASAINYHIVRFLLDHGADPTKKNHQGYSAYCVAVQNASLPLL
LLLAEHRPIDIHERDEMGHALLHWAAYNNSVAICQYLVEVCKMDINVVGKDGRTALVWAA
REGYADVVEYLLCAGADVDVVDTDGYTALNYAKHRNHPEVQYVLENYPLAQRGATGMA
AGDGLLGNQ

>tr|D0A254|

MGFEPFLPFLSTRNSVVKLNTSLNTTDKDLHDGGSR

>tr|D0A254|

RYDGYSAVMDCPVAASNHAWFFIAVFCFMVLQWLIFISGWRQARVMLKCPRSDSLGEYISS
SAWNLLVHALPCRDPMTIDVTGKSYFWVFMFRYIIPTTTNRLG

>tr|C9ZXW1|

MPDSPETGRAPINLEGELCMERVVNYTWENLAFQVPAKDSDGRKIKKTLIHKMSGTALGGR
VLAIMGPSGAGKTTLMSAITGKLHGTEKNLEGCCFLNNAIFTDRYRAVVSFVAQDDIVMAM
DTPYEATYFACRVRLGLGPRESEVLVNEVINRLHLVECQDTVLGIPGLVKGVSGGERKRAN
VATALVANPCVIVLDEPTTGLDSVNALRVGQMLQDLAKKEKRTVIATVHSPSSELVETFDDL
LLLSEGHVVYHGPREEATAYFASIGHQVPPRTNPCEYFMELLQLPKEELEVLWRAWELYLTT
PAASTNPCLLKVSGPITERDPYLEDHLKKKGSSWIVQLVELTKRSYRMYPRHPSA

>tr|C9ZXW1|

NEFSRLEKICDPPDLHCRYPDGQSVIEFFNFDSWSWWKS

>tr|D0A423|

MSFAPTRSLPNEVNEPLPTLQSEESGEATYVHPDARALFNKVPCLRRLPMFGTAAEGYGPKA
VMSISLSYFLCKGLSDYLVRDSLLAMFTQRFGVDVSVYQRLSNVANMGWSVKPLTAVVSDI
FPLFGYSKR

>tr|D0A423|

ELPNREERYGDALLLYKKKREEREKKCDPFSDSLTQPDRAINSESNPLSTEGPDAAGLVPYE
GGAPTGDGDLSSFHFQEPGSCCCGVFEYNKEVVERNG

>tr|D0A423|

KANLFGYLQRATSVSFPGALSNFFIAEADCLADGPHFSFAFYQ

>tr|C9ZVF1|

GFCELFGKGIADNIIRSSLFPMFTYTFGADAKLYQRMSS

>tr|C9ZRE0|

MVSWSRPEFAEAFFGKLVRRRCIYATKAALDSSPFVR

>tr|C9ZRE0|

AMLPVAGSAYTHTSVALGEFASWTVAVCMTLECLVSGCAVSVSWSAYVQAFLKRFSFVLP
QPLRKSPIDVVGGR

>tr|C9ZVS2|

MPLQITWCFFRYASSYAREFSGFLSGHARTL

>tr|D0A3U1|

ATECKFTTEGACGGAKIYGCKWSGTTCKFENPKCSEGSDPSDSCKNEVA

>tr|D0A0N1|

EKSVGGFADVSLMIFLPSLTFVSITKFENAER

>tr|D0A0N1|

PLHFLLLGYEAGRTWKVHATTASERADAAQGDSRDSHGEVSGWETSAGRRDSDDTQIKTF
VL

>tr|C9ZRP4|

MKNKSSKQVLLPLQGNISPSAESPRPSSLMTTEGRTRNYKDLINRTVFSLIM

>tr|C9ZRP4|

KRAFKMKDFGDLIPGHGGITDRMDCQGIMGFFTWVYLQSYVYRDENCPSWHTISSCALQLP
EEQRRSLLSTLNRSLTE

>tr|D0A888|

HHHTHDMTRPPSHHHHHDDHTHGVVDDGIDGDTHGGCESGHGTYS

>tr|D0A0U2|

PARLIGGASVAFDSNTPQLIRKGGAVTTIREDGEGVGKYLPISGTVFVIGLSVPDVKEIARKLE
ERNDLRVIVLFAEFSFLYDLFATALNNTAGAARLVFATSLPHWGDTETSSKTAQLFHDVEKD
SRLWTPLSLLAFATGRLMRVILLHVEEMSPDTLVNFFYADSSIISDDMRYGVFDDTKCDTAE
KLPKDDCASNYGATQVS

>tr|C9ZWK4|

MNGIVPVCSGMSEKINKKRMNATDVHSHSLFKGAHSQCCGFVCALPVPRNSPLFQHLSFSST

>tr|D0A5M0|

SCVVSTGWGPYAPVLVELSDIRYLVWKGEGNTCPAVPAF

>tr|C9ZJ95|

SGIRYPEGSGKVEKHCLNPVDAILGAHEGVFSYSNCGATENTTTYNNVTVAGTSYQSGLKW
QCVEYARRYWMLRGTPQPATFGSVDGAADIWDLKDIQLLNGQKRKPLLKYHNGNATSANS
KPRVGDLLIYPRQPNGFPCGHVAVVVGVTGDRMFVAEQNWENAAWPGPYHNYSRVLNLS
CNPNGTACTVREKDNVTVQGWVRYE

>tr|C9ZZZ3|

DLLKAHGGGELTAFGEKGTAWVFAMYPRDSNG

>tr|D0A148|

YTHSVWHGSTVAARDVKRSTSHQFCKSGVYSS

>tr|D0A6D7|

TIAMELLQVAVPYVLVTLGLQRRPPKGGVDKAMAEAESVEAEVMKKPSKQQRSFRALFIGI
DYKGTPAELRGCQADAVMMAGTMEKIGIPITERCVLMDTDDPRFNAIKPTRANILQHMAWL
VKDAKPGDALFLHYSGYGAQVRAEEDKEEEFDQCIVPCDYEENGCILDNELHEIISTLPRGV
RLTAVFDCSHAGTLLDLPFSLICSSNDCSAVGEMKRIRTGGDVNAHVLMFSACGDDEAAAD
LPNAGDFVEGASGSGGAATQCFISMLLNKTPGTIYSLLSTTRDKLREKGFKQSPQMSASRCLS
LTEKFTLTELFSVAEPCPNILTGEPRWKTTPLGGK

>tr|D0A886|

HHHTHDHGHHHHHHHHDDHTHGADDHEGHGHSHGGCESGHGTYS

>tr|D0A889|

HHHHHDDHTHGVVDDGIDGDTHGGCESGHGTYS

>tr|D0AAF8|

MSPRATEPIINEDEHPTSIPSLSQGNVLELPPDSDKSTAAADGEHRGCLNTVFDPIKGVVPSGG
MASNVFNLESATLGAGIVMLPSGFLNSGI

>tr|C9ZL05|

EARANDHPPGESFQHAGSMGEVSVGVPRRSTRNSRMDTDRSVRNWGAGHG

>tr|C9ZL05|

TENVRDLLYVRPRMLRLGDSYLTEKPPRVGRDVALSYDTPWCLYEVMKTTFFAVTG

>tr|D0A070|

REASAPHSNADSEGSETGGNKKTEEAEKANS

>tr|D0A3E0|

MSVFYPPLIPSEHYFQETIKDKTGAEAMPYACLHGGSLCLLISLLSCLGAQAVQTLHLVQLV
HRHGARSPKVKHNQSQICGEVPCGYLNAAGKMMLINAGEFLRNHYNSNASEPFFPEESYNS
CVTYSRSTDVPRTLQSAGCLLRGMFPNASEFFPAIHTADVSTDWLLRYDVIPQAYAFSHLDE
HWWRNVCNPKLDTLIDTNTLLSVSREVFSEGFCADPQNRCHCAATLFDIGVAMQSDGRIDK
HPLLRENLGRLRDIKFFEDSHRFVYNASDRTHAKMGSLGQHLAQEILKNAENHMNGLTSYK
LYHYSAHDTTIAPLAATLGDSTTTGITPPYGQLYAFELLYDHEVKGYIIRIRRGAPGQKPEEG
YAFSWGEFQMKCMNENHTVYTVEDNKCPYHDFRRFVKSTKPHDPAGLCYLNRRYRELFHC
PGNVGKPPNKQCKVFRRVCPAWSCEEGYTLNSVTLECVCSSRKCMESRDLSISTDTLGRN

>tr|D0A3U3|

EVATNCSLFKTTEACKAVGSYGCEWKDTEVCSWKKECDSDSDGVNPCESLIG

>tr|C9ZPP9|

ARPYLQMHPTRSTLKNIISLCHHNEHPNLKGIGDTSQQRGGTVRRIEQFLKDTFSLNLEGSRG
KTQAVELREQDYEAVASVLHGAGLHN

>tr|C9ZVS3|

MQVAMPVSLVDSCLGMQELLVLVYSCVWQSLLPLYELARFFSFPFQSSSYQAAHTHTHTHT
HVHTQQTHLLNMLSPTEPLGSGKAHTEVVTDEGEGYGAMSAAEEKSHHKNGDTPTTDSKF
MQCINAIIPHGGALSTTFNLGSATLGAGVISLAIAFQM

>tr|D0A1S7|

PLWEVLIDVESELHYSILGIPQGSDARAIKGAYREAVRRWHPDRNPNCDSCRVHMMKIQHA
HDVLLAKGSERYELVDRYGEELAQLRSLVFFRLYNIAFYAAQDIYYLIQAFNGDAHINGSKE

FSWFLQVMCRTLTMGVFTVYDTLFISGFRVIVLLQVLFYCVSCAKSSAEEWEIIGMVKRSYV
DLYREAMFFAGGPLILHCLQMYQSGRILCWADAFEFFLQLAFGVIYVLSHLYHMTPNLLDNI
FMKKCSIPLAYIKLPTRRLSYLNFICTEFGLLLDDLFAFSCRVPSVYRLTVIVVHTVFLCELLW
FPWEPPILSVPDRKDKGKNVHREEEGKHTDMAPHREVTGEPPRAISSEEMTLLKGVDNEAV
NWFDVVSTRFAKQMRSAIHRCAQQRRAYINFDLVPTVNSQEVAFVAVTREHPRAPSKVDVL
FRVRDEFANRMLTTLRPPLEHILGVRLTDATHSEIVSRHARLWEENKRKNPSDTWRRRCTDS
EVKVSFDLPMLSATVISTIVLLYHFL

>tr|C9ZKG2|

MHLCGDEREDDCSAYTGNGPVSGYCFDKGLCDEHMPSQEDEELSEIGAPSSGVNPAATSLP
QRKKNLV

>tr|C9ZKG2|

AYRLLQLLLRGSKFGWTKFWLVTHRACIYLRLPRWVRRSPTRSDDNCDEPWNR

>tr|C9ZY45|

MWVFRDRMMERGGQSFPLAHYLQHEEQNDVPLPEPHMLLLCGRTFHESLGGDWLFPSVVT
FLLGGADVNARDKDGATALHVAVTQGNDIATVCTLGREPFLGECSNEMIIRFLIDNGADINA
RNASGETPLMVAAAKGNITAMRLLLERGAVITQRDDAGYTVLHHASRSPFSLQLLQCFVDS
LLHQVVSEDLLHFVCQKGGKGANFVILFLVEQLGMDVNAREGECVKASTGEELKPEETSVM
SAVAVRSSYTPLHRAVLGGDVALVCALLSCGADVNQTDVIGLTALQLAANNAGVSAVGGS
WLQRFVNIRSLWCPTAAERRVRSKEVYNLLKAYCKETSLSGREALLQRFACGNSPSQNLLSF
EGALVFADGLIVVHALMVLCATLTNE

>tr|D0A564|

MGDTGPKGVPGTNDAGEVHKPQKPQRRQSVLSKAISEHREGDDGSVPLLPPSKGLTSAEAE
ELLLKYGRNELPEKKTPSWLIFLRNLWGPMPIVLWIVIIIQFALQHFADGAVLLGIQLANALIG
WYETIKAGDAVAALKNSLKPIATAYRDGTWQQIDAALLVPGDLVKLGSGSAVPADCTINEG
VIDVDEAALTGESLPVTMGTEHMPKMGSNVVRGEVDATVQYTGQSTFFGKTATLLQSVEA
DIGSIRIILMR

>tr|D0A564|

SKKLSKHKIIVTRLTAIETMSGVNMLCSDKTGTLTLNKMEIQEQCFTFEKGHDLRSLLVLSAL
AAKWREPPRDALDTMVLGAADLDECDNYEQLEFVPFDPTTKRTAATLVDKRSGEKFSVTK
GAPHVIIEMVHNQDEINDSVVDIIDKLASRGIRCLSVAKTDSAGRWHLCGILTFLDPPRPDTK
ETIRRSRQYGVDVKMITGDHVLIAKEMCRMLDLDPNILTAEKLPKVDVNDMPSDLGEKYGD
MMLSVGGFAQVFPEHKFLIVEALRQRGYTCAMTGDGVNDAPALKR

>tr|D0A4W6|

MKGQSSQCRVPLLGAGSSDDCDGDNNANEPVAAVRRCTNRFSTNMPLLEERHDLSLRQKL
QATIPTRHPRFYSSEERRKMERYESVDTFEPATTVYKDHL AGRSQEPR

>tr|D0A4W6|

AFGSAGWSPPSSALNPTDNTTAGGLTTLRWGNSSAVSPHFVDAQSGPCVAVPQTLSDSRDV
SVISFYGANGFFCAAPNNTVIPVTATVHEQQVWIVLHSYASLAFANADSALQTLLSLRTETM

>tr|C9ZPL1|

WFLRHGFSWHDLTTYTACSDMTNGTCLLLANPQ

>tr|C9ZPL1|

FAKLFNIVPLGVDPHVVQQAQPWSILTPTNFDDWK

>tr|C9ZXD7|

MGPTCDRIAKMLPHPILASDDDRQYVQESILSLPDMVENFFTSLERVYTDGKCEEAKQAFAT
YHSIQTIVSVLCRVPASAYRTNDEKRSIDWEIYERIKRMGHLFLGDIVVVGPQSLNWRVPVM
RLAEEVAQRMRCAYVEFPFMSVLLREAVSYS

>tr|C9ZXD7|

TSAKALKVLHTWILEERMYTVLLSSASSEGDLSTTGPSAGAAVPQVTRGHNTKNGYYTIPYG
NALGYIEGKHVDAQVVMIGFDDKYRITRWNMAAEVVTGFLESGCVGKPLSDLVITPTGDIE
RDLAPLQVFSGEVLKLKLRAFATVPVTLFTVAVPILNQEGSLIGRILICANAKDNLGEYRTYIR
DYVVSEVNLSLSEILEGKAVTPRGLSVIGPLQSFLEYGYGKQVEELARGMLAEWEWTSSEQL
LGQMLRLSPLDHKTSVDSLFPGTLCLHPCIPEAVATLLNSLRVPCHLRLQILNSSRNTFALQIV
ATPVAQAAPSTAQISELREKLLPHLRSTCGSIREDDGTVVFRFPCQITAALEDIDDATFRPADA
LNERYVIDQTRAIVNCTVNVLTLITNLVDQHNISMILLRTMFVSLTSVRERCELEKRLQSSPSD
IDVIVCDRGWLNSVQDLVHTLSYDIIVVPISEPGVRLALEGFQYVINTPISSTEVRKVLMAIGT
AVSLRKNAATAQEERERILTLRQDSPWTKGVLLGRGSFGAVYEATSDLTGGKMAVKMFYF
TEDLEESINALLNEIKIMCSLNHPNIVHYFHCERKENSVNLFMELCCCSLGDIIYGRSQKPPDL
TVIKVLRQLLTALTYLHARGVAHRDVKPQNILIKGDVVKITDFGTARQGVGSEDVQGTLRY
MAPEVYRGEDHCSPCDIWSVGCVAAELFDCPPLFMETPHMLADMTDVDYYVDGLTSNPVL
CDFLRMCFCLQPEGRATAADLLLHRLFSSSCSAEVESLPDIFSREKQYVSSLTITTQPHS

>tr|C9ZR23|

STLLPLALTFSLTAIKAARDDIKRHKQDAIYNKKERKVLNREAMTWETRTNHSIRVGDVILL
REGEDIPCDVVVLAATNPIVYIRTDNLDGELDLKPRDVVAPQLSSDHTGGDDVPNAIAHQLL
SVDDSCASIVGKLGQMRVTCSDPSPMINCFDGVAEFFFSRSPAAETVAANNSAPMRVSLSEN
NILPQSCVLKNTKTAICLAVYTGEDTKCCLNKRNPKVKWAQIDRDISK

>tr|C9ZR23|

KFVTDMSKYYFALVIENDVAMHHDGEWCNVRNSSIVEDLGQVDYVLSDKTGTLTQNVMEF
LFATINGERRCLAPVEAEEVQGSCGEHVLHFGRVLSLCNTVEVVYDDVSQEMTQSGLSTGC
GAVGTMRYQAASPDEVALCNGCEKLNVRLVARDATTAAVEVNGIKEEWFVHYVFAFASEF
KTMGVIVEEKSTNAIYYFVKGADDRILEMALDENSSTGGPQWGKGERMSSKAAILAEVEHY
AVFGLRTLLVAEKRLTRNELDEFLEKVREAELSMNNRKEEIYKLRLEMENSVTILGVTAIED
KLQDHVPETIRSFLQAGIKVWMLTGDKVQTAEQIALTCSLCSPGDCVLRVLADKLDAFESW
EGYMESLLQFSKGVMADVQYGDAAFPTGSSSDPAVGEKACAMGVMQKRNGSVTTETNES

ADVNPLSTGSSYVLVIEGGQVLERILTTPSLLKLLTELSENCVSVICARTTPKQKAAVTRLVRS
RGFITLAVGDGGNDVAMIQEAQVGVGITGREGKQAARAADFSISRFSDLRSLVFVHGQLAY
NRTAFVIKYSFYKSVLIGIIQLVHNIFHTHYSGGSFWDGFGLTLWNGLYSLPQTMLYCLDRK
VPRRVLEQTPALYKVTRSGVDLGVCQFFGSF

>tr|C9ZR23|

LIKIWRPDPRDVMRSAELRRQANDPLAVEKRASVSRLSRCLWCVPEEPSTYVTVVLADDELT
IRNANSV

>tr|C9ZN43|

MYDVAYYDPAYVPPALSEDVMTVRVYSLLYNPLIPDNLCDAINAGLNASLASRKWTVAPD
VKVELIPPTSYKIKPVEVLQVALERHKGEFFVVLGPVGDGQALASLPLLERENLVGFAPSTGS
NAMRGWNSHVYFLTASPNAELLTLVRYAVSQLRLLRIGFMYLRGISFGDTEYKMTTSLMSS
VGRELCGVFTVDGLMRGRVNDDDFNTAWEQFAETRPQGVIAFAPPAKDLIKFVTRLLSDSR
THDAYLLSSSSLEFALDTWREALEADGVEFFLGQLMLTRTGPLARDTNYRAIRRFQDHMRS
YLSANPGVTVFNGTDNFDHDDVDGELMVYGWIAGEVLSQALSSREWLTSRKAFMESLYNQ
RRYVIDDLVIGDFGGDCKGGAGERGAACNCNQGGNVLYINVVGNDERFHTVQGGTTVFEP
SRCLAESVRLYSPLNTLMFLILDRELAQVSSEALYYGANVLTGNGRFGQSDRLFISMIPSPSN
ATYLALQSELDTRSVTAVFGVVDDAMLSIAEVAFVDPVMLTPRLHHRGRNVIQLSPTLEQQL
FVVVGYVTNTSASAPMSAIVRGADATIIEVALRKIVWMHGGTLQTVAVLDDNATLVGRLPN
RGNAFVIGLAPGDPSLLAAHLDRNPDVRVLIPFFDVALMYDELVSAFNGNPNAERVQFATSL
PHWADANTSSEIVREFHTALPDSSAWKPLPLLGYAAARFAQAVLPRMEYVTPKTLLDTIYM
QSIITADEMRYGPFEEEEEKECFTANDPVPEQGEVCVVNYGATRISMWSLARALNASVPPLT
SPVTPLIRYADPNAIKLSSAQLAG

>tr|C9ZL71|

PSITWRVLDICGRDAPDMWKYGTWNSNCATIELEDRHTDTYRVQWNGRPYSVDFGDPLLR
FRSLVINLASPDDWEVFHPDERRFTLRVAIPFMEENSNWSTAPLPIVCRRGDDRCVYINLPPG
AVLNDTGGGRVSLSLSDVPGSIARNINNSAVGILFQGRAYALTV

>tr|C9ZL71|

YVLDFRIPGMLAWQPLIQLPALLVWPVLVNVMTLIYTTRHRPETVPIHPRDTRWKESVWPD
DWYRWLARHGGSMYIFHTEKEEASFNWKQLEYRVRRYLVRLKRRGGVHNLSLLLQSIPND
SSSPPVEGPSDPSINYTIMCSVHSRPQDIEFWKGLRESICNRGAAEIVNMDMTSTCFLPYVEAG
RENDLGPERPSGASQVMRQNVGREGGGSRAPLLENDGGALNGSVALRLSSTTGDAPPGDGE
RRLGSVDDHSGGNGDDGGSTNNPKSVWQALRGVISASVRGAGRNLFERPAHAFERAETYLL
DAVQQRMHEPLYLPFFNLETAIDCFNIAFESYNWKGQSHARAQERKVGTSGCCRGSSNYRS
NARESAAYPRETNPDACTTSQGDVEMRKFCPSTEQQSVAATPDHNGTPTIDVKQYGYKPIA
VFEALDVAAVCAVMDTEFLHHRGKAPRIVIAFRGTANMSNVREDIKMRRRAWDEMKNDR
DNASLNSSCCWEPTVHSGFLEIWEAHQTSIEEKLGGFLKENSSTVYRVFCTGHSMGGAVACL
CAYSVRRMLREIEYPLDEVTVYTFGQPPMGNAAFQTAYDKAIPRTFRVVNESDEIATFRLYG
TQVGTEVDINRHGNYICKPTYMEQRCHPMKNKAFGIEGHQVKSYARSLNALALDTSCKIRA
SGDPEASYVAEPGREPDAM

>tr|D0A0U3|

KADIEIEVFSLLHHQKIEKRFVEAVNAGFNASMTSRRWKTAPSVHVKVMHPFTPSASPISGFQ
QAVERNRGKLFVVVGPLGDFGTVSSLITLLAEQDVVAFAPLTGSSGGRGWNPNLYFTRASPS
AELLALIRYALGRLRTIRLGFMYLHNVFFGVEEYDVAQRILRRMGYGFCGVFTLSSSLTGVA
STRHFDSAWNTFVESRPQAVIVFAPPVGDTARFIRNLVADSRTRSAYVLVPSMLQFAIENMW
REALAFAASPFVDGQVVVTGTNPLARDTQYHAIRRFQRDVRSYLKSNPGVTVFNASSNFDH
DDIDGQLLVYGWITGEVLAQALSVPERLTDRKTFMQSLYDQRRYVIDDLVIGDYGGECAGW
AAGQGAMCWCNQGGNTVHVRVIGRGYRLLDAPDGIMMFDSSHCYPSEVEMQAPFNGVSV
LIPDRPTALHAAVEMEEGASLLEVREHNQDSRLFFDTIVSPFSGAAEELQREWSTKITTAVLG
VVDEAMLKTPGVAFIDPVPLAPRLNKMERNVIHLSPTLEQQFYVCTSYLSRNDKKRLHIVIRS
TDAAAIEDVLKKTLATFSVEPQSVVVLDGNATVEGHLPDSGDVYVIGLTAADPVVIAAHLNS
HDSARVFVPFFDVVLLHEEFFNAFEGVSSADRVLFATNLPHWADEITASEVVQKFYTAERNA
SRRTPLSLLGFATAYFMRVIISPMKAMNATALVDSIFAQSVVNVGEMRYGPFADDGCFLKG
VPRLTGCAVNYGATQVSVWSMARALNASIPPLTNPMTPSMRYLDPDAGKLSRGQLA

>tr|C9ZWY7|

MASVGQSAGFGGFAGVITGRHITTYSTGGTMRRRPPAITFFLIPLLLVLPHIVRPLAAKKSIKV
KVYNLLFTNRAPRRSIETMGVGLNASFAARHWATAENVTVEIIPPPPRSVSTLKLLETAADQ
NKGEFFVVVGPMGDTRTLAVLPLLRQEDLVAFAPLTGSSAVRGWDPHFYFLTPAPNAELIAL
LRYAINRLRLLRVGFMYLQGVHFGDREYQETLKLMTRMGRRLCGVFTKMHAATVMRADE
EFDSLWEEFVMTHPQGVIVFTPPIREALKFVRRVAMDARTRGVYVLAPSAMQFVIGATWRT
AVEEAAVPYIPDRVIVAAPNPLASETKYHAVRRFQEDARSYLRYHPGVTAFNASDDFDHDD
ADGALMVSGWVIGEVLSQALSSRTWLESREAFIKSLYNQRRYVVDDLVFGNFGGECHGMA
GERGASCLCNQGGNVVYMNSLGDDHRMIPLRDGITVFNIDRCSTIGVKVPTPLIVVVVLVVD
DSAAVSAFFSLVVGITNADAIRSITNPDRVAFHSVVATSRNTYGRMQGELDTRSVTAVFGVV
DLAMLSVGRVAFIEPITLSPKLSHPGRNVIHFSPTIEQQLFLLVGYIARDKKTTSLHMVTRGGD
VPGIGSVFQKTIESLGGSMNGIVVPDNDTTLNEDLSPHGDTLVFGLTEADITTVAEHLDNHRG
VRVFVSFFDVALLYSEFVKVFKKHPQAAERLLFATSLPHWADNNTTSETVKRFHDEMKDDE
SKWTPLALLGYATARAMESVVSRMGRVNSEELINGIFSQSVIVADDMWYGPFEDSCVPTTG
FAAEGCAVNYGATHISVWSMARVLNPSVPPVTKAATPSMRYANRNRGSLSGKQLAG

>tr|C9ZZQ4|

MSCLRSISLSVPPHALSTFSRTGMLYVMYVLLLLMPYPLQVHGAPANINVKVLLCTWNTRV
PKIFTTAVNAGFNASMESRNWTIADRVKVQVVQSSKSHKTPEEFIKDELSKETDKSGITIVFG
PVGDDTTLDSISELQKHEVVAFGPMTGSGEVRRWVRELYFLRPSPTIETMVLIRHALGHLGV
LRLGFMYLQGHHYGEKEYEVALRVMEEMGYKLCGVFVVLNVNDKPAPDKEFNAVFERFA
ATKPQAVLLFGAPKSDTGRFLRKLVADRRTSGAYVLAPSGAQVFLELMWKRVLLDSQVSPF
PGKLLIAGTNPLAKNEQYVAIKRFQEVMREYLKTHTSETGITDPNYFLKHDTDGEMMVYGW
AAGEVLSQALSVPEWLKDRTTFMDSLYNQRRYVIADFVIGDFGGDCEGEAAKQGAVCYCN
QGGNVVYVREVEDDFSMQTPKDGTVGLAALRCNADSVTLHSPLNGLVIFFGDNTIAVKATA
SWLVGALRHDTGSLEYSGQLFLHAFGTTTSDSAKALKEEKKKRTVTAVFGIVTKALMKMT
NTLFIDPITIEPQLNKFRRHVIHLSPTVEQQIYVLTRYLSNNSGKEANAIVCAAEAHGIMKVIR
KSLEKWGGSLNISLVRRRGAALTGHLPTSGAVFVFGISASDVDVIERHLAAHSELRVLVLFSE
VALLYEKFVTAFNGSAAAPRLVFATNLPHWYDNETSSPTIRKFHATVESREK

>tr|C9ZZQ4|

ITLRDARDNVSAPMDPTDPVTLIFTDIENSTSQWASHPNVMADAVAAHHSLIRTLIGNYDCY
EVKTIGDSFMIASKSATAAVRLARDLQRCFLNYRWGTDSIDNFYRAEEKQVAELNSKAEPPS
AQLDPEVYRKLWNGLRIRAGIHTGLCDIRYDEVTKGYDYYGQTANMAARTESIANGGQVL
LTRATYFSLSTAEREQLDVTALGSVPLRGVPEPVEIYQLDAVPGRTFAPLRLDHEAYVADESS
DVSHTTFNDCMSISSQLGTGGESVSNVLHALLGTFTIVQRQKELTAICERWRVPLPPWKEAV
WNDEYYQEAIHRLAVKVGHIVDVAAVERVGRPTESSDSSSVILISNPAVDQSTEDLEGDAFE
CGWEEKGKQ

>tr|D0A0W7|

MSMLHLSDRNASLAPSGGEHSLPTGGAVCRVAMDTLPVILRAPVALLLLLIVLPQLSVGAEA
NATVKVLSATWNSYMPQEYVNAINAGFNASLESRQWTVAGSVKVEVVYPRNLDLMPQDFI
KEQLELETDQNKIVIVYGPLGDIVTYLALPILMKHNVVAFSPMTGSTFIRQWNPYLYFLRADP
AAETLALIRYALCQLRVLRLGFMYLQGVHYGDEEYALTVNVMSQMGYELHGVFTVMSSD
GEPAPDDEFREVFERFAAALPQAIIVFGAPEKDTAKFLMMMVAEERIARSYILGPSSVQASLA
QMWLHALATAGTSFAPGQLLFTGTNPLAKDSQYIAIKRFQGVMSEYLKAHVSETNITEADY
FLTHGTEGELMVYGWICGEVLSQALSSLEWLKDRTTFVRSLYSQRRYVINDIVIGDYGGTCE
GEAAKHGATCECNQGSKAVYVKEMLENGQTTTMESGFTVVKTSQCYTESSELQGPLNGLA
VFMEDDDTASKAAALWQKGASHLVGKGDLGHSDRFFLHAFNTTIAEAANDLRYEQGERIV
TAVFGPVTEAMLDTPNITFIDPLELKPRLNKFRRHVIHLSPTLEQQLYVLSSYLAGDGVGTVD
AVICSNEADGIADFLRRSLTEFGVSLRSAVIREDGEGVGKYLPISGTVFVIGLSVPNVREIARK
LEERNDLRVIVLFGEFSLLYDLFTTALNNTAGAARLVFATSLPHWGDTETSSKTAQLFHDVE
KDSRLWTPLSLLAFATGRLMRVILLHVEEMSPETLVNFFYADSSIISDDMRYGVFDDTKCDT
AEKLSKDDCASNYGATQVSVWSMARALNASIPPLANPMTPSMSFRDPSEGKLSGASLVG

>tr|C9ZUI4|

KRGLEDPNRDRWKLFLACIQIITAVVPPELPMELTLAVNTALLGLVKQNVFCTEPFRIPYAGK
VDTCCFDKTGTLTTDEMLFSGVDMADGKGLLNTLKTVPPKAELVLVTCHSLLQLEGTDTVA
GDAMEKASLGALGYRVNIDDTVVYDPPAQKEDIKGKSTTGSSKNETSGSSKSQNRKNNSGF
EKQYKILVRFPFLANLRRMPCIVSAPDGKYVVAKGSPEAIAQLCESIPPDFHSVANAHAIKGY
RVIALAYRPLKEEERSKEAIHNMDREDCEKNLIFAGLAVFQCPLKKDAKDTIEMLQSGSHRC
VIITGDSVQTAISVGRDVTILKCRQQLVASSMKKKGNGEDEVDDCIVWTDAATGKEVNLDR
RSILAKTFVQTRRHKVPSDDEWDLCVNAESIPTTTLATLIAQYSEHIAVWARCAPTHKEDIVT
DLKQREHMVLMAGDGTNDVGALKQAHAGIAVLNATSMDASQNVGGSGEHNSNEPHNEPD
VPKDHKIPPGFKLTVVPPAPSADAPFMEQVRHKMAQARRKAEIVQIARWNKQLEESKKSRE
TAEAGKVVSQPEMNAPASDFLMESIFNADDADMGGAPQVKLGDASIAAPFTCRSRALTSVC
DIVRLGRSTLVTTLQMYKILALNCLTSAYSMSVLQMDGVKHGESQMILSGIILTVCFLCMSK
SQPMPTLCPQRPITKVFHPYMMCTIFMQFGLHLYSMVETVRLVEEADAEGVATMRQAGAE
GEFKP

>tr|C9ZNH3|

KVYSLLYDPFLPDAYNNGVNAGLHASFAVRQWSTASNVNVEVIHPLSYEVPPPELLRSIIEEN
KNEFFVVVGPLSDSDTLSVLPSLEEEDLVAFAPFTGSDAVRGWSPNAYFLHVSPAAELLALL
RYAVSQLHLLRIGFMYLQNVHFGDSEYELAVELMSQMGRSLCGVFTLESSFDGEADDAEFT
ATWELFAGTHPQGVMIFAPQVSDAMRFLMKLVTDNRTSSAYILSPTPHVTNIEIAWTLAGTT
SGVKMFPGQVVLSGVLPLVSDNGFRATRRLRADIKAYALSGTGAVEFDPLAFDGDAAYGG
QVMFGWIVGEVLARALECSEFLKSRMTFMDSLYNQRRYVIDDIVIGDFGGECEPLAAAYGA

TCYCNEGGRMIYMMILGDDYLPRPAADGLITFDACDESGVQMLAPLYTLLFSSVNDPFARS
VNGAIHRGALFVSGNGHLGKSERLFIHSVPSTSGNVMSDLRKMLDTRAVTSVLGVVDDATL
STPDVVFIDPITLNPRLRHPGRNVIYLSPTLEQQLFVIAGYLASENASTLHAVMRDDATRLIET
VVNRTLLSFNRSLNSVVSLDRDAPLNAALPTDGSLLLIGLTASDVGGIAAHLSANRNVRVFIP
FFDVALFYDDFIRAFRGLKSAERLIFATNLPHWADHRPSSETIRRFHANRPNASDRTPLALLG
FTSASFLDAVAQHIVGVDPQKVLTTIYTHSVISVDDMQYGAFADEDCSRIVGNHTDAVDGCL
VNYGAMHISLWPLARALNAAVPPLTKPETPSMHYHNLEEDDHPKSV

>tr|C9ZZ34|

MCALRDIASTSPLGDAIRDRVKGGIIVTTGEGAGSSPASTINSSPLGMGRGSDDSAHSPRSGNP
LGWEGLEGQSLPFFGMRSRDGTDTCIHSHSYDQPHVCPISMERNLRPSSSVSWLCDETLSEG
LAGSPKSSFSPLVASPRFVNLRDSVVAASPLDYSEEHSPRFASGPSERGGSFCSQFSDNIALGK
QLQGALANPTLWCSKEESPRSTAPSPHSPRVGYSGNCLESPDLKVASASQPGPAAPSHDPWK
KVLRCLTITT

>tr|C9ZZ34|

HTLRSFSMGTVSLICRVAPLLQQEKDAIVYQLQEATSFSRKEASDAGNKERSETSQPNDKKT
GYAPNRLCDHKGGSTNFVLDSSCNIPKVNTRYENFHELAGPDFAGGRVPLRAVRSHEIIPVSS
SPLLKNLDRSNKFSTPTAVRHNYWSVREARASDSTSLSTQQSDACIDAFALPRPRELKVCCD
VEGHPLKTNLHKHADPDKFDSATTNFSQEVTGRKEDAQESSSCKTKSDFSTSNSDQLLLETF
GSKNAMLSNSSFLAQQVTFLVCRLFLPALDLEMEGVVTTKRLRAVQAMSQQFTEVVLRVA
REEFGVPFDIRLDSVVVTFHTPSGPNSVNLVRPRDCAFRLVSELQKLESQWARTSSLPFVWGI
AMHLSQLLVGVIRTPSGRTSSLYGEEVRLAYRVTELCRILDCPLLMLQPCYDVFRVCVTAVP
VDVIHRRTCGGDVRIYLYDPKAPKERECVGSSREQYAPLMAAFGLMCEKRFSQASEQLEKV
LELDHNAPRLHRLCKYLAQEHEGEKMNSSLSDITRYVREGPQWCAVDREAKKFIRKKIEKA
GMSIDMNATSLSVSQYPFNDVVEPGCAQEISEEYRLLHRVANSCAMMDLSRFGQCECHVSP
QYYAAPGSFQPNEIQSEYALVTLRQNTLMNSSITRDVCIVTHQRSSSACGVMPECNYKSDRTI
AVVAPPHGENTIQNGDCYNKAERLEFDIYGPVGAGSFGRVYRGLHPDGNIVAIKEYPVPSMD
ENNPEIQSTLSEIRMLSASHHKNIVRYVDRCFQNGCLYIITEFVSGGSLAALVETFHGLPCDIIR
RYTGDILRGLQYLHDRQIVHRDISPNNVLVSIDGVCKLSDFGGAVECAMQPPTSEDNVTLTS
DEKSPTRKRTMVLTTESDYCSTGSTTLKTCFGTPVCMSPEACMGVVDPRNDIWGLGITLCFC
VACSYPWSQEDTADVRSFISKLRRGCISPEPPFDLMDVHFADFVRQCLKRDAKDRPSASELL
FHDFMVN

>tr|C9ZMP2|

HYRVGLQKIVLYIRGLLTSELFRCTLCRRKHLFDGRTQGDIMNHLSLDVARVADAASSFNDL
WALPLQLSLA

>tr|C9ZMP2|

MGGELTPGKAVTALALFNSLTMPLNAYPCVINGLVESYVSWCRLSPFLVMRPDACHGDLYG
FASGESDSGPKMFTEITEEYQDMVVSSEEMPCASSLNEIQ

PLLQAKGCVGEMRRQRLAGIHGLPHPFRRNHDLLIDIRSGVIRLNTQGGAGLPRPSFMLHIPT
FQATSSQLIAVVGVSGSGKSTFLDGLAGEHLIDPNDATGHAAVSRFSTAYVEQQPFLMSGTL

RENILMGLMYDSVRYEAVINAVALTQNFQTCFYPDGDTFLVGDRGIRLSGGQRARVALARA
LYAGKELYLIDDILGCLDPTVARHIVINALVGSAKSGSCVIVATHNQELVERADVVYRCVEG
QLLFSQRLVKTAATTVAEPSSPAAKPHATQQHVALANKEKGHGKVGNVLGDVPWPSSDSSP
ARAPAATPESGSIEAAEVLETSKHGTLAWETLACYIGRVGWKLSAFIIISAALMQISRNAGDQ
YVVTWAKSGDGNTTR

>tr|C9ZMP2|

PVSVLYHRIQCPYRASSRELRRLEEAANAPLLDTMRDALDGGVVIRSLGGRVVMSHLRRAS
RNTDLLLRVKFNSM

>tr|C9ZMP2|

ASTETELISVERVRQYFSLASEEPRTLGMFFSPPRSNWPSGGQVVLSNVSMRYDPSGPEVLRS
LSFHVNAGEKVAIVGRTGAGKSSIFSALLRLVEIESGSIHIDGYDTRRLPLEVLRTRLSVLPQQ
PFIFSGSLRQNIDPFGLHSEDAVRDVATSVKLDGVALDYNVTDSSRVSGGQRHLIALARVILQ
RSPLLLLDEPTAQSSAEAEAALWSSLAEHLRSTTVLCITHKLSHIDFFDRVIVIENGHVAGDGT
VAQLRAASVWPFSSPNADASLT

>tr|D0A9H6|

LSTLLPLCIVVGVGMWKDLWEDGKRRKSDKLVNSVGVQVLRGSDFVSVPSRDVRAGDVIL
CGLGDVVPADAVVLNTSLVDGVTYIETSNLDGETNAKTRRAKPETIKALGTVEDIIEGCLPD
AATCAHFLNNGSLVGWKRCDSRGGDNGKGAISQNVLTENGRTSGGTSGQRARASSAFRDV
EVTEVEDGTPQSVELPPPTNVTQSFAEQTSKKGTEGGHGRTSTATPTPLETRGSVCNTLELSS
KERTITECDDRVPSSTVETSDIRDRVRGGPSGLCSSSNPFAAGTASNIYPRAGAPGSAVALAN
DGNGVLNDARGDGDFKGVLLRGATPCPDLHSWIGQLRLRCGSVVSLSIDQFLPRGCIIRNTE
WVLCAVVYTGKNTKMLLNLKSKGEKSSLTSRRINL

>tr|D0A9H6|

MELNKVLQLYLIANDRRMASYDEFKGVLRYSRPKTSCLNSQLAYVRYVFTDKTGTLTENV
MTYVGGCTATERHDEKERPGALGEAFLRLVEARRLSVPPAVGEPIMTTDALERRQVPQQGR
FDFDEEAMEKEPLFRYLRNLSLCHSVVCFDRPEVESAVAAAVEAAAASGHSVLGGSLPPGSQ
HAFTRRPSAADATLPNNNRIMGCSGTTDAVEMVCHRRVSSITPGSGSVFGASPSTGVGRFLH
GHTGSASWRMTCHNDALMHERSLTMSRKVREFRDESKIYEGQSLDEVALVCAARDNLFAL
QGRTSKHVFVKVVQKVMCYEVVAELQFTSQRKLMSVLLSRCPDMDNASTGTQDNVRISYH
RKVQETQTSPSFARAWETPTRSSGQHKTMKVLGDQKGDPTHPVTVEEDRKDRPVDSNARG
KKLPFLLLVKGADSSMMSIMNKQNPRNIDLKDLFEVEIDSVAKKGLRTLVLGQRWVSEEEA
RDWLVKFNEAQCRLNDRDEALHEVYALLEKDVDLIGTTAVSDELQEDVPETVKFLMQADI
VVWMLTGDKRETAVTIACTSGIIESGCEDMVHHLDVCSQLSGTTDLQTELKSERIREVLRSQ
LSAASNKCDSAEEQYGKDTHKMVLVVDGLTLDAIFCDADLTREFFSIGMRCRSAVCCRMTP
LQKAKIVKLFQENTGGVALAIGDGANDVSMIQESSVGIGIMGLEGSQAELASDYAIPKFRFLK
RLLMVHGRFSLYRDAHCLVYSLHKNAFLTSA

>tr|C9ZTG5|

RMDVEGKSLVPSDAREDPSELTYPVEAAWKVFLLHL

>tr|C9ZTG5|

LQLRKLPKHTHHVGCDDVAGKELSRGGDEFNRSSNATTGLSTGETETLPSQSSCGIFRPPPGH
RSAEFGEPGSSGDSSPCHCEVVAKEELGRQNQPKWSPCGLQCSWNIPRGLLDDTRIPFVALD
TTTGCIECASAGFAENVGMPVARLTGREFGGLLSELHVENAATVLDVVRTVASSTVSKPRAI
HTEGNGKEVSGVRRTVFGKFGIRPLFSKQRQVGPLSALVADADKSSMEIEMNFPHIAKDSGG
RRVLVRGCRFQDEMETHSSKSLAAVVDEAKTRGTGTSSSGIERERPMVSEPNSVTGIKPSHFF
SLSLDVYLYQHHDTCNSRYLVLRQPSLHYALDCVPLPCFLVHPQTGHVLHWNEAAERETGL
SAYDMVGWPVCLTSVVDPTGSLGVFKDAQKKHSLMPFNSAPSAVLHPPLASPPLLVSLHLV
AGGGVEAEQGPLLDQIDCLCWPGRHLRDVGECRGYAETDGSVLKDTSACFSSDGDSEEAGG
MLPPLNPASVNLEPLDDYISRKALFLSPAWMPESLTESKANTVTWDALHVPLLFLIHGEAPG
VRECSPLADCCQPKKQRENNGGVEGLSQSFVDIVEEFKESLSCALVKECGISSLPLRDGKETL
PNPDITQYLGELRELFTCLAQKVDSFRECRGVPMSGRGDICGDFSDAEDGDTSSAVRGGRRP
VTAFGSSLDLGRGVTTARYVGDGSRGIKEGNDIFGEEGAPKGIKEQQQRTRYDAPAAGVLA
LAHKNTASHPVSSFAVSQDALVTPPARGAALVDLGNLTRPSGEERFVERGPGSGASKRCDT
HRIPPMQPHSKPLGYSNPAAEIPSRSVTPTSPLRLDHCESPGDLPVWAMLKSNDEATVPSCFIR
VPFGEVFRFGRSSKCHATTSDVFVSSVQFTVSRWVPAQLCAIRSSTPPHQDNGSSCGSCSPTP
SGYGPCNSSALADWRVELCDCSINGTYVNVKRIGKGRTCTLRNNDLITFQLRARRFFLGFRF
VLTDERGVPLKECSSPTCSSFRASGISGRGTPRLWQRTLSGSGSTFALNASQTESAKLNCSDS
ASQAVRSASGVRHGARTPNARRIHTSQRGSGRHQRGTIEWKIGEEMLGKGGNAEVYLGMN
LTNGKLIAVKRVPLPKEAGGGGNGKGLLKRYMSLQEEIKVLSKAVHQNIVQYYGSSQNKDY
FNILLEFVPGGSLRHLLENFGALSPGVICSYLAQTLEGLRYLHENDIVHSDVKAANILVTDKG
RVKLSDFGTAKHLLWQQGQSIDLANISGAAERTADDAACSTHHVAGTLRWMAPELIRASIG
PTKASDIWSVGCALIEMLSGDAPWNEYEIESEEEIINLLKYTTEPPDVPECQVLPELALIAKKC
LALSPRDRPTCEELLQLVEEAREKLGLQEDETQSRCDDSTSLRLSSVLGNAAVEGN

>tr|D0A1Y1|

MAEPPPPQPLRKGFFRADPHGLRPPESPRDAEEPENHDIVEDPPLPASRNLPPPRLRPMGGGPP
LAGEEAAATSHSNNRPSPGEVPPLPRPGTTTAVTSAQYNSLTPRQELFSSPQTAVEENIPAGY
DNQMNVTSNDFSHSQDTGIKRIGDGATGSSAKTRSTIAHAPSGRFSEVVQERPPIESVEQLCQ
LQTLAHPVERFFVEYQPMATRGCRSTQVPVEEDEALFNHLAEYTDDDSFFSSGSAVLEEDTR
RLQNDPLRGRAIRSVFETLSLRSLTKGRIPYELEDQERSSAPWLKQVHQRAIALHHSSFFIFPA
GMKAR

>tr|D0A1Y1|

SGVLQYRCVSPTTKNVTNQLCRFNHSEKNESYYHGATCPSPHLCVADTYGNPHHGYRSFDS
V

>tr|D0A1Y1|

EKIEKTRRLFVQKQLQLFDGMLLEQRQRLNEAIKLRDFVERDESGKLRRHPIELIRSASRRIQR
SKLSNSQTSIATESESGEPAVVVKKPIGDTTKGRSRWTDEQRVQLHLSLTRQRDIASGGERRR
RVVIKNDGEISGGTAGLHSQRKGSESIGERAMMVTGDFALGGRVGVVQHHPLTHTIGAAHG
TDLPLAVRLDNEEEQLRFLKDYQNPIDNDIMRRTNTFEDTNGNLHPSFVLTSTQRRGGSFDE
ASNTIRTTTEAAGVIPSRHSATLNGHNTSRMDSNGSLDEPTSKTQTVSKRGSPMPRLSSGQVP
EVIIHDPEGGDFRFAETRSQKWGIVRNILHMFTEGYPRIITQYIREHRRMQRRFGLTPLNYVN

KYEDDVLRKLRQRRVLQVKEPGAPSQTSRASGDEELVEVNGNKVILTDSDDIGDLSPIQMAT
NIVRNVPVTPFGAVMLVIVIVNGIFNATRYFQQPEYWETALFVLGIIFTSFFVLEIVVRVIGLG
LVSFLLDFNNLLDVTVTILGFVELAYARSNVVTVLNWVRLL

>tr|D0A1Y1|

WAWQSEEEEEENYAAIAKGGSGGRREVTRLHWFDFTVWRSFKHIHGGFERRDVAPDEVFH
LNEDMRKQLRIAEAKERFTKEALAQTDLAMSQRRMGSPMASPSATMGYNTDGCAPAAQV
GTAPRYVNVGGQLQRHINPSVDFVDAQVPPLNAPISQFQAENAQLRFARRYSTVPASVYTPV
IQDDGIDRPSPSARSSPSDAGERSGELGGESQQNGQEEGDGQYSPRGVSPNGTGGRATSTGL
QRKSSVLGRFPRLGYDRAAGKKDGGSRSVSASAMQQTGDGNELRGDYVNEGEANGGSMV
YEHILYPGPRLRYKHVMRNQYVRVFERCLDCNTYQQMPLRAPPNVQQRTPEELHAEHCHM
AAVRSSRQLVLNAIMGYVRLQKDINQPPTRDAVETVLGQAWSCGMLLFETIEYLSCSDIEQR
EYRTWDRTLEALQLQQWLIGLHVGEEQVGRATLAYTLAHRKREKLAVEHKSFELSWRQRS
FFFISPSNPVRRLSTRIIQSR

>tr|D0A1Y1|

NGLTHSCSDPSFVDITACEDAKHEWLPKVRNFDSFFQSLLTMIEVSVGSKWLDVIYTGVNGR
TSEHAPMDDHYLARGF

>tr|D0A1Y1|

SEGMPFNVNVLRMLRLGRFFSAAKVFKPMRKQFSLLHEVLIRSAVSLANVT

>tr|D0A1Y1|

DHYVTAARMNTSITRIEDLRRFRDLWSEFDPNGALVLHTHELPKLLESLRPPLGLTSRHNRV
ELLRLLREYDIPNHRGKVHYHEVLLPLARRVLAMAFSRDTMDYRTTFDTLWRHSEKSLRAL
PTVLGKRSHATAAQHFAASYVQAVCRRKKACREVQRVRSELWHEGRAVCDELGLPYADY
GFGNLLLEGPDPMRDLVPRSASAASSGGKGTQKGASEAENAASSPLWRAGQAESPSSPRSEG
ETIDGRAPAARLPGAYQPAIEEREKRFGPDVPNALRRHETRSEKLRRKDEERMLQSTPDDAV
SSPVSNVRSNSQRVNVGEYQPPLGTDPTSWLGSNVNRGSTVGGPTTESRTSSVMPAPQGPTA
PE

>tr|C9ZN85|

MSRVLDPRVPVLSRGVTPSLEYAVMDGSSLSPSQLAGIIVG

>tr|C9ZQF9|

DLLKQHSGGLVGFGDKGFAGMFSTYPREGNR

>tr|D0A885|

MLGANLGINFHTDDDHGDHGDHEGHEGHDMGGCAPAAGSYS

>tr|D0A887|

HHHTHDHGHHHHHHHHDDHTHGADDHEGHGHSHGGCESGHGTYS

>tr|D0A4L6|

RLLDALGQAFLQPFIRGIRQPTSATPLHVEHLLSIS

>tr|D0A4L6|

CSSFAVQHYEAVNGQVRHRRVDMPAWDSRKCWAEAEALDRPHLRAKDLVVSAQAVAQLS
LFEEFGVCP

>tr|D0A0T8|

QECTRELLKLINPNGESSAASTGAHSHNEHIPNHHQHHGNAMGGHPHEHAHHSHSHYQVAQ
TDEMGRVTIMWTMVALAAASVVCKEALFRWTKRVGERAGSRVVVANAYHHRADAWSGA
IALVGVAGQCIGMPGIDGLAGLFVSASICQIGYALMRDSVLEFFDFQRAEEVAAVRRVLQDY
NKLHLVNVFLIRHGHSYALHVTLLTEMDTAAMVLARTSNELTKLAQRSVRVADTFTTIAPC
DRGSEESLSNILRLVEEFHGLQSIPFDWGTRRISLPQTIDEECMRDVKSIAAFFELEIDIVAGEN
DFKRTAHPSVGCC

>tr|D0A734|

FIQFFVVETKGLTLEEIEEMFDPRARHRGSDGSQCSESCSKVERENEGEGTDHGLNQAETAR
API

>tr|C9ZMM0|

LYKMGSDCMTVTTQSTCPMFIPEKCTWKDNACKFVVDNCSGQLKEACGASSSSSVSQTCY
WNHKTNVCQPMAGFTALQNG

>tr|D0A2X2|

MTSREGIHPPYEVPVDAIFQPEGEGADISRSGTLISGAASARTVASPSQTQPADSRLSLSPPLKS
PKITATPREVFHGERFSGESLDQPLTPGCAKERVGPSSSRGSDLASVGDGFTRFTYGSVQDCD
ELDRHSLGPQLTDALECSFRSGTFVPSSGQSYTSLGRAAFHIFKGNVGTGVFLLPAYYRDAG

>tr|C9ZMW5|

QAAFPELRKWPVRLRISEGGEILTIDHIVVRMHLLSRNASANPLSPPVTSAMTPAHAAFQWG
NTTAFNTDWYASLCAREFHGASVWEVSLLALATYLSTEEEVRQMLHFMNTHMETDWIMRE
RHGMDCVAVDSSTKPTEWNGYFDFYSAKHDLSVVAIRGTDMTSAIDFLVDFNMFFEVVLY
HLLSNFVPGAGILPSHLIADLIGLASLRSDGNFQYGTWESLIAESKADDKNNKLQCVSNNYR
RDFFADVYNHIRYIGSRSKRPKHVILTGHSLGGAVASIVGAKMGIQAVGFGAPGITLARKKF
NVDLRSINKHVGNIISSHDIFPMIGGNVGEQHRIECLATTRELCHAMEFLVGALWRSCGSIRS
RFPSMGSVL

>tr|C9ZJZ0|

SFCFSPAKMYLVQKHRKRKGYWSTNRGHWASSFFFNIDSLASTDTLLCYDPSLLLFLFSQNH
SKGTLSLFVIVVTTTPVVCNAAERERIRRPAAPHSAWGVPFFLKKQLLFVLRSSGPGVAMSL
HVGNGLQQMEKKQCVSSCLLLTSLHVRGPLPLPSARSLVASVCVGTSSITTDVLQPVMSDEV

SGSSVPLSPVFQRASSTHQRLASASSVIGLEPLRIMFPLIDDDACHGDGGSGSAQPHKYEFCIG
LREADSNALLGVAICTRSGAWFPFKPCGGSARITFRYCEQMERKNRILRHLQEAQLYPTSSD
TKMQSDASCKLEASSSFLAPTASSVLSASTSFVDGGGPGKGSRPTHSGLSATDALAVARPHS
ACSSPNFPLLPATDTKVKPENEHSSHIMSVPNVTTEGPRHLASSNVATSQTCKKEPRRNYHC
GLNPISVNGFFTTSDPCPPGGLREREGQPRSLVFSNIHVKGFFPLSLTGSWRLEFLIPHPCIPVT
KHTVTVPATFDTDGSSALRDTLTVDLPLFLRGEVQTRLRATWVDSNNLDDITEVPTVVEFDP
IVIKVGGSEVCQQERRYHKLVRHSAEKFTLSELGVQLLVALKYFAPHREGQNGRPTSAHSDA
RLWRSAMAWREGEKDADAVKTKGNGLGKGGDGADMDVRLPELQTAATPDGKNMSKNSI
FTEEAMSAKALSFGDNASQSNAHRSPPSKEVPPPRRASKEEVRNPGMGHFSTLKPATNAQLV
ASMDDLVPCYTEEQRRAVFALYDTNNRGYMTQEQILHFCRAHVALFDGHQNDANIVRLLQ
SLFPRMPRRRMNATEAAAKATSSYLLQSRKPCAPALEQRLKNRGLQTPSGRPTSGQHTAGG
SPQGALGLPRRCITYDIFEVIALRLAGM

>tr|C9ZUN6|

ALAYSQNQMQKDNNQVRRLCACETMGNATQICSDKTGTLTQNRMTVVQGYIGMRRFRVT
NPGDPSSTVNLEGVSSDAQSLLMLGLALNSSSEKELLPGNVGAESDLLSRWTWRTDKGNKT
DQAILDFVDRVLISVPGSCNDKELPHQKLRMTNRSRGFAIFPFTSERKFMTAVVAGADGVV
MQHVKGGSDRVLGMCNRYLSSEGREEPLTEEVTEMITAQIRSIAGDANRTIGVAYGRIGTDG
AVPEEEPEGPFVWLALLGIQDPLRPEVVDAVRMCQRAGVTVRMCTGDNLDTAVAISRQCGI
YNRLRGDLALTGKDFRNLVYDTYGDEANMEKLWPVLDRMMVMGRSQPLDKQLLVLMLM
LRGEVVAVTGDGTNDAPALRLANVGFVMRSGTDIAVKSGDIVLLDDNFRSVQRAVVWGRT
VNDNIRK

>tr|D0A1S1|

MHESTVGRRAQQRRRFSTRPGTEGSVGTGSVGHLRIFPGVMLHLWILLSLCVPCSLAQGGHE
VRMTVKVLSLMFNSAGATVDIINSLNVGFNASLAAQNWTVVAGIDVTVIRPPSYNVSAAEY
LENYVKNADDGESLLVVFGPMGEGNIRKSYKVLKEHNLVAFAPLTELTESRKFLPNLYFLRP
EPSAELVALIRYAVNHMRVLRLGFMYTESLAGAPSAHSRATELMSQLGYELCCLFTVPNDV
EETASGEAFEAEWEKFAQNLPQAAIMFTRINDYTKQIVGRLVSDQRTATTVLLAPSLLQKSL
VAVWRQALEASNVSFVPHRLIQTGTNPLAKTTYFGAIRRFQNEARDYLTRHPEWSGLSDSN
HFLTNDVDGELMVYGWIAGEVLMRALRSNTKLGDRISFINSLYDQRRYVIDDLVIGDFGGEC
ATGAAAQGAVCDCNRGGKKVFMKEVVKGYHFQHVLPGIFSTSRDHCYSNAIQLHPPLSGVI
MRMSDNLTMLRAALEFYHGISSTASLLNVGELNRLIMLQVGSTTEQSMNDLVELRKNSIITA
VFGVVVEEMLTVKNLTFIDPIVMNPRLNKFRSNVIHLSPTLEQQLYVLVSYLSKNRQGPVHL
AVYSREGAEIAEVLTRTLVTFRANLSSSKIFRDVGELEKYLPAKGDVFLLGIGSGNIPTVKEY
LRTHQDVRIFVQFSEVMLMYDEFVGGFNGSAGADRVLFATNLPHWGDTDSKSKTVRKFHK
VVKPPHRTPLALLGFATERLMQRNIRRMEKVTSQLLVDLFFEEASITVDDMRYGTYDRESCL
ISGFAAAANCISNFGATNISVWSMARVMNSSVPVLQDPVTPQMFYVDPNANGLTAAQ

>tr|C9ZPZ6|

MISIHQSILFGRDVSRVRMENSPVPCINQFRCRLTMSSLSLLLVFLVVVMPPVEALDNITVKV
YSLLYHPFVGRRLIDSMNAGFNASMAARQWTVAPGINVEVIHPASYRIPGPRFLQRAINDNK
DEFFVVVGPMGDGQLAASRPLLQKENLVAFAPSTGASSVRGWSPNIYFLRVSPTVELIALMR
FAVTHLRLLRIGFMYLQGLTFGDSEYEVAIKLMSHLGRELCGVFTVASSHGKGAADSDFDA
VWDKFVVTRPQGVIMFAPPAKDVVKFVVKMLNDSRTQDAYFLASSVLELTIASWSSDIEAA
NTALDLGQIVLSRTNPLATDTQYQAIRRFQDDARSYLSANPGVTIFSGTDDFEHHYVDGKLM

VYGWIVGEVLSQALRSRAWIKDRETFKKSLYSQRRHVVDELVFGDFGGECEGTAGERGAV
CNCNQGGNVVYINVARRDNVLEVIHDGQVVVDSSLCYHDEVRLHSPVNGLLVFMQDDPVA
QNAAEEIYDGAIPLTGDGRLGQTDRFFLHMLTSETAEASSELESELDTRAVTAVFGVAGDAM
LSLQTTAFIDPISLLPGLPHRGRKVIYLSPTLEQQLFALVKYFVGSGSTVVHAVVCRGDVVSIE
GLLYLMLMTFGGHMGTVVGPDSSTNLEGSMPETGDVLVIGLSKDDVAVVASHLDRNPGVR
VAVLFFDIALLYTEFVKTFKSSSSGGRLLFATSLPHWAEANSTSVTVQKFHAAVPDSPRWTP
LSLLGFATGRFIQTLLFHMDKVTPDSIINAIYTLSVVNSDDMRYGPFVEERCPSTKDAPEGGD
HFCGKNYGARRLSVWSMDRALNASVAPLTSGATPSLVYIDPYSKVLSGGR

>tr|D0A7A0|

MNDYRLCGGCGAVLNCRPASRVVRAPPLVASNLPRPFTMAVALLALLSVGVRVAAGAEVT
VNILYLMYNPKFPKVSVDALSTGFEASLAARRGDIPNGVKVSVIRPSSHDQPIEELFESAVEA
SKGKLLIAVGPLGNDNVLWSLEHLKNNDVVAFSPLTYSDEARGWNRHLYFTTAEPDAELLT
LIRYFAVTLRLSRLGFMYLSDSFFGKESHAFTLKILSGMGYELSGTFSLEGTGGLEVVSTAFD
AEWEQFVNTRPLAVLLLGSPNLVTREFVRRMATDDRTTGVYVLAPSSAQVFLINTWRDALE
ESGRELIPGQLIISGVNPLPNHARFAVVKRFREEVDEYLSSSDEENRFAMRQELLEDDTSGEL
MLTGWITGEVVSRALRSTLSLTNSTAFIDSLYEQRRYLIDDLVVGDFGGDCDAFAAWQGAV
CQCNQGGSVVYMKEVVDGFRLQPVVTGFLTWGASECSSAGVVLRAPLNGLIVQLMDNVV
VYRASLRYLNGASALLGNGRIGERDRFFLHPLEATGDEAVEQLEQMRDVKVIPALFGVVNK
EIMATENLAFIDPITSNPRMNRFNRNVIYVSPTLAQELYVLVQYISEHPGGNVRVVIRTHDAD
MILEVLVATLRTYGIFMQSKVIVDNEGSLKPHLPSSGDVFVIGFTALEVYDLAEHLDAKRGL
RIFAIYPDVTLMYEELRAAFSRTPPTTTNRFVFATNQPHWAEENSSSLTVQAYHKAVPEPAM
RTPMSLRGFSTARLMVSVLDHMEKVDAKLLADYFYSESTINVDDMRYGPFSDVDCIVNGVA
LASNCLSNYGGTNISVWSMTRLLDPATPPLQNGVTPTLVYVDENKLTQGQV

>tr|C9ZL70|

MMVCLTIFLDISFRICLPVFNAHYSKRRKGGSGMPIPTVTMGPDPISEPLLHDADRDEIPFCVT
RNIGREMRRGSNSDSGNRWRQVEPVLELQIETLSTTSAGWFLNSWYGALVVALVLQSLVSF
QWGMVNICDSSSVSIRNLDSWRSSCVERYEYQSAELNCSGTKVLVQACDPPPHSSHRAMLP
SLLGCGAVPLSAPSYAEDSPGPNHDGMSMRSGGYIPKQRQQRLFNLRWVDRNVIDIPASKA
NRFPRVVFSLASPQDAISGDIPFKTYQLQVVLEKFPVHAPSEGDINVNSGTSRPATTGVYRYN
TTTTCFRTTPRCSSVVLPQDVVVVGGNSRITLTIIGAVEELASVASMSSVGIAFQRSFYT

>tr|C9ZL70|

DLLYPHIVRIVVYQPFSQLAPVMVSSCFVSHITFVYTPTTASRRVPIRPNNPMWRRVVWSRQ
WYQWLQFHGGILYIFHNEEQERYFNFLQNKQRLAKVLHRRSGRVPVATEHEGVGPICSTAE
CVWNGDGPLGTYDAESSDSSDSSGGESSFSYNSSHNEDDDVASSGRGSRRRNWSRIRRGVST
LFERAECRFVEKSALLLDSLEGAILDPLQSLQLGGNRRVPFFNLEAAIDCLNLSWEAYAPLCS
DVDASRRDADGGGNGTSSYCADCTPCAFPPETRGEGDSDKSTLDERAFLEGAVDSSAVTTV
DNRPIDGDLQSFTVAREGSSTSVGPAMCTKQYGYKPIAVFEALDVVAVCAVMDTEFLHHRG
KAPRIVIAFRGTANMSNARENIRVRQRPWREVDGVRQWWGLTKRARVHSGFLNIWISLKPA
VLHTLHRFLKENSSTVYRVFCTGHSMGGAVACLCAYSVRRMLREIEYPLDEVTVYTFGQPP
MGNAAFQTAYDKAIPRTFRVVNESDAVSLFSLFGGTHVGTEVDVNRHGNYICKPMFIEMLF
RPTGGKGFALKNHTLAAYAQSLNAVADRNSGRECKVRCLQPYVRDVVDPSLSALSSAAAN
VSVEHQAANV

>tr|D0A623|

RGYGKLPHITGCMCGAKSRDLFRTYLSPAIVDYFSARLIVDGRGKAGRGKTVIYSNSEGEEE
NAGPDHNDPSNKYIYSFHPHGVFPGTALWLPMSPQWEELIGRNEETIVTTHGADVIFAVPFM
RDALMSVGTMSVSRKGIENCLKQNNSPIIVTGGMAEMVYQKGSDTEMHIVMHHSGFVRMA
LQHGVPIVPILCFAEQNVMMNVPFPRLQRLTSRKLGFPFPTMPYGRWFLPLPHARPLTVVVG
KPILPDPAMCNADDPDHVVHYRLRYFGELQRLFFKYRDEAGYPNMVLHLHCHNETHIVTEF
RGSESVKDDQNQ

>tr|D0A1K6|

ELLLNVKVAEPRKDVIKLSTRLTLSRLKDMYYDIRRMPQVSEVARASSKVAEKRAALDSVC
SFLRGAPVVLATYSGVNASLTWTHNCSAGDQTDIALPSTMRLSDPTSPTPRISRYRSDLLIVT
EKYGPEADVFVVILRRDITGLMLLGHSLVDDFTTGISPLVAILLPSFSSSRLTVSLYNNAQWLE
DANPQNNPHDDEVLLLFEEWCSSGDTHTWHSVGRLLPGGLANTTVGEDVAHFPAPVPRLA
GGGFFSVTPPLVMCSTFCVDGTSNTCGKDNPSSVWLVTYGSSSYRGLDGLLTTAMVVGYTG
AVLFMVMLCFAYISIDAPVSYLKSLIFSAAGGTERRKEWDHTVHGWRKIWLGDLRALVNTF
QILALCFRLNKKYVPQHILEKQVKNLLDVKDKICSADTDGEAAVEVAEGRHNDDDDAVDD
KANVGAFVCAATVADVKSSISKRMCQGSQSVTSLNRIQPLCEVGDLQATDIAVESGRAAAL
SGAADGREPVNRGMILVENATILTVHLPAVETAYFTDFGLAVEQHRHIMALLLRSVRQYRG
ELFQRSGECISAAWNAFDGCADHAIRAAACALRILDRLEAYRRAGFRVGIVLHQGPFVCGV
VEDRAEAFTTVFGSVPRQAIVFSELAASLTVFDVLISEPVKESLSSHYECIMVDVIKYHEDDPP
ITLYELSKERQLPMTKGMPRGPSAFAEEHARVFFNFRNHEFGLALAGIEKMKRSFSKTELRLL
WRIEQLCKYYMHHEKDLPLPYYRRFPTWRIYEVTESVEGSNDFLALSSRGGTVLCGDIPPSV
MAHKSSFDCDAMKFRQELHDNVLASRRTGSKESGLAFSAVKEADMGTGSPSPAREADGVN
RRMSSMCLLTDAHSGNLESGLERSLGRPTTSLRDTLGKGRPSTVPGGEFGTPEAEGASALNV
AGRNAGSFDAMKSTNPQTKTCADEENYGDVELGVGDERRRFSFTNCRPSIIAEGRRRSYNRC
AESCVSLTSDSMEDAHSFSVVDPGGSLMATYTLPKKIVAKNGITYLRSSRILGKGSFGCVYL
GMDVNSGRMTAIKFLPMPSGEEEVSKVETEVVAMQKVKSGHVVQFISYAFQSNLIIIIMECM
MAGSLKGMLDAFGSIPPATACLFIRDVLRGLHKLHSNGIIHRDVKPQNVLLTLGGTCKISDFG
ASAFLSEVVRREMEGNGLQIQGTPVYLAPEAARGKPVEQSDIWSCGIMFLQLLTGGLPYADH
FLRMPPQVLVYHIGSASAKPIIPDDLDEFCLEFVQICLKSDPNERLSAQQLLALPVFSL

>tr|C9ZZB1|

MSVVPSLLYCPQFALFCIMSRDSCHTTVRRKKKRIKRVRGRDERIGHVWFTFCYSSEVSPR

>tr|C9ZZB1|

CALPSHSSPFSLFFFLCSPLFEIVTCRSPRWGAEPLLLIHSCVHYCYC

>tr|D0AAA6|

MKALKKNITVYSVSLGRLLNEFNNRYFRLKKRSRSEDLKRMPVREEAFLRLSPWHHNVTGS
CESGVSELDSDRVNGDLTRCRFLFGRCQMNVEYQFALSVGTRKDVASSKCTGKGSEGGAVL
LRFTKIPNKSGGSSTVTCSIQVNKEVQYFNREEVVVAMKFCREIYAKLGEANEFLETEKKRV
HMGHYGVDEESNSMGEGSR

>tr|C9ZJ73|

MMIGEIPQAFTDFGLVLFKEGENAGESDNCDGVKFSEHHGTHRRRNVIDCGDEKANKYGDA
SSKTLEDTLKQWESKKPQATGASGGNDVCKAAASSENYPCTMTEEWQTHYKETVKKLKEL
EGAHEKGKKAHDAMLGYANTAYAVNTKVEQEKPLAEVIAAAKEAGKKGAKIIIPAAAPAT
PTNSTKNEDSAPTEHVDRGIATNETQVEVGIDADFDGLLEAAEAAEVTHRHQRTAM

>tr|D0A751|

CRQFLSSAHKQNLFYSRTYDLTNTLQSNMTIPSQRRLVRTKF

>tr|C9ZWE6|

RGSSLSLPFEQLQVLSAAGSLMPLASLVTRFIMEVPSLDAWESFSFPLAIISKPLLTLSMLKLIV
CPETELPMRTSRLRGSVIRADAGLYTRLVLTLLGVVFGLASGRQGPLAWFLGLFSRRVCRPV
LRLLKPILNIVGGQSPTVELKPLKRAGNVRAATDGRYTVDNLVEGGYMNDDEAFGLWSSGG
ARHRRNARPMPQREGRNPTGNQQTTHRPVGGRVGDVEVDERVAQIMELGMGFSAEDIRHA
LSAAGGQVDVAVNVLVGA

>tr|D0A318|

NDTIVTLSSLSMFVHVLLTDYNYLNCYSERYQQNTAVNAATFGIILIASR

>tr|C9ZUV6|

ALNTPSVVPLSVLLEPLLKLCGMVSKAPRQQGSGDGSRAGSGSGGIADTVAVPVLQGAAAG
ALLPGSTEDEAQRRRNIALAALSSRIQQTTADTAATQHDAAVV

>tr|D0A9E1|

MSFVVSNVTINDMNKIDTTIDFITTLPTATDVYEEAQNVRSVLENVAARPPRRLRALKRPSAE
AIVVGSKKDKAYRKEVSKMLMSDLKGLFNRGVWNNLKEVMFPYS

>tr|C9ZWN8|

HSLALLTDASHLLIDVGAYAMSVVSLCTASRSSCGKYNYGWHRAE

>tr|C9ZWN8|

GSHGHSHFGGSHHHSHSGNGEEEDSLCEENTEHNHSHDHGHGYGHSGSEGEGHDHSHSHSG
RGFAVHAA

>tr|C9ZWN8|

RPLLRDLLGILMESTPPGINYSELSSALRSIKGVEGVHDLHVWSIASDYAALSVHLEADDKDA
ALQKAQEVCKRFGITHTTIQVDTVENGAGLCHSTCGTV

>tr|D0A893|

RSCVSQNMGGMDAAAADSKGKGCKAMTPLLYALLVTT

>tr|C9ZXR1|

RKMVFALLELPAGVGLFKVDGNKQKLKALLSFKSTADALATTTQVVNGELAKPVRKFLKK
NFVEKQITEELAVADAKLAKAIKDALAIQCVHGDDTLATFRALRANLDDLLEDVSTEQLNQ
TALGLAHNLNRYKLKFSPDKVDMMVVQAVALLEDLDKEINKYAMRAREWYGWHFPELAK
IVNDNILYAKIVLAAKTRFNVRDTDFSDFLEEELEQKVKDAAMVSMGTEIAEEDIENICRLCS
EVVAASKYRESLAAYLSSRMQTIAPNLTTMVGEQIGARLIQKAGSLLSLAKYPSSTLQILGAE
KALFRALKQRQATPKYGILYNAQVVAKAAATHKGAMSRVLAAKASLSARIDSFGEGDNSA
ALEYRGKVEERLRQFEEGVTYGRTGNVRGRGGGMQLQQKRGPPNGNGGGAPFKRQRLDS
GGFRQRE

>tr|D0A7R4|

DFPGSMGTGSGNTIEQYFRDHNMEYTQEMNQLLYSVYSWPNTVLAFFGGLLIDKY

>tr|D0A7R4|

GKILDAYTPDHNSSSIIDLAFDAELLNGNSNASADGPHPTLEGYE

>tr|D0A3U2|

ATECKFTTEGACGGAKIYGCKWSGTTCKFENPKCSEGSDPSDSCKNEVA

>tr|D0A551|

VARLYTNVPSVAKAVESTMPLVALTHMADSLQLCLQGIFRGAGQPKQA

>tr|C9ZLA0|

RMPMYFPCAWRKQRLSEEEWTKRQQTLQLYMNQPAPPRRMK

>tr|C9ZLA0|

WLGRYTPVRPTDMDTIGEALEDVVTESAVATTKNEVKPLPQYSGSFWQH

>tr|C9ZLA0|

SQDLGKHYHFGFSSGVAATIALNRFMFGGMYDAEAEKLDTKPECKQPSCVKNQ

>tr|C9ZL97|

FLKKKLNLTQTEVTTISTVGNCIMYFSFPSGALFD

>tr|C9ZL97|

RLPMYFPCAWRKQRLSEEEWTKRQQTLQLYMNQPAPPRRMK

>tr|C9ZL97|

WLGRYTPVRPTDMDTIGEALEDVVTESAVATTKNEVKPLPQYSGSFWQH

>tr|D0A939|

RQLAPNENGVNFSFQLYFNTVLFLARESVRSVNARHNLREKSGSGGAALKVMNCAAISLPL
GLLVVLVLELLHGFRITLFPSLAALANVGSVSAASAEAQGPGMDGGTLGLPEVVQVISVIAA
LSIEPCLAVAQSLDNVRTVVTSEFWALLARLTATISILWWYGSLSGHPWITRMCFSVANLSD
ALATVAYFLCLWNAPNQERGRKAAGGGGCEGDEDGEAATSMYQVRVIAARVLWGDTVQT
TTSARSYPLRECLPWCYLSLHSMHDVLLREFRLFLQFFRESCLRLLLTEGEHFALAAMGSAA
AVGQYSVVTNLGSLIVRLVFRVWETACFARWSRDIAAGRMADASVLLFVMLRVS

>tr|D0A939|

IKTPGRPQQESRRFEVQLRDFSAVFDSRIATVWFLLFGCTR

>tr|D0A2F4|

RLWAVSKLCPMLEGDTKDSDGGNALGGNYLSKSMRL

>tr|D0A2F4|

SGDGLLAPYSEPYKVFLALCQAAIVRFGGASFLSCLDYSD

>tr|D0A2F4|

DGPDAPYYDMITRTMFEVVSAFSGCGLSLPPRWS

>tr|C9Z1K0|

MSETQPYAGRPSELVGRQIAGYRIEREIGRGGMAVVYQALDLRLERTVALKLLAPELARND
TFRRRFTH

>tr|D0A3K9|

MRCFRKKVRDEEEFTLTSSVSEELQGDICNDGKPYGNADPQEQVPETGDITQFFGSFVQPNA
SMVGNSFCTTPFPVPVSWHNLTYSLQGRVILHNLTGTALPSRCLAIMGASGAGKSTFLHALS
DHLATSKDRKLEGKIQLGDVEYRHQYRKVMGFVGQDDVLSNISTPKRSLRFSVRVRRNPDP
ETTKQQVSDVMDELGLQHCRDTTVGTPGLVAGLSGGERKRCSMGVDLICDPKILLLDEPTS
GLDHVTSAKVVQLLNTIARKGRTVIYTIHQPSAGVLNHFDDLMLLVRGRCVYHGTMEDSVA
YFESIGYVCPETYTPTDFYMTLMQDSVQAKVLIKQWKRHVKRKRTLHTRVVELNHQPWTS
DTAKFLHGYIQRFKGSAVSQFSELVRRDFTELIRNRT

>tr|D0A3K9|

TDRMRPYWYFLEKLSFFRHAYILVLRNEMKHIDEIECDEKTNASGLCGYIPANGKEVLELNG
LEDGQSEN

>tr|D0A214|

MEGKSVVGGSHKLADGSSSCGTIGANPGYAGTSTPLDASSSLYFGKVVEDAVPVRVEVGET
TTRHTDPQGASVPLTRKATAKDRIAVEKTSDFVFCDECGQNIPVSEWADHREHPRVVENVTS
WVGRLNKLLIRLFIDFFMWVLMNIYFREVVVVNEKSIPKTGGVVFYGNHQNQFIDAMMIRA
NCGRPVRFVMAEKSFQRPIIGLFGHMTDAVPVIRPQDAPLNSGEGRLIRMDGDMIYGEGTKF

TMCLSDRDVIIWYREDVKCTAQVLKINSDTVLQLTMPVAACDAVTKPVGFQVSRRIDHSEM
YASVYQTLQSGQCIGIFPEGGSHDRTSLLPLKAGVALFSLGAAVRGIDVKVVPCGLTYFYGH
KFRSRAHVEFGEPITPSAEVVALFNTDKRKATGIFLEQLNEELRGFTINVPNMSALNFLHGFR
QLYQPQNCILATRDHLRLTRRLSVIMEEQKGNPEFIDYRSRVENYQDYCNALLVRDSQAATL
GKLGSNEARQLHLMFRRCF

>tr|D0A0K9|

PFCKPRFGVSTVEYLLSNAGSKTIGEWNKEGKMTRMRVDAKLLLKSEDDFRRLLELLRPFHL
RTWKYEDFYFDTPNFLLMAKDVQLRLRVPFKSSDGKDEKLAGAQGSNMLCHVSLTLKTNS
SVDVGGQTAGIFEMFPFTPKDVDDMLQEDSLVSVLKNRSSDEPAKTVLEYLTRASEEYGEEL
VLTRFASFETTRRQYKFVPHLGFGSTPSSDKDVASEKNKSDPPRFYVDEVPMGDFKSYEVEM
QGVTDPLADVCQDLMDYLNEKGIEFTHSLSGKLNRFMTRTLELEEMKEESQCVRLRIKGNK
GYEEVCRWQNEENEISLDPIPKRGAPPVDNDKPTFGVTQMASSLLGLGPLTSQASRMTHSQS
VRLKRIREGNHGDEEYFENYFFDDRPNGTLAAKKYTLRLRCCNPPTAFSLELRKEKWSAGG
VKGYERRRAYISGDVARLMLRDPNKFLNSLSSQSSLGHLLRRDMGLQKLTIVGYCKTHRITY
NGKIIERTMVEDSQRTQNGNAGKVAARPPDVEFTLFHGPSSAGCRGEFSIQLNRIMVDTGSD
PVKVAKRASEERCCSIFNPRGCSPFPREPSETESYEVKLAGLPEGLAATAEDWLVSQLHQRQ
VQWEVVMSAGMGQYHPSLAAA

>tr|C9ZNL1|

MTKGGKVAVTKGSAQSDGAGEGGMSKAKSSTTFVAT

>tr|C9ZNL1|

SFSSRVRALFVKHTRTGNPLVDSVAEHRPTTAGAFLRHLHVCYN

>tr|C9ZNL1|

AADCFAASGTEHADSKEHQGKARGKGQKRQITVECGCHNPFYKLWCNSFS

>tr|C9ZN50|

MSSFLQELGPVAVSHRQELEEVKRELDIKRDELDCWIYGFLENKKFDVKETVAKLQRRFAM
EVNEMAKYEFTEYMRTSLRLGIIQVIGEDKCGRTIFYVTVNRDKKAASHRDGKKHTFDLMV
SYGTRLRADNKRCQMVLLVNYEDASMWSNVDMSLQADVALRISKFFPGCISKVYICNMGR
MLCSVAKPLFSQLPSAFSDSIMFFSKSDRASGRLLEFIDESVLPVQLGGTNDCDNQEHWDRH
GDIIEDYYHGMKAAISERGLTVKEWELQCIEQPNVPVRHPSLASETESMVSLLTFGSSLTATP
VEGNLNGMWRHDNDISPPLEEEENEWCLLMKPLPNNLSLFFLEELYRWRVAVSAEEGEMR
CALMDDYAAAWKREFDELPMLDLSNKKWYRFIPESLRE

>tr|C9ZN50|

LGAKWQYWVPHSLPPVNASGPRDPLEEYHQYPICTLRTAGGFGIVDFALLTEVSGASKENVF
HEDFDNWFGSTDVVYNNTVKVYGERNEQWSISRFDSLARNVTVLTLQNGRVYSSIITMTVW
IGYIALAPLQILLPNNWLKQVAFLLSFLTRAIDFTWYDVKDEVVRYLTEMKNTTSNEIVLTA
WGIAGGIASLAGVESHTQTITFGSPGLMDALHYTNYTEGEYHKYVLAVVSELDVLNAVTRY
DPTTYQRIQCSAGSNTCGSMNYISSELVRVCDTTGRRHVGQQVEESVHEM

>tr|C9ZN41|

MIYHSETDGPHTPAASKSAGSAVWHCRLSTVMALLLFSNVLPAHSEGNIKVKVYSFIYSPYV
EYRQVEAINAGLNASFAARQWTVAPNVTVQVVPPPPNNVEVVDALQRVATTEKGLFVVFG
PLTDIETLHALPLLKREDLVAFAPSTGSSIVRGWNPNIYFIRASPTAELIALVRYAVSQLRLLRI
GFMYLQDISFGDSEYKHAVELFSHMGRELCGVFTVKSSMEAFADDRAFEAAWEAFAKTRP
QGVIAVAPPINDTMRFLNKIVADKRTRDAYVLAHSTLEFSIVGAWREALEAAGAPLKFGQVI
LTGTNPLAKNTLYRAIRRFQDHMRSYLSANPGVTVFMARITSTTTTWMGGLWMVYGWIAG
EVLSQALSSREWLTSRKAFMESLYNQRRYVIDDLVIGDFGGDCKGGAAKQGAACNCNQGG
SLVLMNVIGSGYRLFPVNGGVTIFDSKKCYINKPRIPSPMSILSLTLFDTALPVDTYASMSEVL
YASTRGRESALSRRLFFHSMASSSAESARTLQHQLDTRSVTAVFGVVDDAMLSIAEVAFVDP
VMLTPRLHHRGKNVIQLSPTLEQQLFVVVGYVTNTSASAPMSAIVRGTDATIIEVALRKIVW
MHGGTLQTVAVLDDNATLVGRLPNRGNAFVIGLAPGDPSLLAAHLDRNPDVRVLIPFFDVA
LMYDELVSAFNGNPNAERVQFATSLPHWADANTSSEIVREFHTALPDSSAWKPLPLLGYAA
ARFAQAVLPRMEYVTPKTLLDTIYMQSIITADEMRYGPFEEEEEKECFTANDPVPEQGEVCV
VNYGATRISMWSLARALNASVPPLTSPVTPLIRYADPNAIKLSSAQLAG

>tr|C9ZNT1|

MNDRTSLLGSKWRKRGGEKSVHVKGSANGVGVGLRCEGKDNSRVPRLWVSLFMFSLLML
LPMPLRASVHSGDNVTVTVLCLMRGLRAPSPIGDAIISGFNSSLAARNWTAARNVHVVPVS
WGSYGNQSIDALDKKLSNKSELLVLLGPLSDRDVLAVTPLLEKHNVIAFAPVTGSSGLRKWT
PHLYFLGADPVAELIALIYYALSQLRPLRLGLMHLHNTSYGEVQYELTMRLISRMGRDLCGV
FALESSDYGSASGEVFNAMWSRFASLHPQGVLVLGPPNTDTFKFLLAVPSDERTKDMYILAP
SALQPAISIIAEELRRSGKVAFASGQVIITGTNPLATDVKHHAVRRFQKEMRSYLKAQKNESS
PGGEDHFLKHATTGKLAVLGWVAGETLLQALSNREWLTSREEFKKSLYNQRRYVIDDLVIG
DYGGECTGSAAAQGATCRCNQGGHVIYMNRLEENGNLSPLADGLITRDVSGCYSDSGGLH
APLIGLFILTLDDPIALRAATIMRNAASVSVSRGHHEQSNRLFMHTLPALSDGLTGSLQQELN
TRTVTAVFGVVHPTIMRTPGLAFIDPVPLTPQLNRRMKNVIHLSPTLEQQLFVLAAYISSSSGS
GVRAVIRGEDSNAIGDVLTRTLATFGVTPHSLVTARVNETIEGALPVYGNVFIIGLTASDVGS
VVAHMERNPDVHVLVPFFDVVLLYDEVLKAFNGSSSANRLLIATNLPHWAEVHSSSEIVQG
FHAALPDPAQWTPLALVGFAAARVVRTIIPRIEKVTGETVINLFYNNIGVSAGDMYYGPFNH
GECVNDDVDGLGDGGCAVNYGATRISVWSMARVLNASVPALALPVTPSMQYHDPNAAGS
TGSTLIG

>tr|C9ZPJ9|

PWARASVAVMGSLHDVVEVNYVTLSGFTPFLRMTRMAKGIGSMYFSNNTFSNPVMDHAM
PLKDGMVSSLCVTLRDVDKRRMIASIGAFSLTKKQAALCIASKSNPGQYYGHVSEGGIMKDF
YYMDPVTMEYQQPLKRYESTTSGLTITEYTERFHFDSVVRIWNESVREGREMKPSEYWVRP
RFPPTYAAYVYPFFEREDDGTVGVGYIYVGMHTGKISVRWHKASDSGVRVMLVDPNNESG
QFYVFANNWGQPLANVSDEWKSAFMGEPIRFLSPDDVSDPLMSKAIRYLDLQQAARGKNQ
RSWFMYGGLAAVASAHHIVTDSGVEMVVVVVTNPSYYLGPIATYGGIAGLSSFVMYLIVIA
ACYYFVELCLHRPLRSTEEKLRAGLVKSSDEEKQKVIALREVCELHGVCSVLRRRLNAVRTY
MPDRAFDLSGAAASVSRSKDACASDPEAASSPRRYGEELKPVACSVAYVYYTPGRTRNPSD
AVVELMMQIVVSAAAACGGCFEVQRPDYCIVSFGVQSMDREFAAEATRAVEFARQVATKL
MARSEVSGAYRVIVESGAFPSGIVSGGGRSRYVLLCRNIYRRVGDALQSVGVAAAVTEETAL
LVRGHFRLLPFRSVFLEGEDGVCVTLYEVLSGDAGQPTWNEFEGHYNEAYDMMVRGNYVS

ALRLLDKAISVGSLTLSGESPSFMSLQPQRLRDECAARVGRRDRTPFVNQLLLVPDSLNSSAT
SLISSPPLLSGTGKTSGGSSSPCDGGSPRTPSVFVGENAASTLNGEIPRFLEDCHGNSWQRSIDP
IHEGANSVGVAYMGMSATGVLALLKLYPLTELGERLTREELDVALDKVLQVGESVSLVQCL
SYCHVPPHGVLLVWEYVPGGTLRDLKVRYGRKLPAATVKRHVTSLLRGLACLHERGLVQG
CLCPETVVTCVGGHCRLTGVLLDAAPLLKHQMTYCVSPEEARGKPQSWMTDMYALGLLVI
EMLNDDFPWRWTTNAQISRSRNELLAVLSDHEALMESLREGLLEPVPPPEDADPTIQLVVSS
CLRIDPAQRRDAVFLLQRVGGVVAVDA

>tr|D0A0X5|

MNMLHLGDRNASLAPSGGEHSLPTGGAVCRAVMDTLPVILRAPVALLLLLVVVPQLSVVAE
ANVTVKVLSAAWNWYMPRKYVTAINAGFNASLESRQWTVAGSVKVEVVYPSNLHLMPED
FIKEQLQGETNMSSIVIVYGPLGDQSVMHSIPHLMNHRVVAFGLMTGSTFIRQWNPYLYFLR
ADPAAETLVLIRYALCQLRVLRLGFMYLQGVHYGDEEYALTVNVMSRMGYELHGVFTVMS
PDGEPAPDGEFKEVFERFAAALPQAIIVFGAPVDDTAKFLMMMAVDDRIARSYILSPSAVQA
KLPQMWLRAVEAAGASFAPGQLLFTGTNPLAKDSQYIAIKRFQEVMSEYLKAHVSGTNITEP
DYFLTHGTEGELMVYGWICGEVLSQALSSVEWLKDRATFVRSLYNQRRYVINDIVIGDYGG
TCEGDAAKHGATCECNQGNKAVYVKEVLEDGRTTSVRSGFTVLKASRCYTDSSELHGPLN
GLVVFMKDDDIASKAAVLWQKGASHLVGKGDLGHSDRFFLHAFNTTIAEAADDLRNEQGD
RIVTAVFGPVTEAMLDMPNITFIDPLELKPRLNKFRRHVIHLSPTLEQQLYVLSSYLAGAGVG
TVDAVICSNEADGIADFLRRSSNEFAVSLRSAVIREDGEGVGKYLPISGTVFVIGLSVPDVKEI
ARKLEERNDLRVIVLFAEFSFLYDLFATALNNTAGAARLVFATSLPHWGDTETSSKTTQLFH
DVEKDSRLWTPLSFLSFATGRLMREILLRVEEISPETLVNFFYADSSIVSDDMRYGVFDDTKC
VVTAEKLSKNGCASNYGATQISVWSMARALNASIPPLTNPMTPSMTFRNSNAGRISGVALV
G

>tr|D0A513|

MRGSCSEGALSGGCLPSRATTNPLGGPSSAGSLEMAEMLGAEAFYGLPILCSLWSERKLPKR
RGARGRGQHWSCASGESGKKRNCNLPPQLLFTIMSYLLPEGMLKVRLVCRTFNDVFLTYSV
QETFAVSLPRSFMEPTVGFQTLSGLIKEREAVLRNKERSWSKWLSAYVVMWLRKEANNDTI
SKATVEGQALWVQLQRSRVLCRLAMEEWLGHLSDCRRAQYNIRRNWFNPTNLGCTLRTGV
PPVFLPDGTLCINDPPDVVKFYAKRNRGLPLCEDALSHKTVSRKNDSLKDMWAPFEELAFPA
YVCLMYYDPYVDALHISLASGTCTLWRVNSIGIEAAAHRTASEADFESSSHQLWRKTDLPCR
SIPSRIEVVGNYTISSEFLACSVTMTRDGIKLEDYRWSEDEVMSKVRLFGEGANGDTSRIIVAS
CGRDVSASLLPRATAGDAHLTREDVAVLNILQNQLSNASNSVNRPRAGPTRGEASANESYR
PSATNSVDGINGGHNDVESGNNGGNIISIAGGPGNGTFELLVDDIEHEGVGTAASEVTANHL
EGGRGDRITDFRPSSITINSTERIDIKRYVFGVFNEQARLMCTVAITHEVAVFILTQSGCRTLG
VNYSGGIHAASVLSSPVFKRCESPTAEHPCEEGDALFLCASQHNVLICQQKLYRRLEEVEILV
IHQNHREAATSRGALRPSEQRYGTWYFLKGYSYHSPPMILRPLVEVSSLMLQAFVQPRPLLP
EHLEWLSKKSNEGTCKVDCQRQREEHLPPYKSSWWCPQRDGHPYFHKPFVMFISKFVGYLL
AGCDCTSGGYQEVSTDDREHFHSVPLLDGIAVKRLHPSAVALSPSHAFFVLGMENGSILMVS
PSCRKCDQAKDGQGGCTTDAQVNGAHLIEQLDTISIPEGGEGYLASREAPQLLVAGEDLHEE
GADEGESSSSSEDEAPHPRSQHPHTSHLYRTTIISNRVRGSARQKRNLRSFTPQQLFGCAFHE
DPYISALQRCSSSILNSMERLEGEQRRHHPTPIHAVWIHAPRDCYRFSRKSGIWSMHLDDWK
LTVLNRLYELTVYDLSMQSFVGKDNVKGSVVFAPLLTLSPYLSHPFLGVPMKDEEKWLCSK
IRRRMRAETERTASGWEIVWHNGVLVVLGVSKGWRYSVFDFHARFSDPDVKNFSDPAPVA
YSAKRDEYYESISGSQMLVPLDFGKKGTHKLALPRHFPLLRRDEN

>tr|D0A6J5|

DRDHNWFATIAILLYNCGDAIGRFSTSFKCVWPPRRA

>tr|C9ZMC3|

RSAHRMQIRERVANGLDGTAGQTAVVTLAQHYPSLIVGDYRHQGEAVIFNGGSGVGGITDG
NAIPNAADERMARQQIHPLTTDDVSLLHQLAVEGMVVSPTNQMTNSALTETRHFPSWGLSN
RIAVPLPALLLAADEPNAIPFLEGEQQWDEEESSSPAGWTTGTDPQHAGGALSSCFPPTARGG
SRDESHIVYGRSCYFVGPHWDGPRGEVPLECVLRDGCAGSSSESSSDISSVATEPARCIVANR
CAPPTMLSSFPLSLYPFSFSFPSHMHLSVSSSNSLFASDEEEGEEYAAG

>tr|C9ZZW0|

KVYAGVLRPDFLARLEREGYNSTSRVKDWCKVAAEGRRSF

>tr|D0A0B3|

HIDVVAYARSYGFKVGKEDGTSEAPSTDVDSGNGKGEKKYPSFWT

>tr|D0A4Z3|

TRSPISPLLDQHTLSIFPKEGRVKDWGALRSFGALGWGVGSAVSATTVDL

>tr|D0A4Z3|

RYLEKEEAVSATAGDTLACTTDAVVAGEVRLSGESERC

>tr|D0A6J6|

DRDHNWFATIAILLYNCGDAIGRFSTSFKCVWPPRRA

>tr|D0A6J2|

DRDHNWFATIAILLYNCGDAIGRFSTSFKCVWPPRRA

>tr|C9ZUC9|

MALVASCSGLYFPDGESVDNIVTLTWRAPATASAQQPRSVVLFKVKASNPHKFYIVPRYGTI
LVSDDSGRKPPQPPAVSITFGLRPQSDDPLEPPPPSLRRDNSDRFAIEYLLVVPDAATHEHLTQ
ELSRPHRRGSNTVETAARELWEQVSEGKLKATQGPTVFLQAYTRGVVRALHETQGRRGEGF
SDHSHNDGGDEASGTATSNASTTATAMTISNDNKHCNSGAAVADAYGSAVRNNSDKNRSH
RAHNRGISSDEVQRTASSVNSGSHRSSERHEEEQVVVPPNARLVTSMIHRKLSDRLRRDPAC
ATNNDGAAAGPTSSAGGGELQALKFGIKALRSGPRIHPEEAASISQMKTLEADPGMGGGSN
NIASPAGTGTEAMNGIPGESSVGCRRTAVADVIMRIPPATSAVAAVKNQK

>tr|D0A9A1|

DCVDLRRLMSFSVFAAAGCMPVVLLGCTST

>tr|D0A843|

MDVVGGGCYTFWGYKNDCDKSTYTYRTGLLKCVGLRRVLSA

>tr|C9ZIK3|

MGRQEDGSFDARGGDAVLVAELHSEESKEATFVHPEARALFNKVPCLRHIPLFGEAAEGYG
PKPVLSIG

>tr|C9ZLF3|

MMVTNTCIIMWPFRKEQERGGWLIGWNPHGLLVIVVAVVHDPRVENATAALNKLKESEGD
IRATAHRKQHKDVPSATSLLILGWMHSKQATLDKSEYADAWVRSQHSHAELWLELNEGPL
LHQLYCCGSPVQPCHLHVLRSDPTVSYAVTSTAFSAVARHDRGDLVKLVEGKMQPICVEGA
NSTLEGVLLPSHMVHSAGPRTKMRRMVGSDPLTQDEEEKQEGAKQRTEDDRVSVGSSGSA
EGSTESQSVRGSETPGFGGELHPRLDFPTSTDYGELSSLLRASAYGKILRELCENKVSNATDG
PAATARLCAYWKNCGNRFTGFLTKILVVFHLVLKWPGNVSYTAALLEFRLRMVIHWLLLLR
GDTRVCCLHPVLPHIQHNDPALRRFCVVDFLVR

>tr|C9ZLF3|

ASLHLYLVFHSVSLPYRFARFMLRNLFLQFQGKKHNPLRHRTDTYDFPVEQ

>tr|C9ZLF3|

PLFYWALYRRQWPGGVALTGPKVLSTPQHSRALDPFARGSLTVEFGVVSKPLELRVLLADF
LLVAHVVGRLHPFDMIVAVFKVRRWERTNPGKCLVPPLPADIMPLTGMTLPKTAVA

>tr|D0A6T6|

PNSLESFLPVRIARNARMLSSHNYTLCRTEPHTYTCPDLPWSWIRTRNLVMNDEVVLK

>tr|D0A6T6|

AWEYFLPGLTIWFLDWLLRIYRRGSTVDLVSAAASGSFVEIRFRHGSLGALPGQFVFVNVPDI
SLLQWHPFSIMCEHEGYYLLYIKSMGEGTWTEKLGKLVCRRGLKFKLNVEGPCGRALDINE
HQNILFVAGGIGITPCASVYSHICDRMALGLRSPTPMLLWSVRDRELLLLMSQLWQGGDSSA
VSASSTIEEAPSDRVQVFFTGGCNVEDGEYTCVVNERMDISDRIPQVIAGKDPRTVLLFVCGP
PGLVELARSVAHSLGVDFHQETFLL

>tr|C9ZKS6|

MIEYACGWLHAPVSSFNSRGETTSSSEGATWGIALGRFGPTAKCSL

>tr|C9ZKS6|

RHIRSLFMQLDANVEALHRVADALAVPHNGDATGTIDVNPKQSFSHAKSCKGIQNAATPEM
ANDMLMLLHQLRGLLPDSTFEKELTDVAVRQCNGAKLQCEKANLPVGSRLEAHSPRVMSQ
FRVSRRTAAGMVRNREILEHGGTGTTFRKRRVTVVVVCLSDFAHQVEEDLEHCLHFSRQFL
SVVTQIVERYGGIIASVAPNKVVVTWNAFTDSPRHAQNGMQCACDVLVALQPFTQGLSVSP

SLKLFPTIVATSGFVMAGTIRVSDHEDDVLSVYGNCISLSEELPSLLGALRVRCACVGALAHF
CPSNFSCIPRDCVTDANNKRHIIYEMQDKALAQGRRHVEAFQAFQRGEHGVAHHLYTRIHEE
TRSDWNALRMSQICYYLEESHSVYTQRFPEWQLFPVEREHSCVNELQLGRRAYGFMKCEAS
PVEDHIRRAILKQTDTRALPHSYGRNEEESYRTPEVAKSDKCLIVKCNSSSLRYVTEFCDRQG
LVFRMSKQVLGTGACGVAFLGLSQTGALVAIKEIELPLRTRAQNSNLSDLNRRRLRRKGIQV
ESAMEKTLDGIINEVSLLSRLRHANIMGYISSAIWGNKLLIVMELGSGGSLYDLIQKFGSIKES
RARRYLRDVLQGLEYLHRKNIVHRDIKPQNVLLLETGLCKLTDFGTSQNLQKIANSCAPEGT
PPYTAPEAARGKAEKASDIWSFGIMMLYVLSGSLPWPNMNQMTSHAFFYKVGHVESFMPC
VDDKISHDAKQIVKRCCQRDPKERATARELLNNSFFNCTDTSTSCLVKDWPSSCGNISRSEFH
L

>tr|C9ZVY6|

CVLAGTDDYREKSMVSMDEKHCGAQGSNRTGCMELMPYCHYTDADTCVPVPLHGIYDLT
MRNITPNSWR

>tr|C9ZVY6|

QNGMEELNRLITEISRDSIAKLFVTFVTPEGGYWYTK

>tr|C9ZVY6|

TTRRWIATLPNARRLLRNRVPRSVQTQYGTSRSADNVVAPKLPNPKPPARAEEERREADSAV
TFQGATLPVVPRKQYSMETLDLAFDGVAGPTHCAPTTSLLWQSRGMQSSPVGSVCNLQDPD
GTEMEGVVEWDAAPDAEDDMLYHRMSLLPHNPNVDSRYNPRHQQLQRINITHEIERLEETIF
HVERYWDAPYSEVNPEECGIIEQR

>tr|C9ZI17|

TWASRMYVPMFLRCWPMRQYFLFLLDSVAMLSSSSDRLD

>tr|C9ZI17|

SSGGSSPASLWSEEGMDNWKDLYEYSRRATFRTFTVPPYFERRPLRDAVYLLKTFNVLVVG
VEEGRNRMMILDLDYKLALSDTILVLHESGSDSVDLALRALDPTEGSCGSMTGCDRSEQWE
ARMASPAADASAQSGSRGDVPDAEVEELGAVGIDSASLIYPFNPSFEVPTGSNEEVPCLGTEC
DNSPGKTSPVAMQGHKGKANVSLPNSVALPKGTITSVLDMQVERLRRRLLGEDFVVKCFGD
VSLSRLPLGGSLAVLTHLLNWRQAVRSRSEPVESSVERIERQINDILRHVSNEYQCGRSGKAP
GENFLFIDHVSSFKQPLLESMYDHYLNDHASRFELVQMMRCIRGIHSRSRVTLLSWQSLSDSF
LRQWDSSFEFPLRHIRGSSTMESHLNYAIKESGGAANLRGILIYCSQLSHWDFKDVPLVAVE
NNIRAILDCHATSEKQQQGVGGGSSRGAEVEQQVMVELKSFKSCISVTPHHADTEWRQRGE
VHFQYSLAFMMGRCFSANMLQTIFIHAHRNRCIMKFLNNVLCLHRQGSVFDTGGWNSGDK
SDTTLFKVCGNQLLHFQTFGDLFVFLLKHRSWVAIGVFRRFPTSEGLPGVPRYFITNPPMKMP
LRVDDVVYALSGVTGAHNRMGC

>tr|C9ZN45|

MIYHSETDGPHTPAASKSAGSAVWHCRLSTVMALLLFSNVLPAHSEGNIKVKVYSFIYSPYF
EYRQVEAINAGLNASFAARQWTVAPNVTVQVVPPPPNNAEVVDALQRVATTEKGLFVVFG

PLTDIETLHALPLLKREDLVAFAPSTGSSIVRGWNPNIYFIRASPTAELIALVRYAVSQLRLLRI
GFMYLQDISFGDSEYKHAVELFSHMGRELCGVFTVKSSMEAFADDRAFEAAWEAFAKTRP
QGVIAVAPPINDTMRFLNKIVADKRTRDAYVLAHSTLEFSIVGAWREALEAAGAPLKFGQVI
LTGTNPLAKNTLYRAIRRFQDHMRSYLSANPGVTVFNGTDNFDHDDVDGRLMVYGWIAGE
VLSQALSSREWLTSRKAFMESLYNQRRYVIDDLVIGDFGGDCKGGAAKQGAACNCNQGGS
LVLMNVIGSGYRLFPVNGGVTIFDSKKCYINKPRIPSPMSILSLTLFDTALPVDTYASMSEVLY
ASTRGRESALSRRLFFHSMASSSAESARTLQHQLDTRSVTAVFGVVDDAMLSIAEVAFVDPV
MLTPRLHHRGKNVIQLSPTLEQQLFVVVGYVTNTSASAPMSAIVRGTDATIIEVALRKIVWM
HGGTLQTVAVLDDNATLVGRLPNRGNAFVIGLAPGDPSLLAAHLDRNPDVRVLIPFFDVAL
MYDELVSAFNGNPNAERVQFATSLPHWADANTSSEIVREFHTALPDSSAWKPLPLLGYAAA
RFAQAVLPRMEYVTPKTLLDTIYMQSIITADEMRYGPFEEEEEKECFTANDPVPEQGEVCVV
NYGATRISMWSLARALNASVPPLTSPVTPLIRYADPNAIKLSSAQLAG

>tr|C9ZQ51|

MSWQEGGGRGCVYPHGNCRRNLTARSPARRYSMYKHSPVITAMSLLHLLPLLLMWMPPVC
AENGNVTVNVLSMMYSLGFTTPEVNAINAGFDASLSAHSWKTGSGATISVIRPSSPNATIEDI
FQLGVKQSEGKLLVVFGPLGTNHVLKNSDELKKHDLVAIAPVAYSSEVRGWNPHLYFISVEP
NAELLTLIRYAVVYLRVPRIGMMYEKDNTASMGAYEFTVRVLAMLGRHLCGVFVVKDSEN
QNISEDDLNTRWRQFVATRPQAILLFSSLGNTAKWFIKKVAQDNRTANAYLLSTSLQQHFLI
KMWREALVLANRTFTPGQLITTGTVPLANDNQSSLIQHFQRDMNNYLDTNSDWKGFAKPD
HYLEDDGLGEMMVYGWLAGEVLFEALNNAPQLTNRTSFRESLYKQRRYVIDDLVVGDFGG
ECNEAVALQGAMCECNQGGSMVYMKSIMDGFRLRPLWEGFLTWGVSECSSANVQVSAPLS
GLFVILVDNAIVFRATMRWFLGAQALDEAYDVDNRIFFHPLTVSSENVTQSLEQVRDNRDVS
AVFGIVPAAMLDTPNMMFISPMVVGIRQNGFRRNVIHLLPVLAQQLYVLAVYLSNTSSRGV
NAFIRGEQAGEISSLLYKSLVTFGVPLDSSKTLGDGDPISSYLSGNRDVFTIGLTLTDVAAVAR
HLQTHRRARVFVGFNDLAMYYDEFVAAFNASKESIASSERLLFATSFPHWAEKDTKSDVVA
SFHRIVNESHWDPLTFIGFVAARLLQVILPNMKKVNAELLADRIYTESNIKVDDMRFGPFSDV
ECVSGTSVSANECASNFGSTNISVWSMARVLNSSLPRTQVGMTPSMDYVIPQEGQLTRS

>tr|D0A7B9|

TNHIDESPRIDYGALPRTMNPTIYQHFTCSAKLGGVPGLRVCRNKKEVECIKDEITAPFRGVC
VHKRVGATTLVAFFANGFVGRIAPIPPLDTLIMHQWLARHANTRVLIPRGLIPNNRLSAIQSS
GRLYFVGNASVVRGIVQHLGRVSHYFTNVYGGTFETLPEAQEEVRRQTLNWGIVHVRCFEP
GSLDVQIYLNGTALPTLRETVANAYPGGFQHNRAEMYALSGYLTLQKEISEHHMGLFNPGM
GLNITPYIMPQGFVEFTETPLLQTA

>tr|D0A7B9|

QNDWGTKHPLFFITNPIRALFWERRKVGTSACPFTDGRADNGVFEDIGGTKEEATVVMAGL
RKEYQRGGDTFVAVNNFCWSMGRGEISVLLGLNGAGKSTVINMITGMVKPDAGDCYVNGR
SVRRELPAARQQMGFCPQHNILWPQLTCREHLEFFGKIKGLKGKALDLAVRHVLHETGLSE
KSDDLAGHLSGGQKRMLSVGIAFVGGSPLVLLDEPTAGMDASSRRHAWGLLQRMAAHHTI
LLTTHFMDEADILGHRIAILNDGRLQCSGSSMFLKSKLGLGYSLTVVVRSKDNFCFVDDAVK
KHVPGAELLSYCGCEVIYRLPLGGVAAFPSLIEKLEIASDVNLNSYSLAATTLEEVFLRVCGG
QQCSAEKPRDCSSLWGRARHHAAGITQLKAIMLKRIFTALRDRRMHMLQVVCLTSVLLATV
LIGSNPAQVGPLPLTFDLYDEKVIVDSANCGLFWGKSPGVPNVHISEISAKDTRELSIYGMKT

WFAHEYPRYAAIFCGDRMLYNPKLRGMPVVMLYNSSALHQVAITMSMFYQLVLQRVSGV
QANVSWSVGVLEDEATYVGALQL

>tr|D0A7B9|

LEITRETSESLRWPFRFIPSFCVGEAIINISHFRFGKAVGNATSAFDMD

>tr|C9ZM88|

MNADSGEESREPLLRGGHSNDSNNHTDTNDAGTTEVHITADVTPKPTMDILLRSEQQLNTRR
HLWQAQIHALLGPEPPYALRPEDRAGFLFGRLYHTWTGPLMSLAARGVSLVPEDIPLPTRDV
RAFNSGLRLLQTLEEQKFRRFGWDSYTADGDDAAVVRHRRDRQSVGLLRWVGPVQQLRRP
RQMYAGVEWKSAPRHRVREQRRSMRKREKKGEELDTNGEKNVMPFHNGVIDGEHLFSTTD
GAHTATCEAVGDIVFISPFQSQGQERQSNPQGSTRKTYKNGIFMPITSQKPPKHISVARALFDT
FGSSVYILIPIQMLQDACQLAAPVILQKYIEYVQMSDQDWKGGVALVATLCFFSLVQSAAGN
KLTQMSRRVGLTFHNALLTVLFTKCATVARKGLAHPDMSVGRIVNMVSNDVGSARSLPTLL
PIMVGAPLRLAVGAL

>tr|C9ZM88|

VDVGLLSLSLTMAVNISTILTSMVGQAATVEANMNSVERVLHYAHNIEHEDLMEDMEKAIK
KQEERDRQMEKNERKKNKKGVASAGKNRMDGAEVNRSTNGMSGPNGHDFEAARGSCVSI
RVTGGAESDEENGGTDKPSTAHVPSRHFHYTTSSAVEFCNVSMRYRQGQPLVLRDLTFRITT
GQKVGVIGRTGSGKSSLLLTLLRMVDIEGGNILIEGHPIRSYRLRKLRQLFSVIPQDPVLFDGT
LRDNLDPLHTSSDEEIFETLRLVGMQDRITSSAEGLRSRVVDCGANFSVGQRQLLCMARALL
RRDSRFVLMDEATANIDPALDRQLQYAIRHTFVTHTVITVAHRLHTLASYDLLLLLKDGRVV
ETGRPRDLVMDENSRFSRMVAAMGENALKNFMEATERDSFPR
