# Supplementary material for: Immunoinformatic design of a putative multi-epitope vaccine candidate against Trypanosoma brucei gambiense
Source: Comput Struct Biotechnol J. 2022 Oct 7;20:5574–85. doi: 10.1016/j.csbj.2022.10.002 (PMC9576565; doi:10.1016/j.csbj.2022.10.002)
Supplement: Supplementary data 4 [file mmc4.pdf]

NetMHC 4.0 Server - prediction results

Technical University of Denmark - DTU

```
# NetMHC version 4.0

# Input is in FSA format

# Peptide length 9,10,11,12,13,14
# Rank Threshold for Strong binding peptides   0.500
# Rank Threshold for Weak binding peptides   2.000
```

pos	HLA	peptide	Core	Offset	I_pos	I_len	D_pos	D_len	iCore	Identity	1-log50k(aff)	Affinity(nM)	%Rank	BindLevel
47	HLA-A0201	SMKCFTSMI	SMKCFTSMI	0	0	0	0	0	SMKCFTSMI	tr_C9ZMD3	0.429	480.79	3.00	
13	HLA-A0201	VTSPFGSCEV	VTSPFSCEV	0	0	0	5	1	VTSPFGSCEV	tr_C9ZMD3	0.261	2957.20	7.00	
46	HLA-A0201	SSMKCFTSMI	SMKCFTSMI	0	0	0	1	1	SSMKCFTSMI	tr_C9ZMD3	0.254	3203.60	7.50	
46	HLA-A0201	SSMKCFTSM	SSMKCFTSM	0	0	0	0	0	SSMKCFTSM	tr_C9ZMD3	0.240	3741.66	8.00	
22	HLA-A0201	VISGEDEGL	VISGEDEGL	0	0	0	0	0	VISGEDEGL	tr_C9ZMD3	0.213	4976.88	9.50	
29	HLA-A0201	GLKGKNALPA	GLGKNALPA	0	0	0	2	1	GLKGKNALPA	tr_C9ZMD3	0.203	5530.84	11.00	
47	HLA-A0201	SMKCFTSMIP	SMKCFTSMI	0	0	0	0	0	SMKCFTSMI	tr_C9ZMD3	0.169	8046.82	13.00	
14	HLA-A0201	TSPFGSCEV	TSPFGSCEV	0	0	0	0	0	TSPFGSCEV	tr_C9ZMD3	0.136	11422.42	17.00	
45	HLA-A0201	QSSMKCFTSM	SSMKCFTSM	1	0	0	0	0	SSMKCFTSM	tr_C9ZMD3	0.135	11556.93	17.00	
47	HLA-A0201	SMKCFTSMIPP	SMFTSMIPP	0	0	0	2	2	SMKCFTSMIPP	tr_C9ZMD3	0.132	11991.15	17.00	
45	HLA-A0201	QSSMKCFTSMI	SMKCFTSMI	2	0	0	0	0	SMKCFTSMI	tr_C9ZMD3	0.120	13674.64	19.00	
17	HLA-A0201	FGSCEVISGEDEGL	FISGEDEGL	0	0	0	1	5	FGSCEVISGEDEGL	tr_C9ZMD3	0.118	14016.33	20.00	
51	HLA-A0201	FTSMIPPGG	FTSMIPPGG	0	0	0	0	0	FTSMIPPGG	tr_C9ZMD3	0.117	14145.21	20.00	
44	HLA-A0201	EQSSMKCFTSMI	SMKCFTSMI	3	0	0	0	0	SMKCFTSMI	tr_C9ZMD3	0.116	14226.10	20.00	
46	HLA-A0201	SSMKCFTSMIPP	SMFTSMIPP	1	0	0	2	2	SMKCFTSMIPP	tr_C9ZMD3	0.116	14270.04	20.00	
43	HLA-A0201	GEQSSMKCFTSMI	SMKCFTSMI	4	0	0	0	0	SMKCFTSMI	tr_C9ZMD3	0.115	14362.04	20.00	
42	HLA-A0201	EGEQSSMKCFTSMI	SMKCFTSMI	5	0	0	0	0	SMKCFTSMI	tr_C9ZMD3	0.114	14592.78	20.00	
49	HLA-A0201	KCFTSMIPP	KCFTSMIPP	0	0	0	0	0	KCFTSMIPP	tr_C9ZMD3	0.111	15024.07	21.00	
45	HLA-A0201	QSSMKCFTSMIPP	SMFTSMIPP	2	0	0	2	2	SMKCFTSMIPP	tr_C9ZMD3	0.110	15188.98	21.00	
12	HLA-A0201	DVTSPFGSCEV	VTSPFSCEV	1	0	0	5	1	VTSPFGSCEV	tr_C9ZMD3	0.110	15195.57	21.00	
44	HLA-A0201	EQSSMKCFTSMIPP	SMFTSMIPP	3	0	0	2	2	SMKCFTSMIPP	tr_C9ZMD3	0.108	15543.10	22.00	
10	HLA-A0201	KADVTSPFGSCEV	VTSPFSCEV	3	0	0	5	1	VTSPFGSCEV	tr_C9ZMD3	0.105	16020.67	22.00	
21	HLA-A0201	EVISGEDEGL	VISGEDEGL	1	0	0	0	0	VISGEDEGL	tr_C9ZMD3	0.103	16402.69	23.00	
11	HLA-A0201	ADVTSPFGSCEV	VTSPFSCEV	2	0	0	5	1	VTSPFGSCEV	tr_C9ZMD3	0.102	16663.85	23.00	
9	HLA-A0201	NKADVTSPFGSCEV	VTSPFSCEV	4	0	0	5	1	VTSPFGSCEV	tr_C9ZMD3	0.100	16955.39	23.00	
14	HLA-A0201	TSPFGSCEVI	TSFGSCEVI	0	0	0	2	1	TSPFGSCEVI	tr_C9ZMD3	0.100	17002.42	23.00	
48	HLA-A0201	MKCFTSMIPP	MCFTSMIPP	0	0	0	1	1	MKCFTSMIPP	tr_C9ZMD3	0.099	17136.69	23.00	
13	HLA-A0201	VTSPFGSCEVI	VTFGSCEVI	0	0	0	2	2	VTSPFGSCEVI	tr_C9ZMD3	0.098	17249.42	24.00	
15	HLA-A0201	SPFGSCEVI	SPFGSCEVI	0	0	0	0	0	SPFGSCEVI	tr_C9ZMD3	0.095	17926.42	25.00	
10	HLA-A0201	KADVTSPFGSCEVI	KAFGSCEVI	0	0	0	2	5	KADVTSPFGSCEVI	tr_C9ZMD3	0.089	19032.38	26.00	
12	HLA-A0201	DVTSPFGSCEVI	VTFGSCEVI	1	0	0	2	2	VTSPFGSCEVI	tr_C9ZMD3	0.089	19043.08	26.00	
47	HLA-A0201	SMKCFTSMIPPG	SMFTSMIPP	0	0	0	2	2	SMKCFTSMIPP	tr_C9ZMD3	0.089	19140.79	26.00	

28	HLA-A0201	EGLKGKNALPA	GLGKNALPA	1	0	0	2	1	GLGKNALPA	tr_C9ZMD3	0.086	19678.38	27.00
46	HLA-A0201	SSMKCFTSMIP	SMKCFTSMI	0	0	0	1	1	SSMKCFTSMI	tr_C9ZMD3	0.086	19823.69	27.00
11	HLA-A0201	ADVTSPFGSCEVI	VTFGSCEVI	2	0	0	2	2	VTSPFGSCEVI	tr_C9ZMD3	0.085	20027.64	27.00
27	HLA-A0201	DEGLKGKNALPA	GLGKNALPA	2	0	0	2	1	GLGKNALPA	tr_C9ZMD3	0.083	20361.94	28.00
18	HLA-A0201	GSCEVISGEDEGL	GSCEVIEGL	0	0	0	6	4	GSCEVISGEDEGL	tr_C9ZMD3	0.082	20554.30	28.00
45	HLA-A0201	QSSMKCFTSMIP	SMKCFTSMI	2	0	0	0	0	SMKCFTSMI	tr_C9ZMD3	0.082	20645.69	28.00
26	HLA-A0201	EDEGLKGKNALPA	GLGKNALPA	3	0	0	2	1	GLGKNALPA	tr_C9ZMD3	0.081	20766.66	28.00
46	HLA-A0201	SSMKCFTSMIPPG	SMFTSMIPP	1	0	0	2	2	SMKCFTSMIPP	tr_C9ZMD3	0.080	20945.61	29.00
49	HLA-A0201	KCFTSMIPPG	KCFTSMIPG	0	0	0	7	1	KCFTSMIPPG	tr_C9ZMD3	0.080	20988.49	29.00
25	HLA-A0201	GEDEGLKGKNALPA	GLGKNALPA	4	0	0	2	1	GLGKNALPA	tr_C9ZMD3	0.080	21006.88	29.00
44	HLA-A0201	EQSSMKCFTSMIP	SMKCFTSMI	3	0	0	0	0	SMKCFTSMI	tr_C9ZMD3	0.080	21086.36	29.00
43	HLA-A0201	GEQSSMKCFTSMIP	SMKCFTSMI	4	0	0	0	0	SMKCFTSMI	tr_C9ZMD3	0.079	21195.46	29.00
45	HLA-A0201	QSSMKCFTSMIPPG	SMFTSMIPP	2	0	0	2	2	SMKCFTSMIPP	tr_C9ZMD3	0.078	21571.42	30.00
47	HLA-A0201	SMKCFTSMIPPGG	SMFTSMIPP	0	0	0	2	2	SMKCFTSMIPP	tr_C9ZMD3	0.077	21745.54	30.00
44	HLA-A0201	EQSSMKCFTSM	SSMKCFTSM	2	0	0	0	0	SSMKCFTSM	tr_C9ZMD3	0.072	22925.81	32.00
46	HLA-A0201	SSMKCFTSMIPPGG	SMFTSMIPP	1	0	0	2	2	SMKCFTSMIPP	tr_C9ZMD3	0.072	23001.34	32.00
43	HLA-A0201	GEQSSMKCFTSM	SSMKCFTSM	3	0	0	0	0	SSMKCFTSM	tr_C9ZMD3	0.071	23284.03	33.00
42	HLA-A0201	EGEQSSMKCFTSM	SSMKCFTSM	4	0	0	0	0	SSMKCFTSM	tr_C9ZMD3	0.069	23584.49	33.00
41	HLA-A0201	GEGEQSSMKCFTSM	SSMKCFTSM	5	0	0	0	0	SSMKCFTSM	tr_C9ZMD3	0.069	23586.54	33.00
22	HLA-A0201	VISGEDEGLK	VISGEDEGL	0	0	0	0	0	VISGEDEGL	tr_C9ZMD3	0.069	23803.42	33.00
13	HLA-A0201	VTSPFGSCEVIS	VTFGSCEVI	0	0	0	2	2	VTSPFGSCEVI	tr_C9ZMD3	0.068	23851.89	34.00
45	HLA-A0201	QSSMKCFTS	QSSMKCFTS	0	0	0	0	0	QSSMKCFTS	tr_C9ZMD3	0.064	25115.76	36.00
12	HLA-A0201	DVTSPFGSCEVIS	VTFGSCEVI	1	0	0	2	2	VTSPFGSCEVI	tr_C9ZMD3	0.062	25441.79	36.00
13	HLA-A0201	VTSPFGSCEVISG	VTSPFSCEV	0	0	0	5	1	VTSPFGSCEV	tr_C9ZMD3	0.062	25548.81	37.00
17	HLA-A0201	FGSCEVISG	FGSCEVISG	0	0	0	0	0	FGSCEVISG	tr_C9ZMD3	0.062	25688.24	37.00
50	HLA-A0201	CFTSMIPPGG	FTSMIPPGG	1	0	0	0	0	FTSMIPPGG	tr_C9ZMD3	0.061	25828.71	37.00
11	HLA-A0201	ADVTSPFGSCEVIS	VTFGSCEVI	2	0	0	2	2	VTSPFGSCEVI	tr_C9ZMD3	0.060	26239.39	38.00
29	HLA-A0201	GLKGKNALPAN	GLGKNALPA	0	0	0	2	1	GLKGKNALPA	tr_C9ZMD3	0.059	26272.05	38.00
13	HLA-A0201	VTSPFGSCEVISGE	VTSPFSCEV	0	0	0	5	1	VTSPFGSCEV	tr_C9ZMD3	0.059	26426.86	38.00
35	HLA-A0201	ALPANEGEGEQSSM	ALPGEQSSM	0	0	0	3	5	ALPANEGEGEQSSM	tr_C9ZMD3	0.058	26745.87	39.00
19	HLA-A0201	SCEVISGEDEGL	SISGEDEGL	0	0	0	1	3	SCEVISGEDEGL	tr_C9ZMD3	0.058	26803.52	39.00
12	HLA-A0201	DVTSPFGSCEVISG	VTSPFSCEV	1	0	0	5	1	VTSPFGSCEV	tr_C9ZMD3	0.057	27054.38	40.00
17	HLA-A0201	FGSCEVISGE	FSCEVISGE	0	0	0	1	1	FGSCEVISGE	tr_C9ZMD3	0.055	27450.68	41.00
18	HLA-A0201	GSCEVISGEDEGLK	GSCEVIEGL	0	0	0	6	4	GSCEVISGEDEGL	tr_C9ZMD3	0.054	27939.70	42.00
29	HLA-A0201	GLKGKNALP	GLKGKNALP	0	0	0	0	0	GLKGKNALP	tr_C9ZMD3	0.054	28010.84	42.00
20	HLA-A0201	CEVISGEDEGL	VISGEDEGL	2	0	0	0	0	VISGEDEGL	tr_C9ZMD3	0.053	28075.46	42.00
44	HLA-A0201	EQSSMKCFTS	EQSSMCFTS	0	0	0	5	1	EQSSMKCFTS	tr_C9ZMD3	0.053	28231.11	42.00
15	HLA-A0201	SPFGSCEVIS	SPFGSCEVI	0	0	0	0	0	SPFGSCEVI	tr_C9ZMD3	0.052	28475.31	43.00
28	HLA-A0201	EGLKGKNALPAN	GLGKNALPA	1	0	0	2	1	GLKGKNALPA	tr_C9ZMD3	0.052	28528.04	43.00
29	HLA-A0201	GLKGKNALPANE	GLGKNALPA	0	0	0	2	1	GLKGKNALPA	tr_C9ZMD3	0.051	28913.69	44.00
27	HLA-A0201	DEGLKGKNALPAN	GLGKNALPA	2	0	0	2	1	GLKGKNALPA	tr_C9ZMD3	0.050	29037.53	44.00
48	HLA-A0201	MKCFTSMIP	MKCFTSMIP	0	0	0	0	0	MKCFTSMIP	tr_C9ZMD3	0.050	29175.46	45.00
48	HLA-A0201	MKCFTSMIPPG	MCFTSMIPP	0	0	0	1	1	MKCFTSMIPP	tr_C9ZMD3	0.049	29295.35	45.00
26	HLA-A0201	EDEGLKGKNALPAN	GLGKNALPA	3	0	0	2	1	GLKGKNALPA	tr_C9ZMD3	0.049	29335.00	45.00
30	HLA-A0201	LKGKNALPA	LKGKNALPA	0	0	0	0	0	LKGKNALPA	tr_C9ZMD3	0.049	29388.68	45.00
10	HLA-A0201	KADVTSPFG	KADVTSPFG	0	0	0	0	0	KADVTSPFG	tr_C9ZMD3	0.049	29543.95	46.00
10	HLA-A0201	KADVTSPFGS	KADVTSFGS	0	0	0	6	1	KADVTSPFGS	tr_C9ZMD3	0.047	29974.44	47.00
29	HLA-A0201	GLKGKNALPANEG	GLGKNALPA	0	0	0	2	1	GLKGKNALPA	tr_C9ZMD3	0.047	30096.96	47.00
49	HLA-A0201	KCFTSMIPPGG	KCFTSMIPG	0	0	0	8	2	KCFTSMIPPGG	tr_C9ZMD3	0.047	30116.82	47.00
50	HLA-A0201	CFTSMIPPG	CFTSMIPPG	0	0	0	0	0	CFTSMIPPG	tr_C9ZMD3	0.047	30146.82	47.00
48	HLA-A0201	MKCFTSMIPPGG	MCFTSMIPP	0	0	0	1	1	MKCFTSMIPP	tr_C9ZMD3	0.046	30502.14	48.00
29	HLA-A0201	GLKGKNALPANEGE	GLGKNALPA	0	0	0	2	1	GLKGKNALPA	tr_C9ZMD3	0.045	30774.97	49.00
28	HLA-A0201	EGLKGKNALPANE	GLGKNALPA	1	0	0	2	1	GLKGKNALPA	tr_C9ZMD3	0.045	30799.30	49.00
18	HLA-A0201	GSCEVISGE	GSCEVISGE	0	0	0	0	0	GSCEVISGE	tr_C9ZMD3	0.044	30941.92	49.00

35	HLA-A0201	ALPANEGEG	ALPANEGEG	0	0	0	0	0	ALPANEGEG	tr_C9ZMD3	0.044	31147.48	50.00
27	HLA-A0201	DEGLKGKNALPANE	GLGKNALPA	2	0	0	2	1	GLKGKNALPA	tr_C9ZMD3	0.044	31220.02	50.00
28	HLA-A0201	EGLKGKNALPANEG	GLGKNALPA	1	0	0	2	1	GLKGKNALPA	tr_C9ZMD3	0.042	31880.89	55.00
14	HLA-A0201	TSPFGSCEVIS	TSFGSCEVI	0	0	0	2	1	TSPFGSCEVI	tr_C9ZMD3	0.041	32203.31	55.00
9	HLA-A0201	NKADVTSPF	NKADVTSPF	0	0	0	0	0	NKADVTSPF	tr_C9ZMD3	0.040	32375.90	55.00
2	HLA-A0201	TTNREQVNKA	TTNEQVNKA	0	0	0	3	1	TTNREQVNKA	tr_C9ZMD3	0.040	32466.39	55.00
44	HLA-A0201	EQSSMKCFT	EQSSMKCFT	0	0	0	0	0	EQSSMKCFT	tr_C9ZMD3	0.039	32788.71	55.00
19	HLA-A0201	SCEVISGEDEGLK	SISGEDEGL	0	0	0	1	3	SCEVISGEDEGL	tr_C9ZMD3	0.039	32854.77	55.00
16	HLA-A0201	PFGSCEVISG	FGSCEVISG	1	0	0	0	0	FGSCEVISG	tr_C9ZMD3	0.038	33005.11	55.00
14	HLA-A0201	TSPFGSCEVISG	TSFGSCEVI	0	0	0	2	1	TSPFGSCEVI	tr_C9ZMD3	0.037	33412.57	60.00
14	HLA-A0201	TSPFGSCEVISGE	TSFGSCEVI	0	0	0	2	1	TSPFGSCEVI	tr_C9ZMD3	0.037	33419.08	60.00
15	HLA-A0201	SPFGSCEVISGE	FSCEVISGE	2	0	0	1	1	FGSCEVISGE	tr_C9ZMD3	0.037	33486.39	60.00
7	HLA-A0201	QVNKADVTSPF	QVADVTSPF	0	0	0	2	2	QVNKADVTSPF	tr_C9ZMD3	0.037	33487.12	60.00
43	HLA-A0201	GEQSSMKCFT	GQSSMKCFT	0	0	0	1	1	GEQSSMKCFT	tr_C9ZMD3	0.037	33540.79	60.00
15	HLA-A0201	SPFGSCEVISG	SPFGSCEVI	0	0	0	0	0	SPFGSCEVI	tr_C9ZMD3	0.037	33656.76	60.00
7	HLA-A0201	QVNKADVTS	QVNKADVTS	0	0	0	0	0	QVNKADVTS	tr_C9ZMD3	0.036	33779.34	60.00
5	HLA-A0201	REQVNKADVTSPF	REQVVTSPF	0	0	0	4	4	REQVNKADVTSPF	tr_C9ZMD3	0.036	33833.84	60.00
20	HLA-A0201	CEVISGEDEGLK	VISGEDEGL	2	0	0	0	0	VISGEDEGL	tr_C9ZMD3	0.036	33876.33	60.00
43	HLA-A0201	GEQSSMKCFTS	GSSMKCFTS	0	0	0	1	2	GEQSSMKCFTS	tr_C9ZMD3	0.036	33964.77	60.00
22	HLA-A0201	VISGEDEGLKGKNA	VISGEDEGA	0	0	0	8	5	VISGEDEGLKGKNA	tr_C9ZMD3	0.035	34066.74	60.00
19	HLA-A0201	SCEVISGEDEGLKG	SISGEDEGL	0	0	0	1	3	SCEVISGEDEGL	tr_C9ZMD3	0.035	34147.18	60.00
4	HLA-A0201	NREQVNKADVTSPF	REQVVTSPF	1	0	0	4	4	REQVNKADVTSPF	tr_C9ZMD3	0.035	34220.41	60.00
6	HLA-A0201	EQVNKADVTSPF	QVADVTSPF	1	0	0	2	2	QVNKADVTSPF	tr_C9ZMD3	0.035	34258.57	60.00
41	HLA-A0201	GEGEQSSMKCFTS	GSSMKCFTS	0	0	0	1	4	GEGEQSSMKCFTS	tr_C9ZMD3	0.035	34301.59	60.00
42	HLA-A0201	EGEQSSMKCFTS	QSSMKCFTS	3	0	0	0	0	QSSMKCFTS	tr_C9ZMD3	0.034	34475.73	60.00
14	HLA-A0201	TSPFGSCEVISGED	TSFGSCEVI	0	0	0	2	1	TSPFGSCEVI	tr_C9ZMD3	0.034	34485.80	60.00
40	HLA-A0201	EGEGEQSSMKCFTS	QSSMKCFTS	5	0	0	0	0	QSSMKCFTS	tr_C9ZMD3	0.034	34612.89	65.00
15	HLA-A0201	SPFGSCEVISGED	SPFGSCEVI	0	0	0	0	0	SPFGSCEVI	tr_C9ZMD3	0.034	34734.45	65.00
23	HLA-A0201	ISGEDEGLKGKNAL	GEDEGLNAL	2	0	0	6	3	GEDEGLKGKNAL	tr_C9ZMD3	0.033	34815.72	65.00
15	HLA-A0201	SPFGSCEVISGEDE	SPFGSCEVI	0	0	0	0	0	SPFGSCEVI	tr_C9ZMD3	0.033	34922.85	65.00
8	HLA-A0201	VNKADVTSPF	VNADVTSPF	0	0	0	2	1	VNKADVTSPF	tr_C9ZMD3	0.033	34929.30	65.00
16	HLA-A0201	PFGSCEVISGE	FSCEVISGE	1	0	0	1	1	FGSCEVISGE	tr_C9ZMD3	0.033	34965.58	65.00
24	HLA-A0201	SGEDEGLKGKNALP	SLKGKNALP	0	0	0	1	5	SGEDEGLKGKNALP	tr_C9ZMD3	0.033	35073.58	65.00
25	HLA-A0201	GEDEGLKGKNAL	GEDEGLNAL	0	0	0	6	3	GEDEGLKGKNAL	tr_C9ZMD3	0.032	35186.47	65.00
28	HLA-A0201	EGLKGKNALP	GLKGKNALP	1	0	0	0	0	GLKGKNALP	tr_C9ZMD3	0.032	35292.86	65.00
4	HLA-A0201	NREQVNKADV	NREQVNADV	0	0	0	6	1	NREQVNKADV	tr_C9ZMD3	0.032	35392.64	65.00
5	HLA-A0201	REQVNKADV	REQVNKADV	0	0	0	0	0	REQVNKADV	tr_C9ZMD3	0.032	35404.15	65.00
20	HLA-A0201	CEVISGEDEGLKG	VISGEDEGL	2	0	0	0	0	VISGEDEGL	tr_C9ZMD3	0.032	35525.00	65.00
35	HLA-A0201	ALPANEGEGEQSS	ALGEGEQSS	0	0	0	2	4	ALPANEGEGEQSS	tr_C9ZMD3	0.031	35572.32	65.00
17	HLA-A0201	FGSCEVISGEDEG	FGSCEVISG	0	0	0	8	4	FGSCEVISGEDEG	tr_C9ZMD3	0.031	35650.93	65.00
25	HLA-A0201	GEDEGLKGKNALP	GEDEGLNAL	0	0	0	6	3	GEDEGLKGKNAL	tr_C9ZMD3	0.031	35727.76	65.00
18	HLA-A0201	GSCEVISGED	GSCEVISGE	0	0	0	0	0	GSCEVISGE	tr_C9ZMD3	0.031	35766.84	65.00
13	HLA-A0201	VTSPFGSCE	VTSPFGSCE	0	0	0	0	0	VTSPFGSCE	tr_C9ZMD3	0.031	35791.99	65.00
21	HLA-A0201	EVISGEDEGLK	VISGEDEGL	1	0	0	0	0	VISGEDEGL	tr_C9ZMD3	0.031	35866.81	65.00
9	HLA-A0201	NKADVTSPFG	KADVTSPFG	1	0	0	0	0	KADVTSPFG	tr_C9ZMD3	0.031	35888.95	65.00
20	HLA-A0201	CEVISGEDEGLKGK	VISGEDEGL	2	0	0	0	0	VISGEDEGL	tr_C9ZMD3	0.031	35907.98	65.00
22	HLA-A0201	VISGEDEGLKG	VISGEDEGL	0	0	0	0	0	VISGEDEGL	tr_C9ZMD3	0.031	35942.57	70.00
5	HLA-A0201	REQVNKADVTSPFG	REQVVTSPF	0	0	0	4	4	REQVNKADVTSPF	tr_C9ZMD3	0.030	35951.12	70.00
7	HLA-A0201	QVNKADVTSPFGS	QVVTSPFGS	0	0	0	2	4	QVNKADVTSPFGS	tr_C9ZMD3	0.030	35983.04	70.00
24	HLA-A0201	SGEDEGLKGKNAL	GEDEGLNAL	1	0	0	6	3	GEDEGLKGKNAL	tr_C9ZMD3	0.030	36147.32	70.00
6	HLA-A0201	EQVNKADVTSPFGS	KADVTSFGS	4	0	0	6	1	KADVTSPFGS	tr_C9ZMD3	0.030	36166.09	70.00
7	HLA-A0201	QVNKADVTSPFGSC	KADVTSPFC	3	0	0	8	2	KADVTSPFGSC	tr_C9ZMD3	0.030	36320.61	70.00
22	HLA-A0201	VISGEDEGLKGK	VISGEDEGL	0	0	0	0	0	VISGEDEGL	tr_C9ZMD3	0.030	36334.37	70.00
17	HLA-A0201	FGSCEVISGED	FSCEVISGE	0	0	0	1	1	FGSCEVISGE	tr_C9ZMD3	0.029	36400.06	70.00

43	HLA-A0201	GEQSSMKCF	GEQSSMKCF	0	0	0	0	0	GEQSSMKCF	tr_C9ZMD3	0.029	36633.18	70.00
17	HLA-A0201	FGSCEVISGEDE	FGSCEVISE	0	0	0	8	3	FGSCEVISGEDE	tr_C9ZMD3	0.029	36646.65	70.00
8	HLA-A0201	VNKADVTSPFGS	KADVTSFGS	2	0	0	6	1	KADVTSPFGS	tr_C9ZMD3	0.029	36654.19	70.00
7	HLA-A0201	QVNKADVTSPFG	QVADVTSPF	0	0	0	2	2	QVNKADVTSPF	tr_C9ZMD3	0.029	36660.55	70.00
10	HLA-A0201	KADVTSPFGSC	KADVTFGSC	0	0	0	5	2	KADVTSPFGSC	tr_C9ZMD3	0.029	36717.31	70.00
16	HLA-A0201	PFGSCEVISGEDEG	FGSCEVISG	1	0	0	8	4	FGSCEVISGEDEG	tr_C9ZMD3	0.029	36723.66	70.00
6	HLA-A0201	EQVNKADVTSPFG	QVADVTSPF	1	0	0	2	2	QVNKADVTSPF	tr_C9ZMD3	0.029	36728.04	70.00
8	HLA-A0201	VNKADVTSPFGSC	KADVTFGSC	2	0	0	5	2	KADVTSPFGSC	tr_C9ZMD3	0.028	36798.05	70.00
9	HLA-A0201	NKADVTSPFGS	KADVTSFGS	1	0	0	6	1	KADVTSPFGS	tr_C9ZMD3	0.028	36885.75	70.00
34	HLA-A0201	NALPANEGEGEQSS	ALGEGEQSS	1	0	0	2	4	ALPANEGEGEQSS	tr_C9ZMD3	0.028	36951.25	70.00
22	HLA-A0201	VISGEDEGLKGKN	VISGEDEGL	0	0	0	0	0	VISGEDEGL	tr_C9ZMD3	0.028	36972.03	70.00
9	HLA-A0201	NKADVTSPFGSC	KADVTFGSC	1	0	0	5	2	KADVTSPFGSC	tr_C9ZMD3	0.028	37022.47	70.00
18	HLA-A0201	GSCEVISGEDEG	GSCEVISEG	0	0	0	7	3	GSCEVISGEDEG	tr_C9ZMD3	0.027	37217.67	75.00
2	HLA-A0201	TTNREQVNKADV	TTNREQVNV	0	0	0	8	3	TTNREQVNKADV	tr_C9ZMD3	0.027	37238.22	75.00
21	HLA-A0201	EVISGEDEGLKG	VISGEDEGL	1	0	0	0	0	VISGEDEGL	tr_C9ZMD3	0.027	37300.71	75.00
16	HLA-A0201	PFGSCEVISGED	FSCEVISGE	1	0	0	1	1	FGSCEVISGE	tr_C9ZMD3	0.027	37303.94	75.00
16	HLA-A0201	PFGSCEVISGEDE	GSCEVIEDE	2	0	0	6	2	GSCEVISGEDE	tr_C9ZMD3	0.027	37342.30	75.00
11	HLA-A0201	ADVTSPFGS	ADVTSPFGS	0	0	0	0	0	ADVTSPFGS	tr_C9ZMD3	0.027	37426.45	75.00
35	HLA-A0201	ALPANEGEGE	ALANEGEGE	0	0	0	2	1	ALPANEGEGE	tr_C9ZMD3	0.027	37486.43	75.00
21	HLA-A0201	EVISGEDEGLKGK	VISGEDEGL	1	0	0	0	0	VISGEDEGL	tr_C9ZMD3	0.027	37487.64	75.00
18	HLA-A0201	GSCEVISGEDE	GSCEVIEDE	0	0	0	6	2	GSCEVISGEDE	tr_C9ZMD3	0.026	37804.12	75.00
20	HLA-A0201	CEVISGEDE	CEVISGEDE	0	0	0	0	0	CEVISGEDE	tr_C9ZMD3	0.026	37936.89	75.00
8	HLA-A0201	VNKADVTSPFG	KADVTSPFG	2	0	0	0	0	KADVTSPFG	tr_C9ZMD3	0.025	37963.17	75.00
21	HLA-A0201	EVISGEDEGLKGKN	VISGEDEGL	1	0	0	0	0	VISGEDEGL	tr_C9ZMD3	0.025	38087.41	75.00
0	HLA-A0201	VPTTNREQV	VPTTNREQV	0	0	0	0	0	VPTTNREQV	tr_C9ZMD3	0.025	38212.07	75.00
19	HLA-A0201	SCEVISGEDE	SCEVIGEDE	0	0	0	5	1	SCEVISGEDE	tr_C9ZMD3	0.025	38282.02	75.00
0	HLA-A0201	VPTTNREQVNKADV	TTNREQVNV	2	0	0	8	3	TTNREQVNKADV	tr_C9ZMD3	0.025	38294.02	75.00
1	HLA-A0201	PTTNREQVNKADV	TTNREQVNV	1	0	0	8	3	TTNREQVNKADV	tr_C9ZMD3	0.025	38319.71	75.00
7	HLA-A0201	QVNKADVTSP	QVNADVTSP	0	0	0	3	1	QVNKADVTSP	tr_C9ZMD3	0.024	38467.61	80.00
8	HLA-A0201	VNKADVTSPFGSCE	KADVTSPFC	2	0	0	8	2	KADVTSPFGSC	tr_C9ZMD3	0.024	38478.43	80.00
34	HLA-A0201	NALPANEGEG	ALPANEGEG	1	0	0	0	0	ALPANEGEG	tr_C9ZMD3	0.024	38503.00	80.00
3	HLA-A0201	TNREQVNKA	TNREQVNKA	0	0	0	0	0	TNREQVNKA	tr_C9ZMD3	0.024	38534.25	80.00
11	HLA-A0201	ADVTSPFGSC	AVTSPFGSC	0	0	0	1	1	ADVTSPFGSC	tr_C9ZMD3	0.024	38593.91	80.00
35	HLA-A0201	ALPANEGEGEQS	ALEGEGEQS	0	0	0	2	3	ALPANEGEGEQS	tr_C9ZMD3	0.024	38664.99	80.00
23	HLA-A0201	ISGEDEGLKGKNA	ISGEDEGLA	0	0	0	8	4	ISGEDEGLKGKNA	tr_C9ZMD3	0.024	38677.96	80.00
25	HLA-A0201	GEDEGLKGK	GEDEGLKGK	0	0	0	0	0	GEDEGLKGK	tr_C9ZMD3	0.024	38770.14	80.00
42	HLA-A0201	EGEQSSMKCF	EGEQSSMCF	0	0	0	7	1	EGEQSSMKCF	tr_C9ZMD3	0.023	38820.93	80.00
3	HLA-A0201	TNREQVNKADV	TNREQVADV	0	0	0	6	2	TNREQVNKADV	tr_C9ZMD3	0.023	38926.50	80.00
32	HLA-A0201	GKNALPANE	GKNALPANE	0	0	0	0	0	GKNALPANE	tr_C9ZMD3	0.023	39044.18	80.00
10	HLA-A0201	KADVTSPFGSCE	KADVTFGSC	0	0	0	5	2	KADVTSPFGSC	tr_C9ZMD3	0.023	39139.76	80.00
41	HLA-A0201	GEGEQSSMKCFT	GQSSMKCFT	0	0	0	1	3	GEGEQSSMKCFT	tr_C9ZMD3	0.023	39141.45	80.00
9	HLA-A0201	NKADVTSPFGSCE	KADVTSPFC	1	0	0	8	2	KADVTSPFGSC	tr_C9ZMD3	0.023	39163.06	80.00
30	HLA-A0201	LKGKNALPAN	LKGKNALPA	0	0	0	0	0	LKGKNALPA	tr_C9ZMD3	0.023	39174.51	80.00
19	HLA-A0201	SCEVISGED	SCEVISGED	0	0	0	0	0	SCEVISGED	tr_C9ZMD3	0.022	39219.88	80.00
26	HLA-A0201	EDEGLKGKNALP	GLKGKNALP	3	0	0	0	0	GLKGKNALP	tr_C9ZMD3	0.022	39391.26	80.00
27	HLA-A0201	DEGLKGKNALP	GLKGKNALP	2	0	0	0	0	GLKGKNALP	tr_C9ZMD3	0.022	39436.91	80.00
6	HLA-A0201	EQVNKADVTS	QVNKADVTS	1	0	0	0	0	QVNKADVTS	tr_C9ZMD3	0.022	39451.41	80.00
34	HLA-A0201	NALPANEGEGEQS	ALEGEGEQS	1	0	0	2	3	ALPANEGEGEQS	tr_C9ZMD3	0.022	39464.64	80.00
23	HLA-A0201	ISGEDEGLK	ISGEDEGLK	0	0	0	0	0	ISGEDEGLK	tr_C9ZMD3	0.022	39483.43	80.00
42	HLA-A0201	EGEQSSMKCFT	GQSSMKCFT	1	0	0	1	1	GEQSSMKCFT	tr_C9ZMD3	0.022	39549.29	80.00
30	HLA-A0201	LKGKNALPANEGEG	LLPANEGEG	0	0	0	1	5	LKGKNALPANEGEG	tr_C9ZMD3	0.022	39608.38	80.00
5	HLA-A0201	REQVNKADVT	RQVNKADVT	0	0	0	1	1	REQVNKADVT	tr_C9ZMD3	0.021	39637.96	85.00
39	HLA-A0201	NEGEGEQSSMKCFT	NQSSMKCFT	0	0	0	1	5	NEGEGEQSSMKCFT	tr_C9ZMD3	0.021	39680.88	85.00
20	HLA-A0201	CEVISGEDEG	CVISGEDEG	0	0	0	1	1	CEVISGEDEG	tr_C9ZMD3	0.021	39713.09	85.00

33	HLA-A0201	KNALPANEGEGEQS	ALEGEGEQS	2	0	0	2	3	ALPANEGEGEQS	tr_C9ZMD3	0.021	39819.36	85.00
40	HLA-A0201	EGEGEQSSMKCFT	GQSSMKCFT	1	0	0	1	3	GEGEQSSMKCFT	tr_C9ZMD3	0.021	39889.64	85.00
12	HLA-A0201	DVTSPFGSC	DVTSPFGSC	0	0	0	0	0	DVTSPFGSC	tr_C9ZMD3	0.021	39896.55	85.00
31	HLA-A0201	KGKNALPANEGEG	KLPANEGEG	0	0	0	1	4	KGKNALPANEGEG	tr_C9ZMD3	0.021	39960.93	85.00
16	HLA-A0201	PFGSCEVIS	PFGSCEVIS	0	0	0	0	0	PFGSCEVIS	tr_C9ZMD3	0.021	40002.88	85.00
38	HLA-A0201	ANEGEGEQSSMKCF	ANEGEGECF	0	0	0	7	5	ANEGEGEQSSMKCF	tr_C9ZMD3	0.021	40029.72	85.00
34	HLA-A0201	NALPANEGE	NALPANEGE	0	0	0	0	0	NALPANEGE	tr_C9ZMD3	0.021	40040.57	85.00
31	HLA-A0201	KGKNALPANEGEGE	ALPANGEGE	4	0	0	5	1	ALPANEGEGE	tr_C9ZMD3	0.020	40064.82	85.00
6	HLA-A0201	EQVNKADVT	EQVNKADVT	0	0	0	0	0	EQVNKADVT	tr_C9ZMD3	0.020	40091.29	85.00
19	HLA-A0201	SCEVISGEDEG	SCEVIGEDE	0	0	0	5	1	SCEVISGEDE	tr_C9ZMD3	0.020	40098.66	85.00
23	HLA-A0201	ISGEDEGLKGK	ISDEGLKGK	0	0	0	2	2	ISGEDEGLKGK	tr_C9ZMD3	0.020	40201.61	85.00
4	HLA-A0201	NREQVNKADVTSP	NREQVVTSP	0	0	0	5	4	NREQVNKADVTSP	tr_C9ZMD3	0.020	40261.67	85.00
27	HLA-A0201	DEGLKGKNAL	DEGLKGNAL	0	0	0	6	1	DEGLKGKNAL	tr_C9ZMD3	0.020	40308.75	85.00
32	HLA-A0201	GKNALPANEGEGE	ALPANGEGE	3	0	0	5	1	ALPANEGEGE	tr_C9ZMD3	0.020	40351.51	85.00
24	HLA-A0201	SGEDEGLKGKNA	GEDEGLKGA	1	0	0	8	2	GEDEGLKGKNA	tr_C9ZMD3	0.020	40368.12	85.00
23	HLA-A0201	ISGEDEGLKG	ISGEDGLKG	0	0	0	5	1	ISGEDEGLKG	tr_C9ZMD3	0.020	40392.59	85.00
24	HLA-A0201	SGEDEGLKGK	SGDEGLKGK	0	0	0	2	1	SGEDEGLKGK	tr_C9ZMD3	0.020	40397.83	85.00
8	HLA-A0201	VNKADVTSP	VNKADVTSP	0	0	0	0	0	VNKADVTSP	tr_C9ZMD3	0.020	40397.83	85.00
25	HLA-A0201	GEDEGLKGKNA	GEDEGLKGA	0	0	0	8	2	GEDEGLKGKNA	tr_C9ZMD3	0.020	40463.88	85.00
31	HLA-A0201	KGKNALPANE	KGNALPANE	0	0	0	2	1	KGKNALPANE	tr_C9ZMD3	0.020	40467.38	85.00
39	HLA-A0201	NEGEGEQSSMKCF	EGEQSSMCF	3	0	0	7	1	EGEQSSMKCF	tr_C9ZMD3	0.020	40480.09	85.00
41	HLA-A0201	GEGEQSSMKCF	EGEQSSMCF	1	0	0	7	1	EGEQSSMKCF	tr_C9ZMD3	0.019	40500.25	85.00
32	HLA-A0201	GKNALPANEGEG	ALPANEGEG	3	0	0	0	0	ALPANEGEG	tr_C9ZMD3	0.019	40504.19	85.00
30	HLA-A0201	LKGKNALPANE	LKGKNALPA	0	0	0	0	0	LKGKNALPA	tr_C9ZMD3	0.019	40508.55	85.00
2	HLA-A0201	TTNREQVNKADVTS	TTNREQVNV	0	0	0	8	3	TTNREQVNKADV	tr_C9ZMD3	0.019	40520.39	85.00
0	HLA-A0201	VPTTNREQVNKA	TTNEQVNKA	2	0	0	3	1	TTNREQVNKA	tr_C9ZMD3	0.019	40530.92	85.00
12	HLA-A0201	DVTSPFGSCE	VTSPFGSCE	1	0	0	0	0	VTSPFGSCE	tr_C9ZMD3	0.019	40599.38	85.00
40	HLA-A0201	EGEGEQSSMKCF	EGEQSSMCF	2	0	0	7	1	EGEQSSMKCF	tr_C9ZMD3	0.019	40629.27	85.00
2	HLA-A0201	TTNREQVNKADVT	TTNREQVNV	0	0	0	8	3	TTNREQVNKADV	tr_C9ZMD3	0.019	40633.23	85.00
3	HLA-A0201	TNREQVNKADVTSP	NREQVVTSP	1	0	0	5	4	NREQVNKADVTSP	tr_C9ZMD3	0.019	40637.63	85.00
30	HLA-A0201	LKGKNALPANEGE	LKGKNALPA	0	0	0	0	0	LKGKNALPA	tr_C9ZMD3	0.019	40705.40	85.00
3	HLA-A0201	TNREQVNKADVTS	TNREQVVTS	0	0	0	6	4	TNREQVNKADVTS	tr_C9ZMD3	0.019	40720.82	85.00
1	HLA-A0201	PTTNREQVNKA	TTNEQVNKA	1	0	0	3	1	TTNREQVNKA	tr_C9ZMD3	0.019	40824.05	85.00
33	HLA-A0201	KNALPANEG	KNALPANEG	0	0	0	0	0	KNALPANEG	tr_C9ZMD3	0.019	40846.14	85.00
32	HLA-A0201	GKNALPANEGEGEQ	ALNEGEGEQ	3	0	0	2	2	ALPANEGEGEQ	tr_C9ZMD3	0.019	40860.71	90.00
32	HLA-A0201	GKNALPANEG	GKNALPAEG	0	0	0	7	1	GKNALPANEG	tr_C9ZMD3	0.018	40978.94	90.00
30	HLA-A0201	LKGKNALPANEG	LKGKNALPA	0	0	0	0	0	LKGKNALPA	tr_C9ZMD3	0.018	41037.94	90.00
4	HLA-A0201	NREQVNKADVTS	NREQVDVTS	0	0	0	5	3	NREQVNKADVTS	tr_C9ZMD3	0.018	41073.93	90.00
28	HLA-A0201	EGLKGKNAL	EGLKGKNAL	0	0	0	0	0	EGLKGKNAL	tr_C9ZMD3	0.018	41079.25	90.00
1	HLA-A0201	PTTNREQVNKADVT	TTNREQVNV	1	0	0	8	3	TTNREQVNKADV	tr_C9ZMD3	0.018	41127.73	90.00
3	HLA-A0201	TNREQVNKADVT	TNREQVADV	0	0	0	6	2	TNREQVNKADV	tr_C9ZMD3	0.018	41155.32	90.00
31	HLA-A0201	KGKNALPAN	KGKNALPAN	0	0	0	0	0	KGKNALPAN	tr_C9ZMD3	0.018	41262.80	90.00
24	HLA-A0201	SGEDEGLKG	SGEDEGLKG	0	0	0	0	0	SGEDEGLKG	tr_C9ZMD3	0.018	41350.39	90.00
4	HLA-A0201	NREQVNKADVT	NREQVNADV	0	0	0	6	1	NREQVNKADV	tr_C9ZMD3	0.018	41374.55	90.00
34	HLA-A0201	NALPANEGEGE	ALPAEGEGE	1	0	0	4	1	ALPANEGEGE	tr_C9ZMD3	0.017	41384.86	90.00
23	HLA-A0201	ISGEDEGLKGKN	ISDEGLKGK	0	0	0	2	2	ISGEDEGLKGK	tr_C9ZMD3	0.017	41416.66	90.00
5	HLA-A0201	REQVNKADVTSP	QVNADVTSP	2	0	0	3	1	QVNKADVTSP	tr_C9ZMD3	0.017	41457.46	90.00
35	HLA-A0201	ALPANEGEGEQ	ALNEGEGEQ	0	0	0	2	2	ALPANEGEGEQ	tr_C9ZMD3	0.017	41466.42	90.00
6	HLA-A0201	EQVNKADVTSP	QVNKAVTSP	1	0	0	5	1	QVNKADVTSP	tr_C9ZMD3	0.017	41476.29	90.00
31	HLA-A0201	KGKNALPANEGE	KALPANEGE	0	0	0	1	3	KGKNALPANEGE	tr_C9ZMD3	0.017	41519.40	90.00
42	HLA-A0201	EGEQSSMKC	EGEQSSMKC	0	0	0	0	0	EGEQSSMKC	tr_C9ZMD3	0.017	41578.75	90.00
33	HLA-A0201	KNALPANEGEGE	ALPAEGEGE	2	0	0	4	1	ALPANEGEGE	tr_C9ZMD3	0.017	41587.74	90.00
5	HLA-A0201	REQVNKADVTS	RVNKADVTS	0	0	0	1	2	REQVNKADVTS	tr_C9ZMD3	0.017	41634.11	90.00
39	HLA-A0201	NEGEGEQSSM	NEGEGQSSM	0	0	0	5	1	NEGEGEQSSM	tr_C9ZMD3	0.017	41637.28	90.00

33	HLA-A0201	KNALPANEGEG	KLPANEGEG	0	0	0	1	2	KNALPANEGEG	tr_C9ZMD3	0.016	41886.24	90.00
34	HLA-A0201	NALPANEGEGEQ	ALNEGEGEQ	1	0	0	2	2	ALPANEGEGEQ	tr_C9ZMD3	0.016	41888.97	90.00
31	HLA-A0201	KGKNALPANEG	KNALPANEG	2	0	0	0	0	KNALPANEG	tr_C9ZMD3	0.016	41964.71	90.00
27	HLA-A0201	DEGLKGKNA	DEGLKGKNA	0	0	0	0	0	DEGLKGKNA	tr_C9ZMD3	0.016	41977.89	90.00
32	HLA-A0201	GKNALPANEGE	GKNALPEGE	0	0	0	6	2	GKNALPANEGE	tr_C9ZMD3	0.016	42009.68	90.00
33	HLA-A0201	KNALPANEGEGEQ	ALNEGEGEQ	2	0	0	2	2	ALPANEGEGEQ	tr_C9ZMD3	0.016	42049.26	90.00
26	HLA-A0201	EDEGLKGKNAL	DEGLKGNAL	1	0	0	6	1	DEGLKGKNAL	tr_C9ZMD3	0.016	42070.20	90.00
11	HLA-A0201	ADVTSPFGSCE	VTSPFGSCE	2	0	0	0	0	VTSPFGSCE	tr_C9ZMD3	0.016	42129.85	90.00
0	HLA-A0201	VPTTNREQVNKAD	TTNEQVNKA	2	0	0	3	1	TTNREQVNKA	tr_C9ZMD3	0.016	42139.44	90.00
2	HLA-A0201	TTNREQVNK	TTNREQVNK	0	0	0	0	0	TTNREQVNK	tr_C9ZMD3	0.016	42178.21	95.00
2	HLA-A0201	TTNREQVNKAD	TTNEQVNKA	0	0	0	3	1	TTNREQVNKA	tr_C9ZMD3	0.016	42241.23	95.00
33	HLA-A0201	KNALPANEGE	KALPANEGE	0	0	0	1	1	KNALPANEGE	tr_C9ZMD3	0.015	42294.27	95.00
25	HLA-A0201	GEDEGLKGKN	GEDEGLKGK	0	0	0	0	0	GEDEGLKGK	tr_C9ZMD3	0.015	42336.41	95.00
38	HLA-A0201	ANEGEGEQS	ANEGEGEQS	0	0	0	0	0	ANEGEGEQS	tr_C9ZMD3	0.015	42483.23	95.00
38	HLA-A0201	ANEGEGEQSSM	ANEGEGESM	0	0	0	7	2	ANEGEGEQSSM	tr_C9ZMD3	0.015	42590.48	95.00
24	HLA-A0201	SGEDEGLKGKN	SGDEGLKGK	0	0	0	2	1	SGEDEGLKGK	tr_C9ZMD3	0.015	42609.83	95.00
36	HLA-A0201	LPANEGEGE	LPANEGEGE	0	0	0	0	0	LPANEGEGE	tr_C9ZMD3	0.015	42619.06	95.00
36	HLA-A0201	LPANEGEGEQSSM	LPANEGESM	0	0	0	7	4	LPANEGEGEQSSM	tr_C9ZMD3	0.015	42726.64	95.00
1	HLA-A0201	PTTNREQVNKAD	TTNEQVNKA	1	0	0	3	1	TTNREQVNKA	tr_C9ZMD3	0.014	42850.70	95.00
40	HLA-A0201	EGEGEQSSM	EGEGEQSSM	0	0	0	0	0	EGEGEQSSM	tr_C9ZMD3	0.014	42887.36	95.00
21	HLA-A0201	EVISGEDEG	EVISGEDEG	0	0	0	0	0	EVISGEDEG	tr_C9ZMD3	0.014	42927.27	95.00
41	HLA-A0201	GEGEQSSMKC	GEGESSMKC	0	0	0	4	1	GEGEQSSMKC	tr_C9ZMD3	0.014	43017.95	95.00
3	HLA-A0201	TNREQVNKAD	TNREQVNKA	0	0	0	0	0	TNREQVNKA	tr_C9ZMD3	0.014	43075.24	95.00
26	HLA-A0201	EDEGLKGKNA	EDEGLGKNA	0	0	0	5	1	EDEGLKGKNA	tr_C9ZMD3	0.014	43087.80	95.00
41	HLA-A0201	GEGEQSSMK	GEGEQSSMK	0	0	0	0	0	GEGEQSSMK	tr_C9ZMD3	0.014	43194.70	95.00
39	HLA-A0201	NEGEGEQSS	NEGEGEQSS	0	0	0	0	0	NEGEGEQSS	tr_C9ZMD3	0.013	43236.78	95.00
37	HLA-A0201	PANEGEGEQSSM	ANEGEGESM	1	0	0	7	2	ANEGEGEQSSM	tr_C9ZMD3	0.013	43252.23	95.00
4	HLA-A0201	NREQVNKAD	NREQVNKAD	0	0	0	0	0	NREQVNKAD	tr_C9ZMD3	0.013	43435.12	95.00
38	HLA-A0201	ANEGEGEQSSMKC	ANEGSSMKC	0	0	0	4	4	ANEGEGEQSSMKC	tr_C9ZMD3	0.013	43499.57	95.00
36	HLA-A0201	LPANEGEGEQ	LANEGEGEQ	0	0	0	1	1	LPANEGEGEQ	tr_C9ZMD3	0.012	43801.34	99.00
38	HLA-A0201	ANEGEGEQSSMK	ANEGEGEMK	0	0	0	7	3	ANEGEGEQSSMK	tr_C9ZMD3	0.012	43847.80	99.00
36	HLA-A0201	LPANEGEGEQSSMK	LPANEGEMK	0	0	0	7	5	LPANEGEGEQSSMK	tr_C9ZMD3	0.012	43890.07	99.00
37	HLA-A0201	PANEGEGEQSSMKC	ANEGSSMKC	1	0	0	4	4	ANEGEGEQSSMKC	tr_C9ZMD3	0.012	43950.89	99.00
39	HLA-A0201	NEGEGEQSSMKC	NEGEGSMKC	0	0	0	5	3	NEGEGEQSSMKC	tr_C9ZMD3	0.012	44047.54	99.00
37	HLA-A0201	PANEGEGEQSSMK	ANEGEGEMK	1	0	0	7	3	ANEGEGEQSSMK	tr_C9ZMD3	0.011	44306.59	99.00
38	HLA-A0201	ANEGEGEQSS	ANGEGEQSS	0	0	0	2	1	ANEGEGEQSS	tr_C9ZMD3	0.011	44541.63	99.00
0	HLA-A0201	VPTTNREQVN	VPTTNREQV	0	0	0	0	0	VPTTNREQV	tr_C9ZMD3	0.011	44568.64	99.00
40	HLA-A0201	EGEGEQSSMKC	GEGESSMKC	1	0	0	4	1	GEGEQSSMKC	tr_C9ZMD3	0.011	44601.91	99.00
36	HLA-A0201	LPANEGEGEQS	LPEGEGEQS	0	0	0	2	2	LPANEGEGEQS	tr_C9ZMD3	0.010	44716.45	99.00
0	HLA-A0201	VPTTNREQVNK	VPTTNREQV	0	0	0	0	0	VPTTNREQV	tr_C9ZMD3	0.010	44782.77	99.00
39	HLA-A0201	NEGEGEQSSMK	NEGEGEQSM	0	0	0	7	1	NEGEGEQSSM	tr_C9ZMD3	0.010	44893.87	99.00
37	HLA-A0201	PANEGEGEQS	ANEGEGEQS	1	0	0	0	0	ANEGEGEQS	tr_C9ZMD3	0.010	44903.60	99.00
37	HLA-A0201	PANEGEGEQ	PANEGEGEQ	0	0	0	0	0	PANEGEGEQ	tr_C9ZMD3	0.010	45030.09	99.00
1	HLA-A0201	PTTNREQVNK	TTNREQVNK	1	0	0	0	0	TTNREQVNK	tr_C9ZMD3	0.010	45079.32	99.00
36	HLA-A0201	LPANEGEGEQSS	LEGEGEQSS	0	0	0	1	3	LPANEGEGEQSS	tr_C9ZMD3	0.009	45255.75	99.00
40	HLA-A0201	EGEGEQSSMK	GEGEQSSMK	1	0	0	0	0	GEGEQSSMK	tr_C9ZMD3	0.009	45265.06	99.00
26	HLA-A0201	EDEGLKGKN	EDEGLKGKN	0	0	0	0	0	EDEGLKGKN	tr_C9ZMD3	0.008	45869.01	99.00
37	HLA-A0201	PANEGEGEQSS	ANEGEGESS	1	0	0	7	1	ANEGEGEQSS	tr_C9ZMD3	0.008	45917.66	99.00
1	HLA-A0201	PTTNREQVN	PTTNREQVN	0	0	0	0	0	PTTNREQVN	tr_C9ZMD3	0.007	46189.21	99.00

Protein tr_C9ZMD3. Allele HLA-A0201. Number of high binders 0. Number of weak binders 0. Number of peptides 297

Link to Allele Frequencies in Worldwide Populations HLA-A0201

Rank Threshold for Strong binding peptides   0.500
Rank Threshold for Weak binding peptides   2.000
----

pos	HLA	peptide	Core	Offset	I_pos	I_len	D_pos	D_len	iCore	Identity	1-log50k(aff)	Affinity(nM)	%Rank	BindLevel
6	HLA-A0201	GLPDLSQWV	GLPDLSQWV	0	0	0	0	0	GLPDLSQWV	tr_D0A7J0	0.724	19.76	0.30	<= SB
17	HLA-A0201	FWSDVAFSV	FWSDVAFSV	0	0	0	0	0	FWSDVAFSV	tr_D0A7J0	0.617	63.35	0.80	<= WB
5	HLA-A0201	EGLPDLSQWV	GLPDLSQWV	1	0	0	0	0	GLPDLSQWV	tr_D0A7J0	0.474	297.66	2.00	<= WB
23	HLA-A0201	FSVGGVPLRV	FVGGVPLRV	0	0	0	1	1	FSVGGVPLRV	tr_D0A7J0	0.434	457.17	3.00	
16	HLA-A0201	GFWSDVAFSV	GFWDVAFSV	0	0	0	3	1	GFWSDVAFSV	tr_D0A7J0	0.424	508.04	3.00	
24	HLA-A0201	SVGGVPLRV	SVGGVPLRV	0	0	0	0	0	SVGGVPLRV	tr_D0A7J0	0.362	994.76	4.00	
6	HLA-A0201	GLPDLSQWVT	GLPDLSQWV	0	0	0	0	0	GLPDLSQWV	tr_D0A7J0	0.352	1113.78	4.50	
3	HLA-A0201	IREGLPDLSQWV	ILPDLSQWV	0	0	0	1	3	IREGLPDLSQWV	tr_D0A7J0	0.338	1291.72	4.50	
13	HLA-A0201	WVTGFWSDVAFSV	WVSDVAFSV	0	0	0	2	4	WVTGFWSDVAFSV	tr_D0A7J0	0.323	1522.18	5.00	
2	HLA-A0201	SIREGLPDLSQWV	SLPDLSQWV	0	0	0	1	4	SIREGLPDLSQWV	tr_D0A7J0	0.313	1687.66	5.50	
17	HLA-A0201	FWSDVAFSVG	FWSDVAFSV	0	0	0	0	0	FWSDVAFSV	tr_D0A7J0	0.306	1833.02	5.50	
13	HLA-A0201	WVTGFWSDV	WVTGFWSDV	0	0	0	0	0	WVTGFWSDV	tr_D0A7J0	0.300	1954.29	6.00	
1	HLA-A0201	ASIREGLPDLSQWV	ALPDLSQWV	0	0	0	1	5	ASIREGLPDLSQWV	tr_D0A7J0	0.299	1967.62	6.00	
4	HLA-A0201	REGLPDLSQWV	RLPDLSQWV	0	0	0	1	2	REGLPDLSQWV	tr_D0A7J0	0.292	2117.93	6.00	
12	HLA-A0201	QWVTGFWSDVAFSV	WVSDVAFSV	1	0	0	2	4	WVTGFWSDVAFSV	tr_D0A7J0	0.285	2277.91	6.50	
2	HLA-A0201	SIREGLPDL	SIREGLPDL	0	0	0	0	0	SIREGLPDL	tr_D0A7J0	0.267	2789.37	7.00	
11	HLA-A0201	SQWVTGFWSDV	SQWVTGFWV	0	0	0	8	2	SQWVTGFWSDV	tr_D0A7J0	0.257	3108.04	7.50	
14	HLA-A0201	VTGFWSDVAFSV	VTSDVAFSV	0	0	0	2	3	VTGFWSDVAFSV	tr_D0A7J0	0.249	3391.80	8.00	
15	HLA-A0201	TGFWSDVAFSV	GFWDVAFSV	1	0	0	3	1	GFWSDVAFSV	tr_D0A7J0	0.246	3504.27	8.00	
17	HLA-A0201	FWSDVAFSVGGV	FWSDVAFSV	0	0	0	8	3	FWSDVAFSVGGV	tr_D0A7J0	0.232	4045.49	8.50	
10	HLA-A0201	LSQWVTGFWSDV	SQWVTGFWV	1	0	0	8	2	SQWVTGFWSDV	tr_D0A7J0	0.229	4190.69	8.50	
9	HLA-A0201	DLSQWVTGFWSDV	SQWVTGFWV	2	0	0	8	2	SQWVTGFWSDV	tr_D0A7J0	0.224	4427.62	9.00	
22	HLA-A0201	AFSVGGVPLRV	FVGGVPLRV	1	0	0	1	1	FSVGGVPLRV	tr_D0A7J0	0.223	4478.31	9.00	
8	HLA-A0201	PDLSQWVTGFWSDV	SQWVTGFWV	3	0	0	8	2	SQWVTGFWSDV	tr_D0A7J0	0.221	4595.57	9.00	
21	HLA-A0201	VAFSVGGVPL	VAFSVGVPL	0	0	0	5	1	VAFSVGGVPL	tr_D0A7J0	0.216	4819.85	9.50	
21	HLA-A0201	VAFSVGGVPLRV	FVGGVPLRV	2	0	0	1	1	FSVGGVPLRV	tr_D0A7J0	0.210	5139.96	10.00	
16	HLA-A0201	GFWSDVAFSVGGV	FWSDVAFSV	1	0	0	8	3	FWSDVAFSVGGV	tr_D0A7J0	0.207	5346.32	10.00	
20	HLA-A0201	DVAFSVGGVPLRV	FVGGVPLRV	3	0	0	1	1	FSVGGVPLRV	tr_D0A7J0	0.206	5366.89	10.00	
19	HLA-A0201	SDVAFSVGGVPLRV	FVGGVPLRV	4	0	0	1	1	FSVGGVPLRV	tr_D0A7J0	0.204	5503.26	10.00	
13	HLA-A0201	WVTGFWSDVAFSVG	WVSDVAFSV	0	0	0	2	4	WVTGFWSDVAFSV	tr_D0A7J0	0.203	5563.85	11.00	
3	HLA-A0201	IREGLPDLSQWVT	ILPDLSQWV	0	0	0	1	3	IREGLPDLSQWV	tr_D0A7J0	0.196	5980.03	11.00	
15	HLA-A0201	TGFWSDVAFSVGGV	FWSDVAFSV	2	0	0	8	3	FWSDVAFSVGGV	tr_D0A7J0	0.196	5998.11	11.00	
11	HLA-A0201	SQWVTGFWS	SQWVTGFWS	0	0	0	0	0	SQWVTGFWS	tr_D0A7J0	0.195	6093.54	11.00	
17	HLA-A0201	FWSDVAFSVGGVPL	FWSDVAFPL	0	0	0	7	5	FWSDVAFSVGGVPL	tr_D0A7J0	0.190	6376.57	11.00	
11	HLA-A0201	SQWVTGFWSDVA	SQWVTGFWV	0	0	0	8	2	SQWVTGFWSDV	tr_D0A7J0	0.178	7265.19	12.00	
16	HLA-A0201	GFWSDVAFSVG	GFWDVAFSV	0	0	0	3	1	GFWSDVAFSV	tr_D0A7J0	0.178	7284.48	12.00	
2	HLA-A0201	SIREGLPDLSQWVT	SLPDLSQWV	0	0	0	1	4	SIREGLPDLSQWV	tr_D0A7J0	0.178	7302.15	12.00	
4	HLA-A0201	REGLPDLSQWVT	RLPDLSQWV	0	0	0	1	2	REGLPDLSQWV	tr_D0A7J0	0.166	8278.01	13.00	
3	HLA-A0201	IREGLPDLSQWVTG	ILPDLSQWV	0	0	0	1	3	IREGLPDLSQWV	tr_D0A7J0	0.165	8428.95	14.00	
12	HLA-A0201	QWVTGFWSDV	WVTGFWSDV	1	0	0	0	0	WVTGFWSDV	tr_D0A7J0	0.164	8484.03	14.00	
15	HLA-A0201	TGFWSDVAFSVG	GFWDVAFSV	1	0	0	3	1	GFWSDVAFSV	tr_D0A7J0	0.163	8544.74	14.00	
14	HLA-A0201	VTGFWSDVAFSVG	GFWDVAFSV	2	0	0	3	1	GFWSDVAFSV	tr_D0A7J0	0.162	8682.48	14.00	
10	HLA-A0201	LSQWVTGFWSDVA	SQWVTGFWV	1	0	0	8	2	SQWVTGFWSDV	tr_D0A7J0	0.160	8819.01	14.00	
9	HLA-A0201	DLSQWVTGFWSDVA	SQWVTGFWV	2	0	0	8	2	SQWVTGFWSDV	tr_D0A7J0	0.158	9079.46	14.00	
11	HLA-A0201	SQWVTGFWSDVAF	SQWVTGFWV	0	0	0	8	2	SQWVTGFWSDV	tr_D0A7J0	0.157	9119.53	14.00	
16	HLA-A0201	GFWSDVAFSVGG	GFWDVAFSV	0	0	0	3	1	GFWSDVAFSV	tr_D0A7J0	0.156	9232.91	14.00	
11	HLA-A0201	SQWVTGFWSDVAFS	SQWVTGFWV	0	0	0	8	2	SQWVTGFWSDV	tr_D0A7J0	0.152	9639.29	15.00	
6	HLA-A0201	GLPDLSQWVTGFWS	GLWVTGFWS	0	0	0	2	5	GLPDLSQWVTGFWS	tr_D0A7J0	0.149	9994.98	15.00	

19	HLA-A0201	SDVAFSVGGV	SVAFSVGGV	0	0	0	1	1	SDVAFSVGGV	tr_D0A7J0	0.147	10144.35	15.00
17	HLA-A0201	FWSDVAFSVGGVP	FWSDVAFSV	0	0	0	8	3	FWSDVAFSVGGV	tr_D0A7J0	0.147	10176.23	15.00
5	HLA-A0201	EGLPDLSQWVT	GLPDLSQWV	1	0	0	0	0	GLPDLSQWV	tr_D0A7J0	0.147	10203.46	16.00
17	HLA-A0201	FWSDVAFSVGG	FWSDVAFSV	0	0	0	0	0	FWSDVAFSV	tr_D0A7J0	0.145	10371.65	16.00
16	HLA-A0201	GFWSDVAFSVGGVP	GFWDVAFSV	0	0	0	3	1	GFWSDVAFSV	tr_D0A7J0	0.145	10399.29	16.00
15	HLA-A0201	TGFWSDVAFSVGG	GFWDVAFSV	1	0	0	3	1	GFWSDVAFSV	tr_D0A7J0	0.144	10523.46	16.00
1	HLA-A0201	ASIREGLPDL	ASIEGLPDL	0	0	0	3	1	ASIREGLPDL	tr_D0A7J0	0.143	10613.91	16.00
14	HLA-A0201	VTGFWSDVAFSVGG	GFWDVAFSV	2	0	0	3	1	GFWSDVAFSV	tr_D0A7J0	0.143	10679.57	16.00
10	HLA-A0201	LSQWVTGFWSDVAF	SQWVTGFWV	1	0	0	8	2	SQWVTGFWSDV	tr_D0A7J0	0.143	10680.84	16.00
6	HLA-A0201	GLPDLSQWVTG	GLPDLSQWV	0	0	0	0	0	GLPDLSQWV	tr_D0A7J0	0.142	10766.70	16.00
10	HLA-A0201	LSQWVTGFWS	LQWVTGFWS	0	0	0	1	1	LSQWVTGFWS	tr_D0A7J0	0.140	11041.94	16.00
4	HLA-A0201	REGLPDLSQWVTG	RLPDLSQWV	0	0	0	1	2	REGLPDLSQWV	tr_D0A7J0	0.137	11309.52	17.00
4	HLA-A0201	REGLPDLSQWVTGF	RLPDLSQWV	0	0	0	1	2	REGLPDLSQWV	tr_D0A7J0	0.130	12231.88	18.00
6	HLA-A0201	GLPDLSQWVTGF	GLPDLSQWV	0	0	0	0	0	GLPDLSQWV	tr_D0A7J0	0.130	12268.06	18.00
6	HLA-A0201	GLPDLSQWVTGFW	GLPDLSQWV	0	0	0	0	0	GLPDLSQWV	tr_D0A7J0	0.127	12668.55	18.00
16	HLA-A0201	GFWSDVAFS	GFWSDVAFS	0	0	0	0	0	GFWSDVAFS	tr_D0A7J0	0.121	13532.60	19.00
5	HLA-A0201	EGLPDLSQWVTG	GLPDLSQWV	1	0	0	0	0	GLPDLSQWV	tr_D0A7J0	0.120	13698.33	19.00
20	HLA-A0201	DVAFSVGGVPL	VAFSVGVPL	1	0	0	5	1	VAFSVGGVPL	tr_D0A7J0	0.118	13931.81	20.00
19	HLA-A0201	SDVAFSVGGVPL	VAFSVGVPL	2	0	0	5	1	VAFSVGGVPL	tr_D0A7J0	0.114	14616.48	20.00
18	HLA-A0201	WSDVAFSVGGVPL	VAFSVGVPL	3	0	0	5	1	VAFSVGGVPL	tr_D0A7J0	0.113	14776.60	21.00
5	HLA-A0201	EGLPDLSQWVTGF	GLPDLSQWV	1	0	0	0	0	GLPDLSQWV	tr_D0A7J0	0.112	14833.63	21.00
13	HLA-A0201	WVTGFWSDVA	WVTGFWSDV	0	0	0	0	0	WVTGFWSDV	tr_D0A7J0	0.112	14932.99	21.00
11	HLA-A0201	SQWVTGFWSD	SQWVTGFWS	0	0	0	0	0	SQWVTGFWS	tr_D0A7J0	0.110	15149.26	21.00
5	HLA-A0201	EGLPDLSQWVTGFW	GLPDLSQWV	1	0	0	0	0	GLPDLSQWV	tr_D0A7J0	0.110	15284.77	21.00
22	HLA-A0201	AFSVGGVPL	AFSVGGVPL	0	0	0	0	0	AFSVGGVPL	tr_D0A7J0	0.107	15748.78	22.00
14	HLA-A0201	VTGFWSDVA	VTGFWSDVA	0	0	0	0	0	VTGFWSDVA	tr_D0A7J0	0.105	16070.32	22.00
7	HLA-A0201	LPDLSQWVTGF	LLSQWVTGF	0	0	0	1	2	LPDLSQWVTGF	tr_D0A7J0	0.101	16684.24	23.00
14	HLA-A0201	VTGFWSDVAF	VTFWSDVAF	0	0	0	2	1	VTGFWSDVAF	tr_D0A7J0	0.101	16818.19	23.00
9	HLA-A0201	DLSQWVTGF	DLSQWVTGF	0	0	0	0	0	DLSQWVTGF	tr_D0A7J0	0.100	17038.15	23.00
9	HLA-A0201	DLSQWVTGFWS	DLWVTGFWS	0	0	0	2	2	DLSQWVTGFWS	tr_D0A7J0	0.098	17385.08	24.00
7	HLA-A0201	LPDLSQWVTGFWS	LQWVTGFWS	0	0	0	1	4	LPDLSQWVTGFWS	tr_D0A7J0	0.090	18879.58	26.00
21	HLA-A0201	VAFSVGGVPLR	VAFSVGVPL	0	0	0	5	1	VAFSVGGVPL	tr_D0A7J0	0.090	18885.91	26.00
13	HLA-A0201	WVTGFWSDVAFS	WVWSDVAFS	0	0	0	2	3	WVTGFWSDVAFS	tr_D0A7J0	0.089	19014.88	26.00
8	HLA-A0201	PDLSQWVTGFWS	DLWVTGFWS	1	0	0	2	2	DLSQWVTGFWS	tr_D0A7J0	0.089	19091.97	26.00
13	HLA-A0201	WVTGFWSDVAF	WVFWSDVAF	0	0	0	2	2	WVTGFWSDVAF	tr_D0A7J0	0.088	19194.50	26.00
9	HLA-A0201	DLSQWVTGFW	DLSQWVTFW	0	0	0	7	1	DLSQWVTGFW	tr_D0A7J0	0.088	19379.19	27.00
20	HLA-A0201	DVAFSVGGVPLR	VAFSVGVPL	1	0	0	5	1	VAFSVGGVPL	tr_D0A7J0	0.084	20129.97	28.00
20	HLA-A0201	DVAFSVGGV	DVAFSVGGV	0	0	0	0	0	DVAFSVGGV	tr_D0A7J0	0.083	20325.84	28.00
2	HLA-A0201	SIREGLPDLS	SIREGLPDL	0	0	0	0	0	SIREGLPDL	tr_D0A7J0	0.082	20655.74	28.00
12	HLA-A0201	QWVTGFWSDVAF	WVTGFWVAF	1	0	0	6	2	WVTGFWSDVAF	tr_D0A7J0	0.082	20655.96	28.00
18	HLA-A0201	WSDVAFSVGGVPLR	VAFSVGVPL	3	0	0	5	1	VAFSVGGVPL	tr_D0A7J0	0.082	20674.52	28.00
19	HLA-A0201	SDVAFSVGGVPLR	VAFSVGVPL	2	0	0	5	1	VAFSVGGVPL	tr_D0A7J0	0.082	20692.65	28.00
18	HLA-A0201	WSDVAFSVGGV	WVAFSVGGV	0	0	0	1	2	WSDVAFSVGGV	tr_D0A7J0	0.081	20843.64	29.00
12	HLA-A0201	QWVTGFWSDVAFS	WVWSDVAFS	1	0	0	2	3	WVTGFWSDVAFS	tr_D0A7J0	0.080	20996.88	29.00
10	HLA-A0201	LSQWVTGFW	LSQWVTGFW	0	0	0	0	0	LSQWVTGFW	tr_D0A7J0	0.080	21038.73	29.00
23	HLA-A0201	FSVGGVPLR	FSVGGVPLR	0	0	0	0	0	FSVGGVPLR	tr_D0A7J0	0.080	21125.63	29.00
9	HLA-A0201	DLSQWVTGFWSD	LQWVTGFWS	1	0	0	1	1	LSQWVTGFWS	tr_D0A7J0	0.075	22095.60	31.00
7	HLA-A0201	LPDLSQWVTGFWSD	LLSQWVTGF	0	0	0	1	2	LPDLSQWVTGF	tr_D0A7J0	0.075	22121.19	31.00
7	HLA-A0201	LPDLSQWVTGFW	LLSQWVTGF	0	0	0	1	2	LPDLSQWVTGF	tr_D0A7J0	0.074	22343.46	31.00
15	HLA-A0201	TGFWSDVAFS	GFWSDVAFS	1	0	0	0	0	GFWSDVAFS	tr_D0A7J0	0.074	22535.75	31.00
15	HLA-A0201	TGFWSDVAF	TGFWSDVAF	0	0	0	0	0	TGFWSDVAF	tr_D0A7J0	0.072	22827.79	32.00
10	HLA-A0201	LSQWVTGFWSD	LQWVTGFWS	0	0	0	1	1	LSQWVTGFWS	tr_D0A7J0	0.072	23045.44	32.00
8	HLA-A0201	PDLSQWVTGFWSD	SQWVTFWSD	3	0	0	5	1	SQWVTGFWSD	tr_D0A7J0	0.071	23118.11	32.00
8	HLA-A0201	PDLSQWVTGF	DLSQWVTGF	1	0	0	0	0	DLSQWVTGF	tr_D0A7J0	0.071	23189.01	32.00

14	HLA-A0201	VTGFWSDVAFS	VTWSDVAFS	0	0	0	2	2	VTGFWSDVAFS	tr_D0A7J0	0.064	25040.33	36.00
0	HLA-A0201	GASIREGLPDL	SIREGLPDL	2	0	0	0	0	SIREGLPDL	tr_D0A7J0	0.062	25569.28	37.00
8	HLA-A0201	PDLSQWVTGFW	DLSQWVTFW	1	0	0	7	1	DLSQWVTGFW	tr_D0A7J0	0.060	26148.13	38.00
12	HLA-A0201	QWVTGFWSDVA	QWVTGFWSV	0	0	0	8	1	QWVTGFWSDV	tr_D0A7J0	0.060	26229.46	38.00
22	HLA-A0201	AFSVGGVPLR	AFSVGGVPL	0	0	0	0	0	AFSVGGVPL	tr_D0A7J0	0.056	27338.34	40.00
18	HLA-A0201	WSDVAFSVGGVP	WVAFSVGGV	0	0	0	1	2	WSDVAFSVGGV	tr_D0A7J0	0.055	27578.69	41.00
7	HLA-A0201	LPDLSQWVT	LPDLSQWVT	0	0	0	0	0	LPDLSQWVT	tr_D0A7J0	0.051	28891.47	44.00
18	HLA-A0201	WSDVAFSVG	WSDVAFSVG	0	0	0	0	0	WSDVAFSVG	tr_D0A7J0	0.049	29284.25	45.00
21	HLA-A0201	VAFSVGGVP	VAFSVGGVP	0	0	0	0	0	VAFSVGGVP	tr_D0A7J0	0.049	29309.30	45.00
18	HLA-A0201	WSDVAFSVGG	WSVAFSVGG	0	0	0	2	1	WSDVAFSVGG	tr_D0A7J0	0.048	29779.84	46.00
19	HLA-A0201	SDVAFSVGGVP	SVAFSVGGV	0	0	0	1	1	SDVAFSVGGV	tr_D0A7J0	0.046	30545.41	48.00
1	HLA-A0201	ASIREGLPDLS	ASIEGLPDL	0	0	0	3	1	ASIREGLPDL	tr_D0A7J0	0.042	31690.35	55.00
2	HLA-A0201	SIREGLPDLSQ	SIREGLPDL	0	0	0	0	0	SIREGLPDL	tr_D0A7J0	0.042	31869.17	55.00
19	HLA-A0201	SDVAFSVGG	SDVAFSVGG	0	0	0	0	0	SDVAFSVGG	tr_D0A7J0	0.040	32330.04	55.00
0	HLA-A0201	GASIREGLPDLS	SIREGLPDL	2	0	0	0	0	SIREGLPDL	tr_D0A7J0	0.040	32525.47	55.00
1	HLA-A0201	ASIREGLPDLSQ	SIREGLLSQ	1	0	0	6	2	SIREGLPDLSQ	tr_D0A7J0	0.039	32816.40	55.00
2	HLA-A0201	SIREGLPDLSQW	SIREGLPDL	0	0	0	0	0	SIREGLPDL	tr_D0A7J0	0.039	32831.66	55.00
0	HLA-A0201	GASIREGLPDLSQ	SIREGLLSQ	2	0	0	6	2	SIREGLPDLSQ	tr_D0A7J0	0.038	33205.34	60.00
1	HLA-A0201	ASIREGLPDLSQW	ASIEGLPDL	0	0	0	3	1	ASIREGLPDL	tr_D0A7J0	0.038	33271.88	60.00
20	HLA-A0201	DVAFSVGGVP	DVAFSVGGV	0	0	0	0	0	DVAFSVGGV	tr_D0A7J0	0.037	33380.05	60.00
0	HLA-A0201	GASIREGLPDLSQW	ASIEGLPDL	1	0	0	3	1	ASIREGLPDL	tr_D0A7J0	0.037	33646.56	60.00
7	HLA-A0201	LPDLSQWVTG	LPDLSWVTG	0	0	0	5	1	LPDLSQWVTG	tr_D0A7J0	0.036	33892.46	60.00
12	HLA-A0201	QWVTGFWSD	QWVTGFWSD	0	0	0	0	0	QWVTGFWSD	tr_D0A7J0	0.035	34347.28	60.00
4	HLA-A0201	REGLPDLSQW	REGLPDLSW	0	0	0	8	1	REGLPDLSQW	tr_D0A7J0	0.034	34636.50	65.00
5	HLA-A0201	EGLPDLSQW	EGLPDLSQW	0	0	0	0	0	EGLPDLSQW	tr_D0A7J0	0.031	35664.43	65.00
3	HLA-A0201	IREGLPDLSQW	IREGLLSQW	0	0	0	5	2	IREGLPDLSQW	tr_D0A7J0	0.031	35914.98	65.00
3	HLA-A0201	IREGLPDLS	IREGLPDLS	0	0	0	0	0	IREGLPDLS	tr_D0A7J0	0.030	35954.63	70.00
3	HLA-A0201	IREGLPDLSQ	IREGLPLSQ	0	0	0	6	1	IREGLPDLSQ	tr_D0A7J0	0.029	36367.00	70.00
4	HLA-A0201	REGLPDLSQ	REGLPDLSQ	0	0	0	0	0	REGLPDLSQ	tr_D0A7J0	0.029	36387.48	70.00
1	HLA-A0201	ASIREGLPD	ASIREGLPD	0	0	0	0	0	ASIREGLPD	tr_D0A7J0	0.024	38501.76	80.00
0	HLA-A0201	GASIREGLP	GASIREGLP	0	0	0	0	0	GASIREGLP	tr_D0A7J0	0.022	39581.38	80.00
0	HLA-A0201	GASIREGLPD	GASIRGLPD	0	0	0	5	1	GASIREGLPD	tr_D0A7J0	0.021	39824.53	85.00
8	HLA-A0201	PDLSQWVTG	PDLSQWVTG	0	0	0	0	0	PDLSQWVTG	tr_D0A7J0	0.017	41660.24	90.00

Protein tr_D0A7J0. Allele HLA-A0201. Number of high binders 1. Number of weak binders 2. Number of peptides 135

Link to Allele Frequencies in Worldwide Populations HLA-A0201

Rank Threshold for Strong binding peptides 0.500

Rank Threshold for Weak binding peptides 2.000

pos	HLA	peptide	Core	Offset	I_pos	I_len	D_pos	D_len	iCore	Identity	1-log50k(aff)	Affinity(nM)	%Rank	BindLevel
6	HLA-A0201	FFWRWLKYV	FFWRWLKYV	0	0	0	0	0	FFWRWLKYV	tr_D0A3G8	0.547	134.31	1.30	<= WB
23	HLA-A0201	GLVVNEFDGLKFV	GLFDGLKFV	0	0	0	2	4	GLVVNEFDGLKFV	tr_D0A3G8	0.542	142.03	1.30	<= WB
52	HLA-A0201	RLQGYFPEEL	RLQGYFPEL	0	0	0	7	1	RLQGYFPEEL	tr_D0A3G8	0.542	142.62	1.30	<= WB
31	HLA-A0201	GLKFVCEPKV	GLFVCEPKV	0	0	0	2	1	GLKFVCEPKV	tr_D0A3G8	0.536	152.12	1.40	<= WB
3	HLA-A0201	SLPFFWRWLKYV	SLWRWLKYV	0	0	0	2	3	SLPFFWRWLKYV	tr_D0A3G8	0.524	171.69	1.50	<= WB
22	HLA-A0201	AGLVVNEFDGLKFV	GLFDGLKFV	1	0	0	2	4	GLVVNEFDGLKFV	tr_D0A3G8	0.490	249.02	1.90	<= WB
13	HLA-A0201	YVSFVRYGL	YVSFVRYGL	0	0	0	0	0	YVSFVRYGL	tr_D0A3G8	0.488	253.85	1.90	<= WB
2	HLA-A0201	QSLPFFWRWLKYV	SLWRWLKYV	1	0	0	2	3	SLPFFWRWLKYV	tr_D0A3G8	0.484	265.28	1.90	<= WB
1	HLA-A0201	TQSLPFFWRWLKYV	SLWRWLKYV	2	0	0	2	3	SLPFFWRWLKYV	tr_D0A3G8	0.470	310.17	2.50	
16	HLA-A0201	FVRYGLAGLV	FVYGLAGLV	0	0	0	2	1	FVRYGLAGLV	tr_D0A3G8	0.447	395.25	2.50	

3	HLA-A0201	SLPFFWRWL	SLPFFWRWL	0	0	0	0	0	SLPFFWRWL	tr_D0A3G8	0.385	778.37	3.50
16	HLA-A0201	FVRYGLAGL	FVRYGLAGL	0	0	0	0	0	FVRYGLAGL	tr_D0A3G8	0.383	788.94	3.50
3	HLA-A0201	SLPFFWRWLKYVS	SLWRWLKYV	0	0	0	2	3	SLPFFWRWLKYV	tr_D0A3G8	0.373	885.63	4.00
23	HLA-A0201	GLVVNEFDGLKFVC	GLFDGLKFV	0	0	0	2	4	GLVVNEFDGLKFV	tr_D0A3G8	0.370	914.08	4.00
42	HLA-A0201	FCFPDGTTYA	FCFDGTTYA	0	0	0	3	1	FCFPDGTTYA	tr_D0A3G8	0.355	1078.88	4.50
5	HLA-A0201	PFFWRWLKYV	FFWRWLKYV	1	0	0	0	0	FFWRWLKYV	tr_D0A3G8	0.354	1080.42	4.50
2	HLA-A0201	QSLPFFWRWLKYVS	SLWRWLKYV	1	0	0	2	3	SLPFFWRWLKYV	tr_D0A3G8	0.336	1318.59	5.00
23	HLA-A0201	GLVVNEFDGL	GLVVNEFGL	0	0	0	7	1	GLVVNEFDGL	tr_D0A3G8	0.331	1396.12	5.00
20	HLA-A0201	GLAGLVVNEFDGL	GLAGLVVGL	0	0	0	7	4	GLAGLVVNEFDGL	tr_D0A3G8	0.328	1441.82	5.00
3	HLA-A0201	SLPFFWRWLKYVSF	SLWRWLKYV	0	0	0	2	3	SLPFFWRWLKYV	tr_D0A3G8	0.322	1539.36	5.00
9	HLA-A0201	RWLKYVSFV	RWLKYVSFV	0	0	0	0	0	RWLKYVSFV	tr_D0A3G8	0.311	1726.22	5.50
6	HLA-A0201	FFWRWLKYVSFV	FFWRWLSFV	0	0	0	6	3	FFWRWLKYVSFV	tr_D0A3G8	0.305	1839.14	5.50
51	HLA-A0201	ARLQGYFPEEL	RLQGYFPEL	1	0	0	7	1	RLQGYFPEEL	tr_D0A3G8	0.304	1855.89	5.50
30	HLA-A0201	DGLKFVCEPKV	GLFVCEPKV	1	0	0	2	1	GLKFVCEPKV	tr_D0A3G8	0.302	1906.67	6.00
53	HLA-A0201	LQGYFPEEL	LQGYFPEEL	0	0	0	0	0	LQGYFPEEL	tr_D0A3G8	0.294	2073.27	6.00
19	HLA-A0201	YGLAGLVVNEFDGL	GLAGLVVGL	1	0	0	7	4	GLAGLVVNEFDGL	tr_D0A3G8	0.292	2118.37	6.00
50	HLA-A0201	YARLQGYFPEEL	RLQGYFPEL	2	0	0	7	1	RLQGYFPEEL	tr_D0A3G8	0.289	2201.51	6.00
29	HLA-A0201	FDGLKFVCEPKV	GLFVCEPKV	2	0	0	2	1	GLKFVCEPKV	tr_D0A3G8	0.286	2271.24	6.50
6	HLA-A0201	FFWRWLKYVS	FFWRWLKYV	0	0	0	0	0	FFWRWLKYV	tr_D0A3G8	0.283	2335.96	6.50
2	HLA-A0201	QSLPFFWRWL	SLPFFWRWL	1	0	0	0	0	SLPFFWRWL	tr_D0A3G8	0.283	2339.71	6.50
49	HLA-A0201	TYARLQGYFPEEL	RLQGYFPEL	3	0	0	7	1	RLQGYFPEEL	tr_D0A3G8	0.283	2340.69	6.50
28	HLA-A0201	EFDGLKFVCEPKV	GLFVCEPKV	3	0	0	2	1	GLKFVCEPKV	tr_D0A3G8	0.280	2410.81	6.50
48	HLA-A0201	TTYARLQGYFPEEL	RLQGYFPEL	4	0	0	7	1	RLQGYFPEEL	tr_D0A3G8	0.280	2427.12	6.50
27	HLA-A0201	NEFDGLKFVCEPKV	GLFVCEPKV	4	0	0	2	1	GLKFVCEPKV	tr_D0A3G8	0.277	2497.20	6.50
24	HLA-A0201	LVVNEFDGLKFV	LVFDGLKFV	0	0	0	2	3	LVVNEFDGLKFV	tr_D0A3G8	0.274	2565.51	6.50
5	HLA-A0201	PFFWRWLKYVSFV	FFWRWLSFV	1	0	0	6	3	FFWRWLKYVSFV	tr_D0A3G8	0.263	2892.11	7.00
13	HLA-A0201	YVSFVRYGLAGLV	YVYGLAGLV	0	0	0	2	4	YVSFVRYGLAGLV	tr_D0A3G8	0.254	3213.25	7.50
15	HLA-A0201	SFVRYGLAGLV	FVYGLAGLV	1	0	0	2	1	FVRYGLAGLV	tr_D0A3G8	0.253	3245.01	7.50
20	HLA-A0201	GLAGLVVNEF	GLAGLVVEF	0	0	0	7	1	GLAGLVVNEF	tr_D0A3G8	0.251	3320.41	7.50
12	HLA-A0201	KYVSFVRYGL	YVSFVRYGL	1	0	0	0	0	YVSFVRYGL	tr_D0A3G8	0.250	3335.75	7.50
13	HLA-A0201	YVSFVRYGLAGL	YVSFVLAGL	0	0	0	5	3	YVSFVRYGLAGL	tr_D0A3G8	0.249	3363.73	7.50
4	HLA-A0201	LPFFWRWLKYVSFV	FFWRWLSFV	2	0	0	6	3	FFWRWLKYVSFV	tr_D0A3G8	0.249	3368.36	7.50
25	HLA-A0201	VVNEFDGLKFV	VVFDGLKFV	0	0	0	2	2	VVNEFDGLKFV	tr_D0A3G8	0.242	3634.68	8.00
14	HLA-A0201	VSFVRYGLAGLV	FVYGLAGLV	2	0	0	2	1	FVRYGLAGLV	tr_D0A3G8	0.239	3764.60	8.00
12	HLA-A0201	KYVSFVRYGLAGLV	FVYGLAGLV	4	0	0	2	1	FVRYGLAGLV	tr_D0A3G8	0.238	3816.19	8.50
12	HLA-A0201	KYVSFVRYGLAGL	YVSFVLAGL	1	0	0	5	3	YVSFVRYGLAGL	tr_D0A3G8	0.222	4541.79	9.00
13	HLA-A0201	YVSFVRYGLA	YVSFVRYLA	0	0	0	7	1	YVSFVRYGLA	tr_D0A3G8	0.220	4642.60	9.00
34	HLA-A0201	FVCEPKVPFC	FVCEKVPFC	0	0	0	4	1	FVCEPKVPFC	tr_D0A3G8	0.217	4788.51	9.50
24	HLA-A0201	LVVNEFDGL	LVVNEFDGL	0	0	0	0	0	LVVNEFDGL	tr_D0A3G8	0.215	4879.57	9.50
15	HLA-A0201	SFVRYGLAGL	FVRYGLAGL	1	0	0	0	0	FVRYGLAGL	tr_D0A3G8	0.215	4894.37	9.50
52	HLA-A0201	RLQGYFPEELP	RLQGYFPEL	0	0	0	7	1	RLQGYFPEEL	tr_D0A3G8	0.211	5095.94	10.00
8	HLA-A0201	WRWLKYVSFV	WRWLYVSFV	0	0	0	4	1	WRWLKYVSFV	tr_D0A3G8	0.209	5192.84	10.00
10	HLA-A0201	WLKYVSFVRYGL	WLKYVSFGL	0	0	0	7	3	WLKYVSFVRYGL	tr_D0A3G8	0.208	5241.16	10.00
11	HLA-A0201	LKYVSFVRYGLAGL	YVSFVLAGL	2	0	0	5	3	YVSFVRYGLAGL	tr_D0A3G8	0.208	5259.00	10.00
31	HLA-A0201	GLKFVCEPKVP	GLFVCEPKV	0	0	0	2	1	GLKFVCEPKV	tr_D0A3G8	0.208	5272.96	10.00
3	HLA-A0201	SLPFFWRWLK	SLPFFWRWL	0	0	0	0	0	SLPFFWRWL	tr_D0A3G8	0.208	5290.90	10.00
6	HLA-A0201	FFWRWLKYVSFVR	FFWRWLSFV	0	0	0	6	3	FFWRWLKYVSFV	tr_D0A3G8	0.207	5320.18	10.00
20	HLA-A0201	GLAGLVVNEFDGLK	GLAGLVVGL	0	0	0	7	4	GLAGLVVNEFDGL	tr_D0A3G8	0.207	5328.87	10.00
16	HLA-A0201	FVRYGLAGLVV	FVYGLAGLV	0	0	0	2	1	FVRYGLAGLV	tr_D0A3G8	0.198	5862.79	11.00
4	HLA-A0201	LPFFWRWLKYV	FFWRWLKYV	2	0	0	0	0	FFWRWLKYV	tr_D0A3G8	0.194	6106.35	11.00
13	HLA-A0201	YVSFVRYGLAGLVV	YVSFVRYGV	0	0	0	8	5	YVSFVRYGLAGLVV	tr_D0A3G8	0.192	6241.28	11.00
9	HLA-A0201	RWLKYVSFVRYGL	WLKYVSFGL	1	0	0	7	3	WLKYVSFVRYGL	tr_D0A3G8	0.187	6636.59	12.00
30	HLA-A0201	DGLKFVCEPKVP	GLFVCEPKV	1	0	0	2	1	GLKFVCEPKV	tr_D0A3G8	0.186	6661.12	12.00
7	HLA-A0201	FWRWLKYVSFV	FWLKYVSFV	0	0	0	1	2	FWRWLKYVSFV	tr_D0A3G8	0.186	6708.49	12.00

6	HLA-A0201	FFWRWLKYVSFVRY	FFWRWLSFV	0	0	0	6	3	FFWRWLKYVSFV	tr_D0A3G8	0.181	7020.46	12.00
51	HLA-A0201	ARLQGYFPEELP	RLQGYFPEL	1	0	0	7	1	RLQGYFPEEL	tr_D0A3G8	0.181	7086.93	12.00
5	HLA-A0201	PFFWRWLKYVSFVR	FFWRWLSFV	1	0	0	6	3	FFWRWLKYVSFV	tr_D0A3G8	0.180	7097.60	12.00
8	HLA-A0201	WRWLKYVSFVRYGL	WLKYVSFGL	2	0	0	7	3	WLKYVSFVRYGL	tr_D0A3G8	0.180	7132.47	12.00
19	HLA-A0201	YGLAGLVVNE	YLAGLVVNE	0	0	0	1	1	YGLAGLVVNE	tr_D0A3G8	0.178	7310.29	12.00
29	HLA-A0201	FDGLKFVCEPKVP	GLFVCEPKV	2	0	0	2	1	GLKFVCEPKV	tr_D0A3G8	0.177	7344.46	12.00
15	HLA-A0201	SFVRYGLAGLVV	FVYGLAGLV	1	0	0	2	1	FVRYGLAGLV	tr_D0A3G8	0.175	7509.43	13.00
31	HLA-A0201	GLKFVCEPKVPF	GLFVCEPKV	0	0	0	2	1	GLKFVCEPKV	tr_D0A3G8	0.175	7555.24	13.00
28	HLA-A0201	EFDGLKFVCEPKVP	GLFVCEPKV	3	0	0	2	1	GLKFVCEPKV	tr_D0A3G8	0.174	7589.07	13.00
52	HLA-A0201	RLQGYFPEELPE	RLQGYFPEL	0	0	0	7	1	RLQGYFPEEL	tr_D0A3G8	0.172	7763.65	13.00
50	HLA-A0201	YARLQGYFPEELP	RLQGYFPEL	2	0	0	7	1	RLQGYFPEEL	tr_D0A3G8	0.171	7865.95	13.00
31	HLA-A0201	GLKFVCEPKVPFCF	GLFVCEPKV	0	0	0	2	1	GLKFVCEPKV	tr_D0A3G8	0.169	8009.12	13.00
10	HLA-A0201	WLKYVSFVRYGLA	WLKYVSFLA	0	0	0	7	4	WLKYVSFVRYGLA	tr_D0A3G8	0.169	8027.86	13.00
1	HLA-A0201	TQSLPFFWRWL	SLPFFWRWL	2	0	0	0	0	SLPFFWRWL	tr_D0A3G8	0.168	8077.87	13.00
31	HLA-A0201	GLKFVCEPKVPFC	GLFVCEPKV	0	0	0	2	1	GLKFVCEPKV	tr_D0A3G8	0.168	8128.02	13.00
0	HLA-A0201	GTQSLPFFWRWL	SLPFFWRWL	3	0	0	0	0	SLPFFWRWL	tr_D0A3G8	0.168	8132.06	13.00
49	HLA-A0201	TYARLQGYFPEELP	RLQGYFPEL	3	0	0	7	1	RLQGYFPEEL	tr_D0A3G8	0.168	8160.80	13.00
14	HLA-A0201	VSFVRYGLAGLVV	FVYGLAGLV	2	0	0	2	1	FVRYGLAGLV	tr_D0A3G8	0.167	8237.72	13.00
24	HLA-A0201	LVVNEFDGLKFVC	LVFDGLKFV	0	0	0	2	3	LVVNEFDGLKFV	tr_D0A3G8	0.160	8875.10	14.00
16	HLA-A0201	FVRYGLAGLVVN	FVYGLAGLV	0	0	0	2	1	FVRYGLAGLV	tr_D0A3G8	0.159	8973.01	14.00
52	HLA-A0201	RLQGYFPEELPEY	RLQGYFPEL	0	0	0	7	1	RLQGYFPEEL	tr_D0A3G8	0.158	9062.48	14.00
30	HLA-A0201	DGLKFVCEPKVPF	GLFVCEPKV	1	0	0	2	1	GLKFVCEPKV	tr_D0A3G8	0.158	9078.67	14.00
34	HLA-A0201	FVCEPKVPFCF	FVCEVPFCF	0	0	0	4	2	FVCEPKVPFCF	tr_D0A3G8	0.156	9213.25	14.00
34	HLA-A0201	FVCEPKVPF	FVCEPKVPF	0	0	0	0	0	FVCEPKVPF	tr_D0A3G8	0.156	9231.61	14.00
27	HLA-A0201	NEFDGLKFV	NEFDGLKFV	0	0	0	0	0	NEFDGLKFV	tr_D0A3G8	0.155	9363.39	15.00
9	HLA-A0201	RWLKYVSFVRYGLA	WLKYVSFLA	1	0	0	7	4	WLKYVSFVRYGLA	tr_D0A3G8	0.154	9439.27	15.00
30	HLA-A0201	DGLKFVCEPKVPFC	GLFVCEPKV	1	0	0	2	1	GLKFVCEPKV	tr_D0A3G8	0.154	9479.19	15.00
10	HLA-A0201	WLKYVSFVRY	WLYVSFVRY	0	0	0	2	1	WLKYVSFVRY	tr_D0A3G8	0.153	9536.18	15.00
22	HLA-A0201	AGLVVNEFDGL	GLVVNEFGL	1	0	0	7	1	GLVVNEFDGL	tr_D0A3G8	0.151	9754.71	15.00
29	HLA-A0201	FDGLKFVCEPKVPF	GLFVCEPKV	2	0	0	2	1	GLKFVCEPKV	tr_D0A3G8	0.151	9769.49	15.00
16	HLA-A0201	FVRYGLAGLVVNE	FVYGLAGLV	0	0	0	2	1	FVRYGLAGLV	tr_D0A3G8	0.149	9947.08	15.00
51	HLA-A0201	ARLQGYFPEELPE	RLQGYFPEL	1	0	0	7	1	RLQGYFPEEL	tr_D0A3G8	0.148	10101.09	15.00
16	HLA-A0201	FVRYGLAGLVVNEF	FVYGLAGLV	0	0	0	2	1	FVRYGLAGLV	tr_D0A3G8	0.148	10114.43	15.00
41	HLA-A0201	PFCFPDGTTYA	FCFDGTTYA	1	0	0	3	1	FCFPDGTTYA	tr_D0A3G8	0.148	10129.66	15.00
39	HLA-A0201	KVPFCFPDGTTYA	FCFDGTTYA	3	0	0	3	1	FCFPDGTTYA	tr_D0A3G8	0.146	10291.61	16.00
39	HLA-A0201	KVPFCFPDGT	KVFCFPDGT	0	0	0	2	1	KVPFCFPDGT	tr_D0A3G8	0.145	10430.17	16.00
6	HLA-A0201	FFWRWLKYVSF	FFWRWLKYV	0	0	0	0	0	FFWRWLKYV	tr_D0A3G8	0.143	10694.26	16.00
52	HLA-A0201	RLQGYFPEE	RLQGYFPEE	0	0	0	0	0	RLQGYFPEE	tr_D0A3G8	0.142	10746.80	16.00
33	HLA-A0201	KFVCEPKVPFCF	FVCEVPFCF	1	0	0	4	2	FVCEPKVPFCF	tr_D0A3G8	0.141	10875.82	16.00
15	HLA-A0201	SFVRYGLAGLVVN	FVYGLAGLV	1	0	0	2	1	FVRYGLAGLV	tr_D0A3G8	0.141	10929.74	16.00
21	HLA-A0201	LAGLVVNEFDGL	GLVVNEFGL	2	0	0	7	1	GLVVNEFDGL	tr_D0A3G8	0.140	10940.03	16.00
50	HLA-A0201	YARLQGYFPEELPE	RLQGYFPEL	2	0	0	7	1	RLQGYFPEEL	tr_D0A3G8	0.140	10955.07	16.00
25	HLA-A0201	VVNEFDGLKFVC	VVFDGLKFV	0	0	0	2	2	VVNEFDGLKFV	tr_D0A3G8	0.139	11063.47	16.00
19	HLA-A0201	YGLAGLVVNEF	GLAGLVVEF	1	0	0	7	1	GLAGLVVNEF	tr_D0A3G8	0.138	11271.54	17.00
40	HLA-A0201	VPFCFPDGTTYA	FCFDGTTYA	2	0	0	3	1	FCFPDGTTYA	tr_D0A3G8	0.137	11311.48	17.00
5	HLA-A0201	PFFWRWLKYVS	FFWRWLKYV	1	0	0	0	0	FFWRWLKYV	tr_D0A3G8	0.137	11357.47	17.00
17	HLA-A0201	VRYGLAGLVV	VRYGLALVV	0	0	0	6	1	VRYGLAGLVV	tr_D0A3G8	0.136	11446.66	17.00
42	HLA-A0201	FCFPDGTTYARL	FCFPTYARL	0	0	0	4	3	FCFPDGTTYARL	tr_D0A3G8	0.136	11447.28	17.00
7	HLA-A0201	FWRWLKYVSF	FWWLKYVSF	0	0	0	2	1	FWRWLKYVSF	tr_D0A3G8	0.136	11470.10	17.00
51	HLA-A0201	ARLQGYFPEELPEY	RLQGYFPEL	1	0	0	7	1	RLQGYFPEEL	tr_D0A3G8	0.136	11478.04	17.00
38	HLA-A0201	PKVPFCFPDGTTYA	FCFDGTTYA	4	0	0	3	1	FCFPDGTTYA	tr_D0A3G8	0.136	11513.36	17.00
32	HLA-A0201	LKFVCEPKVPFCF	FVCEVPFCF	2	0	0	4	2	FVCEPKVPFCF	tr_D0A3G8	0.135	11550.67	17.00
14	HLA-A0201	VSFVRYGLAGLVVN	FVYGLAGLV	2	0	0	2	1	FVRYGLAGLV	tr_D0A3G8	0.135	11658.15	17.00
20	HLA-A0201	GLAGLVVNE	GLAGLVVNE	0	0	0	0	0	GLAGLVVNE	tr_D0A3G8	0.134	11771.84	17.00

15	HLA-A0201	SFVRYGLAGLVVNE	FVYGLAGLV	1	0	0	2	1	FVRYGLAGLV	tr_D0A3G8	0.133	11884.73	17.00
18	HLA-A0201	RYGLAGLVVNEF	GLAGLVVEF	2	0	0	7	1	GLAGLVVNEF	tr_D0A3G8	0.133	11893.21	17.00
10	HLA-A0201	WLKYVSFVRYGLAG	WLKYVSFLA	0	0	0	7	4	WLKYVSFVRYGLA	tr_D0A3G8	0.132	11979.75	17.00
2	HLA-A0201	QSLPFFWRWLK	SLPFFWRWL	1	0	0	0	0	SLPFFWRWL	tr_D0A3G8	0.131	12099.98	18.00
17	HLA-A0201	VRYGLAGLVVNEF	GLAGLVVEF	3	0	0	7	1	GLAGLVVNEF	tr_D0A3G8	0.131	12136.30	18.00
7	HLA-A0201	FWRWLKYVSFVR	FWLKYVSFV	0	0	0	1	2	FWRWLKYVSFV	tr_D0A3G8	0.131	12166.93	18.00
24	HLA-A0201	LVVNEFDGLKFVCE	LVFDGLKFV	0	0	0	2	3	LVVNEFDGLKFV	tr_D0A3G8	0.130	12198.57	18.00
4	HLA-A0201	LPFFWRWLKYVS	FFWRWLKYV	2	0	0	0	0	FFWRWLKYV	tr_D0A3G8	0.130	12296.63	18.00
1	HLA-A0201	TQSLPFFWRWLK	SLPFFWRWL	2	0	0	0	0	SLPFFWRWL	tr_D0A3G8	0.127	12651.44	18.00
10	HLA-A0201	WLKYVSFVR	WLKYVSFVR	0	0	0	0	0	WLKYVSFVR	tr_D0A3G8	0.126	12758.24	18.00
32	HLA-A0201	LKFVCEPKV	LKFVCEPKV	0	0	0	0	0	LKFVCEPKV	tr_D0A3G8	0.126	12766.25	18.00
0	HLA-A0201	GTQSLPFFWRWLK	SLFFWRWLK	3	0	0	2	1	SLPFFWRWLK	tr_D0A3G8	0.126	12845.65	18.00
7	HLA-A0201	FWRWLKYVSFVRY	WLYVSFVRY	3	0	0	2	1	WLKYVSFVRY	tr_D0A3G8	0.125	12934.63	19.00
7	HLA-A0201	FWRWLKYVSFVRYG	WLVSFVRYG	3	0	0	2	2	WLKYVSFVRYG	tr_D0A3G8	0.124	13076.61	19.00
5	HLA-A0201	PFFWRWLKYVSF	FFWRWLKYV	1	0	0	0	0	FFWRWLKYV	tr_D0A3G8	0.124	13079.43	19.00
34	HLA-A0201	FVCEPKVPFCFPD	FVCEFCFPD	0	0	0	4	4	FVCEPKVPFCFPD	tr_D0A3G8	0.123	13268.15	19.00
9	HLA-A0201	RWLKYVSFVR	RWLKYVSFV	0	0	0	0	0	RWLKYVSFV	tr_D0A3G8	0.120	13603.37	19.00
14	HLA-A0201	VSFVRYGLAGL	FVRYGLAGL	2	0	0	0	0	FVRYGLAGL	tr_D0A3G8	0.120	13630.03	19.00
3	HLA-A0201	SLPFFWRWLKY	SLPFFWRWL	0	0	0	0	0	SLPFFWRWL	tr_D0A3G8	0.119	13737.52	19.00
11	HLA-A0201	LKYVSFVRYGL	YVSFVRYGL	2	0	0	0	0	YVSFVRYGL	tr_D0A3G8	0.119	13868.79	20.00
4	HLA-A0201	LPFFWRWLKYVSF	FFWRWLKYV	2	0	0	0	0	FFWRWLKYV	tr_D0A3G8	0.118	13939.65	20.00
2	HLA-A0201	QSLPFFWRWLKY	SLFWRWLKY	1	0	0	2	2	SLPFFWRWLKY	tr_D0A3G8	0.118	13975.44	20.00
10	HLA-A0201	WLKYVSFVRYG	WLYVSFVRY	0	0	0	2	1	WLKYVSFVRY	tr_D0A3G8	0.118	13986.78	20.00
41	HLA-A0201	PFCFPDGTTYARL	FCFPTYARL	1	0	0	4	3	FCFPDGTTYARL	tr_D0A3G8	0.117	14152.41	20.00
44	HLA-A0201	FPDGTTYARL	FPDGTTYAL	0	0	0	8	1	FPDGTTYARL	tr_D0A3G8	0.117	14160.37	20.00
26	HLA-A0201	VNEFDGLKFV	VEFDGLKFV	0	0	0	1	1	VNEFDGLKFV	tr_D0A3G8	0.115	14427.15	20.00
1	HLA-A0201	TQSLPFFWRWLKY	SLFWRWLKY	2	0	0	2	2	SLPFFWRWLKY	tr_D0A3G8	0.114	14515.45	20.00
25	HLA-A0201	VVNEFDGLKFVCE	VVFDGLKFV	0	0	0	2	2	VVNEFDGLKFV	tr_D0A3G8	0.114	14529.13	20.00
0	HLA-A0201	GTQSLPFFWRWLKY	SLFWRWLKY	3	0	0	2	2	SLPFFWRWLKY	tr_D0A3G8	0.113	14719.32	21.00
8	HLA-A0201	WRWLKYVSFVRYG	WLVSFVRYG	2	0	0	2	2	WLKYVSFVRYG	tr_D0A3G8	0.112	14804.13	21.00
8	HLA-A0201	WRWLKYVSFVRY	WLYVSFVRY	2	0	0	2	1	WLKYVSFVRY	tr_D0A3G8	0.112	14902.97	21.00
31	HLA-A0201	GLKFVCEPK	GLKFVCEPK	0	0	0	0	0	GLKFVCEPK	tr_D0A3G8	0.111	15038.70	21.00
40	HLA-A0201	VPFCFPDGTTYARL	FCFPTYARL	2	0	0	4	3	FCFPDGTTYARL	tr_D0A3G8	0.111	15077.98	21.00
9	HLA-A0201	RWLKYVSFVRY	WLYVSFVRY	1	0	0	2	1	WLKYVSFVRY	tr_D0A3G8	0.110	15197.05	21.00
34	HLA-A0201	FVCEPKVPFCFP	FVCEVPFCF	0	0	0	4	2	FVCEPKVPFCF	tr_D0A3G8	0.110	15209.70	21.00
33	HLA-A0201	KFVCEPKVPFCFPD	FVCEFCFPD	1	0	0	4	4	FVCEPKVPFCFPD	tr_D0A3G8	0.110	15236.55	21.00
9	HLA-A0201	RWLKYVSFVRYG	WLYVSFVRY	1	0	0	2	1	WLKYVSFVRY	tr_D0A3G8	0.110	15266.26	21.00
21	HLA-A0201	LAGLVVNEF	LAGLVVNEF	0	0	0	0	0	LAGLVVNEF	tr_D0A3G8	0.110	15289.57	21.00
23	HLA-A0201	GLVVNEFDGLK	GLVVNEFGL	0	0	0	7	1	GLVVNEFDGL	tr_D0A3G8	0.109	15336.46	21.00
20	HLA-A0201	GLAGLVVNEFD	GLALVVNEF	0	0	0	3	1	GLAGLVVNEF	tr_D0A3G8	0.108	15458.91	21.00
14	HLA-A0201	VSFVRYGLA	VSFVRYGLA	0	0	0	0	0	VSFVRYGLA	tr_D0A3G8	0.108	15584.19	22.00
32	HLA-A0201	LKFVCEPKVPFC	FVCEPVPFC	2	0	0	5	1	FVCEPKVPFC	tr_D0A3G8	0.108	15586.54	22.00
17	HLA-A0201	VRYGLAGLV	VRYGLAGLV	0	0	0	0	0	VRYGLAGLV	tr_D0A3G8	0.107	15760.89	22.00
25	HLA-A0201	VVNEFDGLKFVCEP	VVFDGLKFV	0	0	0	2	2	VVNEFDGLKFV	tr_D0A3G8	0.105	16015.46	22.00
20	HLA-A0201	GLAGLVVNEFDG	GLALVVNEF	0	0	0	3	1	GLAGLVVNEF	tr_D0A3G8	0.105	16020.67	22.00
8	HLA-A0201	WRWLKYVSF	WRWLKYVSF	0	0	0	0	0	WRWLKYVSF	tr_D0A3G8	0.104	16190.40	22.00
33	HLA-A0201	KFVCEPKVPFC	FVCEKVPFC	1	0	0	4	1	FVCEPKVPFC	tr_D0A3G8	0.104	16198.10	22.00
8	HLA-A0201	WRWLKYVSFVR	WRWLYVSFV	0	0	0	4	1	WRWLKYVSFV	tr_D0A3G8	0.104	16226.17	22.00
42	HLA-A0201	FCFPDGTTYAR	FCFDGTTYA	0	0	0	3	1	FCFPDGTTYA	tr_D0A3G8	0.104	16290.03	22.00
12	HLA-A0201	KYVSFVRYGLA	YVSFVRYLA	1	0	0	7	1	YVSFVRYGLA	tr_D0A3G8	0.102	16577.71	23.00
11	HLA-A0201	LKYVSFVRYGLA	YVSFVRYLA	2	0	0	7	1	YVSFVRYGLA	tr_D0A3G8	0.102	16639.89	23.00
32	HLA-A0201	LKFVCEPKVPFCFP	FVCEVPFCF	2	0	0	4	2	FVCEPKVPFCF	tr_D0A3G8	0.101	16755.16	23.00
19	HLA-A0201	YGLAGLVVNEFD	GLALVVNEF	1	0	0	3	1	GLAGLVVNEF	tr_D0A3G8	0.100	16900.63	23.00
53	HLA-A0201	LQGYFPEELP	LQGYFPEEL	0	0	0	0	0	LQGYFPEEL	tr_D0A3G8	0.100	16936.32	23.00

33	HLA-A0201	KFVCEPKVPFCFP	FVCEVPFCF	1	0	0	4	2	FVCEPKVPFCF	tr_D0A3G8	0.099	17078.38	23.00
19	HLA-A0201	YGLAGLVVNEFDG	GLALVVNEF	1	0	0	3	1	GLAGLVVNEF	tr_D0A3G8	0.099	17211.76	24.00
29	HLA-A0201	FDGLKFVCEPK	FLKFVCEPK	0	0	0	1	2	FDGLKFVCEPK	tr_D0A3G8	0.098	17356.51	24.00
18	HLA-A0201	RYGLAGLVVNEFD	GLALVVNEF	2	0	0	3	1	GLAGLVVNEF	tr_D0A3G8	0.097	17441.41	24.00
2	HLA-A0201	QSLPFFWRW	QSLPFFWRW	0	0	0	0	0	QSLPFFWRW	tr_D0A3G8	0.097	17516.69	24.00
17	HLA-A0201	VRYGLAGLVVNEFD	GLALVVNEF	3	0	0	3	1	GLAGLVVNEF	tr_D0A3G8	0.097	17528.05	24.00
34	HLA-A0201	FVCEPKVPFCFPDG	FVCEVPFCF	0	0	0	4	2	FVCEPKVPFCF	tr_D0A3G8	0.097	17551.59	24.00
33	HLA-A0201	KFVCEPKVPF	FVCEPKVPF	1	0	0	0	0	FVCEPKVPF	tr_D0A3G8	0.096	17619.90	24.00
22	HLA-A0201	AGLVVNEFDGLK	GLVVNEFGL	1	0	0	7	1	GLVVNEFDGL	tr_D0A3G8	0.096	17638.40	24.00
42	HLA-A0201	FCFPDGTTYARLQ	FCFPTYARL	0	0	0	4	3	FCFPDGTTYARL	tr_D0A3G8	0.096	17647.56	24.00
18	HLA-A0201	RYGLAGLVVNEFDG	GLALVVNEF	2	0	0	3	1	GLAGLVVNEF	tr_D0A3G8	0.095	17917.88	24.00
23	HLA-A0201	GLVVNEFDGLKF	GLVVNEFGL	0	0	0	7	1	GLVVNEFDGL	tr_D0A3G8	0.094	18003.39	25.00
48	HLA-A0201	TTYARLQGYF	TTYARLQYF	0	0	0	7	1	TTYARLQGYF	tr_D0A3G8	0.094	18143.61	25.00
1	HLA-A0201	TQSLPFFWRW	TQLPFFWRW	0	0	0	2	1	TQSLPFFWRW	tr_D0A3G8	0.093	18293.21	25.00
21	HLA-A0201	LAGLVVNEFDGLK	GLVVNEFGL	2	0	0	7	1	GLVVNEFDGL	tr_D0A3G8	0.091	18625.35	25.00
43	HLA-A0201	CFPDGTTYA	CFPDGTTYA	0	0	0	0	0	CFPDGTTYA	tr_D0A3G8	0.091	18628.38	25.00
28	HLA-A0201	EFDGLKFVCEPK	FLKFVCEPK	1	0	0	1	2	FDGLKFVCEPK	tr_D0A3G8	0.091	18689.14	26.00
18	HLA-A0201	RYGLAGLVV	RYGLAGLVV	0	0	0	0	0	RYGLAGLVV	tr_D0A3G8	0.090	18955.93	26.00
39	HLA-A0201	KVPFCFPDGTTYAR	FCFDGTTYA	3	0	0	3	1	FCFPDGTTYA	tr_D0A3G8	0.090	18974.39	26.00
27	HLA-A0201	NEFDGLKFVCEPK	FLKFVCEPK	2	0	0	1	2	FDGLKFVCEPK	tr_D0A3G8	0.089	19043.29	26.00
26	HLA-A0201	VNEFDGLKFVCEPK	FLKFVCEPK	3	0	0	1	2	FDGLKFVCEPK	tr_D0A3G8	0.088	19269.21	26.00
18	HLA-A0201	RYGLAGLVVNE	YLAGLVVNE	1	0	0	1	1	YGLAGLVVNE	tr_D0A3G8	0.088	19307.61	26.00
0	HLA-A0201	GTQSLPFFW	GTQSLPFFW	0	0	0	0	0	GTQSLPFFW	tr_D0A3G8	0.087	19431.47	27.00
41	HLA-A0201	PFCFPDGTTYAR	FCFDGTTYA	1	0	0	3	1	FCFPDGTTYA	tr_D0A3G8	0.087	19453.55	27.00
42	HLA-A0201	FCFPDGTTYARLQG	FCFDGTTYA	0	0	0	3	1	FCFPDGTTYA	tr_D0A3G8	0.087	19479.25	27.00
23	HLA-A0201	GLVVNEFDG	GLVVNEFDG	0	0	0	0	0	GLVVNEFDG	tr_D0A3G8	0.087	19521.24	27.00
30	HLA-A0201	DGLKFVCEPK	GLKFVCEPK	1	0	0	0	0	GLKFVCEPK	tr_D0A3G8	0.087	19534.77	27.00
4	HLA-A0201	LPFFWRWLKY	LFFWRWLKY	0	0	0	1	1	LPFFWRWLKY	tr_D0A3G8	0.086	19771.22	27.00
21	HLA-A0201	LAGLVVNEFDGLKF	LAGLVVLKF	0	0	0	6	5	LAGLVVNEFDGLKF	tr_D0A3G8	0.086	19792.19	27.00
17	HLA-A0201	VRYGLAGLVVNE	YLAGLVVNE	2	0	0	1	1	YGLAGLVVNE	tr_D0A3G8	0.085	19854.82	27.00
22	HLA-A0201	AGLVVNEFDGLKF	GLVVNEFGL	1	0	0	7	1	GLVVNEFDGL	tr_D0A3G8	0.085	19972.46	27.00
41	HLA-A0201	PFCFPDGTTYARLQ	FCFPTYARL	1	0	0	4	3	FCFPDGTTYARL	tr_D0A3G8	0.084	20237.84	28.00
40	HLA-A0201	VPFCFPDGTTYAR	FCFDGTTYA	2	0	0	3	1	FCFPDGTTYA	tr_D0A3G8	0.083	20407.59	28.00
13	HLA-A0201	YVSFVRYGLAG	YVSFVRYGL	0	0	0	0	0	YVSFVRYGL	tr_D0A3G8	0.082	20594.37	28.00
4	HLA-A0201	LPFFWRWLK	LPFFWRWLK	0	0	0	0	0	LPFFWRWLK	tr_D0A3G8	0.081	20723.56	28.00
51	HLA-A0201	ARLQGYFPEE	ALQGYFPEE	0	0	0	1	1	ARLQGYFPEE	tr_D0A3G8	0.080	21013.94	29.00
11	HLA-A0201	LKYVSFVRY	LKYVSFVRY	0	0	0	0	0	LKYVSFVRY	tr_D0A3G8	0.078	21559.99	30.00
11	HLA-A0201	LKYVSFVRYGLAG	LKYVSFVLA	0	0	0	7	3	LKYVSFVRYGLA	tr_D0A3G8	0.078	21560.46	30.00
25	HLA-A0201	VVNEFDGLKF	VVNEFDLKF	0	0	0	6	1	VVNEFDGLKF	tr_D0A3G8	0.075	22184.24	31.00
1	HLA-A0201	TQSLPFFWR	TQSLPFFWR	0	0	0	0	0	TQSLPFFWR	tr_D0A3G8	0.074	22570.65	31.00
12	HLA-A0201	KYVSFVRYGLAG	YVSFVRYGL	1	0	0	0	0	YVSFVRYGL	tr_D0A3G8	0.073	22595.57	31.00
50	HLA-A0201	YARLQGYFPEE	YLQGYFPEE	0	0	0	1	2	YARLQGYFPEE	tr_D0A3G8	0.072	22881.69	32.00
27	HLA-A0201	NEFDGLKFVC	NEFDGLFVC	0	0	0	6	1	NEFDGLKFVC	tr_D0A3G8	0.071	23177.72	32.00
42	HLA-A0201	FCFPDGTTY	FCFPDGTTY	0	0	0	0	0	FCFPDGTTY	tr_D0A3G8	0.071	23191.76	32.00
32	HLA-A0201	LKFVCEPKVPF	LKFVCEVPF	0	0	0	6	2	LKFVCEPKVPF	tr_D0A3G8	0.071	23303.18	33.00
24	HLA-A0201	LVVNEFDGLK	LVVNEFDGL	0	0	0	0	0	LVVNEFDGL	tr_D0A3G8	0.069	23604.91	33.00
5	HLA-A0201	PFFWRWLKY	PFFWRWLKY	0	0	0	0	0	PFFWRWLKY	tr_D0A3G8	0.069	23691.40	33.00
11	HLA-A0201	LKYVSFVRYG	LKYVSFVYG	0	0	0	7	1	LKYVSFVRYG	tr_D0A3G8	0.068	24036.61	34.00
14	HLA-A0201	VSFVRYGLAG	VSFVYGLAG	0	0	0	4	1	VSFVRYGLAG	tr_D0A3G8	0.067	24161.76	34.00
21	HLA-A0201	LAGLVVNEFD	LAGLVVNEF	0	0	0	0	0	LAGLVVNEF	tr_D0A3G8	0.066	24395.56	34.00
0	HLA-A0201	GTQSLPFFWRW	GTLPFFWRW	0	0	0	2	2	GTQSLPFFWRW	tr_D0A3G8	0.066	24398.72	34.00
17	HLA-A0201	VRYGLAGLVVN	VRYGLALVV	0	0	0	6	1	VRYGLAGLVV	tr_D0A3G8	0.066	24420.38	35.00
49	HLA-A0201	TYARLQGYFPEE	YLQGYFPEE	1	0	0	1	2	YARLQGYFPEE	tr_D0A3G8	0.064	24924.63	35.00
19	HLA-A0201	YGLAGLVVN	YGLAGLVVN	0	0	0	0	0	YGLAGLVVN	tr_D0A3G8	0.063	25163.08	36.00

48	HLA-A0201	TTYARLQGYFPEE	YLQGYFPEE	2	0	0	1	2	YARLQGYFPEE	tr_D0A3G8	0.063	25175.34	36.00
48	HLA-A0201	TTYARLQGY	TTYARLQGY	0	0	0	0	0	TTYARLQGY	tr_D0A3G8	0.062	25442.05	36.00
29	HLA-A0201	FDGLKFVCEP	FGLKFVCEP	0	0	0	1	1	FDGLKFVCEP	tr_D0A3G8	0.062	25455.82	37.00
47	HLA-A0201	GTTYARLQGYFPEE	YLQGYFPEE	3	0	0	1	2	YARLQGYFPEE	tr_D0A3G8	0.062	25469.88	37.00
50	HLA-A0201	YARLQGYFPE	YALQGYFPE	0	0	0	2	1	YARLQGYFPE	tr_D0A3G8	0.061	25807.20	37.00
56	HLA-A0201	YFPEELPEY	YFPEELPEY	0	0	0	0	0	YFPEELPEY	tr_D0A3G8	0.061	25814.17	37.00
22	HLA-A0201	AGLVVNEFDG	ALVVNEFDG	0	0	0	1	1	AGLVVNEFDG	tr_D0A3G8	0.061	25887.17	37.00
7	HLA-A0201	FWRWLKYVS	FWRWLKYVS	0	0	0	0	0	FWRWLKYVS	tr_D0A3G8	0.061	25894.45	37.00
50	HLA-A0201	YARLQGYFP	YARLQGYFP	0	0	0	0	0	YARLQGYFP	tr_D0A3G8	0.061	25977.52	38.00
39	HLA-A0201	KVPFCFPDG	KVPFCFPDG	0	0	0	0	0	KVPFCFPDG	tr_D0A3G8	0.060	26179.84	38.00
44	HLA-A0201	FPDGTTYARLQGYF	TTYARLQYF	4	0	0	7	1	TTYARLQGYF	tr_D0A3G8	0.060	26199.38	38.00
43	HLA-A0201	CFPDGTTYARL	FPDGTTYAL	1	0	0	8	1	FPDGTTYARL	tr_D0A3G8	0.058	26579.99	39.00
35	HLA-A0201	VCEPKVPFCF	VCEPVPFCF	0	0	0	4	1	VCEPKVPFCF	tr_D0A3G8	0.058	26648.81	39.00
38	HLA-A0201	PKVPFCFPDGT	KVFCFPDGT	1	0	0	2	1	KVPFCFPDGT	tr_D0A3G8	0.058	26768.46	39.00
12	HLA-A0201	KYVSFVRYG	KYVSFVRYG	0	0	0	0	0	KYVSFVRYG	tr_D0A3G8	0.057	27094.80	40.00
0	HLA-A0201	GTQSLPFFWR	GQSLPFFWR	0	0	0	1	1	GTQSLPFFWR	tr_D0A3G8	0.057	27126.78	40.00
40	HLA-A0201	VPFCFPDGT	VPFCFPDGT	0	0	0	0	0	VPFCFPDGT	tr_D0A3G8	0.056	27158.80	40.00
47	HLA-A0201	GTTYARLQGYF	TTYARLQYF	1	0	0	7	1	TTYARLQGYF	tr_D0A3G8	0.056	27166.14	40.00
36	HLA-A0201	CEPKVPFCF	CEPKVPFCF	0	0	0	0	0	CEPKVPFCF	tr_D0A3G8	0.056	27172.60	40.00
39	HLA-A0201	KVPFCFPDGTTY	KVPFCFTTY	0	0	0	6	3	KVPFCFPDGTTY	tr_D0A3G8	0.056	27181.44	40.00
21	HLA-A0201	LAGLVVNEFDG	LAGLVVFDG	0	0	0	6	2	LAGLVVNEFDG	tr_D0A3G8	0.056	27321.79	40.00
44	HLA-A0201	FPDGTTYARLQGY	FTYARLQGY	0	0	0	1	4	FPDGTTYARLQGY	tr_D0A3G8	0.056	27388.97	40.00
39	HLA-A0201	KVPFCFPDGTT	KVPFCFPTT	0	0	0	7	2	KVPFCFPDGTT	tr_D0A3G8	0.055	27434.05	41.00
46	HLA-A0201	DGTTYARLQGYF	TTYARLQYF	2	0	0	7	1	TTYARLQGYF	tr_D0A3G8	0.055	27529.20	41.00
37	HLA-A0201	EPKVPFCFPDGT	KVFCFPDGT	2	0	0	2	1	KVPFCFPDGT	tr_D0A3G8	0.055	27654.88	41.00
45	HLA-A0201	PDGTTYARLQGYF	TTYARLQYF	3	0	0	7	1	TTYARLQGYF	tr_D0A3G8	0.054	27745.40	41.00
35	HLA-A0201	VCEPKVPFCFPDGT	KVFCFPDGT	4	0	0	2	1	KVPFCFPDGT	tr_D0A3G8	0.054	27794.67	41.00
36	HLA-A0201	CEPKVPFCFPDGT	KVFCFPDGT	3	0	0	2	1	KVPFCFPDGT	tr_D0A3G8	0.054	27947.87	42.00
48	HLA-A0201	TTYARLQGYFP	TTYARLYFP	0	0	0	6	2	TTYARLQGYFP	tr_D0A3G8	0.054	27975.40	42.00
38	HLA-A0201	PKVPFCFPDGTTY	KVPFCFTTY	1	0	0	6	3	KVPFCFPDGTTY	tr_D0A3G8	0.053	28184.43	42.00
18	HLA-A0201	RYGLAGLVVN	RYGLAGLVV	0	0	0	0	0	RYGLAGLVV	tr_D0A3G8	0.052	28373.83	43.00
37	HLA-A0201	EPKVPFCFPDGTTY	KVPFCFTTY	2	0	0	6	3	KVPFCFPDGTTY	tr_D0A3G8	0.052	28509.53	43.00
35	HLA-A0201	VCEPKVPFC	VCEPKVPFC	0	0	0	0	0	VCEPKVPFC	tr_D0A3G8	0.052	28530.82	43.00
43	HLA-A0201	CFPDGTTYARLQGY	FTYARLQGY	1	0	0	1	4	FPDGTTYARLQGY	tr_D0A3G8	0.052	28575.92	43.00
24	HLA-A0201	LVVNEFDGLKF	LVVNEFLKF	0	0	0	6	2	LVVNEFDGLKF	tr_D0A3G8	0.051	28793.80	44.00
29	HLA-A0201	FDGLKFVCE	FDGLKFVCE	0	0	0	0	0	FDGLKFVCE	tr_D0A3G8	0.050	29142.96	45.00
47	HLA-A0201	GTTYARLQGYFP	TTYARLYFP	1	0	0	6	2	TTYARLQGYFP	tr_D0A3G8	0.050	29258.59	45.00
38	HLA-A0201	PKVPFCFPDGTT	KVPFCFPTT	1	0	0	7	2	KVPFCFPDGTT	tr_D0A3G8	0.050	29264.93	45.00
41	HLA-A0201	PFCFPDGTTY	FCFPDGTTY	1	0	0	0	0	FCFPDGTTY	tr_D0A3G8	0.049	29349.59	45.00
46	HLA-A0201	DGTTYARLQGYFP	TTYARLYFP	2	0	0	6	2	TTYARLQGYFP	tr_D0A3G8	0.049	29537.88	46.00
26	HLA-A0201	VNEFDGLKFVC	NEFDGLFVC	1	0	0	6	1	NEFDGLKFVC	tr_D0A3G8	0.049	29557.37	46.00
45	HLA-A0201	PDGTTYARLQGYFP	TTYARLYFP	3	0	0	6	2	TTYARLQGYFP	tr_D0A3G8	0.048	29701.64	46.00
25	HLA-A0201	VVNEFDGLK	VVNEFDGLK	0	0	0	0	0	VVNEFDGLK	tr_D0A3G8	0.048	29772.10	46.00
48	HLA-A0201	TTYARLQGYFPE	TTYARLFPE	0	0	0	6	3	TTYARLQGYFPE	tr_D0A3G8	0.048	29817.56	46.00
37	HLA-A0201	EPKVPFCFPDGTT	KVPFCFPTT	2	0	0	7	2	KVPFCFPDGTT	tr_D0A3G8	0.048	29852.10	46.00
47	HLA-A0201	GTTYARLQGY	GTYARLQGY	0	0	0	1	1	GTTYARLQGY	tr_D0A3G8	0.047	29945.27	47.00
36	HLA-A0201	CEPKVPFCFPDGTT	KVPFCFPTT	3	0	0	7	2	KVPFCFPDGTT	tr_D0A3G8	0.047	30048.14	47.00
53	HLA-A0201	LQGYFPEELPEY	LQGYFPEEL	0	0	0	0	0	LQGYFPEEL	tr_D0A3G8	0.047	30071.89	47.00
53	HLA-A0201	LQGYFPEELPE	LQGYFPEEL	0	0	0	0	0	LQGYFPEEL	tr_D0A3G8	0.047	30110.96	47.00
32	HLA-A0201	LKFVCEPKVP	LKFVCEPKV	0	0	0	0	0	LKFVCEPKV	tr_D0A3G8	0.046	30390.48	48.00
44	HLA-A0201	FPDGTTYAR	FPDGTTYAR	0	0	0	0	0	FPDGTTYAR	tr_D0A3G8	0.046	30548.72	48.00
47	HLA-A0201	GTTYARLQGYFPE	TTYARLFPE	1	0	0	6	3	TTYARLQGYFPE	tr_D0A3G8	0.045	30617.55	48.00
46	HLA-A0201	DGTTYARLQGYFPE	TTYARLFPE	2	0	0	6	3	TTYARLQGYFPE	tr_D0A3G8	0.045	30800.63	49.00
40	HLA-A0201	VPFCFPDGTTY	VPFCFGTTY	0	0	0	5	2	VPFCFPDGTTY	tr_D0A3G8	0.045	30871.03	49.00

35	HLA-A0201	VCEPKVPFCFPD	VCVPFCFPD	0	0	0	2	3	VCEPKVPFCFPD	tr_D0A3G8	0.044	31164.34	50.00
26	HLA-A0201	VNEFDGLKFVCEP	FDGLFVCEP	3	0	0	4	1	FDGLKFVCEP	tr_D0A3G8	0.042	31647.87	55.00
55	HLA-A0201	GYFPEELPEY	YFPEELPEY	1	0	0	0	0	YFPEELPEY	tr_D0A3G8	0.042	31747.67	55.00
40	HLA-A0201	VPFCFPDGTT	VPFCFPDTT	0	0	0	7	1	VPFCFPDGTT	tr_D0A3G8	0.042	31752.48	55.00
26	HLA-A0201	VNEFDGLKFVCE	VEFDGLKFV	0	0	0	1	1	VNEFDGLKFV	tr_D0A3G8	0.042	31796.15	55.00
27	HLA-A0201	NEFDGLKFVCEP	FDGLFVCEP	2	0	0	4	1	FDGLKFVCEP	tr_D0A3G8	0.041	31929.90	55.00
44	HLA-A0201	FPDGTTYARLQ	FPDGTTYAL	0	0	0	8	1	FPDGTTYARL	tr_D0A3G8	0.041	31982.47	55.00
15	HLA-A0201	SFVRYGLAG	SFVRYGLAG	0	0	0	0	0	SFVRYGLAG	tr_D0A3G8	0.041	32073.96	55.00
22	HLA-A0201	AGLVVNEFD	AGLVVNEFD	0	0	0	0	0	AGLVVNEFD	tr_D0A3G8	0.040	32389.56	55.00
27	HLA-A0201	NEFDGLKFVCE	NEFDGLKFV	0	0	0	0	0	NEFDGLKFV	tr_D0A3G8	0.040	32444.27	55.00
28	HLA-A0201	EFDGLKFVCEP	FDGLFVCEP	1	0	0	4	1	FDGLKFVCEP	tr_D0A3G8	0.039	32651.00	55.00
26	HLA-A0201	VNEFDGLKF	VNEFDGLKF	0	0	0	0	0	VNEFDGLKF	tr_D0A3G8	0.039	32708.26	55.00
43	HLA-A0201	CFPDGTTYARLQ	FPDGTTYAL	1	0	0	8	1	FPDGTTYARL	tr_D0A3G8	0.039	32909.91	55.00
38	HLA-A0201	PKVPFCFPDG	KVPFCFPDG	1	0	0	0	0	KVPFCFPDG	tr_D0A3G8	0.038	33102.76	60.00
28	HLA-A0201	EFDGLKFVC	EFDGLKFVC	0	0	0	0	0	EFDGLKFVC	tr_D0A3G8	0.038	33265.40	60.00
36	HLA-A0201	CEPKVPFCFP	CEPKVPFCF	0	0	0	0	0	CEPKVPFCF	tr_D0A3G8	0.037	33341.07	60.00
35	HLA-A0201	VCEPKVPFCFPDG	VCVPFCFPD	0	0	0	2	3	VCEPKVPFCFPD	tr_D0A3G8	0.037	33543.32	60.00
49	HLA-A0201	TYARLQGYFPE	YALQGYFPE	1	0	0	2	1	YARLQGYFPE	tr_D0A3G8	0.037	33548.06	60.00
44	HLA-A0201	FPDGTTYARLQG	FPDGTTYAL	0	0	0	8	1	FPDGTTYARL	tr_D0A3G8	0.037	33568.02	60.00
36	HLA-A0201	CEPKVPFCFPD	CEVPFCFPD	0	0	0	2	2	CEPKVPFCFPD	tr_D0A3G8	0.037	33576.37	60.00
49	HLA-A0201	TYARLQGYFP	YARLQGYFP	1	0	0	0	0	YARLQGYFP	tr_D0A3G8	0.037	33672.05	60.00
30	HLA-A0201	DGLKFVCEP	DGLKFVCEP	0	0	0	0	0	DGLKFVCEP	tr_D0A3G8	0.036	33692.46	60.00
35	HLA-A0201	VCEPKVPFCFP	VCEPVPFCF	0	0	0	4	1	VCEPKVPFCF	tr_D0A3G8	0.036	33747.55	60.00
43	HLA-A0201	CFPDGTTYAR	CFPDGTTYA	0	0	0	0	0	CFPDGTTYA	tr_D0A3G8	0.036	33810.42	60.00
51	HLA-A0201	ARLQGYFPE	ARLQGYFPE	0	0	0	0	0	ARLQGYFPE	tr_D0A3G8	0.036	33812.25	60.00
46	HLA-A0201	DGTTYARLQGY	GTYARLQGY	1	0	0	1	1	GTTYARLQGY	tr_D0A3G8	0.036	33913.01	60.00
28	HLA-A0201	EFDGLKFVCE	FDGLKFVCE	1	0	0	0	0	FDGLKFVCE	tr_D0A3G8	0.035	34143.85	60.00
38	HLA-A0201	PKVPFCFPD	PKVPFCFPD	0	0	0	0	0	PKVPFCFPD	tr_D0A3G8	0.035	34267.47	60.00
43	HLA-A0201	CFPDGTTYARLQG	FPDGTTYAL	1	0	0	8	1	FPDGTTYARL	tr_D0A3G8	0.035	34276.00	60.00
45	HLA-A0201	PDGTTYARLQGY	TTYARLQGY	3	0	0	0	0	TTYARLQGY	tr_D0A3G8	0.035	34307.54	60.00
37	HLA-A0201	EPKVPFCFPD	EPVPFCFPD	0	0	0	2	1	EPKVPFCFPD	tr_D0A3G8	0.034	34662.73	65.00
36	HLA-A0201	CEPKVPFCFPDG	CEPKVPFCF	0	0	0	0	0	CEPKVPFCF	tr_D0A3G8	0.033	35023.13	65.00
54	HLA-A0201	QGYFPEELPEY	YFPEELPEY	2	0	0	0	0	YFPEELPEY	tr_D0A3G8	0.033	35092.56	65.00
55	HLA-A0201	GYFPEELPE	GYFPEELPE	0	0	0	0	0	GYFPEELPE	tr_D0A3G8	0.031	35586.18	65.00
37	HLA-A0201	EPKVPFCFP	EPKVPFCFP	0	0	0	0	0	EPKVPFCFP	tr_D0A3G8	0.029	36623.66	70.00
37	HLA-A0201	EPKVPFCFPDG	KVPFCFPDG	2	0	0	0	0	KVPFCFPDG	tr_D0A3G8	0.028	36784.91	70.00
54	HLA-A0201	QGYFPEELP	QGYFPEELP	0	0	0	0	0	QGYFPEELP	tr_D0A3G8	0.028	36886.13	70.00
49	HLA-A0201	TYARLQGYF	TYARLQGYF	0	0	0	0	0	TYARLQGYF	tr_D0A3G8	0.027	37419.57	75.00
45	HLA-A0201	PDGTTYARL	PDGTTYARL	0	0	0	0	0	PDGTTYARL	tr_D0A3G8	0.024	38706.83	80.00
54	HLA-A0201	QGYFPEELPE	GYFPEELPE	1	0	0	0	0	GYFPEELPE	tr_D0A3G8	0.023	38917.23	80.00
47	HLA-A0201	GTTYARLQG	GTTYARLQG	0	0	0	0	0	GTTYARLQG	tr_D0A3G8	0.022	39465.92	80.00
33	HLA-A0201	KFVCEPKVP	KFVCEPKVP	0	0	0	0	0	KFVCEPKVP	tr_D0A3G8	0.018	41274.40	90.00
46	HLA-A0201	DGTTYARLQ	DGTTYARLQ	0	0	0	0	0	DGTTYARLQ	tr_D0A3G8	0.016	42191.00	95.00
46	HLA-A0201	DGTTYARLQG	GTTYARLQG	1	0	0	0	0	GTTYARLQG	tr_D0A3G8	0.015	42508.05	95.00
41	HLA-A0201	PFCFPDGTT	PFCFPDGTT	0	0	0	0	0	PFCFPDGTT	tr_D0A3G8	0.015	42613.07	95.00
45	HLA-A0201	PDGTTYARLQ	PDGTTYARL	0	0	0	0	0	PDGTTYARL	tr_D0A3G8	0.013	43207.79	95.00
45	HLA-A0201	PDGTTYARLQG	GTTYARLQG	2	0	0	0	0	GTTYARLQG	tr_D0A3G8	0.013	43414.45	95.00

Protein tr_D0A3G8. Allele HLA-A0201. Number of high binders 0. Number of weak binders 8. Number of peptides 327

Link to Allele Frequencies in Worldwide Populations HLA-A0201

Rank Threshold for Strong binding peptides   0.500

Rank Threshold for Weak binding peptides   2.000

pos	HLA	peptide	Core	Offset	I_pos	I_len	D_pos	D_len	iCore	Identity	1-log50k(aff)	Affinity(nM)	%Rank	BindLevel
9	HLA-A0201	MLRTYLHET	MLRTYLHET	0	0	0	0	0	MLRTYLHET	tr_D0AAR0	0.474	296.43	2.00	<= WB
8	HLA-A0201	GMLRTYLHET	GMLTYLHET	0	0	0	3	1	GMLRTYLHET	tr_D0AAR0	0.429	481.67	3.00	
34	HLA-A0201	FPMRSTPSGV	FMRSTPSGV	0	0	0	1	1	FPMRSTPSGV	tr_D0AAR0	0.387	760.71	3.50	
1	HLA-A0201	YLERRKRGMLRTYL	YLGMLRTYL	0	0	0	2	5	YLERRKRGMLRTYL	tr_D0AAR0	0.302	1908.63	6.00	
13	HLA-A0201	YLHETHGRGGTEA	YLHETHTEA	0	0	0	6	4	YLHETHGRGGTEA	tr_D0AAR0	0.244	3579.69	8.00	
12	HLA-A0201	TYLHETHGRGGTEA	YLHETHTEA	1	0	0	6	4	YLHETHGRGGTEA	tr_D0AAR0	0.209	5227.57	10.00	
7	HLA-A0201	RGMLRTYLHET	GMLTYLHET	1	0	0	3	1	GMLRTYLHET	tr_D0AAR0	0.194	6130.78	11.00	
33	HLA-A0201	GFPMRSTPSGV	FMRSTPSGV	1	0	0	1	1	FPMRSTPSGV	tr_D0AAR0	0.189	6454.24	12.00	
6	HLA-A0201	KRGMLRTYLHET	GMLTYLHET	2	0	0	3	1	GMLRTYLHET	tr_D0AAR0	0.181	7065.80	12.00	
5	HLA-A0201	RKRGMLRTYLHET	GMLTYLHET	3	0	0	3	1	GMLRTYLHET	tr_D0AAR0	0.177	7392.46	13.00	
32	HLA-A0201	AGFPMRSTPSGV	FMRSTPSGV	2	0	0	1	1	FPMRSTPSGV	tr_D0AAR0	0.175	7524.32	13.00	
4	HLA-A0201	RRKRGMLRTYLHET	GMLTYLHET	4	0	0	3	1	GMLRTYLHET	tr_D0AAR0	0.174	7589.56	13.00	
9	HLA-A0201	MLRTYLHETH	MLRTYLHET	0	0	0	0	0	MLRTYLHET	tr_D0AAR0	0.172	7782.32	13.00	
31	HLA-A0201	GAGFPMRSTPSGV	FMRSTPSGV	3	0	0	1	1	FPMRSTPSGV	tr_D0AAR0	0.171	7859.58	13.00	
30	HLA-A0201	GGAGFPMRSTPSGV	FMRSTPSGV	4	0	0	1	1	FPMRSTPSGV	tr_D0AAR0	0.169	8062.94	13.00	
27	HLA-A0201	TVGGGAGFPM	TVGGGAFPM	0	0	0	6	1	TVGGGAGFPM	tr_D0AAR0	0.146	10258.26	16.00	
13	HLA-A0201	YLHETHGRGGTEAQ	YLHETHTEA	0	0	0	6	4	YLHETHGRGGTEA	tr_D0AAR0	0.133	11902.87	17.00	
34	HLA-A0201	FPMRSTPSGVS	FMRSTPSGV	0	0	0	1	1	FPMRSTPSGV	tr_D0AAR0	0.125	12931.41	19.00	
8	HLA-A0201	GMLRTYLHETH	GMLTYLHET	0	0	0	3	1	GMLRTYLHET	tr_D0AAR0	0.120	13681.30	19.00	
33	HLA-A0201	GFPMRSTPSGVS	FMRSTPSGV	1	0	0	1	1	FPMRSTPSGV	tr_D0AAR0	0.106	15856.50	22.00	
7	HLA-A0201	RGMLRTYLHETH	GMLTYLHET	1	0	0	3	1	GMLRTYLHET	tr_D0AAR0	0.104	16224.76	22.00	
34	HLA-A0201	FPMRSTPSGVSW	FMRSTPSGV	0	0	0	1	1	FPMRSTPSGV	tr_D0AAR0	0.102	16596.75	23.00	
13	HLA-A0201	YLHETHGRG	YLHETHGRG	0	0	0	0	0	YLHETHGRG	tr_D0AAR0	0.102	16639.89	23.00	
8	HLA-A0201	GMLRTYLHETHG	GMLTYLHET	0	0	0	3	1	GMLRTYLHET	tr_D0AAR0	0.101	16722.56	23.00	
6	HLA-A0201	KRGMLRTYLHETH	GMLTYLHET	2	0	0	3	1	GMLRTYLHET	tr_D0AAR0	0.099	17126.87	23.00	
32	HLA-A0201	AGFPMRSTPSGVS	FMRSTPSGV	2	0	0	1	1	FPMRSTPSGV	tr_D0AAR0	0.099	17155.06	23.00	
1	HLA-A0201	YLERRKRGM	YLERRKRGM	0	0	0	0	0	YLERRKRGM	tr_D0AAR0	0.098	17308.87	24.00	
5	HLA-A0201	RKRGMLRTYLHETH	GMLTYLHET	3	0	0	3	1	GMLRTYLHET	tr_D0AAR0	0.097	17418.40	24.00	
31	HLA-A0201	GAGFPMRSTPSGVS	FMRSTPSGV	3	0	0	1	1	FPMRSTPSGV	tr_D0AAR0	0.097	17544.76	24.00	
1	HLA-A0201	YLERRKRGML	YLERRRGML	0	0	0	5	1	YLERRKRGML	tr_D0AAR0	0.094	17993.07	25.00	
35	HLA-A0201	PMRSTPSGV	PMRSTPSGV	0	0	0	0	0	PMRSTPSGV	tr_D0AAR0	0.094	18007.67	25.00	
7	HLA-A0201	RGMLRTYLHETHG	GMLTYLHET	1	0	0	3	1	GMLRTYLHET	tr_D0AAR0	0.091	18734.08	26.00	
8	HLA-A0201	GMLRTYLHETHGR	GMLTYLHET	0	0	0	3	1	GMLRTYLHET	tr_D0AAR0	0.090	18927.84	26.00	
33	HLA-A0201	GFPMRSTPSGVSW	FMRSTPSGV	1	0	0	1	1	FPMRSTPSGV	tr_D0AAR0	0.088	19359.06	26.00	
6	HLA-A0201	KRGMLRTYLHETHG	GMLTYLHET	2	0	0	3	1	GMLRTYLHET	tr_D0AAR0	0.088	19374.15	26.00	
8	HLA-A0201	GMLRTYLHE	GMLRTYLHE	0	0	0	0	0	GMLRTYLHE	tr_D0AAR0	0.087	19500.76	27.00	
8	HLA-A0201	GMLRTYLHETHGRG	GMLTYLHET	0	0	0	3	1	GMLRTYLHET	tr_D0AAR0	0.087	19584.49	27.00	
32	HLA-A0201	AGFPMRSTPSGVSW	FMRSTPSGV	2	0	0	1	1	FPMRSTPSGV	tr_D0AAR0	0.082	20501.44	28.00	
9	HLA-A0201	MLRTYLHETHG	MLRTYLHET	0	0	0	0	0	MLRTYLHET	tr_D0AAR0	0.082	20664.23	28.00	
1	HLA-A0201	YLERRKRGMLRT	YLERGMLRT	0	0	0	3	3	YLERRKRGMLRT	tr_D0AAR0	0.081	20765.99	28.00	
7	HLA-A0201	RGMLRTYLHETHGR	GMLTYLHET	1	0	0	3	1	GMLRTYLHET	tr_D0AAR0	0.079	21285.10	29.00	
1	HLA-A0201	YLERRKRGMLRTY	YLEGMLRTY	0	0	0	3	4	YLERRKRGMLRTY	tr_D0AAR0	0.078	21598.29	30.00	
28	HLA-A0201	VGGGAGFPM	VGGGAGFPM	0	0	0	0	0	VGGGAGFPM	tr_D0AAR0	0.077	21740.13	30.00	
25	HLA-A0201	AQTVGGGAGFPM	AQTVAGFPM	0	0	0	4	3	AQTVGGGAGFPM	tr_D0AAR0	0.075	22299.02	31.00	
13	HLA-A0201	YLHETHGRGG	YLHETGRGG	0	0	0	5	1	YLHETHGRGG	tr_D0AAR0	0.074	22548.43	31.00	
0	HLA-A0201	EYLERRKRGMLRTY	YLEGMLRTY	1	0	0	3	4	YLERRKRGMLRTY	tr_D0AAR0	0.072	23007.81	32.00	
0	HLA-A0201	EYLERRKRGMLRT	YLERGMLRT	1	0	0	3	3	YLERRKRGMLRT	tr_D0AAR0	0.071	23265.40	32.00	
9	HLA-A0201	MLRTYLHETHGRG	MLRTYLHEG	0	0	0	8	4	MLRTYLHETHGRG	tr_D0AAR0	0.069	23673.72	33.00	
9	HLA-A0201	MLRTYLHETHGRGG	MLRTYLHEG	0	0	0	8	5	MLRTYLHETHGRGG	tr_D0AAR0	0.069	23812.18	33.00	
24	HLA-A0201	EAQTVGGGAGFPM	AQTVAGFPM	1	0	0	4	3	AQTVGGGAGFPM	tr_D0AAR0	0.068	24053.00	34.00	

9	HLA-A0201	MLRTYLHETHGR	MLRTYLHET	0	0	0	0	0	MLRTYLHET	tr_D0AAR0	0.067	24218.30	34.00
13	HLA-A0201	YLHETHGRGGT	YLHETHGGT	0	0	0	6	2	YLHETHGRGGT	tr_D0AAR0	0.067	24236.12	34.00
23	HLA-A0201	TEAQTVGGGAGFPM	TVGGGAFPM	4	0	0	6	1	TVGGGAGFPM	tr_D0AAR0	0.067	24345.99	34.00
6	HLA-A0201	KRGMLRTYL	KRGMLRTYL	0	0	0	0	0	KRGMLRTYL	tr_D0AAR0	0.065	24845.75	35.00
26	HLA-A0201	QTVGGGAGFPM	TVGGGAFPM	1	0	0	6	1	TVGGGAGFPM	tr_D0AAR0	0.064	25085.62	36.00
7	HLA-A0201	RGMLRTYLHE	RMLRTYLHE	0	0	0	1	1	RGMLRTYLHE	tr_D0AAR0	0.062	25646.03	37.00
5	HLA-A0201	RKRGMLRTYL	RKRGMLTYL	0	0	0	6	1	RKRGMLRTYL	tr_D0AAR0	0.057	26970.50	40.00
12	HLA-A0201	TYLHETHGRGGT	YLHETHGGT	1	0	0	6	2	YLHETHGRGGT	tr_D0AAR0	0.056	27219.10	40.00
12	HLA-A0201	TYLHETHGRG	YLHETHGRG	1	0	0	0	0	YLHETHGRG	tr_D0AAR0	0.053	28098.26	42.00
11	HLA-A0201	RTYLHETHGRGGT	YLHETHGGT	2	0	0	6	2	YLHETHGRGGT	tr_D0AAR0	0.053	28205.16	42.00
10	HLA-A0201	LRTYLHETHGRGGT	YLHETHGGT	3	0	0	6	2	YLHETHGRGGT	tr_D0AAR0	0.052	28608.10	43.00
25	HLA-A0201	AQTVGGGAGFPMR	AQTVAGFPM	0	0	0	4	3	AQTVGGGAGFPM	tr_D0AAR0	0.051	28641.86	43.00
0	HLA-A0201	EYLERRKRGM	YLERRKRGM	1	0	0	0	0	YLERRKRGM	tr_D0AAR0	0.051	28771.38	44.00
25	HLA-A0201	AQTVGGGAGFPMRS	AQAGFPMRS	0	0	0	2	5	AQTVGGGAGFPMRS	tr_D0AAR0	0.051	28779.79	44.00
7	HLA-A0201	RGMLRTYLH	RGMLRTYLH	0	0	0	0	0	RGMLRTYLH	tr_D0AAR0	0.050	28952.50	44.00
20	HLA-A0201	RGGTEAQTV	RGGTEAQTV	0	0	0	0	0	RGGTEAQTV	tr_D0AAR0	0.049	29267.78	45.00
25	HLA-A0201	AQTVGGGAGF	AQVGGGAGF	0	0	0	2	1	AQTVGGGAGF	tr_D0AAR0	0.049	29365.47	45.00
26	HLA-A0201	QTVGGGAGF	QTVGGGAGF	0	0	0	0	0	QTVGGGAGF	tr_D0AAR0	0.049	29407.45	45.00
24	HLA-A0201	EAQTVGGGAGFPMR	AQTVAGFPM	1	0	0	4	3	AQTVGGGAGFPM	tr_D0AAR0	0.048	29689.42	46.00
19	HLA-A0201	GRGGTEAQTV	GRGGTEATV	0	0	0	7	1	GRGGTEAQTV	tr_D0AAR0	0.046	30494.89	48.00
1	HLA-A0201	YLERRKRGMLR	YLRKRGMLR	0	0	0	2	2	YLERRKRGMLR	tr_D0AAR0	0.044	31055.61	49.00
27	HLA-A0201	TVGGGAGFPMR	TVGGGAFPM	0	0	0	6	1	TVGGGAGFPM	tr_D0AAR0	0.043	31238.26	50.00
0	HLA-A0201	EYLERRKRGML	YLERRRGML	1	0	0	5	1	YLERRKRGML	tr_D0AAR0	0.042	31586.64	55.00
26	HLA-A0201	QTVGGGAGFPMR	TVGGGAFPM	1	0	0	6	1	TVGGGAGFPM	tr_D0AAR0	0.041	32042.39	55.00
2	HLA-A0201	LERRKRGMLRTYL	RKRGMLTYL	3	0	0	6	1	RKRGMLRTYL	tr_D0AAR0	0.041	32065.97	55.00
5	HLA-A0201	RKRGMLRTY	RKRGMLRTY	0	0	0	0	0	RKRGMLRTY	tr_D0AAR0	0.041	32198.43	55.00
5	HLA-A0201	RKRGMLRTYLHE	RMLRTYLHE	0	0	0	1	3	RKRGMLRTYLHE	tr_D0AAR0	0.040	32353.13	55.00
0	HLA-A0201	EYLERRKRGMLR	YLRKRGMLR	1	0	0	2	2	YLERRKRGMLR	tr_D0AAR0	0.040	32390.96	55.00
11	HLA-A0201	RTYLHETHG	RTYLHETHG	0	0	0	0	0	RTYLHETHG	tr_D0AAR0	0.040	32472.36	55.00
4	HLA-A0201	RRKRGMLRTYLHE	RMLRTYLHE	0	0	0	1	4	RRKRGMLRTYLHE	tr_D0AAR0	0.040	32546.94	55.00
13	HLA-A0201	YLHETHGRGGTE	YLHETHGGT	0	0	0	6	2	YLHETHGRGGT	tr_D0AAR0	0.039	32612.87	55.00
4	HLA-A0201	RRKRGMLRTYL	RKRGMLTYL	1	0	0	6	1	RKRGMLRTYL	tr_D0AAR0	0.039	32771.69	55.00
6	HLA-A0201	KRGMLRTYLH	KRGMLRTYL	0	0	0	0	0	KRGMLRTYL	tr_D0AAR0	0.039	32792.26	55.00
27	HLA-A0201	TVGGGAGFPMRS	TVGGGAFPM	0	0	0	6	1	TVGGGAGFPM	tr_D0AAR0	0.039	32870.75	55.00
26	HLA-A0201	QTVGGGAGFPMRS	QTVGFPMRS	0	0	0	3	4	QTVGGGAGFPMRS	tr_D0AAR0	0.039	32885.34	55.00
30	HLA-A0201	GGAGFPMRS	GGAGFPMRS	0	0	0	0	0	GGAGFPMRS	tr_D0AAR0	0.038	33068.38	55.00
6	HLA-A0201	KRGMLRTYLHE	KMLRTYLHE	0	0	0	1	2	KRGMLRTYLHE	tr_D0AAR0	0.038	33269.71	60.00
3	HLA-A0201	ERRKRGMLRTYL	RKRGMLTYL	2	0	0	6	1	RKRGMLRTYL	tr_D0AAR0	0.038	33278.36	60.00
3	HLA-A0201	ERRKRGMLRTYLHE	RMLRTYLHE	1	0	0	1	4	RRKRGMLRTYLHE	tr_D0AAR0	0.037	33453.09	60.00
37	HLA-A0201	RSTPSGVSW	RSTPSGVSW	0	0	0	0	0	RSTPSGVSW	tr_D0AAR0	0.036	33908.96	60.00
28	HLA-A0201	VGGGAGFPMR	VGGGAGFPM	0	0	0	0	0	VGGGAGFPM	tr_D0AAR0	0.036	33923.27	60.00
30	HLA-A0201	GGAGFPMRST	GGAGFPMST	0	0	0	7	1	GGAGFPMRST	tr_D0AAR0	0.036	33998.98	60.00
2	HLA-A0201	LERRKRGMLRTYLH	RKRGMLTYL	3	0	0	6	1	RKRGMLRTYL	tr_D0AAR0	0.035	34180.82	60.00
12	HLA-A0201	TYLHETHGRGG	YLHETGRGG	1	0	0	5	1	YLHETHGRGG	tr_D0AAR0	0.035	34195.24	60.00
35	HLA-A0201	PMRSTPSGVS	PMRSTPSGV	0	0	0	0	0	PMRSTPSGV	tr_D0AAR0	0.035	34211.52	60.00
27	HLA-A0201	TVGGGAGFPMRST	TVGGGAFPM	0	0	0	6	1	TVGGGAGFPM	tr_D0AAR0	0.035	34215.60	60.00
31	HLA-A0201	GAGFPMRST	GAGFPMRST	0	0	0	0	0	GAGFPMRST	tr_D0AAR0	0.035	34238.18	60.00
12	HLA-A0201	TYLHETHGRGGTE	YLHETHGGT	1	0	0	6	2	YLHETHGRGGT	tr_D0AAR0	0.035	34260.43	60.00
11	HLA-A0201	RTYLHETHGRGG	YLHETGRGG	2	0	0	5	1	YLHETHGRGG	tr_D0AAR0	0.034	34547.80	60.00
27	HLA-A0201	TVGGGAGFPMRSTP	TVGGGAFPM	0	0	0	6	1	TVGGGAGFPM	tr_D0AAR0	0.034	34555.26	60.00
26	HLA-A0201	QTVGGGAGFPMRST	TVGGGAFPM	1	0	0	6	1	TVGGGAGFPM	tr_D0AAR0	0.034	34560.50	60.00
11	HLA-A0201	RTYLHETHGRGGTE	YLHETHGGT	2	0	0	6	2	YLHETHGRGGT	tr_D0AAR0	0.034	34744.21	65.00
5	HLA-A0201	RKRGMLRTYLH	RKRGMLTYL	0	0	0	6	1	RKRGMLRTYL	tr_D0AAR0	0.034	34752.48	65.00
4	HLA-A0201	RRKRGMLRTY	RRRGMLRTY	0	0	0	2	1	RRKRGMLRTY	tr_D0AAR0	0.033	34817.61	65.00

24	HLA-A0201	EAQTVGGGAGF	EAQTVGAGF	0	0	0	5	2	EAQTVGGGAGF	tr_D0AAR0	0.033	34821.36	65.00
10	HLA-A0201	LRTYLHETHGRGG	YLHETGRGG	3	0	0	5	1	YLHETHGRGG	tr_D0AAR0	0.033	34839.08	65.00
23	HLA-A0201	TEAQTVGGGAGF	AQVGGGAGF	2	0	0	2	1	AQTVGGGAGF	tr_D0AAR0	0.033	34902.84	65.00
4	HLA-A0201	RRKRGMLRTYLH	RKRGMLTYL	1	0	0	6	1	RKRGMLRTYL	tr_D0AAR0	0.033	34982.99	65.00
22	HLA-A0201	GTEAQTVGGGAGF	AQVGGGAGF	3	0	0	2	1	AQTVGGGAGF	tr_D0AAR0	0.033	35101.66	65.00
21	HLA-A0201	GGTEAQTVGGGAGF	AQVGGGAGF	4	0	0	2	1	AQTVGGGAGF	tr_D0AAR0	0.032	35252.00	65.00
3	HLA-A0201	ERRKRGMLRTYLH	RKRGMLTYL	2	0	0	6	1	RKRGMLRTYL	tr_D0AAR0	0.032	35261.16	65.00
36	HLA-A0201	MRSTPSGVSW	MSTPSGVSW	0	0	0	1	1	MRSTPSGVSW	tr_D0AAR0	0.032	35454.73	65.00
24	HLA-A0201	EAQTVGGGA	EAQTVGGGA	0	0	0	0	0	EAQTVGGGA	tr_D0AAR0	0.032	35539.62	65.00
2	HLA-A0201	LERRKRGMLRTY	LERGMLRTY	0	0	0	2	3	LERRKRGMLRTY	tr_D0AAR0	0.031	35610.45	65.00
35	HLA-A0201	PMRSTPSGVSW	PMRSTPSGV	0	0	0	0	0	PMRSTPSGV	tr_D0AAR0	0.031	35749.04	65.00
11	HLA-A0201	RTYLHETHGRG	YLHETHGRG	2	0	0	0	0	YLHETHGRG	tr_D0AAR0	0.031	35796.26	65.00
10	HLA-A0201	LRTYLHETHGRG	YLHETHGRG	3	0	0	0	0	YLHETHGRG	tr_D0AAR0	0.031	35944.52	70.00
32	HLA-A0201	AGFPMRSTPS	AGFMRSTPS	0	0	0	3	1	AGFPMRSTPS	tr_D0AAR0	0.030	36134.41	70.00
3	HLA-A0201	ERRKRGMLRTY	RKRGMLRTY	2	0	0	0	0	RKRGMLRTY	tr_D0AAR0	0.030	36307.24	70.00
11	HLA-A0201	RTYLHETHGR	RTYLHTHGR	0	0	0	5	1	RTYLHETHGR	tr_D0AAR0	0.029	36467.87	70.00
34	HLA-A0201	FPMRSTPSG	FPMRSTPSG	0	0	0	0	0	FPMRSTPSG	tr_D0AAR0	0.029	36495.52	70.00
22	HLA-A0201	GTEAQTVGG	GTEAQTVGG	0	0	0	0	0	GTEAQTVGG	tr_D0AAR0	0.028	36833.90	70.00
29	HLA-A0201	GGGAGFPMR	GGGAGFPMR	0	0	0	0	0	GGGAGFPMR	tr_D0AAR0	0.028	36853.03	70.00
16	HLA-A0201	ETHGRGGTEAQTV	GRGGTEATV	3	0	0	7	1	GRGGTEAQTV	tr_D0AAR0	0.028	36890.12	70.00
18	HLA-A0201	HGRGGTEAQTV	GRGGTEATV	1	0	0	7	1	GRGGTEAQTV	tr_D0AAR0	0.028	36891.72	70.00
29	HLA-A0201	GGGAGFPMRS	GGGAFPMRS	0	0	0	4	1	GGGAGFPMRS	tr_D0AAR0	0.028	36949.65	70.00
24	HLA-A0201	EAQTVGGGAGFP	EAQTVGAGF	0	0	0	5	2	EAQTVGGGAGF	tr_D0AAR0	0.027	37209.63	70.00
23	HLA-A0201	TEAQTVGGGAGFP	EAQTVGAGF	1	0	0	5	2	EAQTVGGGAGF	tr_D0AAR0	0.027	37223.72	75.00
22	HLA-A0201	GTEAQTVGGGAGFP	EAQTVGAGF	2	0	0	5	2	EAQTVGGGAGF	tr_D0AAR0	0.027	37239.02	75.00
27	HLA-A0201	TVGGGAGFP	TVGGGAGFP	0	0	0	0	0	TVGGGAGFP	tr_D0AAR0	0.027	37314.04	75.00
15	HLA-A0201	HETHGRGGTEAQTV	GRGGTEATV	4	0	0	7	1	GRGGTEAQTV	tr_D0AAR0	0.027	37323.72	75.00
33	HLA-A0201	GFPMRSTPS	GFPMRSTPS	0	0	0	0	0	GFPMRSTPS	tr_D0AAR0	0.027	37426.04	75.00
17	HLA-A0201	THGRGGTEAQTV	GRGGTEATV	2	0	0	7	1	GRGGTEAQTV	tr_D0AAR0	0.027	37454.00	75.00
25	HLA-A0201	AQTVGGGAGFP	AQTVGGAGF	0	0	0	4	1	AQTVGGGAGF	tr_D0AAR0	0.027	37521.33	75.00
26	HLA-A0201	QTVGGGAGFP	QTVGGGAGF	0	0	0	0	0	QTVGGGAGF	tr_D0AAR0	0.026	37548.12	75.00
10	HLA-A0201	LRTYLHETHG	LTYLHETHG	0	0	0	1	1	LRTYLHETHG	tr_D0AAR0	0.026	37658.39	75.00
28	HLA-A0201	VGGGAGFPMRS	VGGGAGFPM	0	0	0	0	0	VGGGAGFPM	tr_D0AAR0	0.026	37765.28	75.00
28	HLA-A0201	VGGGAGFPMRST	VGGGAGFPM	0	0	0	0	0	VGGGAGFPM	tr_D0AAR0	0.025	38014.13	75.00
23	HLA-A0201	TEAQTVGGGA	TAQTVGGGA	0	0	0	1	1	TEAQTVGGGA	tr_D0AAR0	0.025	38282.02	75.00
28	HLA-A0201	VGGGAGFPMRSTP	VGGGAGFPM	0	0	0	0	0	VGGGAGFPM	tr_D0AAR0	0.025	38295.68	75.00
28	HLA-A0201	VGGGAGFPMRSTPS	VGGGAGFPM	0	0	0	0	0	VGGGAGFPM	tr_D0AAR0	0.024	38424.76	80.00
25	HLA-A0201	AQTVGGGAG	AQTVGGGAG	0	0	0	0	0	AQTVGGGAG	tr_D0AAR0	0.024	38643.65	80.00
22	HLA-A0201	GTEAQTVGGGA	GTEAQTVGA	0	0	0	7	2	GTEAQTVGGGA	tr_D0AAR0	0.024	38696.37	80.00
4	HLA-A0201	RRKRGMLRT	RRKRGMLRT	0	0	0	0	0	RRKRGMLRT	tr_D0AAR0	0.023	38901.65	80.00
32	HLA-A0201	AGFPMRSTP	AGFPMRSTP	0	0	0	0	0	AGFPMRSTP	tr_D0AAR0	0.023	39095.74	80.00
20	HLA-A0201	RGGTEAQTVG	RGGTEAQTV	0	0	0	0	0	RGGTEAQTV	tr_D0AAR0	0.022	39264.04	80.00
19	HLA-A0201	GRGGTEAQTVGGGA	GTEAQTVGA	3	0	0	7	2	GTEAQTVGGGA	tr_D0AAR0	0.022	39284.03	80.00
29	HLA-A0201	GGGAGFPMRST	GGAGFPMST	1	0	0	7	1	GGAGFPMRST	tr_D0AAR0	0.022	39304.43	80.00
10	HLA-A0201	LRTYLHETH	LRTYLHETH	0	0	0	0	0	LRTYLHETH	tr_D0AAR0	0.022	39379.35	80.00
20	HLA-A0201	RGGTEAQTVGGGA	RAQTVGGGA	0	0	0	1	4	RGGTEAQTVGGGA	tr_D0AAR0	0.022	39383.18	80.00
31	HLA-A0201	GAGFPMRSTP	GAFPMRSTP	0	0	0	2	1	GAGFPMRSTP	tr_D0AAR0	0.022	39550.99	80.00
21	HLA-A0201	GGTEAQTVGGGA	GTEAQTVGA	1	0	0	7	2	GTEAQTVGGGA	tr_D0AAR0	0.022	39621.25	85.00
29	HLA-A0201	GGGAGFPMRSTPS	GGAGFPMPS	1	0	0	7	3	GGAGFPMRSTPS	tr_D0AAR0	0.021	39673.16	85.00
30	HLA-A0201	GGAGFPMRSTPS	GGAGFPMPS	0	0	0	7	3	GGAGFPMRSTPS	tr_D0AAR0	0.021	39683.88	85.00
10	HLA-A0201	LRTYLHETHGR	RTYLETHGR	1	0	0	4	1	RTYLHETHGR	tr_D0AAR0	0.021	39875.41	85.00
29	HLA-A0201	GGGAGFPMRSTP	GGAGFPMTP	1	0	0	7	2	GGAGFPMRSTP	tr_D0AAR0	0.021	39887.50	85.00
3	HLA-A0201	ERRKRGMLR	ERRKRGMLR	0	0	0	0	0	ERRKRGMLR	tr_D0AAR0	0.021	39912.95	85.00
18	HLA-A0201	HGRGGTEAQ	HGRGGTEAQ	0	0	0	0	0	HGRGGTEAQ	tr_D0AAR0	0.021	39964.39	85.00

16	HLA-A0201	ETHGRGGTEA	ETHGGGTEA	0	0	0	4	1	ETHGRGGTEA	tr_D0AAR0	0.021	40037.52	85.00
22	HLA-A0201	GTEAQTVGGG	GTAQTVGGG	0	0	0	2	1	GTEAQTVGGG	tr_D0AAR0	0.021	40051.83	85.00
30	HLA-A0201	GGAGFPMRSTP	GGAGFPMTP	0	0	0	7	2	GGAGFPMRSTP	tr_D0AAR0	0.020	40130.76	85.00
18	HLA-A0201	HGRGGTEAQTVG	GRGGTEATV	1	0	0	7	1	GRGGTEAQTV	tr_D0AAR0	0.020	40159.89	85.00
16	HLA-A0201	ETHGRGGTEAQTVG	GRGGTEATV	3	0	0	7	1	GRGGTEAQTV	tr_D0AAR0	0.020	40171.18	85.00
2	HLA-A0201	LERRKRGMLR	LERKRGMLR	0	0	0	2	1	LERRKRGMLR	tr_D0AAR0	0.019	40516.44	85.00
17	HLA-A0201	THGRGGTEAQTVG	GRGGTEATV	2	0	0	7	1	GRGGTEAQTV	tr_D0AAR0	0.019	40542.76	85.00
2	HLA-A0201	LERRKRGML	LERRKRGML	0	0	0	0	0	LERRKRGML	tr_D0AAR0	0.019	40569.54	85.00
18	HLA-A0201	HGRGGTEAQTVGG	GRGGTEATV	1	0	0	7	1	GRGGTEAQTV	tr_D0AAR0	0.019	40587.52	85.00
29	HLA-A0201	GGGAGFPMRSTPSG	GGAGFPMSG	1	0	0	7	4	GGAGFPMRSTPSG	tr_D0AAR0	0.019	40593.68	85.00
23	HLA-A0201	TEAQTVGGG	TEAQTVGGG	0	0	0	0	0	TEAQTVGGG	tr_D0AAR0	0.019	40645.10	85.00
18	HLA-A0201	HGRGGTEAQTVGGG	GRGGTEATV	1	0	0	7	1	GRGGTEAQTV	tr_D0AAR0	0.019	40678.98	85.00
19	HLA-A0201	GRGGTEAQTVG	GRGGTEATV	0	0	0	7	1	GRGGTEAQTV	tr_D0AAR0	0.019	40884.59	90.00
30	HLA-A0201	GGAGFPMRSTPSG	GGAGFPMSG	0	0	0	7	4	GGAGFPMRSTPSG	tr_D0AAR0	0.019	40887.26	90.00
31	HLA-A0201	GAGFPMRSTPS	AGFMRSTPS	1	0	0	3	1	AGFPMRSTPS	tr_D0AAR0	0.019	40904.51	90.00
17	HLA-A0201	THGRGGTEAQTVGG	GRGGTEATV	2	0	0	7	1	GRGGTEAQTV	tr_D0AAR0	0.019	40909.39	90.00
33	HLA-A0201	GFPMRSTPSG	FPMRSTPSG	1	0	0	0	0	FPMRSTPSG	tr_D0AAR0	0.018	40946.58	90.00
19	HLA-A0201	GRGGTEAQT	GRGGTEAQT	0	0	0	0	0	GRGGTEAQT	tr_D0AAR0	0.018	41023.31	90.00
3	HLA-A0201	ERRKRGMLRT	RRKRGMLRT	1	0	0	0	0	RRKRGMLRT	tr_D0AAR0	0.018	41072.15	90.00
19	HLA-A0201	GRGGTEAQTVGG	GRGGTEATV	0	0	0	7	1	GRGGTEAQTV	tr_D0AAR0	0.018	41088.15	90.00
2	HLA-A0201	LERRKRGMLRT	LERRGMLRT	0	0	0	3	2	LERRKRGMLRT	tr_D0AAR0	0.018	41132.19	90.00
19	HLA-A0201	GRGGTEAQTVGGG	GRGGTEATV	0	0	0	7	1	GRGGTEAQTV	tr_D0AAR0	0.018	41199.43	90.00
22	HLA-A0201	GTEAQTVGGGAG	GTEAQTVGA	0	0	0	7	2	GTEAQTVGGGA	tr_D0AAR0	0.018	41245.82	90.00
20	HLA-A0201	RGGTEAQTVGGGAG	RGGTEAQTV	0	0	0	0	0	RGGTEAQTV	tr_D0AAR0	0.018	41261.02	90.00
18	HLA-A0201	HGRGGTEAQT	HGGGTEAQT	0	0	0	2	1	HGRGGTEAQT	tr_D0AAR0	0.017	41392.92	90.00
24	HLA-A0201	EAQTVGGGAG	EAQTVGGGA	0	0	0	0	0	EAQTVGGGA	tr_D0AAR0	0.017	41539.17	90.00
21	HLA-A0201	GGTEAQTVGG	GTEAQTVGG	0	0	0	1	1	GGTEAQTVGG	tr_D0AAR0	0.017	41709.87	90.00
17	HLA-A0201	THGRGGTEA	THGRGGTEA	0	0	0	0	0	THGRGGTEA	tr_D0AAR0	0.017	41726.55	90.00
21	HLA-A0201	GGTEAQTVGGGAG	GTEAQTVGA	1	0	0	7	2	GTEAQTVGGGA	tr_D0AAR0	0.017	41783.48	90.00
12	HLA-A0201	TYLHETHGR	TYLHETHGR	0	0	0	0	0	TYLHETHGR	tr_D0AAR0	0.016	41830.98	90.00
32	HLA-A0201	AGFPMRSTPSG	AGFMRSTPS	0	0	0	3	1	AGFPMRSTPS	tr_D0AAR0	0.016	41840.05	90.00
31	HLA-A0201	GAGFPMRSTPSG	GAMRSTPSG	0	0	0	2	3	GAGFPMRSTPSG	tr_D0AAR0	0.016	41841.86	90.00
20	HLA-A0201	RGGTEAQTVGG	RGGTEAQTV	0	0	0	0	0	RGGTEAQTV	tr_D0AAR0	0.016	42028.33	90.00
20	HLA-A0201	RGGTEAQTVGGG	RGGTEAQTV	0	0	0	0	0	RGGTEAQTV	tr_D0AAR0	0.016	42040.14	90.00
23	HLA-A0201	TEAQTVGGGAG	TAQTVGGGA	0	0	0	1	1	TEAQTVGGGA	tr_D0AAR0	0.015	42524.62	95.00
14	HLA-A0201	LHETHGRGGTEAQT	LRGGTEAQT	0	0	0	1	5	LHETHGRGGTEAQT	tr_D0AAR0	0.015	42681.80	95.00
36	HLA-A0201	MRSTPSGVS	MRSTPSGVS	0	0	0	0	0	MRSTPSGVS	tr_D0AAR0	0.015	42716.47	95.00
17	HLA-A0201	THGRGGTEAQ	HGRGGTEAQ	1	0	0	0	0	HGRGGTEAQ	tr_D0AAR0	0.014	42770.11	95.00
21	HLA-A0201	GGTEAQTVGGG	GTEATVGGG	1	0	0	4	1	GTEAQTVGGG	tr_D0AAR0	0.014	42835.40	95.00
16	HLA-A0201	ETHGRGGTEAQT	ETGGTEAQT	0	0	0	2	3	ETHGRGGTEAQT	tr_D0AAR0	0.014	42900.81	95.00
14	HLA-A0201	LHETHGRGGTEAQ	LGRGGTEAQ	0	0	0	1	4	LHETHGRGGTEAQ	tr_D0AAR0	0.014	43024.46	95.00
16	HLA-A0201	ETHGRGGTE	ETHGRGGTE	0	0	0	0	0	ETHGRGGTE	tr_D0AAR0	0.014	43047.75	95.00
15	HLA-A0201	HETHGRGGTEAQT	ETGGTEAQT	1	0	0	2	3	ETHGRGGTEAQT	tr_D0AAR0	0.014	43141.94	95.00
16	HLA-A0201	ETHGRGGTEAQ	HGRGGTEAQ	2	0	0	0	0	HGRGGTEAQ	tr_D0AAR0	0.013	43236.33	95.00
14	HLA-A0201	LHETHGRGG	LHETHGRGG	0	0	0	0	0	LHETHGRGG	tr_D0AAR0	0.013	43275.64	95.00
15	HLA-A0201	HETHGRGGTEAQ	HGRGGTEAQ	3	0	0	0	0	HGRGGTEAQ	tr_D0AAR0	0.013	43395.67	95.00
14	HLA-A0201	LHETHGRGGTEA	ETHGGGTEA	2	0	0	4	1	ETHGRGGTEA	tr_D0AAR0	0.013	43420.56	95.00
17	HLA-A0201	THGRGGTEAQT	GRGGTEAQT	2	0	0	0	0	GRGGTEAQT	tr_D0AAR0	0.013	43447.34	95.00
15	HLA-A0201	HETHGRGGTEA	ETHGGGTEA	1	0	0	4	1	ETHGRGGTEA	tr_D0AAR0	0.013	43548.07	95.00
21	HLA-A0201	GGTEAQTVG	GGTEAQTVG	0	0	0	0	0	GGTEAQTVG	tr_D0AAR0	0.012	44137.22	99.00
14	HLA-A0201	LHETHGRGGTE	LTHGRGGTE	0	0	0	1	2	LHETHGRGGTE	tr_D0AAR0	0.010	44686.45	99.00
15	HLA-A0201	HETHGRGGTE	HTHGRGGTE	0	0	0	1	1	HETHGRGGTE	tr_D0AAR0	0.010	44791.99	99.00
14	HLA-A0201	LHETHGRGGT	LHETHGRGT	0	0	0	7	1	LHETHGRGGT	tr_D0AAR0	0.010	45064.20	99.00
15	HLA-A0201	HETHGRGGT	HETHGRGGT	0	0	0	0	0	HETHGRGGT	tr_D0AAR0	0.009	45171.10	99.00

0	HLA-A0201	EYLERRKRG	EYLERRKRG	0	0	0	0	0	EYLERRKRG	tr_D0AAR0	0.008	45751.51	99.00	

Protein tr_D0AAR0. Allele HLA-A0201. Number of high binders 0. Number of weak binders 1. Number of peptides 213

Link to Allele Frequencies in Worldwide Populations HLA-A0201

Rank Threshold for Strong binding peptides   0.500
Rank Threshold for Weak binding peptides   2.000

---

pos	HLA	peptide	Core	Offset	I_pos	I_len	D_pos	D_len	iCore	Identity	1-log50k(aff)	Affinity(nM)	%Rank	BindLevel
35	HLA-A0201	ALMGPYNSI	ALMGPYNSI	0	0	0	0	0	ALMGPYNSI	tr_D0AAR0	0.656	41.23	0.60	<= WB
45	HLA-A0201	VLFSRHTTGL	VLFSHTTGL	0	0	0	4	1	VLFSRHTTGL	tr_D0AAR0	0.556	122.13	1.20	<= WB
36	HLA-A0201	LMGPYNSIRV	LMGPYSIRV	0	0	0	5	1	LMGPYNSIRV	tr_D0AAR0	0.521	177.75	1.50	<= WB
34	HLA-A0201	LALMGPYNSI	LLMGPYNSI	0	0	0	1	1	LALMGPYNSI	tr_D0AAR0	0.465	326.79	2.50	
35	HLA-A0201	ALMGPYNSIRV	ALMGNSIRV	0	0	0	4	2	ALMGPYNSIRV	tr_D0AAR0	0.460	343.01	2.50	
34	HLA-A0201	LALMGPYNSIRV	ALMGNSIRV	1	0	0	4	2	ALMGPYNSIRV	tr_D0AAR0	0.410	593.61	3.00	
33	HLA-A0201	TLALMGPYNSIRV	ALMGNSIRV	2	0	0	4	2	ALMGPYNSIRV	tr_D0AAR0	0.400	659.95	3.50	
32	HLA-A0201	ATLALMGPYNSIRV	ALMGNSIRV	3	0	0	4	2	ALMGPYNSIRV	tr_D0AAR0	0.387	759.09	3.50	
26	HLA-A0201	NTFNPLATL	NTFNPLATL	0	0	0	0	0	NTFNPLATL	tr_D0AAR0	0.323	1511.38	5.00	
35	HLA-A0201	ALMGPYNSIR	ALMGPYNSI	0	0	0	0	0	ALMGPYNSI	tr_D0AAR0	0.313	1689.09	5.50	
35	HLA-A0201	ALMGPYNSIRVL	ALMGNSIRV	0	0	0	4	2	ALMGPYNSIRV	tr_D0AAR0	0.311	1730.26	5.50	
44	HLA-A0201	RVLFSRHTTGL	VLFSHTTGL	1	0	0	4	1	VLFSRHTTGL	tr_D0AAR0	0.297	2007.44	6.00	
43	HLA-A0201	IRVLFSRHTTGL	VLFSHTTGL	2	0	0	4	1	VLFSRHTTGL	tr_D0AAR0	0.275	2556.17	6.50	
42	HLA-A0201	SIRVLFSRHTTGL	VLFSHTTGL	3	0	0	4	1	VLFSRHTTGL	tr_D0AAR0	0.269	2732.22	7.00	
33	HLA-A0201	TLALMGPYNSIRVL	ALMGNSIRV	2	0	0	4	2	ALMGPYNSIRV	tr_D0AAR0	0.267	2770.36	7.00	
34	HLA-A0201	LALMGPYNSIRVL	ALMGNSIRV	1	0	0	4	2	ALMGPYNSIRV	tr_D0AAR0	0.267	2794.44	7.00	
41	HLA-A0201	NSIRVLFSRHTTGL	VLFSHTTGL	4	0	0	4	1	VLFSRHTTGL	tr_D0AAR0	0.265	2837.68	7.00	
31	HLA-A0201	LATLALMGPYNSI	LLMGPYNSI	0	0	0	1	4	LATLALMGPYNSI	tr_D0AAR0	0.260	2988.46	7.50	
35	HLA-A0201	ALMGPYNSIRVLF	ALMGNSIRV	0	0	0	4	2	ALMGPYNSIRV	tr_D0AAR0	0.254	3191.77	7.50	
32	HLA-A0201	ATLALMGPYNSI	ALMGPYNSI	0	0	0	1	3	ATLALMGPYNSI	tr_D0AAR0	0.251	3316.03	7.50	
33	HLA-A0201	TLALMGPYNSI	LLMGPYNSI	1	0	0	1	1	LALMGPYNSI	tr_D0AAR0	0.249	3394.77	8.00	
35	HLA-A0201	ALMGPYNSIRVLFS	ALMGNSIRV	0	0	0	4	2	ALMGPYNSIRV	tr_D0AAR0	0.243	3602.50	8.00	
30	HLA-A0201	PLATLALMGPYNSI	ALMGPYNSI	5	0	0	0	0	ALMGPYNSI	tr_D0AAR0	0.226	4348.10	9.00	
34	HLA-A0201	LALMGPYNSIRVLF	ALMGNSIRV	1	0	0	4	2	ALMGPYNSIRV	tr_D0AAR0	0.217	4775.26	9.50	
36	HLA-A0201	LMGPYNSIRVL	LMYNSIRVL	0	0	0	2	2	LMGPYNSIRVL	tr_D0AAR0	0.217	4778.73	9.50	
13	HLA-A0201	KVELFTEFCA	KVLFTEFCA	0	0	0	2	1	KVELFTEFCA	tr_D0AAR0	0.194	6149.44	11.00	
25	HLA-A0201	ENTFNPLATL	NTFNPLATL	1	0	0	0	0	NTFNPLATL	tr_D0AAR0	0.187	6587.51	12.00	
28	HLA-A0201	FNPLATLALM	FNLATLALM	0	0	0	2	1	FNPLATLALM	tr_D0AAR0	0.181	7037.11	12.00	
26	HLA-A0201	NTFNPLATLA	NTFNPLALA	0	0	0	7	1	NTFNPLATLA	tr_D0AAR0	0.176	7406.63	13.00	
4	HLA-A0201	YVKDHKAEMKVEL	YVAEMKVEL	0	0	0	2	4	YVKDHKAEMKVEL	tr_D0AAR0	0.168	8122.48	13.00	
36	HLA-A0201	LMGPYNSIRVLF	LMGPYSIRV	0	0	0	5	1	LMGPYNSIRV	tr_D0AAR0	0.163	8558.89	14.00	
36	HLA-A0201	LMGPYNSIRVLFS	LMGPYSIRV	0	0	0	5	1	LMGPYNSIRV	tr_D0AAR0	0.157	9133.85	14.00	
34	HLA-A0201	LALMGPYNSIR	LLMGPYNSI	0	0	0	1	1	LALMGPYNSI	tr_D0AAR0	0.154	9435.70	15.00	
36	HLA-A0201	LMGPYNSIRVLFSR	LMGPYSIRV	0	0	0	5	1	LMGPYNSIRV	tr_D0AAR0	0.151	9733.30	15.00	
28	HLA-A0201	FNPLATLALMG	FLATLALMG	0	0	0	1	2	FNPLATLALMG	tr_D0AAR0	0.149	9937.40	15.00	
31	HLA-A0201	LATLALMGPYNSIR	LLMGPYNSI	0	0	0	1	4	LATLALMGPYNSI	tr_D0AAR0	0.148	10038.77	15.00	
4	HLA-A0201	YVKDHKAEM	YVKDHKAEM	0	0	0	0	0	YVKDHKAEM	tr_D0AAR0	0.147	10178.11	15.00	
26	HLA-A0201	NTFNPLATLAL	NTFLATLAL	0	0	0	3	2	NTFNPLATLAL	tr_D0AAR0	0.145	10371.42	16.00	
45	HLA-A0201	VLFSRHTTG	VLFSRHTTG	0	0	0	0	0	VLFSRHTTG	tr_D0AAR0	0.145	10386.92	16.00	
32	HLA-A0201	ATLALMGPYNSIR	ALMGPYNSI	0	0	0	1	3	ATLALMGPYNSI	tr_D0AAR0	0.145	10466.35	16.00	
33	HLA-A0201	TLALMGPYNSIR	LLMGPYNSI	1	0	0	1	1	LALMGPYNSI	tr_D0AAR0	0.144	10563.96	16.00	
22	HLA-A0201	ADRENTFNPLATL	ATFNPLATL	0	0	0	1	4	ADRENTFNPLATL	tr_D0AAR0	0.143	10617.47	16.00	

33	HLA-A0201	TLALMGPYNS	TLLMGPYNS	0	0	0	2	1	TLALMGPYNS	tr_D0AAR0	0.142	10813.52	16.00
26	HLA-A0201	NTFNPLATLALM	NTFATLALM	0	0	0	3	3	NTFNPLATLALM	tr_D0AAR0	0.140	10947.84	16.00
24	HLA-A0201	RENTFNPLATL	RTFNPLATL	0	0	0	1	2	RENTFNPLATL	tr_D0AAR0	0.140	11030.60	16.00
28	HLA-A0201	FNPLATLAL	FNPLATLAL	0	0	0	0	0	FNPLATLAL	tr_D0AAR0	0.138	11178.38	17.00
13	HLA-A0201	KVELFTEFC	KVELFTEFC	0	0	0	0	0	KVELFTEFC	tr_D0AAR0	0.135	11620.62	17.00
27	HLA-A0201	TFNPLATLALMG	FLATLALMG	1	0	0	1	2	FNPLATLALMG	tr_D0AAR0	0.134	11707.70	17.00
26	HLA-A0201	NTFNPLATLALMG	FLATLALMG	2	0	0	1	2	FNPLATLALMG	tr_D0AAR0	0.133	11870.58	17.00
3	HLA-A0201	PYVKDHKAEMKVEL	YVAEMKVEL	1	0	0	2	4	YVKDHKAEMKVEL	tr_D0AAR0	0.132	11923.75	17.00
21	HLA-A0201	CADRENTFNPLATL	ATFNPLATL	1	0	0	1	4	ADRENTFNPLATL	tr_D0AAR0	0.131	12127.11	18.00
25	HLA-A0201	ENTFNPLATLAL	NTFLATLAL	1	0	0	3	2	NTFNPLATLAL	tr_D0AAR0	0.130	12215.61	18.00
25	HLA-A0201	ENTFNPLATLALMG	FLATLALMG	3	0	0	1	2	FNPLATLALMG	tr_D0AAR0	0.130	12247.37	18.00
20	HLA-A0201	FCADRENTFNPL	FCADTFNPL	0	0	0	4	3	FCADRENTFNPL	tr_D0AAR0	0.129	12404.74	18.00
25	HLA-A0201	ENTFNPLATLALM	NTFATLALM	1	0	0	3	3	NTFNPLATLALM	tr_D0AAR0	0.127	12677.20	18.00
11	HLA-A0201	EMKVELFTEF	EMVELFTEF	0	0	0	2	1	EMKVELFTEF	tr_D0AAR0	0.126	12835.92	18.00
24	HLA-A0201	RENTFNPLATLAL	NTFLATLAL	2	0	0	3	2	NTFNPLATLAL	tr_D0AAR0	0.125	12868.04	18.00
23	HLA-A0201	DRENTFNPLATL	RTFNPLATL	1	0	0	1	2	RENTFNPLATL	tr_D0AAR0	0.124	13012.67	19.00
9	HLA-A0201	KAEMKVELFT	KAMKVELFT	0	0	0	2	1	KAEMKVELFT	tr_D0AAR0	0.124	13016.62	19.00
12	HLA-A0201	MKVELFTEFC	MVELFTEFC	0	0	0	1	1	MKVELFTEFC	tr_D0AAR0	0.124	13072.79	19.00
23	HLA-A0201	DRENTFNPLATLAL	NTFLATLAL	3	0	0	3	2	NTFNPLATLAL	tr_D0AAR0	0.124	13124.38	19.00
24	HLA-A0201	RENTFNPLATLALM	NTFATLALM	2	0	0	3	3	NTFNPLATLALM	tr_D0AAR0	0.123	13268.59	19.00
15	HLA-A0201	ELFTEFCADR	ELFEFCADR	0	0	0	3	1	ELFTEFCADR	tr_D0AAR0	0.116	14296.16	20.00
29	HLA-A0201	NPLATLALM	NPLATLALM	0	0	0	0	0	NPLATLALM	tr_D0AAR0	0.114	14503.68	20.00
19	HLA-A0201	EFCADRENTFNPL	FCADTFNPL	1	0	0	4	3	FCADRENTFNPL	tr_D0AAR0	0.114	14562.02	20.00
20	HLA-A0201	FCADRENTFNPLA	FCADFNPLA	0	0	0	4	4	FCADRENTFNPLA	tr_D0AAR0	0.111	15087.93	21.00
18	HLA-A0201	TEFCADRENTFNPL	FCADTFNPL	2	0	0	4	3	FCADRENTFNPL	tr_D0AAR0	0.109	15296.51	21.00
27	HLA-A0201	TFNPLATLALM	FNLATLALM	1	0	0	2	1	FNPLATLALM	tr_D0AAR0	0.107	15674.83	22.00
29	HLA-A0201	NPLATLALMG	NLATLALMG	0	0	0	1	1	NPLATLALMG	tr_D0AAR0	0.107	15722.05	22.00
28	HLA-A0201	FNPLATLALMGPYN	FLALMGPYN	0	0	0	1	5	FNPLATLALMGPYN	tr_D0AAR0	0.107	15726.30	22.00
37	HLA-A0201	MGPYNSIRV	MGPYNSIRV	0	0	0	0	0	MGPYNSIRV	tr_D0AAR0	0.105	16028.99	22.00
40	HLA-A0201	YNSIRVLFSR	YSIRVLFSR	0	0	0	1	1	YNSIRVLFSR	tr_D0AAR0	0.104	16232.32	22.00
28	HLA-A0201	FNPLATLALMGP	FLATLALMG	0	0	0	1	2	FNPLATLALMG	tr_D0AAR0	0.103	16367.23	23.00
11	HLA-A0201	EMKVELFTEFCA	EMLFTEFCA	0	0	0	2	3	EMKVELFTEFCA	tr_D0AAR0	0.103	16463.14	23.00
12	HLA-A0201	MKVELFTEFCA	MKVELFFCA	0	0	0	6	2	MKVELFTEFCA	tr_D0AAR0	0.102	16511.12	23.00
27	HLA-A0201	TFNPLATLAL	FNPLATLAL	1	0	0	0	0	FNPLATLAL	tr_D0AAR0	0.101	16773.66	23.00
26	HLA-A0201	NTFNPLATLALMGP	FLATLALMG	2	0	0	1	2	FNPLATLALMG	tr_D0AAR0	0.099	17056.23	23.00
19	HLA-A0201	EFCADRENTFNPLA	FCADFNPLA	1	0	0	4	4	FCADRENTFNPLA	tr_D0AAR0	0.097	17477.30	24.00
10	HLA-A0201	AEMKVELFTEFCA	MKVELFFCA	2	0	0	6	2	MKVELFTEFCA	tr_D0AAR0	0.095	17909.55	24.00
27	HLA-A0201	TFNPLATLALMGP	FLATLALMG	1	0	0	1	2	FNPLATLALMG	tr_D0AAR0	0.094	18023.66	25.00
33	HLA-A0201	TLALMGPYN	TLALMGPYN	0	0	0	0	0	TLALMGPYN	tr_D0AAR0	0.094	18161.67	25.00
28	HLA-A0201	FNPLATLALMGPY	FLATLALMG	0	0	0	1	2	FNPLATLALMG	tr_D0AAR0	0.093	18197.86	25.00
9	HLA-A0201	KAEMKVELFTEFCA	MKVELFFCA	3	0	0	6	2	MKVELFTEFCA	tr_D0AAR0	0.093	18202.19	25.00
12	HLA-A0201	MKVELFTEF	MKVELFTEF	0	0	0	0	0	MKVELFTEF	tr_D0AAR0	0.092	18388.07	25.00
14	HLA-A0201	VELFTEFCA	VELFTEFCA	0	0	0	0	0	VELFTEFCA	tr_D0AAR0	0.092	18466.23	25.00
12	HLA-A0201	MKVELFTEFCAD	MLFTEFCAD	0	0	0	1	3	MKVELFTEFCAD	tr_D0AAR0	0.092	18567.40	25.00
4	HLA-A0201	YVKDHKAEMKVELF	YVAEMKVEL	0	0	0	2	4	YVKDHKAEMKVEL	tr_D0AAR0	0.091	18726.39	26.00
32	HLA-A0201	ATLALMGPY	ATLALMGPY	0	0	0	0	0	ATLALMGPY	tr_D0AAR0	0.090	18906.97	26.00
22	HLA-A0201	ADRENTFNPLATLA	ATFNPLATL	0	0	0	1	4	ADRENTFNPLATL	tr_D0AAR0	0.089	19050.71	26.00
24	HLA-A0201	RENTFNPLATLA	RTFNPLATL	0	0	0	1	2	RENTFNPLATL	tr_D0AAR0	0.089	19113.47	26.00
44	HLA-A0201	RVLFSRHTTG	RLFSRHTTG	0	0	0	1	1	RVLFSRHTTG	tr_D0AAR0	0.088	19317.84	26.00
40	HLA-A0201	YNSIRVLFS	YNSIRVLFS	0	0	0	0	0	YNSIRVLFS	tr_D0AAR0	0.088	19382.96	27.00
32	HLA-A0201	ATLALMGPYN	ATLALMGPY	0	0	0	0	0	ATLALMGPY	tr_D0AAR0	0.087	19430.42	27.00
10	HLA-A0201	AEMKVELFT	AEMKVELFT	0	0	0	0	0	AEMKVELFT	tr_D0AAR0	0.087	19440.51	27.00
15	HLA-A0201	ELFTEFCADRENTF	ELFTEFCTF	0	0	0	7	5	ELFTEFCADRENTF	tr_D0AAR0	0.087	19465.98	27.00
40	HLA-A0201	YNSIRVLFSRHTTG	YLFSRHTTG	0	0	0	1	5	YNSIRVLFSRHTTG	tr_D0AAR0	0.087	19479.88	27.00

27	HLA-A0201	TFNPLATLALMGPY	FLATLALMG	1	0	0	1	2	FNPLATLALMG	tr_D0AAR0	0.086	19680.72	27.00
14	HLA-A0201	VELFTEFCAD	VLFTEFCAD	0	0	0	1	1	VELFTEFCAD	tr_D0AAR0	0.086	19796.90	27.00
4	HLA-A0201	YVKDHKAEMKV	YVHKAEMKV	0	0	0	2	2	YVKDHKAEMKV	tr_D0AAR0	0.085	20034.15	27.00
11	HLA-A0201	EMKVELFTEFCAD	MLFTEFCAD	1	0	0	1	3	MKVELFTEFCAD	tr_D0AAR0	0.084	20052.36	27.00
25	HLA-A0201	ENTFNPLATLA	NTFNPLALA	1	0	0	7	1	NTFNPLATLA	tr_D0AAR0	0.083	20317.71	28.00
23	HLA-A0201	DRENTFNPLATLA	RTFNPLATL	1	0	0	1	2	RENTFNPLATL	tr_D0AAR0	0.083	20461.99	28.00
34	HLA-A0201	LALMGPYNS	LALMGPYNS	0	0	0	0	0	LALMGPYNS	tr_D0AAR0	0.081	20740.61	28.00
41	HLA-A0201	NSIRVLFSR	NSIRVLFSR	0	0	0	0	0	NSIRVLFSR	tr_D0AAR0	0.081	20818.17	29.00
10	HLA-A0201	AEMKVELFTEFCAD	MLFTEFCAD	2	0	0	1	3	MKVELFTEFCAD	tr_D0AAR0	0.080	20941.98	29.00
12	HLA-A0201	MKVELFTEFCADR	ELFEFCADR	3	0	0	3	1	ELFTEFCADR	tr_D0AAR0	0.080	21127.47	29.00
30	HLA-A0201	PLATLALMG	PLATLALMG	0	0	0	0	0	PLATLALMG	tr_D0AAR0	0.079	21335.59	29.00
42	HLA-A0201	SIRVLFSRHT	SIVLFSRHT	0	0	0	2	1	SIRVLFSRHT	tr_D0AAR0	0.079	21345.98	29.00
11	HLA-A0201	EMKVELFTEFC	MVELFTEFC	1	0	0	1	1	MKVELFTEFC	tr_D0AAR0	0.078	21413.06	29.00
11	HLA-A0201	EMKVELFTEFCADR	ELFEFCADR	4	0	0	3	1	ELFTEFCADR	tr_D0AAR0	0.078	21432.76	29.00
31	HLA-A0201	LATLALMGPY	LATLALMPY	0	0	0	7	1	LATLALMGPY	tr_D0AAR0	0.078	21520.84	30.00
9	HLA-A0201	KAEMKVELF	KAEMKVELF	0	0	0	0	0	KAEMKVELF	tr_D0AAR0	0.077	21645.78	30.00
20	HLA-A0201	FCADRENTFNPLAT	FCADTFNPL	0	0	0	4	3	FCADRENTFNPL	tr_D0AAR0	0.077	21755.43	30.00
10	HLA-A0201	AEMKVELFTEFC	MVELFTEFC	2	0	0	1	1	MKVELFTEFC	tr_D0AAR0	0.075	22180.39	31.00
16	HLA-A0201	LFTEFCADRENTF	LFTEFCATF	0	0	0	7	4	LFTEFCADRENTF	tr_D0AAR0	0.074	22342.26	31.00
9	HLA-A0201	KAEMKVELFTEFC	MVELFTEFC	3	0	0	1	1	MKVELFTEFC	tr_D0AAR0	0.074	22437.46	31.00
13	HLA-A0201	KVELFTEFCADR	ELFEFCADR	2	0	0	3	1	ELFTEFCADR	tr_D0AAR0	0.074	22484.36	31.00
8	HLA-A0201	HKAEMKVELFTEFC	MVELFTEFC	4	0	0	1	1	MKVELFTEFC	tr_D0AAR0	0.074	22551.12	31.00
15	HLA-A0201	ELFTEFCAD	ELFTEFCAD	0	0	0	0	0	ELFTEFCAD	tr_D0AAR0	0.072	23045.68	32.00
13	HLA-A0201	KVELFTEFCAD	KVELFTFCA	0	0	0	6	1	KVELFTEFCA	tr_D0AAR0	0.072	23058.40	32.00
15	HLA-A0201	ELFTEFCADRENT	ELFTEFCAT	0	0	0	8	4	ELFTEFCADRENT	tr_D0AAR0	0.071	23151.14	32.00
44	HLA-A0201	RVLFSRHTT	RVLFSRHTT	0	0	0	0	0	RVLFSRHTT	tr_D0AAR0	0.071	23184.99	32.00
31	HLA-A0201	LATLALMGP	LATLALMGP	0	0	0	0	0	LATLALMGP	tr_D0AAR0	0.070	23322.87	33.00
37	HLA-A0201	MGPYNSIRVL	MGYNSIRVL	0	0	0	2	1	MGPYNSIRVL	tr_D0AAR0	0.070	23333.21	33.00
27	HLA-A0201	TFNPLATLA	TFNPLATLA	0	0	0	0	0	TFNPLATLA	tr_D0AAR0	0.070	23359.99	33.00
10	HLA-A0201	AEMKVELFTEF	EMVELFTEF	1	0	0	2	1	EMKVELFTEF	tr_D0AAR0	0.070	23441.77	33.00
46	HLA-A0201	LFSRHTTGL	LFSRHTTGL	0	0	0	0	0	LFSRHTTGL	tr_D0AAR0	0.070	23463.59	33.00
14	HLA-A0201	VELFTEFCADR	ELFEFCADR	1	0	0	3	1	ELFTEFCADR	tr_D0AAR0	0.070	23516.46	33.00
17	HLA-A0201	FTEFCADRENTF	FTEFCANTF	0	0	0	6	3	FTEFCADRENTF	tr_D0AAR0	0.069	23631.24	33.00
41	HLA-A0201	NSIRVLFSRHTT	NSIRVLFTT	0	0	0	7	3	NSIRVLFSRHTT	tr_D0AAR0	0.069	23805.24	33.00
12	HLA-A0201	MKVELFTEFCADRE	MLFTEFCAD	0	0	0	1	3	MKVELFTEFCAD	tr_D0AAR0	0.068	23942.14	34.00
9	HLA-A0201	KAEMKVELFTEF	EMVELFTEF	2	0	0	2	1	EMKVELFTEF	tr_D0AAR0	0.068	23957.41	34.00
3	HLA-A0201	PYVKDHKAEMKV	YVHKAEMKV	1	0	0	2	2	YVKDHKAEMKV	tr_D0AAR0	0.068	24038.17	34.00
31	HLA-A0201	LATLALMGPYN	LLALMGPYN	0	0	0	1	2	LATLALMGPYN	tr_D0AAR0	0.067	24214.11	34.00
30	HLA-A0201	PLATLALMGP	PLALALMGP	0	0	0	3	1	PLATLALMGP	tr_D0AAR0	0.067	24298.88	34.00
8	HLA-A0201	HKAEMKVELFTEF	EMVELFTEF	3	0	0	2	1	EMKVELFTEF	tr_D0AAR0	0.067	24347.57	34.00
30	HLA-A0201	PLATLALMGPY	LATLALMPY	1	0	0	7	1	LATLALMGPY	tr_D0AAR0	0.066	24408.23	35.00
7	HLA-A0201	DHKAEMKVELFTEF	EMVELFTEF	4	0	0	2	1	EMKVELFTEF	tr_D0AAR0	0.066	24498.46	35.00
40	HLA-A0201	YNSIRVLFSRHTT	NSIRVLFTT	1	0	0	7	3	NSIRVLFSRHTT	tr_D0AAR0	0.064	24921.95	35.00
29	HLA-A0201	NPLATLALMGPY	LATLALMPY	2	0	0	7	1	LATLALMGPY	tr_D0AAR0	0.064	24936.77	36.00
30	HLA-A0201	PLATLALMGPYNS	LATLALMPY	1	0	0	7	1	LATLALMGPY	tr_D0AAR0	0.064	25124.46	36.00
31	HLA-A0201	LATLALMGPYNS	LATLALMPY	0	0	0	7	1	LATLALMGPY	tr_D0AAR0	0.063	25156.29	36.00
37	HLA-A0201	MGPYNSIRVLFSR	YSIRVLFSR	3	0	0	1	1	YNSIRVLFSR	tr_D0AAR0	0.063	25180.52	36.00
14	HLA-A0201	VELFTEFCADRENT	ELFTEFCAT	1	0	0	8	4	ELFTEFCADRENT	tr_D0AAR0	0.063	25247.09	36.00
36	HLA-A0201	LMGPYNSIR	LMGPYNSIR	0	0	0	0	0	LMGPYNSIR	tr_D0AAR0	0.063	25292.75	36.00
10	HLA-A0201	AEMKVELFTE	AMKVELFTE	0	0	0	1	1	AEMKVELFTE	tr_D0AAR0	0.063	25298.77	36.00
29	HLA-A0201	NPLATLALMGPYNS	LATLALMPY	2	0	0	7	1	LATLALMGPY	tr_D0AAR0	0.063	25321.22	36.00
20	HLA-A0201	FCADRENTF	FCADRENTF	0	0	0	0	0	FCADRENTF	tr_D0AAR0	0.063	25348.36	36.00
42	HLA-A0201	SIRVLFSRHTTG	SIRVLFHTT	0	0	0	6	2	SIRVLFSRHTT	tr_D0AAR0	0.062	25604.71	37.00
32	HLA-A0201	ATLALMGPYNS	TLLMGPYNS	1	0	0	2	1	TLALMGPYNS	tr_D0AAR0	0.062	25611.91	37.00

41	HLA-A0201	NSIRVLFSRHT	NSIRVLFST	0	0	0	8	2	NSIRVLFSRHT	tr_D0AAR0	0.061	25704.08	37.00
41	HLA-A0201	NSIRVLFSRHTTG	ILFSRHTTG	2	0	0	1	2	IRVLFSRHTTG	tr_D0AAR0	0.061	25706.32	37.00
39	HLA-A0201	PYNSIRVLFSR	YSIRVLFSR	1	0	0	1	1	YNSIRVLFSR	tr_D0AAR0	0.061	25769.53	37.00
13	HLA-A0201	KVELFTEFCADRE	KVELFTFCA	0	0	0	6	1	KVELFTEFCA	tr_D0AAR0	0.061	25834.00	37.00
38	HLA-A0201	GPYNSIRVLF	GPYNSIVLF	0	0	0	6	1	GPYNSIRVLF	tr_D0AAR0	0.061	25844.07	37.00
2	HLA-A0201	PPYVKDHKAEMKV	YVHKAEMKV	2	0	0	2	2	YVKDHKAEMKV	tr_D0AAR0	0.061	25862.83	37.00
38	HLA-A0201	GPYNSIRVLFSR	YSIRVLFSR	2	0	0	1	1	YNSIRVLFSR	tr_D0AAR0	0.061	25947.46	37.00
40	HLA-A0201	YNSIRVLFSRHT	YNSIRVLFT	0	0	0	8	3	YNSIRVLFSRHT	tr_D0AAR0	0.060	26012.40	38.00
30	HLA-A0201	PLATLALMGPYN	LATLALMPY	1	0	0	7	1	LATLALMGPY	tr_D0AAR0	0.060	26015.49	38.00
38	HLA-A0201	GPYNSIRVL	GPYNSIRVL	0	0	0	0	0	GPYNSIRVL	tr_D0AAR0	0.060	26120.14	38.00
29	HLA-A0201	NPLATLALMGPYN	LATLALMPY	2	0	0	7	1	LATLALMGPY	tr_D0AAR0	0.060	26127.48	38.00
1	HLA-A0201	IPPYVKDHKAEMKV	YVHKAEMKV	3	0	0	2	2	YVKDHKAEMKV	tr_D0AAR0	0.060	26171.33	38.00
24	HLA-A0201	RENTFNPLA	RENTFNPLA	0	0	0	0	0	RENTFNPLA	tr_D0AAR0	0.059	26298.51	38.00
39	HLA-A0201	PYNSIRVLFSRHTT	NSIRVLFTT	2	0	0	7	3	NSIRVLFSRHTT	tr_D0AAR0	0.059	26314.73	38.00
8	HLA-A0201	HKAEMKVEL	HKAEMKVEL	0	0	0	0	0	HKAEMKVEL	tr_D0AAR0	0.058	26640.74	39.00
13	HLA-A0201	KVELFTEFCADREN	KVELFTFCA	0	0	0	6	1	KVELFTEFCA	tr_D0AAR0	0.058	26689.51	39.00
15	HLA-A0201	ELFTEFCADRE	ELFEFCADR	0	0	0	3	1	ELFTEFCADR	tr_D0AAR0	0.057	26916.86	39.00
39	HLA-A0201	PYNSIRVLFS	YNSIRVLFS	1	0	0	0	0	YNSIRVLFS	tr_D0AAR0	0.057	26975.46	40.00
38	HLA-A0201	GPYNSIRVLFSRHT	SIVLFSRHT	4	0	0	2	1	SIRVLFSRHT	tr_D0AAR0	0.057	27049.12	40.00
21	HLA-A0201	CADRENTFNPL	CADNTFNPL	0	0	0	3	2	CADRENTFNPL	tr_D0AAR0	0.057	27074.30	40.00
39	HLA-A0201	PYNSIRVLFSRHT	SIVLFSRHT	3	0	0	2	1	SIRVLFSRHT	tr_D0AAR0	0.057	27090.42	40.00
42	HLA-A0201	SIRVLFSRHTT	SIRVLFHTT	0	0	0	6	2	SIRVLFSRHTT	tr_D0AAR0	0.057	27126.19	40.00
16	HLA-A0201	LFTEFCADR	LFTEFCADR	0	0	0	0	0	LFTEFCADR	tr_D0AAR0	0.056	27323.25	40.00
17	HLA-A0201	FTEFCADRENTFNP	FTEFCAFNP	0	0	0	6	5	FTEFCADRENTFNP	tr_D0AAR0	0.055	27430.49	41.00
11	HLA-A0201	EMKVELFTE	EMKVELFTE	0	0	0	0	0	EMKVELFTE	tr_D0AAR0	0.055	27505.39	41.00
16	HLA-A0201	LFTEFCADRENTFN	LFTEFCATF	0	0	0	7	4	LFTEFCADRENTF	tr_D0AAR0	0.055	27581.68	41.00
8	HLA-A0201	HKAEMKVELFT	KAMKVELFT	1	0	0	2	1	KAEMKVELFT	tr_D0AAR0	0.055	27601.98	41.00
3	HLA-A0201	PYVKDHKAEM	YVKDHKAEM	1	0	0	0	0	YVKDHKAEM	tr_D0AAR0	0.055	27622.29	41.00
43	HLA-A0201	IRVLFSRHT	IRVLFSRHT	0	0	0	0	0	IRVLFSRHT	tr_D0AAR0	0.054	27760.12	41.00
8	HLA-A0201	HKAEMKVELF	KAEMKVELF	1	0	0	0	0	KAEMKVELF	tr_D0AAR0	0.054	27785.35	41.00
38	HLA-A0201	GPYNSIRVLFS	GPYIRVLFS	0	0	0	3	2	GPYNSIRVLFS	tr_D0AAR0	0.054	27911.00	42.00
43	HLA-A0201	IRVLFSRHTTG	ILFSRHTTG	0	0	0	1	2	IRVLFSRHTTG	tr_D0AAR0	0.054	28011.44	42.00
37	HLA-A0201	MGPYNSIRVLFS	MGSIRVLFS	0	0	0	2	3	MGPYNSIRVLFS	tr_D0AAR0	0.053	28071.51	42.00
7	HLA-A0201	DHKAEMKVELFT	KAMKVELFT	2	0	0	2	1	KAEMKVELFT	tr_D0AAR0	0.053	28089.44	42.00
5	HLA-A0201	VKDHKAEMKVELFT	KAMKVELFT	4	0	0	2	1	KAEMKVELFT	tr_D0AAR0	0.053	28197.23	42.00
40	HLA-A0201	YNSIRVLFSRH	YSIRVLFSR	0	0	0	1	1	YNSIRVLFSR	tr_D0AAR0	0.053	28197.54	42.00
42	HLA-A0201	SIRVLFSRH	SIRVLFSRH	0	0	0	0	0	SIRVLFSRH	tr_D0AAR0	0.053	28218.30	42.00
6	HLA-A0201	KDHKAEMKVELFT	KAMKVELFT	3	0	0	2	1	KAEMKVELFT	tr_D0AAR0	0.053	28247.92	42.00
14	HLA-A0201	VELFTEFCADRE	ELFEFCADR	1	0	0	3	1	ELFTEFCADR	tr_D0AAR0	0.053	28260.15	42.00
15	HLA-A0201	ELFTEFCADREN	ELFEFCADR	0	0	0	3	1	ELFTEFCADR	tr_D0AAR0	0.053	28283.71	42.00
41	HLA-A0201	NSIRVLFSRH	NSIRVLFSH	0	0	0	8	1	NSIRVLFSRH	tr_D0AAR0	0.052	28396.23	43.00
43	HLA-A0201	IRVLFSRHTT	IVLFSRHTT	0	0	0	1	1	IRVLFSRHTT	tr_D0AAR0	0.052	28531.45	43.00
17	HLA-A0201	FTEFCADRENTFN	FTEFCANTF	0	0	0	6	3	FTEFCADRENTF	tr_D0AAR0	0.051	28669.14	43.00
29	HLA-A0201	NPLATLALMGP	NLATLALMG	0	0	0	1	1	NPLATLALMG	tr_D0AAR0	0.051	28742.13	44.00
22	HLA-A0201	ADRENTFNPL	ADRENTFPL	0	0	0	7	1	ADRENTFNPL	tr_D0AAR0	0.050	29049.78	44.00
37	HLA-A0201	MGPYNSIRVLFSRH	MSIRVLFSR	0	0	0	1	4	MGPYNSIRVLFSR	tr_D0AAR0	0.050	29103.58	44.00
38	HLA-A0201	GPYNSIRVLFSRH	YSIRVLFSR	2	0	0	1	1	YNSIRVLFSR	tr_D0AAR0	0.049	29447.89	45.00
39	HLA-A0201	PYNSIRVLFSRH	YSIRVLFSR	1	0	0	1	1	YNSIRVLFSR	tr_D0AAR0	0.049	29563.13	46.00
14	HLA-A0201	VELFTEFCADREN	ELFEFCADR	1	0	0	3	1	ELFTEFCADR	tr_D0AAR0	0.048	29616.92	46.00
37	HLA-A0201	MGPYNSIRVLF	GPYNSIVLF	1	0	0	6	1	GPYNSIRVLF	tr_D0AAR0	0.048	29897.03	46.00
4	HLA-A0201	YVKDHKAEMKVE	YVHKAEMKV	0	0	0	2	2	YVKDHKAEMKV	tr_D0AAR0	0.048	29902.52	46.00
16	HLA-A0201	LFTEFCADRENT	LFTEFCANT	0	0	0	7	3	LFTEFCADRENT	tr_D0AAR0	0.047	29977.67	47.00
9	HLA-A0201	KAEMKVELFTE	KAMKVELFT	0	0	0	2	1	KAEMKVELFT	tr_D0AAR0	0.047	30103.46	47.00
17	HLA-A0201	FTEFCADRE	FTEFCADRE	0	0	0	0	0	FTEFCADRE	tr_D0AAR0	0.046	30368.79	48.00

21	HLA-A0201	CADRENTFNPLA	CADTFNPLA	0	0	0	3	3	CADRENTFNPLA	tr_D0AAR0	0.045	30689.85	48.00
6	HLA-A0201	KDHKAEMKV	KDHKAEMKV	0	0	0	0	0	KDHKAEMKV	tr_D0AAR0	0.044	30918.15	49.00
8	HLA-A0201	HKAEMKVELFTE	KAMKVELFT	1	0	0	2	1	KAEMKVELFT	tr_D0AAR0	0.044	30982.78	49.00
5	HLA-A0201	VKDHKAEMKVELF	VAEMKVELF	0	0	0	1	4	VKDHKAEMKVELF	tr_D0AAR0	0.044	31088.56	50.00
6	HLA-A0201	KDHKAEMKVELFTE	KMKVELFTE	0	0	0	1	5	KDHKAEMKVELFTE	tr_D0AAR0	0.044	31124.25	50.00
7	HLA-A0201	DHKAEMKVELFTE	KAMKVELFT	2	0	0	2	1	KAEMKVELFT	tr_D0AAR0	0.043	31257.55	50.00
17	HLA-A0201	FTEFCADREN	FTEFCADRE	0	0	0	0	0	FTEFCADRE	tr_D0AAR0	0.043	31268.70	50.00
6	HLA-A0201	KDHKAEMKVELF	KAEMKVELF	3	0	0	0	0	KAEMKVELF	tr_D0AAR0	0.042	31624.94	55.00
17	HLA-A0201	FTEFCADRENT	FTEFCAENT	0	0	0	6	2	FTEFCADRENT	tr_D0AAR0	0.042	31639.32	55.00
7	HLA-A0201	DHKAEMKVELF	KAEMKVELF	2	0	0	0	0	KAEMKVELF	tr_D0AAR0	0.042	31686.25	55.00
4	HLA-A0201	YVKDHKAEMK	YVKDHKAEM	0	0	0	0	0	YVKDHKAEM	tr_D0AAR0	0.042	31753.86	55.00
39	HLA-A0201	PYNSIRVLF	PYNSIRVLF	0	0	0	0	0	PYNSIRVLF	tr_D0AAR0	0.041	32177.53	55.00
3	HLA-A0201	PYVKDHKAEMKVE	YVHKAEMKV	1	0	0	2	2	YVKDHKAEMKV	tr_D0AAR0	0.040	32342.99	55.00
0	HLA-A0201	EIPPYVKDHKAEM	EIPPYVAEM	0	0	0	6	4	EIPPYVKDHKAEM	tr_D0AAR0	0.040	32494.52	55.00
21	HLA-A0201	CADRENTFNPLAT	CADFNPLAT	0	0	0	3	4	CADRENTFNPLAT	tr_D0AAR0	0.039	32635.11	55.00
5	HLA-A0201	VKDHKAEMKV	VKDHKAEMV	0	0	0	8	1	VKDHKAEMKV	tr_D0AAR0	0.039	32683.87	55.00
16	HLA-A0201	LFTEFCADRE	LFTEFCARE	0	0	0	7	1	LFTEFCADRE	tr_D0AAR0	0.038	33031.91	55.00
19	HLA-A0201	EFCADRENTF	FCADRENTF	1	0	0	0	0	FCADRENTF	tr_D0AAR0	0.038	33087.71	60.00
23	HLA-A0201	DRENTFNPL	DRENTFNPL	0	0	0	0	0	DRENTFNPL	tr_D0AAR0	0.038	33283.75	60.00
2	HLA-A0201	PPYVKDHKAEMKVE	YVHKAEMKV	2	0	0	2	2	YVKDHKAEMKV	tr_D0AAR0	0.037	33374.99	60.00
24	HLA-A0201	RENTFNPLAT	RENTFPLAT	0	0	0	5	1	RENTFNPLAT	tr_D0AAR0	0.036	33709.61	60.00
16	HLA-A0201	LFTEFCADREN	LFTEFCADR	0	0	0	0	0	LFTEFCADR	tr_D0AAR0	0.032	35195.98	65.00
23	HLA-A0201	DRENTFNPLA	RENTFNPLA	1	0	0	0	0	RENTFNPLA	tr_D0AAR0	0.031	35563.46	65.00
7	HLA-A0201	DHKAEMKVEL	HKAEMKVEL	1	0	0	0	0	HKAEMKVEL	tr_D0AAR0	0.031	35645.14	65.00
21	HLA-A0201	CADRENTFNP	CADENTFNP	0	0	0	3	1	CADRENTFNP	tr_D0AAR0	0.031	35709.61	65.00
22	HLA-A0201	ADRENTFNPLA	ADRETFNPL	0	0	0	4	1	ADRENTFNPL	tr_D0AAR0	0.030	36057.87	70.00
0	HLA-A0201	EIPPYVKDHKAEMK	EIPPYVAEM	0	0	0	6	4	EIPPYVKDHKAEM	tr_D0AAR0	0.030	36085.57	70.00
20	HLA-A0201	FCADRENTFNP	FCAENTFNP	0	0	0	3	2	FCADRENTFNP	tr_D0AAR0	0.030	36242.88	70.00
20	HLA-A0201	FCADRENTFN	FCADRENTF	0	0	0	0	0	FCADRENTF	tr_D0AAR0	0.029	36366.60	70.00
22	HLA-A0201	ADRENTFNPLAT	RENTFPLAT	2	0	0	5	1	RENTFNPLAT	tr_D0AAR0	0.029	36462.74	70.00
18	HLA-A0201	TEFCADRENTFNP	FCAENTFNP	2	0	0	3	2	FCADRENTFNP	tr_D0AAR0	0.027	37215.25	75.00
19	HLA-A0201	EFCADRENTFNP	FCAENTFNP	1	0	0	3	2	FCADRENTFNP	tr_D0AAR0	0.027	37300.71	75.00
23	HLA-A0201	DRENTFNPLAT	RENTFPLAT	1	0	0	5	1	RENTFNPLAT	tr_D0AAR0	0.027	37341.51	75.00
0	HLA-A0201	EIPPYVKDHKA	EIPPYVHKA	0	0	0	6	2	EIPPYVKDHKA	tr_D0AAR0	0.027	37447.50	75.00
5	HLA-A0201	VKDHKAEMKVEL	VKDEMKVEL	0	0	0	3	3	VKDHKAEMKVEL	tr_D0AAR0	0.027	37523.75	75.00
18	HLA-A0201	TEFCADRENTF	FCADRENTF	2	0	0	0	0	FCADRENTF	tr_D0AAR0	0.026	37875.77	75.00
6	HLA-A0201	KDHKAEMKVEL	KDAEMKVEL	0	0	0	2	2	KDHKAEMKVEL	tr_D0AAR0	0.025	38138.97	75.00
0	HLA-A0201	EIPPYVKDH	EIPPYVKDH	0	0	0	0	0	EIPPYVKDH	tr_D0AAR0	0.024	38556.35	80.00
1	HLA-A0201	IPPYVKDHKAEMK	IPPYVAEMK	0	0	0	5	4	IPPYVKDHKAEMK	tr_D0AAR0	0.024	38628.18	80.00
1	HLA-A0201	IPPYVKDHKAEM	YVKDHKAEM	3	0	0	0	0	YVKDHKAEM	tr_D0AAR0	0.023	38803.28	80.00
22	HLA-A0201	ADRENTFNP	ADRENTFNP	0	0	0	0	0	ADRENTFNP	tr_D0AAR0	0.023	38989.71	80.00
2	HLA-A0201	PPYVKDHKAEM	YVKDHKAEM	2	0	0	0	0	YVKDHKAEM	tr_D0AAR0	0.023	39013.35	80.00
25	HLA-A0201	ENTFNPLAT	ENTFNPLAT	0	0	0	0	0	ENTFNPLAT	tr_D0AAR0	0.023	39066.15	80.00
0	HLA-A0201	EIPPYVKDHK	EIPPYVDHK	0	0	0	6	1	EIPPYVKDHK	tr_D0AAR0	0.022	39514.64	80.00
5	HLA-A0201	VKDHKAEMK	VKDHKAEMK	0	0	0	0	0	VKDHKAEMK	tr_D0AAR0	0.021	39733.71	85.00
1	HLA-A0201	IPPYVKDHKA	IPPYVDHKA	0	0	0	5	1	IPPYVKDHKA	tr_D0AAR0	0.021	39875.41	85.00
3	HLA-A0201	PYVKDHKAEMK	YVDHKAEMK	1	0	0	2	1	YVKDHKAEMK	tr_D0AAR0	0.020	40123.39	85.00
0	HLA-A0201	EIPPYVKDHKAE	EIPPYVHKA	0	0	0	6	2	EIPPYVKDHKA	tr_D0AAR0	0.020	40194.22	85.00
2	HLA-A0201	PPYVKDHKAEMK	YVDHKAEMK	2	0	0	2	1	YVKDHKAEMK	tr_D0AAR0	0.020	40245.58	85.00
5	HLA-A0201	VKDHKAEMKVE	VKDHKAEMV	0	0	0	8	1	VKDHKAEMKV	tr_D0AAR0	0.020	40303.08	85.00
1	HLA-A0201	IPPYVKDHK	IPPYVKDHK	0	0	0	0	0	IPPYVKDHK	tr_D0AAR0	0.020	40372.49	85.00
6	HLA-A0201	KDHKAEMKVE	KDHKAEMKV	0	0	0	0	0	KDHKAEMKV	tr_D0AAR0	0.019	40514.69	85.00
18	HLA-A0201	TEFCADRENTFN	FCADRENTF	2	0	0	0	0	FCADRENTF	tr_D0AAR0	0.019	40536.62	85.00
19	HLA-A0201	EFCADRENTFN	FCADRENTF	1	0	0	0	0	FCADRENTF	tr_D0AAR0	0.017	41483.03	90.00

18	HLA-A0201	TEFCADRENT	TEFCADENT	0	0	0	6	1	TEFCADRENT	tr_D0AAR0	0.017	41572.00	90.00	
21	HLA-A0201	CADRENTFN	CADRENTFN	0	0	0	0	0	CADRENTFN	tr_D0AAR0	0.017	41593.13	90.00	
2	HLA-A0201	PPYVKDHKA	PPYVKDHKA	0	0	0	0	0	PPYVKDHKA	tr_D0AAR0	0.015	42523.25	95.00	
7	HLA-A0201	DHKAEMKVE	DHKAEMKVE	0	0	0	0	0	DHKAEMKVE	tr_D0AAR0	0.015	42681.80	95.00	
19	HLA-A0201	EFCADRENT	EFCADRENT	0	0	0	0	0	EFCADRENT	tr_D0AAR0	0.015	42703.52	95.00	
18	HLA-A0201	TEFCADREN	TEFCADREN	0	0	0	0	0	TEFCADREN	tr_D0AAR0	0.014	43088.27	95.00	
1	HLA-A0201	IPPYVKDHKAE	IPYVKDHKA	0	0	0	1	1	IPPYVKDHKA	tr_D0AAR0	0.012	43849.73	99.00	
2	HLA-A0201	PPYVKDHKAE	PPYVKDHKA	0	0	0	0	0	PPYVKDHKA	tr_D0AAR0	0.009	45453.01	99.00	
3	HLA-A0201	PYVKDHKAE	PYVKDHKAE	0	0	0	0	0	PYVKDHKAE	tr_D0AAR0	0.007	46497.59	99.00	

Protein tr_D0AAR0. Allele HLA-A0201. Number of high binders 0. Number of weak binders 3. Number of peptides 267

Link to Allele Frequencies in Worldwide Populations HLA-A0201

\# Rank Threshold for Strong binding peptides 0.500
\# Rank Threshold for Weak binding peptides 2.000

---

pos	HLA	peptide	Core	Offset	I_pos	I_len	D_pos	D_len	iCore	Identity	1-log50k(aff)	Affinity(nM)	%Rank	BindLevel
12	HLA-A0201	KLIFMLFLPL	KLIFMLFPL	0	0	0	7	1	KLIFMLFLPL	tr_D0A893	0.757	13.83	0.17	<= SB
18	HLA-A0201	FLPLCGSLWA	FLLCGSLWA	0	0	0	2	1	FLPLCGSLWA	tr_D0A893	0.677	33.05	0.50	<= SB
15	HLA-A0201	FMLFLPLCGSLWAT	FMLGSLWAT	0	0	0	3	5	FMLFLPLCGSLWAT	tr_D0A893	0.612	66.74	0.80	<= WB
16	HLA-A0201	MLFLPLCGSLWATV	MLFSLWATV	0	0	0	3	5	MLFLPLCGSLWATV	tr_D0A893	0.603	73.39	0.90	<= WB
24	HLA-A0201	SLWATVYSDPGYA	SLWATVYYA	0	0	0	7	4	SLWATVYSDPGYA	tr_D0A893	0.586	88.23	1.00	<= WB
15	HLA-A0201	FMLFLPLCGSL	FMLFLPLSL	0	0	0	7	2	FMLFLPLCGSL	tr_D0A893	0.576	98.38	1.00	<= WB
16	HLA-A0201	MLFLPLCGSL	MLFLPLCSL	0	0	0	7	1	MLFLPLCGSL	tr_D0A893	0.576	98.44	1.00	<= WB
13	HLA-A0201	LIFMLFLPL	LIFMLFLPL	0	0	0	0	0	LIFMLFLPL	tr_D0A893	0.570	105.25	1.10	<= WB
11	HLA-A0201	MKLIFMLFLPL	KLIFMLFPL	1	0	0	7	1	KLIFMLFLPL	tr_D0A893	0.564	111.32	1.10	<= WB
10	HLA-A0201	NMKLIFMLFLPL	KLIFMLFPL	2	0	0	7	1	KLIFMLFLPL	tr_D0A893	0.555	123.95	1.20	<= WB
12	HLA-A0201	KLIFMLFLPLCGSL	KLIFMLFSL	0	0	0	7	5	KLIFMLFLPLCGSL	tr_D0A893	0.553	126.09	1.20	<= WB
18	HLA-A0201	FLPLCGSLWATV	FLPSLWATV	0	0	0	3	3	FLPLCGSLWATV	tr_D0A893	0.550	130.26	1.30	<= WB
9	HLA-A0201	HNMKLIFMLFLPL	KLIFMLFPL	3	0	0	7	1	KLIFMLFLPL	tr_D0A893	0.549	132.14	1.30	<= WB
23	HLA-A0201	GSLWATVYSDPGYA	SLWATVYYA	1	0	0	7	4	SLWATVYSDPGYA	tr_D0A893	0.547	134.74	1.30	<= WB
8	HLA-A0201	WHNMKLIFMLFLPL	KLIFMLFPL	4	0	0	7	1	KLIFMLFLPL	tr_D0A893	0.545	137.35	1.30	<= WB
16	HLA-A0201	MLFLPLCGSLWAT	MLFGSLWAT	0	0	0	3	4	MLFLPLCGSLWAT	tr_D0A893	0.540	144.51	1.30	<= WB
14	HLA-A0201	IFMLFLPLCGSL	FMLFLPLSL	1	0	0	7	2	FMLFLPLCGSL	tr_D0A893	0.535	153.10	1.40	<= WB
6	HLA-A0201	HLWHNMKLIFML	HLWHLIFML	0	0	0	4	3	HLWHNMKLIFML	tr_D0A893	0.525	171.33	1.50	<= WB
17	HLA-A0201	LFLPLCGSLWATV	FLPSLWATV	1	0	0	3	3	FLPLCGSLWATV	tr_D0A893	0.514	192.35	1.60	<= WB
13	HLA-A0201	LIFMLFLPLCGSL	FMLFLPLSL	2	0	0	7	2	FMLFLPLCGSL	tr_D0A893	0.513	194.40	1.60	<= WB
15	HLA-A0201	FMLFLPLCGSLWA	FMLCGSLWA	0	0	0	2	4	FMLFLPLCGSLWA	tr_D0A893	0.496	233.35	1.80	<= WB
10	HLA-A0201	NMKLIFMLFL	NMLIFMLFL	0	0	0	2	1	NMKLIFMLFL	tr_D0A893	0.495	236.64	1.80	<= WB
6	HLA-A0201	HLWHNMKLIFMLFL	HLWHFMLFL	0	0	0	4	5	HLWHNMKLIFMLFL	tr_D0A893	0.493	241.25	1.80	<= WB
6	HLA-A0201	HLWHNMKLI	HLWHNMKLI	0	0	0	0	0	HLWHNMKLI	tr_D0A893	0.490	250.14	1.90	<= WB
5	HLA-A0201	PHLWHNMKLIFML	HLWHLIFML	1	0	0	4	3	HLWHNMKLIFML	tr_D0A893	0.475	293.55	2.00	<= WB
12	HLA-A0201	KLIFMLFLPLC	KLIFMLFPL	0	0	0	7	1	KLIFMLFLPL	tr_D0A893	0.462	337.27	2.50	
4	HLA-A0201	EPHLWHNMKLIFML	HLWHLIFML	2	0	0	4	3	HLWHNMKLIFML	tr_D0A893	0.461	339.20	2.50	
24	HLA-A0201	SLWATVYSDPGYAR	SLWATVYYA	0	0	0	7	4	SLWATVYSDPGYA	tr_D0A893	0.449	387.56	2.50	
14	HLA-A0201	IFMLFLPLCGSLWA	FMLCGSLWA	1	0	0	2	4	FMLFLPLCGSLWA	tr_D0A893	0.448	391.24	2.50	
18	HLA-A0201	FLPLCGSLWAT	FLCGSLWAT	0	0	0	1	2	FLPLCGSLWAT	tr_D0A893	0.446	403.01	2.50	
19	HLA-A0201	LPLCGSLWAT	LLCGSLWAT	0	0	0	1	1	LPLCGSLWAT	tr_D0A893	0.439	433.16	2.50	
15	HLA-A0201	FMLFLPLCGS	FMLFLLCGS	0	0	0	5	1	FMLFLPLCGS	tr_D0A893	0.438	437.16	2.50	
15	HLA-A0201	FMLFLPLCGSLW	FMLFLPLSL	0	0	0	7	2	FMLFLPLCGSL	tr_D0A893	0.438	438.49	2.50	
16	HLA-A0201	MLFLPLCGSLWA	MLFCGSLWA	0	0	0	3	3	MLFLPLCGSLWA	tr_D0A893	0.438	438.51	2.50	

18	HLA-A0201	FLPLCGSLWATVY	FLPSLWATV	0	0	0	3	3	FLPLCGSLWATV	tr_D0A893	0.433	463.57	3.00
17	HLA-A0201	LFLPLCGSLWA	FLLCGSLWA	1	0	0	2	1	FLPLCGSLWA	tr_D0A893	0.423	516.92	3.00
12	HLA-A0201	KLIFMLFLPLCG	KLIFMLFPL	0	0	0	7	1	KLIFMLFLPL	tr_D0A893	0.422	520.90	3.00
11	HLA-A0201	MKLIFMLFLPLC	KLIFMLFPL	1	0	0	7	1	KLIFMLFLPL	tr_D0A893	0.420	534.11	3.00
15	HLA-A0201	FMLFLPLCG	FMLFLPLCG	0	0	0	0	0	FMLFLPLCG	tr_D0A893	0.411	588.04	3.00
10	HLA-A0201	NMKLIFMLFLPLC	KLIFMLFPL	2	0	0	7	1	KLIFMLFLPL	tr_D0A893	0.410	591.62	3.00
6	HLA-A0201	HLWHNMKLIFMLF	HLWHLIFML	0	0	0	4	3	HLWHNMKLIFML	tr_D0A893	0.407	611.00	3.00
9	HLA-A0201	HNMKLIFMLFLPLC	KLIFMLFPL	3	0	0	7	1	KLIFMLFLPL	tr_D0A893	0.405	628.39	3.50
12	HLA-A0201	KLIFMLFLPLCGS	KLIFMLFPL	0	0	0	7	1	KLIFMLFLPL	tr_D0A893	0.401	654.49	3.50
17	HLA-A0201	LFLPLCGSLWAT	FLCGSLWAT	1	0	0	1	2	FLPLCGSLWAT	tr_D0A893	0.399	664.83	3.50
17	HLA-A0201	LFLPLCGSLWATVY	FLPSLWATV	1	0	0	3	3	FLPLCGSLWATV	tr_D0A893	0.397	684.65	3.50
14	HLA-A0201	IFMLFLPLCGSLW	FMLFLPLSL	1	0	0	7	2	FMLFLPLCGSL	tr_D0A893	0.396	686.52	3.50
18	HLA-A0201	FLPLCGSLWATVYS	FLGSLWATV	0	0	0	2	3	FLPLCGSLWATV	tr_D0A893	0.394	703.59	3.50
20	HLA-A0201	PLCGSLWATV	PLCGSLWTV	0	0	0	7	1	PLCGSLWATV	tr_D0A893	0.386	767.06	3.50
12	HLA-A0201	KLIFMLFLP	KLIFMLFLP	0	0	0	0	0	KLIFMLFLP	tr_D0A893	0.385	778.40	3.50
13	HLA-A0201	LIFMLFLPLC	LIFMLFLPL	0	0	0	0	0	LIFMLFLPL	tr_D0A893	0.385	779.00	3.50
11	HLA-A0201	MKLIFMLFLPLCG	KLIFMLFPL	1	0	0	7	1	KLIFMLFLPL	tr_D0A893	0.381	807.08	3.50
13	HLA-A0201	LIFMLFLPLCGSLW	FMLFLPLSL	2	0	0	7	2	FMLFLPLCGSL	tr_D0A893	0.376	854.52	4.00
10	HLA-A0201	NMKLIFMLFLPLCG	KLIFMLFPL	2	0	0	7	1	KLIFMLFLPL	tr_D0A893	0.373	887.58	4.00
6	HLA-A0201	HLWHNMKLIFM	HLWHNLIFM	0	0	0	5	2	HLWHNMKLIFM	tr_D0A893	0.369	926.51	4.00
16	HLA-A0201	MLFLPLCGS	MLFLPLCGS	0	0	0	0	0	MLFLPLCGS	tr_D0A893	0.366	951.27	4.00
5	HLA-A0201	PHLWHNMKLIFMLF	HLWHLIFML	1	0	0	4	3	HLWHNMKLIFML	tr_D0A893	0.364	974.30	4.00
11	HLA-A0201	MKLIFMLFLPLCGS	KLIFMLFPL	1	0	0	7	1	KLIFMLFLPL	tr_D0A893	0.362	990.39	4.00
11	HLA-A0201	MKLIFMLFLP	MLIFMLFLP	0	0	0	1	1	MKLIFMLFLP	tr_D0A893	0.337	1309.98	5.00
5	HLA-A0201	PHLWHNMKLIFM	HLWHNLIFM	1	0	0	5	2	HLWHNMKLIFM	tr_D0A893	0.327	1452.43	5.00
9	HLA-A0201	HNMKLIFMLFL	NMLIFMLFL	1	0	0	2	1	NMKLIFMLFL	tr_D0A893	0.318	1604.14	5.50
4	HLA-A0201	EPHLWHNMKLIFM	HLWHNLIFM	2	0	0	5	2	HLWHNMKLIFM	tr_D0A893	0.317	1627.27	5.50
3	HLA-A0201	GEPHLWHNMKLIFM	HLWHNLIFM	3	0	0	5	2	HLWHNMKLIFM	tr_D0A893	0.313	1691.67	5.50
24	HLA-A0201	SLWATVYSDP	SLWATVYSP	0	0	0	8	1	SLWATVYSDP	tr_D0A893	0.310	1756.20	5.50
8	HLA-A0201	WHNMKLIFMLFL	NMLIFMLFL	2	0	0	2	1	NMKLIFMLFL	tr_D0A893	0.309	1756.75	5.50
16	HLA-A0201	MLFLPLCGSLW	MLFLPLCSL	0	0	0	7	1	MLFLPLCGSL	tr_D0A893	0.306	1819.88	5.50
7	HLA-A0201	LWHNMKLIFMLFL	NMLIFMLFL	3	0	0	2	1	NMKLIFMLFL	tr_D0A893	0.306	1828.34	5.50
35	HLA-A0201	YARPTFHSV	YARPTFHSV	0	0	0	0	0	YARPTFHSV	tr_D0A893	0.297	2012.16	6.00
14	HLA-A0201	IFMLFLPLCG	FMLFLPLCG	1	0	0	0	0	FMLFLPLCG	tr_D0A893	0.297	2012.88	6.00
5	HLA-A0201	PHLWHNMKLI	HLWHNMKLI	1	0	0	0	0	HLWHNMKLI	tr_D0A893	0.294	2069.26	6.00
24	HLA-A0201	SLWATVYSD	SLWATVYSD	0	0	0	0	0	SLWATVYSD	tr_D0A893	0.291	2137.43	6.00
45	HLA-A0201	SLAADGPSPREV	SLAADGPEV	0	0	0	7	3	SLAADGPSPREV	tr_D0A893	0.280	2408.78	6.50
10	HLA-A0201	NMKLIFMLFLP	NMLIFMLFL	0	0	0	2	1	NMKLIFMLFL	tr_D0A893	0.261	2973.49	7.00
35	HLA-A0201	YARPTFHSVPSL	YTFHSVPSL	0	0	0	1	3	YARPTFHSVPSL	tr_D0A893	0.259	3046.37	7.50
11	HLA-A0201	MKLIFMLFL	MKLIFMLFL	0	0	0	0	0	MKLIFMLFL	tr_D0A893	0.252	3268.58	7.50
13	HLA-A0201	LIFMLFLPLCGS	LIFMLFLPL	0	0	0	0	0	LIFMLFLPL	tr_D0A893	0.250	3334.42	7.50
6	HLA-A0201	HLWHNMKLIF	HLWHNMKLI	0	0	0	0	0	HLWHNMKLI	tr_D0A893	0.248	3399.44	8.00
24	HLA-A0201	SLWATVYSDPG	SLWATVYPG	0	0	0	7	2	SLWATVYSDPG	tr_D0A893	0.246	3483.06	8.00
94	HLA-A0201	HCYFCGMCV	HCYFCGMCV	0	0	0	0	0	HCYFCGMCV	tr_D0A893	0.245	3515.13	8.00
9	HLA-A0201	HNMKLIFMLFLP	NMLIFMLFL	1	0	0	2	1	NMKLIFMLFL	tr_D0A893	0.245	3537.03	8.00
21	HLA-A0201	LCGSLWATV	LCGSLWATV	0	0	0	0	0	LCGSLWATV	tr_D0A893	0.242	3650.56	8.00
13	HLA-A0201	LIFMLFLPLCG	LIFMLFLPL	0	0	0	0	0	LIFMLFLPL	tr_D0A893	0.241	3678.47	8.00
14	HLA-A0201	IFMLFLPLCGS	FMLFLLCGS	1	0	0	5	1	FMLFLPLCGS	tr_D0A893	0.239	3748.79	8.00
8	HLA-A0201	WHNMKLIFMLFLP	NMLIFMLFL	2	0	0	2	1	NMKLIFMLFL	tr_D0A893	0.239	3749.76	8.00
37	HLA-A0201	RPTFHSVPSL	RTFHSVPSL	0	0	0	1	1	RPTFHSVPSL	tr_D0A893	0.238	3809.96	8.50
7	HLA-A0201	LWHNMKLIFMLFLP	NMLIFMLFL	3	0	0	2	1	NMKLIFMLFL	tr_D0A893	0.237	3845.37	8.50
9	HLA-A0201	HNMKLIFML	HNMKLIFML	0	0	0	0	0	HNMKLIFML	tr_D0A893	0.231	4101.29	8.50
93	HLA-A0201	AHCYFCGMCV	ACYFCGMCV	0	0	0	1	1	AHCYFCGMCV	tr_D0A893	0.225	4389.13	9.00
14	HLA-A0201	IFMLFLPLC	IFMLFLPLC	0	0	0	0	0	IFMLFLPLC	tr_D0A893	0.222	4542.58	9.00

23	HLA-A0201	GSLWATVYSDPG	SLWATVYPG	1	0	0	7	2	SLWATVYSDPG	tr_D0A893	0.221	4562.48	9.00
44	HLA-A0201	PSLAADGPSPREV	SLAADGPEV	1	0	0	7	3	SLAADGPSPREV	tr_D0A893	0.220	4617.05	9.00
34	HLA-A0201	GYARPTFHSVPSL	YTFHSVPSL	1	0	0	1	3	YARPTFHSVPSL	tr_D0A893	0.219	4674.96	9.50
22	HLA-A0201	CGSLWATVYSDPG	SLWATVYPG	2	0	0	7	2	SLWATVYSDPG	tr_D0A893	0.215	4867.17	9.50
21	HLA-A0201	LCGSLWATVYSDPG	SLWATVYPG	3	0	0	7	2	SLWATVYSDPG	tr_D0A893	0.212	5033.85	9.50
20	HLA-A0201	PLCGSLWAT	PLCGSLWAT	0	0	0	0	0	PLCGSLWAT	tr_D0A893	0.209	5201.73	10.00
19	HLA-A0201	LPLCGSLWATV	PLCGSLWTV	1	0	0	7	1	PLCGSLWATV	tr_D0A893	0.207	5333.72	10.00
2	HLA-A0201	VGEPHLWHNMKLI	VLWHNMKLI	0	0	0	1	4	VGEPHLWHNMKLI	tr_D0A893	0.204	5528.26	11.00
43	HLA-A0201	VPSLAADGPSPREV	SLAADGPEV	2	0	0	7	3	SLAADGPSPREV	tr_D0A893	0.203	5573.85	11.00
1	HLA-A0201	VVGEPHLWHNMKLI	VLWHNMKLI	0	0	0	1	5	VVGEPHLWHNMKLI	tr_D0A893	0.202	5624.13	11.00
3	HLA-A0201	GEPHLWHNMKLI	GLWHNMKLI	0	0	0	1	3	GEPHLWHNMKLI	tr_D0A893	0.199	5828.44	11.00
28	HLA-A0201	TVYSDPGYA	TVYSDPGYA	0	0	0	0	0	TVYSDPGYA	tr_D0A893	0.196	5992.53	11.00
8	HLA-A0201	WHNMKLIFML	WNMKLIFML	0	0	0	1	1	WHNMKLIFML	tr_D0A893	0.194	6153.77	11.00
24	HLA-A0201	SLWATVYSDPGY	SLWATVYSY	0	0	0	8	3	SLWATVYSDPGY	tr_D0A893	0.193	6185.41	11.00
33	HLA-A0201	PGYARPTFHSVPSL	YTFHSVPSL	2	0	0	1	3	YARPTFHSVPSL	tr_D0A893	0.190	6367.05	11.00
18	HLA-A0201	FLPLCGSLW	FLPLCGSLW	0	0	0	0	0	FLPLCGSLW	tr_D0A893	0.189	6460.25	12.00
23	HLA-A0201	GSLWATVYSD	SLWATVYSD	1	0	0	0	0	SLWATVYSD	tr_D0A893	0.187	6636.52	12.00
23	HLA-A0201	GSLWATVYSDP	SLWATVYSP	1	0	0	8	1	SLWATVYSDP	tr_D0A893	0.182	6955.58	12.00
99	HLA-A0201	GMCVNEQDHHCGV	GMQDHHCGV	0	0	0	2	4	GMCVNEQDHHCGV	tr_D0A893	0.179	7191.21	12.00
22	HLA-A0201	CGSLWATVYSDP	SLWATVYSP	2	0	0	8	1	SLWATVYSDP	tr_D0A893	0.177	7341.92	12.00
23	HLA-A0201	GSLWATVYSDPGY	SLWATVYSY	1	0	0	8	3	SLWATVYSDPGY	tr_D0A893	0.177	7390.46	13.00
7	HLA-A0201	LWHNMKLIFML	LWMKLIFML	0	0	0	2	2	LWHNMKLIFML	tr_D0A893	0.176	7440.04	13.00
20	HLA-A0201	PLCGSLWATVYSDP	SLWATVYSP	4	0	0	8	1	SLWATVYSDP	tr_D0A893	0.175	7545.92	13.00
21	HLA-A0201	LCGSLWATVYSDP	SLWATVYSP	3	0	0	8	1	SLWATVYSDP	tr_D0A893	0.175	7557.36	13.00
89	HLA-A0201	PLRAAHCYFCGMCV	PLYFCGMCV	0	0	0	2	5	PLRAAHCYFCGMCV	tr_D0A893	0.175	7563.49	13.00
30	HLA-A0201	YSDPGYARPTFHSV	YSDPTFHSV	0	0	0	3	5	YSDPGYARPTFHSV	tr_D0A893	0.173	7700.74	13.00
22	HLA-A0201	CGSLWATVYSDPGY	SLWATVYSY	2	0	0	8	3	SLWATVYSDPGY	tr_D0A893	0.172	7735.06	13.00
92	HLA-A0201	AAHCYFCGM	AAHCYFCGM	0	0	0	0	0	AAHCYFCGM	tr_D0A893	0.169	8047.34	13.00
34	HLA-A0201	GYARPTFHSV	YARPTFHSV	1	0	0	0	0	YARPTFHSV	tr_D0A893	0.166	8280.88	13.00
10	HLA-A0201	NMKLIFMLF	NMKLIFMLF	0	0	0	0	0	NMKLIFMLF	tr_D0A893	0.163	8572.61	14.00
66	HLA-A0201	SSWEDVNGQSV	SSWEDVNSV	0	0	0	7	2	SSWEDVNGQSV	tr_D0A893	0.159	8985.83	14.00
47	HLA-A0201	AADGPSPREV	AADGPSPEV	0	0	0	7	1	AADGPSPREV	tr_D0A893	0.159	8986.61	14.00
92	HLA-A0201	AAHCYFCGMCV	AAYFCGMCV	0	0	0	2	2	AAHCYFCGMCV	tr_D0A893	0.158	9079.46	14.00
4	HLA-A0201	EPHLWHNMKLI	HLWHNMKLI	2	0	0	0	0	HLWHNMKLI	tr_D0A893	0.156	9197.61	14.00
17	HLA-A0201	LFLPLCGSL	LFLPLCGSL	0	0	0	0	0	LFLPLCGSL	tr_D0A893	0.156	9274.76	15.00
98	HLA-A0201	CGMCVNEQDHHCGV	GMQDHHCGV	1	0	0	2	4	GMCVNEQDHHCGV	tr_D0A893	0.155	9330.02	15.00
91	HLA-A0201	RAAHCYFCGMCV	RAYFCGMCV	0	0	0	2	3	RAAHCYFCGMCV	tr_D0A893	0.155	9367.65	15.00
20	HLA-A0201	PLCGSLWATVY	PLCGSLWTV	0	0	0	7	1	PLCGSLWATV	tr_D0A893	0.154	9465.05	15.00
17	HLA-A0201	LFLPLCGSLW	FLPLCGSLW	1	0	0	0	0	FLPLCGSLW	tr_D0A893	0.153	9574.02	15.00
94	HLA-A0201	HCYFCGMCVN	HCYFCGMCV	0	0	0	0	0	HCYFCGMCV	tr_D0A893	0.152	9655.37	15.00
90	HLA-A0201	LRAAHCYFCGMCV	LCYFCGMCV	0	0	0	1	4	LRAAHCYFCGMCV	tr_D0A893	0.151	9779.75	15.00
45	HLA-A0201	SLAADGPSPREVE	SLAADGPEV	0	0	0	7	3	SLAADGPSPREV	tr_D0A893	0.151	9787.58	15.00
19	HLA-A0201	LPLCGSLWATVY	LLCGSLWAT	0	0	0	1	1	LPLCGSLWAT	tr_D0A893	0.150	9830.99	15.00
19	HLA-A0201	LPLCGSLWATVYSD	LLCGSLWAT	0	0	0	1	1	LPLCGSLWAT	tr_D0A893	0.148	10063.78	15.00
9	HLA-A0201	HNMKLIFMLF	HMKLIFMLF	0	0	0	1	1	HNMKLIFMLF	tr_D0A893	0.146	10322.51	16.00
7	HLA-A0201	LWHNMKLIFMLF	LWMKLIFML	0	0	0	2	2	LWHNMKLIFML	tr_D0A893	0.144	10556.42	16.00
35	HLA-A0201	YARPTFHSVPSLA	YTFHSVPSL	0	0	0	1	3	YARPTFHSVPSL	tr_D0A893	0.141	10830.61	16.00
2	HLA-A0201	VGEPHLWHNMKLIF	HLWHNMLIF	4	0	0	6	1	HLWHNMKLIF	tr_D0A893	0.139	11082.88	16.00
75	HLA-A0201	SVERRWCSACGI	SVWCSACGI	0	0	0	2	3	SVERRWCSACGI	tr_D0A893	0.138	11178.02	17.00
3	HLA-A0201	GEPHLWHNMKLIF	HLWHNMLIF	3	0	0	6	1	HLWHNMKLIF	tr_D0A893	0.138	11263.61	17.00
7	HLA-A0201	LWHNMKLIFM	LWHNMLIFM	0	0	0	5	1	LWHNMKLIFM	tr_D0A893	0.137	11309.28	17.00
19	HLA-A0201	LPLCGSLWATVYS	LLCGSLWAT	0	0	0	1	1	LPLCGSLWAT	tr_D0A893	0.136	11475.06	17.00
81	HLA-A0201	CSACGIHRPL	CSACGIHPL	0	0	0	7	1	CSACGIHRPL	tr_D0A893	0.136	11530.32	17.00
65	HLA-A0201	ESSWEDVNGQSV	SSWEDVNSV	1	0	0	7	2	SSWEDVNGQSV	tr_D0A893	0.135	11543.30	17.00

20	HLA-A0201	PLCGSLWATVYSD	PLCGSLWTV	0	0	0	7	1	PLCGSLWATV	tr_D0A893	0.135	11631.56	17.00
101	HLA-A0201	CVNEQDHHCGVIGV	CVNEGVIGV	0	0	0	4	5	CVNEQDHHCGVIGV	tr_D0A893	0.133	11855.05	17.00
20	HLA-A0201	PLCGSLWATVYS	PLCGSLWTV	0	0	0	7	1	PLCGSLWATV	tr_D0A893	0.133	11871.49	17.00
91	HLA-A0201	RAAHCYFCGM	RAAHCYFGM	0	0	0	7	1	RAAHCYFCGM	tr_D0A893	0.132	11935.10	17.00
5	HLA-A0201	PHLWHNMKLIF	HLWHNMLIF	1	0	0	6	1	HLWHNMKLIF	tr_D0A893	0.132	12020.00	17.00
21	HLA-A0201	LCGSLWATVY	LCGSLWATV	0	0	0	0	0	LCGSLWATV	tr_D0A893	0.129	12330.60	18.00
64	HLA-A0201	RESSWEDVNGQSV	SSWEDVNSV	2	0	0	7	2	SSWEDVNGQSV	tr_D0A893	0.128	12540.09	18.00
4	HLA-A0201	EPHLWHNMKLIF	HLWHNMLIF	2	0	0	6	1	HLWHNMKLIF	tr_D0A893	0.128	12553.26	18.00
21	HLA-A0201	LCGSLWATVYSD	LLWATVYSD	0	0	0	1	3	LCGSLWATVYSD	tr_D0A893	0.127	12692.15	18.00
108	HLA-A0201	HCGVIGVCV	HCGVIGVCV	0	0	0	0	0	HCGVIGVCV	tr_D0A893	0.125	12881.28	18.00
38	HLA-A0201	PTFHSVPSL	PTFHSVPSL	0	0	0	0	0	PTFHSVPSL	tr_D0A893	0.125	12893.54	18.00
63	HLA-A0201	ERESSWEDVNGQSV	SSWEDVNSV	3	0	0	7	2	SSWEDVNGQSV	tr_D0A893	0.125	12926.79	18.00
45	HLA-A0201	SLAADGPSPREVEG	SLAADGPEV	0	0	0	7	3	SLAADGPSPREV	tr_D0A893	0.125	12945.27	19.00
73	HLA-A0201	GQSVERRWCSACGI	GQWCSACGI	0	0	0	2	5	GQSVERRWCSACGI	tr_D0A893	0.125	12955.07	19.00
8	HLA-A0201	WHNMKLIFMLF	WMKLIFMLF	0	0	0	1	2	WHNMKLIFMLF	tr_D0A893	0.122	13421.49	19.00
34	HLA-A0201	GYARPTFHSVPSLA	YTFHSVPSL	1	0	0	1	3	YARPTFHSVPSL	tr_D0A893	0.121	13457.26	19.00
36	HLA-A0201	ARPTFHSVPSL	ATFHSVPSL	0	0	0	1	2	ARPTFHSVPSL	tr_D0A893	0.121	13496.92	19.00
74	HLA-A0201	QSVERRWCSACGI	SVWCSACGI	1	0	0	2	3	SVERRWCSACGI	tr_D0A893	0.121	13521.19	19.00
44	HLA-A0201	PSLAADGPSPREVE	SLAADGPEV	1	0	0	7	3	SLAADGPSPREV	tr_D0A893	0.118	13873.00	20.00
27	HLA-A0201	ATVYSDPGYA	AVYSDPGYA	0	0	0	1	1	ATVYSDPGYA	tr_D0A893	0.118	13961.24	20.00
22	HLA-A0201	CGSLWATVYSD	SLWATVYSD	2	0	0	0	0	SLWATVYSD	tr_D0A893	0.117	14084.28	20.00
35	HLA-A0201	YARPTFHSVPSLAA	YTFHSVPSL	0	0	0	1	3	YARPTFHSVPSL	tr_D0A893	0.116	14199.35	20.00
82	HLA-A0201	SACGIHRPL	SACGIHRPL	0	0	0	0	0	SACGIHRPL	tr_D0A893	0.116	14289.66	20.00
107	HLA-A0201	HHCGVIGVCV	HHCGVIVCV	0	0	0	6	1	HHCGVIGVCV	tr_D0A893	0.116	14308.85	20.00
35	HLA-A0201	YARPTFHSVP	YARPTFHSV	0	0	0	0	0	YARPTFHSV	tr_D0A893	0.115	14358.31	20.00
8	HLA-A0201	WHNMKLIFM	WHNMKLIFM	0	0	0	0	0	WHNMKLIFM	tr_D0A893	0.111	15061.18	21.00
90	HLA-A0201	LRAAHCYFCGMCVN	LCYFCGMCV	0	0	0	1	4	LRAAHCYFCGMCV	tr_D0A893	0.111	15071.77	21.00
92	HLA-A0201	AAHCYFCGMCVN	AAYFCGMCV	0	0	0	2	2	AAHCYFCGMCV	tr_D0A893	0.110	15186.36	21.00
91	HLA-A0201	RAAHCYFCGMCVN	RAYFCGMCV	0	0	0	2	3	RAAHCYFCGMCV	tr_D0A893	0.108	15488.54	21.00
89	HLA-A0201	PLRAAHCYFC	PLAAHCYFC	0	0	0	2	1	PLRAAHCYFC	tr_D0A893	0.107	15635.53	22.00
30	HLA-A0201	YSDPGYARPT	YSDPGYAPT	0	0	0	7	1	YSDPGYARPT	tr_D0A893	0.105	16059.72	22.00
99	HLA-A0201	GMCVNEQDHHCGVI	GMCDHHCGV	0	0	0	3	4	GMCVNEQDHHCGV	tr_D0A893	0.105	16126.58	22.00
78	HLA-A0201	RRWCSACGI	RRWCSACGI	0	0	0	0	0	RRWCSACGI	tr_D0A893	0.104	16224.94	22.00
23	HLA-A0201	GSLWATVYS	GSLWATVYS	0	0	0	0	0	GSLWATVYS	tr_D0A893	0.103	16317.73	22.00
93	HLA-A0201	AHCYFCGMCVN	ACYFCGMCV	0	0	0	1	1	AHCYFCGMCV	tr_D0A893	0.103	16465.28	23.00
46	HLA-A0201	LAADGPSPREV	LAADGPSPV	0	0	0	8	2	LAADGPSPREV	tr_D0A893	0.102	16626.22	23.00
110	HLA-A0201	GVIGVCVGR	GVIGVCVGR	0	0	0	0	0	GVIGVCVGR	tr_D0A893	0.101	16701.22	23.00
45	HLA-A0201	SLAADGPSP	SLAADGPSP	0	0	0	0	0	SLAADGPSP	tr_D0A893	0.101	16801.26	23.00
92	HLA-A0201	AAHCYFCGMCVNE	AAYFCGMCV	0	0	0	2	2	AAHCYFCGMCV	tr_D0A893	0.097	17422.74	24.00
91	HLA-A0201	RAAHCYFCGMCVNE	RAYFCGMCV	0	0	0	2	3	RAAHCYFCGMCV	tr_D0A893	0.096	17711.65	24.00
88	HLA-A0201	RPLRAAHCYF	RLRAAHCYF	0	0	0	1	1	RPLRAAHCYF	tr_D0A893	0.093	18249.33	25.00
92	HLA-A0201	AAHCYFCGMCVNEQ	AAYFCGMCV	0	0	0	2	2	AAHCYFCGMCV	tr_D0A893	0.093	18353.88	25.00
19	HLA-A0201	LPLCGSLWA	LPLCGSLWA	0	0	0	0	0	LPLCGSLWA	tr_D0A893	0.092	18445.66	25.00
93	HLA-A0201	AHCYFCGMCVNE	ACYFCGMCV	0	0	0	1	1	AHCYFCGMCV	tr_D0A893	0.092	18509.03	25.00
92	HLA-A0201	AAHCYFCGMC	AAHCYFCMC	0	0	0	7	1	AAHCYFCGMC	tr_D0A893	0.091	18642.69	25.00
66	HLA-A0201	SSWEDVNGQSVE	SSWEDVNSV	0	0	0	7	2	SSWEDVNGQSV	tr_D0A893	0.090	18984.66	26.00
85	HLA-A0201	GIHRPLRAA	GIHRPLRAA	0	0	0	0	0	GIHRPLRAA	tr_D0A893	0.089	19088.67	26.00
93	HLA-A0201	AHCYFCGMCVNEQ	ACYFCGMCV	0	0	0	1	1	AHCYFCGMCV	tr_D0A893	0.088	19355.50	26.00
97	HLA-A0201	FCGMCVNEQ	FCGMCVNEQ	0	0	0	0	0	FCGMCVNEQ	tr_D0A893	0.087	19448.51	27.00
96	HLA-A0201	YFCGMCVNEQDHHC	YFCGMCVHC	0	0	0	7	5	YFCGMCVNEQDHHC	tr_D0A893	0.086	19663.47	27.00
94	HLA-A0201	HCYFCGMCVNE	HCYFCGMCV	0	0	0	0	0	HCYFCGMCV	tr_D0A893	0.085	19911.83	27.00
93	HLA-A0201	AHCYFCGMCVNEQD	ACYFCGMCV	0	0	0	1	1	AHCYFCGMCV	tr_D0A893	0.085	19912.69	27.00
75	HLA-A0201	SVERRWCSACGIH	SVWCSACGI	0	0	0	2	3	SVERRWCSACGI	tr_D0A893	0.083	20345.65	28.00
109	HLA-A0201	CGVIGVCVGR	CVIGVCVGR	0	0	0	1	1	CGVIGVCVGR	tr_D0A893	0.083	20402.74	28.00

97	HLA-A0201	FCGMCVNEQDHHC	FCGMCVHHC	0	0	0	6	4	FCGMCVNEQDHHC	tr_D0A893	0.082	20662.88	28.00
94	HLA-A0201	HCYFCGMCVNEQ	HCYFCGMCV	0	0	0	0	0	HCYFCGMCV	tr_D0A893	0.081	20739.27	28.00
80	HLA-A0201	WCSACGIHRPL	WCSACGIPL	0	0	0	7	2	WCSACGIHRPL	tr_D0A893	0.080	20927.71	29.00
94	HLA-A0201	HCYFCGMCVNEQD	HCYFCGMCV	0	0	0	0	0	HCYFCGMCV	tr_D0A893	0.079	21300.07	29.00
94	HLA-A0201	HCYFCGMCVNEQDH	HCYFCGMCV	0	0	0	0	0	HCYFCGMCV	tr_D0A893	0.078	21569.55	30.00
65	HLA-A0201	ESSWEDVNGQSVE	SSWEDVNSV	1	0	0	7	2	SSWEDVNGQSV	tr_D0A893	0.078	21611.37	30.00
33	HLA-A0201	PGYARPTFHSV	YARPTFHSV	2	0	0	0	0	YARPTFHSV	tr_D0A893	0.078	21612.78	30.00
1	HLA-A0201	VVGEPHLWHNMKL	VVLWHNMKL	0	0	0	2	4	VVGEPHLWHNMKL	tr_D0A893	0.077	21707.69	30.00
75	HLA-A0201	SVERRWCSA	SVERRWCSA	0	0	0	0	0	SVERRWCSA	tr_D0A893	0.076	21882.20	30.00
78	HLA-A0201	RRWCSACGIHRPL	WCSACGIPL	2	0	0	7	2	WCSACGIHRPL	tr_D0A893	0.076	21915.60	30.00
32	HLA-A0201	DPGYARPTFHSV	YARPTFHSV	3	0	0	0	0	YARPTFHSV	tr_D0A893	0.076	21974.73	30.00
104	HLA-A0201	EQDHHCGVIGV	EQDCGVIGV	0	0	0	3	2	EQDHHCGVIGV	tr_D0A893	0.076	22017.81	30.00
79	HLA-A0201	RWCSACGIHRPL	WCSACGIPL	1	0	0	7	2	WCSACGIHRPL	tr_D0A893	0.075	22117.13	31.00
31	HLA-A0201	SDPGYARPTFHSV	YARPTFHSV	4	0	0	0	0	YARPTFHSV	tr_D0A893	0.075	22189.03	31.00
96	HLA-A0201	YFCGMCVNEQ	YFCMCVNEQ	0	0	0	3	1	YFCGMCVNEQ	tr_D0A893	0.075	22235.42	31.00
47	HLA-A0201	AADGPSPREVEGGV	AADGPSPGV	0	0	0	7	5	AADGPSPREVEGGV	tr_D0A893	0.075	22235.66	31.00
21	HLA-A0201	LCGSLWATVYS	LCGSLWATV	0	0	0	0	0	LCGSLWATV	tr_D0A893	0.075	22259.97	31.00
85	HLA-A0201	GIHRPLRAAHCYFC	GIAAHCYFC	0	0	0	2	5	GIHRPLRAAHCYFC	tr_D0A893	0.075	22314.22	31.00
110	HLA-A0201	GVIGVCVGRR	GVIGVCVGR	0	0	0	8	1	GVIGVCVGRR	tr_D0A893	0.074	22333.79	31.00
74	HLA-A0201	QSVERRWCSACGIH	SVWCSACGI	1	0	0	2	3	SVERRWCSACGI	tr_D0A893	0.074	22469.54	31.00
66	HLA-A0201	SSWEDVNGQSVER	SSWEDVNSV	0	0	0	7	2	SSWEDVNGQSV	tr_D0A893	0.074	22485.83	31.00
64	HLA-A0201	RESSWEDVNGQSVE	SSWEDVNSV	2	0	0	7	2	SSWEDVNGQSV	tr_D0A893	0.074	22549.90	31.00
77	HLA-A0201	ERRWCSACGIHRPL	WCSACGIPL	3	0	0	7	2	WCSACGIHRPL	tr_D0A893	0.073	22576.99	31.00
90	HLA-A0201	LRAAHCYFC	LRAAHCYFC	0	0	0	0	0	LRAAHCYFC	tr_D0A893	0.073	22686.21	32.00
40	HLA-A0201	FHSVPSLAA	FHSVPSLAA	0	0	0	0	0	FHSVPSLAA	tr_D0A893	0.073	22754.80	32.00
39	HLA-A0201	TFHSVPSLA	TFHSVPSLA	0	0	0	0	0	TFHSVPSLA	tr_D0A893	0.072	22875.51	32.00
89	HLA-A0201	PLRAAHCYFCGM	PLHCYFCGM	0	0	0	2	3	PLRAAHCYFCGM	tr_D0A893	0.072	22983.93	32.00
86	HLA-A0201	IHRPLRAAHCYF	ILRAAHCYF	0	0	0	1	3	IHRPLRAAHCYF	tr_D0A893	0.072	23037.95	32.00
108	HLA-A0201	HCGVIGVCVG	HCGVIGVCV	0	0	0	0	0	HCGVIGVCV	tr_D0A893	0.071	23088.10	32.00
22	HLA-A0201	CGSLWATVYS	CSLWATVYS	0	0	0	1	1	CGSLWATVYS	tr_D0A893	0.071	23115.35	32.00
75	HLA-A0201	SVERRWCSACGIHR	SVWCSACGI	0	0	0	2	3	SVERRWCSACGI	tr_D0A893	0.071	23262.13	32.00
93	HLA-A0201	AHCYFCGMC	AHCYFCGMC	0	0	0	0	0	AHCYFCGMC	tr_D0A893	0.071	23289.33	33.00
0	HLA-A0201	HVVGEPHLWHNMKL	VVLWHNMKL	1	0	0	2	4	VVGEPHLWHNMKL	tr_D0A893	0.070	23438.47	33.00
105	HLA-A0201	QDHHCGVIGV	QDHHCVIGV	0	0	0	5	1	QDHHCGVIGV	tr_D0A893	0.070	23533.00	33.00
89	HLA-A0201	PLRAAHCYFCGMC	PLCYFCGMC	0	0	0	2	4	PLRAAHCYFCGMC	tr_D0A893	0.069	23593.43	33.00
1	HLA-A0201	VVGEPHLWHNM	VVGELWHNM	0	0	0	4	2	VVGEPHLWHNM	tr_D0A893	0.068	23833.32	33.00
89	HLA-A0201	PLRAAHCYF	PLRAAHCYF	0	0	0	0	0	PLRAAHCYF	tr_D0A893	0.068	23848.80	34.00
101	HLA-A0201	CVNEQDHHCGV	CVNEQDHGV	0	0	0	7	2	CVNEQDHHCGV	tr_D0A893	0.068	23866.35	34.00
45	HLA-A0201	SLAADGPSPR	SLADGPSPR	0	0	0	2	1	SLAADGPSPR	tr_D0A893	0.068	23902.78	34.00
66	HLA-A0201	SSWEDVNGQSVERR	SSWEDVNSV	0	0	0	7	2	SSWEDVNGQSV	tr_D0A893	0.068	23955.61	34.00
106	HLA-A0201	DHHCGVIGVCV	HHCGVIVCV	1	0	0	6	1	HHCGVIGVCV	tr_D0A893	0.067	24121.80	34.00
90	HLA-A0201	LRAAHCYFCGM	LAHCYFCGM	0	0	0	1	2	LRAAHCYFCGM	tr_D0A893	0.067	24153.40	34.00
103	HLA-A0201	NEQDHHCGVIGV	EQDCGVIGV	1	0	0	3	2	EQDHHCGVIGV	tr_D0A893	0.067	24188.19	34.00
39	HLA-A0201	TFHSVPSLAA	TFHSVSLAA	0	0	0	5	1	TFHSVPSLAA	tr_D0A893	0.067	24296.52	34.00
96	HLA-A0201	YFCGMCVNE	YFCGMCVNE	0	0	0	0	0	YFCGMCVNE	tr_D0A893	0.067	24296.78	34.00
104	HLA-A0201	EQDHHCGVIGVCV	HHCGVIVCV	3	0	0	6	1	HHCGVIGVCV	tr_D0A893	0.067	24320.19	34.00
88	HLA-A0201	RPLRAAHCYFCGM	PLHCYFCGM	1	0	0	2	3	PLRAAHCYFCGM	tr_D0A893	0.066	24416.41	35.00
84	HLA-A0201	CGIHRPLRAAHCYF	CLRAAHCYF	0	0	0	1	5	CGIHRPLRAAHCYF	tr_D0A893	0.066	24471.43	35.00
105	HLA-A0201	QDHHCGVIGVCV	HHCGVIVCV	2	0	0	6	1	HHCGVIGVCV	tr_D0A893	0.066	24534.53	35.00
88	HLA-A0201	RPLRAAHCYFCGMC	PLCYFCGMC	1	0	0	2	4	PLRAAHCYFCGMC	tr_D0A893	0.066	24545.15	35.00
36	HLA-A0201	ARPTFHSVPSLA	ATFHSVPSL	0	0	0	1	2	ARPTFHSVPSL	tr_D0A893	0.066	24599.92	35.00
103	HLA-A0201	NEQDHHCGVIGVCV	HHCGVIVCV	4	0	0	6	1	HHCGVIGVCV	tr_D0A893	0.065	24630.28	35.00
0	HLA-A0201	HVVGEPHLWHNM	HVVGLWHNM	0	0	0	4	3	HVVGEPHLWHNM	tr_D0A893	0.065	24663.62	35.00
37	HLA-A0201	RPTFHSVPSLA	RTFHSVPSL	0	0	0	1	1	RPTFHSVPSL	tr_D0A893	0.065	24673.49	35.00

91	HLA-A0201	RAAHCYFCGMC	AAHCYFCMC	1	0	0	7	1	AAHCYFCGMC	tr_D0A893	0.065	24675.90	35.00
85	HLA-A0201	GIHRPLRAAHCYF	ILRAAHCYF	1	0	0	1	3	IHRPLRAAHCYF	tr_D0A893	0.065	24731.50	35.00
67	HLA-A0201	SWEDVNGQSV	SWEDVNQSV	0	0	0	6	1	SWEDVNGQSV	tr_D0A893	0.065	24739.52	35.00
77	HLA-A0201	ERRWCSACGI	RRWCSACGI	1	0	0	0	0	RRWCSACGI	tr_D0A893	0.065	24803.84	35.00
65	HLA-A0201	ESSWEDVNGQSVER	SSWEDVQSV	1	0	0	6	2	SSWEDVNGQSV	tr_D0A893	0.065	24843.33	35.00
87	HLA-A0201	HRPLRAAHCYFCGM	PLHCYFCGM	2	0	0	2	3	PLRAAHCYFCGM	tr_D0A893	0.064	24952.17	36.00
91	HLA-A0201	RAAHCYFCG	RAAHCYFCG	0	0	0	0	0	RAAHCYFCG	tr_D0A893	0.064	25023.27	36.00
109	HLA-A0201	CGVIGVCVG	CGVIGVCVG	0	0	0	0	0	CGVIGVCVG	tr_D0A893	0.064	25029.50	36.00
97	HLA-A0201	FCGMCVNEQDHHCG	FCGMCVHHC	0	0	0	6	4	FCGMCVNEQDHHC	tr_D0A893	0.064	25097.03	36.00
102	HLA-A0201	VNEQDHHCGVIGV	EQDCGVIGV	2	0	0	3	2	EQDHHCGVIGV	tr_D0A893	0.063	25184.87	36.00
80	HLA-A0201	WCSACGIHRPLRA	SACGIPLRA	2	0	0	5	2	SACGIHRPLRA	tr_D0A893	0.063	25353.02	36.00
86	HLA-A0201	IHRPLRAAHCYFC	ILRAAHCYF	0	0	0	1	3	IHRPLRAAHCYF	tr_D0A893	0.063	25418.67	36.00
90	HLA-A0201	LRAAHCYFCGMC	AAHCYFCMC	2	0	0	7	1	AAHCYFCGMC	tr_D0A893	0.062	25689.63	37.00
88	HLA-A0201	RPLRAAHCYFC	PLAAHCYFC	1	0	0	2	1	PLRAAHCYFC	tr_D0A893	0.061	25773.15	37.00
79	HLA-A0201	RWCSACGIHRPLRA	SACGIPLRA	3	0	0	5	2	SACGIHRPLRA	tr_D0A893	0.061	25864.21	37.00
107	HLA-A0201	HHCGVIGVCVGR	HHCGVIVCV	0	0	0	6	1	HHCGVIGVCV	tr_D0A893	0.061	25876.81	37.00
80	HLA-A0201	WCSACGIHRPLRAA	WCSACGIPL	0	0	0	7	2	WCSACGIHRPL	tr_D0A893	0.061	25914.92	37.00
22	HLA-A0201	CGSLWATVY	CGSLWATVY	0	0	0	0	0	CGSLWATVY	tr_D0A893	0.061	25944.93	37.00
60	HLA-A0201	VQQERESSWEDV	VQQESWEDV	0	0	0	4	3	VQQERESSWEDV	tr_D0A893	0.061	25955.61	38.00
108	HLA-A0201	HCGVIGVCVGR	CVIGVCVGR	1	0	0	1	1	CGVIGVCVGR	tr_D0A893	0.060	26046.76	38.00
100	HLA-A0201	MCVNEQDHHCGV	CVNEQDHGV	1	0	0	7	2	CVNEQDHHCGV	tr_D0A893	0.060	26135.41	38.00
106	HLA-A0201	DHHCGVIGVCVGR	CVIGVCVGR	3	0	0	1	1	CGVIGVCVGR	tr_D0A893	0.059	26350.92	38.00
97	HLA-A0201	FCGMCVNEQD	FCGMCVNEQ	0	0	0	0	0	FCGMCVNEQ	tr_D0A893	0.059	26379.44	38.00
87	HLA-A0201	HRPLRAAHCYFC	PLAAHCYFC	2	0	0	2	1	PLRAAHCYFC	tr_D0A893	0.059	26494.14	39.00
105	HLA-A0201	QDHHCGVIGVCVGR	CVIGVCVGR	4	0	0	1	1	CGVIGVCVGR	tr_D0A893	0.059	26525.69	39.00
111	HLA-A0201	VIGVCVGRR	VIGVCVGRR	0	0	0	0	0	VIGVCVGRR	tr_D0A893	0.059	26543.21	39.00
87	HLA-A0201	HRPLRAAHCYF	RLRAAHCYF	1	0	0	1	1	RPLRAAHCYF	tr_D0A893	0.058	26597.54	39.00
81	HLA-A0201	CSACGIHRPLRA	SACGIPLRA	1	0	0	5	2	SACGIHRPLRA	tr_D0A893	0.058	26716.37	39.00
80	HLA-A0201	WCSACGIHRPLR	WCSACGIPL	0	0	0	7	2	WCSACGIHRPL	tr_D0A893	0.057	26850.55	39.00
66	HLA-A0201	SSWEDVNGQ	SSWEDVNGQ	0	0	0	0	0	SSWEDVNGQ	tr_D0A893	0.057	26868.27	39.00
46	HLA-A0201	LAADGPSPREVE	LAADGPSPV	0	0	0	8	2	LAADGPSPREV	tr_D0A893	0.057	26915.99	39.00
68	HLA-A0201	WEDVNGQSV	WEDVNGQSV	0	0	0	0	0	WEDVNGQSV	tr_D0A893	0.057	27055.85	40.00
25	HLA-A0201	LWATVYSDPGYA	LVYSDPGYA	0	0	0	1	3	LWATVYSDPGYA	tr_D0A893	0.057	27109.47	40.00
106	HLA-A0201	DHHCGVIGV	DHHCGVIGV	0	0	0	0	0	DHHCGVIGV	tr_D0A893	0.056	27135.30	40.00
36	HLA-A0201	ARPTFHSVPSLAA	ATFHSVPSL	0	0	0	1	2	ARPTFHSVPSL	tr_D0A893	0.056	27221.75	40.00
37	HLA-A0201	RPTFHSVPSLAA	RTFHSVPSL	0	0	0	1	1	RPTFHSVPSL	tr_D0A893	0.056	27252.40	40.00
107	HLA-A0201	HHCGVIGVCVGRR	HHCGVIVCV	0	0	0	6	1	HHCGVIGVCV	tr_D0A893	0.056	27360.53	40.00
78	HLA-A0201	RRWCSACGIHRPLR	WCSACGIPL	2	0	0	7	2	WCSACGIHRPL	tr_D0A893	0.055	27457.80	41.00
26	HLA-A0201	WATVYSDPGYA	WVYSDPGYA	0	0	0	1	2	WATVYSDPGYA	tr_D0A893	0.055	27464.05	41.00
107	HLA-A0201	HHCGVIGVCVG	HHCGVIVCV	0	0	0	6	1	HHCGVIGVCV	tr_D0A893	0.055	27568.84	41.00
81	HLA-A0201	CSACGIHRPLRAA	SACGILRAA	1	0	0	5	3	SACGIHRPLRAA	tr_D0A893	0.055	27608.55	41.00
95	HLA-A0201	CYFCGMCVNE	YFCGMCVNE	1	0	0	0	0	YFCGMCVNE	tr_D0A893	0.055	27630.96	41.00
7	HLA-A0201	LWHNMKLIF	LWHNMKLIF	0	0	0	0	0	LWHNMKLIF	tr_D0A893	0.055	27683.92	41.00
79	HLA-A0201	RWCSACGIHRPLR	WCSACGIPL	1	0	0	7	2	WCSACGIHRPL	tr_D0A893	0.055	27699.51	41.00
28	HLA-A0201	TVYSDPGYAR	TVYSDPGYA	0	0	0	0	0	TVYSDPGYA	tr_D0A893	0.055	27699.81	41.00
82	HLA-A0201	SACGIHRPLRA	SACGIPLRA	0	0	0	5	2	SACGIHRPLRA	tr_D0A893	0.054	27737.89	41.00
2	HLA-A0201	VGEPHLWHNM	VGEPHLWHM	0	0	0	8	1	VGEPHLWHNM	tr_D0A893	0.054	27753.81	41.00
95	HLA-A0201	CYFCGMCVN	CYFCGMCVN	0	0	0	0	0	CYFCGMCVN	tr_D0A893	0.054	27774.83	41.00
34	HLA-A0201	GYARPTFHSVP	YARPTFHSV	1	0	0	0	0	YARPTFHSV	tr_D0A893	0.054	27844.04	41.00
108	HLA-A0201	HCGVIGVCVGRR	HCGVIGVCV	0	0	0	0	0	HCGVIGVCV	tr_D0A893	0.054	27849.16	41.00
106	HLA-A0201	DHHCGVIGVCVGRR	HHCGVIVCV	1	0	0	6	1	HHCGVIGVCV	tr_D0A893	0.054	27883.83	42.00
104	HLA-A0201	EQDHHCGVI	EQDHHCGVI	0	0	0	0	0	EQDHHCGVI	tr_D0A893	0.054	27933.96	42.00
109	HLA-A0201	CGVIGVCVGRR	GVIGVCVGR	1	0	0	8	1	GVIGVCVGRR	tr_D0A893	0.054	28009.63	42.00
82	HLA-A0201	SACGIHRPLRAA	SACGILRAA	0	0	0	5	3	SACGIHRPLRAA	tr_D0A893	0.053	28031.75	42.00

107	HLA-A0201	HHCGVIGVCVGRRN	HHCGVIVCV	0	0	0	6	1	HHCGVIGVCV	tr_D0A893	0.053	28063.62	42.00
95	HLA-A0201	CYFCGMCVNEQ	CYFMCVNEQ	0	0	0	3	2	CYFCGMCVNEQ	tr_D0A893	0.053	28126.86	42.00
59	HLA-A0201	GVQQERESSWEDV	VQQESWEDV	1	0	0	4	3	VQQERESSWEDV	tr_D0A893	0.053	28128.07	42.00
35	HLA-A0201	YARPTFHSVPS	YARPTFHSV	0	0	0	0	0	YARPTFHSV	tr_D0A893	0.053	28174.67	42.00
108	HLA-A0201	HCGVIGVCVGRRN	GVIGVCVGN	2	0	0	8	2	GVIGVCVGRRN	tr_D0A893	0.053	28276.97	42.00
66	HLA-A0201	SSWEDVNGQS	SSWEDVNGS	0	0	0	8	1	SSWEDVNGQS	tr_D0A893	0.053	28294.12	43.00
109	HLA-A0201	CGVIGVCVGRRN	GVIGVCVGN	1	0	0	8	2	GVIGVCVGRRN	tr_D0A893	0.052	28460.22	43.00
107	HLA-A0201	HHCGVIGVC	HHCGVIGVC	0	0	0	0	0	HHCGVIGVC	tr_D0A893	0.052	28461.76	43.00
86	HLA-A0201	IHRPLRAAHCYFCG	ILRAAHCYF	0	0	0	1	3	IHRPLRAAHCYF	tr_D0A893	0.052	28465.76	43.00
104	HLA-A0201	EQDHHCGVIGVCVG	HHCGVIVCV	3	0	0	6	1	HHCGVIGVCV	tr_D0A893	0.052	28475.62	43.00
106	HLA-A0201	DHHCGVIGVCVG	HHCGVIVCV	1	0	0	6	1	HHCGVIGVCV	tr_D0A893	0.052	28479.31	43.00
104	HLA-A0201	EQDHHCGVIGVC	EQDCGVIGV	0	0	0	3	2	EQDHHCGVIGV	tr_D0A893	0.052	28601.60	43.00
84	HLA-A0201	CGIHRPLRA	CGIHRPLRA	0	0	0	0	0	CGIHRPLRA	tr_D0A893	0.052	28631.33	43.00
38	HLA-A0201	PTFHSVPSLA	PTFHSVPLA	0	0	0	7	1	PTFHSVPSLA	tr_D0A893	0.051	28652.10	43.00
84	HLA-A0201	CGIHRPLRAA	GIHRPLRAA	1	0	0	0	0	GIHRPLRAA	tr_D0A893	0.051	28653.02	43.00
36	HLA-A0201	ARPTFHSVPSLAAD	ATFHSVPSL	0	0	0	1	2	ARPTFHSVPSL	tr_D0A893	0.051	28749.90	44.00
80	HLA-A0201	WCSACGIHR	WCSACGIHR	0	0	0	0	0	WCSACGIHR	tr_D0A893	0.051	28773.87	44.00
105	HLA-A0201	QDHHCGVIGVCVG	HHCGVIVCV	2	0	0	6	1	HHCGVIGVCV	tr_D0A893	0.051	28779.79	44.00
37	HLA-A0201	RPTFHSVPSLAAD	RTFHSVPSL	0	0	0	1	1	RPTFHSVPSL	tr_D0A893	0.051	28786.95	44.00
30	HLA-A0201	YSDPGYARP	YSDPGYARP	0	0	0	0	0	YSDPGYARP	tr_D0A893	0.051	28931.21	44.00
88	HLA-A0201	RPLRAAHCYFCG	RLRAAHCYF	0	0	0	1	1	RPLRAAHCYF	tr_D0A893	0.050	29021.82	44.00
89	HLA-A0201	PLRAAHCYFCG	PLAAHCYFC	0	0	0	2	1	PLRAAHCYFC	tr_D0A893	0.050	29032.49	44.00
74	HLA-A0201	QSVERRWCSA	QSVERWCSA	0	0	0	4	1	QSVERRWCSA	tr_D0A893	0.050	29038.15	44.00
58	HLA-A0201	GGVQQERESSWEDV	VQQESWEDV	2	0	0	4	3	VQQERESSWEDV	tr_D0A893	0.050	29089.73	44.00
100	HLA-A0201	MCVNEQDHHCGVI	MQDHHCGVI	0	0	0	1	4	MCVNEQDHHCGVI	tr_D0A893	0.050	29142.02	45.00
83	HLA-A0201	ACGIHRPLRA	ACIHRPLRA	0	0	0	2	1	ACGIHRPLRA	tr_D0A893	0.050	29221.57	45.00
40	HLA-A0201	FHSVPSLAADGPSP	FLAADGPSP	0	0	0	1	5	FHSVPSLAADGPSP	tr_D0A893	0.050	29234.86	45.00
103	HLA-A0201	NEQDHHCGV	NEQDHHCGV	0	0	0	0	0	NEQDHHCGV	tr_D0A893	0.049	29302.00	45.00
33	HLA-A0201	PGYARPTFHSVP	YARPTFHSV	2	0	0	0	0	YARPTFHSV	tr_D0A893	0.049	29317.86	45.00
40	HLA-A0201	FHSVPSLAAD	FSVPSLAAD	0	0	0	1	1	FHSVPSLAAD	tr_D0A893	0.049	29402.04	45.00
76	HLA-A0201	VERRWCSACGI	VEWCSACGI	0	0	0	2	2	VERRWCSACGI	tr_D0A893	0.049	29442.78	45.00
110	HLA-A0201	GVIGVCVGRRN	GVIGVCVGN	0	0	0	8	2	GVIGVCVGRRN	tr_D0A893	0.049	29453.63	45.00
46	HLA-A0201	LAADGPSPREVEG	LAADGPSPV	0	0	0	8	2	LAADGPSPREV	tr_D0A893	0.049	29503.70	45.00
32	HLA-A0201	DPGYARPTFHSVP	YARPTFHSV	3	0	0	0	0	YARPTFHSV	tr_D0A893	0.049	29566.65	46.00
90	HLA-A0201	LRAAHCYFCG	LAAHCYFCG	0	0	0	1	1	LRAAHCYFCG	tr_D0A893	0.048	29588.42	46.00
78	HLA-A0201	RRWCSACGIH	RRWCSACGI	0	0	0	0	0	RRWCSACGI	tr_D0A893	0.048	29606.99	46.00
81	HLA-A0201	CSACGIHRPLR	CSACGIHPL	0	0	0	7	1	CSACGIHRPL	tr_D0A893	0.048	29711.60	46.00
31	HLA-A0201	SDPGYARPTFHSVP	YARPTFHSV	4	0	0	0	0	YARPTFHSV	tr_D0A893	0.048	29711.93	46.00
87	HLA-A0201	HRPLRAAHCYFCG	RLRAAHCYF	1	0	0	1	1	RPLRAAHCYF	tr_D0A893	0.048	29731.86	46.00
37	HLA-A0201	RPTFHSVPSLAADG	RTFHSVPSL	0	0	0	1	1	RPTFHSVPSL	tr_D0A893	0.048	29740.55	46.00
52	HLA-A0201	SPREVEGGV	SPREVEGGV	0	0	0	0	0	SPREVEGGV	tr_D0A893	0.048	29753.42	46.00
47	HLA-A0201	AADGPSPREVE	AADGPSPEV	0	0	0	7	1	AADGPSPREV	tr_D0A893	0.048	29801.76	46.00
103	HLA-A0201	NEQDHHCGVIGVC	EQDCGVIGV	1	0	0	3	2	EQDHHCGVIGV	tr_D0A893	0.048	29841.11	46.00
30	HLA-A0201	YSDPGYARPTF	YSDPGYATF	0	0	0	7	2	YSDPGYARPTF	tr_D0A893	0.048	29903.18	46.00
101	HLA-A0201	CVNEQDHHC	CVNEQDHHC	0	0	0	0	0	CVNEQDHHC	tr_D0A893	0.047	29907.71	46.00
34	HLA-A0201	GYARPTFHSVPS	YARPTFHSV	1	0	0	0	0	YARPTFHSV	tr_D0A893	0.047	29949.80	47.00
1	HLA-A0201	VVGEPHLWHN	VVGEHLWHN	0	0	0	4	1	VVGEPHLWHN	tr_D0A893	0.047	29959.52	47.00
82	HLA-A0201	SACGIHRPLR	SACGIHRPL	0	0	0	0	0	SACGIHRPL	tr_D0A893	0.047	30048.47	47.00
0	HLA-A0201	HVVGEPHLW	HVVGEPHLW	0	0	0	0	0	HVVGEPHLW	tr_D0A893	0.047	30101.83	47.00
82	HLA-A0201	SACGIHRPLRAAHC	SACGIAAHC	0	0	0	5	5	SACGIHRPLRAAHC	tr_D0A893	0.047	30207.54	47.00
103	HLA-A0201	NEQDHHCGVI	NQDHHCGVI	0	0	0	1	1	NEQDHHCGVI	tr_D0A893	0.046	30238.95	47.00
102	HLA-A0201	VNEQDHHCGVIGVC	EQDCGVIGV	2	0	0	3	2	EQDHHCGVIGV	tr_D0A893	0.046	30257.26	47.00
95	HLA-A0201	CYFCGMCVNEQD	FCMCVNEQD	2	0	0	2	1	FCGMCVNEQD	tr_D0A893	0.046	30267.41	47.00
62	HLA-A0201	QERESSWEDV	QERESSWEV	0	0	0	8	1	QERESSWEDV	tr_D0A893	0.046	30485.98	48.00

95	HLA-A0201	CYFCGMCVNEQDHH	YFCGMCVHH	1	0	0	7	4	YFCGMCVNEQDHH	tr_D0A893	0.046	30555.01	48.00
95	HLA-A0201	CYFCGMCVNEQDH	YFCGMCVNH	1	0	0	8	3	YFCGMCVNEQDH	tr_D0A893	0.045	30627.15	48.00
46	HLA-A0201	LAADGPSPREVEGG	LAADGPSPV	0	0	0	8	2	LAADGPSPREV	tr_D0A893	0.045	30720.74	49.00
1	HLA-A0201	VVGEPHLWHNMK	VVGELWHNM	0	0	0	4	2	VVGEPHLWHNM	tr_D0A893	0.045	30779.30	49.00
38	HLA-A0201	PTFHSVPSLAA	PTFHSVLAA	0	0	0	6	2	PTFHSVPSLAA	tr_D0A893	0.045	30857.67	49.00
28	HLA-A0201	TVYSDPGYARPT	YSDPGYAPT	2	0	0	7	1	YSDPGYARPT	tr_D0A893	0.045	30888.05	49.00
96	HLA-A0201	YFCGMCVNEQD	YFCMCVNEQ	0	0	0	3	1	YFCGMCVNEQ	tr_D0A893	0.044	30938.91	49.00
81	HLA-A0201	CSACGIHRPLRAAH	CSACGIHPL	0	0	0	7	1	CSACGIHRPL	tr_D0A893	0.044	31055.96	49.00
0	HLA-A0201	HVVGEPHLWHNMK	HVVGLWHNM	0	0	0	4	3	HVVGEPHLWHNM	tr_D0A893	0.044	31064.69	49.00
96	HLA-A0201	YFCGMCVNEQDHH	YFCGMCVHH	0	0	0	7	4	YFCGMCVNEQDHH	tr_D0A893	0.044	31077.13	50.00
33	HLA-A0201	PGYARPTFHSVPS	YARPTFHSV	2	0	0	0	0	YARPTFHSV	tr_D0A893	0.044	31089.58	50.00
29	HLA-A0201	VYSDPGYARPT	YSDPGYAPT	1	0	0	7	1	YSDPGYARPT	tr_D0A893	0.043	31230.83	50.00
3	HLA-A0201	GEPHLWHNM	GEPHLWHNM	0	0	0	0	0	GEPHLWHNM	tr_D0A893	0.043	31252.47	50.00
96	HLA-A0201	YFCGMCVNEQDH	YFCMCVNEQ	0	0	0	3	1	YFCGMCVNEQ	tr_D0A893	0.043	31269.38	50.00
32	HLA-A0201	DPGYARPTFHSVPS	YARPTFHSV	3	0	0	0	0	YARPTFHSV	tr_D0A893	0.043	31288.00	50.00
101	HLA-A0201	CVNEQDHHCGVI	CVNEHCGVI	0	0	0	4	3	CVNEQDHHCGVI	tr_D0A893	0.043	31314.41	50.00
100	HLA-A0201	MCVNEQDHHC	MVNEQDHHC	0	0	0	1	1	MCVNEQDHHC	tr_D0A893	0.043	31334.06	50.00
48	HLA-A0201	ADGPSPREV	ADGPSPREV	0	0	0	0	0	ADGPSPREV	tr_D0A893	0.043	31448.53	55.00
2	HLA-A0201	VGEPHLWHNMKL	VGEPHLWHL	0	0	0	8	3	VGEPHLWHNMKL	tr_D0A893	0.043	31561.70	55.00
27	HLA-A0201	ATVYSDPGYARPT	YSDPGYAPT	3	0	0	7	1	YSDPGYARPT	tr_D0A893	0.042	31627.00	55.00
29	HLA-A0201	VYSDPGYARPTF	YSDPGYATF	1	0	0	7	2	YSDPGYARPTF	tr_D0A893	0.042	31721.58	55.00
80	HLA-A0201	WCSACGIHRP	WCSACIHRP	0	0	0	5	1	WCSACGIHRP	tr_D0A893	0.042	31763.83	55.00
73	HLA-A0201	GQSVERRWCSAC	GQSVWCSAC	0	0	0	4	3	GQSVERRWCSAC	tr_D0A893	0.042	31807.49	55.00
28	HLA-A0201	TVYSDPGYARPTF	YSDPGYATF	2	0	0	7	2	YSDPGYARPTF	tr_D0A893	0.042	31824.03	55.00
44	HLA-A0201	PSLAADGPSP	SLAADGPSP	1	0	0	0	0	SLAADGPSP	tr_D0A893	0.042	31834.01	55.00
26	HLA-A0201	WATVYSDPGYARPT	YSDPGYAPT	4	0	0	7	1	YSDPGYARPT	tr_D0A893	0.042	31899.52	55.00
27	HLA-A0201	ATVYSDPGYARPTF	YSDPGYATF	3	0	0	7	2	YSDPGYARPTF	tr_D0A893	0.041	31984.19	55.00
47	HLA-A0201	AADGPSPREVEG	AADGPSPEV	0	0	0	7	1	AADGPSPREV	tr_D0A893	0.041	32046.54	55.00
73	HLA-A0201	GQSVERRWCSA	GQSVRWCSA	0	0	0	4	2	GQSVERRWCSA	tr_D0A893	0.041	32066.66	55.00
75	HLA-A0201	SVERRWCSAC	SVERWCSAC	0	0	0	3	1	SVERRWCSAC	tr_D0A893	0.041	32119.79	55.00
30	HLA-A0201	YSDPGYARPTFHS	YSDPGTFHS	0	0	0	5	4	YSDPGYARPTFHS	tr_D0A893	0.040	32322.35	55.00
79	HLA-A0201	RWCSACGIHR	RWCSACIHR	0	0	0	6	1	RWCSACGIHR	tr_D0A893	0.040	32335.29	55.00
72	HLA-A0201	NGQSVERRWCSAC	GQSVWCSAC	1	0	0	4	3	GQSVERRWCSAC	tr_D0A893	0.040	32437.95	55.00
42	HLA-A0201	SVPSLAADG	SVPSLAADG	0	0	0	0	0	SVPSLAADG	tr_D0A893	0.040	32448.49	55.00
111	HLA-A0201	VIGVCVGRRN	VIGVCVGRR	0	0	0	0	0	VIGVCVGRR	tr_D0A893	0.040	32539.90	55.00
71	HLA-A0201	VNGQSVERRWCSAC	GQSVWCSAC	2	0	0	4	3	GQSVERRWCSAC	tr_D0A893	0.040	32605.44	55.00
72	HLA-A0201	NGQSVERRWCSA	GQSVRWCSA	1	0	0	4	2	GQSVERRWCSA	tr_D0A893	0.039	32693.42	55.00
82	HLA-A0201	SACGIHRPLRAAH	SACGILRAA	0	0	0	5	3	SACGIHRPLRAA	tr_D0A893	0.039	32760.70	55.00
78	HLA-A0201	RRWCSACGIHR	RRWCSACGI	0	0	0	0	0	RRWCSACGI	tr_D0A893	0.039	32812.12	55.00
97	HLA-A0201	FCGMCVNEQDH	FCGMCVNEH	0	0	0	8	2	FCGMCVNEQDH	tr_D0A893	0.039	32820.65	55.00
1	HLA-A0201	VVGEPHLWH	VVGEPHLWH	0	0	0	0	0	VVGEPHLWH	tr_D0A893	0.039	32875.39	55.00
106	HLA-A0201	DHHCGVIGVC	DHHCGVIGV	0	0	0	0	0	DHHCGVIGV	tr_D0A893	0.038	32969.77	55.00
97	HLA-A0201	FCGMCVNEQDHH	FCGMCVNHH	0	0	0	7	3	FCGMCVNEQDHH	tr_D0A893	0.038	32974.06	55.00
60	HLA-A0201	VQQERESSWEDVN	VQQESWEDV	0	0	0	4	3	VQQERESSWEDV	tr_D0A893	0.038	32995.82	55.00
85	HLA-A0201	GIHRPLRAAHC	GIHLRAAHC	0	0	0	3	2	GIHRPLRAAHC	tr_D0A893	0.038	33015.47	55.00
46	HLA-A0201	LAADGPSPR	LAADGPSPR	0	0	0	0	0	LAADGPSPR	tr_D0A893	0.038	33053.71	55.00
99	HLA-A0201	GMCVNEQDHHC	GMCEQDHHC	0	0	0	3	2	GMCVNEQDHHC	tr_D0A893	0.038	33063.01	55.00
81	HLA-A0201	CSACGIHRP	CSACGIHRP	0	0	0	0	0	CSACGIHRP	tr_D0A893	0.038	33069.11	55.00
71	HLA-A0201	VNGQSVERRWCSA	GQSVRWCSA	2	0	0	4	2	GQSVERRWCSA	tr_D0A893	0.038	33069.45	55.00
47	HLA-A0201	AADGPSPREVEGG	AADGPSPEV	0	0	0	7	1	AADGPSPREV	tr_D0A893	0.038	33092.00	60.00
76	HLA-A0201	VERRWCSACGIHR	VEWCSACGI	0	0	0	2	2	VERRWCSACGI	tr_D0A893	0.038	33131.78	60.00
84	HLA-A0201	CGIHRPLRAAHC	GIHLRAAHC	1	0	0	3	2	GIHRPLRAAHC	tr_D0A893	0.038	33132.49	60.00
39	HLA-A0201	TFHSVPSLAAD	FSVPSLAAD	1	0	0	1	1	FHSVPSLAAD	tr_D0A893	0.038	33176.25	60.00
70	HLA-A0201	DVNGQSVERRWCSA	GQSVRWCSA	3	0	0	4	2	GQSVERRWCSA	tr_D0A893	0.038	33211.45	60.00

60	HLA-A0201	VQQERESSW	VQQERESSW	0	0	0	0	0	VQQERESSW	tr_D0A893	0.037	33353.69	60.00
100	HLA-A0201	MCVNEQDHHCGVIG	MQDHHCGVI	0	0	0	1	4	MCVNEQDHHCGVI	tr_D0A893	0.037	33366.69	60.00
25	HLA-A0201	LWATVYSDPGYAR	LVYSDPGYA	0	0	0	1	3	LWATVYSDPGYA	tr_D0A893	0.037	33464.31	60.00
26	HLA-A0201	WATVYSDPG	WATVYSDPG	0	0	0	0	0	WATVYSDPG	tr_D0A893	0.037	33479.16	60.00
83	HLA-A0201	ACGIHRPLRAAHC	GIHLRAAHC	2	0	0	3	2	GIHRPLRAAHC	tr_D0A893	0.037	33552.78	60.00
76	HLA-A0201	VERRWCSACGIH	VEWCSACGI	0	0	0	2	2	VERRWCSACGI	tr_D0A893	0.037	33585.47	60.00
26	HLA-A0201	WATVYSDPGY	WATVYSDPY	0	0	0	8	1	WATVYSDPGY	tr_D0A893	0.037	33637.47	60.00
77	HLA-A0201	ERRWCSACGIHR	RRWCSACGI	1	0	0	0	0	RRWCSACGI	tr_D0A893	0.037	33659.67	60.00
98	HLA-A0201	CGMCVNEQDHHC	GMCEQDHHC	1	0	0	3	2	GMCVNEQDHHC	tr_D0A893	0.036	33694.66	60.00
101	HLA-A0201	CVNEQDHHCGVIG	CVNECGVIG	0	0	0	4	4	CVNEQDHHCGVIG	tr_D0A893	0.036	33757.78	60.00
83	HLA-A0201	ACGIHRPLRAA	AIHRPLRAA	0	0	0	1	2	ACGIHRPLRAA	tr_D0A893	0.036	33785.18	60.00
73	HLA-A0201	GQSVERRWCSACG	GQSVWCSAC	0	0	0	4	3	GQSVERRWCSAC	tr_D0A893	0.036	33796.88	60.00
29	HLA-A0201	VYSDPGYARPTFHS	YSDPGTFHS	1	0	0	5	4	YSDPGYARPTFHS	tr_D0A893	0.036	33865.34	60.00
105	HLA-A0201	QDHHCGVIGVC	QDHHCVIGV	0	0	0	5	1	QDHHCGVIGV	tr_D0A893	0.036	33928.78	60.00
26	HLA-A0201	WATVYSDPGYAR	WVYSDPGYA	0	0	0	1	2	WATVYSDPGYA	tr_D0A893	0.036	33947.89	60.00
38	HLA-A0201	PTFHSVPSLAAD	FSVPSLAAD	2	0	0	1	1	FHSVPSLAAD	tr_D0A893	0.036	33984.62	60.00
86	HLA-A0201	IHRPLRAAHC	IHRPLAAHC	0	0	0	5	1	IHRPLRAAHC	tr_D0A893	0.035	34070.05	60.00
30	HLA-A0201	YSDPGYARPTFH	YSDPGYATF	0	0	0	7	2	YSDPGYARPTF	tr_D0A893	0.035	34113.57	60.00
65	HLA-A0201	ESSWEDVNGQ	SSWEDVNGQ	1	0	0	0	0	SSWEDVNGQ	tr_D0A893	0.035	34148.28	60.00
76	HLA-A0201	VERRWCSACGIHRP	VEWCSACGI	0	0	0	2	2	VERRWCSACGI	tr_D0A893	0.035	34200.05	60.00
85	HLA-A0201	GIHRPLRAAH	GIHRPLRAA	0	0	0	0	0	GIHRPLRAA	tr_D0A893	0.035	34253.38	60.00
2	HLA-A0201	VGEPHLWHN	VGEPHLWHN	0	0	0	0	0	VGEPHLWHN	tr_D0A893	0.035	34261.53	60.00
72	HLA-A0201	NGQSVERRWCSACG	GQSVWCSAC	1	0	0	4	3	GQSVERRWCSAC	tr_D0A893	0.035	34349.14	60.00
59	HLA-A0201	GVQQERESSWEDVN	VQQESWEDV	1	0	0	4	3	VQQERESSWEDV	tr_D0A893	0.035	34403.05	60.00
78	HLA-A0201	RRWCSACGIHRP	RRWCSACGI	0	0	0	0	0	RRWCSACGI	tr_D0A893	0.034	34425.03	60.00
4	HLA-A0201	EPHLWHNMKL	EPHLWHNML	0	0	0	8	1	EPHLWHNMKL	tr_D0A893	0.034	34494.74	60.00
76	HLA-A0201	VERRWCSAC	VERRWCSAC	0	0	0	0	0	VERRWCSAC	tr_D0A893	0.034	34530.22	60.00
60	HLA-A0201	VQQERESSWEDVNG	VQQESWEDV	0	0	0	4	3	VQQERESSWEDV	tr_D0A893	0.034	34543.67	60.00
42	HLA-A0201	SVPSLAADGP	SVPSLAAGP	0	0	0	7	1	SVPSLAADGP	tr_D0A893	0.034	34582.95	65.00
61	HLA-A0201	QQERESSWEDV	QQESSWEDV	0	0	0	2	2	QQERESSWEDV	tr_D0A893	0.034	34592.68	65.00
39	HLA-A0201	TFHSVPSLAADG	FVPSLAADG	1	0	0	1	2	FHSVPSLAADG	tr_D0A893	0.034	34739.33	65.00
85	HLA-A0201	GIHRPLRAAHCY	GIHLRAAHC	0	0	0	3	2	GIHRPLRAAHC	tr_D0A893	0.034	34746.10	65.00
77	HLA-A0201	ERRWCSACGIH	RRWCSACGI	1	0	0	0	0	RRWCSACGI	tr_D0A893	0.034	34777.30	65.00
102	HLA-A0201	VNEQDHHCGVI	VQDHHCGVI	0	0	0	1	2	VNEQDHHCGVI	tr_D0A893	0.033	34805.55	65.00
84	HLA-A0201	CGIHRPLRAAHCY	CGILRAAHC	0	0	0	3	3	CGIHRPLRAAHC	tr_D0A893	0.033	34905.88	65.00
77	HLA-A0201	ERRWCSACGIHRP	RRWCSACGI	1	0	0	0	0	RRWCSACGI	tr_D0A893	0.033	34945.16	65.00
102	HLA-A0201	VNEQDHHCGV	VEQDHHCGV	0	0	0	1	1	VNEQDHHCGV	tr_D0A893	0.033	34996.62	65.00
27	HLA-A0201	ATVYSDPGYAR	AVYSDPGYA	0	0	0	1	1	ATVYSDPGYA	tr_D0A893	0.033	35004.21	65.00
39	HLA-A0201	TFHSVPSLAADGPS	TFHSVPLAA	0	0	0	6	1	TFHSVPSLAA	tr_D0A893	0.033	35097.11	65.00
79	HLA-A0201	RWCSACGIH	RWCSACGIH	0	0	0	0	0	RWCSACGIH	tr_D0A893	0.033	35113.43	65.00
28	HLA-A0201	TVYSDPGYARPTFH	TVYSDPGYA	0	0	0	0	0	TVYSDPGYA	tr_D0A893	0.033	35168.57	65.00
25	HLA-A0201	LWATVYSDPGYARP	LVYSDPGYA	0	0	0	1	3	LWATVYSDPGYA	tr_D0A893	0.033	35174.68	65.00
83	HLA-A0201	ACGIHRPLRAAHCY	IHRPLAHCY	3	0	0	5	2	IHRPLRAAHCY	tr_D0A893	0.032	35223.41	65.00
29	HLA-A0201	VYSDPGYARPTFH	YSDPGYATF	1	0	0	7	2	YSDPGYARPTF	tr_D0A893	0.032	35254.30	65.00
41	HLA-A0201	HSVPSLAAD	HSVPSLAAD	0	0	0	0	0	HSVPSLAAD	tr_D0A893	0.032	35282.91	65.00
38	HLA-A0201	PTFHSVPSLAADG	TFHSVPLAA	1	0	0	6	1	TFHSVPSLAA	tr_D0A893	0.032	35321.89	65.00
2	HLA-A0201	VGEPHLWHNMK	VGEPHLWHM	0	0	0	8	1	VGEPHLWHNM	tr_D0A893	0.032	35352.86	65.00
0	HLA-A0201	HVVGEPHLWHN	HVVGHLWHN	0	0	0	4	2	HVVGEPHLWHN	tr_D0A893	0.032	35465.09	65.00
39	HLA-A0201	TFHSVPSLAADGP	TFHSVPLAA	0	0	0	6	1	TFHSVPSLAA	tr_D0A893	0.031	35622.39	65.00
26	HLA-A0201	WATVYSDPGYARP	WVYSDPGYA	0	0	0	1	2	WATVYSDPGYA	tr_D0A893	0.031	35636.66	65.00
41	HLA-A0201	HSVPSLAADGPSPR	SLADGPSPR	4	0	0	2	1	SLAADGPSPR	tr_D0A893	0.031	35641.28	65.00
41	HLA-A0201	HSVPSLAADG	HSVPSLAAG	0	0	0	8	1	HSVPSLAADG	tr_D0A893	0.031	35649.39	65.00
74	HLA-A0201	QSVERRWCSAC	QSVEWCSAC	0	0	0	4	2	QSVERRWCSAC	tr_D0A893	0.031	35812.54	65.00
40	HLA-A0201	FHSVPSLAADG	FVPSLAADG	0	0	0	1	2	FHSVPSLAADG	tr_D0A893	0.031	35821.06	65.00

79	HLA-A0201	RWCSACGIHRP	WCSACGIHP	1	0	0	8	1	WCSACGIHRP	tr_D0A893	0.031	35877.29	65.00
3	HLA-A0201	GEPHLWHNMKL	GEPHLWMKL	0	0	0	6	2	GEPHLWHNMKL	tr_D0A893	0.031	35881.57	65.00
42	HLA-A0201	SVPSLAADGPSPR	SVPSLAAPR	0	0	0	7	4	SVPSLAADGPSPR	tr_D0A893	0.031	35923.52	65.00
38	HLA-A0201	PTFHSVPSLAADGP	TFHSVPLAA	1	0	0	6	1	TFHSVPSLAA	tr_D0A893	0.030	35953.84	70.00
87	HLA-A0201	HRPLRAAHC	HRPLRAAHC	0	0	0	0	0	HRPLRAAHC	tr_D0A893	0.030	35955.41	70.00
25	HLA-A0201	LWATVYSDPGY	LWATVYSGY	0	0	0	7	2	LWATVYSDPGY	tr_D0A893	0.030	35956.57	70.00
74	HLA-A0201	QSVERRWCS	QSVERRWCS	0	0	0	0	0	QSVERRWCS	tr_D0A893	0.030	36112.53	70.00
83	HLA-A0201	ACGIHRPLRAAH	CIHRPLRAA	1	0	0	1	1	CGIHRPLRAA	tr_D0A893	0.030	36115.65	70.00
73	HLA-A0201	GQSVERRWCS	GQVERRWCS	0	0	0	2	1	GQSVERRWCS	tr_D0A893	0.030	36168.45	70.00
0	HLA-A0201	HVVGEPHLWH	HVVGEPLWH	0	0	0	6	1	HVVGEPHLWH	tr_D0A893	0.030	36186.06	70.00
44	HLA-A0201	PSLAADGPSPR	SLADGPSPR	1	0	0	2	1	SLAADGPSPR	tr_D0A893	0.030	36202.52	70.00
51	HLA-A0201	PSPREVEGGV	SPREVEGGV	1	0	0	0	0	SPREVEGGV	tr_D0A893	0.030	36297.80	70.00
27	HLA-A0201	ATVYSDPGY	ATVYSDPGY	0	0	0	0	0	ATVYSDPGY	tr_D0A893	0.030	36335.94	70.00
63	HLA-A0201	ERESSWEDV	ERESSWEDV	0	0	0	0	0	ERESSWEDV	tr_D0A893	0.029	36337.50	70.00
43	HLA-A0201	VPSLAADGPSPR	SLADGPSPR	2	0	0	2	1	SLAADGPSPR	tr_D0A893	0.029	36376.45	70.00
86	HLA-A0201	IHRPLRAAHCY	IHRPLAHCY	0	0	0	5	2	IHRPLRAAHCY	tr_D0A893	0.029	36405.20	70.00
88	HLA-A0201	RPLRAAHCY	RPLRAAHCY	0	0	0	0	0	RPLRAAHCY	tr_D0A893	0.029	36605.83	70.00
4	HLA-A0201	EPHLWHNMK	EPHLWHNMK	0	0	0	0	0	EPHLWHNMK	tr_D0A893	0.029	36636.75	70.00
84	HLA-A0201	CGIHRPLRAAH	CIHRPLRAA	0	0	0	1	1	CGIHRPLRAA	tr_D0A893	0.029	36653.79	70.00
40	HLA-A0201	FHSVPSLAADGPS	SVPSLAAPS	2	0	0	7	2	SVPSLAADGPS	tr_D0A893	0.029	36696.27	70.00
61	HLA-A0201	QQERESSWEDVNGQ	QQWEDVNGQ	0	0	0	2	5	QQERESSWEDVNGQ	tr_D0A893	0.029	36726.05	70.00
27	HLA-A0201	ATVYSDPGYARP	AVYSDPGYA	0	0	0	1	1	ATVYSDPGYA	tr_D0A893	0.028	36763.42	70.00
65	HLA-A0201	ESSWEDVNGQS	SSWEDVNGS	1	0	0	8	1	SSWEDVNGQS	tr_D0A893	0.028	36773.36	70.00
29	HLA-A0201	VYSDPGYARP	YSDPGYARP	1	0	0	0	0	YSDPGYARP	tr_D0A893	0.028	36802.42	70.00
62	HLA-A0201	QERESSWEDVNGQS	SSWEDVNGS	4	0	0	8	1	SSWEDVNGQS	tr_D0A893	0.028	36835.49	70.00
67	HLA-A0201	SWEDVNGQSVERRW	SWEDVNQSV	0	0	0	6	1	SWEDVNGQSV	tr_D0A893	0.028	36870.57	70.00
75	HLA-A0201	SVERRWCSACG	SVEWCSACG	0	0	0	3	2	SVERRWCSACG	tr_D0A893	0.028	36914.09	70.00
98	HLA-A0201	CGMCVNEQD	CGMCVNEQD	0	0	0	0	0	CGMCVNEQD	tr_D0A893	0.028	36949.24	70.00
64	HLA-A0201	RESSWEDVNGQS	SSWEDVNGS	2	0	0	8	1	SSWEDVNGQS	tr_D0A893	0.028	36969.65	70.00
42	HLA-A0201	SVPSLAADGPS	SVPSLAAPS	0	0	0	7	2	SVPSLAADGPS	tr_D0A893	0.028	36985.65	70.00
86	HLA-A0201	IHRPLRAAH	IHRPLRAAH	0	0	0	0	0	IHRPLRAAH	tr_D0A893	0.028	37077.81	70.00
63	HLA-A0201	ERESSWEDVNGQS	SSWEDVNGS	3	0	0	8	1	SSWEDVNGQS	tr_D0A893	0.027	37148.08	70.00
74	HLA-A0201	QSVERRWCSACG	QSVEWCSAC	0	0	0	4	2	QSVERRWCSAC	tr_D0A893	0.027	37240.24	75.00
87	HLA-A0201	HRPLRAAHCY	HRPLRAAHY	0	0	0	8	1	HRPLRAAHCY	tr_D0A893	0.027	37292.25	75.00
37	HLA-A0201	RPTFHSVPS	RPTFHSVPS	0	0	0	0	0	RPTFHSVPS	tr_D0A893	0.027	37293.86	75.00
72	HLA-A0201	NGQSVERRW	NGQSVERRW	0	0	0	0	0	NGQSVERRW	tr_D0A893	0.027	37310.82	75.00
67	HLA-A0201	SWEDVNGQSVERR	SWEDVNQSV	0	0	0	6	1	SWEDVNGQSV	tr_D0A893	0.027	37350.80	75.00
104	HLA-A0201	EQDHHCGVIG	EQDHHCGVI	0	0	0	0	0	EQDHHCGVI	tr_D0A893	0.027	37353.21	75.00
98	HLA-A0201	CGMCVNEQDH	CGMCVNEQH	0	0	0	8	1	CGMCVNEQDH	tr_D0A893	0.027	37356.06	75.00
68	HLA-A0201	WEDVNGQSVERRWC	WEDVVERRW	0	0	0	4	4	WEDVNGQSVERRW	tr_D0A893	0.027	37377.48	75.00
25	HLA-A0201	LWATVYSDP	LWATVYSDP	0	0	0	0	0	LWATVYSDP	tr_D0A893	0.027	37381.92	75.00
41	HLA-A0201	HSVPSLAADGPS	SVPSLAAPS	1	0	0	7	2	SVPSLAADGPS	tr_D0A893	0.027	37419.16	75.00
99	HLA-A0201	GMCVNEQDH	GMCVNEQDH	0	0	0	0	0	GMCVNEQDH	tr_D0A893	0.027	37454.79	75.00
68	HLA-A0201	WEDVNGQSVERRW	WEDVVERRW	0	0	0	4	4	WEDVNGQSVERRW	tr_D0A893	0.027	37466.15	75.00
40	HLA-A0201	FHSVPSLAADGP	FHSVPSLAA	0	0	0	0	0	FHSVPSLAA	tr_D0A893	0.027	37532.70	75.00
62	HLA-A0201	QERESSWEDVNGQ	SSWEDVNGQ	4	0	0	0	0	SSWEDVNGQ	tr_D0A893	0.026	37568.45	75.00
67	HLA-A0201	SWEDVNGQSVE	SWEDVNQSV	0	0	0	6	1	SWEDVNGQSV	tr_D0A893	0.026	37584.29	75.00
64	HLA-A0201	RESSWEDVNGQ	RSWEDVNGQ	0	0	0	1	2	RESSWEDVNGQ	tr_D0A893	0.026	37605.05	75.00
28	HLA-A0201	TVYSDPGYARP	TVYSDPGYA	0	0	0	0	0	TVYSDPGYA	tr_D0A893	0.026	37686.11	75.00
105	HLA-A0201	QDHHCGVIG	QDHHCGVIG	0	0	0	0	0	QDHHCGVIG	tr_D0A893	0.026	37771.00	75.00
43	HLA-A0201	VPSLAADGP	VPSLAADGP	0	0	0	0	0	VPSLAADGP	tr_D0A893	0.026	37796.76	75.00
25	HLA-A0201	LWATVYSDPG	LWATVYDPG	0	0	0	6	1	LWATVYSDPG	tr_D0A893	0.026	37813.95	75.00
112	HLA-A0201	IGVCVGRRN	IGVCVGRRN	0	0	0	0	0	IGVCVGRRN	tr_D0A893	0.026	37825.00	75.00
31	HLA-A0201	SDPGYARPTF	SDPGYARPF	0	0	0	8	1	SDPGYARPTF	tr_D0A893	0.026	37868.39	75.00

68	HLA-A0201	WEDVNGQSVE	WEDVNGQSV	0	0	0	0	0	WEDVNGQSV	tr_D0A893	0.026	37869.23	75.00
71	HLA-A0201	VNGQSVERR	VNGQSVERR	0	0	0	0	0	VNGQSVERR	tr_D0A893	0.026	37872.08	75.00
45	HLA-A0201	SLAADGPSPRE	SLDGPSPRE	0	0	0	2	2	SLAADGPSPRE	tr_D0A893	0.026	37887.67	75.00
98	HLA-A0201	CGMCVNEQDHHCG	GMCEQDHHC	1	0	0	3	2	GMCVNEQDHHC	tr_D0A893	0.026	37890.11	75.00
42	HLA-A0201	SVPSLAADGPSP	SLAADGPSP	0	0	0	1	3	SVPSLAADGPSP	tr_D0A893	0.026	37929.08	75.00
52	HLA-A0201	SPREVEGGVQ	SPREVEGGV	0	0	0	0	0	SPREVEGGV	tr_D0A893	0.025	37980.83	75.00
63	HLA-A0201	ERESSWEDVNGQ	SSWEDVNGQ	3	0	0	0	0	SSWEDVNGQ	tr_D0A893	0.025	37989.06	75.00
5	HLA-A0201	PHLWHNMKL	PHLWHNMKL	0	0	0	0	0	PHLWHNMKL	tr_D0A893	0.025	38018.67	75.00
67	HLA-A0201	SWEDVNGQSVER	SWEDVNQSV	0	0	0	6	1	SWEDVNGQSV	tr_D0A893	0.025	38035.54	75.00
76	HLA-A0201	VERRWCSACG	VERWCSACG	0	0	0	2	1	VERRWCSACG	tr_D0A893	0.025	38091.53	75.00
102	HLA-A0201	VNEQDHHCGVIG	VQDHHCGVI	0	0	0	1	2	VNEQDHHCGVI	tr_D0A893	0.025	38103.08	75.00
41	HLA-A0201	HSVPSLAADGPSP	HSVPSLAAP	0	0	0	8	4	HSVPSLAADGPSP	tr_D0A893	0.025	38110.08	75.00
47	HLA-A0201	AADGPSPRE	AADGPSPRE	0	0	0	0	0	AADGPSPRE	tr_D0A893	0.025	38174.06	75.00
3	HLA-A0201	GEPHLWHNMK	GEPHLWHNM	0	0	0	0	0	GEPHLWHNM	tr_D0A893	0.025	38217.86	75.00
48	HLA-A0201	ADGPSPREVEGGV	ADGEVEGGV	0	0	0	3	4	ADGPSPREVEGGV	tr_D0A893	0.025	38288.22	75.00
71	HLA-A0201	VNGQSVERRWCS	GQVERRWCS	2	0	0	2	1	GQSVERRWCS	tr_D0A893	0.025	38315.17	75.00
73	HLA-A0201	GQSVERRWC	GQSVERRWC	0	0	0	0	0	GQSVERRWC	tr_D0A893	0.025	38328.02	75.00
50	HLA-A0201	GPSPREVEGGV	SPREVEGGV	2	0	0	0	0	SPREVEGGV	tr_D0A893	0.024	38386.52	75.00
71	HLA-A0201	VNGQSVERRW	VNGQSVERW	0	0	0	7	1	VNGQSVERRW	tr_D0A893	0.024	38442.24	80.00
70	HLA-A0201	DVNGQSVERRWCS	GQVERRWCS	3	0	0	2	1	GQSVERRWCS	tr_D0A893	0.024	38455.53	80.00
99	HLA-A0201	GMCVNEQDHHCG	GMCEQDHHC	0	0	0	3	2	GMCVNEQDHHC	tr_D0A893	0.024	38470.11	80.00
54	HLA-A0201	REVEGGVQQ	REVEGGVQQ	0	0	0	0	0	REVEGGVQQ	tr_D0A893	0.024	38494.66	80.00
59	HLA-A0201	GVQQERESSW	VQQERESSW	1	0	0	0	0	VQQERESSW	tr_D0A893	0.024	38543.02	80.00
34	HLA-A0201	GYARPTFHS	GYARPTFHS	0	0	0	0	0	GYARPTFHS	tr_D0A893	0.024	38589.76	80.00
42	HLA-A0201	SVPSLAADGPSPRE	SVPSLAAPS	0	0	0	7	2	SVPSLAADGPS	tr_D0A893	0.024	38617.32	80.00
69	HLA-A0201	EDVNGQSVERRWCS	GQVERRWCS	4	0	0	2	1	GQSVERRWCS	tr_D0A893	0.024	38630.70	80.00
52	HLA-A0201	SPREVEGGVQQ	SPREVGVQQ	0	0	0	5	2	SPREVEGGVQQ	tr_D0A893	0.024	38667.91	80.00
55	HLA-A0201	EVEGGVQQERESSW	EVEGGVQSW	0	0	0	7	5	EVEGGVQQERESSW	tr_D0A893	0.024	38682.12	80.00
41	HLA-A0201	HSVPSLAADGP	HSVPSLAAP	0	0	0	8	2	HSVPSLAADGP	tr_D0A893	0.024	38685.49	80.00
61	HLA-A0201	QQERESSWEDVN	QQESSWEDV	0	0	0	2	2	QQERESSWEDV	tr_D0A893	0.024	38694.27	80.00
43	HLA-A0201	VPSLAADGPSP	VLAADGPSP	0	0	0	1	2	VPSLAADGPSP	tr_D0A893	0.024	38700.56	80.00
68	HLA-A0201	WEDVNGQSVERR	WEDVNGQSV	0	0	0	0	0	WEDVNGQSV	tr_D0A893	0.024	38708.93	80.00
52	HLA-A0201	SPREVEGGVQQE	SPREVEGGV	0	0	0	0	0	SPREVEGGV	tr_D0A893	0.023	38791.95	80.00
99	HLA-A0201	GMCVNEQDHH	GMCVNEQHH	0	0	0	7	1	GMCVNEQDHH	tr_D0A893	0.023	38818.40	80.00
70	HLA-A0201	DVNGQSVERRWC	DVNGQSVWC	0	0	0	7	3	DVNGQSVERRWC	tr_D0A893	0.023	38939.55	80.00
49	HLA-A0201	DGPSPREVEGGV	SPREVEGGV	3	0	0	0	0	SPREVEGGV	tr_D0A893	0.023	38980.43	80.00
72	HLA-A0201	NGQSVERRWCS	GQVERRWCS	1	0	0	2	1	GQSVERRWCS	tr_D0A893	0.023	38988.02	80.00
71	HLA-A0201	VNGQSVERRWC	VNGQSVEWC	0	0	0	7	2	VNGQSVERRWC	tr_D0A893	0.023	39006.18	80.00
29	HLA-A0201	VYSDPGYAR	VYSDPGYAR	0	0	0	0	0	VYSDPGYAR	tr_D0A893	0.023	39009.98	80.00
43	HLA-A0201	VPSLAADGPSPRE	SLDGPSPRE	2	0	0	2	2	SLAADGPSPRE	tr_D0A893	0.023	39034.46	80.00
52	HLA-A0201	SPREVEGGVQQER	SPREVEGGV	0	0	0	0	0	SPREVEGGV	tr_D0A893	0.023	39054.31	80.00
44	HLA-A0201	PSLAADGPSPRE	SLDGPSPRE	1	0	0	2	2	SLAADGPSPRE	tr_D0A893	0.023	39182.57	80.00
72	HLA-A0201	NGQSVERRWC	NGQSVERRW	0	0	0	0	0	NGQSVERRW	tr_D0A893	0.022	39228.38	80.00
31	HLA-A0201	SDPGYARPT	SDPGYARPT	0	0	0	0	0	SDPGYARPT	tr_D0A893	0.022	39234.73	80.00
103	HLA-A0201	NEQDHHCGVIG	NQDHHCGVI	0	0	0	1	1	NEQDHHCGVI	tr_D0A893	0.022	39238.15	80.00
36	HLA-A0201	ARPTFHSVPS	ARPTFSVPS	0	0	0	5	1	ARPTFHSVPS	tr_D0A893	0.022	39258.95	80.00
69	HLA-A0201	EDVNGQSVERRWC	VNGQSVEWC	2	0	0	7	2	VNGQSVERRWC	tr_D0A893	0.022	39277.64	80.00
52	HLA-A0201	SPREVEGGVQQERE	SPREVEGGV	0	0	0	0	0	SPREVEGGV	tr_D0A893	0.022	39362.31	80.00
61	HLA-A0201	QQERESSWEDVNG	QQESSWEDV	0	0	0	2	2	QQERESSWEDV	tr_D0A893	0.022	39364.42	80.00
70	HLA-A0201	DVNGQSVERRW	DVNGQSVEW	0	0	0	8	2	DVNGQSVERRW	tr_D0A893	0.022	39381.05	80.00
51	HLA-A0201	PSPREVEGGVQQ	SPREVGVQQ	1	0	0	5	2	SPREVEGGVQQ	tr_D0A893	0.022	39392.56	80.00
51	HLA-A0201	PSPREVEGGVQQE	SPREVEGGV	1	0	0	0	0	SPREVEGGV	tr_D0A893	0.022	39410.45	80.00
50	HLA-A0201	GPSPREVEGGVQQE	SPREVVQQE	2	0	0	5	3	SPREVEGGVQQE	tr_D0A893	0.022	39426.23	80.00
50	HLA-A0201	GPSPREVEGGVQQ	SPREVGVQQ	2	0	0	5	2	SPREVEGGVQQ	tr_D0A893	0.022	39453.98	80.00

43	HLA-A0201	VPSLAADGPS	VSLAADGPS	0	0	0	1	1	VPSLAADGPS	tr_D0A893	0.022	39512.06	80.00
98	HLA-A0201	CGMCVNEQDHH	CGMCVNEHH	0	0	0	7	2	CGMCVNEQDHH	tr_D0A893	0.022	39530.46	80.00
49	HLA-A0201	DGPSPREVEGGVQQ	SPREVGVQQ	3	0	0	5	2	SPREVEGGVQQ	tr_D0A893	0.022	39585.24	80.00
51	HLA-A0201	PSPREVEGGVQQER	SPREVEGGV	1	0	0	0	0	SPREVEGGV	tr_D0A893	0.021	39623.37	85.00
83	HLA-A0201	ACGIHRPLR	ACGIHRPLR	0	0	0	0	0	ACGIHRPLR	tr_D0A893	0.021	39633.24	85.00
55	HLA-A0201	EVEGGVQQE	EVEGGVQQE	0	0	0	0	0	EVEGGVQQE	tr_D0A893	0.021	39651.70	85.00
69	HLA-A0201	EDVNGQSVERRW	VNGQSVERW	2	0	0	7	1	VNGQSVERRW	tr_D0A893	0.021	39664.12	85.00
62	HLA-A0201	QERESSWEDVN	QERESSWEV	0	0	0	8	1	QERESSWEDV	tr_D0A893	0.021	39725.54	85.00
56	HLA-A0201	VEGGVQQER	VEGGVQQER	0	0	0	0	0	VEGGVQQER	tr_D0A893	0.021	39778.89	85.00
60	HLA-A0201	VQQERESSWE	VQQERESSW	0	0	0	0	0	VQQERESSW	tr_D0A893	0.021	39903.04	85.00
70	HLA-A0201	DVNGQSVERR	DVNQSVERR	0	0	0	3	1	DVNGQSVERR	tr_D0A893	0.021	39940.61	85.00
77	HLA-A0201	ERRWCSACG	ERRWCSACG	0	0	0	0	0	ERRWCSACG	tr_D0A893	0.021	39970.00	85.00
60	HLA-A0201	VQQERESSWED	VQQESSWED	0	0	0	3	2	VQQERESSWED	tr_D0A893	0.021	40001.16	85.00
33	HLA-A0201	PGYARPTFH	PGYARPTFH	0	0	0	0	0	PGYARPTFH	tr_D0A893	0.020	40119.92	85.00
68	HLA-A0201	WEDVNGQSVER	WEDVNGQSV	0	0	0	0	0	WEDVNGQSV	tr_D0A893	0.020	40223.81	85.00
62	HLA-A0201	QERESSWEDVNG	QERESSWEV	0	0	0	8	1	QERESSWEDV	tr_D0A893	0.020	40236.42	85.00
70	HLA-A0201	DVNGQSVER	DVNGQSVER	0	0	0	0	0	DVNGQSVER	tr_D0A893	0.020	40248.62	85.00
101	HLA-A0201	CVNEQDHHCG	CVNEQDHHC	0	0	0	0	0	CVNEQDHHC	tr_D0A893	0.020	40290.01	85.00
56	HLA-A0201	VEGGVQQERESSW	VQQERESSW	4	0	0	0	0	VQQERESSW	tr_D0A893	0.020	40332.75	85.00
59	HLA-A0201	GVQQERESSWED	GVQESSWED	0	0	0	3	3	GVQQERESSWED	tr_D0A893	0.019	40509.44	85.00
55	HLA-A0201	EVEGGVQQER	EVGGVQQER	0	0	0	2	1	EVEGGVQQER	tr_D0A893	0.019	40526.53	85.00
59	HLA-A0201	GVQQERESS	GVQQERESS	0	0	0	0	0	GVQQERESS	tr_D0A893	0.019	40570.85	85.00
48	HLA-A0201	ADGPSPREVEGGVQ	SPREVEGGV	4	0	0	0	0	SPREVEGGV	tr_D0A893	0.019	40591.06	85.00
31	HLA-A0201	SDPGYARPTFHS	SDPGYAFHS	0	0	0	6	3	SDPGYARPTFHS	tr_D0A893	0.019	40617.84	85.00
54	HLA-A0201	REVEGGVQQE	RVEGGVQQE	0	0	0	1	1	REVEGGVQQE	tr_D0A893	0.019	40647.75	85.00
48	HLA-A0201	ADGPSPREVE	ADGPSPREV	0	0	0	0	0	ADGPSPREV	tr_D0A893	0.019	40669.75	85.00
46	HLA-A0201	LAADGPSPRE	LAADGPSPE	0	0	0	8	1	LAADGPSPRE	tr_D0A893	0.019	40670.16	85.00
50	HLA-A0201	GPSPREVEGGVQ	SPREVEGGV	2	0	0	0	0	SPREVEGGV	tr_D0A893	0.019	40687.35	85.00
67	HLA-A0201	SWEDVNGQS	SWEDVNGQS	0	0	0	0	0	SWEDVNGQS	tr_D0A893	0.019	40756.97	85.00
64	HLA-A0201	RESSWEDVNG	RSSWEDVNG	0	0	0	1	1	RESSWEDVNG	tr_D0A893	0.019	40767.98	85.00
31	HLA-A0201	SDPGYARPTFH	SGYARPTFH	0	0	0	1	2	SDPGYARPTFH	tr_D0A893	0.019	40824.92	85.00
58	HLA-A0201	GGVQQERESSWED	VQQESSWED	2	0	0	3	2	VQQERESSWED	tr_D0A893	0.019	40885.04	90.00
51	HLA-A0201	PSPREVEGGVQ	SPREVEGGV	1	0	0	0	0	SPREVEGGV	tr_D0A893	0.019	40917.80	90.00
33	HLA-A0201	PGYARPTFHS	PGYAPTFHS	0	0	0	4	1	PGYARPTFHS	tr_D0A893	0.019	40927.09	90.00
58	HLA-A0201	GGVQQERESSW	VQQERESSW	2	0	0	0	0	VQQERESSW	tr_D0A893	0.018	40966.97	90.00
49	HLA-A0201	DGPSPREVEGGVQ	SPREVEGGV	3	0	0	0	0	SPREVEGGV	tr_D0A893	0.018	40978.04	90.00
57	HLA-A0201	EGGVQQERESSWED	VQQESSWED	3	0	0	3	2	VQQERESSWED	tr_D0A893	0.018	40993.13	90.00
61	HLA-A0201	QQERESSWED	QQEESSWED	0	0	0	3	1	QQERESSWED	tr_D0A893	0.018	41007.33	90.00
57	HLA-A0201	EGGVQQERESSW	VQQERESSW	3	0	0	0	0	VQQERESSW	tr_D0A893	0.018	41066.38	90.00
62	HLA-A0201	QERESSWED	QERESSWED	0	0	0	0	0	QERESSWED	tr_D0A893	0.018	41231.54	90.00
54	HLA-A0201	REVEGGVQQER	REVEGGVQR	0	0	0	8	2	REVEGGVQQER	tr_D0A893	0.018	41271.26	90.00
56	HLA-A0201	VEGGVQQERESSWE	VEGGVSSWE	0	0	0	5	5	VEGGVQQERESSWE	tr_D0A893	0.018	41295.38	90.00
65	HLA-A0201	ESSWEDVNG	ESSWEDVNG	0	0	0	0	0	ESSWEDVNG	tr_D0A893	0.018	41328.02	90.00
32	HLA-A0201	DPGYARPTF	DPGYARPTF	0	0	0	0	0	DPGYARPTF	tr_D0A893	0.017	41483.03	90.00
69	HLA-A0201	EDVNGQSVERR	VNGQSVERR	2	0	0	0	0	VNGQSVERR	tr_D0A893	0.017	41584.60	90.00
54	HLA-A0201	REVEGGVQQERESS	REVEGGVSS	0	0	0	7	5	REVEGGVQQERESS	tr_D0A893	0.017	41623.75	90.00
53	HLA-A0201	PREVEGGVQQER	REVEGGVQR	1	0	0	8	2	REVEGGVQQER	tr_D0A893	0.017	41648.53	90.00
54	HLA-A0201	REVEGGVQQERES	REVEGGVES	0	0	0	7	4	REVEGGVQQERES	tr_D0A893	0.017	41670.16	90.00
32	HLA-A0201	DPGYARPTFH	DGYARPTFH	0	0	0	1	1	DPGYARPTFH	tr_D0A893	0.017	41761.33	90.00
50	HLA-A0201	GPSPREVEGG	GPSPRVEGG	0	0	0	5	1	GPSPREVEGG	tr_D0A893	0.016	41844.55	90.00
59	HLA-A0201	GVQQERESSWE	VQQERESSW	1	0	0	0	0	VQQERESSW	tr_D0A893	0.016	41858.59	90.00
58	HLA-A0201	GGVQQERESSWE	VQQERESSW	2	0	0	0	0	VQQERESSW	tr_D0A893	0.016	42015.59	90.00
53	HLA-A0201	PREVEGGVQQERES	REVEGGVQS	1	0	0	8	4	REVEGGVQQERES	tr_D0A893	0.016	42051.54	90.00
57	HLA-A0201	EGGVQQERESSWE	VQQERESSW	3	0	0	0	0	VQQERESSW	tr_D0A893	0.016	42076.55	90.00

50	HLA-A0201	GPSPREVEG	GPSPREVEG	0	0	0	0	0	GPSPREVEG	tr_D0A893	0.016	42107.09	90.00
36	HLA-A0201	ARPTFHSVP	ARPTFHSVP	0	0	0	0	0	ARPTFHSVP	tr_D0A893	0.016	42110.26	90.00
55	HLA-A0201	EVEGGVQQERESS	EVEGGVESS	0	0	0	6	4	EVEGGVQQERESS	tr_D0A893	0.016	42140.34	90.00
53	HLA-A0201	PREVEGGVQQ	REVEGGVQQ	1	0	0	0	0	REVEGGVQQ	tr_D0A893	0.016	42198.75	95.00
54	HLA-A0201	REVEGGVQQERE	REVEGGVQE	0	0	0	8	3	REVEGGVQQERE	tr_D0A893	0.016	42246.70	95.00
32	HLA-A0201	DPGYARPTFHS	GYARPTFHS	2	0	0	0	0	GYARPTFHS	tr_D0A893	0.016	42248.99	95.00
100	HLA-A0201	MCVNEQDHHCG	MVNEQDHHC	0	0	0	1	1	MCVNEQDHHC	tr_D0A893	0.015	42296.10	95.00
100	HLA-A0201	MCVNEQDHH	MCVNEQDHH	0	0	0	0	0	MCVNEQDHH	tr_D0A893	0.015	42360.22	95.00
53	HLA-A0201	PREVEGGVQQE	RVEGGVQQE	1	0	0	1	1	REVEGGVQQE	tr_D0A893	0.015	42406.99	95.00
63	HLA-A0201	ERESSWEDVN	ERESSWEDV	0	0	0	0	0	ERESSWEDV	tr_D0A893	0.015	42452.44	95.00
55	HLA-A0201	EVEGGVQQERES	EVEGGVQER	0	0	0	6	1	EVEGGVQQER	tr_D0A893	0.015	42461.64	95.00
53	HLA-A0201	PREVEGGVQQERE	REVEGGVQE	1	0	0	8	3	REVEGGVQQERE	tr_D0A893	0.015	42472.68	95.00
63	HLA-A0201	ERESSWEDVNG	RSSWEDVNG	1	0	0	1	1	RESSWEDVNG	tr_D0A893	0.015	42651.81	95.00
55	HLA-A0201	EVEGGVQQERE	EVEGGVERE	0	0	0	6	2	EVEGGVQQERE	tr_D0A893	0.015	42691.05	95.00
61	HLA-A0201	QQERESSWE	QQERESSWE	0	0	0	0	0	QQERESSWE	tr_D0A893	0.015	42707.68	95.00
48	HLA-A0201	ADGPSPREVEGG	ADGPSPREV	0	0	0	0	0	ADGPSPREV	tr_D0A893	0.015	42713.22	95.00
69	HLA-A0201	EDVNGQSVER	EVNGQSVER	0	0	0	1	1	EDVNGQSVER	tr_D0A893	0.014	43006.29	95.00
51	HLA-A0201	PSPREVEGG	PSPREVEGG	0	0	0	0	0	PSPREVEGG	tr_D0A893	0.014	43023.52	95.00
44	HLA-A0201	PSLAADGPS	PSLAADGPS	0	0	0	0	0	PSLAADGPS	tr_D0A893	0.014	43176.00	95.00
48	HLA-A0201	ADGPSPREVEG	ADGPSPREV	0	0	0	0	0	ADGPSPREV	tr_D0A893	0.013	43375.47	95.00
56	HLA-A0201	VEGGVQQERE	VEGGVQERE	0	0	0	5	1	VEGGVQQERE	tr_D0A893	0.013	43429.49	95.00
49	HLA-A0201	DGPSPREVEGG	GPSPRVEGG	1	0	0	5	1	GPSPREVEGG	tr_D0A893	0.013	43519.80	95.00
56	HLA-A0201	VEGGVQQERESS	VEGGVQQER	0	0	0	0	0	VEGGVQQER	tr_D0A893	0.013	43649.01	95.00
64	HLA-A0201	RESSWEDVN	RESSWEDVN	0	0	0	0	0	RESSWEDVN	tr_D0A893	0.012	43744.04	95.00
53	HLA-A0201	PREVEGGVQ	PREVEGGVQ	0	0	0	0	0	PREVEGGVQ	tr_D0A893	0.012	43842.12	99.00
56	HLA-A0201	VEGGVQQERES	VEGGVQQER	0	0	0	0	0	VEGGVQQER	tr_D0A893	0.011	44201.74	99.00
102	HLA-A0201	VNEQDHHCG	VNEQDHHCG	0	0	0	0	0	VNEQDHHCG	tr_D0A893	0.011	44392.97	99.00
58	HLA-A0201	GGVQQERESS	GVQQERESS	0	0	0	1	1	GGVQQERESS	tr_D0A893	0.011	44452.10	99.00
49	HLA-A0201	DGPSPREVEG	GPSPREVEG	1	0	0	0	0	GPSPREVEG	tr_D0A893	0.010	44824.96	99.00
58	HLA-A0201	GGVQQERES	GGVQQERES	0	0	0	0	0	GGVQQERES	tr_D0A893	0.010	44908.44	99.00
57	HLA-A0201	EGGVQQERE	EGGVQQERE	0	0	0	0	0	EGGVQQERE	tr_D0A893	0.010	45093.94	99.00
49	HLA-A0201	DGPSPREVE	DGPSPREVE	0	0	0	0	0	DGPSPREVE	tr_D0A893	0.009	45422.04	99.00
57	HLA-A0201	EGGVQQERESS	GVQQERESS	2	0	0	0	0	GVQQERESS	tr_D0A893	0.008	45900.29	99.00
57	HLA-A0201	EGGVQQERES	GGVQQERES	1	0	0	0	0	GGVQQERES	tr_D0A893	0.008	46057.47	99.00
69	HLA-A0201	EDVNGQSVE	EDVNGQSVE	0	0	0	0	0	EDVNGQSVE	tr_D0A893	0.007	46435.26	99.00

Protein tr_D0A893. Allele HLA-A0201. Number of high binders 2. Number of weak binders 23. Number of peptides 663

Link to Allele Frequencies in Worldwide Populations HLA-A0201

Rank Threshold for Strong binding peptides   0.500

Rank Threshold for Weak binding peptides   2.000

---

pos	HLA	peptide	Core	Offset	I_pos	I_len	D_pos	D_len	iCore	Identity	1-log50k(aff)	Affinity(nM)	%Rank	BindLevel
16	HLA-A0201	YLNGVHVPKV	YLNGVVPKV	0	0	0	5	1	YLNGVHVPKV	tr_C9ZY92	0.764	12.87	0.17	<= SB
9	HLA-A0201	GIPDVMAYL	GIPDVMAYL	0	0	0	0	0	GIPDVMAYL	tr_C9ZY92	0.655	41.58	0.60	<= WB
24	HLA-A0201	KVMTLRTFV	KVMTLRTFV	0	0	0	0	0	KVMTLRTFV	tr_C9ZY92	0.632	53.36	0.70	<= WB
24	HLA-A0201	KVMTLRTFVV	KVMTLTFVV	0	0	0	5	1	KVMTLRTFVV	tr_C9ZY92	0.630	54.68	0.70	<= WB
25	HLA-A0201	VMTLRTFVV	VMTLRTFVV	0	0	0	0	0	VMTLRTFVV	tr_C9ZY92	0.601	74.57	0.90	<= WB
13	HLA-A0201	VMAYLNGVHV	VMAYLNVHV	0	0	0	6	1	VMAYLNGVHV	tr_C9ZY92	0.565	110.29	1.10	<= WB
15	HLA-A0201	AYLNGVHVPKV	YLNGVVPKV	1	0	0	5	1	YLNGVHVPKV	tr_C9ZY92	0.555	123.97	1.20	<= WB
14	HLA-A0201	MAYLNGVHVPKV	YLNGVVPKV	2	0	0	5	1	YLNGVHVPKV	tr_C9ZY92	0.532	158.55	1.40	<= WB

13	HLA-A0201	VMAYLNGVHVPKV	YLNGVVPKV	3	0	0	5	1	YLNGVHVPKV	tr_C9ZY92	0.525	170.70	1.50	<= WB
16	HLA-A0201	YLNGVHVPKVMTL	YLNGVVMTL	0	0	0	5	4	YLNGVHVPKVMTL	tr_C9ZY92	0.523	174.92	1.50	<= WB
12	HLA-A0201	DVMAYLNGVHVPKV	YLNGVVPKV	4	0	0	5	1	YLNGVHVPKV	tr_C9ZY92	0.521	178.46	1.50	<= WB
15	HLA-A0201	AYLNGVHVPKVMTL	YLNGVVMTL	1	0	0	5	4	YLNGVHVPKVMTL	tr_C9ZY92	0.484	265.71	1.90	<= WB
16	HLA-A0201	YLNGVHVPKVM	YLNGVVPKV	0	0	0	5	1	YLNGVHVPKV	tr_C9ZY92	0.433	461.41	3.00	
9	HLA-A0201	GIPDVMAYLNGV	GIMAYLNGV	0	0	0	2	3	GIPDVMAYLNGV	tr_C9ZY92	0.427	491.53	3.00	
8	HLA-A0201	GGIPDVMAYL	GIPDVMAYL	0	0	0	1	1	GGIPDVMAYL	tr_C9ZY92	0.422	520.46	3.00	
10	HLA-A0201	IPDVMAYLNGV	IVMAYLNGV	0	0	0	1	2	IPDVMAYLNGV	tr_C9ZY92	0.415	560.01	3.00	
16	HLA-A0201	YLNGVHVPKVMTLR	YLNGVVMTL	0	0	0	5	4	YLNGVHVPKVMTL	tr_C9ZY92	0.393	708.71	3.50	
15	HLA-A0201	AYLNGVHVPKVM	YLNGVVPKV	1	0	0	5	1	YLNGVHVPKV	tr_C9ZY92	0.385	773.95	3.50	
8	HLA-A0201	GGIPDVMAYLNGV	GIMAYLNGV	1	0	0	2	3	GIPDVMAYLNGV	tr_C9ZY92	0.377	846.05	4.00	
12	HLA-A0201	DVMAYLNGV	DVMAYLNGV	0	0	0	0	0	DVMAYLNGV	tr_C9ZY92	0.372	895.39	4.00	
16	HLA-A0201	YLNGVHVPKVMT	YLNGVVPKV	0	0	0	5	1	YLNGVHVPKV	tr_C9ZY92	0.372	895.66	4.00	
23	HLA-A0201	PKVMTLRTFVV	KVMTLTFVV	1	0	0	5	1	KVMTLRTFVV	tr_C9ZY92	0.368	935.51	4.00	
14	HLA-A0201	MAYLNGVHVPKVM	YLNGVHVPV	2	0	0	8	1	YLNGVHVPKV	tr_C9ZY92	0.366	955.95	4.00	
7	HLA-A0201	GGGIPDVMAYLNGV	GIMAYLNGV	2	0	0	2	3	GIPDVMAYLNGV	tr_C9ZY92	0.362	993.31	4.00	
13	HLA-A0201	VMAYLNGVHVPKVM	YLNGVHVPV	3	0	0	8	1	YLNGVHVPKV	tr_C9ZY92	0.360	1016.56	4.00	
22	HLA-A0201	VPKVMTLRTFVV	KVMTLTFVV	2	0	0	5	1	KVMTLRTFVV	tr_C9ZY92	0.348	1162.88	4.50	
9	HLA-A0201	GIPDVMAYLNGVHV	GIPDVMAYV	0	0	0	8	5	GIPDVMAYLNGVHV	tr_C9ZY92	0.344	1213.72	4.50	
21	HLA-A0201	HVPKVMTLRTFVV	KVMTLTFVV	3	0	0	5	1	KVMTLRTFVV	tr_C9ZY92	0.340	1261.21	4.50	
20	HLA-A0201	VHVPKVMTLRTFVV	KVMTLTFVV	4	0	0	5	1	KVMTLRTFVV	tr_C9ZY92	0.338	1287.97	4.50	
23	HLA-A0201	PKVMTLRTFV	KVMTLRTFV	1	0	0	0	0	KVMTLRTFV	tr_C9ZY92	0.335	1332.94	5.00	
15	HLA-A0201	AYLNGVHVPKVMT	YLNGVHVPV	1	0	0	8	1	YLNGVHVPKV	tr_C9ZY92	0.330	1412.39	5.00	
14	HLA-A0201	MAYLNGVHVPKVMT	YLNGVHVPV	2	0	0	8	1	YLNGVHVPKV	tr_C9ZY92	0.313	1691.90	5.50	
12	HLA-A0201	DVMAYLNGVHV	VMAYLNVHV	1	0	0	6	1	VMAYLNGVHV	tr_C9ZY92	0.311	1724.71	5.50	
25	HLA-A0201	VMTLRTFVVK	VMTLRTFVV	0	0	0	0	0	VMTLRTFVV	tr_C9ZY92	0.309	1766.89	5.50	
14	HLA-A0201	MAYLNGVHV	MAYLNGVHV	0	0	0	0	0	MAYLNGVHV	tr_C9ZY92	0.297	2020.97	6.00	
9	HLA-A0201	GIPDVMAYLN	GIPDVMAYL	0	0	0	0	0	GIPDVMAYL	tr_C9ZY92	0.294	2066.53	6.00	
27	HLA-A0201	TLRTFVVKSI	TLRTFVVSI	0	0	0	7	1	TLRTFVVKSI	tr_C9ZY92	0.285	2282.37	6.50	
11	HLA-A0201	PDVMAYLNGVHV	VMAYLNVHV	2	0	0	6	1	VMAYLNGVHV	tr_C9ZY92	0.276	2513.74	6.50	
24	HLA-A0201	KVMTLRTFVVK	KVMTLTFVV	0	0	0	5	1	KVMTLRTFVV	tr_C9ZY92	0.276	2528.50	6.50	
10	HLA-A0201	IPDVMAYLNGVHV	VMAYLNVHV	3	0	0	6	1	VMAYLNGVHV	tr_C9ZY92	0.273	2598.47	6.50	
9	HLA-A0201	GIPDVMAYLNGVH	GIMAYLNGV	0	0	0	2	3	GIPDVMAYLNGV	tr_C9ZY92	0.255	3180.60	7.50	
6	HLA-A0201	AGGGIPDVMAYL	AIPDVMAYL	0	0	0	1	3	AGGGIPDVMAYL	tr_C9ZY92	0.252	3282.65	7.50	
10	HLA-A0201	IPDVMAYLNGVH	IVMAYLNGV	0	0	0	1	2	IPDVMAYLNGV	tr_C9ZY92	0.251	3314.99	7.50	
5	HLA-A0201	AAGGGIPDVMAYL	AIPDVMAYL	0	0	0	1	4	AAGGGIPDVMAYL	tr_C9ZY92	0.249	3380.73	7.50	
5	HLA-A0201	AAGGGIPDV	AAGGGIPDV	0	0	0	0	0	AAGGGIPDV	tr_C9ZY92	0.244	3556.06	8.00	
7	HLA-A0201	GGGIPDVMAYL	GIPDVMAYL	0	0	0	1	2	GGGIPDVMAYL	tr_C9ZY92	0.232	4042.08	8.50	
24	HLA-A0201	KVMTLRTFVVKS	KVMTLTFVV	0	0	0	5	1	KVMTLRTFVV	tr_C9ZY92	0.230	4141.51	8.50	
4	HLA-A0201	GAAGGGIPDVMAYL	GIPDVMAYL	0	0	0	1	5	GAAGGGIPDVMAYL	tr_C9ZY92	0.229	4202.27	8.50	
23	HLA-A0201	PKVMTLRTFVVK	KVMTLTFVV	1	0	0	5	1	KVMTLRTFVV	tr_C9ZY92	0.228	4256.36	9.00	
8	HLA-A0201	GGIPDVMAYLNGVH	GIMAYLNGV	1	0	0	2	3	GIPDVMAYLNGV	tr_C9ZY92	0.220	4605.48	9.00	
24	HLA-A0201	KVMTLRTFVVKSI	KVMTLTFVV	0	0	0	5	1	KVMTLRTFVV	tr_C9ZY92	0.218	4743.24	9.50	
22	HLA-A0201	VPKVMTLRTFVVK	KVMTLTFVV	2	0	0	5	1	KVMTLRTFVV	tr_C9ZY92	0.216	4832.59	9.50	
13	HLA-A0201	VMAYLNGVHVP	VMAYLNVHV	0	0	0	6	1	VMAYLNGVHV	tr_C9ZY92	0.212	5064.33	9.50	
21	HLA-A0201	HVPKVMTLRTFVVK	KVMTLTFVV	3	0	0	5	1	KVMTLRTFVV	tr_C9ZY92	0.211	5085.97	10.00	
25	HLA-A0201	VMTLRTFVVKSI	VMTLRTFSI	0	0	0	7	3	VMTLRTFVVKSI	tr_C9ZY92	0.204	5492.49	10.00	
19	HLA-A0201	GVHVPKVMTL	GVVPKVMTL	0	0	0	2	1	GVHVPKVMTL	tr_C9ZY92	0.203	5567.22	11.00	
16	HLA-A0201	YLNGVHVPK	YLNGVHVPK	0	0	0	0	0	YLNGVHVPK	tr_C9ZY92	0.197	5943.90	11.00	
10	HLA-A0201	IPDVMAYLNGVHVP	IVMAYLNGV	0	0	0	1	2	IPDVMAYLNGV	tr_C9ZY92	0.194	6135.49	11.00	
23	HLA-A0201	PKVMTLRTFVVKS	KVMTLTFVV	1	0	0	5	1	KVMTLRTFVV	tr_C9ZY92	0.191	6302.62	11.00	
23	HLA-A0201	PKVMTLRTFVVKSI	KVMTLTFVV	1	0	0	5	1	KVMTLRTFVV	tr_C9ZY92	0.191	6364.64	11.00	
11	HLA-A0201	PDVMAYLNGV	PVMAYLNGV	0	0	0	1	1	PDVMAYLNGV	tr_C9ZY92	0.189	6469.13	12.00	
12	HLA-A0201	DVMAYLNGVHVP	VMAYLNVHV	1	0	0	6	1	VMAYLNGVHV	tr_C9ZY92	0.188	6560.98	12.00	

22	HLA-A0201	VPKVMTLRTFVVKS	KVMTLTFVV	2	0	0	5	1	KVMTLRTFVV	tr_C9ZY92	0.183	6939.35	12.00
4	HLA-A0201	GAAGGGIPDV	GAAGGGIPV	0	0	0	8	1	GAAGGGIPDV	tr_C9ZY92	0.179	7227.09	12.00
13	HLA-A0201	VMAYLNGVHVPK	VMAYLNVHV	0	0	0	6	1	VMAYLNGVHV	tr_C9ZY92	0.175	7504.72	13.00
11	HLA-A0201	PDVMAYLNGVHVP	VMAYLNVHV	2	0	0	6	1	VMAYLNGVHV	tr_C9ZY92	0.169	8070.97	13.00
22	HLA-A0201	VPKVMTLRTFV	KVMTLRTFV	2	0	0	0	0	KVMTLRTFV	tr_C9ZY92	0.163	8582.54	14.00
26	HLA-A0201	MTLRTFVVKSI	TLRTFVVSI	1	0	0	7	1	TLRTFVVKSI	tr_C9ZY92	0.160	8872.32	14.00
20	HLA-A0201	VHVPKVMTLRTFV	VVMTLRTFV	0	0	0	1	4	VHVPKVMTLRTFV	tr_C9ZY92	0.158	9071.31	14.00
12	HLA-A0201	DVMAYLNGVHVPK	VMAYLNVHV	1	0	0	6	1	VMAYLNGVHV	tr_C9ZY92	0.157	9128.31	14.00
14	HLA-A0201	MAYLNGVHVP	MAYLNGVHV	0	0	0	0	0	MAYLNGVHV	tr_C9ZY92	0.153	9555.91	15.00
21	HLA-A0201	HVPKVMTLRTFV	KVMTLRTFV	3	0	0	0	0	KVMTLRTFV	tr_C9ZY92	0.153	9597.77	15.00
19	HLA-A0201	GVHVPKVMTLRTFV	KVMTLRTFV	5	0	0	0	0	KVMTLRTFV	tr_C9ZY92	0.151	9713.00	15.00
6	HLA-A0201	AGGGIPDVMAYLN	AIPDVMAYL	0	0	0	1	3	AGGGIPDVMAYL	tr_C9ZY92	0.146	10277.14	16.00
5	HLA-A0201	AAGGGIPDVMAYLN	AIPDVMAYL	0	0	0	1	4	AAGGGIPDVMAYL	tr_C9ZY92	0.145	10453.33	16.00
11	HLA-A0201	PDVMAYLNGVHVPK	VMAYLNVHV	2	0	0	6	1	VMAYLNGVHV	tr_C9ZY92	0.144	10540.44	16.00
25	HLA-A0201	VMTLRTFVVKS	VMTLRTFVV	0	0	0	0	0	VMTLRTFVV	tr_C9ZY92	0.144	10549.68	16.00
8	HLA-A0201	GGIPDVMAYLN	GIPDVMAYL	0	0	0	1	1	GGIPDVMAYL	tr_C9ZY92	0.138	11285.32	17.00
26	HLA-A0201	MTLRTFVVKS	MLRTFVVKS	0	0	0	1	1	MTLRTFVVKS	tr_C9ZY92	0.137	11296.19	17.00
7	HLA-A0201	GGGIPDVMAYLN	GIPDVMAYL	0	0	0	1	2	GGGIPDVMAYL	tr_C9ZY92	0.133	11858.78	17.00
15	HLA-A0201	AYLNGVHVPK	YLNGVHVPK	1	0	0	0	0	YLNGVHVPK	tr_C9ZY92	0.129	12427.17	18.00
1	HLA-A0201	FEPGAAGGGIPDV	FAGGGIPDV	0	0	0	1	4	FEPGAAGGGIPDV	tr_C9ZY92	0.129	12433.36	18.00
9	HLA-A0201	GIPDVMAYLNG	GIPDVMAYL	0	0	0	0	0	GIPDVMAYL	tr_C9ZY92	0.128	12450.99	18.00
6	HLA-A0201	AGGGIPDVMAYLNG	AIPDVMAYL	0	0	0	1	3	AGGGIPDVMAYL	tr_C9ZY92	0.124	13054.98	19.00
12	HLA-A0201	DVMAYLNGVH	DVMAYLNGV	0	0	0	0	0	DVMAYLNGV	tr_C9ZY92	0.123	13145.12	19.00
27	HLA-A0201	TLRTFVVKS	TLRTFVVKS	0	0	0	0	0	TLRTFVVKS	tr_C9ZY92	0.123	13158.79	19.00
0	HLA-A0201	YFEPGAAGGGIPDV	YAGGGIPDV	0	0	0	1	5	YFEPGAAGGGIPDV	tr_C9ZY92	0.117	14071.03	20.00
8	HLA-A0201	GGIPDVMAYLNG	GIPDVMAYL	0	0	0	1	1	GGIPDVMAYL	tr_C9ZY92	0.116	14191.51	20.00
7	HLA-A0201	GGGIPDVMAYLNG	GIPDVMAYL	0	0	0	1	2	GGGIPDVMAYL	tr_C9ZY92	0.113	14772.29	21.00
18	HLA-A0201	NGVHVPKVMTL	GVVPKVMTL	1	0	0	2	1	GVHVPKVMTL	tr_C9ZY92	0.099	17147.63	23.00
20	HLA-A0201	VHVPKVMTL	VHVPKVMTL	0	0	0	0	0	VHVPKVMTL	tr_C9ZY92	0.094	18045.51	25.00
17	HLA-A0201	LNGVHVPKVMTL	GVVPKVMTL	2	0	0	2	1	GVHVPKVMTL	tr_C9ZY92	0.093	18247.75	25.00
26	HLA-A0201	MTLRTFVVK	MTLRTFVVK	0	0	0	0	0	MTLRTFVVK	tr_C9ZY92	0.091	18675.60	26.00
14	HLA-A0201	MAYLNGVHVPK	MAYLNGVHV	0	0	0	0	0	MAYLNGVHV	tr_C9ZY92	0.089	19106.85	26.00
5	HLA-A0201	AAGGGIPDVM	AAGGGIPDV	0	0	0	0	0	AAGGGIPDV	tr_C9ZY92	0.086	19767.79	27.00
1	HLA-A0201	FEPGAAGGGIPDVM	FAGGGIPDV	0	0	0	1	4	FEPGAAGGGIPDV	tr_C9ZY92	0.076	22022.56	30.00
28	HLA-A0201	LRTFVVKSI	LRTFVVKSI	0	0	0	0	0	LRTFVVKSI	tr_C9ZY92	0.075	22211.14	31.00
19	HLA-A0201	GVHVPKVMTLRTF	GVVMTLRTF	0	0	0	2	4	GVHVPKVMTLRTF	tr_C9ZY92	0.074	22493.60	31.00
19	HLA-A0201	GVHVPKVMTLR	GVVPKVMTL	0	0	0	2	1	GVHVPKVMTL	tr_C9ZY92	0.073	22658.49	31.00
3	HLA-A0201	PGAAGGGIPDV	GAAGGGIPV	1	0	0	8	1	GAAGGGIPDV	tr_C9ZY92	0.070	23534.53	33.00
18	HLA-A0201	NGVHVPKVMTLRTF	GVVMTLRTF	1	0	0	2	4	GVHVPKVMTLRTF	tr_C9ZY92	0.069	23640.69	33.00
18	HLA-A0201	NGVHVPKVMTLR	GVVPKVMTL	1	0	0	2	1	GVHVPKVMTL	tr_C9ZY92	0.068	23881.60	34.00
2	HLA-A0201	EPGAAGGGIPDV	GAAGGGIPV	2	0	0	8	1	GAAGGGIPDV	tr_C9ZY92	0.066	24425.14	35.00
17	HLA-A0201	LNGVHVPKVMTLR	GVVPKVMTL	2	0	0	2	1	GVHVPKVMTL	tr_C9ZY92	0.066	24566.15	35.00
21	HLA-A0201	HVPKVMTLRTF	HVVMTLRTF	0	0	0	2	2	HVPKVMTLRTF	tr_C9ZY92	0.065	24752.91	35.00
22	HLA-A0201	VPKVMTLRTF	VPVMTLRTF	0	0	0	2	1	VPKVMTLRTF	tr_C9ZY92	0.064	25011.08	36.00
19	HLA-A0201	GVHVPKVMTLRT	GVVPKVMTL	0	0	0	2	1	GVHVPKVMTL	tr_C9ZY92	0.064	25093.22	36.00
17	HLA-A0201	LNGVHVPKV	LNGVHVPKV	0	0	0	0	0	LNGVHVPKV	tr_C9ZY92	0.063	25285.09	36.00
18	HLA-A0201	NGVHVPKVMTLRT	GVVPKVMTL	1	0	0	2	1	GVHVPKVMTL	tr_C9ZY92	0.060	26053.52	38.00
20	HLA-A0201	VHVPKVMTLRTF	HVVMTLRTF	1	0	0	2	2	HVPKVMTLRTF	tr_C9ZY92	0.060	26148.40	38.00
21	HLA-A0201	HVPKVMTLRT	HVPKVMLRT	0	0	0	6	1	HVPKVMTLRT	tr_C9ZY92	0.059	26268.93	38.00
17	HLA-A0201	LNGVHVPKVMTLRT	GVHVKVMTL	2	0	0	4	1	GVHVPKVMTL	tr_C9ZY92	0.058	26616.25	39.00
5	HLA-A0201	AAGGGIPDVMA	AAGGGIVMA	0	0	0	6	2	AAGGGIPDVMA	tr_C9ZY92	0.055	27544.10	41.00
6	HLA-A0201	AGGGIPDVMA	AGGGIPVMA	0	0	0	6	1	AGGGIPDVMA	tr_C9ZY92	0.053	28105.86	42.00
8	HLA-A0201	GGIPDVMAY	GGIPDVMAY	0	0	0	0	0	GGIPDVMAY	tr_C9ZY92	0.053	28168.88	42.00
4	HLA-A0201	GAAGGGIPDVM	GAAGGGIPV	0	0	0	8	1	GAAGGGIPDV	tr_C9ZY92	0.052	28605.31	43.00

23	HLA-A0201	PKVMTLRTF	PKVMTLRTF	0	0	0	0	0	PKVMTLRTF	tr_C9ZY92	0.052	28634.42	43.00
11	HLA-A0201	PDVMAYLNGVH	DVMAYLNGV	1	0	0	0	0	DVMAYLNGV	tr_C9ZY92	0.051	28695.83	43.00
21	HLA-A0201	HVPKVMTLR	HVPKVMTLR	0	0	0	0	0	HVPKVMTLR	tr_C9ZY92	0.051	28790.38	44.00
4	HLA-A0201	GAAGGGIPDVMA	AAGGGIVMA	1	0	0	6	2	AAGGGIPDVMA	tr_C9ZY92	0.051	28791.00	44.00
6	HLA-A0201	AGGGIPDVM	AGGGIPDVM	0	0	0	0	0	AGGGIPDVM	tr_C9ZY92	0.048	29634.23	46.00
19	HLA-A0201	GVHVPKVMT	GVHVPKVMT	0	0	0	0	0	GVHVPKVMT	tr_C9ZY92	0.048	29643.85	46.00
20	HLA-A0201	VHVPKVMTLR	VHVPKVMTL	0	0	0	0	0	VHVPKVMTL	tr_C9ZY92	0.047	29949.80	47.00
7	HLA-A0201	GGGIPDVMA	GGGIPDVMA	0	0	0	0	0	GGGIPDVMA	tr_C9ZY92	0.046	30248.76	47.00
3	HLA-A0201	PGAAGGGIPDVMA	AAGGGIVMA	2	0	0	6	2	AAGGGIPDVMA	tr_C9ZY92	0.046	30277.25	47.00
4	HLA-A0201	GAAGGGIPDVMAY	GAAGGGIPV	0	0	0	8	1	GAAGGGIPDV	tr_C9ZY92	0.046	30392.77	48.00
5	HLA-A0201	AAGGGIPDVMAY	AAGGGIVMA	0	0	0	6	2	AAGGGIPDVMA	tr_C9ZY92	0.046	30397.38	48.00
2	HLA-A0201	EPGAAGGGIPDVMA	AAGGGIVMA	3	0	0	6	2	AAGGGIPDVMA	tr_C9ZY92	0.046	30535.84	48.00
10	HLA-A0201	IPDVMAYLN	IPDVMAYLN	0	0	0	0	0	IPDVMAYLN	tr_C9ZY92	0.045	30800.63	49.00
3	HLA-A0201	PGAAGGGIPDVM	GAAGGGIPV	1	0	0	8	1	GAAGGGIPDV	tr_C9ZY92	0.044	30928.86	49.00
3	HLA-A0201	PGAAGGGIPDVMAY	AGGGIVMAY	3	0	0	5	2	AGGGIPDVMAY	tr_C9ZY92	0.043	31406.71	50.00
2	HLA-A0201	EPGAAGGGIPDVM	GAAGGGIPV	2	0	0	8	1	GAAGGGIPDV	tr_C9ZY92	0.043	31426.08	55.00
13	HLA-A0201	VMAYLNGVH	VMAYLNGVH	0	0	0	0	0	VMAYLNGVH	tr_C9ZY92	0.042	31721.93	55.00
7	HLA-A0201	GGGIPDVMAY	GGGIPVMAY	0	0	0	5	1	GGGIPDVMAY	tr_C9ZY92	0.042	31786.86	55.00
6	HLA-A0201	AGGGIPDVMAY	AGGGIVMAY	0	0	0	5	2	AGGGIPDVMAY	tr_C9ZY92	0.041	32168.48	55.00
10	HLA-A0201	IPDVMAYLNG	IPDVMAYLG	0	0	0	8	1	IPDVMAYLNG	tr_C9ZY92	0.038	33031.55	55.00
0	HLA-A0201	YFEPGAAGGGI	YFEPGAAGI	0	0	0	7	2	YFEPGAAGGGI	tr_C9ZY92	0.038	33137.14	60.00
20	HLA-A0201	VHVPKVMTLRT	HVPKVMLRT	1	0	0	6	1	HVPKVMTLRT	tr_C9ZY92	0.037	33539.71	60.00
22	HLA-A0201	VPKVMTLRT	VPKVMTLRT	0	0	0	0	0	VPKVMTLRT	tr_C9ZY92	0.037	33617.82	60.00
18	HLA-A0201	NGVHVPKVM	NGVHVPKVM	0	0	0	0	0	NGVHVPKVM	tr_C9ZY92	0.036	33755.95	60.00
17	HLA-A0201	LNGVHVPKVM	LGVHVPKVM	0	0	0	1	1	LNGVHVPKVM	tr_C9ZY92	0.036	33973.98	60.00
0	HLA-A0201	YFEPGAAGG	YFEPGAAGG	0	0	0	0	0	YFEPGAAGG	tr_C9ZY92	0.035	34125.38	60.00
1	HLA-A0201	FEPGAAGGGI	FEPGAAGGI	0	0	0	6	1	FEPGAAGGGI	tr_C9ZY92	0.032	35452.82	65.00
18	HLA-A0201	NGVHVPKVMT	NGVHVPVMT	0	0	0	6	1	NGVHVPKVMT	tr_C9ZY92	0.032	35493.90	65.00
15	HLA-A0201	AYLNGVHVP	AYLNGVHVP	0	0	0	0	0	AYLNGVHVP	tr_C9ZY92	0.031	35919.24	65.00
17	HLA-A0201	LNGVHVPKVMT	LNGVHVVMT	0	0	0	6	2	LNGVHVPKVMT	tr_C9ZY92	0.028	36888.12	70.00
4	HLA-A0201	GAAGGGIPD	GAAGGGIPD	0	0	0	0	0	GAAGGGIPD	tr_C9ZY92	0.026	37653.48	75.00
0	HLA-A0201	YFEPGAAGGGIP	YFEPGAAGI	0	0	0	7	2	YFEPGAAGGGI	tr_C9ZY92	0.026	37706.90	75.00
0	HLA-A0201	YFEPGAAGGGIPD	YFEPGAAGI	0	0	0	7	2	YFEPGAAGGGI	tr_C9ZY92	0.025	38111.73	75.00
11	HLA-A0201	PDVMAYLNG	PDVMAYLNG	0	0	0	0	0	PDVMAYLNG	tr_C9ZY92	0.023	38981.71	80.00
0	HLA-A0201	YFEPGAAGGG	YFEPGAAGG	0	0	0	7	1	YFEPGAAGGG	tr_C9ZY92	0.022	39502.66	80.00
2	HLA-A0201	EPGAAGGGI	EPGAAGGGI	0	0	0	0	0	EPGAAGGGI	tr_C9ZY92	0.021	40003.32	85.00
1	HLA-A0201	FEPGAAGGGIPD	FAAGGGIPD	0	0	0	1	3	FEPGAAGGGIPD	tr_C9ZY92	0.020	40177.69	85.00
1	HLA-A0201	FEPGAAGGG	FEPGAAGGG	0	0	0	0	0	FEPGAAGGG	tr_C9ZY92	0.020	40435.86	85.00
1	HLA-A0201	FEPGAAGGGIP	FEPGAAGGI	0	0	0	6	1	FEPGAAGGGI	tr_C9ZY92	0.016	42189.61	95.00
3	HLA-A0201	PGAAGGGIPD	GAAGGGIPD	1	0	0	0	0	GAAGGGIPD	tr_C9ZY92	0.014	42840.98	95.00
2	HLA-A0201	EPGAAGGGIPD	GAAGGGIPD	2	0	0	0	0	GAAGGGIPD	tr_C9ZY92	0.013	43551.84	95.00
2	HLA-A0201	EPGAAGGGIP	EPGAAGGGI	0	0	0	0	0	EPGAAGGGI	tr_C9ZY92	0.011	44235.70	99.00
3	HLA-A0201	PGAAGGGIP	PGAAGGGIP	0	0	0	0	0	PGAAGGGIP	tr_C9ZY92	0.010	45091.99	99.00

Protein tr_C9ZY92. Allele HLA-A0201. Number of high binders 1. Number of weak binders 11. Number of peptides 159

Link to Allele Frequencies in Worldwide Populations HLA-A0201

Rank Threshold for Strong binding peptides   0.500
Rank Threshold for Weak binding peptides   2.000

---

pos	HLA	peptide	Core	Offset	I_pos	I_len	D_pos	D_len	iCore	Identity	1-log50k(aff)	Affinity(nM)	%Rank	BindLevel

---

53	HLA-A0201	HLFSRQTIWQYHPV	HLFWQYHPV	0	0	0	3	5	HLFSRQTIWQYHPV	tr_C9ZY92	0.413	574.22	3.00
58	HLA-A0201	QTIWQYHPV	QTIWQYHPV	0	0	0	0	0	QTIWQYHPV	tr_C9ZY92	0.398	674.89	3.50
57	HLA-A0201	RQTIWQYHPV	RQIWQYHPV	0	0	0	2	1	RQTIWQYHPV	tr_C9ZY92	0.352	1112.60	4.50
15	HLA-A0201	LVWGTENTTSLFTA	LVWGTLFTA	0	0	0	5	5	LVWGTENTTSLFTA	tr_C9ZY92	0.346	1186.33	4.50
34	HLA-A0201	NTFSPLATL	NTFSPLATL	0	0	0	0	0	NTFSPLATL	tr_C9ZY92	0.330	1402.40	5.00
34	HLA-A0201	NTFSPLATLV	NTFSPLALV	0	0	0	7	1	NTFSPLATLV	tr_C9ZY92	0.315	1652.14	5.50
14	HLA-A0201	VLVWGTENT	VLVWGTENT	0	0	0	0	0	VLVWGTENT	tr_C9ZY92	0.284	2305.88	6.50
55	HLA-A0201	FSRQTIWQYHPV	FTIWQYHPV	0	0	0	1	3	FSRQTIWQYHPV	tr_C9ZY92	0.275	2545.24	6.50
5	HLA-A0201	FQDMEDRGDVLV	FQDMGDVLV	0	0	0	4	3	FQDMEDRGDVLV	tr_C9ZY92	0.253	3241.89	7.50
14	HLA-A0201	VLVWGTENTTSL	VLVENTTSL	0	0	0	3	3	VLVWGTENTTSL	tr_C9ZY92	0.239	3748.63	8.00
14	HLA-A0201	VLVWGTENTT	VLWGTENTT	0	0	0	2	1	VLVWGTENTT	tr_C9ZY92	0.239	3764.68	8.00
36	HLA-A0201	FSPLATLVLGS	FLATLVLGS	0	0	0	1	2	FSPLATLVLGS	tr_C9ZY92	0.230	4129.83	8.50
54	HLA-A0201	LFSRQTIWQYHPV	FTIWQYHPV	1	0	0	1	3	FSRQTIWQYHPV	tr_C9ZY92	0.230	4143.80	8.50
5	HLA-A0201	FQDMEDRGDV	FQMEDRGDV	0	0	0	2	1	FQDMEDRGDV	tr_C9ZY92	0.229	4214.20	8.50
26	HLA-A0201	FTATCSENNTFSPL	FTATCFSPL	0	0	0	5	5	FTATCSENNTFSPL	tr_C9ZY92	0.222	4519.05	9.00
37	HLA-A0201	SPLATLVLGS	SLATLVLGS	0	0	0	1	1	SPLATLVLGS	tr_C9ZY92	0.216	4806.52	9.50
36	HLA-A0201	FSPLATLVL	FSPLATLVL	0	0	0	0	0	FSPLATLVL	tr_C9ZY92	0.212	5024.93	9.50
15	HLA-A0201	LVWGTENTTSL	LVWENTTSL	0	0	0	3	2	LVWGTENTTSL	tr_C9ZY92	0.212	5034.61	9.50
13	HLA-A0201	DVLVWGTENTTSL	VLVENTTSL	1	0	0	3	3	VLVWGTENTTSL	tr_C9ZY92	0.211	5114.67	10.00
4	HLA-A0201	PFQDMEDRGDVLV	FQDMGDVLV	1	0	0	4	3	FQDMEDRGDVLV	tr_C9ZY92	0.208	5272.16	10.00
35	HLA-A0201	TFSPLATLVLGS	FLATLVLGS	1	0	0	1	2	FSPLATLVLGS	tr_C9ZY92	0.205	5434.26	10.00
34	HLA-A0201	NTFSPLATLVLGS	FLATLVLGS	2	0	0	1	2	FSPLATLVLGS	tr_C9ZY92	0.199	5792.10	11.00
12	HLA-A0201	GDVLVWGTENTTSL	VLVENTTSL	2	0	0	3	3	VLVWGTENTTSL	tr_C9ZY92	0.197	5909.85	11.00
33	HLA-A0201	NNTFSPLATLVLGS	FLATLVLGS	3	0	0	1	2	FSPLATLVLGS	tr_C9ZY92	0.196	6014.35	11.00
3	HLA-A0201	APFQDMEDRGDVLV	FQDMGDVLV	2	0	0	4	3	FQDMEDRGDVLV	tr_C9ZY92	0.195	6071.23	11.00
33	HLA-A0201	NNTFSPLATL	NTFSPLATL	0	0	0	1	1	NNTFSPLATL	tr_C9ZY92	0.195	6091.96	11.00
43	HLA-A0201	VLGSERDTI	VLGSERDTI	0	0	0	0	0	VLGSERDTI	tr_C9ZY92	0.177	7340.89	12.00
14	HLA-A0201	VLVWGTENTTSLFT	VLVWTSLFT	0	0	0	4	5	VLVWGTENTTSLFT	tr_C9ZY92	0.171	7851.16	13.00
15	HLA-A0201	LVWGTENTT	LVWGTENTT	0	0	0	0	0	LVWGTENTT	tr_C9ZY92	0.168	8128.11	13.00
41	HLA-A0201	TLVLGSERDTIRHL	TLVDTIRHL	0	0	0	3	5	TLVLGSERDTIRHL	tr_C9ZY92	0.162	8637.97	14.00
33	HLA-A0201	NNTFSPLATLV	NTFSPLALV	1	0	0	7	1	NTFSPLATLV	tr_C9ZY92	0.161	8728.63	14.00
56	HLA-A0201	SRQTIWQYHPV	RQTIWQYPV	1	0	0	7	1	RQTIWQYHPV	tr_C9ZY92	0.154	9434.06	15.00
24	HLA-A0201	SLFTATCSE	SLFTATCSE	0	0	0	0	0	SLFTATCSE	tr_C9ZY92	0.153	9551.67	15.00
32	HLA-A0201	ENNTFSPLATLV	NTFSPLALV	2	0	0	7	1	NTFSPLATLV	tr_C9ZY92	0.153	9573.09	15.00
24	HLA-A0201	SLFTATCSENNT	SLFTATCST	0	0	0	8	3	SLFTATCSENNT	tr_C9ZY92	0.152	9618.77	15.00
5	HLA-A0201	FQDMEDRGDVLVW	FQDMGDVLV	0	0	0	4	3	FQDMEDRGDVLV	tr_C9ZY92	0.152	9686.24	15.00
15	HLA-A0201	LVWGTENTTSLFT	LVWGTSLFT	0	0	0	4	4	LVWGTENTTSLFT	tr_C9ZY92	0.150	9814.62	15.00
31	HLA-A0201	SENNTFSPLATLV	NTFSPLALV	3	0	0	7	1	NTFSPLATLV	tr_C9ZY92	0.150	9882.83	15.00
30	HLA-A0201	CSENNTFSPLATLV	NTFSPLALV	4	0	0	7	1	NTFSPLATLV	tr_C9ZY92	0.148	10062.59	15.00
35	HLA-A0201	TFSPLATLVL	FSPLATLVL	1	0	0	0	0	FSPLATLVL	tr_C9ZY92	0.148	10100.22	15.00
35	HLA-A0201	TFSPLATLV	TFSPLATLV	0	0	0	0	0	TFSPLATLV	tr_C9ZY92	0.148	10104.80	15.00
36	HLA-A0201	FSPLATLVLGSE	FLATLVLGS	0	0	0	1	2	FSPLATLVLGS	tr_C9ZY92	0.145	10405.14	16.00
24	HLA-A0201	SLFTATCSENNTFS	SLFENNTFS	0	0	0	3	5	SLFTATCSENNTFS	tr_C9ZY92	0.144	10476.77	16.00
34	HLA-A0201	NTFSPLATLVL	NTFLATLVL	0	0	0	3	2	NTFSPLATLVL	tr_C9ZY92	0.141	10879.59	16.00
36	HLA-A0201	FSPLATLVLG	FSLATLVLG	0	0	0	2	1	FSPLATLVLG	tr_C9ZY92	0.140	10962.42	16.00
31	HLA-A0201	SENNTFSPLATL	STFSPLATL	0	0	0	1	3	SENNTFSPLATL	tr_C9ZY92	0.140	11018.19	16.00
7	HLA-A0201	DMEDRGDVLV	DMDRGDVLV	0	0	0	2	1	DMEDRGDVLV	tr_C9ZY92	0.140	11034.89	16.00
43	HLA-A0201	VLGSERDTIRHL	VLGDTIRHL	0	0	0	3	3	VLGSERDTIRHL	tr_C9ZY92	0.139	11163.15	17.00
14	HLA-A0201	VLVWGTENTTSLF	VLVENTTSL	0	0	0	3	3	VLVWGTENTTSL	tr_C9ZY92	0.138	11210.60	17.00
24	HLA-A0201	SLFTATCSENNTF	SLFTATCTF	0	0	0	7	4	SLFTATCSENNTF	tr_C9ZY92	0.138	11277.14	17.00
13	HLA-A0201	DVLVWGTENT	VLVWGTENT	1	0	0	0	0	VLVWGTENT	tr_C9ZY92	0.135	11601.52	17.00
5	HLA-A0201	FQDMEDRGDVLVWG	FQDMGDVLV	0	0	0	4	3	FQDMEDRGDVLV	tr_C9ZY92	0.134	11671.91	17.00
38	HLA-A0201	PLATLVLGSERDTI	PLATLVLTI	0	0	0	7	5	PLATLVLGSERDTI	tr_C9ZY92	0.132	11963.56	17.00
23	HLA-A0201	TSLFTATCSENNT	SLFTATCST	1	0	0	8	3	SLFTATCSENNT	tr_C9ZY92	0.130	12273.50	18.00

35	HLA-A0201	TFSPLATLVLGSE	FLATLVLGS	1	0	0	1	2	FSPLATLVLGS	tr_C9ZY92	0.130	12311.01	18.00
53	HLA-A0201	HLFSRQTIWQY	HLFSTIWQY	0	0	0	4	2	HLFSRQTIWQY	tr_C9ZY92	0.129	12344.22	18.00
34	HLA-A0201	NTFSPLATLVLGSE	FLATLVLGS	2	0	0	1	2	FSPLATLVLGS	tr_C9ZY92	0.129	12344.35	18.00
33	HLA-A0201	NNTFSPLATLVL	NTFLATLVL	1	0	0	3	2	NTFSPLATLVL	tr_C9ZY92	0.129	12399.50	18.00
30	HLA-A0201	CSENNTFSPLATL	STFSPLATL	1	0	0	1	3	SENNTFSPLATL	tr_C9ZY92	0.128	12462.45	18.00
4	HLA-A0201	PFQDMEDRGDVLVW	FQDMGDVLV	1	0	0	4	3	FQDMEDRGDVLV	tr_C9ZY92	0.127	12710.84	18.00
8	HLA-A0201	MEDRGDVLV	MEDRGDVLV	0	0	0	0	0	MEDRGDVLV	tr_C9ZY92	0.125	12989.74	19.00
23	HLA-A0201	TSLFTATCSENNTF	SLFTATCTF	1	0	0	7	4	SLFTATCSENNTF	tr_C9ZY92	0.124	13005.78	19.00
32	HLA-A0201	ENNTFSPLATLVL	NTFLATLVL	2	0	0	3	2	NTFSPLATLVL	tr_C9ZY92	0.124	13038.75	19.00
36	HLA-A0201	FSPLATLVLGSER	FLATLVLGS	0	0	0	1	2	FSPLATLVLGS	tr_C9ZY92	0.123	13154.08	19.00
13	HLA-A0201	DVLVWGTENTTSLF	VLVENTTSL	1	0	0	3	3	VLVWGTENTTSL	tr_C9ZY92	0.123	13231.74	19.00
41	HLA-A0201	TLVLGSERDTI	TLVLGSETI	0	0	0	7	2	TLVLGSERDTI	tr_C9ZY92	0.123	13258.25	19.00
31	HLA-A0201	SENNTFSPLATLVL	NTFLATLVL	3	0	0	3	2	NTFSPLATLVL	tr_C9ZY92	0.123	13262.41	19.00
22	HLA-A0201	TTSLFTATCSENNT	SLFTATCST	2	0	0	8	3	SLFTATCSENNT	tr_C9ZY92	0.122	13303.52	19.00
24	HLA-A0201	SLFTATCSEN	SLFATCSEN	0	0	0	3	1	SLFTATCSEN	tr_C9ZY92	0.121	13432.24	19.00
22	HLA-A0201	TTSLFTATC	TTSLFTATC	0	0	0	0	0	TTSLFTATC	tr_C9ZY92	0.121	13492.54	19.00
42	HLA-A0201	LVLGSERDTIRHL	VLGDTIRHL	1	0	0	3	3	VLGSERDTIRHL	tr_C9ZY92	0.121	13494.89	19.00
29	HLA-A0201	TCSENNTFSPLATL	STFSPLATL	2	0	0	1	3	SENNTFSPLATL	tr_C9ZY92	0.121	13523.38	19.00
53	HLA-A0201	HLFSRQTIWQ	HLFSRTIWQ	0	0	0	5	1	HLFSRQTIWQ	tr_C9ZY92	0.120	13625.46	19.00
52	HLA-A0201	RHLFSRQTIWQY	HLFQTIWQY	1	0	0	3	2	HLFSRQTIWQY	tr_C9ZY92	0.120	13698.19	19.00
15	HLA-A0201	LVWGTENTTSLF	LVWENTTSL	0	0	0	3	2	LVWGTENTTSL	tr_C9ZY92	0.119	13798.74	19.00
53	HLA-A0201	HLFSRQTIW	HLFSRQTIW	0	0	0	0	0	HLFSRQTIW	tr_C9ZY92	0.119	13832.53	20.00
13	HLA-A0201	DVLVWGTENTT	VLWGTENTT	1	0	0	2	1	VLVWGTENTT	tr_C9ZY92	0.118	13917.04	20.00
51	HLA-A0201	IRHLFSRQTIWQY	HLFQTIWQY	2	0	0	3	2	HLFSRQTIWQY	tr_C9ZY92	0.115	14343.41	20.00
36	HLA-A0201	FSPLATLVLGSERD	FLATLVLGS	0	0	0	1	2	FSPLATLVLGS	tr_C9ZY92	0.115	14383.50	20.00
50	HLA-A0201	TIRHLFSRQTIWQY	HLFQTIWQY	3	0	0	3	2	HLFSRQTIWQY	tr_C9ZY92	0.114	14551.62	20.00
34	HLA-A0201	NTFSPLATLVLG	NTFLATLVL	0	0	0	3	2	NTFSPLATLVL	tr_C9ZY92	0.113	14680.99	21.00
42	HLA-A0201	LVLGSERDTI	LLGSERDTI	0	0	0	1	1	LVLGSERDTI	tr_C9ZY92	0.111	14978.94	21.00
12	HLA-A0201	GDVLVWGTENTT	VLWGTENTT	2	0	0	2	1	VLVWGTENTT	tr_C9ZY92	0.111	15024.40	21.00
35	HLA-A0201	TFSPLATLVLGSER	FLATLVLGS	1	0	0	1	2	FSPLATLVLGS	tr_C9ZY92	0.111	15039.20	21.00
53	HLA-A0201	HLFSRQTIWQYHP	HLFIWQYHP	0	0	0	3	4	HLFSRQTIWQYHP	tr_C9ZY92	0.111	15068.02	21.00
11	HLA-A0201	RGDVLVWGTENTT	VLWGTENTT	3	0	0	2	1	VLVWGTENTT	tr_C9ZY92	0.109	15339.11	21.00
40	HLA-A0201	ATLVLGSERDTI	TLVLGSETI	1	0	0	7	2	TLVLGSERDTI	tr_C9ZY92	0.108	15521.25	22.00
10	HLA-A0201	DRGDVLVWGTENTT	VLWGTENTT	4	0	0	2	1	VLVWGTENTT	tr_C9ZY92	0.108	15567.00	22.00
39	HLA-A0201	LATLVLGSERDTI	TLVLGSETI	2	0	0	7	2	TLVLGSERDTI	tr_C9ZY92	0.106	15874.18	22.00
33	HLA-A0201	NNTFSPLATLVLG	NTFLATLVL	1	0	0	3	2	NTFSPLATLVL	tr_C9ZY92	0.104	16194.95	22.00
53	HLA-A0201	HLFSRQTIWQYH	HLFQTIWQY	0	0	0	3	2	HLFSRQTIWQY	tr_C9ZY92	0.104	16239.69	22.00
38	HLA-A0201	PLATLVLGS	PLATLVLGS	0	0	0	0	0	PLATLVLGS	tr_C9ZY92	0.103	16341.22	23.00
52	HLA-A0201	RHLFSRQTIWQYHP	HLFIWQYHP	1	0	0	3	4	HLFSRQTIWQYHP	tr_C9ZY92	0.101	16719.84	23.00
32	HLA-A0201	ENNTFSPLATLVLG	NTFLATLVL	2	0	0	3	2	NTFSPLATLVL	tr_C9ZY92	0.101	16801.63	23.00
32	HLA-A0201	ENNTFSPLATL	NTFSPLATL	2	0	0	0	0	NTFSPLATL	tr_C9ZY92	0.101	16804.35	23.00
5	HLA-A0201	FQDMEDRGDVL	FMEDRGDVL	0	0	0	1	2	FQDMEDRGDVL	tr_C9ZY92	0.098	17256.52	24.00
52	HLA-A0201	RHLFSRQTIWQYH	HLFQTIWQY	1	0	0	3	2	HLFSRQTIWQY	tr_C9ZY92	0.096	17712.41	24.00
26	HLA-A0201	FTATCSENNT	FTATCSENT	0	0	0	7	1	FTATCSENNT	tr_C9ZY92	0.095	17951.46	25.00
51	HLA-A0201	IRHLFSRQTIWQYH	HLFQTIWQY	2	0	0	3	2	HLFSRQTIWQY	tr_C9ZY92	0.093	18304.89	25.00
15	HLA-A0201	LVWGTENTTS	LVWGTNTTS	0	0	0	5	1	LVWGTENTTS	tr_C9ZY92	0.089	19063.91	26.00
52	HLA-A0201	RHLFSRQTIW	RLFSRQTIW	0	0	0	1	1	RHLFSRQTIW	tr_C9ZY92	0.087	19442.62	27.00
4	HLA-A0201	PFQDMEDRGDV	FQMEDRGDV	1	0	0	2	1	FQDMEDRGDV	tr_C9ZY92	0.086	19622.46	27.00
21	HLA-A0201	NTTSLFTATC	TTSLFTATC	1	0	0	0	0	TTSLFTATC	tr_C9ZY92	0.086	19635.84	27.00
43	HLA-A0201	VLGSERDTIRHLF	VLGDTIRHL	0	0	0	3	3	VLGSERDTIRHL	tr_C9ZY92	0.086	19659.43	27.00
55	HLA-A0201	FSRQTIWQY	FSRQTIWQY	0	0	0	0	0	FSRQTIWQY	tr_C9ZY92	0.085	20018.97	27.00
23	HLA-A0201	TSLFTATCSE	SLFTATCSE	1	0	0	0	0	SLFTATCSE	tr_C9ZY92	0.083	20278.17	28.00
35	HLA-A0201	TFSPLATLVLG	FSLATLVLG	1	0	0	2	1	FSPLATLVLG	tr_C9ZY92	0.082	20541.84	28.00
21	HLA-A0201	NTTSLFTAT	NTTSLFTAT	0	0	0	0	0	NTTSLFTAT	tr_C9ZY92	0.080	20953.31	29.00

4	HLA-A0201	PFQDMEDRGDVL	FMEDRGDVL	1	0	0	1	2	FQDMEDRGDVL	tr_C9ZY92	0.080	20999.39	29.00
14	HLA-A0201	VLVWGTENTTS	VLVWGTENTT	0	0	0	2	1	VLVWGTENTT	tr_C9ZY92	0.080	21045.78	29.00
3	HLA-A0201	APFQDMEDRGDV	FQMEDRGDV	2	0	0	2	1	FQDMEDRGDV	tr_C9ZY92	0.080	21050.11	29.00
37	HLA-A0201	SPLATLVLG	SPLATLVLG	0	0	0	0	0	SPLATLVLG	tr_C9ZY92	0.080	21113.29	29.00
11	HLA-A0201	RGDVLVWGT	RGDVLVWGT	0	0	0	0	0	RGDVLVWGT	tr_C9ZY92	0.080	21128.38	29.00
43	HLA-A0201	VLGSERDTIRHLFS	VLGDTIRHL	0	0	0	3	3	VLGSERDTIRHL	tr_C9ZY92	0.079	21240.70	29.00
2	HLA-A0201	CAPFQDMEDRGDV	FQMEDRGDV	3	0	0	2	1	FQDMEDRGDV	tr_C9ZY92	0.078	21468.98	30.00
30	HLA-A0201	CSENNTFSPL	CSENNTFPL	0	0	0	7	1	CSENNTFSPL	tr_C9ZY92	0.078	21552.06	30.00
41	HLA-A0201	TLVLGSERDT	TLVLGSERT	0	0	0	8	1	TLVLGSERDT	tr_C9ZY92	0.077	21648.35	30.00
1	HLA-A0201	GCAPFQDMEDRGDV	FQMEDRGDV	4	0	0	2	1	FQDMEDRGDV	tr_C9ZY92	0.077	21718.97	30.00
42	HLA-A0201	LVLGSERDTIRHLF	VLGDTIRHL	1	0	0	3	3	VLGSERDTIRHL	tr_C9ZY92	0.077	21774.03	30.00
41	HLA-A0201	TLVLGSERDTIR	TLVLGSETI	0	0	0	7	2	TLVLGSERDTI	tr_C9ZY92	0.077	21809.62	30.00
28	HLA-A0201	ATCSENNTFSPL	ATCSTFSPL	0	0	0	4	3	ATCSENNTFSPL	tr_C9ZY92	0.076	21867.04	30.00
18	HLA-A0201	GTENTTSLFTA	GTTTSLFTA	0	0	0	2	2	GTENTTSLFTA	tr_C9ZY92	0.076	22016.86	30.00
3	HLA-A0201	APFQDMEDRGDVL	FMEDRGDVL	2	0	0	1	2	FQDMEDRGDVL	tr_C9ZY92	0.075	22093.93	31.00
2	HLA-A0201	CAPFQDMEDRGDVL	FMEDRGDVL	3	0	0	1	2	FQDMEDRGDVL	tr_C9ZY92	0.074	22397.44	31.00
39	HLA-A0201	LATLVLGSERDTIR	TLVLGSETI	2	0	0	7	2	TLVLGSERDTI	tr_C9ZY92	0.074	22478.77	31.00
31	HLA-A0201	SENNTFSPL	SENNTFSPL	0	0	0	0	0	SENNTFSPL	tr_C9ZY92	0.072	22909.69	32.00
37	HLA-A0201	SPLATLVLGSE	SLATLVLGS	0	0	0	1	1	SPLATLVLGS	tr_C9ZY92	0.072	22970.00	32.00
13	HLA-A0201	DVLVWGTENTTS	VLWGTENTT	1	0	0	2	1	VLVWGTENTT	tr_C9ZY92	0.071	23130.37	32.00
55	HLA-A0201	FSRQTIWQYH	FSRQTIWYH	0	0	0	7	1	FSRQTIWQYH	tr_C9ZY92	0.071	23239.24	32.00
40	HLA-A0201	ATLVLGSERDTIR	TLVLGSETI	1	0	0	7	2	TLVLGSERDTI	tr_C9ZY92	0.069	23658.86	33.00
17	HLA-A0201	WGTENTTSLFTA	GTENTLFTA	1	0	0	5	2	GTENTTSLFTA	tr_C9ZY92	0.068	23994.26	34.00
27	HLA-A0201	TATCSENNTFSPL	ATCSTFSPL	1	0	0	4	3	ATCSENNTFSPL	tr_C9ZY92	0.068	24024.66	34.00
17	HLA-A0201	WGTENTTSL	WGTENTTSL	0	0	0	0	0	WGTENTTSL	tr_C9ZY92	0.068	24027.77	34.00
11	HLA-A0201	RGDVLVWGTENTTS	VLWGTENTT	3	0	0	2	1	VLVWGTENTT	tr_C9ZY92	0.068	24065.50	34.00
12	HLA-A0201	GDVLVWGTENTTS	VLWGTENTT	2	0	0	2	1	VLVWGTENTT	tr_C9ZY92	0.067	24137.20	34.00
11	HLA-A0201	RGDVLVWGTENT	RLVWGTENT	0	0	0	1	3	RGDVLVWGTENT	tr_C9ZY92	0.067	24309.40	34.00
51	HLA-A0201	IRHLFSRQTIW	ILFSRQTIW	0	0	0	1	2	IRHLFSRQTIW	tr_C9ZY92	0.067	24313.09	34.00
7	HLA-A0201	DMEDRGDVLVWGT	DMDVLVWGT	0	0	0	2	4	DMEDRGDVLVWGT	tr_C9ZY92	0.066	24464.82	35.00
51	HLA-A0201	IRHLFSRQTI	IRHLFSQTI	0	0	0	6	1	IRHLFSRQTI	tr_C9ZY92	0.066	24496.06	35.00
22	HLA-A0201	TTSLFTATCS	TTSLFTATC	0	0	0	0	0	TTSLFTATC	tr_C9ZY92	0.066	24538.25	35.00
54	HLA-A0201	LFSRQTIWQY	FSRQTIWQY	1	0	0	0	0	FSRQTIWQY	tr_C9ZY92	0.066	24565.61	35.00
41	HLA-A0201	TLVLGSERDTIRH	TLVLGSETI	0	0	0	7	2	TLVLGSERDTI	tr_C9ZY92	0.065	24621.48	35.00
28	HLA-A0201	ATCSENNTFSPLA	ATCSFSPLA	0	0	0	4	4	ATCSENNTFSPLA	tr_C9ZY92	0.065	24668.96	35.00
16	HLA-A0201	VWGTENTTSLFTA	GTENTLFTA	2	0	0	5	2	GTENTTSLFTA	tr_C9ZY92	0.065	24725.87	35.00
17	HLA-A0201	WGTENTTSLFTATC	WTSLFTATC	0	0	0	1	5	WGTENTTSLFTATC	tr_C9ZY92	0.065	24766.04	35.00
51	HLA-A0201	IRHLFSRQTIWQ	HLFSRTIWQ	2	0	0	5	1	HLFSRQTIWQ	tr_C9ZY92	0.064	24886.65	35.00
52	HLA-A0201	RHLFSRQTIWQ	HLFSRTIWQ	1	0	0	5	1	HLFSRQTIWQ	tr_C9ZY92	0.064	25023.27	36.00
26	HLA-A0201	FTATCSENNTF	FTATCSETF	0	0	0	7	2	FTATCSENNTF	tr_C9ZY92	0.064	25043.04	36.00
50	HLA-A0201	TIRHLFSRQTIWQ	HLFSRTIWQ	3	0	0	5	1	HLFSRQTIWQ	tr_C9ZY92	0.064	25073.41	36.00
37	HLA-A0201	SPLATLVLGSER	SLATLVLGS	0	0	0	1	1	SPLATLVLGS	tr_C9ZY92	0.063	25166.09	36.00
6	HLA-A0201	QDMEDRGDVL	QMEDRGDVL	0	0	0	1	1	QDMEDRGDVL	tr_C9ZY92	0.063	25196.33	36.00
49	HLA-A0201	DTIRHLFSRQTIWQ	HLFSRTIWQ	4	0	0	5	1	HLFSRQTIWQ	tr_C9ZY92	0.063	25236.43	36.00
10	HLA-A0201	DRGDVLVWGTENT	VLVWGTENT	4	0	0	0	0	VLVWGTENT	tr_C9ZY92	0.063	25359.88	36.00
23	HLA-A0201	TSLFTATCSEN	SLFATCSEN	1	0	0	3	1	SLFTATCSEN	tr_C9ZY92	0.062	25576.75	37.00
9	HLA-A0201	EDRGDVLVWGTENT	VLVWGTENT	5	0	0	0	0	VLVWGTENT	tr_C9ZY92	0.062	25654.91	37.00
57	HLA-A0201	RQTIWQYHP	RQTIWQYHP	0	0	0	0	0	RQTIWQYHP	tr_C9ZY92	0.062	25661.02	37.00
24	HLA-A0201	SLFTATCSENN	SLFTATCSE	0	0	0	0	0	SLFTATCSE	tr_C9ZY92	0.061	25707.97	37.00
12	HLA-A0201	GDVLVWGTENT	VLVWGTENT	2	0	0	0	0	VLVWGTENT	tr_C9ZY92	0.061	25760.88	37.00
50	HLA-A0201	TIRHLFSRQTIW	ILFSRQTIW	1	0	0	1	2	IRHLFSRQTIW	tr_C9ZY92	0.061	25818.37	37.00
6	HLA-A0201	QDMEDRGDVLV	DMDRGDVLV	1	0	0	2	1	DMEDRGDVLV	tr_C9ZY92	0.061	25893.05	37.00
26	HLA-A0201	FTATCSENNTFSP	FTATCTFSP	0	0	0	5	4	FTATCSENNTFSP	tr_C9ZY92	0.060	26079.19	38.00
39	HLA-A0201	LATLVLGSE	LATLVLGSE	0	0	0	0	0	LATLVLGSE	tr_C9ZY92	0.060	26088.51	38.00

48	HLA-A0201	RDTIRHLFSRQTIW	ILFSRQTIW	3	0	0	1	2	IRHLFSRQTIW	tr_C9ZY92	0.060	26102.90	38.00
43	HLA-A0201	VLGSERDTIR	VLGSERDTI	0	0	0	0	0	VLGSERDTI	tr_C9ZY92	0.060	26145.59	38.00
6	HLA-A0201	QDMEDRGDVLVWGT	DMDVLVWGT	1	0	0	2	4	DMEDRGDVLVWGT	tr_C9ZY92	0.060	26173.04	38.00
22	HLA-A0201	TTSLFTATCSEN	SLFATCSEN	2	0	0	3	1	SLFTATCSEN	tr_C9ZY92	0.060	26216.40	38.00
49	HLA-A0201	DTIRHLFSRQTIW	ILFSRQTIW	2	0	0	1	2	IRHLFSRQTIW	tr_C9ZY92	0.060	26221.79	38.00
40	HLA-A0201	ATLVLGSERDTIRH	TLVLGSETI	1	0	0	7	2	TLVLGSERDTI	tr_C9ZY92	0.059	26297.65	38.00
37	HLA-A0201	SPLATLVLGSERDT	SLATLVLGS	0	0	0	1	1	SPLATLVLGS	tr_C9ZY92	0.059	26356.05	38.00
37	HLA-A0201	SPLATLVLGSERD	SLATLVLGS	0	0	0	1	1	SPLATLVLGS	tr_C9ZY92	0.059	26495.29	39.00
21	HLA-A0201	NTTSLFTATCSEN	SLFATCSEN	3	0	0	3	1	SLFTATCSEN	tr_C9ZY92	0.059	26511.92	39.00
25	HLA-A0201	LFTATCSENNTF	FTATCSETF	1	0	0	7	2	FTATCSENNTF	tr_C9ZY92	0.058	26607.62	39.00
20	HLA-A0201	ENTTSLFTATCSEN	SLFATCSEN	4	0	0	3	1	SLFTATCSEN	tr_C9ZY92	0.058	26769.61	39.00
27	HLA-A0201	TATCSENNTFSPLA	ATCSFSPLA	1	0	0	4	4	ATCSENNTFSPLA	tr_C9ZY92	0.058	26829.93	39.00
38	HLA-A0201	PLATLVLGSE	PLATLVLGS	0	0	0	0	0	PLATLVLGS	tr_C9ZY92	0.057	26978.38	40.00
18	HLA-A0201	GTENTTSLFT	GTNTTSLFT	0	0	0	2	1	GTENTTSLFT	tr_C9ZY92	0.056	27173.50	40.00
49	HLA-A0201	DTIRHLFSRQTI	DTIRHLFTI	0	0	0	7	3	DTIRHLFSRQTI	tr_C9ZY92	0.056	27341.30	40.00
50	HLA-A0201	TIRHLFSRQTI	TIRHLFQTI	0	0	0	6	2	TIRHLFSRQTI	tr_C9ZY92	0.055	27507.16	41.00
38	HLA-A0201	PLATLVLGSERDT	PLATLVLGT	0	0	0	8	4	PLATLVLGSERDT	tr_C9ZY92	0.055	27525.03	41.00
25	HLA-A0201	LFTATCSENNTFSP	FTATCTFSP	1	0	0	5	4	FTATCSENNTFSP	tr_C9ZY92	0.055	27718.70	41.00
10	HLA-A0201	DRGDVLVWGT	RGDVLVWGT	1	0	0	0	0	RGDVLVWGT	tr_C9ZY92	0.054	27847.65	41.00
18	HLA-A0201	GTENTTSLFTATC	GTSLFTATC	0	0	0	1	4	GTENTTSLFTATC	tr_C9ZY92	0.054	27874.48	42.00
23	HLA-A0201	TSLFTATCSENN	SLFTATCSE	1	0	0	0	0	SLFTATCSE	tr_C9ZY92	0.054	27999.93	42.00
19	HLA-A0201	TENTTSLFTA	TENTTLFTA	0	0	0	5	1	TENTTSLFTA	tr_C9ZY92	0.054	28016.89	42.00
8	HLA-A0201	MEDRGDVLVW	MEDRGDVLV	0	0	0	0	0	MEDRGDVLV	tr_C9ZY92	0.053	28233.57	42.00
22	HLA-A0201	TTSLFTATCSENN	SLFTCSENN	2	0	0	3	2	SLFTATCSENN	tr_C9ZY92	0.052	28378.73	43.00
26	HLA-A0201	FTATCSENNTFS	FTATCSTFS	0	0	0	6	3	FTATCSENNTFS	tr_C9ZY92	0.052	28484.55	43.00
48	HLA-A0201	RDTIRHLFSRQTI	DTIRHLFTI	1	0	0	7	3	DTIRHLFSRQTI	tr_C9ZY92	0.052	28524.35	43.00
7	HLA-A0201	DMEDRGDVL	DMEDRGDVL	0	0	0	0	0	DMEDRGDVL	tr_C9ZY92	0.052	28542.25	43.00
21	HLA-A0201	NTTSLFTATCSENN	SLFTCSENN	3	0	0	3	2	SLFTATCSENN	tr_C9ZY92	0.051	28654.26	43.00
7	HLA-A0201	DMEDRGDVLVWGTE	DMDVLVWGT	0	0	0	2	4	DMEDRGDVLVWGT	tr_C9ZY92	0.051	28806.58	44.00
8	HLA-A0201	MEDRGDVLVWGT	MGDVLVWGT	0	0	0	1	3	MEDRGDVLVWGT	tr_C9ZY92	0.051	28813.75	44.00
47	HLA-A0201	ERDTIRHLFSRQTI	DTIRHLFTI	2	0	0	7	3	DTIRHLFSRQTI	tr_C9ZY92	0.051	28834.65	44.00
55	HLA-A0201	FSRQTIWQYHP	FQTIWQYHP	0	0	0	1	2	FSRQTIWQYHP	tr_C9ZY92	0.050	28962.53	44.00
29	HLA-A0201	TCSENNTFSPL	TCSETFSPL	0	0	0	4	2	TCSENNTFSPL	tr_C9ZY92	0.050	29042.55	44.00
26	HLA-A0201	FTATCSENN	FTATCSENN	0	0	0	0	0	FTATCSENN	tr_C9ZY92	0.050	29096.65	44.00
19	HLA-A0201	TENTTSLFTATC	TTSLFTATC	0	0	0	1	3	TENTTSLFTATC	tr_C9ZY92	0.050	29252.27	45.00
7	HLA-A0201	DMEDRGDVLVW	DMDRGDVLV	0	0	0	2	1	DMEDRGDVLV	tr_C9ZY92	0.049	29308.03	45.00
41	HLA-A0201	TLVLGSERD	TLVLGSERD	0	0	0	0	0	TLVLGSERD	tr_C9ZY92	0.049	29361.99	45.00
20	HLA-A0201	ENTTSLFTATC	TTSLFTATC	2	0	0	0	0	TTSLFTATC	tr_C9ZY92	0.049	29408.08	45.00
22	HLA-A0201	TTSLFTATCSE	SLFTATCSE	2	0	0	0	0	SLFTATCSE	tr_C9ZY92	0.048	29594.81	46.00
39	HLA-A0201	LATLVLGSER	LATLVLSER	0	0	0	6	1	LATLVLGSER	tr_C9ZY92	0.048	29685.90	46.00
54	HLA-A0201	LFSRQTIWQYH	FSRQTIWYH	1	0	0	7	1	FSRQTIWQYH	tr_C9ZY92	0.048	29780.79	46.00
21	HLA-A0201	NTTSLFTATCSE	SLFTATCSE	3	0	0	0	0	SLFTATCSE	tr_C9ZY92	0.048	29783.06	46.00
0	HLA-A0201	SGCAPFQDM	SGCAPFQDM	0	0	0	0	0	SGCAPFQDM	tr_C9ZY92	0.048	29799.82	46.00
28	HLA-A0201	ATCSENNTFSPLAT	ATCFSPLAT	0	0	0	3	5	ATCSENNTFSPLAT	tr_C9ZY92	0.048	29814.00	46.00
20	HLA-A0201	ENTTSLFTATCSE	SLFTATCSE	4	0	0	0	0	SLFTATCSE	tr_C9ZY92	0.047	29929.40	47.00
19	HLA-A0201	TENTTSLFTATCSE	SLFTATCSE	5	0	0	0	0	SLFTATCSE	tr_C9ZY92	0.047	30003.64	47.00
25	HLA-A0201	LFTATCSENNTFS	FTATCSTFS	1	0	0	6	3	FTATCSENNTFS	tr_C9ZY92	0.047	30018.26	47.00
18	HLA-A0201	GTENTTSLFTAT	GTENTLFTA	0	0	0	5	2	GTENTTSLFTA	tr_C9ZY92	0.047	30199.05	47.00
18	HLA-A0201	GTENTTSLF	GTENTTSLF	0	0	0	0	0	GTENTTSLF	tr_C9ZY92	0.047	30232.06	47.00
46	HLA-A0201	SERDTIRHL	SERDTIRHL	0	0	0	0	0	SERDTIRHL	tr_C9ZY92	0.046	30251.06	47.00
54	HLA-A0201	LFSRQTIWQYHP	FQTIWQYHP	1	0	0	1	2	FSRQTIWQYHP	tr_C9ZY92	0.046	30275.61	47.00
11	HLA-A0201	RGDVLVWGTE	RGDVLVWGT	0	0	0	0	0	RGDVLVWGT	tr_C9ZY92	0.046	30275.94	47.00
28	HLA-A0201	ATCSENNTF	ATCSENNTF	0	0	0	0	0	ATCSENNTF	tr_C9ZY92	0.046	30323.46	48.00
7	HLA-A0201	DMEDRGDVLVWG	DMGDVLVWG	0	0	0	2	3	DMEDRGDVLVWG	tr_C9ZY92	0.046	30335.28	48.00

40	HLA-A0201	ATLVLGSER	ATLVLGSER	0	0	0	0	0	ATLVLGSER	tr_C9ZY92	0.046	30340.21	48.00
45	HLA-A0201	GSERDTIRHL	GSEDTIRHL	0	0	0	3	1	GSERDTIRHL	tr_C9ZY92	0.046	30377.98	48.00
16	HLA-A0201	VWGTENTTSL	VWGENTTSL	0	0	0	3	1	VWGTENTTSL	tr_C9ZY92	0.046	30480.38	48.00
39	HLA-A0201	LATLVLGSERDT	LATLVLGST	0	0	0	8	3	LATLVLGSERDT	tr_C9ZY92	0.046	30535.50	48.00
51	HLA-A0201	IRHLFSRQT	IRHLFSRQT	0	0	0	0	0	IRHLFSRQT	tr_C9ZY92	0.045	30568.57	48.00
6	HLA-A0201	QDMEDRGDVLVW	DMDRGDVLV	1	0	0	2	1	DMEDRGDVLV	tr_C9ZY92	0.045	30653.02	48.00
20	HLA-A0201	ENTTSLFTAT	NTTSLFTAT	1	0	0	0	0	NTTSLFTAT	tr_C9ZY92	0.045	30670.26	48.00
12	HLA-A0201	GDVLVWGTE	GDVLVWGTE	0	0	0	0	0	GDVLVWGTE	tr_C9ZY92	0.045	30698.14	48.00
20	HLA-A0201	ENTTSLFTA	ENTTSLFTA	0	0	0	0	0	ENTTSLFTA	tr_C9ZY92	0.045	30730.05	49.00
48	HLA-A0201	RDTIRHLFSR	RTIRHLFSR	0	0	0	1	1	RDTIRHLFSR	tr_C9ZY92	0.045	30865.01	49.00
17	HLA-A0201	WGTENTTSLFTAT	GTENTLFTA	1	0	0	5	2	GTENTTSLFTA	tr_C9ZY92	0.044	30920.84	49.00
8	HLA-A0201	MEDRGDVLVWGTE	MGDVLVWGT	0	0	0	1	3	MEDRGDVLVWGT	tr_C9ZY92	0.044	30980.10	49.00
16	HLA-A0201	VWGTENTTSLFTAT	GTENTLFTA	2	0	0	5	2	GTENTTSLFTA	tr_C9ZY92	0.044	31046.20	49.00
40	HLA-A0201	ATLVLGSERD	ATLVLGSER	0	0	0	0	0	ATLVLGSER	tr_C9ZY92	0.044	31098.66	50.00
50	HLA-A0201	TIRHLFSRQT	TIHLFSRQT	0	0	0	2	1	TIRHLFSRQT	tr_C9ZY92	0.044	31103.04	50.00
6	HLA-A0201	QDMEDRGDVLVWG	DMGDVLVWG	1	0	0	2	3	DMEDRGDVLVWG	tr_C9ZY92	0.043	31381.91	50.00
29	HLA-A0201	TCSENNTFSPLA	TCSEFSPLA	0	0	0	4	3	TCSENNTFSPLA	tr_C9ZY92	0.043	31415.20	50.00
38	HLA-A0201	PLATLVLGSER	PLATLVLER	0	0	0	7	2	PLATLVLGSER	tr_C9ZY92	0.041	31935.79	55.00
31	HLA-A0201	SENNTFSPLA	SENNTFSPL	0	0	0	0	0	SENNTFSPL	tr_C9ZY92	0.041	32036.50	55.00
8	HLA-A0201	MEDRGDVLVWGTEN	MGDVLVWGT	0	0	0	1	3	MEDRGDVLVWGT	tr_C9ZY92	0.041	32136.49	55.00
18	HLA-A0201	GTENTTSLFTATCS	GTENTLFTA	0	0	0	5	2	GTENTTSLFTA	tr_C9ZY92	0.041	32142.39	55.00
39	HLA-A0201	LATLVLGSERD	LLVLGSERD	0	0	0	1	2	LATLVLGSERD	tr_C9ZY92	0.041	32147.27	55.00
49	HLA-A0201	DTIRHLFSR	DTIRHLFSR	0	0	0	0	0	DTIRHLFSR	tr_C9ZY92	0.040	32325.85	55.00
30	HLA-A0201	CSENNTFSPLA	CSNTFSPLA	0	0	0	2	2	CSENNTFSPLA	tr_C9ZY92	0.040	32454.46	55.00
56	HLA-A0201	SRQTIWQYHP	SQTIWQYHP	0	0	0	1	1	SRQTIWQYHP	tr_C9ZY92	0.040	32518.78	55.00
38	HLA-A0201	PLATLVLGSERD	LLVLGSERD	1	0	0	1	2	LATLVLGSERD	tr_C9ZY92	0.039	32632.62	55.00
9	HLA-A0201	EDRGDVLVWGT	RGDVLVWGT	2	0	0	0	0	RGDVLVWGT	tr_C9ZY92	0.039	32866.85	55.00
40	HLA-A0201	ATLVLGSERDT	TLVLGSERT	1	0	0	8	1	TLVLGSERDT	tr_C9ZY92	0.039	32948.75	55.00
23	HLA-A0201	TSLFTATCS	TSLFTATCS	0	0	0	0	0	TSLFTATCS	tr_C9ZY92	0.038	33034.04	55.00
42	HLA-A0201	LVLGSERDT	LVLGSERDT	0	0	0	0	0	LVLGSERDT	tr_C9ZY92	0.038	33040.14	55.00
21	HLA-A0201	NTTSLFTATCS	TTSLFTATC	1	0	0	0	0	TTSLFTATC	tr_C9ZY92	0.038	33087.71	60.00
52	HLA-A0201	RHLFSRQTI	RHLFSRQTI	0	0	0	0	0	RHLFSRQTI	tr_C9ZY92	0.038	33220.08	60.00
8	HLA-A0201	MEDRGDVLVWG	MEDDVLVWG	0	0	0	3	2	MEDRGDVLVWG	tr_C9ZY92	0.038	33301.05	60.00
17	HLA-A0201	WGTENTTSLF	WTENTTSLF	0	0	0	1	1	WGTENTTSLF	tr_C9ZY92	0.038	33301.05	60.00
56	HLA-A0201	SRQTIWQYH	SRQTIWQYH	0	0	0	0	0	SRQTIWQYH	tr_C9ZY92	0.038	33305.74	60.00
25	HLA-A0201	LFTATCSENNT	FTATCSENT	1	0	0	7	1	FTATCSENNT	tr_C9ZY92	0.037	33477.34	60.00
19	HLA-A0201	TENTTSLFT	TENTTSLFT	0	0	0	0	0	TENTTSLFT	tr_C9ZY92	0.037	33491.84	60.00
19	HLA-A0201	TENTTSLFTATCS	TTSLFTATS	3	0	0	8	1	TTSLFTATCS	tr_C9ZY92	0.037	33601.80	60.00
20	HLA-A0201	ENTTSLFTATCS	TTSLFTATC	2	0	0	0	0	TTSLFTATC	tr_C9ZY92	0.036	33733.69	60.00
10	HLA-A0201	DRGDVLVWGTE	RGDVLVWGT	1	0	0	0	0	RGDVLVWGT	tr_C9ZY92	0.036	33755.23	60.00
50	HLA-A0201	TIRHLFSRQ	TIRHLFSRQ	0	0	0	0	0	TIRHLFSRQ	tr_C9ZY92	0.036	33877.07	60.00
28	HLA-A0201	ATCSENNTFS	ATCENNTFS	0	0	0	3	1	ATCSENNTFS	tr_C9ZY92	0.036	33884.39	60.00
29	HLA-A0201	TCSENNTFSPLAT	TCSETFSPL	0	0	0	4	2	TCSENNTFSPL	tr_C9ZY92	0.036	34024.36	60.00
46	HLA-A0201	SERDTIRHLF	SERDTIHLF	0	0	0	6	1	SERDTIRHLF	tr_C9ZY92	0.036	34024.36	60.00
10	HLA-A0201	DRGDVLVWG	DRGDVLVWG	0	0	0	0	0	DRGDVLVWG	tr_C9ZY92	0.035	34054.94	60.00
9	HLA-A0201	EDRGDVLVWGTE	RGDVLVWGT	2	0	0	0	0	RGDVLVWGT	tr_C9ZY92	0.035	34060.83	60.00
44	HLA-A0201	LGSERDTIRHL	LGSETIRHL	0	0	0	4	2	LGSERDTIRHL	tr_C9ZY92	0.035	34091.07	60.00
11	HLA-A0201	RGDVLVWGTEN	RGDVLVWGT	0	0	0	0	0	RGDVLVWGT	tr_C9ZY92	0.035	34184.88	60.00
30	HLA-A0201	CSENNTFSPLAT	CSEFSPLAT	0	0	0	3	3	CSENNTFSPLAT	tr_C9ZY92	0.035	34276.73	60.00
44	HLA-A0201	LGSERDTIRHLFSR	LTIRHLFSR	0	0	0	1	5	LGSERDTIRHLFSR	tr_C9ZY92	0.034	34513.05	60.00
9	HLA-A0201	EDRGDVLVW	EDRGDVLVW	0	0	0	0	0	EDRGDVLVW	tr_C9ZY92	0.034	34602.78	65.00
10	HLA-A0201	DRGDVLVWGTEN	RGDVLVWGT	1	0	0	0	0	RGDVLVWGT	tr_C9ZY92	0.034	34734.07	65.00
47	HLA-A0201	ERDTIRHLF	ERDTIRHLF	0	0	0	0	0	ERDTIRHLF	tr_C9ZY92	0.033	34857.94	65.00
42	HLA-A0201	LVLGSERDTIR	LLGSERDTI	0	0	0	1	1	LVLGSERDTI	tr_C9ZY92	0.033	34912.29	65.00

9	HLA-A0201	EDRGDVLVWGTEN	RGDVLVWGT	2	0	0	0	0	RGDVLVWGT	tr_C9ZY92	0.033	34951.60	65.00
46	HLA-A0201	SERDTIRHLFSR	STIRHLFSR	0	0	0	1	3	SERDTIRHLFSR	tr_C9ZY92	0.033	34986.79	65.00
32	HLA-A0201	ENNTFSPLA	ENNTFSPLA	0	0	0	0	0	ENNTFSPLA	tr_C9ZY92	0.033	35114.21	65.00
3	HLA-A0201	APFQDMEDR	APFQDMEDR	0	0	0	0	0	APFQDMEDR	tr_C9ZY92	0.032	35400.70	65.00
48	HLA-A0201	RDTIRHLFS	RDTIRHLFS	0	0	0	0	0	RDTIRHLFS	tr_C9ZY92	0.032	35466.63	65.00
47	HLA-A0201	ERDTIRHLFSR	RTIRHLFSR	1	0	0	1	1	RDTIRHLFSR	tr_C9ZY92	0.032	35545.01	65.00
46	HLA-A0201	SERDTIRHLFSRQT	TIHLFSRQT	4	0	0	2	1	TIRHLFSRQT	tr_C9ZY92	0.032	35550.39	65.00
45	HLA-A0201	GSERDTIRHLFSR	ERDTILFSR	2	0	0	5	2	ERDTIRHLFSR	tr_C9ZY92	0.031	35607.37	65.00
19	HLA-A0201	TENTTSLFTAT	TTTSLFTAT	0	0	0	1	2	TENTTSLFTAT	tr_C9ZY92	0.031	35630.50	65.00
47	HLA-A0201	ERDTIRHLFSRQT	ERDTILFSR	0	0	0	5	2	ERDTIRHLFSR	tr_C9ZY92	0.031	35633.58	65.00
43	HLA-A0201	VLGSERDTIRH	VLGSERDTI	0	0	0	0	0	VLGSERDTI	tr_C9ZY92	0.031	35638.59	65.00
42	HLA-A0201	LVLGSERDTIRH	LLGSERDTI	0	0	0	1	1	LVLGSERDTI	tr_C9ZY92	0.031	35642.06	65.00
44	HLA-A0201	LGSERDTIRHLF	LGSEIRHLF	0	0	0	4	3	LGSERDTIRHLF	tr_C9ZY92	0.031	35706.13	65.00
30	HLA-A0201	CSENNTFSP	CSENNTFSP	0	0	0	0	0	CSENNTFSP	tr_C9ZY92	0.031	35737.44	65.00
9	HLA-A0201	EDRGDVLVWG	EDRGVLVWG	0	0	0	4	1	EDRGDVLVWG	tr_C9ZY92	0.031	35744.79	65.00
17	HLA-A0201	WGTENTTSLFT	GTNTTSLFT	1	0	0	2	1	GTENTTSLFT	tr_C9ZY92	0.031	35750.19	65.00
45	HLA-A0201	GSERDTIRHLFS	GSEIRHLFS	0	0	0	3	3	GSERDTIRHLFS	tr_C9ZY92	0.031	35765.29	65.00
45	HLA-A0201	GSERDTIRHLF	SERDTIHLF	1	0	0	6	1	SERDTIRHLF	tr_C9ZY92	0.031	35801.68	65.00
16	HLA-A0201	VWGTENTTSLFT	GTNTTSLFT	2	0	0	2	1	GTENTTSLFT	tr_C9ZY92	0.031	35833.07	65.00
44	HLA-A0201	LGSERDTIRHLFS	LGSIRHLFS	0	0	0	3	4	LGSERDTIRHLFS	tr_C9ZY92	0.031	35861.37	65.00
2	HLA-A0201	CAPFQDMEDR	CAFQDMEDR	0	0	0	2	1	CAPFQDMEDR	tr_C9ZY92	0.030	35946.84	70.00
46	HLA-A0201	SERDTIRHLFS	SERDTILFS	0	0	0	6	2	SERDTIRHLFS	tr_C9ZY92	0.030	35974.85	70.00
54	HLA-A0201	LFSRQTIWQ	LFSRQTIWQ	0	0	0	0	0	LFSRQTIWQ	tr_C9ZY92	0.030	35990.45	70.00
25	HLA-A0201	LFTATCSENN	FTATCSENN	1	0	0	0	0	FTATCSENN	tr_C9ZY92	0.030	36050.84	70.00
12	HLA-A0201	GDVLVWGTEN	GDVLVWGTE	0	0	0	0	0	GDVLVWGTE	tr_C9ZY92	0.030	36059.03	70.00
49	HLA-A0201	DTIRHLFSRQT	TIHLFSRQT	1	0	0	2	1	TIRHLFSRQT	tr_C9ZY92	0.030	36231.90	70.00
48	HLA-A0201	RDTIRHLFSRQT	TIHLFSRQT	2	0	0	2	1	TIRHLFSRQT	tr_C9ZY92	0.030	36248.75	70.00
33	HLA-A0201	NNTFSPLAT	NNTFSPLAT	0	0	0	0	0	NNTFSPLAT	tr_C9ZY92	0.029	36372.51	70.00
5	HLA-A0201	FQDMEDRGD	FQDMEDRGD	0	0	0	0	0	FQDMEDRGD	tr_C9ZY92	0.029	36431.20	70.00
31	HLA-A0201	SENNTFSPLAT	SENNTFLAT	0	0	0	6	2	SENNTFSPLAT	tr_C9ZY92	0.029	36511.69	70.00
46	HLA-A0201	SERDTIRHLFSRQ	SIRHLFSRQ	0	0	0	1	4	SERDTIRHLFSRQ	tr_C9ZY92	0.029	36612.58	70.00
27	HLA-A0201	TATCSENNTF	ATCSENNTF	1	0	0	0	0	ATCSENNTF	tr_C9ZY92	0.028	36781.73	70.00
45	HLA-A0201	GSERDTIRHLFSRQ	ERDTILFSR	2	0	0	5	2	ERDTIRHLFSR	tr_C9ZY92	0.028	36917.28	70.00
47	HLA-A0201	ERDTIRHLFSRQ	ERDTILFSR	0	0	0	5	2	ERDTIRHLFSR	tr_C9ZY92	0.028	37021.68	70.00
49	HLA-A0201	DTIRHLFSRQ	DTIRHLFSQ	0	0	0	8	1	DTIRHLFSRQ	tr_C9ZY92	0.027	37134.00	70.00
16	HLA-A0201	VWGTENTTSLF	WTENTTSLF	1	0	0	1	1	WGTENTTSLF	tr_C9ZY92	0.027	37150.88	70.00
47	HLA-A0201	ERDTIRHLFS	RDTIRHLFS	1	0	0	0	0	RDTIRHLFS	tr_C9ZY92	0.027	37385.97	75.00
0	HLA-A0201	SGCAPFQDME	SGCAPFQDM	0	0	0	0	0	SGCAPFQDM	tr_C9ZY92	0.027	37421.59	75.00
48	HLA-A0201	RDTIRHLFSRQ	RIRHLFSRQ	0	0	0	1	2	RDTIRHLFSRQ	tr_C9ZY92	0.027	37533.91	75.00
29	HLA-A0201	TCSENNTFS	TCSENNTFS	0	0	0	0	0	TCSENNTFS	tr_C9ZY92	0.026	37588.37	75.00
6	HLA-A0201	QDMEDRGDV	QDMEDRGDV	0	0	0	0	0	QDMEDRGDV	tr_C9ZY92	0.026	37742.00	75.00
32	HLA-A0201	ENNTFSPLAT	ENNTFPLAT	0	0	0	5	1	ENNTFSPLAT	tr_C9ZY92	0.026	37769.79	75.00
1	HLA-A0201	GCAPFQDMEDR	CAFQDMEDR	1	0	0	2	1	CAPFQDMEDR	tr_C9ZY92	0.026	37823.36	75.00
0	HLA-A0201	SGCAPFQDMEDR	CAFQDMEDR	2	0	0	2	1	CAPFQDMEDR	tr_C9ZY92	0.026	37874.96	75.00
13	HLA-A0201	DVLVWGTEN	DVLVWGTEN	0	0	0	0	0	DVLVWGTEN	tr_C9ZY92	0.024	38437.65	80.00
25	HLA-A0201	LFTATCSEN	LFTATCSEN	0	0	0	0	0	LFTATCSEN	tr_C9ZY92	0.024	38617.32	80.00
29	HLA-A0201	TCSENNTFSP	TCSENTFSP	0	0	0	4	1	TCSENNTFSP	tr_C9ZY92	0.024	38765.51	80.00
0	HLA-A0201	SGCAPFQDMEDRG	SGCAPFQDM	0	0	0	0	0	SGCAPFQDM	tr_C9ZY92	0.023	39036.98	80.00
0	HLA-A0201	SGCAPFQDMEDRGD	SGCAPFQDM	0	0	0	0	0	SGCAPFQDM	tr_C9ZY92	0.023	39166.02	80.00
1	HLA-A0201	GCAPFQDMEDRG	GCAPFMEDR	0	0	0	5	2	GCAPFQDMEDR	tr_C9ZY92	0.022	39264.04	80.00
28	HLA-A0201	ATCSENNTFSP	ATCNNTFSP	0	0	0	3	2	ATCSENNTFSP	tr_C9ZY92	0.022	39314.62	80.00
2	HLA-A0201	CAPFQDMEDRG	CAFQDMEDR	0	0	0	2	1	CAPFQDMEDR	tr_C9ZY92	0.022	39387.86	80.00
1	HLA-A0201	GCAPFQDME	GCAPFQDME	0	0	0	0	0	GCAPFQDME	tr_C9ZY92	0.022	39427.94	80.00
1	HLA-A0201	GCAPFQDMEDRGD	FQDMEDRGD	4	0	0	0	0	FQDMEDRGD	tr_C9ZY92	0.022	39523.61	80.00

27	HLA-A0201	TATCSENNT	TATCSENNT	0	0	0	0	0	TATCSENNT	tr_C9ZY92	0.022	39545.87	80.00
2	HLA-A0201	CAPFQDMEDRGD	CAFQDMEDR	0	0	0	2	1	CAPFQDMEDR	tr_C9ZY92	0.021	39781.89	85.00
3	HLA-A0201	APFQDMEDRG	APFQDMERG	0	0	0	7	1	APFQDMEDRG	tr_C9ZY92	0.021	39815.92	85.00
0	HLA-A0201	SGCAPFQDMED	SGCAPFQDM	0	0	0	0	0	SGCAPFQDM	tr_C9ZY92	0.021	39850.40	85.00
45	HLA-A0201	GSERDTIRH	GSERDTIRH	0	0	0	0	0	GSERDTIRH	tr_C9ZY92	0.021	39922.89	85.00
27	HLA-A0201	TATCSENNTFSP	ATCNNTFSP	1	0	0	3	2	ATCSENNTFSP	tr_C9ZY92	0.020	40153.80	85.00
44	HLA-A0201	LGSERDTIRH	LGSERTIRH	0	0	0	5	1	LGSERDTIRH	tr_C9ZY92	0.020	40413.12	85.00
2	HLA-A0201	CAPFQDMED	CAPFQDMED	0	0	0	0	0	CAPFQDMED	tr_C9ZY92	0.020	40413.12	85.00
1	HLA-A0201	GCAPFQDMED	GCAPFDMED	0	0	0	5	1	GCAPFQDMED	tr_C9ZY92	0.019	40822.29	85.00
44	HLA-A0201	LGSERDTIR	LGSERDTIR	0	0	0	0	0	LGSERDTIR	tr_C9ZY92	0.018	41145.98	90.00
27	HLA-A0201	TATCSENNTFS	ATCENNTFS	1	0	0	3	1	ATCSENNTFS	tr_C9ZY92	0.018	41171.81	90.00
3	HLA-A0201	APFQDMEDRGD	FQDMEDRGD	2	0	0	0	0	FQDMEDRGD	tr_C9ZY92	0.017	41576.50	90.00
4	HLA-A0201	PFQDMEDRGD	FQDMEDRGD	1	0	0	0	0	FQDMEDRGD	tr_C9ZY92	0.016	41963.36	90.00
16	HLA-A0201	VWGTENTTS	VWGTENTTS	0	0	0	0	0	VWGTENTTS	tr_C9ZY92	0.016	42012.42	90.00
4	HLA-A0201	PFQDMEDRG	PFQDMEDRG	0	0	0	0	0	PFQDMEDRG	tr_C9ZY92	0.010	44853.09	99.00

Protein tr_C9ZY92. Allele HLA-A0201. Number of high binders 0. Number of weak binders 0. Number of peptides 339

Link to Allele Frequencies in Worldwide Populations HLA-A0201

\# Rank Threshold for Strong binding peptides 0.500
\# Rank Threshold for Weak binding peptides 2.000

---

pos	HLA	peptide	Core	Offset	I_pos	I_len	D_pos	D_len	iCore	Identity	1-log50k(aff)	Affinity(nM)	%Rank	BindLevel
318	HLA-A0201	AMAHYLPMV	AMAHYLPMV	0	0	0	0	0	AMAHYLPMV	tr_D0A9V0	0.888	3.37	0.01	<= SB
712	HLA-A0201	SLLSYAFLL	SLLSYAFLL	0	0	0	0	0	SLLSYAFLL	tr_D0A9V0	0.862	4.46	0.03	<= SB
49	HLA-A0201	VLLKFIPEA	VLLKFIPEA	0	0	0	0	0	VLLKFIPEA	tr_D0A9V0	0.818	7.19	0.07	<= SB
663	HLA-A0201	SLFNRDLLFV	SLFNDLLFV	0	0	0	4	1	SLFNRDLLFV	tr_D0A9V0	0.810	7.84	0.08	<= SB
236	HLA-A0201	HLFQLSYAV	HLFQLSYAV	0	0	0	0	0	HLFQLSYAV	tr_D0A9V0	0.780	10.78	0.12	<= SB
315	HLA-A0201	YGEAMAHYLPMV	YMAHYLPMV	0	0	0	1	3	YGEAMAHYLPMV	tr_D0A9V0	0.779	10.91	0.12	<= SB
209	HLA-A0201	MLVDTNEFL	MLVDTNEFL	0	0	0	0	0	MLVDTNEFL	tr_D0A9V0	0.778	11.02	0.15	<= SB
209	HLA-A0201	MLVDTNEFLV	MLVDTNFLV	0	0	0	6	1	MLVDTNEFLV	tr_D0A9V0	0.775	11.42	0.15	<= SB
264	HLA-A0201	FLAFPVDCV	FLAFPVDCV	0	0	0	0	0	FLAFPVDCV	tr_D0A9V0	0.768	12.27	0.15	<= SB
260	HLA-A0201	HMQGFLAFPV	HMQGFLFPV	0	0	0	6	1	HMQGFLAFPV	tr_D0A9V0	0.766	12.61	0.15	<= SB
317	HLA-A0201	EAMAHYLPMV	AMAHYLPMV	1	0	0	0	0	AMAHYLPMV	tr_D0A9V0	0.756	13.97	0.17	<= SB
674	HLA-A0201	SLALGHLEPV	SLLGHLEPV	0	0	0	2	1	SLALGHLEPV	tr_D0A9V0	0.755	14.23	0.17	<= SB
91	HLA-A0201	RIMGASISV	RIMGASISV	0	0	0	0	0	RIMGASISV	tr_D0A9V0	0.753	14.49	0.20	<= SB
314	HLA-A0201	RYGEAMAHYLPMV	YMAHYLPMV	1	0	0	1	3	YGEAMAHYLPMV	tr_D0A9V0	0.748	15.33	0.20	<= SB
313	HLA-A0201	GRYGEAMAHYLPMV	YMAHYLPMV	2	0	0	1	3	YGEAMAHYLPMV	tr_D0A9V0	0.731	18.35	0.25	<= SB
711	HLA-A0201	SSLLSYAFLL	SLLSYAFLL	0	0	0	1	1	SSLLSYAFLL	tr_D0A9V0	0.709	23.36	0.40	<= SB
24	HLA-A0201	FLGRSRIVEV	FLGSRIVEV	0	0	0	3	1	FLGRSRIVEV	tr_D0A9V0	0.694	27.29	0.40	<= SB
261	HLA-A0201	MQGFLAFPV	MQGFLAFPV	0	0	0	0	0	MQGFLAFPV	tr_D0A9V0	0.692	28.07	0.40	<= SB
272	HLA-A0201	VLLPCSTLPI	VLLPCTLPI	0	0	0	5	1	VLLPCSTLPI	tr_D0A9V0	0.690	28.74	0.40	<= SB
582	HLA-A0201	KLSDFGSTI	KLSDFGSTI	0	0	0	0	0	KLSDFGSTI	tr_D0A9V0	0.689	29.04	0.40	<= SB
273	HLA-A0201	LLPCSTLPI	LLPCSTLPI	0	0	0	0	0	LLPCSTLPI	tr_D0A9V0	0.688	29.32	0.40	<= SB
232	HLA-A0201	FGADHLFQLSYAV	FLFQLSYAV	0	0	0	1	4	FGADHLFQLSYAV	tr_D0A9V0	0.677	33.00	0.50	<= SB
53	HLA-A0201	FIPEAILARV	FIPEAILAV	0	0	0	8	1	FIPEAILARV	tr_D0A9V0	0.673	34.57	0.50	<= SB
437	HLA-A0201	FLTDSNRNV	FLTDSNRNV	0	0	0	0	0	FLTDSNRNV	tr_D0A9V0	0.672	34.68	0.50	<= SB
547	HLA-A0201	FTVDILQGL	FTVDILQGL	0	0	0	0	0	FTVDILQGL	tr_D0A9V0	0.672	34.71	0.50	<= SB
264	HLA-A0201	FLAFPVDCVL	FLFPVDCVL	0	0	0	2	1	FLAFPVDCVL	tr_D0A9V0	0.668	36.28	0.50	<= SB
519	HLA-A0201	IIMEYVPGGSLHSV	IIMEYLHSV	0	0	0	5	5	IIMEYVPGGSLHSV	tr_D0A9V0	0.665	37.61	0.50	<= SB
318	HLA-A0201	AMAHYLPMVK	AMAHYLPMV	0	0	0	0	0	AMAHYLPMV	tr_D0A9V0	0.659	40.05	0.60	<= WB

49	HLA-A0201	VLLKFIPEAI	VLLKFIPEI	0	0	0	8	1	VLLKFIPEAI	tr_D0A9V0	0.656	41.55	0.60 <= WB
662	HLA-A0201	RSLFNRDLLFV	SLFNDLLFV	1	0	0	4	1	SLFNRDLLFV	tr_D0A9V0	0.652	43.30	0.60 <= WB
646	HLA-A0201	WQWSSTAPA	WQWSSTAPA	0	0	0	0	0	WQWSSTAPA	tr_D0A9V0	0.646	46.28	0.60 <= WB
315	HLA-A0201	YGEAMAHYLPMVK	YMAHYLPMV	0	0	0	1	3	YGEAMAHYLPMV	tr_D0A9V0	0.640	48.99	0.60 <= WB
273	HLA-A0201	LLPCSTLPIV	LLPCSTLPV	0	0	0	8	1	LLPCSTLPIV	tr_D0A9V0	0.639	49.49	0.60 <= WB
231	HLA-A0201	LFGADHLFQLSYAV	FLFQLSYAV	1	0	0	1	4	FGADHLFQLSYAV	tr_D0A9V0	0.639	49.68	0.60 <= WB
191	HLA-A0201	FAFDLFAKL	FAFDLFAKL	0	0	0	0	0	FAFDLFAKL	tr_D0A9V0	0.632	53.72	0.70 <= WB
661	HLA-A0201	LRSLFNRDLLFV	SLFNDLLFV	2	0	0	4	1	SLFNRDLLFV	tr_D0A9V0	0.631	54.18	0.70 <= WB
660	HLA-A0201	RLRSLFNRDLLFV	SLFNDLLFV	3	0	0	4	1	SLFNRDLLFV	tr_D0A9V0	0.626	57.42	0.70 <= WB
278	HLA-A0201	TLPIVLFEL	TLPIVLFEL	0	0	0	0	0	TLPIVLFEL	tr_D0A9V0	0.626	57.43	0.70 <= WB
230	HLA-A0201	VLFGADHLFQL	VLFDHLFQL	0	0	0	3	2	VLFGADHLFQL	tr_D0A9V0	0.624	58.43	0.70 <= WB
516	HLA-A0201	HLSIIMEYV	HLSIIMEYV	0	0	0	0	0	HLSIIMEYV	tr_D0A9V0	0.624	58.45	0.70 <= WB
659	HLA-A0201	TRLRSLFNRDLLFV	SLFNDLLFV	4	0	0	4	1	SLFNRDLLFV	tr_D0A9V0	0.622	59.48	0.70 <= WB
48	HLA-A0201	AVLLKFIPEA	ALLKFIPEA	0	0	0	1	1	AVLLKFIPEA	tr_D0A9V0	0.621	60.10	0.70 <= WB
605	HLA-A0201	MLRGTALYMAPEV	MLLYMAPEV	0	0	0	2	4	MLRGTALYMAPEV	tr_D0A9V0	0.610	67.74	0.80 <= WB
235	HLA-A0201	DHLFQLSYAV	HLFQLSYAV	1	0	0	0	0	HLFQLSYAV	tr_D0A9V0	0.608	69.25	0.80 <= WB
669	HLA-A0201	LLFVQSLAL	LLFVQSLAL	0	0	0	0	0	LLFVQSLAL	tr_D0A9V0	0.608	69.35	0.80 <= WB
208	HLA-A0201	TMLVDTNEFLV	MLVDTNFLV	1	0	0	6	1	MLVDTNEFLV	tr_D0A9V0	0.606	71.36	0.80 <= WB
89	HLA-A0201	FVRIMGASISV	FIMGASISV	0	0	0	1	2	FVRIMGASISV	tr_D0A9V0	0.595	80.29	0.90 <= WB
314	HLA-A0201	RYGEAMAHYLPMVK	YMAHYLPMV	1	0	0	1	3	YGEAMAHYLPMV	tr_D0A9V0	0.594	81.20	0.90 <= WB
49	HLA-A0201	VLLKFIPEAILARV	VLLKFIPEV	0	0	0	8	5	VLLKFIPEAILARV	tr_D0A9V0	0.592	82.20	0.90 <= WB
0	HLA-A0201	KPLMLLVSAL	KLMLLVSAL	0	0	0	1	1	KPLMLLVSAL	tr_D0A9V0	0.586	87.74	1.00 <= WB
263	HLA-A0201	GFLAFPVDCV	FLAFPVDCV	1	0	0	0	0	FLAFPVDCV	tr_D0A9V0	0.586	88.18	1.00 <= WB
207	HLA-A0201	VTMLVDTNEFLV	MLVDTNFLV	2	0	0	6	1	MLVDTNEFLV	tr_D0A9V0	0.585	89.32	1.00 <= WB
316	HLA-A0201	GEAMAHYLPMV	AMAHYLPMV	2	0	0	0	0	AMAHYLPMV	tr_D0A9V0	0.582	92.01	1.00 <= WB
229	HLA-A0201	RVLFGADHLFQL	VLFDHLFQL	1	0	0	3	2	VLFGADHLFQL	tr_D0A9V0	0.581	93.47	1.00 <= WB
206	HLA-A0201	SVTMLVDTNEFLV	MLVDTNFLV	3	0	0	6	1	MLVDTNEFLV	tr_D0A9V0	0.578	95.66	1.00 <= WB
315	HLA-A0201	YGEAMAHYLPMVKM	YMAHYLPMV	0	0	0	1	3	YGEAMAHYLPMV	tr_D0A9V0	0.578	95.91	1.00 <= WB
205	HLA-A0201	DSVTMLVDTNEFLV	MLVDTNFLV	4	0	0	6	1	MLVDTNEFLV	tr_D0A9V0	0.575	99.72	1.10 <= WB
210	HLA-A0201	LVDTNEFLV	LVDTNEFLV	0	0	0	0	0	LVDTNEFLV	tr_D0A9V0	0.571	103.20	1.10 <= WB
208	HLA-A0201	TMLVDTNEFL	MLVDTNEFL	1	0	0	0	0	MLVDTNEFL	tr_D0A9V0	0.571	104.04	1.10 <= WB
515	HLA-A0201	SHLSIIMEYV	SLSIIMEYV	0	0	0	1	1	SHLSIIMEYV	tr_D0A9V0	0.570	104.60	1.10 <= WB
259	HLA-A0201	SHMQGFLAFPV	HMQGFLFPV	1	0	0	6	1	HMQGFLAFPV	tr_D0A9V0	0.567	107.90	1.10 <= WB
90	HLA-A0201	VRIMGASISV	VIMGASISV	0	0	0	1	1	VRIMGASISV	tr_D0A9V0	0.566	109.94	1.10 <= WB
171	HLA-A0201	ALISFGAHA	ALISFGAHA	0	0	0	0	0	ALISFGAHA	tr_D0A9V0	0.563	113.06	1.10 <= WB
604	HLA-A0201	VMLRGTALYMAPEV	MLLYMAPEV	1	0	0	2	4	MLRGTALYMAPEV	tr_D0A9V0	0.560	116.21	1.20 <= WB
232	HLA-A0201	FGADHLFQLSYAVR	FLFQLSYAV	0	0	0	1	4	FGADHLFQLSYAV	tr_D0A9V0	0.557	120.07	1.20 <= WB
228	HLA-A0201	ARVLFGADHLFQL	VLFDHLFQL	2	0	0	3	2	VLFGADHLFQL	tr_D0A9V0	0.557	120.27	1.20 <= WB
663	HLA-A0201	SLFNRDLLFVQ	SLFNDLLFV	0	0	0	4	1	SLFNRDLLFV	tr_D0A9V0	0.554	124.58	1.20 <= WB
675	HLA-A0201	LALGHLEPV	LALGHLEPV	0	0	0	0	0	LALGHLEPV	tr_D0A9V0	0.554	125.12	1.20 <= WB
227	HLA-A0201	NARVLFGADHLFQL	VLFDHLFQL	3	0	0	3	2	VLFGADHLFQL	tr_D0A9V0	0.550	129.58	1.30 <= WB
88	HLA-A0201	AFVRIMGASISV	FIMGASISV	1	0	0	1	2	FVRIMGASISV	tr_D0A9V0	0.547	134.93	1.30 <= WB
258	HLA-A0201	ASHMQGFLAFPV	HMQGFLFPV	2	0	0	6	1	HMQGFLAFPV	tr_D0A9V0	0.546	135.22	1.30 <= WB
272	HLA-A0201	VLLPCSTLPIV	VLLPCLPIV	0	0	0	5	2	VLLPCSTLPIV	tr_D0A9V0	0.542	142.39	1.30 <= WB
257	HLA-A0201	GASHMQGFLAFPV	HMQGFLFPV	3	0	0	6	1	HMQGFLAFPV	tr_D0A9V0	0.541	144.25	1.30 <= WB
673	HLA-A0201	QSLALGHLEPV	SLLGHLEPV	1	0	0	2	1	SLALGHLEPV	tr_D0A9V0	0.540	145.68	1.40 <= WB
353	HLA-A0201	RVLDNHYCV	RVLDNHYCV	0	0	0	0	0	RVLDNHYCV	tr_D0A9V0	0.538	148.20	1.40 <= WB
256	HLA-A0201	GGASHMQGFLAFPV	HMQGFLFPV	4	0	0	6	1	HMQGFLAFPV	tr_D0A9V0	0.537	149.98	1.40 <= WB
198	HLA-A0201	KLPLDHRDSVTMLV	KLDSVTMLV	0	0	0	2	5	KLPLDHRDSVTMLV	tr_D0A9V0	0.531	160.36	1.40 <= WB
282	HLA-A0201	VLFELRPGESSSEV	VLFELSSEV	0	0	0	5	5	VLFELRPGESSSEV	tr_D0A9V0	0.530	161.25	1.40 <= WB
87	HLA-A0201	TAFVRIMGASISV	FIMGASISV	2	0	0	1	2	FVRIMGASISV	tr_D0A9V0	0.529	163.36	1.50 <= WB
595	HLA-A0201	VMARTLADGV	VMARTLAGV	0	0	0	7	1	VMARTLADGV	tr_D0A9V0	0.527	167.74	1.50 <= WB
234	HLA-A0201	ADHLFQLSYAV	ALFQLSYAV	0	0	0	1	2	ADHLFQLSYAV	tr_D0A9V0	0.526	168.04	1.50 <= WB
604	HLA-A0201	VMLRGTALYM	VMLGTALYM	0	0	0	3	1	VMLRGTALYM	tr_D0A9V0	0.524	171.90	1.50 <= WB

277	HLA-A0201	STLPIVLFEL	SLPIVLFEL	0	0	0	1	1	STLPIVLFEL	tr_D0A9V0	0.524	172.07	1.50	<= WB
86	HLA-A0201	STAFVRIMGASISV	FIMGASISV	3	0	0	1	2	FVRIMGASISV	tr_D0A9V0	0.522	176.11	1.50	<= WB
709	HLA-A0201	SASSLLSYAFLL	SLLSYAFLL	0	0	0	1	3	SASSLLSYAFLL	tr_D0A9V0	0.522	176.13	1.50	<= WB
671	HLA-A0201	FVQSLALGHLEPV	SLLGHLEPV	3	0	0	2	1	SLALGHLEPV	tr_D0A9V0	0.520	179.87	1.50	<= WB
710	HLA-A0201	ASSLLSYAFLL	ALLSYAFLL	0	0	0	1	2	ASSLLSYAFLL	tr_D0A9V0	0.520	180.09	1.50	<= WB
672	HLA-A0201	VQSLALGHLEPV	SLLGHLEPV	2	0	0	2	1	SLALGHLEPV	tr_D0A9V0	0.517	186.78	1.60	<= WB
236	HLA-A0201	HLFQLSYAVR	HLFQLSYAV	0	0	0	0	0	HLFQLSYAV	tr_D0A9V0	0.516	187.94	1.60	<= WB
662	HLA-A0201	RSLFNRDLLFVQ	SLFNDLLFV	1	0	0	4	1	SLFNRDLLFV	tr_D0A9V0	0.515	189.11	1.60	<= WB
151	HLA-A0201	ALIEVATEHSGCV	ALIEVATEV	0	0	0	8	4	ALIEVATEHSGCV	tr_D0A9V0	0.514	191.90	1.60	<= WB
523	HLA-A0201	YVPGGSLHSV	YVPGGLHSV	0	0	0	5	1	YVPGGSLHSV	tr_D0A9V0	0.514	192.55	1.60	<= WB
519	HLA-A0201	IIMEYVPGGSL	IIMEYVPSL	0	0	0	7	2	IIMEYVPGGSL	tr_D0A9V0	0.513	194.16	1.60	<= WB
663	HLA-A0201	SLFNRDLLFVQSL	SLFNDLLFV	0	0	0	4	1	SLFNRDLLFV	tr_D0A9V0	0.511	198.57	1.60	<= WB
233	HLA-A0201	GADHLFQLSYAV	GLFQLSYAV	0	0	0	1	3	GADHLFQLSYAV	tr_D0A9V0	0.508	205.22	1.70	<= WB
209	HLA-A0201	MLVDTNEFLVG	MLVDTNFLV	0	0	0	6	1	MLVDTNEFLV	tr_D0A9V0	0.507	207.85	1.70	<= WB
707	HLA-A0201	RPSASSLLSYAFLL	RLLSYAFLL	0	0	0	1	5	RPSASSLLSYAFLL	tr_D0A9V0	0.506	209.59	1.70	<= WB
670	HLA-A0201	LFVQSLALGHLEPV	SLLGHLEPV	4	0	0	2	1	SLALGHLEPV	tr_D0A9V0	0.506	209.84	1.70	<= WB
271	HLA-A0201	CVLLPCSTLPIV	VLLPCLPIV	1	0	0	5	2	VLLPCSTLPIV	tr_D0A9V0	0.505	210.98	1.70	<= WB
663	HLA-A0201	SLFNRDLLFVQS	SLFNDLLFV	0	0	0	4	1	SLFNRDLLFV	tr_D0A9V0	0.503	215.69	1.70	<= WB
605	HLA-A0201	MLRGTALYMA	MLGTALYMA	0	0	0	2	1	MLRGTALYMA	tr_D0A9V0	0.499	226.22	1.70	<= WB
111	HLA-A0201	ALNCGIFKEKPNV	ALNCGIFNV	0	0	0	7	4	ALNCGIFKEKPNV	tr_D0A9V0	0.498	229.43	1.80	<= WB
708	HLA-A0201	PSASSLLSYAFLL	SLLSYAFLL	4	0	0	0	0	SLLSYAFLL	tr_D0A9V0	0.497	229.97	1.80	<= WB
23	HLA-A0201	IFLGRSRIVEV	FLGSRIVEV	1	0	0	3	1	FLGRSRIVEV	tr_D0A9V0	0.497	231.64	1.80	<= WB
270	HLA-A0201	DCVLLPCSTLPIV	VLLPCLPIV	2	0	0	5	2	VLLPCSTLPIV	tr_D0A9V0	0.494	237.83	1.80	<= WB
661	HLA-A0201	LRSLFNRDLLFVQ	SLFNDLLFV	2	0	0	4	1	SLFNRDLLFV	tr_D0A9V0	0.493	240.41	1.80	<= WB
551	HLA-A0201	ILQGLNYLHNL	ILLNYLHNL	0	0	0	2	2	ILQGLNYLHNL	tr_D0A9V0	0.491	247.33	1.80	<= WB
679	HLA-A0201	HLEPVIPASV	HLEPVIPSV	0	0	0	7	1	HLEPVIPASV	tr_D0A9V0	0.490	248.91	1.90	<= WB
269	HLA-A0201	VDCVLLPCSTLPIV	VLLPCLPIV	3	0	0	5	2	VLLPCSTLPIV	tr_D0A9V0	0.488	254.87	1.90	<= WB
660	HLA-A0201	RLRSLFNRDLLFVQ	SLFNDLLFV	3	0	0	4	1	SLFNRDLLFV	tr_D0A9V0	0.488	255.80	1.90	<= WB
22	HLA-A0201	DIFLGRSRIVEV	FLGSRIVEV	2	0	0	3	1	FLGRSRIVEV	tr_D0A9V0	0.486	259.30	1.90	<= WB
582	HLA-A0201	KLSDFGSTISEASV	KLSDFEASV	0	0	0	5	5	KLSDFGSTISEASV	tr_D0A9V0	0.486	259.48	1.90	<= WB
21	HLA-A0201	ADIFLGRSRIVEV	FLGSRIVEV	3	0	0	3	1	FLGRSRIVEV	tr_D0A9V0	0.480	276.43	2.00	<= WB
456	HLA-A0201	GAFSTIFLGV	GAFSTIFLV	0	0	0	8	1	GAFSTIFLGV	tr_D0A9V0	0.478	282.58	2.00	<= WB
20	HLA-A0201	NADIFLGRSRIVEV	FLGSRIVEV	4	0	0	3	1	FLGRSRIVEV	tr_D0A9V0	0.477	287.24	2.00	<= WB
92	HLA-A0201	IMGASISVTWNA	IMISVTWNA	0	0	0	2	3	IMGASISVTWNA	tr_D0A9V0	0.471	305.41	2.50	
189	HLA-A0201	CQFAFDLFA	CQFAFDLFA	0	0	0	0	0	CQFAFDLFA	tr_D0A9V0	0.471	306.15	2.50	
662	HLA-A0201	RSLFNRDLLFVQSL	SLFNDLLFV	1	0	0	4	1	SLFNRDLLFV	tr_D0A9V0	0.471	306.70	2.50	
263	HLA-A0201	GFLAFPVDCVL	FLFPVDCVL	1	0	0	2	1	FLAFPVDCVL	tr_D0A9V0	0.470	309.15	2.50	
49	HLA-A0201	VLLKFIPEAIL	VLLKFIPEL	0	0	0	8	2	VLLKFIPEAIL	tr_D0A9V0	0.470	309.17	2.50	
260	HLA-A0201	HMQGFLAFPVDCV	HMQGFLAFV	0	0	0	8	4	HMQGFLAFPVDCV	tr_D0A9V0	0.469	311.60	2.50	
668	HLA-A0201	DLLFVQSLAL	LLFVQSLAL	1	0	0	0	0	LLFVQSLAL	tr_D0A9V0	0.468	316.16	2.50	
663	HLA-A0201	SLFNRDLLFVQSLA	SLFNDLLFV	0	0	0	4	1	SLFNRDLLFV	tr_D0A9V0	0.468	316.36	2.50	
150	HLA-A0201	AALIEVATEHSGCV	ALIEVATEV	1	0	0	8	4	ALIEVATEHSGCV	tr_D0A9V0	0.468	317.72	2.50	
662	HLA-A0201	RSLFNRDLLFVQS	SLFNDLLFV	1	0	0	4	1	SLFNRDLLFV	tr_D0A9V0	0.465	326.68	2.50	
554	HLA-A0201	GLNYLHNLGI	GLYLHNLGI	0	0	0	2	1	GLNYLHNLGI	tr_D0A9V0	0.465	327.11	2.50	
462	HLA-A0201	FLGVAEDGAQV	FLAEDGAQV	0	0	0	2	2	FLGVAEDGAQV	tr_D0A9V0	0.464	329.80	2.50	
273	HLA-A0201	LLPCSTLPIVLFEL	LLPCVLFEL	0	0	0	4	5	LLPCSTLPIVLFEL	tr_D0A9V0	0.463	332.58	2.50	
232	HLA-A0201	FGADHLFQL	FGADHLFQL	0	0	0	0	0	FGADHLFQL	tr_D0A9V0	0.463	333.00	2.50	
208	HLA-A0201	TMLVDTNEFLVG	MLVDTNFLV	1	0	0	6	1	MLVDTNEFLV	tr_D0A9V0	0.463	333.73	2.50	
171	HLA-A0201	ALISFGAHAA	ALISFAHAA	0	0	0	5	1	ALISFGAHAA	tr_D0A9V0	0.462	336.27	2.50	
518	HLA-A0201	SIIMEYVPGGSL	IIMEYVPSL	1	0	0	7	2	IIMEYVPGGSL	tr_D0A9V0	0.462	336.64	2.50	
230	HLA-A0201	VLFGADHLFQLS	VLFDHLFQL	0	0	0	3	2	VLFGADHLFQL	tr_D0A9V0	0.460	343.50	2.50	
271	HLA-A0201	CVLLPCSTLPI	VLLPCTLPI	1	0	0	5	1	VLLPCSTLPI	tr_D0A9V0	0.459	346.91	2.50	
3	HLA-A0201	MLLVSALKSA	MLLVSALSA	0	0	0	7	1	MLLVSALKSA	tr_D0A9V0	0.459	347.55	2.50	
296	HLA-A0201	SLSISKQFRL	SLISKQFRL	0	0	0	2	1	SLSISKQFRL	tr_D0A9V0	0.457	355.03	2.50	

262	HLA-A0201	QGFLAFPVDCVL	FLFPVDCVL	2	0	0	2	1	FLAFPVDCVL	tr_D0A9V0	0.456	359.68	2.50
665	HLA-A0201	FNRDLLFVQSLAL	FLFVQSLAL	0	0	0	1	4	FNRDLLFVQSLAL	tr_D0A9V0	0.453	371.20	2.50
209	HLA-A0201	MLVDTNEFLVGT	MLVDTNFLV	0	0	0	6	1	MLVDTNEFLV	tr_D0A9V0	0.453	372.18	2.50
519	HLA-A0201	IIMEYVPGG	IIMEYVPGG	0	0	0	0	0	IIMEYVPGG	tr_D0A9V0	0.452	376.96	2.50
261	HLA-A0201	MQGFLAFPVDCVL	FLFPVDCVL	3	0	0	2	1	FLAFPVDCVL	tr_D0A9V0	0.451	379.96	2.50
550	HLA-A0201	DILQGLNYLHNL	ILLNYLHNL	1	0	0	2	2	ILQGLNYLHNL	tr_D0A9V0	0.450	382.38	2.50
301	HLA-A0201	KQFRLGFAA	KQFRLGFAA	0	0	0	0	0	KQFRLGFAA	tr_D0A9V0	0.449	386.97	2.50
45	HLA-A0201	RQLAVLLKFI	RQLAVLLFI	0	0	0	7	1	RQLAVLLKFI	tr_D0A9V0	0.449	387.12	2.50
260	HLA-A0201	HMQGFLAFPVDCVL	FLFPVDCVL	4	0	0	2	1	FLAFPVDCVL	tr_D0A9V0	0.449	387.15	2.50
270	HLA-A0201	DCVLLPCSTLPI	VLLPCTLPI	2	0	0	5	1	VLLPCSTLPI	tr_D0A9V0	0.449	389.79	2.50
110	HLA-A0201	VALNCGIFKEKPNV	ALNCGIFNV	1	0	0	7	4	ALNCGIFKEKPNV	tr_D0A9V0	0.448	392.16	2.50
260	HLA-A0201	HMQGFLAFPVD	HMQGFLFPV	0	0	0	6	1	HMQGFLAFPV	tr_D0A9V0	0.448	392.51	2.50
330	HLA-A0201	MQAKRLMRL	MQAKRLMRL	0	0	0	0	0	MQAKRLMRL	tr_D0A9V0	0.446	401.21	2.50
452	HLA-A0201	KISEGAFSTI	KISEGAFSI	0	0	0	8	1	KISEGAFSTI	tr_D0A9V0	0.446	402.04	2.50
661	HLA-A0201	LRSLFNRDLLFVQS	SLFNDLLFV	2	0	0	4	1	SLFNRDLLFV	tr_D0A9V0	0.444	410.49	2.50
269	HLA-A0201	VDCVLLPCSTLPI	VLLPCTLPI	3	0	0	5	1	VLLPCSTLPI	tr_D0A9V0	0.442	416.97	2.50
207	HLA-A0201	VTMLVDTNEFLVG	MLVDTNFLV	2	0	0	6	1	MLVDTNEFLV	tr_D0A9V0	0.441	422.50	2.50
230	HLA-A0201	VLFGADHLFQLSYA	VLFGALSYA	0	0	0	5	5	VLFGADHLFQLSYA	tr_D0A9V0	0.441	424.50	2.50
52	HLA-A0201	KFIPEAILARV	FIPEAILAV	1	0	0	8	1	FIPEAILARV	tr_D0A9V0	0.440	430.24	2.50
268	HLA-A0201	PVDCVLLPCSTLPI	VLLPCTLPI	4	0	0	5	1	VLLPCSTLPI	tr_D0A9V0	0.439	434.14	2.50
264	HLA-A0201	FLAFPVDCVLL	FLAFDCVLL	0	0	0	4	2	FLAFPVDCVLL	tr_D0A9V0	0.439	434.45	2.50
452	HLA-A0201	KISEGAFST	KISEGAFST	0	0	0	0	0	KISEGAFST	tr_D0A9V0	0.437	440.76	2.50
165	HLA-A0201	ALDPDRALI	ALDPDRALI	0	0	0	0	0	ALDPDRALI	tr_D0A9V0	0.437	444.48	2.50
206	HLA-A0201	SVTMLVDTNEFLVG	MLVDTNFLV	3	0	0	6	1	MLVDTNEFLV	tr_D0A9V0	0.435	453.65	3.00
517	HLA-A0201	LSIIMEYVPGGSL	IIMEYVPSL	2	0	0	7	2	IIMEYVPGGSL	tr_D0A9V0	0.435	453.84	3.00
47	HLA-A0201	LAVLLKFIPEA	LLLKFIPEA	0	0	0	1	2	LAVLLKFIPEA	tr_D0A9V0	0.434	455.35	3.00
605	HLA-A0201	MLRGTALYMAPEVA	MLLYMAPEV	0	0	0	2	4	MLRGTALYMAPEV	tr_D0A9V0	0.434	458.89	3.00
259	HLA-A0201	SHMQGFLAFPVDCV	HMQGFLAFV	1	0	0	8	4	HMQGFLAFPVDCV	tr_D0A9V0	0.432	467.26	3.00
551	HLA-A0201	ILQGLNYLHNLGIV	ILQGLNYLV	0	0	0	8	5	ILQGLNYLHNLGIV	tr_D0A9V0	0.431	472.70	3.00
581	HLA-A0201	CKLSDFGSTI	KLSDFGSTI	1	0	0	0	0	KLSDFGSTI	tr_D0A9V0	0.431	473.19	3.00
191	HLA-A0201	FAFDLFAKLPL	FAFDLFLPL	0	0	0	6	2	FAFDLFAKLPL	tr_D0A9V0	0.430	475.45	3.00
48	HLA-A0201	AVLLKFIPEAI	VLLKFIPEI	1	0	0	8	1	VLLKFIPEAI	tr_D0A9V0	0.429	484.25	3.00
516	HLA-A0201	HLSIIMEYVPGGSL	IIMEYVPSL	3	0	0	7	2	IIMEYVPGGSL	tr_D0A9V0	0.427	489.97	3.00
91	HLA-A0201	RIMGASISVTWNA	IMISVTWNA	1	0	0	2	3	IMGASISVTWNA	tr_D0A9V0	0.427	490.61	3.00
549	HLA-A0201	VDILQGLNYLHNL	ILLNYLHNL	2	0	0	2	2	ILQGLNYLHNL	tr_D0A9V0	0.427	494.26	3.00
547	HLA-A0201	FTVDILQGLNYL	FILQGLNYL	0	0	0	1	3	FTVDILQGLNYL	tr_D0A9V0	0.426	498.66	3.00
209	HLA-A0201	MLVDTNEFLVGTC	MLVDTNFLV	0	0	0	6	1	MLVDTNEFLV	tr_D0A9V0	0.425	501.78	3.00
89	HLA-A0201	FVRIMGASISVT	FIMGASISV	0	0	0	1	2	FVRIMGASISV	tr_D0A9V0	0.423	513.43	3.00
610	HLA-A0201	ALYMAPEVA	ALYMAPEVA	0	0	0	0	0	ALYMAPEVA	tr_D0A9V0	0.423	515.39	3.00
8	HLA-A0201	ALKSASNLEI	ALSASNLEI	0	0	0	2	1	ALKSASNLEI	tr_D0A9V0	0.422	519.21	3.00
610	HLA-A0201	ALYMAPEVAA	ALYMAPVAA	0	0	0	6	1	ALYMAPEVAA	tr_D0A9V0	0.421	528.12	3.00
48	HLA-A0201	AVLLKFIPEAIL	VLLKFIPEL	1	0	0	8	2	VLLKFIPEAIL	tr_D0A9V0	0.420	530.81	3.00
548	HLA-A0201	TVDILQGLNYLHNL	ILLNYLHNL	3	0	0	2	2	ILQGLNYLHNL	tr_D0A9V0	0.420	532.59	3.00
627	HLA-A0201	SDIFSFGISL	SIFSFGISL	0	0	0	1	1	SDIFSFGISL	tr_D0A9V0	0.419	537.78	3.00
229	HLA-A0201	RVLFGADHLFQLS	VLFDHLFQL	1	0	0	3	2	VLFGADHLFQL	tr_D0A9V0	0.417	551.89	3.00
51	HLA-A0201	LKFIPEAILARV	FIPEAILAV	2	0	0	8	1	FIPEAILARV	tr_D0A9V0	0.416	553.94	3.00
208	HLA-A0201	TMLVDTNEFLVGT	MLVDTNFLV	1	0	0	6	1	MLVDTNEFLV	tr_D0A9V0	0.415	560.38	3.00
664	HLA-A0201	LFNRDLLFVQSLAL	FLFVQSLAL	1	0	0	1	4	FNRDLLFVQSLAL	tr_D0A9V0	0.415	560.62	3.00
632	HLA-A0201	FGISLLEMLL	FISLLEMLL	0	0	0	1	1	FGISLLEMLL	tr_D0A9V0	0.415	561.50	3.00
674	HLA-A0201	SLALGHLEPVI	SLLGHLEPV	0	0	0	2	1	SLALGHLEPV	tr_D0A9V0	0.415	562.39	3.00
546	HLA-A0201	RFTVDILQGL	FTVDILQGL	1	0	0	0	0	FTVDILQGL	tr_D0A9V0	0.413	575.85	3.00
461	HLA-A0201	IFLGVAEDGAQV	FLAEDGAQV	1	0	0	2	2	FLGVAEDGAQV	tr_D0A9V0	0.412	576.63	3.00
91	HLA-A0201	RIMGASISVT	RIMGASISV	0	0	0	0	0	RIMGASISV	tr_D0A9V0	0.412	580.20	3.00
317	HLA-A0201	EAMAHYLPMVK	AMAHYLPMV	1	0	0	0	0	AMAHYLPMV	tr_D0A9V0	0.411	583.06	3.00

406	HLA-A0201	SLFKMYAPS	SLFKMYAPS	0	0	0	0	0	SLFKMYAPS	tr_D0A9V0	0.411	587.80	3.00
209	HLA-A0201	MLVDTNEFLVGTCG	MLVDTNFLV	0	0	0	6	1	MLVDTNEFLV	tr_D0A9V0	0.411	587.83	3.00
50	HLA-A0201	LLKFIPEAILARV	FIPEAILAV	3	0	0	8	1	FIPEAILARV	tr_D0A9V0	0.410	590.66	3.00
630	HLA-A0201	FSFGISLLEM	FSFGILLEM	0	0	0	5	1	FSFGISLLEM	tr_D0A9V0	0.410	594.32	3.00
674	HLA-A0201	SLALGHLEPVIPA	SLLEPVIPA	0	0	0	2	4	SLALGHLEPVIPA	tr_D0A9V0	0.409	595.54	3.00
599	HLA-A0201	TLADGVMLRGTAL	TLADGVMLL	0	0	0	8	4	TLADGVMLRGTAL	tr_D0A9V0	0.409	600.13	3.00
436	HLA-A0201	LFLTDSNRNV	FLTDSNRNV	1	0	0	0	0	FLTDSNRNV	tr_D0A9V0	0.406	616.01	3.00
259	HLA-A0201	SHMQGFLAFPVD	HMQGFLFPV	1	0	0	6	1	HMQGFLAFPV	tr_D0A9V0	0.406	617.82	3.00
4	HLA-A0201	LLVSALKSA	LLVSALKSA	0	0	0	0	0	LLVSALKSA	tr_D0A9V0	0.406	618.96	3.00
676	HLA-A0201	ALGHLEPVI	ALGHLEPVI	0	0	0	0	0	ALGHLEPVI	tr_D0A9V0	0.405	628.13	3.50
230	HLA-A0201	VLFGADHLFQLSY	VLFDHLFQL	0	0	0	3	2	VLFGADHLFQL	tr_D0A9V0	0.403	637.85	3.50
318	HLA-A0201	AMAHYLPMVKM	AMAHYLPMV	0	0	0	0	0	AMAHYLPMV	tr_D0A9V0	0.403	640.48	3.50
47	HLA-A0201	LAVLLKFIPEAI	VLLKFIPEI	2	0	0	8	1	VLLKFIPEAI	tr_D0A9V0	0.402	642.90	3.50
263	HLA-A0201	GFLAFPVDCVLL	FLAFDCVLL	1	0	0	4	2	FLAFPVDCVLL	tr_D0A9V0	0.402	644.51	3.50
676	HLA-A0201	ALGHLEPVIPA	ALLEPVIPA	0	0	0	2	2	ALGHLEPVIPA	tr_D0A9V0	0.402	648.00	3.50
90	HLA-A0201	VRIMGASISVTWNA	IMISVTWNA	2	0	0	2	3	IMGASISVTWNA	tr_D0A9V0	0.401	649.15	3.50
49	HLA-A0201	VLLKFIPEAILA	VLLKFIPEA	0	0	0	8	3	VLLKFIPEAILA	tr_D0A9V0	0.401	655.52	3.50
45	HLA-A0201	RQLAVLLKFIPEA	RLLKFIPEA	0	0	0	1	4	RQLAVLLKFIPEA	tr_D0A9V0	0.400	660.08	3.50
609	HLA-A0201	TALYMAPEV	TALYMAPEV	0	0	0	0	0	TALYMAPEV	tr_D0A9V0	0.398	673.45	3.50
605	HLA-A0201	MLRGTALYM	MLRGTALYM	0	0	0	0	0	MLRGTALYM	tr_D0A9V0	0.397	678.94	3.50
460	HLA-A0201	TIFLGVAEDGAQV	FLAEDGAQV	2	0	0	2	2	FLGVAEDGAQV	tr_D0A9V0	0.397	681.32	3.50
44	HLA-A0201	RRQLAVLLKFIPEA	RLLKFIPEA	0	0	0	1	5	RRQLAVLLKFIPEA	tr_D0A9V0	0.397	684.51	3.50
216	HLA-A0201	FLVGTCGAFS	FLVGTCAFS	0	0	0	6	1	FLVGTCGAFS	tr_D0A9V0	0.396	686.03	3.50
639	HLA-A0201	MLLGRPPWQWSSTA	MLWQWSSTA	0	0	0	2	5	MLLGRPPWQWSSTA	tr_D0A9V0	0.396	689.15	3.50
198	HLA-A0201	KLPLDHRDSV	KLLDHRDSV	0	0	0	2	1	KLPLDHRDSV	tr_D0A9V0	0.396	690.31	3.50
46	HLA-A0201	QLAVLLKFIPEAI	VLLKFIPEI	3	0	0	8	1	VLLKFIPEAI	tr_D0A9V0	0.396	690.79	3.50
228	HLA-A0201	ARVLFGADHLFQLS	VLFDHLFQL	2	0	0	3	2	VLFGADHLFQL	tr_D0A9V0	0.396	692.26	3.50
190	HLA-A0201	QFAFDLFAKLPL	FAFDLFLPL	1	0	0	6	2	FAFDLFAKLPL	tr_D0A9V0	0.396	692.57	3.50
516	HLA-A0201	HLSIIMEYVP	HLSIIMEYV	0	0	0	0	0	HLSIIMEYV	tr_D0A9V0	0.395	693.71	3.50
24	HLA-A0201	FLGRSRIVEVR	FLGSRIVEV	0	0	0	3	1	FLGRSRIVEV	tr_D0A9V0	0.395	694.83	3.50
272	HLA-A0201	VLLPCSTLPIVL	VLLPCLPIV	0	0	0	5	2	VLLPCSTLPIV	tr_D0A9V0	0.395	695.59	3.50
47	HLA-A0201	LAVLLKFIPEAIL	VLLKFIPEL	2	0	0	8	2	VLLKFIPEAIL	tr_D0A9V0	0.395	697.19	3.50
316	HLA-A0201	GEAMAHYLPMVK	AMAHYLPMV	2	0	0	0	0	AMAHYLPMV	tr_D0A9V0	0.394	707.33	3.50
322	HLA-A0201	YLPMVKMDM	YLPMVKMDM	0	0	0	0	0	YLPMVKMDM	tr_D0A9V0	0.393	709.21	3.50
45	HLA-A0201	RQLAVLLKFIPEAI	VLLKFIPEI	4	0	0	8	1	VLLKFIPEAI	tr_D0A9V0	0.392	720.95	3.50
549	HLA-A0201	VDILQGLNYL	VILQGLNYL	0	0	0	1	1	VDILQGLNYL	tr_D0A9V0	0.392	721.04	3.50
552	HLA-A0201	LQGLNYLHNL	LQLNYLHNL	0	0	0	2	1	LQGLNYLHNL	tr_D0A9V0	0.392	722.16	3.50
234	HLA-A0201	ADHLFQLSYAVR	ALFQLSYAV	0	0	0	1	2	ADHLFQLSYAV	tr_D0A9V0	0.392	722.25	3.50
260	HLA-A0201	HMQGFLAFPVDC	HMQGFLFPV	0	0	0	6	1	HMQGFLAFPV	tr_D0A9V0	0.391	725.64	3.50
46	HLA-A0201	QLAVLLKFI	QLAVLLKFI	0	0	0	0	0	QLAVLLKFI	tr_D0A9V0	0.391	726.05	3.50
207	HLA-A0201	VTMLVDTNEFLVGT	MLVDTNFLV	2	0	0	6	1	MLVDTNEFLV	tr_D0A9V0	0.391	727.41	3.50
46	HLA-A0201	QLAVLLKFIPEA	LLLKFIPEA	1	0	0	1	2	LAVLLKFIPEA	tr_D0A9V0	0.391	727.70	3.50
687	HLA-A0201	SVDHEAAAFV	SVDEAAAFV	0	0	0	3	1	SVDHEAAAFV	tr_D0A9V0	0.390	732.69	3.50
459	HLA-A0201	STIFLGVAEDGAQV	FLAEDGAQV	3	0	0	2	2	FLGVAEDGAQV	tr_D0A9V0	0.389	745.03	3.50
46	HLA-A0201	QLAVLLKFIPEAIL	VLLKFIPEL	3	0	0	8	2	VLLKFIPEAIL	tr_D0A9V0	0.389	745.18	3.50
278	HLA-A0201	TLPIVLFELR	TLPIVLFEL	0	0	0	0	0	TLPIVLFEL	tr_D0A9V0	0.389	746.61	3.50
262	HLA-A0201	QGFLAFPVDCVLL	FLAFDCVLL	2	0	0	4	2	FLAFPVDCVLL	tr_D0A9V0	0.388	747.53	3.50
190	HLA-A0201	QFAFDLFAKL	FAFDLFAKL	1	0	0	0	0	FAFDLFAKL	tr_D0A9V0	0.388	750.26	3.50
189	HLA-A0201	CQFAFDLFAKLPL	FAFDLFLPL	2	0	0	6	2	FAFDLFAKLPL	tr_D0A9V0	0.388	750.91	3.50
512	HLA-A0201	ITPSHLSIIMEYV	ILSIIMEYV	0	0	0	1	4	ITPSHLSIIMEYV	tr_D0A9V0	0.388	751.70	3.50
462	HLA-A0201	FLGVAEDGAQVAI	FLDGAQVAI	0	0	0	2	4	FLGVAEDGAQVAI	tr_D0A9V0	0.387	761.96	3.50
258	HLA-A0201	ASHMQGFLAFPVD	HMQGFLFPV	2	0	0	6	1	HMQGFLAFPV	tr_D0A9V0	0.387	762.44	3.50
669	HLA-A0201	LLFVQSLALG	LLFVQSLAL	0	0	0	0	0	LLFVQSLAL	tr_D0A9V0	0.386	764.12	3.50
261	HLA-A0201	MQGFLAFPVD	MQGFLAFPV	0	0	0	0	0	MQGFLAFPV	tr_D0A9V0	0.385	776.46	3.50

557	HLA-A0201	YLHNLGIVHCDV	YLHNLGIVV	0	0	0	8	3	YLHNLGIVHCDV	tr_D0A9V0	0.384	783.42	3.50
608	HLA-A0201	GTALYMAPEV	GTALYMAPV	0	0	0	8	1	GTALYMAPEV	tr_D0A9V0	0.384	784.20	3.50
208	HLA-A0201	TMLVDTNEFLVGTC	MLVDTNFLV	1	0	0	6	1	MLVDTNEFLV	tr_D0A9V0	0.384	786.24	3.50
261	HLA-A0201	MQGFLAFPVDCVLL	FLAFDCVLL	3	0	0	4	2	FLAFPVDCVLL	tr_D0A9V0	0.384	788.20	3.50
546	HLA-A0201	RFTVDILQGLNYL	FILQGLNYL	1	0	0	1	3	FTVDILQGLNYL	tr_D0A9V0	0.383	797.06	3.50
574	HLA-A0201	LLGTDGVCKL	LLTDGVCKL	0	0	0	2	1	LLGTDGVCKL	tr_D0A9V0	0.382	797.68	3.50
188	HLA-A0201	CCQFAFDLFAKLPL	FAFDLFLPL	3	0	0	6	2	FAFDLFAKLPL	tr_D0A9V0	0.382	799.24	3.50
406	HLA-A0201	SLFKMYAPSSSSSL	SLFKMYAPL	0	0	0	8	5	SLFKMYAPSSSSSL	tr_D0A9V0	0.382	799.65	3.50
511	HLA-A0201	SITPSHLSIIMEYV	SLSIIMEYV	0	0	0	1	5	SITPSHLSIIMEYV	tr_D0A9V0	0.381	806.10	3.50
257	HLA-A0201	GASHMQGFLAFPVD	HMQGFLFPV	3	0	0	6	1	HMQGFLAFPV	tr_D0A9V0	0.381	809.60	3.50
582	HLA-A0201	KLSDFGSTISEA	KLSDFISEA	0	0	0	5	3	KLSDFGSTISEA	tr_D0A9V0	0.380	814.82	3.50
612	HLA-A0201	YMAPEVAAG	YMAPEVAAG	0	0	0	0	0	YMAPEVAAG	tr_D0A9V0	0.380	816.07	3.50
264	HLA-A0201	FLAFPVDCVLLPC	FLACVLLPC	0	0	0	3	4	FLAFPVDCVLLPC	tr_D0A9V0	0.374	871.07	4.00
520	HLA-A0201	IMEYVPGGSLHSV	IMGGSLHSV	0	0	0	2	4	IMEYVPGGSLHSV	tr_D0A9V0	0.373	879.25	4.00
88	HLA-A0201	AFVRIMGASISVT	FIMGASISV	1	0	0	1	2	FVRIMGASISV	tr_D0A9V0	0.373	881.44	4.00
462	HLA-A0201	FLGVAEDGA	FLGVAEDGA	0	0	0	0	0	FLGVAEDGA	tr_D0A9V0	0.372	893.19	4.00
233	HLA-A0201	GADHLFQLSYAVR	GLFQLSYAV	0	0	0	1	3	GADHLFQLSYAV	tr_D0A9V0	0.372	893.46	4.00
498	HLA-A0201	KLHHPNIVRYV	KLHHPNIYV	0	0	0	7	2	KLHHPNIVRYV	tr_D0A9V0	0.372	897.74	4.00
673	HLA-A0201	QSLALGHLEPVI	SLLGHLEPV	1	0	0	2	1	SLALGHLEPV	tr_D0A9V0	0.371	902.29	4.00
318	HLA-A0201	AMAHYLPMVKMDM	AMAHYLPMV	0	0	0	0	0	AMAHYLPMV	tr_D0A9V0	0.370	912.00	4.00
673	HLA-A0201	QSLALGHLEPVIPA	SLLEPVIPA	1	0	0	2	4	SLALGHLEPVIPA	tr_D0A9V0	0.369	925.47	4.00
452	HLA-A0201	KISEGAFSTIFLGV	KISEGAFSV	0	0	0	8	5	KISEGAFSTIFLGV	tr_D0A9V0	0.368	937.16	4.00
318	HLA-A0201	AMAHYLPMVKMD	AMAHYLPMV	0	0	0	0	0	AMAHYLPMV	tr_D0A9V0	0.366	948.34	4.00
545	HLA-A0201	RRFTVDILQGLNYL	FILQGLNYL	2	0	0	1	3	FTVDILQGLNYL	tr_D0A9V0	0.366	953.59	4.00
271	HLA-A0201	CVLLPCSTLPIVL	VLLPCLPIV	1	0	0	5	2	VLLPCSTLPIV	tr_D0A9V0	0.364	970.59	4.00
89	HLA-A0201	FVRIMGASISVTW	FIMGASISV	0	0	0	1	2	FVRIMGASISV	tr_D0A9V0	0.363	979.86	4.00
229	HLA-A0201	RVLFGADHLFQLSY	VLFDHLFQL	1	0	0	3	2	VLFGADHLFQL	tr_D0A9V0	0.363	985.37	4.00
170	HLA-A0201	RALISFGAHA	ALISFGAHA	1	0	0	0	0	ALISFGAHA	tr_D0A9V0	0.363	986.08	4.00
437	HLA-A0201	FLTDSNRNVWTRSL	FLTDWTRSL	0	0	0	4	5	FLTDSNRNVWTRSL	tr_D0A9V0	0.362	991.75	4.00
598	HLA-A0201	RTLADGVMLRGTAL	TLADGVMLL	1	0	0	8	4	TLADGVMLRGTAL	tr_D0A9V0	0.362	992.51	4.00
274	HLA-A0201	LPCSTLPIVLFEL	LLPIVLFEL	0	0	0	1	4	LPCSTLPIVLFEL	tr_D0A9V0	0.362	995.34	4.00
115	HLA-A0201	GIFKEKPNV	GIFKEKPNV	0	0	0	0	0	GIFKEKPNV	tr_D0A9V0	0.361	1003.87	4.00
262	HLA-A0201	QGFLAFPVDCV	FLAFPVDCV	2	0	0	0	0	FLAFPVDCV	tr_D0A9V0	0.360	1016.11	4.00
261	HLA-A0201	MQGFLAFPVDCV	FLAFPVDCV	3	0	0	0	0	FLAFPVDCV	tr_D0A9V0	0.359	1027.05	4.00
674	HLA-A0201	SLALGHLEPVIP	SLLGHLEPV	0	0	0	2	1	SLALGHLEPV	tr_D0A9V0	0.358	1034.08	4.00
207	HLA-A0201	VTMLVDTNEFL	MLVDTNEFL	2	0	0	0	0	MLVDTNEFL	tr_D0A9V0	0.357	1049.36	4.00
264	HLA-A0201	FLAFPVDCVLLP	FLFPVDCVL	0	0	0	2	1	FLAFPVDCVL	tr_D0A9V0	0.357	1051.59	4.00
48	HLA-A0201	AVLLKFIPEAILA	VLLKFIPEA	1	0	0	8	3	VLLKFIPEAILA	tr_D0A9V0	0.357	1052.01	4.00
87	HLA-A0201	TAFVRIMGASISVT	FIMGASISV	2	0	0	1	2	FVRIMGASISV	tr_D0A9V0	0.357	1054.87	4.00
151	HLA-A0201	ALIEVATEHSGCVE	ALIEVATEV	0	0	0	8	4	ALIEVATEHSGCV	tr_D0A9V0	0.357	1056.03	4.50
556	HLA-A0201	NYLHNLGIVHCDV	YLHNLGIVV	1	0	0	8	3	YLHNLGIVHCDV	tr_D0A9V0	0.356	1063.77	4.50
531	HLA-A0201	SVIKNFGCVSPYV	SVICVSPYV	0	0	0	3	4	SVIKNFGCVSPYV	tr_D0A9V0	0.356	1066.58	4.50
270	HLA-A0201	DCVLLPCSTLPIVL	VLLPCLPIV	2	0	0	5	2	VLLPCSTLPIV	tr_D0A9V0	0.355	1068.99	4.50
488	HLA-A0201	SLEKEMEVASKL	SLMEVASKL	0	0	0	2	3	SLEKEMEVASKL	tr_D0A9V0	0.355	1071.47	4.50
675	HLA-A0201	LALGHLEPVIPA	ALLEPVIPA	1	0	0	2	2	ALGHLEPVIPA	tr_D0A9V0	0.355	1073.19	4.50
317	HLA-A0201	EAMAHYLPMVKM	AMAHYLPMV	1	0	0	0	0	AMAHYLPMV	tr_D0A9V0	0.355	1074.45	4.50
23	HLA-A0201	IFLGRSRIVEVR	FLGSRIVEV	1	0	0	3	1	FLGRSRIVEV	tr_D0A9V0	0.355	1074.75	4.50
1	HLA-A0201	PLMLLVSAL	PLMLLVSAL	0	0	0	0	0	PLMLLVSAL	tr_D0A9V0	0.354	1080.46	4.50
557	HLA-A0201	YLHNLGIVHC	YLHNLIVHC	0	0	0	5	1	YLHNLGIVHC	tr_D0A9V0	0.354	1080.55	4.50
671	HLA-A0201	FVQSLALGHLEPVI	SLLGHLEPV	3	0	0	2	1	SLALGHLEPV	tr_D0A9V0	0.353	1101.27	4.50
259	HLA-A0201	SHMQGFLAFPVDC	HMQGFLFPV	1	0	0	6	1	HMQGFLAFPV	tr_D0A9V0	0.352	1103.35	4.50
206	HLA-A0201	SVTMLVDTNEFL	MLVDTNEFL	3	0	0	0	0	MLVDTNEFL	tr_D0A9V0	0.351	1124.30	4.50
672	HLA-A0201	VQSLALGHLEPVI	SLLGHLEPV	2	0	0	2	1	SLALGHLEPV	tr_D0A9V0	0.350	1132.44	4.50
276	HLA-A0201	CSTLPIVLFEL	SLPIVLFEL	1	0	0	1	1	STLPIVLFEL	tr_D0A9V0	0.350	1133.88	4.50

205	HLA-A0201	DSVTMLVDTNEFL	MLVDTNEFL	4	0	0	0	0	MLVDTNEFL	tr_D0A9V0	0.347	1170.87	4.50
263	HLA-A0201	GFLAFPVDCVLLPC	FLACVLLPC	1	0	0	3	4	FLAFPVDCVLLPC	tr_D0A9V0	0.346	1180.92	4.50
22	HLA-A0201	DIFLGRSRIVEVR	FLGSRIVEV	2	0	0	3	1	FLGRSRIVEV	tr_D0A9V0	0.346	1184.99	4.50
188	HLA-A0201	CCQFAFDLFA	CQFAFDLFA	0	0	0	1	1	CCQFAFDLFA	tr_D0A9V0	0.345	1190.95	4.50
24	HLA-A0201	FLGRSRIVEVRK	FLGSRIVEV	0	0	0	3	1	FLGRSRIVEV	tr_D0A9V0	0.345	1194.24	4.50
234	HLA-A0201	ADHLFQLSYAVRD	ALFQLSYAV	0	0	0	1	2	ADHLFQLSYAV	tr_D0A9V0	0.345	1200.47	4.50
204	HLA-A0201	RDSVTMLVDTNEFL	MLVDTNEFL	5	0	0	0	0	MLVDTNEFL	tr_D0A9V0	0.344	1202.79	4.50
665	HLA-A0201	FNRDLLFVQSLALG	FLFVQSLAL	0	0	0	1	4	FNRDLLFVQSLAL	tr_D0A9V0	0.343	1222.56	4.50
513	HLA-A0201	TPSHLSIIMEYV	SLSIIMEYV	2	0	0	1	1	SHLSIIMEYV	tr_D0A9V0	0.343	1226.64	4.50
645	HLA-A0201	PWQWSSTAPA	WQWSSTAPA	1	0	0	0	0	WQWSSTAPA	tr_D0A9V0	0.343	1228.90	4.50
461	HLA-A0201	IFLGVAEDGAQVAI	FLDGAQVAI	1	0	0	2	4	FLGVAEDGAQVAI	tr_D0A9V0	0.342	1230.47	4.50
551	HLA-A0201	ILQGLNYLHNLGI	ILQGLNYLI	0	0	0	8	4	ILQGLNYLHNLGI	tr_D0A9V0	0.342	1235.23	4.50
581	HLA-A0201	CKLSDFGSTISEA	KLSDFISEA	1	0	0	5	3	KLSDFGSTISEA	tr_D0A9V0	0.341	1244.39	4.50
21	HLA-A0201	ADIFLGRSRIVEVR	FLGSRIVEV	3	0	0	3	1	FLGRSRIVEV	tr_D0A9V0	0.341	1252.25	4.50
514	HLA-A0201	PSHLSIIMEYV	SLSIIMEYV	1	0	0	1	1	SHLSIIMEYV	tr_D0A9V0	0.340	1265.55	4.50
318	HLA-A0201	AMAHYLPMVKMDMQ	AMAHYLPMV	0	0	0	0	0	AMAHYLPMV	tr_D0A9V0	0.339	1270.94	4.50
95	HLA-A0201	ASISVTWNA	ASISVTWNA	0	0	0	0	0	ASISVTWNA	tr_D0A9V0	0.338	1290.93	4.50
89	HLA-A0201	FVRIMGASISVTWN	FIMGASISV	0	0	0	1	2	FVRIMGASISV	tr_D0A9V0	0.338	1294.20	4.50
555	HLA-A0201	LNYLHNLGIVHCDV	YLHNLGIVV	2	0	0	8	3	YLHNLGIVHCDV	tr_D0A9V0	0.337	1301.25	4.50
316	HLA-A0201	GEAMAHYLPMVKM	AMAHYLPMV	2	0	0	0	0	AMAHYLPMV	tr_D0A9V0	0.337	1302.70	4.50
47	HLA-A0201	LAVLLKFIPEAILA	VLLKFIPEA	2	0	0	8	3	VLLKFIPEAILA	tr_D0A9V0	0.337	1311.34	5.00
272	HLA-A0201	VLLPCSTLPIVLF	VLLPCLPIV	0	0	0	5	2	VLLPCSTLPIV	tr_D0A9V0	0.336	1311.52	5.00
171	HLA-A0201	ALISFGAHAALPM	ALISFALPM	0	0	0	5	4	ALISFGAHAALPM	tr_D0A9V0	0.336	1320.25	5.00
551	HLA-A0201	ILQGLNYLHNLG	ILLNYLHNL	0	0	0	2	2	ILQGLNYLHNL	tr_D0A9V0	0.336	1322.47	5.00
4	HLA-A0201	LLVSALKSASNLEI	LLVSALLEI	0	0	0	6	5	LLVSALKSASNLEI	tr_D0A9V0	0.335	1328.39	5.00
258	HLA-A0201	ASHMQGFLAFPVDC	HMQGFLFPV	2	0	0	6	1	HMQGFLAFPV	tr_D0A9V0	0.335	1333.33	5.00
519	HLA-A0201	IIMEYVPGGSLH	IIMEYVPSL	0	0	0	7	2	IIMEYVPGGSL	tr_D0A9V0	0.334	1351.40	5.00
275	HLA-A0201	PCSTLPIVLFEL	SLPIVLFEL	2	0	0	1	1	STLPIVLFEL	tr_D0A9V0	0.333	1364.55	5.00
663	HLA-A0201	SLFNRDLLF	SLFNRDLLF	0	0	0	0	0	SLFNRDLLF	tr_D0A9V0	0.331	1395.98	5.00
235	HLA-A0201	DHLFQLSYAVR	HLFQLSYAV	1	0	0	0	0	HLFQLSYAV	tr_D0A9V0	0.330	1401.63	5.00
497	HLA-A0201	SKLHHPNIVRYV	KLHHPNIYV	1	0	0	7	2	KLHHPNIVRYV	tr_D0A9V0	0.330	1402.77	5.00
111	HLA-A0201	ALNCGIFKEKPNVF	ALNCGIFNV	0	0	0	7	4	ALNCGIFKEKPNV	tr_D0A9V0	0.330	1413.93	5.00
264	HLA-A0201	FLAFPVDCVLLPCS	FLFPVDCVL	0	0	0	2	1	FLAFPVDCVL	tr_D0A9V0	0.329	1421.25	5.00
604	HLA-A0201	VMLRGTALYMA	VMLTALYMA	0	0	0	3	2	VMLRGTALYMA	tr_D0A9V0	0.328	1432.83	5.00
633	HLA-A0201	GISLLEMLL	GISLLEMLL	0	0	0	0	0	GISLLEMLL	tr_D0A9V0	0.328	1435.48	5.00
2	HLA-A0201	LMLLVSALKSASNL	LMLLVSALL	0	0	0	8	5	LMLLVSALKSASNL	tr_D0A9V0	0.327	1450.60	5.00
263	HLA-A0201	GFLAFPVDCVLLP	FLFPVDCVL	1	0	0	2	1	FLAFPVDCVL	tr_D0A9V0	0.326	1461.41	5.00
233	HLA-A0201	GADHLFQLSYAVRD	GLFQLSYAV	0	0	0	1	3	GADHLFQLSYAV	tr_D0A9V0	0.326	1470.44	5.00
234	HLA-A0201	ADHLFQLSYAVRDG	ALFQLSYAV	0	0	0	1	2	ADHLFQLSYAV	tr_D0A9V0	0.326	1471.08	5.00
24	HLA-A0201	FLGRSRIVEVRKI	FLGSRIVEV	0	0	0	3	1	FLGRSRIVEV	tr_D0A9V0	0.325	1479.70	5.00
53	HLA-A0201	FIPEAILARVE	FIPEAILAV	0	0	0	8	1	FIPEAILARV	tr_D0A9V0	0.325	1489.52	5.00
317	HLA-A0201	EAMAHYLPMVKMDM	AMAHYLPMV	1	0	0	0	0	AMAHYLPMV	tr_D0A9V0	0.324	1502.74	5.00
171	HLA-A0201	ALISFGAHAAL	ALISFHAAL	0	0	0	5	2	ALISFGAHAAL	tr_D0A9V0	0.323	1511.81	5.00
671	HLA-A0201	FVQSLALGHL	FVQSLALGL	0	0	0	8	1	FVQSLALGHL	tr_D0A9V0	0.323	1517.71	5.00
317	HLA-A0201	EAMAHYLPMVKMD	AMAHYLPMV	1	0	0	0	0	AMAHYLPMV	tr_D0A9V0	0.322	1525.99	5.00
674	HLA-A0201	SLALGHLEPVIPAS	SLLGHLEPV	0	0	0	2	1	SLALGHLEPV	tr_D0A9V0	0.321	1554.56	5.00
50	HLA-A0201	LLKFIPEAIL	LLFIPEAIL	0	0	0	2	1	LLKFIPEAIL	tr_D0A9V0	0.320	1565.00	5.00
580	HLA-A0201	VCKLSDFGSTISEA	KLSDFISEA	2	0	0	5	3	KLSDFGSTISEA	tr_D0A9V0	0.320	1574.46	5.00
267	HLA-A0201	FPVDCVLLPC	FVDCVLLPC	0	0	0	1	1	FPVDCVLLPC	tr_D0A9V0	0.319	1588.56	5.00
573	HLA-A0201	VLLGTDGVCKL	VLLDGVCKL	0	0	0	3	2	VLLGTDGVCKL	tr_D0A9V0	0.318	1604.28	5.50
673	HLA-A0201	QSLALGHLEPVIP	SLLGHLEPV	1	0	0	2	1	SLALGHLEPV	tr_D0A9V0	0.318	1606.64	5.50
88	HLA-A0201	AFVRIMGASISVTW	FIMGASISV	1	0	0	1	2	FVRIMGASISV	tr_D0A9V0	0.317	1612.23	5.50
676	HLA-A0201	ALGHLEPVIPASV	ALGHLPASV	0	0	0	5	4	ALGHLEPVIPASV	tr_D0A9V0	0.317	1615.23	5.50
464	HLA-A0201	GVAEDGAQV	GVAEDGAQV	0	0	0	0	0	GVAEDGAQV	tr_D0A9V0	0.317	1624.38	5.50

272	HLA-A0201	VLLPCSTLPIVLFE	VLLPCLPIV	0	0	0	5	2	VLLPCSTLPIV	tr_D0A9V0	0.316	1635.76	5.50
262	HLA-A0201	QGFLAFPVDCVLLP	FLFPVDCVL	2	0	0	2	1	FLAFPVDCVL	tr_D0A9V0	0.316	1645.56	5.50
530	HLA-A0201	HSVIKNFGCVSPYV	SVICVSPYV	1	0	0	3	4	SVIKNFGCVSPYV	tr_D0A9V0	0.314	1675.06	5.50
236	HLA-A0201	HLFQLSYAVRD	HLFQLSYAV	0	0	0	0	0	HLFQLSYAV	tr_D0A9V0	0.314	1677.21	5.50
230	HLA-A0201	VLFGADHLF	VLFGADHLF	0	0	0	0	0	VLFGADHLF	tr_D0A9V0	0.313	1683.61	5.50
550	HLA-A0201	DILQGLNYLHNLGI	ILYLHNLGI	1	0	0	2	4	ILQGLNYLHNLGI	tr_D0A9V0	0.313	1688.14	5.50
273	HLA-A0201	LLPCSTLPIVL	LLPCSTLPL	0	0	0	8	2	LLPCSTLPIVL	tr_D0A9V0	0.312	1702.87	5.50
24	HLA-A0201	FLGRSRIVEVRKIQ	FLGSRIVEV	0	0	0	3	1	FLGRSRIVEV	tr_D0A9V0	0.312	1706.89	5.50
487	HLA-A0201	ESLEKEMEVASKL	SLMEVASKL	1	0	0	2	3	SLEKEMEVASKL	tr_D0A9V0	0.312	1718.68	5.50
496	HLA-A0201	ASKLHHPNIVRYV	KLHHPNIYV	2	0	0	7	2	KLHHPNIVRYV	tr_D0A9V0	0.311	1727.81	5.50
599	HLA-A0201	TLADGVMLRGTA	TLADGVMLA	0	0	0	8	3	TLADGVMLRGTA	tr_D0A9V0	0.310	1751.53	5.50
92	HLA-A0201	IMGASISVTWNAG	IMISVTWNA	0	0	0	2	3	IMGASISVTWNA	tr_D0A9V0	0.309	1756.94	5.50
23	HLA-A0201	IFLGRSRIVEVRK	FLGSRIVEV	1	0	0	3	1	FLGRSRIVEV	tr_D0A9V0	0.308	1778.18	5.50
452	HLA-A0201	KISEGAFSTIFL	KISEGAFFL	0	0	0	7	3	KISEGAFSTIFL	tr_D0A9V0	0.308	1783.77	5.50
271	HLA-A0201	CVLLPCSTLPIVLF	VLLPCLPIV	1	0	0	5	2	VLLPCSTLPIV	tr_D0A9V0	0.307	1796.85	5.50
316	HLA-A0201	GEAMAHYLPMVKMD	AMAHYLPMV	2	0	0	0	0	AMAHYLPMV	tr_D0A9V0	0.307	1806.81	5.50
170	HLA-A0201	RALISFGAHAALPM	ALISFALPM	1	0	0	5	4	ALISFGAHAALPM	tr_D0A9V0	0.307	1812.66	5.50
705	HLA-A0201	SMRPSASSLLSYA	SMSSLLSYA	0	0	0	2	4	SMRPSASSLLSYA	tr_D0A9V0	0.306	1814.55	5.50
495	HLA-A0201	VASKLHHPNIVRYV	KLHHPNIYV	3	0	0	7	2	KLHHPNIVRYV	tr_D0A9V0	0.305	1836.25	5.50
667	HLA-A0201	RDLLFVQSLA	RLLFVQSLA	0	0	0	1	1	RDLLFVQSLA	tr_D0A9V0	0.304	1855.61	5.50
191	HLA-A0201	FAFDLFAKLPLD	FAFDLFLPL	0	0	0	6	2	FAFDLFAKLPL	tr_D0A9V0	0.304	1856.47	5.50
582	HLA-A0201	KLSDFGSTIS	KLSDFGSTI	0	0	0	0	0	KLSDFGSTI	tr_D0A9V0	0.304	1864.89	5.50
572	HLA-A0201	NVLLGTDGV	NVLLGTDGV	0	0	0	0	0	NVLLGTDGV	tr_D0A9V0	0.304	1867.03	5.50
165	HLA-A0201	ALDPDRALISFGA	ALDPISFGA	0	0	0	4	4	ALDPDRALISFGA	tr_D0A9V0	0.303	1876.65	5.50
472	HLA-A0201	VAIKCIPRL	VAIKCIPRL	0	0	0	0	0	VAIKCIPRL	tr_D0A9V0	0.303	1880.16	5.50
589	HLA-A0201	TISEASVMA	TISEASVMA	0	0	0	0	0	TISEASVMA	tr_D0A9V0	0.303	1884.46	5.50
265	HLA-A0201	LAFPVDCVLL	LAFPVCVLL	0	0	0	5	1	LAFPVDCVLL	tr_D0A9V0	0.302	1897.99	5.50
665	HLA-A0201	FNRDLLFVQSLA	FLLFVQSLA	0	0	0	1	3	FNRDLLFVQSLA	tr_D0A9V0	0.302	1902.74	6.00
594	HLA-A0201	SVMARTLADGV	VMARTLAGV	1	0	0	7	1	VMARTLADGV	tr_D0A9V0	0.302	1905.54	6.00
639	HLA-A0201	MLLGRPPWQWSS	MLLGWQWSS	0	0	0	4	3	MLLGRPPWQWSS	tr_D0A9V0	0.302	1909.97	6.00
550	HLA-A0201	DILQGLNYLHNLG	ILLNYLHNL	1	0	0	2	2	ILQGLNYLHNL	tr_D0A9V0	0.302	1911.73	6.00
484	HLA-A0201	VVAESLEKEMEV	VVAESLMEV	0	0	0	6	3	VVAESLEKEMEV	tr_D0A9V0	0.301	1918.32	6.00
462	HLA-A0201	FLGVAEDGAQVA	FLAEDGAQV	0	0	0	2	2	FLGVAEDGAQV	tr_D0A9V0	0.301	1922.00	6.00
640	HLA-A0201	LLGRPPWQWSSTA	LLWQWSSTA	0	0	0	2	4	LLGRPPWQWSSTA	tr_D0A9V0	0.301	1924.29	6.00
437	HLA-A0201	FLTDSNRNVW	FLTDSNRNV	0	0	0	0	0	FLTDSNRNV	tr_D0A9V0	0.301	1926.04	6.00
534	HLA-A0201	KNFGCVSPYV	KNFGCVPYV	0	0	0	6	1	KNFGCVSPYV	tr_D0A9V0	0.300	1936.30	6.00
22	HLA-A0201	DIFLGRSRIVEVRK	FLGSRIVEV	2	0	0	3	1	FLGRSRIVEV	tr_D0A9V0	0.300	1939.92	6.00
0	HLA-A0201	KPLMLLVSALK	KLMLLVSAL	0	0	0	1	1	KPLMLLVSAL	tr_D0A9V0	0.300	1949.84	6.00
296	HLA-A0201	SLSISKQFRLGFAA	SLFRLGFAA	0	0	0	2	5	SLSISKQFRLGFAA	tr_D0A9V0	0.299	1958.08	6.00
547	HLA-A0201	FTVDILQGLN	FTVDILQGL	0	0	0	0	0	FTVDILQGL	tr_D0A9V0	0.299	1966.19	6.00
672	HLA-A0201	VQSLALGHLEPVIP	SLLGHLEPV	2	0	0	2	1	SLALGHLEPV	tr_D0A9V0	0.299	1970.81	6.00
191	HLA-A0201	FAFDLFAKLP	FAFDLFAKL	0	0	0	0	0	FAFDLFAKL	tr_D0A9V0	0.296	2022.75	6.00
236	HLA-A0201	HLFQLSYAVRDG	HLFQLSYAV	0	0	0	0	0	HLFQLSYAV	tr_D0A9V0	0.296	2023.97	6.00
669	HLA-A0201	LLFVQSLALGHL	LLFVQSLAL	0	0	0	8	3	LLFVQSLALGHL	tr_D0A9V0	0.296	2024.80	6.00
351	HLA-A0201	YVRVLDNHYCV	YVLDNHYCV	0	0	0	1	2	YVRVLDNHYCV	tr_D0A9V0	0.295	2048.63	6.00
3	HLA-A0201	MLLVSALKSASNL	MLLVSALNL	0	0	0	7	4	MLLVSALKSASNL	tr_D0A9V0	0.295	2055.29	6.00
603	HLA-A0201	GVMLRGTALYMA	VMLTALYMA	1	0	0	3	2	VMLRGTALYMA	tr_D0A9V0	0.294	2078.55	6.00
518	HLA-A0201	SIIMEYVPGGSLH	IIMEYVPSL	1	0	0	7	2	IIMEYVPGGSL	tr_D0A9V0	0.293	2100.91	6.00
486	HLA-A0201	AESLEKEMEVASKL	SLMEVASKL	2	0	0	2	3	SLEKEMEVASKL	tr_D0A9V0	0.291	2136.34	6.00
170	HLA-A0201	RALISFGAHAAL	ALISFHAAL	1	0	0	5	2	ALISFGAHAAL	tr_D0A9V0	0.291	2154.85	6.00
667	HLA-A0201	RDLLFVQSLAL	LLFVQSLAL	2	0	0	0	0	LLFVQSLAL	tr_D0A9V0	0.290	2159.89	6.00
23	HLA-A0201	IFLGRSRIVEVRKI	FLGSRIVEV	1	0	0	3	1	FLGRSRIVEV	tr_D0A9V0	0.290	2167.87	6.00
2	HLA-A0201	LMLLVSALKSA	LMLLVSASA	0	0	0	7	2	LMLLVSALKSA	tr_D0A9V0	0.290	2170.36	6.00
216	HLA-A0201	FLVGTCGAF	FLVGTCGAF	0	0	0	0	0	FLVGTCGAF	tr_D0A9V0	0.290	2172.24	6.00

235	HLA-A0201	DHLFQLSYAVRD	HLFQLSYAV	1	0	0	0	0	HLFQLSYAV	tr_D0A9V0	0.289	2186.44	6.00
52	HLA-A0201	KFIPEAILARVE	FIPEAILAV	1	0	0	8	1	FIPEAILARV	tr_D0A9V0	0.289	2196.63	6.00
133	HLA-A0201	AIQVYHPDV	AIQVYHPDV	0	0	0	0	0	AIQVYHPDV	tr_D0A9V0	0.289	2203.84	6.00
542	HLA-A0201	YVARRFTVDILQGL	YVADILQGL	0	0	0	3	5	YVARRFTVDILQGL	tr_D0A9V0	0.288	2204.89	6.00
406	HLA-A0201	SLFKMYAPSS	SLFMYAPSS	0	0	0	3	1	SLFKMYAPSS	tr_D0A9V0	0.288	2212.23	6.00
512	HLA-A0201	ITPSHLSIIMEYVP	ILSIIMEYV	0	0	0	1	4	ITPSHLSIIMEYV	tr_D0A9V0	0.288	2222.57	6.00
560	HLA-A0201	NLGIVHCDV	NLGIVHCDV	0	0	0	0	0	NLGIVHCDV	tr_D0A9V0	0.288	2223.31	6.00
172	HLA-A0201	LISFGAHAAL	LIFGAHAAL	0	0	0	2	1	LISFGAHAAL	tr_D0A9V0	0.287	2240.65	6.50
602	HLA-A0201	DGVMLRGTALYMA	VMLTALYMA	2	0	0	3	2	VMLRGTALYMA	tr_D0A9V0	0.286	2255.88	6.50
236	HLA-A0201	HLFQLSYAVRDGP	HLFQLSYAV	0	0	0	0	0	HLFQLSYAV	tr_D0A9V0	0.286	2257.37	6.50
49	HLA-A0201	VLLKFIPEAILAR	VLLKFIILA	0	0	0	6	3	VLLKFIPEAILA	tr_D0A9V0	0.286	2262.29	6.50
612	HLA-A0201	YMAPEVAAGG	YMAEVAAGG	0	0	0	3	1	YMAPEVAAGG	tr_D0A9V0	0.285	2280.35	6.50
675	HLA-A0201	LALGHLEPVIPASV	ALGHLPASV	1	0	0	5	4	ALGHLEPVIPASV	tr_D0A9V0	0.285	2286.60	6.50
42	HLA-A0201	KLRRQLAVL	KLRRQLAVL	0	0	0	0	0	KLRRQLAVL	tr_D0A9V0	0.285	2287.15	6.50
635	HLA-A0201	SLLEMLLGRP	SLLEMLLGP	0	0	0	8	1	SLLEMLLGRP	tr_D0A9V0	0.285	2289.89	6.50
572	HLA-A0201	NVLLGTDGVCKL	VLLDGVCKL	1	0	0	3	2	VLLGTDGVCKL	tr_D0A9V0	0.285	2292.03	6.50
455	HLA-A0201	EGAFSTIFLGV	GAFSTIFLV	1	0	0	8	1	GAFSTIFLGV	tr_D0A9V0	0.285	2295.35	6.50
50	HLA-A0201	LLKFIPEAI	LLKFIPEAI	0	0	0	0	0	LLKFIPEAI	tr_D0A9V0	0.285	2301.67	6.50
92	HLA-A0201	IMGASISVT	IMGASISVT	0	0	0	0	0	IMGASISVT	tr_D0A9V0	0.283	2327.91	6.50
549	HLA-A0201	VDILQGLNYLHNLG	ILLNYLHNL	2	0	0	2	2	ILQGLNYLHNL	tr_D0A9V0	0.283	2329.25	6.50
507	HLA-A0201	YVSCSITPS	YVSCSITPS	0	0	0	0	0	YVSCSITPS	tr_D0A9V0	0.283	2337.18	6.50
169	HLA-A0201	DRALISFGAHAAL	ALISFHAAL	2	0	0	5	2	ALISFGAHAAL	tr_D0A9V0	0.283	2347.29	6.50
601	HLA-A0201	ADGVMLRGTALYMA	VMLTALYMA	3	0	0	3	2	VMLRGTALYMA	tr_D0A9V0	0.282	2352.45	6.50
603	HLA-A0201	GVMLRGTALYM	VMLGTALYM	1	0	0	3	1	VMLRGTALYM	tr_D0A9V0	0.282	2356.93	6.50
635	HLA-A0201	SLLEMLLGR	SLLEMLLGR	0	0	0	0	0	SLLEMLLGR	tr_D0A9V0	0.282	2373.93	6.50
666	HLA-A0201	NRDLLFVQSLAL	LLFVQSLAL	3	0	0	0	0	LLFVQSLAL	tr_D0A9V0	0.282	2375.91	6.50
132	HLA-A0201	IAIQVYHPDV	IAIQVYHPV	0	0	0	8	1	IAIQVYHPDV	tr_D0A9V0	0.281	2387.45	6.50
701	HLA-A0201	HMNPSMRPSA	HMNPSMPSA	0	0	0	6	1	HMNPSMRPSA	tr_D0A9V0	0.281	2399.68	6.50
519	HLA-A0201	IIMEYVPGGSLHS	IIMEYVPSL	0	0	0	7	2	IIMEYVPGGSL	tr_D0A9V0	0.280	2405.99	6.50
340	HLA-A0201	RQRCRRNDTVPYV	RQNDTVPYV	0	0	0	2	4	RQRCRRNDTVPYV	tr_D0A9V0	0.280	2408.57	6.50
515	HLA-A0201	SHLSIIMEYVP	SLSIIMEYV	0	0	0	1	1	SHLSIIMEYV	tr_D0A9V0	0.280	2413.53	6.50
236	HLA-A0201	HLFQLSYAVRDGPC	HLFQLSYAV	0	0	0	0	0	HLFQLSYAV	tr_D0A9V0	0.280	2422.27	6.50
632	HLA-A0201	FGISLLEML	FGISLLEML	0	0	0	0	0	FGISLLEML	tr_D0A9V0	0.279	2430.82	6.50
352	HLA-A0201	VRVLDNHYCV	VVLDNHYCV	0	0	0	1	1	VRVLDNHYCV	tr_D0A9V0	0.279	2451.98	6.50
168	HLA-A0201	PDRALISFGAHAAL	ALISFHAAL	3	0	0	5	2	ALISFGAHAAL	tr_D0A9V0	0.278	2468.94	6.50
609	HLA-A0201	TALYMAPEVA	ALYMAPEVA	1	0	0	0	0	ALYMAPEVA	tr_D0A9V0	0.277	2501.45	6.50
678	HLA-A0201	GHLEPVIPASV	HLEPVIASV	1	0	0	6	1	HLEPVIPASV	tr_D0A9V0	0.277	2509.69	6.50
190	HLA-A0201	QFAFDLFAKLPLD	FAFDLFLPL	1	0	0	6	2	FAFDLFAKLPL	tr_D0A9V0	0.276	2516.52	6.50
53	HLA-A0201	FIPEAILARVES	FIPEAILAV	0	0	0	8	1	FIPEAILARV	tr_D0A9V0	0.276	2521.45	6.50
547	HLA-A0201	FTVDILQGLNYLH	FILQGLNYL	0	0	0	1	3	FTVDILQGLNYL	tr_D0A9V0	0.276	2523.77	6.50
668	HLA-A0201	DLLFVQSLALGHL	LLFVQSLAL	1	0	0	8	3	LLFVQSLALGHL	tr_D0A9V0	0.276	2528.14	6.50
277	HLA-A0201	STLPIVLFELR	SLPIVLFEL	0	0	0	1	1	STLPIVLFEL	tr_D0A9V0	0.276	2531.45	6.50
91	HLA-A0201	RIMGASISVTWNAG	IMISVTWNA	1	0	0	2	3	IMGASISVTWNA	tr_D0A9V0	0.275	2544.52	6.50
35	HLA-A0201	KIQGHYAKL	KIQGHYAKL	0	0	0	0	0	KIQGHYAKL	tr_D0A9V0	0.275	2558.63	6.50
602	HLA-A0201	DGVMLRGTALYM	VMLGTALYM	2	0	0	3	1	VMLRGTALYM	tr_D0A9V0	0.275	2558.96	6.50
593	HLA-A0201	ASVMARTLADGV	VMARTLAGV	2	0	0	7	1	VMARTLADGV	tr_D0A9V0	0.275	2559.91	6.50
630	HLA-A0201	FSFGISLLEMLL	FSFGISMLL	0	0	0	6	3	FSFGISLLEMLL	tr_D0A9V0	0.274	2576.10	6.50
519	HLA-A0201	IIMEYVPGGS	IIMEYVPGS	0	0	0	7	1	IIMEYVPGGS	tr_D0A9V0	0.274	2577.81	6.50
571	HLA-A0201	HNVLLGTDGVCKL	VLLDGVCKL	2	0	0	3	2	VLLGTDGVCKL	tr_D0A9V0	0.274	2581.80	6.50
301	HLA-A0201	KQFRLGFAAM	KQFLGFAAM	0	0	0	3	1	KQFRLGFAAM	tr_D0A9V0	0.274	2590.39	6.50
517	HLA-A0201	LSIIMEYVPGGSLH	IIMEYVPSL	2	0	0	7	2	IIMEYVPGGSL	tr_D0A9V0	0.273	2606.05	7.00
235	HLA-A0201	DHLFQLSYAVRDG	HLFQLSYAV	1	0	0	0	0	HLFQLSYAV	tr_D0A9V0	0.273	2606.98	7.00
454	HLA-A0201	SEGAFSTIFLGV	GAFSTIFLV	2	0	0	8	1	GAFSTIFLGV	tr_D0A9V0	0.272	2627.54	7.00
409	HLA-A0201	KMYAPSSSSSL	KMYASSSSL	0	0	0	4	2	KMYAPSSSSSL	tr_D0A9V0	0.271	2665.10	7.00

51	HLA-A0201	LKFIPEAILARVE	FIPEAILAV	2	0	0	8	1	FIPEAILARV	tr_D0A9V0	0.271	2670.99	7.00
274	HLA-A0201	LPCSTLPIVLFELR	LLPIVLFEL	0	0	0	1	4	LPCSTLPIVLFEL	tr_D0A9V0	0.271	2674.89	7.00
189	HLA-A0201	CQFAFDLFAKLPLD	FAFDLFLPL	2	0	0	6	2	FAFDLFAKLPL	tr_D0A9V0	0.270	2679.67	7.00
601	HLA-A0201	ADGVMLRGTALYM	VMLGTALYM	3	0	0	3	1	VMLRGTALYM	tr_D0A9V0	0.270	2683.94	7.00
265	HLA-A0201	LAFPVDCVL	LAFPVDCVL	0	0	0	0	0	LAFPVDCVL	tr_D0A9V0	0.270	2687.83	7.00
321	HLA-A0201	HYLPMVKMDM	YLPMVKMDM	1	0	0	0	0	YLPMVKMDM	tr_D0A9V0	0.270	2705.63	7.00
507	HLA-A0201	YVSCSITPSHL	YVSCSITPL	0	0	0	8	2	YVSCSITPSHL	tr_D0A9V0	0.269	2711.93	7.00
0	HLA-A0201	KPLMLLVSALKSA	KLMLLVSAL	0	0	0	1	1	KPLMLLVSAL	tr_D0A9V0	0.269	2716.10	7.00
598	HLA-A0201	RTLADGVMLRGTA	TLADGVMLA	1	0	0	8	3	TLADGVMLRGTA	tr_D0A9V0	0.269	2722.99	7.00
570	HLA-A0201	PHNVLLGTDGVCKL	VLLDGVCKL	3	0	0	3	2	VLLGTDGVCKL	tr_D0A9V0	0.269	2727.15	7.00
592	HLA-A0201	EASVMARTLADGV	VMARTLAGV	3	0	0	7	1	VMARTLADGV	tr_D0A9V0	0.269	2729.39	7.00
453	HLA-A0201	ISEGAFSTIFLGV	GAFSTIFLV	3	0	0	8	1	GAFSTIFLGV	tr_D0A9V0	0.268	2748.44	7.00
600	HLA-A0201	LADGVMLRGTALYM	VMLGTALYM	4	0	0	3	1	VMLRGTALYM	tr_D0A9V0	0.268	2760.87	7.00
630	HLA-A0201	FSFGISLLEML	FSFGISLLL	0	0	0	8	2	FSFGISLLEML	tr_D0A9V0	0.268	2761.14	7.00
50	HLA-A0201	LLKFIPEAILARVE	FIPEAILAV	3	0	0	8	1	FIPEAILARV	tr_D0A9V0	0.267	2782.16	7.00
164	HLA-A0201	EALDPDRALISFGA	ALDPISFGA	1	0	0	4	4	ALDPDRALISFGA	tr_D0A9V0	0.267	2783.70	7.00
667	HLA-A0201	RDLLFVQSLALGHL	LLFVQSLAL	2	0	0	8	3	LLFVQSLALGHL	tr_D0A9V0	0.267	2788.61	7.00
191	HLA-A0201	FAFDLFAKLPLDH	FAFDLFLPL	0	0	0	6	2	FAFDLFAKLPL	tr_D0A9V0	0.267	2796.14	7.00
483	HLA-A0201	DVVAESLEKEMEV	VVAESLMEV	1	0	0	6	3	VVAESLEKEMEV	tr_D0A9V0	0.266	2799.56	7.00
709	HLA-A0201	SASSLLSYA	SASSLLSYA	0	0	0	0	0	SASSLLSYA	tr_D0A9V0	0.266	2825.42	7.00
591	HLA-A0201	SEASVMARTLADGV	VMARTLAGV	4	0	0	7	1	VMARTLADGV	tr_D0A9V0	0.265	2827.50	7.00
664	HLA-A0201	LFNRDLLFVQSLA	FLLFVQSLA	1	0	0	1	3	FNRDLLFVQSLA	tr_D0A9V0	0.265	2833.72	7.00
557	HLA-A0201	YLHNLGIVHCDVK	YLHNLGIVV	0	0	0	8	3	YLHNLGIVHCDV	tr_D0A9V0	0.265	2833.90	7.00
507	HLA-A0201	YVSCSITPSHLSI	YVSCSILSI	0	0	0	6	4	YVSCSITPSHLSI	tr_D0A9V0	0.265	2833.90	7.00
291	HLA-A0201	SSSEVSLSI	SSSEVSLSI	0	0	0	0	0	SSSEVSLSI	tr_D0A9V0	0.265	2835.34	7.00
451	HLA-A0201	KKISEGAFSTIFL	KISEGAFFL	1	0	0	7	3	KISEGAFSTIFL	tr_D0A9V0	0.265	2837.77	7.00
235	HLA-A0201	DHLFQLSYAVRDGP	HLFQLSYAV	1	0	0	0	0	HLFQLSYAV	tr_D0A9V0	0.264	2885.92	7.00
42	HLA-A0201	KLRRQLAVLL	KLRQLAVLL	0	0	0	2	1	KLRRQLAVLL	tr_D0A9V0	0.263	2898.53	7.00
0	HLA-A0201	KPLMLLVSALKS	KLMLLVSAL	0	0	0	1	1	KPLMLLVSAL	tr_D0A9V0	0.262	2928.98	7.00
522	HLA-A0201	EYVPGGSLHSV	YVPGGLHSV	1	0	0	5	1	YVPGGSLHSV	tr_D0A9V0	0.262	2935.30	7.00
222	HLA-A0201	GAFSRNARV	GAFSRNARV	0	0	0	0	0	GAFSRNARV	tr_D0A9V0	0.262	2935.84	7.00
638	HLA-A0201	EMLLGRPPWQWSS	MLLGWQWSS	1	0	0	4	3	MLLGRPPWQWSS	tr_D0A9V0	0.261	2957.16	7.00
189	HLA-A0201	CQFAFDLFAKL	CQFAFDLFL	0	0	0	8	2	CQFAFDLFAKL	tr_D0A9V0	0.261	2980.71	7.50
44	HLA-A0201	RRQLAVLLKFI	RQLAVLLFI	1	0	0	7	1	RQLAVLLKFI	tr_D0A9V0	0.260	3011.93	7.50
677	HLA-A0201	LGHLEPVIPASV	HLEPVIASV	2	0	0	6	1	HLEPVIPASV	tr_D0A9V0	0.260	3013.01	7.50
48	HLA-A0201	AVLLKFIPEAILAR	VLLKFIILA	1	0	0	6	3	VLLKFIPEAILA	tr_D0A9V0	0.259	3049.31	7.50
461	HLA-A0201	IFLGVAEDGAQVA	FLAEDGAQV	1	0	0	2	2	FLGVAEDGAQV	tr_D0A9V0	0.258	3059.62	7.50
172	HLA-A0201	LISFGAHAA	LISFGAHAA	0	0	0	0	0	LISFGAHAA	tr_D0A9V0	0.258	3081.08	7.50
462	HLA-A0201	FLGVAEDGAQVAIK	FLDGAQVAI	0	0	0	2	4	FLGVAEDGAQVAI	tr_D0A9V0	0.257	3089.59	7.50
646	HLA-A0201	WQWSSTAPAG	WQWSSTAPA	0	0	0	0	0	WQWSSTAPA	tr_D0A9V0	0.257	3095.72	7.50
276	HLA-A0201	CSTLPIVLFELR	SLPIVLFEL	1	0	0	1	1	STLPIVLFEL	tr_D0A9V0	0.257	3096.19	7.50
676	HLA-A0201	ALGHLEPVIPAS	ALLEPVIPA	0	0	0	2	2	ALGHLEPVIPA	tr_D0A9V0	0.257	3108.14	7.50
92	HLA-A0201	IMGASISVTWNAGD	IMISVTWNA	0	0	0	2	3	IMGASISVTWNA	tr_D0A9V0	0.256	3120.94	7.50
53	HLA-A0201	FIPEAILARVESK	FIPEAILAV	0	0	0	8	1	FIPEAILARV	tr_D0A9V0	0.256	3122.66	7.50
189	HLA-A0201	CQFAFDLFAK	CQFAFDLFA	0	0	0	0	0	CQFAFDLFA	tr_D0A9V0	0.256	3127.53	7.50
532	HLA-A0201	VIKNFGCVSPYV	VIGCVSPYV	0	0	0	2	3	VIKNFGCVSPYV	tr_D0A9V0	0.256	3141.61	7.50
322	HLA-A0201	YLPMVKMDMQA	YLPMVDMQA	0	0	0	5	2	YLPMVKMDMQA	tr_D0A9V0	0.256	3143.51	7.50
662	HLA-A0201	RSLFNRDLLF	SLFNRDLLF	1	0	0	0	0	SLFNRDLLF	tr_D0A9V0	0.255	3156.84	7.50
198	HLA-A0201	KLPLDHRDSVTML	KLPDSVTML	0	0	0	3	4	KLPLDHRDSVTML	tr_D0A9V0	0.255	3170.19	7.50
675	HLA-A0201	LALGHLEPVI	LLGHLEPVI	0	0	0	1	1	LALGHLEPVI	tr_D0A9V0	0.255	3174.34	7.50
300	HLA-A0201	SKQFRLGFAA	KQFRLGFAA	1	0	0	0	0	KQFRLGFAA	tr_D0A9V0	0.254	3206.79	7.50
515	HLA-A0201	SHLSIIMEYVPG	SLSIIMEYV	0	0	0	1	1	SHLSIIMEYV	tr_D0A9V0	0.254	3209.81	7.50
538	HLA-A0201	CVSPYVARRFTV	CVSPYVFTV	0	0	0	6	3	CVSPYVARRFTV	tr_D0A9V0	0.254	3218.65	7.50
151	HLA-A0201	ALIEVATEHS	ALIEVATES	0	0	0	8	1	ALIEVATEHS	tr_D0A9V0	0.253	3248.42	7.50

1	HLA-A0201	PLMLLVSALKSA	LMLLVSASA	1	0	0	7	2	LMLLVSALKSA	tr_D0A9V0	0.252	3264.34	7.50
188	HLA-A0201	CCQFAFDLFAKL	FAFDLFAKL	3	0	0	0	0	FAFDLFAKL	tr_D0A9V0	0.252	3264.59	7.50
482	HLA-A0201	RDVVAESLEKEMEV	VVAESLMEV	2	0	0	6	3	VVAESLEKEMEV	tr_D0A9V0	0.251	3290.09	7.50
542	HLA-A0201	YVARRFTVDI	YVARFTVDI	0	0	0	3	1	YVARRFTVDI	tr_D0A9V0	0.251	3293.43	7.50
191	HLA-A0201	FAFDLFAKLPLDHR	FAFDLFLPL	0	0	0	6	2	FAFDLFAKLPL	tr_D0A9V0	0.251	3316.82	7.50
42	HLA-A0201	KLRRQLAVLLKFI	RQLAVLLFI	3	0	0	7	1	RQLAVLLKFI	tr_D0A9V0	0.251	3319.73	7.50
43	HLA-A0201	LRRQLAVLLKFI	RQLAVLLFI	2	0	0	7	1	RQLAVLLKFI	tr_D0A9V0	0.251	3323.83	7.50
597	HLA-A0201	ARTLADGVMLRGTA	TLADGVMLA	2	0	0	8	3	TLADGVMLRGTA	tr_D0A9V0	0.250	3336.29	7.50
511	HLA-A0201	SITPSHLSI	SITPSHLSI	0	0	0	0	0	SITPSHLSI	tr_D0A9V0	0.250	3342.18	7.50
430	HLA-A0201	AADVVPLFL	AADVVPLFL	0	0	0	0	0	AADVVPLFL	tr_D0A9V0	0.250	3344.68	7.50
507	HLA-A0201	YVSCSITPSHLSII	YVSCSITPI	0	0	0	8	5	YVSCSITPSHLSII	tr_D0A9V0	0.250	3350.18	7.50
595	HLA-A0201	VMARTLADGVML	VMARTLAML	0	0	0	7	3	VMARTLADGVML	tr_D0A9V0	0.250	3354.21	7.50
460	HLA-A0201	TIFLGVAEDGAQVA	FLAEDGAQV	2	0	0	2	2	FLGVAEDGAQV	tr_D0A9V0	0.249	3368.72	7.50
429	HLA-A0201	NAADVVPLFL	NAADVVLFL	0	0	0	6	1	NAADVVPLFL	tr_D0A9V0	0.248	3401.87	8.00
711	HLA-A0201	SSLLSYAFL	SSLLSYAFL	0	0	0	0	0	SSLLSYAFL	tr_D0A9V0	0.248	3404.04	8.00
187	HLA-A0201	RCCQFAFDLFAKL	FAFDLFAKL	4	0	0	0	0	FAFDLFAKL	tr_D0A9V0	0.248	3408.80	8.00
2	HLA-A0201	LMLLVSALKS	LMLLVSALS	0	0	0	8	1	LMLLVSALKS	tr_D0A9V0	0.248	3415.77	8.00
629	HLA-A0201	IFSFGISLLEMLL	FSFGISMLL	1	0	0	6	3	FSFGISLLEMLL	tr_D0A9V0	0.248	3421.51	8.00
705	HLA-A0201	SMRPSASSL	SMRPSASSL	0	0	0	0	0	SMRPSASSL	tr_D0A9V0	0.248	3422.47	8.00
164	HLA-A0201	EALDPDRALI	ALDPDRALI	1	0	0	0	0	ALDPDRALI	tr_D0A9V0	0.248	3423.54	8.00
339	HLA-A0201	CRQRCRRNDTVPYV	RQNDTVPYV	1	0	0	2	4	RQRCRRNDTVPYV	tr_D0A9V0	0.247	3444.72	8.00
277	HLA-A0201	STLPIVLFELRP	SLPIVLFEL	0	0	0	1	1	STLPIVLFEL	tr_D0A9V0	0.247	3451.03	8.00
216	HLA-A0201	FLVGTCGAFSRNA	FLVGTCGAA	0	0	0	8	4	FLVGTCGAFSRNA	tr_D0A9V0	0.247	3466.07	8.00
130	HLA-A0201	TVIAIQVYHPDV	TVIAIQVYV	0	0	0	8	3	TVIAIQVYHPDV	tr_D0A9V0	0.246	3473.39	8.00
186	HLA-A0201	RRCCQFAFDLFAKL	FAFDLFAKL	5	0	0	0	0	FAFDLFAKL	tr_D0A9V0	0.246	3480.31	8.00
518	HLA-A0201	SIIMEYVPGGSLHS	IIMEYVPSL	1	0	0	7	2	IIMEYVPGGSL	tr_D0A9V0	0.246	3484.95	8.00
704	HLA-A0201	PSMRPSASSLLSYA	SMSSLLSYA	1	0	0	2	4	SMRPSASSLLSYA	tr_D0A9V0	0.246	3492.72	8.00
599	HLA-A0201	TLADGVMLRGTALY	TLADGVMLL	0	0	0	8	4	TLADGVMLRGTAL	tr_D0A9V0	0.246	3495.52	8.00
193	HLA-A0201	FDLFAKLPLD	FLFAKLPLD	0	0	0	1	1	FDLFAKLPLD	tr_D0A9V0	0.246	3509.89	8.00
546	HLA-A0201	RFTVDILQGLNYLH	FILQGLNYL	1	0	0	1	3	FTVDILQGLNYL	tr_D0A9V0	0.245	3513.99	8.00
514	HLA-A0201	PSHLSIIMEYVP	SLSIIMEYV	1	0	0	1	1	SHLSIIMEYV	tr_D0A9V0	0.245	3525.60	8.00
53	HLA-A0201	FIPEAILARVESKN	FIPEAILAV	0	0	0	8	1	FIPEAILARV	tr_D0A9V0	0.245	3528.12	8.00
41	HLA-A0201	AKLRRQLAVLLKFI	RQLAVLLFI	4	0	0	7	1	RQLAVLLKFI	tr_D0A9V0	0.245	3529.00	8.00
52	HLA-A0201	KFIPEAILARVES	FIPEAILAV	1	0	0	8	1	FIPEAILARV	tr_D0A9V0	0.245	3547.38	8.00
513	HLA-A0201	TPSHLSIIMEYVP	SLSIIMEYV	2	0	0	1	1	SHLSIIMEYV	tr_D0A9V0	0.244	3551.26	8.00
629	HLA-A0201	IFSFGISLLEML	FSFGISLLL	1	0	0	8	2	FSFGISLLEML	tr_D0A9V0	0.244	3561.22	8.00
450	HLA-A0201	LKKISEGAFSTIFL	KISEGAFFL	2	0	0	7	3	KISEGAFSTIFL	tr_D0A9V0	0.244	3571.60	8.00
238	HLA-A0201	FQLSYAVRDGPCRI	FQLSYAVRI	0	0	0	7	5	FQLSYAVRDGPCRI	tr_D0A9V0	0.244	3583.41	8.00
521	HLA-A0201	MEYVPGGSLHSV	YVPGGLHSV	2	0	0	5	1	YVPGGSLHSV	tr_D0A9V0	0.244	3585.93	8.00
628	HLA-A0201	DIFSFGISLLEMLL	FSFGISMLL	2	0	0	6	3	FSFGISLLEMLL	tr_D0A9V0	0.243	3591.02	8.00
556	HLA-A0201	NYLHNLGIVHCDVK	YLHNLGIVV	1	0	0	8	3	YLHNLGIVHCDV	tr_D0A9V0	0.243	3594.28	8.00
327	HLA-A0201	KMDMQAKRL	KMDMQAKRL	0	0	0	0	0	KMDMQAKRL	tr_D0A9V0	0.243	3599.73	8.00
531	HLA-A0201	SVIKNFGCV	SVIKNFGCV	0	0	0	0	0	SVIKNFGCV	tr_D0A9V0	0.243	3609.21	8.00
327	HLA-A0201	KMDMQAKRLMRL	KMDMQLMRL	0	0	0	5	3	KMDMQAKRLMRL	tr_D0A9V0	0.243	3616.36	8.00
275	HLA-A0201	PCSTLPIVLFELR	SLPIVLFEL	2	0	0	1	1	STLPIVLFEL	tr_D0A9V0	0.242	3634.80	8.00
190	HLA-A0201	QFAFDLFAKLPLDH	FAFDLFLPL	1	0	0	6	2	FAFDLFAKLPL	tr_D0A9V0	0.242	3635.50	8.00
273	HLA-A0201	LLPCSTLPIVLF	LLPCSTLPV	0	0	0	8	1	LLPCSTLPIV	tr_D0A9V0	0.242	3664.17	8.00
637	HLA-A0201	LEMLLGRPPWQWSS	MLLGWQWSS	2	0	0	4	3	MLLGRPPWQWSS	tr_D0A9V0	0.241	3698.15	8.00
86	HLA-A0201	STAFVRIMGA	STFVRIMGA	0	0	0	2	1	STAFVRIMGA	tr_D0A9V0	0.241	3700.15	8.00
405	HLA-A0201	ESLFKMYAPS	SLFKMYAPS	1	0	0	0	0	SLFKMYAPS	tr_D0A9V0	0.240	3712.26	8.00
515	HLA-A0201	SHLSIIMEYVPGG	SLSIIMEYV	0	0	0	1	1	SHLSIIMEYV	tr_D0A9V0	0.240	3717.81	8.00
0	HLA-A0201	KPLMLLVSALKSAS	KLMLLVSAL	0	0	0	1	1	KPLMLLVSAL	tr_D0A9V0	0.240	3739.92	8.00
628	HLA-A0201	DIFSFGISLLEML	FSFGISLLL	2	0	0	8	2	FSFGISLLEML	tr_D0A9V0	0.239	3752.56	8.00
215	HLA-A0201	EFLVGTCGAF	FLVGTCGAF	1	0	0	0	0	FLVGTCGAF	tr_D0A9V0	0.239	3778.15	8.00

194	HLA-A0201	DLFAKLPLDHRDSV	DLFAKLPLV	0	0	0	8	5	DLFAKLPLDHRDSV	tr_D0A9V0	0.239	3785.68	8.50
260	HLA-A0201	HMQGFLAFP	HMQGFLAFP	0	0	0	0	0	HMQGFLAFP	tr_D0A9V0	0.238	3789.61	8.50
498	HLA-A0201	KLHHPNIVRYVS	KLHHPNIYV	0	0	0	7	2	KLHHPNIVRYV	tr_D0A9V0	0.238	3816.19	8.50
553	HLA-A0201	QGLNYLHNLGI	GLYLHNLGI	1	0	0	2	1	GLNYLHNLGI	tr_D0A9V0	0.238	3824.13	8.50
627	HLA-A0201	SDIFSFGISLLEML	FSFGISLLL	3	0	0	8	2	FSFGISLLEML	tr_D0A9V0	0.236	3873.60	8.50
272	HLA-A0201	VLLPCSTLP	VLLPCSTLP	0	0	0	0	0	VLLPCSTLP	tr_D0A9V0	0.236	3880.90	8.50
215	HLA-A0201	EFLVGTCGAFS	FLVGTCAFS	1	0	0	6	1	FLVGTCGAFS	tr_D0A9V0	0.236	3883.59	8.50
520	HLA-A0201	IMEYVPGGSLHSVI	IMGGSLHSV	0	0	0	2	4	IMEYVPGGSLHSV	tr_D0A9V0	0.236	3883.67	8.50
457	HLA-A0201	AFSTIFLGV	AFSTIFLGV	0	0	0	0	0	AFSTIFLGV	tr_D0A9V0	0.235	3948.15	8.50
277	HLA-A0201	STLPIVLFELRPG	SLPIVLFEL	0	0	0	1	1	STLPIVLFEL	tr_D0A9V0	0.235	3951.06	8.50
171	HLA-A0201	ALISFGAHAALPMH	ALISFALPM	0	0	0	5	4	ALISFGAHAALPM	tr_D0A9V0	0.234	3959.45	8.50
350	HLA-A0201	PYVRVLDNHYCV	YVLDNHYCV	1	0	0	1	2	YVRVLDNHYCV	tr_D0A9V0	0.234	3967.73	8.50
547	HLA-A0201	FTVDILQGLNYLHN	FILQGLNYL	0	0	0	1	3	FTVDILQGLNYL	tr_D0A9V0	0.234	3973.10	8.50
528	HLA-A0201	SLHSVIKNFGCV	SLHSVIGCV	0	0	0	6	3	SLHSVIKNFGCV	tr_D0A9V0	0.234	3976.45	8.50
170	HLA-A0201	RALISFGAHAA	ALISFAHAA	1	0	0	5	1	ALISFGAHAA	tr_D0A9V0	0.233	4008.07	8.50
629	HLA-A0201	IFSFGISLLEM	FSFGILLEM	1	0	0	5	1	FSFGISLLEM	tr_D0A9V0	0.233	4025.63	8.50
515	HLA-A0201	SHLSIIMEYVPGGS	SLSIIMEYV	0	0	0	1	1	SHLSIIMEYV	tr_D0A9V0	0.232	4046.76	8.50
506	HLA-A0201	RYVSCSITPSHL	YVSCSITPL	1	0	0	8	2	YVSCSITPSHL	tr_D0A9V0	0.232	4048.12	8.50
187	HLA-A0201	RCCQFAFDLFA	RQFAFDLFA	0	0	0	1	2	RCCQFAFDLFA	tr_D0A9V0	0.232	4063.61	8.50
628	HLA-A0201	DIFSFGISL	DIFSFGISL	0	0	0	0	0	DIFSFGISL	tr_D0A9V0	0.232	4067.22	8.50
598	HLA-A0201	RTLADGVML	RTLADGVML	0	0	0	0	0	RTLADGVML	tr_D0A9V0	0.232	4071.67	8.50
557	HLA-A0201	YLHNLGIVHCDVKP	YLHNLGIVV	0	0	0	8	3	YLHNLGIVHCDV	tr_D0A9V0	0.231	4087.29	8.50
603	HLA-A0201	GVMLRGTAL	GVMLRGTAL	0	0	0	0	0	GVMLRGTAL	tr_D0A9V0	0.231	4101.25	8.50
506	HLA-A0201	RYVSCSITPSHLSI	YVSCSILSI	1	0	0	6	4	YVSCSITPSHLSI	tr_D0A9V0	0.231	4102.84	8.50
321	HLA-A0201	HYLPMVKMDMQA	YLPMVDMQA	1	0	0	5	2	YLPMVKMDMQA	tr_D0A9V0	0.231	4116.32	8.50
535	HLA-A0201	NFGCVSPYV	NFGCVSPYV	0	0	0	0	0	NFGCVSPYV	tr_D0A9V0	0.231	4116.81	8.50
214	HLA-A0201	NEFLVGTCGAFS	FLVGTCAFS	2	0	0	6	1	FLVGTCGAFS	tr_D0A9V0	0.231	4120.28	8.50
276	HLA-A0201	CSTLPIVLFELRP	SLPIVLFEL	1	0	0	1	1	STLPIVLFEL	tr_D0A9V0	0.230	4158.53	8.50
229	HLA-A0201	RVLFGADHL	RVLFGADHL	0	0	0	0	0	RVLFGADHL	tr_D0A9V0	0.230	4162.81	8.50
408	HLA-A0201	FKMYAPSSSSSL	KMYASSSSL	1	0	0	4	2	KMYAPSSSSSL	tr_D0A9V0	0.230	4163.48	8.50
626	HLA-A0201	QSDIFSFGISL	SIFSFGISL	1	0	0	1	1	SDIFSFGISL	tr_D0A9V0	0.230	4172.41	8.50
322	HLA-A0201	YLPMVKMDMQAKRL	YLPMVKMRL	0	0	0	7	5	YLPMVKMDMQAKRL	tr_D0A9V0	0.229	4175.17	8.50
51	HLA-A0201	LKFIPEAILARVES	FIPEAILAV	2	0	0	8	1	FIPEAILARV	tr_D0A9V0	0.229	4188.65	8.50
186	HLA-A0201	RRCCQFAFDLFA	RQFAFDLFA	0	0	0	1	3	RRCCQFAFDLFA	tr_D0A9V0	0.229	4190.46	8.50
705	HLA-A0201	SMRPSASSLL	SMPSASSLL	0	0	0	2	1	SMRPSASSLL	tr_D0A9V0	0.229	4205.18	8.50
323	HLA-A0201	LPMVKMDMQA	LMVKMDMQA	0	0	0	1	1	LPMVKMDMQA	tr_D0A9V0	0.229	4208.50	8.50
634	HLA-A0201	ISLLEMLLGR	ILLEMLLGR	0	0	0	1	1	ISLLEMLLGR	tr_D0A9V0	0.228	4219.77	8.50
3	HLA-A0201	MLLVSALKS	MLLVSALKS	0	0	0	0	0	MLLVSALKS	tr_D0A9V0	0.228	4223.88	8.50
628	HLA-A0201	DIFSFGISLLEM	FSFGILLEM	2	0	0	5	1	FSFGISLLEM	tr_D0A9V0	0.228	4226.21	8.50
87	HLA-A0201	TAFVRIMGA	TAFVRIMGA	0	0	0	0	0	TAFVRIMGA	tr_D0A9V0	0.228	4245.32	9.00
213	HLA-A0201	TNEFLVGTCGAFS	FLVGTCAFS	3	0	0	6	1	FLVGTCGAFS	tr_D0A9V0	0.227	4266.41	9.00
277	HLA-A0201	STLPIVLFELRPGE	SLPIVLFEL	0	0	0	1	1	STLPIVLFEL	tr_D0A9V0	0.227	4273.24	9.00
52	HLA-A0201	KFIPEAILARVESK	FIPEAILAV	1	0	0	8	1	FIPEAILARV	tr_D0A9V0	0.227	4295.59	9.00
582	HLA-A0201	KLSDFGSTISEAS	KLSDFISEA	0	0	0	5	3	KLSDFGSTISEA	tr_D0A9V0	0.227	4298.38	9.00
631	HLA-A0201	SFGISLLEMLL	FISLLEMLL	1	0	0	1	1	FGISLLEMLL	tr_D0A9V0	0.226	4316.98	9.00
594	HLA-A0201	SVMARTLADGVML	VMARTLVML	1	0	0	6	3	VMARTLADGVML	tr_D0A9V0	0.226	4345.84	9.00
627	HLA-A0201	SDIFSFGISLLEM	FSFGILLEM	3	0	0	5	1	FSFGISLLEM	tr_D0A9V0	0.226	4353.80	9.00
273	HLA-A0201	LLPCSTLPIVLFE	LLPCSTLPV	0	0	0	8	1	LLPCSTLPIV	tr_D0A9V0	0.226	4354.22	9.00
212	HLA-A0201	DTNEFLVGTCGAFS	FLVGTCAFS	4	0	0	6	1	FLVGTCGAFS	tr_D0A9V0	0.226	4355.73	9.00
169	HLA-A0201	DRALISFGAHAA	ALISFAHAA	2	0	0	5	1	ALISFGAHAA	tr_D0A9V0	0.225	4360.35	9.00
580	HLA-A0201	VCKLSDFGSTI	KLSDFGSTI	2	0	0	0	0	KLSDFGSTI	tr_D0A9V0	0.225	4364.32	9.00
278	HLA-A0201	TLPIVLFELRP	TLPIVLFEL	0	0	0	0	0	TLPIVLFEL	tr_D0A9V0	0.224	4409.93	9.00
676	HLA-A0201	ALGHLEPVIPASVD	ALLEPVIPA	0	0	0	2	2	ALGHLEPVIPA	tr_D0A9V0	0.224	4413.56	9.00
626	HLA-A0201	QSDIFSFGISLLEM	FSFGILLEM	4	0	0	5	1	FSFGISLLEM	tr_D0A9V0	0.224	4430.93	9.00

40	HLA-A0201	YAKLRRQLAV	YALRRQLAV	0	0	0	2	1	YAKLRRQLAV	tr_D0A9V0	0.223	4458.82	9.00
675	HLA-A0201	LALGHLEPVIPAS	ALLEPVIPA	1	0	0	2	2	ALGHLEPVIPA	tr_D0A9V0	0.223	4488.01	9.00
612	HLA-A0201	YMAPEVAAGGRCT	YMAPEVAAT	0	0	0	8	4	YMAPEVAAGGRCT	tr_D0A9V0	0.223	4498.26	9.00
329	HLA-A0201	DMQAKRLMRL	MQAKRLMRL	1	0	0	0	0	MQAKRLMRL	tr_D0A9V0	0.223	4501.87	9.00
604	HLA-A0201	VMLRGTALYMAP	VMLTALYMA	0	0	0	3	2	VMLRGTALYMA	tr_D0A9V0	0.222	4519.00	9.00
537	HLA-A0201	GCVSPYVARRFTV	CVSPYVFTV	1	0	0	6	3	CVSPYVARRFTV	tr_D0A9V0	0.222	4532.22	9.00
215	HLA-A0201	EFLVGTCGAFSRNA	FLVGTCGAA	1	0	0	8	4	FLVGTCGAFSRNA	tr_D0A9V0	0.222	4538.60	9.00
552	HLA-A0201	LQGLNYLHNLGI	GLYLHNLGI	2	0	0	2	1	GLNYLHNLGI	tr_D0A9V0	0.221	4552.47	9.00
514	HLA-A0201	PSHLSIIMEYVPG	SLSIIMEYV	1	0	0	1	1	SHLSIIMEYV	tr_D0A9V0	0.221	4556.26	9.00
168	HLA-A0201	PDRALISFGAHAA	ALISFAHAA	3	0	0	5	1	ALISFGAHAA	tr_D0A9V0	0.221	4574.93	9.00
409	HLA-A0201	KMYAPSSSS	KMYAPSSSS	0	0	0	0	0	KMYAPSSSS	tr_D0A9V0	0.221	4575.33	9.00
14	HLA-A0201	NLEIPDNADIFL	NLDNADIFL	0	0	0	2	3	NLEIPDNADIFL	tr_D0A9V0	0.220	4602.69	9.00
513	HLA-A0201	TPSHLSIIMEYVPG	SLSIIMEYV	2	0	0	1	1	SHLSIIMEYV	tr_D0A9V0	0.220	4614.11	9.00
173	HLA-A0201	ISFGAHAAL	ISFGAHAAL	0	0	0	0	0	ISFGAHAAL	tr_D0A9V0	0.220	4630.26	9.00
295	HLA-A0201	VSLSISKQFRL	SLISKQFRL	1	0	0	2	1	SLSISKQFRL	tr_D0A9V0	0.220	4637.98	9.00
559	HLA-A0201	HNLGIVHCDV	HLGIVHCDV	0	0	0	1	1	HNLGIVHCDV	tr_D0A9V0	0.220	4641.90	9.00
231	HLA-A0201	LFGADHLFQL	FGADHLFQL	1	0	0	0	0	FGADHLFQL	tr_D0A9V0	0.219	4660.82	9.50
197	HLA-A0201	AKLPLDHRDSVTML	KLPDSVTML	1	0	0	3	4	KLPLDHRDSVTML	tr_D0A9V0	0.219	4664.91	9.50
579	HLA-A0201	GVCKLSDFGSTI	KLSDFGSTI	3	0	0	0	0	KLSDFGSTI	tr_D0A9V0	0.219	4669.50	9.50
668	HLA-A0201	DLLFVQSLALG	LLFVQSLAL	1	0	0	0	0	LLFVQSLAL	tr_D0A9V0	0.219	4689.65	9.50
167	HLA-A0201	DPDRALISFGAHAA	ALISFAHAA	4	0	0	5	1	ALISFGAHAA	tr_D0A9V0	0.218	4705.92	9.50
609	HLA-A0201	TALYMAPEVAA	ALYMAPVAA	1	0	0	6	1	ALYMAPEVAA	tr_D0A9V0	0.218	4715.86	9.50
129	HLA-A0201	CTVIAIQVYHPDV	TVIAIQVYV	1	0	0	8	3	TVIAIQVYHPDV	tr_D0A9V0	0.218	4716.37	9.50
276	HLA-A0201	CSTLPIVLFELRPG	SLPIVLFEL	1	0	0	1	1	STLPIVLFEL	tr_D0A9V0	0.218	4721.07	9.50
89	HLA-A0201	FVRIMGASI	FVRIMGASI	0	0	0	0	0	FVRIMGASI	tr_D0A9V0	0.218	4723.32	9.50
320	HLA-A0201	AHYLPMVKMDMQA	YLPMVDMQA	2	0	0	5	2	YLPMVKMDMQA	tr_D0A9V0	0.218	4728.02	9.50
46	HLA-A0201	QLAVLLKFIP	QLAVLLKFI	0	0	0	0	0	QLAVLLKFI	tr_D0A9V0	0.218	4736.82	9.50
516	HLA-A0201	HLSIIMEYVPG	HLSIIMEYV	0	0	0	0	0	HLSIIMEYV	tr_D0A9V0	0.218	4749.71	9.50
630	HLA-A0201	FSFGISLLEMLLGR	FLLEMLLGR	0	0	0	1	5	FSFGISLLEMLLGR	tr_D0A9V0	0.217	4799.30	9.50
349	HLA-A0201	VPYVRVLDNHYCV	YVLDNHYCV	2	0	0	1	2	YVRVLDNHYCV	tr_D0A9V0	0.217	4801.95	9.50
50	HLA-A0201	LLKFIPEAILA	LLIPEAILA	0	0	0	2	2	LLKFIPEAILA	tr_D0A9V0	0.216	4818.39	9.50
275	HLA-A0201	PCSTLPIVLFELRP	SLPIVLFEL	2	0	0	1	1	STLPIVLFEL	tr_D0A9V0	0.216	4819.18	9.50
578	HLA-A0201	DGVCKLSDFGSTI	KLSDFGSTI	4	0	0	0	0	KLSDFGSTI	tr_D0A9V0	0.216	4832.86	9.50
545	HLA-A0201	RRFTVDILQGL	FTVDILQGL	2	0	0	0	0	FTVDILQGL	tr_D0A9V0	0.216	4844.58	9.50
667	HLA-A0201	RDLLFVQSLALG	LLFVQSLAL	2	0	0	0	0	LLFVQSLAL	tr_D0A9V0	0.215	4857.13	9.50
505	HLA-A0201	VRYVSCSITPSHL	YVSCSITPL	2	0	0	8	2	YVSCSITPSHL	tr_D0A9V0	0.215	4857.81	9.50
229	HLA-A0201	RVLFGADHLF	RLFGADHLF	0	0	0	1	1	RVLFGADHLF	tr_D0A9V0	0.215	4880.57	9.50
319	HLA-A0201	MAHYLPMVKMDMQA	YLPMVDMQA	3	0	0	5	2	YLPMVKMDMQA	tr_D0A9V0	0.215	4883.21	9.50
259	HLA-A0201	SHMQGFLAFP	SMQGFLAFP	0	0	0	1	1	SHMQGFLAFP	tr_D0A9V0	0.215	4894.43	9.50
456	HLA-A0201	GAFSTIFLGVA	GAFSTIFLV	0	0	0	8	1	GAFSTIFLGV	tr_D0A9V0	0.214	4939.22	9.50
577	HLA-A0201	TDGVCKLSDFGSTI	KLSDFGSTI	5	0	0	0	0	KLSDFGSTI	tr_D0A9V0	0.214	4940.23	9.50
632	HLA-A0201	FGISLLEMLLGR	FLLEMLLGR	0	0	0	1	3	FGISLLEMLLGR	tr_D0A9V0	0.214	4951.42	9.50
669	HLA-A0201	LLFVQSLALGHLE	LLFVQSLAL	0	0	0	8	3	LLFVQSLALGHL	tr_D0A9V0	0.214	4951.90	9.50
210	HLA-A0201	LVDTNEFLVG	LVDTNEFLV	0	0	0	0	0	LVDTNEFLV	tr_D0A9V0	0.213	4970.85	9.50
664	HLA-A0201	LFNRDLLFV	LFNRDLLFV	0	0	0	0	0	LFNRDLLFV	tr_D0A9V0	0.213	4994.14	9.50
624	HLA-A0201	TPQSDIFSFGISL	SIFSFGISL	3	0	0	1	1	SDIFSFGISL	tr_D0A9V0	0.213	5012.38	9.50
488	HLA-A0201	SLEKEMEVASKLH	SLMEVASKL	0	0	0	2	3	SLEKEMEVASKL	tr_D0A9V0	0.212	5019.33	9.50
278	HLA-A0201	TLPIVLFELRPG	TLPIVLFEL	0	0	0	0	0	TLPIVLFEL	tr_D0A9V0	0.212	5028.51	9.50
171	HLA-A0201	ALISFGAHAALP	ALISFHAAL	0	0	0	5	2	ALISFGAHAAL	tr_D0A9V0	0.212	5032.27	9.50
411	HLA-A0201	YAPSSSSSL	YAPSSSSSL	0	0	0	0	0	YAPSSSSSL	tr_D0A9V0	0.212	5049.73	9.50
348	HLA-A0201	TVPYVRVLDNHYCV	YVLDNHYCV	3	0	0	1	2	YVRVLDNHYCV	tr_D0A9V0	0.212	5056.12	9.50
451	HLA-A0201	KKISEGAFST	KISEGAFST	0	0	0	1	1	KKISEGAFST	tr_D0A9V0	0.211	5074.04	9.50
185	HLA-A0201	CRRCCQFAFDLFA	RQFAFDLFA	1	0	0	1	3	RRCCQFAFDLFA	tr_D0A9V0	0.211	5088.28	10.00
544	HLA-A0201	ARRFTVDILQGL	FTVDILQGL	3	0	0	0	0	FTVDILQGL	tr_D0A9V0	0.211	5091.58	10.00

297	HLA-A0201	LSISKQFRL	LSISKQFRL	0	0	0	0	0	LSISKQFRL	tr_D0A9V0	0.211	5095.77	10.00
504	HLA-A0201	IVRYVSCSITPSHL	YVSCSITPL	3	0	0	8	2	YVSCSITPSHL	tr_D0A9V0	0.211	5104.44	10.00
536	HLA-A0201	FGCVSPYVARRFTV	CVSPYVFTV	2	0	0	6	3	CVSPYVARRFTV	tr_D0A9V0	0.211	5123.86	10.00
623	HLA-A0201	CTPQSDIFSFGISL	SIFSFGISL	4	0	0	1	1	SDIFSFGISL	tr_D0A9V0	0.210	5135.01	10.00
550	HLA-A0201	DILQGLNYL	DILQGLNYL	0	0	0	0	0	DILQGLNYL	tr_D0A9V0	0.210	5160.41	10.00
514	HLA-A0201	PSHLSIIMEYVPGG	SLSIIMEYV	1	0	0	1	1	SHLSIIMEYV	tr_D0A9V0	0.210	5177.08	10.00
666	HLA-A0201	NRDLLFVQSLALG	LLFVQSLAL	3	0	0	0	0	LLFVQSLAL	tr_D0A9V0	0.210	5178.48	10.00
407	HLA-A0201	LFKMYAPSSSSSL	KMYASSSSL	2	0	0	4	2	KMYAPSSSSSL	tr_D0A9V0	0.209	5186.95	10.00
166	HLA-A0201	LDPDRALISFGAHA	LLISFGAHA	0	0	0	1	5	LDPDRALISFGAHA	tr_D0A9V0	0.209	5200.27	10.00
128	HLA-A0201	HCTVIAIQVYHPDV	TVIAIQVYV	2	0	0	8	3	TVIAIQVYHPDV	tr_D0A9V0	0.209	5209.84	10.00
527	HLA-A0201	GSLHSVIKNFGCV	SLHSVIGCV	1	0	0	6	3	SLHSVIKNFGCV	tr_D0A9V0	0.209	5211.42	10.00
625	HLA-A0201	PQSDIFSFGISL	SIFSFGISL	2	0	0	1	1	SDIFSFGISL	tr_D0A9V0	0.209	5238.67	10.00
543	HLA-A0201	VARRFTVDILQGL	FTVDILQGL	4	0	0	0	0	FTVDILQGL	tr_D0A9V0	0.208	5242.41	10.00
593	HLA-A0201	ASVMARTLADGVML	VMARTLAML	2	0	0	7	3	VMARTLADGVML	tr_D0A9V0	0.208	5259.11	10.00
516	HLA-A0201	HLSIIMEYVPGG	HLSIIMEYV	0	0	0	0	0	HLSIIMEYV	tr_D0A9V0	0.208	5285.69	10.00
326	HLA-A0201	VKMDMQAKRLMRL	KMDMQLMRL	1	0	0	5	3	KMDMQAKRLMRL	tr_D0A9V0	0.208	5291.65	10.00
608	HLA-A0201	GTALYMAPEVAA	ALYMAPVAA	2	0	0	6	1	ALYMAPEVAA	tr_D0A9V0	0.207	5310.52	10.00
184	HLA-A0201	HCRRCCQFAFDLFA	RQFAFDLFA	2	0	0	1	3	RRCCQFAFDLFA	tr_D0A9V0	0.207	5310.75	10.00
408	HLA-A0201	FKMYAPSSSS	FMYAPSSSS	0	0	0	1	1	FKMYAPSSSS	tr_D0A9V0	0.207	5323.40	10.00
497	HLA-A0201	SKLHHPNIVRYVS	KLHHPNIYV	1	0	0	7	2	KLHHPNIVRYV	tr_D0A9V0	0.207	5343.13	10.00
635	HLA-A0201	SLLEMLLGRPP	SLLEMLLPP	0	0	0	7	2	SLLEMLLGRPP	tr_D0A9V0	0.207	5348.75	10.00
435	HLA-A0201	PLFLTDSNRNV	FLTDSNRNV	2	0	0	0	0	FLTDSNRNV	tr_D0A9V0	0.207	5352.68	10.00
554	HLA-A0201	GLNYLHNLGIV	GLNYLHNLV	0	0	0	8	2	GLNYLHNLGIV	tr_D0A9V0	0.206	5388.13	10.00
294	HLA-A0201	EVSLSISKQFRL	SLISKQFRL	2	0	0	2	1	SLSISKQFRL	tr_D0A9V0	0.206	5398.34	10.00
278	HLA-A0201	TLPIVLFELRPGE	TLPIVLFEL	0	0	0	0	0	TLPIVLFEL	tr_D0A9V0	0.205	5422.87	10.00
451	HLA-A0201	KKISEGAFSTI	KISEGAFSI	1	0	0	8	1	KISEGAFSTI	tr_D0A9V0	0.205	5427.21	10.00
669	HLA-A0201	LLFVQSLALGH	LLFVQSLAL	0	0	0	0	0	LLFVQSLAL	tr_D0A9V0	0.205	5453.70	10.00
3	HLA-A0201	MLLVSALKSASNLE	MLLVSALNL	0	0	0	7	4	MLLVSALKSASNL	tr_D0A9V0	0.205	5466.05	10.00
45	HLA-A0201	RQLAVLLKFIP	RQLAVLLFI	0	0	0	7	1	RQLAVLLKFI	tr_D0A9V0	0.204	5488.63	10.00
607	HLA-A0201	RGTALYMAPEVAA	ALYMAPVAA	3	0	0	6	1	ALYMAPEVAA	tr_D0A9V0	0.204	5510.41	10.00
4	HLA-A0201	LLVSALKSASNL	LLVSALSNL	0	0	0	6	3	LLVSALKSASNL	tr_D0A9V0	0.204	5515.84	10.00
531	HLA-A0201	SVIKNFGCVSPYVA	SVICVSPYV	0	0	0	3	4	SVIKNFGCVSPYV	tr_D0A9V0	0.204	5522.88	10.00
271	HLA-A0201	CVLLPCSTL	CVLLPCSTL	0	0	0	0	0	CVLLPCSTL	tr_D0A9V0	0.203	5533.23	11.00
122	HLA-A0201	NVFNRRHCTVIAI	NVFCTVIAI	0	0	0	3	4	NVFNRRHCTVIAI	tr_D0A9V0	0.203	5545.15	11.00
282	HLA-A0201	VLFELRPGES	VLFELRPES	0	0	0	7	1	VLFELRPGES	tr_D0A9V0	0.203	5552.66	11.00
556	HLA-A0201	NYLHNLGIVHC	YLHNLIVHC	1	0	0	5	1	YLHNLGIVHC	tr_D0A9V0	0.203	5556.81	11.00
433	HLA-A0201	VVPLFLTDSNRNV	FLTDSNRNV	4	0	0	0	0	FLTDSNRNV	tr_D0A9V0	0.203	5563.97	11.00
603	HLA-A0201	GVMLRGTALYMAP	VMLTALYMA	1	0	0	3	2	VMLRGTALYMA	tr_D0A9V0	0.203	5585.02	11.00
315	HLA-A0201	YGEAMAHYLPM	YAMAHYLPM	0	0	0	1	2	YGEAMAHYLPM	tr_D0A9V0	0.203	5586.41	11.00
631	HLA-A0201	SFGISLLEML	FGISLLEML	1	0	0	0	0	FGISLLEML	tr_D0A9V0	0.203	5587.74	11.00
216	HLA-A0201	FLVGTCGAFSR	FLVGTCFSR	0	0	0	6	2	FLVGTCGAFSR	tr_D0A9V0	0.202	5595.18	11.00
1	HLA-A0201	PLMLLVSALK	PLMLLVSAL	0	0	0	0	0	PLMLLVSAL	tr_D0A9V0	0.202	5606.88	11.00
683	HLA-A0201	VIPASVDHEAAAFV	VIPASVAFV	0	0	0	6	5	VIPASVDHEAAAFV	tr_D0A9V0	0.202	5608.81	11.00
605	HLA-A0201	MLRGTALYMAP	MLGTALYMA	0	0	0	2	1	MLRGTALYMA	tr_D0A9V0	0.202	5613.37	11.00
606	HLA-A0201	LRGTALYMAPEVAA	ALYMAPVAA	4	0	0	6	1	ALYMAPEVAA	tr_D0A9V0	0.202	5630.16	11.00
2	HLA-A0201	LMLLVSALK	LMLLVSALK	0	0	0	0	0	LMLLVSALK	tr_D0A9V0	0.202	5645.71	11.00
434	HLA-A0201	VPLFLTDSNRNV	FLTDSNRNV	3	0	0	0	0	FLTDSNRNV	tr_D0A9V0	0.202	5646.63	11.00
679	HLA-A0201	HLEPVIPASVD	HLEPVIASV	0	0	0	6	1	HLEPVIPASV	tr_D0A9V0	0.201	5665.91	11.00
293	HLA-A0201	SEVSLSISKQFRL	SLISKQFRL	3	0	0	2	1	SLSISKQFRL	tr_D0A9V0	0.201	5689.31	11.00
581	HLA-A0201	CKLSDFGSTISEAS	KLSDFISEA	1	0	0	5	3	KLSDFGSTISEA	tr_D0A9V0	0.201	5694.55	11.00
278	HLA-A0201	TLPIVLFELRPGES	TLPIVLFEL	0	0	0	0	0	TLPIVLFEL	tr_D0A9V0	0.201	5694.92	11.00
8	HLA-A0201	ALKSASNLEIPDNA	ALLEIPDNA	0	0	0	2	5	ALKSASNLEIPDNA	tr_D0A9V0	0.200	5715.66	11.00
2	HLA-A0201	LMLLVSALKSAS	LMLLVSALA	0	0	0	8	2	LMLLVSALKSA	tr_D0A9V0	0.200	5743.61	11.00
432	HLA-A0201	DVVPLFLTDSNRNV	FLTDSNRNV	5	0	0	0	0	FLTDSNRNV	tr_D0A9V0	0.200	5748.59	11.00

516	HLA-A0201	HLSIIMEYVPGGS	HLSIIMEYV	0	0	0	0	0	HLSIIMEYV	tr_D0A9V0	0.200	5769.96	11.00
322	HLA-A0201	YLPMVKMDMQ	YLPMVKMDM	0	0	0	0	0	YLPMVKMDM	tr_D0A9V0	0.199	5787.09	11.00
595	HLA-A0201	VMARTLADGVM	VMARTLAGV	0	0	0	7	1	VMARTLADGV	tr_D0A9V0	0.199	5793.55	11.00
449	HLA-A0201	SLKKISEGAFST	SLSEGAFST	0	0	0	2	3	SLKKISEGAFST	tr_D0A9V0	0.199	5794.99	11.00
668	HLA-A0201	DLLFVQSLALGHLE	LLFVQSLAL	1	0	0	8	3	LLFVQSLALGHL	tr_D0A9V0	0.199	5795.12	11.00
526	HLA-A0201	GGSLHSVIKNFGCV	SLHSVIGCV	2	0	0	6	3	SLHSVIKNFGCV	tr_D0A9V0	0.199	5795.62	11.00
498	HLA-A0201	KLHHPNIVRYVSC	KLHNIVRYV	0	0	0	3	2	KLHHPNIVRYV	tr_D0A9V0	0.199	5809.37	11.00
90	HLA-A0201	VRIMGASISVT	VIMGASISV	0	0	0	1	1	VRIMGASISV	tr_D0A9V0	0.199	5815.34	11.00
639	HLA-A0201	MLLGRPPWQ	MLLGRPPWQ	0	0	0	0	0	MLLGRPPWQ	tr_D0A9V0	0.199	5833.55	11.00
602	HLA-A0201	DGVMLRGTALYMAP	VMLTALYMA	2	0	0	3	2	VMLRGTALYMA	tr_D0A9V0	0.199	5835.32	11.00
604	HLA-A0201	VMLRGTALYMAPE	VMLTALYMA	0	0	0	3	2	VMLRGTALYMA	tr_D0A9V0	0.198	5853.85	11.00
292	HLA-A0201	SSEVSLSISKQFRL	SLISKQFRL	4	0	0	2	1	SLSISKQFRL	tr_D0A9V0	0.198	5867.42	11.00
630	HLA-A0201	FSFGISLLEMLLG	FSFGISMLL	0	0	0	6	3	FSFGISLLEMLL	tr_D0A9V0	0.198	5897.33	11.00
147	HLA-A0201	KYHAALIEV	KYHAALIEV	0	0	0	0	0	KYHAALIEV	tr_D0A9V0	0.197	5901.87	11.00
449	HLA-A0201	SLKKISEGA	SLKKISEGA	0	0	0	0	0	SLKKISEGA	tr_D0A9V0	0.197	5919.71	11.00
471	HLA-A0201	QVAIKCIPRL	QVAIKCIPL	0	0	0	8	1	QVAIKCIPRL	tr_D0A9V0	0.197	5925.35	11.00
669	HLA-A0201	LLFVQSLALGHLEP	LLFVQSLAL	0	0	0	8	3	LLFVQSLALGHL	tr_D0A9V0	0.197	5947.96	11.00
461	HLA-A0201	IFLGVAEDGA	FLGVAEDGA	1	0	0	0	0	FLGVAEDGA	tr_D0A9V0	0.197	5958.78	11.00
631	HLA-A0201	SFGISLLEMLLGR	FLLEMLLGR	1	0	0	1	3	FGISLLEMLLGR	tr_D0A9V0	0.196	6012.01	11.00
639	HLA-A0201	MLLGRPPWQW	MLLGRPWQW	0	0	0	5	1	MLLGRPPWQW	tr_D0A9V0	0.195	6034.49	11.00
626	HLA-A0201	QSDIFSFGI	QSDIFSFGI	0	0	0	0	0	QSDIFSFGI	tr_D0A9V0	0.195	6065.32	11.00
14	HLA-A0201	NLEIPDNADI	NLIPDNADI	0	0	0	2	1	NLEIPDNADI	tr_D0A9V0	0.195	6069.00	11.00
660	HLA-A0201	RLRSLFNRDL	RLRSLFNRL	0	0	0	8	1	RLRSLFNRDL	tr_D0A9V0	0.195	6082.34	11.00
518	HLA-A0201	SIIMEYVPGG	IIMEYVPGG	1	0	0	0	0	IIMEYVPGG	tr_D0A9V0	0.195	6086.23	11.00
555	HLA-A0201	LNYLHNLGIVHC	YLHNLIVHC	2	0	0	5	1	YLHNLGIVHC	tr_D0A9V0	0.194	6131.24	11.00
325	HLA-A0201	MVKMDMQAKRLMRL	KMDMQLMRL	2	0	0	5	3	KMDMQAKRLMRL	tr_D0A9V0	0.194	6134.83	11.00
599	HLA-A0201	TLADGVMLR	TLADGVMLR	0	0	0	0	0	TLADGVMLR	tr_D0A9V0	0.194	6140.14	11.00
7	HLA-A0201	SALKSASNLEI	ALSASNLEI	1	0	0	2	1	ALKSASNLEI	tr_D0A9V0	0.194	6144.66	11.00
554	HLA-A0201	GLNYLHNLGIVHC	YLHNLIVHC	3	0	0	5	1	YLHNLGIVHC	tr_D0A9V0	0.194	6145.45	11.00
496	HLA-A0201	ASKLHHPNIVRYVS	KLHNIVRYV	2	0	0	3	2	KLHHPNIVRYV	tr_D0A9V0	0.194	6157.57	11.00
456	HLA-A0201	GAFSTIFLG	GAFSTIFLG	0	0	0	0	0	GAFSTIFLG	tr_D0A9V0	0.193	6190.43	11.00
455	HLA-A0201	EGAFSTIFLGVA	GAFSTIFLV	1	0	0	8	1	GAFSTIFLGV	tr_D0A9V0	0.193	6192.11	11.00
635	HLA-A0201	SLLEMLLGRPPW	SLLEMLLPW	0	0	0	7	3	SLLEMLLGRPPW	tr_D0A9V0	0.193	6203.38	11.00
169	HLA-A0201	DRALISFGAHA	ALISFGAHA	2	0	0	0	0	ALISFGAHA	tr_D0A9V0	0.193	6211.84	11.00
197	HLA-A0201	AKLPLDHRDSV	KLLDHRDSV	1	0	0	2	1	KLPLDHRDSV	tr_D0A9V0	0.192	6234.19	11.00
528	HLA-A0201	SLHSVIKNFGC	SLHSVIFGC	0	0	0	6	2	SLHSVIKNFGC	tr_D0A9V0	0.192	6236.28	11.00
520	HLA-A0201	IMEYVPGGSL	IMEYVPGSL	0	0	0	6	1	IMEYVPGGSL	tr_D0A9V0	0.192	6262.86	11.00
628	HLA-A0201	DIFSFGISLL	DIFSFISLL	0	0	0	5	1	DIFSFGISLL	tr_D0A9V0	0.192	6280.09	11.00
437	HLA-A0201	FLTDSNRNVWT	FLTDSNVWT	0	0	0	5	2	FLTDSNRNVWT	tr_D0A9V0	0.192	6296.35	11.00
639	HLA-A0201	MLLGRPPWQWSST	MLLGRPPST	0	0	0	7	4	MLLGRPPWQWSST	tr_D0A9V0	0.191	6309.45	11.00
553	HLA-A0201	QGLNYLHNLGIVHC	YLHNLIVHC	4	0	0	5	1	YLHNLGIVHC	tr_D0A9V0	0.191	6340.65	11.00
573	HLA-A0201	VLLGTDGVCKLS	VLLDGVCKL	0	0	0	3	2	VLLGTDGVCKL	tr_D0A9V0	0.191	6340.86	11.00
640	HLA-A0201	LLGRPPWQWSSTAP	LLWQWSSTA	0	0	0	2	4	LLGRPPWQWSSTA	tr_D0A9V0	0.191	6341.82	11.00
668	HLA-A0201	DLLFVQSLALGH	LLFVQSLAL	1	0	0	0	0	LLFVQSLAL	tr_D0A9V0	0.190	6401.59	11.00
261	HLA-A0201	MQGFLAFPVDC	MQGFLAFPV	0	0	0	0	0	MQGFLAFPV	tr_D0A9V0	0.190	6420.53	11.00
334	HLA-A0201	RLMRLCRQRC	RLMRLCQRC	0	0	0	6	1	RLMRLCRQRC	tr_D0A9V0	0.189	6437.64	11.00
13	HLA-A0201	SNLEIPDNADIFL	NLDNADIFL	1	0	0	2	3	NLEIPDNADIFL	tr_D0A9V0	0.189	6447.68	12.00
168	HLA-A0201	PDRALISFGAHA	ALISFGAHA	3	0	0	0	0	ALISFGAHA	tr_D0A9V0	0.189	6482.23	12.00
180	HLA-A0201	ALPMHCRRCCQFA	ALPMCCQFA	0	0	0	4	4	ALPMHCRRCCQFA	tr_D0A9V0	0.189	6503.80	12.00
44	HLA-A0201	RRQLAVLLKFIP	RQLAVLLFI	1	0	0	7	1	RQLAVLLKFI	tr_D0A9V0	0.188	6524.17	12.00
679	HLA-A0201	HLEPVIPAS	HLEPVIPAS	0	0	0	0	0	HLEPVIPAS	tr_D0A9V0	0.188	6555.94	12.00
170	HLA-A0201	RALISFGAHAALP	ALISFHAAL	1	0	0	5	2	ALISFGAHAAL	tr_D0A9V0	0.188	6560.55	12.00
165	HLA-A0201	ALDPDRALISFGAH	ALDPISFGA	0	0	0	4	4	ALDPDRALISFGA	tr_D0A9V0	0.188	6561.76	12.00
548	HLA-A0201	TVDILQGLNYL	VILQGLNYL	1	0	0	1	1	VDILQGLNYL	tr_D0A9V0	0.187	6590.36	12.00

667	HLA-A0201	RDLLFVQSLALGH	LLFVQSLAL	2	0	0	0	0	LLFVQSLAL	tr_D0A9V0	0.187	6620.66	12.00
316	HLA-A0201	GEAMAHYLPM	GAMAHYLPM	0	0	0	1	1	GEAMAHYLPM	tr_D0A9V0	0.187	6624.18	12.00
450	HLA-A0201	LKKISEGAFSTI	KISEGAFTI	2	0	0	7	1	KISEGAFSTI	tr_D0A9V0	0.187	6632.78	12.00
167	HLA-A0201	DPDRALISFGAHA	ALISFGAHA	4	0	0	0	0	ALISFGAHA	tr_D0A9V0	0.187	6640.47	12.00
344	HLA-A0201	RRNDTVPYV	RRNDTVPYV	0	0	0	0	0	RRNDTVPYV	tr_D0A9V0	0.186	6659.17	12.00
452	HLA-A0201	KISEGAFSTIFLG	KISEGAFFL	0	0	0	7	3	KISEGAFSTIFL	tr_D0A9V0	0.186	6666.89	12.00
599	HLA-A0201	TLADGVMLRGT	TLADGVMLT	0	0	0	8	2	TLADGVMLRGT	tr_D0A9V0	0.186	6669.41	12.00
710	HLA-A0201	ASSLLSYAFL	ASSLLSYFL	0	0	0	7	1	ASSLLSYAFL	tr_D0A9V0	0.186	6695.80	12.00
215	HLA-A0201	EFLVGTCGAFSR	FLVGTCFSR	1	0	0	6	2	FLVGTCGAFSR	tr_D0A9V0	0.186	6715.97	12.00
454	HLA-A0201	SEGAFSTIFLGVA	GAFSTIFLV	2	0	0	8	1	GAFSTIFLGV	tr_D0A9V0	0.185	6727.18	12.00
634	HLA-A0201	ISLLEMLLGRPP	SLLEMLLPP	1	0	0	7	2	SLLEMLLGRPP	tr_D0A9V0	0.185	6785.88	12.00
456	HLA-A0201	GAFSTIFLGVAE	GAFSTIFLV	0	0	0	8	1	GAFSTIFLGV	tr_D0A9V0	0.185	6791.75	12.00
487	HLA-A0201	ESLEKEMEVASKLH	SLMEVASKL	1	0	0	2	3	SLEKEMEVASKL	tr_D0A9V0	0.184	6807.57	12.00
611	HLA-A0201	LYMAPEVAAGGRCT	YMAPEVAAT	1	0	0	8	4	YMAPEVAAGGRCT	tr_D0A9V0	0.184	6822.32	12.00
463	HLA-A0201	LGVAEDGAQV	LVAEDGAQV	0	0	0	1	1	LGVAEDGAQV	tr_D0A9V0	0.184	6825.05	12.00
45	HLA-A0201	RQLAVLLKF	RQLAVLLKF	0	0	0	0	0	RQLAVLLKF	tr_D0A9V0	0.184	6836.58	12.00
449	HLA-A0201	SLKKISEGAFSTI	KISEGAFSI	3	0	0	8	1	KISEGAFSTI	tr_D0A9V0	0.184	6845.54	12.00
498	HLA-A0201	KLHHPNIVRYVSCS	KLHHPNIYV	0	0	0	7	2	KLHHPNIVRYV	tr_D0A9V0	0.184	6851.46	12.00
265	HLA-A0201	LAFPVDCVLLPC	FVDCVLLPC	2	0	0	1	1	FPVDCVLLPC	tr_D0A9V0	0.184	6864.89	12.00
632	HLA-A0201	FGISLLEMLLGRPP	SLLEMLLPP	3	0	0	7	2	SLLEMLLGRPP	tr_D0A9V0	0.183	6881.26	12.00
479	HLA-A0201	RLRRDVVAESL	RLDVVAESL	0	0	0	2	2	RLRRDVVAESL	tr_D0A9V0	0.183	6881.78	12.00
94	HLA-A0201	GASISVTWNA	GAISVTWNA	0	0	0	2	1	GASISVTWNA	tr_D0A9V0	0.183	6884.68	12.00
611	HLA-A0201	LYMAPEVAAG	YMAPEVAAG	1	0	0	0	0	YMAPEVAAG	tr_D0A9V0	0.183	6902.73	12.00
453	HLA-A0201	ISEGAFSTIFLGVA	GAFSTIFLV	3	0	0	8	1	GAFSTIFLGV	tr_D0A9V0	0.183	6909.68	12.00
504	HLA-A0201	IVRYVSCSI	IVRYVSCSI	0	0	0	0	0	IVRYVSCSI	tr_D0A9V0	0.183	6914.99	12.00
668	HLA-A0201	DLLFVQSLA	DLLFVQSLA	0	0	0	0	0	DLLFVQSLA	tr_D0A9V0	0.182	6941.60	12.00
458	HLA-A0201	FSTIFLGVA	FSTIFLGVA	0	0	0	0	0	FSTIFLGVA	tr_D0A9V0	0.182	6949.42	12.00
169	HLA-A0201	DRALISFGAHAALP	ALISFHAAL	2	0	0	5	2	ALISFGAHAAL	tr_D0A9V0	0.182	6967.79	12.00
646	HLA-A0201	WQWSSTAPAGSDA	WQWSSTAPA	0	0	0	8	4	WQWSSTAPAGSDA	tr_D0A9V0	0.182	6987.94	12.00
553	HLA-A0201	QGLNYLHNLGIV	GLNYLHNLV	1	0	0	8	2	GLNYLHNLGIV	tr_D0A9V0	0.182	6995.51	12.00
666	HLA-A0201	NRDLLFVQSLALGH	LLFVQSLAL	3	0	0	0	0	LLFVQSLAL	tr_D0A9V0	0.182	6996.87	12.00
214	HLA-A0201	NEFLVGTCGAFSR	FLVGTCFSR	2	0	0	6	2	FLVGTCGAFSR	tr_D0A9V0	0.181	7003.31	12.00
43	HLA-A0201	LRRQLAVLLKFIP	RQLAVLLFI	2	0	0	7	1	RQLAVLLKFI	tr_D0A9V0	0.181	7022.43	12.00
45	HLA-A0201	RQLAVLLKFIPE	RQLAVLLFI	0	0	0	7	1	RQLAVLLKFI	tr_D0A9V0	0.181	7036.12	12.00
189	HLA-A0201	CQFAFDLFAKLP	CQFAFDLFL	0	0	0	8	2	CQFAFDLFAKL	tr_D0A9V0	0.181	7067.63	12.00
42	HLA-A0201	KLRRQLAVLLKFIP	RQLAVLLFI	3	0	0	7	1	RQLAVLLKFI	tr_D0A9V0	0.181	7069.24	12.00
486	HLA-A0201	AESLEKEMEV	ASLEKEMEV	0	0	0	1	1	AESLEKEMEV	tr_D0A9V0	0.181	7073.60	12.00
448	HLA-A0201	RSLKKISEGAFSTI	KISEGAFTI	4	0	0	7	1	KISEGAFSTI	tr_D0A9V0	0.180	7127.53	12.00
603	HLA-A0201	GVMLRGTALYMAPE	VMLTALYMA	1	0	0	3	2	VMLRGTALYMA	tr_D0A9V0	0.180	7138.34	12.00
586	HLA-A0201	FGSTISEASVMA	FISEASVMA	0	0	0	1	3	FGSTISEASVMA	tr_D0A9V0	0.180	7144.52	12.00
629	HLA-A0201	IFSFGISLLEMLLG	FSFGISMLL	1	0	0	6	3	FSFGISLLEMLL	tr_D0A9V0	0.180	7152.02	12.00
213	HLA-A0201	TNEFLVGTCGAFSR	FLVGTCFSR	3	0	0	6	2	FLVGTCGAFSR	tr_D0A9V0	0.179	7178.61	12.00
672	HLA-A0201	VQSLALGHL	VQSLALGHL	0	0	0	0	0	VQSLALGHL	tr_D0A9V0	0.179	7182.89	12.00
187	HLA-A0201	RCCQFAFDL	RCCQFAFDL	0	0	0	0	0	RCCQFAFDL	tr_D0A9V0	0.179	7195.26	12.00
41	HLA-A0201	AKLRRQLAVL	KLRRQLAVL	1	0	0	0	0	KLRRQLAVL	tr_D0A9V0	0.179	7196.35	12.00
6	HLA-A0201	VSALKSASNLEI	ALSASNLEI	2	0	0	2	1	ALKSASNLEI	tr_D0A9V0	0.179	7198.91	12.00
196	HLA-A0201	FAKLPLDHRDSV	KLLDHRDSV	2	0	0	2	1	KLPLDHRDSV	tr_D0A9V0	0.179	7199.30	12.00
484	HLA-A0201	VVAESLEKEM	VVAESLEEM	0	0	0	7	1	VVAESLEKEM	tr_D0A9V0	0.179	7217.16	12.00
267	HLA-A0201	FPVDCVLLPCSTL	FVLLPCSTL	0	0	0	1	4	FPVDCVLLPCSTL	tr_D0A9V0	0.179	7221.93	12.00
607	HLA-A0201	RGTALYMAPEV	GTALYMAPV	1	0	0	8	1	GTALYMAPEV	tr_D0A9V0	0.179	7235.54	12.00
629	HLA-A0201	IFSFGISLL	IFSFGISLL	0	0	0	0	0	IFSFGISLL	tr_D0A9V0	0.178	7257.41	12.00
3	HLA-A0201	MLLVSALKSAS	MLLVSALSA	0	0	0	7	1	MLLVSALKSA	tr_D0A9V0	0.178	7263.15	12.00
266	HLA-A0201	AFPVDCVLLPC	FVDCVLLPC	1	0	0	1	1	FPVDCVLLPC	tr_D0A9V0	0.178	7282.74	12.00
678	HLA-A0201	GHLEPVIPASVD	HLEPVIASV	1	0	0	6	1	HLEPVIPASV	tr_D0A9V0	0.178	7293.70	12.00

594	HLA-A0201	SVMARTLADGVM	VMARTLAGV	1	0	0	7	1	VMARTLADGV	tr_D0A9V0	0.178	7295.12	12.00
641	HLA-A0201	LGRPPWQWSSTAPA	LQWSSTAPA	0	0	0	1	5	LGRPPWQWSSTAPA	tr_D0A9V0	0.178	7298.99	12.00
121	HLA-A0201	PNVFNRRHCTVIAI	NVFCTVIAI	1	0	0	3	4	NVFNRRHCTVIAI	tr_D0A9V0	0.178	7317.97	12.00
354	HLA-A0201	VLDNHYCVNS	VLDNYCVNS	0	0	0	4	1	VLDNHYCVNS	tr_D0A9V0	0.178	7325.10	12.00
588	HLA-A0201	STISEASVMA	SISEASVMA	0	0	0	1	1	STISEASVMA	tr_D0A9V0	0.177	7376.00	12.00
512	HLA-A0201	ITPSHLSII	ITPSHLSII	0	0	0	0	0	ITPSHLSII	tr_D0A9V0	0.177	7381.35	12.00
91	HLA-A0201	RIMGASISVTW	RIMGASISV	0	0	0	0	0	RIMGASISV	tr_D0A9V0	0.177	7389.74	13.00
605	HLA-A0201	MLRGTALYMAPE	MLGTALYMA	0	0	0	2	1	MLRGTALYMA	tr_D0A9V0	0.177	7394.14	13.00
633	HLA-A0201	GISLLEMLLGRPP	SLLEMLLPP	2	0	0	7	2	SLLEMLLGRPP	tr_D0A9V0	0.177	7402.62	13.00
42	HLA-A0201	KLRRQLAVLLKF	KLLAVLLKF	0	0	0	2	3	KLRRQLAVLLKF	tr_D0A9V0	0.177	7405.27	13.00
1	HLA-A0201	PLMLLVSALKSAS	LMLLVSALA	1	0	0	8	2	LMLLVSALKSA	tr_D0A9V0	0.176	7424.84	13.00
12	HLA-A0201	ASNLEIPDNADIFL	NLDNADIFL	2	0	0	2	3	NLEIPDNADIFL	tr_D0A9V0	0.176	7439.96	13.00
632	HLA-A0201	FGISLLEMLLG	FISLLEMLL	0	0	0	1	1	FGISLLEMLL	tr_D0A9V0	0.175	7498.15	13.00
586	HLA-A0201	FGSTISEASV	FGSTISASV	0	0	0	6	1	FGSTISEASV	tr_D0A9V0	0.175	7500.01	13.00
686	HLA-A0201	ASVDHEAAAFV	SVDEAAAFV	1	0	0	3	1	SVDHEAAAFV	tr_D0A9V0	0.175	7500.42	13.00
230	HLA-A0201	VLFGADHLFQ	VLFGADLFQ	0	0	0	6	1	VLFGADHLFQ	tr_D0A9V0	0.175	7508.37	13.00
5	HLA-A0201	LVSALKSASNLEI	ALSASNLEI	3	0	0	2	1	ALKSASNLEI	tr_D0A9V0	0.175	7518.78	13.00
527	HLA-A0201	GSLHSVIKNFGC	SLHSVIFGC	1	0	0	6	2	SLHSVIKNFGC	tr_D0A9V0	0.175	7521.95	13.00
503	HLA-A0201	NIVRYVSCSI	NIVYVSCSI	0	0	0	3	1	NIVRYVSCSI	tr_D0A9V0	0.175	7529.28	13.00
488	HLA-A0201	SLEKEMEVASKLHH	SLMEVASKL	0	0	0	2	3	SLEKEMEVASKL	tr_D0A9V0	0.175	7552.05	13.00
195	HLA-A0201	LFAKLPLDHRDSV	KLLDHRDSV	3	0	0	2	1	KLPLDHRDSV	tr_D0A9V0	0.175	7555.07	13.00
2	HLA-A0201	LMLLVSALKSASN	LMLLVSALA	0	0	0	8	2	LMLLVSALKSA	tr_D0A9V0	0.175	7559.81	13.00
165	HLA-A0201	ALDPDRALIS	ALDPDRALI	0	0	0	0	0	ALDPDRALI	tr_D0A9V0	0.174	7568.40	13.00
634	HLA-A0201	ISLLEMLLGRPPW	SLLEMLLPW	1	0	0	7	3	SLLEMLLGRPPW	tr_D0A9V0	0.174	7575.62	13.00
488	HLA-A0201	SLEKEMEVA	SLEKEMEVA	0	0	0	0	0	SLEKEMEVA	tr_D0A9V0	0.174	7594.17	13.00
314	HLA-A0201	RYGEAMAHYLPM	YAMAHYLPM	1	0	0	1	2	YGEAMAHYLPM	tr_D0A9V0	0.173	7655.29	13.00
456	HLA-A0201	GAFSTIFLGVAED	GAFSTIFLV	0	0	0	8	1	GAFSTIFLGV	tr_D0A9V0	0.173	7656.12	13.00
705	HLA-A0201	SMRPSASSLLSYAF	SMSSLLSYA	0	0	0	2	4	SMRPSASSLLSYA	tr_D0A9V0	0.173	7670.55	13.00
122	HLA-A0201	NVFNRRHCTV	NVFNRHCTV	0	0	0	4	1	NVFNRRHCTV	tr_D0A9V0	0.173	7694.07	13.00
572	HLA-A0201	NVLLGTDGVCKLS	VLLDGVCKL	1	0	0	3	2	VLLGTDGVCKL	tr_D0A9V0	0.173	7703.74	13.00
497	HLA-A0201	SKLHHPNIVRYVSC	KLHNIVRYV	1	0	0	3	2	KLHHPNIVRYV	tr_D0A9V0	0.173	7705.15	13.00
188	HLA-A0201	CCQFAFDLFAKLP	CQFAFDLFL	1	0	0	8	2	CQFAFDLFAKL	tr_D0A9V0	0.173	7707.82	13.00
267	HLA-A0201	FPVDCVLLPCSTLP	FLLPCSTLP	0	0	0	1	5	FPVDCVLLPCSTLP	tr_D0A9V0	0.172	7786.78	13.00
599	HLA-A0201	TLADGVMLRG	TLADGVMLG	0	0	0	8	1	TLADGVMLRG	tr_D0A9V0	0.172	7797.23	13.00
208	HLA-A0201	TMLVDTNEF	TMLVDTNEF	0	0	0	0	0	TMLVDTNEF	tr_D0A9V0	0.172	7800.95	13.00
523	HLA-A0201	YVPGGSLHSVI	YVPGGLHSV	0	0	0	5	1	YVPGGSLHSV	tr_D0A9V0	0.172	7803.65	13.00
222	HLA-A0201	GAFSRNARVLFGA	GAFSVLFGA	0	0	0	4	4	GAFSRNARVLFGA	tr_D0A9V0	0.172	7805.00	13.00
679	HLA-A0201	HLEPVIPASVDH	HLEPVIASV	0	0	0	6	1	HLEPVIPASV	tr_D0A9V0	0.171	7832.75	13.00
610	HLA-A0201	ALYMAPEVAAGG	ALYEVAAGG	0	0	0	3	3	ALYMAPEVAAGG	tr_D0A9V0	0.171	7858.47	13.00
552	HLA-A0201	LQGLNYLHNLGIV	GLNYLHNLV	2	0	0	8	2	GLNYLHNLGIV	tr_D0A9V0	0.171	7901.19	13.00
131	HLA-A0201	VIAIQVYHPDV	VIAIQVYHV	0	0	0	8	2	VIAIQVYHPDV	tr_D0A9V0	0.170	7946.88	13.00
187	HLA-A0201	RCCQFAFDLFAKLP	CQFAFDLFL	2	0	0	8	2	CQFAFDLFAKL	tr_D0A9V0	0.169	7995.18	13.00
632	HLA-A0201	FGISLLEMLLGRP	SLLEMLLGP	3	0	0	8	1	SLLEMLLGRP	tr_D0A9V0	0.169	8014.32	13.00
216	HLA-A0201	FLVGTCGAFSRNAR	FLVGTCGAA	0	0	0	8	4	FLVGTCGAFSRNA	tr_D0A9V0	0.169	8044.73	13.00
53	HLA-A0201	FIPEAILAR	FIPEAILAR	0	0	0	0	0	FIPEAILAR	tr_D0A9V0	0.169	8072.45	13.00
179	HLA-A0201	AALPMHCRRCCQFA	ALPMCCQFA	1	0	0	4	4	ALPMHCRRCCQFA	tr_D0A9V0	0.168	8077.43	13.00
216	HLA-A0201	FLVGTCGAFSRN	FLVGTCAFS	0	0	0	6	1	FLVGTCGAFS	tr_D0A9V0	0.168	8078.05	13.00
526	HLA-A0201	GGSLHSVIKNFGC	SLHSVIFGC	2	0	0	6	2	SLHSVIKNFGC	tr_D0A9V0	0.168	8091.08	13.00
627	HLA-A0201	SDIFSFGISLL	SIFSFGISL	0	0	0	1	1	SDIFSFGISL	tr_D0A9V0	0.168	8096.42	13.00
633	HLA-A0201	GISLLEMLLGRPPW	SLLEMLLPW	2	0	0	7	3	SLLEMLLGRPPW	tr_D0A9V0	0.168	8115.28	13.00
448	HLA-A0201	RSLKKISEGAFST	SLSEGAFST	1	0	0	2	3	SLKKISEGAFST	tr_D0A9V0	0.168	8133.03	13.00
353	HLA-A0201	RVLDNHYCVN	RVLDNHYCV	0	0	0	0	0	RVLDNHYCV	tr_D0A9V0	0.168	8136.03	13.00
610	HLA-A0201	ALYMAPEVAAG	ALYMAPVAA	0	0	0	6	1	ALYMAPEVAA	tr_D0A9V0	0.168	8154.09	13.00
44	HLA-A0201	RRQLAVLLKFIPE	RQLAVLLFI	1	0	0	7	1	RQLAVLLKFI	tr_D0A9V0	0.168	8156.65	13.00

455	HLA-A0201	EGAFSTIFLGVAE	GAFSTIFLV	1	0	0	8	1	GAFSTIFLGV	tr_D0A9V0	0.167	8172.64	13.00
635	HLA-A0201	SLLEMLLGRPPWQW	SLLEMLWQW	0	0	0	6	5	SLLEMLLGRPPWQW	tr_D0A9V0	0.167	8174.59	13.00
484	HLA-A0201	VVAESLEKEMEVA	VVAESLMEV	0	0	0	6	3	VVAESLEKEMEV	tr_D0A9V0	0.167	8180.69	13.00
456	HLA-A0201	GAFSTIFLGVAEDG	GAFSTIFLV	0	0	0	8	1	GAFSTIFLGV	tr_D0A9V0	0.167	8193.45	13.00
571	HLA-A0201	HNVLLGTDGVCKLS	VLLDGVCKL	2	0	0	3	2	VLLGTDGVCKL	tr_D0A9V0	0.167	8201.52	13.00
677	HLA-A0201	LGHLEPVIPASVD	HLEPVIASV	2	0	0	6	1	HLEPVIPASV	tr_D0A9V0	0.167	8235.40	13.00
683	HLA-A0201	VIPASVDHEA	VIASVDHEA	0	0	0	2	1	VIPASVDHEA	tr_D0A9V0	0.167	8245.30	13.00
525	HLA-A0201	PGGSLHSVIKNFGC	SLHSVIFGC	3	0	0	6	2	SLHSVIKNFGC	tr_D0A9V0	0.166	8282.94	13.00
151	HLA-A0201	ALIEVATEH	ALIEVATEH	0	0	0	0	0	ALIEVATEH	tr_D0A9V0	0.166	8295.95	13.00
606	HLA-A0201	LRGTALYMAPEV	GTALYMAPV	2	0	0	8	1	GTALYMAPEV	tr_D0A9V0	0.166	8320.67	13.00
643	HLA-A0201	RPPWQWSSTAPA	RQWSSTAPA	0	0	0	1	3	RPPWQWSSTAPA	tr_D0A9V0	0.166	8324.54	13.00
528	HLA-A0201	SLHSVIKNFG	SLHSVINFG	0	0	0	6	1	SLHSVIKNFG	tr_D0A9V0	0.165	8342.93	14.00
679	HLA-A0201	HLEPVIPASVDHEA	HLEPVIASV	0	0	0	6	1	HLEPVIPASV	tr_D0A9V0	0.165	8344.92	14.00
193	HLA-A0201	FDLFAKLPL	FDLFAKLPL	0	0	0	0	0	FDLFAKLPL	tr_D0A9V0	0.165	8359.47	14.00
631	HLA-A0201	SFGISLLEMLLGRP	SLLEMLLGP	4	0	0	8	1	SLLEMLLGRP	tr_D0A9V0	0.164	8514.46	14.00
606	HLA-A0201	LRGTALYMAPEVA	LLYMAPEVA	0	0	0	1	4	LRGTALYMAPEVA	tr_D0A9V0	0.164	8522.02	14.00
41	HLA-A0201	AKLRRQLAVLLKF	KLLAVLLKF	1	0	0	2	3	KLRRQLAVLLKF	tr_D0A9V0	0.163	8541.78	14.00
418	HLA-A0201	SLDGSGCAEGA	SLDGCAEGA	0	0	0	3	2	SLDGSGCAEGA	tr_D0A9V0	0.163	8549.18	14.00
560	HLA-A0201	NLGIVHCDVKPHNV	NLGIVPHNV	0	0	0	5	5	NLGIVHCDVKPHNV	tr_D0A9V0	0.163	8577.53	14.00
192	HLA-A0201	AFDLFAKLPL	AFDLFALPL	0	0	0	6	1	AFDLFAKLPL	tr_D0A9V0	0.163	8583.19	14.00
595	HLA-A0201	VMARTLADGVMLR	VMARTLAGV	0	0	0	7	1	VMARTLADGV	tr_D0A9V0	0.163	8594.44	14.00
638	HLA-A0201	EMLLGRPPWQWSST	MLLGRPPST	1	0	0	7	4	MLLGRPPWQWSST	tr_D0A9V0	0.163	8600.11	14.00
180	HLA-A0201	ALPMHCRRCCQFAF	ALPCCQFAF	0	0	0	3	5	ALPMHCRRCCQFAF	tr_D0A9V0	0.163	8611.93	14.00
90	HLA-A0201	VRIMGASISVTW	VIMGASISV	0	0	0	1	1	VRIMGASISV	tr_D0A9V0	0.162	8655.09	14.00
313	HLA-A0201	GRYGEAMAHYLPM	YAMAHYLPM	2	0	0	1	2	YGEAMAHYLPM	tr_D0A9V0	0.162	8657.44	14.00
351	HLA-A0201	YVRVLDNHYCVN	YVLDNHYCV	0	0	0	1	2	YVRVLDNHYCV	tr_D0A9V0	0.162	8694.80	14.00
43	HLA-A0201	LRRQLAVLLKFIPE	RQLAVLLFI	2	0	0	7	1	RQLAVLLKFI	tr_D0A9V0	0.162	8694.89	14.00
114	HLA-A0201	CGIFKEKPNV	GIFKEKPNV	1	0	0	0	0	GIFKEKPNV	tr_D0A9V0	0.162	8708.83	14.00
593	HLA-A0201	ASVMARTLADGVM	VMARTLAGV	2	0	0	7	1	VMARTLADGV	tr_D0A9V0	0.161	8719.38	14.00
322	HLA-A0201	YLPMVKMDMQAK	YLPMVDMQA	0	0	0	5	2	YLPMVKMDMQA	tr_D0A9V0	0.161	8722.12	14.00
451	HLA-A0201	KKISEGAFSTIFLG	KISEGAFFL	1	0	0	7	3	KISEGAFSTIFL	tr_D0A9V0	0.161	8758.34	14.00
454	HLA-A0201	SEGAFSTIFLGVAE	GAFSTIFLV	2	0	0	8	1	GAFSTIFLGV	tr_D0A9V0	0.161	8760.33	14.00
528	HLA-A0201	SLHSVIKNFGCVS	SLHSVIGCV	0	0	0	6	3	SLHSVIKNFGCV	tr_D0A9V0	0.161	8784.44	14.00
91	HLA-A0201	RIMGASISVTWN	RIMGASISV	0	0	0	0	0	RIMGASISV	tr_D0A9V0	0.160	8809.66	14.00
266	HLA-A0201	AFPVDCVLLPCSTL	FVLLPCSTL	1	0	0	1	4	FPVDCVLLPCSTL	tr_D0A9V0	0.160	8844.33	14.00
557	HLA-A0201	YLHNLGIVHCD	YLHNLIVHC	0	0	0	5	1	YLHNLGIVHC	tr_D0A9V0	0.160	8857.73	14.00
40	HLA-A0201	YAKLRRQLAVLLKF	KLLAVLLKF	2	0	0	2	3	KLRRQLAVLLKF	tr_D0A9V0	0.160	8883.84	14.00
573	HLA-A0201	VLLGTDGVCKLSD	VLLDGVCKL	0	0	0	3	2	VLLGTDGVCKL	tr_D0A9V0	0.160	8897.50	14.00
679	HLA-A0201	HLEPVIPASVDHE	HLEPVIASV	0	0	0	6	1	HLEPVIPASV	tr_D0A9V0	0.159	8909.35	14.00
598	HLA-A0201	RTLADGVMLRGT	TLADGVMLT	1	0	0	8	2	TLADGVMLRGT	tr_D0A9V0	0.159	8943.16	14.00
340	HLA-A0201	RQRCRRNDTVPYVR	RQNDTVPYV	0	0	0	2	4	RQRCRRNDTVPYV	tr_D0A9V0	0.159	8967.67	14.00
585	HLA-A0201	DFGSTISEASVMA	FISEASVMA	1	0	0	1	3	FGSTISEASVMA	tr_D0A9V0	0.159	8969.22	14.00
312	HLA-A0201	QGRYGEAMAHYLPM	YAMAHYLPM	3	0	0	1	2	YGEAMAHYLPM	tr_D0A9V0	0.158	8999.07	14.00
418	HLA-A0201	SLDGSGCAEGANAA	SLAEGANAA	0	0	0	2	5	SLDGSGCAEGANAA	tr_D0A9V0	0.158	9000.13	14.00
631	HLA-A0201	SFGISLLEMLLG	FISLLEMLL	1	0	0	1	1	FGISLLEMLL	tr_D0A9V0	0.158	9032.43	14.00
635	HLA-A0201	SLLEMLLGRPPWQ	SLLEMLLWQ	0	0	0	7	4	SLLEMLLGRPPWQ	tr_D0A9V0	0.158	9036.14	14.00
271	HLA-A0201	CVLLPCSTLP	VLLPCSTLP	1	0	0	0	0	VLLPCSTLP	tr_D0A9V0	0.158	9062.28	14.00
670	HLA-A0201	LFVQSLALGHL	FVQSLALHL	1	0	0	7	1	FVQSLALGHL	tr_D0A9V0	0.158	9076.71	14.00
455	HLA-A0201	EGAFSTIFLGVAED	GAFSTIFLV	1	0	0	8	1	GAFSTIFLGV	tr_D0A9V0	0.158	9078.48	14.00
436	HLA-A0201	LFLTDSNRNVWT	FLTDSNVWT	1	0	0	5	2	FLTDSNRNVWT	tr_D0A9V0	0.158	9081.52	14.00
558	HLA-A0201	LHNLGIVHCDV	LLGIVHCDV	0	0	0	1	2	LHNLGIVHCDV	tr_D0A9V0	0.157	9107.60	14.00
592	HLA-A0201	EASVMARTLADGVM	VMARTLAGV	3	0	0	7	1	VMARTLADGV	tr_D0A9V0	0.157	9109.67	14.00
447	HLA-A0201	TRSLKKISEGAFST	SLSEGAFST	2	0	0	2	3	SLKKISEGAFST	tr_D0A9V0	0.157	9119.04	14.00
661	HLA-A0201	LRSLFNRDLLF	LLFNRDLLF	0	0	0	1	2	LRSLFNRDLLF	tr_D0A9V0	0.157	9120.61	14.00

298	HLA-A0201	SISKQFRLGFAA	SIFRLGFAA	0	0	0	2	3	SISKQFRLGFAA	tr_D0A9V0	0.157	9152.35	14.00
478	HLA-A0201	PRLRRDVVAESL	RLDVVAESL	1	0	0	2	2	RLRRDVVAESL	tr_D0A9V0	0.157	9183.00	14.00
1	HLA-A0201	PLMLLVSALKSASN	LMLLVSALA	1	0	0	8	2	LMLLVSALKSA	tr_D0A9V0	0.156	9216.84	14.00
221	HLA-A0201	CGAFSRNARVLFGA	GAFSVLFGA	1	0	0	4	4	GAFSRNARVLFGA	tr_D0A9V0	0.156	9217.24	14.00
538	HLA-A0201	CVSPYVARRFTVD	CVSPYVFTV	0	0	0	6	3	CVSPYVARRFTV	tr_D0A9V0	0.156	9255.92	15.00
200	HLA-A0201	PLDHRDSVTMLV	PLDSVTMLV	0	0	0	2	3	PLDHRDSVTMLV	tr_D0A9V0	0.155	9310.35	15.00
4	HLA-A0201	LLVSALKSAS	LLVSALKSA	0	0	0	0	0	LLVSALKSA	tr_D0A9V0	0.155	9320.13	15.00
607	HLA-A0201	RGTALYMAPEVA	RLYMAPEVA	0	0	0	1	3	RGTALYMAPEVA	tr_D0A9V0	0.155	9338.50	15.00
40	HLA-A0201	YAKLRRQLAVL	YLRRQLAVL	0	0	0	1	2	YAKLRRQLAVL	tr_D0A9V0	0.155	9349.52	15.00
427	HLA-A0201	GANAADVVPL	GAAADVVPL	0	0	0	2	1	GANAADVVPL	tr_D0A9V0	0.155	9361.06	15.00
460	HLA-A0201	TIFLGVAEDGA	TIFLGVAEA	0	0	0	8	2	TIFLGVAEDGA	tr_D0A9V0	0.155	9371.91	15.00
187	HLA-A0201	RCCQFAFDLFAK	RQFAFDLFA	0	0	0	1	2	RCCQFAFDLFA	tr_D0A9V0	0.155	9372.31	15.00
3	HLA-A0201	MLLVSALKSASN	MLLVSALSA	0	0	0	7	1	MLLVSALKSA	tr_D0A9V0	0.155	9385.30	15.00
554	HLA-A0201	GLNYLHNLGIVHCD	GLYLHNLGI	0	0	0	2	1	GLNYLHNLGI	tr_D0A9V0	0.155	9390.58	15.00
642	HLA-A0201	GRPPWQWSSTAPA	GQWSSTAPA	0	0	0	1	4	GRPPWQWSSTAPA	tr_D0A9V0	0.154	9415.82	15.00
609	HLA-A0201	TALYMAPEVAAGG	ALYEVAAGG	1	0	0	3	3	ALYMAPEVAAGG	tr_D0A9V0	0.154	9434.88	15.00
232	HLA-A0201	FGADHLFQLSYA	FGADHLFYA	0	0	0	7	3	FGADHLFQLSYA	tr_D0A9V0	0.154	9444.08	15.00
608	HLA-A0201	GTALYMAPEVA	ALYMAPEVA	2	0	0	0	0	ALYMAPEVA	tr_D0A9V0	0.154	9444.38	15.00
215	HLA-A0201	EFLVGTCGAFSRN	FLVGTCAFS	1	0	0	6	1	FLVGTCGAFS	tr_D0A9V0	0.154	9444.49	15.00
571	HLA-A0201	HNVLLGTDGV	HVLLGTDGV	0	0	0	1	1	HNVLLGTDGV	tr_D0A9V0	0.154	9444.49	15.00
92	HLA-A0201	IMGASISVTW	IMASISVTW	0	0	0	2	1	IMGASISVTW	tr_D0A9V0	0.154	9446.73	15.00
327	HLA-A0201	KMDMQAKRLM	KMMQAKRLM	0	0	0	2	1	KMDMQAKRLM	tr_D0A9V0	0.154	9458.60	15.00
633	HLA-A0201	GISLLEMLLG	GISLLEMLL	0	0	0	0	0	GISLLEMLL	tr_D0A9V0	0.154	9487.19	15.00
0	HLA-A0201	KPLMLLVSA	KPLMLLVSA	0	0	0	0	0	KPLMLLVSA	tr_D0A9V0	0.154	9496.95	15.00
685	HLA-A0201	PASVDHEAAAFV	SVDEAAAFV	2	0	0	3	1	SVDHEAAAFV	tr_D0A9V0	0.153	9528.96	15.00
573	HLA-A0201	VLLGTDGVCKLSDF	VLLDGVCKL	0	0	0	3	2	VLLGTDGVCKL	tr_D0A9V0	0.153	9531.54	15.00
658	HLA-A0201	ATRLRSLFNRDLLF	ALFNRDLLF	0	0	0	1	5	ATRLRSLFNRDLLF	tr_D0A9V0	0.153	9547.44	15.00
290	HLA-A0201	ESSSEVSLSI	SSSEVSLSI	1	0	0	0	0	SSSEVSLSI	tr_D0A9V0	0.153	9590.81	15.00
186	HLA-A0201	RRCCQFAFDLFAK	RQFAFDLFA	0	0	0	1	3	RRCCQFAFDLFA	tr_D0A9V0	0.153	9599.12	15.00
430	HLA-A0201	AADVVPLFLT	AADVVLFLT	0	0	0	5	1	AADVVPLFLT	tr_D0A9V0	0.152	9637.62	15.00
528	HLA-A0201	SLHSVIKNF	SLHSVIKNF	0	0	0	0	0	SLHSVIKNF	tr_D0A9V0	0.152	9661.22	15.00
660	HLA-A0201	RLRSLFNRDLLF	LLFNRDLLF	1	0	0	1	2	LRSLFNRDLLF	tr_D0A9V0	0.152	9665.82	15.00
626	HLA-A0201	QSDIFSFGISLL	SIFSFGISL	1	0	0	1	1	SDIFSFGISL	tr_D0A9V0	0.152	9666.03	15.00
88	HLA-A0201	AFVRIMGASI	FVRIMGASI	1	0	0	0	0	FVRIMGASI	tr_D0A9V0	0.152	9670.64	15.00
609	HLA-A0201	TALYMAPEVAAG	ALYMAPVAA	1	0	0	6	1	ALYMAPEVAA	tr_D0A9V0	0.152	9706.06	15.00
678	HLA-A0201	GHLEPVIPASVDH	HLEPVIASV	1	0	0	6	1	HLEPVIPASV	tr_D0A9V0	0.151	9717.52	15.00
666	HLA-A0201	NRDLLFVQSLA	RLLFVQSLA	1	0	0	1	1	RDLLFVQSLA	tr_D0A9V0	0.151	9740.25	15.00
507	HLA-A0201	YVSCSITPSHLS	YVSCSITPL	0	0	0	8	2	YVSCSITPSHL	tr_D0A9V0	0.151	9750.38	15.00
232	HLA-A0201	FGADHLFQLS	FGADHLFQL	0	0	0	0	0	FGADHLFQL	tr_D0A9V0	0.151	9771.29	15.00
604	HLA-A0201	VMLRGTALY	VMLRGTALY	0	0	0	0	0	VMLRGTALY	tr_D0A9V0	0.151	9771.81	15.00
214	HLA-A0201	NEFLVGTCGAFSRN	FLVGTCAFS	2	0	0	6	1	FLVGTCGAFS	tr_D0A9V0	0.151	9787.16	15.00
175	HLA-A0201	FGAHAALPM	FGAHAALPM	0	0	0	0	0	FGAHAALPM	tr_D0A9V0	0.151	9809.42	15.00
560	HLA-A0201	NLGIVHCDVK	NLGIVHCDV	0	0	0	0	0	NLGIVHCDV	tr_D0A9V0	0.151	9810.48	15.00
190	HLA-A0201	QFAFDLFAKLP	FAFDLFAKL	1	0	0	0	0	FAFDLFAKL	tr_D0A9V0	0.150	9819.30	15.00
630	HLA-A0201	FSFGISLLE	FSFGISLLE	0	0	0	0	0	FSFGISLLE	tr_D0A9V0	0.150	9844.72	15.00
151	HLA-A0201	ALIEVATEHSGC	ALIEVATEC	0	0	0	8	3	ALIEVATEHSGC	tr_D0A9V0	0.150	9845.58	15.00
684	HLA-A0201	IPASVDHEAAAFV	SVDEAAAFV	3	0	0	3	1	SVDHEAAAFV	tr_D0A9V0	0.150	9889.56	15.00
128	HLA-A0201	HCTVIAIQV	HCTVIAIQV	0	0	0	0	0	HCTVIAIQV	tr_D0A9V0	0.150	9891.38	15.00
676	HLA-A0201	ALGHLEPVIP	ALGHLEPVI	0	0	0	0	0	ALGHLEPVI	tr_D0A9V0	0.150	9894.59	15.00
330	HLA-A0201	MQAKRLMRLC	MQAKRLMRL	0	0	0	0	0	MQAKRLMRL	tr_D0A9V0	0.150	9894.91	15.00
644	HLA-A0201	PPWQWSSTAPA	WQWSSTAPA	2	0	0	0	0	WQWSSTAPA	tr_D0A9V0	0.150	9908.41	15.00
556	HLA-A0201	NYLHNLGIVHCD	YLHNLIVHC	1	0	0	5	1	YLHNLGIVHC	tr_D0A9V0	0.149	9938.05	15.00
584	HLA-A0201	SDFGSTISEASVMA	FISEASVMA	2	0	0	1	3	FGSTISEASVMA	tr_D0A9V0	0.149	9945.47	15.00
595	HLA-A0201	VMARTLADGVMLRG	VMARTLAGV	0	0	0	7	1	VMARTLADGV	tr_D0A9V0	0.149	9948.38	15.00

317	HLA-A0201	EAMAHYLPM	EAMAHYLPM	0	0	0	0	0	EAMAHYLPM	tr_D0A9V0	0.149	9964.53	15.00
477	HLA-A0201	IPRLRRDVVAESL	RLDVVAESL	2	0	0	2	2	RLRRDVVAESL	tr_D0A9V0	0.149	10015.76	15.00
296	HLA-A0201	SLSISKQFRLG	SLISKQFRL	0	0	0	2	1	SLSISKQFRL	tr_D0A9V0	0.149	10016.53	15.00
224	HLA-A0201	FSRNARVLFGA	FSARVLFGA	0	0	0	2	2	FSRNARVLFGA	tr_D0A9V0	0.148	10035.94	15.00
612	HLA-A0201	YMAPEVAAGGRC	YMAPEVAAC	0	0	0	8	3	YMAPEVAAGGRC	tr_D0A9V0	0.148	10085.69	15.00
594	HLA-A0201	SVMARTLADGVMLR	VMARTLVML	1	0	0	6	3	VMARTLADGVML	tr_D0A9V0	0.148	10091.15	15.00
608	HLA-A0201	GTALYMAPEVAAGG	ALYMAPVAA	2	0	0	6	1	ALYMAPEVAA	tr_D0A9V0	0.148	10112.90	15.00
479	HLA-A0201	RLRRDVVAES	RLRDVVAES	0	0	0	2	1	RLRRDVVAES	tr_D0A9V0	0.148	10113.23	15.00
300	HLA-A0201	SKQFRLGFAAM	KQFLGFAAM	1	0	0	3	1	KQFRLGFAAM	tr_D0A9V0	0.148	10117.94	15.00
242	HLA-A0201	YAVRDGPCRI	YAVDGPCRI	0	0	0	3	1	YAVRDGPCRI	tr_D0A9V0	0.148	10133.49	15.00
483	HLA-A0201	DVVAESLEKEMEVA	VVAESLMEV	1	0	0	6	3	VVAESLEKEMEV	tr_D0A9V0	0.147	10154.35	15.00
265	HLA-A0201	LAFPVDCVLLPCS	LAFPVCVLL	0	0	0	5	1	LAFPVDCVLL	tr_D0A9V0	0.147	10162.48	15.00
597	HLA-A0201	ARTLADGVMLRGT	TLADGVMLT	2	0	0	8	2	TLADGVMLRGT	tr_D0A9V0	0.147	10167.65	15.00
90	HLA-A0201	VRIMGASISVTWN	VIMGASISV	0	0	0	1	1	VRIMGASISV	tr_D0A9V0	0.147	10168.86	15.00
522	HLA-A0201	EYVPGGSLHSVI	YVPGGLHSV	1	0	0	5	1	YVPGGSLHSV	tr_D0A9V0	0.147	10194.64	16.00
145	HLA-A0201	STKYHAALIEV	STHAALIEV	0	0	0	2	2	STKYHAALIEV	tr_D0A9V0	0.147	10197.29	16.00
634	HLA-A0201	ISLLEMLLGRP	SLLEMLLGP	1	0	0	8	1	SLLEMLLGRP	tr_D0A9V0	0.147	10239.18	16.00
299	HLA-A0201	ISKQFRLGFAA	IQFRLGFAA	0	0	0	1	2	ISKQFRLGFAA	tr_D0A9V0	0.146	10264.47	16.00
476	HLA-A0201	CIPRLRRDVVAESL	RLDVVAESL	3	0	0	2	2	RLRRDVVAESL	tr_D0A9V0	0.146	10268.58	16.00
321	HLA-A0201	HYLPMVKMDMQAK	YLPMVDMQA	1	0	0	5	2	YLPMVKMDMQA	tr_D0A9V0	0.146	10316.58	16.00
659	HLA-A0201	TRLRSLFNRDLLF	LLFNRDLLF	2	0	0	1	2	LRSLFNRDLLF	tr_D0A9V0	0.146	10323.17	16.00
572	HLA-A0201	NVLLGTDGVCKLSD	VLLDGVCKL	1	0	0	3	2	VLLGTDGVCKL	tr_D0A9V0	0.145	10366.82	16.00
265	HLA-A0201	LAFPVDCVLLP	LAFPVCVLL	0	0	0	5	1	LAFPVDCVLL	tr_D0A9V0	0.145	10373.56	16.00
185	HLA-A0201	CRRCCQFAFDLFAK	CQFAFDLFA	0	0	0	1	4	CRRCCQFAFDLFA	tr_D0A9V0	0.145	10386.70	16.00
608	HLA-A0201	GTALYMAPEVAAG	ALYMAPVAA	2	0	0	6	1	ALYMAPEVAA	tr_D0A9V0	0.145	10402.90	16.00
146	HLA-A0201	TKYHAALIEV	TKYAALIEV	0	0	0	3	1	TKYHAALIEV	tr_D0A9V0	0.145	10409.42	16.00
50	HLA-A0201	LLKFIPEAILAR	LLIPEAILA	0	0	0	2	2	LLKFIPEAILA	tr_D0A9V0	0.145	10418.21	16.00
270	HLA-A0201	DCVLLPCSTL	CVLLPCSTL	1	0	0	0	0	CVLLPCSTL	tr_D0A9V0	0.145	10427.23	16.00
296	HLA-A0201	SLSISKQFRLGFA	SLSISLGFA	0	0	0	5	4	SLSISKQFRLGFA	tr_D0A9V0	0.145	10434.57	16.00
188	HLA-A0201	CCQFAFDLFAK	CQFAFDLFA	0	0	0	1	1	CCQFAFDLFA	tr_D0A9V0	0.145	10436.15	16.00
457	HLA-A0201	AFSTIFLGVA	FSTIFLGVA	1	0	0	0	0	FSTIFLGVA	tr_D0A9V0	0.145	10441.35	16.00
409	HLA-A0201	KMYAPSSSSSLD	KMYASSSSL	0	0	0	4	2	KMYAPSSSSSL	tr_D0A9V0	0.145	10445.52	16.00
527	HLA-A0201	GSLHSVIKNFGCVS	SLHSVIGCV	1	0	0	6	3	SLHSVIKNFGCV	tr_D0A9V0	0.145	10451.74	16.00
554	HLA-A0201	GLNYLHNLGIVH	GLYLHNLGI	0	0	0	2	1	GLNYLHNLGI	tr_D0A9V0	0.145	10456.83	16.00
130	HLA-A0201	TVIAIQVYHPDVW	TVIAIQVYV	0	0	0	8	3	TVIAIQVYHPDV	tr_D0A9V0	0.144	10514.58	16.00
596	HLA-A0201	MARTLADGVMLRGT	TLADGVMLT	3	0	0	8	2	TLADGVMLRGT	tr_D0A9V0	0.144	10523.23	16.00
627	HLA-A0201	SDIFSFGISLLE	SIFSFGISL	0	0	0	1	1	SDIFSFGISL	tr_D0A9V0	0.144	10528.13	16.00
667	HLA-A0201	RDLLFVQSL	RDLLFVQSL	0	0	0	0	0	RDLLFVQSL	tr_D0A9V0	0.144	10544.20	16.00
532	HLA-A0201	VIKNFGCVSPYVA	VIGCVSPYV	0	0	0	2	3	VIKNFGCVSPYV	tr_D0A9V0	0.144	10554.71	16.00
267	HLA-A0201	FPVDCVLLPCS	FVDCVLLPC	0	0	0	1	1	FPVDCVLLPC	tr_D0A9V0	0.144	10576.77	16.00
645	HLA-A0201	PWQWSSTAPAGSDA	WQWSSTAPA	1	0	0	8	4	WQWSSTAPAGSDA	tr_D0A9V0	0.143	10585.47	16.00
41	HLA-A0201	AKLRRQLAVLL	KLRQLAVLL	1	0	0	2	1	KLRRQLAVLL	tr_D0A9V0	0.143	10592.00	16.00
634	HLA-A0201	ISLLEMLLGRPPWQ	SLLEMLLWQ	1	0	0	7	4	SLLEMLLGRPPWQ	tr_D0A9V0	0.143	10613.22	16.00
528	HLA-A0201	SLHSVIKNFGCVSP	SLHSVIGCV	0	0	0	6	3	SLHSVIKNFGCV	tr_D0A9V0	0.143	10630.23	16.00
555	HLA-A0201	LNYLHNLGIVHCD	YLHNLIVHC	2	0	0	5	1	YLHNLGIVHC	tr_D0A9V0	0.143	10632.65	16.00
214	HLA-A0201	NEFLVGTCGAF	FLVGTCGAF	2	0	0	0	0	FLVGTCGAF	tr_D0A9V0	0.143	10648.77	16.00
607	HLA-A0201	RGTALYMAPEVAAG	ALYMAPVAA	3	0	0	6	1	ALYMAPEVAA	tr_D0A9V0	0.143	10650.26	16.00
327	HLA-A0201	KMDMQAKRLMRLC	KMDMRLMRL	0	0	0	4	3	KMDMQAKRLMRL	tr_D0A9V0	0.143	10671.14	16.00
437	HLA-A0201	FLTDSNRNVWTR	FLTDSVWTR	0	0	0	5	3	FLTDSNRNVWTR	tr_D0A9V0	0.143	10682.23	16.00
660	HLA-A0201	RLRSLFNRDLL	RLRSLFNLL	0	0	0	7	2	RLRSLFNRDLL	tr_D0A9V0	0.143	10691.83	16.00
677	HLA-A0201	LGHLEPVIPASVDH	HLEPVIASV	2	0	0	6	1	HLEPVIPASV	tr_D0A9V0	0.142	10765.43	16.00
297	HLA-A0201	LSISKQFRLGFAA	SIFRLGFAA	1	0	0	2	3	SISKQFRLGFAA	tr_D0A9V0	0.141	10819.14	16.00
299	HLA-A0201	ISKQFRLGFAAM	KQFLGFAAM	2	0	0	3	1	KQFRLGFAAM	tr_D0A9V0	0.141	10823.58	16.00
533	HLA-A0201	IKNFGCVSPYV	KNFGCVPYV	1	0	0	6	1	KNFGCVSPYV	tr_D0A9V0	0.141	10853.02	16.00

213	HLA-A0201	TNEFLVGTCGAF	FLVGTCGAF	3	0	0	0	0	FLVGTCGAF	tr_D0A9V0	0.141	10855.25	16.00
678	HLA-A0201	GHLEPVIPASVDHE	HLEPVIASV	1	0	0	6	1	HLEPVIPASV	tr_D0A9V0	0.141	10888.54	16.00
418	HLA-A0201	SLDGSGCAEG	SLDGGCAEG	0	0	0	4	1	SLDGSGCAEG	tr_D0A9V0	0.141	10900.09	16.00
319	HLA-A0201	MAHYLPMVKMDM	YLPMVKMDM	3	0	0	0	0	YLPMVKMDM	tr_D0A9V0	0.141	10925.83	16.00
610	HLA-A0201	ALYMAPEVAAGGRC	ALYMAPVAA	0	0	0	6	1	ALYMAPEVAA	tr_D0A9V0	0.140	10938.14	16.00
238	HLA-A0201	FQLSYAVRDGPC	FQLSYAVPC	0	0	0	7	3	FQLSYAVRDGPC	tr_D0A9V0	0.140	10942.52	16.00
212	HLA-A0201	DTNEFLVGTCGAF	FLVGTCGAF	4	0	0	0	0	FLVGTCGAF	tr_D0A9V0	0.140	10951.99	16.00
127	HLA-A0201	RHCTVIAIQV	RHCTVIAIV	0	0	0	8	1	RHCTVIAIQV	tr_D0A9V0	0.140	10958.15	16.00
417	HLA-A0201	SSLDGSGCAEGA	SLDGCAEGA	1	0	0	3	2	SLDGSGCAEGA	tr_D0A9V0	0.140	10966.22	16.00
52	HLA-A0201	KFIPEAILA	KFIPEAILA	0	0	0	0	0	KFIPEAILA	tr_D0A9V0	0.140	10977.85	16.00
624	HLA-A0201	TPQSDIFSFGISLL	SIFSFGISL	3	0	0	1	1	SDIFSFGISL	tr_D0A9V0	0.140	11013.66	16.00
633	HLA-A0201	GISLLEMLLGRP	SLLEMLLGP	2	0	0	8	1	SLLEMLLGRP	tr_D0A9V0	0.140	11015.09	16.00
211	HLA-A0201	VDTNEFLVGTCGAF	FLVGTCGAF	5	0	0	0	0	FLVGTCGAF	tr_D0A9V0	0.140	11018.19	16.00
122	HLA-A0201	NVFNRRHCTVIAIQ	NVFCTVIAI	0	0	0	3	4	NVFNRRHCTVIAI	tr_D0A9V0	0.140	11029.29	16.00
470	HLA-A0201	AQVAIKCIPRL	AQVACIPRL	0	0	0	4	2	AQVAIKCIPRL	tr_D0A9V0	0.140	11037.52	16.00
320	HLA-A0201	AHYLPMVKMDM	YLPMVKMDM	2	0	0	0	0	YLPMVKMDM	tr_D0A9V0	0.140	11044.69	16.00
36	HLA-A0201	IQGHYAKLRRQLAV	IQGHYALAV	0	0	0	6	5	IQGHYAKLRRQLAV	tr_D0A9V0	0.139	11066.22	16.00
625	HLA-A0201	PQSDIFSFGISLL	SIFSFGISL	2	0	0	1	1	SDIFSFGISL	tr_D0A9V0	0.139	11069.33	16.00
678	HLA-A0201	GHLEPVIPAS	GLEPVIPAS	0	0	0	1	1	GHLEPVIPAS	tr_D0A9V0	0.139	11078.56	16.00
298	HLA-A0201	SISKQFRLGFAAM	KQFLGFAAM	3	0	0	3	1	KQFRLGFAAM	tr_D0A9V0	0.139	11080.72	16.00
40	HLA-A0201	YAKLRRQLAVLL	KLRQLAVLL	2	0	0	2	1	KLRRQLAVLL	tr_D0A9V0	0.139	11085.87	16.00
265	HLA-A0201	LAFPVDCVLLPCST	LAFPVCVLL	0	0	0	5	1	LAFPVDCVLL	tr_D0A9V0	0.139	11093.31	16.00
435	HLA-A0201	PLFLTDSNRNVWT	FLTDSNVWT	2	0	0	5	2	FLTDSNRNVWT	tr_D0A9V0	0.139	11120.36	16.00
693	HLA-A0201	AAFVRACCHM	AAFVACCHM	0	0	0	4	1	AAFVRACCHM	tr_D0A9V0	0.139	11147.58	17.00
124	HLA-A0201	FNRRHCTVIAIQV	FCTVIAIQV	0	0	0	1	4	FNRRHCTVIAIQV	tr_D0A9V0	0.139	11157.47	17.00
315	HLA-A0201	YGEAMAHYL	YGEAMAHYL	0	0	0	0	0	YGEAMAHYL	tr_D0A9V0	0.139	11171.13	17.00
39	HLA-A0201	HYAKLRRQLAVL	YLRRQLAVL	1	0	0	1	2	YAKLRRQLAVL	tr_D0A9V0	0.138	11174.39	17.00
320	HLA-A0201	AHYLPMVKMDMQAK	YLPMVDMQA	2	0	0	5	2	YLPMVKMDMQA	tr_D0A9V0	0.138	11189.75	17.00
523	HLA-A0201	YVPGGSLHSVIK	YVPGGLHSV	0	0	0	5	1	YVPGGSLHSV	tr_D0A9V0	0.138	11191.09	17.00
444	HLA-A0201	NVWTRSLKKI	NVWTRSLKI	0	0	0	7	1	NVWTRSLKKI	tr_D0A9V0	0.138	11203.33	17.00
186	HLA-A0201	RRCCQFAFDL	RCCQFAFDL	0	0	0	1	1	RRCCQFAFDL	tr_D0A9V0	0.138	11240.35	17.00
405	HLA-A0201	ESLFKMYAPSS	SLFMYAPSS	1	0	0	3	1	SLFKMYAPSS	tr_D0A9V0	0.138	11243.40	17.00
551	HLA-A0201	ILQGLNYLH	ILQGLNYLH	0	0	0	0	0	ILQGLNYLH	tr_D0A9V0	0.138	11244.24	17.00
297	HLA-A0201	LSISKQFRLGFAAM	KQFLGFAAM	4	0	0	3	1	KQFRLGFAAM	tr_D0A9V0	0.138	11251.19	17.00
537	HLA-A0201	GCVSPYVARRFTVD	CVSPYVFTV	1	0	0	6	3	CVSPYVARRFTV	tr_D0A9V0	0.138	11255.69	17.00
1	HLA-A0201	PLMLLVSALKS	LMLLVSALS	1	0	0	8	1	LMLLVSALKS	tr_D0A9V0	0.138	11277.14	17.00
322	HLA-A0201	YLPMVKMDMQAKR	YLPMVDMQA	0	0	0	5	2	YLPMVKMDMQA	tr_D0A9V0	0.137	11315.77	17.00
610	HLA-A0201	ALYMAPEVAAGGR	ALYMAPVAA	0	0	0	6	1	ALYMAPEVAA	tr_D0A9V0	0.137	11322.75	17.00
39	HLA-A0201	HYAKLRRQLAVLL	KLRQLAVLL	3	0	0	2	1	KLRRQLAVLL	tr_D0A9V0	0.137	11328.38	17.00
459	HLA-A0201	STIFLGVAEDGA	TIFLGVAEA	1	0	0	8	2	TIFLGVAEDGA	tr_D0A9V0	0.137	11335.74	17.00
521	HLA-A0201	MEYVPGGSLHSVI	YVPGGLHSV	2	0	0	5	1	YVPGGSLHSV	tr_D0A9V0	0.137	11351.94	17.00
636	HLA-A0201	LLEMLLGRPP	LLMLLGRPP	0	0	0	2	1	LLEMLLGRPP	tr_D0A9V0	0.136	11456.70	17.00
38	HLA-A0201	GHYAKLRRQLAVLL	KLRQLAVLL	4	0	0	2	1	KLRRQLAVLL	tr_D0A9V0	0.136	11471.46	17.00
538	HLA-A0201	CVSPYVARRFTVDI	CVSPYVFTV	0	0	0	6	3	CVSPYVARRFTV	tr_D0A9V0	0.136	11524.46	17.00
434	HLA-A0201	VPLFLTDSNRNVWT	FLTDSNVWT	3	0	0	5	2	FLTDSNRNVWT	tr_D0A9V0	0.135	11541.81	17.00
199	HLA-A0201	LPLDHRDSVTMLV	PLDSVTMLV	1	0	0	2	3	PLDHRDSVTMLV	tr_D0A9V0	0.135	11556.05	17.00
437	HLA-A0201	FLTDSNRNVWTRS	FLTDVWTRS	0	0	0	4	4	FLTDSNRNVWTRS	tr_D0A9V0	0.135	11564.18	17.00
44	HLA-A0201	RRQLAVLLKF	RQLAVLLKF	0	0	0	1	1	RRQLAVLLKF	tr_D0A9V0	0.135	11616.22	17.00
484	HLA-A0201	VVAESLEKEMEVAS	VVAESLMEV	0	0	0	6	3	VVAESLEKEMEV	tr_D0A9V0	0.135	11623.14	17.00
581	HLA-A0201	CKLSDFGSTIS	KLSDFGSTI	1	0	0	0	0	KLSDFGSTI	tr_D0A9V0	0.135	11632.45	17.00
406	HLA-A0201	SLFKMYAPSSS	SLFKMYAPS	0	0	0	8	2	SLFKMYAPSSS	tr_D0A9V0	0.135	11662.19	17.00
471	HLA-A0201	QVAIKCIPRLRRDV	QVAIKCIPV	0	0	0	8	5	QVAIKCIPRLRRDV	tr_D0A9V0	0.134	11699.09	17.00
221	HLA-A0201	CGAFSRNARV	CAFSRNARV	0	0	0	1	1	CGAFSRNARV	tr_D0A9V0	0.134	11719.49	17.00
160	HLA-A0201	SGCVEALDPDRALI	SLDPDRALI	0	0	0	1	5	SGCVEALDPDRALI	tr_D0A9V0	0.134	11741.83	17.00

462	HLA-A0201	FLGVAEDGAQ	FLGVAEDGA	0	0	0	0	0	FLGVAEDGA	tr_D0A9V0	0.134	11744.87	17.00
458	HLA-A0201	FSTIFLGVAEDGA	TIFLGVAEA	2	0	0	8	2	TIFLGVAEDGA	tr_D0A9V0	0.134	11754.03	17.00
546	HLA-A0201	RFTVDILQGLN	FTVDILQGL	1	0	0	0	0	FTVDILQGL	tr_D0A9V0	0.134	11763.95	17.00
523	HLA-A0201	YVPGGSLHS	YVPGGSLHS	0	0	0	0	0	YVPGGSLHS	tr_D0A9V0	0.134	11771.72	17.00
217	HLA-A0201	LVGTCGAFSRNARV	LVGTCGAFV	0	0	0	8	5	LVGTCGAFSRNARV	tr_D0A9V0	0.133	11850.06	17.00
111	HLA-A0201	ALNCGIFKE	ALNCGIFKE	0	0	0	0	0	ALNCGIFKE	tr_D0A9V0	0.133	11879.97	17.00
631	HLA-A0201	SFGISLLEM	SFGISLLEM	0	0	0	0	0	SFGISLLEM	tr_D0A9V0	0.133	11881.90	17.00
625	HLA-A0201	PQSDIFSFGI	QSDIFSFGI	1	0	0	0	0	QSDIFSFGI	tr_D0A9V0	0.133	11887.55	17.00
709	HLA-A0201	SASSLLSYAFL	SASSLLAFL	0	0	0	6	2	SASSLLSYAFL	tr_D0A9V0	0.133	11911.89	17.00
416	HLA-A0201	SSSLDGSGCAEGA	SLDGCAEGA	2	0	0	3	2	SLDGSGCAEGA	tr_D0A9V0	0.132	12006.73	17.00
266	HLA-A0201	AFPVDCVLLPCS	FVDCVLLPC	1	0	0	1	1	FPVDCVLLPC	tr_D0A9V0	0.132	12014.40	17.00
511	HLA-A0201	SITPSHLSII	SITPSHLSI	0	0	0	8	1	SITPSHLSII	tr_D0A9V0	0.132	12019.60	17.00
211	HLA-A0201	VDTNEFLVGT	VTNEFLVGT	0	0	0	1	1	VDTNEFLVGT	tr_D0A9V0	0.132	12032.87	17.00
404	HLA-A0201	SESLFKMYAPS	SLFKMYAPS	0	0	0	1	2	SESLFKMYAPS	tr_D0A9V0	0.131	12062.72	18.00
150	HLA-A0201	AALIEVATEHSGC	ALIEVATEC	1	0	0	8	3	ALIEVATEHSGC	tr_D0A9V0	0.131	12069.38	18.00
14	HLA-A0201	NLEIPDNADIFLG	NLDNADIFL	0	0	0	2	3	NLEIPDNADIFL	tr_D0A9V0	0.131	12072.25	18.00
506	HLA-A0201	RYVSCSITPSHLS	YVSCSITPL	1	0	0	8	2	YVSCSITPSHL	tr_D0A9V0	0.131	12095.13	18.00
38	HLA-A0201	GHYAKLRRQLAVL	YLRRQLAVL	2	0	0	1	2	YAKLRRQLAVL	tr_D0A9V0	0.131	12098.15	18.00
267	HLA-A0201	FPVDCVLLPCST	FVDCVLLPC	0	0	0	1	1	FPVDCVLLPC	tr_D0A9V0	0.131	12157.33	18.00
406	HLA-A0201	SLFKMYAPSSSS	SLFKMYAPS	0	0	0	8	3	SLFKMYAPSSSS	tr_D0A9V0	0.131	12171.54	18.00
457	HLA-A0201	AFSTIFLGVAEDGA	TIFLGVAEA	3	0	0	8	2	TIFLGVAEDGA	tr_D0A9V0	0.130	12209.66	18.00
351	HLA-A0201	YVRVLDNHYCVNS	YVLDNHYCV	0	0	0	1	2	YVRVLDNHYCV	tr_D0A9V0	0.130	12210.32	18.00
597	HLA-A0201	ARTLADGVML	ATLADGVML	0	0	0	1	1	ARTLADGVML	tr_D0A9V0	0.130	12235.06	18.00
626	HLA-A0201	QSDIFSFGISLLE	SIFSFGISL	1	0	0	1	1	SDIFSFGISL	tr_D0A9V0	0.130	12247.77	18.00
452	HLA-A0201	KISEGAFSTIF	KISEGAFTI	0	0	0	7	1	KISEGAFSTI	tr_D0A9V0	0.130	12256.65	18.00
250	HLA-A0201	RIAATSGGA	RIAATSGGA	0	0	0	0	0	RIAATSGGA	tr_D0A9V0	0.130	12260.76	18.00
404	HLA-A0201	SESLFKMYAPSS	SLFMYAPSS	2	0	0	3	1	SLFKMYAPSS	tr_D0A9V0	0.130	12275.49	18.00
231	HLA-A0201	LFGADHLFQLSYA	FGADHLFYA	1	0	0	7	3	FGADHLFQLSYA	tr_D0A9V0	0.130	12288.38	18.00
223	HLA-A0201	AFSRNARVLFGA	FSARVLFGA	1	0	0	2	2	FSRNARVLFGA	tr_D0A9V0	0.129	12328.74	18.00
266	HLA-A0201	AFPVDCVLL	AFPVDCVLL	0	0	0	0	0	AFPVDCVLL	tr_D0A9V0	0.129	12345.16	18.00
436	HLA-A0201	LFLTDSNRNVW	FLTDSNRNV	1	0	0	0	0	FLTDSNRNV	tr_D0A9V0	0.129	12345.69	18.00
415	HLA-A0201	SSSSLDGSGCAEGA	SLDGCAEGA	3	0	0	3	2	SLDGSGCAEGA	tr_D0A9V0	0.129	12370.43	18.00
129	HLA-A0201	CTVIAIQVYHPDVW	TVIAIQVYV	1	0	0	8	3	TVIAIQVYHPDV	tr_D0A9V0	0.129	12399.37	18.00
37	HLA-A0201	QGHYAKLRRQLAVL	YLRRQLAVL	3	0	0	1	2	YAKLRRQLAVL	tr_D0A9V0	0.129	12411.58	18.00
553	HLA-A0201	QGLNYLHNL	QGLNYLHNL	0	0	0	0	0	QGLNYLHNL	tr_D0A9V0	0.129	12413.06	18.00
553	HLA-A0201	QGLNYLHNLGIVH	GLYLHNLGI	1	0	0	2	1	GLNYLHNLGI	tr_D0A9V0	0.129	12418.57	18.00
282	HLA-A0201	VLFELRPGE	VLFELRPGE	0	0	0	0	0	VLFELRPGE	tr_D0A9V0	0.129	12423.67	18.00
406	HLA-A0201	SLFKMYAPSSSSS	SLFKMYAPS	0	0	0	8	4	SLFKMYAPSSSSS	tr_D0A9V0	0.129	12428.79	18.00
123	HLA-A0201	VFNRRHCTVIAIQV	FCTVIAIQV	1	0	0	1	4	FNRRHCTVIAIQV	tr_D0A9V0	0.128	12475.94	18.00
295	HLA-A0201	VSLSISKQFRLGFA	SLSISLGFA	1	0	0	5	4	SLSISKQFRLGFA	tr_D0A9V0	0.128	12483.91	18.00
111	HLA-A0201	ALNCGIFKEK	ALNCGIFEK	0	0	0	7	1	ALNCGIFKEK	tr_D0A9V0	0.128	12487.56	18.00
706	HLA-A0201	MRPSASSLLSYAFL	MSLLSYAFL	0	0	0	1	5	MRPSASSLLSYAFL	tr_D0A9V0	0.128	12492.42	18.00
659	HLA-A0201	TRLRSLFNRDLL	RLRSLFNLL	1	0	0	7	2	RLRSLFNRDLL	tr_D0A9V0	0.128	12492.83	18.00
547	HLA-A0201	FTVDILQGLNY	FTVDILQGL	0	0	0	0	0	FTVDILQGL	tr_D0A9V0	0.128	12510.01	18.00
350	HLA-A0201	PYVRVLDNHYCVN	YVLDNHYCV	1	0	0	1	2	YVRVLDNHYCV	tr_D0A9V0	0.128	12549.73	18.00
602	HLA-A0201	DGVMLRGTAL	GVMLRGTAL	1	0	0	0	0	GVMLRGTAL	tr_D0A9V0	0.128	12567.26	18.00
239	HLA-A0201	QLSYAVRDGPCRI	QLSDGPCRI	0	0	0	3	4	QLSYAVRDGPCRI	tr_D0A9V0	0.128	12575.15	18.00
165	HLA-A0201	ALDPDRALISF	ALDPALISF	0	0	0	4	2	ALDPDRALISF	tr_D0A9V0	0.128	12580.73	18.00
633	HLA-A0201	GISLLEMLLGR	ILLEMLLGR	1	0	0	1	1	ISLLEMLLGR	tr_D0A9V0	0.128	12581.81	18.00
403	HLA-A0201	GSESLFKMYAPSS	SLFMYAPSS	3	0	0	3	1	SLFKMYAPSS	tr_D0A9V0	0.127	12633.79	18.00
523	HLA-A0201	YVPGGSLHSVIKNF	YVPGGLHSV	0	0	0	5	1	YVPGGSLHSV	tr_D0A9V0	0.127	12638.99	18.00
277	HLA-A0201	STLPIVLFE	STLPIVLFE	0	0	0	0	0	STLPIVLFE	tr_D0A9V0	0.127	12649.51	18.00
207	HLA-A0201	VTMLVDTNEF	VMLVDTNEF	0	0	0	1	1	VTMLVDTNEF	tr_D0A9V0	0.127	12652.53	18.00
545	HLA-A0201	RRFTVDILQGLN	FTVDILQGL	2	0	0	0	0	FTVDILQGL	tr_D0A9V0	0.127	12659.93	18.00

149	HLA-A0201	HAALIEVATEHSGC	ALIEVATEC	2	0	0	8	3	ALIEVATEHSGC	tr_D0A9V0	0.127	12719.65	18.00
203	HLA-A0201	HRDSVTMLV	HRDSVTMLV	0	0	0	0	0	HRDSVTMLV	tr_D0A9V0	0.126	12735.07	18.00
198	HLA-A0201	KLPLDHRDSVTM	KLPLDSVTM	0	0	0	4	3	KLPLDHRDSVTM	tr_D0A9V0	0.126	12738.24	18.00
523	HLA-A0201	YVPGGSLHSVIKN	YVPGGLHSV	0	0	0	5	1	YVPGGSLHSV	tr_D0A9V0	0.126	12768.60	18.00
695	HLA-A0201	FVRACCHMNPSM	FVRACCHMM	0	0	0	8	3	FVRACCHMNPSM	tr_D0A9V0	0.126	12796.68	18.00
8	HLA-A0201	ALKSASNLEIP	ALSASNLEI	0	0	0	2	1	ALKSASNLEI	tr_D0A9V0	0.126	12801.10	18.00
612	HLA-A0201	YMAPEVAAGGRCTP	YMAPEVAAT	0	0	0	8	4	YMAPEVAAGGRCT	tr_D0A9V0	0.126	12806.37	18.00
144	HLA-A0201	SSTKYHAALIEV	STHAALIEV	1	0	0	2	2	STKYHAALIEV	tr_D0A9V0	0.126	12816.77	18.00
192	HLA-A0201	AFDLFAKLPLD	FLFAKLPLD	1	0	0	1	1	FDLFAKLPLD	tr_D0A9V0	0.126	12840.09	18.00
319	HLA-A0201	MAHYLPMVKM	MAYLPMVKM	0	0	0	2	1	MAHYLPMVKM	tr_D0A9V0	0.126	12845.23	18.00
402	HLA-A0201	QGSESLFKMYAPSS	SLFMYAPSS	4	0	0	3	1	SLFKMYAPSS	tr_D0A9V0	0.126	12846.90	18.00
87	HLA-A0201	TAFVRIMGASI	TAFVRIMSI	0	0	0	7	2	TAFVRIMGASI	tr_D0A9V0	0.126	12854.41	18.00
611	HLA-A0201	LYMAPEVAAGGRC	YMAPEVAAC	1	0	0	8	3	YMAPEVAAGGRC	tr_D0A9V0	0.126	12858.86	18.00
580	HLA-A0201	VCKLSDFGSTIS	KLSDFGSTI	2	0	0	0	0	KLSDFGSTI	tr_D0A9V0	0.125	12873.47	18.00
296	HLA-A0201	SLSISKQFRLGF	SLISKQFRL	0	0	0	2	1	SLSISKQFRL	tr_D0A9V0	0.125	12900.38	18.00
487	HLA-A0201	ESLEKEMEV	ESLEKEMEV	0	0	0	0	0	ESLEKEMEV	tr_D0A9V0	0.125	12928.47	19.00
409	HLA-A0201	KMYAPSSSSS	KMYASSSSS	0	0	0	4	1	KMYAPSSSSS	tr_D0A9V0	0.125	12942.32	19.00
609	HLA-A0201	TALYMAPEVAAGGR	ALYMAPVAA	1	0	0	6	1	ALYMAPEVAA	tr_D0A9V0	0.125	12985.66	19.00
544	HLA-A0201	ARRFTVDILQGLN	FTVDILQGL	3	0	0	0	0	FTVDILQGL	tr_D0A9V0	0.125	12991.85	19.00
243	HLA-A0201	AVRDGPCRI	AVRDGPCRI	0	0	0	0	0	AVRDGPCRI	tr_D0A9V0	0.124	13007.61	19.00
321	HLA-A0201	HYLPMVKMDMQAKR	YLPMVDMQA	1	0	0	5	2	YLPMVKMDMQA	tr_D0A9V0	0.124	13009.72	19.00
161	HLA-A0201	GCVEALDPDRALI	ALDPDRALI	4	0	0	0	0	ALDPDRALI	tr_D0A9V0	0.124	13038.75	19.00
664	HLA-A0201	LFNRDLLFVQSL	LFLLFVQSL	0	0	0	2	3	LFNRDLLFVQSL	tr_D0A9V0	0.124	13042.13	19.00
295	HLA-A0201	VSLSISKQFRLG	SLISKQFRL	1	0	0	2	1	SLSISKQFRL	tr_D0A9V0	0.124	13052.71	19.00
512	HLA-A0201	ITPSHLSIIM	ITPSHLIIM	0	0	0	6	1	ITPSHLSIIM	tr_D0A9V0	0.124	13070.10	19.00
549	HLA-A0201	VDILQGLNYLH	VILQGLNYL	0	0	0	1	1	VDILQGLNYL	tr_D0A9V0	0.124	13071.52	19.00
326	HLA-A0201	VKMDMQAKRLMRLC	KMDMRLMRL	1	0	0	4	3	KMDMQAKRLMRL	tr_D0A9V0	0.124	13101.54	19.00
237	HLA-A0201	LFQLSYAVRDGPC	FQLSYAVPC	1	0	0	7	3	FQLSYAVRDGPC	tr_D0A9V0	0.124	13109.33	19.00
428	HLA-A0201	ANAADVVPL	ANAADVVPL	0	0	0	0	0	ANAADVVPL	tr_D0A9V0	0.124	13110.89	19.00
674	HLA-A0201	SLALGHLEP	SLALGHLEP	0	0	0	0	0	SLALGHLEP	tr_D0A9V0	0.124	13136.73	19.00
143	HLA-A0201	KSSTKYHAALIEV	STHAALIEV	2	0	0	2	2	STKYHAALIEV	tr_D0A9V0	0.123	13146.40	19.00
552	HLA-A0201	LQGLNYLHNLGIVH	GLYLHNLGI	2	0	0	2	1	GLNYLHNLGI	tr_D0A9V0	0.123	13153.81	19.00
224	HLA-A0201	FSRNARVLFGADHL	FVLFGADHL	0	0	0	1	5	FSRNARVLFGADHL	tr_D0A9V0	0.123	13163.06	19.00
518	HLA-A0201	SIIMEYVPG	SIIMEYVPG	0	0	0	0	0	SIIMEYVPG	tr_D0A9V0	0.123	13166.62	19.00
408	HLA-A0201	FKMYAPSSSSSLD	KMYASSSSL	1	0	0	4	2	KMYAPSSSSSL	tr_D0A9V0	0.123	13175.74	19.00
557	HLA-A0201	YLHNLGIVH	YLHNLGIVH	0	0	0	0	0	YLHNLGIVH	tr_D0A9V0	0.123	13181.30	19.00
543	HLA-A0201	VARRFTVDILQGLN	FTVDILQGL	4	0	0	0	0	FTVDILQGL	tr_D0A9V0	0.123	13188.44	19.00
530	HLA-A0201	HSVIKNFGCV	SVIKNFGCV	1	0	0	0	0	SVIKNFGCV	tr_D0A9V0	0.123	13203.57	19.00
86	HLA-A0201	STAFVRIMGASI	TAFVRIMSI	1	0	0	7	2	TAFVRIMGASI	tr_D0A9V0	0.123	13219.01	19.00
472	HLA-A0201	VAIKCIPRLRRDVV	VAIKCIPVV	0	0	0	7	5	VAIKCIPRLRRDVV	tr_D0A9V0	0.123	13229.88	19.00
505	HLA-A0201	VRYVSCSITPSHLS	YVSCSITPL	2	0	0	8	2	YVSCSITPSHL	tr_D0A9V0	0.123	13234.46	19.00
228	HLA-A0201	ARVLFGADHLF	ALFGADHLF	0	0	0	1	2	ARVLFGADHLF	tr_D0A9V0	0.123	13252.65	19.00
506	HLA-A0201	RYVSCSITPS	YVSCSITPS	1	0	0	0	0	YVSCSITPS	tr_D0A9V0	0.123	13253.66	19.00
436	HLA-A0201	LFLTDSNRNVWTR	FLTDSVWTR	1	0	0	5	3	FLTDSNRNVWTR	tr_D0A9V0	0.123	13261.26	19.00
542	HLA-A0201	YVARRFTVDIL	YVAFTVDIL	0	0	0	3	2	YVARRFTVDIL	tr_D0A9V0	0.123	13272.17	19.00
93	HLA-A0201	MGASISVTWNA	MSISVTWNA	0	0	0	1	2	MGASISVTWNA	tr_D0A9V0	0.123	13272.60	19.00
479	HLA-A0201	RLRRDVVAESLE	RLDVVAESL	0	0	0	2	2	RLRRDVVAESL	tr_D0A9V0	0.123	13277.63	19.00
163	HLA-A0201	VEALDPDRALI	VLDPDRALI	0	0	0	1	2	VEALDPDRALI	tr_D0A9V0	0.122	13292.01	19.00
573	HLA-A0201	VLLGTDGVC	VLLGTDGVC	0	0	0	0	0	VLLGTDGVC	tr_D0A9V0	0.122	13325.70	19.00
582	HLA-A0201	KLSDFGSTISE	KLSDFGSTI	0	0	0	0	0	KLSDFGSTI	tr_D0A9V0	0.122	13330.31	19.00
658	HLA-A0201	ATRLRSLFNRDLL	RLRSLFNLL	2	0	0	7	2	RLRSLFNRDLL	tr_D0A9V0	0.122	13387.99	19.00
469	HLA-A0201	GAQVAIKCIPRL	AQVACIPRL	1	0	0	4	2	AQVAIKCIPRL	tr_D0A9V0	0.122	13389.14	19.00
579	HLA-A0201	GVCKLSDFGSTIS	KLSDFGSTI	3	0	0	0	0	KLSDFGSTI	tr_D0A9V0	0.122	13393.93	19.00
665	HLA-A0201	FNRDLLFVQSL	FNLLFVQSL	0	0	0	2	2	FNRDLLFVQSL	tr_D0A9V0	0.122	13417.28	19.00

435	HLA-A0201	PLFLTDSNRNVWTR	PLFLTVWTR	0	0	0	5	5	PLFLTDSNRNVWTR	tr_D0A9V0	0.121	13441.55	19.00
351	HLA-A0201	YVRVLDNHYCVNSS	YVLDNHYCV	0	0	0	1	2	YVRVLDNHYCV	tr_D0A9V0	0.121	13461.49	19.00
198	HLA-A0201	KLPLDHRDSVT	KLLDHRDSV	0	0	0	2	1	KLPLDHRDSV	tr_D0A9V0	0.121	13465.86	19.00
225	HLA-A0201	SRNARVLFGADHLF	SLFGADHLF	0	0	0	1	5	SRNARVLFGADHLF	tr_D0A9V0	0.121	13470.96	19.00
625	HLA-A0201	PQSDIFSFGISLLE	SIFSFGISL	2	0	0	1	1	SDIFSFGISL	tr_D0A9V0	0.121	13483.35	19.00
4	HLA-A0201	LLVSALKSASNLE	LLVSALSNL	0	0	0	6	3	LLVSALKSASNL	tr_D0A9V0	0.121	13498.53	19.00
187	HLA-A0201	RCCQFAFDLF	RCCQFAFLF	0	0	0	7	1	RCCQFAFDLF	tr_D0A9V0	0.121	13546.82	19.00
403	HLA-A0201	GSESLFKMYAPS	SLFKMYAPS	3	0	0	0	0	SLFKMYAPS	tr_D0A9V0	0.121	13550.77	19.00
85	HLA-A0201	SSTAFVRIMGASI	TAFVRIMSI	2	0	0	7	2	TAFVRIMGASI	tr_D0A9V0	0.121	13568.08	19.00
266	HLA-A0201	AFPVDCVLLPCST	FVDCVLLPC	1	0	0	1	1	FPVDCVLLPC	tr_D0A9V0	0.120	13576.16	19.00
574	HLA-A0201	LLGTDGVCKLS	LLGDGVCKL	0	0	0	3	1	LLGTDGVCKL	tr_D0A9V0	0.120	13611.47	19.00
455	HLA-A0201	EGAFSTIFLG	GAFSTIFLG	1	0	0	0	0	GAFSTIFLG	tr_D0A9V0	0.120	13621.93	19.00
409	HLA-A0201	KMYAPSSSSSLDG	KMYASSSSL	0	0	0	4	2	KMYAPSSSSSL	tr_D0A9V0	0.120	13638.88	19.00
578	HLA-A0201	DGVCKLSDFGSTIS	KLSDFGSTI	4	0	0	0	0	KLSDFGSTI	tr_D0A9V0	0.120	13642.28	19.00
491	HLA-A0201	KEMEVASKL	KEMEVASKL	0	0	0	0	0	KEMEVASKL	tr_D0A9V0	0.120	13651.43	19.00
688	HLA-A0201	VDHEAAAFV	VDHEAAAFV	0	0	0	0	0	VDHEAAAFV	tr_D0A9V0	0.120	13658.82	19.00
349	HLA-A0201	VPYVRVLDNHYCVN	YVLDNHYCV	2	0	0	1	2	YVRVLDNHYCV	tr_D0A9V0	0.120	13668.87	19.00
539	HLA-A0201	VSPYVARRFTV	VSPYVAFTV	0	0	0	6	2	VSPYVARRFTV	tr_D0A9V0	0.120	13671.98	19.00
708	HLA-A0201	PSASSLLSYA	SASSLLSYA	1	0	0	0	0	SASSLLSYA	tr_D0A9V0	0.120	13675.82	19.00
657	HLA-A0201	DATRLRSLFNRDLL	RLRSLFNLL	3	0	0	7	2	RLRSLFNRDLL	tr_D0A9V0	0.120	13676.71	19.00
600	HLA-A0201	LADGVMLRGTAL	LADGVMLAL	0	0	0	7	3	LADGVMLRGTAL	tr_D0A9V0	0.120	13677.74	19.00
228	HLA-A0201	ARVLFGADHL	AVLFGADHL	0	0	0	1	1	ARVLFGADHL	tr_D0A9V0	0.120	13691.37	19.00
552	HLA-A0201	LQGLNYLHNLG	LQLNYLHNL	0	0	0	2	1	LQGLNYLHNL	tr_D0A9V0	0.119	13722.81	19.00
85	HLA-A0201	SSTAFVRIMGA	STFVRIMGA	1	0	0	2	1	STAFVRIMGA	tr_D0A9V0	0.119	13746.14	19.00
611	HLA-A0201	LYMAPEVAAGG	YMAEVAAGG	1	0	0	3	1	YMAPEVAAGG	tr_D0A9V0	0.119	13766.83	19.00
522	HLA-A0201	EYVPGGSLHSVIK	YVPGGLHSV	1	0	0	5	1	YVPGGSLHSV	tr_D0A9V0	0.119	13772.49	19.00
598	HLA-A0201	RTLADGVMLR	RLADGVMLR	0	0	0	1	1	RTLADGVMLR	tr_D0A9V0	0.119	13773.24	19.00
130	HLA-A0201	TVIAIQVYHPDVWK	TVIAIQVYV	0	0	0	8	3	TVIAIQVYHPDV	tr_D0A9V0	0.119	13805.91	19.00
327	HLA-A0201	KMDMQAKRLMRLCR	KMDMRLMRL	0	0	0	4	3	KMDMQAKRLMRL	tr_D0A9V0	0.119	13846.90	20.00
562	HLA-A0201	GIVHCDVKPHNVLL	GIVHCNVLL	0	0	0	5	5	GIVHCDVKPHNVLL	tr_D0A9V0	0.119	13847.35	20.00
640	HLA-A0201	LLGRPPWQWSS	LLGPWQWSS	0	0	0	3	2	LLGRPPWQWSS	tr_D0A9V0	0.119	13856.94	20.00
468	HLA-A0201	DGAQVAIKCIPRL	AQVACIPRL	2	0	0	4	2	AQVAIKCIPRL	tr_D0A9V0	0.118	13906.06	20.00
268	HLA-A0201	PVDCVLLPC	PVDCVLLPC	0	0	0	0	0	PVDCVLLPC	tr_D0A9V0	0.118	13906.66	20.00
162	HLA-A0201	CVEALDPDRALI	ALDPDRALI	3	0	0	0	0	ALDPDRALI	tr_D0A9V0	0.118	13931.66	20.00
399	HLA-A0201	SSDQGSESL	SSDQGSESL	0	0	0	0	0	SSDQGSESL	tr_D0A9V0	0.118	13934.67	20.00
428	HLA-A0201	ANAADVVPLFL	NAADVVLFL	1	0	0	6	1	NAADVVPLFL	tr_D0A9V0	0.118	13968.64	20.00
562	HLA-A0201	GIVHCDVKPHNV	GIVHCDVNV	0	0	0	7	3	GIVHCDVKPHNV	tr_D0A9V0	0.118	13979.37	20.00
226	HLA-A0201	RNARVLFGADHLF	RLFGADHLF	0	0	0	1	4	RNARVLFGADHLF	tr_D0A9V0	0.118	13987.85	20.00
282	HLA-A0201	VLFELRPGESS	VLFELGESS	0	0	0	5	2	VLFELRPGESS	tr_D0A9V0	0.118	13991.02	20.00
700	HLA-A0201	CHMNPSMRPSA	HMNPSMPSA	1	0	0	6	1	HMNPSMRPSA	tr_D0A9V0	0.118	13993.75	20.00
84	HLA-A0201	ESSTAFVRIMGASI	TAFVRIMSI	3	0	0	7	2	TAFVRIMGASI	tr_D0A9V0	0.117	14029.07	20.00
701	HLA-A0201	HMNPSMRPSASSLL	HMNPSMSLL	0	0	0	6	5	HMNPSMRPSASSLL	tr_D0A9V0	0.117	14033.93	20.00
402	HLA-A0201	QGSESLFKMYAPS	SLFKMYAPS	4	0	0	0	0	SLFKMYAPS	tr_D0A9V0	0.117	14036.82	20.00
536	HLA-A0201	FGCVSPYVA	FGCVSPYVA	0	0	0	0	0	FGCVSPYVA	tr_D0A9V0	0.117	14042.59	20.00
532	HLA-A0201	VIKNFGCVSPYVAR	VIGCVSPYV	0	0	0	2	3	VIKNFGCVSPYV	tr_D0A9V0	0.117	14087.02	20.00
449	HLA-A0201	SLKKISEGAFSTIF	SLGAFSTIF	0	0	0	2	5	SLKKISEGAFSTIF	tr_D0A9V0	0.117	14111.28	20.00
596	HLA-A0201	MARTLADGV	MARTLADGV	0	0	0	0	0	MARTLADGV	tr_D0A9V0	0.117	14116.78	20.00
405	HLA-A0201	ESLFKMYAPSSS	SLFKMYAPS	1	0	0	8	2	SLFKMYAPSSS	tr_D0A9V0	0.117	14154.10	20.00
148	HLA-A0201	YHAALIEVA	YHAALIEVA	0	0	0	0	0	YHAALIEVA	tr_D0A9V0	0.116	14180.00	20.00
274	HLA-A0201	LPCSTLPIV	LPCSTLPIV	0	0	0	0	0	LPCSTLPIV	tr_D0A9V0	0.116	14188.45	20.00
294	HLA-A0201	EVSLSISKQFRLG	SLISKQFRL	2	0	0	2	1	SLSISKQFRL	tr_D0A9V0	0.116	14200.27	20.00
467	HLA-A0201	EDGAQVAIKCIPRL	AQVACIPRL	3	0	0	4	2	AQVAIKCIPRL	tr_D0A9V0	0.116	14201.80	20.00
401	HLA-A0201	DQGSESLFKMYAPS	SLFKMYAPS	5	0	0	0	0	SLFKMYAPS	tr_D0A9V0	0.116	14215.64	20.00
258	HLA-A0201	ASHMQGFLAFP	AMQGFLAFP	0	0	0	1	2	ASHMQGFLAFP	tr_D0A9V0	0.116	14223.95	20.00

510	HLA-A0201	CSITPSHLSI	SITPSHLSI	1	0	0	0	0	SITPSHLSI	tr_D0A9V0	0.116	14227.95	20.00
150	HLA-A0201	AALIEVATEH	ALIEVATEH	0	0	0	1	1	AALIEVATEH	tr_D0A9V0	0.116	14228.72	20.00
142	HLA-A0201	WKSSTKYHAALIEV	STHAALIEV	3	0	0	2	2	STKYHAALIEV	tr_D0A9V0	0.116	14266.64	20.00
555	HLA-A0201	LNYLHNLGIV	LNYLHNLGV	0	0	0	8	1	LNYLHNLGIV	tr_D0A9V0	0.116	14279.15	20.00
433	HLA-A0201	VVPLFLTDSNRNVW	FLTDSNRNV	4	0	0	0	0	FLTDSNRNV	tr_D0A9V0	0.115	14340.30	20.00
46	HLA-A0201	QLAVLLKFIPE	QLAVLLKFI	0	0	0	0	0	QLAVLLKFI	tr_D0A9V0	0.115	14401.42	20.00
687	HLA-A0201	SVDHEAAAFVR	SVDEAAAFV	0	0	0	3	1	SVDHEAAAFV	tr_D0A9V0	0.115	14431.36	20.00
227	HLA-A0201	NARVLFGADHLF	ALFGADHLF	1	0	0	1	2	ARVLFGADHLF	tr_D0A9V0	0.115	14432.77	20.00
16	HLA-A0201	EIPDNADIFL	EIDNADIFL	0	0	0	2	1	EIPDNADIFL	tr_D0A9V0	0.115	14481.57	20.00
255	HLA-A0201	SGGASHMQGFLAFP	SMQGFLAFP	0	0	0	1	5	SGGASHMQGFLAFP	tr_D0A9V0	0.115	14483.61	20.00
671	HLA-A0201	FVQSLALGHLE	FVQSLALHL	0	0	0	7	1	FVQSLALGHL	tr_D0A9V0	0.114	14492.08	20.00
563	HLA-A0201	IVHCDVKPHNVLL	IVHCDVVLL	0	0	0	6	4	IVHCDVKPHNVLL	tr_D0A9V0	0.114	14492.39	20.00
301	HLA-A0201	KQFRLGFAAMR	KQFLGFAAM	0	0	0	3	1	KQFRLGFAAM	tr_D0A9V0	0.114	14526.62	20.00
436	HLA-A0201	LFLTDSNRNVWTRS	FLTDVWTRS	1	0	0	4	4	FLTDSNRNVWTRS	tr_D0A9V0	0.114	14551.78	20.00
548	HLA-A0201	TVDILQGLNYLH	VILQGLNYL	1	0	0	1	1	VDILQGLNYL	tr_D0A9V0	0.114	14592.62	20.00
293	HLA-A0201	SEVSLSISKQFRLG	SLISKQFRL	3	0	0	2	1	SLSISKQFRL	tr_D0A9V0	0.114	14616.95	20.00
405	HLA-A0201	ESLFKMYAPSSSS	SLFKMYAPS	1	0	0	8	3	SLFKMYAPSSSS	tr_D0A9V0	0.114	14625.34	20.00
407	HLA-A0201	LFKMYAPSSSSSLD	KMYASSSSL	2	0	0	4	2	KMYAPSSSSSL	tr_D0A9V0	0.114	14629.45	20.00
409	HLA-A0201	KMYAPSSSSSLDGS	KMYASSSSL	0	0	0	4	2	KMYAPSSSSSL	tr_D0A9V0	0.113	14647.82	20.00
425	HLA-A0201	AEGANAADVVPLFL	NAADVVLFL	4	0	0	6	1	NAADVVPLFL	tr_D0A9V0	0.113	14648.14	20.00
13	HLA-A0201	SNLEIPDNADIFLG	NLDNADIFL	1	0	0	2	3	NLEIPDNADIFL	tr_D0A9V0	0.113	14684.96	21.00
84	HLA-A0201	ESSTAFVRIMGA	STFVRIMGA	2	0	0	2	1	STAFVRIMGA	tr_D0A9V0	0.113	14709.29	21.00
699	HLA-A0201	CCHMNPSMRPSA	HMNPSMPSA	2	0	0	6	1	HMNPSMRPSA	tr_D0A9V0	0.113	14729.68	21.00
435	HLA-A0201	PLFLTDSNRNVW	FLTDSNRNV	2	0	0	0	0	FLTDSNRNV	tr_D0A9V0	0.113	14785.24	21.00
42	HLA-A0201	KLRRQLAVLLK	KLRQLAVLL	0	0	0	2	1	KLRRQLAVLL	tr_D0A9V0	0.113	14789.08	21.00
546	HLA-A0201	RFTVDILQGLNY	FTVDILQGL	1	0	0	0	0	FTVDILQGL	tr_D0A9V0	0.113	14790.68	21.00
183	HLA-A0201	MHCRRCCQFAFDL	MCCQFAFDL	0	0	0	1	4	MHCRRCCQFAFDL	tr_D0A9V0	0.113	14792.44	21.00
427	HLA-A0201	GANAADVVPLFL	NAADVVLFL	2	0	0	6	1	NAADVVPLFL	tr_D0A9V0	0.112	14804.44	21.00
89	HLA-A0201	FVRIMGASIS	FVRIMGASI	0	0	0	0	0	FVRIMGASI	tr_D0A9V0	0.112	14809.26	21.00
448	HLA-A0201	RSLKKISEGA	RLKKISEGA	0	0	0	1	1	RSLKKISEGA	tr_D0A9V0	0.112	14835.08	21.00
551	HLA-A0201	ILQGLNYLHN	ILQGLYLHN	0	0	0	5	1	ILQGLNYLHN	tr_D0A9V0	0.112	14839.57	21.00
150	HLA-A0201	AALIEVATEHS	ALIEVATES	1	0	0	8	1	ALIEVATEHS	tr_D0A9V0	0.112	14861.10	21.00
701	HLA-A0201	HMNPSMRPSASSL	HMNPSMSSL	0	0	0	6	4	HMNPSMRPSASSL	tr_D0A9V0	0.112	14865.12	21.00
708	HLA-A0201	PSASSLLSYAFL	SASSLLAFL	1	0	0	6	2	SASSLLSYAFL	tr_D0A9V0	0.112	14873.97	21.00
210	HLA-A0201	LVDTNEFLVGT	LVDEFLVGT	0	0	0	3	2	LVDTNEFLVGT	tr_D0A9V0	0.112	14876.39	21.00
222	HLA-A0201	GAFSRNARVLFGAD	GAFSVLFGA	0	0	0	4	4	GAFSRNARVLFGA	tr_D0A9V0	0.112	14910.87	21.00
164	HLA-A0201	EALDPDRALISF	ALDPALISF	1	0	0	4	2	ALDPDRALISF	tr_D0A9V0	0.112	14912.00	21.00
29	HLA-A0201	RIVEVRKIQGHYA	RIVEVGHYA	0	0	0	5	4	RIVEVRKIQGHYA	tr_D0A9V0	0.112	14918.78	21.00
550	HLA-A0201	DILQGLNYLH	DILQGLNYL	0	0	0	0	0	DILQGLNYL	tr_D0A9V0	0.112	14922.49	21.00
54	HLA-A0201	IPEAILARV	IPEAILARV	0	0	0	0	0	IPEAILARV	tr_D0A9V0	0.112	14941.39	21.00
34	HLA-A0201	RKIQGHYAKL	KIQGHYAKL	1	0	0	0	0	KIQGHYAKL	tr_D0A9V0	0.112	14943.82	21.00
521	HLA-A0201	MEYVPGGSLHSVIK	YVPGGLHSV	2	0	0	5	1	YVPGGSLHSV	tr_D0A9V0	0.112	14952.39	21.00
267	HLA-A0201	FPVDCVLLP	FPVDCVLLP	0	0	0	0	0	FPVDCVLLP	tr_D0A9V0	0.112	14958.86	21.00
83	HLA-A0201	GESSTAFVRIMGA	STFVRIMGA	3	0	0	2	1	STAFVRIMGA	tr_D0A9V0	0.112	14962.59	21.00
405	HLA-A0201	ESLFKMYAPSSSSS	SLFKMYAPS	1	0	0	8	4	SLFKMYAPSSSSS	tr_D0A9V0	0.111	14967.93	21.00
639	HLA-A0201	MLLGRPPWQWS	MLLGRPPWS	0	0	0	7	2	MLLGRPPWQWS	tr_D0A9V0	0.111	14969.38	21.00
418	HLA-A0201	SLDGSGCAEGANA	SLDGSGCAA	0	0	0	8	4	SLDGSGCAEGANA	tr_D0A9V0	0.111	14970.04	21.00
303	HLA-A0201	FRLGFAAMRQ	FLGFAAMRQ	0	0	0	1	1	FRLGFAAMRQ	tr_D0A9V0	0.111	14976.03	21.00
694	HLA-A0201	AFVRACCHMNPSM	FVRACCHMM	1	0	0	8	3	FVRACCHMNPSM	tr_D0A9V0	0.111	15019.84	21.00
295	HLA-A0201	VSLSISKQFRLGF	SLISKQFRL	1	0	0	2	1	SLSISKQFRL	tr_D0A9V0	0.111	15056.12	21.00
518	HLA-A0201	SIIMEYVPGGS	IIMEYVPGS	1	0	0	7	1	IIMEYVPGGS	tr_D0A9V0	0.111	15064.27	21.00
426	HLA-A0201	EGANAADVVPLFL	NAADVVLFL	3	0	0	6	1	NAADVVPLFL	tr_D0A9V0	0.111	15079.11	21.00
623	HLA-A0201	CTPQSDIFSFGI	CTDIFSFGI	0	0	0	2	3	CTPQSDIFSFGI	tr_D0A9V0	0.111	15109.50	21.00
274	HLA-A0201	LPCSTLPIVL	LCSTLPIVL	0	0	0	1	1	LPCSTLPIVL	tr_D0A9V0	0.110	15136.81	21.00

698	HLA-A0201	ACCHMNPSMRPSA	HMNPSMPSA	3	0	0	6	1	HMNPSMRPSA	tr_D0A9V0	0.110	15146.65	21.00
454	HLA-A0201	SEGAFSTIFL	SEGAFTIFL	0	0	0	5	1	SEGAFSTIFL	tr_D0A9V0	0.110	15164.02	21.00
549	HLA-A0201	VDILQGLNYLHN	VILQGLNYL	0	0	0	1	1	VDILQGLNYL	tr_D0A9V0	0.110	15169.93	21.00
82	HLA-A0201	PGESSTAFVRIMGA	STFVRIMGA	4	0	0	2	1	STAFVRIMGA	tr_D0A9V0	0.110	15172.07	21.00
48	HLA-A0201	AVLLKFIPE	AVLLKFIPE	0	0	0	0	0	AVLLKFIPE	tr_D0A9V0	0.110	15174.86	21.00
99	HLA-A0201	VTWNAGDQQRDVAL	VTWNADVAL	0	0	0	5	5	VTWNAGDQQRDVAL	tr_D0A9V0	0.110	15180.61	21.00
429	HLA-A0201	NAADVVPLF	NAADVVPLF	0	0	0	0	0	NAADVVPLF	tr_D0A9V0	0.110	15196.06	21.00
404	HLA-A0201	SESLFKMYAPSSS	SLFKMYAPS	2	0	0	8	2	SLFKMYAPSSS	tr_D0A9V0	0.110	15204.45	21.00
434	HLA-A0201	VPLFLTDSNRNVW	FLTDSNRNV	3	0	0	0	0	FLTDSNRNV	tr_D0A9V0	0.110	15206.08	21.00
182	HLA-A0201	PMHCRRCCQFAFDL	MCCQFAFDL	1	0	0	1	4	MHCRRCCQFAFDL	tr_D0A9V0	0.110	15207.90	21.00
172	HLA-A0201	LISFGAHAALPM	LISFAALPM	0	0	0	4	3	LISFGAHAALPM	tr_D0A9V0	0.110	15207.90	21.00
629	HLA-A0201	IFSFGISLLE	FSFGISLLE	1	0	0	0	0	FSFGISLLE	tr_D0A9V0	0.110	15220.90	21.00
451	HLA-A0201	KKISEGAFSTIF	KISEGAFTI	1	0	0	7	1	KISEGAFSTI	tr_D0A9V0	0.110	15250.41	21.00
180	HLA-A0201	ALPMHCRRC	ALPMHCRRC	0	0	0	0	0	ALPMHCRRC	tr_D0A9V0	0.110	15251.89	21.00
458	HLA-A0201	FSTIFLGVAE	FSTIFLGVA	0	0	0	0	0	FSTIFLGVA	tr_D0A9V0	0.110	15273.86	21.00
258	HLA-A0201	ASHMQGFLA	ASHMQGFLA	0	0	0	0	0	ASHMQGFLA	tr_D0A9V0	0.109	15314.57	21.00
707	HLA-A0201	RPSASSLLSYAFL	SASSLLAFL	2	0	0	6	2	SASSLLSYAFL	tr_D0A9V0	0.109	15319.70	21.00
7	HLA-A0201	SALKSASNL	SALKSASNL	0	0	0	0	0	SALKSASNL	tr_D0A9V0	0.109	15321.03	21.00
634	HLA-A0201	ISLLEMLLG	ISLLEMLLG	0	0	0	0	0	ISLLEMLLG	tr_D0A9V0	0.109	15346.75	21.00
671	HLA-A0201	FVQSLALGHLEP	FVQSLALHL	0	0	0	7	1	FVQSLALGHL	tr_D0A9V0	0.109	15348.40	21.00
646	HLA-A0201	WQWSSTAPAGSDAT	WQWSSTAPA	0	0	0	8	4	WQWSSTAPAGSDA	tr_D0A9V0	0.109	15358.05	21.00
581	HLA-A0201	CKLSDFGSTISE	KLSDFGSTI	1	0	0	0	0	KLSDFGSTI	tr_D0A9V0	0.109	15366.85	21.00
522	HLA-A0201	EYVPGGSLHSVIKN	YVPGGLHSV	1	0	0	5	1	YVPGGSLHSV	tr_D0A9V0	0.109	15369.51	21.00
704	HLA-A0201	PSMRPSASSL	SMRPSASSL	1	0	0	0	0	SMRPSASSL	tr_D0A9V0	0.109	15380.82	21.00
253	HLA-A0201	ATSGGASHM	ATSGGASHM	0	0	0	0	0	ATSGGASHM	tr_D0A9V0	0.109	15384.48	21.00
77	HLA-A0201	SATETPGESSTAFV	SATETTAFV	0	0	0	5	5	SATETPGESSTAFV	tr_D0A9V0	0.109	15388.15	21.00
697	HLA-A0201	RACCHMNPSMRPSA	HMNPSMPSA	4	0	0	6	1	HMNPSMRPSA	tr_D0A9V0	0.109	15394.98	21.00
95	HLA-A0201	ASISVTWNAG	ASISVTWNA	0	0	0	0	0	ASISVTWNA	tr_D0A9V0	0.109	15420.48	21.00
328	HLA-A0201	MDMQAKRLMRL	MQAKRLMRL	0	0	0	1	2	MDMQAKRLMRL	tr_D0A9V0	0.109	15427.50	21.00
14	HLA-A0201	NLEIPDNADIFLGR	NLDNADIFL	0	0	0	2	3	NLEIPDNADIFL	tr_D0A9V0	0.109	15431.17	21.00
197	HLA-A0201	AKLPLDHRDSVTM	KLPLDSVTM	1	0	0	4	3	KLPLDHRDSVTM	tr_D0A9V0	0.109	15433.67	21.00
326	HLA-A0201	VKMDMQAKRL	KMDMQAKRL	1	0	0	0	0	KMDMQAKRL	tr_D0A9V0	0.109	15435.51	21.00
636	HLA-A0201	LLEMLLGRPPWQW	LLEMLLWQW	0	0	0	6	4	LLEMLLGRPPWQW	tr_D0A9V0	0.109	15444.20	21.00
555	HLA-A0201	LNYLHNLGI	LNYLHNLGI	0	0	0	0	0	LNYLHNLGI	tr_D0A9V0	0.108	15474.14	21.00
545	HLA-A0201	RRFTVDILQGLNY	FTVDILQGL	2	0	0	0	0	FTVDILQGL	tr_D0A9V0	0.108	15481.17	21.00
640	HLA-A0201	LLGRPPWQWS	LLGRPPWWS	0	0	0	7	1	LLGRPPWQWS	tr_D0A9V0	0.108	15523.78	22.00
351	HLA-A0201	YVRVLDNHYC	YVVLDNHYC	0	0	0	2	1	YVRVLDNHYC	tr_D0A9V0	0.108	15531.84	22.00
238	HLA-A0201	FQLSYAVRDG	FQLSYAVRG	0	0	0	8	1	FQLSYAVRDG	tr_D0A9V0	0.108	15537.89	22.00
131	HLA-A0201	VIAIQVYHPDVW	VIAIQVYHV	0	0	0	8	2	VIAIQVYHPDV	tr_D0A9V0	0.108	15561.95	22.00
403	HLA-A0201	GSESLFKMYAPSSS	SLFKMYAPS	3	0	0	8	2	SLFKMYAPSSS	tr_D0A9V0	0.108	15570.87	22.00
472	HLA-A0201	VAIKCIPRLRRDV	VAIKCIPRV	0	0	0	8	4	VAIKCIPRLRRDV	tr_D0A9V0	0.108	15592.11	22.00
404	HLA-A0201	SESLFKMYAPSSSS	SLFKMYAPS	2	0	0	8	3	SLFKMYAPSSSS	tr_D0A9V0	0.108	15602.07	22.00
51	HLA-A0201	LKFIPEAILA	LKFIEAILA	0	0	0	4	1	LKFIPEAILA	tr_D0A9V0	0.108	15604.27	22.00
563	HLA-A0201	IVHCDVKPHNV	IVHCDVHNV	0	0	0	6	2	IVHCDVKPHNV	tr_D0A9V0	0.108	15616.93	22.00
693	HLA-A0201	AAFVRACCHMNPSM	FVRACCHMM	2	0	0	8	3	FVRACCHMNPSM	tr_D0A9V0	0.107	15677.20	22.00
7	HLA-A0201	SALKSASNLEIP	ALSASNLEI	1	0	0	2	1	ALKSASNLEI	tr_D0A9V0	0.107	15680.93	22.00
149	HLA-A0201	HAALIEVATEHS	ALIEVATES	2	0	0	8	1	ALIEVATEHS	tr_D0A9V0	0.107	15693.33	22.00
508	HLA-A0201	VSCSITPSHL	VSCSITPSL	0	0	0	8	1	VSCSITPSHL	tr_D0A9V0	0.107	15698.59	22.00
558	HLA-A0201	LHNLGIVHCDVK	LLGIVHCDV	0	0	0	1	2	LHNLGIVHCDV	tr_D0A9V0	0.107	15707.43	22.00
636	HLA-A0201	LLEMLLGRP	LLEMLLGRP	0	0	0	0	0	LLEMLLGRP	tr_D0A9V0	0.107	15722.73	22.00
678	HLA-A0201	GHLEPVIPA	GHLEPVIPA	0	0	0	0	0	GHLEPVIPA	tr_D0A9V0	0.107	15734.64	22.00
595	HLA-A0201	VMARTLADG	VMARTLADG	0	0	0	0	0	VMARTLADG	tr_D0A9V0	0.107	15755.08	22.00
350	HLA-A0201	PYVRVLDNHYCVNS	YVLDNHYCV	1	0	0	1	2	YVRVLDNHYCV	tr_D0A9V0	0.107	15763.27	22.00
257	HLA-A0201	GASHMQGFLAFP	GMQGFLAFP	0	0	0	1	3	GASHMQGFLAFP	tr_D0A9V0	0.107	15785.12	22.00

43	HLA-A0201	LRRQLAVLLKF	LQLAVLLKF	0	0	0	1	2	LRRQLAVLLKF	tr_D0A9V0	0.107	15785.97	22.00
321	HLA-A0201	HYLPMVKMDMQ	YLPMVKMDM	1	0	0	0	0	YLPMVKMDM	tr_D0A9V0	0.106	15809.05	22.00
479	HLA-A0201	RLRRDVVAESLEK	RLDVVAESL	0	0	0	2	2	RLRRDVVAESL	tr_D0A9V0	0.106	15824.61	22.00
226	HLA-A0201	RNARVLFGA	RNARVLFGA	0	0	0	0	0	RNARVLFGA	tr_D0A9V0	0.106	15824.61	22.00
163	HLA-A0201	VEALDPDRALISF	ALDPALISF	2	0	0	4	2	ALDPDRALISF	tr_D0A9V0	0.106	15838.49	22.00
294	HLA-A0201	EVSLSISKQFRLGF	SLISKQFRL	2	0	0	2	1	SLSISKQFRL	tr_D0A9V0	0.106	15842.09	22.00
544	HLA-A0201	ARRFTVDILQGLNY	FTVDILQGL	3	0	0	0	0	FTVDILQGL	tr_D0A9V0	0.106	15853.76	22.00
148	HLA-A0201	YHAALIEVATEHS	ALIEVATES	3	0	0	8	1	ALIEVATEHS	tr_D0A9V0	0.106	15873.14	22.00
52	HLA-A0201	KFIPEAILAR	FIPEAILAR	1	0	0	0	0	FIPEAILAR	tr_D0A9V0	0.106	15897.38	22.00
87	HLA-A0201	TAFVRIMGAS	TAFVRIMGA	0	0	0	0	0	TAFVRIMGA	tr_D0A9V0	0.106	15927.34	22.00
124	HLA-A0201	FNRRHCTVIAIQVY	FCTVIAIQV	0	0	0	1	4	FNRRHCTVIAIQV	tr_D0A9V0	0.106	15934.75	22.00
527	HLA-A0201	GSLHSVIKNF	SLHSVIKNF	1	0	0	0	0	SLHSVIKNF	tr_D0A9V0	0.106	15938.02	22.00
478	HLA-A0201	PRLRRDVVAESLE	RLDVVAESL	1	0	0	2	2	RLRRDVVAESL	tr_D0A9V0	0.106	15941.83	22.00
541	HLA-A0201	PYVARRFTVDIL	YVAFTVDIL	1	0	0	3	2	YVARRFTVDIL	tr_D0A9V0	0.105	16022.41	22.00
151	HLA-A0201	ALIEVATEHSG	ALIEVAHSG	0	0	0	6	2	ALIEVATEHSG	tr_D0A9V0	0.105	16048.94	22.00
256	HLA-A0201	GGASHMQGFLAFP	GMQGFLAFP	0	0	0	1	4	GGASHMQGFLAFP	tr_D0A9V0	0.105	16069.62	22.00
17	HLA-A0201	IPDNADIFL	IPDNADIFL	0	0	0	0	0	IPDNADIFL	tr_D0A9V0	0.105	16075.01	22.00
291	HLA-A0201	SSSEVSLSIS	SSSEVSLSI	0	0	0	0	0	SSSEVSLSI	tr_D0A9V0	0.105	16076.76	22.00
622	HLA-A0201	RCTPQSDIFSFGI	CTDIFSFGI	1	0	0	2	3	CTPQSDIFSFGI	tr_D0A9V0	0.105	16091.03	22.00
58	HLA-A0201	ILARVESKNEA	ILARVENEA	0	0	0	6	2	ILARVESKNEA	tr_D0A9V0	0.105	16091.55	22.00
162	HLA-A0201	CVEALDPDRALISF	ALDPALISF	3	0	0	4	2	ALDPDRALISF	tr_D0A9V0	0.105	16124.14	22.00
147	HLA-A0201	KYHAALIEVATEHS	ALIEVATES	4	0	0	8	1	ALIEVATEHS	tr_D0A9V0	0.105	16134.09	22.00
548	HLA-A0201	TVDILQGLNYLHN	VILQGLNYL	1	0	0	1	1	VDILQGLNYL	tr_D0A9V0	0.104	16190.57	22.00
300	HLA-A0201	SKQFRLGFAAMR	KQFLGFAAM	1	0	0	3	1	KQFRLGFAAM	tr_D0A9V0	0.104	16230.21	22.00
126	HLA-A0201	RRHCTVIAI	RRHCTVIAI	0	0	0	0	0	RRHCTVIAI	tr_D0A9V0	0.104	16245.50	22.00
403	HLA-A0201	GSESLFKMYA	GSESLFMYA	0	0	0	6	1	GSESLFKMYA	tr_D0A9V0	0.104	16253.41	22.00
680	HLA-A0201	LEPVIPASV	LEPVIPASV	0	0	0	0	0	LEPVIPASV	tr_D0A9V0	0.104	16275.40	22.00
40	HLA-A0201	YAKLRRQLAVLLK	KLRQLAVLL	2	0	0	2	1	KLRRQLAVLL	tr_D0A9V0	0.104	16278.57	22.00
572	HLA-A0201	NVLLGTDGVC	NVLLGTDGV	0	0	0	0	0	NVLLGTDGV	tr_D0A9V0	0.104	16296.38	22.00
600	HLA-A0201	LADGVMLRGT	LADGVMLGT	0	0	0	7	1	LADGVMLRGT	tr_D0A9V0	0.103	16319.66	22.00
677	HLA-A0201	LGHLEPVIPA	LGLEPVIPA	0	0	0	2	1	LGHLEPVIPA	tr_D0A9V0	0.103	16328.67	22.00
282	HLA-A0201	VLFELRPGESSS	VLFELESSS	0	0	0	5	3	VLFELRPGESSS	tr_D0A9V0	0.103	16340.69	23.00
337	HLA-A0201	RLCRQRCRRNDTV	RLCRRNDTV	0	0	0	2	4	RLCRQRCRRNDTV	tr_D0A9V0	0.103	16348.65	23.00
173	HLA-A0201	ISFGAHAALPM	ISFGAALPM	0	0	0	4	2	ISFGAHAALPM	tr_D0A9V0	0.103	16384.95	23.00
710	HLA-A0201	ASSLLSYAF	ASSLLSYAF	0	0	0	0	0	ASSLLSYAF	tr_D0A9V0	0.103	16392.93	23.00
408	HLA-A0201	FKMYAPSSSSSLDG	KMYASSSSL	1	0	0	4	2	KMYAPSSSSSL	tr_D0A9V0	0.103	16413.16	23.00
636	HLA-A0201	LLEMLLGRPPWQWS	LLEMLLWQW	0	0	0	6	4	LLEMLLGRPPWQW	tr_D0A9V0	0.103	16427.73	23.00
41	HLA-A0201	AKLRRQLAVLLK	KLRQLAVLL	1	0	0	2	1	KLRRQLAVLL	tr_D0A9V0	0.103	16435.38	23.00
196	HLA-A0201	FAKLPLDHRDSVTM	KLPLDSVTM	2	0	0	4	3	KLPLDHRDSVTM	tr_D0A9V0	0.103	16444.26	23.00
115	HLA-A0201	GIFKEKPNVF	GIFKEKPNV	0	0	0	0	0	GIFKEKPNV	tr_D0A9V0	0.103	16448.36	23.00
268	HLA-A0201	PVDCVLLPCSTL	PVDCVLLTL	0	0	0	7	3	PVDCVLLPCSTL	tr_D0A9V0	0.103	16457.26	23.00
319	HLA-A0201	MAHYLPMVKMDMQ	YLPMVKMDM	3	0	0	0	0	YLPMVKMDM	tr_D0A9V0	0.103	16472.05	23.00
122	HLA-A0201	NVFNRRHCTVIA	NVFNCTVIA	0	0	0	4	3	NVFNRRHCTVIA	tr_D0A9V0	0.102	16544.93	23.00
298	HLA-A0201	SISKQFRLGFAAMR	KQFLGFAAM	3	0	0	3	1	KQFRLGFAAM	tr_D0A9V0	0.102	16546.00	23.00
299	HLA-A0201	ISKQFRLGFAAMR	KQFLGFAAM	2	0	0	3	1	KQFRLGFAAM	tr_D0A9V0	0.102	16546.89	23.00
320	HLA-A0201	AHYLPMVKMDMQ	YLPMVKMDM	2	0	0	0	0	YLPMVKMDM	tr_D0A9V0	0.102	16554.95	23.00
309	HLA-A0201	AMRQGRYGEA	AMQGRYGEA	0	0	0	2	1	AMRQGRYGEA	tr_D0A9V0	0.102	16561.94	23.00
580	HLA-A0201	VCKLSDFGSTISE	KLSDFGSTI	2	0	0	0	0	KLSDFGSTI	tr_D0A9V0	0.102	16575.20	23.00
586	HLA-A0201	FGSTISEASVMAR	FISEASVMA	0	0	0	1	3	FGSTISEASVMA	tr_D0A9V0	0.102	16605.36	23.00
659	HLA-A0201	TRLRSLFNRDL	RLRSLFNRL	1	0	0	8	1	RLRSLFNRDL	tr_D0A9V0	0.102	16615.07	23.00
460	HLA-A0201	TIFLGVAEDG	TIFLGVAEG	0	0	0	8	1	TIFLGVAEDG	tr_D0A9V0	0.102	16636.65	23.00
640	HLA-A0201	LLGRPPWQW	LLGRPPWQW	0	0	0	0	0	LLGRPPWQW	tr_D0A9V0	0.102	16646.91	23.00
540	HLA-A0201	SPYVARRFTVDIL	YVAFTVDIL	2	0	0	3	2	YVARRFTVDIL	tr_D0A9V0	0.102	16657.54	23.00
539	HLA-A0201	VSPYVARRFTVDIL	YVAFTVDIL	3	0	0	3	2	YVARRFTVDIL	tr_D0A9V0	0.101	16678.83	23.00

670	HLA-A0201	LFVQSLALGHLE	FVQSLALHL	1	0	0	7	1	FVQSLALGHL	tr_D0A9V0	0.101	16748.82	23.00
148	HLA-A0201	YHAALIEVATEH	YLIEVATEH	0	0	0	1	3	YHAALIEVATEH	tr_D0A9V0	0.101	16752.80	23.00
450	HLA-A0201	LKKISEGAFSTIF	KISEGAFTI	2	0	0	7	1	KISEGAFSTI	tr_D0A9V0	0.101	16760.24	23.00
281	HLA-A0201	IVLFELRPGESS	VLFELGESS	1	0	0	5	2	VLFELRPGESS	tr_D0A9V0	0.101	16760.96	23.00
666	HLA-A0201	NRDLLFVQSL	RDLLFVQSL	1	0	0	0	0	RDLLFVQSL	tr_D0A9V0	0.101	16766.94	23.00
342	HLA-A0201	RCRRNDTVPYV	RCNDTVPYV	0	0	0	2	2	RCRRNDTVPYV	tr_D0A9V0	0.101	16778.02	23.00
603	HLA-A0201	GVMLRGTALY	GVMLRGTAL	0	0	0	0	0	GVMLRGTAL	tr_D0A9V0	0.101	16795.63	23.00
301	HLA-A0201	KQFRLGFAAMRQ	KQFLGFAAM	0	0	0	3	1	KQFRLGFAAM	tr_D0A9V0	0.101	16800.72	23.00
504	HLA-A0201	IVRYVSCSIT	IVYVSCSIT	0	0	0	2	1	IVRYVSCSIT	tr_D0A9V0	0.101	16806.00	23.00
477	HLA-A0201	IPRLRRDVVAESLE	RLDVVAESL	2	0	0	2	2	RLRRDVVAESL	tr_D0A9V0	0.101	16811.81	23.00
165	HLA-A0201	ALDPDRALISFG	ALDALISFG	0	0	0	3	3	ALDPDRALISFG	tr_D0A9V0	0.101	16813.81	23.00
148	HLA-A0201	YHAALIEVAT	YAALIEVAT	0	0	0	1	1	YHAALIEVAT	tr_D0A9V0	0.101	16834.39	23.00
23	HLA-A0201	IFLGRSRIV	IFLGRSRIV	0	0	0	0	0	IFLGRSRIV	tr_D0A9V0	0.101	16839.85	23.00
39	HLA-A0201	HYAKLRRQLAVLLK	KLRQLAVLL	3	0	0	2	1	KLRRQLAVLL	tr_D0A9V0	0.101	16844.04	23.00
129	HLA-A0201	CTVIAIQVY	CTVIAIQVY	0	0	0	0	0	CTVIAIQVY	tr_D0A9V0	0.100	16855.53	23.00
706	HLA-A0201	MRPSASSLLSYA	MASSLLSYA	0	0	0	1	3	MRPSASSLLSYA	tr_D0A9V0	0.100	16860.08	23.00
197	HLA-A0201	AKLPLDHRDSVT	KLLDHRDSV	1	0	0	2	1	KLPLDHRDSV	tr_D0A9V0	0.100	16860.82	23.00
8	HLA-A0201	ALKSASNLEIPD	ALSASNLEI	0	0	0	2	1	ALKSASNLEI	tr_D0A9V0	0.100	16867.21	23.00
517	HLA-A0201	LSIIMEYVPGGS	IIMEYVPGS	2	0	0	7	1	IIMEYVPGGS	tr_D0A9V0	0.100	16873.59	23.00
541	HLA-A0201	PYVARRFTVDI	YVARFTVDI	1	0	0	3	1	YVARRFTVDI	tr_D0A9V0	0.100	16876.15	23.00
155	HLA-A0201	VATEHSGCV	VATEHSGCV	0	0	0	0	0	VATEHSGCV	tr_D0A9V0	0.100	16876.69	23.00
701	HLA-A0201	HMNPSMRPS	HMNPSMRPS	0	0	0	0	0	HMNPSMRPS	tr_D0A9V0	0.100	16878.34	23.00
569	HLA-A0201	KPHNVLLGTDGV	KVLLGTDGV	0	0	0	1	3	KPHNVLLGTDGV	tr_D0A9V0	0.100	16907.03	23.00
258	HLA-A0201	ASHMQGFLAF	ASMQGFLAF	0	0	0	2	1	ASHMQGFLAF	tr_D0A9V0	0.100	16912.16	23.00
638	HLA-A0201	EMLLGRPPWQ	MLLGRPPWQ	1	0	0	0	0	MLLGRPPWQ	tr_D0A9V0	0.100	16926.99	23.00
479	HLA-A0201	RLRRDVVAESLEKE	RLDVVAESL	0	0	0	2	2	RLRRDVVAESL	tr_D0A9V0	0.100	16949.52	23.00
542	HLA-A0201	YVARRFTVDILQ	YVFTVDILQ	0	0	0	2	3	YVARRFTVDILQ	tr_D0A9V0	0.100	16959.07	23.00
145	HLA-A0201	STKYHAALI	STKYHAALI	0	0	0	0	0	STKYHAALI	tr_D0A9V0	0.100	16969.34	23.00
561	HLA-A0201	LGIVHCDVKPHNV	GIVHCDVNV	1	0	0	7	3	GIVHCDVKPHNV	tr_D0A9V0	0.100	16987.71	23.00
709	HLA-A0201	SASSLLSYAF	SASSLLYAF	0	0	0	6	1	SASSLLSYAF	tr_D0A9V0	0.100	17011.81	23.00
6	HLA-A0201	VSALKSASNLEIP	ALSASNLEI	2	0	0	2	1	ALKSASNLEI	tr_D0A9V0	0.100	17029.86	23.00
700	HLA-A0201	CHMNPSMRPSASSL	HMNPSMSSL	1	0	0	6	4	HMNPSMRPSASSL	tr_D0A9V0	0.099	17039.62	23.00
579	HLA-A0201	GVCKLSDFGSTISE	KLSDFGSTI	3	0	0	0	0	KLSDFGSTI	tr_D0A9V0	0.099	17066.93	23.00
314	HLA-A0201	RYGEAMAHYL	RYGEAMAYL	0	0	0	7	1	RYGEAMAHYL	tr_D0A9V0	0.099	17089.85	23.00
238	HLA-A0201	FQLSYAVRD	FQLSYAVRD	0	0	0	0	0	FQLSYAVRD	tr_D0A9V0	0.099	17114.46	23.00
646	HLA-A0201	WQWSSTAPAGS	WQWSSTAPA	0	0	0	0	0	WQWSSTAPA	tr_D0A9V0	0.099	17129.09	23.00
196	HLA-A0201	FAKLPLDHRDSVT	KLLDHRDSV	2	0	0	2	1	KLPLDHRDSV	tr_D0A9V0	0.099	17135.76	23.00
540	HLA-A0201	SPYVARRFTV	SPYVARFTV	0	0	0	5	1	SPYVARRFTV	tr_D0A9V0	0.099	17157.66	23.00
472	HLA-A0201	VAIKCIPRLR	VAIKCIPRL	0	0	0	0	0	VAIKCIPRL	tr_D0A9V0	0.099	17199.67	24.00
621	HLA-A0201	GRCTPQSDIFSFGI	CTDIFSFGI	2	0	0	2	3	CTPQSDIFSFGI	tr_D0A9V0	0.099	17212.14	24.00
600	HLA-A0201	LADGVMLRGTA	LADGVMLTA	0	0	0	7	2	LADGVMLRGTA	tr_D0A9V0	0.098	17230.02	24.00
524	HLA-A0201	VPGGSLHSV	VPGGSLHSV	0	0	0	0	0	VPGGSLHSV	tr_D0A9V0	0.098	17293.90	24.00
658	HLA-A0201	ATRLRSLFNRDL	RLRSLFNRL	2	0	0	8	1	RLRSLFNRDL	tr_D0A9V0	0.098	17339.99	24.00
636	HLA-A0201	LLEMLLGRPPW	LLEMLLPPW	0	0	0	6	2	LLEMLLGRPPW	tr_D0A9V0	0.098	17379.44	24.00
638	HLA-A0201	EMLLGRPPWQW	MLLGRPWQW	1	0	0	5	1	MLLGRPPWQW	tr_D0A9V0	0.097	17416.52	24.00
5	HLA-A0201	LVSALKSASNLEIP	ALSASNLEI	3	0	0	2	1	ALKSASNLEI	tr_D0A9V0	0.097	17419.92	24.00
180	HLA-A0201	ALPMHCRRCC	ALMHCRRCC	0	0	0	2	1	ALPMHCRRCC	tr_D0A9V0	0.097	17423.87	24.00
132	HLA-A0201	IAIQVYHPDVW	IAIQVYHPV	0	0	0	8	1	IAIQVYHPDV	tr_D0A9V0	0.097	17432.55	24.00
131	HLA-A0201	VIAIQVYHP	VIAIQVYHP	0	0	0	0	0	VIAIQVYHP	tr_D0A9V0	0.097	17445.75	24.00
589	HLA-A0201	TISEASVMARTL	TISEASVTL	0	0	0	7	3	TISEASVMARTL	tr_D0A9V0	0.097	17455.58	24.00
460	HLA-A0201	TIFLGVAEDGAQ	TIFLGVAEA	0	0	0	8	2	TIFLGVAEDGA	tr_D0A9V0	0.097	17471.82	24.00
453	HLA-A0201	ISEGAFSTI	ISEGAFSTI	0	0	0	0	0	ISEGAFSTI	tr_D0A9V0	0.097	17527.86	24.00
324	HLA-A0201	PMVKMDMQA	PMVKMDMQA	0	0	0	0	0	PMVKMDMQA	tr_D0A9V0	0.097	17537.17	24.00
686	HLA-A0201	ASVDHEAAAFVR	SVDEAAAFV	1	0	0	3	1	SVDHEAAAFV	tr_D0A9V0	0.097	17546.27	24.00

488	HLA-A0201	SLEKEMEVAS	SLEEMEVAS	0	0	0	3	1	SLEKEMEVAS	tr_D0A9V0	0.097	17566.98	24.00
645	HLA-A0201	PWQWSSTAPAG	WQWSSTAPA	1	0	0	0	0	WQWSSTAPA	tr_D0A9V0	0.097	17574.39	24.00
556	HLA-A0201	NYLHNLGIVH	YLHNLGIVH	1	0	0	0	0	YLHNLGIVH	tr_D0A9V0	0.097	17582.00	24.00
670	HLA-A0201	LFVQSLALGHLEP	FLALGHLEP	1	0	0	1	3	FVQSLALGHLEP	tr_D0A9V0	0.097	17588.09	24.00
200	HLA-A0201	PLDHRDSVTMLVD	PLDSVTMLV	0	0	0	2	3	PLDHRDSVTMLV	tr_D0A9V0	0.096	17613.61	24.00
333	HLA-A0201	KRLMRLCRQRC	RLMRLCQRC	1	0	0	6	1	RLMRLCRQRC	tr_D0A9V0	0.096	17619.90	24.00
657	HLA-A0201	DATRLRSLFNRDL	RLRSLFNRL	3	0	0	8	1	RLRSLFNRDL	tr_D0A9V0	0.096	17664.75	24.00
574	HLA-A0201	LLGTDGVCKLSDF	LLVCKLSDF	0	0	0	2	4	LLGTDGVCKLSDF	tr_D0A9V0	0.096	17668.19	24.00
540	HLA-A0201	SPYVARRFTVDI	YVARFTVDI	2	0	0	3	1	YVARRFTVDI	tr_D0A9V0	0.096	17676.80	24.00
276	HLA-A0201	CSTLPIVLF	CSTLPIVLF	0	0	0	0	0	CSTLPIVLF	tr_D0A9V0	0.096	17682.16	24.00
539	HLA-A0201	VSPYVARRFTVDI	YVARFTVDI	3	0	0	3	1	YVARRFTVDI	tr_D0A9V0	0.096	17696.51	24.00
638	HLA-A0201	EMLLGRPPWQWS	MLLGRPPWS	1	0	0	7	2	MLLGRPPWQWS	tr_D0A9V0	0.096	17706.85	24.00
57	HLA-A0201	AILARVESKNEA	ILARVENEA	1	0	0	6	2	ILARVESKNEA	tr_D0A9V0	0.096	17737.15	24.00
85	HLA-A0201	SSTAFVRIM	SSTAFVRIM	0	0	0	0	0	SSTAFVRIM	tr_D0A9V0	0.096	17751.93	24.00
534	HLA-A0201	KNFGCVSPYVA	KNFGCVPYV	0	0	0	6	1	KNFGCVSPYV	tr_D0A9V0	0.096	17754.82	24.00
28	HLA-A0201	SRIVEVRKIQGHYA	RIVEVGHYA	1	0	0	5	4	RIVEVRKIQGHYA	tr_D0A9V0	0.096	17763.85	24.00
487	HLA-A0201	ESLEKEMEVA	SLEKEMEVA	1	0	0	0	0	SLEKEMEVA	tr_D0A9V0	0.096	17790.97	24.00
417	HLA-A0201	SSLDGSGCAEGANA	SLDGSGCAA	1	0	0	8	4	SLDGSGCAEGANA	tr_D0A9V0	0.095	17795.58	24.00
429	HLA-A0201	NAADVVPLFLT	AADVVLFLT	1	0	0	5	1	AADVVPLFLT	tr_D0A9V0	0.095	17799.44	24.00
596	HLA-A0201	MARTLADGVML	MTLADGVML	0	0	0	1	2	MARTLADGVML	tr_D0A9V0	0.095	17820.06	24.00
418	HLA-A0201	SLDGSGCAEGAN	SLDGCAEGA	0	0	0	3	2	SLDGSGCAEGA	tr_D0A9V0	0.095	17831.24	24.00
656	HLA-A0201	SDATRLRSLFNRDL	RLRSLFNRL	4	0	0	8	1	RLRSLFNRDL	tr_D0A9V0	0.095	17857.11	24.00
269	HLA-A0201	VDCVLLPCSTL	VVLLPCSTL	0	0	0	1	2	VDCVLLPCSTL	tr_D0A9V0	0.095	17921.37	25.00
470	HLA-A0201	AQVAIKCIPR	AQVAICIPR	0	0	0	5	1	AQVAIKCIPR	tr_D0A9V0	0.095	17945.24	25.00
281	HLA-A0201	IVLFELRPGE	ILFELRPGE	0	0	0	1	1	IVLFELRPGE	tr_D0A9V0	0.095	17947.96	25.00
433	HLA-A0201	VVPLFLTDS	VVPLFLTDS	0	0	0	0	0	VVPLFLTDS	tr_D0A9V0	0.095	17960.58	25.00
301	HLA-A0201	KQFRLGFAAMRQG	KQFLGFAAM	0	0	0	3	1	KQFRLGFAAM	tr_D0A9V0	0.095	17964.28	25.00
354	HLA-A0201	VLDNHYCVNSS	VLDNCVNSS	0	0	0	4	2	VLDNHYCVNSS	tr_D0A9V0	0.095	17969.92	25.00
574	HLA-A0201	LLGTDGVCKLSD	LLGDGVCKL	0	0	0	3	1	LLGTDGVCKL	tr_D0A9V0	0.095	17984.31	25.00
453	HLA-A0201	ISEGAFSTIFL	ISAFSTIFL	0	0	0	2	2	ISEGAFSTIFL	tr_D0A9V0	0.094	17988.59	25.00
695	HLA-A0201	FVRACCHMNP	FVRACCHMP	0	0	0	8	1	FVRACCHMNP	tr_D0A9V0	0.094	17989.18	25.00
47	HLA-A0201	LAVLLKFIPE	LAVLLFIPE	0	0	0	5	1	LAVLLKFIPE	tr_D0A9V0	0.094	18005.93	25.00
587	HLA-A0201	GSTISEASV	GSTISEASV	0	0	0	0	0	GSTISEASV	tr_D0A9V0	0.094	18026.97	25.00
675	HLA-A0201	LALGHLEPVIP	LALGHLEPV	0	0	0	0	0	LALGHLEPV	tr_D0A9V0	0.094	18096.75	25.00
499	HLA-A0201	LHHPNIVRYV	LHHPNIVYV	0	0	0	7	1	LHHPNIVRYV	tr_D0A9V0	0.094	18100.66	25.00
130	HLA-A0201	TVIAIQVYHP	TVIAIVYHP	0	0	0	5	1	TVIAIQVYHP	tr_D0A9V0	0.094	18103.60	25.00
643	HLA-A0201	RPPWQWSSTAPAG	RQWSSTAPA	0	0	0	1	3	RPPWQWSSTAPA	tr_D0A9V0	0.094	18106.93	25.00
299	HLA-A0201	ISKQFRLGFAAMRQ	KQFLGFAAM	2	0	0	3	1	KQFRLGFAAM	tr_D0A9V0	0.094	18112.22	25.00
124	HLA-A0201	FNRRHCTVIAI	FRHCTVIAI	0	0	0	1	2	FNRRHCTVIAI	tr_D0A9V0	0.094	18118.69	25.00
535	HLA-A0201	NFGCVSPYVA	NFGCVSPYV	0	0	0	0	0	NFGCVSPYV	tr_D0A9V0	0.094	18118.69	25.00
224	HLA-A0201	FSRNARVLFGAD	FSARVLFGA	0	0	0	2	2	FSRNARVLFGA	tr_D0A9V0	0.094	18126.93	25.00
300	HLA-A0201	SKQFRLGFAAMRQ	KQFLGFAAM	1	0	0	3	1	KQFRLGFAAM	tr_D0A9V0	0.094	18152.63	25.00
195	HLA-A0201	LFAKLPLDHRDSVT	KLLDHRDSV	3	0	0	2	1	KLPLDHRDSV	tr_D0A9V0	0.094	18154.60	25.00
558	HLA-A0201	LHNLGIVHCDVKP	LLGIVHCDV	0	0	0	1	2	LHNLGIVHCDV	tr_D0A9V0	0.094	18177.79	25.00
687	HLA-A0201	SVDHEAAAFVRA	SVDEAAAFV	0	0	0	3	1	SVDHEAAAFV	tr_D0A9V0	0.093	18192.75	25.00
128	HLA-A0201	HCTVIAIQVY	HCTVIAIQV	0	0	0	0	0	HCTVIAIQV	tr_D0A9V0	0.093	18237.68	25.00
554	HLA-A0201	GLNYLHNLG	GLNYLHNLG	0	0	0	0	0	GLNYLHNLG	tr_D0A9V0	0.093	18259.00	25.00
25	HLA-A0201	LGRSRIVEV	LGRSRIVEV	0	0	0	0	0	LGRSRIVEV	tr_D0A9V0	0.093	18266.32	25.00
596	HLA-A0201	MARTLADGVMLR	MLADGVMLR	0	0	0	1	3	MARTLADGVMLR	tr_D0A9V0	0.093	18270.26	25.00
637	HLA-A0201	LEMLLGRPPWQW	MLLGRPWQW	2	0	0	5	1	MLLGRPPWQW	tr_D0A9V0	0.093	18290.05	25.00
21	HLA-A0201	ADIFLGRSRI	AIFLGRSRI	0	0	0	1	1	ADIFLGRSRI	tr_D0A9V0	0.093	18298.96	25.00
222	HLA-A0201	GAFSRNARVL	GAFSRNAVL	0	0	0	7	1	GAFSRNARVL	tr_D0A9V0	0.093	18305.49	25.00
281	HLA-A0201	IVLFELRPG	IVLFELRPG	0	0	0	0	0	IVLFELRPG	tr_D0A9V0	0.093	18328.48	25.00
238	HLA-A0201	FQLSYAVRDGPCR	FQLSYAVPC	0	0	0	7	3	FQLSYAVRDGPC	tr_D0A9V0	0.093	18336.21	25.00

453	HLA-A0201	ISEGAFSTIFLG	IAFSTIFLG	0	0	0	1	3	ISEGAFSTIFLG	tr_D0A9V0	0.093	18376.33	25.00
131	HLA-A0201	VIAIQVYHPDVWK	VIAIQVYHV	0	0	0	8	2	VIAIQVYHPDV	tr_D0A9V0	0.093	18378.53	25.00
156	HLA-A0201	ATEHSGCVEA	ATHSGCVEA	0	0	0	2	1	ATEHSGCVEA	tr_D0A9V0	0.092	18380.50	25.00
478	HLA-A0201	PRLRRDVVAESLEK	RLDVVAESL	1	0	0	2	2	RLRRDVVAESL	tr_D0A9V0	0.092	18402.00	25.00
269	HLA-A0201	VDCVLLPCSTLP	VLLPCSTLP	3	0	0	0	0	VLLPCSTLP	tr_D0A9V0	0.092	18427.91	25.00
39	HLA-A0201	HYAKLRRQLAV	YALRRQLAV	1	0	0	2	1	YAKLRRQLAV	tr_D0A9V0	0.092	18444.46	25.00
156	HLA-A0201	ATEHSGCVEAL	ATSGCVEAL	0	0	0	2	2	ATEHSGCVEAL	tr_D0A9V0	0.092	18446.46	25.00
485	HLA-A0201	VAESLEKEMEV	VALEKEMEV	0	0	0	2	2	VAESLEKEMEV	tr_D0A9V0	0.092	18471.02	25.00
664	HLA-A0201	LFNRDLLFVQ	LFNRDLLFV	0	0	0	0	0	LFNRDLLFV	tr_D0A9V0	0.092	18478.82	25.00
112	HLA-A0201	LNCGIFKEKPNV	LIFKEKPNV	0	0	0	1	3	LNCGIFKEKPNV	tr_D0A9V0	0.092	18483.42	25.00
330	HLA-A0201	MQAKRLMRLCRQRC	RLMRLCQRC	4	0	0	6	1	RLMRLCRQRC	tr_D0A9V0	0.092	18487.21	25.00
573	HLA-A0201	VLLGTDGVCK	VLLGTDVCK	0	0	0	6	1	VLLGTDGVCK	tr_D0A9V0	0.092	18499.02	25.00
585	HLA-A0201	DFGSTISEASVMAR	FISEASVMA	1	0	0	1	3	FGSTISEASVMA	tr_D0A9V0	0.092	18521.65	25.00
276	HLA-A0201	CSTLPIVLFE	STLPIVLFE	1	0	0	0	0	STLPIVLFE	tr_D0A9V0	0.092	18525.86	25.00
270	HLA-A0201	DCVLLPCSTLP	VLLPCSTLP	2	0	0	0	0	VLLPCSTLP	tr_D0A9V0	0.092	18539.29	25.00
334	HLA-A0201	RLMRLCRQR	RLMRLCRQR	0	0	0	0	0	RLMRLCRQR	tr_D0A9V0	0.092	18540.50	25.00
185	HLA-A0201	CRRCCQFAFDL	RCCQFAFDL	2	0	0	0	0	RCCQFAFDL	tr_D0A9V0	0.092	18540.70	25.00
268	HLA-A0201	PVDCVLLPCSTLP	VLLPCSTLP	4	0	0	0	0	VLLPCSTLP	tr_D0A9V0	0.092	18559.16	25.00
332	HLA-A0201	AKRLMRLCRQRC	RLMRLCQRC	2	0	0	6	1	RLMRLCRQRC	tr_D0A9V0	0.092	18566.60	25.00
301	HLA-A0201	KQFRLGFAAMRQGR	KQFLGFAAM	0	0	0	3	1	KQFRLGFAAM	tr_D0A9V0	0.092	18574.03	25.00
229	HLA-A0201	RVLFGADHLFQ	VLFGADLFQ	1	0	0	6	1	VLFGADHLFQ	tr_D0A9V0	0.092	18575.43	25.00
343	HLA-A0201	CRRNDTVPYV	RRNDTVPYV	1	0	0	0	0	RRNDTVPYV	tr_D0A9V0	0.091	18582.48	25.00
8	HLA-A0201	ALKSASNLEIPDN	ALSASNLEI	0	0	0	2	1	ALKSASNLEI	tr_D0A9V0	0.091	18591.93	25.00
428	HLA-A0201	ANAADVVPLFLT	AADVVLFLT	2	0	0	5	1	AADVVPLFLT	tr_D0A9V0	0.091	18654.19	25.00
692	HLA-A0201	AAAFVRACCHM	AAAFVCCHM	0	0	0	5	2	AAAFVRACCHM	tr_D0A9V0	0.091	18665.09	25.00
628	HLA-A0201	DIFSFGISLLE	DIFSFGILL	0	0	0	7	1	DIFSFGISLL	tr_D0A9V0	0.091	18696.82	26.00
51	HLA-A0201	LKFIPEAIL	LKFIPEAIL	0	0	0	0	0	LKFIPEAIL	tr_D0A9V0	0.091	18737.94	26.00
144	HLA-A0201	SSTKYHAAL	SSTKYHAAL	0	0	0	0	0	SSTKYHAAL	tr_D0A9V0	0.091	18753.55	26.00
280	HLA-A0201	PIVLFELRPGESS	VLFELGESS	2	0	0	5	2	VLFELRPGESS	tr_D0A9V0	0.091	18763.50	26.00
184	HLA-A0201	HCRRCCQFAFDL	RCCQFAFDL	3	0	0	0	0	RCCQFAFDL	tr_D0A9V0	0.090	18823.28	26.00
460	HLA-A0201	TIFLGVAED	TIFLGVAED	0	0	0	0	0	TIFLGVAED	tr_D0A9V0	0.090	18866.71	26.00
331	HLA-A0201	QAKRLMRLCRQRC	RLMRLCQRC	3	0	0	6	1	RLMRLCRQRC	tr_D0A9V0	0.090	18867.94	26.00
228	HLA-A0201	ARVLFGADHLFQ	VLFGADLFQ	2	0	0	6	1	VLFGADHLFQ	tr_D0A9V0	0.090	18897.76	26.00
637	HLA-A0201	LEMLLGRPPWQWS	MLLGRWQWS	2	0	0	5	2	MLLGRPPWQWS	tr_D0A9V0	0.090	18909.22	26.00
588	HLA-A0201	STISEASVM	STISEASVM	0	0	0	0	0	STISEASVM	tr_D0A9V0	0.090	18909.42	26.00
86	HLA-A0201	STAFVRIMGASIS	TAFVRIMSI	1	0	0	7	2	TAFVRIMGASI	tr_D0A9V0	0.090	18963.10	26.00
626	HLA-A0201	QSDIFSFGIS	QSDIFSFGI	0	0	0	0	0	QSDIFSFGI	tr_D0A9V0	0.090	18975.83	26.00
257	HLA-A0201	GASHMQGFL	GASHMQGFL	0	0	0	0	0	GASHMQGFL	tr_D0A9V0	0.089	19007.47	26.00
87	HLA-A0201	TAFVRIMGASIS	TAFVRIMSI	0	0	0	7	2	TAFVRIMGASI	tr_D0A9V0	0.089	19024.54	26.00
123	HLA-A0201	VFNRRHCTVIAI	VFHCTVIAI	0	0	0	2	3	VFNRRHCTVIAI	tr_D0A9V0	0.089	19026.61	26.00
173	HLA-A0201	ISFGAHAALP	ISFGAHAAL	0	0	0	0	0	ISFGAHAAL	tr_D0A9V0	0.089	19030.10	26.00
646	HLA-A0201	WQWSSTAPAGSD	WQWSSTAPA	0	0	0	0	0	WQWSSTAPA	tr_D0A9V0	0.089	19037.32	26.00
311	HLA-A0201	RQGRYGEAMAHYL	RQGAMAHYL	0	0	0	3	4	RQGRYGEAMAHYL	tr_D0A9V0	0.089	19041.03	26.00
38	HLA-A0201	GHYAKLRRQLAV	YALRRQLAV	2	0	0	2	1	YAKLRRQLAV	tr_D0A9V0	0.089	19042.26	26.00
427	HLA-A0201	GANAADVVPLFLT	AADVVLFLT	3	0	0	5	1	AADVVPLFLT	tr_D0A9V0	0.089	19055.03	26.00
192	HLA-A0201	AFDLFAKLPLDH	FLFAKLPLD	1	0	0	1	1	FDLFAKLPLD	tr_D0A9V0	0.089	19057.10	26.00
458	HLA-A0201	FSTIFLGVAEDGAQ	TIFLGVAEA	2	0	0	8	2	TIFLGVAEDGA	tr_D0A9V0	0.089	19069.06	26.00
279	HLA-A0201	LPIVLFELRPGESS	VLFELGESS	3	0	0	5	2	VLFELRPGESS	tr_D0A9V0	0.089	19070.51	26.00
150	HLA-A0201	AALIEVATEHSG	ALIEVAHSG	1	0	0	6	2	ALIEVATEHSG	tr_D0A9V0	0.089	19097.35	26.00
172	HLA-A0201	LISFGAHAALP	LIFGAHAAL	0	0	0	2	1	LISFGAHAAL	tr_D0A9V0	0.089	19134.17	26.00
558	HLA-A0201	LHNLGIVHCDVKPH	LLGIVHCDV	0	0	0	1	2	LHNLGIVHCDV	tr_D0A9V0	0.089	19147.00	26.00
541	HLA-A0201	PYVARRFTVDILQ	YVFTVDILQ	1	0	0	2	3	YVARRFTVDILQ	tr_D0A9V0	0.089	19147.42	26.00
694	HLA-A0201	AFVRACCHM	AFVRACCHM	0	0	0	0	0	AFVRACCHM	tr_D0A9V0	0.089	19151.15	26.00
219	HLA-A0201	GTCGAFSRNARV	GTCGAFARV	0	0	0	6	3	GTCGAFSRNARV	tr_D0A9V0	0.089	19154.67	26.00

304	HLA-A0201	RLGFAAMRQG	RLFAAMRQG	0	0	0	2	1	RLGFAAMRQG	tr_D0A9V0	0.088	19193.67	26.00
426	HLA-A0201	EGANAADVVPLFLT	AADVVLFLT	4	0	0	5	1	AADVVPLFLT	tr_D0A9V0	0.088	19204.89	26.00
164	HLA-A0201	EALDPDRALISFG	ALDALISFG	1	0	0	3	3	ALDPDRALISFG	tr_D0A9V0	0.088	19204.89	26.00
459	HLA-A0201	STIFLGVAEDGAQ	TIFLGVAEA	1	0	0	8	2	TIFLGVAEDGA	tr_D0A9V0	0.088	19207.80	26.00
263	HLA-A0201	GFLAFPVDC	GFLAFPVDC	0	0	0	0	0	GFLAFPVDC	tr_D0A9V0	0.088	19211.13	26.00
568	HLA-A0201	VKPHNVLLGTDGV	VVLLGTDGV	0	0	0	1	4	VKPHNVLLGTDGV	tr_D0A9V0	0.088	19255.45	26.00
266	HLA-A0201	AFPVDCVLLP	FPVDCVLLP	1	0	0	0	0	FPVDCVLLP	tr_D0A9V0	0.088	19280.68	26.00
183	HLA-A0201	MHCRRCCQFAFDLF	MCCQFAFDL	0	0	0	1	4	MHCRRCCQFAFDL	tr_D0A9V0	0.088	19281.51	26.00
147	HLA-A0201	KYHAALIEVATEH	YLIEVATEH	1	0	0	1	3	YHAALIEVATEH	tr_D0A9V0	0.088	19281.51	26.00
227	HLA-A0201	NARVLFGADHLFQ	VLFGADLFQ	3	0	0	6	1	VLFGADHLFQ	tr_D0A9V0	0.088	19283.81	26.00
239	HLA-A0201	QLSYAVRDGPCRIA	QLDGPCRIA	0	0	0	2	5	QLSYAVRDGPCRIA	tr_D0A9V0	0.088	19292.36	26.00
702	HLA-A0201	MNPSMRPSASSL	MMRPSASSL	0	0	0	1	3	MNPSMRPSASSL	tr_D0A9V0	0.088	19296.96	26.00
56	HLA-A0201	EAILARVESKNEA	ILARVENEA	2	0	0	6	2	ILARVESKNEA	tr_D0A9V0	0.088	19312.41	26.00
677	HLA-A0201	LGHLEPVIPAS	LLEPVIPAS	0	0	0	1	2	LGHLEPVIPAS	tr_D0A9V0	0.088	19315.96	26.00
226	HLA-A0201	RNARVLFGADHLFQ	VLFGADLFQ	4	0	0	6	1	VLFGADHLFQ	tr_D0A9V0	0.088	19333.11	26.00
300	HLA-A0201	SKQFRLGFAAMRQG	KQFLGFAAM	1	0	0	3	1	KQFRLGFAAM	tr_D0A9V0	0.088	19334.78	26.00
121	HLA-A0201	PNVFNRRHCTVIA	NVFNCTVIA	1	0	0	4	3	NVFNRRHCTVIA	tr_D0A9V0	0.088	19339.81	26.00
450	HLA-A0201	LKKISEGAFST	LISEGAFST	0	0	0	1	2	LKKISEGAFST	tr_D0A9V0	0.088	19352.37	26.00
464	HLA-A0201	GVAEDGAQVA	GVAEDGAQV	0	0	0	0	0	GVAEDGAQV	tr_D0A9V0	0.088	19383.80	27.00
341	HLA-A0201	QRCRRNDTVPYV	RCNDTVPYV	1	0	0	2	2	RCRRNDTVPYV	tr_D0A9V0	0.088	19398.90	27.00
174	HLA-A0201	SFGAHAALPM	FGAHAALPM	1	0	0	0	0	FGAHAALPM	tr_D0A9V0	0.087	19431.47	27.00
517	HLA-A0201	LSIIMEYVPGG	LIMEYVPGG	0	0	0	1	2	LSIIMEYVPGG	tr_D0A9V0	0.087	19446.19	27.00
404	HLA-A0201	SESLFKMYA	SESLFKMYA	0	0	0	0	0	SESLFKMYA	tr_D0A9V0	0.087	19455.23	27.00
353	HLA-A0201	RVLDNHYCVNSS	VLDNCVNSS	1	0	0	4	2	VLDNHYCVNSS	tr_D0A9V0	0.087	19470.61	27.00
304	HLA-A0201	RLGFAAMRQ	RLGFAAMRQ	0	0	0	0	0	RLGFAAMRQ	tr_D0A9V0	0.087	19478.41	27.00
86	HLA-A0201	STAFVRIMGAS	STFVRIMGA	0	0	0	2	1	STAFVRIMGA	tr_D0A9V0	0.087	19482.42	27.00
458	HLA-A0201	FSTIFLGVAED	FIFLGVAED	0	0	0	1	2	FSTIFLGVAED	tr_D0A9V0	0.087	19492.96	27.00
642	HLA-A0201	GRPPWQWSSTAPAG	WQWSSTAPA	4	0	0	0	0	WQWSSTAPA	tr_D0A9V0	0.087	19498.01	27.00
336	HLA-A0201	MRLCRQRCRRNDTV	RLCRRNDTV	1	0	0	2	4	RLCRQRCRRNDTV	tr_D0A9V0	0.087	19502.87	27.00
281	HLA-A0201	IVLFELRPGES	VLFELRPES	1	0	0	7	1	VLFELRPGES	tr_D0A9V0	0.087	19506.67	27.00
311	HLA-A0201	RQGRYGEAM	RQGRYGEAM	0	0	0	0	0	RQGRYGEAM	tr_D0A9V0	0.087	19512.58	27.00
131	HLA-A0201	VIAIQVYHPDVWKS	VIAIQVYHV	0	0	0	8	2	VIAIQVYHPDV	tr_D0A9V0	0.087	19522.08	27.00
85	HLA-A0201	SSTAFVRIMGASIS	TAFVRIMSI	2	0	0	7	2	TAFVRIMGASI	tr_D0A9V0	0.087	19526.52	27.00
145	HLA-A0201	STKYHAALIEVA	STHAALIEV	0	0	0	2	2	STKYHAALIEV	tr_D0A9V0	0.087	19563.74	27.00
37	HLA-A0201	QGHYAKLRRQLAV	YALRRQLAV	3	0	0	2	1	YAKLRRQLAV	tr_D0A9V0	0.087	19576.87	27.00
199	HLA-A0201	LPLDHRDSVTMLVD	PLDSVTMLV	1	0	0	2	3	PLDHRDSVTMLV	tr_D0A9V0	0.087	19579.62	27.00
217	HLA-A0201	LVGTCGAFS	LVGTCGAFS	0	0	0	0	0	LVGTCGAFS	tr_D0A9V0	0.087	19587.46	27.00
470	HLA-A0201	AQVAIKCIPRLR	AQVAIIPRL	0	0	0	5	2	AQVAIKCIPRL	tr_D0A9V0	0.087	19591.49	27.00
640	HLA-A0201	LLGRPPWQWSST	LLGRPPWST	0	0	0	7	3	LLGRPPWQWSST	tr_D0A9V0	0.087	19601.66	27.00
234	HLA-A0201	ADHLFQLSYA	ADHLFLSYA	0	0	0	5	1	ADHLFQLSYA	tr_D0A9V0	0.087	19603.79	27.00
686	HLA-A0201	ASVDHEAAA	ASVDHEAAA	0	0	0	0	0	ASVDHEAAA	tr_D0A9V0	0.087	19610.58	27.00
637	HLA-A0201	LEMLLGRPPW	LMLLGRPPW	0	0	0	1	1	LEMLLGRPPW	tr_D0A9V0	0.086	19623.52	27.00
303	HLA-A0201	FRLGFAAMR	FRLGFAAMR	0	0	0	0	0	FRLGFAAMR	tr_D0A9V0	0.086	19648.81	27.00
281	HLA-A0201	IVLFELRPGESSS	VLFELESSS	1	0	0	5	3	VLFELRPGESSS	tr_D0A9V0	0.086	19649.23	27.00
335	HLA-A0201	LMRLCRQRC	LMRLCRQRC	0	0	0	0	0	LMRLCRQRC	tr_D0A9V0	0.086	19661.56	27.00
540	HLA-A0201	SPYVARRFTVDILQ	YVFTVDILQ	2	0	0	2	3	YVARRFTVDILQ	tr_D0A9V0	0.086	19670.72	27.00
559	HLA-A0201	HNLGIVHCDVK	HLGIVHCDV	0	0	0	1	1	HNLGIVHCDV	tr_D0A9V0	0.086	19671.57	27.00
527	HLA-A0201	GSLHSVIKNFG	SLHSVINFG	1	0	0	6	1	SLHSVIKNFG	tr_D0A9V0	0.086	19681.15	27.00
7	HLA-A0201	SALKSASNLEIPD	ALSASNLEI	1	0	0	2	1	ALKSASNLEI	tr_D0A9V0	0.086	19686.90	27.00
691	HLA-A0201	EAAAFVRACCHM	AAFVACCHM	2	0	0	4	1	AAFVRACCHM	tr_D0A9V0	0.086	19699.89	27.00
685	HLA-A0201	PASVDHEAAAFVR	SVDEAAAFV	2	0	0	3	1	SVDHEAAAFV	tr_D0A9V0	0.086	19721.22	27.00
146	HLA-A0201	TKYHAALIEVATEH	YLIEVATEH	2	0	0	1	3	YHAALIEVATEH	tr_D0A9V0	0.086	19731.25	27.00
574	HLA-A0201	LLGTDGVCKLSDFG	LLGDGVCKL	0	0	0	3	1	LLGTDGVCKL	tr_D0A9V0	0.086	19747.69	27.00
126	HLA-A0201	RRHCTVIAIQV	RCTVIAIQV	0	0	0	1	2	RRHCTVIAIQV	tr_D0A9V0	0.086	19767.15	27.00

498	HLA-A0201	KLHHPNIVRY	KLHPNIVRY	0	0	0	2	1	KLHHPNIVRY	tr_D0A9V0	0.086	19780.85	27.00
644	HLA-A0201	PPWQWSSTAPAG	WQWSSTAPA	2	0	0	0	0	WQWSSTAPA	tr_D0A9V0	0.086	19822.62	27.00
147	HLA-A0201	KYHAALIEVA	KYHAALIEV	0	0	0	0	0	KYHAALIEV	tr_D0A9V0	0.085	19845.16	27.00
302	HLA-A0201	QFRLGFAAM	QFRLGFAAM	0	0	0	0	0	QFRLGFAAM	tr_D0A9V0	0.085	19885.78	27.00
257	HLA-A0201	GASHMQGFLA	GASHMQFLA	0	0	0	6	1	GASHMQGFLA	tr_D0A9V0	0.085	19886.64	27.00
129	HLA-A0201	CTVIAIQVYH	CTVIAIVYH	0	0	0	6	1	CTVIAIQVYH	tr_D0A9V0	0.085	19912.91	27.00
155	HLA-A0201	VATEHSGCVEAL	ATSGCVEAL	1	0	0	2	2	ATEHSGCVEAL	tr_D0A9V0	0.085	19927.13	27.00
188	HLA-A0201	CCQFAFDLF	CCQFAFDLF	0	0	0	0	0	CCQFAFDLF	tr_D0A9V0	0.085	19937.48	27.00
149	HLA-A0201	HAALIEVATEHSG	ALIEVAHSG	2	0	0	6	2	ALIEVATEHSG	tr_D0A9V0	0.085	19943.30	27.00
591	HLA-A0201	SEASVMARTL	SEASVMATL	0	0	0	7	1	SEASVMARTL	tr_D0A9V0	0.085	19952.16	27.00
286	HLA-A0201	LRPGESSSEVSLSI	LSSEVSLSI	0	0	0	1	5	LRPGESSSEVSLSI	tr_D0A9V0	0.085	19955.62	27.00
431	HLA-A0201	ADVVPLFLT	ADVVPLFLT	0	0	0	0	0	ADVVPLFLT	tr_D0A9V0	0.085	19963.60	27.00
55	HLA-A0201	PEAILARVESKNEA	ILARVENEA	3	0	0	6	2	ILARVESKNEA	tr_D0A9V0	0.085	19968.14	27.00
520	HLA-A0201	IMEYVPGGS	IMEYVPGGS	0	0	0	0	0	IMEYVPGGS	tr_D0A9V0	0.085	19970.09	27.00
533	HLA-A0201	IKNFGCVSPYVA	KNFGCVPYV	1	0	0	6	1	KNFGCVSPYV	tr_D0A9V0	0.085	19975.27	27.00
690	HLA-A0201	HEAAAFVRACCHM	AAFVACCHM	3	0	0	4	1	AAFVRACCHM	tr_D0A9V0	0.085	19986.72	27.00
687	HLA-A0201	SVDHEAAAFVRAC	SVDEAAAFV	0	0	0	3	1	SVDHEAAAFV	tr_D0A9V0	0.085	19988.02	27.00
43	HLA-A0201	LRRQLAVLL	LRRQLAVLL	0	0	0	0	0	LRRQLAVLL	tr_D0A9V0	0.085	19996.89	27.00
120	HLA-A0201	KPNVFNRRHCTVIA	NVFNCTVIA	2	0	0	4	3	NVFNRRHCTVIA	tr_D0A9V0	0.085	20010.53	27.00
695	HLA-A0201	FVRACCHMNPSMR	FVRACCHMM	0	0	0	8	3	FVRACCHMNPSM	tr_D0A9V0	0.085	20011.61	27.00
509	HLA-A0201	SCSITPSHL	SCSITPSHL	0	0	0	0	0	SCSITPSHL	tr_D0A9V0	0.085	20028.72	27.00
684	HLA-A0201	IPASVDHEAAAFVR	SVDEAAAFV	3	0	0	3	1	SVDHEAAAFV	tr_D0A9V0	0.084	20046.08	27.00
449	HLA-A0201	SLKKISEGAF	SLKKISEAF	0	0	0	7	1	SLKKISEGAF	tr_D0A9V0	0.084	20050.83	27.00
193	HLA-A0201	FDLFAKLPLDH	FLFAKLPLD	0	0	0	1	1	FDLFAKLPLD	tr_D0A9V0	0.084	20080.16	27.00
148	HLA-A0201	YHAALIEVATEHSG	ALIEVAHSG	3	0	0	6	2	ALIEVATEHSG	tr_D0A9V0	0.084	20083.85	28.00
465	HLA-A0201	VAEDGAQVAI	VADGAQVAI	0	0	0	2	1	VAEDGAQVAI	tr_D0A9V0	0.084	20084.50	28.00
586	HLA-A0201	FGSTISEASVMART	FISEASVMA	0	0	0	1	3	FGSTISEASVMA	tr_D0A9V0	0.084	20124.52	28.00
689	HLA-A0201	DHEAAAFVRACCHM	AAFVACCHM	4	0	0	4	1	AAFVRACCHM	tr_D0A9V0	0.084	20125.61	28.00
163	HLA-A0201	VEALDPDRALISFG	ALDALISFG	2	0	0	3	3	ALDPDRALISFG	tr_D0A9V0	0.084	20173.79	28.00
122	HLA-A0201	NVFNRRHCTVI	NVFNRCTVI	0	0	0	5	2	NVFNRRHCTVI	tr_D0A9V0	0.084	20179.03	28.00
279	HLA-A0201	LPIVLFELRP	LIVLFELRP	0	0	0	1	1	LPIVLFELRP	tr_D0A9V0	0.084	20192.79	28.00
223	HLA-A0201	AFSRNARVLFGAD	FSARVLFGA	1	0	0	2	2	FSRNARVLFGA	tr_D0A9V0	0.084	20220.11	28.00
237	HLA-A0201	LFQLSYAVRDGPCR	FQLSYAVPC	1	0	0	7	3	FQLSYAVRDGPC	tr_D0A9V0	0.083	20263.05	28.00
636	HLA-A0201	LLEMLLGRPPWQ	LLEMLLPPW	0	0	0	6	2	LLEMLLGRPPW	tr_D0A9V0	0.083	20268.31	28.00
670	HLA-A0201	LFVQSLALG	LFVQSLALG	0	0	0	0	0	LFVQSLALG	tr_D0A9V0	0.083	20277.29	28.00
428	HLA-A0201	ANAADVVPLF	AAADVVPLF	0	0	0	1	1	ANAADVVPLF	tr_D0A9V0	0.083	20301.23	28.00
611	HLA-A0201	LYMAPEVAA	LYMAPEVAA	0	0	0	0	0	LYMAPEVAA	tr_D0A9V0	0.083	20306.73	28.00
224	HLA-A0201	FSRNARVLFGADH	FSARVLFGA	0	0	0	2	2	FSRNARVLFGA	tr_D0A9V0	0.083	20311.77	28.00
704	HLA-A0201	PSMRPSASSLL	SMPSASSLL	1	0	0	2	1	SMRPSASSLL	tr_D0A9V0	0.083	20337.29	28.00
588	HLA-A0201	STISEASVMARTL	TISEASVTL	1	0	0	7	3	TISEASVMARTL	tr_D0A9V0	0.083	20372.96	28.00
695	HLA-A0201	FVRACCHMN	FVRACCHMN	0	0	0	0	0	FVRACCHMN	tr_D0A9V0	0.083	20401.86	28.00
149	HLA-A0201	HAALIEVAT	HAALIEVAT	0	0	0	0	0	HAALIEVAT	tr_D0A9V0	0.083	20407.38	28.00
417	HLA-A0201	SSLDGSGCAEGAN	SLDGCAEGA	1	0	0	3	2	SLDGSGCAEGA	tr_D0A9V0	0.083	20417.10	28.00
697	HLA-A0201	RACCHMNPSM	RACCHMNPM	0	0	0	8	1	RACCHMNPSM	tr_D0A9V0	0.083	20442.74	28.00
132	HLA-A0201	IAIQVYHPDVWK	IAIQVYHPV	0	0	0	8	1	IAIQVYHPDV	tr_D0A9V0	0.083	20466.19	28.00
353	HLA-A0201	RVLDNHYCVNS	VLDNYCVNS	1	0	0	4	1	VLDNHYCVNS	tr_D0A9V0	0.083	20474.84	28.00
180	HLA-A0201	ALPMHCRRCCQF	ALPMRCCQF	0	0	0	4	3	ALPMHCRRCCQF	tr_D0A9V0	0.082	20479.26	28.00
185	HLA-A0201	CRRCCQFAFDLF	RCCQFAFLF	2	0	0	7	1	RCCQFAFDLF	tr_D0A9V0	0.082	20480.60	28.00
259	HLA-A0201	SHMQGFLAF	SHMQGFLAF	0	0	0	0	0	SHMQGFLAF	tr_D0A9V0	0.082	20481.71	28.00
565	HLA-A0201	HCDVKPHNV	HCDVKPHNV	0	0	0	0	0	HCDVKPHNV	tr_D0A9V0	0.082	20493.45	28.00
585	HLA-A0201	DFGSTISEASV	FGSTISASV	1	0	0	6	1	FGSTISEASV	tr_D0A9V0	0.082	20517.64	28.00
279	HLA-A0201	LPIVLFELR	LPIVLFELR	0	0	0	0	0	LPIVLFELR	tr_D0A9V0	0.082	20534.52	28.00
22	HLA-A0201	DIFLGRSRIV	DIFLGSRIV	0	0	0	5	1	DIFLGRSRIV	tr_D0A9V0	0.082	20544.07	28.00
526	HLA-A0201	GGSLHSVIKNFG	SLHSVINFG	2	0	0	6	1	SLHSVIKNFG	tr_D0A9V0	0.082	20547.18	28.00

567	HLA-A0201	DVKPHNVLLGTDGV	KVLLGTDGV	2	0	0	1	3	KPHNVLLGTDGV	tr_D0A9V0	0.082	20551.85	28.00
200	HLA-A0201	PLDHRDSVTMLVDT	PLDSVTMLV	0	0	0	2	3	PLDHRDSVTMLV	tr_D0A9V0	0.082	20554.96	28.00
600	HLA-A0201	LADGVMLRGTALY	LADGVMLAL	0	0	0	7	3	LADGVMLRGTAL	tr_D0A9V0	0.082	20595.70	28.00
47	HLA-A0201	LAVLLKFIP	LAVLLKFIP	0	0	0	0	0	LAVLLKFIP	tr_D0A9V0	0.082	20599.04	28.00
184	HLA-A0201	HCRRCCQFAFDLF	RCCQFAFLF	3	0	0	7	1	RCCQFAFDLF	tr_D0A9V0	0.082	20621.35	28.00
475	HLA-A0201	KCIPRLRRDV	KCIPRLRRV	0	0	0	8	1	KCIPRLRRDV	tr_D0A9V0	0.082	20683.24	28.00
589	HLA-A0201	TISEASVMAR	TISEASVMA	0	0	0	0	0	TISEASVMA	tr_D0A9V0	0.082	20687.72	28.00
125	HLA-A0201	NRRHCTVIAIQV	RCTVIAIQV	1	0	0	1	2	RRHCTVIAIQV	tr_D0A9V0	0.082	20700.25	28.00
687	HLA-A0201	SVDHEAAAF	SVDHEAAAF	0	0	0	0	0	SVDHEAAAF	tr_D0A9V0	0.081	20705.63	28.00
612	HLA-A0201	YMAPEVAAGGR	YMAEVAAGG	0	0	0	3	1	YMAPEVAAGG	tr_D0A9V0	0.081	20728.05	28.00
454	HLA-A0201	SEGAFSTIFLG	SAFSTIFLG	0	0	0	1	2	SEGAFSTIFLG	tr_D0A9V0	0.081	20728.27	28.00
172	HLA-A0201	LISFGAHAALPMHC	LISFAALPM	0	0	0	4	3	LISFGAHAALPM	tr_D0A9V0	0.081	20743.98	28.00
186	HLA-A0201	RRCCQFAFDLF	RCCQFAFLF	1	0	0	7	1	RCCQFAFDLF	tr_D0A9V0	0.081	20765.09	28.00
511	HLA-A0201	SITPSHLSIIMEY	SILSIIMEY	0	0	0	2	4	SITPSHLSIIMEY	tr_D0A9V0	0.081	20794.09	29.00
511	HLA-A0201	SITPSHLSIIM	SISHLSIIM	0	0	0	2	2	SITPSHLSIIM	tr_D0A9V0	0.081	20823.81	29.00
352	HLA-A0201	VRVLDNHYCVNSS	VLDNCVNSS	2	0	0	4	2	VLDNHYCVNSS	tr_D0A9V0	0.081	20828.54	29.00
430	HLA-A0201	AADVVPLFLTD	AADVVLFLT	0	0	0	5	1	AADVVPLFLT	tr_D0A9V0	0.081	20836.20	29.00
153	HLA-A0201	IEVATEHSGCVEAL	ATSGCVEAL	3	0	0	2	2	ATEHSGCVEAL	tr_D0A9V0	0.081	20836.43	29.00
525	HLA-A0201	PGGSLHSVIKNFG	SLHSVINFG	3	0	0	6	1	SLHSVIKNFG	tr_D0A9V0	0.081	20836.88	29.00
13	HLA-A0201	SNLEIPDNADI	NLIPDNADI	1	0	0	2	1	NLEIPDNADI	tr_D0A9V0	0.081	20840.94	29.00
606	HLA-A0201	LRGTALYMA	LRGTALYMA	0	0	0	0	0	LRGTALYMA	tr_D0A9V0	0.081	20849.06	29.00
600	HLA-A0201	LADGVMLRG	LADGVMLRG	0	0	0	0	0	LADGVMLRG	tr_D0A9V0	0.081	20863.72	29.00
402	HLA-A0201	QGSESLFKM	QGSESLFKM	0	0	0	0	0	QGSESLFKM	tr_D0A9V0	0.081	20865.76	29.00
212	HLA-A0201	DTNEFLVGT	DTNEFLVGT	0	0	0	0	0	DTNEFLVGT	tr_D0A9V0	0.081	20873.44	29.00
172	HLA-A0201	LISFGAHAALPMH	LIFGAHAAL	0	0	0	2	1	LISFGAHAAL	tr_D0A9V0	0.081	20877.05	29.00
192	HLA-A0201	AFDLFAKLPLDHR	AFDLFALPL	0	0	0	6	1	AFDLFAKLPL	tr_D0A9V0	0.081	20882.25	29.00
200	HLA-A0201	PLDHRDSVTM	PLDHDSVTM	0	0	0	4	1	PLDHRDSVTM	tr_D0A9V0	0.081	20889.92	29.00
517	HLA-A0201	LSIIMEYVPG	LIIMEYVPG	0	0	0	1	1	LSIIMEYVPG	tr_D0A9V0	0.081	20891.74	29.00
671	HLA-A0201	FVQSLALGH	FVQSLALGH	0	0	0	0	0	FVQSLALGH	tr_D0A9V0	0.081	20918.42	29.00
6	HLA-A0201	VSALKSASNLEIPD	ALSASNLEI	2	0	0	2	1	ALKSASNLEI	tr_D0A9V0	0.080	20950.37	29.00
562	HLA-A0201	GIVHCDVKPHNVL	GIVHCDVVL	0	0	0	7	4	GIVHCDVKPHNVL	tr_D0A9V0	0.080	20958.53	29.00
532	HLA-A0201	VIKNFGCVSPY	VIFGCVSPY	0	0	0	2	2	VIKNFGCVSPY	tr_D0A9V0	0.080	20960.80	29.00
687	HLA-A0201	SVDHEAAAFVRACC	SVDEAAAFV	0	0	0	3	1	SVDHEAAAFV	tr_D0A9V0	0.080	20983.71	29.00
542	HLA-A0201	YVARRFTVDILQG	YVAFTVDIL	0	0	0	3	2	YVARRFTVDIL	tr_D0A9V0	0.080	20995.98	29.00
524	HLA-A0201	VPGGSLHSVIKNFG	SLHSVINFG	4	0	0	6	1	SLHSVIKNFG	tr_D0A9V0	0.080	21008.94	29.00
510	HLA-A0201	CSITPSHLSIIM	SISHLSIIM	1	0	0	2	2	SITPSHLSIIM	tr_D0A9V0	0.080	21014.62	29.00
194	HLA-A0201	DLFAKLPLD	DLFAKLPLD	0	0	0	0	0	DLFAKLPLD	tr_D0A9V0	0.080	21024.85	29.00
143	HLA-A0201	KSSTKYHAA	KSSTKYHAA	0	0	0	0	0	KSSTKYHAA	tr_D0A9V0	0.080	21029.39	29.00
85	HLA-A0201	SSTAFVRIMGAS	STFVRIMGA	1	0	0	2	1	STAFVRIMGA	tr_D0A9V0	0.080	21044.20	29.00
455	HLA-A0201	EGAFSTIFL	EGAFSTIFL	0	0	0	0	0	EGAFSTIFL	tr_D0A9V0	0.080	21057.17	29.00
686	HLA-A0201	ASVDHEAAAFVRA	SVDEAAAFV	1	0	0	3	1	SVDHEAAAFV	tr_D0A9V0	0.080	21075.19	29.00
703	HLA-A0201	NPSMRPSASSLL	SMPSASSLL	2	0	0	2	1	SMRPSASSLL	tr_D0A9V0	0.080	21103.01	29.00
161	HLA-A0201	GCVEALDPDRALIS	GCVEALALI	0	0	0	6	4	GCVEALDPDRALI	tr_D0A9V0	0.080	21109.64	29.00
584	HLA-A0201	SDFGSTISEASV	FGSTISASV	2	0	0	6	1	FGSTISEASV	tr_D0A9V0	0.080	21119.24	29.00
502	HLA-A0201	PNIVRYVSCSI	NIVYVSCSI	1	0	0	3	1	NIVRYVSCSI	tr_D0A9V0	0.080	21130.66	29.00
429	HLA-A0201	NAADVVPLFLTD	NAADVVLFL	0	0	0	6	1	NAADVVPLFL	tr_D0A9V0	0.080	21143.94	29.00
563	HLA-A0201	IVHCDVKPHNVLLG	IVHCDVVLL	0	0	0	6	4	IVHCDVKPHNVLL	tr_D0A9V0	0.079	21159.72	29.00
41	HLA-A0201	AKLRRQLAV	AKLRRQLAV	0	0	0	0	0	AKLRRQLAV	tr_D0A9V0	0.079	21165.67	29.00
154	HLA-A0201	EVATEHSGCVEAL	ATSGCVEAL	2	0	0	2	2	ATEHSGCVEAL	tr_D0A9V0	0.079	21179.19	29.00
424	HLA-A0201	CAEGANAADV	CAEGANAAV	0	0	0	8	1	CAEGANAADV	tr_D0A9V0	0.079	21218.18	29.00
287	HLA-A0201	RPGESSSEVSLSI	RSSEVSLSI	0	0	0	1	4	RPGESSSEVSLSI	tr_D0A9V0	0.079	21231.50	29.00
457	HLA-A0201	AFSTIFLGVAED	FIFLGVAED	1	0	0	1	2	FSTIFLGVAED	tr_D0A9V0	0.079	21248.74	29.00
7	HLA-A0201	SALKSASNLEIPDN	ALSASNLEI	1	0	0	2	1	ALKSASNLEI	tr_D0A9V0	0.079	21254.72	29.00
130	HLA-A0201	TVIAIQVYH	TVIAIQVYH	0	0	0	0	0	TVIAIQVYH	tr_D0A9V0	0.079	21275.19	29.00

509	HLA-A0201	SCSITPSHLSIIM	SISHLSIIM	2	0	0	2	2	SITPSHLSIIM	tr_D0A9V0	0.079	21285.55	29.00
507	HLA-A0201	YVSCSITPSH	YVSCSITPS	0	0	0	0	0	YVSCSITPS	tr_D0A9V0	0.079	21288.55	29.00
469	HLA-A0201	GAQVAIKCIPRLR	AQVAIIPRL	1	0	0	5	2	AQVAIKCIPRL	tr_D0A9V0	0.079	21290.40	29.00
645	HLA-A0201	PWQWSSTAPAGS	WQWSSTAPA	1	0	0	0	0	WQWSSTAPA	tr_D0A9V0	0.079	21292.24	29.00
210	HLA-A0201	LVDTNEFLVGTC	LVDTNEFLV	0	0	0	0	0	LVDTNEFLV	tr_D0A9V0	0.079	21301.46	29.00
214	HLA-A0201	NEFLVGTCGA	NEFLVTCGA	0	0	0	5	1	NEFLVGTCGA	tr_D0A9V0	0.079	21326.60	29.00
407	HLA-A0201	LFKMYAPSSSS	FMYAPSSSS	1	0	0	1	1	FKMYAPSSSS	tr_D0A9V0	0.078	21385.28	29.00
298	HLA-A0201	SISKQFRLGF	SISQFRLGF	0	0	0	3	1	SISKQFRLGF	tr_D0A9V0	0.078	21408.20	29.00
416	HLA-A0201	SSSLDGSGCAEGAN	SLDGCAEGA	2	0	0	3	2	SLDGSGCAEGA	tr_D0A9V0	0.078	21408.66	29.00
534	HLA-A0201	KNFGCVSPYVAR	KNFGCVPYV	0	0	0	6	1	KNFGCVSPYV	tr_D0A9V0	0.078	21410.05	29.00
218	HLA-A0201	VGTCGAFSRNARV	GTCGAFARV	1	0	0	6	3	GTCGAFSRNARV	tr_D0A9V0	0.078	21428.37	29.00
20	HLA-A0201	NADIFLGRSRIV	NADIFLGIV	0	0	0	7	3	NADIFLGRSRIV	tr_D0A9V0	0.078	21430.44	29.00
132	HLA-A0201	IAIQVYHPDVWKS	IAIQVYHPV	0	0	0	8	1	IAIQVYHPDV	tr_D0A9V0	0.078	21437.17	29.00
202	HLA-A0201	DHRDSVTMLV	HRDSVTMLV	1	0	0	0	0	HRDSVTMLV	tr_D0A9V0	0.078	21439.95	30.00
702	HLA-A0201	MNPSMRPSASSLL	SMPSASSLL	3	0	0	2	1	SMRPSASSLL	tr_D0A9V0	0.078	21453.19	30.00
111	HLA-A0201	ALNCGIFKEKPN	ALNCGIFPN	0	0	0	7	3	ALNCGIFKEKPN	tr_D0A9V0	0.078	21475.02	30.00
543	HLA-A0201	VARRFTVDI	VARRFTVDI	0	0	0	0	0	VARRFTVDI	tr_D0A9V0	0.078	21483.38	30.00
673	HLA-A0201	QSLALGHLEP	SLALGHLEP	1	0	0	0	0	SLALGHLEP	tr_D0A9V0	0.078	21485.01	30.00
587	HLA-A0201	GSTISEASVMARTL	TISEASVTL	2	0	0	7	3	TISEASVMARTL	tr_D0A9V0	0.078	21486.86	30.00
110	HLA-A0201	VALNCGIFKE	VLNCGIFKE	0	0	0	1	1	VALNCGIFKE	tr_D0A9V0	0.078	21512.92	30.00
583	HLA-A0201	LSDFGSTISEASV	FGSTISASV	3	0	0	6	1	FGSTISEASV	tr_D0A9V0	0.078	21513.86	30.00
695	HLA-A0201	FVRACCHMNPSMRP	FVRACCHMM	0	0	0	8	3	FVRACCHMNPSM	tr_D0A9V0	0.078	21554.40	30.00
539	HLA-A0201	VSPYVARRFTVD	VSPYVAFTV	0	0	0	6	2	VSPYVARRFTV	tr_D0A9V0	0.078	21559.05	30.00
345	HLA-A0201	RNDTVPYVRV	RNDTVPYVV	0	0	0	8	1	RNDTVPYVRV	tr_D0A9V0	0.078	21567.22	30.00
508	HLA-A0201	VSCSITPSHLSIIM	SISHLSIIM	3	0	0	2	2	SITPSHLSIIM	tr_D0A9V0	0.078	21579.60	30.00
598	HLA-A0201	RTLADGVMLRG	TLADGVMLG	1	0	0	8	1	TLADGVMLRG	tr_D0A9V0	0.078	21608.80	30.00
594	HLA-A0201	SVMARTLADG	SVMARTLAG	0	0	0	8	1	SVMARTLADG	tr_D0A9V0	0.077	21639.46	30.00
694	HLA-A0201	AFVRACCHMNPSMR	FVRACCHMM	1	0	0	8	3	FVRACCHMNPSM	tr_D0A9V0	0.077	21648.35	30.00
468	HLA-A0201	DGAQVAIKCIPRLR	AQVAIIPRL	2	0	0	5	2	AQVAIKCIPRL	tr_D0A9V0	0.077	21650.69	30.00
11	HLA-A0201	SASNLEIPDNADI	NLIPDNADI	3	0	0	2	1	NLEIPDNADI	tr_D0A9V0	0.077	21672.25	30.00
125	HLA-A0201	NRRHCTVIAI	RRHCTVIAI	1	0	0	0	0	RRHCTVIAI	tr_D0A9V0	0.077	21674.36	30.00
601	HLA-A0201	ADGVMLRGTAL	AVMLRGTAL	0	0	0	1	2	ADGVMLRGTAL	tr_D0A9V0	0.077	21675.07	30.00
428	HLA-A0201	ANAADVVPLFLTD	AADVVLFLT	2	0	0	5	1	AADVVPLFLT	tr_D0A9V0	0.077	21680.23	30.00
501	HLA-A0201	HPNIVRYVSCSI	NIVYVSCSI	2	0	0	3	1	NIVRYVSCSI	tr_D0A9V0	0.077	21681.87	30.00
225	HLA-A0201	SRNARVLFGA	SRNAVLFGA	0	0	0	4	1	SRNARVLFGA	tr_D0A9V0	0.077	21695.71	30.00
192	HLA-A0201	AFDLFAKLPLDHRD	AFDLFALPL	0	0	0	6	1	AFDLFAKLPL	tr_D0A9V0	0.077	21705.34	30.00
110	HLA-A0201	VALNCGIFK	VALNCGIFK	0	0	0	0	0	VALNCGIFK	tr_D0A9V0	0.077	21724.61	30.00
531	HLA-A0201	SVIKNFGCVSPY	VIFGCVSPY	1	0	0	2	2	VIKNFGCVSPY	tr_D0A9V0	0.077	21736.83	30.00
560	HLA-A0201	NLGIVHCDVKP	NLGIVHCDV	0	0	0	0	0	NLGIVHCDV	tr_D0A9V0	0.077	21739.19	30.00
173	HLA-A0201	ISFGAHAALPMHC	ISFGAALPM	0	0	0	4	2	ISFGAHAALPM	tr_D0A9V0	0.077	21741.07	30.00
285	HLA-A0201	ELRPGESSSEV	ELGESSSEV	0	0	0	2	2	ELRPGESSSEV	tr_D0A9V0	0.077	21765.31	30.00
624	HLA-A0201	TPQSDIFSFGI	TSDIFSFGI	0	0	0	1	2	TPQSDIFSFGI	tr_D0A9V0	0.077	21771.43	30.00
643	HLA-A0201	RPPWQWSSTAPAGS	RQWSSTAPA	0	0	0	1	3	RPPWQWSSTAPA	tr_D0A9V0	0.077	21774.97	30.00
325	HLA-A0201	MVKMDMQAKRL	MMDMQAKRL	0	0	0	1	2	MVKMDMQAKRL	tr_D0A9V0	0.077	21782.03	30.00
84	HLA-A0201	ESSTAFVRIMGAS	STFVRIMGA	2	0	0	2	1	STAFVRIMGA	tr_D0A9V0	0.077	21786.05	30.00
498	HLA-A0201	KLHHPNIVR	KLHHPNIVR	0	0	0	0	0	KLHHPNIVR	tr_D0A9V0	0.077	21787.46	30.00
410	HLA-A0201	MYAPSSSSSL	YAPSSSSSL	1	0	0	0	0	YAPSSSSSL	tr_D0A9V0	0.077	21801.13	30.00
210	HLA-A0201	LVDTNEFLVGTCGA	LVDTNEFLA	0	0	0	8	5	LVDTNEFLVGTCGA	tr_D0A9V0	0.077	21833.94	30.00
57	HLA-A0201	AILARVESK	AILARVESK	0	0	0	0	0	AILARVESK	tr_D0A9V0	0.076	21872.25	30.00
430	HLA-A0201	AADVVPLFLTDS	AADVVLFLT	0	0	0	5	1	AADVVPLFLT	tr_D0A9V0	0.076	21892.85	30.00
144	HLA-A0201	SSTKYHAALIEVA	STHAALIEV	1	0	0	2	2	STKYHAALIEV	tr_D0A9V0	0.076	21893.80	30.00
499	HLA-A0201	LHHPNIVRYVSCSI	NIVRYVCSI	4	0	0	6	1	NIVRYVSCSI	tr_D0A9V0	0.076	21908.73	30.00
423	HLA-A0201	GCAEGANAADVVPL	GCAEGVVPL	0	0	0	5	5	GCAEGANAADVVPL	tr_D0A9V0	0.076	21920.11	30.00
596	HLA-A0201	MARTLADGVMLRG	TLDGVMLRG	3	0	0	2	1	TLADGVMLRG	tr_D0A9V0	0.076	21936.48	30.00

430	HLA-A0201	AADVVPLFLTDSNR	AADVVLFLT	0	0	0	5	1	AADVVPLFLT	tr_D0A9V0	0.076	21936.72	30.00
500	HLA-A0201	HHPNIVRYVSCSI	NIVYVSCSI	3	0	0	3	1	NIVRYVSCSI	tr_D0A9V0	0.076	21938.62	30.00
175	HLA-A0201	FGAHAALPMHCRRC	FLPMHCRRC	0	0	0	1	5	FGAHAALPMHCRRC	tr_D0A9V0	0.076	21945.03	30.00
83	HLA-A0201	GESSTAFVRIMGAS	STFVRIMGA	3	0	0	2	1	STAFVRIMGA	tr_D0A9V0	0.076	21947.41	30.00
279	HLA-A0201	LPIVLFELRPGES	VLFELRPES	3	0	0	7	1	VLFELRPGES	tr_D0A9V0	0.076	21955.23	30.00
429	HLA-A0201	NAADVVPLFLTDS	NAADVVLFL	0	0	0	6	1	NAADVVPLFL	tr_D0A9V0	0.076	21956.66	30.00
280	HLA-A0201	PIVLFELRPGES	VLFELRPES	2	0	0	7	1	VLFELRPGES	tr_D0A9V0	0.076	21976.62	30.00
623	HLA-A0201	CTPQSDIFSFGIS	CTDIFSFGI	0	0	0	2	3	CTPQSDIFSFGI	tr_D0A9V0	0.076	21977.35	30.00
289	HLA-A0201	GESSSEVSLSI	SSSEVSLSI	2	0	0	0	0	SSSEVSLSI	tr_D0A9V0	0.076	21982.58	30.00
262	HLA-A0201	QGFLAFPVDC	QGFLFPVDC	0	0	0	4	1	QGFLAFPVDC	tr_D0A9V0	0.076	21985.19	30.00
352	HLA-A0201	VRVLDNHYCVNS	VLDNYCVNS	2	0	0	4	1	VLDNHYCVNS	tr_D0A9V0	0.076	21993.29	30.00
280	HLA-A0201	PIVLFELRPGESSS	VLFELESSS	2	0	0	5	3	VLFELRPGESSS	tr_D0A9V0	0.076	21996.84	30.00
434	HLA-A0201	VPLFLTDSNR	VLFLTDSNR	0	0	0	1	1	VPLFLTDSNR	tr_D0A9V0	0.076	21998.99	30.00
152	HLA-A0201	LIEVATEHSGCVEA	LIHSGCVEA	0	0	0	2	5	LIEVATEHSGCVEA	tr_D0A9V0	0.076	22010.42	30.00
457	HLA-A0201	AFSTIFLGVAE	AFSTIFLGV	0	0	0	0	0	AFSTIFLGV	tr_D0A9V0	0.076	22022.81	30.00
481	HLA-A0201	RRDVVAESL	RRDVVAESL	0	0	0	0	0	RRDVVAESL	tr_D0A9V0	0.076	22024.47	30.00
510	HLA-A0201	CSITPSHLSIIMEY	SILSIIMEY	1	0	0	2	4	SITPSHLSIIMEY	tr_D0A9V0	0.076	22024.71	30.00
12	HLA-A0201	ASNLEIPDNADI	NLIPDNADI	2	0	0	2	1	NLEIPDNADI	tr_D0A9V0	0.076	22030.19	30.00
623	HLA-A0201	CTPQSDIFSF	CTPQSIFSF	0	0	0	5	1	CTPQSDIFSF	tr_D0A9V0	0.076	22058.10	30.00
240	HLA-A0201	LSYAVRDGPCRIAA	LSYAVRIAA	0	0	0	5	5	LSYAVRDGPCRIAA	tr_D0A9V0	0.076	22064.07	30.00
531	HLA-A0201	SVIKNFGCVS	SVIKNFGCV	0	0	0	0	0	SVIKNFGCV	tr_D0A9V0	0.076	22086.28	31.00
556	HLA-A0201	NYLHNLGIV	NYLHNLGIV	0	0	0	0	0	NYLHNLGIV	tr_D0A9V0	0.075	22091.78	31.00
179	HLA-A0201	AALPMHCRRCCQF	ALPMRCCQF	1	0	0	4	3	ALPMHCRRCCQF	tr_D0A9V0	0.075	22096.09	31.00
698	HLA-A0201	ACCHMNPSM	ACCHMNPSM	0	0	0	0	0	ACCHMNPSM	tr_D0A9V0	0.075	22099.19	31.00
471	HLA-A0201	QVAIKCIPRLR	QVAIKCIPL	0	0	0	8	1	QVAIKCIPRL	tr_D0A9V0	0.075	22103.01	31.00
310	HLA-A0201	MRQGRYGEAMAHYL	RQGAMAHYL	1	0	0	3	4	RQGRYGEAMAHYL	tr_D0A9V0	0.075	22108.03	31.00
608	HLA-A0201	GTALYMAPE	GTALYMAPE	0	0	0	0	0	GTALYMAPE	tr_D0A9V0	0.075	22151.86	31.00
223	HLA-A0201	AFSRNARVLFGADH	FSARVLFGA	1	0	0	2	2	FSRNARVLFGA	tr_D0A9V0	0.075	22160.25	31.00
470	HLA-A0201	AQVAIKCIPRLRR	AQVAIIPRL	0	0	0	5	2	AQVAIKCIPRL	tr_D0A9V0	0.075	22162.65	31.00
143	HLA-A0201	KSSTKYHAALIEVA	STHAALIEV	2	0	0	2	2	STKYHAALIEV	tr_D0A9V0	0.075	22172.96	31.00
613	HLA-A0201	MAPEVAAGG	MAPEVAAGG	0	0	0	0	0	MAPEVAAGG	tr_D0A9V0	0.075	22174.16	31.00
559	HLA-A0201	HNLGIVHCDVKP	HLGIVHCDV	0	0	0	1	1	HNLGIVHCDV	tr_D0A9V0	0.075	22196.96	31.00
320	HLA-A0201	AHYLPMVKM	AHYLPMVKM	0	0	0	0	0	AHYLPMVKM	tr_D0A9V0	0.075	22213.78	31.00
10	HLA-A0201	KSASNLEIPDNADI	NLIPDNADI	4	0	0	2	1	NLEIPDNADI	tr_D0A9V0	0.075	22230.37	31.00
93	HLA-A0201	MGASISVTWNAG	MSISVTWNA	0	0	0	1	2	MGASISVTWNA	tr_D0A9V0	0.075	22232.29	31.00
705	HLA-A0201	SMRPSASSLLSY	SMASSLLSY	0	0	0	2	3	SMRPSASSLLSY	tr_D0A9V0	0.075	22242.40	31.00
200	HLA-A0201	PLDHRDSVTML	PLDDSVTML	0	0	0	3	2	PLDHRDSVTML	tr_D0A9V0	0.075	22244.08	31.00
194	HLA-A0201	DLFAKLPLDH	DLFAKLLDH	0	0	0	6	1	DLFAKLPLDH	tr_D0A9V0	0.075	22250.58	31.00
12	HLA-A0201	ASNLEIPDNA	ASLEIPDNA	0	0	0	2	1	ASNLEIPDNA	tr_D0A9V0	0.075	22282.39	31.00
183	HLA-A0201	MHCRRCCQFA	MCRRCCQFA	0	0	0	1	1	MHCRRCCQFA	tr_D0A9V0	0.075	22297.09	31.00
133	HLA-A0201	AIQVYHPDVW	AIQVYHPDV	0	0	0	0	0	AIQVYHPDV	tr_D0A9V0	0.075	22299.02	31.00
86	HLA-A0201	STAFVRIMG	STAFVRIMG	0	0	0	0	0	STAFVRIMG	tr_D0A9V0	0.075	22328.72	31.00
164	HLA-A0201	EALDPDRALIS	ALDPDRALI	1	0	0	0	0	ALDPDRALI	tr_D0A9V0	0.074	22348.05	31.00
427	HLA-A0201	GANAADVVPLFLTD	AADVVPLFL	3	0	0	0	0	AADVVPLFL	tr_D0A9V0	0.074	22359.43	31.00
543	HLA-A0201	VARRFTVDIL	VARRFTVIL	0	0	0	7	1	VARRFTVDIL	tr_D0A9V0	0.074	22376.86	31.00
662	HLA-A0201	RSLFNRDLL	RSLFNRDLL	0	0	0	0	0	RSLFNRDLL	tr_D0A9V0	0.074	22378.30	31.00
693	HLA-A0201	AAFVRACCHMN	AAFVACCHM	0	0	0	4	1	AAFVRACCHM	tr_D0A9V0	0.074	22403.74	31.00
520	HLA-A0201	IMEYVPGGSLHS	IMEYVSLHS	0	0	0	5	3	IMEYVPGGSLHS	tr_D0A9V0	0.074	22417.80	31.00
132	HLA-A0201	IAIQVYHPDVWKSS	IAIQVYHPV	0	0	0	8	1	IAIQVYHPDV	tr_D0A9V0	0.074	22454.95	31.00
206	HLA-A0201	SVTMLVDTNEF	VTMLVTNEF	1	0	0	5	1	VTMLVDTNEF	tr_D0A9V0	0.074	22458.35	31.00
672	HLA-A0201	VQSLALGHLE	VQSLALGHL	0	0	0	0	0	VQSLALGHL	tr_D0A9V0	0.074	22461.02	31.00
428	HLA-A0201	ANAADVVPLFLTDS	AADVVLFLT	2	0	0	5	1	AADVVPLFLT	tr_D0A9V0	0.074	22468.79	31.00
288	HLA-A0201	PGESSSEVSLSI	SSSEVSLSI	3	0	0	0	0	SSSEVSLSI	tr_D0A9V0	0.074	22485.83	31.00
560	HLA-A0201	NLGIVHCDVKPH	NLGIVHCDV	0	0	0	0	0	NLGIVHCDV	tr_D0A9V0	0.074	22526.48	31.00

24	HLA-A0201	FLGRSRIVE	FLGRSRIVE	0	0	0	0	0	FLGRSRIVE	tr_D0A9V0	0.074	22537.95	31.00
340	HLA-A0201	RQRCRRNDTV	RQCRRNDTV	0	0	0	2	1	RQRCRRNDTV	tr_D0A9V0	0.074	22539.42	31.00
333	HLA-A0201	KRLMRLCRQR	KLMRLCRQR	0	0	0	1	1	KRLMRLCRQR	tr_D0A9V0	0.074	22540.62	31.00
530	HLA-A0201	HSVIKNFGCVSPY	VIFGCVSPY	2	0	0	2	2	VIKNFGCVSPY	tr_D0A9V0	0.074	22560.63	31.00
224	HLA-A0201	FSRNARVLFG	FSNARVLFG	0	0	0	2	1	FSRNARVLFG	tr_D0A9V0	0.073	22585.56	31.00
178	HLA-A0201	HAALPMHCRRCCQF	ALPMRCCQF	2	0	0	4	3	ALPMHCRRCCQF	tr_D0A9V0	0.073	22600.21	31.00
97	HLA-A0201	ISVTWNAGDQQRDV	ISVTWNAGV	0	0	0	8	5	ISVTWNAGDQQRDV	tr_D0A9V0	0.073	22613.92	31.00
686	HLA-A0201	ASVDHEAAAFVRAC	SVDEAAAFV	1	0	0	3	1	SVDHEAAAFV	tr_D0A9V0	0.073	22615.38	31.00
4	HLA-A0201	LLVSALKSASN	LLVSALKSA	0	0	0	0	0	LLVSALKSA	tr_D0A9V0	0.073	22616.60	31.00
701	HLA-A0201	HMNPSMRPSAS	HMNPSMPSA	0	0	0	6	1	HMNPSMRPSA	tr_D0A9V0	0.073	22620.03	31.00
508	HLA-A0201	VSCSITPSHLSI	VSCSITLSI	0	0	0	6	3	VSCSITPSHLSI	tr_D0A9V0	0.073	22640.11	31.00
210	HLA-A0201	LVDTNEFLVGTCG	LVDTNEFLV	0	0	0	0	0	LVDTNEFLV	tr_D0A9V0	0.073	22646.48	31.00
19	HLA-A0201	DNADIFLGRSRIV	NADIFLGIV	1	0	0	7	3	NADIFLGRSRIV	tr_D0A9V0	0.073	22647.71	31.00
299	HLA-A0201	ISKQFRLGFA	ISQFRLGFA	0	0	0	2	1	ISKQFRLGFA	tr_D0A9V0	0.073	22653.83	31.00
597	HLA-A0201	ARTLADGVMLRG	TLDGVMLRG	2	0	0	2	1	TLADGVMLRG	tr_D0A9V0	0.073	22669.28	31.00
469	HLA-A0201	GAQVAIKCI	GAQVAIKCI	0	0	0	0	0	GAQVAIKCI	tr_D0A9V0	0.073	22709.54	32.00
622	HLA-A0201	RCTPQSDIFSFGIS	CTDIFSFGI	1	0	0	2	3	CTPQSDIFSFGI	tr_D0A9V0	0.073	22721.83	32.00
483	HLA-A0201	DVVAESLEKEM	VVAESLEEM	1	0	0	7	1	VVAESLEKEM	tr_D0A9V0	0.073	22724.53	32.00
500	HLA-A0201	HHPNIVRYV	HHPNIVRYV	0	0	0	0	0	HHPNIVRYV	tr_D0A9V0	0.073	22737.33	32.00
430	HLA-A0201	AADVVPLFLTDSN	AADVVLFLT	0	0	0	5	1	AADVVPLFLT	tr_D0A9V0	0.073	22738.31	32.00
193	HLA-A0201	FDLFAKLPLDHR	FLFAKLPLD	0	0	0	1	1	FDLFAKLPLD	tr_D0A9V0	0.073	22752.33	32.00
334	HLA-A0201	RLMRLCRQRCR	RLMRLCQRC	0	0	0	6	1	RLMRLCRQRC	tr_D0A9V0	0.073	22774.00	32.00
417	HLA-A0201	SSLDGSGCA	SSLDGSGCA	0	0	0	0	0	SSLDGSGCA	tr_D0A9V0	0.073	22779.67	32.00
127	HLA-A0201	RHCTVIAIQVY	CTVIAIQVY	2	0	0	0	0	CTVIAIQVY	tr_D0A9V0	0.073	22783.88	32.00
163	HLA-A0201	VEALDPDRALIS	VLDPDRALI	0	0	0	1	2	VEALDPDRALI	tr_D0A9V0	0.073	22807.79	32.00
159	HLA-A0201	HSGCVEALDPDRAL	GCVEALDPL	2	0	0	8	3	GCVEALDPDRAL	tr_D0A9V0	0.072	22825.57	32.00
534	HLA-A0201	KNFGCVSPYVARR	KNFGCVPYV	0	0	0	6	1	KNFGCVSPYV	tr_D0A9V0	0.072	22829.26	32.00
515	HLA-A0201	SHLSIIMEY	SHLSIIMEY	0	0	0	0	0	SHLSIIMEY	tr_D0A9V0	0.072	22844.60	32.00
424	HLA-A0201	CAEGANAADVVPL	CAAADVVPL	0	0	0	2	4	CAEGANAADVVPL	tr_D0A9V0	0.072	22847.31	32.00
692	HLA-A0201	AAAFVRACCHMN	AAAFVCCHM	0	0	0	5	2	AAAFVRACCHM	tr_D0A9V0	0.072	22882.93	32.00
145	HLA-A0201	STKYHAALIEVAT	STHAALIEV	0	0	0	2	2	STKYHAALIEV	tr_D0A9V0	0.072	22898.79	32.00
705	HLA-A0201	SMRPSASSLLS	SMSASSLLS	0	0	0	2	2	SMRPSASSLLS	tr_D0A9V0	0.072	22910.93	32.00
541	HLA-A0201	PYVARRFTVDILQG	YVAFTVDIL	1	0	0	3	2	YVARRFTVDIL	tr_D0A9V0	0.072	22915.64	32.00
432	HLA-A0201	DVVPLFLTDS	VVPLFLTDS	1	0	0	0	0	VVPLFLTDS	tr_D0A9V0	0.072	22917.88	32.00
429	HLA-A0201	NAADVVPLFLTDSN	AADVVPLFL	1	0	0	0	0	AADVVPLFL	tr_D0A9V0	0.072	22919.86	32.00
268	HLA-A0201	PVDCVLLPCS	PVDCVLLPC	0	0	0	0	0	PVDCVLLPC	tr_D0A9V0	0.072	22925.31	32.00
173	HLA-A0201	ISFGAHAALPMH	ISFGAALPM	0	0	0	4	2	ISFGAHAALPM	tr_D0A9V0	0.072	22928.28	32.00
162	HLA-A0201	CVEALDPDRALIS	ALDPDRALI	3	0	0	0	0	ALDPDRALI	tr_D0A9V0	0.072	22933.50	32.00
529	HLA-A0201	LHSVIKNFGCVSPY	VIFGCVSPY	3	0	0	2	2	VIKNFGCVSPY	tr_D0A9V0	0.072	22942.19	32.00
458	HLA-A0201	FSTIFLGVAEDG	FSTIFLGVA	0	0	0	0	0	FSTIFLGVA	tr_D0A9V0	0.072	22951.61	32.00
587	HLA-A0201	GSTISEASVMA	SISEASVMA	1	0	0	1	1	STISEASVMA	tr_D0A9V0	0.072	22953.36	32.00
559	HLA-A0201	HNLGIVHCDVKPH	HLGIVHCDV	0	0	0	1	1	HNLGIVHCDV	tr_D0A9V0	0.072	22955.09	32.00
685	HLA-A0201	PASVDHEAAAFVRA	SVDEAAAFV	2	0	0	3	1	SVDHEAAAFV	tr_D0A9V0	0.072	22997.35	32.00
645	HLA-A0201	PWQWSSTAPAGSD	WQWSSTAPA	1	0	0	0	0	WQWSSTAPA	tr_D0A9V0	0.072	23003.58	32.00
459	HLA-A0201	STIFLGVAED	SIFLGVAED	0	0	0	1	1	STIFLGVAED	tr_D0A9V0	0.072	23003.83	32.00
423	HLA-A0201	GCAEGANAA	GCAEGANAA	0	0	0	0	0	GCAEGANAA	tr_D0A9V0	0.072	23022.00	32.00
199	HLA-A0201	LPLDHRDSVT	LLDHRDSVT	0	0	0	1	1	LPLDHRDSVT	tr_D0A9V0	0.072	23040.95	32.00
179	HLA-A0201	AALPMHCRRC	ALPMHCRRC	0	0	0	1	1	AALPMHCRRC	tr_D0A9V0	0.072	23046.93	32.00
143	HLA-A0201	KSSTKYHAAL	KSSTYHAAL	0	0	0	4	1	KSSTKYHAAL	tr_D0A9V0	0.072	23063.15	32.00
240	HLA-A0201	LSYAVRDGPCRI	LSYAVPCRI	0	0	0	5	3	LSYAVRDGPCRI	tr_D0A9V0	0.071	23072.62	32.00
561	HLA-A0201	LGIVHCDVKPHNVL	GIVHCDVVL	1	0	0	7	4	GIVHCDVKPHNVL	tr_D0A9V0	0.071	23087.61	32.00
655	HLA-A0201	GSDATRLRSL	GSDATLRSL	0	0	0	5	1	GSDATRLRSL	tr_D0A9V0	0.071	23104.10	32.00
51	HLA-A0201	LKFIPEAILAR	FIPEAILAR	2	0	0	0	0	FIPEAILAR	tr_D0A9V0	0.071	23108.09	32.00
310	HLA-A0201	MRQGRYGEAM	MQGRYGEAM	0	0	0	1	1	MRQGRYGEAM	tr_D0A9V0	0.071	23142.38	32.00

126	HLA-A0201	RRHCTVIAIQVY	CTVIAIQVY	3	0	0	0	0	CTVIAIQVY	tr_D0A9V0	0.071	23191.25	32.00
470	HLA-A0201	AQVAIKCIPRLRRD	AQVAIIPRL	0	0	0	5	2	AQVAIKCIPRL	tr_D0A9V0	0.071	23196.53	32.00
431	HLA-A0201	ADVVPLFLTD	AVVPLFLTD	0	0	0	1	1	ADVVPLFLTD	tr_D0A9V0	0.071	23199.54	32.00
693	HLA-A0201	AAFVRACCHMNP	AAFVACCHM	0	0	0	4	1	AAFVRACCHM	tr_D0A9V0	0.071	23201.54	32.00
693	HLA-A0201	AAFVRACCHMNPS	AAFVACCHM	0	0	0	4	1	AAFVRACCHM	tr_D0A9V0	0.071	23208.08	32.00
9	HLA-A0201	LKSASNLEI	LKSASNLEI	0	0	0	0	0	LKSASNLEI	tr_D0A9V0	0.071	23226.66	32.00
204	HLA-A0201	RDSVTMLVDTNEF	VTMLVTNEF	3	0	0	5	1	VTMLVDTNEF	tr_D0A9V0	0.071	23235.22	32.00
121	HLA-A0201	PNVFNRRHCTVI	NVFNRCTVI	1	0	0	5	2	NVFNRRHCTVI	tr_D0A9V0	0.071	23235.97	32.00
534	HLA-A0201	KNFGCVSPYVARRF	KNFGCVPYV	0	0	0	6	1	KNFGCVSPYV	tr_D0A9V0	0.071	23249.55	32.00
533	HLA-A0201	IKNFGCVSPYVAR	KNFGCVPYV	1	0	0	6	1	KNFGCVSPYV	tr_D0A9V0	0.071	23270.43	32.00
644	HLA-A0201	PPWQWSSTAPAGS	WQWSSTAPA	2	0	0	0	0	WQWSSTAPA	tr_D0A9V0	0.071	23286.06	33.00
560	HLA-A0201	NLGIVHCDVKPHN	NLGIVHCDV	0	0	0	0	0	NLGIVHCDV	tr_D0A9V0	0.071	23308.23	33.00
349	HLA-A0201	VPYVRVLDNHYC	VPYVRVLYC	0	0	0	7	3	VPYVRVLDNHYC	tr_D0A9V0	0.070	23319.84	33.00
512	HLA-A0201	ITPSHLSIIMEY	ITLSIIMEY	0	0	0	2	3	ITPSHLSIIMEY	tr_D0A9V0	0.070	23324.88	33.00
346	HLA-A0201	NDTVPYVRVL	NTVPYVRVL	0	0	0	1	1	NDTVPYVRVL	tr_D0A9V0	0.070	23334.22	33.00
444	HLA-A0201	NVWTRSLKKISEGA	NVWTISEGA	0	0	0	4	5	NVWTRSLKKISEGA	tr_D0A9V0	0.070	23338.51	33.00
106	HLA-A0201	QQRDVALNC	QQRDVALNC	0	0	0	0	0	QQRDVALNC	tr_D0A9V0	0.070	23348.61	33.00
329	HLA-A0201	DMQAKRLMRLC	MQAKRLMRL	1	0	0	0	0	MQAKRLMRL	tr_D0A9V0	0.070	23375.91	33.00
35	HLA-A0201	KIQGHYAKLR	KIQGHYAKL	0	0	0	0	0	KIQGHYAKL	tr_D0A9V0	0.070	23394.65	33.00
113	HLA-A0201	NCGIFKEKPNV	GIFKEKPNV	2	0	0	0	0	GIFKEKPNV	tr_D0A9V0	0.070	23411.10	33.00
205	HLA-A0201	DSVTMLVDTNEF	VTMLVTNEF	2	0	0	5	1	VTMLVDTNEF	tr_D0A9V0	0.070	23413.12	33.00
182	HLA-A0201	PMHCRRCCQFA	PMHCCCQFA	0	0	0	4	2	PMHCRRCCQFA	tr_D0A9V0	0.070	23414.90	33.00
665	HLA-A0201	FNRDLLFVQS	FRDLLFVQS	0	0	0	1	1	FNRDLLFVQS	tr_D0A9V0	0.070	23416.43	33.00
471	HLA-A0201	QVAIKCIPR	QVAIKCIPR	0	0	0	0	0	QVAIKCIPR	tr_D0A9V0	0.070	23422.24	33.00
459	HLA-A0201	STIFLGVAE	STIFLGVAE	0	0	0	0	0	STIFLGVAE	tr_D0A9V0	0.070	23435.43	33.00
544	HLA-A0201	ARRFTVDIL	ARRFTVDIL	0	0	0	0	0	ARRFTVDIL	tr_D0A9V0	0.070	23438.23	33.00
457	HLA-A0201	AFSTIFLGVAEDG	AFSTIFLGV	0	0	0	0	0	AFSTIFLGV	tr_D0A9V0	0.070	23452.43	33.00
550	HLA-A0201	DILQGLNYLHN	DILQGLNYL	0	0	0	0	0	DILQGLNYL	tr_D0A9V0	0.070	23483.91	33.00
534	HLA-A0201	KNFGCVSPY	KNFGCVSPY	0	0	0	0	0	KNFGCVSPY	tr_D0A9V0	0.070	23571.49	33.00
469	HLA-A0201	GAQVAIKCIPRLRR	QVAIKCIPL	2	0	0	8	1	QVAIKCIPRL	tr_D0A9V0	0.069	23574.80	33.00
334	HLA-A0201	RLMRLCRQRCRR	RLMRLCCRR	0	0	0	6	3	RLMRLCRQRCRR	tr_D0A9V0	0.069	23580.41	33.00
212	HLA-A0201	DTNEFLVGTC	DTNEFLVTC	0	0	0	7	1	DTNEFLVGTC	tr_D0A9V0	0.069	23581.95	33.00
691	HLA-A0201	EAAAFVRACCHMN	AAFVACCHM	2	0	0	4	1	AAFVRACCHM	tr_D0A9V0	0.069	23593.43	33.00
18	HLA-A0201	PDNADIFLGRSRIV	NADIFLGIV	2	0	0	7	3	NADIFLGRSRIV	tr_D0A9V0	0.069	23594.19	33.00
589	HLA-A0201	TISEASVMARTLA	TISEASVLA	0	0	0	7	4	TISEASVMARTLA	tr_D0A9V0	0.069	23599.81	33.00
253	HLA-A0201	ATSGGASHMQGFLA	ATSGGAFLA	0	0	0	6	5	ATSGGASHMQGFLA	tr_D0A9V0	0.069	23603.64	33.00
275	HLA-A0201	PCSTLPIVLF	CSTLPIVLF	1	0	0	0	0	CSTLPIVLF	tr_D0A9V0	0.069	23610.28	33.00
478	HLA-A0201	PRLRRDVVAES	RLRDVVAES	1	0	0	2	1	RLRRDVVAES	tr_D0A9V0	0.069	23626.64	33.00
692	HLA-A0201	AAAFVRACCHMNPS	AAFVACCHM	1	0	0	4	1	AAFVRACCHM	tr_D0A9V0	0.069	23639.93	33.00
120	HLA-A0201	KPNVFNRRHCTVI	NVFNRCTVI	2	0	0	5	2	NVFNRRHCTVI	tr_D0A9V0	0.069	23658.62	33.00
611	HLA-A0201	LYMAPEVAAGGR	YMAEVAAGG	1	0	0	3	1	YMAPEVAAGG	tr_D0A9V0	0.069	23663.73	33.00
92	HLA-A0201	IMGASISVTWN	IMGASIVTW	0	0	0	6	1	IMGASISVTW	tr_D0A9V0	0.069	23668.08	33.00
464	HLA-A0201	GVAEDGAQVAI	GVAEAQVAI	0	0	0	4	2	GVAEDGAQVAI	tr_D0A9V0	0.069	23670.90	33.00
692	HLA-A0201	AAAFVRACCHMNP	AAFVACCHM	1	0	0	4	1	AAFVRACCHM	tr_D0A9V0	0.069	23675.51	33.00
449	HLA-A0201	SLKKISEGAFS	SLISEGAFS	0	0	0	2	2	SLKKISEGAFS	tr_D0A9V0	0.069	23678.84	33.00
466	HLA-A0201	AEDGAQVAI	AEDGAQVAI	0	0	0	0	0	AEDGAQVAI	tr_D0A9V0	0.069	23685.25	33.00
328	HLA-A0201	MDMQAKRLMRLC	MQAKRLMRL	0	0	0	1	2	MDMQAKRLMRL	tr_D0A9V0	0.069	23692.68	33.00
559	HLA-A0201	HNLGIVHCDVKPHN	HLGIVHCDV	0	0	0	1	1	HNLGIVHCDV	tr_D0A9V0	0.069	23749.14	33.00
695	HLA-A0201	FVRACCHMNPS	FVCCHMNPS	0	0	0	2	2	FVRACCHMNPS	tr_D0A9V0	0.069	23771.76	33.00
274	HLA-A0201	LPCSTLPIVLFE	LTLPIVLFE	0	0	0	1	3	LPCSTLPIVLFE	tr_D0A9V0	0.069	23784.38	33.00
482	HLA-A0201	RDVVAESLEKEM	VVAESLEEM	2	0	0	7	1	VVAESLEKEM	tr_D0A9V0	0.069	23792.10	33.00
690	HLA-A0201	HEAAAFVRACCHMN	AAFVACCHM	3	0	0	4	1	AAFVRACCHM	tr_D0A9V0	0.069	23792.62	33.00
347	HLA-A0201	DTVPYVRVL	DTVPYVRVL	0	0	0	0	0	DTVPYVRVL	tr_D0A9V0	0.069	23815.79	33.00
232	HLA-A0201	FGADHLFQLSY	FGADHLFQL	0	0	0	0	0	FGADHLFQL	tr_D0A9V0	0.068	23828.43	33.00

330	HLA-A0201	MQAKRLMRLCRQR	MLMRLCRQR	0	0	0	1	4	MQAKRLMRLCRQR	tr_D0A9V0	0.068	23854.47	34.00
564	HLA-A0201	VHCDVKPHNV	VHCDVPHNV	0	0	0	5	1	VHCDVKPHNV	tr_D0A9V0	0.068	23880.04	34.00
125	HLA-A0201	NRRHCTVIAIQVY	CTVIAIQVY	4	0	0	0	0	CTVIAIQVY	tr_D0A9V0	0.068	23884.96	34.00
496	HLA-A0201	ASKLHHPNIV	ASLHHPNIV	0	0	0	2	1	ASKLHHPNIV	tr_D0A9V0	0.068	23885.72	34.00
203	HLA-A0201	HRDSVTMLVDTNEF	VTMLVTNEF	4	0	0	5	1	VTMLVDTNEF	tr_D0A9V0	0.068	23891.15	34.00
538	HLA-A0201	CVSPYVARRF	CVSPYVARF	0	0	0	7	1	CVSPYVARRF	tr_D0A9V0	0.068	23907.18	34.00
354	HLA-A0201	VLDNHYCVNSSD	VLYCVNSSD	0	0	0	2	3	VLDNHYCVNSSD	tr_D0A9V0	0.068	23910.03	34.00
508	HLA-A0201	VSCSITPSHLSII	VSCSILSII	0	0	0	5	4	VSCSITPSHLSII	tr_D0A9V0	0.068	23923.49	34.00
569	HLA-A0201	KPHNVLLGTDGVC	KVLLGTDGV	0	0	0	1	3	KPHNVLLGTDGV	tr_D0A9V0	0.068	23944.46	34.00
233	HLA-A0201	GADHLFQLSYA	GADHLLSYA	0	0	0	5	2	GADHLFQLSYA	tr_D0A9V0	0.068	23970.12	34.00
418	HLA-A0201	SLDGSGCAE	SLDGSGCAE	0	0	0	0	0	SLDGSGCAE	tr_D0A9V0	0.068	23970.64	34.00
145	HLA-A0201	STKYHAALIEVATE	STHAALIEV	0	0	0	2	2	STKYHAALIEV	tr_D0A9V0	0.068	23988.80	34.00
78	HLA-A0201	ATETPGESSTAFV	ATESSTAFV	0	0	0	2	4	ATETPGESSTAFV	tr_D0A9V0	0.068	23995.04	34.00
706	HLA-A0201	MRPSASSLLSYAF	MSSLLSYAF	0	0	0	1	4	MRPSASSLLSYAF	tr_D0A9V0	0.068	24005.95	34.00
701	HLA-A0201	HMNPSMRPSASS	HMNPSMPSA	0	0	0	6	1	HMNPSMRPSA	tr_D0A9V0	0.068	24018.94	34.00
298	HLA-A0201	SISKQFRLGFA	SISFRLGFA	0	0	0	3	2	SISKQFRLGFA	tr_D0A9V0	0.068	24030.11	34.00
304	HLA-A0201	RLGFAAMRQGRYG	RLGFAAMYG	0	0	0	7	4	RLGFAAMRQGRYG	tr_D0A9V0	0.068	24041.81	34.00
224	HLA-A0201	FSRNARVLF	FSRNARVLF	0	0	0	0	0	FSRNARVLF	tr_D0A9V0	0.068	24052.22	34.00
307	HLA-A0201	FAAMRQGRYGEAM	FAAMYGEAM	0	0	0	4	4	FAAMRQGRYGEAM	tr_D0A9V0	0.068	24053.00	34.00
353	HLA-A0201	RVLDNHYCVNSSD	RVLDNHYCV	0	0	0	0	0	RVLDNHYCV	tr_D0A9V0	0.068	24053.52	34.00
93	HLA-A0201	MGASISVTW	MGASISVTW	0	0	0	0	0	MGASISVTW	tr_D0A9V0	0.068	24054.04	34.00
376	HLA-A0201	IKDRVPETI	IKDRVPETI	0	0	0	0	0	IKDRVPETI	tr_D0A9V0	0.068	24071.48	34.00
119	HLA-A0201	EKPNVFNRRHCTVI	NVFNRCTVI	3	0	0	5	2	NVFNRRHCTVI	tr_D0A9V0	0.068	24073.57	34.00
284	HLA-A0201	FELRPGESSS	FLRPGESSS	0	0	0	1	1	FELRPGESSS	tr_D0A9V0	0.068	24076.17	34.00
352	HLA-A0201	VRVLDNHYCVN	VVLDNHYCV	0	0	0	1	1	VRVLDNHYCV	tr_D0A9V0	0.067	24100.42	34.00
495	HLA-A0201	VASKLHHPNI	VASKLHHPI	0	0	0	8	1	VASKLHHPNI	tr_D0A9V0	0.067	24107.71	34.00
691	HLA-A0201	EAAAFVRACCHMNP	AAFVACCHM	2	0	0	4	1	AAFVRACCHM	tr_D0A9V0	0.067	24110.58	34.00
240	HLA-A0201	LSYAVRDGPCRIA	LSYAVCRIA	0	0	0	5	4	LSYAVRDGPCRIA	tr_D0A9V0	0.067	24117.36	34.00
526	HLA-A0201	GGSLHSVIKNF	SLHSVIKNF	2	0	0	0	0	SLHSVIKNF	tr_D0A9V0	0.067	24133.29	34.00
96	HLA-A0201	SISVTWNAG	SISVTWNAG	0	0	0	0	0	SISVTWNAG	tr_D0A9V0	0.067	24141.39	34.00
426	HLA-A0201	EGANAADVVPL	GAAADVVPL	1	0	0	2	1	GANAADVVPL	tr_D0A9V0	0.067	24162.29	34.00
348	HLA-A0201	TVPYVRVLDNHYC	VPYVRVLYC	1	0	0	7	3	VPYVRVLDNHYC	tr_D0A9V0	0.067	24164.38	34.00
481	HLA-A0201	RRDVVAESLEKEM	VVAESLEEM	3	0	0	7	1	VVAESLEKEM	tr_D0A9V0	0.067	24169.09	34.00
569	HLA-A0201	KPHNVLLGT	KPHNVLLGT	0	0	0	0	0	KPHNVLLGT	tr_D0A9V0	0.067	24173.79	34.00
193	HLA-A0201	FDLFAKLPLDHRD	FLFAKLPLD	0	0	0	1	1	FDLFAKLPLD	tr_D0A9V0	0.067	24201.02	34.00
110	HLA-A0201	VALNCGIFKEKPN	ALNCGIFPN	1	0	0	7	3	ALNCGIFKEKPN	tr_D0A9V0	0.067	24218.30	34.00
473	HLA-A0201	AIKCIPRLRRDVV	AIKCIPRLV	0	0	0	8	4	AIKCIPRLRRDVV	tr_D0A9V0	0.067	24230.36	34.00
231	HLA-A0201	LFGADHLFQLS	FGADHLFQL	1	0	0	0	0	FGADHLFQL	tr_D0A9V0	0.067	24231.94	34.00
563	HLA-A0201	IVHCDVKPHNVL	IVHCDVNVL	0	0	0	6	3	IVHCDVKPHNVL	tr_D0A9V0	0.067	24248.20	34.00
85	HLA-A0201	SSTAFVRIMG	SSTAFVIMG	0	0	0	6	1	SSTAFVRIMG	tr_D0A9V0	0.067	24252.65	34.00
683	HLA-A0201	VIPASVDHEAAA	VIPASVAAA	0	0	0	6	3	VIPASVDHEAAA	tr_D0A9V0	0.067	24262.63	34.00
480	HLA-A0201	LRRDVVAESL	LRDVVAESL	0	0	0	1	1	LRRDVVAESL	tr_D0A9V0	0.067	24265.78	34.00
524	HLA-A0201	VPGGSLHSVIKNF	VLHSVIKNF	0	0	0	1	4	VPGGSLHSVIKNF	tr_D0A9V0	0.067	24277.33	34.00
13	HLA-A0201	SNLEIPDNA	SNLEIPDNA	0	0	0	0	0	SNLEIPDNA	tr_D0A9V0	0.067	24301.25	34.00
471	HLA-A0201	QVAIKCIPRLRR	QVAIKCIPL	0	0	0	8	1	QVAIKCIPRL	tr_D0A9V0	0.067	24313.35	34.00
638	HLA-A0201	EMLLGRPPW	EMLLGRPPW	0	0	0	0	0	EMLLGRPPW	tr_D0A9V0	0.066	24357.06	34.00
284	HLA-A0201	FELRPGESSSEV	ELGESSSEV	1	0	0	2	2	ELRPGESSSEV	tr_D0A9V0	0.066	24368.65	34.00
121	HLA-A0201	PNVFNRRHCTV	NVFNRHCTV	1	0	0	4	1	NVFNRRHCTV	tr_D0A9V0	0.066	24373.67	34.00
161	HLA-A0201	GCVEALDPDRAL	GCVEALDPL	0	0	0	8	3	GCVEALDPDRAL	tr_D0A9V0	0.066	24381.31	34.00
347	HLA-A0201	DTVPYVRVLDNHYC	VPYVRVLYC	2	0	0	7	3	VPYVRVLDNHYC	tr_D0A9V0	0.066	24384.74	34.00
184	HLA-A0201	HCRRCCQFA	HCRRCCQFA	0	0	0	0	0	HCRRCCQFA	tr_D0A9V0	0.066	24386.33	34.00
475	HLA-A0201	KCIPRLRRDVVAES	RLRDVVAES	4	0	0	2	1	RLRRDVVAES	tr_D0A9V0	0.066	24393.97	34.00
555	HLA-A0201	LNYLHNLGIVH	YLHNLGIVH	2	0	0	0	0	YLHNLGIVH	tr_D0A9V0	0.066	24405.33	35.00
144	HLA-A0201	SSTKYHAALI	SSTKYAALI	0	0	0	5	1	SSTKYHAALI	tr_D0A9V0	0.066	24409.82	35.00

152	HLA-A0201	LIEVATEHSGCV	LIEVATEHV	0	0	0	8	3	LIEVATEHSGCV	tr_D0A9V0	0.066	24416.41	35.00
480	HLA-A0201	LRRDVVAESLEKEM	VVAESLEEM	4	0	0	7	1	VVAESLEKEM	tr_D0A9V0	0.066	24424.08	35.00
6	HLA-A0201	VSALKSASNL	VSALKSASL	0	0	0	8	1	VSALKSASNL	tr_D0A9V0	0.066	24427.52	35.00
354	HLA-A0201	VLDNHYCVN	VLDNHYCVN	0	0	0	0	0	VLDNHYCVN	tr_D0A9V0	0.066	24439.41	35.00
242	HLA-A0201	YAVRDGPCRIAA	YAVPCRIAA	0	0	0	3	3	YAVRDGPCRIAA	tr_D0A9V0	0.066	24447.61	35.00
242	HLA-A0201	YAVRDGPCRIAAT	YAVCRIAAT	0	0	0	3	4	YAVRDGPCRIAAT	tr_D0A9V0	0.066	24473.81	35.00
124	HLA-A0201	FNRRHCTVIAIQ	FRHCTVIAI	0	0	0	1	2	FNRRHCTVIAI	tr_D0A9V0	0.066	24475.41	35.00
14	HLA-A0201	NLEIPDNADIF	NLIPDNADI	0	0	0	2	1	NLEIPDNADI	tr_D0A9V0	0.066	24485.46	35.00
127	HLA-A0201	RHCTVIAIQVYH	CTVIAIQVY	2	0	0	0	0	CTVIAIQVY	tr_D0A9V0	0.066	24487.33	35.00
590	HLA-A0201	ISEASVMARTL	ISEASVMTL	0	0	0	7	2	ISEASVMARTL	tr_D0A9V0	0.066	24487.33	35.00
469	HLA-A0201	GAQVAIKCIPR	AQVAICIPR	1	0	0	5	1	AQVAIKCIPR	tr_D0A9V0	0.066	24489.71	35.00
707	HLA-A0201	RPSASSLLSYA	RASSLLSYA	0	0	0	1	2	RPSASSLLSYA	tr_D0A9V0	0.066	24492.37	35.00
533	HLA-A0201	IKNFGCVSPYVARR	KNFGCVPYV	1	0	0	6	1	KNFGCVSPYV	tr_D0A9V0	0.066	24493.16	35.00
525	HLA-A0201	PGGSLHSVIKNF	SLHSVIKNF	3	0	0	0	0	SLHSVIKNF	tr_D0A9V0	0.066	24497.39	35.00
333	HLA-A0201	KRLMRLCRQRCR	RLMRLCQRC	1	0	0	6	1	RLMRLCRQRC	tr_D0A9V0	0.066	24497.39	35.00
529	HLA-A0201	LHSVIKNFGCV	LVIKNFGCV	0	0	0	1	2	LHSVIKNFGCV	tr_D0A9V0	0.066	24508.26	35.00
575	HLA-A0201	LGTDGVCKL	LGTDGVCKL	0	0	0	0	0	LGTDGVCKL	tr_D0A9V0	0.066	24517.29	35.00
510	HLA-A0201	CSITPSHLSIIME	CSILSIIME	0	0	0	3	4	CSITPSHLSIIME	tr_D0A9V0	0.066	24537.46	35.00
150	HLA-A0201	AALIEVATE	AALIEVATE	0	0	0	0	0	AALIEVATE	tr_D0A9V0	0.066	24540.64	35.00
509	HLA-A0201	SCSITPSHLSII	SCSITLSII	0	0	0	5	3	SCSITPSHLSII	tr_D0A9V0	0.066	24589.00	35.00
476	HLA-A0201	CIPRLRRDVV	CIRLRRDVV	0	0	0	2	1	CIPRLRRDVV	tr_D0A9V0	0.066	24598.33	35.00
326	HLA-A0201	VKMDMQAKRLM	KMDMQARLM	1	0	0	6	1	KMDMQAKRLM	tr_D0A9V0	0.066	24610.31	35.00
22	HLA-A0201	DIFLGRSRI	DIFLGRSRI	0	0	0	0	0	DIFLGRSRI	tr_D0A9V0	0.066	24613.23	35.00
509	HLA-A0201	SCSITPSHLSIIME	SCSILSIIM	0	0	0	4	4	SCSITPSHLSIIM	tr_D0A9V0	0.065	24626.82	35.00
584	HLA-A0201	SDFGSTISEA	SDFGSTISA	0	0	0	8	1	SDFGSTISEA	tr_D0A9V0	0.065	24643.61	35.00
193	HLA-A0201	FDLFAKLPLDHRDS	FLFAKLPLD	0	0	0	1	1	FDLFAKLPLD	tr_D0A9V0	0.065	24645.47	35.00
311	HLA-A0201	RQGRYGEAMA	RQGYGEAMA	0	0	0	3	1	RQGRYGEAMA	tr_D0A9V0	0.065	24649.48	35.00
302	HLA-A0201	QFRLGFAAMR	FRLGFAAMR	1	0	0	0	0	FRLGFAAMR	tr_D0A9V0	0.065	24654.80	35.00
568	HLA-A0201	VKPHNVLLGTDGVC	VLLGTDGVC	0	0	0	1	5	VKPHNVLLGTDGVC	tr_D0A9V0	0.065	24669.49	35.00
477	HLA-A0201	IPRLRRDVVAES	RLRDVVAES	2	0	0	2	1	RLRRDVVAES	tr_D0A9V0	0.065	24677.49	35.00
109	HLA-A0201	DVALNCGIFKEKPN	ALNCGIFPN	2	0	0	7	3	ALNCGIFKEKPN	tr_D0A9V0	0.065	24679.37	35.00
231	HLA-A0201	LFGADHLFQLSY	FGADHLFQL	1	0	0	0	0	FGADHLFQL	tr_D0A9V0	0.065	24692.72	35.00
201	HLA-A0201	LDHRDSVTMLV	LRDSVTMLV	0	0	0	1	2	LDHRDSVTMLV	tr_D0A9V0	0.065	24711.16	35.00
522	HLA-A0201	EYVPGGSLHS	YVPGGSLHS	1	0	0	0	0	YVPGGSLHS	tr_D0A9V0	0.065	24719.46	35.00
144	HLA-A0201	SSTKYHAALIEVAT	STHAALIEV	1	0	0	2	2	STKYHAALIEV	tr_D0A9V0	0.065	24723.73	35.00
351	HLA-A0201	YVRVLDNHY	YVRVLDNHY	0	0	0	0	0	YVRVLDNHY	tr_D0A9V0	0.065	24735.23	35.00
160	HLA-A0201	SGCVEALDPDRAL	GCVEALDPL	1	0	0	8	3	GCVEALDPDRAL	tr_D0A9V0	0.065	24747.28	35.00
468	HLA-A0201	DGAQVAIKCIPR	AQVAICIPR	2	0	0	5	1	AQVAIKCIPR	tr_D0A9V0	0.065	24752.64	35.00
700	HLA-A0201	CHMNPSMRPSAS	HMNPSMPSA	1	0	0	6	1	HMNPSMRPSA	tr_D0A9V0	0.065	24755.32	35.00
597	HLA-A0201	ARTLADGVMLR	ALADGVMLR	0	0	0	1	2	ARTLADGVMLR	tr_D0A9V0	0.065	24780.51	35.00
511	HLA-A0201	SITPSHLSIIME	SISHLSIIM	0	0	0	2	2	SITPSHLSIIM	tr_D0A9V0	0.065	24784.54	35.00
533	HLA-A0201	IKNFGCVSPY	INFGCVSPY	0	0	0	1	1	IKNFGCVSPY	tr_D0A9V0	0.065	24804.38	35.00
704	HLA-A0201	PSMRPSASSLLSY	SMASSLLSY	1	0	0	2	3	SMRPSASSLLSY	tr_D0A9V0	0.065	24811.90	35.00
323	HLA-A0201	LPMVKMDMQAKRL	MMDMQAKRL	2	0	0	1	2	MVKMDMQAKRL	tr_D0A9V0	0.065	24822.64	35.00
538	HLA-A0201	CVSPYVARRFT	CVSPYVAFT	0	0	0	7	2	CVSPYVARRFT	tr_D0A9V0	0.065	24839.30	35.00
199	HLA-A0201	LPLDHRDSVTML	PLDDSVTML	1	0	0	3	2	PLDHRDSVTML	tr_D0A9V0	0.065	24849.78	35.00
220	HLA-A0201	TCGAFSRNARV	GAFSRNARV	2	0	0	0	0	GAFSRNARV	tr_D0A9V0	0.065	24861.61	35.00
128	HLA-A0201	HCTVIAIQVYH	HCTVIAIQV	0	0	0	0	0	HCTVIAIQV	tr_D0A9V0	0.065	24862.42	35.00
644	HLA-A0201	PPWQWSSTAPAGSD	WQWSSTAPA	2	0	0	0	0	WQWSSTAPA	tr_D0A9V0	0.065	24862.70	35.00
126	HLA-A0201	RRHCTVIAIQVYH	CTVIAIVYH	3	0	0	6	1	CTVIAIQVYH	tr_D0A9V0	0.065	24874.26	35.00
296	HLA-A0201	SLSISKQFR	SLSISKQFR	0	0	0	0	0	SLSISKQFR	tr_D0A9V0	0.065	24879.65	35.00
467	HLA-A0201	EDGAQVAIKCIPR	AQVAICIPR	3	0	0	5	1	AQVAIKCIPR	tr_D0A9V0	0.064	24913.05	35.00
29	HLA-A0201	RIVEVRKIQGHYAK	RIVEVGHYA	0	0	0	5	4	RIVEVRKIQGHYA	tr_D0A9V0	0.064	24932.99	36.00
123	HLA-A0201	VFNRRHCTVIAIQ	VFHCTVIAI	0	0	0	2	3	VFNRRHCTVIAI	tr_D0A9V0	0.064	24933.81	36.00

303	HLA-A0201	FRLGFAAMRQGRYG	RLGFAAMYG	1	0	0	7	4	RLGFAAMRQGRYG	tr_D0A9V0	0.064	24957.83	36.00
297	HLA-A0201	LSISKQFRLGFA	LSISKQFFA	0	0	0	7	3	LSISKQFRLGFA	tr_D0A9V0	0.064	24971.61	36.00
476	HLA-A0201	CIPRLRRDVVAES	RLRDVVAES	3	0	0	2	1	RLRRDVVAES	tr_D0A9V0	0.064	24973.77	36.00
110	HLA-A0201	VALNCGIFKEK	ALNCGIFEK	1	0	0	7	1	ALNCGIFKEK	tr_D0A9V0	0.064	24998.91	36.00
466	HLA-A0201	AEDGAQVAIKCIPR	AQVAICIPR	4	0	0	5	1	AQVAIKCIPR	tr_D0A9V0	0.064	25008.38	36.00
287	HLA-A0201	RPGESSSEV	RPGESSSEV	0	0	0	0	0	RPGESSSEV	tr_D0A9V0	0.064	25038.44	36.00
274	HLA-A0201	LPCSTLPIVLF	LSTLPIVLF	0	0	0	1	2	LPCSTLPIVLF	tr_D0A9V0	0.064	25040.05	36.00
576	HLA-A0201	GTDGVCKLS	GTDGVCKLS	0	0	0	0	0	GTDGVCKLS	tr_D0A9V0	0.064	25041.14	36.00
282	HLA-A0201	VLFELRPGESSSE	VLFELGESS	0	0	0	5	2	VLFELRPGESS	tr_D0A9V0	0.064	25041.41	36.00
233	HLA-A0201	GADHLFQLS	GADHLFQLS	0	0	0	0	0	GADHLFQLS	tr_D0A9V0	0.064	25042.49	36.00
323	HLA-A0201	LPMVKMDMQAKRLM	KMDMQARLM	4	0	0	6	1	KMDMQAKRLM	tr_D0A9V0	0.064	25049.55	36.00
593	HLA-A0201	ASVMARTLA	ASVMARTLA	0	0	0	0	0	ASVMARTLA	tr_D0A9V0	0.064	25067.72	36.00
120	HLA-A0201	KPNVFNRRHCTV	NVFNRHCTV	2	0	0	4	1	NVFNRRHCTV	tr_D0A9V0	0.064	25070.14	36.00
324	HLA-A0201	PMVKMDMQAKRL	MMDMQAKRL	1	0	0	1	2	MVKMDMQAKRL	tr_D0A9V0	0.064	25072.32	36.00
425	HLA-A0201	AEGANAADVVPL	GAAADVVPL	2	0	0	2	1	GANAADVVPL	tr_D0A9V0	0.064	25078.29	36.00
492	HLA-A0201	EMEVASKLHHPNIV	EMLHHPNIV	0	0	0	2	5	EMEVASKLHHPNIV	tr_D0A9V0	0.064	25081.82	36.00
275	HLA-A0201	PCSTLPIVLFE	STLPIVLFE	2	0	0	0	0	STLPIVLFE	tr_D0A9V0	0.064	25083.98	36.00
127	HLA-A0201	RHCTVIAIQVYHP	CTVIAIQVY	2	0	0	0	0	CTVIAIQVY	tr_D0A9V0	0.064	25092.40	36.00
269	HLA-A0201	VDCVLLPCST	VCVLLPCST	0	0	0	1	1	VDCVLLPCST	tr_D0A9V0	0.064	25119.84	36.00
226	HLA-A0201	RNARVLFGADHL	RNARVLFHL	0	0	0	7	3	RNARVLFGADHL	tr_D0A9V0	0.064	25122.55	36.00
325	HLA-A0201	MVKMDMQAKRLM	KMDMQARLM	2	0	0	6	1	KMDMQAKRLM	tr_D0A9V0	0.064	25132.61	36.00
88	HLA-A0201	AFVRIMGASIS	FVRIMGASI	1	0	0	0	0	FVRIMGASI	tr_D0A9V0	0.064	25138.33	36.00
503	HLA-A0201	NIVRYVSCSIT	NIVYVSCSI	0	0	0	3	1	NIVRYVSCSI	tr_D0A9V0	0.064	25151.92	36.00
332	HLA-A0201	AKRLMRLCRQRCR	RLMRLCQRC	2	0	0	6	1	RLMRLCRQRC	tr_D0A9V0	0.063	25169.90	36.00
54	HLA-A0201	IPEAILARVE	IPEAILARV	0	0	0	0	0	IPEAILARV	tr_D0A9V0	0.063	25173.16	36.00
520	HLA-A0201	IMEYVPGGSLH	IMEYVPGSL	0	0	0	6	1	IMEYVPGGSL	tr_D0A9V0	0.063	25178.07	36.00
185	HLA-A0201	CRRCCQFAF	CRRCCQFAF	0	0	0	0	0	CRRCCQFAF	tr_D0A9V0	0.063	25182.97	36.00
302	HLA-A0201	QFRLGFAAMRQ	FLGFAAMRQ	1	0	0	1	1	FRLGFAAMRQ	tr_D0A9V0	0.063	25214.06	36.00
417	HLA-A0201	SSLDGSGCAEG	SLDGGCAEG	1	0	0	4	1	SLDGSGCAEG	tr_D0A9V0	0.063	25226.06	36.00
219	HLA-A0201	GTCGAFSRNA	GTCGAFSNA	0	0	0	7	1	GTCGAFSRNA	tr_D0A9V0	0.063	25229.61	36.00
703	HLA-A0201	NPSMRPSASSLLSY	SMASSLLSY	2	0	0	2	3	SMRPSASSLLSY	tr_D0A9V0	0.063	25238.62	36.00
27	HLA-A0201	RSRIVEVRKI	RSRIVEVRI	0	0	0	8	1	RSRIVEVRKI	tr_D0A9V0	0.063	25242.17	36.00
471	HLA-A0201	QVAIKCIPRLRRD	QVAIKCIPL	0	0	0	8	1	QVAIKCIPRL	tr_D0A9V0	0.063	25242.43	36.00
192	HLA-A0201	AFDLFAKLP	AFDLFAKLP	0	0	0	0	0	AFDLFAKLP	tr_D0A9V0	0.063	25249.83	36.00
401	HLA-A0201	DQGSESLFKM	DQSESLFKM	0	0	0	2	1	DQGSESLFKM	tr_D0A9V0	0.063	25250.09	36.00
173	HLA-A0201	ISFGAHAALPMHCR	ISFGAALPM	0	0	0	4	2	ISFGAHAALPM	tr_D0A9V0	0.063	25259.92	36.00
181	HLA-A0201	LPMHCRRCCQFA	PMHCCCQFA	1	0	0	4	2	PMHCRRCCQFA	tr_D0A9V0	0.063	25261.57	36.00
517	HLA-A0201	LSIIMEYVP	LSIIMEYVP	0	0	0	0	0	LSIIMEYVP	tr_D0A9V0	0.063	25263.75	36.00
323	HLA-A0201	LPMVKMDMQAK	LMVKMDMQA	0	0	0	1	1	LPMVKMDMQA	tr_D0A9V0	0.063	25265.66	36.00
184	HLA-A0201	HCRRCCQFAF	HCRRCCFAF	0	0	0	6	1	HCRRCCQFAF	tr_D0A9V0	0.063	25274.41	36.00
234	HLA-A0201	ADHLFQLSY	ADHLFQLSY	0	0	0	0	0	ADHLFQLSY	tr_D0A9V0	0.063	25274.69	36.00
233	HLA-A0201	GADHLFQLSY	GAHLFQLSY	0	0	0	2	1	GADHLFQLSY	tr_D0A9V0	0.063	25277.15	36.00
333	HLA-A0201	KRLMRLCRQRCRR	RLMRLCCRR	1	0	0	6	3	RLMRLCRQRCRR	tr_D0A9V0	0.063	25281.79	36.00
330	HLA-A0201	MQAKRLMRLCR	MQAKRLMRL	0	0	0	0	0	MQAKRLMRL	tr_D0A9V0	0.063	25302.33	36.00
149	HLA-A0201	HAALIEVATEH	ALIEVATEH	2	0	0	0	0	ALIEVATEH	tr_D0A9V0	0.063	25327.24	36.00
353	HLA-A0201	RVLDNHYCVNSSDC	RVLDNHYCV	0	0	0	0	0	RVLDNHYCV	tr_D0A9V0	0.063	25335.20	36.00
334	HLA-A0201	RLMRLCRQRCRRN	RLMRLCQRC	0	0	0	6	1	RLMRLCRQRC	tr_D0A9V0	0.063	25342.05	36.00
504	HLA-A0201	IVRYVSCSITP	IVVSCSITP	0	0	0	2	2	IVRYVSCSITP	tr_D0A9V0	0.063	25356.04	36.00
545	HLA-A0201	RRFTVDILQ	RRFTVDILQ	0	0	0	0	0	RRFTVDILQ	tr_D0A9V0	0.063	25357.14	36.00
243	HLA-A0201	AVRDGPCRIA	AVDGPCRIA	0	0	0	2	1	AVRDGPCRIA	tr_D0A9V0	0.063	25359.07	36.00
399	HLA-A0201	SSDQGSESLFKMYA	SSDLFKMYA	0	0	0	3	5	SSDQGSESLFKMYA	tr_D0A9V0	0.063	25372.77	36.00
225	HLA-A0201	SRNARVLFGADHL	SVLFGADHL	0	0	0	1	4	SRNARVLFGADHL	tr_D0A9V0	0.063	25373.89	36.00
699	HLA-A0201	CCHMNPSMRPSAS	HMNPSMPSA	2	0	0	6	1	HMNPSMRPSA	tr_D0A9V0	0.063	25374.98	36.00
125	HLA-A0201	NRRHCTVIAIQVYH	CTVIAIVYH	4	0	0	6	1	CTVIAIQVYH	tr_D0A9V0	0.063	25395.30	36.00

109	HLA-A0201	DVALNCGIFKEK	ALNCGIFEK	2	0	0	7	1	ALNCGIFKEK	tr_D0A9V0	0.063	25399.71	36.00
331	HLA-A0201	QAKRLMRLCRQRCR	RLMRLCRQRC	3	0	0	6	1	RLMRLCRQRC	tr_D0A9V0	0.063	25410.42	36.00
118	HLA-A0201	KEKPNVFNRRHCTV	NVFNRHCTV	4	0	0	4	1	NVFNRRHCTV	tr_D0A9V0	0.063	25422.52	36.00
324	HLA-A0201	PMVKMDMQAKRLM	KMDMQARLM	3	0	0	6	1	KMDMQAKRLM	tr_D0A9V0	0.063	25425.55	36.00
329	HLA-A0201	DMQAKRLMRLCRQR	MLMRLCRQR	1	0	0	1	4	MQAKRLMRLCRQR	tr_D0A9V0	0.062	25428.03	36.00
257	HLA-A0201	GASHMQGFLAF	GAMQGFLAF	0	0	0	2	2	GASHMQGFLAF	tr_D0A9V0	0.062	25436.28	36.00
665	HLA-A0201	FNRDLLFVQ	FNRDLLFVQ	0	0	0	0	0	FNRDLLFVQ	tr_D0A9V0	0.062	25437.93	36.00
590	HLA-A0201	ISEASVMART	ISASVMART	0	0	0	2	1	ISEASVMART	tr_D0A9V0	0.062	25440.67	36.00
84	HLA-A0201	ESSTAFVRIM	SSTAFVRIM	1	0	0	0	0	SSTAFVRIM	tr_D0A9V0	0.062	25455.00	37.00
443	HLA-A0201	RNVWTRSLKKI	NVWTRSLKI	1	0	0	7	1	NVWTRSLKKI	tr_D0A9V0	0.062	25465.20	37.00
126	HLA-A0201	RRHCTVIAIQVYHP	RCTVIAIQV	0	0	0	1	2	RRHCTVIAIQV	tr_D0A9V0	0.062	25475.66	37.00
303	HLA-A0201	FRLGFAAMRQGRY	FRLGFAAMY	0	0	0	8	4	FRLGFAAMRQGRY	tr_D0A9V0	0.062	25476.48	37.00
249	HLA-A0201	CRIAATSGGA	RIAATSGGA	1	0	0	0	0	RIAATSGGA	tr_D0A9V0	0.062	25478.14	37.00
330	HLA-A0201	MQAKRLMRLCRQ	MQAKRLMRL	0	0	0	0	0	MQAKRLMRL	tr_D0A9V0	0.062	25483.66	37.00
307	HLA-A0201	FAAMRQGRYGEAMA	FAAMRQAMA	0	0	0	6	5	FAAMRQGRYGEAMA	tr_D0A9V0	0.062	25487.53	37.00
35	HLA-A0201	KIQGHYAKLRRQL	KIQGHYAQL	0	0	0	7	4	KIQGHYAKLRRQL	tr_D0A9V0	0.062	25490.55	37.00
352	HLA-A0201	VRVLDNHYCVNSSD	VLYCVNSSD	2	0	0	2	3	VLDNHYCVNSSD	tr_D0A9V0	0.062	25502.14	37.00
93	HLA-A0201	MGASISVTWNAGD	MSISVTWNA	0	0	0	1	2	MGASISVTWNA	tr_D0A9V0	0.062	25525.89	37.00
660	HLA-A0201	RLRSLFNRD	RLRSLFNRD	0	0	0	0	0	RLRSLFNRD	tr_D0A9V0	0.062	25528.09	37.00
119	HLA-A0201	EKPNVFNRRHCTV	NVFNRHCTV	3	0	0	4	1	NVFNRRHCTV	tr_D0A9V0	0.062	25534.71	37.00
227	HLA-A0201	NARVLFGADHL	AVLFGADHL	1	0	0	1	1	ARVLFGADHL	tr_D0A9V0	0.062	25551.58	37.00
342	HLA-A0201	RCRRNDTVPYVRVL	RTVPYVRVL	0	0	0	1	5	RCRRNDTVPYVRVL	tr_D0A9V0	0.062	25573.42	37.00
108	HLA-A0201	RDVALNCGIFKEK	ALNCGIFEK	3	0	0	7	1	ALNCGIFKEK	tr_D0A9V0	0.062	25579.79	37.00
344	HLA-A0201	RRNDTVPYVR	RRNDTVPYV	0	0	0	0	0	RRNDTVPYV	tr_D0A9V0	0.062	25601.11	37.00
670	HLA-A0201	LFVQSLALGH	FVQSLALGH	1	0	0	0	0	FVQSLALGH	tr_D0A9V0	0.062	25609.98	37.00
128	HLA-A0201	HCTVIAIQVYHP	HCTVIAIQV	0	0	0	0	0	HCTVIAIQV	tr_D0A9V0	0.062	25624.67	37.00
239	HLA-A0201	QLSYAVRDGP	QLYAVRDGP	0	0	0	2	1	QLSYAVRDGP	tr_D0A9V0	0.062	25643.81	37.00
299	HLA-A0201	ISKQFRLGF	ISKQFRLGF	0	0	0	0	0	ISKQFRLGF	tr_D0A9V0	0.062	25675.73	37.00
588	HLA-A0201	STISEASVMARTLA	TISEASVLA	1	0	0	7	4	TISEASVMARTLA	tr_D0A9V0	0.062	25688.80	37.00
698	HLA-A0201	ACCHMNPSMRPSAS	HMNPSMPSA	3	0	0	6	1	HMNPSMRPSA	tr_D0A9V0	0.062	25700.18	37.00
175	HLA-A0201	FGAHAALPMH	FGAHAALPM	0	0	0	0	0	FGAHAALPM	tr_D0A9V0	0.061	25709.09	37.00
570	HLA-A0201	PHNVLLGTDGV	HVLLGTDGV	1	0	0	1	1	HNVLLGTDGV	tr_D0A9V0	0.061	25715.77	37.00
497	HLA-A0201	SKLHHPNIV	SKLHHPNIV	0	0	0	0	0	SKLHHPNIV	tr_D0A9V0	0.061	25730.80	37.00
509	HLA-A0201	SCSITPSHLSI	SCSITPLSI	0	0	0	6	2	SCSITPSHLSI	tr_D0A9V0	0.061	25738.60	37.00
475	HLA-A0201	KCIPRLRRDVV	KCIPRLDVV	0	0	0	6	2	KCIPRLRRDVV	tr_D0A9V0	0.061	25740.54	37.00
354	HLA-A0201	VLDNHYCVNSSDC	VLDNHYCVC	0	0	0	8	4	VLDNHYCVNSSDC	tr_D0A9V0	0.061	25744.72	37.00
503	HLA-A0201	NIVRYVSCSITP	IVVSCSITP	1	0	0	2	2	IVRYVSCSITP	tr_D0A9V0	0.061	25751.40	37.00
684	HLA-A0201	IPASVDHEA	IPASVDHEA	0	0	0	0	0	IPASVDHEA	tr_D0A9V0	0.061	25755.59	37.00
129	HLA-A0201	CTVIAIQVYHP	TVIAIVYHP	1	0	0	5	1	TVIAIQVYHP	tr_D0A9V0	0.061	25767.85	37.00
558	HLA-A0201	LHNLGIVHC	LHNLGIVHC	0	0	0	0	0	LHNLGIVHC	tr_D0A9V0	0.061	25772.60	37.00
107	HLA-A0201	QRDVALNCGIFKEK	ALNCGIFEK	4	0	0	7	1	ALNCGIFKEK	tr_D0A9V0	0.061	25786.81	37.00
569	HLA-A0201	KPHNVLLGTDGVCK	VLLGTDVCK	4	0	0	6	1	VLLGTDGVCK	tr_D0A9V0	0.061	25790.44	37.00
694	HLA-A0201	AFVRACCHMNPS	FVRACCHMS	1	0	0	8	2	FVRACCHMNPS	tr_D0A9V0	0.061	25794.63	37.00
601	HLA-A0201	ADGVMLRGTALY	AVMLRGTAL	0	0	0	1	2	ADGVMLRGTAL	tr_D0A9V0	0.061	25812.77	37.00
111	HLA-A0201	ALNCGIFKEKP	ALNCGIFKP	0	0	0	7	2	ALNCGIFKEKP	tr_D0A9V0	0.061	25830.11	37.00
219	HLA-A0201	GTCGAFSRNARVLF	GTCGAFVLF	0	0	0	6	5	GTCGAFSRNARVLF	tr_D0A9V0	0.061	25843.52	37.00
20	HLA-A0201	NADIFLGRS	NADIFLGRS	0	0	0	0	0	NADIFLGRS	tr_D0A9V0	0.061	25843.52	37.00
190	HLA-A0201	QFAFDLFAK	QFAFDLFAK	0	0	0	0	0	QFAFDLFAK	tr_D0A9V0	0.061	25848.55	37.00
217	HLA-A0201	LVGTCGAFSRNA	LVGTCGAFA	0	0	0	8	3	LVGTCGAFSRNA	tr_D0A9V0	0.061	25854.14	37.00
44	HLA-A0201	RRQLAVLLK	RRQLAVLLK	0	0	0	0	0	RRQLAVLLK	tr_D0A9V0	0.061	25862.54	37.00
300	HLA-A0201	SKQFRLGFA	SKQFRLGFA	0	0	0	0	0	SKQFRLGFA	tr_D0A9V0	0.061	25874.02	37.00
661	HLA-A0201	LRSLFNRDLL	LRSLFNDLL	0	0	0	6	1	LRSLFNRDLL	tr_D0A9V0	0.061	25897.83	37.00
337	HLA-A0201	RLCRQRCRRNDTVP	RLCRRNDTV	0	0	0	2	4	RLCRQRCRRNDTV	tr_D0A9V0	0.061	25898.66	37.00
503	HLA-A0201	NIVRYVSCSITPS	YVSCSITPS	4	0	0	0	0	YVSCSITPS	tr_D0A9V0	0.061	25904.82	37.00

123	HLA-A0201	VFNRRHCTV	VFNRRHCTV	0	0	0	0	0	VFNRRHCTV	tr_D0A9V0	0.061	25907.92	37.00
309	HLA-A0201	AMRQGRYGEAM	AMGRYGEAM	0	0	0	2	2	AMRQGRYGEAM	tr_D0A9V0	0.061	25929.51	37.00
35	HLA-A0201	KIQGHYAKLRRQLA	KIQGHYALA	0	0	0	7	5	KIQGHYAKLRRQLA	tr_D0A9V0	0.061	25940.73	37.00
453	HLA-A0201	ISEGAFSTIF	ISGAFSTIF	0	0	0	2	1	ISEGAFSTIF	tr_D0A9V0	0.061	25941.29	37.00
342	HLA-A0201	RCRRNDTVPYVR	RCNDTVPYV	0	0	0	2	2	RCRRNDTVPYV	tr_D0A9V0	0.061	25948.03	37.00
135	HLA-A0201	QVYHPDVWKS	QVYPDVWKS	0	0	0	3	1	QVYHPDVWKS	tr_D0A9V0	0.061	25949.15	37.00
504	HLA-A0201	IVRYVSCSITPS	YVSCSITPS	3	0	0	0	0	YVSCSITPS	tr_D0A9V0	0.061	25953.37	38.00
473	HLA-A0201	AIKCIPRLRRDV	AIKCIPRLV	0	0	0	8	3	AIKCIPRLRRDV	tr_D0A9V0	0.061	25957.86	38.00
399	HLA-A0201	SSDQGSESLFKM	SSDESLFKM	0	0	0	3	3	SSDQGSESLFKM	tr_D0A9V0	0.061	25959.26	38.00
553	HLA-A0201	QGLNYLHNLG	QGLNYLHNL	0	0	0	0	0	QGLNYLHNL	tr_D0A9V0	0.061	25963.75	38.00
579	HLA-A0201	GVCKLSDFGS	GVCKLSFGS	0	0	0	6	1	GVCKLSDFGS	tr_D0A9V0	0.061	25978.92	38.00
574	HLA-A0201	LLGTDGVCK	LLGTDGVCK	0	0	0	0	0	LLGTDGVCK	tr_D0A9V0	0.061	25979.21	38.00
283	HLA-A0201	LFELRPGESSSEV	ELGESSSEV	2	0	0	2	2	ELRPGESSSEV	tr_D0A9V0	0.060	25999.74	38.00
442	HLA-A0201	NRNVWTRSLKKI	NVWTRSLKI	2	0	0	7	1	NVWTRSLKKI	tr_D0A9V0	0.060	26027.61	38.00
306	HLA-A0201	GFAAMRQGRYGEAM	FAAMYGEAM	1	0	0	4	4	FAAMRQGRYGEAM	tr_D0A9V0	0.060	26033.51	38.00
448	HLA-A0201	RSLKKISEGAFS	SLISEGAFS	1	0	0	2	2	SLKKISEGAFS	tr_D0A9V0	0.060	26036.34	38.00
332	HLA-A0201	AKRLMRLCRQRCRR	RLMRLCCRR	2	0	0	6	3	RLMRLCRQRCRR	tr_D0A9V0	0.060	26038.59	38.00
127	HLA-A0201	RHCTVIAIQVYHPD	RHCTVIIQV	0	0	0	6	1	RHCTVIAIQV	tr_D0A9V0	0.060	26040.00	38.00
510	HLA-A0201	CSITPSHLSII	SITPSHLSI	1	0	0	8	1	SITPSHLSII	tr_D0A9V0	0.060	26052.11	38.00
700	HLA-A0201	CHMNPSMRPSASS	HMNPSMPSA	1	0	0	6	1	HMNPSMRPSA	tr_D0A9V0	0.060	26074.11	38.00
17	HLA-A0201	IPDNADIFLGRSRI	IIFLGRSRI	0	0	0	1	5	IPDNADIFLGRSRI	tr_D0A9V0	0.060	26089.06	38.00
694	HLA-A0201	AFVRACCHMN	FVRACCHMN	1	0	0	0	0	FVRACCHMN	tr_D0A9V0	0.060	26149.26	38.00
319	HLA-A0201	MAHYLPMVK	MAHYLPMVK	0	0	0	0	0	MAHYLPMVK	tr_D0A9V0	0.060	26150.68	38.00
13	HLA-A0201	SNLEIPDNADIF	NLIPDNADI	1	0	0	2	1	NLEIPDNADI	tr_D0A9V0	0.060	26188.05	38.00
99	HLA-A0201	VTWNAGDQQRDV	VTWNAGDQV	0	0	0	8	3	VTWNAGDQQRDV	tr_D0A9V0	0.060	26234.84	38.00
302	HLA-A0201	QFRLGFAAMRQGRY	FRLGFAAMY	1	0	0	8	4	FRLGFAAMRQGRY	tr_D0A9V0	0.060	26247.05	38.00
83	HLA-A0201	GESSTAFVRI	GSSTAFVRI	0	0	0	1	1	GESSTAFVRI	tr_D0A9V0	0.060	26248.19	38.00
416	HLA-A0201	SSSLDGSGCAEG	SLDGGCAEG	2	0	0	4	1	SLDGSGCAEG	tr_D0A9V0	0.060	26255.86	38.00
182	HLA-A0201	PMHCRRCCQFAF	PMHCCQFAF	0	0	0	3	3	PMHCRRCCQFAF	tr_D0A9V0	0.059	26270.92	38.00
672	HLA-A0201	VQSLALGHLEP	VQSLALGHL	0	0	0	0	0	VQSLALGHL	tr_D0A9V0	0.059	26271.49	38.00
84	HLA-A0201	ESSTAFVRI	ESSTAFVRI	0	0	0	0	0	ESSTAFVRI	tr_D0A9V0	0.059	26272.34	38.00
20	HLA-A0201	NADIFLGRSRIVE	NADIFLGIV	0	0	0	7	3	NADIFLGRSRIV	tr_D0A9V0	0.059	26291.97	38.00
637	HLA-A0201	LEMLLGRPPWQ	MLLGRPPWQ	2	0	0	0	0	MLLGRPPWQ	tr_D0A9V0	0.059	26298.51	38.00
502	HLA-A0201	PNIVRYVSCSITPS	YVSCSITPS	5	0	0	0	0	YVSCSITPS	tr_D0A9V0	0.059	26303.63	38.00
441	HLA-A0201	SNRNVWTRSLKKI	NVWTRSLKI	3	0	0	7	1	NVWTRSLKKI	tr_D0A9V0	0.059	26307.89	38.00
566	HLA-A0201	CDVKPHNVLL	CVKPHNVLL	0	0	0	1	1	CDVKPHNVLL	tr_D0A9V0	0.059	26311.03	38.00
194	HLA-A0201	DLFAKLPLDHRDS	DLFAKLPLS	0	0	0	8	4	DLFAKLPLDHRDS	tr_D0A9V0	0.059	26311.32	38.00
109	HLA-A0201	DVALNCGIF	DVALNCGIF	0	0	0	0	0	DVALNCGIF	tr_D0A9V0	0.059	26314.44	38.00
106	HLA-A0201	QQRDVALNCGI	QQVALNCGI	0	0	0	2	2	QQRDVALNCGI	tr_D0A9V0	0.059	26328.97	38.00
375	HLA-A0201	DIKDRVPETI	DIDRVPETI	0	0	0	2	1	DIKDRVPETI	tr_D0A9V0	0.059	26330.96	38.00
5	HLA-A0201	LVSALKSASNL	LVSALASNL	0	0	0	5	2	LVSALKSASNL	tr_D0A9V0	0.059	26340.66	38.00
692	HLA-A0201	AAAFVRACC	AAAFVRACC	0	0	0	0	0	AAAFVRACC	tr_D0A9V0	0.059	26392.28	38.00
334	HLA-A0201	RLMRLCRQRCRRND	RLMRLCQRC	0	0	0	6	1	RLMRLCRQRC	tr_D0A9V0	0.059	26417.71	38.00
126	HLA-A0201	RRHCTVIAIQ	RRHCTVIAI	0	0	0	0	0	RRHCTVIAI	tr_D0A9V0	0.059	26431.73	38.00
256	HLA-A0201	GGASHMQGFLAF	ASMQGFLAF	2	0	0	2	1	ASHMQGFLAF	tr_D0A9V0	0.059	26435.45	38.00
440	HLA-A0201	DSNRNVWTRSLKKI	NVWTRSLKI	4	0	0	7	1	NVWTRSLKKI	tr_D0A9V0	0.059	26442.88	38.00
446	HLA-A0201	WTRSLKKISEGA	WLKKISEGA	0	0	0	1	3	WTRSLKKISEGA	tr_D0A9V0	0.059	26478.09	39.00
280	HLA-A0201	PIVLFELRP	PIVLFELRP	0	0	0	0	0	PIVLFELRP	tr_D0A9V0	0.059	26483.82	39.00
181	HLA-A0201	LPMHCRRCCQFAF	LPMCCQFAF	0	0	0	3	4	LPMHCRRCCQFAF	tr_D0A9V0	0.059	26505.03	39.00
476	HLA-A0201	CIPRLRRDV	CIPRLRRDV	0	0	0	0	0	CIPRLRRDV	tr_D0A9V0	0.059	26505.03	39.00
108	HLA-A0201	RDVALNCGI	RDVALNCGI	0	0	0	0	0	RDVALNCGI	tr_D0A9V0	0.059	26507.05	39.00
81	HLA-A0201	TPGESSTAFV	TPGESTAFV	0	0	0	4	1	TPGESSTAFV	tr_D0A9V0	0.059	26510.48	39.00
176	HLA-A0201	GAHAALPMHC	GAHAALMHC	0	0	0	6	1	GAHAALPMHC	tr_D0A9V0	0.059	26517.96	39.00
594	HLA-A0201	SVMARTLAD	SVMARTLAD	0	0	0	0	0	SVMARTLAD	tr_D0A9V0	0.059	26520.25	39.00

319	HLA-A0201	MAHYLPMVKMD	MAYLPMVKM	0	0	0	2	1	MAHYLPMVKM	tr_D0A9V0	0.059	26531.72	39.00
424	HLA-A0201	CAEGANAADVVPLF	CAEGVVPLF	0	0	0	4	5	CAEGANAADVVPLF	tr_D0A9V0	0.059	26533.73	39.00
586	HLA-A0201	FGSTISEASVM	FGSTISASV	0	0	0	6	1	FGSTISEASV	tr_D0A9V0	0.059	26537.46	39.00
521	HLA-A0201	MEYVPGGSL	MEYVPGGSL	0	0	0	0	0	MEYVPGGSL	tr_D0A9V0	0.059	26538.33	39.00
241	HLA-A0201	SYAVRDGPCRIAA	YAVPCRIAA	1	0	0	3	3	YAVRDGPCRIAA	tr_D0A9V0	0.059	26550.11	39.00
415	HLA-A0201	SSSSLDGSGCAEG	SLDGGCAEG	3	0	0	4	1	SLDGSGCAEG	tr_D0A9V0	0.058	26557.28	39.00
699	HLA-A0201	CCHMNPSMRPSASS	HMNPSMPSA	2	0	0	6	1	HMNPSMRPSA	tr_D0A9V0	0.058	26559.87	39.00
58	HLA-A0201	ILARVESKNEAG	ILARVENEA	0	0	0	6	2	ILARVESKNEA	tr_D0A9V0	0.058	26571.37	39.00
255	HLA-A0201	SGGASHMQGFLAF	ASMQGFLAF	3	0	0	2	1	ASHMQGFLAF	tr_D0A9V0	0.058	26576.54	39.00
431	HLA-A0201	ADVVPLFLTDSNR	ALFLTDSNR	0	0	0	1	4	ADVVPLFLTDSNR	tr_D0A9V0	0.058	26586.04	39.00
704	HLA-A0201	PSMRPSASSLLS	SMSASSLLS	1	0	0	2	2	SMRPSASSLLS	tr_D0A9V0	0.058	26590.34	39.00
682	HLA-A0201	PVIPASVDHEA	VIASVDHEA	1	0	0	2	1	VIPASVDHEA	tr_D0A9V0	0.058	26609.35	39.00
254	HLA-A0201	TSGGASHMQGFLAF	ASMQGFLAF	4	0	0	2	1	ASHMQGFLAF	tr_D0A9V0	0.058	26617.40	39.00
602	HLA-A0201	DGVMLRGTALY	VMLRGTALY	2	0	0	0	0	VMLRGTALY	tr_D0A9V0	0.058	26617.98	39.00
140	HLA-A0201	DVWKSSTKYHAALI	DVWYHAALI	0	0	0	3	5	DVWKSSTKYHAALI	tr_D0A9V0	0.058	26646.22	39.00
463	HLA-A0201	LGVAEDGAQVAI	GVAEAQVAI	1	0	0	4	2	GVAEDGAQVAI	tr_D0A9V0	0.058	26651.41	39.00
212	HLA-A0201	DTNEFLVGTCGA	DTNEFLVGA	0	0	0	7	3	DTNEFLVGTCGA	tr_D0A9V0	0.058	26653.71	39.00
479	HLA-A0201	RLRRDVVAE	RLRRDVVAE	0	0	0	0	0	RLRRDVVAE	tr_D0A9V0	0.058	26687.48	39.00
280	HLA-A0201	PIVLFELRPG	PIVLFLRPG	0	0	0	5	1	PIVLFELRPG	tr_D0A9V0	0.058	26701.35	39.00
241	HLA-A0201	SYAVRDGPCRIAAT	YAVCRIAAT	1	0	0	3	4	YAVRDGPCRIAAT	tr_D0A9V0	0.058	26723.59	39.00
505	HLA-A0201	VRYVSCSITP	VRYVSCITP	0	0	0	6	1	VRYVSCSITP	tr_D0A9V0	0.058	26758.32	39.00
93	HLA-A0201	MGASISVTWNAGDQ	MSISVTWNA	0	0	0	1	2	MGASISVTWNA	tr_D0A9V0	0.058	26764.69	39.00
414	HLA-A0201	SSSSSLDGSGCAEG	SLDGGCAEG	4	0	0	4	1	SLDGSGCAEG	tr_D0A9V0	0.058	26770.77	39.00
505	HLA-A0201	VRYVSCSIT	VRYVSCSIT	0	0	0	0	0	VRYVSCSIT	tr_D0A9V0	0.058	26774.24	39.00
502	HLA-A0201	PNIVRYVSCSIT	IVYVSCSIT	2	0	0	2	1	IVRYVSCSIT	tr_D0A9V0	0.058	26776.28	39.00
12	HLA-A0201	ASNLEIPDNADIF	NLIPDNADI	2	0	0	2	1	NLEIPDNADI	tr_D0A9V0	0.058	26777.43	39.00
128	HLA-A0201	HCTVIAIQVYHPD	HCTVIAIQV	0	0	0	0	0	HCTVIAIQV	tr_D0A9V0	0.058	26792.22	39.00
304	HLA-A0201	RLGFAAMRQGRY	RLGFAAMRY	0	0	0	7	3	RLGFAAMRQGRY	tr_D0A9V0	0.058	26798.60	39.00
545	HLA-A0201	RRFTVDILQG	RRFTVILQG	0	0	0	5	1	RRFTVDILQG	tr_D0A9V0	0.058	26800.32	39.00
512	HLA-A0201	ITPSHLSIIME	ITHLSIIME	0	0	0	2	2	ITPSHLSIIME	tr_D0A9V0	0.058	26802.66	39.00
461	HLA-A0201	IFLGVAEDGAQ	FLGVAEDGA	1	0	0	0	0	FLGVAEDGA	tr_D0A9V0	0.058	26811.35	39.00
329	HLA-A0201	DMQAKRLMRLCR	MQAKRLMRL	1	0	0	0	0	MQAKRLMRL	tr_D0A9V0	0.058	26812.22	39.00
398	HLA-A0201	DSSDQGSESL	SSDQGSESL	1	0	0	0	0	SSDQGSESL	tr_D0A9V0	0.058	26823.82	39.00
11	HLA-A0201	SASNLEIPDNADIF	NLEINADIF	3	0	0	4	2	NLEIPDNADIF	tr_D0A9V0	0.058	26831.66	39.00
379	HLA-A0201	RVPETIDSP	RVPETIDSP	0	0	0	0	0	RVPETIDSP	tr_D0A9V0	0.057	26843.57	39.00
342	HLA-A0201	RCRRNDTVPYVRV	RCNDTVPYV	0	0	0	2	2	RCRRNDTVPYV	tr_D0A9V0	0.057	26849.96	39.00
108	HLA-A0201	RDVALNCGIF	RVALNCGIF	0	0	0	1	1	RDVALNCGIF	tr_D0A9V0	0.057	26851.71	39.00
238	HLA-A0201	FQLSYAVRDGP	FQLSYAVGP	0	0	0	7	2	FQLSYAVRDGP	tr_D0A9V0	0.057	26861.88	39.00
374	HLA-A0201	SDIKDRVPET	SIKDRVPET	0	0	0	1	1	SDIKDRVPET	tr_D0A9V0	0.057	26877.87	39.00
57	HLA-A0201	AILARVESKNEAG	AILARVESA	0	0	0	8	3	AILARVESKNEA	tr_D0A9V0	0.057	26887.18	39.00
447	HLA-A0201	TRSLKKISEGAFS	SLISEGAFS	2	0	0	2	2	SLKKISEGAFS	tr_D0A9V0	0.057	26887.75	39.00
459	HLA-A0201	STIFLGVAEDG	TIFLGVAEG	1	0	0	8	1	TIFLGVAEDG	tr_D0A9V0	0.057	26898.52	39.00
333	HLA-A0201	KRLMRLCRQRCRRN	RLMRLCQRC	1	0	0	6	1	RLMRLCRQRC	tr_D0A9V0	0.057	26901.73	39.00
433	HLA-A0201	VVPLFLTDSNR	VLFLTDSNR	0	0	0	1	2	VVPLFLTDSNR	tr_D0A9V0	0.057	26913.95	39.00
402	HLA-A0201	QGSESLFKMYA	GSESLFMYA	1	0	0	6	1	GSESLFKMYA	tr_D0A9V0	0.057	26922.97	39.00
329	HLA-A0201	DMQAKRLMRLCRQ	MQAKRLMRL	1	0	0	0	0	MQAKRLMRL	tr_D0A9V0	0.057	26943.68	40.00
490	HLA-A0201	EKEMEVASKL	KEMEVASKL	1	0	0	0	0	KEMEVASKL	tr_D0A9V0	0.057	26952.99	40.00
315	HLA-A0201	YGEAMAHYLP	YGEAMAHYL	0	0	0	0	0	YGEAMAHYL	tr_D0A9V0	0.057	26964.68	40.00
285	HLA-A0201	ELRPGESSSEVSL	ELSSSEVSL	0	0	0	2	4	ELRPGESSSEVSL	tr_D0A9V0	0.057	26976.92	40.00
328	HLA-A0201	MDMQAKRLMRLCR	MQAKRLMRL	0	0	0	1	2	MDMQAKRLMRL	tr_D0A9V0	0.057	26994.45	40.00
693	HLA-A0201	AAFVRACCH	AAFVRACCH	0	0	0	0	0	AAFVRACCH	tr_D0A9V0	0.057	27002.04	40.00
502	HLA-A0201	PNIVRYVSCSITP	IVVSCSITP	2	0	0	2	2	IVRYVSCSITP	tr_D0A9V0	0.057	27022.49	40.00
438	HLA-A0201	LTDSNRNVWTRSL	LTDSWTRSL	0	0	0	4	4	LTDSNRNVWTRSL	tr_D0A9V0	0.057	27029.51	40.00
20	HLA-A0201	NADIFLGRSRI	NADIFLSRI	0	0	0	6	2	NADIFLGRSRI	tr_D0A9V0	0.057	27032.15	40.00

537	HLA-A0201	GCVSPYVARRFT	CVSPYVAFT	1	0	0	7	2	CVSPYVARRFT	tr_D0A9V0	0.057	27047.65	40.00
548	HLA-A0201	TVDILQGLNY	TVDILGLNY	0	0	0	5	1	TVDILQGLNY	tr_D0A9V0	0.057	27048.24	40.00
445	HLA-A0201	VWTRSLKKISEGA	SLKKISEGA	4	0	0	0	0	SLKKISEGA	tr_D0A9V0	0.057	27049.41	40.00
109	HLA-A0201	DVALNCGIFK	VALNCGIFK	1	0	0	0	0	VALNCGIFK	tr_D0A9V0	0.057	27056.44	40.00
222	HLA-A0201	GAFSRNARVLFG	GAFARVLFG	0	0	0	3	3	GAFSRNARVLFG	tr_D0A9V0	0.057	27069.32	40.00
146	HLA-A0201	TKYHAALIEVA	TKYAALIEV	0	0	0	3	1	TKYHAALIEV	tr_D0A9V0	0.057	27069.32	40.00
501	HLA-A0201	HPNIVRYVSCSIT	IVYVSCSIT	3	0	0	2	1	IVRYVSCSIT	tr_D0A9V0	0.057	27085.72	40.00
536	HLA-A0201	FGCVSPYVARRFT	CVSPYVAFT	2	0	0	7	2	CVSPYVARRFT	tr_D0A9V0	0.057	27117.98	40.00
702	HLA-A0201	MNPSMRPSASSLLS	SMSASSLLS	3	0	0	2	2	SMRPSASSLLS	tr_D0A9V0	0.057	27118.56	40.00
156	HLA-A0201	ATEHSGCVEALD	ATSGCVEAL	0	0	0	2	2	ATEHSGCVEAL	tr_D0A9V0	0.056	27137.95	40.00
664	HLA-A0201	LFNRDLLFVQS	LFNRDLLFV	0	0	0	0	0	LFNRDLLFV	tr_D0A9V0	0.056	27150.87	40.00
297	HLA-A0201	LSISKQFRLG	LSISKQFRL	0	0	0	0	0	LSISKQFRL	tr_D0A9V0	0.056	27150.87	40.00
571	HLA-A0201	HNVLLGTDGVC	VLLGTDGVC	2	0	0	0	0	VLLGTDGVC	tr_D0A9V0	0.056	27155.28	40.00
446	HLA-A0201	WTRSLKKISEGAFS	SLISEGAFS	3	0	0	2	2	SLKKISEGAFS	tr_D0A9V0	0.056	27160.85	40.00
530	HLA-A0201	HSVIKNFGC	HSVIKNFGC	0	0	0	0	0	HSVIKNFGC	tr_D0A9V0	0.056	27161.74	40.00
536	HLA-A0201	FGCVSPYVAR	FGCVSPYVA	0	0	0	0	0	FGCVSPYVA	tr_D0A9V0	0.056	27164.09	40.00
328	HLA-A0201	MDMQAKRLMRLCRQ	MQAKRLMRL	0	0	0	1	2	MDMQAKRLMRL	tr_D0A9V0	0.056	27179.07	40.00
703	HLA-A0201	NPSMRPSASSLLS	SMSASSLLS	2	0	0	2	2	SMRPSASSLLS	tr_D0A9V0	0.056	27184.37	40.00
343	HLA-A0201	CRRNDTVPYVRVL	CTVPYVRVL	0	0	0	1	4	CRRNDTVPYVRVL	tr_D0A9V0	0.056	27199.38	40.00
572	HLA-A0201	NVLLGTDGVCK	VLLGTDVCK	1	0	0	6	1	VLLGTDGVCK	tr_D0A9V0	0.056	27199.38	40.00
219	HLA-A0201	GTCGAFSRNARVL	GTCGAFSVL	0	0	0	7	4	GTCGAFSRNARVL	tr_D0A9V0	0.056	27202.62	40.00
474	HLA-A0201	IKCIPRLRRDVV	KCIPRLDVV	1	0	0	6	2	KCIPRLRRDVV	tr_D0A9V0	0.056	27204.08	40.00
19	HLA-A0201	DNADIFLGRSRIVE	NADIFLGIV	1	0	0	7	3	NADIFLGRSRIV	tr_D0A9V0	0.056	27207.03	40.00
596	HLA-A0201	MARTLADGVM	MARTLADGV	0	0	0	0	0	MARTLADGV	tr_D0A9V0	0.056	27213.80	40.00
501	HLA-A0201	HPNIVRYVSCSITP	IVVSCSITP	3	0	0	2	2	IVRYVSCSITP	tr_D0A9V0	0.056	27221.17	40.00
268	HLA-A0201	PVDCVLLPCST	PVDCVLLPT	0	0	0	8	2	PVDCVLLPCST	tr_D0A9V0	0.056	27227.05	40.00
309	HLA-A0201	AMRQGRYGEAMAHY	AMGEAMAHY	0	0	0	2	5	AMRQGRYGEAMAHY	tr_D0A9V0	0.056	27233.23	40.00
650	HLA-A0201	STAPAGSDA	STAPAGSDA	0	0	0	0	0	STAPAGSDA	tr_D0A9V0	0.056	27238.26	40.00
500	HLA-A0201	HHPNIVRYVSCSIT	IVYVSCSIT	4	0	0	2	1	IVRYVSCSIT	tr_D0A9V0	0.056	27243.56	40.00
279	HLA-A0201	LPIVLFELRPGE	LLFELRPGE	0	0	0	1	3	LPIVLFELRPGE	tr_D0A9V0	0.056	27261.84	40.00
588	HLA-A0201	STISEASVMART	STISVMART	0	0	0	3	3	STISEASVMART	tr_D0A9V0	0.056	27297.26	40.00
505	HLA-A0201	VRYVSCSITPS	YVSCSITPS	2	0	0	0	0	YVSCSITPS	tr_D0A9V0	0.056	27297.26	40.00
427	HLA-A0201	GANAADVVPLF	GAAADVVPL	0	0	0	2	1	GANAADVVPL	tr_D0A9V0	0.056	27301.39	40.00
281	HLA-A0201	IVLFELRPGESSSE	VLFELGESS	1	0	0	5	2	VLFELRPGESS	tr_D0A9V0	0.056	27303.17	40.00
513	HLA-A0201	TPSHLSIIM	TPSHLSIIM	0	0	0	0	0	TPSHLSIIM	tr_D0A9V0	0.056	27308.49	40.00
241	HLA-A0201	SYAVRDGPCRI	YAVDGPCRI	1	0	0	3	1	YAVRDGPCRI	tr_D0A9V0	0.056	27318.23	40.00
707	HLA-A0201	RPSASSLLSYAF	RSSLLSYAF	0	0	0	1	3	RPSASSLLSYAF	tr_D0A9V0	0.056	27321.49	40.00
203	HLA-A0201	HRDSVTMLVD	HRDSVTMLV	0	0	0	0	0	HRDSVTMLV	tr_D0A9V0	0.056	27338.64	40.00
194	HLA-A0201	DLFAKLPLDHR	DLFLPLDHR	0	0	0	3	2	DLFAKLPLDHR	tr_D0A9V0	0.056	27349.87	40.00
157	HLA-A0201	TEHSGCVEAL	TESGCVEAL	0	0	0	2	1	TEHSGCVEAL	tr_D0A9V0	0.056	27370.61	40.00
30	HLA-A0201	IVEVRKIQGHYAKL	IIQGHYAKL	0	0	0	1	5	IVEVRKIQGHYAKL	tr_D0A9V0	0.056	27380.37	40.00
401	HLA-A0201	DQGSESLFKMYA	GSESLFMYA	2	0	0	6	1	GSESLFKMYA	tr_D0A9V0	0.056	27381.57	40.00
303	HLA-A0201	FRLGFAAMRQG	FLGFAAMRQ	0	0	0	1	1	FRLGFAAMRQ	tr_D0A9V0	0.056	27384.24	40.00
535	HLA-A0201	NFGCVSPYVARRFT	CVSPYVAFT	3	0	0	7	2	CVSPYVARRFT	tr_D0A9V0	0.056	27394.90	40.00
454	HLA-A0201	SEGAFSTIF	SEGAFSTIF	0	0	0	0	0	SEGAFSTIF	tr_D0A9V0	0.056	27403.49	40.00
447	HLA-A0201	TRSLKKISEGA	SLKKISEGA	2	0	0	0	0	SLKKISEGA	tr_D0A9V0	0.056	27407.35	41.00
211	HLA-A0201	VDTNEFLVGTCGA	NEFLVTCGA	3	0	0	5	1	NEFLVGTCGA	tr_D0A9V0	0.056	27408.25	41.00
169	HLA-A0201	DRALISFGA	DRALISFGA	0	0	0	0	0	DRALISFGA	tr_D0A9V0	0.055	27469.99	41.00
313	HLA-A0201	GRYGEAMAHYL	RYGEAMAYL	1	0	0	7	1	RYGEAMAHYL	tr_D0A9V0	0.055	27499.43	41.00
112	HLA-A0201	LNCGIFKEKPNVF	LIFKEKPNV	0	0	0	1	3	LNCGIFKEKPNV	tr_D0A9V0	0.055	27513.43	41.00
473	HLA-A0201	AIKCIPRLRRDVVA	AIKCIPVVA	0	0	0	6	5	AIKCIPRLRRDVVA	tr_D0A9V0	0.055	27521.46	41.00
708	HLA-A0201	PSASSLLSYAF	SASSLLYAF	1	0	0	6	1	SASSLLSYAF	tr_D0A9V0	0.055	27546.18	41.00
571	HLA-A0201	HNVLLGTDGVCK	VLLGTDVCK	2	0	0	6	1	VLLGTDGVCK	tr_D0A9V0	0.055	27579.58	41.00
433	HLA-A0201	VVPLFLTDSN	VVPLFLTSN	0	0	0	7	1	VVPLFLTDSN	tr_D0A9V0	0.055	27601.08	41.00

503	HLA-A0201	NIVRYVSCS	NIVRYVSCS	0	0	0	0	0	NIVRYVSCS	tr_D0A9V0	0.055	27610.94	41.00
589	HLA-A0201	TISEASVMART	TISEVMART	0	0	0	4	2	TISEASVMART	tr_D0A9V0	0.055	27613.63	41.00
155	HLA-A0201	VATEHSGCVEALD	ATSGCVEAL	1	0	0	2	2	ATEHSGCVEAL	tr_D0A9V0	0.055	27639.62	41.00
681	HLA-A0201	EPVIPASVDHEA	VIASVDHEA	2	0	0	2	1	VIPASVDHEA	tr_D0A9V0	0.055	27666.55	41.00
425	HLA-A0201	AEGANAADVVPLF	AAADVVPLF	0	0	0	1	4	AEGANAADVVPLF	tr_D0A9V0	0.055	27674.35	41.00
341	HLA-A0201	QRCRRNDTVPYVRV	RCNDTVPYV	1	0	0	2	2	RCRRNDTVPYV	tr_D0A9V0	0.055	27685.13	41.00
697	HLA-A0201	RACCHMNPS	RACCHMNPS	0	0	0	0	0	RACCHMNPS	tr_D0A9V0	0.055	27687.83	41.00
690	HLA-A0201	HEAAAFVRA	HEAAAFVRA	0	0	0	0	0	HEAAAFVRA	tr_D0A9V0	0.055	27694.42	41.00
682	HLA-A0201	PVIPASVDHEAAA	VIPASVAAA	1	0	0	6	3	VIPASVDHEAAA	tr_D0A9V0	0.055	27706.40	41.00
40	HLA-A0201	YAKLRRQLA	YAKLRRQLA	0	0	0	0	0	YAKLRRQLA	tr_D0A9V0	0.055	27712.11	41.00
214	HLA-A0201	NEFLVGTCG	NEFLVGTCG	0	0	0	0	0	NEFLVGTCG	tr_D0A9V0	0.054	27749.61	41.00
485	HLA-A0201	VAESLEKEMEVA	VALEKEMEV	0	0	0	2	2	VAESLEKEMEV	tr_D0A9V0	0.054	27762.51	41.00
110	HLA-A0201	VALNCGIFKEKP	ALNCGIFKP	1	0	0	7	2	ALNCGIFKEKP	tr_D0A9V0	0.054	27763.41	41.00
341	HLA-A0201	QRCRRNDTVPYVR	RCNDTVPYV	1	0	0	2	2	RCRRNDTVPYV	tr_D0A9V0	0.054	27773.63	41.00
432	HLA-A0201	DVVPLFLTDSNR	VLFLTDSNR	1	0	0	1	2	VVPLFLTDSNR	tr_D0A9V0	0.054	27775.44	41.00
532	HLA-A0201	VIKNFGCVSP	VIKNFCVSP	0	0	0	5	1	VIKNFGCVSP	tr_D0A9V0	0.054	27776.93	41.00
538	HLA-A0201	CVSPYVARR	CVSPYVARR	0	0	0	0	0	CVSPYVARR	tr_D0A9V0	0.054	27786.86	41.00
146	HLA-A0201	TKYHAALIEVAT	YAALIEVAT	2	0	0	1	1	YHAALIEVAT	tr_D0A9V0	0.054	27787.46	41.00
552	HLA-A0201	LQGLNYLHN	LQGLNYLHN	0	0	0	0	0	LQGLNYLHN	tr_D0A9V0	0.054	27789.56	41.00
275	HLA-A0201	PCSTLPIVL	PCSTLPIVL	0	0	0	0	0	PCSTLPIVL	tr_D0A9V0	0.054	27795.58	41.00
302	HLA-A0201	QFRLGFAAMRQG	RLFAAMRQG	2	0	0	2	1	RLGFAAMRQG	tr_D0A9V0	0.054	27811.53	41.00
127	HLA-A0201	RHCTVIAIQ	RHCTVIAIQ	0	0	0	0	0	RHCTVIAIQ	tr_D0A9V0	0.054	27832.59	41.00
570	HLA-A0201	PHNVLLGTDGVC	VLLGTDGVC	3	0	0	0	0	VLLGTDGVC	tr_D0A9V0	0.054	27846.14	41.00
706	HLA-A0201	MRPSASSLL	MRPSASSLL	0	0	0	0	0	MRPSASSLL	tr_D0A9V0	0.054	27849.16	41.00
570	HLA-A0201	PHNVLLGTDGVCK	VLLGTDVCK	3	0	0	6	1	VLLGTDGVCK	tr_D0A9V0	0.054	27876.60	42.00
461	HLA-A0201	IFLGVAEDG	IFLGVAEDG	0	0	0	0	0	IFLGVAEDG	tr_D0A9V0	0.054	27879.60	42.00
398	HLA-A0201	DSSDQGSESLFKM	SSDESLFKM	1	0	0	3	3	SSDQGSESLFKM	tr_D0A9V0	0.054	27881.72	42.00
700	HLA-A0201	CHMNPSMRPS	CMNPSMRPS	0	0	0	1	1	CHMNPSMRPS	tr_D0A9V0	0.054	27887.46	42.00
400	HLA-A0201	SDQGSESLFKMYA	GSESLFMYA	3	0	0	6	1	GSESLFKMYA	tr_D0A9V0	0.054	27894.39	42.00
132	HLA-A0201	IAIQVYHPD	IAIQVYHPD	0	0	0	0	0	IAIQVYHPD	tr_D0A9V0	0.054	27907.07	42.00
15	HLA-A0201	LEIPDNADIFL	EIDNADIFL	1	0	0	2	1	EIPDNADIFL	tr_D0A9V0	0.054	27912.51	42.00
680	HLA-A0201	LEPVIPASVDHEA	VIASVDHEA	3	0	0	2	1	VIPASVDHEA	tr_D0A9V0	0.054	27920.36	42.00
470	HLA-A0201	AQVAIKCIP	AQVAIKCIP	0	0	0	0	0	AQVAIKCIP	tr_D0A9V0	0.054	27930.33	42.00
237	HLA-A0201	LFQLSYAVR	LFQLSYAVR	0	0	0	0	0	LFQLSYAVR	tr_D0A9V0	0.054	27930.64	42.00
661	HLA-A0201	LRSLFNRDL	LRSLFNRDL	0	0	0	0	0	LRSLFNRDL	tr_D0A9V0	0.054	27933.36	42.00
217	HLA-A0201	LVGTCGAFSR	LVGTCAFSR	0	0	0	5	1	LVGTCGAFSR	tr_D0A9V0	0.054	27946.96	42.00
129	HLA-A0201	CTVIAIQVYHPD	CTVIAIQVY	0	0	0	0	0	CTVIAIQVY	tr_D0A9V0	0.054	27952.10	42.00
295	HLA-A0201	VSLSISKQF	VSLSISKQF	0	0	0	0	0	VSLSISKQF	tr_D0A9V0	0.054	27954.52	42.00
94	HLA-A0201	GASISVTWNAG	GAISVTWNA	0	0	0	2	1	GASISVTWNA	tr_D0A9V0	0.054	27960.58	42.00
213	HLA-A0201	TNEFLVGTCGA	NEFLVTCGA	1	0	0	5	1	NEFLVGTCGA	tr_D0A9V0	0.054	27961.17	42.00
183	HLA-A0201	MHCRRCCQFAF	MHCCCQFAF	0	0	0	3	2	MHCRRCCQFAF	tr_D0A9V0	0.054	27969.03	42.00
291	HLA-A0201	SSSEVSLSISKQF	SSSEVSLSF	0	0	0	8	4	SSSEVSLSISKQF	tr_D0A9V0	0.054	27973.27	42.00
497	HLA-A0201	SKLHHPNIVRY	KLHPNIVRY	1	0	0	2	1	KLHHPNIVRY	tr_D0A9V0	0.054	27978.72	42.00
475	HLA-A0201	KCIPRLRRDVVA	KCIPRLVVA	0	0	0	6	3	KCIPRLRRDVVA	tr_D0A9V0	0.054	27982.06	42.00
590	HLA-A0201	ISEASVMARTLA	ISEASVMLA	0	0	0	7	3	ISEASVMARTLA	tr_D0A9V0	0.054	27999.93	42.00
683	HLA-A0201	VIPASVDHEAAAF	VIPASVAAF	0	0	0	6	4	VIPASVDHEAAAF	tr_D0A9V0	0.054	28009.02	42.00
206	HLA-A0201	SVTMLVDTN	SVTMLVDTN	0	0	0	0	0	SVTMLVDTN	tr_D0A9V0	0.054	28012.36	42.00
544	HLA-A0201	ARRFTVDILQ	ARFTVDILQ	0	0	0	1	1	ARRFTVDILQ	tr_D0A9V0	0.054	28013.88	42.00
34	HLA-A0201	RKIQGHYAKLRRQL	KIQGHYAQL	1	0	0	7	4	KIQGHYAKLRRQL	tr_D0A9V0	0.054	28021.75	42.00
163	HLA-A0201	VEALDPDRAL	VALDPDRAL	0	0	0	1	1	VEALDPDRAL	tr_D0A9V0	0.053	28027.21	42.00
432	HLA-A0201	DVVPLFLTD	DVVPLFLTD	0	0	0	0	0	DVVPLFLTD	tr_D0A9V0	0.053	28032.67	42.00
625	HLA-A0201	PQSDIFSFGIS	QSDIFSFGI	1	0	0	0	0	QSDIFSFGI	tr_D0A9V0	0.053	28057.25	42.00
543	HLA-A0201	VARRFTVDILQ	VAFTVDILQ	0	0	0	2	2	VARRFTVDILQ	tr_D0A9V0	0.053	28076.38	42.00
152	HLA-A0201	LIEVATEHS	LIEVATEHS	0	0	0	0	0	LIEVATEHS	tr_D0A9V0	0.053	28076.38	42.00

624	HLA-A0201	TPQSDIFSFGIS	TSDIFSFGI	0	0	0	1	2	TPQSDIFSFGI	tr_D0A9V0	0.053	28081.85	42.00
56	HLA-A0201	EAILARVESKNEAG	ILARVENEA	2	0	0	6	2	ILARVESKNEA	tr_D0A9V0	0.053	28085.49	42.00
221	HLA-A0201	CGAFSRNARVLFG	GAFARVLFG	1	0	0	3	3	GAFSRNARVLFG	tr_D0A9V0	0.053	28087.92	42.00
19	HLA-A0201	DNADIFLGRSRI	NADIFLSRI	1	0	0	6	2	NADIFLGRSRI	tr_D0A9V0	0.053	28099.17	42.00
105	HLA-A0201	DQQRDVALNCGI	QQVALNCGI	1	0	0	2	2	QQRDVALNCGI	tr_D0A9V0	0.053	28111.02	42.00
147	HLA-A0201	KYHAALIEVAT	YAALIEVAT	1	0	0	1	1	YHAALIEVAT	tr_D0A9V0	0.053	28113.16	42.00
323	HLA-A0201	LPMVKMDMQAKR	LMVKMDMQA	0	0	0	1	1	LPMVKMDMQA	tr_D0A9V0	0.053	28117.42	42.00
43	HLA-A0201	LRRQLAVLLK	LRRQLAVLL	0	0	0	0	0	LRRQLAVLL	tr_D0A9V0	0.053	28136.60	42.00
565	HLA-A0201	HCDVKPHNVLLGT	HCDVVLLGT	0	0	0	4	4	HCDVKPHNVLLGT	tr_D0A9V0	0.053	28139.63	42.00
703	HLA-A0201	NPSMRPSASSL	SMRPSASSL	2	0	0	0	0	SMRPSASSL	tr_D0A9V0	0.053	28157.91	42.00
109	HLA-A0201	DVALNCGIFKEKP	ALNCGIFKP	2	0	0	7	2	ALNCGIFKEKP	tr_D0A9V0	0.053	28159.74	42.00
354	HLA-A0201	VLDNHYCVNSSDCK	VLDNYCVNS	0	0	0	4	1	VLDNHYCVNS	tr_D0A9V0	0.053	28161.56	42.00
162	HLA-A0201	CVEALDPDRA	CVEALDPRA	0	0	0	7	1	CVEALDPDRA	tr_D0A9V0	0.053	28163.99	42.00
486	HLA-A0201	AESLEKEMEVA	ALEKEMEVA	0	0	0	1	2	AESLEKEMEVA	tr_D0A9V0	0.053	28181.38	42.00
220	HLA-A0201	TCGAFSRNARVLFG	GAFARVLFG	2	0	0	3	3	GAFSRNARVLFG	tr_D0A9V0	0.053	28193.58	42.00
218	HLA-A0201	VGTCGAFSRNARVL	GTCGAFARV	1	0	0	6	3	GTCGAFSRNARV	tr_D0A9V0	0.053	28209.44	42.00
536	HLA-A0201	FGCVSPYVARRF	FGCVVARRF	0	0	0	4	3	FGCVSPYVARRF	tr_D0A9V0	0.053	28211.58	42.00
311	HLA-A0201	RQGRYGEAMAHYLP	RQGAMAHYL	0	0	0	3	4	RQGRYGEAMAHYL	tr_D0A9V0	0.053	28212.18	42.00
576	HLA-A0201	GTDGVCKLSDFGS	GTDGVCFGS	0	0	0	6	4	GTDGVCKLSDFGS	tr_D0A9V0	0.053	28213.71	42.00
514	HLA-A0201	PSHLSIIMEY	SHLSIIMEY	1	0	0	0	0	SHLSIIMEY	tr_D0A9V0	0.053	28231.42	42.00
309	HLA-A0201	AMRQGRYGEAMA	AMRYGEAMA	0	0	0	2	3	AMRQGRYGEAMA	tr_D0A9V0	0.053	28236.92	42.00
108	HLA-A0201	RDVALNCGIFKEKP	ALNCGIFKP	3	0	0	7	2	ALNCGIFKEKP	tr_D0A9V0	0.053	28256.18	42.00
297	HLA-A0201	LSISKQFRLGF	LSIQFRLGF	0	0	0	3	2	LSISKQFRLGF	tr_D0A9V0	0.053	28282.48	42.00
686	HLA-A0201	ASVDHEAAAF	AVDHEAAAF	0	0	0	1	1	ASVDHEAAAF	tr_D0A9V0	0.053	28297.17	43.00
683	HLA-A0201	VIPASVDHEAA	VIPASVEAA	0	0	0	6	2	VIPASVDHEAA	tr_D0A9V0	0.053	28299.01	43.00
158	HLA-A0201	EHSGCVEAL	EHSGCVEAL	0	0	0	0	0	EHSGCVEAL	tr_D0A9V0	0.052	28331.79	43.00
586	HLA-A0201	FGSTISEAS	FGSTISEAS	0	0	0	0	0	FGSTISEAS	tr_D0A9V0	0.052	28341.29	43.00
408	HLA-A0201	FKMYAPSSSSS	FMYAPSSSS	0	0	0	1	1	FKMYAPSSSS	tr_D0A9V0	0.052	28357.86	43.00
11	HLA-A0201	SASNLEIPDNA	SASNLEIPA	0	0	0	8	2	SASNLEIPDNA	tr_D0A9V0	0.052	28359.71	43.00
308	HLA-A0201	AAMRQGRYGEAM	AMGRYGEAM	1	0	0	2	2	AMRQGRYGEAM	tr_D0A9V0	0.052	28363.38	43.00
643	HLA-A0201	RPPWQWSSTA	RPWQWSSTA	0	0	0	1	1	RPPWQWSSTA	tr_D0A9V0	0.052	28368.60	43.00
666	HLA-A0201	NRDLLFVQS	NRDLLFVQS	0	0	0	0	0	NRDLLFVQS	tr_D0A9V0	0.052	28375.66	43.00
681	HLA-A0201	EPVIPASVDHEAAA	VIPASVAAA	2	0	0	6	3	VIPASVDHEAAA	tr_D0A9V0	0.052	28391.33	43.00
425	HLA-A0201	AEGANAADV	AEGANAADV	0	0	0	0	0	AEGANAADV	tr_D0A9V0	0.052	28400.85	43.00
133	HLA-A0201	AIQVYHPDVWKS	AIQVYVWKS	0	0	0	5	3	AIQVYHPDVWKS	tr_D0A9V0	0.052	28406.07	43.00
349	HLA-A0201	VPYVRVLDNHY	VPYVRVLHY	0	0	0	7	2	VPYVRVLDNHY	tr_D0A9V0	0.052	28416.83	43.00
350	HLA-A0201	PYVRVLDNHYC	YVRVLNHYC	1	0	0	5	1	YVRVLDNHYC	tr_D0A9V0	0.052	28426.38	43.00
691	HLA-A0201	EAAAFVRAC	EAAAFVRAC	0	0	0	0	0	EAAAFVRAC	tr_D0A9V0	0.052	28448.53	43.00
312	HLA-A0201	QGRYGEAMAHYL	RYGEAMAYL	2	0	0	7	1	RYGEAMAHYL	tr_D0A9V0	0.052	28449.45	43.00
279	HLA-A0201	LPIVLFELRPG	LIVLFELRP	0	0	0	1	1	LPIVLFELRP	tr_D0A9V0	0.052	28458.99	43.00
575	HLA-A0201	LGTDGVCKLS	LTDGVCKLS	0	0	0	1	1	LGTDGVCKLS	tr_D0A9V0	0.052	28478.40	43.00
240	HLA-A0201	LSYAVRDGPC	LSYAVDGPC	0	0	0	5	1	LSYAVRDGPC	tr_D0A9V0	0.052	28493.18	43.00
207	HLA-A0201	VTMLVDTNE	VTMLVDTNE	0	0	0	0	0	VTMLVDTNE	tr_D0A9V0	0.052	28504.60	43.00
496	HLA-A0201	ASKLHHPNIVRY	KLHPNIVRY	2	0	0	2	1	KLHHPNIVRY	tr_D0A9V0	0.052	28514.47	43.00
426	HLA-A0201	EGANAADVVPLF	AAADVVPLF	2	0	0	1	1	ANAADVVPLF	tr_D0A9V0	0.052	28518.17	43.00
58	HLA-A0201	ILARVESKNEAGG	ILARVEAGG	0	0	0	5	4	ILARVESKNEAGG	tr_D0A9V0	0.052	28526.19	43.00
215	HLA-A0201	EFLVGTCGA	EFLVGTCGA	0	0	0	0	0	EFLVGTCGA	tr_D0A9V0	0.052	28550.59	43.00
170	HLA-A0201	RALISFGAH	RALISFGAH	0	0	0	0	0	RALISFGAH	tr_D0A9V0	0.052	28552.75	43.00
98	HLA-A0201	SVTWNAGDQQRDV	VTWNAGDQV	1	0	0	8	3	VTWNAGDQQRDV	tr_D0A9V0	0.052	28563.87	43.00
694	HLA-A0201	AFVRACCHMNP	FVRACCHMP	1	0	0	8	1	FVRACCHMNP	tr_D0A9V0	0.052	28564.79	43.00
211	HLA-A0201	VDTNEFLVGTC	VTNEFLVGT	0	0	0	1	1	VDTNEFLVGT	tr_D0A9V0	0.052	28568.83	43.00
535	HLA-A0201	NFGCVSPYVARRF	FGCVVARRF	1	0	0	4	3	FGCVSPYVARRF	tr_D0A9V0	0.052	28577.47	43.00
585	HLA-A0201	DFGSTISEASVM	FGSTISASV	1	0	0	6	1	FGSTISEASV	tr_D0A9V0	0.052	28591.09	43.00
539	HLA-A0201	VSPYVARRF	VSPYVARRF	0	0	0	0	0	VSPYVARRF	tr_D0A9V0	0.052	28607.17	43.00

154	HLA-A0201	EVATEHSGCVEALD	ATSGCVEAL	2	0	0	2	2	ATEHSGCVEAL	tr_D0A9V0	0.052	28613.37	43.00
370	HLA-A0201	EQDESDIKDRV	EQDESDIRV	0	0	0	7	2	EQDESDIKDRV	tr_D0A9V0	0.052	28615.53	43.00
495	HLA-A0201	VASKLHHPNIV	VASKLHHPV	0	0	0	8	2	VASKLHHPNIV	tr_D0A9V0	0.052	28635.97	43.00
239	HLA-A0201	QLSYAVRDG	QLSYAVRDG	0	0	0	0	0	QLSYAVRDG	tr_D0A9V0	0.051	28642.49	43.00
344	HLA-A0201	RRNDTVPYVRV	RRNDTVPYV	0	0	0	8	2	RRNDTVPYVRV	tr_D0A9V0	0.051	28675.98	43.00
623	HLA-A0201	CTPQSDIFS	CTPQSDIFS	0	0	0	0	0	CTPQSDIFS	tr_D0A9V0	0.051	28692.11	43.00
23	HLA-A0201	IFLGRSRIVE	FLGRSRIVE	1	0	0	0	0	FLGRSRIVE	tr_D0A9V0	0.051	28698.62	43.00
495	HLA-A0201	VASKLHHPNIVRY	KLHPNIVRY	3	0	0	2	1	KLHHPNIVRY	tr_D0A9V0	0.051	28708.56	43.00
584	HLA-A0201	SDFGSTISEASVM	FGSTISASV	2	0	0	6	1	FGSTISEASV	tr_D0A9V0	0.051	28712.60	43.00
243	HLA-A0201	AVRDGPCRIAA	AVGPCRIAA	0	0	0	2	2	AVRDGPCRIAA	tr_D0A9V0	0.051	28717.88	43.00
404	HLA-A0201	SESLFKMYAP	SESLFMYAP	0	0	0	5	1	SESLFKMYAP	tr_D0A9V0	0.051	28728.46	44.00
397	HLA-A0201	SDSSDQGSESLFKM	SSDESLFKM	2	0	0	3	3	SSDQGSESLFKM	tr_D0A9V0	0.051	28735.29	44.00
57	HLA-A0201	AILARVESKNEAGG	ILARVEAGG	1	0	0	5	4	ILARVESKNEAGG	tr_D0A9V0	0.051	28740.26	44.00
18	HLA-A0201	PDNADIFLGRSRI	NADIFLSRI	2	0	0	6	2	NADIFLGRSRI	tr_D0A9V0	0.051	28752.40	44.00
106	HLA-A0201	QQRDVALNCGIF	QQRDVALIF	0	0	0	7	3	QQRDVALNCGIF	tr_D0A9V0	0.051	28759.56	44.00
438	HLA-A0201	LTDSNRNVWT	LTDSNNVWT	0	0	0	5	1	LTDSNRNVWT	tr_D0A9V0	0.051	28770.13	44.00
124	HLA-A0201	FNRRHCTVI	FNRRHCTVI	0	0	0	0	0	FNRRHCTVI	tr_D0A9V0	0.051	28773.57	44.00
592	HLA-A0201	EASVMARTL	EASVMARTL	0	0	0	0	0	EASVMARTL	tr_D0A9V0	0.051	28805.65	44.00
588	HLA-A0201	STISEASVMAR	SISEASVMA	0	0	0	1	1	STISEASVMA	tr_D0A9V0	0.051	28815.61	44.00
494	HLA-A0201	EVASKLHHPNIVRY	KLHPNIVRY	4	0	0	2	1	KLHHPNIVRY	tr_D0A9V0	0.051	28823.42	44.00
591	HLA-A0201	SEASVMART	SEASVMART	0	0	0	0	0	SEASVMART	tr_D0A9V0	0.051	28823.73	44.00
549	HLA-A0201	VDILQGLNY	VDILQGLNY	0	0	0	0	0	VDILQGLNY	tr_D0A9V0	0.051	28837.46	44.00
465	HLA-A0201	VAEDGAQVA	VAEDGAQVA	0	0	0	0	0	VAEDGAQVA	tr_D0A9V0	0.051	28844.00	44.00
532	HLA-A0201	VIKNFGCVS	VIKNFGCVS	0	0	0	0	0	VIKNFGCVS	tr_D0A9V0	0.051	28867.73	44.00
344	HLA-A0201	RRNDTVPYVRVL	RTVPYVRVL	0	0	0	1	3	RRNDTVPYVRVL	tr_D0A9V0	0.051	28906.79	44.00
680	HLA-A0201	LEPVIPASVD	LEPVIPASV	0	0	0	0	0	LEPVIPASV	tr_D0A9V0	0.051	28914.31	44.00
448	HLA-A0201	RSLKKISEGAF	SLKKISEAF	1	0	0	7	1	SLKKISEGAF	tr_D0A9V0	0.051	28922.44	44.00
252	HLA-A0201	AATSGGASHM	ATSGGASHM	0	0	0	1	1	AATSGGASHM	tr_D0A9V0	0.051	28922.44	44.00
104	HLA-A0201	GDQQRDVALNCGI	QQRDVALGI	2	0	0	7	2	QQRDVALNCGI	tr_D0A9V0	0.051	28930.89	44.00
468	HLA-A0201	DGAQVAIKCI	GAQVAIKCI	1	0	0	0	0	GAQVAIKCI	tr_D0A9V0	0.051	28939.04	44.00
345	HLA-A0201	RNDTVPYVRVL	RTVPYVRVL	0	0	0	1	2	RNDTVPYVRVL	tr_D0A9V0	0.051	28946.87	44.00
568	HLA-A0201	VKPHNVLLGT	VPHNVLLGT	0	0	0	1	1	VKPHNVLLGT	tr_D0A9V0	0.051	28950.32	44.00
182	HLA-A0201	PMHCRRCCQF	PMHCRCCQF	0	0	0	4	1	PMHCRRCCQF	tr_D0A9V0	0.050	28955.64	44.00
488	HLA-A0201	SLEKEMEVASK	SLEMEVASK	0	0	0	2	2	SLEKEMEVASK	tr_D0A9V0	0.050	28967.22	44.00
348	HLA-A0201	TVPYVRVLDNHY	VPYVRVLHY	1	0	0	7	2	VPYVRVLDNHY	tr_D0A9V0	0.050	29003.30	44.00
183	HLA-A0201	MHCRRCCQF	MHCRRCCQF	0	0	0	0	0	MHCRRCCQF	tr_D0A9V0	0.050	29004.23	44.00
29	HLA-A0201	RIVEVRKIQG	RIVEVRIQG	0	0	0	6	1	RIVEVRKIQG	tr_D0A9V0	0.050	29062.03	44.00
27	HLA-A0201	RSRIVEVRK	RSRIVEVRK	0	0	0	0	0	RSRIVEVRK	tr_D0A9V0	0.050	29062.67	44.00
162	HLA-A0201	CVEALDPDRAL	CVEALDPAL	0	0	0	7	2	CVEALDPDRAL	tr_D0A9V0	0.050	29089.09	44.00
529	HLA-A0201	LHSVIKNFGC	LSVIKNFGC	0	0	0	1	1	LHSVIKNFGC	tr_D0A9V0	0.050	29114.28	44.00
347	HLA-A0201	DTVPYVRVLDNHY	VPYVRVLHY	2	0	0	7	2	VPYVRVLDNHY	tr_D0A9V0	0.050	29126.58	44.00
658	HLA-A0201	ATRLRSLFNR	ATLRSLFNR	0	0	0	2	1	ATRLRSLFNR	tr_D0A9V0	0.050	29131.61	45.00
444	HLA-A0201	NVWTRSLKK	NVWTRSLKK	0	0	0	0	0	NVWTRSLKK	tr_D0A9V0	0.050	29139.19	45.00
237	HLA-A0201	LFQLSYAVRDG	FQLSYAVRG	1	0	0	8	1	FQLSYAVRDG	tr_D0A9V0	0.050	29154.32	45.00
237	HLA-A0201	LFQLSYAVRD	FQLSYAVRD	1	0	0	0	0	FQLSYAVRD	tr_D0A9V0	0.050	29154.64	45.00
290	HLA-A0201	ESSSEVSLSIS	SSSEVSLSI	1	0	0	0	0	SSSEVSLSI	tr_D0A9V0	0.050	29171.36	45.00
239	HLA-A0201	QLSYAVRDGPC	QLSYAVRPC	0	0	0	7	2	QLSYAVRDGPC	tr_D0A9V0	0.050	29173.24	45.00
253	HLA-A0201	ATSGGASHMQGFL	ATSGGASFL	0	0	0	7	4	ATSGGASHMQGFL	tr_D0A9V0	0.050	29178.92	45.00
327	HLA-A0201	KMDMQAKRLMR	KMDMQALMR	0	0	0	6	2	KMDMQAKRLMR	tr_D0A9V0	0.050	29184.61	45.00
103	HLA-A0201	AGDQQRDVALNCGI	QQRDVALGI	3	0	0	7	2	QQRDVALNCGI	tr_D0A9V0	0.050	29204.21	45.00
284	HLA-A0201	FELRPGESSSEVSL	ELSSSEVSL	1	0	0	2	4	ELRPGESSSEVSL	tr_D0A9V0	0.050	29212.42	45.00
242	HLA-A0201	YAVRDGPCRIAATS	YAVCRIAAT	0	0	0	3	4	YAVRDGPCRIAAT	tr_D0A9V0	0.050	29221.27	45.00
583	HLA-A0201	LSDFGSTISEASVM	FGSTISASV	3	0	0	6	1	FGSTISEASV	tr_D0A9V0	0.050	29229.81	45.00
124	HLA-A0201	FNRRHCTVIA	FNRHCTVIA	0	0	0	2	1	FNRRHCTVIA	tr_D0A9V0	0.050	29237.09	45.00

287	HLA-A0201	RPGESSSEVSLSIS	RSSEVSLSI	0	0	0	1	4	RPGESSSEVSLSI	tr_D0A9V0	0.050	29238.97	45.00
564	HLA-A0201	VHCDVKPHNVLLGT	HCDVVLLGT	1	0	0	4	4	HCDVKPHNVLLGT	tr_D0A9V0	0.050	29241.50	45.00
269	HLA-A0201	VDCVLLPCS	VDCVLLPCS	0	0	0	0	0	VDCVLLPCS	tr_D0A9V0	0.050	29247.52	45.00
237	HLA-A0201	LFQLSYAVRDGP	FQLSYAVGP	1	0	0	7	2	FQLSYAVRDGP	tr_D0A9V0	0.050	29259.88	45.00
303	HLA-A0201	FRLGFAAMRQGR	FLGFAAMRQ	0	0	0	1	1	FRLGFAAMRQ	tr_D0A9V0	0.049	29281.71	45.00
587	HLA-A0201	GSTISEASVMART	STISVMART	1	0	0	3	3	STISEASVMART	tr_D0A9V0	0.049	29288.06	45.00
346	HLA-A0201	NDTVPYVRVLDNHY	VPYVRVLHY	3	0	0	7	2	VPYVRVLDNHY	tr_D0A9V0	0.049	29293.12	45.00
149	HLA-A0201	HAALIEVATE	HAALIVATE	0	0	0	5	1	HAALIEVATE	tr_D0A9V0	0.049	29301.36	45.00
474	HLA-A0201	IKCIPRLRRDVVA	KCIPRLVVA	1	0	0	6	3	KCIPRLRRDVVA	tr_D0A9V0	0.049	29322.61	45.00
33	HLA-A0201	VRKIQGHYAKL	KIQGHYAKL	2	0	0	0	0	KIQGHYAKL	tr_D0A9V0	0.049	29332.14	45.00
589	HLA-A0201	TISEASVMARTLAD	TISEASVML	0	0	0	8	3	TISEASVMARTL	tr_D0A9V0	0.049	29335.30	45.00
506	HLA-A0201	RYVSCSITP	RYVSCSITP	0	0	0	0	0	RYVSCSITP	tr_D0A9V0	0.049	29347.05	45.00
156	HLA-A0201	ATEHSGCVEALDP	ATSGCVEAL	0	0	0	2	2	ATEHSGCVEAL	tr_D0A9V0	0.049	29350.55	45.00
113	HLA-A0201	NCGIFKEKPNVFNR	NCGIFVFNR	0	0	0	5	5	NCGIFKEKPNVFNR	tr_D0A9V0	0.049	29351.50	45.00
155	HLA-A0201	VATEHSGCVEALDP	ATSGCVEAL	1	0	0	2	2	ATEHSGCVEAL	tr_D0A9V0	0.049	29353.73	45.00
21	HLA-A0201	ADIFLGRSRIV	DIFLGSRIV	1	0	0	5	1	DIFLGRSRIV	tr_D0A9V0	0.049	29362.61	45.00
220	HLA-A0201	TCGAFSRNARVLF	TCGAFSVLF	0	0	0	6	4	TCGAFSRNARVLF	tr_D0A9V0	0.049	29363.58	45.00
304	HLA-A0201	RLGFAAMRQGRYGE	RLGFAAMYG	0	0	0	7	4	RLGFAAMRQGRYG	tr_D0A9V0	0.049	29363.88	45.00
624	HLA-A0201	TPQSDIFSFG	TQSDIFSFG	0	0	0	1	1	TPQSDIFSFG	tr_D0A9V0	0.049	29367.38	45.00
122	HLA-A0201	NVFNRRHCT	NVFNRRHCT	0	0	0	0	0	NVFNRRHCT	tr_D0A9V0	0.049	29399.18	45.00
131	HLA-A0201	VIAIQVYHPD	VIIQVYHPD	0	0	0	2	1	VIAIQVYHPD	tr_D0A9V0	0.049	29426.86	45.00
314	HLA-A0201	RYGEAMAHY	RYGEAMAHY	0	0	0	0	0	RYGEAMAHY	tr_D0A9V0	0.049	29430.69	45.00
199	HLA-A0201	LPLDHRDSV	LPLDHRDSV	0	0	0	0	0	LPLDHRDSV	tr_D0A9V0	0.049	29438.64	45.00
146	HLA-A0201	TKYHAALIEVATE	TKYAALIEV	0	0	0	3	1	TKYHAALIEV	tr_D0A9V0	0.049	29452.98	45.00
179	HLA-A0201	AALPMHCRRCC	ALMHCRRCC	1	0	0	2	1	ALPMHCRRCC	tr_D0A9V0	0.049	29467.33	45.00
590	HLA-A0201	ISEASVMARTLADG	IMARTLADG	0	0	0	1	5	ISEASVMARTLADG	tr_D0A9V0	0.049	29468.60	45.00
435	HLA-A0201	PLFLTDSNR	PLFLTDSNR	0	0	0	0	0	PLFLTDSNR	tr_D0A9V0	0.049	29484.55	45.00
242	HLA-A0201	YAVRDGPCRIA	YAVGPCRIA	0	0	0	3	2	YAVRDGPCRIA	tr_D0A9V0	0.049	29494.76	45.00
309	HLA-A0201	AMRQGRYGEAMAH	AMYGEAMAH	0	0	0	2	4	AMRQGRYGEAMAH	tr_D0A9V0	0.049	29499.55	45.00
328	HLA-A0201	MDMQAKRLMR	MMQAKRLMR	0	0	0	1	1	MDMQAKRLMR	tr_D0A9V0	0.049	29503.06	45.00
658	HLA-A0201	ATRLRSLFN	ATRLRSLFN	0	0	0	0	0	ATRLRSLFN	tr_D0A9V0	0.049	29512.32	45.00
291	HLA-A0201	SSSEVSLSISK	SSSEVSLSI	0	0	0	0	0	SSSEVSLSI	tr_D0A9V0	0.049	29513.90	45.00
447	HLA-A0201	TRSLKKISEGAF	SLKKISEAF	2	0	0	7	1	SLKKISEGAF	tr_D0A9V0	0.049	29519.02	45.00
290	HLA-A0201	ESSSEVSLSISKQF	SSSEVSLSF	1	0	0	8	4	SSSEVSLSISKQF	tr_D0A9V0	0.049	29532.45	46.00
270	HLA-A0201	DCVLLPCST	DCVLLPCST	0	0	0	0	0	DCVLLPCST	tr_D0A9V0	0.049	29537.55	46.00
464	HLA-A0201	GVAEDGAQVAIKCI	GVAEDGACI	0	0	0	7	5	GVAEDGAQVAIKCI	tr_D0A9V0	0.049	29544.58	46.00
503	HLA-A0201	NIVRYVSCSITPSH	NIVYVSCSI	0	0	0	3	1	NIVRYVSCSI	tr_D0A9V0	0.049	29573.04	46.00
115	HLA-A0201	GIFKEKPNVFNR	GIFPNVFNR	0	0	0	3	3	GIFKEKPNVFNR	tr_D0A9V0	0.049	29574.97	46.00
620	HLA-A0201	GGRCTPQSDIFSF	CTPQSIFSF	3	0	0	5	1	CTPQSDIFSF	tr_D0A9V0	0.048	29595.14	46.00
619	HLA-A0201	AGGRCTPQSDIFSF	CTPQSIFSF	4	0	0	5	1	CTPQSDIFSF	tr_D0A9V0	0.048	29605.39	46.00
622	HLA-A0201	RCTPQSDIFSF	CTPQSIFSF	1	0	0	5	1	CTPQSDIFSF	tr_D0A9V0	0.048	29610.18	46.00
302	HLA-A0201	QFRLGFAAMRQGR	FLGFAAMRQ	1	0	0	1	1	FRLGFAAMRQ	tr_D0A9V0	0.048	29616.92	46.00
114	HLA-A0201	CGIFKEKPNVFNR	GIFPNVFNR	1	0	0	3	3	GIFKEKPNVFNR	tr_D0A9V0	0.048	29625.25	46.00
446	HLA-A0201	WTRSLKKISEGAF	SLKKISEAF	3	0	0	7	1	SLKKISEGAF	tr_D0A9V0	0.048	29626.53	46.00
590	HLA-A0201	ISEASVMAR	ISEASVMAR	0	0	0	0	0	ISEASVMAR	tr_D0A9V0	0.048	29652.18	46.00
108	HLA-A0201	RDVALNCGIFKE	RLNCGIFKE	0	0	0	1	3	RDVALNCGIFKE	tr_D0A9V0	0.048	29666.62	46.00
688	HLA-A0201	VDHEAAAFVR	VDHEAAAFV	0	0	0	0	0	VDHEAAAFV	tr_D0A9V0	0.048	29676.90	46.00
250	HLA-A0201	RIAATSGGASHM	RIAATASHM	0	0	0	5	3	RIAATSGGASHM	tr_D0A9V0	0.048	29682.36	46.00
153	HLA-A0201	IEVATEHSGCVEA	IEVATCVEA	0	0	0	5	4	IEVATEHSGCVEA	tr_D0A9V0	0.048	29684.94	46.00
134	HLA-A0201	IQVYHPDVW	IQVYHPDVW	0	0	0	0	0	IQVYHPDVW	tr_D0A9V0	0.048	29689.42	46.00
105	HLA-A0201	DQQRDVALNCGIF	QQRDVALIF	1	0	0	7	3	QQRDVALNCGIF	tr_D0A9V0	0.048	29693.92	46.00
73	HLA-A0201	RANGSATET	RANGSATET	0	0	0	0	0	RANGSATET	tr_D0A9V0	0.048	29700.03	46.00
31	HLA-A0201	VEVRKIQGHYAKL	KIQGHYAKL	4	0	0	0	0	KIQGHYAKL	tr_D0A9V0	0.048	29713.84	46.00
204	HLA-A0201	RDSVTMLVDT	RSVTMLVDT	0	0	0	1	1	RDSVTMLVDT	tr_D0A9V0	0.048	29720.61	46.00

445	HLA-A0201	VWTRSLKKISEGAF	SLKKISEAF	4	0	0	7	1	SLKKISEGAF	tr_D0A9V0	0.048	29731.21	46.00
17	HLA-A0201	IPDNADIFLG	IPDNADIFL	0	0	0	0	0	IPDNADIFL	tr_D0A9V0	0.048	29736.37	46.00
194	HLA-A0201	DLFAKLPLDHRD	DLFAKLPLD	0	0	0	8	3	DLFAKLPLDHRD	tr_D0A9V0	0.048	29745.05	46.00
494	HLA-A0201	EVASKLHHPNIV	VASKLHHPV	1	0	0	8	2	VASKLHHPNIV	tr_D0A9V0	0.048	29770.18	46.00
104	HLA-A0201	GDQQRDVALNCGIF	QQRDVALIF	2	0	0	7	3	QQRDVALNCGIF	tr_D0A9V0	0.048	29784.98	46.00
343	HLA-A0201	CRRNDTVPYVRV	RRNDTVPYV	1	0	0	8	2	RRNDTVPYVRV	tr_D0A9V0	0.048	29792.40	46.00
491	HLA-A0201	KEMEVASKLHHPNI	KEMEVAPNI	0	0	0	6	5	KEMEVASKLHHPNI	tr_D0A9V0	0.048	29810.77	46.00
109	HLA-A0201	DVALNCGIFKE	ALNCGIFKE	2	0	0	0	0	ALNCGIFKE	tr_D0A9V0	0.048	29820.14	46.00
178	HLA-A0201	HAALPMHCRRCC	ALMHCRRCC	2	0	0	2	1	ALPMHCRRCC	tr_D0A9V0	0.048	29868.56	46.00
682	HLA-A0201	PVIPASVDHEAAAF	VIPASVAAF	1	0	0	6	4	VIPASVDHEAAAF	tr_D0A9V0	0.048	29869.22	46.00
607	HLA-A0201	RGTALYMAPE	RTALYMAPE	0	0	0	1	1	RGTALYMAPE	tr_D0A9V0	0.048	29870.18	46.00
624	HLA-A0201	TPQSDIFSF	TPQSDIFSF	0	0	0	0	0	TPQSDIFSF	tr_D0A9V0	0.048	29877.94	46.00
182	HLA-A0201	PMHCRRCCQFAFD	PMHCCCQFA	0	0	0	4	2	PMHCRRCCQFA	tr_D0A9V0	0.048	29880.20	46.00
220	HLA-A0201	TCGAFSRNA	TCGAFSRNA	0	0	0	0	0	TCGAFSRNA	tr_D0A9V0	0.048	29886.35	46.00
106	HLA-A0201	QQRDVALNCGIFKE	ALNCGIFKE	5	0	0	0	0	ALNCGIFKE	tr_D0A9V0	0.048	29888.62	46.00
349	HLA-A0201	VPYVRVLDN	VPYVRVLDN	0	0	0	0	0	VPYVRVLDN	tr_D0A9V0	0.048	29890.55	46.00
256	HLA-A0201	GGASHMQGFL	GGASHMQFL	0	0	0	7	1	GGASHMQGFL	tr_D0A9V0	0.048	29891.84	46.00
196	HLA-A0201	FAKLPLDHR	FAKLPLDHR	0	0	0	0	0	FAKLPLDHR	tr_D0A9V0	0.048	29893.46	46.00
32	HLA-A0201	EVRKIQGHYAKL	KIQGHYAKL	3	0	0	0	0	KIQGHYAKL	tr_D0A9V0	0.048	29899.28	46.00
497	HLA-A0201	SKLHHPNIVR	KLHHPNIVR	1	0	0	0	0	KLHHPNIVR	tr_D0A9V0	0.047	29907.38	46.00
333	HLA-A0201	KRLMRLCRQ	KRLMRLCRQ	0	0	0	0	0	KRLMRLCRQ	tr_D0A9V0	0.047	29914.50	46.00
289	HLA-A0201	GESSSEVSLSIS	SSSEVSLSI	2	0	0	0	0	SSSEVSLSI	tr_D0A9V0	0.047	29915.13	46.00
567	HLA-A0201	DVKPHNVLL	DVKPHNVLL	0	0	0	0	0	DVKPHNVLL	tr_D0A9V0	0.047	29919.03	46.00
176	HLA-A0201	GAHAALPMHCRRCC	ALMHCRRCC	4	0	0	2	1	ALPMHCRRCC	tr_D0A9V0	0.047	29922.28	46.00
147	HLA-A0201	KYHAALIEVATE	KYHAALIEV	0	0	0	0	0	KYHAALIEV	tr_D0A9V0	0.047	29922.91	46.00
226	HLA-A0201	RNARVLFGAD	RNARVLFGA	0	0	0	0	0	RNARVLFGA	tr_D0A9V0	0.047	29927.12	47.00
28	HLA-A0201	SRIVEVRKI	SRIVEVRKI	0	0	0	0	0	SRIVEVRKI	tr_D0A9V0	0.047	29959.52	47.00
159	HLA-A0201	HSGCVEALDPDRA	HSGCVEALA	0	0	0	8	4	HSGCVEALDPDRA	tr_D0A9V0	0.047	29960.16	47.00
134	HLA-A0201	IQVYHPDVWKS	IQVYHVWKS	0	0	0	5	2	IQVYHPDVWKS	tr_D0A9V0	0.047	29961.47	47.00
493	HLA-A0201	MEVASKLHHPNIV	VASKLHHPV	2	0	0	8	2	VASKLHHPNIV	tr_D0A9V0	0.047	29972.16	47.00
691	HLA-A0201	EAAAFVRACC	EAAAFVACC	0	0	0	6	1	EAAAFVRACC	tr_D0A9V0	0.047	29974.75	47.00
106	HLA-A0201	QQRDVALNCGIFK	VALNCGIFK	4	0	0	0	0	VALNCGIFK	tr_D0A9V0	0.047	29975.74	47.00
484	HLA-A0201	VVAESLEKEME	VVAESLEEM	0	0	0	7	1	VVAESLEKEM	tr_D0A9V0	0.047	29987.09	47.00
244	HLA-A0201	VRDGPCRIAA	VRDGPCIAA	0	0	0	6	1	VRDGPCRIAA	tr_D0A9V0	0.047	29989.68	47.00
223	HLA-A0201	AFSRNARVLF	FSRNARVLF	1	0	0	0	0	FSRNARVLF	tr_D0A9V0	0.047	29990.97	47.00
411	HLA-A0201	YAPSSSSSLD	YAPSSSSSL	0	0	0	0	0	YAPSSSSSL	tr_D0A9V0	0.047	29998.77	47.00
308	HLA-A0201	AAMRQGRYGEAMA	AAMYGEAMA	0	0	0	3	4	AAMRQGRYGEAMA	tr_D0A9V0	0.047	30002.35	47.00
707	HLA-A0201	RPSASSLLSY	RSASSLLSY	0	0	0	1	1	RPSASSLLSY	tr_D0A9V0	0.047	30009.16	47.00
95	HLA-A0201	ASISVTWNAGD	ASISVTWNA	0	0	0	0	0	ASISVTWNA	tr_D0A9V0	0.047	30016.30	47.00
112	HLA-A0201	LNCGIFKEKPNVFN	LNCGIFVFN	0	0	0	6	5	LNCGIFKEKPNVFN	tr_D0A9V0	0.047	30021.82	47.00
472	HLA-A0201	VAIKCIPRLRR	VAIKCIPRL	0	0	0	0	0	VAIKCIPRL	tr_D0A9V0	0.047	30048.47	47.00
105	HLA-A0201	DQQRDVALNC	QQRDVALNC	1	0	0	0	0	QQRDVALNC	tr_D0A9V0	0.047	30053.03	47.00
114	HLA-A0201	CGIFKEKPNVF	GIFEKPNVF	1	0	0	3	1	GIFKEKPNVF	tr_D0A9V0	0.047	30075.79	47.00
177	HLA-A0201	AHAALPMHCRRCC	ALMHCRRCC	3	0	0	2	1	ALPMHCRRCC	tr_D0A9V0	0.047	30085.55	47.00
431	HLA-A0201	ADVVPLFLTDS	VVPLFLTDS	2	0	0	0	0	VVPLFLTDS	tr_D0A9V0	0.047	30096.30	47.00
181	HLA-A0201	LPMHCRRCCQFAFD	PMHCCCQFA	1	0	0	4	2	PMHCRRCCQFA	tr_D0A9V0	0.047	30100.20	47.00
206	HLA-A0201	SVTMLVDTNE	SVMLVDTNE	0	0	0	2	1	SVTMLVDTNE	tr_D0A9V0	0.047	30103.46	47.00
114	HLA-A0201	CGIFKEKPNVFNRR	CGIFVFNRR	0	0	0	4	5	CGIFKEKPNVFNRR	tr_D0A9V0	0.047	30112.59	47.00
107	HLA-A0201	QRDVALNCGIFKE	ALNCGIFKE	4	0	0	0	0	ALNCGIFKE	tr_D0A9V0	0.047	30118.12	47.00
576	HLA-A0201	GTDGVCKLSD	GTDGVCLSD	0	0	0	6	1	GTDGVCKLSD	tr_D0A9V0	0.047	30119.42	47.00
535	HLA-A0201	NFGCVSPYVARR	NFGCVSPYV	0	0	0	0	0	NFGCVSPYV	tr_D0A9V0	0.047	30122.36	47.00
325	HLA-A0201	MVKMDMQAKRLMR	MMDMQAKRL	0	0	0	1	2	MVKMDMQAKRL	tr_D0A9V0	0.047	30125.95	47.00
155	HLA-A0201	VATEHSGCVEA	VATEGCVEA	0	0	0	4	2	VATEHSGCVEA	tr_D0A9V0	0.047	30134.75	47.00
656	HLA-A0201	SDATRLRSL	SDATRLRSL	0	0	0	0	0	SDATRLRSL	tr_D0A9V0	0.047	30155.95	47.00

403	HLA-A0201	GSESLFKMY	GSESLFKMY	0	0	0	0	0	GSESLFKMY	tr_D0A9V0	0.047	30160.52	47.00
157	HLA-A0201	TEHSGCVEA	TEHSGCVEA	0	0	0	0	0	TEHSGCVEA	tr_D0A9V0	0.047	30174.87	47.00
621	HLA-A0201	GRCTPQSDIFSF	CTPQSIFSF	2	0	0	5	1	CTPQSDIFSF	tr_D0A9V0	0.047	30194.81	47.00
575	HLA-A0201	LGTDGVCKLSDFGS	GTDGVCFGS	1	0	0	6	4	GTDGVCKLSDFGS	tr_D0A9V0	0.047	30204.27	47.00
280	HLA-A0201	PIVLFELRPGE	ILFELRPGE	1	0	0	1	1	IVLFELRPGE	tr_D0A9V0	0.047	30231.75	47.00
514	HLA-A0201	PSHLSIIME	PSHLSIIME	0	0	0	0	0	PSHLSIIME	tr_D0A9V0	0.046	30232.39	47.00
504	HLA-A0201	IVRYVSCSITPSH	IVRYVSCSI	0	0	0	0	0	IVRYVSCSI	tr_D0A9V0	0.046	30235.67	47.00
579	HLA-A0201	GVCKLSDFG	GVCKLSDFG	0	0	0	0	0	GVCKLSDFG	tr_D0A9V0	0.046	30242.87	47.00
332	HLA-A0201	AKRLMRLCRQR	ALMRLCRQR	0	0	0	1	2	AKRLMRLCRQR	tr_D0A9V0	0.046	30245.81	47.00
25	HLA-A0201	LGRSRIVEVR	LGRSRIVEV	0	0	0	0	0	LGRSRIVEV	tr_D0A9V0	0.046	30253.01	47.00
335	HLA-A0201	LMRLCRQRCR	LMRLCRQRC	0	0	0	0	0	LMRLCRQRC	tr_D0A9V0	0.046	30253.34	47.00
10	HLA-A0201	KSASNLEIPDNA	SASNLEIPA	1	0	0	8	2	SASNLEIPDNA	tr_D0A9V0	0.046	30265.13	47.00
637	HLA-A0201	LEMLLGRPP	LEMLLGRPP	0	0	0	0	0	LEMLLGRPP	tr_D0A9V0	0.046	30267.10	47.00
288	HLA-A0201	PGESSSEVSLSIS	SSSEVSLSI	3	0	0	0	0	SSSEVSLSI	tr_D0A9V0	0.046	30274.30	47.00
15	HLA-A0201	LEIPDNADI	LEIPDNADI	0	0	0	0	0	LEIPDNADI	tr_D0A9V0	0.046	30289.69	47.00
543	HLA-A0201	VARRFTVDILQG	VARFTVDIL	0	0	0	2	1	VARRFTVDIL	tr_D0A9V0	0.046	30294.62	47.00
113	HLA-A0201	NCGIFKEKPNVF	GIFEKPNVF	2	0	0	3	1	GIFKEKPNVF	tr_D0A9V0	0.046	30295.59	47.00
291	HLA-A0201	SSSEVSLSISKQFR	SSSEVSLSI	0	0	0	0	0	SSSEVSLSI	tr_D0A9V0	0.046	30310.01	47.00
485	HLA-A0201	VAESLEKEMEVAS	SLEEMEVAS	3	0	0	3	1	SLEKEMEVAS	tr_D0A9V0	0.046	30310.68	47.00
402	HLA-A0201	QGSESLFKMY	QGSESLFMY	0	0	0	7	1	QGSESLFKMY	tr_D0A9V0	0.046	30330.68	48.00
94	HLA-A0201	GASISVTWNAGD	GAISVTWNA	0	0	0	2	1	GASISVTWNA	tr_D0A9V0	0.046	30334.63	48.00
166	HLA-A0201	LDPDRALISFGA	LRALISFGA	0	0	0	1	3	LDPDRALISFGA	tr_D0A9V0	0.046	30336.92	48.00
350	HLA-A0201	PYVRVLDNHY	YVRVLDNHY	1	0	0	0	0	YVRVLDNHY	tr_D0A9V0	0.046	30366.81	48.00
105	HLA-A0201	DQQRDVALNCGIFK	VALNCGIFK	5	0	0	0	0	VALNCGIFK	tr_D0A9V0	0.046	30373.71	48.00
235	HLA-A0201	DHLFQLSYA	DHLFQLSYA	0	0	0	0	0	DHLFQLSYA	tr_D0A9V0	0.046	30379.29	48.00
444	HLA-A0201	NVWTRSLKKIS	NVWTRSLKI	0	0	0	7	1	NVWTRSLKKI	tr_D0A9V0	0.046	30414.50	48.00
221	HLA-A0201	CGAFSRNARVLF	CGAFARVLF	0	0	0	4	3	CGAFSRNARVLF	tr_D0A9V0	0.046	30420.09	48.00
156	HLA-A0201	ATEHSGCVEALDPD	ATSGCVEAL	0	0	0	2	2	ATEHSGCVEAL	tr_D0A9V0	0.046	30441.81	48.00
56	HLA-A0201	EAILARVESK	AILARVESK	1	0	0	0	0	AILARVESK	tr_D0A9V0	0.046	30460.92	48.00
531	HLA-A0201	SVIKNFGCVSP	SVIFGCVSP	0	0	0	3	2	SVIKNFGCVSP	tr_D0A9V0	0.046	30461.59	48.00
431	HLA-A0201	ADVVPLFLTDSNRN	VVPLFLTNR	2	0	0	7	2	VVPLFLTDSNR	tr_D0A9V0	0.046	30468.17	48.00
291	HLA-A0201	SSSEVSLSISKQ	SSSEVSLSI	0	0	0	0	0	SSSEVSLSI	tr_D0A9V0	0.046	30476.74	48.00
304	HLA-A0201	RLGFAAMRQGR	RLGFAAMGR	0	0	0	7	2	RLGFAAMRQGR	tr_D0A9V0	0.046	30476.74	48.00
535	HLA-A0201	NFGCVSPYVAR	NFGCVSPYV	0	0	0	0	0	NFGCVSPYV	tr_D0A9V0	0.046	30486.98	48.00
221	HLA-A0201	CGAFSRNARVL	GAFSRNAVL	1	0	0	7	1	GAFSRNARVL	tr_D0A9V0	0.046	30491.27	48.00
245	HLA-A0201	RDGPCRIAA	RDGPCRIAA	0	0	0	0	0	RDGPCRIAA	tr_D0A9V0	0.046	30495.89	48.00
618	HLA-A0201	AAGGRCTPQSDI	AAGGRCTPI	0	0	0	8	3	AAGGRCTPQSDI	tr_D0A9V0	0.046	30500.18	48.00
485	HLA-A0201	VAESLEKEMEVASK	SLEMEVASK	3	0	0	2	2	SLEKEMEVASK	tr_D0A9V0	0.046	30502.81	48.00
576	HLA-A0201	GTDGVCKLSDF	GTDGVCKLF	0	0	0	8	2	GTDGVCKLSDF	tr_D0A9V0	0.046	30511.06	48.00
546	HLA-A0201	RFTVDILQG	RFTVDILQG	0	0	0	0	0	RFTVDILQG	tr_D0A9V0	0.046	30511.72	48.00
690	HLA-A0201	HEAAAFVRAC	HAAAFVRAC	0	0	0	1	1	HEAAAFVRAC	tr_D0A9V0	0.046	30519.32	48.00
180	HLA-A0201	ALPMHCRRCCQ	ALPMHCCCQ	0	0	0	6	2	ALPMHCRRCCQ	tr_D0A9V0	0.046	30532.87	48.00
587	HLA-A0201	GSTISEASVMAR	SISEASVMA	1	0	0	1	1	STISEASVMA	tr_D0A9V0	0.046	30539.46	48.00
652	HLA-A0201	APAGSDATRL	AAGSDATRL	0	0	0	1	1	APAGSDATRL	tr_D0A9V0	0.046	30541.45	48.00
289	HLA-A0201	GESSSEVSL	GESSSEVSL	0	0	0	0	0	GESSSEVSL	tr_D0A9V0	0.046	30545.41	48.00
222	HLA-A0201	GAFSRNARVLF	GAFSARVLF	0	0	0	4	2	GAFSRNARVLF	tr_D0A9V0	0.045	30565.90	48.00
529	HLA-A0201	LHSVIKNFGCVS	LHSVIFGCV	0	0	0	5	2	LHSVIKNFGCV	tr_D0A9V0	0.045	30591.38	48.00
562	HLA-A0201	GIVHCDVKP	GIVHCDVKP	0	0	0	0	0	GIVHCDVKP	tr_D0A9V0	0.045	30596.02	48.00
107	HLA-A0201	QRDVALNCGI	QRDVALCGI	0	0	0	6	1	QRDVALNCGI	tr_D0A9V0	0.045	30598.66	48.00
220	HLA-A0201	TCGAFSRNARVL	GAFSRNAVL	2	0	0	7	1	GAFSRNARVL	tr_D0A9V0	0.045	30607.94	48.00
93	HLA-A0201	MGASISVTWN	MGASISVTW	0	0	0	0	0	MGASISVTW	tr_D0A9V0	0.045	30613.25	48.00
613	HLA-A0201	MAPEVAAGGRCT	MAPEVAAGT	0	0	0	8	3	MAPEVAAGGRCT	tr_D0A9V0	0.045	30616.88	48.00
201	HLA-A0201	LDHRDSVTMLVD	LRDSVTMLV	0	0	0	1	2	LDHRDSVTMLV	tr_D0A9V0	0.045	30638.10	48.00
83	HLA-A0201	GESSTAFVRIM	SSTAFVRIM	2	0	0	0	0	SSTAFVRIM	tr_D0A9V0	0.045	30638.43	48.00

338	HLA-A0201	LCRQRCRRNDTV	LCRQRCDTV	0	0	0	6	3	LCRQRCRRNDTV	tr_D0A9V0	0.045	30640.41	48.00
432	HLA-A0201	DVVPLFLTDSNRN	VVPLFLTNR	1	0	0	7	2	VVPLFLTDSNR	tr_D0A9V0	0.045	30653.02	48.00
487	HLA-A0201	ESLEKEMEVASK	SLEMEVASK	1	0	0	2	2	SLEKEMEVASK	tr_D0A9V0	0.045	30659.64	48.00
441	HLA-A0201	SNRNVWTRSL	SNRNVWTSL	0	0	0	7	1	SNRNVWTRSL	tr_D0A9V0	0.045	30674.24	48.00
148	HLA-A0201	YHAALIEVATE	YALIEVATE	0	0	0	1	2	YHAALIEVATE	tr_D0A9V0	0.045	30675.90	48.00
211	HLA-A0201	VDTNEFLVGTCG	VTNEFLVGT	0	0	0	1	1	VDTNEFLVGT	tr_D0A9V0	0.045	30684.55	48.00
473	HLA-A0201	AIKCIPRLR	AIKCIPRLR	0	0	0	0	0	AIKCIPRLR	tr_D0A9V0	0.045	30695.49	48.00
133	HLA-A0201	AIQVYHPDVWKSS	AIQVYVWKS	0	0	0	5	3	AIQVYHPDVWKS	tr_D0A9V0	0.045	30714.76	49.00
477	HLA-A0201	IPRLRRDVV	IPRLRRDVV	0	0	0	0	0	IPRLRRDVV	tr_D0A9V0	0.045	30754.34	49.00
407	HLA-A0201	LFKMYAPSSSSS	FMYAPSSSS	1	0	0	1	1	FKMYAPSSSS	tr_D0A9V0	0.045	30754.68	49.00
536	HLA-A0201	FGCVSPYVARR	FVSPYVARR	0	0	0	1	2	FGCVSPYVARR	tr_D0A9V0	0.045	30755.00	49.00
487	HLA-A0201	ESLEKEMEVAS	SLEEMEVAS	1	0	0	3	1	SLEKEMEVAS	tr_D0A9V0	0.045	30759.00	49.00
324	HLA-A0201	PMVKMDMQAKRLMR	KMDMQALMR	3	0	0	6	2	KMDMQAKRLMR	tr_D0A9V0	0.045	30762.00	49.00
583	HLA-A0201	LSDFGSTISEA	LSDFTISEA	0	0	0	4	2	LSDFGSTISEA	tr_D0A9V0	0.045	30775.97	49.00
115	HLA-A0201	GIFKEKPNVFNRR	GIFNVFNRR	0	0	0	3	4	GIFKEKPNVFNRR	tr_D0A9V0	0.045	30776.64	49.00
205	HLA-A0201	DSVTMLVDT	DSVTMLVDT	0	0	0	0	0	DSVTMLVDT	tr_D0A9V0	0.045	30802.95	49.00
433	HLA-A0201	VVPLFLTDSNRN	VVPLFLTNR	0	0	0	7	2	VVPLFLTDSNR	tr_D0A9V0	0.045	30804.62	49.00
58	HLA-A0201	ILARVESKNEAGGH	ILARVENEA	0	0	0	6	2	ILARVESKNEA	tr_D0A9V0	0.045	30806.63	49.00
326	HLA-A0201	VKMDMQAKRLMR	KMDMQALMR	1	0	0	6	2	KMDMQAKRLMR	tr_D0A9V0	0.045	30835.31	49.00
405	HLA-A0201	ESLFKMYAP	ESLFKMYAP	0	0	0	0	0	ESLFKMYAP	tr_D0A9V0	0.045	30840.98	49.00
369	HLA-A0201	EEQDESDIKDRV	EQDESDIRV	1	0	0	7	2	EQDESDIKDRV	tr_D0A9V0	0.045	30859.66	49.00
102	HLA-A0201	NAGDQQRDV	NAGDQQRDV	0	0	0	0	0	NAGDQQRDV	tr_D0A9V0	0.045	30886.72	49.00
486	HLA-A0201	AESLEKEMEVASK	SLEMEVASK	2	0	0	2	2	SLEKEMEVASK	tr_D0A9V0	0.044	30899.43	49.00
403	HLA-A0201	GSESLFKMYAP	GSESLFMYA	0	0	0	6	1	GSESLFKMYA	tr_D0A9V0	0.044	30914.14	49.00
107	HLA-A0201	QRDVALNCGIFK	VALNCGIFK	3	0	0	0	0	VALNCGIFK	tr_D0A9V0	0.044	30960.33	49.00
537	HLA-A0201	GCVSPYVARRF	CVSPYVARF	1	0	0	7	1	CVSPYVARRF	tr_D0A9V0	0.044	30963.34	49.00
95	HLA-A0201	ASISVTWNAGDQ	ASISVTWNA	0	0	0	0	0	ASISVTWNA	tr_D0A9V0	0.044	30965.70	49.00
463	HLA-A0201	LGVAEDGAQVAIKC	GVAEVAIKC	1	0	0	4	4	GVAEDGAQVAIKC	tr_D0A9V0	0.044	30972.73	49.00
463	HLA-A0201	LGVAEDGAQVA	LVAEDGAQV	0	0	0	1	1	LGVAEDGAQV	tr_D0A9V0	0.044	30980.44	49.00
464	HLA-A0201	GVAEDGAQVAIKC	GVAEVAIKC	0	0	0	4	4	GVAEDGAQVAIKC	tr_D0A9V0	0.044	30984.79	49.00
154	HLA-A0201	EVATEHSGCV	VATEHSGCV	1	0	0	0	0	VATEHSGCV	tr_D0A9V0	0.044	30990.15	49.00
166	HLA-A0201	LDPDRALISF	LPDRALISF	0	0	0	1	1	LDPDRALISF	tr_D0A9V0	0.044	31010.62	49.00
501	HLA-A0201	HPNIVRYVSC	HPNIVYVSC	0	0	0	5	1	HPNIVRYVSC	tr_D0A9V0	0.044	31026.39	49.00
134	HLA-A0201	IQVYHPDVWK	IVYHPDVWK	0	0	0	1	1	IQVYHPDVWK	tr_D0A9V0	0.044	31037.13	49.00
346	HLA-A0201	NDTVPYVRV	NDTVPYVRV	0	0	0	0	0	NDTVPYVRV	tr_D0A9V0	0.044	31037.13	49.00
36	HLA-A0201	IQGHYAKLRRQLA	IQGHYAQLA	0	0	0	6	4	IQGHYAKLRRQLA	tr_D0A9V0	0.044	31050.25	49.00
82	HLA-A0201	PGESSTAFVRIM	SSTAFVRIM	3	0	0	0	0	SSTAFVRIM	tr_D0A9V0	0.044	31073.08	50.00
529	HLA-A0201	LHSVIKNFGCVSP	SVIFGCVSP	2	0	0	3	2	SVIKNFGCVSP	tr_D0A9V0	0.044	31074.45	50.00
513	HLA-A0201	TPSHLSIIME	TSHLSIIME	0	0	0	1	1	TPSHLSIIME	tr_D0A9V0	0.044	31078.81	50.00
564	HLA-A0201	VHCDVKPHNVLL	VHCDVNVLL	0	0	0	5	3	VHCDVKPHNVLL	tr_D0A9V0	0.044	31091.93	50.00
158	HLA-A0201	EHSGCVEALDPDRA	HSGCVEALA	1	0	0	8	4	HSGCVEALDPDRA	tr_D0A9V0	0.044	31093.27	50.00
137	HLA-A0201	YHPDVWKSSTKYHA	YHPDVWYHA	0	0	0	6	5	YHPDVWKSSTKYHA	tr_D0A9V0	0.044	31093.27	50.00
331	HLA-A0201	QAKRLMRLCRQR	RLMRLCRQR	3	0	0	0	0	RLMRLCRQR	tr_D0A9V0	0.044	31099.33	50.00
243	HLA-A0201	AVRDGPCRIAAT	AVPCRIAAT	0	0	0	2	3	AVRDGPCRIAAT	tr_D0A9V0	0.044	31121.88	50.00
108	HLA-A0201	RDVALNCGIFK	RVALNCGIF	0	0	0	1	1	RDVALNCGIF	tr_D0A9V0	0.044	31133.66	50.00
591	HLA-A0201	SEASVMARTLA	SEASVMALA	0	0	0	7	2	SEASVMARTLA	tr_D0A9V0	0.044	31134.69	50.00
590	HLA-A0201	ISEASVMARTLAD	ISEASVMTL	0	0	0	7	2	ISEASVMARTL	tr_D0A9V0	0.044	31147.14	50.00
241	HLA-A0201	SYAVRDGPCRIA	YAVGPCRIA	1	0	0	3	2	YAVRDGPCRIA	tr_D0A9V0	0.044	31155.23	50.00
587	HLA-A0201	GSTISEASVM	STISEASVM	1	0	0	0	0	STISEASVM	tr_D0A9V0	0.044	31158.61	50.00
493	HLA-A0201	MEVASKLHHP	MVASKLHHP	0	0	0	1	1	MEVASKLHHP	tr_D0A9V0	0.044	31169.06	50.00
152	HLA-A0201	LIEVATEHSGCVE	LIEVATEHV	0	0	0	8	3	LIEVATEHSGCV	tr_D0A9V0	0.044	31172.44	50.00
682	HLA-A0201	PVIPASVDHEAA	VIPASVEAA	1	0	0	6	2	VIPASVDHEAA	tr_D0A9V0	0.044	31175.81	50.00
292	HLA-A0201	SSEVSLSISK	SSEVSLISK	0	0	0	6	1	SSEVSLSISK	tr_D0A9V0	0.044	31183.56	50.00
81	HLA-A0201	TPGESSTAFVRIM	SSTAFVRIM	4	0	0	0	0	SSTAFVRIM	tr_D0A9V0	0.044	31187.96	50.00

305	HLA-A0201	LGFAAMRQGRYGEA	AMQGRYGEA	4	0	0	2	1	AMRQGRYGEA	tr_D0A9V0	0.044	31190.66	50.00
486	HLA-A0201	AESLEKEMEVAS	SLEEMEVAS	2	0	0	3	1	SLEKEMEVAS	tr_D0A9V0	0.044	31196.05	50.00
94	HLA-A0201	GASISVTWNAGDQ	ASISVTWNA	1	0	0	0	0	ASISVTWNA	tr_D0A9V0	0.044	31205.16	50.00
659	HLA-A0201	TRLRSLFNRD	RLRSLFNRD	1	0	0	0	0	RLRSLFNRD	tr_D0A9V0	0.044	31205.50	50.00
499	HLA-A0201	LHHPNIVRY	LHHPNIVRY	0	0	0	0	0	LHHPNIVRY	tr_D0A9V0	0.044	31210.23	50.00
223	HLA-A0201	AFSRNARVLFG	FSNARVLFG	1	0	0	2	1	FSRNARVLFG	tr_D0A9V0	0.044	31218.67	50.00
88	HLA-A0201	AFVRIMGAS	AFVRIMGAS	0	0	0	0	0	AFVRIMGAS	tr_D0A9V0	0.044	31219.70	50.00
472	HLA-A0201	VAIKCIPRLRRD	VAIKCIPRL	0	0	0	0	0	VAIKCIPRL	tr_D0A9V0	0.044	31224.08	50.00
140	HLA-A0201	DVWKSSTKYHAAL	DVWKYHAAL	0	0	0	3	4	DVWKSSTKYHAAL	tr_D0A9V0	0.043	31229.48	50.00
544	HLA-A0201	ARRFTVDILQG	RRFTVILQG	1	0	0	5	1	RRFTVDILQG	tr_D0A9V0	0.043	31237.94	50.00
255	HLA-A0201	SGGASHMQGFLA	SSHMQGFLA	0	0	0	1	3	SGGASHMQGFLA	tr_D0A9V0	0.043	31238.95	50.00
26	HLA-A0201	GRSRIVEVRKI	RSRIVEVRI	1	0	0	8	1	RSRIVEVRKI	tr_D0A9V0	0.043	31250.78	50.00
54	HLA-A0201	IPEAILARVESK	IPEAILARV	0	0	0	0	0	IPEAILARV	tr_D0A9V0	0.043	31260.92	50.00
290	HLA-A0201	ESSSEVSLSISK	SSSEVSLSI	1	0	0	0	0	SSSEVSLSI	tr_D0A9V0	0.043	31263.64	50.00
84	HLA-A0201	ESSTAFVRIMG	SSTAFVIMG	1	0	0	6	1	SSTAFVRIMG	tr_D0A9V0	0.043	31265.65	50.00
256	HLA-A0201	GGASHMQGFLA	ASHMQGFLA	2	0	0	0	0	ASHMQGFLA	tr_D0A9V0	0.043	31267.01	50.00
80	HLA-A0201	ETPGESSTAFVRIM	SSTAFVRIM	5	0	0	0	0	SSTAFVRIM	tr_D0A9V0	0.043	31271.74	50.00
706	HLA-A0201	MRPSASSLLSY	MSASSLLSY	0	0	0	1	2	MRPSASSLLSY	tr_D0A9V0	0.043	31309.00	50.00
164	HLA-A0201	EALDPDRAL	EALDPDRAL	0	0	0	0	0	EALDPDRAL	tr_D0A9V0	0.043	31330.00	50.00
30	HLA-A0201	IVEVRKIQGHYA	IVEVRKIYA	0	0	0	7	3	IVEVRKIQGHYA	tr_D0A9V0	0.043	31337.80	50.00
54	HLA-A0201	IPEAILARVES	IPEAILARV	0	0	0	0	0	IPEAILARV	tr_D0A9V0	0.043	31353.06	50.00
252	HLA-A0201	AATSGGASHMQGFL	ATSGGASFL	1	0	0	7	4	ATSGGASHMQGFL	tr_D0A9V0	0.043	31358.14	50.00
308	HLA-A0201	AAMRQGRYGEA	AMQGRYGEA	1	0	0	2	1	AMRQGRYGEA	tr_D0A9V0	0.043	31359.52	50.00
107	HLA-A0201	QRDVALNCGIF	RVALNCGIF	1	0	0	1	1	RDVALNCGIF	tr_D0A9V0	0.043	31382.92	50.00
308	HLA-A0201	AAMRQGRYGEAMAH	AMYGEAMAH	1	0	0	2	4	AMRQGRYGEAMAH	tr_D0A9V0	0.043	31384.28	50.00
566	HLA-A0201	CDVKPHNVLLGT	CDVKVLLGT	0	0	0	4	3	CDVKPHNVLLGT	tr_D0A9V0	0.043	31388.69	50.00
481	HLA-A0201	RRDVVAESLE	RRDVVAESL	0	0	0	0	0	RRDVVAESL	tr_D0A9V0	0.043	31394.80	50.00
161	HLA-A0201	GCVEALDPDRA	GCVEALDPA	0	0	0	8	2	GCVEALDPDRA	tr_D0A9V0	0.043	31395.49	50.00
177	HLA-A0201	AHAALPMHC	AHAALPMHC	0	0	0	0	0	AHAALPMHC	tr_D0A9V0	0.043	31401.61	50.00
117	HLA-A0201	FKEKPNVFNRRHCT	FVFNRRHCT	0	0	0	1	5	FKEKPNVFNRRHCT	tr_D0A9V0	0.043	31409.77	50.00
399	HLA-A0201	SSDQGSESLFKMY	SSDESLFKM	0	0	0	3	3	SSDQGSESLFKM	tr_D0A9V0	0.043	31422.01	55.00
325	HLA-A0201	MVKMDMQAK	MVKMDMQAK	0	0	0	0	0	MVKMDMQAK	tr_D0A9V0	0.043	31423.02	55.00
25	HLA-A0201	LGRSRIVEVRKI	RSRIVEVRI	2	0	0	8	1	RSRIVEVRKI	tr_D0A9V0	0.043	31432.87	55.00
419	HLA-A0201	LDGSGCAEGA	LGSGCAEGA	0	0	0	1	1	LDGSGCAEGA	tr_D0A9V0	0.043	31440.03	55.00
83	HLA-A0201	GESSTAFVRIMG	SSTAFVIMG	2	0	0	6	1	SSTAFVRIMG	tr_D0A9V0	0.043	31445.12	55.00
254	HLA-A0201	TSGGASHMQGFLA	ASHMQGFLA	4	0	0	0	0	ASHMQGFLA	tr_D0A9V0	0.043	31451.59	55.00
513	HLA-A0201	TPSHLSIIMEY	SHLSIIMEY	2	0	0	0	0	SHLSIIMEY	tr_D0A9V0	0.043	31465.89	55.00
591	HLA-A0201	SEASVMARTLADG	SEASVMATL	0	0	0	7	1	SEASVMARTL	tr_D0A9V0	0.043	31466.91	55.00
613	HLA-A0201	MAPEVAAGGRC	MAPEVAAGC	0	0	0	8	2	MAPEVAAGGRC	tr_D0A9V0	0.043	31470.66	55.00
228	HLA-A0201	ARVLFGADH	ARVLFGADH	0	0	0	0	0	ARVLFGADH	tr_D0A9V0	0.043	31472.37	55.00
425	HLA-A0201	AEGANAADVV	AEGANAAVV	0	0	0	7	1	AEGANAADVV	tr_D0A9V0	0.043	31473.04	55.00
325	HLA-A0201	MVKMDMQAKR	MVMDMQAKR	0	0	0	2	1	MVKMDMQAKR	tr_D0A9V0	0.043	31475.43	55.00
15	HLA-A0201	LEIPDNADIFLGRS	LADIFLGRS	0	0	0	1	5	LEIPDNADIFLGRS	tr_D0A9V0	0.043	31481.89	55.00
154	HLA-A0201	EVATEHSGCVEA	ATHSGCVEA	2	0	0	2	1	ATEHSGCVEA	tr_D0A9V0	0.043	31481.89	55.00
530	HLA-A0201	HSVIKNFGCVSP	SVIFGCVSP	1	0	0	3	2	SVIKNFGCVSP	tr_D0A9V0	0.043	31488.36	55.00
249	HLA-A0201	CRIAATSGGASHM	RIAATASHM	1	0	0	5	3	RIAATSGGASHM	tr_D0A9V0	0.043	31489.38	55.00
95	HLA-A0201	ASISVTWNAGDQQ	ASISVTWNA	0	0	0	0	0	ASISVTWNA	tr_D0A9V0	0.043	31511.54	55.00
9	HLA-A0201	LKSASNLEIPDNA	SASNLEIPA	2	0	0	8	2	SASNLEIPDNA	tr_D0A9V0	0.043	31522.80	55.00
540	HLA-A0201	SPYVARRFTVD	SPYVARFTV	0	0	0	5	1	SPYVARRFTV	tr_D0A9V0	0.043	31526.54	55.00
474	HLA-A0201	IKCIPRLRRDV	KCIPRLRRV	1	0	0	8	1	KCIPRLRRDV	tr_D0A9V0	0.043	31542.59	55.00
680	HLA-A0201	LEPVIPASVDHEAA	VIPASVEAA	3	0	0	6	2	VIPASVDHEAA	tr_D0A9V0	0.043	31554.19	55.00
95	HLA-A0201	ASISVTWNAGDQQR	ASISVTWNA	0	0	0	0	0	ASISVTWNA	tr_D0A9V0	0.043	31558.62	55.00
167	HLA-A0201	DPDRALISF	DPDRALISF	0	0	0	0	0	DPDRALISF	tr_D0A9V0	0.043	31558.62	55.00
423	HLA-A0201	GCAEGANAADV	GCAEGANAV	0	0	0	8	2	GCAEGANAADV	tr_D0A9V0	0.043	31566.13	55.00

8	HLA-A0201	ALKSASNLE	ALKSASNLE	0	0	0	0	0	ALKSASNLE	tr_D0A9V0	0.042	31577.07	55.00
474	HLA-A0201	IKCIPRLRRDVVAE	ILRRDVVAE	0	0	0	1	5	IKCIPRLRRDVVAE	tr_D0A9V0	0.042	31589.72	55.00
285	HLA-A0201	ELRPGESSSEVS	ELGESSSEV	0	0	0	2	2	ELRPGESSSEV	tr_D0A9V0	0.042	31600.66	55.00
177	HLA-A0201	AHAALPMHCRRC	ALPMHCRRC	0	0	0	1	3	AHAALPMHCRRC	tr_D0A9V0	0.042	31609.89	55.00
483	HLA-A0201	DVVAESLEKEME	VVAESLEEM	1	0	0	7	1	VVAESLEKEM	tr_D0A9V0	0.042	31623.23	55.00
181	HLA-A0201	LPMHCRRCCQF	LPMHCCCQF	0	0	0	5	2	LPMHCRRCCQF	tr_D0A9V0	0.042	31646.85	55.00
201	HLA-A0201	LDHRDSVTMLVDT	LRDSVTMLV	0	0	0	1	2	LDHRDSVTMLV	tr_D0A9V0	0.042	31647.87	55.00
225	HLA-A0201	SRNARVLFG	SRNARVLFG	0	0	0	0	0	SRNARVLFG	tr_D0A9V0	0.042	31670.47	55.00
323	HLA-A0201	LPMVKMDMQ	LPMVKMDMQ	0	0	0	0	0	LPMVKMDMQ	tr_D0A9V0	0.042	31672.88	55.00
201	HLA-A0201	LDHRDSVTML	LDHDSVTML	0	0	0	3	1	LDHRDSVTML	tr_D0A9V0	0.042	31675.62	55.00
94	HLA-A0201	GASISVTWNAGDQQ	ASISVTWNA	1	0	0	0	0	ASISVTWNA	tr_D0A9V0	0.042	31679.73	55.00
541	HLA-A0201	PYVARRFTV	PYVARRFTV	0	0	0	0	0	PYVARRFTV	tr_D0A9V0	0.042	31686.94	55.00
499	HLA-A0201	LHHPNIVRYVS	LHHPNIVYV	0	0	0	7	1	LHHPNIVRYV	tr_D0A9V0	0.042	31693.11	55.00
175	HLA-A0201	FGAHAALPMHC	FGAALPMHC	0	0	0	2	2	FGAHAALPMHC	tr_D0A9V0	0.042	31697.22	55.00
343	HLA-A0201	CRRNDTVPYVRVLD	CTVPYVRVL	0	0	0	1	4	CRRNDTVPYVRVL	tr_D0A9V0	0.042	31697.92	55.00
575	HLA-A0201	LGTDGVCKLSDF	GTDGVCKLF	1	0	0	8	2	GTDGVCKLSDF	tr_D0A9V0	0.042	31697.92	55.00
443	HLA-A0201	RNVWTRSLKKIS	NVWTRSLKI	1	0	0	7	1	NVWTRSLKKI	tr_D0A9V0	0.042	31701.00	55.00
681	HLA-A0201	EPVIPASVDHEAA	VIPASVEAA	2	0	0	6	2	VIPASVDHEAA	tr_D0A9V0	0.042	31713.01	55.00
27	HLA-A0201	RSRIVEVRKIQGHY	RSRIVEVHY	0	0	0	7	5	RSRIVEVRKIQGHY	tr_D0A9V0	0.042	31714.72	55.00
82	HLA-A0201	PGESSTAFVRIMG	SSTAFVIMG	3	0	0	6	1	SSTAFVRIMG	tr_D0A9V0	0.042	31719.18	55.00
133	HLA-A0201	AIQVYHPDVWKSST	AIQVYVWKS	0	0	0	5	3	AIQVYHPDVWKS	tr_D0A9V0	0.042	31719.86	55.00
176	HLA-A0201	GAHAALPMHCRRC	ALPMHCRRC	4	0	0	0	0	ALPMHCRRC	tr_D0A9V0	0.042	31722.27	55.00
57	HLA-A0201	AILARVESKN	AILARVESK	0	0	0	0	0	AILARVESK	tr_D0A9V0	0.042	31724.67	55.00
524	HLA-A0201	VPGGSLHSVI	VPGGSLHSV	0	0	0	0	0	VPGGSLHSV	tr_D0A9V0	0.042	31725.01	55.00
475	HLA-A0201	KCIPRLRRDVVAE	KCIPRLVVA	0	0	0	6	3	KCIPRLRRDVVA	tr_D0A9V0	0.042	31726.04	55.00
36	HLA-A0201	IQGHYAKLRRQL	IQGHYAKLL	0	0	0	8	3	IQGHYAKLRRQL	tr_D0A9V0	0.042	31733.26	55.00
58	HLA-A0201	ILARVESKN	ILARVESKN	0	0	0	0	0	ILARVESKN	tr_D0A9V0	0.042	31733.26	55.00
402	HLA-A0201	QGSESLFKMYAP	GSESLFMYA	1	0	0	6	1	GSESLFKMYA	tr_D0A9V0	0.042	31734.96	55.00
530	HLA-A0201	HSVIKNFGCVS	SVIKNFGCV	1	0	0	0	0	SVIKNFGCV	tr_D0A9V0	0.042	31763.83	55.00
565	HLA-A0201	HCDVKPHNVLLGTD	HCDVVLLGT	0	0	0	4	4	HCDVKPHNVLLGT	tr_D0A9V0	0.042	31765.20	55.00
183	HLA-A0201	MHCRRCCQFAFD	MHCCCQFAF	0	0	0	3	2	MHCRRCCQFAF	tr_D0A9V0	0.042	31775.16	55.00
613	HLA-A0201	MAPEVAAGGR	MAPEVAAGR	0	0	0	7	1	MAPEVAAGGR	tr_D0A9V0	0.042	31777.93	55.00
499	HLA-A0201	LHHPNIVRYVSC	LHHPNIVYV	0	0	0	7	1	LHHPNIVRYV	tr_D0A9V0	0.042	31779.65	55.00
298	HLA-A0201	SISKQFRLG	SISKQFRLG	0	0	0	0	0	SISKQFRLG	tr_D0A9V0	0.042	31779.98	55.00
295	HLA-A0201	VSLSISKQFR	VLSISKQFR	0	0	0	1	1	VSLSISKQFR	tr_D0A9V0	0.042	31802.34	55.00
401	HLA-A0201	DQGSESLFKMYAP	GSESLFMYA	2	0	0	6	1	GSESLFKMYA	tr_D0A9V0	0.042	31808.54	55.00
565	HLA-A0201	HCDVKPHNVL	HCDVKPHNL	0	0	0	8	1	HCDVKPHNVL	tr_D0A9V0	0.042	31821.61	55.00
178	HLA-A0201	HAALPMHCRRC	ALPMHCRRC	2	0	0	0	0	ALPMHCRRC	tr_D0A9V0	0.042	31826.08	55.00
81	HLA-A0201	TPGESSTAFVRIMG	SSTAFVIMG	4	0	0	6	1	SSTAFVRIMG	tr_D0A9V0	0.042	31837.46	55.00
320	HLA-A0201	AHYLPMVKMD	AHYLPMVKM	0	0	0	0	0	AHYLPMVKM	tr_D0A9V0	0.042	31840.21	55.00
509	HLA-A0201	SCSITPSHLS	SCSITPSHL	0	0	0	0	0	SCSITPSHL	tr_D0A9V0	0.042	31843.66	55.00
307	HLA-A0201	FAAMRQGRYGEA	AMQGRYGEA	2	0	0	2	1	AMRQGRYGEA	tr_D0A9V0	0.042	31845.38	55.00
561	HLA-A0201	LGIVHCDVK	LGIVHCDVK	0	0	0	0	0	LGIVHCDVK	tr_D0A9V0	0.042	31856.06	55.00
289	HLA-A0201	GESSSEVSLSISK	SSSEVSLSI	2	0	0	0	0	SSSEVSLSI	tr_D0A9V0	0.042	31862.97	55.00
399	HLA-A0201	SSDQGSESLF	SSDQGSESL	0	0	0	0	0	SSDQGSESL	tr_D0A9V0	0.042	31863.29	55.00
424	HLA-A0201	CAEGANAADVV	CAEGANAAV	0	0	0	8	2	CAEGANAADVV	tr_D0A9V0	0.042	31865.02	55.00
198	HLA-A0201	KLPLDHRDS	KLPLDHRDS	0	0	0	0	0	KLPLDHRDS	tr_D0A9V0	0.042	31869.17	55.00
565	HLA-A0201	HCDVKPHNVLL	HCDVKPVLL	0	0	0	6	2	HCDVKPHNVLL	tr_D0A9V0	0.042	31907.46	55.00
15	HLA-A0201	LEIPDNADIFLGR	LEIPIFLGR	0	0	0	4	4	LEIPDNADIFLGR	tr_D0A9V0	0.041	31924.38	55.00
442	HLA-A0201	NRNVWTRSLKKIS	NVWTRSLKI	2	0	0	7	1	NVWTRSLKKI	tr_D0A9V0	0.041	31965.18	55.00
373	HLA-A0201	ESDIKDRVPETI	ESDIVPETI	0	0	0	4	3	ESDIKDRVPETI	tr_D0A9V0	0.041	31967.24	55.00
625	HLA-A0201	PQSDIFSFG	PQSDIFSFG	0	0	0	0	0	PQSDIFSFG	tr_D0A9V0	0.041	31986.97	55.00
708	HLA-A0201	PSASSLLSY	PSASSLLSY	0	0	0	0	0	PSASSLLSY	tr_D0A9V0	0.041	31998.05	55.00
292	HLA-A0201	SSEVSLSIS	SSEVSLSIS	0	0	0	0	0	SSEVSLSIS	tr_D0A9V0	0.041	31999.43	55.00

592	HLA-A0201	EASVMARTLA	ASVMARTLA	1	0	0	0	0	ASVMARTLA	tr_D0A9V0	0.041	32023.32	55.00
481	HLA-A0201	RRDVVAESLEKEME	VVAESLEEM	3	0	0	7	1	VVAESLEKEM	tr_D0A9V0	0.041	32025.41	55.00
349	HLA-A0201	VPYVRVLDNH	VPYVRVLNH	0	0	0	7	1	VPYVRVLDNH	tr_D0A9V0	0.041	32052.45	55.00
125	HLA-A0201	NRRHCTVIAIQ	RRHCTVIAI	1	0	0	0	0	RRHCTVIAI	tr_D0A9V0	0.041	32052.78	55.00
422	HLA-A0201	SGCAEGANAA	SCAEGANAA	0	0	0	1	1	SGCAEGANAA	tr_D0A9V0	0.041	32059.03	55.00
400	HLA-A0201	SDQGSESLFKMYAP	GSESLFMYA	3	0	0	6	1	GSESLFKMYA	tr_D0A9V0	0.041	32075.34	55.00
179	HLA-A0201	AALPMHCRRCCQ	ALPMHCCCQ	1	0	0	6	2	ALPMHCRRCCQ	tr_D0A9V0	0.041	32090.26	55.00
441	HLA-A0201	SNRNVWTRSLKKIS	NVWTRSLKI	3	0	0	7	1	NVWTRSLKKI	tr_D0A9V0	0.041	32105.54	55.00
123	HLA-A0201	VFNRRHCTVI	VFNRRCTVI	0	0	0	5	1	VFNRRHCTVI	tr_D0A9V0	0.041	32113.53	55.00
484	HLA-A0201	VVAESLEKE	VVAESLEKE	0	0	0	0	0	VVAESLEKE	tr_D0A9V0	0.041	32128.49	55.00
622	HLA-A0201	RCTPQSDIFSFG	RQSDIFSFG	0	0	0	1	3	RCTPQSDIFSFG	tr_D0A9V0	0.041	32129.88	55.00
288	HLA-A0201	PGESSSEVSLSISK	SSSEVSLSI	3	0	0	0	0	SSSEVSLSI	tr_D0A9V0	0.041	32134.04	55.00
101	HLA-A0201	WNAGDQQRDVALNC	WQRDVALNC	0	0	0	1	5	WNAGDQQRDVALNC	tr_D0A9V0	0.041	32155.95	55.00
623	HLA-A0201	CTPQSDIFSFG	CTPQSIFSF	0	0	0	5	1	CTPQSDIFSF	tr_D0A9V0	0.041	32158.05	55.00
476	HLA-A0201	CIPRLRRDVVA	CIPRLDVVA	0	0	0	5	2	CIPRLRRDVVA	tr_D0A9V0	0.041	32166.39	55.00
417	HLA-A0201	SSLDGSGCAE	SLDGSGCAE	0	0	0	1	1	SSLDGSGCAE	tr_D0A9V0	0.041	32167.80	55.00
491	HLA-A0201	KEMEVASKLH	KEMEVASKL	0	0	0	0	0	KEMEVASKL	tr_D0A9V0	0.041	32181.72	55.00
174	HLA-A0201	SFGAHAALPMHC	GAHAALMHC	2	0	0	6	1	GAHAALPMHC	tr_D0A9V0	0.041	32182.76	55.00
78	HLA-A0201	ATETPGESSTAFVR	ATESSTAFV	0	0	0	2	4	ATETPGESSTAFV	tr_D0A9V0	0.041	32185.19	55.00
561	HLA-A0201	LGIVHCDVKP	LIVHCDVKP	0	0	0	1	1	LGIVHCDVKP	tr_D0A9V0	0.041	32192.16	55.00
620	HLA-A0201	GGRCTPQSDIFSFG	GQSDIFSFG	0	0	0	1	5	GGRCTPQSDIFSFG	tr_D0A9V0	0.041	32194.94	55.00
442	HLA-A0201	NRNVWTRSL	NRNVWTRSL	0	0	0	0	0	NRNVWTRSL	tr_D0A9V0	0.041	32198.78	55.00
103	HLA-A0201	AGDQQRDVAL	AGDQRDVAL	0	0	0	3	1	AGDQQRDVAL	tr_D0A9V0	0.041	32199.47	55.00
135	HLA-A0201	QVYHPDVWK	QVYHPDVWK	0	0	0	0	0	QVYHPDVWK	tr_D0A9V0	0.041	32199.82	55.00
294	HLA-A0201	EVSLSISKQF	EVSLSISQF	0	0	0	7	1	EVSLSISKQF	tr_D0A9V0	0.041	32202.25	55.00
104	HLA-A0201	GDQQRDVAL	GDQQRDVAL	0	0	0	0	0	GDQQRDVAL	tr_D0A9V0	0.041	32204.70	55.00
290	HLA-A0201	ESSSEVSLSISKQ	SSSEVSLSI	1	0	0	0	0	SSSEVSLSI	tr_D0A9V0	0.041	32214.46	55.00
262	HLA-A0201	QGFLAFPVD	QGFLAFPVD	0	0	0	0	0	QGFLAFPVD	tr_D0A9V0	0.041	32226.66	55.00
160	HLA-A0201	SGCVEALDPDRA	GCVEALDPA	1	0	0	8	2	GCVEALDPDRA	tr_D0A9V0	0.041	32230.49	55.00
20	HLA-A0201	NADIFLGRSR	NADIFLRSR	0	0	0	6	1	NADIFLGRSR	tr_D0A9V0	0.041	32234.69	55.00
398	HLA-A0201	DSSDQGSESLFKMY	SSDESLFKM	1	0	0	3	3	SSDQGSESLFKM	tr_D0A9V0	0.041	32238.51	55.00
133	HLA-A0201	AIQVYHPDVWK	AIQVYPDVW	0	0	0	5	1	AIQVYHPDVW	tr_D0A9V0	0.041	32249.68	55.00
464	HLA-A0201	GVAEDGAQVAIK	GVAEAQVAI	0	0	0	4	2	GVAEDGAQVAI	tr_D0A9V0	0.041	32257.02	55.00
563	HLA-A0201	IVHCDVKPH	IVHCDVKPH	0	0	0	0	0	IVHCDVKPH	tr_D0A9V0	0.040	32273.77	55.00
482	HLA-A0201	RDVVAESLEKEME	VVAESLEEM	2	0	0	7	1	VVAESLEKEM	tr_D0A9V0	0.040	32280.41	55.00
485	HLA-A0201	VAESLEKEM	VAESLEKEM	0	0	0	0	0	VAESLEKEM	tr_D0A9V0	0.040	32282.49	55.00
306	HLA-A0201	GFAAMRQGRYGEA	AMQGRYGEA	3	0	0	2	1	AMRQGRYGEA	tr_D0A9V0	0.040	32309.76	55.00
176	HLA-A0201	GAHAALPMH	GAHAALPMH	0	0	0	0	0	GAHAALPMH	tr_D0A9V0	0.040	32316.41	55.00
54	HLA-A0201	IPEAILARVESKN	IPEAILARV	0	0	0	0	0	IPEAILARV	tr_D0A9V0	0.040	32317.80	55.00
213	HLA-A0201	TNEFLVGTC	TNEFLVGTC	0	0	0	0	0	TNEFLVGTC	tr_D0A9V0	0.040	32325.85	55.00
185	HLA-A0201	CRRCCQFAFD	CRRCCQFAF	0	0	0	0	0	CRRCCQFAF	tr_D0A9V0	0.040	32335.63	55.00
567	HLA-A0201	DVKPHNVLLGT	DVHNVLLGT	0	0	0	2	2	DVKPHNVLLGT	tr_D0A9V0	0.040	32347.18	55.00
106	HLA-A0201	QQRDVALNCG	QQRDVALNC	0	0	0	0	0	QQRDVALNC	tr_D0A9V0	0.040	32348.58	55.00
227	HLA-A0201	NARVLFGADH	NAVLFGADH	0	0	0	2	1	NARVLFGADH	tr_D0A9V0	0.040	32361.18	55.00
348	HLA-A0201	TVPYVRVLDN	TVYVRVLDN	0	0	0	2	1	TVPYVRVLDN	tr_D0A9V0	0.040	32362.58	55.00
134	HLA-A0201	IQVYHPDVWKSS	IQVYVWKSS	0	0	0	4	3	IQVYHPDVWKSS	tr_D0A9V0	0.040	32367.84	55.00
593	HLA-A0201	ASVMARTLADG	SVMARTLAG	1	0	0	8	1	SVMARTLADG	tr_D0A9V0	0.040	32368.53	55.00
677	HLA-A0201	LGHLEPVIP	LGHLEPVIP	0	0	0	0	0	LGHLEPVIP	tr_D0A9V0	0.040	32376.25	55.00
368	HLA-A0201	PEEQDESDIKDRV	EQDESDIRV	2	0	0	7	2	EQDESDIKDRV	tr_D0A9V0	0.040	32377.99	55.00
684	HLA-A0201	IPASVDHEAA	IPASVDEAA	0	0	0	6	1	IPASVDHEAA	tr_D0A9V0	0.040	32379.41	55.00
423	HLA-A0201	GCAEGANAADVV	GCAEGANAV	0	0	0	8	3	GCAEGANAADVV	tr_D0A9V0	0.040	32379.74	55.00
696	HLA-A0201	VRACCHMNPSM	RACCHMNPM	1	0	0	8	1	RACCHMNPSM	tr_D0A9V0	0.040	32380.81	55.00
338	HLA-A0201	LCRQRCRRNDTVPY	LCRQRCVPY	0	0	0	6	5	LCRQRCRRNDTVPY	tr_D0A9V0	0.040	32397.62	55.00
19	HLA-A0201	DNADIFLGRS	NADIFLGRS	1	0	0	0	0	NADIFLGRS	tr_D0A9V0	0.040	32409.53	55.00

17	HLA-A0201	IPDNADIFLGRS	IADIFLGRS	0	0	0	1	3	IPDNADIFLGRS	tr_D0A9V0	0.040	32436.19	55.00
201	HLA-A0201	LDHRDSVTMLVDTN	LRDSVTMLV	0	0	0	1	2	LDHRDSVTMLV	tr_D0A9V0	0.040	32438.30	55.00
569	HLA-A0201	KPHNVLLGTD	KPHNVLLGT	0	0	0	0	0	KPHNVLLGT	tr_D0A9V0	0.040	32450.59	55.00
305	HLA-A0201	LGFAAMRQG	LGFAAMRQG	0	0	0	0	0	LGFAAMRQG	tr_D0A9V0	0.040	32451.30	55.00
480	HLA-A0201	LRRDVVAES	LRRDVVAES	0	0	0	0	0	LRRDVVAES	tr_D0A9V0	0.040	32479.40	55.00
422	HLA-A0201	SGCAEGANAADV	CAEGANAAV	2	0	0	8	1	CAEGANAADV	tr_D0A9V0	0.040	32486.44	55.00
174	HLA-A0201	SFGAHAALPMH	FGAHAALPM	1	0	0	0	0	FGAHAALPM	tr_D0A9V0	0.040	32487.48	55.00
641	HLA-A0201	LGRPPWQWSSTA	LGWQWSSTA	0	0	0	2	3	LGRPPWQWSSTA	tr_D0A9V0	0.040	32490.65	55.00
130	HLA-A0201	TVIAIQVYHPD	TVIAIVYHP	0	0	0	5	1	TVIAIQVYHP	tr_D0A9V0	0.040	32494.86	55.00
61	HLA-A0201	RVESKNEAGGHIRA	RVAGGHIRA	0	0	0	2	5	RVESKNEAGGHIRA	tr_D0A9V0	0.040	32497.33	55.00
469	HLA-A0201	GAQVAIKCIP	AQVAIKCIP	1	0	0	0	0	AQVAIKCIP	tr_D0A9V0	0.040	32498.02	55.00
145	HLA-A0201	STKYHAALIE	STKYHAALI	0	0	0	0	0	STKYHAALI	tr_D0A9V0	0.040	32500.50	55.00
178	HLA-A0201	HAALPMHCRRCCQ	ALPMHCCCQ	2	0	0	6	2	ALPMHCRRCCQ	tr_D0A9V0	0.040	32504.71	55.00
22	HLA-A0201	DIFLGRSRIVE	DIFLGSRIV	0	0	0	5	1	DIFLGRSRIV	tr_D0A9V0	0.040	32515.97	55.00
617	HLA-A0201	VAAGGRCTPQSDI	AAGGRCTPI	1	0	0	8	3	AAGGRCTPQSDI	tr_D0A9V0	0.040	32523.71	55.00
217	HLA-A0201	LVGTCGAFSRNAR	LVGTCGAFA	0	0	0	8	3	LVGTCGAFSRNA	tr_D0A9V0	0.040	32523.71	55.00
576	HLA-A0201	GTDGVCKLSDFGST	GTDGVCFGS	0	0	0	6	4	GTDGVCKLSDFGS	tr_D0A9V0	0.040	32546.58	55.00
400	HLA-A0201	SDQGSESLFKM	SGSESLFKM	0	0	0	1	2	SDQGSESLFKM	tr_D0A9V0	0.040	32549.41	55.00
16	HLA-A0201	EIPDNADIFLGRS	IADIFLGRS	1	0	0	1	3	IPDNADIFLGRS	tr_D0A9V0	0.040	32553.63	55.00
463	HLA-A0201	LGVAEDGAQVAIK	LVAEDGAQV	0	0	0	1	1	LGVAEDGAQV	tr_D0A9V0	0.040	32559.62	55.00
29	HLA-A0201	RIVEVRKIQGH	RIVEVIQGH	0	0	0	5	2	RIVEVRKIQGH	tr_D0A9V0	0.040	32564.19	55.00
658	HLA-A0201	ATRLRSLFNRD	ATRLSLFNR	0	0	0	4	1	ATRLRSLFNR	tr_D0A9V0	0.040	32572.30	55.00
54	HLA-A0201	IPEAILARVESKNE	IPEAILARV	0	0	0	0	0	IPEAILARV	tr_D0A9V0	0.040	32574.42	55.00
421	HLA-A0201	GSGCAEGANAADV	GCAEGANAV	2	0	0	8	2	GCAEGANAADV	tr_D0A9V0	0.040	32574.42	55.00
431	HLA-A0201	ADVVPLFLTDSN	ADVVPLFLT	0	0	0	0	0	ADVVPLFLT	tr_D0A9V0	0.040	32584.99	55.00
432	HLA-A0201	DVVPLFLTDSN	DVVPLFLTN	0	0	0	8	2	DVVPLFLTDSN	tr_D0A9V0	0.040	32587.81	55.00
115	HLA-A0201	GIFKEKPNVFN	GIFKEKPNV	0	0	0	0	0	GIFKEKPNV	tr_D0A9V0	0.040	32591.34	55.00
199	HLA-A0201	LPLDHRDSVTM	PLDHRSVTM	1	0	0	5	1	PLDHRDSVTM	tr_D0A9V0	0.040	32595.92	55.00
367	HLA-A0201	KPEEQDESDIKDRV	EQDESDIRV	3	0	0	7	2	EQDESDIKDRV	tr_D0A9V0	0.040	32601.57	55.00
328	HLA-A0201	MDMQAKRLM	MDMQAKRLM	0	0	0	0	0	MDMQAKRLM	tr_D0A9V0	0.039	32611.10	55.00
684	HLA-A0201	IPASVDHEAAAF	IVDHEAAAF	0	0	0	1	3	IPASVDHEAAAF	tr_D0A9V0	0.039	32613.20	55.00
248	HLA-A0201	PCRIAATSGGASHM	RIAATASHM	2	0	0	5	3	RIAATSGGASHM	tr_D0A9V0	0.039	32622.04	55.00
27	HLA-A0201	RSRIVEVRKIQG	RIVEVRIQG	2	0	0	6	1	RIVEVRKIQG	tr_D0A9V0	0.039	32635.44	55.00
245	HLA-A0201	RDGPCRIAAT	RDGPCIAAT	0	0	0	5	1	RDGPCRIAAT	tr_D0A9V0	0.039	32643.93	55.00
115	HLA-A0201	GIFKEKPNVFNRRH	GIFKEKPNV	0	0	0	0	0	GIFKEKPNV	tr_D0A9V0	0.039	32649.57	55.00
613	HLA-A0201	MAPEVAAGGRCTP	MAPEVAATP	0	0	0	7	4	MAPEVAAGGRCTP	tr_D0A9V0	0.039	32664.78	55.00
692	HLA-A0201	AAAFVRACCH	AAAFVACCH	0	0	0	5	1	AAAFVRACCH	tr_D0A9V0	0.039	32672.19	55.00
438	HLA-A0201	LTDSNRNVWTRSLK	LTDSWTRSL	0	0	0	4	4	LTDSNRNVWTRSL	tr_D0A9V0	0.039	32673.60	55.00
177	HLA-A0201	AHAALPMHCRRCCQ	ALPMHCCCQ	3	0	0	6	2	ALPMHCRRCCQ	tr_D0A9V0	0.039	32674.68	55.00
508	HLA-A0201	VSCSITPSHLS	VSCSITPSL	0	0	0	8	1	VSCSITPSHL	tr_D0A9V0	0.039	32674.68	55.00
5	HLA-A0201	LVSALKSAS	LVSALKSAS	0	0	0	0	0	LVSALKSAS	tr_D0A9V0	0.039	32683.87	55.00
473	HLA-A0201	AIKCIPRLRR	AIKCIPLRR	0	0	0	6	1	AIKCIPRLRR	tr_D0A9V0	0.039	32689.16	55.00
201	HLA-A0201	LDHRDSVTM	LDHRDSVTM	0	0	0	0	0	LDHRDSVTM	tr_D0A9V0	0.039	32730.23	55.00
621	HLA-A0201	GRCTPQSDIFSFG	GQSDIFSFG	0	0	0	1	4	GRCTPQSDIFSFG	tr_D0A9V0	0.039	32732.00	55.00
250	HLA-A0201	RIAATSGGAS	RIAATSGGA	0	0	0	0	0	RIAATSGGA	tr_D0A9V0	0.039	32752.88	55.00
113	HLA-A0201	NCGIFKEKPNVFN	GIFKEKPNV	2	0	0	0	0	GIFKEKPNV	tr_D0A9V0	0.039	32785.16	55.00
292	HLA-A0201	SSEVSLSISKQF	SSEVSLSIF	0	0	0	8	3	SSEVSLSISKQF	tr_D0A9V0	0.039	32785.16	55.00
25	HLA-A0201	LGRSRIVEVRKIQG	RIVEVRIQG	4	0	0	6	1	RIVEVRKIQG	tr_D0A9V0	0.039	32786.93	55.00
15	HLA-A0201	LEIPDNADIFLG	LEIADIFLG	0	0	0	3	3	LEIPDNADIFLG	tr_D0A9V0	0.039	32788.35	55.00
27	HLA-A0201	RSRIVEVRKIQGH	RIVEVIQGH	2	0	0	5	2	RIVEVRKIQGH	tr_D0A9V0	0.039	32795.79	55.00
289	HLA-A0201	GESSSEVSLSISKQ	SSSEVSLSI	2	0	0	0	0	SSSEVSLSI	tr_D0A9V0	0.039	32805.38	55.00
533	HLA-A0201	IKNFGCVSP	IKNFGCVSP	0	0	0	0	0	IKNFGCVSP	tr_D0A9V0	0.039	32810.35	55.00
499	HLA-A0201	LHHPNIVRYVSCS	LHHPNIVYV	0	0	0	7	1	LHHPNIVRYV	tr_D0A9V0	0.039	32812.48	55.00
562	HLA-A0201	GIVHCDVKPH	GIVHCDVPH	0	0	0	7	1	GIVHCDVKPH	tr_D0A9V0	0.039	32820.65	55.00

493	HLA-A0201	MEVASKLHHPNIVR	MLHHPNIVR	0	0	0	1	5	MEVASKLHHPNIVR	tr_D0A9V0	0.039	32822.43	55.00
314	HLA-A0201	RYGEAMAHYLP	RYGEAMAYL	0	0	0	7	1	RYGEAMAHYL	tr_D0A9V0	0.039	32856.19	55.00
656	HLA-A0201	SDATRLRSLFNRD	SLRSLFNRD	0	0	0	1	4	SDATRLRSLFNRD	tr_D0A9V0	0.039	32861.15	55.00
293	HLA-A0201	SEVSLSISK	SEVSLSISK	0	0	0	0	0	SEVSLSISK	tr_D0A9V0	0.039	32892.46	55.00
337	HLA-A0201	RLCRQRCRRN	RLCQRCRRN	0	0	0	3	1	RLCRQRCRRN	tr_D0A9V0	0.039	32901.36	55.00
601	HLA-A0201	ADGVMLRGTA	ADGVMLRTA	0	0	0	7	1	ADGVMLRGTA	tr_D0A9V0	0.039	32910.63	55.00
593	HLA-A0201	ASVMARTLAD	AVMARTLAD	0	0	0	1	1	ASVMARTLAD	tr_D0A9V0	0.039	32913.11	55.00
313	HLA-A0201	GRYGEAMAHY	GRYEAMAHY	0	0	0	3	1	GRYGEAMAHY	tr_D0A9V0	0.039	32923.79	55.00
195	HLA-A0201	LFAKLPLDH	LFAKLPLDH	0	0	0	0	0	LFAKLPLDH	tr_D0A9V0	0.039	32929.50	55.00
136	HLA-A0201	VYHPDVWKS	VYHPDVWKS	0	0	0	0	0	VYHPDVWKS	tr_D0A9V0	0.039	32929.84	55.00
313	HLA-A0201	GRYGEAMAHYLP	RYGEAMAYL	1	0	0	7	1	RYGEAMAHYL	tr_D0A9V0	0.039	32930.93	55.00
444	HLA-A0201	NVWTRSLKKISE	NVWTRSLKI	0	0	0	7	1	NVWTRSLKKI	tr_D0A9V0	0.039	32936.98	55.00
285	HLA-A0201	ELRPGESSSEVSLS	ELGESSSEV	0	0	0	2	2	ELRPGESSSEV	tr_D0A9V0	0.039	32939.12	55.00
21	HLA-A0201	ADIFLGRSRIVE	FLGRSRIVE	3	0	0	0	0	FLGRSRIVE	tr_D0A9V0	0.039	32948.75	55.00
80	HLA-A0201	ETPGESSTAFV	ETPGSTAFV	0	0	0	4	2	ETPGESSTAFV	tr_D0A9V0	0.039	32950.17	55.00
114	HLA-A0201	CGIFKEKPNVFN	GIFKEKPNV	1	0	0	0	0	GIFKEKPNV	tr_D0A9V0	0.038	32971.21	55.00
143	HLA-A0201	KSSTKYHAALI	KSSYHAALI	0	0	0	3	2	KSSTKYHAALI	tr_D0A9V0	0.038	32982.27	55.00
125	HLA-A0201	NRRHCTVIA	NRRHCTVIA	0	0	0	0	0	NRRHCTVIA	tr_D0A9V0	0.038	32995.46	55.00
116	HLA-A0201	IFKEKPNVF	IFKEKPNVF	0	0	0	0	0	IFKEKPNVF	tr_D0A9V0	0.038	32995.82	55.00
10	HLA-A0201	KSASNLEIP	KSASNLEIP	0	0	0	0	0	KSASNLEIP	tr_D0A9V0	0.038	33001.19	55.00
478	HLA-A0201	PRLRRDVVAE	RLRRDVVAE	1	0	0	0	0	RLRRDVVAE	tr_D0A9V0	0.038	33008.32	55.00
654	HLA-A0201	AGSDATRLRSL	GSDATLRSL	1	0	0	5	1	GSDATRLRSL	tr_D0A9V0	0.038	33021.54	55.00
613	HLA-A0201	MAPEVAAGGRCTPQ	MAPEVCTPQ	0	0	0	5	5	MAPEVAAGGRCTPQ	tr_D0A9V0	0.038	33035.83	55.00
372	HLA-A0201	DESDIKDRVPETI	ESDIVPETI	1	0	0	4	3	ESDIKDRVPETI	tr_D0A9V0	0.038	33037.62	55.00
142	HLA-A0201	WKSSTKYHAALI	WTKYHAALI	0	0	0	1	3	WKSSTKYHAALI	tr_D0A9V0	0.038	33039.78	55.00
16	HLA-A0201	EIPDNADIFLG	EIDNADIFL	0	0	0	2	1	EIPDNADIFL	tr_D0A9V0	0.038	33061.94	55.00
26	HLA-A0201	GRSRIVEVRKIQG	RIVEVRIQG	3	0	0	6	1	RIVEVRKIQG	tr_D0A9V0	0.038	33067.32	55.00
212	HLA-A0201	DTNEFLVGTCG	DTNEFLVTC	0	0	0	7	1	DTNEFLVGTC	tr_D0A9V0	0.038	33067.66	55.00
505	HLA-A0201	VRYVSCSITPSH	YVSCSITPS	2	0	0	0	0	YVSCSITPS	tr_D0A9V0	0.038	33068.75	55.00
655	HLA-A0201	GSDATRLRSLFNRD	RLRSLFNRD	5	0	0	0	0	RLRSLFNRD	tr_D0A9V0	0.038	33076.62	55.00
657	HLA-A0201	DATRLRSLFNRD	RLRSLFNRD	3	0	0	0	0	RLRSLFNRD	tr_D0A9V0	0.038	33086.98	60.00
408	HLA-A0201	FKMYAPSSS	FKMYAPSSS	0	0	0	0	0	FKMYAPSSS	tr_D0A9V0	0.038	33087.35	60.00
414	HLA-A0201	SSSSSLDGS	SSSSSLDGS	0	0	0	0	0	SSSSSLDGS	tr_D0A9V0	0.038	33088.07	60.00
583	HLA-A0201	LSDFGSTIS	LSDFGSTIS	0	0	0	0	0	LSDFGSTIS	tr_D0A9V0	0.038	33089.14	60.00
521	HLA-A0201	MEYVPGGSLHS	YVPGGSLHS	2	0	0	0	0	YVPGGSLHS	tr_D0A9V0	0.038	33110.64	60.00
606	HLA-A0201	LRGTALYMAP	LRGTALYMA	0	0	0	0	0	LRGTALYMA	tr_D0A9V0	0.038	33114.20	60.00
496	HLA-A0201	ASKLHHPNI	ASKLHHPNI	0	0	0	0	0	ASKLHHPNI	tr_D0A9V0	0.038	33115.65	60.00
243	HLA-A0201	AVRDGPCRIAATS	AVCRIAATS	0	0	0	2	4	AVRDGPCRIAATS	tr_D0A9V0	0.038	33120.65	60.00
699	HLA-A0201	CCHMNPSMR	CCHMNPSMR	0	0	0	0	0	CCHMNPSMR	tr_D0A9V0	0.038	33126.04	60.00
29	HLA-A0201	RIVEVRKIQGHY	RIVEVQGHY	0	0	0	5	3	RIVEVRKIQGHY	tr_D0A9V0	0.038	33127.10	60.00
435	HLA-A0201	PLFLTDSNRN	PLFLTDSNR	0	0	0	0	0	PLFLTDSNR	tr_D0A9V0	0.038	33130.35	60.00
655	HLA-A0201	GSDATRLRSLF	GSDATRLLF	0	0	0	7	2	GSDATRLRSLF	tr_D0A9V0	0.038	33133.55	60.00
284	HLA-A0201	FELRPGESSSEVS	ELGESSSEV	1	0	0	2	2	ELRPGESSSEV	tr_D0A9V0	0.038	33146.83	60.00
616	HLA-A0201	EVAAGGRCTPQSDI	AAGGRCTPI	2	0	0	8	3	AAGGRCTPQSDI	tr_D0A9V0	0.038	33157.60	60.00
420	HLA-A0201	DGSGCAEGANAADV	CAEGANAAV	4	0	0	8	1	CAEGANAADV	tr_D0A9V0	0.038	33161.90	60.00
196	HLA-A0201	FAKLPLDHRD	FAKLPLDHR	0	0	0	0	0	FAKLPLDHR	tr_D0A9V0	0.038	33167.63	60.00
26	HLA-A0201	GRSRIVEVRKIQGH	RIVEVIQGH	3	0	0	5	2	RIVEVRKIQGH	tr_D0A9V0	0.038	33178.42	60.00
592	HLA-A0201	EASVMARTLADG	SVMATLADG	2	0	0	4	1	SVMARTLADG	tr_D0A9V0	0.038	33178.42	60.00
401	HLA-A0201	DQGSESLFKMY	QGSESLFMY	1	0	0	7	1	QGSESLFKMY	tr_D0A9V0	0.038	33188.45	60.00
96	HLA-A0201	SISVTWNAGD	SISVTWNAG	0	0	0	0	0	SISVTWNAG	tr_D0A9V0	0.038	33194.21	60.00
195	HLA-A0201	LFAKLPLDHR	LFAKLPLHR	0	0	0	7	1	LFAKLPLDHR	tr_D0A9V0	0.038	33195.28	60.00
444	HLA-A0201	NVWTRSLKKISEG	NVWTRSLKI	0	0	0	7	1	NVWTRSLKKI	tr_D0A9V0	0.038	33201.04	60.00
618	HLA-A0201	AAGGRCTPQSDIFS	AAGGRCIFS	0	0	0	6	5	AAGGRCTPQSDIFS	tr_D0A9V0	0.038	33207.51	60.00
38	HLA-A0201	GHYAKLRRQL	GHYAKLRQL	0	0	0	6	1	GHYAKLRRQL	tr_D0A9V0	0.038	33208.94	60.00

443	HLA-A0201	RNVWTRSLKK	RVWTRSLKK	0	0	0	1	1	RNVWTRSLKK	tr_D0A9V0	0.038	33210.38	60.00
506	HLA-A0201	RYVSCSITPSH	YVSCSITPS	1	0	0	0	0	YVSCSITPS	tr_D0A9V0	0.038	33213.98	60.00
606	HLA-A0201	LRGTALYMAPE	LTALYMAPE	0	0	0	1	2	LRGTALYMAPE	tr_D0A9V0	0.038	33216.85	60.00
184	HLA-A0201	HCRRCCQFAFD	HCRRCCFAF	0	0	0	6	1	HCRRCCQFAF	tr_D0A9V0	0.038	33257.12	60.00
13	HLA-A0201	SNLEIPDNAD	SLEIPDNAD	0	0	0	1	1	SNLEIPDNAD	tr_D0A9V0	0.038	33257.83	60.00
335	HLA-A0201	LMRLCRQRCRRN	LMRLCCRRN	0	0	0	5	3	LMRLCRQRCRRN	tr_D0A9V0	0.038	33263.96	60.00
293	HLA-A0201	SEVSLSISKQF	SEVSLSISF	0	0	0	8	2	SEVSLSISKQF	tr_D0A9V0	0.038	33264.67	60.00
422	HLA-A0201	SGCAEGANAADVV	CAEGANAAV	2	0	0	8	2	CAEGANAADVV	tr_D0A9V0	0.038	33270.79	60.00
16	HLA-A0201	EIPDNADIFLGR	EIDNADIFL	0	0	0	2	1	EIPDNADIFL	tr_D0A9V0	0.038	33275.83	60.00
225	HLA-A0201	SRNARVLFGADH	SRNAVLFGA	0	0	0	4	1	SRNARVLFGA	tr_D0A9V0	0.038	33276.93	60.00
500	HLA-A0201	HHPNIVRYVS	HHPNIVRYV	0	0	0	0	0	HHPNIVRYV	tr_D0A9V0	0.037	33327.72	60.00
476	HLA-A0201	CIPRLRRDVVAE	CIPRLVVAE	0	0	0	5	3	CIPRLRRDVVAE	tr_D0A9V0	0.037	33352.98	60.00
231	HLA-A0201	LFGADHLFQ	LFGADHLFQ	0	0	0	0	0	LFGADHLFQ	tr_D0A9V0	0.037	33358.02	60.00
226	HLA-A0201	RNARVLFGADH	RNARVLFGA	0	0	0	0	0	RNARVLFGA	tr_D0A9V0	0.037	33366.32	60.00
345	HLA-A0201	RNDTVPYVRVLDNH	VPYVVLDNH	4	0	0	4	1	VPYVRVLDNH	tr_D0A9V0	0.037	33384.01	60.00
312	HLA-A0201	QGRYGEAMAHYLP	RYGEAMAYL	2	0	0	7	1	RYGEAMAHYL	tr_D0A9V0	0.037	33394.13	60.00
166	HLA-A0201	LDPDRALISFGAH	LRALISFGA	0	0	0	1	3	LDPDRALISFGA	tr_D0A9V0	0.037	33397.02	60.00
627	HLA-A0201	SDIFSFGIS	SDIFSFGIS	0	0	0	0	0	SDIFSFGIS	tr_D0A9V0	0.037	33410.40	60.00
27	HLA-A0201	RSRIVEVRKIQ	RSRIVEVRI	0	0	0	8	1	RSRIVEVRKI	tr_D0A9V0	0.037	33420.53	60.00
112	HLA-A0201	LNCGIFKEK	LNCGIFKEK	0	0	0	0	0	LNCGIFKEK	tr_D0A9V0	0.037	33424.15	60.00
371	HLA-A0201	QDESDIKDRVPETI	ESDIVPETI	2	0	0	4	3	ESDIKDRVPETI	tr_D0A9V0	0.037	33430.29	60.00
159	HLA-A0201	HSGCVEALDP	HSGCVALDP	0	0	0	5	1	HSGCVEALDP	tr_D0A9V0	0.037	33437.17	60.00
168	HLA-A0201	PDRALISFGA	DRALISFGA	1	0	0	0	0	DRALISFGA	tr_D0A9V0	0.037	33441.50	60.00
344	HLA-A0201	RRNDTVPYVRVLDN	RTVPYVRVL	0	0	0	1	3	RRNDTVPYVRVL	tr_D0A9V0	0.037	33445.84	60.00
29	HLA-A0201	RIVEVRKIQ	RIVEVRKIQ	0	0	0	0	0	RIVEVRKIQ	tr_D0A9V0	0.037	33464.31	60.00
307	HLA-A0201	FAAMRQGRY	FAAMRQGRY	0	0	0	0	0	FAAMRQGRY	tr_D0A9V0	0.037	33466.49	60.00
15	HLA-A0201	LEIPDNADIF	LIPDNADIF	0	0	0	1	1	LEIPDNADIF	tr_D0A9V0	0.037	33468.28	60.00
607	HLA-A0201	RGTALYMAP	RGTALYMAP	0	0	0	0	0	RGTALYMAP	tr_D0A9V0	0.037	33482.79	60.00
379	HLA-A0201	RVPETIDSPR	RVPETISPR	0	0	0	6	1	RVPETIDSPR	tr_D0A9V0	0.037	33492.20	60.00
583	HLA-A0201	LSDFGSTISE	LSFGSTISE	0	0	0	2	1	LSDFGSTISE	tr_D0A9V0	0.037	33499.80	60.00
400	HLA-A0201	SDQGSESLFKMY	QGSESLFMY	2	0	0	7	1	QGSESLFKMY	tr_D0A9V0	0.037	33500.89	60.00
203	HLA-A0201	HRDSVTMLVDT	HRDSVTMLV	0	0	0	0	0	HRDSVTMLV	tr_D0A9V0	0.037	33508.52	60.00
564	HLA-A0201	VHCDVKPHNVLLG	VHCDVVLLG	0	0	0	5	4	VHCDVKPHNVLLG	tr_D0A9V0	0.037	33512.86	60.00
537	HLA-A0201	GCVSPYVARR	CVSPYVARR	1	0	0	0	0	CVSPYVARR	tr_D0A9V0	0.037	33525.91	60.00
657	HLA-A0201	DATRLRSLFNR	ATRLSLFNR	1	0	0	4	1	ATRLRSLFNR	tr_D0A9V0	0.037	33550.96	60.00
480	HLA-A0201	LRRDVVAESLEK	LVVAESLEK	0	0	0	1	3	LRRDVVAESLEK	tr_D0A9V0	0.037	33579.27	60.00
576	HLA-A0201	GTDGVCKLSDFG	GTDGVCKLF	0	0	0	8	2	GTDGVCKLSDF	tr_D0A9V0	0.037	33582.91	60.00
36	HLA-A0201	IQGHYAKLR	IQGHYAKLR	0	0	0	0	0	IQGHYAKLR	tr_D0A9V0	0.037	33612.36	60.00
644	HLA-A0201	PPWQWSSTA	PPWQWSSTA	0	0	0	0	0	PPWQWSSTA	tr_D0A9V0	0.037	33628.36	60.00
468	HLA-A0201	DGAQVAIKC	DGAQVAIKC	0	0	0	0	0	DGAQVAIKC	tr_D0A9V0	0.037	33636.02	60.00
655	HLA-A0201	GSDATRLRSLFNR	ATRLSLFNR	3	0	0	4	1	ATRLRSLFNR	tr_D0A9V0	0.037	33642.57	60.00
480	HLA-A0201	LRRDVVAESLE	LRDVVAESL	0	0	0	1	1	LRRDVVAESL	tr_D0A9V0	0.037	33643.65	60.00
345	HLA-A0201	RNDTVPYVRVLDN	RTVPYVRVL	0	0	0	1	2	RNDTVPYVRVL	tr_D0A9V0	0.037	33645.11	60.00
225	HLA-A0201	SRNARVLFGAD	SRNAVLFGA	0	0	0	4	1	SRNARVLFGA	tr_D0A9V0	0.037	33653.11	60.00
141	HLA-A0201	VWKSSTKYHAALI	WTKYHAALI	1	0	0	1	3	WKSSTKYHAALI	tr_D0A9V0	0.037	33660.41	60.00
344	HLA-A0201	RRNDTVPYVRVLD	RTVPYVRVL	0	0	0	1	3	RRNDTVPYVRVL	tr_D0A9V0	0.037	33662.23	60.00
240	HLA-A0201	LSYAVRDGP	LSYAVRDGP	0	0	0	0	0	LSYAVRDGP	tr_D0A9V0	0.037	33683.71	60.00
335	HLA-A0201	LMRLCRQRCRRNDT	LMRLCRQRT	0	0	0	8	5	LMRLCRQRCRRNDT	tr_D0A9V0	0.036	33700.11	60.00
134	HLA-A0201	IQVYHPDVWKSST	QVYHPDVWT	1	0	0	8	3	QVYHPDVWKSST	tr_D0A9V0	0.036	33701.22	60.00
123	HLA-A0201	VFNRRHCTVIA	VFNHCTVIA	0	0	0	3	2	VFNRRHCTVIA	tr_D0A9V0	0.036	33712.52	60.00
239	HLA-A0201	QLSYAVRDGPCR	QLSYAVRPC	0	0	0	7	2	QLSYAVRDGPC	tr_D0A9V0	0.036	33713.24	60.00
292	HLA-A0201	SSEVSLSISKQFR	SSEVSLSIF	0	0	0	8	3	SSEVSLSISKQF	tr_D0A9V0	0.036	33715.44	60.00
591	HLA-A0201	SEASVMARTLAD	SEASVMATL	0	0	0	7	1	SEASVMARTL	tr_D0A9V0	0.036	33717.27	60.00
345	HLA-A0201	RNDTVPYVRVLD	RTVPYVRVL	0	0	0	1	2	RNDTVPYVRVL	tr_D0A9V0	0.036	33717.27	60.00

28	HLA-A0201	SRIVEVRKIQGH	RIVEVIQGH	1	0	0	5	2	RIVEVRKIQGH	tr_D0A9V0	0.036	33722.75	60.00
203	HLA-A0201	HRDSVTMLVDTNE	HRDSVTMLV	0	0	0	0	0	HRDSVTMLV	tr_D0A9V0	0.036	33726.03	60.00
139	HLA-A0201	PDVWKSSTKYHAAL	DVWKYHAAL	1	0	0	3	4	DVWKSSTKYHAAL	tr_D0A9V0	0.036	33736.60	60.00
103	HLA-A0201	AGDQQRDVALNC	AQRDVALNC	0	0	0	1	3	AGDQQRDVALNC	tr_D0A9V0	0.036	33740.98	60.00
286	HLA-A0201	LRPGESSSEV	LPGESSSEV	0	0	0	1	1	LRPGESSSEV	tr_D0A9V0	0.036	33745.37	60.00
673	HLA-A0201	QSLALGHLE	QSLALGHLE	0	0	0	0	0	QSLALGHLE	tr_D0A9V0	0.036	33747.55	60.00
5	HLA-A0201	LVSALKSASNLE	LVSALASNL	0	0	0	5	2	LVSALKSASNL	tr_D0A9V0	0.036	33752.66	60.00
99	HLA-A0201	VTWNAGDQQRDVA	VTWNAGDQV	0	0	0	8	3	VTWNAGDQQRDV	tr_D0A9V0	0.036	33758.52	60.00
489	HLA-A0201	LEKEMEVASKL	LEMEVASKL	0	0	0	1	2	LEKEMEVASKL	tr_D0A9V0	0.036	33765.09	60.00
618	HLA-A0201	AAGGRCTPQ	AAGGRCTPQ	0	0	0	0	0	AAGGRCTPQ	tr_D0A9V0	0.036	33768.73	60.00
16	HLA-A0201	EIPDNADIFLGRSR	EIDNADIFL	0	0	0	2	1	EIPDNADIFL	tr_D0A9V0	0.036	33770.93	60.00
374	HLA-A0201	SDIKDRVPETI	SDIKVPETI	0	0	0	4	2	SDIKDRVPETI	tr_D0A9V0	0.036	33773.13	60.00
324	HLA-A0201	PMVKMDMQAK	PMVKMDMQA	0	0	0	0	0	PMVKMDMQA	tr_D0A9V0	0.036	33783.71	60.00
501	HLA-A0201	HPNIVRYVS	HPNIVRYVS	0	0	0	0	0	HPNIVRYVS	tr_D0A9V0	0.036	33787.75	60.00
683	HLA-A0201	VIPASVDHE	VIPASVDHE	0	0	0	0	0	VIPASVDHE	tr_D0A9V0	0.036	33793.24	60.00
656	HLA-A0201	SDATRLRSLFNR	ATRLSLFNR	2	0	0	4	1	ATRLRSLFNR	tr_D0A9V0	0.036	33795.05	60.00
510	HLA-A0201	CSITPSHLS	CSITPSHLS	0	0	0	0	0	CSITPSHLS	tr_D0A9V0	0.036	33806.04	60.00
622	HLA-A0201	RCTPQSDIFS	RTPQSDIFS	0	0	0	1	1	RCTPQSDIFS	tr_D0A9V0	0.036	33818.09	60.00
421	HLA-A0201	GSGCAEGANAADVV	CAEGANAAV	3	0	0	8	2	CAEGANAADVV	tr_D0A9V0	0.036	33818.46	60.00
465	HLA-A0201	VAEDGAQVAIKCI	VAQVAIKCI	0	0	0	1	4	VAEDGAQVAIKCI	tr_D0A9V0	0.036	33823.96	60.00
688	HLA-A0201	VDHEAAAFVRAC	VAAAFVRAC	0	0	0	1	3	VDHEAAAFVRAC	tr_D0A9V0	0.036	33836.39	60.00
205	HLA-A0201	DSVTMLVDTN	SVTMLVDTN	1	0	0	0	0	SVTMLVDTN	tr_D0A9V0	0.036	33836.77	60.00
223	HLA-A0201	AFSRNARVL	AFSRNARVL	0	0	0	0	0	AFSRNARVL	tr_D0A9V0	0.036	33838.97	60.00
283	HLA-A0201	LFELRPGESSSEVS	ELGESSSEV	2	0	0	2	2	ELRPGESSSEV	tr_D0A9V0	0.036	33845.19	60.00
202	HLA-A0201	DHRDSVTMLVD	HRDSVTMLV	1	0	0	0	0	HRDSVTMLV	tr_D0A9V0	0.036	33846.28	60.00
169	HLA-A0201	DRALISFGAH	RALISFGAH	1	0	0	0	0	RALISFGAH	tr_D0A9V0	0.036	33852.16	60.00
697	HLA-A0201	RACCHMNPSMRPS	RMNPSMRPS	0	0	0	1	4	RACCHMNPSMRPS	tr_D0A9V0	0.036	33856.54	60.00
539	HLA-A0201	VSPYVARRFT	VSPYVARFT	0	0	0	6	1	VSPYVARRFT	tr_D0A9V0	0.036	33861.30	60.00
340	HLA-A0201	RQRCRRNDTVPY	RQRCRTVPY	0	0	0	5	3	RQRCRRNDTVPY	tr_D0A9V0	0.036	33872.66	60.00
443	HLA-A0201	RNVWTRSLKKISE	NVWTRSLKI	1	0	0	7	1	NVWTRSLKKI	tr_D0A9V0	0.036	33881.09	60.00
17	HLA-A0201	IPDNADIFLGRSR	IPDNADIFL	0	0	0	0	0	IPDNADIFL	tr_D0A9V0	0.036	33887.70	60.00
311	HLA-A0201	RQGRYGEAMAHY	RQGEAMAHY	0	0	0	2	3	RQGRYGEAMAHY	tr_D0A9V0	0.036	33906.04	60.00
244	HLA-A0201	VRDGPCRIA	VRDGPCRIA	0	0	0	0	0	VRDGPCRIA	tr_D0A9V0	0.036	33917.41	60.00
654	HLA-A0201	AGSDATRLRSLFNR	ATRLSLFNR	4	0	0	4	1	ATRLRSLFNR	tr_D0A9V0	0.036	33920.71	60.00
25	HLA-A0201	LGRSRIVEVRKIQ	LGRSRIVEV	0	0	0	0	0	LGRSRIVEV	tr_D0A9V0	0.036	33933.55	60.00
601	HLA-A0201	ADGVMLRGT	ADGVMLRGT	0	0	0	0	0	ADGVMLRGT	tr_D0A9V0	0.036	33939.44	60.00
442	HLA-A0201	NRNVWTRSLKKISE	NVWTRSLKI	2	0	0	7	1	NVWTRSLKKI	tr_D0A9V0	0.036	33955.97	60.00
378	HLA-A0201	DRVPETIDSP	RVPETIDSP	1	0	0	0	0	RVPETIDSP	tr_D0A9V0	0.036	33967.36	60.00
698	HLA-A0201	ACCHMNPSMRPS	AMNPSMRPS	0	0	0	1	3	ACCHMNPSMRPS	tr_D0A9V0	0.036	33983.16	60.00
685	HLA-A0201	PASVDHEAAA	ASVDHEAAA	1	0	0	0	0	ASVDHEAAA	tr_D0A9V0	0.036	33991.25	60.00
196	HLA-A0201	FAKLPLDHRDS	FLPLDHRDS	0	0	0	1	2	FAKLPLDHRDS	tr_D0A9V0	0.036	33993.09	60.00
25	HLA-A0201	LGRSRIVEVRK	LGRSRIVEV	0	0	0	0	0	LGRSRIVEV	tr_D0A9V0	0.036	34009.27	60.00
202	HLA-A0201	DHRDSVTMLVDT	HRDSVTMLV	1	0	0	0	0	HRDSVTMLV	tr_D0A9V0	0.036	34031.36	60.00
618	HLA-A0201	AAGGRCTPQSDIF	AAGGRCTPF	0	0	0	8	4	AAGGRCTPQSDIF	tr_D0A9V0	0.035	34053.47	60.00
28	HLA-A0201	SRIVEVRKIQGHY	RIVEVQGHY	1	0	0	5	3	RIVEVRKIQGHY	tr_D0A9V0	0.035	34056.04	60.00
585	HLA-A0201	DFGSTISEA	DFGSTISEA	0	0	0	0	0	DFGSTISEA	tr_D0A9V0	0.035	34057.89	60.00
175	HLA-A0201	FGAHAALPMHCR	FGAHAALPM	0	0	0	0	0	FGAHAALPM	tr_D0A9V0	0.035	34058.61	60.00
14	HLA-A0201	NLEIPDNAD	NLEIPDNAD	0	0	0	0	0	NLEIPDNAD	tr_D0A9V0	0.035	34061.20	60.00
575	HLA-A0201	LGTDGVCKLSDFG	GTDGVCKLF	1	0	0	8	2	GTDGVCKLSDF	tr_D0A9V0	0.035	34068.21	60.00
434	HLA-A0201	VPLFLTDSNRN	VLFLTDSNR	0	0	0	1	1	VPLFLTDSNR	tr_D0A9V0	0.035	34068.93	60.00
652	HLA-A0201	APAGSDATRLRSL	GSDATLRSL	3	0	0	5	1	GSDATRLRSL	tr_D0A9V0	0.035	34085.15	60.00
213	HLA-A0201	TNEFLVGTCG	NEFLVGTCG	1	0	0	0	0	NEFLVGTCG	tr_D0A9V0	0.035	34086.27	60.00
26	HLA-A0201	GRSRIVEVRK	RSRIVEVRK	1	0	0	0	0	RSRIVEVRK	tr_D0A9V0	0.035	34094.39	60.00
477	HLA-A0201	IPRLRRDVVAE	ILRRDVVAE	0	0	0	1	2	IPRLRRDVVAE	tr_D0A9V0	0.035	34095.86	60.00

81	HLA-A0201	TPGESSTAFVRI	TSSTAFVRI	0	0	0	1	3	TPGESSTAFVRI	tr_D0A9V0	0.035	34106.20	60.00
443	HLA-A0201	RNVWTRSLKKISEG	NVWTRSLKI	1	0	0	7	1	NVWTRSLKKI	tr_D0A9V0	0.035	34108.39	60.00
174	HLA-A0201	SFGAHAALP	SFGAHAALP	0	0	0	0	0	SFGAHAALP	tr_D0A9V0	0.035	34108.77	60.00
203	HLA-A0201	HRDSVTMLVDTN	HRDSVTMLV	0	0	0	0	0	HRDSVTMLV	tr_D0A9V0	0.035	34113.21	60.00
346	HLA-A0201	NDTVPYVRVLDNH	NTVPYVRVL	0	0	0	1	1	NDTVPYVRVL	tr_D0A9V0	0.035	34116.16	60.00
653	HLA-A0201	PAGSDATRLRSL	GSDATLRSL	2	0	0	5	1	GSDATRLRSL	tr_D0A9V0	0.035	34116.52	60.00
558	HLA-A0201	LHNLGIVHCD	LHNLGIVHC	0	0	0	0	0	LHNLGIVHC	tr_D0A9V0	0.035	34136.45	60.00
332	HLA-A0201	AKRLMRLCRQ	AKRLMLCRQ	0	0	0	5	1	AKRLMRLCRQ	tr_D0A9V0	0.035	34137.20	60.00
696	HLA-A0201	VRACCHMNPSMRPS	VMNPSMRPS	0	0	0	1	5	VRACCHMNPSMRPS	tr_D0A9V0	0.035	34142.75	60.00
566	HLA-A0201	CDVKPHNVLLGTD	CDVKVLLGT	0	0	0	4	3	CDVKPHNVLLGT	tr_D0A9V0	0.035	34142.75	60.00
466	HLA-A0201	AEDGAQVAIKCI	AAQVAIKCI	0	0	0	1	3	AEDGAQVAIKCI	tr_D0A9V0	0.035	34146.07	60.00
492	HLA-A0201	EMEVASKLHHPNI	EMEVASKLI	0	0	0	8	4	EMEVASKLHHPNI	tr_D0A9V0	0.035	34163.81	60.00
651	HLA-A0201	TAPAGSDATRLRSL	GSDATLRSL	4	0	0	5	1	GSDATRLRSL	tr_D0A9V0	0.035	34170.84	60.00
227	HLA-A0201	NARVLFGAD	NARVLFGAD	0	0	0	0	0	NARVLFGAD	tr_D0A9V0	0.035	34173.04	60.00
422	HLA-A0201	SGCAEGANA	SGCAEGANA	0	0	0	0	0	SGCAEGANA	tr_D0A9V0	0.035	34180.07	60.00
407	HLA-A0201	LFKMYAPSS	LFKMYAPSS	0	0	0	0	0	LFKMYAPSS	tr_D0A9V0	0.035	34180.82	60.00
707	HLA-A0201	RPSASSLLS	RPSASSLLS	0	0	0	0	0	RPSASSLLS	tr_D0A9V0	0.035	34184.52	60.00
578	HLA-A0201	DGVCKLSDF	DGVCKLSDF	0	0	0	0	0	DGVCKLSDF	tr_D0A9V0	0.035	34198.20	60.00
343	HLA-A0201	CRRNDTVPY	CRRNDTVPY	0	0	0	0	0	CRRNDTVPY	tr_D0A9V0	0.035	34201.90	60.00
32	HLA-A0201	EVRKIQGHYA	EVRKIGHYA	0	0	0	5	1	EVRKIQGHYA	tr_D0A9V0	0.035	34223.37	60.00
343	HLA-A0201	CRRNDTVPYVR	RRNDTVPYV	1	0	0	0	0	RRNDTVPYV	tr_D0A9V0	0.035	34226.33	60.00
293	HLA-A0201	SEVSLSISKQFR	SLSISKQFR	0	0	0	1	3	SEVSLSISKQFR	tr_D0A9V0	0.035	34237.08	60.00
339	HLA-A0201	CRQRCRRNDTVPY	RQRCRTVPY	1	0	0	5	3	RQRCRRNDTVPY	tr_D0A9V0	0.035	34244.12	60.00
650	HLA-A0201	STAPAGSDATRL	STAPAATRL	0	0	0	5	3	STAPAGSDATRL	tr_D0A9V0	0.035	34258.95	60.00
565	HLA-A0201	HCDVKPHNVLLG	HCDVNVLLG	0	0	0	4	3	HCDVKPHNVLLG	tr_D0A9V0	0.035	34266.35	60.00
80	HLA-A0201	ETPGESSTAFVRI	GSSTAFVRI	3	0	0	1	1	GESSTAFVRI	tr_D0A9V0	0.035	34281.18	60.00
142	HLA-A0201	WKSSTKYHAA	WSSTKYHAA	0	0	0	1	1	WKSSTKYHAA	tr_D0A9V0	0.035	34288.98	60.00
335	HLA-A0201	LMRLCRQRCRR	LMRLCRCRR	0	0	0	5	2	LMRLCRQRCRR	tr_D0A9V0	0.035	34291.20	60.00
17	HLA-A0201	IPDNADIFLGR	IPDNADIFL	0	0	0	0	0	IPDNADIFL	tr_D0A9V0	0.035	34299.00	60.00
329	HLA-A0201	DMQAKRLMR	DMQAKRLMR	0	0	0	0	0	DMQAKRLMR	tr_D0A9V0	0.035	34311.61	60.00
580	HLA-A0201	VCKLSDFGS	VCKLSDFGS	0	0	0	0	0	VCKLSDFGS	tr_D0A9V0	0.035	34316.06	60.00
202	HLA-A0201	DHRDSVTMLVDTNE	HRDSVTMLV	1	0	0	0	0	HRDSVTMLV	tr_D0A9V0	0.035	34322.74	60.00
654	HLA-A0201	AGSDATRLRSLF	GSDATRLLF	1	0	0	7	2	GSDATRLRSLF	tr_D0A9V0	0.035	34324.25	60.00
79	HLA-A0201	TETPGESSTAFVRI	TSSTAFVRI	0	0	0	1	5	TETPGESSTAFVRI	tr_D0A9V0	0.035	34325.73	60.00
66	HLA-A0201	NEAGGHIRA	NEAGGHIRA	0	0	0	0	0	NEAGGHIRA	tr_D0A9V0	0.035	34329.43	60.00
355	HLA-A0201	LDNHYCVNS	LDNHYCVNS	0	0	0	0	0	LDNHYCVNS	tr_D0A9V0	0.035	34330.91	60.00
82	HLA-A0201	PGESSTAFV	PGESSTAFV	0	0	0	0	0	PGESSTAFV	tr_D0A9V0	0.035	34340.21	60.00
174	HLA-A0201	SFGAHAALPMHCR	FGAHAALPM	1	0	0	0	0	FGAHAALPM	tr_D0A9V0	0.035	34345.42	60.00
583	HLA-A0201	LSDFGSTISEAS	LSDFTISEA	0	0	0	4	2	LSDFGSTISEA	tr_D0A9V0	0.035	34353.21	60.00
294	HLA-A0201	EVSLSISKQFR	SLSISKQFR	2	0	0	0	0	SLSISKQFR	tr_D0A9V0	0.035	34353.97	60.00
26	HLA-A0201	GRSRIVEVRKIQ	RSRIVEVRI	1	0	0	8	1	RSRIVEVRKI	tr_D0A9V0	0.035	34358.80	60.00
175	HLA-A0201	FGAHAALPMHCRR	FGAHAALPM	0	0	0	0	0	FGAHAALPM	tr_D0A9V0	0.035	34372.18	60.00
256	HLA-A0201	GGASHMQGF	GGASHMQGF	0	0	0	0	0	GGASHMQGF	tr_D0A9V0	0.035	34374.40	60.00
699	HLA-A0201	CCHMNPSMRPS	CMNPSMRPS	0	0	0	1	2	CCHMNPSMRPS	tr_D0A9V0	0.035	34380.37	60.00
102	HLA-A0201	NAGDQQRDVALNC	AQRDVALNC	1	0	0	1	3	AGDQQRDVALNC	tr_D0A9V0	0.035	34386.68	60.00
481	HLA-A0201	RRDVVAESLEK	RRDVVAESL	0	0	0	0	0	RRDVVAESL	tr_D0A9V0	0.035	34395.62	60.00
195	HLA-A0201	LFAKLPLDHRDS	FLPLDHRDS	1	0	0	1	2	FAKLPLDHRDS	tr_D0A9V0	0.035	34408.27	60.00
493	HLA-A0201	MEVASKLHHPNI	MEVASKLHI	0	0	0	8	3	MEVASKLHHPNI	tr_D0A9V0	0.035	34409.02	60.00
495	HLA-A0201	VASKLHHPNIVR	VASKLHHPV	0	0	0	8	2	VASKLHHPNIV	tr_D0A9V0	0.035	34423.16	60.00
688	HLA-A0201	VDHEAAAFVRACC	AAAFVRACC	4	0	0	0	0	AAAFVRACC	tr_D0A9V0	0.034	34444.04	60.00
698	HLA-A0201	ACCHMNPSMR	ACCHMNPSM	0	0	0	0	0	ACCHMNPSM	tr_D0A9V0	0.034	34444.77	60.00
467	HLA-A0201	EDGAQVAIKCI	DGAQVAICI	1	0	0	7	1	DGAQVAIKCI	tr_D0A9V0	0.034	34445.90	60.00
348	HLA-A0201	TVPYVRVLDNH	VPYVRVLNH	1	0	0	7	1	VPYVRVLDNH	tr_D0A9V0	0.034	34447.01	60.00
157	HLA-A0201	TEHSGCVEALDP	HSGCVALDP	2	0	0	5	1	HSGCVEALDP	tr_D0A9V0	0.034	34453.72	60.00

11	HLA-A0201	SASNLEIPDNAD	SASNLEIPA	0	0	0	8	2	SASNLEIPDNA	tr_D0A9V0	0.034	34458.57	60.00
648	HLA-A0201	WSSTAPAGS	WSSTAPAGS	0	0	0	0	0	WSSTAPAGS	tr_D0A9V0	0.034	34462.68	60.00
310	HLA-A0201	MRQGRYGEAMAHY	RQGEAMAHY	1	0	0	2	3	RQGRYGEAMAHY	tr_D0A9V0	0.034	34474.97	60.00
94	HLA-A0201	GASISVTWN	GASISVTWN	0	0	0	0	0	GASISVTWN	tr_D0A9V0	0.034	34486.18	60.00
36	HLA-A0201	IQGHYAKLRR	IQGHYALRR	0	0	0	6	1	IQGHYAKLRR	tr_D0A9V0	0.034	34491.41	60.00
79	HLA-A0201	TETPGESSTAFV	ETPGSTAFV	1	0	0	4	2	ETPGESSTAFV	tr_D0A9V0	0.034	34491.76	60.00
332	HLA-A0201	AKRLMRLCR	AKRLMRLCR	0	0	0	0	0	AKRLMRLCR	tr_D0A9V0	0.034	34498.12	60.00
650	HLA-A0201	STAPAGSDAT	STAPGSDAT	0	0	0	4	1	STAPAGSDAT	tr_D0A9V0	0.034	34501.11	60.00
135	HLA-A0201	QVYHPDVWKSSTKY	QVYHPDVWY	0	0	0	8	5	QVYHPDVWKSSTKY	tr_D0A9V0	0.034	34504.84	60.00
82	HLA-A0201	PGESSTAFVRI	GSSTAFVRI	1	0	0	1	1	GESSTAFVRI	tr_D0A9V0	0.034	34509.32	60.00
339	HLA-A0201	CRQRCRRNDTV	RQCRRNDTV	1	0	0	2	1	RQRCRRNDTV	tr_D0A9V0	0.034	34515.66	60.00
465	HLA-A0201	VAEDGAQVAIKC	VAAQVAIKC	0	0	0	2	3	VAEDGAQVAIKC	tr_D0A9V0	0.034	34541.07	60.00
345	HLA-A0201	RNDTVPYVR	RNDTVPYVR	0	0	0	0	0	RNDTVPYVR	tr_D0A9V0	0.034	34544.05	60.00
347	HLA-A0201	DTVPYVRVLDNH	VPYVVLDNH	2	0	0	4	1	VPYVRVLDNH	tr_D0A9V0	0.034	34556.02	60.00
659	HLA-A0201	TRLRSLFNR	TRLRSLFNR	0	0	0	0	0	TRLRSLFNR	tr_D0A9V0	0.034	34566.85	60.00
500	HLA-A0201	HHPNIVRYVSC	HPNIVYVSC	1	0	0	5	1	HPNIVRYVSC	tr_D0A9V0	0.034	34601.29	65.00
477	HLA-A0201	IPRLRRDVVA	IPRLRDVVA	0	0	0	4	1	IPRLRRDVVA	tr_D0A9V0	0.034	34606.91	65.00
134	HLA-A0201	IQVYHPDVWKSSTK	QVYHPDVTK	1	0	0	7	4	QVYHPDVWKSSTK	tr_D0A9V0	0.034	34618.90	65.00
200	HLA-A0201	PLDHRDSVT	PLDHRDSVT	0	0	0	0	0	PLDHRDSVT	tr_D0A9V0	0.034	34626.76	65.00
331	HLA-A0201	QAKRLMRLC	QAKRLMRLC	0	0	0	0	0	QAKRLMRLC	tr_D0A9V0	0.034	34628.63	65.00
376	HLA-A0201	IKDRVPETID	IKDRVPETI	0	0	0	0	0	IKDRVPETI	tr_D0A9V0	0.034	34633.12	65.00
202	HLA-A0201	DHRDSVTMLVDTN	HRDSVTMLV	1	0	0	0	0	HRDSVTMLV	tr_D0A9V0	0.034	34647.38	65.00
655	HLA-A0201	GSDATRLRSLFN	GSDATLRSL	0	0	0	5	1	GSDATRLRSL	tr_D0A9V0	0.034	34663.87	65.00
174	HLA-A0201	SFGAHAALPMHCRR	FGAHAALPM	1	0	0	0	0	FGAHAALPM	tr_D0A9V0	0.034	34672.12	65.00
696	HLA-A0201	VRACCHMNPS	VACCHMNPS	0	0	0	1	1	VRACCHMNPS	tr_D0A9V0	0.034	34684.87	65.00
577	HLA-A0201	TDGVCKLSDFGS	GVCLSDFGS	2	0	0	3	1	GVCKLSDFGS	tr_D0A9V0	0.034	34698.76	65.00
688	HLA-A0201	VDHEAAAFVRACCH	VDHEAAAFV	0	0	0	0	0	VDHEAAAFV	tr_D0A9V0	0.034	34700.26	65.00
494	HLA-A0201	EVASKLHHPNI	VASKLHHPI	1	0	0	8	1	VASKLHHPNI	tr_D0A9V0	0.034	34704.40	65.00
152	HLA-A0201	LIEVATEHSG	LIVATEHSG	0	0	0	2	1	LIEVATEHSG	tr_D0A9V0	0.034	34711.90	65.00
104	HLA-A0201	GDQQRDVALNC	GQRDVALNC	0	0	0	1	2	GDQQRDVALNC	tr_D0A9V0	0.034	34712.66	65.00
702	HLA-A0201	MNPSMRPSA	MNPSMRPSA	0	0	0	0	0	MNPSMRPSA	tr_D0A9V0	0.034	34713.40	65.00
566	HLA-A0201	CDVKPHNVLLGTDG	CDVKVLLGT	0	0	0	4	3	CDVKPHNVLLGT	tr_D0A9V0	0.034	34732.57	65.00
580	HLA-A0201	VCKLSDFGST	VCKLSFGST	0	0	0	5	1	VCKLSDFGST	tr_D0A9V0	0.034	34754.75	65.00
577	HLA-A0201	TDGVCKLSDF	TDGVCLSDF	0	0	0	5	1	TDGVCKLSDF	tr_D0A9V0	0.034	34771.68	65.00
346	HLA-A0201	NDTVPYVRVLDN	NTVPYVRVL	0	0	0	1	1	NDTVPYVRVL	tr_D0A9V0	0.034	34795.00	65.00
688	HLA-A0201	VDHEAAAFVRA	VDHEAAAFV	0	0	0	0	0	VDHEAAAFV	tr_D0A9V0	0.033	34809.68	65.00
480	HLA-A0201	LRRDVVAESLEKE	LRDVVAESL	0	0	0	1	1	LRRDVVAESL	tr_D0A9V0	0.033	34810.07	65.00
416	HLA-A0201	SSSLDGSGCA	SSLDGSGCA	0	0	0	1	1	SSSLDGSGCA	tr_D0A9V0	0.033	34839.85	65.00
508	HLA-A0201	VSCSITPSH	VSCSITPSH	0	0	0	0	0	VSCSITPSH	tr_D0A9V0	0.033	34847.38	65.00
9	HLA-A0201	LKSASNLEIPDNAD	LLEIPDNAD	0	0	0	1	5	LKSASNLEIPDNAD	tr_D0A9V0	0.033	34853.04	65.00
689	HLA-A0201	DHEAAAFVRA	HEAAAFVRA	1	0	0	0	0	HEAAAFVRA	tr_D0A9V0	0.033	34856.79	65.00
648	HLA-A0201	WSSTAPAGSDATRL	WSSTAPAGL	0	0	0	8	5	WSSTAPAGSDATRL	tr_D0A9V0	0.033	34859.45	65.00
99	HLA-A0201	VTWNAGDQQ	VTWNAGDQQ	0	0	0	0	0	VTWNAGDQQ	tr_D0A9V0	0.033	34868.86	65.00
142	HLA-A0201	WKSSTKYHA	WKSSTKYHA	0	0	0	0	0	WKSSTKYHA	tr_D0A9V0	0.033	34888.12	65.00
247	HLA-A0201	GPCRIAATS	GPCRIAATS	0	0	0	0	0	GPCRIAATS	tr_D0A9V0	0.033	34915.31	65.00
158	HLA-A0201	EHSGCVEALD	EHSGCVEAL	0	0	0	0	0	EHSGCVEAL	tr_D0A9V0	0.033	34918.33	65.00
656	HLA-A0201	SDATRLRSLF	SDATRLSLF	0	0	0	6	1	SDATRLRSLF	tr_D0A9V0	0.033	34921.73	65.00
494	HLA-A0201	EVASKLHHPNIVR	KLHHPNIVR	4	0	0	0	0	KLHHPNIVR	tr_D0A9V0	0.033	34932.70	65.00
352	HLA-A0201	VRVLDNHYC	VRVLDNHYC	0	0	0	0	0	VRVLDNHYC	tr_D0A9V0	0.033	34952.35	65.00
11	HLA-A0201	SASNLEIPD	SASNLEIPD	0	0	0	0	0	SASNLEIPD	tr_D0A9V0	0.033	34953.50	65.00
584	HLA-A0201	SDFGSTISE	SDFGSTISE	0	0	0	0	0	SDFGSTISE	tr_D0A9V0	0.033	34961.05	65.00
347	HLA-A0201	DTVPYVRVLD	DTVPYVRVL	0	0	0	0	0	DTVPYVRVL	tr_D0A9V0	0.033	34965.22	65.00
653	HLA-A0201	PAGSDATRLRSLF	GSDATRLLF	2	0	0	7	2	GSDATRLRSLF	tr_D0A9V0	0.033	35005.34	65.00
178	HLA-A0201	HAALPMHCR	HAALPMHCR	0	0	0	0	0	HAALPMHCR	tr_D0A9V0	0.033	35007.62	65.00

652	HLA-A0201	APAGSDATRLRSLF	GSDATRLLF	3	0	0	7	2	GSDATRLRSLF	tr_D0A9V0	0.033	35021.25	65.00
438	HLA-A0201	LTDSNRNVWTR	LTDSNVWTR	0	0	0	4	2	LTDSNRNVWTR	tr_D0A9V0	0.033	35022.39	65.00
253	HLA-A0201	ATSGGASHMQ	ATSGGASHM	0	0	0	0	0	ATSGGASHM	tr_D0A9V0	0.033	35023.52	65.00
697	HLA-A0201	RACCHMNPSMR	RACCHMNPM	0	0	0	8	1	RACCHMNPSM	tr_D0A9V0	0.033	35027.31	65.00
568	HLA-A0201	VKPHNVLLG	VKPHNVLLG	0	0	0	0	0	VKPHNVLLG	tr_D0A9V0	0.033	35049.68	65.00
567	HLA-A0201	DVKPHNVLLGTD	DVHNVLLGT	0	0	0	2	2	DVKPHNVLLGT	tr_D0A9V0	0.033	35056.12	65.00
204	HLA-A0201	RDSVTMLVD	RDSVTMLVD	0	0	0	0	0	RDSVTMLVD	tr_D0A9V0	0.033	35060.67	65.00
680	HLA-A0201	LEPVIPASVDH	LEPVIPASV	0	0	0	0	0	LEPVIPASV	tr_D0A9V0	0.033	35064.85	65.00
654	HLA-A0201	AGSDATRLRSLFN	GSDATLRSL	1	0	0	5	1	GSDATRLRSL	tr_D0A9V0	0.033	35072.81	65.00
98	HLA-A0201	SVTWNAGDQQRDVA	VTWNAGDQV	1	0	0	8	3	VTWNAGDQQRDV	tr_D0A9V0	0.033	35073.58	65.00
30	HLA-A0201	IVEVRKIQGHYAK	IVEVRKIYA	0	0	0	7	3	IVEVRKIQGHYA	tr_D0A9V0	0.033	35084.57	65.00
245	HLA-A0201	RDGPCRIAATSGGA	RIAATSGGA	0	0	0	1	5	RDGPCRIAATSGGA	tr_D0A9V0	0.033	35113.43	65.00
19	HLA-A0201	DNADIFLGRSR	NADIFLRSR	1	0	0	6	1	NADIFLGRSR	tr_D0A9V0	0.033	35116.49	65.00
16	HLA-A0201	EIPDNADIF	EIPDNADIF	0	0	0	0	0	EIPDNADIF	tr_D0A9V0	0.033	35118.01	65.00
135	HLA-A0201	QVYHPDVWKSST	QVYHPDVWT	0	0	0	8	3	QVYHPDVWKSST	tr_D0A9V0	0.033	35127.50	65.00
30	HLA-A0201	IVEVRKIQG	IVEVRKIQG	0	0	0	0	0	IVEVRKIQG	tr_D0A9V0	0.033	35128.66	65.00
28	HLA-A0201	SRIVEVRKIQG	RIVEVRIQG	1	0	0	6	1	RIVEVRKIQG	tr_D0A9V0	0.033	35130.54	65.00
657	HLA-A0201	DATRLRSLFN	ATRLRSLFN	1	0	0	0	0	ATRLRSLFN	tr_D0A9V0	0.033	35149.16	65.00
254	HLA-A0201	TSGGASHMQGFL	TSGGASHFL	0	0	0	7	3	TSGGASHMQGFL	tr_D0A9V0	0.033	35156.78	65.00
167	HLA-A0201	DPDRALISFGA	DPDLISFGA	0	0	0	3	2	DPDRALISFGA	tr_D0A9V0	0.033	35158.30	65.00
585	HLA-A0201	DFGSTISEAS	FGSTISEAS	1	0	0	0	0	FGSTISEAS	tr_D0A9V0	0.033	35160.59	65.00
338	HLA-A0201	LCRQRCRRNDTVP	LCRQRCDTV	0	0	0	6	3	LCRQRCRRNDTV	tr_D0A9V0	0.033	35169.71	65.00
337	HLA-A0201	RLCRQRCRR	RLCRQRCRR	0	0	0	0	0	RLCRQRCRR	tr_D0A9V0	0.032	35185.70	65.00
617	HLA-A0201	VAAGGRCTPQSDIF	AAGGRCTPF	1	0	0	8	4	AAGGRCTPQSDIF	tr_D0A9V0	0.032	35193.33	65.00
38	HLA-A0201	GHYAKLRRQLA	GHYAKLQLA	0	0	0	6	2	GHYAKLRRQLA	tr_D0A9V0	0.032	35202.45	65.00
542	HLA-A0201	YVARRFTVD	YVARRFTVD	0	0	0	0	0	YVARRFTVD	tr_D0A9V0	0.032	35209.31	65.00
64	HLA-A0201	SKNEAGGHI	SKNEAGGHI	0	0	0	0	0	SKNEAGGHI	tr_D0A9V0	0.032	35223.03	65.00
30	HLA-A0201	IVEVRKIQGHY	IVEVRKIHY	0	0	0	7	2	IVEVRKIQGHY	tr_D0A9V0	0.032	35225.71	65.00
440	HLA-A0201	DSNRNVWTRSL	DSNRNVWTL	0	0	0	8	2	DSNRNVWTRSL	tr_D0A9V0	0.032	35227.23	65.00
421	HLA-A0201	GSGCAEGANA	GSCAEGANA	0	0	0	2	1	GSGCAEGANA	tr_D0A9V0	0.032	35230.27	65.00
312	HLA-A0201	QGRYGEAMAHY	GRYEAMAHY	1	0	0	3	1	GRYGEAMAHY	tr_D0A9V0	0.032	35237.14	65.00
10	HLA-A0201	KSASNLEIPDNAD	SASNLEIPA	1	0	0	8	2	SASNLEIPDNA	tr_D0A9V0	0.032	35241.34	65.00
370	HLA-A0201	EQDESDIKDRVP	EQDESDIRV	0	0	0	7	2	EQDESDIKDRV	tr_D0A9V0	0.032	35241.34	65.00
59	HLA-A0201	LARVESKNEA	LAVESKNEA	0	0	0	2	1	LARVESKNEA	tr_D0A9V0	0.032	35248.20	65.00
651	HLA-A0201	TAPAGSDAT	TAPAGSDAT	0	0	0	0	0	TAPAGSDAT	tr_D0A9V0	0.032	35258.87	65.00
537	HLA-A0201	GCVSPYVAR	GCVSPYVAR	0	0	0	0	0	GCVSPYVAR	tr_D0A9V0	0.032	35266.51	65.00
445	HLA-A0201	VWTRSLKKI	VWTRSLKKI	0	0	0	0	0	VWTRSLKKI	tr_D0A9V0	0.032	35271.86	65.00
161	HLA-A0201	GCVEALDPD	GCVEALDPD	0	0	0	0	0	GCVEALDPD	tr_D0A9V0	0.032	35279.86	65.00
19	HLA-A0201	DNADIFLGR	DNADIFLGR	0	0	0	0	0	DNADIFLGR	tr_D0A9V0	0.032	35285.21	65.00
218	HLA-A0201	VGTCGAFSRNA	GTCGAFSNA	1	0	0	7	1	GTCGAFSRNA	tr_D0A9V0	0.032	35293.61	65.00
310	HLA-A0201	MRQGRYGEAMA	RQGYGEAMA	1	0	0	3	1	RQGRYGEAMA	tr_D0A9V0	0.032	35311.93	65.00
255	HLA-A0201	SGGASHMQGFL	SGGASHMFL	0	0	0	7	2	SGGASHMQGFL	tr_D0A9V0	0.032	35315.37	65.00
653	HLA-A0201	PAGSDATRLRSLFN	ATRLRSLFN	5	0	0	0	0	ATRLRSLFN	tr_D0A9V0	0.032	35334.49	65.00
569	HLA-A0201	KPHNVLLGTDG	KPHNVLLGG	0	0	0	8	2	KPHNVLLGTDG	tr_D0A9V0	0.032	35342.14	65.00
9	HLA-A0201	LKSASNLEIP	LKSASNLEI	0	0	0	0	0	LKSASNLEI	tr_D0A9V0	0.032	35346.73	65.00
309	HLA-A0201	AMRQGRYGE	AMRQGRYGE	0	0	0	0	0	AMRQGRYGE	tr_D0A9V0	0.032	35359.74	65.00
348	HLA-A0201	TVPYVRVLD	TVPYVRVLD	0	0	0	0	0	TVPYVRVLD	tr_D0A9V0	0.032	35367.38	65.00
618	HLA-A0201	AAGGRCTPQS	AAGGRCTPS	0	0	0	8	1	AAGGRCTPQS	tr_D0A9V0	0.032	35369.30	65.00
438	HLA-A0201	LTDSNRNVWTRS	LTDSNVWTR	0	0	0	4	2	LTDSNRNVWTR	tr_D0A9V0	0.032	35385.00	65.00
699	HLA-A0201	CCHMNPSMRP	CCMNPSMRP	0	0	0	2	1	CCHMNPSMRP	tr_D0A9V0	0.032	35393.03	65.00
102	HLA-A0201	NAGDQQRDVAL	NAGDRDVAL	0	0	0	4	2	NAGDQQRDVAL	tr_D0A9V0	0.032	35395.72	65.00
58	HLA-A0201	ILARVESKNE	ILARVESNE	0	0	0	7	1	ILARVESKNE	tr_D0A9V0	0.032	35396.87	65.00
140	HLA-A0201	DVWKSSTKY	DVWKSSTKY	0	0	0	0	0	DVWKSSTKY	tr_D0A9V0	0.032	35405.68	65.00
568	HLA-A0201	VKPHNVLLGTD	VPHNVLLGT	0	0	0	1	1	VKPHNVLLGT	tr_D0A9V0	0.032	35412.19	65.00

179	HLA-A0201	AALPMHCRR	AALPMHCRR	0	0	0	0	0	AALPMHCRR	tr_D0A9V0	0.032	35412.57	65.00
346	HLA-A0201	NDTVPYVRVLD	NTVPYVRVL	0	0	0	1	1	NDTVPYVRVL	tr_D0A9V0	0.032	35418.70	65.00
642	HLA-A0201	GRPPWQWSSTA	RPWQWSSTA	1	0	0	1	1	RPPWQWSSTA	tr_D0A9V0	0.032	35422.14	65.00
157	HLA-A0201	TEHSGCVEALD	HSGCVEALD	2	0	0	0	0	HSGCVEALD	tr_D0A9V0	0.032	35429.43	65.00
12	HLA-A0201	ASNLEIPDNAD	ALEIPDNAD	0	0	0	1	2	ASNLEIPDNAD	tr_D0A9V0	0.032	35431.35	65.00
142	HLA-A0201	WKSSTKYHAAL	KSSTYHAAL	1	0	0	4	1	KSSTKYHAAL	tr_D0A9V0	0.032	35438.64	65.00
202	HLA-A0201	DHRDSVTML	DHRDSVTML	0	0	0	0	0	DHRDSVTML	tr_D0A9V0	0.032	35451.67	65.00
35	HLA-A0201	KIQGHYAKLRRQ	KIQGHYAKL	0	0	0	0	0	KIQGHYAKL	tr_D0A9V0	0.032	35454.37	65.00
375	HLA-A0201	DIKDRVPET	DIKDRVPET	0	0	0	0	0	DIKDRVPET	tr_D0A9V0	0.032	35455.90	65.00
204	HLA-A0201	RDSVTMLVDTNE	SVTMLVDTE	2	0	0	8	1	SVTMLVDTNE	tr_D0A9V0	0.032	35463.56	65.00
137	HLA-A0201	YHPDVWKSSTKY	YVWKSSTKY	0	0	0	1	3	YHPDVWKSSTKY	tr_D0A9V0	0.032	35467.02	65.00
465	HLA-A0201	VAEDGAQVAIKCIP	VAQVAIKCI	0	0	0	1	4	VAEDGAQVAIKCI	tr_D0A9V0	0.032	35468.55	65.00
159	HLA-A0201	HSGCVEALD	HSGCVEALD	0	0	0	0	0	HSGCVEALD	tr_D0A9V0	0.032	35473.93	65.00
474	HLA-A0201	IKCIPRLRR	IKCIPRLRR	0	0	0	0	0	IKCIPRLRR	tr_D0A9V0	0.032	35476.62	65.00
526	HLA-A0201	GGSLHSVIK	GGSLHSVIK	0	0	0	0	0	GGSLHSVIK	tr_D0A9V0	0.032	35516.55	65.00
30	HLA-A0201	IVEVRKIQGH	IVEVRIQGH	0	0	0	5	1	IVEVRKIQGH	tr_D0A9V0	0.032	35523.10	65.00
34	HLA-A0201	RKIQGHYAKLR	KIQGHYAKL	1	0	0	0	0	KIQGHYAKL	tr_D0A9V0	0.032	35529.24	65.00
401	HLA-A0201	DQGSESLFK	DQGSESLFK	0	0	0	0	0	DQGSESLFK	tr_D0A9V0	0.032	35536.16	65.00
491	HLA-A0201	KEMEVASKLHHP	KEMEVLHHP	0	0	0	5	3	KEMEVASKLHHP	tr_D0A9V0	0.032	35540.40	65.00
136	HLA-A0201	VYHPDVWKSSTKY	YVWKSSTKY	1	0	0	1	3	YHPDVWKSSTKY	tr_D0A9V0	0.032	35544.62	65.00
491	HLA-A0201	KEMEVASKLHH	KEMEVALHH	0	0	0	6	2	KEMEVASKLHH	tr_D0A9V0	0.032	35555.78	65.00
205	HLA-A0201	DSVTMLVDTNE	SVMLVDTNE	1	0	0	2	1	SVTMLVDTNE	tr_D0A9V0	0.031	35561.17	65.00
160	HLA-A0201	SGCVEALDP	SGCVEALDP	0	0	0	0	0	SGCVEALDP	tr_D0A9V0	0.031	35567.32	65.00
642	HLA-A0201	GRPPWQWSS	GRPPWQWSS	0	0	0	0	0	GRPPWQWSS	tr_D0A9V0	0.031	35570.00	65.00
481	HLA-A0201	RRDVVAESLEKE	RRDVVAESL	0	0	0	0	0	RRDVVAESL	tr_D0A9V0	0.031	35578.49	65.00
161	HLA-A0201	GCVEALDPDR	GCVEALDPR	0	0	0	8	1	GCVEALDPDR	tr_D0A9V0	0.031	35578.49	65.00
540	HLA-A0201	SPYVARRFT	SPYVARRFT	0	0	0	0	0	SPYVARRFT	tr_D0A9V0	0.031	35579.64	65.00
680	HLA-A0201	LEPVIPASVDHE	LEPVIPASV	0	0	0	0	0	LEPVIPASV	tr_D0A9V0	0.031	35581.96	65.00
217	HLA-A0201	LVGTCGAFSRN	LVGTCGAFN	0	0	0	8	2	LVGTCGAFSRN	tr_D0A9V0	0.031	35599.26	65.00
18	HLA-A0201	PDNADIFLGRSR	NADIFLRSR	2	0	0	6	1	NADIFLGRSR	tr_D0A9V0	0.031	35603.12	65.00
567	HLA-A0201	DVKPHNVLLGTDG	DVHNVLLGT	0	0	0	2	2	DVKPHNVLLGT	tr_D0A9V0	0.031	35623.93	65.00
527	HLA-A0201	GSLHSVIKN	GSLHSVIKN	0	0	0	0	0	GSLHSVIKN	tr_D0A9V0	0.031	35625.10	65.00
311	HLA-A0201	RQGRYGEAMAH	RQYGEAMAH	0	0	0	2	2	RQGRYGEAMAH	tr_D0A9V0	0.031	35640.13	65.00
690	HLA-A0201	HEAAAFVRACC	AAAFVRACC	2	0	0	0	0	AAAFVRACC	tr_D0A9V0	0.031	35641.67	65.00
140	HLA-A0201	DVWKSSTKYHA	DVWKSSYHA	0	0	0	6	2	DVWKSSTKYHA	tr_D0A9V0	0.031	35644.75	65.00
492	HLA-A0201	EMEVASKLH	EMEVASKLH	0	0	0	0	0	EMEVASKLH	tr_D0A9V0	0.031	35645.14	65.00
5	HLA-A0201	LVSALKSASN	LVSALSASN	0	0	0	5	1	LVSALKSASN	tr_D0A9V0	0.031	35646.29	65.00
468	HLA-A0201	DGAQVAIKCIP	AQVAIKCIP	2	0	0	0	0	AQVAIKCIP	tr_D0A9V0	0.031	35647.07	65.00
135	HLA-A0201	QVYHPDVWKSS	QVYHVWKSS	0	0	0	4	2	QVYHPDVWKSS	tr_D0A9V0	0.031	35647.46	65.00
578	HLA-A0201	DGVCKLSDFGS	GVCKLSFGS	1	0	0	6	1	GVCKLSDFGS	tr_D0A9V0	0.031	35649.39	65.00
655	HLA-A0201	GSDATRLRS	GSDATRLRS	0	0	0	0	0	GSDATRLRS	tr_D0A9V0	0.031	35653.62	65.00
218	HLA-A0201	VGTCGAFSR	VGTCGAFSR	0	0	0	0	0	VGTCGAFSR	tr_D0A9V0	0.031	35659.42	65.00
35	HLA-A0201	KIQGHYAKLRR	KIQGHYAKL	0	0	0	0	0	KIQGHYAKL	tr_D0A9V0	0.031	35663.65	65.00
26	HLA-A0201	GRSRIVEVR	GRSRIVEVR	0	0	0	0	0	GRSRIVEVR	tr_D0A9V0	0.031	35664.43	65.00
373	HLA-A0201	ESDIKDRVPETID	ESDIVPETI	0	0	0	4	3	ESDIKDRVPETI	tr_D0A9V0	0.031	35676.79	65.00
146	HLA-A0201	TKYHAALIE	TKYHAALIE	0	0	0	0	0	TKYHAALIE	tr_D0A9V0	0.031	35678.72	65.00
247	HLA-A0201	GPCRIAATSGGA	RIAATSGGA	3	0	0	0	0	RIAATSGGA	tr_D0A9V0	0.031	35685.67	65.00
689	HLA-A0201	DHEAAAFVRACC	AAAFVRACC	3	0	0	0	0	AAAFVRACC	tr_D0A9V0	0.031	35703.05	65.00
690	HLA-A0201	HEAAAFVRACCH	AAFVRACCH	3	0	0	0	0	AAFVRACCH	tr_D0A9V0	0.031	35704.96	65.00
448	HLA-A0201	RSLKKISEG	RSLKKISEG	0	0	0	0	0	RSLKKISEG	tr_D0A9V0	0.031	35704.96	65.00
305	HLA-A0201	LGFAAMRQGRY	LGFAAMRQY	0	0	0	8	2	LGFAAMRQGRY	tr_D0A9V0	0.031	35723.52	65.00
575	HLA-A0201	LGTDGVCKLSD	GTDGVCLSD	1	0	0	6	1	GTDGVCKLSD	tr_D0A9V0	0.031	35736.27	65.00
568	HLA-A0201	VKPHNVLLGTDG	KPHNVLLGG	1	0	0	8	2	KPHNVLLGTDG	tr_D0A9V0	0.031	35738.20	65.00
251	HLA-A0201	IAATSGGASHM	ITSGGASHM	0	0	0	1	2	IAATSGGASHM	tr_D0A9V0	0.031	35747.10	65.00

186	HLA-A0201	RRCCQFAFD	RRCCQFAFD	0	0	0	0	0	RRCCQFAFD	tr_D0A9V0	0.031	35763.36	65.00
691	HLA-A0201	EAAAFVRACCH	AAFVRACCH	2	0	0	0	0	AAFVRACCH	tr_D0A9V0	0.031	35775.35	65.00
689	HLA-A0201	DHEAAAFVRACCH	AAFVRACCH	4	0	0	0	0	AAFVRACCH	tr_D0A9V0	0.031	35791.23	65.00
331	HLA-A0201	QAKRLMRLCRQ	QALMRLCRQ	0	0	0	2	2	QAKRLMRLCRQ	tr_D0A9V0	0.031	35802.84	65.00
31	HLA-A0201	VEVRKIQGHYAKLR	KIQGHYAKL	4	0	0	0	0	KIQGHYAKL	tr_D0A9V0	0.031	35805.56	65.00
18	HLA-A0201	PDNADIFLGRS	NADIFLGRS	2	0	0	0	0	NADIFLGRS	tr_D0A9V0	0.031	35817.57	65.00
335	HLA-A0201	LMRLCRQRCRRND	LMRLCCRRN	0	0	0	5	3	LMRLCRQRCRRN	tr_D0A9V0	0.031	35819.88	65.00
307	HLA-A0201	FAAMRQGRYG	FAAMRGRYG	0	0	0	5	1	FAAMRQGRYG	tr_D0A9V0	0.031	35821.43	65.00
141	HLA-A0201	VWKSSTKYHAAL	KSSTYHAAL	2	0	0	4	1	KSSTKYHAAL	tr_D0A9V0	0.031	35831.53	65.00
656	HLA-A0201	SDATRLRSLFN	ATRLRSLFN	2	0	0	0	0	ATRLRSLFN	tr_D0A9V0	0.031	35848.59	65.00
176	HLA-A0201	GAHAALPMHCR	GAHAALMHC	0	0	0	6	1	GAHAALPMHC	tr_D0A9V0	0.031	35850.53	65.00
649	HLA-A0201	SSTAPAGSDATRL	STAPAATRL	1	0	0	5	3	STAPAGSDATRL	tr_D0A9V0	0.031	35854.02	65.00
439	HLA-A0201	TDSNRNVWTRSL	SNRNVWTSL	2	0	0	7	1	SNRNVWTRSL	tr_D0A9V0	0.031	35856.33	65.00
33	HLA-A0201	VRKIQGHYAKLR	KIQGHYAKL	2	0	0	0	0	KIQGHYAKL	tr_D0A9V0	0.031	35861.37	65.00
342	HLA-A0201	RCRRNDTVPY	RCRNDTVPY	0	0	0	2	1	RCRRNDTVPY	tr_D0A9V0	0.031	35864.10	65.00
136	HLA-A0201	VYHPDVWKSS	VYHPDVWSS	0	0	0	7	1	VYHPDVWKSS	tr_D0A9V0	0.031	35867.20	65.00
548	HLA-A0201	TVDILQGLN	TVDILQGLN	0	0	0	0	0	TVDILQGLN	tr_D0A9V0	0.031	35873.79	65.00
158	HLA-A0201	EHSGCVEALDP	EHSGCVEAL	0	0	0	0	0	EHSGCVEAL	tr_D0A9V0	0.031	35878.84	65.00
563	HLA-A0201	IVHCDVKPHN	IVHCDVKPH	0	0	0	0	0	IVHCDVKPH	tr_D0A9V0	0.031	35895.55	65.00
400	HLA-A0201	SDQGSESLFK	SQGSESLFK	0	0	0	1	1	SDQGSESLFK	tr_D0A9V0	0.031	35897.10	65.00
204	HLA-A0201	RDSVTMLVDTN	SVTMLVDTN	2	0	0	0	0	SVTMLVDTN	tr_D0A9V0	0.031	35900.59	65.00
441	HLA-A0201	SNRNVWTRSLKK	SVWTRSLKK	0	0	0	1	3	SNRNVWTRSLKK	tr_D0A9V0	0.031	35906.80	65.00
466	HLA-A0201	AEDGAQVAIKCIP	AQVAIKCIP	4	0	0	0	0	AQVAIKCIP	tr_D0A9V0	0.031	35918.47	65.00
101	HLA-A0201	WNAGDQQRDV	WAGDQQRDV	0	0	0	1	1	WNAGDQQRDV	tr_D0A9V0	0.031	35919.63	65.00
7	HLA-A0201	SALKSASNLE	SALKSASNL	0	0	0	0	0	SALKSASNL	tr_D0A9V0	0.031	35921.18	65.00
243	HLA-A0201	AVRDGPCRIAATSG	AVGPCRIAA	0	0	0	2	2	AVRDGPCRIAA	tr_D0A9V0	0.031	35930.91	65.00
496	HLA-A0201	ASKLHHPNIVR	ALHHPNIVR	0	0	0	1	2	ASKLHHPNIVR	tr_D0A9V0	0.031	35940.62	65.00
397	HLA-A0201	SDSSDQGSESL	SSDQGSESL	0	0	0	1	2	SDSSDQGSESL	tr_D0A9V0	0.030	35953.07	70.00
696	HLA-A0201	VRACCHMNPSMR	RACCHMNPM	1	0	0	8	1	RACCHMNPSM	tr_D0A9V0	0.030	35974.85	70.00
71	HLA-A0201	HIRANGSAT	HIRANGSAT	0	0	0	0	0	HIRANGSAT	tr_D0A9V0	0.030	35981.09	70.00
579	HLA-A0201	GVCKLSDFGST	GVCKLFGST	0	0	0	5	2	GVCKLSDFGST	tr_D0A9V0	0.030	35983.81	70.00
83	HLA-A0201	GESSTAFVR	GESSTAFVR	0	0	0	0	0	GESSTAFVR	tr_D0A9V0	0.030	35989.66	70.00
420	HLA-A0201	DGSGCAEGA	DGSGCAEGA	0	0	0	0	0	DGSGCAEGA	tr_D0A9V0	0.030	35993.93	70.00
167	HLA-A0201	DPDRALISFGAH	RALISFGAH	3	0	0	0	0	RALISFGAH	tr_D0A9V0	0.030	35999.01	70.00
492	HLA-A0201	EMEVASKLHH	EMEVASLHH	0	0	0	6	1	EMEVASKLHH	tr_D0A9V0	0.030	36011.09	70.00
32	HLA-A0201	EVRKIQGHYAKLR	KIQGHYAKL	3	0	0	0	0	KIQGHYAKL	tr_D0A9V0	0.030	36014.96	70.00
37	HLA-A0201	QGHYAKLRRQLA	GHYAKLQLA	1	0	0	6	2	GHYAKLRRQLA	tr_D0A9V0	0.030	36015.36	70.00
253	HLA-A0201	ATSGGASHMQGF	ATSGGMQGF	0	0	0	5	3	ATSGGASHMQGF	tr_D0A9V0	0.030	36018.87	70.00
641	HLA-A0201	LGRPPWQWSS	LGPPWQWSS	0	0	0	2	1	LGRPPWQWSS	tr_D0A9V0	0.030	36035.25	70.00
440	HLA-A0201	DSNRNVWTR	DSNRNVWTR	0	0	0	0	0	DSNRNVWTR	tr_D0A9V0	0.030	36039.15	70.00
697	HLA-A0201	RACCHMNPSMRP	RACCHMNPM	0	0	0	8	1	RACCHMNPSM	tr_D0A9V0	0.030	36080.11	70.00
313	HLA-A0201	GRYGEAMAH	GRYGEAMAH	0	0	0	0	0	GRYGEAMAH	tr_D0A9V0	0.030	36085.57	70.00
246	HLA-A0201	DGPCRIAATSGGA	RIAATSGGA	4	0	0	0	0	RIAATSGGA	tr_D0A9V0	0.030	36091.44	70.00
685	HLA-A0201	PASVDHEAAAF	SVDHEAAAF	2	0	0	0	0	SVDHEAAAF	tr_D0A9V0	0.030	36098.07	70.00
162	HLA-A0201	CVEALDPDR	CVEALDPDR	0	0	0	0	0	CVEALDPDR	tr_D0A9V0	0.030	36100.42	70.00
577	HLA-A0201	TDGVCKLSDFGST	TDGVCLSDF	0	0	0	5	1	TDGVCKLSDF	tr_D0A9V0	0.030	36105.50	70.00
622	HLA-A0201	RCTPQSDIF	RCTPQSDIF	0	0	0	0	0	RCTPQSDIF	tr_D0A9V0	0.030	36105.89	70.00
438	HLA-A0201	LTDSNRNVW	LTDSNRNVW	0	0	0	0	0	LTDSNRNVW	tr_D0A9V0	0.030	36108.22	70.00
166	HLA-A0201	LDPDRALISFG	LPDRALISF	0	0	0	1	1	LDPDRALISF	tr_D0A9V0	0.030	36112.93	70.00
157	HLA-A0201	TEHSGCVEALDPD	HSGCVALDP	2	0	0	5	1	HSGCVEALDP	tr_D0A9V0	0.030	36116.82	70.00
564	HLA-A0201	VHCDVKPHNVL	VHCDVPHNV	0	0	0	5	1	VHCDVKPHNV	tr_D0A9V0	0.030	36123.08	70.00
331	HLA-A0201	QAKRLMRLCR	QARLMRLCR	0	0	0	2	1	QAKRLMRLCR	tr_D0A9V0	0.030	36130.52	70.00
467	HLA-A0201	EDGAQVAIKCIP	AQVAIKCIP	3	0	0	0	0	AQVAIKCIP	tr_D0A9V0	0.030	36130.89	70.00
521	HLA-A0201	MEYVPGGSLH	MEYVPGGSL	0	0	0	0	0	MEYVPGGSL	tr_D0A9V0	0.030	36141.84	70.00

490	HLA-A0201	EKEMEVASKLHH	KEMEVALHH	1	0	0	6	2	KEMEVASKLHH	tr_D0A9V0	0.030	36148.11	70.00
28	HLA-A0201	SRIVEVRKIQ	SRIVEVRKI	0	0	0	0	0	SRIVEVRKI	tr_D0A9V0	0.030	36150.84	70.00
138	HLA-A0201	HPDVWKSSTKYHA	DVWKSSYHA	2	0	0	6	2	DVWKSSTKYHA	tr_D0A9V0	0.030	36161.40	70.00
157	HLA-A0201	TEHSGCVEALDPDR	HSGCVEALR	2	0	0	8	3	HSGCVEALDPDR	tr_D0A9V0	0.030	36164.93	70.00
153	HLA-A0201	IEVATEHSGCV	IATEHSGCV	0	0	0	1	2	IEVATEHSGCV	tr_D0A9V0	0.030	36173.14	70.00
490	HLA-A0201	EKEMEVASKLHHP	KEMEVLHHP	1	0	0	5	3	KEMEVASKLHHP	tr_D0A9V0	0.030	36175.89	70.00
168	HLA-A0201	PDRALISFGAH	RALISFGAH	2	0	0	0	0	RALISFGAH	tr_D0A9V0	0.030	36175.89	70.00
101	HLA-A0201	WNAGDQQRDVAL	NAGDRDVAL	1	0	0	4	2	NAGDQQRDVAL	tr_D0A9V0	0.030	36177.45	70.00
255	HLA-A0201	SGGASHMQGF	SGGASMQGF	0	0	0	5	1	SGGASHMQGF	tr_D0A9V0	0.030	36184.50	70.00
363	HLA-A0201	SSDCKPEEQDESDI	SSDCKPEEI	0	0	0	8	5	SSDCKPEEQDESDI	tr_D0A9V0	0.030	36187.62	70.00
466	HLA-A0201	AEDGAQVAIK	AEDGAQVAI	0	0	0	0	0	AEDGAQVAI	tr_D0A9V0	0.030	36229.14	70.00
395	HLA-A0201	SESDSSDQGSESL	SSDQGSESL	0	0	0	1	4	SESDSSDQGSESL	tr_D0A9V0	0.030	36229.54	70.00
370	HLA-A0201	EQDESDIKDRVPET	EQDESDIRV	0	0	0	7	2	EQDESDIKDRV	tr_D0A9V0	0.030	36230.34	70.00
96	HLA-A0201	SISVTWNAGDQQR	SISVTWNAR	0	0	0	8	4	SISVTWNAGDQQR	tr_D0A9V0	0.030	36231.50	70.00
440	HLA-A0201	DSNRNVWTRSLKK	RVWTRSLKK	3	0	0	1	1	RNVWTRSLKK	tr_D0A9V0	0.030	36232.67	70.00
581	HLA-A0201	CKLSDFGST	CKLSDFGST	0	0	0	0	0	CKLSDFGST	tr_D0A9V0	0.030	36239.73	70.00
135	HLA-A0201	QVYHPDVWKSSTK	QVYHPDVTK	0	0	0	7	4	QVYHPDVWKSSTK	tr_D0A9V0	0.030	36240.52	70.00
55	HLA-A0201	PEAILARVESK	AILARVESK	2	0	0	0	0	AILARVESK	tr_D0A9V0	0.030	36242.88	70.00
489	HLA-A0201	LEKEMEVASKLHHP	KEMEVLHHP	2	0	0	5	3	KEMEVASKLHHP	tr_D0A9V0	0.030	36249.15	70.00
137	HLA-A0201	YHPDVWKSS	YHPDVWKSS	0	0	0	0	0	YHPDVWKSS	tr_D0A9V0	0.030	36253.85	70.00
347	HLA-A0201	DTVPYVRVLDN	TVYVRVLDN	1	0	0	2	1	TVPYVRVLDN	tr_D0A9V0	0.030	36286.43	70.00
137	HLA-A0201	YHPDVWKSST	YHPDVWSST	0	0	0	6	1	YHPDVWKSST	tr_D0A9V0	0.030	36287.20	70.00
501	HLA-A0201	HPNIVRYVSCS	HPNIVYVSC	0	0	0	5	1	HPNIVRYVSC	tr_D0A9V0	0.030	36288.40	70.00
34	HLA-A0201	RKIQGHYAKLRRQ	IQYAKLRRQ	2	0	0	2	2	IQGHYAKLRRQ	tr_D0A9V0	0.030	36294.67	70.00
310	HLA-A0201	MRQGRYGEA	MRQGRYGEA	0	0	0	0	0	MRQGRYGEA	tr_D0A9V0	0.030	36303.71	70.00
81	HLA-A0201	TPGESSTAF	TPGESSTAF	0	0	0	0	0	TPGESSTAF	tr_D0A9V0	0.030	36304.50	70.00
337	HLA-A0201	RLCRQRCRRNDT	RLCCRRNDT	0	0	0	3	3	RLCRQRCRRNDT	tr_D0A9V0	0.030	36317.45	70.00
500	HLA-A0201	HHPNIVRYVSCS	HPNIVYVSC	1	0	0	5	1	HPNIVRYVSC	tr_D0A9V0	0.030	36318.25	70.00
689	HLA-A0201	DHEAAAFVRAC	HAAAFVRAC	1	0	0	1	1	HEAAAFVRAC	tr_D0A9V0	0.030	36323.36	70.00
465	HLA-A0201	VAEDGAQVAIK	VADGAQVAI	0	0	0	2	1	VAEDGAQVAI	tr_D0A9V0	0.030	36327.29	70.00
310	HLA-A0201	MRQGRYGEAMAH	RQYGEAMAH	1	0	0	2	2	RQGRYGEAMAH	tr_D0A9V0	0.030	36327.66	70.00
21	HLA-A0201	ADIFLGRSR	ADIFLGRSR	0	0	0	0	0	ADIFLGRSR	tr_D0A9V0	0.030	36336.34	70.00
489	HLA-A0201	LEKEMEVASKLHH	KEMEVALHH	2	0	0	6	2	KEMEVASKLHH	tr_D0A9V0	0.029	36340.64	70.00
163	HLA-A0201	VEALDPDRA	VEALDPDRA	0	0	0	0	0	VEALDPDRA	tr_D0A9V0	0.029	36345.36	70.00
248	HLA-A0201	PCRIAATSGGA	RIAATSGGA	2	0	0	0	0	RIAATSGGA	tr_D0A9V0	0.029	36352.44	70.00
72	HLA-A0201	IRANGSATET	IANGSATET	0	0	0	1	1	IRANGSATET	tr_D0A9V0	0.029	36359.52	70.00
107	HLA-A0201	QRDVALNCG	QRDVALNCG	0	0	0	0	0	QRDVALNCG	tr_D0A9V0	0.029	36363.46	70.00
305	HLA-A0201	LGFAAMRQGRYG	LGFAAMRYG	0	0	0	6	3	LGFAAMRQGRYG	tr_D0A9V0	0.029	36391.02	70.00
251	HLA-A0201	IAATSGGASHMQGF	ATSGGMQGF	2	0	0	5	3	ATSGGASHMQGF	tr_D0A9V0	0.029	36394.17	70.00
529	HLA-A0201	LHSVIKNFG	LHSVIKNFG	0	0	0	0	0	LHSVIKNFG	tr_D0A9V0	0.029	36396.14	70.00
372	HLA-A0201	DESDIKDRVPETID	ESDIVPETI	1	0	0	4	3	ESDIKDRVPETI	tr_D0A9V0	0.029	36407.55	70.00
103	HLA-A0201	AGDQQRDVALNCG	AQRDVALNC	0	0	0	1	3	AGDQQRDVALNC	tr_D0A9V0	0.029	36409.52	70.00
90	HLA-A0201	VRIMGASIS	VRIMGASIS	0	0	0	0	0	VRIMGASIS	tr_D0A9V0	0.029	36413.07	70.00
33	HLA-A0201	VRKIQGHYA	VRKIQGHYA	0	0	0	0	0	VRKIQGHYA	tr_D0A9V0	0.029	36413.46	70.00
439	HLA-A0201	TDSNRNVWTRSLKK	RVWTRSLKK	4	0	0	1	1	RNVWTRSLKK	tr_D0A9V0	0.029	36432.38	70.00
442	HLA-A0201	NRNVWTRSLKK	RVWTRSLKK	1	0	0	1	1	RNVWTRSLKK	tr_D0A9V0	0.029	36442.25	70.00
561	HLA-A0201	LGIVHCDVKPH	GIVHCDVPH	1	0	0	7	1	GIVHCDVKPH	tr_D0A9V0	0.029	36442.62	70.00
250	HLA-A0201	RIAATSGGASHMQ	RIAATASHM	0	0	0	5	3	RIAATSGGASHM	tr_D0A9V0	0.029	36444.59	70.00
39	HLA-A0201	HYAKLRRQLA	YAKLRRQLA	1	0	0	0	0	YAKLRRQLA	tr_D0A9V0	0.029	36454.84	70.00
33	HLA-A0201	VRKIQGHYAKLRRQ	IQYAKLRRQ	3	0	0	2	2	IQGHYAKLRRQ	tr_D0A9V0	0.029	36478.53	70.00
373	HLA-A0201	ESDIKDRVPETIDS	ESDIVPETI	0	0	0	4	3	ESDIKDRVPETI	tr_D0A9V0	0.029	36494.71	70.00
369	HLA-A0201	EEQDESDIKDRVP	EQDESDIRV	1	0	0	7	2	EQDESDIKDRV	tr_D0A9V0	0.029	36494.71	70.00
100	HLA-A0201	TWNAGDQQRDVAL	NAGDRDVAL	2	0	0	4	2	NAGDQQRDVAL	tr_D0A9V0	0.029	36505.38	70.00
176	HLA-A0201	GAHAALPMHCRR	GAHAALMHC	0	0	0	6	1	GAHAALPMHC	tr_D0A9V0	0.029	36509.33	70.00

34	HLA-A0201	RKIQGHYAKLRR	KIQGHYAKL	1	0	0	0	0	KIQGHYAKL	tr_D0A9V0	0.029	36531.85	70.00
305	HLA-A0201	LGFAAMRQGR	LGFAAMQGR	0	0	0	6	1	LGFAAMRQGR	tr_D0A9V0	0.029	36545.68	70.00
152	HLA-A0201	LIEVATEHSGC	LIEVATEHC	0	0	0	8	2	LIEVATEHSGC	tr_D0A9V0	0.029	36565.06	70.00
584	HLA-A0201	SDFGSTISEAS	SDFGISEAS	0	0	0	4	2	SDFGSTISEAS	tr_D0A9V0	0.029	36571.40	70.00
105	HLA-A0201	DQQRDVALNCG	QQRDVALNC	1	0	0	0	0	QQRDVALNC	tr_D0A9V0	0.029	36580.89	70.00
56	HLA-A0201	EAILARVES	EAILARVES	0	0	0	0	0	EAILARVES	tr_D0A9V0	0.029	36581.29	70.00
138	HLA-A0201	HPDVWKSSTKYHAA	HPDVWYHAA	0	0	0	5	5	HPDVWKSSTKYHAA	tr_D0A9V0	0.029	36586.83	70.00
597	HLA-A0201	ARTLADGVM	ARTLADGVM	0	0	0	0	0	ARTLADGVM	tr_D0A9V0	0.029	36587.63	70.00
450	HLA-A0201	LKKISEGAF	LKKISEGAF	0	0	0	0	0	LKKISEGAF	tr_D0A9V0	0.029	36628.81	70.00
167	HLA-A0201	DPDRALISFG	DPDRALISF	0	0	0	0	0	DPDRALISF	tr_D0A9V0	0.029	36630.79	70.00
706	HLA-A0201	MRPSASSLLS	MPSASSLLS	0	0	0	1	1	MRPSASSLLS	tr_D0A9V0	0.029	36633.98	70.00
141	HLA-A0201	VWKSSTKYHAALIE	VWYHAALIE	0	0	0	2	5	VWKSSTKYHAALIE	tr_D0A9V0	0.029	36640.71	70.00
566	HLA-A0201	CDVKPHNVLLG	CVKPHNVLL	0	0	0	1	1	CDVKPHNVLL	tr_D0A9V0	0.029	36658.96	70.00
396	HLA-A0201	ESDSSDQGSESL	SSDQGSESL	3	0	0	0	0	SSDQGSESL	tr_D0A9V0	0.029	36669.68	70.00
117	HLA-A0201	FKEKPNVFNR	FKEPNVFNR	0	0	0	3	1	FKEKPNVFNR	tr_D0A9V0	0.029	36680.77	70.00
102	HLA-A0201	NAGDQQRDVALNCG	AQRDVALNC	1	0	0	1	3	AGDQQRDVALNC	tr_D0A9V0	0.029	36695.46	70.00
80	HLA-A0201	ETPGESSTA	ETPGESSTA	0	0	0	0	0	ETPGESSTA	tr_D0A9V0	0.029	36718.09	70.00
136	HLA-A0201	VYHPDVWKSSTKYH	VYHPDVWYH	0	0	0	7	5	VYHPDVWKSSTKYH	tr_D0A9V0	0.029	36726.05	70.00
619	HLA-A0201	AGGRCTPQSDIFS	ATPQSDIFS	0	0	0	1	4	AGGRCTPQSDIFS	tr_D0A9V0	0.029	36726.05	70.00
155	HLA-A0201	VATEHSGCVE	VATEHSGCV	0	0	0	0	0	VATEHSGCV	tr_D0A9V0	0.028	36744.34	70.00
32	HLA-A0201	EVRKIQGHYAKLRR	KIQGHYAKL	3	0	0	0	0	KIQGHYAKL	tr_D0A9V0	0.028	36758.65	70.00
394	HLA-A0201	GSESDSSDQGSESL	SSDQGSESL	5	0	0	0	0	SSDQGSESL	tr_D0A9V0	0.028	36762.61	70.00
355	HLA-A0201	LDNHYCVNSS	LDNHYCVSS	0	0	0	7	1	LDNHYCVNSS	tr_D0A9V0	0.028	36763.02	70.00
376	HLA-A0201	IKDRVPETIDSP	IVPETIDSP	0	0	0	1	3	IKDRVPETIDSP	tr_D0A9V0	0.028	36763.42	70.00
33	HLA-A0201	VRKIQGHYAKLRR	IQGHYALRR	3	0	0	6	1	IQGHYAKLRR	tr_D0A9V0	0.028	36765.01	70.00
559	HLA-A0201	HNLGIVHCD	HNLGIVHCD	0	0	0	0	0	HNLGIVHCD	tr_D0A9V0	0.028	36766.19	70.00
494	HLA-A0201	EVASKLHHP	EVASKLHHP	0	0	0	0	0	EVASKLHHP	tr_D0A9V0	0.028	36791.67	70.00
104	HLA-A0201	GDQQRDVALNCG	GQRDVALNC	0	0	0	1	2	GDQQRDVALNC	tr_D0A9V0	0.028	36793.66	70.00
141	HLA-A0201	VWKSSTKYHA	VWSSTKYHA	0	0	0	2	1	VWKSSTKYHA	tr_D0A9V0	0.028	36802.82	70.00
502	HLA-A0201	PNIVRYVSC	PNIVRYVSC	0	0	0	0	0	PNIVRYVSC	tr_D0A9V0	0.028	36802.82	70.00
177	HLA-A0201	AHAALPMHCR	AHAALPMHC	0	0	0	0	0	AHAALPMHC	tr_D0A9V0	0.028	36804.81	70.00
195	HLA-A0201	LFAKLPLDHRD	FALPLDHRD	1	0	0	2	1	FAKLPLDHRD	tr_D0A9V0	0.028	36810.77	70.00
370	HLA-A0201	EQDESDIKDRVPE	EQDESDIRV	0	0	0	7	2	EQDESDIKDRV	tr_D0A9V0	0.028	36811.98	70.00
578	HLA-A0201	DGVCKLSDFGST	GVCKLFGST	1	0	0	5	2	GVCKLSDFGST	tr_D0A9V0	0.028	36817.56	70.00
424	HLA-A0201	CAEGANAADVVP	CAEGANAAV	0	0	0	8	2	CAEGANAADVV	tr_D0A9V0	0.028	36832.29	70.00
305	HLA-A0201	LGFAAMRQGRYGE	LGFAAMYGE	0	0	0	6	4	LGFAAMRQGRYGE	tr_D0A9V0	0.028	36843.45	70.00
696	HLA-A0201	VRACCHMNPSMRP	RACCHMNPM	1	0	0	8	1	RACCHMNPSM	tr_D0A9V0	0.028	36865.79	70.00
252	HLA-A0201	AATSGGASHMQGF	ATSGGMQGF	1	0	0	5	3	ATSGGASHMQGF	tr_D0A9V0	0.028	36868.98	70.00
489	HLA-A0201	LEKEMEVASKLH	LEMEVASKL	0	0	0	1	2	LEKEMEVASKL	tr_D0A9V0	0.028	36875.35	70.00
144	HLA-A0201	SSTKYHAALIE	SSTKYAALI	0	0	0	5	1	SSTKYHAALI	tr_D0A9V0	0.028	36878.95	70.00
181	HLA-A0201	LPMHCRRCCQ	LMHCRRCCQ	0	0	0	1	1	LPMHCRRCCQ	tr_D0A9V0	0.028	36881.75	70.00
116	HLA-A0201	IFKEKPNVFNRRHC	IFVFNRRHC	0	0	0	2	5	IFKEKPNVFNRRHC	tr_D0A9V0	0.028	36882.94	70.00
38	HLA-A0201	GHYAKLRRQ	GHYAKLRRQ	0	0	0	0	0	GHYAKLRRQ	tr_D0A9V0	0.028	36888.53	70.00
61	HLA-A0201	RVESKNEAGGHI	RVNEAGGHI	0	0	0	2	3	RVESKNEAGGHI	tr_D0A9V0	0.028	36890.53	70.00
492	HLA-A0201	EMEVASKLHHP	EMEVALHHP	0	0	0	5	2	EMEVASKLHHP	tr_D0A9V0	0.028	36906.50	70.00
592	HLA-A0201	EASVMARTLAD	EASVMARTL	0	0	0	0	0	EASVMARTL	tr_D0A9V0	0.028	36913.68	70.00
620	HLA-A0201	GGRCTPQSDIFS	CTPQSDIFS	3	0	0	0	0	CTPQSDIFS	tr_D0A9V0	0.028	36916.88	70.00
482	HLA-A0201	RDVVAESLEK	RVVAESLEK	0	0	0	1	1	RDVVAESLEK	tr_D0A9V0	0.028	36924.07	70.00
36	HLA-A0201	IQGHYAKLRRQ	IQYAKLRRQ	0	0	0	2	2	IQGHYAKLRRQ	tr_D0A9V0	0.028	36925.28	70.00
294	HLA-A0201	EVSLSISKQ	EVSLSISKQ	0	0	0	0	0	EVSLSISKQ	tr_D0A9V0	0.028	36943.24	70.00
641	HLA-A0201	LGRPPWQWSSTAP	LGWQWSSTA	0	0	0	2	3	LGRPPWQWSSTA	tr_D0A9V0	0.028	36943.64	70.00
32	HLA-A0201	EVRKIQGHY	EVRKIQGHY	0	0	0	0	0	EVRKIQGHY	tr_D0A9V0	0.028	36945.64	70.00
112	HLA-A0201	LNCGIFKEKP	LNCGIFEKP	0	0	0	6	1	LNCGIFKEKP	tr_D0A9V0	0.028	36946.86	70.00
79	HLA-A0201	TETPGESSTA	TTPGESSTA	0	0	0	1	1	TETPGESSTA	tr_D0A9V0	0.028	36948.84	70.00

293	HLA-A0201	SEVSLSISKQ	SEVSLSISQ	0	0	0	8	1	SEVSLSISKQ	tr_D0A9V0	0.028	36969.24	70.00
142	HLA-A0201	WKSSTKYHAALIE	WTKYHAALI	0	0	0	1	3	WKSSTKYHAALI	tr_D0A9V0	0.028	36980.86	70.00
116	HLA-A0201	IFKEKPNVFNRR	IFKEVFNRR	0	0	0	4	3	IFKEKPNVFNRR	tr_D0A9V0	0.028	36988.44	70.00
684	HLA-A0201	IPASVDHEAAA	ISVDHEAAA	0	0	0	1	2	IPASVDHEAAA	tr_D0A9V0	0.028	37005.26	70.00
143	HLA-A0201	KSSTKYHAALIE	KSSYHAALI	0	0	0	3	2	KSSTKYHAALI	tr_D0A9V0	0.028	37043.32	70.00
77	HLA-A0201	SATETPGESSTAF	SATETSTAF	0	0	0	5	4	SATETPGESSTAF	tr_D0A9V0	0.028	37050.54	70.00
577	HLA-A0201	TDGVCKLSDFG	GVCKLSDFG	2	0	0	0	0	GVCKLSDFG	tr_D0A9V0	0.028	37062.96	70.00
363	HLA-A0201	SSDCKPEEQ	SSDCKPEEQ	0	0	0	0	0	SSDCKPEEQ	tr_D0A9V0	0.028	37065.38	70.00
374	HLA-A0201	SDIKDRVPETIDSP	SVPETIDSP	0	0	0	1	5	SDIKDRVPETIDSP	tr_D0A9V0	0.028	37079.00	70.00
578	HLA-A0201	DGVCKLSDFG	GVCKLSDFG	1	0	0	0	0	GVCKLSDFG	tr_D0A9V0	0.028	37099.48	70.00
566	HLA-A0201	CDVKPHNVL	CDVKPHNVL	0	0	0	0	0	CDVKPHNVL	tr_D0A9V0	0.028	37103.88	70.00
118	HLA-A0201	KEKPNVFNRRHCT	KVFNRRHCT	0	0	0	1	4	KEKPNVFNRRHCT	tr_D0A9V0	0.028	37122.77	70.00
427	HLA-A0201	GANAADVVP	GANAADVVP	0	0	0	0	0	GANAADVVP	tr_D0A9V0	0.028	37125.57	70.00
375	HLA-A0201	DIKDRVPETIDSP	IVPETIDSP	1	0	0	1	3	IKDRVPETIDSP	tr_D0A9V0	0.028	37130.79	70.00
118	HLA-A0201	KEKPNVFNR	KEKPNVFNR	0	0	0	0	0	KEKPNVFNR	tr_D0A9V0	0.028	37131.60	70.00
617	HLA-A0201	VAAGGRCTPQ	VAAGGCTPQ	0	0	0	5	1	VAAGGRCTPQ	tr_D0A9V0	0.027	37150.48	70.00
283	HLA-A0201	LFELRPGESSS	FLRPGESSS	1	0	0	1	1	FELRPGESSS	tr_D0A9V0	0.027	37160.13	70.00
96	HLA-A0201	SISVTWNAGDQ	SISVTWNAQ	0	0	0	8	2	SISVTWNAGDQ	tr_D0A9V0	0.027	37182.26	70.00
466	HLA-A0201	AEDGAQVAIKC	AEDGVAIKC	0	0	0	4	2	AEDGAQVAIKC	tr_D0A9V0	0.027	37183.46	70.00
96	HLA-A0201	SISVTWNAGDQQ	SISVTWNAQ	0	0	0	8	3	SISVTWNAGDQQ	tr_D0A9V0	0.027	37188.28	70.00
426	HLA-A0201	EGANAADVV	EGANAADVV	0	0	0	0	0	EGANAADVV	tr_D0A9V0	0.027	37191.90	70.00
249	HLA-A0201	CRIAATSGGASHMQ	RIAATASHM	1	0	0	5	3	RIAATSGGASHM	tr_D0A9V0	0.027	37209.22	70.00
561	HLA-A0201	LGIVHCDVKPHN	GIVHCDVHN	1	0	0	7	2	GIVHCDVKPHN	tr_D0A9V0	0.027	37214.45	75.00
244	HLA-A0201	VRDGPCRIAAT	VRDGPCIAA	0	0	0	6	1	VRDGPCRIAA	tr_D0A9V0	0.027	37216.47	75.00
562	HLA-A0201	GIVHCDVKPHN	GIVHCDVHN	0	0	0	7	2	GIVHCDVKPHN	tr_D0A9V0	0.027	37221.70	75.00
140	HLA-A0201	DVWKSSTKYHAA	DVWTKYHAA	0	0	0	3	3	DVWKSSTKYHAA	tr_D0A9V0	0.027	37237.41	75.00
65	HLA-A0201	KNEAGGHIRA	KNAGGHIRA	0	0	0	2	1	KNEAGGHIRA	tr_D0A9V0	0.027	37241.44	75.00
641	HLA-A0201	LGRPPWQWS	LGRPPWQWS	0	0	0	0	0	LGRPPWQWS	tr_D0A9V0	0.027	37247.09	75.00
246	HLA-A0201	DGPCRIAAT	DGPCRIAAT	0	0	0	0	0	DGPCRIAAT	tr_D0A9V0	0.027	37263.21	75.00
483	HLA-A0201	DVVAESLEK	DVVAESLEK	0	0	0	0	0	DVVAESLEK	tr_D0A9V0	0.027	37274.50	75.00
442	HLA-A0201	NRNVWTRSLK	NRNVWTRSL	0	0	0	0	0	NRNVWTRSL	tr_D0A9V0	0.027	37279.34	75.00
285	HLA-A0201	ELRPGESSS	ELRPGESSS	0	0	0	0	0	ELRPGESSS	tr_D0A9V0	0.027	37280.54	75.00
158	HLA-A0201	EHSGCVEALDPDR	HSGCVEALR	1	0	0	8	3	HSGCVEALDPDR	tr_D0A9V0	0.027	37292.25	75.00
31	HLA-A0201	VEVRKIQGHYA	VEVIQGHYA	0	0	0	3	2	VEVRKIQGHYA	tr_D0A9V0	0.027	37294.27	75.00
368	HLA-A0201	PEEQDESDIKDRVP	EQDESDIRV	2	0	0	7	2	EQDESDIKDRV	tr_D0A9V0	0.027	37295.88	75.00
78	HLA-A0201	ATETPGESSTAF	ATGESSTAF	0	0	0	2	3	ATETPGESSTAF	tr_D0A9V0	0.027	37296.67	75.00
441	HLA-A0201	SNRNVWTRS	SNRNVWTRS	0	0	0	0	0	SNRNVWTRS	tr_D0A9V0	0.027	37296.67	75.00
473	HLA-A0201	AIKCIPRLRRD	AIKCIPLRR	0	0	0	6	1	AIKCIPRLRR	tr_D0A9V0	0.027	37336.26	75.00
59	HLA-A0201	LARVESKNEAGGHI	LARVAGGHI	0	0	0	4	5	LARVESKNEAGGHI	tr_D0A9V0	0.027	37340.69	75.00
423	HLA-A0201	GCAEGANAADVVP	GCAEGANAV	0	0	0	8	3	GCAEGANAADVV	tr_D0A9V0	0.027	37356.85	75.00
37	HLA-A0201	QGHYAKLRRQL	GHYAKLRQL	1	0	0	6	1	GHYAKLRRQL	tr_D0A9V0	0.027	37369.79	75.00
136	HLA-A0201	VYHPDVWKSST	VYHPDVWST	0	0	0	7	2	VYHPDVWKSST	tr_D0A9V0	0.027	37371.41	75.00
250	HLA-A0201	RIAATSGGASHMQG	RIAATSMQG	0	0	0	6	5	RIAATSGGASHMQG	tr_D0A9V0	0.027	37395.68	75.00
567	HLA-A0201	DVKPHNVLLG	DVPHNVLLG	0	0	0	2	1	DVKPHNVLLG	tr_D0A9V0	0.027	37406.61	75.00
240	HLA-A0201	LSYAVRDGPCR	LSYAVDGPC	0	0	0	5	1	LSYAVRDGPC	tr_D0A9V0	0.027	37407.82	75.00
489	HLA-A0201	LEKEMEVAS	LEKEMEVAS	0	0	0	0	0	LEKEMEVAS	tr_D0A9V0	0.027	37407.82	75.00
80	HLA-A0201	ETPGESSTAFVR	ETPGSTAFV	0	0	0	4	2	ETPGESSTAFV	tr_D0A9V0	0.027	37409.43	75.00
211	HLA-A0201	VDTNEFLVG	VDTNEFLVG	0	0	0	0	0	VDTNEFLVG	tr_D0A9V0	0.027	37414.31	75.00
336	HLA-A0201	MRLCRQRCRR	MLCRQRCRR	0	0	0	1	1	MRLCRQRCRR	tr_D0A9V0	0.027	37421.18	75.00
244	HLA-A0201	VRDGPCRIAATS	VRDGPCIAA	0	0	0	6	1	VRDGPCRIAA	tr_D0A9V0	0.027	37421.98	75.00
414	HLA-A0201	SSSSSLDGSGCAE	SLDGSGCAE	0	0	0	1	4	SSSSSLDGSGCAE	tr_D0A9V0	0.027	37431.71	75.00
77	HLA-A0201	SATETPGES	SATETPGES	0	0	0	0	0	SATETPGES	tr_D0A9V0	0.027	37437.38	75.00
139	HLA-A0201	PDVWKSSTKYHA	DVWKSSYHA	1	0	0	6	2	DVWKSSTKYHA	tr_D0A9V0	0.027	37468.59	75.00
375	HLA-A0201	DIKDRVPETID	DIDRVPETI	0	0	0	2	1	DIKDRVPETI	tr_D0A9V0	0.027	37469.00	75.00

159	HLA-A0201	HSGCVEALDPDR	HSGCVEALR	0	0	0	8	3	HSGCVEALDPDR	tr_D0A9V0	0.027	37473.04	75.00
489	HLA-A0201	LEKEMEVASK	LEEMEVASK	0	0	0	2	1	LEKEMEVASK	tr_D0A9V0	0.027	37495.76	75.00
416	HLA-A0201	SSSLDGSGC	SSSLDGSGC	0	0	0	0	0	SSSLDGSGC	tr_D0A9V0	0.027	37513.61	75.00
374	HLA-A0201	SDIKDRVPETID	SDIKVPETI	0	0	0	4	2	SDIKDRVPETI	tr_D0A9V0	0.026	37538.78	75.00
524	HLA-A0201	VPGGSLHSVIK	VPGGSLHSV	0	0	0	0	0	VPGGSLHSV	tr_D0A9V0	0.026	37541.22	75.00
375	HLA-A0201	DIKDRVPETIDSPR	DIDRVPETI	0	0	0	2	1	DIKDRVPETI	tr_D0A9V0	0.026	37542.43	75.00
399	HLA-A0201	SSDQGSESLFK	SQGSESLFK	0	0	0	1	2	SSDQGSESLFK	tr_D0A9V0	0.026	37546.91	75.00
490	HLA-A0201	EKEMEVASKLH	KEMEVASKL	1	0	0	0	0	KEMEVASKL	tr_D0A9V0	0.026	37556.26	75.00
117	HLA-A0201	FKEKPNVFNRRHC	FNVFNRRHC	0	0	0	1	4	FKEKPNVFNRRHC	tr_D0A9V0	0.026	37569.66	75.00
336	HLA-A0201	MRLCRQRCRRNDT	RLCCRRNDT	1	0	0	3	3	RLCRQRCRRNDT	tr_D0A9V0	0.026	37576.18	75.00
316	HLA-A0201	GEAMAHYLP	GEAMAHYLP	0	0	0	0	0	GEAMAHYLP	tr_D0A9V0	0.026	37596.51	75.00
292	HLA-A0201	SSEVSLSISKQ	SSEVSLISK	0	0	0	6	1	SSEVSLSISK	tr_D0A9V0	0.026	37600.97	75.00
324	HLA-A0201	PMVKMDMQAKR	PMVKMDMQA	0	0	0	0	0	PMVKMDMQA	tr_D0A9V0	0.026	37605.05	75.00
136	HLA-A0201	VYHPDVWKSSTK	VYHPDVWTK	0	0	0	7	3	VYHPDVWKSSTK	tr_D0A9V0	0.026	37608.71	75.00
440	HLA-A0201	DSNRNVWTRSLK	DSNRNVWTL	0	0	0	8	2	DSNRNVWTRSL	tr_D0A9V0	0.026	37623.36	75.00
441	HLA-A0201	SNRNVWTRSLK	SNRNVWTSL	0	0	0	7	1	SNRNVWTRSL	tr_D0A9V0	0.026	37635.57	75.00
31	HLA-A0201	VEVRKIQGHY	VVRKIQGHY	0	0	0	1	1	VEVRKIQGHY	tr_D0A9V0	0.026	37640.86	75.00
113	HLA-A0201	NCGIFKEKP	NCGIFKEKP	0	0	0	0	0	NCGIFKEKP	tr_D0A9V0	0.026	37657.15	75.00
39	HLA-A0201	HYAKLRRQL	HYAKLRRQL	0	0	0	0	0	HYAKLRRQL	tr_D0A9V0	0.026	37670.61	75.00
376	HLA-A0201	IKDRVPETIDSPR	IKDRVPETI	0	0	0	0	0	IKDRVPETI	tr_D0A9V0	0.026	37681.20	75.00
493	HLA-A0201	MEVASKLHH	MEVASKLHH	0	0	0	0	0	MEVASKLHH	tr_D0A9V0	0.026	37683.25	75.00
158	HLA-A0201	EHSGCVEALDPD	EHSGCVEAL	0	0	0	0	0	EHSGCVEAL	tr_D0A9V0	0.026	37698.34	75.00
689	HLA-A0201	DHEAAAFVR	DHEAAAFVR	0	0	0	0	0	DHEAAAFVR	tr_D0A9V0	0.026	37698.34	75.00
415	HLA-A0201	SSSSLDGSGCAE	SLDGSGCAE	3	0	0	0	0	SLDGSGCAE	tr_D0A9V0	0.026	37730.98	75.00
422	HLA-A0201	SGCAEGANAADVVP	CAEGANAAV	2	0	0	8	2	CAEGANAADVV	tr_D0A9V0	0.026	37750.57	75.00
407	HLA-A0201	LFKMYAPSSS	LFMYAPSSS	0	0	0	2	1	LFKMYAPSSS	tr_D0A9V0	0.026	37755.88	75.00
11	HLA-A0201	SASNLEIPDN	SASNLEIPN	0	0	0	8	1	SASNLEIPDN	tr_D0A9V0	0.026	37761.21	75.00
10	HLA-A0201	KSASNLEIPD	KSASNLIPD	0	0	0	6	1	KSASNLEIPD	tr_D0A9V0	0.026	37769.79	75.00
160	HLA-A0201	SGCVEALDPD	SGCVALDPD	0	0	0	4	1	SGCVEALDPD	tr_D0A9V0	0.026	37771.00	75.00
400	HLA-A0201	SDQGSESLF	SDQGSESLF	0	0	0	0	0	SDQGSESLF	tr_D0A9V0	0.026	37775.93	75.00
369	HLA-A0201	EEQDESDIKDRVPE	EQDESDIRV	1	0	0	7	2	EQDESDIKDRV	tr_D0A9V0	0.026	37778.78	75.00
341	HLA-A0201	QRCRRNDTV	QRCRRNDTV	0	0	0	0	0	QRCRRNDTV	tr_D0A9V0	0.026	37780.82	75.00
398	HLA-A0201	DSSDQGSESLF	SSDQGSELF	1	0	0	7	1	SSDQGSESLF	tr_D0A9V0	0.026	37786.55	75.00
99	HLA-A0201	VTWNAGDQQR	VTWAGDQQR	0	0	0	3	1	VTWNAGDQQR	tr_D0A9V0	0.026	37791.04	75.00
97	HLA-A0201	ISVTWNAGD	ISVTWNAGD	0	0	0	0	0	ISVTWNAGD	tr_D0A9V0	0.026	37798.81	75.00
491	HLA-A0201	KEMEVASKLHHPN	KEMEVAHPN	0	0	0	6	4	KEMEVASKLHHPN	tr_D0A9V0	0.026	37799.22	75.00
197	HLA-A0201	AKLPLDHRDS	KLPLDHRDS	1	0	0	0	0	KLPLDHRDS	tr_D0A9V0	0.026	37804.12	75.00
96	HLA-A0201	SISVTWNAGDQQRD	SISVTWNAD	0	0	0	8	5	SISVTWNAGDQQRD	tr_D0A9V0	0.026	37837.68	75.00
416	HLA-A0201	SSSLDGSGCAE	SLDGSGCAE	0	0	0	1	2	SSSLDGSGCAE	tr_D0A9V0	0.026	37847.91	75.00
434	HLA-A0201	VPLFLTDSN	VPLFLTDSN	0	0	0	0	0	VPLFLTDSN	tr_D0A9V0	0.026	37854.89	75.00
116	HLA-A0201	IFKEKPNVFNR	IFKENVFNR	0	0	0	4	2	IFKEKPNVFNR	tr_D0A9V0	0.026	37856.92	75.00
76	HLA-A0201	GSATETPGESSTAF	SATETPTAF	1	0	0	6	4	SATETPGESSTAF	tr_D0A9V0	0.026	37862.27	75.00
341	HLA-A0201	QRCRRNDTVPY	CRRNDTVPY	2	0	0	0	0	CRRNDTVPY	tr_D0A9V0	0.026	37867.59	75.00
419	HLA-A0201	LDGSGCAEGANAA	LCAEGANAA	0	0	0	1	4	LDGSGCAEGANAA	tr_D0A9V0	0.026	37874.96	75.00
413	HLA-A0201	PSSSSSLDGSGCAE	SLDGSGCAE	5	0	0	0	0	SLDGSGCAE	tr_D0A9V0	0.026	37900.79	75.00
79	HLA-A0201	TETPGESSTAFVR	ETPGSTAFV	1	0	0	4	2	ETPGESSTAFV	tr_D0A9V0	0.026	37902.43	75.00
283	HLA-A0201	LFELRPGES	LFELRPGES	0	0	0	0	0	LFELRPGES	tr_D0A9V0	0.026	37908.98	75.00
602	HLA-A0201	DGVMLRGTA	DGVMLRGTA	0	0	0	0	0	DGVMLRGTA	tr_D0A9V0	0.026	37911.45	75.00
439	HLA-A0201	TDSNRNVWTR	TSNRNVWTR	0	0	0	1	1	TDSNRNVWTR	tr_D0A9V0	0.026	37917.20	75.00
375	HLA-A0201	DIKDRVPETIDS	DIDRVPETI	0	0	0	2	1	DIKDRVPETI	tr_D0A9V0	0.026	37922.53	75.00
651	HLA-A0201	TAPAGSDATRL	APAGSDATL	1	0	0	8	1	APAGSDATRL	tr_D0A9V0	0.026	37927.03	75.00
116	HLA-A0201	IFKEKPNVFN	IFKEKPNVF	0	0	0	0	0	IFKEKPNVF	tr_D0A9V0	0.026	37930.72	75.00
18	HLA-A0201	PDNADIFLGR	PDNAIFLGR	0	0	0	4	1	PDNADIFLGR	tr_D0A9V0	0.026	37936.47	75.00
168	HLA-A0201	PDRALISFG	PDRALISFG	0	0	0	0	0	PDRALISFG	tr_D0A9V0	0.026	37937.30	75.00

696	HLA-A0201	VRACCHMNP	VRACCHMNP	0	0	0	0	0	VRACCHMNP	tr_D0A9V0	0.025	37946.33	75.00
137	HLA-A0201	YHPDVWKSSTKYH	YVWKSSTKY	0	0	0	1	3	YHPDVWKSSTKY	tr_D0A9V0	0.025	37955.36	75.00
117	HLA-A0201	FKEKPNVFNRR	FKEKVFNRR	0	0	0	4	2	FKEKPNVFNRR	tr_D0A9V0	0.025	37966.45	75.00
414	HLA-A0201	SSSSSLDGSGCA	SSSSSLDGA	0	0	0	8	3	SSSSSLDGSGCA	tr_D0A9V0	0.025	37968.51	75.00
439	HLA-A0201	TDSNRNVWTRSLK	SNRNVWTSL	2	0	0	7	1	SNRNVWTRSL	tr_D0A9V0	0.025	37968.93	75.00
653	HLA-A0201	PAGSDATRL	PAGSDATRL	0	0	0	0	0	PAGSDATRL	tr_D0A9V0	0.025	37975.07	75.00
116	HLA-A0201	IFKEKPNVFNRRH	IFKEVFNRR	0	0	0	4	3	IFKEKPNVFNRR	tr_D0A9V0	0.025	37977.13	75.00
312	HLA-A0201	QGRYGEAMA	QGRYGEAMA	0	0	0	0	0	QGRYGEAMA	tr_D0A9V0	0.025	37982.89	75.00
419	HLA-A0201	LDGSGCAEG	LDGSGCAEG	0	0	0	0	0	LDGSGCAEG	tr_D0A9V0	0.025	37994.40	75.00
620	HLA-A0201	GGRCTPQSDI	GGRCTPQSI	0	0	0	8	1	GGRCTPQSDI	tr_D0A9V0	0.025	37999.32	75.00
254	HLA-A0201	TSGGASHMQGF	TSGGAMQGF	0	0	0	5	2	TSGGASHMQGF	tr_D0A9V0	0.025	38007.14	75.00
642	HLA-A0201	GRPPWQWSST	GRPPWQWST	0	0	0	7	1	GRPPWQWSST	tr_D0A9V0	0.025	38019.48	75.00
374	HLA-A0201	SDIKDRVPETIDS	SDIKVPETI	0	0	0	4	2	SDIKDRVPETI	tr_D0A9V0	0.025	38036.77	75.00
377	HLA-A0201	KDRVPETIDSP	KVPETIDSP	0	0	0	1	2	KDRVPETIDSP	tr_D0A9V0	0.025	38041.70	75.00
490	HLA-A0201	EKEMEVASK	EKEMEVASK	0	0	0	0	0	EKEMEVASK	tr_D0A9V0	0.025	38044.18	75.00
698	HLA-A0201	ACCHMNPSMRP	CCHMNPSMR	1	0	0	0	0	CCHMNPSMR	tr_D0A9V0	0.025	38045.82	75.00
490	HLA-A0201	EKEMEVASKLHHPN	KEMEVAHPN	1	0	0	6	4	KEMEVASKLHHPN	tr_D0A9V0	0.025	38048.30	75.00
356	HLA-A0201	DNHYCVNSS	DNHYCVNSS	0	0	0	0	0	DNHYCVNSS	tr_D0A9V0	0.025	38068.05	75.00
56	HLA-A0201	EAILARVESKN	AILARVESN	1	0	0	8	1	AILARVESKN	tr_D0A9V0	0.025	38074.63	75.00
357	HLA-A0201	NHYCVNSSDCKPE	NHYCVCKPE	0	0	0	5	4	NHYCVNSSDCKPE	tr_D0A9V0	0.025	38094.43	75.00
245	HLA-A0201	RDGPCRIAATS	RDGPCIAAT	0	0	0	5	1	RDGPCRIAAT	tr_D0A9V0	0.025	38103.91	75.00
178	HLA-A0201	HAALPMHCRR	HAALPMCRR	0	0	0	6	1	HAALPMHCRR	tr_D0A9V0	0.025	38111.73	75.00
380	HLA-A0201	VPETIDSPR	VPETIDSPR	0	0	0	0	0	VPETIDSPR	tr_D0A9V0	0.025	38127.00	75.00
321	HLA-A0201	HYLPMVKMD	HYLPMVKMD	0	0	0	0	0	HYLPMVKMD	tr_D0A9V0	0.025	38128.64	75.00
219	HLA-A0201	GTCGAFSRNAR	GTCAFSRNA	0	0	0	3	1	GTCGAFSRNA	tr_D0A9V0	0.025	38133.19	75.00
397	HLA-A0201	SDSSDQGSESLF	SSDQGSELF	2	0	0	7	1	SSDQGSESLF	tr_D0A9V0	0.025	38140.20	75.00
219	HLA-A0201	GTCGAFSRN	GTCGAFSRN	0	0	0	0	0	GTCGAFSRN	tr_D0A9V0	0.025	38158.78	75.00
57	HLA-A0201	AILARVESKNE	AILARVESE	0	0	0	8	2	AILARVESKNE	tr_D0A9V0	0.025	38162.91	75.00
55	HLA-A0201	PEAILARVESKN	AILARVESN	2	0	0	8	1	AILARVESKN	tr_D0A9V0	0.025	38166.63	75.00
138	HLA-A0201	HPDVWKSSTKY	HVWKSSTKY	0	0	0	1	2	HPDVWKSSTKY	tr_D0A9V0	0.025	38176.13	75.00
120	HLA-A0201	KPNVFNRRH	KPNVFNRRH	0	0	0	0	0	KPNVFNRRH	tr_D0A9V0	0.025	38176.13	75.00
649	HLA-A0201	SSTAPAGSDA	STAPAGSDA	0	0	0	1	1	SSTAPAGSDA	tr_D0A9V0	0.025	38177.36	75.00
287	HLA-A0201	RPGESSSEVSL	RPGESSVSL	0	0	0	6	2	RPGESSSEVSL	tr_D0A9V0	0.025	38179.43	75.00
397	HLA-A0201	SDSSDQGSESLFK	SQGSESLFK	0	0	0	1	4	SDSSDQGSESLFK	tr_D0A9V0	0.025	38184.38	75.00
359	HLA-A0201	YCVNSSDCK	YCVNSSDCK	0	0	0	0	0	YCVNSSDCK	tr_D0A9V0	0.025	38200.09	75.00
118	HLA-A0201	KEKPNVFNRR	KEKPVFNRR	0	0	0	4	1	KEKPNVFNRR	tr_D0A9V0	0.025	38217.05	75.00
467	HLA-A0201	EDGAQVAIKC	DGAQVAIKC	1	0	0	0	0	DGAQVAIKC	tr_D0A9V0	0.025	38241.43	75.00
371	HLA-A0201	QDESDIKDRV	QDESDIDRV	0	0	0	6	1	QDESDIKDRV	tr_D0A9V0	0.025	38241.85	75.00
373	HLA-A0201	ESDIKDRVPET	SIKDRVPET	1	0	0	1	1	SDIKDRVPET	tr_D0A9V0	0.025	38243.08	75.00
166	HLA-A0201	LDPDRALIS	LDPDRALIS	0	0	0	0	0	LDPDRALIS	tr_D0A9V0	0.025	38253.86	75.00
378	HLA-A0201	DRVPETIDSPR	RVPETIDPR	1	0	0	7	1	RVPETIDSPR	tr_D0A9V0	0.025	38255.93	75.00
18	HLA-A0201	PDNADIFLG	PDNADIFLG	0	0	0	0	0	PDNADIFLG	tr_D0A9V0	0.025	38269.16	75.00
160	HLA-A0201	SGCVEALDPDR	GCVEALDPR	1	0	0	8	1	GCVEALDPDR	tr_D0A9V0	0.025	38270.00	75.00
81	HLA-A0201	TPGESSTAFVR	TPGESTAFV	0	0	0	4	1	TPGESSTAFV	tr_D0A9V0	0.025	38273.31	75.00
371	HLA-A0201	QDESDIKDRVPET	SIKDRVPET	3	0	0	1	1	SDIKDRVPET	tr_D0A9V0	0.025	38275.80	75.00
396	HLA-A0201	ESDSSDQGSESLF	SSDQGSELF	3	0	0	7	1	SSDQGSESLF	tr_D0A9V0	0.025	38294.86	75.00
372	HLA-A0201	DESDIKDRVPET	SIKDRVPET	2	0	0	1	1	SDIKDRVPET	tr_D0A9V0	0.025	38296.52	75.00
379	HLA-A0201	RVPETIDSPRRP	RVPETIDSP	0	0	0	8	3	RVPETIDSPRRP	tr_D0A9V0	0.025	38297.75	75.00
69	HLA-A0201	GGHIRANGSA	GGHIRANSA	0	0	0	7	1	GGHIRANGSA	tr_D0A9V0	0.025	38302.71	75.00
12	HLA-A0201	ASNLEIPDN	ASNLEIPDN	0	0	0	0	0	ASNLEIPDN	tr_D0A9V0	0.025	38311.86	75.00
502	HLA-A0201	PNIVRYVSCS	NIVRYVSCS	1	0	0	0	0	NIVRYVSCS	tr_D0A9V0	0.025	38312.67	75.00
395	HLA-A0201	SESDSSDQGSESLF	SSDQGSELF	4	0	0	7	1	SSDQGSESLF	tr_D0A9V0	0.025	38316.40	75.00
60	HLA-A0201	ARVESKNEAGGHI	RVNEAGGHI	1	0	0	2	3	RVESKNEAGGHI	tr_D0A9V0	0.025	38320.97	75.00
376	HLA-A0201	IKDRVPETIDSPRR	IKDRVPETI	0	0	0	0	0	IKDRVPETI	tr_D0A9V0	0.025	38324.29	75.00

360	HLA-A0201	CVNSSDCKP	CVNSSDCKP	0	0	0	0	0	CVNSSDCKP	tr_D0A9V0	0.025	38330.49	75.00
356	HLA-A0201	DNHYCVNSSDCKPE	NHYCVCKPE	1	0	0	5	4	NHYCVNSSDCKPE	tr_D0A9V0	0.025	38338.38	75.00
620	HLA-A0201	GGRCTPQSDIF	GGRCTPQIF	0	0	0	7	2	GGRCTPQSDIF	tr_D0A9V0	0.025	38341.69	75.00
398	HLA-A0201	DSSDQGSESLFK	SSDQGSLFK	1	0	0	5	2	SSDQGSESLFK	tr_D0A9V0	0.024	38366.18	75.00
376	HLA-A0201	IKDRVPETIDS	IKDRVPETI	0	0	0	0	0	IKDRVPETI	tr_D0A9V0	0.024	38366.18	75.00
377	HLA-A0201	KDRVPETIDSPR	RVPETISPR	2	0	0	6	1	RVPETIDSPR	tr_D0A9V0	0.024	38416.86	75.00
283	HLA-A0201	LFELRPGESS	LFELRPESS	0	0	0	6	1	LFELRPGESS	tr_D0A9V0	0.024	38432.25	80.00
218	HLA-A0201	VGTCGAFSRNAR	GTCAFSRNA	1	0	0	3	1	GTCGAFSRNA	tr_D0A9V0	0.024	38437.23	80.00
571	HLA-A0201	HNVLLGTDG	HNVLLGTDG	0	0	0	0	0	HNVLLGTDG	tr_D0A9V0	0.024	38458.03	80.00
619	HLA-A0201	AGGRCTPQSDIF	GGRCTPQIF	1	0	0	7	2	GGRCTPQSDIF	tr_D0A9V0	0.024	38470.93	80.00
220	HLA-A0201	TCGAFSRNAR	TCGAFSRNA	0	0	0	0	0	TCGAFSRNA	tr_D0A9V0	0.024	38481.75	80.00
440	HLA-A0201	DSNRNVWTRS	DSNRNVWTR	0	0	0	0	0	DSNRNVWTR	tr_D0A9V0	0.024	38509.66	80.00
119	HLA-A0201	EKPNVFNRR	EKPNVFNRR	0	0	0	0	0	EKPNVFNRR	tr_D0A9V0	0.024	38514.25	80.00
312	HLA-A0201	QGRYGEAMAH	QGYGEAMAH	0	0	0	2	1	QGRYGEAMAH	tr_D0A9V0	0.024	38523.00	80.00
284	HLA-A0201	FELRPGESS	FELRPGESS	0	0	0	0	0	FELRPGESS	tr_D0A9V0	0.024	38530.92	80.00
77	HLA-A0201	SATETPGESSTA	SATETPSTA	0	0	0	6	3	SATETPGESSTA	tr_D0A9V0	0.024	38531.77	80.00
244	HLA-A0201	VRDGPCRIAATSG	VRDGPCIAA	0	0	0	6	1	VRDGPCRIAA	tr_D0A9V0	0.024	38544.69	80.00
451	HLA-A0201	KKISEGAFS	KKISEGAFS	0	0	0	0	0	KKISEGAFS	tr_D0A9V0	0.024	38560.13	80.00
159	HLA-A0201	HSGCVEALDPD	HSGCVALDP	0	0	0	5	1	HSGCVEALDP	tr_D0A9V0	0.024	38577.22	80.00
69	HLA-A0201	GGHIRANGS	GGHIRANGS	0	0	0	0	0	GGHIRANGS	tr_D0A9V0	0.024	38581.40	80.00
246	HLA-A0201	DGPCRIAATS	GPCRIAATS	1	0	0	0	0	GPCRIAATS	tr_D0A9V0	0.024	38593.09	80.00
524	HLA-A0201	VPGGSLHSVIKN	VPGGSLHSV	0	0	0	0	0	VPGGSLHSV	tr_D0A9V0	0.024	38594.76	80.00
118	HLA-A0201	KEKPNVFNRRHC	KEKPNVFHC	0	0	0	7	3	KEKPNVFNRRHC	tr_D0A9V0	0.024	38598.52	80.00
483	HLA-A0201	DVVAESLEKE	VVAESLEKE	1	0	0	0	0	VVAESLEKE	tr_D0A9V0	0.024	38621.93	80.00
121	HLA-A0201	PNVFNRRHCT	NVFNRRHCT	1	0	0	0	0	NVFNRRHCT	tr_D0A9V0	0.024	38639.04	80.00
6	HLA-A0201	VSALKSASN	VSALKSASN	0	0	0	0	0	VSALKSASN	tr_D0A9V0	0.024	38657.46	80.00
357	HLA-A0201	NHYCVNSSDCKPEE	NHYCVCKPE	0	0	0	5	4	NHYCVNSSDCKPE	tr_D0A9V0	0.024	38666.24	80.00
336	HLA-A0201	MRLCRQRCRRN	RLCQRCRRN	1	0	0	3	1	RLCRQRCRRN	tr_D0A9V0	0.024	38668.33	80.00
654	HLA-A0201	AGSDATRLRS	ASDATRLRS	0	0	0	1	1	AGSDATRLRS	tr_D0A9V0	0.024	38684.64	80.00
350	HLA-A0201	PYVRVLDNH	PYVRVLDNH	0	0	0	0	0	PYVRVLDNH	tr_D0A9V0	0.024	38703.06	80.00
614	HLA-A0201	APEVAAGGR	APEVAAGGR	0	0	0	0	0	APEVAAGGR	tr_D0A9V0	0.024	38707.25	80.00
338	HLA-A0201	LCRQRCRRN	LCRQRCRRN	0	0	0	0	0	LCRQRCRRN	tr_D0A9V0	0.024	38709.35	80.00
139	HLA-A0201	PDVWKSSTKYHAA	DVWTKYHAA	1	0	0	3	3	DVWKSSTKYHAA	tr_D0A9V0	0.024	38729.05	80.00
396	HLA-A0201	ESDSSDQGSESLFK	SQGSESLFK	4	0	0	1	1	SDQGSESLFK	tr_D0A9V0	0.024	38750.41	80.00
360	HLA-A0201	CVNSSDCKPE	CVNSSDCPE	0	0	0	7	1	CVNSSDCKPE	tr_D0A9V0	0.024	38754.61	80.00
80	HLA-A0201	ETPGESSTAF	ETGESSTAF	0	0	0	2	1	ETPGESSTAF	tr_D0A9V0	0.024	38757.96	80.00
117	HLA-A0201	FKEKPNVFNRRH	FPNVFNRRH	0	0	0	1	3	FKEKPNVFNRRH	tr_D0A9V0	0.024	38760.06	80.00
247	HLA-A0201	GPCRIAATSG	GPCRIAATG	0	0	0	8	1	GPCRIAATSG	tr_D0A9V0	0.023	38774.31	80.00
56	HLA-A0201	EAILARVESKNE	AILARVESE	1	0	0	8	2	AILARVESKNE	tr_D0A9V0	0.023	38775.16	80.00
614	HLA-A0201	APEVAAGGRCTPQ	APEVACTPQ	0	0	0	5	4	APEVAAGGRCTPQ	tr_D0A9V0	0.023	38778.94	80.00
340	HLA-A0201	RQRCRRNDTVP	RQCRRNDTV	0	0	0	2	1	RQRCRRNDTV	tr_D0A9V0	0.023	38782.29	80.00
379	HLA-A0201	RVPETIDSPRRPDG	RVPETIDSG	0	0	0	8	5	RVPETIDSPRRPDG	tr_D0A9V0	0.023	38787.74	80.00
306	HLA-A0201	GFAAMRQGRY	FAAMRQGRY	1	0	0	0	0	FAAMRQGRY	tr_D0A9V0	0.023	38796.57	80.00
77	HLA-A0201	SATETPGESS	SATETPESS	0	0	0	6	1	SATETPGESS	tr_D0A9V0	0.023	38797.82	80.00
97	HLA-A0201	ISVTWNAGDQ	ISVTWNAGQ	0	0	0	8	1	ISVTWNAGDQ	tr_D0A9V0	0.023	38814.19	80.00
6	HLA-A0201	VSALKSASNLE	VSALKSASL	0	0	0	8	1	VSALKSASNL	tr_D0A9V0	0.023	38836.05	80.00
78	HLA-A0201	ATETPGESS	ATETPGESS	0	0	0	0	0	ATETPGESS	tr_D0A9V0	0.023	38838.15	80.00
55	HLA-A0201	PEAILARVESKNE	AILARVESE	2	0	0	8	2	AILARVESKNE	tr_D0A9V0	0.023	38845.72	80.00
378	HLA-A0201	DRVPETIDSPRRP	RVPETIDSP	1	0	0	8	3	RVPETIDSPRRP	tr_D0A9V0	0.023	38850.33	80.00
73	HLA-A0201	RANGSATETP	RANGSATET	0	0	0	0	0	RANGSATET	tr_D0A9V0	0.023	38877.67	80.00
61	HLA-A0201	RVESKNEAG	RVESKNEAG	0	0	0	0	0	RVESKNEAG	tr_D0A9V0	0.023	38882.29	80.00
105	HLA-A0201	DQQRDVALN	DQQRDVALN	0	0	0	0	0	DQQRDVALN	tr_D0A9V0	0.023	38883.57	80.00
657	HLA-A0201	DATRLRSLF	DATRLRSLF	0	0	0	0	0	DATRLRSLF	tr_D0A9V0	0.023	38900.80	80.00
177	HLA-A0201	AHAALPMHCRR	AHAALPMHC	0	0	0	0	0	AHAALPMHC	tr_D0A9V0	0.023	38911.76	80.00

423	HLA-A0201	GCAEGANAAD	GCAEGANAA	0	0	0	0	0	GCAEGANAA	tr_D0A9V0	0.023	38917.63	80.00
98	HLA-A0201	SVTWNAGDQQ	SVWNAGDQQ	0	0	0	2	1	SVTWNAGDQQ	tr_D0A9V0	0.023	38934.08	80.00
379	HLA-A0201	RVPETIDSPRR	RVPETIDSR	0	0	0	8	2	RVPETIDSPRR	tr_D0A9V0	0.023	38940.81	80.00
286	HLA-A0201	LRPGESSSEVSL	RPGESSVSL	1	0	0	6	2	RPGESSSEVSL	tr_D0A9V0	0.023	38957.26	80.00
421	HLA-A0201	GSGCAEGANAA	GSAEGANAA	0	0	0	2	2	GSGCAEGANAA	tr_D0A9V0	0.023	38966.93	80.00
244	HLA-A0201	VRDGPCRIAATSGG	VRDGPCIAA	0	0	0	6	1	VRDGPCRIAA	tr_D0A9V0	0.023	38974.95	80.00
377	HLA-A0201	KDRVPETIDSPRRP	RVPETIDSP	2	0	0	8	3	RVPETIDSPRRP	tr_D0A9V0	0.023	39028.55	80.00
475	HLA-A0201	KCIPRLRRD	KCIPRLRRD	0	0	0	0	0	KCIPRLRRD	tr_D0A9V0	0.023	39046.70	80.00
370	HLA-A0201	EQDESDIKDR	EQDESDIDR	0	0	0	7	1	EQDESDIKDR	tr_D0A9V0	0.023	39047.99	80.00
650	HLA-A0201	STAPAGSDATRLR	STAPAATRL	0	0	0	5	3	STAPAGSDATRL	tr_D0A9V0	0.023	39090.24	80.00
419	HLA-A0201	LDGSGCAEGANA	LDGSGCAEA	0	0	0	8	3	LDGSGCAEGANA	tr_D0A9V0	0.023	39098.71	80.00
138	HLA-A0201	HPDVWKSSTKYH	DVWKSSTYH	2	0	0	7	1	DVWKSSTKYH	tr_D0A9V0	0.023	39102.95	80.00
492	HLA-A0201	EMEVASKLHHPN	EMEVALHHP	0	0	0	5	2	EMEVASKLHHP	tr_D0A9V0	0.023	39110.14	80.00
650	HLA-A0201	STAPAGSDATRLRS	STAPAATRL	0	0	0	5	3	STAPAGSDATRL	tr_D0A9V0	0.023	39110.57	80.00
614	HLA-A0201	APEVAAGGRCTPQS	APEVACTPQ	0	0	0	5	4	APEVAAGGRCTPQ	tr_D0A9V0	0.023	39112.25	80.00
700	HLA-A0201	CHMNPSMRP	CHMNPSMRP	0	0	0	0	0	CHMNPSMRP	tr_D0A9V0	0.023	39137.23	80.00
643	HLA-A0201	RPPWQWSSTAP	RPWQWSSTA	0	0	0	1	1	RPPWQWSSTA	tr_D0A9V0	0.023	39162.20	80.00
120	HLA-A0201	KPNVFNRRHCT	KVFNRRHCT	0	0	0	1	2	KPNVFNRRHCT	tr_D0A9V0	0.023	39166.45	80.00
355	HLA-A0201	LDNHYCVNSSDC	LDNHYCVNC	0	0	0	8	3	LDNHYCVNSSDC	tr_D0A9V0	0.023	39179.59	80.00
247	HLA-A0201	GPCRIAATSGGASH	RIAATSASH	3	0	0	6	2	RIAATSGGASH	tr_D0A9V0	0.023	39188.91	80.00
450	HLA-A0201	LKKISEGAFS	LKISEGAFS	0	0	0	1	1	LKKISEGAFS	tr_D0A9V0	0.023	39192.73	80.00
119	HLA-A0201	EKPNVFNRRHCT	KVFNRRHCT	1	0	0	1	2	KPNVFNRRHCT	tr_D0A9V0	0.023	39193.59	80.00
526	HLA-A0201	GGSLHSVIKN	GGSLSVIKN	0	0	0	4	1	GGSLHSVIKN	tr_D0A9V0	0.023	39194.85	80.00
621	HLA-A0201	GRCTPQSDIFS	CTPQSDIFS	2	0	0	0	0	CTPQSDIFS	tr_D0A9V0	0.022	39196.97	80.00
288	HLA-A0201	PGESSSEVSL	GESSSEVSL	1	0	0	0	0	GESSSEVSL	tr_D0A9V0	0.022	39207.59	80.00
339	HLA-A0201	CRQRCRRNDTVP	RQCRRNDTV	1	0	0	2	1	RQRCRRNDTV	tr_D0A9V0	0.022	39211.41	80.00
642	HLA-A0201	GRPPWQWSSTAP	RPWQWSSTA	1	0	0	1	1	RPPWQWSSTA	tr_D0A9V0	0.022	39214.80	80.00
355	HLA-A0201	LDNHYCVNSSDCK	YCVNSSDCK	4	0	0	0	0	YCVNSSDCK	tr_D0A9V0	0.022	39225.39	80.00
410	HLA-A0201	MYAPSSSSS	MYAPSSSSS	0	0	0	0	0	MYAPSSSSS	tr_D0A9V0	0.022	39251.31	80.00
78	HLA-A0201	ATETPGESST	ATTPGESST	0	0	0	2	1	ATETPGESST	tr_D0A9V0	0.022	39262.78	80.00
218	HLA-A0201	VGTCGAFSRN	VGTCGAFSR	0	0	0	0	0	VGTCGAFSR	tr_D0A9V0	0.022	39264.47	80.00
378	HLA-A0201	DRVPETIDSPRR	RVPETIDSR	1	0	0	8	2	RVPETIDSPRR	tr_D0A9V0	0.022	39299.33	80.00
648	HLA-A0201	WSSTAPAGSDA	WSSTAPAGA	0	0	0	8	2	WSSTAPAGSDA	tr_D0A9V0	0.022	39303.13	80.00
570	HLA-A0201	PHNVLLGTD	PHNVLLGTD	0	0	0	0	0	PHNVLLGTD	tr_D0A9V0	0.022	39304.43	80.00
379	HLA-A0201	RVPETIDSPRRPD	RVPETIDPD	0	0	0	7	4	RVPETIDSPRRPD	tr_D0A9V0	0.022	39324.41	80.00
31	HLA-A0201	VEVRKIQGH	VEVRKIQGH	0	0	0	0	0	VEVRKIQGH	tr_D0A9V0	0.022	39335.06	80.00
287	HLA-A0201	RPGESSSEVS	RPGESSSEV	0	0	0	0	0	RPGESSSEV	tr_D0A9V0	0.022	39344.42	80.00
140	HLA-A0201	DVWKSSTKYH	DVWKSSTYH	0	0	0	7	1	DVWKSSTKYH	tr_D0A9V0	0.022	39347.39	80.00
102	HLA-A0201	NAGDQQRDVALN	NAGQRDVAL	0	0	0	3	2	NAGDQQRDVAL	tr_D0A9V0	0.022	39350.82	80.00
411	HLA-A0201	YAPSSSSSLDGS	YASSSLDGS	0	0	0	2	3	YAPSSSSSLDGS	tr_D0A9V0	0.022	39366.55	80.00
412	HLA-A0201	APSSSSSLDGSGCA	SSSSSLDGA	2	0	0	8	3	SSSSSLDGSGCA	tr_D0A9V0	0.022	39366.98	80.00
31	HLA-A0201	VEVRKIQGHYAK	VEVIQGHYA	0	0	0	3	2	VEVRKIQGHYA	tr_D0A9V0	0.022	39388.29	80.00
247	HLA-A0201	GPCRIAATSGGAS	RIAATSGGA	3	0	0	0	0	RIAATSGGA	tr_D0A9V0	0.022	39390.43	80.00
245	HLA-A0201	RDGPCRIAATSG	RDGPCIAAT	0	0	0	5	1	RDGPCRIAAT	tr_D0A9V0	0.022	39400.23	80.00
377	HLA-A0201	KDRVPETIDSPRR	RVPETIDSR	2	0	0	8	2	RVPETIDSPRR	tr_D0A9V0	0.022	39401.50	80.00
413	HLA-A0201	PSSSSSLDGSGCA	SSSSSLDGA	1	0	0	8	3	SSSSSLDGSGCA	tr_D0A9V0	0.022	39404.91	80.00
570	HLA-A0201	PHNVLLGTDG	HNVLLGTDG	1	0	0	0	0	HNVLLGTDG	tr_D0A9V0	0.022	39405.34	80.00
617	HLA-A0201	VAAGGRCTP	VAAGGRCTP	0	0	0	0	0	VAAGGRCTP	tr_D0A9V0	0.022	39410.45	80.00
248	HLA-A0201	PCRIAATSGGASH	RIAATSASH	2	0	0	6	2	RIAATSGGASH	tr_D0A9V0	0.022	39428.80	80.00
76	HLA-A0201	GSATETPGESSTA	SATETPSTA	1	0	0	6	3	SATETPGESSTA	tr_D0A9V0	0.022	39443.73	80.00
359	HLA-A0201	YCVNSSDCKPEEQ	YCVCKPEEQ	0	0	0	3	4	YCVNSSDCKPEEQ	tr_D0A9V0	0.022	39460.37	80.00
446	HLA-A0201	WTRSLKKISE	WTRSLKISE	0	0	0	5	1	WTRSLKKISE	tr_D0A9V0	0.022	39470.20	80.00
120	HLA-A0201	KPNVFNRRHC	KPNVFNRHC	0	0	0	6	1	KPNVFNRRHC	tr_D0A9V0	0.022	39487.71	80.00
117	HLA-A0201	FKEKPNVFN	FKEKPNVFN	0	0	0	0	0	FKEKPNVFN	tr_D0A9V0	0.022	39527.46	80.00

446	HLA-A0201	WTRSLKKISEG	WTRSLISEG	0	0	0	5	2	WTRSLKKISEG	tr_D0A9V0	0.022	39533.02	80.00
357	HLA-A0201	NHYCVNSSDC	NHYCVNSSC	0	0	0	8	1	NHYCVNSSDC	tr_D0A9V0	0.022	39534.32	80.00
495	HLA-A0201	VASKLHHPN	VASKLHHPN	0	0	0	0	0	VASKLHHPN	tr_D0A9V0	0.022	39537.30	80.00
104	HLA-A0201	GDQQRDVALN	GDQQRDVAL	0	0	0	0	0	GDQQRDVAL	tr_D0A9V0	0.022	39543.73	80.00
682	HLA-A0201	PVIPASVDHE	VIPASVDHE	1	0	0	0	0	VIPASVDHE	tr_D0A9V0	0.022	39571.53	80.00
101	HLA-A0201	WNAGDQQRDVALN	WQQRDVALN	0	0	0	1	4	WNAGDQQRDVALN	tr_D0A9V0	0.022	39578.40	80.00
82	HLA-A0201	PGESSTAFVR	GESSTAFVR	1	0	0	0	0	GESSTAFVR	tr_D0A9V0	0.022	39592.11	80.00
420	HLA-A0201	DGSGCAEGANAA	GSAEGANAA	1	0	0	2	2	GSGCAEGANAA	tr_D0A9V0	0.022	39592.95	80.00
415	HLA-A0201	SSSSLDGSGC	SSSSLDSGC	0	0	0	6	1	SSSSLDGSGC	tr_D0A9V0	0.021	39633.67	85.00
307	HLA-A0201	FAAMRQGRYGE	FMRQGRYGE	0	0	0	1	2	FAAMRQGRYGE	tr_D0A9V0	0.021	39641.39	85.00
359	HLA-A0201	YCVNSSDCKP	YVNSSDCKP	0	0	0	1	1	YCVNSSDCKP	tr_D0A9V0	0.021	39659.42	85.00
474	HLA-A0201	IKCIPRLRRD	ICIPRLRRD	0	0	0	1	1	IKCIPRLRRD	tr_D0A9V0	0.021	39660.70	85.00
100	HLA-A0201	TWNAGDQQRDVALN	TWNAGDVAL	0	0	0	5	4	TWNAGDQQRDVAL	tr_D0A9V0	0.021	39661.98	85.00
372	HLA-A0201	DESDIKDRV	DESDIKDRV	0	0	0	0	0	DESDIKDRV	tr_D0A9V0	0.021	39673.57	85.00
290	HLA-A0201	ESSSEVSLS	ESSSEVSLS	0	0	0	0	0	ESSSEVSLS	tr_D0A9V0	0.021	39677.43	85.00
71	HLA-A0201	HIRANGSATE	HIANGSATE	0	0	0	2	1	HIRANGSATE	tr_D0A9V0	0.021	39679.58	85.00
246	HLA-A0201	DGPCRIAATSGGAS	RIAATSGGA	4	0	0	0	0	RIAATSGGA	tr_D0A9V0	0.021	39682.16	85.00
415	HLA-A0201	SSSSLDGSG	SSSSLDGSG	0	0	0	0	0	SSSSLDGSG	tr_D0A9V0	0.021	39691.17	85.00
362	HLA-A0201	NSSDCKPEEQ	NSSDCPEEQ	0	0	0	5	1	NSSDCKPEEQ	tr_D0A9V0	0.021	39695.04	85.00
181	HLA-A0201	LPMHCRRCC	LPMHCRRCC	0	0	0	0	0	LPMHCRRCC	tr_D0A9V0	0.021	39705.36	85.00
378	HLA-A0201	DRVPETIDSPRRPD	RVPETIDPD	1	0	0	7	4	RVPETIDSPRRPD	tr_D0A9V0	0.021	39714.80	85.00
443	HLA-A0201	RNVWTRSLK	RNVWTRSLK	0	0	0	0	0	RNVWTRSLK	tr_D0A9V0	0.021	39725.97	85.00
685	HLA-A0201	PASVDHEAA	PASVDHEAA	0	0	0	0	0	PASVDHEAA	tr_D0A9V0	0.021	39755.65	85.00
249	HLA-A0201	CRIAATSGGASH	RIAATSASH	1	0	0	6	2	RIAATSGGASH	tr_D0A9V0	0.021	39757.80	85.00
118	HLA-A0201	KEKPNVFNRRH	KEKPVFNRR	0	0	0	4	1	KEKPNVFNRR	tr_D0A9V0	0.021	39759.96	85.00
251	HLA-A0201	IAATSGGAS	IAATSGGAS	0	0	0	0	0	IAATSGGAS	tr_D0A9V0	0.021	39775.45	85.00
649	HLA-A0201	SSTAPAGSDATRLR	STAPAATRL	1	0	0	5	3	STAPAGSDATRL	tr_D0A9V0	0.021	39779.32	85.00
355	HLA-A0201	LDNHYCVNSSDCKP	YCVNSSDCP	4	0	0	8	1	YCVNSSDCKP	tr_D0A9V0	0.021	39781.04	85.00
250	HLA-A0201	RIAATSGGASH	RIAATSGGA	0	0	0	0	0	RIAATSGGA	tr_D0A9V0	0.021	39791.36	85.00
75	HLA-A0201	NGSATETPGESSTA	SATETPSTA	2	0	0	6	3	SATETPGESSTA	tr_D0A9V0	0.021	39798.67	85.00
358	HLA-A0201	HYCVNSSDCKPEEQ	YCVCKPEEQ	1	0	0	3	4	YCVNSSDCKPEEQ	tr_D0A9V0	0.021	39806.01	85.00
241	HLA-A0201	SYAVRDGPC	SYAVRDGPC	0	0	0	0	0	SYAVRDGPC	tr_D0A9V0	0.021	39815.92	85.00
245	HLA-A0201	RDGPCRIAATSGG	RDGPCIAAT	0	0	0	5	1	RDGPCRIAAT	tr_D0A9V0	0.021	39825.40	85.00
340	HLA-A0201	RQRCRRNDT	RQRCRRNDT	0	0	0	0	0	RQRCRRNDT	tr_D0A9V0	0.021	39840.48	85.00
37	HLA-A0201	QGHYAKLRR	QGHYAKLRR	0	0	0	0	0	QGHYAKLRR	tr_D0A9V0	0.021	39847.37	85.00
467	HLA-A0201	EDGAQVAIK	EDGAQVAIK	0	0	0	0	0	EDGAQVAIK	tr_D0A9V0	0.021	39859.88	85.00
439	HLA-A0201	TDSNRNVWTRS	TSNRNVWTR	0	0	0	1	1	TDSNRNVWTR	tr_D0A9V0	0.021	39862.04	85.00
32	HLA-A0201	EVRKIQGHYAK	EVRKIGHYA	0	0	0	5	1	EVRKIQGHYA	tr_D0A9V0	0.021	39862.89	85.00
445	HLA-A0201	VWTRSLKKISEG	WTRSLISEG	1	0	0	5	2	WTRSLKKISEG	tr_D0A9V0	0.021	39887.50	85.00
367	HLA-A0201	KPEEQDESDI	KPEEQDESI	0	0	0	8	1	KPEEQDESDI	tr_D0A9V0	0.021	39888.35	85.00
248	HLA-A0201	PCRIAATSG	PCRIAATSG	0	0	0	0	0	PCRIAATSG	tr_D0A9V0	0.021	39905.18	85.00
60	HLA-A0201	ARVESKNEA	ARVESKNEA	0	0	0	0	0	ARVESKNEA	tr_D0A9V0	0.021	39922.04	85.00
138	HLA-A0201	HPDVWKSST	HPDVWKSST	0	0	0	0	0	HPDVWKSST	tr_D0A9V0	0.021	39930.25	85.00
37	HLA-A0201	QGHYAKLRRQ	QGYAKLRRQ	0	0	0	2	1	QGHYAKLRRQ	tr_D0A9V0	0.021	39931.10	85.00
55	HLA-A0201	PEAILARVE	PEAILARVE	0	0	0	0	0	PEAILARVE	tr_D0A9V0	0.021	39951.84	85.00
359	HLA-A0201	YCVNSSDCKPE	CVNSSCKPE	1	0	0	5	1	CVNSSDCKPE	tr_D0A9V0	0.021	39957.89	85.00
308	HLA-A0201	AAMRQGRYG	AAMRQGRYG	0	0	0	0	0	AAMRQGRYG	tr_D0A9V0	0.021	39968.28	85.00
643	HLA-A0201	RPPWQWSST	RPPWQWSST	0	0	0	0	0	RPPWQWSST	tr_D0A9V0	0.021	40000.28	85.00
249	HLA-A0201	CRIAATSGG	CRIAATSGG	0	0	0	0	0	CRIAATSGG	tr_D0A9V0	0.021	40021.93	85.00
411	HLA-A0201	YAPSSSSSLDG	YAPSSSSSL	0	0	0	0	0	YAPSSSSSL	tr_D0A9V0	0.020	40055.29	85.00
242	HLA-A0201	YAVRDGPCR	YAVRDGPCR	0	0	0	0	0	YAVRDGPCR	tr_D0A9V0	0.020	40065.26	85.00
249	HLA-A0201	CRIAATSGGAS	RIAATSGGA	1	0	0	0	0	RIAATSGGA	tr_D0A9V0	0.020	40072.19	85.00
153	HLA-A0201	IEVATEHSGCVE	IATEHSGCV	0	0	0	1	2	IEVATEHSGCV	tr_D0A9V0	0.020	40075.23	85.00
55	HLA-A0201	PEAILARVES	EAILARVES	1	0	0	0	0	EAILARVES	tr_D0A9V0	0.020	40083.89	85.00

358	HLA-A0201	HYCVNSSDCKPE	CVNSSCKPE	2	0	0	5	1	CVNSSDCKPE	tr_D0A9V0	0.020	40100.83	85.00
410	HLA-A0201	MYAPSSSSSLDGS	YASSSLDGS	1	0	0	2	3	YAPSSSSSLDGS	tr_D0A9V0	0.020	40102.56	85.00
485	HLA-A0201	VAESLEKEME	VAESLEKEM	0	0	0	0	0	VAESLEKEM	tr_D0A9V0	0.020	40103.42	85.00
647	HLA-A0201	QWSSTAPAGSDA	WSSTAPAGA	1	0	0	8	2	WSSTAPAGSDA	tr_D0A9V0	0.020	40109.08	85.00
78	HLA-A0201	ATETPGESSTA	ATPGESSTA	0	0	0	1	2	ATETPGESSTA	tr_D0A9V0	0.020	40130.35	85.00
419	HLA-A0201	LDGSGCAEGAN	LGSGCAEGA	0	0	0	1	1	LDGSGCAEGA	tr_D0A9V0	0.020	40134.26	85.00
9	HLA-A0201	LKSASNLEIPD	LASNLEIPD	0	0	0	1	2	LKSASNLEIPD	tr_D0A9V0	0.020	40138.58	85.00
289	HLA-A0201	GESSSEVSLS	GESSSEVSL	0	0	0	0	0	GESSSEVSL	tr_D0A9V0	0.020	40148.13	85.00
248	HLA-A0201	PCRIAATSGGAS	RIAATSGGA	2	0	0	0	0	RIAATSGGA	tr_D0A9V0	0.020	40168.12	85.00
71	HLA-A0201	HIRANGSATET	HINGSATET	0	0	0	2	2	HIRANGSATET	tr_D0A9V0	0.020	40175.09	85.00
482	HLA-A0201	RDVVAESLEKE	RVVAESLEK	0	0	0	1	1	RDVVAESLEK	tr_D0A9V0	0.020	40189.87	85.00
112	HLA-A0201	LNCGIFKEKPN	LNCGIFKPN	0	0	0	6	2	LNCGIFKEKPN	tr_D0A9V0	0.020	40209.44	85.00
97	HLA-A0201	ISVTWNAGDQQR	VTWNAGQQR	2	0	0	6	1	VTWNAGDQQR	tr_D0A9V0	0.020	40220.75	85.00
182	HLA-A0201	PMHCRRCCQ	PMHCRRCCQ	0	0	0	0	0	PMHCRRCCQ	tr_D0A9V0	0.020	40221.63	85.00
251	HLA-A0201	IAATSGGASHMQ	ITSGGASHM	0	0	0	1	2	IAATSGGASHM	tr_D0A9V0	0.020	40226.40	85.00
359	HLA-A0201	YCVNSSDCKPEE	CVNSSCKPE	1	0	0	5	1	CVNSSDCKPE	tr_D0A9V0	0.020	40229.91	85.00
424	HLA-A0201	CAEGANAAD	CAEGANAAD	0	0	0	0	0	CAEGANAAD	tr_D0A9V0	0.020	40235.56	85.00
119	HLA-A0201	EKPNVFNRRHC	KPNVFNRHC	1	0	0	6	1	KPNVFNRRHC	tr_D0A9V0	0.020	40263.00	85.00
360	HLA-A0201	CVNSSDCKPEEQ	CVDCKPEEQ	0	0	0	2	3	CVNSSDCKPEEQ	tr_D0A9V0	0.020	40283.46	85.00
641	HLA-A0201	LGRPPWQWSST	LGRPPWWSS	0	0	0	6	1	LGRPPWQWSS	tr_D0A9V0	0.020	40284.35	85.00
682	HLA-A0201	PVIPASVDH	PVIPASVDH	0	0	0	0	0	PVIPASVDH	tr_D0A9V0	0.020	40286.09	85.00
370	HLA-A0201	EQDESDIKD	EQDESDIKD	0	0	0	0	0	EQDESDIKD	tr_D0A9V0	0.020	40286.95	85.00
62	HLA-A0201	VESKNEAGGHIRA	SKNEAGIRA	2	0	0	6	2	SKNEAGGHIRA	tr_D0A9V0	0.020	40287.39	85.00
358	HLA-A0201	HYCVNSSDCKPEE	CVNSSCKPE	2	0	0	5	1	CVNSSDCKPE	tr_D0A9V0	0.020	40287.83	85.00
64	HLA-A0201	SKNEAGGHIRA	SKNEAGIRA	0	0	0	6	2	SKNEAGGHIRA	tr_D0A9V0	0.020	40289.57	85.00
153	HLA-A0201	IEVATEHSGC	IVATEHSGC	0	0	0	1	1	IEVATEHSGC	tr_D0A9V0	0.020	40311.82	85.00
306	HLA-A0201	GFAAMRQGRYGE	FMRQGRYGE	1	0	0	1	2	FAAMRQGRYGE	tr_D0A9V0	0.020	40317.93	85.00
358	HLA-A0201	HYCVNSSDC	HYCVNSSDC	0	0	0	0	0	HYCVNSSDC	tr_D0A9V0	0.020	40338.42	85.00
359	HLA-A0201	YCVNSSDCKPEEQD	YCVNSSEQD	0	0	0	6	5	YCVNSSDCKPEEQD	tr_D0A9V0	0.020	40339.72	85.00
541	HLA-A0201	PYVARRFTVD	PYVARRFTV	0	0	0	0	0	PYVARRFTV	tr_D0A9V0	0.020	40341.91	85.00
525	HLA-A0201	PGGSLHSVIK	GGSLHSVIK	1	0	0	0	0	GGSLHSVIK	tr_D0A9V0	0.020	40348.02	85.00
34	HLA-A0201	RKIQGHYAK	RKIQGHYAK	0	0	0	0	0	RKIQGHYAK	tr_D0A9V0	0.020	40357.21	85.00
141	HLA-A0201	VWKSSTKYHAA	KSSTKYHAA	2	0	0	0	0	KSSTKYHAA	tr_D0A9V0	0.020	40359.37	85.00
411	HLA-A0201	YAPSSSSSLDGSGC	YAPSSSSSL	0	0	0	0	0	YAPSSSSSL	tr_D0A9V0	0.020	40361.55	85.00
103	HLA-A0201	AGDQQRDVALN	AGDQRDVAL	0	0	0	3	1	AGDQQRDVAL	tr_D0A9V0	0.020	40369.42	85.00
284	HLA-A0201	FELRPGESSSE	FLRPGESSS	0	0	0	1	1	FELRPGESSS	tr_D0A9V0	0.020	40369.86	85.00
61	HLA-A0201	RVESKNEAGGHIR	RVNEAGGHI	0	0	0	2	3	RVESKNEAGGHI	tr_D0A9V0	0.020	40371.16	85.00
139	HLA-A0201	PDVWKSSTKY	DVWKSSTKY	1	0	0	0	0	DVWKSSTKY	tr_D0A9V0	0.020	40374.23	85.00
619	HLA-A0201	AGGRCTPQSDI	AGGRCTPQI	0	0	0	8	2	AGGRCTPQSDI	tr_D0A9V0	0.020	40375.09	85.00
621	HLA-A0201	GRCTPQSDIF	RCTPQSDIF	1	0	0	0	0	RCTPQSDIF	tr_D0A9V0	0.020	40379.91	85.00
254	HLA-A0201	TSGGASHMQ	TSGGASHMQ	0	0	0	0	0	TSGGASHMQ	tr_D0A9V0	0.020	40391.27	85.00
119	HLA-A0201	EKPNVFNRRH	KPNVFNRRH	1	0	0	0	0	KPNVFNRRH	tr_D0A9V0	0.020	40426.68	85.00
63	HLA-A0201	ESKNEAGGHI	SKNEAGGHI	1	0	0	0	0	SKNEAGGHI	tr_D0A9V0	0.020	40427.57	85.00
410	HLA-A0201	MYAPSSSSSLD	YAPSSSSSL	1	0	0	0	0	YAPSSSSSL	tr_D0A9V0	0.020	40442.43	85.00
102	HLA-A0201	NAGDQQRDVA	NAGDQQDVA	0	0	0	6	1	NAGDQQRDVA	tr_D0A9V0	0.020	40447.26	85.00
413	HLA-A0201	PSSSSSLDGS	SSSSSLDGS	1	0	0	0	0	SSSSSLDGS	tr_D0A9V0	0.020	40452.95	85.00
63	HLA-A0201	ESKNEAGGHIRA	SKNEAGIRA	1	0	0	6	2	SKNEAGGHIRA	tr_D0A9V0	0.020	40469.57	85.00
221	HLA-A0201	CGAFSRNAR	CGAFSRNAR	0	0	0	0	0	CGAFSRNAR	tr_D0A9V0	0.020	40475.70	85.00
647	HLA-A0201	QWSSTAPAGS	WSSTAPAGS	1	0	0	0	0	WSSTAPAGS	tr_D0A9V0	0.020	40487.53	85.00
617	HLA-A0201	VAAGGRCTPQS	AAGGRCTPS	1	0	0	8	1	AAGGRCTPQS	tr_D0A9V0	0.019	40494.11	85.00
306	HLA-A0201	GFAAMRQGRYG	FAAMRGRYG	1	0	0	5	1	FAAMRQGRYG	tr_D0A9V0	0.019	40499.36	85.00
414	HLA-A0201	SSSSSLDGSG	SSSSSLDSG	0	0	0	7	1	SSSSSLDGSG	tr_D0A9V0	0.019	40505.94	85.00
415	HLA-A0201	SSSSLDGSGCA	SSSSLDGSA	0	0	0	8	2	SSSSLDGSGCA	tr_D0A9V0	0.019	40512.08	85.00
439	HLA-A0201	TDSNRNVWT	TDSNRNVWT	0	0	0	0	0	TDSNRNVWT	tr_D0A9V0	0.019	40517.33	85.00

703	HLA-A0201	NPSMRPSASS	NSMRPSASS	0	0	0	1	1	NPSMRPSASS	tr_D0A9V0	0.019	40526.53	85.00
419	HLA-A0201	LDGSGCAEGANAAD	LCAEGANAA	0	0	0	1	4	LDGSGCAEGANAA	tr_D0A9V0	0.019	40545.85	85.00
337	HLA-A0201	RLCRQRCRRND	RLCQRCRRN	0	0	0	3	1	RLCRQRCRRN	tr_D0A9V0	0.019	40550.66	85.00
9	HLA-A0201	LKSASNLEIPDN	SASNLEIPN	2	0	0	8	1	SASNLEIPDN	tr_D0A9V0	0.019	40555.50	85.00
356	HLA-A0201	DNHYCVNSSDCKP	DNHYCVCKP	0	0	0	6	4	DNHYCVNSSDCKP	tr_D0A9V0	0.019	40575.67	85.00
647	HLA-A0201	QWSSTAPAG	QWSSTAPAG	0	0	0	0	0	QWSSTAPAG	tr_D0A9V0	0.019	40583.15	85.00
308	HLA-A0201	AAMRQGRYGE	AMRQGRYGE	0	0	0	1	1	AAMRQGRYGE	tr_D0A9V0	0.019	40583.57	85.00
616	HLA-A0201	EVAAGGRCTPQ	AAGGRCTPQ	2	0	0	0	0	AAGGRCTPQ	tr_D0A9V0	0.019	40590.17	85.00
681	HLA-A0201	EPVIPASVDH	EPVIPAVDH	0	0	0	6	1	EPVIPASVDH	tr_D0A9V0	0.019	40600.27	85.00
74	HLA-A0201	ANGSATETP	ANGSATETP	0	0	0	0	0	ANGSATETP	tr_D0A9V0	0.019	40602.47	85.00
156	HLA-A0201	ATEHSGCVE	ATEHSGCVE	0	0	0	0	0	ATEHSGCVE	tr_D0A9V0	0.019	40614.78	85.00
445	HLA-A0201	VWTRSLKKISE	VWTRSLISE	0	0	0	6	2	VWTRSLKKISE	tr_D0A9V0	0.019	40622.69	85.00
138	HLA-A0201	HPDVWKSSTK	HPDVWSSTK	0	0	0	5	1	HPDVWKSSTK	tr_D0A9V0	0.019	40622.69	85.00
252	HLA-A0201	AATSGGASH	AATSGGASH	0	0	0	0	0	AATSGGASH	tr_D0A9V0	0.019	40637.63	85.00
137	HLA-A0201	YHPDVWKSSTK	YHPDVWSTK	0	0	0	6	2	YHPDVWKSSTK	tr_D0A9V0	0.019	40651.26	85.00
141	HLA-A0201	VWKSSTKYH	VWKSSTKYH	0	0	0	0	0	VWKSSTKYH	tr_D0A9V0	0.019	40655.21	85.00
356	HLA-A0201	DNHYCVNSSDCK	YCVNSSDCK	3	0	0	0	0	YCVNSSDCK	tr_D0A9V0	0.019	40660.07	85.00
355	HLA-A0201	LDNHYCVNSSD	LDHYCVNSS	0	0	0	2	1	LDNHYCVNSS	tr_D0A9V0	0.019	40671.50	85.00
654	HLA-A0201	AGSDATRLR	AGSDATRLR	0	0	0	0	0	AGSDATRLR	tr_D0A9V0	0.019	40690.42	85.00
283	HLA-A0201	LFELRPGESSSE	FLRPGESSS	1	0	0	1	1	FELRPGESSS	tr_D0A9V0	0.019	40693.52	85.00
361	HLA-A0201	VNSSDCKPE	VNSSDCKPE	0	0	0	0	0	VNSSDCKPE	tr_D0A9V0	0.019	40698.79	85.00
97	HLA-A0201	ISVTWNAGDQQ	ITWNAGDQQ	0	0	0	1	2	ISVTWNAGDQQ	tr_D0A9V0	0.019	40713.75	85.00
338	HLA-A0201	LCRQRCRRNDT	LCRQRCNDT	0	0	0	6	2	LCRQRCRRNDT	tr_D0A9V0	0.019	40739.34	85.00
616	HLA-A0201	EVAAGGRCTPQS	AAGGRCTPS	2	0	0	8	1	AAGGRCTPQS	tr_D0A9V0	0.019	40741.09	85.00
420	HLA-A0201	DGSGCAEGANA	DGSGCAEGA	0	0	0	8	2	DGSGCAEGANA	tr_D0A9V0	0.019	40753.87	85.00
651	HLA-A0201	TAPAGSDATRLR	APAGSDATL	1	0	0	8	1	APAGSDATRL	tr_D0A9V0	0.019	40759.60	85.00
68	HLA-A0201	AGGHIRANGSA	AGGHIRASA	0	0	0	7	2	AGGHIRANGSA	tr_D0A9V0	0.019	40760.49	85.00
652	HLA-A0201	APAGSDATRLR	APAGSDATL	0	0	0	8	1	APAGSDATRL	tr_D0A9V0	0.019	40762.70	85.00
446	HLA-A0201	WTRSLKKIS	WTRSLKKIS	0	0	0	0	0	WTRSLKKIS	tr_D0A9V0	0.019	40768.88	85.00
369	HLA-A0201	EEQDESDIKDR	EQDESDIDR	1	0	0	7	1	EQDESDIKDR	tr_D0A9V0	0.019	40779.89	85.00
410	HLA-A0201	MYAPSSSSSLDG	YAPSSSSSL	1	0	0	0	0	YAPSSSSSL	tr_D0A9V0	0.019	40793.12	85.00
69	HLA-A0201	GGHIRANGSATET	HINGSATET	2	0	0	2	2	HIRANGSATET	tr_D0A9V0	0.019	40793.57	85.00
647	HLA-A0201	QWSSTAPAGSDATR	STAGSDATR	3	0	0	2	2	STAPAGSDATR	tr_D0A9V0	0.019	40798.44	85.00
619	HLA-A0201	AGGRCTPQS	AGGRCTPQS	0	0	0	0	0	AGGRCTPQS	tr_D0A9V0	0.019	40809.02	85.00
68	HLA-A0201	AGGHIRANGSATET	HINGSATET	3	0	0	2	2	HIRANGSATET	tr_D0A9V0	0.019	40810.36	85.00
644	HLA-A0201	PPWQWSSTAP	PPWQWSSTA	0	0	0	0	0	PPWQWSSTA	tr_D0A9V0	0.019	40810.78	85.00
616	HLA-A0201	EVAAGGRCT	EVAAGGRCT	0	0	0	0	0	EVAAGGRCT	tr_D0A9V0	0.019	40822.71	85.00
98	HLA-A0201	SVTWNAGDQQR	VTWNAGQQR	1	0	0	6	1	VTWNAGDQQR	tr_D0A9V0	0.019	40831.11	85.00
411	HLA-A0201	YAPSSSSSLDGSG	YAPSSSSSL	0	0	0	0	0	YAPSSSSSL	tr_D0A9V0	0.019	40840.38	85.00
702	HLA-A0201	MNPSMRPSASS	MSMRPSASS	0	0	0	1	2	MNPSMRPSASS	tr_D0A9V0	0.019	40851.44	90.00
287	HLA-A0201	RPGESSSEVSLS	RPGESSVSL	0	0	0	6	2	RPGESSSEVSL	tr_D0A9V0	0.019	40854.97	90.00
615	HLA-A0201	PEVAAGGRCTPQ	AAGGRCTPQ	3	0	0	0	0	AAGGRCTPQ	tr_D0A9V0	0.019	40859.39	90.00
648	HLA-A0201	WSSTAPAGSDATR	WSSTAPAGA	0	0	0	8	2	WSSTAPAGSDA	tr_D0A9V0	0.019	40874.87	90.00
366	HLA-A0201	CKPEEQDESDIKDR	EQDESIKDR	4	0	0	5	1	EQDESDIKDR	tr_D0A9V0	0.019	40889.02	90.00
425	HLA-A0201	AEGANAADVVP	AEGANAAVV	0	0	0	7	1	AEGANAADVV	tr_D0A9V0	0.019	40890.79	90.00
614	HLA-A0201	APEVAAGGRC	APEVAAGGC	0	0	0	8	1	APEVAAGGRC	tr_D0A9V0	0.019	40918.23	90.00
286	HLA-A0201	LRPGESSSEVSLS	LSSSEVSLS	0	0	0	1	4	LRPGESSSEVSLS	tr_D0A9V0	0.019	40920.00	90.00
69	HLA-A0201	GGHIRANGSATETP	RANGSAETP	4	0	0	6	1	RANGSATETP	tr_D0A9V0	0.019	40920.89	90.00
251	HLA-A0201	IAATSGGASHMQG	ITSGGASHM	0	0	0	1	2	IAATSGGASHM	tr_D0A9V0	0.019	40924.43	90.00
615	HLA-A0201	PEVAAGGRCTPQS	AAGGRCTPS	3	0	0	8	1	AAGGRCTPQS	tr_D0A9V0	0.018	40962.08	90.00
360	HLA-A0201	CVNSSDCKPEEQD	CVNSSCKPE	0	0	0	5	1	CVNSSDCKPE	tr_D0A9V0	0.018	40965.62	90.00
367	HLA-A0201	KPEEQDESDIKDR	EQDESIKDR	3	0	0	5	1	EQDESDIKDR	tr_D0A9V0	0.018	40967.42	90.00
356	HLA-A0201	DNHYCVNSSDC	DNHYCVNSC	0	0	0	8	2	DNHYCVNSSDC	tr_D0A9V0	0.018	40970.06	90.00
652	HLA-A0201	APAGSDATRLRS	ASDATRLRS	0	0	0	1	3	APAGSDATRLRS	tr_D0A9V0	0.018	40972.28	90.00

71	HLA-A0201	HIRANGSATETP	HIRAATETP	0	0	0	4	3	HIRANGSATETP	tr_D0A9V0	0.018	40972.28	90.00
67	HLA-A0201	EAGGHIRANGSA	GGHIRANSA	2	0	0	7	1	GGHIRANGSA	tr_D0A9V0	0.018	40991.36	90.00
253	HLA-A0201	ATSGGASHMQG	ATSGGASHM	0	0	0	0	0	ATSGGASHM	tr_D0A9V0	0.018	40992.23	90.00
252	HLA-A0201	AATSGGASHMQ	ATSGGASHM	0	0	0	1	1	AATSGGASHM	tr_D0A9V0	0.018	41003.34	90.00
651	HLA-A0201	TAPAGSDATRLRS	TAPAGATRL	0	0	0	5	2	TAPAGSDATRL	tr_D0A9V0	0.018	41003.76	90.00
380	HLA-A0201	VPETIDSPRR	VPETIDSPR	0	0	0	8	1	VPETIDSPRR	tr_D0A9V0	0.018	41011.30	90.00
360	HLA-A0201	CVNSSDCKPEE	CVNSSCKPE	0	0	0	5	1	CVNSSDCKPE	tr_D0A9V0	0.018	41012.20	90.00
368	HLA-A0201	PEEQDESDIKDR	EQDESDIDR	2	0	0	7	1	EQDESDIKDR	tr_D0A9V0	0.018	41025.95	90.00
357	HLA-A0201	NHYCVNSSD	NHYCVNSSD	0	0	0	0	0	NHYCVNSSD	tr_D0A9V0	0.018	41036.62	90.00
306	HLA-A0201	GFAAMRQGR	GFAAMRQGR	0	0	0	0	0	GFAAMRQGR	tr_D0A9V0	0.018	41041.94	90.00
255	HLA-A0201	SGGASHMQG	SGGASHMQG	0	0	0	0	0	SGGASHMQG	tr_D0A9V0	0.018	41043.71	90.00
652	HLA-A0201	APAGSDATR	APAGSDATR	0	0	0	0	0	APAGSDATR	tr_D0A9V0	0.018	41051.71	90.00
60	HLA-A0201	ARVESKNEAGGHIR	RVNEAGGHI	1	0	0	2	3	RVESKNEAGGHI	tr_D0A9V0	0.018	41070.38	90.00
98	HLA-A0201	SVTWNAGDQ	SVTWNAGDQ	0	0	0	0	0	SVTWNAGDQ	tr_D0A9V0	0.018	41074.38	90.00
33	HLA-A0201	VRKIQGHYAK	VRKIQGYAK	0	0	0	6	1	VRKIQGHYAK	tr_D0A9V0	0.018	41082.38	90.00
326	HLA-A0201	VKMDMQAKR	VKMDMQAKR	0	0	0	0	0	VKMDMQAKR	tr_D0A9V0	0.018	41091.70	90.00
65	HLA-A0201	KNEAGGHIRANGSA	GGHIRANSA	4	0	0	7	1	GGHIRANGSA	tr_D0A9V0	0.018	41092.15	90.00
66	HLA-A0201	NEAGGHIRANGSA	GGHIRANSA	3	0	0	7	1	GGHIRANGSA	tr_D0A9V0	0.018	41098.83	90.00
113	HLA-A0201	NCGIFKEKPN	NCGIFKEPN	0	0	0	7	1	NCGIFKEKPN	tr_D0A9V0	0.018	41114.84	90.00
70	HLA-A0201	GHIRANGSATET	HINGSATET	1	0	0	2	2	HIRANGSATET	tr_D0A9V0	0.018	41128.63	90.00
153	HLA-A0201	IEVATEHSG	IEVATEHSG	0	0	0	0	0	IEVATEHSG	tr_D0A9V0	0.018	41130.86	90.00
681	HLA-A0201	EPVIPASVDHE	VIPASVDHE	2	0	0	0	0	VIPASVDHE	tr_D0A9V0	0.018	41132.64	90.00
525	HLA-A0201	PGGSLHSVIKN	GSLHSVIKN	2	0	0	0	0	GSLHSVIKN	tr_D0A9V0	0.018	41158.91	90.00
525	HLA-A0201	PGGSLHSVI	PGGSLHSVI	0	0	0	0	0	PGGSLHSVI	tr_D0A9V0	0.018	41178.05	90.00
68	HLA-A0201	AGGHIRANG	AGGHIRANG	0	0	0	0	0	AGGHIRANG	tr_D0A9V0	0.018	41182.96	90.00
100	HLA-A0201	TWNAGDQQRDV	TWNAGDQQV	0	0	0	8	2	TWNAGDQQRDV	tr_D0A9V0	0.018	41186.98	90.00
377	HLA-A0201	KDRVPETIDS	KDRVPTIDS	0	0	0	5	1	KDRVPETIDS	tr_D0A9V0	0.018	41217.72	90.00
59	HLA-A0201	LARVESKNE	LARVESKNE	0	0	0	0	0	LARVESKNE	tr_D0A9V0	0.018	41217.72	90.00
614	HLA-A0201	APEVAAGGRCTP	APEVAACTP	0	0	0	6	3	APEVAAGGRCTP	tr_D0A9V0	0.018	41222.18	90.00
648	HLA-A0201	WSSTAPAGSDAT	WSSTAPAAT	0	0	0	7	3	WSSTAPAGSDAT	tr_D0A9V0	0.018	41232.00	90.00
103	HLA-A0201	AGDQQRDVA	AGDQQRDVA	0	0	0	0	0	AGDQQRDVA	tr_D0A9V0	0.018	41236.46	90.00
246	HLA-A0201	DGPCRIAATSG	GPCRIAATG	1	0	0	8	1	GPCRIAATSG	tr_D0A9V0	0.018	41248.06	90.00
577	HLA-A0201	TDGVCKLSD	TDGVCKLSD	0	0	0	0	0	TDGVCKLSD	tr_D0A9V0	0.018	41258.33	90.00
371	HLA-A0201	QDESDIKDR	QDESDIKDR	0	0	0	0	0	QDESDIKDR	tr_D0A9V0	0.018	41258.33	90.00
447	HLA-A0201	TRSLKKISEG	RSLKKISEG	1	0	0	0	0	RSLKKISEG	tr_D0A9V0	0.018	41265.91	90.00
703	HLA-A0201	NPSMRPSAS	NPSMRPSAS	0	0	0	0	0	NPSMRPSAS	tr_D0A9V0	0.018	41267.24	90.00
68	HLA-A0201	AGGHIRANGS	AGHIRANGS	0	0	0	1	1	AGGHIRANGS	tr_D0A9V0	0.018	41283.32	90.00
336	HLA-A0201	MRLCRQRCRRND	RLCQRCRRN	1	0	0	3	1	RLCRQRCRRN	tr_D0A9V0	0.018	41285.11	90.00
360	HLA-A0201	CVNSSDCKPEEQDE	CVDCKPEEQ	0	0	0	2	3	CVNSSDCKPEEQ	tr_D0A9V0	0.018	41320.88	90.00
76	HLA-A0201	GSATETPGE	GSATETPGE	0	0	0	0	0	GSATETPGE	tr_D0A9V0	0.018	41322.66	90.00
97	HLA-A0201	ISVTWNAGDQQRD	ISVTWNAGD	0	0	0	8	4	ISVTWNAGDQQRD	tr_D0A9V0	0.018	41336.07	90.00
410	HLA-A0201	MYAPSSSSSLDGSG	YAPSSSSSL	1	0	0	0	0	YAPSSSSSL	tr_D0A9V0	0.018	41357.54	90.00
79	HLA-A0201	TETPGESSTAF	ETGESSTAF	1	0	0	2	1	ETPGESSTAF	tr_D0A9V0	0.018	41366.50	90.00
362	HLA-A0201	NSSDCKPEE	NSSDCKPEE	0	0	0	0	0	NSSDCKPEE	tr_D0A9V0	0.018	41372.32	90.00
371	HLA-A0201	QDESDIKDRVPE	QDESDIVPE	0	0	0	6	3	QDESDIKDRVPE	tr_D0A9V0	0.017	41375.46	90.00
247	HLA-A0201	GPCRIAATSGG	GPCRIAATG	0	0	0	8	2	GPCRIAATSGG	tr_D0A9V0	0.017	41385.74	90.00
65	HLA-A0201	KNEAGGHIR	KNEAGGHIR	0	0	0	0	0	KNEAGGHIR	tr_D0A9V0	0.017	41407.25	90.00
10	HLA-A0201	KSASNLEIPDN	SASNLEIPN	1	0	0	8	1	SASNLEIPDN	tr_D0A9V0	0.017	41479.45	90.00
76	HLA-A0201	GSATETPGES	SATETPGES	1	0	0	0	0	SATETPGES	tr_D0A9V0	0.017	41500.53	90.00
357	HLA-A0201	NHYCVNSSDCK	NHYCVSDCK	0	0	0	5	2	NHYCVNSSDCK	tr_D0A9V0	0.017	41529.74	90.00
445	HLA-A0201	VWTRSLKKIS	VWTRSLKKI	0	0	0	0	0	VWTRSLKKI	tr_D0A9V0	0.017	41543.21	90.00
420	HLA-A0201	DGSGCAEGAN	DGSGCAEGA	0	0	0	0	0	DGSGCAEGA	tr_D0A9V0	0.017	41564.36	90.00
62	HLA-A0201	VESKNEAGGHIRAN	SKNEAGIRA	2	0	0	6	2	SKNEAGGHIRA	tr_D0A9V0	0.017	41570.20	90.00
650	HLA-A0201	STAPAGSDATR	STAGSDATR	0	0	0	2	2	STAPAGSDATR	tr_D0A9V0	0.017	41571.09	90.00

70	HLA-A0201	GHIRANGSATETP	HIRAATETP	1	0	0	4	3	HIRANGSATETP	tr_D0A9V0	0.017	41577.84	90.00
70	HLA-A0201	GHIRANGSAT	GIRANGSAT	0	0	0	1	1	GHIRANGSAT	tr_D0A9V0	0.017	41599.00	90.00
651	HLA-A0201	TAPAGSDATR	TAAGSDATR	0	0	0	2	1	TAPAGSDATR	tr_D0A9V0	0.017	41605.28	90.00
62	HLA-A0201	VESKNEAGGHI	VENEAGGHI	0	0	0	2	2	VESKNEAGGHI	tr_D0A9V0	0.017	41613.84	90.00
380	HLA-A0201	VPETIDSPRRP	VTIDSPRRP	0	0	0	1	2	VPETIDSPRRP	tr_D0A9V0	0.017	41615.64	90.00
380	HLA-A0201	VPETIDSPRRPDGE	VPETIDSPE	0	0	0	8	5	VPETIDSPRRPDGE	tr_D0A9V0	0.017	41632.77	90.00
252	HLA-A0201	AATSGGASHMQG	ATSGGASHM	0	0	0	1	1	AATSGGASHM	tr_D0A9V0	0.017	41640.88	90.00
246	HLA-A0201	DGPCRIAATSGG	GPCRIAATG	1	0	0	8	2	GPCRIAATSGG	tr_D0A9V0	0.017	41669.27	90.00
64	HLA-A0201	SKNEAGGHIRAN	SAGGHIRAN	0	0	0	1	3	SKNEAGGHIRAN	tr_D0A9V0	0.017	41670.62	90.00
361	HLA-A0201	VNSSDCKPEEQ	SSDCKPEEQ	2	0	0	0	0	SSDCKPEEQ	tr_D0A9V0	0.017	41678.27	90.00
357	HLA-A0201	NHYCVNSSDCKP	NHYCVNCKP	0	0	0	6	3	NHYCVNSSDCKP	tr_D0A9V0	0.017	41721.14	90.00
378	HLA-A0201	DRVPETIDS	DRVPETIDS	0	0	0	0	0	DRVPETIDS	tr_D0A9V0	0.017	41725.20	90.00
64	HLA-A0201	SKNEAGGHIR	SKNEAGGHI	0	0	0	0	0	SKNEAGGHI	tr_D0A9V0	0.017	41750.51	90.00
647	HLA-A0201	QWSSTAPAGSDAT	WSSTAPAAT	1	0	0	7	3	WSSTAPAGSDAT	tr_D0A9V0	0.017	41763.14	90.00
373	HLA-A0201	ESDIKDRVPE	ESDIKDVPE	0	0	0	6	1	ESDIKDRVPE	tr_D0A9V0	0.017	41764.51	90.00
493	HLA-A0201	MEVASKLHHPN	MVASKLHHP	0	0	0	1	1	MEVASKLHHP	tr_D0A9V0	0.017	41772.64	90.00
414	HLA-A0201	SSSSSLDGSGC	SSSSSLSGC	0	0	0	6	2	SSSSSLDGSGC	tr_D0A9V0	0.017	41786.20	90.00
621	HLA-A0201	GRCTPQSDI	GRCTPQSDI	0	0	0	0	0	GRCTPQSDI	tr_D0A9V0	0.017	41788.46	90.00
649	HLA-A0201	SSTAPAGSDATR	SSTAPAATR	0	0	0	6	3	SSTAPAGSDATR	tr_D0A9V0	0.017	41798.87	90.00
373	HLA-A0201	ESDIKDRVP	ESDIKDRVP	0	0	0	0	0	ESDIKDRVP	tr_D0A9V0	0.017	41819.68	90.00
447	HLA-A0201	TRSLKKISE	TRSLKKISE	0	0	0	0	0	TRSLKKISE	tr_D0A9V0	0.016	41845.02	90.00
486	HLA-A0201	AESLEKEME	AESLEKEME	0	0	0	0	0	AESLEKEME	tr_D0A9V0	0.016	41854.08	90.00
100	HLA-A0201	TWNAGDQQR	TWNAGDQQR	0	0	0	0	0	TWNAGDQQR	tr_D0A9V0	0.016	41858.59	90.00
426	HLA-A0201	EGANAADVVP	GANAADVVP	1	0	0	0	0	GANAADVVP	tr_D0A9V0	0.016	41860.43	90.00
63	HLA-A0201	ESKNEAGGHIRAN	SKNEAGIRA	1	0	0	6	2	SKNEAGGHIRA	tr_D0A9V0	0.016	41864.04	90.00
436	HLA-A0201	LFLTDSNRN	LFLTDSNRN	0	0	0	0	0	LFLTDSNRN	tr_D0A9V0	0.016	41864.93	90.00
702	HLA-A0201	MNPSMRPSAS	MNPSMRPSA	0	0	0	0	0	MNPSMRPSA	tr_D0A9V0	0.016	41868.12	90.00
614	HLA-A0201	APEVAAGGRCT	AVAAGGRCT	0	0	0	1	2	APEVAAGGRCT	tr_D0A9V0	0.016	41876.73	90.00
64	HLA-A0201	SKNEAGGHIRANGS	SKNEAGIRA	0	0	0	6	2	SKNEAGGHIRA	tr_D0A9V0	0.016	41936.11	90.00
69	HLA-A0201	GGHIRANGSATE	GGHIRAATE	0	0	0	6	3	GGHIRANGSATE	tr_D0A9V0	0.016	41936.11	90.00
99	HLA-A0201	VTWNAGDQQRD	VTWNAGQQR	0	0	0	6	1	VTWNAGDQQR	tr_D0A9V0	0.016	41938.38	90.00
285	HLA-A0201	ELRPGESSSE	ELRPGSSSE	0	0	0	5	1	ELRPGESSSE	tr_D0A9V0	0.016	41947.01	90.00
68	HLA-A0201	AGGHIRANGSAT	AIRANGSAT	0	0	0	1	3	AGGHIRANGSAT	tr_D0A9V0	0.016	41950.18	90.00
59	HLA-A0201	LARVESKNEAGGH	LARVEAGGH	0	0	0	4	4	LARVESKNEAGGH	tr_D0A9V0	0.016	41953.37	90.00
356	HLA-A0201	DNHYCVNSSD	DNHYCVNSS	0	0	0	0	0	DNHYCVNSS	tr_D0A9V0	0.016	41958.35	90.00
71	HLA-A0201	HIRANGSATETPG	HISATETPG	0	0	0	2	4	HIRANGSATETPG	tr_D0A9V0	0.016	41999.23	90.00
68	HLA-A0201	AGGHIRANGSATE	GGHIRAATE	1	0	0	6	3	GGHIRANGSATE	tr_D0A9V0	0.016	41999.23	90.00
358	HLA-A0201	HYCVNSSDCK	YCVNSSDCK	1	0	0	0	0	YCVNSSDCK	tr_D0A9V0	0.016	42000.61	90.00
139	HLA-A0201	PDVWKSSTKYH	DVWKSSTYH	1	0	0	7	1	DVWKSSTKYH	tr_D0A9V0	0.016	42001.07	90.00
361	HLA-A0201	VNSSDCKPEE	VSSDCKPEE	0	0	0	1	1	VNSSDCKPEE	tr_D0A9V0	0.016	42016.97	90.00
61	HLA-A0201	RVESKNEAGG	RVESKNAGG	0	0	0	6	1	RVESKNEAGG	tr_D0A9V0	0.016	42043.34	90.00
363	HLA-A0201	SSDCKPEEQD	SSDCKPEEQ	0	0	0	0	0	SSDCKPEEQ	tr_D0A9V0	0.016	42044.24	90.00
64	HLA-A0201	SKNEAGGHIRANG	SKNEAGIRA	0	0	0	6	2	SKNEAGGHIRA	tr_D0A9V0	0.016	42075.19	90.00
67	HLA-A0201	EAGGHIRANGSATE	GGHIRAATE	2	0	0	6	3	GGHIRANGSATE	tr_D0A9V0	0.016	42089.76	90.00
482	HLA-A0201	RDVVAESLE	RDVVAESLE	0	0	0	0	0	RDVVAESLE	tr_D0A9V0	0.016	42089.76	90.00
69	HLA-A0201	GGHIRANGSAT	GGHIRANSA	0	0	0	7	1	GGHIRANGSA	tr_D0A9V0	0.016	42093.40	90.00
154	HLA-A0201	EVATEHSGCVE	VATEHSGCV	1	0	0	0	0	VATEHSGCV	tr_D0A9V0	0.016	42095.22	90.00
63	HLA-A0201	ESKNEAGGHIRANG	SKNEAGIRA	1	0	0	6	2	SKNEAGGHIRA	tr_D0A9V0	0.016	42111.62	90.00
396	HLA-A0201	ESDSSDQGS	ESDSSDQGS	0	0	0	0	0	ESDSSDQGS	tr_D0A9V0	0.016	42117.54	90.00
383	HLA-A0201	TIDSPRRPDGEGS	TIDPDGEGS	0	0	0	3	4	TIDSPRRPDGEGS	tr_D0A9V0	0.016	42121.21	90.00
251	HLA-A0201	IAATSGGASH	IAATGGASH	0	0	0	4	1	IAATSGGASH	tr_D0A9V0	0.016	42121.64	90.00
421	HLA-A0201	GSGCAEGANAAD	GSAEGANAA	0	0	0	2	2	GSGCAEGANAA	tr_D0A9V0	0.016	42125.31	90.00
617	HLA-A0201	VAAGGRCTPQSD	AAGGRCTPD	1	0	0	8	2	AAGGRCTPQSD	tr_D0A9V0	0.016	42127.59	90.00
412	HLA-A0201	APSSSSSLDG	ASSSSSLDG	0	0	0	1	1	APSSSSSLDG	tr_D0A9V0	0.016	42128.03	90.00

67	HLA-A0201	EAGGHIRAN	EAGGHIRAN	0	0	0	0	0	EAGGHIRAN	tr_D0A9V0	0.016	42144.90	90.00
616	HLA-A0201	EVAAGGRCTPQSD	AAGGRCTPD	2	0	0	8	2	AAGGRCTPQSD	tr_D0A9V0	0.016	42160.86	90.00
371	HLA-A0201	QDESDIKDRVP	QDESDIDRV	0	0	0	6	1	QDESDIKDRV	tr_D0A9V0	0.016	42162.25	90.00
98	HLA-A0201	SVTWNAGDQQRD	VTWNAGQQR	1	0	0	6	1	VTWNAGDQQR	tr_D0A9V0	0.016	42166.35	90.00
336	HLA-A0201	MRLCRQRCR	MRLCRQRCR	0	0	0	0	0	MRLCRQRCR	tr_D0A9V0	0.016	42174.57	95.00
75	HLA-A0201	NGSATETPG	NGSATETPG	0	0	0	0	0	NGSATETPG	tr_D0A9V0	0.016	42175.93	95.00
67	HLA-A0201	EAGGHIRANGSAT	HIRANGSAT	4	0	0	0	0	HIRANGSAT	tr_D0A9V0	0.016	42185.96	95.00
66	HLA-A0201	NEAGGHIRANGSAT	HIRANGSAT	5	0	0	0	0	HIRANGSAT	tr_D0A9V0	0.016	42196.00	95.00
72	HLA-A0201	IRANGSATE	IRANGSATE	0	0	0	0	0	IRANGSATE	tr_D0A9V0	0.016	42196.93	95.00
66	HLA-A0201	NEAGGHIRAN	NEAGGHIRA	0	0	0	0	0	NEAGGHIRA	tr_D0A9V0	0.016	42198.75	95.00
380	HLA-A0201	VPETIDSPRRPD	VIDSPRRPD	0	0	0	1	3	VPETIDSPRRPD	tr_D0A9V0	0.016	42227.06	95.00
420	HLA-A0201	DGSGCAEGANAAD	DGSGCAEAA	0	0	0	7	3	DGSGCAEGANAA	tr_D0A9V0	0.016	42228.43	95.00
653	HLA-A0201	PAGSDATRLRS	ASDATRLRS	1	0	0	1	1	AGSDATRLRS	tr_D0A9V0	0.016	42232.54	95.00
618	HLA-A0201	AAGGRCTPQSD	AAGGRCTPQ	0	0	0	0	0	AAGGRCTPQ	tr_D0A9V0	0.016	42264.55	95.00
649	HLA-A0201	SSTAPAGSDAT	STAPGSDAT	1	0	0	4	1	STAPAGSDAT	tr_D0A9V0	0.016	42279.19	95.00
422	HLA-A0201	SGCAEGANAAD	SCAEGANAA	0	0	0	1	1	SGCAEGANAA	tr_D0A9V0	0.015	42281.46	95.00
380	HLA-A0201	VPETIDSPRRPDG	VPETIDSPG	0	0	0	8	4	VPETIDSPRRPDG	tr_D0A9V0	0.015	42283.31	95.00
478	HLA-A0201	PRLRRDVVA	PRLRRDVVA	0	0	0	0	0	PRLRRDVVA	tr_D0A9V0	0.015	42284.67	95.00
522	HLA-A0201	EYVPGGSLH	EYVPGGSLH	0	0	0	0	0	EYVPGGSLH	tr_D0A9V0	0.015	42298.39	95.00
620	HLA-A0201	GGRCTPQSD	GGRCTPQSD	0	0	0	0	0	GGRCTPQSD	tr_D0A9V0	0.015	42298.86	95.00
65	HLA-A0201	KNEAGGHIRAN	KNEAGGIRA	0	0	0	6	1	KNEAGGHIRA	tr_D0A9V0	0.015	42327.70	95.00
248	HLA-A0201	PCRIAATSGG	CRIAATSGG	1	0	0	0	0	CRIAATSGG	tr_D0A9V0	0.015	42337.77	95.00
615	HLA-A0201	PEVAAGGRCTPQSD	AAGGRCTPD	3	0	0	8	2	AAGGRCTPQSD	tr_D0A9V0	0.015	42340.53	95.00
65	HLA-A0201	KNEAGGHIRANGS	KNEAGGIRA	0	0	0	6	1	KNEAGGHIRA	tr_D0A9V0	0.015	42389.57	95.00
67	HLA-A0201	EAGGHIRANG	EAGGHIANG	0	0	0	6	1	EAGGHIRANG	tr_D0A9V0	0.015	42393.70	95.00
389	HLA-A0201	RPDGEGSES	RPDGEGSES	0	0	0	0	0	RPDGEGSES	tr_D0A9V0	0.015	42399.66	95.00
412	HLA-A0201	APSSSSSLDGSGC	SSSSSLDGC	2	0	0	7	2	SSSSSLDGSGC	tr_D0A9V0	0.015	42400.13	95.00
413	HLA-A0201	PSSSSSLDG	PSSSSSLDG	0	0	0	0	0	PSSSSSLDG	tr_D0A9V0	0.015	42401.96	95.00
361	HLA-A0201	VNSSDCKPEEQDES	SSDCKPEES	2	0	0	7	3	SSDCKPEEQDES	tr_D0A9V0	0.015	42405.62	95.00
338	HLA-A0201	LCRQRCRRND	LCRQRCRRD	0	0	0	8	1	LCRQRCRRND	tr_D0A9V0	0.015	42461.64	95.00
362	HLA-A0201	NSSDCKPEEQDES	SSDCKPEES	1	0	0	7	3	SSDCKPEEQDES	tr_D0A9V0	0.015	42463.94	95.00
395	HLA-A0201	SESDSSDQGS	SSDSSDQGS	0	0	0	1	1	SESDSSDQGS	tr_D0A9V0	0.015	42514.50	95.00
70	HLA-A0201	GHIRANGSA	GHIRANGSA	0	0	0	0	0	GHIRANGSA	tr_D0A9V0	0.015	42519.57	95.00
413	HLA-A0201	PSSSSSLDGSGC	SSSSSLSGC	1	0	0	6	2	SSSSSLDGSGC	tr_D0A9V0	0.015	42520.04	95.00
71	HLA-A0201	HIRANGSATETPGE	HINGSATET	0	0	0	2	2	HIRANGSATET	tr_D0A9V0	0.015	42536.14	95.00
77	HLA-A0201	SATETPGESST	SATETPSST	0	0	0	6	2	SATETPGESST	tr_D0A9V0	0.015	42537.51	95.00
154	HLA-A0201	EVATEHSGC	EVATEHSGC	0	0	0	0	0	EVATEHSGC	tr_D0A9V0	0.015	42555.45	95.00
72	HLA-A0201	IRANGSATETP	RANGSAETP	1	0	0	6	1	RANGSATETP	tr_D0A9V0	0.015	42578.95	95.00
361	HLA-A0201	VNSSDCKPEEQD	NSSDCPEEQ	1	0	0	5	1	NSSDCKPEEQ	tr_D0A9V0	0.015	42582.19	95.00
463	HLA-A0201	LGVAEDGAQ	LGVAEDGAQ	0	0	0	0	0	LGVAEDGAQ	tr_D0A9V0	0.015	42587.71	95.00
254	HLA-A0201	TSGGASHMQG	TSGGASMQG	0	0	0	6	1	TSGGASHMQG	tr_D0A9V0	0.015	42600.16	95.00
339	HLA-A0201	CRQRCRRNDT	RQRCRRNDT	1	0	0	0	0	RQRCRRNDT	tr_D0A9V0	0.015	42602.46	95.00
616	HLA-A0201	EVAAGGRCTP	EVAAGGCTP	0	0	0	6	1	EVAAGGRCTP	tr_D0A9V0	0.015	42617.22	95.00
65	HLA-A0201	KNEAGGHIRANG	KNEAGGIRA	0	0	0	6	1	KNEAGGHIRA	tr_D0A9V0	0.015	42631.52	95.00
59	HLA-A0201	LARVESKNEAG	LARVESEAG	0	0	0	6	2	LARVESKNEAG	tr_D0A9V0	0.015	42637.51	95.00
70	HLA-A0201	GHIRANGSATETPG	HISATETPG	1	0	0	2	4	HIRANGSATETPG	tr_D0A9V0	0.015	42638.88	95.00
494	HLA-A0201	EVASKLHHPN	EVASKLHHP	0	0	0	0	0	EVASKLHHP	tr_D0A9V0	0.015	42641.19	95.00
412	HLA-A0201	APSSSSSLDGS	ASSSSLDGS	0	0	0	1	2	APSSSSSLDGS	tr_D0A9V0	0.015	42643.50	95.00
62	HLA-A0201	VESKNEAGGHIR	SKNEAGGHI	2	0	0	0	0	SKNEAGGHI	tr_D0A9V0	0.015	42644.88	95.00
382	HLA-A0201	ETIDSPRRPDGEGS	TIDPDGEGS	1	0	0	3	4	TIDSPRRPDGEGS	tr_D0A9V0	0.015	42646.72	95.00
197	HLA-A0201	AKLPLDHRD	AKLPLDHRD	0	0	0	0	0	AKLPLDHRD	tr_D0A9V0	0.015	42651.81	95.00
372	HLA-A0201	DESDIKDRVPE	ESDIKDVPE	1	0	0	6	1	ESDIKDRVPE	tr_D0A9V0	0.015	42678.11	95.00
241	HLA-A0201	SYAVRDGPCR	YAVRDGPCR	1	0	0	0	0	YAVRDGPCR	tr_D0A9V0	0.015	42691.99	95.00
653	HLA-A0201	PAGSDATRLR	PAGSDATRL	0	0	0	0	0	PAGSDATRL	tr_D0A9V0	0.014	42740.50	95.00

374	HLA-A0201	SDIKDRVPE	SDIKDRVPE	0	0	0	0	0	SDIKDRVPE	tr_D0A9V0	0.014	42802.51	95.00
362	HLA-A0201	NSSDCKPEEQD	NSSDCPEEQ	0	0	0	5	1	NSSDCKPEEQ	tr_D0A9V0	0.014	42809.46	95.00
366	HLA-A0201	CKPEEQDESDIKD	CQDESDIKD	0	0	0	1	4	CKPEEQDESDIKD	tr_D0A9V0	0.014	42813.63	95.00
73	HLA-A0201	RANGSATETPGESS	RANGSAESS	0	0	0	6	5	RANGSATETPGESS	tr_D0A9V0	0.014	42835.40	95.00
114	HLA-A0201	CGIFKEKPN	CGIFKEKPN	0	0	0	0	0	CGIFKEKPN	tr_D0A9V0	0.014	42854.88	95.00
383	HLA-A0201	TIDSPRRPDGE	TIDSPPDGE	0	0	0	5	2	TIDSPRRPDGE	tr_D0A9V0	0.014	42884.57	95.00
76	HLA-A0201	GSATETPGESST	SATETPSST	1	0	0	6	2	SATETPGESST	tr_D0A9V0	0.014	42918.91	95.00
358	HLA-A0201	HYCVNSSDCKP	YVNSSDCKP	1	0	0	1	1	YCVNSSDCKP	tr_D0A9V0	0.014	42958.39	95.00
361	HLA-A0201	VNSSDCKPEEQDE	SSDCKPEEE	2	0	0	8	2	SSDCKPEEQDE	tr_D0A9V0	0.014	42976.07	95.00
681	HLA-A0201	EPVIPASVD	EPVIPASVD	0	0	0	0	0	EPVIPASVD	tr_D0A9V0	0.014	42983.97	95.00
70	HLA-A0201	GHIRANGSATE	HIRAGSATE	1	0	0	4	1	HIRANGSATE	tr_D0A9V0	0.014	43030.04	95.00
365	HLA-A0201	DCKPEEQDESDIKD	CQDESDIKD	1	0	0	1	4	CKPEEQDESDIKD	tr_D0A9V0	0.014	43042.14	95.00
383	HLA-A0201	TIDSPRRPD	TIDSPRRPD	0	0	0	0	0	TIDSPRRPD	tr_D0A9V0	0.014	43072.91	95.00
648	HLA-A0201	WSSTAPAGSD	WSSTAPAGS	0	0	0	0	0	WSSTAPAGS	tr_D0A9V0	0.014	43077.55	95.00
59	HLA-A0201	LARVESKNEAGG	LARVESAGG	0	0	0	6	3	LARVESKNEAGG	tr_D0A9V0	0.014	43082.21	95.00
362	HLA-A0201	NSSDCKPEEQDESD	NSSDCPEEQ	0	0	0	5	1	NSSDCKPEEQ	tr_D0A9V0	0.014	43104.14	95.00
75	HLA-A0201	NGSATETPGESST	SATETPSST	2	0	0	6	2	SATETPGESST	tr_D0A9V0	0.014	43106.48	95.00
363	HLA-A0201	SSDCKPEEQDES	SSDCKPEES	0	0	0	7	3	SSDCKPEEQDES	tr_D0A9V0	0.014	43131.20	95.00
63	HLA-A0201	ESKNEAGGH	ESKNEAGGH	0	0	0	0	0	ESKNEAGGH	tr_D0A9V0	0.014	43151.73	95.00
63	HLA-A0201	ESKNEAGGHIR	SKNEAGGHI	1	0	0	0	0	SKNEAGGHI	tr_D0A9V0	0.014	43158.27	95.00
704	HLA-A0201	PSMRPSASS	PSMRPSASS	0	0	0	0	0	PSMRPSASS	tr_D0A9V0	0.014	43171.36	95.00
366	HLA-A0201	CKPEEQDESDI	KPEEQDESI	1	0	0	8	1	KPEEQDESDI	tr_D0A9V0	0.014	43183.97	95.00
66	HLA-A0201	NEAGGHIRANGS	NEAGGHIRA	0	0	0	0	0	NEAGGHIRA	tr_D0A9V0	0.014	43193.77	95.00
286	HLA-A0201	LRPGESSSEVS	LPGESSSEV	0	0	0	1	1	LRPGESSSEV	tr_D0A9V0	0.014	43197.04	95.00
75	HLA-A0201	NGSATETPGESS	SATETPESS	2	0	0	6	1	SATETPGESS	tr_D0A9V0	0.013	43206.39	95.00
364	HLA-A0201	SDCKPEEQDESDI	KPEEQDESI	3	0	0	8	1	KPEEQDESDI	tr_D0A9V0	0.013	43213.86	95.00
365	HLA-A0201	DCKPEEQDESDI	KPEEQDESI	2	0	0	8	1	KPEEQDESDI	tr_D0A9V0	0.013	43214.34	95.00
74	HLA-A0201	ANGSATETPGESST	SATETPSST	3	0	0	6	2	SATETPGESST	tr_D0A9V0	0.013	43219.02	95.00
382	HLA-A0201	ETIDSPRRPDGE	TIDSPPDGE	1	0	0	5	2	TIDSPRRPDGE	tr_D0A9V0	0.013	43221.84	95.00
342	HLA-A0201	RCRRNDTVP	RCRRNDTVP	0	0	0	0	0	RCRRNDTVP	tr_D0A9V0	0.013	43224.15	95.00
362	HLA-A0201	NSSDCKPEEQDE	SSDCKPEEE	1	0	0	8	2	SSDCKPEEQDE	tr_D0A9V0	0.013	43229.78	95.00
67	HLA-A0201	EAGGHIRANGS	AGHIRANGS	1	0	0	1	1	AGGHIRANGS	tr_D0A9V0	0.013	43233.52	95.00
645	HLA-A0201	PWQWSSTAP	PWQWSSTAP	0	0	0	0	0	PWQWSSTAP	tr_D0A9V0	0.013	43246.15	95.00
100	HLA-A0201	TWNAGDQQRDVA	TWNAGDQQV	0	0	0	8	2	TWNAGDQQRDV	tr_D0A9V0	0.013	43252.23	95.00
372	HLA-A0201	DESDIKDRVP	DESDIKDRV	0	0	0	0	0	DESDIKDRV	tr_D0A9V0	0.013	43265.34	95.00
79	HLA-A0201	TETPGESST	TETPGESST	0	0	0	0	0	TETPGESST	tr_D0A9V0	0.013	43273.77	95.00
76	HLA-A0201	GSATETPGESS	SATETPESS	1	0	0	6	1	SATETPGESS	tr_D0A9V0	0.013	43276.11	95.00
383	HLA-A0201	TIDSPRRPDG	TIDSPRRPG	0	0	0	8	1	TIDSPRRPDG	tr_D0A9V0	0.013	43277.03	95.00
383	HLA-A0201	TIDSPRRPDGEGSE	TIDPDGEGS	0	0	0	3	4	TIDSPRRPDGEGS	tr_D0A9V0	0.013	43292.97	95.00
412	HLA-A0201	APSSSSSLDGSG	SSSSSLDGG	2	0	0	8	1	SSSSSLDGSG	tr_D0A9V0	0.013	43303.75	95.00
382	HLA-A0201	ETIDSPRRP	ETIDSPRRP	0	0	0	0	0	ETIDSPRRP	tr_D0A9V0	0.013	43308.89	95.00
74	HLA-A0201	ANGSATETPGESS	SATETPESS	3	0	0	6	1	SATETPGESS	tr_D0A9V0	0.013	43316.85	95.00
383	HLA-A0201	TIDSPRRPDGEG	TIDSPDGEG	0	0	0	4	3	TIDSPRRPDGEG	tr_D0A9V0	0.013	43317.80	95.00
288	HLA-A0201	PGESSSEVSLS	GSSSEVSLS	1	0	0	1	1	GESSSEVSLS	tr_D0A9V0	0.013	43336.07	95.00
73	HLA-A0201	RANGSATETPG	RASATETPG	0	0	0	2	2	RANGSATETPG	tr_D0A9V0	0.013	43357.18	95.00
60	HLA-A0201	ARVESKNEAG	RVESKNEAG	1	0	0	0	0	RVESKNEAG	tr_D0A9V0	0.013	43374.07	95.00
564	HLA-A0201	VHCDVKPHN	VHCDVKPHN	0	0	0	0	0	VHCDVKPHN	tr_D0A9V0	0.013	43392.84	95.00
381	HLA-A0201	PETIDSPRRPDGE	TIDSPPDGE	2	0	0	5	2	TIDSPRRPDGE	tr_D0A9V0	0.013	43396.59	95.00
367	HLA-A0201	KPEEQDESDIKD	KQDESDIKD	0	0	0	1	3	KPEEQDESDIKD	tr_D0A9V0	0.013	43422.91	95.00
72	HLA-A0201	IRANGSATETPGES	IATETPGES	0	0	0	1	5	IRANGSATETPGES	tr_D0A9V0	0.013	43428.54	95.00
73	HLA-A0201	RANGSATETPGES	RANGSATES	0	0	0	7	4	RANGSATETPGES	tr_D0A9V0	0.013	43431.84	95.00
413	HLA-A0201	PSSSSSLDGSG	SSSSSLDGG	1	0	0	8	1	SSSSSLDGSG	tr_D0A9V0	0.013	43464.27	95.00
73	HLA-A0201	RANGSATETPGE	RANGSATET	0	0	0	0	0	RANGSATET	tr_D0A9V0	0.013	43498.61	95.00
62	HLA-A0201	VESKNEAGG	VESKNEAGG	0	0	0	0	0	VESKNEAGG	tr_D0A9V0	0.013	43549.95	95.00

369	HLA-A0201	EEQDESDIKD	EQDESDIKD	1	0	0	0	0	EQDESDIKD	tr_D0A9V0	0.013	43641.47	95.00
421	HLA-A0201	GSGCAEGAN	GSGCAEGAN	0	0	0	0	0	GSGCAEGAN	tr_D0A9V0	0.013	43663.65	95.00
386	HLA-A0201	SPRRPDGEGS	SPRPDGEGS	0	0	0	2	1	SPRRPDGEGS	tr_D0A9V0	0.013	43667.91	95.00
382	HLA-A0201	ETIDSPRRPDGEG	TIDSPPDGE	1	0	0	5	2	TIDSPRRPDGE	tr_D0A9V0	0.012	43684.44	95.00
72	HLA-A0201	IRANGSATETPG	IANGSATET	0	0	0	1	1	IRANGSATET	tr_D0A9V0	0.012	43709.52	95.00
377	HLA-A0201	KDRVPETID	KDRVPETID	0	0	0	0	0	KDRVPETID	tr_D0A9V0	0.012	43715.66	95.00
74	HLA-A0201	ANGSATETPG	ANGSATEPG	0	0	0	7	1	ANGSATETPG	tr_D0A9V0	0.012	43730.81	95.00
72	HLA-A0201	IRANGSATETPGE	IANGSATET	0	0	0	1	1	IRANGSATET	tr_D0A9V0	0.012	43741.21	95.00
619	HLA-A0201	AGGRCTPQSD	AGRCTPQSD	0	0	0	1	1	AGGRCTPQSD	tr_D0A9V0	0.012	43746.89	95.00
75	HLA-A0201	NGSATETPGE	NGSATTPGE	0	0	0	5	1	NGSATETPGE	tr_D0A9V0	0.012	43756.34	99.00
394	HLA-A0201	GSESDSSDQ	GSESDSSDQ	0	0	0	0	0	GSESDSSDQ	tr_D0A9V0	0.012	43765.34	99.00
649	HLA-A0201	SSTAPAGSD	SSTAPAGSD	0	0	0	0	0	SSTAPAGSD	tr_D0A9V0	0.012	43773.39	99.00
384	HLA-A0201	IDSPRRPDGE	ISPRRPDGE	0	0	0	1	1	IDSPRRPDGE	tr_D0A9V0	0.012	43815.10	99.00
341	HLA-A0201	QRCRRNDTVP	QRCRRNDTV	0	0	0	0	0	QRCRRNDTV	tr_D0A9V0	0.012	43824.12	99.00
363	HLA-A0201	SSDCKPEEQDE	SSDCKPEEE	0	0	0	8	2	SSDCKPEEQDE	tr_D0A9V0	0.012	43831.21	99.00
381	HLA-A0201	PETIDSPRRPDGEG	TIDSPPDGE	2	0	0	5	2	TIDSPRRPDGE	tr_D0A9V0	0.012	43874.88	99.00
363	HLA-A0201	SSDCKPEEQDESD	SSDCKPEEQ	0	0	0	0	0	SSDCKPEEQ	tr_D0A9V0	0.012	43892.45	99.00
395	HLA-A0201	SESDSSDQG	SESDSSDQG	0	0	0	0	0	SESDSSDQG	tr_D0A9V0	0.012	43906.21	99.00
61	HLA-A0201	RVESKNEAGGH	RVESKNEAH	0	0	0	8	2	RVESKNEAGGH	tr_D0A9V0	0.012	43909.55	99.00
366	HLA-A0201	CKPEEQDES	CKPEEQDES	0	0	0	0	0	CKPEEQDES	tr_D0A9V0	0.012	43926.18	99.00
66	HLA-A0201	NEAGGHIRANG	NEAGGHIRA	0	0	0	0	0	NEAGGHIRA	tr_D0A9V0	0.012	43928.07	99.00
387	HLA-A0201	PRRPDGEGS	PRRPDGEGS	0	0	0	0	0	PRRPDGEGS	tr_D0A9V0	0.012	43978.96	99.00
101	HLA-A0201	WNAGDQQRDVA	WAGDQQRDV	0	0	0	1	1	WNAGDQQRDV	tr_D0A9V0	0.012	43984.66	99.00
365	HLA-A0201	DCKPEEQDESDIK	KPEEQDESI	2	0	0	8	1	KPEEQDESDI	tr_D0A9V0	0.012	44013.23	99.00
366	HLA-A0201	CKPEEQDESDIK	KPEEQDESI	1	0	0	8	1	KPEEQDESDI	tr_D0A9V0	0.012	44015.62	99.00
62	HLA-A0201	VESKNEAGGH	VSKNEAGGH	0	0	0	1	1	VESKNEAGGH	tr_D0A9V0	0.012	44064.70	99.00
364	HLA-A0201	SDCKPEEQDESDIK	KPEEQDESI	3	0	0	8	1	KPEEQDESDI	tr_D0A9V0	0.012	44084.25	99.00
367	HLA-A0201	KPEEQDESD	KPEEQDESD	0	0	0	0	0	KPEEQDESD	tr_D0A9V0	0.012	44089.48	99.00
369	HLA-A0201	EEQDESDIK	EEQDESDIK	0	0	0	0	0	EEQDESDIK	tr_D0A9V0	0.012	44145.82	99.00
412	HLA-A0201	APSSSSSLD	APSSSSSLD	0	0	0	0	0	APSSSSSLD	tr_D0A9V0	0.012	44149.15	99.00
74	HLA-A0201	ANGSATETPGES	SATETPGES	3	0	0	0	0	SATETPGES	tr_D0A9V0	0.011	44191.71	99.00
381	HLA-A0201	PETIDSPRR	PETIDSPRR	0	0	0	0	0	PETIDSPRR	tr_D0A9V0	0.011	44200.77	99.00
367	HLA-A0201	KPEEQDESDIK	KPEEQDESI	0	0	0	8	1	KPEEQDESDI	tr_D0A9V0	0.011	44214.17	99.00
75	HLA-A0201	NGSATETPGES	SATETPGES	2	0	0	0	0	SATETPGES	tr_D0A9V0	0.011	44228.06	99.00
368	HLA-A0201	PEEQDESDI	PEEQDESDI	0	0	0	0	0	PEEQDESDI	tr_D0A9V0	0.011	44233.30	99.00
60	HLA-A0201	ARVESKNEAGGH	RVESKNEAH	1	0	0	8	2	RVESKNEAGGH	tr_D0A9V0	0.011	44261.55	99.00
121	HLA-A0201	PNVFNRRHC	PNVFNRRHC	0	0	0	0	0	PNVFNRRHC	tr_D0A9V0	0.011	44279.77	99.00
384	HLA-A0201	IDSPRRPDGEGS	IDSPRGEGS	0	0	0	5	3	IDSPRRPDGEGS	tr_D0A9V0	0.011	44296.05	99.00
397	HLA-A0201	SDSSDQGSES	SSSDQGSES	0	0	0	1	1	SDSSDQGSES	tr_D0A9V0	0.011	44318.11	99.00
339	HLA-A0201	CRQRCRRND	CRQRCRRND	0	0	0	0	0	CRQRCRRND	tr_D0A9V0	0.011	44370.42	99.00
615	HLA-A0201	PEVAAGGRCTP	EVAAGGCTP	1	0	0	6	1	EVAAGGRCTP	tr_D0A9V0	0.011	44377.60	99.00
392	HLA-A0201	GEGSESDSS	GEGSESDSS	0	0	0	0	0	GEGSESDSS	tr_D0A9V0	0.011	44384.32	99.00
286	HLA-A0201	LRPGESSSE	LRPGESSSE	0	0	0	0	0	LRPGESSSE	tr_D0A9V0	0.011	44433.33	99.00
615	HLA-A0201	PEVAAGGRC	PEVAAGGRC	0	0	0	0	0	PEVAAGGRC	tr_D0A9V0	0.011	44433.82	99.00
389	HLA-A0201	RPDGEGSESD	RPDEGSESD	0	0	0	3	1	RPDGEGSESD	tr_D0A9V0	0.011	44576.35	99.00
647	HLA-A0201	QWSSTAPAGSD	WSSTAPAGS	1	0	0	0	0	WSSTAPAGS	tr_D0A9V0	0.011	44625.10	99.00
365	HLA-A0201	DCKPEEQDE	DCKPEEQDE	0	0	0	0	0	DCKPEEQDE	tr_D0A9V0	0.010	44641.51	99.00
385	HLA-A0201	DSPRRPDGE	DSPRRPDGE	0	0	0	0	0	DSPRRPDGE	tr_D0A9V0	0.010	44645.86	99.00
139	HLA-A0201	PDVWKSSTK	PDVWKSSTK	0	0	0	0	0	PDVWKSSTK	tr_D0A9V0	0.010	44647.30	99.00
288	HLA-A0201	PGESSSEVS	PGESSSEVS	0	0	0	0	0	PGESSSEVS	tr_D0A9V0	0.010	44710.14	99.00
384	HLA-A0201	IDSPRRPDGEGSES	IPDGEGSES	0	0	0	1	5	IDSPRRPDGEGSES	tr_D0A9V0	0.010	44743.05	99.00
386	HLA-A0201	SPRRPDGEG	SPRRPDGEG	0	0	0	0	0	SPRRPDGEG	tr_D0A9V0	0.010	44788.58	99.00
384	HLA-A0201	IDSPRRPDG	IDSPRRPDG	0	0	0	0	0	IDSPRRPDG	tr_D0A9V0	0.010	44801.20	99.00
74	HLA-A0201	ANGSATETPGE	ANGSATETP	0	0	0	0	0	ANGSATETP	tr_D0A9V0	0.010	44819.12	99.00

385	HLA-A0201	DSPRRPDGEGS	SPRPDGEGS	1	0	0	2	1	SPRRPDGEGS	tr_D0A9V0	0.010	44838.05	99.00
615	HLA-A0201	PEVAAGGRCT	EVAAGGRCT	1	0	0	0	0	EVAAGGRCT	tr_D0A9V0	0.010	44846.79	99.00
364	HLA-A0201	SDCKPEEQD	SDCKPEEQD	0	0	0	0	0	SDCKPEEQD	tr_D0A9V0	0.010	44851.16	99.00
388	HLA-A0201	RRPDGEGSES	RPDGEGSES	1	0	0	0	0	RPDGEGSES	tr_D0A9V0	0.010	44913.30	99.00
368	HLA-A0201	PEEQDESDIKD	EQDESDIKD	2	0	0	0	0	EQDESDIKD	tr_D0A9V0	0.010	44913.79	99.00
398	HLA-A0201	DSSDQGSES	DSSDQGSES	0	0	0	0	0	DSSDQGSES	tr_D0A9V0	0.010	44926.92	99.00
100	HLA-A0201	TWNAGDQQRD	TWNAGDQQR	0	0	0	0	0	TWNAGDQQR	tr_D0A9V0	0.010	44931.79	99.00
384	HLA-A0201	IDSPRRPDGEGSE	IDSPRGEGS	0	0	0	5	3	IDSPRRPDGEGS	tr_D0A9V0	0.010	44932.25	99.00
387	HLA-A0201	PRRPDGEGSE	PRRPDGEGE	0	0	0	8	1	PRRPDGEGSE	tr_D0A9V0	0.010	44939.55	99.00
386	HLA-A0201	SPRRPDGEGSES	RPDGEGSES	3	0	0	0	0	RPDGEGSES	tr_D0A9V0	0.010	44963.88	99.00
381	HLA-A0201	PETIDSPRRP	ETIDSPRRP	1	0	0	0	0	ETIDSPRRP	tr_D0A9V0	0.010	44995.49	99.00
366	HLA-A0201	CKPEEQDESD	CPEEQDESD	0	0	0	1	1	CKPEEQDESD	tr_D0A9V0	0.010	45084.20	99.00
385	HLA-A0201	DSPRRPDGEGSES	RPDGEGSES	4	0	0	0	0	RPDGEGSES	tr_D0A9V0	0.010	45085.66	99.00
386	HLA-A0201	SPRRPDGEGSESD	SPRPDGEGS	0	0	0	2	1	SPRRPDGEGS	tr_D0A9V0	0.010	45102.26	99.00
60	HLA-A0201	ARVESKNEAGG	RVESKNAGG	1	0	0	6	1	RVESKNEAGG	tr_D0A9V0	0.010	45109.56	99.00
394	HLA-A0201	GSESDSSDQG	GSSDSSDQG	0	0	0	2	1	GSESDSSDQG	tr_D0A9V0	0.010	45114.45	99.00
386	HLA-A0201	SPRRPDGEGSE	SPRPDGEGS	0	0	0	2	1	SPRRPDGEGS	tr_D0A9V0	0.009	45116.89	99.00
384	HLA-A0201	IDSPRRPDGEG	IDSPRPDGE	0	0	0	4	1	IDSPRRPDGE	tr_D0A9V0	0.009	45124.69	99.00
385	HLA-A0201	DSPRRPDGEGSESD	RPDGESESD	4	0	0	5	1	RPDGEGSESD	tr_D0A9V0	0.009	45197.01	99.00
386	HLA-A0201	SPRRPDGEGSESDS	SPRPDGEGS	0	0	0	2	1	SPRRPDGEGS	tr_D0A9V0	0.009	45201.91	99.00
364	HLA-A0201	SDCKPEEQDE	SDCKPEEQE	0	0	0	8	1	SDCKPEEQDE	tr_D0A9V0	0.009	45235.18	99.00
394	HLA-A0201	GSESDSSDQGS	SSDSSDQGS	1	0	0	1	1	SESDSSDQGS	tr_D0A9V0	0.009	45240.58	99.00
396	HLA-A0201	ESDSSDQGSE	ESDSSDQGS	0	0	0	0	0	ESDSSDQGS	tr_D0A9V0	0.009	45247.90	99.00
385	HLA-A0201	DSPRRPDGEGSE	DSRPDGEGS	0	0	0	2	2	DSPRRPDGEGS	tr_D0A9V0	0.009	45270.43	99.00
392	HLA-A0201	GEGSESDSSDQGS	SSDSSDQGS	3	0	0	1	1	SESDSSDQGS	tr_D0A9V0	0.009	45294.44	99.00
388	HLA-A0201	RRPDGEGSE	RRPDGEGSE	0	0	0	0	0	RRPDGEGSE	tr_D0A9V0	0.009	45303.26	99.00
387	HLA-A0201	PRRPDGEGSES	RPDGEGSES	2	0	0	0	0	RPDGEGSES	tr_D0A9V0	0.009	45311.59	99.00
365	HLA-A0201	DCKPEEQDES	CKPEEQDES	1	0	0	0	0	CKPEEQDES	tr_D0A9V0	0.009	45354.27	99.00
391	HLA-A0201	DGEGSESDSSDQGS	SSDSSDQGS	4	0	0	1	1	SESDSSDQGS	tr_D0A9V0	0.009	45371.45	99.00
393	HLA-A0201	EGSESDSSDQGS	SSDSSDQGS	2	0	0	1	1	SESDSSDQGS	tr_D0A9V0	0.009	45424.00	99.00
381	HLA-A0201	PETIDSPRRPDG	TIDSPRRPG	2	0	0	8	1	TIDSPRRPDG	tr_D0A9V0	0.009	45446.62	99.00
389	HLA-A0201	RPDGEGSESDSSDQ	RPDGEGSEQ	0	0	0	8	5	RPDGEGSESDSSDQ	tr_D0A9V0	0.009	45463.82	99.00
387	HLA-A0201	PRRPDGEGSESD	RPDEGSESD	2	0	0	3	1	RPDGEGSESD	tr_D0A9V0	0.009	45481.54	99.00
397	HLA-A0201	SDSSDQGSE	SDSSDQGSE	0	0	0	0	0	SDSSDQGSE	tr_D0A9V0	0.009	45550.49	99.00
391	HLA-A0201	DGEGSESDS	DGEGSESDS	0	0	0	0	0	DGEGSESDS	tr_D0A9V0	0.009	45551.49	99.00
382	HLA-A0201	ETIDSPRRPDG	TIDSPRRPG	1	0	0	8	1	TIDSPRRPDG	tr_D0A9V0	0.009	45561.83	99.00
389	HLA-A0201	RPDGEGSESDSS	RPDGESDSS	0	0	0	4	3	RPDGEGSESDSS	tr_D0A9V0	0.009	45593.40	99.00
387	HLA-A0201	PRRPDGEGSESDSS	RPDGESDSS	2	0	0	4	3	RPDGEGSESDSS	tr_D0A9V0	0.008	45613.13	99.00
395	HLA-A0201	SESDSSDQGSE	SSDSSDQGS	0	0	0	1	1	SESDSSDQGS	tr_D0A9V0	0.008	45615.11	99.00
368	HLA-A0201	PEEQDESDIK	EEQDESDIK	1	0	0	0	0	EEQDESDIK	tr_D0A9V0	0.008	45622.02	99.00
382	HLA-A0201	ETIDSPRRPD	TIDSPRRPD	1	0	0	0	0	TIDSPRRPD	tr_D0A9V0	0.008	45624.49	99.00
387	HLA-A0201	PRRPDGEGSESDS	PRRPDGEGE	0	0	0	8	1	PRRPDGEGSE	tr_D0A9V0	0.008	45629.90	99.00
388	HLA-A0201	RRPDGEGSESD	RPDEGSESD	1	0	0	3	1	RPDGEGSESD	tr_D0A9V0	0.008	45675.83	99.00
395	HLA-A0201	SESDSSDQGSES	SSSDQGSES	0	0	0	1	3	SESDSSDQGSES	tr_D0A9V0	0.008	45740.62	99.00
389	HLA-A0201	RPDGEGSESDS	RPDGEGSES	0	0	0	8	2	RPDGEGSESDS	tr_D0A9V0	0.008	45759.45	99.00
101	HLA-A0201	WNAGDQQRD	WNAGDQQRD	0	0	0	0	0	WNAGDQQRD	tr_D0A9V0	0.008	45770.34	99.00
396	HLA-A0201	ESDSSDQGSES	SSSDQGSES	1	0	0	1	1	SDSSDQGSES	tr_D0A9V0	0.008	45793.13	99.00
393	HLA-A0201	EGSESDSSD	EGSESDSSD	0	0	0	0	0	EGSESDSSD	tr_D0A9V0	0.008	45794.61	99.00
388	HLA-A0201	RRPDGEGSESDSS	RPDGESDSS	1	0	0	4	3	RPDGEGSESDSS	tr_D0A9V0	0.008	45799.57	99.00
364	HLA-A0201	SDCKPEEQDES	SDCEEQDES	0	0	0	3	2	SDCKPEEQDES	tr_D0A9V0	0.008	45809.97	99.00
390	HLA-A0201	PDGEGSESD	PDGEGSESD	0	0	0	0	0	PDGEGSESD	tr_D0A9V0	0.008	45830.80	99.00
394	HLA-A0201	GSESDSSDQGSE	SSDSSDQGS	1	0	0	1	1	SESDSSDQGS	tr_D0A9V0	0.008	45838.22	99.00
394	HLA-A0201	GSESDSSDQGSES	GSSDQGSES	0	0	0	1	4	GSESDSSDQGSES	tr_D0A9V0	0.008	45856.58	99.00
364	HLA-A0201	SDCKPEEQDESD	SDCEQDESD	0	0	0	3	3	SDCKPEEQDESD	tr_D0A9V0	0.008	45857.09	99.00

381	HLA-A0201	PETIDSPRRPD	TIDSPRRPD	2	0	0	0	0	TIDSPRRPD	tr_D0A9V0	0.008	45931.08	99.00	
392	HLA-A0201	GEGSESDSSDQGSE	SSDSSDQGS	3	0	0	1	1	SESDSSDQGS	tr_D0A9V0	0.008	45932.56	99.00	
393	HLA-A0201	EGSESDSSDQGSE	SSDSSDQGS	2	0	0	1	1	SESDSSDQGS	tr_D0A9V0	0.008	45935.05	99.00	
388	HLA-A0201	RRPDGEGSESDS	RPDGEGSES	1	0	0	8	2	RPDGEGSESDS	tr_D0A9V0	0.008	45936.53	99.00	
393	HLA-A0201	EGSESDSSDQ	GSESDSSDQ	1	0	0	0	0	GSESDSSDQ	tr_D0A9V0	0.008	45947.97	99.00	
393	HLA-A0201	EGSESDSSDQGSES	SSSDQGSES	4	0	0	1	1	SDSSDQGSES	tr_D0A9V0	0.008	45961.39	99.00	
365	HLA-A0201	DCKPEEQDESD	KPEEQDESD	2	0	0	0	0	KPEEQDESD	tr_D0A9V0	0.008	45964.39	99.00	
389	HLA-A0201	RPDGEGSESDSSD	RPDEGSESD	0	0	0	3	1	RPDGEGSESD	tr_D0A9V0	0.008	45974.32	99.00	
385	HLA-A0201	DSPRRPDGEG	SPRRPDGEG	1	0	0	0	0	SPRRPDGEG	tr_D0A9V0	0.008	46060.98	99.00	
388	HLA-A0201	RRPDGEGSESDSSD	RGSESDSSD	0	0	0	1	5	RRPDGEGSESDSSD	tr_D0A9V0	0.007	46105.85	99.00	
391	HLA-A0201	DGEGSESDSS	GEGSESDSS	1	0	0	0	0	GEGSESDSS	tr_D0A9V0	0.007	46131.28	99.00	
390	HLA-A0201	PDGEGSESDSSDQ	GSESDSSDQ	4	0	0	0	0	GSESDSSDQ	tr_D0A9V0	0.007	46275.26	99.00	
390	HLA-A0201	PDGEGSESDSSDQG	GSSDSSDQG	4	0	0	2	1	GSESDSSDQG	tr_D0A9V0	0.007	46291.80	99.00	
392	HLA-A0201	GEGSESDSSDQG	GSSDSSDQG	2	0	0	2	1	GSESDSSDQG	tr_D0A9V0	0.007	46359.46	99.00	
391	HLA-A0201	DGEGSESDSSDQG	GSSDSSDQG	3	0	0	2	1	GSESDSSDQG	tr_D0A9V0	0.007	46371.99	99.00	
392	HLA-A0201	GEGSESDSSDQ	GSESDSSDQ	2	0	0	0	0	GSESDSSDQ	tr_D0A9V0	0.007	46390.57	99.00	
391	HLA-A0201	DGEGSESDSSDQ	GSESDSSDQ	3	0	0	0	0	GSESDSSDQ	tr_D0A9V0	0.007	46427.23	99.00	
390	HLA-A0201	PDGEGSESDS	PDGEGSESS	0	0	0	8	1	PDGEGSESDS	tr_D0A9V0	0.007	46432.74	99.00	
393	HLA-A0201	EGSESDSSDQG	GSSDSSDQG	1	0	0	2	1	GSESDSSDQG	tr_D0A9V0	0.007	46433.25	99.00	
392	HLA-A0201	GEGSESDSSD	GEGSESDSS	0	0	0	0	0	GEGSESDSS	tr_D0A9V0	0.007	46486.52	99.00	
390	HLA-A0201	PDGEGSESDSS	GEGSESDSS	2	0	0	0	0	GEGSESDSS	tr_D0A9V0	0.006	46756.91	99.00	
390	HLA-A0201	PDGEGSESDSSD	EGSESDSSD	3	0	0	0	0	EGSESDSSD	tr_D0A9V0	0.006	46969.35	99.00	
391	HLA-A0201	DGEGSESDSSD	DGEGSSDSS	0	0	0	5	1	DGEGSESDSS	tr_D0A9V0	0.006	46978.01	99.00	

Protein tr_D0A9V0. Allele HLA-A0201. Number of high binders 27. Number of weak binders 88. Number of peptides 4263

Link to Allele Frequencies in Worldwide Populations HLA-A0201

Rank Threshold for Strong binding peptides 0.500

Rank Threshold for Weak binding peptides 2.000

----

pos	HLA	peptide	Core	Offset	I_pos	I_len	D_pos	D_len	iCore	Identity	1-log50k(aff)	Affinity(nM)	%Rank	BindLevel
236	HLA-A0201	FMAKVSDAV	FMAKVSDAV	0	0	0	0	0	FMAKVSDAV	tr_C9ZP82	0.824	6.75	0.06	<= SB
537	HLA-A0201	ALDNLLELYV	ALDNLLLYV	0	0	0	6	1	ALDNLLELYV	tr_C9ZP82	0.777	11.21	0.15	<= SB
302	HLA-A0201	GLDADLIFL	GLDADLIFL	0	0	0	0	0	GLDADLIFL	tr_C9ZP82	0.774	11.55	0.15	<= SB
114	HLA-A0201	YMPNKSGMAIVEEV	YMMAIVEEV	0	0	0	2	5	YMPNKSGMAIVEEV	tr_C9ZP82	0.773	11.67	0.15	<= SB
722	HLA-A0201	ALLPMVLGEV	ALLPMVLEV	0	0	0	7	1	ALLPMVLGEV	tr_C9ZP82	0.759	13.50	0.17	<= SB
743	HLA-A0201	STFPLQWNV	STFPLQWNV	0	0	0	0	0	STFPLQWNV	tr_C9ZP82	0.739	16.83	0.25	<= SB
817	HLA-A0201	KLSDALDTI	KLSDALDTI	0	0	0	0	0	KLSDALDTI	tr_C9ZP82	0.738	16.95	0.25	<= SB
765	HLA-A0201	MLPFADMDVLQQV	MLMDVLQQV	0	0	0	2	4	MLPFADMDVLQQV	tr_C9ZP82	0.737	17.25	0.25	<= SB
554	HLA-A0201	FLTKGPHEI	FLTKGPHEI	0	0	0	0	0	FLTKGPHEI	tr_C9ZP82	0.724	19.83	0.30	<= SB
711	HLA-A0201	QLLSILPPT	QLLSILPPT	0	0	0	0	0	QLLSILPPT	tr_C9ZP82	0.723	20.10	0.30	<= SB
508	HLA-A0201	KIIDDIIVL	KIIDDIIVL	0	0	0	0	0	KIIDDIIVL	tr_C9ZP82	0.721	20.45	0.30	<= SB
540	HLA-A0201	NLLELYVSEV	NLLEYVSEV	0	0	0	4	1	NLLELYVSEV	tr_C9ZP82	0.718	21.07	0.30	<= SB
569	HLA-A0201	RLFEAYAKI	RLFEAYAKI	0	0	0	0	0	RLFEAYAKI	tr_C9ZP82	0.713	22.28	0.30	<= SB
302	HLA-A0201	GLDADLIFLA	GLADLIFLA	0	0	0	2	1	GLDADLIFLA	tr_C9ZP82	0.705	24.35	0.40	<= SB
764	HLA-A0201	VMLPFADMDVLQQV	MLMDVLQQV	1	0	0	2	4	MLPFADMDVLQQV	tr_C9ZP82	0.701	25.54	0.40	<= SB
421	HLA-A0201	SLISEVSAL	SLISEVSAL	0	0	0	0	0	SLISEVSAL	tr_C9ZP82	0.689	29.06	0.40	<= SB
311	HLA-A0201	ALSLHITQV	ALSLHITQV	0	0	0	0	0	ALSLHITQV	tr_C9ZP82	0.683	31.03	0.40	<= SB
351	HLA-A0201	FIDDTCIISL	FIDDCIISL	0	0	0	4	1	FIDDTCIISL	tr_C9ZP82	0.677	32.96	0.50	<= SB
76	HLA-A0201	VMYIAVDGV	VMYIAVDGV	0	0	0	0	0	VMYIAVDGV	tr_C9ZP82	0.656	41.50	0.60	<= WB
851	HLA-A0201	RTYSCTVEV	RTYSCTVEV	0	0	0	0	0	RTYSCTVEV	tr_C9ZP82	0.654	42.29	0.60	<= WB

235	HLA-A0201	DFMAKVSDAV	FMAKVSDAV	1	0	0	0	0	FMAKVSDAV	tr_C9ZP82	0.636	51.38	0.70	<= WB
301	HLA-A0201	VGLDADLIFL	VLDADLIFL	0	0	0	1	1	VGLDADLIFL	tr_C9ZP82	0.625	57.58	0.70	<= WB
768	HLA-A0201	FADMDVLQQV	FAMDVLQQV	0	0	0	2	1	FADMDVLQQV	tr_C9ZP82	0.621	60.65	0.70	<= WB
764	HLA-A0201	VMLPFADMDV	VMLPFAMDV	0	0	0	6	1	VMLPFADMDV	tr_C9ZP82	0.616	63.41	0.80	<= WB
541	HLA-A0201	LLELYVSEV	LLELYVSEV	0	0	0	0	0	LLELYVSEV	tr_C9ZP82	0.616	63.81	0.80	<= WB
519	HLA-A0201	LLGNDFLPHA	LLNDFLPHA	0	0	0	2	1	LLGNDFLPHA	tr_C9ZP82	0.614	65.32	0.80	<= WB
765	HLA-A0201	MLPFADMDVLQQVV	MLMDVLQQV	0	0	0	2	4	MLPFADMDVLQQV	tr_C9ZP82	0.602	74.44	0.90	<= WB
518	HLA-A0201	ILLGNDFLPHA	ILLDFLPHA	0	0	0	3	2	ILLGNDFLPHA	tr_C9ZP82	0.587	87.61	1.00	<= WB
536	HLA-A0201	SALDNLLELYV	ALDNLLLYV	1	0	0	6	1	ALDNLLELYV	tr_C9ZP82	0.586	87.76	1.00	<= WB
710	HLA-A0201	VQLLSILPPT	VLLSILPPT	0	0	0	1	1	VQLLSILPPT	tr_C9ZP82	0.584	90.42	1.00	<= WB
723	HLA-A0201	LLPMVLGEV	LLPMVLGEV	0	0	0	0	0	LLPMVLGEV	tr_C9ZP82	0.580	94.51	1.00	<= WB
765	HLA-A0201	MLPFADMDV	MLPFADMDV	0	0	0	0	0	MLPFADMDV	tr_C9ZP82	0.577	96.68	1.00	<= WB
715	HLA-A0201	ILPPTSHAL	ILPPTSHAL	0	0	0	0	0	ILPPTSHAL	tr_C9ZP82	0.577	96.96	1.00	<= WB
27	HLA-A0201	GLYIDLNGI	GLYIDLNGI	0	0	0	0	0	GLYIDLNGI	tr_C9ZP82	0.576	98.46	1.00	<= WB
760	HLA-A0201	HLATVMLPFA	HLATVLPFA	0	0	0	5	1	HLATVMLPFA	tr_C9ZP82	0.575	99.13	1.00	<= WB
821	HLA-A0201	ALDTILRRV	ALDTILRRV	0	0	0	0	0	ALDTILRRV	tr_C9ZP82	0.575	99.86	1.10	<= WB
236	HLA-A0201	FMAKVSDAVL	FMAKVSAVL	0	0	0	6	1	FMAKVSDAVL	tr_C9ZP82	0.574	100.05	1.10	<= WB
535	HLA-A0201	ESALDNLLELYV	ALDNLLLYV	2	0	0	6	1	ALDNLLELYV	tr_C9ZP82	0.572	103.10	1.10	<= WB
534	HLA-A0201	GESALDNLLELYV	ALDNLLLYV	3	0	0	6	1	ALDNLLELYV	tr_C9ZP82	0.566	109.12	1.10	<= WB
721	HLA-A0201	HALLPMVLGEV	ALLPMVLEV	1	0	0	7	1	ALLPMVLGEV	tr_C9ZP82	0.565	111.12	1.10	<= WB
533	HLA-A0201	SGESALDNLLELYV	ALDNLLLYV	4	0	0	6	1	ALDNLLELYV	tr_C9ZP82	0.563	112.93	1.10	<= WB
545	HLA-A0201	YVSEVLPYGFL	YVSEVLPYL	0	0	0	8	2	YVSEVLPYGFL	tr_C9ZP82	0.560	117.10	1.20	<= WB
309	HLA-A0201	FLALSLHITQV	FLALSLHQV	0	0	0	7	2	FLALSLHITQV	tr_C9ZP82	0.556	122.02	1.20	<= WB
569	HLA-A0201	RLFEAYAKIEAV	RLFEAIEAV	0	0	0	5	3	RLFEAYAKIEAV	tr_C9ZP82	0.555	122.89	1.20	<= WB
765	HLA-A0201	MLPFADMDVL	MLFADMDVL	0	0	0	2	1	MLPFADMDVL	tr_C9ZP82	0.547	134.76	1.30	<= WB
720	HLA-A0201	SHALLPMVLGEV	ALLPMVLEV	2	0	0	7	1	ALLPMVLGEV	tr_C9ZP82	0.546	135.64	1.30	<= WB
309	HLA-A0201	FLALSLHIT	FLALSLHIT	0	0	0	0	0	FLALSLHIT	tr_C9ZP82	0.546	135.91	1.30	<= WB
722	HLA-A0201	ALLPMVLGEVMV	ALLPMVLGV	0	0	0	8	3	ALLPMVLGEVMV	tr_C9ZP82	0.546	136.25	1.30	<= WB
719	HLA-A0201	TSHALLPMVLGEV	ALLPMVLEV	3	0	0	7	1	ALLPMVLGEV	tr_C9ZP82	0.540	144.96	1.30	<= WB
517	HLA-A0201	SILLGNDFLPHA	ILLDFLPHA	1	0	0	3	2	ILLGNDFLPHA	tr_C9ZP82	0.539	146.82	1.40	<= WB
236	HLA-A0201	FMAKVSDAVLNML	FMAAVLNML	0	0	0	3	4	FMAKVSDAVLNML	tr_C9ZP82	0.539	146.94	1.40	<= WB
536	HLA-A0201	SALDNLLEL	SALDNLLEL	0	0	0	0	0	SALDNLLEL	tr_C9ZP82	0.538	148.58	1.40	<= WB
718	HLA-A0201	PTSHALLPMVLGEV	ALLPMVLEV	4	0	0	7	1	ALLPMVLGEV	tr_C9ZP82	0.536	150.89	1.40	<= WB
309	HLA-A0201	FLALSLHITQVFI	FLALSLHFI	0	0	0	7	4	FLALSLHITQVFI	tr_C9ZP82	0.522	176.01	1.50	<= WB
364	HLA-A0201	YIDEEESDGVIGV	YIDEGVIGV	0	0	0	4	4	YIDEEESDGVIGV	tr_C9ZP82	0.522	176.08	1.50	<= WB
687	HLA-A0201	YLKEANIKRMPLPV	YLKEMPLPV	0	0	0	4	5	YLKEANIKRMPLPV	tr_C9ZP82	0.521	178.03	1.50	<= WB
817	HLA-A0201	KLSDALDTIL	KLSDALDTL	0	0	0	8	1	KLSDALDTIL	tr_C9ZP82	0.518	184.78	1.60	<= WB
568	HLA-A0201	QRLFEAYAKIEAV	RLFEAIEAV	1	0	0	5	3	RLFEAYAKIEAV	tr_C9ZP82	0.517	186.06	1.60	<= WB
539	HLA-A0201	DNLLELYVSEV	NLLEYVSEV	1	0	0	4	1	NLLELYVSEV	tr_C9ZP82	0.512	196.02	1.60	<= WB
516	HLA-A0201	LSILLGNDFLPHA	ILLDFLPHA	2	0	0	3	2	ILLGNDFLPHA	tr_C9ZP82	0.512	196.73	1.60	<= WB
313	HLA-A0201	SLHITQVFIL	SLITQVFIL	0	0	0	2	1	SLHITQVFIL	tr_C9ZP82	0.510	201.20	1.60	<= WB
721	HLA-A0201	HALLPMVLGEVMV	ALLPMVLGV	1	0	0	8	3	ALLPMVLGEVMV	tr_C9ZP82	0.509	201.89	1.60	<= WB
56	HLA-A0201	KLECICSEL	KLECICSEL	0	0	0	0	0	KLECICSEL	tr_C9ZP82	0.509	202.49	1.60	<= WB
308	HLA-A0201	IFLALSLHITQV	FLALSLHQV	1	0	0	7	2	FLALSLHITQV	tr_C9ZP82	0.508	205.16	1.70	<= WB
515	HLA-A0201	VLSILLGNDFLPHA	ILLDFLPHA	3	0	0	3	2	ILLGNDFLPHA	tr_C9ZP82	0.506	209.19	1.70	<= WB
675	HLA-A0201	FYHAPPVIDLAIYL	FVIDLAIYL	0	0	0	1	5	FYHAPPVIDLAIYL	tr_C9ZP82	0.506	210.58	1.70	<= WB
544	HLA-A0201	LYVSEVLPYGFL	YVSEVLPYL	1	0	0	8	2	YVSEVLPYGFL	tr_C9ZP82	0.504	213.51	1.70	<= WB
235	HLA-A0201	DFMAKVSDAVLNML	FMAAVLNML	1	0	0	3	4	FMAKVSDAVLNML	tr_C9ZP82	0.504	213.62	1.70	<= WB
309	HLA-A0201	FLALSLHITQVFIL	FLITQVFIL	0	0	0	2	5	FLALSLHITQVFIL	tr_C9ZP82	0.504	214.36	1.70	<= WB
310	HLA-A0201	LALSLHITQV	LLSLHITQV	0	0	0	1	1	LALSLHITQV	tr_C9ZP82	0.504	214.57	1.70	<= WB
715	HLA-A0201	ILPPTSHALLPMV	ILPALLPMV	0	0	0	3	4	ILPPTSHALLPMV	tr_C9ZP82	0.504	214.76	1.70	<= WB
537	HLA-A0201	ALDNLLELYVSEV	NLLEYVSEV	3	0	0	4	1	NLLELYVSEV	tr_C9ZP82	0.503	215.80	1.70	<= WB
114	HLA-A0201	YMPNKSGMAI	YMPNKSMAI	0	0	0	6	1	YMPNKSGMAI	tr_C9ZP82	0.496	233.07	1.80	<= WB
676	HLA-A0201	YHAPPVIDLAIYL	YVIDLAIYL	0	0	0	1	4	YHAPPVIDLAIYL	tr_C9ZP82	0.496	234.41	1.80	<= WB

742	HLA-A0201	ESTFPLQWNV	STFPLQWNV	1	0	0	0	0	STFPLQWNV	tr_C9ZP82	0.495	235.11	1.80	<= WB
509	HLA-A0201	IIDDIIVLSI	IIDDIILSI	0	0	0	6	1	IIDDIIVLSI	tr_C9ZP82	0.495	235.45	1.80	<= WB
655	HLA-A0201	FVWRYYTTT	FVWRYYTTT	0	0	0	0	0	FVWRYYTTT	tr_C9ZP82	0.495	236.45	1.80	<= WB
567	HLA-A0201	LQRLFEAYAKIEAV	RLFEAIEAV	2	0	0	5	3	RLFEAYAKIEAV	tr_C9ZP82	0.494	237.79	1.80	<= WB
149	HLA-A0201	TLLEDPLYSGAL	TLLEDPLYL	0	0	0	8	3	TLLEDPLYSGAL	tr_C9ZP82	0.493	240.22	1.80	<= WB
305	HLA-A0201	ADLIFLALSL	ALIFLALSL	0	0	0	1	1	ADLIFLALSL	tr_C9ZP82	0.492	243.85	1.80	<= WB
120	HLA-A0201	GMAIVEEVV	GMAIVEEVV	0	0	0	0	0	GMAIVEEVV	tr_C9ZP82	0.492	244.79	1.80	<= WB
720	HLA-A0201	SHALLPMVLGEVMV	ALLPMVLGV	2	0	0	8	3	ALLPMVLGEVMV	tr_C9ZP82	0.491	246.38	1.80	<= WB
56	HLA-A0201	KLECICSELELLV	KLSELELLV	0	0	0	2	4	KLECICSELELLV	tr_C9ZP82	0.488	253.84	1.90	<= WB
711	HLA-A0201	QLLSILPPTSHAL	QLLSILPPL	0	0	0	8	4	QLLSILPPTSHAL	tr_C9ZP82	0.488	254.36	1.90	<= WB
711	HLA-A0201	QLLSILPPTSHALL	QLLSILPPL	0	0	0	8	5	QLLSILPPTSHALL	tr_C9ZP82	0.486	259.07	1.90	<= WB
816	HLA-A0201	RKLSDALDTI	KLSDALDTI	1	0	0	0	0	KLSDALDTI	tr_C9ZP82	0.485	262.27	1.90	<= WB
538	HLA-A0201	LDNLLELYVSEV	NLLEYVSEV	2	0	0	4	1	NLLELYVSEV	tr_C9ZP82	0.485	262.53	1.90	<= WB
568	HLA-A0201	QRLFEAYAKI	RLFEAYAKI	1	0	0	0	0	RLFEAYAKI	tr_C9ZP82	0.485	263.49	1.90	<= WB
301	HLA-A0201	VGLDADLIFLA	GLADLIFLA	1	0	0	2	1	GLDADLIFLA	tr_C9ZP82	0.484	265.96	1.90	<= WB
543	HLA-A0201	ELYVSEVLPYGFL	YVSEVLPYL	2	0	0	8	2	YVSEVLPYGFL	tr_C9ZP82	0.484	266.39	1.90	<= WB
307	HLA-A0201	LIFLALSLHITQV	FLALSLHQV	2	0	0	7	2	FLALSLHITQV	tr_C9ZP82	0.483	268.65	1.90	<= WB
536	HLA-A0201	SALDNLLELYVSEV	NLLEYVSEV	4	0	0	4	1	NLLELYVSEV	tr_C9ZP82	0.479	279.37	2.00	<= WB
236	HLA-A0201	FMAKVSDAVLNM	FMADAVLNM	0	0	0	3	3	FMAKVSDAVLNM	tr_C9ZP82	0.479	280.56	2.00	<= WB
537	HLA-A0201	ALDNLLELYVS	ALDNLLLYV	0	0	0	6	1	ALDNLLELYV	tr_C9ZP82	0.479	280.80	2.00	<= WB
306	HLA-A0201	DLIFLALSLHITQV	FLALSLHQV	3	0	0	7	2	FLALSLHITQV	tr_C9ZP82	0.477	288.24	2.00	<= WB
542	HLA-A0201	LELYVSEVLPYGFL	YVSEVLPYL	3	0	0	8	2	YVSEVLPYGFL	tr_C9ZP82	0.476	288.39	2.00	<= WB
308	HLA-A0201	IFLALSLHITQVFI	FLALSLHFI	1	0	0	7	4	FLALSLHITQVFI	tr_C9ZP82	0.476	288.53	2.00	<= WB
313	HLA-A0201	SLHITQVFI	SLHITQVFI	0	0	0	0	0	SLHITQVFI	tr_C9ZP82	0.476	288.67	2.00	<= WB
350	HLA-A0201	NFIDDTCIISL	FIDDCIISL	1	0	0	4	1	FIDDTCIISL	tr_C9ZP82	0.476	288.77	2.00	<= WB
637	HLA-A0201	GLSNSVQEA	GLSNSVQEA	0	0	0	0	0	GLSNSVQEA	tr_C9ZP82	0.476	290.44	2.00	<= WB
363	HLA-A0201	SYIDEEESDGVIGV	YIDEGVIGV	1	0	0	4	4	YIDEEESDGVIGV	tr_C9ZP82	0.473	298.94	2.00	<= WB
545	HLA-A0201	YVSEVLPYG	YVSEVLPYG	0	0	0	0	0	YVSEVLPYG	tr_C9ZP82	0.472	303.02	2.50	
149	HLA-A0201	TLLEDPLYS	TLLEDPLYS	0	0	0	0	0	TLLEDPLYS	tr_C9ZP82	0.468	316.24	2.50	
714	HLA-A0201	SILPPTSHALLPMV	ILPALLPMV	1	0	0	3	4	ILPPTSHALLPMV	tr_C9ZP82	0.466	323.11	2.50	
300	HLA-A0201	IVGLDADLIFLA	GLADLIFLA	2	0	0	2	1	GLDADLIFLA	tr_C9ZP82	0.462	338.30	2.50	
349	HLA-A0201	RNFIDDTCIISL	FIDDCIISL	2	0	0	4	1	FIDDTCIISL	tr_C9ZP82	0.460	346.53	2.50	
760	HLA-A0201	HLATVMLPFADMDV	HLATVMLPV	0	0	0	8	5	HLATVMLPFADMDV	tr_C9ZP82	0.459	347.40	2.50	
364	HLA-A0201	YIDEEESDGV	YIDEESDGV	0	0	0	3	1	YIDEEESDGV	tr_C9ZP82	0.455	362.18	2.50	
553	HLA-A0201	GFLTKGPHEI	FLTKGPHEI	1	0	0	0	0	FLTKGPHEI	tr_C9ZP82	0.455	362.99	2.50	
299	HLA-A0201	VIVGLDADLIFLA	GLADLIFLA	3	0	0	2	1	GLDADLIFLA	tr_C9ZP82	0.454	366.79	2.50	
348	HLA-A0201	KRNFIDDTCIISL	FIDDCIISL	3	0	0	4	1	FIDDTCIISL	tr_C9ZP82	0.453	371.08	2.50	
507	HLA-A0201	SKIIDDIIVL	KIIDDIIVL	1	0	0	0	0	KIIDDIIVL	tr_C9ZP82	0.451	378.31	2.50	
298	HLA-A0201	HVIVGLDADLIFLA	GLADLIFLA	4	0	0	2	1	GLDADLIFLA	tr_C9ZP82	0.450	382.95	2.50	
347	HLA-A0201	RKRNFIDDTCIISL	FIDDCIISL	4	0	0	4	1	FIDDTCIISL	tr_C9ZP82	0.449	386.64	2.50	
75	HLA-A0201	EVMYIAVDGV	VMYIAVDGV	1	0	0	0	0	VMYIAVDGV	tr_C9ZP82	0.448	391.34	2.50	
612	HLA-A0201	TVADVAVAL	TVADVAVAL	0	0	0	0	0	TVADVAVAL	tr_C9ZP82	0.448	392.91	2.50	
148	HLA-A0201	QTLLEDPLYSGAL	TLLEDPLYL	1	0	0	8	3	TLLEDPLYSGAL	tr_C9ZP82	0.447	398.32	2.50	
632	HLA-A0201	YLKSTGLSNSV	YLTGLSNSV	0	0	0	2	2	YLKSTGLSNSV	tr_C9ZP82	0.447	398.64	2.50	
848	HLA-A0201	YRPRTYSCTVEV	YTYSCTVEV	0	0	0	1	3	YRPRTYSCTVEV	tr_C9ZP82	0.447	398.88	2.50	
55	HLA-A0201	EKLECICSELELLV	KLSELELLV	1	0	0	2	4	KLECICSELELLV	tr_C9ZP82	0.446	403.02	2.50	
722	HLA-A0201	ALLPMVLGEVM	ALLPMVLEV	0	0	0	7	1	ALLPMVLGEV	tr_C9ZP82	0.445	403.83	2.50	
171	HLA-A0201	ALNDGDGVNSFCV	ALNDGSFCV	0	0	0	5	4	ALNDGDGVNSFCV	tr_C9ZP82	0.444	409.67	2.50	
300	HLA-A0201	IVGLDADLIFL	ILDADLIFL	0	0	0	1	2	IVGLDADLIFL	tr_C9ZP82	0.444	410.55	2.50	
114	HLA-A0201	YMPNKSGMAIV	YMPSGMAIV	0	0	0	3	2	YMPNKSGMAIV	tr_C9ZP82	0.444	410.66	2.50	
571	HLA-A0201	FEAYAKIEAV	FAYAKIEAV	0	0	0	1	1	FEAYAKIEAV	tr_C9ZP82	0.441	421.68	2.50	
307	HLA-A0201	LIFLALSLHI	LIFALSLHI	0	0	0	3	1	LIFLALSLHI	tr_C9ZP82	0.441	423.45	2.50	
536	HLA-A0201	SALDNLLELYVS	ALDNLLLYV	1	0	0	6	1	ALDNLLELYV	tr_C9ZP82	0.440	428.12	2.50	
49	HLA-A0201	ALLPEEEKL	ALLPEEEKL	0	0	0	0	0	ALLPEEEKL	tr_C9ZP82	0.439	433.81	2.50	

235	HLA-A0201	DFMAKVSDAVLNM	FMADAVLNM	1	0	0	3	3	FMAKVSDAVLNM	tr_C9ZP82	0.438	437.06	2.50
8	HLA-A0201	WLRNKYPAIV	WLNKYPAIV	0	0	0	2	1	WLRNKYPAIV	tr_C9ZP82	0.437	441.99	2.50
710	HLA-A0201	VQLLSILPPTSHAL	QLLSILPPL	1	0	0	8	4	QLLSILPPTSHAL	tr_C9ZP82	0.436	445.20	2.50
517	HLA-A0201	SILLGNDFL	SILLGNDFL	0	0	0	0	0	SILLGNDFL	tr_C9ZP82	0.432	469.08	3.00
817	HLA-A0201	KLSDALDTILRRV	KLSDALDTV	0	0	0	8	4	KLSDALDTILRRV	tr_C9ZP82	0.430	477.00	3.00
234	HLA-A0201	TDFMAKVSDAV	FMAKVSDAV	2	0	0	0	0	FMAKVSDAV	tr_C9ZP82	0.429	484.35	3.00
147	HLA-A0201	RQTLLEDPLYSGAL	TLLEDPLYL	2	0	0	8	3	TLLEDPLYSGAL	tr_C9ZP82	0.428	488.83	3.00
537	HLA-A0201	ALDNLLELYVSE	ALDNLLLYV	0	0	0	6	1	ALDNLLELYV	tr_C9ZP82	0.424	508.01	3.00
535	HLA-A0201	ESALDNLLELYVS	ALDNLLLYV	2	0	0	6	1	ALDNLLELYV	tr_C9ZP82	0.424	508.79	3.00
420	HLA-A0201	GSLISEVSAL	SLISEVSAL	1	0	0	0	0	SLISEVSAL	tr_C9ZP82	0.424	511.43	3.00
233	HLA-A0201	GTDFMAKVSDAV	FMAKVSDAV	3	0	0	0	0	FMAKVSDAV	tr_C9ZP82	0.422	521.37	3.00
234	HLA-A0201	TDFMAKVSDAVLNM	FMADAVLNM	2	0	0	3	3	FMAKVSDAVLNM	tr_C9ZP82	0.420	532.46	3.00
534	HLA-A0201	GESALDNLLELYVS	ALDNLLLYV	3	0	0	6	1	ALDNLLELYV	tr_C9ZP82	0.418	540.54	3.00
518	HLA-A0201	ILLGNDFLPHAP	ILLDFLPHA	0	0	0	3	2	ILLGNDFLPHA	tr_C9ZP82	0.418	541.21	3.00
232	HLA-A0201	PGTDFMAKVSDAV	FMAKVSDAV	4	0	0	0	0	FMAKVSDAV	tr_C9ZP82	0.418	544.92	3.00
569	HLA-A0201	RLFEAYAKIEA	RLFEAYIEA	0	0	0	6	2	RLFEAYAKIEA	tr_C9ZP82	0.417	551.20	3.00
231	HLA-A0201	SPGTDFMAKVSDAV	FMAKVSDAV	5	0	0	0	0	FMAKVSDAV	tr_C9ZP82	0.415	561.19	3.00
159	HLA-A0201	ALYNEGEEEA	ALYEGEEEA	0	0	0	3	1	ALYNEGEEEA	tr_C9ZP82	0.414	564.20	3.00
302	HLA-A0201	GLDADLIFLAL	GLDADLIFL	0	0	0	8	2	GLDADLIFLAL	tr_C9ZP82	0.414	565.51	3.00
708	HLA-A0201	TLVQLLSIL	TLVQLLSIL	0	0	0	0	0	TLVQLLSIL	tr_C9ZP82	0.414	569.60	3.00
711	HLA-A0201	QLLSILPPTSHA	QLLSILPPA	0	0	0	8	3	QLLSILPPTSHA	tr_C9ZP82	0.411	586.45	3.00
569	HLA-A0201	RLFEAYAKIEAVK	RLFEAIEAV	0	0	0	5	3	RLFEAYAKIEAV	tr_C9ZP82	0.410	593.91	3.00
27	HLA-A0201	GLYIDLNGII	GLYIDLNGI	0	0	0	8	1	GLYIDLNGII	tr_C9ZP82	0.409	598.86	3.00
721	HLA-A0201	HALLPMVLGEVM	ALLPMVLEV	1	0	0	7	1	ALLPMVLGEV	tr_C9ZP82	0.409	600.11	3.00
299	HLA-A0201	VIVGLDADLIFL	ILDADLIFL	1	0	0	1	2	IVGLDADLIFL	tr_C9ZP82	0.407	610.14	3.00
170	HLA-A0201	DALNDGDGVNSFCV	ALNDGSFCV	1	0	0	5	4	ALNDGDGVNSFCV	tr_C9ZP82	0.405	621.74	3.50
537	HLA-A0201	ALDNLLELYVSEVL	ALDNLLLYV	0	0	0	6	1	ALDNLLELYV	tr_C9ZP82	0.405	626.77	3.50
541	HLA-A0201	LLELYVSEVL	LLLYVSEVL	0	0	0	2	1	LLELYVSEVL	tr_C9ZP82	0.404	631.77	3.50
540	HLA-A0201	NLLELYVSEVL	NLLEYVSEV	0	0	0	4	1	NLLELYVSEV	tr_C9ZP82	0.404	632.45	3.50
631	HLA-A0201	TYLKSTGLSNSV	YLTGLSNSV	1	0	0	2	2	YLKSTGLSNSV	tr_C9ZP82	0.402	646.33	3.50
847	HLA-A0201	RYRPRTYSCTVEV	YTYSCTVEV	1	0	0	1	3	YRPRTYSCTVEV	tr_C9ZP82	0.400	662.95	3.50
795	HLA-A0201	KLDFHLVIT	KLDFHLVIT	0	0	0	0	0	KLDFHLVIT	tr_C9ZP82	0.399	669.87	3.50
26	HLA-A0201	HGLYIDLNGI	GLYIDLNGI	1	0	0	0	0	GLYIDLNGI	tr_C9ZP82	0.399	670.07	3.50
709	HLA-A0201	LVQLLSILPPT	LLLSILPPT	0	0	0	1	2	LVQLLSILPPT	tr_C9ZP82	0.399	670.14	3.50
795	HLA-A0201	KLDFHLVITS	KLFHLVITS	0	0	0	2	1	KLDFHLVITS	tr_C9ZP82	0.398	672.01	3.50
309	HLA-A0201	FLALSLHITQVF	FLALHITQV	0	0	0	3	2	FLALSLHITQV	tr_C9ZP82	0.398	673.09	3.50
113	HLA-A0201	TYMPNKSGMAIV	YMPSGMAIV	1	0	0	3	2	YMPNKSGMAIV	tr_C9ZP82	0.398	674.97	3.50
669	HLA-A0201	WSWYYPFYHA	WSWYYPFYA	0	0	0	8	1	WSWYYPFYHA	tr_C9ZP82	0.398	676.31	3.50
724	HLA-A0201	LPMVLGEVMV	LMVLGEVMV	0	0	0	1	1	LPMVLGEVMV	tr_C9ZP82	0.397	684.81	3.50
722	HLA-A0201	ALLPMVLGEVMVR	ALLPMVLGV	0	0	0	8	3	ALLPMVLGEVMV	tr_C9ZP82	0.396	690.81	3.50
302	HLA-A0201	GLDADLIFLALSL	GLDADLIFL	0	0	0	8	4	GLDADLIFLALSL	tr_C9ZP82	0.394	706.20	3.50
298	HLA-A0201	HVIVGLDADLIFL	ILDADLIFL	2	0	0	1	2	IVGLDADLIFL	tr_C9ZP82	0.393	711.69	3.50
236	HLA-A0201	FMAKVSDAVLNMLN	FMAAVLNML	0	0	0	3	4	FMAKVSDAVLNML	tr_C9ZP82	0.392	718.91	3.50
518	HLA-A0201	ILLGNDFLPHAPSA	ILLDFLPHA	0	0	0	3	2	ILLGNDFLPHA	tr_C9ZP82	0.392	722.06	3.50
715	HLA-A0201	ILPPTSHALL	ILPPTSALL	0	0	0	6	1	ILPPTSHALL	tr_C9ZP82	0.392	722.93	3.50
720	HLA-A0201	SHALLPMVLGEVM	ALLPMVLEV	2	0	0	7	1	ALLPMVLGEV	tr_C9ZP82	0.391	724.31	3.50
850	HLA-A0201	PRTYSCTVEV	RTYSCTVEV	1	0	0	0	0	RTYSCTVEV	tr_C9ZP82	0.391	727.01	3.50
669	HLA-A0201	WSWYYPFYHAPPV	WSWYYPFYV	0	0	0	8	4	WSWYYPFYHAPPV	tr_C9ZP82	0.390	733.72	3.50
630	HLA-A0201	DTYLKSTGLSNSV	YLTGLSNSV	2	0	0	2	2	YLKSTGLSNSV	tr_C9ZP82	0.390	735.72	3.50
297	HLA-A0201	NHVIVGLDADLIFL	ILDADLIFL	3	0	0	1	2	IVGLDADLIFL	tr_C9ZP82	0.388	748.60	3.50
719	HLA-A0201	TSHALLPMVLGEVM	ALLPMVLEV	3	0	0	7	1	ALLPMVLGEV	tr_C9ZP82	0.386	770.43	3.50
536	HLA-A0201	SALDNLLELYVSE	ALDNLLLYV	1	0	0	6	1	ALDNLLELYV	tr_C9ZP82	0.386	770.89	3.50
629	HLA-A0201	RDTYLKSTGLSNSV	YLTGLSNSV	3	0	0	2	2	YLKSTGLSNSV	tr_C9ZP82	0.383	791.55	3.50
816	HLA-A0201	RKLSDALDTILRRV	KLSDALDTV	1	0	0	8	4	KLSDALDTILRRV	tr_C9ZP82	0.383	795.88	3.50

846	HLA-A0201	ERYRPRTYSCTVEV	YTYSCTVEV	2	0	0	1	3	YRPRTYSCTVEV	tr_C9ZP82	0.380	814.82	3.50
545	HLA-A0201	YVSEVLPYGFLT	YVSEVLPYL	0	0	0	8	2	YVSEVLPYGFL	tr_C9ZP82	0.379	829.95	4.00
802	HLA-A0201	ITSNKTSFV	ITSNKTSFV	0	0	0	0	0	ITSNKTSFV	tr_C9ZP82	0.378	834.43	4.00
112	HLA-A0201	STYMPNKSGMAIV	YMPSGMAIV	2	0	0	3	2	YMPNKSGMAIV	tr_C9ZP82	0.378	838.13	4.00
655	HLA-A0201	FVWRYYTTTT	FVWYYTTTT	0	0	0	3	1	FVWRYYTTTT	tr_C9ZP82	0.377	843.21	4.00
554	HLA-A0201	FLTKGPHEINLPQL	FLTKGLPQL	0	0	0	5	5	FLTKGPHEINLPQL	tr_C9ZP82	0.377	846.79	4.00
568	HLA-A0201	QRLFEAYAKIEA	RLFEAYIEA	1	0	0	6	2	RLFEAYAKIEA	tr_C9ZP82	0.375	866.03	4.00
149	HLA-A0201	TLLEDPLYSGA	TLLEDPLYA	0	0	0	8	2	TLLEDPLYSGA	tr_C9ZP82	0.373	878.90	4.00
768	HLA-A0201	FADMDVLQQVV	FMDVLQQVV	0	0	0	1	2	FADMDVLQQVV	tr_C9ZP82	0.373	880.50	4.00
517	HLA-A0201	SILLGNDFLPHAP	ILLDFLPHA	1	0	0	3	2	ILLGNDFLPHA	tr_C9ZP82	0.373	881.24	4.00
66	HLA-A0201	LLVQTVQPKEVMYI	LLVQTVMYI	0	0	0	5	5	LLVQTVQPKEVMYI	tr_C9ZP82	0.373	883.78	4.00
568	HLA-A0201	QRLFEAYAKIEAVK	RLFEAIEAV	1	0	0	5	3	RLFEAYAKIEAV	tr_C9ZP82	0.373	886.82	4.00
763	HLA-A0201	TVMLPFADMDV	VMLPFAMDV	1	0	0	6	1	VMLPFADMDV	tr_C9ZP82	0.372	897.04	4.00
111	HLA-A0201	ASTYMPNKSGMAIV	YMPSGMAIV	3	0	0	3	2	YMPNKSGMAIV	tr_C9ZP82	0.371	901.45	4.00
637	HLA-A0201	GLSNSVQEACEKYV	GLSNSVQYV	0	0	0	7	5	GLSNSVQEACEKYV	tr_C9ZP82	0.370	908.47	4.00
535	HLA-A0201	ESALDNLLELYVSE	ALDNLLLYV	2	0	0	6	1	ALDNLLELYV	tr_C9ZP82	0.370	910.14	4.00
820	HLA-A0201	DALDTILRRV	ALDTILRRV	1	0	0	0	0	ALDTILRRV	tr_C9ZP82	0.370	912.06	4.00
301	HLA-A0201	VGLDADLIFLAL	GLDADLIFL	1	0	0	8	2	GLDADLIFLAL	tr_C9ZP82	0.370	915.01	4.00
569	HLA-A0201	RLFEAYAKIE	RLFEAYAKI	0	0	0	0	0	RLFEAYAKI	tr_C9ZP82	0.369	925.00	4.00
743	HLA-A0201	STFPLQWNVD	STFPLQWNV	0	0	0	0	0	STFPLQWNV	tr_C9ZP82	0.368	930.13	4.00
308	HLA-A0201	IFLALSLHIT	FLALSLHIT	1	0	0	0	0	FLALSLHIT	tr_C9ZP82	0.368	930.13	4.00
539	HLA-A0201	DNLLELYVSEVL	NLLELYVSL	1	0	0	8	2	NLLELYVSEVL	tr_C9ZP82	0.368	936.86	4.00
119	HLA-A0201	SGMAIVEEVV	SMAIVEEVV	0	0	0	1	1	SGMAIVEEVV	tr_C9ZP82	0.367	943.89	4.00
356	HLA-A0201	CIISLNPSYI	CIISLNPYI	0	0	0	7	1	CIISLNPSYI	tr_C9ZP82	0.366	948.65	4.00
687	HLA-A0201	YLKEANIKRMPL	YLKEANMPL	0	0	0	6	3	YLKEANIKRMPL	tr_C9ZP82	0.365	967.73	4.00
118	HLA-A0201	KSGMAIVEEV	KSMAIVEEV	0	0	0	2	1	KSGMAIVEEV	tr_C9ZP82	0.364	973.75	4.00
711	HLA-A0201	QLLSILPPTS	QLLSILPPT	0	0	0	0	0	QLLSILPPT	tr_C9ZP82	0.364	974.49	4.00
721	HLA-A0201	HALLPMVLGEVMVR	ALLPMVLGV	1	0	0	8	3	ALLPMVLGEVMV	tr_C9ZP82	0.363	979.91	4.00
518	HLA-A0201	ILLGNDFLPHAPS	ILLDFLPHA	0	0	0	3	2	ILLGNDFLPHA	tr_C9ZP82	0.363	980.18	4.00
767	HLA-A0201	PFADMDVLQQV	FAMDVLQQV	1	0	0	2	1	FADMDVLQQV	tr_C9ZP82	0.363	984.38	4.00
137	HLA-A0201	AQANDDLDEV	AQADDLDEV	0	0	0	3	1	AQANDDLDEV	tr_C9ZP82	0.363	984.80	4.00
62	HLA-A0201	SELELLVQTV	SLELLVQTV	0	0	0	1	1	SELELLVQTV	tr_C9ZP82	0.363	987.81	4.00
508	HLA-A0201	KIIDDIIVLSI	KIIDIVLSI	0	0	0	4	2	KIIDDIIVLSI	tr_C9ZP82	0.363	988.83	4.00
235	HLA-A0201	DFMAKVSDAVL	FMAKVSAVL	1	0	0	6	1	FMAKVSDAVL	tr_C9ZP82	0.362	990.23	4.00
150	HLA-A0201	LLEDPLYSGA	LLEDPLYSA	0	0	0	8	1	LLEDPLYSGA	tr_C9ZP82	0.362	998.33	4.00
239	HLA-A0201	KVSDAVLNM	KVSDAVLNM	0	0	0	0	0	KVSDAVLNM	tr_C9ZP82	0.361	1008.59	4.00
710	HLA-A0201	VQLLSILPPTSHA	QLLSILPPA	1	0	0	8	3	QLLSILPPTSHA	tr_C9ZP82	0.361	1008.77	4.00
59	HLA-A0201	CICSELELLV	CISELELLV	0	0	0	2	1	CICSELELLV	tr_C9ZP82	0.359	1024.51	4.00
569	HLA-A0201	RLFEAYAKIEAVKF	RLFEAIEAV	0	0	0	5	3	RLFEAYAKIEAV	tr_C9ZP82	0.359	1032.40	4.00
722	HLA-A0201	ALLPMVLGEVMVRS	ALLPMVLEV	0	0	0	7	1	ALLPMVLGEV	tr_C9ZP82	0.358	1037.03	4.00
673	HLA-A0201	YPFYHAPPV	YPFYHAPPV	0	0	0	0	0	YPFYHAPPV	tr_C9ZP82	0.358	1037.86	4.00
312	HLA-A0201	LSLHITQVFI	LLHITQVFI	0	0	0	1	1	LSLHITQVFI	tr_C9ZP82	0.356	1058.93	4.50
301	HLA-A0201	VGLDADLIFLALSL	GLDADLIFL	1	0	0	8	4	GLDADLIFLALSL	tr_C9ZP82	0.356	1060.06	4.50
708	HLA-A0201	TLVQLLSILPPT	LLLSILPPT	1	0	0	1	2	LVQLLSILPPT	tr_C9ZP82	0.356	1060.46	4.50
773	HLA-A0201	VLQQVVDAA	VLQQVVDAA	0	0	0	0	0	VLQQVVDAA	tr_C9ZP82	0.356	1061.73	4.50
723	HLA-A0201	LLPMVLGEVM	LLPMVLGEV	0	0	0	0	0	LLPMVLGEV	tr_C9ZP82	0.356	1066.15	4.50
311	HLA-A0201	ALSLHITQVF	ALSLHITQV	0	0	0	0	0	ALSLHITQV	tr_C9ZP82	0.355	1068.58	4.50
308	HLA-A0201	IFLALSLHITQVF	FLALHITQV	1	0	0	3	2	FLALSLHITQV	tr_C9ZP82	0.355	1071.67	4.50
715	HLA-A0201	ILPPTSHALLPMVL	ILHALLPMV	0	0	0	2	4	ILPPTSHALLPMV	tr_C9ZP82	0.355	1078.65	4.50
668	HLA-A0201	DWSWYYPFYHAPPV	WSWYYPFYV	1	0	0	8	4	WSWYYPFYHAPPV	tr_C9ZP82	0.354	1080.93	4.50
8	HLA-A0201	WLRNKYPAI	WLRNKYPAI	0	0	0	0	0	WLRNKYPAI	tr_C9ZP82	0.354	1081.21	4.50
351	HLA-A0201	FIDDTCIISLN	FIDDCIISL	0	0	0	4	1	FIDDTCIISL	tr_C9ZP82	0.353	1099.67	4.50
762	HLA-A0201	ATVMLPFADMDV	VMLPFAMDV	2	0	0	6	1	VMLPFADMDV	tr_C9ZP82	0.352	1106.72	4.50
567	HLA-A0201	LQRLFEAYAKIEA	RLFEAYIEA	2	0	0	6	2	RLFEAYAKIEA	tr_C9ZP82	0.352	1110.42	4.50

516	HLA-A0201	LSILLGNDFLPHAP	ILLDFLPHA	2	0	0	3	2	ILLGNDFLPHA	tr_C9ZP82	0.351	1124.93	4.50
747	HLA-A0201	LQWNVDYHDSNV	LQWNVDYHV	0	0	0	8	3	LQWNVDYHDSNV	tr_C9ZP82	0.350	1127.44	4.50
300	HLA-A0201	IVGLDADLIFLAL	GLDADLIFL	2	0	0	8	2	GLDADLIFLAL	tr_C9ZP82	0.350	1128.05	4.50
76	HLA-A0201	VMYIAVDGVA	VMYIAVDGV	0	0	0	0	0	VMYIAVDGV	tr_C9ZP82	0.350	1131.13	4.50
364	HLA-A0201	YIDEEESDGVIGVP	YIDEGVIGV	0	0	0	4	4	YIDEEESDGVIGV	tr_C9ZP82	0.348	1154.53	4.50
234	HLA-A0201	TDFMAKVSDAVL	FMAKVSAVL	2	0	0	6	1	FMAKVSDAVL	tr_C9ZP82	0.348	1160.44	4.50
761	HLA-A0201	LATVMLPFADMDV	VMLPFAMDV	3	0	0	6	1	VMLPFADMDV	tr_C9ZP82	0.347	1174.32	4.50
299	HLA-A0201	VIVGLDADLIFLAL	GLDADLIFL	3	0	0	8	2	GLDADLIFLAL	tr_C9ZP82	0.346	1181.68	4.50
566	HLA-A0201	QLQRLFEAYAKIEA	RLFEAYIEA	3	0	0	6	2	RLFEAYAKIEA	tr_C9ZP82	0.346	1182.13	4.50
747	HLA-A0201	LQWNVDYHDSNVV	LQWNVDYHV	0	0	0	8	4	LQWNVDYHDSNVV	tr_C9ZP82	0.345	1192.50	4.50
538	HLA-A0201	LDNLLELYVSEVL	NLLELYVSL	2	0	0	8	2	NLLELYVSEVL	tr_C9ZP82	0.345	1193.75	4.50
71	HLA-A0201	VQPKEVMYI	VQPKEVMYI	0	0	0	0	0	VQPKEVMYI	tr_C9ZP82	0.345	1193.75	4.50
421	HLA-A0201	SLISEVSALC	SLISEVSAL	0	0	0	0	0	SLISEVSAL	tr_C9ZP82	0.344	1207.35	4.50
769	HLA-A0201	ADMDVLQQVV	AMDVLQQVV	0	0	0	1	1	ADMDVLQQVV	tr_C9ZP82	0.344	1207.76	4.50
714	HLA-A0201	SILPPTSHAL	ILPPTSHAL	1	0	0	0	0	ILPPTSHAL	tr_C9ZP82	0.344	1210.36	4.50
707	HLA-A0201	DTLVQLLSILPPT	LLLSILPPT	2	0	0	1	2	LVQLLSILPPT	tr_C9ZP82	0.343	1221.40	4.50
233	HLA-A0201	GTDFMAKVSDAVL	FMAKVSAVL	3	0	0	6	1	FMAKVSDAVL	tr_C9ZP82	0.342	1233.94	4.50
740	HLA-A0201	ELESTFPLQWNV	ELFPLQWNV	0	0	0	2	3	ELESTFPLQWNV	tr_C9ZP82	0.342	1236.70	4.50
282	HLA-A0201	FLRTQSSLA	FLRTQSSLA	0	0	0	0	0	FLRTQSSLA	tr_C9ZP82	0.342	1239.58	4.50
303	HLA-A0201	LDADLIFLALSL	LLIFLALSL	0	0	0	1	3	LDADLIFLALSL	tr_C9ZP82	0.341	1250.99	4.50
766	HLA-A0201	LPFADMDVLQQV	FAMDVLQQV	2	0	0	2	1	FADMDVLQQV	tr_C9ZP82	0.340	1264.75	4.50
861	HLA-A0201	RLSYGADGQV	RLSYGADGV	0	0	0	8	1	RLSYGADGQV	tr_C9ZP82	0.340	1265.62	4.50
232	HLA-A0201	PGTDFMAKVSDAVL	FMAKVSAVL	4	0	0	6	1	FMAKVSDAVL	tr_C9ZP82	0.339	1279.86	4.50
777	HLA-A0201	VVDAAWPQL	VVDAAWPQL	0	0	0	0	0	VVDAAWPQL	tr_C9ZP82	0.338	1289.46	4.50
567	HLA-A0201	LQRLFEAYAKI	LLFEAYAKI	0	0	0	1	2	LQRLFEAYAKI	tr_C9ZP82	0.338	1294.03	4.50
545	HLA-A0201	YVSEVLPYGF	YVSEVLPYF	0	0	0	8	1	YVSEVLPYGF	tr_C9ZP82	0.337	1301.66	4.50
66	HLA-A0201	LLVQTVQPKEV	LLVQTVQPV	0	0	0	8	2	LLVQTVQPKEV	tr_C9ZP82	0.337	1310.89	5.00
706	HLA-A0201	PDTLVQLLSILPPT	LLLSILPPT	3	0	0	1	2	LVQLLSILPPT	tr_C9ZP82	0.336	1311.61	5.00
709	HLA-A0201	LVQLLSILPPTSHA	QLLSILPPA	2	0	0	8	3	QLLSILPPTSHA	tr_C9ZP82	0.336	1313.65	5.00
554	HLA-A0201	FLTKGPHEIN	FLTKGPHEI	0	0	0	0	0	FLTKGPHEI	tr_C9ZP82	0.336	1322.54	5.00
357	HLA-A0201	IISLNPSYI	IISLNPSYI	0	0	0	0	0	IISLNPSYI	tr_C9ZP82	0.336	1324.59	5.00
307	HLA-A0201	LIFLALSLHITQVF	FLALHITQV	2	0	0	3	2	FLALSLHITQV	tr_C9ZP82	0.335	1327.74	5.00
676	HLA-A0201	YHAPPVIDLAIYLK	YVIDLAIYL	0	0	0	1	4	YHAPPVIDLAIYL	tr_C9ZP82	0.335	1331.92	5.00
508	HLA-A0201	KIIDDIIVLSILL	KIIDDIILL	0	0	0	6	4	KIIDDIIVLSILL	tr_C9ZP82	0.334	1349.70	5.00
56	HLA-A0201	KLECICSELELLVQ	KLSELELLV	0	0	0	2	4	KLECICSELELLV	tr_C9ZP82	0.331	1387.70	5.00
278	HLA-A0201	KIIDFLRTQSSL	KIIDFLSSL	0	0	0	6	3	KIIDFLRTQSSL	tr_C9ZP82	0.331	1392.90	5.00
441	HLA-A0201	SAFDKKTLV	SAFDKKTLV	0	0	0	0	0	SAFDKKTLV	tr_C9ZP82	0.330	1406.44	5.00
540	HLA-A0201	NLLELYVSEVLP	NLLEYVSEV	0	0	0	4	1	NLLELYVSEV	tr_C9ZP82	0.330	1406.98	5.00
508	HLA-A0201	KIIDDIIVLS	KIIDDIIVL	0	0	0	0	0	KIIDDIIVL	tr_C9ZP82	0.330	1409.11	5.00
148	HLA-A0201	QTLLEDPLYSGA	TLLEDPLYA	1	0	0	8	2	TLLEDPLYSGA	tr_C9ZP82	0.329	1419.24	5.00
119	HLA-A0201	SGMAIVEEV	SGMAIVEEV	0	0	0	0	0	SGMAIVEEV	tr_C9ZP82	0.329	1429.36	5.00
417	HLA-A0201	VVGGSLISEV	VVGGSLIEV	0	0	0	7	1	VVGGSLISEV	tr_C9ZP82	0.328	1433.47	5.00
564	HLA-A0201	LPQLQRLFEAYAKI	LLFEAYAKI	0	0	0	1	5	LPQLQRLFEAYAKI	tr_C9ZP82	0.328	1434.61	5.00
74	HLA-A0201	KEVMYIAVDGV	KMYIAVDGV	0	0	0	1	2	KEVMYIAVDGV	tr_C9ZP82	0.328	1443.85	5.00
641	HLA-A0201	SVQEACEKYV	SVQEACEYV	0	0	0	7	1	SVQEACEKYV	tr_C9ZP82	0.327	1446.52	5.00
544	HLA-A0201	LYVSEVLPYGFLT	YVSEVLPYL	1	0	0	8	2	YVSEVLPYGFL	tr_C9ZP82	0.327	1450.33	5.00
564	HLA-A0201	LPQLQRLFEA	LQLQRLFEA	0	0	0	1	1	LPQLQRLFEA	tr_C9ZP82	0.327	1452.50	5.00
428	HLA-A0201	ALCDIKKFTPA	ALCDIFTPA	0	0	0	5	2	ALCDIKKFTPA	tr_C9ZP82	0.327	1453.27	5.00
566	HLA-A0201	QLQRLFEAYA	QLQLFEAYA	0	0	0	3	1	QLQRLFEAYA	tr_C9ZP82	0.326	1466.92	5.00
55	HLA-A0201	EKLECICSEL	KLECICSEL	1	0	0	0	0	KLECICSEL	tr_C9ZP82	0.326	1473.57	5.00
554	HLA-A0201	FLTKGPHEINL	FLTPHEINL	0	0	0	3	2	FLTKGPHEINL	tr_C9ZP82	0.326	1475.55	5.00
682	HLA-A0201	IDLAIYLKEA	ILAIYLKEA	0	0	0	1	1	IDLAIYLKEA	tr_C9ZP82	0.325	1478.69	5.00
56	HLA-A0201	KLECICSELEL	KLICSELEL	0	0	0	2	2	KLECICSELEL	tr_C9ZP82	0.324	1498.63	5.00
723	HLA-A0201	LLPMVLGEVMV	LLPMVLVMV	0	0	0	6	2	LLPMVLGEVMV	tr_C9ZP82	0.324	1498.96	5.00

114	HLA-A0201	YMPNKSGMA	YMPNKSGMA	0	0	0	0	0	YMPNKSGMA	tr_C9ZP82	0.324	1504.16	5.00
764	HLA-A0201	VMLPFADMDVL	MLFADMDVL	1	0	0	2	1	MLPFADMDVL	tr_C9ZP82	0.323	1513.66	5.00
517	HLA-A0201	SILLGNDFLPHAPS	ILLDFLPHA	1	0	0	3	2	ILLGNDFLPHA	tr_C9ZP82	0.323	1514.22	5.00
302	HLA-A0201	GLDADLIFLALS	GLADLIFLA	0	0	0	2	1	GLDADLIFLA	tr_C9ZP82	0.322	1538.04	5.00
149	HLA-A0201	TLLEDPLYSGALY	TLLEDPLYL	0	0	0	8	3	TLLEDPLYSGAL	tr_C9ZP82	0.321	1545.13	5.00
507	HLA-A0201	SKIIDDIIVLSI	KIIDIVLSI	1	0	0	4	2	KIIDDIIVLSI	tr_C9ZP82	0.320	1564.44	5.00
350	HLA-A0201	NFIDDTCIISLN	FIDDCIISL	1	0	0	4	1	FIDDTCIISL	tr_C9ZP82	0.320	1568.80	5.00
686	HLA-A0201	IYLKEANIKRMPL	YLKEANMPL	1	0	0	6	3	YLKEANIKRMPL	tr_C9ZP82	0.318	1604.37	5.50
767	HLA-A0201	PFADMDVLQQVV	FMDVLQQVV	1	0	0	1	2	FADMDVLQQVV	tr_C9ZP82	0.318	1607.29	5.50
309	HLA-A0201	FLALSLHITQ	FLALSLHIT	0	0	0	0	0	FLALSLHIT	tr_C9ZP82	0.317	1627.67	5.50
311	HLA-A0201	ALSLHITQVFIL	ALITQVFIL	0	0	0	2	3	ALSLHITQVFIL	tr_C9ZP82	0.314	1679.12	5.50
545	HLA-A0201	YVSEVLPYGFLTK	YVSEVLPYL	0	0	0	8	2	YVSEVLPYGFL	tr_C9ZP82	0.314	1679.57	5.50
290	HLA-A0201	AQFGGKANHV	AQFGGANHV	0	0	0	5	1	AQFGGKANHV	tr_C9ZP82	0.314	1681.61	5.50
304	HLA-A0201	DADLIFLALSL	ALIFLALSL	1	0	0	1	1	ADLIFLALSL	tr_C9ZP82	0.313	1686.51	5.50
147	HLA-A0201	RQTLLEDPLYSGA	TLLEDPLYA	2	0	0	8	2	TLLEDPLYSGA	tr_C9ZP82	0.312	1705.62	5.50
49	HLA-A0201	ALLPEEEKLECI	ALLEKLECI	0	0	0	3	3	ALLPEEEKLECI	tr_C9ZP82	0.312	1713.31	5.50
759	HLA-A0201	VHLATVMLPFA	HLATVLPFA	1	0	0	5	1	HLATVMLPFA	tr_C9ZP82	0.311	1731.41	5.50
506	HLA-A0201	NSKIIDDIIVLSI	KIIDIVLSI	2	0	0	4	2	KIIDDIIVLSI	tr_C9ZP82	0.311	1732.25	5.50
30	HLA-A0201	IDLNGIIHPC	ILNGIIHPC	0	0	0	1	1	IDLNGIIHPC	tr_C9ZP82	0.311	1732.45	5.50
741	HLA-A0201	LESTFPLQWNV	LTFPLQWNV	0	0	0	1	2	LESTFPLQWNV	tr_C9ZP82	0.311	1734.52	5.50
543	HLA-A0201	ELYVSEVLPYGFLT	YVSEVLPYL	2	0	0	8	2	YVSEVLPYGFL	tr_C9ZP82	0.311	1736.02	5.50
738	HLA-A0201	QAELESTFPL	QALESTFPL	0	0	0	2	1	QAELESTFPL	tr_C9ZP82	0.310	1739.31	5.50
239	HLA-A0201	KVSDAVLNML	KVSDVLNML	0	0	0	4	1	KVSDAVLNML	tr_C9ZP82	0.310	1751.09	5.50
763	HLA-A0201	TVMLPFADMDVL	MLFADMDVL	2	0	0	2	1	MLPFADMDVL	tr_C9ZP82	0.308	1783.12	5.50
739	HLA-A0201	AELESTFPL	AELESTFPL	0	0	0	0	0	AELESTFPL	tr_C9ZP82	0.308	1793.27	5.50
540	HLA-A0201	NLLELYVSEVLPY	NLLEYVSEV	0	0	0	4	1	NLLELYVSEV	tr_C9ZP82	0.307	1795.43	5.50
739	HLA-A0201	AELESTFPLQWNV	ELFPLQWNV	1	0	0	2	3	ELESTFPLQWNV	tr_C9ZP82	0.307	1795.45	5.50
131	HLA-A0201	FTSTEMAQA	FTSTEMAQA	0	0	0	0	0	FTSTEMAQA	tr_C9ZP82	0.307	1802.89	5.50
509	HLA-A0201	IIDDIIVLS	IIDDIIVLS	0	0	0	0	0	IIDDIIVLS	tr_C9ZP82	0.307	1814.33	5.50
98	HLA-A0201	YMGRAAPAQTGEV	YMGRAAPEV	0	0	0	7	4	YMGRAAPAQTGEV	tr_C9ZP82	0.306	1818.30	5.50
65	HLA-A0201	ELLVQTVQPKEV	LLVQTVQPV	1	0	0	8	2	LLVQTVQPKEV	tr_C9ZP82	0.306	1821.31	5.50
146	HLA-A0201	VRQTLLEDPLYSGA	TLLEDPLYA	3	0	0	8	2	TLLEDPLYSGA	tr_C9ZP82	0.306	1823.80	5.50
349	HLA-A0201	RNFIDDTCIISLN	FIDDCIISL	2	0	0	4	1	FIDDTCIISL	tr_C9ZP82	0.305	1834.82	5.50
505	HLA-A0201	ANSKIIDDIIVLSI	KIIDIVLSI	3	0	0	4	2	KIIDDIIVLSI	tr_C9ZP82	0.305	1835.48	5.50
532	HLA-A0201	FSGESALDNLLEL	FALDNLLEL	0	0	0	1	4	FSGESALDNLLEL	tr_C9ZP82	0.304	1860.92	5.50
566	HLA-A0201	QLQRLFEAYAKI	LLFEAYAKI	1	0	0	1	2	LQRLFEAYAKI	tr_C9ZP82	0.303	1878.03	5.50
351	HLA-A0201	FIDDTCIISLNP	FIDDCIISL	0	0	0	4	1	FIDDTCIISL	tr_C9ZP82	0.303	1884.21	5.50
851	HLA-A0201	RTYSCTVEVP	RTYSCTVEV	0	0	0	0	0	RTYSCTVEV	tr_C9ZP82	0.303	1884.24	5.50
514	HLA-A0201	IVLSILLGNDFL	IVLSILLFL	0	0	0	7	3	IVLSILLGNDFL	tr_C9ZP82	0.303	1894.25	5.50
762	HLA-A0201	ATVMLPFADMDVL	MLFADMDVL	3	0	0	2	1	MLPFADMDVL	tr_C9ZP82	0.302	1898.83	5.50
760	HLA-A0201	HLATVMLPF	HLATVMLPF	0	0	0	0	0	HLATVMLPF	tr_C9ZP82	0.301	1926.25	6.00
351	HLA-A0201	FIDDTCIIS	FIDDTCIIS	0	0	0	0	0	FIDDTCIIS	tr_C9ZP82	0.300	1938.87	6.00
348	HLA-A0201	KRNFIDDTCIISLN	FIDDCIISL	3	0	0	4	1	FIDDTCIISL	tr_C9ZP82	0.300	1941.41	6.00
761	HLA-A0201	LATVMLPFADMDVL	MLFADMDVL	4	0	0	2	1	MLPFADMDVL	tr_C9ZP82	0.299	1962.11	6.00
540	HLA-A0201	NLLELYVSEVLPYG	NLLEYVSEV	0	0	0	4	1	NLLELYVSEV	tr_C9ZP82	0.299	1970.49	6.00
312	HLA-A0201	LSLHITQVFIL	SLITQVFIL	1	0	0	2	1	SLHITQVFIL	tr_C9ZP82	0.299	1971.73	6.00
766	HLA-A0201	LPFADMDVLQQVV	FMDVLQQVV	2	0	0	1	2	FADMDVLQQVV	tr_C9ZP82	0.299	1975.49	6.00
685	HLA-A0201	AIYLKEANIKRMPL	YLKEANMPL	2	0	0	6	3	YLKEANIKRMPL	tr_C9ZP82	0.298	1983.99	6.00
539	HLA-A0201	DNLLELYVSEVLP	NLLEYVSEV	1	0	0	4	1	NLLELYVSEV	tr_C9ZP82	0.298	1990.94	6.00
509	HLA-A0201	IIDDIIVLSILL	IIDDIIILL	0	0	0	6	3	IIDDIIVLSILL	tr_C9ZP82	0.297	2008.46	6.00
707	HLA-A0201	DTLVQLLSIL	TLVQLLSIL	1	0	0	0	0	TLVQLLSIL	tr_C9ZP82	0.297	2012.73	6.00
678	HLA-A0201	APPVIDLAIYL	AVIDLAIYL	0	0	0	1	2	APPVIDLAIYL	tr_C9ZP82	0.297	2016.35	6.00
60	HLA-A0201	ICSELELLV	ICSELELLV	0	0	0	0	0	ICSELELLV	tr_C9ZP82	0.296	2025.77	6.00
507	HLA-A0201	SKIIDDIIVLSILL	KIIDDIILL	1	0	0	6	4	KIIDDIIVLSILL	tr_C9ZP82	0.296	2035.64	6.00

648	HLA-A0201	KYVEGVCFV	KYVEGVCFV	0	0	0	0	0	KYVEGVCFV	tr_C9ZP82	0.295	2044.64	6.00
302	HLA-A0201	GLDADLIFLALSLH	GLADLIFLA	0	0	0	2	1	GLDADLIFLA	tr_C9ZP82	0.295	2064.81	6.00
776	HLA-A0201	QVVDAAWPQL	QVVDAAWPL	0	0	0	8	1	QVVDAAWPQL	tr_C9ZP82	0.294	2079.02	6.00
306	HLA-A0201	DLIFLALSL	DLIFLALSL	0	0	0	0	0	DLIFLALSL	tr_C9ZP82	0.294	2079.11	6.00
738	HLA-A0201	QAELESTFPLQWNV	ELFPLQWNV	2	0	0	2	3	ELESTFPLQWNV	tr_C9ZP82	0.294	2083.84	6.00
746	HLA-A0201	PLQWNVDYHDSNV	LQWNVDYHV	1	0	0	8	3	LQWNVDYHDSNV	tr_C9ZP82	0.293	2089.66	6.00
758	HLA-A0201	VVHLATVMLPFA	HLATVLPFA	2	0	0	5	1	HLATVMLPFA	tr_C9ZP82	0.292	2113.63	6.00
277	HLA-A0201	HKIIDFLRTQSSL	KIIDFLSSL	1	0	0	6	3	KIIDFLRTQSSL	tr_C9ZP82	0.292	2116.26	6.00
687	HLA-A0201	YLKEANIKRM	YLKEANIRM	0	0	0	7	1	YLKEANIKRM	tr_C9ZP82	0.292	2130.76	6.00
311	HLA-A0201	ALSLHITQVFI	ALSLHIVFI	0	0	0	6	2	ALSLHITQVFI	tr_C9ZP82	0.291	2149.61	6.00
746	HLA-A0201	PLQWNVDYHDSNVV	LQWNVDYHV	1	0	0	8	4	LQWNVDYHDSNVV	tr_C9ZP82	0.291	2152.26	6.00
427	HLA-A0201	SALCDIKKFTPA	ALCDIFTPA	1	0	0	5	2	ALCDIKKFTPA	tr_C9ZP82	0.291	2153.43	6.00
64	HLA-A0201	LELLVQTVQPKEV	LLVQTVQPV	2	0	0	8	2	LLVQTVQPKEV	tr_C9ZP82	0.290	2163.54	6.00
545	HLA-A0201	YVSEVLPYGFLTKG	YVSEVLPYL	0	0	0	8	2	YVSEVLPYGFL	tr_C9ZP82	0.289	2187.93	6.00
73	HLA-A0201	PKEVMYIAVDGV	KMYIAVDGV	1	0	0	1	2	KEVMYIAVDGV	tr_C9ZP82	0.288	2217.09	6.00
565	HLA-A0201	PQLQRLFEAYAKI	LLFEAYAKI	2	0	0	1	2	LQRLFEAYAKI	tr_C9ZP82	0.288	2218.32	6.00
301	HLA-A0201	VGLDADLIFLALS	GLADLIFLA	1	0	0	2	1	GLDADLIFLA	tr_C9ZP82	0.288	2219.25	6.00
310	HLA-A0201	LALSLHITQVFIL	SLITQVFIL	3	0	0	2	1	SLHITQVFIL	tr_C9ZP82	0.287	2229.05	6.00
757	HLA-A0201	NVVHLATVMLPFA	HLATVLPFA	3	0	0	5	1	HLATVMLPFA	tr_C9ZP82	0.287	2232.65	6.50
848	HLA-A0201	YRPRTYSCTVEVP	YTYSCTVEV	0	0	0	1	3	YRPRTYSCTVEV	tr_C9ZP82	0.287	2249.47	6.50
71	HLA-A0201	VQPKEVMYIAVDGV	VMYIAVDGV	0	0	0	1	5	VQPKEVMYIAVDGV	tr_C9ZP82	0.286	2254.52	6.50
63	HLA-A0201	ELELLVQTVQPKEV	LLVQTVQPV	3	0	0	8	2	LLVQTVQPKEV	tr_C9ZP82	0.286	2259.87	6.50
535	HLA-A0201	ESALDNLLEL	SALDNLLEL	1	0	0	0	0	SALDNLLEL	tr_C9ZP82	0.286	2265.50	6.50
680	HLA-A0201	PVIDLAIYL	PVIDLAIYL	0	0	0	0	0	PVIDLAIYL	tr_C9ZP82	0.284	2303.93	6.50
563	HLA-A0201	NLPQLQRLFEA	NLLQRLFEA	0	0	0	2	2	NLPQLQRLFEA	tr_C9ZP82	0.284	2305.31	6.50
756	HLA-A0201	SNVVHLATVMLPFA	HLATVLPFA	4	0	0	5	1	HLATVMLPFA	tr_C9ZP82	0.284	2305.76	6.50
55	HLA-A0201	EKLECICSELEL	KLICSELEL	1	0	0	2	2	KLECICSELEL	tr_C9ZP82	0.284	2320.17	6.50
632	HLA-A0201	YLKSTGLSNSVQ	YLTGLSNSV	0	0	0	2	2	YLKSTGLSNSV	tr_C9ZP82	0.284	2322.81	6.50
300	HLA-A0201	IVGLDADLIFLALS	GLADLIFLA	2	0	0	2	1	GLDADLIFLA	tr_C9ZP82	0.284	2322.83	6.50
171	HLA-A0201	ALNDGDGVNSFCVR	ALNDGSFCV	0	0	0	5	4	ALNDGDGVNSFCV	tr_C9ZP82	0.283	2331.87	6.50
351	HLA-A0201	FIDDTCIISLNPS	FIDDCIISL	0	0	0	4	1	FIDDTCIISL	tr_C9ZP82	0.283	2337.03	6.50
685	HLA-A0201	AIYLKEANI	AIYLKEANI	0	0	0	0	0	AIYLKEANI	tr_C9ZP82	0.283	2337.63	6.50
553	HLA-A0201	GFLTKGPHEINL	FLTPHEINL	1	0	0	3	2	FLTKGPHEINL	tr_C9ZP82	0.282	2353.98	6.50
408	HLA-A0201	TGFEYFDISV	TGFEYFISV	0	0	0	6	1	TGFEYFDISV	tr_C9ZP82	0.281	2378.53	6.50
236	HLA-A0201	FMAKVSDAVLN	FMAKVSDAV	0	0	0	0	0	FMAKVSDAV	tr_C9ZP82	0.281	2389.80	6.50
72	HLA-A0201	QPKEVMYIAVDGV	KMYIAVDGV	2	0	0	1	2	KEVMYIAVDGV	tr_C9ZP82	0.281	2390.22	6.50
148	HLA-A0201	QTLLEDPLYSGALY	TLLEDPLYL	1	0	0	8	3	TLLEDPLYSGAL	tr_C9ZP82	0.281	2393.32	6.50
632	HLA-A0201	YLKSTGLSNSVQEA	YLSNSVQEA	0	0	0	1	5	YLKSTGLSNSVQEA	tr_C9ZP82	0.280	2406.25	6.50
719	HLA-A0201	TSHALLPMV	TSHALLPMV	0	0	0	0	0	TSHALLPMV	tr_C9ZP82	0.280	2407.48	6.50
812	HLA-A0201	KGDLRKLSDALDTI	KLSDALDTI	0	0	0	1	5	KGDLRKLSDALDTI	tr_C9ZP82	0.280	2425.41	6.50
114	HLA-A0201	YMPNKSGMAIVE	YMPSGMAIV	0	0	0	3	2	YMPNKSGMAIV	tr_C9ZP82	0.279	2432.19	6.50
538	HLA-A0201	LDNLLELYVSEVLP	NLLEYVSEV	2	0	0	4	1	NLLELYVSEV	tr_C9ZP82	0.279	2440.13	6.50
745	HLA-A0201	FPLQWNVDYHDSNV	LQWNVDYHV	2	0	0	8	3	LQWNVDYHDSNV	tr_C9ZP82	0.279	2445.94	6.50
715	HLA-A0201	ILPPTSHALLPM	ILSHALLPM	0	0	0	2	3	ILPPTSHALLPM	tr_C9ZP82	0.278	2471.67	6.50
288	HLA-A0201	SLAQFGGKANHV	SLAQFANHV	0	0	0	5	3	SLAQFGGKANHV	tr_C9ZP82	0.278	2482.79	6.50
539	HLA-A0201	DNLLELYVSEVLPY	NLLEYVSEV	1	0	0	4	1	NLLELYVSEV	tr_C9ZP82	0.277	2487.33	6.50
513	HLA-A0201	IIVLSILLGNDFL	IVLSILLFL	1	0	0	7	3	IVLSILLGNDFL	tr_C9ZP82	0.277	2494.64	6.50
737	HLA-A0201	QQAELESTFPL	QQLESTFPL	0	0	0	2	2	QQAELESTFPL	tr_C9ZP82	0.276	2525.98	6.50
276	HLA-A0201	EHKIIDFLRTQSSL	KIIDFLSSL	2	0	0	6	3	KIIDFLRTQSSL	tr_C9ZP82	0.276	2530.17	6.50
815	HLA-A0201	LRKLSDALDTI	LLSDALDTI	0	0	0	1	2	LRKLSDALDTI	tr_C9ZP82	0.276	2532.47	6.50
48	HLA-A0201	IALLPEEEKLECI	ALLEKLECI	1	0	0	3	3	ALLPEEEKLECI	tr_C9ZP82	0.275	2560.82	6.50
426	HLA-A0201	VSALCDIKKFTPA	ALCDIFTPA	2	0	0	5	2	ALCDIKKFTPA	tr_C9ZP82	0.274	2568.73	6.50
251	HLA-A0201	KMSGNDPTWTRLCV	KMSGNDPTV	0	0	0	8	5	KMSGNDPTWTRLCV	tr_C9ZP82	0.274	2582.05	6.50
508	HLA-A0201	KIIDDIIVLSIL	KIIDIVLSI	0	0	0	4	2	KIIDDIIVLSI	tr_C9ZP82	0.274	2585.29	6.50

350	HLA-A0201	NFIDDTCIISLNP	FIDDCIISL	1	0	0	4	1	FIDDTCIISL	tr_C9ZP82	0.274	2588.82	6.50
611	HLA-A0201	VTVADVAVAL	VVADVAVAL	0	0	0	1	1	VTVADVAVAL	tr_C9ZP82	0.274	2589.10	6.50
816	HLA-A0201	RKLSDALDTIL	KLSDALDTL	1	0	0	8	1	KLSDALDTIL	tr_C9ZP82	0.272	2632.86	7.00
512	HLA-A0201	DIIVLSILLGNDFL	IVLSILLFL	2	0	0	7	3	IVLSILLGNDFL	tr_C9ZP82	0.271	2652.18	7.00
148	HLA-A0201	QTLLEDPLYS	TLLEDPLYS	1	0	0	0	0	TLLEDPLYS	tr_C9ZP82	0.271	2653.22	7.00
712	HLA-A0201	LLSILPPTSHALL	LLSILPALL	0	0	0	6	4	LLSILPPTSHALL	tr_C9ZP82	0.271	2660.09	7.00
115	HLA-A0201	MPNKSGMAIVEEVV	MMAIVEEVV	0	0	0	1	5	MPNKSGMAIVEEVV	tr_C9ZP82	0.270	2692.75	7.00
425	HLA-A0201	EVSALCDIKKFTPA	ALCDIFTPA	3	0	0	5	2	ALCDIKKFTPA	tr_C9ZP82	0.270	2700.48	7.00
351	HLA-A0201	FIDDTCIISLNPSY	FIDDCIISL	0	0	0	4	1	FIDDTCIISL	tr_C9ZP82	0.270	2706.92	7.00
544	HLA-A0201	LYVSEVLPYGFLTK	YVSEVLPYL	1	0	0	8	2	YVSEVLPYGFL	tr_C9ZP82	0.269	2717.22	7.00
54	HLA-A0201	EEKLECICSELEL	KLICSELEL	2	0	0	2	2	KLECICSELEL	tr_C9ZP82	0.268	2742.95	7.00
821	HLA-A0201	ALDTILRRVP	ALDTILRRV	0	0	0	0	0	ALDTILRRV	tr_C9ZP82	0.268	2760.36	7.00
149	HLA-A0201	TLLEDPLYSGALYN	TLLEDPLYL	0	0	0	8	3	TLLEDPLYSGAL	tr_C9ZP82	0.268	2760.72	7.00
509	HLA-A0201	IIDDIIVLSIL	IIDDIIVLL	0	0	0	8	2	IIDDIIVLSIL	tr_C9ZP82	0.267	2774.38	7.00
518	HLA-A0201	ILLGNDFLP	ILLGNDFLP	0	0	0	0	0	ILLGNDFLP	tr_C9ZP82	0.267	2774.59	7.00
669	HLA-A0201	WSWYYPFYHAPPVI	WSWYYPFYI	0	0	0	8	5	WSWYYPFYHAPPVI	tr_C9ZP82	0.267	2774.77	7.00
552	HLA-A0201	YGFLTKGPHEINL	FLTPHEINL	2	0	0	3	2	FLTKGPHEINL	tr_C9ZP82	0.267	2774.95	7.00
677	HLA-A0201	HAPPVIDLAIYL	AVIDLAIYL	1	0	0	1	2	APPVIDLAIYL	tr_C9ZP82	0.266	2808.26	7.00
817	HLA-A0201	KLSDALDTILRRVP	KLSDALDTV	0	0	0	8	4	KLSDALDTILRRV	tr_C9ZP82	0.266	2822.24	7.00
759	HLA-A0201	VHLATVMLPF	VLATVMLPF	0	0	0	1	1	VHLATVMLPF	tr_C9ZP82	0.265	2830.78	7.00
97	HLA-A0201	RYMGRAAPAQTGEV	YMGRAAPEV	1	0	0	7	4	YMGRAAPAQTGEV	tr_C9ZP82	0.265	2846.47	7.00
149	HLA-A0201	TLLEDPLYSG	TLLEDLYSG	0	0	0	5	1	TLLEDPLYSG	tr_C9ZP82	0.265	2852.79	7.00
29	HLA-A0201	YIDLNGIIHPC	YLNGIIHPC	0	0	0	1	2	YIDLNGIIHPC	tr_C9ZP82	0.263	2897.43	7.00
531	HLA-A0201	AFSGESALDNLLEL	FALDNLLEL	1	0	0	1	4	FSGESALDNLLEL	tr_C9ZP82	0.263	2900.35	7.00
727	HLA-A0201	VLGEVMVRS	VLGEVMVRS	0	0	0	0	0	VLGEVMVRS	tr_C9ZP82	0.263	2905.19	7.00
53	HLA-A0201	EEEKLECICSELEL	KLICSELEL	3	0	0	2	2	KLECICSELEL	tr_C9ZP82	0.263	2907.04	7.00
551	HLA-A0201	PYGFLTKGPHEINL	FLTPHEINL	3	0	0	3	2	FLTKGPHEINL	tr_C9ZP82	0.262	2944.11	7.00
349	HLA-A0201	RNFIDDTCIISLNP	FIDDCIISL	2	0	0	4	1	FIDDTCIISL	tr_C9ZP82	0.261	2969.77	7.00
295	HLA-A0201	KANHVIVGL	KANHVIVGL	0	0	0	0	0	KANHVIVGL	tr_C9ZP82	0.260	2987.62	7.50
654	HLA-A0201	CFVWRYYTTT	FVWRYYTTT	1	0	0	0	0	FVWRYYTTT	tr_C9ZP82	0.259	3020.68	7.50
47	HLA-A0201	AIALLPEEEKLECI	ALLEKLECI	2	0	0	3	3	ALLPEEEKLECI	tr_C9ZP82	0.259	3025.12	7.50
278	HLA-A0201	KIIDFLRTQSSLA	KIIDFLSLA	0	0	0	6	4	KIIDFLRTQSSLA	tr_C9ZP82	0.259	3029.64	7.50
712	HLA-A0201	LLSILPPTSHAL	LLSILPPTL	0	0	0	8	3	LLSILPPTSHAL	tr_C9ZP82	0.259	3047.00	7.50
310	HLA-A0201	LALSLHITQVFI	ALSLHIVFI	1	0	0	6	2	ALSLHITQVFI	tr_C9ZP82	0.258	3051.82	7.50
814	HLA-A0201	DLRKLSDALDTI	KLSDALDTI	3	0	0	0	0	KLSDALDTI	tr_C9ZP82	0.258	3074.99	7.50
24	HLA-A0201	SVHGLYIDLNGI	SLYIDLNGI	0	0	0	1	3	SVHGLYIDLNGI	tr_C9ZP82	0.257	3083.28	7.50
519	HLA-A0201	LLGNDFLPHAPSA	LLLPHAPSA	0	0	0	2	4	LLGNDFLPHAPSA	tr_C9ZP82	0.257	3104.04	7.50
80	HLA-A0201	AVDGVAPRA	AVDGVAPRA	0	0	0	0	0	AVDGVAPRA	tr_C9ZP82	0.256	3124.86	7.50
23	HLA-A0201	SSVHGLYIDLNGI	SLYIDLNGI	0	0	0	1	4	SSVHGLYIDLNGI	tr_C9ZP82	0.256	3141.51	7.50
290	HLA-A0201	AQFGGKANHVIVGL	AQFGVIVGL	0	0	0	4	5	AQFGGKANHVIVGL	tr_C9ZP82	0.256	3142.87	7.50
306	HLA-A0201	DLIFLALSLHI	LIFALSLHI	1	0	0	3	1	LIFLALSLHI	tr_C9ZP82	0.256	3144.09	7.50
515	HLA-A0201	VLSILLGNDFL	VLSILLDFL	0	0	0	6	2	VLSILLGNDFL	tr_C9ZP82	0.256	3150.19	7.50
244	HLA-A0201	VLNMLNEKM	VLNMLNEKM	0	0	0	0	0	VLNMLNEKM	tr_C9ZP82	0.255	3151.79	7.50
350	HLA-A0201	NFIDDTCIISLNPS	FIDDCIISL	1	0	0	4	1	FIDDTCIISL	tr_C9ZP82	0.255	3155.92	7.50
27	HLA-A0201	GLYIDLNGIIHPC	GLYGIIHPC	0	0	0	3	4	GLYIDLNGIIHPC	tr_C9ZP82	0.255	3159.85	7.50
113	HLA-A0201	TYMPNKSGMAI	YMPNKSMAI	1	0	0	6	1	YMPNKSGMAI	tr_C9ZP82	0.255	3174.65	7.50
793	HLA-A0201	TQKLDFHLVI	TQLDFHLVI	0	0	0	2	1	TQKLDFHLVI	tr_C9ZP82	0.255	3183.60	7.50
519	HLA-A0201	LLGNDFLPHAP	LLNDFLPHA	0	0	0	2	1	LLGNDFLPHA	tr_C9ZP82	0.254	3185.69	7.50
813	HLA-A0201	GDLRKLSDALDTI	KLSDALDTI	4	0	0	0	0	KLSDALDTI	tr_C9ZP82	0.253	3226.42	7.50
552	HLA-A0201	YGFLTKGPHEI	YLTKGPHEI	0	0	0	1	2	YGFLTKGPHEI	tr_C9ZP82	0.253	3227.68	7.50
59	HLA-A0201	CICSELELL	CICSELELL	0	0	0	0	0	CICSELELL	tr_C9ZP82	0.253	3239.12	7.50
303	HLA-A0201	LDADLIFLALSLH	LLIFLALSL	0	0	0	1	3	LDADLIFLALSL	tr_C9ZP82	0.253	3240.73	7.50
305	HLA-A0201	ADLIFLALSLHI	LIFALSLHI	2	0	0	3	1	LIFLALSLHI	tr_C9ZP82	0.253	3243.68	7.50
792	HLA-A0201	RTQKLDFHL	RTQKLDFHL	0	0	0	0	0	RTQKLDFHL	tr_C9ZP82	0.253	3246.52	7.50

138	HLA-A0201	QANDDLDEV	QANDDLDEV	0	0	0	0	0	QANDDLDEV	tr_C9ZP82	0.253	3249.37	7.50
760	HLA-A0201	HLATVMLPFADM	HLATVMLPM	0	0	0	8	3	HLATVMLPFADM	tr_C9ZP82	0.252	3255.52	7.50
672	HLA-A0201	YYPFYHAPPV	YYFYHAPPV	0	0	0	2	1	YYPFYHAPPV	tr_C9ZP82	0.252	3261.27	7.50
847	HLA-A0201	RYRPRTYSCTVEVP	YTYSCTVEV	1	0	0	1	3	YRPRTYSCTVEV	tr_C9ZP82	0.252	3261.94	7.50
714	HLA-A0201	SILPPTSHA	SILPPTSHA	0	0	0	0	0	SILPPTSHA	tr_C9ZP82	0.252	3263.35	7.50
48	HLA-A0201	IALLPEEEKL	ILLPEEEKL	0	0	0	1	1	IALLPEEEKL	tr_C9ZP82	0.252	3281.45	7.50
562	HLA-A0201	INLPQLQRLFEA	NLLQRLFEA	1	0	0	2	2	NLPQLQRLFEA	tr_C9ZP82	0.252	3282.44	7.50
235	HLA-A0201	DFMAKVSDAVLN	FMAKVSDAV	1	0	0	0	0	FMAKVSDAV	tr_C9ZP82	0.251	3302.71	7.50
726	HLA-A0201	MVLGEVMVRS	MLGEVMVRS	0	0	0	1	1	MVLGEVMVRS	tr_C9ZP82	0.251	3304.50	7.50
308	HLA-A0201	IFLALSLHI	IFLALSLHI	0	0	0	0	0	IFLALSLHI	tr_C9ZP82	0.251	3306.39	7.50
60	HLA-A0201	ICSELELLVQTV	ILELLVQTV	0	0	0	1	3	ICSELELLVQTV	tr_C9ZP82	0.251	3307.72	7.50
815	HLA-A0201	LRKLSDALDTIL	KLSDALDTL	2	0	0	8	1	KLSDALDTIL	tr_C9ZP82	0.251	3324.44	7.50
288	HLA-A0201	SLAQFGGKANHVIV	SLAQFHVIV	0	0	0	5	5	SLAQFGGKANHVIV	tr_C9ZP82	0.251	3325.12	7.50
769	HLA-A0201	ADMDVLQQV	ADMDVLQQV	0	0	0	0	0	ADMDVLQQV	tr_C9ZP82	0.250	3327.57	7.50
714	HLA-A0201	SILPPTSHALLPM	ILSHALLPM	1	0	0	2	3	ILPPTSHALLPM	tr_C9ZP82	0.250	3344.21	7.50
768	HLA-A0201	FADMDVLQQVVDAA	FLQQVVDAA	0	0	0	1	5	FADMDVLQQVVDAA	tr_C9ZP82	0.250	3357.73	7.50
549	HLA-A0201	VLPYGFLTKGPHEI	FLTKGPHEI	5	0	0	0	0	FLTKGPHEI	tr_C9ZP82	0.249	3387.65	8.00
410	HLA-A0201	FEYFDISVV	FEYFDISVV	0	0	0	0	0	FEYFDISVV	tr_C9ZP82	0.249	3396.06	8.00
303	HLA-A0201	LDADLIFLALSLHI	LLIFLALSL	0	0	0	1	3	LDADLIFLALSL	tr_C9ZP82	0.248	3417.07	8.00
723	HLA-A0201	LLPMVLGEVMVR	LLPMVLVMV	0	0	0	6	2	LLPMVLGEVMV	tr_C9ZP82	0.247	3440.25	8.00
507	HLA-A0201	SKIIDDIIVLSIL	KIIDIVLSI	1	0	0	4	2	KIIDDIIVLSI	tr_C9ZP82	0.247	3447.07	8.00
768	HLA-A0201	FADMDVLQQVVD	FMDVLQQVV	0	0	0	1	2	FADMDVLQQVV	tr_C9ZP82	0.247	3450.17	8.00
631	HLA-A0201	TYLKSTGLSNSVQ	YLTGLSNSV	1	0	0	2	2	YLKSTGLSNSV	tr_C9ZP82	0.247	3458.73	8.00
709	HLA-A0201	LVQLLSILPPTS	LLLSILPPT	0	0	0	1	2	LVQLLSILPPT	tr_C9ZP82	0.247	3472.04	8.00
524	HLA-A0201	FLPHAPSAFS	FLHAPSAFS	0	0	0	2	1	FLPHAPSAFS	tr_C9ZP82	0.246	3481.93	8.00
305	HLA-A0201	ADLIFLALSLH	ALIFLALSL	0	0	0	1	1	ADLIFLALSL	tr_C9ZP82	0.246	3495.48	8.00
814	HLA-A0201	DLRKLSDALDTIL	KLSDALDTL	3	0	0	8	1	KLSDALDTIL	tr_C9ZP82	0.245	3519.20	8.00
150	HLA-A0201	LLEDPLYSGAL	LLEDPLYSL	0	0	0	8	2	LLEDPLYSGAL	tr_C9ZP82	0.245	3520.42	8.00
287	HLA-A0201	SSLAQFGGKANHV	SLAQFANHV	1	0	0	5	3	SLAQFGGKANHV	tr_C9ZP82	0.245	3524.54	8.00
544	HLA-A0201	LYVSEVLPYG	YVSEVLPYG	1	0	0	0	0	YVSEVLPYG	tr_C9ZP82	0.245	3547.95	8.00
304	HLA-A0201	DADLIFLALSLHI	LIFALSLHI	3	0	0	3	1	LIFLALSLHI	tr_C9ZP82	0.244	3555.68	8.00
113	HLA-A0201	TYMPNKSGMAIVE	YMPSGMAIV	1	0	0	3	2	YMPNKSGMAIV	tr_C9ZP82	0.244	3557.37	8.00
801	HLA-A0201	VITSNKTSFV	VISNKTSFV	0	0	0	2	1	VITSNKTSFV	tr_C9ZP82	0.244	3574.89	8.00
63	HLA-A0201	ELELLVQTV	ELELLVQTV	0	0	0	0	0	ELELLVQTV	tr_C9ZP82	0.244	3576.82	8.00
524	HLA-A0201	FLPHAPSAF	FLPHAPSAF	0	0	0	0	0	FLPHAPSAF	tr_C9ZP82	0.243	3594.94	8.00
761	HLA-A0201	LATVMLPFA	LATVMLPFA	0	0	0	0	0	LATVMLPFA	tr_C9ZP82	0.243	3616.75	8.00
25	HLA-A0201	VHGLYIDLNGI	VLYIDLNGI	0	0	0	1	2	VHGLYIDLNGI	tr_C9ZP82	0.243	3620.94	8.00
636	HLA-A0201	TGLSNSVQEA	GLSNSVQEA	1	0	0	0	0	GLSNSVQEA	tr_C9ZP82	0.242	3635.86	8.00
813	HLA-A0201	GDLRKLSDALDTIL	KLSDALDTL	4	0	0	8	1	KLSDALDTIL	tr_C9ZP82	0.242	3638.93	8.00
506	HLA-A0201	NSKIIDDIIVLSIL	KIIDIVLSI	2	0	0	4	2	KIIDDIIVLSI	tr_C9ZP82	0.242	3664.17	8.00
551	HLA-A0201	PYGFLTKGPHEI	FLTKGPHEI	3	0	0	0	0	FLTKGPHEI	tr_C9ZP82	0.241	3683.81	8.00
764	HLA-A0201	VMLPFADMDVLQ	VMLPFAMDV	0	0	0	6	1	VMLPFADMDV	tr_C9ZP82	0.241	3696.51	8.00
561	HLA-A0201	EINLPQLQRLFEA	NLLQRLFEA	2	0	0	2	2	NLPQLQRLFEA	tr_C9ZP82	0.241	3697.31	8.00
848	HLA-A0201	YRPRTYSCTVEVPR	YTYSCTVEV	0	0	0	1	3	YRPRTYSCTVEV	tr_C9ZP82	0.240	3725.22	8.00
506	HLA-A0201	NSKIIDDIIVL	KIIDDIIVL	2	0	0	0	0	KIIDDIIVL	tr_C9ZP82	0.240	3739.55	8.00
234	HLA-A0201	TDFMAKVSDAVLN	FMAKVSDAV	2	0	0	0	0	FMAKVSDAV	tr_C9ZP82	0.239	3759.72	8.00
422	HLA-A0201	LISEVSALC	LISEVSALC	0	0	0	0	0	LISEVSALC	tr_C9ZP82	0.239	3769.45	8.00
792	HLA-A0201	RTQKLDFHLV	RTQKLDFLV	0	0	0	7	1	RTQKLDFHLV	tr_C9ZP82	0.239	3775.25	8.00
59	HLA-A0201	CICSELELLVQTV	ILELLVQTV	1	0	0	1	3	ICSELELLVQTV	tr_C9ZP82	0.238	3793.30	8.50
56	HLA-A0201	KLECICSELE	KLECICSEL	0	0	0	0	0	KLECICSEL	tr_C9ZP82	0.238	3802.51	8.50
630	HLA-A0201	DTYLKSTGLSNSVQ	YLTGLSNSV	2	0	0	2	2	YLKSTGLSNSV	tr_C9ZP82	0.238	3810.95	8.50
112	HLA-A0201	STYMPNKSGMAI	YMPNKSMAI	2	0	0	6	1	YMPNKSGMAI	tr_C9ZP82	0.238	3811.90	8.50
120	HLA-A0201	GMAIVEEVVR	GMAIVEEVV	0	0	0	0	0	GMAIVEEVV	tr_C9ZP82	0.238	3819.37	8.50
26	HLA-A0201	HGLYIDLNGIIHPC	GLYGIIHPC	1	0	0	3	4	GLYIDLNGIIHPC	tr_C9ZP82	0.238	3819.58	8.50

550	HLA-A0201	LPYGFLTKGPHEI	FLTKGPHEI	4	0	0	0	0	FLTKGPHEI	tr_C9ZP82	0.238	3825.08	8.50
560	HLA-A0201	HEINLPQLQRLFEA	NLLQRLFEA	3	0	0	2	2	NLPQLQRLFEA	tr_C9ZP82	0.237	3851.49	8.50
708	HLA-A0201	TLVQLLSILP	TLVQLLSIL	0	0	0	0	0	TLVQLLSIL	tr_C9ZP82	0.237	3860.67	8.50
421	HLA-A0201	SLISEVSALCDI	SLISALCDI	0	0	0	3	3	SLISEVSALCDI	tr_C9ZP82	0.237	3868.74	8.50
505	HLA-A0201	ANSKIIDDIIVL	KIIDDIIVL	3	0	0	0	0	KIIDDIIVL	tr_C9ZP82	0.236	3877.54	8.50
56	HLA-A0201	KLECICSELELL	KLCSELELL	0	0	0	2	3	KLECICSELELL	tr_C9ZP82	0.236	3886.07	8.50
794	HLA-A0201	QKLDFHLVITS	KLFHLVITS	1	0	0	2	1	KLDFHLVITS	tr_C9ZP82	0.236	3905.71	8.50
713	HLA-A0201	LSILPPTSHALLPM	ILPPTLLPM	2	0	0	5	3	ILPPTSHALLPM	tr_C9ZP82	0.235	3930.42	8.50
66	HLA-A0201	LLVQTVQPKEVM	LLVQTVQPV	0	0	0	8	2	LLVQTVQPKEV	tr_C9ZP82	0.235	3939.58	8.50
28	HLA-A0201	LYIDLNGIIHPC	YLNGIIHPC	1	0	0	1	2	YIDLNGIIHPC	tr_C9ZP82	0.235	3940.39	8.50
233	HLA-A0201	GTDFMAKVSDAVLN	FMAKVSDAV	3	0	0	0	0	FMAKVSDAV	tr_C9ZP82	0.235	3950.29	8.50
849	HLA-A0201	RPRTYSCTVEV	RTYSCTVEV	0	0	0	1	2	RPRTYSCTVEV	tr_C9ZP82	0.235	3952.90	8.50
516	HLA-A0201	LSILLGNDFL	LILLGNDFL	0	0	0	1	1	LSILLGNDFL	tr_C9ZP82	0.234	3978.26	8.50
286	HLA-A0201	QSSLAQFGGKANHV	SLAQFANHV	2	0	0	5	3	SLAQFGGKANHV	tr_C9ZP82	0.234	3980.76	8.50
799	HLA-A0201	HLVITSNKTSFV	HLVITTSFV	0	0	0	5	3	HLVITSNKTSFV	tr_C9ZP82	0.233	4002.87	8.50
503	HLA-A0201	SPANSKIIDDIIVL	KIIDDIIVL	5	0	0	0	0	KIIDDIIVL	tr_C9ZP82	0.233	4023.84	8.50
632	HLA-A0201	YLKSTGLSNSVQE	YLTGLSNSV	0	0	0	2	2	YLKSTGLSNSV	tr_C9ZP82	0.233	4033.39	8.50
313	HLA-A0201	SLHITQVFILR	SLITQVFIL	0	0	0	2	1	SLHITQVFIL	tr_C9ZP82	0.232	4044.18	8.50
111	HLA-A0201	ASTYMPNKSGMAI	YMPNKSMAI	3	0	0	6	1	YMPNKSGMAI	tr_C9ZP82	0.232	4045.49	8.50
794	HLA-A0201	QKLDFHLVIT	KLDFHLVIT	1	0	0	0	0	KLDFHLVIT	tr_C9ZP82	0.232	4055.31	8.50
416	HLA-A0201	SVVGGSLISEVSAL	SLISEVSAL	0	0	0	1	5	SVVGGSLISEVSAL	tr_C9ZP82	0.232	4072.55	8.50
58	HLA-A0201	ECICSELELLVQTV	ILELLVQTV	2	0	0	1	3	ICSELELLVQTV	tr_C9ZP82	0.232	4074.31	8.50
428	HLA-A0201	ALCDIKKFTPAT	ALCDIFTPA	0	0	0	5	2	ALCDIKKFTPA	tr_C9ZP82	0.232	4079.21	8.50
504	HLA-A0201	PANSKIIDDIIVL	KIIDDIIVL	4	0	0	0	0	KIIDDIIVL	tr_C9ZP82	0.231	4095.61	8.50
299	HLA-A0201	VIVGLDADLI	VIVGLDALI	0	0	0	7	1	VIVGLDADLI	tr_C9ZP82	0.231	4101.91	8.50
67	HLA-A0201	LVQTVQPKEV	LVQTVQPEV	0	0	0	7	1	LVQTVQPKEV	tr_C9ZP82	0.231	4114.71	8.50
112	HLA-A0201	STYMPNKSGMAIVE	YMPSGMAIV	2	0	0	3	2	YMPNKSGMAIV	tr_C9ZP82	0.230	4132.34	8.50
114	HLA-A0201	YMPNKSGMAIVEE	YMPSGMAIV	0	0	0	3	2	YMPNKSGMAIV	tr_C9ZP82	0.230	4139.99	8.50
110	HLA-A0201	VASTYMPNKSGMAI	YMPNKSMAI	4	0	0	6	1	YMPNKSGMAI	tr_C9ZP82	0.229	4189.47	8.50
277	HLA-A0201	HKIIDFLRTQSSLA	KIIDFLSLA	1	0	0	6	4	KIIDFLRTQSSLA	tr_C9ZP82	0.228	4223.46	8.50
76	HLA-A0201	VMYIAVDGVAPRA	VMYGVAPRA	0	0	0	3	4	VMYIAVDGVAPRA	tr_C9ZP82	0.228	4237.43	9.00
304	HLA-A0201	DADLIFLALSLH	ALIFLALSL	1	0	0	1	1	ADLIFLALSL	tr_C9ZP82	0.228	4244.77	9.00
765	HLA-A0201	MLPFADMDVLQ	MLFADMDVL	0	0	0	2	1	MLPFADMDVL	tr_C9ZP82	0.228	4249.82	9.00
793	HLA-A0201	TQKLDFHLVITS	KLFHLVITS	2	0	0	2	1	KLDFHLVITS	tr_C9ZP82	0.228	4256.36	9.00
305	HLA-A0201	ADLIFLALSLHIT	ALIFLALSL	0	0	0	1	1	ADLIFLALSL	tr_C9ZP82	0.227	4273.34	9.00
570	HLA-A0201	LFEAYAKIEAV	FAYAKIEAV	1	0	0	1	1	FEAYAKIEAV	tr_C9ZP82	0.227	4274.87	9.00
759	HLA-A0201	VHLATVMLPFADM	HLATVMLPM	1	0	0	8	3	HLATVMLPFADM	tr_C9ZP82	0.226	4345.66	9.00
712	HLA-A0201	LLSILPPTS	LLSILPPTS	0	0	0	0	0	LLSILPPTS	tr_C9ZP82	0.226	4351.82	9.00
772	HLA-A0201	DVLQQVVDAA	VLQQVVDAA	1	0	0	0	0	VLQQVVDAA	tr_C9ZP82	0.226	4352.29	9.00
22	HLA-A0201	PSSVHGLYIDLNGI	SLYIDLNGI	1	0	0	1	4	SSVHGLYIDLNGI	tr_C9ZP82	0.225	4372.63	9.00
723	HLA-A0201	LLPMVLGEVMVRS	LLPMVLVMV	0	0	0	6	2	LLPMVLGEVMV	tr_C9ZP82	0.225	4382.34	9.00
792	HLA-A0201	RTQKLDFHLVITS	KLFHLVITS	3	0	0	2	1	KLDFHLVITS	tr_C9ZP82	0.225	4400.92	9.00
839	HLA-A0201	QLYDVPLERYRPRT	QLYDVPLET	0	0	0	8	5	QLYDVPLERYRPRT	tr_C9ZP82	0.224	4423.41	9.00
418	HLA-A0201	VGGSLISEVSAL	VLISEVSAL	0	0	0	1	3	VGGSLISEVSAL	tr_C9ZP82	0.224	4425.04	9.00
710	HLA-A0201	VQLLSILPPTS	VLLSILPPT	0	0	0	1	1	VQLLSILPPT	tr_C9ZP82	0.224	4450.05	9.00
363	HLA-A0201	SYIDEEESDGV	YIDEESDGV	1	0	0	3	1	YIDEEESDGV	tr_C9ZP82	0.223	4476.61	9.00
764	HLA-A0201	VMLPFADMDVLQQ	VMLPFAMDV	0	0	0	6	1	VMLPFADMDV	tr_C9ZP82	0.223	4480.15	9.00
791	HLA-A0201	WRTQKLDFHLVITS	KLFHLVITS	4	0	0	2	1	KLDFHLVITS	tr_C9ZP82	0.223	4489.03	9.00
758	HLA-A0201	VVHLATVML	VVHLATVML	0	0	0	0	0	VVHLATVML	tr_C9ZP82	0.223	4491.50	9.00
417	HLA-A0201	VVGGSLISEVSAL	VLISEVSAL	0	0	0	1	4	VVGGSLISEVSAL	tr_C9ZP82	0.222	4540.61	9.00
61	HLA-A0201	CSELELLVQTV	SLELLVQTV	1	0	0	1	1	SELELLVQTV	tr_C9ZP82	0.222	4543.66	9.00
24	HLA-A0201	SVHGLYIDL	SVHGLYIDL	0	0	0	0	0	SVHGLYIDL	tr_C9ZP82	0.221	4555.97	9.00
419	HLA-A0201	GGSLISEVSAL	SLISEVSAL	2	0	0	0	0	SLISEVSAL	tr_C9ZP82	0.221	4566.13	9.00
514	HLA-A0201	IVLSILLGNDFLP	IVLSILLFL	0	0	0	7	3	IVLSILLGNDFL	tr_C9ZP82	0.221	4587.13	9.00

307	HLA-A0201	LIFLALSLHIT	FLALSLHIT	2	0	0	0	0	FLALSLHIT	tr_C9ZP82	0.220	4607.92	9.00
747	HLA-A0201	LQWNVDYHDSNVVH	LQWNVDYHV	0	0	0	8	4	LQWNVDYHDSNVV	tr_C9ZP82	0.220	4617.00	9.00
763	HLA-A0201	TVMLPFADMDVLQ	VMLPFAMDV	1	0	0	6	1	VMLPFADMDV	tr_C9ZP82	0.220	4626.65	9.00
687	HLA-A0201	YLKEANIKRMPLP	YLKEANMPL	0	0	0	6	3	YLKEANIKRMPL	tr_C9ZP82	0.220	4633.82	9.00
736	HLA-A0201	PQQAELESTFPL	QQLESTFPL	1	0	0	2	2	QQAELESTFPL	tr_C9ZP82	0.219	4652.86	9.50
26	HLA-A0201	HGLYIDLNGII	GLYIDLNGI	1	0	0	8	1	GLYIDLNGII	tr_C9ZP82	0.219	4672.23	9.50
455	HLA-A0201	YFFGQPNHI	YFFGQPNHI	0	0	0	0	0	YFFGQPNHI	tr_C9ZP82	0.219	4672.79	9.50
304	HLA-A0201	DADLIFLALSLHIT	FLALSLHIT	5	0	0	0	0	FLALSLHIT	tr_C9ZP82	0.219	4689.70	9.50
288	HLA-A0201	SLAQFGGKA	SLAQFGGKA	0	0	0	0	0	SLAQFGGKA	tr_C9ZP82	0.219	4690.31	9.50
760	HLA-A0201	HLATVMLPFAD	HLATVLPFA	0	0	0	5	1	HLATVMLPFA	tr_C9ZP82	0.218	4719.33	9.50
768	HLA-A0201	FADMDVLQQVVDA	FAMDVLQQV	0	0	0	2	1	FADMDVLQQV	tr_C9ZP82	0.218	4729.09	9.50
708	HLA-A0201	TLVQLLSILPPTS	LLLSILPPT	1	0	0	1	2	LVQLLSILPPT	tr_C9ZP82	0.218	4749.66	9.50
299	HLA-A0201	VIVGLDADL	VIVGLDADL	0	0	0	0	0	VIVGLDADL	tr_C9ZP82	0.217	4757.22	9.50
311	HLA-A0201	ALSLHITQVFILR	ALITQVFIL	0	0	0	2	3	ALSLHITQVFIL	tr_C9ZP82	0.217	4757.88	9.50
773	HLA-A0201	VLQQVVDAAWPQL	VLDAAWPQL	0	0	0	2	4	VLQQVVDAAWPQL	tr_C9ZP82	0.217	4774.69	9.50
247	HLA-A0201	MLNEKMSGNDPT	MLMSGNDPT	0	0	0	2	3	MLNEKMSGNDPT	tr_C9ZP82	0.217	4795.15	9.50
229	HLA-A0201	CISPGTDFM	CISPGTDFM	0	0	0	0	0	CISPGTDFM	tr_C9ZP82	0.217	4801.90	9.50
508	HLA-A0201	KIIDDIIVLSILLG	KIIDDIILL	0	0	0	6	4	KIIDDIIVLSILL	tr_C9ZP82	0.216	4819.80	9.50
723	HLA-A0201	LLPMVLGEVMVRSP	LLPMVLVMV	0	0	0	6	2	LLPMVLGEVMV	tr_C9ZP82	0.216	4827.32	9.50
306	HLA-A0201	DLIFLALSLHIT	FLALSLHIT	3	0	0	0	0	FLALSLHIT	tr_C9ZP82	0.216	4848.83	9.50
758	HLA-A0201	VVHLATVMLPFADM	HLATVMLPM	2	0	0	8	3	HLATVMLPFADM	tr_C9ZP82	0.215	4882.36	9.50
118	HLA-A0201	KSGMAIVEEVV	KMAIVEEVV	0	0	0	1	2	KSGMAIVEEVV	tr_C9ZP82	0.215	4889.29	9.50
519	HLA-A0201	LLGNDFLPHAPS	LLNDFLPHA	0	0	0	2	1	LLGNDFLPHA	tr_C9ZP82	0.214	4913.48	9.50
818	HLA-A0201	LSDALDTILRRV	LLDTILRRV	0	0	0	1	3	LSDALDTILRRV	tr_C9ZP82	0.214	4959.78	9.50
65	HLA-A0201	ELLVQTVQPKEVM	LLVQTVQPV	1	0	0	8	2	LLVQTVQPKEV	tr_C9ZP82	0.213	4984.64	9.50
762	HLA-A0201	ATVMLPFADMDVLQ	MLFADMDVL	3	0	0	2	1	MLPFADMDVL	tr_C9ZP82	0.213	4985.77	9.50
549	HLA-A0201	VLPYGFLTKG	VLYGFLTKG	0	0	0	2	1	VLPYGFLTKG	tr_C9ZP82	0.213	4991.82	9.50
798	HLA-A0201	FHLVITSNKTSFV	HLVITTSFV	1	0	0	5	3	HLVITSNKTSFV	tr_C9ZP82	0.213	5006.09	9.50
303	HLA-A0201	LDADLIFLA	LDADLIFLA	0	0	0	0	0	LDADLIFLA	tr_C9ZP82	0.211	5074.31	9.50
530	HLA-A0201	SAFSGESAL	SAFSGESAL	0	0	0	0	0	SAFSGESAL	tr_C9ZP82	0.211	5082.23	10.00
510	HLA-A0201	IDDIIVLSILL	IIIVLSILL	0	0	0	1	2	IDDIIVLSILL	tr_C9ZP82	0.211	5086.35	10.00
703	HLA-A0201	STPPDTLVQLLSIL	SLVQLLSIL	0	0	0	1	5	STPPDTLVQLLSIL	tr_C9ZP82	0.211	5094.23	10.00
55	HLA-A0201	EKLECICSELELL	KLCSELELL	1	0	0	2	3	KLECICSELELL	tr_C9ZP82	0.211	5101.46	10.00
541	HLA-A0201	LLELYVSEVLPYG	LLSEVLPYG	0	0	0	2	4	LLELYVSEVLPYG	tr_C9ZP82	0.211	5105.60	10.00
203	HLA-A0201	QVVDDVFGHRGEV	QVVDDVFEV	0	0	0	7	4	QVVDDVFGHRGEV	tr_C9ZP82	0.211	5109.69	10.00
447	HLA-A0201	TLVSSAGYYF	TLVSSAYYF	0	0	0	6	1	TLVSSAGYYF	tr_C9ZP82	0.210	5162.93	10.00
74	HLA-A0201	KEVMYIAVDGVA	KMYIAVDGV	0	0	0	1	2	KEVMYIAVDGV	tr_C9ZP82	0.210	5164.10	10.00
536	HLA-A0201	SALDNLLELY	SALDNLLEL	0	0	0	0	0	SALDNLLEL	tr_C9ZP82	0.210	5174.95	10.00
819	HLA-A0201	SDALDTILRRV	SLDTILRRV	0	0	0	1	2	SDALDTILRRV	tr_C9ZP82	0.210	5182.18	10.00
513	HLA-A0201	IIVLSILLGNDFLP	IVLSILLFL	1	0	0	7	3	IVLSILLGNDFL	tr_C9ZP82	0.209	5183.30	10.00
305	HLA-A0201	ADLIFLALSLHITQ	ALIFLALSL	0	0	0	1	1	ADLIFLALSL	tr_C9ZP82	0.209	5183.75	10.00
707	HLA-A0201	DTLVQLLSILPPTS	LLLSILPPT	2	0	0	1	2	LVQLLSILPPT	tr_C9ZP82	0.209	5198.91	10.00
740	HLA-A0201	ELESTFPLQWNVD	ELFPLQWNV	0	0	0	2	3	ELESTFPLQWNV	tr_C9ZP82	0.209	5222.48	10.00
240	HLA-A0201	VSDAVLNML	VSDAVLNML	0	0	0	0	0	VSDAVLNML	tr_C9ZP82	0.208	5246.10	10.00
98	HLA-A0201	YMGRAAPAQT	YMGAAPAQT	0	0	0	3	1	YMGRAAPAQT	tr_C9ZP82	0.208	5256.15	10.00
692	HLA-A0201	NIKRMPLPV	NIKRMPLPV	0	0	0	0	0	NIKRMPLPV	tr_C9ZP82	0.208	5272.61	10.00
567	HLA-A0201	LQRLFEAYAKIE	LLFEAYAKI	0	0	0	1	2	LQRLFEAYAKI	tr_C9ZP82	0.208	5274.44	10.00
312	HLA-A0201	LSLHITQVFILR	SLITQVFIL	1	0	0	2	1	SLHITQVFIL	tr_C9ZP82	0.208	5288.27	10.00
734	HLA-A0201	RSPQQAELESTFPL	QQLESTFPL	3	0	0	2	2	QQAELESTFPL	tr_C9ZP82	0.207	5300.13	10.00
767	HLA-A0201	PFADMDVLQQVVD	FMDVLQQVV	1	0	0	1	2	FADMDVLQQVV	tr_C9ZP82	0.207	5319.37	10.00
25	HLA-A0201	VHGLYIDLNGII	GLYIDLNGI	2	0	0	8	1	GLYIDLNGII	tr_C9ZP82	0.207	5326.80	10.00
243	HLA-A0201	AVLNMLNEKM	AVLNMLNEM	0	0	0	8	1	AVLNMLNEKM	tr_C9ZP82	0.207	5343.66	10.00
7	HLA-A0201	SWLRNKYPAIV	WLNKYPAIV	1	0	0	2	1	WLRNKYPAIV	tr_C9ZP82	0.207	5351.06	10.00
251	HLA-A0201	KMSGNDPTWTRL	KMSGNDPTL	0	0	0	8	3	KMSGNDPTWTRL	tr_C9ZP82	0.206	5358.54	10.00

735	HLA-A0201	SPQQAELESTFPL	QQLESTFPL	2	0	0	2	2	QQAELESTFPL	tr_C9ZP82	0.206	5395.13	10.00
725	HLA-A0201	PMVLGEVMV	PMVLGEVMV	0	0	0	0	0	PMVLGEVMV	tr_C9ZP82	0.205	5428.10	10.00
709	HLA-A0201	LVQLLSILPPTSH	LLLSILPPT	0	0	0	1	2	LVQLLSILPPT	tr_C9ZP82	0.205	5440.85	10.00
834	HLA-A0201	GVEIRQLYDV	GVIRQLYDV	0	0	0	2	1	GVEIRQLYDV	tr_C9ZP82	0.205	5444.15	10.00
420	HLA-A0201	GSLISEVSALCDI	SLISALCDI	1	0	0	3	3	SLISEVSALCDI	tr_C9ZP82	0.205	5446.68	10.00
428	HLA-A0201	ALCDIKKFT	ALCDIKKFT	0	0	0	0	0	ALCDIKKFT	tr_C9ZP82	0.205	5455.59	10.00
50	HLA-A0201	LLPEEEKLECI	LLPEELECI	0	0	0	5	2	LLPEEEKLECI	tr_C9ZP82	0.204	5477.89	10.00
517	HLA-A0201	SILLGNDFLP	SILLGNDFL	0	0	0	0	0	SILLGNDFL	tr_C9ZP82	0.204	5477.89	10.00
5	HLA-A0201	FASWLRNKYPAIV	WLNKYPAIV	3	0	0	2	1	WLRNKYPAIV	tr_C9ZP82	0.204	5483.82	10.00
306	HLA-A0201	DLIFLALSLH	DLIFLALSL	0	0	0	0	0	DLIFLALSL	tr_C9ZP82	0.204	5484.06	10.00
762	HLA-A0201	ATVMLPFADM	ATVMLPFAM	0	0	0	8	1	ATVMLPFADM	tr_C9ZP82	0.204	5498.02	10.00
313	HLA-A0201	SLHITQVFILRD	SLITQVFIL	0	0	0	2	1	SLHITQVFIL	tr_C9ZP82	0.204	5522.76	10.00
24	HLA-A0201	SVHGLYIDLNGII	GLYIDLNGI	3	0	0	8	1	GLYIDLNGII	tr_C9ZP82	0.204	5528.50	11.00
66	HLA-A0201	LLVQTVQPKEVMY	LLVQTVQPV	0	0	0	8	2	LLVQTVQPKEV	tr_C9ZP82	0.203	5550.32	11.00
16	HLA-A0201	IVTKNCPSSV	IVTNCPSSV	0	0	0	3	1	IVTKNCPSSV	tr_C9ZP82	0.203	5563.12	11.00
797	HLA-A0201	DFHLVITSNKTSFV	HLVITTSFV	2	0	0	5	3	HLVITSNKTSFV	tr_C9ZP82	0.203	5571.56	11.00
229	HLA-A0201	CISPGTDFMA	CISPGTFMA	0	0	0	6	1	CISPGTDFMA	tr_C9ZP82	0.203	5573.31	11.00
64	HLA-A0201	LELLVQTVQPKEVM	LLVQTVQPV	2	0	0	8	2	LLVQTVQPKEV	tr_C9ZP82	0.202	5596.21	11.00
763	HLA-A0201	TVMLPFADMDVLQQ	VMLPFAMDV	1	0	0	6	1	VMLPFADMDV	tr_C9ZP82	0.202	5624.01	11.00
54	HLA-A0201	EEKLECICSELELL	KLCSELELL	2	0	0	2	3	KLECICSELELL	tr_C9ZP82	0.202	5628.21	11.00
427	HLA-A0201	SALCDIKKFTPAT	ALCDIFTPA	1	0	0	5	2	ALCDIKKFTPA	tr_C9ZP82	0.202	5633.27	11.00
563	HLA-A0201	NLPQLQRLFEAYA	NLLQRLFEA	0	0	0	2	2	NLPQLQRLFEA	tr_C9ZP82	0.202	5639.97	11.00
631	HLA-A0201	TYLKSTGLSNSVQE	YLTGLSNSV	1	0	0	2	2	YLKSTGLSNSV	tr_C9ZP82	0.202	5642.78	11.00
23	HLA-A0201	SSVHGLYIDLNGII	GLYIDLNGI	4	0	0	8	1	GLYIDLNGII	tr_C9ZP82	0.202	5650.66	11.00
310	HLA-A0201	LALSLHITQVFILR	SLHITVFIL	3	0	0	5	1	SLHITQVFIL	tr_C9ZP82	0.201	5656.41	11.00
113	HLA-A0201	TYMPNKSGMAIVEE	YMPSGMAIV	1	0	0	3	2	YMPNKSGMAIV	tr_C9ZP82	0.201	5668.79	11.00
839	HLA-A0201	QLYDVPLER	QLYDVPLER	0	0	0	0	0	QLYDVPLER	tr_C9ZP82	0.201	5669.46	11.00
512	HLA-A0201	DIIVLSILL	DIIVLSILL	0	0	0	0	0	DIIVLSILL	tr_C9ZP82	0.201	5672.96	11.00
509	HLA-A0201	IIDDIIVLSILLG	IIDDIIILL	0	0	0	6	3	IIDDIIVLSILL	tr_C9ZP82	0.201	5674.92	11.00
714	HLA-A0201	SILPPTSHALL	ILPPTSALL	1	0	0	6	1	ILPPTSHALL	tr_C9ZP82	0.200	5713.62	11.00
288	HLA-A0201	SLAQFGGKANHVI	SLAQFNHVI	0	0	0	5	4	SLAQFGGKANHVI	tr_C9ZP82	0.200	5729.96	11.00
853	HLA-A0201	YSCTVEVPRL	YSCTVEVPL	0	0	0	8	1	YSCTVEVPRL	tr_C9ZP82	0.200	5740.45	11.00
159	HLA-A0201	ALYNEGEEEAGDAL	ALYEAGDAL	0	0	0	3	5	ALYNEGEEEAGDAL	tr_C9ZP82	0.199	5793.99	11.00
727	HLA-A0201	VLGEVMVRSPQQA	VLGEVMVQA	0	0	0	7	4	VLGEVMVRSPQQA	tr_C9ZP82	0.198	5841.00	11.00
695	HLA-A0201	RMLPPVVKST	RMLPVVKST	0	0	0	2	1	RMLPPVVKST	tr_C9ZP82	0.198	5876.06	11.00
142	HLA-A0201	DLDEVRQTLL	DLDEVQTLL	0	0	0	5	1	DLDEVRQTLL	tr_C9ZP82	0.198	5892.93	11.00
75	HLA-A0201	EVMYIAVDGVAPRA	VMYGVAPRA	1	0	0	3	4	VMYIAVDGVAPRA	tr_C9ZP82	0.198	5899.44	11.00
532	HLA-A0201	FSGESALDNLLELY	FALDNLLEL	0	0	0	1	4	FSGESALDNLLEL	tr_C9ZP82	0.197	5955.81	11.00
49	HLA-A0201	ALLPEEEKLECIC	ALLEKLECI	0	0	0	3	3	ALLPEEEKLECI	tr_C9ZP82	0.196	5987.47	11.00
668	HLA-A0201	DWSWYYPFYHA	WSWYYPFYA	1	0	0	8	1	WSWYYPFYHA	tr_C9ZP82	0.196	6000.25	11.00
652	HLA-A0201	GVCFVWRYYT	GVCFVWYYT	0	0	0	6	1	GVCFVWRYYT	tr_C9ZP82	0.196	6012.86	11.00
519	HLA-A0201	LLGNDFLPHAPSAF	LLNDFLPHA	0	0	0	2	1	LLGNDFLPHA	tr_C9ZP82	0.196	6016.70	11.00
514	HLA-A0201	IVLSILLGNDFLPH	IVLSILLFL	0	0	0	7	3	IVLSILLGNDFL	tr_C9ZP82	0.195	6049.40	11.00
731	HLA-A0201	VMVRSPQQA	VMVRSPQQA	0	0	0	0	0	VMVRSPQQA	tr_C9ZP82	0.195	6077.15	11.00
554	HLA-A0201	FLTKGPHEINLP	FLGPHEINL	0	0	0	2	2	FLTKGPHEINL	tr_C9ZP82	0.195	6082.81	11.00
150	HLA-A0201	LLEDPLYSG	LLEDPLYSG	0	0	0	0	0	LLEDPLYSG	tr_C9ZP82	0.195	6084.52	11.00
766	HLA-A0201	LPFADMDVLQQVVD	FMDVLQQVV	2	0	0	1	2	FADMDVLQQVV	tr_C9ZP82	0.195	6089.52	11.00
772	HLA-A0201	DVLQQVVDAAWPQL	VLDAAWPQL	1	0	0	2	4	VLQQVVDAAWPQL	tr_C9ZP82	0.195	6094.53	11.00
117	HLA-A0201	NKSGMAIVEEVV	KMAIVEEVV	1	0	0	1	2	KSGMAIVEEVV	tr_C9ZP82	0.194	6118.32	11.00
362	HLA-A0201	PSYIDEEESDGV	YIDEESDGV	2	0	0	3	1	YIDEEESDGV	tr_C9ZP82	0.194	6133.83	11.00
6	HLA-A0201	ASWLRNKYPAIV	WLNKYPAIV	2	0	0	2	1	WLRNKYPAIV	tr_C9ZP82	0.194	6143.26	11.00
311	HLA-A0201	ALSLHITQVFILRD	SLHITVFIL	2	0	0	5	1	SLHITQVFIL	tr_C9ZP82	0.194	6155.24	11.00
422	HLA-A0201	LISEVSALCDI	LISEVSALI	0	0	0	8	2	LISEVSALCDI	tr_C9ZP82	0.193	6177.58	11.00
765	HLA-A0201	MLPFADMDVLQQ	MLFADMDVL	0	0	0	2	1	MLPFADMDVL	tr_C9ZP82	0.193	6178.12	11.00

313	HLA-A0201	SLHITQVFILRDT	SLITQVFIL	0	0	0	2	1	SLHITQVFIL	tr_C9ZP82	0.193	6179.73	11.00
419	HLA-A0201	GGSLISEVSALCDI	SLISALCDI	2	0	0	3	3	SLISEVSALCDI	tr_C9ZP82	0.193	6195.32	11.00
158	HLA-A0201	GALYNEGEEEA	ALYEGEEEA	1	0	0	3	1	ALYNEGEEEA	tr_C9ZP82	0.193	6195.32	11.00
71	HLA-A0201	VQPKEVMYIA	VQPEVMYIA	0	0	0	3	1	VQPKEVMYIA	tr_C9ZP82	0.193	6206.06	11.00
712	HLA-A0201	LLSILPPTSHA	LLSILPPTA	0	0	0	8	2	LLSILPPTSHA	tr_C9ZP82	0.192	6239.32	11.00
303	HLA-A0201	LDADLIFLAL	LADLIFLAL	0	0	0	1	1	LDADLIFLAL	tr_C9ZP82	0.192	6254.66	11.00
567	HLA-A0201	LQRLFEAYA	LQRLFEAYA	0	0	0	0	0	LQRLFEAYA	tr_C9ZP82	0.192	6255.41	11.00
307	HLA-A0201	LIFLALSLHITQ	FLLSLHITQ	2	0	0	2	1	FLALSLHITQ	tr_C9ZP82	0.192	6264.35	11.00
759	HLA-A0201	VHLATVMLPFAD	HLATVLPFA	1	0	0	5	1	HLATVMLPFA	tr_C9ZP82	0.192	6268.14	11.00
413	HLA-A0201	FDISVVGGSL	FISVVGGSL	0	0	0	1	1	FDISVVGGSL	tr_C9ZP82	0.192	6290.97	11.00
750	HLA-A0201	NVDYHDSNV	NVDYHDSNV	0	0	0	0	0	NVDYHDSNV	tr_C9ZP82	0.191	6302.55	11.00
4	HLA-A0201	KFASWLRNKYPAIV	WLNKYPAIV	4	0	0	2	1	WLRNKYPAIV	tr_C9ZP82	0.191	6350.40	11.00
686	HLA-A0201	IYLKEANIKRMPLP	YLKEANIPL	1	0	0	7	3	YLKEANIKRMPL	tr_C9ZP82	0.191	6351.78	11.00
634	HLA-A0201	KSTGLSNSV	KSTGLSNSV	0	0	0	0	0	KSTGLSNSV	tr_C9ZP82	0.191	6355.56	11.00
649	HLA-A0201	YVEGVCFVWRYYT	YVFVWRYYT	0	0	0	2	4	YVEGVCFVWRYYT	tr_C9ZP82	0.190	6396.40	11.00
793	HLA-A0201	TQKLDFHLV	TQKLDFHLV	0	0	0	0	0	TQKLDFHLV	tr_C9ZP82	0.190	6397.85	11.00
70	HLA-A0201	TVQPKEVMYI	TVQPEVMYI	0	0	0	4	1	TVQPKEVMYI	tr_C9ZP82	0.190	6401.11	11.00
632	HLA-A0201	YLKSTGLSNS	YLSTGLSNS	0	0	0	2	1	YLKSTGLSNS	tr_C9ZP82	0.190	6404.50	11.00
798	HLA-A0201	FHLVITSNKT	FLVITSNKT	0	0	0	1	1	FHLVITSNKT	tr_C9ZP82	0.190	6408.24	11.00
361	HLA-A0201	NPSYIDEEESDGV	YIDEESDGV	3	0	0	3	1	YIDEEESDGV	tr_C9ZP82	0.189	6435.34	11.00
313	HLA-A0201	SLHITQVFILRDTT	SLITQVFIL	0	0	0	2	1	SLHITQVFIL	tr_C9ZP82	0.189	6446.07	12.00
426	HLA-A0201	VSALCDIKKFTPAT	ALCDIFTPA	2	0	0	5	2	ALCDIKKFTPA	tr_C9ZP82	0.189	6455.21	12.00
654	HLA-A0201	CFVWRYYTTTT	FVWYYTTTT	1	0	0	3	1	FVWRYYTTTT	tr_C9ZP82	0.189	6458.29	12.00
515	HLA-A0201	VLSILLGNDFLP	VLSILLDFL	0	0	0	6	2	VLSILLGNDFL	tr_C9ZP82	0.189	6467.66	12.00
246	HLA-A0201	NMLNEKMSGNDPT	MLMSGNDPT	1	0	0	2	3	MLNEKMSGNDPT	tr_C9ZP82	0.189	6471.30	12.00
261	HLA-A0201	RLCVIFSGS	RLCVIFSGS	0	0	0	0	0	RLCVIFSGS	tr_C9ZP82	0.189	6488.27	12.00
642	HLA-A0201	VQEACEKYV	VQEACEKYV	0	0	0	0	0	VQEACEKYV	tr_C9ZP82	0.189	6489.18	12.00
27	HLA-A0201	GLYIDLNGIIHPCC	YLNGIIHPC	2	0	0	1	2	YIDLNGIIHPC	tr_C9ZP82	0.188	6511.61	12.00
306	HLA-A0201	DLIFLALSLHITQ	FLLSLHITQ	3	0	0	2	1	FLALSLHITQ	tr_C9ZP82	0.188	6557.50	12.00
722	HLA-A0201	ALLPMVLGE	ALLPMVLGE	0	0	0	0	0	ALLPMVLGE	tr_C9ZP82	0.187	6576.47	12.00
58	HLA-A0201	ECICSELELLV	CICELELLV	1	0	0	3	1	CICSELELLV	tr_C9ZP82	0.187	6600.93	12.00
360	HLA-A0201	LNPSYIDEEESDGV	YIDEESDGV	4	0	0	3	1	YIDEEESDGV	tr_C9ZP82	0.187	6618.52	12.00
763	HLA-A0201	TVMLPFADM	TVMLPFADM	0	0	0	0	0	TVMLPFADM	tr_C9ZP82	0.187	6642.05	12.00
310	HLA-A0201	LALSLHITQVF	LLSLHITQV	0	0	0	1	1	LALSLHITQV	tr_C9ZP82	0.186	6647.01	12.00
712	HLA-A0201	LLSILPPTSHALLP	LLSILPALL	0	0	0	6	4	LLSILPPTSHALL	tr_C9ZP82	0.186	6670.42	12.00
713	HLA-A0201	LSILPPTSHALL	ILPPTSALL	2	0	0	6	1	ILPPTSHALL	tr_C9ZP82	0.186	6670.64	12.00
73	HLA-A0201	PKEVMYIAVDGVA	KMYIAVDGV	1	0	0	1	2	KEVMYIAVDGV	tr_C9ZP82	0.186	6671.36	12.00
566	HLA-A0201	QLQRLFEAYAKIE	LLFEAYAKI	1	0	0	1	2	LQRLFEAYAKI	tr_C9ZP82	0.186	6688.78	12.00
60	HLA-A0201	ICSELELLVQTVQ	ILELLVQTV	0	0	0	1	3	ICSELELLVQTV	tr_C9ZP82	0.186	6689.86	12.00
563	HLA-A0201	NLPQLQRLFEAY	NLLQRLFEA	0	0	0	2	2	NLPQLQRLFEA	tr_C9ZP82	0.186	6702.54	12.00
350	HLA-A0201	NFIDDTCIIS	FIDDTCIIS	1	0	0	0	0	FIDDTCIIS	tr_C9ZP82	0.186	6702.76	12.00
202	HLA-A0201	NQVVDDVFGHRGEV	QVVDDVFEV	1	0	0	7	4	QVVDDVFGHRGEV	tr_C9ZP82	0.185	6720.70	12.00
75	HLA-A0201	EVMYIAVDGVA	VMYIAVDGV	1	0	0	0	0	VMYIAVDGV	tr_C9ZP82	0.185	6721.50	12.00
416	HLA-A0201	SVVGGSLISEV	SVVGLISEV	0	0	0	4	2	SVVGGSLISEV	tr_C9ZP82	0.185	6731.54	12.00
667	HLA-A0201	SDWSWYYPFYHA	WSWYYPFYA	2	0	0	8	1	WSWYYPFYHA	tr_C9ZP82	0.185	6769.16	12.00
710	HLA-A0201	VQLLSILPPTSH	VLLSILPPT	0	0	0	1	1	VQLLSILPPT	tr_C9ZP82	0.185	6770.33	12.00
51	HLA-A0201	LPEEEKLECICSEL	LLECICSEL	0	0	0	1	5	LPEEEKLECICSEL	tr_C9ZP82	0.185	6771.43	12.00
726	HLA-A0201	MVLGEVMVRSPQQA	VLGEVMVQA	1	0	0	7	4	VLGEVMVRSPQQA	tr_C9ZP82	0.184	6795.87	12.00
65	HLA-A0201	ELLVQTVQPKEVMY	LLVQTVQPV	1	0	0	8	2	LLVQTVQPKEV	tr_C9ZP82	0.184	6813.98	12.00
312	HLA-A0201	LSLHITQVFILRD	SLHITVFIL	1	0	0	5	1	SLHITQVFIL	tr_C9ZP82	0.184	6830.96	12.00
116	HLA-A0201	PNKSGMAIVEEVV	KMAIVEEVV	2	0	0	1	2	KSGMAIVEEVV	tr_C9ZP82	0.184	6855.61	12.00
562	HLA-A0201	INLPQLQRLFEAYA	NLLQRLFEA	1	0	0	2	2	NLPQLQRLFEA	tr_C9ZP82	0.183	6875.15	12.00
767	HLA-A0201	PFADMDVLQQVVDA	FAMDVLQQV	1	0	0	2	1	FADMDVLQQV	tr_C9ZP82	0.183	6910.65	12.00
760	HLA-A0201	HLATVMLPFADMD	HLATVMLPM	0	0	0	8	3	HLATVMLPFADM	tr_C9ZP82	0.183	6932.82	12.00

1	HLA-A0201	GVPKFASWL	GVPKFASWL	0	0	0	0	0	GVPKFASWL	tr_C9ZP82	0.183	6935.75	12.00
259	HLA-A0201	WTRLCVIFSG	WTLCVIFSG	0	0	0	2	1	WTRLCVIFSG	tr_C9ZP82	0.183	6935.82	12.00
547	HLA-A0201	SEVLPYGFLT	SVLPYGFLT	0	0	0	1	1	SEVLPYGFLT	tr_C9ZP82	0.182	6943.56	12.00
72	HLA-A0201	QPKEVMYIAVDGVA	KMYIAVDGV	2	0	0	1	2	KEVMYIAVDGV	tr_C9ZP82	0.182	6963.34	12.00
251	HLA-A0201	KMSGNDPTWT	KMSGNDPTT	0	0	0	8	1	KMSGNDPTWT	tr_C9ZP82	0.182	6966.28	12.00
739	HLA-A0201	AELESTFPLQWNVD	ELFPLQWNV	1	0	0	2	3	ELESTFPLQWNV	tr_C9ZP82	0.182	6972.99	12.00
758	HLA-A0201	VVHLATVMLPFAD	HLATVLPFA	2	0	0	5	1	HLATVMLPFA	tr_C9ZP82	0.182	6984.69	12.00
139	HLA-A0201	ANDDLDEVRQTL	ALDEVRQTL	0	0	0	1	3	ANDDLDEVRQTL	tr_C9ZP82	0.182	6994.00	12.00
5	HLA-A0201	FASWLRNKYPAI	FLRNKYPAI	0	0	0	1	3	FASWLRNKYPAI	tr_C9ZP82	0.182	7003.16	12.00
708	HLA-A0201	TLVQLLSILPPTSH	LLLSILPPT	1	0	0	1	2	LVQLLSILPPT	tr_C9ZP82	0.182	7006.80	12.00
666	HLA-A0201	DSDWSWYYPFYHA	WSWYYPFYA	3	0	0	8	1	WSWYYPFYHA	tr_C9ZP82	0.181	7038.56	12.00
678	HLA-A0201	APPVIDLAIYLK	AVIDLAIYL	0	0	0	1	2	APPVIDLAIYL	tr_C9ZP82	0.180	7096.44	12.00
524	HLA-A0201	FLPHAPSAFSGESA	FLAFSGESA	0	0	0	2	5	FLPHAPSAFSGESA	tr_C9ZP82	0.180	7097.60	12.00
7	HLA-A0201	SWLRNKYPAI	SLRNKYPAI	0	0	0	1	1	SWLRNKYPAI	tr_C9ZP82	0.180	7105.36	12.00
795	HLA-A0201	KLDFHLVITSN	KLFHLVITS	0	0	0	2	1	KLDFHLVITS	tr_C9ZP82	0.180	7153.49	12.00
771	HLA-A0201	MDVLQQVVDAA	MLQQVVDAA	0	0	0	1	2	MDVLQQVVDAA	tr_C9ZP82	0.180	7160.46	12.00
157	HLA-A0201	SGALYNEGEEEA	ALYEGEEEA	2	0	0	3	1	ALYNEGEEEA	tr_C9ZP82	0.180	7166.20	12.00
757	HLA-A0201	NVVHLATVMLPFAD	HLATVLPFA	3	0	0	5	1	HLATVMLPFA	tr_C9ZP82	0.179	7190.27	12.00
420	HLA-A0201	GSLISEVSALC	SLIEVSALC	1	0	0	3	1	SLISEVSALC	tr_C9ZP82	0.179	7197.98	12.00
665	HLA-A0201	TDSDWSWYYPFYHA	WSWYYPFYA	4	0	0	8	1	WSWYYPFYHA	tr_C9ZP82	0.179	7201.17	12.00
74	HLA-A0201	KEVMYIAVDGVAP	KMYIAVDGV	0	0	0	1	2	KEVMYIAVDGV	tr_C9ZP82	0.179	7210.76	12.00
76	HLA-A0201	VMYIAVDGVAP	VMYIAVDGV	0	0	0	0	0	VMYIAVDGV	tr_C9ZP82	0.179	7218.96	12.00
817	HLA-A0201	KLSDALDTILR	KLSDALDTL	0	0	0	8	1	KLSDALDTIL	tr_C9ZP82	0.179	7226.78	12.00
838	HLA-A0201	RQLYDVPLER	RLYDVPLER	0	0	0	1	1	RQLYDVPLER	tr_C9ZP82	0.179	7235.54	12.00
137	HLA-A0201	AQANDDLDEVRQTL	ALDEVRQTL	0	0	0	1	5	AQANDDLDEVRQTL	tr_C9ZP82	0.179	7243.53	12.00
29	HLA-A0201	YIDLNGIIHPCC	YLNGIIHPC	0	0	0	1	2	YIDLNGIIHPC	tr_C9ZP82	0.178	7251.06	12.00
568	HLA-A0201	QRLFEAYAKIE	RLFEAYAKI	1	0	0	0	0	RLFEAYAKI	tr_C9ZP82	0.178	7256.47	12.00
704	HLA-A0201	TPPDTLVQLLSIL	TLVQLLSIL	0	0	0	1	4	TPPDTLVQLLSIL	tr_C9ZP82	0.178	7262.99	12.00
541	HLA-A0201	LLELYVSEVLP	LLLYVSEVL	0	0	0	2	1	LLELYVSEVL	tr_C9ZP82	0.178	7264.41	12.00
428	HLA-A0201	ALCDIKKFTPATE	ALCDIFTPA	0	0	0	5	2	ALCDIKKFTPA	tr_C9ZP82	0.178	7274.47	12.00
809	HLA-A0201	FVPKGDLRKL	FVPKGDLRL	0	0	0	8	1	FVPKGDLRKL	tr_C9ZP82	0.178	7310.06	12.00
59	HLA-A0201	CICSELELLVQTVQ	ILELLVQTV	1	0	0	1	3	ICSELELLVQTV	tr_C9ZP82	0.177	7353.61	12.00
117	HLA-A0201	NKSGMAIVEEV	KSMAIVEEV	1	0	0	2	1	KSGMAIVEEV	tr_C9ZP82	0.177	7360.93	12.00
741	HLA-A0201	LESTFPLQWNVD	LTFPLQWNV	0	0	0	1	2	LESTFPLQWNV	tr_C9ZP82	0.177	7368.58	12.00
565	HLA-A0201	PQLQRLFEAYAKIE	LLFEAYAKI	2	0	0	1	2	LQRLFEAYAKI	tr_C9ZP82	0.177	7373.53	12.00
250	HLA-A0201	EKMSGNDPTWTRL	KMSGNDPTL	1	0	0	8	3	KMSGNDPTWTRL	tr_C9ZP82	0.177	7387.26	12.00
610	HLA-A0201	NVTVADVAV	NVTVADVAV	0	0	0	0	0	NVTVADVAV	tr_C9ZP82	0.177	7388.46	13.00
229	HLA-A0201	CISPGTDFMAKV	CISDFMAKV	0	0	0	3	3	CISPGTDFMAKV	tr_C9ZP82	0.177	7402.06	13.00
757	HLA-A0201	NVVHLATVML	NVVHLTVML	0	0	0	5	1	NVVHLATVML	tr_C9ZP82	0.176	7416.17	13.00
67	HLA-A0201	LVQTVQPKEVMYI	LVQTVVMYI	0	0	0	5	4	LVQTVQPKEVMYI	tr_C9ZP82	0.176	7416.25	13.00
98	HLA-A0201	YMGRAAPAQTGEVA	YMGRAAPEV	0	0	0	7	4	YMGRAAPAQTGEV	tr_C9ZP82	0.176	7424.52	13.00
554	HLA-A0201	FLTKGPHEINLPQ	FLHEINLPQ	0	0	0	2	4	FLTKGPHEINLPQ	tr_C9ZP82	0.176	7432.32	13.00
57	HLA-A0201	LECICSELELLV	CICELELLV	2	0	0	3	1	CICSELELLV	tr_C9ZP82	0.176	7434.49	13.00
532	HLA-A0201	FSGESALDNL	FSGESALNL	0	0	0	7	1	FSGESALDNL	tr_C9ZP82	0.176	7449.30	13.00
653	HLA-A0201	VCFVWRYYTTTT	FVWYYTTTT	2	0	0	3	1	FVWRYYTTTT	tr_C9ZP82	0.176	7460.43	13.00
312	HLA-A0201	LSLHITQVFILRDT	SLHITVFIL	1	0	0	5	1	SLHITQVFIL	tr_C9ZP82	0.176	7483.64	13.00
509	HLA-A0201	IIDDIIVLSILLGN	IIDDIIILL	0	0	0	6	3	IIDDIIVLSILL	tr_C9ZP82	0.175	7492.06	13.00
142	HLA-A0201	DLDEVRQTL	DLDEVRQTL	0	0	0	0	0	DLDEVRQTL	tr_C9ZP82	0.175	7505.53	13.00
156	HLA-A0201	YSGALYNEGEEEA	ALYEGEEEA	3	0	0	3	1	ALYNEGEEEA	tr_C9ZP82	0.175	7514.96	13.00
287	HLA-A0201	SSLAQFGGKANHVI	SLAQFNHVI	1	0	0	5	4	SLAQFGGKANHVI	tr_C9ZP82	0.174	7571.93	13.00
611	HLA-A0201	VTVADVAVA	VTVADVAVA	0	0	0	0	0	VTVADVAVA	tr_C9ZP82	0.174	7637.83	13.00
54	HLA-A0201	EEKLECICSEL	KLECICSEL	2	0	0	0	0	KLECICSEL	tr_C9ZP82	0.174	7638.33	13.00
773	HLA-A0201	VLQQVVDAAW	VLQQVVDAA	0	0	0	0	0	VLQQVVDAA	tr_C9ZP82	0.174	7644.20	13.00
740	HLA-A0201	ELESTFPLQWNVDY	ELFPLQWNV	0	0	0	2	3	ELESTFPLQWNV	tr_C9ZP82	0.173	7652.06	13.00

756	HLA-A0201	SNVVHLATVMLPF	SLATVMLPF	0	0	0	1	4	SNVVHLATVMLPF	tr_C9ZP82	0.173	7683.42	13.00
713	HLA-A0201	LSILPPTSHAL	LLPPTSHAL	0	0	0	1	2	LSILPPTSHAL	tr_C9ZP82	0.173	7693.07	13.00
48	HLA-A0201	IALLPEEEKLECIC	ALLEKLECI	1	0	0	3	3	ALLPEEEKLECI	tr_C9ZP82	0.173	7710.58	13.00
245	HLA-A0201	LNMLNEKMSGNDPT	MLMSGNDPT	2	0	0	2	3	MLNEKMSGNDPT	tr_C9ZP82	0.173	7726.52	13.00
155	HLA-A0201	LYSGALYNEGEEEA	ALYEGEEEA	4	0	0	3	1	ALYNEGEEEA	tr_C9ZP82	0.173	7727.44	13.00
298	HLA-A0201	HVIVGLDADL	HVIVGLADL	0	0	0	6	1	HVIVGLDADL	tr_C9ZP82	0.172	7735.81	13.00
653	HLA-A0201	VCFVWRYYT	VCFVWRYYT	0	0	0	0	0	VCFVWRYYT	tr_C9ZP82	0.172	7757.44	13.00
882	HLA-A0201	QLKGNGEGV	QLKGNGEGV	0	0	0	0	0	QLKGNGEGV	tr_C9ZP82	0.172	7759.20	13.00
281	HLA-A0201	DFLRTQSSLA	FLRTQSSLA	1	0	0	0	0	FLRTQSSLA	tr_C9ZP82	0.172	7767.60	13.00
540	HLA-A0201	NLLELYVSE	NLLELYVSE	0	0	0	0	0	NLLELYVSE	tr_C9ZP82	0.172	7773.57	13.00
221	HLA-A0201	TAFEFDSNCI	TAFEFSNCI	0	0	0	5	1	TAFEFDSNCI	tr_C9ZP82	0.172	7775.84	13.00
147	HLA-A0201	RQTLLEDPL	RQTLLEDPL	0	0	0	0	0	RQTLLEDPL	tr_C9ZP82	0.172	7776.09	13.00
652	HLA-A0201	GVCFVWRYYTTTT	FVWYYTTTT	3	0	0	3	1	FVWRYYTTTT	tr_C9ZP82	0.172	7785.35	13.00
770	HLA-A0201	DMDVLQQVV	DMDVLQQVV	0	0	0	0	0	DMDVLQQVV	tr_C9ZP82	0.172	7796.31	13.00
79	HLA-A0201	IAVDGVAPRA	IAVDGVAPA	0	0	0	8	1	IAVDGVAPRA	tr_C9ZP82	0.172	7803.31	13.00
204	HLA-A0201	VVDDVFGHRGEVIV	VVDDVFVIV	0	0	0	6	5	VVDDVFGHRGEVIV	tr_C9ZP82	0.172	7805.25	13.00
839	HLA-A0201	QLYDVPLERY	QLYDVPLEY	0	0	0	8	1	QLYDVPLERY	tr_C9ZP82	0.172	7810.07	13.00
679	HLA-A0201	PPVIDLAIYL	PVIDLAIYL	0	0	0	1	1	PPVIDLAIYL	tr_C9ZP82	0.172	7812.26	13.00
418	HLA-A0201	VGGSLISEVSALC	SLIEVSALC	3	0	0	3	1	SLISEVSALC	tr_C9ZP82	0.172	7816.58	13.00
62	HLA-A0201	SELELLVQTVQ	SLELLVQTV	0	0	0	1	1	SELELLVQTV	tr_C9ZP82	0.171	7859.75	13.00
419	HLA-A0201	GGSLISEVSALC	SLIEVSALC	2	0	0	3	1	SLISEVSALC	tr_C9ZP82	0.171	7873.28	13.00
53	HLA-A0201	EEEKLECICSEL	KLECICSEL	3	0	0	0	0	KLECICSEL	tr_C9ZP82	0.170	7917.44	13.00
249	HLA-A0201	NEKMSGNDPTWTRL	KMSGNDPTL	2	0	0	8	3	KMSGNDPTWTRL	tr_C9ZP82	0.170	7927.82	13.00
417	HLA-A0201	VVGGSLISEVSALC	SLIEVSALC	4	0	0	3	1	SLISEVSALC	tr_C9ZP82	0.170	7947.91	13.00
651	HLA-A0201	EGVCFVWRYYTTTT	FVWYYTTTT	4	0	0	3	1	FVWRYYTTTT	tr_C9ZP82	0.170	7975.48	13.00
252	HLA-A0201	MSGNDPTWTRLCVI	MTWTRLCVI	0	0	0	1	5	MSGNDPTWTRLCVI	tr_C9ZP82	0.170	7978.50	13.00
49	HLA-A0201	ALLPEEEKLECICS	ALLEKLECI	0	0	0	3	3	ALLPEEEKLECI	tr_C9ZP82	0.169	8004.18	13.00
637	HLA-A0201	GLSNSVQEAC	GLSNSVQEA	0	0	0	0	0	GLSNSVQEA	tr_C9ZP82	0.169	8022.65	13.00
710	HLA-A0201	VQLLSILPP	VQLLSILPP	0	0	0	0	0	VQLLSILPP	tr_C9ZP82	0.169	8074.81	13.00
52	HLA-A0201	PEEEKLECICSEL	KLECICSEL	4	0	0	0	0	KLECICSEL	tr_C9ZP82	0.168	8086.44	13.00
684	HLA-A0201	LAIYLKEANI	LIYLKEANI	0	0	0	1	1	LAIYLKEANI	tr_C9ZP82	0.168	8096.51	13.00
513	HLA-A0201	IIVLSILLG	IIVLSILLG	0	0	0	0	0	IIVLSILLG	tr_C9ZP82	0.168	8117.74	13.00
706	HLA-A0201	PDTLVQLLSIL	TLVQLLSIL	2	0	0	0	0	TLVQLLSIL	tr_C9ZP82	0.168	8127.31	13.00
648	HLA-A0201	KYVEGVCFVWRYYT	YVEGVCFYT	1	0	0	7	4	YVEGVCFVWRYYT	tr_C9ZP82	0.168	8153.65	13.00
553	HLA-A0201	GFLTKGPHEINLP	FLGPHEINL	1	0	0	2	2	FLTKGPHEINL	tr_C9ZP82	0.168	8156.03	13.00
541	HLA-A0201	LLELYVSEVLPYGF	YVSEVLPYF	4	0	0	8	1	YVSEVLPYGF	tr_C9ZP82	0.167	8166.72	13.00
518	HLA-A0201	ILLGNDFLPH	ILLGNFLPH	0	0	0	5	1	ILLGNDFLPH	tr_C9ZP82	0.167	8182.90	13.00
428	HLA-A0201	ALCDIKKFTPATES	ALCDIFTPA	0	0	0	5	2	ALCDIKKFTPA	tr_C9ZP82	0.167	8184.50	13.00
515	HLA-A0201	VLSILLGNDFLPH	VLSILLDFL	0	0	0	6	2	VLSILLGNDFL	tr_C9ZP82	0.167	8197.00	13.00
76	HLA-A0201	VMYIAVDGVAPR	VMYIAVDGV	0	0	0	0	0	VMYIAVDGV	tr_C9ZP82	0.167	8204.45	13.00
60	HLA-A0201	ICSELELLVQTVQP	ILELLVQTV	0	0	0	1	3	ICSELELLVQTV	tr_C9ZP82	0.167	8215.47	13.00
565	HLA-A0201	PQLQRLFEA	PQLQRLFEA	0	0	0	0	0	PQLQRLFEA	tr_C9ZP82	0.167	8223.74	13.00
74	HLA-A0201	KEVMYIAVDGVAPR	KMYIAVDGV	0	0	0	1	2	KEVMYIAVDGV	tr_C9ZP82	0.166	8257.17	13.00
308	HLA-A0201	IFLALSLHITQ	FLLSLHITQ	1	0	0	2	1	FLALSLHITQ	tr_C9ZP82	0.166	8282.40	13.00
562	HLA-A0201	INLPQLQRLFEAY	NLLQRLFEA	1	0	0	2	2	NLPQLQRLFEA	tr_C9ZP82	0.166	8282.94	13.00
705	HLA-A0201	PPDTLVQLLSIL	TLVQLLSIL	3	0	0	0	0	TLVQLLSIL	tr_C9ZP82	0.166	8323.01	13.00
727	HLA-A0201	VLGEVMVRSP	VLGEVMVSP	0	0	0	7	1	VLGEVMVRSP	tr_C9ZP82	0.165	8349.34	14.00
278	HLA-A0201	KIIDFLRTQS	KIIDFLTQS	0	0	0	6	1	KIIDFLRTQS	tr_C9ZP82	0.165	8352.59	14.00
770	HLA-A0201	DMDVLQQVVDAA	MLQQVVDAA	1	0	0	1	2	MDVLQQVVDAA	tr_C9ZP82	0.165	8358.38	14.00
758	HLA-A0201	VVHLATVMLPF	VLATVMLPF	0	0	0	1	2	VVHLATVMLPF	tr_C9ZP82	0.165	8375.77	14.00
537	HLA-A0201	ALDNLLELY	ALDNLLELY	0	0	0	0	0	ALDNLLELY	tr_C9ZP82	0.164	8453.97	14.00
681	HLA-A0201	VIDLAIYLKEANI	VIDLAIYLI	0	0	0	8	4	VIDLAIYLKEANI	tr_C9ZP82	0.164	8471.74	14.00
759	HLA-A0201	VHLATVMLPFADMD	HLATVMLPM	1	0	0	8	3	HLATVMLPFADM	tr_C9ZP82	0.164	8506.54	14.00
135	HLA-A0201	EMAQANDDLDEV	EMADDLDEV	0	0	0	3	3	EMAQANDDLDEV	tr_C9ZP82	0.164	8523.50	14.00

355	HLA-A0201	TCIISLNPSYI	CIISLNPYI	1	0	0	7	1	CIISLNPSYI	tr_C9ZP82	0.163	8559.91	14.00
742	HLA-A0201	ESTFPLQWNVD	STFPLQWNV	1	0	0	0	0	STFPLQWNV	tr_C9ZP82	0.163	8561.30	14.00
881	HLA-A0201	GQLKGNGEGV	GQLKGNEGV	0	0	0	6	1	GQLKGNGEGV	tr_C9ZP82	0.163	8564.18	14.00
794	HLA-A0201	QKLDFHLVITSN	KLFHLVITS	1	0	0	2	1	KLDFHLVITS	tr_C9ZP82	0.163	8581.89	14.00
61	HLA-A0201	CSELELLVQTVQ	SLELLVQTV	1	0	0	1	1	SELELLVQTV	tr_C9ZP82	0.163	8612.03	14.00
541	HLA-A0201	LLELYVSEVLPY	LLLYVSEVL	0	0	0	2	1	LLELYVSEVL	tr_C9ZP82	0.162	8628.72	14.00
511	HLA-A0201	DDIIVLSILL	DIIVLSILL	0	0	0	1	1	DDIIVLSILL	tr_C9ZP82	0.162	8644.05	14.00
28	HLA-A0201	LYIDLNGIIHPCC	YLNGIIHPC	1	0	0	1	2	YIDLNGIIHPC	tr_C9ZP82	0.162	8649.85	14.00
605	HLA-A0201	GSNNDNVTV	GSNNDNVTV	0	0	0	0	0	GSNNDNVTV	tr_C9ZP82	0.162	8650.69	14.00
238	HLA-A0201	AKVSDAVLNM	KVSDAVLNM	1	0	0	0	0	KVSDAVLNM	tr_C9ZP82	0.162	8655.00	14.00
895	HLA-A0201	KSNSRVTVV	KSNSRVTVV	0	0	0	0	0	KSNSRVTVV	tr_C9ZP82	0.162	8678.25	14.00
571	HLA-A0201	FEAYAKIEAVK	FAYAKIEAV	0	0	0	1	1	FEAYAKIEAV	tr_C9ZP82	0.162	8704.59	14.00
677	HLA-A0201	HAPPVIDLAIYLK	AVIDLAIYL	1	0	0	1	2	APPVIDLAIYL	tr_C9ZP82	0.162	8704.96	14.00
691	HLA-A0201	ANIKRMPLPV	AIKRMPLPV	0	0	0	1	1	ANIKRMPLPV	tr_C9ZP82	0.162	8707.22	14.00
655	HLA-A0201	FVWRYYTTTTT	FVWYTTTTT	0	0	0	3	2	FVWRYYTTTTT	tr_C9ZP82	0.162	8707.51	14.00
415	HLA-A0201	ISVVGGSLISEV	SVVGLISEV	1	0	0	4	2	SVVGGSLISEV	tr_C9ZP82	0.161	8717.02	14.00
78	HLA-A0201	YIAVDGVAPRA	YIAGVAPRA	0	0	0	3	2	YIAVDGVAPRA	tr_C9ZP82	0.161	8717.69	14.00
27	HLA-A0201	GLYIDLNGIIH	GLYIDLNGI	0	0	0	8	1	GLYIDLNGII	tr_C9ZP82	0.161	8734.77	14.00
755	HLA-A0201	DSNVVHLATVMLPF	SLATVMLPF	1	0	0	1	4	SNVVHLATVMLPF	tr_C9ZP82	0.161	8753.88	14.00
278	HLA-A0201	KIIDFLRTQSSLAQ	KIIDFLSSL	0	0	0	6	3	KIIDFLRTQSSL	tr_C9ZP82	0.161	8759.38	14.00
29	HLA-A0201	YIDLNGIIHP	YILNGIIHP	0	0	0	2	1	YIDLNGIIHP	tr_C9ZP82	0.161	8788.91	14.00
681	HLA-A0201	VIDLAIYLKEA	ILAIYLKEA	1	0	0	1	1	IDLAIYLKEA	tr_C9ZP82	0.160	8825.78	14.00
572	HLA-A0201	EAYAKIEAV	EAYAKIEAV	0	0	0	0	0	EAYAKIEAV	tr_C9ZP82	0.160	8833.81	14.00
544	HLA-A0201	LYVSEVLPYGF	YVSEVLPYF	1	0	0	8	1	YVSEVLPYGF	tr_C9ZP82	0.160	8844.90	14.00
561	HLA-A0201	EINLPQLQRLFEAY	NLLQRLFEA	2	0	0	2	2	NLPQLQRLFEA	tr_C9ZP82	0.160	8856.30	14.00
75	HLA-A0201	EVMYIAVDGVAP	VMYIAVDGV	1	0	0	0	0	VMYIAVDGV	tr_C9ZP82	0.160	8877.41	14.00
73	HLA-A0201	PKEVMYIAVDGVAP	KMYIAVDGV	1	0	0	1	2	KEVMYIAVDGV	tr_C9ZP82	0.160	8898.46	14.00
427	HLA-A0201	SALCDIKKFTPATE	ALCDIFTPA	1	0	0	5	2	ALCDIKKFTPA	tr_C9ZP82	0.160	8899.43	14.00
510	HLA-A0201	IDDIIVLSILLG	IIIVLSILL	0	0	0	1	2	IDDIIVLSILL	tr_C9ZP82	0.160	8899.81	14.00
409	HLA-A0201	GFEYFDISVV	FEYFDISVV	1	0	0	0	0	FEYFDISVV	tr_C9ZP82	0.159	8930.10	14.00
678	HLA-A0201	APPVIDLAIYLKEA	ALAIYLKEA	0	0	0	1	5	APPVIDLAIYLKEA	tr_C9ZP82	0.159	8932.51	14.00
552	HLA-A0201	YGFLTKGPHEINLP	FLGPHEINL	2	0	0	2	2	FLTKGPHEINL	tr_C9ZP82	0.159	8948.87	14.00
683	HLA-A0201	DLAIYLKEA	DLAIYLKEA	0	0	0	0	0	DLAIYLKEA	tr_C9ZP82	0.159	8964.37	14.00
58	HLA-A0201	ECICSELELL	CICSELELL	1	0	0	0	0	CICSELELL	tr_C9ZP82	0.159	8972.53	14.00
732	HLA-A0201	MVRSPQQAEL	MVSPQQAEL	0	0	0	2	1	MVRSPQQAEL	tr_C9ZP82	0.159	8980.87	14.00
136	HLA-A0201	MAQANDDLDEV	AQADDLDEV	1	0	0	3	1	AQANDDLDEV	tr_C9ZP82	0.159	8992.55	14.00
76	HLA-A0201	VMYIAVDGVAPRAK	VMYIAVDGV	0	0	0	0	0	VMYIAVDGV	tr_C9ZP82	0.159	8998.67	14.00
585	HLA-A0201	FLLFKSTNK	FLLFKSTNK	0	0	0	0	0	FLLFKSTNK	tr_C9ZP82	0.158	9005.98	14.00
138	HLA-A0201	QANDDLDEVRQTL	ALDEVRQTL	1	0	0	1	3	ANDDLDEVRQTL	tr_C9ZP82	0.158	9047.20	14.00
31	HLA-A0201	DLNGIIHPC	DLNGIIHPC	0	0	0	0	0	DLNGIIHPC	tr_C9ZP82	0.158	9072.58	14.00
769	HLA-A0201	ADMDVLQQVVDAA	MLQQVVDAA	2	0	0	1	2	MDVLQQVVDAA	tr_C9ZP82	0.158	9075.23	14.00
115	HLA-A0201	MPNKSGMAIVEEV	KSMAIVEEV	3	0	0	2	1	KSGMAIVEEV	tr_C9ZP82	0.158	9090.56	14.00
793	HLA-A0201	TQKLDFHLVITSN	KLFHLVITS	2	0	0	2	1	KLDFHLVITS	tr_C9ZP82	0.157	9122.09	14.00
63	HLA-A0201	ELELLVQTVQ	ELELLVQTV	0	0	0	0	0	ELELLVQTV	tr_C9ZP82	0.157	9171.28	14.00
414	HLA-A0201	DISVVGGSLISEV	SVVGLISEV	2	0	0	4	2	SVVGGSLISEV	tr_C9ZP82	0.157	9172.77	14.00
228	HLA-A0201	NCISPGTDFMAKV	CISDFMAKV	1	0	0	3	3	CISPGTDFMAKV	tr_C9ZP82	0.157	9175.35	14.00
851	HLA-A0201	RTYSCTVEVPRL	RTYSCTVEL	0	0	0	8	3	RTYSCTVEVPRL	tr_C9ZP82	0.156	9196.42	14.00
354	HLA-A0201	DTCIISLNPSYI	CIISLNPYI	2	0	0	7	1	CIISLNPSYI	tr_C9ZP82	0.156	9207.97	14.00
147	HLA-A0201	RQTLLEDPLYS	RLLEDPLYS	0	0	0	1	2	RQTLLEDPLYS	tr_C9ZP82	0.156	9216.34	14.00
116	HLA-A0201	PNKSGMAIVEEV	KSMAIVEEV	2	0	0	2	1	KSGMAIVEEV	tr_C9ZP82	0.156	9219.34	14.00
795	HLA-A0201	KLDFHLVITSNK	KLFHLVITS	0	0	0	2	1	KLDFHLVITS	tr_C9ZP82	0.156	9239.20	14.00
563	HLA-A0201	NLPQLQRLFEAYAK	NLLQRLFEA	0	0	0	2	2	NLPQLQRLFEA	tr_C9ZP82	0.156	9281.79	15.00
29	HLA-A0201	YIDLNGIIHPCCH	YLNGIIHPC	0	0	0	1	2	YIDLNGIIHPC	tr_C9ZP82	0.155	9300.79	15.00
6	HLA-A0201	ASWLRNKYPA	ASWLRNYPA	0	0	0	6	1	ASWLRNKYPA	tr_C9ZP82	0.155	9317.71	15.00

757	HLA-A0201	NVVHLATVMLPF	VLATVMLPF	1	0	0	1	2	VVHLATVMLPF	tr_C9ZP82	0.155	9340.93	15.00
792	HLA-A0201	RTQKLDFHLVITSN	KLFHLVITS	3	0	0	2	1	KLDFHLVITS	tr_C9ZP82	0.155	9341.53	15.00
799	HLA-A0201	HLVITSNKTSFVP	HLVITTSFV	0	0	0	5	3	HLVITSNKTSFV	tr_C9ZP82	0.155	9342.95	15.00
641	HLA-A0201	SVQEACEKYVEGV	SVQEYVEGV	0	0	0	4	4	SVQEACEKYVEGV	tr_C9ZP82	0.155	9354.38	15.00
413	HLA-A0201	FDISVVGGSLISEV	SVVGLISEV	3	0	0	4	2	SVVGGSLISEV	tr_C9ZP82	0.155	9359.44	15.00
553	HLA-A0201	GFLTKGPHEINLPQ	FLHEINLPQ	1	0	0	2	4	FLTKGPHEINLPQ	tr_C9ZP82	0.155	9368.76	15.00
565	HLA-A0201	PQLQRLFEAYA	QLQLFEAYA	1	0	0	3	1	QLQRLFEAYA	tr_C9ZP82	0.155	9379.11	15.00
743	HLA-A0201	STFPLQWNVDY	STFPLQWNV	0	0	0	0	0	STFPLQWNV	tr_C9ZP82	0.155	9380.12	15.00
146	HLA-A0201	VRQTLLEDPLYS	VLLEDPLYS	0	0	0	1	3	VRQTLLEDPLYS	tr_C9ZP82	0.154	9404.72	15.00
737	HLA-A0201	QQAELESTFPLQ	QQLESTFPL	0	0	0	2	2	QQAELESTFPL	tr_C9ZP82	0.154	9416.22	15.00
816	HLA-A0201	RKLSDALDTILR	KLSDALDTL	1	0	0	8	1	KLSDALDTIL	tr_C9ZP82	0.154	9422.84	15.00
647	HLA-A0201	EKYVEGVCFV	KYVEGVCFV	1	0	0	0	0	KYVEGVCFV	tr_C9ZP82	0.154	9442.65	15.00
670	HLA-A0201	SWYYPFYHA	SWYYPFYHA	0	0	0	0	0	SWYYPFYHA	tr_C9ZP82	0.154	9461.57	15.00
634	HLA-A0201	KSTGLSNSVQEA	KLSNSVQEA	0	0	0	1	3	KSTGLSNSVQEA	tr_C9ZP82	0.154	9472.53	15.00
4	HLA-A0201	KFASWLRNKYPAI	FLRNKYPAI	1	0	0	1	3	FASWLRNKYPAI	tr_C9ZP82	0.154	9485.65	15.00
791	HLA-A0201	WRTQKLDFHL	WTQKLDFHL	0	0	0	1	1	WRTQKLDFHL	tr_C9ZP82	0.154	9495.51	15.00
428	HLA-A0201	ALCDIKKFTP	ALCDIKFTP	0	0	0	5	1	ALCDIKKFTP	tr_C9ZP82	0.153	9506.61	15.00
27	HLA-A0201	GLYIDLNGIIHP	GLYIDLNII	0	0	0	7	1	GLYIDLNGII	tr_C9ZP82	0.153	9544.02	15.00
404	HLA-A0201	TVPNTGFEYFDISV	TGFEYFISV	4	0	0	6	1	TGFEYFDISV	tr_C9ZP82	0.153	9549.50	15.00
640	HLA-A0201	NSVQEACEKYV	SVQEACEYV	1	0	0	7	1	SVQEACEKYV	tr_C9ZP82	0.153	9588.85	15.00
353	HLA-A0201	DDTCIISLNPSYI	CIISLNPYI	3	0	0	7	1	CIISLNPSYI	tr_C9ZP82	0.153	9594.97	15.00
543	HLA-A0201	ELYVSEVLPYGF	YVSEVLPYF	2	0	0	8	1	YVSEVLPYGF	tr_C9ZP82	0.152	9605.56	15.00
62	HLA-A0201	SELELLVQTVQP	SLELLVQTV	0	0	0	1	1	SELELLVQTV	tr_C9ZP82	0.152	9627.20	15.00
409	HLA-A0201	GFEYFDISV	GFEYFDISV	0	0	0	0	0	GFEYFDISV	tr_C9ZP82	0.152	9638.36	15.00
703	HLA-A0201	STPPDTLVQL	STPDTLVQL	0	0	0	2	1	STPPDTLVQL	tr_C9ZP82	0.151	9708.27	15.00
564	HLA-A0201	LPQLQRLFEAYA	QLQRLFAYA	2	0	0	6	1	QLQRLFEAYA	tr_C9ZP82	0.151	9710.58	15.00
23	HLA-A0201	SSVHGLYIDL	SVHGLYIDL	0	0	0	1	1	SSVHGLYIDL	tr_C9ZP82	0.151	9714.26	15.00
795	HLA-A0201	KLDFHLVITSNKT	KLFHLVITS	0	0	0	2	1	KLDFHLVITS	tr_C9ZP82	0.151	9720.88	15.00
622	HLA-A0201	STIDDRWRDTYL	STIDDRWYL	0	0	0	7	3	STIDDRWRDTYL	tr_C9ZP82	0.151	9721.51	15.00
113	HLA-A0201	TYMPNKSGMA	YMPNKSGMA	1	0	0	0	0	YMPNKSGMA	tr_C9ZP82	0.151	9724.56	15.00
515	HLA-A0201	VLSILLGNDF	VLSILLNDF	0	0	0	6	1	VLSILLGNDF	tr_C9ZP82	0.151	9747.21	15.00
352	HLA-A0201	IDDTCIISL	IDDTCIISL	0	0	0	0	0	IDDTCIISL	tr_C9ZP82	0.151	9764.84	15.00
0	HLA-A0201	MGVPKFASWL	MVPKFASWL	0	0	0	1	1	MGVPKFASWL	tr_C9ZP82	0.151	9781.13	15.00
352	HLA-A0201	IDDTCIISLNPSYI	CIISLNPYI	4	0	0	7	1	CIISLNPSYI	tr_C9ZP82	0.150	9828.34	15.00
676	HLA-A0201	YHAPPVIDLA	YAPPVIDLA	0	0	0	1	1	YHAPPVIDLA	tr_C9ZP82	0.150	9836.85	15.00
247	HLA-A0201	MLNEKMSGNDPTWT	MLNEKMTWT	0	0	0	6	5	MLNEKMSGNDPTWT	tr_C9ZP82	0.150	9841.85	15.00
524	HLA-A0201	FLPHAPSAFSG	FLAPSAFSG	0	0	0	2	2	FLPHAPSAFSG	tr_C9ZP82	0.150	9856.98	15.00
300	HLA-A0201	IVGLDADLI	IVGLDADLI	0	0	0	0	0	IVGLDADLI	tr_C9ZP82	0.150	9858.26	15.00
407	HLA-A0201	NTGFEYFDISV	TGFEYFISV	1	0	0	6	1	TGFEYFDISV	tr_C9ZP82	0.150	9860.18	15.00
75	HLA-A0201	EVMYIAVDGVAPR	VMYIAVDGV	1	0	0	0	0	VMYIAVDGV	tr_C9ZP82	0.150	9912.92	15.00
542	HLA-A0201	LELYVSEVLPYGF	YVSEVLPYF	3	0	0	8	1	YVSEVLPYGF	tr_C9ZP82	0.149	9932.13	15.00
678	HLA-A0201	APPVIDLAIYLKE	AVIDLAIYL	0	0	0	1	2	APPVIDLAIYL	tr_C9ZP82	0.149	9933.96	15.00
809	HLA-A0201	FVPKGDLRKLSDAL	FLRKLSDAL	0	0	0	1	5	FVPKGDLRKLSDAL	tr_C9ZP82	0.149	9938.37	15.00
680	HLA-A0201	PVIDLAIYLKEANI	VIDLAIYLI	1	0	0	8	4	VIDLAIYLKEANI	tr_C9ZP82	0.149	9952.03	15.00
134	HLA-A0201	TEMAQANDDLDEV	AQADDLDEV	3	0	0	3	1	AQANDDLDEV	tr_C9ZP82	0.149	10007.32	15.00
612	HLA-A0201	TVADVAVALH	TVADVAVAL	0	0	0	0	0	TVADVAVAL	tr_C9ZP82	0.148	10030.30	15.00
771	HLA-A0201	MDVLQQVVDA	MVLQQVVDA	0	0	0	1	1	MDVLQQVVDA	tr_C9ZP82	0.148	10033.67	15.00
747	HLA-A0201	LQWNVDYHDS	LQWNVDYHS	0	0	0	8	1	LQWNVDYHDS	tr_C9ZP82	0.148	10065.42	15.00
817	HLA-A0201	KLSDALDTILRR	KLSDALDTL	0	0	0	8	1	KLSDALDTIL	tr_C9ZP82	0.148	10075.44	15.00
24	HLA-A0201	SVHGLYIDLNGIIH	SLYIDLNGI	0	0	0	1	3	SVHGLYIDLNGI	tr_C9ZP82	0.148	10101.96	15.00
421	HLA-A0201	SLISEVSALCD	SLIEVSALC	0	0	0	3	1	SLISEVSALC	tr_C9ZP82	0.148	10122.75	15.00
227	HLA-A0201	SNCISPGTDFMAKV	CISDFMAKV	2	0	0	3	3	CISPGTDFMAKV	tr_C9ZP82	0.147	10140.84	15.00
60	HLA-A0201	ICSELELLVQ	ICSELELLV	0	0	0	0	0	ICSELELLV	tr_C9ZP82	0.147	10153.90	15.00
520	HLA-A0201	LGNDFLPHA	LGNDFLPHA	0	0	0	0	0	LGNDFLPHA	tr_C9ZP82	0.147	10185.93	15.00

17	HLA-A0201	VTKNCPSSV	VTKNCPSSV	0	0	0	0	0	VTKNCPSSV	tr_C9ZP82	0.147	10187.47	15.00
756	HLA-A0201	SNVVHLATV	SNVVHLATV	0	0	0	0	0	SNVVHLATV	tr_C9ZP82	0.147	10224.24	16.00
29	HLA-A0201	YIDLNGIIHPCCHN	YLNGIIHPC	0	0	0	1	2	YIDLNGIIHPC	tr_C9ZP82	0.146	10248.94	16.00
795	HLA-A0201	KLDFHLVITSNKTS	KLFHLVITS	0	0	0	2	1	KLDFHLVITS	tr_C9ZP82	0.146	10255.82	16.00
26	HLA-A0201	HGLYIDLNGIIH	GLYIDLNGI	1	0	0	8	1	GLYIDLNGII	tr_C9ZP82	0.146	10289.27	16.00
708	HLA-A0201	TLVQLLSILPP	TLLLSILPP	0	0	0	2	2	TLVQLLSILPP	tr_C9ZP82	0.146	10301.30	16.00
633	HLA-A0201	LKSTGLSNSVQEA	LLSNSVQEA	0	0	0	1	4	LKSTGLSNSVQEA	tr_C9ZP82	0.146	10301.64	16.00
61	HLA-A0201	CSELELLVQTVQP	SLELLVQTV	1	0	0	1	1	SELELLVQTV	tr_C9ZP82	0.146	10312.12	16.00
133	HLA-A0201	STEMAQANDDLDEV	AQADDLDEV	4	0	0	3	1	AQANDDLDEV	tr_C9ZP82	0.145	10365.37	16.00
649	HLA-A0201	YVEGVCFVWRYYTT	YVEGVCFTT	0	0	0	7	5	YVEGVCFVWRYYTT	tr_C9ZP82	0.145	10367.61	16.00
711	HLA-A0201	QLLSILPPTSH	QLLSILPPT	0	0	0	0	0	QLLSILPPT	tr_C9ZP82	0.145	10382.77	16.00
702	HLA-A0201	KSTPPDTLV	KSTPPDTLV	0	0	0	0	0	KSTPPDTLV	tr_C9ZP82	0.145	10399.86	16.00
421	HLA-A0201	SLISEVSALCDIK	SLISALCDI	0	0	0	3	3	SLISEVSALCDI	tr_C9ZP82	0.145	10410.33	16.00
510	HLA-A0201	IDDIIVLSILLGN	IIIVLSILL	0	0	0	1	2	IDDIIVLSILL	tr_C9ZP82	0.145	10419.57	16.00
773	HLA-A0201	VLQQVVDAAWPQLT	VLDAAWPQL	0	0	0	2	4	VLQQVVDAAWPQL	tr_C9ZP82	0.145	10420.92	16.00
815	HLA-A0201	LRKLSDALDTILR	KLSDALDTL	2	0	0	8	1	KLSDALDTIL	tr_C9ZP82	0.145	10427.23	16.00
364	HLA-A0201	YIDEEESDGVI	YIDEESDGV	0	0	0	3	1	YIDEEESDGV	tr_C9ZP82	0.145	10448.02	16.00
680	HLA-A0201	PVIDLAIYLKEA	ILAIYLKEA	2	0	0	1	1	IDLAIYLKEA	tr_C9ZP82	0.145	10453.33	16.00
150	HLA-A0201	LLEDPLYSGALY	LLEDPLYSL	0	0	0	8	2	LLEDPLYSGAL	tr_C9ZP82	0.145	10459.32	16.00
507	HLA-A0201	SKIIDDIIVLS	KIIDDIIVL	1	0	0	0	0	KIIDDIIVL	tr_C9ZP82	0.144	10473.14	16.00
510	HLA-A0201	IDDIIVLSI	IDDIIVLSI	0	0	0	0	0	IDDIIVLSI	tr_C9ZP82	0.144	10502.07	16.00
62	HLA-A0201	SELELLVQTVQPK	SLELLVQTV	0	0	0	1	1	SELELLVQTV	tr_C9ZP82	0.144	10505.94	16.00
553	HLA-A0201	GFLTKGPHEIN	FLTKGPHEI	1	0	0	0	0	FLTKGPHEI	tr_C9ZP82	0.144	10507.99	16.00
741	HLA-A0201	LESTFPLQWNVDY	LTFPLQWNV	0	0	0	1	2	LESTFPLQWNV	tr_C9ZP82	0.144	10510.94	16.00
25	HLA-A0201	VHGLYIDLNGIIH	VLYIDLNGI	0	0	0	1	2	VHGLYIDLNGI	tr_C9ZP82	0.144	10537.02	16.00
649	HLA-A0201	YVEGVCFVW	YVEGVCFVW	0	0	0	0	0	YVEGVCFVW	tr_C9ZP82	0.144	10553.10	16.00
282	HLA-A0201	FLRTQSSLAQF	FLQSSLAQF	0	0	0	2	2	FLRTQSSLAQF	tr_C9ZP82	0.144	10573.45	16.00
66	HLA-A0201	LLVQTVQPK	LLVQTVQPK	0	0	0	0	0	LLVQTVQPK	tr_C9ZP82	0.144	10576.08	16.00
860	HLA-A0201	PRLSYGADGQV	RLYGADGQV	1	0	0	2	1	RLSYGADGQV	tr_C9ZP82	0.144	10576.66	16.00
512	HLA-A0201	DIIVLSILLG	IIVLSILLG	1	0	0	0	0	IIVLSILLG	tr_C9ZP82	0.143	10593.83	16.00
653	HLA-A0201	VCFVWRYYTT	VCFVWYYTT	0	0	0	5	1	VCFVWRYYTT	tr_C9ZP82	0.143	10621.96	16.00
654	HLA-A0201	CFVWRYYTTTT	FVWYTTTTT	1	0	0	3	2	FVWRYYTTTT	tr_C9ZP82	0.143	10633.80	16.00
724	HLA-A0201	LPMVLGEVMVR	LMVLGEVMV	0	0	0	1	1	LPMVLGEVMV	tr_C9ZP82	0.143	10642.20	16.00
639	HLA-A0201	SNSVQEACEKYV	SVQEACEYV	2	0	0	7	1	SVQEACEKYV	tr_C9ZP82	0.143	10667.33	16.00
798	HLA-A0201	FHLVITSNKTSFVP	HLVITTSFV	1	0	0	5	3	HLVITSNKTSFV	tr_C9ZP82	0.142	10770.32	16.00
679	HLA-A0201	PPVIDLAIYLKEA	ILAIYLKEA	3	0	0	1	1	IDLAIYLKEA	tr_C9ZP82	0.142	10770.66	16.00
28	HLA-A0201	LYIDLNGIIHPCCH	YLNGIIHPC	1	0	0	1	2	YIDLNGIIHPC	tr_C9ZP82	0.142	10775.10	16.00
794	HLA-A0201	QKLDFHLVITSNK	KLFHLVITS	1	0	0	2	1	KLDFHLVITS	tr_C9ZP82	0.142	10780.46	16.00
821	HLA-A0201	ALDTILRRVPS	ALDTILVPS	0	0	0	6	2	ALDTILRRVPS	tr_C9ZP82	0.141	10826.86	16.00
731	HLA-A0201	VMVRSPQQAEL	VMSPQQAEL	0	0	0	2	2	VMVRSPQQAEL	tr_C9ZP82	0.141	10830.38	16.00
225	HLA-A0201	FDSNCISPGTDFM	FISPGTDFM	0	0	0	1	4	FDSNCISPGTDFM	tr_C9ZP82	0.141	10873.00	16.00
824	HLA-A0201	TILRRVPSREGV	TILRRVPSV	0	0	0	8	3	TILRRVPSREGV	tr_C9ZP82	0.141	10874.18	16.00
715	HLA-A0201	ILPPTSHALLP	ILPPTSALL	0	0	0	6	1	ILPPTSHALL	tr_C9ZP82	0.141	10878.18	16.00
743	HLA-A0201	STFPLQWNVDYH	STFPLQWNV	0	0	0	0	0	STFPLQWNV	tr_C9ZP82	0.141	10903.39	16.00
800	HLA-A0201	LVITSNKTSFV	LVITSTSFV	0	0	0	5	2	LVITSNKTSFV	tr_C9ZP82	0.140	10956.26	16.00
523	HLA-A0201	DFLPHAPSAF	FLPHAPSAF	1	0	0	0	0	FLPHAPSAF	tr_C9ZP82	0.140	10965.03	16.00
718	HLA-A0201	PTSHALLPMV	TSHALLPMV	1	0	0	0	0	TSHALLPMV	tr_C9ZP82	0.140	10966.69	16.00
814	HLA-A0201	DLRKLSDALDTILR	KLSDALDTL	3	0	0	8	1	KLSDALDTIL	tr_C9ZP82	0.140	10978.21	16.00
26	HLA-A0201	HGLYIDLNGIIHP	GLYIDLNII	1	0	0	7	1	GLYIDLNGII	tr_C9ZP82	0.140	11032.51	16.00
638	HLA-A0201	LSNSVQEACEKYV	SVQEACEYV	3	0	0	7	1	SVQEACEKYV	tr_C9ZP82	0.140	11043.26	16.00
62	HLA-A0201	SELELLVQTVQPKE	SLELLVQTV	0	0	0	1	1	SELELLVQTV	tr_C9ZP82	0.140	11048.52	16.00
533	HLA-A0201	SGESALDNLLEL	SALDNLLEL	0	0	0	1	3	SGESALDNLLEL	tr_C9ZP82	0.139	11068.86	16.00
707	HLA-A0201	DTLVQLLSILP	TLVQLLSIL	1	0	0	0	0	TLVQLLSIL	tr_C9ZP82	0.139	11099.56	16.00
552	HLA-A0201	YGFLTKGPHEIN	FLTKGPHEI	2	0	0	0	0	FLTKGPHEI	tr_C9ZP82	0.139	11101.12	16.00

543	HLA-A0201	ELYVSEVLPY	ELYVSVLPY	0	0	0	5	1	ELYVSEVLPY	tr_C9ZP82	0.139	11101.12	16.00
59	HLA-A0201	CICSELELLVQ	CICELELLV	0	0	0	3	1	CICSELELLV	tr_C9ZP82	0.139	11103.16	16.00
61	HLA-A0201	CSELELLVQTVQPK	CLELLVQTV	0	0	0	1	2	CSELELLVQTV	tr_C9ZP82	0.139	11111.21	16.00
543	HLA-A0201	ELYVSEVLPYG	ELYEVLPYG	0	0	0	3	2	ELYVSEVLPYG	tr_C9ZP82	0.139	11116.14	16.00
570	HLA-A0201	LFEAYAKIEAVK	FAYAKIEAV	1	0	0	1	1	FEAYAKIEAV	tr_C9ZP82	0.139	11133.12	16.00
230	HLA-A0201	ISPGTDFMA	ISPGTDFMA	0	0	0	0	0	ISPGTDFMA	tr_C9ZP82	0.139	11146.37	17.00
579	HLA-A0201	AVKFRRFLLF	AVFRRFLLF	0	0	0	2	1	AVKFRRFLLF	tr_C9ZP82	0.139	11154.09	17.00
825	HLA-A0201	ILRRVPSREGV	ILVPSREGV	0	0	0	2	2	ILRRVPSREGV	tr_C9ZP82	0.139	11154.22	17.00
290	HLA-A0201	AQFGGKANHVIV	AQFGGHVIV	0	0	0	5	3	AQFGGKANHVIV	tr_C9ZP82	0.138	11187.82	17.00
548	HLA-A0201	EVLPYGFLT	EVLPYGFLT	0	0	0	0	0	EVLPYGFLT	tr_C9ZP82	0.138	11222.13	17.00
506	HLA-A0201	NSKIIDDIIVLS	KIIDDIIVL	2	0	0	0	0	KIIDDIIVL	tr_C9ZP82	0.138	11224.44	17.00
259	HLA-A0201	WTRLCVIFS	WTRLCVIFS	0	0	0	0	0	WTRLCVIFS	tr_C9ZP82	0.138	11269.09	17.00
635	HLA-A0201	STGLSNSVQEA	SLSNSVQEA	0	0	0	1	2	STGLSNSVQEA	tr_C9ZP82	0.138	11271.16	17.00
369	HLA-A0201	ESDGVIGVPV	ESDGVIVPV	0	0	0	6	1	ESDGVIGVPV	tr_C9ZP82	0.138	11290.94	17.00
25	HLA-A0201	VHGLYIDLNGIIHP	VLYIDLNGI	0	0	0	1	2	VHGLYIDLNGI	tr_C9ZP82	0.137	11302.30	17.00
794	HLA-A0201	QKLDFHLVITSNKT	KLFHLVITS	1	0	0	2	1	KLDFHLVITS	tr_C9ZP82	0.137	11303.65	17.00
8	HLA-A0201	WLRNKYPAIVT	WLNKYPAIV	0	0	0	2	1	WLRNKYPAIV	tr_C9ZP82	0.137	11305.37	17.00
71	HLA-A0201	VQPKEVMYIAV	VQPKEVMYV	0	0	0	8	2	VQPKEVMYIAV	tr_C9ZP82	0.137	11310.99	17.00
510	HLA-A0201	IDDIIVLSILLGND	IIIVLSILL	0	0	0	1	2	IDDIIVLSILL	tr_C9ZP82	0.137	11328.38	17.00
260	HLA-A0201	TRLCVIFSGS	RLCVIFSGS	1	0	0	0	0	RLCVIFSGS	tr_C9ZP82	0.137	11337.83	17.00
709	HLA-A0201	LVQLLSILPP	LVLLSILPP	0	0	0	2	1	LVQLLSILPP	tr_C9ZP82	0.137	11342.00	17.00
793	HLA-A0201	TQKLDFHLVITSNK	KLFHLVITS	2	0	0	2	1	KLDFHLVITS	tr_C9ZP82	0.137	11359.07	17.00
505	HLA-A0201	ANSKIIDDIIVLS	KIIDDIIVL	3	0	0	0	0	KIIDDIIVL	tr_C9ZP82	0.137	11371.86	17.00
707	HLA-A0201	DTLVQLLSILPP	TLLLSILPP	1	0	0	2	2	TLVQLLSILPP	tr_C9ZP82	0.137	11376.29	17.00
677	HLA-A0201	HAPPVIDLAI	HAPPVILAI	0	0	0	6	1	HAPPVIDLAI	tr_C9ZP82	0.137	11389.10	17.00
542	HLA-A0201	LELYVSEVLP	LLYVSEVLP	0	0	0	1	1	LELYVSEVLP	tr_C9ZP82	0.137	11400.94	17.00
307	HLA-A0201	LIFLALSLH	LIFLALSLH	0	0	0	0	0	LIFLALSLH	tr_C9ZP82	0.137	11406.36	17.00
294	HLA-A0201	GKANHVIVGL	KANHVIVGL	1	0	0	0	0	KANHVIVGL	tr_C9ZP82	0.137	11407.35	17.00
799	HLA-A0201	HLVITSNKTSFVPK	HLVITTSFV	0	0	0	5	3	HLVITSNKTSFV	tr_C9ZP82	0.136	11466.12	17.00
653	HLA-A0201	VCFVWRYYTTTTT	FVWYTTTTT	2	0	0	3	2	FVWRYYTTTTT	tr_C9ZP82	0.136	11466.25	17.00
513	HLA-A0201	IIVLSILLGNDF	IIVLSILLF	0	0	0	8	3	IIVLSILLGNDF	tr_C9ZP82	0.136	11476.30	17.00
440	HLA-A0201	ESAFDKKTLV	SAFDKKTLV	1	0	0	0	0	SAFDKKTLV	tr_C9ZP82	0.136	11481.27	17.00
640	HLA-A0201	NSVQEACEKYVEGV	SVQEYVEGV	1	0	0	4	4	SVQEACEKYVEGV	tr_C9ZP82	0.136	11485.00	17.00
510	HLA-A0201	IDDIIVLSIL	IDIIVLSIL	0	0	0	1	1	IDDIIVLSIL	tr_C9ZP82	0.136	11486.49	17.00
849	HLA-A0201	RPRTYSCTVEVP	RTYSCTVEV	0	0	0	1	2	RPRTYSCTVEV	tr_C9ZP82	0.136	11494.57	17.00
753	HLA-A0201	YHDSNVVHLATVML	YVHLATVML	0	0	0	1	5	YHDSNVVHLATVML	tr_C9ZP82	0.136	11503.53	17.00
237	HLA-A0201	MAKVSDAVLNML	MADAVLNML	0	0	0	2	3	MAKVSDAVLNML	tr_C9ZP82	0.135	11561.80	17.00
35	HLA-A0201	IIHPCCHNDSDTAI	IIHPCCHAI	0	0	0	7	5	IIHPCCHNDSDTAI	tr_C9ZP82	0.135	11574.44	17.00
669	HLA-A0201	WSWYYPFYHAP	WSWYYPFYA	0	0	0	8	1	WSWYYPFYHA	tr_C9ZP82	0.135	11585.60	17.00
314	HLA-A0201	LHITQVFIL	LHITQVFIL	0	0	0	0	0	LHITQVFIL	tr_C9ZP82	0.135	11603.28	17.00
677	HLA-A0201	HAPPVIDLAIYLKE	AVIDLAIYL	1	0	0	1	2	APPVIDLAIYL	tr_C9ZP82	0.135	11635.84	17.00
238	HLA-A0201	AKVSDAVLNML	KVSDVLNML	1	0	0	4	1	KVSDAVLNML	tr_C9ZP82	0.135	11654.11	17.00
77	HLA-A0201	MYIAVDGVAPRA	YIAGVAPRA	1	0	0	3	2	YIAVDGVAPRA	tr_C9ZP82	0.134	11677.59	17.00
292	HLA-A0201	FGGKANHVIVGL	FANHVIVGL	0	0	0	1	3	FGGKANHVIVGL	tr_C9ZP82	0.134	11684.67	17.00
818	HLA-A0201	LSDALDTILRRVP	LLDTILRRV	0	0	0	1	3	LSDALDTILRRV	tr_C9ZP82	0.134	11691.62	17.00
49	HLA-A0201	ALLPEEEKLE	ALLPEEEKL	0	0	0	0	0	ALLPEEEKL	tr_C9ZP82	0.134	11692.89	17.00
571	HLA-A0201	FEAYAKIEAVKF	FAYAKIEAV	0	0	0	1	1	FEAYAKIEAV	tr_C9ZP82	0.134	11695.54	17.00
406	HLA-A0201	PNTGFEYFDISV	TGFEYFISV	2	0	0	6	1	TGFEYFDISV	tr_C9ZP82	0.134	11720.37	17.00
3	HLA-A0201	PKFASWLRNKYPAI	FLRNKYPAI	2	0	0	1	3	FASWLRNKYPAI	tr_C9ZP82	0.134	11731.54	17.00
704	HLA-A0201	TPPDTLVQLLSILP	TLVQLLSIL	0	0	0	1	4	TPPDTLVQLLSIL	tr_C9ZP82	0.134	11737.89	17.00
140	HLA-A0201	NDDLDEVRQTL	NLDEVRQTL	0	0	0	1	2	NDDLDEVRQTL	tr_C9ZP82	0.134	11742.21	17.00
145	HLA-A0201	EVRQTLLEDPLYS	VLLEDPLYS	1	0	0	1	3	VRQTLLEDPLYS	tr_C9ZP82	0.134	11759.88	17.00
859	HLA-A0201	VPRLSYGADGQV	RLYGADGQV	2	0	0	2	1	RLSYGADGQV	tr_C9ZP82	0.134	11760.89	17.00
504	HLA-A0201	PANSKIIDDIIVLS	KIIDDIIVL	4	0	0	0	0	KIIDDIIVL	tr_C9ZP82	0.134	11764.46	17.00

448	HLA-A0201	LVSSAGYYF	LVSSAGYYF	0	0	0	0	0	LVSSAGYYF	tr_C9ZP82	0.134	11770.06	17.00
150	HLA-A0201	LLEDPLYSGALYN	LLYSGALYN	0	0	0	1	4	LLEDPLYSGALYN	tr_C9ZP82	0.134	11781.66	17.00
818	HLA-A0201	LSDALDTIL	LSDALDTIL	0	0	0	0	0	LSDALDTIL	tr_C9ZP82	0.133	11801.31	17.00
652	HLA-A0201	GVCFVWRYYTTTTT	FVWYTTTTT	3	0	0	3	2	FVWRYYTTTTT	tr_C9ZP82	0.133	11824.05	17.00
130	HLA-A0201	EFTSTEMAQA	FTSTEMAQA	1	0	0	0	0	FTSTEMAQA	tr_C9ZP82	0.133	11830.07	17.00
185	HLA-A0201	STDDDYEST	STDDDYEST	0	0	0	0	0	STDDDYEST	tr_C9ZP82	0.133	11854.80	17.00
538	HLA-A0201	LDNLLELYV	LDNLLELYV	0	0	0	0	0	LDNLLELYV	tr_C9ZP82	0.133	11860.06	17.00
118	HLA-A0201	KSGMAIVEEVVR	KMAIVEEVV	0	0	0	1	2	KSGMAIVEEVV	tr_C9ZP82	0.133	11876.37	17.00
251	HLA-A0201	KMSGNDPTW	KMSGNDPTW	0	0	0	0	0	KMSGNDPTW	tr_C9ZP82	0.133	11892.44	17.00
743	HLA-A0201	STFPLQWNVDYHD	STFPLQWNV	0	0	0	0	0	STFPLQWNV	tr_C9ZP82	0.133	11895.27	17.00
59	HLA-A0201	CICSELELLVQT	CILELLVQT	0	0	0	2	3	CICSELELLVQT	tr_C9ZP82	0.133	11908.03	17.00
745	HLA-A0201	FPLQWNVDYH	FLQWNVDYH	0	0	0	1	1	FPLQWNVDYH	tr_C9ZP82	0.133	11918.98	17.00
823	HLA-A0201	DTILRRVPSREGV	TILRRVPSV	1	0	0	8	3	TILRRVPSREGV	tr_C9ZP82	0.132	11933.30	17.00
512	HLA-A0201	DIIVLSILLGNDF	IIVLSILLF	1	0	0	8	3	IIVLSILLGNDF	tr_C9ZP82	0.132	11950.23	17.00
513	HLA-A0201	IIVLSILLGN	IIVLSILLG	0	0	0	0	0	IIVLSILLG	tr_C9ZP82	0.132	11956.69	17.00
551	HLA-A0201	PYGFLTKGPHEIN	FLTKGPHEI	3	0	0	0	0	FLTKGPHEI	tr_C9ZP82	0.132	11957.34	17.00
741	HLA-A0201	LESTFPLQWNVDYH	LTFPLQWNV	0	0	0	1	2	LESTFPLQWNV	tr_C9ZP82	0.132	11980.78	17.00
742	HLA-A0201	ESTFPLQWNVDY	STFPLQWNV	1	0	0	0	0	STFPLQWNV	tr_C9ZP82	0.132	12047.99	18.00
405	HLA-A0201	VPNTGFEYFDISV	TGFEYFISV	3	0	0	6	1	TGFEYFDISV	tr_C9ZP82	0.131	12057.11	18.00
123	HLA-A0201	IVEEVVREFT	IVEEVVEFT	0	0	0	6	1	IVEEVVREFT	tr_C9ZP82	0.131	12086.64	18.00
534	HLA-A0201	GESALDNLLEL	SALDNLLEL	2	0	0	0	0	SALDNLLEL	tr_C9ZP82	0.131	12095.53	18.00
706	HLA-A0201	PDTLVQLLSILP	TLVQLLSIL	2	0	0	0	0	TLVQLLSIL	tr_C9ZP82	0.131	12100.50	18.00
511	HLA-A0201	DDIIVLSILLGNDF	IIVLSILLF	2	0	0	8	3	IIVLSILLGNDF	tr_C9ZP82	0.131	12115.83	18.00
858	HLA-A0201	EVPRLSYGADGQV	RLYGADGQV	3	0	0	2	1	RLSYGADGQV	tr_C9ZP82	0.131	12119.24	18.00
281	HLA-A0201	DFLRTQSSLAQF	FLQSSLAQF	1	0	0	2	2	FLRTQSSLAQF	tr_C9ZP82	0.131	12122.25	18.00
421	HLA-A0201	SLISEVSALCDIKK	SLISALCDI	0	0	0	3	3	SLISEVSALCDI	tr_C9ZP82	0.131	12143.66	18.00
724	HLA-A0201	LPMVLGEVMVRS	LMVLGEVMV	0	0	0	1	1	LPMVLGEVMV	tr_C9ZP82	0.131	12149.04	18.00
119	HLA-A0201	SGMAIVEEVVR	SMAIVEEVV	0	0	0	1	1	SGMAIVEEVV	tr_C9ZP82	0.131	12154.70	18.00
550	HLA-A0201	LPYGFLTKGPHEIN	FLTKGPHEI	4	0	0	0	0	FLTKGPHEI	tr_C9ZP82	0.131	12156.80	18.00
707	HLA-A0201	DTLVQLLSI	DTLVQLLSI	0	0	0	0	0	DTLVQLLSI	tr_C9ZP82	0.130	12189.99	18.00
144	HLA-A0201	DEVRQTLLEDPLYS	VLLEDPLYS	2	0	0	1	3	VRQTLLEDPLYS	tr_C9ZP82	0.130	12208.60	18.00
523	HLA-A0201	DFLPHAPSAFSG	FLAPSAFSG	1	0	0	2	2	FLPHAPSAFSG	tr_C9ZP82	0.130	12229.63	18.00
705	HLA-A0201	PPDTLVQLLSILP	TLVQLLSIL	3	0	0	0	0	TLVQLLSIL	tr_C9ZP82	0.130	12285.86	18.00
621	HLA-A0201	HSTIDDRWRDTYL	STIDDRWYL	1	0	0	7	3	STIDDRWRDTYL	tr_C9ZP82	0.130	12297.03	18.00
447	HLA-A0201	TLVSSAGYYFFG	TLAGYYFFG	0	0	0	2	3	TLVSSAGYYFFG	tr_C9ZP82	0.130	12301.69	18.00
216	HLA-A0201	IVDDPTAFEF	IVDDTAFEF	0	0	0	4	1	IVDDPTAFEF	tr_C9ZP82	0.130	12310.21	18.00
683	HLA-A0201	DLAIYLKEANI	DLAIYLANI	0	0	0	6	2	DLAIYLKEANI	tr_C9ZP82	0.129	12330.20	18.00
758	HLA-A0201	VVHLATVMLP	VVLATVMLP	0	0	0	2	1	VVHLATVMLP	tr_C9ZP82	0.129	12330.74	18.00
857	HLA-A0201	VEVPRLSYGADGQV	RLYGADGQV	4	0	0	2	1	RLSYGADGQV	tr_C9ZP82	0.129	12334.21	18.00
653	HLA-A0201	VCFVWRYYTTT	FVWRYYTTT	2	0	0	0	0	FVWRYYTTT	tr_C9ZP82	0.129	12349.83	18.00
450	HLA-A0201	SSAGYYFFGQPNHI	SSAGYYFFI	0	0	0	8	5	SSAGYYFFGQPNHI	tr_C9ZP82	0.129	12352.90	18.00
623	HLA-A0201	TIDDRWRDTYL	TIDDRWTYL	0	0	0	6	2	TIDDRWRDTYL	tr_C9ZP82	0.129	12362.00	18.00
257	HLA-A0201	PTWTRLCVI	PTWTRLCVI	0	0	0	0	0	PTWTRLCVI	tr_C9ZP82	0.129	12371.77	18.00
816	HLA-A0201	RKLSDALDTILRR	KLSDALDTL	1	0	0	8	1	KLSDALDTIL	tr_C9ZP82	0.129	12373.91	18.00
727	HLA-A0201	VLGEVMVRSPQQAE	VLGEVMVQA	0	0	0	7	4	VLGEVMVRSPQQA	tr_C9ZP82	0.129	12376.71	18.00
813	HLA-A0201	GDLRKLSDAL	GLRKLSDAL	0	0	0	1	1	GDLRKLSDAL	tr_C9ZP82	0.129	12385.16	18.00
706	HLA-A0201	PDTLVQLLSILPP	TLLLSILPP	2	0	0	2	2	TLVQLLSILPP	tr_C9ZP82	0.129	12395.89	18.00
819	HLA-A0201	SDALDTILRRVP	SLDTILRRV	0	0	0	1	2	SDALDTILRRV	tr_C9ZP82	0.129	12397.76	18.00
579	HLA-A0201	AVKFRRFLL	AVKFRRFLL	0	0	0	0	0	AVKFRRFLL	tr_C9ZP82	0.129	12412.66	18.00
850	HLA-A0201	PRTYSCTVEVPRL	RTYSCTVEL	1	0	0	8	3	RTYSCTVEVPRL	tr_C9ZP82	0.129	12437.66	18.00
420	HLA-A0201	GSLISEVSALCD	SLIEVSALC	1	0	0	3	1	SLISEVSALC	tr_C9ZP82	0.129	12448.17	18.00
15	HLA-A0201	AIVTKNCPSSV	AIVNCPSSV	0	0	0	3	2	AIVTKNCPSSV	tr_C9ZP82	0.128	12480.80	18.00
822	HLA-A0201	LDTILRRVPSREGV	TILRRVPSV	2	0	0	8	3	TILRRVPSREGV	tr_C9ZP82	0.128	12493.91	18.00
514	HLA-A0201	IVLSILLGN	IVLSILLGN	0	0	0	0	0	IVLSILLGN	tr_C9ZP82	0.128	12533.17	18.00

743	HLA-A0201	STFPLQWNVDYHDS	STFPLQWNV	0	0	0	0	0	STFPLQWNV	tr_C9ZP82	0.128	12560.46	18.00
50	HLA-A0201	LLPEEEKLECICSE	LLLECICSE	0	0	0	2	5	LLPEEEKLECICSE	tr_C9ZP82	0.128	12567.39	18.00
705	HLA-A0201	PPDTLVQLLSILPP	TLLLSILPP	3	0	0	2	2	TLVQLLSILPP	tr_C9ZP82	0.128	12582.90	18.00
652	HLA-A0201	GVCFVWRYYTTT	FVWRYYTTT	3	0	0	0	0	FVWRYYTTT	tr_C9ZP82	0.128	12584.40	18.00
549	HLA-A0201	VLPYGFLTK	VLPYGFLTK	0	0	0	0	0	VLPYGFLTK	tr_C9ZP82	0.127	12587.26	18.00
131	HLA-A0201	FTSTEMAQANDDL	FMAQANDDL	0	0	0	1	4	FTSTEMAQANDDL	tr_C9ZP82	0.127	12592.71	18.00
737	HLA-A0201	QQAELESTFPLQW	QQLESTFPL	0	0	0	2	2	QQAELESTFPL	tr_C9ZP82	0.127	12612.76	18.00
420	HLA-A0201	GSLISEVSALCDIK	SLISALCDI	1	0	0	3	3	SLISEVSALCDI	tr_C9ZP82	0.127	12616.85	18.00
542	HLA-A0201	LELYVSEVLPYG	YVSEVLPYG	3	0	0	0	0	YVSEVLPYG	tr_C9ZP82	0.127	12627.23	18.00
820	HLA-A0201	DALDTILRRVPS	ALDTILVPS	1	0	0	6	2	ALDTILRRVPS	tr_C9ZP82	0.127	12638.99	18.00
851	HLA-A0201	RTYSCTVEVPR	RTYSCTVEV	0	0	0	0	0	RTYSCTVEV	tr_C9ZP82	0.127	12640.22	18.00
791	HLA-A0201	WRTQKLDFHLVIT	WLDFHLVIT	0	0	0	1	4	WRTQKLDFHLVIT	tr_C9ZP82	0.127	12663.08	18.00
820	HLA-A0201	DALDTILRRVP	ALDTILRRV	1	0	0	0	0	ALDTILRRV	tr_C9ZP82	0.127	12689.41	18.00
736	HLA-A0201	PQQAELESTFPLQ	QQLESTFPL	1	0	0	2	2	QQAELESTFPL	tr_C9ZP82	0.126	12722.40	18.00
304	HLA-A0201	DADLIFLAL	DADLIFLAL	0	0	0	0	0	DADLIFLAL	tr_C9ZP82	0.126	12725.99	18.00
282	HLA-A0201	FLRTQSSLAQ	FLRTQSSLA	0	0	0	0	0	FLRTQSSLA	tr_C9ZP82	0.126	12757.69	18.00
682	HLA-A0201	IDLAIYLKEANI	DLAIYLANI	1	0	0	6	2	DLAIYLKEANI	tr_C9ZP82	0.126	12777.86	18.00
159	HLA-A0201	ALYNEGEEEAG	ALYEGEEEA	0	0	0	3	1	ALYNEGEEEA	tr_C9ZP82	0.126	12780.35	18.00
58	HLA-A0201	ECICSELELLVQ	CICELELLV	1	0	0	3	1	CICSELELLV	tr_C9ZP82	0.126	12804.57	18.00
454	HLA-A0201	YYFFGQPNHI	YFFGQPNHI	0	0	0	1	1	YYFFGQPNHI	tr_C9ZP82	0.126	12843.14	18.00
305	HLA-A0201	ADLIFLALS	ADLIFLALS	0	0	0	0	0	ADLIFLALS	tr_C9ZP82	0.126	12845.92	18.00
610	HLA-A0201	NVTVADVAVAL	VVADVAVAL	1	0	0	1	1	VTVADVAVAL	tr_C9ZP82	0.126	12851.76	18.00
784	HLA-A0201	QLTEEERWRT	QLTEEEWRT	0	0	0	6	1	QLTEEERWRT	tr_C9ZP82	0.125	12862.89	18.00
651	HLA-A0201	EGVCFVWRYYTTT	FVWRYYTTT	4	0	0	0	0	FVWRYYTTT	tr_C9ZP82	0.125	12865.39	18.00
253	HLA-A0201	SGNDPTWTRLCVI	STWTRLCVI	0	0	0	1	4	SGNDPTWTRLCVI	tr_C9ZP82	0.125	12871.94	18.00
815	HLA-A0201	LRKLSDALDTILRR	LLSDALDTI	0	0	0	1	2	LRKLSDALDTI	tr_C9ZP82	0.125	12921.90	18.00
522	HLA-A0201	NDFLPHAPSAFSG	FLAPSAFSG	2	0	0	2	2	FLPHAPSAFSG	tr_C9ZP82	0.125	12930.01	19.00
850	HLA-A0201	PRTYSCTVEVP	RTYSCTVEV	1	0	0	0	0	RTYSCTVEV	tr_C9ZP82	0.125	12965.73	19.00
50	HLA-A0201	LLPEEEKLEC	LLPEEELEC	0	0	0	6	1	LLPEEEKLEC	tr_C9ZP82	0.125	12980.33	19.00
650	HLA-A0201	VEGVCFVWRYYTTT	FVWRYYTTT	5	0	0	0	0	FVWRYYTTT	tr_C9ZP82	0.125	12991.71	19.00
280	HLA-A0201	IDFLRTQSSLAQF	FLQSSLAQF	2	0	0	2	2	FLRTQSSLAQF	tr_C9ZP82	0.125	12992.97	19.00
753	HLA-A0201	YHDSNVVHL	YHDSNVVHL	0	0	0	0	0	YHDSNVVHL	tr_C9ZP82	0.125	12995.09	19.00
702	HLA-A0201	KSTPPDTLVQLLSI	KTLVQLLSI	0	0	0	1	5	KSTPPDTLVQLLSI	tr_C9ZP82	0.124	13015.21	19.00
849	HLA-A0201	RPRTYSCTVEVPRL	RTYSCTVEL	2	0	0	8	3	RTYSCTVEVPRL	tr_C9ZP82	0.124	13018.02	19.00
289	HLA-A0201	LAQFGGKANHV	AQFGGANHV	1	0	0	5	1	AQFGGKANHV	tr_C9ZP82	0.124	13019.01	19.00
278	HLA-A0201	KIIDFLRTQ	KIIDFLRTQ	0	0	0	0	0	KIIDFLRTQ	tr_C9ZP82	0.124	13020.98	19.00
648	HLA-A0201	KYVEGVCFVW	YVEGVCFVW	1	0	0	0	0	YVEGVCFVW	tr_C9ZP82	0.124	13051.59	19.00
418	HLA-A0201	VGGSLISEVSALCD	SLIEVSALC	3	0	0	3	1	SLISEVSALC	tr_C9ZP82	0.124	13074.91	19.00
571	HLA-A0201	FEAYAKIEAVKFR	FAYAKIEAV	0	0	0	1	1	FEAYAKIEAV	tr_C9ZP82	0.124	13083.54	19.00
67	HLA-A0201	LVQTVQPKEVMYIA	LVQEVMYIA	0	0	0	3	5	LVQTVQPKEVMYIA	tr_C9ZP82	0.124	13084.82	19.00
721	HLA-A0201	HALLPMVLGE	ALLPMVLGE	1	0	0	0	0	ALLPMVLGE	tr_C9ZP82	0.124	13100.83	19.00
670	HLA-A0201	SWYYPFYHAPPV	SWYYPFYPV	0	0	0	7	3	SWYYPFYHAPPV	tr_C9ZP82	0.124	13105.08	19.00
749	HLA-A0201	WNVDYHDSNV	WVDYHDSNV	0	0	0	1	1	WNVDYHDSNV	tr_C9ZP82	0.124	13108.20	19.00
106	HLA-A0201	QTGEVASTYM	QTGEVSTYM	0	0	0	5	1	QTGEVASTYM	tr_C9ZP82	0.124	13115.00	19.00
790	HLA-A0201	RWRTQKLDFHLVIT	KLDFHLVIT	5	0	0	0	0	KLDFHLVIT	tr_C9ZP82	0.124	13118.27	19.00
418	HLA-A0201	VGGSLISEV	VGGSLISEV	0	0	0	0	0	VGGSLISEV	tr_C9ZP82	0.124	13128.92	19.00
204	HLA-A0201	VVDDVFGHRGEV	VVDDVFGEV	0	0	0	6	3	VVDDVFGHRGEV	tr_C9ZP82	0.123	13158.93	19.00
279	HLA-A0201	IIDFLRTQSSLAQF	FLQSSLAQF	3	0	0	2	2	FLRTQSSLAQF	tr_C9ZP82	0.123	13160.78	19.00
447	HLA-A0201	TLVSSAGYYFF	TLVAGYYFF	0	0	0	3	2	TLVSSAGYYFF	tr_C9ZP82	0.123	13185.15	19.00
57	HLA-A0201	LECICSELEL	LCICSELEL	0	0	0	1	1	LECICSELEL	tr_C9ZP82	0.123	13206.57	19.00
703	HLA-A0201	STPPDTLVQLLSI	STLVQLLSI	0	0	0	1	4	STPPDTLVQLLSI	tr_C9ZP82	0.123	13222.01	19.00
669	HLA-A0201	WSWYYPFYH	WSWYYPFYH	0	0	0	0	0	WSWYYPFYH	tr_C9ZP82	0.123	13230.31	19.00
818	HLA-A0201	LSDALDTILRRVPS	ALDTILVPS	3	0	0	6	2	ALDTILRRVPS	tr_C9ZP82	0.123	13230.45	19.00
771	HLA-A0201	MDVLQQVVDAAW	MLQQVVDAA	0	0	0	1	2	MDVLQQVVDAA	tr_C9ZP82	0.123	13250.51	19.00

68	HLA-A0201	VQTVQPKEVMYIAV	VQTVMYIAV	0	0	0	3	5	VQTVQPKEVMYIAV	tr_C9ZP82	0.123	13256.10	19.00
363	HLA-A0201	SYIDEEESDGVI	YIDEESDGV	1	0	0	3	1	YIDEEESDGV	tr_C9ZP82	0.123	13258.68	19.00
5	HLA-A0201	FASWLRNKYPAIVT	WLNKYPAIV	3	0	0	2	1	WLRNKYPAIV	tr_C9ZP82	0.123	13264.85	19.00
825	HLA-A0201	ILRRVPSREGVEI	ILRRVPVEI	0	0	0	6	4	ILRRVPSREGVEI	tr_C9ZP82	0.123	13266.72	19.00
686	HLA-A0201	IYLKEANIKRM	YLKEANIRM	1	0	0	7	1	YLKEANIKRM	tr_C9ZP82	0.123	13271.74	19.00
576	HLA-A0201	KIEAVKFRRFL	KIEAVKFFL	0	0	0	7	2	KIEAVKFRRFL	tr_C9ZP82	0.122	13287.69	19.00
819	HLA-A0201	SDALDTILRRVPS	ALDTILVPS	2	0	0	6	2	ALDTILRRVPS	tr_C9ZP82	0.122	13311.87	19.00
521	HLA-A0201	GNDFLPHAPSAFSG	FLAPSAFSG	3	0	0	2	2	FLPHAPSAFSG	tr_C9ZP82	0.122	13352.69	19.00
419	HLA-A0201	GGSLISEVSALCD	SLIEVSALC	2	0	0	3	1	SLISEVSALC	tr_C9ZP82	0.122	13355.15	19.00
668	HLA-A0201	DWSWYYPFYHAP	WSWYYPFYA	1	0	0	8	1	WSWYYPFYHA	tr_C9ZP82	0.122	13374.38	19.00
609	HLA-A0201	DNVTVADVAVAL	VVADVAVAL	2	0	0	1	1	VTVADVAVAL	tr_C9ZP82	0.122	13379.01	19.00
141	HLA-A0201	DDLDEVRQTL	DLDEVRQTL	0	0	0	1	1	DDLDEVRQTL	tr_C9ZP82	0.122	13379.59	19.00
30	HLA-A0201	IDLNGIIHPCC	ILNGIIHPC	0	0	0	1	1	IDLNGIIHPC	tr_C9ZP82	0.122	13382.77	19.00
714	HLA-A0201	SILPPTSHALLP	ILPPTSALL	1	0	0	6	1	ILPPTSHALL	tr_C9ZP82	0.122	13407.27	19.00
70	HLA-A0201	TVQPKEVMYIAV	VQPKEVMYV	1	0	0	8	2	VQPKEVMYIAV	tr_C9ZP82	0.121	13436.31	19.00
793	HLA-A0201	TQKLDFHLVIT	KLDFHLVIT	2	0	0	0	0	KLDFHLVIT	tr_C9ZP82	0.121	13440.82	19.00
58	HLA-A0201	ECICSELELLVQT	CILELLVQT	1	0	0	2	3	CICSELELLVQT	tr_C9ZP82	0.121	13445.77	19.00
673	HLA-A0201	YPFYHAPPVI	YFYHAPPVI	0	0	0	1	1	YPFYHAPPVI	tr_C9ZP82	0.121	13478.83	19.00
792	HLA-A0201	RTQKLDFHLVIT	KLDFHLVIT	3	0	0	0	0	KLDFHLVIT	tr_C9ZP82	0.121	13487.73	19.00
117	HLA-A0201	NKSGMAIVEEVVR	KMAIVEEVV	1	0	0	1	2	KSGMAIVEEVV	tr_C9ZP82	0.121	13490.94	19.00
50	HLA-A0201	LLPEEEKLECIC	LLPEELECI	0	0	0	5	2	LLPEEEKLECI	tr_C9ZP82	0.121	13501.89	19.00
105	HLA-A0201	AQTGEVAST	AQTGEVAST	0	0	0	0	0	AQTGEVAST	tr_C9ZP82	0.121	13503.50	19.00
735	HLA-A0201	SPQQAELESTFPLQ	QQLESTFPL	2	0	0	2	2	QQAELESTFPL	tr_C9ZP82	0.121	13511.54	19.00
692	HLA-A0201	NIKRMPLPVV	NIKRMPLPV	0	0	0	8	1	NIKRMPLPVV	tr_C9ZP82	0.121	13516.22	19.00
719	HLA-A0201	TSHALLPMVL	TSHALLMVL	0	0	0	6	1	TSHALLPMVL	tr_C9ZP82	0.121	13545.79	19.00
203	HLA-A0201	QVVDDVFGHRGEVI	QVVDDVFEV	0	0	0	7	4	QVVDDVFGHRGEV	tr_C9ZP82	0.120	13580.43	19.00
247	HLA-A0201	MLNEKMSGNDPTW	MLMSGNDPT	0	0	0	2	3	MLNEKMSGNDPT	tr_C9ZP82	0.120	13591.15	19.00
742	HLA-A0201	ESTFPLQWNVDYH	STFPLQWNV	1	0	0	0	0	STFPLQWNV	tr_C9ZP82	0.120	13592.04	19.00
251	HLA-A0201	KMSGNDPTWTRLC	KMSGNDPTL	0	0	0	8	3	KMSGNDPTWTRL	tr_C9ZP82	0.120	13614.26	19.00
681	HLA-A0201	VIDLAIYLK	VIDLAIYLK	0	0	0	0	0	VIDLAIYLK	tr_C9ZP82	0.120	13657.05	19.00
224	HLA-A0201	EFDSNCISPGTDFM	FISPGTDFM	1	0	0	1	4	FDSNCISPGTDFM	tr_C9ZP82	0.120	13669.01	19.00
862	HLA-A0201	LSYGADGQV	LSYGADGQV	0	0	0	0	0	LSYGADGQV	tr_C9ZP82	0.120	13671.09	19.00
608	HLA-A0201	NDNVTVADVAVAL	VVADVAVAL	3	0	0	1	1	VTVADVAVAL	tr_C9ZP82	0.120	13673.61	19.00
730	HLA-A0201	EVMVRSPQQAEL	VMSPQQAEL	1	0	0	2	2	VMVRSPQQAEL	tr_C9ZP82	0.120	13678.34	19.00
57	HLA-A0201	LECICSELELLVQ	CICELELLV	2	0	0	3	1	CICSELELLV	tr_C9ZP82	0.120	13688.26	19.00
244	HLA-A0201	VLNMLNEKMS	VLNMLNEMS	0	0	0	7	1	VLNMLNEKMS	tr_C9ZP82	0.120	13690.78	19.00
8	HLA-A0201	WLRNKYPAIVTK	WLNKYPAIV	0	0	0	2	1	WLRNKYPAIV	tr_C9ZP82	0.120	13695.22	19.00
410	HLA-A0201	FEYFDISVVG	FEYFDISVV	0	0	0	0	0	FEYFDISVV	tr_C9ZP82	0.120	13701.15	19.00
364	HLA-A0201	YIDEEESDGVIG	YIDEESDGV	0	0	0	3	1	YIDEEESDGV	tr_C9ZP82	0.119	13738.71	19.00
298	HLA-A0201	HVIVGLDADLI	VIVGLDALI	1	0	0	7	1	VIVGLDADLI	tr_C9ZP82	0.119	13774.58	19.00
519	HLA-A0201	LLGNDFLPH	LLGNDFLPH	0	0	0	0	0	LLGNDFLPH	tr_C9ZP82	0.119	13796.06	19.00
620	HLA-A0201	LHSTIDDRWRDTYL	STIDDRWYL	2	0	0	7	3	STIDDRWRDTYL	tr_C9ZP82	0.119	13810.54	20.00
799	HLA-A0201	HLVITSNKT	HLVITSNKT	0	0	0	0	0	HLVITSNKT	tr_C9ZP82	0.119	13829.24	20.00
775	HLA-A0201	QQVVDAAWPQL	QVVDAAWPL	1	0	0	8	1	QVVDAAWPQL	tr_C9ZP82	0.119	13835.82	20.00
792	HLA-A0201	RTQKLDFHLVI	RTLDFHLVI	0	0	0	2	2	RTQKLDFHLVI	tr_C9ZP82	0.119	13837.62	20.00
122	HLA-A0201	AIVEEVVREF	AIVEEVVEF	0	0	0	7	1	AIVEEVVREF	tr_C9ZP82	0.119	13838.07	20.00
607	HLA-A0201	NNDNVTVADVAVAL	VVADVAVAL	4	0	0	1	1	VTVADVAVAL	tr_C9ZP82	0.119	13848.85	20.00
69	HLA-A0201	QTVQPKEVMYIAV	VQPKEVMYV	2	0	0	8	2	VQPKEVMYIAV	tr_C9ZP82	0.119	13872.10	20.00
566	HLA-A0201	QLQRLFEAYAK	QLQLFEAYA	0	0	0	3	1	QLQRLFEAYA	tr_C9ZP82	0.118	13874.80	20.00
671	HLA-A0201	WYYPFYHAPPV	WYYPFYPPV	0	0	0	6	2	WYYPFYHAPPV	tr_C9ZP82	0.118	13879.90	20.00
291	HLA-A0201	QFGGKANHVIVGL	FANHVIVGL	1	0	0	1	3	FGGKANHVIVGL	tr_C9ZP82	0.118	13882.91	20.00
159	HLA-A0201	ALYNEGEEEAGDA	ALYNEGEEA	0	0	0	8	4	ALYNEGEEEAGDA	tr_C9ZP82	0.118	13892.67	20.00
57	HLA-A0201	LECICSELELL	LICSELELL	0	0	0	1	2	LECICSELELL	tr_C9ZP82	0.118	13901.25	20.00
774	HLA-A0201	LQQVVDAAWPQL	QVVDAAWPL	2	0	0	8	1	QVVDAAWPQL	tr_C9ZP82	0.118	13911.78	20.00

7	HLA-A0201	SWLRNKYPAIVT	WLNKYPAIV	1	0	0	2	1	WLRNKYPAIV	tr_C9ZP82	0.118	13930.00	20.00
571	HLA-A0201	FEAYAKIEAVKFRR	FAYAKIEAV	0	0	0	1	1	FEAYAKIEAV	tr_C9ZP82	0.118	13932.26	20.00
750	HLA-A0201	NVDYHDSNVV	NVYHDSNVV	0	0	0	2	1	NVDYHDSNVV	tr_C9ZP82	0.118	13976.96	20.00
725	HLA-A0201	PMVLGEVMVRS	MLGEVMVRS	1	0	0	1	1	MVLGEVMVRS	tr_C9ZP82	0.118	14012.84	20.00
824	HLA-A0201	TILRRVPSREGVEI	ILRRVPVEI	1	0	0	6	4	ILRRVPSREGVEI	tr_C9ZP82	0.117	14078.34	20.00
316	HLA-A0201	ITQVFILRDT	ITQVFILRT	0	0	0	8	1	ITQVFILRDT	tr_C9ZP82	0.117	14089.47	20.00
289	HLA-A0201	LAQFGGKANHVIV	AQFGGHVIV	1	0	0	5	3	AQFGGKANHVIV	tr_C9ZP82	0.117	14122.89	20.00
724	HLA-A0201	LPMVLGEVMVRSP	LMVLGEVMV	0	0	0	1	1	LPMVLGEVMV	tr_C9ZP82	0.117	14127.02	20.00
737	HLA-A0201	QQAELESTFPLQWN	QQLESTFPL	0	0	0	2	2	QQAELESTFPL	tr_C9ZP82	0.117	14127.94	20.00
645	HLA-A0201	ACEKYVEGVCFV	ACVEGVCFV	0	0	0	2	3	ACEKYVEGVCFV	tr_C9ZP82	0.117	14158.99	20.00
542	HLA-A0201	LELYVSEVL	LELYVSEVL	0	0	0	0	0	LELYVSEVL	tr_C9ZP82	0.116	14195.81	20.00
57	HLA-A0201	LECICSELELLVQT	CILELLVQT	2	0	0	2	3	CICSELELLVQT	tr_C9ZP82	0.116	14208.72	20.00
667	HLA-A0201	SDWSWYYPFYHAP	WSWYYPFYA	2	0	0	8	1	WSWYYPFYHA	tr_C9ZP82	0.116	14228.26	20.00
261	HLA-A0201	RLCVIFSGSN	RLCVIFSGS	0	0	0	0	0	RLCVIFSGS	tr_C9ZP82	0.116	14239.35	20.00
514	HLA-A0201	IVLSILLGNDF	IVLSILLGF	0	0	0	8	2	IVLSILLGNDF	tr_C9ZP82	0.116	14247.83	20.00
570	HLA-A0201	LFEAYAKIEAVKF	FAYAKIEAV	1	0	0	1	1	FEAYAKIEAV	tr_C9ZP82	0.116	14268.04	20.00
677	HLA-A0201	HAPPVIDLA	HAPPVIDLA	0	0	0	0	0	HAPPVIDLA	tr_C9ZP82	0.116	14271.89	20.00
681	HLA-A0201	VIDLAIYLKEANIK	VIDLAIYLI	0	0	0	8	4	VIDLAIYLKEANI	tr_C9ZP82	0.116	14285.02	20.00
422	HLA-A0201	LISEVSALCDIK	LISEVSALI	0	0	0	8	2	LISEVSALCDI	tr_C9ZP82	0.116	14318.45	20.00
116	HLA-A0201	PNKSGMAIVEEVVR	SMAIVEEVV	3	0	0	1	1	SGMAIVEEVV	tr_C9ZP82	0.116	14320.46	20.00
669	HLA-A0201	WSWYYPFYHAPP	WSWYYPFYA	0	0	0	8	1	WSWYYPFYHA	tr_C9ZP82	0.115	14331.00	20.00
297	HLA-A0201	NHVIVGLDADLI	VIVGLDALI	2	0	0	7	1	VIVGLDADLI	tr_C9ZP82	0.115	14355.53	20.00
685	HLA-A0201	AIYLKEANIKRM	YLKEANIRM	2	0	0	7	1	YLKEANIKRM	tr_C9ZP82	0.115	14357.54	20.00
837	HLA-A0201	IRQLYDVPLER	ILYDVPLER	0	0	0	1	2	IRQLYDVPLER	tr_C9ZP82	0.115	14366.56	20.00
821	HLA-A0201	ALDTILRRVPSR	ALDTILRRV	0	0	0	0	0	ALDTILRRV	tr_C9ZP82	0.115	14369.98	20.00
532	HLA-A0201	FSGESALDNLL	FSGESALLL	0	0	0	7	2	FSGESALDNLL	tr_C9ZP82	0.115	14392.69	20.00
78	HLA-A0201	YIAVDGVAPR	YIADGVAPR	0	0	0	3	1	YIAVDGVAPR	tr_C9ZP82	0.115	14415.13	20.00
851	HLA-A0201	RTYSCTVEVPRLS	RTYSCTVEV	0	0	0	0	0	RTYSCTVEV	tr_C9ZP82	0.115	14438.08	20.00
412	HLA-A0201	YFDISVVGGSLI	YFDISVVLI	0	0	0	7	3	YFDISVVGGSLI	tr_C9ZP82	0.115	14448.08	20.00
755	HLA-A0201	DSNVVHLATV	DSNVVLATV	0	0	0	5	1	DSNVVHLATV	tr_C9ZP82	0.115	14448.40	20.00
770	HLA-A0201	DMDVLQQVVDAAW	MLQQVVDAA	1	0	0	1	2	MDVLQQVVDAA	tr_C9ZP82	0.115	14454.49	20.00
645	HLA-A0201	ACEKYVEGV	ACEKYVEGV	0	0	0	0	0	ACEKYVEGV	tr_C9ZP82	0.115	14466.70	20.00
604	HLA-A0201	KGSNNDNVTV	KSNNDNVTV	0	0	0	1	1	KGSNNDNVTV	tr_C9ZP82	0.115	14476.71	20.00
667	HLA-A0201	SDWSWYYPF	SDWSWYYPF	0	0	0	0	0	SDWSWYYPF	tr_C9ZP82	0.115	14483.30	20.00
666	HLA-A0201	DSDWSWYYPFYHAP	WSWYYPFYA	3	0	0	8	1	WSWYYPFYHA	tr_C9ZP82	0.114	14514.20	20.00
6	HLA-A0201	ASWLRNKYPAIVT	WLNKYPAIV	2	0	0	2	1	WLRNKYPAIV	tr_C9ZP82	0.114	14519.39	20.00
198	HLA-A0201	YVESNQVVDDV	YVNQVVDDV	0	0	0	2	2	YVESNQVVDDV	tr_C9ZP82	0.114	14529.92	20.00
713	HLA-A0201	LSILPPTSHA	LILPPTSHA	0	0	0	1	1	LSILPPTSHA	tr_C9ZP82	0.114	14542.96	20.00
680	HLA-A0201	PVIDLAIYLK	PVIDLAIYL	0	0	0	0	0	PVIDLAIYL	tr_C9ZP82	0.114	14545.65	20.00
739	HLA-A0201	AELESTFPLQ	AELESTFPL	0	0	0	0	0	AELESTFPL	tr_C9ZP82	0.114	14592.15	20.00
629	HLA-A0201	RDTYLKSTGL	RTYLKSTGL	0	0	0	1	1	RDTYLKSTGL	tr_C9ZP82	0.114	14594.67	20.00
55	HLA-A0201	EKLECICSELE	KLECICSEL	1	0	0	0	0	KLECICSEL	tr_C9ZP82	0.114	14606.20	20.00
742	HLA-A0201	ESTFPLQWNVDYHD	STFPLQWNV	1	0	0	0	0	STFPLQWNV	tr_C9ZP82	0.114	14617.43	20.00
761	HLA-A0201	LATVMLPFADM	ATVMLPFAM	1	0	0	8	1	ATVMLPFADM	tr_C9ZP82	0.114	14622.65	20.00
539	HLA-A0201	DNLLELYVSE	NLLELYVSE	1	0	0	0	0	NLLELYVSE	tr_C9ZP82	0.114	14639.59	20.00
282	HLA-A0201	FLRTQSSLAQFG	FLSSLAQFG	0	0	0	2	3	FLRTQSSLAQFG	tr_C9ZP82	0.113	14650.83	20.00
446	HLA-A0201	KTLVSSAGYYFFG	TLAGYYFFG	1	0	0	2	3	TLVSSAGYYFFG	tr_C9ZP82	0.113	14657.97	20.00
791	HLA-A0201	WRTQKLDFHLV	RTQKLDFLV	1	0	0	7	1	RTQKLDFHLV	tr_C9ZP82	0.113	14700.22	21.00
296	HLA-A0201	ANHVIVGLDADLI	VIVGLDALI	3	0	0	7	1	VIVGLDADLI	tr_C9ZP82	0.113	14756.96	21.00
682	HLA-A0201	IDLAIYLKEAN	ILAIYLKEA	0	0	0	1	1	IDLAIYLKEA	tr_C9ZP82	0.113	14760.47	21.00
713	HLA-A0201	LSILPPTSHALLP	ILPPTSALL	2	0	0	6	1	ILPPTSHALL	tr_C9ZP82	0.113	14763.02	21.00
147	HLA-A0201	RQTLLEDPLYSG	TLLEDLYSG	2	0	0	5	1	TLLEDPLYSG	tr_C9ZP82	0.113	14769.41	21.00
684	HLA-A0201	LAIYLKEANIKRM	YLKEANIRM	3	0	0	7	1	YLKEANIKRM	tr_C9ZP82	0.113	14772.29	21.00
408	HLA-A0201	TGFEYFDISVV	TGFEYFISV	0	0	0	6	1	TGFEYFDISV	tr_C9ZP82	0.113	14792.12	21.00

724	HLA-A0201	LPMVLGEVMVRSPQ	LMVLGEVMV	0	0	0	1	1	LPMVLGEVMV	tr_C9ZP82	0.113	14799.64	21.00
295	HLA-A0201	KANHVIVGLDADLI	VIVGLDALI	4	0	0	7	1	VIVGLDADLI	tr_C9ZP82	0.112	14809.74	21.00
118	HLA-A0201	KSGMAIVEEVVRE	KMAIVEEVV	0	0	0	1	2	KSGMAIVEEVV	tr_C9ZP82	0.112	14836.04	21.00
616	HLA-A0201	VAVALHSTI	VAVALHSTI	0	0	0	0	0	VAVALHSTI	tr_C9ZP82	0.112	14857.57	21.00
649	HLA-A0201	YVEGVCFVWRYY	YVEGVCFYY	0	0	0	7	3	YVEGVCFVWRYY	tr_C9ZP82	0.112	14861.10	21.00
254	HLA-A0201	GNDPTWTRLCVI	GTWTRLCVI	0	0	0	1	3	GNDPTWTRLCVI	tr_C9ZP82	0.112	14869.79	21.00
148	HLA-A0201	QTLLEDPLYSG	TLLEDLYSG	1	0	0	5	1	TLLEDPLYSG	tr_C9ZP82	0.112	14870.27	21.00
849	HLA-A0201	RPRTYSCTVEVPR	RTYSCTVEV	0	0	0	1	2	RPRTYSCTVEV	tr_C9ZP82	0.112	14888.94	21.00
35	HLA-A0201	IIHPCCHNDSDTA	IIHPCCHTA	0	0	0	7	4	IIHPCCHNDSDTA	tr_C9ZP82	0.112	14897.16	21.00
729	HLA-A0201	GEVMVRSPQQAEL	VMSPQQAEL	2	0	0	2	2	VMVRSPQQAEL	tr_C9ZP82	0.112	14917.01	21.00
261	HLA-A0201	RLCVIFSGSNT	RLCVIFSGT	0	0	0	8	2	RLCVIFSGSNT	tr_C9ZP82	0.112	14947.54	21.00
683	HLA-A0201	DLAIYLKEANIKRM	YLKEANIRM	4	0	0	7	1	YLKEANIKRM	tr_C9ZP82	0.112	14957.08	21.00
287	HLA-A0201	SSLAQFGGKA	SLAQFGGKA	0	0	0	1	1	SSLAQFGGKA	tr_C9ZP82	0.111	14964.53	21.00
150	HLA-A0201	LLEDPLYSGALYNE	LLEDPLYSL	0	0	0	8	2	LLEDPLYSGAL	tr_C9ZP82	0.111	14970.52	21.00
412	HLA-A0201	YFDISVVGGSL	YFDISVVSL	0	0	0	7	2	YFDISVVGGSL	tr_C9ZP82	0.111	14975.38	21.00
716	HLA-A0201	LPPTSHALLPMV	LSHALLPMV	0	0	0	1	3	LPPTSHALLPMV	tr_C9ZP82	0.111	14987.70	21.00
769	HLA-A0201	ADMDVLQQVVDAAW	MLQQVVDAA	2	0	0	1	2	MDVLQQVVDAA	tr_C9ZP82	0.111	15016.60	21.00
564	HLA-A0201	LPQLQRLFEAY	LQLQRLFEA	0	0	0	1	1	LPQLQRLFEA	tr_C9ZP82	0.111	15021.47	21.00
516	HLA-A0201	LSILLGNDFLP	LLLGNDFLP	0	0	0	1	2	LSILLGNDFLP	tr_C9ZP82	0.111	15098.05	21.00
407	HLA-A0201	NTGFEYFDI	NTGFEYFDI	0	0	0	0	0	NTGFEYFDI	tr_C9ZP82	0.111	15104.75	21.00
446	HLA-A0201	KTLVSSAGYYFF	TLVAGYYFF	1	0	0	3	2	TLVSSAGYYFF	tr_C9ZP82	0.111	15108.51	21.00
407	HLA-A0201	NTGFEYFDISVV	TGFEYFISV	1	0	0	6	1	TGFEYFDISV	tr_C9ZP82	0.111	15113.91	21.00
450	HLA-A0201	SSAGYYFFG	SSAGYYFFG	0	0	0	0	0	SSAGYYFFG	tr_C9ZP82	0.111	15118.48	21.00
46	HLA-A0201	TAIALLPEEEKL	TAIALLPEL	0	0	0	8	3	TAIALLPEEEKL	tr_C9ZP82	0.110	15156.15	21.00
68	HLA-A0201	VQTVQPKEVMYIA	VQTEVMYIA	0	0	0	3	4	VQTVQPKEVMYIA	tr_C9ZP82	0.110	15161.74	21.00
119	HLA-A0201	SGMAIVEEVVRE	SMAIVEEVV	0	0	0	1	1	SGMAIVEEVV	tr_C9ZP82	0.110	15181.60	21.00
649	HLA-A0201	YVEGVCFVWR	YVEGVCFWR	0	0	0	7	1	YVEGVCFVWR	tr_C9ZP82	0.110	15212.67	21.00
523	HLA-A0201	DFLPHAPSAFS	FLHAPSAFS	1	0	0	2	1	FLPHAPSAFS	tr_C9ZP82	0.110	15221.56	21.00
851	HLA-A0201	RTYSCTVEVPRLSY	RTYSCTVEV	0	0	0	0	0	RTYSCTVEV	tr_C9ZP82	0.110	15223.87	21.00
709	HLA-A0201	LVQLLSILP	LVQLLSILP	0	0	0	0	0	LVQLLSILP	tr_C9ZP82	0.110	15242.99	21.00
139	HLA-A0201	ANDDLDEVRQTLL	ALDEVRQTL	0	0	0	1	3	ANDDLDEVRQTL	tr_C9ZP82	0.110	15243.15	21.00
282	HLA-A0201	FLRTQSSLAQFGG	FLSLAQFGG	0	0	0	2	4	FLRTQSSLAQFGG	tr_C9ZP82	0.110	15258.49	21.00
410	HLA-A0201	FEYFDISVVGGSL	FISVVGGSL	0	0	0	1	4	FEYFDISVVGGSL	tr_C9ZP82	0.110	15277.16	21.00
652	HLA-A0201	GVCFVWRYYTT	GVCFVWYTT	0	0	0	6	2	GVCFVWRYYTT	tr_C9ZP82	0.109	15319.87	21.00
652	HLA-A0201	GVCFVWRYY	GVCFVWRYY	0	0	0	0	0	GVCFVWRYY	tr_C9ZP82	0.109	15326.17	21.00
564	HLA-A0201	LPQLQRLFEAYAK	LQLQRLFEA	0	0	0	1	1	LPQLQRLFEA	tr_C9ZP82	0.109	15328.66	21.00
146	HLA-A0201	VRQTLLEDPLYSG	TLLEDLYSG	3	0	0	5	1	TLLEDPLYSG	tr_C9ZP82	0.109	15331.64	21.00
247	HLA-A0201	MLNEKMSGN	MLNEKMSGN	0	0	0	0	0	MLNEKMSGN	tr_C9ZP82	0.109	15333.14	21.00
728	HLA-A0201	LGEVMVRSPQQAEL	VMSPQQAEL	3	0	0	2	2	VMVRSPQQAEL	tr_C9ZP82	0.109	15336.46	21.00
821	HLA-A0201	ALDTILRRVPSRE	ALDTILRRV	0	0	0	0	0	ALDTILRRV	tr_C9ZP82	0.109	15362.53	21.00
225	HLA-A0201	FDSNCISPGTDFMA	FISPGTDFM	0	0	0	1	4	FDSNCISPGTDFM	tr_C9ZP82	0.109	15376.67	21.00
31	HLA-A0201	DLNGIIHPCC	DLNGIIHPC	0	0	0	8	1	DLNGIIHPCC	tr_C9ZP82	0.109	15389.81	21.00
237	HLA-A0201	MAKVSDAVLNM	MVSDAVLNM	0	0	0	1	2	MAKVSDAVLNM	tr_C9ZP82	0.109	15391.65	21.00
362	HLA-A0201	PSYIDEEESDGVI	YIDEESDGV	2	0	0	3	1	YIDEEESDGV	tr_C9ZP82	0.109	15414.31	21.00
130	HLA-A0201	EFTSTEMAQANDDL	FMAQANDDL	1	0	0	1	4	FTSTEMAQANDDL	tr_C9ZP82	0.109	15414.97	21.00
839	HLA-A0201	QLYDVPLERYR	QLYDVPLYR	0	0	0	7	2	QLYDVPLERYR	tr_C9ZP82	0.109	15455.06	21.00
258	HLA-A0201	TWTRLCVIFS	WTRLCVIFS	1	0	0	0	0	WTRLCVIFS	tr_C9ZP82	0.108	15460.74	21.00
54	HLA-A0201	EEKLECICSELE	KLECICSEL	2	0	0	0	0	KLECICSEL	tr_C9ZP82	0.108	15486.03	21.00
61	HLA-A0201	CSELELLVQT	CSLELLVQT	0	0	0	2	1	CSELELLVQT	tr_C9ZP82	0.108	15512.52	21.00
393	HLA-A0201	SPIESREAHV	SIESREAHV	0	0	0	1	1	SPIESREAHV	tr_C9ZP82	0.108	15530.66	22.00
736	HLA-A0201	PQQAELESTFPLQW	QQAELTFPL	1	0	0	5	2	QQAELESTFPL	tr_C9ZP82	0.108	15562.62	22.00
445	HLA-A0201	KKTLVSSAGYYFFG	TLAGYYFFG	2	0	0	2	3	TLVSSAGYYFFG	tr_C9ZP82	0.108	15568.18	22.00
649	HLA-A0201	YVEGVCFVWRY	YVVCFVWRY	0	0	0	2	2	YVEGVCFVWRY	tr_C9ZP82	0.108	15569.53	22.00
118	HLA-A0201	KSGMAIVEEVVREF	KMAIVEEVV	0	0	0	1	2	KSGMAIVEEVV	tr_C9ZP82	0.108	15579.97	22.00

612	HLA-A0201	TVADVAVALHSTI	TVADVAVTI	0	0	0	7	4	TVADVAVALHSTI	tr_C9ZP82	0.108	15605.28	22.00
790	HLA-A0201	RWRTQKLDFHLV	RTQKLDFLV	2	0	0	7	1	RTQKLDFHLV	tr_C9ZP82	0.107	15631.47	22.00
839	HLA-A0201	QLYDVPLERYRP	QLYDVPLEP	0	0	0	8	3	QLYDVPLERYRP	tr_C9ZP82	0.107	15631.81	22.00
861	HLA-A0201	RLSYGADGQVI	RLYGADGQV	0	0	0	2	1	RLSYGADGQV	tr_C9ZP82	0.107	15634.86	22.00
771	HLA-A0201	MDVLQQVVDAAWP	MLQQVVDAA	0	0	0	1	2	MDVLQQVVDAA	tr_C9ZP82	0.107	15649.58	22.00
44	HLA-A0201	SDTAIALLPEEEKL	SLLPEEEKL	0	0	0	1	5	SDTAIALLPEEEKL	tr_C9ZP82	0.107	15667.87	22.00
416	HLA-A0201	SVVGGSLISEVS	SVVGLISEV	0	0	0	4	2	SVVGGSLISEV	tr_C9ZP82	0.107	15693.33	22.00
570	HLA-A0201	LFEAYAKIEAVKFR	FAYAKIEAV	1	0	0	1	1	FEAYAKIEAV	tr_C9ZP82	0.107	15697.57	22.00
47	HLA-A0201	AIALLPEEEKL	AIALLPEEL	0	0	0	8	2	AIALLPEEEKL	tr_C9ZP82	0.107	15707.26	22.00
119	HLA-A0201	SGMAIVEEVVREF	SMAIVEEVV	0	0	0	1	1	SGMAIVEEVV	tr_C9ZP82	0.107	15707.94	22.00
7	HLA-A0201	SWLRNKYPAIVTK	WLYPAIVTK	1	0	0	2	3	WLRNKYPAIVTK	tr_C9ZP82	0.107	15708.45	22.00
644	HLA-A0201	EACEKYVEGVCFV	ACVEGVCFV	1	0	0	2	3	ACEKYVEGVCFV	tr_C9ZP82	0.107	15710.15	22.00
681	HLA-A0201	VIDLAIYLKEAN	ILAIYLKEA	1	0	0	1	1	IDLAIYLKEA	tr_C9ZP82	0.107	15728.01	22.00
411	HLA-A0201	EYFDISVVGGSLI	YFDISVVLI	1	0	0	7	3	YFDISVVGGSLI	tr_C9ZP82	0.107	15732.09	22.00
575	HLA-A0201	AKIEAVKFRRFL	KIEAVKFFL	1	0	0	7	2	KIEAVKFRRFL	tr_C9ZP82	0.107	15745.37	22.00
120	HLA-A0201	GMAIVEEVVREFTS	GMAIVEFTS	0	0	0	5	5	GMAIVEEVVREFTS	tr_C9ZP82	0.107	15748.09	22.00
50	HLA-A0201	LLPEEEKLECICS	LLPEKLECI	0	0	0	3	2	LLPEEEKLECI	tr_C9ZP82	0.107	15752.02	22.00
361	HLA-A0201	NPSYIDEEESDGVI	YIDEESDGV	3	0	0	3	1	YIDEEESDGV	tr_C9ZP82	0.107	15791.44	22.00
53	HLA-A0201	EEEKLECICSELE	KLECICSEL	3	0	0	0	0	KLECICSEL	tr_C9ZP82	0.106	15803.57	22.00
837	HLA-A0201	IRQLYDVPL	IRQLYDVPL	0	0	0	0	0	IRQLYDVPL	tr_C9ZP82	0.106	15807.33	22.00
576	HLA-A0201	KIEAVKFRRFLL	KIEAVRFLL	0	0	0	5	3	KIEAVKFRRFLL	tr_C9ZP82	0.106	15819.99	22.00
445	HLA-A0201	KKTLVSSAGYYFF	TLVAGYYFF	2	0	0	3	2	TLVSSAGYYFF	tr_C9ZP82	0.106	15852.55	22.00
575	HLA-A0201	AKIEAVKFRRFLL	AKIEAVFLL	0	0	0	6	4	AKIEAVKFRRFLL	tr_C9ZP82	0.106	15859.93	22.00
246	HLA-A0201	NMLNEKMSGNDPTW	MLMSGNDPT	1	0	0	2	3	MLNEKMSGNDPT	tr_C9ZP82	0.106	15865.76	22.00
791	HLA-A0201	WRTQKLDFHLVI	TQLDFHLVI	2	0	0	2	1	TQKLDFHLVI	tr_C9ZP82	0.106	15879.15	22.00
317	HLA-A0201	TQVFILRDTT	TQVFILDTT	0	0	0	6	1	TQVFILRDTT	tr_C9ZP82	0.106	15886.72	22.00
446	HLA-A0201	KTLVSSAGYYF	TLVSSAYYF	1	0	0	6	1	TLVSSAGYYF	tr_C9ZP82	0.106	15900.99	22.00
259	HLA-A0201	WTRLCVIFSGSNT	RLCVIFSGT	2	0	0	8	2	RLCVIFSGSNT	tr_C9ZP82	0.106	15910.28	22.00
261	HLA-A0201	RLCVIFSGSNTPG	RLCVIFTPG	0	0	0	6	4	RLCVIFSGSNTPG	tr_C9ZP82	0.106	15912.70	22.00
171	HLA-A0201	ALNDGDGVNS	ALNDGDVNS	0	0	0	6	1	ALNDGDGVNS	tr_C9ZP82	0.106	15914.59	22.00
757	HLA-A0201	NVVHLATVM	NVVHLATVM	0	0	0	0	0	NVVHLATVM	tr_C9ZP82	0.106	15917.35	22.00
522	HLA-A0201	NDFLPHAPSAFS	FLHAPSAFS	2	0	0	2	1	FLPHAPSAFS	tr_C9ZP82	0.106	15936.47	22.00
821	HLA-A0201	ALDTILRRVPSREG	ALDTILRRV	0	0	0	0	0	ALDTILRRV	tr_C9ZP82	0.106	15939.24	22.00
769	HLA-A0201	ADMDVLQQVVD	AMDVLQQVV	0	0	0	1	1	ADMDVLQQVV	tr_C9ZP82	0.106	15946.13	22.00
120	HLA-A0201	GMAIVEEVVREF	GMAIVEEVV	0	0	0	0	0	GMAIVEEVV	tr_C9ZP82	0.106	15953.55	22.00
30	HLA-A0201	IDLNGIIHPCCH	ILNGIIHPC	0	0	0	1	1	IDLNGIIHPC	tr_C9ZP82	0.106	15965.64	22.00
230	HLA-A0201	ISPGTDFMAKV	ISPGTDFMV	0	0	0	8	2	ISPGTDFMAKV	tr_C9ZP82	0.105	15978.78	22.00
410	HLA-A0201	FEYFDISVVGGSLI	YFDISVVLI	2	0	0	7	3	YFDISVVGGSLI	tr_C9ZP82	0.105	15979.29	22.00
889	HLA-A0201	GVIEGKKSNSRVTV	GVIEGKVTV	0	0	0	6	5	GVIEGKKSNSRVTV	tr_C9ZP82	0.105	15990.54	22.00
52	HLA-A0201	PEEEKLECICSELE	KLECICSEL	4	0	0	0	0	KLECICSEL	tr_C9ZP82	0.105	15991.92	22.00
8	HLA-A0201	WLRNKYPAIVTKN	WLNKYPAIV	0	0	0	2	1	WLRNKYPAIV	tr_C9ZP82	0.105	15995.38	22.00
789	HLA-A0201	ERWRTQKLDFHLV	RTQKLDFLV	3	0	0	7	1	RTQKLDFHLV	tr_C9ZP82	0.105	15995.55	22.00
668	HLA-A0201	DWSWYYPFYHAPP	WSWYYPFYA	1	0	0	8	1	WSWYYPFYHA	tr_C9ZP82	0.105	16013.21	22.00
357	HLA-A0201	IISLNPSYID	IISLNPSYI	0	0	0	0	0	IISLNPSYI	tr_C9ZP82	0.105	16024.31	22.00
102	HLA-A0201	AAPAQTGEV	AAPAQTGEV	0	0	0	0	0	AAPAQTGEV	tr_C9ZP82	0.105	16031.59	22.00
250	HLA-A0201	EKMSGNDPTWTRLC	KMSGNDPTL	1	0	0	8	3	KMSGNDPTWTRL	tr_C9ZP82	0.105	16043.04	22.00
726	HLA-A0201	MVLGEVMVR	MVLGEVMVR	0	0	0	0	0	MVLGEVMVR	tr_C9ZP82	0.105	16047.39	22.00
158	HLA-A0201	GALYNEGEEEAG	ALYEGEEEA	1	0	0	3	1	ALYNEGEEEA	tr_C9ZP82	0.105	16050.86	22.00
777	HLA-A0201	VVDAAWPQLT	VVDAAWPQL	0	0	0	0	0	VVDAAWPQL	tr_C9ZP82	0.105	16051.73	22.00
120	HLA-A0201	GMAIVEEVVRE	GMAIVEEVV	0	0	0	0	0	GMAIVEEVV	tr_C9ZP82	0.105	16054.50	22.00
633	HLA-A0201	LKSTGLSNSV	LSTGLSNSV	0	0	0	1	1	LKSTGLSNSV	tr_C9ZP82	0.105	16067.19	22.00
68	HLA-A0201	VQTVQPKEVMYI	VQTVEVMYI	0	0	0	4	3	VQTVQPKEVMYI	tr_C9ZP82	0.105	16069.28	22.00
62	HLA-A0201	SELELLVQT	SELELLVQT	0	0	0	0	0	SELELLVQT	tr_C9ZP82	0.105	16095.73	22.00
127	HLA-A0201	VVREFTSTEM	VVREFTTEM	0	0	0	6	1	VVREFTSTEM	tr_C9ZP82	0.105	16108.28	22.00

444	HLA-A0201	DKKTLVSSAGYYFF	TLVAGYYFF	3	0	0	3	2	TLVSSAGYYFF	tr_C9ZP82	0.105	16123.44	22.00
417	HLA-A0201	VVGGSLISEVS	VVGGSLISV	0	0	0	8	1	VVGGSLISEV	tr_C9ZP82	0.105	16140.72	22.00
68	HLA-A0201	VQTVQPKEV	VQTVQPKEV	0	0	0	0	0	VQTVQPKEV	tr_C9ZP82	0.104	16144.92	22.00
177	HLA-A0201	GVNSFCVRST	GVNSFCVST	0	0	0	7	1	GVNSFCVRST	tr_C9ZP82	0.104	16153.65	22.00
370	HLA-A0201	SDGVIGVPV	SDGVIGVPV	0	0	0	0	0	SDGVIGVPV	tr_C9ZP82	0.104	16154.53	22.00
648	HLA-A0201	KYVEGVCFVWRYY	YVEGVCFYY	1	0	0	7	3	YVEGVCFVWRYY	tr_C9ZP82	0.104	16160.29	22.00
4	HLA-A0201	KFASWLRNKYPA	KFASWLYPA	0	0	0	6	3	KFASWLRNKYPA	tr_C9ZP82	0.104	16171.13	22.00
406	HLA-A0201	PNTGFEYFDISVV	FEYFDISVV	4	0	0	0	0	FEYFDISVV	tr_C9ZP82	0.104	16172.19	22.00
643	HLA-A0201	QEACEKYVEGVCFV	ACVEGVCFV	2	0	0	2	3	ACEKYVEGVCFV	tr_C9ZP82	0.104	16172.36	22.00
788	HLA-A0201	EERWRTQKLDFHLV	RTQKLDFLV	4	0	0	7	1	RTQKLDFHLV	tr_C9ZP82	0.104	16202.14	22.00
506	HLA-A0201	NSKIIDDIIV	NSIIDDIIV	0	0	0	2	1	NSKIIDDIIV	tr_C9ZP82	0.104	16212.13	22.00
543	HLA-A0201	ELYVSEVLP	ELYVSEVLP	0	0	0	0	0	ELYVSEVLP	tr_C9ZP82	0.104	16219.68	22.00
209	HLA-A0201	FGHRGEVIV	FGHRGEVIV	0	0	0	0	0	FGHRGEVIV	tr_C9ZP82	0.104	16241.11	22.00
448	HLA-A0201	LVSSAGYYFF	LVSAGYYFF	0	0	0	2	1	LVSSAGYYFF	tr_C9ZP82	0.104	16246.02	22.00
548	HLA-A0201	EVLPYGFLTKG	VLYGFLTKG	1	0	0	2	1	VLPYGFLTKG	tr_C9ZP82	0.104	16269.06	22.00
120	HLA-A0201	GMAIVEEVVREFT	GMAIVEEVV	0	0	0	0	0	GMAIVEEVV	tr_C9ZP82	0.104	16272.23	22.00
655	HLA-A0201	FVWRYYTTTTTD	FVWYYTTTT	0	0	0	3	1	FVWRYYTTTT	tr_C9ZP82	0.104	16280.51	22.00
651	HLA-A0201	EGVCFVWRYYT	GVCFVWYYT	1	0	0	6	1	GVCFVWRYYT	tr_C9ZP82	0.104	16282.45	22.00
655	HLA-A0201	FVWRYYTTTTTDS	FVWYYTTTT	0	0	0	3	1	FVWRYYTTTT	tr_C9ZP82	0.104	16282.80	22.00
819	HLA-A0201	SDALDTILRRVPSR	SLDTILRRV	0	0	0	1	2	SDALDTILRRV	tr_C9ZP82	0.104	16290.20	22.00
850	HLA-A0201	PRTYSCTVEVPR	RTYSCTVEV	1	0	0	0	0	RTYSCTVEV	tr_C9ZP82	0.104	16292.67	22.00
258	HLA-A0201	TWTRLCVIFSGSNT	RLCVIFSGT	3	0	0	8	2	RLCVIFSGSNT	tr_C9ZP82	0.103	16319.14	22.00
405	HLA-A0201	VPNTGFEYFDISVV	FEYFDISVV	5	0	0	0	0	FEYFDISVV	tr_C9ZP82	0.103	16334.68	22.00
820	HLA-A0201	DALDTILRRVPSR	ALDTILRRV	1	0	0	0	0	ALDTILRRV	tr_C9ZP82	0.103	16336.63	22.00
533	HLA-A0201	SGESALDNLLELY	SLDNLLELY	0	0	0	1	4	SGESALDNLLELY	tr_C9ZP82	0.103	16378.57	23.00
145	HLA-A0201	EVRQTLLEDPLYSG	TLLEDLYSG	4	0	0	5	1	TLLEDPLYSG	tr_C9ZP82	0.103	16379.98	23.00
6	HLA-A0201	ASWLRNKYPAIVTK	WLYPAIVTK	2	0	0	2	3	WLRNKYPAIVTK	tr_C9ZP82	0.103	16382.65	23.00
521	HLA-A0201	GNDFLPHAPSAFS	FLHAPSAFS	3	0	0	2	1	FLPHAPSAFS	tr_C9ZP82	0.103	16384.24	23.00
411	HLA-A0201	EYFDISVVGGSL	YFDISVVSL	1	0	0	7	2	YFDISVVGGSL	tr_C9ZP82	0.103	16385.66	23.00
511	HLA-A0201	DDIIVLSIL	DDIIVLSIL	0	0	0	0	0	DDIIVLSIL	tr_C9ZP82	0.103	16386.02	23.00
673	HLA-A0201	YPFYHAPPVIDLAI	YPFVIDLAI	0	0	0	3	5	YPFYHAPPVIDLAI	tr_C9ZP82	0.103	16390.10	23.00
101	HLA-A0201	RAAPAQTGEV	RAAPAQTEV	0	0	0	7	1	RAAPAQTGEV	tr_C9ZP82	0.103	16391.68	23.00
562	HLA-A0201	INLPQLQRLF	ILPQLQRLF	0	0	0	1	1	INLPQLQRLF	tr_C9ZP82	0.103	16408.90	23.00
756	HLA-A0201	SNVVHLATVML	SVHLATVML	0	0	0	1	2	SNVVHLATVML	tr_C9ZP82	0.103	16410.85	23.00
507	HLA-A0201	SKIIDDIIV	SKIIDDIIV	0	0	0	0	0	SKIIDDIIV	tr_C9ZP82	0.103	16416.53	23.00
117	HLA-A0201	NKSGMAIVEEVVRE	KMAIVEEVV	1	0	0	1	2	KSGMAIVEEVV	tr_C9ZP82	0.103	16421.87	23.00
119	HLA-A0201	SGMAIVEEVVREFT	SMAIVEEVV	0	0	0	1	1	SGMAIVEEVV	tr_C9ZP82	0.103	16424.18	23.00
65	HLA-A0201	ELLVQTVQPK	LLVQTVQPK	1	0	0	0	0	LLVQTVQPK	tr_C9ZP82	0.103	16435.20	23.00
137	HLA-A0201	AQANDDLDEVR	AQADDLDEV	0	0	0	3	1	AQANDDLDEV	tr_C9ZP82	0.103	16478.82	23.00
574	HLA-A0201	YAKIEAVKFRRFLL	AKIEAVFLL	1	0	0	6	4	AKIEAVKFRRFLL	tr_C9ZP82	0.102	16494.34	23.00
790	HLA-A0201	RWRTQKLDFHLVI	TQLDFHLVI	3	0	0	2	1	TQKLDFHLVI	tr_C9ZP82	0.102	16499.34	23.00
45	HLA-A0201	DTAIALLPEEEKL	TAIALLPEL	1	0	0	8	3	TAIALLPEEEKL	tr_C9ZP82	0.102	16539.20	23.00
685	HLA-A0201	AIYLKEANIK	AIYLKEANI	0	0	0	0	0	AIYLKEANI	tr_C9ZP82	0.102	16560.50	23.00
802	HLA-A0201	ITSNKTSFVP	ITSNKTSFV	0	0	0	0	0	ITSNKTSFV	tr_C9ZP82	0.102	16568.39	23.00
836	HLA-A0201	EIRQLYDVPLER	ILYDVPLER	1	0	0	1	2	IRQLYDVPLER	tr_C9ZP82	0.102	16573.59	23.00
520	HLA-A0201	LGNDFLPHAPSAFS	FLHAPSAFS	4	0	0	2	1	FLPHAPSAFS	tr_C9ZP82	0.102	16581.84	23.00
315	HLA-A0201	HITQVFILR	HITQVFILR	0	0	0	0	0	HITQVFILR	tr_C9ZP82	0.102	16584.54	23.00
574	HLA-A0201	YAKIEAVKFRRFL	KIEAVKFFL	2	0	0	7	2	KIEAVKFRRFL	tr_C9ZP82	0.102	16594.77	23.00
427	HLA-A0201	SALCDIKKFT	SLCDIKKFT	0	0	0	1	1	SALCDIKKFT	tr_C9ZP82	0.102	16595.30	23.00
530	HLA-A0201	SAFSGESALDNLL	FSGESALLL	2	0	0	7	2	FSGESALDNLL	tr_C9ZP82	0.102	16597.63	23.00
197	HLA-A0201	RYVESNQVVDDV	YVNQVVDDV	1	0	0	2	2	YVESNQVVDDV	tr_C9ZP82	0.102	16597.63	23.00
356	HLA-A0201	CIISLNPSYID	CIISLNPYI	0	0	0	7	1	CIISLNPSYI	tr_C9ZP82	0.102	16635.57	23.00
771	HLA-A0201	MDVLQQVVDAAWPQ	MLQQVVDAA	0	0	0	1	2	MDVLQQVVDAA	tr_C9ZP82	0.102	16652.14	23.00
647	HLA-A0201	EKYVEGVCFVWRYY	YVEGVCFYY	2	0	0	7	3	YVEGVCFVWRYY	tr_C9ZP82	0.102	16665.12	23.00

281	HLA-A0201	DFLRTQSSLAQFG	FLSSLAQFG	1	0	0	2	3	FLRTQSSLAQFG	tr_C9ZP82	0.102	16669.99	23.00
363	HLA-A0201	SYIDEEESDGVIG	YIDEESDGV	1	0	0	3	1	YIDEEESDGV	tr_C9ZP82	0.102	16671.25	23.00
511	HLA-A0201	DDIIVLSILLG	IIVLSILLG	2	0	0	0	0	IIVLSILLG	tr_C9ZP82	0.101	16688.76	23.00
259	HLA-A0201	WTRLCVIFSGS	WLCVIFSGS	0	0	0	1	2	WTRLCVIFSGS	tr_C9ZP82	0.101	16690.56	23.00
229	HLA-A0201	CISPGTDFMAKVS	CISDFMAKV	0	0	0	3	3	CISPGTDFMAKV	tr_C9ZP82	0.101	16694.90	23.00
260	HLA-A0201	TRLCVIFSGSNT	RLCVIFSGT	1	0	0	8	2	RLCVIFSGSNT	tr_C9ZP82	0.101	16709.35	23.00
644	HLA-A0201	EACEKYVEGV	EACEYVEGV	0	0	0	4	1	EACEKYVEGV	tr_C9ZP82	0.101	16709.90	23.00
409	HLA-A0201	GFEYFDISVVGGSL	FISVVGGSL	1	0	0	1	4	FEYFDISVVGGSL	tr_C9ZP82	0.101	16750.80	23.00
648	HLA-A0201	KYVEGVCFVWRY	YVVCFVWRY	1	0	0	2	2	YVEGVCFVWRY	tr_C9ZP82	0.101	16763.86	23.00
789	HLA-A0201	ERWRTQKLDFHLVI	TQLDFHLVI	4	0	0	2	1	TQKLDFHLVI	tr_C9ZP82	0.101	16774.21	23.00
704	HLA-A0201	TPPDTLVQLLSI	TTLVQLLSI	0	0	0	1	3	TPPDTLVQLLSI	tr_C9ZP82	0.101	16774.93	23.00
576	HLA-A0201	KIEAVKFRRFLLF	KIFRRFLLF	0	0	0	2	4	KIEAVKFRRFLLF	tr_C9ZP82	0.101	16777.83	23.00
8	HLA-A0201	WLRNKYPAIVTKNC	WLNKYPAIV	0	0	0	2	1	WLRNKYPAIV	tr_C9ZP82	0.101	16781.83	23.00
138	HLA-A0201	QANDDLDEVRQTLL	ALDEVRQTL	1	0	0	1	3	ANDDLDEVRQTL	tr_C9ZP82	0.101	16782.55	23.00
445	HLA-A0201	KKTLVSSAGYYF	TLVSSAYYF	2	0	0	6	1	TLVSSAGYYF	tr_C9ZP82	0.101	16783.83	23.00
667	HLA-A0201	SDWSWYYPFYHAPP	WSWYYPFYA	2	0	0	8	1	WSWYYPFYHA	tr_C9ZP82	0.101	16791.09	23.00
258	HLA-A0201	TWTRLCVIFSG	WTLCVIFSG	1	0	0	2	1	WTRLCVIFSG	tr_C9ZP82	0.101	16797.99	23.00
422	HLA-A0201	LISEVSALCD	LISEVSALC	0	0	0	0	0	LISEVSALC	tr_C9ZP82	0.101	16799.45	23.00
651	HLA-A0201	EGVCFVWRYYTT	GVCFVWYTT	1	0	0	6	2	GVCFVWRYYTT	tr_C9ZP82	0.101	16812.36	23.00
770	HLA-A0201	DMDVLQQVVDAAWP	MLQQVVDAA	1	0	0	1	2	MDVLQQVVDAA	tr_C9ZP82	0.101	16846.59	23.00
261	HLA-A0201	RLCVIFSGSNTP	RLCVIFNTP	0	0	0	6	3	RLCVIFSGSNTP	tr_C9ZP82	0.100	16857.71	23.00
836	HLA-A0201	EIRQLYDVPL	EIQLYDVPL	0	0	0	2	1	EIRQLYDVPL	tr_C9ZP82	0.100	16864.10	23.00
316	HLA-A0201	ITQVFILRDTT	ITQVFILTT	0	0	0	7	2	ITQVFILRDTT	tr_C9ZP82	0.100	16868.11	23.00
650	HLA-A0201	VEGVCFVWRYYT	GVCFVWYYT	2	0	0	6	1	GVCFVWRYYT	tr_C9ZP82	0.100	16876.51	23.00
546	HLA-A0201	VSEVLPYGFL	VSEVLPYFL	0	0	0	7	1	VSEVLPYGFL	tr_C9ZP82	0.100	16901.73	23.00
602	HLA-A0201	FRKGSNNDNVTV	FSNNDNVTV	0	0	0	1	3	FRKGSNNDNVTV	tr_C9ZP82	0.100	16922.04	23.00
573	HLA-A0201	AYAKIEAVKFRRFL	KIEAVKFFL	3	0	0	7	2	KIEAVKFRRFL	tr_C9ZP82	0.100	16923.13	23.00
565	HLA-A0201	PQLQRLFEAYAK	QLQRLFAYA	1	0	0	6	1	QLQRLFEAYA	tr_C9ZP82	0.100	16927.35	23.00
755	HLA-A0201	DSNVVHLATVML	VVHLATVML	3	0	0	0	0	VVHLATVML	tr_C9ZP82	0.100	16941.27	23.00
215	HLA-A0201	VIVDDPTAF	VIVDDPTAF	0	0	0	0	0	VIVDDPTAF	tr_C9ZP82	0.100	16952.82	23.00
542	HLA-A0201	LELYVSEVLPY	ELYVSVLPY	1	0	0	5	1	ELYVSEVLPY	tr_C9ZP82	0.100	16957.04	23.00
372	HLA-A0201	GVIGVPVCES	GVIGVVCES	0	0	0	5	1	GVIGVPVCES	tr_C9ZP82	0.100	16964.94	23.00
14	HLA-A0201	PAIVTKNCPSSV	AIVNCPSSV	1	0	0	3	2	AIVTKNCPSSV	tr_C9ZP82	0.100	16966.04	23.00
449	HLA-A0201	VSSAGYYFF	VSSAGYYFF	0	0	0	0	0	VSSAGYYFF	tr_C9ZP82	0.100	16973.21	23.00
712	HLA-A0201	LLSILPPTSH	LLSILPPTS	0	0	0	0	0	LLSILPPTS	tr_C9ZP82	0.100	17008.13	23.00
852	HLA-A0201	TYSCTVEVPRL	YSCTVEVPL	1	0	0	8	1	YSCTVEVPRL	tr_C9ZP82	0.100	17029.48	23.00
531	HLA-A0201	AFSGESALDNLL	FSGESALLL	1	0	0	7	2	FSGESALDNLL	tr_C9ZP82	0.100	17030.23	23.00
646	HLA-A0201	CEKYVEGVCFVWRY	YVVCFVWRY	3	0	0	2	2	YVEGVCFVWRY	tr_C9ZP82	0.099	17039.26	23.00
580	HLA-A0201	VKFRRFLLF	VKFRRFLLF	0	0	0	0	0	VKFRRFLLF	tr_C9ZP82	0.099	17041.28	23.00
647	HLA-A0201	EKYVEGVCFVWRY	YVVCFVWRY	2	0	0	2	2	YVEGVCFVWRY	tr_C9ZP82	0.099	17047.56	23.00
535	HLA-A0201	ESALDNLLELY	SALDNLLEL	1	0	0	0	0	SALDNLLEL	tr_C9ZP82	0.099	17064.91	23.00
443	HLA-A0201	FDKKTLVSSAGYYF	TLVSSAYYF	4	0	0	6	1	TLVSSAGYYF	tr_C9ZP82	0.099	17075.62	23.00
97	HLA-A0201	RYMGRAAPA	RYMGRAAPA	0	0	0	0	0	RYMGRAAPA	tr_C9ZP82	0.099	17086.15	23.00
835	HLA-A0201	VEIRQLYDV	VEIRQLYDV	0	0	0	0	0	VEIRQLYDV	tr_C9ZP82	0.099	17091.14	23.00
444	HLA-A0201	DKKTLVSSAGYYF	TLVSSAYYF	3	0	0	6	1	TLVSSAGYYF	tr_C9ZP82	0.099	17093.92	23.00
584	HLA-A0201	RFLLFKSTNK	FLLFKSTNK	1	0	0	0	0	FLLFKSTNK	tr_C9ZP82	0.099	17099.46	23.00
30	HLA-A0201	IDLNGIIHPCCHN	ILNGIIHPC	0	0	0	1	1	IDLNGIIHPC	tr_C9ZP82	0.099	17101.68	23.00
835	HLA-A0201	VEIRQLYDVPLER	ILYDVPLER	2	0	0	1	2	IRQLYDVPLER	tr_C9ZP82	0.099	17132.61	23.00
794	HLA-A0201	QKLDFHLVI	QKLDFHLVI	0	0	0	0	0	QKLDFHLVI	tr_C9ZP82	0.099	17147.27	23.00
196	HLA-A0201	NRYVESNQVVDDV	YVNQVVDDV	2	0	0	2	2	YVESNQVVDDV	tr_C9ZP82	0.099	17149.49	23.00
547	HLA-A0201	SEVLPYGFLTKG	VLYGFLTKG	2	0	0	2	1	VLPYGFLTKG	tr_C9ZP82	0.099	17153.76	23.00
34	HLA-A0201	GIIHPCCHNDSDTA	IIHPCCHTA	1	0	0	7	4	IIHPCCHNDSDTA	tr_C9ZP82	0.099	17160.81	23.00
300	HLA-A0201	IVGLDADLIF	IVLDADLIF	0	0	0	2	1	IVGLDADLIF	tr_C9ZP82	0.099	17162.86	23.00
524	HLA-A0201	FLPHAPSAFSGES	FLSAFSGES	0	0	0	2	4	FLPHAPSAFSGES	tr_C9ZP82	0.099	17166.01	24.00

680	HLA-A0201	PVIDLAIYLKEAN	ILAIYLKEA	2	0	0	1	1	IDLAIYLKEA	tr_C9ZP82	0.099	17201.90	24.00
611	HLA-A0201	VTVADVAVALHSTI	TVADVAVTI	1	0	0	7	4	TVADVAVALHSTI	tr_C9ZP82	0.099	17208.41	24.00
695	HLA-A0201	RMPLPVVKS	RMPLPVVKS	0	0	0	0	0	RMPLPVVKS	tr_C9ZP82	0.099	17211.76	24.00
159	HLA-A0201	ALYNEGEEEAGD	ALYEGEEEA	0	0	0	3	1	ALYNEGEEEA	tr_C9ZP82	0.099	17212.70	24.00
754	HLA-A0201	HDSNVVHLATVML	VVHLATVML	4	0	0	0	0	VVHLATVML	tr_C9ZP82	0.099	17217.72	24.00
641	HLA-A0201	SVQEACEKYVE	SVQEACEYV	0	0	0	7	1	SVQEACEKYV	tr_C9ZP82	0.098	17225.74	24.00
747	HLA-A0201	LQWNVDYHD	LQWNVDYHD	0	0	0	0	0	LQWNVDYHD	tr_C9ZP82	0.098	17265.29	24.00
516	HLA-A0201	LSILLGNDFLPH	ILLGNFLPH	2	0	0	5	1	ILLGNDFLPH	tr_C9ZP82	0.098	17271.46	24.00
563	HLA-A0201	NLPQLQRLF	NLPQLQRLF	0	0	0	0	0	NLPQLQRLF	tr_C9ZP82	0.098	17275.38	24.00
5	HLA-A0201	FASWLRNKYPA	FASWLKYPA	0	0	0	5	2	FASWLRNKYPA	tr_C9ZP82	0.098	17283.80	24.00
158	HLA-A0201	GALYNEGEEEAGDA	ALYNEGEEA	1	0	0	8	4	ALYNEGEEEAGDA	tr_C9ZP82	0.098	17284.73	24.00
281	HLA-A0201	DFLRTQSSLAQFGG	FLSLAQFGG	1	0	0	2	4	FLRTQSSLAQFGG	tr_C9ZP82	0.098	17302.32	24.00
451	HLA-A0201	SAGYYFFGQPNHI	SAGYYFFHI	0	0	0	7	4	SAGYYFFGQPNHI	tr_C9ZP82	0.098	17320.50	24.00
422	HLA-A0201	LISEVSALCDIKKF	LISEVSALF	0	0	0	8	5	LISEVSALCDIKKF	tr_C9ZP82	0.098	17326.31	24.00
304	HLA-A0201	DADLIFLALS	ADLIFLALS	1	0	0	0	0	ADLIFLALS	tr_C9ZP82	0.098	17342.25	24.00
157	HLA-A0201	SGALYNEGEEEAG	ALYEGEEEA	2	0	0	3	1	ALYNEGEEEA	tr_C9ZP82	0.098	17370.41	24.00
141	HLA-A0201	DDLDEVRQTLL	DLDEVQTLL	1	0	0	5	1	DLDEVRQTLL	tr_C9ZP82	0.098	17382.83	24.00
820	HLA-A0201	DALDTILRRVPSRE	ALDTILRRV	1	0	0	0	0	ALDTILRRV	tr_C9ZP82	0.098	17387.71	24.00
546	HLA-A0201	VSEVLPYGFLTKG	VLYGFLTKG	3	0	0	2	1	VLPYGFLTKG	tr_C9ZP82	0.098	17390.91	24.00
415	HLA-A0201	ISVVGGSLISEVS	SVVGLISEV	1	0	0	4	2	SVVGGSLISEV	tr_C9ZP82	0.098	17393.74	24.00
296	HLA-A0201	ANHVIVGLDADL	AIVGLDADL	0	0	0	1	3	ANHVIVGLDADL	tr_C9ZP82	0.098	17403.71	24.00
534	HLA-A0201	GESALDNLLELY	SALDNLLEL	2	0	0	0	0	SALDNLLEL	tr_C9ZP82	0.098	17407.66	24.00
650	HLA-A0201	VEGVCFVWRYYTT	GVCFVWYTT	2	0	0	6	2	GVCFVWRYYTT	tr_C9ZP82	0.097	17417.65	24.00
447	HLA-A0201	TLVSSAGYYFFGQ	TLVYYFFGQ	0	0	0	3	4	TLVSSAGYYFFGQ	tr_C9ZP82	0.097	17419.53	24.00
303	HLA-A0201	LDADLIFLALS	LADLIFLAL	0	0	0	1	1	LDADLIFLAL	tr_C9ZP82	0.097	17426.89	24.00
295	HLA-A0201	KANHVIVGLDADL	HVIVGLDAL	3	0	0	8	1	HVIVGLDADL	tr_C9ZP82	0.097	17428.58	24.00
517	HLA-A0201	SILLGNDFLPH	ILLGNFLPH	1	0	0	5	1	ILLGNDFLPH	tr_C9ZP82	0.097	17431.79	24.00
567	HLA-A0201	LQRLFEAYAK	LQRLFEAYA	0	0	0	0	0	LQRLFEAYA	tr_C9ZP82	0.097	17432.74	24.00
764	HLA-A0201	VMLPFADMD	VMLPFADMD	0	0	0	0	0	VMLPFADMD	tr_C9ZP82	0.097	17437.08	24.00
195	HLA-A0201	ENRYVESNQVVDDV	YVNQVVDDV	3	0	0	2	2	YVESNQVVDDV	tr_C9ZP82	0.097	17457.84	24.00
259	HLA-A0201	WTRLCVIFSGSNTP	RLCVIFNTP	2	0	0	6	3	RLCVIFSGSNTP	tr_C9ZP82	0.097	17458.03	24.00
260	HLA-A0201	TRLCVIFSGSNTPG	RLCVIFTPG	1	0	0	6	4	RLCVIFSGSNTPG	tr_C9ZP82	0.097	17460.29	24.00
753	HLA-A0201	YHDSNVVHLATV	YNVVHLATV	0	0	0	1	3	YHDSNVVHLATV	tr_C9ZP82	0.097	17461.05	24.00
838	HLA-A0201	RQLYDVPLERYR	QLYDVPLYR	1	0	0	7	2	QLYDVPLERYR	tr_C9ZP82	0.097	17473.71	24.00
582	HLA-A0201	FRRFLLFKST	FRRFLLFST	0	0	0	7	1	FRRFLLFKST	tr_C9ZP82	0.097	17477.87	24.00
228	HLA-A0201	NCISPGTDFM	CISPGTDFM	1	0	0	0	0	CISPGTDFM	tr_C9ZP82	0.097	17501.71	24.00
679	HLA-A0201	PPVIDLAIYLKEAN	ILAIYLKEA	3	0	0	1	1	IDLAIYLKEA	tr_C9ZP82	0.097	17516.11	24.00
69	HLA-A0201	QTVQPKEVMYIA	QTVEVMYIA	0	0	0	3	3	QTVQPKEVMYIA	tr_C9ZP82	0.097	17519.52	24.00
682	HLA-A0201	IDLAIYLKEANIK	ILAIYLKEA	0	0	0	1	1	IDLAIYLKEA	tr_C9ZP82	0.097	17559.95	24.00
575	HLA-A0201	AKIEAVKFRRFLLF	KIFRRFLLF	1	0	0	2	4	KIEAVKFRRFLLF	tr_C9ZP82	0.097	17562.22	24.00
548	HLA-A0201	EVLPYGFLTK	VLPYGFLTK	1	0	0	0	0	VLPYGFLTK	tr_C9ZP82	0.097	17562.22	24.00
140	HLA-A0201	NDDLDEVRQTLL	DLDEVQTLL	2	0	0	5	1	DLDEVRQTLL	tr_C9ZP82	0.097	17567.16	24.00
240	HLA-A0201	VSDAVLNMLN	VSDAVLNML	0	0	0	0	0	VSDAVLNML	tr_C9ZP82	0.097	17568.69	24.00
258	HLA-A0201	TWTRLCVIFSGS	RLCVIFSGS	3	0	0	0	0	RLCVIFSGS	tr_C9ZP82	0.097	17571.35	24.00
24	HLA-A0201	SVHGLYIDLN	SVHGLYIDL	0	0	0	0	0	SVHGLYIDL	tr_C9ZP82	0.097	17578.38	24.00
408	HLA-A0201	TGFEYFDISVVG	TGFEYFISV	0	0	0	6	1	TGFEYFDISV	tr_C9ZP82	0.097	17596.66	24.00
834	HLA-A0201	GVEIRQLYDVPLER	ILYDVPLER	3	0	0	1	2	IRQLYDVPLER	tr_C9ZP82	0.096	17602.56	24.00
773	HLA-A0201	VLQQVVDAAWPQ	VLQQVVWPQ	0	0	0	6	3	VLQQVVDAAWPQ	tr_C9ZP82	0.096	17605.79	24.00
422	HLA-A0201	LISEVSALCDIKK	LISEVSALI	0	0	0	8	2	LISEVSALCDI	tr_C9ZP82	0.096	17606.55	24.00
13	HLA-A0201	YPAIVTKNCPSSV	AIVNCPSSV	2	0	0	3	2	AIVTKNCPSSV	tr_C9ZP82	0.096	17610.56	24.00
761	HLA-A0201	LATVMLPFAD	LATVMLPFA	0	0	0	0	0	LATVMLPFA	tr_C9ZP82	0.096	17623.33	24.00
239	HLA-A0201	KVSDAVLNMLNEKM	KLNMLNEKM	0	0	0	1	5	KVSDAVLNMLNEKM	tr_C9ZP82	0.096	17625.43	24.00
511	HLA-A0201	DDIIVLSILLGN	IIVLILLGN	2	0	0	4	1	IIVLSILLGN	tr_C9ZP82	0.096	17627.14	24.00
850	HLA-A0201	PRTYSCTVEVPRLS	RTYSCTVEV	1	0	0	0	0	RTYSCTVEV	tr_C9ZP82	0.096	17645.84	24.00

134	HLA-A0201	TEMAQANDDL	TMAQANDDL	0	0	0	1	1	TEMAQANDDL	tr_C9ZP82	0.096	17654.82	24.00
146	HLA-A0201	VRQTLLEDPL	VQTLLEDPL	0	0	0	1	1	VRQTLLEDPL	tr_C9ZP82	0.096	17669.34	24.00
123	HLA-A0201	IVEEVVREF	IVEEVVREF	0	0	0	0	0	IVEEVVREF	tr_C9ZP82	0.096	17672.98	24.00
317	HLA-A0201	TQVFILRDT	TQVFILRDT	0	0	0	0	0	TQVFILRDT	tr_C9ZP82	0.096	17687.52	24.00
171	HLA-A0201	ALNDGDGVNSFC	ALDGVNSFC	0	0	0	2	3	ALNDGDGVNSFC	tr_C9ZP82	0.096	17699.38	24.00
98	HLA-A0201	YMGRAAPAQ	YMGRAAPAQ	0	0	0	0	0	YMGRAAPAQ	tr_C9ZP82	0.096	17704.75	24.00
255	HLA-A0201	NDPTWTRLCVI	NTWTRLCVI	0	0	0	1	2	NDPTWTRLCVI	tr_C9ZP82	0.096	17737.34	24.00
676	HLA-A0201	YHAPPVIDL	YHAPPVIDL	0	0	0	0	0	YHAPPVIDL	tr_C9ZP82	0.096	17748.67	24.00
30	HLA-A0201	IDLNGIIHPCCHND	ILNGIIHPC	0	0	0	1	1	IDLNGIIHPC	tr_C9ZP82	0.096	17764.81	24.00
280	HLA-A0201	IDFLRTQSSLAQFG	FLSSLAQFG	2	0	0	2	3	FLRTQSSLAQFG	tr_C9ZP82	0.096	17780.96	24.00
796	HLA-A0201	LDFHLVITS	LDFHLVITS	0	0	0	0	0	LDFHLVITS	tr_C9ZP82	0.096	17783.46	24.00
315	HLA-A0201	HITQVFILRDTT	ITQVFILTT	1	0	0	7	2	ITQVFILRDTT	tr_C9ZP82	0.096	17787.70	24.00
156	HLA-A0201	YSGALYNEGEEEAG	ALYEGEEEA	3	0	0	3	1	ALYNEGEEEA	tr_C9ZP82	0.095	17821.60	24.00
257	HLA-A0201	PTWTRLCVIFSGS	RLCVIFSGS	4	0	0	0	0	RLCVIFSGS	tr_C9ZP82	0.095	17828.74	24.00
350	HLA-A0201	NFIDDTCII	NFIDDTCII	0	0	0	0	0	NFIDDTCII	tr_C9ZP82	0.095	17865.43	24.00
414	HLA-A0201	DISVVGGSLISEVS	SVVGLISEV	2	0	0	4	2	SVVGGSLISEV	tr_C9ZP82	0.095	17872.00	24.00
239	HLA-A0201	KVSDAVLNMLN	KVDAVLNML	0	0	0	2	1	KVSDAVLNML	tr_C9ZP82	0.095	17875.10	24.00
610	HLA-A0201	NVTVADVAVA	VTVADVAVA	1	0	0	0	0	VTVADVAVA	tr_C9ZP82	0.095	17893.67	24.00
349	HLA-A0201	RNFIDDTCIIS	FIDDTCIIS	2	0	0	0	0	FIDDTCIIS	tr_C9ZP82	0.095	17913.43	24.00
257	HLA-A0201	PTWTRLCVIFSG	WTLCVIFSG	2	0	0	2	1	WTRLCVIFSG	tr_C9ZP82	0.095	17925.26	25.00
645	HLA-A0201	ACEKYVEGVCFVW	ACVEGVCFV	0	0	0	2	3	ACEKYVEGVCFV	tr_C9ZP82	0.095	17934.96	25.00
256	HLA-A0201	DPTWTRLCVIFSGS	RLCVIFSGS	5	0	0	0	0	RLCVIFSGS	tr_C9ZP82	0.095	17947.57	25.00
726	HLA-A0201	MVLGEVMVRSP	MLGEVMVRS	0	0	0	1	1	MVLGEVMVRS	tr_C9ZP82	0.095	17965.65	25.00
420	HLA-A0201	GSLISEVSA	GSLISEVSA	0	0	0	0	0	GSLISEVSA	tr_C9ZP82	0.095	17969.15	25.00
538	HLA-A0201	LDNLLELYVSE	LLLELYVSE	0	0	0	1	2	LDNLLELYVSE	tr_C9ZP82	0.095	17977.70	25.00
773	HLA-A0201	VLQQVVDAAWP	VLQQVVAWP	0	0	0	6	2	VLQQVVDAAWP	tr_C9ZP82	0.094	17991.51	25.00
609	HLA-A0201	DNVTVADVAV	NVTVADVAV	1	0	0	0	0	NVTVADVAV	tr_C9ZP82	0.094	18012.55	25.00
70	HLA-A0201	TVQPKEVMYIA	VQPEVMYIA	1	0	0	3	1	VQPKEVMYIA	tr_C9ZP82	0.094	18013.53	25.00
295	HLA-A0201	KANHVIVGLD	KANHVIVGL	0	0	0	0	0	KANHVIVGL	tr_C9ZP82	0.094	18048.25	25.00
529	HLA-A0201	PSAFSGESALDNLL	FSGESALLL	3	0	0	7	2	FSGESALDNLL	tr_C9ZP82	0.094	18051.96	25.00
415	HLA-A0201	ISVVGGSLI	ISVVGGSLI	0	0	0	0	0	ISVVGGSLI	tr_C9ZP82	0.094	18059.78	25.00
512	HLA-A0201	DIIVLSILLGN	DIIVLSILL	0	0	0	0	0	DIIVLSILL	tr_C9ZP82	0.094	18062.71	25.00
838	HLA-A0201	RQLYDVPLERYRP	QLYDVPLEP	1	0	0	8	3	QLYDVPLERYRP	tr_C9ZP82	0.094	18068.77	25.00
772	HLA-A0201	DVLQQVVDAAW	VLQQVVDAA	1	0	0	0	0	VLQQVVDAA	tr_C9ZP82	0.094	18076.79	25.00
566	HLA-A0201	QLQRLFEAY	QLQRLFEAY	0	0	0	0	0	QLQRLFEAY	tr_C9ZP82	0.094	18111.83	25.00
209	HLA-A0201	FGHRGEVIVDDPTA	FVIVDDPTA	0	0	0	1	5	FGHRGEVIVDDPTA	tr_C9ZP82	0.094	18119.87	25.00
756	HLA-A0201	SNVVHLATVM	SVVHLATVM	0	0	0	1	1	SNVVHLATVM	tr_C9ZP82	0.094	18146.94	25.00
256	HLA-A0201	DPTWTRLCVIFSG	WTLCVIFSG	3	0	0	2	1	WTRLCVIFSG	tr_C9ZP82	0.093	18185.26	25.00
654	HLA-A0201	CFVWRYYTTTTD	FVWYYTTTT	1	0	0	3	1	FVWRYYTTTT	tr_C9ZP82	0.093	18188.22	25.00
799	HLA-A0201	HLVITSNKTS	HLVITSNTS	0	0	0	7	1	HLVITSNKTS	tr_C9ZP82	0.093	18191.17	25.00
838	HLA-A0201	RQLYDVPLERY	QLYDVPLEY	1	0	0	8	1	QLYDVPLERY	tr_C9ZP82	0.093	18222.30	25.00
122	HLA-A0201	AIVEEVVREFT	AIVEEVVFT	0	0	0	7	2	AIVEEVVREFT	tr_C9ZP82	0.093	18231.76	25.00
12	HLA-A0201	KYPAIVTKNCPSSV	AIVNCPSSV	3	0	0	3	2	AIVTKNCPSSV	tr_C9ZP82	0.093	18235.12	25.00
290	HLA-A0201	AQFGGKANHVI	AQFGGANHV	0	0	0	5	1	AQFGGKANHV	tr_C9ZP82	0.093	18243.21	25.00
673	HLA-A0201	YPFYHAPPVIDLA	YPFYVIDLA	0	0	0	4	4	YPFYHAPPVIDLA	tr_C9ZP82	0.093	18258.41	25.00
228	HLA-A0201	NCISPGTDFMAKVS	CISDFMAKV	1	0	0	3	3	CISPGTDFMAKV	tr_C9ZP82	0.093	18266.71	25.00
462	HLA-A0201	HIEDGTLPS	HIEDGTLPS	0	0	0	0	0	HIEDGTLPS	tr_C9ZP82	0.093	18266.71	25.00
675	HLA-A0201	FYHAPPVIDL	FYAPPVIDL	0	0	0	2	1	FYHAPPVIDL	tr_C9ZP82	0.093	18298.96	25.00
135	HLA-A0201	EMAQANDDL	EMAQANDDL	0	0	0	0	0	EMAQANDDL	tr_C9ZP82	0.093	18307.87	25.00
314	HLA-A0201	LHITQVFILR	LITQVFILR	0	0	0	1	1	LHITQVFILR	tr_C9ZP82	0.093	18318.16	25.00
255	HLA-A0201	NDPTWTRLCVIFSG	WTLCVIFSG	4	0	0	2	1	WTRLCVIFSG	tr_C9ZP82	0.093	18320.35	25.00
7	HLA-A0201	SWLRNKYPAIVTKN	WLNKYPAIV	1	0	0	2	1	WLRNKYPAIV	tr_C9ZP82	0.093	18330.66	25.00
654	HLA-A0201	CFVWRYYTTTTDS	FVWYYTTTT	1	0	0	3	1	FVWRYYTTTT	tr_C9ZP82	0.093	18336.01	25.00
204	HLA-A0201	VVDDVFGHRGEVI	VVDDVFEVI	0	0	0	6	4	VVDDVFGHRGEVI	tr_C9ZP82	0.093	18336.62	25.00

585	HLA-A0201	FLLFKSTNKE	FLLFKSTNE	0	0	0	8	1	FLLFKSTNKE	tr_C9ZP82	0.093	18336.82	25.00
644	HLA-A0201	EACEKYVEGVCFVW	YVEGVCFVW	5	0	0	0	0	YVEGVCFVW	tr_C9ZP82	0.093	18343.36	25.00
66	HLA-A0201	LLVQTVQPKE	LLVQTVQPE	0	0	0	8	1	LLVQTVQPKE	tr_C9ZP82	0.093	18350.71	25.00
653	HLA-A0201	VCFVWRYYTTTTTD	FVWYYTTTT	2	0	0	3	1	FVWRYYTTTT	tr_C9ZP82	0.093	18358.05	25.00
654	HLA-A0201	CFVWRYYTT	CFVWRYYTT	0	0	0	0	0	CFVWRYYTT	tr_C9ZP82	0.093	18360.03	25.00
260	HLA-A0201	TRLCVIFSGSNTP	RLCVIFNTP	1	0	0	6	3	RLCVIFSGSNTP	tr_C9ZP82	0.093	18365.80	25.00
348	HLA-A0201	KRNFIDDTCIIS	FIDDTCIIS	3	0	0	0	0	FIDDTCIIS	tr_C9ZP82	0.092	18392.85	25.00
555	HLA-A0201	LTKGPHEINL	LTGPHEINL	0	0	0	2	1	LTKGPHEINL	tr_C9ZP82	0.092	18402.20	25.00
298	HLA-A0201	HVIVGLDADLIF	HVIVGLLIF	0	0	0	6	3	HVIVGLDADLIF	tr_C9ZP82	0.092	18415.54	25.00
177	HLA-A0201	GVNSFCVRS	GVNSFCVRS	0	0	0	0	0	GVNSFCVRS	tr_C9ZP82	0.092	18433.69	25.00
853	HLA-A0201	YSCTVEVPRLSYGA	YVPRLSYGA	0	0	0	1	5	YSCTVEVPRLSYGA	tr_C9ZP82	0.092	18440.67	25.00
720	HLA-A0201	SHALLPMVL	SHALLPMVL	0	0	0	0	0	SHALLPMVL	tr_C9ZP82	0.092	18453.44	25.00
373	HLA-A0201	VIGVPVCESS	VIVPVCESS	0	0	0	2	1	VIGVPVCESS	tr_C9ZP82	0.092	18471.02	25.00
837	HLA-A0201	IRQLYDVPLERYR	QLYDVPLYR	2	0	0	7	2	QLYDVPLERYR	tr_C9ZP82	0.092	18473.42	25.00
355	HLA-A0201	TCIISLNPSYID	CIISLNPYI	1	0	0	7	1	CIISLNPSYI	tr_C9ZP82	0.092	18486.82	25.00
256	HLA-A0201	DPTWTRLCVI	DTWTRLCVI	0	0	0	1	1	DPTWTRLCVI	tr_C9ZP82	0.092	18504.22	25.00
228	HLA-A0201	NCISPGTDFMA	CISPGTFMA	1	0	0	6	1	CISPGTDFMA	tr_C9ZP82	0.092	18510.04	25.00
221	HLA-A0201	TAFEFDSNC	TAFEFDSNC	0	0	0	0	0	TAFEFDSNC	tr_C9ZP82	0.092	18510.63	25.00
135	HLA-A0201	EMAQANDDLDEVR	EMADDLDEV	0	0	0	3	3	EMAQANDDLDEV	tr_C9ZP82	0.092	18521.05	25.00
675	HLA-A0201	FYHAPPVIDLA	FAPPVIDLA	0	0	0	1	2	FYHAPPVIDLA	tr_C9ZP82	0.092	18526.26	25.00
687	HLA-A0201	YLKEANIKR	YLKEANIKR	0	0	0	0	0	YLKEANIKR	tr_C9ZP82	0.092	18543.11	25.00
413	HLA-A0201	FDISVVGGSLI	FDISVVSLI	0	0	0	6	2	FDISVVGGSLI	tr_C9ZP82	0.092	18576.44	25.00
15	HLA-A0201	AIVTKNCPSSVHGL	AIVSSVHGL	0	0	0	3	5	AIVTKNCPSSVHGL	tr_C9ZP82	0.091	18583.08	25.00
407	HLA-A0201	NTGFEYFDISVVG	TGFEYFISV	1	0	0	6	1	TGFEYFDISV	tr_C9ZP82	0.091	18625.76	25.00
347	HLA-A0201	RKRNFIDDTCIIS	FIDDTCIIS	4	0	0	0	0	FIDDTCIIS	tr_C9ZP82	0.091	18653.58	25.00
241	HLA-A0201	SDAVLNMLNEKM	SLNMLNEKM	0	0	0	1	3	SDAVLNMLNEKM	tr_C9ZP82	0.091	18666.10	25.00
60	HLA-A0201	ICSELELLVQT	ICLELLVQT	0	0	0	2	2	ICSELELLVQT	tr_C9ZP82	0.091	18669.33	25.00
260	HLA-A0201	TRLCVIFSG	TRLCVIFSG	0	0	0	0	0	TRLCVIFSG	tr_C9ZP82	0.091	18683.27	26.00
279	HLA-A0201	IIDFLRTQSSL	IIDFLQSSL	0	0	0	5	2	IIDFLRTQSSL	tr_C9ZP82	0.091	18687.12	26.00
797	HLA-A0201	DFHLVITSNKT	FLVITSNKT	1	0	0	1	1	FHLVITSNKT	tr_C9ZP82	0.091	18688.94	26.00
837	HLA-A0201	IRQLYDVPLERY	QLYDVPLEY	2	0	0	8	1	QLYDVPLERY	tr_C9ZP82	0.091	18694.40	26.00
682	HLA-A0201	IDLAIYLKEANIKR	ILAIYLKEA	0	0	0	1	1	IDLAIYLKEA	tr_C9ZP82	0.091	18698.24	26.00
297	HLA-A0201	NHVIVGLDADL	HVIVGLDAL	1	0	0	8	1	HVIVGLDADL	tr_C9ZP82	0.091	18699.26	26.00
279	HLA-A0201	IIDFLRTQSSLA	IIDFLSSLA	0	0	0	5	3	IIDFLRTQSSLA	tr_C9ZP82	0.091	18752.54	26.00
408	HLA-A0201	TGFEYFDISVVGG	TGFEYFISV	0	0	0	6	1	TGFEYFDISV	tr_C9ZP82	0.091	18774.47	26.00
836	HLA-A0201	EIRQLYDVPLERYR	QLYDVPLYR	3	0	0	7	2	QLYDVPLERYR	tr_C9ZP82	0.091	18774.87	26.00
839	HLA-A0201	QLYDVPLERYRPR	QLYDVPLPR	0	0	0	7	4	QLYDVPLERYRPR	tr_C9ZP82	0.091	18777.11	26.00
63	HLA-A0201	ELELLVQTVQP	ELELLVQTV	0	0	0	0	0	ELELLVQTV	tr_C9ZP82	0.091	18777.11	26.00
725	HLA-A0201	PMVLGEVMVR	PMVLGEVMV	0	0	0	0	0	PMVLGEVMV	tr_C9ZP82	0.090	18781.18	26.00
641	HLA-A0201	SVQEACEKYVEGVC	SVQEYVEGV	0	0	0	4	4	SVQEACEKYVEGV	tr_C9ZP82	0.090	18789.91	26.00
642	HLA-A0201	VQEACEKYVEGV	VQEACVEGV	0	0	0	5	3	VQEACEKYVEGV	tr_C9ZP82	0.090	18799.47	26.00
362	HLA-A0201	PSYIDEEESDGVIG	YIDEESDGV	2	0	0	3	1	YIDEEESDGV	tr_C9ZP82	0.090	18801.50	26.00
346	HLA-A0201	RRKRNFIDDTCIIS	FIDDTCIIS	5	0	0	0	0	FIDDTCIIS	tr_C9ZP82	0.090	18819.00	26.00
294	HLA-A0201	GKANHVIVGLDADL	HVIVGLDAL	4	0	0	8	1	HVIVGLDADL	tr_C9ZP82	0.090	18835.10	26.00
416	HLA-A0201	SVVGGSLISEVSA	SVVGLISEV	0	0	0	4	2	SVVGGSLISEV	tr_C9ZP82	0.090	18846.30	26.00
585	HLA-A0201	FLLFKSTNKENS	FLLFKSTNS	0	0	0	7	3	FLLFKSTNKENS	tr_C9ZP82	0.090	18851.00	26.00
314	HLA-A0201	LHITQVFILRDTT	ITQVFILTT	2	0	0	7	2	ITQVFILRDTT	tr_C9ZP82	0.090	18852.43	26.00
242	HLA-A0201	DAVLNMLNEKM	AVLNMLNEM	1	0	0	8	1	AVLNMLNEKM	tr_C9ZP82	0.090	18866.71	26.00
297	HLA-A0201	NHVIVGLDADLIF	HVIVGLLIF	1	0	0	6	3	HVIVGLDADLIF	tr_C9ZP82	0.090	18878.76	26.00
354	HLA-A0201	DTCIISLNPSYID	CIISLNPYI	2	0	0	7	1	CIISLNPSYI	tr_C9ZP82	0.090	18897.15	26.00
412	HLA-A0201	YFDISVVGGSLIS	YFDISVVLI	0	0	0	7	3	YFDISVVGGSLI	tr_C9ZP82	0.090	18918.43	26.00
834	HLA-A0201	GVEIRQLYDVPL	GVQLYDVPL	0	0	0	2	3	GVEIRQLYDVPL	tr_C9ZP82	0.090	18926.00	26.00
672	HLA-A0201	YYPFYHAPPVIDLA	YPFYVIDLA	1	0	0	4	4	YPFYHAPPVIDLA	tr_C9ZP82	0.090	18930.50	26.00
727	HLA-A0201	VLGEVMVRSPQ	VLGEVMVPQ	0	0	0	7	2	VLGEVMVRSPQ	tr_C9ZP82	0.090	18944.04	26.00

772	HLA-A0201	DVLQQVVDAAWPQ	VLQQVVWPQ	1	0	0	6	3	VLQQVVDAAWPQ	tr_C9ZP82	0.090	18963.10	26.00
296	HLA-A0201	ANHVIVGLDADLIF	HVIVGLLIF	2	0	0	6	3	HVIVGLDADLIF	tr_C9ZP82	0.090	18966.59	26.00
151	HLA-A0201	LEDPLYSGALYN	LLYSGALYN	0	0	0	1	3	LEDPLYSGALYN	tr_C9ZP82	0.090	18976.85	26.00
417	HLA-A0201	VVGGSLISEVSA	VVGGSLISA	0	0	0	7	3	VVGGSLISEVSA	tr_C9ZP82	0.089	19013.24	26.00
769	HLA-A0201	ADMDVLQQVVDA	AMDVLQQVV	0	0	0	1	1	ADMDVLQQVV	tr_C9ZP82	0.089	19018.17	26.00
646	HLA-A0201	CEKYVEGVCFV	KYVEGVCFV	2	0	0	0	0	KYVEGVCFV	tr_C9ZP82	0.089	19030.52	26.00
651	HLA-A0201	EGVCFVWRYY	GVCFVWRYY	1	0	0	0	0	GVCFVWRYY	tr_C9ZP82	0.089	19053.18	26.00
611	HLA-A0201	VTVADVAVALH	VTVAVAVAL	0	0	0	4	1	VTVADVAVAL	tr_C9ZP82	0.089	19067.83	26.00
752	HLA-A0201	DYHDSNVVHLATV	YNVVHLATV	1	0	0	1	3	YHDSNVVHLATV	tr_C9ZP82	0.089	19092.18	26.00
799	HLA-A0201	HLVITSNKTSF	HLVITSTSF	0	0	0	6	2	HLVITSNKTSF	tr_C9ZP82	0.089	19094.87	26.00
837	HLA-A0201	IRQLYDVPLERYRP	QLYDVPLEP	2	0	0	8	3	QLYDVPLERYRP	tr_C9ZP82	0.089	19098.59	26.00
836	HLA-A0201	EIRQLYDVPLERY	QLYDVPLEY	3	0	0	8	1	QLYDVPLERY	tr_C9ZP82	0.089	19116.79	26.00
69	HLA-A0201	QTVQPKEVMYI	TVQPEVMYI	1	0	0	4	1	TVQPKEVMYI	tr_C9ZP82	0.089	19133.76	26.00
225	HLA-A0201	FDSNCISPGT	FSNCISPGT	0	0	0	1	1	FDSNCISPGT	tr_C9ZP82	0.089	19147.21	26.00
237	HLA-A0201	MAKVSDAVLNMLN	MADAVLNML	0	0	0	2	3	MAKVSDAVLNML	tr_C9ZP82	0.089	19153.02	26.00
42	HLA-A0201	NDSDTAIALL	NSDTAIALL	0	0	0	1	1	NDSDTAIALL	tr_C9ZP82	0.089	19169.39	26.00
700	HLA-A0201	VVKSTPPDTLVQLL	VVDTLVQLL	0	0	0	2	5	VVKSTPPDTLVQLL	tr_C9ZP82	0.089	19181.42	26.00
706	HLA-A0201	PDTLVQLLSI	DTLVQLLSI	1	0	0	0	0	DTLVQLLSI	tr_C9ZP82	0.088	19200.32	26.00
683	HLA-A0201	DLAIYLKEANIK	DLAIYLANI	0	0	0	6	2	DLAIYLKEANI	tr_C9ZP82	0.088	19216.74	26.00
136	HLA-A0201	MAQANDDLDEVR	AQADDLDEV	1	0	0	3	1	AQANDDLDEV	tr_C9ZP82	0.088	19218.82	26.00
716	HLA-A0201	LPPTSHALLPM	LTSHALLPM	0	0	0	1	2	LPPTSHALLPM	tr_C9ZP82	0.088	19238.58	26.00
3	HLA-A0201	PKFASWLRNKYPA	KFASWLYPA	1	0	0	6	3	KFASWLRNKYPA	tr_C9ZP82	0.088	19241.29	26.00
446	HLA-A0201	KTLVSSAGYYFFGQ	TLVYYFFGQ	1	0	0	3	4	TLVSSAGYYFFGQ	tr_C9ZP82	0.088	19246.50	26.00
447	HLA-A0201	TLVSSAGYY	TLVSSAGYY	0	0	0	0	0	TLVSSAGYY	tr_C9ZP82	0.088	19250.25	26.00
655	HLA-A0201	FVWRYYTTTTTDSD	FVWYYTTTT	0	0	0	3	1	FVWRYYTTTT	tr_C9ZP82	0.088	19255.24	26.00
753	HLA-A0201	YHDSNVVHLA	YHDSVVHLA	0	0	0	4	1	YHDSNVVHLA	tr_C9ZP82	0.088	19257.12	26.00
671	HLA-A0201	WYYPFYHAPPVIDL	WYYPFVIDL	0	0	0	5	5	WYYPFYHAPPVIDL	tr_C9ZP82	0.088	19260.04	26.00
751	HLA-A0201	VDYHDSNVVHLATV	VDYVHLATV	0	0	0	3	5	VDYHDSNVVHLATV	tr_C9ZP82	0.088	19265.45	26.00
578	HLA-A0201	EAVKFRRFLLF	AVFRRFLLF	1	0	0	2	1	AVKFRRFLLF	tr_C9ZP82	0.088	19292.36	26.00
833	HLA-A0201	EGVEIRQLYDV	GVIRQLYDV	1	0	0	2	1	GVEIRQLYDV	tr_C9ZP82	0.088	19295.29	26.00
447	HLA-A0201	TLVSSAGYYFFGQP	TLYYFFGQP	0	0	0	2	5	TLVSSAGYYFFGQP	tr_C9ZP82	0.088	19296.75	26.00
835	HLA-A0201	VEIRQLYDVPLERY	QLYDVPLEY	4	0	0	8	1	QLYDVPLERY	tr_C9ZP82	0.088	19313.88	26.00
675	HLA-A0201	FYHAPPVIDLAI	FYHVIDLAI	0	0	0	3	3	FYHAPPVIDLAI	tr_C9ZP82	0.088	19327.25	26.00
97	HLA-A0201	RYMGRAAPAQT	YMGAAPAQT	1	0	0	3	1	YMGRAAPAQT	tr_C9ZP82	0.088	19367.65	26.00
63	HLA-A0201	ELELLVQTVQPK	ELELLVQTV	0	0	0	0	0	ELELLVQTV	tr_C9ZP82	0.088	19372.06	26.00
240	HLA-A0201	VSDAVLNMLNEKM	VLNMLNEKM	0	0	0	1	4	VSDAVLNMLNEKM	tr_C9ZP82	0.088	19398.69	27.00
441	HLA-A0201	SAFDKKTLVSSA	SAFDLVSSA	0	0	0	4	3	SAFDKKTLVSSA	tr_C9ZP82	0.087	19404.79	27.00
353	HLA-A0201	DDTCIISLNPSYID	CIISLNPSYI	3	0	0	7	1	CIISLNPSYI	tr_C9ZP82	0.087	19418.02	27.00
408	HLA-A0201	TGFEYFDISVVGGS	TGFEYFISV	0	0	0	6	1	TGFEYFDISV	tr_C9ZP82	0.087	19439.24	27.00
814	HLA-A0201	DLRKLSDAL	DLRKLSDAL	0	0	0	0	0	DLRKLSDAL	tr_C9ZP82	0.087	19440.94	27.00
78	HLA-A0201	YIAVDGVAPRAK	YIDGVAPRA	0	0	0	2	2	YIAVDGVAPRA	tr_C9ZP82	0.087	19444.29	27.00
772	HLA-A0201	DVLQQVVDAAWP	VLQQVVAWP	1	0	0	6	2	VLQQVVDAAWP	tr_C9ZP82	0.087	19449.98	27.00
190	HLA-A0201	YESTHENRYV	YSTHENRYV	0	0	0	1	1	YESTHENRYV	tr_C9ZP82	0.087	19462.81	27.00
718	HLA-A0201	PTSHALLPM	PTSHALLPM	0	0	0	0	0	PTSHALLPM	tr_C9ZP82	0.087	19465.76	27.00
860	HLA-A0201	PRLSYGADGQVI	RLYGADGQV	1	0	0	2	1	RLSYGADGQV	tr_C9ZP82	0.087	19472.09	27.00
640	HLA-A0201	NSVQEACEKYVE	SVQEACEYV	1	0	0	7	1	SVQEACEKYV	tr_C9ZP82	0.087	19490.22	27.00
64	HLA-A0201	LELLVQTVQP	LLLVQTVQP	0	0	0	1	1	LELLVQTVQP	tr_C9ZP82	0.087	19494.22	27.00
282	HLA-A0201	FLRTQSSLAQFGGK	FLQSSLAQF	0	0	0	2	2	FLRTQSSLAQF	tr_C9ZP82	0.087	19495.27	27.00
515	HLA-A0201	VLSILLGND	VLSILLGND	0	0	0	0	0	VLSILLGND	tr_C9ZP82	0.087	19501.18	27.00
585	HLA-A0201	FLLFKSTNKENSNS	FLLFKSTNS	0	0	0	7	5	FLLFKSTNKENSNS	tr_C9ZP82	0.087	19514.70	27.00
798	HLA-A0201	FHLVITSNKTSF	HLVITSTSF	1	0	0	6	2	HLVITSNKTSF	tr_C9ZP82	0.087	19521.66	27.00
766	HLA-A0201	LPFADMDVL	LPFADMDVL	0	0	0	0	0	LPFADMDVL	tr_C9ZP82	0.087	19523.14	27.00
139	HLA-A0201	ANDDLDEVRQTLLE	ALDEVRQTL	0	0	0	1	3	ANDDLDEVRQTL	tr_C9ZP82	0.087	19525.67	27.00
254	HLA-A0201	GNDPTWTRLCVIFS	PTWLCVIFS	3	0	0	3	2	PTWTRLCVIFS	tr_C9ZP82	0.087	19532.85	27.00

227	HLA-A0201	SNCISPGTDFMA	CISPGTFMA	2	0	0	6	1	CISPGTDFMA	tr_C9ZP82	0.087	19556.75	27.00
523	HLA-A0201	DFLPHAPSAFSGES	FLSAFSGES	1	0	0	2	4	FLPHAPSAFSGES	tr_C9ZP82	0.087	19562.25	27.00
511	HLA-A0201	DDIIVLSILLGND	DDIIVLILL	0	0	0	6	1	DDIIVLSILL	tr_C9ZP82	0.087	19580.46	27.00
257	HLA-A0201	PTWTRLCVIFS	PTWLCVIFS	0	0	0	3	2	PTWTRLCVIFS	tr_C9ZP82	0.087	19589.15	27.00
825	HLA-A0201	ILRRVPSREGVE	ILRRVPEGV	0	0	0	6	2	ILRRVPSREGV	tr_C9ZP82	0.086	19616.94	27.00
78	HLA-A0201	YIAVDGVAPRAKM	YIAVDGVAM	0	0	0	8	4	YIAVDGVAPRAKM	tr_C9ZP82	0.086	19647.95	27.00
170	HLA-A0201	DALNDGDGVNSFC	ALDGVNSFC	1	0	0	2	3	ALNDGDGVNSFC	tr_C9ZP82	0.086	19700.53	27.00
407	HLA-A0201	NTGFEYFDISVVGG	TGFEYFISV	1	0	0	6	1	TGFEYFDISV	tr_C9ZP82	0.086	19742.99	27.00
855	HLA-A0201	CTVEVPRLSYGA	CTVEVSYGA	0	0	0	5	3	CTVEVPRLSYGA	tr_C9ZP82	0.086	19759.87	27.00
726	HLA-A0201	MVLGEVMVRSPQ	VLGEVMVPQ	1	0	0	7	2	VLGEVMVRSPQ	tr_C9ZP82	0.086	19773.78	27.00
857	HLA-A0201	VEVPRLSYGA	VVPRLSYGA	0	0	0	1	1	VEVPRLSYGA	tr_C9ZP82	0.086	19785.12	27.00
121	HLA-A0201	MAIVEEVVREFT	AIVEEVVFT	1	0	0	7	2	AIVEEVVREFT	tr_C9ZP82	0.086	19787.48	27.00
731	HLA-A0201	VMVRSPQQAELEST	VMVAELEST	0	0	0	3	5	VMVRSPQQAELEST	tr_C9ZP82	0.086	19793.04	27.00
577	HLA-A0201	IEAVKFRRFLLF	AVFRRFLLF	2	0	0	2	1	AVKFRRFLLF	tr_C9ZP82	0.086	19794.76	27.00
576	HLA-A0201	KIEAVKFRRFLLFK	KIFRRFLLF	0	0	0	2	4	KIEAVKFRRFLLF	tr_C9ZP82	0.086	19797.76	27.00
753	HLA-A0201	YHDSNVVHLATVM	YVVHLATVM	0	0	0	1	4	YHDSNVVHLATVM	tr_C9ZP82	0.086	19799.26	27.00
724	HLA-A0201	LPMVLGEVM	LPMVLGEVM	0	0	0	0	0	LPMVLGEVM	tr_C9ZP82	0.086	19800.32	27.00
123	HLA-A0201	IVEEVVREFTS	IVEEVVFTS	0	0	0	6	2	IVEEVVREFTS	tr_C9ZP82	0.086	19815.12	27.00
694	HLA-A0201	KRMPLPVVKST	RMLPVVKST	1	0	0	2	1	RMPLPVVKST	tr_C9ZP82	0.086	19815.33	27.00
861	HLA-A0201	RLSYGADGQVIK	RLYGADGQV	0	0	0	2	1	RLSYGADGQV	tr_C9ZP82	0.085	19825.20	27.00
634	HLA-A0201	KSTGLSNSVQEAC	KLSNSVQEA	0	0	0	1	3	KSTGLSNSVQEA	tr_C9ZP82	0.085	19825.62	27.00
290	HLA-A0201	AQFGGKANHVIVG	AQFGGHVIV	0	0	0	5	3	AQFGGKANHVIV	tr_C9ZP82	0.085	19833.78	27.00
253	HLA-A0201	SGNDPTWTRLCVIF	STWTRLCVI	0	0	0	1	4	SGNDPTWTRLCVI	tr_C9ZP82	0.085	19834.00	27.00
796	HLA-A0201	LDFHLVITSNKTSF	HLVITSTSF	3	0	0	6	2	HLVITSNKTSF	tr_C9ZP82	0.085	19841.71	27.00
689	HLA-A0201	KEANIKRMPLPV	KIKRMPLPV	0	0	0	1	3	KEANIKRMPLPV	tr_C9ZP82	0.085	19896.97	27.00
406	HLA-A0201	PNTGFEYFDISVVG	TGFEYFISV	2	0	0	6	1	TGFEYFDISV	tr_C9ZP82	0.085	19900.84	27.00
6	HLA-A0201	ASWLRNKYPAI	ALRNKYPAI	0	0	0	1	2	ASWLRNKYPAI	tr_C9ZP82	0.085	19913.55	27.00
2	HLA-A0201	VPKFASWLRNKYPA	KFASWLYPA	2	0	0	6	3	KFASWLRNKYPA	tr_C9ZP82	0.085	19917.65	27.00
226	HLA-A0201	DSNCISPGTDFMA	CISPGTFMA	3	0	0	6	1	CISPGTDFMA	tr_C9ZP82	0.085	19917.65	27.00
546	HLA-A0201	VSEVLPYGFLT	VVLPYGFLT	0	0	0	1	2	VSEVLPYGFLT	tr_C9ZP82	0.085	19923.69	27.00
796	HLA-A0201	LDFHLVITSNKT	FLVITSNKT	2	0	0	1	1	FHLVITSNKT	tr_C9ZP82	0.085	19934.68	27.00
259	HLA-A0201	WTRLCVIFSGSN	WTLCVIFSG	0	0	0	2	1	WTRLCVIFSG	tr_C9ZP82	0.085	19941.36	27.00
411	HLA-A0201	EYFDISVVGGSLIS	YFDISVVLI	1	0	0	7	3	YFDISVVGGSLI	tr_C9ZP82	0.085	19946.77	27.00
774	HLA-A0201	LQQVVDAAW	LQQVVDAAW	0	0	0	0	0	LQQVVDAAW	tr_C9ZP82	0.085	19948.27	27.00
797	HLA-A0201	DFHLVITSNKTSF	HLVITSTSF	2	0	0	6	2	HLVITSNKTSF	tr_C9ZP82	0.085	19975.92	27.00
687	HLA-A0201	YLKEANIKRMP	YLKEANIRM	0	0	0	7	1	YLKEANIKRM	tr_C9ZP82	0.085	19977.87	27.00
725	HLA-A0201	PMVLGEVMVRSP	VLGEVMVSP	2	0	0	7	1	VLGEVMVRSP	tr_C9ZP82	0.085	19983.27	27.00
122	HLA-A0201	AIVEEVVREFTS	IVEEVVFTS	1	0	0	6	2	IVEEVVREFTS	tr_C9ZP82	0.085	19995.38	27.00
255	HLA-A0201	NDPTWTRLCVIFS	PTWLCVIFS	2	0	0	3	2	PTWTRLCVIFS	tr_C9ZP82	0.085	20008.15	27.00
229	HLA-A0201	CISPGTDFMAKVSD	CISDFMAKV	0	0	0	3	3	CISPGTDFMAKV	tr_C9ZP82	0.085	20008.58	27.00
688	HLA-A0201	LKEANIKRMPLPV	LIKRMPLPV	0	0	0	1	4	LKEANIKRMPLPV	tr_C9ZP82	0.084	20049.98	27.00
63	HLA-A0201	ELELLVQTVQPKE	ELELLVQTV	0	0	0	0	0	ELELLVQTV	tr_C9ZP82	0.084	20059.95	27.00
134	HLA-A0201	TEMAQANDDLDEVR	AQADDLDEV	3	0	0	3	1	AQANDDLDEV	tr_C9ZP82	0.084	20075.37	27.00
824	HLA-A0201	TILRRVPSREGVE	ILRRVPEGV	1	0	0	6	2	ILRRVPSREGV	tr_C9ZP82	0.084	20109.29	28.00
512	HLA-A0201	DIIVLSILLGND	DIIVLSILL	0	0	0	0	0	DIIVLSILL	tr_C9ZP82	0.084	20110.59	28.00
318	HLA-A0201	QVFILRDTT	QVFILRDTT	0	0	0	0	0	QVFILRDTT	tr_C9ZP82	0.084	20130.18	28.00
641	HLA-A0201	SVQEACEKYVEG	SVQEACEYV	0	0	0	7	1	SVQEACEKYV	tr_C9ZP82	0.084	20133.45	28.00
646	HLA-A0201	CEKYVEGVCFVW	YVEGVCFVW	3	0	0	0	0	YVEGVCFVW	tr_C9ZP82	0.084	20134.54	28.00
238	HLA-A0201	AKVSDAVLNMLN	KVDAVLNML	1	0	0	2	1	KVSDAVLNML	tr_C9ZP82	0.084	20138.24	28.00
441	HLA-A0201	SAFDKKTLVS	SAFDKKTLV	0	0	0	0	0	SAFDKKTLV	tr_C9ZP82	0.084	20142.39	28.00
415	HLA-A0201	ISVVGGSLISEVSA	SVVGLISEV	1	0	0	4	2	SVVGGSLISEV	tr_C9ZP82	0.084	20159.38	28.00
832	HLA-A0201	REGVEIRQLYDV	GVEIRQLYV	2	0	0	8	1	GVEIRQLYDV	tr_C9ZP82	0.084	20184.05	28.00
601	HLA-A0201	EFRKGSNNDNVTV	FSNNDNVTV	1	0	0	1	3	FRKGSNNDNVTV	tr_C9ZP82	0.084	20186.02	28.00
256	HLA-A0201	DPTWTRLCVIFS	PTWLCVIFS	1	0	0	3	2	PTWTRLCVIFS	tr_C9ZP82	0.084	20186.24	28.00

131	HLA-A0201	FTSTEMAQAN	FTSTEMAQA	0	0	0	0	0	FTSTEMAQA	tr_C9ZP82	0.084	20192.36	28.00
292	HLA-A0201	FGGKANHVIVGLD	FANHVIVGL	0	0	0	1	3	FGGKANHVIVGL	tr_C9ZP82	0.084	20198.04	28.00
264	HLA-A0201	VIFSGSNTP	VIFSGSNTP	0	0	0	0	0	VIFSGSNTP	tr_C9ZP82	0.084	20200.65	28.00
514	HLA-A0201	IVLSILLGND	IVLSILLGD	0	0	0	8	1	IVLSILLGND	tr_C9ZP82	0.084	20201.75	28.00
660	HLA-A0201	YTTTTTDSDWSWYY	YTSDWSWYY	0	0	0	2	5	YTTTTTDSDWSWYY	tr_C9ZP82	0.084	20203.06	28.00
429	HLA-A0201	LCDIKKFTPA	LCDIKFTPA	0	0	0	4	1	LCDIKKFTPA	tr_C9ZP82	0.084	20206.35	28.00
237	HLA-A0201	MAKVSDAVL	MAKVSDAVL	0	0	0	0	0	MAKVSDAVL	tr_C9ZP82	0.084	20207.43	28.00
71	HLA-A0201	VQPKEVMYIAVD	VQPVMYIAV	0	0	0	3	2	VQPKEVMYIAV	tr_C9ZP82	0.084	20208.30	28.00
252	HLA-A0201	MSGNDPTWTRLCV	MSGWTRLCV	0	0	0	3	4	MSGNDPTWTRLCV	tr_C9ZP82	0.084	20237.41	28.00
757	HLA-A0201	NVVHLATVMLP	VVHLATVML	1	0	0	0	0	VVHLATVML	tr_C9ZP82	0.084	20250.77	28.00
647	HLA-A0201	EKYVEGVCFVW	YVEGVCFVW	2	0	0	0	0	YVEGVCFVW	tr_C9ZP82	0.083	20261.29	28.00
142	HLA-A0201	DLDEVRQTLLEDPL	DLDEVEDPL	0	0	0	5	5	DLDEVRQTLLEDPL	tr_C9ZP82	0.083	20262.82	28.00
645	HLA-A0201	ACEKYVEGVCFVWR	ACVEGVCFV	0	0	0	2	3	ACEKYVEGVCFV	tr_C9ZP82	0.083	20266.33	28.00
137	HLA-A0201	AQANDDLDEVRQ	AQADDLDEV	0	0	0	3	1	AQANDDLDEV	tr_C9ZP82	0.083	20270.94	28.00
412	HLA-A0201	YFDISVVGG	YFDISVVGG	0	0	0	0	0	YFDISVVGG	tr_C9ZP82	0.083	20282.79	28.00
890	HLA-A0201	VIEGKKSNSRVTVV	VINSRVTVV	0	0	0	2	5	VIEGKKSNSRVTVV	tr_C9ZP82	0.083	20339.26	28.00
275	HLA-A0201	GEHKIIDFL	GEHKIIDFL	0	0	0	0	0	GEHKIIDFL	tr_C9ZP82	0.083	20340.36	28.00
738	HLA-A0201	QAELESTFPLQ	QALESTFPL	0	0	0	2	1	QAELESTFPL	tr_C9ZP82	0.083	20363.71	28.00
762	HLA-A0201	ATVMLPFADMD	ATVMLPFAM	0	0	0	8	1	ATVMLPFADM	tr_C9ZP82	0.083	20391.92	28.00
299	HLA-A0201	VIVGLDADLIF	VIVGLDALI	0	0	0	7	1	VIVGLDADLI	tr_C9ZP82	0.083	20436.32	28.00
231	HLA-A0201	SPGTDFMAKV	SGTDFMAKV	0	0	0	1	1	SPGTDFMAKV	tr_C9ZP82	0.083	20444.50	28.00
562	HLA-A0201	INLPQLQRL	INLPQLQRL	0	0	0	0	0	INLPQLQRL	tr_C9ZP82	0.083	20458.67	28.00
158	HLA-A0201	GALYNEGEEEAGD	ALYEGEEEA	1	0	0	3	1	ALYNEGEEEA	tr_C9ZP82	0.083	20459.11	28.00
96	HLA-A0201	RRYMGRAAPAQT	YMGAAPAQT	2	0	0	3	1	YMGRAAPAQT	tr_C9ZP82	0.083	20463.31	28.00
43	HLA-A0201	DSDTAIALL	DSDTAIALL	0	0	0	0	0	DSDTAIALL	tr_C9ZP82	0.083	20472.84	28.00
633	HLA-A0201	LKSTGLSNSVQEAC	LLSNSVQEA	0	0	0	1	4	LKSTGLSNSVQEA	tr_C9ZP82	0.083	20473.50	28.00
257	HLA-A0201	PTWTRLCVIFSGSN	WTLCVIFSG	2	0	0	2	1	WTRLCVIFSG	tr_C9ZP82	0.083	20473.73	28.00
258	HLA-A0201	TWTRLCVIFSGSN	WTLCVIFSG	1	0	0	2	1	WTRLCVIFSG	tr_C9ZP82	0.082	20480.15	28.00
700	HLA-A0201	VVKSTPPDTLVQL	VVPDTLVQL	0	0	0	2	4	VVKSTPPDTLVQL	tr_C9ZP82	0.082	20499.66	28.00
315	HLA-A0201	HITQVFILRDT	HITQVFILT	0	0	0	8	2	HITQVFILRDT	tr_C9ZP82	0.082	20507.65	28.00
279	HLA-A0201	IIDFLRTQSS	IIFLRTQSS	0	0	0	2	1	IIDFLRTQSS	tr_C9ZP82	0.082	20508.76	28.00
549	HLA-A0201	VLPYGFLTKGP	VLYGFLTKG	0	0	0	2	1	VLPYGFLTKG	tr_C9ZP82	0.082	20510.31	28.00
582	HLA-A0201	FRRFLLFKS	FRRFLLFKS	0	0	0	0	0	FRRFLLFKS	tr_C9ZP82	0.082	20510.31	28.00
703	HLA-A0201	STPPDTLVQLL	STDTLVQLL	0	0	0	2	2	STPPDTLVQLL	tr_C9ZP82	0.082	20515.19	28.00
639	HLA-A0201	SNSVQEACEKYVE	SVQEACEYV	2	0	0	7	1	SVQEACEKYV	tr_C9ZP82	0.082	20558.96	28.00
610	HLA-A0201	NVTVADVAVALH	VTVAVAVAL	1	0	0	4	1	VTVADVAVAL	tr_C9ZP82	0.082	20568.31	28.00
524	HLA-A0201	FLPHAPSAFSGE	FLAPSAFSG	0	0	0	2	2	FLPHAPSAFSG	tr_C9ZP82	0.082	20588.58	28.00
423	HLA-A0201	ISEVSALCDI	ISVSALCDI	0	0	0	2	1	ISEVSALCDI	tr_C9ZP82	0.082	20590.58	28.00
356	HLA-A0201	CIISLNPSYIDE	CIISLNPYI	0	0	0	7	1	CIISLNPSYI	tr_C9ZP82	0.082	20615.55	28.00
854	HLA-A0201	SCTVEVPRL	SCTVEVPRL	0	0	0	0	0	SCTVEVPRL	tr_C9ZP82	0.082	20633.63	28.00
756	HLA-A0201	SNVVHLATVMLP	VVLATVMLP	2	0	0	2	1	VVHLATVMLP	tr_C9ZP82	0.082	20641.21	28.00
78	HLA-A0201	YIAVDGVAP	YIAVDGVAP	0	0	0	0	0	YIAVDGVAP	tr_C9ZP82	0.082	20643.22	28.00
831	HLA-A0201	SREGVEIRQLYDV	GVEIRQLYV	3	0	0	8	1	GVEIRQLYDV	tr_C9ZP82	0.082	20649.03	28.00
825	HLA-A0201	ILRRVPSREGVEIR	ILRRVPVEI	0	0	0	6	4	ILRRVPSREGVEI	tr_C9ZP82	0.082	20649.25	28.00
438	HLA-A0201	ATESAFDKKTLV	ATFDKKTLV	0	0	0	2	3	ATESAFDKKTLV	tr_C9ZP82	0.082	20649.71	28.00
859	HLA-A0201	VPRLSYGADGQVI	RLYGADGQV	2	0	0	2	1	RLSYGADGQV	tr_C9ZP82	0.082	20664.68	28.00
670	HLA-A0201	SWYYPFYHAPPVI	SWYYPFYPV	0	0	0	7	3	SWYYPFYHAPPV	tr_C9ZP82	0.082	20668.48	28.00
312	HLA-A0201	LSLHITQVF	LSLHITQVF	0	0	0	0	0	LSLHITQVF	tr_C9ZP82	0.082	20671.39	28.00
768	HLA-A0201	FADMDVLQQ	FADMDVLQQ	0	0	0	0	0	FADMDVLQQ	tr_C9ZP82	0.082	20682.57	28.00
355	HLA-A0201	TCIISLNPS	TCIISLNPS	0	0	0	0	0	TCIISLNPS	tr_C9ZP82	0.082	20693.09	28.00
169	HLA-A0201	GDALNDGDGVNSFC	ALDGVNSFC	2	0	0	2	3	ALNDGDGVNSFC	tr_C9ZP82	0.081	20714.37	28.00
565	HLA-A0201	PQLQRLFEAY	QLQRLFEAY	1	0	0	0	0	QLQRLFEAY	tr_C9ZP82	0.081	20728.50	28.00
763	HLA-A0201	TVMLPFADMD	TVMLPFADM	0	0	0	0	0	TVMLPFADM	tr_C9ZP82	0.081	20732.53	28.00
755	HLA-A0201	DSNVVHLATVMLP	VVLATVMLP	3	0	0	2	1	VVHLATVMLP	tr_C9ZP82	0.081	20742.40	28.00

664	HLA-A0201	TTDSDWSWYYPF	TTWSWYYPF	0	0	0	2	3	TTDSDWSWYYPF	tr_C9ZP82	0.081	20750.48	28.00
257	HLA-A0201	PTWTRLCVIF	PTWTLCVIF	0	0	0	4	1	PTWTRLCVIF	tr_C9ZP82	0.081	20752.50	28.00
674	HLA-A0201	PFYHAPPVIDLAI	FYHVIDLAI	1	0	0	3	3	FYHAPPVIDLAI	tr_C9ZP82	0.081	20754.98	28.00
123	HLA-A0201	IVEEVVREFTSTEM	IVEEVVTEM	0	0	0	6	5	IVEEVVREFTSTEM	tr_C9ZP82	0.081	20759.25	28.00
838	HLA-A0201	RQLYDVPLERYRPR	QLYDVPLPR	1	0	0	7	4	QLYDVPLERYRPR	tr_C9ZP82	0.081	20794.09	29.00
261	HLA-A0201	RLCVIFSGSNTPGE	RLCVIFTPG	0	0	0	6	4	RLCVIFSGSNTPG	tr_C9ZP82	0.081	20794.09	29.00
614	HLA-A0201	ADVAVALHST	AVAVALHST	0	0	0	1	1	ADVAVALHST	tr_C9ZP82	0.081	20795.21	29.00
513	HLA-A0201	IIVLSILLGND	IIVLSILLG	0	0	0	0	0	IIVLSILLG	tr_C9ZP82	0.081	20796.34	29.00
121	HLA-A0201	MAIVEEVVREFTS	IVEEVVFTS	2	0	0	6	2	IVEEVVREFTS	tr_C9ZP82	0.081	20820.20	29.00
278	HLA-A0201	KIIDFLRTQSS	KIIDFLQSS	0	0	0	6	2	KIIDFLRTQSS	tr_C9ZP82	0.081	20821.56	29.00
239	HLA-A0201	KVSDAVLNMLNE	KVDAVLNML	0	0	0	2	1	KVSDAVLNML	tr_C9ZP82	0.081	20827.87	29.00
668	HLA-A0201	DWSWYYPFYH	WSWYYPFYH	1	0	0	0	0	WSWYYPFYH	tr_C9ZP82	0.081	20828.54	29.00
95	HLA-A0201	ARRYMGRAAPAQT	YMGAAPAQT	3	0	0	3	1	YMGRAAPAQT	tr_C9ZP82	0.081	20878.85	29.00
530	HLA-A0201	SAFSGESALDNL	FSGESALNL	2	0	0	7	1	FSGESALDNL	tr_C9ZP82	0.081	20908.24	29.00
121	HLA-A0201	MAIVEEVVREF	MAIVVVREF	0	0	0	4	2	MAIVEEVVREF	tr_C9ZP82	0.081	20910.96	29.00
638	HLA-A0201	LSNSVQEACEKYVE	SVQEACEYV	3	0	0	7	1	SVQEACEKYV	tr_C9ZP82	0.081	20919.56	29.00
830	HLA-A0201	PSREGVEIRQLYDV	GVEIRQLYV	4	0	0	8	1	GVEIRQLYDV	tr_C9ZP82	0.080	20927.26	29.00
412	HLA-A0201	YFDISVVGGSLISE	YFDISVVLI	0	0	0	7	3	YFDISVVGGSLI	tr_C9ZP82	0.080	20932.92	29.00
894	HLA-A0201	KKSNSRVTVV	KSNSRVTVV	0	0	0	1	1	KKSNSRVTVV	tr_C9ZP82	0.080	20937.45	29.00
823	HLA-A0201	DTILRRVPSREGVE	ILRRVPEGV	2	0	0	6	2	ILRRVPSREGV	tr_C9ZP82	0.080	20937.90	29.00
19	HLA-A0201	KNCPSSVHGLYIDL	KVHGLYIDL	0	0	0	1	5	KNCPSSVHGLYIDL	tr_C9ZP82	0.080	20940.84	29.00
812	HLA-A0201	KGDLRKLSDAL	KLRKLSDAL	0	0	0	1	2	KGDLRKLSDAL	tr_C9ZP82	0.080	20950.81	29.00
292	HLA-A0201	FGGKANHVIVGLDA	FANHVIVGL	0	0	0	1	3	FGGKANHVIVGL	tr_C9ZP82	0.080	20961.25	29.00
29	HLA-A0201	YIDLNGIIH	YIDLNGIIH	0	0	0	0	0	YIDLNGIIH	tr_C9ZP82	0.080	20965.11	29.00
663	HLA-A0201	TTTDSDWSWYYPF	TTWSWYYPF	0	0	0	2	4	TTTDSDWSWYYPF	tr_C9ZP82	0.080	20968.06	29.00
882	HLA-A0201	QLKGNGEGVI	QLGNGEGVI	0	0	0	2	1	QLKGNGEGVI	tr_C9ZP82	0.080	20976.68	29.00
609	HLA-A0201	DNVTVADVAVALH	VTVAVAVAL	2	0	0	4	1	VTVADVAVAL	tr_C9ZP82	0.080	20981.22	29.00
697	HLA-A0201	PLPVVKSTPPDTLV	PLPVVDTLV	0	0	0	5	5	PLPVVKSTPPDTLV	tr_C9ZP82	0.080	20992.12	29.00
284	HLA-A0201	RTQSSLAQF	RTQSSLAQF	0	0	0	0	0	RTQSSLAQF	tr_C9ZP82	0.080	21000.06	29.00
693	HLA-A0201	IKRMPLPVVKST	RMLPVVKST	2	0	0	2	1	RMPLPVVKST	tr_C9ZP82	0.080	21008.71	29.00
858	HLA-A0201	EVPRLSYGADGQVI	RLYGADGQV	3	0	0	2	1	RLSYGADGQV	tr_C9ZP82	0.080	21011.20	29.00
531	HLA-A0201	AFSGESALDNL	FSGESALNL	1	0	0	7	1	FSGESALDNL	tr_C9ZP82	0.080	21015.29	29.00
578	HLA-A0201	EAVKFRRFLL	AVKFRRFLL	1	0	0	0	0	AVKFRRFLL	tr_C9ZP82	0.080	21015.75	29.00
215	HLA-A0201	VIVDDPTAFEF	VIVDTAFEF	0	0	0	4	2	VIVDDPTAFEF	tr_C9ZP82	0.080	21022.57	29.00
662	HLA-A0201	TTTTDSDWSWYYPF	TTWSWYYPF	0	0	0	2	5	TTTTDSDWSWYYPF	tr_C9ZP82	0.080	21087.95	29.00
754	HLA-A0201	HDSNVVHLATVMLP	VVLATVMLP	4	0	0	2	1	VVHLATVMLP	tr_C9ZP82	0.080	21093.21	29.00
761	HLA-A0201	LATVMLPFADMD	ATVMLPFAM	1	0	0	8	1	ATVMLPFADM	tr_C9ZP82	0.080	21109.41	29.00
94	HLA-A0201	RARRYMGRAAPAQT	YMGAAPAQT	4	0	0	3	1	YMGRAAPAQT	tr_C9ZP82	0.080	21124.27	29.00
678	HLA-A0201	APPVIDLAI	APPVIDLAI	0	0	0	0	0	APPVIDLAI	tr_C9ZP82	0.080	21145.08	29.00
721	HLA-A0201	HALLPMVLG	HALLPMVLG	0	0	0	0	0	HALLPMVLG	tr_C9ZP82	0.080	21150.10	29.00
861	HLA-A0201	RLSYGADGQVIKR	RLYGADGQV	0	0	0	2	1	RLSYGADGQV	tr_C9ZP82	0.080	21154.00	29.00
46	HLA-A0201	TAIALLPEEEKLEC	TAIALLLEC	0	0	0	6	5	TAIALLPEEEKLEC	tr_C9ZP82	0.080	21154.22	29.00
833	HLA-A0201	EGVEIRQLYDVPL	GVQLYDVPL	1	0	0	2	3	GVEIRQLYDVPL	tr_C9ZP82	0.079	21154.69	29.00
21	HLA-A0201	CPSSVHGLYI	CSSVHGLYI	0	0	0	1	1	CPSSVHGLYI	tr_C9ZP82	0.079	21208.31	29.00
608	HLA-A0201	NDNVTVADVAVALH	VTVAVAVAL	3	0	0	4	1	VTVADVAVAL	tr_C9ZP82	0.079	21218.18	29.00
622	HLA-A0201	STIDDRWRDTYLK	STIDDRWYL	0	0	0	7	3	STIDDRWRDTYL	tr_C9ZP82	0.079	21225.99	29.00
611	HLA-A0201	VTVADVAVALHS	VTVAVAVAL	0	0	0	4	1	VTVADVAVAL	tr_C9ZP82	0.079	21257.02	29.00
69	HLA-A0201	QTVQPKEVMYIAVD	QTVVMYIAV	0	0	0	3	4	QTVQPKEVMYIAV	tr_C9ZP82	0.079	21264.38	29.00
692	HLA-A0201	NIKRMPLPVVKST	RMLPVVKST	3	0	0	2	1	RMPLPVVKST	tr_C9ZP82	0.079	21274.27	29.00
664	HLA-A0201	TTDSDWSWYY	TTDDWSWYY	0	0	0	3	1	TTDSDWSWYY	tr_C9ZP82	0.079	21302.38	29.00
750	HLA-A0201	NVDYHDSNVVHLA	NVDYVVHLA	0	0	0	4	4	NVDYHDSNVVHLA	tr_C9ZP82	0.079	21320.59	29.00
254	HLA-A0201	GNDPTWTRLCVIF	GTWTRLCVI	0	0	0	1	3	GNDPTWTRLCVI	tr_C9ZP82	0.079	21322.21	29.00
632	HLA-A0201	YLKSTGLSN	YLKSTGLSN	0	0	0	0	0	YLKSTGLSN	tr_C9ZP82	0.079	21327.05	29.00
613	HLA-A0201	VADVAVALHSTI	VADVAVALI	0	0	0	8	3	VADVAVALHSTI	tr_C9ZP82	0.079	21353.14	29.00

725	HLA-A0201	PMVLGEVMVRSPQ	VLGEVMVPQ	2	0	0	7	2	VLGEVMVRSPQ	tr_C9ZP82	0.079	21359.84	29.00
674	HLA-A0201	PFYHAPPVIDLA	FAPPVIDLA	1	0	0	1	2	FYHAPPVIDLA	tr_C9ZP82	0.079	21368.16	29.00
529	HLA-A0201	PSAFSGESAL	SAFSGESAL	1	0	0	0	0	SAFSGESAL	tr_C9ZP82	0.078	21442.51	30.00
538	HLA-A0201	LDNLLELYVS	LDNLLELYV	0	0	0	0	0	LDNLLELYV	tr_C9ZP82	0.078	21454.12	30.00
730	HLA-A0201	EVMVRSPQQA	VMVRSPQQA	1	0	0	0	0	VMVRSPQQA	tr_C9ZP82	0.078	21476.40	30.00
691	HLA-A0201	ANIKRMPLPVVKST	RMLPVVKST	4	0	0	2	1	RMPLPVVKST	tr_C9ZP82	0.078	21490.59	30.00
435	HLA-A0201	FTPATESAF	FTPATESAF	0	0	0	0	0	FTPATESAF	tr_C9ZP82	0.078	21503.15	30.00
237	HLA-A0201	MAKVSDAVLNMLNE	MADAVLNML	0	0	0	2	3	MAKVSDAVLNML	tr_C9ZP82	0.078	21511.29	30.00
776	HLA-A0201	QVVDAAWPQLT	QVVDAAWPL	0	0	0	8	1	QVVDAAWPQL	tr_C9ZP82	0.078	21518.97	30.00
516	HLA-A0201	LSILLGNDF	LSILLGNDF	0	0	0	0	0	LSILLGNDF	tr_C9ZP82	0.078	21535.52	30.00
105	HLA-A0201	AQTGEVASTYM	AQTGEVAYM	0	0	0	7	2	AQTGEVASTYM	tr_C9ZP82	0.078	21557.66	30.00
731	HLA-A0201	VMVRSPQQAELE	VMSPQQAEL	0	0	0	2	2	VMVRSPQQAEL	tr_C9ZP82	0.078	21580.30	30.00
683	HLA-A0201	DLAIYLKEANIKR	DLAIYLANI	0	0	0	6	2	DLAIYLKEANI	tr_C9ZP82	0.078	21581.46	30.00
250	HLA-A0201	EKMSGNDPTWT	KMSGNDPTT	1	0	0	8	1	KMSGNDPTWT	tr_C9ZP82	0.078	21583.33	30.00
774	HLA-A0201	LQQVVDAAWPQLT	QVVDAAWPL	2	0	0	8	1	QVVDAAWPQL	tr_C9ZP82	0.078	21595.71	30.00
586	HLA-A0201	LLFKSTNKE	LLFKSTNKE	0	0	0	0	0	LLFKSTNKE	tr_C9ZP82	0.078	21607.16	30.00
316	HLA-A0201	ITQVFILRD	ITQVFILRD	0	0	0	0	0	ITQVFILRD	tr_C9ZP82	0.078	21614.41	30.00
579	HLA-A0201	AVKFRRFLLFKST	AVFLLFKST	0	0	0	2	4	AVKFRRFLLFKST	tr_C9ZP82	0.077	21622.37	30.00
77	HLA-A0201	MYIAVDGVAPRAKM	YIAVDGVAM	1	0	0	8	4	YIAVDGVAPRAKM	tr_C9ZP82	0.077	21630.32	30.00
292	HLA-A0201	FGGKANHVIV	FGGANHVIV	0	0	0	3	1	FGGKANHVIV	tr_C9ZP82	0.077	21630.79	30.00
355	HLA-A0201	TCIISLNPSYIDE	CIISLNPYI	1	0	0	7	1	CIISLNPSYI	tr_C9ZP82	0.077	21658.65	30.00
600	HLA-A0201	AEFRKGSNNDNVTV	FSNNDNVTV	2	0	0	1	3	FRKGSNNDNVTV	tr_C9ZP82	0.077	21660.30	30.00
752	HLA-A0201	DYHDSNVVHLATVM	YVVHLATVM	1	0	0	1	4	YHDSNVVHLATVM	tr_C9ZP82	0.077	21666.16	30.00
70	HLA-A0201	TVQPKEVMYIAVD	VQPVMYIAV	1	0	0	3	2	VQPKEVMYIAV	tr_C9ZP82	0.077	21683.04	30.00
280	HLA-A0201	IDFLRTQSSLA	FLRTQSSLA	2	0	0	0	0	FLRTQSSLA	tr_C9ZP82	0.077	21694.31	30.00
157	HLA-A0201	SGALYNEGEEEAGD	ALYEGEEEA	2	0	0	3	1	ALYNEGEEEA	tr_C9ZP82	0.077	21706.28	30.00
443	HLA-A0201	FDKKTLVSSAGYY	FLVSSAGYY	0	0	0	1	4	FDKKTLVSSAGYY	tr_C9ZP82	0.077	21715.68	30.00
502	HLA-A0201	SSPANSKIIDDIIV	SSIIDDIIV	0	0	0	2	5	SSPANSKIIDDIIV	tr_C9ZP82	0.077	21729.07	30.00
289	HLA-A0201	LAQFGGKANHVI	AQFGGANHV	1	0	0	5	1	AQFGGKANHV	tr_C9ZP82	0.077	21737.77	30.00
683	HLA-A0201	DLAIYLKEAN	DLAIYLKEA	0	0	0	0	0	DLAIYLKEA	tr_C9ZP82	0.077	21747.90	30.00
356	HLA-A0201	CIISLNPSY	CIISLNPSY	0	0	0	0	0	CIISLNPSY	tr_C9ZP82	0.077	21760.37	30.00
584	HLA-A0201	RFLLFKSTNKENS	FLLFKSTNS	1	0	0	7	3	FLLFKSTNKENS	tr_C9ZP82	0.077	21761.77	30.00
635	HLA-A0201	STGLSNSVQEAC	SLSNSVQEA	0	0	0	1	2	STGLSNSVQEA	tr_C9ZP82	0.077	21763.19	30.00
316	HLA-A0201	ITQVFILRDTTR	ITQVFILTT	0	0	0	7	2	ITQVFILRDTT	tr_C9ZP82	0.077	21765.31	30.00
213	HLA-A0201	GEVIVDDPTAFEF	GEVIVAFEF	0	0	0	5	4	GEVIVDDPTAFEF	tr_C9ZP82	0.077	21791.24	30.00
314	HLA-A0201	LHITQVFILRDT	HITQVFILT	1	0	0	8	2	HITQVFILRDT	tr_C9ZP82	0.077	21820.96	30.00
579	HLA-A0201	AVKFRRFLLFK	AVFRRFLLF	0	0	0	2	1	AVKFRRFLLF	tr_C9ZP82	0.077	21849.78	30.00
21	HLA-A0201	CPSSVHGLYIDL	CVHGLYIDL	0	0	0	1	3	CPSSVHGLYIDL	tr_C9ZP82	0.077	21852.14	30.00
354	HLA-A0201	DTCIISLNPSYIDE	CIISLNPYI	2	0	0	7	1	CIISLNPSYI	tr_C9ZP82	0.076	21867.99	30.00
726	HLA-A0201	MVLGEVMVRSPQQ	MLGEVMVRS	0	0	0	1	1	MVLGEVMVRS	tr_C9ZP82	0.076	21868.47	30.00
137	HLA-A0201	AQANDDLDEVRQT	AQADDLDEV	0	0	0	3	1	AQANDDLDEV	tr_C9ZP82	0.076	21868.47	30.00
35	HLA-A0201	IIHPCCHNDS	IIHPCCHNS	0	0	0	8	1	IIHPCCHNDS	tr_C9ZP82	0.076	21871.54	30.00
832	HLA-A0201	REGVEIRQLYDVPL	GVQLYDVPL	2	0	0	2	3	GVEIRQLYDVPL	tr_C9ZP82	0.076	21892.61	30.00
20	HLA-A0201	NCPSSVHGL	NCPSSVHGL	0	0	0	0	0	NCPSSVHGL	tr_C9ZP82	0.076	21910.86	30.00
790	HLA-A0201	RWRTQKLDFHL	RTQKLDFHL	0	0	0	1	2	RWRTQKLDFHL	tr_C9ZP82	0.076	21920.11	30.00
416	HLA-A0201	SVVGGSLIS	SVVGGSLIS	0	0	0	0	0	SVVGGSLIS	tr_C9ZP82	0.076	21940.27	30.00
239	HLA-A0201	KVSDAVLNMLNEK	KVDAVLNML	0	0	0	2	1	KVSDAVLNML	tr_C9ZP82	0.076	21943.13	30.00
79	HLA-A0201	IAVDGVAPRAKM	IAVDGVAPM	0	0	0	8	3	IAVDGVAPRAKM	tr_C9ZP82	0.076	21948.11	30.00
529	HLA-A0201	PSAFSGESALDNL	FSGESALNL	3	0	0	7	1	FSGESALDNL	tr_C9ZP82	0.076	21949.54	30.00
246	HLA-A0201	NMLNEKMSG	NMLNEKMSG	0	0	0	0	0	NMLNEKMSG	tr_C9ZP82	0.076	21969.96	30.00
413	HLA-A0201	FDISVVGGSLIS	FISVVGGSL	0	0	0	1	1	FDISVVGGSL	tr_C9ZP82	0.076	21979.49	30.00
720	HLA-A0201	SHALLPMVLGE	SLLPMVLGE	0	0	0	1	2	SHALLPMVLGE	tr_C9ZP82	0.076	21981.61	30.00
577	HLA-A0201	IEAVKFRRFLL	IEAVKFFLL	0	0	0	6	2	IEAVKFRRFLL	tr_C9ZP82	0.076	21986.86	30.00
80	HLA-A0201	AVDGVAPRAK	AVDGVAPRA	0	0	0	0	0	AVDGVAPRA	tr_C9ZP82	0.076	22001.13	30.00

848	HLA-A0201	YRPRTYSCTV	YRPTYSCTV	0	0	0	3	1	YRPRTYSCTV	tr_C9ZP82	0.076	22016.13	30.00
404	HLA-A0201	TVPNTGFEYF	TVPNTFEYF	0	0	0	5	1	TVPNTGFEYF	tr_C9ZP82	0.076	22018.05	30.00
135	HLA-A0201	EMAQANDDLDEVRQ	EMADDLDEV	0	0	0	3	3	EMAQANDDLDEV	tr_C9ZP82	0.076	22043.78	30.00
854	HLA-A0201	SCTVEVPRLSYGA	CTVEVSYGA	1	0	0	5	3	CTVEVPRLSYGA	tr_C9ZP82	0.076	22050.70	30.00
72	HLA-A0201	QPKEVMYIAV	QPKEVMIAV	0	0	0	6	1	QPKEVMYIAV	tr_C9ZP82	0.076	22051.66	30.00
440	HLA-A0201	ESAFDKKTLVSSA	SAFDLVSSA	1	0	0	4	3	SAFDKKTLVSSA	tr_C9ZP82	0.076	22066.45	30.00
171	HLA-A0201	ALNDGDGVNSF	ALGDGVNSF	0	0	0	2	2	ALNDGDGVNSF	tr_C9ZP82	0.076	22072.90	31.00
744	HLA-A0201	TFPLQWNVDYH	FLQWNVDYH	1	0	0	1	1	FPLQWNVDYH	tr_C9ZP82	0.076	22074.58	31.00
577	HLA-A0201	IEAVKFRRFLLFK	AVFRRFLLF	2	0	0	2	1	AVKFRRFLLF	tr_C9ZP82	0.076	22075.05	31.00
611	HLA-A0201	VTVADVAVALHST	VTVAVAVAL	0	0	0	4	1	VTVADVAVAL	tr_C9ZP82	0.076	22087.00	31.00
577	HLA-A0201	IEAVKFRRFL	IAVKFRRFL	0	0	0	1	1	IEAVKFRRFL	tr_C9ZP82	0.075	22108.27	31.00
212	HLA-A0201	RGEVIVDDPTAFEF	GEVIVAFEF	1	0	0	5	4	GEVIVDDPTAFEF	tr_C9ZP82	0.075	22111.15	31.00
861	HLA-A0201	RLSYGADGQVIKRQ	RLYGADGQV	0	0	0	2	1	RLSYGADGQV	tr_C9ZP82	0.075	22113.31	31.00
631	HLA-A0201	TYLKSTGLSNS	YLSTGLSNS	1	0	0	2	1	YLKSTGLSNS	tr_C9ZP82	0.075	22121.92	31.00
291	HLA-A0201	QFGGKANHVIVGLD	FANHVIVGL	1	0	0	1	3	FGGKANHVIVGL	tr_C9ZP82	0.075	22129.57	31.00
651	HLA-A0201	EGVCFVWRY	EGVCFVWRY	0	0	0	0	0	EGVCFVWRY	tr_C9ZP82	0.075	22155.22	31.00
77	HLA-A0201	MYIAVDGVAPRAK	YIDGVAPRA	1	0	0	2	2	YIAVDGVAPRA	tr_C9ZP82	0.075	22178.95	31.00
249	HLA-A0201	NEKMSGNDPTWT	KMSGNDPTT	2	0	0	8	1	KMSGNDPTWT	tr_C9ZP82	0.075	22190.48	31.00
446	HLA-A0201	KTLVSSAGYY	KLVSSAGYY	0	0	0	1	1	KTLVSSAGYY	tr_C9ZP82	0.075	22204.65	31.00
528	HLA-A0201	APSAFSGESALDNL	FSGESALNL	4	0	0	7	1	FSGESALDNL	tr_C9ZP82	0.075	22211.61	31.00
580	HLA-A0201	VKFRRFLLFK	VKFRFLLFK	0	0	0	3	1	VKFRRFLLFK	tr_C9ZP82	0.075	22214.51	31.00
754	HLA-A0201	HDSNVVHLATV	DSNVVLATV	1	0	0	5	1	DSNVVHLATV	tr_C9ZP82	0.075	22231.33	31.00
673	HLA-A0201	YPFYHAPPVIDL	YPFYHVIDL	0	0	0	5	3	YPFYHAPPVIDL	tr_C9ZP82	0.075	22232.05	31.00
356	HLA-A0201	CIISLNPSYIDEE	CIISLNPYI	0	0	0	7	1	CIISLNPSYI	tr_C9ZP82	0.075	22243.84	31.00
49	HLA-A0201	ALLPEEEKLEC	ALLPEELEC	0	0	0	6	2	ALLPEEEKLEC	tr_C9ZP82	0.075	22245.77	31.00
646	HLA-A0201	CEKYVEGVCFVWR	YVEGVCFWR	3	0	0	7	1	YVEGVCFVWR	tr_C9ZP82	0.075	22252.75	31.00
71	HLA-A0201	VQPKEVMYIAVDG	VQPVMYIAV	0	0	0	3	2	VQPKEVMYIAV	tr_C9ZP82	0.075	22255.88	31.00
666	HLA-A0201	DSDWSWYYPF	SDWSWYYPF	1	0	0	0	0	SDWSWYYPF	tr_C9ZP82	0.075	22266.00	31.00
154	HLA-A0201	PLYSGALYN	PLYSGALYN	0	0	0	0	0	PLYSGALYN	tr_C9ZP82	0.075	22266.24	31.00
855	HLA-A0201	CTVEVPRLSY	CTVEVPLSY	0	0	0	6	1	CTVEVPRLSY	tr_C9ZP82	0.075	22278.05	31.00
640	HLA-A0201	NSVQEACEKYVEG	SVQEACEYV	1	0	0	7	1	SVQEACEKYV	tr_C9ZP82	0.075	22278.77	31.00
648	HLA-A0201	KYVEGVCFVWR	YVEGVCFWR	1	0	0	7	1	YVEGVCFVWR	tr_C9ZP82	0.075	22285.03	31.00
462	HLA-A0201	HIEDGTLPSS	HIDGTLPSS	0	0	0	2	1	HIEDGTLPSS	tr_C9ZP82	0.075	22296.37	31.00
880	HLA-A0201	SGQLKGNGEGV	SLKGNGEGV	0	0	0	1	2	SGQLKGNGEGV	tr_C9ZP82	0.075	22300.23	31.00
647	HLA-A0201	EKYVEGVCFVWR	YVEGVCFWR	2	0	0	7	1	YVEGVCFVWR	tr_C9ZP82	0.074	22331.37	31.00
607	HLA-A0201	NNDNVTVADV	NNDNVTVAV	0	0	0	8	1	NNDNVTVADV	tr_C9ZP82	0.074	22360.62	31.00
223	HLA-A0201	FEFDSNCISP	FEFDSCISP	0	0	0	5	1	FEFDSNCISP	tr_C9ZP82	0.074	22360.87	31.00
39	HLA-A0201	CCHNDSDTAIALL	CCHTAIALL	0	0	0	3	4	CCHNDSDTAIALL	tr_C9ZP82	0.074	22372.97	31.00
213	HLA-A0201	GEVIVDDPTA	GVIVDDPTA	0	0	0	1	1	GEVIVDDPTA	tr_C9ZP82	0.074	22387.75	31.00
610	HLA-A0201	NVTVADVAVALHS	VTVAVAVAL	1	0	0	4	1	VTVADVAVAL	tr_C9ZP82	0.074	22392.84	31.00
58	HLA-A0201	ECICSELEL	ECICSELEL	0	0	0	0	0	ECICSELEL	tr_C9ZP82	0.074	22394.05	31.00
889	HLA-A0201	GVIEGKKSNSRV	GVIEGNSRV	0	0	0	5	3	GVIEGKKSNSRV	tr_C9ZP82	0.074	22395.26	31.00
676	HLA-A0201	YHAPPVIDLAI	HAPPVILAI	1	0	0	6	1	HAPPVIDLAI	tr_C9ZP82	0.074	22431.15	31.00
675	HLA-A0201	FYHAPPVIDLAIY	FYVIDLAIY	0	0	0	2	4	FYHAPPVIDLAIY	tr_C9ZP82	0.074	22436.74	31.00
745	HLA-A0201	FPLQWNVDYHD	FQWNVDYHD	0	0	0	1	2	FPLQWNVDYHD	tr_C9ZP82	0.074	22437.71	31.00
853	HLA-A0201	YSCTVEVPRLS	YSCTVEVPL	0	0	0	8	1	YSCTVEVPRL	tr_C9ZP82	0.074	22447.17	31.00
581	HLA-A0201	KFRRFLLFK	KFRRFLLFK	0	0	0	0	0	KFRRFLLFK	tr_C9ZP82	0.074	22451.30	31.00
315	HLA-A0201	HITQVFILRDTTR	ITQVFILTT	1	0	0	7	2	ITQVFILRDTT	tr_C9ZP82	0.074	22459.07	31.00
615	HLA-A0201	DVAVALHSTI	DVAVALHTI	0	0	0	7	1	DVAVALHSTI	tr_C9ZP82	0.074	22470.51	31.00
138	HLA-A0201	QANDDLDEVR	QANDDLDEV	0	0	0	0	0	QANDDLDEV	tr_C9ZP82	0.074	22471.71	31.00
776	HLA-A0201	QVVDAAWPQ	QVVDAAWPQ	0	0	0	0	0	QVVDAAWPQ	tr_C9ZP82	0.074	22473.42	31.00
77	HLA-A0201	MYIAVDGVA	MYIAVDGVA	0	0	0	0	0	MYIAVDGVA	tr_C9ZP82	0.074	22485.09	31.00
548	HLA-A0201	EVLPYGFLTKGP	VLYGFLTKG	1	0	0	2	1	VLPYGFLTKG	tr_C9ZP82	0.074	22487.77	31.00
828	HLA-A0201	RVPSREGVEI	RVPSREVEI	0	0	0	6	1	RVPSREGVEI	tr_C9ZP82	0.074	22488.99	31.00

199	HLA-A0201	VESNQVVDDV	VSNQVVDDV	0	0	0	1	1	VESNQVVDDV	tr_C9ZP82	0.074	22490.21	31.00
455	HLA-A0201	YFFGQPNHIE	YFFGQPNHI	0	0	0	0	0	YFFGQPNHI	tr_C9ZP82	0.074	22496.78	31.00
220	HLA-A0201	PTAFEFDSNCI	TAFEFSNCI	1	0	0	5	1	TAFEFDSNCI	tr_C9ZP82	0.074	22516.01	31.00
289	HLA-A0201	LAQFGGKANHVIVG	AQFGGHVIV	1	0	0	5	3	AQFGGKANHVIV	tr_C9ZP82	0.074	22516.51	31.00
686	HLA-A0201	IYLKEANIKRMP	YLKEANIRM	1	0	0	7	1	YLKEANIKRM	tr_C9ZP82	0.074	22521.37	31.00
671	HLA-A0201	WYYPFYHAPPVI	WYYPFYPPV	0	0	0	6	2	WYYPFYHAPPV	tr_C9ZP82	0.074	22544.53	31.00
89	HLA-A0201	KMNQQRARRYM	KMNQQRAYM	0	0	0	7	2	KMNQQRARRYM	tr_C9ZP82	0.074	22565.52	31.00
681	HLA-A0201	VIDLAIYLKE	VIDLAIYLE	0	0	0	8	1	VIDLAIYLKE	tr_C9ZP82	0.074	22569.92	31.00
583	HLA-A0201	RRFLLFKSTNKENS	FLLFKSTNS	2	0	0	7	3	FLLFKSTNKENS	tr_C9ZP82	0.073	22576.76	31.00
248	HLA-A0201	LNEKMSGNDPTWT	KMSGNDPTT	3	0	0	8	1	KMSGNDPTWT	tr_C9ZP82	0.073	22586.53	31.00
672	HLA-A0201	YYPFYHAPPVIDL	YYPFYVIDL	0	0	0	5	4	YYPFYHAPPVIDL	tr_C9ZP82	0.073	22590.68	31.00
702	HLA-A0201	KSTPPDTLVQLL	STDTLVQLL	1	0	0	2	2	STPPDTLVQLL	tr_C9ZP82	0.073	22637.18	31.00
198	HLA-A0201	YVESNQVVDDVF	YVNQVVDDV	0	0	0	2	2	YVESNQVVDDV	tr_C9ZP82	0.073	22649.18	31.00
719	HLA-A0201	TSHALLPMVLGE	ALLPMVLGE	3	0	0	0	0	ALLPMVLGE	tr_C9ZP82	0.073	22658.74	31.00
547	HLA-A0201	SEVLPYGFL	SEVLPYGFL	0	0	0	0	0	SEVLPYGFL	tr_C9ZP82	0.073	22669.03	31.00
214	HLA-A0201	EVIVDDPTAFEF	VIVDTAFEF	1	0	0	4	2	VIVDDPTAFEF	tr_C9ZP82	0.073	22671.00	31.00
578	HLA-A0201	EAVKFRRFLLFK	AVFRRFLLF	1	0	0	2	1	AVKFRRFLLF	tr_C9ZP82	0.073	22691.36	32.00
693	HLA-A0201	IKRMPLPVV	IKRMPLPVV	0	0	0	0	0	IKRMPLPVV	tr_C9ZP82	0.073	22696.03	32.00
544	HLA-A0201	LYVSEVLPY	LYVSEVLPY	0	0	0	0	0	LYVSEVLPY	tr_C9ZP82	0.073	22697.76	32.00
546	HLA-A0201	VSEVLPYGFLTK	VLPYGFLTK	0	0	0	1	3	VSEVLPYGFLTK	tr_C9ZP82	0.073	22698.24	32.00
810	HLA-A0201	VPKGDLRKLSDAL	VLRKLSDAL	0	0	0	1	4	VPKGDLRKLSDAL	tr_C9ZP82	0.073	22699.22	32.00
238	HLA-A0201	AKVSDAVLNMLNE	KVDAVLNML	1	0	0	2	1	KVSDAVLNML	tr_C9ZP82	0.073	22708.31	32.00
547	HLA-A0201	SEVLPYGFLTK	SLPYGFLTK	0	0	0	1	2	SEVLPYGFLTK	tr_C9ZP82	0.073	22722.07	32.00
136	HLA-A0201	MAQANDDLDEVRQ	AQADDLDEV	1	0	0	3	1	AQANDDLDEV	tr_C9ZP82	0.073	22743.47	32.00
623	HLA-A0201	TIDDRWRDTYLK	TIDDRWTYL	0	0	0	6	2	TIDDRWRDTYL	tr_C9ZP82	0.073	22755.78	32.00
650	HLA-A0201	VEGVCFVWRYY	VVCFVWRYY	0	0	0	1	2	VEGVCFVWRYY	tr_C9ZP82	0.073	22769.57	32.00
424	HLA-A0201	SEVSALCDI	SEVSALCDI	0	0	0	0	0	SEVSALCDI	tr_C9ZP82	0.073	22786.83	32.00
853	HLA-A0201	YSCTVEVPR	YSCTVEVPR	0	0	0	0	0	YSCTVEVPR	tr_C9ZP82	0.073	22787.08	32.00
20	HLA-A0201	NCPSSVHGLYIDL	SVHGLYIDL	4	0	0	0	0	SVHGLYIDL	tr_C9ZP82	0.073	22797.93	32.00
373	HLA-A0201	VIGVPVCES	VIGVPVCES	0	0	0	0	0	VIGVPVCES	tr_C9ZP82	0.073	22805.57	32.00
716	HLA-A0201	LPPTSHALLPMVL	LSHALLPMV	0	0	0	1	3	LPPTSHALLPMV	tr_C9ZP82	0.072	22823.10	32.00
288	HLA-A0201	SLAQFGGKAN	SLAQFGGKA	0	0	0	0	0	SLAQFGGKA	tr_C9ZP82	0.072	22841.87	32.00
800	HLA-A0201	LVITSNKTSFVP	LVITSTSFV	0	0	0	5	2	LVITSNKTSFV	tr_C9ZP82	0.072	22848.79	32.00
115	HLA-A0201	MPNKSGMAIV	MPNSGMAIV	0	0	0	3	1	MPNKSGMAIV	tr_C9ZP82	0.072	22852.51	32.00
700	HLA-A0201	VVKSTPPDTL	VVKSTPPTL	0	0	0	7	1	VVKSTPPDTL	tr_C9ZP82	0.072	22855.22	32.00
523	HLA-A0201	DFLPHAPSAFSGE	FLAPSAFSG	1	0	0	2	2	FLPHAPSAFSG	tr_C9ZP82	0.072	22855.72	32.00
630	HLA-A0201	DTYLKSTGL	DTYLKSTGL	0	0	0	0	0	DTYLKSTGL	tr_C9ZP82	0.072	22860.66	32.00
578	HLA-A0201	EAVKFRRFLLFKST	AVFLLFKST	1	0	0	2	4	AVKFRRFLLFKST	tr_C9ZP82	0.072	22865.11	32.00
630	HLA-A0201	DTYLKSTGLSNS	YLSTGLSNS	2	0	0	2	1	YLKSTGLSNS	tr_C9ZP82	0.072	22865.11	32.00
321	HLA-A0201	ILRDTTRNPY	ILRDTTNPY	0	0	0	6	1	ILRDTTRNPY	tr_C9ZP82	0.072	22868.83	32.00
22	HLA-A0201	PSSVHGLYIDL	SVHGLYIDL	2	0	0	0	0	SVHGLYIDL	tr_C9ZP82	0.072	22872.53	32.00
452	HLA-A0201	AGYYFFGQPNHI	AGYYFFNHI	0	0	0	6	3	AGYYFFGQPNHI	tr_C9ZP82	0.072	22880.70	32.00
355	HLA-A0201	TCIISLNPSYIDEE	CIISLNPYI	1	0	0	7	1	CIISLNPSYI	tr_C9ZP82	0.072	22882.93	32.00
175	HLA-A0201	GDGVNSFCV	GDGVNSFCV	0	0	0	0	0	GDGVNSFCV	tr_C9ZP82	0.072	22905.48	32.00
609	HLA-A0201	DNVTVADVAVALHS	VTVAVAVAL	2	0	0	4	1	VTVADVAVAL	tr_C9ZP82	0.072	22920.59	32.00
221	HLA-A0201	TAFEFDSNCISP	TAFEFCISP	0	0	0	5	3	TAFEFDSNCISP	tr_C9ZP82	0.072	22929.78	32.00
64	HLA-A0201	LELLVQTVQPK	LLVQTVQPK	0	0	0	1	2	LELLVQTVQPK	tr_C9ZP82	0.072	22961.80	32.00
439	HLA-A0201	TESAFDKKTLVSSA	SAFDLVSSA	2	0	0	4	3	SAFDKKTLVSSA	tr_C9ZP82	0.072	22962.30	32.00
770	HLA-A0201	DMDVLQQVVD	DMDVLQQVV	0	0	0	0	0	DMDVLQQVV	tr_C9ZP82	0.072	22962.55	32.00
368	HLA-A0201	EESDGVIGVPV	ESDGVIVPV	1	0	0	6	1	ESDGVIGVPV	tr_C9ZP82	0.072	22964.79	32.00
493	HLA-A0201	RSWKNFRPC	RSWKNFRPC	0	0	0	0	0	RSWKNFRPC	tr_C9ZP82	0.072	22969.26	32.00
532	HLA-A0201	FSGESALDNLLE	FSGESALLL	0	0	0	7	2	FSGESALDNLL	tr_C9ZP82	0.072	22970.74	32.00
737	HLA-A0201	QQAELESTF	QQAELESTF	0	0	0	0	0	QQAELESTF	tr_C9ZP82	0.072	22980.69	32.00
427	HLA-A0201	SALCDIKKFTP	ALCDIKFTP	1	0	0	5	1	ALCDIKKFTP	tr_C9ZP82	0.072	23001.09	32.00

727	HLA-A0201	VLGEVMVRSPQQ	VLGEVMVPQ	0	0	0	7	2	VLGEVMVRSPQ	tr_C9ZP82	0.072	23013.03	32.00
718	HLA-A0201	PTSHALLPMVLGE	ALLPMVLGE	4	0	0	0	0	ALLPMVLGE	tr_C9ZP82	0.072	23027.73	32.00
650	HLA-A0201	VEGVCFVWRY	VGVCFVWRY	0	0	0	1	1	VEGVCFVWRY	tr_C9ZP82	0.072	23034.21	32.00
789	HLA-A0201	ERWRTQKLDFHL	RTQKLDFHL	3	0	0	0	0	RTQKLDFHL	tr_C9ZP82	0.072	23054.66	32.00
759	HLA-A0201	VHLATVMLP	VHLATVMLP	0	0	0	0	0	VHLATVMLP	tr_C9ZP82	0.072	23055.65	32.00
219	HLA-A0201	DPTAFEFDSNCI	TAFEFSNCI	2	0	0	5	1	TAFEFDSNCI	tr_C9ZP82	0.072	23062.15	32.00
685	HLA-A0201	AIYLKEANIKRMP	YLKEANIRM	2	0	0	7	1	YLKEANIKRM	tr_C9ZP82	0.071	23081.87	32.00
808	HLA-A0201	SFVPKGDLRKL	FVPKGDLRL	1	0	0	8	1	FVPKGDLRKL	tr_C9ZP82	0.071	23085.86	32.00
277	HLA-A0201	HKIIDFLRTQ	KIIDFLRTQ	1	0	0	0	0	KIIDFLRTQ	tr_C9ZP82	0.071	23091.11	32.00
455	HLA-A0201	YFFGQPNHIEDGTL	YFFGQPNTL	0	0	0	7	5	YFFGQPNHIEDGTL	tr_C9ZP82	0.071	23113.36	32.00
749	HLA-A0201	WNVDYHDSNVVHLA	NVDYVVHLA	1	0	0	4	4	NVDYHDSNVVHLA	tr_C9ZP82	0.071	23114.10	32.00
522	HLA-A0201	NDFLPHAPSAF	FLPHAPSAF	2	0	0	0	0	FLPHAPSAF	tr_C9ZP82	0.071	23132.37	32.00
547	HLA-A0201	SEVLPYGFLTKGP	VLPYGFLTP	2	0	0	8	2	VLPYGFLTKGP	tr_C9ZP82	0.071	23137.88	32.00
314	HLA-A0201	LHITQVFILRDTTR	ITQVFILTT	2	0	0	7	2	ITQVFILRDTT	tr_C9ZP82	0.071	23139.62	32.00
806	HLA-A0201	KTSFVPKGDL	KTSFVPKGL	0	0	0	8	1	KTSFVPKGDL	tr_C9ZP82	0.071	23146.38	32.00
717	HLA-A0201	PPTSHALLPMVLGE	ALLPMVLGE	5	0	0	0	0	ALLPMVLGE	tr_C9ZP82	0.071	23146.63	32.00
409	HLA-A0201	GFEYFDISVVG	FEYFDISVV	1	0	0	0	0	FEYFDISVV	tr_C9ZP82	0.071	23171.19	32.00
28	HLA-A0201	LYIDLNGIIHP	YILNGIIHP	1	0	0	2	1	YIDLNGIIHP	tr_C9ZP82	0.071	23172.95	32.00
628	HLA-A0201	WRDTYLKSTGL	WTYLKSTGL	0	0	0	1	2	WRDTYLKSTGL	tr_C9ZP82	0.071	23173.21	32.00
356	HLA-A0201	CIISLNPSYIDEEE	CIISLNPYI	0	0	0	7	1	CIISLNPSYI	tr_C9ZP82	0.071	23189.76	32.00
293	HLA-A0201	GGKANHVIVGL	KANHVIVGL	2	0	0	0	0	KANHVIVGL	tr_C9ZP82	0.071	23195.28	32.00
855	HLA-A0201	CTVEVPRLS	CTVEVPRLS	0	0	0	0	0	CTVEVPRLS	tr_C9ZP82	0.071	23196.02	32.00
690	HLA-A0201	EANIKRMPLPV	AIKRMPLPV	1	0	0	1	1	ANIKRMPLPV	tr_C9ZP82	0.071	23196.53	32.00
610	HLA-A0201	NVTVADVAVALHST	VTVAVAVAL	1	0	0	4	1	VTVADVAVAL	tr_C9ZP82	0.071	23199.79	32.00
9	HLA-A0201	LRNKYPAIV	LRNKYPAIV	0	0	0	0	0	LRNKYPAIV	tr_C9ZP82	0.071	23200.79	32.00
878	HLA-A0201	RHSGQLKGNGEGV	RLKGNGEGV	0	0	0	1	4	RHSGQLKGNGEGV	tr_C9ZP82	0.071	23219.62	32.00
317	HLA-A0201	TQVFILRDTTRNPY	TQVFILNPY	0	0	0	6	5	TQVFILRDTTRNPY	tr_C9ZP82	0.071	23222.14	32.00
546	HLA-A0201	VSEVLPYGFLTKGP	VLPYGFLTP	3	0	0	8	2	VLPYGFLTKGP	tr_C9ZP82	0.071	23255.34	32.00
639	HLA-A0201	SNSVQEACEKYVEG	SVQEACEYV	2	0	0	7	1	SVQEACEKYV	tr_C9ZP82	0.071	23264.14	32.00
619	HLA-A0201	ALHSTIDDRW	ALHSTIDRW	0	0	0	6	1	ALHSTIDDRW	tr_C9ZP82	0.071	23264.89	32.00
762	HLA-A0201	ATVMLPFAD	ATVMLPFAD	0	0	0	0	0	ATVMLPFAD	tr_C9ZP82	0.071	23275.22	33.00
365	HLA-A0201	IDEEESDGVIGVPV	ESDGVIVPV	4	0	0	6	1	ESDGVIGVPV	tr_C9ZP82	0.071	23286.06	33.00
629	HLA-A0201	RDTYLKSTGLSNS	YLSTGLSNS	3	0	0	2	1	YLKSTGLSNS	tr_C9ZP82	0.071	23289.57	33.00
255	HLA-A0201	NDPTWTRLCVIF	NTWTRLCVI	0	0	0	1	2	NDPTWTRLCVI	tr_C9ZP82	0.071	23297.89	33.00
738	HLA-A0201	QAELESTFPLQW	QALESTFPL	0	0	0	2	1	QAELESTFPL	tr_C9ZP82	0.071	23306.46	33.00
558	HLA-A0201	GPHEINLPQL	GPHEINLPL	0	0	0	8	1	GPHEINLPQL	tr_C9ZP82	0.071	23306.46	33.00
860	HLA-A0201	PRLSYGADGQVIK	RLYGADGQV	1	0	0	2	1	RLSYGADGQV	tr_C9ZP82	0.070	23320.08	33.00
571	HLA-A0201	FEAYAKIEA	FEAYAKIEA	0	0	0	0	0	FEAYAKIEA	tr_C9ZP82	0.070	23324.38	33.00
621	HLA-A0201	HSTIDDRWRDTYLK	STIDDRWYL	1	0	0	7	3	STIDDRWRDTYL	tr_C9ZP82	0.070	23328.67	33.00
619	HLA-A0201	ALHSTIDDR	ALHSTIDDR	0	0	0	0	0	ALHSTIDDR	tr_C9ZP82	0.070	23334.48	33.00
788	HLA-A0201	EERWRTQKLDFHL	RTQKLDFHL	4	0	0	0	0	RTQKLDFHL	tr_C9ZP82	0.070	23343.83	33.00
699	HLA-A0201	PVVKSTPPDTLVQL	VVPDTLVQL	1	0	0	2	4	VVKSTPPDTLVQL	tr_C9ZP82	0.070	23375.67	33.00
218	HLA-A0201	DDPTAFEFDSNCI	TAFEFSNCI	3	0	0	5	1	TAFEFDSNCI	tr_C9ZP82	0.070	23387.30	33.00
277	HLA-A0201	HKIIDFLRTQSS	KIIDFLQSS	1	0	0	6	2	KIIDFLRTQSS	tr_C9ZP82	0.070	23389.58	33.00
451	HLA-A0201	SAGYYFFGQ	SAGYYFFGQ	0	0	0	0	0	SAGYYFFGQ	tr_C9ZP82	0.070	23391.36	33.00
549	HLA-A0201	VLPYGFLTKGPH	VLYGFLTKG	0	0	0	2	1	VLPYGFLTKG	tr_C9ZP82	0.070	23394.39	33.00
628	HLA-A0201	WRDTYLKSTGLSNS	YLSTGLSNS	4	0	0	2	1	YLKSTGLSNS	tr_C9ZP82	0.070	23423.27	33.00
352	HLA-A0201	IDDTCIISLN	IDDTCIISL	0	0	0	0	0	IDDTCIISL	tr_C9ZP82	0.070	23423.51	33.00
561	HLA-A0201	EINLPQLQRL	EILPQLQRL	0	0	0	2	1	EINLPQLQRL	tr_C9ZP82	0.070	23433.15	33.00
280	HLA-A0201	IDFLRTQSSL	IDFLRTQSL	0	0	0	7	1	IDFLRTQSSL	tr_C9ZP82	0.070	23439.23	33.00
355	HLA-A0201	TCIISLNPSY	TCIISLNPY	0	0	0	8	1	TCIISLNPSY	tr_C9ZP82	0.070	23455.72	33.00
740	HLA-A0201	ELESTFPLQW	ELSTFPLQW	0	0	0	2	1	ELESTFPLQW	tr_C9ZP82	0.070	23463.59	33.00
522	HLA-A0201	NDFLPHAPSAFSGE	FLAPSAFSG	2	0	0	2	2	FLPHAPSAFSG	tr_C9ZP82	0.070	23472.47	33.00
701	HLA-A0201	VKSTPPDTLV	KSTPPDTLV	1	0	0	0	0	KSTPPDTLV	tr_C9ZP82	0.070	23475.02	33.00

386	HLA-A0201	SSDTDNESPI	SSDTDNEPI	0	0	0	7	1	SSDTDNESPI	tr_C9ZP82	0.070	23487.72	33.00
131	HLA-A0201	FTSTEMAQANDDLD	FMAQANDDL	0	0	0	1	4	FTSTEMAQANDDL	tr_C9ZP82	0.070	23491.28	33.00
217	HLA-A0201	VDDPTAFEFDSNCI	TAFEFSNCI	4	0	0	5	1	TAFEFDSNCI	tr_C9ZP82	0.070	23498.40	33.00
47	HLA-A0201	AIALLPEEEKLEC	AIALLPLEC	0	0	0	6	4	AIALLPEEEKLEC	tr_C9ZP82	0.070	23500.69	33.00
787	HLA-A0201	EEERWRTQKLDFHL	RTQKLDFHL	5	0	0	0	0	RTQKLDFHL	tr_C9ZP82	0.070	23506.29	33.00
260	HLA-A0201	TRLCVIFSGSN	TLCVIFSGS	0	0	0	1	1	TRLCVIFSGS	tr_C9ZP82	0.070	23514.43	33.00
521	HLA-A0201	GNDFLPHAPSAF	FLPHAPSAF	3	0	0	0	0	FLPHAPSAF	tr_C9ZP82	0.070	23515.95	33.00
414	HLA-A0201	DISVVGGSL	DISVVGGSL	0	0	0	0	0	DISVVGGSL	tr_C9ZP82	0.070	23526.64	33.00
520	HLA-A0201	LGNDFLPHAPSAF	FLPHAPSAF	4	0	0	0	0	FLPHAPSAF	tr_C9ZP82	0.070	23527.66	33.00
636	HLA-A0201	TGLSNSVQEAC	TLSNSVQEA	0	0	0	1	1	TGLSNSVQEA	tr_C9ZP82	0.070	23538.60	33.00
684	HLA-A0201	LAIYLKEANIKRMP	YLKEANIRM	3	0	0	7	1	YLKEANIKRM	tr_C9ZP82	0.070	23554.15	33.00
15	HLA-A0201	AIVTKNCPS	AIVTKNCPS	0	0	0	0	0	AIVTKNCPS	tr_C9ZP82	0.070	23562.56	33.00
70	HLA-A0201	TVQPKEVMYIAVDG	VQPVMYIAV	1	0	0	3	2	VQPKEVMYIAV	tr_C9ZP82	0.070	23569.19	33.00
96	HLA-A0201	RRYMGRAAPA	RRYMGAAPA	0	0	0	5	1	RRYMGRAAPA	tr_C9ZP82	0.070	23569.19	33.00
579	HLA-A0201	AVKFRRFLLFKS	AVFRRFLLF	0	0	0	2	1	AVKFRRFLLF	tr_C9ZP82	0.070	23570.21	33.00
775	HLA-A0201	QQVVDAAWPQLT	QVVDAAWPL	1	0	0	8	1	QVVDAAWPQL	tr_C9ZP82	0.069	23571.99	33.00
670	HLA-A0201	SWYYPFYHAPPVID	SWYYPFYPV	0	0	0	7	3	SWYYPFYHAPPV	tr_C9ZP82	0.069	23587.04	33.00
243	HLA-A0201	AVLNMLNEK	AVLNMLNEK	0	0	0	0	0	AVLNMLNEK	tr_C9ZP82	0.069	23603.38	33.00
583	HLA-A0201	RRFLLFKST	RRFLLFKST	0	0	0	0	0	RRFLLFKST	tr_C9ZP82	0.069	23664.75	33.00
197	HLA-A0201	RYVESNQVV	RYVESNQVV	0	0	0	0	0	RYVESNQVV	tr_C9ZP82	0.069	23673.97	33.00
442	HLA-A0201	AFDKKTLVSSAGYY	FLVSSAGYY	1	0	0	1	4	FDKKTLVSSAGYY	tr_C9ZP82	0.069	23676.03	33.00
238	HLA-A0201	AKVSDAVLNMLNEK	KVDAVLNML	1	0	0	2	1	KVSDAVLNML	tr_C9ZP82	0.069	23684.99	33.00
702	HLA-A0201	KSTPPDTLVQL	STPDTLVQL	1	0	0	2	1	STPPDTLVQL	tr_C9ZP82	0.069	23686.02	33.00
770	HLA-A0201	DMDVLQQVVDA	MVLQQVVDA	1	0	0	1	1	MDVLQQVVDA	tr_C9ZP82	0.069	23690.89	33.00
16	HLA-A0201	IVTKNCPSSVHGL	IVPSSVHGL	0	0	0	2	4	IVTKNCPSSVHGL	tr_C9ZP82	0.069	23704.98	33.00
253	HLA-A0201	SGNDPTWTRL	SGNDPTWTL	0	0	0	8	1	SGNDPTWTRL	tr_C9ZP82	0.069	23714.22	33.00
426	HLA-A0201	VSALCDIKKFTP	ALCDIKFTP	2	0	0	5	1	ALCDIKKFTP	tr_C9ZP82	0.069	23725.00	33.00
367	HLA-A0201	EEESDGVIGVPV	ESDGVIVPV	2	0	0	6	1	ESDGVIGVPV	tr_C9ZP82	0.069	23736.30	33.00
65	HLA-A0201	ELLVQTVQP	ELLVQTVQP	0	0	0	0	0	ELLVQTVQP	tr_C9ZP82	0.069	23754.54	33.00
15	HLA-A0201	AIVTKNCPSSVH	AIVNCPSSV	0	0	0	3	2	AIVTKNCPSSV	tr_C9ZP82	0.069	23773.57	33.00
129	HLA-A0201	REFTSTEMA	REFTSTEMA	0	0	0	0	0	REFTSTEMA	tr_C9ZP82	0.069	23782.57	33.00
725	HLA-A0201	PMVLGEVMVRSPQQ	MLGEVMVRS	1	0	0	1	1	MVLGEVMVRS	tr_C9ZP82	0.069	23785.65	33.00
696	HLA-A0201	MPLPVVKSTP	MLPVVKSTP	0	0	0	1	1	MPLPVVKSTP	tr_C9ZP82	0.069	23811.67	33.00
122	HLA-A0201	AIVEEVVREFTST	AIVEEVVST	0	0	0	7	4	AIVEEVVREFTST	tr_C9ZP82	0.069	23818.89	33.00
672	HLA-A0201	YYPFYHAPPVI	YYFYHAPPV	0	0	0	2	1	YYPFYHAPPV	tr_C9ZP82	0.068	23829.71	33.00
577	HLA-A0201	IEAVKFRRFLLFKS	AVFRRFLLF	2	0	0	2	1	AVKFRRFLLF	tr_C9ZP82	0.068	23838.99	33.00
582	HLA-A0201	FRRFLLFKSTNK	FRRFLLFST	0	0	0	7	1	FRRFLLFKST	tr_C9ZP82	0.068	23868.15	34.00
780	HLA-A0201	AAWPQLTEEERWRT	AAWPQLWRT	0	0	0	6	5	AAWPQLTEEERWRT	tr_C9ZP82	0.068	23884.17	34.00
806	HLA-A0201	KTSFVPKGDLRKL	FVPKGDLRL	3	0	0	8	1	FVPKGDLRKL	tr_C9ZP82	0.068	23920.12	34.00
701	HLA-A0201	VKSTPPDTLVQLL	STDTLVQLL	2	0	0	2	2	STPPDTLVQLL	tr_C9ZP82	0.068	23939.28	34.00
364	HLA-A0201	YIDEEESDG	YIDEEESDG	0	0	0	0	0	YIDEEESDG	tr_C9ZP82	0.068	23942.14	34.00
717	HLA-A0201	PPTSHALLPMV	TSHALLPMV	2	0	0	0	0	TSHALLPMV	tr_C9ZP82	0.068	23957.15	34.00
410	HLA-A0201	FEYFDISVVGG	FEYFDISVV	0	0	0	0	0	FEYFDISVV	tr_C9ZP82	0.068	23973.24	34.00
807	HLA-A0201	TSFVPKGDLRKL	FVPKGDLRL	2	0	0	8	1	FVPKGDLRKL	tr_C9ZP82	0.068	23981.02	34.00
250	HLA-A0201	EKMSGNDPTW	KMSGNDPTW	1	0	0	0	0	KMSGNDPTW	tr_C9ZP82	0.068	23985.69	34.00
397	HLA-A0201	SREAHVTTV	SREAHVTTV	0	0	0	0	0	SREAHVTTV	tr_C9ZP82	0.068	23989.33	34.00
695	HLA-A0201	RMPLPVVKSTP	RMLPVVKST	0	0	0	2	1	RMPLPVVKST	tr_C9ZP82	0.068	24010.36	34.00
315	HLA-A0201	HITQVFILRD	HITQVFILR	0	0	0	0	0	HITQVFILR	tr_C9ZP82	0.068	24010.87	34.00
366	HLA-A0201	DEEESDGVIGVPV	ESDGVIVPV	3	0	0	6	1	ESDGVIGVPV	tr_C9ZP82	0.068	24018.68	34.00
674	HLA-A0201	PFYHAPPVIDLAIY	FYVIDLAIY	1	0	0	2	4	FYHAPPVIDLAIY	tr_C9ZP82	0.068	24027.00	34.00
270	HLA-A0201	NTPGEGEHKIIDFL	NTPGIIDFL	0	0	0	4	5	NTPGEGEHKIIDFL	tr_C9ZP82	0.068	24050.14	34.00
852	HLA-A0201	TYSCTVEVPRLS	YSCTVEVPL	1	0	0	8	1	YSCTVEVPRL	tr_C9ZP82	0.068	24057.17	34.00
425	HLA-A0201	EVSALCDIKKFTP	ALCDIKFTP	3	0	0	5	1	ALCDIKKFTP	tr_C9ZP82	0.068	24065.50	34.00
17	HLA-A0201	VTKNCPSSVHGLYI	VTKNCPLYI	0	0	0	6	5	VTKNCPSSVHGLYI	tr_C9ZP82	0.068	24071.74	34.00

127	HLA-A0201	VVREFTSTEMA	VVFTSTEMA	0	0	0	2	2	VVREFTSTEMA	tr_C9ZP82	0.068	24084.77	34.00
38	HLA-A0201	PCCHNDSDTAIALL	CCHTAIALL	1	0	0	3	4	CCHNDSDTAIALL	tr_C9ZP82	0.068	24085.55	34.00
170	HLA-A0201	DALNDGDGVNSF	ALGDGVNSF	1	0	0	2	2	ALNDGDGVNSF	tr_C9ZP82	0.067	24090.24	34.00
853	HLA-A0201	YSCTVEVPRLSY	YSCTVEVPL	0	0	0	8	1	YSCTVEVPRL	tr_C9ZP82	0.067	24093.64	34.00
613	HLA-A0201	VADVAVALHS	VAVAVALHS	0	0	0	2	1	VADVAVALHS	tr_C9ZP82	0.067	24096.50	34.00
752	HLA-A0201	DYHDSNVVHL	YHDSNVVHL	1	0	0	0	0	YHDSNVVHL	tr_C9ZP82	0.067	24097.80	34.00
754	HLA-A0201	HDSNVVHLA	HDSNVVHLA	0	0	0	0	0	HDSNVVHLA	tr_C9ZP82	0.067	24113.97	34.00
580	HLA-A0201	VKFRRFLLFKSTNK	FLLFKSTNK	5	0	0	0	0	FLLFKSTNK	tr_C9ZP82	0.067	24116.32	34.00
316	HLA-A0201	ITQVFILRDTTRN	ITQVFILTT	0	0	0	7	2	ITQVFILRDTT	tr_C9ZP82	0.067	24140.60	34.00
28	HLA-A0201	LYIDLNGII	LYIDLNGII	0	0	0	0	0	LYIDLNGII	tr_C9ZP82	0.067	24152.10	34.00
578	HLA-A0201	EAVKFRRFLLFKS	AVFRRFLLF	1	0	0	2	1	AVKFRRFLLF	tr_C9ZP82	0.067	24158.10	34.00
368	HLA-A0201	EESDGVIGV	EESDGVIGV	0	0	0	0	0	EESDGVIGV	tr_C9ZP82	0.067	24171.44	34.00
586	HLA-A0201	LLFKSTNKENSNSG	LLFENSNSG	0	0	0	3	5	LLFKSTNKENSNSG	tr_C9ZP82	0.067	24173.53	34.00
136	HLA-A0201	MAQANDDLDEVRQT	AQADDLDEV	1	0	0	3	1	AQANDDLDEV	tr_C9ZP82	0.067	24196.30	34.00
279	HLA-A0201	IIDFLRTQSSLAQ	IIDFLSSLA	0	0	0	5	3	IIDFLRTQSSLA	tr_C9ZP82	0.067	24218.30	34.00
730	HLA-A0201	EVMVRSPQQAELE	VMSPQQAEL	1	0	0	2	2	VMVRSPQQAEL	tr_C9ZP82	0.067	24232.45	34.00
197	HLA-A0201	RYVESNQVVDDVF	YVNQVVDDV	1	0	0	2	2	YVESNQVVDDV	tr_C9ZP82	0.067	24244.25	34.00
862	HLA-A0201	LSYGADGQVI	LSYGADQVI	0	0	0	6	1	LSYGADGQVI	tr_C9ZP82	0.067	24247.66	34.00
142	HLA-A0201	DLDEVRQTLLE	DLDEVQTLL	0	0	0	5	1	DLDEVRQTLL	tr_C9ZP82	0.067	24249.24	34.00
811	HLA-A0201	PKGDLRKLSDAL	KLRKLSDAL	1	0	0	1	2	KGDLRKLSDAL	tr_C9ZP82	0.067	24250.29	34.00
581	HLA-A0201	KFRRFLLFKS	KFRRFLLFS	0	0	0	8	1	KFRRFLLFKS	tr_C9ZP82	0.067	24257.11	34.00
315	HLA-A0201	HITQVFILRDTTRN	ITQVFILTT	1	0	0	7	2	ITQVFILRDTT	tr_C9ZP82	0.067	24267.61	34.00
424	HLA-A0201	SEVSALCDIKKFTP	ALCDIKFTP	4	0	0	5	1	ALCDIKKFTP	tr_C9ZP82	0.067	24288.11	34.00
349	HLA-A0201	RNFIDDTCII	RNFIDTCII	0	0	0	4	1	RNFIDDTCII	tr_C9ZP82	0.067	24294.94	34.00
750	HLA-A0201	NVDYHDSNVVHL	NVDYHVVHL	0	0	0	5	3	NVDYHDSNVVHL	tr_C9ZP82	0.067	24296.52	34.00
879	HLA-A0201	HSGQLKGNGEGV	SLKGNGEGV	1	0	0	1	2	SGQLKGNGEGV	tr_C9ZP82	0.067	24297.57	34.00
78	HLA-A0201	YIAVDGVAPRAKMN	YIDGVAPRA	0	0	0	2	2	YIAVDGVAPRA	tr_C9ZP82	0.067	24302.57	34.00
572	HLA-A0201	EAYAKIEAVK	EAYAKIEAV	0	0	0	0	0	EAYAKIEAV	tr_C9ZP82	0.067	24312.30	34.00
297	HLA-A0201	NHVIVGLDA	NHVIVGLDA	0	0	0	0	0	NHVIVGLDA	tr_C9ZP82	0.067	24323.08	34.00
805	HLA-A0201	NKTSFVPKGDLRKL	FVPKGDLRL	4	0	0	8	1	FVPKGDLRKL	tr_C9ZP82	0.067	24331.77	34.00
745	HLA-A0201	FPLQWNVDYHDS	LQWNVDYHS	2	0	0	8	1	LQWNVDYHDS	tr_C9ZP82	0.067	24341.78	34.00
140	HLA-A0201	NDDLDEVRQTLLE	NLDEVRQTL	0	0	0	1	2	NDDLDEVRQTL	tr_C9ZP82	0.067	24342.56	34.00
453	HLA-A0201	GYYFFGQPNHI	GYYFFGQPI	0	0	0	8	2	GYYFFGQPNHI	tr_C9ZP82	0.066	24352.83	34.00
859	HLA-A0201	VPRLSYGADGQVIK	RLYGADGQV	2	0	0	2	1	RLSYGADGQV	tr_C9ZP82	0.066	24354.69	34.00
627	HLA-A0201	RWRDTYLKSTGL	RTYLKSTGL	0	0	0	1	3	RWRDTYLKSTGL	tr_C9ZP82	0.066	24357.58	34.00
581	HLA-A0201	KFRRFLLFKSTNK	FLLFKSTNK	4	0	0	0	0	FLLFKSTNK	tr_C9ZP82	0.066	24367.33	34.00
316	HLA-A0201	ITQVFILRDTTRNP	ITQVFILTT	0	0	0	7	2	ITQVFILRDTT	tr_C9ZP82	0.066	24377.10	34.00
774	HLA-A0201	LQQVVDAAWPQLTE	LQQVVDAAL	0	0	0	8	3	LQQVVDAAWPQL	tr_C9ZP82	0.066	24406.92	35.00
579	HLA-A0201	AVKFRRFLLFKSTN	AVFRRFLLF	0	0	0	2	1	AVKFRRFLLF	tr_C9ZP82	0.066	24413.52	35.00
612	HLA-A0201	TVADVAVALHST	TVADVLHST	0	0	0	5	3	TVADVAVALHST	tr_C9ZP82	0.066	24415.63	35.00
153	HLA-A0201	DPLYSGALYN	DLYSGALYN	0	0	0	1	1	DPLYSGALYN	tr_C9ZP82	0.066	24417.74	35.00
276	HLA-A0201	EHKIIDFLRTQSS	KIIDFLQSS	2	0	0	6	2	KIIDFLRTQSS	tr_C9ZP82	0.066	24422.24	35.00
860	HLA-A0201	PRLSYGADGQVIKR	RLYGADGQV	1	0	0	2	1	RLSYGADGQV	tr_C9ZP82	0.066	24428.84	35.00
774	HLA-A0201	LQQVVDAAWP	LQQVVAAWP	0	0	0	5	1	LQQVVDAAWP	tr_C9ZP82	0.066	24432.81	35.00
744	HLA-A0201	TFPLQWNVDYHD	FQWNVDYHD	1	0	0	1	2	FPLQWNVDYHD	tr_C9ZP82	0.066	24442.85	35.00
159	HLA-A0201	ALYNEGEEE	ALYNEGEEE	0	0	0	0	0	ALYNEGEEE	tr_C9ZP82	0.066	24448.15	35.00
277	HLA-A0201	HKIIDFLRTQS	KIIDFLTQS	1	0	0	6	1	KIIDFLRTQS	tr_C9ZP82	0.066	24449.21	35.00
252	HLA-A0201	MSGNDPTWT	MSGNDPTWT	0	0	0	0	0	MSGNDPTWT	tr_C9ZP82	0.066	24453.97	35.00
412	HLA-A0201	YFDISVVGGS	YFDISVVGS	0	0	0	7	1	YFDISVVGGS	tr_C9ZP82	0.066	24498.72	35.00
634	HLA-A0201	KSTGLSNSVQEACE	KLSNSVQEA	0	0	0	1	3	KSTGLSNSVQEA	tr_C9ZP82	0.066	24527.09	35.00
296	HLA-A0201	ANHVIVGLDA	ANVIVGLDA	0	0	0	2	1	ANHVIVGLDA	tr_C9ZP82	0.066	24527.36	35.00
121	HLA-A0201	MAIVEEVVREFTST	AIVEEVVST	1	0	0	7	4	AIVEEVVREFTST	tr_C9ZP82	0.066	24528.69	35.00
853	HLA-A0201	YSCTVEVPRLSYG	YSCTVLSYG	0	0	0	5	4	YSCTVEVPRLSYG	tr_C9ZP82	0.066	24553.65	35.00
784	HLA-A0201	QLTEEERWRTQKL	QLTEETQKL	0	0	0	5	4	QLTEEERWRTQKL	tr_C9ZP82	0.066	24556.58	35.00

244	HLA-A0201	VLNMLNEKMSG	VLNMLNMSG	0	0	0	6	2	VLNMLNEKMSG	tr_C9ZP82	0.066	24557.10	35.00
48	HLA-A0201	IALLPEEEKLEC	ALLPEELEC	1	0	0	6	2	ALLPEEEKLEC	tr_C9ZP82	0.066	24560.03	35.00
451	HLA-A0201	SAGYYFFGQPNHIE	SAGYYFFHI	0	0	0	7	4	SAGYYFFGQPNHI	tr_C9ZP82	0.066	24563.75	35.00
606	HLA-A0201	SNNDNVTVADVAV	SVTVADVAV	0	0	0	1	4	SNNDNVTVADVAV	tr_C9ZP82	0.066	24575.72	35.00
570	HLA-A0201	LFEAYAKIEA	LFEAYAIEA	0	0	0	6	1	LFEAYAKIEA	tr_C9ZP82	0.066	24582.90	35.00
890	HLA-A0201	VIEGKKSNSRVTV	VISNSRVTV	0	0	0	2	4	VIEGKKSNSRVTV	tr_C9ZP82	0.066	24605.25	35.00
251	HLA-A0201	KMSGNDPTWTR	KMSGNDPTT	0	0	0	8	1	KMSGNDPTWT	tr_C9ZP82	0.065	24614.56	35.00
586	HLA-A0201	LLFKSTNKEN	LLFKSTNKE	0	0	0	0	0	LLFKSTNKE	tr_C9ZP82	0.065	24627.35	35.00
92	HLA-A0201	QQRARRYMGRAAPA	QQMGRAAPA	0	0	0	2	5	QQRARRYMGRAAPA	tr_C9ZP82	0.065	24629.49	35.00
79	HLA-A0201	IAVDGVAPR	IAVDGVAPR	0	0	0	0	0	IAVDGVAPR	tr_C9ZP82	0.065	24632.14	35.00
585	HLA-A0201	FLLFKSTNKENSN	FLLFKSTSN	0	0	0	7	4	FLLFKSTNKENSN	tr_C9ZP82	0.065	24639.88	35.00
694	HLA-A0201	KRMPLPVVKS	KMPLPVVKS	0	0	0	1	1	KRMPLPVVKS	tr_C9ZP82	0.065	24650.54	35.00
858	HLA-A0201	EVPRLSYGA	EVPRLSYGA	0	0	0	0	0	EVPRLSYGA	tr_C9ZP82	0.065	24654.28	35.00
295	HLA-A0201	KANHVIVGLDA	KANHVIVGA	0	0	0	8	2	KANHVIVGLDA	tr_C9ZP82	0.065	24664.41	35.00
409	HLA-A0201	GFEYFDISVVGG	FEYFDISVV	1	0	0	0	0	FEYFDISVV	tr_C9ZP82	0.065	24664.95	35.00
112	HLA-A0201	STYMPNKSGMA	YMPNKSGMA	2	0	0	0	0	YMPNKSGMA	tr_C9ZP82	0.065	24669.49	35.00
437	HLA-A0201	PATESAFDKKTLV	ATFDKKTLV	1	0	0	2	3	ATESAFDKKTLV	tr_C9ZP82	0.065	24671.89	35.00
581	HLA-A0201	KFRRFLLFKST	FRRFLLFST	1	0	0	7	1	FRRFLLFKST	tr_C9ZP82	0.065	24694.86	35.00
19	HLA-A0201	KNCPSSVHGL	KCPSSVHGL	0	0	0	1	1	KNCPSSVHGL	tr_C9ZP82	0.065	24703.68	35.00
196	HLA-A0201	NRYVESNQVVDDVF	YVNQVVDDV	2	0	0	2	2	YVESNQVVDDV	tr_C9ZP82	0.065	24706.88	35.00
689	HLA-A0201	KEANIKRMPL	KANIKRMPL	0	0	0	1	1	KEANIKRMPL	tr_C9ZP82	0.065	24719.71	35.00
576	HLA-A0201	KIEAVKFRRF	KIEAVKFRF	0	0	0	7	1	KIEAVKFRRF	tr_C9ZP82	0.065	24730.41	35.00
359	HLA-A0201	SLNPSYIDEE	SLNPYIDEE	0	0	0	4	1	SLNPSYIDEE	tr_C9ZP82	0.065	24731.50	35.00
310	HLA-A0201	LALSLHITQ	LALSLHITQ	0	0	0	0	0	LALSLHITQ	tr_C9ZP82	0.065	24733.37	35.00
718	HLA-A0201	PTSHALLPMVL	TSHALLMVL	1	0	0	6	1	TSHALLPMVL	tr_C9ZP82	0.065	24749.69	35.00
744	HLA-A0201	TFPLQWNVDYHDS	LQWNVDYHS	3	0	0	8	1	LQWNVDYHDS	tr_C9ZP82	0.065	24750.23	35.00
198	HLA-A0201	YVESNQVVDDVFG	YVNQVVDDV	0	0	0	2	2	YVESNQVVDDV	tr_C9ZP82	0.065	24764.97	35.00
275	HLA-A0201	GEHKIIDFLRTQSS	KIIDFLQSS	3	0	0	6	2	KIIDFLRTQSS	tr_C9ZP82	0.065	24782.65	35.00
716	HLA-A0201	LPPTSHALLPMVLG	LSHALLPMV	0	0	0	1	3	LPPTSHALLPMV	tr_C9ZP82	0.065	24787.21	35.00
530	HLA-A0201	SAFSGESALDNLLE	FSGESALLL	2	0	0	7	2	FSGESALDNLL	tr_C9ZP82	0.065	24795.00	35.00
216	HLA-A0201	IVDDPTAFE	IVDDPTAFE	0	0	0	0	0	IVDDPTAFE	tr_C9ZP82	0.065	24827.21	35.00
493	HLA-A0201	RSWKNFRPCSSPA	RSWPCSSPA	0	0	0	3	4	RSWKNFRPCSSPA	tr_C9ZP82	0.065	24833.66	35.00
110	HLA-A0201	VASTYMPNKSGMA	YMPNKSGMA	4	0	0	0	0	YMPNKSGMA	tr_C9ZP82	0.065	24840.37	35.00
127	HLA-A0201	VVREFTSTEMAQA	VVREFMAQA	0	0	0	5	4	VVREFTSTEMAQA	tr_C9ZP82	0.065	24842.52	35.00
798	HLA-A0201	FHLVITSNKTS	FLVITSNKT	0	0	0	1	1	FHLVITSNKT	tr_C9ZP82	0.065	24847.90	35.00
46	HLA-A0201	TAIALLPEEEKLE	TAIALLPEL	0	0	0	8	3	TAIALLPEEEKL	tr_C9ZP82	0.065	24849.78	35.00
122	HLA-A0201	AIVEEVVRE	AIVEEVVRE	0	0	0	0	0	AIVEEVVRE	tr_C9ZP82	0.065	24853.01	35.00
731	HLA-A0201	VMVRSPQQAELES	VMSPQQAEL	0	0	0	2	2	VMVRSPQQAEL	tr_C9ZP82	0.065	24855.43	35.00
200	HLA-A0201	ESNQVVDDV	ESNQVVDDV	0	0	0	0	0	ESNQVVDDV	tr_C9ZP82	0.065	24866.99	35.00
622	HLA-A0201	STIDDRWRDTYLKS	STIDDRWYL	0	0	0	7	3	STIDDRWRDTYL	tr_C9ZP82	0.065	24868.34	35.00
413	HLA-A0201	FDISVVGGSLISE	FISVVGGSL	0	0	0	1	1	FDISVVGGSL	tr_C9ZP82	0.065	24876.68	35.00
772	HLA-A0201	DVLQQVVDA	DVLQQVVDA	0	0	0	0	0	DVLQQVVDA	tr_C9ZP82	0.065	24879.11	35.00
877	HLA-A0201	HRHSGQLKGNGEGV	RLKGNGEGV	1	0	0	1	4	RHSGQLKGNGEGV	tr_C9ZP82	0.064	24890.42	35.00
614	HLA-A0201	ADVAVALHSTI	ADVAVALTI	0	0	0	7	2	ADVAVALHSTI	tr_C9ZP82	0.064	24901.72	35.00
243	HLA-A0201	AVLNMLNEKMS	VLNMLNEMS	1	0	0	7	1	VLNMLNEKMS	tr_C9ZP82	0.064	24905.22	35.00
704	HLA-A0201	TPPDTLVQL	TPPDTLVQL	0	0	0	0	0	TPPDTLVQL	tr_C9ZP82	0.064	24916.02	35.00
292	HLA-A0201	FGGKANHVI	FGGKANHVI	0	0	0	0	0	FGGKANHVI	tr_C9ZP82	0.064	24922.21	35.00
612	HLA-A0201	TVADVAVALHSTID	TVADVAVTI	0	0	0	7	4	TVADVAVALHSTI	tr_C9ZP82	0.064	24924.91	36.00
230	HLA-A0201	ISPGTDFMAKVS	ISPGTDFMV	0	0	0	8	2	ISPGTDFMAKV	tr_C9ZP82	0.064	24934.62	36.00
548	HLA-A0201	EVLPYGFLTKGPH	VLYGFLTKG	1	0	0	2	1	VLPYGFLTKG	tr_C9ZP82	0.064	24954.86	36.00
670	HLA-A0201	SWYYPFYHAP	SWYYPFYHA	0	0	0	0	0	SWYYPFYHA	tr_C9ZP82	0.064	24954.86	36.00
776	HLA-A0201	QVVDAAWPQLTE	QVVDAAWPL	0	0	0	8	1	QVVDAAWPQL	tr_C9ZP82	0.064	24956.75	36.00
89	HLA-A0201	KMNQQRARRYMGRA	KMNQYMGRA	0	0	0	4	5	KMNQQRARRYMGRA	tr_C9ZP82	0.064	24973.50	36.00
112	HLA-A0201	STYMPNKSGM	STYMPNSGM	0	0	0	6	1	STYMPNKSGM	tr_C9ZP82	0.064	24984.03	36.00

582	HLA-A0201	FRRFLLFKSTNKEN	FLLFKSTEN	3	0	0	7	2	FLLFKSTNKEN	tr_C9ZP82	0.064	24984.86	36.00
546	HLA-A0201	VSEVLPYGF	VSEVLPYGF	0	0	0	0	0	VSEVLPYGF	tr_C9ZP82	0.064	25002.15	36.00
667	HLA-A0201	SDWSWYYPFY	SDWSWYYFY	0	0	0	7	1	SDWSWYYPFY	tr_C9ZP82	0.064	25014.60	36.00
410	HLA-A0201	FEYFDISVVGGS	FEYFDISVV	0	0	0	0	0	FEYFDISVV	tr_C9ZP82	0.064	25028.95	36.00
169	HLA-A0201	GDALNDGDGVNSF	ALGDGVNSF	2	0	0	2	2	ALNDGDGVNSF	tr_C9ZP82	0.064	25045.74	36.00
407	HLA-A0201	NTGFEYFDIS	NTFEYFDIS	0	0	0	2	1	NTGFEYFDIS	tr_C9ZP82	0.064	25050.62	36.00
372	HLA-A0201	GVIGVPVCE	GVIGVPVCE	0	0	0	0	0	GVIGVPVCE	tr_C9ZP82	0.064	25059.57	36.00
104	HLA-A0201	PAQTGEVASTYM	AQTGEVAYM	1	0	0	7	2	AQTGEVASTYM	tr_C9ZP82	0.064	25059.84	36.00
701	HLA-A0201	VKSTPPDTLVQL	STPDTLVQL	2	0	0	2	1	STPPDTLVQL	tr_C9ZP82	0.064	25061.73	36.00
729	HLA-A0201	GEVMVRSPQQAELE	VMSPQQAEL	2	0	0	2	2	VMVRSPQQAEL	tr_C9ZP82	0.064	25066.08	36.00
147	HLA-A0201	RQTLLEDPLY	RQTLLEDPL	0	0	0	0	0	RQTLLEDPL	tr_C9ZP82	0.064	25074.77	36.00
240	HLA-A0201	VSDAVLNMLNEKMS	VSDAVLNML	0	0	0	0	0	VSDAVLNML	tr_C9ZP82	0.064	25080.73	36.00
838	HLA-A0201	RQLYDVPLE	RQLYDVPLE	0	0	0	0	0	RQLYDVPLE	tr_C9ZP82	0.064	25082.90	36.00
258	HLA-A0201	TWTRLCVIF	TWTRLCVIF	0	0	0	0	0	TWTRLCVIF	tr_C9ZP82	0.064	25083.98	36.00
738	HLA-A0201	QAELESTFPLQWN	QALESTFPL	0	0	0	2	1	QAELESTFPL	tr_C9ZP82	0.064	25091.04	36.00
585	HLA-A0201	FLLFKSTNKEN	FLLFKSTEN	0	0	0	7	2	FLLFKSTNKEN	tr_C9ZP82	0.064	25105.98	36.00
852	HLA-A0201	TYSCTVEVPRLSY	YSCTVEVPL	1	0	0	8	1	YSCTVEVPRL	tr_C9ZP82	0.064	25106.53	36.00
359	HLA-A0201	SLNPSYIDEEES	SLNPSYIES	0	0	0	7	3	SLNPSYIDEEES	tr_C9ZP82	0.064	25106.53	36.00
111	HLA-A0201	ASTYMPNKSGMA	YMPNKSGMA	3	0	0	0	0	YMPNKSGMA	tr_C9ZP82	0.064	25111.68	36.00
229	HLA-A0201	CISPGTDFMAK	CISPGTFMA	0	0	0	6	1	CISPGTDFMA	tr_C9ZP82	0.064	25119.02	36.00
45	HLA-A0201	DTAIALLPEEEKLE	TAIALLPEL	1	0	0	8	3	TAIALLPEEEKL	tr_C9ZP82	0.064	25130.99	36.00
220	HLA-A0201	PTAFEFDSNCISP	TAFEFCISP	1	0	0	5	3	TAFEFDSNCISP	tr_C9ZP82	0.064	25147.30	36.00
109	HLA-A0201	EVASTYMPNKSGMA	YMPNKSGMA	5	0	0	0	0	YMPNKSGMA	tr_C9ZP82	0.063	25184.61	36.00
449	HLA-A0201	VSSAGYYFFG	VSSAYYFFG	0	0	0	4	1	VSSAGYYFFG	tr_C9ZP82	0.063	25191.14	36.00
746	HLA-A0201	PLQWNVDYHDS	LQWNVDYHS	1	0	0	8	1	LQWNVDYHDS	tr_C9ZP82	0.063	25197.97	36.00
264	HLA-A0201	VIFSGSNTPG	VIFSGSTPG	0	0	0	6	1	VIFSGSNTPG	tr_C9ZP82	0.063	25226.60	36.00
320	HLA-A0201	FILRDTTRNP	FLRDTTRNP	0	0	0	1	1	FILRDTTRNP	tr_C9ZP82	0.063	25226.88	36.00
531	HLA-A0201	AFSGESALDNLLE	FSGESALLL	1	0	0	7	2	FSGESALDNLL	tr_C9ZP82	0.063	25238.07	36.00
359	HLA-A0201	SLNPSYIDE	SLNPSYIDE	0	0	0	0	0	SLNPSYIDE	tr_C9ZP82	0.063	25250.92	36.00
612	HLA-A0201	TVADVAVALHS	TVADVAVAL	0	0	0	0	0	TVADVAVAL	tr_C9ZP82	0.063	25258.29	36.00
505	HLA-A0201	ANSKIIDDIIV	ANSKIIIIV	0	0	0	6	2	ANSKIIDDIIV	tr_C9ZP82	0.063	25258.83	36.00
642	HLA-A0201	VQEACEKYVE	VQEACEKYV	0	0	0	0	0	VQEACEKYV	tr_C9ZP82	0.063	25268.13	36.00
436	HLA-A0201	TPATESAFDKKTLV	ATFDKKTLV	2	0	0	2	3	ATESAFDKKTLV	tr_C9ZP82	0.063	25293.02	36.00
74	HLA-A0201	KEVMYIAVDG	KVMYIAVDG	0	0	0	1	1	KEVMYIAVDG	tr_C9ZP82	0.063	25295.76	36.00
608	HLA-A0201	NDNVTVADV	NDNVTVADV	0	0	0	0	0	NDNVTVADV	tr_C9ZP82	0.063	25301.24	36.00
549	HLA-A0201	VLPYGFLTKGPHE	VLYGFLTKG	0	0	0	2	1	VLPYGFLTKG	tr_C9ZP82	0.063	25310.26	36.00
279	HLA-A0201	IIDFLRTQS	IIDFLRTQS	0	0	0	0	0	IIDFLRTQS	tr_C9ZP82	0.063	25310.54	36.00
145	HLA-A0201	EVRQTLLEDPL	EVRQTLLPL	0	0	0	7	2	EVRQTLLEDPL	tr_C9ZP82	0.063	25322.05	36.00
720	HLA-A0201	SHALLPMVLG	SALLPMVLG	0	0	0	1	1	SHALLPMVLG	tr_C9ZP82	0.063	25331.92	36.00
696	HLA-A0201	MPLPVVKSTPPDTL	MPLPVVDTL	0	0	0	6	5	MPLPVVKSTPPDTL	tr_C9ZP82	0.063	25334.11	36.00
42	HLA-A0201	NDSDTAIAL	NDSDTAIAL	0	0	0	0	0	NDSDTAIAL	tr_C9ZP82	0.063	25346.72	36.00
168	HLA-A0201	AGDALNDGDGVNSF	ALGDGVNSF	3	0	0	2	2	ALNDGDGVNSF	tr_C9ZP82	0.063	25357.69	36.00
650	HLA-A0201	VEGVCFVWR	VEGVCFVWR	0	0	0	0	0	VEGVCFVWR	tr_C9ZP82	0.063	25387.88	36.00
48	HLA-A0201	IALLPEEEKLE	ILLPEEEKL	0	0	0	1	1	IALLPEEEKL	tr_C9ZP82	0.063	25391.17	36.00
563	HLA-A0201	NLPQLQRLFE	NLPQLQLFE	0	0	0	6	1	NLPQLQRLFE	tr_C9ZP82	0.063	25396.12	36.00
61	HLA-A0201	CSELELLVQ	CSELELLVQ	0	0	0	0	0	CSELELLVQ	tr_C9ZP82	0.063	25399.97	36.00
547	HLA-A0201	SEVLPYGFLTKGPH	VLYGFLTKG	2	0	0	2	1	VLPYGFLTKG	tr_C9ZP82	0.063	25400.80	36.00
448	HLA-A0201	LVSSAGYYFFG	LVAGYYFFG	0	0	0	2	2	LVSSAGYYFFG	tr_C9ZP82	0.063	25401.07	36.00
140	HLA-A0201	NDDLDEVRQTLLED	NLDEVRQTL	0	0	0	1	2	NDDLDEVRQTL	tr_C9ZP82	0.063	25401.62	36.00
679	HLA-A0201	PPVIDLAIYLK	PVIDLAIYL	0	0	0	1	1	PPVIDLAIYL	tr_C9ZP82	0.063	25410.42	36.00
446	HLA-A0201	KTLVSSAGY	KTLVSSAGY	0	0	0	0	0	KTLVSSAGY	tr_C9ZP82	0.063	25412.62	36.00
746	HLA-A0201	PLQWNVDYHD	PLWNVDYHD	0	0	0	2	1	PLQWNVDYHD	tr_C9ZP82	0.062	25441.23	36.00
98	HLA-A0201	YMGRAAPAQTG	YMGAAPAQT	0	0	0	3	1	YMGRAAPAQT	tr_C9ZP82	0.062	25444.53	37.00
374	HLA-A0201	IGVPVCESS	IGVPVCESS	0	0	0	0	0	IGVPVCESS	tr_C9ZP82	0.062	25450.04	37.00

216	HLA-A0201	IVDDPTAFEFDSNC	IVFEFDSNC	0	0	0	2	5	IVDDPTAFEFDSNC	tr_C9ZP82	0.062	25467.12	37.00
219	HLA-A0201	DPTAFEFDSNCISP	TAFEFCISP	2	0	0	5	3	TAFEFDSNCISP	tr_C9ZP82	0.062	25489.17	37.00
77	HLA-A0201	MYIAVDGVAPR	MYIAVVAPR	0	0	0	5	2	MYIAVDGVAPR	tr_C9ZP82	0.062	25495.79	37.00
580	HLA-A0201	VKFRRFLLFKST	FRRFLLFST	2	0	0	7	1	FRRFLLFKST	tr_C9ZP82	0.062	25499.66	37.00
276	HLA-A0201	EHKIIDFLRTQS	KIIDFLTQS	2	0	0	6	1	KIIDFLRTQS	tr_C9ZP82	0.062	25521.46	37.00
797	HLA-A0201	DFHLVITSNKTS	FLVITSNKT	1	0	0	1	1	FHLVITSNKT	tr_C9ZP82	0.062	25525.61	37.00
5	HLA-A0201	FASWLRNKYP	FAWLRNKYP	0	0	0	2	1	FASWLRNKYP	tr_C9ZP82	0.062	25527.81	37.00
227	HLA-A0201	SNCISPGTDFM	SISPGTDFM	0	0	0	1	2	SNCISPGTDFM	tr_C9ZP82	0.062	25564.02	37.00
242	HLA-A0201	DAVLNMLNEKMS	VLNMLNEMS	2	0	0	7	1	VLNMLNEKMS	tr_C9ZP82	0.062	25574.53	37.00
613	HLA-A0201	VADVAVALH	VADVAVALH	0	0	0	0	0	VADVAVALH	tr_C9ZP82	0.062	25575.91	37.00
142	HLA-A0201	DLDEVRQTLLED	DLDEVQTLL	0	0	0	5	1	DLDEVRQTLL	tr_C9ZP82	0.062	25595.85	37.00
676	HLA-A0201	YHAPPVIDLAIY	HAPPVILAI	1	0	0	6	1	HAPPVIDLAI	tr_C9ZP82	0.062	25603.61	37.00
47	HLA-A0201	AIALLPEEEKLE	AIALLPEEL	0	0	0	8	2	AIALLPEEEKL	tr_C9ZP82	0.062	25617.18	37.00
574	HLA-A0201	YAKIEAVKF	YAKIEAVKF	0	0	0	0	0	YAKIEAVKF	tr_C9ZP82	0.062	25618.29	37.00
354	HLA-A0201	DTCIISLNPS	DTCIISLPS	0	0	0	7	1	DTCIISLNPS	tr_C9ZP82	0.062	25619.40	37.00
749	HLA-A0201	WNVDYHDSNVVHL	NVDYHVVHL	1	0	0	5	3	NVDYHDSNVVHL	tr_C9ZP82	0.062	25646.03	37.00
888	HLA-A0201	EGVIEGKKSNSRV	GVIEGNSRV	1	0	0	5	3	GVIEGKKSNSRV	tr_C9ZP82	0.062	25652.41	37.00
40	HLA-A0201	CHNDSDTAIALL	CSDTAIALL	0	0	0	1	3	CHNDSDTAIALL	tr_C9ZP82	0.062	25654.35	37.00
619	HLA-A0201	ALHSTIDDRWRDTY	ALHSTIDTY	0	0	0	6	5	ALHSTIDDRWRDTY	tr_C9ZP82	0.062	25666.01	37.00
467	HLA-A0201	TLPSSTECG	TLPSSTECG	0	0	0	0	0	TLPSSTECG	tr_C9ZP82	0.062	25672.67	37.00
409	HLA-A0201	GFEYFDISVVGGS	FEYFDISVV	1	0	0	0	0	FEYFDISVV	tr_C9ZP82	0.062	25675.19	37.00
634	HLA-A0201	KSTGLSNSVQ	KSTGLSNSV	0	0	0	0	0	KSTGLSNSV	tr_C9ZP82	0.062	25675.19	37.00
835	HLA-A0201	VEIRQLYDVPL	VEILYDVPL	0	0	0	3	2	VEIRQLYDVPL	tr_C9ZP82	0.062	25676.84	37.00
80	HLA-A0201	AVDGVAPRAKM	AVDGVAPRM	0	0	0	8	2	AVDGVAPRAKM	tr_C9ZP82	0.062	25702.97	37.00
852	HLA-A0201	TYSCTVEVPRLSYG	CTVEVLSYG	3	0	0	5	2	CTVEVPRLSYG	tr_C9ZP82	0.061	25726.07	37.00
740	HLA-A0201	ELESTFPLQ	ELESTFPLQ	0	0	0	0	0	ELESTFPLQ	tr_C9ZP82	0.061	25727.46	37.00
466	HLA-A0201	GTLPSSTEC	GTLPSSTEC	0	0	0	0	0	GTLPSSTEC	tr_C9ZP82	0.061	25756.42	37.00
626	HLA-A0201	DRWRDTYLKSTGL	WTYLKSTGL	2	0	0	1	2	WRDTYLKSTGL	tr_C9ZP82	0.061	25757.54	37.00
143	HLA-A0201	LDEVRQTLLEDPL	LQTLLEDPL	0	0	0	1	4	LDEVRQTLLEDPL	tr_C9ZP82	0.061	25767.30	37.00
174	HLA-A0201	DGDGVNSFCV	DGDGVSFCV	0	0	0	5	1	DGDGVNSFCV	tr_C9ZP82	0.061	25775.66	37.00
834	HLA-A0201	GVEIRQLYDVP	GVEIRQLYV	0	0	0	8	1	GVEIRQLYDV	tr_C9ZP82	0.061	25785.98	37.00
291	HLA-A0201	QFGGKANHV	QFGGKANHV	0	0	0	0	0	QFGGKANHV	tr_C9ZP82	0.061	25787.93	37.00
677	HLA-A0201	HAPPVIDLAIY	HAPPVILAI	0	0	0	6	1	HAPPVIDLAI	tr_C9ZP82	0.061	25811.11	37.00
671	HLA-A0201	WYYPFYHAPPVID	WYYPFYHPV	0	0	0	7	2	WYYPFYHAPPV	tr_C9ZP82	0.061	25811.94	37.00
694	HLA-A0201	KRMPLPVVKSTP	RMLPVVKST	1	0	0	2	1	RMPLPVVKST	tr_C9ZP82	0.061	25824.23	37.00
243	HLA-A0201	AVLNMLNEKMSG	VLNMLNMSG	1	0	0	6	2	VLNMLNEKMSG	tr_C9ZP82	0.061	25826.19	37.00
580	HLA-A0201	VKFRRFLLFKS	VKFFLLFKS	0	0	0	3	2	VKFRRFLLFKS	tr_C9ZP82	0.061	25828.71	37.00
414	HLA-A0201	DISVVGGSLI	DISVVGSLI	0	0	0	5	1	DISVVGGSLI	tr_C9ZP82	0.061	25835.14	37.00
401	HLA-A0201	HVTTVPNTGFEYF	HVTTVFEYF	0	0	0	5	4	HVTTVPNTGFEYF	tr_C9ZP82	0.061	25845.47	37.00
796	HLA-A0201	LDFHLVITSNKTS	HLVITSNTS	3	0	0	7	1	HLVITSNKTS	tr_C9ZP82	0.061	25848.27	37.00
834	HLA-A0201	GVEIRQLYDVPLE	GVQLYDVPL	0	0	0	2	3	GVEIRQLYDVPL	tr_C9ZP82	0.061	25853.30	37.00
745	HLA-A0201	FPLQWNVDY	FPLQWNVDY	0	0	0	0	0	FPLQWNVDY	tr_C9ZP82	0.061	25859.46	37.00
275	HLA-A0201	GEHKIIDFLRTQS	KIIDFLTQS	3	0	0	6	1	KIIDFLRTQS	tr_C9ZP82	0.061	25862.54	37.00
849	HLA-A0201	RPRTYSCTV	RPRTYSCTV	0	0	0	0	0	RPRTYSCTV	tr_C9ZP82	0.061	25870.65	37.00
241	HLA-A0201	SDAVLNMLNEKMS	VLNMLNEMS	3	0	0	7	1	VLNMLNEKMS	tr_C9ZP82	0.061	25876.81	37.00
88	HLA-A0201	AKMNQQRARRYM	KMNQQRAYM	1	0	0	7	2	KMNQQRARRYM	tr_C9ZP82	0.061	25883.82	37.00
434	HLA-A0201	KFTPATESA	KFTPATESA	0	0	0	0	0	KFTPATESA	tr_C9ZP82	0.061	25892.50	37.00
664	HLA-A0201	TTDSDWSWYYPFYH	WSWYYPFYH	5	0	0	0	0	WSWYYPFYH	tr_C9ZP82	0.061	25896.71	37.00
785	HLA-A0201	LTEEERWRT	LTEEERWRT	0	0	0	0	0	LTEEERWRT	tr_C9ZP82	0.061	25918.56	37.00
717	HLA-A0201	PPTSHALLPMVL	TSHALLMVL	2	0	0	6	1	TSHALLPMVL	tr_C9ZP82	0.061	25919.96	37.00
844	HLA-A0201	PLERYRPRTYSCTV	PLETYSCTV	0	0	0	3	5	PLERYRPRTYSCTV	tr_C9ZP82	0.061	25926.70	37.00
623	HLA-A0201	TIDDRWRDTYLKS	TIDDRWTYL	0	0	0	6	2	TIDDRWRDTYL	tr_C9ZP82	0.061	25926.70	37.00
198	HLA-A0201	YVESNQVVDDVFGH	YVNQVVDDV	0	0	0	2	2	YVESNQVVDDV	tr_C9ZP82	0.061	25927.82	37.00
39	HLA-A0201	CCHNDSDTAIAL	CCSDTAIAL	0	0	0	2	3	CCHNDSDTAIAL	tr_C9ZP82	0.061	25937.07	37.00

671	HLA-A0201	WYYPFYHAP	WYYPFYHAP	0	0	0	0	0	WYYPFYHAP	tr_C9ZP82	0.061	25952.80	38.00
123	HLA-A0201	IVEEVVREFTST	IVEEVVFTS	0	0	0	6	2	IVEEVVREFTS	tr_C9ZP82	0.061	25965.72	38.00
252	HLA-A0201	MSGNDPTWTRL	MSDPTWTRL	0	0	0	2	2	MSGNDPTWTRL	tr_C9ZP82	0.061	25971.33	38.00
642	HLA-A0201	VQEACEKYVEGVCF	VQYVEGVCF	0	0	0	2	5	VQEACEKYVEGVCF	tr_C9ZP82	0.060	25990.74	38.00
457	HLA-A0201	FGQPNHIEDGTLPS	FIEDGTLPS	0	0	0	1	5	FGQPNHIEDGTLPS	tr_C9ZP82	0.060	25990.74	38.00
689	HLA-A0201	KEANIKRMPLPVV	KIKRMPLPV	0	0	0	1	3	KEANIKRMPLPV	tr_C9ZP82	0.060	25997.21	38.00
751	HLA-A0201	VDYHDSNVV	VDYHDSNVV	0	0	0	0	0	VDYHDSNVV	tr_C9ZP82	0.060	26019.72	38.00
696	HLA-A0201	MPLPVVKST	MPLPVVKST	0	0	0	0	0	MPLPVVKST	tr_C9ZP82	0.060	26032.39	38.00
103	HLA-A0201	APAQTGEVASTYM	AQTGEVAYM	2	0	0	7	2	AQTGEVASTYM	tr_C9ZP82	0.060	26034.93	38.00
413	HLA-A0201	FDISVVGGS	FDISVVGGS	0	0	0	0	0	FDISVVGGS	tr_C9ZP82	0.060	26039.16	38.00
534	HLA-A0201	GESALDNLL	GESALDNLL	0	0	0	0	0	GESALDNLL	tr_C9ZP82	0.060	26042.82	38.00
126	HLA-A0201	EVVREFTSTEMAQA	FTSTEMAQA	5	0	0	0	0	FTSTEMAQA	tr_C9ZP82	0.060	26044.78	38.00
281	HLA-A0201	DFLRTQSSLAQ	FLTQSSLAQ	1	0	0	2	1	FLRTQSSLAQ	tr_C9ZP82	0.060	26044.78	38.00
691	HLA-A0201	ANIKRMPLPVV	NIRMPLPVV	1	0	0	2	1	NIKRMPLPVV	tr_C9ZP82	0.060	26052.68	38.00
230	HLA-A0201	ISPGTDFMAK	ISPGTDFMA	0	0	0	0	0	ISPGTDFMA	tr_C9ZP82	0.060	26054.09	38.00
735	HLA-A0201	SPQQAELEST	SQQAELEST	0	0	0	1	1	SPQQAELEST	tr_C9ZP82	0.060	26079.76	38.00
625	HLA-A0201	DDRWRDTYLKSTGL	WTYLKSTGL	3	0	0	1	2	WRDTYLKSTGL	tr_C9ZP82	0.060	26082.30	38.00
274	HLA-A0201	EGEHKIIDFLRTQS	KIIDFLTQS	4	0	0	6	1	KIIDFLRTQS	tr_C9ZP82	0.060	26091.60	38.00
417	HLA-A0201	VVGGSLISE	VVGGSLISE	0	0	0	0	0	VVGGSLISE	tr_C9ZP82	0.060	26102.63	38.00
695	HLA-A0201	RMPLPVVKSTPP	RMLPVVKST	0	0	0	2	1	RMPLPVVKST	tr_C9ZP82	0.060	26117.88	38.00
301	HLA-A0201	VGLDADLIF	VGLDADLIF	0	0	0	0	0	VGLDADLIF	tr_C9ZP82	0.060	26120.41	38.00
141	HLA-A0201	DDLDEVRQTLLE	DLDEVQTLL	1	0	0	5	1	DLDEVRQTLL	tr_C9ZP82	0.060	26139.08	38.00
678	HLA-A0201	APPVIDLAIY	APPVILAIY	0	0	0	5	1	APPVIDLAIY	tr_C9ZP82	0.060	26146.72	38.00
800	HLA-A0201	LVITSNKTSFVPK	LVITSTSFV	0	0	0	5	2	LVITSNKTSFV	tr_C9ZP82	0.060	26147.00	38.00
72	HLA-A0201	QPKEVMYIA	QPKEVMYIA	0	0	0	0	0	QPKEVMYIA	tr_C9ZP82	0.060	26154.64	38.00
317	HLA-A0201	TQVFILRDTTR	TQVFILTTR	0	0	0	6	2	TQVFILRDTTR	tr_C9ZP82	0.060	26180.11	38.00
688	HLA-A0201	LKEANIKRMPLPVV	LIKRMPLPV	0	0	0	1	4	LKEANIKRMPLPV	tr_C9ZP82	0.060	26195.98	38.00
796	HLA-A0201	LDFHLVITSN	LDFHLVITN	0	0	0	8	1	LDFHLVITSN	tr_C9ZP82	0.060	26210.72	38.00
277	HLA-A0201	HKIIDFLRT	HKIIDFLRT	0	0	0	0	0	HKIIDFLRT	tr_C9ZP82	0.060	26238.25	38.00
151	HLA-A0201	LEDPLYSGALYNE	LLYSGALYN	0	0	0	1	3	LEDPLYSGALYN	tr_C9ZP82	0.060	26239.10	38.00
776	HLA-A0201	QVVDAAWPQLTEE	QVVDAAWPL	0	0	0	8	1	QVVDAAWPQL	tr_C9ZP82	0.060	26240.52	38.00
719	HLA-A0201	TSHALLPMVLG	TSHALLPMV	0	0	0	0	0	TSHALLPMV	tr_C9ZP82	0.059	26270.63	38.00
294	HLA-A0201	GKANHVIVGLDA	KAVIVGLDA	1	0	0	2	2	KANHVIVGLDA	tr_C9ZP82	0.059	26294.80	38.00
426	HLA-A0201	VSALCDIKKF	VSALCDIKF	0	0	0	7	1	VSALCDIKKF	tr_C9ZP82	0.059	26298.22	38.00
423	HLA-A0201	ISEVSALCDIKKFT	ILCDIKKFT	0	0	0	1	5	ISEVSALCDIKKFT	tr_C9ZP82	0.059	26307.61	38.00
184	HLA-A0201	RSTDDDYEST	RTDDDYEST	0	0	0	1	1	RSTDDDYEST	tr_C9ZP82	0.059	26307.89	38.00
39	HLA-A0201	CCHNDSDTAIALLP	CCHTAIALL	0	0	0	3	4	CCHNDSDTAIALL	tr_C9ZP82	0.059	26314.44	38.00
102	HLA-A0201	AAPAQTGEVASTYM	AQTGEVAYM	3	0	0	7	2	AQTGEVASTYM	tr_C9ZP82	0.059	26328.69	38.00
755	HLA-A0201	DSNVVHLATVM	DSNVVLATV	0	0	0	5	1	DSNVVHLATV	tr_C9ZP82	0.059	26349.21	38.00
748	HLA-A0201	QWNVDYHDSNVVHL	NVDYHVVHL	2	0	0	5	3	NVDYHDSNVVHL	tr_C9ZP82	0.059	26349.49	38.00
1	HLA-A0201	GVPKFASWLR	GVPKFASWL	0	0	0	0	0	GVPKFASWL	tr_C9ZP82	0.059	26352.35	38.00
441	HLA-A0201	SAFDKKTLVSS	SAFDTLVSS	0	0	0	4	2	SAFDKKTLVSS	tr_C9ZP82	0.059	26352.62	38.00
256	HLA-A0201	DPTWTRLCVIF	PTWTLCVIF	1	0	0	4	1	PTWTRLCVIF	tr_C9ZP82	0.059	26355.76	38.00
680	HLA-A0201	PVIDLAIYLKE	PVIDLAIYL	0	0	0	0	0	PVIDLAIYL	tr_C9ZP82	0.059	26375.73	38.00
126	HLA-A0201	EVVREFTSTEMA	VVFTSTEMA	1	0	0	2	2	VVREFTSTEMA	tr_C9ZP82	0.059	26379.16	38.00
894	HLA-A0201	KKSNSRVTV	KKSNSRVTV	0	0	0	0	0	KKSNSRVTV	tr_C9ZP82	0.059	26379.16	38.00
809	HLA-A0201	FVPKGDLRKLSDA	FVLRKLSDA	0	0	0	2	4	FVPKGDLRKLSDA	tr_C9ZP82	0.059	26406.28	38.00
197	HLA-A0201	RYVESNQVVDDVFG	YVNQVVDDV	1	0	0	2	2	YVESNQVVDDV	tr_C9ZP82	0.059	26410.28	38.00
151	HLA-A0201	LEDPLYSGA	LEDPLYSGA	0	0	0	0	0	LEDPLYSGA	tr_C9ZP82	0.059	26414.28	38.00
207	HLA-A0201	DVFGHRGEV	DVFGHRGEV	0	0	0	0	0	DVFGHRGEV	tr_C9ZP82	0.059	26416.00	38.00
693	HLA-A0201	IKRMPLPVVKSTP	RMLPVVKST	2	0	0	2	1	RMPLPVVKST	tr_C9ZP82	0.059	26426.00	38.00
280	HLA-A0201	IDFLRTQSSLAQ	FLTQSSLAQ	2	0	0	2	1	FLRTQSSLAQ	tr_C9ZP82	0.059	26431.15	38.00
605	HLA-A0201	GSNNDNVTVADVAV	GVTVADVAV	0	0	0	1	5	GSNNDNVTVADVAV	tr_C9ZP82	0.059	26479.81	39.00
430	HLA-A0201	CDIKKFTPA	CDIKKFTPA	0	0	0	0	0	CDIKKFTPA	tr_C9ZP82	0.059	26503.88	39.00

561	HLA-A0201	EINLPQLQRLF	ILPQLQRLF	1	0	0	1	1	INLPQLQRLF	tr_C9ZP82	0.059	26508.48	39.00
503	HLA-A0201	SPANSKIIDDIIV	SPIIDDIIV	0	0	0	2	4	SPANSKIIDDIIV	tr_C9ZP82	0.059	26513.64	39.00
144	HLA-A0201	DEVRQTLLEDPL	EVRQTLLPL	1	0	0	7	2	EVRQTLLEDPL	tr_C9ZP82	0.059	26528.00	39.00
67	HLA-A0201	LVQTVQPKEVM	LVQTVQPEV	0	0	0	7	1	LVQTVQPKEV	tr_C9ZP82	0.059	26530.00	39.00
586	HLA-A0201	LLFKSTNKENS	LLFKSTENS	0	0	0	6	2	LLFKSTNKENS	tr_C9ZP82	0.059	26542.92	39.00
408	HLA-A0201	TGFEYFDIS	TGFEYFDIS	0	0	0	0	0	TGFEYFDIS	tr_C9ZP82	0.059	26548.38	39.00
35	HLA-A0201	IIHPCCHNDSDT	IIHPCCHNT	0	0	0	8	3	IIHPCCHNDSDT	tr_C9ZP82	0.058	26571.95	39.00
221	HLA-A0201	TAFEFDSNCISPGT	TAFEFSPGT	0	0	0	5	5	TAFEFDSNCISPGT	tr_C9ZP82	0.058	26576.25	39.00
254	HLA-A0201	GNDPTWTRL	GNDPTWTRL	0	0	0	0	0	GNDPTWTRL	tr_C9ZP82	0.058	26577.98	39.00
283	HLA-A0201	LRTQSSLAQFGGKA	LLAQFGGKA	0	0	0	1	5	LRTQSSLAQFGGKA	tr_C9ZP82	0.058	26596.10	39.00
695	HLA-A0201	RMPLPVVKSTPPDT	RMPLPVVDT	0	0	0	7	5	RMPLPVVKSTPPDT	tr_C9ZP82	0.058	26601.29	39.00
635	HLA-A0201	STGLSNSVQEACE	SLSNSVQEA	0	0	0	1	2	STGLSNSVQEA	tr_C9ZP82	0.058	26606.17	39.00
245	HLA-A0201	LNMLNEKMSG	LMLNEKMSG	0	0	0	1	1	LNMLNEKMSG	tr_C9ZP82	0.058	26608.47	39.00
775	HLA-A0201	QQVVDAAWPQLTE	QVVDAAWPL	1	0	0	8	1	QVVDAAWPQL	tr_C9ZP82	0.058	26621.43	39.00
242	HLA-A0201	DAVLNMLNEKMSG	VLNMLNMSG	2	0	0	6	2	VLNMLNEKMSG	tr_C9ZP82	0.058	26630.65	39.00
818	HLA-A0201	LSDALDTILR	LSDALDTIL	0	0	0	0	0	LSDALDTIL	tr_C9ZP82	0.058	26632.39	39.00
419	HLA-A0201	GGSLISEVSA	GGSLISVSA	0	0	0	6	1	GGSLISEVSA	tr_C9ZP82	0.058	26633.83	39.00
692	HLA-A0201	NIKRMPLPVVKSTP	RMLPVVKST	3	0	0	2	1	RMPLPVVKST	tr_C9ZP82	0.058	26634.69	39.00
192	HLA-A0201	STHENRYVESNQV	STYVESNQV	0	0	0	2	4	STHENRYVESNQV	tr_C9ZP82	0.058	26642.47	39.00
774	HLA-A0201	LQQVVDAAWPQ	LQQVVAWPQ	0	0	0	5	2	LQQVVDAAWPQ	tr_C9ZP82	0.058	26647.08	39.00
208	HLA-A0201	VFGHRGEVIV	FGHRGEVIV	1	0	0	0	0	FGHRGEVIV	tr_C9ZP82	0.058	26667.57	39.00
214	HLA-A0201	EVIVDDPTA	EVIVDDPTA	0	0	0	0	0	EVIVDDPTA	tr_C9ZP82	0.058	26669.88	39.00
882	HLA-A0201	QLKGNGEGVIEG	QLGEGVIEG	0	0	0	2	3	QLKGNGEGVIEG	tr_C9ZP82	0.058	26677.38	39.00
642	HLA-A0201	VQEACEKYVEGVC	VQEACVEGV	0	0	0	5	3	VQEACEKYVEGV	tr_C9ZP82	0.058	26679.11	39.00
250	HLA-A0201	EKMSGNDPTWTR	KMSGNDPTT	1	0	0	8	1	KMSGNDPTWT	tr_C9ZP82	0.058	26679.40	39.00
94	HLA-A0201	RARRYMGRAAPA	RAMGRAAPA	0	0	0	2	3	RARRYMGRAAPA	tr_C9ZP82	0.058	26684.02	39.00
614	HLA-A0201	ADVAVALHS	ADVAVALHS	0	0	0	0	0	ADVAVALHS	tr_C9ZP82	0.058	26719.83	39.00
887	HLA-A0201	GEGVIEGKKSNSRV	GVIEGNSRV	2	0	0	5	3	GVIEGKKSNSRV	tr_C9ZP82	0.058	26739.80	39.00
115	HLA-A0201	MPNKSGMAI	MPNKSGMAI	0	0	0	0	0	MPNKSGMAI	tr_C9ZP82	0.058	26741.81	39.00
700	HLA-A0201	VVKSTPPDTLV	VVTPPDTLV	0	0	0	2	2	VVKSTPPDTLV	tr_C9ZP82	0.058	26746.75	39.00
240	HLA-A0201	VSDAVLNMLNE	VSDAVLNML	0	0	0	0	0	VSDAVLNML	tr_C9ZP82	0.058	26758.89	39.00
15	HLA-A0201	AIVTKNCPSSVHG	AIVNCPSSV	0	0	0	3	2	AIVTKNCPSSV	tr_C9ZP82	0.058	26763.54	39.00
213	HLA-A0201	GEVIVDDPTAFEFD	GEVIVAFEF	0	0	0	5	4	GEVIVDDPTAFEF	tr_C9ZP82	0.058	26789.89	39.00
582	HLA-A0201	FRRFLLFKSTN	FRRFLLFTN	0	0	0	7	2	FRRFLLFKSTN	tr_C9ZP82	0.058	26806.72	39.00
314	HLA-A0201	LHITQVFILRD	LITQVFILR	0	0	0	1	1	LHITQVFILR	tr_C9ZP82	0.058	26816.28	39.00
690	HLA-A0201	EANIKRMPLPVV	NIRMPLPVV	2	0	0	2	1	NIKRMPLPVV	tr_C9ZP82	0.058	26816.58	39.00
583	HLA-A0201	RRFLLFKSTNK	FLLFKSTNK	2	0	0	0	0	FLLFKSTNK	tr_C9ZP82	0.058	26823.55	39.00
228	HLA-A0201	NCISPGTDFMAK	CISPGTFMA	1	0	0	6	1	CISPGTDFMA	tr_C9ZP82	0.058	26825.28	39.00
548	HLA-A0201	EVLPYGFLTKGPHE	VLYGFLTKG	1	0	0	2	1	VLPYGFLTKG	tr_C9ZP82	0.058	26826.16	39.00
285	HLA-A0201	TQSSLAQFG	TQSSLAQFG	0	0	0	0	0	TQSSLAQFG	tr_C9ZP82	0.058	26831.09	39.00
496	HLA-A0201	KNFRPCSSPA	KNFPCSSPA	0	0	0	3	1	KNFRPCSSPA	tr_C9ZP82	0.058	26838.64	39.00
667	HLA-A0201	SDWSWYYPFYH	WSWYYPFYH	2	0	0	0	0	WSWYYPFYH	tr_C9ZP82	0.057	26849.09	39.00
504	HLA-A0201	PANSKIIDDIIV	ANSKIIIIV	1	0	0	6	2	ANSKIIDDIIV	tr_C9ZP82	0.057	26857.53	39.00
293	HLA-A0201	GGKANHVIVGLDA	KAVIVGLDA	2	0	0	2	2	KANHVIVGLDA	tr_C9ZP82	0.057	26876.12	39.00
221	HLA-A0201	TAFEFDSNCISPG	TAFEFCISP	0	0	0	5	3	TAFEFDSNCISP	tr_C9ZP82	0.057	26879.32	39.00
435	HLA-A0201	FTPATESAFDKKTL	FTPATESTL	0	0	0	7	5	FTPATESAFDKKTL	tr_C9ZP82	0.057	26879.90	39.00
861	HLA-A0201	RLSYGADGQ	RLSYGADGQ	0	0	0	0	0	RLSYGADGQ	tr_C9ZP82	0.057	26902.60	39.00
0	HLA-A0201	MGVPKFASW	MGVPKFASW	0	0	0	0	0	MGVPKFASW	tr_C9ZP82	0.057	26922.97	39.00
87	HLA-A0201	RAKMNQQRARRYM	KMNQQRAYM	2	0	0	7	2	KMNQQRARRYM	tr_C9ZP82	0.057	26933.46	39.00
855	HLA-A0201	CTVEVPRLSYG	CTVEVLSYG	0	0	0	5	2	CTVEVPRLSYG	tr_C9ZP82	0.057	26946.58	40.00
154	HLA-A0201	PLYSGALYNE	PLYSGALYE	0	0	0	8	1	PLYSGALYNE	tr_C9ZP82	0.057	26947.17	40.00
349	HLA-A0201	RNFIDDTCI	RNFIDDTCI	0	0	0	0	0	RNFIDDTCI	tr_C9ZP82	0.057	26952.12	40.00
415	HLA-A0201	ISVVGGSLIS	IVVGGSLIS	0	0	0	1	1	ISVVGGSLIS	tr_C9ZP82	0.057	26954.45	40.00
623	HLA-A0201	TIDDRWRDTYLKST	TIDDRWTYL	0	0	0	6	2	TIDDRWRDTYL	tr_C9ZP82	0.057	26956.78	40.00

703	HLA-A0201	STPPDTLVQLLS	STDTLVQLL	0	0	0	2	2	STPPDTLVQLL	tr_C9ZP82	0.057	26957.96	40.00
776	HLA-A0201	QVVDAAWPQLTEEE	QVVDAAWPL	0	0	0	8	1	QVVDAAWPQL	tr_C9ZP82	0.057	26970.50	40.00
664	HLA-A0201	TTDSDWSWYYPFY	TTWSWYYPF	0	0	0	2	3	TTDSDWSWYYPF	tr_C9ZP82	0.057	26971.37	40.00
181	HLA-A0201	FCVRSTDDDYEST	FTDDDYEST	0	0	0	1	4	FCVRSTDDDYEST	tr_C9ZP82	0.057	26978.68	40.00
679	HLA-A0201	PPVIDLAIYLKE	PVIDLAIYL	0	0	0	1	1	PPVIDLAIYL	tr_C9ZP82	0.057	26980.71	40.00
241	HLA-A0201	SDAVLNMLNEKMSG	VLNMLNMSG	3	0	0	6	2	VLNMLNEKMSG	tr_C9ZP82	0.057	26992.69	40.00
458	HLA-A0201	GQPNHIEDGTL	GQPNHIETL	0	0	0	7	2	GQPNHIEDGTL	tr_C9ZP82	0.057	26998.24	40.00
283	HLA-A0201	LRTQSSLAQF	LTQSSLAQF	0	0	0	1	1	LRTQSSLAQF	tr_C9ZP82	0.057	27000.28	40.00
142	HLA-A0201	DLDEVRQTLLEDP	DLDEVQTLL	0	0	0	5	1	DLDEVRQTLL	tr_C9ZP82	0.057	27042.68	40.00
129	HLA-A0201	REFTSTEMAQA	FTSTEMAQA	2	0	0	0	0	FTSTEMAQA	tr_C9ZP82	0.057	27054.98	40.00
704	HLA-A0201	TPPDTLVQLL	TPPDTLVLL	0	0	0	7	1	TPPDTLVQLL	tr_C9ZP82	0.057	27057.61	40.00
400	HLA-A0201	AHVTTVPNTGFEYF	HVTTVFEYF	1	0	0	5	4	HVTTVPNTGFEYF	tr_C9ZP82	0.057	27058.78	40.00
16	HLA-A0201	IVTKNCPSSVH	IVTNCPSSV	0	0	0	3	1	IVTKNCPSSV	tr_C9ZP82	0.057	27083.08	40.00
37	HLA-A0201	HPCCHNDSDTAIAL	HPCCTAIAL	0	0	0	4	5	HPCCHNDSDTAIAL	tr_C9ZP82	0.057	27086.61	40.00
44	HLA-A0201	SDTAIALLPE	STAIALLPE	0	0	0	1	1	SDTAIALLPE	tr_C9ZP82	0.057	27096.57	40.00
613	HLA-A0201	VADVAVALHSTID	VADVAVALI	0	0	0	8	3	VADVAVALHSTI	tr_C9ZP82	0.057	27098.33	40.00
730	HLA-A0201	EVMVRSPQQAELES	VMSPQQAEL	1	0	0	2	2	VMVRSPQQAEL	tr_C9ZP82	0.057	27111.23	40.00
801	HLA-A0201	VITSNKTSFVP	VISNKTSFV	0	0	0	2	1	VITSNKTSFV	tr_C9ZP82	0.057	27119.73	40.00
125	HLA-A0201	EEVVREFTSTEMA	VVFTSTEMA	2	0	0	2	2	VVREFTSTEMA	tr_C9ZP82	0.057	27120.33	40.00
13	HLA-A0201	YPAIVTKNCPSSVH	AIVNCPSSV	2	0	0	3	2	AIVTKNCPSSV	tr_C9ZP82	0.056	27138.23	40.00
663	HLA-A0201	TTTDSDWSWYYPFY	TTWSWYYPF	0	0	0	2	4	TTTDSDWSWYYPF	tr_C9ZP82	0.056	27138.82	40.00
624	HLA-A0201	IDDRWRDTYL	IDDRWDTYL	0	0	0	5	1	IDDRWRDTYL	tr_C9ZP82	0.056	27144.41	40.00
560	HLA-A0201	HEINLPQLQRLF	ILPQLQRLF	2	0	0	1	1	INLPQLQRLF	tr_C9ZP82	0.056	27150.27	40.00
439	HLA-A0201	TESAFDKKTLV	SAFDKKTLV	2	0	0	0	0	SAFDKKTLV	tr_C9ZP82	0.056	27150.57	40.00
558	HLA-A0201	GPHEINLPQLQRLF	ILPQLQRLF	4	0	0	1	1	INLPQLQRLF	tr_C9ZP82	0.056	27153.21	40.00
739	HLA-A0201	AELESTFPLQW	AELESTFPL	0	0	0	0	0	AELESTFPL	tr_C9ZP82	0.056	27154.68	40.00
750	HLA-A0201	NVDYHDSNVVHLAT	NVDYVVHLA	0	0	0	4	4	NVDYHDSNVVHLA	tr_C9ZP82	0.056	27161.44	40.00
205	HLA-A0201	VDDVFGHRGEVIV	VDDVFGVIV	0	0	0	6	4	VDDVFGHRGEVIV	tr_C9ZP82	0.056	27162.92	40.00
249	HLA-A0201	NEKMSGNDPTWTR	KMSGNDPTT	2	0	0	8	1	KMSGNDPTWT	tr_C9ZP82	0.056	27168.21	40.00
369	HLA-A0201	ESDGVIGVPVC	ESDGVIVPV	0	0	0	6	1	ESDGVIGVPV	tr_C9ZP82	0.056	27169.38	40.00
754	HLA-A0201	HDSNVVHLATVM	DSNVVLATV	1	0	0	5	1	DSNVVHLATV	tr_C9ZP82	0.056	27170.55	40.00
370	HLA-A0201	SDGVIGVPVC	SDGVIGVPV	0	0	0	0	0	SDGVIGVPV	tr_C9ZP82	0.056	27187.01	40.00
426	HLA-A0201	VSALCDIKKFT	VSALCDIFT	0	0	0	7	2	VSALCDIKKFT	tr_C9ZP82	0.056	27189.66	40.00
141	HLA-A0201	DDLDEVRQTLLED	DLDEVQTLL	1	0	0	5	1	DLDEVRQTLL	tr_C9ZP82	0.056	27199.97	40.00
14	HLA-A0201	PAIVTKNCPSSVH	AIVNCPSSV	1	0	0	3	2	AIVTKNCPSSV	tr_C9ZP82	0.056	27203.20	40.00
637	HLA-A0201	GLSNSVQEACE	GLSNSVQEA	0	0	0	0	0	GLSNSVQEA	tr_C9ZP82	0.056	27206.15	40.00
581	HLA-A0201	KFRRFLLFKSTN	FRRFLLFTN	1	0	0	7	2	FRRFLLFKSTN	tr_C9ZP82	0.056	27215.27	40.00
584	HLA-A0201	RFLLFKSTNKENSN	FLLFKSTSN	1	0	0	7	4	FLLFKSTNKENSN	tr_C9ZP82	0.056	27217.92	40.00
35	HLA-A0201	IIHPCCHND	IIHPCCHND	0	0	0	0	0	IIHPCCHND	tr_C9ZP82	0.056	27222.93	40.00
492	HLA-A0201	ERSWKNFRPCSSPA	RSWPCSSPA	1	0	0	3	4	RSWKNFRPCSSPA	tr_C9ZP82	0.056	27234.13	40.00
20	HLA-A0201	NCPSSVHGLYI	NCPSSVLYI	0	0	0	6	2	NCPSSVHGLYI	tr_C9ZP82	0.056	27250.33	40.00
41	HLA-A0201	HNDSDTAIAL	HNDDTAIAL	0	0	0	3	1	HNDSDTAIAL	tr_C9ZP82	0.056	27260.07	40.00
441	HLA-A0201	SAFDKKTLVSSAG	SAFDLVSSA	0	0	0	4	3	SAFDKKTLVSSA	tr_C9ZP82	0.056	27264.20	40.00
227	HLA-A0201	SNCISPGTDFMAK	CISPGTFMA	2	0	0	6	1	CISPGTDFMA	tr_C9ZP82	0.056	27274.22	40.00
321	HLA-A0201	ILRDTTRNP	ILRDTTRNP	0	0	0	0	0	ILRDTTRNP	tr_C9ZP82	0.056	27326.81	40.00
666	HLA-A0201	DSDWSWYYPFYH	WSWYYPFYH	3	0	0	0	0	WSWYYPFYH	tr_C9ZP82	0.056	27333.02	40.00
86	HLA-A0201	PRAKMNQQRARRYM	KMNQQRAYM	3	0	0	7	2	KMNQQRARRYM	tr_C9ZP82	0.056	27346.93	40.00
19	HLA-A0201	KNCPSSVHGLYI	KSSVHGLYI	0	0	0	1	3	KNCPSSVHGLYI	tr_C9ZP82	0.056	27354.32	40.00
354	HLA-A0201	DTCIISLNPSY	TCIISLNPY	1	0	0	8	1	TCIISLNPSY	tr_C9ZP82	0.056	27357.28	40.00
580	HLA-A0201	VKFRRFLLFKSTN	FRRFLLFTN	2	0	0	7	2	FRRFLLFKSTN	tr_C9ZP82	0.056	27368.82	40.00
230	HLA-A0201	ISPGTDFMAKVSDA	ISPGTDFMA	0	0	0	8	5	ISPGTDFMAKVSDA	tr_C9ZP82	0.056	27370.61	40.00
705	HLA-A0201	PPDTLVQLLSI	DTLVQLLSI	2	0	0	0	0	DTLVQLLSI	tr_C9ZP82	0.056	27371.79	40.00
352	HLA-A0201	IDDTCIISLNPSY	TCIISLNPY	3	0	0	8	1	TCIISLNPSY	tr_C9ZP82	0.056	27389.57	40.00
833	HLA-A0201	EGVEIRQLYDVPLE	GVQLYDVPL	1	0	0	2	3	GVEIRQLYDVPL	tr_C9ZP82	0.056	27392.82	40.00

393	HLA-A0201	SPIESREAHVTTV	SPIESVTTV	0	0	0	5	4	SPIESREAHVTTV	tr_C9ZP82	0.056	27407.35	41.00
372	HLA-A0201	GVIGVPVCESS	GVIGVCESS	0	0	0	4	2	GVIGVPVCESS	tr_C9ZP82	0.056	27410.62	41.00
169	HLA-A0201	GDALNDGDGV	GALNDGDGV	0	0	0	1	1	GDALNDGDGV	tr_C9ZP82	0.056	27416.54	41.00
263	HLA-A0201	CVIFSGSNT	CVIFSGSNT	0	0	0	0	0	CVIFSGSNT	tr_C9ZP82	0.056	27420.69	41.00
248	HLA-A0201	LNEKMSGNDPTWTR	KMSGNDPTT	3	0	0	8	1	KMSGNDPTWT	tr_C9ZP82	0.055	27434.95	41.00
284	HLA-A0201	RTQSSLAQFGGKA	RLAQFGGKA	0	0	0	1	4	RTQSSLAQFGGKA	tr_C9ZP82	0.055	27438.81	41.00
559	HLA-A0201	PHEINLPQLQRLF	ILPQLQRLF	3	0	0	1	1	INLPQLQRLF	tr_C9ZP82	0.055	27452.46	41.00
128	HLA-A0201	VREFTSTEMAQA	FTSTEMAQA	3	0	0	0	0	FTSTEMAQA	tr_C9ZP82	0.055	27469.10	41.00
64	HLA-A0201	LELLVQTVQPKE	LLVQTVQPK	0	0	0	1	2	LELLVQTVQPK	tr_C9ZP82	0.055	27474.15	41.00
223	HLA-A0201	FEFDSNCIS	FEFDSNCIS	0	0	0	0	0	FEFDSNCIS	tr_C9ZP82	0.055	27478.31	41.00
124	HLA-A0201	VEEVVREFTSTEMA	VVFTSTEMA	3	0	0	2	2	VVREFTSTEMA	tr_C9ZP82	0.055	27478.90	41.00
240	HLA-A0201	VSDAVLNMLNEK	VSDAVLNML	0	0	0	0	0	VSDAVLNML	tr_C9ZP82	0.055	27480.39	41.00
833	HLA-A0201	EGVEIRQLYDVP	GVEIRQLYV	1	0	0	8	1	GVEIRQLYDV	tr_C9ZP82	0.055	27489.32	41.00
176	HLA-A0201	DGVNSFCVRS	GVNSFCVRS	1	0	0	0	0	GVNSFCVRS	tr_C9ZP82	0.055	27508.65	41.00
23	HLA-A0201	SSVHGLYIDLN	SVHGLYIDL	0	0	0	1	1	SSVHGLYIDL	tr_C9ZP82	0.055	27513.71	41.00
467	HLA-A0201	TLPSSTECGS	TLSSTECGS	0	0	0	2	1	TLPSSTECGS	tr_C9ZP82	0.055	27517.29	41.00
775	HLA-A0201	QQVVDAAWPQ	QQVVAAWPQ	0	0	0	4	1	QQVVDAAWPQ	tr_C9ZP82	0.055	27526.22	41.00
619	HLA-A0201	ALHSTIDDRWRDT	ALHSTIDDT	0	0	0	7	4	ALHSTIDDRWRDT	tr_C9ZP82	0.055	27529.20	41.00
855	HLA-A0201	CTVEVPRLSYGAD	CTVEVSYGA	0	0	0	5	3	CTVEVPRLSYGA	tr_C9ZP82	0.055	27531.00	41.00
665	HLA-A0201	TDSDWSWYYPFYH	WSWYYPFYH	4	0	0	0	0	WSWYYPFYH	tr_C9ZP82	0.055	27534.26	41.00
583	HLA-A0201	RRFLLFKSTNKEN	FLLFKSTEN	2	0	0	7	2	FLLFKSTNKEN	tr_C9ZP82	0.055	27535.45	41.00
406	HLA-A0201	PNTGFEYFDI	NTGFEYFDI	1	0	0	0	0	NTGFEYFDI	tr_C9ZP82	0.055	27537.25	41.00
285	HLA-A0201	TQSSLAQFGGKA	SLAQFGGKA	3	0	0	0	0	SLAQFGGKA	tr_C9ZP82	0.055	27556.91	41.00
226	HLA-A0201	DSNCISPGTDFM	SISPGTDFM	1	0	0	1	2	SNCISPGTDFM	tr_C9ZP82	0.055	27568.25	41.00
226	HLA-A0201	DSNCISPGTDFMAK	CISPGTFMA	3	0	0	6	1	CISPGTDFMA	tr_C9ZP82	0.055	27575.71	41.00
663	HLA-A0201	TTTDSDWSW	TTTDSDWSW	0	0	0	0	0	TTTDSDWSW	tr_C9ZP82	0.055	27586.45	41.00
672	HLA-A0201	YYPFYHAPPVID	YYFYHAPPV	0	0	0	2	1	YYPFYHAPPV	tr_C9ZP82	0.055	27587.94	41.00
411	HLA-A0201	EYFDISVVGG	YFDISVVGG	1	0	0	0	0	YFDISVVGG	tr_C9ZP82	0.055	27593.32	41.00
746	HLA-A0201	PLQWNVDYH	PLQWNVDYH	0	0	0	0	0	PLQWNVDYH	tr_C9ZP82	0.055	27601.08	41.00
800	HLA-A0201	LVITSNKTSFVPKG	LVITSTSFV	0	0	0	5	2	LVITSNKTSFV	tr_C9ZP82	0.055	27630.66	41.00
205	HLA-A0201	VDDVFGHRGEV	VVFGHRGEV	0	0	0	1	2	VDDVFGHRGEV	tr_C9ZP82	0.055	27643.22	41.00
246	HLA-A0201	NMLNEKMSGN	MLNEKMSGN	1	0	0	0	0	MLNEKMSGN	tr_C9ZP82	0.055	27645.61	41.00
354	HLA-A0201	DTCIISLNP	DTCIISLNP	0	0	0	0	0	DTCIISLNP	tr_C9ZP82	0.055	27659.97	41.00
584	HLA-A0201	RFLLFKSTNKEN	FLLFKSTEN	1	0	0	7	2	FLLFKSTNKEN	tr_C9ZP82	0.055	27662.07	41.00
45	HLA-A0201	DTAIALLPE	DTAIALLPE	0	0	0	0	0	DTAIALLPE	tr_C9ZP82	0.055	27671.65	41.00
631	HLA-A0201	TYLKSTGLSN	YLKSTGLSN	1	0	0	0	0	YLKSTGLSN	tr_C9ZP82	0.055	27672.24	41.00
133	HLA-A0201	STEMAQANDDL	SMAQANDDL	0	0	0	1	2	STEMAQANDDL	tr_C9ZP82	0.055	27692.92	41.00
602	HLA-A0201	FRKGSNNDNVTVA	FSNNDNVTV	0	0	0	1	3	FRKGSNNDNVTV	tr_C9ZP82	0.055	27714.50	41.00
215	HLA-A0201	VIVDDPTAFEFDS	VIVAFEFDS	0	0	0	3	4	VIVDDPTAFEFDS	tr_C9ZP82	0.055	27717.79	41.00
47	HLA-A0201	AIALLPEEE	AIALLPEEE	0	0	0	0	0	AIALLPEEE	tr_C9ZP82	0.054	27733.99	41.00
684	HLA-A0201	LAIYLKEANIK	LIYLKEANI	0	0	0	1	1	LAIYLKEANI	tr_C9ZP82	0.054	27740.00	41.00
97	HLA-A0201	RYMGRAAPAQTG	YMGAAPAQT	1	0	0	3	1	YMGRAAPAQT	tr_C9ZP82	0.054	27747.19	41.00
882	HLA-A0201	QLKGNGEGVIEGK	QLEGVIEGK	0	0	0	2	4	QLKGNGEGVIEGK	tr_C9ZP82	0.054	27748.41	41.00
28	HLA-A0201	LYIDLNGIIH	YIDLNGIIH	1	0	0	0	0	YIDLNGIIH	tr_C9ZP82	0.054	27751.70	41.00
253	HLA-A0201	SGNDPTWTRLCV	SGNDPTWTV	0	0	0	8	3	SGNDPTWTRLCV	tr_C9ZP82	0.054	27752.60	41.00
775	HLA-A0201	QQVVDAAWPQLTEE	QVVDAAWPL	1	0	0	8	1	QVVDAAWPQL	tr_C9ZP82	0.054	27753.81	41.00
65	HLA-A0201	ELLVQTVQPKE	LLVQTVQPE	1	0	0	8	1	LLVQTVQPKE	tr_C9ZP82	0.054	27766.11	41.00
221	HLA-A0201	TAFEFDSNCIS	TAFEFSNCI	0	0	0	5	1	TAFEFDSNCI	tr_C9ZP82	0.054	27767.03	41.00
530	HLA-A0201	SAFSGESALD	SAFSGESAL	0	0	0	0	0	SAFSGESAL	tr_C9ZP82	0.054	27772.13	41.00
105	HLA-A0201	AQTGEVASTY	AQGEVASTY	0	0	0	2	1	AQTGEVASTY	tr_C9ZP82	0.054	27803.71	41.00
697	HLA-A0201	PLPVVKSTPPDTL	PLPVVPDTL	0	0	0	5	4	PLPVVKSTPPDTL	tr_C9ZP82	0.054	27811.53	41.00
427	HLA-A0201	SALCDIKKF	SALCDIKKF	0	0	0	0	0	SALCDIKKF	tr_C9ZP82	0.054	27814.54	41.00
365	HLA-A0201	IDEEESDGVIGV	IESDGVIGV	0	0	0	1	3	IDEEESDGVIGV	tr_C9ZP82	0.054	27823.56	41.00
21	HLA-A0201	CPSSVHGLYIDLN	CVHGLYIDL	0	0	0	1	3	CPSSVHGLYIDL	tr_C9ZP82	0.054	27832.29	41.00

77	HLA-A0201	MYIAVDGVAP	MYIAVGVAP	0	0	0	5	1	MYIAVDGVAP	tr_C9ZP82	0.054	27833.79	41.00
745	HLA-A0201	FPLQWNVDYHDSN	FLQWNVDYH	0	0	0	1	1	FPLQWNVDYH	tr_C9ZP82	0.054	27842.84	41.00
41	HLA-A0201	HNDSDTAIALL	HSDTAIALL	0	0	0	1	2	HNDSDTAIALL	tr_C9ZP82	0.054	27845.84	41.00
582	HLA-A0201	FRRFLLFKSTNKE	FRRFLLFST	0	0	0	7	1	FRRFLLFKST	tr_C9ZP82	0.054	27852.47	42.00
506	HLA-A0201	NSKIIDDII	NSKIIDDII	0	0	0	0	0	NSKIIDDII	tr_C9ZP82	0.054	27856.99	42.00
156	HLA-A0201	YSGALYNEG	YSGALYNEG	0	0	0	0	0	YSGALYNEG	tr_C9ZP82	0.054	27862.72	42.00
854	HLA-A0201	SCTVEVPRLS	SCTVEVPRL	0	0	0	0	0	SCTVEVPRL	tr_C9ZP82	0.054	27866.03	42.00
230	HLA-A0201	ISPGTDFMAKVSD	ISPGTDFMV	0	0	0	8	2	ISPGTDFMAKV	tr_C9ZP82	0.054	27866.34	42.00
394	HLA-A0201	PIESREAHV	PIESREAHV	0	0	0	0	0	PIESREAHV	tr_C9ZP82	0.054	27881.11	42.00
780	HLA-A0201	AAWPQLTEE	AAWPQLTEE	0	0	0	0	0	AAWPQLTEE	tr_C9ZP82	0.054	27881.72	42.00
204	HLA-A0201	VVDDVFGHRG	VVDDVFHRG	0	0	0	6	1	VVDDVFGHRG	tr_C9ZP82	0.054	27882.62	42.00
215	HLA-A0201	VIVDDPTAFEFD	VIVPTAFEF	0	0	0	3	2	VIVDDPTAFEF	tr_C9ZP82	0.054	27893.49	42.00
694	HLA-A0201	KRMPLPVVKSTPP	RMPLPVVPP	1	0	0	7	3	RMPLPVVKSTPP	tr_C9ZP82	0.054	27907.07	42.00
405	HLA-A0201	VPNTGFEYF	VPNTGFEYF	0	0	0	0	0	VPNTGFEYF	tr_C9ZP82	0.054	27914.63	42.00
358	HLA-A0201	ISLNPSYIDEEES	SLNPSYIES	1	0	0	7	3	SLNPSYIDEEES	tr_C9ZP82	0.054	27922.18	42.00
619	HLA-A0201	ALHSTIDDRWRD	ALHSTIWRD	0	0	0	6	3	ALHSTIDDRWRD	tr_C9ZP82	0.054	27923.69	42.00
122	HLA-A0201	AIVEEVVREFTSTE	IVEEVVFTS	1	0	0	6	2	IVEEVVREFTS	tr_C9ZP82	0.054	27926.41	42.00
837	HLA-A0201	IRQLYDVPLE	IRQLYDVPL	0	0	0	0	0	IRQLYDVPL	tr_C9ZP82	0.054	27944.24	42.00
635	HLA-A0201	STGLSNSVQEACEK	SLSNSVQEA	0	0	0	1	2	STGLSNSVQEA	tr_C9ZP82	0.054	27951.20	42.00
451	HLA-A0201	SAGYYFFGQP	SAGYYFFGP	0	0	0	8	1	SAGYYFFGQP	tr_C9ZP82	0.054	27953.31	42.00
7	HLA-A0201	SWLRNKYPA	SWLRNKYPA	0	0	0	0	0	SWLRNKYPA	tr_C9ZP82	0.054	27967.23	42.00
106	HLA-A0201	QTGEVASTY	QTGEVASTY	0	0	0	0	0	QTGEVASTY	tr_C9ZP82	0.054	27974.19	42.00
832	HLA-A0201	REGVEIRQLYDVP	GVEIRQLYV	2	0	0	8	1	GVEIRQLYDV	tr_C9ZP82	0.054	27977.82	42.00
402	HLA-A0201	VTTVPNTGFEYF	VTTVGFEYF	0	0	0	4	3	VTTVPNTGFEYF	tr_C9ZP82	0.054	27988.41	42.00
424	HLA-A0201	SEVSALCDIKKFT	SLCDIKKFT	0	0	0	1	4	SEVSALCDIKKFT	tr_C9ZP82	0.054	28006.90	42.00
608	HLA-A0201	NDNVTVADVAV	NVTVADVAV	0	0	0	1	2	NDNVTVADVAV	tr_C9ZP82	0.054	28012.05	42.00
606	HLA-A0201	SNNDNVTVADVAVA	STVADVAVA	0	0	0	1	5	SNNDNVTVADVAVA	tr_C9ZP82	0.053	28027.21	42.00
75	HLA-A0201	EVMYIAVDG	EVMYIAVDG	0	0	0	0	0	EVMYIAVDG	tr_C9ZP82	0.053	28027.21	42.00
151	HLA-A0201	LEDPLYSGALYNEG	LLYSGALYN	0	0	0	1	3	LEDPLYSGALYN	tr_C9ZP82	0.053	28029.63	42.00
31	HLA-A0201	DLNGIIHPCCHNDS	DLNGIIHPS	0	0	0	8	5	DLNGIIHPCCHNDS	tr_C9ZP82	0.053	28033.57	42.00
581	HLA-A0201	KFRRFLLFKSTNKE	FLLFKSTNK	4	0	0	0	0	FLLFKSTNK	tr_C9ZP82	0.053	28039.04	42.00
605	HLA-A0201	GSNNDNVTVADV	GSNVTVADV	0	0	0	2	3	GSNNDNVTVADV	tr_C9ZP82	0.053	28047.54	42.00
402	HLA-A0201	VTTVPNTGFEYFDI	VTGFEYFDI	0	0	0	1	5	VTTVPNTGFEYFDI	tr_C9ZP82	0.053	28060.89	42.00
645	HLA-A0201	ACEKYVEGVC	ACEKYVEGV	0	0	0	0	0	ACEKYVEGV	tr_C9ZP82	0.053	28063.00	42.00
718	HLA-A0201	PTSHALLPMVLG	TSHALLMVL	1	0	0	6	1	TSHALLPMVL	tr_C9ZP82	0.053	28066.04	42.00
550	HLA-A0201	LPYGFLTKG	LPYGFLTKG	0	0	0	0	0	LPYGFLTKG	tr_C9ZP82	0.053	28067.57	42.00
747	HLA-A0201	LQWNVDYHDSN	LQWNVDYHS	0	0	0	8	1	LQWNVDYHDS	tr_C9ZP82	0.053	28074.25	42.00
353	HLA-A0201	DDTCIISLNPSY	TCIISLNPY	2	0	0	8	1	TCIISLNPSY	tr_C9ZP82	0.053	28078.50	42.00
637	HLA-A0201	GLSNSVQEACEKY	GLSNSVQEA	0	0	0	0	0	GLSNSVQEA	tr_C9ZP82	0.053	28101.29	42.00
204	HLA-A0201	VVDDVFGHR	VVDDVFGHR	0	0	0	0	0	VVDDVFGHR	tr_C9ZP82	0.053	28111.33	42.00
192	HLA-A0201	STHENRYVESNQVV	STVESNQVV	0	0	0	2	5	STHENRYVESNQVV	tr_C9ZP82	0.053	28115.28	42.00
690	HLA-A0201	EANIKRMPL	EANIKRMPL	0	0	0	0	0	EANIKRMPL	tr_C9ZP82	0.053	28121.67	42.00
403	HLA-A0201	TTVPNTGFEYFDI	TTVFEYFDI	0	0	0	3	4	TTVPNTGFEYFDI	tr_C9ZP82	0.053	28137.81	42.00
38	HLA-A0201	PCCHNDSDTAIAL	CCSDTAIAL	1	0	0	2	3	CCHNDSDTAIAL	tr_C9ZP82	0.053	28141.15	42.00
702	HLA-A0201	KSTPPDTLVQLLS	STDTLVQLL	1	0	0	2	2	STPPDTLVQLL	tr_C9ZP82	0.053	28163.08	42.00
404	HLA-A0201	TVPNTGFEYFDI	TVPNTGFEI	0	0	0	8	3	TVPNTGFEYFDI	tr_C9ZP82	0.053	28175.89	42.00
34	HLA-A0201	GIIHPCCHNDSDT	IIHPCCHNT	1	0	0	8	3	IIHPCCHNDSDT	tr_C9ZP82	0.053	28177.71	42.00
73	HLA-A0201	PKEVMYIAV	PKEVMYIAV	0	0	0	0	0	PKEVMYIAV	tr_C9ZP82	0.053	28178.95	42.00
607	HLA-A0201	NNDNVTVADVAV	NVTVADVAV	0	0	0	1	3	NNDNVTVADVAV	tr_C9ZP82	0.053	28190.21	42.00
217	HLA-A0201	VDDPTAFEF	VDDPTAFEF	0	0	0	0	0	VDDPTAFEF	tr_C9ZP82	0.053	28213.40	42.00
456	HLA-A0201	FFGQPNHIEDGTL	GQPNHIETL	2	0	0	7	2	GQPNHIEDGTL	tr_C9ZP82	0.053	28234.79	42.00
450	HLA-A0201	SSAGYYFFGQ	SSAGYYFFQ	0	0	0	8	1	SSAGYYFFGQ	tr_C9ZP82	0.053	28234.79	42.00
67	HLA-A0201	LVQTVQPKEVMY	LVQTVQPEV	0	0	0	7	1	LVQTVQPKEV	tr_C9ZP82	0.053	28235.70	42.00
16	HLA-A0201	IVTKNCPSS	IVTKNCPSS	0	0	0	0	0	IVTKNCPSS	tr_C9ZP82	0.053	28235.70	42.00

831	HLA-A0201	SREGVEIRQLYDVP	GVEIRQLYV	3	0	0	8	1	GVEIRQLYDV	tr_C9ZP82	0.053	28251.89	42.00
18	HLA-A0201	TKNCPSSVHGLYI	CPSSVGLYI	3	0	0	5	1	CPSSVHGLYI	tr_C9ZP82	0.053	28254.04	42.00
622	HLA-A0201	STIDDRWRDT	STIDDRWRT	0	0	0	8	1	STIDDRWRDT	tr_C9ZP82	0.053	28266.58	42.00
357	HLA-A0201	IISLNPSYIDEEES	SLNPSYIES	2	0	0	7	3	SLNPSYIDEEES	tr_C9ZP82	0.053	28269.63	42.00
854	HLA-A0201	SCTVEVPRLSY	CTVEVPLSY	1	0	0	6	1	CTVEVPRLSY	tr_C9ZP82	0.053	28277.60	42.00
671	HLA-A0201	WYYPFYHAPP	WYYPFYAPP	0	0	0	6	1	WYYPFYHAPP	tr_C9ZP82	0.053	28295.04	43.00
895	HLA-A0201	KSNSRVTVVN	KSNSRVTVV	0	0	0	0	0	KSNSRVTVV	tr_C9ZP82	0.053	28305.75	43.00
783	HLA-A0201	PQLTEEERWRTQKL	QLTEETQKL	1	0	0	5	4	QLTEEERWRTQKL	tr_C9ZP82	0.053	28318.93	43.00
457	HLA-A0201	FGQPNHIEDGTL	GQPNHIETL	1	0	0	7	2	GQPNHIEDGTL	tr_C9ZP82	0.053	28321.38	43.00
609	HLA-A0201	DNVTVADVAVA	VTVADVAVA	2	0	0	0	0	VTVADVAVA	tr_C9ZP82	0.053	28322.29	43.00
96	HLA-A0201	RRYMGRAAPAQTG	YMGAAPAQT	2	0	0	3	1	YMGRAAPAQT	tr_C9ZP82	0.052	28339.16	43.00
452	HLA-A0201	AGYYFFGQP	AGYYFFGQP	0	0	0	0	0	AGYYFFGQP	tr_C9ZP82	0.052	28343.74	43.00
854	HLA-A0201	SCTVEVPRLSYG	CTVEVLSYG	1	0	0	5	2	CTVEVPRLSYG	tr_C9ZP82	0.052	28344.68	43.00
425	HLA-A0201	EVSALCDIKKFT	VSALCDIFT	1	0	0	7	2	VSALCDIKKFT	tr_C9ZP82	0.052	28368.60	43.00
151	HLA-A0201	LEDPLYSGAL	LEDPLYSAL	0	0	0	7	1	LEDPLYSGAL	tr_C9ZP82	0.052	28374.12	43.00
22	HLA-A0201	PSSVHGLYI	PSSVHGLYI	0	0	0	0	0	PSSVHGLYI	tr_C9ZP82	0.052	28379.36	43.00
744	HLA-A0201	TFPLQWNVDYHDSN	FLQWNVDYH	1	0	0	1	1	FPLQWNVDYH	tr_C9ZP82	0.052	28384.57	43.00
440	HLA-A0201	ESAFDKKTLVSS	SAFDTLVSS	1	0	0	4	2	SAFDKKTLVSS	tr_C9ZP82	0.052	28389.48	43.00
520	HLA-A0201	LGNDFLPHAP	LGNDFLPHA	0	0	0	0	0	LGNDFLPHA	tr_C9ZP82	0.052	28400.85	43.00
881	HLA-A0201	GQLKGNGEGVIEG	QLGEGVIEG	1	0	0	2	3	QLKGNGEGVIEG	tr_C9ZP82	0.052	28409.46	43.00
0	HLA-A0201	MGVPKFASWLR	MVPKFASWL	0	0	0	1	1	MGVPKFASWL	tr_C9ZP82	0.052	28438.68	43.00
741	HLA-A0201	LESTFPLQW	LESTFPLQW	0	0	0	0	0	LESTFPLQW	tr_C9ZP82	0.052	28440.20	43.00
243	HLA-A0201	AVLNMLNEKMSGN	VLNMLNEKM	1	0	0	0	0	VLNMLNEKM	tr_C9ZP82	0.052	28448.82	43.00
321	HLA-A0201	ILRDTTRNPYKRRA	ILRDTTRNA	0	0	0	8	5	ILRDTTRNPYKRRA	tr_C9ZP82	0.052	28451.59	43.00
196	HLA-A0201	NRYVESNQV	NRYVESNQV	0	0	0	0	0	NRYVESNQV	tr_C9ZP82	0.052	28457.13	43.00
369	HLA-A0201	ESDGVIGVPVCESS	ESDGVIVPV	0	0	0	6	1	ESDGVIGVPV	tr_C9ZP82	0.052	28459.61	43.00
266	HLA-A0201	FSGSNTPGEGEHKI	FSGSNTPGI	0	0	0	8	5	FSGSNTPGEGEHKI	tr_C9ZP82	0.052	28461.14	43.00
286	HLA-A0201	QSSLAQFGGKA	SLAQFGGKA	2	0	0	0	0	SLAQFGGKA	tr_C9ZP82	0.052	28462.99	43.00
798	HLA-A0201	FHLVITSNK	FHLVITSNK	0	0	0	0	0	FHLVITSNK	tr_C9ZP82	0.052	28469.16	43.00
501	HLA-A0201	CSSPANSKI	CSSPANSKI	0	0	0	0	0	CSSPANSKI	tr_C9ZP82	0.052	28472.85	43.00
214	HLA-A0201	EVIVDDPTAFEFDS	VIVAFEFDS	1	0	0	3	4	VIVDDPTAFEFDS	tr_C9ZP82	0.052	28498.42	43.00
141	HLA-A0201	DDLDEVRQTLLEDP	DLDEVQTLL	1	0	0	5	1	DLDEVRQTLL	tr_C9ZP82	0.052	28513.85	43.00
369	HLA-A0201	ESDGVIGVPVCES	GVIGVVCES	3	0	0	5	1	GVIGVPVCES	tr_C9ZP82	0.052	28514.78	43.00
13	HLA-A0201	YPAIVTKNC	YPAIVTKNC	0	0	0	0	0	YPAIVTKNC	tr_C9ZP82	0.052	28517.86	43.00
244	HLA-A0201	VLNMLNEKMSGN	VLNMLNEKM	0	0	0	0	0	VLNMLNEKM	tr_C9ZP82	0.052	28537.61	43.00
605	HLA-A0201	GSNNDNVTVA	GSNNDNVTV	0	0	0	0	0	GSNNDNVTV	tr_C9ZP82	0.052	28546.27	43.00
202	HLA-A0201	NQVVDDVFG	NQVVDDVFG	0	0	0	0	0	NQVVDDVFG	tr_C9ZP82	0.052	28557.70	43.00
24	HLA-A0201	SVHGLYIDLNG	SVHGLYIDL	0	0	0	0	0	SVHGLYIDL	tr_C9ZP82	0.052	28562.03	43.00
555	HLA-A0201	LTKGPHEINLPQL	LTEINLPQL	0	0	0	2	4	LTKGPHEINLPQL	tr_C9ZP82	0.052	28567.28	43.00
693	HLA-A0201	IKRMPLPVVKSTPP	RMPLPVVPP	2	0	0	7	3	RMPLPVVKSTPP	tr_C9ZP82	0.052	28567.59	43.00
533	HLA-A0201	SGESALDNLL	SGSALDNLL	0	0	0	2	1	SGESALDNLL	tr_C9ZP82	0.052	28567.89	43.00
812	HLA-A0201	KGDLRKLSDALD	KLRKLSDAL	0	0	0	1	2	KGDLRKLSDAL	tr_C9ZP82	0.052	28573.45	43.00
615	HLA-A0201	DVAVALHST	DVAVALHST	0	0	0	0	0	DVAVALHST	tr_C9ZP82	0.052	28586.45	43.00
20	HLA-A0201	NCPSSVHGLYIDLN	CVHGLYIDL	1	0	0	1	3	CPSSVHGLYIDL	tr_C9ZP82	0.052	28595.41	43.00
371	HLA-A0201	DGVIGVPVCESS	VIVPVCESS	2	0	0	2	1	VIGVPVCESS	tr_C9ZP82	0.052	28598.19	43.00
576	HLA-A0201	KIEAVKFRR	KIEAVKFRR	0	0	0	0	0	KIEAVKFRR	tr_C9ZP82	0.052	28604.39	43.00
220	HLA-A0201	PTAFEFDSNCISPG	TAFEFCISP	1	0	0	5	3	TAFEFDSNCISP	tr_C9ZP82	0.052	28605.02	43.00
840	HLA-A0201	LYDVPLERY	LYDVPLERY	0	0	0	0	0	LYDVPLERY	tr_C9ZP82	0.052	28616.16	43.00
274	HLA-A0201	EGEHKIIDFL	GEHKIIDFL	1	0	0	0	0	GEHKIIDFL	tr_C9ZP82	0.052	28617.08	43.00
637	HLA-A0201	GLSNSVQEACEK	GLSNSVQEA	0	0	0	0	0	GLSNSVQEA	tr_C9ZP82	0.052	28622.97	43.00
608	HLA-A0201	NDNVTVADVAVA	VTVADVAVA	3	0	0	0	0	VTVADVAVA	tr_C9ZP82	0.052	28625.45	43.00
317	HLA-A0201	TQVFILRDTTRNP	TQVFILTTR	0	0	0	6	2	TQVFILRDTTR	tr_C9ZP82	0.052	28628.84	43.00
452	HLA-A0201	AGYYFFGQPNHIE	AGYYFFNHI	0	0	0	6	3	AGYYFFGQPNHI	tr_C9ZP82	0.052	28638.15	43.00
751	HLA-A0201	VDYHDSNVVHLA	VDYNVVHLA	0	0	0	3	3	VDYHDSNVVHLA	tr_C9ZP82	0.051	28659.23	43.00

214	HLA-A0201	EVIVDDPTAFEFD	VIVPTAFEF	1	0	0	3	2	VIVDDPTAFEF	tr_C9ZP82	0.051	28660.47	43.00
679	HLA-A0201	PPVIDLAIY	PPVIDLAIY	0	0	0	0	0	PPVIDLAIY	tr_C9ZP82	0.051	28660.47	43.00
717	HLA-A0201	PPTSHALLPMVLG	TSHALLMVL	2	0	0	6	1	TSHALLPMVL	tr_C9ZP82	0.051	28665.74	43.00
244	HLA-A0201	VLNMLNEKMSGNDP	VLNMLNEKM	0	0	0	0	0	VLNMLNEKM	tr_C9ZP82	0.051	28670.39	43.00
57	HLA-A0201	LECICSELE	LECICSELE	0	0	0	0	0	LECICSELE	tr_C9ZP82	0.051	28675.67	43.00
370	HLA-A0201	SDGVIGVPVCESS	VIVPVCESS	3	0	0	2	1	VIGVPVCESS	tr_C9ZP82	0.051	28679.07	43.00
95	HLA-A0201	ARRYMGRAAPAQTG	YMGAAPAQT	3	0	0	3	1	YMGRAAPAQT	tr_C9ZP82	0.051	28689.32	43.00
368	HLA-A0201	EESDGVIGVPVC	ESDGVIVPV	1	0	0	6	1	ESDGVIGVPV	tr_C9ZP82	0.051	28696.14	43.00
586	HLA-A0201	LLFKSTNKENSNS	LLFKSTNNS	0	0	0	7	4	LLFKSTNKENSNS	tr_C9ZP82	0.051	28705.77	43.00
121	HLA-A0201	MAIVEEVVRE	MIVEEVVRE	0	0	0	1	1	MAIVEEVVRE	tr_C9ZP82	0.051	28707.32	43.00
352	HLA-A0201	IDDTCIISLNP	ITCIISLNP	0	0	0	1	2	IDDTCIISLNP	tr_C9ZP82	0.051	28741.82	44.00
505	HLA-A0201	ANSKIIDDI	ANSKIIDDI	0	0	0	0	0	ANSKIIDDI	tr_C9ZP82	0.051	28744.93	44.00
33	HLA-A0201	NGIIHPCCHNDSDT	IIHPCCHNT	2	0	0	8	3	IIHPCCHNDSDT	tr_C9ZP82	0.051	28748.36	44.00
225	HLA-A0201	FDSNCISPG	FDSNCISPG	0	0	0	0	0	FDSNCISPG	tr_C9ZP82	0.051	28752.09	44.00
318	HLA-A0201	QVFILRDTTR	QVFILDTTR	0	0	0	5	1	QVFILRDTTR	tr_C9ZP82	0.051	28753.02	44.00
646	HLA-A0201	CEKYVEGVCF	CEYVEGVCF	0	0	0	2	1	CEKYVEGVCF	tr_C9ZP82	0.051	28759.86	44.00
808	HLA-A0201	SFVPKGDLRKLSDA	FVLRKLSDA	1	0	0	2	4	FVPKGDLRKLSDA	tr_C9ZP82	0.051	28768.89	44.00
767	HLA-A0201	PFADMDVLQQ	FADMDVLQQ	1	0	0	0	0	FADMDVLQQ	tr_C9ZP82	0.051	28769.21	44.00
613	HLA-A0201	VADVAVALHSTIDD	VADVAVALI	0	0	0	8	3	VADVAVALHSTI	tr_C9ZP82	0.051	28769.52	44.00
104	HLA-A0201	PAQTGEVAST	AQTGEVAST	1	0	0	0	0	AQTGEVAST	tr_C9ZP82	0.051	28772.31	44.00
176	HLA-A0201	DGVNSFCVRST	GVNSFCVST	1	0	0	7	1	GVNSFCVRST	tr_C9ZP82	0.051	28775.11	44.00
262	HLA-A0201	LCVIFSGSNT	LCVIFSGST	0	0	0	8	1	LCVIFSGSNT	tr_C9ZP82	0.051	28778.23	44.00
46	HLA-A0201	TAIALLPEE	TAIALLPEE	0	0	0	0	0	TAIALLPEE	tr_C9ZP82	0.051	28786.95	44.00
607	HLA-A0201	NNDNVTVADVAVA	VTVADVAVA	4	0	0	0	0	VTVADVAVA	tr_C9ZP82	0.051	28796.92	44.00
445	HLA-A0201	KKTLVSSAGYY	KLVSSAGYY	0	0	0	1	2	KKTLVSSAGYY	tr_C9ZP82	0.051	28818.73	44.00
665	HLA-A0201	TDSDWSWYYPF	SDWSWYYPF	2	0	0	0	0	SDWSWYYPF	tr_C9ZP82	0.051	28839.63	44.00
126	HLA-A0201	EVVREFTSTEM	VVREFTTEM	1	0	0	6	1	VVREFTSTEM	tr_C9ZP82	0.051	28842.76	44.00
657	HLA-A0201	WRYYTTTTT	WRYYTTTTT	0	0	0	0	0	WRYYTTTTT	tr_C9ZP82	0.051	28864.60	44.00
695	HLA-A0201	RMPLPVVKSTPPD	RMLPVVKST	0	0	0	2	1	RMPLPVVKST	tr_C9ZP82	0.051	28879.29	44.00
438	HLA-A0201	ATESAFDKKTLVSS	SAFDTLVSS	3	0	0	4	2	SAFDKKTLVSS	tr_C9ZP82	0.051	28884.92	44.00
623	HLA-A0201	TIDDRWRDT	TIDDRWRDT	0	0	0	0	0	TIDDRWRDT	tr_C9ZP82	0.051	28897.74	44.00
17	HLA-A0201	VTKNCPSSVH	VTKNCPSSV	0	0	0	0	0	VTKNCPSSV	tr_C9ZP82	0.051	28902.73	44.00
352	HLA-A0201	IDDTCIISLNPS	ICIISLNPS	0	0	0	1	3	IDDTCIISLNPS	tr_C9ZP82	0.051	28906.18	44.00
440	HLA-A0201	ESAFDKKTLVSSAG	SAFDLVSSA	1	0	0	4	3	SAFDKKTLVSSA	tr_C9ZP82	0.051	28908.36	44.00
717	HLA-A0201	PPTSHALLPM	PTSHALLPM	0	0	0	1	1	PPTSHALLPM	tr_C9ZP82	0.051	28913.69	44.00
317	HLA-A0201	TQVFILRDTTRN	TQVFILTTR	0	0	0	6	2	TQVFILRDTTR	tr_C9ZP82	0.051	28927.46	44.00
881	HLA-A0201	GQLKGNGEGVIEGK	QLEGVIEGK	1	0	0	2	4	QLKGNGEGVIEGK	tr_C9ZP82	0.051	28928.39	44.00
203	HLA-A0201	QVVDDVFGH	QVVDDVFGH	0	0	0	0	0	QVVDDVFGH	tr_C9ZP82	0.051	28932.14	44.00
574	HLA-A0201	YAKIEAVKFR	YAIEAVKFR	0	0	0	2	1	YAKIEAVKFR	tr_C9ZP82	0.051	28933.09	44.00
578	HLA-A0201	EAVKFRRFL	EAVKFRRFL	0	0	0	0	0	EAVKFRRFL	tr_C9ZP82	0.051	28943.11	44.00
418	HLA-A0201	VGGSLISEVS	VGGSLISEV	0	0	0	0	0	VGGSLISEV	tr_C9ZP82	0.051	28944.37	44.00
441	HLA-A0201	SAFDKKTLVSSAGY	SAFDLVSSA	0	0	0	4	3	SAFDKKTLVSSA	tr_C9ZP82	0.050	28961.28	44.00
403	HLA-A0201	TTVPNTGFEYF	TTVPGFEYF	0	0	0	4	2	TTVPNTGFEYF	tr_C9ZP82	0.050	28992.94	44.00
643	HLA-A0201	QEACEKYVEGV	EACEYVEGV	1	0	0	4	1	EACEKYVEGV	tr_C9ZP82	0.050	28996.08	44.00
854	HLA-A0201	SCTVEVPRLSYGAD	CTVEVSYGA	1	0	0	5	3	CTVEVPRLSYGA	tr_C9ZP82	0.050	29013.96	44.00
701	HLA-A0201	VKSTPPDTLVQLLS	STDTLVQLL	2	0	0	2	2	STPPDTLVQLL	tr_C9ZP82	0.050	29018.68	44.00
628	HLA-A0201	WRDTYLKSTGLSN	YLKSTGLSN	4	0	0	0	0	YLKSTGLSN	tr_C9ZP82	0.050	29035.64	44.00
368	HLA-A0201	EESDGVIGVPVCES	GVIGVVCES	4	0	0	5	1	GVIGVPVCES	tr_C9ZP82	0.050	29039.40	44.00
295	HLA-A0201	KANHVIVGLDAD	KANHVIVGL	0	0	0	0	0	KANHVIVGL	tr_C9ZP82	0.050	29043.48	44.00
636	HLA-A0201	TGLSNSVQEACE	GLSNSVQEA	1	0	0	0	0	GLSNSVQEA	tr_C9ZP82	0.050	29050.10	44.00
855	HLA-A0201	CTVEVPRLSYGADG	CTVEVSYGA	0	0	0	5	3	CTVEVPRLSYGA	tr_C9ZP82	0.050	29053.54	44.00
439	HLA-A0201	TESAFDKKTLVSS	SAFDTLVSS	2	0	0	4	2	SAFDKKTLVSS	tr_C9ZP82	0.050	29054.18	44.00
242	HLA-A0201	DAVLNMLNEKMSGN	VLNMLNEKM	2	0	0	0	0	VLNMLNEKM	tr_C9ZP82	0.050	29055.44	44.00
23	HLA-A0201	SSVHGLYIDLNG	SVHGLYIDL	0	0	0	1	1	SSVHGLYIDL	tr_C9ZP82	0.050	29056.69	44.00

638	HLA-A0201	LSNSVQEAC	LSNSVQEAC	0	0	0	0	0	LSNSVQEAC	tr_C9ZP82	0.050	29071.48	44.00
396	HLA-A0201	ESREAHVTTV	ESREAVTTV	0	0	0	5	1	ESREAHVTTV	tr_C9ZP82	0.050	29080.60	44.00
416	HLA-A0201	SVVGGSLISE	SVVGSLISE	0	0	0	3	1	SVVGGSLISE	tr_C9ZP82	0.050	29081.23	44.00
145	HLA-A0201	EVRQTLLEDPLY	EVRQTLLPL	0	0	0	7	2	EVRQTLLEDPL	tr_C9ZP82	0.050	29081.53	44.00
533	HLA-A0201	SGESALDNL	SGESALDNL	0	0	0	0	0	SGESALDNL	tr_C9ZP82	0.050	29108.61	44.00
641	HLA-A0201	SVQEACEKY	SVQEACEKY	0	0	0	0	0	SVQEACEKY	tr_C9ZP82	0.050	29112.38	44.00
123	HLA-A0201	IVEEVVREFTSTE	IVEEVVFTS	0	0	0	6	2	IVEEVVREFTS	tr_C9ZP82	0.050	29158.74	45.00
125	HLA-A0201	EEVVREFTSTEM	VVREFTTEM	2	0	0	6	1	VVREFTSTEM	tr_C9ZP82	0.050	29166.30	45.00
369	HLA-A0201	ESDGVIGVPVCE	ESDGVIVPV	0	0	0	6	1	ESDGVIGVPV	tr_C9ZP82	0.050	29176.10	45.00
180	HLA-A0201	SFCVRSTDDDYEST	FTDDDYEST	1	0	0	1	4	FCVRSTDDDYEST	tr_C9ZP82	0.050	29186.51	45.00
21	HLA-A0201	CPSSVHGLYIDLNG	CVHGLYIDL	0	0	0	1	3	CPSSVHGLYIDL	tr_C9ZP82	0.050	29188.09	45.00
686	HLA-A0201	IYLKEANIKR	YLKEANIKR	1	0	0	0	0	YLKEANIKR	tr_C9ZP82	0.050	29189.35	45.00
753	HLA-A0201	YHDSNVVHLAT	YSNVVHLAT	0	0	0	1	2	YHDSNVVHLAT	tr_C9ZP82	0.050	29189.67	45.00
191	HLA-A0201	ESTHENRYVESNQV	STYVESNQV	1	0	0	2	4	STHENRYVESNQV	tr_C9ZP82	0.050	29210.83	45.00
448	HLA-A0201	LVSSAGYYFFGQ	LVSSYFFGQ	0	0	0	4	3	LVSSAGYYFFGQ	tr_C9ZP82	0.050	29214.32	45.00
64	HLA-A0201	LELLVQTVQ	LELLVQTVQ	0	0	0	0	0	LELLVQTVQ	tr_C9ZP82	0.050	29215.88	45.00
93	HLA-A0201	QRARRYMGRAAPA	RAMGRAAPA	1	0	0	2	3	RARRYMGRAAPA	tr_C9ZP82	0.050	29219.69	45.00
577	HLA-A0201	IEAVKFRRF	IEAVKFRRF	0	0	0	0	0	IEAVKFRRF	tr_C9ZP82	0.050	29221.27	45.00
367	HLA-A0201	EEESDGVIGVPVC	ESDGVIVPV	2	0	0	6	1	ESDGVIGVPV	tr_C9ZP82	0.050	29222.21	45.00
207	HLA-A0201	DVFGHRGEVIV	DVFGGEVIV	0	0	0	4	2	DVFGHRGEVIV	tr_C9ZP82	0.050	29226.00	45.00
17	HLA-A0201	VTKNCPSSVHGL	VTPSSVHGL	0	0	0	2	3	VTKNCPSSVHGL	tr_C9ZP82	0.050	29241.20	45.00
604	HLA-A0201	KGSNNDNVTVADV	KGSVTVADV	0	0	0	3	4	KGSNNDNVTVADV	tr_C9ZP82	0.050	29252.89	45.00
216	HLA-A0201	IVDDPTAFEFDS	IVDAFEFDS	0	0	0	3	3	IVDDPTAFEFDS	tr_C9ZP82	0.050	29257.01	45.00
160	HLA-A0201	LYNEGEEEA	LYNEGEEEA	0	0	0	0	0	LYNEGEEEA	tr_C9ZP82	0.050	29260.50	45.00
627	HLA-A0201	RWRDTYLKSTGLSN	YLKSTGLSN	5	0	0	0	0	YLKSTGLSN	tr_C9ZP82	0.049	29269.05	45.00
243	HLA-A0201	AVLNMLNEKMSGND	VLNMLNEKM	1	0	0	0	0	VLNMLNEKM	tr_C9ZP82	0.049	29271.58	45.00
880	HLA-A0201	SGQLKGNGEGVIEG	QLGEGVIEG	2	0	0	2	3	QLKGNGEGVIEG	tr_C9ZP82	0.049	29280.45	45.00
731	HLA-A0201	VMVRSPQQAE	VMVRSPQQA	0	0	0	0	0	VMVRSPQQA	tr_C9ZP82	0.049	29313.10	45.00
448	HLA-A0201	LVSSAGYYFFGQP	LVYYFFGQP	0	0	0	2	4	LVSSAGYYFFGQP	tr_C9ZP82	0.049	29320.40	45.00
98	HLA-A0201	YMGRAAPAQTGE	YMGAAPAQT	0	0	0	3	1	YMGRAAPAQT	tr_C9ZP82	0.049	29324.52	45.00
97	HLA-A0201	RYMGRAAPAQ	YMGRAAPAQ	1	0	0	0	0	YMGRAAPAQ	tr_C9ZP82	0.049	29359.45	45.00
674	HLA-A0201	PFYHAPPVIDL	FYAPPVIDL	1	0	0	2	1	FYHAPPVIDL	tr_C9ZP82	0.049	29367.38	45.00
366	HLA-A0201	DEEESDGVIGVPVC	ESDGVIVPV	3	0	0	6	1	ESDGVIGVPV	tr_C9ZP82	0.049	29367.71	45.00
22	HLA-A0201	PSSVHGLYIDLN	SVHGLYIDL	2	0	0	0	0	SVHGLYIDL	tr_C9ZP82	0.049	29372.78	45.00
810	HLA-A0201	VPKGDLRKLSDALD	VLRKLSDAL	0	0	0	1	4	VPKGDLRKLSDAL	tr_C9ZP82	0.049	29377.56	45.00
124	HLA-A0201	VEEVVREFTSTEM	VVREFTTEM	3	0	0	6	1	VVREFTSTEM	tr_C9ZP82	0.049	29387.73	45.00
31	HLA-A0201	DLNGIIHPCCH	DLNGIIHPC	0	0	0	8	1	DLNGIIHPCC	tr_C9ZP82	0.049	29409.67	45.00
214	HLA-A0201	EVIVDDPTAF	VIVDDPTAF	1	0	0	0	0	VIVDDPTAF	tr_C9ZP82	0.049	29409.67	45.00
223	HLA-A0201	FEFDSNCISPGT	FEFCISPGT	0	0	0	3	3	FEFDSNCISPGT	tr_C9ZP82	0.049	29422.72	45.00
370	HLA-A0201	SDGVIGVPVCES	GVIGVVCES	2	0	0	5	1	GVIGVPVCES	tr_C9ZP82	0.049	29426.55	45.00
809	HLA-A0201	FVPKGDLRKLS	FVPKGDLRL	0	0	0	8	1	FVPKGDLRKL	tr_C9ZP82	0.049	29438.97	45.00
752	HLA-A0201	DYHDSNVVHLA	YHDSVVHLA	1	0	0	4	1	YHDSNVVHLA	tr_C9ZP82	0.049	29444.38	45.00
244	HLA-A0201	VLNMLNEKMSGND	VLNMLNEKM	0	0	0	0	0	VLNMLNEKM	tr_C9ZP82	0.049	29451.06	45.00
685	HLA-A0201	AIYLKEANIKR	AIYLKEANI	0	0	0	0	0	AIYLKEANI	tr_C9ZP82	0.049	29489.64	45.00
294	HLA-A0201	GKANHVIVGLD	KANHVIVGL	1	0	0	0	0	KANHVIVGL	tr_C9ZP82	0.049	29489.97	45.00
392	HLA-A0201	ESPIESREAHVTTV	SPIESVTTV	1	0	0	5	4	SPIESREAHVTTV	tr_C9ZP82	0.049	29500.51	45.00
6	HLA-A0201	ASWLRNKYP	ASWLRNKYP	0	0	0	0	0	ASWLRNKYP	tr_C9ZP82	0.049	29508.80	45.00
320	HLA-A0201	FILRDTTRN	FILRDTTRN	0	0	0	0	0	FILRDTTRN	tr_C9ZP82	0.049	29529.25	46.00
802	HLA-A0201	ITSNKTSFVPKGDL	ITSNKTSFL	0	0	0	8	5	ITSNKTSFVPKGDL	tr_C9ZP82	0.049	29546.18	46.00
175	HLA-A0201	GDGVNSFCVRST	GVNSFCVST	2	0	0	7	1	GVNSFCVRST	tr_C9ZP82	0.049	29564.73	46.00
371	HLA-A0201	DGVIGVPVCES	GVIGVVCES	1	0	0	5	1	GVIGVPVCES	tr_C9ZP82	0.049	29580.73	46.00
121	HLA-A0201	MAIVEEVVR	MAIVEEVVR	0	0	0	0	0	MAIVEEVVR	tr_C9ZP82	0.049	29582.96	46.00
320	HLA-A0201	FILRDTTRNPY	ILRDTTNPY	1	0	0	6	1	ILRDTTRNPY	tr_C9ZP82	0.048	29588.42	46.00
434	HLA-A0201	KFTPATESAF	FTPATESAF	1	0	0	0	0	FTPATESAF	tr_C9ZP82	0.048	29590.00	46.00

404	HLA-A0201	TVPNTGFEY	TVPNTGFEY	0	0	0	0	0	TVPNTGFEY	tr_C9ZP82	0.048	29597.06	46.00
448	HLA-A0201	LVSSAGYYFFGQPN	LVYFFGQPN	0	0	0	2	5	LVSSAGYYFFGQPN	tr_C9ZP82	0.048	29602.83	46.00
618	HLA-A0201	VALHSTIDDRWRDT	ALHSTIDDT	1	0	0	7	4	ALHSTIDDRWRDT	tr_C9ZP82	0.048	29603.46	46.00
636	HLA-A0201	TGLSNSVQEACEKY	GLSNSVQEA	1	0	0	0	0	GLSNSVQEA	tr_C9ZP82	0.048	29623.97	46.00
16	HLA-A0201	IVTKNCPSSVHG	IVTNCPSSV	0	0	0	3	1	IVTKNCPSSV	tr_C9ZP82	0.048	29625.88	46.00
30	HLA-A0201	IDLNGIIHP	IDLNGIIHP	0	0	0	0	0	IDLNGIIHP	tr_C9ZP82	0.048	29625.88	46.00
40	HLA-A0201	CHNDSDTAIALLPE	CTAIALLPE	0	0	0	1	5	CHNDSDTAIALLPE	tr_C9ZP82	0.048	29638.07	46.00
318	HLA-A0201	QVFILRDTTRNPY	QVFILRNPY	0	0	0	5	4	QVFILRDTTRNPY	tr_C9ZP82	0.048	29638.72	46.00
16	HLA-A0201	IVTKNCPSSVHGLY	IVTNCPSSV	0	0	0	3	1	IVTKNCPSSV	tr_C9ZP82	0.048	29673.69	46.00
684	HLA-A0201	LAIYLKEANIKR	LIYLKEANI	0	0	0	1	1	LAIYLKEANI	tr_C9ZP82	0.048	29683.96	46.00
255	HLA-A0201	NDPTWTRLCV	NDPTWTLCV	0	0	0	6	1	NDPTWTRLCV	tr_C9ZP82	0.048	29688.14	46.00
44	HLA-A0201	SDTAIALLP	SDTAIALLP	0	0	0	0	0	SDTAIALLP	tr_C9ZP82	0.048	29698.10	46.00
223	HLA-A0201	FEFDSNCISPG	FEFDCISPG	0	0	0	4	2	FEFDSNCISPG	tr_C9ZP82	0.048	29706.46	46.00
444	HLA-A0201	DKKTLVSSAGYY	KLVSSAGYY	1	0	0	1	2	KKTLVSSAGYY	tr_C9ZP82	0.048	29719.96	46.00
143	HLA-A0201	LDEVRQTLLEDPLY	LQTLLEDPL	0	0	0	1	4	LDEVRQTLLEDPL	tr_C9ZP82	0.048	29730.90	46.00
603	HLA-A0201	RKGSNNDNVTV	KSNNDNVTV	1	0	0	1	1	KGSNNDNVTV	tr_C9ZP82	0.048	29740.88	46.00
825	HLA-A0201	ILRRVPSREG	ILRRVPSEG	0	0	0	7	1	ILRRVPSREG	tr_C9ZP82	0.048	29746.67	46.00
814	HLA-A0201	DLRKLSDALD	DLRKLSDAL	0	0	0	0	0	DLRKLSDAL	tr_C9ZP82	0.048	29754.38	46.00
689	HLA-A0201	KEANIKRMPLPVVK	KIKRMPLPV	0	0	0	1	3	KEANIKRMPLPV	tr_C9ZP82	0.048	29756.32	46.00
14	HLA-A0201	PAIVTKNCPSSVHG	AIVNCPSSV	1	0	0	3	2	AIVTKNCPSSV	tr_C9ZP82	0.048	29769.20	46.00
423	HLA-A0201	ISEVSALCDIKKF	ISEVSALCF	0	0	0	8	4	ISEVSALCDIKKF	tr_C9ZP82	0.048	29770.81	46.00
739	HLA-A0201	AELESTFPLQWN	AELESTFPL	0	0	0	0	0	AELESTFPL	tr_C9ZP82	0.048	29776.61	46.00
665	HLA-A0201	TDSDWSWYY	TDSDWSWYY	0	0	0	0	0	TDSDWSWYY	tr_C9ZP82	0.048	29779.18	46.00
443	HLA-A0201	FDKKTLVSSA	FDKKTLVSA	0	0	0	7	1	FDKKTLVSSA	tr_C9ZP82	0.048	29782.41	46.00
618	HLA-A0201	VALHSTIDDRWRD	ALHSTIWRD	1	0	0	6	3	ALHSTIDDRWRD	tr_C9ZP82	0.048	29784.66	46.00
5	HLA-A0201	FASWLRNKY	FASWLRNKY	0	0	0	0	0	FASWLRNKY	tr_C9ZP82	0.048	29784.66	46.00
284	HLA-A0201	RTQSSLAQFG	RTQSSLAFG	0	0	0	7	1	RTQSSLAQFG	tr_C9ZP82	0.048	29788.54	46.00
831	HLA-A0201	SREGVEIRQL	SREGVEIQL	0	0	0	7	1	SREGVEIRQL	tr_C9ZP82	0.048	29801.76	46.00
222	HLA-A0201	AFEFDSNCI	AFEFDSNCI	0	0	0	0	0	AFEFDSNCI	tr_C9ZP82	0.048	29808.52	46.00
174	HLA-A0201	DGDGVNSFCVRST	GVNSFCVST	3	0	0	7	1	GVNSFCVRST	tr_C9ZP82	0.048	29816.27	46.00
819	HLA-A0201	SDALDTILRR	SALDTILRR	0	0	0	1	1	SDALDTILRR	tr_C9ZP82	0.048	29833.37	46.00
173	HLA-A0201	NDGDGVNSFCVRST	GVNSFCVST	4	0	0	7	1	GVNSFCVRST	tr_C9ZP82	0.048	29842.72	46.00
31	HLA-A0201	DLNGIIHPCCHN	DLNGIICHN	0	0	0	6	3	DLNGIIHPCCHN	tr_C9ZP82	0.048	29845.63	46.00
152	HLA-A0201	EDPLYSGALYN	ELYSGALYN	0	0	0	1	2	EDPLYSGALYN	tr_C9ZP82	0.048	29860.49	46.00
645	HLA-A0201	ACEKYVEGVCF	ACYVEGVCF	0	0	0	2	2	ACEKYVEGVCF	tr_C9ZP82	0.048	29885.38	46.00
220	HLA-A0201	PTAFEFDSNC	TAFEFDSNC	1	0	0	0	0	TAFEFDSNC	tr_C9ZP82	0.048	29887.00	46.00
682	HLA-A0201	IDLAIYLKE	IDLAIYLKE	0	0	0	0	0	IDLAIYLKE	tr_C9ZP82	0.048	29889.58	46.00
852	HLA-A0201	TYSCTVEVPR	YSCTVEVPR	1	0	0	0	0	YSCTVEVPR	tr_C9ZP82	0.048	29890.24	46.00
144	HLA-A0201	DEVRQTLLEDPLY	EVRQTLLPL	1	0	0	7	2	EVRQTLLEDPL	tr_C9ZP82	0.047	29910.29	46.00
825	HLA-A0201	ILRRVPSRE	ILRRVPSRE	0	0	0	0	0	ILRRVPSRE	tr_C9ZP82	0.047	29922.28	46.00
105	HLA-A0201	AQTGEVASTYMP	AQTGEVAYM	0	0	0	7	2	AQTGEVASTYM	tr_C9ZP82	0.047	29967.31	47.00
691	HLA-A0201	ANIKRMPLPVVKS	AIKRMPLPV	0	0	0	1	1	ANIKRMPLPV	tr_C9ZP82	0.047	29972.49	47.00
666	HLA-A0201	DSDWSWYYPFY	SDWSWYYFY	1	0	0	7	1	SDWSWYYPFY	tr_C9ZP82	0.047	29974.11	47.00
293	HLA-A0201	GGKANHVIVGLD	KANHVIVGL	2	0	0	0	0	KANHVIVGL	tr_C9ZP82	0.047	29975.41	47.00
801	HLA-A0201	VITSNKTSFVPK	VISNKTSFV	0	0	0	2	1	VITSNKTSFV	tr_C9ZP82	0.047	29977.36	47.00
618	HLA-A0201	VALHSTIDDR	VLHSTIDDR	0	0	0	1	1	VALHSTIDDR	tr_C9ZP82	0.047	29986.43	47.00
613	HLA-A0201	VADVAVALHST	VADVAVALT	0	0	0	8	2	VADVAVALHST	tr_C9ZP82	0.047	29989.68	47.00
132	HLA-A0201	TSTEMAQANDDL	SMAQANDDL	1	0	0	1	2	STEMAQANDDL	tr_C9ZP82	0.047	30019.88	47.00
699	HLA-A0201	PVVKSTPPDTLV	VVTPPDTLV	1	0	0	2	2	VVKSTPPDTLV	tr_C9ZP82	0.047	30026.37	47.00
263	HLA-A0201	CVIFSGSNTP	VIFSGSNTP	1	0	0	0	0	VIFSGSNTP	tr_C9ZP82	0.047	30029.29	47.00
358	HLA-A0201	ISLNPSYIDE	ILNPSYIDE	0	0	0	1	1	ISLNPSYIDE	tr_C9ZP82	0.047	30037.41	47.00
216	HLA-A0201	IVDDPTAFEFD	IVDPTAFEF	0	0	0	2	1	IVDDPTAFEF	tr_C9ZP82	0.047	30044.89	47.00
744	HLA-A0201	TFPLQWNVDY	FPLQWNVDY	1	0	0	0	0	FPLQWNVDY	tr_C9ZP82	0.047	30064.08	47.00
348	HLA-A0201	KRNFIDDTCII	KRNFITCII	0	0	0	5	2	KRNFIDDTCII	tr_C9ZP82	0.047	30064.41	47.00

172	HLA-A0201	LNDGDGVNSFCV	LNDGNSFCV	0	0	0	4	3	LNDGDGVNSFCV	tr_C9ZP82	0.047	30066.04	47.00
497	HLA-A0201	NFRPCSSPA	NFRPCSSPA	0	0	0	0	0	NFRPCSSPA	tr_C9ZP82	0.047	30080.02	47.00
691	HLA-A0201	ANIKRMPLPVVK	AIKRMPLPV	0	0	0	1	1	ANIKRMPLPV	tr_C9ZP82	0.047	30091.74	47.00
452	HLA-A0201	AGYYFFGQPNHIED	AGYYFFNHI	0	0	0	6	3	AGYYFFGQPNHI	tr_C9ZP82	0.047	30099.23	47.00
601	HLA-A0201	EFRKGSNNDNVTVA	FSNNDNVTV	1	0	0	1	3	FRKGSNNDNVTV	tr_C9ZP82	0.047	30124.32	47.00
732	HLA-A0201	MVRSPQQAELESTF	MVAELESTF	0	0	0	2	5	MVRSPQQAELESTF	tr_C9ZP82	0.047	30126.28	47.00
614	HLA-A0201	ADVAVALHSTID	ADVAVALTI	0	0	0	7	2	ADVAVALHSTI	tr_C9ZP82	0.047	30133.12	47.00
603	HLA-A0201	RKGSNNDNVTVADV	GSNNTVADV	2	0	0	4	3	GSNNDNVTVADV	tr_C9ZP82	0.047	30134.09	47.00
692	HLA-A0201	NIKRMPLPVVKS	IMPLPVVKS	1	0	0	1	2	IKRMPLPVVKS	tr_C9ZP82	0.047	30159.87	47.00
617	HLA-A0201	AVALHSTIDDRWRD	ALHSTIWRD	2	0	0	6	3	ALHSTIDDRWRD	tr_C9ZP82	0.047	30169.66	47.00
812	HLA-A0201	KGDLRKLSDALDT	KLRKLSDAL	0	0	0	1	2	KGDLRKLSDAL	tr_C9ZP82	0.047	30177.50	47.00
636	HLA-A0201	TGLSNSVQEACEK	GLSNSVQEA	1	0	0	0	0	GLSNSVQEA	tr_C9ZP82	0.047	30193.81	47.00
211	HLA-A0201	HRGEVIVDDPTAF	HRGEVIVAF	0	0	0	7	4	HRGEVIVDDPTAF	tr_C9ZP82	0.047	30202.32	47.00
809	HLA-A0201	FVPKGDLRK	FVPKGDLRK	0	0	0	0	0	FVPKGDLRK	tr_C9ZP82	0.047	30204.94	47.00
275	HLA-A0201	GEHKIIDFLR	GEHKIIDFL	0	0	0	0	0	GEHKIIDFL	tr_C9ZP82	0.047	30209.18	47.00
357	HLA-A0201	IISLNPSYIDEE	IISLNPSYI	0	0	0	0	0	IISLNPSYI	tr_C9ZP82	0.047	30212.12	47.00
201	HLA-A0201	SNQVVDDVF	SNQVVDDVF	0	0	0	0	0	SNQVVDDVF	tr_C9ZP82	0.047	30212.12	47.00
154	HLA-A0201	PLYSGALYNEG	PLYSGALYG	0	0	0	8	2	PLYSGALYNEG	tr_C9ZP82	0.047	30218.34	47.00
749	HLA-A0201	WNVDYHDSNVV	NVYHDSNVV	1	0	0	2	1	NVDYHDSNVV	tr_C9ZP82	0.047	30230.44	47.00
365	HLA-A0201	IDEEESDGV	IDEEESDGV	0	0	0	0	0	IDEEESDGV	tr_C9ZP82	0.047	30231.75	47.00
207	HLA-A0201	DVFGHRGEVI	DVFGRGEVI	0	0	0	4	1	DVFGHRGEVI	tr_C9ZP82	0.046	30233.06	47.00
74	HLA-A0201	KEVMYIAVD	KEVMYIAVD	0	0	0	0	0	KEVMYIAVD	tr_C9ZP82	0.046	30233.06	47.00
698	HLA-A0201	LPVVKSTPPDTLV	VVKSTPPLV	2	0	0	7	2	VVKSTPPDTLV	tr_C9ZP82	0.046	30234.69	47.00
692	HLA-A0201	NIKRMPLPVVK	NIRMPLPVV	0	0	0	2	1	NIKRMPLPVV	tr_C9ZP82	0.046	30247.12	47.00
220	HLA-A0201	PTAFEFDSNCIS	TAFEFSNCI	1	0	0	5	1	TAFEFDSNCI	tr_C9ZP82	0.046	30252.37	47.00
443	HLA-A0201	FDKKTLVSSAGY	FTLVSSAGY	0	0	0	1	3	FDKKTLVSSAGY	tr_C9ZP82	0.046	30260.87	47.00
732	HLA-A0201	MVRSPQQAELEST	MQQAELEST	0	0	0	1	4	MVRSPQQAELEST	tr_C9ZP82	0.046	30264.46	47.00
215	HLA-A0201	VIVDDPTAFEFDSN	VIVPTAFEF	0	0	0	3	2	VIVDDPTAFEF	tr_C9ZP82	0.046	30271.02	47.00
391	HLA-A0201	NESPIESREAHV	SIESREAHV	2	0	0	1	1	SPIESREAHV	tr_C9ZP82	0.046	30282.48	47.00
688	HLA-A0201	LKEANIKRMPL	LKEANIMPL	0	0	0	6	2	LKEANIKRMPL	tr_C9ZP82	0.046	30287.07	47.00
644	HLA-A0201	EACEKYVEGVCF	ACYVEGVCF	1	0	0	2	2	ACEKYVEGVCF	tr_C9ZP82	0.046	30321.18	47.00
389	HLA-A0201	TDNESPIESREAHV	SIESREAHV	4	0	0	1	1	SPIESREAHV	tr_C9ZP82	0.046	30328.73	48.00
79	HLA-A0201	IAVDGVAPRAK	IAVDGVAPA	0	0	0	8	1	IAVDGVAPRA	tr_C9ZP82	0.046	30331.35	48.00
401	HLA-A0201	HVTTVPNTGFEYFD	HVTTVFEYF	0	0	0	5	4	HVTTVPNTGFEYF	tr_C9ZP82	0.046	30334.97	48.00
294	HLA-A0201	GKANHVIVGLDAD	KANHVIVGL	1	0	0	0	0	KANHVIVGL	tr_C9ZP82	0.046	30335.61	48.00
796	HLA-A0201	LDFHLVITSNK	LDFHLVITK	0	0	0	8	2	LDFHLVITSNK	tr_C9ZP82	0.046	30337.59	48.00
247	HLA-A0201	MLNEKMSGND	MLNEKMSGD	0	0	0	8	1	MLNEKMSGND	tr_C9ZP82	0.046	30338.23	48.00
705	HLA-A0201	PPDTLVQLL	PPDTLVQLL	0	0	0	0	0	PPDTLVQLL	tr_C9ZP82	0.046	30338.23	48.00
392	HLA-A0201	ESPIESREAHV	SIESREAHV	1	0	0	1	1	SPIESREAHV	tr_C9ZP82	0.046	30340.88	48.00
583	HLA-A0201	RRFLLFKSTN	RRFLLFKST	0	0	0	0	0	RRFLLFKST	tr_C9ZP82	0.046	30341.85	48.00
647	HLA-A0201	EKYVEGVCF	EKYVEGVCF	0	0	0	0	0	EKYVEGVCF	tr_C9ZP82	0.046	30346.45	48.00
751	HLA-A0201	VDYHDSNVVHL	VDYSNVVHL	0	0	0	3	2	VDYHDSNVVHL	tr_C9ZP82	0.046	30348.74	48.00
813	HLA-A0201	GDLRKLSDALD	GLRKLSDAL	0	0	0	1	1	GDLRKLSDAL	tr_C9ZP82	0.046	30353.67	48.00
453	HLA-A0201	GYYFFGQPNHIE	GYYFFGQPI	0	0	0	8	2	GYYFFGQPNHI	tr_C9ZP82	0.046	30358.59	48.00
775	HLA-A0201	QQVVDAAWP	QQVVDAAWP	0	0	0	0	0	QQVVDAAWP	tr_C9ZP82	0.046	30368.46	48.00
148	HLA-A0201	QTLLEDPLY	QTLLEDPLY	0	0	0	0	0	QTLLEDPLY	tr_C9ZP82	0.046	30370.10	48.00
401	HLA-A0201	HVTTVPNTGF	HVTTVPNTF	0	0	0	8	1	HVTTVPNTGF	tr_C9ZP82	0.046	30379.96	48.00
357	HLA-A0201	IISLNPSYIDE	IISLNPSYI	0	0	0	0	0	IISLNPSYI	tr_C9ZP82	0.046	30391.12	48.00
694	HLA-A0201	KRMPLPVVKSTPPD	RMLPVVKST	1	0	0	2	1	RMPLPVVKST	tr_C9ZP82	0.046	30395.40	48.00
751	HLA-A0201	VDYHDSNVVHLAT	YSNVVHLAT	2	0	0	1	2	YHDSNVVHLAT	tr_C9ZP82	0.046	30397.71	48.00
40	HLA-A0201	CHNDSDTAIALLP	CSDTAIALL	0	0	0	1	3	CHNDSDTAIALL	tr_C9ZP82	0.046	30401.32	48.00
891	HLA-A0201	IEGKKSNSRVTVV	ISNSRVTVV	0	0	0	1	4	IEGKKSNSRVTVV	tr_C9ZP82	0.046	30401.65	48.00
347	HLA-A0201	RKRNFIDDTCII	KRNFITCII	1	0	0	5	2	KRNFIDDTCII	tr_C9ZP82	0.046	30407.24	48.00
275	HLA-A0201	GEHKIIDFLRTQ	KIIDFLRTQ	3	0	0	0	0	KIIDFLRTQ	tr_C9ZP82	0.046	30412.18	48.00

368	HLA-A0201	EESDGVIGVPVCE	ESDGVIVPV	1	0	0	6	1	ESDGVIGVPV	tr_C9ZP82	0.046	30417.77	48.00
318	HLA-A0201	QVFILRDTTRNPYK	QVFILNPYK	0	0	0	5	5	QVFILRDTTRNPYK	tr_C9ZP82	0.046	30421.40	48.00
405	HLA-A0201	VPNTGFEYFDI	VTGFEYFDI	0	0	0	1	2	VPNTGFEYFDI	tr_C9ZP82	0.046	30422.38	48.00
0	HLA-A0201	MGVPKFASWLRN	MVPKFASWL	0	0	0	1	1	MGVPKFASWL	tr_C9ZP82	0.046	30428.64	48.00
835	HLA-A0201	VEIRQLYDVPLE	VEILYDVPL	0	0	0	3	2	VEIRQLYDVPL	tr_C9ZP82	0.046	30433.25	48.00
728	HLA-A0201	LGEVMVRSPQQA	LMVRSPQQA	0	0	0	1	3	LGEVMVRSPQQA	tr_C9ZP82	0.046	30441.17	48.00
79	HLA-A0201	IAVDGVAPRAKMN	IAVDGVAPM	0	0	0	8	3	IAVDGVAPRAKM	tr_C9ZP82	0.046	30447.74	48.00
212	HLA-A0201	RGEVIVDDPTAF	RGEVIVTAF	0	0	0	6	3	RGEVIVDDPTAF	tr_C9ZP82	0.046	30452.69	48.00
170	HLA-A0201	DALNDGDGVNS	ALNDGDVNS	1	0	0	6	1	ALNDGDGVNS	tr_C9ZP82	0.046	30453.67	48.00
663	HLA-A0201	TTTDSDWSWYY	TTDDWSWYY	1	0	0	3	1	TTDSDWSWYY	tr_C9ZP82	0.046	30454.01	48.00
690	HLA-A0201	EANIKRMPLPVVKS	AIKRMPLPV	1	0	0	1	1	ANIKRMPLPV	tr_C9ZP82	0.046	30466.21	48.00
752	HLA-A0201	DYHDSNVVHLAT	YSNVVHLAT	1	0	0	1	2	YHDSNVVHLAT	tr_C9ZP82	0.046	30472.14	48.00
11	HLA-A0201	NKYPAIVTK	NKYPAIVTK	0	0	0	0	0	NKYPAIVTK	tr_C9ZP82	0.046	30485.33	48.00
390	HLA-A0201	DNESPIESREAHV	SIESREAHV	3	0	0	1	1	SPIESREAHV	tr_C9ZP82	0.046	30485.64	48.00
693	HLA-A0201	IKRMPLPVVKS	IMPLPVVKS	0	0	0	1	2	IKRMPLPVVKS	tr_C9ZP82	0.046	30486.31	48.00
665	HLA-A0201	TDSDWSWYYPFY	SDWSWYYFY	2	0	0	7	1	SDWSWYYPFY	tr_C9ZP82	0.046	30490.26	48.00
702	HLA-A0201	KSTPPDTLVQ	KSTPPDTLV	0	0	0	0	0	KSTPPDTLV	tr_C9ZP82	0.046	30498.86	48.00
755	HLA-A0201	DSNVVHLAT	DSNVVHLAT	0	0	0	0	0	DSNVVHLAT	tr_C9ZP82	0.046	30507.10	48.00
832	HLA-A0201	REGVEIRQL	REGVEIRQL	0	0	0	0	0	REGVEIRQL	tr_C9ZP82	0.046	30514.03	48.00
716	HLA-A0201	LPPTSHALL	LPPTSHALL	0	0	0	0	0	LPPTSHALL	tr_C9ZP82	0.046	30516.35	48.00
558	HLA-A0201	GPHEINLPQLQRL	GPHEINLPL	0	0	0	8	4	GPHEINLPQLQRL	tr_C9ZP82	0.046	30518.32	48.00
285	HLA-A0201	TQSSLAQFGG	TQSSLAFGG	0	0	0	6	1	TQSSLAQFGG	tr_C9ZP82	0.046	30525.60	48.00
211	HLA-A0201	HRGEVIVDDPTA	HRGEVIVTA	0	0	0	7	3	HRGEVIVDDPTA	tr_C9ZP82	0.046	30528.24	48.00
811	HLA-A0201	PKGDLRKLSDALD	KLRKLSDAL	1	0	0	1	2	KGDLRKLSDAL	tr_C9ZP82	0.046	30528.89	48.00
766	HLA-A0201	LPFADMDVLQ	LPFADMVLQ	0	0	0	6	1	LPFADMDVLQ	tr_C9ZP82	0.046	30532.87	48.00
254	HLA-A0201	GNDPTWTRLCV	GNDPTWLCV	0	0	0	6	2	GNDPTWTRLCV	tr_C9ZP82	0.046	30534.83	48.00
242	HLA-A0201	DAVLNMLNEK	AVLNMLNEK	1	0	0	0	0	AVLNMLNEK	tr_C9ZP82	0.046	30543.76	48.00
619	HLA-A0201	ALHSTIDDRWR	ALHSTIDWR	0	0	0	7	2	ALHSTIDDRWR	tr_C9ZP82	0.046	30549.06	48.00
273	HLA-A0201	GEGEHKIIDFLRTQ	KIIDFLRTQ	5	0	0	0	0	KIIDFLRTQ	tr_C9ZP82	0.045	30568.88	48.00
438	HLA-A0201	ATESAFDKKTLVS	ATFDKKTLV	0	0	0	2	3	ATESAFDKKTLV	tr_C9ZP82	0.045	30579.47	48.00
662	HLA-A0201	TTTTDSDWSWYY	TTSDWSWYY	0	0	0	2	3	TTTTDSDWSWYY	tr_C9ZP82	0.045	30598.66	48.00
661	HLA-A0201	TTTTTDSDWSWYY	TTSDWSWYY	0	0	0	2	4	TTTTTDSDWSWYY	tr_C9ZP82	0.045	30599.34	48.00
656	HLA-A0201	VWRYYTTTTT	VWYYTTTTT	0	0	0	2	1	VWRYYTTTTT	tr_C9ZP82	0.045	30602.97	48.00
806	HLA-A0201	KTSFVPKGDLRKLS	FVPKGDLRL	3	0	0	8	1	FVPKGDLRKL	tr_C9ZP82	0.045	30607.29	48.00
628	HLA-A0201	WRDTYLKSTGLS	WTYLKSTGL	0	0	0	1	2	WRDTYLKSTGL	tr_C9ZP82	0.045	30610.92	48.00
562	HLA-A0201	INLPQLQRLFE	ILPQLQRLF	0	0	0	1	1	INLPQLQRLF	tr_C9ZP82	0.045	30612.92	48.00
836	HLA-A0201	EIRQLYDVPLE	EIQLYDVPL	0	0	0	2	1	EIRQLYDVPL	tr_C9ZP82	0.045	30622.85	48.00
583	HLA-A0201	RRFLLFKSTNKE	FLLFKSTNK	2	0	0	0	0	FLLFKSTNK	tr_C9ZP82	0.045	30624.51	48.00
219	HLA-A0201	DPTAFEFDSNCIS	TAFEFSNCI	2	0	0	5	1	TAFEFDSNCI	tr_C9ZP82	0.045	30625.49	48.00
319	HLA-A0201	VFILRDTTRNPY	ILRDTTNPY	2	0	0	6	1	ILRDTTRNPY	tr_C9ZP82	0.045	30636.10	48.00
274	HLA-A0201	EGEHKIIDFLRTQ	KIIDFLRTQ	4	0	0	0	0	KIIDFLRTQ	tr_C9ZP82	0.045	30649.70	48.00
321	HLA-A0201	ILRDTTRNPYK	ILRDTTNPY	0	0	0	6	1	ILRDTTRNPY	tr_C9ZP82	0.045	30650.04	48.00
346	HLA-A0201	RRKRNFIDDTCII	KRNFITCII	2	0	0	5	2	KRNFIDDTCII	tr_C9ZP82	0.045	30653.02	48.00
232	HLA-A0201	PGTDFMAKV	PGTDFMAKV	0	0	0	0	0	PGTDFMAKV	tr_C9ZP82	0.045	30663.30	48.00
664	HLA-A0201	TTDSDWSWY	TTDSDWSWY	0	0	0	0	0	TTDSDWSWY	tr_C9ZP82	0.045	30673.25	48.00
293	HLA-A0201	GGKANHVIVGLDAD	KAVIVGLDA	2	0	0	2	2	KANHVIVGLDA	tr_C9ZP82	0.045	30675.58	48.00
39	HLA-A0201	CCHNDSDTAI	CCHDSDTAI	0	0	0	3	1	CCHNDSDTAI	tr_C9ZP82	0.045	30676.91	48.00
404	HLA-A0201	TVPNTGFEYFDIS	TVFEYFDIS	0	0	0	2	4	TVPNTGFEYFDIS	tr_C9ZP82	0.045	30681.56	48.00
584	HLA-A0201	RFLLFKSTNKE	FLLFKSTNK	1	0	0	0	0	FLLFKSTNK	tr_C9ZP82	0.045	30692.17	48.00
212	HLA-A0201	RGEVIVDDPTA	RVIVDDPTA	0	0	0	1	2	RGEVIVDDPTA	tr_C9ZP82	0.045	30697.15	48.00
754	HLA-A0201	HDSNVVHLAT	HSNVVHLAT	0	0	0	1	1	HDSNVVHLAT	tr_C9ZP82	0.045	30698.82	48.00
22	HLA-A0201	PSSVHGLYIDLNG	SVHGLYIDL	2	0	0	0	0	SVHGLYIDL	tr_C9ZP82	0.045	30715.43	49.00
426	HLA-A0201	VSALCDIKK	VSALCDIKK	0	0	0	0	0	VSALCDIKK	tr_C9ZP82	0.045	30734.38	49.00
797	HLA-A0201	DFHLVITSN	DFHLVITSN	0	0	0	0	0	DFHLVITSN	tr_C9ZP82	0.045	30737.03	49.00

345	HLA-A0201	SRRKRNFIDDTCII	KRNFITCII	3	0	0	5	2	KRNFIDDTCII	tr_C9ZP82	0.045	30758.67	49.00
748	HLA-A0201	QWNVDYHDSNV	WVDYHDSNV	1	0	0	1	1	WNVDYHDSNV	tr_C9ZP82	0.045	30764.99	49.00
618	HLA-A0201	VALHSTIDDRW	ALHSTIDRW	1	0	0	6	1	ALHSTIDDRW	tr_C9ZP82	0.045	30775.65	49.00
880	HLA-A0201	SGQLKGNGEGVI	SLKGNGEGV	0	0	0	1	2	SGQLKGNGEGV	tr_C9ZP82	0.045	30775.97	49.00
273	HLA-A0201	GEGEHKIIDFL	GEGEIIDFL	0	0	0	4	2	GEGEHKIIDFL	tr_C9ZP82	0.045	30798.62	49.00
606	HLA-A0201	SNNDNVTVADV	NNDNVTVAV	1	0	0	8	1	NNDNVTVADV	tr_C9ZP82	0.045	30799.30	49.00
746	HLA-A0201	PLQWNVDYHDSN	LQWNVDYHS	1	0	0	8	1	LQWNVDYHDS	tr_C9ZP82	0.045	30810.62	49.00
210	HLA-A0201	GHRGEVIVDDPTAF	HRGEVIVAF	1	0	0	7	4	HRGEVIVDDPTAF	tr_C9ZP82	0.045	30813.62	49.00
575	HLA-A0201	AKIEAVKFRRF	KIEAVFRRF	1	0	0	5	1	KIEAVKFRRF	tr_C9ZP82	0.045	30831.97	49.00
206	HLA-A0201	DDVFGHRGEVIV	DVFGGEVIV	1	0	0	4	2	DVFGHRGEVIV	tr_C9ZP82	0.045	30832.31	49.00
218	HLA-A0201	DDPTAFEFDSNCIS	TAFEFSNCI	3	0	0	5	1	TAFEFDSNCI	tr_C9ZP82	0.045	30832.62	49.00
572	HLA-A0201	EAYAKIEAVKFRRF	KIEAVFRRF	4	0	0	5	1	KIEAVKFRRF	tr_C9ZP82	0.045	30834.63	49.00
673	HLA-A0201	YPFYHAPPVID	YPFYHAPPV	0	0	0	0	0	YPFYHAPPV	tr_C9ZP82	0.045	30849.31	49.00
461	HLA-A0201	NHIEDGTLPS	HIEDGTLPS	1	0	0	0	0	HIEDGTLPS	tr_C9ZP82	0.045	30856.65	49.00
429	HLA-A0201	LCDIKKFTPAT	LCDIFTPAT	0	0	0	4	2	LCDIKKFTPAT	tr_C9ZP82	0.045	30858.01	49.00
367	HLA-A0201	EEESDGVIGVPVCE	ESDGVIVPV	2	0	0	6	1	ESDGVIGVPV	tr_C9ZP82	0.045	30863.34	49.00
643	HLA-A0201	QEACEKYVEGVCF	ACYVEGVCF	2	0	0	2	2	ACEKYVEGVCF	tr_C9ZP82	0.045	30865.01	49.00
458	HLA-A0201	GQPNHIEDGT	GQPNHIEGT	0	0	0	7	1	GQPNHIEDGT	tr_C9ZP82	0.045	30886.40	49.00
690	HLA-A0201	EANIKRMPLPVVK	AIKRMPLPV	1	0	0	1	1	ANIKRMPLPV	tr_C9ZP82	0.044	30899.09	49.00
550	HLA-A0201	LPYGFLTKGP	LPYGFLTGP	0	0	0	7	1	LPYGFLTKGP	tr_C9ZP82	0.044	30903.77	49.00
118	HLA-A0201	KSGMAIVEE	KSGMAIVEE	0	0	0	0	0	KSGMAIVEE	tr_C9ZP82	0.044	30908.12	49.00
107	HLA-A0201	TGEVASTYM	TGEVASTYM	0	0	0	0	0	TGEVASTYM	tr_C9ZP82	0.044	30909.79	49.00
247	HLA-A0201	MLNEKMSGNDP	MLNEKMSGP	0	0	0	8	2	MLNEKMSGNDP	tr_C9ZP82	0.044	30930.19	49.00
615	HLA-A0201	DVAVALHSTIDDRW	ALHSTIDRW	4	0	0	6	1	ALHSTIDDRW	tr_C9ZP82	0.044	30931.21	49.00
222	HLA-A0201	AFEFDSNCISPG	FEFDCISPG	1	0	0	4	2	FEFDSNCISPG	tr_C9ZP82	0.044	30934.88	49.00
748	HLA-A0201	QWNVDYHDSNVV	NVYHDSNVV	2	0	0	2	1	NVDYHDSNVV	tr_C9ZP82	0.044	30935.90	49.00
878	HLA-A0201	RHSGQLKGNGEGVI	RLKGNGEGV	0	0	0	1	4	RHSGQLKGNGEGV	tr_C9ZP82	0.044	30946.27	49.00
270	HLA-A0201	NTPGEGEHKI	NTGEGEHKI	0	0	0	2	1	NTPGEGEHKI	tr_C9ZP82	0.044	30949.95	49.00
196	HLA-A0201	NRYVESNQVV	RYVESNQVV	1	0	0	0	0	RYVESNQVV	tr_C9ZP82	0.044	30951.96	49.00
276	HLA-A0201	EHKIIDFLRTQ	KIIDFLRTQ	2	0	0	0	0	KIIDFLRTQ	tr_C9ZP82	0.044	30952.96	49.00
574	HLA-A0201	YAKIEAVKFRRF	KIEAVFRRF	2	0	0	5	1	KIEAVKFRRF	tr_C9ZP82	0.044	30953.64	49.00
617	HLA-A0201	AVALHSTIDDRW	ALHSTIDRW	2	0	0	6	1	ALHSTIDDRW	tr_C9ZP82	0.044	30955.63	49.00
348	HLA-A0201	KRNFIDDTCI	KNFIDDTCI	0	0	0	1	1	KRNFIDDTCI	tr_C9ZP82	0.044	30968.38	49.00
897	HLA-A0201	NSRVTVVNK	NSRVTVVNK	0	0	0	0	0	NSRVTVVNK	tr_C9ZP82	0.044	30971.73	49.00
505	HLA-A0201	ANSKIIDDII	ASKIIDDII	0	0	0	1	1	ANSKIIDDII	tr_C9ZP82	0.044	30982.78	49.00
665	HLA-A0201	TDSDWSWYYP	TSDWSWYYP	0	0	0	1	1	TDSDWSWYYP	tr_C9ZP82	0.044	30986.13	49.00
425	HLA-A0201	EVSALCDIKKF	VSALCDIKF	1	0	0	7	1	VSALCDIKKF	tr_C9ZP82	0.044	30989.83	49.00
660	HLA-A0201	YTTTTTDSDWSW	YTTTTTWSW	0	0	0	6	3	YTTTTTDSDWSW	tr_C9ZP82	0.044	31003.59	49.00
344	HLA-A0201	RSRRKRNFIDDTCI	RSFIDDTCI	0	0	0	2	5	RSRRKRNFIDDTCI	tr_C9ZP82	0.044	31015.98	49.00
740	HLA-A0201	ELESTFPLQWN	ELSTFPLQW	0	0	0	2	1	ELESTFPLQW	tr_C9ZP82	0.044	31020.69	49.00
208	HLA-A0201	VFGHRGEVI	VFGHRGEVI	0	0	0	0	0	VFGHRGEVI	tr_C9ZP82	0.044	31025.73	49.00
602	HLA-A0201	FRKGSNNDNVTVAD	FSNNDNVTV	0	0	0	1	3	FRKGSNNDNVTV	tr_C9ZP82	0.044	31030.43	49.00
539	HLA-A0201	DNLLELYVS	DNLLELYVS	0	0	0	0	0	DNLLELYVS	tr_C9ZP82	0.044	31054.27	49.00
124	HLA-A0201	VEEVVREFT	VEEVVREFT	0	0	0	0	0	VEEVVREFT	tr_C9ZP82	0.044	31064.35	49.00
43	HLA-A0201	DSDTAIALLP	DSDTAIALL	0	0	0	0	0	DSDTAIALL	tr_C9ZP82	0.044	31065.37	49.00
184	HLA-A0201	RSTDDDYES	RSTDDDYES	0	0	0	0	0	RSTDDDYES	tr_C9ZP82	0.044	31070.40	49.00
403	HLA-A0201	TTVPNTGFEYFDIS	TVFEYFDIS	1	0	0	2	4	TVPNTGFEYFDIS	tr_C9ZP82	0.044	31097.98	50.00
372	HLA-A0201	GVIGVPVCESSENS	GVIGVPVCS	0	0	0	8	5	GVIGVPVCESSENS	tr_C9ZP82	0.044	31103.70	50.00
668	HLA-A0201	DWSWYYPFY	DWSWYYPFY	0	0	0	0	0	DWSWYYPFY	tr_C9ZP82	0.044	31104.04	50.00
222	HLA-A0201	AFEFDSNCISPGT	FEFCISPGT	1	0	0	3	3	FEFDSNCISPGT	tr_C9ZP82	0.044	31107.07	50.00
572	HLA-A0201	EAYAKIEAVKF	EAYAKIEAV	0	0	0	0	0	EAYAKIEAV	tr_C9ZP82	0.044	31121.21	50.00
430	HLA-A0201	CDIKKFTPAT	CIKKFTPAT	0	0	0	1	1	CDIKKFTPAT	tr_C9ZP82	0.044	31124.25	50.00
210	HLA-A0201	GHRGEVIVDDPTA	HRGEVIVTA	1	0	0	7	3	HRGEVIVDDPTA	tr_C9ZP82	0.044	31137.72	50.00
463	HLA-A0201	IEDGTLPSS	IEDGTLPSS	0	0	0	0	0	IEDGTLPSS	tr_C9ZP82	0.044	31147.14	50.00

732	HLA-A0201	MVRSPQQAELE	MVSPQQAEL	0	0	0	2	1	MVRSPQQAEL	tr_C9ZP82	0.044	31156.92	50.00
97	HLA-A0201	RYMGRAAPAQTGE	YMGAAPAQT	1	0	0	3	1	YMGRAAPAQT	tr_C9ZP82	0.044	31159.61	50.00
811	HLA-A0201	PKGDLRKLSDALDT	KLRKLSDAL	1	0	0	1	2	KGDLRKLSDAL	tr_C9ZP82	0.044	31160.30	50.00
573	HLA-A0201	AYAKIEAVKFRRF	KIEAVFRRF	3	0	0	5	1	KIEAVKFRRF	tr_C9ZP82	0.044	31174.79	50.00
616	HLA-A0201	VAVALHSTIDDRW	ALHSTIDRW	3	0	0	6	1	ALHSTIDDRW	tr_C9ZP82	0.044	31175.47	50.00
414	HLA-A0201	DISVVGGSLISE	DISVVLISE	0	0	0	5	3	DISVVGGSLISE	tr_C9ZP82	0.044	31181.54	50.00
127	HLA-A0201	VVREFTSTEMAQ	VVFTSTEMA	0	0	0	2	2	VVREFTSTEMA	tr_C9ZP82	0.044	31187.62	50.00
357	HLA-A0201	IISLNPSYIDEEE	IISLNPSYI	0	0	0	0	0	IISLNPSYI	tr_C9ZP82	0.044	31197.05	50.00
320	HLA-A0201	FILRDTTRNPYK	ILRDTTNPY	1	0	0	6	1	ILRDTTRNPY	tr_C9ZP82	0.044	31207.20	50.00
435	HLA-A0201	FTPATESAFD	FTPATESAF	0	0	0	0	0	FTPATESAF	tr_C9ZP82	0.044	31211.24	50.00
31	HLA-A0201	DLNGIIHPCCHND	DLNGIIHPC	0	0	0	0	0	DLNGIIHPC	tr_C9ZP82	0.044	31214.28	50.00
288	HLA-A0201	SLAQFGGKANH	SLAQFGANH	0	0	0	6	2	SLAQFGGKANH	tr_C9ZP82	0.044	31226.78	50.00
520	HLA-A0201	LGNDFLPHAPSA	LGNDFLPSA	0	0	0	6	3	LGNDFLPHAPSA	tr_C9ZP82	0.043	31233.53	50.00
501	HLA-A0201	CSSPANSKIIDDII	CSSIIDDII	0	0	0	3	5	CSSPANSKIIDDII	tr_C9ZP82	0.043	31234.90	50.00
801	HLA-A0201	VITSNKTSFVPKG	VISNKTSFV	0	0	0	2	1	VITSNKTSFV	tr_C9ZP82	0.043	31236.25	50.00
51	HLA-A0201	LPEEEKLECI	LPEEELECI	0	0	0	5	1	LPEEEKLECI	tr_C9ZP82	0.043	31237.94	50.00
881	HLA-A0201	GQLKGNGEGVI	GQLKGNEGV	0	0	0	6	1	GQLKGNGEGV	tr_C9ZP82	0.043	31241.31	50.00
808	HLA-A0201	SFVPKGDLRKLS	FVPKGDLRL	1	0	0	8	1	FVPKGDLRKL	tr_C9ZP82	0.043	31250.09	50.00
627	HLA-A0201	RWRDTYLKSTGLS	RTYLKSTGL	0	0	0	1	3	RWRDTYLKSTGL	tr_C9ZP82	0.043	31256.18	50.00
411	HLA-A0201	EYFDISVVG	EYFDISVVG	0	0	0	0	0	EYFDISVVG	tr_C9ZP82	0.043	31275.13	50.00
453	HLA-A0201	GYYFFGQPN	GYYFFGQPN	0	0	0	0	0	GYYFFGQPN	tr_C9ZP82	0.043	31293.41	50.00
47	HLA-A0201	AIALLPEEEK	AIALLPEEK	0	0	0	6	1	AIALLPEEEK	tr_C9ZP82	0.043	31310.69	50.00
411	HLA-A0201	EYFDISVVGGS	YFDIVVGGS	1	0	0	4	1	YFDISVVGGS	tr_C9ZP82	0.043	31325.94	50.00
213	HLA-A0201	GEVIVDDPTAF	GEVIVPTAF	0	0	0	5	2	GEVIVDDPTAF	tr_C9ZP82	0.043	31335.76	50.00
205	HLA-A0201	VDDVFGHRGEVIVD	VDDVFGVIV	0	0	0	6	4	VDDVFGHRGEVIV	tr_C9ZP82	0.043	31336.45	50.00
807	HLA-A0201	TSFVPKGDLRKLS	FVPKGDLRL	2	0	0	8	1	FVPKGDLRKL	tr_C9ZP82	0.043	31343.22	50.00
169	HLA-A0201	GDALNDGDGVNS	ALNDGDVNS	2	0	0	6	1	ALNDGDGVNS	tr_C9ZP82	0.043	31343.56	50.00
813	HLA-A0201	GDLRKLSDALDT	GLRKLSDAL	0	0	0	1	1	GDLRKLSDAL	tr_C9ZP82	0.043	31354.76	50.00
800	HLA-A0201	LVITSNKTSF	LVITSNTSF	0	0	0	6	1	LVITSNKTSF	tr_C9ZP82	0.043	31364.59	50.00
424	HLA-A0201	SEVSALCDIKKF	VSALCDIKF	2	0	0	7	1	VSALCDIKKF	tr_C9ZP82	0.043	31368.68	50.00
525	HLA-A0201	LPHAPSAFSGESAL	LAFSGESAL	0	0	0	1	5	LPHAPSAFSGESAL	tr_C9ZP82	0.043	31378.51	50.00
573	HLA-A0201	AYAKIEAVKF	YAKIEAVKF	1	0	0	0	0	YAKIEAVKF	tr_C9ZP82	0.043	31392.78	50.00
280	HLA-A0201	IDFLRTQSS	IDFLRTQSS	0	0	0	0	0	IDFLRTQSS	tr_C9ZP82	0.043	31394.80	50.00
21	HLA-A0201	CPSSVHGLYID	CPSSVGLYI	0	0	0	5	1	CPSSVHGLYI	tr_C9ZP82	0.043	31422.01	55.00
561	HLA-A0201	EINLPQLQRLFE	ILPQLQRLF	1	0	0	1	1	INLPQLQRLF	tr_C9ZP82	0.043	31425.07	55.00
178	HLA-A0201	VNSFCVRST	VNSFCVRST	0	0	0	0	0	VNSFCVRST	tr_C9ZP82	0.043	31425.07	55.00
168	HLA-A0201	AGDALNDGDGVNS	ALNDGDVNS	3	0	0	6	1	ALNDGDGVNS	tr_C9ZP82	0.043	31434.92	55.00
0	HLA-A0201	MGVPKFASWLRNKY	MVPKFASWL	0	0	0	1	1	MGVPKFASWL	tr_C9ZP82	0.043	31463.17	55.00
212	HLA-A0201	RGEVIVDDPT	RGEVIVDPT	0	0	0	6	1	RGEVIVDDPT	tr_C9ZP82	0.043	31472.37	55.00
96	HLA-A0201	RRYMGRAAPAQTGE	YMGAAPAQT	2	0	0	3	1	YMGRAAPAQT	tr_C9ZP82	0.043	31482.92	55.00
146	HLA-A0201	VRQTLLEDPLY	VQTLLEDPL	0	0	0	1	1	VRQTLLEDPL	tr_C9ZP82	0.043	31488.02	55.00
109	HLA-A0201	EVASTYMPNKSGM	EVASTYMPM	0	0	0	8	4	EVASTYMPNKSGM	tr_C9ZP82	0.043	31489.38	55.00
127	HLA-A0201	VVREFTSTEMAQAN	VVREFMAQA	0	0	0	5	4	VVREFTSTEMAQA	tr_C9ZP82	0.043	31499.62	55.00
418	HLA-A0201	VGGSLISEVSA	VGGSLIVSA	0	0	0	6	2	VGGSLISEVSA	tr_C9ZP82	0.043	31539.86	55.00
403	HLA-A0201	TTVPNTGFEY	TTVPTGFEY	0	0	0	4	1	TTVPNTGFEY	tr_C9ZP82	0.043	31552.82	55.00
172	HLA-A0201	LNDGDGVNSFCVRS	LVNSFCVRS	0	0	0	1	5	LNDGDGVNSFCVRS	tr_C9ZP82	0.043	31556.24	55.00
202	HLA-A0201	NQVVDDVFGH	NQVVDVFGH	0	0	0	4	1	NQVVDDVFGH	tr_C9ZP82	0.043	31560.33	55.00
367	HLA-A0201	EEESDGVIGV	EESDGVIGV	0	0	0	1	1	EEESDGVIGV	tr_C9ZP82	0.043	31565.81	55.00
15	HLA-A0201	AIVTKNCPSS	AIVTNCPSS	0	0	0	4	1	AIVTKNCPSS	tr_C9ZP82	0.043	31567.50	55.00
557	HLA-A0201	KGPHEINLPQL	GPHEINLPL	1	0	0	8	1	GPHEINLPQL	tr_C9ZP82	0.042	31571.96	55.00
777	HLA-A0201	VVDAAWPQLTE	VVDAAWPQL	0	0	0	0	0	VVDAAWPQL	tr_C9ZP82	0.042	31579.80	55.00
177	HLA-A0201	GVNSFCVRSTDDDY	GVNSFCVRY	0	0	0	8	5	GVNSFCVRSTDDDY	tr_C9ZP82	0.042	31586.64	55.00
492	HLA-A0201	ERSWKNFRPC	RSWKNFRPC	1	0	0	0	0	RSWKNFRPC	tr_C9ZP82	0.042	31597.23	55.00
99	HLA-A0201	MGRAAPAQTGEV	RAAPAQTEV	2	0	0	7	1	RAAPAQTGEV	tr_C9ZP82	0.042	31598.95	55.00

414	HLA-A0201	DISVVGGSLIS	DISVVGSLI	0	0	0	5	1	DISVVGGSLI	tr_C9ZP82	0.042	31602.37	55.00
629	HLA-A0201	RDTYLKSTGLSN	YLKSTGLSN	3	0	0	0	0	YLKSTGLSN	tr_C9ZP82	0.042	31616.72	55.00
557	HLA-A0201	KGPHEINLPQLQRL	GPHEINLPL	1	0	0	8	4	GPHEINLPQLQRL	tr_C9ZP82	0.042	31624.94	55.00
820	HLA-A0201	DALDTILRR	DALDTILRR	0	0	0	0	0	DALDTILRR	tr_C9ZP82	0.042	31625.96	55.00
556	HLA-A0201	TKGPHEINL	TKGPHEINL	0	0	0	0	0	TKGPHEINL	tr_C9ZP82	0.042	31627.00	55.00
879	HLA-A0201	HSGQLKGNGEGVI	SLKGNGEGV	1	0	0	1	2	SGQLKGNGEGV	tr_C9ZP82	0.042	31628.37	55.00
110	HLA-A0201	VASTYMPNKSGM	VASTYMPGM	0	0	0	7	3	VASTYMPNKSGM	tr_C9ZP82	0.042	31628.37	55.00
167	HLA-A0201	EAGDALNDGDGVNS	ALNDGDVNS	4	0	0	6	1	ALNDGDGVNS	tr_C9ZP82	0.042	31631.10	55.00
0	HLA-A0201	MGVPKFASWLRNK	MVPKFASWL	0	0	0	1	1	MGVPKFASWL	tr_C9ZP82	0.042	31644.79	55.00
532	HLA-A0201	FSGESALDN	FSGESALDN	0	0	0	0	0	FSGESALDN	tr_C9ZP82	0.042	31649.24	55.00
693	HLA-A0201	IKRMPLPVVK	IKRMPLPVV	0	0	0	0	0	IKRMPLPVV	tr_C9ZP82	0.042	31649.59	55.00
493	HLA-A0201	RSWKNFRPCS	RSWNFRPCS	0	0	0	3	1	RSWKNFRPCS	tr_C9ZP82	0.042	31654.38	55.00
222	HLA-A0201	AFEFDSNCISP	FEFDSCISP	1	0	0	5	1	FEFDSNCISP	tr_C9ZP82	0.042	31654.73	55.00
89	HLA-A0201	KMNQQRARRYMG	KMNQQRAYM	0	0	0	7	2	KMNQQRARRYM	tr_C9ZP82	0.042	31670.15	55.00
262	HLA-A0201	LCVIFSGSN	LCVIFSGSN	0	0	0	0	0	LCVIFSGSN	tr_C9ZP82	0.042	31681.79	55.00
153	HLA-A0201	DPLYSGALYNEG	PLYSGALYG	1	0	0	8	2	PLYSGALYNEG	tr_C9ZP82	0.042	31690.03	55.00
658	HLA-A0201	RYYTTTTTDSDWSW	YTTTTTWSW	2	0	0	6	3	YTTTTTDSDWSW	tr_C9ZP82	0.042	31691.74	55.00
824	HLA-A0201	TILRRVPSR	TILRRVPSR	0	0	0	0	0	TILRRVPSR	tr_C9ZP82	0.042	31693.11	55.00
443	HLA-A0201	FDKKTLVSS	FDKKTLVSS	0	0	0	0	0	FDKKTLVSS	tr_C9ZP82	0.042	31694.13	55.00
402	HLA-A0201	VTTVPNTGFEYFD	VTTVGFEYF	0	0	0	4	3	VTTVPNTGFEYF	tr_C9ZP82	0.042	31711.64	55.00
271	HLA-A0201	TPGEGEHKIIDFL	TPGEIIDFL	0	0	0	4	4	TPGEGEHKIIDFL	tr_C9ZP82	0.042	31727.08	55.00
882	HLA-A0201	QLKGNGEGVIEGKK	QLEGVIEGK	0	0	0	2	4	QLKGNGEGVIEGK	tr_C9ZP82	0.042	31728.10	55.00
68	HLA-A0201	VQTVQPKEVM	VQTVQPEVM	0	0	0	6	1	VQTVQPKEVM	tr_C9ZP82	0.042	31732.91	55.00
41	HLA-A0201	HNDSDTAIALLPE	STAIALLPE	3	0	0	1	1	SDTAIALLPE	tr_C9ZP82	0.042	31736.00	55.00
70	HLA-A0201	TVQPKEVMY	TVQPKEVMY	0	0	0	0	0	TVQPKEVMY	tr_C9ZP82	0.042	31736.35	55.00
34	HLA-A0201	GIIHPCCHN	GIIHPCCHN	0	0	0	0	0	GIIHPCCHN	tr_C9ZP82	0.042	31750.09	55.00
433	HLA-A0201	KKFTPATESA	KKFPATESA	0	0	0	3	1	KKFTPATESA	tr_C9ZP82	0.042	31750.76	55.00
442	HLA-A0201	AFDKKTLVSSAGY	FTLVSSAGY	1	0	0	1	3	FDKKTLVSSAGY	tr_C9ZP82	0.042	31752.48	55.00
423	HLA-A0201	ISEVSALCDIK	ISVSALCDI	0	0	0	2	1	ISEVSALCDI	tr_C9ZP82	0.042	31753.86	55.00
209	HLA-A0201	FGHRGEVIVDDPT	FGVIVDDPT	0	0	0	2	4	FGHRGEVIVDDPT	tr_C9ZP82	0.042	31758.32	55.00
213	HLA-A0201	GEVIVDDPT	GEVIVDDPT	0	0	0	0	0	GEVIVDDPT	tr_C9ZP82	0.042	31758.67	55.00
856	HLA-A0201	TVEVPRLSYGA	TVEVLSYGA	0	0	0	4	2	TVEVPRLSYGA	tr_C9ZP82	0.042	31763.83	55.00
897	HLA-A0201	NSRVTVVNKGPC	NSRVTVVPC	0	0	0	7	3	NSRVTVVNKGPC	tr_C9ZP82	0.042	31770.37	55.00
783	HLA-A0201	PQLTEEERWRT	QLTEEEWRT	1	0	0	6	1	QLTEEERWRT	tr_C9ZP82	0.042	31774.13	55.00
223	HLA-A0201	FEFDSNCISPGTDF	FEFDSNCIF	0	0	0	8	5	FEFDSNCISPGTDF	tr_C9ZP82	0.042	31782.39	55.00
20	HLA-A0201	NCPSSVHGLYID	NCPSSVLYI	0	0	0	6	2	NCPSSVHGLYI	tr_C9ZP82	0.042	31790.31	55.00
666	HLA-A0201	DSDWSWYYP	DSDWSWYYP	0	0	0	0	0	DSDWSWYYP	tr_C9ZP82	0.042	31792.03	55.00
424	HLA-A0201	SEVSALCDIK	SVSALCDIK	0	0	0	1	1	SEVSALCDIK	tr_C9ZP82	0.042	31793.05	55.00
614	HLA-A0201	ADVAVALHSTIDD	ADVAVALTI	0	0	0	7	2	ADVAVALHSTI	tr_C9ZP82	0.042	31793.40	55.00
782	HLA-A0201	WPQLTEEERWRT	QLTEEEWRT	2	0	0	6	1	QLTEEERWRT	tr_C9ZP82	0.042	31804.07	55.00
766	HLA-A0201	LPFADMDVLQQ	FADMDVLQQ	2	0	0	0	0	FADMDVLQQ	tr_C9ZP82	0.042	31813.01	55.00
21	HLA-A0201	CPSSVHGLY	CPSSVHGLY	0	0	0	0	0	CPSSVHGLY	tr_C9ZP82	0.042	31813.69	55.00
205	HLA-A0201	VDDVFGHRGEVI	VVFGHRGEV	0	0	0	1	2	VDDVFGHRGEV	tr_C9ZP82	0.042	31828.85	55.00
560	HLA-A0201	HEINLPQLQRLFE	ILPQLQRLF	2	0	0	1	1	INLPQLQRLF	tr_C9ZP82	0.042	31840.21	55.00
445	HLA-A0201	KKTLVSSAGY	KTLVSSAGY	0	0	0	1	1	KKTLVSSAGY	tr_C9ZP82	0.042	31844.01	55.00
618	HLA-A0201	VALHSTIDDRWR	ALHSTIDWR	1	0	0	7	2	ALHSTIDDRWR	tr_C9ZP82	0.042	31857.09	55.00
659	HLA-A0201	YYTTTTTDSDWSW	YTTTTTWSW	1	0	0	6	3	YTTTTTDSDWSW	tr_C9ZP82	0.042	31862.27	55.00
419	HLA-A0201	GGSLISEVS	GGSLISEVS	0	0	0	0	0	GGSLISEVS	tr_C9ZP82	0.042	31883.99	55.00
19	HLA-A0201	KNCPSSVHGLYID	CPSSVGLYI	2	0	0	5	1	CPSSVHGLYI	tr_C9ZP82	0.042	31885.37	55.00
127	HLA-A0201	VVREFTSTE	VVREFTSTE	0	0	0	0	0	VVREFTSTE	tr_C9ZP82	0.042	31892.96	55.00
453	HLA-A0201	GYYFFGQPNHIEDG	GYYFFGQPI	0	0	0	8	2	GYYFFGQPNHI	tr_C9ZP82	0.042	31900.55	55.00
143	HLA-A0201	LDEVRQTLL	LDEVRQTLL	0	0	0	0	0	LDEVRQTLL	tr_C9ZP82	0.042	31908.14	55.00
781	HLA-A0201	AWPQLTEEERWRT	QLTEEEWRT	3	0	0	6	1	QLTEEERWRT	tr_C9ZP82	0.041	31915.41	55.00
298	HLA-A0201	HVIVGLDAD	HVIVGLDAD	0	0	0	0	0	HVIVGLDAD	tr_C9ZP82	0.041	31931.63	55.00

736	HLA-A0201	PQQAELESTF	QQAELESTF	1	0	0	0	0	QQAELESTF	tr_C9ZP82	0.041	31931.98	55.00
614	HLA-A0201	ADVAVALHSTIDDR	ADVAVALTI	0	0	0	7	2	ADVAVALHSTI	tr_C9ZP82	0.041	31935.09	55.00
13	HLA-A0201	YPAIVTKNCPS	YPAIVTCPS	0	0	0	6	2	YPAIVTKNCPS	tr_C9ZP82	0.041	31948.56	55.00
453	HLA-A0201	GYYFFGQPNHIED	GYYFFGQPI	0	0	0	8	2	GYYFFGQPNHI	tr_C9ZP82	0.041	31957.90	55.00
353	HLA-A0201	DDTCIISLNPS	DTCIISLPS	1	0	0	7	1	DTCIISLNPS	tr_C9ZP82	0.041	31963.79	55.00
42	HLA-A0201	NDSDTAIALLPE	STAIALLPE	2	0	0	1	1	SDTAIALLPE	tr_C9ZP82	0.041	31965.18	55.00
128	HLA-A0201	VREFTSTEMA	VEFTSTEMA	0	0	0	1	1	VREFTSTEMA	tr_C9ZP82	0.041	31985.92	55.00
560	HLA-A0201	HEINLPQLQRL	EILPQLQRL	1	0	0	2	1	EINLPQLQRL	tr_C9ZP82	0.041	31992.85	55.00
617	HLA-A0201	AVALHSTIDDRWR	ALHSTIDWR	2	0	0	7	2	ALHSTIDDRWR	tr_C9ZP82	0.041	31995.27	55.00
814	HLA-A0201	DLRKLSDALDT	DLLSDALDT	0	0	0	2	2	DLRKLSDALDT	tr_C9ZP82	0.041	32000.47	55.00
72	HLA-A0201	QPKEVMYIAVD	QPKEVMIAV	0	0	0	6	1	QPKEVMYIAV	tr_C9ZP82	0.041	32013.96	55.00
670	HLA-A0201	SWYYPFYHAPP	SWYYPFYHA	0	0	0	0	0	SWYYPFYHA	tr_C9ZP82	0.041	32015.02	55.00
100	HLA-A0201	GRAAPAQTGEV	RAAPAQTEV	1	0	0	7	1	RAAPAQTGEV	tr_C9ZP82	0.041	32035.80	55.00
559	HLA-A0201	PHEINLPQLQRLFE	ILPQLQRLF	3	0	0	1	1	INLPQLQRLF	tr_C9ZP82	0.041	32041.00	55.00
72	HLA-A0201	QPKEVMYIAVDG	QPKEVMIAV	0	0	0	6	1	QPKEVMYIAV	tr_C9ZP82	0.041	32070.13	55.00
626	HLA-A0201	DRWRDTYLKSTGLS	WTYLKSTGL	2	0	0	1	2	WRDTYLKSTGL	tr_C9ZP82	0.041	32081.24	55.00
802	HLA-A0201	ITSNKTSFVPK	ITSNKTSFV	0	0	0	0	0	ITSNKTSFV	tr_C9ZP82	0.041	32085.75	55.00
572	HLA-A0201	EAYAKIEAVKFR	EAYAKIEAV	0	0	0	0	0	EAYAKIEAV	tr_C9ZP82	0.041	32087.49	55.00
104	HLA-A0201	PAQTGEVASTYMP	AQTGEVAYM	1	0	0	7	2	AQTGEVASTYM	tr_C9ZP82	0.041	32095.12	55.00
556	HLA-A0201	TKGPHEINLPQL	GPHEINLPL	2	0	0	8	1	GPHEINLPQL	tr_C9ZP82	0.041	32096.16	55.00
152	HLA-A0201	EDPLYSGALYNEG	PLYSGALYG	2	0	0	8	2	PLYSGALYNEG	tr_C9ZP82	0.041	32103.12	55.00
664	HLA-A0201	TTDSDWSWYYP	TTDWSWYYP	0	0	0	2	2	TTDSDWSWYYP	tr_C9ZP82	0.041	32106.59	55.00
606	HLA-A0201	SNNDNVTVA	SNNDNVTVA	0	0	0	0	0	SNNDNVTVA	tr_C9ZP82	0.041	32106.95	55.00
272	HLA-A0201	PGEGEHKIIDFL	GEGEIIDFL	1	0	0	4	2	GEGEHKIIDFL	tr_C9ZP82	0.041	32107.98	55.00
105	HLA-A0201	AQTGEVASTYMPN	AQTGEVAYM	0	0	0	7	2	AQTGEVASTYM	tr_C9ZP82	0.041	32111.46	55.00
106	HLA-A0201	QTGEVASTYMP	QTGEVATYM	0	0	0	6	1	QTGEVASTYM	tr_C9ZP82	0.041	32114.23	55.00
801	HLA-A0201	VITSNKTSFVPKGD	VISNKTSFV	0	0	0	2	1	VITSNKTSFV	tr_C9ZP82	0.041	32124.30	55.00
203	HLA-A0201	QVVDDVFGHR	QVVDDVFHR	0	0	0	7	1	QVVDDVFGHR	tr_C9ZP82	0.041	32125.01	55.00
558	HLA-A0201	GPHEINLPQ	GPHEINLPQ	0	0	0	0	0	GPHEINLPQ	tr_C9ZP82	0.041	32135.43	55.00
276	HLA-A0201	EHKIIDFLRT	HKIIDFLRT	1	0	0	0	0	HKIIDFLRT	tr_C9ZP82	0.041	32144.13	55.00
1	HLA-A0201	GVPKFASWLRNKYP	FAWLRNKYP	4	0	0	2	1	FASWLRNKYP	tr_C9ZP82	0.041	32151.43	55.00
415	HLA-A0201	ISVVGGSLISE	ISVVSLISE	0	0	0	4	2	ISVVGGSLISE	tr_C9ZP82	0.041	32159.44	55.00
91	HLA-A0201	NQQRARRYM	NQQRARRYM	0	0	0	0	0	NQQRARRYM	tr_C9ZP82	0.041	32160.47	55.00
95	HLA-A0201	ARRYMGRAAPA	RRYMGAAPA	1	0	0	5	1	RRYMGRAAPA	tr_C9ZP82	0.041	32168.83	55.00
616	HLA-A0201	VAVALHSTIDDRWR	ALHSTIDWR	3	0	0	7	2	ALHSTIDDRWR	tr_C9ZP82	0.041	32177.53	55.00
402	HLA-A0201	VTTVPNTGF	VTTVPNTGF	0	0	0	0	0	VTTVPNTGF	tr_C9ZP82	0.041	32195.29	55.00
34	HLA-A0201	GIIHPCCHNDS	IIHPCCHNS	1	0	0	8	1	IIHPCCHNDS	tr_C9ZP82	0.041	32195.65	55.00
733	HLA-A0201	VRSPQQAEL	VRSPQQAEL	0	0	0	0	0	VRSPQQAEL	tr_C9ZP82	0.041	32204.00	55.00
586	HLA-A0201	LLFKSTNKENSN	LLFKSTENS	0	0	0	6	2	LLFKSTNKENS	tr_C9ZP82	0.041	32206.09	55.00
359	HLA-A0201	SLNPSYIDEEESD	SLNPSYIES	0	0	0	7	3	SLNPSYIDEEES	tr_C9ZP82	0.041	32220.74	55.00
2	HLA-A0201	VPKFASWLR	VPKFASWLR	0	0	0	0	0	VPKFASWLR	tr_C9ZP82	0.041	32227.70	55.00
216	HLA-A0201	IVDDPTAFEFDSN	IVDPTAFEF	0	0	0	2	1	IVDDPTAFEF	tr_C9ZP82	0.041	32238.51	55.00
429	HLA-A0201	LCDIKKFTPATESA	LCDIKKFTA	0	0	0	8	5	LCDIKKFTPATESA	tr_C9ZP82	0.041	32248.29	55.00
202	HLA-A0201	NQVVDDVFGHR	NQVVVFGHR	0	0	0	4	2	NQVVDDVFGHR	tr_C9ZP82	0.040	32279.02	55.00
296	HLA-A0201	ANHVIVGLDAD	ANVIVGLDA	0	0	0	2	1	ANHVIVGLDA	tr_C9ZP82	0.040	32285.99	55.00
319	HLA-A0201	VFILRDTTRNPYK	ILRDTTNPY	2	0	0	6	1	ILRDTTRNPY	tr_C9ZP82	0.040	32302.07	55.00
373	HLA-A0201	VIGVPVCESSE	VIVPVCESS	0	0	0	2	1	VIGVPVCESS	tr_C9ZP82	0.040	32306.61	55.00
10	HLA-A0201	RNKYPAIVTK	RNYPAIVTK	0	0	0	2	1	RNKYPAIVTK	tr_C9ZP82	0.040	32313.26	55.00
293	HLA-A0201	GGKANHVIV	GGKANHVIV	0	0	0	0	0	GGKANHVIV	tr_C9ZP82	0.040	32313.59	55.00
201	HLA-A0201	SNQVVDDVFG	SQVVDDVFG	0	0	0	1	1	SNQVVDDVFG	tr_C9ZP82	0.040	32313.95	55.00
847	HLA-A0201	RYRPRTYSCTV	YRPTYSCTV	1	0	0	3	1	YRPRTYSCTV	tr_C9ZP82	0.040	32320.24	55.00
43	HLA-A0201	DSDTAIALLPE	STAIALLPE	1	0	0	1	1	SDTAIALLPE	tr_C9ZP82	0.040	32321.64	55.00
737	HLA-A0201	QQAELESTFP	QQAELSTFP	0	0	0	5	1	QQAELESTFP	tr_C9ZP82	0.040	32328.64	55.00
1	HLA-A0201	GVPKFASWLRN	GVFASWLRN	0	0	0	2	2	GVPKFASWLRN	tr_C9ZP82	0.040	32336.69	55.00

18	HLA-A0201	TKNCPSSVHGLYID	CPSSVGLYI	3	0	0	5	1	CPSSVHGLYI	tr_C9ZP82	0.040	32337.73	55.00
803	HLA-A0201	TSNKTSFVPKGDL	KTSFVPKGL	3	0	0	8	1	KTSFVPKGDL	tr_C9ZP82	0.040	32356.28	55.00
242	HLA-A0201	DAVLNMLNE	DAVLNMLNE	0	0	0	0	0	DAVLNMLNE	tr_C9ZP82	0.040	32356.99	55.00
370	HLA-A0201	SDGVIGVPVCESSE	SDGVIGVPV	0	0	0	0	0	SDGVIGVPV	tr_C9ZP82	0.040	32357.68	55.00
809	HLA-A0201	FVPKGDLRKLSD	FVPKGDLRL	0	0	0	8	1	FVPKGDLRKL	tr_C9ZP82	0.040	32369.95	55.00
663	HLA-A0201	TTTDSDWSWYYP	TTDWSWYYP	0	0	0	2	3	TTTDSDWSWYYP	tr_C9ZP82	0.040	32371.68	55.00
822	HLA-A0201	LDTILRRVPS	LTILRRVPS	0	0	0	1	1	LDTILRRVPS	tr_C9ZP82	0.040	32373.10	55.00
778	HLA-A0201	VDAAWPQLT	VDAAWPQLT	0	0	0	0	0	VDAAWPQLT	tr_C9ZP82	0.040	32381.50	55.00
429	HLA-A0201	LCDIKKFTP	LCDIKKFTP	0	0	0	0	0	LCDIKKFTP	tr_C9ZP82	0.040	32382.89	55.00
777	HLA-A0201	VVDAAWPQLTEE	VVDAAWPQL	0	0	0	0	0	VVDAAWPQL	tr_C9ZP82	0.040	32395.18	55.00
401	HLA-A0201	HVTTVPNTGFEY	HVTTVPFEY	0	0	0	6	3	HVTTVPNTGFEY	tr_C9ZP82	0.040	32395.51	55.00
493	HLA-A0201	RSWKNFRPCSSPAN	RSWPCSSPA	0	0	0	3	4	RSWKNFRPCSSPA	tr_C9ZP82	0.040	32398.31	55.00
39	HLA-A0201	CCHNDSDTA	CCHNDSDTA	0	0	0	0	0	CCHNDSDTA	tr_C9ZP82	0.040	32403.22	55.00
797	HLA-A0201	DFHLVITSNK	DFHLVITSK	0	0	0	8	1	DFHLVITSNK	tr_C9ZP82	0.040	32408.84	55.00
662	HLA-A0201	TTTTDSDWSWYYP	TTDWSWYYP	0	0	0	2	4	TTTTDSDWSWYYP	tr_C9ZP82	0.040	32417.25	55.00
856	HLA-A0201	TVEVPRLSY	TVEVPRLSY	0	0	0	0	0	TVEVPRLSY	tr_C9ZP82	0.040	32420.42	55.00
2	HLA-A0201	VPKFASWLRNKYP	FAWLRNKYP	3	0	0	2	1	FASWLRNKYP	tr_C9ZP82	0.040	32421.11	55.00
602	HLA-A0201	FRKGSNNDNV	FKGSNNDNV	0	0	0	1	1	FRKGSNNDNV	tr_C9ZP82	0.040	32425.67	55.00
126	HLA-A0201	EVVREFTSTEMAQ	VVFTSTEMA	1	0	0	2	2	VVREFTSTEMA	tr_C9ZP82	0.040	32426.74	55.00
215	HLA-A0201	VIVDDPTAFE	VIVDDPTAF	0	0	0	0	0	VIVDDPTAF	tr_C9ZP82	0.040	32433.39	55.00
454	HLA-A0201	YYFFGQPNHIE	YFFGQPNHI	0	0	0	1	1	YYFFGQPNHI	tr_C9ZP82	0.040	32436.55	55.00
395	HLA-A0201	IESREAHVTTV	IESAHVTTV	0	0	0	3	2	IESREAHVTTV	tr_C9ZP82	0.040	32452.35	55.00
777	HLA-A0201	VVDAAWPQLTEEER	VVDAAWPQL	0	0	0	0	0	VVDAAWPQL	tr_C9ZP82	0.040	32453.06	55.00
353	HLA-A0201	DDTCIISLNP	DTCIISLNP	1	0	0	0	0	DTCIISLNP	tr_C9ZP82	0.040	32455.86	55.00
372	HLA-A0201	GVIGVPVCESSE	GVIGVCESS	0	0	0	4	2	GVIGVPVCESS	tr_C9ZP82	0.040	32466.06	55.00
173	HLA-A0201	NDGDGVNSFCV	DGDGVSFCV	1	0	0	5	1	DGDGVNSFCV	tr_C9ZP82	0.040	32471.67	55.00
501	HLA-A0201	CSSPANSKIIDDI	CSSPIIDDI	0	0	0	4	4	CSSPANSKIIDDI	tr_C9ZP82	0.040	32474.47	55.00
79	HLA-A0201	IAVDGVAPRAKMNQ	IAVDGVAPM	0	0	0	8	3	IAVDGVAPRAKM	tr_C9ZP82	0.040	32476.23	55.00
661	HLA-A0201	TTTTTDSDWSWYYP	TTDWSWYYP	0	0	0	2	5	TTTTTDSDWSWYYP	tr_C9ZP82	0.040	32483.61	55.00
291	HLA-A0201	QFGGKANHVIV	QFGGKAVIV	0	0	0	6	2	QFGGKANHVIV	tr_C9ZP82	0.040	32497.33	55.00
399	HLA-A0201	EAHVTTVPNTGF	EAHVTTVPF	0	0	0	8	3	EAHVTTVPNTGF	tr_C9ZP82	0.040	32505.42	55.00
528	HLA-A0201	APSAFSGESAL	AAFSGESAL	0	0	0	1	2	APSAFSGESAL	tr_C9ZP82	0.040	32505.76	55.00
4	HLA-A0201	KFASWLRNKYP	FAWLRNKYP	1	0	0	2	1	FASWLRNKYP	tr_C9ZP82	0.040	32517.37	55.00
108	HLA-A0201	GEVASTYMPNKSGM	VASTYMPGM	2	0	0	7	3	VASTYMPNKSGM	tr_C9ZP82	0.040	32538.14	55.00
656	HLA-A0201	VWRYYTTTT	VWRYYTTTT	0	0	0	0	0	VWRYYTTTT	tr_C9ZP82	0.040	32539.55	55.00
153	HLA-A0201	DPLYSGALYNE	DLYSGALYN	0	0	0	1	1	DPLYSGALYN	tr_C9ZP82	0.040	32558.56	55.00
297	HLA-A0201	NHVIVGLDAD	NHVIVGLDA	0	0	0	0	0	NHVIVGLDA	tr_C9ZP82	0.040	32568.44	55.00
177	HLA-A0201	GVNSFCVRSTD	GVNSFCVST	0	0	0	7	1	GVNSFCVRST	tr_C9ZP82	0.040	32574.78	55.00
780	HLA-A0201	AAWPQLTEEE	AAWQLTEEE	0	0	0	3	1	AAWPQLTEEE	tr_C9ZP82	0.040	32585.71	55.00
26	HLA-A0201	HGLYIDLNG	HGLYIDLNG	0	0	0	0	0	HGLYIDLNG	tr_C9ZP82	0.040	32588.52	55.00
701	HLA-A0201	VKSTPPDTL	VKSTPPDTL	0	0	0	0	0	VKSTPPDTL	tr_C9ZP82	0.040	32594.87	55.00
103	HLA-A0201	APAQTGEVASTYMP	AQTGEVAYM	2	0	0	7	2	AQTGEVASTYM	tr_C9ZP82	0.040	32599.81	55.00
896	HLA-A0201	SNSRVTVVNKGPC	NSRVTVVPC	1	0	0	7	3	NSRVTVVNKGPC	tr_C9ZP82	0.040	32602.63	55.00
522	HLA-A0201	NDFLPHAPSA	NDFLPHAPA	0	0	0	8	1	NDFLPHAPSA	tr_C9ZP82	0.040	32602.98	55.00
616	HLA-A0201	VAVALHSTID	VAVALHSTI	0	0	0	0	0	VAVALHSTI	tr_C9ZP82	0.040	32609.33	55.00
41	HLA-A0201	HNDSDTAIALLP	HSDTAIALL	0	0	0	1	2	HNDSDTAIALL	tr_C9ZP82	0.039	32626.27	55.00
256	HLA-A0201	DPTWTRLCV	DPTWTRLCV	0	0	0	0	0	DPTWTRLCV	tr_C9ZP82	0.039	32645.34	55.00
818	HLA-A0201	LSDALDTILRR	LSDALILRR	0	0	0	5	2	LSDALDTILRR	tr_C9ZP82	0.039	32654.52	55.00
285	HLA-A0201	TQSSLAQFGGKANH	SLAQFGANH	3	0	0	6	2	SLAQFGGKANH	tr_C9ZP82	0.039	32667.60	55.00
252	HLA-A0201	MSGNDPTWTRLC	MSDPTWTRL	0	0	0	2	2	MSGNDPTWTRL	tr_C9ZP82	0.039	32671.14	55.00
828	HLA-A0201	RVPSREGVEIRQL	RVGVEIRQL	0	0	0	2	4	RVPSREGVEIRQL	tr_C9ZP82	0.039	32671.83	55.00
784	HLA-A0201	QLTEEERWRTQKLD	QLTEETQKL	0	0	0	5	4	QLTEEERWRTQKL	tr_C9ZP82	0.039	32674.32	55.00
372	HLA-A0201	GVIGVPVCESSEN	GVIGVPVEN	0	0	0	7	4	GVIGVPVCESSEN	tr_C9ZP82	0.039	32677.14	55.00
400	HLA-A0201	AHVTTVPNTGFEY	AHVTTVFEY	0	0	0	6	4	AHVTTVPNTGFEY	tr_C9ZP82	0.039	32678.55	55.00

437	HLA-A0201	PATESAFDKKTLVS	ATFDKKTLV	1	0	0	2	3	ATESAFDKKTLV	tr_C9ZP82	0.039	32679.63	55.00
694	HLA-A0201	KRMPLPVVK	KRMPLPVVK	0	0	0	0	0	KRMPLPVVK	tr_C9ZP82	0.039	32681.38	55.00
237	HLA-A0201	MAKVSDAVLN	MAKVSDAVL	0	0	0	0	0	MAKVSDAVL	tr_C9ZP82	0.039	32692.36	55.00
370	HLA-A0201	SDGVIGVPVCE	SDGVIGVPV	0	0	0	0	0	SDGVIGVPV	tr_C9ZP82	0.039	32699.43	55.00
208	HLA-A0201	VFGHRGEVIVDDPT	FGVIVDDPT	1	0	0	2	4	FGHRGEVIVDDPT	tr_C9ZP82	0.039	32701.89	55.00
399	HLA-A0201	EAHVTTVPNTGFEY	HVTTVPFEY	2	0	0	6	3	HVTTVPNTGFEY	tr_C9ZP82	0.039	32706.15	55.00
33	HLA-A0201	NGIIHPCCHNDS	IIHPCCHNS	2	0	0	8	1	IIHPCCHNDS	tr_C9ZP82	0.039	32707.21	55.00
241	HLA-A0201	SDAVLNMLNE	SAVLNMLNE	0	0	0	1	1	SDAVLNMLNE	tr_C9ZP82	0.039	32710.39	55.00
246	HLA-A0201	NMLNEKMSGNDP	MLNEKMSGP	1	0	0	8	2	MLNEKMSGNDP	tr_C9ZP82	0.039	32717.48	55.00
90	HLA-A0201	MNQQRARRYM	MQQRARRYM	0	0	0	1	1	MNQQRARRYM	tr_C9ZP82	0.039	32723.50	55.00
211	HLA-A0201	HRGEVIVDDPTAFE	HRGEVIVAF	0	0	0	7	4	HRGEVIVDDPTAF	tr_C9ZP82	0.039	32732.70	55.00
672	HLA-A0201	YYPFYHAPP	YYPFYHAPP	0	0	0	0	0	YYPFYHAPP	tr_C9ZP82	0.039	32758.57	55.00
125	HLA-A0201	EEVVREFTSTEMAQ	VVFTSTEMA	2	0	0	2	2	VVREFTSTEMA	tr_C9ZP82	0.039	32762.46	55.00
449	HLA-A0201	VSSAGYYFFGQP	VSSAGYYFF	0	0	0	0	0	VSSAGYYFF	tr_C9ZP82	0.039	32770.97	55.00
442	HLA-A0201	AFDKKTLVSS	AFDKTLVSS	0	0	0	3	1	AFDKKTLVSS	tr_C9ZP82	0.039	32775.94	55.00
572	HLA-A0201	EAYAKIEAVKFRR	EAYAKIEAV	0	0	0	0	0	EAYAKIEAV	tr_C9ZP82	0.039	32792.26	55.00
893	HLA-A0201	GKKSNSRVTVV	KSNSRVTVV	2	0	0	0	0	KSNSRVTVV	tr_C9ZP82	0.039	32793.32	55.00
359	HLA-A0201	SLNPSYIDEEESDG	SLNPSYIDG	0	0	0	7	5	SLNPSYIDEEESDG	tr_C9ZP82	0.039	32800.07	55.00
895	HLA-A0201	KSNSRVTVVNKGPC	NSRVTVVPC	2	0	0	7	3	NSRVTVVNKGPC	tr_C9ZP82	0.039	32803.26	55.00
845	HLA-A0201	LERYRPRTYSCTV	YRPTYSCTV	3	0	0	3	1	YRPRTYSCTV	tr_C9ZP82	0.039	32808.57	55.00
201	HLA-A0201	SNQVVDDVFGHR	NQVVVFGHR	1	0	0	4	2	NQVVDDVFGHR	tr_C9ZP82	0.039	32808.57	55.00
209	HLA-A0201	FGHRGEVIVD	FGHRGEVIV	0	0	0	0	0	FGHRGEVIV	tr_C9ZP82	0.039	32812.12	55.00
457	HLA-A0201	FGQPNHIEDG	FGQPNHIEDG	0	0	0	1	1	FGQPNHIEDG	tr_C9ZP82	0.039	32812.84	55.00
804	HLA-A0201	SNKTSFVPKGDL	KTSFVPKGL	2	0	0	8	1	KTSFVPKGDL	tr_C9ZP82	0.039	32813.54	55.00
199	HLA-A0201	VESNQVVDDVFGHR	NQVVVFGHR	3	0	0	4	2	NQVVDDVFGHR	tr_C9ZP82	0.039	32814.96	55.00
700	HLA-A0201	VVKSTPPDT	VVKSTPPDT	0	0	0	0	0	VVKSTPPDT	tr_C9ZP82	0.039	32825.27	55.00
886	HLA-A0201	NGEGVIEGK	NGEGVIEGK	0	0	0	0	0	NGEGVIEGK	tr_C9ZP82	0.039	32830.60	55.00
805	HLA-A0201	NKTSFVPKGDL	KTSFVPKGL	1	0	0	8	1	KTSFVPKGDL	tr_C9ZP82	0.039	32848.01	55.00
777	HLA-A0201	VVDAAWPQLTEEE	VVDAAWPQL	0	0	0	0	0	VVDAAWPQL	tr_C9ZP82	0.039	32866.85	55.00
450	HLA-A0201	SSAGYYFFGQP	SSAGYYFFP	0	0	0	8	2	SSAGYYFFGQP	tr_C9ZP82	0.039	32867.55	55.00
736	HLA-A0201	PQQAELEST	PQQAELEST	0	0	0	0	0	PQQAELEST	tr_C9ZP82	0.039	32869.69	55.00
152	HLA-A0201	EDPLYSGALYNE	ELYSGALYN	0	0	0	1	2	EDPLYSGALYN	tr_C9ZP82	0.039	32883.55	55.00
225	HLA-A0201	FDSNCISPGTDF	FDSNCISPF	0	0	0	8	3	FDSNCISPGTDF	tr_C9ZP82	0.039	32892.82	55.00
423	HLA-A0201	ISEVSALCDIKK	ISVSALCDI	0	0	0	2	1	ISEVSALCDI	tr_C9ZP82	0.039	32898.87	55.00
42	HLA-A0201	NDSDTAIALLP	NSDTAIALL	0	0	0	1	1	NDSDTAIALL	tr_C9ZP82	0.039	32912.05	55.00
835	HLA-A0201	VEIRQLYDVP	VEIRQLYDV	0	0	0	0	0	VEIRQLYDV	tr_C9ZP82	0.039	32913.47	55.00
559	HLA-A0201	PHEINLPQLQRL	EILPQLQRL	2	0	0	2	1	EINLPQLQRL	tr_C9ZP82	0.039	32920.22	55.00
80	HLA-A0201	AVDGVAPRAKMN	AVDGVAAKM	0	0	0	6	2	AVDGVAPRAKM	tr_C9ZP82	0.039	32920.95	55.00
287	HLA-A0201	SSLAQFGGKANH	SLAQFGANH	1	0	0	6	2	SLAQFGGKANH	tr_C9ZP82	0.039	32925.57	55.00
128	HLA-A0201	VREFTSTEM	VREFTSTEM	0	0	0	0	0	VREFTSTEM	tr_C9ZP82	0.039	32927.36	55.00
32	HLA-A0201	LNGIIHPCCHNDS	IIHPCCHNS	3	0	0	8	1	IIHPCCHNDS	tr_C9ZP82	0.039	32928.78	55.00
200	HLA-A0201	ESNQVVDDVF	ESNQVVDDV	0	0	0	0	0	ESNQVVDDV	tr_C9ZP82	0.039	32947.32	55.00
212	HLA-A0201	RGEVIVDDPTAFE	RGEVIVTAF	0	0	0	6	3	RGEVIVDDPTAF	tr_C9ZP82	0.038	32966.58	55.00
371	HLA-A0201	DGVIGVPVCE	GVIGVPVCE	1	0	0	0	0	GVIGVPVCE	tr_C9ZP82	0.038	32966.92	55.00
276	HLA-A0201	EHKIIDFLR	EHKIIDFLR	0	0	0	0	0	EHKIIDFLR	tr_C9ZP82	0.038	32967.64	55.00
273	HLA-A0201	GEGEHKIIDFLR	GEGEIIDFL	0	0	0	4	2	GEGEHKIIDFL	tr_C9ZP82	0.038	32969.77	55.00
170	HLA-A0201	DALNDGDGV	DALNDGDGV	0	0	0	0	0	DALNDGDGV	tr_C9ZP82	0.038	32975.13	55.00
185	HLA-A0201	STDDDYESTH	STDDDYEST	0	0	0	0	0	STDDDYEST	tr_C9ZP82	0.038	32975.85	55.00
105	HLA-A0201	AQTGEVASTYMPNK	AQTGEVAYM	0	0	0	7	2	AQTGEVASTYM	tr_C9ZP82	0.038	32992.27	55.00
398	HLA-A0201	REAHVTTVPNTGF	EAHVTTVPF	1	0	0	8	3	EAHVTTVPNTGF	tr_C9ZP82	0.038	32999.40	55.00
527	HLA-A0201	HAPSAFSGESAL	SAFSGESAL	3	0	0	0	0	SAFSGESAL	tr_C9ZP82	0.038	32999.76	55.00
365	HLA-A0201	IDEEESDGVIGVP	IESDGVIGV	0	0	0	1	3	IDEEESDGVIGV	tr_C9ZP82	0.038	33001.19	55.00
45	HLA-A0201	DTAIALLPEE	DTAIALLPE	0	0	0	8	1	DTAIALLPEE	tr_C9ZP82	0.038	33022.27	55.00
832	HLA-A0201	REGVEIRQLY	REGVEIQLY	0	0	0	6	1	REGVEIRQLY	tr_C9ZP82	0.038	33037.26	55.00

462	HLA-A0201	HIEDGTLPSST	HIEDGTLPT	0	0	0	8	2	HIEDGTLPSST	tr_C9ZP82	0.038	33040.84	55.00
252	HLA-A0201	MSGNDPTWTR	MSGNPTWTR	0	0	0	4	1	MSGNDPTWTR	tr_C9ZP82	0.038	33040.84	55.00
44	HLA-A0201	SDTAIALLPEEEK	AIALLPEEK	3	0	0	6	1	AIALLPEEEK	tr_C9ZP82	0.038	33045.49	55.00
217	HLA-A0201	VDDPTAFEFDSNC	VAFEFDSNC	0	0	0	1	4	VDDPTAFEFDSNC	tr_C9ZP82	0.038	33048.70	55.00
846	HLA-A0201	ERYRPRTYSCTV	YRPTYSCTV	2	0	0	3	1	YRPRTYSCTV	tr_C9ZP82	0.038	33056.93	55.00
403	HLA-A0201	TTVPNTGFEYFD	TTVTGFEYF	0	0	0	3	2	TTVPNTGFEYF	tr_C9ZP82	0.038	33062.67	55.00
200	HLA-A0201	ESNQVVDDVFGHR	NQVVVFGHR	2	0	0	4	2	NQVVDDVFGHR	tr_C9ZP82	0.038	33071.60	55.00
234	HLA-A0201	TDFMAKVSDA	TFMAKVSDA	0	0	0	1	1	TDFMAKVSDA	tr_C9ZP82	0.038	33088.07	60.00
275	HLA-A0201	GEHKIIDFLRT	GEIIDFLRT	0	0	0	2	2	GEHKIIDFLRT	tr_C9ZP82	0.038	33094.88	60.00
34	HLA-A0201	GIIHPCCHND	IIHPCCHND	1	0	0	0	0	IIHPCCHND	tr_C9ZP82	0.038	33094.88	60.00
528	HLA-A0201	APSAFSGESA	APSAFSESA	0	0	0	6	1	APSAFSGESA	tr_C9ZP82	0.038	33100.60	60.00
25	HLA-A0201	VHGLYIDLNG	VHGLYILNG	0	0	0	6	1	VHGLYIDLNG	tr_C9ZP82	0.038	33107.41	60.00
158	HLA-A0201	GALYNEGEEE	ALYNEGEEE	1	0	0	0	0	ALYNEGEEE	tr_C9ZP82	0.038	33107.75	60.00
780	HLA-A0201	AAWPQLTEEERW	AAWPQLTEW	0	0	0	8	3	AAWPQLTEEERW	tr_C9ZP82	0.038	33117.79	60.00
296	HLA-A0201	ANHVIVGLD	ANHVIVGLD	0	0	0	0	0	ANHVIVGLD	tr_C9ZP82	0.038	33129.26	60.00
660	HLA-A0201	YTTTTTDSDWSWY	YTTTTTWSW	0	0	0	6	3	YTTTTTDSDWSW	tr_C9ZP82	0.038	33129.62	60.00
892	HLA-A0201	EGKKSNSRVTVV	KSNSRVTVV	3	0	0	0	0	KSNSRVTVV	tr_C9ZP82	0.038	33137.87	60.00
630	HLA-A0201	DTYLKSTGLSN	YLKSTGLSN	2	0	0	0	0	YLKSTGLSN	tr_C9ZP82	0.038	33138.58	60.00
284	HLA-A0201	RTQSSLAQFGGKAN	RLAQFGGKA	0	0	0	1	4	RTQSSLAQFGGKA	tr_C9ZP82	0.038	33140.01	60.00
12	HLA-A0201	KYPAIVTKNCPS	YPAIVTCPS	1	0	0	6	2	YPAIVTKNCPS	tr_C9ZP82	0.038	33142.89	60.00
11	HLA-A0201	NKYPAIVTKNCPS	YIVTKNCPS	2	0	0	1	2	YPAIVTKNCPS	tr_C9ZP82	0.038	33143.60	60.00
449	HLA-A0201	VSSAGYYFFGQPN	VSSAGYYFF	0	0	0	0	0	VSSAGYYFF	tr_C9ZP82	0.038	33148.26	60.00
425	HLA-A0201	EVSALCDIK	EVSALCDIK	0	0	0	0	0	EVSALCDIK	tr_C9ZP82	0.038	33156.88	60.00
889	HLA-A0201	GVIEGKKSNSRVT	GVIEGNSRV	0	0	0	5	3	GVIEGKKSNSRV	tr_C9ZP82	0.038	33160.47	60.00
4	HLA-A0201	KFASWLRNKY	KFASWLRNY	0	0	0	8	1	KFASWLRNKY	tr_C9ZP82	0.038	33164.06	60.00
43	HLA-A0201	DSDTAIALLPEEEK	AIALLPEEK	4	0	0	6	1	AIALLPEEEK	tr_C9ZP82	0.038	33173.39	60.00
438	HLA-A0201	ATESAFDKKTL	ATESAFDTL	0	0	0	7	2	ATESAFDKKTL	tr_C9ZP82	0.038	33174.46	60.00
359	HLA-A0201	SLNPSYIDEEE	SLNPSYIEE	0	0	0	7	2	SLNPSYIDEEE	tr_C9ZP82	0.038	33175.18	60.00
644	HLA-A0201	EACEKYVEGVC	EACEYVEGV	0	0	0	4	1	EACEKYVEGV	tr_C9ZP82	0.038	33179.85	60.00
452	HLA-A0201	AGYYFFGQPN	AGYYFFQPN	0	0	0	6	1	AGYYFFGQPN	tr_C9ZP82	0.038	33184.88	60.00
25	HLA-A0201	VHGLYIDLN	VHGLYIDLN	0	0	0	0	0	VHGLYIDLN	tr_C9ZP82	0.038	33192.78	60.00
371	HLA-A0201	DGVIGVPVCESSE	VIGVVCESS	2	0	0	4	1	VIGVPVCESS	tr_C9ZP82	0.038	33197.45	60.00
819	HLA-A0201	SDALDTILR	SDALDTILR	0	0	0	0	0	SDALDTILR	tr_C9ZP82	0.038	33203.18	60.00
4	HLA-A0201	KFASWLRNK	KFASWLRNK	0	0	0	0	0	KFASWLRNK	tr_C9ZP82	0.038	33204.62	60.00
802	HLA-A0201	ITSNKTSFVPKG	ITSNKTSFV	0	0	0	0	0	ITSNKTSFV	tr_C9ZP82	0.038	33210.74	60.00
840	HLA-A0201	LYDVPLERYRPRTY	LYDVPLETY	0	0	0	7	5	LYDVPLERYRPRTY	tr_C9ZP82	0.038	33221.15	60.00
405	HLA-A0201	VPNTGFEYFDIS	VTGFEYFDI	0	0	0	1	2	VPNTGFEYFDI	tr_C9ZP82	0.038	33221.15	60.00
397	HLA-A0201	SREAHVTTVPNTGF	EAHVTTVPF	2	0	0	8	3	EAHVTTVPNTGF	tr_C9ZP82	0.038	33221.88	60.00
463	HLA-A0201	IEDGTLPSST	IEDGTLPST	0	0	0	7	1	IEDGTLPSST	tr_C9ZP82	0.038	33240.20	60.00
224	HLA-A0201	EFDSNCISPGT	FSNCISPGT	1	0	0	1	1	FDSNCISPGT	tr_C9ZP82	0.038	33242.00	60.00
273	HLA-A0201	GEGEHKIIDFLRT	GEIIDFLRT	0	0	0	2	4	GEGEHKIIDFLRT	tr_C9ZP82	0.038	33252.45	60.00
40	HLA-A0201	CHNDSDTAI	CHNDSDTAI	0	0	0	0	0	CHNDSDTAI	tr_C9ZP82	0.038	33262.16	60.00
46	HLA-A0201	TAIALLPEEE	AIALLPEEE	1	0	0	0	0	AIALLPEEE	tr_C9ZP82	0.038	33265.40	60.00
202	HLA-A0201	NQVVDDVFGHRG	NQVVVFGHR	0	0	0	4	2	NQVVDDVFGHR	tr_C9ZP82	0.038	33270.08	60.00
828	HLA-A0201	RVPSREGVEIRQLY	RVVEIRQLY	0	0	0	2	5	RVPSREGVEIRQLY	tr_C9ZP82	0.038	33278.71	60.00
843	HLA-A0201	VPLERYRPRT	VLERYRPRT	0	0	0	1	1	VPLERYRPRT	tr_C9ZP82	0.038	33294.91	60.00
20	HLA-A0201	NCPSSVHGLY	NCPSSVHGL	0	0	0	0	0	NCPSSVHGL	tr_C9ZP82	0.038	33316.18	60.00
41	HLA-A0201	HNDSDTAIALLPEE	HSDTAIALL	0	0	0	1	2	HNDSDTAIALL	tr_C9ZP82	0.037	33327.35	60.00
603	HLA-A0201	RKGSNNDNV	RKGSNNDNV	0	0	0	0	0	RKGSNNDNV	tr_C9ZP82	0.037	33336.38	60.00
608	HLA-A0201	NDNVTVADVA	NDNVTVAVA	0	0	0	7	1	NDNVTVADVA	tr_C9ZP82	0.037	33337.09	60.00
285	HLA-A0201	TQSSLAQFGGKAN	TQSSLAQFA	0	0	0	8	3	TQSSLAQFGGKA	tr_C9ZP82	0.037	33341.43	60.00
3	HLA-A0201	PKFASWLRNKYP	FAWLRNKYP	2	0	0	2	1	FASWLRNKYP	tr_C9ZP82	0.037	33342.14	60.00
552	HLA-A0201	YGFLTKGPH	YGFLTKGPH	0	0	0	0	0	YGFLTKGPH	tr_C9ZP82	0.037	33350.45	60.00
697	HLA-A0201	PLPVVKSTPP	PLPVVSTPP	0	0	0	5	1	PLPVVKSTPP	tr_C9ZP82	0.037	33356.94	60.00

271	HLA-A0201	TPGEGEHKIIDFLR	GEHKIIFLR	4	0	0	6	1	GEHKIIDFLR	tr_C9ZP82	0.037	33378.98	60.00
224	HLA-A0201	EFDSNCISPGTDF	FDSNCISPF	1	0	0	8	3	FDSNCISPGTDF	tr_C9ZP82	0.037	33395.23	60.00
840	HLA-A0201	LYDVPLERYR	LYDVPLEYR	0	0	0	7	1	LYDVPLERYR	tr_C9ZP82	0.037	33400.64	60.00
201	HLA-A0201	SNQVVDDVFGH	SNQVVVFGH	0	0	0	5	2	SNQVVDDVFGH	tr_C9ZP82	0.037	33415.46	60.00
827	HLA-A0201	RRVPSREGVEIRQL	RVGVEIRQL	1	0	0	2	4	RVPSREGVEIRQL	tr_C9ZP82	0.037	33415.83	60.00
10	HLA-A0201	RNKYPAIVTKNCPS	YIVTKNCPS	3	0	0	1	2	YPAIVTKNCPS	tr_C9ZP82	0.037	33419.45	60.00
401	HLA-A0201	HVTTVPNTG	HVTTVPNTG	0	0	0	0	0	HVTTVPNTG	tr_C9ZP82	0.037	33436.07	60.00
151	HLA-A0201	LEDPLYSGALY	LEDPLYALY	0	0	0	6	2	LEDPLYSGALY	tr_C9ZP82	0.037	33438.25	60.00
502	HLA-A0201	SSPANSKIIDDII	SSPIIDDII	0	0	0	3	4	SSPANSKIIDDII	tr_C9ZP82	0.037	33438.98	60.00
248	HLA-A0201	LNEKMSGNDPTW	LMSGNDPTW	0	0	0	1	3	LNEKMSGNDPTW	tr_C9ZP82	0.037	33450.54	60.00
223	HLA-A0201	FEFDSNCISPGTD	FEFDCISPG	0	0	0	4	2	FEFDSNCISPG	tr_C9ZP82	0.037	33462.50	60.00
449	HLA-A0201	VSSAGYYFFGQ	VSSAYFFGQ	0	0	0	4	2	VSSAGYYFFGQ	tr_C9ZP82	0.037	33463.94	60.00
42	HLA-A0201	NDSDTAIALLPEE	NSDTAIALL	0	0	0	1	1	NDSDTAIALL	tr_C9ZP82	0.037	33488.21	60.00
451	HLA-A0201	SAGYYFFGQPN	SAGYYFFPN	0	0	0	7	2	SAGYYFFGQPN	tr_C9ZP82	0.037	33490.75	60.00
550	HLA-A0201	LPYGFLTKGPH	LPYGFLTPH	0	0	0	7	2	LPYGFLTKGPH	tr_C9ZP82	0.037	33510.68	60.00
856	HLA-A0201	TVEVPRLSYG	TVVPRLSYG	0	0	0	2	1	TVEVPRLSYG	tr_C9ZP82	0.037	33511.78	60.00
48	HLA-A0201	IALLPEEEK	IALLPEEEK	0	0	0	0	0	IALLPEEEK	tr_C9ZP82	0.037	33512.86	60.00
176	HLA-A0201	DGVNSFCVRSTD	GVNSFCVST	1	0	0	7	1	GVNSFCVRST	tr_C9ZP82	0.037	33513.59	60.00
659	HLA-A0201	YYTTTTTDSDWSWY	YTTTTTWSW	1	0	0	6	3	YTTTTTDSDWSW	tr_C9ZP82	0.037	33518.30	60.00
371	HLA-A0201	DGVIGVPVC	DGVIGVPVC	0	0	0	0	0	DGVIGVPVC	tr_C9ZP82	0.037	33520.83	60.00
371	HLA-A0201	DGVIGVPVCESSEN	VIGVVCESS	2	0	0	4	1	VIGVPVCESS	tr_C9ZP82	0.037	33525.91	60.00
425	HLA-A0201	EVSALCDIKK	VSALCDIKK	1	0	0	0	0	VSALCDIKK	tr_C9ZP82	0.037	33526.65	60.00
286	HLA-A0201	QSSLAQFGGKANH	SLAQFGANH	2	0	0	6	2	SLAQFGGKANH	tr_C9ZP82	0.037	33528.46	60.00
429	HLA-A0201	LCDIKKFTPATE	LCDIKFTPA	0	0	0	4	1	LCDIKKFTPA	tr_C9ZP82	0.037	33531.36	60.00
402	HLA-A0201	VTTVPNTGFEY	VTTVTGFEY	0	0	0	4	2	VTTVPNTGFEY	tr_C9ZP82	0.037	33534.26	60.00
40	HLA-A0201	CHNDSDTAIAL	CHNDTAIAL	0	0	0	3	2	CHNDSDTAIAL	tr_C9ZP82	0.037	33535.34	60.00
262	HLA-A0201	LCVIFSGSNTPG	LCVIFSTPG	0	0	0	6	3	LCVIFSGSNTPG	tr_C9ZP82	0.037	33537.16	60.00
605	HLA-A0201	GSNNDNVTVADVA	GSNVTVADV	0	0	0	2	3	GSNNDNVTVADV	tr_C9ZP82	0.037	33538.24	60.00
807	HLA-A0201	TSFVPKGDLRKLSD	FVPKGDLRL	2	0	0	8	1	FVPKGDLRKL	tr_C9ZP82	0.037	33540.79	60.00
89	HLA-A0201	KMNQQRARRYMGR	KMNQQRAYM	0	0	0	7	2	KMNQQRARRYM	tr_C9ZP82	0.037	33545.88	60.00
455	HLA-A0201	YFFGQPNHIEDGT	YFFGIEDGT	0	0	0	4	4	YFFGQPNHIEDGT	tr_C9ZP82	0.037	33554.94	60.00
502	HLA-A0201	SSPANSKIIDDI	SSPAIIDDI	0	0	0	4	3	SSPANSKIIDDI	tr_C9ZP82	0.037	33563.30	60.00
199	HLA-A0201	VESNQVVDDVF	VSNQVVDDV	0	0	0	1	1	VESNQVVDDV	tr_C9ZP82	0.037	33568.02	60.00
880	HLA-A0201	SGQLKGNGEGVIE	SLKGNGEGV	0	0	0	1	2	SGQLKGNGEGV	tr_C9ZP82	0.037	33572.02	60.00
200	HLA-A0201	ESNQVVDDVFGHRG	NQVVVFGHR	2	0	0	4	2	NQVVDDVFGHR	tr_C9ZP82	0.037	33580.38	60.00
102	HLA-A0201	AAPAQTGEVA	AAAQTGEVA	0	0	0	2	1	AAPAQTGEVA	tr_C9ZP82	0.037	33586.92	60.00
1	HLA-A0201	GVPKFASWLRNKY	VPKFASWLY	1	0	0	8	3	VPKFASWLRNKY	tr_C9ZP82	0.037	33588.37	60.00
831	HLA-A0201	SREGVEIRQLY	SREGVEILY	0	0	0	7	2	SREGVEIRQLY	tr_C9ZP82	0.037	33589.82	60.00
430	HLA-A0201	CDIKKFTPATESAF	CDIKKFTPF	0	0	0	8	5	CDIKKFTPATESAF	tr_C9ZP82	0.037	33598.18	60.00
101	HLA-A0201	RAAPAQTGEVASTY	RTGEVASTY	0	0	0	1	5	RAAPAQTGEVASTY	tr_C9ZP82	0.037	33614.55	60.00
186	HLA-A0201	TDDDYESTHENRYV	YSTHENRYV	4	0	0	1	1	YESTHENRYV	tr_C9ZP82	0.037	33615.63	60.00
274	HLA-A0201	EGEHKIIDFLRT	HKIIDFLRT	3	0	0	0	0	HKIIDFLRT	tr_C9ZP82	0.037	33624.00	60.00
17	HLA-A0201	VTKNCPSSVHGLY	VTPSSVHGL	0	0	0	2	3	VTKNCPSSVHGL	tr_C9ZP82	0.037	33626.91	60.00
42	HLA-A0201	NDSDTAIALLPEEE	NSDTAIALL	0	0	0	1	1	NDSDTAIALL	tr_C9ZP82	0.037	33630.18	60.00
199	HLA-A0201	VESNQVVDDVFGH	VSNQVVDDV	0	0	0	1	1	VESNQVVDDV	tr_C9ZP82	0.037	33635.28	60.00
272	HLA-A0201	PGEGEHKIIDFLR	GEGEIIDFL	1	0	0	4	2	GEGEHKIIDFL	tr_C9ZP82	0.037	33640.01	60.00
442	HLA-A0201	AFDKKTLVSSA	AFDTLVSSA	0	0	0	3	2	AFDKKTLVSSA	tr_C9ZP82	0.037	33645.11	60.00
373	HLA-A0201	VIGVPVCESSENS	VIGVVCESS	0	0	0	4	1	VIGVPVCESS	tr_C9ZP82	0.037	33649.84	60.00
729	HLA-A0201	GEVMVRSPQQA	VMVRSPQQA	2	0	0	0	0	VMVRSPQQA	tr_C9ZP82	0.037	33652.75	60.00
833	HLA-A0201	EGVEIRQLY	EGVEIRQLY	0	0	0	0	0	EGVEIRQLY	tr_C9ZP82	0.037	33667.31	60.00
168	HLA-A0201	AGDALNDGDGV	AALNDGDGV	0	0	0	1	2	AGDALNDGDGV	tr_C9ZP82	0.037	33670.24	60.00
450	HLA-A0201	SSAGYYFFGQPN	SSYFFGQPN	0	0	0	2	3	SSAGYYFFGQPN	tr_C9ZP82	0.037	33675.69	60.00
211	HLA-A0201	HRGEVIVDDPT	HRGEVIVPT	0	0	0	7	2	HRGEVIVDDPT	tr_C9ZP82	0.037	33679.71	60.00
808	HLA-A0201	SFVPKGDLRKLSD	FVPKGDLRL	1	0	0	8	1	FVPKGDLRKL	tr_C9ZP82	0.037	33685.90	60.00

629	HLA-A0201	RDTYLKSTGLS	RTYLKSTGL	0	0	0	1	1	RDTYLKSTGL	tr_C9ZP82	0.036	33686.64	60.00
201	HLA-A0201	SNQVVDDVFGHRG	NQVVVFGHR	1	0	0	4	2	NQVVDDVFGHR	tr_C9ZP82	0.036	33687.36	60.00
526	HLA-A0201	PHAPSAFSGESAL	SAFSGESAL	4	0	0	0	0	SAFSGESAL	tr_C9ZP82	0.036	33688.81	60.00
404	HLA-A0201	TVPNTGFEYFD	TVPNTFEYF	0	0	0	5	1	TVPNTGFEYF	tr_C9ZP82	0.036	33692.46	60.00
575	HLA-A0201	AKIEAVKFRR	AKIEAVFRR	0	0	0	6	1	AKIEAVKFRR	tr_C9ZP82	0.036	33696.48	60.00
373	HLA-A0201	VIGVPVCESSEN	VIVPVCESS	0	0	0	2	1	VIGVPVCESS	tr_C9ZP82	0.036	33700.11	60.00
555	HLA-A0201	LTKGPHEINLPQLQ	LTEINLPQL	0	0	0	2	4	LTKGPHEINLPQL	tr_C9ZP82	0.036	33703.05	60.00
245	HLA-A0201	LNMLNEKMSGNDP	MLNEKMSGP	2	0	0	8	2	MLNEKMSGNDP	tr_C9ZP82	0.036	33707.05	60.00
156	HLA-A0201	YSGALYNEGEEE	YLYNEGEEE	0	0	0	1	3	YSGALYNEGEEE	tr_C9ZP82	0.036	33713.24	60.00
274	HLA-A0201	EGEHKIIDFLR	GEHIIDFLR	1	0	0	3	1	GEHKIIDFLR	tr_C9ZP82	0.036	33716.18	60.00
207	HLA-A0201	DVFGHRGEVIVDD	DVFEVIVDD	0	0	0	3	4	DVFGHRGEVIVDD	tr_C9ZP82	0.036	33719.46	60.00
145	HLA-A0201	EVRQTLLEDP	EVRQTLLEP	0	0	0	8	1	EVRQTLLEDP	tr_C9ZP82	0.036	33720.55	60.00
262	HLA-A0201	LCVIFSGSNTP	LCVIFSNTP	0	0	0	5	2	LCVIFSGSNTP	tr_C9ZP82	0.036	33725.66	60.00
274	HLA-A0201	EGEHKIIDF	EGEHKIIDF	0	0	0	0	0	EGEHKIIDF	tr_C9ZP82	0.036	33730.40	60.00
732	HLA-A0201	MVRSPQQAELES	MVSPQQAEL	0	0	0	2	1	MVRSPQQAEL	tr_C9ZP82	0.036	33735.14	60.00
502	HLA-A0201	SSPANSKII	SSPANSKII	0	0	0	0	0	SSPANSKII	tr_C9ZP82	0.036	33740.62	60.00
94	HLA-A0201	RARRYMGRA	RARRYMGRA	0	0	0	0	0	RARRYMGRA	tr_C9ZP82	0.036	33747.92	60.00
145	HLA-A0201	EVRQTLLED	EVRQTLLED	0	0	0	0	0	EVRQTLLED	tr_C9ZP82	0.036	33761.80	60.00
189	HLA-A0201	DYESTHENRYV	YSTHENRYV	1	0	0	1	1	YESTHENRYV	tr_C9ZP82	0.036	33773.13	60.00
23	HLA-A0201	SSVHGLYID	SSVHGLYID	0	0	0	0	0	SSVHGLYID	tr_C9ZP82	0.036	33773.13	60.00
212	HLA-A0201	RGEVIVDDP	RGEVIVDDP	0	0	0	0	0	RGEVIVDDP	tr_C9ZP82	0.036	33775.31	60.00
272	HLA-A0201	PGEGEHKIIDFLRT	HKIIDFLRT	5	0	0	0	0	HKIIDFLRT	tr_C9ZP82	0.036	33793.24	60.00
827	HLA-A0201	RRVPSREGV	RRVPSREGV	0	0	0	0	0	RRVPSREGV	tr_C9ZP82	0.036	33793.96	60.00
50	HLA-A0201	LLPEEEKLE	LLPEEEKLE	0	0	0	0	0	LLPEEEKLE	tr_C9ZP82	0.036	33799.08	60.00
213	HLA-A0201	GEVIVDDPTAFE	GEVIVPTAF	0	0	0	5	2	GEVIVDDPTAF	tr_C9ZP82	0.036	33807.87	60.00
104	HLA-A0201	PAQTGEVASTYMPN	AQTGEVAYM	1	0	0	7	2	AQTGEVASTYM	tr_C9ZP82	0.036	33808.59	60.00
628	HLA-A0201	WRDTYLKST	WRDTYLKST	0	0	0	0	0	WRDTYLKST	tr_C9ZP82	0.036	33811.51	60.00
640	HLA-A0201	NSVQEACEKY	SVQEACEKY	1	0	0	0	0	SVQEACEKY	tr_C9ZP82	0.036	33813.34	60.00
358	HLA-A0201	ISLNPSYIDEE	SLNPYIDEE	1	0	0	4	1	SLNPSYIDEE	tr_C9ZP82	0.036	33827.62	60.00
88	HLA-A0201	AKMNQQRARRYMG	KMNQQRAYM	1	0	0	7	2	KMNQQRARRYM	tr_C9ZP82	0.036	33845.91	60.00
454	HLA-A0201	YYFFGQPNHIEDGT	YFFGIEDGT	1	0	0	4	4	YFFGQPNHIEDGT	tr_C9ZP82	0.036	33848.12	60.00
206	HLA-A0201	DDVFGHRGEV	DVFGHRGEV	0	0	0	1	1	DDVFGHRGEV	tr_C9ZP82	0.036	33852.88	60.00
175	HLA-A0201	GDGVNSFCVRS	GVNSFCVRS	0	0	0	1	2	GDGVNSFCVRS	tr_C9ZP82	0.036	33855.45	60.00
741	HLA-A0201	LESTFPLQWN	LESTFPLQW	0	0	0	0	0	LESTFPLQW	tr_C9ZP82	0.036	33855.80	60.00
500	HLA-A0201	PCSSPANSKIIDDI	CSSPIIDDI	1	0	0	4	4	CSSPANSKIIDDI	tr_C9ZP82	0.036	33857.64	60.00
735	HLA-A0201	SPQQAELESTF	SQAELESTF	0	0	0	1	2	SPQQAELESTF	tr_C9ZP82	0.036	33866.06	60.00
200	HLA-A0201	ESNQVVDDVFGH	SNQVVVFGH	1	0	0	5	2	SNQVVDDVFGH	tr_C9ZP82	0.036	33870.48	60.00
287	HLA-A0201	SSLAQFGGKAN	SLAQFGGKA	0	0	0	1	1	SSLAQFGGKA	tr_C9ZP82	0.036	33879.63	60.00
896	HLA-A0201	SNSRVTVVNK	SSRVTVVNK	0	0	0	1	1	SNSRVTVVNK	tr_C9ZP82	0.036	33881.46	60.00
175	HLA-A0201	GDGVNSFCVRSTD	GVNSFCVST	2	0	0	7	1	GVNSFCVRST	tr_C9ZP82	0.036	33887.70	60.00
394	HLA-A0201	PIESREAHVTTV	IESAHVTTV	1	0	0	3	2	IESREAHVTTV	tr_C9ZP82	0.036	33898.69	60.00
173	HLA-A0201	NDGDGVNSFCVRS	GVNSFCVRS	4	0	0	0	0	GVNSFCVRS	tr_C9ZP82	0.036	33900.52	60.00
249	HLA-A0201	NEKMSGNDPTW	KMSGNDPTW	2	0	0	0	0	KMSGNDPTW	tr_C9ZP82	0.036	33904.20	60.00
623	HLA-A0201	TIDDRWRDTY	TIDDRWDTY	0	0	0	6	1	TIDDRWRDTY	tr_C9ZP82	0.036	33905.66	60.00
89	HLA-A0201	KMNQQRARRY	KMQQRARRY	0	0	0	2	1	KMNQQRARRY	tr_C9ZP82	0.036	33910.43	60.00
779	HLA-A0201	DAAWPQLTEEERW	AAWPQLTEW	1	0	0	8	3	AAWPQLTEEERW	tr_C9ZP82	0.036	33911.89	60.00
106	HLA-A0201	QTGEVASTYMPN	QTGEVATYM	0	0	0	6	1	QTGEVASTYM	tr_C9ZP82	0.036	33914.48	60.00
174	HLA-A0201	DGDGVNSFCVRSTD	GVNSFCVST	3	0	0	7	1	GVNSFCVRST	tr_C9ZP82	0.036	33917.41	60.00
358	HLA-A0201	ISLNPSYIDEEESD	SLYIDEEES	1	0	0	2	3	SLNPSYIDEEES	tr_C9ZP82	0.036	33926.95	60.00
575	HLA-A0201	AKIEAVKFR	AKIEAVKFR	0	0	0	0	0	AKIEAVKFR	tr_C9ZP82	0.036	33928.06	60.00
785	HLA-A0201	LTEEERWRTQKL	LTEEERWRL	0	0	0	8	3	LTEEERWRTQKL	tr_C9ZP82	0.036	33929.16	60.00
321	HLA-A0201	ILRDTTRNPYKR	ILRDTTNPY	0	0	0	6	1	ILRDTTRNPY	tr_C9ZP82	0.036	33941.28	60.00
153	HLA-A0201	DPLYSGALY	DPLYSGALY	0	0	0	0	0	DPLYSGALY	tr_C9ZP82	0.036	33972.51	60.00
458	HLA-A0201	GQPNHIEDGTLPS	GQPNHILPS	0	0	0	6	4	GQPNHIEDGTLPS	tr_C9ZP82	0.036	33973.60	60.00

154	HLA-A0201	PLYSGALYNEGE	PLYSGALYE	0	0	0	8	3	PLYSGALYNEGE	tr_C9ZP82	0.036	33974.70	60.00
373	HLA-A0201	VIGVPVCESSENSS	VIGVVCESS	0	0	0	4	1	VIGVPVCESS	tr_C9ZP82	0.036	33984.27	60.00
178	HLA-A0201	VNSFCVRSTDDDY	VNSFCVRSY	0	0	0	8	4	VNSFCVRSTDDDY	tr_C9ZP82	0.036	33986.09	60.00
207	HLA-A0201	DVFGHRGEVIVDDP	DVFGGEVIV	0	0	0	4	2	DVFGHRGEVIV	tr_C9ZP82	0.036	33994.55	60.00
73	HLA-A0201	PKEVMYIAVDG	KVMYIAVDG	1	0	0	1	1	KEVMYIAVDG	tr_C9ZP82	0.036	33997.52	60.00
319	HLA-A0201	VFILRDTTR	VFILRDTTR	0	0	0	0	0	VFILRDTTR	tr_C9ZP82	0.036	33997.87	60.00
574	HLA-A0201	YAKIEAVKFRR	YIEAVKFRR	0	0	0	1	2	YAKIEAVKFRR	tr_C9ZP82	0.036	34015.89	60.00
13	HLA-A0201	YPAIVTKNCPSS	YPAIVCPSS	0	0	0	5	3	YPAIVTKNCPSS	tr_C9ZP82	0.036	34022.52	60.00
94	HLA-A0201	RARRYMGRAAPAQ	RAMGRAAPA	0	0	0	2	3	RARRYMGRAAPA	tr_C9ZP82	0.036	34024.36	60.00
80	HLA-A0201	AVDGVAPRAKMNQ	AVDGVAAKM	0	0	0	6	2	AVDGVAPRAKM	tr_C9ZP82	0.036	34024.36	60.00
615	HLA-A0201	DVAVALHSTIDDR	ALHSTIDDR	4	0	0	0	0	ALHSTIDDR	tr_C9ZP82	0.036	34028.42	60.00
102	HLA-A0201	AAPAQTGEVASTY	AQGEVASTY	3	0	0	2	1	AQTGEVASTY	tr_C9ZP82	0.036	34032.46	60.00
454	HLA-A0201	YYFFGQPNHIEDG	YFFGQPNHI	0	0	0	1	1	YYFFGQPNHI	tr_C9ZP82	0.036	34035.42	60.00
45	HLA-A0201	DTAIALLPEEEK	DTAIALLPK	0	0	0	8	3	DTAIALLPEEEK	tr_C9ZP82	0.036	34037.99	60.00
802	HLA-A0201	ITSNKTSFVPKGD	ITSNKTSFV	0	0	0	0	0	ITSNKTSFV	tr_C9ZP82	0.036	34039.46	60.00
347	HLA-A0201	RKRNFIDDTCI	RKRNFITCI	0	0	0	6	2	RKRNFIDDTCI	tr_C9ZP82	0.036	34040.56	60.00
2	HLA-A0201	VPKFASWLRNKY	VPKFASWLY	0	0	0	8	3	VPKFASWLRNKY	tr_C9ZP82	0.036	34041.30	60.00
103	HLA-A0201	APAQTGEVASTY	ATGEVASTY	0	0	0	1	3	APAQTGEVASTY	tr_C9ZP82	0.036	34043.15	60.00
801	HLA-A0201	VITSNKTSF	VITSNKTSF	0	0	0	0	0	VITSNKTSF	tr_C9ZP82	0.035	34059.73	60.00
466	HLA-A0201	GTLPSSTECG	GLPSSTECG	0	0	0	1	1	GTLPSSTECG	tr_C9ZP82	0.035	34074.47	60.00
834	HLA-A0201	GVEIRQLYD	GVEIRQLYD	0	0	0	0	0	GVEIRQLYD	tr_C9ZP82	0.035	34078.89	60.00
155	HLA-A0201	LYSGALYNE	LYSGALYNE	0	0	0	0	0	LYSGALYNE	tr_C9ZP82	0.035	34081.11	60.00
188	HLA-A0201	DDYESTHENRYV	YSTHENRYV	2	0	0	1	1	YESTHENRYV	tr_C9ZP82	0.035	34086.65	60.00
374	HLA-A0201	IGVPVCESSE	IGVPVCESS	0	0	0	0	0	IGVPVCESS	tr_C9ZP82	0.035	34093.29	60.00
154	HLA-A0201	PLYSGALYNEGEEE	PLYSGALYE	0	0	0	8	5	PLYSGALYNEGEEE	tr_C9ZP82	0.035	34093.64	60.00
199	HLA-A0201	VESNQVVDDVFG	VSNQVVDDV	0	0	0	1	1	VESNQVVDDV	tr_C9ZP82	0.035	34094.76	60.00
841	HLA-A0201	YDVPLERYRPRT	YLERYRPRT	0	0	0	1	3	YDVPLERYRPRT	tr_C9ZP82	0.035	34095.11	60.00
369	HLA-A0201	ESDGVIGVP	ESDGVIGVP	0	0	0	0	0	ESDGVIGVP	tr_C9ZP82	0.035	34095.49	60.00
291	HLA-A0201	QFGGKANHVI	FGGKANHVI	1	0	0	0	0	FGGKANHVI	tr_C9ZP82	0.035	34096.96	60.00
174	HLA-A0201	DGDGVNSFCVRS	GVNSFCVRS	3	0	0	0	0	GVNSFCVRS	tr_C9ZP82	0.035	34105.07	60.00
555	HLA-A0201	LTKGPHEINLPQ	LTHEINLPQ	0	0	0	2	3	LTKGPHEINLPQ	tr_C9ZP82	0.035	34110.24	60.00
734	HLA-A0201	RSPQQAELESTF	RQAELESTF	0	0	0	1	3	RSPQQAELESTF	tr_C9ZP82	0.035	34110.62	60.00
455	HLA-A0201	YFFGQPNHIEDG	YFFGQPNHI	0	0	0	0	0	YFFGQPNHI	tr_C9ZP82	0.035	34114.69	60.00
458	HLA-A0201	GQPNHIEDGTLPSS	GQDGTLPSS	0	0	0	2	5	GQPNHIEDGTLPSS	tr_C9ZP82	0.035	34123.54	60.00
806	HLA-A0201	KTSFVPKGDLRK	KTSFVPLRK	0	0	0	6	3	KTSFVPKGDLRK	tr_C9ZP82	0.035	34137.58	60.00
11	HLA-A0201	NKYPAIVTKNC	NKYPAIVTC	0	0	0	8	2	NKYPAIVTKNC	tr_C9ZP82	0.035	34139.05	60.00
273	HLA-A0201	GEGEHKIIDF	GEGEHIIDF	0	0	0	5	1	GEGEHKIIDF	tr_C9ZP82	0.035	34157.90	60.00
385	HLA-A0201	SSSDTDNESPI	SSSDTDNPI	0	0	0	7	2	SSSDTDNESPI	tr_C9ZP82	0.035	34158.25	60.00
643	HLA-A0201	QEACEKYVEGVC	EACEYVEGV	1	0	0	4	1	EACEKYVEGV	tr_C9ZP82	0.035	34169.36	60.00
700	HLA-A0201	VVKSTPPDTLVQ	VVTPPDTLV	0	0	0	2	2	VVKSTPPDTLV	tr_C9ZP82	0.035	34170.46	60.00
840	HLA-A0201	LYDVPLERYRPRT	YLERYRPRT	1	0	0	1	3	YDVPLERYRPRT	tr_C9ZP82	0.035	34174.54	60.00
520	HLA-A0201	LGNDFLPHAPS	LGNDFLPHA	0	0	0	0	0	LGNDFLPHA	tr_C9ZP82	0.035	34175.27	60.00
181	HLA-A0201	FCVRSTDDDYESTH	FTDDDYEST	0	0	0	1	4	FCVRSTDDDYEST	tr_C9ZP82	0.035	34183.40	60.00
604	HLA-A0201	KGSNNDNVTVADVA	KGSVTVADV	0	0	0	3	4	KGSNNDNVTVADV	tr_C9ZP82	0.035	34184.52	60.00
104	HLA-A0201	PAQTGEVASTY	AQGEVASTY	1	0	0	2	1	AQTGEVASTY	tr_C9ZP82	0.035	34191.54	60.00
429	HLA-A0201	LCDIKKFTPATES	LCDIKFTPA	0	0	0	4	1	LCDIKKFTPA	tr_C9ZP82	0.035	34194.86	60.00
879	HLA-A0201	HSGQLKGNGEGVIE	SLKGNGEGV	1	0	0	1	2	SGQLKGNGEGV	tr_C9ZP82	0.035	34195.61	60.00
44	HLA-A0201	SDTAIALLPEE	STAIALLPE	0	0	0	1	1	SDTAIALLPE	tr_C9ZP82	0.035	34197.47	60.00
346	HLA-A0201	RRKRNFIDDTCI	RNFIDDTCI	0	0	0	1	3	RRKRNFIDDTCI	tr_C9ZP82	0.035	34197.84	60.00
175	HLA-A0201	GDGVNSFCVR	GDGVNSFCV	0	0	0	0	0	GDGVNSFCV	tr_C9ZP82	0.035	34200.42	60.00
393	HLA-A0201	SPIESREAHVTTVP	SPIESVTTV	0	0	0	5	4	SPIESREAHVTTV	tr_C9ZP82	0.035	34205.61	60.00
881	HLA-A0201	GQLKGNGEGVIE	GLKGNGEGV	0	0	0	1	1	GQLKGNGEGV	tr_C9ZP82	0.035	34208.56	60.00
68	HLA-A0201	VQTVQPKEVMY	VQTVQPVMY	0	0	0	6	2	VQTVQPKEVMY	tr_C9ZP82	0.035	34209.66	60.00
807	HLA-A0201	TSFVPKGDL	TSFVPKGDL	0	0	0	0	0	TSFVPKGDL	tr_C9ZP82	0.035	34214.85	60.00

43	HLA-A0201	DSDTAIALLPEEE	DSDTAIALL	0	0	0	0	0	DSDTAIALL	tr_C9ZP82	0.035	34215.23	60.00
43	HLA-A0201	DSDTAIALLPEE	STAIALLPE	1	0	0	1	1	SDTAIALLPE	tr_C9ZP82	0.035	34216.33	60.00
345	HLA-A0201	SRRKRNFIDDTCI	SNFIDDTCI	0	0	0	1	4	SRRKRNFIDDTCI	tr_C9ZP82	0.035	34217.83	60.00
573	HLA-A0201	AYAKIEAVKFR	YAIEAVKFR	1	0	0	2	1	YAKIEAVKFR	tr_C9ZP82	0.035	34237.80	60.00
154	HLA-A0201	PLYSGALYNEGEE	PLYSGALYE	0	0	0	8	4	PLYSGALYNEGEE	tr_C9ZP82	0.035	34246.71	60.00
179	HLA-A0201	NSFCVRSTDDDYES	NSFCVDYES	0	0	0	5	5	NSFCVRSTDDDYES	tr_C9ZP82	0.035	34248.94	60.00
449	HLA-A0201	VSSAGYYFFGQPNH	VSSAGYYFF	0	0	0	0	0	VSSAGYYFF	tr_C9ZP82	0.035	34269.68	60.00
2	HLA-A0201	VPKFASWLRN	VPFASWLRN	0	0	0	2	1	VPKFASWLRN	tr_C9ZP82	0.035	34286.00	60.00
187	HLA-A0201	DDDYESTHENRYV	YSTHENRYV	3	0	0	1	1	YESTHENRYV	tr_C9ZP82	0.035	34288.23	60.00
44	HLA-A0201	SDTAIALLPEEE	STAIALLPE	0	0	0	1	1	SDTAIALLPE	tr_C9ZP82	0.035	34296.39	60.00
41	HLA-A0201	HNDSDTAIA	HNDSDTAIA	0	0	0	0	0	HNDSDTAIA	tr_C9ZP82	0.035	34302.34	60.00
192	HLA-A0201	STHENRYVES	STHERYVES	0	0	0	4	1	STHENRYVES	tr_C9ZP82	0.035	34303.07	60.00
12	HLA-A0201	KYPAIVTKNC	KYPAIVTNC	0	0	0	7	1	KYPAIVTKNC	tr_C9ZP82	0.035	34308.65	60.00
222	HLA-A0201	AFEFDSNCISPGTD	FEFDCISPG	1	0	0	4	2	FEFDSNCISPG	tr_C9ZP82	0.035	34311.61	60.00
233	HLA-A0201	GTDFMAKVSDA	GTDFMVSDA	0	0	0	5	2	GTDFMAKVSDA	tr_C9ZP82	0.035	34311.61	60.00
863	HLA-A0201	SYGADGQVI	SYGADGQVI	0	0	0	0	0	SYGADGQVI	tr_C9ZP82	0.035	34314.95	60.00
172	HLA-A0201	LNDGDGVNSFCVR	LNDGNSFCV	0	0	0	4	3	LNDGDGVNSFCV	tr_C9ZP82	0.035	34319.78	60.00
778	HLA-A0201	VDAAWPQLTEEERW	AAWPQLTEW	2	0	0	8	3	AAWPQLTEEERW	tr_C9ZP82	0.035	34323.50	60.00
286	HLA-A0201	QSSLAQFGGKAN	SLAQFGGKA	2	0	0	0	0	SLAQFGGKA	tr_C9ZP82	0.035	34334.28	60.00
167	HLA-A0201	EAGDALNDGDGV	AALNDGDGV	1	0	0	1	2	AGDALNDGDGV	tr_C9ZP82	0.035	34340.21	60.00
437	HLA-A0201	PATESAFDKKTL	ATESAFDTL	1	0	0	7	2	ATESAFDKKTL	tr_C9ZP82	0.035	34341.70	60.00
865	HLA-A0201	GADGQVIKR	GADGQVIKR	0	0	0	0	0	GADGQVIKR	tr_C9ZP82	0.035	34348.76	60.00
454	HLA-A0201	YYFFGQPNHIED	YYFFGPNHI	0	0	0	5	1	YYFFGQPNHI	tr_C9ZP82	0.035	34355.45	60.00
146	HLA-A0201	VRQTLLEDP	VRQTLLEDP	0	0	0	0	0	VRQTLLEDP	tr_C9ZP82	0.035	34377.02	60.00
87	HLA-A0201	RAKMNQQRARRYMG	KMNQQRAYM	2	0	0	7	2	KMNQQRARRYM	tr_C9ZP82	0.035	34387.79	60.00
442	HLA-A0201	AFDKKTLVS	AFDKKTLVS	0	0	0	0	0	AFDKKTLVS	tr_C9ZP82	0.035	34394.14	60.00
584	HLA-A0201	RFLLFKSTN	RFLLFKSTN	0	0	0	0	0	RFLLFKSTN	tr_C9ZP82	0.035	34399.71	60.00
210	HLA-A0201	GHRGEVIVDDPT	HRGEVIVPT	1	0	0	7	2	HRGEVIVDDPT	tr_C9ZP82	0.035	34407.16	60.00
320	HLA-A0201	FILRDTTRNPYKR	ILRDTTNPY	1	0	0	6	1	ILRDTTRNPY	tr_C9ZP82	0.034	34423.54	60.00
890	HLA-A0201	VIEGKKSNSRV	VIEGSNSRV	0	0	0	4	2	VIEGKKSNSRV	tr_C9ZP82	0.034	34438.82	60.00
688	HLA-A0201	LKEANIKRMPLP	LKEANIMPL	0	0	0	6	2	LKEANIKRMPL	tr_C9ZP82	0.034	34439.93	60.00
728	HLA-A0201	LGEVMVRSP	LGEVMVRSP	0	0	0	0	0	LGEVMVRSP	tr_C9ZP82	0.034	34445.52	60.00
864	HLA-A0201	YGADGQVIKR	YADGQVIKR	0	0	0	1	1	YGADGQVIKR	tr_C9ZP82	0.034	34446.64	60.00
200	HLA-A0201	ESNQVVDDVFG	ESNQVVVFG	0	0	0	6	2	ESNQVVDDVFG	tr_C9ZP82	0.034	34447.01	60.00
177	HLA-A0201	GVNSFCVRSTDD	GVNSFCVST	0	0	0	7	1	GVNSFCVRST	tr_C9ZP82	0.034	34448.50	60.00
130	HLA-A0201	EFTSTEMAQAN	FTSTEMAQA	1	0	0	0	0	FTSTEMAQA	tr_C9ZP82	0.034	34453.72	60.00
55	HLA-A0201	EKLECICSE	EKLECICSE	0	0	0	0	0	EKLECICSE	tr_C9ZP82	0.034	34469.39	60.00
461	HLA-A0201	NHIEDGTLPSST	HIEDGTLPT	1	0	0	8	2	HIEDGTLPSST	tr_C9ZP82	0.034	34476.84	60.00
456	HLA-A0201	FFGQPNHIEDGTLP	GQPNHIETL	2	0	0	7	2	GQPNHIEDGTL	tr_C9ZP82	0.034	34481.69	60.00
698	HLA-A0201	LPVVKSTPPDTL	VVKSTPPTL	2	0	0	7	1	VVKSTPPDTL	tr_C9ZP82	0.034	34482.82	60.00
423	HLA-A0201	ISEVSALCD	ISEVSALCD	0	0	0	0	0	ISEVSALCD	tr_C9ZP82	0.034	34484.69	60.00
164	HLA-A0201	GEEEAGDAL	GEEEAGDAL	0	0	0	0	0	GEEEAGDAL	tr_C9ZP82	0.034	34486.91	60.00
461	HLA-A0201	NHIEDGTLPSS	HIDGTLPSS	1	0	0	2	1	HIEDGTLPSS	tr_C9ZP82	0.034	34490.65	60.00
211	HLA-A0201	HRGEVIVDD	HRGEVIVDD	0	0	0	0	0	HRGEVIVDD	tr_C9ZP82	0.034	34496.99	60.00
458	HLA-A0201	GQPNHIEDGTLP	GQPNHIETL	0	0	0	7	2	GQPNHIEDGTL	tr_C9ZP82	0.034	34502.60	60.00
882	HLA-A0201	QLKGNGEGVIE	QLKGNGEGV	0	0	0	0	0	QLKGNGEGV	tr_C9ZP82	0.034	34504.84	60.00
823	HLA-A0201	DTILRRVPS	DTILRRVPS	0	0	0	0	0	DTILRRVPS	tr_C9ZP82	0.034	34508.56	60.00
450	HLA-A0201	SSAGYYFFGQPNH	SSAGYYFFH	0	0	0	8	4	SSAGYYFFGQPNH	tr_C9ZP82	0.034	34530.60	60.00
153	HLA-A0201	DPLYSGALYNEGE	DLYSGALYN	0	0	0	1	1	DPLYSGALYN	tr_C9ZP82	0.034	34537.70	60.00
696	HLA-A0201	MPLPVVKSTPP	MPLPVVTPP	0	0	0	6	2	MPLPVVKSTPP	tr_C9ZP82	0.034	34538.44	60.00
318	HLA-A0201	QVFILRDTTRNP	FLRDTTRNP	2	0	0	1	1	FILRDTTRNP	tr_C9ZP82	0.034	34546.66	60.00
358	HLA-A0201	ISLNPSYIDEEE	SLNPSYIEE	1	0	0	7	2	SLNPSYIDEEE	tr_C9ZP82	0.034	34559.00	60.00
862	HLA-A0201	LSYGADGQVIKR	LSYGAVIKR	0	0	0	5	3	LSYGADGQVIKR	tr_C9ZP82	0.034	34563.86	60.00
455	HLA-A0201	YFFGQPNHIED	YFFGQPNHI	0	0	0	0	0	YFFGQPNHI	tr_C9ZP82	0.034	34566.85	60.00

857	HLA-A0201	VEVPRLSYG	VEVPRLSYG	0	0	0	0	0	VEVPRLSYG	tr_C9ZP82	0.034	34568.72	60.00
155	HLA-A0201	LYSGALYNEG	YSGALYNEG	1	0	0	0	0	YSGALYNEG	tr_C9ZP82	0.034	34569.86	60.00
852	HLA-A0201	TYSCTVEVP	TYSCTVEVP	0	0	0	0	0	TYSCTVEVP	tr_C9ZP82	0.034	34570.97	60.00
206	HLA-A0201	DDVFGHRGEVIVDD	DVFEVIVDD	1	0	0	3	4	DVFGHRGEVIVDD	tr_C9ZP82	0.034	34575.84	65.00
171	HLA-A0201	ALNDGDGVN	ALNDGDGVN	0	0	0	0	0	ALNDGDGVN	tr_C9ZP82	0.034	34576.59	65.00
830	HLA-A0201	PSREGVEIRQLY	SREGVEILY	1	0	0	7	2	SREGVEIRQLY	tr_C9ZP82	0.034	34584.06	65.00
124	HLA-A0201	VEEVVREFTS	VEEVVEFTS	0	0	0	5	1	VEEVVREFTS	tr_C9ZP82	0.034	34587.81	65.00
829	HLA-A0201	VPSREGVEI	VPSREGVEI	0	0	0	0	0	VPSREGVEI	tr_C9ZP82	0.034	34613.27	65.00
688	HLA-A0201	LKEANIKRM	LKEANIKRM	0	0	0	0	0	LKEANIKRM	tr_C9ZP82	0.034	34615.88	65.00
233	HLA-A0201	GTDFMAKVS	GTDFMAKVS	0	0	0	0	0	GTDFMAKVS	tr_C9ZP82	0.034	34616.26	65.00
69	HLA-A0201	QTVQPKEVM	QTVQPKEVM	0	0	0	0	0	QTVQPKEVM	tr_C9ZP82	0.034	34619.25	65.00
607	HLA-A0201	NNDNVTVADVA	NNDNVTVAV	0	0	0	8	1	NNDNVTVADV	tr_C9ZP82	0.034	34619.63	65.00
241	HLA-A0201	SDAVLNMLNEK	SDAVLMLNE	0	0	0	5	1	SDAVLNMLNE	tr_C9ZP82	0.034	34627.88	65.00
733	HLA-A0201	VRSPQQAELESTF	VQAELESTF	0	0	0	1	4	VRSPQQAELESTF	tr_C9ZP82	0.034	34628.25	65.00
10	HLA-A0201	RNKYPAIVTKNC	NKYPAIVTC	1	0	0	8	2	NKYPAIVTKNC	tr_C9ZP82	0.034	34644.00	65.00
89	HLA-A0201	KMNQQRARR	KMNQQRARR	0	0	0	0	0	KMNQQRARR	tr_C9ZP82	0.034	34648.49	65.00
172	HLA-A0201	LNDGDGVNSF	LNDGGVNSF	0	0	0	4	1	LNDGDGVNSF	tr_C9ZP82	0.034	34671.00	65.00
106	HLA-A0201	QTGEVASTYMPNK	QTGEVATYM	0	0	0	6	1	QTGEVASTYM	tr_C9ZP82	0.034	34674.00	65.00
617	HLA-A0201	AVALHSTIDDR	ALHSTIDDR	0	0	0	1	2	AVALHSTIDDR	tr_C9ZP82	0.034	34678.49	65.00
899	HLA-A0201	RVTVVNKGPC	RVTVVNGPC	0	0	0	6	1	RVTVVNKGPC	tr_C9ZP82	0.034	34686.75	65.00
224	HLA-A0201	EFDSNCISPG	FDSNCISPG	1	0	0	0	0	FDSNCISPG	tr_C9ZP82	0.034	34686.75	65.00
804	HLA-A0201	SNKTSFVPKGDLRK	KTSFVDLRK	2	0	0	5	3	KTSFVPKGDLRK	tr_C9ZP82	0.034	34704.76	65.00
558	HLA-A0201	GPHEINLPQLQ	GPHEINLPL	0	0	0	8	1	GPHEINLPQL	tr_C9ZP82	0.034	34706.28	65.00
573	HLA-A0201	AYAKIEAVKFRR	YIEAVKFRR	1	0	0	1	2	YAKIEAVKFRR	tr_C9ZP82	0.034	34711.16	65.00
457	HLA-A0201	FGQPNHIEDGTLP	GQPNHIETL	1	0	0	7	2	GQPNHIEDGTL	tr_C9ZP82	0.034	34711.52	65.00
290	HLA-A0201	AQFGGKANH	AQFGGKANH	0	0	0	0	0	AQFGGKANH	tr_C9ZP82	0.034	34728.80	65.00
827	HLA-A0201	RRVPSREGVEI	RVPSREVEI	1	0	0	6	1	RVPSREGVEI	tr_C9ZP82	0.034	34733.33	65.00
399	HLA-A0201	EAHVTTVPNT	EAHVTTVPT	0	0	0	8	1	EAHVTTVPNT	tr_C9ZP82	0.034	34744.21	65.00
616	HLA-A0201	VAVALHSTIDDR	ALHSTIDDR	3	0	0	0	0	ALHSTIDDR	tr_C9ZP82	0.034	34765.64	65.00
18	HLA-A0201	TKNCPSSVHGL	KCPSSVHGL	1	0	0	1	1	KNCPSSVHGL	tr_C9ZP82	0.034	34765.64	65.00
69	HLA-A0201	QTVQPKEVMY	QTVQPEVMY	0	0	0	5	1	QTVQPKEVMY	tr_C9ZP82	0.034	34770.53	65.00
713	HLA-A0201	LSILPPTSH	LSILPPTSH	0	0	0	0	0	LSILPPTSH	tr_C9ZP82	0.034	34772.06	65.00
286	HLA-A0201	QSSLAQFGG	QSSLAQFGG	0	0	0	0	0	QSSLAQFGG	tr_C9ZP82	0.034	34772.41	65.00
826	HLA-A0201	LRRVPSREGV	LRRVPSEGV	0	0	0	6	1	LRRVPSREGV	tr_C9ZP82	0.034	34776.95	65.00
453	HLA-A0201	GYYFFGQPNH	GYYFFGQPH	0	0	0	8	1	GYYFFGQPNH	tr_C9ZP82	0.034	34780.71	65.00
406	HLA-A0201	PNTGFEYFDIS	NTFEYFDIS	1	0	0	2	1	NTGFEYFDIS	tr_C9ZP82	0.034	34783.34	65.00
207	HLA-A0201	DVFGHRGEVIVD	DVFGGEVIV	0	0	0	4	2	DVFGHRGEVIV	tr_C9ZP82	0.034	34786.34	65.00
829	HLA-A0201	VPSREGVEIRQLY	SREGVEILY	2	0	0	7	2	SREGVEIRQLY	tr_C9ZP82	0.034	34788.99	65.00
704	HLA-A0201	TPPDTLVQLLS	TPPDTLVLL	0	0	0	7	1	TPPDTLVQLL	tr_C9ZP82	0.034	34792.74	65.00
129	HLA-A0201	REFTSTEMAQAN	FTSTEMAQA	2	0	0	0	0	FTSTEMAQA	tr_C9ZP82	0.034	34794.27	65.00
319	HLA-A0201	VFILRDTTRNPYKR	ILRDTTNPY	2	0	0	6	1	ILRDTTRNPY	tr_C9ZP82	0.033	34807.82	65.00
431	HLA-A0201	DIKKFTPAT	DIKKFTPAT	0	0	0	0	0	DIKKFTPAT	tr_C9ZP82	0.033	34811.19	65.00
642	HLA-A0201	VQEACEKYVEG	VQEACEKYV	0	0	0	0	0	VQEACEKYV	tr_C9ZP82	0.033	34814.96	65.00
203	HLA-A0201	QVVDDVFGHRG	VVDDVFHRG	1	0	0	6	1	VVDDVFGHRG	tr_C9ZP82	0.033	34819.11	65.00
674	HLA-A0201	PFYHAPPVI	PFYHAPPVI	0	0	0	0	0	PFYHAPPVI	tr_C9ZP82	0.033	34828.52	65.00
451	HLA-A0201	SAGYYFFGQPNH	SAGYYFFGH	0	0	0	8	3	SAGYYFFGQPNH	tr_C9ZP82	0.033	34837.20	65.00
152	HLA-A0201	EDPLYSGALYNEGE	PLYSGALYE	2	0	0	8	3	PLYSGALYNEGE	tr_C9ZP82	0.033	34837.58	65.00
805	HLA-A0201	NKTSFVPKGDLRK	KTSFVPLRK	1	0	0	6	3	KTSFVPKGDLRK	tr_C9ZP82	0.033	34838.70	65.00
606	HLA-A0201	SNNDNVTVADVA	NNDNVTVAV	1	0	0	8	1	NNDNVTVADV	tr_C9ZP82	0.033	34841.35	65.00
9	HLA-A0201	LRNKYPAIVTKNC	NKYPAIVTC	2	0	0	8	2	NKYPAIVTKNC	tr_C9ZP82	0.033	34842.86	65.00
734	HLA-A0201	RSPQQAELEST	RQQAELEST	0	0	0	1	2	RSPQQAELEST	tr_C9ZP82	0.033	34844.36	65.00
504	HLA-A0201	PANSKIIDDI	PANSKIIDI	0	0	0	7	1	PANSKIIDDI	tr_C9ZP82	0.033	34850.39	65.00
1	HLA-A0201	GVPKFASWLRNK	GVPKFASWL	0	0	0	0	0	GVPKFASWL	tr_C9ZP82	0.033	34876.80	65.00
522	HLA-A0201	NDFLPHAPS	NDFLPHAPS	0	0	0	0	0	NDFLPHAPS	tr_C9ZP82	0.033	34891.90	65.00

177	HLA-A0201	GVNSFCVRSTDDD	GVNSFCVST	0	0	0	7	1	GVNSFCVRST	tr_C9ZP82	0.033	34895.68	65.00
675	HLA-A0201	FYHAPPVID	FYHAPPVID	0	0	0	0	0	FYHAPPVID	tr_C9ZP82	0.033	34898.31	65.00
488	HLA-A0201	KAEAERSWKNFRPC	KAWKNFRPC	0	0	0	2	5	KAEAERSWKNFRPC	tr_C9ZP82	0.033	34900.20	65.00
321	HLA-A0201	ILRDTTRNPYKRR	ILRDTTNPY	0	0	0	6	1	ILRDTTRNPY	tr_C9ZP82	0.033	34904.34	65.00
166	HLA-A0201	EEAGDALNDGDGV	AALNDGDGV	2	0	0	1	2	AGDALNDGDGV	tr_C9ZP82	0.033	34916.82	65.00
153	HLA-A0201	DPLYSGALYNEGEE	PLYSGALYE	1	0	0	8	4	PLYSGALYNEGEE	tr_C9ZP82	0.033	34917.95	65.00
9	HLA-A0201	LRNKYPAIVTK	LKYPAIVTK	0	0	0	1	2	LRNKYPAIVTK	tr_C9ZP82	0.033	34918.33	65.00
110	HLA-A0201	VASTYMPNK	VASTYMPNK	0	0	0	0	0	VASTYMPNK	tr_C9ZP82	0.033	34943.65	65.00
888	HLA-A0201	EGVIEGKKSNSRVT	GVIEGNSRV	1	0	0	5	3	GVIEGKKSNSRV	tr_C9ZP82	0.033	34950.84	65.00
559	HLA-A0201	PHEINLPQL	PHEINLPQL	0	0	0	0	0	PHEINLPQL	tr_C9ZP82	0.033	34961.05	65.00
638	HLA-A0201	LSNSVQEACEKY	LVQEACEKY	0	0	0	1	3	LSNSVQEACEKY	tr_C9ZP82	0.033	34970.14	65.00
432	HLA-A0201	IKKFTPATESAF	FTPATESAF	3	0	0	0	0	FTPATESAF	tr_C9ZP82	0.033	34983.38	65.00
431	HLA-A0201	DIKKFTPATESAF	FTPATESAF	4	0	0	0	0	FTPATESAF	tr_C9ZP82	0.033	34984.12	65.00
780	HLA-A0201	AAWPQLTEEERWR	AAWPQLTWR	0	0	0	7	4	AAWPQLTEEERWR	tr_C9ZP82	0.033	34987.91	65.00
779	HLA-A0201	DAAWPQLTEE	AAWPQLTEE	1	0	0	0	0	AAWPQLTEE	tr_C9ZP82	0.033	34990.94	65.00
436	HLA-A0201	TPATESAFDKKTL	ATESAFDTL	2	0	0	7	2	ATESAFDKKTL	tr_C9ZP82	0.033	34995.50	65.00
12	HLA-A0201	KYPAIVTKNCPSS	YPAIVCPSS	1	0	0	5	3	YPAIVTKNCPSS	tr_C9ZP82	0.033	34998.14	65.00
283	HLA-A0201	LRTQSSLAQFG	LTQSSLAQF	0	0	0	1	1	LRTQSSLAQF	tr_C9ZP82	0.033	35003.82	65.00
503	HLA-A0201	SPANSKIIDDII	SSKIIDDII	0	0	0	1	3	SPANSKIIDDII	tr_C9ZP82	0.033	35004.95	65.00
630	HLA-A0201	DTYLKSTGLS	DTYLKSTGL	0	0	0	0	0	DTYLKSTGL	tr_C9ZP82	0.033	35005.72	65.00
202	HLA-A0201	NQVVDDVFGHRGE	NQVVVFGHR	0	0	0	4	2	NQVVDDVFGHR	tr_C9ZP82	0.033	35019.36	65.00
703	HLA-A0201	STPPDTLVQ	STPPDTLVQ	0	0	0	0	0	STPPDTLVQ	tr_C9ZP82	0.033	35022.00	65.00
32	HLA-A0201	LNGIIHPCC	LNGIIHPCC	0	0	0	0	0	LNGIIHPCC	tr_C9ZP82	0.033	35023.52	65.00
11	HLA-A0201	NKYPAIVTKNCPSS	YPAIVCPSS	2	0	0	5	3	YPAIVTKNCPSS	tr_C9ZP82	0.033	35028.08	65.00
826	HLA-A0201	LRRVPSREGVEI	RVPSREVEI	2	0	0	6	1	RVPSREGVEI	tr_C9ZP82	0.033	35032.23	65.00
155	HLA-A0201	LYSGALYNEGEEE	YLYNEGEEE	1	0	0	1	3	YSGALYNEGEEE	tr_C9ZP82	0.033	35059.15	65.00
491	HLA-A0201	AERSWKNFRPC	ASWKNFRPC	0	0	0	1	2	AERSWKNFRPC	tr_C9ZP82	0.033	35059.54	65.00
165	HLA-A0201	EEEAGDALNDGDGV	AALNDGDGV	3	0	0	1	2	AGDALNDGDGV	tr_C9ZP82	0.033	35060.67	65.00
399	HLA-A0201	EAHVTTVPNTGFE	EAHVTTVPF	0	0	0	8	3	EAHVTTVPNTGF	tr_C9ZP82	0.033	35071.68	65.00
231	HLA-A0201	SPGTDFMAK	SPGTDFMAK	0	0	0	0	0	SPGTDFMAK	tr_C9ZP82	0.033	35072.45	65.00
662	HLA-A0201	TTTTDSDWSW	TTTDSDWSW	0	0	0	1	1	TTTTDSDWSW	tr_C9ZP82	0.033	35073.96	65.00
728	HLA-A0201	LGEVMVRSPQ	LGVMVRSPQ	0	0	0	2	1	LGEVMVRSPQ	tr_C9ZP82	0.033	35086.11	65.00
128	HLA-A0201	VREFTSTEMAQAN	FTSTEMAQA	3	0	0	0	0	FTSTEMAQA	tr_C9ZP82	0.033	35087.25	65.00
35	HLA-A0201	IIHPCCHNDSD	IIHPCCHNS	0	0	0	8	1	IIHPCCHNDS	tr_C9ZP82	0.033	35091.05	65.00
182	HLA-A0201	CVRSTDDDY	CVRSTDDDY	0	0	0	0	0	CVRSTDDDY	tr_C9ZP82	0.033	35108.50	65.00
444	HLA-A0201	DKKTLVSSAGY	KTLVSSAGY	2	0	0	0	0	KTLVSSAGY	tr_C9ZP82	0.033	35118.39	65.00
174	HLA-A0201	DGDGVNSFCVR	DGDGVSFCV	0	0	0	5	1	DGDGVNSFCV	tr_C9ZP82	0.033	35127.12	65.00
124	HLA-A0201	VEEVVREFTST	VEEVVFTST	0	0	0	5	2	VEEVVREFTST	tr_C9ZP82	0.033	35127.89	65.00
862	HLA-A0201	LSYGADGQVIKRQ	LSYGADGQV	0	0	0	0	0	LSYGADGQV	tr_C9ZP82	0.033	35128.27	65.00
830	HLA-A0201	PSREGVEIRQL	SREGVEIQL	1	0	0	7	1	SREGVEIRQL	tr_C9ZP82	0.033	35137.02	65.00
895	HLA-A0201	KSNSRVTVVNK	KSNSRVTVV	0	0	0	0	0	KSNSRVTVV	tr_C9ZP82	0.033	35138.15	65.00
201	HLA-A0201	SNQVVDDVFGHRGE	NQVVVFGHR	1	0	0	4	2	NQVVDDVFGHR	tr_C9ZP82	0.033	35139.28	65.00
385	HLA-A0201	SSSDTDNES	SSSDTDNES	0	0	0	0	0	SSSDTDNES	tr_C9ZP82	0.033	35149.93	65.00
103	HLA-A0201	APAQTGEVA	APAQTGEVA	0	0	0	0	0	APAQTGEVA	tr_C9ZP82	0.033	35157.91	65.00
699	HLA-A0201	PVVKSTPPDTL	VVKSTPPTL	1	0	0	7	1	VVKSTPPDTL	tr_C9ZP82	0.033	35158.69	65.00
627	HLA-A0201	RWRDTYLKST	RWRDTYLST	0	0	0	7	1	RWRDTYLKST	tr_C9ZP82	0.033	35161.34	65.00
733	HLA-A0201	VRSPQQAELEST	VQQAELEST	0	0	0	1	3	VRSPQQAELEST	tr_C9ZP82	0.033	35161.73	65.00
179	HLA-A0201	NSFCVRSTDDDY	NSFCVRSTY	0	0	0	8	3	NSFCVRSTDDDY	tr_C9ZP82	0.033	35162.86	65.00
176	HLA-A0201	DGVNSFCVRSTDD	GVNSFCVST	1	0	0	7	1	GVNSFCVRST	tr_C9ZP82	0.033	35172.38	65.00
106	HLA-A0201	QTGEVASTYMPNKS	QTGEVATYM	0	0	0	6	1	QTGEVASTYM	tr_C9ZP82	0.033	35175.04	65.00
80	HLA-A0201	AVDGVAPRAKMNQQ	AVDGVAAKM	0	0	0	6	2	AVDGVAPRAKM	tr_C9ZP82	0.032	35176.56	65.00
615	HLA-A0201	DVAVALHSTID	DVAVALHTI	0	0	0	7	1	DVAVALHSTI	tr_C9ZP82	0.032	35180.75	65.00
131	HLA-A0201	FTSTEMAQAND	FTSTEMAQA	0	0	0	0	0	FTSTEMAQA	tr_C9ZP82	0.032	35187.61	65.00
224	HLA-A0201	EFDSNCISP	EFDSNCISP	0	0	0	0	0	EFDSNCISP	tr_C9ZP82	0.032	35191.42	65.00

88	HLA-A0201	AKMNQQRARRYMGR	KMNQQRAYM	1	0	0	7	2	KMNQQRARRYM	tr_C9ZP82	0.032	35198.66	65.00
194	HLA-A0201	HENRYVESNQVV	RYVESNQVV	3	0	0	0	0	RYVESNQVV	tr_C9ZP82	0.032	35218.46	65.00
270	HLA-A0201	NTPGEGEHKIIDF	NTPGEIIDF	0	0	0	5	4	NTPGEGEHKIIDF	tr_C9ZP82	0.032	35225.71	65.00
784	HLA-A0201	QLTEEERWRTQ	QLTEEEWRT	0	0	0	6	1	QLTEEERWRT	tr_C9ZP82	0.032	35227.98	65.00
829	HLA-A0201	VPSREGVEIRQL	SREGVEIQL	2	0	0	7	1	SREGVEIRQL	tr_C9ZP82	0.032	35229.50	65.00
231	HLA-A0201	SPGTDFMAKVS	SPGTDFMAV	0	0	0	8	1	SPGTDFMAKV	tr_C9ZP82	0.032	35230.27	65.00
73	HLA-A0201	PKEVMYIAVD	PKEVMYIAV	0	0	0	0	0	PKEVMYIAV	tr_C9ZP82	0.032	35237.91	65.00
368	HLA-A0201	EESDGVIGVP	EESDGVIGV	0	0	0	0	0	EESDGVIGV	tr_C9ZP82	0.032	35248.57	65.00
181	HLA-A0201	FCVRSTDDDY	FVRSTDDDY	0	0	0	1	1	FCVRSTDDDY	tr_C9ZP82	0.032	35251.64	65.00
812	HLA-A0201	KGDLRKLSDA	KGDLRLSDA	0	0	0	5	1	KGDLRKLSDA	tr_C9ZP82	0.032	35256.21	65.00
633	HLA-A0201	LKSTGLSNSVQ	LSTGLSNSV	0	0	0	1	1	LKSTGLSNSV	tr_C9ZP82	0.032	35270.33	65.00
93	HLA-A0201	QRARRYMGRAAPAQ	RAMGRAAPA	1	0	0	2	3	RARRYMGRAAPA	tr_C9ZP82	0.032	35270.70	65.00
800	HLA-A0201	LVITSNKTS	LVITSNKTS	0	0	0	0	0	LVITSNKTS	tr_C9ZP82	0.032	35272.61	65.00
405	HLA-A0201	VPNTGFEYFD	VPNTGFEYF	0	0	0	0	0	VPNTGFEYF	tr_C9ZP82	0.032	35287.87	65.00
489	HLA-A0201	AEAERSWKNFRPC	ASWKNFRPC	0	0	0	1	4	AEAERSWKNFRPC	tr_C9ZP82	0.032	35290.95	65.00
424	HLA-A0201	SEVSALCDIKK	EVSALCIKK	1	0	0	6	1	EVSALCDIKK	tr_C9ZP82	0.032	35292.08	65.00
231	HLA-A0201	SPGTDFMAKVSDA	SFMAKVSDA	0	0	0	1	4	SPGTDFMAKVSDA	tr_C9ZP82	0.032	35294.38	65.00
885	HLA-A0201	GNGEGVIEG	GNGEGVIEG	0	0	0	0	0	GNGEGVIEG	tr_C9ZP82	0.032	35296.66	65.00
705	HLA-A0201	PPDTLVQLLS	PPDLVQLLS	0	0	0	3	1	PPDTLVQLLS	tr_C9ZP82	0.032	35305.83	65.00
440	HLA-A0201	ESAFDKKTLVS	SAFDKKTLV	1	0	0	0	0	SAFDKKTLV	tr_C9ZP82	0.032	35310.80	65.00
115	HLA-A0201	MPNKSGMAIVEE	MSGMAIVEE	0	0	0	1	3	MPNKSGMAIVEE	tr_C9ZP82	0.032	35316.90	65.00
564	HLA-A0201	LPQLQRLFE	LPQLQRLFE	0	0	0	0	0	LPQLQRLFE	tr_C9ZP82	0.032	35316.90	65.00
831	HLA-A0201	SREGVEIRQLYD	SVEIRQLYD	0	0	0	1	3	SREGVEIRQLYD	tr_C9ZP82	0.032	35318.06	65.00
99	HLA-A0201	MGRAAPAQT	MGRAAPAQT	0	0	0	0	0	MGRAAPAQT	tr_C9ZP82	0.032	35336.40	65.00
462	HLA-A0201	HIEDGTLPSSTEC	HIEDGTLPC	0	0	0	8	4	HIEDGTLPSSTEC	tr_C9ZP82	0.032	35337.54	65.00
193	HLA-A0201	THENRYVESNQVV	RYVESNQVV	4	0	0	0	0	RYVESNQVV	tr_C9ZP82	0.032	35344.06	65.00
320	HLA-A0201	FILRDTTRNPYKRR	ILRDTTNPY	1	0	0	6	1	ILRDTTRNPY	tr_C9ZP82	0.032	35358.97	65.00
191	HLA-A0201	ESTHENRYV	ESTHENRYV	0	0	0	0	0	ESTHENRYV	tr_C9ZP82	0.032	35360.50	65.00
557	HLA-A0201	KGPHEINLPQLQ	GPHEINLPL	1	0	0	8	1	GPHEINLPQL	tr_C9ZP82	0.032	35366.24	65.00
45	HLA-A0201	DTAIALLPEEE	DTAIALLPE	0	0	0	8	2	DTAIALLPEEE	tr_C9ZP82	0.032	35377.33	65.00
347	HLA-A0201	RKRNFIDDT	RKRNFIDDT	0	0	0	0	0	RKRNFIDDT	tr_C9ZP82	0.032	35382.69	65.00
173	HLA-A0201	NDGDGVNSFCVR	DGDGVSFCV	1	0	0	5	1	DGDGVNSFCV	tr_C9ZP82	0.032	35386.14	65.00
501	HLA-A0201	CSSPANSKII	CSSPANSII	0	0	0	7	1	CSSPANSKII	tr_C9ZP82	0.032	35403.37	65.00
292	HLA-A0201	FGGKANHVIVG	FGGANHVIV	0	0	0	3	1	FGGKANHVIV	tr_C9ZP82	0.032	35409.12	65.00
138	HLA-A0201	QANDDLDEVRQ	QANDDLDEV	0	0	0	0	0	QANDDLDEV	tr_C9ZP82	0.032	35413.33	65.00
387	HLA-A0201	SDTDNESPI	SDTDNESPI	0	0	0	0	0	SDTDNESPI	tr_C9ZP82	0.032	35414.49	65.00
175	HLA-A0201	GDGVNSFCVRSTDD	GVNSFCVST	2	0	0	7	1	GVNSFCVRST	tr_C9ZP82	0.032	35422.92	65.00
398	HLA-A0201	REAHVTTVPNTGFE	EAHVTTVPF	1	0	0	8	3	EAHVTTVPNTGF	tr_C9ZP82	0.032	35429.43	65.00
806	HLA-A0201	KTSFVPKGDLR	KTSFVPKGL	0	0	0	8	1	KTSFVPKGDL	tr_C9ZP82	0.032	35439.01	65.00
550	HLA-A0201	LPYGFLTKGPHE	LPYGFLTHE	0	0	0	7	3	LPYGFLTKGPHE	tr_C9ZP82	0.032	35439.79	65.00
13	HLA-A0201	YPAIVTKNCP	YPAIVTNCP	0	0	0	6	1	YPAIVTKNCP	tr_C9ZP82	0.032	35445.54	65.00
698	HLA-A0201	LPVVKSTPPDTLVQ	VVKSTPPLV	2	0	0	7	2	VVKSTPPDTLV	tr_C9ZP82	0.032	35448.22	65.00
235	HLA-A0201	DFMAKVSDA	DFMAKVSDA	0	0	0	0	0	DFMAKVSDA	tr_C9ZP82	0.032	35449.00	65.00
3	HLA-A0201	PKFASWLRNKY	FASWLRNKY	2	0	0	0	0	FASWLRNKY	tr_C9ZP82	0.032	35460.11	65.00
781	HLA-A0201	AWPQLTEEERWRTQ	QLEERWRTQ	3	0	0	2	2	QLTEEERWRTQ	tr_C9ZP82	0.032	35468.94	65.00
52	HLA-A0201	PEEEKLECI	PEEEKLECI	0	0	0	0	0	PEEEKLECI	tr_C9ZP82	0.032	35474.70	65.00
598	HLA-A0201	SGAEFRKGSNNDNV	SGAEFNDNV	0	0	0	5	5	SGAEFRKGSNNDNV	tr_C9ZP82	0.032	35501.18	65.00
284	HLA-A0201	RTQSSLAQFGG	RTQSSLFGG	0	0	0	6	2	RTQSSLAQFGG	tr_C9ZP82	0.032	35505.03	65.00
433	HLA-A0201	KKFTPATESAF	FTPATESAF	2	0	0	0	0	FTPATESAF	tr_C9ZP82	0.032	35508.10	65.00
832	HLA-A0201	REGVEIRQLYD	REGVEIQLY	0	0	0	6	1	REGVEIRQLY	tr_C9ZP82	0.032	35509.27	65.00
460	HLA-A0201	PNHIEDGTLPSST	HIEDGTLPT	2	0	0	8	2	HIEDGTLPSST	tr_C9ZP82	0.032	35511.55	65.00
810	HLA-A0201	VPKGDLRKL	VPKGDLRKL	0	0	0	0	0	VPKGDLRKL	tr_C9ZP82	0.032	35528.48	65.00
849	HLA-A0201	RPRTYSCTVE	RPRTYSCTV	0	0	0	0	0	RPRTYSCTV	tr_C9ZP82	0.032	35530.39	65.00
227	HLA-A0201	SNCISPGTDF	SNCISPGTF	0	0	0	8	1	SNCISPGTDF	tr_C9ZP82	0.032	35533.84	65.00

490	HLA-A0201	EAERSWKNFRPC	RSWKNFRPC	3	0	0	0	0	RSWKNFRPC	tr_C9ZP82	0.032	35534.23	65.00
374	HLA-A0201	IGVPVCESSENS	IGVPVCESS	0	0	0	8	3	IGVPVCESSENS	tr_C9ZP82	0.032	35537.30	65.00
779	HLA-A0201	DAAWPQLTEEERWR	AAWPQLTWR	1	0	0	7	4	AAWPQLTEEERWR	tr_C9ZP82	0.032	35540.77	65.00
397	HLA-A0201	SREAHVTTVP	SREAHVTTV	0	0	0	0	0	SREAHVTTV	tr_C9ZP82	0.032	35540.77	65.00
897	HLA-A0201	NSRVTVVNKG	NSRVTVVNG	0	0	0	8	1	NSRVTVVNKG	tr_C9ZP82	0.032	35542.69	65.00
780	HLA-A0201	AAWPQLTEEER	AAWPQLEER	0	0	0	6	2	AAWPQLTEEER	tr_C9ZP82	0.032	35544.23	65.00
750	HLA-A0201	NVDYHDSNVVH	NVYHDSNVV	0	0	0	2	1	NVDYHDSNVV	tr_C9ZP82	0.032	35546.15	65.00
384	HLA-A0201	NSSSDTDNESPI	SSSDTDNPI	1	0	0	7	2	SSSDTDNESPI	tr_C9ZP82	0.031	35566.17	65.00
399	HLA-A0201	EAHVTTVPN	EAHVTTVPN	0	0	0	0	0	EAHVTTVPN	tr_C9ZP82	0.031	35566.54	65.00
557	HLA-A0201	KGPHEINLPQ	KGHEINLPQ	0	0	0	2	1	KGPHEINLPQ	tr_C9ZP82	0.031	35572.32	65.00
706	HLA-A0201	PDTLVQLLS	PDTLVQLLS	0	0	0	0	0	PDTLVQLLS	tr_C9ZP82	0.031	35573.10	65.00
495	HLA-A0201	WKNFRPCSSPA	KNFPCSSPA	1	0	0	3	1	KNFRPCSSPA	tr_C9ZP82	0.031	35578.86	65.00
525	HLA-A0201	LPHAPSAFS	LPHAPSAFS	0	0	0	0	0	LPHAPSAFS	tr_C9ZP82	0.031	35580.39	65.00
656	HLA-A0201	VWRYYTTTTDSDW	VWRYYTTTW	0	0	0	8	5	VWRYYTTTTDSDW	tr_C9ZP82	0.031	35581.18	65.00
19	HLA-A0201	KNCPSSVHGLY	KCPSSVHGL	0	0	0	1	1	KNCPSSVHGL	tr_C9ZP82	0.031	35589.64	65.00
663	HLA-A0201	TTTDSDWSWY	TTTDSDWWY	0	0	0	7	1	TTTDSDWSWY	tr_C9ZP82	0.031	35594.26	65.00
176	HLA-A0201	DGVNSFCVRSTDDD	GVNSFCVST	1	0	0	7	1	GVNSFCVRST	tr_C9ZP82	0.031	35595.04	65.00
697	HLA-A0201	PLPVVKSTP	PLPVVKSTP	0	0	0	0	0	PLPVVKSTP	tr_C9ZP82	0.031	35599.65	65.00
366	HLA-A0201	DEEESDGVIGV	DEEESVIGV	0	0	0	5	2	DEEESDGVIGV	tr_C9ZP82	0.031	35606.20	65.00
287	HLA-A0201	SSLAQFGGK	SSLAQFGGK	0	0	0	0	0	SSLAQFGGK	tr_C9ZP82	0.031	35618.92	65.00
894	HLA-A0201	KKSNSRVTVVNK	KSNSRVTVV	0	0	0	1	1	KKSNSRVTVV	tr_C9ZP82	0.031	35631.26	65.00
728	HLA-A0201	LGEVMVRSPQQAE	LMVRSPQQA	0	0	0	1	3	LGEVMVRSPQQA	tr_C9ZP82	0.031	35633.58	65.00
400	HLA-A0201	AHVTTVPNTGF	HVTTVPNTF	1	0	0	8	1	HVTTVPNTGF	tr_C9ZP82	0.031	35637.81	65.00
439	HLA-A0201	TESAFDKKTLVS	SAFDKKTLV	2	0	0	0	0	SAFDKKTLV	tr_C9ZP82	0.031	35643.60	65.00
558	HLA-A0201	GPHEINLPQLQR	GPHEINLPL	0	0	0	8	1	GPHEINLPQL	tr_C9ZP82	0.031	35651.30	65.00
816	HLA-A0201	RKLSDALDT	RKLSDALDT	0	0	0	0	0	RKLSDALDT	tr_C9ZP82	0.031	35653.23	65.00
489	HLA-A0201	AEAERSWKNF	AEAERSWNF	0	0	0	7	1	AEAERSWKNF	tr_C9ZP82	0.031	35659.03	65.00
459	HLA-A0201	QPNHIEDGTLPSST	HIEDGTLPT	3	0	0	8	2	HIEDGTLPSST	tr_C9ZP82	0.031	35664.43	65.00
460	HLA-A0201	PNHIEDGTLPSS	HIDGTLPSS	2	0	0	2	1	HIEDGTLPSS	tr_C9ZP82	0.031	35664.43	65.00
269	HLA-A0201	SNTPGEGEHKIIDF	NTPGEIIDF	1	0	0	5	4	NTPGEGEHKIIDF	tr_C9ZP82	0.031	35667.12	65.00
217	HLA-A0201	VDDPTAFEFD	VDDPTAFEF	0	0	0	0	0	VDDPTAFEF	tr_C9ZP82	0.031	35674.85	65.00
803	HLA-A0201	TSNKTSFVPKGDLR	KTSFVPKGL	3	0	0	8	1	KTSFVPKGDL	tr_C9ZP82	0.031	35677.18	65.00
129	HLA-A0201	REFTSTEMAQ	REFTSTEMA	0	0	0	0	0	REFTSTEMA	tr_C9ZP82	0.031	35685.28	65.00
556	HLA-A0201	TKGPHEINLPQLQ	GPHEINLPL	2	0	0	8	1	GPHEINLPQL	tr_C9ZP82	0.031	35686.43	65.00
9	HLA-A0201	LRNKYPAIVT	LRNKYPAIV	0	0	0	0	0	LRNKYPAIV	tr_C9ZP82	0.031	35690.29	65.00
782	HLA-A0201	WPQLTEEERWRTQ	QLTEEEWRT	2	0	0	6	1	QLTEEERWRT	tr_C9ZP82	0.031	35710.76	65.00
893	HLA-A0201	GKKSNSRVTV	KKSNSRVTV	1	0	0	0	0	KKSNSRVTV	tr_C9ZP82	0.031	35716.57	65.00
824	HLA-A0201	TILRRVPSRE	ILRRVPSRE	1	0	0	0	0	ILRRVPSRE	tr_C9ZP82	0.031	35716.57	65.00
784	HLA-A0201	QLTEEERWR	QLTEEERWR	0	0	0	0	0	QLTEEERWR	tr_C9ZP82	0.031	35719.65	65.00
885	HLA-A0201	GNGEGVIEGK	NGEGVIEGK	1	0	0	0	0	NGEGVIEGK	tr_C9ZP82	0.031	35720.80	65.00
561	HLA-A0201	EINLPQLQR	EINLPQLQR	0	0	0	0	0	EINLPQLQR	tr_C9ZP82	0.031	35726.61	65.00
898	HLA-A0201	SRVTVVNKG	SRVTVVNKG	0	0	0	0	0	SRVTVVNKG	tr_C9ZP82	0.031	35727.00	65.00
493	HLA-A0201	RSWKNFRPCSS	RSWFRPCSS	0	0	0	3	2	RSWKNFRPCSS	tr_C9ZP82	0.031	35740.14	65.00
211	HLA-A0201	HRGEVIVDDP	HRGEVIVDP	0	0	0	7	1	HRGEVIVDDP	tr_C9ZP82	0.031	35742.86	65.00
348	HLA-A0201	KRNFIDDTC	KRNFIDDTC	0	0	0	0	0	KRNFIDDTC	tr_C9ZP82	0.031	35752.52	65.00
393	HLA-A0201	SPIESREAHVT	SIESREAHV	0	0	0	1	1	SPIESREAHV	tr_C9ZP82	0.031	35762.57	65.00
804	HLA-A0201	SNKTSFVPKGDLR	KTSFVPKGL	2	0	0	8	1	KTSFVPKGDL	tr_C9ZP82	0.031	35768.38	65.00
462	HLA-A0201	HIEDGTLPSSTECG	ILPSSTECG	1	0	0	1	4	IEDGTLPSSTECG	tr_C9ZP82	0.031	35769.93	65.00
504	HLA-A0201	PANSKIIDDII	PANSKIIII	0	0	0	7	2	PANSKIIDDII	tr_C9ZP82	0.031	35778.44	65.00
604	HLA-A0201	KGSNNDNVTVA	KSNNDNVTV	0	0	0	1	1	KGSNNDNVTV	tr_C9ZP82	0.031	35780.38	65.00
206	HLA-A0201	DDVFGHRGEVIVD	DVFGGEVIV	1	0	0	4	2	DVFGHRGEVIV	tr_C9ZP82	0.031	35784.26	65.00
152	HLA-A0201	EDPLYSGAL	EDPLYSGAL	0	0	0	0	0	EDPLYSGAL	tr_C9ZP82	0.031	35799.75	65.00
115	HLA-A0201	MPNKSGMAIVE	MPNSGMAIV	0	0	0	3	1	MPNKSGMAIV	tr_C9ZP82	0.031	35806.32	65.00
152	HLA-A0201	EDPLYSGALY	EDPLYGALY	0	0	0	5	1	EDPLYSGALY	tr_C9ZP82	0.031	35812.54	65.00

831	HLA-A0201	SREGVEIRQ	SREGVEIRQ	0	0	0	0	0	SREGVEIRQ	tr_C9ZP82	0.031	35815.24	65.00
459	HLA-A0201	QPNHIEDGTLPSS	HIDGTLPSS	3	0	0	2	1	HIEDGTLPSS	tr_C9ZP82	0.031	35828.80	65.00
209	HLA-A0201	FGHRGEVIVDD	FGHEVIVDD	0	0	0	3	2	FGHRGEVIVDD	tr_C9ZP82	0.031	35832.68	65.00
46	HLA-A0201	TAIALLPEEEK	TAIALLEEK	0	0	0	6	2	TAIALLPEEEK	tr_C9ZP82	0.031	35843.55	65.00
458	HLA-A0201	GQPNHIEDG	GQPNHIEDG	0	0	0	0	0	GQPNHIEDG	tr_C9ZP82	0.031	35843.91	65.00
178	HLA-A0201	VNSFCVRSTD	VSFCVRSTD	0	0	0	1	1	VNSFCVRSTD	tr_C9ZP82	0.031	35844.70	65.00
790	HLA-A0201	RWRTQKLDF	RWRTQKLDF	0	0	0	0	0	RWRTQKLDF	tr_C9ZP82	0.031	35859.45	65.00
228	HLA-A0201	NCISPGTDF	NCISPGTDF	0	0	0	0	0	NCISPGTDF	tr_C9ZP82	0.031	35871.48	65.00
96	HLA-A0201	RRYMGRAAPAQ	YMGRAAPAQ	2	0	0	0	0	YMGRAAPAQ	tr_C9ZP82	0.031	35881.18	65.00
133	HLA-A0201	STEMAQANDDLD	SMAQANDDL	0	0	0	1	2	STEMAQANDDL	tr_C9ZP82	0.031	35883.51	65.00
829	HLA-A0201	VPSREGVEIRQLYD	REGVEIQLY	3	0	0	6	1	REGVEIRQLY	tr_C9ZP82	0.031	35899.81	65.00
822	HLA-A0201	LDTILRRVPSREG	LDTILVPSR	0	0	0	5	2	LDTILRRVPSR	tr_C9ZP82	0.031	35910.30	65.00
432	HLA-A0201	IKKFTPATESAFDK	FTPATAFDK	3	0	0	5	2	FTPATESAFDK	tr_C9ZP82	0.031	35916.53	65.00
34	HLA-A0201	GIIHPCCHNDSD	IIHPCCHNS	1	0	0	8	1	IIHPCCHNDS	tr_C9ZP82	0.031	35922.36	65.00
262	HLA-A0201	LCVIFSGSNTPGE	LCVIFSTPG	0	0	0	6	3	LCVIFSGSNTPG	tr_C9ZP82	0.031	35925.07	65.00
463	HLA-A0201	IEDGTLPSSTECG	ILPSSTECG	0	0	0	1	4	IEDGTLPSSTECG	tr_C9ZP82	0.031	35925.47	65.00
836	HLA-A0201	EIRQLYDVP	EIRQLYDVP	0	0	0	0	0	EIRQLYDVP	tr_C9ZP82	0.030	35949.57	70.00
11	HLA-A0201	NKYPAIVTKNCP	NKYPAIVTC	0	0	0	8	2	NKYPAIVTKNC	tr_C9ZP82	0.030	35953.07	70.00
435	HLA-A0201	FTPATESAFDK	FTPATAFDK	0	0	0	5	2	FTPATESAFDK	tr_C9ZP82	0.030	35953.07	70.00
205	HLA-A0201	VDDVFGHRG	VDDVFGHRG	0	0	0	0	0	VDDVFGHRG	tr_C9ZP82	0.030	35962.79	70.00
845	HLA-A0201	LERYRPRTY	LERYRPRTY	0	0	0	0	0	LERYRPRTY	tr_C9ZP82	0.030	35963.58	70.00
660	HLA-A0201	YTTTTTDSDW	YTTTTTDSW	0	0	0	8	1	YTTTTTDSDW	tr_C9ZP82	0.030	35967.08	70.00
862	HLA-A0201	LSYGADGQVIKRQR	LSYGADGQV	0	0	0	0	0	LSYGADGQV	tr_C9ZP82	0.030	35979.93	70.00
699	HLA-A0201	PVVKSTPPDTLVQ	VVTPPDTLV	1	0	0	2	2	VVKSTPPDTLV	tr_C9ZP82	0.030	35990.82	70.00
497	HLA-A0201	NFRPCSSPANSKI	NFRPCSSPI	0	0	0	8	4	NFRPCSSPANSKI	tr_C9ZP82	0.030	36003.29	70.00
568	HLA-A0201	QRLFEAYAK	QRLFEAYAK	0	0	0	0	0	QRLFEAYAK	tr_C9ZP82	0.030	36003.29	70.00
374	HLA-A0201	IGVPVCESSENSS	IGVPVCESS	0	0	0	7	4	IGVPVCESSENSS	tr_C9ZP82	0.030	36011.46	70.00
126	HLA-A0201	EVVREFTST	EVVREFTST	0	0	0	0	0	EVVREFTST	tr_C9ZP82	0.030	36024.33	70.00
266	HLA-A0201	FSGSNTPGE	FSGSNTPGE	0	0	0	0	0	FSGSNTPGE	tr_C9ZP82	0.030	36045.78	70.00
609	HLA-A0201	DNVTVADVA	DNVTVADVA	0	0	0	0	0	DNVTVADVA	tr_C9ZP82	0.030	36046.17	70.00
291	HLA-A0201	QFGGKANHVIVG	FGGKAVIVG	1	0	0	5	2	FGGKANHVIVG	tr_C9ZP82	0.030	36050.05	70.00
182	HLA-A0201	CVRSTDDDYEST	CVDDDYEST	0	0	0	2	3	CVRSTDDDYEST	tr_C9ZP82	0.030	36058.26	70.00
830	HLA-A0201	PSREGVEIRQLYD	REGVEIQLY	2	0	0	6	1	REGVEIRQLY	tr_C9ZP82	0.030	36059.03	70.00
283	HLA-A0201	LRTQSSLAQFGG	LTQSSLAQF	0	0	0	1	1	LRTQSSLAQF	tr_C9ZP82	0.030	36059.43	70.00
494	HLA-A0201	SWKNFRPCSSPA	KNFPCSSPA	2	0	0	3	1	KNFRPCSSPA	tr_C9ZP82	0.030	36064.50	70.00
891	HLA-A0201	IEGKKSNSRVTVVN	ISNSRVTVV	0	0	0	1	4	IEGKKSNSRVTVV	tr_C9ZP82	0.030	36066.45	70.00
32	HLA-A0201	LNGIIHPCCHN	LNGIICCHN	0	0	0	5	2	LNGIIHPCCHN	tr_C9ZP82	0.030	36076.59	70.00
131	HLA-A0201	FTSTEMAQANDD	FTSTEMAQA	0	0	0	0	0	FTSTEMAQA	tr_C9ZP82	0.030	36077.78	70.00
18	HLA-A0201	TKNCPSSVHGLY	KCPSSVHGL	1	0	0	1	1	KNCPSSVHGL	tr_C9ZP82	0.030	36077.78	70.00
657	HLA-A0201	WRYYTTTTTDSDW	WRYYTTTTW	0	0	0	8	4	WRYYTTTTTDSDW	tr_C9ZP82	0.030	36080.11	70.00
319	HLA-A0201	VFILRDTTRNP	FLRDTTRNP	1	0	0	1	1	FILRDTTRNP	tr_C9ZP82	0.030	36089.88	70.00
434	HLA-A0201	KFTPATESAFDK	FTPATAFDK	1	0	0	5	2	FTPATESAFDK	tr_C9ZP82	0.030	36096.12	70.00
117	HLA-A0201	NKSGMAIVEE	KSGMAIVEE	1	0	0	0	0	KSGMAIVEE	tr_C9ZP82	0.030	36096.51	70.00
374	HLA-A0201	IGVPVCESSENSSS	IGVPVCESS	0	0	0	7	5	IGVPVCESSENSSS	tr_C9ZP82	0.030	36105.10	70.00
497	HLA-A0201	NFRPCSSPANSKII	NFRPCSSPI	0	0	0	8	5	NFRPCSSPANSKII	tr_C9ZP82	0.030	36105.50	70.00
557	HLA-A0201	KGPHEINLPQLQR	GPHEINLPL	1	0	0	8	1	GPHEINLPQL	tr_C9ZP82	0.030	36106.66	70.00
893	HLA-A0201	GKKSNSRVTVVNK	KSNSRVTVV	2	0	0	0	0	KSNSRVTVV	tr_C9ZP82	0.030	36110.18	70.00
178	HLA-A0201	VNSFCVRSTDDDYE	VNSFCVRSY	0	0	0	8	4	VNSFCVRSTDDDY	tr_C9ZP82	0.030	36120.34	70.00
209	HLA-A0201	FGHRGEVIVDDP	FGHRGEVIV	0	0	0	0	0	FGHRGEVIV	tr_C9ZP82	0.030	36130.12	70.00
552	HLA-A0201	YGFLTKGPHE	YGFLTGPHE	0	0	0	5	1	YGFLTKGPHE	tr_C9ZP82	0.030	36133.24	70.00
805	HLA-A0201	NKTSFVPKGDLR	KTSFVPKGL	1	0	0	8	1	KTSFVPKGDL	tr_C9ZP82	0.030	36134.41	70.00
461	HLA-A0201	NHIEDGTLPSSTEC	HIEDGTLPC	1	0	0	8	4	HIEDGTLPSSTEC	tr_C9ZP82	0.030	36135.20	70.00
232	HLA-A0201	PGTDFMAKVSDA	GTDFMVSDA	1	0	0	5	2	GTDFMAKVSDA	tr_C9ZP82	0.030	36136.76	70.00
848	HLA-A0201	YRPRTYSCTVE	YRPTYSCTV	0	0	0	3	1	YRPRTYSCTV	tr_C9ZP82	0.030	36138.32	70.00

101	HLA-A0201	RAAPAQTGEVAST	RQTGEVAST	0	0	0	1	4	RAAPAQTGEVAST	tr_C9ZP82	0.030	36142.63	70.00
138	HLA-A0201	QANDDLDEVRQT	QANDDLDEV	0	0	0	0	0	QANDDLDEV	tr_C9ZP82	0.030	36142.63	70.00
442	HLA-A0201	AFDKKTLVSSAG	AFDTLVSSA	0	0	0	3	2	AFDKKTLVSSA	tr_C9ZP82	0.030	36144.57	70.00
803	HLA-A0201	TSNKTSFVPK	TSNKTFVPK	0	0	0	5	1	TSNKTSFVPK	tr_C9ZP82	0.030	36144.57	70.00
383	HLA-A0201	ENSSSDTDNESPI	SSSDTDNPI	2	0	0	7	2	SSSDTDNESPI	tr_C9ZP82	0.030	36151.61	70.00
195	HLA-A0201	ENRYVESNQV	NRYVESNQV	1	0	0	0	0	NRYVESNQV	tr_C9ZP82	0.030	36153.20	70.00
803	HLA-A0201	TSNKTSFVP	TSNKTSFVP	0	0	0	0	0	TSNKTSFVP	tr_C9ZP82	0.030	36154.36	70.00
860	HLA-A0201	PRLSYGADGQ	RLSYGADGQ	1	0	0	0	0	RLSYGADGQ	tr_C9ZP82	0.030	36157.09	70.00
433	HLA-A0201	KKFTPATESAFDK	FTPATAFDK	2	0	0	5	2	FTPATESAFDK	tr_C9ZP82	0.030	36160.61	70.00
898	HLA-A0201	SRVTVVNKGPC	SRVTVVNPC	0	0	0	7	2	SRVTVVNKGPC	tr_C9ZP82	0.030	36171.97	70.00
156	HLA-A0201	YSGALYNEGE	YSGALYEGE	0	0	0	6	1	YSGALYNEGE	tr_C9ZP82	0.030	36173.14	70.00
51	HLA-A0201	LPEEEKLECICSE	LKLECICSE	0	0	0	1	4	LPEEEKLECICSE	tr_C9ZP82	0.030	36173.93	70.00
253	HLA-A0201	SGNDPTWTR	SGNDPTWTR	0	0	0	0	0	SGNDPTWTR	tr_C9ZP82	0.030	36175.89	70.00
208	HLA-A0201	VFGHRGEVIVDDP	FGHVIVDDP	1	0	0	3	3	FGHRGEVIVDDP	tr_C9ZP82	0.030	36177.45	70.00
841	HLA-A0201	YDVPLERYRPRTY	YLERYRPRT	0	0	0	1	3	YDVPLERYRPRT	tr_C9ZP82	0.030	36186.46	70.00
10	HLA-A0201	RNKYPAIVTKNCP	NKYPAIVTC	1	0	0	8	2	NKYPAIVTKNC	tr_C9ZP82	0.030	36188.02	70.00
347	HLA-A0201	RKRNFIDDTC	RKRNFIDTC	0	0	0	6	1	RKRNFIDDTC	tr_C9ZP82	0.030	36195.47	70.00
262	HLA-A0201	LCVIFSGSNTPGEG	LCVIFSTPG	0	0	0	6	3	LCVIFSGSNTPG	tr_C9ZP82	0.030	36202.52	70.00
33	HLA-A0201	NGIIHPCCHNDSD	IIHPCCHNS	2	0	0	8	1	IIHPCCHNDS	tr_C9ZP82	0.030	36207.21	70.00
804	HLA-A0201	SNKTSFVPK	SNKTSFVPK	0	0	0	0	0	SNKTSFVPK	tr_C9ZP82	0.030	36215.83	70.00
208	HLA-A0201	VFGHRGEVIVDD	FGHEVIVDD	1	0	0	3	2	FGHRGEVIVDD	tr_C9ZP82	0.030	36222.09	70.00
496	HLA-A0201	KNFRPCSSPANSKI	FSSPANSKI	2	0	0	1	3	FRPCSSPANSKI	tr_C9ZP82	0.030	36231.11	70.00
892	HLA-A0201	EGKKSNSRVTVVNK	KSNSRVTVV	3	0	0	0	0	KSNSRVTVV	tr_C9ZP82	0.030	36242.09	70.00
828	HLA-A0201	RVPSREGVEIRQ	RVEGVEIRQ	0	0	0	2	3	RVPSREGVEIRQ	tr_C9ZP82	0.030	36248.75	70.00
431	HLA-A0201	DIKKFTPATESAFD	FTPATSAFD	4	0	0	5	1	FTPATESAFD	tr_C9ZP82	0.030	36256.61	70.00
173	HLA-A0201	NDGDGVNSF	NDGDGVNSF	0	0	0	0	0	NDGDGVNSF	tr_C9ZP82	0.030	36259.74	70.00
785	HLA-A0201	LTEEERWRTQKLDF	LTEEERLDF	0	0	0	6	5	LTEEERWRTQKLDF	tr_C9ZP82	0.030	36261.31	70.00
143	HLA-A0201	LDEVRQTLLED	LVRQTLLED	0	0	0	1	2	LDEVRQTLLED	tr_C9ZP82	0.030	36267.60	70.00
101	HLA-A0201	RAAPAQTGEVA	RAAPAQTEV	0	0	0	7	1	RAAPAQTGEV	tr_C9ZP82	0.030	36269.16	70.00
9	HLA-A0201	LRNKYPAIVTKNCP	NKYPAIVTC	2	0	0	8	2	NKYPAIVTKNC	tr_C9ZP82	0.030	36281.33	70.00
194	HLA-A0201	HENRYVESNQV	HENRYVESV	0	0	0	8	2	HENRYVESNQV	tr_C9ZP82	0.030	36286.83	70.00
841	HLA-A0201	YDVPLERYR	YDVPLERYR	0	0	0	0	0	YDVPLERYR	tr_C9ZP82	0.030	36293.10	70.00
856	HLA-A0201	TVEVPRLSYGAD	TVEVLSYGA	0	0	0	4	2	TVEVPRLSYGA	tr_C9ZP82	0.030	36304.50	70.00
90	HLA-A0201	MNQQRARRY	MNQQRARRY	0	0	0	0	0	MNQQRARRY	tr_C9ZP82	0.030	36305.67	70.00
895	HLA-A0201	KSNSRVTVVNKG	KSNSRVTVV	0	0	0	0	0	KSNSRVTVV	tr_C9ZP82	0.030	36308.41	70.00
32	HLA-A0201	LNGIIHPCCHNDSD	IIHPCCHNS	3	0	0	8	1	IIHPCCHNDS	tr_C9ZP82	0.030	36319.02	70.00
254	HLA-A0201	GNDPTWTRLC	GNDPTWTRL	0	0	0	0	0	GNDPTWTRL	tr_C9ZP82	0.030	36322.56	70.00
779	HLA-A0201	DAAWPQLTEEER	AAWPQLEER	1	0	0	6	2	AAWPQLTEEER	tr_C9ZP82	0.030	36324.13	70.00
615	HLA-A0201	DVAVALHSTIDD	DVAVALHTI	0	0	0	7	1	DVAVALHSTI	tr_C9ZP82	0.030	36328.86	70.00
400	HLA-A0201	AHVTTVPNT	AHVTTVPNT	0	0	0	0	0	AHVTTVPNT	tr_C9ZP82	0.030	36333.97	70.00
840	HLA-A0201	LYDVPLERYRP	LYDVPLERY	0	0	0	0	0	LYDVPLERY	tr_C9ZP82	0.029	36348.12	70.00
862	HLA-A0201	LSYGADGQVIK	LSYGAGQVI	0	0	0	5	1	LSYGADGQVI	tr_C9ZP82	0.029	36351.27	70.00
382	HLA-A0201	SENSSSDTDNESPI	SSSDTDNPI	3	0	0	7	2	SSSDTDNESPI	tr_C9ZP82	0.029	36363.06	70.00
241	HLA-A0201	SDAVLNMLN	SDAVLNMLN	0	0	0	0	0	SDAVLNMLN	tr_C9ZP82	0.029	36383.94	70.00
696	HLA-A0201	MPLPVVKSTPPDT	MPLPVVPDT	0	0	0	6	4	MPLPVVKSTPPDT	tr_C9ZP82	0.029	36394.57	70.00
498	HLA-A0201	FRPCSSPANSKI	FSSPANSKI	0	0	0	1	3	FRPCSSPANSKI	tr_C9ZP82	0.029	36398.49	70.00
99	HLA-A0201	MGRAAPAQTGEVA	MGRAAPAEV	0	0	0	7	3	MGRAAPAQTGEV	tr_C9ZP82	0.029	36399.29	70.00
748	HLA-A0201	QWNVDYHDSNVVH	NVYHDSNVV	2	0	0	2	1	NVDYHDSNVV	tr_C9ZP82	0.029	36400.06	70.00
620	HLA-A0201	LHSTIDDRW	LHSTIDDRW	0	0	0	0	0	LHSTIDDRW	tr_C9ZP82	0.029	36401.26	70.00
390	HLA-A0201	DNESPIESREAHVT	SIESREAHV	3	0	0	1	1	SPIESREAHV	tr_C9ZP82	0.029	36402.83	70.00
94	HLA-A0201	RARRYMGRAA	RARRYMRAA	0	0	0	6	1	RARRYMGRAA	tr_C9ZP82	0.029	36402.83	70.00
840	HLA-A0201	LYDVPLERYRPR	LYDVPLERY	0	0	0	0	0	LYDVPLERY	tr_C9ZP82	0.029	36405.57	70.00
808	HLA-A0201	SFVPKGDLRK	FVPKGDLRK	1	0	0	0	0	FVPKGDLRK	tr_C9ZP82	0.029	36406.38	70.00
782	HLA-A0201	WPQLTEEERW	WQLTEEERW	0	0	0	1	1	WPQLTEEERW	tr_C9ZP82	0.029	36406.38	70.00

639	HLA-A0201	SNSVQEACEKY	SVQEACEKY	2	0	0	0	0	SVQEACEKY	tr_C9ZP82	0.029	36412.67	70.00
556	HLA-A0201	TKGPHEINLPQLQR	GPHEINLPL	2	0	0	8	1	GPHEINLPQL	tr_C9ZP82	0.029	36421.33	70.00
395	HLA-A0201	IESREAHVTT	ISREAHVTT	0	0	0	1	1	IESREAHVTT	tr_C9ZP82	0.029	36431.98	70.00
95	HLA-A0201	ARRYMGRAAPAQ	YMGRAAPAQ	3	0	0	0	0	YMGRAAPAQ	tr_C9ZP82	0.029	36434.35	70.00
521	HLA-A0201	GNDFLPHAPS	GNDFLPAPS	0	0	0	6	1	GNDFLPHAPS	tr_C9ZP82	0.029	36434.75	70.00
523	HLA-A0201	DFLPHAPSA	DFLPHAPSA	0	0	0	0	0	DFLPHAPSA	tr_C9ZP82	0.029	36436.32	70.00
222	HLA-A0201	AFEFDSNCIS	FEFDSNCIS	1	0	0	0	0	FEFDSNCIS	tr_C9ZP82	0.029	36436.72	70.00
179	HLA-A0201	NSFCVRSTD	NSFCVRSTD	0	0	0	0	0	NSFCVRSTD	tr_C9ZP82	0.029	36447.77	70.00
432	HLA-A0201	IKKFTPATESAFD	FTPATSAFD	3	0	0	5	1	FTPATESAFD	tr_C9ZP82	0.029	36448.54	70.00
253	HLA-A0201	SGNDPTWTRLC	SGNDPTWTL	0	0	0	8	1	SGNDPTWTRL	tr_C9ZP82	0.029	36449.72	70.00
376	HLA-A0201	VPVCESSENS	VVCESSENS	0	0	0	1	1	VPVCESSENS	tr_C9ZP82	0.029	36451.32	70.00
284	HLA-A0201	RTQSSLAQFGGK	RTQSSLAQF	0	0	0	0	0	RTQSSLAQF	tr_C9ZP82	0.029	36468.27	70.00
231	HLA-A0201	SPGTDFMAKVSD	SPGTDFMAV	0	0	0	8	1	SPGTDFMAKV	tr_C9ZP82	0.029	36468.67	70.00
111	HLA-A0201	ASTYMPNKSGM	STYMPNSGM	1	0	0	6	1	STYMPNKSGM	tr_C9ZP82	0.029	36470.25	70.00
54	HLA-A0201	EEKLECICS	EEKLECICS	0	0	0	0	0	EEKLECICS	tr_C9ZP82	0.029	36474.57	70.00
263	HLA-A0201	CVIFSGSNTPG	VIFGSNTPG	1	0	0	3	1	VIFSGSNTPG	tr_C9ZP82	0.029	36506.96	70.00
828	HLA-A0201	RVPSREGVEIR	RVPSRGVEI	0	0	0	5	1	RVPSREGVEI	tr_C9ZP82	0.029	36508.93	70.00
521	HLA-A0201	GNDFLPHAP	GNDFLPHAP	0	0	0	0	0	GNDFLPHAP	tr_C9ZP82	0.029	36513.66	70.00
791	HLA-A0201	WRTQKLDFH	WRTQKLDFH	0	0	0	0	0	WRTQKLDFH	tr_C9ZP82	0.029	36536.21	70.00
847	HLA-A0201	RYRPRTYSC	RYRPRTYSC	0	0	0	0	0	RYRPRTYSC	tr_C9ZP82	0.029	36540.14	70.00
826	HLA-A0201	LRRVPSREGVEIRQ	RVEGVEIRQ	2	0	0	2	3	RVPSREGVEIRQ	tr_C9ZP82	0.029	36543.70	70.00
826	HLA-A0201	LRRVPSREGVEIR	LRRVPVEIR	0	0	0	5	4	LRRVPSREGVEIR	tr_C9ZP82	0.029	36548.86	70.00
391	HLA-A0201	NESPIESREAHVT	SIESREAHV	2	0	0	1	1	SPIESREAHV	tr_C9ZP82	0.029	36549.24	70.00
865	HLA-A0201	GADGQVIKRQ	GADGQVIRQ	0	0	0	7	1	GADGQVIKRQ	tr_C9ZP82	0.029	36550.82	70.00
226	HLA-A0201	DSNCISPGT	DSNCISPGT	0	0	0	0	0	DSNCISPGT	tr_C9ZP82	0.029	36564.28	70.00
11	HLA-A0201	NKYPAIVTKN	NKYPAIVTK	0	0	0	0	0	NKYPAIVTK	tr_C9ZP82	0.029	36565.86	70.00
137	HLA-A0201	AQANDDLDE	AQANDDLDE	0	0	0	0	0	AQANDDLDE	tr_C9ZP82	0.029	36565.86	70.00
622	HLA-A0201	STIDDRWRDTY	STIDDRWTY	0	0	0	7	2	STIDDRWRDTY	tr_C9ZP82	0.029	36568.62	70.00
33	HLA-A0201	NGIIHPCCHN	GIIHPCCHN	1	0	0	0	0	GIIHPCCHN	tr_C9ZP82	0.029	36586.05	70.00
847	HLA-A0201	RYRPRTYSCTVE	YRPTYSCTV	1	0	0	3	1	YRPRTYSCTV	tr_C9ZP82	0.029	36605.05	70.00
555	HLA-A0201	LTKGPHEINLP	LTGPHEINL	0	0	0	2	1	LTKGPHEINL	tr_C9ZP82	0.029	36607.04	70.00
627	HLA-A0201	RWRDTYLKS	RWRDTYLKS	0	0	0	0	0	RWRDTYLKS	tr_C9ZP82	0.029	36607.44	70.00
824	HLA-A0201	TILRRVPSREG	ILRRVPSEG	1	0	0	7	1	ILRRVPSREG	tr_C9ZP82	0.029	36608.21	70.00
856	HLA-A0201	TVEVPRLSYGADGQ	RLSYGADGQ	5	0	0	0	0	RLSYGADGQ	tr_C9ZP82	0.029	36625.27	70.00
822	HLA-A0201	LDTILRRVPSR	LTILRRVPS	0	0	0	1	1	LDTILRRVPS	tr_C9ZP82	0.029	36625.64	70.00
778	HLA-A0201	VDAAWPQLTEEER	AAWPQLEER	2	0	0	6	2	AAWPQLTEEER	tr_C9ZP82	0.029	36628.03	70.00
603	HLA-A0201	RKGSNNDNVTVA	KSNNDNVTV	1	0	0	1	1	KGSNNDNVTV	tr_C9ZP82	0.029	36629.61	70.00
527	HLA-A0201	HAPSAFSGESALDN	FSGESALDN	5	0	0	0	0	FSGESALDN	tr_C9ZP82	0.029	36644.29	70.00
696	HLA-A0201	MPLPVVKSTPPD	MLPVVKSTP	0	0	0	1	1	MPLPVVKSTP	tr_C9ZP82	0.029	36646.65	70.00
283	HLA-A0201	LRTQSSLAQFGGK	LTQSSLAQF	0	0	0	1	1	LRTQSSLAQF	tr_C9ZP82	0.029	36654.99	70.00
470	HLA-A0201	SSTECGSPK	SSTECGSPK	0	0	0	0	0	SSTECGSPK	tr_C9ZP82	0.029	36656.57	70.00
533	HLA-A0201	SGESALDNLLE	SGESALNLL	0	0	0	6	1	SGESALDNLL	tr_C9ZP82	0.029	36661.33	70.00
749	HLA-A0201	WNVDYHDSNVVH	NVYHDSNVV	1	0	0	2	1	NVDYHDSNVV	tr_C9ZP82	0.029	36677.60	70.00
318	HLA-A0201	QVFILRDTTRN	QVFILTTRN	0	0	0	5	2	QVFILRDTTRN	tr_C9ZP82	0.029	36679.18	70.00
735	HLA-A0201	SPQQAELESTFP	SQAELESTF	0	0	0	1	2	SPQQAELESTF	tr_C9ZP82	0.029	36698.23	70.00
344	HLA-A0201	RSRRKRNFI	RSRRKRNFI	0	0	0	0	0	RSRRKRNFI	tr_C9ZP82	0.029	36702.61	70.00
845	HLA-A0201	LERYRPRTYSCTVE	YRPTYSCTV	3	0	0	3	1	YRPRTYSCTV	tr_C9ZP82	0.029	36705.00	70.00
434	HLA-A0201	KFTPATESAFD	FTPATSAFD	1	0	0	5	1	FTPATESAFD	tr_C9ZP82	0.029	36716.91	70.00
108	HLA-A0201	GEVASTYMP	GEVASTYMP	0	0	0	0	0	GEVASTYMP	tr_C9ZP82	0.029	36727.64	70.00
143	HLA-A0201	LDEVRQTLLEDP	LDEVLLEDP	0	0	0	4	3	LDEVRQTLLEDP	tr_C9ZP82	0.028	36733.20	70.00
245	HLA-A0201	LNMLNEKMSGN	MLNEKMSGN	2	0	0	0	0	MLNEKMSGN	tr_C9ZP82	0.028	36743.93	70.00
729	HLA-A0201	GEVMVRSPQ	GEVMVRSPQ	0	0	0	0	0	GEVMVRSPQ	tr_C9ZP82	0.028	36745.12	70.00
130	HLA-A0201	EFTSTEMAQAND	FTSTEMAQA	1	0	0	0	0	FTSTEMAQA	tr_C9ZP82	0.028	36745.93	70.00
465	HLA-A0201	DGTLPSSTEC	GTLPSSTEC	1	0	0	0	0	GTLPSSTEC	tr_C9ZP82	0.028	36752.28	70.00

823	HLA-A0201	DTILRRVPSREG	ILRRVPSEG	2	0	0	7	1	ILRRVPSREG	tr_C9ZP82	0.028	36767.00	70.00
883	HLA-A0201	LKGNGEGVIEGK	LGEGVIEGK	0	0	0	1	3	LKGNGEGVIEGK	tr_C9ZP82	0.028	36770.58	70.00
734	HLA-A0201	RSPQQAELESTFP	PQQAELETF	2	0	0	7	1	PQQAELESTF	tr_C9ZP82	0.028	36772.57	70.00
491	HLA-A0201	AERSWKNFRPCSS	RSWFRPCSS	2	0	0	3	2	RSWKNFRPCSS	tr_C9ZP82	0.028	36790.48	70.00
433	HLA-A0201	KKFTPATESAFD	FTPATSAFD	2	0	0	5	1	FTPATESAFD	tr_C9ZP82	0.028	36794.06	70.00
103	HLA-A0201	APAQTGEVAST	AQTGEVAST	0	0	0	1	2	APAQTGEVAST	tr_C9ZP82	0.028	36795.25	70.00
193	HLA-A0201	THENRYVESNQV	HENRYVESV	1	0	0	8	2	HENRYVESNQV	tr_C9ZP82	0.028	36799.24	70.00
844	HLA-A0201	PLERYRPRTY	PLEYRPRTY	0	0	0	3	1	PLERYRPRTY	tr_C9ZP82	0.028	36802.01	70.00
530	HLA-A0201	SAFSGESALDN	SAFSGESAL	0	0	0	0	0	SAFSGESAL	tr_C9ZP82	0.028	36819.56	70.00
895	HLA-A0201	KSNSRVTVVNKGP	KSNSRVTVV	0	0	0	0	0	KSNSRVTVV	tr_C9ZP82	0.028	36822.34	70.00
894	HLA-A0201	KKSNSRVTVVNKG	KSNSRVTVV	0	0	0	1	1	KKSNSRVTVV	tr_C9ZP82	0.028	36828.32	70.00
503	HLA-A0201	SPANSKIIDDI	SPANIIDDI	0	0	0	4	2	SPANSKIIDDI	tr_C9ZP82	0.028	36829.51	70.00
452	HLA-A0201	AGYYFFGQPNH	GYYFFGQPH	1	0	0	8	1	GYYFFGQPNH	tr_C9ZP82	0.028	36829.51	70.00
827	HLA-A0201	RRVPSREGVEIR	RVPSRGVEI	1	0	0	5	1	RVPSREGVEI	tr_C9ZP82	0.028	36842.27	70.00
783	HLA-A0201	PQLTEEERWRTQ	QLTEEEWRT	1	0	0	6	1	QLTEEERWRT	tr_C9ZP82	0.028	36851.44	70.00
463	HLA-A0201	IEDGTLPSSTECGS	TLSSTECGS	4	0	0	2	1	TLPSSTECGS	tr_C9ZP82	0.028	36854.62	70.00
827	HLA-A0201	RRVPSREGVEIRQ	RVEGVEIRQ	1	0	0	2	3	RVPSREGVEIRQ	tr_C9ZP82	0.028	36856.62	70.00
633	HLA-A0201	LKSTGLSNSVQE	LSTGLSNSV	0	0	0	1	1	LKSTGLSNSV	tr_C9ZP82	0.028	36858.21	70.00
322	HLA-A0201	LRDTTRNPY	LRDTTRNPY	0	0	0	0	0	LRDTTRNPY	tr_C9ZP82	0.028	36863.39	70.00
51	HLA-A0201	LPEEEKLECICS	LPELECICS	0	0	0	3	3	LPEEEKLECICS	tr_C9ZP82	0.028	36870.57	70.00
886	HLA-A0201	NGEGVIEGKK	NGEGVIEGK	0	0	0	8	1	NGEGVIEGKK	tr_C9ZP82	0.028	36879.75	70.00
218	HLA-A0201	DDPTAFEFDSNC	TAFEFDSNC	3	0	0	0	0	TAFEFDSNC	tr_C9ZP82	0.028	36898.12	70.00
129	HLA-A0201	REFTSTEMAQAND	FTSTEMAQA	2	0	0	0	0	FTSTEMAQA	tr_C9ZP82	0.028	36899.71	70.00
657	HLA-A0201	WRYYTTTTTDSDWS	WRYYTTTTW	0	0	0	8	4	WRYYTTTTTDSDW	tr_C9ZP82	0.028	36918.88	70.00
857	HLA-A0201	VEVPRLSYGAD	VVPRLSYGA	0	0	0	1	1	VEVPRLSYGA	tr_C9ZP82	0.028	36925.66	70.00
183	HLA-A0201	VRSTDDDYEST	STDDDYEST	2	0	0	0	0	STDDDYEST	tr_C9ZP82	0.028	36925.66	70.00
172	HLA-A0201	LNDGDGVNSFC	LGDGVNSFC	0	0	0	1	2	LNDGDGVNSFC	tr_C9ZP82	0.028	36938.45	70.00
492	HLA-A0201	ERSWKNFRPCSS	RSWFRPCSS	1	0	0	3	2	RSWKNFRPCSS	tr_C9ZP82	0.028	36944.05	70.00
736	HLA-A0201	PQQAELESTFP	QQAELSTFP	1	0	0	5	1	QQAELESTFP	tr_C9ZP82	0.028	36950.43	70.00
135	HLA-A0201	EMAQANDDLD	EMAQANDDL	0	0	0	0	0	EMAQANDDL	tr_C9ZP82	0.028	36958.04	70.00
463	HLA-A0201	IEDGTLPSSTEC	ITLPSSTEC	0	0	0	1	3	IEDGTLPSSTEC	tr_C9ZP82	0.028	36966.05	70.00
22	HLA-A0201	PSSVHGLYID	PSSVHGLYI	0	0	0	0	0	PSSVHGLYI	tr_C9ZP82	0.028	36966.45	70.00
102	HLA-A0201	AAPAQTGEVAST	AQTGEVAST	0	0	0	1	3	AAPAQTGEVAST	tr_C9ZP82	0.028	36967.64	70.00
219	HLA-A0201	DPTAFEFDSNC	TAFEFDSNC	2	0	0	0	0	TAFEFDSNC	tr_C9ZP82	0.028	36967.64	70.00
490	HLA-A0201	EAERSWKNFRPCSS	RSWFRPCSS	3	0	0	3	2	RSWKNFRPCSS	tr_C9ZP82	0.028	36982.84	70.00
621	HLA-A0201	HSTIDDRWRDTY	STIDDRWTY	1	0	0	7	2	STIDDRWRDTY	tr_C9ZP82	0.028	36987.65	70.00
144	HLA-A0201	DEVRQTLLED	EVRQTLLED	1	0	0	0	0	EVRQTLLED	tr_C9ZP82	0.028	36988.06	70.00
271	HLA-A0201	TPGEGEHKI	TPGEGEHKI	0	0	0	0	0	TPGEGEHKI	tr_C9ZP82	0.028	36994.05	70.00
733	HLA-A0201	VRSPQQAELESTFP	QQAELSTFP	4	0	0	5	1	QQAELESTFP	tr_C9ZP82	0.028	37003.26	70.00
846	HLA-A0201	ERYRPRTYSCTVE	YRPTYSCTV	2	0	0	3	1	YRPRTYSCTV	tr_C9ZP82	0.028	37006.05	70.00
124	HLA-A0201	VEEVVREFTSTE	VEEVVFTST	0	0	0	5	2	VEEVVREFTST	tr_C9ZP82	0.028	37009.27	70.00
374	HLA-A0201	IGVPVCESSEN	IGVPVCESE	0	0	0	7	1	IGVPVCESSE	tr_C9ZP82	0.028	37012.47	70.00
109	HLA-A0201	EVASTYMPN	EVASTYMPN	0	0	0	0	0	EVASTYMPN	tr_C9ZP82	0.028	37027.68	70.00
32	HLA-A0201	LNGIIHPCCH	LNGIIPCCH	0	0	0	5	1	LNGIIHPCCH	tr_C9ZP82	0.028	37028.08	70.00
534	HLA-A0201	GESALDNLLE	GESALDNLL	0	0	0	0	0	GESALDNLL	tr_C9ZP82	0.028	37030.09	70.00
857	HLA-A0201	VEVPRLSYGADGQ	VVPRLSYGA	0	0	0	1	1	VEVPRLSYGA	tr_C9ZP82	0.028	37034.10	70.00
467	HLA-A0201	TLPSSTECGSPK	TLPECGSPK	0	0	0	3	3	TLPSSTECGSPK	tr_C9ZP82	0.028	37034.51	70.00
271	HLA-A0201	TPGEGEHKIIDF	TPGEGIIDF	0	0	0	5	3	TPGEGEHKIIDF	tr_C9ZP82	0.028	37038.11	70.00
331	HLA-A0201	KRRAVARQQ	KRRAVARQQ	0	0	0	0	0	KRRAVARQQ	tr_C9ZP82	0.028	37038.50	70.00
856	HLA-A0201	TVEVPRLSYGADG	TVEVLSYGA	0	0	0	4	2	TVEVPRLSYGA	tr_C9ZP82	0.028	37038.90	70.00
132	HLA-A0201	TSTEMAQANDDLD	SMAQANDDL	1	0	0	1	2	STEMAQANDDL	tr_C9ZP82	0.028	37051.32	70.00
144	HLA-A0201	DEVRQTLLEDP	EVRQTLLEP	1	0	0	8	1	EVRQTLLEDP	tr_C9ZP82	0.028	37052.54	70.00
398	HLA-A0201	REAHVTTVPN	RAHVTTVPN	0	0	0	1	1	REAHVTTVPN	tr_C9ZP82	0.028	37058.95	70.00
454	HLA-A0201	YYFFGQPNH	YYFFGQPNH	0	0	0	0	0	YYFFGQPNH	tr_C9ZP82	0.028	37060.14	70.00

884	HLA-A0201	KGNGEGVIEG	KGGEGVIEG	0	0	0	2	1	KGNGEGVIEG	tr_C9ZP82	0.028	37063.37	70.00
833	HLA-A0201	EGVEIRQLYD	GVEIRQLYD	1	0	0	0	0	GVEIRQLYD	tr_C9ZP82	0.028	37074.58	70.00
128	HLA-A0201	VREFTSTEMAQAND	FTSTEMAQA	3	0	0	0	0	FTSTEMAQA	tr_C9ZP82	0.028	37076.59	70.00
195	HLA-A0201	ENRYVESNQVV	RYVESNQVV	2	0	0	0	0	RYVESNQVV	tr_C9ZP82	0.028	37082.62	70.00
95	HLA-A0201	ARRYMGRAA	ARRYMGRAA	0	0	0	0	0	ARRYMGRAA	tr_C9ZP82	0.028	37090.63	70.00
823	HLA-A0201	DTILRRVPSR	TILRRVPSR	1	0	0	0	0	TILRRVPSR	tr_C9ZP82	0.028	37092.25	70.00
822	HLA-A0201	LDTILRRVPSRE	LDTILVPSR	0	0	0	5	2	LDTILRRVPSR	tr_C9ZP82	0.028	37105.08	70.00
782	HLA-A0201	WPQLTEEER	WPQLTEEER	0	0	0	0	0	WPQLTEEER	tr_C9ZP82	0.028	37107.50	70.00
225	HLA-A0201	FDSNCISPGTD	FSNCISPGT	0	0	0	1	1	FDSNCISPGT	tr_C9ZP82	0.028	37121.95	70.00
181	HLA-A0201	FCVRSTDDDYES	FSTDDDYES	0	0	0	1	3	FCVRSTDDDYES	tr_C9ZP82	0.028	37122.77	70.00
36	HLA-A0201	IHPCCHNDSDTAIA	IHPCCTAIA	0	0	0	5	5	IHPCCHNDSDTAIA	tr_C9ZP82	0.028	37123.96	70.00
525	HLA-A0201	LPHAPSAFSGESA	LSAFSGESA	0	0	0	1	4	LPHAPSAFSGESA	tr_C9ZP82	0.027	37135.22	70.00
226	HLA-A0201	DSNCISPGTDF	SNCISPGTF	1	0	0	8	1	SNCISPGTDF	tr_C9ZP82	0.027	37137.23	70.00
462	HLA-A0201	HIEDGTLPSSTE	HIEDGTLPT	0	0	0	8	2	HIEDGTLPSST	tr_C9ZP82	0.027	37138.02	70.00
110	HLA-A0201	VASTYMPNKS	VASTYMPNS	0	0	0	8	1	VASTYMPNKS	tr_C9ZP82	0.027	37140.85	70.00
899	HLA-A0201	RVTVVNKGP	RVTVVNKGP	0	0	0	0	0	RVTVVNKGP	tr_C9ZP82	0.027	37141.23	70.00
894	HLA-A0201	KKSNSRVTVVN	KSNSRVTVV	0	0	0	1	1	KKSNSRVTVV	tr_C9ZP82	0.027	37148.46	70.00
560	HLA-A0201	HEINLPQLQR	HINLPQLQR	0	0	0	1	1	HEINLPQLQR	tr_C9ZP82	0.027	37148.87	70.00
54	HLA-A0201	EEKLECICSE	EELECICSE	0	0	0	2	1	EEKLECICSE	tr_C9ZP82	0.027	37152.08	70.00
395	HLA-A0201	IESREAHVTTVP	IESAHVTTV	0	0	0	3	2	IESREAHVTTV	tr_C9ZP82	0.027	37173.00	70.00
738	HLA-A0201	QAELESTFP	QAELESTFP	0	0	0	0	0	QAELESTFP	tr_C9ZP82	0.027	37182.64	70.00
435	HLA-A0201	FTPATESAFDKKT	FTPATESAT	0	0	0	8	4	FTPATESAFDKKT	tr_C9ZP82	0.027	37185.88	70.00
9	HLA-A0201	LRNKYPAIVTKN	LRNKYPAIV	0	0	0	0	0	LRNKYPAIV	tr_C9ZP82	0.027	37185.88	70.00
445	HLA-A0201	KKTLVSSAG	KKTLVSSAG	0	0	0	0	0	KKTLVSSAG	tr_C9ZP82	0.027	37192.71	70.00
363	HLA-A0201	SYIDEEESDG	YIDEEESDG	1	0	0	0	0	YIDEEESDG	tr_C9ZP82	0.027	37194.73	70.00
100	HLA-A0201	GRAAPAQTGEVAST	GQTGEVAST	0	0	0	1	5	GRAAPAQTGEVAST	tr_C9ZP82	0.027	37198.35	70.00
595	HLA-A0201	NSNSGAEFR	NSNSGAEFR	0	0	0	0	0	NSNSGAEFR	tr_C9ZP82	0.027	37199.96	70.00
893	HLA-A0201	GKKSNSRVTVVNKG	KSNSRVTVV	2	0	0	0	0	KSNSRVTVV	tr_C9ZP82	0.027	37209.22	70.00
866	HLA-A0201	ADGQVIKRQ	ADGQVIKRQ	0	0	0	0	0	ADGQVIKRQ	tr_C9ZP82	0.027	37211.24	70.00
392	HLA-A0201	ESPIESREAHVT	SIESREAHV	1	0	0	1	1	SPIESREAHV	tr_C9ZP82	0.027	37222.50	75.00
894	HLA-A0201	KKSNSRVTVVNKGP	KSNSRVTVV	0	0	0	1	1	KKSNSRVTVV	tr_C9ZP82	0.027	37223.72	75.00
891	HLA-A0201	IEGKKSNSRVTV	IEGKKSVTV	0	0	0	6	3	IEGKKSNSRVTV	tr_C9ZP82	0.027	37228.54	75.00
498	HLA-A0201	FRPCSSPANSKII	FRPCSSPAI	0	0	0	8	4	FRPCSSPANSKII	tr_C9ZP82	0.027	37229.36	75.00
900	HLA-A0201	VTVVNKGPC	VTVVNKGPC	0	0	0	0	0	VTVVNKGPC	tr_C9ZP82	0.027	37231.76	75.00
36	HLA-A0201	IHPCCHNDSDTAI	IHPCCDTAI	0	0	0	5	4	IHPCCHNDSDTAI	tr_C9ZP82	0.027	37248.70	75.00
521	HLA-A0201	GNDFLPHAPSA	NDFLPHAPA	1	0	0	8	1	NDFLPHAPSA	tr_C9ZP82	0.027	37249.51	75.00
782	HLA-A0201	WPQLTEEERWRTQK	QLTEEEWRT	2	0	0	6	1	QLTEEERWRT	tr_C9ZP82	0.027	37252.32	75.00
206	HLA-A0201	DDVFGHRGEVI	DVFGRGEVI	1	0	0	4	1	DVFGHRGEVI	tr_C9ZP82	0.027	37252.32	75.00
527	HLA-A0201	HAPSAFSGESA	HAPSAFESA	0	0	0	6	2	HAPSAFSGESA	tr_C9ZP82	0.027	37253.52	75.00
684	HLA-A0201	LAIYLKEAN	LAIYLKEAN	0	0	0	0	0	LAIYLKEAN	tr_C9ZP82	0.027	37256.36	75.00
570	HLA-A0201	LFEAYAKIE	LFEAYAKIE	0	0	0	0	0	LFEAYAKIE	tr_C9ZP82	0.027	37257.15	75.00
531	HLA-A0201	AFSGESALDN	FSGESALDN	1	0	0	0	0	FSGESALDN	tr_C9ZP82	0.027	37259.58	75.00
527	HLA-A0201	HAPSAFSGESALD	HAPSAFSGL	0	0	0	8	3	HAPSAFSGESAL	tr_C9ZP82	0.027	37259.98	75.00
620	HLA-A0201	LHSTIDDRWRDTY	STIDDRWTY	2	0	0	7	2	STIDDRWRDTY	tr_C9ZP82	0.027	37262.39	75.00
112	HLA-A0201	STYMPNKSG	STYMPNKSG	0	0	0	0	0	STYMPNKSG	tr_C9ZP82	0.027	37262.80	75.00
560	HLA-A0201	HEINLPQLQ	HEINLPQLQ	0	0	0	0	0	HEINLPQLQ	tr_C9ZP82	0.027	37264.82	75.00
646	HLA-A0201	CEKYVEGVC	CEKYVEGVC	0	0	0	0	0	CEKYVEGVC	tr_C9ZP82	0.027	37274.89	75.00
443	HLA-A0201	FDKKTLVSSAG	FDKKTLVSA	0	0	0	7	1	FDKKTLVSSA	tr_C9ZP82	0.027	37284.17	75.00
528	HLA-A0201	APSAFSGESALDN	FSGESALDN	4	0	0	0	0	FSGESALDN	tr_C9ZP82	0.027	37299.92	75.00
491	HLA-A0201	AERSWKNFRPCS	RSWNFRPCS	2	0	0	3	1	RSWKNFRPCS	tr_C9ZP82	0.027	37307.98	75.00
17	HLA-A0201	VTKNCPSSVHG	VTKNCPSSV	0	0	0	0	0	VTKNCPSSV	tr_C9ZP82	0.027	37316.06	75.00
395	HLA-A0201	IESREAHVTTVPN	IAHVTTVPN	0	0	0	1	4	IESREAHVTTVPN	tr_C9ZP82	0.027	37341.51	75.00
204	HLA-A0201	VVDDVFGHRGE	VVDDVFHRG	0	0	0	6	1	VVDDVFGHRG	tr_C9ZP82	0.027	37343.12	75.00
393	HLA-A0201	SPIESREAHVTT	SIESREAHV	0	0	0	1	1	SPIESREAHV	tr_C9ZP82	0.027	37349.17	75.00

203	HLA-A0201	QVVDDVFGHRGE	VVDDVFRGE	1	0	0	6	2	VVDDVFGHRGE	tr_C9ZP82	0.027	37350.80	75.00
116	HLA-A0201	PNKSGMAIV	PNKSGMAIV	0	0	0	0	0	PNKSGMAIV	tr_C9ZP82	0.027	37354.04	75.00
358	HLA-A0201	ISLNPSYID	ISLNPSYID	0	0	0	0	0	ISLNPSYID	tr_C9ZP82	0.027	37362.52	75.00
897	HLA-A0201	NSRVTVVNKGP	NSRVTVVGP	0	0	0	7	2	NSRVTVVNKGP	tr_C9ZP82	0.027	37400.12	75.00
625	HLA-A0201	DDRWRDTYL	DDRWRDTYL	0	0	0	0	0	DDRWRDTYL	tr_C9ZP82	0.027	37415.93	75.00
330	HLA-A0201	YKRRAVARQ	YKRRAVARQ	0	0	0	0	0	YKRRAVARQ	tr_C9ZP82	0.027	37419.98	75.00
344	HLA-A0201	RSRRKRNFIDDTC	RSNFIDDTC	0	0	0	2	4	RSRRKRNFIDDTC	tr_C9ZP82	0.027	37432.91	75.00
53	HLA-A0201	EEEKLECICS	EEEKLCICS	0	0	0	5	1	EEEKLECICS	tr_C9ZP82	0.027	37436.97	75.00
781	HLA-A0201	AWPQLTEEERW	AWPQLTEEW	0	0	0	8	2	AWPQLTEEERW	tr_C9ZP82	0.027	37437.38	75.00
319	HLA-A0201	VFILRDTTRN	FILRDTTRN	1	0	0	0	0	FILRDTTRN	tr_C9ZP82	0.027	37437.79	75.00
786	HLA-A0201	TEEERWRTQKLDF	TEEERWLDF	0	0	0	6	4	TEEERWRTQKLDF	tr_C9ZP82	0.027	37438.59	75.00
493	HLA-A0201	RSWKNFRPCSSP	RSWKNFRPP	0	0	0	8	3	RSWKNFRPCSSP	tr_C9ZP82	0.027	37443.85	75.00
884	HLA-A0201	KGNGEGVIEGK	KGNGVIEGK	0	0	0	3	2	KGNGEGVIEGK	tr_C9ZP82	0.027	37448.73	75.00
489	HLA-A0201	AEAERSWKNFRPCS	RSWNFRPCS	4	0	0	3	1	RSWKNFRPCS	tr_C9ZP82	0.027	37456.03	75.00
14	HLA-A0201	PAIVTKNCPS	AIVTKNCPS	1	0	0	0	0	AIVTKNCPS	tr_C9ZP82	0.027	37469.80	75.00
179	HLA-A0201	NSFCVRSTDDDYE	NSFCVDDYE	0	0	0	5	4	NSFCVRSTDDDYE	tr_C9ZP82	0.027	37487.64	75.00
829	HLA-A0201	VPSREGVEIR	VSREGVEIR	0	0	0	1	1	VPSREGVEIR	tr_C9ZP82	0.027	37490.08	75.00
130	HLA-A0201	EFTSTEMAQANDD	FTSTEMAQA	1	0	0	0	0	FTSTEMAQA	tr_C9ZP82	0.027	37496.17	75.00
686	HLA-A0201	IYLKEANIK	IYLKEANIK	0	0	0	0	0	IYLKEANIK	tr_C9ZP82	0.027	37501.83	75.00
784	HLA-A0201	QLTEEERWRTQK	QLTEEEWRT	0	0	0	6	1	QLTEEERWRT	tr_C9ZP82	0.027	37504.27	75.00
624	HLA-A0201	IDDRWRDTY	IDDRWRDTY	0	0	0	0	0	IDDRWRDTY	tr_C9ZP82	0.027	37514.02	75.00
689	HLA-A0201	KEANIKRMPLP	KANIKRMPL	0	0	0	1	1	KEANIKRMPL	tr_C9ZP82	0.027	37518.47	75.00
658	HLA-A0201	RYYTTTTTDSDW	RYYTTTTTW	0	0	0	8	3	RYYTTTTTDSDW	tr_C9ZP82	0.027	37520.09	75.00
490	HLA-A0201	EAERSWKNFRPCS	RSWNFRPCS	3	0	0	3	1	RSWKNFRPCS	tr_C9ZP82	0.027	37527.81	75.00
208	HLA-A0201	VFGHRGEVIVD	VFGHRGVIV	0	0	0	6	1	VFGHRGEVIV	tr_C9ZP82	0.027	37528.64	75.00
129	HLA-A0201	REFTSTEMAQANDD	FTSTEMAQA	2	0	0	0	0	FTSTEMAQA	tr_C9ZP82	0.027	37529.05	75.00
285	HLA-A0201	TQSSLAQFGGK	TQSSLAQFK	0	0	0	8	2	TQSSLAQFGGK	tr_C9ZP82	0.027	37532.29	75.00
266	HLA-A0201	FSGSNTPGEG	FSGSNTPEG	0	0	0	7	1	FSGSNTPGEG	tr_C9ZP82	0.026	37540.40	75.00
32	HLA-A0201	LNGIIHPCCHND	IIHPCCHND	3	0	0	0	0	IIHPCCHND	tr_C9ZP82	0.026	37541.64	75.00
173	HLA-A0201	NDGDGVNSFC	NGDGVNSFC	0	0	0	1	1	NDGDGVNSFC	tr_C9ZP82	0.026	37556.65	75.00
400	HLA-A0201	AHVTTVPNTG	AVTTVPNTG	0	0	0	1	1	AHVTTVPNTG	tr_C9ZP82	0.026	37561.13	75.00
781	HLA-A0201	AWPQLTEEERWR	ALTEEERWR	0	0	0	1	3	AWPQLTEEERWR	tr_C9ZP82	0.026	37561.95	75.00
634	HLA-A0201	KSTGLSNSVQE	KSTGLSNSV	0	0	0	0	0	KSTGLSNSV	tr_C9ZP82	0.026	37561.95	75.00
430	HLA-A0201	CDIKKFTPATESA	CDIKKFTPA	0	0	0	8	4	CDIKKFTPATESA	tr_C9ZP82	0.026	37564.79	75.00
113	HLA-A0201	TYMPNKSGM	TYMPNKSGM	0	0	0	0	0	TYMPNKSGM	tr_C9ZP82	0.026	37566.00	75.00
272	HLA-A0201	PGEGEHKIIDF	GEGEHIIDF	1	0	0	5	1	GEGEHKIIDF	tr_C9ZP82	0.026	37572.52	75.00
841	HLA-A0201	YDVPLERYRPRTYS	YLERYRPRT	0	0	0	1	3	YDVPLERYRPRT	tr_C9ZP82	0.026	37572.90	75.00
108	HLA-A0201	GEVASTYMPN	GVASTYMPN	0	0	0	1	1	GEVASTYMPN	tr_C9ZP82	0.026	37574.55	75.00
100	HLA-A0201	GRAAPAQTGEVA	RAAPAQTEV	1	0	0	7	1	RAAPAQTGEV	tr_C9ZP82	0.026	37577.80	75.00
133	HLA-A0201	STEMAQANDDLDE	SMAQANDDL	0	0	0	1	2	STEMAQANDDL	tr_C9ZP82	0.026	37589.99	75.00
618	HLA-A0201	VALHSTIDD	VALHSTIDD	0	0	0	0	0	VALHSTIDD	tr_C9ZP82	0.026	37621.32	75.00
2	HLA-A0201	VPKFASWLRNK	VPFASWLRN	0	0	0	2	1	VPKFASWLRN	tr_C9ZP82	0.026	37631.11	75.00
896	HLA-A0201	SNSRVTVVNKG	SSRVTVVNK	0	0	0	1	1	SNSRVTVVNK	tr_C9ZP82	0.026	37633.95	75.00
180	HLA-A0201	SFCVRSTDDDYES	FSTDDDYES	1	0	0	1	3	FCVRSTDDDYES	tr_C9ZP82	0.026	37648.19	75.00
217	HLA-A0201	VDDPTAFEFDS	VDDPTAFEF	0	0	0	0	0	VDDPTAFEF	tr_C9ZP82	0.026	37652.68	75.00
843	HLA-A0201	VPLERYRPR	VPLERYRPR	0	0	0	0	0	VPLERYRPR	tr_C9ZP82	0.026	37660.43	75.00
117	HLA-A0201	NKSGMAIVE	NKSGMAIVE	0	0	0	0	0	NKSGMAIVE	tr_C9ZP82	0.026	37662.06	75.00
180	HLA-A0201	SFCVRSTDDDY	FCVSTDDDY	1	0	0	3	1	FCVRSTDDDY	tr_C9ZP82	0.026	37664.10	75.00
10	HLA-A0201	RNKYPAIVTKN	RNYPAIVTK	0	0	0	2	1	RNKYPAIVTK	tr_C9ZP82	0.026	37666.94	75.00
526	HLA-A0201	PHAPSAFSGESALD	SAFSGESAL	4	0	0	0	0	SAFSGESAL	tr_C9ZP82	0.026	37675.91	75.00
790	HLA-A0201	RWRTQKLDFH	RWRQKLDFH	0	0	0	3	1	RWRTQKLDFH	tr_C9ZP82	0.026	37678.36	75.00
459	HLA-A0201	QPNHIEDGTLPS	QIEDGTLPS	0	0	0	1	3	QPNHIEDGTLPS	tr_C9ZP82	0.026	37683.25	75.00
434	HLA-A0201	KFTPATESAFDKKT	FTPATESAT	1	0	0	8	4	FTPATESAFDKKT	tr_C9ZP82	0.026	37687.74	75.00
644	HLA-A0201	EACEKYVEG	EACEKYVEG	0	0	0	0	0	EACEKYVEG	tr_C9ZP82	0.026	37693.04	75.00

604	HLA-A0201	KGSNNDNVTVAD	KSNNDNVTV	0	0	0	1	1	KGSNNDNVTV	tr_C9ZP82	0.026	37695.08	75.00
535	HLA-A0201	ESALDNLLE	ESALDNLLE	0	0	0	0	0	ESALDNLLE	tr_C9ZP82	0.026	37698.75	75.00
779	HLA-A0201	DAAWPQLTEEE	AAWPLTEEE	1	0	0	4	1	AAWPQLTEEE	tr_C9ZP82	0.026	37715.87	75.00
690	HLA-A0201	EANIKRMPLP	EANIKRMPL	0	0	0	0	0	EANIKRMPL	tr_C9ZP82	0.026	37718.33	75.00
438	HLA-A0201	ATESAFDKK	ATESAFDKK	0	0	0	0	0	ATESAFDKK	tr_C9ZP82	0.026	37729.35	75.00
33	HLA-A0201	NGIIHPCCH	NGIIHPCCH	0	0	0	0	0	NGIIHPCCH	tr_C9ZP82	0.026	37736.70	75.00
176	HLA-A0201	DGVNSFCVR	DGVNSFCVR	0	0	0	0	0	DGVNSFCVR	tr_C9ZP82	0.026	37743.64	75.00
528	HLA-A0201	APSAFSGESALD	AAFSGESAL	0	0	0	1	2	APSAFSGESAL	tr_C9ZP82	0.026	37749.77	75.00
607	HLA-A0201	NNDNVTVAD	NNDNVTVAD	0	0	0	0	0	NNDNVTVAD	tr_C9ZP82	0.026	37749.77	75.00
857	HLA-A0201	VEVPRLSYGADG	VVPRLSYGA	0	0	0	1	1	VEVPRLSYGA	tr_C9ZP82	0.026	37759.17	75.00
807	HLA-A0201	TSFVPKGDLRK	TSFVPDLRK	0	0	0	5	2	TSFVPKGDLRK	tr_C9ZP82	0.026	37762.43	75.00
728	HLA-A0201	LGEVMVRSPQQ	LVMVRSPQQ	0	0	0	1	2	LGEVMVRSPQQ	tr_C9ZP82	0.026	37768.57	75.00
224	HLA-A0201	EFDSNCISPGTD	FSNCISPGT	1	0	0	1	1	FDSNCISPGT	tr_C9ZP82	0.026	37772.25	75.00
789	HLA-A0201	ERWRTQKLDF	ERWRTQLDF	0	0	0	6	1	ERWRTQKLDF	tr_C9ZP82	0.026	37777.56	75.00
807	HLA-A0201	TSFVPKGDLR	TSFVPGDLR	0	0	0	5	1	TSFVPKGDLR	tr_C9ZP82	0.026	37780.41	75.00
391	HLA-A0201	NESPIESREAHVTT	SIESREAHV	2	0	0	1	1	SPIESREAHV	tr_C9ZP82	0.026	37785.30	75.00
624	HLA-A0201	IDDRWRDTYLK	IDDRWDTYL	0	0	0	5	1	IDDRWRDTYL	tr_C9ZP82	0.026	37793.08	75.00
432	HLA-A0201	IKKFTPATESA	IFTPATESA	0	0	0	1	2	IKKFTPATESA	tr_C9ZP82	0.026	37816.00	75.00
85	HLA-A0201	APRAKMNQQRARRY	KMQQRARRY	4	0	0	2	1	KMNQQRARRY	tr_C9ZP82	0.026	37816.39	75.00
294	HLA-A0201	GKANHVIVG	GKANHVIVG	0	0	0	0	0	GKANHVIVG	tr_C9ZP82	0.026	37827.85	75.00
286	HLA-A0201	QSSLAQFGGK	QSSLAFGGK	0	0	0	5	1	QSSLAQFGGK	tr_C9ZP82	0.026	37829.90	75.00
33	HLA-A0201	NGIIHPCCHND	IIHPCCHND	2	0	0	0	0	IIHPCCHND	tr_C9ZP82	0.026	37837.27	75.00
461	HLA-A0201	NHIEDGTLPSSTE	HIEDGTLPT	1	0	0	8	2	HIEDGTLPSST	tr_C9ZP82	0.026	37838.10	75.00
366	HLA-A0201	DEEESDGVIGVP	DEEESVIGV	0	0	0	5	2	DEEESDGVIGV	tr_C9ZP82	0.026	37838.49	75.00
126	HLA-A0201	EVVREFTSTE	VVREFTSTE	1	0	0	0	0	VVREFTSTE	tr_C9ZP82	0.026	37839.73	75.00
778	HLA-A0201	VDAAWPQLTEEE	AAWPLTEEE	2	0	0	4	1	AAWPQLTEEE	tr_C9ZP82	0.026	37855.28	75.00
396	HLA-A0201	ESREAHVTT	ESREAHVTT	0	0	0	0	0	ESREAHVTT	tr_C9ZP82	0.026	37870.03	75.00
439	HLA-A0201	TESAFDKKTL	TESAFDKTL	0	0	0	6	1	TESAFDKKTL	tr_C9ZP82	0.026	37871.67	75.00
893	HLA-A0201	GKKSNSRVTVVN	KSNSRVTVV	2	0	0	0	0	KSNSRVTVV	tr_C9ZP82	0.026	37872.08	75.00
896	HLA-A0201	SNSRVTVVNKGP	NSRVTVVGP	1	0	0	7	2	NSRVTVVNKGP	tr_C9ZP82	0.026	37874.96	75.00
96	HLA-A0201	RRYMGRAAP	RRYMGRAAP	0	0	0	0	0	RRYMGRAAP	tr_C9ZP82	0.026	37892.16	75.00
174	HLA-A0201	DGDGVNSFC	DGDGVNSFC	0	0	0	0	0	DGDGVNSFC	tr_C9ZP82	0.026	37894.22	75.00
432	HLA-A0201	IKKFTPATE	IKKFTPATE	0	0	0	0	0	IKKFTPATE	tr_C9ZP82	0.026	37897.49	75.00
3	HLA-A0201	PKFASWLRNK	KFASWLRNK	1	0	0	0	0	KFASWLRNK	tr_C9ZP82	0.026	37914.31	75.00
12	HLA-A0201	KYPAIVTKNCP	YPAIVTNCP	1	0	0	6	1	YPAIVTKNCP	tr_C9ZP82	0.026	37934.83	75.00
265	HLA-A0201	IFSGSNTPG	IFSGSNTPG	0	0	0	0	0	IFSGSNTPG	tr_C9ZP82	0.026	37939.75	75.00
395	HLA-A0201	IESREAHVTTVPNT	IESAHVTTV	0	0	0	3	2	IESREAHVTTV	tr_C9ZP82	0.025	37944.69	75.00
891	HLA-A0201	IEGKKSNSRV	IEGKKSNSV	0	0	0	8	1	IEGKKSNSRV	tr_C9ZP82	0.025	37948.80	75.00
431	HLA-A0201	DIKKFTPATESA	DIKKFTESA	0	0	0	5	3	DIKKFTPATESA	tr_C9ZP82	0.025	37952.89	75.00
491	HLA-A0201	AERSWKNFRPCSSP	RSWKNFRPP	2	0	0	8	3	RSWKNFRPCSSP	tr_C9ZP82	0.025	37955.78	75.00
751	HLA-A0201	VDYHDSNVVH	VDYHDSNVV	0	0	0	0	0	VDYHDSNVV	tr_C9ZP82	0.025	37983.72	75.00
830	HLA-A0201	PSREGVEIR	PSREGVEIR	0	0	0	0	0	PSREGVEIR	tr_C9ZP82	0.025	37994.40	75.00
788	HLA-A0201	EERWRTQKL	EERWRTQKL	0	0	0	0	0	EERWRTQKL	tr_C9ZP82	0.025	37995.62	75.00
786	HLA-A0201	TEEERWRTQKLDFH	TEEERWLDF	0	0	0	6	4	TEEERWRTQKLDF	tr_C9ZP82	0.025	38000.16	75.00
781	HLA-A0201	AWPQLTEEE	AWPQLTEEE	0	0	0	0	0	AWPQLTEEE	tr_C9ZP82	0.025	38000.55	75.00
92	HLA-A0201	QQRARRYMG	QQRARRYMG	0	0	0	0	0	QQRARRYMG	tr_C9ZP82	0.025	38006.31	75.00
92	HLA-A0201	QQRARRYMGRA	QQRAYMGRA	0	0	0	4	2	QQRARRYMGRA	tr_C9ZP82	0.025	38011.26	75.00
620	HLA-A0201	LHSTIDDRWRDT	STIDDRWRT	2	0	0	8	1	STIDDRWRDT	tr_C9ZP82	0.025	38018.67	75.00
40	HLA-A0201	CHNDSDTAIA	CNDSDTAIA	0	0	0	1	1	CHNDSDTAIA	tr_C9ZP82	0.025	38019.48	75.00
492	HLA-A0201	ERSWKNFRPCS	RSWNFRPCS	1	0	0	3	1	RSWKNFRPCS	tr_C9ZP82	0.025	38021.95	75.00
10	HLA-A0201	RNKYPAIVT	RNKYPAIVT	0	0	0	0	0	RNKYPAIVT	tr_C9ZP82	0.025	38033.48	75.00
435	HLA-A0201	FTPATESAFDKK	FTPATAFDK	0	0	0	5	2	FTPATESAFDK	tr_C9ZP82	0.025	38040.89	75.00
394	HLA-A0201	PIESREAHVTTVP	IESAHVTTV	1	0	0	3	2	IESREAHVTTV	tr_C9ZP82	0.025	38041.70	75.00
892	HLA-A0201	EGKKSNSRVTVVN	KSNSRVTVV	3	0	0	0	0	KSNSRVTVV	tr_C9ZP82	0.025	38042.53	75.00

51	HLA-A0201	LPEEEKLECIC	LPEEELECI	0	0	0	5	1	LPEEEKLECI	tr_C9ZP82	0.025	38042.95	75.00
444	HLA-A0201	DKKTLVSSA	DKKTLVSSA	0	0	0	0	0	DKKTLVSSA	tr_C9ZP82	0.025	38043.34	75.00
198	HLA-A0201	YVESNQVVDD	YVSNQVVDD	0	0	0	2	1	YVESNQVVDD	tr_C9ZP82	0.025	38050.36	75.00
12	HLA-A0201	KYPAIVTKN	KYPAIVTKN	0	0	0	0	0	KYPAIVTKN	tr_C9ZP82	0.025	38068.47	75.00
401	HLA-A0201	HVTTVPNTGFE	HVTTVPNTF	0	0	0	8	1	HVTTVPNTGF	tr_C9ZP82	0.025	38070.54	75.00
813	HLA-A0201	GDLRKLSDA	GDLRKLSDA	0	0	0	0	0	GDLRKLSDA	tr_C9ZP82	0.025	38078.34	75.00
858	HLA-A0201	EVPRLSYGAD	EVPRLSYGA	0	0	0	0	0	EVPRLSYGA	tr_C9ZP82	0.025	38079.59	75.00
167	HLA-A0201	EAGDALNDGDGVN	EAGDALNGV	0	0	0	7	3	EAGDALNDGDGV	tr_C9ZP82	0.025	38079.99	75.00
658	HLA-A0201	RYYTTTTTDSDWS	RYYTTTTTS	0	0	0	8	4	RYYTTTTTDSDWS	tr_C9ZP82	0.025	38086.18	75.00
283	HLA-A0201	LRTQSSLAQ	LRTQSSLAQ	0	0	0	0	0	LRTQSSLAQ	tr_C9ZP82	0.025	38088.66	75.00
844	HLA-A0201	PLERYRPRTYSC	PLEPRTYSC	0	0	0	3	3	PLERYRPRTYSC	tr_C9ZP82	0.025	38090.72	75.00
785	HLA-A0201	LTEEERWRTQ	LTEEERWTQ	0	0	0	7	1	LTEEERWRTQ	tr_C9ZP82	0.025	38091.95	75.00
168	HLA-A0201	AGDALNDGDGVN	AALNDGDGV	0	0	0	1	2	AGDALNDGDGV	tr_C9ZP82	0.025	38100.20	75.00
210	HLA-A0201	GHRGEVIVDD	GHREVIVDD	0	0	0	3	1	GHRGEVIVDD	tr_C9ZP82	0.025	38105.56	75.00
264	HLA-A0201	VIFSGSNTPGEG	VIFSGSNTG	0	0	0	8	3	VIFSGSNTPGEG	tr_C9ZP82	0.025	38107.62	75.00
573	HLA-A0201	AYAKIEAVK	AYAKIEAVK	0	0	0	0	0	AYAKIEAVK	tr_C9ZP82	0.025	38109.66	75.00
529	HLA-A0201	PSAFSGESALDN	SAFSGESAL	1	0	0	0	0	SAFSGESAL	tr_C9ZP82	0.025	38127.83	75.00
39	HLA-A0201	CCHNDSDTAIA	CCDSDTAIA	0	0	0	2	2	CCHNDSDTAIA	tr_C9ZP82	0.025	38143.50	75.00
166	HLA-A0201	EEAGDALNDGDGVN	EAGDALNGV	1	0	0	7	3	EAGDALNDGDGV	tr_C9ZP82	0.025	38159.59	75.00
197	HLA-A0201	RYVESNQVVD	RYVESNQVV	0	0	0	0	0	RYVESNQVV	tr_C9ZP82	0.025	38160.01	75.00
843	HLA-A0201	VPLERYRPRTYSC	VPLERYYSC	0	0	0	6	4	VPLERYRPRTYSC	tr_C9ZP82	0.025	38163.30	75.00
210	HLA-A0201	GHRGEVIVDDP	HRGEVIVDP	1	0	0	7	1	HRGEVIVDDP	tr_C9ZP82	0.025	38164.56	75.00
433	HLA-A0201	KKFTPATES	KKFTPATES	0	0	0	0	0	KKFTPATES	tr_C9ZP82	0.025	38165.79	75.00
843	HLA-A0201	VPLERYRPRTY	VPLERYRPY	0	0	0	8	2	VPLERYRPRTY	tr_C9ZP82	0.025	38171.58	75.00
841	HLA-A0201	YDVPLERYRP	YVPLERYRP	0	0	0	1	1	YDVPLERYRP	tr_C9ZP82	0.025	38174.46	75.00
466	HLA-A0201	GTLPSSTECGS	TLSSTECGS	1	0	0	2	1	TLPSSTECGS	tr_C9ZP82	0.025	38178.59	75.00
806	HLA-A0201	KTSFVPKGD	KTSFVPKGD	0	0	0	0	0	KTSFVPKGD	tr_C9ZP82	0.025	38187.68	75.00
438	HLA-A0201	ATESAFDKKT	ATSAFDKKT	0	0	0	2	1	ATESAFDKKT	tr_C9ZP82	0.025	38189.75	75.00
87	HLA-A0201	RAKMNQQRA	RAKMNQQRA	0	0	0	0	0	RAKMNQQRA	tr_C9ZP82	0.025	38194.72	75.00
160	HLA-A0201	LYNEGEEEAGDAL	LYNEGEEAL	0	0	0	7	4	LYNEGEEEAGDAL	tr_C9ZP82	0.025	38195.12	75.00
778	HLA-A0201	VDAAWPQLTEE	AAWPQLTEE	2	0	0	0	0	AAWPQLTEE	tr_C9ZP82	0.025	38201.75	75.00
459	HLA-A0201	QPNHIEDGTL	QPNHIEDGL	0	0	0	8	1	QPNHIEDGTL	tr_C9ZP82	0.025	38206.28	75.00
161	HLA-A0201	YNEGEEEAGDAL	YEEEAGDAL	0	0	0	1	3	YNEGEEEAGDAL	tr_C9ZP82	0.025	38209.61	75.00
343	HLA-A0201	RRSRRKRNFIDDTC	RSNFIDDTC	1	0	0	2	4	RSRRKRNFIDDTC	tr_C9ZP82	0.025	38210.00	75.00
624	HLA-A0201	IDDRWRDTYLKST	RWRDTYLST	3	0	0	7	1	RWRDTYLKST	tr_C9ZP82	0.025	38217.44	75.00
400	HLA-A0201	AHVTTVPNTGFE	HVTTVPNTF	1	0	0	8	1	HVTTVPNTGF	tr_C9ZP82	0.025	38221.16	75.00
434	HLA-A0201	KFTPATESAFDKK	FTPATAFDK	1	0	0	5	2	FTPATESAFDK	tr_C9ZP82	0.025	38222.82	75.00
526	HLA-A0201	PHAPSAFSGESA	HAPSAFESA	1	0	0	6	2	HAPSAFSGESA	tr_C9ZP82	0.025	38226.96	75.00
281	HLA-A0201	DFLRTQSSL	DFLRTQSSL	0	0	0	0	0	DFLRTQSSL	tr_C9ZP82	0.025	38230.26	75.00
788	HLA-A0201	EERWRTQKLDF	EERWRTLDF	0	0	0	6	2	EERWRTQKLDF	tr_C9ZP82	0.025	38235.24	75.00
198	HLA-A0201	YVESNQVVD	YVESNQVVD	0	0	0	0	0	YVESNQVVD	tr_C9ZP82	0.025	38235.24	75.00
626	HLA-A0201	DRWRDTYLK	DRWRDTYLK	0	0	0	0	0	DRWRDTYLK	tr_C9ZP82	0.025	38240.20	75.00
886	HLA-A0201	NGEGVIEGKKSNSR	NGEGVIEGR	0	0	0	8	5	NGEGVIEGKKSNSR	tr_C9ZP82	0.025	38245.57	75.00
433	HLA-A0201	KKFTPATESAFDKK	FTPATAFDK	2	0	0	5	2	FTPATESAFDK	tr_C9ZP82	0.025	38247.64	75.00
389	HLA-A0201	TDNESPIES	TDNESPIES	0	0	0	0	0	TDNESPIES	tr_C9ZP82	0.025	38247.64	75.00
782	HLA-A0201	WPQLTEEERWR	WLTEEERWR	0	0	0	1	2	WPQLTEEERWR	tr_C9ZP82	0.025	38258.40	75.00
617	HLA-A0201	AVALHSTID	AVALHSTID	0	0	0	0	0	AVALHSTID	tr_C9ZP82	0.025	38259.24	75.00
289	HLA-A0201	LAQFGGKANH	LQFGGKANH	0	0	0	1	1	LAQFGGKANH	tr_C9ZP82	0.025	38259.66	75.00
808	HLA-A0201	SFVPKGDLR	SFVPKGDLR	0	0	0	0	0	SFVPKGDLR	tr_C9ZP82	0.025	38265.04	75.00
367	HLA-A0201	EEESDGVIGVP	ESDGVIGVP	2	0	0	0	0	ESDGVIGVP	tr_C9ZP82	0.025	38265.43	75.00
87	HLA-A0201	RAKMNQQRARRY	KMQQRARRY	2	0	0	2	1	KMNQQRARRY	tr_C9ZP82	0.025	38268.77	75.00
466	HLA-A0201	GTLPSSTECGSPK	TLPECGSPK	1	0	0	3	3	TLPSSTECGSPK	tr_C9ZP82	0.025	38271.65	75.00
661	HLA-A0201	TTTTTDSDWSW	TTTTTDWSW	0	0	0	5	2	TTTTTDSDWSW	tr_C9ZP82	0.025	38272.49	75.00
492	HLA-A0201	ERSWKNFRPCSSP	RSWKNFRPP	1	0	0	8	3	RSWKNFRPCSSP	tr_C9ZP82	0.025	38286.14	75.00

460	HLA-A0201	PNHIEDGTLPSSTE	HIEDGTLPT	2	0	0	8	2	HIEDGTLPSST	tr_C9ZP82	0.025	38295.26	75.00
99	HLA-A0201	MGRAAPAQTGEVAS	RAAPAQTEV	2	0	0	7	1	RAAPAQTGEV	tr_C9ZP82	0.025	38311.44	75.00
109	HLA-A0201	EVASTYMPNK	EVASTYMPK	0	0	0	8	1	EVASTYMPNK	tr_C9ZP82	0.025	38311.44	75.00
139	HLA-A0201	ANDDLDEVR	ANDDLDEVR	0	0	0	0	0	ANDDLDEVR	tr_C9ZP82	0.025	38337.96	75.00
88	HLA-A0201	AKMNQQRARRY	KMQQRARRY	1	0	0	2	1	KMNQQRARRY	tr_C9ZP82	0.025	38340.88	75.00
134	HLA-A0201	TEMAQANDDLD	TMAQANDDL	0	0	0	1	1	TEMAQANDDL	tr_C9ZP82	0.025	38345.43	75.00
67	HLA-A0201	LVQTVQPKE	LVQTVQPKE	0	0	0	0	0	LVQTVQPKE	tr_C9ZP82	0.025	38345.43	75.00
81	HLA-A0201	VDGVAPRAKM	VDGVAPAKM	0	0	0	6	1	VDGVAPRAKM	tr_C9ZP82	0.025	38347.92	75.00
37	HLA-A0201	HPCCHNDSDTAI	CCHDSDTAI	2	0	0	3	1	CCHNDSDTAI	tr_C9ZP82	0.025	38350.84	75.00
863	HLA-A0201	SYGADGQVIKR	YADGQVIKR	1	0	0	1	1	YGADGQVIKR	tr_C9ZP82	0.025	38350.84	75.00
525	HLA-A0201	LPHAPSAFSG	LPHASAFSG	0	0	0	4	1	LPHAPSAFSG	tr_C9ZP82	0.024	38359.97	75.00
385	HLA-A0201	SSSDTDNESPIESR	SSSDTDNPI	0	0	0	7	2	SSSDTDNESPI	tr_C9ZP82	0.024	38362.44	75.00
464	HLA-A0201	EDGTLPSSTECGS	TLSSTECGS	3	0	0	2	1	TLPSSTECGS	tr_C9ZP82	0.024	38367.84	75.00
19	HLA-A0201	KNCPSSVHG	KNCPSSVHG	0	0	0	0	0	KNCPSSVHG	tr_C9ZP82	0.024	38371.99	75.00
603	HLA-A0201	RKGSNNDNVTVAD	KSNNDNVTV	1	0	0	1	1	KGSNNDNVTV	tr_C9ZP82	0.024	38385.70	75.00
233	HLA-A0201	GTDFMAKVSD	GTDFMAVSD	0	0	0	6	1	GTDFMAKVSD	tr_C9ZP82	0.024	38391.10	75.00
556	HLA-A0201	TKGPHEINLPQ	GPHEINLPQ	2	0	0	0	0	GPHEINLPQ	tr_C9ZP82	0.024	38396.08	75.00
896	HLA-A0201	SNSRVTVVN	SNSRVTVVN	0	0	0	0	0	SNSRVTVVN	tr_C9ZP82	0.024	38402.32	75.00
661	HLA-A0201	TTTTTDSDWSWY	TTTTTWSWY	0	0	0	5	3	TTTTTDSDWSWY	tr_C9ZP82	0.024	38402.74	75.00
396	HLA-A0201	ESREAHVTTVP	ESREAVTTV	0	0	0	5	1	ESREAHVTTV	tr_C9ZP82	0.024	38404.82	75.00
787	HLA-A0201	EEERWRTQKLDF	EERWRTLDF	1	0	0	6	2	EERWRTQKLDF	tr_C9ZP82	0.024	38406.48	75.00
328	HLA-A0201	NPYKRRAVA	NPYKRRAVA	0	0	0	0	0	NPYKRRAVA	tr_C9ZP82	0.024	38407.72	75.00
778	HLA-A0201	VDAAWPQLTE	VAAWPQLTE	0	0	0	1	1	VDAAWPQLTE	tr_C9ZP82	0.024	38413.12	75.00
394	HLA-A0201	PIESREAHVTTVPN	IAHVTTVPN	1	0	0	1	4	IESREAHVTTVPN	tr_C9ZP82	0.024	38414.36	75.00
787	HLA-A0201	EEERWRTQKLDFH	EEERWLDFH	0	0	0	5	4	EEERWRTQKLDFH	tr_C9ZP82	0.024	38417.28	75.00
842	HLA-A0201	DVPLERYRPRTY	PLEYRPRTY	2	0	0	3	1	PLERYRPRTY	tr_C9ZP82	0.024	38426.00	80.00
399	HLA-A0201	EAHVTTVPNTG	EAHVTTVPT	0	0	0	8	1	EAHVTTVPNT	tr_C9ZP82	0.024	38428.50	80.00
214	HLA-A0201	EVIVDDPTAFE	VIVDDPTAF	1	0	0	0	0	VIVDDPTAF	tr_C9ZP82	0.024	38432.67	80.00
194	HLA-A0201	HENRYVESNQVVD	RYVESNQVV	3	0	0	0	0	RYVESNQVV	tr_C9ZP82	0.024	38433.49	80.00
397	HLA-A0201	SREAHVTTVPNT	EAHVTTVPT	2	0	0	8	1	EAHVTTVPNT	tr_C9ZP82	0.024	38436.41	80.00
551	HLA-A0201	PYGFLTKGPH	YGFLTKGPH	1	0	0	0	0	YGFLTKGPH	tr_C9ZP82	0.024	38436.81	80.00
499	HLA-A0201	RPCSSPANSKII	RPCSSPAII	0	0	0	7	3	RPCSSPANSKII	tr_C9ZP82	0.024	38444.71	80.00
392	HLA-A0201	ESPIESREAHVTT	SIESREAHV	1	0	0	1	1	SPIESREAHV	tr_C9ZP82	0.024	38446.38	80.00
785	HLA-A0201	LTEEERWRTQKLD	LTEEERWRL	0	0	0	8	3	LTEEERWRTQKL	tr_C9ZP82	0.024	38453.47	80.00
386	HLA-A0201	SSDTDNESPIESR	SSDESPIES	0	0	0	3	3	SSDTDNESPIES	tr_C9ZP82	0.024	38453.87	80.00
217	HLA-A0201	VDDPTAFEFDSN	VDDPTAFEF	0	0	0	0	0	VDDPTAFEF	tr_C9ZP82	0.024	38459.70	80.00
624	HLA-A0201	IDDRWRDTYLKS	IDDWRDTYL	0	0	0	3	1	IDDRWRDTYL	tr_C9ZP82	0.024	38463.02	80.00
456	HLA-A0201	FFGQPNHIEDGT	FFGQIEDGT	0	0	0	4	3	FFGQPNHIEDGT	tr_C9ZP82	0.024	38468.03	80.00
90	HLA-A0201	MNQQRARRYMGRA	QQRAYMGRA	2	0	0	4	2	QQRARRYMGRA	tr_C9ZP82	0.024	38470.11	80.00
132	HLA-A0201	TSTEMAQANDDLDE	SMAQANDDL	1	0	0	1	2	STEMAQANDDL	tr_C9ZP82	0.024	38472.59	80.00
883	HLA-A0201	LKGNGEGVIEGKK	NGEGVIEGK	3	0	0	8	1	NGEGVIEGKK	tr_C9ZP82	0.024	38473.02	80.00
823	HLA-A0201	DTILRRVPSRE	ILRRVPSRE	2	0	0	0	0	ILRRVPSRE	tr_C9ZP82	0.024	38480.51	80.00
815	HLA-A0201	LRKLSDALDT	LRKLSALDT	0	0	0	5	1	LRKLSDALDT	tr_C9ZP82	0.024	38481.75	80.00
396	HLA-A0201	ESREAHVTTVPNT	EAHVTTVPT	3	0	0	8	1	EAHVTTVPNT	tr_C9ZP82	0.024	38483.02	80.00
865	HLA-A0201	GADGQVIKRQRRHR	GADGQVIHR	0	0	0	7	5	GADGQVIKRQRRHR	tr_C9ZP82	0.024	38483.84	80.00
157	HLA-A0201	SGALYNEGEEE	SLYNEGEEE	0	0	0	1	2	SGALYNEGEEE	tr_C9ZP82	0.024	38486.77	80.00
495	HLA-A0201	WKNFRPCSS	WKNFRPCSS	0	0	0	0	0	WKNFRPCSS	tr_C9ZP82	0.024	38490.09	80.00
92	HLA-A0201	QQRARRYMGRAAP	QQYMGRAAP	0	0	0	2	4	QQRARRYMGRAAP	tr_C9ZP82	0.024	38495.93	80.00
842	HLA-A0201	DVPLERYRPRTYSC	PLERYTYSC	2	0	0	5	3	PLERYRPRTYSC	tr_C9ZP82	0.024	38497.16	80.00
86	HLA-A0201	PRAKMNQQRARRY	KMQQRARRY	3	0	0	2	1	KMNQQRARRY	tr_C9ZP82	0.024	38499.25	80.00
193	HLA-A0201	THENRYVESNQVVD	RYVESNQVV	4	0	0	0	0	RYVESNQVV	tr_C9ZP82	0.024	38503.42	80.00
107	HLA-A0201	TGEVASTYMP	TGEVASTYM	0	0	0	0	0	TGEVASTYM	tr_C9ZP82	0.024	38505.91	80.00
92	HLA-A0201	QQRARRYMGRAA	QQRARRYMA	0	0	0	8	3	QQRARRYMGRAA	tr_C9ZP82	0.024	38508.84	80.00
465	HLA-A0201	DGTLPSSTECGSPK	TLPECGSPK	2	0	0	3	3	TLPSSTECGSPK	tr_C9ZP82	0.024	38514.67	80.00

465	HLA-A0201	DGTLPSSTECGS	TLSSTECGS	2	0	0	2	1	TLPSSTECGS	tr_C9ZP82	0.024	38515.49	80.00
53	HLA-A0201	EEEKLECICSE	EEKLCICSE	1	0	0	4	1	EEKLECICSE	tr_C9ZP82	0.024	38518.00	80.00
788	HLA-A0201	EERWRTQKLDFH	EERWRTLDF	0	0	0	6	2	EERWRTQKLDF	tr_C9ZP82	0.024	38527.17	80.00
859	HLA-A0201	VPRLSYGADGQ	VLSYGADGQ	0	0	0	1	2	VPRLSYGADGQ	tr_C9ZP82	0.024	38527.17	80.00
353	HLA-A0201	DDTCIISLN	DDTCIISLN	0	0	0	0	0	DDTCIISLN	tr_C9ZP82	0.024	38530.10	80.00
91	HLA-A0201	NQQRARRYMGRA	QQRAYMGRA	1	0	0	4	2	QQRARRYMGRA	tr_C9ZP82	0.024	38534.25	80.00
460	HLA-A0201	PNHIEDGTLPS	HIEDGTLPS	2	0	0	0	0	HIEDGTLPS	tr_C9ZP82	0.024	38545.11	80.00
398	HLA-A0201	REAHVTTVPNT	EAHVTTVPT	1	0	0	8	1	EAHVTTVPNT	tr_C9ZP82	0.024	38558.44	80.00
698	HLA-A0201	LPVVKSTPP	LPVVKSTPP	0	0	0	0	0	LPVVKSTPP	tr_C9ZP82	0.024	38563.86	80.00
735	HLA-A0201	SPQQAELES	SPQQAELES	0	0	0	0	0	SPQQAELES	tr_C9ZP82	0.024	38574.73	80.00
528	HLA-A0201	APSAFSGES	APSAFSGES	0	0	0	0	0	APSAFSGES	tr_C9ZP82	0.024	38583.49	80.00
624	HLA-A0201	IDDRWRDTYLKSTG	IDDRWDTYL	0	0	0	5	1	IDDRWRDTYL	tr_C9ZP82	0.024	38588.09	80.00
884	HLA-A0201	KGNGEGVIEGKK	NGEGVIEGK	2	0	0	8	1	NGEGVIEGKK	tr_C9ZP82	0.024	38592.67	80.00
496	HLA-A0201	KNFRPCSSP	KNFRPCSSP	0	0	0	0	0	KNFRPCSSP	tr_C9ZP82	0.024	38596.85	80.00
457	HLA-A0201	FGQPNHIEDGT	GQPNHIEGT	1	0	0	7	1	GQPNHIEDGT	tr_C9ZP82	0.024	38598.10	80.00
658	HLA-A0201	RYYTTTTTDS	RYYTTTTTS	0	0	0	8	1	RYYTTTTTDS	tr_C9ZP82	0.024	38598.94	80.00
91	HLA-A0201	NQQRARRYMGRAAP	NQYMGRAAP	0	0	0	2	5	NQQRARRYMGRAAP	tr_C9ZP82	0.024	38600.19	80.00
881	HLA-A0201	GQLKGNGEG	GQLKGNGEG	0	0	0	0	0	GQLKGNGEG	tr_C9ZP82	0.024	38601.86	80.00
101	HLA-A0201	RAAPAQTGEVAS	RAAPAQTEV	0	0	0	7	1	RAAPAQTGEV	tr_C9ZP82	0.024	38606.88	80.00
633	HLA-A0201	LKSTGLSNS	LKSTGLSNS	0	0	0	0	0	LKSTGLSNS	tr_C9ZP82	0.024	38617.74	80.00
490	HLA-A0201	EAERSWKNF	EAERSWKNF	0	0	0	0	0	EAERSWKNF	tr_C9ZP82	0.024	38618.56	80.00
92	HLA-A0201	QQRARRYMGR	QQRARYMGR	0	0	0	4	1	QQRARRYMGR	tr_C9ZP82	0.024	38631.12	80.00
125	HLA-A0201	EEVVREFTS	EEVVREFTS	0	0	0	0	0	EEVVREFTS	tr_C9ZP82	0.024	38634.88	80.00
396	HLA-A0201	ESREAHVTTVPNTG	ESREAVTTV	0	0	0	5	1	ESREAHVTTV	tr_C9ZP82	0.024	38641.13	80.00
172	HLA-A0201	LNDGDGVNS	LNDGDGVNS	0	0	0	0	0	LNDGDGVNS	tr_C9ZP82	0.024	38641.98	80.00
883	HLA-A0201	LKGNGEGVI	LKGNGEGVI	0	0	0	0	0	LKGNGEGVI	tr_C9ZP82	0.024	38646.99	80.00
456	HLA-A0201	FFGQPNHIEDG	FQPNHIEDG	0	0	0	1	2	FFGQPNHIEDG	tr_C9ZP82	0.024	38651.18	80.00
396	HLA-A0201	ESREAHVTTVPN	ESREAVTTV	0	0	0	5	1	ESREAHVTTV	tr_C9ZP82	0.024	38670.82	80.00
864	HLA-A0201	YGADGQVIKRQRR	YQVIKRQRR	0	0	0	1	4	YGADGQVIKRQRR	tr_C9ZP82	0.024	38677.54	80.00
263	HLA-A0201	CVIFSGSNTPGEG	VIFSGSNTG	1	0	0	8	3	VIFSGSNTPGEG	tr_C9ZP82	0.024	38680.88	80.00
864	HLA-A0201	YGADGQVIKRQ	GADGQVIRQ	1	0	0	7	1	GADGQVIKRQ	tr_C9ZP82	0.024	38686.32	80.00
488	HLA-A0201	KAEAERSWKNF	AEAERSWNF	1	0	0	7	1	AEAERSWKNF	tr_C9ZP82	0.024	38688.41	80.00
38	HLA-A0201	PCCHNDSDTAI	CCHDSDTAI	1	0	0	3	1	CCHNDSDTAI	tr_C9ZP82	0.024	38690.51	80.00
789	HLA-A0201	ERWRTQKLDFH	ERWQKLDFH	0	0	0	3	2	ERWRTQKLDFH	tr_C9ZP82	0.024	38692.18	80.00
194	HLA-A0201	HENRYVESNQVVDD	RYVESNQVV	3	0	0	0	0	RYVESNQVV	tr_C9ZP82	0.024	38701.81	80.00
864	HLA-A0201	YGADGQVIK	YGADGQVIK	0	0	0	0	0	YGADGQVIK	tr_C9ZP82	0.024	38718.56	80.00
430	HLA-A0201	CDIKKFTPATE	CIKKFTPAT	0	0	0	1	1	CDIKKFTPAT	tr_C9ZP82	0.024	38722.76	80.00
398	HLA-A0201	REAHVTTVPNTG	RVTTVPNTG	0	0	0	1	3	REAHVTTVPNTG	tr_C9ZP82	0.024	38729.88	80.00
783	HLA-A0201	PQLTEEERWRTQK	QLTEEEWRT	1	0	0	6	1	QLTEEERWRT	tr_C9ZP82	0.024	38730.73	80.00
185	HLA-A0201	STDDDYESTHENRY	STDDDYESY	0	0	0	8	5	STDDDYESTHENRY	tr_C9ZP82	0.024	38731.12	80.00
485	HLA-A0201	VSKKAEAERSWKNF	AEAERSWNF	4	0	0	7	1	AEAERSWKNF	tr_C9ZP82	0.024	38738.27	80.00
397	HLA-A0201	SREAHVTTVPNTG	SVTTVPNTG	0	0	0	1	4	SREAHVTTVPNTG	tr_C9ZP82	0.024	38738.67	80.00
91	HLA-A0201	NQQRARRYMGRAA	QQRARRYMA	1	0	0	8	3	QQRARRYMGRAA	tr_C9ZP82	0.024	38743.29	80.00
386	HLA-A0201	SSDTDNESPIES	SSDESPIES	0	0	0	3	3	SSDTDNESPIES	tr_C9ZP82	0.024	38744.96	80.00
486	HLA-A0201	SKKAEAERSWKNF	AEAERSWNF	3	0	0	7	1	AEAERSWKNF	tr_C9ZP82	0.024	38749.59	80.00
529	HLA-A0201	PSAFSGESALD	SAFSGESAL	1	0	0	0	0	SAFSGESAL	tr_C9ZP82	0.024	38755.86	80.00
858	HLA-A0201	EVPRLSYGADGQ	RLSYGADGQ	3	0	0	0	0	RLSYGADGQ	tr_C9ZP82	0.024	38756.29	80.00
484	HLA-A0201	RVSKKAEAE	RVSKKAEAE	0	0	0	0	0	RVSKKAEAE	tr_C9ZP82	0.024	38757.96	80.00
830	HLA-A0201	PSREGVEIRQ	SREGVEIRQ	1	0	0	0	0	SREGVEIRQ	tr_C9ZP82	0.023	38778.51	80.00
330	HLA-A0201	YKRRAVARQQ	YRRAVARQQ	0	0	0	1	1	YKRRAVARQQ	tr_C9ZP82	0.023	38779.36	80.00
346	HLA-A0201	RRKRNFIDDTC	RKRNFIDTC	1	0	0	6	1	RKRNFIDDTC	tr_C9ZP82	0.023	38780.61	80.00
38	HLA-A0201	PCCHNDSDTA	CCHNDSDTA	1	0	0	0	0	CCHNDSDTA	tr_C9ZP82	0.023	38782.71	80.00
190	HLA-A0201	YESTHENRYVE	YSTHENRYV	0	0	0	1	1	YESTHENRYV	tr_C9ZP82	0.023	38785.64	80.00
863	HLA-A0201	SYGADGQVIKRQ	GADGQVIRQ	2	0	0	7	1	GADGQVIKRQ	tr_C9ZP82	0.023	38798.25	80.00

658	HLA-A0201	RYYTTTTTD	RYYTTTTTD	0	0	0	0	0	RYYTTTTTD	tr_C9ZP82	0.023	38799.93	80.00
467	HLA-A0201	TLPSSTECGSP	TLPSSTESP	0	0	0	7	2	TLPSSTECGSP	tr_C9ZP82	0.023	38800.35	80.00
729	HLA-A0201	GEVMVRSPQQAE	VMVRSPQQA	2	0	0	0	0	VMVRSPQQA	tr_C9ZP82	0.023	38802.03	80.00
90	HLA-A0201	MNQQRARRYMGRAA	QQRARRYMA	2	0	0	8	3	QQRARRYMGRAA	tr_C9ZP82	0.023	38807.91	80.00
246	HLA-A0201	NMLNEKMSGND	MLNEKMSGD	1	0	0	8	1	MLNEKMSGND	tr_C9ZP82	0.023	38822.18	80.00
52	HLA-A0201	PEEEKLECICSE	EEKLCICSE	2	0	0	4	1	EEKLECICSE	tr_C9ZP82	0.023	38822.60	80.00
701	HLA-A0201	VKSTPPDTLVQ	KSTPPDTLV	1	0	0	0	0	KSTPPDTLV	tr_C9ZP82	0.023	38825.53	80.00
116	HLA-A0201	PNKSGMAIVEE	KSGMAIVEE	2	0	0	0	0	KSGMAIVEE	tr_C9ZP82	0.023	38829.74	80.00
219	HLA-A0201	DPTAFEFDS	DPTAFEFDS	0	0	0	0	0	DPTAFEFDS	tr_C9ZP82	0.023	38830.16	80.00
322	HLA-A0201	LRDTTRNPYKRRAV	TTRNPYRAV	3	0	0	6	2	TTRNPYKRRAV	tr_C9ZP82	0.023	38833.09	80.00
810	HLA-A0201	VPKGDLRKLSDA	KGDLRLSDA	2	0	0	5	1	KGDLRKLSDA	tr_C9ZP82	0.023	38835.20	80.00
662	HLA-A0201	TTTTDSDWSWY	TTTDSDWWY	1	0	0	7	1	TTTDSDWSWY	tr_C9ZP82	0.023	38842.34	80.00
128	HLA-A0201	VREFTSTEMAQ	REFTSTEMA	1	0	0	0	0	REFTSTEMA	tr_C9ZP82	0.023	38847.80	80.00
885	HLA-A0201	GNGEGVIEGKK	NGEGVIEGK	1	0	0	8	1	NGEGVIEGKK	tr_C9ZP82	0.023	38850.33	80.00
886	HLA-A0201	NGEGVIEGKKS	NGEGVIEGS	0	0	0	8	2	NGEGVIEGKKS	tr_C9ZP82	0.023	38856.22	80.00
345	HLA-A0201	SRRKRNFIDDTC	RKRNFIDTC	2	0	0	6	1	RKRNFIDDTC	tr_C9ZP82	0.023	38864.21	80.00
500	HLA-A0201	PCSSPANSKI	CSSPANSKI	1	0	0	0	0	CSSPANSKI	tr_C9ZP82	0.023	38864.64	80.00
640	HLA-A0201	NSVQEACEK	NSVQEACEK	0	0	0	0	0	NSVQEACEK	tr_C9ZP82	0.023	38864.64	80.00
883	HLA-A0201	LKGNGEGVIEGKKS	NGEGVIEGS	3	0	0	8	2	NGEGVIEGKKS	tr_C9ZP82	0.023	38869.25	80.00
398	HLA-A0201	REAHVTTVP	REAHVTTVP	0	0	0	0	0	REAHVTTVP	tr_C9ZP82	0.023	38893.22	80.00
884	HLA-A0201	KGNGEGVIEGKKS	NGEGVIEGS	2	0	0	8	2	NGEGVIEGKKS	tr_C9ZP82	0.023	38903.76	80.00
898	HLA-A0201	SRVTVVNKGP	SRVTVVNGP	0	0	0	7	1	SRVTVVNKGP	tr_C9ZP82	0.023	38915.55	80.00
346	HLA-A0201	RRKRNFIDDT	RRRNFIDDT	0	0	0	2	1	RRKRNFIDDT	tr_C9ZP82	0.023	38916.80	80.00
559	HLA-A0201	PHEINLPQLQ	HEINLPQLQ	1	0	0	0	0	HEINLPQLQ	tr_C9ZP82	0.023	38918.91	80.00
605	HLA-A0201	GSNNDNVTVAD	GSNNDNVTV	0	0	0	0	0	GSNNDNVTV	tr_C9ZP82	0.023	38921.02	80.00
3	HLA-A0201	PKFASWLRN	PKFASWLRN	0	0	0	0	0	PKFASWLRN	tr_C9ZP82	0.023	38926.92	80.00
843	HLA-A0201	VPLERYRPRTYS	PLYRPRTYS	1	0	0	2	2	PLERYRPRTYS	tr_C9ZP82	0.023	38939.98	80.00
264	HLA-A0201	VIFSGSNTPGE	VIFGSNTPG	0	0	0	3	1	VIFSGSNTPG	tr_C9ZP82	0.023	38940.38	80.00
657	HLA-A0201	WRYYTTTTTD	WRYYTTTTT	0	0	0	0	0	WRYYTTTTT	tr_C9ZP82	0.023	38943.34	80.00
210	HLA-A0201	GHRGEVIVD	GHRGEVIVD	0	0	0	0	0	GHRGEVIVD	tr_C9ZP82	0.023	38950.93	80.00
328	HLA-A0201	NPYKRRAVAR	NPYKRAVAR	0	0	0	4	1	NPYKRRAVAR	tr_C9ZP82	0.023	38954.72	80.00
638	HLA-A0201	LSNSVQEACE	LSNSVQEAC	0	0	0	0	0	LSNSVQEAC	tr_C9ZP82	0.023	38976.64	80.00
263	HLA-A0201	CVIFSGSNTPGE	VIFGSNTPG	1	0	0	3	1	VIFSGSNTPG	tr_C9ZP82	0.023	38984.65	80.00
559	HLA-A0201	PHEINLPQLQR	PHEINLLQR	0	0	0	6	2	PHEINLPQLQR	tr_C9ZP82	0.023	38989.30	80.00
829	HLA-A0201	VPSREGVEIRQ	VPSGVEIRQ	0	0	0	3	2	VPSREGVEIRQ	tr_C9ZP82	0.023	38991.82	80.00
527	HLA-A0201	HAPSAFSGE	HAPSAFSGE	0	0	0	0	0	HAPSAFSGE	tr_C9ZP82	0.023	38998.99	80.00
497	HLA-A0201	NFRPCSSPAN	NFRPCSSPA	0	0	0	0	0	NFRPCSSPA	tr_C9ZP82	0.023	38999.41	80.00
385	HLA-A0201	SSSDTDNESPIES	SSDESPIES	1	0	0	3	3	SSDTDNESPIES	tr_C9ZP82	0.023	39000.27	80.00
863	HLA-A0201	SYGADGQVIKRQRR	GADGQVIRR	2	0	0	7	3	GADGQVIKRQRR	tr_C9ZP82	0.023	39007.87	80.00
691	HLA-A0201	ANIKRMPLP	ANIKRMPLP	0	0	0	0	0	ANIKRMPLP	tr_C9ZP82	0.023	39009.55	80.00
487	HLA-A0201	KKAEAERSWKNF	AEAERSWNF	2	0	0	7	1	AEAERSWKNF	tr_C9ZP82	0.023	39010.38	80.00
125	HLA-A0201	EEVVREFTST	EEVVRFTST	0	0	0	5	1	EEVVREFTST	tr_C9ZP82	0.023	39011.66	80.00
593	HLA-A0201	KENSNSGAEF	KENSSGAEF	0	0	0	4	1	KENSNSGAEF	tr_C9ZP82	0.023	39014.61	80.00
481	HLA-A0201	RSKRVSKKA	RSKRVSKKA	0	0	0	0	0	RSKRVSKKA	tr_C9ZP82	0.023	39037.41	80.00
622	HLA-A0201	STIDDRWRD	STIDDRWRD	0	0	0	0	0	STIDDRWRD	tr_C9ZP82	0.023	39039.10	80.00
495	HLA-A0201	WKNFRPCSSPAN	KNFPCSSPA	1	0	0	3	1	KNFRPCSSPA	tr_C9ZP82	0.023	39045.02	80.00
136	HLA-A0201	MAQANDDLDE	MQANDDLDE	0	0	0	1	1	MAQANDDLDE	tr_C9ZP82	0.023	39053.91	80.00
742	HLA-A0201	ESTFPLQWN	ESTFPLQWN	0	0	0	0	0	ESTFPLQWN	tr_C9ZP82	0.023	39055.59	80.00
865	HLA-A0201	GADGQVIKRQR	GADGQVIQR	0	0	0	7	2	GADGQVIKRQR	tr_C9ZP82	0.023	39056.00	80.00
844	HLA-A0201	PLERYRPRTYS	PLYRPRTYS	0	0	0	2	2	PLERYRPRTYS	tr_C9ZP82	0.023	39058.11	80.00
865	HLA-A0201	GADGQVIKRQRR	GADGQVIRR	0	0	0	7	3	GADGQVIKRQRR	tr_C9ZP82	0.023	39061.51	80.00
498	HLA-A0201	FRPCSSPANS	FPCSSPANS	0	0	0	1	1	FRPCSSPANS	tr_C9ZP82	0.023	39065.72	80.00
557	HLA-A0201	KGPHEINLP	KGPHEINLP	0	0	0	0	0	KGPHEINLP	tr_C9ZP82	0.023	39065.72	80.00
885	HLA-A0201	GNGEGVIEGKKS	NGEGVIEGS	1	0	0	8	2	NGEGVIEGKKS	tr_C9ZP82	0.023	39069.09	80.00

484	HLA-A0201	RVSKKAEAERSW	RVSKKAEAW	0	0	0	8	3	RVSKKAEAERSW	tr_C9ZP82	0.023	39073.33	80.00
886	HLA-A0201	NGEGVIEGKKSNS	NGEGVIEGS	0	0	0	8	4	NGEGVIEGKKSNS	tr_C9ZP82	0.023	39077.13	80.00
85	HLA-A0201	APRAKMNQQ	APRAKMNQQ	0	0	0	0	0	APRAKMNQQ	tr_C9ZP82	0.023	39086.03	80.00
471	HLA-A0201	STECGSPKG	STECGSPKG	0	0	0	0	0	STECGSPKG	tr_C9ZP82	0.023	39087.29	80.00
626	HLA-A0201	DRWRDTYLKST	RWRDTYLST	1	0	0	7	1	RWRDTYLKST	tr_C9ZP82	0.023	39091.93	80.00
238	HLA-A0201	AKVSDAVLN	AKVSDAVLN	0	0	0	0	0	AKVSDAVLN	tr_C9ZP82	0.023	39092.36	80.00
143	HLA-A0201	LDEVRQTLLE	LDEVRTLLE	0	0	0	5	1	LDEVRQTLLE	tr_C9ZP82	0.023	39113.52	80.00
868	HLA-A0201	GQVIKRQRR	GQVIKRQRR	0	0	0	0	0	GQVIKRQRR	tr_C9ZP82	0.023	39117.75	80.00
716	HLA-A0201	LPPTSHALLP	LPPTSHALL	0	0	0	0	0	LPPTSHALL	tr_C9ZP82	0.023	39124.95	80.00
293	HLA-A0201	GGKANHVIVG	GGKANHVIV	0	0	0	0	0	GGKANHVIV	tr_C9ZP82	0.023	39124.95	80.00
187	HLA-A0201	DDDYESTHENRYVE	YSTHENRYV	3	0	0	1	1	YESTHENRYV	tr_C9ZP82	0.023	39132.57	80.00
37	HLA-A0201	HPCCHNDSDTAIA	CCDSDTAIA	2	0	0	2	2	CCHNDSDTAIA	tr_C9ZP82	0.023	39135.54	80.00
189	HLA-A0201	DYESTHENRYVE	YSTHENRYV	1	0	0	1	1	YESTHENRYV	tr_C9ZP82	0.023	39141.45	80.00
781	HLA-A0201	AWPQLTEEER	AWPQLTEER	0	0	0	6	1	AWPQLTEEER	tr_C9ZP82	0.023	39152.48	80.00
638	HLA-A0201	LSNSVQEACEK	LSNSVACEK	0	0	0	5	2	LSNSVQEACEK	tr_C9ZP82	0.023	39162.63	80.00
188	HLA-A0201	DDYESTHENRYVE	YSTHENRYV	2	0	0	1	1	YESTHENRYV	tr_C9ZP82	0.023	39164.75	80.00
590	HLA-A0201	STNKENSNSGAEF	STSNSGAEF	0	0	0	2	4	STNKENSNSGAEF	tr_C9ZP82	0.023	39175.36	80.00
397	HLA-A0201	SREAHVTTVPN	SREAHVTTV	0	0	0	0	0	SREAHVTTV	tr_C9ZP82	0.023	39177.89	80.00
51	HLA-A0201	LPEEEKLEC	LPEEEKLEC	0	0	0	0	0	LPEEEKLEC	tr_C9ZP82	0.023	39179.18	80.00
467	HLA-A0201	TLPSSTECGSPKG	TLPSSTECG	0	0	0	8	4	TLPSSTECGSPKG	tr_C9ZP82	0.023	39184.26	80.00
494	HLA-A0201	SWKNFRPCSSPAN	KNFPCSSPA	2	0	0	3	1	KNFRPCSSPA	tr_C9ZP82	0.022	39200.36	80.00
155	HLA-A0201	LYSGALYNEGE	YSGALYEGE	1	0	0	6	1	YSGALYNEGE	tr_C9ZP82	0.022	39205.04	80.00
182	HLA-A0201	CVRSTDDDYESTH	CVDDDYEST	0	0	0	2	3	CVRSTDDDYEST	tr_C9ZP82	0.022	39210.55	80.00
842	HLA-A0201	DVPLERYRPRTYS	PLYRPRTYS	2	0	0	2	2	PLERYRPRTYS	tr_C9ZP82	0.022	39216.49	80.00
660	HLA-A0201	YTTTTTDSDWS	YTTTTTDSW	0	0	0	8	1	YTTTTTDSDW	tr_C9ZP82	0.022	39220.74	80.00
344	HLA-A0201	RSRRKRNFIDDT	RSRNFIDDT	0	0	0	2	3	RSRRKRNFIDDT	tr_C9ZP82	0.022	39222.01	80.00
864	HLA-A0201	YGADGQVIKRQR	GADGQVIQR	1	0	0	7	2	GADGQVIKRQR	tr_C9ZP82	0.022	39224.99	80.00
628	HLA-A0201	WRDTYLKSTG	WRDTYLSTG	0	0	0	6	1	WRDTYLKSTG	tr_C9ZP82	0.022	39225.82	80.00
386	HLA-A0201	SSDTDNESPIESRE	SSDTDNEPI	0	0	0	7	1	SSDTDNESPI	tr_C9ZP82	0.022	39226.25	80.00
365	HLA-A0201	IDEEESDGVI	IEEESDGVI	0	0	0	1	1	IDEEESDGVI	tr_C9ZP82	0.022	39233.04	80.00
621	HLA-A0201	HSTIDDRWRDT	STIDDRWRT	1	0	0	8	1	STIDDRWRDT	tr_C9ZP82	0.022	39234.73	80.00
866	HLA-A0201	ADGQVIKRQR	ADGQVIRQR	0	0	0	6	1	ADGQVIKRQR	tr_C9ZP82	0.022	39240.25	80.00
885	HLA-A0201	GNGEGVIEGKKSNS	NGEGVIEGS	1	0	0	8	4	NGEGVIEGKKSNS	tr_C9ZP82	0.022	39249.61	80.00
232	HLA-A0201	PGTDFMAKVS	PGTDFMAKV	0	0	0	0	0	PGTDFMAKV	tr_C9ZP82	0.022	39250.45	80.00
626	HLA-A0201	DRWRDTYLKS	RWRDTYLKS	1	0	0	0	0	RWRDTYLKS	tr_C9ZP82	0.022	39253.84	80.00
388	HLA-A0201	DTDNESPIES	DTDESPIES	0	0	0	3	1	DTDNESPIES	tr_C9ZP82	0.022	39256.83	80.00
625	HLA-A0201	DDRWRDTYLKST	RWRDTYLST	2	0	0	7	1	RWRDTYLKST	tr_C9ZP82	0.022	39264.04	80.00
863	HLA-A0201	SYGADGQVIKRQR	GADGQVIQR	2	0	0	7	2	GADGQVIKRQR	tr_C9ZP82	0.022	39272.12	80.00
94	HLA-A0201	RARRYMGRAAP	RAYMGRAAP	0	0	0	2	2	RARRYMGRAAP	tr_C9ZP82	0.022	39286.99	80.00
495	HLA-A0201	WKNFRPCSSPANS	FPCSSPANS	3	0	0	1	1	FRPCSSPANS	tr_C9ZP82	0.022	39287.42	80.00
496	HLA-A0201	KNFRPCSSPAN	KNFPCSSPA	0	0	0	3	1	KNFRPCSSPA	tr_C9ZP82	0.022	39289.55	80.00
600	HLA-A0201	AEFRKGSNNDNV	AEFSNNDNV	0	0	0	3	3	AEFRKGSNNDNV	tr_C9ZP82	0.022	39294.21	80.00
730	HLA-A0201	EVMVRSPQQAE	VMVRSPQQA	1	0	0	0	0	VMVRSPQQA	tr_C9ZP82	0.022	39310.79	80.00
391	HLA-A0201	NESPIESREA	NSPIESREA	0	0	0	1	1	NESPIESREA	tr_C9ZP82	0.022	39313.78	80.00
495	HLA-A0201	WKNFRPCSSPANSK	KNFPCSSPA	1	0	0	3	1	KNFRPCSSPA	tr_C9ZP82	0.022	39321.44	80.00
38	HLA-A0201	PCCHNDSDTAIA	CCDSDTAIA	1	0	0	2	2	CCHNDSDTAIA	tr_C9ZP82	0.022	39326.11	80.00
90	HLA-A0201	MNQQRARRYMGR	MQQRARRYM	0	0	0	1	1	MNQQRARRYM	tr_C9ZP82	0.022	39332.93	80.00
456	HLA-A0201	FFGQPNHIE	FFGQPNHIE	0	0	0	0	0	FFGQPNHIE	tr_C9ZP82	0.022	39340.59	80.00
494	HLA-A0201	SWKNFRPCSSPANS	FPCSSPANS	4	0	0	1	1	FRPCSSPANS	tr_C9ZP82	0.022	39344.42	80.00
83	HLA-A0201	GVAPRAKMNQQRA	GVAPRAQRA	0	0	0	6	4	GVAPRAKMNQQRA	tr_C9ZP82	0.022	39356.35	80.00
390	HLA-A0201	DNESPIESR	DNESPIESR	0	0	0	0	0	DNESPIESR	tr_C9ZP82	0.022	39364.01	80.00
771	HLA-A0201	MDVLQQVVD	MDVLQQVVD	0	0	0	0	0	MDVLQQVVD	tr_C9ZP82	0.022	39364.85	80.00
190	HLA-A0201	YESTHENRYVES	YSTHENRYV	0	0	0	1	1	YESTHENRYV	tr_C9ZP82	0.022	39373.38	80.00
457	HLA-A0201	FGQPNHIED	FGQPNHIED	0	0	0	0	0	FGQPNHIED	tr_C9ZP82	0.022	39405.77	80.00

527	HLA-A0201	HAPSAFSGES	HASAFSGES	0	0	0	2	1	HAPSAFSGES	tr_C9ZP82	0.022	39407.04	80.00
674	HLA-A0201	PFYHAPPVID	FYHAPPVID	1	0	0	0	0	FYHAPPVID	tr_C9ZP82	0.022	39407.47	80.00
183	HLA-A0201	VRSTDDDYES	VSTDDDYES	0	0	0	1	1	VRSTDDDYES	tr_C9ZP82	0.022	39409.61	80.00
430	HLA-A0201	CDIKKFTPATES	CIKKFTPAT	0	0	0	1	1	CDIKKFTPAT	tr_C9ZP82	0.022	39412.58	80.00
659	HLA-A0201	YYTTTTTDSDW	YTTTTTDSW	1	0	0	8	1	YTTTTTDSDW	tr_C9ZP82	0.022	39413.02	80.00
499	HLA-A0201	RPCSSPANS	RPCSSPANS	0	0	0	0	0	RPCSSPANS	tr_C9ZP82	0.022	39415.15	80.00
529	HLA-A0201	PSAFSGESA	PSAFSGESA	0	0	0	0	0	PSAFSGESA	tr_C9ZP82	0.022	39416.42	80.00
865	HLA-A0201	GADGQVIKRQRRH	GADGQVIRH	0	0	0	7	4	GADGQVIKRQRRH	tr_C9ZP82	0.022	39418.12	80.00
245	HLA-A0201	LNMLNEKMSGND	MLNEKMSGD	2	0	0	8	1	MLNEKMSGND	tr_C9ZP82	0.022	39422.83	80.00
81	HLA-A0201	VDGVAPRAK	VDGVAPRAK	0	0	0	0	0	VDGVAPRAK	tr_C9ZP82	0.022	39423.26	80.00
185	HLA-A0201	STDDDYESTHEN	STYESTHEN	0	0	0	2	3	STDDDYESTHEN	tr_C9ZP82	0.022	39426.66	80.00
360	HLA-A0201	LNPSYIDEE	LNPSYIDEE	0	0	0	0	0	LNPSYIDEE	tr_C9ZP82	0.022	39431.78	80.00
635	HLA-A0201	STGLSNSVQ	STGLSNSVQ	0	0	0	0	0	STGLSNSVQ	tr_C9ZP82	0.022	39449.71	80.00
386	HLA-A0201	SSDTDNESP	SSDTDNESP	0	0	0	0	0	SSDTDNESP	tr_C9ZP82	0.022	39459.94	80.00
734	HLA-A0201	RSPQQAELES	RSPQQALES	0	0	0	6	1	RSPQQAELES	tr_C9ZP82	0.022	39462.51	80.00
864	HLA-A0201	YGADGQVIKRQRRH	GADGQVIRH	1	0	0	7	4	GADGQVIKRQRRH	tr_C9ZP82	0.022	39465.08	80.00
327	HLA-A0201	RNPYKRRAV	RNPYKRRAV	0	0	0	0	0	RNPYKRRAV	tr_C9ZP82	0.022	39468.49	80.00
843	HLA-A0201	VPLERYRPRTYSCT	VPLETYSCT	0	0	0	4	5	VPLERYRPRTYSCT	tr_C9ZP82	0.022	39485.14	80.00
487	HLA-A0201	KKAEAERSW	KKAEAERSW	0	0	0	0	0	KKAEAERSW	tr_C9ZP82	0.022	39486.84	80.00
375	HLA-A0201	GVPVCESSE	GVPVCESSE	0	0	0	0	0	GVPVCESSE	tr_C9ZP82	0.022	39491.56	80.00
734	HLA-A0201	RSPQQAELE	RSPQQAELE	0	0	0	0	0	RSPQQAELE	tr_C9ZP82	0.022	39498.38	80.00
431	HLA-A0201	DIKKFTPATE	DIKFTPATE	0	0	0	2	1	DIKKFTPATE	tr_C9ZP82	0.022	39501.39	80.00
255	HLA-A0201	NDPTWTRLC	NDPTWTRLC	0	0	0	0	0	NDPTWTRLC	tr_C9ZP82	0.022	39501.39	80.00
189	HLA-A0201	DYESTHENRYVES	YSTHENRYV	1	0	0	1	1	YESTHENRYV	tr_C9ZP82	0.022	39512.50	80.00
163	HLA-A0201	EGEEEAGDAL	GEEEAGDAL	1	0	0	0	0	GEEEAGDAL	tr_C9ZP82	0.022	39513.36	80.00
190	HLA-A0201	YESTHENRYVESNQ	YSTHENRYV	0	0	0	1	1	YESTHENRYV	tr_C9ZP82	0.022	39522.77	80.00
467	HLA-A0201	TLPSSTECGSPKGR	TLPSSTEGR	0	0	0	7	5	TLPSSTECGSPKGR	tr_C9ZP82	0.022	39531.74	80.00
884	HLA-A0201	KGNGEGVIEGKKSN	KGNGVIEGK	0	0	0	3	2	KGNGEGVIEGK	tr_C9ZP82	0.022	39535.16	80.00
100	HLA-A0201	GRAAPAQTGEVAS	RAAPAQTEV	1	0	0	7	1	RAAPAQTGEV	tr_C9ZP82	0.022	39538.60	80.00
188	HLA-A0201	DDYESTHENRYVES	YSTHENRYV	2	0	0	1	1	YESTHENRYV	tr_C9ZP82	0.022	39543.29	80.00
264	HLA-A0201	VIFSGSNTPGEGEH	VIFSGSNTH	0	0	0	8	5	VIFSGSNTPGEGEH	tr_C9ZP82	0.022	39547.14	80.00
599	HLA-A0201	GAEFRKGSNNDNV	GAGSNNDNV	0	0	0	2	4	GAEFRKGSNNDNV	tr_C9ZP82	0.022	39553.14	80.00
180	HLA-A0201	SFCVRSTDDDYE	SFCVRSTYE	0	0	0	7	3	SFCVRSTDDDYE	tr_C9ZP82	0.022	39557.42	80.00
886	HLA-A0201	NGEGVIEGKKSN	NGEGVIEGK	0	0	0	0	0	NGEGVIEGK	tr_C9ZP82	0.022	39558.27	80.00
263	HLA-A0201	CVIFSGSNTPGEGE	CVIFSGEGE	0	0	0	5	5	CVIFSGSNTPGEGE	tr_C9ZP82	0.022	39559.13	80.00
323	HLA-A0201	RDTTRNPYKRRAV	TTRNPYRAV	2	0	0	6	2	TTRNPYKRRAV	tr_C9ZP82	0.022	39560.84	80.00
627	HLA-A0201	RWRDTYLKSTG	RWRDTYLTG	0	0	0	7	2	RWRDTYLKSTG	tr_C9ZP82	0.022	39565.97	80.00
488	HLA-A0201	KAEAERSWKNFRP	KASWKNFRP	0	0	0	2	4	KAEAERSWKNFRP	tr_C9ZP82	0.022	39586.11	80.00
93	HLA-A0201	QRARRYMGRA	RARRYMGRA	1	0	0	0	0	RARRYMGRA	tr_C9ZP82	0.022	39586.95	80.00
844	HLA-A0201	PLERYRPRT	PLERYRPRT	0	0	0	0	0	PLERYRPRT	tr_C9ZP82	0.022	39595.96	80.00
134	HLA-A0201	TEMAQANDDLDE	TMAQANDDL	0	0	0	1	1	TEMAQANDDL	tr_C9ZP82	0.022	39597.67	80.00
494	HLA-A0201	SWKNFRPCSS	SWKNFPCSS	0	0	0	5	1	SWKNFRPCSS	tr_C9ZP82	0.022	39602.81	80.00
325	HLA-A0201	TTRNPYKRRAV	TTRNPYRAV	0	0	0	6	2	TTRNPYKRRAV	tr_C9ZP82	0.022	39607.10	80.00
616	HLA-A0201	VAVALHSTIDD	VAVALHSTI	0	0	0	0	0	VAVALHSTI	tr_C9ZP82	0.022	39609.68	80.00
482	HLA-A0201	SKRVSKKAEAERSW	RVSKKAEAW	2	0	0	8	3	RVSKKAEAERSW	tr_C9ZP82	0.022	39612.23	80.00
483	HLA-A0201	KRVSKKAEAERSW	RVSKKAEAW	1	0	0	8	3	RVSKKAEAERSW	tr_C9ZP82	0.022	39621.66	85.00
496	HLA-A0201	KNFRPCSSPANS	KNFPCSSPA	0	0	0	3	1	KNFRPCSSPA	tr_C9ZP82	0.022	39622.09	85.00
182	HLA-A0201	CVRSTDDDYES	CVRSTDDDY	0	0	0	0	0	CVRSTDDDY	tr_C9ZP82	0.022	39622.53	85.00
491	HLA-A0201	AERSWKNFR	AERSWKNFR	0	0	0	0	0	AERSWKNFR	tr_C9ZP82	0.021	39625.52	85.00
53	HLA-A0201	EEEKLECIC	EEEKLECIC	0	0	0	0	0	EEEKLECIC	tr_C9ZP82	0.021	39631.09	85.00
324	HLA-A0201	DTTRNPYKRRAV	TTRNPYRAV	1	0	0	6	2	TTRNPYKRRAV	tr_C9ZP82	0.021	39643.11	85.00
729	HLA-A0201	GEVMVRSPQQ	GVMVRSPQQ	0	0	0	1	1	GEVMVRSPQQ	tr_C9ZP82	0.021	39644.41	85.00
325	HLA-A0201	TTRNPYKRRAVAR	TTYKRRAVA	0	0	0	2	3	TTRNPYKRRAVA	tr_C9ZP82	0.021	39646.96	85.00
805	HLA-A0201	NKTSFVPKG	NKTSFVPKG	0	0	0	0	0	NKTSFVPKG	tr_C9ZP82	0.021	39652.11	85.00

811	HLA-A0201	PKGDLRKLSDA	KGDLRLSDA	1	0	0	5	1	KGDLRKLSDA	tr_C9ZP82	0.021	39678.73	85.00
499	HLA-A0201	RPCSSPANSKI	RPCSSPANI	0	0	0	8	2	RPCSSPANSKI	tr_C9ZP82	0.021	39695.91	85.00
643	HLA-A0201	QEACEKYVEG	QACEKYVEG	0	0	0	1	1	QEACEKYVEG	tr_C9ZP82	0.021	39698.06	85.00
107	HLA-A0201	TGEVASTYMPN	TGEVASTYM	0	0	0	0	0	TGEVASTYM	tr_C9ZP82	0.021	39698.90	85.00
732	HLA-A0201	MVRSPQQAE	MVRSPQQAE	0	0	0	0	0	MVRSPQQAE	tr_C9ZP82	0.021	39703.20	85.00
195	HLA-A0201	ENRYVESNQVVDD	ENRYVVVDD	0	0	0	5	4	ENRYVESNQVVDD	tr_C9ZP82	0.021	39704.07	85.00
384	HLA-A0201	NSSSDTDNESPIES	SSDESPIES	2	0	0	3	3	SSDTDNESPIES	tr_C9ZP82	0.021	39704.48	85.00
385	HLA-A0201	SSSDTDNESPIE	SSSDTDNPI	0	0	0	7	2	SSSDTDNESPI	tr_C9ZP82	0.021	39710.07	85.00
890	HLA-A0201	VIEGKKSNSRVT	VIEGSNSRV	0	0	0	4	2	VIEGKKSNSRV	tr_C9ZP82	0.021	39718.67	85.00
488	HLA-A0201	KAEAERSWKNFR	AEAERSWFR	1	0	0	7	2	AEAERSWKNFR	tr_C9ZP82	0.021	39719.54	85.00
182	HLA-A0201	CVRSTDDDYE	CVRSTDDDY	0	0	0	0	0	CVRSTDDDY	tr_C9ZP82	0.021	39731.12	85.00
52	HLA-A0201	PEEEKLECICS	EEEKLCICS	1	0	0	5	1	EEEKLECICS	tr_C9ZP82	0.021	39733.71	85.00
456	HLA-A0201	FFGQPNHIED	FFGQPNIED	0	0	0	6	1	FFGQPNHIED	tr_C9ZP82	0.021	39739.30	85.00
620	HLA-A0201	LHSTIDDRWR	LSTIDDRWR	0	0	0	1	1	LHSTIDDRWR	tr_C9ZP82	0.021	39742.32	85.00
463	HLA-A0201	IEDGTLPSSTE	IEDGTLPST	0	0	0	7	1	IEDGTLPSST	tr_C9ZP82	0.021	39743.17	85.00
845	HLA-A0201	LERYRPRTYS	LERYRPRTY	0	0	0	0	0	LERYRPRTY	tr_C9ZP82	0.021	39744.04	85.00
387	HLA-A0201	SDTDNESPIESREA	SSPIESREA	0	0	0	1	5	SDTDNESPIESREA	tr_C9ZP82	0.021	39744.48	85.00
205	HLA-A0201	VDDVFGHRGE	VDDVFGHGE	0	0	0	7	1	VDDVFGHRGE	tr_C9ZP82	0.021	39748.76	85.00
659	HLA-A0201	YYTTTTTDSDWS	YTTTTTDSW	1	0	0	8	1	YTTTTTDSDW	tr_C9ZP82	0.021	39750.91	85.00
190	HLA-A0201	YESTHENRY	YESTHENRY	0	0	0	0	0	YESTHENRY	tr_C9ZP82	0.021	39758.65	85.00
596	HLA-A0201	SNSGAEFRK	SNSGAEFRK	0	0	0	0	0	SNSGAEFRK	tr_C9ZP82	0.021	39763.83	85.00
268	HLA-A0201	GSNTPGEGEHKI	NTPGEGEHI	2	0	0	8	1	NTPGEGEHKI	tr_C9ZP82	0.021	39773.30	85.00
844	HLA-A0201	PLERYRPRTYSCT	PLERYTYSC	0	0	0	5	3	PLERYRPRTYSC	tr_C9ZP82	0.021	39774.14	85.00
551	HLA-A0201	PYGFLTKGPHE	YGFLTGPHE	1	0	0	5	1	YGFLTKGPHE	tr_C9ZP82	0.021	39775.45	85.00
264	HLA-A0201	VIFSGSNTPGEGE	VIFSGSNTE	0	0	0	8	4	VIFSGSNTPGEGE	tr_C9ZP82	0.021	39778.89	85.00
107	HLA-A0201	TGEVASTYMPNK	EVASTYMPK	2	0	0	8	1	EVASTYMPNK	tr_C9ZP82	0.021	39779.32	85.00
324	HLA-A0201	DTTRNPYKRRAVAR	TRNPYAVAR	2	0	0	5	3	TRNPYKRRAVAR	tr_C9ZP82	0.021	39780.17	85.00
190	HLA-A0201	YESTHENRYVESN	YSTHENRYV	0	0	0	1	1	YESTHENRYV	tr_C9ZP82	0.021	39782.32	85.00
803	HLA-A0201	TSNKTSFVPKG	TSNKTFVPK	0	0	0	5	1	TSNKTSFVPK	tr_C9ZP82	0.021	39785.33	85.00
804	HLA-A0201	SNKTSFVPKG	SNKTSFVPG	0	0	0	8	1	SNKTSFVPKG	tr_C9ZP82	0.021	39785.33	85.00
484	HLA-A0201	RVSKKAEAERSWK	RVSKKAEAK	0	0	0	8	4	RVSKKAEAERSWK	tr_C9ZP82	0.021	39795.67	85.00
402	HLA-A0201	VTTVPNTGFE	VTTVPNTGF	0	0	0	0	0	VTTVPNTGF	tr_C9ZP82	0.021	39798.67	85.00
466	HLA-A0201	GTLPSSTECGSP	TLSTECGSP	1	0	0	2	2	TLPSSTECGSP	tr_C9ZP82	0.021	39803.42	85.00
496	HLA-A0201	KNFRPCSSPANSK	KNFPCSSPA	0	0	0	3	1	KNFRPCSSPA	tr_C9ZP82	0.021	39809.02	85.00
697	HLA-A0201	PLPVVKSTPPDT	PLPVVPPDT	0	0	0	5	3	PLPVVKSTPPDT	tr_C9ZP82	0.021	39811.17	85.00
660	HLA-A0201	YTTTTTDSD	YTTTTTDSD	0	0	0	0	0	YTTTTTDSD	tr_C9ZP82	0.021	39813.77	85.00
841	HLA-A0201	YDVPLERYRPR	YDVPLYRPR	0	0	0	5	2	YDVPLERYRPR	tr_C9ZP82	0.021	39814.18	85.00
486	HLA-A0201	SKKAEAERSW	SKKAEAESW	0	0	0	7	1	SKKAEAERSW	tr_C9ZP82	0.021	39816.77	85.00
606	HLA-A0201	SNNDNVTVAD	SNDNVTVAD	0	0	0	1	1	SNNDNVTVAD	tr_C9ZP82	0.021	39823.68	85.00
885	HLA-A0201	GNGEGVIEGKKSN	NGEGVIEGK	1	0	0	0	0	NGEGVIEGK	tr_C9ZP82	0.021	39826.25	85.00
484	HLA-A0201	RVSKKAEAERSWKN	RVAERSWKN	0	0	0	2	5	RVSKKAEAERSWKN	tr_C9ZP82	0.021	39835.32	85.00
656	HLA-A0201	VWRYYTTTTTDS	VWYYTTTTT	0	0	0	2	1	VWRYYTTTTT	tr_C9ZP82	0.021	39838.32	85.00
157	HLA-A0201	SGALYNEGE	SGALYNEGE	0	0	0	0	0	SGALYNEGE	tr_C9ZP82	0.021	39840.92	85.00
170	HLA-A0201	DALNDGDGVN	ALNDGDGVN	1	0	0	0	0	ALNDGDGVN	tr_C9ZP82	0.021	39841.33	85.00
783	HLA-A0201	PQLTEEERW	PQLTEEERW	0	0	0	0	0	PQLTEEERW	tr_C9ZP82	0.021	39852.12	85.00
389	HLA-A0201	TDNESPIESREA	TDNESPIEA	0	0	0	7	3	TDNESPIESREA	tr_C9ZP82	0.021	39862.89	85.00
388	HLA-A0201	DTDNESPIESREA	TDNESPIEA	1	0	0	7	3	TDNESPIESREA	tr_C9ZP82	0.021	39863.33	85.00
626	HLA-A0201	DRWRDTYLKSTG	RWRDTYLTG	1	0	0	7	2	RWRDTYLKSTG	tr_C9ZP82	0.021	39870.66	85.00
343	HLA-A0201	RRSRRKRNFIDDT	RSRNFIDDT	1	0	0	2	3	RSRRKRNFIDDT	tr_C9ZP82	0.021	39879.30	85.00
617	HLA-A0201	AVALHSTIDD	AVALSTIDD	0	0	0	4	1	AVALHSTIDD	tr_C9ZP82	0.021	39888.79	85.00
486	HLA-A0201	SKKAEAERSWKNFR	AEAERSWFR	3	0	0	7	2	AEAERSWKNFR	tr_C9ZP82	0.021	39895.68	85.00
483	HLA-A0201	KRVSKKAEA	KRVSKKAEA	0	0	0	0	0	KRVSKKAEA	tr_C9ZP82	0.021	39901.32	85.00
866	HLA-A0201	ADGQVIKRQRRHR	ADGQVIRHR	0	0	0	6	4	ADGQVIKRQRRHR	tr_C9ZP82	0.021	39902.60	85.00
192	HLA-A0201	STHENRYVE	STHENRYVE	0	0	0	0	0	STHENRYVE	tr_C9ZP82	0.021	39903.89	85.00

866	HLA-A0201	ADGQVIKRQRR	ADGQVIQRR	0	0	0	6	2	ADGQVIKRQRR	tr_C9ZP82	0.021	39905.61	85.00
659	HLA-A0201	YYTTTTTDS	YYTTTTTDS	0	0	0	0	0	YYTTTTTDS	tr_C9ZP82	0.021	39908.65	85.00
525	HLA-A0201	LPHAPSAFSGES	LPHAPSAFS	0	0	0	8	3	LPHAPSAFSGES	tr_C9ZP82	0.021	39909.94	85.00
267	HLA-A0201	SGSNTPGEGEHKI	NTPGEGEHI	3	0	0	8	1	NTPGEGEHKI	tr_C9ZP82	0.021	39912.54	85.00
140	HLA-A0201	NDDLDEVRQ	NDDLDEVRQ	0	0	0	0	0	NDDLDEVRQ	tr_C9ZP82	0.021	39914.70	85.00
810	HLA-A0201	VPKGDLRKLS	VPKGDLRKL	0	0	0	0	0	VPKGDLRKL	tr_C9ZP82	0.021	39919.88	85.00
589	HLA-A0201	KSTNKENSNSGAEF	STSNSGAEF	1	0	0	2	4	STNKENSNSGAEF	tr_C9ZP82	0.021	39921.16	85.00
656	HLA-A0201	VWRYYTTTTTD	VWYYTTTTT	0	0	0	2	1	VWRYYTTTTT	tr_C9ZP82	0.021	39923.76	85.00
328	HLA-A0201	NPYKRRAVARQQ	NPYAVARQQ	0	0	0	3	3	NPYKRRAVARQQ	tr_C9ZP82	0.021	39925.05	85.00
189	HLA-A0201	DYESTHENRYVESN	YSTHENRYV	1	0	0	1	1	YESTHENRYV	tr_C9ZP82	0.021	39928.94	85.00
465	HLA-A0201	DGTLPSSTECGSP	TLSTECGSP	2	0	0	2	2	TLPSSTECGSP	tr_C9ZP82	0.021	39934.14	85.00
489	HLA-A0201	AEAERSWKNFR	AEAERSWFR	0	0	0	7	2	AEAERSWKNFR	tr_C9ZP82	0.021	39935.43	85.00
432	HLA-A0201	IKKFTPATES	IKKFTPATS	0	0	0	8	1	IKKFTPATES	tr_C9ZP82	0.021	39943.64	85.00
82	HLA-A0201	DGVAPRAKMNQQRA	GVAPRAQRA	1	0	0	6	4	GVAPRAKMNQQRA	tr_C9ZP82	0.021	39944.93	85.00
464	HLA-A0201	EDGTLPSSTECGSP	TLPSSTCGS	3	0	0	6	1	TLPSSTECGS	tr_C9ZP82	0.021	39947.54	85.00
389	HLA-A0201	TDNESPIESR	TDNEPIESR	0	0	0	4	1	TDNESPIESR	tr_C9ZP82	0.021	39948.82	85.00
107	HLA-A0201	TGEVASTYMPNKS	EVASTYMPS	2	0	0	8	2	EVASTYMPNKS	tr_C9ZP82	0.021	39954.00	85.00
326	HLA-A0201	TRNPYKRRAVAR	TRNPYAVAR	0	0	0	5	3	TRNPYKRRAVAR	tr_C9ZP82	0.021	39974.34	85.00
182	HLA-A0201	CVRSTDDDYESTHE	CVDDDYEST	0	0	0	2	3	CVRSTDDDYEST	tr_C9ZP82	0.021	39989.03	85.00
487	HLA-A0201	KKAEAERSWKNFR	AEAERSWFR	2	0	0	7	2	AEAERSWKNFR	tr_C9ZP82	0.021	39989.03	85.00
594	HLA-A0201	ENSNSGAEF	ENSNSGAEF	0	0	0	0	0	ENSNSGAEF	tr_C9ZP82	0.021	39994.66	85.00
99	HLA-A0201	MGRAAPAQTG	MGRAAPATG	0	0	0	7	1	MGRAAPAQTG	tr_C9ZP82	0.021	40000.28	85.00
196	HLA-A0201	NRYVESNQVVDD	RYVESNQVV	1	0	0	0	0	RYVESNQVV	tr_C9ZP82	0.021	40009.39	85.00
181	HLA-A0201	FCVRSTDDDYE	CVRSTDDDY	1	0	0	0	0	CVRSTDDDY	tr_C9ZP82	0.021	40024.09	85.00
625	HLA-A0201	DDRWRDTYLKSTG	RWRDTYLTG	2	0	0	7	2	RWRDTYLKSTG	tr_C9ZP82	0.021	40026.27	85.00
155	HLA-A0201	LYSGALYNEGEE	LYSGALYEE	0	0	0	7	3	LYSGALYNEGEE	tr_C9ZP82	0.021	40027.99	85.00
327	HLA-A0201	RNPYKRRAVARQQ	NPYAVARQQ	1	0	0	3	3	NPYKRRAVARQQ	tr_C9ZP82	0.021	40047.05	85.00
326	HLA-A0201	TRNPYKRRAVARQQ	YRRAVARQQ	4	0	0	1	1	YKRRAVARQQ	tr_C9ZP82	0.021	40052.27	85.00
487	HLA-A0201	KKAEAERSWKNFRP	KASWKNFRP	1	0	0	2	4	KAEAERSWKNFRP	tr_C9ZP82	0.020	40060.07	85.00
386	HLA-A0201	SSDTDNESPIE	SSDTDNEPI	0	0	0	7	1	SSDTDNESPI	tr_C9ZP82	0.020	40065.26	85.00
167	HLA-A0201	EAGDALNDG	EAGDALNDG	0	0	0	0	0	EAGDALNDG	tr_C9ZP82	0.020	40066.55	85.00
786	HLA-A0201	TEEERWRTQKL	TEEERWRTL	0	0	0	8	2	TEEERWRTQKL	tr_C9ZP82	0.020	40070.89	85.00
179	HLA-A0201	NSFCVRSTDD	NSFCVSTDD	0	0	0	5	1	NSFCVRSTDD	tr_C9ZP82	0.020	40072.19	85.00
531	HLA-A0201	AFSGESALD	AFSGESALD	0	0	0	0	0	AFSGESALD	tr_C9ZP82	0.020	40095.19	85.00
90	HLA-A0201	MNQQRARRYMG	MQQRARRYM	0	0	0	1	1	MNQQRARRYM	tr_C9ZP82	0.020	40095.61	85.00
245	HLA-A0201	LNMLNEKMS	LNMLNEKMS	0	0	0	0	0	LNMLNEKMS	tr_C9ZP82	0.020	40102.56	85.00
103	HLA-A0201	APAQTGEVAS	AAQTGEVAS	0	0	0	1	1	APAQTGEVAS	tr_C9ZP82	0.020	40107.32	85.00
688	HLA-A0201	LKEANIKRMP	LKEANIKRM	0	0	0	0	0	LKEANIKRM	tr_C9ZP82	0.020	40110.38	85.00
269	HLA-A0201	SNTPGEGEHKI	NTPGEGEHI	1	0	0	8	1	NTPGEGEHKI	tr_C9ZP82	0.020	40110.81	85.00
342	HLA-A0201	ERRSRRKRNFIDDT	RSRNFIDDT	2	0	0	2	3	RSRRKRNFIDDT	tr_C9ZP82	0.020	40121.21	85.00
125	HLA-A0201	EEVVREFTSTE	VVREFTSTE	2	0	0	0	0	VVREFTSTE	tr_C9ZP82	0.020	40121.21	85.00
803	HLA-A0201	TSNKTSFVPKGD	TSNKTFVPK	0	0	0	5	1	TSNKTSFVPK	tr_C9ZP82	0.020	40125.56	85.00
196	HLA-A0201	NRYVESNQVVD	RYVESNQVV	1	0	0	0	0	RYVESNQVV	tr_C9ZP82	0.020	40126.86	85.00
392	HLA-A0201	ESPIESREA	ESPIESREA	0	0	0	0	0	ESPIESREA	tr_C9ZP82	0.020	40129.91	85.00
95	HLA-A0201	ARRYMGRAAP	ARRYMGRAA	0	0	0	0	0	ARRYMGRAA	tr_C9ZP82	0.020	40133.82	85.00
444	HLA-A0201	DKKTLVSSAG	KKTLVSSAG	1	0	0	0	0	KKTLVSSAG	tr_C9ZP82	0.020	40140.31	85.00
495	HLA-A0201	WKNFRPCSSP	WNFRPCSSP	0	0	0	1	1	WKNFRPCSSP	tr_C9ZP82	0.020	40141.20	85.00
822	HLA-A0201	LDTILRRVP	LDTILRRVP	0	0	0	0	0	LDTILRRVP	tr_C9ZP82	0.020	40150.31	85.00
360	HLA-A0201	LNPSYIDEEES	LSYIDEEES	0	0	0	1	2	LNPSYIDEEES	tr_C9ZP82	0.020	40151.63	85.00
331	HLA-A0201	KRRAVARQQQ	KRRAVAQQQ	0	0	0	6	1	KRRAVARQQQ	tr_C9ZP82	0.020	40154.22	85.00
466	HLA-A0201	GTLPSSTECGSPKG	TLPSSTECG	1	0	0	8	4	TLPSSTECGSPKG	tr_C9ZP82	0.020	40155.95	85.00
590	HLA-A0201	STNKENSNS	STNKENSNS	0	0	0	0	0	STNKENSNS	tr_C9ZP82	0.020	40156.39	85.00
883	HLA-A0201	LKGNGEGVIEG	LNGEGVIEG	0	0	0	1	2	LKGNGEGVIEG	tr_C9ZP82	0.020	40175.51	85.00
339	HLA-A0201	QQRERRSRRKRNFI	QQRERRNFI	0	0	0	5	5	QQRERRSRRKRNFI	tr_C9ZP82	0.020	40186.84	85.00

184	HLA-A0201	RSTDDDYESTHEN	STYESTHEN	1	0	0	2	3	STDDDYESTHEN	tr_C9ZP82	0.020	40188.13	85.00
859	HLA-A0201	VPRLSYGAD	VPRLSYGAD	0	0	0	0	0	VPRLSYGAD	tr_C9ZP82	0.020	40189.01	85.00
36	HLA-A0201	IHPCCHNDSDTA	IHPCCSDTA	0	0	0	5	3	IHPCCHNDSDTA	tr_C9ZP82	0.020	40189.87	85.00
93	HLA-A0201	QRARRYMGRAAP	RAYMGRAAP	1	0	0	2	2	RARRYMGRAAP	tr_C9ZP82	0.020	40191.16	85.00
91	HLA-A0201	NQQRARRYMGR	QQRARYMGR	1	0	0	4	1	QQRARRYMGR	tr_C9ZP82	0.020	40205.52	85.00
460	HLA-A0201	PNHIEDGTL	PNHIEDGTL	0	0	0	0	0	PNHIEDGTL	tr_C9ZP82	0.020	40206.82	85.00
184	HLA-A0201	RSTDDDYESTH	STDDDYEST	1	0	0	0	0	STDDDYEST	tr_C9ZP82	0.020	40214.24	85.00
491	HLA-A0201	AERSWKNFRP	AERSWNFRP	0	0	0	5	1	AERSWKNFRP	tr_C9ZP82	0.020	40219.01	85.00
325	HLA-A0201	TTRNPYKRRAVA	TTYKRRAVA	0	0	0	2	3	TTRNPYKRRAVA	tr_C9ZP82	0.020	40225.99	85.00
139	HLA-A0201	ANDDLDEVRQ	ANDDLDVRQ	0	0	0	6	1	ANDDLDEVRQ	tr_C9ZP82	0.020	40226.40	85.00
139	HLA-A0201	ANDDLDEVRQT	ANDDLVRQT	0	0	0	5	2	ANDDLDEVRQT	tr_C9ZP82	0.020	40235.12	85.00
498	HLA-A0201	FRPCSSPANSKIID	CSSPASKII	3	0	0	5	1	CSSPANSKII	tr_C9ZP82	0.020	40238.16	85.00
183	HLA-A0201	VRSTDDDYESTH	STDDDYEST	2	0	0	0	0	STDDDYEST	tr_C9ZP82	0.020	40254.71	85.00
325	HLA-A0201	TTRNPYKRRAVARQ	TTRNPYRAV	0	0	0	6	2	TTRNPYKRRAV	tr_C9ZP82	0.020	40258.64	85.00
525	HLA-A0201	LPHAPSAFSGE	LPHAPSAFS	0	0	0	0	0	LPHAPSAFS	tr_C9ZP82	0.020	40268.66	85.00
730	HLA-A0201	EVMVRSPQQ	EVMVRSPQQ	0	0	0	0	0	EVMVRSPQQ	tr_C9ZP82	0.020	40269.96	85.00
111	HLA-A0201	ASTYMPNKS	ASTYMPNKS	0	0	0	0	0	ASTYMPNKS	tr_C9ZP82	0.020	40273.88	85.00
268	HLA-A0201	GSNTPGEGEHKII	GSNTPGEII	0	0	0	7	4	GSNTPGEGEHKII	tr_C9ZP82	0.020	40281.72	85.00
744	HLA-A0201	TFPLQWNVD	TFPLQWNVD	0	0	0	0	0	TFPLQWNVD	tr_C9ZP82	0.020	40287.39	85.00
635	HLA-A0201	STGLSNSVQE	STGLSSVQE	0	0	0	5	1	STGLSNSVQE	tr_C9ZP82	0.020	40294.38	85.00
845	HLA-A0201	LERYRPRTYSC	LYRPRTYSC	0	0	0	1	2	LERYRPRTYSC	tr_C9ZP82	0.020	40297.42	85.00
136	HLA-A0201	MAQANDDLD	MAQANDDLD	0	0	0	0	0	MAQANDDLD	tr_C9ZP82	0.020	40309.19	85.00
394	HLA-A0201	PIESREAHVT	PIESREAHV	0	0	0	0	0	PIESREAHV	tr_C9ZP82	0.020	40310.49	85.00
270	HLA-A0201	NTPGEGEHKII	NTPGEGEHI	0	0	0	8	2	NTPGEGEHKII	tr_C9ZP82	0.020	40314.00	85.00
499	HLA-A0201	RPCSSPANSKIIDD	CSSPAIIDD	2	0	0	5	3	CSSPANSKIIDD	tr_C9ZP82	0.020	40315.30	85.00
501	HLA-A0201	CSSPANSKIIDD	CSSPAIIDD	0	0	0	5	3	CSSPANSKIIDD	tr_C9ZP82	0.020	40320.53	85.00
483	HLA-A0201	KRVSKKAEAERSWK	RVSKKAEAK	1	0	0	8	4	RVSKKAEAERSWK	tr_C9ZP82	0.020	40322.27	85.00
267	HLA-A0201	SGSNTPGEGEHKII	SGSNTPGEI	0	0	0	8	5	SGSNTPGEGEHKII	tr_C9ZP82	0.020	40334.05	85.00
884	HLA-A0201	KGNGEGVIE	KGNGEGVIE	0	0	0	0	0	KGNGEGVIE	tr_C9ZP82	0.020	40334.94	85.00
388	HLA-A0201	DTDNESPIESR	DTDESPIES	0	0	0	3	1	DTDNESPIES	tr_C9ZP82	0.020	40345.42	85.00
595	HLA-A0201	NSNSGAEFRK	NSNSGAFRK	0	0	0	6	1	NSNSGAEFRK	tr_C9ZP82	0.020	40360.70	85.00
192	HLA-A0201	STHENRYVESN	STHEYVESN	0	0	0	4	2	STHENRYVESN	tr_C9ZP82	0.020	40363.74	85.00
107	HLA-A0201	TGEVASTYMPNKSG	TGEVASTYM	0	0	0	0	0	TGEVASTYM	tr_C9ZP82	0.020	40365.93	85.00
468	HLA-A0201	LPSSTECGS	LPSSTECGS	0	0	0	0	0	LPSSTECGS	tr_C9ZP82	0.020	40368.12	85.00
384	HLA-A0201	NSSSDTDNESPIE	SSSDTDNPI	1	0	0	7	2	SSSDTDNESPI	tr_C9ZP82	0.020	40376.42	85.00
52	HLA-A0201	PEEEKLECIC	PEEEKLECI	0	0	0	0	0	PEEEKLECI	tr_C9ZP82	0.020	40389.08	85.00
767	HLA-A0201	PFADMDVLQ	PFADMDVLQ	0	0	0	0	0	PFADMDVLQ	tr_C9ZP82	0.020	40397.39	85.00
195	HLA-A0201	ENRYVESNQVVD	RYVESNQVV	2	0	0	0	0	RYVESNQVV	tr_C9ZP82	0.020	40400.44	85.00
331	HLA-A0201	KRRAVARQQQRERR	KRRAVAERR	0	0	0	6	5	KRRAVARQQQRERR	tr_C9ZP82	0.020	40402.21	85.00
330	HLA-A0201	YKRRAVARQQQR	YAVARQQQR	0	0	0	1	3	YKRRAVARQQQR	tr_C9ZP82	0.020	40412.68	85.00
178	HLA-A0201	VNSFCVRSTDD	VNSFCVRST	0	0	0	0	0	VNSFCVRST	tr_C9ZP82	0.020	40425.38	85.00
866	HLA-A0201	ADGQVIKRQRRHRH	ADGQVIHRH	0	0	0	6	5	ADGQVIKRQRRHRH	tr_C9ZP82	0.020	40430.18	85.00
494	HLA-A0201	SWKNFRPCSSP	SWFRPCSSP	0	0	0	2	2	SWKNFRPCSSP	tr_C9ZP82	0.020	40435.86	85.00
93	HLA-A0201	QRARRYMGRAA	RARYMGRAA	1	0	0	2	1	RARRYMGRAA	tr_C9ZP82	0.020	40438.05	85.00
183	HLA-A0201	VRSTDDDYESTHEN	STYESTHEN	2	0	0	2	3	STDDDYESTHEN	tr_C9ZP82	0.020	40438.94	85.00
489	HLA-A0201	AEAERSWKNFRP	AEAERSWNF	0	0	0	7	1	AEAERSWKNF	tr_C9ZP82	0.020	40445.07	85.00
787	HLA-A0201	EEERWRTQKL	EEERWTQKL	0	0	0	5	1	EEERWRTQKL	tr_C9ZP82	0.020	40447.68	85.00
497	HLA-A0201	NFRPCSSPANSK	FCSSPANSK	1	0	0	1	2	FRPCSSPANSK	tr_C9ZP82	0.020	40449.87	85.00
859	HLA-A0201	VPRLSYGADG	VPRLYGADG	0	0	0	4	1	VPRLSYGADG	tr_C9ZP82	0.020	40450.75	85.00
185	HLA-A0201	STDDDYESTHENR	STDDDYEST	0	0	0	0	0	STDDDYEST	tr_C9ZP82	0.020	40465.62	85.00
875	HLA-A0201	RRHRHSGQL	RRHRHSGQL	0	0	0	0	0	RRHRHSGQL	tr_C9ZP82	0.020	40465.62	85.00
826	HLA-A0201	LRRVPSREGVE	LRRVPSEGV	0	0	0	6	1	LRRVPSREGV	tr_C9ZP82	0.020	40466.07	85.00
83	HLA-A0201	GVAPRAKMNQ	GVAPRAMNQ	0	0	0	6	1	GVAPRAKMNQ	tr_C9ZP82	0.020	40470.90	85.00
661	HLA-A0201	TTTTTDSDW	TTTTTDSDW	0	0	0	0	0	TTTTTDSDW	tr_C9ZP82	0.020	40482.28	85.00

699	HLA-A0201	PVVKSTPPDT	VVKSTPPDT	1	0	0	0	0	VVKSTPPDT	tr_C9ZP82	0.020	40483.14	85.00
234	HLA-A0201	TDFMAKVSD	TDFMAKVSD	0	0	0	0	0	TDFMAKVSD	tr_C9ZP82	0.019	40491.47	85.00
497	HLA-A0201	NFRPCSSPANS	FPCSSPANS	1	0	0	1	1	FRPCSSPANS	tr_C9ZP82	0.019	40495.86	85.00
161	HLA-A0201	YNEGEEEAGDALN	YEEEAGDAL	0	0	0	1	3	YNEGEEEAGDAL	tr_C9ZP82	0.019	40496.75	85.00
388	HLA-A0201	DTDNESPIESREAH	SPIESREAH	5	0	0	0	0	SPIESREAH	tr_C9ZP82	0.019	40519.97	85.00
597	HLA-A0201	NSGAEFRKG	NSGAEFRKG	0	0	0	0	0	NSGAEFRKG	tr_C9ZP82	0.019	40519.97	85.00
140	HLA-A0201	NDDLDEVRQT	NDDLEVRQT	0	0	0	4	1	NDDLDEVRQT	tr_C9ZP82	0.019	40535.75	85.00
326	HLA-A0201	TRNPYKRRAVARQ	TRNPYVARQ	0	0	0	5	4	TRNPYKRRAVARQ	tr_C9ZP82	0.019	40541.01	85.00
387	HLA-A0201	SDTDNESPIESR	DTDESPIES	1	0	0	3	1	DTDNESPIES	tr_C9ZP82	0.019	40546.71	85.00
361	HLA-A0201	NPSYIDEEES	NSYIDEEES	0	0	0	1	1	NPSYIDEEES	tr_C9ZP82	0.019	40547.16	85.00
156	HLA-A0201	YSGALYNEGEE	YSGALYEGE	0	0	0	6	1	YSGALYNEGE	tr_C9ZP82	0.019	40548.46	85.00
556	HLA-A0201	TKGPHEINLP	TKGPHEINL	0	0	0	0	0	TKGPHEINL	tr_C9ZP82	0.019	40553.75	85.00
185	HLA-A0201	STDDDYESTHE	STDDDYEST	0	0	0	0	0	STDDDYEST	tr_C9ZP82	0.019	40556.80	85.00
866	HLA-A0201	ADGQVIKRQRRH	ADGQVIRRH	0	0	0	6	3	ADGQVIKRQRRH	tr_C9ZP82	0.019	40559.87	85.00
662	HLA-A0201	TTTTDSDWS	TTTTDSDWS	0	0	0	0	0	TTTTDSDWS	tr_C9ZP82	0.019	40563.82	85.00
867	HLA-A0201	DGQVIKRQR	DGQVIKRQR	0	0	0	0	0	DGQVIKRQR	tr_C9ZP82	0.019	40566.01	85.00
327	HLA-A0201	RNPYKRRAVAR	NPYKRAVAR	1	0	0	4	1	NPYKRRAVAR	tr_C9ZP82	0.019	40576.11	85.00
812	HLA-A0201	KGDLRKLSD	KGDLRKLSD	0	0	0	0	0	KGDLRKLSD	tr_C9ZP82	0.019	40578.31	85.00
345	HLA-A0201	SRRKRNFIDDT	RKRNFIDDT	2	0	0	0	0	RKRNFIDDT	tr_C9ZP82	0.019	40578.75	85.00
361	HLA-A0201	NPSYIDEEE	NPSYIDEEE	0	0	0	0	0	NPSYIDEEE	tr_C9ZP82	0.019	40579.20	85.00
389	HLA-A0201	TDNESPIESREAH	SPIESREAH	4	0	0	0	0	SPIESREAH	tr_C9ZP82	0.019	40595.88	85.00
601	HLA-A0201	EFRKGSNNDNV	FKGSNNDNV	1	0	0	1	1	FRKGSNNDNV	tr_C9ZP82	0.019	40598.52	85.00
786	HLA-A0201	TEEERWRTQ	TEEERWRTQ	0	0	0	0	0	TEEERWRTQ	tr_C9ZP82	0.019	40601.14	85.00
697	HLA-A0201	PLPVVKSTPPD	PLPVVSTPP	0	0	0	5	1	PLPVVKSTPP	tr_C9ZP82	0.019	40601.58	85.00
384	HLA-A0201	NSSSDTDNES	SSSDTDNES	1	0	0	0	0	SSSDTDNES	tr_C9ZP82	0.019	40603.34	85.00
328	HLA-A0201	NPYKRRAVARQQQR	YKRRAVARR	2	0	0	8	3	YKRRAVARQQQR	tr_C9ZP82	0.019	40610.38	85.00
500	HLA-A0201	PCSSPANSKII	CSSPASKII	1	0	0	5	1	CSSPANSKII	tr_C9ZP82	0.019	40610.82	85.00
14	HLA-A0201	PAIVTKNCPSS	AIVTNCPSS	1	0	0	4	1	AIVTKNCPSS	tr_C9ZP82	0.019	40631.47	85.00
325	HLA-A0201	TTRNPYKRRA	TTNPYKRRA	0	0	0	2	1	TTRNPYKRRA	tr_C9ZP82	0.019	40636.32	85.00
160	HLA-A0201	LYNEGEEEAGDALN	LYNEGEEAL	0	0	0	7	4	LYNEGEEEAGDAL	tr_C9ZP82	0.019	40636.74	85.00
109	HLA-A0201	EVASTYMPNKS	EVASTYMPS	0	0	0	8	2	EVASTYMPNKS	tr_C9ZP82	0.019	40646.43	85.00
88	HLA-A0201	AKMNQQRARR	KMNQQRARR	1	0	0	0	0	KMNQQRARR	tr_C9ZP82	0.019	40648.61	85.00
620	HLA-A0201	LHSTIDDRWRD	LHSTIDDRW	0	0	0	0	0	LHSTIDDRW	tr_C9ZP82	0.019	40654.77	85.00
162	HLA-A0201	NEGEEEAGDAL	NEGEEEAAL	0	0	0	7	2	NEGEEEAGDAL	tr_C9ZP82	0.019	40656.53	85.00
481	HLA-A0201	RSKRVSKKAEA	RSKRVSAEA	0	0	0	6	2	RSKRVSKKAEA	tr_C9ZP82	0.019	40673.26	85.00
842	HLA-A0201	DVPLERYRPRT	VLERYRPRT	1	0	0	1	1	VPLERYRPRT	tr_C9ZP82	0.019	40684.25	85.00
383	HLA-A0201	ENSSSDTDNESPIE	SSSDTDNPI	2	0	0	7	2	SSSDTDNESPI	tr_C9ZP82	0.019	40688.66	85.00
485	HLA-A0201	VSKKAEAERSW	VSAEAERSW	0	0	0	2	2	VSKKAEAERSW	tr_C9ZP82	0.019	40688.66	85.00
590	HLA-A0201	STNKENSNSGA	STNNSNSGA	0	0	0	3	2	STNKENSNSGA	tr_C9ZP82	0.019	40693.93	85.00
484	HLA-A0201	RVSKKAEAER	RVSKKAEAR	0	0	0	8	1	RVSKKAEAER	tr_C9ZP82	0.019	40694.38	85.00
324	HLA-A0201	DTTRNPYKRRAVA	TTYKRRAVA	1	0	0	2	3	TTRNPYKRRAVA	tr_C9ZP82	0.019	40699.68	85.00
440	HLA-A0201	ESAFDKKTL	ESAFDKKTL	0	0	0	0	0	ESAFDKKTL	tr_C9ZP82	0.019	40705.40	85.00
403	HLA-A0201	TTVPNTGFE	TTVPNTGFE	0	0	0	0	0	TTVPNTGFE	tr_C9ZP82	0.019	40708.92	85.00
331	HLA-A0201	KRRAVARQQQR	KRRAVAQQR	0	0	0	6	2	KRRAVARQQQR	tr_C9ZP82	0.019	40709.79	85.00
842	HLA-A0201	DVPLERYRPR	DVLERYRPR	0	0	0	2	1	DVPLERYRPR	tr_C9ZP82	0.019	40711.13	85.00
880	HLA-A0201	SGQLKGNGE	SGQLKGNGE	0	0	0	0	0	SGQLKGNGE	tr_C9ZP82	0.019	40730.51	85.00
328	HLA-A0201	NPYKRRAVARQ	YKRRAVARQ	2	0	0	0	0	YKRRAVARQ	tr_C9ZP82	0.019	40731.82	85.00
132	HLA-A0201	TSTEMAQAN	TSTEMAQAN	0	0	0	0	0	TSTEMAQAN	tr_C9ZP82	0.019	40742.41	85.00
847	HLA-A0201	RYRPRTYSCT	RYRPRTYST	0	0	0	8	1	RYRPRTYSCT	tr_C9ZP82	0.019	40747.24	85.00
323	HLA-A0201	RDTTRNPYKRRAVA	TTYKRRAVA	2	0	0	2	3	TTRNPYKRRAVA	tr_C9ZP82	0.019	40752.55	85.00
377	HLA-A0201	PVCESSENS	PVCESSENS	0	0	0	0	0	PVCESSENS	tr_C9ZP82	0.019	40752.55	85.00
327	HLA-A0201	RNPYKRRAVARQ	YKRRAVARQ	3	0	0	0	0	YKRRAVARQ	tr_C9ZP82	0.019	40756.97	85.00
437	HLA-A0201	PATESAFDK	PATESAFDK	0	0	0	0	0	PATESAFDK	tr_C9ZP82	0.019	40762.70	85.00
178	HLA-A0201	VNSFCVRSTDDD	VNSFCVRST	0	0	0	0	0	VNSFCVRST	tr_C9ZP82	0.019	40769.75	85.00

890	HLA-A0201	VIEGKKSNS	VIEGKKSNS	0	0	0	0	0	VIEGKKSNS	tr_C9ZP82	0.019	40769.75	85.00
329	HLA-A0201	PYKRRAVARQQQR	YKRRAVARR	1	0	0	8	3	YKRRAVARQQQR	tr_C9ZP82	0.019	40778.58	85.00
329	HLA-A0201	PYKRRAVARQQ	YRRAVARQQ	1	0	0	1	1	YKRRAVARQQ	tr_C9ZP82	0.019	40781.21	85.00
590	HLA-A0201	STNKENSNSGAEFR	STSNSGAEF	0	0	0	2	4	STNKENSNSGAEF	tr_C9ZP82	0.019	40783.87	85.00
876	HLA-A0201	RHRHSGQLK	RHRHSGQLK	0	0	0	0	0	RHRHSGQLK	tr_C9ZP82	0.019	40788.29	85.00
130	HLA-A0201	EFTSTEMAQ	EFTSTEMAQ	0	0	0	0	0	EFTSTEMAQ	tr_C9ZP82	0.019	40790.91	85.00
892	HLA-A0201	EGKKSNSRVTV	KKSNSRVTV	2	0	0	0	0	KKSNSRVTV	tr_C9ZP82	0.019	40795.34	85.00
848	HLA-A0201	YRPRTYSCT	YRPRTYSCT	0	0	0	0	0	YRPRTYSCT	tr_C9ZP82	0.019	40805.04	85.00
360	HLA-A0201	LNPSYIDEEE	LNPSYIEEE	0	0	0	6	1	LNPSYIDEEE	tr_C9ZP82	0.019	40808.15	85.00
503	HLA-A0201	SPANSKIIDD	SANSKIIDD	0	0	0	1	1	SPANSKIIDD	tr_C9ZP82	0.019	40812.57	85.00
470	HLA-A0201	SSTECGSPKG	SSTECGSPG	0	0	0	8	1	SSTECGSPKG	tr_C9ZP82	0.019	40830.24	85.00
191	HLA-A0201	ESTHENRYVES	STHERYVES	1	0	0	4	1	STHENRYVES	tr_C9ZP82	0.019	40839.51	85.00
116	HLA-A0201	PNKSGMAIVE	NKSGMAIVE	1	0	0	0	0	NKSGMAIVE	tr_C9ZP82	0.019	40865.14	90.00
437	HLA-A0201	PATESAFDKK	ATESAFDKK	1	0	0	0	0	ATESAFDKK	tr_C9ZP82	0.019	40869.12	90.00
270	HLA-A0201	NTPGEGEHK	NTPGEGEHK	0	0	0	0	0	NTPGEGEHK	tr_C9ZP82	0.019	40873.55	90.00
779	HLA-A0201	DAAWPQLTE	DAAWPQLTE	0	0	0	0	0	DAAWPQLTE	tr_C9ZP82	0.019	40875.76	90.00
464	HLA-A0201	EDGTLPSSTECG	GLPSSTECG	2	0	0	1	1	GTLPSSTECG	tr_C9ZP82	0.019	40881.96	90.00
337	HLA-A0201	RQQQRERRS	RQQQRERRS	0	0	0	0	0	RQQQRERRS	tr_C9ZP82	0.019	40887.26	90.00
459	HLA-A0201	QPNHIEDGT	QPNHIEDGT	0	0	0	0	0	QPNHIEDGT	tr_C9ZP82	0.019	40908.94	90.00
815	HLA-A0201	LRKLSDALD	LRKLSDALD	0	0	0	0	0	LRKLSDALD	tr_C9ZP82	0.019	40912.47	90.00
863	HLA-A0201	SYGADGQVIK	SYGADGQVI	0	0	0	0	0	SYGADGQVI	tr_C9ZP82	0.019	40918.23	90.00
84	HLA-A0201	VAPRAKMNQQ	VARAKMNQQ	0	0	0	2	1	VAPRAKMNQQ	tr_C9ZP82	0.019	40918.23	90.00
329	HLA-A0201	PYKRRAVARQ	YKRRAVARQ	1	0	0	0	0	YKRRAVARQ	tr_C9ZP82	0.019	40926.64	90.00
184	HLA-A0201	RSTDDDYESTHENR	STDDDYEST	1	0	0	0	0	STDDDYEST	tr_C9ZP82	0.019	40928.41	90.00
810	HLA-A0201	VPKGDLRKLSD	VPKGDLRKL	0	0	0	0	0	VPKGDLRKL	tr_C9ZP82	0.018	40934.17	90.00
101	HLA-A0201	RAAPAQTGE	RAAPAQTGE	0	0	0	0	0	RAAPAQTGE	tr_C9ZP82	0.018	40935.52	90.00
330	HLA-A0201	YKRRAVARQQQRER	YKRRAVARR	0	0	0	8	5	YKRRAVARQQQRER	tr_C9ZP82	0.018	40941.27	90.00
327	HLA-A0201	RNPYKRRAVA	RPYKRRAVA	0	0	0	1	1	RNPYKRRAVA	tr_C9ZP82	0.018	40942.14	90.00
327	HLA-A0201	RNPYKRRAVARQQQ	RPYKRRAVA	0	0	0	1	1	RNPYKRRAVA	tr_C9ZP82	0.018	40949.67	90.00
218	HLA-A0201	DDPTAFEFDS	DPTAFEFDS	1	0	0	0	0	DPTAFEFDS	tr_C9ZP82	0.018	40958.54	90.00
194	HLA-A0201	HENRYVESN	HENRYVESN	0	0	0	0	0	HENRYVESN	tr_C9ZP82	0.018	40971.41	90.00
500	HLA-A0201	PCSSPANSKIIDD	CSSPAIIDD	1	0	0	5	3	CSSPANSKIIDD	tr_C9ZP82	0.018	40975.40	90.00
83	HLA-A0201	GVAPRAKMNQQRAR	GVAPRAQRA	0	0	0	6	4	GVAPRAKMNQQRA	tr_C9ZP82	0.018	40978.94	90.00
160	HLA-A0201	LYNEGEEEAG	LYNEGEEEA	0	0	0	0	0	LYNEGEEEA	tr_C9ZP82	0.018	40978.94	90.00
783	HLA-A0201	PQLTEEERWR	QLTEEERWR	1	0	0	0	0	QLTEEERWR	tr_C9ZP82	0.018	40985.57	90.00
639	HLA-A0201	SNSVQEACEK	SSVQEACEK	0	0	0	1	1	SNSVQEACEK	tr_C9ZP82	0.018	40991.79	90.00
657	HLA-A0201	WRYYTTTTDS	RYYTTTTTS	1	0	0	8	1	RYYTTTTDS	tr_C9ZP82	0.018	40996.23	90.00
161	HLA-A0201	YNEGEEEAGDALND	YEEEAGDAL	0	0	0	1	3	YNEGEEEAGDAL	tr_C9ZP82	0.018	41002.89	90.00
486	HLA-A0201	SKKAEAERSWKN	KAAERSWKN	2	0	0	2	1	KAEAERSWKN	tr_C9ZP82	0.018	41006.88	90.00
485	HLA-A0201	VSKKAEAERSWKN	VSAERSWKN	0	0	0	2	4	VSKKAEAERSWKN	tr_C9ZP82	0.018	41007.75	90.00
431	HLA-A0201	DIKKFTPATES	DIFTPATES	0	0	0	2	2	DIKKFTPATES	tr_C9ZP82	0.018	41016.64	90.00
845	HLA-A0201	LERYRPRTYSCT	LERYRPRTY	0	0	0	0	0	LERYRPRTY	tr_C9ZP82	0.018	41022.86	90.00
164	HLA-A0201	GEEEAGDALN	GEEEAGDAL	0	0	0	0	0	GEEEAGDAL	tr_C9ZP82	0.018	41029.50	90.00
656	HLA-A0201	VWRYYTTTTDSD	VWYYTTTTT	0	0	0	2	1	VWRYYTTTTT	tr_C9ZP82	0.018	41043.71	90.00
381	HLA-A0201	SSENSSSDT	SSENSSSDT	0	0	0	0	0	SSENSSSDT	tr_C9ZP82	0.018	41044.61	90.00
328	HLA-A0201	NPYKRRAVARQQQ	NPYKRRAVA	0	0	0	0	0	NPYKRRAVA	tr_C9ZP82	0.018	41049.06	90.00
486	HLA-A0201	SKKAEAERSWK	SKKAEAESW	0	0	0	7	1	SKKAEAERSW	tr_C9ZP82	0.018	41054.38	90.00
621	HLA-A0201	HSTIDDRWR	HSTIDDRWR	0	0	0	0	0	HSTIDDRWR	tr_C9ZP82	0.018	41059.70	90.00
388	HLA-A0201	DTDNESPIESRE	DTDESPIES	0	0	0	3	1	DTDNESPIES	tr_C9ZP82	0.018	41072.15	90.00
322	HLA-A0201	LRDTTRNPYK	LRDTTRNPY	0	0	0	0	0	LRDTTRNPY	tr_C9ZP82	0.018	41075.70	90.00
199	HLA-A0201	VESNQVVDD	VESNQVVDD	0	0	0	0	0	VESNQVVDD	tr_C9ZP82	0.018	41075.70	90.00
161	HLA-A0201	YNEGEEEAG	YNEGEEEAG	0	0	0	0	0	YNEGEEEAG	tr_C9ZP82	0.018	41086.38	90.00
192	HLA-A0201	STHENRYVESNQ	STHEYVESN	0	0	0	4	2	STHENRYVESN	tr_C9ZP82	0.018	41086.83	90.00
108	HLA-A0201	GEVASTYMPNK	EVASTYMPK	1	0	0	8	1	EVASTYMPNK	tr_C9ZP82	0.018	41087.70	90.00

91	HLA-A0201	NQQRARRYMG	NQQRARRYM	0	0	0	0	0	NQQRARRYM	tr_C9ZP82	0.018	41088.15	90.00
867	HLA-A0201	DGQVIKRQRR	GQVIKRQRR	1	0	0	0	0	GQVIKRQRR	tr_C9ZP82	0.018	41089.48	90.00
499	HLA-A0201	RPCSSPANSKIID	RPCSSPAII	0	0	0	7	3	RPCSSPANSKII	tr_C9ZP82	0.018	41089.93	90.00
184	HLA-A0201	RSTDDDYESTHE	STDDDYEST	1	0	0	0	0	STDDDYEST	tr_C9ZP82	0.018	41104.61	90.00
289	HLA-A0201	LAQFGGKAN	LAQFGGKAN	0	0	0	0	0	LAQFGGKAN	tr_C9ZP82	0.018	41111.71	90.00
183	HLA-A0201	VRSTDDDYESTHE	STDDDYEST	2	0	0	0	0	STDDDYEST	tr_C9ZP82	0.018	41115.72	90.00
85	HLA-A0201	APRAKMNQQRARR	AMNQQRARR	0	0	0	1	4	APRAKMNQQRARR	tr_C9ZP82	0.018	41116.17	90.00
846	HLA-A0201	ERYRPRTYSC	RYRPRTYSC	1	0	0	0	0	RYRPRTYSC	tr_C9ZP82	0.018	41125.50	90.00
232	HLA-A0201	PGTDFMAKVSD	GTDFMAVSD	1	0	0	6	1	GTDFMAKVSD	tr_C9ZP82	0.018	41135.29	90.00
393	HLA-A0201	SPIESREAH	SPIESREAH	0	0	0	0	0	SPIESREAH	tr_C9ZP82	0.018	41135.74	90.00
266	HLA-A0201	FSGSNTPGEGEHK	FSNTPGEGE	0	0	0	1	2	FSGSNTPGEGE	tr_C9ZP82	0.018	41142.43	90.00
465	HLA-A0201	DGTLPSSTECG	GLPSSTECG	1	0	0	1	1	GTLPSSTECG	tr_C9ZP82	0.018	41161.11	90.00
269	HLA-A0201	SNTPGEGEHKII	NTPGEGEHI	1	0	0	8	2	NTPGEGEHKII	tr_C9ZP82	0.018	41162.02	90.00
471	HLA-A0201	STECGSPKGR	STCGSPKGR	0	0	0	2	1	STECGSPKGR	tr_C9ZP82	0.018	41178.05	90.00
594	HLA-A0201	ENSNSGAEFR	NSNSGAEFR	1	0	0	0	0	NSNSGAEFR	tr_C9ZP82	0.018	41180.73	90.00
133	HLA-A0201	STEMAQAND	STEMAQAND	0	0	0	0	0	STEMAQAND	tr_C9ZP82	0.018	41186.07	90.00
485	HLA-A0201	VSKKAEAERSWK	SKKAEAESW	1	0	0	7	1	SKKAEAERSW	tr_C9ZP82	0.018	41191.41	90.00
504	HLA-A0201	PANSKIIDD	PANSKIIDD	0	0	0	0	0	PANSKIIDD	tr_C9ZP82	0.018	41198.56	90.00
194	HLA-A0201	HENRYVESNQ	HENRYVESQ	0	0	0	8	1	HENRYVESNQ	tr_C9ZP82	0.018	41202.57	90.00
625	HLA-A0201	DDRWRDTYLKS	RWRDTYLKS	2	0	0	0	0	RWRDTYLKS	tr_C9ZP82	0.018	41208.36	90.00
330	HLA-A0201	YKRRAVARQQQ	YKRRAVARQ	0	0	0	8	2	YKRRAVARQQQ	tr_C9ZP82	0.018	41208.82	90.00
870	HLA-A0201	VIKRQRRHRHSGQL	VIHRHSGQL	0	0	0	2	5	VIKRQRRHRHSGQL	tr_C9ZP82	0.018	41213.71	90.00
625	HLA-A0201	DDRWRDTYLK	DRWRDTYLK	1	0	0	0	0	DRWRDTYLK	tr_C9ZP82	0.018	41214.61	90.00
108	HLA-A0201	GEVASTYMPNKS	EVASTYMPS	1	0	0	8	2	EVASTYMPNKS	tr_C9ZP82	0.018	41215.94	90.00
390	HLA-A0201	DNESPIESREA	NSPIESREA	1	0	0	1	1	NESPIESREA	tr_C9ZP82	0.018	41216.39	90.00
733	HLA-A0201	VRSPQQAELE	VRSPQQAEL	0	0	0	0	0	VRSPQQAEL	tr_C9ZP82	0.018	41216.84	90.00
395	HLA-A0201	IESREAHVT	IESREAHVT	0	0	0	0	0	IESREAHVT	tr_C9ZP82	0.018	41222.18	90.00
197	HLA-A0201	RYVESNQVVDD	RYVESNQVV	0	0	0	0	0	RYVESNQVV	tr_C9ZP82	0.018	41224.42	90.00
436	HLA-A0201	TPATESAFDK	TATESAFDK	0	0	0	1	1	TPATESAFDK	tr_C9ZP82	0.018	41238.70	90.00
191	HLA-A0201	ESTHENRYVESN	STHEYVESN	1	0	0	4	2	STHENRYVESN	tr_C9ZP82	0.018	41239.57	90.00
480	HLA-A0201	RRSKRVSKKAEA	RSKRVSAEA	1	0	0	6	2	RSKRVSKKAEA	tr_C9ZP82	0.018	41244.50	90.00
390	HLA-A0201	DNESPIESREAH	SPIESREAH	3	0	0	0	0	SPIESREAH	tr_C9ZP82	0.018	41254.31	90.00
81	HLA-A0201	VDGVAPRAKMNQ	GVAPRAMNQ	2	0	0	6	1	GVAPRAKMNQ	tr_C9ZP82	0.018	41256.09	90.00
387	HLA-A0201	SDTDNESPIESRE	DTDESPIES	1	0	0	3	1	DTDNESPIES	tr_C9ZP82	0.018	41267.70	90.00
36	HLA-A0201	IHPCCHNDS	IHPCCHNDS	0	0	0	0	0	IHPCCHNDS	tr_C9ZP82	0.018	41267.70	90.00
85	HLA-A0201	APRAKMNQQR	APRAKMQQR	0	0	0	6	1	APRAKMNQQR	tr_C9ZP82	0.018	41268.60	90.00
858	HLA-A0201	EVPRLSYGADG	EVPRLSYGA	0	0	0	0	0	EVPRLSYGA	tr_C9ZP82	0.018	41276.19	90.00
804	HLA-A0201	SNKTSFVPKGD	STSFVPKGD	0	0	0	1	2	SNKTSFVPKGD	tr_C9ZP82	0.018	41297.62	90.00
490	HLA-A0201	EAERSWKNFR	EAERSWNFR	0	0	0	6	1	EAERSWKNFR	tr_C9ZP82	0.018	41301.19	90.00
331	HLA-A0201	KRRAVARQQQRER	KRRAVARQR	0	0	0	8	4	KRRAVARQQQRER	tr_C9ZP82	0.018	41302.55	90.00
326	HLA-A0201	TRNPYKRRAV	TRNPYRRAV	0	0	0	5	1	TRNPYKRRAV	tr_C9ZP82	0.018	41308.36	90.00
265	HLA-A0201	IFSGSNTPGE	FSGSNTPGE	1	0	0	0	0	FSGSNTPGE	tr_C9ZP82	0.018	41309.23	90.00
469	HLA-A0201	PSSTECGSPK	SSTECGSPK	1	0	0	0	0	SSTECGSPK	tr_C9ZP82	0.018	41313.26	90.00
375	HLA-A0201	GVPVCESSENS	GVPVCSENS	0	0	0	5	2	GVPVCESSENS	tr_C9ZP82	0.018	41316.40	90.00
218	HLA-A0201	DDPTAFEFD	DDPTAFEFD	0	0	0	0	0	DDPTAFEFD	tr_C9ZP82	0.018	41320.43	90.00
631	HLA-A0201	TYLKSTGLS	TYLKSTGLS	0	0	0	0	0	TYLKSTGLS	tr_C9ZP82	0.018	41322.21	90.00
93	HLA-A0201	QRARRYMGR	QRARRYMGR	0	0	0	0	0	QRARRYMGR	tr_C9ZP82	0.018	41325.78	90.00
268	HLA-A0201	GSNTPGEGE	GSNTPGEGE	0	0	0	0	0	GSNTPGEGE	tr_C9ZP82	0.018	41329.80	90.00
265	HLA-A0201	IFSGSNTPGEGEHK	IFNTPGEGE	0	0	0	2	3	IFSGSNTPGEGE	tr_C9ZP82	0.018	41344.12	90.00
330	HLA-A0201	YKRRAVARQQQRE	YRRAVARQQ	0	0	0	1	1	YKRRAVARQQ	tr_C9ZP82	0.018	41347.27	90.00
362	HLA-A0201	PSYIDEEES	PSYIDEEES	0	0	0	0	0	PSYIDEEES	tr_C9ZP82	0.018	41350.84	90.00
385	HLA-A0201	SSSDTDNESP	SSSDTDESP	0	0	0	6	1	SSSDTDNESP	tr_C9ZP82	0.018	41353.96	90.00
887	HLA-A0201	GEGVIEGKK	GEGVIEGKK	0	0	0	0	0	GEGVIEGKK	tr_C9ZP82	0.018	41362.02	90.00
109	HLA-A0201	EVASTYMPNKSG	EVASTYMPG	0	0	0	8	3	EVASTYMPNKSG	tr_C9ZP82	0.018	41371.41	90.00

81	HLA-A0201	VDGVAPRAKMNQQ	VDGVAPRAM	0	0	0	8	1	VDGVAPRAKM	tr_C9ZP82	0.017	41379.04	90.00
461	HLA-A0201	NHIEDGTLP	NHIEDGTLP	0	0	0	0	0	NHIEDGTLP	tr_C9ZP82	0.017	41382.61	90.00
84	HLA-A0201	VAPRAKMNQQRARR	APRAKMARR	1	0	0	6	4	APRAKMNQQRARR	tr_C9ZP82	0.017	41390.68	90.00
394	HLA-A0201	PIESREAHVTT	ISREAHVTT	1	0	0	1	1	IESREAHVTT	tr_C9ZP82	0.017	41392.92	90.00
84	HLA-A0201	VAPRAKMNQQRA	VAPMNQQRA	0	0	0	3	3	VAPRAKMNQQRA	tr_C9ZP82	0.017	41401.86	90.00
193	HLA-A0201	THENRYVES	THENRYVES	0	0	0	0	0	THENRYVES	tr_C9ZP82	0.017	41407.68	90.00
322	HLA-A0201	LRDTTRNPYKRRA	LRDTTRNPA	0	0	0	8	4	LRDTTRNPYKRRA	tr_C9ZP82	0.017	41409.04	90.00
490	HLA-A0201	EAERSWKNFRP	AERSWNFRP	1	0	0	5	1	AERSWKNFRP	tr_C9ZP82	0.017	41417.11	90.00
598	HLA-A0201	SGAEFRKGS	SGAEFRKGS	0	0	0	0	0	SGAEFRKGS	tr_C9ZP82	0.017	41435.49	90.00
717	HLA-A0201	PPTSHALLP	PPTSHALLP	0	0	0	0	0	PPTSHALLP	tr_C9ZP82	0.017	41438.62	90.00
271	HLA-A0201	TPGEGEHKII	TPGEGEHII	0	0	0	7	1	TPGEGEHKII	tr_C9ZP82	0.017	41441.32	90.00
748	HLA-A0201	QWNVDYHDS	QWNVDYHDS	0	0	0	0	0	QWNVDYHDS	tr_C9ZP82	0.017	41443.99	90.00
189	HLA-A0201	DYESTHENRY	YESTHENRY	1	0	0	0	0	YESTHENRY	tr_C9ZP82	0.017	41448.48	90.00
227	HLA-A0201	SNCISPGTD	SNCISPGTD	0	0	0	0	0	SNCISPGTD	tr_C9ZP82	0.017	41449.82	90.00
805	HLA-A0201	NKTSFVPKGD	KTSFVPKGD	1	0	0	0	0	KTSFVPKGD	tr_C9ZP82	0.017	41450.27	90.00
436	HLA-A0201	TPATESAFDKKT	ATSAFDKKT	2	0	0	2	1	ATESAFDKKT	tr_C9ZP82	0.017	41452.52	90.00
191	HLA-A0201	ESTHENRYVESNQ	ESTHENRYV	0	0	0	0	0	ESTHENRYV	tr_C9ZP82	0.017	41453.43	90.00
85	HLA-A0201	APRAKMNQQRA	APRAKMQRA	0	0	0	6	2	APRAKMNQQRA	tr_C9ZP82	0.017	41470.46	90.00
325	HLA-A0201	TTRNPYKRR	TTRNPYKRR	0	0	0	0	0	TTRNPYKRR	tr_C9ZP82	0.017	41483.94	90.00
389	HLA-A0201	TDNESPIESRE	TDNESPIES	0	0	0	0	0	TDNESPIES	tr_C9ZP82	0.017	41493.81	90.00
468	HLA-A0201	LPSSTECGSP	LSSTECGSP	0	0	0	1	1	LPSSTECGSP	tr_C9ZP82	0.017	41503.69	90.00
346	HLA-A0201	RRKRNFIDD	RRKRNFIDD	0	0	0	0	0	RRKRNFIDD	tr_C9ZP82	0.017	41508.18	90.00
752	HLA-A0201	DYHDSNVVH	DYHDSNVVH	0	0	0	0	0	DYHDSNVVH	tr_C9ZP82	0.017	41508.61	90.00
366	HLA-A0201	DEEESDGVI	DEEESDGVI	0	0	0	0	0	DEEESDGVI	tr_C9ZP82	0.017	41525.24	90.00
81	HLA-A0201	VDGVAPRAKMNQQR	VDGVAPRAM	0	0	0	8	1	VDGVAPRAKM	tr_C9ZP82	0.017	41539.17	90.00
636	HLA-A0201	TGLSNSVQE	TGLSNSVQE	0	0	0	0	0	TGLSNSVQE	tr_C9ZP82	0.017	41545.46	90.00
485	HLA-A0201	VSKKAEAER	VSKKAEAER	0	0	0	0	0	VSKKAEAER	tr_C9ZP82	0.017	41548.17	90.00
329	HLA-A0201	PYKRRAVARQQQ	KRRAVAQQQ	2	0	0	6	1	KRRAVARQQQ	tr_C9ZP82	0.017	41564.79	90.00
887	HLA-A0201	GEGVIEGKKSNSR	GEGVISNSR	0	0	0	5	4	GEGVIEGKKSNSR	tr_C9ZP82	0.017	41566.15	90.00
479	HLA-A0201	GRRSKRVSKKAEA	RSKRVSAEA	2	0	0	6	2	RSKRVSKKAEA	tr_C9ZP82	0.017	41569.75	90.00
474	HLA-A0201	CGSPKGRRSKRV	CGSPKGRRV	0	0	0	7	3	CGSPKGRRSKRV	tr_C9ZP82	0.017	41571.09	90.00
144	HLA-A0201	DEVRQTLLE	DEVRQTLLE	0	0	0	0	0	DEVRQTLLE	tr_C9ZP82	0.017	41576.50	90.00
437	HLA-A0201	PATESAFDKKT	ATSAFDKKT	1	0	0	2	1	ATESAFDKKT	tr_C9ZP82	0.017	41583.69	90.00
186	HLA-A0201	TDDDYESTHENRY	YESTHENRY	4	0	0	0	0	YESTHENRY	tr_C9ZP82	0.017	41592.25	90.00
81	HLA-A0201	VDGVAPRAKMN	VDGVAPRAM	0	0	0	8	1	VDGVAPRAKM	tr_C9ZP82	0.017	41593.13	90.00
593	HLA-A0201	KENSNSGAEFR	KSNSGAEFR	0	0	0	1	2	KENSNSGAEFR	tr_C9ZP82	0.017	41594.04	90.00
478	HLA-A0201	KGRRSKRVSKKAEA	RSKRVSAEA	3	0	0	6	2	RSKRVSKKAEA	tr_C9ZP82	0.017	41598.09	90.00
472	HLA-A0201	TECGSPKGRRSKRV	TECGSPKGV	0	0	0	8	5	TECGSPKGRRSKRV	tr_C9ZP82	0.017	41611.16	90.00
589	HLA-A0201	KSTNKENSNSGA	STNNSNSGA	1	0	0	3	2	STNKENSNSGA	tr_C9ZP82	0.017	41624.66	90.00
604	HLA-A0201	KGSNNDNVT	KGSNNDNVT	0	0	0	0	0	KGSNNDNVT	tr_C9ZP82	0.017	41645.39	90.00
169	HLA-A0201	GDALNDGDGVN	GALNDGDGV	0	0	0	1	1	GDALNDGDGV	tr_C9ZP82	0.017	41646.73	90.00
489	HLA-A0201	AEAERSWKN	AEAERSWKN	0	0	0	0	0	AEAERSWKN	tr_C9ZP82	0.017	41649.88	90.00
108	HLA-A0201	GEVASTYMPNKSG	VASTYMPSG	2	0	0	7	2	VASTYMPNKSG	tr_C9ZP82	0.017	41655.73	90.00
501	HLA-A0201	CSSPANSKIID	CSSPASKII	0	0	0	5	1	CSSPANSKII	tr_C9ZP82	0.017	41656.19	90.00
472	HLA-A0201	TECGSPKGR	TECGSPKGR	0	0	0	0	0	TECGSPKGR	tr_C9ZP82	0.017	41660.24	90.00
102	HLA-A0201	AAPAQTGEVAS	AAPAQTGEV	0	0	0	0	0	AAPAQTGEV	tr_C9ZP82	0.017	41662.96	90.00
329	HLA-A0201	PYKRRAVARQQQRE	YRRAVARQQ	1	0	0	1	1	YKRRAVARQQ	tr_C9ZP82	0.017	41670.16	90.00
591	HLA-A0201	TNKENSNSGAEF	KENSSGAEF	2	0	0	4	1	KENSNSGAEF	tr_C9ZP82	0.017	41683.25	90.00
464	HLA-A0201	EDGTLPSST	EDGTLPSST	0	0	0	0	0	EDGTLPSST	tr_C9ZP82	0.017	41700.83	90.00
331	HLA-A0201	KRRAVARQQQRE	KRRAVAQQR	0	0	0	6	2	KRRAVARQQQR	tr_C9ZP82	0.017	41708.96	90.00
439	HLA-A0201	TESAFDKKT	TESAFDKKT	0	0	0	0	0	TESAFDKKT	tr_C9ZP82	0.017	41709.87	90.00
481	HLA-A0201	RSKRVSKKAEAE	RSKRVSAEA	0	0	0	6	2	RSKRVSKKAEA	tr_C9ZP82	0.017	41711.67	90.00
592	HLA-A0201	NKENSNSGAEF	KENSSGAEF	1	0	0	4	1	KENSNSGAEF	tr_C9ZP82	0.017	41713.93	90.00
360	HLA-A0201	LNPSYIDEEESDG	YIDEEESDG	4	0	0	0	0	YIDEEESDG	tr_C9ZP82	0.017	41715.28	90.00

322	HLA-A0201	LRDTTRNPYKR	LRDTTPYKR	0	0	0	5	2	LRDTTRNPYKR	tr_C9ZP82	0.017	41720.25	90.00
110	HLA-A0201	VASTYMPNKSG	VASTYMPSG	0	0	0	7	2	VASTYMPNKSG	tr_C9ZP82	0.017	41735.59	90.00
464	HLA-A0201	EDGTLPSSTEC	GTLPSSTEC	2	0	0	0	0	GTLPSSTEC	tr_C9ZP82	0.017	41755.00	90.00
111	HLA-A0201	ASTYMPNKSG	ATYMPNKSG	0	0	0	1	1	ASTYMPNKSG	tr_C9ZP82	0.017	41755.46	90.00
168	HLA-A0201	AGDALNDGD	AGDALNDGD	0	0	0	0	0	AGDALNDGD	tr_C9ZP82	0.017	41759.07	90.00
249	HLA-A0201	NEKMSGNDPT	NKMSGNDPT	0	0	0	1	1	NEKMSGNDPT	tr_C9ZP82	0.017	41764.05	90.00
873	HLA-A0201	RQRRHRHSGQL	RQHRHSGQL	0	0	0	2	2	RQRRHRHSGQL	tr_C9ZP82	0.017	41775.34	90.00
391	HLA-A0201	NESPIESREAH	SPIESREAH	2	0	0	0	0	SPIESREAH	tr_C9ZP82	0.017	41775.80	90.00
135	HLA-A0201	EMAQANDDLDE	EMAQANDDL	0	0	0	0	0	EMAQANDDL	tr_C9ZP82	0.017	41778.06	90.00
889	HLA-A0201	GVIEGKKSNS	GVIEGKSNS	0	0	0	5	1	GVIEGKKSNS	tr_C9ZP82	0.017	41779.41	90.00
879	HLA-A0201	HSGQLKGNGE	HSGQLGNGE	0	0	0	5	1	HSGQLKGNGE	tr_C9ZP82	0.017	41779.41	90.00
83	HLA-A0201	GVAPRAKMNQQ	GVAAKMNQQ	0	0	0	3	2	GVAPRAKMNQQ	tr_C9ZP82	0.017	41798.87	90.00
162	HLA-A0201	NEGEEEAGDA	NEGEEEAGA	0	0	0	8	1	NEGEEEAGDA	tr_C9ZP82	0.017	41800.67	90.00
826	HLA-A0201	LRRVPSREG	LRRVPSREG	0	0	0	0	0	LRRVPSREG	tr_C9ZP82	0.017	41804.74	90.00
180	HLA-A0201	SFCVRSTDD	SFCVRSTDD	0	0	0	0	0	SFCVRSTDD	tr_C9ZP82	0.017	41807.90	90.00
85	HLA-A0201	APRAKMNQQRAR	APRAKMNAR	0	0	0	7	3	APRAKMNQQRAR	tr_C9ZP82	0.017	41811.08	90.00
18	HLA-A0201	TKNCPSSVHG	KNCPSSVHG	1	0	0	0	0	KNCPSSVHG	tr_C9ZP82	0.017	41819.68	90.00
499	HLA-A0201	RPCSSPANSK	RCSSPANSK	0	0	0	1	1	RPCSSPANSK	tr_C9ZP82	0.016	41828.26	90.00
488	HLA-A0201	KAEAERSWKN	KAAERSWKN	0	0	0	2	1	KAEAERSWKN	tr_C9ZP82	0.016	41837.78	90.00
436	HLA-A0201	TPATESAFDKK	ATESAFDKK	2	0	0	0	0	ATESAFDKK	tr_C9ZP82	0.016	41856.78	90.00
786	HLA-A0201	TEEERWRTQKLD	TEEERWRTL	0	0	0	8	2	TEEERWRTQKL	tr_C9ZP82	0.016	41865.85	90.00
367	HLA-A0201	EEESDGVIG	EEESDGVIG	0	0	0	0	0	EEESDGVIG	tr_C9ZP82	0.016	41868.58	90.00
591	HLA-A0201	TNKENSNSGAEFR	NSNSGAEFR	4	0	0	0	0	NSNSGAEFR	tr_C9ZP82	0.016	41869.47	90.00
187	HLA-A0201	DDDYESTHENRY	YESTHENRY	3	0	0	0	0	YESTHENRY	tr_C9ZP82	0.016	41870.39	90.00
83	HLA-A0201	GVAPRAKMNQQR	APRAKMQQR	2	0	0	6	1	APRAKMNQQR	tr_C9ZP82	0.016	41871.28	90.00
588	HLA-A0201	FKSTNKENSNSGA	STNNSNSGA	2	0	0	3	2	STNKENSNSGA	tr_C9ZP82	0.016	41881.25	90.00
592	HLA-A0201	NKENSNSGAEFR	NSNSGAEFR	3	0	0	0	0	NSNSGAEFR	tr_C9ZP82	0.016	41891.67	90.00
375	HLA-A0201	GVPVCESSEN	GVPVCESSE	0	0	0	0	0	GVPVCESSE	tr_C9ZP82	0.016	41901.18	90.00
82	HLA-A0201	DGVAPRAKM	DGVAPRAKM	0	0	0	0	0	DGVAPRAKM	tr_C9ZP82	0.016	41907.54	90.00
592	HLA-A0201	NKENSNSGA	NKENSNSGA	0	0	0	0	0	NKENSNSGA	tr_C9ZP82	0.016	41909.34	90.00
343	HLA-A0201	RRSRRKRNFI	RSRRKRNFI	1	0	0	0	0	RSRRKRNFI	tr_C9ZP82	0.016	41910.26	90.00
890	HLA-A0201	VIEGKKSNSR	VIEGKSNSR	0	0	0	4	1	VIEGKKSNSR	tr_C9ZP82	0.016	41914.34	90.00
84	HLA-A0201	VAPRAKMNQQRAR	VAPMNQQRA	0	0	0	3	3	VAPRAKMNQQRA	tr_C9ZP82	0.016	41920.24	90.00
880	HLA-A0201	SGQLKGNGEG	SQLKGNGEG	0	0	0	1	1	SGQLKGNGEG	tr_C9ZP82	0.016	41925.24	90.00
329	HLA-A0201	PYKRRAVAR	PYKRRAVAR	0	0	0	0	0	PYKRRAVAR	tr_C9ZP82	0.016	41925.67	90.00
587	HLA-A0201	LFKSTNKENSNSGA	STNNSNSGA	3	0	0	3	2	STNKENSNSGA	tr_C9ZP82	0.016	41929.32	90.00
785	HLA-A0201	LTEEERWRTQK	LTEEERWQK	0	0	0	7	2	LTEEERWRTQK	tr_C9ZP82	0.016	41936.11	90.00
37	HLA-A0201	HPCCHNDSDTA	CCHNDSDTA	2	0	0	0	0	CCHNDSDTA	tr_C9ZP82	0.016	41945.20	90.00
468	HLA-A0201	LPSSTECGSPK	LSTECGSPK	0	0	0	1	2	LPSSTECGSPK	tr_C9ZP82	0.016	41955.18	90.00
226	HLA-A0201	DSNCISPGTD	DSNCISPGT	0	0	0	0	0	DSNCISPGT	tr_C9ZP82	0.016	41956.54	90.00
593	HLA-A0201	KENSNSGAEFRK	KENSSGAEF	0	0	0	4	1	KENSNSGAEF	tr_C9ZP82	0.016	41965.62	90.00
83	HLA-A0201	GVAPRAKMN	GVAPRAKMN	0	0	0	0	0	GVAPRAKMN	tr_C9ZP82	0.016	41969.25	90.00
390	HLA-A0201	DNESPIESRE	DNESPIESR	0	0	0	0	0	DNESPIESR	tr_C9ZP82	0.016	41973.34	90.00
82	HLA-A0201	DGVAPRAKMNQQR	APRAKMQQR	3	0	0	6	1	APRAKMNQQR	tr_C9ZP82	0.016	41978.78	90.00
470	HLA-A0201	SSTECGSPKGR	SSTECGSPR	0	0	0	8	2	SSTECGSPKGR	tr_C9ZP82	0.016	41983.79	90.00
82	HLA-A0201	DGVAPRAKMNQQ	GVAAKMNQQ	1	0	0	3	2	GVAPRAKMNQQ	tr_C9ZP82	0.016	42009.68	90.00
597	HLA-A0201	NSGAEFRKGS	NSGAEFRGS	0	0	0	7	1	NSGAEFRKGS	tr_C9ZP82	0.016	42011.07	90.00
551	HLA-A0201	PYGFLTKGP	PYGFLTKGP	0	0	0	0	0	PYGFLTKGP	tr_C9ZP82	0.016	42013.31	90.00
480	HLA-A0201	RRSKRVSKKAEAE	RSKRVSAEA	1	0	0	6	2	RSKRVSKKAEA	tr_C9ZP82	0.016	42021.06	90.00
473	HLA-A0201	ECGSPKGRRSKRV	CGSPKGRRV	1	0	0	7	3	CGSPKGRRSKRV	tr_C9ZP82	0.016	42031.52	90.00
498	HLA-A0201	FRPCSSPAN	FRPCSSPAN	0	0	0	0	0	FRPCSSPAN	tr_C9ZP82	0.016	42034.70	90.00
883	HLA-A0201	LKGNGEGVIE	LKGNGEGVI	0	0	0	0	0	LKGNGEGVI	tr_C9ZP82	0.016	42036.97	90.00
591	HLA-A0201	TNKENSNSGAEFRK	SNSGAEFRK	5	0	0	0	0	SNSGAEFRK	tr_C9ZP82	0.016	42041.53	90.00
333	HLA-A0201	RAVARQQQR	RAVARQQQR	0	0	0	0	0	RAVARQQQR	tr_C9ZP82	0.016	42045.16	90.00

470	HLA-A0201	SSTECGSPKGRRS	SSTECGSPS	0	0	0	8	4	SSTECGSPKGRRS	tr_C9ZP82	0.016	42057.45	90.00
842	HLA-A0201	DVPLERYRP	DVPLERYRP	0	0	0	0	0	DVPLERYRP	tr_C9ZP82	0.016	42059.73	90.00
526	HLA-A0201	PHAPSAFSGE	HAPSAFSGE	1	0	0	0	0	HAPSAFSGE	tr_C9ZP82	0.016	42079.29	90.00
378	HLA-A0201	VCESSENSS	VCESSENSS	0	0	0	0	0	VCESSENSS	tr_C9ZP82	0.016	42085.66	90.00
481	HLA-A0201	RSKRVSKKAEAERS	RSKRVAERS	0	0	0	5	5	RSKRVSKKAEAERS	tr_C9ZP82	0.016	42088.87	90.00
592	HLA-A0201	NKENSNSGAEFRK	SNSGAEFRK	4	0	0	0	0	SNSGAEFRK	tr_C9ZP82	0.016	42105.70	90.00
468	HLA-A0201	LPSSTECGSPKGR	SSTECGSPR	2	0	0	8	2	SSTECGSPKGR	tr_C9ZP82	0.016	42107.09	90.00
268	HLA-A0201	GSNTPGEGEHKIID	GSNTPGEII	0	0	0	7	4	GSNTPGEGEHKII	tr_C9ZP82	0.016	42119.82	90.00
391	HLA-A0201	NESPIESRE	NESPIESRE	0	0	0	0	0	NESPIESRE	tr_C9ZP82	0.016	42124.39	90.00
104	HLA-A0201	PAQTGEVAS	PAQTGEVAS	0	0	0	0	0	PAQTGEVAS	tr_C9ZP82	0.016	42124.39	90.00
481	HLA-A0201	RSKRVSKKAEAER	RSKRVSAEA	0	0	0	6	2	RSKRVSKKAEA	tr_C9ZP82	0.016	42131.70	90.00
188	HLA-A0201	DDYESTHENRY	DDYESTHEY	0	0	0	8	2	DDYESTHENRY	tr_C9ZP82	0.016	42134.88	90.00
219	HLA-A0201	DPTAFEFDSN	DPTAFFDSN	0	0	0	5	1	DPTAFEFDSN	tr_C9ZP82	0.016	42145.82	90.00
322	HLA-A0201	LRDTTRNPYKRR	LRDTTRNPY	0	0	0	0	0	LRDTTRNPY	tr_C9ZP82	0.016	42146.72	90.00
360	HLA-A0201	LNPSYIDEEESD	LSYIDEEES	0	0	0	1	2	LNPSYIDEEES	tr_C9ZP82	0.016	42156.32	90.00
189	HLA-A0201	DYESTHENR	DYESTHENR	0	0	0	0	0	DYESTHENR	tr_C9ZP82	0.016	42156.32	90.00
600	HLA-A0201	AEFRKGSNNDNVT	AEFSNNDNV	0	0	0	3	3	AEFRKGSNNDNV	tr_C9ZP82	0.016	42183.21	95.00
480	HLA-A0201	RRSKRVSKK	RRSKRVSKK	0	0	0	0	0	RRSKRVSKK	tr_C9ZP82	0.016	42188.71	95.00
827	HLA-A0201	RRVPSREGVE	RRVPSREGV	0	0	0	0	0	RRVPSREGV	tr_C9ZP82	0.016	42206.05	95.00
494	HLA-A0201	SWKNFRPCS	SWKNFRPCS	0	0	0	0	0	SWKNFRPCS	tr_C9ZP82	0.016	42211.98	95.00
484	HLA-A0201	RVSKKAEAERS	RVSAEAERS	0	0	0	3	2	RVSKKAEAERS	tr_C9ZP82	0.016	42213.37	95.00
502	HLA-A0201	SSPANSKIIDD	SSPANIIDD	0	0	0	5	2	SSPANSKIIDD	tr_C9ZP82	0.016	42214.27	95.00
479	HLA-A0201	GRRSKRVSKKAEAE	RSKRVSAEA	2	0	0	6	2	RSKRVSKKAEA	tr_C9ZP82	0.016	42229.82	95.00
555	HLA-A0201	LTKGPHEIN	LTKGPHEIN	0	0	0	0	0	LTKGPHEIN	tr_C9ZP82	0.016	42232.10	95.00
590	HLA-A0201	STNKENSNSG	STNENSNSG	0	0	0	3	1	STNKENSNSG	tr_C9ZP82	0.016	42237.58	95.00
459	HLA-A0201	QPNHIEDGTLP	QPNHIGTLP	0	0	0	5	2	QPNHIEDGTLP	tr_C9ZP82	0.016	42239.87	95.00
84	HLA-A0201	VAPRAKMNQ	VAPRAKMNQ	0	0	0	0	0	VAPRAKMNQ	tr_C9ZP82	0.016	42252.21	95.00
339	HLA-A0201	QQRERRSRR	QQRERRSRR	0	0	0	0	0	QQRERRSRR	tr_C9ZP82	0.016	42255.86	95.00
867	HLA-A0201	DGQVIKRQRRHRHS	GQVIRHRHS	1	0	0	4	4	GQVIKRQRRHRHS	tr_C9ZP82	0.016	42258.61	95.00
502	HLA-A0201	SSPANSKIID	SSPANSIID	0	0	0	6	1	SSPANSKIID	tr_C9ZP82	0.016	42268.67	95.00
500	HLA-A0201	PCSSPANSKIID	CSSPASKII	1	0	0	5	1	CSSPANSKII	tr_C9ZP82	0.016	42275.98	95.00
553	HLA-A0201	GFLTKGPHE	GFLTKGPHE	0	0	0	0	0	GFLTKGPHE	tr_C9ZP82	0.015	42282.85	95.00
163	HLA-A0201	EGEEEAGDA	EGEEEAGDA	0	0	0	0	0	EGEEEAGDA	tr_C9ZP82	0.015	42287.86	95.00
474	HLA-A0201	CGSPKGRRS	CGSPKGRRS	0	0	0	0	0	CGSPKGRRS	tr_C9ZP82	0.015	42293.38	95.00
891	HLA-A0201	IEGKKSNSR	IEGKKSNSR	0	0	0	0	0	IEGKKSNSR	tr_C9ZP82	0.015	42299.79	95.00
99	HLA-A0201	MGRAAPAQTGE	MGRAAPAGE	0	0	0	7	2	MGRAAPAQTGE	tr_C9ZP82	0.015	42306.63	95.00
698	HLA-A0201	LPVVKSTPPDT	LPVVKSTPT	0	0	0	8	2	LPVVKSTPPDT	tr_C9ZP82	0.015	42307.09	95.00
599	HLA-A0201	GAEFRKGSNNDNVT	GAGSNNDNV	0	0	0	2	4	GAEFRKGSNNDNV	tr_C9ZP82	0.015	42307.56	95.00
498	HLA-A0201	FRPCSSPANSK	FCSSPANSK	0	0	0	1	2	FRPCSSPANSK	tr_C9ZP82	0.015	42318.09	95.00
250	HLA-A0201	EKMSGNDPT	EKMSGNDPT	0	0	0	0	0	EKMSGNDPT	tr_C9ZP82	0.015	42320.82	95.00
162	HLA-A0201	NEGEEEAGDALN	NEGEEEAAL	0	0	0	7	2	NEGEEEAGDAL	tr_C9ZP82	0.015	42324.04	95.00
526	HLA-A0201	PHAPSAFSG	PHAPSAFSG	0	0	0	0	0	PHAPSAFSG	tr_C9ZP82	0.015	42330.89	95.00
480	HLA-A0201	RRSKRVSKKAEAER	RVSKKAEAR	4	0	0	8	1	RVSKKAEAER	tr_C9ZP82	0.015	42333.65	95.00
266	HLA-A0201	FSGSNTPGEGE	FSNTPGEGE	0	0	0	1	2	FSGSNTPGEGE	tr_C9ZP82	0.015	42344.66	95.00
100	HLA-A0201	GRAAPAQTG	GRAAPAQTG	0	0	0	0	0	GRAAPAQTG	tr_C9ZP82	0.015	42352.90	95.00
193	HLA-A0201	THENRYVESNQ	HENRYVESQ	1	0	0	8	1	HENRYVESNQ	tr_C9ZP82	0.015	42354.27	95.00
469	HLA-A0201	PSSTECGSPKGR	SSTECGSPR	1	0	0	8	2	SSTECGSPKGR	tr_C9ZP82	0.015	42357.93	95.00
733	HLA-A0201	VRSPQQAELES	RSPQQALES	1	0	0	6	1	RSPQQAELES	tr_C9ZP82	0.015	42384.52	95.00
470	HLA-A0201	SSTECGSPKGRR	SSTECGSPR	0	0	0	8	3	SSTECGSPKGRR	tr_C9ZP82	0.015	42388.64	95.00
84	HLA-A0201	VAPRAKMNQQR	APRAKMQQR	1	0	0	6	1	APRAKMNQQR	tr_C9ZP82	0.015	42390.04	95.00
345	HLA-A0201	SRRKRNFIDD	SRRKRFIDD	0	0	0	5	1	SRRKRNFIDD	tr_C9ZP82	0.015	42391.41	95.00
179	HLA-A0201	NSFCVRSTDDD	NSFCVTDDD	0	0	0	5	2	NSFCVRSTDDD	tr_C9ZP82	0.015	42397.37	95.00
593	HLA-A0201	KENSNSGAEFRKGS	KENSSGAEF	0	0	0	4	1	KENSNSGAEF	tr_C9ZP82	0.015	42401.96	95.00
265	HLA-A0201	IFSGSNTPGEGE	IFNTPGEGE	0	0	0	2	3	IFSGSNTPGEGE	tr_C9ZP82	0.015	42408.39	95.00

168	HLA-A0201	AGDALNDGDG	AGDALNDGG	0	0	0	8	1	AGDALNDGDG	tr_C9ZP82	0.015	42422.14	95.00
160	HLA-A0201	LYNEGEEEAGDA	LYNEGEEEA	0	0	0	8	3	LYNEGEEEAGDA	tr_C9ZP82	0.015	42434.07	95.00
594	HLA-A0201	ENSNSGAEFRK	NSNSGAFRK	1	0	0	6	1	NSNSGAEFRK	tr_C9ZP82	0.015	42435.91	95.00
480	HLA-A0201	RRSKRVSKKA	RSKRVSKKA	1	0	0	0	0	RSKRVSKKA	tr_C9ZP82	0.015	42437.77	95.00
593	HLA-A0201	KENSNSGAEFRKG	NSNSGAEFG	2	0	0	8	2	NSNSGAEFRKG	tr_C9ZP82	0.015	42438.67	95.00
470	HLA-A0201	SSTECGSPKGRRSK	SSTECGSPK	0	0	0	8	5	SSTECGSPKGRRSK	tr_C9ZP82	0.015	42446.01	95.00
162	HLA-A0201	NEGEEEAGDALNDG	NEEEAGDAL	0	0	0	1	2	NEGEEEAGDAL	tr_C9ZP82	0.015	42449.24	95.00
468	HLA-A0201	LPSSTECGSPKGRR	SSTECGSPR	2	0	0	8	3	SSTECGSPKGRR	tr_C9ZP82	0.015	42452.44	95.00
474	HLA-A0201	CGSPKGRRSKRVSK	CGSPKGVSK	0	0	0	6	5	CGSPKGRRSKRVSK	tr_C9ZP82	0.015	42452.91	95.00
365	HLA-A0201	IDEEESDGVIG	IDEEESDGV	0	0	0	0	0	IDEEESDGV	tr_C9ZP82	0.015	42472.68	95.00
867	HLA-A0201	DGQVIKRQRRHR	GQVIKRQHR	1	0	0	7	2	GQVIKRQRRHR	tr_C9ZP82	0.015	42480.95	95.00
392	HLA-A0201	ESPIESREAH	SPIESREAH	1	0	0	0	0	SPIESREAH	tr_C9ZP82	0.015	42480.95	95.00
878	HLA-A0201	RHSGQLKGN	RHSGQLKGN	0	0	0	0	0	RHSGQLKGN	tr_C9ZP82	0.015	42489.66	95.00
871	HLA-A0201	IKRQRRHRHSGQL	RQHRHSGQL	2	0	0	2	2	RQRRHRHSGQL	tr_C9ZP82	0.015	42492.43	95.00
603	HLA-A0201	RKGSNNDNVT	RKGSNNDNV	0	0	0	0	0	RKGSNNDNV	tr_C9ZP82	0.015	42503.92	95.00
487	HLA-A0201	KKAEAERSWK	KKAEAERSW	0	0	0	0	0	KKAEAERSW	tr_C9ZP82	0.015	42511.29	95.00
657	HLA-A0201	WRYYTTTTTDSD	WRYYTTTTT	0	0	0	0	0	WRYYTTTTT	tr_C9ZP82	0.015	42536.14	95.00
270	HLA-A0201	NTPGEGEHKIID	NTPGEGEHI	0	0	0	8	2	NTPGEGEHKII	tr_C9ZP82	0.015	42536.60	95.00
595	HLA-A0201	NSNSGAEFRKGS	NSNSGAEFS	0	0	0	8	3	NSNSGAEFRKGS	tr_C9ZP82	0.015	42548.10	95.00
195	HLA-A0201	ENRYVESNQ	ENRYVESNQ	0	0	0	0	0	ENRYVESNQ	tr_C9ZP82	0.015	42554.08	95.00
526	HLA-A0201	PHAPSAFSGES	HASAFSGES	1	0	0	2	1	HAPSAFSGES	tr_C9ZP82	0.015	42557.76	95.00
872	HLA-A0201	KRQRRHRHSGQL	RQHRHSGQL	1	0	0	2	2	RQRRHRHSGQL	tr_C9ZP82	0.015	42558.69	95.00
486	HLA-A0201	SKKAEAERS	SKKAEAERS	0	0	0	0	0	SKKAEAERS	tr_C9ZP82	0.015	42578.05	95.00
469	HLA-A0201	PSSTECGSPKGRRS	SSTECGSPS	1	0	0	8	4	SSTECGSPKGRRS	tr_C9ZP82	0.015	42581.73	95.00
181	HLA-A0201	FCVRSTDDD	FCVRSTDDD	0	0	0	0	0	FCVRSTDDD	tr_C9ZP82	0.015	42582.19	95.00
873	HLA-A0201	RQRRHRHSGQLK	RQHRHSGQL	0	0	0	2	2	RQRRHRHSGQL	tr_C9ZP82	0.015	42584.03	95.00
482	HLA-A0201	SKRVSKKAEAERS	RVSKKAEAS	2	0	0	8	2	RVSKKAEAERS	tr_C9ZP82	0.015	42584.47	95.00
592	HLA-A0201	NKENSNSGAEFRKG	NSNSGAEFG	3	0	0	8	2	NSNSGAEFRKG	tr_C9ZP82	0.015	42588.62	95.00
787	HLA-A0201	EEERWRTQKLD	EEERWTQKL	0	0	0	5	1	EEERWRTQKL	tr_C9ZP82	0.015	42595.07	95.00
186	HLA-A0201	TDDDYESTH	TDDDYESTH	0	0	0	0	0	TDDDYESTH	tr_C9ZP82	0.015	42601.06	95.00
267	HLA-A0201	SGSNTPGEG	SGSNTPGEG	0	0	0	0	0	SGSNTPGEG	tr_C9ZP82	0.015	42610.29	95.00
788	HLA-A0201	EERWRTQKLD	EERWRTQKL	0	0	0	0	0	EERWRTQKL	tr_C9ZP82	0.015	42613.07	95.00
877	HLA-A0201	HRHSGQLKG	HRHSGQLKG	0	0	0	0	0	HRHSGQLKG	tr_C9ZP82	0.015	42615.38	95.00
141	HLA-A0201	DDLDEVRQT	DDLDEVRQT	0	0	0	0	0	DDLDEVRQT	tr_C9ZP82	0.015	42615.38	95.00
162	HLA-A0201	NEGEEEAGDALND	NEEEAGDAL	0	0	0	1	2	NEGEEEAGDAL	tr_C9ZP82	0.015	42622.28	95.00
166	HLA-A0201	EEAGDALNDG	EAGDALNDG	1	0	0	0	0	EAGDALNDG	tr_C9ZP82	0.015	42623.68	95.00
595	HLA-A0201	NSNSGAEFRKG	NSNSGAEFG	0	0	0	8	2	NSNSGAEFRKG	tr_C9ZP82	0.015	42631.52	95.00
164	HLA-A0201	GEEEAGDALNDG	GEEEAGDAL	0	0	0	0	0	GEEEAGDAL	tr_C9ZP82	0.015	42638.41	95.00
596	HLA-A0201	SNSGAEFRKG	SNSGAEFRG	0	0	0	8	1	SNSGAEFRKG	tr_C9ZP82	0.015	42642.13	95.00
163	HLA-A0201	EGEEEAGDALN	GEEEAGDAL	1	0	0	0	0	GEEEAGDAL	tr_C9ZP82	0.015	42651.34	95.00
488	HLA-A0201	KAEAERSWK	KAEAERSWK	0	0	0	0	0	KAEAERSWK	tr_C9ZP82	0.015	42663.34	95.00
361	HLA-A0201	NPSYIDEEESDG	YIDEEESDG	3	0	0	0	0	YIDEEESDG	tr_C9ZP82	0.015	42670.74	95.00
266	HLA-A0201	FSGSNTPGEGEH	FSGSNTPEH	0	0	0	7	3	FSGSNTPGEGEH	tr_C9ZP82	0.015	42670.74	95.00
387	HLA-A0201	SDTDNESPIES	DTDESPIES	1	0	0	3	1	DTDNESPIES	tr_C9ZP82	0.015	42681.36	95.00
265	HLA-A0201	IFSGSNTPGEGEH	IFSGSNTPH	0	0	0	8	4	IFSGSNTPGEGEH	tr_C9ZP82	0.015	42684.11	95.00
492	HLA-A0201	ERSWKNFRP	ERSWKNFRP	0	0	0	0	0	ERSWKNFRP	tr_C9ZP82	0.015	42686.89	95.00
163	HLA-A0201	EGEEEAGDALNDG	GAGDALNDG	1	0	0	1	3	GEEEAGDALNDG	tr_C9ZP82	0.015	42688.74	95.00
339	HLA-A0201	QQRERRSRRKRNF	QQRERRRNF	0	0	0	6	4	QQRERRSRRKRNF	tr_C9ZP82	0.015	42693.36	95.00
698	HLA-A0201	LPVVKSTPPD	LVVKSTPPD	0	0	0	1	1	LPVVKSTPPD	tr_C9ZP82	0.015	42694.27	95.00
191	HLA-A0201	ESTHENRYVE	ESTHENRYV	0	0	0	0	0	ESTHENRYV	tr_C9ZP82	0.015	42735.43	95.00
479	HLA-A0201	GRRSKRVSK	GRRSKRVSK	0	0	0	0	0	GRRSKRVSK	tr_C9ZP82	0.014	42744.66	95.00
345	HLA-A0201	SRRKRNFID	SRRKRNFID	0	0	0	0	0	SRRKRNFID	tr_C9ZP82	0.014	42746.04	95.00
469	HLA-A0201	PSSTECGSPKGRR	SSTECGSPR	1	0	0	8	3	SSTECGSPKGRR	tr_C9ZP82	0.014	42752.52	95.00
483	HLA-A0201	KRVSKKAEAE	KVSKKAEAE	0	0	0	1	1	KRVSKKAEAE	tr_C9ZP82	0.014	42753.46	95.00

476	HLA-A0201	SPKGRRSKRV	SPKGRSKRV	0	0	0	4	1	SPKGRRSKRV	tr_C9ZP82	0.014	42771.02	95.00
384	HLA-A0201	NSSSDTDNE	NSSSDTDNE	0	0	0	0	0	NSSSDTDNE	tr_C9ZP82	0.014	42786.31	95.00
602	HLA-A0201	FRKGSNNDNVT	FKGSNNDNV	0	0	0	1	1	FRKGSNNDNV	tr_C9ZP82	0.014	42788.16	95.00
482	HLA-A0201	SKRVSKKAEAE	SKRVSAEAE	0	0	0	5	2	SKRVSKKAEAE	tr_C9ZP82	0.014	42796.49	95.00
161	HLA-A0201	YNEGEEEAGDA	YNEGEEEAA	0	0	0	8	2	YNEGEEEAGDA	tr_C9ZP82	0.014	42796.96	95.00
621	HLA-A0201	HSTIDDRWRD	STIDDRWRD	1	0	0	0	0	STIDDRWRD	tr_C9ZP82	0.014	42799.28	95.00
218	HLA-A0201	DDPTAFEFDSN	DDPTAFFDS	0	0	0	6	1	DDPTAFEFDS	tr_C9ZP82	0.014	42804.36	95.00
323	HLA-A0201	RDTTRNPYKRRA	RDTTRNPYA	0	0	0	8	3	RDTTRNPYKRRA	tr_C9ZP82	0.014	42806.23	95.00
867	HLA-A0201	DGQVIKRQRRH	GQVIRQRRH	1	0	0	4	1	GQVIKRQRRH	tr_C9ZP82	0.014	42811.32	95.00
326	HLA-A0201	TRNPYKRRA	TRNPYKRRA	0	0	0	0	0	TRNPYKRRA	tr_C9ZP82	0.014	42816.86	95.00
482	HLA-A0201	SKRVSKKAEA	SRVSKKAEA	0	0	0	1	1	SKRVSKKAEA	tr_C9ZP82	0.014	42821.50	95.00
469	HLA-A0201	PSSTECGSP	PSSTECGSP	0	0	0	0	0	PSSTECGSP	tr_C9ZP82	0.014	42825.20	95.00
166	HLA-A0201	EEAGDALND	EEAGDALND	0	0	0	0	0	EEAGDALND	tr_C9ZP82	0.014	42842.36	95.00
18	HLA-A0201	TKNCPSSVH	TKNCPSSVH	0	0	0	0	0	TKNCPSSVH	tr_C9ZP82	0.014	42842.83	95.00
661	HLA-A0201	TTTTTDSDWS	TTTTTDSDW	0	0	0	0	0	TTTTTDSDW	tr_C9ZP82	0.014	42848.38	95.00
483	HLA-A0201	KRVSKKAEAERS	RVSAEAERS	1	0	0	3	2	RVSKKAEAERS	tr_C9ZP82	0.014	42855.35	95.00
482	HLA-A0201	SKRVSKKAEAER	RVSKKAEAR	2	0	0	8	1	RVSKKAEAER	tr_C9ZP82	0.014	42857.67	95.00
879	HLA-A0201	HSGQLKGNG	HSGQLKGNG	0	0	0	0	0	HSGQLKGNG	tr_C9ZP82	0.014	42864.61	95.00
249	HLA-A0201	NEKMSGNDP	NEKMSGNDP	0	0	0	0	0	NEKMSGNDP	tr_C9ZP82	0.014	42868.34	95.00
265	HLA-A0201	IFSGSNTPGEG	FSGSNTPEG	1	0	0	7	1	FSGSNTPGEG	tr_C9ZP82	0.014	42869.25	95.00
163	HLA-A0201	EGEEEAGDALND	GEEEAGDAL	1	0	0	0	0	GEEEAGDAL	tr_C9ZP82	0.014	42876.22	95.00
594	HLA-A0201	ENSNSGAEFRKGS	NSNSGAEFS	1	0	0	8	3	NSNSGAEFRKGS	tr_C9ZP82	0.014	42884.57	95.00
164	HLA-A0201	GEEEAGDALND	GEEEAGDAL	0	0	0	0	0	GEEEAGDAL	tr_C9ZP82	0.014	42893.84	95.00
689	HLA-A0201	KEANIKRMP	KEANIKRMP	0	0	0	0	0	KEANIKRMP	tr_C9ZP82	0.014	42896.63	95.00
87	HLA-A0201	RAKMNQQRAR	RAMNQQRAR	0	0	0	2	1	RAKMNQQRAR	tr_C9ZP82	0.014	42897.10	95.00
873	HLA-A0201	RQRRHRHSGQLKGN	RQSGQLKGN	0	0	0	2	5	RQRRHRHSGQLKGN	tr_C9ZP82	0.014	42911.49	95.00
335	HLA-A0201	VARQQQRER	VARQQQRER	0	0	0	0	0	VARQQQRER	tr_C9ZP82	0.014	42912.43	95.00
876	HLA-A0201	RHRHSGQLKGNGEG	RQLKGNGEG	0	0	0	1	5	RHRHSGQLKGNGEG	tr_C9ZP82	0.014	42913.81	95.00
588	HLA-A0201	FKSTNKENS	FKSTNKENS	0	0	0	0	0	FKSTNKENS	tr_C9ZP82	0.014	42914.73	95.00
871	HLA-A0201	IKRQRRHRHSGQLK	RQHRHSGQL	2	0	0	2	2	RQRRHRHSGQL	tr_C9ZP82	0.014	42926.35	95.00
876	HLA-A0201	RHRHSGQLKGNGE	HSGQLGNGE	3	0	0	5	1	HSGQLKGNGE	tr_C9ZP82	0.014	42929.59	95.00
344	HLA-A0201	RSRRKRNFIDD	RSRRNFIDD	0	0	0	3	2	RSRRKRNFIDD	tr_C9ZP82	0.014	42929.59	95.00
786	HLA-A0201	TEEERWRTQK	TEEERWRTK	0	0	0	8	1	TEEERWRTQK	tr_C9ZP82	0.014	42931.45	95.00
485	HLA-A0201	VSKKAEAERS	VSKKAAERS	0	0	0	5	1	VSKKAEAERS	tr_C9ZP82	0.014	42936.10	95.00
878	HLA-A0201	RHSGQLKGNGE	HSGQLGNGE	1	0	0	5	1	HSGQLKGNGE	tr_C9ZP82	0.014	42940.28	95.00
873	HLA-A0201	RQRRHRHSGQLKG	RQHSGQLKG	0	0	0	2	4	RQRRHRHSGQLKG	tr_C9ZP82	0.014	42946.32	95.00
248	HLA-A0201	LNEKMSGNDPT	LNMSGNDPT	0	0	0	2	2	LNEKMSGNDPT	tr_C9ZP82	0.014	42953.77	95.00
601	HLA-A0201	EFRKGSNNDNVT	FKGSNNDNV	1	0	0	1	1	FRKGSNNDNV	tr_C9ZP82	0.014	42957.95	95.00
594	HLA-A0201	ENSNSGAEFRKG	NSNSGAEFG	1	0	0	8	2	NSNSGAEFRKG	tr_C9ZP82	0.014	42957.95	95.00
220	HLA-A0201	PTAFEFDSN	PTAFEFDSN	0	0	0	0	0	PTAFEFDSN	tr_C9ZP82	0.014	42960.28	95.00
188	HLA-A0201	DDYESTHENR	DDYESTENR	0	0	0	6	1	DDYESTHENR	tr_C9ZP82	0.014	42985.83	95.00
267	HLA-A0201	SGSNTPGEGE	SGSNTPEGE	0	0	0	6	1	SGSNTPGEGE	tr_C9ZP82	0.014	42987.69	95.00
867	HLA-A0201	DGQVIKRQRRHRH	GQVIRRHRH	1	0	0	4	3	GQVIKRQRRHRH	tr_C9ZP82	0.014	42993.29	95.00
269	HLA-A0201	SNTPGEGEHKIID	NTPGEGEHI	1	0	0	8	2	NTPGEGEHKII	tr_C9ZP82	0.014	43003.99	95.00
375	HLA-A0201	GVPVCESSENSS	GVPVCENSS	0	0	0	5	3	GVPVCESSENSS	tr_C9ZP82	0.014	43007.71	95.00
362	HLA-A0201	PSYIDEEESDG	YIDEEESDG	2	0	0	0	0	YIDEEESDG	tr_C9ZP82	0.014	43009.57	95.00
474	HLA-A0201	CGSPKGRRSKRVS	CGSPKGRRV	0	0	0	7	3	CGSPKGRRSKRV	tr_C9ZP82	0.014	43010.48	95.00
850	HLA-A0201	PRTYSCTVE	PRTYSCTVE	0	0	0	0	0	PRTYSCTVE	tr_C9ZP82	0.014	43016.09	95.00
875	HLA-A0201	RRHRHSGQLKGNGE	HSGQLGNGE	4	0	0	5	1	HSGQLKGNGE	tr_C9ZP82	0.014	43017.47	95.00
878	HLA-A0201	RHSGQLKGNGEG	RQLKGNGEG	0	0	0	1	3	RHSGQLKGNGEG	tr_C9ZP82	0.014	43023.99	95.00
133	HLA-A0201	STEMAQANDD	STEMAANDD	0	0	0	5	1	STEMAQANDD	tr_C9ZP82	0.014	43024.46	95.00
877	HLA-A0201	HRHSGQLKGNGE	HSGQLGNGE	2	0	0	5	1	HSGQLKGNGE	tr_C9ZP82	0.014	43039.81	95.00
164	HLA-A0201	GEEEAGDALNDGDG	GEEEAGDAL	0	0	0	0	0	GEEEAGDAL	tr_C9ZP82	0.014	43047.28	95.00
193	HLA-A0201	THENRYVESN	THENYVESN	0	0	0	4	1	THENRYVESN	tr_C9ZP82	0.014	43051.47	95.00

468	HLA-A0201	LPSSTECGSPKG	SSTECGSPG	2	0	0	8	1	SSTECGSPKG	tr_C9ZP82	0.014	43060.33	95.00
338	HLA-A0201	QQQRERRSRRKRNF	QQRERRRNF	1	0	0	6	4	QQRERRSRRKRNF	tr_C9ZP82	0.014	43077.11	95.00
163	HLA-A0201	EGEEEAGDALNDGD	AGDALNDGD	5	0	0	0	0	AGDALNDGD	tr_C9ZP82	0.014	43086.41	95.00
595	HLA-A0201	NSNSGAEFRKGSN	NSNSGAEFN	0	0	0	8	4	NSNSGAEFRKGSN	tr_C9ZP82	0.014	43098.53	95.00
268	HLA-A0201	GSNTPGEGEH	GSNTPGEGH	0	0	0	8	1	GSNTPGEGEH	tr_C9ZP82	0.014	43119.99	95.00
889	HLA-A0201	GVIEGKKSNSR	GVIEGSNSR	0	0	0	5	2	GVIEGKKSNSR	tr_C9ZP82	0.014	43121.86	95.00
471	HLA-A0201	STECGSPKGRRS	STECGSPRS	0	0	0	7	3	STECGSPKGRRS	tr_C9ZP82	0.014	43125.14	95.00
877	HLA-A0201	HRHSGQLKGNGEG	RQLKGNGEG	1	0	0	1	3	RHSGQLKGNGEG	tr_C9ZP82	0.014	43130.73	95.00
326	HLA-A0201	TRNPYKRRAVA	RPYKRRAVA	1	0	0	1	1	RNPYKRRAVA	tr_C9ZP82	0.014	43131.67	95.00
595	HLA-A0201	NSNSGAEFRKGSNN	NSNSGAEFN	0	0	0	8	5	NSNSGAEFRKGSNN	tr_C9ZP82	0.014	43147.53	95.00
164	HLA-A0201	GEEEAGDALNDGD	GEEEAGDAL	0	0	0	0	0	GEEEAGDAL	tr_C9ZP82	0.014	43148.00	95.00
888	HLA-A0201	EGVIEGKKSNSR	GVIEGSNSR	1	0	0	5	2	GVIEGKKSNSR	tr_C9ZP82	0.014	43156.41	95.00
872	HLA-A0201	KRQRRHRHSGQLK	RQHRHSGQL	1	0	0	2	2	RQRRHRHSGQL	tr_C9ZP82	0.014	43161.53	95.00
500	HLA-A0201	PCSSPANSK	PCSSPANSK	0	0	0	0	0	PCSSPANSK	tr_C9ZP82	0.014	43181.16	95.00
269	HLA-A0201	SNTPGEGEHK	STPGEGEHK	0	0	0	1	1	SNTPGEGEHK	tr_C9ZP82	0.014	43190.49	95.00
846	HLA-A0201	ERYRPRTYSCT	RYRPRTYST	1	0	0	8	1	RYRPRTYSCT	tr_C9ZP82	0.014	43193.30	95.00
879	HLA-A0201	HSGQLKGNGEG	HSGQLGNGE	0	0	0	5	1	HSGQLKGNGE	tr_C9ZP82	0.014	43198.45	95.00
366	HLA-A0201	DEEESDGVIG	DEEESGVIG	0	0	0	5	1	DEEESDGVIG	tr_C9ZP82	0.014	43204.05	95.00
343	HLA-A0201	RRSRRKRNFIDD	RSRRNFIDD	1	0	0	3	2	RSRRKRNFIDD	tr_C9ZP82	0.013	43208.26	95.00
82	HLA-A0201	DGVAPRAKMNQ	GVAPRAMNQ	1	0	0	6	1	GVAPRAKMNQ	tr_C9ZP82	0.013	43217.15	95.00
887	HLA-A0201	GEGVIEGKKS	GEGVIGKKS	0	0	0	5	1	GEGVIEGKKS	tr_C9ZP82	0.013	43224.15	95.00
186	HLA-A0201	TDDDYESTHENR	DDYETHENR	2	0	0	4	1	DDYESTHENR	tr_C9ZP82	0.013	43233.52	95.00
375	HLA-A0201	GVPVCESSENSSS	GVPVCNSSS	0	0	0	5	4	GVPVCESSENSSS	tr_C9ZP82	0.013	43245.67	95.00
341	HLA-A0201	RERRSRRKRNFIDD	RSRRNFIDD	3	0	0	3	2	RSRRKRNFIDD	tr_C9ZP82	0.013	43248.02	95.00
324	HLA-A0201	DTTRNPYKRRA	TTRNPYRRA	1	0	0	6	1	TTRNPYKRRA	tr_C9ZP82	0.013	43249.41	95.00
87	HLA-A0201	RAKMNQQRARR	RMNQQRARR	0	0	0	1	2	RAKMNQQRARR	tr_C9ZP82	0.013	43256.44	95.00
487	HLA-A0201	KKAEAERSWKN	KAAERSWKN	1	0	0	2	1	KAEAERSWKN	tr_C9ZP82	0.013	43260.65	95.00
387	HLA-A0201	SDTDNESPIE	STDNESPIE	0	0	0	1	1	SDTDNESPIE	tr_C9ZP82	0.013	43282.67	95.00
471	HLA-A0201	STECGSPKGRRSK	STECGSPSK	0	0	0	7	4	STECGSPKGRRSK	tr_C9ZP82	0.013	43283.12	95.00
868	HLA-A0201	GQVIKRQRRHRHS	GQVIRHRHS	0	0	0	4	4	GQVIKRQRRHRHS	tr_C9ZP82	0.013	43287.81	95.00
267	HLA-A0201	SGSNTPGEGEHK	STPGEGEHK	0	0	0	1	3	SGSNTPGEGEHK	tr_C9ZP82	0.013	43295.77	95.00
873	HLA-A0201	RQRRHRHSG	RQRRHRHSG	0	0	0	0	0	RQRRHRHSG	tr_C9ZP82	0.013	43298.11	95.00
483	HLA-A0201	KRVSKKAEAER	RVSKKAEAR	1	0	0	8	1	RVSKKAEAER	tr_C9ZP82	0.013	43308.41	95.00
342	HLA-A0201	ERRSRRKRNFIDD	RSRRNFIDD	2	0	0	3	2	RSRRKRNFIDD	tr_C9ZP82	0.013	43314.06	95.00
473	HLA-A0201	ECGSPKGRRSKRVS	CGSPKGRRV	1	0	0	7	3	CGSPKGRRSKRV	tr_C9ZP82	0.013	43316.85	95.00
167	HLA-A0201	EAGDALNDGD	AGDALNDGD	1	0	0	0	0	AGDALNDGD	tr_C9ZP82	0.013	43317.32	95.00
892	HLA-A0201	EGKKSNSRV	EGKKSNSRV	0	0	0	0	0	EGKKSNSRV	tr_C9ZP82	0.013	43319.67	95.00
332	HLA-A0201	RRAVARQQQ	RRAVARQQQ	0	0	0	0	0	RRAVARQQQ	tr_C9ZP82	0.013	43319.67	95.00
871	HLA-A0201	IKRQRRHRH	IKRQRRHRH	0	0	0	0	0	IKRQRRHRH	tr_C9ZP82	0.013	43321.54	95.00
594	HLA-A0201	ENSNSGAEFRKGSN	NSNSGAEFN	1	0	0	8	4	NSNSGAEFRKGSN	tr_C9ZP82	0.013	43336.07	95.00
343	HLA-A0201	RRSRRKRNF	RRSRRKRNF	0	0	0	0	0	RRSRRKRNF	tr_C9ZP82	0.013	43364.70	95.00
340	HLA-A0201	QRERRSRRKRNFI	RSRRKRNFI	4	0	0	0	0	RSRRKRNFI	tr_C9ZP82	0.013	43376.42	95.00
183	HLA-A0201	VRSTDDDYE	VRSTDDDYE	0	0	0	0	0	VRSTDDDYE	tr_C9ZP82	0.013	43382.52	95.00
157	HLA-A0201	SGALYNEGEE	SGALYNEGE	0	0	0	8	1	SGALYNEGEE	tr_C9ZP82	0.013	43396.59	95.00
872	HLA-A0201	KRQRRHRHSGQLKG	RQHSGQLKG	1	0	0	2	4	RQRRHRHSGQLKG	tr_C9ZP82	0.013	43405.52	95.00
596	HLA-A0201	SNSGAEFRKGS	SNSGAEFGS	0	0	0	7	2	SNSGAEFRKGS	tr_C9ZP82	0.013	43411.62	95.00
324	HLA-A0201	DTTRNPYKR	DTTRNPYKR	0	0	0	0	0	DTTRNPYKR	tr_C9ZP82	0.013	43421.04	95.00
406	HLA-A0201	PNTGFEYFD	PNTGFEYFD	0	0	0	0	0	PNTGFEYFD	tr_C9ZP82	0.013	43429.49	95.00
876	HLA-A0201	RHRHSGQLKG	RHRHSGLKG	0	0	0	6	1	RHRHSGQLKG	tr_C9ZP82	0.013	43437.95	95.00
878	HLA-A0201	RHSGQLKGNG	RHSGQLKGG	0	0	0	8	1	RHSGQLKGNG	tr_C9ZP82	0.013	43438.88	95.00
889	HLA-A0201	GVIEGKKSN	GVIEGKKSN	0	0	0	0	0	GVIEGKKSN	tr_C9ZP82	0.013	43444.06	95.00
86	HLA-A0201	PRAKMNQQRA	RAKMNQQRA	1	0	0	0	0	RAKMNQQRA	tr_C9ZP82	0.013	43456.75	95.00
165	HLA-A0201	EEEAGDALNDG	EEEAALNDG	0	0	0	4	2	EEEAGDALNDG	tr_C9ZP82	0.013	43464.74	95.00
324	HLA-A0201	DTTRNPYKRR	TTRNPYKRR	1	0	0	0	0	TTRNPYKRR	tr_C9ZP82	0.013	43466.14	95.00

465	HLA-A0201	DGTLPSSTE	DGTLPSSTE	0	0	0	0	0	DGTLPSSTE	tr_C9ZP82	0.013	43468.05	95.00
248	HLA-A0201	LNEKMSGNDP	LNEMSGNDP	0	0	0	3	1	LNEKMSGNDP	tr_C9ZP82	0.013	43479.32	95.00
186	HLA-A0201	TDDDYESTHE	TDDDYSTHE	0	0	0	5	1	TDDDYESTHE	tr_C9ZP82	0.013	43495.33	95.00
337	HLA-A0201	RQQQRERRSR	RQQQRERSR	0	0	0	6	1	RQQQRERRSR	tr_C9ZP82	0.013	43507.57	95.00
344	HLA-A0201	RSRRKRNFID	RSRRKRNFI	0	0	0	0	0	RSRRKRNFI	tr_C9ZP82	0.013	43513.68	95.00
590	HLA-A0201	STNKENSNSGAE	STNNSNSGA	0	0	0	3	2	STNKENSNSGA	tr_C9ZP82	0.013	43516.52	95.00
388	HLA-A0201	DTDNESPIE	DTDNESPIE	0	0	0	0	0	DTDNESPIE	tr_C9ZP82	0.013	43521.20	95.00
268	HLA-A0201	GSNTPGEGEHK	GSNTPGEGH	0	0	0	8	1	GSNTPGEGEH	tr_C9ZP82	0.013	43522.16	95.00
639	HLA-A0201	SNSVQEACE	SNSVQEACE	0	0	0	0	0	SNSVQEACE	tr_C9ZP82	0.013	43524.52	95.00
471	HLA-A0201	STECGSPKGRR	STECGSGRR	0	0	0	6	2	STECGSPKGRR	tr_C9ZP82	0.013	43524.99	95.00
868	HLA-A0201	GQVIKRQRRH	GQVIRQRRH	0	0	0	4	1	GQVIKRQRRH	tr_C9ZP82	0.013	43531.59	95.00
338	HLA-A0201	QQQRERRSR	QQQRERRSR	0	0	0	0	0	QQQRERRSR	tr_C9ZP82	0.013	43534.40	95.00
379	HLA-A0201	CESSENSSS	CESSENSSS	0	0	0	0	0	CESSENSSS	tr_C9ZP82	0.013	43540.07	95.00
475	HLA-A0201	GSPKGRRSKRV	GSPKGSKRV	0	0	0	5	2	GSPKGRRSKRV	tr_C9ZP82	0.013	43546.64	95.00
471	HLA-A0201	STECGSPKGRRSKR	STECGSPSK	0	0	0	7	4	STECGSPKGRRSK	tr_C9ZP82	0.013	43551.84	95.00
659	HLA-A0201	YYTTTTTDSD	YTTTTTDSD	1	0	0	0	0	YTTTTTDSD	tr_C9ZP82	0.013	43568.80	95.00
341	HLA-A0201	RERRSRRKRNFI	RSRRKRNFI	3	0	0	0	0	RSRRKRNFI	tr_C9ZP82	0.013	43577.29	95.00
875	HLA-A0201	RRHRHSGQLK	RHRHSGQLK	1	0	0	0	0	RHRHSGQLK	tr_C9ZP82	0.013	43580.13	95.00
503	HLA-A0201	SPANSKIID	SPANSKIID	0	0	0	0	0	SPANSKIID	tr_C9ZP82	0.013	43612.66	95.00
877	HLA-A0201	HRHSGQLKGN	HRHSGLKGN	0	0	0	5	1	HRHSGQLKGN	tr_C9ZP82	0.013	43619.75	95.00
165	HLA-A0201	EEEAGDALN	EEEAGDALN	0	0	0	0	0	EEEAGDALN	tr_C9ZP82	0.013	43620.68	95.00
336	HLA-A0201	ARQQQRERR	ARQQQRERR	0	0	0	0	0	ARQQQRERR	tr_C9ZP82	0.013	43635.33	95.00
481	HLA-A0201	RSKRVSKKAE	RSKRVSKKA	0	0	0	0	0	RSKRVSKKA	tr_C9ZP82	0.013	43638.62	95.00
478	HLA-A0201	KGRRSKRVSKKA	RSKRVSKKA	3	0	0	0	0	RSKRVSKKA	tr_C9ZP82	0.013	43640.04	95.00
787	HLA-A0201	EEERWRTQK	EEERWRTQK	0	0	0	0	0	EEERWRTQK	tr_C9ZP82	0.013	43653.26	95.00
476	HLA-A0201	SPKGRRSKRVSKKA	RSKRVSKKA	5	0	0	0	0	RSKRVSKKA	tr_C9ZP82	0.013	43656.11	95.00
460	HLA-A0201	PNHIEDGTLP	NHIEDGTLP	1	0	0	0	0	NHIEDGTLP	tr_C9ZP82	0.013	43659.40	95.00
658	HLA-A0201	RYYTTTTTDSD	RYYTTTTTS	0	0	0	8	1	RYYTTTTTDS	tr_C9ZP82	0.013	43660.81	95.00
377	HLA-A0201	PVCESSENSS	PVCESSESS	0	0	0	7	1	PVCESSENSS	tr_C9ZP82	0.012	43678.30	95.00
14	HLA-A0201	PAIVTKNCP	PAIVTKNCP	0	0	0	0	0	PAIVTKNCP	tr_C9ZP82	0.012	43689.65	95.00
811	HLA-A0201	PKGDLRKLSD	KGDLRKLSD	1	0	0	0	0	KGDLRKLSD	tr_C9ZP82	0.012	43694.39	95.00
86	HLA-A0201	PRAKMNQQRARR	KMNQQRARR	3	0	0	0	0	KMNQQRARR	tr_C9ZP82	0.012	43697.68	95.00
474	HLA-A0201	CGSPKGRRSK	CGSPKGRSK	0	0	0	6	1	CGSPKGRRSK	tr_C9ZP82	0.012	43701.46	95.00
165	HLA-A0201	EEEAGDALNDGDG	AGDALNDGG	3	0	0	8	1	AGDALNDGDG	tr_C9ZP82	0.012	43718.04	95.00
436	HLA-A0201	TPATESAFD	TPATESAFD	0	0	0	0	0	TPATESAFD	tr_C9ZP82	0.012	43738.84	95.00
273	HLA-A0201	GEGEHKIID	GEGEHKIID	0	0	0	0	0	GEGEHKIID	tr_C9ZP82	0.012	43738.84	95.00
339	HLA-A0201	QQRERRSRRK	QQRERSRRK	0	0	0	4	1	QQRERRSRRK	tr_C9ZP82	0.012	43757.30	99.00
342	HLA-A0201	ERRSRRKRNFI	RSRRKRNFI	2	0	0	0	0	RSRRKRNFI	tr_C9ZP82	0.012	43769.12	99.00
166	HLA-A0201	EEAGDALNDGDG	AGDALNDGG	2	0	0	8	1	AGDALNDGDG	tr_C9ZP82	0.012	43781.46	99.00
132	HLA-A0201	TSTEMAQAND	STEMAQAND	1	0	0	0	0	STEMAQAND	tr_C9ZP82	0.012	43786.18	99.00
323	HLA-A0201	RDTTRNPYKR	RTTRNPYKR	0	0	0	1	1	RDTTRNPYKR	tr_C9ZP82	0.012	43803.71	99.00
479	HLA-A0201	GRRSKRVSKKA	RSKRVSKKA	2	0	0	0	0	RSKRVSKKA	tr_C9ZP82	0.012	43805.61	99.00
464	HLA-A0201	EDGTLPSSTE	EDGTLPSST	0	0	0	0	0	EDGTLPSST	tr_C9ZP82	0.012	43805.61	99.00
165	HLA-A0201	EEEAGDALND	EEEAGALND	0	0	0	5	1	EEEAGDALND	tr_C9ZP82	0.012	43807.98	99.00
870	HLA-A0201	VIKRQRRHR	VIKRQRRHR	0	0	0	0	0	VIKRQRRHR	tr_C9ZP82	0.012	43823.64	99.00
272	HLA-A0201	PGEGEHKII	PGEGEHKII	0	0	0	0	0	PGEGEHKII	tr_C9ZP82	0.012	43827.42	99.00
187	HLA-A0201	DDDYESTHENR	DDYESTENR	1	0	0	6	1	DDYESTHENR	tr_C9ZP82	0.012	43830.73	99.00
469	HLA-A0201	PSSTECGSPKG	SSTECGSPG	1	0	0	8	1	SSTECGSPKG	tr_C9ZP82	0.012	43832.65	99.00
323	HLA-A0201	RDTTRNPYK	RDTTRNPYK	0	0	0	0	0	RDTTRNPYK	tr_C9ZP82	0.012	43844.02	99.00
37	HLA-A0201	HPCCHNDSDT	HCCHNDSDT	0	0	0	1	1	HPCCHNDSDT	tr_C9ZP82	0.012	43844.98	99.00
477	HLA-A0201	PKGRRSKRVSKKA	RSKRVSKKA	4	0	0	0	0	RSKRVSKKA	tr_C9ZP82	0.012	43846.39	99.00
629	HLA-A0201	RDTYLKSTG	RDTYLKSTG	0	0	0	0	0	RDTYLKSTG	tr_C9ZP82	0.012	43851.14	99.00
828	HLA-A0201	RVPSREGVE	RVPSREGVE	0	0	0	0	0	RVPSREGVE	tr_C9ZP82	0.012	43867.27	99.00
876	HLA-A0201	RHRHSGQLKGN	RHSGQLKGN	2	0	0	0	0	RHSGQLKGN	tr_C9ZP82	0.012	43868.71	99.00

165	HLA-A0201	EEEAGDALNDGD	AGDALNDGD	3	0	0	0	0	AGDALNDGD	tr_C9ZP82	0.012	43881.98	99.00
479	HLA-A0201	GRRSKRVSKK	GRRSKRVSK	0	0	0	8	1	GRRSKRVSKK	tr_C9ZP82	0.012	43884.84	99.00
323	HLA-A0201	RDTTRNPYKRR	RTRNPYKRR	0	0	0	1	2	RDTTRNPYKRR	tr_C9ZP82	0.012	43885.32	99.00
874	HLA-A0201	QRRHRHSGQL	RRHRHSGQL	1	0	0	0	0	RRHRHSGQL	tr_C9ZP82	0.012	43897.18	99.00
588	HLA-A0201	FKSTNKENSNSGAE	STNNSNSGA	2	0	0	3	2	STNKENSNSGA	tr_C9ZP82	0.012	43920.94	99.00
167	HLA-A0201	EAGDALNDGDG	AGDALNDGG	1	0	0	8	1	AGDALNDGDG	tr_C9ZP82	0.012	43946.12	99.00
868	HLA-A0201	GQVIKRQRRHR	GQVIKRQHR	0	0	0	7	2	GQVIKRQRRHR	tr_C9ZP82	0.012	43954.23	99.00
158	HLA-A0201	GALYNEGEE	GALYNEGEE	0	0	0	0	0	GALYNEGEE	tr_C9ZP82	0.012	43955.17	99.00
876	HLA-A0201	RHRHSGQLKGNG	RSGQLKGNG	0	0	0	1	3	RHRHSGQLKGNG	tr_C9ZP82	0.012	43975.64	99.00
375	HLA-A0201	GVPVCESSENSSSD	GVPVCSENS	0	0	0	5	2	GVPVCESSENS	tr_C9ZP82	0.012	43980.86	99.00
589	HLA-A0201	KSTNKENSNSGAE	STNNSNSGA	1	0	0	3	2	STNKENSNSGA	tr_C9ZP82	0.012	43992.77	99.00
187	HLA-A0201	DDDYESTHE	DDDYESTHE	0	0	0	0	0	DDDYESTHE	tr_C9ZP82	0.012	43998.47	99.00
891	HLA-A0201	IEGKKSNSRVT	IEGKKSNSV	0	0	0	8	1	IEGKKSNSRV	tr_C9ZP82	0.012	44005.14	99.00
887	HLA-A0201	GEGVIEGKKSNS	GVIEGKSNS	2	0	0	5	1	GVIEGKKSNS	tr_C9ZP82	0.012	44007.98	99.00
875	HLA-A0201	RRHRHSGQLKGN	RHSGQLKGN	3	0	0	0	0	RHSGQLKGN	tr_C9ZP82	0.012	44017.04	99.00
376	HLA-A0201	VPVCESSEN	VPVCESSEN	0	0	0	0	0	VPVCESSEN	tr_C9ZP82	0.012	44027.51	99.00
474	HLA-A0201	CGSPKGRRSKR	CGSPKGRRR	0	0	0	8	2	CGSPKGRRSKR	tr_C9ZP82	0.012	44027.99	99.00
589	HLA-A0201	KSTNKENSNS	KTNKENSNS	0	0	0	1	1	KSTNKENSNS	tr_C9ZP82	0.012	44045.63	99.00
472	HLA-A0201	TECGSPKGRR	TECGSPKGR	0	0	0	8	1	TECGSPKGRR	tr_C9ZP82	0.012	44059.92	99.00
188	HLA-A0201	DDYESTHEN	DDYESTHEN	0	0	0	0	0	DDYESTHEN	tr_C9ZP82	0.012	44072.79	99.00
874	HLA-A0201	QRRHRHSGQLKGN	RHSGQLKGN	4	0	0	0	0	RHSGQLKGN	tr_C9ZP82	0.012	44075.18	99.00
267	HLA-A0201	SGSNTPGEGEH	GSNTPGEGH	1	0	0	8	1	GSNTPGEGEH	tr_C9ZP82	0.012	44082.80	99.00
875	HLA-A0201	RRHRHSGQLKGNG	RSGQLKGNG	0	0	0	1	4	RRHRHSGQLKGNG	tr_C9ZP82	0.012	44116.22	99.00
472	HLA-A0201	TECGSPKGRRSKR	CGSPKGRRR	2	0	0	8	2	CGSPKGRRSKR	tr_C9ZP82	0.012	44116.22	99.00
166	HLA-A0201	EEAGDALNDGD	AGDALNDGD	2	0	0	0	0	AGDALNDGD	tr_C9ZP82	0.012	44131.95	99.00
846	HLA-A0201	ERYRPRTYS	ERYRPRTYS	0	0	0	0	0	ERYRPRTYS	tr_C9ZP82	0.012	44140.09	99.00
475	HLA-A0201	GSPKGRRSKRVSKK	KGRKRVSKK	3	0	0	3	2	KGRRSKRVSKK	tr_C9ZP82	0.012	44142.00	99.00
868	HLA-A0201	GQVIKRQRRHRHSG	GQVIHRHSG	0	0	0	4	5	GQVIKRQRRHRHSG	tr_C9ZP82	0.011	44155.84	99.00
593	HLA-A0201	KENSNSGAE	KENSNSGAE	0	0	0	0	0	KENSNSGAE	tr_C9ZP82	0.011	44161.59	99.00
596	HLA-A0201	SNSGAEFRKGSN	SNSGAEFSN	0	0	0	7	3	SNSGAEFRKGSN	tr_C9ZP82	0.011	44185.95	99.00
874	HLA-A0201	QRRHRHSGQLKGNG	HRHSGLKGN	3	0	0	5	1	HRHSGQLKGN	tr_C9ZP82	0.011	44196.46	99.00
382	HLA-A0201	SENSSSDTDNES	SSSDTDNES	3	0	0	0	0	SSSDTDNES	tr_C9ZP82	0.011	44201.25	99.00
100	HLA-A0201	GRAAPAQTGE	RAAPAQTGE	1	0	0	0	0	RAAPAQTGE	tr_C9ZP82	0.011	44211.32	99.00
381	HLA-A0201	SSENSSSDTDNES	SSSDTDNES	4	0	0	0	0	SSSDTDNES	tr_C9ZP82	0.011	44217.05	99.00
869	HLA-A0201	QVIKRQRRH	QVIKRQRRH	0	0	0	0	0	QVIKRQRRH	tr_C9ZP82	0.011	44235.70	99.00
334	HLA-A0201	AVARQQQRER	AVARQQQER	0	0	0	7	1	AVARQQQRER	tr_C9ZP82	0.011	44245.75	99.00
476	HLA-A0201	SPKGRRSKRVSKK	KGRKRVSKK	2	0	0	3	2	KGRRSKRVSKK	tr_C9ZP82	0.011	44248.14	99.00
376	HLA-A0201	VPVCESSENSS	VVCESSENS	0	0	0	1	1	VPVCESSENS	tr_C9ZP82	0.011	44251.02	99.00
482	HLA-A0201	SKRVSKKAE	SKRVSKKAE	0	0	0	0	0	SKRVSKKAE	tr_C9ZP82	0.011	44254.36	99.00
868	HLA-A0201	GQVIKRQRRHRH	GQVIRRHRH	0	0	0	4	3	GQVIKRQRRHRH	tr_C9ZP82	0.011	44256.30	99.00
473	HLA-A0201	ECGSPKGRRSKR	CGSPKGRRR	1	0	0	8	2	CGSPKGRRSKR	tr_C9ZP82	0.011	44262.98	99.00
336	HLA-A0201	ARQQQRERRS	RQQQRERRS	1	0	0	0	0	RQQQRERRS	tr_C9ZP82	0.011	44266.35	99.00
600	HLA-A0201	AEFRKGSNN	AEFRKGSNN	0	0	0	0	0	AEFRKGSNN	tr_C9ZP82	0.011	44267.78	99.00
591	HLA-A0201	TNKENSNSGA	TNKENSNSA	0	0	0	8	1	TNKENSNSGA	tr_C9ZP82	0.011	44283.11	99.00
888	HLA-A0201	EGVIEGKKS	EGVIEGKKS	0	0	0	0	0	EGVIEGKKS	tr_C9ZP82	0.011	44286.48	99.00
887	HLA-A0201	GEGVIEGKKSN	GEGVIGKKS	0	0	0	5	1	GEGVIEGKKS	tr_C9ZP82	0.011	44287.43	99.00
335	HLA-A0201	VARQQQRERR	VARQQQERR	0	0	0	6	1	VARQQQRERR	tr_C9ZP82	0.011	44290.31	99.00
478	HLA-A0201	KGRRSKRVSKK	KGRKRVSKK	0	0	0	3	2	KGRRSKRVSKK	tr_C9ZP82	0.011	44306.11	99.00
186	HLA-A0201	TDDDYESTHEN	TDDDYSTHE	0	0	0	5	1	TDDDYESTHE	tr_C9ZP82	0.011	44317.14	99.00
870	HLA-A0201	VIKRQRRHRH	VIRQRRHRH	0	0	0	2	1	VIKRQRRHRH	tr_C9ZP82	0.011	44327.69	99.00
248	HLA-A0201	LNEKMSGND	LNEKMSGND	0	0	0	0	0	LNEKMSGND	tr_C9ZP82	0.011	44332.00	99.00
596	HLA-A0201	SNSGAEFRKGSNN	NSGAEFRGS	1	0	0	7	1	NSGAEFRKGS	tr_C9ZP82	0.011	44344.96	99.00
383	HLA-A0201	ENSSSDTDNES	SSSDTDNES	2	0	0	0	0	SSSDTDNES	tr_C9ZP82	0.011	44347.84	99.00
597	HLA-A0201	NSGAEFRKGSN	NSGAEFRGS	0	0	0	7	1	NSGAEFRKGS	tr_C9ZP82	0.011	44349.76	99.00

36	HLA-A0201	IHPCCHNDSDT	ICCHNDSDT	0	0	0	1	2	IHPCCHNDSDT	tr_C9ZP82	0.011	44355.05	99.00
380	HLA-A0201	ESSENSSSDTDNES	SSSDTDNES	5	0	0	0	0	SSSDTDNES	tr_C9ZP82	0.011	44356.00	99.00
877	HLA-A0201	HRHSGQLKGNG	RHSGQLGNG	1	0	0	6	1	RHSGQLKGNG	tr_C9ZP82	0.011	44357.46	99.00
363	HLA-A0201	SYIDEEESD	SYIDEEESD	0	0	0	0	0	SYIDEEESD	tr_C9ZP82	0.011	44361.29	99.00
643	HLA-A0201	QEACEKYVE	QEACEKYVE	0	0	0	0	0	QEACEKYVE	tr_C9ZP82	0.011	44367.53	99.00
361	HLA-A0201	NPSYIDEEESD	NSYIDEEES	0	0	0	1	1	NPSYIDEEES	tr_C9ZP82	0.011	44374.25	99.00
587	HLA-A0201	LFKSTNKENS	LFKSTNENS	0	0	0	6	1	LFKSTNKENS	tr_C9ZP82	0.011	44378.57	99.00
180	HLA-A0201	SFCVRSTDDD	SFCVRSTDD	0	0	0	7	1	SFCVRSTDDD	tr_C9ZP82	0.011	44378.57	99.00
875	HLA-A0201	RRHRHSGQLKG	RHRHSGLKG	1	0	0	6	1	RHRHSGQLKG	tr_C9ZP82	0.011	44386.73	99.00
597	HLA-A0201	NSGAEFRKGSNNDN	NSGAEFRGS	0	0	0	7	1	NSGAEFRKGS	tr_C9ZP82	0.011	44390.08	99.00
169	HLA-A0201	GDALNDGDG	GDALNDGDG	0	0	0	0	0	GDALNDGDG	tr_C9ZP82	0.011	44400.64	99.00
597	HLA-A0201	NSGAEFRKGSNND	SGAEFSNND	1	0	0	5	3	SGAEFRKGSNND	tr_C9ZP82	0.011	44404.51	99.00
472	HLA-A0201	TECGSPKGRRSK	TECGSPRSK	0	0	0	6	3	TECGSPKGRRSK	tr_C9ZP82	0.011	44428.54	99.00
160	HLA-A0201	LYNEGEEEAGD	LYNEGEEEA	0	0	0	0	0	LYNEGEEEA	tr_C9ZP82	0.011	44436.71	99.00
597	HLA-A0201	NSGAEFRKGSNN	NSGAEFRGS	0	0	0	7	1	NSGAEFRKGS	tr_C9ZP82	0.011	44437.66	99.00
332	HLA-A0201	RRAVARQQQR	RRAVAQQQR	0	0	0	5	1	RRAVARQQQR	tr_C9ZP82	0.011	44443.45	99.00
596	HLA-A0201	SNSGAEFRKGSNND	SGAEFSNND	2	0	0	5	3	SGAEFRKGSNND	tr_C9ZP82	0.011	44450.64	99.00
478	HLA-A0201	KGRRSKRVSKKAE	RSKRVSKKA	3	0	0	0	0	RSKRVSKKA	tr_C9ZP82	0.011	44467.50	99.00
340	HLA-A0201	QRERRSRRKRNFID	RSRRKRNFI	4	0	0	0	0	RSRRKRNFI	tr_C9ZP82	0.011	44473.26	99.00
382	HLA-A0201	SENSSSDTD	SENSSSDTD	0	0	0	0	0	SENSSSDTD	tr_C9ZP82	0.011	44482.41	99.00
384	HLA-A0201	NSSSDTDNESP	SSSDTDESP	1	0	0	6	1	SSSDTDNESP	tr_C9ZP82	0.011	44498.28	99.00
874	HLA-A0201	QRRHRHSGQLKG	RHRHSGLKG	2	0	0	6	1	RHRHSGQLKG	tr_C9ZP82	0.011	44505.51	99.00
860	HLA-A0201	PRLSYGADG	PRLSYGADG	0	0	0	0	0	PRLSYGADG	tr_C9ZP82	0.011	44505.51	99.00
380	HLA-A0201	ESSENSSSDT	SSENSSSDT	1	0	0	0	0	SSENSSSDT	tr_C9ZP82	0.011	44507.92	99.00
333	HLA-A0201	RAVARQQQRERRSR	RAVARQQSR	0	0	0	7	5	RAVARQQQRERRSR	tr_C9ZP82	0.011	44510.79	99.00
478	HLA-A0201	KGRRSKRVSK	KGRSKRVSK	0	0	0	2	1	KGRRSKRVSK	tr_C9ZP82	0.011	44512.26	99.00
477	HLA-A0201	PKGRRSKRVSKK	KGRKRVSKK	1	0	0	3	2	KGRRSKRVSKK	tr_C9ZP82	0.011	44527.19	99.00
334	HLA-A0201	AVARQQQRE	AVARQQQRE	0	0	0	0	0	AVARQQQRE	tr_C9ZP82	0.011	44536.35	99.00
337	HLA-A0201	RQQQRERRSRRK	RQQQRSRRK	0	0	0	4	3	RQQQRERRSRRK	tr_C9ZP82	0.011	44546.95	99.00
480	HLA-A0201	RRSKRVSKKAE	RSKRVSKKA	1	0	0	0	0	RSKRVSKKA	tr_C9ZP82	0.011	44571.06	99.00
378	HLA-A0201	VCESSENSSS	VCSSENSSS	0	0	0	2	1	VCESSENSSS	tr_C9ZP82	0.011	44595.64	99.00
479	HLA-A0201	GRRSKRVSKKAE	RSKRVSKKA	2	0	0	0	0	RSKRVSKKA	tr_C9ZP82	0.011	44599.50	99.00
88	HLA-A0201	AKMNQQRAR	AKMNQQRAR	0	0	0	0	0	AKMNQQRAR	tr_C9ZP82	0.011	44599.50	99.00
591	HLA-A0201	TNKENSNSG	TNKENSNSG	0	0	0	0	0	TNKENSNSG	tr_C9ZP82	0.011	44602.40	99.00
162	HLA-A0201	NEGEEEAGD	NEGEEEAGD	0	0	0	0	0	NEGEEEAGD	tr_C9ZP82	0.011	44615.92	99.00
475	HLA-A0201	GSPKGRRSKRVSK	GSPKGSKRV	0	0	0	5	2	GSPKGRRSKRV	tr_C9ZP82	0.011	44616.87	99.00
342	HLA-A0201	ERRSRRKRNF	RRSRRKRNF	1	0	0	0	0	RRSRRKRNF	tr_C9ZP82	0.011	44618.34	99.00
333	HLA-A0201	RAVARQQQRERR	RAVARRERR	0	0	0	5	3	RAVARQQQRERR	tr_C9ZP82	0.011	44619.78	99.00
477	HLA-A0201	PKGRRSKRVSKKAE	RSKRVSKKA	4	0	0	0	0	RSKRVSKKA	tr_C9ZP82	0.011	44626.05	99.00
477	HLA-A0201	PKGRRSKRV	PKGRRSKRV	0	0	0	0	0	PKGRRSKRV	tr_C9ZP82	0.010	44632.32	99.00
472	HLA-A0201	TECGSPKGRRS	CGSPKGRRS	2	0	0	0	0	CGSPKGRRS	tr_C9ZP82	0.010	44651.18	99.00
382	HLA-A0201	SENSSSDTDNESP	SSSDTDESP	3	0	0	6	1	SSSDTDNESP	tr_C9ZP82	0.010	44660.83	99.00
335	HLA-A0201	VARQQQRERRSRRK	RQQQRSRRK	2	0	0	4	3	RQQQRERRSRRK	tr_C9ZP82	0.010	44670.51	99.00
333	HLA-A0201	RAVARQQQRERRS	RAVARQQQS	0	0	0	8	4	RAVARQQQRERRS	tr_C9ZP82	0.010	44676.30	99.00
340	HLA-A0201	QRERRSRRKRNF	QRERRSRRF	0	0	0	8	3	QRERRSRRKRNF	tr_C9ZP82	0.010	44690.80	99.00
332	HLA-A0201	RRAVARQQQRERR	RAVARRERR	1	0	0	5	3	RAVARQQQRERR	tr_C9ZP82	0.010	44693.21	99.00
381	HLA-A0201	SSENSSSDTDNESP	SSSDTDESP	4	0	0	6	1	SSSDTDNESP	tr_C9ZP82	0.010	44694.69	99.00
383	HLA-A0201	ENSSSDTDNESP	SSSDTDESP	2	0	0	6	1	SSSDTDNESP	tr_C9ZP82	0.010	44696.62	99.00
874	HLA-A0201	QRRHRHSGQLK	RHRHSGQLK	2	0	0	0	0	RHRHSGQLK	tr_C9ZP82	0.010	44698.06	99.00
338	HLA-A0201	QQQRERRSRR	QQQERRSRR	0	0	0	3	1	QQQRERRSRR	tr_C9ZP82	0.010	44699.99	99.00
748	HLA-A0201	QWNVDYHDSN	QWNVDYHDS	0	0	0	0	0	QWNVDYHDS	tr_C9ZP82	0.010	44716.91	99.00
341	HLA-A0201	RERRSRRKRNFID	RSRRKRNFI	3	0	0	0	0	RSRRKRNFI	tr_C9ZP82	0.010	44719.34	99.00
602	HLA-A0201	FRKGSNNDN	FRKGSNNDN	0	0	0	0	0	FRKGSNNDN	tr_C9ZP82	0.010	44729.03	99.00
473	HLA-A0201	ECGSPKGRRS	CGSPKGRRS	1	0	0	0	0	CGSPKGRRS	tr_C9ZP82	0.010	44744.52	99.00

789	HLA-A0201	ERWRTQKLD	ERWRTQKLD	0	0	0	0	0	ERWRTQKLD	tr_C9ZP82	0.010	44748.88	99.00
38	HLA-A0201	PCCHNDSDT	PCCHNDSDT	0	0	0	0	0	PCCHNDSDT	tr_C9ZP82	0.010	44759.53	99.00
588	HLA-A0201	FKSTNKENSNS	FTNKENSNS	0	0	0	1	2	FKSTNKENSNS	tr_C9ZP82	0.010	44760.48	99.00
893	HLA-A0201	GKKSNSRVT	GKKSNSRVT	0	0	0	0	0	GKKSNSRVT	tr_C9ZP82	0.010	44764.84	99.00
473	HLA-A0201	ECGSPKGRRSK	CGSPKGRSK	1	0	0	6	1	CGSPKGRRSK	tr_C9ZP82	0.010	44778.90	99.00
161	HLA-A0201	YNEGEEEAGD	YNEGEEEAG	0	0	0	0	0	YNEGEEEAG	tr_C9ZP82	0.010	44783.27	99.00
82	HLA-A0201	DGVAPRAKMN	GVAPRAKMN	1	0	0	0	0	GVAPRAKMN	tr_C9ZP82	0.010	44802.16	99.00
589	HLA-A0201	KSTNKENSN	KSTNKENSN	0	0	0	0	0	KSTNKENSN	tr_C9ZP82	0.010	44815.25	99.00
337	HLA-A0201	RQQQRERRSRR	RQQQRERRR	0	0	0	7	2	RQQQRERRSRR	tr_C9ZP82	0.010	44815.74	99.00
699	HLA-A0201	PVVKSTPPD	PVVKSTPPD	0	0	0	0	0	PVVKSTPPD	tr_C9ZP82	0.010	44825.42	99.00
476	HLA-A0201	SPKGRRSKRVSK	SPRSKRVSK	0	0	0	2	3	SPKGRRSKRVSK	tr_C9ZP82	0.010	44829.79	99.00
334	HLA-A0201	AVARQQQRERRSR	AVARQQQSR	0	0	0	7	4	AVARQQQRERRSR	tr_C9ZP82	0.010	44832.71	99.00
332	HLA-A0201	RRAVARQQQRERRS	RAVARQQQS	1	0	0	8	4	RAVARQQQRERRS	tr_C9ZP82	0.010	44838.05	99.00
342	HLA-A0201	ERRSRRKRNFID	RSRRKRNFI	2	0	0	0	0	RSRRKRNFI	tr_C9ZP82	0.010	44849.22	99.00
336	HLA-A0201	ARQQQRERRSRRK	RQQQRSRRK	1	0	0	4	3	RQQQRERRSRRK	tr_C9ZP82	0.010	44853.09	99.00
749	HLA-A0201	WNVDYHDSN	WNVDYHDSN	0	0	0	0	0	WNVDYHDSN	tr_C9ZP82	0.010	44854.54	99.00
335	HLA-A0201	VARQQQRERRSR	RQQQRERSR	2	0	0	6	1	RQQQRERRSR	tr_C9ZP82	0.010	44859.40	99.00
340	HLA-A0201	QRERRSRRK	QRERRSRRK	0	0	0	0	0	QRERRSRRK	tr_C9ZP82	0.010	44864.26	99.00
134	HLA-A0201	TEMAQANDD	TEMAQANDD	0	0	0	0	0	TEMAQANDD	tr_C9ZP82	0.010	44868.14	99.00
269	HLA-A0201	SNTPGEGEH	SNTPGEGEH	0	0	0	0	0	SNTPGEGEH	tr_C9ZP82	0.010	44878.32	99.00
379	HLA-A0201	CESSENSSSDTDNE	CSSSDTDNE	0	0	0	1	5	CESSENSSSDTDNE	tr_C9ZP82	0.010	44880.27	99.00
376	HLA-A0201	VPVCESSENSSS	VVCESSENS	0	0	0	1	1	VPVCESSENS	tr_C9ZP82	0.010	44883.68	99.00
341	HLA-A0201	RERRSRRKR	RERRSRRKR	0	0	0	0	0	RERRSRRKR	tr_C9ZP82	0.010	44888.05	99.00
873	HLA-A0201	RQRRHRHSGQ	RQRHRHSGQ	0	0	0	2	1	RQRRHRHSGQ	tr_C9ZP82	0.010	44889.01	99.00
587	HLA-A0201	LFKSTNKEN	LFKSTNKEN	0	0	0	0	0	LFKSTNKEN	tr_C9ZP82	0.010	44889.99	99.00
37	HLA-A0201	HPCCHNDSD	HPCCHNDSD	0	0	0	0	0	HPCCHNDSD	tr_C9ZP82	0.010	44896.30	99.00
36	HLA-A0201	IHPCCHNDSD	IHPCCHNDS	0	0	0	0	0	IHPCCHNDS	tr_C9ZP82	0.010	44896.79	99.00
334	HLA-A0201	AVARQQQRERRSRR	AVAERRSRR	0	0	0	3	5	AVARQQQRERRSRR	tr_C9ZP82	0.010	44901.17	99.00
336	HLA-A0201	ARQQQRERRSR	RQQQRERSR	1	0	0	6	1	RQQQRERRSR	tr_C9ZP82	0.010	44921.56	99.00
588	HLA-A0201	FKSTNKENSN	FSTNKENSN	0	0	0	1	1	FKSTNKENSN	tr_C9ZP82	0.010	44930.80	99.00
86	HLA-A0201	PRAKMNQQRAR	RAMNQQRAR	1	0	0	2	1	RAKMNQQRAR	tr_C9ZP82	0.010	44931.29	99.00
335	HLA-A0201	VARQQQRERRSRR	RQQQRERRR	2	0	0	7	2	RQQQRERRSRR	tr_C9ZP82	0.010	44938.10	99.00
475	HLA-A0201	GSPKGRRSK	GSPKGRRSK	0	0	0	0	0	GSPKGRRSK	tr_C9ZP82	0.010	44939.55	99.00
334	HLA-A0201	AVARQQQRERRS	AVARQQQRS	0	0	0	7	3	AVARQQQRERRS	tr_C9ZP82	0.010	44972.63	99.00
376	HLA-A0201	VPVCESSENSSSDT	VVCESSENS	0	0	0	1	1	VPVCESSENS	tr_C9ZP82	0.010	44975.07	99.00
206	HLA-A0201	DDVFGHRGE	DDVFGHRGE	0	0	0	0	0	DDVFGHRGE	tr_C9ZP82	0.010	44975.07	99.00
132	HLA-A0201	TSTEMAQANDD	STEMAANDD	1	0	0	5	1	STEMAQANDD	tr_C9ZP82	0.010	44982.84	99.00
343	HLA-A0201	RRSRRKRNFID	RSRRKRNFI	1	0	0	0	0	RSRRKRNFI	tr_C9ZP82	0.010	45020.34	99.00
341	HLA-A0201	RERRSRRKRNF	RERRSRRNF	0	0	0	6	2	RERRSRRKRNF	tr_C9ZP82	0.010	45032.04	99.00
337	HLA-A0201	RQQQRERRSRRKR	RQQQRERRR	0	0	0	8	4	RQQQRERRSRRKR	tr_C9ZP82	0.010	45036.43	99.00
475	HLA-A0201	GSPKGRRSKRVS	GSPKGSKRV	0	0	0	5	2	GSPKGRRSKRV	tr_C9ZP82	0.010	45036.92	99.00
335	HLA-A0201	VARQQQRERRS	RQQQRERRS	2	0	0	0	0	RQQQRERRS	tr_C9ZP82	0.010	45038.37	99.00
338	HLA-A0201	QQQRERRSRRK	QQRERSRRK	1	0	0	4	1	QQRERRSRRK	tr_C9ZP82	0.010	45053.48	99.00
599	HLA-A0201	GAEFRKGSN	GAEFRKGSN	0	0	0	0	0	GAEFRKGSN	tr_C9ZP82	0.010	45053.97	99.00
601	HLA-A0201	EFRKGSNND	EFRKGSNND	0	0	0	0	0	EFRKGSNND	tr_C9ZP82	0.010	45060.31	99.00
336	HLA-A0201	ARQQQRERRSRR	RQQQRERRR	1	0	0	7	2	RQQQRERRSRR	tr_C9ZP82	0.010	45064.70	99.00
872	HLA-A0201	KRQRRHRHS	KRQRRHRHS	0	0	0	0	0	KRQRRHRHS	tr_C9ZP82	0.010	45065.66	99.00
874	HLA-A0201	QRRHRHSGQ	QRRHRHSGQ	0	0	0	0	0	QRRHRHSGQ	tr_C9ZP82	0.010	45068.59	99.00
476	HLA-A0201	SPKGRRSKR	SPKGRRSKR	0	0	0	0	0	SPKGRRSKR	tr_C9ZP82	0.010	45070.54	99.00
271	HLA-A0201	TPGEGEHKIID	TPGEGEHII	0	0	0	7	1	TPGEGEHKII	tr_C9ZP82	0.010	45098.83	99.00
380	HLA-A0201	ESSENSSSD	ESSENSSSD	0	0	0	0	0	ESSENSSSD	tr_C9ZP82	0.009	45116.89	99.00
376	HLA-A0201	VPVCESSENSSSD	VVCESSENS	0	0	0	1	1	VPVCESSENS	tr_C9ZP82	0.009	45122.28	99.00
598	HLA-A0201	SGAEFRKGSN	SGAEFRGSN	0	0	0	6	1	SGAEFRKGSN	tr_C9ZP82	0.009	45126.18	99.00
869	HLA-A0201	QVIKRQRRHR	QVIKRQRHR	0	0	0	6	1	QVIKRQRRHR	tr_C9ZP82	0.009	45128.62	99.00

187	HLA-A0201	DDDYESTHEN	DDYESTHEN	1	0	0	0	0	DDYESTHEN	tr_C9ZP82	0.009	45143.76	99.00
587	HLA-A0201	LFKSTNKENSNSG	STNENSNSG	3	0	0	3	1	STNKENSNSG	tr_C9ZP82	0.009	45147.66	99.00
871	HLA-A0201	IKRQRRHRHS	IKRQRRHRH	0	0	0	0	0	IKRQRRHRH	tr_C9ZP82	0.009	45148.15	99.00
383	HLA-A0201	ENSSSDTDNE	NSSSDTDNE	1	0	0	0	0	NSSSDTDNE	tr_C9ZP82	0.009	45151.56	99.00
381	HLA-A0201	SSENSSSDTD	SSENSSSDT	0	0	0	0	0	SSENSSSDT	tr_C9ZP82	0.009	45159.86	99.00
588	HLA-A0201	FKSTNKENSNSG	FKSENSNSG	0	0	0	3	3	FKSTNKENSNSG	tr_C9ZP82	0.009	45160.85	99.00
334	HLA-A0201	AVARQQQRERR	AVQQQRERR	0	0	0	2	2	AVARQQQRERR	tr_C9ZP82	0.009	45175.01	99.00
869	HLA-A0201	QVIKRQRRHRHS	QVIKRHRHS	0	0	0	4	3	QVIKRQRRHRHS	tr_C9ZP82	0.009	45195.05	99.00
587	HLA-A0201	LFKSTNKENSNS	FTNKENSNS	1	0	0	1	2	FKSTNKENSNS	tr_C9ZP82	0.009	45203.86	99.00
362	HLA-A0201	PSYIDEEESD	PSYIDEEES	0	0	0	0	0	PSYIDEEES	tr_C9ZP82	0.009	45218.06	99.00
332	HLA-A0201	RRAVARQQQRER	RAVARQQER	1	0	0	7	2	RAVARQQQRER	tr_C9ZP82	0.009	45225.88	99.00
377	HLA-A0201	PVCESSENSSSDT	PVCESSENT	0	0	0	8	4	PVCESSENSSSDT	tr_C9ZP82	0.009	45228.32	99.00
888	HLA-A0201	EGVIEGKKSNS	GVIEGKSNS	1	0	0	5	1	GVIEGKKSNS	tr_C9ZP82	0.009	45238.62	99.00
333	HLA-A0201	RAVARQQQRER	RAVARQQER	0	0	0	7	2	RAVARQQQRER	tr_C9ZP82	0.009	45266.02	99.00
337	HLA-A0201	RQQQRERRSRRKRN	RQQQRERRN	0	0	0	7	5	RQQQRERRSRRKRN	tr_C9ZP82	0.009	45280.24	99.00
336	HLA-A0201	ARQQQRERRSRRKR	RQQQRERRR	1	0	0	8	4	RQQQRERRSRRKR	tr_C9ZP82	0.009	45283.66	99.00
869	HLA-A0201	QVIKRQRRHRHSG	QVIKRQHSG	0	0	0	6	4	QVIKRQRRHRHSG	tr_C9ZP82	0.009	45287.57	99.00
869	HLA-A0201	QVIKRQRRHRH	VIRQRRHRH	1	0	0	2	1	VIKRQRRHRH	tr_C9ZP82	0.009	45304.25	99.00
870	HLA-A0201	VIKRQRRHRHS	VIQRRHRHS	0	0	0	2	2	VIKRQRRHRHS	tr_C9ZP82	0.009	45306.71	99.00
339	HLA-A0201	QQRERRSRRKR	QQRERSRRK	0	0	0	4	1	QQRERRSRRK	tr_C9ZP82	0.009	45314.54	99.00
473	HLA-A0201	ECGSPKGRR	ECGSPKGRR	0	0	0	0	0	ECGSPKGRR	tr_C9ZP82	0.009	45335.15	99.00
86	HLA-A0201	PRAKMNQQR	PRAKMNQQR	0	0	0	0	0	PRAKMNQQR	tr_C9ZP82	0.009	45361.64	99.00
379	HLA-A0201	CESSENSSSD	CSSENSSSD	0	0	0	1	1	CESSENSSSD	tr_C9ZP82	0.009	45367.52	99.00
589	HLA-A0201	KSTNKENSNSG	STNENSNSG	1	0	0	3	1	STNKENSNSG	tr_C9ZP82	0.009	45393.54	99.00
378	HLA-A0201	VCESSENSSSDT	SSENSSSDT	3	0	0	0	0	SSENSSSDT	tr_C9ZP82	0.009	45433.34	99.00
600	HLA-A0201	AEFRKGSNND	AEFKGSNND	0	0	0	3	1	AEFRKGSNND	tr_C9ZP82	0.009	45441.23	99.00
869	HLA-A0201	QVIKRQRRHRHSGQ	QVIHRHSGQ	0	0	0	3	5	QVIKRQRRHRHSGQ	tr_C9ZP82	0.009	45450.08	99.00
599	HLA-A0201	GAEFRKGSNN	GAFRKGSNN	0	0	0	2	1	GAEFRKGSNN	tr_C9ZP82	0.009	45477.61	99.00
592	HLA-A0201	NKENSNSGAE	NKENSNSGA	0	0	0	0	0	NKENSNSGA	tr_C9ZP82	0.009	45480.07	99.00
888	HLA-A0201	EGVIEGKKSN	GVIEGKKSN	1	0	0	0	0	GVIEGKKSN	tr_C9ZP82	0.009	45486.47	99.00
379	HLA-A0201	CESSENSSSDT	SSENSSSDT	2	0	0	0	0	SSENSSSDT	tr_C9ZP82	0.009	45493.36	99.00
598	HLA-A0201	SGAEFRKGSNND	SGAEFSNND	0	0	0	5	3	SGAEFRKGSNND	tr_C9ZP82	0.009	45513.55	99.00
381	HLA-A0201	SSENSSSDTDNE	SSENSSSDT	0	0	0	0	0	SSENSSSDT	tr_C9ZP82	0.009	45516.48	99.00
476	HLA-A0201	SPKGRRSKRVS	SPKGRSKRV	0	0	0	4	1	SPKGRRSKRV	tr_C9ZP82	0.009	45530.29	99.00
477	HLA-A0201	PKGRRSKRVSK	GRRSKRVSK	2	0	0	0	0	GRRSKRVSK	tr_C9ZP82	0.009	45533.75	99.00
811	HLA-A0201	PKGDLRKLS	PKGDLRKLS	0	0	0	0	0	PKGDLRKLS	tr_C9ZP82	0.009	45537.18	99.00
338	HLA-A0201	QQQRERRSRRKR	QQRERSRRK	1	0	0	4	1	QQRERRSRRK	tr_C9ZP82	0.009	45541.12	99.00
339	HLA-A0201	QQRERRSRRKRN	QQRERRSRN	0	0	0	7	3	QQRERRSRRKRN	tr_C9ZP82	0.009	45570.71	99.00
598	HLA-A0201	SGAEFRKGSNNDN	SGAEFNNDN	0	0	0	5	4	SGAEFRKGSNNDN	tr_C9ZP82	0.009	45573.18	99.00
870	HLA-A0201	VIKRQRRHRHSGQ	VIRHRHSGQ	0	0	0	2	4	VIKRQRRHRHSGQ	tr_C9ZP82	0.009	45579.58	99.00
380	HLA-A0201	ESSENSSSDTDNE	SSENSSSDT	1	0	0	0	0	SSENSSSDT	tr_C9ZP82	0.009	45601.28	99.00
382	HLA-A0201	SENSSSDTDNE	SSSSDTDNE	0	0	0	1	2	SENSSSDTDNE	tr_C9ZP82	0.009	45604.25	99.00
478	HLA-A0201	KGRRSKRVS	KGRRSKRVS	0	0	0	0	0	KGRRSKRVS	tr_C9ZP82	0.008	45642.73	99.00
377	HLA-A0201	PVCESSENSSS	PVCESSESS	0	0	0	7	2	PVCESSENSSS	tr_C9ZP82	0.008	45643.74	99.00
333	HLA-A0201	RAVARQQQRE	RAVARQQQE	0	0	0	8	1	RAVARQQQRE	tr_C9ZP82	0.008	45650.66	99.00
598	HLA-A0201	SGAEFRKGSNN	SGAEFGSNN	0	0	0	5	2	SGAEFRKGSNN	tr_C9ZP82	0.008	45680.78	99.00
272	HLA-A0201	PGEGEHKIID	GEGEHKIID	1	0	0	0	0	GEGEHKIID	tr_C9ZP82	0.008	45708.98	99.00
338	HLA-A0201	QQQRERRSRRKRN	QQRERSRRK	1	0	0	4	1	QQRERRSRRK	tr_C9ZP82	0.008	45762.90	99.00
377	HLA-A0201	PVCESSENSSSDTD	PVCESSETD	0	0	0	7	5	PVCESSENSSSDTD	tr_C9ZP82	0.008	45774.80	99.00
871	HLA-A0201	IKRQRRHRHSGQ	RQRHRHSGQ	2	0	0	2	1	RQRRHRHSGQ	tr_C9ZP82	0.008	45787.17	99.00
872	HLA-A0201	KRQRRHRHSG	KQRRHRHSG	0	0	0	1	1	KRQRRHRHSG	tr_C9ZP82	0.008	45795.61	99.00
870	HLA-A0201	VIKRQRRHRHSG	VIRQRRHRH	0	0	0	2	1	VIKRQRRHRH	tr_C9ZP82	0.008	45802.53	99.00
600	HLA-A0201	AEFRKGSNNDN	AEFGSNNDN	0	0	0	3	2	AEFRKGSNNDN	tr_C9ZP82	0.008	45824.83	99.00
599	HLA-A0201	GAEFRKGSNNDN	AEFGSNNDN	1	0	0	3	2	AEFRKGSNNDN	tr_C9ZP82	0.008	45882.41	99.00

378	HLA-A0201	VCESSENSSSDTD	SSENSSSTD	3	0	0	7	1	SSENSSSDTD	tr_C9ZP82	0.008	45908.22	99.00	
379	HLA-A0201	CESSENSSSDTD	SSENSSSTD	2	0	0	7	1	SSENSSSDTD	tr_C9ZP82	0.008	45917.16	99.00	
871	HLA-A0201	IKRQRRHRHSG	IQRRHRHSG	0	0	0	1	2	IKRQRRHRHSG	tr_C9ZP82	0.008	45964.89	99.00	
383	HLA-A0201	ENSSSDTDN	ENSSSDTDN	0	0	0	0	0	ENSSSDTDN	tr_C9ZP82	0.008	45991.75	99.00	
599	HLA-A0201	GAEFRKGSNND	GAEFGSNND	0	0	0	4	2	GAEFRKGSNND	tr_C9ZP82	0.008	46000.71	99.00	
332	HLA-A0201	RRAVARQQQRE	RAVARQQQE	1	0	0	8	1	RAVARQQQRE	tr_C9ZP82	0.008	46013.65	99.00	
872	HLA-A0201	KRQRRHRHSGQ	RQRHRHSGQ	1	0	0	2	1	RQRRHRHSGQ	tr_C9ZP82	0.008	46029.08	99.00	
587	HLA-A0201	LFKSTNKENSN	LFKSTNENS	0	0	0	6	1	LFKSTNKENS	tr_C9ZP82	0.008	46031.58	99.00	
601	HLA-A0201	EFRKGSNNDN	FRKGSNNDN	1	0	0	0	0	FRKGSNNDN	tr_C9ZP82	0.008	46066.44	99.00	
340	HLA-A0201	QRERRSRRKR	QRERRSRRR	0	0	0	8	1	QRERRSRRKR	tr_C9ZP82	0.007	46111.32	99.00	
892	HLA-A0201	EGKKSNSRVT	EGKKSNSRV	0	0	0	0	0	EGKKSNSRV	tr_C9ZP82	0.007	46118.31	99.00	
475	HLA-A0201	GSPKGRRSKR	GSPGRRSKR	0	0	0	3	1	GSPKGRRSKR	tr_C9ZP82	0.007	46126.29	99.00	
378	HLA-A0201	VCESSENSSSDTDN	SSENSDTDN	3	0	0	4	2	SSENSSSDTDN	tr_C9ZP82	0.007	46135.77	99.00	
379	HLA-A0201	CESSENSSSDTDN	CSENSSSDT	0	0	0	1	2	CESSENSSSDT	tr_C9ZP82	0.007	46146.25	99.00	
591	HLA-A0201	TNKENSNSGAE	TNKENSNGA	0	0	0	7	1	TNKENSNSGA	tr_C9ZP82	0.007	46179.23	99.00	
382	HLA-A0201	SENSSSDTDN	SENSSSDTN	0	0	0	8	1	SENSSSDTDN	tr_C9ZP82	0.007	46235.73	99.00	
378	HLA-A0201	VCESSENSSSD	VSSENSSSD	0	0	0	1	2	VCESSENSSSD	tr_C9ZP82	0.007	46290.27	99.00	
377	HLA-A0201	PVCESSENSSSD	PVCESSESD	0	0	0	7	3	PVCESSENSSSD	tr_C9ZP82	0.007	46334.89	99.00	
381	HLA-A0201	SSENSSSDTDN	SSENSSSDT	0	0	0	0	0	SSENSSSDT	tr_C9ZP82	0.007	46416.16	99.00	
380	HLA-A0201	ESSENSSSDTD	SSENSSSDT	1	0	0	0	0	SSENSSSDT	tr_C9ZP82	0.007	46470.94	99.00	
380	HLA-A0201	ESSENSSSDTDN	SSENSSSDT	1	0	0	0	0	SSENSSSDT	tr_C9ZP82	0.007	46575.14	99.00	
341	HLA-A0201	RERRSRRKRN	RERRSRRKR	0	0	0	0	0	RERRSRRKR	tr_C9ZP82	0.006	46615.98	99.00	
477	HLA-A0201	PKGRRSKRVS	PKGRRSKRV	0	0	0	0	0	PKGRRSKRV	tr_C9ZP82	0.006	46645.24	99.00	
340	HLA-A0201	QRERRSRRKRN	QRERRSRRR	0	0	0	8	1	QRERRSRRKR	tr_C9ZP82	0.006	46771.09	99.00	
342	HLA-A0201	ERRSRRKRN	ERRSRRKRN	0	0	0	0	0	ERRSRRKRN	tr_C9ZP82	0.006	46808.02	99.00	

Protein tr_C9ZP82. Allele HLA-A0201. Number of high binders 18. Number of weak binders 83. Number of peptides 5391

Link to Allele Frequencies in Worldwide Populations HLA-A0201

Rank Threshold for Strong binding peptides 0.500
Rank Threshold for Weak binding peptides 2.000

pos	HLA	peptide	Core	Offset	I_pos	I_len	D_pos	D_len	iCore	Identity	1-log50k(aff)	Affinity(nM)	%Rank	BindLevel
60	HLA-A0201	SLYDPDHSPHIEAV	SLYDHIEAV	0	0	0	4	5	SLYDPDHSPHIEAV	tr_C9ZP82	0.571	103.67	1.10	<= WB
19	HLA-A0201	WLCTECLSL	WLCTECLSL	0	0	0	0	0	WLCTECLSL	tr_C9ZP82	0.538	148.98	1.40	<= WB
16	HLA-A0201	FVDWLCTECL	FVWLCTECL	0	0	0	2	1	FVDWLCTECL	tr_C9ZP82	0.472	302.16	2.50	
49	HLA-A0201	AVFSCTQSA	AVFSCTQSA	0	0	0	0	0	AVFSCTQSA	tr_C9ZP82	0.450	385.40	2.50	
16	HLA-A0201	FVDWLCTECLSL	FLCTECLSL	0	0	0	1	3	FVDWLCTECLSL	tr_C9ZP82	0.409	596.09	3.00	
18	HLA-A0201	DWLCTECLSL	WLCTECLSL	1	0	0	0	0	WLCTECLSL	tr_C9ZP82	0.403	641.33	3.50	
60	HLA-A0201	SLYDPDHSPHI	SLYDHSPHI	0	0	0	3	2	SLYDPDHSPHI	tr_C9ZP82	0.401	653.73	3.50	
15	HLA-A0201	TFVDWLCTECLSL	FLCTECLSL	1	0	0	1	3	FVDWLCTECLSL	tr_C9ZP82	0.377	841.66	4.00	
14	HLA-A0201	QTFVDWLCTECLSL	FLCTECLSL	2	0	0	1	3	FVDWLCTECLSL	tr_C9ZP82	0.365	968.16	4.00	
59	HLA-A0201	QSLYDPDHSPHI	SLYDHSPHI	1	0	0	3	2	SLYDPDHSPHI	tr_C9ZP82	0.352	1103.35	4.50	
58	HLA-A0201	DQSLYDPDHSPHI	SLYDHSPHI	2	0	0	3	2	SLYDPDHSPHI	tr_C9ZP82	0.340	1256.63	4.50	
57	HLA-A0201	ADQSLYDPDHSPHI	SLYDHSPHI	3	0	0	3	2	SLYDPDHSPHI	tr_C9ZP82	0.334	1351.92	5.00	
19	HLA-A0201	WLCTECLSLN	WLCTECLSL	0	0	0	0	0	WLCTECLSL	tr_C9ZP82	0.310	1745.61	5.50	
16	HLA-A0201	FVDWLCTEC	FVDWLCTEC	0	0	0	0	0	FVDWLCTEC	tr_C9ZP82	0.310	1755.28	5.50	
60	HLA-A0201	SLYDPDHSPHIEA	SLYDPDIEA	0	0	0	6	4	SLYDPDHSPHIEA	tr_C9ZP82	0.300	1948.57	6.00	
15	HLA-A0201	TFVDWLCTECL	FVWLCTECL	1	0	0	2	1	FVDWLCTECL	tr_C9ZP82	0.300	1955.52	6.00	
16	HLA-A0201	FVDWLCTECLSLN	FLCTECLSL	0	0	0	1	3	FVDWLCTECLSL	tr_C9ZP82	0.296	2022.09	6.00	
14	HLA-A0201	QTFVDWLCTECL	FVWLCTECL	2	0	0	2	1	FVDWLCTECL	tr_C9ZP82	0.289	2195.49	6.00	

13	HLA-A0201	RQTFVDWLCTECL	FVWLCTECL	3	0	0	2	1	FVDWLCTECL	tr_C9ZP82	0.285	2290.41	6.50
12	HLA-A0201	IRQTFVDWLCTECL	FVWLCTECL	4	0	0	2	1	FVDWLCTECL	tr_C9ZP82	0.283	2348.61	6.50
15	HLA-A0201	TFVDWLCTECLSLN	FLCTECLSL	1	0	0	1	3	FVDWLCTECLSL	tr_C9ZP82	0.270	2683.71	7.00
26	HLA-A0201	SLNFSSRTRCFV	SLNFSSCFV	0	0	0	6	3	SLNFSSRTRCFV	tr_C9ZP82	0.269	2735.78	7.00
59	HLA-A0201	QSLYDPDHSPHIEA	SLYDPDIEA	1	0	0	6	4	SLYDPDHSPHIEA	tr_C9ZP82	0.263	2894.42	7.00
16	HLA-A0201	FVDWLCTECLSLNF	FLCTECLSL	0	0	0	1	3	FVDWLCTECLSL	tr_C9ZP82	0.262	2936.98	7.00
47	HLA-A0201	CLAVFSCTQSA	CLFSCTQSA	0	0	0	2	2	CLAVFSCTQSA	tr_C9ZP82	0.261	2983.65	7.50
24	HLA-A0201	CLSLNFSSRTRCFV	CLSLNFCFV	0	0	0	6	5	CLSLNFSSRTRCFV	tr_C9ZP82	0.255	3176.85	7.50
48	HLA-A0201	LAVFSCTQSA	LVFSCTQSA	0	0	0	1	1	LAVFSCTQSA	tr_C9ZP82	0.251	3321.99	7.50
6	HLA-A0201	GMSRNNIRQTFV	GMSRNNIFV	0	0	0	7	3	GMSRNNIRQTFV	tr_C9ZP82	0.240	3713.83	8.00
60	HLA-A0201	SLYDPDHSPHIE	SLYDHSPHI	0	0	0	3	2	SLYDPDHSPHI	tr_C9ZP82	0.238	3801.44	8.50
17	HLA-A0201	VDWLCTECLSL	WLCTECLSL	2	0	0	0	0	WLCTECLSL	tr_C9ZP82	0.235	3932.76	8.50
16	HLA-A0201	FVDWLCTECLS	FVWLCTECL	0	0	0	2	1	FVDWLCTECL	tr_C9ZP82	0.232	4044.05	8.50
25	HLA-A0201	LSLNFSSRTRCFV	SLNFSSCFV	1	0	0	6	3	SLNFSSRTRCFV	tr_C9ZP82	0.232	4046.72	8.50
46	HLA-A0201	RCLAVFSCTQSA	CLFSCTQSA	1	0	0	2	2	CLAVFSCTQSA	tr_C9ZP82	0.232	4069.86	8.50
29	HLA-A0201	FSSRTRCFV	FSSRTRCFV	0	0	0	0	0	FSSRTRCFV	tr_C9ZP82	0.223	4489.37	9.00
45	HLA-A0201	RRCLAVFSCTQSA	CLFSCTQSA	2	0	0	2	2	CLAVFSCTQSA	tr_C9ZP82	0.222	4544.05	9.00
44	HLA-A0201	NRRCLAVFSCTQSA	CLFSCTQSA	3	0	0	2	2	CLAVFSCTQSA	tr_C9ZP82	0.218	4727.86	9.50
15	HLA-A0201	TFVDWLCTECLS	FVWLCTECL	1	0	0	2	1	FVDWLCTECL	tr_C9ZP82	0.213	4998.79	9.50
5	HLA-A0201	CGMSRNNIRQTFV	GMSRNNIFV	1	0	0	7	3	GMSRNNIRQTFV	tr_C9ZP82	0.210	5161.98	10.00
14	HLA-A0201	QTFVDWLCTECLS	FVWLCTECL	2	0	0	2	1	FVDWLCTECL	tr_C9ZP82	0.206	5389.59	10.00
59	HLA-A0201	QSLYDPDHSPHIE	SLYDHSPHI	1	0	0	3	2	SLYDPDHSPHI	tr_C9ZP82	0.206	5390.52	10.00
15	HLA-A0201	TFVDWLCTEC	FVDWLCTEC	1	0	0	0	0	FVDWLCTEC	tr_C9ZP82	0.205	5418.76	10.00
13	HLA-A0201	RQTFVDWLCTECLS	FVWLCTECL	3	0	0	2	1	FVDWLCTECL	tr_C9ZP82	0.204	5509.21	10.00
58	HLA-A0201	DQSLYDPDHSPHIE	SLYDHSPHI	2	0	0	3	2	SLYDPDHSPHI	tr_C9ZP82	0.198	5851.76	11.00
4	HLA-A0201	GCGMSRNNIRQTFV	GMSRNNIFV	2	0	0	7	3	GMSRNNIRQTFV	tr_C9ZP82	0.195	6031.42	11.00
60	HLA-A0201	SLYDPDHSP	SLYDPDHSP	0	0	0	0	0	SLYDPDHSP	tr_C9ZP82	0.190	6414.62	11.00
24	HLA-A0201	CLSLNFSSRT	CLLNFSSRT	0	0	0	2	1	CLSLNFSSRT	tr_C9ZP82	0.180	7104.90	12.00
19	HLA-A0201	WLCTECLSLNFSS	WLLSLNFSS	0	0	0	2	4	WLCTECLSLNFSS	tr_C9ZP82	0.178	7284.32	12.00
26	HLA-A0201	SLNFSSRTRCFVC	SLNFSSCFV	0	0	0	6	3	SLNFSSRTRCFV	tr_C9ZP82	0.170	7923.02	13.00
13	HLA-A0201	RQTFVDWLC	RQTFVDWLC	0	0	0	0	0	RQTFVDWLC	tr_C9ZP82	0.170	7980.74	13.00
47	HLA-A0201	CLAVFSCTQS	CLAVFSCTS	0	0	0	8	1	CLAVFSCTQS	tr_C9ZP82	0.169	8057.36	13.00
47	HLA-A0201	CLAVFSCTQSAD	CLFSCTQSA	0	0	0	2	2	CLAVFSCTQSA	tr_C9ZP82	0.165	8415.64	14.00
18	HLA-A0201	DWLCTECLSLN	WLCTECLSL	1	0	0	0	0	WLCTECLSL	tr_C9ZP82	0.161	8795.75	14.00
19	HLA-A0201	WLCTECLSLNF	WLCTECLSL	0	0	0	0	0	WLCTECLSL	tr_C9ZP82	0.159	8919.67	14.00
18	HLA-A0201	DWLCTECLSLNFSS	WLLSLNFSS	1	0	0	2	4	WLCTECLSLNFSS	tr_C9ZP82	0.159	8946.93	14.00
17	HLA-A0201	VDWLCTECLSLN	WLCTECLSL	2	0	0	0	0	WLCTECLSL	tr_C9ZP82	0.157	9114.89	14.00
25	HLA-A0201	LSLNFSSRTRCFVC	SLNFSSCFV	1	0	0	6	3	SLNFSSRTRCFV	tr_C9ZP82	0.154	9425.60	15.00
17	HLA-A0201	VDWLCTECL	VDWLCTECL	0	0	0	0	0	VDWLCTECL	tr_C9ZP82	0.149	10010.35	15.00
46	HLA-A0201	RCLAVFSCTQSAD	CLFSCTQSA	1	0	0	2	2	CLAVFSCTQSA	tr_C9ZP82	0.148	10027.58	15.00
47	HLA-A0201	CLAVFSCTQ	CLAVFSCTQ	0	0	0	0	0	CLAVFSCTQ	tr_C9ZP82	0.148	10092.46	15.00
18	HLA-A0201	DWLCTECLSLNF	WLCTECLSL	1	0	0	0	0	WLCTECLSL	tr_C9ZP82	0.148	10124.18	15.00
19	HLA-A0201	WLCTECLSLNFS	WLCTECLSL	0	0	0	0	0	WLCTECLSL	tr_C9ZP82	0.147	10193.20	16.00
49	HLA-A0201	AVFSCTQSADQSL	AVFSCTQSL	0	0	0	6	4	AVFSCTQSADQSL	tr_C9ZP82	0.146	10286.38	16.00
17	HLA-A0201	VDWLCTECLSLNF	WLCTECLSL	2	0	0	0	0	WLCTECLSL	tr_C9ZP82	0.144	10549.34	16.00
49	HLA-A0201	AVFSCTQSAD	AVFSCTQSA	0	0	0	0	0	AVFSCTQSA	tr_C9ZP82	0.143	10597.04	16.00
45	HLA-A0201	RRCLAVFSCTQSAD	CLFSCTQSA	2	0	0	2	2	CLAVFSCTQSA	tr_C9ZP82	0.143	10648.53	16.00
26	HLA-A0201	SLNFSSRTRCFVCR	SLNFSSCFV	0	0	0	6	3	SLNFSSRTRCFV	tr_C9ZP82	0.141	10841.17	16.00
47	HLA-A0201	CLAVFSCTQSADQ	CLFSCTQSA	0	0	0	2	2	CLAVFSCTQSA	tr_C9ZP82	0.141	10888.07	16.00
36	HLA-A0201	FVCRAPFDNRRCL	FVCRAPFCL	0	0	0	7	4	FVCRAPFDNRRCL	tr_C9ZP82	0.140	10934.23	16.00
19	HLA-A0201	WLCTECLSLNFSSR	WLCTECLSL	0	0	0	0	0	WLCTECLSL	tr_C9ZP82	0.136	11534.81	17.00
18	HLA-A0201	DWLCTECLSLNFS	WLCTECLSL	1	0	0	0	0	WLCTECLSL	tr_C9ZP82	0.136	11537.68	17.00
47	HLA-A0201	CLAVFSCTQSADQS	CLFSCTQSA	0	0	0	2	2	CLAVFSCTQSA	tr_C9ZP82	0.135	11542.43	17.00
11	HLA-A0201	NIRQTFVDWL	NIQTFVDWL	0	0	0	2	1	NIRQTFVDWL	tr_C9ZP82	0.134	11705.42	17.00

13	HLA-A0201	RQTFVDWLCT	RQTFVDWLT	0	0	0	8	1	RQTFVDWLCT	tr_C9ZP82	0.134	11752.25	17.00
17	HLA-A0201	VDWLCTECLSLNFS	WLCTECLSL	2	0	0	0	0	WLCTECLSL	tr_C9ZP82	0.133	11908.41	17.00
12	HLA-A0201	IRQTFVDWLC	IQTFVDWLC	0	0	0	1	1	IRQTFVDWLC	tr_C9ZP82	0.133	11915.37	17.00
42	HLA-A0201	FDNRRCLAV	FDNRRCLAV	0	0	0	0	0	FDNRRCLAV	tr_C9ZP82	0.130	12228.83	18.00
6	HLA-A0201	GMSRNNIRQTFVD	GMSRNNIFV	0	0	0	7	3	GMSRNNIRQTFV	tr_C9ZP82	0.129	12344.89	18.00
14	HLA-A0201	QTFVDWLCT	QTFVDWLCT	0	0	0	0	0	QTFVDWLCT	tr_C9ZP82	0.129	12392.40	18.00
46	HLA-A0201	RCLAVFSCTQSADQ	CLFSCTQSA	1	0	0	2	2	CLAVFSCTQSA	tr_C9ZP82	0.128	12463.93	18.00
32	HLA-A0201	RTRCFVCRA	RTRCFVCRA	0	0	0	0	0	RTRCFVCRA	tr_C9ZP82	0.126	12780.62	18.00
35	HLA-A0201	CFVCRAPFDNRRCL	FVCRAPFCL	1	0	0	7	4	FVCRAPFDNRRCL	tr_C9ZP82	0.125	12883.09	18.00
69	HLA-A0201	HIEAVKLQI	HIEAVKLQI	0	0	0	0	0	HIEAVKLQI	tr_C9ZP82	0.124	13036.77	19.00
48	HLA-A0201	LAVFSCTQSADQSL	AVFSCTQSL	1	0	0	6	4	AVFSCTQSADQSL	tr_C9ZP82	0.124	13043.68	19.00
36	HLA-A0201	FVCRAPFDNRRCLA	FVCRAPFLA	0	0	0	7	5	FVCRAPFDNRRCLA	tr_C9ZP82	0.123	13173.03	19.00
34	HLA-A0201	RCFVCRAPF	RCFVCRAPF	0	0	0	0	0	RCFVCRAPF	tr_C9ZP82	0.123	13206.57	19.00
46	HLA-A0201	RCLAVFSCT	RCLAVFSCT	0	0	0	0	0	RCLAVFSCT	tr_C9ZP82	0.121	13489.19	19.00
28	HLA-A0201	NFSSRTRCFV	FSSRTRCFV	1	0	0	0	0	FSSRTRCFV	tr_C9ZP82	0.121	13533.63	19.00
29	HLA-A0201	FSSRTRCFVC	FSSRTCFVC	0	0	0	5	1	FSSRTRCFVC	tr_C9ZP82	0.120	13657.19	19.00
46	HLA-A0201	RCLAVFSCTQ	RLAVFSCTQ	0	0	0	1	1	RCLAVFSCTQ	tr_C9ZP82	0.120	13719.69	19.00
42	HLA-A0201	FDNRRCLAVFSCTQ	FLAVFSCTQ	0	0	0	1	5	FDNRRCLAVFSCTQ	tr_C9ZP82	0.119	13806.51	20.00
14	HLA-A0201	QTFVDWLCTEC	FVDWLCTEC	2	0	0	0	0	FVDWLCTEC	tr_C9ZP82	0.116	14189.67	20.00
13	HLA-A0201	RQTFVDWLCTEC	FVDWLCTEC	3	0	0	0	0	FVDWLCTEC	tr_C9ZP82	0.116	14315.19	20.00
12	HLA-A0201	IRQTFVDWLCTEC	FVDWLCTEC	4	0	0	0	0	FVDWLCTEC	tr_C9ZP82	0.114	14550.83	20.00
11	HLA-A0201	NIRQTFVDWLCTEC	FVDWLCTEC	5	0	0	0	0	FVDWLCTEC	tr_C9ZP82	0.114	14596.41	20.00
62	HLA-A0201	YDPDHSPHI	YDPDHSPHI	0	0	0	0	0	YDPDHSPHI	tr_C9ZP82	0.113	14787.00	21.00
5	HLA-A0201	CGMSRNNIRQTFVD	GMSRNNIFV	1	0	0	7	3	GMSRNNIRQTFV	tr_C9ZP82	0.113	14787.80	21.00
29	HLA-A0201	FSSRTRCFVCRAPF	FSFVCRAPF	0	0	0	2	5	FSSRTRCFVCRAPF	tr_C9ZP82	0.112	14919.42	21.00
6	HLA-A0201	GMSRNNIRQTFVDW	GMSRNNIFV	0	0	0	7	3	GMSRNNIRQTFV	tr_C9ZP82	0.104	16151.73	22.00
53	HLA-A0201	CTQSADQSL	CTQSADQSL	0	0	0	0	0	CTQSADQSL	tr_C9ZP82	0.100	16950.63	23.00
29	HLA-A0201	FSSRTRCFVCRA	FSSCFVCRA	0	0	0	3	3	FSSRTRCFVCRA	tr_C9ZP82	0.099	17090.22	23.00
20	HLA-A0201	LCTECLSLNF	LCTECLLNF	0	0	0	6	1	LCTECLSLNF	tr_C9ZP82	0.099	17091.52	23.00
24	HLA-A0201	CLSLNFSSR	CLSLNFSSR	0	0	0	0	0	CLSLNFSSR	tr_C9ZP82	0.099	17167.32	24.00
33	HLA-A0201	TRCFVCRAPF	RCFVCRAPF	1	0	0	0	0	RCFVCRAPF	tr_C9ZP82	0.098	17389.22	24.00
46	HLA-A0201	RCLAVFSCTQS	CLAVFSCTS	1	0	0	8	1	CLAVFSCTQS	tr_C9ZP82	0.096	17617.99	24.00
59	HLA-A0201	QSLYDPDHSP	SLYDPDHSP	1	0	0	0	0	SLYDPDHSP	tr_C9ZP82	0.096	17621.99	24.00
45	HLA-A0201	RRCLAVFSC	RRCLAVFSC	0	0	0	0	0	RRCLAVFSC	tr_C9ZP82	0.095	17860.21	24.00
45	HLA-A0201	RRCLAVFSCTQS	CLAVFSCTS	2	0	0	8	1	CLAVFSCTQS	tr_C9ZP82	0.093	18210.86	25.00
44	HLA-A0201	NRRCLAVFSCTQS	CLAVFSCTS	3	0	0	8	1	CLAVFSCTQS	tr_C9ZP82	0.092	18445.46	25.00
42	HLA-A0201	FDNRRCLAVFSCT	FCLAVFSCT	0	0	0	1	4	FDNRRCLAVFSCT	tr_C9ZP82	0.091	18584.28	25.00
43	HLA-A0201	DNRRCLAVFSCTQS	CLAVFSCTS	4	0	0	8	1	CLAVFSCTQS	tr_C9ZP82	0.091	18585.29	25.00
12	HLA-A0201	IRQTFVDWL	IRQTFVDWL	0	0	0	0	0	IRQTFVDWL	tr_C9ZP82	0.090	18805.16	26.00
28	HLA-A0201	NFSSRTRCFVCRA	FSSCFVCRA	1	0	0	3	3	FSSRTRCFVCRA	tr_C9ZP82	0.090	18811.88	26.00
24	HLA-A0201	CLSLNFSSRTRCF	CLSLNFSSF	0	0	0	8	4	CLSLNFSSRTRCF	tr_C9ZP82	0.089	19034.84	26.00
39	HLA-A0201	RAPFDNRRCLAV	RAPFRCLAV	0	0	0	4	3	RAPFDNRRCLAV	tr_C9ZP82	0.088	19297.79	26.00
26	HLA-A0201	SLNFSSRTRC	SLFSSRTRC	0	0	0	2	1	SLNFSSRTRC	tr_C9ZP82	0.087	19526.73	27.00
27	HLA-A0201	LNFSSRTRCFVCRA	FSSCFVCRA	2	0	0	3	3	FSSRTRCFVCRA	tr_C9ZP82	0.087	19548.07	27.00
31	HLA-A0201	SRTRCFVCRA	STRCFVCRA	0	0	0	1	1	SRTRCFVCRA	tr_C9ZP82	0.087	19601.45	27.00
40	HLA-A0201	APFDNRRCLAV	APFDRCLAV	0	0	0	4	2	APFDNRRCLAV	tr_C9ZP82	0.086	19628.41	27.00
41	HLA-A0201	PFDNRRCLAV	FDNRRCLAV	1	0	0	0	0	FDNRRCLAV	tr_C9ZP82	0.086	19748.76	27.00
38	HLA-A0201	CRAPFDNRRCLAV	RAPFRCLAV	1	0	0	4	3	RAPFDNRRCLAV	tr_C9ZP82	0.084	20124.96	28.00
11	HLA-A0201	NIRQTFVDWLC	IQTFVDWLC	1	0	0	1	1	IRQTFVDWLC	tr_C9ZP82	0.084	20158.08	28.00
49	HLA-A0201	AVFSCTQSADQSLY	AVFSCTQSL	0	0	0	6	4	AVFSCTQSADQSL	tr_C9ZP82	0.084	20181.65	28.00
17	HLA-A0201	VDWLCTECLS	VDWLCTECL	0	0	0	0	0	VDWLCTECL	tr_C9ZP82	0.084	20214.22	28.00
23	HLA-A0201	ECLSLNFSSRTRCF	CLSLNFSSF	1	0	0	8	4	CLSLNFSSRTRCF	tr_C9ZP82	0.083	20372.75	28.00
12	HLA-A0201	IRQTFVDWLCT	ITFVDWLCT	0	0	0	1	2	IRQTFVDWLCT	tr_C9ZP82	0.083	20413.78	28.00
41	HLA-A0201	PFDNRRCLAVFSCT	FCLAVFSCT	1	0	0	1	4	FDNRRCLAVFSCT	tr_C9ZP82	0.083	20460.21	28.00

10	HLA-A0201	NNIRQTFVDWLC	IQTFVDWLC	2	0	0	1	1	IRQTFVDWLC	tr_C9ZP82	0.082	20613.54	28.00
40	HLA-A0201	APFDNRRCLAVFSC	APFLAVFSC	0	0	0	3	5	APFDNRRCLAVFSC	tr_C9ZP82	0.082	20623.58	28.00
21	HLA-A0201	CTECLSLNFS	CTCLSLNFS	0	0	0	2	1	CTECLSLNFS	tr_C9ZP82	0.081	20717.74	28.00
9	HLA-A0201	RNNIRQTFVDWLC	IQTFVDWLC	3	0	0	1	1	IRQTFVDWLC	tr_C9ZP82	0.081	20739.04	28.00
37	HLA-A0201	VCRAPFDNRRCLAV	RAPFRCLAV	2	0	0	4	3	RAPFDNRRCLAV	tr_C9ZP82	0.081	20765.99	28.00
8	HLA-A0201	SRNNIRQTFVDWLC	IQTFVDWLC	4	0	0	1	1	IRQTFVDWLC	tr_C9ZP82	0.081	20844.54	29.00
11	HLA-A0201	NIRQTFVDWLCT	ITFVDWLCT	1	0	0	1	2	IRQTFVDWLCT	tr_C9ZP82	0.081	20848.61	29.00
24	HLA-A0201	CLSLNFSSRTRC	CLSLNFSSC	0	0	0	8	3	CLSLNFSSRTRC	tr_C9ZP82	0.081	20858.99	29.00
23	HLA-A0201	ECLSLNFSSRT	CLLNFSSRT	1	0	0	2	1	CLSLNFSSRT	tr_C9ZP82	0.080	20929.30	29.00
32	HLA-A0201	RTRCFVCRAPF	RTRCFVAPF	0	0	0	6	2	RTRCFVCRAPF	tr_C9ZP82	0.080	21027.58	29.00
42	HLA-A0201	FDNRRCLAVFSC	FRCLAVFSC	0	0	0	1	3	FDNRRCLAVFSC	tr_C9ZP82	0.080	21039.87	29.00
45	HLA-A0201	RRCLAVFSCT	RCLAVFSCT	0	0	0	1	1	RRCLAVFSCT	tr_C9ZP82	0.079	21241.62	29.00
45	HLA-A0201	RRCLAVFSCTQ	CLAVFSCTQ	2	0	0	0	0	CLAVFSCTQ	tr_C9ZP82	0.078	21502.68	30.00
10	HLA-A0201	NNIRQTFVDWLCT	ITFVDWLCT	2	0	0	1	2	IRQTFVDWLCT	tr_C9ZP82	0.078	21506.63	30.00
21	HLA-A0201	CTECLSLNF	CTECLSLNF	0	0	0	0	0	CTECLSLNF	tr_C9ZP82	0.078	21525.03	30.00
14	HLA-A0201	QTFVDWLCTE	QTFVWLCTE	0	0	0	4	1	QTFVDWLCTE	tr_C9ZP82	0.078	21596.88	30.00
9	HLA-A0201	RNNIRQTFVDWLCT	ITFVDWLCT	3	0	0	1	2	IRQTFVDWLCT	tr_C9ZP82	0.078	21616.29	30.00
31	HLA-A0201	SRTRCFVCRAPF	RTRCFVAPF	1	0	0	6	2	RTRCFVCRAPF	tr_C9ZP82	0.077	21791.70	30.00
22	HLA-A0201	TECLSLNFSSRT	CLLNFSSRT	2	0	0	2	1	CLSLNFSSRT	tr_C9ZP82	0.077	21791.70	30.00
20	HLA-A0201	LCTECLSLNFSSRT	CLLNFSSRT	4	0	0	2	1	CLSLNFSSRT	tr_C9ZP82	0.076	21870.36	30.00
60	HLA-A0201	SLYDPDHSPH	SLYDPDHSP	0	0	0	0	0	SLYDPDHSP	tr_C9ZP82	0.076	21978.06	30.00
30	HLA-A0201	SSRTRCFVCRAPF	RTRCFVAPF	2	0	0	6	2	RTRCFVCRAPF	tr_C9ZP82	0.076	21981.61	30.00
25	HLA-A0201	LSLNFSSRT	LSLNFSSRT	0	0	0	0	0	LSLNFSSRT	tr_C9ZP82	0.075	22113.54	31.00
21	HLA-A0201	CTECLSLNFSSRT	CLLNFSSRT	3	0	0	2	1	CLSLNFSSRT	tr_C9ZP82	0.075	22132.45	31.00
42	HLA-A0201	FDNRRCLAVF	FDNRRCLAV	0	0	0	0	0	FDNRRCLAV	tr_C9ZP82	0.074	22361.36	31.00
44	HLA-A0201	NRRCLAVFSCTQ	CLAVFSCTQ	3	0	0	0	0	CLAVFSCTQ	tr_C9ZP82	0.074	22370.32	31.00
23	HLA-A0201	ECLSLNFSSRTRC	CLSLNFSSC	1	0	0	8	3	CLSLNFSSRTRC	tr_C9ZP82	0.074	22371.76	31.00
41	HLA-A0201	PFDNRRCLAVFSC	FRCLAVFSC	1	0	0	1	3	FDNRRCLAVFSC	tr_C9ZP82	0.074	22435.03	31.00
6	HLA-A0201	GMSRNNIRQT	GMSRNNIQT	0	0	0	7	1	GMSRNNIRQT	tr_C9ZP82	0.074	22450.33	31.00
43	HLA-A0201	DNRRCLAVFSCTQ	CLAVFSCTQ	4	0	0	0	0	CLAVFSCTQ	tr_C9ZP82	0.074	22558.93	31.00
7	HLA-A0201	MSRNNIRQTFVDWL	NIQTFVDWL	4	0	0	2	1	NIRQTFVDWL	tr_C9ZP82	0.073	22577.74	31.00
7	HLA-A0201	MSRNNIRQTFV	MSRNNITFV	0	0	0	6	2	MSRNNIRQTFV	tr_C9ZP82	0.073	22644.03	31.00
22	HLA-A0201	TECLSLNFSSRTRC	CLSLNFSSC	2	0	0	8	3	CLSLNFSSRTRC	tr_C9ZP82	0.072	22879.71	32.00
34	HLA-A0201	RCFVCRAPFD	RCFVCRAPF	0	0	0	0	0	RCFVCRAPF	tr_C9ZP82	0.072	22933.00	32.00
10	HLA-A0201	NNIRQTFVDWL	NIQTFVDWL	1	0	0	2	1	NIRQTFVDWL	tr_C9ZP82	0.072	22941.20	32.00
68	HLA-A0201	PHIEAVKLQI	HIEAVKLQI	1	0	0	0	0	HIEAVKLQI	tr_C9ZP82	0.071	23147.38	32.00
51	HLA-A0201	FSCTQSADQSL	FTQSADQSL	0	0	0	1	2	FSCTQSADQSL	tr_C9ZP82	0.070	23391.60	33.00
29	HLA-A0201	FSSRTRCFVCRAP	FSSCFVCRA	0	0	0	3	3	FSSRTRCFVCRA	tr_C9ZP82	0.070	23460.04	33.00
9	HLA-A0201	RNNIRQTFVDWL	NIQTFVDWL	2	0	0	2	1	NIRQTFVDWL	tr_C9ZP82	0.070	23506.79	33.00
48	HLA-A0201	LAVFSCTQSAD	LVFSCTQSA	0	0	0	1	1	LAVFSCTQSA	tr_C9ZP82	0.070	23540.91	33.00
8	HLA-A0201	SRNNIRQTFVDWL	NIQTFVDWL	3	0	0	2	1	NIRQTFVDWL	tr_C9ZP82	0.069	23774.60	33.00
39	HLA-A0201	RAPFDNRRCLAVF	RAPFCLAVF	0	0	0	4	4	RAPFDNRRCLAVF	tr_C9ZP82	0.069	23823.00	33.00
23	HLA-A0201	ECLSLNFSSR	CLSLNFSSR	1	0	0	0	0	CLSLNFSSR	tr_C9ZP82	0.068	23918.84	34.00
44	HLA-A0201	NRRCLAVFSC	RRCLAVFSC	1	0	0	0	0	RRCLAVFSC	tr_C9ZP82	0.067	24104.32	34.00
38	HLA-A0201	CRAPFDNRRCLAVF	RAPFCLAVF	1	0	0	4	4	RAPFDNRRCLAVF	tr_C9ZP82	0.067	24291.79	34.00
30	HLA-A0201	SSRTRCFVC	SSRTRCFVC	0	0	0	0	0	SSRTRCFVC	tr_C9ZP82	0.066	24377.10	34.00
11	HLA-A0201	NIRQTFVDWLCTE	NIFVDWLCT	0	0	0	2	3	NIRQTFVDWLCT	tr_C9ZP82	0.065	24678.30	35.00
28	HLA-A0201	NFSSRTRCFVCRAP	FSSCFVCRA	1	0	0	3	3	FSSRTRCFVCRA	tr_C9ZP82	0.065	24765.49	35.00
12	HLA-A0201	IRQTFVDWLCTE	IQTFVDWLC	0	0	0	1	1	IRQTFVDWLC	tr_C9ZP82	0.065	24771.93	35.00
32	HLA-A0201	RTRCFVCRAP	RTRCFVCRA	0	0	0	0	0	RTRCFVCRA	tr_C9ZP82	0.064	25004.05	36.00
24	HLA-A0201	CLSLNFSSRTR	CLLNFSSRT	0	0	0	2	1	CLSLNFSSRT	tr_C9ZP82	0.064	25007.02	36.00
40	HLA-A0201	APFDNRRCLAVF	APFDRCLAV	0	0	0	4	2	APFDNRRCLAV	tr_C9ZP82	0.064	25058.21	36.00
28	HLA-A0201	NFSSRTRCFVC	FSSRTCFVC	1	0	0	5	1	FSSRTRCFVC	tr_C9ZP82	0.064	25101.64	36.00
13	HLA-A0201	RQTFVDWLCTE	QTFVWLCTE	1	0	0	4	1	QTFVDWLCTE	tr_C9ZP82	0.063	25171.53	36.00

10	HLA-A0201	NNIRQTFVDWLCTE	ITFVDWLCT	2	0	0	1	2	IRQTFVDWLCT	tr_C9ZP82	0.063	25190.33	36.00
27	HLA-A0201	LNFSSRTRCFVC	FSSRTCFVC	2	0	0	5	1	FSSRTRCFVC	tr_C9ZP82	0.063	25190.61	36.00
9	HLA-A0201	RNNIRQTFV	RNNIRQTFV	0	0	0	0	0	RNNIRQTFV	tr_C9ZP82	0.063	25330.54	36.00
52	HLA-A0201	SCTQSADQSL	STQSADQSL	0	0	0	1	1	SCTQSADQSL	tr_C9ZP82	0.062	25555.45	37.00
20	HLA-A0201	LCTECLSLNFSS	LCTECLFSS	0	0	0	6	3	LCTECLSLNFSS	tr_C9ZP82	0.062	25646.31	37.00
32	HLA-A0201	RTRCFVCRAPFD	RTRCFVAPF	0	0	0	6	2	RTRCFVCRAPF	tr_C9ZP82	0.060	26159.17	38.00
48	HLA-A0201	LAVFSCTQSADQ	LVFSCTQSA	0	0	0	1	1	LAVFSCTQSA	tr_C9ZP82	0.060	26255.28	38.00
50	HLA-A0201	VFSCTQSADQSL	FTQSADQSL	1	0	0	1	2	FSCTQSADQSL	tr_C9ZP82	0.059	26464.92	39.00
11	HLA-A0201	NIRQTFVDW	NIRQTFVDW	0	0	0	0	0	NIRQTFVDW	tr_C9ZP82	0.059	26472.66	39.00
27	HLA-A0201	LNFSSRTRCFV	FSSRTRCFV	2	0	0	0	0	FSSRTRCFV	tr_C9ZP82	0.059	26478.09	39.00
31	HLA-A0201	SRTRCFVCRAPFD	SCFVCRAPF	0	0	0	1	3	SRTRCFVCRAPF	tr_C9ZP82	0.059	26531.43	39.00
23	HLA-A0201	ECLSLNFSSRTR	CLLNFSSRT	1	0	0	2	1	CLSLNFSSRT	tr_C9ZP82	0.058	26561.60	39.00
48	HLA-A0201	LAVFSCTQS	LAVFSCTQS	0	0	0	0	0	LAVFSCTQS	tr_C9ZP82	0.058	26562.75	39.00
26	HLA-A0201	SLNFSSRTR	SLNFSSRTR	0	0	0	0	0	SLNFSSRTR	tr_C9ZP82	0.058	26610.21	39.00
39	HLA-A0201	RAPFDNRRCLAVFS	RAPFRCLAV	0	0	0	4	3	RAPFDNRRCLAV	tr_C9ZP82	0.058	26652.85	39.00
30	HLA-A0201	SSRTRCFVCRAPFD	SCFVCRAPF	0	0	0	1	4	SSRTRCFVCRAPF	tr_C9ZP82	0.058	26701.06	39.00
20	HLA-A0201	LCTECLSLNFS	LCCLSLNFS	0	0	0	2	2	LCTECLSLNFS	tr_C9ZP82	0.058	26755.43	39.00
34	HLA-A0201	RCFVCRAPFDNRRC	RCFVCRAPC	0	0	0	8	5	RCFVCRAPFDNRRC	tr_C9ZP82	0.058	26819.19	39.00
20	HLA-A0201	LCTECLSLNFSSR	LLSLNFSSR	0	0	0	1	4	LCTECLSLNFSSR	tr_C9ZP82	0.057	26926.48	39.00
22	HLA-A0201	TECLSLNFS	TECLSLNFS	0	0	0	0	0	TECLSLNFS	tr_C9ZP82	0.057	26983.93	40.00
40	HLA-A0201	APFDNRRCLAVFS	APFCLAVFS	0	0	0	3	4	APFDNRRCLAVFS	tr_C9ZP82	0.057	27041.21	40.00
22	HLA-A0201	TECLSLNFSSRTR	CLSLNFSTR	2	0	0	7	2	CLSLNFSSRTR	tr_C9ZP82	0.057	27060.53	40.00
67	HLA-A0201	SPHIEAVKL	SPHIEAVKL	0	0	0	0	0	SPHIEAVKL	tr_C9ZP82	0.057	27079.28	40.00
21	HLA-A0201	CTECLSLNFSSRTR	CLLNFSSRT	3	0	0	2	1	CLSLNFSSRT	tr_C9ZP82	0.056	27183.49	40.00
67	HLA-A0201	SPHIEAVKLQI	SIEAVKLQI	0	0	0	1	2	SPHIEAVKLQI	tr_C9ZP82	0.056	27258.59	40.00
33	HLA-A0201	TRCFVCRAPFD	RCFVCRAPF	1	0	0	0	0	RCFVCRAPF	tr_C9ZP82	0.056	27334.50	40.00
32	HLA-A0201	RTRCFVCRAPFDN	RTRCFVCPF	0	0	0	7	2	RTRCFVCRAPF	tr_C9ZP82	0.056	27351.36	40.00
31	HLA-A0201	SRTRCFVCRAPFDN	SCFVCRAPF	0	0	0	1	3	SRTRCFVCRAPF	tr_C9ZP82	0.055	27567.65	41.00
62	HLA-A0201	YDPDHSPHIEAV	YDSPHIEAV	0	0	0	2	3	YDPDHSPHIEAV	tr_C9ZP82	0.055	27650.11	41.00
48	HLA-A0201	LAVFSCTQSADQS	LVFSCTQSA	0	0	0	1	1	LAVFSCTQSA	tr_C9ZP82	0.055	27653.39	41.00
49	HLA-A0201	AVFSCTQSADQ	AVFSCTQSA	0	0	0	0	0	AVFSCTQSA	tr_C9ZP82	0.055	27659.97	41.00
30	HLA-A0201	SSRTRCFVCRA	STRCFVCRA	0	0	0	1	2	SSRTRCFVCRA	tr_C9ZP82	0.055	27681.53	41.00
26	HLA-A0201	SLNFSSRTRCF	SLNFSSRTF	0	0	0	8	2	SLNFSSRTRCF	tr_C9ZP82	0.055	27688.72	41.00
32	HLA-A0201	RTRCFVCRAPFDNR	RTRCFVAPF	0	0	0	6	2	RTRCFVCRAPF	tr_C9ZP82	0.054	27829.88	41.00
36	HLA-A0201	FVCRAPFDN	FVCRAPFDN	0	0	0	0	0	FVCRAPFDN	tr_C9ZP82	0.054	27878.11	42.00
22	HLA-A0201	TECLSLNFSS	TCLSLNFSS	0	0	0	1	1	TECLSLNFSS	tr_C9ZP82	0.054	27983.27	42.00
61	HLA-A0201	LYDPDHSPHI	LYDDHSPHI	0	0	0	3	1	LYDPDHSPHI	tr_C9ZP82	0.053	28027.50	42.00
8	HLA-A0201	SRNNIRQTFV	SRNNIQTFV	0	0	0	5	1	SRNNIRQTFV	tr_C9ZP82	0.053	28040.55	42.00
29	HLA-A0201	FSSRTRCFVCR	FSSRTRCFV	0	0	0	0	0	FSSRTRCFV	tr_C9ZP82	0.053	28139.32	42.00
66	HLA-A0201	HSPHIEAVKL	HSPHIEAVL	0	0	0	8	1	HSPHIEAVKL	tr_C9ZP82	0.053	28146.63	42.00
33	HLA-A0201	TRCFVCRAPFDN	TRCFVCAPF	0	0	0	6	1	TRCFVCRAPF	tr_C9ZP82	0.053	28193.58	42.00
61	HLA-A0201	LYDPDHSPHIEAV	LYDPDIEAV	0	0	0	5	4	LYDPDHSPHIEAV	tr_C9ZP82	0.052	28390.71	43.00
33	HLA-A0201	TRCFVCRAPFDNRR	TRCFVCAPF	0	0	0	6	1	TRCFVCRAPF	tr_C9ZP82	0.052	28454.07	43.00
33	HLA-A0201	TRCFVCRAPFDNR	TRCFVCAPF	0	0	0	6	1	TRCFVCRAPF	tr_C9ZP82	0.052	28566.34	43.00
21	HLA-A0201	CTECLSLNFSSR	CLSLNFSSR	3	0	0	0	0	CLSLNFSSR	tr_C9ZP82	0.051	28697.70	43.00
15	HLA-A0201	TFVDWLCTE	TFVDWLCTE	0	0	0	0	0	TFVDWLCTE	tr_C9ZP82	0.051	28710.75	43.00
49	HLA-A0201	AVFSCTQSADQS	AVFSCTQSA	0	0	0	0	0	AVFSCTQSA	tr_C9ZP82	0.051	28713.23	43.00
66	HLA-A0201	HSPHIEAVKLQI	SIEAVKLQI	1	0	0	1	2	SPHIEAVKLQI	tr_C9ZP82	0.051	28775.42	44.00
20	HLA-A0201	LCTECLSLN	LCTECLSLN	0	0	0	0	0	LCTECLSLN	tr_C9ZP82	0.051	28781.03	44.00
27	HLA-A0201	LNFSSRTRCFVCR	FSSRTRCFV	2	0	0	0	0	FSSRTRCFV	tr_C9ZP82	0.051	28804.39	44.00
21	HLA-A0201	CTECLSLNFSS	CTLSLNFSS	0	0	0	2	2	CTECLSLNFSS	tr_C9ZP82	0.051	28822.17	44.00
36	HLA-A0201	FVCRAPFDNR	FVCRAPFNR	0	0	0	7	1	FVCRAPFDNR	tr_C9ZP82	0.050	29119.95	44.00
22	HLA-A0201	TECLSLNFSSR	CLSLNFSSR	2	0	0	0	0	CLSLNFSSR	tr_C9ZP82	0.050	29142.64	45.00
36	HLA-A0201	FVCRAPFDNRRC	FVCRAPFRC	0	0	0	7	3	FVCRAPFDNRRC	tr_C9ZP82	0.050	29182.40	45.00

7	HLA-A0201	MSRNNIRQTFVDW	MIRQTFVDW	0	0	0	1	4	MSRNNIRQTFVDW	tr_C9ZP82	0.049	29322.61	45.00
65	HLA-A0201	DHSPHIEAVKLQI	SIEAVKLQI	2	0	0	1	2	SPHIEAVKLQI	tr_C9ZP82	0.049	29343.57	45.00
25	HLA-A0201	LSLNFSSRTRCF	SLNFSSRTF	1	0	0	8	2	SLNFSSRTRCF	tr_C9ZP82	0.049	29415.09	45.00
6	HLA-A0201	GMSRNNIRQTF	GMSRNNITF	0	0	0	7	2	GMSRNNIRQTF	tr_C9ZP82	0.049	29432.91	45.00
28	HLA-A0201	NFSSRTRCFVCR	FSSRTRCFV	1	0	0	0	0	FSSRTRCFV	tr_C9ZP82	0.049	29459.37	45.00
34	HLA-A0201	RCFVCRAPFDNR	RCFVCRAPF	0	0	0	0	0	RCFVCRAPF	tr_C9ZP82	0.049	29479.13	45.00
34	HLA-A0201	RCFVCRAPFDNRR	RCFVCRAPF	0	0	0	0	0	RCFVCRAPF	tr_C9ZP82	0.049	29534.35	46.00
34	HLA-A0201	RCFVCRAPFDN	RCFVCRAPF	0	0	0	0	0	RCFVCRAPF	tr_C9ZP82	0.049	29554.17	46.00
64	HLA-A0201	PDHSPHIEAVKLQI	SIEAVKLQI	3	0	0	1	2	SPHIEAVKLQI	tr_C9ZP82	0.049	29568.89	46.00
44	HLA-A0201	NRRCLAVFSCT	RCLAVFSCT	2	0	0	0	0	RCLAVFSCT	tr_C9ZP82	0.048	29597.06	46.00
56	HLA-A0201	SADQSLYDP	SADQSLYDP	0	0	0	0	0	SADQSLYDP	tr_C9ZP82	0.048	29672.40	46.00
43	HLA-A0201	DNRRCLAVFSC	RRCLAVFSC	2	0	0	0	0	RRCLAVFSC	tr_C9ZP82	0.048	29771.77	46.00
35	HLA-A0201	CFVCRAPFDNRRC	FVCRAPFRC	1	0	0	7	3	FVCRAPFDNRRC	tr_C9ZP82	0.048	29782.74	46.00
57	HLA-A0201	ADQSLYDPDHSP	ALYDPDHSP	0	0	0	1	3	ADQSLYDPDHSP	tr_C9ZP82	0.048	29817.87	46.00
43	HLA-A0201	DNRRCLAVFSCT	RCLAVFSCT	3	0	0	0	0	RCLAVFSCT	tr_C9ZP82	0.048	29829.18	46.00
56	HLA-A0201	SADQSLYDPDHSP	SLYDPDHSP	0	0	0	1	4	SADQSLYDPDHSP	tr_C9ZP82	0.047	29979.95	47.00
23	HLA-A0201	ECLSLNFSS	ECLSLNFSS	0	0	0	0	0	ECLSLNFSS	tr_C9ZP82	0.047	29980.59	47.00
42	HLA-A0201	FDNRRCLAVFS	FDNRRCLAV	0	0	0	0	0	FDNRRCLAV	tr_C9ZP82	0.046	30331.68	48.00
41	HLA-A0201	PFDNRRCLAVF	FDNRRCLAV	1	0	0	0	0	FDNRRCLAV	tr_C9ZP82	0.046	30383.89	48.00
58	HLA-A0201	DQSLYDPDHSP	SLYDPDHSP	2	0	0	0	0	SLYDPDHSP	tr_C9ZP82	0.045	30627.15	48.00
7	HLA-A0201	MSRNNIRQTF	MSRNNIQTF	0	0	0	6	1	MSRNNIRQTF	tr_C9ZP82	0.045	30647.05	48.00
33	HLA-A0201	TRCFVCRAP	TRCFVCRAP	0	0	0	0	0	TRCFVCRAP	tr_C9ZP82	0.045	30716.09	49.00
7	HLA-A0201	MSRNNIRQTFVD	MSRNNITFV	0	0	0	6	2	MSRNNIRQTFV	tr_C9ZP82	0.045	30725.40	49.00
6	HLA-A0201	GMSRNNIRQ	GMSRNNIRQ	0	0	0	0	0	GMSRNNIRQ	tr_C9ZP82	0.045	30753.01	49.00
65	HLA-A0201	DHSPHIEAV	DHSPHIEAV	0	0	0	0	0	DHSPHIEAV	tr_C9ZP82	0.045	30774.97	49.00
55	HLA-A0201	QSADQSLYDPDHSP	SLYDPDHSP	5	0	0	0	0	SLYDPDHSP	tr_C9ZP82	0.044	30950.62	49.00
5	HLA-A0201	CGMSRNNIRQTF	GMSRNNITF	1	0	0	7	2	GMSRNNIRQTF	tr_C9ZP82	0.044	31039.84	49.00
10	HLA-A0201	NNIRQTFVDW	NIRQTFVDW	1	0	0	0	0	NIRQTFVDW	tr_C9ZP82	0.044	31049.23	49.00
30	HLA-A0201	SSRTRCFVCRAP	STRCFVCRA	0	0	0	1	2	SSRTRCFVCRA	tr_C9ZP82	0.044	31142.76	50.00
31	HLA-A0201	SRTRCFVCRAP	STRCFVCRA	0	0	0	1	1	SRTRCFVCRA	tr_C9ZP82	0.043	31255.17	50.00
44	HLA-A0201	NRRCLAVFS	NRRCLAVFS	0	0	0	0	0	NRRCLAVFS	tr_C9ZP82	0.043	31400.25	50.00
51	HLA-A0201	FSCTQSADQSLY	FTQSADQSL	0	0	0	1	2	FSCTQSADQSL	tr_C9ZP82	0.043	31425.74	55.00
43	HLA-A0201	DNRRCLAVF	DNRRCLAVF	0	0	0	0	0	DNRRCLAVF	tr_C9ZP82	0.043	31540.19	55.00
4	HLA-A0201	GCGMSRNNIRQTF	GMSRNNITF	2	0	0	7	2	GMSRNNIRQTF	tr_C9ZP82	0.042	31609.20	55.00
25	HLA-A0201	LSLNFSSRTRC	SLFSSRTRC	1	0	0	2	1	SLNFSSRTRC	tr_C9ZP82	0.042	31736.35	55.00
3	HLA-A0201	SGCGMSRNNIRQTF	GMSRNNITF	3	0	0	7	2	GMSRNNIRQTF	tr_C9ZP82	0.042	31756.95	55.00
41	HLA-A0201	PFDNRRCLAVFS	FDNRRCLAV	1	0	0	0	0	FDNRRCLAV	tr_C9ZP82	0.042	31782.39	55.00
25	HLA-A0201	LSLNFSSRTR	LLNFSSRTR	0	0	0	1	1	LSLNFSSRTR	tr_C9ZP82	0.042	31791.68	55.00
9	HLA-A0201	RNNIRQTFVDW	RIRQTFVDW	0	0	0	1	2	RNNIRQTFVDW	tr_C9ZP82	0.041	32070.83	55.00
8	HLA-A0201	SRNNIRQTFVDW	SIRQTFVDW	0	0	0	1	3	SRNNIRQTFVDW	tr_C9ZP82	0.041	32123.27	55.00
62	HLA-A0201	YDPDHSPHIEAVKL	YPHIEAVKL	0	0	0	1	5	YDPDHSPHIEAVKL	tr_C9ZP82	0.039	32611.79	55.00
51	HLA-A0201	FSCTQSADQSLYDP	FSCQSLYDP	0	0	0	3	5	FSCTQSADQSLYDP	tr_C9ZP82	0.039	32682.79	55.00
39	HLA-A0201	RAPFDNRRCL	RAFDNRRCL	0	0	0	2	1	RAPFDNRRCL	tr_C9ZP82	0.039	32744.04	55.00
30	HLA-A0201	SSRTRCFVCR	SSRTRCFVC	0	0	0	0	0	SSRTRCFVC	tr_C9ZP82	0.039	32872.19	55.00
50	HLA-A0201	VFSCTQSADQSLY	FTQSADQSL	1	0	0	1	2	FSCTQSADQSL	tr_C9ZP82	0.038	33145.03	60.00
37	HLA-A0201	VCRAPFDNRRCLA	VCRAPFCLA	0	0	0	6	4	VCRAPFDNRRCLA	tr_C9ZP82	0.036	33771.30	60.00
36	HLA-A0201	FVCRAPFDNRR	FVCPFDNRR	0	0	0	3	2	FVCRAPFDNRR	tr_C9ZP82	0.036	33880.00	60.00
51	HLA-A0201	FSCTQSADQSLYD	FTQSADQSL	0	0	0	1	2	FSCTQSADQSL	tr_C9ZP82	0.036	33906.78	60.00
35	HLA-A0201	CFVCRAPFDN	FVCRAPFDN	1	0	0	0	0	FVCRAPFDN	tr_C9ZP82	0.036	33943.47	60.00
56	HLA-A0201	SADQSLYDPDHSPH	SLYDPDHPH	4	0	0	7	1	SLYDPDHSPH	tr_C9ZP82	0.036	34042.05	60.00
61	HLA-A0201	LYDPDHSPHIEAVK	LYDPDIEAV	0	0	0	5	4	LYDPDHSPHIEAV	tr_C9ZP82	0.035	34053.47	60.00
35	HLA-A0201	CFVCRAPFDNRR	FVCPFDNRR	1	0	0	3	2	FVCRAPFDNRR	tr_C9ZP82	0.035	34053.82	60.00
53	HLA-A0201	CTQSADQSLY	CTQSADQSL	0	0	0	0	0	CTQSADQSL	tr_C9ZP82	0.035	34081.84	60.00
62	HLA-A0201	YDPDHSPHIEAVK	YHSPHIEAV	0	0	0	1	3	YDPDHSPHIEAV	tr_C9ZP82	0.035	34126.13	60.00

43	HLA-A0201	DNRRCLAVFS	NRRCLAVFS	1	0	0	0	0	NRRCLAVFS	tr_C9ZP82	0.035	34237.08	60.00
65	HLA-A0201	DHSPHIEAVKL	HSHIEAVKL	1	0	0	2	1	HSPHIEAVKL	tr_C9ZP82	0.035	34410.51	60.00
54	HLA-A0201	TQSADQSLY	TQSADQSLY	0	0	0	0	0	TQSADQSLY	tr_C9ZP82	0.034	34523.88	60.00
7	HLA-A0201	MSRNNIRQT	MSRNNIRQT	0	0	0	0	0	MSRNNIRQT	tr_C9ZP82	0.034	34563.13	60.00
3	HLA-A0201	SGCGMSRNNI	SGCGMSNNI	0	0	0	6	1	SGCGMSRNNI	tr_C9ZP82	0.034	34634.24	65.00
4	HLA-A0201	GCGMSRNNI	GCGMSRNNI	0	0	0	0	0	GCGMSRNNI	tr_C9ZP82	0.034	34782.57	65.00
67	HLA-A0201	SPHIEAVKLQ	SPHIEAVKL	0	0	0	0	0	SPHIEAVKL	tr_C9ZP82	0.033	34860.21	65.00
50	HLA-A0201	VFSCTQSADQSLYD	FTQSADQSL	1	0	0	1	2	FSCTQSADQSL	tr_C9ZP82	0.033	34934.95	65.00
35	HLA-A0201	CFVCRAPFDNR	FVCRAPFNR	1	0	0	7	1	FVCRAPFDNR	tr_C9ZP82	0.033	34936.46	65.00
64	HLA-A0201	PDHSPHIEAVKL	SPHIEAVKL	3	0	0	0	0	SPHIEAVKL	tr_C9ZP82	0.033	34978.07	65.00
57	HLA-A0201	ADQSLYDPDHSPH	SLYDPDHPH	3	0	0	7	1	SLYDPDHSPH	tr_C9ZP82	0.033	35027.69	65.00
31	HLA-A0201	SRTRCFVCR	SRTRCFVCR	0	0	0	0	0	SRTRCFVCR	tr_C9ZP82	0.033	35069.39	65.00
63	HLA-A0201	DPDHSPHIEAVKL	SPHIEAVKL	4	0	0	0	0	SPHIEAVKL	tr_C9ZP82	0.033	35076.23	65.00
37	HLA-A0201	VCRAPFDNRRCL	VCRAPFRCL	0	0	0	6	3	VCRAPFDNRRCL	tr_C9ZP82	0.033	35102.43	65.00
59	HLA-A0201	QSLYDPDHSPH	SLYDPDHPH	1	0	0	7	1	SLYDPDHSPH	tr_C9ZP82	0.032	35199.41	65.00
8	HLA-A0201	SRNNIRQTF	SRNNIRQTF	0	0	0	0	0	SRNNIRQTF	tr_C9ZP82	0.032	35337.18	65.00
58	HLA-A0201	DQSLYDPDHSPH	SLYDPDHPH	2	0	0	7	1	SLYDPDHSPH	tr_C9ZP82	0.032	35511.95	65.00
5	HLA-A0201	CGMSRNNIRQT	GMSRNNIQT	1	0	0	7	1	GMSRNNIRQT	tr_C9ZP82	0.031	35579.25	65.00
55	HLA-A0201	QSADQSLYDP	SADQSLYDP	1	0	0	0	0	SADQSLYDP	tr_C9ZP82	0.031	35636.66	65.00
40	HLA-A0201	APFDNRRCL	APFDNRRCL	0	0	0	0	0	APFDNRRCL	tr_C9ZP82	0.031	35659.03	65.00
51	HLA-A0201	FSCTQSADQS	FSCTQSAQS	0	0	0	7	1	FSCTQSADQS	tr_C9ZP82	0.030	36000.57	70.00
40	HLA-A0201	APFDNRRCLA	AFDNRRCLA	0	0	0	1	1	APFDNRRCLA	tr_C9ZP82	0.030	36105.50	70.00
62	HLA-A0201	YDPDHSPHIE	YDPDHSPHI	0	0	0	0	0	YDPDHSPHI	tr_C9ZP82	0.030	36128.93	70.00
4	HLA-A0201	GCGMSRNNIRQT	GMSRNNIQT	2	0	0	7	1	GMSRNNIRQT	tr_C9ZP82	0.030	36170.80	70.00
56	HLA-A0201	SADQSLYDPD	SADQSLYPD	0	0	0	7	1	SADQSLYDPD	tr_C9ZP82	0.030	36203.29	70.00
2	HLA-A0201	SSGCGMSRNNIRQT	GMSRNNIQT	4	0	0	7	1	GMSRNNIRQT	tr_C9ZP82	0.030	36315.88	70.00
66	HLA-A0201	HSPHIEAVKLQ	HSPHIEAVL	0	0	0	8	1	HSPHIEAVKL	tr_C9ZP82	0.029	36339.87	70.00
51	HLA-A0201	FSCTQSADQ	FSCTQSADQ	0	0	0	0	0	FSCTQSADQ	tr_C9ZP82	0.029	36363.46	70.00
3	HLA-A0201	SGCGMSRNNIRQT	GMSRNNIQT	3	0	0	7	1	GMSRNNIRQT	tr_C9ZP82	0.029	36368.57	70.00
2	HLA-A0201	SSGCGMSRNNI	SSGCGMNNI	0	0	0	6	2	SSGCGMSRNNI	tr_C9ZP82	0.029	36377.23	70.00
37	HLA-A0201	VCRAPFDNR	VCRAPFDNR	0	0	0	0	0	VCRAPFDNR	tr_C9ZP82	0.029	36534.63	70.00
28	HLA-A0201	NFSSRTRCF	NFSSRTRCF	0	0	0	0	0	NFSSRTRCF	tr_C9ZP82	0.029	36555.18	70.00
5	HLA-A0201	CGMSRNNIRQ	CMSRNNIRQ	0	0	0	1	1	CGMSRNNIRQ	tr_C9ZP82	0.029	36618.91	70.00
38	HLA-A0201	CRAPFDNRRCLA	CRAPFDCLA	0	0	0	6	3	CRAPFDNRRCLA	tr_C9ZP82	0.029	36640.31	70.00
64	HLA-A0201	PDHSPHIEAV	DHSPHIEAV	1	0	0	0	0	DHSPHIEAV	tr_C9ZP82	0.029	36699.84	70.00
35	HLA-A0201	CFVCRAPFD	CFVCRAPFD	0	0	0	0	0	CFVCRAPFD	tr_C9ZP82	0.029	36715.72	70.00
65	HLA-A0201	DHSPHIEAVKLQ	HSPHIEAVL	1	0	0	8	1	HSPHIEAVKL	tr_C9ZP82	0.028	36759.84	70.00
37	HLA-A0201	VCRAPFDNRR	VCAPFDNRR	0	0	0	2	1	VCRAPFDNRR	tr_C9ZP82	0.028	36805.22	70.00
56	HLA-A0201	SADQSLYDPDHS	SADQSLYHS	0	0	0	7	3	SADQSLYDPDHS	tr_C9ZP82	0.028	36857.40	70.00
27	HLA-A0201	LNFSSRTRCF	LFSSRTRCF	0	0	0	1	1	LNFSSRTRCF	tr_C9ZP82	0.028	36992.04	70.00
18	HLA-A0201	DWLCTECLS	DWLCTECLS	0	0	0	0	0	DWLCTECLS	tr_C9ZP82	0.028	36997.24	70.00
64	HLA-A0201	PDHSPHIEAVKLQ	SPHIEAVLQ	3	0	0	7	1	SPHIEAVKLQ	tr_C9ZP82	0.028	37044.92	70.00
63	HLA-A0201	DPDHSPHIEAVKLQ	SPHIEAVLQ	4	0	0	7	1	SPHIEAVKLQ	tr_C9ZP82	0.028	37068.99	70.00
0	HLA-A0201	DPSSGCGMSRNNI	SSGCGMNNI	2	0	0	6	2	SSGCGMSRNNI	tr_C9ZP82	0.027	37367.36	75.00
38	HLA-A0201	CRAPFDNRRCL	RAFDNRRCL	1	0	0	2	1	RAPFDNRRCL	tr_C9ZP82	0.027	37393.25	75.00
63	HLA-A0201	DPDHSPHIEAV	DPDPHIEAV	0	0	0	3	2	DPDHSPHIEAV	tr_C9ZP82	0.027	37422.80	75.00
53	HLA-A0201	CTQSADQSLYDP	CTDQSLYDP	0	0	0	2	3	CTQSADQSLYDP	tr_C9ZP82	0.027	37448.73	75.00
1	HLA-A0201	PSSGCGMSRNNI	SSGCGMNNI	1	0	0	6	2	SSGCGMSRNNI	tr_C9ZP82	0.026	37577.80	75.00
52	HLA-A0201	SCTQSADQSLYDP	SADQSLYDP	4	0	0	0	0	SADQSLYDP	tr_C9ZP82	0.026	37684.07	75.00
52	HLA-A0201	SCTQSADQSLY	STQSADQSL	0	0	0	1	1	SCTQSADQSL	tr_C9ZP82	0.026	37722.41	75.00
55	HLA-A0201	QSADQSLYDPDHS	SADQSLYHS	1	0	0	7	3	SADQSLYDPDHS	tr_C9ZP82	0.026	37726.09	75.00
9	HLA-A0201	RNNIRQTFVD	RNNIRQTFV	0	0	0	0	0	RNNIRQTFV	tr_C9ZP82	0.026	37728.52	75.00
38	HLA-A0201	CRAPFDNRR	CRAPFDNRR	0	0	0	0	0	CRAPFDNRR	tr_C9ZP82	0.026	37730.15	75.00
54	HLA-A0201	TQSADQSLYDPDHS	SADQSLYHS	2	0	0	7	3	SADQSLYDPDHS	tr_C9ZP82	0.025	38022.76	75.00

62	HLA-A0201	YDPDHSPHIEA	YDPDHSIEA	0	0	0	6	2	YDPDHSPHIEA	tr_C9ZP82	0.025	38037.57	75.00
61	HLA-A0201	LYDPDHSPHIEA	YDPDHSIEA	1	0	0	6	2	YDPDHSPHIEA	tr_C9ZP82	0.025	38056.52	75.00
53	HLA-A0201	CTQSADQSLYD	CTQSASLYD	0	0	0	5	2	CTQSADQSLYD	tr_C9ZP82	0.025	38238.54	75.00
52	HLA-A0201	SCTQSADQSLYD	SCTQSALYD	0	0	0	6	3	SCTQSADQSLYD	tr_C9ZP82	0.025	38295.68	75.00
54	HLA-A0201	TQSADQSLYD	TQSADSLYD	0	0	0	5	1	TQSADQSLYD	tr_C9ZP82	0.024	38393.18	75.00
65	HLA-A0201	DHSPHIEAVK	DHSPHIEAV	0	0	0	0	0	DHSPHIEAV	tr_C9ZP82	0.024	38547.18	80.00
54	HLA-A0201	TQSADQSLYDP	TQDQSLYDP	0	0	0	2	2	TQSADQSLYDP	tr_C9ZP82	0.024	38547.60	80.00
66	HLA-A0201	HSPHIEAVK	HSPHIEAVK	0	0	0	0	0	HSPHIEAVK	tr_C9ZP82	0.024	38746.64	80.00
52	HLA-A0201	SCTQSADQSLYDPD	SADQSLYPD	4	0	0	7	1	SADQSLYDPD	tr_C9ZP82	0.023	38775.16	80.00
53	HLA-A0201	CTQSADQSLYDPD	SADQSLYPD	3	0	0	7	1	SADQSLYDPD	tr_C9ZP82	0.023	38775.59	80.00
39	HLA-A0201	RAPFDNRRCLA	RAPFDNCLA	0	0	0	6	2	RAPFDNRRCLA	tr_C9ZP82	0.023	38803.70	80.00
1	HLA-A0201	PSSGCGMSR	PSSGCGMSR	0	0	0	0	0	PSSGCGMSR	tr_C9ZP82	0.023	38813.77	80.00
37	HLA-A0201	VCRAPFDNRRC	VCRAPFRRC	0	0	0	6	2	VCRAPFDNRRC	tr_C9ZP82	0.023	38865.46	80.00
2	HLA-A0201	SSGCGMSRNNIRQ	SMSRNNIRQ	0	0	0	1	4	SSGCGMSRNNIRQ	tr_C9ZP82	0.023	38875.14	80.00
55	HLA-A0201	QSADQSLYD	QSADQSLYD	0	0	0	0	0	QSADQSLYD	tr_C9ZP82	0.023	38981.29	80.00
53	HLA-A0201	CTQSADQSLYDPDH	SADQSLYDH	3	0	0	7	2	SADQSLYDPDH	tr_C9ZP82	0.023	39039.10	80.00
57	HLA-A0201	ADQSLYDPDH	AQSLYDPDH	0	0	0	1	1	ADQSLYDPDH	tr_C9ZP82	0.022	39246.20	80.00
56	HLA-A0201	SADQSLYDPDH	SADQSLYDH	0	0	0	7	2	SADQSLYDPDH	tr_C9ZP82	0.022	39330.37	80.00
59	HLA-A0201	QSLYDPDHS	QSLYDPDHS	0	0	0	0	0	QSLYDPDHS	tr_C9ZP82	0.022	39349.09	80.00
8	HLA-A0201	SRNNIRQTFVD	SRNNIQTFV	0	0	0	5	1	SRNNIRQTFV	tr_C9ZP82	0.022	39349.52	80.00
27	HLA-A0201	LNFSSRTRC	LNFSSRTRC	0	0	0	0	0	LNFSSRTRC	tr_C9ZP82	0.022	39412.15	80.00
1	HLA-A0201	PSSGCGMSRNNIRQ	GMSRNNIRQ	5	0	0	0	0	GMSRNNIRQ	tr_C9ZP82	0.022	39463.38	80.00
0	HLA-A0201	DPSSGCGMSR	DSSGCGMSR	0	0	0	1	1	DPSSGCGMSR	tr_C9ZP82	0.022	39513.77	80.00
55	HLA-A0201	QSADQSLYDPDH	SADQSLYDH	1	0	0	7	2	SADQSLYDPDH	tr_C9ZP82	0.022	39571.96	80.00
63	HLA-A0201	DPDHSPHIEAVK	DPDPHIEAV	0	0	0	3	2	DPDHSPHIEAV	tr_C9ZP82	0.022	39572.40	80.00
54	HLA-A0201	TQSADQSLYDPDH	SADQSLYDH	2	0	0	7	2	SADQSLYDPDH	tr_C9ZP82	0.021	39714.37	85.00
2	HLA-A0201	SSGCGMSRNNIR	SSGCGMNNI	0	0	0	6	2	SSGCGMSRNNI	tr_C9ZP82	0.021	39811.61	85.00
0	HLA-A0201	DPSSGCGMSRNNIR	SSGCGMNNI	2	0	0	6	2	SSGCGMSRNNI	tr_C9ZP82	0.021	39832.29	85.00
38	HLA-A0201	CRAPFDNRRC	CRAPFDNRC	0	0	0	7	1	CRAPFDNRRC	tr_C9ZP82	0.021	39833.57	85.00
54	HLA-A0201	TQSADQSLYDPD	SADQSLYPD	2	0	0	7	1	SADQSLYDPD	tr_C9ZP82	0.021	40018.91	85.00
1	HLA-A0201	PSSGCGMSRNNIR	SSGCGMNNI	1	0	0	6	2	SSGCGMSRNNI	tr_C9ZP82	0.020	40061.36	85.00
3	HLA-A0201	SGCGMSRNNIRQ	SMSRNNIRQ	0	0	0	1	3	SGCGMSRNNIRQ	tr_C9ZP82	0.020	40256.02	85.00
50	HLA-A0201	VFSCTQSADQ	VFSCTSADQ	0	0	0	5	1	VFSCTQSADQ	tr_C9ZP82	0.020	40281.72	85.00
58	HLA-A0201	DQSLYDPDH	DQSLYDPDH	0	0	0	0	0	DQSLYDPDH	tr_C9ZP82	0.020	40360.70	85.00
58	HLA-A0201	DQSLYDPDHS	DQSLYPDHS	0	0	0	5	1	DQSLYDPDHS	tr_C9ZP82	0.020	40411.82	85.00
61	HLA-A0201	LYDPDHSPHIE	LYDDHSPHI	0	0	0	3	1	LYDPDHSPHI	tr_C9ZP82	0.020	40469.57	85.00
55	HLA-A0201	QSADQSLYDPD	SADQSLYPD	1	0	0	7	1	SADQSLYDPD	tr_C9ZP82	0.020	40477.45	85.00
41	HLA-A0201	PFDNRRCLA	PFDNRRCLA	0	0	0	0	0	PFDNRRCLA	tr_C9ZP82	0.019	40526.11	85.00
5	HLA-A0201	CGMSRNNIR	CGMSRNNIR	0	0	0	0	0	CGMSRNNIR	tr_C9ZP82	0.019	40582.71	85.00
68	HLA-A0201	PHIEAVKLQ	PHIEAVKLQ	0	0	0	0	0	PHIEAVKLQ	tr_C9ZP82	0.019	40665.34	85.00
64	HLA-A0201	PDHSPHIEAVK	DHSPHIEAV	1	0	0	0	0	DHSPHIEAV	tr_C9ZP82	0.019	40674.57	85.00
57	HLA-A0201	ADQSLYDPD	ADQSLYDPD	0	0	0	0	0	ADQSLYDPD	tr_C9ZP82	0.019	40818.73	85.00
57	HLA-A0201	ADQSLYDPDHS	ASLYDPDHS	0	0	0	1	2	ADQSLYDPDHS	tr_C9ZP82	0.019	40839.95	85.00
61	HLA-A0201	LYDPDHSPH	LYDPDHSPH	0	0	0	0	0	LYDPDHSPH	tr_C9ZP82	0.019	40856.29	90.00
2	HLA-A0201	SSGCGMSRN	SSGCGMSRN	0	0	0	0	0	SSGCGMSRN	tr_C9ZP82	0.019	40866.04	90.00
63	HLA-A0201	DPDHSPHIEA	DPDHSPIEA	0	0	0	6	1	DPDHSPHIEA	tr_C9ZP82	0.018	41046.39	90.00
64	HLA-A0201	PDHSPHIEA	PDHSPHIEA	0	0	0	0	0	PDHSPHIEA	tr_C9ZP82	0.018	41142.88	90.00
4	HLA-A0201	GCGMSRNNIRQ	CMSRNNIRQ	1	0	0	1	1	CGMSRNNIRQ	tr_C9ZP82	0.018	41249.84	90.00
39	HLA-A0201	RAPFDNRRC	RAPFDNRRC	0	0	0	0	0	RAPFDNRRC	tr_C9ZP82	0.018	41253.86	90.00
52	HLA-A0201	SCTQSADQS	SCTQSADQS	0	0	0	0	0	SCTQSADQS	tr_C9ZP82	0.018	41269.48	90.00
0	HLA-A0201	DPSSGCGMSRN	SSGCGMSRN	2	0	0	0	0	SSGCGMSRN	tr_C9ZP82	0.017	41484.82	90.00
0	HLA-A0201	DPSSGCGMS	DPSSGCGMS	0	0	0	0	0	DPSSGCGMS	tr_C9ZP82	0.017	41489.77	90.00
0	HLA-A0201	DPSSGCGMSRNN	SSCGMSRNN	2	0	0	2	1	SSGCGMSRNN	tr_C9ZP82	0.017	41680.53	90.00
50	HLA-A0201	VFSCTQSADQS	FSCTQSAQS	1	0	0	7	1	FSCTQSADQS	tr_C9ZP82	0.017	41754.11	90.00

1	HLA-A0201	PSSGCGMSRN	PSSGCGMSR	0	0	0	0	0	PSSGCGMSR	tr_C9ZP82	0.016	41868.58	90.00
4	HLA-A0201	GCGMSRNNIR	GCGMSRNNI	0	0	0	0	0	GCGMSRNNI	tr_C9ZP82	0.016	41921.16	90.00
3	HLA-A0201	SGCGMSRNNIR	SGCGMSNNI	0	0	0	6	1	SGCGMSRNNI	tr_C9ZP82	0.016	42026.05	90.00
50	HLA-A0201	VFSCTQSAD	VFSCTQSAD	0	0	0	0	0	VFSCTQSAD	tr_C9ZP82	0.014	42821.04	95.00
3	HLA-A0201	SGCGMSRNN	SGCGMSRNN	0	0	0	0	0	SGCGMSRNN	tr_C9ZP82	0.014	42865.08	95.00
1	HLA-A0201	PSSGCGMSRNN	PSSGCGMSR	0	0	0	0	0	PSSGCGMSR	tr_C9ZP82	0.013	43257.84	95.00
10	HLA-A0201	NNIRQTFVD	NNIRQTFVD	0	0	0	0	0	NNIRQTFVD	tr_C9ZP82	0.013	43264.39	95.00
63	HLA-A0201	DPDHSPHIE	DPDHSPHIE	0	0	0	0	0	DPDHSPHIE	tr_C9ZP82	0.013	43293.42	95.00
2	HLA-A0201	SSGCGMSRNN	SSCGMSRNN	0	0	0	2	1	SSGCGMSRNN	tr_C9ZP82	0.013	43494.38	95.00

Protein tr_C9ZP82. Allele HLA-A0201. Number of high binders 0. Number of weak binders 2. Number of peptides 405

Link to Allele Frequencies in Worldwide Populations HLA-A0201

Rank Threshold for Strong binding peptides 0.500
Rank Threshold for Weak binding peptides 2.000

pos	HLA	peptide	Core	Offset	I_pos	I_len	D_pos	D_len	iCore	Identity	1-log50k(aff)	Affinity(nM)	%Rank	BindLevel
485	HLA-A0201	YMWDLLTQHVIWPL	YMWDLLWPL	0	0	0	6	5	YMWDLLTQHVIWPL	tr_C9ZJU1	0.889	3.34	0.01	<= SB
485	HLA-A0201	YMWDLLTQHV	YMWDLLTHV	0	0	0	7	1	YMWDLLTQHV	tr_C9ZJU1	0.883	3.54	0.01	<= SB
603	HLA-A0201	FQMADTLPL	FQMADTLPL	0	0	0	0	0	FQMADTLPL	tr_C9ZJU1	0.875	3.86	0.01	<= SB
70	HLA-A0201	MQLPSVFAV	MQLPSVFAV	0	0	0	0	0	MQLPSVFAV	tr_C9ZJU1	0.861	4.52	0.03	<= SB
286	HLA-A0201	ILSDIITSI	ILSDIITSI	0	0	0	0	0	ILSDIITSI	tr_C9ZJU1	0.830	6.26	0.05	<= SB
494	HLA-A0201	VIWPLVPSV	VIWPLVPSV	0	0	0	0	0	VIWPLVPSV	tr_C9ZJU1	0.826	6.61	0.06	<= SB
603	HLA-A0201	FQMADTLPLL	FMADTLPLL	0	0	0	1	1	FQMADTLPLL	tr_C9ZJU1	0.820	7.03	0.06	<= SB
69	HLA-A0201	IMQLPSVFAV	IMLPSVFAV	0	0	0	2	1	IMQLPSVFAV	tr_C9ZJU1	0.812	7.64	0.07	<= SB
186	HLA-A0201	KLMCSSCSI	KLMCSSCSI	0	0	0	0	0	KLMCSSCSI	tr_C9ZJU1	0.808	7.97	0.08	<= SB
484	HLA-A0201	GYMWDLLTQHV	YMWDLLTHV	1	0	0	7	1	YMWDLLTQHV	tr_C9ZJU1	0.806	8.13	0.08	<= SB
604	HLA-A0201	QMADTLPLL	QMADTLPLL	0	0	0	0	0	QMADTLPLL	tr_C9ZJU1	0.796	9.06	0.10	<= SB
370	HLA-A0201	FLYFLNESL	FLYFLNESL	0	0	0	0	0	FLYFLNESL	tr_C9ZJU1	0.794	9.33	0.10	<= SB
483	HLA-A0201	LGYMWDLLTQHV	YMWDLLTHV	2	0	0	7	1	YMWDLLTQHV	tr_C9ZJU1	0.793	9.38	0.10	<= SB
482	HLA-A0201	PLGYMWDLLTQHV	YMWDLLTHV	3	0	0	7	1	YMWDLLTQHV	tr_C9ZJU1	0.789	9.75	0.12	<= SB
481	HLA-A0201	RPLGYMWDLLTQHV	YMWDLLTHV	4	0	0	7	1	YMWDLLTQHV	tr_C9ZJU1	0.787	9.99	0.12	<= SB
270	HLA-A0201	FAFESLYCI	FAFESLYCI	0	0	0	0	0	FAFESLYCI	tr_C9ZJU1	0.754	14.25	0.17	<= SB
485	HLA-A0201	YMWDLLTQHVI	YMWDLLTHV	0	0	0	7	1	YMWDLLTQHV	tr_C9ZJU1	0.753	14.45	0.20	<= SB
249	HLA-A0201	FIIKQLMGL	FIIKQLMGL	0	0	0	0	0	FIIKQLMGL	tr_C9ZJU1	0.752	14.67	0.20	<= SB
687	HLA-A0201	YQVLEGLQSV	YQLEGLQSV	0	0	0	2	1	YQVLEGLQSV	tr_C9ZJU1	0.744	15.94	0.20	<= SB
111	HLA-A0201	ILLDDGVAL	ILLDDGVAL	0	0	0	0	0	ILLDDGVAL	tr_C9ZJU1	0.739	16.89	0.25	<= SB
253	HLA-A0201	QLMGLPATFV	QLMGLPAFV	0	0	0	7	1	QLMGLPATFV	tr_C9ZJU1	0.734	17.71	0.25	<= SB
602	HLA-A0201	LFQMADTLPL	FQMADTLPL	1	0	0	0	0	FQMADTLPL	tr_C9ZJU1	0.730	18.57	0.25	<= SB
484	HLA-A0201	GYMWDLLTQHVI	YMWDLLTHV	1	0	0	7	1	YMWDLLTQHV	tr_C9ZJU1	0.720	20.67	0.30	<= SB
135	HLA-A0201	MLLRGKYSI	MLLRGKYSI	0	0	0	0	0	MLLRGKYSI	tr_C9ZJU1	0.712	22.50	0.30	<= SB
489	HLA-A0201	LLTQHVIWPL	LLTHVIWPL	0	0	0	3	1	LLTQHVIWPL	tr_C9ZJU1	0.707	23.91	0.40	<= SB
483	HLA-A0201	LGYMWDLLTQHVI	YMWDLLTHV	2	0	0	7	1	YMWDLLTQHV	tr_C9ZJU1	0.699	25.90	0.40	<= SB
733	HLA-A0201	YVLRRVSFV	YVLRRVSFV	0	0	0	0	0	YVLRRVSFV	tr_C9ZJU1	0.695	27.04	0.40	<= SB
482	HLA-A0201	PLGYMWDLLTQHVI	YMWDLLTHV	3	0	0	7	1	YMWDLLTQHV	tr_C9ZJU1	0.694	27.54	0.40	<= SB
485	HLA-A0201	YMWDLLTQHVIW	YMWDLLTHV	0	0	0	7	1	YMWDLLTQHV	tr_C9ZJU1	0.692	28.06	0.40	<= SB
936	HLA-A0201	VLNEGMVAEV	VLNEMVAEV	0	0	0	4	1	VLNEGMVAEV	tr_C9ZJU1	0.688	29.11	0.40	<= SB
330	HLA-A0201	VMDELQSRV	VMDELQSRV	0	0	0	0	0	VMDELQSRV	tr_C9ZJU1	0.687	29.51	0.40	<= SB
602	HLA-A0201	LFQMADTLPLL	FMADTLPLL	1	0	0	1	1	FQMADTLPLL	tr_C9ZJU1	0.680	31.77	0.50	<= SB
489	HLA-A0201	LLTQHVIWPLVPSV	LLWPLVPSV	0	0	0	2	5	LLTQHVIWPLVPSV	tr_C9ZJU1	0.678	32.74	0.50	<= SB
603	HLA-A0201	FQMADTLPLLLV	FQMADTLPV	0	0	0	8	3	FQMADTLPLLLV	tr_C9ZJU1	0.674	34.07	0.50	<= SB

485	HLA-A0201	YMWDLLTQHVIWP	YMWDLLTHV	0	0	0	7	1	YMWDLLTQHV	tr_C9ZJU1	0.670	35.44	0.50	<= SB
601	HLA-A0201	GLFQMADTLPLL	FMADTLPLL	2	0	0	1	1	FQMADTLPLL	tr_C9ZJU1	0.670	35.58	0.50	<= SB
91	HLA-A0201	LLVCDIWNCL	LLVCDIWNL	0	0	0	8	1	LLVCDIWNCL	tr_C9ZJU1	0.662	38.88	0.50	<= SB
600	HLA-A0201	HGLFQMADTLPLL	FMADTLPLL	3	0	0	1	1	FQMADTLPLL	tr_C9ZJU1	0.657	40.80	0.60	<= WB
68	HLA-A0201	NIMQLPSVFAV	IMLPSVFAV	1	0	0	2	1	IMQLPSVFAV	tr_C9ZJU1	0.655	41.96	0.60	<= WB
484	HLA-A0201	GYMWDLLTQHVIW	YMWDLLTHV	1	0	0	7	1	YMWDLLTQHV	tr_C9ZJU1	0.652	42.97	0.60	<= WB
599	HLA-A0201	LHGLFQMADTLPLL	FMADTLPLL	4	0	0	1	1	FQMADTLPLL	tr_C9ZJU1	0.652	43.32	0.60	<= WB
243	HLA-A0201	KLSPFRFII	KLSPFRFII	0	0	0	0	0	KLSPFRFII	tr_C9ZJU1	0.650	44.12	0.60	<= WB
322	HLA-A0201	ALWQASGSV	ALWQASGSV	0	0	0	0	0	ALWQASGSV	tr_C9ZJU1	0.650	44.23	0.60	<= WB
688	HLA-A0201	QVLEGLQSV	QVLEGLQSV	0	0	0	0	0	QVLEGLQSV	tr_C9ZJU1	0.645	46.35	0.60	<= WB
67	HLA-A0201	HNIMQLPSVFAV	IMLPSVFAV	2	0	0	2	1	IMQLPSVFAV	tr_C9ZJU1	0.642	48.10	0.60	<= WB
66	HLA-A0201	NHNIMQLPSVFAV	IMLPSVFAV	3	0	0	2	1	IMQLPSVFAV	tr_C9ZJU1	0.636	51.46	0.70	<= WB
65	HLA-A0201	FNHNIMQLPSVFAV	IMLPSVFAV	4	0	0	2	1	IMQLPSVFAV	tr_C9ZJU1	0.634	52.34	0.70	<= WB
281	HLA-A0201	FVIQSILSDIITSI	FLSDIITSI	0	0	0	1	5	FVIQSILSDIITSI	tr_C9ZJU1	0.634	52.64	0.70	<= WB
121	HLA-A0201	ILDDVKAEEV	ILDDVAEEV	0	0	0	5	1	ILDDVKAEEV	tr_C9ZJU1	0.633	53.01	0.70	<= WB
369	HLA-A0201	NFLYFLNESL	FLYFLNESL	1	0	0	0	0	FLYFLNESL	tr_C9ZJU1	0.633	53.15	0.70	<= WB
285	HLA-A0201	SILSDIITSI	ILSDIITSI	1	0	0	0	0	ILSDIITSI	tr_C9ZJU1	0.633	53.24	0.70	<= WB
100	HLA-A0201	LMALVNCSI	LMALVNCSI	0	0	0	0	0	LMALVNCSI	tr_C9ZJU1	0.629	55.29	0.70	<= WB
484	HLA-A0201	GYMWDLLTQHVIWP	YMWDLLTHV	1	0	0	7	1	YMWDLLTQHV	tr_C9ZJU1	0.629	55.47	0.70	<= WB
483	HLA-A0201	LGYMWDLLTQHVIW	YMWDLLTHV	2	0	0	7	1	YMWDLLTQHV	tr_C9ZJU1	0.629	55.52	0.70	<= WB
493	HLA-A0201	HVIWPLVPSV	VIWPLVPSV	1	0	0	0	0	VIWPLVPSV	tr_C9ZJU1	0.628	55.86	0.70	<= WB
468	HLA-A0201	GLVLFSMVFV	GLLFSMVFV	0	0	0	2	1	GLVLFSMVFV	tr_C9ZJU1	0.628	56.15	0.70	<= WB
49	HLA-A0201	FLNCNGVESI	FLNCGVESI	0	0	0	4	1	FLNCNGVESI	tr_C9ZJU1	0.626	57.11	0.70	<= WB
602	HLA-A0201	LFQMADTLPLLLV	FQMADTLPV	1	0	0	8	3	FQMADTLPLLLV	tr_C9ZJU1	0.626	57.30	0.70	<= WB
352	HLA-A0201	FSFPVVSLV	FSFPVVSLV	0	0	0	0	0	FSFPVVSLV	tr_C9ZJU1	0.625	57.89	0.70	<= WB
340	HLA-A0201	ALLEVMHGCRV	ALLEVMHGV	0	0	0	8	2	ALLEVMHGCRV	tr_C9ZJU1	0.625	58.06	0.70	<= WB
558	HLA-A0201	YLQGFAALRL	YLQGFAALL	0	0	0	8	1	YLQGFAALRL	tr_C9ZJU1	0.624	58.40	0.70	<= WB
112	HLA-A0201	LLDDGVALCI	LLDDGVALI	0	0	0	8	1	LLDDGVALCI	tr_C9ZJU1	0.621	60.40	0.70	<= WB
365	HLA-A0201	TQWSNFLYFL	TQWNFLYFL	0	0	0	3	1	TQWSNFLYFL	tr_C9ZJU1	0.620	60.91	0.70	<= WB
340	HLA-A0201	ALLEVMHGCRVV	ALLEVMHGV	0	0	0	8	3	ALLEVMHGCRVV	tr_C9ZJU1	0.619	61.39	0.80	<= WB
185	HLA-A0201	WKLMCSSCSI	WLMCSSCSI	0	0	0	1	1	WKLMCSSCSI	tr_C9ZJU1	0.617	62.87	0.80	<= WB
469	HLA-A0201	LVLFSMVFV	LVLFSMVFV	0	0	0	0	0	LVLFSMVFV	tr_C9ZJU1	0.616	63.91	0.80	<= WB
470	HLA-A0201	VLFSMVFVESGRPL	VLFSMVFPL	0	0	0	7	5	VLFSMVFVESGRPL	tr_C9ZJU1	0.612	66.79	0.80	<= WB
274	HLA-A0201	SLYCIRGFV	SLYCIRGFV	0	0	0	0	0	SLYCIRGFV	tr_C9ZJU1	0.611	67.51	0.80	<= WB
604	HLA-A0201	QMADTLPLLL	QMADTLLLL	0	0	0	6	1	QMADTLPLLL	tr_C9ZJU1	0.608	69.78	0.80	<= WB
186	HLA-A0201	KLMCSSCSIPQLV	KLMCIPQLV	0	0	0	4	4	KLMCSSCSIPQLV	tr_C9ZJU1	0.607	70.61	0.80	<= WB
683	HLA-A0201	SLGCYQVLEGLQSV	SLLEGLQSV	0	0	0	2	5	SLGCYQVLEGLQSV	tr_C9ZJU1	0.606	70.89	0.80	<= WB
601	HLA-A0201	GLFQMADTLPLLLV	FQMADTLPV	2	0	0	8	3	FQMADTLPLLLV	tr_C9ZJU1	0.606	71.06	0.80	<= WB
270	HLA-A0201	FAFESLYCIRGFV	FAFESLYFV	0	0	0	7	4	FAFESLYCIRGFV	tr_C9ZJU1	0.605	71.48	0.80	<= WB
344	HLA-A0201	VMHGCRVVFSFPV	VMHGFSFPV	0	0	0	4	4	VMHGCRVVFSFPV	tr_C9ZJU1	0.603	73.32	0.90	<= WB
184	HLA-A0201	FWKLMCSSCSI	FLMCSSCSI	0	0	0	1	2	FWKLMCSSCSI	tr_C9ZJU1	0.600	75.90	0.90	<= WB
603	HLA-A0201	FQMADTLPLLL	FMADTLPLL	0	0	0	1	1	FQMADTLPLLL	tr_C9ZJU1	0.596	78.85	0.90	<= WB
99	HLA-A0201	CLMALVNCSI	CLMALVCSI	0	0	0	6	1	CLMALVNCSI	tr_C9ZJU1	0.595	80.04	0.90	<= WB
912	HLA-A0201	LLRDSTIIIV	LLDSTIIIV	0	0	0	2	1	LLRDSTIIIV	tr_C9ZJU1	0.590	84.75	0.90	<= WB
370	HLA-A0201	FLYFLNESLYM	FLYNESLYM	0	0	0	3	2	FLYFLNESLYM	tr_C9ZJU1	0.589	85.32	1.00	<= WB
91	HLA-A0201	LLVCDIWNCLMALV	LLVCDIWLV	0	0	0	7	5	LLVCDIWNCLMALV	tr_C9ZJU1	0.585	88.76	1.00	<= WB
91	HLA-A0201	LLVCDIWNCLMAL	LLWNCLMAL	0	0	0	2	4	LLVCDIWNCLMAL	tr_C9ZJU1	0.584	90.39	1.00	<= WB
490	HLA-A0201	LTQHVIWPL	LTQHVIWPL	0	0	0	0	0	LTQHVIWPL	tr_C9ZJU1	0.581	93.26	1.00	<= WB
70	HLA-A0201	MQLPSVFAVH	MQLPSVFAV	0	0	0	0	0	MQLPSVFAV	tr_C9ZJU1	0.580	93.86	1.00	<= WB
214	HLA-A0201	CILGMHAFV	CILGMHAFV	0	0	0	0	0	CILGMHAFV	tr_C9ZJU1	0.578	96.28	1.00	<= WB
570	HLA-A0201	WELMAATAAA	WLMAATAAA	0	0	0	1	1	WELMAATAAA	tr_C9ZJU1	0.578	96.42	1.00	<= WB
420	HLA-A0201	SLTSSPLTL	SLTSSPLTL	0	0	0	0	0	SLTSSPLTL	tr_C9ZJU1	0.568	107.20	1.10	<= WB
185	HLA-A0201	WKLMCSSCSIPQLV	KLMCIPQLV	1	0	0	4	4	KLMCSSCSIPQLV	tr_C9ZJU1	0.566	109.82	1.10	<= WB
269	HLA-A0201	TFAFESLYCIRGFV	FAFESLYFV	1	0	0	7	4	FAFESLYCIRGFV	tr_C9ZJU1	0.565	110.47	1.10	<= WB

601	HLA-A0201	GLFQMADTLPL	GLMADTLPL	0	0	0	2	2	GLFQMADTLPL	tr_C9ZJU1	0.562	114.28	1.10	<= WB
339	HLA-A0201	PALLEVMHGCRV	ALLEVMHGV	1	0	0	8	2	ALLEVMHGCRV	tr_C9ZJU1	0.561	115.35	1.20	<= WB
598	HLA-A0201	VLHGLFQMADTLPL	VLMADTLPL	0	0	0	2	5	VLHGLFQMADTLPL	tr_C9ZJU1	0.560	116.29	1.20	<= WB
17	HLA-A0201	YEILEMLTRA	YILEMLTRA	0	0	0	1	1	YEILEMLTRA	tr_C9ZJU1	0.560	116.73	1.20	<= WB
362	HLA-A0201	WLGTQWSNFLYFL	WLSNFLYFL	0	0	0	2	4	WLGTQWSNFLYFL	tr_C9ZJU1	0.559	117.75	1.20	<= WB
343	HLA-A0201	EVMHGCRVVFSFPV	VMHGFSFPV	1	0	0	4	4	VMHGCRVVFSFPV	tr_C9ZJU1	0.559	117.85	1.20	<= WB
253	HLA-A0201	QLMGLPATFVSMAV	QLMGVSMAV	0	0	0	4	5	QLMGLPATFVSMAV	tr_C9ZJU1	0.559	118.13	1.20	<= WB
369	HLA-A0201	NFLYFLNESLYM	FLYNESLYM	1	0	0	3	2	FLYFLNESLYM	tr_C9ZJU1	0.558	118.84	1.20	<= WB
90	HLA-A0201	HLLVCDIWNCLMAL	LLWNCLMAL	1	0	0	2	4	LLVCDIWNCLMAL	tr_C9ZJU1	0.557	120.82	1.20	<= WB
186	HLA-A0201	KLMCSSCSIPQL	KLMCSIPQL	0	0	0	3	3	KLMCSSCSIPQL	tr_C9ZJU1	0.556	122.39	1.20	<= WB
339	HLA-A0201	PALLEVMHGCRVV	ALLEVMHGV	1	0	0	8	3	ALLEVMHGCRVV	tr_C9ZJU1	0.556	122.59	1.20	<= WB
566	HLA-A0201	RLSGWELMAA	RLSGWLMAA	0	0	0	5	1	RLSGWELMAA	tr_C9ZJU1	0.554	124.54	1.20	<= WB
183	HLA-A0201	SFWKLMCSSCSI	FLMCSSCSI	1	0	0	1	2	FWKLMCSSCSI	tr_C9ZJU1	0.554	124.89	1.20	<= WB
733	HLA-A0201	YVLRRVSFVV	YVLRVSFVV	0	0	0	3	1	YVLRRVSFVV	tr_C9ZJU1	0.552	126.91	1.20	<= WB
196	HLA-A0201	QLVTLAATV	QLVTLAATV	0	0	0	0	0	QLVTLAATV	tr_C9ZJU1	0.546	135.43	1.30	<= WB
252	HLA-A0201	KQLMGLPATFV	QLMGLPAFV	1	0	0	7	1	QLMGLPATFV	tr_C9ZJU1	0.545	137.78	1.30	<= WB
370	HLA-A0201	FLYFLNESLY	FLYFLNESL	0	0	0	0	0	FLYFLNESL	tr_C9ZJU1	0.544	139.51	1.30	<= WB
368	HLA-A0201	SNFLYFLNESLYM	FLYNESLYM	2	0	0	3	2	FLYFLNESLYM	tr_C9ZJU1	0.542	142.41	1.30	<= WB
249	HLA-A0201	FIIKQLMGLPATFV	QLMGLPAFV	4	0	0	7	1	QLMGLPATFV	tr_C9ZJU1	0.541	143.11	1.30	<= WB
602	HLA-A0201	LFQMADTLPLLL	FMADTLPLL	1	0	0	1	1	FQMADTLPLL	tr_C9ZJU1	0.539	146.33	1.40	<= WB
338	HLA-A0201	VPALLEVMHGCRV	ALLEVMHGV	2	0	0	8	2	ALLEVMHGCRV	tr_C9ZJU1	0.539	146.66	1.40	<= WB
367	HLA-A0201	WSNFLYFLNESLYM	FLYNESLYM	3	0	0	3	2	FLYFLNESLYM	tr_C9ZJU1	0.536	151.04	1.40	<= WB
182	HLA-A0201	TSFWKLMCSSCSI	FLMCSSCSI	2	0	0	1	2	FWKLMCSSCSI	tr_C9ZJU1	0.536	151.72	1.40	<= WB
269	HLA-A0201	TFAFESLYCI	FAFESLYCI	1	0	0	0	0	FAFESLYCI	tr_C9ZJU1	0.534	154.05	1.40	<= WB
338	HLA-A0201	VPALLEVMHGCRVV	ALLEVMHGV	2	0	0	8	3	ALLEVMHGCRVV	tr_C9ZJU1	0.533	155.87	1.40	<= WB
337	HLA-A0201	RVPALLEVMHGCRV	ALLEVMHGV	3	0	0	8	2	ALLEVMHGCRV	tr_C9ZJU1	0.532	157.39	1.40	<= WB
112	HLA-A0201	LLDDGVALCILDDV	LLDDGVALV	0	0	0	8	5	LLDDGVALCILDDV	tr_C9ZJU1	0.531	160.64	1.40	<= WB
69	HLA-A0201	IMQLPSVFAVH	IMLPSVFAV	0	0	0	2	1	IMQLPSVFAV	tr_C9ZJU1	0.530	161.37	1.40	<= WB
608	HLA-A0201	TLPLLLVSAV	TLLLLVSAV	0	0	0	2	1	TLPLLLVSAV	tr_C9ZJU1	0.530	161.87	1.40	<= WB
181	HLA-A0201	ITSFWKLMCSSCSI	FLMCSSCSI	3	0	0	1	2	FWKLMCSSCSI	tr_C9ZJU1	0.529	163.80	1.50	<= WB
254	HLA-A0201	LMGLPATFV	LMGLPATFV	0	0	0	0	0	LMGLPATFV	tr_C9ZJU1	0.529	164.08	1.50	<= WB
329	HLA-A0201	SVMDELQSRV	SVMDELQSV	0	0	0	8	1	SVMDELQSRV	tr_C9ZJU1	0.528	164.86	1.50	<= WB
604	HLA-A0201	QMADTLPLLLV	QMADTLPLV	0	0	0	7	2	QMADTLPLLLV	tr_C9ZJU1	0.527	167.52	1.50	<= WB
221	HLA-A0201	FVHWRPIYA	FVHWRPIYA	0	0	0	0	0	FVHWRPIYA	tr_C9ZJU1	0.526	168.87	1.50	<= WB
600	HLA-A0201	HGLFQMADTLPL	GLMADTLPL	1	0	0	2	2	GLFQMADTLPL	tr_C9ZJU1	0.525	170.51	1.50	<= WB
566	HLA-A0201	RLSGWELMA	RLSGWELMA	0	0	0	0	0	RLSGWELMA	tr_C9ZJU1	0.525	170.68	1.50	<= WB
274	HLA-A0201	SLYCIRGFVI	SLYCIGFVI	0	0	0	5	1	SLYCIRGFVI	tr_C9ZJU1	0.525	170.78	1.50	<= WB
603	HLA-A0201	FQMADTLPLLLVS	FQMADTLPV	0	0	0	8	3	FQMADTLPLLLV	tr_C9ZJU1	0.525	170.97	1.50	<= WB
251	HLA-A0201	IKQLMGLPATFV	QLMGLPAFV	2	0	0	7	1	QLMGLPATFV	tr_C9ZJU1	0.524	171.93	1.50	<= WB
821	HLA-A0201	NLWDCSDENI	NLWDCSDEI	0	0	0	8	1	NLWDCSDENI	tr_C9ZJU1	0.524	172.13	1.50	<= WB
373	HLA-A0201	FLNESLYMESQFT	FLNESLYFT	0	0	0	7	4	FLNESLYMESQFT	tr_C9ZJU1	0.524	172.55	1.50	<= WB
204	HLA-A0201	VLFSPEVIL	VLFSPEVIL	0	0	0	0	0	VLFSPEVIL	tr_C9ZJU1	0.523	174.69	1.50	<= WB
202	HLA-A0201	ATVLFSPEV	ATVLFSPEV	0	0	0	0	0	ATVLFSPEV	tr_C9ZJU1	0.523	174.90	1.50	<= WB
601	HLA-A0201	GLFQMADTLPLLL	FMADTLPLL	2	0	0	1	1	FQMADTLPLL	tr_C9ZJU1	0.522	175.96	1.50	<= WB
248	HLA-A0201	RFIIKQLMGL	FIIKQLMGL	1	0	0	0	0	FIIKQLMGL	tr_C9ZJU1	0.522	176.37	1.50	<= WB
110	HLA-A0201	DILLDDGVAL	ILLDDGVAL	1	0	0	0	0	ILLDDGVAL	tr_C9ZJU1	0.520	179.77	1.50	<= WB
572	HLA-A0201	LMAATAAAA	LMAATAAAA	0	0	0	0	0	LMAATAAAA	tr_C9ZJU1	0.519	182.55	1.60	<= WB
263	HLA-A0201	SMAVASTFA	SMAVASTFA	0	0	0	0	0	SMAVASTFA	tr_C9ZJU1	0.519	182.66	1.60	<= WB
250	HLA-A0201	IIKQLMGLPATFV	QLMGLPAFV	3	0	0	7	1	QLMGLPATFV	tr_C9ZJU1	0.518	183.41	1.60	<= WB
611	HLA-A0201	LLLVSAVAL	LLLVSAVAL	0	0	0	0	0	LLLVSAVAL	tr_C9ZJU1	0.517	185.21	1.60	<= WB
601	HLA-A0201	GLFQMADTL	GLFQMADTL	0	0	0	0	0	GLFQMADTL	tr_C9ZJU1	0.516	187.79	1.60	<= WB
603	HLA-A0201	FQMADTLPLLLVSA	FMADTLPLL	0	0	0	1	1	FQMADTLPLL	tr_C9ZJU1	0.516	188.23	1.60	<= WB
199	HLA-A0201	TLAATVLFSPEV	TLAAFSPEV	0	0	0	4	3	TLAATVLFSPEV	tr_C9ZJU1	0.514	192.67	1.60	<= WB
361	HLA-A0201	MWLGTQWSNFLYFL	WLSNFLYFL	1	0	0	2	4	WLGTQWSNFLYFL	tr_C9ZJU1	0.513	194.56	1.60	<= WB

286	HLA-A0201	ILSDIITSIH	ILSDIITSI	0	0	0	0	0	ILSDIITSI	tr_C9ZJU1	0.512	196.66	1.60	<= WB
600	HLA-A0201	HGLFQMADTLPLLL	FMADTLPLL	3	0	0	1	1	FQMADTLPLL	tr_C9ZJU1	0.509	202.25	1.60	<= WB
185	HLA-A0201	WKLMCSSCSIPQL	KLMCSIPQL	1	0	0	3	3	KLMCSSCSIPQL	tr_C9ZJU1	0.509	202.49	1.60	<= WB
599	HLA-A0201	LHGLFQMADTLPL	GLMADTLPL	2	0	0	2	2	GLFQMADTLPL	tr_C9ZJU1	0.508	205.22	1.70	<= WB
373	HLA-A0201	FLNESLYMES	FLNESLYES	0	0	0	7	1	FLNESLYMES	tr_C9ZJU1	0.507	208.30	1.70	<= WB
686	HLA-A0201	CYQVLEGLQSV	YQLEGLQSV	1	0	0	2	1	YQVLEGLQSV	tr_C9ZJU1	0.506	210.67	1.70	<= WB
494	HLA-A0201	VIWPLVPSVF	VIWPLVPSV	0	0	0	0	0	VIWPLVPSV	tr_C9ZJU1	0.505	211.08	1.70	<= WB
340	HLA-A0201	ALLEVMHGC	ALLEVMHGC	0	0	0	0	0	ALLEVMHGC	tr_C9ZJU1	0.504	213.72	1.70	<= WB
213	HLA-A0201	KCILGMHAFV	KILGMHAFV	0	0	0	1	1	KCILGMHAFV	tr_C9ZJU1	0.504	213.95	1.70	<= WB
790	HLA-A0201	MLRRRIANV	MLRRRIANV	0	0	0	0	0	MLRRRIANV	tr_C9ZJU1	0.503	216.79	1.70	<= WB
604	HLA-A0201	QMADTLPLLLVSAV	QMADTLPLV	0	0	0	8	5	QMADTLPLLLVSAV	tr_C9ZJU1	0.502	218.35	1.70	<= WB
8	HLA-A0201	VLLDCERKAYEI	VLLDCAYEI	0	0	0	5	3	VLLDCERKAYEI	tr_C9ZJU1	0.499	226.15	1.70	<= WB
490	HLA-A0201	LTQHVIWPLV	LTQHVIWPV	0	0	0	8	1	LTQHVIWPLV	tr_C9ZJU1	0.495	235.24	1.80	<= WB
295	HLA-A0201	HIDAYLALA	HIDAYLALA	0	0	0	0	0	HIDAYLALA	tr_C9ZJU1	0.495	235.88	1.80	<= WB
159	HLA-A0201	FPEELYLGLITA	FLYLGLITA	0	0	0	1	3	FPEELYLGLITA	tr_C9ZJU1	0.494	239.64	1.80	<= WB
488	HLA-A0201	DLLTQHVIWPL	LLTHVIWPL	1	0	0	3	1	LLTQHVIWPL	tr_C9ZJU1	0.492	243.68	1.80	<= WB
68	HLA-A0201	NIMQLPSVFAVH	IMLPSVFAV	1	0	0	2	1	IMQLPSVFAV	tr_C9ZJU1	0.491	247.47	1.80	<= WB
354	HLA-A0201	FPVVSLVMWL	FVVSLVMWL	0	0	0	1	1	FPVVSLVMWL	tr_C9ZJU1	0.490	249.88	1.90	<= WB
184	HLA-A0201	FWKLMCSSCSIPQL	KLMCSIPQL	2	0	0	3	3	KLMCSSCSIPQL	tr_C9ZJU1	0.488	254.30	1.90	<= WB
211	HLA-A0201	ILKCILGMHAFV	ILLGMHAFV	0	0	0	2	3	ILKCILGMHAFV	tr_C9ZJU1	0.488	254.38	1.90	<= WB
49	HLA-A0201	FLNCNGVESIHV	FLNCNSIHV	0	0	0	5	3	FLNCNGVESIHV	tr_C9ZJU1	0.487	256.69	1.90	<= WB
47	HLA-A0201	AIFLNCNGV	AIFLNCNGV	0	0	0	0	0	AIFLNCNGV	tr_C9ZJU1	0.487	258.49	1.90	<= WB
281	HLA-A0201	FVIQSILSDI	FVIQSILSI	0	0	0	8	1	FVIQSILSDI	tr_C9ZJU1	0.486	259.29	1.90	<= WB
821	HLA-A0201	NLWDCSDENICAAL	NLWDCCAAL	0	0	0	5	5	NLWDCSDENICAAL	tr_C9ZJU1	0.482	270.54	1.90	<= WB
685	HLA-A0201	GCYQVLEGLQSV	YQLEGLQSV	2	0	0	2	1	YQVLEGLQSV	tr_C9ZJU1	0.482	271.94	1.90	<= WB
186	HLA-A0201	KLMCSSCSIP	KLMCSSCSI	0	0	0	0	0	KLMCSSCSI	tr_C9ZJU1	0.480	276.18	2.00	<= WB
94	HLA-A0201	CDIWNCLMAL	CIWNCLMAL	0	0	0	1	1	CDIWNCLMAL	tr_C9ZJU1	0.480	277.59	2.00	<= WB
487	HLA-A0201	WDLLTQHVIWPL	LLTHVIWPL	2	0	0	3	1	LLTQHVIWPL	tr_C9ZJU1	0.478	282.80	2.00	<= WB
789	HLA-A0201	RMLRRRIANV	RMLRRIANV	0	0	0	3	1	RMLRRRIANV	tr_C9ZJU1	0.477	286.96	2.00	<= WB
67	HLA-A0201	HNIMQLPSVFAVH	IMLPSVFAV	2	0	0	2	1	IMQLPSVFAV	tr_C9ZJU1	0.476	288.71	2.00	<= WB
372	HLA-A0201	YFLNESLYMESQFT	FLNESLYFT	1	0	0	7	4	FLNESLYMESQFT	tr_C9ZJU1	0.476	289.12	2.00	<= WB
286	HLA-A0201	ILSDIITSIHI	ILSDIIIHI	0	0	0	6	2	ILSDIITSIHI	tr_C9ZJU1	0.476	291.47	2.00	<= WB
112	HLA-A0201	LLDDGVALC	LLDDGVALC	0	0	0	0	0	LLDDGVALC	tr_C9ZJU1	0.474	295.18	2.00	<= WB
684	HLA-A0201	LGCYQVLEGLQSV	YQLEGLQSV	3	0	0	2	1	YQVLEGLQSV	tr_C9ZJU1	0.474	295.58	2.00	<= WB
490	HLA-A0201	LTQHVIWPLVPSV	LIWPLVPSV	0	0	0	1	4	LTQHVIWPLVPSV	tr_C9ZJU1	0.474	295.68	2.00	<= WB
486	HLA-A0201	MWDLLTQHVIWPL	LLTHVIWPL	3	0	0	3	1	LLTQHVIWPL	tr_C9ZJU1	0.472	301.81	2.50	
911	HLA-A0201	NLLRDSTIII	NLLDSTIII	0	0	0	3	1	NLLRDSTIII	tr_C9ZJU1	0.472	304.11	2.50	
66	HLA-A0201	NHNIMQLPSVFAVH	IMLPSVFAV	3	0	0	2	1	IMQLPSVFAV	tr_C9ZJU1	0.470	310.94	2.50	
602	HLA-A0201	LFQMADTLPLLLVS	FQMADTLPV	1	0	0	8	3	FQMADTLPLLLV	tr_C9ZJU1	0.469	311.95	2.50	
612	HLA-A0201	LLVSAVALRL	LLVSAVALL	0	0	0	8	1	LLVSAVALRL	tr_C9ZJU1	0.469	312.20	2.50	
69	HLA-A0201	IMQLPSVFAVHC	IMLPSVFAV	0	0	0	2	1	IMQLPSVFAV	tr_C9ZJU1	0.468	315.50	2.50	
387	HLA-A0201	SQLASVTNI	SQLASVTNI	0	0	0	0	0	SQLASVTNI	tr_C9ZJU1	0.464	330.04	2.50	
198	HLA-A0201	VTLAATVLFSPEV	TLAAFSPEV	1	0	0	4	3	TLAATVLFSPEV	tr_C9ZJU1	0.463	332.55	2.50	
158	HLA-A0201	EFPEELYLGLITA	FLYLGLITA	1	0	0	1	3	FPEELYLGLITA	tr_C9ZJU1	0.462	336.95	2.50	
270	HLA-A0201	FAFESLYCIRGFVI	FAFESLYFV	0	0	0	7	4	FAFESLYCIRGFV	tr_C9ZJU1	0.461	339.95	2.50	
164	HLA-A0201	YLGLITAYNRKRSL	YLGLITAYL	0	0	0	8	5	YLGLITAYNRKRSL	tr_C9ZJU1	0.461	340.22	2.50	
368	HLA-A0201	SNFLYFLNESL	FLYFLNESL	2	0	0	0	0	FLYFLNESL	tr_C9ZJU1	0.461	342.79	2.50	
571	HLA-A0201	ELMAATAAA	ELMAATAAA	0	0	0	0	0	ELMAATAAA	tr_C9ZJU1	0.460	344.47	2.50	
313	HLA-A0201	WLLSRCVSQA	WLLSRCVSA	0	0	0	8	1	WLLSRCVSQA	tr_C9ZJU1	0.458	351.23	2.50	
340	HLA-A0201	ALLEVMHGCRVVF	ALLEVMHGV	0	0	0	8	3	ALLEVMHGCRVV	tr_C9ZJU1	0.458	352.80	2.50	
734	HLA-A0201	VLRRVSFVV	VLRRVSFVV	0	0	0	0	0	VLRRVSFVV	tr_C9ZJU1	0.456	358.11	2.50	
367	HLA-A0201	WSNFLYFLNESL	FLYFLNESL	3	0	0	0	0	FLYFLNESL	tr_C9ZJU1	0.455	362.23	2.50	
365	HLA-A0201	TQWSNFLYFLNESL	FLYFLNESL	5	0	0	0	0	FLYFLNESL	tr_C9ZJU1	0.454	366.92	2.50	
935	HLA-A0201	VVLNEGMVAEV	VLNEMVAEV	1	0	0	4	1	VLNEGMVAEV	tr_C9ZJU1	0.454	369.27	2.50	

235	HLA-A0201	SLNGDVSPKL	SLNDVSPKL	0	0	0	3	1	SLNGDVSPKL	tr_C9ZJU1	0.453	370.72	2.50
91	HLA-A0201	LLVCDIWNC	LLVCDIWNC	0	0	0	0	0	LLVCDIWNC	tr_C9ZJU1	0.453	371.27	2.50
366	HLA-A0201	QWSNFLYFLNESL	FLYFLNESL	4	0	0	0	0	FLYFLNESL	tr_C9ZJU1	0.452	374.49	2.50
121	HLA-A0201	ILDDVKAEEVLAL	ILDDVVLAL	0	0	0	5	4	ILDDVKAEEVLAL	tr_C9ZJU1	0.452	376.31	2.50
420	HLA-A0201	SLTSSPLTLL	SLTSSLTLL	0	0	0	5	1	SLTSSPLTLL	tr_C9ZJU1	0.451	378.21	2.50
81	HLA-A0201	GLMKNSHPDHLLV	GLMPDHLLV	0	0	0	3	4	GLMKNSHPDHLLV	tr_C9ZJU1	0.451	379.66	2.50
370	HLA-A0201	FLYFLNESLYME	FLYFLNLYM	0	0	0	6	2	FLYFLNESLYM	tr_C9ZJU1	0.451	381.06	2.50
157	HLA-A0201	QEFPEELYLGLITA	FLYLGLITA	2	0	0	1	3	FPEELYLGLITA	tr_C9ZJU1	0.450	386.09	2.50
210	HLA-A0201	VILKCILGMHAFV	ILLGMHAFV	1	0	0	2	3	ILKCILGMHAFV	tr_C9ZJU1	0.448	391.12	2.50
221	HLA-A0201	FVHWRPIYAA	FVWRPIYAA	0	0	0	2	1	FVHWRPIYAA	tr_C9ZJU1	0.448	391.69	2.50
7	HLA-A0201	AVLLDCERKAYEI	VLLDCAYEI	1	0	0	5	3	VLLDCERKAYEI	tr_C9ZJU1	0.448	393.86	2.50
321	HLA-A0201	QALWQASGSV	ALWQASGSV	1	0	0	0	0	ALWQASGSV	tr_C9ZJU1	0.447	398.50	2.50
470	HLA-A0201	VLFSMVFVES	VLFSMVFES	0	0	0	7	1	VLFSMVFVES	tr_C9ZJU1	0.446	400.31	2.50
15	HLA-A0201	KAYEILEML	KAYEILEML	0	0	0	0	0	KAYEILEML	tr_C9ZJU1	0.445	403.57	2.50
272	HLA-A0201	FESLYCIRGFV	FLYCIRGFV	0	0	0	1	2	FESLYCIRGFV	tr_C9ZJU1	0.445	404.16	2.50
362	HLA-A0201	WLGTQWSNFL	WLGTWSNFL	0	0	0	4	1	WLGTQWSNFL	tr_C9ZJU1	0.445	405.47	2.50
74	HLA-A0201	SVFAVHCGL	SVFAVHCGL	0	0	0	0	0	SVFAVHCGL	tr_C9ZJU1	0.443	413.42	2.50
283	HLA-A0201	IQSILSDIITSI	ILSDIITSI	0	0	0	1	3	IQSILSDIITSI	tr_C9ZJU1	0.443	416.04	2.50
69	HLA-A0201	IMQLPSVFAVHCG	IMLPSVFAV	0	0	0	2	1	IMQLPSVFAV	tr_C9ZJU1	0.442	420.63	2.50
256	HLA-A0201	GLPATFVSM	GLPATFVSM	0	0	0	0	0	GLPATFVSM	tr_C9ZJU1	0.441	423.91	2.50
344	HLA-A0201	VMHGCRVVFSFPVV	VMHGFSFPV	0	0	0	4	4	VMHGCRVVFSFPV	tr_C9ZJU1	0.441	425.04	2.50
48	HLA-A0201	IFLNCNGVESIHV	FLNCNSIHV	1	0	0	5	3	FLNCNGVESIHV	tr_C9ZJU1	0.441	425.39	2.50
134	HLA-A0201	CMLLRGKYSI	MLLRGKYSI	1	0	0	0	0	MLLRGKYSI	tr_C9ZJU1	0.440	425.68	2.50
866	HLA-A0201	RLCIARAFV	RLCIARAFV	0	0	0	0	0	RLCIARAFV	tr_C9ZJU1	0.440	428.70	2.50
351	HLA-A0201	VFSFPVVSLV	FSFPVVSLV	1	0	0	0	0	FSFPVVSLV	tr_C9ZJU1	0.438	436.51	2.50
273	HLA-A0201	ESLYCIRGFV	SLYCIRGFV	1	0	0	0	0	SLYCIRGFV	tr_C9ZJU1	0.438	437.21	2.50
833	HLA-A0201	ALSASRALNFV	ALSASLNFV	0	0	0	5	2	ALSASRALNFV	tr_C9ZJU1	0.438	439.04	2.50
384	HLA-A0201	FTKSQLASV	FTKSQLASV	0	0	0	0	0	FTKSQLASV	tr_C9ZJU1	0.437	440.86	2.50
69	HLA-A0201	IMQLPSVFAVHCGL	IMLPSVFAV	0	0	0	2	1	IMQLPSVFAV	tr_C9ZJU1	0.437	442.03	2.50
197	HLA-A0201	LVTLAATVLFSPEV	TLAAFSPEV	2	0	0	4	3	TLAATVLFSPEV	tr_C9ZJU1	0.437	442.21	2.50
253	HLA-A0201	QLMGLPATFVS	QLMGLPAFV	0	0	0	7	1	QLMGLPATFV	tr_C9ZJU1	0.436	445.65	3.00
377	HLA-A0201	SLYMESQFT	SLYMESQFT	0	0	0	0	0	SLYMESQFT	tr_C9ZJU1	0.436	445.98	3.00
255	HLA-A0201	MGLPATFVSM	MLPATFVSM	0	0	0	1	1	MGLPATFVSM	tr_C9ZJU1	0.436	448.38	3.00
769	HLA-A0201	YAPTEGDIFV	YATEGDIFV	0	0	0	2	1	YAPTEGDIFV	tr_C9ZJU1	0.436	448.97	3.00
186	HLA-A0201	KLMCSSCSIPQLVT	KLMCIPQLV	0	0	0	4	4	KLMCSSCSIPQLV	tr_C9ZJU1	0.435	449.63	3.00
476	HLA-A0201	FVESGRPLGYMWDL	FVLGYMWDL	0	0	0	2	5	FVESGRPLGYMWDL	tr_C9ZJU1	0.435	451.43	3.00
465	HLA-A0201	VQYGLVLFSM	VQYGVLFSM	0	0	0	4	1	VQYGLVLFSM	tr_C9ZJU1	0.435	452.10	3.00
285	HLA-A0201	SILSDIITSIHI	ILSDIIIHI	1	0	0	6	2	ILSDIITSIHI	tr_C9ZJU1	0.433	462.36	3.00
184	HLA-A0201	FWKLMCSSCSIP	FLMCSSCSI	0	0	0	1	2	FWKLMCSSCSI	tr_C9ZJU1	0.432	464.66	3.00
210	HLA-A0201	VILKCILGM	VILKCILGM	0	0	0	0	0	VILKCILGM	tr_C9ZJU1	0.432	467.46	3.00
209	HLA-A0201	EVILKCILGMHAFV	ILLGMHAFV	2	0	0	2	3	ILKCILGMHAFV	tr_C9ZJU1	0.431	470.25	3.00
934	HLA-A0201	IVVLNEGMVAEV	VLNEMVAEV	2	0	0	4	1	VLNEGMVAEV	tr_C9ZJU1	0.431	470.70	3.00
494	HLA-A0201	VIWPLVPSVFSGCV	VIWPLVPSV	0	0	0	8	5	VIWPLVPSVFSGCV	tr_C9ZJU1	0.430	476.46	3.00
68	HLA-A0201	NIMQLPSVFAVHC	IMLPSVFAV	1	0	0	2	1	IMQLPSVFAV	tr_C9ZJU1	0.430	477.99	3.00
314	HLA-A0201	LLSRCVSQAL	LLSCVSQAL	0	0	0	3	1	LLSRCVSQAL	tr_C9ZJU1	0.429	483.52	3.00
270	HLA-A0201	FAFESLYCIR	FAFESLYCI	0	0	0	0	0	FAFESLYCI	tr_C9ZJU1	0.427	491.21	3.00
111	HLA-A0201	ILLDDGVALCI	ILLDVALCI	0	0	0	4	2	ILLDDGVALCI	tr_C9ZJU1	0.427	491.69	3.00
821	HLA-A0201	NLWDCSDENICAA	NLWDCICAA	0	0	0	5	4	NLWDCSDENICAA	tr_C9ZJU1	0.426	498.64	3.00
598	HLA-A0201	VLHGLFQMA	VLHGLFQMA	0	0	0	0	0	VLHGLFQMA	tr_C9ZJU1	0.426	500.40	3.00
360	HLA-A0201	VMWLGTQWSNFL	VMWQWSNFL	0	0	0	3	3	VMWLGTQWSNFL	tr_C9ZJU1	0.425	502.44	3.00
933	HLA-A0201	KIVVLNEGMVAEV	VLNEMVAEV	3	0	0	4	1	VLNEGMVAEV	tr_C9ZJU1	0.425	504.74	3.00
62	HLA-A0201	TLVFNHNIMQLPSV	TLIMQLPSV	0	0	0	2	5	TLVFNHNIMQLPSV	tr_C9ZJU1	0.424	506.97	3.00
608	HLA-A0201	TLPLLLVSA	TLPLLLVSA	0	0	0	0	0	TLPLLLVSA	tr_C9ZJU1	0.424	510.39	3.00
492	HLA-A0201	QHVIWPLVPSV	VIWPLVPSV	2	0	0	0	0	VIWPLVPSV	tr_C9ZJU1	0.424	510.92	3.00

369	HLA-A0201	NFLYFLNESLYME	FLYFLNLYM	1	0	0	6	2	FLYFLNESLYM	tr_C9ZJU1	0.423	516.21	3.00
408	HLA-A0201	QVSPTCSFPV	QVSPCSFPV	0	0	0	4	1	QVSPTCSFPV	tr_C9ZJU1	0.423	517.10	3.00
314	HLA-A0201	LLSRCVSQA	LLSRCVSQA	0	0	0	0	0	LLSRCVSQA	tr_C9ZJU1	0.422	520.80	3.00
350	HLA-A0201	VVFSFPVVSL	VVFSFVVSL	0	0	0	5	1	VVFSFPVVSL	tr_C9ZJU1	0.422	521.63	3.00
932	HLA-A0201	DKIVVLNEGMVAEV	VLNEMVAEV	4	0	0	4	1	VLNEGMVAEV	tr_C9ZJU1	0.421	526.23	3.00
120	HLA-A0201	CILDDVKAEEVLAL	ILDDVVLAL	1	0	0	5	4	ILDDVKAEEVLAL	tr_C9ZJU1	0.420	529.77	3.00
491	HLA-A0201	TQHVIWPLVPSV	VIWPLVPSV	3	0	0	0	0	VIWPLVPSV	tr_C9ZJU1	0.420	529.79	3.00
90	HLA-A0201	HLLVCDIWNCL	LLVCDIWNL	1	0	0	8	1	LLVCDIWNCL	tr_C9ZJU1	0.420	531.31	3.00
284	HLA-A0201	QSILSDIITSI	ILSDIITSI	2	0	0	0	0	ILSDIITSI	tr_C9ZJU1	0.420	534.09	3.00
47	HLA-A0201	AIFLNCNGVESIHV	FLNCNSIHV	2	0	0	5	3	FLNCNGVESIHV	tr_C9ZJU1	0.419	535.50	3.00
379	HLA-A0201	YMESQFTKSQLASV	YMESQLASV	0	0	0	3	5	YMESQFTKSQLASV	tr_C9ZJU1	0.419	537.51	3.00
330	HLA-A0201	VMDELQSRVPAL	VMDELVPAL	0	0	0	5	3	VMDELQSRVPAL	tr_C9ZJU1	0.418	545.03	3.00
912	HLA-A0201	LLRDSTIII	LLRDSTIII	0	0	0	0	0	LLRDSTIII	tr_C9ZJU1	0.418	545.93	3.00
282	HLA-A0201	VIQSILSDIITSI	ILSDIITSI	1	0	0	1	3	IQSILSDIITSI	tr_C9ZJU1	0.417	546.99	3.00
67	HLA-A0201	HNIMQLPSVFAVHC	IMLPSVFAV	2	0	0	2	1	IMQLPSVFAV	tr_C9ZJU1	0.416	552.71	3.00
284	HLA-A0201	QSILSDIITSIHI	ILSDIIIHI	2	0	0	6	2	ILSDIITSIHI	tr_C9ZJU1	0.416	553.82	3.00
252	HLA-A0201	KQLMGLPAT	KQLMGLPAT	0	0	0	0	0	KQLMGLPAT	tr_C9ZJU1	0.416	554.02	3.00
120	HLA-A0201	CILDDVKAEEV	ILDDVAEEV	1	0	0	5	1	ILDDVKAEEV	tr_C9ZJU1	0.415	559.57	3.00
102	HLA-A0201	ALVNCSIRDI	ALVNCSIRI	0	0	0	8	1	ALVNCSIRDI	tr_C9ZJU1	0.415	563.32	3.00
92	HLA-A0201	LVCDIWNCLMAL	LIWNCLMAL	0	0	0	1	3	LVCDIWNCLMAL	tr_C9ZJU1	0.414	564.89	3.00
920	HLA-A0201	IVAHRLATL	IVAHRLATL	0	0	0	0	0	IVAHRLATL	tr_C9ZJU1	0.414	568.51	3.00
111	HLA-A0201	ILLDDGVALC	ILLDDGVAL	0	0	0	0	0	ILLDDGVAL	tr_C9ZJU1	0.413	572.66	3.00
653	HLA-A0201	FLGSFYDEC	FLGSFYDEC	0	0	0	0	0	FLGSFYDEC	tr_C9ZJU1	0.412	576.96	3.00
409	HLA-A0201	VSPTCSFPV	VSPTCSFPV	0	0	0	0	0	VSPTCSFPV	tr_C9ZJU1	0.411	582.72	3.00
80	HLA-A0201	CGLMKNSHPDHLLV	GLMPDHLLV	1	0	0	3	4	GLMKNSHPDHLLV	tr_C9ZJU1	0.411	583.69	3.00
112	HLA-A0201	LLDDGVALCIL	LLDDGVALL	0	0	0	8	2	LLDDGVALCIL	tr_C9ZJU1	0.411	586.50	3.00
557	HLA-A0201	DYLQGFAALRL	YLQGFAALL	1	0	0	8	1	YLQGFAALRL	tr_C9ZJU1	0.411	587.08	3.00
283	HLA-A0201	IQSILSDIITSIHI	ILSDIIIHI	3	0	0	6	2	ILSDIITSIHI	tr_C9ZJU1	0.410	591.46	3.00
370	HLA-A0201	FLYFLNESLYMES	FLYFLNLYM	0	0	0	6	2	FLYFLNESLYM	tr_C9ZJU1	0.410	591.58	3.00
274	HLA-A0201	SLYCIRGFVIQSI	SLYCVIQSI	0	0	0	4	4	SLYCIRGFVIQSI	tr_C9ZJU1	0.409	595.91	3.00
89	HLA-A0201	DHLLVCDIWNCL	LLVCDIWNL	2	0	0	8	1	LLVCDIWNCL	tr_C9ZJU1	0.409	596.95	3.00
368	HLA-A0201	SNFLYFLNESLYME	FLYFLNLYM	2	0	0	6	2	FLYFLNESLYM	tr_C9ZJU1	0.408	602.32	3.00
565	HLA-A0201	LRLSGWELMA	LLSGWELMA	0	0	0	1	1	LRLSGWELMA	tr_C9ZJU1	0.408	603.14	3.00
6	HLA-A0201	PAVLLDCERKAYEI	VLLDCAYEI	2	0	0	5	3	VLLDCERKAYEI	tr_C9ZJU1	0.408	604.87	3.00
271	HLA-A0201	AFESLYCIRGFV	FLYCIRGFV	1	0	0	1	2	FESLYCIRGFV	tr_C9ZJU1	0.407	611.18	3.00
732	HLA-A0201	PYVLRRVSFV	YVLRRVSFV	1	0	0	0	0	YVLRRVSFV	tr_C9ZJU1	0.407	612.19	3.00
362	HLA-A0201	WLGTQWSNFLYFLN	WLSNFLYFL	0	0	0	2	4	WLGTQWSNFLYFL	tr_C9ZJU1	0.406	621.59	3.50
618	HLA-A0201	ALRLSPNFFT	ALLSPNFFT	0	0	0	2	1	ALRLSPNFFT	tr_C9ZJU1	0.405	625.27	3.50
313	HLA-A0201	WLLSRCVSQALWQA	WLLSALWQA	0	0	0	4	5	WLLSRCVSQALWQA	tr_C9ZJU1	0.405	625.92	3.50
68	HLA-A0201	NIMQLPSVFAVHCG	IMLPSVFAV	1	0	0	2	1	IMQLPSVFAV	tr_C9ZJU1	0.404	629.91	3.50
88	HLA-A0201	PDHLLVCDIWNCL	LLVCDIWNL	3	0	0	8	1	LLVCDIWNCL	tr_C9ZJU1	0.403	637.84	3.50
242	HLA-A0201	PKLSPFRFII	KLSPFRFII	1	0	0	0	0	KLSPFRFII	tr_C9ZJU1	0.403	640.16	3.50
256	HLA-A0201	GLPATFVSMA	GLATFVSMA	0	0	0	2	1	GLPATFVSMA	tr_C9ZJU1	0.402	647.38	3.50
340	HLA-A0201	ALLEVMHGCRVVFS	ALLEVMHGV	0	0	0	8	3	ALLEVMHGCRVV	tr_C9ZJU1	0.400	659.83	3.50
111	HLA-A0201	ILLDDGVALCIL	ILLDDLCIL	0	0	0	5	3	ILLDDGVALCIL	tr_C9ZJU1	0.400	660.11	3.50
467	HLA-A0201	YGLVLFSMVFV	GLLFSMVFV	1	0	0	2	1	GLVLFSMVFV	tr_C9ZJU1	0.400	660.50	3.50
87	HLA-A0201	HPDHLLVCDIWNCL	LLVCDIWNL	4	0	0	8	1	LLVCDIWNCL	tr_C9ZJU1	0.399	663.53	3.50
249	HLA-A0201	FIIKQLMGLP	FIIKQLMGL	0	0	0	0	0	FIIKQLMGL	tr_C9ZJU1	0.399	664.20	3.50
110	HLA-A0201	DILLDDGVALCI	ILLDVALCI	1	0	0	4	2	ILLDDGVALCI	tr_C9ZJU1	0.399	664.70	3.50
322	HLA-A0201	ALWQASGSVMDEL	ALWSVMDEL	0	0	0	3	4	ALWQASGSVMDEL	tr_C9ZJU1	0.399	666.30	3.50
19	HLA-A0201	ILEMLTRATI	ILMLTRATI	0	0	0	2	1	ILEMLTRATI	tr_C9ZJU1	0.398	673.36	3.50
556	HLA-A0201	QDYLQGFAALRL	YLQGFAALL	2	0	0	8	1	YLQGFAALRL	tr_C9ZJU1	0.397	681.34	3.50
489	HLA-A0201	LLTQHVIWPLV	LLTHVIWPL	0	0	0	3	1	LLTQHVIWPL	tr_C9ZJU1	0.397	681.50	3.50
465	HLA-A0201	VQYGLVLFSMVFV	VQYGLVLFV	0	0	0	7	4	VQYGLVLFSMVFV	tr_C9ZJU1	0.397	682.49	3.50

562	HLA-A0201	FAALRLSGWELMA	FLSGWELMA	0	0	0	1	4	FAALRLSGWELMA	tr_C9ZJU1	0.397	683.43	3.50
253	HLA-A0201	QLMGLPATFVSM	QLMGLPAFV	0	0	0	7	1	QLMGLPATFV	tr_C9ZJU1	0.396	688.71	3.50
905	HLA-A0201	RLREGNNLL	RLREGNNLL	0	0	0	0	0	RLREGNNLL	tr_C9ZJU1	0.395	692.88	3.50
339	HLA-A0201	PALLEVMHGCRVVF	ALLEVMHGV	1	0	0	8	3	ALLEVMHGCRVV	tr_C9ZJU1	0.395	693.36	3.50
364	HLA-A0201	GTQWSNFLYFL	TQWNFLYFL	1	0	0	3	1	TQWSNFLYFL	tr_C9ZJU1	0.395	695.06	3.50
252	HLA-A0201	KQLMGLPATFVS	QLMGLPAFV	1	0	0	7	1	QLMGLPATFV	tr_C9ZJU1	0.395	699.40	3.50
294	HLA-A0201	IHIDAYLALA	IIDAYLALA	0	0	0	1	1	IHIDAYLALA	tr_C9ZJU1	0.394	701.60	3.50
564	HLA-A0201	ALRLSGWELMAAT	ALWELMAAT	0	0	0	2	4	ALRLSGWELMAAT	tr_C9ZJU1	0.394	704.01	3.50
349	HLA-A0201	RVVFSFPVV	RVVFSFPVV	0	0	0	0	0	RVVFSFPVV	tr_C9ZJU1	0.394	706.88	3.50
883	HLA-A0201	CIFDEPTSA	CIFDEPTSA	0	0	0	0	0	CIFDEPTSA	tr_C9ZJU1	0.393	708.46	3.50
63	HLA-A0201	LVFNHNIMQL	LVFNHIMQL	0	0	0	5	1	LVFNHNIMQL	tr_C9ZJU1	0.393	710.27	3.50
941	HLA-A0201	MVAEVGSWET	MVAEVSWET	0	0	0	5	1	MVAEVGSWET	tr_C9ZJU1	0.393	714.56	3.50
555	HLA-A0201	RQDYLQGFAALRL	YLQGFAALL	3	0	0	8	1	YLQGFAALRL	tr_C9ZJU1	0.392	719.12	3.50
98	HLA-A0201	NCLMALVNCSI	CLMALVCSI	1	0	0	6	1	CLMALVNCSI	tr_C9ZJU1	0.392	720.00	3.50
911	HLA-A0201	NLLRDSTIIIV	LLDSTIIIV	1	0	0	2	1	LLRDSTIIIV	tr_C9ZJU1	0.392	721.41	3.50
119	HLA-A0201	LCILDDVKAEEV	ILDDVAEEV	2	0	0	5	1	ILDDVKAEEV	tr_C9ZJU1	0.392	722.06	3.50
491	HLA-A0201	TQHVIWPLV	TQHVIWPLV	0	0	0	0	0	TQHVIWPLV	tr_C9ZJU1	0.391	728.84	3.50
832	HLA-A0201	AALSASRALNFV	ALSASLNFV	1	0	0	5	2	ALSASRALNFV	tr_C9ZJU1	0.390	737.27	3.50
554	HLA-A0201	CRQDYLQGFAALRL	YLQGFAALL	4	0	0	8	1	YLQGFAALRL	tr_C9ZJU1	0.389	742.64	3.50
358	HLA-A0201	SLVMWLGTQWSNFL	SLVMWLNFL	0	0	0	6	5	SLVMWLGTQWSNFL	tr_C9ZJU1	0.389	744.53	3.50
293	HLA-A0201	SIHIDAYLAL	SIIDAYLAL	0	0	0	2	1	SIHIDAYLAL	tr_C9ZJU1	0.388	748.31	3.50
48	HLA-A0201	IFLNCNGVESI	FLNCGVESI	1	0	0	4	1	FLNCNGVESI	tr_C9ZJU1	0.388	751.45	3.50
118	HLA-A0201	ALCILDDVKAEEV	ILDDVAEEV	3	0	0	5	1	ILDDVKAEEV	tr_C9ZJU1	0.387	756.89	3.50
370	HLA-A0201	FLYFLNESLYMESQ	FLYFLNLYM	0	0	0	6	2	FLYFLNESLYM	tr_C9ZJU1	0.387	757.71	3.50
109	HLA-A0201	RDILLDDGVALCI	ILLDVALCI	2	0	0	4	2	ILLDDGVALCI	tr_C9ZJU1	0.387	758.94	3.50
820	HLA-A0201	GNLWDCSDENICAA	NLWDCICAA	1	0	0	5	4	NLWDCSDENICAA	tr_C9ZJU1	0.385	773.03	3.50
804	HLA-A0201	NLRFIEDLSI	NLFIEDLSI	0	0	0	2	1	NLRFIEDLSI	tr_C9ZJU1	0.385	777.50	3.50
8	HLA-A0201	VLLDCERKA	VLLDCERKA	0	0	0	0	0	VLLDCERKA	tr_C9ZJU1	0.385	778.92	3.50
415	HLA-A0201	FPVKNSLTSSPLTL	FLTSSPLTL	0	0	0	1	5	FPVKNSLTSSPLTL	tr_C9ZJU1	0.384	781.13	3.50
183	HLA-A0201	SFWKLMCSSCSIP	FLMCSSCSI	1	0	0	1	2	FWKLMCSSCSI	tr_C9ZJU1	0.384	785.40	3.50
93	HLA-A0201	VCDIWNCLMAL	VIWNCLMAL	0	0	0	1	2	VCDIWNCLMAL	tr_C9ZJU1	0.383	790.83	3.50
117	HLA-A0201	VALCILDDVKAEEV	ILDDVAEEV	4	0	0	5	1	ILDDVKAEEV	tr_C9ZJU1	0.383	792.40	3.50
159	HLA-A0201	FPEELYLGLITAY	FLYLGLITA	0	0	0	1	3	FPEELYLGLITA	tr_C9ZJU1	0.383	793.88	3.50
369	HLA-A0201	NFLYFLNESLYMES	FLYFLNLYM	1	0	0	6	2	FLYFLNESLYM	tr_C9ZJU1	0.383	794.01	3.50
195	HLA-A0201	PQLVTLAATV	QLVTLAATV	1	0	0	0	0	QLVTLAATV	tr_C9ZJU1	0.382	798.02	3.50
108	HLA-A0201	IRDILLDDGVALCI	ILLDVALCI	3	0	0	4	2	ILLDDGVALCI	tr_C9ZJU1	0.382	798.50	3.50
566	HLA-A0201	RLSGWELMAAT	RLWELMAAT	0	0	0	2	2	RLSGWELMAAT	tr_C9ZJU1	0.382	802.18	3.50
388	HLA-A0201	QLASVTNIA	QLASVTNIA	0	0	0	0	0	QLASVTNIA	tr_C9ZJU1	0.381	812.31	3.50
359	HLA-A0201	LVMWLGTQWSNFL	VMWLWSNFL	1	0	0	4	3	VMWLGTQWSNFL	tr_C9ZJU1	0.380	821.36	3.50
132	HLA-A0201	ALCMLLRGKYSI	ALCMLLYSI	0	0	0	6	3	ALCMLLRGKYSI	tr_C9ZJU1	0.380	823.14	4.00
97	HLA-A0201	WNCLMALVNCSI	CLMALVCSI	2	0	0	6	1	CLMALVNCSI	tr_C9ZJU1	0.380	823.22	4.00
740	HLA-A0201	FVVNKGEFL	FVVNKGEFL	0	0	0	0	0	FVVNKGEFL	tr_C9ZJU1	0.379	826.11	4.00
466	HLA-A0201	QYGLVLFSMVFV	GLLFSMVFV	2	0	0	2	1	GLVLFSMVFV	tr_C9ZJU1	0.379	826.54	4.00
910	HLA-A0201	NNLLRDSTIIIV	LLDSTIIIV	2	0	0	2	1	LLRDSTIIIV	tr_C9ZJU1	0.379	826.78	4.00
464	HLA-A0201	SVQYGLVLFSMVFV	GLLFSMVFV	4	0	0	2	1	GLVLFSMVFV	tr_C9ZJU1	0.379	827.14	4.00
831	HLA-A0201	CAALSASRALNFV	ALSASLNFV	2	0	0	5	2	ALSASRALNFV	tr_C9ZJU1	0.378	833.39	4.00
95	HLA-A0201	DIWNCLMALV	DIWNCLMLV	0	0	0	7	1	DIWNCLMALV	tr_C9ZJU1	0.378	834.26	4.00
155	HLA-A0201	VVQEFPEEL	VVQEFPEEL	0	0	0	0	0	VVQEFPEEL	tr_C9ZJU1	0.378	839.70	4.00
69	HLA-A0201	IMQLPSVFA	IMQLPSVFA	0	0	0	0	0	IMQLPSVFA	tr_C9ZJU1	0.377	848.65	4.00
187	HLA-A0201	LMCSSCSIPQLVTL	LMIPQLVTL	0	0	0	2	5	LMCSSCSIPQLVTL	tr_C9ZJU1	0.376	856.29	4.00
440	HLA-A0201	MLSSTDNKML	MLSSTDNML	0	0	0	7	1	MLSSTDNKML	tr_C9ZJU1	0.376	858.93	4.00
251	HLA-A0201	IKQLMGLPATFVS	QLMGLPAFV	2	0	0	7	1	QLMGLPATFV	tr_C9ZJU1	0.375	867.60	4.00
141	HLA-A0201	YSIARYPPA	YSIARYPPA	0	0	0	0	0	YSIARYPPA	tr_C9ZJU1	0.374	870.44	4.00
96	HLA-A0201	IWNCLMALVNCSI	CLMALVCSI	3	0	0	6	1	CLMALVNCSI	tr_C9ZJU1	0.374	871.23	4.00

465	HLA-A0201	VQYGLVLFSMV	VQYGLVLFV	0	0	0	8	2	VQYGLVLFSMV	tr_C9ZJU1	0.374	872.66	4.00
273	HLA-A0201	ESLYCIRGFVIQSI	SLYCVIQSI	1	0	0	4	4	SLYCIRGFVIQSI	tr_C9ZJU1	0.374	876.56	4.00
643	HLA-A0201	IQHWCPLYHA	IQWCPLYHA	0	0	0	2	1	IQHWCPLYHA	tr_C9ZJU1	0.374	878.36	4.00
830	HLA-A0201	ICAALSASRALNFV	ALSASLNFV	3	0	0	5	2	ALSASRALNFV	tr_C9ZJU1	0.373	886.70	4.00
909	HLA-A0201	GNNLLRDSTIIIV	LLDSTIIIV	3	0	0	2	1	LLRDSTIIIV	tr_C9ZJU1	0.373	887.54	4.00
373	HLA-A0201	FLNESLYME	FLNESLYME	0	0	0	0	0	FLNESLYME	tr_C9ZJU1	0.372	891.56	4.00
46	HLA-A0201	IAIFLNCNGV	IIFLNCNGV	0	0	0	1	1	IAIFLNCNGV	tr_C9ZJU1	0.372	893.11	4.00
363	HLA-A0201	LGTQWSNFLYFL	TQWNFLYFL	2	0	0	3	1	TQWSNFLYFL	tr_C9ZJU1	0.372	893.33	4.00
95	HLA-A0201	DIWNCLMALVNCSI	CLMALVCSI	4	0	0	6	1	CLMALVNCSI	tr_C9ZJU1	0.371	901.15	4.00
253	HLA-A0201	QLMGLPATFVSMA	QLMGLPAFV	0	0	0	7	1	QLMGLPATFV	tr_C9ZJU1	0.371	901.73	4.00
329	HLA-A0201	SVMDELQSRVPAL	VMDELVPAL	1	0	0	5	3	VMDELQSRVPAL	tr_C9ZJU1	0.371	902.78	4.00
184	HLA-A0201	FWKLMCSSCSIPQ	FLMCSSCSI	0	0	0	1	2	FWKLMCSSCSI	tr_C9ZJU1	0.371	905.84	4.00
110	HLA-A0201	DILLDDGVALCIL	LLDDGVALL	2	0	0	8	2	LLDDGVALCIL	tr_C9ZJU1	0.371	906.81	4.00
130	HLA-A0201	VLALCMLLRGKYSI	VLALCMYSI	0	0	0	6	5	VLALCMLLRGKYSI	tr_C9ZJU1	0.370	909.15	4.00
609	HLA-A0201	LPLLLVSAVA	LLLLVSAVA	0	0	0	1	1	LPLLLVSAVA	tr_C9ZJU1	0.370	911.25	4.00
821	HLA-A0201	NLWDCSDENICA	NLWDCSICA	0	0	0	6	3	NLWDCSDENICA	tr_C9ZJU1	0.370	916.01	4.00
908	HLA-A0201	EGNNLLRDSTIIIV	LLDSTIIIV	4	0	0	2	1	LLRDSTIIIV	tr_C9ZJU1	0.369	917.77	4.00
605	HLA-A0201	MADTLPLLLV	MADTLLLLV	0	0	0	5	1	MADTLPLLLV	tr_C9ZJU1	0.369	920.13	4.00
250	HLA-A0201	IIKQLMGLPATFVS	QLMGLPAFV	3	0	0	7	1	QLMGLPATFV	tr_C9ZJU1	0.369	923.50	4.00
176	HLA-A0201	SLQSGITSFWKL	SLITSFWKL	0	0	0	2	3	SLQSGITSFWKL	tr_C9ZJU1	0.369	924.09	4.00
47	HLA-A0201	AIFLNCNGVESI	FLNCGVESI	2	0	0	4	1	FLNCNGVESI	tr_C9ZJU1	0.368	930.98	4.00
610	HLA-A0201	PLLLVSAVAL	LLLVSAVAL	1	0	0	0	0	LLLVSAVAL	tr_C9ZJU1	0.367	945.78	4.00
182	HLA-A0201	TSFWKLMCSSCSIP	FLMCSSCSI	2	0	0	1	2	FWKLMCSSCSI	tr_C9ZJU1	0.367	945.86	4.00
91	HLA-A0201	LLVCDIWNCLMA	LLVCDIWMA	0	0	0	7	3	LLVCDIWNCLMA	tr_C9ZJU1	0.366	951.01	4.00
653	HLA-A0201	FLGSFYDECGMSEA	FLGSFMSEA	0	0	0	5	5	FLGSFYDECGMSEA	tr_C9ZJU1	0.366	955.81	4.00
488	HLA-A0201	DLLTQHVIWPLV	LLTHVIWPL	1	0	0	3	1	LLTQHVIWPL	tr_C9ZJU1	0.365	958.91	4.00
162	HLA-A0201	ELYLGLITA	ELYLGLITA	0	0	0	0	0	ELYLGLITA	tr_C9ZJU1	0.365	962.36	4.00
330	HLA-A0201	VMDELQSRVPALL	VMDEVPALL	0	0	0	4	4	VMDELQSRVPALL	tr_C9ZJU1	0.364	979.10	4.00
419	HLA-A0201	NSLTSSPLTL	SLTSSPLTL	1	0	0	0	0	SLTSSPLTL	tr_C9ZJU1	0.363	983.22	4.00
46	HLA-A0201	IAIFLNCNGVESI	FLNCGVESI	3	0	0	4	1	FLNCNGVESI	tr_C9ZJU1	0.362	990.22	4.00
286	HLA-A0201	ILSDIITSIHIDA	ILSDIITSA	0	0	0	8	4	ILSDIITSIHIDA	tr_C9ZJU1	0.362	996.80	4.00
321	HLA-A0201	QALWQASGSVMDEL	ALWSVMDEL	1	0	0	3	4	ALWQASGSVMDEL	tr_C9ZJU1	0.362	999.29	4.00
217	HLA-A0201	GMHAFVHWRPIYA	GMHAFVIYA	0	0	0	6	4	GMHAFVHWRPIYA	tr_C9ZJU1	0.362	1000.41	4.00
373	HLA-A0201	FLNESLYMESQFTK	FLNESLYFT	0	0	0	7	4	FLNESLYMESQFT	tr_C9ZJU1	0.362	1000.66	4.00
199	HLA-A0201	TLAATVLFSPEVI	TLAATVLFI	0	0	0	8	4	TLAATVLFSPEVI	tr_C9ZJU1	0.360	1012.69	4.00
109	HLA-A0201	RDILLDDGVALCIL	LLDDGVALL	3	0	0	8	2	LLDDGVALCIL	tr_C9ZJU1	0.360	1015.14	4.00
199	HLA-A0201	TLAATVLFSPEVIL	TLAATVLFL	0	0	0	8	5	TLAATVLFSPEVIL	tr_C9ZJU1	0.360	1016.52	4.00
564	HLA-A0201	ALRLSGWELM	ALLSGWELM	0	0	0	2	1	ALRLSGWELM	tr_C9ZJU1	0.360	1019.89	4.00
45	HLA-A0201	SIAIFLNCNGVESI	FLNCGVESI	4	0	0	4	1	FLNCNGVESI	tr_C9ZJU1	0.359	1027.28	4.00
763	HLA-A0201	RLLNRTYAPT	RLLNRTYPT	0	0	0	7	1	RLLNRTYAPT	tr_C9ZJU1	0.358	1036.97	4.00
142	HLA-A0201	SIARYPPAA	SIARYPPAA	0	0	0	0	0	SIARYPPAA	tr_C9ZJU1	0.358	1040.34	4.00
91	HLA-A0201	LLVCDIWNCLM	LLVCDIWLM	0	0	0	7	2	LLVCDIWNCLM	tr_C9ZJU1	0.357	1045.33	4.00
135	HLA-A0201	MLLRGKYSIA	MLLRGKYSA	0	0	0	8	1	MLLRGKYSIA	tr_C9ZJU1	0.357	1047.22	4.00
604	HLA-A0201	QMADTLPLLLVS	QMADTLLLV	0	0	0	5	2	QMADTLPLLLV	tr_C9ZJU1	0.357	1048.87	4.00
252	HLA-A0201	KQLMGLPATFVSM	QLMGLPAFV	1	0	0	7	1	QLMGLPATFV	tr_C9ZJU1	0.357	1050.61	4.00
352	HLA-A0201	FSFPVVSLVM	FSFPVVSLV	0	0	0	0	0	FSFPVVSLV	tr_C9ZJU1	0.356	1056.57	4.50
194	HLA-A0201	IPQLVTLAATV	ILVTLAATV	0	0	0	1	2	IPQLVTLAATV	tr_C9ZJU1	0.356	1058.76	4.50
600	HLA-A0201	HGLFQMADTL	GLFQMADTL	1	0	0	0	0	GLFQMADTL	tr_C9ZJU1	0.356	1060.69	4.50
687	HLA-A0201	YQVLEGLQSVQ	YQLEGLQSV	0	0	0	2	1	YQVLEGLQSV	tr_C9ZJU1	0.356	1061.71	4.50
563	HLA-A0201	AALRLSGWELMAAT	ALWELMAAT	1	0	0	2	4	ALRLSGWELMAAT	tr_C9ZJU1	0.356	1064.34	4.50
561	HLA-A0201	GFAALRLSGWELMA	FLSGWELMA	1	0	0	1	4	FAALRLSGWELMA	tr_C9ZJU1	0.355	1077.92	4.50
485	HLA-A0201	YMWDLLTQH	YMWDLLTQH	0	0	0	0	0	YMWDLLTQH	tr_C9ZJU1	0.355	1077.98	4.50
158	HLA-A0201	EFPEELYLGLITAY	FLYLGLITA	1	0	0	1	3	FPEELYLGLITA	tr_C9ZJU1	0.354	1085.39	4.50
487	HLA-A0201	WDLLTQHVIWPLV	LLTHVIWPL	2	0	0	3	1	LLTQHVIWPL	tr_C9ZJU1	0.353	1093.60	4.50

199	HLA-A0201	TLAATVLFS	TLAATVLFS	0	0	0	0	0	TLAATVLFS	tr_C9ZJU1	0.353	1093.93	4.50
706	HLA-A0201	GVAPPSLDGPWSV	GVLDGPWSV	0	0	0	2	4	GVAPPSLDGPWSV	tr_C9ZJU1	0.353	1093.96	4.50
256	HLA-A0201	GLPATFVSMAV	GLPAVSMAV	0	0	0	4	2	GLPATFVSMAV	tr_C9ZJU1	0.353	1097.39	4.50
249	HLA-A0201	FIIKQLMGLPATF	FLMGLPATF	0	0	0	1	4	FIIKQLMGLPATF	tr_C9ZJU1	0.353	1100.73	4.50
620	HLA-A0201	RLSPNFFTQSAL	RLSPNFFTL	0	0	0	8	3	RLSPNFFTQSAL	tr_C9ZJU1	0.352	1103.48	4.50
63	HLA-A0201	LVFNHNIMQLPSV	LVFNHLPSV	0	0	0	5	4	LVFNHNIMQLPSV	tr_C9ZJU1	0.352	1112.32	4.50
254	HLA-A0201	LMGLPATFVSMAV	LMTFVSMAV	0	0	0	2	4	LMGLPATFVSMAV	tr_C9ZJU1	0.352	1112.38	4.50
272	HLA-A0201	FESLYCIRGFVI	FLYCIRGFV	0	0	0	1	2	FESLYCIRGFV	tr_C9ZJU1	0.352	1112.81	4.50
268	HLA-A0201	STFAFESLYCI	FAFESLYCI	2	0	0	0	0	FAFESLYCI	tr_C9ZJU1	0.351	1116.30	4.50
95	HLA-A0201	DIWNCLMAL	DIWNCLMAL	0	0	0	0	0	DIWNCLMAL	tr_C9ZJU1	0.351	1118.16	4.50
328	HLA-A0201	GSVMDELQSRVPAL	VMDELVPAL	2	0	0	5	3	VMDELQSRVPAL	tr_C9ZJU1	0.351	1124.75	4.50
369	HLA-A0201	NFLYFLNESLY	FLYFLNESL	1	0	0	0	0	FLYFLNESL	tr_C9ZJU1	0.351	1125.37	4.50
567	HLA-A0201	LSGWELMAATAAA	LLMAATAAA	0	0	0	1	4	LSGWELMAATAAA	tr_C9ZJU1	0.350	1128.48	4.50
252	HLA-A0201	KQLMGLPATF	KLMGLPATF	0	0	0	1	1	KQLMGLPATF	tr_C9ZJU1	0.350	1130.73	4.50
261	HLA-A0201	FVSMAVAST	FVSMAVAST	0	0	0	0	0	FVSMAVAST	tr_C9ZJU1	0.350	1131.45	4.50
322	HLA-A0201	ALWQASGSVM	ALWQASGSV	0	0	0	0	0	ALWQASGSV	tr_C9ZJU1	0.350	1132.94	4.50
919	HLA-A0201	IIVAHRLATL	IIVAHLATL	0	0	0	5	1	IIVAHRLATL	tr_C9ZJU1	0.349	1139.77	4.50
253	HLA-A0201	QLMGLPATF	QLMGLPATF	0	0	0	0	0	QLMGLPATF	tr_C9ZJU1	0.349	1148.28	4.50
514	HLA-A0201	KLQAGGHTCAVSV	KLQACAVSV	0	0	0	4	4	KLQAGGHTCAVSV	tr_C9ZJU1	0.349	1151.66	4.50
217	HLA-A0201	GMHAFVHWRPI	GMHAFVHPI	0	0	0	7	2	GMHAFVHWRPI	tr_C9ZJU1	0.348	1152.02	4.50
131	HLA-A0201	LALCMLLRGKYSI	ALCMLLYSI	1	0	0	6	3	ALCMLLRGKYSI	tr_C9ZJU1	0.348	1153.38	4.50
8	HLA-A0201	VLLDCERKAYEIL	VLLDCAYEI	0	0	0	5	3	VLLDCERKAYEI	tr_C9ZJU1	0.348	1153.78	4.50
486	HLA-A0201	MWDLLTQHVIWPLV	LLTHVIWPL	3	0	0	3	1	LLTQHVIWPL	tr_C9ZJU1	0.348	1156.43	4.50
90	HLA-A0201	HLLVCDIWNC	HLLVCIWNC	0	0	0	5	1	HLLVCDIWNC	tr_C9ZJU1	0.348	1156.89	4.50
156	HLA-A0201	VQEFPEELYL	VQFPEELYL	0	0	0	2	1	VQEFPEELYL	tr_C9ZJU1	0.348	1160.98	4.50
873	HLA-A0201	FVKAGSAVGL	FVAGSAVGL	0	0	0	2	1	FVKAGSAVGL	tr_C9ZJU1	0.347	1172.22	4.50
936	HLA-A0201	VLNEGMVAEVG	VLNEMVAEV	0	0	0	4	1	VLNEGMVAEV	tr_C9ZJU1	0.346	1177.12	4.50
159	HLA-A0201	FPEELYLGLITAYN	FLYLGLITA	0	0	0	1	3	FPEELYLGLITA	tr_C9ZJU1	0.346	1178.98	4.50
964	HLA-A0201	RQSQQLPHFV	RQSQQLPFV	0	0	0	7	1	RQSQQLPHFV	tr_C9ZJU1	0.346	1187.40	4.50
267	HLA-A0201	ASTFAFESLYCI	FAFESLYCI	3	0	0	0	0	FAFESLYCI	tr_C9ZJU1	0.346	1188.21	4.50
92	HLA-A0201	LVCDIWNCL	LVCDIWNCL	0	0	0	0	0	LVCDIWNCL	tr_C9ZJU1	0.345	1191.18	4.50
440	HLA-A0201	MLSSTDNKM	MLSSTDNKM	0	0	0	0	0	MLSSTDNKM	tr_C9ZJU1	0.344	1207.12	4.50
313	HLA-A0201	WLLSRCVSQAL	WLLSRCVSL	0	0	0	8	2	WLLSRCVSQAL	tr_C9ZJU1	0.343	1222.19	4.50
565	HLA-A0201	LRLSGWELMAAT	RLWELMAAT	1	0	0	2	2	RLSGWELMAAT	tr_C9ZJU1	0.342	1231.24	4.50
266	HLA-A0201	VASTFAFESLYCI	FAFESLYCI	4	0	0	0	0	FAFESLYCI	tr_C9ZJU1	0.342	1232.29	4.50
568	HLA-A0201	SGWELMAATAAA	SLMAATAAA	0	0	0	1	3	SGWELMAATAAA	tr_C9ZJU1	0.342	1236.76	4.50
211	HLA-A0201	ILKCILGMHAFVH	ILLGMHAFV	0	0	0	2	3	ILKCILGMHAFV	tr_C9ZJU1	0.341	1248.51	4.50
464	HLA-A0201	SVQYGLVLFSMV	VQYGLVLFV	1	0	0	8	2	VQYGLVLFSMV	tr_C9ZJU1	0.341	1249.35	4.50
70	HLA-A0201	MQLPSVFAVHCGL	MQLPSVFAL	0	0	0	8	4	MQLPSVFAVHCGL	tr_C9ZJU1	0.340	1259.99	4.50
265	HLA-A0201	AVASTFAFESLYCI	FAFESLYCI	5	0	0	0	0	FAFESLYCI	tr_C9ZJU1	0.340	1261.28	4.50
99	HLA-A0201	CLMALVNCS	CLMALVNCS	0	0	0	0	0	CLMALVNCS	tr_C9ZJU1	0.340	1265.23	4.50
251	HLA-A0201	IKQLMGLPATFVSM	QLMGLPAFV	2	0	0	7	1	QLMGLPATFV	tr_C9ZJU1	0.339	1276.15	4.50
193	HLA-A0201	SIPQLVTLAATV	SLVTLAATV	0	0	0	1	3	SIPQLVTLAATV	tr_C9ZJU1	0.337	1299.97	4.50
273	HLA-A0201	ESLYCIRGFVI	SLYCIGFVI	1	0	0	5	1	SLYCIRGFVI	tr_C9ZJU1	0.337	1301.64	4.50
191	HLA-A0201	SCSIPQLVTLAATV	SLVTLAATV	0	0	0	1	5	SCSIPQLVTLAATV	tr_C9ZJU1	0.337	1305.97	4.50
368	HLA-A0201	SNFLYFLNESLY	FLYFLNESL	2	0	0	0	0	FLYFLNESL	tr_C9ZJU1	0.336	1314.07	5.00
99	HLA-A0201	CLMALVNCSIRDIL	CLMALVNCL	0	0	0	8	5	CLMALVNCSIRDIL	tr_C9ZJU1	0.336	1317.55	5.00
49	HLA-A0201	FLNCNGVESIHVR	FLNCNSIHV	0	0	0	5	3	FLNCNGVESIHV	tr_C9ZJU1	0.336	1320.64	5.00
271	HLA-A0201	AFESLYCIRGFVI	SLYCIGFVI	3	0	0	5	1	SLYCIRGFVI	tr_C9ZJU1	0.336	1323.03	5.00
90	HLA-A0201	HLLVCDIWNCLMA	LLVCDIWMA	1	0	0	7	3	LLVCDIWNCLMA	tr_C9ZJU1	0.335	1332.88	5.00
564	HLA-A0201	ALRLSGWEL	ALRLSGWEL	0	0	0	0	0	ALRLSGWEL	tr_C9ZJU1	0.334	1347.31	5.00
653	HLA-A0201	FLGSFYDECGM	FLFYDECGM	0	0	0	2	2	FLGSFYDECGM	tr_C9ZJU1	0.334	1348.66	5.00
607	HLA-A0201	DTLPLLLVSAV	TLLLLVSAV	1	0	0	2	1	TLPLLLVSAV	tr_C9ZJU1	0.334	1354.71	5.00
99	HLA-A0201	CLMALVNCSIRDI	CLMALVNCI	0	0	0	8	4	CLMALVNCSIRDI	tr_C9ZJU1	0.333	1355.16	5.00

196	HLA-A0201	QLVTLAATVL	QLVTLAATL	0	0	0	8	1	QLVTLAATVL	tr_C9ZJU1	0.333	1357.27	5.00
252	HLA-A0201	KQLMGLPATFVSMA	QLMGLPAFV	1	0	0	7	1	QLMGLPATFV	tr_C9ZJU1	0.333	1358.60	5.00
367	HLA-A0201	WSNFLYFLNESLY	FLYFLNESL	3	0	0	0	0	FLYFLNESL	tr_C9ZJU1	0.332	1382.17	5.00
683	HLA-A0201	SLGCYQVLEGL	SLYQVLEGL	0	0	0	2	2	SLGCYQVLEGL	tr_C9ZJU1	0.331	1390.74	5.00
329	HLA-A0201	SVMDELQSRVPALL	VMDEVPALL	1	0	0	4	4	VMDELQSRVPALL	tr_C9ZJU1	0.331	1395.82	5.00
820	HLA-A0201	GNLWDCSDENICA	NLWDCSICA	1	0	0	6	3	NLWDCSDENICA	tr_C9ZJU1	0.331	1398.07	5.00
90	HLA-A0201	HLLVCDIWNCLM	LLVCDIWLM	1	0	0	7	2	LLVCDIWNCLM	tr_C9ZJU1	0.330	1411.66	5.00
566	HLA-A0201	RLSGWELMAATAAA	RLMAATAAA	0	0	0	1	5	RLSGWELMAATAAA	tr_C9ZJU1	0.329	1414.87	5.00
366	HLA-A0201	QWSNFLYFLNESLY	FLYFLNESL	4	0	0	0	0	FLYFLNESL	tr_C9ZJU1	0.329	1427.04	5.00
911	HLA-A0201	NLLRDSTII	NLLRDSTII	0	0	0	0	0	NLLRDSTII	tr_C9ZJU1	0.328	1432.64	5.00
203	HLA-A0201	TVLFSPEVIL	VLFSPEVIL	1	0	0	0	0	VLFSPEVIL	tr_C9ZJU1	0.328	1434.20	5.00
760	HLA-A0201	TLLRLLNRTYA	TLLRLLTYA	0	0	0	6	2	TLLRLLNRTYA	tr_C9ZJU1	0.328	1444.91	5.00
463	HLA-A0201	SSVQYGLVLFSMV	VQYGLVLFV	2	0	0	8	2	VQYGLVLFSMV	tr_C9ZJU1	0.327	1445.99	5.00
807	HLA-A0201	FIEDLSIGGNIAL	FIIGGNIAL	0	0	0	2	4	FIEDLSIGGNIAL	tr_C9ZJU1	0.327	1448.70	5.00
100	HLA-A0201	LMALVNCSIR	LMALVNCSI	0	0	0	0	0	LMALVNCSI	tr_C9ZJU1	0.327	1448.70	5.00
489	HLA-A0201	LLTQHVIWPLVP	LLTHVIWPL	0	0	0	3	1	LLTQHVIWPL	tr_C9ZJU1	0.327	1449.69	5.00
611	HLA-A0201	LLLVSAVALRL	LLLAVALRL	0	0	0	3	2	LLLVSAVALRL	tr_C9ZJU1	0.327	1455.72	5.00
676	HLA-A0201	HLAFSGSSL	HLAFSGSSL	0	0	0	0	0	HLAFSGSSL	tr_C9ZJU1	0.327	1459.21	5.00
175	HLA-A0201	RSLQSGITSFWKL	SLITSFWKL	1	0	0	2	3	SLQSGITSFWKL	tr_C9ZJU1	0.326	1465.08	5.00
102	HLA-A0201	ALVNCSIRDILL	ALVNCSILL	0	0	0	6	3	ALVNCSIRDILL	tr_C9ZJU1	0.326	1466.16	5.00
89	HLA-A0201	DHLLVCDIWNCLMA	LLVCDIWMA	2	0	0	7	3	LLVCDIWNCLMA	tr_C9ZJU1	0.326	1467.79	5.00
605	HLA-A0201	MADTLPLLL	MADTLPLLL	0	0	0	0	0	MADTLPLLL	tr_C9ZJU1	0.326	1470.08	5.00
183	HLA-A0201	SFWKLMCSSCSIPQ	FLMCSSCSI	1	0	0	1	2	FWKLMCSSCSI	tr_C9ZJU1	0.326	1471.78	5.00
360	HLA-A0201	VMWLGTQWS	VMWLGTQWS	0	0	0	0	0	VMWLGTQWS	tr_C9ZJU1	0.326	1475.55	5.00
220	HLA-A0201	AFVHWRPIYA	FVHWRPIYA	1	0	0	0	0	FVHWRPIYA	tr_C9ZJU1	0.326	1475.66	5.00
286	HLA-A0201	ILSDIITSIHID	ILSDIIIHI	0	0	0	6	2	ILSDIITSIHI	tr_C9ZJU1	0.325	1482.47	5.00
248	HLA-A0201	RFIIKQLMGLPATF	FLMGLPATF	1	0	0	1	4	FIIKQLMGLPATF	tr_C9ZJU1	0.325	1483.95	5.00
192	HLA-A0201	CSIPQLVTLAATV	ILVTLAATV	2	0	0	1	2	IPQLVTLAATV	tr_C9ZJU1	0.325	1487.44	5.00
604	HLA-A0201	QMADTLPLLLVSA	QMADTLPLV	0	0	0	7	2	QMADTLPLLLV	tr_C9ZJU1	0.324	1502.04	5.00
865	HLA-A0201	ERLCIARAFV	RLCIARAFV	1	0	0	0	0	RLCIARAFV	tr_C9ZJU1	0.323	1514.89	5.00
462	HLA-A0201	KSSVQYGLVLFSMV	VQYGLVLFV	3	0	0	8	2	VQYGLVLFSMV	tr_C9ZJU1	0.323	1516.99	5.00
569	HLA-A0201	GWELMAATAAA	WLMAATAAA	1	0	0	1	1	WELMAATAAA	tr_C9ZJU1	0.322	1528.54	5.00
89	HLA-A0201	DHLLVCDIWNCLM	LLVCDIWLM	2	0	0	7	2	LLVCDIWNCLM	tr_C9ZJU1	0.322	1540.32	5.00
217	HLA-A0201	GMHAFVHWRPIYAA	GMHAFIYAA	0	0	0	5	5	GMHAFVHWRPIYAA	tr_C9ZJU1	0.321	1543.16	5.00
732	HLA-A0201	PYVLRRVSFVV	YVLRVSFVV	1	0	0	3	1	YVLRRVSFVV	tr_C9ZJU1	0.321	1548.23	5.00
211	HLA-A0201	ILKCILGMHA	ILKCILMHA	0	0	0	6	1	ILKCILGMHA	tr_C9ZJU1	0.321	1554.61	5.00
514	HLA-A0201	KLQAGGHTCAV	KLQAGTCAV	0	0	0	5	2	KLQAGGHTCAV	tr_C9ZJU1	0.321	1557.03	5.00
198	HLA-A0201	VTLAATVLFSPEVI	TLAATVLFI	1	0	0	8	4	TLAATVLFSPEVI	tr_C9ZJU1	0.320	1562.90	5.00
330	HLA-A0201	VMDELQSRVP	VMDELQSRV	0	0	0	0	0	VMDELQSRV	tr_C9ZJU1	0.320	1563.78	5.00
515	HLA-A0201	LQAGGHTCAV	LQAGGTCAV	0	0	0	5	1	LQAGGHTCAV	tr_C9ZJU1	0.320	1565.49	5.00
339	HLA-A0201	PALLEVMHGC	ALLEVMHGC	1	0	0	0	0	ALLEVMHGC	tr_C9ZJU1	0.320	1566.36	5.00
285	HLA-A0201	SILSDIITSIHIDA	ILSDIITSA	1	0	0	8	4	ILSDIITSIHIDA	tr_C9ZJU1	0.320	1575.71	5.00
606	HLA-A0201	ADTLPLLLVSAV	TLLLLVSAV	2	0	0	2	1	TLPLLLVSAV	tr_C9ZJU1	0.319	1586.19	5.00
469	HLA-A0201	LVLFSMVFVE	LVLFSMVFV	0	0	0	0	0	LVLFSMVFV	tr_C9ZJU1	0.319	1587.17	5.00
216	HLA-A0201	LGMHAFVHWRPIYA	GMHAFVIYA	1	0	0	6	4	GMHAFVHWRPIYA	tr_C9ZJU1	0.319	1590.37	5.00
352	HLA-A0201	FSFPVVSLVMWL	FVVSLVMWL	0	0	0	1	3	FSFPVVSLVMWL	tr_C9ZJU1	0.319	1591.73	5.50
100	HLA-A0201	LMALVNCSIRDILL	LMALVNILL	0	0	0	6	5	LMALVNCSIRDILL	tr_C9ZJU1	0.318	1594.71	5.50
61	HLA-A0201	RTLVFNHNI	RTLVFNHNI	0	0	0	0	0	RTLVFNHNI	tr_C9ZJU1	0.318	1596.00	5.50
607	HLA-A0201	DTLPLLLVSA	TLPLLLVSA	1	0	0	0	0	TLPLLLVSA	tr_C9ZJU1	0.318	1602.70	5.50
210	HLA-A0201	VILKCILGMHAFVH	ILLGMHAFV	1	0	0	2	3	ILKCILGMHAFV	tr_C9ZJU1	0.318	1604.28	5.50
92	HLA-A0201	LVCDIWNCLMALV	LIWNCLMAL	0	0	0	1	3	LVCDIWNCLMAL	tr_C9ZJU1	0.317	1618.40	5.50
686	HLA-A0201	CYQVLEGLQSVQ	YQLEGLQSV	1	0	0	2	1	YQVLEGLQSV	tr_C9ZJU1	0.317	1619.75	5.50
88	HLA-A0201	PDHLLVCDIWNCLM	LLVCDIWLM	3	0	0	7	2	LLVCDIWNCLM	tr_C9ZJU1	0.317	1619.82	5.50
215	HLA-A0201	ILGMHAFVHWRPI	ILGMHAFPI	0	0	0	7	4	ILGMHAFVHWRPI	tr_C9ZJU1	0.316	1631.52	5.50

372	HLA-A0201	YFLNESLYM	YFLNESLYM	0	0	0	0	0	YFLNESLYM	tr_C9ZJU1	0.316	1634.40	5.50
312	HLA-A0201	DWLLSRCVSQAL	WLLSRCVSL	1	0	0	8	2	WLLSRCVSQAL	tr_C9ZJU1	0.316	1640.32	5.50
121	HLA-A0201	ILDDVKAEEVLALC	ILDDVVLAL	0	0	0	5	4	ILDDVKAEEVLAL	tr_C9ZJU1	0.315	1646.89	5.50
206	HLA-A0201	FSPEVILKCI	FSPEVILCI	0	0	0	7	1	FSPEVILKCI	tr_C9ZJU1	0.315	1652.20	5.50
760	HLA-A0201	TLLRLLNRT	TLLRLLNRT	0	0	0	0	0	TLLRLLNRT	tr_C9ZJU1	0.315	1658.21	5.50
605	HLA-A0201	MADTLPLLLVSAV	TLLLLVSAV	3	0	0	2	1	TLPLLLVSAV	tr_C9ZJU1	0.315	1659.74	5.50
255	HLA-A0201	MGLPATFVSMAV	GLPAVSMAV	1	0	0	4	2	GLPATFVSMAV	tr_C9ZJU1	0.315	1663.80	5.50
571	HLA-A0201	ELMAATAAAA	LMAATAAAA	1	0	0	0	0	LMAATAAAA	tr_C9ZJU1	0.314	1675.22	5.50
558	HLA-A0201	YLQGFAALRLS	YLQGFAALL	0	0	0	8	1	YLQGFAALRL	tr_C9ZJU1	0.314	1675.85	5.50
249	HLA-A0201	FIIKQLMGLPA	FIIKQLMPA	0	0	0	7	2	FIIKQLMGLPA	tr_C9ZJU1	0.314	1680.16	5.50
243	HLA-A0201	KLSPFRFIIK	KLSPFRFII	0	0	0	0	0	KLSPFRFII	tr_C9ZJU1	0.314	1681.41	5.50
281	HLA-A0201	FVIQSILSDII	FVIQSILSI	0	0	0	8	2	FVIQSILSDII	tr_C9ZJU1	0.313	1683.70	5.50
705	HLA-A0201	RGVAPPSLDGPWSV	GVLDGPWSV	1	0	0	2	4	GVAPPSLDGPWSV	tr_C9ZJU1	0.313	1684.89	5.50
247	HLA-A0201	FRFIIKQLMGL	FIIKQLMGL	0	0	0	1	2	FRFIIKQLMGL	tr_C9ZJU1	0.313	1698.21	5.50
350	HLA-A0201	VVFSFPVVSLV	VVFSFPVLV	0	0	0	7	2	VVFSFPVVSLV	tr_C9ZJU1	0.312	1700.93	5.50
731	HLA-A0201	HPYVLRRVSFVV	YVLRVSFVV	2	0	0	3	1	YVLRRVSFVV	tr_C9ZJU1	0.312	1707.91	5.50
513	HLA-A0201	RKLQAGGHTCAVSV	KLQACAVSV	1	0	0	4	4	KLQAGGHTCAVSV	tr_C9ZJU1	0.312	1713.22	5.50
819	HLA-A0201	LGNLWDCSDENICA	NLWDCSICA	2	0	0	6	3	NLWDCSDENICA	tr_C9ZJU1	0.311	1730.54	5.50
141	HLA-A0201	YSIARYPPAA	YIARYPPAA	0	0	0	1	1	YSIARYPPAA	tr_C9ZJU1	0.311	1733.84	5.50
206	HLA-A0201	FSPEVILKCILGM	FILKCILGM	0	0	0	1	4	FSPEVILKCILGM	tr_C9ZJU1	0.311	1735.19	5.50
820	HLA-A0201	GNLWDCSDENI	NLWDCSDEI	1	0	0	8	1	NLWDCSDENI	tr_C9ZJU1	0.310	1740.63	5.50
68	HLA-A0201	NIMQLPSVFA	NIMQLPVFA	0	0	0	6	1	NIMQLPSVFA	tr_C9ZJU1	0.310	1745.04	5.50
174	HLA-A0201	KRSLQSGITSFWKL	SLITSFWKL	2	0	0	2	3	SLQSGITSFWKL	tr_C9ZJU1	0.309	1768.25	5.50
7	HLA-A0201	AVLLDCERKAYEIL	VLLDCAYEI	1	0	0	5	3	VLLDCERKAYEI	tr_C9ZJU1	0.309	1772.23	5.50
98	HLA-A0201	NCLMALVNCSIRDI	CLMALVNCI	1	0	0	8	4	CLMALVNCSIRDI	tr_C9ZJU1	0.309	1775.32	5.50
619	HLA-A0201	LRLSPNFFTQSAL	RLSPNFFTL	1	0	0	8	3	RLSPNFFTQSAL	tr_C9ZJU1	0.308	1778.49	5.50
311	HLA-A0201	DDWLLSRCVSQAL	WLLSRCVSL	2	0	0	8	2	WLLSRCVSQAL	tr_C9ZJU1	0.308	1779.80	5.50
730	HLA-A0201	RHPYVLRRVSFVV	YVLRVSFVV	3	0	0	3	1	YVLRRVSFVV	tr_C9ZJU1	0.308	1783.29	5.50
490	HLA-A0201	LTQHVIWPLVPSVF	LIWPLVPSV	0	0	0	1	4	LTQHVIWPLVPSV	tr_C9ZJU1	0.308	1793.39	5.50
489	HLA-A0201	LLTQHVIWPLVPS	LLTHVIWPL	0	0	0	3	1	LLTQHVIWPL	tr_C9ZJU1	0.307	1804.58	5.50
121	HLA-A0201	ILDDVKAEEVL	ILDDVAEEV	0	0	0	5	1	ILDDVKAEEV	tr_C9ZJU1	0.306	1825.24	5.50
639	HLA-A0201	QLRSIQHWCPLYHA	QLWCPLYHA	0	0	0	2	5	QLRSIQHWCPLYHA	tr_C9ZJU1	0.306	1828.15	5.50
379	HLA-A0201	YMESQFTKSQLA	YMFTKSQLA	0	0	0	2	3	YMESQFTKSQLA	tr_C9ZJU1	0.306	1829.27	5.50
291	HLA-A0201	ITSIHIDAYL	ITIHIDAYL	0	0	0	2	1	ITSIHIDAYL	tr_C9ZJU1	0.306	1833.30	5.50
764	HLA-A0201	LLNRTYAPT	LLNRTYAPT	0	0	0	0	0	LLNRTYAPT	tr_C9ZJU1	0.305	1835.54	5.50
81	HLA-A0201	GLMKNSHPDHL	GLMKNSHPL	0	0	0	8	2	GLMKNSHPDHL	tr_C9ZJU1	0.305	1837.62	5.50
729	HLA-A0201	DRHPYVLRRVSFVV	YVLRVSFVV	4	0	0	3	1	YVLRRVSFVV	tr_C9ZJU1	0.305	1841.81	5.50
116	HLA-A0201	GVALCILDDV	GVALCILDV	0	0	0	7	1	GVALCILDDV	tr_C9ZJU1	0.305	1842.14	5.50
676	HLA-A0201	HLAFSGSSLGCYQV	HLAFGCYQV	0	0	0	4	5	HLAFSGSSLGCYQV	tr_C9ZJU1	0.305	1846.02	5.50
310	HLA-A0201	SDDWLLSRCVSQAL	WLLSRCVSL	3	0	0	8	2	WLLSRCVSQAL	tr_C9ZJU1	0.304	1866.96	5.50
334	HLA-A0201	LQSRVPALL	LQSRVPALL	0	0	0	0	0	LQSRVPALL	tr_C9ZJU1	0.304	1867.75	5.50
935	HLA-A0201	VVLNEGMVAEVG	VLNEMVAEV	1	0	0	4	1	VLNEGMVAEV	tr_C9ZJU1	0.303	1877.72	5.50
611	HLA-A0201	LLLVSAVALR	LLLVSAVAL	0	0	0	0	0	LLLVSAVAL	tr_C9ZJU1	0.303	1893.45	5.50
70	HLA-A0201	MQLPSVFAVHC	MQLPSVFAV	0	0	0	0	0	MQLPSVFAV	tr_C9ZJU1	0.302	1901.52	6.00
216	HLA-A0201	LGMHAFVHWRPI	GMHAFVHPI	1	0	0	7	2	GMHAFVHWRPI	tr_C9ZJU1	0.302	1903.15	6.00
161	HLA-A0201	EELYLGLITA	ELYLGLITA	0	0	0	1	1	EELYLGLITA	tr_C9ZJU1	0.302	1910.55	6.00
613	HLA-A0201	LVSAVALRL	LVSAVALRL	0	0	0	0	0	LVSAVALRL	tr_C9ZJU1	0.301	1921.23	6.00
682	HLA-A0201	SSLGCYQVLEGL	SLYQVLEGL	1	0	0	2	2	SLGCYQVLEGL	tr_C9ZJU1	0.301	1925.56	6.00
81	HLA-A0201	GLMKNSHPDHLL	GLMKNSHPL	0	0	0	8	3	GLMKNSHPDHLL	tr_C9ZJU1	0.301	1927.16	6.00
488	HLA-A0201	DLLTQHVIWPLVP	LLTHVIWPL	1	0	0	3	1	LLTQHVIWPL	tr_C9ZJU1	0.300	1949.67	6.00
187	HLA-A0201	LMCSSCSIPQLV	LMCSIPQLV	0	0	0	2	3	LMCSSCSIPQLV	tr_C9ZJU1	0.300	1953.97	6.00
924	HLA-A0201	RLATLKHADKIVVL	RLATLIVVL	0	0	0	5	5	RLATLKHADKIVVL	tr_C9ZJU1	0.300	1956.60	6.00
117	HLA-A0201	VALCILDDV	VALCILDDV	0	0	0	0	0	VALCILDDV	tr_C9ZJU1	0.299	1957.93	6.00
387	HLA-A0201	SQLASVTNIA	SLASVTNIA	0	0	0	1	1	SQLASVTNIA	tr_C9ZJU1	0.299	1959.31	6.00

384	HLA-A0201	FTKSQLASVTNIA	FLASVTNIA	0	0	0	1	4	FTKSQLASVTNIA	tr_C9ZJU1	0.299	1965.34	6.00
468	HLA-A0201	GLVLFSMVFVE	GLLFSMVFV	0	0	0	2	1	GLVLFSMVFV	tr_C9ZJU1	0.299	1966.32	6.00
662	HLA-A0201	GMSEAQVDEA	GMSEAVDEA	0	0	0	5	1	GMSEAQVDEA	tr_C9ZJU1	0.299	1975.57	6.00
365	HLA-A0201	TQWSNFLYFLN	TQWNFLYFL	0	0	0	3	1	TQWSNFLYFL	tr_C9ZJU1	0.298	1979.27	6.00
685	HLA-A0201	GCYQVLEGLQSVQ	YQLEGLQSV	2	0	0	2	1	YQVLEGLQSV	tr_C9ZJU1	0.298	1983.09	6.00
767	HLA-A0201	RTYAPTEGDIFV	RTYEGDIFV	0	0	0	3	3	RTYAPTEGDIFV	tr_C9ZJU1	0.298	1988.63	6.00
353	HLA-A0201	SFPVVSLVMWL	FVVSLVMWL	1	0	0	1	1	FPVVSLVMWL	tr_C9ZJU1	0.298	1989.39	6.00
99	HLA-A0201	CLMALVNCSIR	CLMALVCSI	0	0	0	6	1	CLMALVNCSI	tr_C9ZJU1	0.298	1994.60	6.00
330	HLA-A0201	VMDELQSRVPA	VMDELQVPA	0	0	0	6	2	VMDELQSRVPA	tr_C9ZJU1	0.297	2001.07	6.00
565	HLA-A0201	LRLSGWELMAA	RLSGWLMAA	1	0	0	5	1	RLSGWELMAA	tr_C9ZJU1	0.297	2004.32	6.00
272	HLA-A0201	FESLYCIRGFVIQ	FLYCIRGFV	0	0	0	1	2	FESLYCIRGFV	tr_C9ZJU1	0.297	2015.58	6.00
349	HLA-A0201	RVVFSFPVVSLV	VVFSFPVLV	1	0	0	7	2	VVFSFPVVSLV	tr_C9ZJU1	0.297	2015.86	6.00
350	HLA-A0201	VVFSFPVVSLVMWL	VVFSFVMWL	0	0	0	5	5	VVFSFPVVSLVMWL	tr_C9ZJU1	0.297	2021.06	6.00
16	HLA-A0201	AYEILEMLTRA	YILEMLTRA	1	0	0	1	1	YEILEMLTRA	tr_C9ZJU1	0.296	2024.94	6.00
48	HLA-A0201	IFLNCNGVESIHVR	FLNCNSIHV	1	0	0	5	3	FLNCNGVESIHV	tr_C9ZJU1	0.296	2035.55	6.00
187	HLA-A0201	LMCSSCSIPQL	LMSCSIPQL	0	0	0	2	2	LMCSSCSIPQL	tr_C9ZJU1	0.296	2041.28	6.00
201	HLA-A0201	AATVLFSPEV	ATVLFSPEV	0	0	0	1	1	AATVLFSPEV	tr_C9ZJU1	0.295	2044.22	6.00
211	HLA-A0201	ILKCILGMHAFVHW	ILLGMHAFV	0	0	0	2	3	ILKCILGMHAFV	tr_C9ZJU1	0.295	2047.25	6.00
108	HLA-A0201	IRDILLDDGVAL	ILLDDGVAL	0	0	0	1	3	IRDILLDDGVAL	tr_C9ZJU1	0.295	2047.59	6.00
9	HLA-A0201	LLDCERKAYEI	LLDCEAYEI	0	0	0	5	2	LLDCERKAYEI	tr_C9ZJU1	0.295	2054.64	6.00
687	HLA-A0201	YQVLEGLQSVQR	YQLEGLQSV	0	0	0	2	1	YQVLEGLQSV	tr_C9ZJU1	0.295	2060.99	6.00
214	HLA-A0201	CILGMHAFVHWRPI	GMHAFVHPI	3	0	0	7	2	GMHAFVHWRPI	tr_C9ZJU1	0.295	2061.86	6.00
341	HLA-A0201	LLEVMHGCRVV	LLMHGCRVV	0	0	0	2	2	LLEVMHGCRVV	tr_C9ZJU1	0.295	2065.52	6.00
101	HLA-A0201	MALVNCSIRDILL	ALVNCSILL	1	0	0	6	3	ALVNCSIRDILL	tr_C9ZJU1	0.294	2068.40	6.00
917	HLA-A0201	TIIIVAHRL	TIIIVAHRL	0	0	0	0	0	TIIIVAHRL	tr_C9ZJU1	0.294	2069.21	6.00
274	HLA-A0201	SLYCIRGFVIQSIL	SLYCVIQSI	0	0	0	4	4	SLYCIRGFVIQSI	tr_C9ZJU1	0.294	2079.40	6.00
618	HLA-A0201	ALRLSPNFFTQSAL	RLSPNFFTL	2	0	0	8	3	RLSPNFFTQSAL	tr_C9ZJU1	0.294	2081.79	6.00
261	HLA-A0201	FVSMAVASTFA	FMAVASTFA	0	0	0	1	2	FVSMAVASTFA	tr_C9ZJU1	0.294	2085.21	6.00
763	HLA-A0201	RLLNRTYAPTEGDI	RLLNRTYAI	0	0	0	8	5	RLLNRTYAPTEGDI	tr_C9ZJU1	0.293	2092.01	6.00
331	HLA-A0201	MDELQSRVPAL	MLQSRVPAL	0	0	0	1	2	MDELQSRVPAL	tr_C9ZJU1	0.293	2101.63	6.00
936	HLA-A0201	VLNEGMVAEVGS	VLNEMVAEV	0	0	0	4	1	VLNEGMVAEV	tr_C9ZJU1	0.293	2110.20	6.00
684	HLA-A0201	LGCYQVLEGLQSVQ	YQLEGLQSV	3	0	0	2	1	YQVLEGLQSV	tr_C9ZJU1	0.292	2112.05	6.00
652	HLA-A0201	AFLGSFYDECGM	FLFYDECGM	1	0	0	2	2	FLGSFYDECGM	tr_C9ZJU1	0.292	2128.39	6.00
610	HLA-A0201	PLLLVSAVALRL	LLLAVALRL	1	0	0	3	2	LLLVSAVALRL	tr_C9ZJU1	0.292	2129.49	6.00
608	HLA-A0201	TLPLLLVSAVALRL	LLLAVALRL	3	0	0	3	2	LLLVSAVALRL	tr_C9ZJU1	0.292	2131.59	6.00
759	HLA-A0201	TTLLRLLNRTYA	TLLRLLTYA	1	0	0	6	2	TLLRLLNRTYA	tr_C9ZJU1	0.291	2138.13	6.00
333	HLA-A0201	ELQSRVPAL	ELQSRVPAL	0	0	0	0	0	ELQSRVPAL	tr_C9ZJU1	0.291	2142.57	6.00
597	HLA-A0201	DVLHGLFQMA	VLHGLFQMA	1	0	0	0	0	VLHGLFQMA	tr_C9ZJU1	0.291	2149.96	6.00
328	HLA-A0201	GSVMDELQSRV	SVMDELQSV	1	0	0	8	1	SVMDELQSRV	tr_C9ZJU1	0.291	2155.76	6.00
360	HLA-A0201	VMWLGTQWSNFLY	VMWLWSNFL	0	0	0	4	3	VMWLGTQWSNFL	tr_C9ZJU1	0.290	2159.77	6.00
382	HLA-A0201	SQFTKSQLASV	SQFSQLASV	0	0	0	3	2	SQFTKSQLASV	tr_C9ZJU1	0.290	2160.66	6.00
557	HLA-A0201	DYLQGFAALRLS	YLQGFAALL	1	0	0	8	1	YLQGFAALRL	tr_C9ZJU1	0.290	2178.52	6.00
819	HLA-A0201	LGNLWDCSDENI	NLWDCSDEI	2	0	0	8	1	NLWDCSDENI	tr_C9ZJU1	0.290	2180.60	6.00
285	HLA-A0201	SILSDIITSIHID	ILSDIIIHI	1	0	0	6	2	ILSDIITSIHI	tr_C9ZJU1	0.289	2181.73	6.00
487	HLA-A0201	WDLLTQHVIWPLVP	LLTHVIWPL	2	0	0	3	1	LLTQHVIWPL	tr_C9ZJU1	0.289	2183.95	6.00
501	HLA-A0201	SVFSGCVVDA	SVFGCVVDA	0	0	0	3	1	SVFSGCVVDA	tr_C9ZJU1	0.289	2194.23	6.00
280	HLA-A0201	GFVIQSILSDI	FVIQSILSI	1	0	0	8	1	FVIQSILSDI	tr_C9ZJU1	0.289	2194.33	6.00
629	HLA-A0201	SALDQTEIV	SALDQTEIV	0	0	0	0	0	SALDQTEIV	tr_C9ZJU1	0.289	2195.87	6.00
8	HLA-A0201	VLLDCERKAYEILE	VLLDCAYEI	0	0	0	5	3	VLLDCERKAYEI	tr_C9ZJU1	0.288	2205.39	6.00
681	HLA-A0201	GSSLGCYQVLEGL	SLYQVLEGL	2	0	0	2	2	SLGCYQVLEGL	tr_C9ZJU1	0.288	2205.59	6.00
382	HLA-A0201	SQFTKSQLA	SQFTKSQLA	0	0	0	0	0	SQFTKSQLA	tr_C9ZJU1	0.288	2205.85	6.00
351	HLA-A0201	VFSFPVVSLVMWL	FVVSLVMWL	1	0	0	1	3	FSFPVVSLVMWL	tr_C9ZJU1	0.288	2213.52	6.00
49	HLA-A0201	FLNCNGVESIHVRT	FLNCNSIHV	0	0	0	5	3	FLNCNGVESIHV	tr_C9ZJU1	0.288	2215.73	6.00
81	HLA-A0201	GLMKNSHPDHLLVC	GLMPDHLLV	0	0	0	3	4	GLMKNSHPDHLLV	tr_C9ZJU1	0.288	2215.99	6.00

246	HLA-A0201	PFRFIIKQLMGL	FIIKQLMGL	3	0	0	0	0	FIIKQLMGL	tr_C9ZJU1	0.288	2219.61	6.00
348	HLA-A0201	CRVVFSFPVVSLV	VVFSFPVLV	2	0	0	7	2	VVFSFPVVSLV	tr_C9ZJU1	0.288	2219.69	6.00
912	HLA-A0201	LLRDSTIIIVA	LLDSTIIIV	0	0	0	2	1	LLRDSTIIIV	tr_C9ZJU1	0.288	2224.01	6.00
806	HLA-A0201	RFIEDLSIGGNIAL	FIIGGNIAL	1	0	0	2	4	FIEDLSIGGNIAL	tr_C9ZJU1	0.287	2235.25	6.50
605	HLA-A0201	MADTLPLLLVSAVA	MLLLVSAVA	0	0	0	1	5	MADTLPLLLVSAVA	tr_C9ZJU1	0.287	2236.44	6.50
881	HLA-A0201	GLCIFDEPT	GLCIFDEPT	0	0	0	0	0	GLCIFDEPT	tr_C9ZJU1	0.287	2241.31	6.50
360	HLA-A0201	VMWLGTQWSNFLYF	VMWSNFLYF	0	0	0	2	5	VMWLGTQWSNFLYF	tr_C9ZJU1	0.287	2244.85	6.50
911	HLA-A0201	NLLRDSTIIIVA	NLLSTIIIV	0	0	0	3	2	NLLRDSTIIIV	tr_C9ZJU1	0.287	2247.04	6.50
962	HLA-A0201	RMRQSQQLPHFV	RMQQLPHFV	0	0	0	2	3	RMRQSQQLPHFV	tr_C9ZJU1	0.287	2249.40	6.50
818	HLA-A0201	ALGNLWDCSDENI	NLWDCSDEI	3	0	0	8	1	NLWDCSDENI	tr_C9ZJU1	0.287	2250.20	6.50
503	HLA-A0201	FSGCVVDAL	FSGCVVDAL	0	0	0	0	0	FSGCVVDAL	tr_C9ZJU1	0.287	2250.54	6.50
129	HLA-A0201	EVLALCMLL	EVLALCMLL	0	0	0	0	0	EVLALCMLL	tr_C9ZJU1	0.287	2251.71	6.50
835	HLA-A0201	SASRALNFV	SASRALNFV	0	0	0	0	0	SASRALNFV	tr_C9ZJU1	0.286	2254.15	6.50
465	HLA-A0201	VQYGLVLFSMVFVE	VQYGLVLFV	0	0	0	7	4	VQYGLVLFSMVFV	tr_C9ZJU1	0.286	2257.69	6.50
327	HLA-A0201	SGSVMDELQSRV	SMDELQSRV	0	0	0	1	3	SGSVMDELQSRV	tr_C9ZJU1	0.286	2263.24	6.50
49	HLA-A0201	FLNCNGVESIH	FLCNGVESI	0	0	0	2	1	FLNCNGVESI	tr_C9ZJU1	0.286	2267.16	6.50
562	HLA-A0201	FAALRLSGWELMAA	RLSGWLMAA	4	0	0	5	1	RLSGWELMAA	tr_C9ZJU1	0.286	2269.89	6.50
109	HLA-A0201	RDILLDDGVAL	ILLDDGVAL	2	0	0	0	0	ILLDDGVAL	tr_C9ZJU1	0.285	2285.14	6.50
934	HLA-A0201	IVVLNEGMVAEVG	VLNEMVAEV	2	0	0	4	1	VLNEGMVAEV	tr_C9ZJU1	0.285	2285.86	6.50
209	HLA-A0201	EVILKCILGM	VILKCILGM	1	0	0	0	0	VILKCILGM	tr_C9ZJU1	0.285	2290.29	6.50
467	HLA-A0201	YGLVLFSMVFVE	GLLFSMVFV	1	0	0	2	1	GLVLFSMVFV	tr_C9ZJU1	0.285	2290.41	6.50
329	HLA-A0201	SVMDELQSRVPA	SVMDELVPA	0	0	0	6	3	SVMDELQSRVPA	tr_C9ZJU1	0.285	2292.42	6.50
347	HLA-A0201	GCRVVFSFPVVSLV	FSFPVVSLV	5	0	0	0	0	FSFPVVSLV	tr_C9ZJU1	0.285	2294.03	6.50
680	HLA-A0201	SGSSLGCYQVLEGL	SLYQVLEGL	3	0	0	2	2	SLGCYQVLEGL	tr_C9ZJU1	0.284	2303.81	6.50
245	HLA-A0201	SPFRFIIKQLMGL	FIIKQLMGL	4	0	0	0	0	FIIKQLMGL	tr_C9ZJU1	0.284	2324.74	6.50
107	HLA-A0201	SIRDILLDDGVAL	ILLDDGVAL	4	0	0	0	0	ILLDDGVAL	tr_C9ZJU1	0.283	2337.83	6.50
605	HLA-A0201	MADTLPLLLVSA	MLPLLLVSA	0	0	0	1	3	MADTLPLLLVSA	tr_C9ZJU1	0.283	2349.68	6.50
448	HLA-A0201	MLFQQTFSHP	MLFQQTFSP	0	0	0	8	1	MLFQQTFSHP	tr_C9ZJU1	0.282	2361.73	6.50
817	HLA-A0201	IALGNLWDCSDENI	NLWDCSDEI	4	0	0	8	1	NLWDCSDENI	tr_C9ZJU1	0.282	2366.36	6.50
14	HLA-A0201	RKAYEILEML	KAYEILEML	1	0	0	0	0	KAYEILEML	tr_C9ZJU1	0.282	2367.46	6.50
421	HLA-A0201	LTSSPLTLL	LTSSPLTLL	0	0	0	0	0	LTSSPLTLL	tr_C9ZJU1	0.282	2373.42	6.50
609	HLA-A0201	LPLLLVSAVALRL	LLLAVALRL	2	0	0	3	2	LLLVSAVALRL	tr_C9ZJU1	0.282	2377.42	6.50
467	HLA-A0201	YGLVLFSMVF	YLVLFSMVF	0	0	0	1	1	YGLVLFSMVF	tr_C9ZJU1	0.282	2377.58	6.50
283	HLA-A0201	IQSILSDIITSIH	ILSDIITSI	0	0	0	1	3	IQSILSDIITSI	tr_C9ZJU1	0.281	2381.75	6.50
244	HLA-A0201	LSPFRFIIKQLMGL	FIIKQLMGL	5	0	0	0	0	FIIKQLMGL	tr_C9ZJU1	0.281	2381.77	6.50
488	HLA-A0201	DLLTQHVIWPLVPS	LLTHVIWPL	1	0	0	3	1	LLTQHVIWPL	tr_C9ZJU1	0.281	2386.70	6.50
833	HLA-A0201	ALSASRALNFVQ	ALSASLNFV	0	0	0	5	2	ALSASRALNFV	tr_C9ZJU1	0.281	2391.72	6.50
280	HLA-A0201	GFVIQSILSDII	FVIQSILSI	1	0	0	8	2	FVIQSILSDII	tr_C9ZJU1	0.281	2392.34	6.50
558	HLA-A0201	YLQGFAALRLSG	YLQGFAALL	0	0	0	8	1	YLQGFAALRL	tr_C9ZJU1	0.281	2392.39	6.50
132	HLA-A0201	ALCMLLRGKYSIA	ALCMLLYSI	0	0	0	6	3	ALCMLLRGKYSI	tr_C9ZJU1	0.281	2393.76	6.50
468	HLA-A0201	GLVLFSMVFVES	GLLFSMVFV	0	0	0	2	1	GLVLFSMVFV	tr_C9ZJU1	0.281	2394.00	6.50
272	HLA-A0201	FESLYCIRGFVIQS	FLYCIRGFV	0	0	0	1	2	FESLYCIRGFV	tr_C9ZJU1	0.281	2394.93	6.50
651	HLA-A0201	HAFLGSFYDECGM	FLFYDECGM	2	0	0	2	2	FLGSFYDECGM	tr_C9ZJU1	0.281	2396.59	6.50
933	HLA-A0201	KIVVLNEGMVAEVG	VLNEMVAEV	3	0	0	4	1	VLNEGMVAEV	tr_C9ZJU1	0.280	2410.11	6.50
564	HLA-A0201	ALRLSGWELMAA	RLSGWLMAA	2	0	0	5	1	RLSGWELMAA	tr_C9ZJU1	0.280	2412.67	6.50
513	HLA-A0201	RKLQAGGHTCAV	KLQAGTCAV	1	0	0	5	2	KLQAGGHTCAV	tr_C9ZJU1	0.280	2420.64	6.50
493	HLA-A0201	HVIWPLVPSVF	VIWPLVPSV	1	0	0	0	0	VIWPLVPSV	tr_C9ZJU1	0.280	2422.32	6.50
70	HLA-A0201	MQLPSVFAVHCG	MQLPSVFAV	0	0	0	0	0	MQLPSVFAV	tr_C9ZJU1	0.280	2425.60	6.50
314	HLA-A0201	LLSRCVSQALWQA	LLSRCLWQA	0	0	0	5	4	LLSRCVSQALWQA	tr_C9ZJU1	0.280	2429.51	6.50
15	HLA-A0201	KAYEILEMLTRA	YILEMLTRA	2	0	0	1	1	YEILEMLTRA	tr_C9ZJU1	0.280	2429.59	6.50
70	HLA-A0201	MQLPSVFAVHCGLM	MQLPSVFAV	0	0	0	0	0	MQLPSVFAV	tr_C9ZJU1	0.279	2434.59	6.50
121	HLA-A0201	ILDDVKAEEVLA	ILDDVEVLA	0	0	0	5	3	ILDDVKAEEVLA	tr_C9ZJU1	0.279	2435.04	6.50
470	HLA-A0201	VLFSMVFVE	VLFSMVFVE	0	0	0	0	0	VLFSMVFVE	tr_C9ZJU1	0.279	2435.48	6.50
285	HLA-A0201	SILSDIITSIH	ILSDIITSI	1	0	0	0	0	ILSDIITSI	tr_C9ZJU1	0.279	2441.87	6.50

341	HLA-A0201	LLEVMHGCRV	LLVMHGCRV	0	0	0	2	1	LLEVMHGCRV	tr_C9ZJU1	0.279	2444.41	6.50
556	HLA-A0201	QDYLQGFAALRLS	YLQGFAALL	2	0	0	8	1	YLQGFAALRL	tr_C9ZJU1	0.279	2445.17	6.50
326	HLA-A0201	ASGSVMDELQSRV	AMDELQSRV	0	0	0	1	4	ASGSVMDELQSRV	tr_C9ZJU1	0.279	2455.06	6.50
320	HLA-A0201	SQALWQASGSV	SLWQASGSV	0	0	0	1	2	SQALWQASGSV	tr_C9ZJU1	0.278	2457.69	6.50
450	HLA-A0201	FQQTFSHPS	FQQTFSHPS	0	0	0	0	0	FQQTFSHPS	tr_C9ZJU1	0.278	2462.70	6.50
279	HLA-A0201	RGFVIQSILSDI	FVIQSILSI	2	0	0	8	1	FVIQSILSDI	tr_C9ZJU1	0.277	2488.55	6.50
205	HLA-A0201	LFSPEVILKCILGM	FILKCILGM	1	0	0	1	4	FSPEVILKCILGM	tr_C9ZJU1	0.277	2491.62	6.50
193	HLA-A0201	SIPQLVTLA	SIPQLVTLA	0	0	0	0	0	SIPQLVTLA	tr_C9ZJU1	0.277	2504.35	6.50
284	HLA-A0201	QSILSDIITSIHID	ILSDIIIHI	2	0	0	6	2	ILSDIITSIHI	tr_C9ZJU1	0.277	2504.86	6.50
620	HLA-A0201	RLSPNFFTQSA	RLSPNFFSA	0	0	0	7	2	RLSPNFFTQSA	tr_C9ZJU1	0.277	2505.00	6.50
22	HLA-A0201	MLTRATIKT	MLTRATIKT	0	0	0	0	0	MLTRATIKT	tr_C9ZJU1	0.277	2505.73	6.50
758	HLA-A0201	KTTLLRLLNRTYA	TLLRLLTYA	2	0	0	6	2	TLLRLLNRTYA	tr_C9ZJU1	0.276	2515.07	6.50
481	HLA-A0201	RPLGYMWDLL	RLGYMWDLL	0	0	0	1	1	RPLGYMWDLL	tr_C9ZJU1	0.276	2526.69	6.50
248	HLA-A0201	RFIIKQLMGLPA	FIIKQLMPA	1	0	0	7	2	FIIKQLMGLPA	tr_C9ZJU1	0.276	2527.49	6.50
106	HLA-A0201	CSIRDILLDDGVAL	ILLDDGVAL	5	0	0	0	0	ILLDDGVAL	tr_C9ZJU1	0.276	2531.02	6.50
49	HLA-A0201	FLNCNGVES	FLNCNGVES	0	0	0	0	0	FLNCNGVES	tr_C9ZJU1	0.276	2533.51	6.50
650	HLA-A0201	YHAFLGSFYDECGM	FLFYDECGM	3	0	0	2	2	FLGSFYDECGM	tr_C9ZJU1	0.276	2536.33	6.50
910	HLA-A0201	NNLLRDSTIIIVA	LLDSTIIIV	2	0	0	2	1	LLRDSTIIIV	tr_C9ZJU1	0.275	2546.26	6.50
555	HLA-A0201	RQDYLQGFAALRLS	YLQGFAALL	3	0	0	8	1	YLQGFAALRL	tr_C9ZJU1	0.275	2549.46	6.50
563	HLA-A0201	AALRLSGWELMAA	RLSGWLMAA	3	0	0	5	1	RLSGWELMAA	tr_C9ZJU1	0.275	2555.89	6.50
254	HLA-A0201	LMGLPATFVS	LMGLPATFV	0	0	0	0	0	LMGLPATFV	tr_C9ZJU1	0.274	2566.76	6.50
214	HLA-A0201	CILGMHAFVH	CILGMHAFV	0	0	0	0	0	CILGMHAFV	tr_C9ZJU1	0.274	2566.95	6.50
465	HLA-A0201	VQYGLVLFS	VQYGLVLFS	0	0	0	0	0	VQYGLVLFS	tr_C9ZJU1	0.274	2572.46	6.50
388	HLA-A0201	QLASVTNIAT	QLASVTIAT	0	0	0	6	1	QLASVTNIAT	tr_C9ZJU1	0.274	2576.41	6.50
14	HLA-A0201	RKAYEILEMLTRA	YILEMLTRA	3	0	0	1	1	YEILEMLTRA	tr_C9ZJU1	0.274	2578.56	6.50
466	HLA-A0201	QYGLVLFSMVFVE	GLLFSMVFV	2	0	0	2	1	GLVLFSMVFV	tr_C9ZJU1	0.274	2580.54	6.50
111	HLA-A0201	ILLDDGVALCILD	ILLDVALCI	0	0	0	4	2	ILLDDGVALCI	tr_C9ZJU1	0.274	2580.99	6.50
120	HLA-A0201	CILDDVKAEEVL	ILDDVAEEV	1	0	0	5	1	ILDDVKAEEV	tr_C9ZJU1	0.274	2591.96	6.50
262	HLA-A0201	VSMAVASTFA	SMAVASTFA	1	0	0	0	0	SMAVASTFA	tr_C9ZJU1	0.273	2593.47	6.50
98	HLA-A0201	NCLMALVNCSIR	CLMALVCSI	1	0	0	6	1	CLMALVNCSI	tr_C9ZJU1	0.273	2593.67	6.50
278	HLA-A0201	IRGFVIQSILSDI	FVIQSILSI	3	0	0	8	1	FVIQSILSDI	tr_C9ZJU1	0.273	2606.75	7.00
761	HLA-A0201	LLRLLNRTYA	LLRLLNTYA	0	0	0	6	1	LLRLLNRTYA	tr_C9ZJU1	0.273	2607.54	7.00
601	HLA-A0201	GLFQMADTLP	GLFQMADTL	0	0	0	0	0	GLFQMADTL	tr_C9ZJU1	0.272	2629.47	7.00
271	HLA-A0201	AFESLYCIRGFVIQ	FLYCIRGFV	1	0	0	1	2	FESLYCIRGFV	tr_C9ZJU1	0.272	2633.65	7.00
212	HLA-A0201	LKCILGMHAFV	LILGMHAFV	0	0	0	1	2	LKCILGMHAFV	tr_C9ZJU1	0.272	2639.76	7.00
198	HLA-A0201	VTLAATVLFS	VLAATVLFS	0	0	0	1	1	VTLAATVLFS	tr_C9ZJU1	0.271	2652.13	7.00
757	HLA-A0201	GKTTLLRLLNRTYA	TLLRLLTYA	3	0	0	6	2	TLLRLLNRTYA	tr_C9ZJU1	0.271	2653.53	7.00
93	HLA-A0201	VCDIWNCLMALV	VIWNCLMAL	0	0	0	1	2	VCDIWNCLMAL	tr_C9ZJU1	0.271	2667.93	7.00
13	HLA-A0201	ERKAYEILEMLTRA	YILEMLTRA	4	0	0	1	1	YEILEMLTRA	tr_C9ZJU1	0.271	2670.35	7.00
377	HLA-A0201	SLYMESQFTKSQL	SLYMESQFL	0	0	0	8	4	SLYMESQFTKSQL	tr_C9ZJU1	0.271	2670.56	7.00
936	HLA-A0201	VLNEGMVAEVGSW	VLNEMVAEV	0	0	0	4	1	VLNEGMVAEV	tr_C9ZJU1	0.271	2676.05	7.00
277	HLA-A0201	CIRGFVIQSILSDI	FVIQSILSI	4	0	0	8	1	FVIQSILSDI	tr_C9ZJU1	0.270	2679.30	7.00
687	HLA-A0201	YQVLEGLQSVQRK	YQLEGLQSV	0	0	0	2	1	YQVLEGLQSV	tr_C9ZJU1	0.270	2686.09	7.00
279	HLA-A0201	RGFVIQSILSDII	FVIQSILSI	2	0	0	8	2	FVIQSILSDII	tr_C9ZJU1	0.270	2703.09	7.00
564	HLA-A0201	ALRLSGWELMAATA	ALWELMAAT	0	0	0	2	4	ALRLSGWELMAAT	tr_C9ZJU1	0.270	2703.38	7.00
909	HLA-A0201	GNNLLRDSTIIIVA	LLDSTIIIV	3	0	0	2	1	LLRDSTIIIV	tr_C9ZJU1	0.270	2706.60	7.00
566	HLA-A0201	RLSGWELMAATA	RLWELMAAT	0	0	0	2	2	RLSGWELMAAT	tr_C9ZJU1	0.269	2719.25	7.00
630	HLA-A0201	ALDQTEIVNQL	ALDEIVNQL	0	0	0	3	2	ALDQTEIVNQL	tr_C9ZJU1	0.269	2729.01	7.00
322	HLA-A0201	ALWQASGSVMDELQ	ALWSVMDEL	0	0	0	3	4	ALWQASGSVMDEL	tr_C9ZJU1	0.269	2730.24	7.00
788	HLA-A0201	ARMLRRRIANV	RMLRRIANV	1	0	0	3	1	RMLRRRIANV	tr_C9ZJU1	0.268	2746.24	7.00
528	HLA-A0201	RLGWIKDVI	RLGWIKDVI	0	0	0	0	0	RLGWIKDVI	tr_C9ZJU1	0.268	2748.12	7.00
274	HLA-A0201	SLYCIRGFVIQ	SLYCIGFVI	0	0	0	5	1	SLYCIRGFVI	tr_C9ZJU1	0.268	2749.07	7.00
596	HLA-A0201	RDVLHGLFQM	RVLHGLFQM	0	0	0	1	1	RDVLHGLFQM	tr_C9ZJU1	0.267	2767.69	7.00
372	HLA-A0201	YFLNESLYMES	FLNESLYES	1	0	0	7	1	FLNESLYMES	tr_C9ZJU1	0.267	2781.29	7.00

472	HLA-A0201	FSMVFVESGRPL	FSMVFVEPL	0	0	0	7	3	FSMVFVESGRPL	tr_C9ZJU1	0.267	2781.77	7.00
325	HLA-A0201	QASGSVMDELQSRV	SVMDELQSV	4	0	0	8	1	SVMDELQSRV	tr_C9ZJU1	0.267	2785.29	7.00
204	HLA-A0201	VLFSPEVILKCI	VLFSILKCI	0	0	0	4	3	VLFSPEVILKCI	tr_C9ZJU1	0.267	2792.11	7.00
80	HLA-A0201	CGLMKNSHPDHL	GLMKNSHPL	1	0	0	8	2	GLMKNSHPDHL	tr_C9ZJU1	0.266	2801.50	7.00
572	HLA-A0201	LMAATAAAAFATA	LMAATFATA	0	0	0	5	4	LMAATAAAAFATA	tr_C9ZJU1	0.266	2804.59	7.00
364	HLA-A0201	GTQWSNFLYFLN	TQWNFLYFL	1	0	0	3	1	TQWSNFLYFL	tr_C9ZJU1	0.266	2810.45	7.00
882	HLA-A0201	LCIFDEPTSA	LIFDEPTSA	0	0	0	1	1	LCIFDEPTSA	tr_C9ZJU1	0.266	2811.18	7.00
278	HLA-A0201	IRGFVIQSILSDII	FVIQSILSI	3	0	0	8	2	FVIQSILSDII	tr_C9ZJU1	0.266	2827.44	7.00
286	HLA-A0201	ILSDIITSIHIDAY	ILSDIIIHI	0	0	0	6	2	ILSDIITSIHI	tr_C9ZJU1	0.265	2835.77	7.00
102	HLA-A0201	ALVNCSIRDIL	ALVNCSIIL	0	0	0	7	2	ALVNCSIRDIL	tr_C9ZJU1	0.265	2835.87	7.00
467	HLA-A0201	YGLVLFSMVFVES	VLFSMVFES	3	0	0	7	1	VLFSMVFVES	tr_C9ZJU1	0.265	2855.60	7.00
9	HLA-A0201	LLDCERKAYEILEM	LLDCEILEM	0	0	0	4	5	LLDCERKAYEILEM	tr_C9ZJU1	0.265	2855.85	7.00
15	HLA-A0201	KAYEILEMLT	KAYEILMLT	0	0	0	6	1	KAYEILEMLT	tr_C9ZJU1	0.265	2856.71	7.00
126	HLA-A0201	KAEEVLALCMLL	KVLALCMLL	0	0	0	1	3	KAEEVLALCMLL	tr_C9ZJU1	0.265	2857.39	7.00
448	HLA-A0201	MLFQQTFSHPS	MLFQQTFPS	0	0	0	7	2	MLFQQTFSHPS	tr_C9ZJU1	0.265	2857.98	7.00
558	HLA-A0201	YLQGFAALRLSGW	YLQGFAALL	0	0	0	8	1	YLQGFAALRL	tr_C9ZJU1	0.264	2859.78	7.00
263	HLA-A0201	SMAVASTFAF	SMAASTFAF	0	0	0	3	1	SMAVASTFAF	tr_C9ZJU1	0.264	2859.99	7.00
766	HLA-A0201	NRTYAPTEGDIFV	RTYEGDIFV	1	0	0	3	3	RTYAPTEGDIFV	tr_C9ZJU1	0.264	2863.21	7.00
492	HLA-A0201	QHVIWPLVPSVF	VIWPLVPSV	2	0	0	0	0	VIWPLVPSV	tr_C9ZJU1	0.264	2866.28	7.00
97	HLA-A0201	WNCLMALVNCSIR	CLMALVCSI	2	0	0	6	1	CLMALVNCSI	tr_C9ZJU1	0.264	2869.14	7.00
811	HLA-A0201	LSIGGNIAL	LSIGGNIAL	0	0	0	0	0	LSIGGNIAL	tr_C9ZJU1	0.264	2877.12	7.00
383	HLA-A0201	QFTKSQLASVTNIA	FLASVTNIA	1	0	0	1	4	FTKSQLASVTNIA	tr_C9ZJU1	0.264	2886.60	7.00
465	HLA-A0201	VQYGLVLFSMVF	VQYGLVLFV	0	0	0	8	2	VQYGLVLFSMV	tr_C9ZJU1	0.264	2887.92	7.00
512	HLA-A0201	KRKLQAGGHTCAV	KLQAGTCAV	2	0	0	5	2	KLQAGGHTCAV	tr_C9ZJU1	0.263	2890.42	7.00
100	HLA-A0201	LMALVNCSIRDIL	LMALVNCSL	0	0	0	8	4	LMALVNCSIRDIL	tr_C9ZJU1	0.263	2905.50	7.00
284	HLA-A0201	QSILSDIITSIH	ILSDIITSI	2	0	0	0	0	ILSDIITSI	tr_C9ZJU1	0.263	2908.46	7.00
80	HLA-A0201	CGLMKNSHPDHLL	GLMKNSHPL	1	0	0	8	3	GLMKNSHPDHLL	tr_C9ZJU1	0.263	2910.00	7.00
200	HLA-A0201	LAATVLFSPEV	LAATVLFEV	0	0	0	7	2	LAATVLFSPEV	tr_C9ZJU1	0.263	2910.91	7.00
494	HLA-A0201	VIWPLVPSVFS	VIWPLVPSV	0	0	0	0	0	VIWPLVPSV	tr_C9ZJU1	0.263	2917.69	7.00
933	HLA-A0201	KIVVLNEGMV	KIVVLNEMV	0	0	0	7	1	KIVVLNEGMV	tr_C9ZJU1	0.263	2917.69	7.00
92	HLA-A0201	LVCDIWNCLMALVN	LIWNCLMAL	0	0	0	1	3	LVCDIWNCLMAL	tr_C9ZJU1	0.262	2930.09	7.00
608	HLA-A0201	TLPLLLVSAVA	TLLLLVSAV	0	0	0	2	1	TLPLLLVSAV	tr_C9ZJU1	0.262	2936.50	7.00
373	HLA-A0201	FLNESLYMESQF	FLNESLYMF	0	0	0	8	3	FLNESLYMESQF	tr_C9ZJU1	0.262	2943.98	7.00
204	HLA-A0201	VLFSPEVILKC	VLFEVILKC	0	0	0	3	2	VLFSPEVILKC	tr_C9ZJU1	0.262	2949.62	7.00
608	HLA-A0201	TLPLLLVSAVAL	TLPLLLVSL	0	0	0	8	3	TLPLLLVSAVAL	tr_C9ZJU1	0.261	2958.80	7.00
468	HLA-A0201	GLVLFSMVFVESG	GLLFSMVFV	0	0	0	2	1	GLVLFSMVFV	tr_C9ZJU1	0.261	2959.89	7.00
686	HLA-A0201	CYQVLEGLQSVQR	YQLEGLQSV	1	0	0	2	1	YQVLEGLQSV	tr_C9ZJU1	0.261	2960.27	7.00
247	HLA-A0201	FRFIIKQLMGLPA	FIIKQLMPA	2	0	0	7	2	FIIKQLMGLPA	tr_C9ZJU1	0.261	2963.03	7.00
96	HLA-A0201	IWNCLMALV	IWNCLMALV	0	0	0	0	0	IWNCLMALV	tr_C9ZJU1	0.261	2966.17	7.00
282	HLA-A0201	VIQSILSDIITSIH	ILSDIITSI	1	0	0	1	3	IQSILSDIITSI	tr_C9ZJU1	0.261	2969.80	7.00
366	HLA-A0201	QWSNFLYFL	QWSNFLYFL	0	0	0	0	0	QWSNFLYFL	tr_C9ZJU1	0.261	2976.07	7.00
118	HLA-A0201	ALCILDDVKAEEVL	ILDDVAEEV	3	0	0	5	1	ILDDVKAEEV	tr_C9ZJU1	0.261	2982.48	7.50
199	HLA-A0201	TLAATVLFSP	TLAAVLFSP	0	0	0	4	1	TLAATVLFSP	tr_C9ZJU1	0.260	2989.95	7.50
96	HLA-A0201	IWNCLMALVNCSIR	CLMALVCSI	3	0	0	6	1	CLMALVNCSI	tr_C9ZJU1	0.260	2991.02	7.50
467	HLA-A0201	YGLVLFSMV	YGLVLFSMV	0	0	0	0	0	YGLVLFSMV	tr_C9ZJU1	0.260	2991.63	7.50
491	HLA-A0201	TQHVIWPLVPSVF	VIWPLVPSV	3	0	0	0	0	VIWPLVPSV	tr_C9ZJU1	0.260	2994.03	7.50
317	HLA-A0201	RCVSQALWQASGSV	RLWQASGSV	0	0	0	1	5	RCVSQALWQASGSV	tr_C9ZJU1	0.260	2999.96	7.50
112	HLA-A0201	LLDDGVALCILD	LLDDGVALL	0	0	0	8	2	LLDDGVALCIL	tr_C9ZJU1	0.260	3007.76	7.50
254	HLA-A0201	LMGLPATFVSMAVA	LMFVSMAVA	0	0	0	2	5	LMGLPATFVSMAVA	tr_C9ZJU1	0.259	3031.22	7.50
99	HLA-A0201	CLMALVNCSIRD	CLMALVCSI	0	0	0	6	1	CLMALVNCSI	tr_C9ZJU1	0.259	3041.14	7.50
359	HLA-A0201	LVMWLGTQWSNFLY	VMWLWSNFL	1	0	0	4	3	VMWLGTQWSNFL	tr_C9ZJU1	0.259	3041.44	7.50
557	HLA-A0201	DYLQGFAALRLSG	YLQGFAALL	1	0	0	8	1	YLQGFAALRL	tr_C9ZJU1	0.259	3044.73	7.50
319	HLA-A0201	VSQALWQASGSV	VLWQASGSV	0	0	0	1	3	VSQALWQASGSV	tr_C9ZJU1	0.258	3050.43	7.50
466	HLA-A0201	QYGLVLFSMVFVES	VLFSMVFES	4	0	0	7	1	VLFSMVFVES	tr_C9ZJU1	0.258	3052.45	7.50

243	HLA-A0201	KLSPFRFIIKQL	KLSPFRFQL	0	0	0	7	3	KLSPFRFIIKQL	tr_C9ZJU1	0.258	3056.05	7.50
883	HLA-A0201	CIFDEPTSAI	CIFDEPTSI	0	0	0	8	1	CIFDEPTSAI	tr_C9ZJU1	0.258	3057.54	7.50
259	HLA-A0201	ATFVSMAVA	ATFVSMAVA	0	0	0	0	0	ATFVSMAVA	tr_C9ZJU1	0.258	3062.60	7.50
511	HLA-A0201	LKRKLQAGGHTCAV	KLQAGTCAV	3	0	0	5	2	KLQAGGHTCAV	tr_C9ZJU1	0.258	3063.93	7.50
936	HLA-A0201	VLNEGMVAEVGSWE	VLNEMVAEV	0	0	0	4	1	VLNEGMVAEV	tr_C9ZJU1	0.258	3065.72	7.50
176	HLA-A0201	SLQSGITSF	SLQSGITSF	0	0	0	0	0	SLQSGITSF	tr_C9ZJU1	0.258	3072.79	7.50
274	HLA-A0201	SLYCIRGFVIQS	SLYCIGFVI	0	0	0	5	1	SLYCIRGFVI	tr_C9ZJU1	0.258	3082.42	7.50
927	HLA-A0201	TLKHADKIVV	TLHADKIVV	0	0	0	2	1	TLKHADKIVV	tr_C9ZJU1	0.257	3084.68	7.50
786	HLA-A0201	YPARMLRRRIANV	YLRRRIANV	0	0	0	1	4	YPARMLRRRIANV	tr_C9ZJU1	0.257	3106.59	7.50
916	HLA-A0201	STIIIVAHRL	STIIIVAHL	0	0	0	8	1	STIIIVAHRL	tr_C9ZJU1	0.257	3107.70	7.50
378	HLA-A0201	LYMESQFTKSQLA	YMFTKSQLA	1	0	0	2	3	YMESQFTKSQLA	tr_C9ZJU1	0.257	3114.09	7.50
927	HLA-A0201	TLKHADKIVVL	TLADKIVVL	0	0	0	2	2	TLKHADKIVVL	tr_C9ZJU1	0.256	3117.87	7.50
131	HLA-A0201	LALCMLLRGKYSIA	ALCMLLYSI	1	0	0	6	3	ALCMLLRGKYSI	tr_C9ZJU1	0.256	3119.32	7.50
687	HLA-A0201	YQVLEGLQSVQRKI	YQLEGLQSV	0	0	0	2	1	YQVLEGLQSV	tr_C9ZJU1	0.256	3120.44	7.50
866	HLA-A0201	RLCIARAFVK	RLCIARAFV	0	0	0	0	0	RLCIARAFV	tr_C9ZJU1	0.256	3120.44	7.50
246	HLA-A0201	PFRFIIKQLMGLPA	FIIKQLMPA	3	0	0	7	2	FIIKQLMGLPA	tr_C9ZJU1	0.256	3125.50	7.50
365	HLA-A0201	TQWSNFLYFLNE	TQWNFLYFL	0	0	0	3	1	TQWSNFLYFL	tr_C9ZJU1	0.256	3129.19	7.50
260	HLA-A0201	TFVSMAVASTFA	FMAVASTFA	1	0	0	1	2	FVSMAVASTFA	tr_C9ZJU1	0.256	3129.73	7.50
550	HLA-A0201	CVWQCRQDYL	CVWQCQDYL	0	0	0	5	1	CVWQCRQDYL	tr_C9ZJU1	0.256	3130.82	7.50
340	HLA-A0201	ALLEVMHGCR	ALLEVMHGC	0	0	0	0	0	ALLEVMHGC	tr_C9ZJU1	0.256	3135.97	7.50
790	HLA-A0201	MLRRRIANVW	MLRRRIANV	0	0	0	0	0	MLRRRIANV	tr_C9ZJU1	0.256	3136.95	7.50
318	HLA-A0201	CVSQALWQASGSV	ALWQASGSV	4	0	0	0	0	ALWQASGSV	tr_C9ZJU1	0.256	3150.08	7.50
733	HLA-A0201	YVLRRVSFVVN	YVLRVSFVV	0	0	0	3	1	YVLRRVSFVV	tr_C9ZJU1	0.255	3166.21	7.50
558	HLA-A0201	YLQGFAALRLSGWE	YLQGFAALL	0	0	0	8	1	YLQGFAALRL	tr_C9ZJU1	0.255	3169.67	7.50
734	HLA-A0201	VLRRVSFVVN	VLRRVSFVV	0	0	0	0	0	VLRRVSFVV	tr_C9ZJU1	0.255	3170.84	7.50
935	HLA-A0201	VVLNEGMVAEVGS	VLNEMVAEV	1	0	0	4	1	VLNEGMVAEV	tr_C9ZJU1	0.255	3176.02	7.50
194	HLA-A0201	IPQLVTLAATVL	ILVTLAATV	0	0	0	1	2	IPQLVTLAATV	tr_C9ZJU1	0.255	3178.71	7.50
119	HLA-A0201	LCILDDVKAEEVL	ILDDVAEEV	2	0	0	5	1	ILDDVKAEEV	tr_C9ZJU1	0.254	3190.11	7.50
111	HLA-A0201	ILLDDGVALCILDD	ILLDVALCI	0	0	0	4	2	ILLDDGVALCI	tr_C9ZJU1	0.254	3190.63	7.50
469	HLA-A0201	LVLFSMVFVES	VLFSMVFES	1	0	0	7	1	VLFSMVFVES	tr_C9ZJU1	0.254	3203.77	7.50
110	HLA-A0201	DILLDDGVALCILD	ILLDVALCI	1	0	0	4	2	ILLDDGVALCI	tr_C9ZJU1	0.254	3205.85	7.50
599	HLA-A0201	LHGLFQMADTL	LLFQMADTL	0	0	0	1	2	LHGLFQMADTL	tr_C9ZJU1	0.254	3216.97	7.50
79	HLA-A0201	HCGLMKNSHPDHL	GLMKNSHPL	2	0	0	8	2	GLMKNSHPDHL	tr_C9ZJU1	0.253	3223.07	7.50
328	HLA-A0201	GSVMDELQSRVPA	VMDELQVPA	2	0	0	6	2	VMDELQSRVPA	tr_C9ZJU1	0.253	3229.84	7.50
381	HLA-A0201	ESQFTKSQLASV	SQFSQLASV	1	0	0	3	2	SQFTKSQLASV	tr_C9ZJU1	0.253	3246.98	7.50
699	HLA-A0201	KIDRPVRGV	KIDRPVRGV	0	0	0	0	0	KIDRPVRGV	tr_C9ZJU1	0.252	3267.42	7.50
386	HLA-A0201	KSQLASVTNI	KQLASVTNI	0	0	0	1	1	KSQLASVTNI	tr_C9ZJU1	0.252	3277.93	7.50
688	HLA-A0201	QVLEGLQSVQ	QVLEGLQSV	0	0	0	0	0	QVLEGLQSV	tr_C9ZJU1	0.252	3280.03	7.50
606	HLA-A0201	ADTLPLLLVSAVA	ALLLVSAVA	0	0	0	1	4	ADTLPLLLVSAVA	tr_C9ZJU1	0.251	3297.28	7.50
132	HLA-A0201	ALCMLLRGKYSIAR	ALCMLLYSI	0	0	0	6	3	ALCMLLRGKYSI	tr_C9ZJU1	0.251	3299.96	7.50
285	HLA-A0201	SILSDIITS	SILSDIITS	0	0	0	0	0	SILSDIITS	tr_C9ZJU1	0.251	3308.83	7.50
347	HLA-A0201	GCRVVFSFPV	GCVVFSFPV	0	0	0	2	1	GCRVVFSFPV	tr_C9ZJU1	0.251	3310.29	7.50
48	HLA-A0201	IFLNCNGVESIH	FLNCGVESI	1	0	0	4	1	FLNCNGVESI	tr_C9ZJU1	0.251	3313.16	7.50
363	HLA-A0201	LGTQWSNFLYFLN	TQWNFLYFL	2	0	0	3	1	TQWSNFLYFL	tr_C9ZJU1	0.250	3325.66	7.50
120	HLA-A0201	CILDDVKAEEVLA	ILDDVEVLA	1	0	0	5	3	ILDDVKAEEVLA	tr_C9ZJU1	0.250	3330.96	7.50
268	HLA-A0201	STFAFESLYC	STFAFSLYC	0	0	0	5	1	STFAFESLYC	tr_C9ZJU1	0.250	3332.80	7.50
79	HLA-A0201	HCGLMKNSHPDHLL	GLMKNSHPL	2	0	0	8	3	GLMKNSHPDHLL	tr_C9ZJU1	0.250	3338.17	7.50
711	HLA-A0201	SLDGPWSVEFREV	SLDGPWSEV	0	0	0	7	4	SLDGPWSVEFREV	tr_C9ZJU1	0.250	3345.62	7.50
961	HLA-A0201	SRMRQSQQLPHFV	RMQQLPHFV	1	0	0	2	3	RMRQSQQLPHFV	tr_C9ZJU1	0.250	3352.29	7.50
100	HLA-A0201	LMALVNCSIRDI	LMALVNCSI	0	0	0	8	3	LMALVNCSIRDI	tr_C9ZJU1	0.250	3354.54	7.50
727	HLA-A0201	YSDRHPYVL	YSDRHPYVL	0	0	0	0	0	YSDRHPYVL	tr_C9ZJU1	0.250	3354.75	7.50
17	HLA-A0201	YEILEMLTRATI	ILMLTRATI	2	0	0	2	1	ILEMLTRATI	tr_C9ZJU1	0.249	3367.01	7.50
293	HLA-A0201	SIHIDAYLA	SIHIDAYLA	0	0	0	0	0	SIHIDAYLA	tr_C9ZJU1	0.249	3369.92	7.50
468	HLA-A0201	GLVLFSMVFVESGR	GLLFSMVFV	0	0	0	2	1	GLVLFSMVFV	tr_C9ZJU1	0.249	3378.47	7.50

556	HLA-A0201	QDYLQGFAALRLSG	YLQGFAALL	2	0	0	8	1	YLQGFAALRL	tr_C9ZJU1	0.249	3383.26	8.00
282	HLA-A0201	VIQSILSDI	VIQSILSDI	0	0	0	0	0	VIQSILSDI	tr_C9ZJU1	0.249	3386.99	8.00
598	HLA-A0201	VLHGLFQMADTL	VLHGLFQTL	0	0	0	7	3	VLHGLFQMADTL	tr_C9ZJU1	0.249	3387.51	8.00
607	HLA-A0201	DTLPLLLVSAVA	TLLLLVSAV	1	0	0	2	1	TLPLLLVSAV	tr_C9ZJU1	0.249	3395.07	8.00
464	HLA-A0201	SVQYGLVLFSM	VQYGVLFSM	1	0	0	4	1	VQYGLVLFSM	tr_C9ZJU1	0.249	3397.49	8.00
349	HLA-A0201	RVVFSFPVVSL	VVFSFVVSL	1	0	0	5	1	VVFSFPVVSL	tr_C9ZJU1	0.248	3399.40	8.00
318	HLA-A0201	CVSQALWQA	CVSQALWQA	0	0	0	0	0	CVSQALWQA	tr_C9ZJU1	0.248	3407.03	8.00
652	HLA-A0201	AFLGSFYDEC	FLGSFYDEC	1	0	0	0	0	FLGSFYDEC	tr_C9ZJU1	0.248	3417.07	8.00
47	HLA-A0201	AIFLNCNGVE	AIFLNCNGV	0	0	0	0	0	AIFLNCNGV	tr_C9ZJU1	0.248	3423.03	8.00
203	HLA-A0201	TVLFSPEVILKCI	VLFSILKCI	1	0	0	4	3	VLFSPEVILKCI	tr_C9ZJU1	0.248	3423.06	8.00
376	HLA-A0201	ESLYMESQFT	SLYMESQFT	1	0	0	0	0	SLYMESQFT	tr_C9ZJU1	0.247	3438.39	8.00
78	HLA-A0201	VHCGLMKNSHPDHL	GLMKNSHPL	3	0	0	8	2	GLMKNSHPDHL	tr_C9ZJU1	0.247	3440.89	8.00
911	HLA-A0201	NLLRDSTIIIVAH	NLLSTIIIV	0	0	0	3	2	NLLRDSTIIIV	tr_C9ZJU1	0.247	3448.00	8.00
371	HLA-A0201	LYFLNESLYMES	FLNESLYES	2	0	0	7	1	FLNESLYMES	tr_C9ZJU1	0.247	3449.53	8.00
384	HLA-A0201	FTKSQLASVTNI	FQLASVTNI	0	0	0	1	3	FTKSQLASVTNI	tr_C9ZJU1	0.247	3470.57	8.00
912	HLA-A0201	LLRDSTIIIVAH	LLDSTIIIV	0	0	0	2	1	LLRDSTIIIV	tr_C9ZJU1	0.246	3483.59	8.00
94	HLA-A0201	CDIWNCLMALV	CIWNCLMAL	0	0	0	1	1	CDIWNCLMAL	tr_C9ZJU1	0.246	3487.29	8.00
447	HLA-A0201	KMLFQQTFS	KMLFQQTFS	0	0	0	0	0	KMLFQQTFS	tr_C9ZJU1	0.246	3490.91	8.00
881	HLA-A0201	GLCIFDEPTSA	GLFDEPTSA	0	0	0	2	2	GLCIFDEPTSA	tr_C9ZJU1	0.246	3491.02	8.00
912	HLA-A0201	LLRDSTIIIVAHRL	LLIIVAHRL	0	0	0	2	5	LLRDSTIIIVAHRL	tr_C9ZJU1	0.246	3495.45	8.00
273	HLA-A0201	ESLYCIRGFVIQ	SLYCIGFVI	1	0	0	5	1	SLYCIRGFVI	tr_C9ZJU1	0.246	3498.13	8.00
203	HLA-A0201	TVLFSPEVILKC	VLFEVILKC	1	0	0	3	2	VLFSPEVILKC	tr_C9ZJU1	0.246	3499.84	8.00
562	HLA-A0201	FAALRLSGWEL	FLRLSGWEL	0	0	0	1	2	FAALRLSGWEL	tr_C9ZJU1	0.246	3501.28	8.00
685	HLA-A0201	GCYQVLEGLQSVQR	YQLEGLQSV	2	0	0	2	1	YQVLEGLQSV	tr_C9ZJU1	0.246	3502.83	8.00
832	HLA-A0201	AALSASRALNFVQ	ALSASLNFV	1	0	0	5	2	ALSASRALNFV	tr_C9ZJU1	0.245	3519.43	8.00
312	HLA-A0201	DWLLSRCVSQA	WLLSRCVSA	1	0	0	8	1	WLLSRCVSQA	tr_C9ZJU1	0.245	3531.22	8.00
18	HLA-A0201	EILEMLTRATI	ILMLTRATI	1	0	0	2	1	ILEMLTRATI	tr_C9ZJU1	0.245	3534.93	8.00
118	HLA-A0201	ALCILDDVKA	ALCILDVKA	0	0	0	5	1	ALCILDDVKA	tr_C9ZJU1	0.245	3535.46	8.00
71	HLA-A0201	QLPSVFAVHCGL	QLFAVHCGL	0	0	0	2	3	QLPSVFAVHCGL	tr_C9ZJU1	0.245	3542.47	8.00
185	HLA-A0201	WKLMCSSCSIP	KLMCSSCSI	1	0	0	0	0	KLMCSSCSI	tr_C9ZJU1	0.245	3548.64	8.00
327	HLA-A0201	SGSVMDELQSRVPA	VMDELQVPA	3	0	0	6	2	VMDELQSRVPA	tr_C9ZJU1	0.244	3553.79	8.00
269	HLA-A0201	TFAFESLYCIR	FAFESLYCI	1	0	0	0	0	FAFESLYCI	tr_C9ZJU1	0.244	3562.92	8.00
904	HLA-A0201	LRLREGNNLL	LLREGNNLL	0	0	0	1	1	LRLREGNNLL	tr_C9ZJU1	0.244	3564.65	8.00
365	HLA-A0201	TQWSNFLYF	TQWSNFLYF	0	0	0	0	0	TQWSNFLYF	tr_C9ZJU1	0.244	3568.74	8.00
127	HLA-A0201	AEEVLALCMLL	AVLALCMLL	0	0	0	1	2	AEEVLALCMLL	tr_C9ZJU1	0.244	3568.94	8.00
607	HLA-A0201	DTLPLLLVSAVAL	TLPLLLVSL	1	0	0	8	3	TLPLLLVSAVAL	tr_C9ZJU1	0.244	3575.47	8.00
563	HLA-A0201	AALRLSGWEL	ALRLSGWEL	0	0	0	1	1	AALRLSGWEL	tr_C9ZJU1	0.244	3577.36	8.00
765	HLA-A0201	LNRTYAPTEGDIFV	RTYEGDIFV	2	0	0	3	3	RTYAPTEGDIFV	tr_C9ZJU1	0.244	3580.62	8.00
377	HLA-A0201	SLYMESQFTKSQLA	YMFTKSQLA	2	0	0	2	3	YMESQFTKSQLA	tr_C9ZJU1	0.244	3586.90	8.00
467	HLA-A0201	YGLVLFSMVFVESG	GLVLFMVFV	1	0	0	5	1	GLVLFSMVFV	tr_C9ZJU1	0.243	3588.06	8.00
557	HLA-A0201	DYLQGFAALRLSGW	YLQGFAALL	1	0	0	8	1	YLQGFAALRL	tr_C9ZJU1	0.243	3591.44	8.00
787	HLA-A0201	PARMLRRRIANV	RMLRRIANV	2	0	0	3	1	RMLRRRIANV	tr_C9ZJU1	0.243	3606.16	8.00
785	HLA-A0201	QYPARMLRRRIANV	RMLRRIANV	4	0	0	3	1	RMLRRRIANV	tr_C9ZJU1	0.243	3607.92	8.00
136	HLA-A0201	LLRGKYSIARYPPA	LLIARYPPA	0	0	0	2	5	LLRGKYSIARYPPA	tr_C9ZJU1	0.243	3612.68	8.00
747	HLA-A0201	FLGIAGYSGS	FLIAGYSGS	0	0	0	2	1	FLGIAGYSGS	tr_C9ZJU1	0.243	3613.97	8.00
16	HLA-A0201	AYEILEMLTRATI	ILMLTRATI	3	0	0	2	1	ILEMLTRATI	tr_C9ZJU1	0.243	3618.63	8.00
494	HLA-A0201	VIWPLVPSVFSG	VIWPLVPSV	0	0	0	0	0	VIWPLVPSV	tr_C9ZJU1	0.242	3638.02	8.00
204	HLA-A0201	VLFSPEVILKCIL	VLFEVILKC	0	0	0	3	2	VLFSPEVILKC	tr_C9ZJU1	0.242	3640.86	8.00
15	HLA-A0201	KAYEILEMLTRATI	ILMLTRATI	4	0	0	2	1	ILEMLTRATI	tr_C9ZJU1	0.242	3646.10	8.00
204	HLA-A0201	VLFSPEVILK	VLFSPEVIL	0	0	0	0	0	VLFSPEVIL	tr_C9ZJU1	0.242	3653.09	8.00
270	HLA-A0201	FAFESLYCIRG	FAFESLYCI	0	0	0	0	0	FAFESLYCI	tr_C9ZJU1	0.242	3657.16	8.00
18	HLA-A0201	EILEMLTRA	EILEMLTRA	0	0	0	0	0	EILEMLTRA	tr_C9ZJU1	0.242	3664.17	8.00
125	HLA-A0201	VKAEEVLALCMLL	KVLALCMLL	1	0	0	1	3	KAEEVLALCMLL	tr_C9ZJU1	0.242	3665.36	8.00
348	HLA-A0201	CRVVFSFPVVSL	VVFSFVVSL	2	0	0	5	1	VVFSFPVVSL	tr_C9ZJU1	0.241	3670.05	8.00

471	HLA-A0201	LFSMVFVESGRPL	FSMVFVEPL	1	0	0	7	3	FSMVFVESGRPL	tr_C9ZJU1	0.241	3675.65	8.00
301	HLA-A0201	ALATADYDPSDDWL	ALATADYWL	0	0	0	7	5	ALATADYDPSDDWL	tr_C9ZJU1	0.241	3685.69	8.00
292	HLA-A0201	TSIHIDAYL	TSIHIDAYL	0	0	0	0	0	TSIHIDAYL	tr_C9ZJU1	0.241	3695.11	8.00
474	HLA-A0201	MVFVESGRPL	MVFESGRPL	0	0	0	3	1	MVFVESGRPL	tr_C9ZJU1	0.241	3697.71	8.00
407	HLA-A0201	NQVSPTCSFPV	NQVSCSFPV	0	0	0	4	2	NQVSPTCSFPV	tr_C9ZJU1	0.241	3700.07	8.00
254	HLA-A0201	LMGLPATFVSMA	LMATFVSMA	0	0	0	2	3	LMGLPATFVSMA	tr_C9ZJU1	0.241	3703.63	8.00
365	HLA-A0201	TQWSNFLYFLNES	TQWNFLYFL	0	0	0	3	1	TQWSNFLYFL	tr_C9ZJU1	0.240	3720.10	8.00
362	HLA-A0201	WLGTQWSNFLYF	WLWSNFLYF	0	0	0	2	3	WLGTQWSNFLYF	tr_C9ZJU1	0.240	3725.78	8.00
330	HLA-A0201	VMDELQSRVPALLE	VMDEVPALL	0	0	0	4	4	VMDELQSRVPALL	tr_C9ZJU1	0.240	3732.52	8.00
98	HLA-A0201	NCLMALVNCS	CLMALVNCS	1	0	0	0	0	CLMALVNCS	tr_C9ZJU1	0.240	3736.11	8.00
596	HLA-A0201	RDVLHGLFQMADTL	RLFQMADTL	0	0	0	1	5	RDVLHGLFQMADTL	tr_C9ZJU1	0.240	3737.08	8.00
202	HLA-A0201	ATVLFSPEVILKCI	VLFSILKCI	2	0	0	4	3	VLFSPEVILKCI	tr_C9ZJU1	0.240	3742.42	8.00
686	HLA-A0201	CYQVLEGLQSVQRK	YQLEGLQSV	1	0	0	2	1	YQVLEGLQSV	tr_C9ZJU1	0.239	3750.37	8.00
934	HLA-A0201	IVVLNEGMVAEVGS	VLNEMVAEV	2	0	0	4	1	VLNEGMVAEV	tr_C9ZJU1	0.239	3750.41	8.00
376	HLA-A0201	ESLYMESQFTKSQL	SLYMESQFL	1	0	0	8	4	SLYMESQFTKSQL	tr_C9ZJU1	0.239	3750.82	8.00
256	HLA-A0201	GLPATFVSMAVA	GLFVSMAVA	0	0	0	2	3	GLPATFVSMAVA	tr_C9ZJU1	0.239	3753.74	8.00
228	HLA-A0201	YAAEIGKSL	YAAEIGKSL	0	0	0	0	0	YAAEIGKSL	tr_C9ZJU1	0.239	3765.05	8.00
290	HLA-A0201	IITSIHIDAYL	IIIHIDAYL	0	0	0	2	2	IITSIHIDAYL	tr_C9ZJU1	0.239	3767.04	8.00
259	HLA-A0201	ATFVSMAVASTFA	FMAVASTFA	2	0	0	1	2	FVSMAVASTFA	tr_C9ZJU1	0.239	3770.92	8.00
261	HLA-A0201	FVSMAVASTF	FVMAVASTF	0	0	0	2	1	FVSMAVASTF	tr_C9ZJU1	0.239	3776.19	8.00
606	HLA-A0201	ADTLPLLLVSAVAL	ALLVSAVAL	0	0	0	1	5	ADTLPLLLVSAVAL	tr_C9ZJU1	0.239	3781.46	8.00
464	HLA-A0201	SVQYGLVLFSMVF	VQYGLVLFV	1	0	0	8	2	VQYGLVLFSMV	tr_C9ZJU1	0.239	3783.67	8.50
611	HLA-A0201	LLLVSAVALRLS	LLLAVALRL	0	0	0	3	2	LLLVSAVALRL	tr_C9ZJU1	0.239	3786.13	8.50
202	HLA-A0201	ATVLFSPEVILKC	VLFEVILKC	2	0	0	3	2	VLFSPEVILKC	tr_C9ZJU1	0.238	3790.84	8.50
273	HLA-A0201	ESLYCIRGFVIQS	SLYCIGFVI	1	0	0	5	1	SLYCIRGFVI	tr_C9ZJU1	0.238	3800.66	8.50
311	HLA-A0201	DDWLLSRCVSQA	WLLSRCVSA	2	0	0	8	1	WLLSRCVSQA	tr_C9ZJU1	0.238	3804.98	8.50
98	HLA-A0201	NCLMALVNCSIRD	CLMALVCSI	1	0	0	6	1	CLMALVNCSI	tr_C9ZJU1	0.238	3812.35	8.50
463	HLA-A0201	SSVQYGLVLFSM	VQYGVLFSM	2	0	0	4	1	VQYGLVLFSM	tr_C9ZJU1	0.238	3815.94	8.50
347	HLA-A0201	GCRVVFSFPVVSL	VVFSFVVSL	3	0	0	5	1	VVFSFPVVSL	tr_C9ZJU1	0.238	3816.07	8.50
910	HLA-A0201	NNLLRDSTIIIVAH	LLDSTIIIV	2	0	0	2	1	LLRDSTIIIV	tr_C9ZJU1	0.238	3824.54	8.50
372	HLA-A0201	YFLNESLYMESQF	FLNESLYMF	1	0	0	8	3	FLNESLYMESQF	tr_C9ZJU1	0.237	3829.92	8.50
566	HLA-A0201	RLSGWELMAATAA	RLWELMAAT	0	0	0	2	2	RLSGWELMAAT	tr_C9ZJU1	0.237	3835.77	8.50
73	HLA-A0201	PSVFAVHCGL	SVFAVHCGL	1	0	0	0	0	SVFAVHCGL	tr_C9ZJU1	0.237	3839.67	8.50
831	HLA-A0201	CAALSASRALNFVQ	ALSASLNFV	2	0	0	5	2	ALSASRALNFV	tr_C9ZJU1	0.237	3842.83	8.50
565	HLA-A0201	LRLSGWELMAATA	RLWELMAAT	1	0	0	2	2	RLSGWELMAAT	tr_C9ZJU1	0.237	3855.12	8.50
47	HLA-A0201	AIFLNCNGVESIH	FLNCGVESI	2	0	0	4	1	FLNCNGVESI	tr_C9ZJU1	0.237	3862.93	8.50
193	HLA-A0201	SIPQLVTLAATVL	ILVTLAATV	1	0	0	1	2	IPQLVTLAATV	tr_C9ZJU1	0.237	3865.69	8.50
572	HLA-A0201	LMAATAAAAFA	LMAATAAFA	0	0	0	5	2	LMAATAAAAFA	tr_C9ZJU1	0.236	3869.66	8.50
960	HLA-A0201	FSRMRQSQQLPHFV	RMQQLPHFV	2	0	0	2	3	RMRQSQQLPHFV	tr_C9ZJU1	0.236	3869.87	8.50
82	HLA-A0201	LMKNSHPDHLLV	LMHPDHLLV	0	0	0	2	3	LMKNSHPDHLLV	tr_C9ZJU1	0.236	3869.96	8.50
629	HLA-A0201	SALDQTEIVNQL	ALDEIVNQL	1	0	0	3	2	ALDQTEIVNQL	tr_C9ZJU1	0.236	3871.46	8.50
101	HLA-A0201	MALVNCSIRDIL	ALVNCSIIL	1	0	0	7	2	ALVNCSIRDIL	tr_C9ZJU1	0.236	3872.30	8.50
498	HLA-A0201	LVPSVFSGCV	LVPSVFSGV	0	0	0	8	1	LVPSVFSGCV	tr_C9ZJU1	0.236	3872.38	8.50
619	HLA-A0201	LRLSPNFFTQSA	RLSPNFFTA	1	0	0	8	2	RLSPNFFTQSA	tr_C9ZJU1	0.236	3881.28	8.50
344	HLA-A0201	VMHGCRVVFS	VMHGCVVFS	0	0	0	5	1	VMHGCRVVFS	tr_C9ZJU1	0.236	3881.49	8.50
201	HLA-A0201	AATVLFSPEVILKC	VLFEVILKC	3	0	0	3	2	VLFSPEVILKC	tr_C9ZJU1	0.236	3881.91	8.50
380	HLA-A0201	MESQFTKSQLASV	SQFSQLASV	2	0	0	3	2	SQFTKSQLASV	tr_C9ZJU1	0.236	3889.26	8.50
609	HLA-A0201	LPLLLVSAVAL	LLLVSAVAL	0	0	0	1	2	LPLLLVSAVAL	tr_C9ZJU1	0.236	3896.89	8.50
162	HLA-A0201	ELYLGLITAY	ELYLGLITA	0	0	0	0	0	ELYLGLITA	tr_C9ZJU1	0.236	3898.41	8.50
346	HLA-A0201	HGCRVVFSFPVVSL	VVFSFVVSL	4	0	0	5	1	VVFSFPVVSL	tr_C9ZJU1	0.236	3900.51	8.50
571	HLA-A0201	ELMAATAAAAFATA	LMAATFATA	1	0	0	5	4	LMAATAAAAFATA	tr_C9ZJU1	0.236	3905.96	8.50
448	HLA-A0201	MLFQQTFSH	MLFQQTFSH	0	0	0	0	0	MLFQQTFSH	tr_C9ZJU1	0.236	3908.25	8.50
910	HLA-A0201	NNLLRDSTIII	NLLDSTIII	1	0	0	3	1	NLLRDSTIII	tr_C9ZJU1	0.235	3913.49	8.50
935	HLA-A0201	VVLNEGMVAEVGSW	VLNEMVAEV	1	0	0	4	1	VLNEGMVAEV	tr_C9ZJU1	0.235	3917.64	8.50

313	HLA-A0201	WLLSRCVSQALW	WLLSRCVSL	0	0	0	8	2	WLLSRCVSQAL	tr_C9ZJU1	0.235	3926.77	8.50
833	HLA-A0201	ALSASRALNFVQK	ALSASLNFV	0	0	0	5	2	ALSASRALNFV	tr_C9ZJU1	0.235	3930.89	8.50
268	HLA-A0201	STFAFESLYCIR	FAFESLYCI	2	0	0	0	0	FAFESLYCI	tr_C9ZJU1	0.235	3935.11	8.50
45	HLA-A0201	SIAIFLNCNGV	SIAIFLNGV	0	0	0	6	2	SIAIFLNCNGV	tr_C9ZJU1	0.235	3943.42	8.50
462	HLA-A0201	KSSVQYGLVLFSM	VQYGVLFSM	3	0	0	4	1	VQYGLVLFSM	tr_C9ZJU1	0.234	3963.56	8.50
258	HLA-A0201	PATFVSMAVASTFA	FMAVASTFA	3	0	0	1	2	FVSMAVASTFA	tr_C9ZJU1	0.234	3970.65	8.50
310	HLA-A0201	SDDWLLSRCVSQA	WLLSRCVSA	3	0	0	8	1	WLLSRCVSQA	tr_C9ZJU1	0.234	3972.45	8.50
220	HLA-A0201	AFVHWRPIYAA	FVWRPIYAA	1	0	0	2	1	FVHWRPIYAA	tr_C9ZJU1	0.234	3973.91	8.50
558	HLA-A0201	YLQGFAALR	YLQGFAALR	0	0	0	0	0	YLQGFAALR	tr_C9ZJU1	0.234	3975.46	8.50
154	HLA-A0201	VVVQEFPEEL	VVVEFPEEL	0	0	0	3	1	VVVQEFPEEL	tr_C9ZJU1	0.234	3978.21	8.50
82	HLA-A0201	LMKNSHPDHL	LMNSHPDHL	0	0	0	2	1	LMKNSHPDHL	tr_C9ZJU1	0.234	3993.83	8.50
119	HLA-A0201	LCILDDVKAEEVLA	ILDDVEVLA	2	0	0	5	3	ILDDVKAEEVLA	tr_C9ZJU1	0.234	3995.21	8.50
354	HLA-A0201	FPVVSLVMWLG	FVVSLVMWL	0	0	0	1	1	FPVVSLVMWL	tr_C9ZJU1	0.233	4002.61	8.50
642	HLA-A0201	SIQHWCPLYHA	SIWCPLYHA	0	0	0	2	2	SIQHWCPLYHA	tr_C9ZJU1	0.233	4007.51	8.50
176	HLA-A0201	SLQSGITSFWKLM	SLITSFWKL	0	0	0	2	3	SLQSGITSFWKL	tr_C9ZJU1	0.233	4011.24	8.50
711	HLA-A0201	SLDGPWSVEF	SLDGWSVEF	0	0	0	4	1	SLDGPWSVEF	tr_C9ZJU1	0.233	4016.97	8.50
707	HLA-A0201	VAPPSLDGPWSV	VALDGPWSV	0	0	0	2	3	VAPPSLDGPWSV	tr_C9ZJU1	0.233	4023.19	8.50
133	HLA-A0201	LCMLLRGKYSI	MLLRGKYSI	2	0	0	0	0	MLLRGKYSI	tr_C9ZJU1	0.233	4024.98	8.50
911	HLA-A0201	NLLRDSTIIIVAHR	NLLSTIIIV	0	0	0	3	2	NLLRDSTIIIV	tr_C9ZJU1	0.233	4025.50	8.50
46	HLA-A0201	IAIFLNCNGVESIH	FLNCGVESI	3	0	0	4	1	FLNCNGVESI	tr_C9ZJU1	0.233	4034.26	8.50
461	HLA-A0201	DKSSVQYGLVLFSM	VQYGVLFSM	4	0	0	4	1	VQYGLVLFSM	tr_C9ZJU1	0.232	4053.34	8.50
842	HLA-A0201	FVQKRSSGI	FVQKRSSGI	0	0	0	0	0	FVQKRSSGI	tr_C9ZJU1	0.232	4055.09	8.50
597	HLA-A0201	DVLHGLFQMADTL	LLFQMADTL	2	0	0	1	2	LHGLFQMADTL	tr_C9ZJU1	0.232	4055.58	8.50
493	HLA-A0201	HVIWPLVPSVFS	VIWPLVPSV	1	0	0	0	0	VIWPLVPSV	tr_C9ZJU1	0.232	4059.13	8.50
447	HLA-A0201	KMLFQQTFSHPS	MLFQQTFPS	1	0	0	7	2	MLFQQTFSHPS	tr_C9ZJU1	0.232	4059.44	8.50
93	HLA-A0201	VCDIWNCLMALVN	VIWNCLMAL	0	0	0	1	2	VCDIWNCLMAL	tr_C9ZJU1	0.232	4060.01	8.50
74	HLA-A0201	SVFAVHCGLM	SVFAVCGLM	0	0	0	5	1	SVFAVHCGLM	tr_C9ZJU1	0.232	4063.31	8.50
309	HLA-A0201	PSDDWLLSRCVSQA	WLLSRCVSA	4	0	0	8	1	WLLSRCVSQA	tr_C9ZJU1	0.232	4074.49	8.50
124	HLA-A0201	DVKAEEVLALCMLL	KVLALCMLL	2	0	0	1	3	KAEEVLALCMLL	tr_C9ZJU1	0.232	4079.56	8.50
388	HLA-A0201	QLASVTNIATSTYA	QLASVTTYA	0	0	0	6	5	QLASVTNIATSTYA	tr_C9ZJU1	0.231	4086.98	8.50
494	HLA-A0201	VIWPLVPSVFSGC	VIWPLVPSV	0	0	0	0	0	VIWPLVPSV	tr_C9ZJU1	0.231	4091.81	8.50
912	HLA-A0201	LLRDSTIIIVAHR	LLDSTIIIV	0	0	0	2	1	LLRDSTIIIV	tr_C9ZJU1	0.231	4102.98	8.50
683	HLA-A0201	SLGCYQVLEGLQ	SLYQVLEGL	0	0	0	2	2	SLGCYQVLEGL	tr_C9ZJU1	0.231	4109.86	8.50
352	HLA-A0201	FSFPVVSLVMWLG	FVVSLVMWL	0	0	0	1	3	FSFPVVSLVMWL	tr_C9ZJU1	0.231	4110.35	8.50
880	HLA-A0201	VGLCIFDEPT	VLCIFDEPT	0	0	0	1	1	VGLCIFDEPT	tr_C9ZJU1	0.231	4120.59	8.50
267	HLA-A0201	ASTFAFESLYCIR	FAFESLYCI	3	0	0	0	0	FAFESLYCI	tr_C9ZJU1	0.231	4126.17	8.50
221	HLA-A0201	FVHWRPIYAAEI	FVHWRPIYI	0	0	0	8	3	FVHWRPIYAAEI	tr_C9ZJU1	0.231	4127.06	8.50
251	HLA-A0201	IKQLMGLPATF	ILMGLPATF	0	0	0	1	2	IKQLMGLPATF	tr_C9ZJU1	0.231	4128.54	8.50
192	HLA-A0201	CSIPQLVTLAATVL	ILVTLAATV	2	0	0	1	2	IPQLVTLAATV	tr_C9ZJU1	0.230	4129.66	8.50
692	HLA-A0201	GLQSVQRKI	GLQSVQRKI	0	0	0	0	0	GLQSVQRKI	tr_C9ZJU1	0.230	4138.51	8.50
97	HLA-A0201	WNCLMALVNCSIRD	CLMALVCSI	2	0	0	6	1	CLMALVNCSI	tr_C9ZJU1	0.230	4149.90	8.50
254	HLA-A0201	LMGLPATFVSM	MLPATFVSM	1	0	0	1	1	MGLPATFVSM	tr_C9ZJU1	0.230	4154.39	8.50
901	HLA-A0201	FDTLRLREGNNLL	FLREGNNLL	0	0	0	1	4	FDTLRLREGNNLL	tr_C9ZJU1	0.230	4156.42	8.50
543	HLA-A0201	ATASPSTCV	ATASPSTCV	0	0	0	0	0	ATASPSTCV	tr_C9ZJU1	0.230	4162.00	8.50
364	HLA-A0201	GTQWSNFLYFLNE	TQWNFLYFL	1	0	0	3	1	TQWSNFLYFL	tr_C9ZJU1	0.229	4185.84	8.50
463	HLA-A0201	SSVQYGLVLFSMVF	VQYGLVLFV	2	0	0	8	2	VQYGLVLFSMV	tr_C9ZJU1	0.229	4187.56	8.50
164	HLA-A0201	YLGLITAYN	YLGLITAYN	0	0	0	0	0	YLGLITAYN	tr_C9ZJU1	0.229	4188.79	8.50
250	HLA-A0201	IIKQLMGLPATF	ILMGLPATF	0	0	0	1	3	IIKQLMGLPATF	tr_C9ZJU1	0.229	4216.07	8.50
243	HLA-A0201	KLSPFRFIIKQLM	KLSPFRFLM	0	0	0	7	4	KLSPFRFIIKQLM	tr_C9ZJU1	0.229	4216.80	8.50
383	HLA-A0201	QFTKSQLASV	FTKSQLASV	1	0	0	0	0	FTKSQLASV	tr_C9ZJU1	0.229	4219.26	8.50
419	HLA-A0201	NSLTSSPLTLL	SLTSSLTLL	1	0	0	5	1	SLTSSPLTLL	tr_C9ZJU1	0.228	4234.77	9.00
164	HLA-A0201	YLGLITAYNR	YLGLITAYR	0	0	0	8	1	YLGLITAYNR	tr_C9ZJU1	0.228	4235.32	9.00
266	HLA-A0201	VASTFAFESLYCIR	FAFESLYCI	4	0	0	0	0	FAFESLYCI	tr_C9ZJU1	0.228	4235.82	9.00
747	HLA-A0201	FLGIAGYSG	FLGIAGYSG	0	0	0	0	0	FLGIAGYSG	tr_C9ZJU1	0.228	4239.99	9.00

186	HLA-A0201	KLMCSSCSIPQ	KLMCSSCSI	0	0	0	0	0	KLMCSSCSI	tr_C9ZJU1	0.228	4252.86	9.00
130	HLA-A0201	VLALCMLLRG	VLLCMLLRG	0	0	0	2	1	VLALCMLLRG	tr_C9ZJU1	0.227	4275.23	9.00
470	HLA-A0201	VLFSMVFVESG	VLFSMVFSG	0	0	0	7	2	VLFSMVFVESG	tr_C9ZJU1	0.227	4297.73	9.00
372	HLA-A0201	YFLNESLYME	FLNESLYME	1	0	0	0	0	FLNESLYME	tr_C9ZJU1	0.227	4307.13	9.00
249	HLA-A0201	FIIKQLMGLPAT	FILMGLPAT	0	0	0	2	3	FIIKQLMGLPAT	tr_C9ZJU1	0.227	4309.84	9.00
917	HLA-A0201	TIIIVAHRLATL	TIIIVLATL	0	0	0	5	3	TIIIVAHRLATL	tr_C9ZJU1	0.226	4315.90	9.00
270	HLA-A0201	FAFESLYCIRGF	FAFESLYCI	0	0	0	0	0	FAFESLYCI	tr_C9ZJU1	0.226	4342.84	9.00
219	HLA-A0201	HAFVHWRPIYAA	FVWRPIYAA	2	0	0	2	1	FVHWRPIYAA	tr_C9ZJU1	0.226	4353.19	9.00
203	HLA-A0201	TVLFSPEVILKCIL	VLFEVILKC	1	0	0	3	2	VLFSPEVILKC	tr_C9ZJU1	0.225	4367.62	9.00
628	HLA-A0201	QSALDQTEIVNQL	ALDEIVNQL	2	0	0	3	2	ALDQTEIVNQL	tr_C9ZJU1	0.225	4373.86	9.00
446	HLA-A0201	NKMLFQQTFSHPS	MLFQQTFPS	2	0	0	7	2	MLFQQTFSHPS	tr_C9ZJU1	0.225	4393.64	9.00
371	HLA-A0201	LYFLNESLYMESQF	FLNESLYMF	2	0	0	8	3	FLNESLYMESQF	tr_C9ZJU1	0.225	4397.07	9.00
234	HLA-A0201	KSLNGDVSPKL	SLNDVSPKL	1	0	0	3	1	SLNGDVSPKL	tr_C9ZJU1	0.224	4414.75	9.00
514	HLA-A0201	KLQAGGHTCAVSVR	KLQACAVSV	0	0	0	4	4	KLQAGGHTCAVSV	tr_C9ZJU1	0.224	4414.99	9.00
62	HLA-A0201	TLVFNHNIMQL	TLVFNIMQL	0	0	0	4	2	TLVFNHNIMQL	tr_C9ZJU1	0.224	4417.91	9.00
572	HLA-A0201	LMAATAAAAF	LMAATAAAA	0	0	0	0	0	LMAATAAAA	tr_C9ZJU1	0.224	4421.16	9.00
732	HLA-A0201	PYVLRRVSFVVN	YLRRVSFVV	1	0	0	1	1	YVLRRVSFVV	tr_C9ZJU1	0.224	4435.82	9.00
334	HLA-A0201	LQSRVPALLEV	LQVPALLEV	0	0	0	2	2	LQSRVPALLEV	tr_C9ZJU1	0.224	4439.42	9.00
881	HLA-A0201	GLCIFDEPTSAI	GLCIFDEPI	0	0	0	8	3	GLCIFDEPTSAI	tr_C9ZJU1	0.224	4442.16	9.00
102	HLA-A0201	ALVNCSIRDILLD	ALVNCSILL	0	0	0	6	3	ALVNCSIRDILL	tr_C9ZJU1	0.223	4456.75	9.00
92	HLA-A0201	LVCDIWNCLM	LVCDIWCLM	0	0	0	6	1	LVCDIWNCLM	tr_C9ZJU1	0.223	4468.04	9.00
733	HLA-A0201	YVLRRVSFVVNK	YLRRVSFVV	0	0	0	1	1	YVLRRVSFVV	tr_C9ZJU1	0.223	4474.24	9.00
194	HLA-A0201	IPQLVTLAATVLF	ILVTLAATV	0	0	0	1	2	IPQLVTLAATV	tr_C9ZJU1	0.223	4488.35	9.00
620	HLA-A0201	RLSPNFFTQSALD	RLSPNFFTL	0	0	0	8	3	RLSPNFFTQSAL	tr_C9ZJU1	0.222	4537.62	9.00
618	HLA-A0201	ALRLSPNFFTQSA	RLSPNFFTA	2	0	0	8	2	RLSPNFFTQSA	tr_C9ZJU1	0.222	4541.64	9.00
218	HLA-A0201	MHAFVHWRPIYAA	FVWRPIYAA	3	0	0	2	1	FVHWRPIYAA	tr_C9ZJU1	0.222	4544.05	9.00
612	HLA-A0201	LLVSAVALRLS	LLVSAVALL	0	0	0	8	1	LLVSAVALRL	tr_C9ZJU1	0.222	4549.71	9.00
627	HLA-A0201	TQSALDQTEIVNQL	ALDEIVNQL	3	0	0	3	2	ALDQTEIVNQL	tr_C9ZJU1	0.221	4564.95	9.00
348	HLA-A0201	CRVVFSFPVV	RVVFSFPVV	1	0	0	0	0	RVVFSFPVV	tr_C9ZJU1	0.221	4574.19	9.00
361	HLA-A0201	MWLGTQWSNFLYF	WLWSNFLYF	1	0	0	2	3	WLGTQWSNFLYF	tr_C9ZJU1	0.221	4581.77	9.00
418	HLA-A0201	KNSLTSSPLTL	KLTSSPLTL	0	0	0	1	2	KNSLTSSPLTL	tr_C9ZJU1	0.221	4582.07	9.00
468	HLA-A0201	GLVLFSMVF	GLVLFSMVF	0	0	0	0	0	GLVLFSMVF	tr_C9ZJU1	0.221	4584.15	9.00
561	HLA-A0201	GFAALRLSGWEL	FLRLSGWEL	1	0	0	1	2	FAALRLSGWEL	tr_C9ZJU1	0.221	4585.74	9.00
909	HLA-A0201	GNNLLRDSTIII	NLLDSTIII	2	0	0	3	1	NLLRDSTIII	tr_C9ZJU1	0.221	4591.64	9.00
445	HLA-A0201	DNKMLFQQTFSHPS	MLFQQTFPS	3	0	0	7	2	MLFQQTFSHPS	tr_C9ZJU1	0.221	4597.61	9.00
361	HLA-A0201	MWLGTQWSNFL	WLGTWSNFL	1	0	0	4	1	WLGTQWSNFL	tr_C9ZJU1	0.221	4597.91	9.00
570	HLA-A0201	WELMAATAAAA	WLMAATAAA	0	0	0	1	1	WELMAATAAA	tr_C9ZJU1	0.220	4603.88	9.00
303	HLA-A0201	ATADYDPSDDWLL	ATADYDWLL	0	0	0	5	4	ATADYDPSDDWLL	tr_C9ZJU1	0.220	4618.00	9.00
710	HLA-A0201	PSLDGPWSV	PSLDGPWSV	0	0	0	0	0	PSLDGPWSV	tr_C9ZJU1	0.220	4623.95	9.00
464	HLA-A0201	SVQYGLVLFS	SVYGLVLFS	0	0	0	2	1	SVQYGLVLFS	tr_C9ZJU1	0.220	4640.39	9.00
241	HLA-A0201	SPKLSPFRFII	SLSPFRFII	0	0	0	1	2	SPKLSPFRFII	tr_C9ZJU1	0.220	4643.10	9.00
941	HLA-A0201	MVAEVGSWETL	MVAEVWETL	0	0	0	5	2	MVAEVGSWETL	tr_C9ZJU1	0.220	4643.50	9.00
821	HLA-A0201	NLWDCSDENIC	NLWDCSDEI	0	0	0	8	1	NLWDCSDENI	tr_C9ZJU1	0.220	4650.54	9.00
112	HLA-A0201	LLDDGVALCILDD	LLDDGVALL	0	0	0	8	2	LLDDGVALCIL	tr_C9ZJU1	0.219	4662.48	9.50
918	HLA-A0201	IIIVAHRLATL	IIIVALATL	0	0	0	5	2	IIIVAHRLATL	tr_C9ZJU1	0.219	4666.67	9.50
926	HLA-A0201	ATLKHADKIVVL	TLADKIVVL	1	0	0	2	2	TLKHADKIVVL	tr_C9ZJU1	0.219	4683.31	9.50
492	HLA-A0201	QHVIWPLVPSVFS	VIWPLVPSV	2	0	0	0	0	VIWPLVPSV	tr_C9ZJU1	0.219	4684.53	9.50
342	HLA-A0201	LEVMHGCRVV	LVMHGCRVV	0	0	0	1	1	LEVMHGCRVV	tr_C9ZJU1	0.219	4692.19	9.50
731	HLA-A0201	HPYVLRRVSFVVN	YLRRVSFVV	2	0	0	1	1	YVLRRVSFVV	tr_C9ZJU1	0.219	4699.30	9.50
296	HLA-A0201	IDAYLALATA	IAYLALATA	0	0	0	1	1	IDAYLALATA	tr_C9ZJU1	0.218	4708.98	9.50
916	HLA-A0201	STIIIVAHRLATL	TIIIVLATL	1	0	0	5	3	TIIIVAHRLATL	tr_C9ZJU1	0.218	4723.01	9.50
93	HLA-A0201	VCDIWNCLMALVNC	VIWNCLMAL	0	0	0	1	2	VCDIWNCLMAL	tr_C9ZJU1	0.218	4727.51	9.50
89	HLA-A0201	DHLLVCDIWNC	HLLVCIWNC	1	0	0	5	1	HLLVCDIWNC	tr_C9ZJU1	0.218	4731.70	9.50
350	HLA-A0201	VVFSFPVVSLVM	VVFSFPVLV	0	0	0	7	2	VVFSFPVVSLV	tr_C9ZJU1	0.218	4740.11	9.50

406	HLA-A0201	ENQVSPTCSFPV	NQVSCSFPV	1	0	0	4	2	NQVSPTCSFPV	tr_C9ZJU1	0.218	4745.65	9.50
833	HLA-A0201	ALSASRALNFVQKR	ALSASLNFV	0	0	0	5	2	ALSASRALNFV	tr_C9ZJU1	0.217	4768.66	9.50
44	HLA-A0201	ASIAIFLNCNGV	SIAIFLNGV	1	0	0	6	2	SIAIFLNCNGV	tr_C9ZJU1	0.217	4772.94	9.50
514	HLA-A0201	KLQAGGHTCA	KLQAGGHTA	0	0	0	8	1	KLQAGGHTCA	tr_C9ZJU1	0.217	4775.99	9.50
269	HLA-A0201	TFAFESLYCIRG	FAFESLYCI	1	0	0	0	0	FAFESLYCI	tr_C9ZJU1	0.217	4779.30	9.50
842	HLA-A0201	FVQKRSSGIHAPL	FVSGIHAPL	0	0	0	2	4	FVQKRSSGIHAPL	tr_C9ZJU1	0.217	4784.78	9.50
363	HLA-A0201	LGTQWSNFLYFLNE	TQWNFLYFL	2	0	0	3	1	TQWSNFLYFL	tr_C9ZJU1	0.217	4794.26	9.50
312	HLA-A0201	DWLLSRCVSQALW	WLLSRCVSL	1	0	0	8	2	WLLSRCVSQAL	tr_C9ZJU1	0.217	4796.65	9.50
606	HLA-A0201	ADTLPLLLVSA	ALPLLLVSA	0	0	0	1	2	ADTLPLLLVSA	tr_C9ZJU1	0.217	4804.18	9.50
617	HLA-A0201	VALRLSPNFFTQSA	RLSPNFFTA	3	0	0	8	2	RLSPNFFTQSA	tr_C9ZJU1	0.216	4812.30	9.50
377	HLA-A0201	SLYMESQFTK	SLMESQFTK	0	0	0	2	1	SLYMESQFTK	tr_C9ZJU1	0.216	4815.22	9.50
730	HLA-A0201	RHPYVLRRVSFVVN	YLRRVSFVV	3	0	0	1	1	YVLRRVSFVV	tr_C9ZJU1	0.216	4816.26	9.50
908	HLA-A0201	EGNNLLRDSTIII	NLLDSTIII	3	0	0	3	1	NLLRDSTIII	tr_C9ZJU1	0.216	4826.85	9.50
128	HLA-A0201	EEVLALCMLL	EVLALCMLL	0	0	0	1	1	EEVLALCMLL	tr_C9ZJU1	0.216	4839.82	9.50
364	HLA-A0201	GTQWSNFLYFLNES	TQWNFLYFL	1	0	0	3	1	TQWSNFLYFL	tr_C9ZJU1	0.215	4857.86	9.50
496	HLA-A0201	WPLVPSVFSG	WLVPSVFSG	0	0	0	1	1	WPLVPSVFSG	tr_C9ZJU1	0.215	4860.07	9.50
101	HLA-A0201	MALVNCSIRDI	ALVNCSIRI	1	0	0	8	1	ALVNCSIRDI	tr_C9ZJU1	0.215	4862.65	9.50
491	HLA-A0201	TQHVIWPLVPSVFS	VIWPLVPSV	3	0	0	0	0	VIWPLVPSV	tr_C9ZJU1	0.215	4864.75	9.50
567	HLA-A0201	LSGWELMAATAAAA	LLMAATAAA	0	0	0	1	4	LSGWELMAATAAA	tr_C9ZJU1	0.215	4884.16	9.50
891	HLA-A0201	AIDTITEGEI	AIDTITEEI	0	0	0	7	1	AIDTITEGEI	tr_C9ZJU1	0.215	4897.92	9.50
418	HLA-A0201	KNSLTSSPLTLL	SLTSSLTLL	2	0	0	5	1	SLTSSPLTLL	tr_C9ZJU1	0.215	4900.42	9.50
217	HLA-A0201	GMHAFVHWRPIY	GMHAFVHPI	0	0	0	7	2	GMHAFVHWRPI	tr_C9ZJU1	0.215	4906.52	9.50
86	HLA-A0201	SHPDHLLVCDIWNC	HLLVCIWNC	4	0	0	5	1	HLLVCDIWNC	tr_C9ZJU1	0.214	4915.55	9.50
608	HLA-A0201	TLPLLLVSAVALR	TLLLLVSAV	0	0	0	2	1	TLPLLLVSAV	tr_C9ZJU1	0.214	4917.83	9.50
383	HLA-A0201	QFTKSQLASVTNI	FQLASVTNI	1	0	0	1	3	FTKSQLASVTNI	tr_C9ZJU1	0.214	4919.06	9.50
611	HLA-A0201	LLLVSAVALRLSP	LLLAVALRL	0	0	0	3	2	LLLVSAVALRL	tr_C9ZJU1	0.214	4926.52	9.50
924	HLA-A0201	RLATLKHADKIVV	RLATLKIVV	0	0	0	5	4	RLATLKHADKIVV	tr_C9ZJU1	0.214	4930.14	9.50
610	HLA-A0201	PLLLVSAVALRLS	LLLAVALRL	1	0	0	3	2	LLLVSAVALRL	tr_C9ZJU1	0.214	4931.42	9.50
263	HLA-A0201	SMAVASTFAFESL	SMAFAFESL	0	0	0	3	4	SMAVASTFAFESL	tr_C9ZJU1	0.214	4937.35	9.50
620	HLA-A0201	RLSPNFFTQS	RLSPNFFTS	0	0	0	8	1	RLSPNFFTQS	tr_C9ZJU1	0.214	4941.62	9.50
493	HLA-A0201	HVIWPLVPSVFSG	VIWPLVPSV	1	0	0	0	0	VIWPLVPSV	tr_C9ZJU1	0.214	4944.62	9.50
610	HLA-A0201	PLLLVSAVA	PLLLVSAVA	0	0	0	0	0	PLLLVSAVA	tr_C9ZJU1	0.214	4952.54	9.50
242	HLA-A0201	PKLSPFRFIIKQL	KLSPFRFQL	1	0	0	7	3	KLSPFRFIIKQL	tr_C9ZJU1	0.213	4963.70	9.50
731	HLA-A0201	HPYVLRRVSFV	YVLRRVSFV	2	0	0	0	0	YVLRRVSFV	tr_C9ZJU1	0.213	4965.80	9.50
206	HLA-A0201	FSPEVILKCILGMH	FILKCILGM	0	0	0	1	4	FSPEVILKCILGM	tr_C9ZJU1	0.213	4968.97	9.50
907	HLA-A0201	REGNNLLRDSTIII	NLLDSTIII	4	0	0	3	1	NLLRDSTIII	tr_C9ZJU1	0.213	4970.42	9.50
484	HLA-A0201	GYMWDLLTQH	YMWDLLTQH	1	0	0	0	0	YMWDLLTQH	tr_C9ZJU1	0.213	4984.69	9.50
210	HLA-A0201	VILKCILGMH	VILKCILGM	0	0	0	0	0	VILKCILGM	tr_C9ZJU1	0.213	4989.28	9.50
110	HLA-A0201	DILLDDGVALC	ILLDGVALC	1	0	0	3	1	ILLDDGVALC	tr_C9ZJU1	0.213	4990.52	9.50
289	HLA-A0201	DIITSIHIDAYL	IIIHIDAYL	1	0	0	2	2	IITSIHIDAYL	tr_C9ZJU1	0.213	5001.87	9.50
653	HLA-A0201	FLGSFYDECG	FLGSFYDEC	0	0	0	0	0	FLGSFYDEC	tr_C9ZJU1	0.213	5003.93	9.50
501	HLA-A0201	SVFSGCVVDAL	SVFCVVDAL	0	0	0	3	2	SVFSGCVVDAL	tr_C9ZJU1	0.213	5005.39	9.50
760	HLA-A0201	TLLRLLNRTYAPT	TLLRLLAPT	0	0	0	6	4	TLLRLLNRTYAPT	tr_C9ZJU1	0.212	5026.39	9.50
560	HLA-A0201	QGFAALRLSGWEL	FLRLSGWEL	2	0	0	1	2	FAALRLSGWEL	tr_C9ZJU1	0.212	5034.50	9.50
88	HLA-A0201	PDHLLVCDIWNC	HLLVCIWNC	2	0	0	5	1	HLLVCDIWNC	tr_C9ZJU1	0.212	5037.83	9.50
194	HLA-A0201	IPQLVTLAATVLFS	ILVTLAATV	0	0	0	1	2	IPQLVTLAATV	tr_C9ZJU1	0.212	5041.59	9.50
290	HLA-A0201	IITSIHIDAYLAL	IIIDAYLAL	0	0	0	2	4	IITSIHIDAYLAL	tr_C9ZJU1	0.212	5043.56	9.50
281	HLA-A0201	FVIQSILSDIIT	FVIQSILSI	0	0	0	8	2	FVIQSILSDII	tr_C9ZJU1	0.212	5050.11	9.50
255	HLA-A0201	MGLPATFVSMAVA	GLFVSMAVA	1	0	0	2	3	GLPATFVSMAVA	tr_C9ZJU1	0.212	5054.70	9.50
311	HLA-A0201	DDWLLSRCVSQALW	WLLSRCVSL	2	0	0	8	2	WLLSRCVSQAL	tr_C9ZJU1	0.212	5056.29	9.50
353	HLA-A0201	SFPVVSLVMWLG	FVVSLVMWL	1	0	0	1	1	FPVVSLVMWL	tr_C9ZJU1	0.211	5110.63	10.00
405	HLA-A0201	EENQVSPTCSFPV	NQVSCSFPV	2	0	0	4	2	NQVSPTCSFPV	tr_C9ZJU1	0.211	5116.55	10.00
87	HLA-A0201	HPDHLLVCDIWNC	HLLVCIWNC	3	0	0	5	1	HLLVCDIWNC	tr_C9ZJU1	0.211	5119.26	10.00
733	HLA-A0201	YVLRRVSFVVNKG	YLRRVSFVV	0	0	0	1	1	YVLRRVSFVV	tr_C9ZJU1	0.211	5122.81	10.00

351	HLA-A0201	VFSFPVVSLVMWLG	FVVSLVMWL	1	0	0	1	3	FSFPVVSLVMWL	tr_C9ZJU1	0.211	5124.30	10.00
352	HLA-A0201	FSFPVVSLVMWLGT	FVVSLVMWL	0	0	0	1	3	FSFPVVSLVMWL	tr_C9ZJU1	0.210	5128.19	10.00
598	HLA-A0201	VLHGLFQMAD	VLHGLFQMA	0	0	0	0	0	VLHGLFQMA	tr_C9ZJU1	0.210	5134.52	10.00
417	HLA-A0201	VKNSLTSSPLTLL	SLTSSLTLL	3	0	0	5	1	SLTSSPLTLL	tr_C9ZJU1	0.210	5144.58	10.00
564	HLA-A0201	ALRLSGWELMA	ALSGWELMA	0	0	0	1	2	ALRLSGWELMA	tr_C9ZJU1	0.210	5171.26	10.00
233	HLA-A0201	GKSLNGDVSPKL	SLNDVSPKL	2	0	0	3	1	SLNGDVSPKL	tr_C9ZJU1	0.210	5174.73	10.00
777	HLA-A0201	FVNGVHISQYPA	FVNGVQYPA	0	0	0	5	3	FVNGVHISQYPA	tr_C9ZJU1	0.210	5175.29	10.00
349	HLA-A0201	RVVFSFPVVSLVM	FSFPVVLVM	3	0	0	6	1	FSFPVVSLVM	tr_C9ZJU1	0.209	5184.70	10.00
653	HLA-A0201	FLGSFYDECGMS	FLGSFYCGM	0	0	0	6	2	FLGSFYDECGM	tr_C9ZJU1	0.209	5194.53	10.00
740	HLA-A0201	FVVNKGEFLGI	FVVGEFLGI	0	0	0	3	2	FVVNKGEFLGI	tr_C9ZJU1	0.209	5195.99	10.00
559	HLA-A0201	LQGFAALRLSGWEL	FLRLSGWEL	3	0	0	1	2	FAALRLSGWEL	tr_C9ZJU1	0.209	5205.33	10.00
730	HLA-A0201	RHPYVLRRVSFV	YVLRRVSFV	3	0	0	0	0	YVLRRVSFV	tr_C9ZJU1	0.209	5205.89	10.00
473	HLA-A0201	SMVFVESGRPLGYM	SMVFVLGYM	0	0	0	5	5	SMVFVESGRPLGYM	tr_C9ZJU1	0.209	5206.46	10.00
466	HLA-A0201	QYGLVLFSMV	YGLVLFSMV	1	0	0	0	0	YGLVLFSMV	tr_C9ZJU1	0.209	5210.29	10.00
681	HLA-A0201	GSSLGCYQV	GSSLGCYQV	0	0	0	0	0	GSSLGCYQV	tr_C9ZJU1	0.209	5213.28	10.00
682	HLA-A0201	SSLGCYQVLEGLQ	SLYQVLEGL	1	0	0	2	2	SLGCYQVLEGL	tr_C9ZJU1	0.209	5213.33	10.00
915	HLA-A0201	DSTIIIVAHRLATL	TIIIVLATL	2	0	0	5	3	TIIIVAHRLATL	tr_C9ZJU1	0.209	5222.59	10.00
333	HLA-A0201	ELQSRVPALL	ELQSVPALL	0	0	0	4	1	ELQSRVPALL	tr_C9ZJU1	0.209	5223.05	10.00
268	HLA-A0201	STFAFESLYCIRG	FAFESLYCI	2	0	0	0	0	FAFESLYCI	tr_C9ZJU1	0.209	5225.64	10.00
215	HLA-A0201	ILGMHAFVHWRPIY	ILGMHAFPI	0	0	0	7	4	ILGMHAFVHWRPI	tr_C9ZJU1	0.209	5232.60	10.00
61	HLA-A0201	RTLVFNHNIMQL	TLVFNIMQL	1	0	0	4	2	TLVFNHNIMQL	tr_C9ZJU1	0.209	5233.51	10.00
880	HLA-A0201	VGLCIFDEPTSA	GLFDEPTSA	1	0	0	2	2	GLCIFDEPTSA	tr_C9ZJU1	0.208	5245.24	10.00
807	HLA-A0201	FIEDLSIGGNI	FIEDLSIGI	0	0	0	8	2	FIEDLSIGGNI	tr_C9ZJU1	0.208	5251.83	10.00
404	HLA-A0201	GEENQVSPTCSFPV	NQVSCSFPV	3	0	0	4	2	NQVSPTCSFPV	tr_C9ZJU1	0.208	5280.83	10.00
43	HLA-A0201	DASIAIFLNCNGV	SIAIFLNGV	2	0	0	6	2	SIAIFLNCNGV	tr_C9ZJU1	0.208	5285.69	10.00
609	HLA-A0201	LPLLLVSAVALRLS	LLLAVALRL	2	0	0	3	2	LLLVSAVALRL	tr_C9ZJU1	0.208	5292.16	10.00
416	HLA-A0201	PVKNSLTSSPLTLL	SLTSSLTLL	4	0	0	5	1	SLTSSPLTLL	tr_C9ZJU1	0.208	5294.16	10.00
706	HLA-A0201	GVAPPSLDGPWSVE	GVLDGPWSV	0	0	0	2	4	GVAPPSLDGPWSV	tr_C9ZJU1	0.207	5319.83	10.00
565	HLA-A0201	LRLSGWELMAATAA	RLWELMAAT	1	0	0	2	2	RLSGWELMAAT	tr_C9ZJU1	0.207	5338.22	10.00
641	HLA-A0201	RSIQHWCPLYHA	SIWCPLYHA	1	0	0	2	2	SIQHWCPLYHA	tr_C9ZJU1	0.207	5338.57	10.00
729	HLA-A0201	DRHPYVLRRVSFV	YVLRRVSFV	4	0	0	0	0	YVLRRVSFV	tr_C9ZJU1	0.207	5352.05	10.00
240	HLA-A0201	VSPKLSPFRFII	KLSPFRFII	3	0	0	0	0	KLSPFRFII	tr_C9ZJU1	0.206	5356.39	10.00
65	HLA-A0201	FNHNIMQLPSVFA	FMQLPSVFA	0	0	0	1	4	FNHNIMQLPSVFA	tr_C9ZJU1	0.206	5360.74	10.00
348	HLA-A0201	CRVVFSFPVVSLVM	FSFPVVLVM	4	0	0	6	1	FSFPVVSLVM	tr_C9ZJU1	0.206	5372.30	10.00
780	HLA-A0201	GVHISQYPA	GVHISQYPA	0	0	0	0	0	GVHISQYPA	tr_C9ZJU1	0.206	5374.45	10.00
193	HLA-A0201	SIPQLVTLAATVLF	ILVTLAATV	1	0	0	1	2	IPQLVTLAATV	tr_C9ZJU1	0.206	5386.20	10.00
568	HLA-A0201	SGWELMAATAAAA	SLMAATAAA	0	0	0	1	3	SGWELMAATAAA	tr_C9ZJU1	0.206	5391.16	10.00
175	HLA-A0201	RSLQSGITSFWKLM	SLITSFWKL	1	0	0	2	3	SLQSGITSFWKL	tr_C9ZJU1	0.206	5405.77	10.00
204	HLA-A0201	VLFSPEVILKCILG	VLFEVILKC	0	0	0	3	2	VLFSPEVILKC	tr_C9ZJU1	0.205	5412.67	10.00
469	HLA-A0201	LVLFSMVFVESG	VLFSMVFSG	1	0	0	7	2	VLFSMVFVESG	tr_C9ZJU1	0.205	5415.36	10.00
571	HLA-A0201	ELMAATAAAAFA	LMAATAAFA	1	0	0	5	2	LMAATAAAAFA	tr_C9ZJU1	0.205	5417.83	10.00
834	HLA-A0201	LSASRALNFV	LSASALNFV	0	0	0	4	1	LSASRALNFV	tr_C9ZJU1	0.205	5424.75	10.00
832	HLA-A0201	AALSASRALNFVQK	ALSASLNFV	1	0	0	5	2	ALSASRALNFV	tr_C9ZJU1	0.205	5431.32	10.00
42	HLA-A0201	DDASIAIFLNCNGV	SIAIFLNGV	3	0	0	6	2	SIAIFLNCNGV	tr_C9ZJU1	0.205	5434.26	10.00
641	HLA-A0201	RSIQHWCPL	RSIQHWCPL	0	0	0	0	0	RSIQHWCPL	tr_C9ZJU1	0.205	5435.79	10.00
257	HLA-A0201	LPATFVSMAV	LATFVSMAV	0	0	0	1	1	LPATFVSMAV	tr_C9ZJU1	0.205	5436.62	10.00
232	HLA-A0201	IGKSLNGDVSPKL	SLNDVSPKL	3	0	0	3	1	SLNGDVSPKL	tr_C9ZJU1	0.205	5438.73	10.00
313	HLA-A0201	WLLSRCVSQALWQ	WLLSRCVSL	0	0	0	8	2	WLLSRCVSQAL	tr_C9ZJU1	0.205	5441.27	10.00
277	HLA-A0201	CIRGFVIQSI	CIRGFVISI	0	0	0	7	1	CIRGFVIQSI	tr_C9ZJU1	0.205	5444.68	10.00
803	HLA-A0201	NNLRFIEDLSI	NLFIEDLSI	1	0	0	2	1	NLRFIEDLSI	tr_C9ZJU1	0.205	5450.10	10.00
728	HLA-A0201	SDRHPYVLRRVSFV	YVLRRVSFV	5	0	0	0	0	YVLRRVSFV	tr_C9ZJU1	0.205	5450.34	10.00
193	HLA-A0201	SIPQLVTLAA	SIQLVTLAA	0	0	0	2	1	SIPQLVTLAA	tr_C9ZJU1	0.205	5451.05	10.00
760	HLA-A0201	TLLRLLNRTYAP	TLLRLLTYA	0	0	0	6	2	TLLRLLNRTYA	tr_C9ZJU1	0.205	5451.81	10.00
267	HLA-A0201	ASTFAFESLYCIRG	FAFESLYCI	3	0	0	0	0	FAFESLYCI	tr_C9ZJU1	0.205	5459.25	10.00

563	HLA-A0201	AALRLSGWELMA	ALSGWELMA	0	0	0	1	3	AALRLSGWELMA	tr_C9ZJU1	0.205	5460.79	10.00
493	HLA-A0201	HVIWPLVPSVFSGC	VIWPLVPSV	1	0	0	0	0	VIWPLVPSV	tr_C9ZJU1	0.205	5467.11	10.00
710	HLA-A0201	PSLDGPWSVEFREV	SLDGPWSEV	1	0	0	7	4	SLDGPWSVEFREV	tr_C9ZJU1	0.204	5479.13	10.00
333	HLA-A0201	ELQSRVPALLEV	ELVPALLEV	0	0	0	2	3	ELQSRVPALLEV	tr_C9ZJU1	0.204	5481.92	10.00
108	HLA-A0201	IRDILLDDGVALC	ILLDGVALC	3	0	0	3	1	ILLDDGVALC	tr_C9ZJU1	0.204	5481.98	10.00
763	HLA-A0201	RLLNRTYAP	RLLNRTYAP	0	0	0	0	0	RLLNRTYAP	tr_C9ZJU1	0.204	5494.27	10.00
611	HLA-A0201	LLLVSAVALRLSPN	LLLAVALRL	0	0	0	3	2	LLLVSAVALRL	tr_C9ZJU1	0.204	5494.45	10.00
895	HLA-A0201	ITEGEIFDTL	ITEGEIFTL	0	0	0	7	1	ITEGEIFDTL	tr_C9ZJU1	0.204	5500.64	10.00
94	HLA-A0201	CDIWNCLMALVN	CIWNCLMAL	0	0	0	1	1	CDIWNCLMAL	tr_C9ZJU1	0.204	5507.90	10.00
555	HLA-A0201	RQDYLQGFAA	RQDYLQFAA	0	0	0	6	1	RQDYLQGFAA	tr_C9ZJU1	0.204	5509.63	10.00
188	HLA-A0201	MCSSCSIPQL	MCSSCSIPL	0	0	0	8	1	MCSSCSIPQL	tr_C9ZJU1	0.204	5521.69	10.00
291	HLA-A0201	ITSIHIDAYLALA	IIDAYLALA	0	0	0	1	4	ITSIHIDAYLALA	tr_C9ZJU1	0.204	5527.90	11.00
733	HLA-A0201	YVLRRVSFVVNKGE	YLRRVSFVV	0	0	0	1	1	YVLRRVSFVV	tr_C9ZJU1	0.203	5535.56	11.00
879	HLA-A0201	AVGLCIFDEPTSAI	AVGLCIFAI	0	0	0	7	5	AVGLCIFDEPTSAI	tr_C9ZJU1	0.203	5538.86	11.00
109	HLA-A0201	RDILLDDGVALC	ILLDGVALC	2	0	0	3	1	ILLDDGVALC	tr_C9ZJU1	0.203	5542.82	11.00
351	HLA-A0201	VFSFPVVSLVM	FSFPVVSLV	1	0	0	0	0	FSFPVVSLV	tr_C9ZJU1	0.203	5546.96	11.00
266	HLA-A0201	VASTFAFESL	VASTFAFEL	0	0	0	8	1	VASTFAFESL	tr_C9ZJU1	0.203	5550.80	11.00
382	HLA-A0201	SQFTKSQLASVTNI	FQLASVTNI	2	0	0	1	3	FTKSQLASVTNI	tr_C9ZJU1	0.203	5553.38	11.00
269	HLA-A0201	TFAFESLYCIRGF	FAFESLYCI	1	0	0	0	0	FAFESLYCI	tr_C9ZJU1	0.203	5569.03	11.00
354	HLA-A0201	FPVVSLVMWLGT	FVVSLVMWL	0	0	0	1	1	FPVVSLVMWL	tr_C9ZJU1	0.203	5577.47	11.00
675	HLA-A0201	RHLAFSGSSL	RLAFSGSSL	0	0	0	1	1	RHLAFSGSSL	tr_C9ZJU1	0.203	5582.30	11.00
940	HLA-A0201	GMVAEVGSWETL	MVAEVWETL	1	0	0	5	2	MVAEVGSWETL	tr_C9ZJU1	0.203	5583.45	11.00
185	HLA-A0201	WKLMCSSCSIPQ	WLMCSSCSI	0	0	0	1	1	WKLMCSSCSI	tr_C9ZJU1	0.203	5586.47	11.00
200	HLA-A0201	LAATVLFSPEVIL	LLFSPEVIL	0	0	0	1	4	LAATVLFSPEVIL	tr_C9ZJU1	0.202	5590.52	11.00
290	HLA-A0201	IITSIHIDAYLALA	IIDAYLALA	0	0	0	1	5	IITSIHIDAYLALA	tr_C9ZJU1	0.202	5599.84	11.00
231	HLA-A0201	EIGKSLNGDVSPKL	SLNDVSPKL	4	0	0	3	1	SLNGDVSPKL	tr_C9ZJU1	0.202	5599.84	11.00
683	HLA-A0201	SLGCYQVLEGLQS	SLYQVLEGL	0	0	0	2	2	SLGCYQVLEGL	tr_C9ZJU1	0.202	5608.57	11.00
241	HLA-A0201	SPKLSPFRFIIKQL	KLSPFRFQL	2	0	0	7	3	KLSPFRFIIKQL	tr_C9ZJU1	0.202	5610.33	11.00
348	HLA-A0201	CRVVFSFPV	CRVVFSFPV	0	0	0	0	0	CRVVFSFPV	tr_C9ZJU1	0.202	5613.49	11.00
925	HLA-A0201	LATLKHADKIVVL	TLADKIVVL	2	0	0	2	2	TLKHADKIVVL	tr_C9ZJU1	0.202	5615.13	11.00
880	HLA-A0201	VGLCIFDEPTSAI	GLCIFDEPI	1	0	0	8	3	GLCIFDEPTSAI	tr_C9ZJU1	0.202	5616.83	11.00
492	HLA-A0201	QHVIWPLVPSVFSG	VIWPLVPSV	2	0	0	0	0	VIWPLVPSV	tr_C9ZJU1	0.202	5626.87	11.00
864	HLA-A0201	VERLCIARAFV	VLCIARAFV	0	0	0	1	2	VERLCIARAFV	tr_C9ZJU1	0.202	5642.72	11.00
472	HLA-A0201	FSMVFVESG	FSMVFVESG	0	0	0	0	0	FSMVFVESG	tr_C9ZJU1	0.202	5647.30	11.00
101	HLA-A0201	MALVNCSIRDILLD	ALVNCSILL	1	0	0	6	3	ALVNCSIRDILL	tr_C9ZJU1	0.201	5651.89	11.00
239	HLA-A0201	DVSPKLSPFRFII	KLSPFRFII	4	0	0	0	0	KLSPFRFII	tr_C9ZJU1	0.201	5659.72	11.00
810	HLA-A0201	DLSIGGNIAL	DLIGGNIAL	0	0	0	2	1	DLSIGGNIAL	tr_C9ZJU1	0.201	5661.62	11.00
60	HLA-A0201	VRTLVFNHNIMQL	LVFNHIMQL	3	0	0	5	1	LVFNHNIMQL	tr_C9ZJU1	0.201	5662.78	11.00
439	HLA-A0201	CMLSSTDNKM	MLSSTDNKM	1	0	0	0	0	MLSSTDNKM	tr_C9ZJU1	0.201	5663.39	11.00
359	HLA-A0201	LVMWLGTQWS	LMWLGTQWS	0	0	0	1	1	LVMWLGTQWS	tr_C9ZJU1	0.201	5669.09	11.00
192	HLA-A0201	CSIPQLVTL	CSIPQLVTL	0	0	0	0	0	CSIPQLVTL	tr_C9ZJU1	0.201	5671.37	11.00
238	HLA-A0201	GDVSPKLSPFRFII	KLSPFRFII	5	0	0	0	0	KLSPFRFII	tr_C9ZJU1	0.201	5674.92	11.00
807	HLA-A0201	FIEDLSIGGNIALG	FIIGGNIAL	0	0	0	2	4	FIEDLSIGGNIAL	tr_C9ZJU1	0.201	5675.66	11.00
190	HLA-A0201	SSCSIPQLV	SSCSIPQLV	0	0	0	0	0	SSCSIPQLV	tr_C9ZJU1	0.201	5697.81	11.00
202	HLA-A0201	ATVLFSPEVI	ATVLFSPEV	0	0	0	0	0	ATVLFSPEV	tr_C9ZJU1	0.201	5702.62	11.00
17	HLA-A0201	YEILEMLTRAT	YILEMLTRA	0	0	0	1	1	YEILEMLTRA	tr_C9ZJU1	0.201	5703.05	11.00
63	HLA-A0201	LVFNHNIMQLPSVF	LVFNHLPSV	0	0	0	5	4	LVFNHNIMQLPSV	tr_C9ZJU1	0.201	5707.75	11.00
288	HLA-A0201	SDIITSIHIDAYL	IIIHIDAYL	2	0	0	2	2	IITSIHIDAYL	tr_C9ZJU1	0.200	5714.11	11.00
681	HLA-A0201	GSSLGCYQVLEGLQ	SLYQVLEGL	2	0	0	2	2	SLGCYQVLEGL	tr_C9ZJU1	0.200	5720.17	11.00
708	HLA-A0201	APPSLDGPWSV	ASLDGPWSV	0	0	0	1	2	APPSLDGPWSV	tr_C9ZJU1	0.200	5743.43	11.00
768	HLA-A0201	TYAPTEGDIFV	YATEGDIFV	1	0	0	2	1	YAPTEGDIFV	tr_C9ZJU1	0.200	5745.67	11.00
107	HLA-A0201	SIRDILLDDGVALC	ILLDGVALC	4	0	0	3	1	ILLDDGVALC	tr_C9ZJU1	0.200	5772.59	11.00
439	HLA-A0201	CMLSSTDNKML	MLSSTDNML	1	0	0	7	1	MLSSTDNKML	tr_C9ZJU1	0.200	5773.90	11.00
220	HLA-A0201	AFVHWRPIYAAEI	FVHWRPIYI	1	0	0	8	3	FVHWRPIYAAEI	tr_C9ZJU1	0.199	5781.52	11.00

607	HLA-A0201	DTLPLLLVSAVALR	TLLLLVSAV	1	0	0	2	1	TLPLLLVSAV	tr_C9ZJU1	0.199	5784.71	11.00
59	HLA-A0201	HVRTLVFNHNIMQL	LVFNHIMQL	4	0	0	5	1	LVFNHNIMQL	tr_C9ZJU1	0.199	5813.14	11.00
879	HLA-A0201	AVGLCIFDEPTSA	GLFDEPTSA	2	0	0	2	2	GLCIFDEPTSA	tr_C9ZJU1	0.199	5821.01	11.00
281	HLA-A0201	FVIQSILSDIITS	FVIQSILSI	0	0	0	8	2	FVIQSILSDII	tr_C9ZJU1	0.199	5829.32	11.00
639	HLA-A0201	QLRSIQHWCPL	QLIQHWCPL	0	0	0	2	2	QLRSIQHWCPL	tr_C9ZJU1	0.199	5834.37	11.00
210	HLA-A0201	VILKCILGMHA	ILCILGMHA	1	0	0	2	1	ILKCILGMHA	tr_C9ZJU1	0.198	5858.09	11.00
155	HLA-A0201	VVQEFPEELYL	VVQEFPLYL	0	0	0	6	2	VVQEFPEELYL	tr_C9ZJU1	0.198	5862.98	11.00
295	HLA-A0201	HIDAYLALAT	HIDAYLALA	0	0	0	0	0	HIDAYLALA	tr_C9ZJU1	0.198	5864.88	11.00
293	HLA-A0201	SIHIDAYLALA	SIDAYLALA	0	0	0	1	2	SIHIDAYLALA	tr_C9ZJU1	0.198	5873.20	11.00
336	HLA-A0201	SRVPALLEVMHGC	SLLEVMHGC	0	0	0	1	4	SRVPALLEVMHGC	tr_C9ZJU1	0.198	5878.28	11.00
256	HLA-A0201	GLPATFVSMAVAS	GLPATFMAV	0	0	0	6	2	GLPATFVSMAV	tr_C9ZJU1	0.198	5891.97	11.00
248	HLA-A0201	RFIIKQLMGLPAT	FILMGLPAT	1	0	0	2	3	FIIKQLMGLPAT	tr_C9ZJU1	0.197	5901.61	11.00
255	HLA-A0201	MGLPATFVSMA	GLATFVSMA	1	0	0	2	1	GLPATFVSMA	tr_C9ZJU1	0.197	5909.28	11.00
732	HLA-A0201	PYVLRRVSFVVNK	YLRRVSFVV	1	0	0	1	1	YVLRRVSFVV	tr_C9ZJU1	0.197	5914.71	11.00
612	HLA-A0201	LLVSAVALRLSP	LLVSAVALL	0	0	0	8	1	LLVSAVALRL	tr_C9ZJU1	0.197	5918.43	11.00
900	HLA-A0201	IFDTLRLREGNNLL	FLREGNNLL	1	0	0	1	4	FDTLRLREGNNLL	tr_C9ZJU1	0.197	5921.89	11.00
417	HLA-A0201	VKNSLTSSPLTL	KLTSSPLTL	1	0	0	1	2	KNSLTSSPLTL	tr_C9ZJU1	0.197	5922.85	11.00
570	HLA-A0201	WELMAATAAAAFA	LMAATAAFA	2	0	0	5	2	LMAATAAAAFA	tr_C9ZJU1	0.197	5924.26	11.00
933	HLA-A0201	KIVVLNEGM	KIVVLNEGM	0	0	0	0	0	KIVVLNEGM	tr_C9ZJU1	0.197	5930.41	11.00
685	HLA-A0201	GCYQVLEGL	GCYQVLEGL	0	0	0	0	0	GCYQVLEGL	tr_C9ZJU1	0.197	5930.86	11.00
643	HLA-A0201	IQHWCPLYHAFL	IQHWCPLYL	0	0	0	8	3	IQHWCPLYHAFL	tr_C9ZJU1	0.197	5932.40	11.00
739	HLA-A0201	SFVVNKGEFL	FVVNKGEFL	1	0	0	0	0	FVVNKGEFL	tr_C9ZJU1	0.197	5944.93	11.00
287	HLA-A0201	LSDIITSIHIDAYL	IIIHIDAYL	3	0	0	2	2	IITSIHIDAYL	tr_C9ZJU1	0.196	5973.11	11.00
164	HLA-A0201	YLGLITAYNRKRS	YLGLITAYS	0	0	0	8	4	YLGLITAYNRKRS	tr_C9ZJU1	0.196	5984.17	11.00
268	HLA-A0201	STFAFESLYCIRGF	FAFESLYCI	2	0	0	0	0	FAFESLYCI	tr_C9ZJU1	0.196	5984.69	11.00
802	HLA-A0201	ENNLRFIEDLSI	NLFIEDLSI	2	0	0	2	1	NLRFIEDLSI	tr_C9ZJU1	0.196	5985.66	11.00
642	HLA-A0201	SIQHWCPLYHAFL	SIQHWCPFL	0	0	0	7	4	SIQHWCPLYHAFL	tr_C9ZJU1	0.196	6010.65	11.00
215	HLA-A0201	ILGMHAFVHW	ILMHAFVHW	0	0	0	2	1	ILGMHAFVHW	tr_C9ZJU1	0.196	6012.66	11.00
354	HLA-A0201	FPVVSLVMWLGTQ	FVVSLVMWL	0	0	0	1	1	FPVVSLVMWL	tr_C9ZJU1	0.195	6047.89	11.00
569	HLA-A0201	GWELMAATAAAA	WLMAATAAA	1	0	0	1	1	WELMAATAAA	tr_C9ZJU1	0.195	6055.82	11.00
94	HLA-A0201	CDIWNCLMALVNCS	CIWNCLMAL	0	0	0	1	1	CDIWNCLMAL	tr_C9ZJU1	0.195	6057.78	11.00
640	HLA-A0201	LRSIQHWCPLYHA	SIWCPLYHA	2	0	0	2	2	SIQHWCPLYHA	tr_C9ZJU1	0.194	6108.79	11.00
251	HLA-A0201	IKQLMGLPAT	IQLMGLPAT	0	0	0	1	1	IKQLMGLPAT	tr_C9ZJU1	0.194	6115.14	11.00
247	HLA-A0201	FRFIIKQLMGLPAT	FQLMGLPAT	0	0	0	1	5	FRFIIKQLMGLPAT	tr_C9ZJU1	0.194	6119.31	11.00
862	HLA-A0201	GEVERLCIARAFV	VLCIARAFV	2	0	0	1	2	VERLCIARAFV	tr_C9ZJU1	0.194	6125.47	11.00
176	HLA-A0201	SLQSGITSFWKLMC	SLITSFWKL	0	0	0	2	3	SLQSGITSFWKL	tr_C9ZJU1	0.194	6134.03	11.00
472	HLA-A0201	FSMVFVESGRPLG	FSMVFVEPL	0	0	0	7	3	FSMVFVESGRPL	tr_C9ZJU1	0.194	6136.02	11.00
878	HLA-A0201	SAVGLCIFDEPTSA	GLFDEPTSA	3	0	0	2	2	GLCIFDEPTSA	tr_C9ZJU1	0.194	6139.94	11.00
352	HLA-A0201	FSFPVVSLVMW	FSFPVVSLV	0	0	0	0	0	FSFPVVSLV	tr_C9ZJU1	0.194	6145.32	11.00
486	HLA-A0201	MWDLLTQHV	MWDLLTQHV	0	0	0	0	0	MWDLLTQHV	tr_C9ZJU1	0.194	6156.17	11.00
332	HLA-A0201	DELQSRVPALLEV	LQVPALLEV	2	0	0	2	2	LQSRVPALLEV	tr_C9ZJU1	0.193	6177.99	11.00
801	HLA-A0201	EENNLRFIEDLSI	NLFIEDLSI	3	0	0	2	1	NLRFIEDLSI	tr_C9ZJU1	0.193	6183.14	11.00
337	HLA-A0201	RVPALLEVMHGC	RLLEVMHGC	0	0	0	1	3	RVPALLEVMHGC	tr_C9ZJU1	0.193	6189.23	11.00
428	HLA-A0201	LLRKQKDDGIKCML	LLDGIKCML	0	0	0	2	5	LLRKQKDDGIKCML	tr_C9ZJU1	0.193	6189.29	11.00
102	HLA-A0201	ALVNCSIRDILLDD	ALVNCSILL	0	0	0	6	3	ALVNCSIRDILL	tr_C9ZJU1	0.193	6192.51	11.00
187	HLA-A0201	LMCSSCSIPQLVT	LMCSIPQLV	0	0	0	2	3	LMCSSCSIPQLV	tr_C9ZJU1	0.193	6198.01	11.00
515	HLA-A0201	LQAGGHTCAVSV	LQAGCAVSV	0	0	0	4	3	LQAGGHTCAVSV	tr_C9ZJU1	0.193	6199.95	11.00
360	HLA-A0201	VMWLGTQWSN	VMWLGTQWS	0	0	0	0	0	VMWLGTQWS	tr_C9ZJU1	0.193	6200.42	11.00
861	HLA-A0201	GGEVERLCIARAFV	VLCIARAFV	3	0	0	1	2	VERLCIARAFV	tr_C9ZJU1	0.193	6206.33	11.00
341	HLA-A0201	LLEVMHGCRVVFSF	LLCRVVFSF	0	0	0	2	5	LLEVMHGCRVVFSF	tr_C9ZJU1	0.193	6206.40	11.00
610	HLA-A0201	PLLLVSAVALRLSP	LLLAVALRL	1	0	0	3	2	LLLVSAVALRL	tr_C9ZJU1	0.193	6208.95	11.00
731	HLA-A0201	HPYVLRRVSFVVNK	YLRRVSFVV	2	0	0	1	1	YVLRRVSFVV	tr_C9ZJU1	0.193	6223.81	11.00
94	HLA-A0201	CDIWNCLMALVNC	CIWNCLMAL	0	0	0	1	1	CDIWNCLMAL	tr_C9ZJU1	0.193	6225.02	11.00
242	HLA-A0201	PKLSPFRFIIKQLM	KLSPFRFLM	1	0	0	7	4	KLSPFRFIIKQLM	tr_C9ZJU1	0.192	6232.84	11.00

331	HLA-A0201	MDELQSRVPALLEV	LQVPALLEV	3	0	0	2	2	LQSRVPALLEV	tr_C9ZJU1	0.192	6237.09	11.00
820	HLA-A0201	GNLWDCSDENIC	NLWDCSDEI	1	0	0	8	1	NLWDCSDENI	tr_C9ZJU1	0.192	6242.49	11.00
219	HLA-A0201	HAFVHWRPIYAAEI	FVHWRPIYI	2	0	0	8	3	FVHWRPIYAAEI	tr_C9ZJU1	0.192	6273.77	11.00
924	HLA-A0201	RLATLKHADKIV	RLATLKHAV	0	0	0	8	3	RLATLKHADKIV	tr_C9ZJU1	0.192	6273.98	11.00
800	HLA-A0201	QEENNLRFIEDLSI	NLFIEDLSI	4	0	0	2	1	NLRFIEDLSI	tr_C9ZJU1	0.191	6303.10	11.00
619	HLA-A0201	LRLSPNFFTQSALD	RLSPNFFTL	1	0	0	8	3	RLSPNFFTQSAL	tr_C9ZJU1	0.191	6314.71	11.00
569	HLA-A0201	GWELMAATAAAAFA	LMAATAAFA	3	0	0	5	2	LMAATAAAAFA	tr_C9ZJU1	0.191	6316.69	11.00
338	HLA-A0201	VPALLEVMHGC	VLLEVMHGC	0	0	0	1	2	VPALLEVMHGC	tr_C9ZJU1	0.191	6324.55	11.00
559	HLA-A0201	LQGFAALRL	LQGFAALRL	0	0	0	0	0	LQGFAALRL	tr_C9ZJU1	0.191	6325.51	11.00
19	HLA-A0201	ILEMLTRATIK	ILMLTRATI	0	0	0	2	1	ILEMLTRATI	tr_C9ZJU1	0.191	6326.81	11.00
364	HLA-A0201	GTQWSNFLYF	GQWSNFLYF	0	0	0	1	1	GTQWSNFLYF	tr_C9ZJU1	0.191	6329.14	11.00
209	HLA-A0201	EVILKCILGMHA	ILCILGMHA	2	0	0	2	1	ILKCILGMHA	tr_C9ZJU1	0.191	6329.27	11.00
841	HLA-A0201	NFVQKRSSGIHAPL	FVSGIHAPL	1	0	0	2	4	FVQKRSSGIHAPL	tr_C9ZJU1	0.191	6339.69	11.00
647	HLA-A0201	CPLYHAFLGS	CLYHAFLGS	0	0	0	1	1	CPLYHAFLGS	tr_C9ZJU1	0.191	6341.89	11.00
260	HLA-A0201	TFVSMAVAST	FVSMAVAST	1	0	0	0	0	FVSMAVAST	tr_C9ZJU1	0.191	6352.94	11.00
202	HLA-A0201	ATVLFSPEVIL	ALFSPEVIL	0	0	0	1	2	ATVLFSPEVIL	tr_C9ZJU1	0.191	6356.66	11.00
356	HLA-A0201	VVSLVMWLGT	VVSLVMWLT	0	0	0	8	1	VVSLVMWLGT	tr_C9ZJU1	0.191	6358.03	11.00
863	HLA-A0201	EVERLCIARAFV	VLCIARAFV	1	0	0	1	2	VERLCIARAFV	tr_C9ZJU1	0.191	6363.47	11.00
17	HLA-A0201	YEILEMLTRATIK	ILMLTRATI	2	0	0	2	1	ILEMLTRATI	tr_C9ZJU1	0.190	6379.74	11.00
567	HLA-A0201	LSGWELMAAT	LSWELMAAT	0	0	0	2	1	LSGWELMAAT	tr_C9ZJU1	0.190	6392.39	11.00
247	HLA-A0201	FRFIIKQLMGLP	FIIKQLMGL	0	0	0	1	2	FRFIIKQLMGL	tr_C9ZJU1	0.190	6397.78	11.00
329	HLA-A0201	SVMDELQSRVP	SVMDELQSV	0	0	0	8	1	SVMDELQSRV	tr_C9ZJU1	0.190	6400.48	11.00
130	HLA-A0201	VLALCMLLR	VLALCMLLR	0	0	0	0	0	VLALCMLLR	tr_C9ZJU1	0.190	6412.47	11.00
280	HLA-A0201	GFVIQSILSDIIT	FVIQSILSI	1	0	0	8	2	FVIQSILSDII	tr_C9ZJU1	0.190	6414.83	11.00
350	HLA-A0201	VVFSFPVVSLVMW	VVFSFPVLV	0	0	0	7	2	VVFSFPVVSLV	tr_C9ZJU1	0.190	6420.53	11.00
312	HLA-A0201	DWLLSRCVSQALWQ	WLLSRCVSL	1	0	0	8	2	WLLSRCVSQAL	tr_C9ZJU1	0.190	6424.00	11.00
497	HLA-A0201	PLVPSVFSGCV	PLVPSVFSV	0	0	0	8	2	PLVPSVFSGCV	tr_C9ZJU1	0.189	6441.82	11.00
630	HLA-A0201	ALDQTEIVNQLRSI	ALDQTEISI	0	0	0	7	5	ALDQTEIVNQLRSI	tr_C9ZJU1	0.189	6450.89	12.00
939	HLA-A0201	EGMVAEVGSWETL	MVAEVWETL	2	0	0	5	2	MVAEVGSWETL	tr_C9ZJU1	0.189	6454.94	12.00
502	HLA-A0201	VFSGCVVDAL	FSGCVVDAL	1	0	0	0	0	FSGCVVDAL	tr_C9ZJU1	0.189	6480.55	12.00
677	HLA-A0201	LAFSGSSLGCYQV	LAFLGCYQV	0	0	0	3	4	LAFSGSSLGCYQV	tr_C9ZJU1	0.189	6503.87	12.00
789	HLA-A0201	RMLRRRIANVW	RMLRRIANV	0	0	0	3	1	RMLRRRIANV	tr_C9ZJU1	0.188	6537.52	12.00
289	HLA-A0201	DIITSIHIDAYLAL	IIIDAYLAL	1	0	0	2	4	IITSIHIDAYLAL	tr_C9ZJU1	0.188	6538.02	12.00
923	HLA-A0201	HRLATLKHADKIVV	RLATLKIVV	1	0	0	5	4	RLATLKHADKIVV	tr_C9ZJU1	0.188	6549.28	12.00
514	HLA-A0201	KLQAGGHTCAVS	KLQAGTCAV	0	0	0	5	2	KLQAGGHTCAV	tr_C9ZJU1	0.188	6555.30	12.00
248	HLA-A0201	RFIIKQLMGLP	FIIKQLMGL	1	0	0	0	0	FIIKQLMGL	tr_C9ZJU1	0.188	6555.87	12.00
201	HLA-A0201	AATVLFSPEVIL	ALFSPEVIL	0	0	0	1	3	AATVLFSPEVIL	tr_C9ZJU1	0.187	6586.30	12.00
759	HLA-A0201	TTLLRLLNRTYAPT	TLLRLLAPT	1	0	0	6	4	TLLRLLNRTYAPT	tr_C9ZJU1	0.187	6597.14	12.00
917	HLA-A0201	TIIIVAHRLA	TIIIVAHLA	0	0	0	7	1	TIIIVAHRLA	tr_C9ZJU1	0.187	6609.29	12.00
575	HLA-A0201	ATAAAAFAT	ATAAAAFAT	0	0	0	0	0	ATAAAAFAT	tr_C9ZJU1	0.187	6612.08	12.00
208	HLA-A0201	PEVILKCILGMHA	ILCILGMHA	3	0	0	2	1	ILKCILGMHA	tr_C9ZJU1	0.187	6614.79	12.00
497	HLA-A0201	PLVPSVFSGCVV	PLVPSVFSV	0	0	0	8	3	PLVPSVFSGCVV	tr_C9ZJU1	0.187	6616.58	12.00
470	HLA-A0201	VLFSMVFVESGRP	VLFSMVFVP	0	0	0	8	4	VLFSMVFVESGRP	tr_C9ZJU1	0.187	6621.53	12.00
438	HLA-A0201	KCMLSSTDNKML	MLSSTDNML	2	0	0	7	1	MLSSTDNKML	tr_C9ZJU1	0.187	6632.43	12.00
612	HLA-A0201	LLVSAVALRLSPN	LLVSAVALL	0	0	0	8	1	LLVSAVALRL	tr_C9ZJU1	0.187	6634.29	12.00
216	HLA-A0201	LGMHAFVHWRPIY	GMHAFVHPI	1	0	0	7	2	GMHAFVHWRPI	tr_C9ZJU1	0.187	6642.05	12.00
293	HLA-A0201	SIHIDAYLALATA	SIYLALATA	0	0	0	2	4	SIHIDAYLALATA	tr_C9ZJU1	0.187	6642.33	12.00
732	HLA-A0201	PYVLRRVSFVVNKG	YLRRVSFVV	1	0	0	1	1	YVLRRVSFVV	tr_C9ZJU1	0.186	6653.63	12.00
262	HLA-A0201	VSMAVASTFAFESL	SMAFAFESL	1	0	0	3	4	SMAVASTFAFESL	tr_C9ZJU1	0.186	6659.25	12.00
194	HLA-A0201	IPQLVTLAAT	IQLVTLAAT	0	0	0	1	1	IPQLVTLAAT	tr_C9ZJU1	0.186	6666.02	12.00
354	HLA-A0201	FPVVSLVMWLGTQW	FVVSLVMWL	0	0	0	1	1	FPVVSLVMWL	tr_C9ZJU1	0.186	6674.83	12.00
150	HLA-A0201	AQADVVVQEFPEEL	AQADVVVEL	0	0	0	7	5	AQADVVVQEFPEEL	tr_C9ZJU1	0.186	6678.22	12.00
7	HLA-A0201	AVLLDCERKA	ALLDCERKA	0	0	0	1	1	AVLLDCERKA	tr_C9ZJU1	0.186	6681.26	12.00
620	HLA-A0201	RLSPNFFTQSALDQ	RLSPNFFTL	0	0	0	8	3	RLSPNFFTQSAL	tr_C9ZJU1	0.186	6686.97	12.00

207	HLA-A0201	SPEVILKCILGMHA	ILCILGMHA	4	0	0	2	1	ILKCILGMHA	tr_C9ZJU1	0.185	6719.61	12.00
938	HLA-A0201	NEGMVAEVGSWETL	MVAEVWETL	3	0	0	5	2	MVAEVGSWETL	tr_C9ZJU1	0.185	6725.57	12.00
416	HLA-A0201	PVKNSLTSSPLTL	SLTSSPLTL	4	0	0	0	0	SLTSSPLTL	tr_C9ZJU1	0.185	6728.85	12.00
612	HLA-A0201	LLVSAVALRLSPNF	LLVSAVALL	0	0	0	8	1	LLVSAVALRL	tr_C9ZJU1	0.185	6738.90	12.00
612	HLA-A0201	LLVSAVALR	LLVSAVALR	0	0	0	0	0	LLVSAVALR	tr_C9ZJU1	0.185	6757.16	12.00
71	HLA-A0201	QLPSVFAVHC	QLSVFAVHC	0	0	0	2	1	QLPSVFAVHC	tr_C9ZJU1	0.185	6768.94	12.00
846	HLA-A0201	RSSGIHAPL	RSSGIHAPL	0	0	0	0	0	RSSGIHAPL	tr_C9ZJU1	0.185	6771.13	12.00
206	HLA-A0201	FSPEVILKC	FSPEVILKC	0	0	0	0	0	FSPEVILKC	tr_C9ZJU1	0.185	6776.78	12.00
353	HLA-A0201	SFPVVSLVMWLGT	FVVSLVMWL	1	0	0	1	1	FPVVSLVMWL	tr_C9ZJU1	0.184	6799.54	12.00
71	HLA-A0201	QLPSVFAVHCGLM	QLFAVHCGL	0	0	0	2	3	QLPSVFAVHCGL	tr_C9ZJU1	0.184	6799.84	12.00
940	HLA-A0201	GMVAEVGSWET	GMVAEVWET	0	0	0	6	2	GMVAEVGSWET	tr_C9ZJU1	0.184	6803.59	12.00
373	HLA-A0201	FLNESLYMESQ	FLNESLYMS	0	0	0	8	1	FLNESLYMES	tr_C9ZJU1	0.184	6808.68	12.00
313	HLA-A0201	WLLSRCVSQ	WLLSRCVSQ	0	0	0	0	0	WLLSRCVSQ	tr_C9ZJU1	0.184	6824.09	12.00
350	HLA-A0201	VVFSFPVVS	VVFSFPVVS	0	0	0	0	0	VVFSFPVVS	tr_C9ZJU1	0.184	6854.43	12.00
302	HLA-A0201	LATADYDPSDDWLL	ATADYDWLL	1	0	0	5	4	ATADYDPSDDWLL	tr_C9ZJU1	0.184	6856.87	12.00
682	HLA-A0201	SSLGCYQVLEGLQS	SLYQVLEGL	1	0	0	2	2	SLGCYQVLEGL	tr_C9ZJU1	0.183	6870.54	12.00
16	HLA-A0201	AYEILEMLTRATIK	ILMLTRATI	3	0	0	2	1	ILEMLTRATI	tr_C9ZJU1	0.183	6880.06	12.00
256	HLA-A0201	GLPATFVSMAVAST	GLPATFMAV	0	0	0	6	2	GLPATFVSMAV	tr_C9ZJU1	0.183	6884.16	12.00
739	HLA-A0201	SFVVNKGEFLGI	FVVGEFLGI	1	0	0	3	2	FVVNKGEFLGI	tr_C9ZJU1	0.183	6891.16	12.00
682	HLA-A0201	SSLGCYQVL	SSLGCYQVL	0	0	0	0	0	SSLGCYQVL	tr_C9ZJU1	0.183	6895.12	12.00
683	HLA-A0201	SLGCYQVLEG	SLGCYVLEG	0	0	0	5	1	SLGCYQVLEG	tr_C9ZJU1	0.183	6930.94	12.00
349	HLA-A0201	RVVFSFPVVSLVMW	FSFPVVSLV	3	0	0	0	0	FSFPVVSLV	tr_C9ZJU1	0.183	6931.77	12.00
725	HLA-A0201	FRYSDRHPYV	FRYDRHPYV	0	0	0	3	1	FRYSDRHPYV	tr_C9ZJU1	0.183	6933.57	12.00
466	HLA-A0201	QYGLVLFSMVF	YLVLFSMVF	1	0	0	1	1	YGLVLFSMVF	tr_C9ZJU1	0.183	6936.12	12.00
279	HLA-A0201	RGFVIQSILSDIIT	FVIQSILSI	2	0	0	8	2	FVIQSILSDII	tr_C9ZJU1	0.182	6944.08	12.00
9	HLA-A0201	LLDCERKAYEIL	LLDCEAYEI	0	0	0	5	2	LLDCERKAYEI	tr_C9ZJU1	0.182	6958.82	12.00
384	HLA-A0201	FTKSQLASVTNIAT	FLASVTNIA	0	0	0	1	4	FTKSQLASVTNIA	tr_C9ZJU1	0.182	6960.40	12.00
335	HLA-A0201	QSRVPALLEVMHGC	SLLEVMHGC	1	0	0	1	4	SRVPALLEVMHGC	tr_C9ZJU1	0.182	6960.78	12.00
126	HLA-A0201	KAEEVLALCMLLR	KVLALCMLL	0	0	0	1	3	KAEEVLALCMLL	tr_C9ZJU1	0.182	6964.55	12.00
437	HLA-A0201	IKCMLSSTDNKML	MLSSTDNML	3	0	0	7	1	MLSSTDNKML	tr_C9ZJU1	0.182	6970.20	12.00
283	HLA-A0201	IQSILSDII	IQSILSDII	0	0	0	0	0	IQSILSDII	tr_C9ZJU1	0.182	6973.67	12.00
500	HLA-A0201	PSVFSGCVVDAL	SVFCVVDAL	1	0	0	3	2	SVFSGCVVDAL	tr_C9ZJU1	0.182	6987.19	12.00
562	HLA-A0201	FAALRLSGWELM	ALLSGWELM	2	0	0	2	1	ALRLSGWELM	tr_C9ZJU1	0.182	6991.88	12.00
818	HLA-A0201	ALGNLWDCSDENIC	NLWDCSDEI	3	0	0	8	1	NLWDCSDENI	tr_C9ZJU1	0.182	7000.20	12.00
290	HLA-A0201	IITSIHIDA	IITSIHIDA	0	0	0	0	0	IITSIHIDA	tr_C9ZJU1	0.182	7009.75	12.00
64	HLA-A0201	VFNHNIMQLPSVFA	FMQLPSVFA	1	0	0	1	4	FNHNIMQLPSVFA	tr_C9ZJU1	0.181	7026.46	12.00
292	HLA-A0201	TSIHIDAYLALA	IIDAYLALA	2	0	0	1	1	IHIDAYLALA	tr_C9ZJU1	0.181	7029.05	12.00
129	HLA-A0201	EVLALCMLLR	EVLALCMLL	0	0	0	0	0	EVLALCMLL	tr_C9ZJU1	0.181	7029.35	12.00
759	HLA-A0201	TTLLRLLNRTYAP	TLLRLLTYA	1	0	0	6	2	TLLRLLNRTYA	tr_C9ZJU1	0.181	7030.19	12.00
652	HLA-A0201	AFLGSFYDECGMS	FLGSFYCGM	1	0	0	6	2	FLGSFYDECGM	tr_C9ZJU1	0.181	7035.97	12.00
626	HLA-A0201	FTQSALDQTEIV	FTLDQTEIV	0	0	0	2	3	FTQSALDQTEIV	tr_C9ZJU1	0.181	7036.04	12.00
490	HLA-A0201	LTQHVIWPLVP	LTQHVIWPV	0	0	0	8	1	LTQHVIWPLV	tr_C9ZJU1	0.181	7060.99	12.00
819	HLA-A0201	LGNLWDCSDENIC	NLWDCSDEI	2	0	0	8	1	NLWDCSDENI	tr_C9ZJU1	0.180	7109.82	12.00
662	HLA-A0201	GMSEAQVDEAHL	GMSEAQVHL	0	0	0	7	3	GMSEAQVDEAHL	tr_C9ZJU1	0.180	7129.23	12.00
436	HLA-A0201	GIKCMLSSTDNKML	MLSSTDNML	4	0	0	7	1	MLSSTDNKML	tr_C9ZJU1	0.180	7149.62	12.00
806	HLA-A0201	RFIEDLSIGGNI	FIEDLSIGI	1	0	0	8	2	FIEDLSIGGNI	tr_C9ZJU1	0.180	7155.12	12.00
473	HLA-A0201	SMVFVESGRPL	SMVFVESPL	0	0	0	7	2	SMVFVESGRPL	tr_C9ZJU1	0.179	7171.62	12.00
344	HLA-A0201	VMHGCRVVFSF	VMHGCVFSF	0	0	0	5	2	VMHGCRVVFSF	tr_C9ZJU1	0.179	7185.22	12.00
759	HLA-A0201	TTLLRLLNRT	TLLRLLNRT	0	0	0	1	1	TTLLRLLNRT	tr_C9ZJU1	0.179	7200.55	12.00
353	HLA-A0201	SFPVVSLVMWLGTQ	FVVSLVMWL	1	0	0	1	1	FPVVSLVMWL	tr_C9ZJU1	0.179	7205.46	12.00
641	HLA-A0201	RSIQHWCPLYHAFL	IQHWCPLYL	2	0	0	8	3	IQHWCPLYHAFL	tr_C9ZJU1	0.179	7239.06	12.00
154	HLA-A0201	VVVQEFPEELYL	VVQEFPLYL	1	0	0	6	2	VVQEFPEELYL	tr_C9ZJU1	0.179	7242.35	12.00
776	HLA-A0201	IFVNGVHISQYPA	FVNGVQYPA	1	0	0	5	3	FVNGVHISQYPA	tr_C9ZJU1	0.179	7243.84	12.00
631	HLA-A0201	LDQTEIVNQL	LQTEIVNQL	0	0	0	1	1	LDQTEIVNQL	tr_C9ZJU1	0.178	7267.47	12.00

341	HLA-A0201	LLEVMHGCRVVF	LLMHGCRVV	0	0	0	2	2	LLEVMHGCRVV	tr_C9ZJU1	0.178	7294.02	12.00
617	HLA-A0201	VALRLSPNFFT	ALLSPNFFT	1	0	0	2	1	ALRLSPNFFT	tr_C9ZJU1	0.178	7296.54	12.00
469	HLA-A0201	LVLFSMVFVESGRP	VLFSMVFVP	1	0	0	8	4	VLFSMVFVESGRP	tr_C9ZJU1	0.178	7300.49	12.00
470	HLA-A0201	VLFSMVFVESGR	VLFSMVFES	0	0	0	7	1	VLFSMVFVES	tr_C9ZJU1	0.178	7306.50	12.00
355	HLA-A0201	PVVSLVMWL	PVVSLVMWL	0	0	0	0	0	PVVSLVMWL	tr_C9ZJU1	0.178	7311.01	12.00
280	HLA-A0201	GFVIQSILSDIITS	FVIQSILSI	1	0	0	8	2	FVIQSILSDII	tr_C9ZJU1	0.178	7311.09	12.00
345	HLA-A0201	MHGCRVVFSFPVV	MVVFSFPVV	0	0	0	1	4	MHGCRVVFSFPVV	tr_C9ZJU1	0.177	7338.43	12.00
638	HLA-A0201	NQLRSIQHWCPL	QLIQHWCPL	1	0	0	2	2	QLRSIQHWCPL	tr_C9ZJU1	0.177	7352.65	12.00
371	HLA-A0201	LYFLNESLYM	YFLNESLYM	1	0	0	0	0	YFLNESLYM	tr_C9ZJU1	0.177	7394.30	13.00
572	HLA-A0201	LMAATAAAAFAT	LMAATAFAT	0	0	0	5	3	LMAATAAAAFAT	tr_C9ZJU1	0.177	7403.18	13.00
206	HLA-A0201	FSPEVILKCIL	FSPEVILIL	0	0	0	7	2	FSPEVILKCIL	tr_C9ZJU1	0.176	7413.60	13.00
738	HLA-A0201	VSFVVNKGEFLGI	FVVGEFLGI	2	0	0	3	2	FVVNKGEFLGI	tr_C9ZJU1	0.176	7417.62	13.00
351	HLA-A0201	VFSFPVVSLVMW	FSFPVVSLV	1	0	0	0	0	FSFPVVSLV	tr_C9ZJU1	0.176	7418.98	13.00
314	HLA-A0201	LLSRCVSQALWQAS	LLSRCLWQA	0	0	0	5	4	LLSRCVSQALWQA	tr_C9ZJU1	0.176	7456.56	13.00
331	HLA-A0201	MDELQSRVPALL	MLQSRVPAL	0	0	0	1	2	MDELQSRVPAL	tr_C9ZJU1	0.176	7460.84	13.00
203	HLA-A0201	TVLFSPEVI	TVLFSPEVI	0	0	0	0	0	TVLFSPEVI	tr_C9ZJU1	0.176	7463.26	13.00
471	HLA-A0201	LFSMVFVESGRPLG	FSMVFVEPL	1	0	0	7	3	FSMVFVESGRPL	tr_C9ZJU1	0.176	7464.31	13.00
177	HLA-A0201	LQSGITSFWKL	LQITSFWKL	0	0	0	2	2	LQSGITSFWKL	tr_C9ZJU1	0.175	7492.63	13.00
246	HLA-A0201	PFRFIIKQLMGLP	FIIKQLMGL	3	0	0	0	0	FIIKQLMGL	tr_C9ZJU1	0.175	7500.66	13.00
570	HLA-A0201	WELMAATAAAAF	WLMAATAAA	0	0	0	1	1	WELMAATAAA	tr_C9ZJU1	0.175	7507.48	13.00
859	HLA-A0201	FSGGEVERL	FSGGEVERL	0	0	0	0	0	FSGGEVERL	tr_C9ZJU1	0.175	7514.22	13.00
163	HLA-A0201	LYLGLITAYN	YLGLITAYN	1	0	0	0	0	YLGLITAYN	tr_C9ZJU1	0.175	7517.23	13.00
881	HLA-A0201	GLCIFDEPTS	GLCIFDEPT	0	0	0	0	0	GLCIFDEPT	tr_C9ZJU1	0.175	7518.86	13.00
160	HLA-A0201	PEELYLGLITA	ELYLGLITA	2	0	0	0	0	ELYLGLITA	tr_C9ZJU1	0.175	7532.05	13.00
195	HLA-A0201	PQLVTLAATVL	QLVTLAATL	1	0	0	8	1	QLVTLAATVL	tr_C9ZJU1	0.175	7539.39	13.00
684	HLA-A0201	LGCYQVLEGL	LCYQVLEGL	0	0	0	1	1	LGCYQVLEGL	tr_C9ZJU1	0.175	7553.11	13.00
737	HLA-A0201	RVSFVVNKGEFLGI	FVVGEFLGI	3	0	0	3	2	FVVNKGEFLGI	tr_C9ZJU1	0.175	7562.27	13.00
134	HLA-A0201	CMLLRGKYSIA	CMLLRGYSI	0	0	0	6	1	CMLLRGKYSI	tr_C9ZJU1	0.174	7569.22	13.00
651	HLA-A0201	HAFLGSFYDECGMS	FLGSFYCGM	2	0	0	6	2	FLGSFYDECGM	tr_C9ZJU1	0.174	7578.32	13.00
472	HLA-A0201	FSMVFVESGRPLGY	FSMVFVEPL	0	0	0	7	3	FSMVFVESGRPL	tr_C9ZJU1	0.174	7579.14	13.00
130	HLA-A0201	VLALCMLLRGKYS	VLALCMLYS	0	0	0	7	4	VLALCMLLRGKYS	tr_C9ZJU1	0.174	7582.34	13.00
620	HLA-A0201	RLSPNFFTQ	RLSPNFFTQ	0	0	0	0	0	RLSPNFFTQ	tr_C9ZJU1	0.174	7582.34	13.00
599	HLA-A0201	LHGLFQMADTLP	LLFQMADTL	0	0	0	1	2	LHGLFQMADTL	tr_C9ZJU1	0.174	7597.61	13.00
255	HLA-A0201	MGLPATFVSMAVAS	GLPATFMAV	1	0	0	6	2	GLPATFVSMAV	tr_C9ZJU1	0.174	7598.44	13.00
261	HLA-A0201	FVSMAVASTFAF	FMAVASTFA	0	0	0	1	2	FVSMAVASTFA	tr_C9ZJU1	0.174	7602.47	13.00
609	HLA-A0201	LPLLLVSAVALR	LLLVSAVAL	0	0	0	1	2	LPLLLVSAVAL	tr_C9ZJU1	0.174	7613.91	13.00
163	HLA-A0201	LYLGLITAYNRKRS	YLGLITAYS	1	0	0	8	4	YLGLITAYNRKRS	tr_C9ZJU1	0.174	7628.91	13.00
107	HLA-A0201	SIRDILLDDGV	SIRDILLGV	0	0	0	7	2	SIRDILLDDGV	tr_C9ZJU1	0.174	7634.04	13.00
320	HLA-A0201	SQALWQASGSVM	SLWQASGSV	0	0	0	1	2	SQALWQASGSV	tr_C9ZJU1	0.174	7640.15	13.00
692	HLA-A0201	GLQSVQRKIDRPV	GLQSVDRPV	0	0	0	5	4	GLQSVQRKIDRPV	tr_C9ZJU1	0.174	7641.56	13.00
499	HLA-A0201	VPSVFSGCVVDAL	SVFCVVDAL	2	0	0	3	2	SVFSGCVVDAL	tr_C9ZJU1	0.174	7645.03	13.00
16	HLA-A0201	AYEILEMLTRAT	YILEMLTRA	1	0	0	1	1	YEILEMLTRA	tr_C9ZJU1	0.173	7657.12	13.00
92	HLA-A0201	LVCDIWNCLMA	LVCDICLMA	0	0	0	5	2	LVCDIWNCLMA	tr_C9ZJU1	0.173	7657.53	13.00
469	HLA-A0201	LVLFSMVFVESGR	LVLFSMVFV	0	0	0	0	0	LVLFSMVFV	tr_C9ZJU1	0.173	7658.94	13.00
127	HLA-A0201	AEEVLALCMLLR	AVLALCMLL	0	0	0	1	2	AEEVLALCMLL	tr_C9ZJU1	0.173	7660.02	13.00
292	HLA-A0201	TSIHIDAYLAL	SIIDAYLAL	1	0	0	2	1	SIHIDAYLAL	tr_C9ZJU1	0.173	7661.84	13.00
18	HLA-A0201	EILEMLTRATIK	ILMLTRATI	1	0	0	2	1	ILEMLTRATI	tr_C9ZJU1	0.173	7666.15	13.00
568	HLA-A0201	SGWELMAATA	SGWELMATA	0	0	0	6	1	SGWELMAATA	tr_C9ZJU1	0.173	7668.14	13.00
125	HLA-A0201	VKAEEVLALCMLLR	VVLALCMLL	0	0	0	1	4	VKAEEVLALCMLL	tr_C9ZJU1	0.173	7687.00	13.00
200	HLA-A0201	LAATVLFSPEVI	LAATVLFSI	0	0	0	8	3	LAATVLFSPEVI	tr_C9ZJU1	0.173	7698.57	13.00
207	HLA-A0201	SPEVILKCILGM	SILKCILGM	0	0	0	1	3	SPEVILKCILGM	tr_C9ZJU1	0.173	7700.24	13.00
653	HLA-A0201	FLGSFYDECGMSE	FLGSFYCGM	0	0	0	6	2	FLGSFYDECGM	tr_C9ZJU1	0.173	7708.24	13.00
561	HLA-A0201	GFAALRLSGWELM	ALLSGWELM	3	0	0	2	1	ALRLSGWELM	tr_C9ZJU1	0.173	7718.92	13.00
356	HLA-A0201	VVSLVMWLG	VVSLVMWLG	0	0	0	0	0	VVSLVMWLG	tr_C9ZJU1	0.173	7724.35	13.00

245	HLA-A0201	SPFRFIIKQLMGLP	FIIKQLMGL	4	0	0	0	0	FIIKQLMGL	tr_C9ZJU1	0.173	7724.69	13.00
764	HLA-A0201	LLNRTYAPTEGDI	LLNRTYAPI	0	0	0	8	4	LLNRTYAPTEGDI	tr_C9ZJU1	0.173	7728.28	13.00
17	HLA-A0201	YEILEMLTRATIKT	ILMLTRATI	2	0	0	2	1	ILEMLTRATI	tr_C9ZJU1	0.172	7737.23	13.00
276	HLA-A0201	YCIRGFVIQSI	YCIFVIQSI	0	0	0	3	2	YCIRGFVIQSI	tr_C9ZJU1	0.172	7747.03	13.00
96	HLA-A0201	IWNCLMALVNCS	ILMALVNCS	0	0	0	1	3	IWNCLMALVNCS	tr_C9ZJU1	0.172	7748.29	13.00
758	HLA-A0201	KTTLLRLLNRTYAP	TLLRLLTYA	2	0	0	6	2	TLLRLLNRTYA	tr_C9ZJU1	0.172	7752.73	13.00
775	HLA-A0201	DIFVNGVHISQYPA	FVNGVQYPA	2	0	0	5	3	FVNGVHISQYPA	tr_C9ZJU1	0.172	7758.95	13.00
95	HLA-A0201	DIWNCLMALVN	DIWNCLMLV	0	0	0	7	1	DIWNCLMALV	tr_C9ZJU1	0.172	7765.67	13.00
141	HLA-A0201	YSIARYPPAAQADV	YSIARYPPV	0	0	0	8	5	YSIARYPPAAQADV	tr_C9ZJU1	0.172	7769.03	13.00
482	HLA-A0201	PLGYMWDLLT	PLYMWDLLT	0	0	0	2	1	PLGYMWDLLT	tr_C9ZJU1	0.172	7776.09	13.00
563	HLA-A0201	AALRLSGWELM	ALLSGWELM	1	0	0	2	1	ALRLSGWELM	tr_C9ZJU1	0.172	7788.80	13.00
498	HLA-A0201	LVPSVFSGCVVDAL	SVFCVVDAL	3	0	0	3	2	SVFSGCVVDAL	tr_C9ZJU1	0.171	7844.71	13.00
382	HLA-A0201	SQFTKSQLASVT	SQFSQLASV	0	0	0	3	2	SQFTKSQLASV	tr_C9ZJU1	0.171	7848.44	13.00
962	HLA-A0201	RMRQSQQLPHFVK	RMQQLPHFV	0	0	0	2	3	RMRQSQQLPHFV	tr_C9ZJU1	0.171	7855.58	13.00
358	HLA-A0201	SLVMWLGTQW	SLVMWLGTW	0	0	0	8	1	SLVMWLGTQW	tr_C9ZJU1	0.171	7861.79	13.00
557	HLA-A0201	DYLQGFAALR	YLQGFAALR	1	0	0	0	0	YLQGFAALR	tr_C9ZJU1	0.171	7876.00	13.00
191	HLA-A0201	SCSIPQLVTL	SSIPQLVTL	0	0	0	1	1	SCSIPQLVTL	tr_C9ZJU1	0.171	7886.84	13.00
598	HLA-A0201	VLHGLFQMADTLP	LLFQMADTL	1	0	0	1	2	LHGLFQMADTL	tr_C9ZJU1	0.170	7913.94	13.00
126	HLA-A0201	KAEEVLALCMLLRG	KVLALCMLL	0	0	0	1	3	KAEEVLALCMLL	tr_C9ZJU1	0.170	7914.45	13.00
767	HLA-A0201	RTYAPTEGDIFVN	RTYEGDIFV	0	0	0	3	3	RTYAPTEGDIFV	tr_C9ZJU1	0.170	7916.42	13.00
555	HLA-A0201	RQDYLQGFA	RQDYLQGFA	0	0	0	0	0	RQDYLQGFA	tr_C9ZJU1	0.170	7919.84	13.00
609	HLA-A0201	LPLLLVSAV	LPLLLVSAV	0	0	0	0	0	LPLLLVSAV	tr_C9ZJU1	0.170	7926.02	13.00
379	HLA-A0201	YMESQFTKSQLAS	YMFTKSQLA	0	0	0	2	3	YMESQFTKSQLA	tr_C9ZJU1	0.170	7930.74	13.00
292	HLA-A0201	TSIHIDAYLALATA	SIYLALATA	1	0	0	2	4	SIHIDAYLALATA	tr_C9ZJU1	0.170	7944.99	13.00
560	HLA-A0201	QGFAALRLSGWELM	ALLSGWELM	4	0	0	2	1	ALRLSGWELM	tr_C9ZJU1	0.170	7974.02	13.00
496	HLA-A0201	WPLVPSVFSGCV	PLVPSVFSV	1	0	0	8	2	PLVPSVFSGCV	tr_C9ZJU1	0.169	8033.68	13.00
95	HLA-A0201	DIWNCLMALVNCS	ILMALVNCS	1	0	0	1	3	IWNCLMALVNCS	tr_C9ZJU1	0.169	8034.64	13.00
917	HLA-A0201	TIIIVAHRLATLK	TIIIVLATL	0	0	0	5	3	TIIIVAHRLATL	tr_C9ZJU1	0.169	8035.33	13.00
321	HLA-A0201	QALWQASGSVM	ALWQASGSV	1	0	0	0	0	ALWQASGSV	tr_C9ZJU1	0.169	8068.44	13.00
213	HLA-A0201	KCILGMHAFVH	KILGMHAFV	0	0	0	1	1	KCILGMHAFV	tr_C9ZJU1	0.168	8148.19	13.00
496	HLA-A0201	WPLVPSVFSGCVV	PLVPSVFSV	1	0	0	8	3	PLVPSVFSGCVV	tr_C9ZJU1	0.168	8162.83	13.00
740	HLA-A0201	FVVNKGEFLGIA	FVVEFLGIA	0	0	0	3	3	FVVNKGEFLGIA	tr_C9ZJU1	0.167	8167.87	13.00
219	HLA-A0201	HAFVHWRPIYA	FVHWRPIYA	2	0	0	0	0	FVHWRPIYA	tr_C9ZJU1	0.167	8168.13	13.00
19	HLA-A0201	ILEMLTRATIKT	ILMLTRATI	0	0	0	2	1	ILEMLTRATI	tr_C9ZJU1	0.167	8169.46	13.00
774	HLA-A0201	GDIFVNGVHI	GIFVNGVHI	0	0	0	1	1	GDIFVNGVHI	tr_C9ZJU1	0.167	8171.67	13.00
15	HLA-A0201	KAYEILEMLTRAT	YILEMLTRA	2	0	0	1	1	YEILEMLTRA	tr_C9ZJU1	0.167	8172.90	13.00
153	HLA-A0201	DVVVQEFPEELYL	VVQEFPLYL	2	0	0	6	2	VVQEFPEELYL	tr_C9ZJU1	0.167	8200.19	13.00
797	HLA-A0201	NVWQEENNL	NVWQEENNL	0	0	0	0	0	NVWQEENNL	tr_C9ZJU1	0.167	8212.53	13.00
489	HLA-A0201	LLTQHVIWP	LLTQHVIWP	0	0	0	0	0	LLTQHVIWP	tr_C9ZJU1	0.166	8260.12	13.00
337	HLA-A0201	RVPALLEVM	RVPALLEVM	0	0	0	0	0	RVPALLEVM	tr_C9ZJU1	0.166	8287.96	13.00
299	HLA-A0201	YLALATADY	YLALATADY	0	0	0	0	0	YLALATADY	tr_C9ZJU1	0.166	8303.40	13.00
10	HLA-A0201	LDCERKAYEILEML	LAYEILEML	0	0	0	1	5	LDCERKAYEILEML	tr_C9ZJU1	0.166	8311.94	13.00
637	HLA-A0201	VNQLRSIQHWCPL	QLIQHWCPL	2	0	0	2	2	QLRSIQHWCPL	tr_C9ZJU1	0.166	8320.30	13.00
514	HLA-A0201	KLQAGGHTC	KLQAGGHTC	0	0	0	0	0	KLQAGGHTC	tr_C9ZJU1	0.166	8323.01	13.00
250	HLA-A0201	IIKQLMGLPA	IIQLMGLPA	0	0	0	2	1	IIKQLMGLPA	tr_C9ZJU1	0.166	8327.33	14.00
630	HLA-A0201	ALDQTEIVNQLR	ALDEIVNQL	0	0	0	3	2	ALDQTEIVNQL	tr_C9ZJU1	0.166	8327.96	14.00
596	HLA-A0201	RDVLHGLFQMA	RLHGLFQMA	0	0	0	1	2	RDVLHGLFQMA	tr_C9ZJU1	0.166	8335.89	14.00
799	HLA-A0201	WQEENNLRFI	WQEENNLFI	0	0	0	7	1	WQEENNLRFI	tr_C9ZJU1	0.166	8337.70	14.00
482	HLA-A0201	PLGYMWDLL	PLGYMWDLL	0	0	0	0	0	PLGYMWDLL	tr_C9ZJU1	0.165	8343.56	14.00
805	HLA-A0201	LRFIEDLSIGGNI	FIEDLSIGI	2	0	0	8	2	FIEDLSIGGNI	tr_C9ZJU1	0.165	8347.17	14.00
616	HLA-A0201	AVALRLSPNFFT	ALLSPNFFT	2	0	0	2	1	ALRLSPNFFT	tr_C9ZJU1	0.165	8354.04	14.00
264	HLA-A0201	MAVASTFAF	MAVASTFAF	0	0	0	0	0	MAVASTFAF	tr_C9ZJU1	0.165	8363.99	14.00
769	HLA-A0201	YAPTEGDIFVNGV	YADIFVNGV	0	0	0	2	4	YAPTEGDIFVNGV	tr_C9ZJU1	0.165	8388.73	14.00
576	HLA-A0201	TAAAAFATA	TAAAAFATA	0	0	0	0	0	TAAAAFATA	tr_C9ZJU1	0.165	8397.27	14.00

60	HLA-A0201	VRTLVFNHNI	VTLVFNHNI	0	0	0	1	1	VRTLVFNHNI	tr_C9ZJU1	0.165	8402.18	14.00
782	HLA-A0201	HISQYPARM	HISQYPARM	0	0	0	0	0	HISQYPARM	tr_C9ZJU1	0.165	8415.82	14.00
127	HLA-A0201	AEEVLALCMLLRG	AVLALCMLL	0	0	0	1	2	AEEVLALCMLL	tr_C9ZJU1	0.165	8430.77	14.00
873	HLA-A0201	FVKAGSAVGLCI	FVSAVGLCI	0	0	0	2	3	FVKAGSAVGLCI	tr_C9ZJU1	0.164	8446.29	14.00
358	HLA-A0201	SLVMWLGTQ	SLVMWLGTQ	0	0	0	0	0	SLVMWLGTQ	tr_C9ZJU1	0.164	8446.38	14.00
923	HLA-A0201	HRLATLKHADKIV	RLATLKHAV	1	0	0	8	3	RLATLKHADKIV	tr_C9ZJU1	0.164	8447.75	14.00
636	HLA-A0201	IVNQLRSIQHWCPL	QLIQHWCPL	3	0	0	2	2	QLRSIQHWCPL	tr_C9ZJU1	0.164	8457.26	14.00
197	HLA-A0201	LVTLAATVLFS	LLAATVLFS	0	0	0	1	2	LVTLAATVLFS	tr_C9ZJU1	0.164	8463.86	14.00
205	HLA-A0201	LFSPEVILKCI	FSPEVILCI	1	0	0	7	1	FSPEVILKCI	tr_C9ZJU1	0.164	8473.20	14.00
804	HLA-A0201	NLRFIEDLSIGGNI	FIEDLSIGI	3	0	0	8	2	FIEDLSIGGNI	tr_C9ZJU1	0.164	8474.03	14.00
379	HLA-A0201	YMESQFTKSQL	YMESQFTQL	0	0	0	7	2	YMESQFTKSQL	tr_C9ZJU1	0.164	8479.90	14.00
196	HLA-A0201	QLVTLAATVLF	QLVTLAATV	0	0	0	0	0	QLVTLAATV	tr_C9ZJU1	0.164	8480.45	14.00
196	HLA-A0201	QLVTLAATVLFS	LLAATVLFS	1	0	0	1	2	LVTLAATVLFS	tr_C9ZJU1	0.164	8486.14	14.00
939	HLA-A0201	EGMVAEVGSWET	MVAEVSWET	2	0	0	5	1	MVAEVGSWET	tr_C9ZJU1	0.164	8494.96	14.00
568	HLA-A0201	SGWELMAATAAAAF	SLMAATAAA	0	0	0	1	3	SGWELMAATAAA	tr_C9ZJU1	0.164	8501.03	14.00
221	HLA-A0201	FVHWRPIYAAE	FVWRPIYAA	0	0	0	2	1	FVHWRPIYAA	tr_C9ZJU1	0.164	8502.50	14.00
295	HLA-A0201	HIDAYLALATA	HIYLALATA	0	0	0	2	2	HIDAYLALATA	tr_C9ZJU1	0.164	8507.00	14.00
327	HLA-A0201	SGSVMDELQSRVP	SMDELQSRV	0	0	0	1	3	SGSVMDELQSRV	tr_C9ZJU1	0.164	8509.77	14.00
328	HLA-A0201	GSVMDELQSRVP	SVMDELQSV	1	0	0	8	1	SVMDELQSRV	tr_C9ZJU1	0.163	8539.93	14.00
218	HLA-A0201	MHAFVHWRPIYA	FVHWRPIYA	3	0	0	0	0	FVHWRPIYA	tr_C9ZJU1	0.163	8542.33	14.00
152	HLA-A0201	ADVVVQEFPEELYL	VVQEFPLYL	3	0	0	6	2	VVQEFPEELYL	tr_C9ZJU1	0.163	8558.15	14.00
786	HLA-A0201	YPARMLRRRIANVW	YLRRRIANV	0	0	0	1	4	YPARMLRRRIANV	tr_C9ZJU1	0.163	8561.30	14.00
610	HLA-A0201	PLLLVSAVALR	LLLVSAVAL	1	0	0	0	0	LLLVSAVAL	tr_C9ZJU1	0.163	8580.03	14.00
14	HLA-A0201	RKAYEILEMLTRAT	YILEMLTRA	3	0	0	1	1	YEILEMLTRA	tr_C9ZJU1	0.163	8581.15	14.00
567	HLA-A0201	LSGWELMAA	LSGWELMAA	0	0	0	0	0	LSGWELMAA	tr_C9ZJU1	0.163	8588.95	14.00
916	HLA-A0201	STIIIVAHRLATLK	TIIIVLATL	1	0	0	5	3	TIIIVAHRLATL	tr_C9ZJU1	0.163	8614.17	14.00
513	HLA-A0201	RKLQAGGHTCAVS	KLQAGTCAV	1	0	0	5	2	KLQAGGHTCAV	tr_C9ZJU1	0.163	8617.53	14.00
915	HLA-A0201	DSTIIIVAHRL	STIIIVAHL	1	0	0	8	1	STIIIVAHRL	tr_C9ZJU1	0.162	8618.92	14.00
343	HLA-A0201	EVMHGCRVVFSF	VMHGVVFSF	1	0	0	4	2	VMHGCRVVFSF	tr_C9ZJU1	0.162	8620.79	14.00
142	HLA-A0201	SIARYPPAAQADV	SIARYPPAV	0	0	0	8	4	SIARYPPAAQADV	tr_C9ZJU1	0.162	8620.98	14.00
318	HLA-A0201	CVSQALWQASGSVM	ALWQASGSV	4	0	0	0	0	ALWQASGSV	tr_C9ZJU1	0.162	8627.98	14.00
448	HLA-A0201	MLFQQTFSHPSR	MLFQQTFPS	0	0	0	7	2	MLFQQTFSHPS	tr_C9ZJU1	0.162	8633.67	14.00
319	HLA-A0201	VSQALWQASGSVM	VLWQASGSV	0	0	0	1	3	VSQALWQASGSV	tr_C9ZJU1	0.162	8665.96	14.00
180	HLA-A0201	GITSFWKLM	GITSFWKLM	0	0	0	0	0	GITSFWKLM	tr_C9ZJU1	0.162	8668.21	14.00
572	HLA-A0201	LMAATAAAAFATAS	LMAATFATA	0	0	0	5	4	LMAATAAAAFATA	tr_C9ZJU1	0.162	8676.94	14.00
479	HLA-A0201	SGRPLGYMWDLL	SLGYMWDLL	0	0	0	1	3	SGRPLGYMWDLL	tr_C9ZJU1	0.162	8680.79	14.00
275	HLA-A0201	LYCIRGFVIQSI	YCIFVIQSI	1	0	0	3	2	YCIRGFVIQSI	tr_C9ZJU1	0.162	8698.09	14.00
615	HLA-A0201	SAVALRLSPNFFT	ALLSPNFFT	3	0	0	2	1	ALRLSPNFFT	tr_C9ZJU1	0.162	8706.37	14.00
192	HLA-A0201	CSIPQLVTLA	SIPQLVTLA	1	0	0	0	0	SIPQLVTLA	tr_C9ZJU1	0.162	8708.54	14.00
714	HLA-A0201	GPWSVEFREV	GPWSVEFEV	0	0	0	7	1	GPWSVEFREV	tr_C9ZJU1	0.161	8716.65	14.00
570	HLA-A0201	WELMAATAAAAFAT	WLMAATAAA	0	0	0	1	1	WELMAATAAA	tr_C9ZJU1	0.161	8717.87	14.00
788	HLA-A0201	ARMLRRRIANVW	RMLRRIANV	1	0	0	3	1	RMLRRRIANV	tr_C9ZJU1	0.161	8720.61	14.00
291	HLA-A0201	ITSIHIDAYLAL	SIIDAYLAL	2	0	0	2	1	SIHIDAYLAL	tr_C9ZJU1	0.161	8727.50	14.00
646	HLA-A0201	WCPLYHAFL	WCPLYHAFL	0	0	0	0	0	WCPLYHAFL	tr_C9ZJU1	0.161	8734.02	14.00
372	HLA-A0201	YFLNESLYMESQ	FLNESLYMS	1	0	0	8	1	FLNESLYMES	tr_C9ZJU1	0.161	8738.65	14.00
142	HLA-A0201	SIARYPPAAQADVV	SIARYPPAV	0	0	0	8	5	SIARYPPAAQADVV	tr_C9ZJU1	0.161	8743.47	14.00
883	HLA-A0201	CIFDEPTSAIDTI	CIFDAIDTI	0	0	0	4	4	CIFDEPTSAIDTI	tr_C9ZJU1	0.161	8750.85	14.00
129	HLA-A0201	EVLALCMLLRGKYS	VLALCMLYS	1	0	0	7	4	VLALCMLLRGKYS	tr_C9ZJU1	0.161	8759.19	14.00
920	HLA-A0201	IVAHRLATLK	IVAHRLATL	0	0	0	0	0	IVAHRLATL	tr_C9ZJU1	0.161	8764.41	14.00
166	HLA-A0201	GLITAYNRKRSL	GLITAYNSL	0	0	0	7	3	GLITAYNRKRSL	tr_C9ZJU1	0.161	8785.77	14.00
133	HLA-A0201	LCMLLRGKYSIA	CMLLRGYSI	1	0	0	6	1	CMLLRGKYSI	tr_C9ZJU1	0.161	8789.95	14.00
568	HLA-A0201	SGWELMAAT	SGWELMAAT	0	0	0	0	0	SGWELMAAT	tr_C9ZJU1	0.161	8796.90	14.00
741	HLA-A0201	VVNKGEFLGI	VVNGEFLGI	0	0	0	3	1	VVNKGEFLGI	tr_C9ZJU1	0.161	8802.33	14.00
345	HLA-A0201	MHGCRVVFSFPV	GCVVFSFPV	2	0	0	2	1	GCRVVFSFPV	tr_C9ZJU1	0.160	8814.90	14.00

317	HLA-A0201	RCVSQALWQA	RVSQALWQA	0	0	0	1	1	RCVSQALWQA	tr_C9ZJU1	0.160	8828.27	14.00
916	HLA-A0201	STIIIVAHRLA	STIIIVALA	0	0	0	7	2	STIIIVAHRLA	tr_C9ZJU1	0.160	8848.35	14.00
97	HLA-A0201	WNCLMALVNCS	WLMALVNCS	0	0	0	1	2	WNCLMALVNCS	tr_C9ZJU1	0.160	8862.53	14.00
326	HLA-A0201	ASGSVMDELQSRVP	AMDELQSRV	0	0	0	1	4	ASGSVMDELQSRV	tr_C9ZJU1	0.160	8872.60	14.00
189	HLA-A0201	CSSCSIPQLV	CSSCSIPLV	0	0	0	7	1	CSSCSIPQLV	tr_C9ZJU1	0.160	8877.89	14.00
762	HLA-A0201	LRLLNRTYAPT	RLLNRTYPT	1	0	0	7	1	RLLNRTYAPT	tr_C9ZJU1	0.160	8879.23	14.00
575	HLA-A0201	ATAAAAFATA	ATAAAAFTA	0	0	0	7	1	ATAAAAFATA	tr_C9ZJU1	0.160	8886.24	14.00
197	HLA-A0201	LVTLAATVL	LVTLAATVL	0	0	0	0	0	LVTLAATVL	tr_C9ZJU1	0.160	8897.89	14.00
597	HLA-A0201	DVLHGLFQMADTLP	LLFQMADTL	2	0	0	1	2	LHGLFQMADTL	tr_C9ZJU1	0.159	8902.70	14.00
910	HLA-A0201	NNLLRDSTII	NLLRDSTII	0	0	0	1	1	NNLLRDSTII	tr_C9ZJU1	0.159	8913.40	14.00
614	HLA-A0201	VSAVALRLSPNFFT	ALLSPNFFT	4	0	0	2	1	ALRLSPNFFT	tr_C9ZJU1	0.159	8916.78	14.00
938	HLA-A0201	NEGMVAEVGSWET	MVAEVSWET	3	0	0	5	1	MVAEVGSWET	tr_C9ZJU1	0.159	8917.07	14.00
495	HLA-A0201	IWPLVPSVFSGCV	PLVPSVFSV	2	0	0	8	2	PLVPSVFSGCV	tr_C9ZJU1	0.159	8933.39	14.00
807	HLA-A0201	FIEDLSIGG	FIEDLSIGG	0	0	0	0	0	FIEDLSIGG	tr_C9ZJU1	0.159	8934.55	14.00
913	HLA-A0201	LRDSTIIIVAHRL	STIIIVAHL	3	0	0	8	1	STIIIVAHRL	tr_C9ZJU1	0.159	8951.48	14.00
212	HLA-A0201	LKCILGMHAFVH	LILGMHAFV	0	0	0	1	2	LKCILGMHAFV	tr_C9ZJU1	0.159	8958.16	14.00
595	HLA-A0201	GRDVLHGLFQMA	GLHGLFQMA	0	0	0	1	3	GRDVLHGLFQMA	tr_C9ZJU1	0.159	8970.68	14.00
965	HLA-A0201	QSQQLPHFV	QSQQLPHFV	0	0	0	0	0	QSQQLPHFV	tr_C9ZJU1	0.159	8978.15	14.00
760	HLA-A0201	TLLRLLNRTYAPTE	TLLRLLTYA	0	0	0	6	2	TLLRLLNRTYA	tr_C9ZJU1	0.159	8987.29	14.00
914	HLA-A0201	RDSTIIIVAHRL	STIIIVAHL	2	0	0	8	1	STIIIVAHRL	tr_C9ZJU1	0.159	8989.62	14.00
495	HLA-A0201	IWPLVPSVFSGCVV	PLVPSVFSV	2	0	0	8	3	PLVPSVFSGCVV	tr_C9ZJU1	0.159	8996.05	14.00
839	HLA-A0201	ALNFVQKRSSGIHA	ALNFVQIHA	0	0	0	6	5	ALNFVQKRSSGIHA	tr_C9ZJU1	0.158	9005.49	14.00
782	HLA-A0201	HISQYPARML	HISQYPAML	0	0	0	7	1	HISQYPARML	tr_C9ZJU1	0.158	9025.40	14.00
19	HLA-A0201	ILEMLTRATIKTD	ILMLTRATI	0	0	0	2	1	ILEMLTRATI	tr_C9ZJU1	0.158	9054.93	14.00
179	HLA-A0201	SGITSFWKL	SGITSFWKL	0	0	0	0	0	SGITSFWKL	tr_C9ZJU1	0.158	9065.03	14.00
821	HLA-A0201	NLWDCSDEN	NLWDCSDEN	0	0	0	0	0	NLWDCSDEN	tr_C9ZJU1	0.158	9083.48	14.00
447	HLA-A0201	KMLFQQTFSH	MLFQQTFSH	1	0	0	0	0	MLFQQTFSH	tr_C9ZJU1	0.158	9092.33	14.00
807	HLA-A0201	FIEDLSIGGNIA	FIEDLSIGA	0	0	0	8	3	FIEDLSIGGNIA	tr_C9ZJU1	0.157	9112.53	14.00
45	HLA-A0201	SIAIFLNCNGVE	SIAIFLNGV	0	0	0	6	2	SIAIFLNCNGV	tr_C9ZJU1	0.157	9119.23	14.00
115	HLA-A0201	DGVALCILDDV	GVALCILDV	1	0	0	7	1	GVALCILDDV	tr_C9ZJU1	0.157	9126.34	14.00
265	HLA-A0201	AVASTFAFESL	AVASTFAFL	0	0	0	8	2	AVASTFAFESL	tr_C9ZJU1	0.157	9126.83	14.00
205	HLA-A0201	LFSPEVILKCIL	FSPEVILIL	1	0	0	7	2	FSPEVILKCIL	tr_C9ZJU1	0.157	9146.80	14.00
322	HLA-A0201	ALWQASGSVMD	ALWQASGSV	0	0	0	0	0	ALWQASGSV	tr_C9ZJU1	0.157	9154.23	14.00
937	HLA-A0201	LNEGMVAEVGSWET	MVAEVSWET	4	0	0	5	1	MVAEVGSWET	tr_C9ZJU1	0.157	9165.13	14.00
661	HLA-A0201	CGMSEAQVDEAHL	GMSEAQVHL	1	0	0	7	3	GMSEAQVDEAHL	tr_C9ZJU1	0.157	9180.02	14.00
643	HLA-A0201	IQHWCPLYHAFLGS	ILYHAFLGS	0	0	0	1	5	IQHWCPLYHAFLGS	tr_C9ZJU1	0.157	9185.48	14.00
287	HLA-A0201	LSDIITSIHI	LSIITSIHI	0	0	0	2	1	LSDIITSIHI	tr_C9ZJU1	0.157	9194.13	14.00
314	HLA-A0201	LLSRCVSQALW	LLSCVSQAL	0	0	0	3	1	LLSRCVSQAL	tr_C9ZJU1	0.156	9202.69	14.00
594	HLA-A0201	CGRDVLHGLFQMA	VLHGLFQMA	4	0	0	0	0	VLHGLFQMA	tr_C9ZJU1	0.156	9213.45	14.00
44	HLA-A0201	ASIAIFLNC	ASIAIFLNC	0	0	0	0	0	ASIAIFLNC	tr_C9ZJU1	0.156	9218.34	14.00
260	HLA-A0201	TFVSMAVASTFAF	FMAVASTFA	1	0	0	1	2	FVSMAVASTFA	tr_C9ZJU1	0.156	9222.03	14.00
491	HLA-A0201	TQHVIWPLVP	TQHVIWPLV	0	0	0	0	0	TQHVIWPLV	tr_C9ZJU1	0.156	9231.21	14.00
12	HLA-A0201	CERKAYEILEML	KAYEILEML	3	0	0	0	0	KAYEILEML	tr_C9ZJU1	0.156	9239.91	14.00
817	HLA-A0201	IALGNLWDC	IALGNLWDC	0	0	0	0	0	IALGNLWDC	tr_C9ZJU1	0.156	9255.01	14.00
13	HLA-A0201	ERKAYEILEML	KAYEILEML	2	0	0	0	0	KAYEILEML	tr_C9ZJU1	0.156	9268.94	15.00
679	HLA-A0201	FSGSSLGCYQV	FSGSSLYQV	0	0	0	6	2	FSGSSLGCYQV	tr_C9ZJU1	0.156	9272.25	15.00
19	HLA-A0201	ILEMLTRAT	ILEMLTRAT	0	0	0	0	0	ILEMLTRAT	tr_C9ZJU1	0.156	9273.56	15.00
881	HLA-A0201	GLCIFDEPTSAID	GLCIFDEPI	0	0	0	8	3	GLCIFDEPTSAI	tr_C9ZJU1	0.156	9287.01	15.00
527	HLA-A0201	RRLGWIKDVI	RLGWIKDVI	0	0	0	1	1	RRLGWIKDVI	tr_C9ZJU1	0.155	9299.48	15.00
258	HLA-A0201	PATFVSMAVA	ATFVSMAVA	1	0	0	0	0	ATFVSMAVA	tr_C9ZJU1	0.155	9300.39	15.00
307	HLA-A0201	YDPSDDWLL	YDPSDDWLL	0	0	0	0	0	YDPSDDWLL	tr_C9ZJU1	0.155	9314.08	15.00
211	HLA-A0201	ILKCILGMHAF	ILILGMHAF	0	0	0	2	2	ILKCILGMHAF	tr_C9ZJU1	0.155	9346.89	15.00
276	HLA-A0201	YCIRGFVIQS	YCIGFVIQS	0	0	0	3	1	YCIRGFVIQS	tr_C9ZJU1	0.155	9352.25	15.00
342	HLA-A0201	LEVMHGCRVVFSF	VMHGVVFSF	2	0	0	4	2	VMHGCRVVFSF	tr_C9ZJU1	0.155	9363.19	15.00

789	HLA-A0201	RMLRRRIANVWQ	RMLRRIANV	0	0	0	3	1	RMLRRRIANV	tr_C9ZJU1	0.155	9371.19	15.00
106	HLA-A0201	CSIRDILLDDGV	SIRDILLGV	1	0	0	7	2	SIRDILLDDGV	tr_C9ZJU1	0.155	9372.51	15.00
95	HLA-A0201	DIWNCLMALVNC	DIWNCLMLV	0	0	0	7	1	DIWNCLMALV	tr_C9ZJU1	0.154	9401.76	15.00
386	HLA-A0201	KSQLASVTNIA	KLASVTNIA	0	0	0	1	2	KSQLASVTNIA	tr_C9ZJU1	0.154	9403.50	15.00
346	HLA-A0201	HGCRVVFSFPV	GCVVFSFPV	1	0	0	2	1	GCRVVFSFPV	tr_C9ZJU1	0.154	9404.00	15.00
196	HLA-A0201	QLVTLAATVLFSP	QLVTLAATV	0	0	0	0	0	QLVTLAATV	tr_C9ZJU1	0.154	9427.43	15.00
918	HLA-A0201	IIIVAHRLATLK	IIIVALATL	0	0	0	5	2	IIIVAHRLATL	tr_C9ZJU1	0.154	9455.12	15.00
493	HLA-A0201	HVIWPLVPS	HVIWPLVPS	0	0	0	0	0	HVIWPLVPS	tr_C9ZJU1	0.154	9475.91	15.00
11	HLA-A0201	DCERKAYEILEML	KAYEILEML	4	0	0	0	0	KAYEILEML	tr_C9ZJU1	0.154	9480.73	15.00
198	HLA-A0201	VTLAATVLF	VTLAATVLF	0	0	0	0	0	VTLAATVLF	tr_C9ZJU1	0.154	9491.71	15.00
569	HLA-A0201	GWELMAATAAAAF	WLMAATAAA	1	0	0	1	1	WELMAATAAA	tr_C9ZJU1	0.154	9492.74	15.00
625	HLA-A0201	FFTQSALDQTEIV	FTLDQTEIV	1	0	0	2	3	FTQSALDQTEIV	tr_C9ZJU1	0.154	9493.77	15.00
114	HLA-A0201	DDGVALCILDDV	GVALCILDV	2	0	0	7	1	GVALCILDDV	tr_C9ZJU1	0.153	9524.63	15.00
935	HLA-A0201	VVLNEGMVA	VVLNEGMVA	0	0	0	0	0	VVLNEGMVA	tr_C9ZJU1	0.153	9529.37	15.00
362	HLA-A0201	WLGTQWSNFLY	WLGTWSNFL	0	0	0	4	1	WLGTQWSNFL	tr_C9ZJU1	0.153	9531.13	15.00
512	HLA-A0201	KRKLQAGGHTCAVS	KLQAGTCAV	2	0	0	5	2	KLQAGGHTCAV	tr_C9ZJU1	0.153	9534.53	15.00
282	HLA-A0201	VIQSILSDII	VIQSILSII	0	0	0	7	1	VIQSILSDII	tr_C9ZJU1	0.153	9541.34	15.00
127	HLA-A0201	AEEVLALCMLLRGK	AVLALCMLL	0	0	0	1	2	AEEVLALCMLL	tr_C9ZJU1	0.153	9551.88	15.00
481	HLA-A0201	RPLGYMWDL	RPLGYMWDL	0	0	0	0	0	RPLGYMWDL	tr_C9ZJU1	0.153	9553.43	15.00
490	HLA-A0201	LTQHVIWPLVPS	LTQHVIWPV	0	0	0	8	1	LTQHVIWPLV	tr_C9ZJU1	0.153	9562.74	15.00
515	HLA-A0201	LQAGGHTCA	LQAGGHTCA	0	0	0	0	0	LQAGGHTCA	tr_C9ZJU1	0.153	9570.60	15.00
261	HLA-A0201	FVSMAVASTFAFES	FVSMFAFES	0	0	0	4	5	FVSMAVASTFAFES	tr_C9ZJU1	0.153	9572.15	15.00
420	HLA-A0201	SLTSSPLTLLR	SLTSSLTLL	0	0	0	5	1	SLTSSPLTLL	tr_C9ZJU1	0.153	9592.68	15.00
243	HLA-A0201	KLSPFRFIIKQLMG	KLSPFRFLM	0	0	0	7	4	KLSPFRFIIKQLM	tr_C9ZJU1	0.153	9593.31	15.00
93	HLA-A0201	VCDIWNCLMA	VCDIWCLMA	0	0	0	5	1	VCDIWNCLMA	tr_C9ZJU1	0.153	9600.68	15.00
19	HLA-A0201	ILEMLTRATIKTDG	ILMLTRATI	0	0	0	2	1	ILEMLTRATI	tr_C9ZJU1	0.152	9607.43	15.00
804	HLA-A0201	NLRFIEDLSIG	NLFIEDLSI	0	0	0	2	1	NLRFIEDLSI	tr_C9ZJU1	0.152	9609.72	15.00
917	HLA-A0201	TIIIVAHRLATLKH	TIIIVLATL	0	0	0	5	3	TIIIVAHRLATL	tr_C9ZJU1	0.152	9620.23	15.00
341	HLA-A0201	LLEVMHGCRVVFS	LLMHGCRVV	0	0	0	2	2	LLEVMHGCRVV	tr_C9ZJU1	0.152	9632.21	15.00
593	HLA-A0201	QCGRDVLHGLFQMA	VLHGLFQMA	5	0	0	0	0	VLHGLFQMA	tr_C9ZJU1	0.152	9636.69	15.00
711	HLA-A0201	SLDGPWSVEFREVS	SLDGPWSEV	0	0	0	7	4	SLDGPWSVEFREV	tr_C9ZJU1	0.152	9644.61	15.00
384	HLA-A0201	FTKSQLASVT	FTKSQLASV	0	0	0	0	0	FTKSQLASV	tr_C9ZJU1	0.152	9664.77	15.00
571	HLA-A0201	ELMAATAAAAFAT	LMAATAFAT	1	0	0	5	3	LMAATAAAAFAT	tr_C9ZJU1	0.152	9670.53	15.00
221	HLA-A0201	FVHWRPIYAAEIG	FVHWRPIYI	0	0	0	8	3	FVHWRPIYAAEI	tr_C9ZJU1	0.152	9673.67	15.00
18	HLA-A0201	EILEMLTRATIKT	ILMLTRATI	1	0	0	2	1	ILEMLTRATI	tr_C9ZJU1	0.152	9680.26	15.00
208	HLA-A0201	PEVILKCILGM	VILKCILGM	2	0	0	0	0	VILKCILGM	tr_C9ZJU1	0.152	9687.18	15.00
335	HLA-A0201	QSRVPALLEV	QSVPALLEV	0	0	0	2	1	QSRVPALLEV	tr_C9ZJU1	0.151	9713.84	15.00
259	HLA-A0201	ATFVSMAVASTFAF	FMAVASTFA	2	0	0	1	2	FVSMAVASTFA	tr_C9ZJU1	0.151	9715.52	15.00
922	HLA-A0201	AHRLATLKHADKIV	RLATLKHAV	2	0	0	8	3	RLATLKHADKIV	tr_C9ZJU1	0.151	9745.11	15.00
294	HLA-A0201	IHIDAYLALATA	IAYLALATA	0	0	0	1	3	IHIDAYLALATA	tr_C9ZJU1	0.151	9747.63	15.00
516	HLA-A0201	QAGGHTCAV	QAGGHTCAV	0	0	0	0	0	QAGGHTCAV	tr_C9ZJU1	0.151	9749.01	15.00
210	HLA-A0201	VILKCILGMHAF	ILCILGMHA	1	0	0	2	1	ILKCILGMHA	tr_C9ZJU1	0.151	9749.53	15.00
879	HLA-A0201	AVGLCIFDEPT	AVGLCIFPT	0	0	0	7	2	AVGLCIFDEPT	tr_C9ZJU1	0.151	9755.97	15.00
113	HLA-A0201	LDDGVALCILDDV	GVALCILDV	3	0	0	7	1	GVALCILDDV	tr_C9ZJU1	0.151	9768.65	15.00
825	HLA-A0201	CSDENICAAL	CSDEICAAL	0	0	0	4	1	CSDENICAAL	tr_C9ZJU1	0.151	9783.13	15.00
787	HLA-A0201	PARMLRRRIANVW	RMLRRIANV	2	0	0	3	1	RMLRRRIANV	tr_C9ZJU1	0.151	9785.68	15.00
652	HLA-A0201	AFLGSFYDECGMSE	FLGSFYCGM	1	0	0	6	2	FLGSFYDECGM	tr_C9ZJU1	0.151	9805.07	15.00
739	HLA-A0201	SFVVNKGEFLGIA	FVVEFLGIA	1	0	0	3	3	FVVNKGEFLGIA	tr_C9ZJU1	0.151	9809.00	15.00
868	HLA-A0201	CIARAFVKA	CIARAFVKA	0	0	0	0	0	CIARAFVKA	tr_C9ZJU1	0.151	9810.69	15.00
477	HLA-A0201	VESGRPLGYMWDLL	VLGYMWDLL	0	0	0	1	5	VESGRPLGYMWDLL	tr_C9ZJU1	0.150	9820.89	15.00
878	HLA-A0201	SAVGLCIFDEPT	SLCIFDEPT	0	0	0	1	3	SAVGLCIFDEPT	tr_C9ZJU1	0.150	9836.31	15.00
371	HLA-A0201	LYFLNESLYMESQ	FLNESLYMS	2	0	0	8	1	FLNESLYMES	tr_C9ZJU1	0.150	9853.46	15.00
379	HLA-A0201	YMESQFTKS	YMESQFTKS	0	0	0	0	0	YMESQFTKS	tr_C9ZJU1	0.150	9854.10	15.00
46	HLA-A0201	IAIFLNCNGVE	IIFLNCNGV	0	0	0	1	1	IAIFLNCNGV	tr_C9ZJU1	0.150	9873.85	15.00

62	HLA-A0201	TLVFNHNIMQLP	TLVFNIMQL	0	0	0	4	2	TLVFNHNIMQL	tr_C9ZJU1	0.150	9888.38	15.00
218	HLA-A0201	MHAFVHWRPI	MAFVHWRPI	0	0	0	1	1	MHAFVHWRPI	tr_C9ZJU1	0.150	9893.42	15.00
103	HLA-A0201	LVNCSIRDILL	LVNCSIILL	0	0	0	6	2	LVNCSIRDILL	tr_C9ZJU1	0.150	9917.63	15.00
105	HLA-A0201	NCSIRDILLDDGV	SIRDILLGV	2	0	0	7	2	SIRDILLDDGV	tr_C9ZJU1	0.149	9923.00	15.00
915	HLA-A0201	DSTIIIVAHRLA	STIIIVALA	1	0	0	7	2	STIIIVAHRLA	tr_C9ZJU1	0.149	9941.92	15.00
961	HLA-A0201	SRMRQSQQLPHFVK	RMQQLPHFV	1	0	0	2	3	RMRQSQQLPHFV	tr_C9ZJU1	0.149	9976.18	15.00
161	HLA-A0201	EELYLGLITAY	ELYLGLITA	0	0	0	1	1	EELYLGLITA	tr_C9ZJU1	0.149	9976.29	15.00
480	HLA-A0201	GRPLGYMWDLL	GLGYMWDLL	0	0	0	1	2	GRPLGYMWDLL	tr_C9ZJU1	0.149	9986.88	15.00
766	HLA-A0201	NRTYAPTEGDIFVN	RTYEGDIFV	1	0	0	3	3	RTYAPTEGDIFV	tr_C9ZJU1	0.149	9989.36	15.00
162	HLA-A0201	ELYLGLITAYN	ELYLGLITA	0	0	0	0	0	ELYLGLITA	tr_C9ZJU1	0.149	9990.33	15.00
409	HLA-A0201	VSPTCSFPVK	VSPTCSFPV	0	0	0	0	0	VSPTCSFPV	tr_C9ZJU1	0.149	9995.95	15.00
140	HLA-A0201	KYSIARYPPA	YSIARYPPA	1	0	0	0	0	YSIARYPPA	tr_C9ZJU1	0.149	10004.94	15.00
301	HLA-A0201	ALATADYDPS	ALATADYPS	0	0	0	7	1	ALATADYDPS	tr_C9ZJU1	0.149	10005.69	15.00
48	HLA-A0201	IFLNCNGVES	FLNCNGVES	1	0	0	0	0	FLNCNGVES	tr_C9ZJU1	0.149	10007.00	15.00
761	HLA-A0201	LLRLLNRTYAPT	RLLNRTYPT	2	0	0	7	1	RLLNRTYAPT	tr_C9ZJU1	0.148	10032.79	15.00
188	HLA-A0201	MCSSCSIPQLV	MCSSCSIPV	0	0	0	8	2	MCSSCSIPQLV	tr_C9ZJU1	0.148	10038.55	15.00
175	HLA-A0201	RSLQSGITSF	SLQSGITSF	1	0	0	0	0	SLQSGITSF	tr_C9ZJU1	0.148	10048.98	15.00
463	HLA-A0201	SSVQYGLVL	SSVQYGLVL	0	0	0	0	0	SSVQYGLVL	tr_C9ZJU1	0.148	10062.59	15.00
154	HLA-A0201	VVVQEFPEELYLGL	VVVELYLGL	0	0	0	3	5	VVVQEFPEELYLGL	tr_C9ZJU1	0.148	10068.90	15.00
381	HLA-A0201	ESQFTKSQLASVT	SQFSQLASV	1	0	0	3	2	SQFTKSQLASV	tr_C9ZJU1	0.148	10072.39	15.00
768	HLA-A0201	TYAPTEGDIFVNGV	YADIFVNGV	1	0	0	2	4	YAPTEGDIFVNGV	tr_C9ZJU1	0.148	10075.77	15.00
691	HLA-A0201	EGLQSVQRKIDRPV	GLQSVDRPV	1	0	0	5	4	GLQSVQRKIDRPV	tr_C9ZJU1	0.148	10077.40	15.00
642	HLA-A0201	SIQHWCPLYHAF	SIWCPLYHA	0	0	0	2	2	SIQHWCPLYHA	tr_C9ZJU1	0.148	10093.99	15.00
881	HLA-A0201	GLCIFDEPTSAIDT	GLCIFTSAI	0	0	0	5	3	GLCIFDEPTSAI	tr_C9ZJU1	0.148	10094.53	15.00
747	HLA-A0201	FLGIAGYSGSGKTT	FLGIAGYTT	0	0	0	7	5	FLGIAGYSGSGKTT	tr_C9ZJU1	0.148	10095.84	15.00
290	HLA-A0201	IITSIHIDAYLA	IIIHIDAYL	0	0	0	2	2	IITSIHIDAYL	tr_C9ZJU1	0.148	10098.25	15.00
235	HLA-A0201	SLNGDVSPKLS	SLNDVSPKL	0	0	0	3	1	SLNGDVSPKL	tr_C9ZJU1	0.147	10170.07	15.00
299	HLA-A0201	YLALATADYD	YLALATADY	0	0	0	0	0	YLALATADY	tr_C9ZJU1	0.147	10172.49	15.00
276	HLA-A0201	YCIRGFVIQSIL	CIRGFVIIL	1	0	0	7	2	CIRGFVIQSIL	tr_C9ZJU1	0.147	10178.55	15.00
963	HLA-A0201	MRQSQQLPHFV	RQSQQLPFV	1	0	0	7	1	RQSQQLPHFV	tr_C9ZJU1	0.147	10180.86	15.00
660	HLA-A0201	ECGMSEAQVDEAHL	GMSEAQVHL	2	0	0	7	3	GMSEAQVDEAHL	tr_C9ZJU1	0.147	10189.35	16.00
187	HLA-A0201	LMCSSCSIPQ	LMCSCSIPQ	0	0	0	3	1	LMCSSCSIPQ	tr_C9ZJU1	0.147	10189.35	16.00
600	HLA-A0201	HGLFQMADTLP	GLFQMADTL	1	0	0	0	0	GLFQMADTL	tr_C9ZJU1	0.147	10202.25	16.00
217	HLA-A0201	GMHAFVHWRP	GMHAFVWRP	0	0	0	6	1	GMHAFVHWRP	tr_C9ZJU1	0.147	10203.02	16.00
44	HLA-A0201	ASIAIFLNCNGVE	SIAIFLNCV	1	0	0	8	2	SIAIFLNCNGV	tr_C9ZJU1	0.147	10203.13	16.00
777	HLA-A0201	FVNGVHISQYPARM	FVNGVHISM	0	0	0	8	5	FVNGVHISQYPARM	tr_C9ZJU1	0.147	10203.25	16.00
891	HLA-A0201	AIDTITEGEIFDTL	AIDEIFDTL	0	0	0	3	5	AIDTITEGEIFDTL	tr_C9ZJU1	0.147	10211.31	16.00
934	HLA-A0201	IVVLNEGMV	IVVLNEGMV	0	0	0	0	0	IVVLNEGMV	tr_C9ZJU1	0.147	10213.40	16.00
217	HLA-A0201	GMHAFVHWR	GMHAFVHWR	0	0	0	0	0	GMHAFVHWR	tr_C9ZJU1	0.147	10216.28	16.00
927	HLA-A0201	TLKHADKIVVLN	TLADKIVVL	0	0	0	2	2	TLKHADKIVVL	tr_C9ZJU1	0.147	10218.93	16.00
358	HLA-A0201	SLVMWLGTQWSNF	SLVMWLGTF	0	0	0	8	4	SLVMWLGTQWSNF	tr_C9ZJU1	0.147	10221.59	16.00
336	HLA-A0201	SRVPALLEV	SRVPALLEV	0	0	0	0	0	SRVPALLEV	tr_C9ZJU1	0.147	10228.45	16.00
738	HLA-A0201	VSFVVNKGEFLGIA	FVVEFLGIA	2	0	0	3	3	FVVNKGEFLGIA	tr_C9ZJU1	0.146	10249.27	16.00
264	HLA-A0201	MAVASTFAFESL	AVASTFAFL	1	0	0	8	2	AVASTFAFESL	tr_C9ZJU1	0.146	10254.26	16.00
347	HLA-A0201	GCRVVFSFPVV	RVVFSFPVV	2	0	0	0	0	RVVFSFPVV	tr_C9ZJU1	0.146	10259.81	16.00
136	HLA-A0201	LLRGKYSIA	LLRGKYSIA	0	0	0	0	0	LLRGKYSIA	tr_C9ZJU1	0.146	10260.59	16.00
104	HLA-A0201	VNCSIRDILLDDGV	SIRDILLGV	3	0	0	7	2	SIRDILLDDGV	tr_C9ZJU1	0.146	10264.14	16.00
597	HLA-A0201	DVLHGLFQM	DVLHGLFQM	0	0	0	0	0	DVLHGLFQM	tr_C9ZJU1	0.146	10272.70	16.00
872	HLA-A0201	AFVKAGSAVGL	FVAGSAVGL	1	0	0	2	1	FVKAGSAVGL	tr_C9ZJU1	0.146	10273.14	16.00
629	HLA-A0201	SALDQTEIVNQLR	ALDEIVNQL	1	0	0	3	2	ALDQTEIVNQL	tr_C9ZJU1	0.146	10274.26	16.00
624	HLA-A0201	NFFTQSALDQTEIV	FTLDQTEIV	2	0	0	2	3	FTQSALDQTEIV	tr_C9ZJU1	0.146	10286.04	16.00
392	HLA-A0201	VTNIATSTYA	VTIATSTYA	0	0	0	2	1	VTNIATSTYA	tr_C9ZJU1	0.146	10317.04	16.00
209	HLA-A0201	EVILKCILGMHAF	ILILGMHAF	2	0	0	2	2	ILKCILGMHAF	tr_C9ZJU1	0.146	10325.52	16.00
737	HLA-A0201	RVSFVVNKGEFL	RVSFVVEFL	0	0	0	6	3	RVSFVVNKGEFL	tr_C9ZJU1	0.146	10333.68	16.00

195	HLA-A0201	PQLVTLAATVLFS	LLAATVLFS	2	0	0	1	2	LVTLAATVLFS	tr_C9ZJU1	0.146	10338.48	16.00
100	HLA-A0201	LMALVNCSIRD	LMALVNCSI	0	0	0	0	0	LMALVNCSI	tr_C9ZJU1	0.146	10340.72	16.00
126	HLA-A0201	KAEEVLALCM	KAEEVLALM	0	0	0	8	1	KAEEVLALCM	tr_C9ZJU1	0.146	10344.42	16.00
876	HLA-A0201	AGSAVGLCIFDEPT	ALCIFDEPT	0	0	0	1	5	AGSAVGLCIFDEPT	tr_C9ZJU1	0.145	10367.95	16.00
62	HLA-A0201	TLVFNHNIM	TLVFNHNIM	0	0	0	0	0	TLVFNHNIM	tr_C9ZJU1	0.145	10370.75	16.00
161	HLA-A0201	EELYLGLITAYN	YLGLITAYN	3	0	0	0	0	YLGLITAYN	tr_C9ZJU1	0.145	10380.29	16.00
872	HLA-A0201	AFVKAGSAVGLCI	FVSAVGLCI	1	0	0	2	3	FVKAGSAVGLCI	tr_C9ZJU1	0.145	10391.64	16.00
626	HLA-A0201	FTQSALDQT	FTQSALDQT	0	0	0	0	0	FTQSALDQT	tr_C9ZJU1	0.145	10409.65	16.00
914	HLA-A0201	RDSTIIIVAHRLA	STIIIVALA	2	0	0	7	2	STIIIVAHRLA	tr_C9ZJU1	0.145	10424.08	16.00
67	HLA-A0201	HNIMQLPSVFA	NIMQLSVFA	1	0	0	5	1	NIMQLPSVFA	tr_C9ZJU1	0.145	10429.38	16.00
866	HLA-A0201	RLCIARAFVKA	RLCIAFVKA	0	0	0	4	2	RLCIARAFVKA	tr_C9ZJU1	0.145	10445.98	16.00
839	HLA-A0201	ALNFVQKRSSGI	ALNFVSSGI	0	0	0	5	3	ALNFVQKRSSGI	tr_C9ZJU1	0.145	10458.53	16.00
322	HLA-A0201	ALWQASGSVMDE	ALWQASGSV	0	0	0	0	0	ALWQASGSV	tr_C9ZJU1	0.144	10474.39	16.00
196	HLA-A0201	QLVTLAATVLFSPE	QLVTLAATV	0	0	0	0	0	QLVTLAATV	tr_C9ZJU1	0.144	10488.56	16.00
474	HLA-A0201	MVFVESGRPLGYM	MVFVELGYM	0	0	0	5	4	MVFVESGRPLGYM	tr_C9ZJU1	0.144	10488.90	16.00
220	HLA-A0201	AFVHWRPIYAAE	FVWRPIYAA	1	0	0	2	1	FVHWRPIYAA	tr_C9ZJU1	0.144	10498.21	16.00
219	HLA-A0201	HAFVHWRPI	HAFVHWRPI	0	0	0	0	0	HAFVHWRPI	tr_C9ZJU1	0.144	10520.39	16.00
61	HLA-A0201	RTLVFNHNIMQLP	TLVFNIMQL	1	0	0	4	2	TLVFNHNIMQL	tr_C9ZJU1	0.144	10536.90	16.00
349	HLA-A0201	RVVFSFPVVS	RVVFSFPVV	0	0	0	0	0	RVVFSFPVV	tr_C9ZJU1	0.144	10538.62	16.00
913	HLA-A0201	LRDSTIIIVAHRLA	STIIIVALA	3	0	0	7	2	STIIIVAHRLA	tr_C9ZJU1	0.144	10562.70	16.00
913	HLA-A0201	LRDSTIIIV	LRDSTIIIV	0	0	0	0	0	LRDSTIIIV	tr_C9ZJU1	0.144	10569.22	16.00
478	HLA-A0201	ESGRPLGYMWDLL	SLGYMWDLL	1	0	0	1	3	SGRPLGYMWDLL	tr_C9ZJU1	0.144	10582.38	16.00
746	HLA-A0201	EFLGIAGYSG	FLGIAGYSG	1	0	0	0	0	FLGIAGYSG	tr_C9ZJU1	0.143	10586.04	16.00
344	HLA-A0201	VMHGCRVVF	VMHGCRVVF	0	0	0	0	0	VMHGCRVVF	tr_C9ZJU1	0.143	10610.81	16.00
871	HLA-A0201	RAFVKAGSAVGLCI	FVSAVGLCI	2	0	0	2	3	FVKAGSAVGLCI	tr_C9ZJU1	0.143	10612.30	16.00
213	HLA-A0201	KCILGMHAFVHW	KILGMHAFV	0	0	0	1	1	KCILGMHAFV	tr_C9ZJU1	0.143	10615.29	16.00
332	HLA-A0201	DELQSRVPAL	ELQSRVPAL	1	0	0	0	0	ELQSRVPAL	tr_C9ZJU1	0.143	10617.24	16.00
208	HLA-A0201	PEVILKCILGMHAF	ILILGMHAF	3	0	0	2	2	ILKCILGMHAF	tr_C9ZJU1	0.143	10617.35	16.00
605	HLA-A0201	MADTLPLLLVS	MADTLLLLV	0	0	0	5	1	MADTLPLLLV	tr_C9ZJU1	0.143	10622.53	16.00
723	HLA-A0201	VSFRYSDRHPYV	VSFRYSPYV	0	0	0	6	3	VSFRYSDRHPYV	tr_C9ZJU1	0.143	10623.45	16.00
18	HLA-A0201	EILEMLTRATIKTD	ILMLTRATI	1	0	0	2	1	ILEMLTRATI	tr_C9ZJU1	0.143	10629.77	16.00
126	HLA-A0201	KAEEVLALC	KAEEVLALC	0	0	0	0	0	KAEEVLALC	tr_C9ZJU1	0.143	10636.33	16.00
439	HLA-A0201	CMLSSTDNKMLF	MLSSTDMLF	1	0	0	6	2	MLSSTDNKMLF	tr_C9ZJU1	0.143	10661.79	16.00
200	HLA-A0201	LAATVLFSPEVILK	LLFSPEVIL	0	0	0	1	4	LAATVLFSPEVIL	tr_C9ZJU1	0.143	10662.60	16.00
447	HLA-A0201	KMLFQQTFSHPSR	MLFQQTFPS	1	0	0	7	2	MLFQQTFSHPS	tr_C9ZJU1	0.143	10665.94	16.00
407	HLA-A0201	NQVSPTCSFPVK	NQVSCSFPV	0	0	0	4	2	NQVSPTCSFPV	tr_C9ZJU1	0.143	10671.25	16.00
263	HLA-A0201	SMAVASTFAFES	SMAVFAFES	0	0	0	4	3	SMAVASTFAFES	tr_C9ZJU1	0.143	10682.57	16.00
72	HLA-A0201	LPSVFAVHCGL	LVFAVHCGL	0	0	0	1	2	LPSVFAVHCGL	tr_C9ZJU1	0.143	10691.48	16.00
320	HLA-A0201	SQALWQASGSVMD	SLWQASGSV	0	0	0	1	2	SQALWQASGSV	tr_C9ZJU1	0.142	10700.51	16.00
789	HLA-A0201	RMLRRRIANVWQE	RMLRRIANV	0	0	0	3	1	RMLRRRIANV	tr_C9ZJU1	0.142	10714.18	16.00
160	HLA-A0201	PEELYLGLITAYN	YLGLITAYN	4	0	0	0	0	YLGLITAYN	tr_C9ZJU1	0.142	10715.69	16.00
378	HLA-A0201	LYMESQFTKSQLAS	YMFTKSQLA	1	0	0	2	3	YMESQFTKSQLA	tr_C9ZJU1	0.142	10717.89	16.00
195	HLA-A0201	PQLVTLAATVLF	QLVTLAATV	1	0	0	0	0	QLVTLAATV	tr_C9ZJU1	0.142	10730.77	16.00
346	HLA-A0201	HGCRVVFSFPVV	RVVFSFPVV	3	0	0	0	0	RVVFSFPVV	tr_C9ZJU1	0.142	10733.67	16.00
767	HLA-A0201	RTYAPTEGDIFVNG	RTYEGDIFV	0	0	0	3	3	RTYAPTEGDIFV	tr_C9ZJU1	0.142	10740.06	16.00
501	HLA-A0201	SVFSGCVVDALK	SVFCVVDAL	0	0	0	3	2	SVFSGCVVDAL	tr_C9ZJU1	0.142	10741.81	16.00
440	HLA-A0201	MLSSTDNKMLF	MLSSTDMLF	0	0	0	6	2	MLSSTDNKMLF	tr_C9ZJU1	0.142	10774.63	16.00
503	HLA-A0201	FSGCVVDALK	FSGCVVDAL	0	0	0	0	0	FSGCVVDAL	tr_C9ZJU1	0.142	10776.96	16.00
331	HLA-A0201	MDELQSRVPALLE	MLQSRVPAL	0	0	0	1	2	MDELQSRVPAL	tr_C9ZJU1	0.142	10780.69	16.00
818	HLA-A0201	ALGNLWDCS	ALGNLWDCS	0	0	0	0	0	ALGNLWDCS	tr_C9ZJU1	0.142	10783.84	16.00
261	HLA-A0201	FVSMAVASTFAFE	FMAVASTFA	0	0	0	1	2	FVSMAVASTFA	tr_C9ZJU1	0.142	10805.10	16.00
962	HLA-A0201	RMRQSQQLPHFVKK	RMQQLPHFV	0	0	0	2	3	RMRQSQQLPHFV	tr_C9ZJU1	0.142	10810.36	16.00
275	HLA-A0201	LYCIRGFVIQSIL	CIRGFVIIL	2	0	0	7	2	CIRGFVIQSIL	tr_C9ZJU1	0.141	10820.78	16.00
299	HLA-A0201	YLALATADYDPS	YLALATAPS	0	0	0	7	3	YLALATADYDPS	tr_C9ZJU1	0.141	10823.82	16.00

43	HLA-A0201	DASIAIFLNCNGVE	IIFLNCNGV	3	0	0	1	1	IAIFLNCNGV	tr_C9ZJU1	0.141	10838.71	16.00
382	HLA-A0201	SQFTKSQLASVTN	SQFSQLASV	0	0	0	3	2	SQFTKSQLASV	tr_C9ZJU1	0.141	10865.01	16.00
882	HLA-A0201	LCIFDEPTSAIDTI	CIFDAIDTI	1	0	0	4	4	CIFDEPTSAIDTI	tr_C9ZJU1	0.141	10865.71	16.00
66	HLA-A0201	NHNIMQLPSVFA	NIMQLSVFA	2	0	0	5	1	NIMQLPSVFA	tr_C9ZJU1	0.141	10868.06	16.00
680	HLA-A0201	SGSSLGCYQV	SSSLGCYQV	0	0	0	1	1	SGSSLGCYQV	tr_C9ZJU1	0.141	10891.72	16.00
96	HLA-A0201	IWNCLMALVN	IWNCLMALV	0	0	0	0	0	IWNCLMALV	tr_C9ZJU1	0.141	10894.43	16.00
833	HLA-A0201	ALSASRALNF	ALSASALNF	0	0	0	5	1	ALSASRALNF	tr_C9ZJU1	0.141	10895.73	16.00
946	HLA-A0201	GSWETLSRT	GSWETLSRT	0	0	0	0	0	GSWETLSRT	tr_C9ZJU1	0.141	10925.36	16.00
762	HLA-A0201	LRLLNRTYAP	LLLNRTYAP	0	0	0	1	1	LRLLNRTYAP	tr_C9ZJU1	0.141	10928.32	16.00
500	HLA-A0201	PSVFSGCVVDA	SVFGCVVDA	1	0	0	3	1	SVFSGCVVDA	tr_C9ZJU1	0.141	10931.51	16.00
189	HLA-A0201	CSSCSIPQL	CSSCSIPQL	0	0	0	0	0	CSSCSIPQL	tr_C9ZJU1	0.141	10932.46	16.00
726	HLA-A0201	RYSDRHPYV	RYSDRHPYV	0	0	0	0	0	RYSDRHPYV	tr_C9ZJU1	0.140	10938.72	16.00
573	HLA-A0201	MAATAAAAFA	MAAAAAAFA	0	0	0	3	1	MAATAAAAFA	tr_C9ZJU1	0.140	10941.57	16.00
695	HLA-A0201	SVQRKIDRPV	SVQRKIDPV	0	0	0	7	1	SVQRKIDRPV	tr_C9ZJU1	0.140	10963.14	16.00
181	HLA-A0201	ITSFWKLMCS	ITFWKLMCS	0	0	0	2	1	ITSFWKLMCS	tr_C9ZJU1	0.140	10963.72	16.00
440	HLA-A0201	MLSSTDNKMLFQQT	MLSMLFQQT	0	0	0	3	5	MLSSTDNKMLFQQT	tr_C9ZJU1	0.140	10969.78	16.00
877	HLA-A0201	GSAVGLCIFDEPT	AVGLCIFPT	2	0	0	7	2	AVGLCIFDEPT	tr_C9ZJU1	0.140	10992.11	16.00
880	HLA-A0201	VGLCIFDEPTSAID	GLCIFDEPI	1	0	0	8	3	GLCIFDEPTSAI	tr_C9ZJU1	0.140	11000.80	16.00
464	HLA-A0201	SVQYGLVLF	SVQYGLVLF	0	0	0	0	0	SVQYGLVLF	tr_C9ZJU1	0.140	11011.87	16.00
219	HLA-A0201	HAFVHWRPIYAAE	FVWRPIYAA	2	0	0	2	1	FVHWRPIYAA	tr_C9ZJU1	0.140	11017.00	16.00
71	HLA-A0201	QLPSVFAVHCGLMK	QLFAVHCGL	0	0	0	2	3	QLPSVFAVHCGL	tr_C9ZJU1	0.140	11017.96	16.00
630	HLA-A0201	ALDQTEIVNQLRS	ALDEIVNQL	0	0	0	3	2	ALDQTEIVNQL	tr_C9ZJU1	0.140	11019.50	16.00
677	HLA-A0201	LAFSGSSLGCYQVL	LAFLGCYQV	0	0	0	3	4	LAFSGSSLGCYQV	tr_C9ZJU1	0.140	11033.22	16.00
628	HLA-A0201	QSALDQTEIVNQLR	ALDEIVNQL	2	0	0	3	2	ALDQTEIVNQL	tr_C9ZJU1	0.140	11033.71	16.00
643	HLA-A0201	IQHWCPLYHAF	IQWCPLYHA	0	0	0	2	1	IQHWCPLYHA	tr_C9ZJU1	0.139	11055.57	16.00
619	HLA-A0201	LRLSPNFFTQ	LLSPNFFTQ	0	0	0	1	1	LRLSPNFFTQ	tr_C9ZJU1	0.139	11065.74	16.00
162	HLA-A0201	ELYLGLITAYNR	ELYLGLITA	0	0	0	0	0	ELYLGLITA	tr_C9ZJU1	0.139	11073.53	16.00
643	HLA-A0201	IQHWCPLYHAFLG	IQHWCPLYL	0	0	0	8	3	IQHWCPLYHAFL	tr_C9ZJU1	0.139	11100.76	16.00
803	HLA-A0201	NNLRFIEDLSIG	NLFIEDLSI	1	0	0	2	1	NLRFIEDLSI	tr_C9ZJU1	0.139	11101.60	16.00
864	HLA-A0201	VERLCIARAFVK	VLCIARAFV	0	0	0	1	2	VERLCIARAFV	tr_C9ZJU1	0.139	11123.97	16.00
380	HLA-A0201	MESQFTKSQLASVT	SQFSQLASV	2	0	0	3	2	SQFTKSQLASV	tr_C9ZJU1	0.139	11146.74	17.00
292	HLA-A0201	TSIHIDAYLA	SIHIDAYLA	1	0	0	0	0	SIHIDAYLA	tr_C9ZJU1	0.139	11156.27	17.00
642	HLA-A0201	SIQHWCPLYHAFLG	SIWCPLYHA	0	0	0	2	2	SIQHWCPLYHA	tr_C9ZJU1	0.139	11169.79	17.00
446	HLA-A0201	NKMLFQQTFSHPSR	MLFQQTFPS	2	0	0	7	2	MLFQQTFSHPS	tr_C9ZJU1	0.139	11170.15	17.00
448	HLA-A0201	MLFQQTFSHPSRH	MLFQQTFPS	0	0	0	7	2	MLFQQTFSHPS	tr_C9ZJU1	0.138	11211.93	17.00
552	HLA-A0201	WQCRQDYLQGFAAL	WQLQGFAAL	0	0	0	2	5	WQCRQDYLQGFAAL	tr_C9ZJU1	0.138	11214.36	17.00
212	HLA-A0201	LKCILGMHAFVHW	LILGMHAFV	0	0	0	1	2	LKCILGMHAFV	tr_C9ZJU1	0.138	11217.39	17.00
471	HLA-A0201	LFSMVFVESG	FSMVFVESG	1	0	0	0	0	FSMVFVESG	tr_C9ZJU1	0.138	11218.85	17.00
447	HLA-A0201	KMLFQQTFSHP	MLFQQTFSP	1	0	0	8	1	MLFQQTFSHP	tr_C9ZJU1	0.138	11221.52	17.00
941	HLA-A0201	MVAEVGSWETLS	MVAEVWETL	0	0	0	5	2	MVAEVGSWETL	tr_C9ZJU1	0.138	11230.26	17.00
378	HLA-A0201	LYMESQFTKSQL	YMESQFTQL	1	0	0	7	2	YMESQFTKSQL	tr_C9ZJU1	0.138	11232.21	17.00
60	HLA-A0201	VRTLVFNHNIMQLP	TLVFNIMQL	2	0	0	4	2	TLVFNHNIMQL	tr_C9ZJU1	0.138	11235.73	17.00
188	HLA-A0201	MCSSCSIPQLVTL	MCSSCLVTL	0	0	0	5	4	MCSSCSIPQLVTL	tr_C9ZJU1	0.138	11241.93	17.00
812	HLA-A0201	SIGGNIALGNL	SIGGNIALL	0	0	0	8	2	SIGGNIALGNL	tr_C9ZJU1	0.138	11255.32	17.00
247	HLA-A0201	FRFIIKQLM	FRFIIKQLM	0	0	0	0	0	FRFIIKQLM	tr_C9ZJU1	0.138	11257.39	17.00
825	HLA-A0201	CSDENICAA	CSDENICAA	0	0	0	0	0	CSDENICAA	tr_C9ZJU1	0.138	11258.86	17.00
134	HLA-A0201	CMLLRGKYSIARYP	MLYSIARYP	1	0	0	2	4	MLLRGKYSIARYP	tr_C9ZJU1	0.138	11263.73	17.00
740	HLA-A0201	FVVNKGEFLG	FVVNKGEFL	0	0	0	0	0	FVVNKGEFL	tr_C9ZJU1	0.138	11272.39	17.00
45	HLA-A0201	SIAIFLNCNGVES	SIAIFLNGV	0	0	0	6	2	SIAIFLNCNGV	tr_C9ZJU1	0.138	11286.18	17.00
321	HLA-A0201	QALWQASGSVMD	ALWQASGSV	1	0	0	0	0	ALWQASGSV	tr_C9ZJU1	0.138	11290.45	17.00
160	HLA-A0201	PEELYLGLITAY	ELYLGLITA	2	0	0	0	0	ELYLGLITA	tr_C9ZJU1	0.137	11299.25	17.00
82	HLA-A0201	LMKNSHPDHLLVC	LMHPDHLLV	0	0	0	2	3	LMKNSHPDHLLV	tr_C9ZJU1	0.137	11305.85	17.00
218	HLA-A0201	MHAFVHWRPIYAAE	FVWRPIYAA	3	0	0	2	1	FVHWRPIYAA	tr_C9ZJU1	0.137	11308.42	17.00
871	HLA-A0201	RAFVKAGSAVGL	FVAGSAVGL	2	0	0	2	1	FVKAGSAVGL	tr_C9ZJU1	0.137	11310.62	17.00

243	HLA-A0201	KLSPFRFIIKQ	KLSPFRFII	0	0	0	0	0	KLSPFRFII	tr_C9ZJU1	0.137	11316.26	17.00
498	HLA-A0201	LVPSVFSGCVV	LVPSVFCVV	0	0	0	6	2	LVPSVFSGCVV	tr_C9ZJU1	0.137	11321.28	17.00
905	HLA-A0201	RLREGNNLLR	RLREGNNLL	0	0	0	0	0	RLREGNNLL	tr_C9ZJU1	0.137	11329.49	17.00
162	HLA-A0201	ELYLGLITAYNRK	ELYLGLITA	0	0	0	0	0	ELYLGLITA	tr_C9ZJU1	0.137	11362.39	17.00
613	HLA-A0201	LVSAVALRLS	LVSAVALRL	0	0	0	0	0	LVSAVALRL	tr_C9ZJU1	0.137	11371.12	17.00
358	HLA-A0201	SLVMWLGTQWSN	SLVMWLWSN	0	0	0	6	3	SLVMWLGTQWSN	tr_C9ZJU1	0.137	11374.07	17.00
343	HLA-A0201	EVMHGCRVV	EVMHGCRVV	0	0	0	0	0	EVMHGCRVV	tr_C9ZJU1	0.137	11382.32	17.00
9	HLA-A0201	LLDCERKAYEILE	LLDCEAYEI	0	0	0	5	2	LLDCERKAYEI	tr_C9ZJU1	0.137	11382.94	17.00
847	HLA-A0201	SSGIHAPLNV	SSIHAPLNV	0	0	0	2	1	SSGIHAPLNV	tr_C9ZJU1	0.137	11395.26	17.00
438	HLA-A0201	KCMLSSTDNKMLF	MLSSTDMLF	2	0	0	6	2	MLSSTDNKMLF	tr_C9ZJU1	0.137	11399.58	17.00
555	HLA-A0201	RQDYLQGFAAL	RQDYLFAAL	0	0	0	5	2	RQDYLQGFAAL	tr_C9ZJU1	0.137	11414.51	17.00
241	HLA-A0201	SPKLSPFRFIIK	SLSPFRFII	0	0	0	1	2	SPKLSPFRFII	tr_C9ZJU1	0.136	11419.94	17.00
918	HLA-A0201	IIIVAHRLATLKH	IIIVALATL	0	0	0	5	2	IIIVAHRLATL	tr_C9ZJU1	0.136	11432.06	17.00
195	HLA-A0201	PQLVTLAATVLFSP	QLVTLAATV	1	0	0	0	0	QLVTLAATV	tr_C9ZJU1	0.136	11453.35	17.00
281	HLA-A0201	FVIQSILSD	FVIQSILSD	0	0	0	0	0	FVIQSILSD	tr_C9ZJU1	0.136	11491.21	17.00
326	HLA-A0201	ASGSVMDEL	ASGSVMDEL	0	0	0	0	0	ASGSVMDEL	tr_C9ZJU1	0.136	11494.20	17.00
357	HLA-A0201	VSLVMWLGTQWSNF	SLVMWLGTF	1	0	0	8	4	SLVMWLGTQWSNF	tr_C9ZJU1	0.136	11507.51	17.00
806	HLA-A0201	RFIEDLSIGGNIA	FIEDLSIGA	1	0	0	8	3	FIEDLSIGGNIA	tr_C9ZJU1	0.136	11509.50	17.00
334	HLA-A0201	LQSRVPALLEVM	LQVPALLEV	0	0	0	2	2	LQSRVPALLEV	tr_C9ZJU1	0.136	11523.58	17.00
408	HLA-A0201	QVSPTCSFPVK	QVSPTCFPV	0	0	0	6	1	QVSPTCSFPV	tr_C9ZJU1	0.136	11525.83	17.00
678	HLA-A0201	AFSGSSLGCYQV	FSGSSLYQV	1	0	0	6	2	FSGSSLGCYQV	tr_C9ZJU1	0.135	11561.43	17.00
36	HLA-A0201	KQTECKDDASIAI	KQTECSIAI	0	0	0	5	4	KQTECKDDASIAI	tr_C9ZJU1	0.135	11566.18	17.00
789	HLA-A0201	RMLRRRIANVWQEE	RMLRRRIANV	0	0	0	3	1	RMLRRRIANV	tr_C9ZJU1	0.135	11581.33	17.00
497	HLA-A0201	PLVPSVFSGCVVDA	SVFGCVVDA	4	0	0	3	1	SVFSGCVVDA	tr_C9ZJU1	0.135	11606.17	17.00
361	HLA-A0201	MWLGTQWSNFLY	WLGTWSNFL	1	0	0	4	1	WLGTQWSNFL	tr_C9ZJU1	0.135	11610.06	17.00
419	HLA-A0201	NSLTSSPLTLLR	SLTSSLTLL	1	0	0	5	1	SLTSSPLTLL	tr_C9ZJU1	0.135	11612.71	17.00
749	HLA-A0201	GIAGYSGSGKTTLL	GIAGYTTLL	0	0	0	5	5	GIAGYSGSGKTTLL	tr_C9ZJU1	0.135	11620.24	17.00
63	HLA-A0201	LVFNHNIMQLP	LVFNHIMQL	0	0	0	5	1	LVFNHNIMQL	tr_C9ZJU1	0.135	11620.87	17.00
117	HLA-A0201	VALCILDDVK	VALCILDDV	0	0	0	0	0	VALCILDDV	tr_C9ZJU1	0.135	11628.41	17.00
753	HLA-A0201	YSGSGKTTLL	YSSGKTTLL	0	0	0	2	1	YSGSGKTTLL	tr_C9ZJU1	0.135	11629.17	17.00
466	HLA-A0201	QYGLVLFSM	QYGLVLFSM	0	0	0	0	0	QYGLVLFSM	tr_C9ZJU1	0.135	11632.57	17.00
134	HLA-A0201	CMLLRGKYSIARY	MLLYSIARY	1	0	0	3	3	MLLRGKYSIARY	tr_C9ZJU1	0.135	11633.96	17.00
804	HLA-A0201	NLRFIEDLSIGG	NLFIEDLSI	0	0	0	2	1	NLRFIEDLSI	tr_C9ZJU1	0.135	11658.15	17.00
770	HLA-A0201	APTEGDIFV	APTEGDIFV	0	0	0	0	0	APTEGDIFV	tr_C9ZJU1	0.134	11671.28	17.00
14	HLA-A0201	RKAYEILEMLT	KAYEILMLT	1	0	0	6	1	KAYEILEMLT	tr_C9ZJU1	0.134	11686.44	17.00
747	HLA-A0201	FLGIAGYSGSGKT	FLGIAGYST	0	0	0	8	4	FLGIAGYSGSGKT	tr_C9ZJU1	0.134	11687.07	17.00
788	HLA-A0201	ARMLRRRIANVWQ	RMLRRRIANV	1	0	0	3	1	RMLRRRIANV	tr_C9ZJU1	0.134	11687.96	17.00
374	HLA-A0201	LNESLYMESQFT	LLYMESQFT	0	0	0	1	3	LNESLYMESQFT	tr_C9ZJU1	0.134	11697.95	17.00
870	HLA-A0201	ARAFVKAGSAVGL	FVAGSAVGL	3	0	0	2	1	FVKAGSAVGL	tr_C9ZJU1	0.134	11707.20	17.00
641	HLA-A0201	RSIQHWCPLYHAF	SIWCPLYHA	1	0	0	2	2	SIQHWCPLYHA	tr_C9ZJU1	0.134	11708.59	17.00
499	HLA-A0201	VPSVFSGCVVDA	SVFGCVVDA	2	0	0	3	1	SVFSGCVVDA	tr_C9ZJU1	0.134	11709.85	17.00
437	HLA-A0201	IKCMLSSTDNKMLF	MLSSTDMLF	3	0	0	6	2	MLSSTDNKMLF	tr_C9ZJU1	0.134	11722.02	17.00
446	HLA-A0201	NKMLFQQTFSHP	MLFQQTFSP	2	0	0	8	1	MLFQQTFSHP	tr_C9ZJU1	0.134	11729.00	17.00
161	HLA-A0201	EELYLGLITAYNR	YLGLITAYR	3	0	0	8	1	YLGLITAYNR	tr_C9ZJU1	0.134	11730.91	17.00
736	HLA-A0201	RRVSFVVNKGEFL	RVSFVVEFL	1	0	0	6	3	RVSFVVNKGEFL	tr_C9ZJU1	0.134	11736.24	17.00
130	HLA-A0201	VLALCMLLRGKY	VLALCMLLY	0	0	0	8	3	VLALCMLLRGKY	tr_C9ZJU1	0.134	11737.13	17.00
549	HLA-A0201	TCVWQCRQDYL	CVWQCQDYL	1	0	0	5	1	CVWQCRQDYL	tr_C9ZJU1	0.134	11760.64	17.00
126	HLA-A0201	KAEEVLALCML	KAVLALCML	0	0	0	2	2	KAEEVLALCML	tr_C9ZJU1	0.134	11765.36	17.00
135	HLA-A0201	MLLRGKYSIARYP	MLLRGKYSI	0	0	0	0	0	MLLRGKYSI	tr_C9ZJU1	0.134	11766.24	17.00
657	HLA-A0201	FYDECGMSEA	FYDECMSEA	0	0	0	5	1	FYDECGMSEA	tr_C9ZJU1	0.134	11780.38	17.00
242	HLA-A0201	PKLSPFRFIIK	KLSPFRFII	1	0	0	0	0	KLSPFRFII	tr_C9ZJU1	0.134	11785.23	17.00
319	HLA-A0201	VSQALWQASGSVMD	VLWQASGSV	0	0	0	1	3	VSQALWQASGSV	tr_C9ZJU1	0.133	11807.18	17.00
162	HLA-A0201	ELYLGLITAYNRKR	ELYLGLITA	0	0	0	0	0	ELYLGLITA	tr_C9ZJU1	0.133	11819.20	17.00
129	HLA-A0201	EVLALCMLLRG	VLALCMLLG	1	0	0	8	1	VLALCMLLRG	tr_C9ZJU1	0.133	11820.86	17.00

862	HLA-A0201	GEVERLCIARAFVK	RLCIARAFV	4	0	0	0	0	RLCIARAFV	tr_C9ZJU1	0.133	11826.11	17.00
267	HLA-A0201	ASTFAFESLYC	STFAFSLYC	1	0	0	5	1	STFAFESLYC	tr_C9ZJU1	0.133	11841.21	17.00
802	HLA-A0201	ENNLRFIEDLSIG	NLFIEDLSI	2	0	0	2	1	NLRFIEDLSI	tr_C9ZJU1	0.133	11842.49	17.00
220	HLA-A0201	AFVHWRPIYAAEIG	FVHWRPIYI	1	0	0	8	3	FVHWRPIYAAEI	tr_C9ZJU1	0.133	11846.08	17.00
358	HLA-A0201	SLVMWLGTQWS	SLVMWLQWS	0	0	0	6	2	SLVMWLGTQWS	tr_C9ZJU1	0.133	11849.54	17.00
865	HLA-A0201	ERLCIARAFVK	RLCIARAFV	1	0	0	0	0	RLCIARAFV	tr_C9ZJU1	0.133	11873.29	17.00
385	HLA-A0201	TKSQLASVTNIA	KLASVTNIA	1	0	0	1	2	KSQLASVTNIA	tr_C9ZJU1	0.133	11874.57	17.00
918	HLA-A0201	IIIVAHRLA	IIIVAHRLA	0	0	0	0	0	IIIVAHRLA	tr_C9ZJU1	0.133	11897.20	17.00
498	HLA-A0201	LVPSVFSGCVVDA	SVFGCVVDA	3	0	0	3	1	SVFSGCVVDA	tr_C9ZJU1	0.133	11899.26	17.00
869	HLA-A0201	IARAFVKAGSAVGL	FVAGSAVGL	4	0	0	2	1	FVKAGSAVGL	tr_C9ZJU1	0.133	11908.28	17.00
61	HLA-A0201	RTLVFNHNIM	RLVFNHNIM	0	0	0	1	1	RTLVFNHNIM	tr_C9ZJU1	0.133	11916.78	17.00
62	HLA-A0201	TLVFNHNIMQLPS	TLVFNIMQL	0	0	0	4	2	TLVFNHNIMQL	tr_C9ZJU1	0.132	11923.36	17.00
336	HLA-A0201	SRVPALLEVMHGCR	SLLEVMHGC	0	0	0	1	4	SRVPALLEVMHGC	tr_C9ZJU1	0.132	11927.88	17.00
135	HLA-A0201	MLLRGKYSIARY	MLLYSIARY	0	0	0	3	3	MLLRGKYSIARY	tr_C9ZJU1	0.132	11936.14	17.00
161	HLA-A0201	EELYLGLITAYNRK	YLGLITAYK	3	0	0	8	2	YLGLITAYNRK	tr_C9ZJU1	0.132	11938.34	17.00
164	HLA-A0201	YLGLITAYNRK	YLGLITAYK	0	0	0	8	2	YLGLITAYNRK	tr_C9ZJU1	0.132	11942.34	17.00
333	HLA-A0201	ELQSRVPALLEVM	ELVPALLEV	0	0	0	2	3	ELQSRVPALLEV	tr_C9ZJU1	0.132	11949.71	17.00
165	HLA-A0201	LGLITAYNRKRSL	GLITAYNSL	1	0	0	7	3	GLITAYNRKRSL	tr_C9ZJU1	0.132	11950.62	17.00
760	HLA-A0201	TLLRLLNRTY	TLLRLLNTY	0	0	0	7	1	TLLRLLNRTY	tr_C9ZJU1	0.132	11955.66	17.00
206	HLA-A0201	FSPEVILKCILG	FSPEVILIL	0	0	0	7	2	FSPEVILKCIL	tr_C9ZJU1	0.132	11964.46	17.00
221	HLA-A0201	FVHWRPIYAAEIGK	FVHWRPIYI	0	0	0	8	3	FVHWRPIYAAEI	tr_C9ZJU1	0.132	11964.97	17.00
916	HLA-A0201	STIIIVAHRLAT	STIIIVLAT	0	0	0	6	3	STIIIVAHRLAT	tr_C9ZJU1	0.132	11976.76	17.00
314	HLA-A0201	LLSRCVSQALWQ	LLSCVSQAL	0	0	0	3	1	LLSRCVSQAL	tr_C9ZJU1	0.132	11977.02	17.00
863	HLA-A0201	EVERLCIARAFVK	RLCIARAFV	3	0	0	0	0	RLCIARAFV	tr_C9ZJU1	0.132	11994.39	17.00
901	HLA-A0201	FDTLRLREGNNLLR	FLREGNNLL	0	0	0	1	4	FDTLRLREGNNLL	tr_C9ZJU1	0.132	12002.58	17.00
176	HLA-A0201	SLQSGITSFW	SLSGITSFW	0	0	0	2	1	SLQSGITSFW	tr_C9ZJU1	0.132	12006.08	17.00
213	HLA-A0201	KCILGMHAFVHWR	KILGMHAFV	0	0	0	1	1	KCILGMHAFV	tr_C9ZJU1	0.132	12022.99	17.00
320	HLA-A0201	SQALWQASGSVMDE	SLWQASGSV	0	0	0	1	2	SQALWQASGSV	tr_C9ZJU1	0.132	12030.92	17.00
294	HLA-A0201	IHIDAYLAL	IHIDAYLAL	0	0	0	0	0	IHIDAYLAL	tr_C9ZJU1	0.132	12034.05	17.00
445	HLA-A0201	DNKMLFQQTFSHP	MLFQQTFSP	3	0	0	8	1	MLFQQTFSHP	tr_C9ZJU1	0.132	12040.17	18.00
618	HLA-A0201	ALRLSPNFF	ALRLSPNFF	0	0	0	0	0	ALRLSPNFF	tr_C9ZJU1	0.132	12042.78	18.00
289	HLA-A0201	DIITSIHIDAYLA	IIIHIDAYL	1	0	0	2	2	IITSIHIDAYL	tr_C9ZJU1	0.132	12050.07	18.00
357	HLA-A0201	VSLVMWLGTQ	VLVMWLGTQ	0	0	0	1	1	VSLVMWLGTQ	tr_C9ZJU1	0.131	12057.38	18.00
46	HLA-A0201	IAIFLNCNGVES	IIFLNCNGV	0	0	0	1	1	IAIFLNCNGV	tr_C9ZJU1	0.131	12064.81	18.00
82	HLA-A0201	LMKNSHPDHLL	LMSHPDHLL	0	0	0	2	2	LMKNSHPDHLL	tr_C9ZJU1	0.131	12066.25	18.00
263	HLA-A0201	SMAVASTFAFESLY	SMAFAFESL	0	0	0	3	4	SMAVASTFAFESL	tr_C9ZJU1	0.131	12066.51	18.00
337	HLA-A0201	RVPALLEVMHGCR	RLLEVMHGC	0	0	0	1	3	RVPALLEVMHGC	tr_C9ZJU1	0.131	12079.44	18.00
646	HLA-A0201	WCPLYHAFLGS	WLYHAFLGS	0	0	0	1	2	WCPLYHAFLGS	tr_C9ZJU1	0.131	12080.49	18.00
293	HLA-A0201	SIHIDAYLALATAD	SIYLALATA	0	0	0	2	4	SIHIDAYLALATA	tr_C9ZJU1	0.131	12087.68	18.00
801	HLA-A0201	EENNLRFIEDLSIG	NLFIEDLSI	3	0	0	2	1	NLRFIEDLSI	tr_C9ZJU1	0.131	12112.29	18.00
941	HLA-A0201	MVAEVGSWETLSRT	MVWETLSRT	0	0	0	2	5	MVAEVGSWETLSRT	tr_C9ZJU1	0.131	12112.56	18.00
198	HLA-A0201	VTLAATVLFSP	TLAAVLFSP	1	0	0	4	1	TLAATVLFSP	tr_C9ZJU1	0.131	12132.23	18.00
357	HLA-A0201	VSLVMWLGT	VSLVMWLGT	0	0	0	0	0	VSLVMWLGT	tr_C9ZJU1	0.131	12134.20	18.00
284	HLA-A0201	QSILSDIITS	SILSDIITS	1	0	0	0	0	SILSDIITS	tr_C9ZJU1	0.131	12138.40	18.00
936	HLA-A0201	VLNEGMVAE	VLNEGMVAE	0	0	0	0	0	VLNEGMVAE	tr_C9ZJU1	0.131	12146.68	18.00
260	HLA-A0201	TFVSMAVASTF	FVMAVASTF	1	0	0	2	1	FVSMAVASTF	tr_C9ZJU1	0.131	12151.67	18.00
338	HLA-A0201	VPALLEVMHGCR	VLLEVMHGC	0	0	0	1	2	VPALLEVMHGC	tr_C9ZJU1	0.131	12161.67	18.00
918	HLA-A0201	IIIVAHRLATLKHA	IIIVALATL	0	0	0	5	2	IIIVAHRLATL	tr_C9ZJU1	0.131	12161.93	18.00
406	HLA-A0201	ENQVSPTCSFPVK	NQVSCSFPV	1	0	0	4	2	NQVSPTCSFPV	tr_C9ZJU1	0.131	12168.12	18.00
865	HLA-A0201	ERLCIARAFVKA	RLCIAFVKA	1	0	0	4	2	RLCIARAFVKA	tr_C9ZJU1	0.131	12168.12	18.00
905	HLA-A0201	RLREGNNLLRDSTI	RLLLRDSTI	0	0	0	2	5	RLREGNNLLRDSTI	tr_C9ZJU1	0.130	12198.18	18.00
159	HLA-A0201	FPEELYLGL	FPEELYLGL	0	0	0	0	0	FPEELYLGL	tr_C9ZJU1	0.130	12203.46	18.00
200	HLA-A0201	LAATVLFSP	LAATVLFSP	0	0	0	0	0	LAATVLFSP	tr_C9ZJU1	0.130	12225.65	18.00
444	HLA-A0201	TDNKMLFQQTFSHP	MLFQQTFSP	4	0	0	8	1	MLFQQTFSHP	tr_C9ZJU1	0.130	12226.84	18.00

128	HLA-A0201	EEVLALCMLLRG	VLALCMLLG	2	0	0	8	1	VLALCMLLRG	tr_C9ZJU1	0.130	12228.83	18.00
44	HLA-A0201	ASIAIFLNCNGVES	SIAIFLNCV	1	0	0	8	2	SIAIFLNCNGV	tr_C9ZJU1	0.130	12250.15	18.00
277	HLA-A0201	CIRGFVIQSIL	CIRGFVIIL	0	0	0	7	2	CIRGFVIQSIL	tr_C9ZJU1	0.130	12259.43	18.00
294	HLA-A0201	IHIDAYLALAT	IIDAYLALA	0	0	0	1	1	IHIDAYLALA	tr_C9ZJU1	0.130	12273.37	18.00
240	HLA-A0201	VSPKLSPFRFIIK	VLSPFRFII	0	0	0	1	3	VSPKLSPFRFII	tr_C9ZJU1	0.130	12292.37	18.00
140	HLA-A0201	KYSIARYPPAA	YIARYPPAA	1	0	0	1	1	YSIARYPPAA	tr_C9ZJU1	0.130	12299.56	18.00
134	HLA-A0201	CMLLRGKYSIAR	MLLRGKYSI	1	0	0	0	0	MLLRGKYSI	tr_C9ZJU1	0.130	12302.48	18.00
129	HLA-A0201	EVLALCMLLRGKY	VLALCMLLY	1	0	0	8	3	VLALCMLLRGKY	tr_C9ZJU1	0.130	12306.61	18.00
363	HLA-A0201	LGTQWSNFLYF	LQWSNFLYF	0	0	0	1	2	LGTQWSNFLYF	tr_C9ZJU1	0.130	12311.54	18.00
651	HLA-A0201	HAFLGSFYDEC	FLGSFYDEC	2	0	0	0	0	FLGSFYDEC	tr_C9ZJU1	0.130	12313.01	18.00
345	HLA-A0201	MHGCRVVFSFPVVS	MVVFSFPVV	0	0	0	1	4	MHGCRVVFSFPVV	tr_C9ZJU1	0.130	12313.94	18.00
448	HLA-A0201	MLFQQTFSHPSRHD	MLFQQTFPS	0	0	0	7	2	MLFQQTFSHPS	tr_C9ZJU1	0.129	12325.13	18.00
93	HLA-A0201	VCDIWNCLM	VCDIWNCLM	0	0	0	0	0	VCDIWNCLM	tr_C9ZJU1	0.129	12336.88	18.00
919	HLA-A0201	IIVAHRLATLK	IIVAHLATL	0	0	0	5	1	IIVAHRLATL	tr_C9ZJU1	0.129	12343.42	18.00
159	HLA-A0201	FPEELYLGLI	FPEELYLLI	0	0	0	7	1	FPEELYLGLI	tr_C9ZJU1	0.129	12344.09	18.00
90	HLA-A0201	HLLVCDIWN	HLLVCDIWN	0	0	0	0	0	HLLVCDIWN	tr_C9ZJU1	0.129	12345.16	18.00
418	HLA-A0201	KNSLTSSPLTLLR	SLTSSLTLL	2	0	0	5	1	SLTSSPLTLL	tr_C9ZJU1	0.129	12355.98	18.00
160	HLA-A0201	PEELYLGLITAYNR	YLGLITAYR	4	0	0	8	1	YLGLITAYNR	tr_C9ZJU1	0.129	12374.58	18.00
267	HLA-A0201	ASTFAFESL	ASTFAFESL	0	0	0	0	0	ASTFAFESL	tr_C9ZJU1	0.129	12386.90	18.00
61	HLA-A0201	RTLVFNHNIMQLPS	TLVFNIMQL	1	0	0	4	2	TLVFNHNIMQL	tr_C9ZJU1	0.129	12392.40	18.00
420	HLA-A0201	SLTSSPLTLLRK	SLTSSLTLL	0	0	0	5	1	SLTSSPLTLL	tr_C9ZJU1	0.129	12394.81	18.00
116	HLA-A0201	GVALCILDDVKA	ALCILDVKA	2	0	0	5	1	ALCILDDVKA	tr_C9ZJU1	0.129	12397.36	18.00
67	HLA-A0201	HNIMQLPSV	HNIMQLPSV	0	0	0	0	0	HNIMQLPSV	tr_C9ZJU1	0.129	12416.02	18.00
735	HLA-A0201	LRRVSFVVNKGEFL	RVSFVVEFL	2	0	0	6	3	RVSFVVNKGEFL	tr_C9ZJU1	0.129	12428.11	18.00
769	HLA-A0201	YAPTEGDIFVN	YAPEGDIFV	0	0	0	3	1	YAPTEGDIFV	tr_C9ZJU1	0.129	12437.12	18.00
864	HLA-A0201	VERLCIARAFVKA	VLCIARAFV	0	0	0	1	2	VERLCIARAFV	tr_C9ZJU1	0.128	12453.29	18.00
640	HLA-A0201	LRSIQHWCPLYHAF	SIWCPLYHA	2	0	0	2	2	SIQHWCPLYHA	tr_C9ZJU1	0.128	12458.14	18.00
13	HLA-A0201	ERKAYEILEMLT	KAYEILMLT	2	0	0	6	1	KAYEILEMLT	tr_C9ZJU1	0.128	12465.42	18.00
413	HLA-A0201	CSFPVKNSL	CSFPVKNSL	0	0	0	0	0	CSFPVKNSL	tr_C9ZJU1	0.128	12465.95	18.00
117	HLA-A0201	VALCILDDVKA	ALCILDVKA	1	0	0	5	1	ALCILDDVKA	tr_C9ZJU1	0.128	12469.33	18.00
574	HLA-A0201	AATAAAAFA	AATAAAAFA	0	0	0	0	0	AATAAAAFA	tr_C9ZJU1	0.128	12471.22	18.00
155	HLA-A0201	VVQEFPEELYLGL	VVQEFYLGL	0	0	0	5	4	VVQEFPEELYLGL	tr_C9ZJU1	0.128	12478.91	18.00
866	HLA-A0201	RLCIARAFVKAGSA	RLCIARAFA	0	0	0	8	5	RLCIARAFVKAGSA	tr_C9ZJU1	0.128	12486.62	18.00
661	HLA-A0201	CGMSEAQVDEA	GMSEAQVEA	1	0	0	7	1	GMSEAQVDEA	tr_C9ZJU1	0.128	12488.64	18.00
197	HLA-A0201	LVTLAATVLFSP	LLAATVLFS	0	0	0	1	2	LVTLAATVLFS	tr_C9ZJU1	0.128	12489.45	18.00
171	HLA-A0201	YNRKRSLQSGITSF	YLQSGITSF	0	0	0	1	5	YNRKRSLQSGITSF	tr_C9ZJU1	0.128	12495.94	18.00
205	HLA-A0201	LFSPEVILKC	FSPEVILKC	1	0	0	0	0	FSPEVILKC	tr_C9ZJU1	0.128	12505.81	18.00
548	HLA-A0201	STCVWQCRQDYL	CVWQCQDYL	2	0	0	5	1	CVWQCRQDYL	tr_C9ZJU1	0.128	12507.71	18.00
463	HLA-A0201	SSVQYGLVLFS	SQYGLVLFS	0	0	0	1	2	SSVQYGLVLFS	tr_C9ZJU1	0.128	12524.36	18.00
734	HLA-A0201	VLRRVSFVVNK	VLRRVSFVV	0	0	0	0	0	VLRRVSFVV	tr_C9ZJU1	0.128	12542.94	18.00
128	HLA-A0201	EEVLALCMLLRGKY	VLALCMLLY	2	0	0	8	3	VLALCMLLRGKY	tr_C9ZJU1	0.128	12550.27	18.00
212	HLA-A0201	LKCILGMHAFVHWR	LILGMHAFV	0	0	0	1	2	LKCILGMHAFV	tr_C9ZJU1	0.128	12557.88	18.00
297	HLA-A0201	DAYLALATA	DAYLALATA	0	0	0	0	0	DAYLALATA	tr_C9ZJU1	0.128	12558.29	18.00
866	HLA-A0201	RLCIARAFVKAG	RLCIARAFV	0	0	0	0	0	RLCIARAFV	tr_C9ZJU1	0.128	12561.41	18.00
164	HLA-A0201	YLGLITAYNRKR	YLGLITAYR	0	0	0	8	3	YLGLITAYNRKR	tr_C9ZJU1	0.128	12566.98	18.00
362	HLA-A0201	WLGTQWSNF	WLGTQWSNF	0	0	0	0	0	WLGTQWSNF	tr_C9ZJU1	0.128	12568.21	18.00
650	HLA-A0201	YHAFLGSFYDEC	FLGSFYDEC	3	0	0	0	0	FLGSFYDEC	tr_C9ZJU1	0.128	12574.20	18.00
266	HLA-A0201	VASTFAFESLYC	STFAFSLYC	2	0	0	5	1	STFAFESLYC	tr_C9ZJU1	0.128	12582.63	18.00
265	HLA-A0201	AVASTFAFESLYC	STFAFSLYC	3	0	0	5	1	STFAFESLYC	tr_C9ZJU1	0.127	12586.03	18.00
202	HLA-A0201	ATVLFSPEVILK	ALFSPEVIL	0	0	0	1	2	ATVLFSPEVIL	tr_C9ZJU1	0.127	12592.30	18.00
528	HLA-A0201	RLGWIKDVIQ	RLWIKDVIQ	0	0	0	2	1	RLGWIKDVIQ	tr_C9ZJU1	0.127	12594.62	18.00
926	HLA-A0201	ATLKHADKIVVLN	TLADKIVVL	1	0	0	2	2	TLKHADKIVVL	tr_C9ZJU1	0.127	12600.89	18.00
640	HLA-A0201	LRSIQHWCPL	LSIQHWCPL	0	0	0	1	1	LRSIQHWCPL	tr_C9ZJU1	0.127	12603.07	18.00
848	HLA-A0201	SGIHAPLNV	SGIHAPLNV	0	0	0	0	0	SGIHAPLNV	tr_C9ZJU1	0.127	12605.11	18.00

462	HLA-A0201	KSSVQYGLVLFS	KQYGLVLFS	0	0	0	1	3	KSSVQYGLVLFS	tr_C9ZJU1	0.127	12614.66	18.00
838	HLA-A0201	RALNFVQKRSSGI	ALNFVSSGI	1	0	0	5	3	ALNFVQKRSSGI	tr_C9ZJU1	0.127	12616.30	18.00
133	HLA-A0201	LCMLLRGKYSIARY	MLLYSIARY	2	0	0	3	3	MLLRGKYSIARY	tr_C9ZJU1	0.127	12620.80	18.00
698	HLA-A0201	RKIDRPVRGV	KIDRPVRGV	1	0	0	0	0	KIDRPVRGV	tr_C9ZJU1	0.127	12621.63	18.00
12	HLA-A0201	CERKAYEILEMLT	KAYEILMLT	3	0	0	6	1	KAYEILEMLT	tr_C9ZJU1	0.127	12624.77	18.00
303	HLA-A0201	ATADYDPSDDWL	ATADYDPSL	0	0	0	8	3	ATADYDPSDDWL	tr_C9ZJU1	0.127	12626.95	18.00
321	HLA-A0201	QALWQASGSVMDE	ALWQASGSV	1	0	0	0	0	ALWQASGSV	tr_C9ZJU1	0.127	12637.21	18.00
260	HLA-A0201	TFVSMAVASTFAFE	FMAVASTFA	1	0	0	1	2	FVSMAVASTFA	tr_C9ZJU1	0.127	12649.79	18.00
214	HLA-A0201	CILGMHAFVHW	CILGMHAFV	0	0	0	0	0	CILGMHAFV	tr_C9ZJU1	0.127	12652.12	18.00
707	HLA-A0201	VAPPSLDGPWSVE	VALDGPWSV	0	0	0	2	3	VAPPSLDGPWSV	tr_C9ZJU1	0.127	12663.49	18.00
787	HLA-A0201	PARMLRRRIANVWQ	RMLRRIANV	2	0	0	3	1	RMLRRRIANV	tr_C9ZJU1	0.127	12667.19	18.00
132	HLA-A0201	ALCMLLRGKYS	ALCMLLRYS	0	0	0	7	2	ALCMLLRGKYS	tr_C9ZJU1	0.127	12680.89	18.00
916	HLA-A0201	STIIIVAHR	STIIIVAHR	0	0	0	0	0	STIIIVAHR	tr_C9ZJU1	0.127	12688.30	18.00
405	HLA-A0201	EENQVSPTCSFPVK	NQVSCSFPV	2	0	0	4	2	NQVSPTCSFPV	tr_C9ZJU1	0.127	12692.15	18.00
163	HLA-A0201	LYLGLITAYNR	YLGLITAYR	1	0	0	8	1	YLGLITAYNR	tr_C9ZJU1	0.127	12709.20	18.00
336	HLA-A0201	SRVPALLEVM	SVPALLEVM	0	0	0	1	1	SRVPALLEVM	tr_C9ZJU1	0.127	12712.22	18.00
940	HLA-A0201	GMVAEVGSWETLS	MVAEVWETL	1	0	0	5	2	MVAEVGSWETL	tr_C9ZJU1	0.126	12726.12	18.00
234	HLA-A0201	KSLNGDVSPKLS	SLNDVSPKL	1	0	0	3	1	SLNGDVSPKL	tr_C9ZJU1	0.126	12728.04	18.00
483	HLA-A0201	LGYMWDLLT	LGYMWDLLT	0	0	0	0	0	LGYMWDLLT	tr_C9ZJU1	0.126	12739.48	18.00
259	HLA-A0201	ATFVSMAVASTF	FVMAVASTF	2	0	0	2	1	FVSMAVASTF	tr_C9ZJU1	0.126	12750.65	18.00
115	HLA-A0201	DGVALCILDDVKA	ALCILDVKA	3	0	0	5	1	ALCILDDVKA	tr_C9ZJU1	0.126	12760.04	18.00
291	HLA-A0201	ITSIHIDAYLALAT	IIDAYLALA	0	0	0	1	4	ITSIHIDAYLALA	tr_C9ZJU1	0.126	12761.42	18.00
867	HLA-A0201	LCIARAFVKAGSAV	LCIARAFAV	0	0	0	7	5	LCIARAFVKAGSAV	tr_C9ZJU1	0.126	12771.91	18.00
85	HLA-A0201	NSHPDHLLV	NSHPDHLLV	0	0	0	0	0	NSHPDHLLV	tr_C9ZJU1	0.126	12779.11	18.00
871	HLA-A0201	RAFVKAGSAV	RAFVKASAV	0	0	0	6	1	RAFVKAGSAV	tr_C9ZJU1	0.126	12781.59	18.00
351	HLA-A0201	VFSFPVVSL	VFSFPVVSL	0	0	0	0	0	VFSFPVVSL	tr_C9ZJU1	0.126	12785.60	18.00
11	HLA-A0201	DCERKAYEILEMLT	KAYEILMLT	4	0	0	6	1	KAYEILEMLT	tr_C9ZJU1	0.126	12809.69	18.00
804	HLA-A0201	NLRFIEDLSIGGN	NLFIEDLSI	0	0	0	2	1	NLRFIEDLSI	tr_C9ZJU1	0.126	12816.21	18.00
264	HLA-A0201	MAVASTFAFESLYC	STFAFSLYC	4	0	0	5	1	STFAFESLYC	tr_C9ZJU1	0.126	12831.89	18.00
648	HLA-A0201	PLYHAFLGSF	PLYAFLGSF	0	0	0	3	1	PLYHAFLGSF	tr_C9ZJU1	0.126	12834.39	18.00
547	HLA-A0201	PSTCVWQCRQDYL	CVWQCQDYL	3	0	0	5	1	CVWQCRQDYL	tr_C9ZJU1	0.126	12838.84	18.00
155	HLA-A0201	VVQEFPEELY	VVQEFPEEL	0	0	0	0	0	VVQEFPEEL	tr_C9ZJU1	0.126	12849.12	18.00
312	HLA-A0201	DWLLSRCVSQ	WLLSRCVSQ	1	0	0	0	0	WLLSRCVSQ	tr_C9ZJU1	0.125	12863.58	18.00
805	HLA-A0201	LRFIEDLSIGGNIA	FIEDLSIGA	2	0	0	8	3	FIEDLSIGGNIA	tr_C9ZJU1	0.125	12869.99	18.00
239	HLA-A0201	DVSPKLSPFRFIIK	KLSPFRFII	4	0	0	0	0	KLSPFRFII	tr_C9ZJU1	0.125	12871.66	18.00
417	HLA-A0201	VKNSLTSSPLTLLR	SLTSSLTLL	3	0	0	5	1	SLTSSPLTLL	tr_C9ZJU1	0.125	12877.65	18.00
649	HLA-A0201	LYHAFLGSFYDEC	FLGSFYDEC	4	0	0	0	0	FLGSFYDEC	tr_C9ZJU1	0.125	12884.90	18.00
201	HLA-A0201	AATVLFSPEVILK	ALFSPEVIL	0	0	0	1	3	AATVLFSPEVIL	tr_C9ZJU1	0.125	12888.80	18.00
339	HLA-A0201	PALLEVMHGCR	ALLEVMHGC	1	0	0	0	0	ALLEVMHGC	tr_C9ZJU1	0.125	12903.73	18.00
672	HLA-A0201	HLRRHLAFSGSSL	HLRRHLAFL	0	0	0	8	4	HLRRHLAFSGSSL	tr_C9ZJU1	0.125	12904.29	18.00
121	HLA-A0201	ILDDVKAEE	ILDDVKAEE	0	0	0	0	0	ILDDVKAEE	tr_C9ZJU1	0.125	12906.95	18.00
344	HLA-A0201	VMHGCRVVFSFP	VMHGVVFSF	0	0	0	4	2	VMHGCRVVFSF	tr_C9ZJU1	0.125	12917.42	18.00
356	HLA-A0201	VVSLVMWLGTQWSN	SLVMWLWSN	2	0	0	6	3	SLVMWLGTQWSN	tr_C9ZJU1	0.125	12928.05	19.00
500	HLA-A0201	PSVFSGCVVDALK	SVFCVVDAL	1	0	0	3	2	SVFSGCVVDAL	tr_C9ZJU1	0.125	12937.85	19.00
753	HLA-A0201	YSGSGKTTL	YSGSGKTTL	0	0	0	0	0	YSGSGKTTL	tr_C9ZJU1	0.125	12937.85	19.00
114	HLA-A0201	DDGVALCILDDVKA	ALCILDVKA	4	0	0	5	1	ALCILDDVKA	tr_C9ZJU1	0.125	12942.60	19.00
788	HLA-A0201	ARMLRRRIANVWQE	RMLRRIANV	1	0	0	3	1	RMLRRRIANV	tr_C9ZJU1	0.125	12943.31	19.00
648	HLA-A0201	PLYHAFLGSFYDEC	FLGSFYDEC	5	0	0	0	0	FLGSFYDEC	tr_C9ZJU1	0.125	12955.92	19.00
903	HLA-A0201	TLRLREGNNL	TLLREGNNL	0	0	0	2	1	TLRLREGNNL	tr_C9ZJU1	0.125	12956.19	19.00
259	HLA-A0201	ATFVSMAVAS	ATFVSMAVA	0	0	0	0	0	ATFVSMAVA	tr_C9ZJU1	0.125	12959.42	19.00
863	HLA-A0201	EVERLCIARAFVKA	RLCIAFVKA	3	0	0	4	2	RLCIARAFVKA	tr_C9ZJU1	0.125	12969.66	19.00
135	HLA-A0201	MLLRGKYSIAR	MLLRGKYSI	0	0	0	0	0	MLLRGKYSI	tr_C9ZJU1	0.125	12971.77	19.00
495	HLA-A0201	IWPLVPSVFSG	ILVPSVFSG	0	0	0	1	2	IWPLVPSVFSG	tr_C9ZJU1	0.125	12973.45	19.00
213	HLA-A0201	KCILGMHAFVHWRP	KILGMHAFV	0	0	0	1	1	KCILGMHAFV	tr_C9ZJU1	0.124	13013.24	19.00

546	HLA-A0201	SPSTCVWQCRQDYL	CVWQCQDYL	4	0	0	5	1	CVWQCRQDYL	tr_C9ZJU1	0.124	13015.48	19.00
932	HLA-A0201	DKIVVLNEGMV	KIVVLNEMV	1	0	0	7	1	KIVVLNEGMV	tr_C9ZJU1	0.124	13028.45	19.00
288	HLA-A0201	SDIITSIHIDAYLA	IIIHIDAYL	2	0	0	2	2	IITSIHIDAYL	tr_C9ZJU1	0.124	13028.59	19.00
639	HLA-A0201	QLRSIQHWCPLY	QLIQHWCPL	0	0	0	2	2	QLRSIQHWCPL	tr_C9ZJU1	0.124	13050.17	19.00
501	HLA-A0201	SVFSGCVVDALKR	SVFCVVDAL	0	0	0	3	2	SVFSGCVVDAL	tr_C9ZJU1	0.124	13056.25	19.00
629	HLA-A0201	SALDQTEIVNQLRS	ALDEIVNQL	1	0	0	3	2	ALDQTEIVNQL	tr_C9ZJU1	0.124	13056.81	19.00
842	HLA-A0201	FVQKRSSGIHAPLN	FVSGIHAPL	0	0	0	2	4	FVQKRSSGIHAPL	tr_C9ZJU1	0.124	13061.62	19.00
740	HLA-A0201	FVVNKGEFLGIAGY	FVFLGIAGY	0	0	0	2	5	FVVNKGEFLGIAGY	tr_C9ZJU1	0.124	13067.84	19.00
357	HLA-A0201	VSLVMWLGTQWSN	SLVMWLWSN	1	0	0	6	3	SLVMWLGTQWSN	tr_C9ZJU1	0.124	13077.59	19.00
332	HLA-A0201	DELQSRVPALLEVM	ELVPALLEV	1	0	0	2	3	ELQSRVPALLEV	tr_C9ZJU1	0.124	13079.86	19.00
21	HLA-A0201	EMLTRATIKT	MLTRATIKT	1	0	0	0	0	MLTRATIKT	tr_C9ZJU1	0.124	13083.83	19.00
679	HLA-A0201	FSGSSLGCYQVL	FSLGCYQVL	0	0	0	1	3	FSGSSLGCYQVL	tr_C9ZJU1	0.124	13089.21	19.00
293	HLA-A0201	SIHIDAYLALAT	IIDAYLALA	1	0	0	1	1	IHIDAYLALA	tr_C9ZJU1	0.124	13090.19	19.00
626	HLA-A0201	FTQSALDQTEI	FTQSALTEI	0	0	0	6	2	FTQSALDQTEI	tr_C9ZJU1	0.124	13090.34	19.00
262	HLA-A0201	VSMAVASTFAF	SMVASTFAF	1	0	0	2	1	SMAVASTFAF	tr_C9ZJU1	0.124	13090.62	19.00
683	HLA-A0201	SLGCYQVLE	SLGCYQVLE	0	0	0	0	0	SLGCYQVLE	tr_C9ZJU1	0.124	13102.95	19.00
245	HLA-A0201	SPFRFIIKQL	SPFRFIIQL	0	0	0	7	1	SPFRFIIKQL	tr_C9ZJU1	0.124	13104.22	19.00
360	HLA-A0201	VMWLGTQWSNF	VMWLGTQWF	0	0	0	8	2	VMWLGTQWSNF	tr_C9ZJU1	0.124	13105.93	19.00
915	HLA-A0201	DSTIIIVAHRLAT	STIIIVLAT	1	0	0	6	3	STIIIVAHRLAT	tr_C9ZJU1	0.124	13114.01	19.00
188	HLA-A0201	MCSSCSIPQLVTLA	MIPQLVTLA	0	0	0	1	5	MCSSCSIPQLVTLA	tr_C9ZJU1	0.124	13129.49	19.00
627	HLA-A0201	TQSALDQTEI	TQSALDTEI	0	0	0	6	1	TQSALDQTEI	tr_C9ZJU1	0.124	13137.88	19.00
867	HLA-A0201	LCIARAFVKA	LCIARAFVA	0	0	0	8	1	LCIARAFVKA	tr_C9ZJU1	0.123	13145.69	19.00
258	HLA-A0201	PATFVSMAVASTF	FVMAVASTF	3	0	0	2	1	FVSMAVASTF	tr_C9ZJU1	0.123	13146.27	19.00
235	HLA-A0201	SLNGDVSPKLSP	SLNDVSPKL	0	0	0	3	1	SLNGDVSPKL	tr_C9ZJU1	0.123	13156.94	19.00
73	HLA-A0201	PSVFAVHCGLM	SVFAVCGLM	1	0	0	5	1	SVFAVHCGLM	tr_C9ZJU1	0.123	13185.87	19.00
155	HLA-A0201	VVQEFPEELYLG	VVQEFPLYL	0	0	0	6	2	VVQEFPEELYL	tr_C9ZJU1	0.123	13209.85	19.00
132	HLA-A0201	ALCMLLRGKY	ALCMLLRGY	0	0	0	8	1	ALCMLLRGKY	tr_C9ZJU1	0.123	13220.72	19.00
884	HLA-A0201	IFDEPTSAI	IFDEPTSAI	0	0	0	0	0	IFDEPTSAI	tr_C9ZJU1	0.123	13227.88	19.00
98	HLA-A0201	NCLMALVNC	NCLMALVNC	0	0	0	0	0	NCLMALVNC	tr_C9ZJU1	0.123	13236.89	19.00
381	HLA-A0201	ESQFTKSQLASVTN	SQFSQLASV	1	0	0	3	2	SQFTKSQLASV	tr_C9ZJU1	0.123	13240.34	19.00
57	HLA-A0201	SIHVRTLVFNHNI	SILVFNHNI	0	0	0	2	4	SIHVRTLVFNHNI	tr_C9ZJU1	0.123	13248.36	19.00
740	HLA-A0201	FVVNKGEFLGIAG	FVVEFLGIA	0	0	0	3	3	FVVNKGEFLGIA	tr_C9ZJU1	0.123	13252.65	19.00
197	HLA-A0201	LVTLAATVLF	LVLAATVLF	0	0	0	2	1	LVTLAATVLF	tr_C9ZJU1	0.123	13253.24	19.00
47	HLA-A0201	AIFLNCNGVES	AIFLNCNGV	0	0	0	0	0	AIFLNCNGV	tr_C9ZJU1	0.123	13256.54	19.00
803	HLA-A0201	NNLRFIEDLSIGG	NLFIEDLSI	1	0	0	2	1	NLRFIEDLSI	tr_C9ZJU1	0.123	13266.43	19.00
128	HLA-A0201	EEVLALCMLLR	VLALCMLLR	2	0	0	0	0	VLALCMLLR	tr_C9ZJU1	0.122	13297.47	19.00
103	HLA-A0201	LVNCSIRDI	LVNCSIRDI	0	0	0	0	0	LVNCSIRDI	tr_C9ZJU1	0.122	13311.29	19.00
447	HLA-A0201	KMLFQQTFSHPSRH	MLFQQTFPS	1	0	0	7	2	MLFQQTFSHPS	tr_C9ZJU1	0.122	13319.21	19.00
215	HLA-A0201	ILGMHAFVH	ILGMHAFVH	0	0	0	0	0	ILGMHAFVH	tr_C9ZJU1	0.122	13331.04	19.00
257	HLA-A0201	LPATFVSMAVASTF	FVMAVASTF	4	0	0	2	1	FVSMAVASTF	tr_C9ZJU1	0.122	13346.77	19.00
817	HLA-A0201	IALGNLWDCS	ILGNLWDCS	0	0	0	1	1	IALGNLWDCS	tr_C9ZJU1	0.122	13387.12	19.00
276	HLA-A0201	YCIRGFVIQSILS	YCIFVIQSI	0	0	0	3	2	YCIRGFVIQSI	tr_C9ZJU1	0.122	13389.58	19.00
866	HLA-A0201	RLCIARAFVKAGS	RLCIARAFV	0	0	0	0	0	RLCIARAFV	tr_C9ZJU1	0.122	13411.77	19.00
696	HLA-A0201	VQRKIDRPV	VQRKIDRPV	0	0	0	0	0	VQRKIDRPV	tr_C9ZJU1	0.122	13424.69	19.00
738	HLA-A0201	VSFVVNKGEFL	VSFVVGEFL	0	0	0	5	2	VSFVVNKGEFL	tr_C9ZJU1	0.121	13436.03	19.00
775	HLA-A0201	DIFVNGVHI	DIFVNGVHI	0	0	0	0	0	DIFVNGVHI	tr_C9ZJU1	0.121	13442.27	19.00
645	HLA-A0201	HWCPLYHAFL	WCPLYHAFL	1	0	0	0	0	WCPLYHAFL	tr_C9ZJU1	0.121	13443.29	19.00
845	HLA-A0201	KRSSGIHAPL	KSSGIHAPL	0	0	0	1	1	KRSSGIHAPL	tr_C9ZJU1	0.121	13445.04	19.00
262	HLA-A0201	VSMAVASTFAFES	SMAVFAFES	1	0	0	4	3	SMAVASTFAFES	tr_C9ZJU1	0.121	13468.77	19.00
520	HLA-A0201	HTCAVSVRRL	HTCAVSVRL	0	0	0	7	1	HTCAVSVRRL	tr_C9ZJU1	0.121	13471.25	19.00
790	HLA-A0201	MLRRRIANVWQE	MLRRRIANV	0	0	0	0	0	MLRRRIANV	tr_C9ZJU1	0.121	13473.58	19.00
125	HLA-A0201	VKAEEVLAL	VKAEEVLAL	0	0	0	0	0	VKAEEVLAL	tr_C9ZJU1	0.121	13478.39	19.00
400	HLA-A0201	YAGNGEENQV	YAGNGEENV	0	0	0	8	1	YAGNGEENQV	tr_C9ZJU1	0.121	13494.59	19.00
133	HLA-A0201	LCMLLRGKYSIAR	MLLRGKYSI	2	0	0	0	0	MLLRGKYSI	tr_C9ZJU1	0.121	13500.14	19.00

125	HLA-A0201	VKAEEVLALCML	KAVLALCML	1	0	0	2	2	KAEEVLALCML	tr_C9ZJU1	0.121	13501.31	19.00
353	HLA-A0201	SFPVVSLVM	SFPVVSLVM	0	0	0	0	0	SFPVVSLVM	tr_C9ZJU1	0.121	13522.80	19.00
894	HLA-A0201	TITEGEIFDTL	TITEGEIFL	0	0	0	8	2	TITEGEIFDTL	tr_C9ZJU1	0.121	13523.83	19.00
150	HLA-A0201	AQADVVVQEF	AQADVVVEF	0	0	0	7	1	AQADVVVQEF	tr_C9ZJU1	0.121	13547.26	19.00
554	HLA-A0201	CRQDYLQGFAAL	RQDYLFAAL	1	0	0	5	2	RQDYLQGFAAL	tr_C9ZJU1	0.121	13566.18	19.00
767	HLA-A0201	RTYAPTEGDI	RTYAPTEGI	0	0	0	8	1	RTYAPTEGDI	tr_C9ZJU1	0.121	13570.58	19.00
388	HLA-A0201	QLASVTNIATS	QLASVIATS	0	0	0	5	2	QLASVTNIATS	tr_C9ZJU1	0.121	13570.88	19.00
499	HLA-A0201	VPSVFSGCVVDALK	SVFCVVDAL	2	0	0	3	2	SVFSGCVVDAL	tr_C9ZJU1	0.121	13573.08	19.00
927	HLA-A0201	TLKHADKIVVLNEG	TLADKIVVL	0	0	0	2	2	TLKHADKIVVL	tr_C9ZJU1	0.120	13580.57	19.00
927	HLA-A0201	TLKHADKIVVLNE	TLADKIVVL	0	0	0	2	2	TLKHADKIVVL	tr_C9ZJU1	0.120	13585.12	19.00
914	HLA-A0201	RDSTIIIVAHRLAT	STIIIVLAT	2	0	0	6	3	STIIIVAHRLAT	tr_C9ZJU1	0.120	13587.63	19.00
298	HLA-A0201	AYLALATADYDPS	YLALATAPS	1	0	0	7	3	YLALATADYDPS	tr_C9ZJU1	0.120	13615.73	19.00
660	HLA-A0201	ECGMSEAQVDEA	GMSEAQVEA	2	0	0	7	1	GMSEAQVDEA	tr_C9ZJU1	0.120	13616.47	19.00
357	HLA-A0201	VSLVMWLGTQWS	SLVMWLQWS	1	0	0	6	2	SLVMWLGTQWS	tr_C9ZJU1	0.120	13619.12	19.00
734	HLA-A0201	VLRRVSFVVNKG	VLRRVSFVV	0	0	0	0	0	VLRRVSFVV	tr_C9ZJU1	0.120	13622.65	19.00
707	HLA-A0201	VAPPSLDGPWSVEF	VALDGPWSV	0	0	0	2	3	VAPPSLDGPWSV	tr_C9ZJU1	0.120	13626.05	19.00
924	HLA-A0201	RLATLKHADKI	RLATLKHAI	0	0	0	8	2	RLATLKHADKI	tr_C9ZJU1	0.120	13626.34	19.00
131	HLA-A0201	LALCMLLRG	LALCMLLRG	0	0	0	0	0	LALCMLLRG	tr_C9ZJU1	0.120	13630.48	19.00
132	HLA-A0201	ALCMLLRGK	ALCMLLRGK	0	0	0	0	0	ALCMLLRGK	tr_C9ZJU1	0.120	13631.80	19.00
139	HLA-A0201	GKYSIARYPPAA	YIARYPPAA	2	0	0	1	1	YSIARYPPAA	tr_C9ZJU1	0.120	13639.47	19.00
648	HLA-A0201	PLYHAFLGS	PLYHAFLGS	0	0	0	0	0	PLYHAFLGS	tr_C9ZJU1	0.120	13640.06	19.00
777	HLA-A0201	FVNGVHISQYPAR	FVNGVQYPA	0	0	0	5	3	FVNGVHISQYPA	tr_C9ZJU1	0.120	13645.96	19.00
657	HLA-A0201	FYDECGMSEAQV	FYDECGMSV	0	0	0	8	3	FYDECGMSEAQV	tr_C9ZJU1	0.120	13648.47	19.00
461	HLA-A0201	DKSSVQYGLVLFS	VQYGLVLFS	4	0	0	0	0	VQYGLVLFS	tr_C9ZJU1	0.120	13649.07	19.00
356	HLA-A0201	VVSLVMWLGTQWS	SLVMWLQWS	2	0	0	6	2	SLVMWLGTQWS	tr_C9ZJU1	0.120	13656.90	19.00
163	HLA-A0201	LYLGLITAYNRK	YLGLITAYK	1	0	0	8	2	YLGLITAYNRK	tr_C9ZJU1	0.120	13658.08	19.00
235	HLA-A0201	SLNGDVSPKLSPF	SLNDVSPKL	0	0	0	3	1	SLNGDVSPKL	tr_C9ZJU1	0.120	13658.67	19.00
837	HLA-A0201	SRALNFVQKRSSGI	ALNFVSSGI	2	0	0	5	3	ALNFVQKRSSGI	tr_C9ZJU1	0.120	13671.54	19.00
130	HLA-A0201	VLALCMLLRGK	VLALCMLLK	0	0	0	8	2	VLALCMLLRGK	tr_C9ZJU1	0.120	13684.86	19.00
375	HLA-A0201	NESLYMESQFT	SLYMESQFT	2	0	0	0	0	SLYMESQFT	tr_C9ZJU1	0.120	13686.63	19.00
407	HLA-A0201	NQVSPTCSFPVKN	NQVSCSFPV	0	0	0	4	2	NQVSPTCSFPV	tr_C9ZJU1	0.120	13719.84	19.00
672	HLA-A0201	HLRRHLAFS	HLRRHLAFS	0	0	0	0	0	HLRRHLAFS	tr_C9ZJU1	0.120	13720.29	19.00
618	HLA-A0201	ALRLSPNFFTQ	ALLSPNFFT	0	0	0	2	1	ALRLSPNFFT	tr_C9ZJU1	0.119	13747.93	19.00
925	HLA-A0201	LATLKHADKIVVLN	TLADKIVVL	2	0	0	2	2	TLKHADKIVVL	tr_C9ZJU1	0.119	13749.57	19.00
628	HLA-A0201	QSALDQTEIV	SALDQTEIV	1	0	0	0	0	SALDQTEIV	tr_C9ZJU1	0.119	13792.03	19.00
233	HLA-A0201	GKSLNGDVSPKLS	SLNDVSPKL	2	0	0	3	1	SLNGDVSPKL	tr_C9ZJU1	0.119	13798.74	19.00
645	HLA-A0201	HWCPLYHAFLGS	WLYHAFLGS	1	0	0	1	2	WCPLYHAFLGS	tr_C9ZJU1	0.119	13812.49	20.00
354	HLA-A0201	FPVVSLVMW	FPVVSLVMW	0	0	0	0	0	FPVVSLVMW	tr_C9ZJU1	0.119	13828.03	20.00
598	HLA-A0201	VLHGLFQMADT	VLHGLFQMA	0	0	0	0	0	VLHGLFQMA	tr_C9ZJU1	0.119	13828.49	20.00
722	HLA-A0201	EVSFRYSDRHPYV	VSFRYSPYV	1	0	0	6	3	VSFRYSDRHPYV	tr_C9ZJU1	0.119	13844.20	20.00
347	HLA-A0201	GCRVVFSFPVVS	GCVVFSFPV	0	0	0	2	1	GCRVVFSFPV	tr_C9ZJU1	0.119	13844.95	20.00
103	HLA-A0201	LVNCSIRDIL	LVNCSIDIL	0	0	0	6	1	LVNCSIRDIL	tr_C9ZJU1	0.119	13846.16	20.00
595	HLA-A0201	GRDVLHGLFQM	GVLHGLFQM	0	0	0	1	2	GRDVLHGLFQM	tr_C9ZJU1	0.119	13857.54	20.00
72	HLA-A0201	LPSVFAVHCGLM	SVFAVCGLM	2	0	0	5	1	SVFAVHCGLM	tr_C9ZJU1	0.119	13863.39	20.00
460	HLA-A0201	HDKSSVQYGLVLFS	VQYGLVLFS	5	0	0	0	0	VQYGLVLFS	tr_C9ZJU1	0.119	13864.30	20.00
178	HLA-A0201	QSGITSFWKL	QSITSFWKL	0	0	0	2	1	QSGITSFWKL	tr_C9ZJU1	0.119	13865.04	20.00
939	HLA-A0201	EGMVAEVGSWETLS	MVAEVWETL	2	0	0	5	2	MVAEVGSWETL	tr_C9ZJU1	0.119	13867.74	20.00
203	HLA-A0201	TVLFSPEVILK	VLFSPEVIL	1	0	0	0	0	VLFSPEVIL	tr_C9ZJU1	0.118	13878.25	20.00
205	HLA-A0201	LFSPEVILKCILG	FSPEVILIL	1	0	0	7	2	FSPEVILKCIL	tr_C9ZJU1	0.118	13893.57	20.00
129	HLA-A0201	EVLALCMLLRGK	VLALCMLLK	1	0	0	8	2	VLALCMLLRGK	tr_C9ZJU1	0.118	13899.89	20.00
758	HLA-A0201	KTTLLRLLNRT	KLLRLLNRT	0	0	0	1	2	KTTLLRLLNRT	tr_C9ZJU1	0.118	13908.02	20.00
124	HLA-A0201	DVKAEEVLALCML	KAVLALCML	2	0	0	2	2	KAEEVLALCML	tr_C9ZJU1	0.118	13908.91	20.00
420	HLA-A0201	SLTSSPLTLLRKQ	SLTSSLTLL	0	0	0	5	1	SLTSSPLTLL	tr_C9ZJU1	0.118	13919.30	20.00
811	HLA-A0201	LSIGGNIALGNL	SIGGNIALL	1	0	0	8	2	SIGGNIALGNL	tr_C9ZJU1	0.118	13931.66	20.00

343	HLA-A0201	EVMHGCRVVFS	VMHGCVVFS	1	0	0	5	1	VMHGCRVVFS	tr_C9ZJU1	0.118	13937.54	20.00
790	HLA-A0201	MLRRRIANVWQ	MLRRRIANV	0	0	0	0	0	MLRRRIANV	tr_C9ZJU1	0.118	13959.27	20.00
472	HLA-A0201	FSMVFVESGR	FSMVFVESR	0	0	0	8	1	FSMVFVESGR	tr_C9ZJU1	0.118	13984.52	20.00
179	HLA-A0201	SGITSFWKLM	SGITSFWLM	0	0	0	7	1	SGITSFWKLM	tr_C9ZJU1	0.118	13989.66	20.00
746	HLA-A0201	EFLGIAGYSGSGKT	FLGIAGYST	1	0	0	8	4	FLGIAGYSGSGKT	tr_C9ZJU1	0.118	13997.08	20.00
931	HLA-A0201	ADKIVVLNEGMV	KIVVLNEMV	2	0	0	7	1	KIVVLNEGMV	tr_C9ZJU1	0.118	14007.08	20.00
606	HLA-A0201	ADTLPLLLV	ADTLPLLLV	0	0	0	0	0	ADTLPLLLV	tr_C9ZJU1	0.118	14010.87	20.00
241	HLA-A0201	SPKLSPFRFIIKQ	SLSPFRFII	0	0	0	1	2	SPKLSPFRFII	tr_C9ZJU1	0.118	14014.51	20.00
659	HLA-A0201	DECGMSEAQVDEA	GMSEAQVEA	3	0	0	7	1	GMSEAQVDEA	tr_C9ZJU1	0.118	14017.39	20.00
355	HLA-A0201	PVVSLVMWLGTQWS	SLVMWLQWS	3	0	0	6	2	SLVMWLGTQWS	tr_C9ZJU1	0.118	14017.54	20.00
272	HLA-A0201	FESLYCIRGF	FSLYCIRGF	0	0	0	1	1	FESLYCIRGF	tr_C9ZJU1	0.117	14025.58	20.00
303	HLA-A0201	ATADYDPSDDWLLS	ATADYDWLL	0	0	0	5	4	ATADYDPSDDWLL	tr_C9ZJU1	0.117	14027.10	20.00
192	HLA-A0201	CSIPQLVTLAA	CSILVTLAA	0	0	0	3	2	CSIPQLVTLAA	tr_C9ZJU1	0.117	14031.35	20.00
180	HLA-A0201	GITSFWKLMC	GITSFWLMC	0	0	0	6	1	GITSFWKLMC	tr_C9ZJU1	0.117	14033.17	20.00
802	HLA-A0201	ENNLRFIEDLSIGG	NLFIEDLSI	2	0	0	2	1	NLRFIEDLSI	tr_C9ZJU1	0.117	14046.84	20.00
480	HLA-A0201	GRPLGYMWDLLTQH	YMWDLLTQH	5	0	0	0	0	YMWDLLTQH	tr_C9ZJU1	0.117	14053.53	20.00
94	HLA-A0201	CDIWNCLMA	CDIWNCLMA	0	0	0	0	0	CDIWNCLMA	tr_C9ZJU1	0.117	14070.88	20.00
138	HLA-A0201	RGKYSIARYPPAA	YIARYPPAA	3	0	0	1	1	YSIARYPPAA	tr_C9ZJU1	0.117	14085.20	20.00
941	HLA-A0201	MVAEVGSWETLSR	MVAEVWETL	0	0	0	5	2	MVAEVGSWETL	tr_C9ZJU1	0.117	14095.41	20.00
128	HLA-A0201	EEVLALCMLLRGK	VLALCMLLK	2	0	0	8	2	VLALCMLLRGK	tr_C9ZJU1	0.117	14104.26	20.00
481	HLA-A0201	RPLGYMWDLLTQH	YMWDLLTQH	4	0	0	0	0	YMWDLLTQH	tr_C9ZJU1	0.117	14105.79	20.00
501	HLA-A0201	SVFSGCVVDALKRK	SVFCVVDAL	0	0	0	3	2	SVFSGCVVDAL	tr_C9ZJU1	0.117	14107.46	20.00
275	HLA-A0201	LYCIRGFVIQSILS	YCIFVIQSI	1	0	0	3	2	YCIRGFVIQSI	tr_C9ZJU1	0.117	14107.46	20.00
497	HLA-A0201	PLVPSVFSGCVVD	PLVPSVFSV	0	0	0	8	3	PLVPSVFSGCVV	tr_C9ZJU1	0.117	14124.26	20.00
387	HLA-A0201	SQLASVTNIAT	QLASVTIAT	1	0	0	6	1	QLASVTNIAT	tr_C9ZJU1	0.117	14147.67	20.00
232	HLA-A0201	IGKSLNGDVSPKLS	SLNDVSPKL	3	0	0	3	1	SLNGDVSPKL	tr_C9ZJU1	0.117	14149.96	20.00
123	HLA-A0201	DDVKAEEVLALCML	KAVLALCML	3	0	0	2	2	KAEEVLALCML	tr_C9ZJU1	0.117	14163.60	20.00
864	HLA-A0201	VERLCIARAFVKAG	VLCIARAFV	0	0	0	1	2	VERLCIARAFV	tr_C9ZJU1	0.117	14174.94	20.00
207	HLA-A0201	SPEVILKCILGMH	SILKCILGM	0	0	0	1	3	SPEVILKCILGM	tr_C9ZJU1	0.116	14183.07	20.00
419	HLA-A0201	NSLTSSPLTLLRK	SLTSSLTLL	1	0	0	5	1	SLTSSPLTLL	tr_C9ZJU1	0.116	14218.56	20.00
297	HLA-A0201	DAYLALATADYDPS	YLALATAPS	2	0	0	7	3	YLALATADYDPS	tr_C9ZJU1	0.116	14222.72	20.00
681	HLA-A0201	GSSLGCYQVL	SSLGCYQVL	1	0	0	0	0	SSLGCYQVL	tr_C9ZJU1	0.116	14240.28	20.00
131	HLA-A0201	LALCMLLRGKYS	ALCMLLRYS	1	0	0	7	2	ALCMLLRGKYS	tr_C9ZJU1	0.116	14240.73	20.00
116	HLA-A0201	GVALCILDDVK	GVALCILDV	0	0	0	7	1	GVALCILDDV	tr_C9ZJU1	0.116	14244.74	20.00
930	HLA-A0201	HADKIVVLNEGMV	KIVVLNEMV	3	0	0	7	1	KIVVLNEGMV	tr_C9ZJU1	0.116	14253.99	20.00
734	HLA-A0201	VLRRVSFVVNKGE	VLRRVSFVV	0	0	0	0	0	VLRRVSFVV	tr_C9ZJU1	0.116	14255.53	20.00
658	HLA-A0201	YDECGMSEAQVDEA	GMSEAQVEA	4	0	0	7	1	GMSEAQVDEA	tr_C9ZJU1	0.116	14256.93	20.00
292	HLA-A0201	TSIHIDAYLALAT	IIDAYLALA	2	0	0	1	1	IHIDAYLALA	tr_C9ZJU1	0.116	14269.73	20.00
214	HLA-A0201	CILGMHAFVHWR	CILGMHAFV	0	0	0	0	0	CILGMHAFV	tr_C9ZJU1	0.116	14271.74	20.00
163	HLA-A0201	LYLGLITAYNRKR	YLGLITAYR	1	0	0	8	3	YLGLITAYNRKR	tr_C9ZJU1	0.116	14274.52	20.00
283	HLA-A0201	IQSILSDIIT	IQSILSIIT	0	0	0	6	1	IQSILSDIIT	tr_C9ZJU1	0.116	14286.73	20.00
355	HLA-A0201	PVVSLVMWLG	VVSLVMWLG	1	0	0	0	0	VVSLVMWLG	tr_C9ZJU1	0.116	14304.20	20.00
439	HLA-A0201	CMLSSTDNKMLFQ	MLSSTMLFQ	1	0	0	5	3	MLSSTDNKMLFQ	tr_C9ZJU1	0.116	14310.86	20.00
276	HLA-A0201	YCIRGFVIQSILSD	YCIFVIQSI	0	0	0	3	2	YCIRGFVIQSI	tr_C9ZJU1	0.116	14322.63	20.00
193	HLA-A0201	SIPQLVTLAAT	SILVTLAAT	0	0	0	2	2	SIPQLVTLAAT	tr_C9ZJU1	0.115	14332.71	20.00
209	HLA-A0201	EVILKCILGMH	VILKCILGM	1	0	0	0	0	VILKCILGM	tr_C9ZJU1	0.115	14334.88	20.00
242	HLA-A0201	PKLSPFRFIIKQ	KLSPFRFII	1	0	0	0	0	KLSPFRFII	tr_C9ZJU1	0.115	14337.51	20.00
291	HLA-A0201	ITSIHIDAYLA	ITSIHIYLA	0	0	0	6	2	ITSIHIDAYLA	tr_C9ZJU1	0.115	14339.69	20.00
137	HLA-A0201	LRGKYSIARYPPAA	YIARYPPAA	4	0	0	1	1	YSIARYPPAA	tr_C9ZJU1	0.115	14351.18	20.00
182	HLA-A0201	TSFWKLMCS	TSFWKLMCS	0	0	0	0	0	TSFWKLMCS	tr_C9ZJU1	0.115	14385.84	20.00
18	HLA-A0201	EILEMLTRAT	ILEMLTRAT	1	0	0	0	0	ILEMLTRAT	tr_C9ZJU1	0.115	14393.16	20.00
606	HLA-A0201	ADTLPLLLVS	ATLPLLLVS	0	0	0	1	1	ADTLPLLLVS	tr_C9ZJU1	0.115	14407.49	20.00
371	HLA-A0201	LYFLNESLYME	FLNESLYME	2	0	0	0	0	FLNESLYME	tr_C9ZJU1	0.115	14412.48	20.00
35	HLA-A0201	SKQTECKDDASIAI	KQTECSIAI	1	0	0	5	4	KQTECKDDASIAI	tr_C9ZJU1	0.115	14435.26	20.00

810	HLA-A0201	DLSIGGNIALGNL	SIGGNIALL	2	0	0	8	2	SIGGNIALGNL	tr_C9ZJU1	0.115	14439.96	20.00
486	HLA-A0201	MWDLLTQHVI	MWDLLTHVI	0	0	0	6	1	MWDLLTQHVI	tr_C9ZJU1	0.115	14440.89	20.00
865	HLA-A0201	ERLCIARAFVKAG	RLCIARAFV	1	0	0	0	0	RLCIARAFV	tr_C9ZJU1	0.115	14442.15	20.00
288	HLA-A0201	SDIITSIHI	SDIITSIHI	0	0	0	0	0	SDIITSIHI	tr_C9ZJU1	0.115	14447.30	20.00
63	HLA-A0201	LVFNHNIMQLPS	LVFNHIMQL	0	0	0	5	1	LVFNHNIMQL	tr_C9ZJU1	0.115	14450.27	20.00
366	HLA-A0201	QWSNFLYFLN	QWSNFLYFL	0	0	0	0	0	QWSNFLYFL	tr_C9ZJU1	0.115	14455.12	20.00
803	HLA-A0201	NNLRFIEDLSIGGN	NLFIEDLSI	1	0	0	2	1	NLRFIEDLSI	tr_C9ZJU1	0.115	14455.90	20.00
346	HLA-A0201	HGCRVVFSFPVVS	GCVVFSFPV	1	0	0	2	1	GCRVVFSFPV	tr_C9ZJU1	0.115	14465.60	20.00
644	HLA-A0201	QHWCPLYHAFLGS	WLYHAFLGS	2	0	0	1	2	WCPLYHAFLGS	tr_C9ZJU1	0.115	14466.86	20.00
440	HLA-A0201	MLSSTDNKMLFQ	MLSSTMLFQ	0	0	0	5	3	MLSSTDNKMLFQ	tr_C9ZJU1	0.115	14480.63	20.00
553	HLA-A0201	QCRQDYLQGFAAL	RQDYLFAAL	2	0	0	5	2	RQDYLQGFAAL	tr_C9ZJU1	0.115	14482.04	20.00
334	HLA-A0201	LQSRVPALLEVMH	LQVPALLEV	0	0	0	2	2	LQSRVPALLEV	tr_C9ZJU1	0.114	14491.60	20.00
594	HLA-A0201	CGRDVLHGLFQM	CVLHGLFQM	0	0	0	1	3	CGRDVLHGLFQM	tr_C9ZJU1	0.114	14494.12	20.00
879	HLA-A0201	AVGLCIFDEPTS	AVGLCIFPT	0	0	0	7	2	AVGLCIFDEPT	tr_C9ZJU1	0.114	14498.98	20.00
298	HLA-A0201	AYLALATADY	YLALATADY	1	0	0	0	0	YLALATADY	tr_C9ZJU1	0.114	14508.87	20.00
929	HLA-A0201	KHADKIVVLNEGMV	KIVVLNEMV	4	0	0	7	1	KIVVLNEGMV	tr_C9ZJU1	0.114	14509.02	20.00
832	HLA-A0201	AALSASRAL	AALSASRAL	0	0	0	0	0	AALSASRAL	tr_C9ZJU1	0.114	14532.43	20.00
343	HLA-A0201	EVMHGCRVVFSFP	VMHGVVFSF	1	0	0	4	2	VMHGCRVVFSF	tr_C9ZJU1	0.114	14535.58	20.00
324	HLA-A0201	WQASGSVMDEL	WQASVMDEL	0	0	0	3	2	WQASGSVMDEL	tr_C9ZJU1	0.114	14542.65	20.00
843	HLA-A0201	VQKRSSGIHAPLNV	VQIHAPLNV	0	0	0	2	5	VQKRSSGIHAPLNV	tr_C9ZJU1	0.114	14566.28	20.00
294	HLA-A0201	IHIDAYLALATAD	IIDAYLALA	0	0	0	1	1	IHIDAYLALA	tr_C9ZJU1	0.114	14574.47	20.00
43	HLA-A0201	DASIAIFLNC	ASIAIFLNC	1	0	0	0	0	ASIAIFLNC	tr_C9ZJU1	0.114	14582.52	20.00
734	HLA-A0201	VLRRVSFVVNKGEF	VLRRVSFVV	0	0	0	0	0	VLRRVSFVV	tr_C9ZJU1	0.114	14600.51	20.00
356	HLA-A0201	VVSLVMWLGTQW	SLVMWLGTW	2	0	0	8	1	SLVMWLGTQW	tr_C9ZJU1	0.114	14619.33	20.00
381	HLA-A0201	ESQFTKSQLA	SQFTKSQLA	1	0	0	0	0	SQFTKSQLA	tr_C9ZJU1	0.114	14619.64	20.00
418	HLA-A0201	KNSLTSSPLTLLRK	SLTSSLTLL	2	0	0	5	1	SLTSSPLTLL	tr_C9ZJU1	0.114	14625.50	20.00
528	HLA-A0201	RLGWIKDVIQS	RLGWIVIQS	0	0	0	5	2	RLGWIKDVIQS	tr_C9ZJU1	0.113	14649.73	20.00
873	HLA-A0201	FVKAGSAVGLCIF	FVSAVGLCI	0	0	0	2	3	FVKAGSAVGLCI	tr_C9ZJU1	0.113	14650.83	20.00
794	HLA-A0201	RIANVWQEENNL	RIANVWQEL	0	0	0	8	3	RIANVWQEENNL	tr_C9ZJU1	0.113	14667.65	20.00
359	HLA-A0201	LVMWLGTQWSNF	VMWLGTQWF	1	0	0	8	2	VMWLGTQWSNF	tr_C9ZJU1	0.113	14706.74	21.00
816	HLA-A0201	NIALGNLWDC	NILGNLWDC	0	0	0	2	1	NIALGNLWDC	tr_C9ZJU1	0.113	14708.18	21.00
64	HLA-A0201	VFNHNIMQL	VFNHNIMQL	0	0	0	0	0	VFNHNIMQL	tr_C9ZJU1	0.113	14712.63	21.00
932	HLA-A0201	DKIVVLNEGM	KIVVLNEGM	1	0	0	0	0	KIVVLNEGM	tr_C9ZJU1	0.113	14723.94	21.00
474	HLA-A0201	MVFVESGRPLGYMW	MVFVLGYMW	0	0	0	4	5	MVFVESGRPLGYMW	tr_C9ZJU1	0.113	14724.58	21.00
427	HLA-A0201	TLLRKQKDDGIKCM	TLLDGIKCM	0	0	0	3	5	TLLRKQKDDGIKCM	tr_C9ZJU1	0.113	14743.07	21.00
515	HLA-A0201	LQAGGHTCAVSVR	LQAGCAVSV	0	0	0	4	3	LQAGGHTCAVSV	tr_C9ZJU1	0.113	14747.38	21.00
420	HLA-A0201	SLTSSPLTLLRKQK	SLTSSLTLL	0	0	0	5	1	SLTSSPLTLL	tr_C9ZJU1	0.113	14748.17	21.00
183	HLA-A0201	SFWKLMCSSC	SFWLMCSSC	0	0	0	3	1	SFWKLMCSSC	tr_C9ZJU1	0.113	14759.19	21.00
57	HLA-A0201	SIHVRTLVF	SIHVRTLVF	0	0	0	0	0	SIHVRTLVF	tr_C9ZJU1	0.113	14762.38	21.00
769	HLA-A0201	YAPTEGDIFVNGVH	YADIFVNGV	0	0	0	2	4	YAPTEGDIFVNGV	tr_C9ZJU1	0.113	14770.37	21.00
240	HLA-A0201	VSPKLSPFRFIIKQ	VLSPFRFII	0	0	0	1	3	VSPKLSPFRFII	tr_C9ZJU1	0.113	14776.93	21.00
191	HLA-A0201	SCSIPQLVTLAA	SIQLVTLAA	2	0	0	2	1	SIPQLVTLAA	tr_C9ZJU1	0.113	14779.97	21.00
796	HLA-A0201	ANVWQEENNL	AVWQEENNL	0	0	0	1	1	ANVWQEENNL	tr_C9ZJU1	0.113	14784.92	21.00
671	HLA-A0201	AHLRRHLAFSGSSL	HLRRHLAFL	1	0	0	8	4	HLRRHLAFSGSSL	tr_C9ZJU1	0.113	14787.48	21.00
262	HLA-A0201	VSMAVASTF	VSMAVASTF	0	0	0	0	0	VSMAVASTF	tr_C9ZJU1	0.113	14792.29	21.00
333	HLA-A0201	ELQSRVPALLEVMH	ELVPALLEV	0	0	0	2	3	ELQSRVPALLEV	tr_C9ZJU1	0.112	14803.80	21.00
873	HLA-A0201	FVKAGSAVGLCIFD	FVSAVGLCI	0	0	0	2	3	FVKAGSAVGLCI	tr_C9ZJU1	0.112	14804.61	21.00
449	HLA-A0201	LFQQTFSHPS	FQQTFSHPS	1	0	0	0	0	FQQTFSHPS	tr_C9ZJU1	0.112	14809.74	21.00
439	HLA-A0201	CMLSSTDNKMLFQQ	MLSSTLFQQ	1	0	0	5	4	MLSSTDNKMLFQQ	tr_C9ZJU1	0.112	14824.97	21.00
177	HLA-A0201	LQSGITSFWKLM	LQITSFWKL	0	0	0	2	2	LQSGITSFWKL	tr_C9ZJU1	0.112	14828.50	21.00
408	HLA-A0201	QVSPTCSFPVKN	QVSPTCFPV	0	0	0	6	1	QVSPTCSFPV	tr_C9ZJU1	0.112	14837.32	21.00
192	HLA-A0201	CSIPQLVTLAAT	SILVTLAAT	1	0	0	2	2	SIPQLVTLAAT	tr_C9ZJU1	0.112	14843.58	21.00
275	HLA-A0201	LYCIRGFVI	LYCIRGFVI	0	0	0	0	0	LYCIRGFVI	tr_C9ZJU1	0.112	14849.37	21.00
638	HLA-A0201	NQLRSIQHWCPLY	QLIQHWCPL	1	0	0	2	2	QLRSIQHWCPL	tr_C9ZJU1	0.112	14850.81	21.00

428	HLA-A0201	LLRKQKDDGIKCM	LLDDGIKCM	0	0	0	2	4	LLRKQKDDGIKCM	tr_C9ZJU1	0.112	14853.87	21.00
919	HLA-A0201	IIVAHRLAT	IIVAHRLAT	0	0	0	0	0	IIVAHRLAT	tr_C9ZJU1	0.112	14868.18	21.00
893	HLA-A0201	DTITEGEIFDTL	ITEGEIFTL	2	0	0	7	1	ITEGEIFDTL	tr_C9ZJU1	0.112	14880.41	21.00
809	HLA-A0201	EDLSIGGNIALGNL	SIGGNIALL	3	0	0	8	2	SIGGNIALGNL	tr_C9ZJU1	0.112	14885.56	21.00
357	HLA-A0201	VSLVMWLGTQW	SLVMWLGTW	1	0	0	8	1	SLVMWLGTQW	tr_C9ZJU1	0.112	14904.75	21.00
154	HLA-A0201	VVVQEFPEELYLG	VVQEFPLYL	1	0	0	6	2	VVQEFPEELYL	tr_C9ZJU1	0.112	14915.06	21.00
875	HLA-A0201	KAGSAVGLCI	KASAVGLCI	0	0	0	2	1	KAGSAVGLCI	tr_C9ZJU1	0.112	14918.29	21.00
155	HLA-A0201	VVQEFPEELYLGLI	VVQEFPLYL	0	0	0	6	2	VVQEFPEELYL	tr_C9ZJU1	0.112	14920.07	21.00
295	HLA-A0201	HIDAYLALATAD	HIYLALATA	0	0	0	2	2	HIDAYLALATA	tr_C9ZJU1	0.112	14923.13	21.00
342	HLA-A0201	LEVMHGCRVVFS	VMHGCVVFS	2	0	0	5	1	VMHGCRVVFS	tr_C9ZJU1	0.112	14947.21	21.00
265	HLA-A0201	AVASTFAFES	AVASFAFES	0	0	0	4	1	AVASTFAFES	tr_C9ZJU1	0.112	14958.05	21.00
355	HLA-A0201	PVVSLVMWLGTQW	SLVMWLGTW	3	0	0	8	1	SLVMWLGTQW	tr_C9ZJU1	0.112	14963.07	21.00
868	HLA-A0201	CIARAFVKAGSAV	CIARAFVAV	0	0	0	7	4	CIARAFVKAGSAV	tr_C9ZJU1	0.111	14965.34	21.00
135	HLA-A0201	MLLRGKYSIARYPP	MLLRGKYSI	0	0	0	0	0	MLLRGKYSI	tr_C9ZJU1	0.111	14973.92	21.00
190	HLA-A0201	SSCSIPQLVTLAA	SIQLVTLAA	3	0	0	2	1	SIPQLVTLAA	tr_C9ZJU1	0.111	14981.71	21.00
483	HLA-A0201	LGYMWDLLTQH	YMWDLLTQH	2	0	0	0	0	YMWDLLTQH	tr_C9ZJU1	0.111	14981.86	21.00
250	HLA-A0201	IIKQLMGLPAT	IILMGLPAT	0	0	0	2	2	IIKQLMGLPAT	tr_C9ZJU1	0.111	14988.03	21.00
190	HLA-A0201	SSCSIPQLVTL	SSCSILVTL	0	0	0	5	2	SSCSIPQLVTL	tr_C9ZJU1	0.111	15002.30	21.00
82	HLA-A0201	LMKNSHPDHLLVCD	LMHPDHLLV	0	0	0	2	3	LMKNSHPDHLLV	tr_C9ZJU1	0.111	15021.63	21.00
386	HLA-A0201	KSQLASVTNIAT	QLASVTIAT	2	0	0	6	1	QLASVTNIAT	tr_C9ZJU1	0.111	15023.91	21.00
878	HLA-A0201	SAVGLCIFDEPTS	AVGLCIFPT	1	0	0	7	2	AVGLCIFDEPT	tr_C9ZJU1	0.111	15030.41	21.00
214	HLA-A0201	CILGMHAFVHWRP	CILGMHAFV	0	0	0	0	0	CILGMHAFV	tr_C9ZJU1	0.111	15037.57	21.00
471	HLA-A0201	LFSMVFVES	LFSMVFVES	0	0	0	0	0	LFSMVFVES	tr_C9ZJU1	0.111	15052.05	21.00
927	HLA-A0201	TLKHADKIV	TLKHADKIV	0	0	0	0	0	TLKHADKIV	tr_C9ZJU1	0.111	15053.69	21.00
502	HLA-A0201	VFSGCVVDA	VFSGCVVDA	0	0	0	0	0	VFSGCVVDA	tr_C9ZJU1	0.111	15064.44	21.00
488	HLA-A0201	DLLTQHVIWP	LLTQHVIWP	1	0	0	0	0	LLTQHVIWP	tr_C9ZJU1	0.111	15072.10	21.00
407	HLA-A0201	NQVSPTCSFPVKNS	NQVSCSFPV	0	0	0	4	2	NQVSPTCSFPV	tr_C9ZJU1	0.111	15085.15	21.00
721	HLA-A0201	REVSFRYSDRHPYV	VSFRYSPYV	2	0	0	6	3	VSFRYSDRHPYV	tr_C9ZJU1	0.111	15095.27	21.00
685	HLA-A0201	GCYQVLEGLQ	GCYQVLEGL	0	0	0	0	0	GCYQVLEGL	tr_C9ZJU1	0.111	15102.79	21.00
482	HLA-A0201	PLGYMWDLLTQH	YMWDLLTQH	3	0	0	0	0	YMWDLLTQH	tr_C9ZJU1	0.111	15104.92	21.00
894	HLA-A0201	TITEGEIFDT	TITEGIFDT	0	0	0	5	1	TITEGEIFDT	tr_C9ZJU1	0.111	15114.56	21.00
127	HLA-A0201	AEEVLALCML	AEVLALCML	0	0	0	1	1	AEEVLALCML	tr_C9ZJU1	0.111	15124.87	21.00
51	HLA-A0201	NCNGVESIHV	NCNGVSIHV	0	0	0	5	1	NCNGVESIHV	tr_C9ZJU1	0.110	15136.66	21.00
189	HLA-A0201	CSSCSIPQLVTLAA	SIQLVTLAA	4	0	0	2	1	SIPQLVTLAA	tr_C9ZJU1	0.110	15145.17	21.00
919	HLA-A0201	IIVAHRLATLKH	IIVAHLATL	0	0	0	5	1	IIVAHRLATL	tr_C9ZJU1	0.110	15153.53	21.00
756	HLA-A0201	SGKTTLLRLLNRT	SLLRLLNRT	0	0	0	1	4	SGKTTLLRLLNRT	tr_C9ZJU1	0.110	15155.17	21.00
440	HLA-A0201	MLSSTDNKMLFQQ	MLSSMLFQQ	0	0	0	4	4	MLSSTDNKMLFQQ	tr_C9ZJU1	0.110	15161.89	21.00
518	HLA-A0201	GGHTCAVSV	GGHTCAVSV	0	0	0	0	0	GGHTCAVSV	tr_C9ZJU1	0.110	15174.86	21.00
342	HLA-A0201	LEVMHGCRVVFSFP	VMHGVVFSF	2	0	0	4	2	VMHGCRVVFSF	tr_C9ZJU1	0.110	15176.17	21.00
438	HLA-A0201	KCMLSSTDNKMLFQ	MLSSTMLFQ	2	0	0	5	3	MLSSTDNKMLFQ	tr_C9ZJU1	0.110	15182.58	21.00
768	HLA-A0201	TYAPTEGDIFVN	YAPEGDIFV	1	0	0	3	1	YAPTEGDIFV	tr_C9ZJU1	0.110	15196.88	21.00
406	HLA-A0201	ENQVSPTCSFPVKN	NQVSCSFPV	1	0	0	4	2	NQVSPTCSFPV	tr_C9ZJU1	0.110	15197.70	21.00
859	HLA-A0201	FSGGEVERLCIARA	FSGLCIARA	0	0	0	3	5	FSGGEVERLCIARA	tr_C9ZJU1	0.110	15200.16	21.00
8	HLA-A0201	VLLDCERKAY	VLLDCERKA	0	0	0	0	0	VLLDCERKA	tr_C9ZJU1	0.110	15207.90	21.00
942	HLA-A0201	VAEVGSWETL	VAVGSWETL	0	0	0	2	1	VAEVGSWETL	tr_C9ZJU1	0.110	15212.67	21.00
376	HLA-A0201	ESLYMESQFTK	SLMESQFTK	1	0	0	2	1	SLYMESQFTK	tr_C9ZJU1	0.110	15215.80	21.00
708	HLA-A0201	APPSLDGPWSVEF	ASLDGPWSV	0	0	0	1	2	APPSLDGPWSV	tr_C9ZJU1	0.110	15219.92	21.00
500	HLA-A0201	PSVFSGCVVDALKR	SVFCVVDAL	1	0	0	3	2	SVFSGCVVDAL	tr_C9ZJU1	0.110	15240.68	21.00
656	HLA-A0201	SFYDECGMSEAQV	FYDECGMSV	1	0	0	8	3	FYDECGMSEAQV	tr_C9ZJU1	0.110	15240.68	21.00
739	HLA-A0201	SFVVNKGEFLGIAG	FVVEFLGIA	1	0	0	3	3	FVVNKGEFLGIA	tr_C9ZJU1	0.110	15252.55	21.00
473	HLA-A0201	SMVFVESGRPLG	SMVFVESPL	0	0	0	7	2	SMVFVESGRPL	tr_C9ZJU1	0.110	15255.36	21.00
15	HLA-A0201	KAYEILEMLTR	KAYEILEML	0	0	0	0	0	KAYEILEML	tr_C9ZJU1	0.110	15255.86	21.00
497	HLA-A0201	PLVPSVFSGC	PLVPSVFSC	0	0	0	8	1	PLVPSVFSGC	tr_C9ZJU1	0.110	15273.36	21.00
879	HLA-A0201	AVGLCIFDEP	AVGLCIFEP	0	0	0	7	1	AVGLCIFDEP	tr_C9ZJU1	0.109	15294.70	21.00

593	HLA-A0201	QCGRDVLHGLFQM	RVLHGLFQM	3	0	0	1	1	RDVLHGLFQM	tr_C9ZJU1	0.109	15301.98	21.00
213	HLA-A0201	KCILGMHAF	KCILGMHAF	0	0	0	0	0	KCILGMHAF	tr_C9ZJU1	0.109	15322.69	21.00
678	HLA-A0201	AFSGSSLGCYQVL	FSLGCYQVL	1	0	0	1	3	FSGSSLGCYQVL	tr_C9ZJU1	0.109	15342.76	21.00
865	HLA-A0201	ERLCIARAFVKAGS	RLCIARAFV	1	0	0	0	0	RLCIARAFV	tr_C9ZJU1	0.109	15352.73	21.00
639	HLA-A0201	QLRSIQHWCPLYH	QLIQHWCPL	0	0	0	2	2	QLRSIQHWCPL	tr_C9ZJU1	0.109	15358.71	21.00
596	HLA-A0201	RDVLHGLFQMAD	VLHGLFQMA	2	0	0	0	0	VLHGLFQMA	tr_C9ZJU1	0.109	15366.02	21.00
97	HLA-A0201	WNCLMALVNC	WCLMALVNC	0	0	0	1	1	WNCLMALVNC	tr_C9ZJU1	0.109	15390.15	21.00
361	HLA-A0201	MWLGTQWSNF	MLGTQWSNF	0	0	0	1	1	MWLGTQWSNF	tr_C9ZJU1	0.109	15390.82	21.00
374	HLA-A0201	LNESLYMESQFTK	LLYMESQFT	0	0	0	1	3	LNESLYMESQFT	tr_C9ZJU1	0.109	15396.64	21.00
355	HLA-A0201	PVVSLVMWLGT	VVSLVMWLT	1	0	0	8	1	VVSLVMWLGT	tr_C9ZJU1	0.109	15400.97	21.00
55	HLA-A0201	VESIHVRTLV	VSIHVRTLV	0	0	0	1	1	VESIHVRTLV	tr_C9ZJU1	0.109	15433.83	21.00
279	HLA-A0201	RGFVIQSIL	RGFVIQSIL	0	0	0	0	0	RGFVIQSIL	tr_C9ZJU1	0.109	15434.34	21.00
479	HLA-A0201	SGRPLGYMWDLLT	SLGYMWDLL	0	0	0	1	3	SGRPLGYMWDLL	tr_C9ZJU1	0.109	15440.68	21.00
191	HLA-A0201	SCSIPQLVTLAAT	SILVTLAAT	2	0	0	2	2	SIPQLVTLAAT	tr_C9ZJU1	0.109	15445.86	21.00
294	HLA-A0201	IHIDAYLALATADY	IIDAYLALA	0	0	0	1	1	IHIDAYLALA	tr_C9ZJU1	0.109	15453.38	21.00
892	HLA-A0201	IDTITEGEIFDTL	ITEGEIFTL	3	0	0	7	1	ITEGEIFDTL	tr_C9ZJU1	0.109	15453.38	21.00
708	HLA-A0201	APPSLDGPWSVE	ASLDGPWSV	0	0	0	1	2	APPSLDGPWSV	tr_C9ZJU1	0.108	15467.27	21.00
334	HLA-A0201	LQSRVPALLE	LQSRVPALL	0	0	0	0	0	LQSRVPALL	tr_C9ZJU1	0.108	15472.29	21.00
790	HLA-A0201	MLRRRIANVWQEE	MLRRRIANV	0	0	0	0	0	MLRRRIANV	tr_C9ZJU1	0.108	15478.66	21.00
481	HLA-A0201	RPLGYMWDLLT	RLGYMWDLL	0	0	0	1	1	RPLGYMWDLL	tr_C9ZJU1	0.108	15488.88	21.00
131	HLA-A0201	LALCMLLRGK	LLCMLLRGK	0	0	0	1	1	LALCMLLRGK	tr_C9ZJU1	0.108	15525.62	22.00
592	HLA-A0201	EQCGRDVLHGLFQM	RVLHGLFQM	4	0	0	1	1	RDVLHGLFQM	tr_C9ZJU1	0.108	15550.50	22.00
216	HLA-A0201	LGMHAFVHWR	LMHAFVHWR	0	0	0	1	1	LGMHAFVHWR	tr_C9ZJU1	0.108	15561.78	22.00
597	HLA-A0201	DVLHGLFQMADT	VLHGLFQMA	1	0	0	0	0	VLHGLFQMA	tr_C9ZJU1	0.108	15563.63	22.00
385	HLA-A0201	TKSQLASVTNIAT	QLASVTIAT	3	0	0	6	1	QLASVTNIAT	tr_C9ZJU1	0.108	15565.99	22.00
115	HLA-A0201	DGVALCILDDVK	GVALCILDV	1	0	0	7	1	GVALCILDDV	tr_C9ZJU1	0.108	15566.49	22.00
257	HLA-A0201	LPATFVSMAVA	LTFVSMAVA	0	0	0	1	2	LPATFVSMAVA	tr_C9ZJU1	0.108	15567.17	22.00
302	HLA-A0201	LATADYDPSDDWL	ATADYDPSL	1	0	0	8	3	ATADYDPSDDWL	tr_C9ZJU1	0.108	15574.25	22.00
906	HLA-A0201	LREGNNLLRDSTII	LLLRDSTII	0	0	0	1	5	LREGNNLLRDSTII	tr_C9ZJU1	0.108	15586.38	22.00
208	HLA-A0201	PEVILKCILGMH	VILKCILGM	2	0	0	0	0	VILKCILGM	tr_C9ZJU1	0.108	15593.97	22.00
463	HLA-A0201	SSVQYGLVLF	SSVQYLVLF	0	0	0	5	1	SSVQYGLVLF	tr_C9ZJU1	0.108	15596.67	22.00
56	HLA-A0201	ESIHVRTLVFNHNI	SILVFNHNI	1	0	0	2	4	SIHVRTLVFNHNI	tr_C9ZJU1	0.108	15603.76	22.00
940	HLA-A0201	GMVAEVGSWETLSR	MVAEVWETL	1	0	0	5	2	MVAEVGSWETL	tr_C9ZJU1	0.108	15609.34	22.00
618	HLA-A0201	ALRLSPNFFTQS	ALLSPNFFT	0	0	0	2	1	ALRLSPNFFT	tr_C9ZJU1	0.108	15615.92	22.00
777	HLA-A0201	FVNGVHISQ	FVNGVHISQ	0	0	0	0	0	FVNGVHISQ	tr_C9ZJU1	0.107	15627.25	22.00
498	HLA-A0201	LVPSVFSGC	LVPSVFSGC	0	0	0	0	0	LVPSVFSGC	tr_C9ZJU1	0.107	15630.62	22.00
385	HLA-A0201	TKSQLASVTNI	KQLASVTNI	1	0	0	1	1	KSQLASVTNI	tr_C9ZJU1	0.107	15631.14	22.00
710	HLA-A0201	PSLDGPWSVEF	SLDGWSVEF	1	0	0	4	1	SLDGPWSVEF	tr_C9ZJU1	0.107	15639.25	22.00
676	HLA-A0201	HLAFSGSSLG	HLAFSGSSL	0	0	0	0	0	HLAFSGSSL	tr_C9ZJU1	0.107	15644.33	22.00
107	HLA-A0201	SIRDILLDDGVA	SIRDILLGV	0	0	0	7	2	SIRDILLDDGV	tr_C9ZJU1	0.107	15653.82	22.00
877	HLA-A0201	GSAVGLCIFDEPTS	AVGLCIFPT	2	0	0	7	2	AVGLCIFDEPT	tr_C9ZJU1	0.107	15664.66	22.00
128	HLA-A0201	EEVLALCML	EEVLALCML	0	0	0	0	0	EEVLALCML	tr_C9ZJU1	0.107	15670.25	22.00
769	HLA-A0201	YAPTEGDIFVNG	YAPEGDIFV	0	0	0	3	1	YAPTEGDIFV	tr_C9ZJU1	0.107	15671.95	22.00
234	HLA-A0201	KSLNGDVSPKLSP	SLNDVSPKL	1	0	0	3	1	SLNGDVSPKL	tr_C9ZJU1	0.107	15677.38	22.00
190	HLA-A0201	SSCSIPQLVTLAAT	SILVTLAAT	3	0	0	2	2	SIPQLVTLAAT	tr_C9ZJU1	0.107	15685.18	22.00
763	HLA-A0201	RLLNRTYAPTE	RLLNRTYPT	0	0	0	7	1	RLLNRTYAPT	tr_C9ZJU1	0.107	15713.71	22.00
266	HLA-A0201	VASTFAFES	VASTFAFES	0	0	0	0	0	VASTFAFES	tr_C9ZJU1	0.107	15719.33	22.00
62	HLA-A0201	TLVFNHNIMQ	TLFNHNIMQ	0	0	0	2	1	TLVFNHNIMQ	tr_C9ZJU1	0.107	15729.70	22.00
113	HLA-A0201	LDDGVALCILDDVK	GVALCILDV	3	0	0	7	1	GVALCILDDV	tr_C9ZJU1	0.107	15741.11	22.00
926	HLA-A0201	ATLKHADKIVVLNE	TLADKIVVL	1	0	0	2	2	TLKHADKIVVL	tr_C9ZJU1	0.107	15742.64	22.00
625	HLA-A0201	FFTQSALDQTEI	FTQSALTEI	1	0	0	6	2	FTQSALDQTEI	tr_C9ZJU1	0.107	15744.86	22.00
882	HLA-A0201	LCIFDEPTSAI	CIFDEPTSI	1	0	0	8	1	CIFDEPTSAI	tr_C9ZJU1	0.107	15765.65	22.00
673	HLA-A0201	LRRHLAFSGSSL	LLAFSGSSL	0	0	0	1	3	LRRHLAFSGSSL	tr_C9ZJU1	0.107	15770.78	22.00
375	HLA-A0201	NESLYMESQFTK	SLMESQFTK	2	0	0	2	1	SLYMESQFTK	tr_C9ZJU1	0.107	15773.33	22.00

597	HLA-A0201	DVLHGLFQMAD	VLHGLFQMA	1	0	0	0	0	VLHGLFQMA	tr_C9ZJU1	0.107	15779.14	22.00
334	HLA-A0201	LQSRVPALLEVMHG	LQVPALLEV	0	0	0	2	2	LQSRVPALLEV	tr_C9ZJU1	0.106	15804.43	22.00
387	HLA-A0201	SQLASVTNIATS	QLASVIATS	1	0	0	5	2	QLASVTNIATS	tr_C9ZJU1	0.106	15805.97	22.00
65	HLA-A0201	FNHNIMQLPSV	FNIMQLPSV	0	0	0	1	2	FNHNIMQLPSV	tr_C9ZJU1	0.106	15823.07	22.00
71	HLA-A0201	QLPSVFAVH	QLPSVFAVH	0	0	0	0	0	QLPSVFAVH	tr_C9ZJU1	0.106	15829.76	22.00
637	HLA-A0201	VNQLRSIQHWCPLY	QLIQHWCPL	2	0	0	2	2	QLRSIQHWCPL	tr_C9ZJU1	0.106	15840.21	22.00
496	HLA-A0201	WPLVPSVFSGCVVD	PLVPSVFSV	1	0	0	8	3	PLVPSVFSGCVV	tr_C9ZJU1	0.106	15842.09	22.00
153	HLA-A0201	DVVVQEFPEELYLG	VVQEFPLYL	2	0	0	6	2	VVQEFPEELYL	tr_C9ZJU1	0.106	15842.94	22.00
332	HLA-A0201	DELQSRVPALL	ELQSVPALL	1	0	0	4	1	ELQSRVPALL	tr_C9ZJU1	0.106	15849.12	22.00
737	HLA-A0201	RVSFVVNKGEFLG	RVSFVVEFL	0	0	0	6	3	RVSFVVNKGEFL	tr_C9ZJU1	0.106	15854.95	22.00
617	HLA-A0201	VALRLSPNFFTQ	ALLSPNFFT	1	0	0	2	1	ALRLSPNFFT	tr_C9ZJU1	0.106	15865.08	22.00
446	HLA-A0201	NKMLFQQTFS	KMLFQQTFS	1	0	0	0	0	KMLFQQTFS	tr_C9ZJU1	0.106	15887.75	22.00
325	HLA-A0201	QASGSVMDEL	QASGSVMEL	0	0	0	7	1	QASGSVMDEL	tr_C9ZJU1	0.106	15888.27	22.00
153	HLA-A0201	DVVVQEFPEEL	VVVEFPEEL	1	0	0	3	1	VVVQEFPEEL	tr_C9ZJU1	0.106	15889.47	22.00
356	HLA-A0201	VVSLVMWLGTQ	VVSLVMWLT	0	0	0	8	1	VVSLVMWLGT	tr_C9ZJU1	0.106	15899.62	22.00
497	HLA-A0201	PLVPSVFSG	PLVPSVFSG	0	0	0	0	0	PLVPSVFSG	tr_C9ZJU1	0.106	15907.36	22.00
419	HLA-A0201	NSLTSSPLTLLRKQ	SLTSSLTLL	1	0	0	5	1	SLTSSPLTLL	tr_C9ZJU1	0.106	15907.71	22.00
235	HLA-A0201	SLNGDVSPKLSPFR	SLNDVSPKL	0	0	0	3	1	SLNGDVSPKL	tr_C9ZJU1	0.106	15909.77	22.00
797	HLA-A0201	NVWQEENNLRFI	NVWNNLRFI	0	0	0	3	3	NVWQEENNLRFI	tr_C9ZJU1	0.106	15909.95	22.00
919	HLA-A0201	IIVAHRLATLKHA	IIVAHLATL	0	0	0	5	1	IIVAHRLATL	tr_C9ZJU1	0.106	15914.59	22.00
114	HLA-A0201	DDGVALCILDDVK	GVALCILDV	2	0	0	7	1	GVALCILDDV	tr_C9ZJU1	0.106	15914.59	22.00
926	HLA-A0201	ATLKHADKIVV	TLHADKIVV	1	0	0	2	1	TLKHADKIVV	tr_C9ZJU1	0.106	15939.58	22.00
699	HLA-A0201	KIDRPVRGVAPPSL	KIDVAPPSL	0	0	0	3	5	KIDRPVRGVAPPSL	tr_C9ZJU1	0.106	15942.51	22.00
726	HLA-A0201	RYSDRHPYVL	YSDRHPYVL	1	0	0	0	0	YSDRHPYVL	tr_C9ZJU1	0.106	15964.43	22.00
84	HLA-A0201	KNSHPDHLLV	KSHPDHLLV	0	0	0	1	1	KNSHPDHLLV	tr_C9ZJU1	0.105	15967.89	22.00
594	HLA-A0201	CGRDVLHGLFQMAD	VLHGLFQMA	4	0	0	0	0	VLHGLFQMA	tr_C9ZJU1	0.105	15975.49	22.00
278	HLA-A0201	IRGFVIQSI	IRGFVIQSI	0	0	0	0	0	IRGFVIQSI	tr_C9ZJU1	0.105	15977.22	22.00
917	HLA-A0201	TIIIVAHRLAT	TIIIVALAT	0	0	0	6	2	TIIIVAHRLAT	tr_C9ZJU1	0.105	15986.38	22.00
234	HLA-A0201	KSLNGDVSPKLSPF	SLNDVSPKL	1	0	0	3	1	SLNGDVSPKL	tr_C9ZJU1	0.105	15988.63	22.00
894	HLA-A0201	TITEGEIFDTLRL	TITEGEIFL	0	0	0	8	4	TITEGEIFDTLRL	tr_C9ZJU1	0.105	16030.90	22.00
359	HLA-A0201	LVMWLGTQW	LVMWLGTQW	0	0	0	0	0	LVMWLGTQW	tr_C9ZJU1	0.105	16035.76	22.00
503	HLA-A0201	FSGCVVDALKRKL	FSGCVVDAL	0	0	0	8	4	FSGCVVDALKRKL	tr_C9ZJU1	0.105	16040.27	22.00
639	HLA-A0201	QLRSIQHWC	QLRSIQHWC	0	0	0	0	0	QLRSIQHWC	tr_C9ZJU1	0.105	16048.94	22.00
277	HLA-A0201	CIRGFVIQS	CIRGFVIQS	0	0	0	0	0	CIRGFVIQS	tr_C9ZJU1	0.105	16065.28	22.00
258	HLA-A0201	PATFVSMAV	PATFVSMAV	0	0	0	0	0	PATFVSMAV	tr_C9ZJU1	0.105	16068.06	22.00
542	HLA-A0201	AATASPSTCV	ATASPSTCV	0	0	0	1	1	AATASPSTCV	tr_C9ZJU1	0.105	16086.33	22.00
595	HLA-A0201	GRDVLHGLFQMAD	VLHGLFQMA	3	0	0	0	0	VLHGLFQMA	tr_C9ZJU1	0.105	16126.06	22.00
227	HLA-A0201	IYAAEIGKSL	YAAEIGKSL	1	0	0	0	0	YAAEIGKSL	tr_C9ZJU1	0.105	16135.13	22.00
215	HLA-A0201	ILGMHAFVHWRP	ILAFVHWRP	0	0	0	2	3	ILGMHAFVHWRP	tr_C9ZJU1	0.104	16144.74	22.00
903	HLA-A0201	TLRLREGNNLL	LLREGNNLL	1	0	0	1	1	LRLREGNNLL	tr_C9ZJU1	0.104	16149.46	22.00
89	HLA-A0201	DHLLVCDIWN	HLLVCDIWN	1	0	0	0	0	HLLVCDIWN	tr_C9ZJU1	0.104	16150.67	22.00
616	HLA-A0201	AVALRLSPNFFTQ	ALLSPNFFT	2	0	0	2	1	ALRLSPNFFT	tr_C9ZJU1	0.104	16163.62	22.00
596	HLA-A0201	RDVLHGLFQMADT	VLHGLFQMA	2	0	0	0	0	VLHGLFQMA	tr_C9ZJU1	0.104	16209.67	22.00
247	HLA-A0201	FRFIIKQLMG	FRFIIQLMG	0	0	0	5	1	FRFIIKQLMG	tr_C9ZJU1	0.104	16221.08	22.00
883	HLA-A0201	CIFDEPTSAIDTIT	CIFDAIDTI	0	0	0	4	4	CIFDEPTSAIDTI	tr_C9ZJU1	0.104	16227.75	22.00
776	HLA-A0201	IFVNGVHISQYPAR	FVNGVQYPA	1	0	0	5	3	FVNGVHISQYPA	tr_C9ZJU1	0.104	16246.90	22.00
101	HLA-A0201	MALVNCSIRD	MLVNCSIRD	0	0	0	1	1	MALVNCSIRD	tr_C9ZJU1	0.104	16253.05	22.00
152	HLA-A0201	ADVVVQEFPEEL	AVQEFPEEL	0	0	0	1	3	ADVVVQEFPEEL	tr_C9ZJU1	0.104	16267.84	22.00
790	HLA-A0201	MLRRRIANVWQEEN	MLRRRIANV	0	0	0	0	0	MLRRRIANV	tr_C9ZJU1	0.104	16301.13	22.00
555	HLA-A0201	RQDYLQGFAALR	YLQGFAALR	3	0	0	0	0	YLQGFAALR	tr_C9ZJU1	0.104	16305.02	22.00
644	HLA-A0201	QHWCPLYHA	QHWCPLYHA	0	0	0	0	0	QHWCPLYHA	tr_C9ZJU1	0.104	16307.66	22.00
157	HLA-A0201	QEFPEELYL	QEFPEELYL	0	0	0	0	0	QEFPEELYL	tr_C9ZJU1	0.104	16309.95	22.00
408	HLA-A0201	QVSPTCSFPVKNS	QVSPTCFPV	0	0	0	6	1	QVSPTCSFPV	tr_C9ZJU1	0.103	16316.84	22.00
764	HLA-A0201	LLNRTYAPTEGDIF	LLNRTYAPI	0	0	0	8	4	LLNRTYAPTEGDI	tr_C9ZJU1	0.103	16327.79	22.00

764	HLA-A0201	LLNRTYAPTE	LLNRTYAPT	0	0	0	0	0	LLNRTYAPT	tr_C9ZJU1	0.103	16334.15	22.00
615	HLA-A0201	SAVALRLSPNFFTQ	ALLSPNFFT	3	0	0	2	1	ALRLSPNFFT	tr_C9ZJU1	0.103	16336.27	22.00
923	HLA-A0201	HRLATLKHADKI	RLATLKHAI	1	0	0	8	2	RLATLKHADKI	tr_C9ZJU1	0.103	16336.45	22.00
473	HLA-A0201	SMVFVESGRPLGY	SMVFVPLGY	0	0	0	5	4	SMVFVESGRPLGY	tr_C9ZJU1	0.103	16352.72	23.00
480	HLA-A0201	GRPLGYMWDLLT	GLGYMWDLL	0	0	0	1	2	GRPLGYMWDLL	tr_C9ZJU1	0.103	16352.89	23.00
535	HLA-A0201	VIQSSPRAA	VIQSSPRAA	0	0	0	0	0	VIQSSPRAA	tr_C9ZJU1	0.103	16378.39	23.00
757	HLA-A0201	GKTTLLRLLNRT	KLLRLLNRT	1	0	0	1	2	KTTLLRLLNRT	tr_C9ZJU1	0.103	16391.15	23.00
805	HLA-A0201	LRFIEDLSI	LRFIEDLSI	0	0	0	0	0	LRFIEDLSI	tr_C9ZJU1	0.103	16391.15	23.00
812	HLA-A0201	SIGGNIALG	SIGGNIALG	0	0	0	0	0	SIGGNIALG	tr_C9ZJU1	0.103	16405.53	23.00
181	HLA-A0201	ITSFWKLMC	ITSFWKLMC	0	0	0	0	0	ITSFWKLMC	tr_C9ZJU1	0.103	16406.60	23.00
189	HLA-A0201	CSSCSIPQLVTL	SSCSILVTL	1	0	0	5	2	SSCSIPQLVTL	tr_C9ZJU1	0.103	16412.28	23.00
527	HLA-A0201	RRLGWIKDVIQS	RLGWIVIQS	1	0	0	5	2	RLGWIKDVIQS	tr_C9ZJU1	0.103	16418.49	23.00
194	HLA-A0201	IPQLVTLAA	IPQLVTLAA	0	0	0	0	0	IPQLVTLAA	tr_C9ZJU1	0.103	16421.33	23.00
156	HLA-A0201	VQEFPEELYLG	VQFPEELYL	0	0	0	2	1	VQEFPEELYL	tr_C9ZJU1	0.103	16441.95	23.00
595	HLA-A0201	GRDVLHGLFQMADT	VLHGLFQMA	3	0	0	0	0	VLHGLFQMA	tr_C9ZJU1	0.103	16455.13	23.00
624	HLA-A0201	NFFTQSALDQTEI	FTQSALTEI	2	0	0	6	2	FTQSALDQTEI	tr_C9ZJU1	0.103	16461.90	23.00
691	HLA-A0201	EGLQSVQRKI	GLQSVQRKI	1	0	0	0	0	GLQSVQRKI	tr_C9ZJU1	0.103	16468.31	23.00
552	HLA-A0201	WQCRQDYLQGFAA	WQYLQGFAA	0	0	0	2	4	WQCRQDYLQGFAA	tr_C9ZJU1	0.103	16476.15	23.00
536	HLA-A0201	IQSSPRAATA	IQSPRAATA	0	0	0	2	1	IQSSPRAATA	tr_C9ZJU1	0.102	16504.33	23.00
197	HLA-A0201	LVTLAATVLFSPE	LLAATVLFS	0	0	0	1	2	LVTLAATVLFS	tr_C9ZJU1	0.102	16512.37	23.00
937	HLA-A0201	LNEGMVAEV	LNEGMVAEV	0	0	0	0	0	LNEGMVAEV	tr_C9ZJU1	0.102	16517.74	23.00
386	HLA-A0201	KSQLASVTNIATS	QLASVIATS	2	0	0	5	2	QLASVTNIATS	tr_C9ZJU1	0.102	16518.09	23.00
183	HLA-A0201	SFWKLMCSS	SFWKLMCSS	0	0	0	0	0	SFWKLMCSS	tr_C9ZJU1	0.102	16522.38	23.00
525	HLA-A0201	SVRRLGWIKDV	SVLGWIKDV	0	0	0	2	2	SVRRLGWIKDV	tr_C9ZJU1	0.102	16537.23	23.00
655	HLA-A0201	GSFYDECGMSEAQV	FYDECGMSV	2	0	0	8	3	FYDECGMSEAQV	tr_C9ZJU1	0.102	16552.27	23.00
215	HLA-A0201	ILGMHAFVHWR	ILGMFVHWR	0	0	0	4	2	ILGMHAFVHWR	tr_C9ZJU1	0.102	16556.74	23.00
295	HLA-A0201	HIDAYLALATADYD	HIYLALATA	0	0	0	2	2	HIDAYLALATA	tr_C9ZJU1	0.102	16558.54	23.00
626	HLA-A0201	FTQSALDQTEIVN	FTLDQTEIV	0	0	0	2	3	FTQSALDQTEIV	tr_C9ZJU1	0.102	16559.97	23.00
295	HLA-A0201	HIDAYLALATADY	HIYLALATA	0	0	0	2	2	HIDAYLALATA	tr_C9ZJU1	0.102	16565.17	23.00
872	HLA-A0201	AFVKAGSAVGLCIF	FVSAVGLCI	1	0	0	2	3	FVKAGSAVGLCI	tr_C9ZJU1	0.102	16571.26	23.00
74	HLA-A0201	SVFAVHCGLMK	SVFAVCGLM	0	0	0	5	1	SVFAVHCGLM	tr_C9ZJU1	0.102	16571.80	23.00
125	HLA-A0201	VKAEEVLALC	KAEEVLALC	1	0	0	0	0	KAEEVLALC	tr_C9ZJU1	0.102	16615.42	23.00
950	HLA-A0201	TLSRTGPHSCFSRM	TLSSCFSRM	0	0	0	3	5	TLSRTGPHSCFSRM	tr_C9ZJU1	0.102	16639.36	23.00
478	HLA-A0201	ESGRPLGYMWDLLT	SLGYMWDLL	1	0	0	1	3	SGRPLGYMWDLL	tr_C9ZJU1	0.102	16647.28	23.00
617	HLA-A0201	VALRLSPNFFTQS	RLSPNFFTS	3	0	0	8	1	RLSPNFFTQS	tr_C9ZJU1	0.102	16647.81	23.00
616	HLA-A0201	AVALRLSPNFFTQS	RLSPNFFTS	4	0	0	8	1	RLSPNFFTQS	tr_C9ZJU1	0.102	16652.68	23.00
770	HLA-A0201	APTEGDIFVNGVHI	AIFVNGVHI	0	0	0	1	5	APTEGDIFVNGVHI	tr_C9ZJU1	0.102	16662.96	23.00
528	HLA-A0201	RLGWIKDVIQSS	RLGWVIQSS	0	0	0	4	3	RLGWIKDVIQSS	tr_C9ZJU1	0.102	16664.40	23.00
355	HLA-A0201	PVVSLVMWLGTQ	SLVMWLGTQ	3	0	0	0	0	SLVMWLGTQ	tr_C9ZJU1	0.102	16667.46	23.00
14	HLA-A0201	RKAYEILEMLTR	KAYEILMLT	1	0	0	6	1	KAYEILEMLT	tr_C9ZJU1	0.101	16674.86	23.00
746	HLA-A0201	EFLGIAGYSGS	FLIAGYSGS	1	0	0	2	1	FLGIAGYSGS	tr_C9ZJU1	0.101	16697.79	23.00
554	HLA-A0201	CRQDYLQGFAALR	YLQGFAALR	4	0	0	0	0	YLQGFAALR	tr_C9ZJU1	0.101	16701.04	23.00
233	HLA-A0201	GKSLNGDVSPKLSP	SLNDVSPKL	2	0	0	3	1	SLNGDVSPKL	tr_C9ZJU1	0.101	16705.02	23.00
617	HLA-A0201	VALRLSPNFF	ALRLSPNFF	1	0	0	0	0	ALRLSPNFF	tr_C9ZJU1	0.101	16723.10	23.00
187	HLA-A0201	LMCSSCSIP	LMCSSCSIP	0	0	0	0	0	LMCSSCSIP	tr_C9ZJU1	0.101	16726.18	23.00
343	HLA-A0201	EVMHGCRVVF	VMHGCRVVF	1	0	0	0	0	VMHGCRVVF	tr_C9ZJU1	0.101	16741.20	23.00
277	HLA-A0201	CIRGFVIQSILS	CIRGFVIIL	0	0	0	7	2	CIRGFVIQSIL	tr_C9ZJU1	0.101	16745.55	23.00
934	HLA-A0201	IVVLNEGMVA	IVLNEGMVA	0	0	0	1	1	IVVLNEGMVA	tr_C9ZJU1	0.101	16748.09	23.00
688	HLA-A0201	QVLEGLQSVQRKI	QVLEGLQSI	0	0	0	8	4	QVLEGLQSVQRKI	tr_C9ZJU1	0.101	16754.97	23.00
571	HLA-A0201	ELMAATAAAAF	LMAATAAAA	1	0	0	0	0	LMAATAAAA	tr_C9ZJU1	0.101	16758.42	23.00
820	HLA-A0201	GNLWDCSDEN	GLWDCSDEN	0	0	0	1	1	GNLWDCSDEN	tr_C9ZJU1	0.101	16761.14	23.00
553	HLA-A0201	QCRQDYLQGFAALR	YLQGFAALR	5	0	0	0	0	YLQGFAALR	tr_C9ZJU1	0.101	16807.81	23.00
902	HLA-A0201	DTLRLREGNNLL	LLREGNNLL	2	0	0	1	1	LRLREGNNLL	tr_C9ZJU1	0.101	16811.64	23.00
177	HLA-A0201	LQSGITSFW	LQSGITSFW	0	0	0	0	0	LQSGITSFW	tr_C9ZJU1	0.101	16811.99	23.00

918	HLA-A0201	IIIVAHRLAT	IIIVAHLAT	0	0	0	6	1	IIIVAHRLAT	tr_C9ZJU1	0.101	16813.81	23.00
177	HLA-A0201	LQSGITSFWKLMCS	LQFWKLMCS	0	0	0	2	5	LQSGITSFWKLMCS	tr_C9ZJU1	0.101	16821.82	23.00
353	HLA-A0201	SFPVVSLVMW	FPVVSLVMW	1	0	0	0	0	FPVVSLVMW	tr_C9ZJU1	0.101	16837.67	23.00
105	HLA-A0201	NCSIRDILL	NCSIRDILL	0	0	0	0	0	NCSIRDILL	tr_C9ZJU1	0.101	16846.22	23.00
749	HLA-A0201	GIAGYSGSGKTTL	GIAGYSTTL	0	0	0	6	4	GIAGYSGSGKTTL	tr_C9ZJU1	0.100	16860.82	23.00
517	HLA-A0201	AGGHTCAVSV	AGHTCAVSV	0	0	0	1	1	AGGHTCAVSV	tr_C9ZJU1	0.100	16876.33	23.00
408	HLA-A0201	QVSPTCSFPVKNSL	QVSPTCFPV	0	0	0	6	1	QVSPTCSFPV	tr_C9ZJU1	0.100	16893.14	23.00
623	HLA-A0201	PNFFTQSALDQTEI	FTQSALTEI	3	0	0	6	2	FTQSALDQTEI	tr_C9ZJU1	0.100	16894.97	23.00
54	HLA-A0201	GVESIHVRTL	GVESIHVTL	0	0	0	7	1	GVESIHVRTL	tr_C9ZJU1	0.100	16906.30	23.00
36	HLA-A0201	KQTECKDDASI	KQTECDASI	0	0	0	5	2	KQTECKDDASI	tr_C9ZJU1	0.100	16929.73	23.00
858	HLA-A0201	EFSGGEVERL	FSGGEVERL	1	0	0	0	0	FSGGEVERL	tr_C9ZJU1	0.100	16934.50	23.00
556	HLA-A0201	QDYLQGFAALR	YLQGFAALR	2	0	0	0	0	YLQGFAALR	tr_C9ZJU1	0.100	16952.65	23.00
643	HLA-A0201	IQHWCPLYH	IQHWCPLYH	0	0	0	0	0	IQHWCPLYH	tr_C9ZJU1	0.100	16955.95	23.00
462	HLA-A0201	KSSVQYGLVL	KSVQYGLVL	0	0	0	1	1	KSSVQYGLVL	tr_C9ZJU1	0.100	16976.69	23.00
755	HLA-A0201	GSGKTTLLRLLNRT	KLLRLLNRT	3	0	0	1	2	KTTLLRLLNRT	tr_C9ZJU1	0.100	16982.94	23.00
638	HLA-A0201	NQLRSIQHWCPLYH	QLIQHWCPL	1	0	0	2	2	QLRSIQHWCPL	tr_C9ZJU1	0.100	17019.54	23.00
781	HLA-A0201	VHISQYPARM	VISQYPARM	0	0	0	1	1	VHISQYPARM	tr_C9ZJU1	0.099	17041.83	23.00
116	HLA-A0201	GVALCILDDVKAE	GVALCILDV	0	0	0	7	1	GVALCILDDV	tr_C9ZJU1	0.099	17053.64	23.00
211	HLA-A0201	ILKCILGMH	ILKCILGMH	0	0	0	0	0	ILKCILGMH	tr_C9ZJU1	0.099	17056.79	23.00
385	HLA-A0201	TKSQLASVTNIATS	QLASVIATS	3	0	0	5	2	QLASVTNIATS	tr_C9ZJU1	0.099	17065.08	23.00
662	HLA-A0201	GMSEAQVDEAHLR	GMSEAQVHL	0	0	0	7	3	GMSEAQVDEAHL	tr_C9ZJU1	0.099	17080.78	23.00
296	HLA-A0201	IDAYLALATAD	IAYLALATA	0	0	0	1	1	IDAYLALATA	tr_C9ZJU1	0.099	17097.43	23.00
20	HLA-A0201	LEMLTRATI	LEMLTRATI	0	0	0	0	0	LEMLTRATI	tr_C9ZJU1	0.099	17119.46	23.00
890	HLA-A0201	SAIDTITEGEI	SAIDTITEI	0	0	0	7	2	SAIDTITEGEI	tr_C9ZJU1	0.099	17132.06	23.00
158	HLA-A0201	EFPEELYLGL	FPEELYLGL	1	0	0	0	0	FPEELYLGL	tr_C9ZJU1	0.099	17138.36	23.00
799	HLA-A0201	WQEENNLRFIEDL	WQLRFIEDL	0	0	0	2	4	WQEENNLRFIEDL	tr_C9ZJU1	0.099	17145.78	23.00
96	HLA-A0201	IWNCLMALVNC	IWNCLMALV	0	0	0	0	0	IWNCLMALV	tr_C9ZJU1	0.099	17152.46	23.00
919	HLA-A0201	IIVAHRLATLKHAD	IIVAHLATL	0	0	0	5	1	IIVAHRLATL	tr_C9ZJU1	0.099	17190.18	24.00
235	HLA-A0201	SLNGDVSPK	SLNGDVSPK	0	0	0	0	0	SLNGDVSPK	tr_C9ZJU1	0.099	17196.32	24.00
102	HLA-A0201	ALVNCSIRD	ALVNCSIRD	0	0	0	0	0	ALVNCSIRD	tr_C9ZJU1	0.099	17201.34	24.00
315	HLA-A0201	LSRCVSQAL	LSRCVSQAL	0	0	0	0	0	LSRCVSQAL	tr_C9ZJU1	0.098	17231.89	24.00
727	HLA-A0201	YSDRHPYVLRRV	YSDYVLRRV	0	0	0	3	3	YSDRHPYVLRRV	tr_C9ZJU1	0.098	17235.24	24.00
68	HLA-A0201	NIMQLPSVF	NIMQLPSVF	0	0	0	0	0	NIMQLPSVF	tr_C9ZJU1	0.098	17235.62	24.00
526	HLA-A0201	VRRLGWIKDVIQS	RLGWIVIQS	2	0	0	5	2	RLGWIKDVIQS	tr_C9ZJU1	0.098	17238.79	24.00
37	HLA-A0201	QTECKDDASIAIFL	QTASIAIFL	0	0	0	2	5	QTECKDDASIAIFL	tr_C9ZJU1	0.098	17249.05	24.00
151	HLA-A0201	QADVVVQEFPEEL	VVVEFPEEL	3	0	0	3	1	VVVQEFPEEL	tr_C9ZJU1	0.098	17260.25	24.00
736	HLA-A0201	RRVSFVVNKGEFLG	RVSFVVEFL	1	0	0	6	3	RVSFVVNKGEFL	tr_C9ZJU1	0.098	17263.24	24.00
671	HLA-A0201	AHLRRHLAFS	ALRRHLAFS	0	0	0	1	1	AHLRRHLAFS	tr_C9ZJU1	0.098	17277.45	24.00
41	HLA-A0201	KDDASIAIFL	KDDASIIFL	0	0	0	6	1	KDDASIAIFL	tr_C9ZJU1	0.098	17279.87	24.00
391	HLA-A0201	SVTNIATST	SVTNIATST	0	0	0	0	0	SVTNIATST	tr_C9ZJU1	0.098	17288.67	24.00
761	HLA-A0201	LLRLLNRTYAP	LLRLLNTYA	0	0	0	6	1	LLRLLNRTYA	tr_C9ZJU1	0.098	17292.96	24.00
44	HLA-A0201	ASIAIFLNCN	ASIAIFLNC	0	0	0	0	0	ASIAIFLNC	tr_C9ZJU1	0.098	17308.87	24.00
259	HLA-A0201	ATFVSMAVAST	FVSMAVAST	2	0	0	0	0	FVSMAVAST	tr_C9ZJU1	0.098	17319.74	24.00
777	HLA-A0201	FVNGVHISQY	FVNGVHISY	0	0	0	8	1	FVNGVHISQY	tr_C9ZJU1	0.098	17323.30	24.00
45	HLA-A0201	SIAIFLNCNG	SIAIFLCNG	0	0	0	6	1	SIAIFLNCNG	tr_C9ZJU1	0.098	17328.55	24.00
761	HLA-A0201	LLRLLNRTY	LLRLLNRTY	0	0	0	0	0	LLRLLNRTY	tr_C9ZJU1	0.098	17341.87	24.00
632	HLA-A0201	DQTEIVNQL	DQTEIVNQL	0	0	0	0	0	DQTEIVNQL	tr_C9ZJU1	0.098	17351.63	24.00
177	HLA-A0201	LQSGITSFWKLMC	LQITSFWKL	0	0	0	2	2	LQSGITSFWKL	tr_C9ZJU1	0.098	17362.89	24.00
431	HLA-A0201	KQKDDGIKCM	KQDDGIKCM	0	0	0	2	1	KQKDDGIKCM	tr_C9ZJU1	0.098	17389.22	24.00
12	HLA-A0201	CERKAYEILEMLTR	KAYEILEML	3	0	0	0	0	KAYEILEML	tr_C9ZJU1	0.098	17400.51	24.00
345	HLA-A0201	MHGCRVVFSF	MGCRVVFSF	0	0	0	1	1	MHGCRVVFSF	tr_C9ZJU1	0.097	17413.13	24.00
687	HLA-A0201	YQVLEGLQS	YQVLEGLQS	0	0	0	0	0	YQVLEGLQS	tr_C9ZJU1	0.097	17435.75	24.00
692	HLA-A0201	GLQSVQRKIDRPVR	GLQSVDRPV	0	0	0	5	4	GLQSVQRKIDRPV	tr_C9ZJU1	0.097	17456.14	24.00
310	HLA-A0201	SDDWLLSRCV	SDDWLLSRCV	0	0	0	1	1	SDDWLLSRCV	tr_C9ZJU1	0.097	17462.56	24.00

268	HLA-A0201	STFAFESLY	STFAFESLY	0	0	0	0	0	STFAFESLY	tr_C9ZJU1	0.097	17466.90	24.00
64	HLA-A0201	VFNHNIMQLPSV	VFIMQLPSV	0	0	0	2	3	VFNHNIMQLPSV	tr_C9ZJU1	0.097	17479.76	24.00
652	HLA-A0201	AFLGSFYDECG	FLGSFYDEC	1	0	0	0	0	FLGSFYDEC	tr_C9ZJU1	0.097	17482.97	24.00
103	HLA-A0201	LVNCSIRDILLD	LVNCSIILL	0	0	0	6	2	LVNCSIRDILL	tr_C9ZJU1	0.097	17502.47	24.00
525	HLA-A0201	SVRRLGWIKDVIQS	RLGWIVIQS	3	0	0	5	2	RLGWIKDVIQS	tr_C9ZJU1	0.097	17522.37	24.00
679	HLA-A0201	FSGSSLGCYQVLEG	SLGCYVLEG	4	0	0	5	1	SLGCYQVLEG	tr_C9ZJU1	0.097	17554.63	24.00
625	HLA-A0201	FFTQSALDQTEIVN	FTLDQTEIV	1	0	0	2	3	FTQSALDQTEIV	tr_C9ZJU1	0.097	17560.33	24.00
709	HLA-A0201	PPSLDGPWSVEF	SLDGWSVEF	2	0	0	4	1	SLDGPWSVEF	tr_C9ZJU1	0.097	17568.50	24.00
199	HLA-A0201	TLAATVLFSPE	TLAAVLFSP	0	0	0	4	1	TLAATVLFSP	tr_C9ZJU1	0.097	17573.07	24.00
630	HLA-A0201	ALDQTEIVNQ	ALDQTIVNQ	0	0	0	5	1	ALDQTEIVNQ	tr_C9ZJU1	0.097	17573.07	24.00
198	HLA-A0201	VTLAATVLFSPE	VLAATVLFS	0	0	0	1	1	VTLAATVLFS	tr_C9ZJU1	0.097	17584.10	24.00
793	HLA-A0201	RRIANVWQEENNL	RIANVWQEL	1	0	0	8	3	RIANVWQEENNL	tr_C9ZJU1	0.097	17588.09	24.00
106	HLA-A0201	CSIRDILLDDGVA	SIRDILLGV	1	0	0	7	2	SIRDILLDDGV	tr_C9ZJU1	0.096	17608.27	24.00
651	HLA-A0201	HAFLGSFYDECG	FLGSFYDEC	2	0	0	0	0	FLGSFYDEC	tr_C9ZJU1	0.096	17612.85	24.00
265	HLA-A0201	AVASTFAFESLY	AVASTFAFL	0	0	0	8	2	AVASTFAFESL	tr_C9ZJU1	0.096	17618.95	24.00
323	HLA-A0201	LWQASGSVMDEL	WQASVMDEL	1	0	0	3	2	WQASGSVMDEL	tr_C9ZJU1	0.096	17634.77	24.00
725	HLA-A0201	FRYSDRHPYVLRRV	FRYYVLRRV	0	0	0	3	5	FRYSDRHPYVLRRV	tr_C9ZJU1	0.096	17663.81	24.00
925	HLA-A0201	LATLKHADKIVV	TLHADKIVV	2	0	0	2	1	TLKHADKIVV	tr_C9ZJU1	0.096	17677.56	24.00
13	HLA-A0201	ERKAYEILEMLTR	KAYEILEML	2	0	0	0	0	KAYEILEML	tr_C9ZJU1	0.096	17677.76	24.00
462	HLA-A0201	KSSVQYGLV	KSSVQYGLV	0	0	0	0	0	KSSVQYGLV	tr_C9ZJU1	0.096	17688.28	24.00
550	HLA-A0201	CVWQCRQDYLQ	CVWQCQDYL	0	0	0	5	1	CVWQCRQDYL	tr_C9ZJU1	0.096	17691.16	24.00
289	HLA-A0201	DIITSIHIDA	IITSIHIDA	1	0	0	0	0	IITSIHIDA	tr_C9ZJU1	0.096	17702.44	24.00
195	HLA-A0201	PQLVTLAAT	PQLVTLAAT	0	0	0	0	0	PQLVTLAAT	tr_C9ZJU1	0.096	17708.20	24.00
621	HLA-A0201	LSPNFFTQSA	LSPNFFTSA	0	0	0	7	1	LSPNFFTQSA	tr_C9ZJU1	0.096	17723.14	24.00
551	HLA-A0201	VWQCRQDYLQGFAA	WQYLQGFAA	1	0	0	2	4	WQCRQDYLQGFAA	tr_C9ZJU1	0.096	17741.56	24.00
873	HLA-A0201	FVKAGSAVGLC	FVAGSAVGL	0	0	0	2	1	FVKAGSAVGL	tr_C9ZJU1	0.096	17745.59	24.00
964	HLA-A0201	RQSQQLPHFVK	RQSQQLPFV	0	0	0	7	1	RQSQQLPHFV	tr_C9ZJU1	0.096	17746.94	24.00
922	HLA-A0201	AHRLATLKHADKI	RLATLKHAI	2	0	0	8	2	RLATLKHADKI	tr_C9ZJU1	0.096	17756.16	24.00
502	HLA-A0201	VFSGCVVDALKRKL	FSGCVVDAL	1	0	0	8	4	FSGCVVDALKRKL	tr_C9ZJU1	0.096	17760.97	24.00
156	HLA-A0201	VQEFPEELYLGL	VQEELYLGL	0	0	0	2	3	VQEFPEELYLGL	tr_C9ZJU1	0.096	17771.15	24.00
134	HLA-A0201	CMLLRGKYS	CMLLRGKYS	0	0	0	0	0	CMLLRGKYS	tr_C9ZJU1	0.096	17771.53	24.00
283	HLA-A0201	IQSILSDIITS	IQSILIITS	0	0	0	5	2	IQSILSDIITS	tr_C9ZJU1	0.095	17796.94	24.00
613	HLA-A0201	LVSAVALRLSPNFF	LVSAVALFF	0	0	0	7	5	LVSAVALRLSPNFF	tr_C9ZJU1	0.095	17805.41	24.00
115	HLA-A0201	DGVALCILDDVKAE	GVALCILDV	1	0	0	7	1	GVALCILDDV	tr_C9ZJU1	0.095	17830.47	24.00
523	HLA-A0201	AVSVRRLGWIKDV	AVLGWIKDV	0	0	0	2	4	AVSVRRLGWIKDV	tr_C9ZJU1	0.095	17841.66	24.00
880	HLA-A0201	VGLCIFDEPTS	VLCIFDEPT	0	0	0	1	1	VGLCIFDEPT	tr_C9ZJU1	0.095	17850.74	24.00
619	HLA-A0201	LRLSPNFFTQS	RLSPNFFTS	1	0	0	8	1	RLSPNFFTQS	tr_C9ZJU1	0.095	17851.52	24.00
188	HLA-A0201	MCSSCSIPQLVT	MCSSCSIPV	0	0	0	8	2	MCSSCSIPQLV	tr_C9ZJU1	0.095	17853.06	24.00
841	HLA-A0201	NFVQKRSSGI	FVQKRSSGI	1	0	0	0	0	FVQKRSSGI	tr_C9ZJU1	0.095	17869.88	24.00
650	HLA-A0201	YHAFLGSFYDECG	FLGSFYDEC	3	0	0	0	0	FLGSFYDEC	tr_C9ZJU1	0.095	17898.51	24.00
348	HLA-A0201	CRVVFSFPVVS	CRVVFSFPV	0	0	0	8	1	CRVVFSFPVV	tr_C9ZJU1	0.095	17920.41	25.00
745	HLA-A0201	GEFLGIAGYSGS	FLIAGYSGS	2	0	0	2	1	FLGIAGYSGS	tr_C9ZJU1	0.095	17927.00	25.00
113	HLA-A0201	LDDGVALCI	LDDGVALCI	0	0	0	0	0	LDDGVALCI	tr_C9ZJU1	0.095	17940.00	25.00
190	HLA-A0201	SSCSIPQLVTLA	SIPQLVTLA	0	0	0	1	3	SSCSIPQLVTLA	tr_C9ZJU1	0.095	17952.23	25.00
843	HLA-A0201	VQKRSSGIHAPL	VQSGIHAPL	0	0	0	2	3	VQKRSSGIHAPL	tr_C9ZJU1	0.095	17952.81	25.00
646	HLA-A0201	WCPLYHAFLGSF	WLYHAFLGS	0	0	0	1	2	WCPLYHAFLGS	tr_C9ZJU1	0.095	17959.23	25.00
258	HLA-A0201	PATFVSMAVAST	FVSMAVAST	3	0	0	0	0	FVSMAVAST	tr_C9ZJU1	0.094	17990.74	25.00
709	HLA-A0201	PPSLDGPWSV	PSLDGPWSV	0	0	0	1	1	PPSLDGPWSV	tr_C9ZJU1	0.094	18000.66	25.00
811	HLA-A0201	LSIGGNIALG	LSIGGNIAL	0	0	0	0	0	LSIGGNIAL	tr_C9ZJU1	0.094	18005.14	25.00
761	HLA-A0201	LLRLLNRTYAPTE	LLRLLYAPT	0	0	0	5	3	LLRLLNRTYAPT	tr_C9ZJU1	0.094	18010.99	25.00
436	HLA-A0201	GIKCMLSST	GIKCMLSST	0	0	0	0	0	GIKCMLSST	tr_C9ZJU1	0.094	18018.99	25.00
674	HLA-A0201	RRHLAFSGSSL	RLAFSGSSL	0	0	0	1	2	RRHLAFSGSSL	tr_C9ZJU1	0.094	18030.48	25.00
359	HLA-A0201	LVMWLGTQWSN	LMWLGTQWS	0	0	0	1	1	LVMWLGTQWS	tr_C9ZJU1	0.094	18032.05	25.00
515	HLA-A0201	LQAGGHTCAVSVRR	LQAGCAVSV	0	0	0	4	3	LQAGGHTCAVSV	tr_C9ZJU1	0.094	18040.84	25.00

562	HLA-A0201	FAALRLSGW	FAALRLSGW	0	0	0	0	0	FAALRLSGW	tr_C9ZJU1	0.094	18050.01	25.00
116	HLA-A0201	GVALCILDDVKAEE	GVALCILDV	0	0	0	7	1	GVALCILDDV	tr_C9ZJU1	0.094	18073.27	25.00
559	HLA-A0201	LQGFAALRLS	LQFAALRLS	0	0	0	2	1	LQGFAALRLS	tr_C9ZJU1	0.094	18077.37	25.00
893	HLA-A0201	DTITEGEIFDTLRL	TITEGEIFL	1	0	0	8	4	TITEGEIFDTLRL	tr_C9ZJU1	0.094	18089.30	25.00
191	HLA-A0201	SCSIPQLVTLA	SIPQLVTLA	0	0	0	1	2	SCSIPQLVTLA	tr_C9ZJU1	0.094	18111.83	25.00
45	HLA-A0201	SIAIFLNCN	SIAIFLNCN	0	0	0	0	0	SIAIFLNCN	tr_C9ZJU1	0.094	18112.03	25.00
257	HLA-A0201	LPATFVSMAVAST	FVSMAVAST	4	0	0	0	0	FVSMAVAST	tr_C9ZJU1	0.094	18133.21	25.00
753	HLA-A0201	YSGSGKTTLLRL	YSGSTLLRL	0	0	0	4	3	YSGSGKTTLLRL	tr_C9ZJU1	0.094	18153.61	25.00
921	HLA-A0201	VAHRLATLKHADKI	RLATLKHAI	3	0	0	8	2	RLATLKHADKI	tr_C9ZJU1	0.094	18172.68	25.00
649	HLA-A0201	LYHAFLGSFYDECG	FLGSFYDEC	4	0	0	0	0	FLGSFYDEC	tr_C9ZJU1	0.094	18176.22	25.00
246	HLA-A0201	PFRFIIKQLM	FRFIIKQLM	1	0	0	0	0	FRFIIKQLM	tr_C9ZJU1	0.093	18184.48	25.00
105	HLA-A0201	NCSIRDILLDDGVA	SIRDILLGV	2	0	0	7	2	SIRDILLDDGV	tr_C9ZJU1	0.093	18184.68	25.00
960	HLA-A0201	FSRMRQSQQL	FSMRQSQQL	0	0	0	2	1	FSRMRQSQQL	tr_C9ZJU1	0.093	18190.19	25.00
907	HLA-A0201	REGNNLLRDSTII	RLLRDSTII	0	0	0	1	4	REGNNLLRDSTII	tr_C9ZJU1	0.093	18201.21	25.00
438	HLA-A0201	KCMLSSTDNKM	MLSSTDNKM	2	0	0	0	0	MLSSTDNKM	tr_C9ZJU1	0.093	18204.17	25.00
201	HLA-A0201	AATVLFSPEVI	ATVLFSPEV	0	0	0	1	1	AATVLFSPEV	tr_C9ZJU1	0.093	18210.07	25.00
118	HLA-A0201	ALCILDDVK	ALCILDDVK	0	0	0	0	0	ALCILDDVK	tr_C9ZJU1	0.093	18220.13	25.00
679	HLA-A0201	FSGSSLGCYQVLE	FSSLGCYQV	0	0	0	1	2	FSGSSLGCYQV	tr_C9ZJU1	0.093	18227.43	25.00
277	HLA-A0201	CIRGFVIQSILSD	CIRGFVIIL	0	0	0	7	2	CIRGFVIQSIL	tr_C9ZJU1	0.093	18232.35	25.00
113	HLA-A0201	LDDGVALCIL	LDDGVLCIL	0	0	0	5	1	LDDGVALCIL	tr_C9ZJU1	0.093	18241.03	25.00
964	HLA-A0201	RQSQQLPHF	RQSQQLPHF	0	0	0	0	0	RQSQQLPHF	tr_C9ZJU1	0.093	18249.53	25.00
142	HLA-A0201	SIARYPPAAQ	SIARYPPAA	0	0	0	0	0	SIARYPPAA	tr_C9ZJU1	0.093	18260.78	25.00
933	HLA-A0201	KIVVLNEGMVA	KIVVLNEMV	0	0	0	7	1	KIVVLNEGMV	tr_C9ZJU1	0.093	18263.55	25.00
926	HLA-A0201	ATLKHADKI	ATLKHADKI	0	0	0	0	0	ATLKHADKI	tr_C9ZJU1	0.093	18269.88	25.00
222	HLA-A0201	VHWRPIYAA	VHWRPIYAA	0	0	0	0	0	VHWRPIYAA	tr_C9ZJU1	0.093	18276.00	25.00
296	HLA-A0201	IDAYLALATADYD	IAYLALATA	0	0	0	1	1	IDAYLALATA	tr_C9ZJU1	0.093	18288.06	25.00
806	HLA-A0201	RFIEDLSIGG	FIEDLSIGG	1	0	0	0	0	FIEDLSIGG	tr_C9ZJU1	0.093	18289.06	25.00
58	HLA-A0201	IHVRTLVFNHNI	ITLVFNHNI	0	0	0	1	3	IHVRTLVFNHNI	tr_C9ZJU1	0.093	18290.64	25.00
895	HLA-A0201	ITEGEIFDT	ITEGEIFDT	0	0	0	0	0	ITEGEIFDT	tr_C9ZJU1	0.093	18306.29	25.00
744	HLA-A0201	KGEFLGIAGYSGS	FLIAGYSGS	3	0	0	2	1	FLGIAGYSGS	tr_C9ZJU1	0.093	18356.07	25.00
796	HLA-A0201	ANVWQEENNLRFI	NVWNNLRFI	1	0	0	3	3	NVWQEENNLRFI	tr_C9ZJU1	0.093	18364.01	25.00
808	HLA-A0201	IEDLSIGGNIAL	DLIGGNIAL	2	0	0	2	1	DLSIGGNIAL	tr_C9ZJU1	0.093	18370.57	25.00
74	HLA-A0201	SVFAVHCGLMKN	SVFAVCGLM	0	0	0	5	1	SVFAVHCGLM	tr_C9ZJU1	0.093	18370.96	25.00
377	HLA-A0201	SLYMESQFTKS	SLYMESQFS	0	0	0	8	2	SLYMESQFTKS	tr_C9ZJU1	0.092	18380.11	25.00
363	HLA-A0201	LGTQWSNFL	LGTQWSNFL	0	0	0	0	0	LGTQWSNFL	tr_C9ZJU1	0.092	18380.11	25.00
104	HLA-A0201	VNCSIRDILL	VCSIRDILL	0	0	0	1	1	VNCSIRDILL	tr_C9ZJU1	0.092	18382.90	25.00
523	HLA-A0201	AVSVRRLGWI	AVSVRLGWI	0	0	0	4	1	AVSVRRLGWI	tr_C9ZJU1	0.092	18385.69	25.00
855	HLA-A0201	NVREFSGGEV	NVREFSGEV	0	0	0	6	1	NVREFSGGEV	tr_C9ZJU1	0.092	18391.85	25.00
655	HLA-A0201	GSFYDECGM	GSFYDECGM	0	0	0	0	0	GSFYDECGM	tr_C9ZJU1	0.092	18401.20	25.00
650	HLA-A0201	YHAFLGSFYD	YAFLGSFYD	0	0	0	1	1	YHAFLGSFYD	tr_C9ZJU1	0.092	18401.81	25.00
278	HLA-A0201	IRGFVIQSIL	IGFVIQSIL	0	0	0	1	1	IRGFVIQSIL	tr_C9ZJU1	0.092	18458.43	25.00
738	HLA-A0201	VSFVVNKGEFLG	VSFVVGEFL	0	0	0	5	2	VSFVVNKGEFL	tr_C9ZJU1	0.092	18469.42	25.00
768	HLA-A0201	TYAPTEGDIFVNG	YAPEGDIFV	1	0	0	3	1	YAPTEGDIFV	tr_C9ZJU1	0.092	18480.62	25.00
479	HLA-A0201	SGRPLGYMWDLLTQ	SLGYMWDLL	0	0	0	1	3	SGRPLGYMWDLL	tr_C9ZJU1	0.092	18488.42	25.00
795	HLA-A0201	IANVWQEENNLRFI	NVWNNLRFI	2	0	0	3	3	NVWQEENNLRFI	tr_C9ZJU1	0.092	18494.41	25.00
108	HLA-A0201	IRDILLDDGV	IRDILLDGV	0	0	0	6	1	IRDILLDDGV	tr_C9ZJU1	0.092	18496.62	25.00
57	HLA-A0201	SIHVRTLVFNHNIM	SLVFNHNIM	0	0	0	1	5	SIHVRTLVFNHNIM	tr_C9ZJU1	0.092	18502.42	25.00
73	HLA-A0201	PSVFAVHCGLMK	SVFAVCGLM	1	0	0	5	1	SVFAVHCGLM	tr_C9ZJU1	0.092	18506.43	25.00
642	HLA-A0201	SIQHWCPLY	SIQHWCPLY	0	0	0	0	0	SIQHWCPLY	tr_C9ZJU1	0.092	18531.88	25.00
550	HLA-A0201	CVWQCRQDYLQGFA	CVWYLQGFA	0	0	0	3	5	CVWQCRQDYLQGFA	tr_C9ZJU1	0.092	18533.28	25.00
156	HLA-A0201	VQEFPEELYLGLI	VQELYLGLI	0	0	0	2	4	VQEFPEELYLGLI	tr_C9ZJU1	0.092	18566.00	25.00
245	HLA-A0201	SPFRFIIKQLM	SPFRFIILM	0	0	0	7	2	SPFRFIIKQLM	tr_C9ZJU1	0.092	18571.22	25.00
550	HLA-A0201	CVWQCRQDYLQGF	CVWQCLQGF	0	0	0	5	4	CVWQCRQDYLQGF	tr_C9ZJU1	0.092	18576.44	25.00
915	HLA-A0201	DSTIIIVAHR	STIIIVAHR	1	0	0	0	0	STIIIVAHR	tr_C9ZJU1	0.092	18577.65	25.00

567	HLA-A0201	LSGWELMAATA	SGWELMATA	1	0	0	6	1	SGWELMAATA	tr_C9ZJU1	0.091	18584.28	25.00
895	HLA-A0201	ITEGEIFDTLRL	ITIFDTLRL	0	0	0	2	3	ITEGEIFDTLRL	tr_C9ZJU1	0.091	18588.10	25.00
189	HLA-A0201	CSSCSIPQLVTLA	SIPQLVTLA	4	0	0	0	0	SIPQLVTLA	tr_C9ZJU1	0.091	18598.96	25.00
568	HLA-A0201	SGWELMAATAA	SGWELMAAA	0	0	0	7	2	SGWELMAATAA	tr_C9ZJU1	0.091	18606.82	25.00
743	HLA-A0201	NKGEFLGIAGYSGS	FLIAGYSGS	4	0	0	2	1	FLGIAGYSGS	tr_C9ZJU1	0.091	18609.03	25.00
72	HLA-A0201	LPSVFAVHCGLMK	SVFAVCGLM	2	0	0	5	1	SVFAVHCGLM	tr_C9ZJU1	0.091	18620.71	25.00
264	HLA-A0201	MAVASTFAFESLY	AVASTFAFL	1	0	0	8	2	AVASTFAFESL	tr_C9ZJU1	0.091	18633.61	25.00
296	HLA-A0201	IDAYLALATADY	IAYLALATA	0	0	0	1	1	IDAYLALATA	tr_C9ZJU1	0.091	18641.08	25.00
822	HLA-A0201	LWDCSDENICAAL	LWDCICAAL	0	0	0	4	4	LWDCSDENICAAL	tr_C9ZJU1	0.091	18667.11	25.00
792	HLA-A0201	RRRIANVWQEENNL	RIANVWQEL	2	0	0	8	3	RIANVWQEENNL	tr_C9ZJU1	0.091	18674.79	26.00
65	HLA-A0201	FNHNIMQLPSVF	FIMQLPSVF	0	0	0	1	3	FNHNIMQLPSVF	tr_C9ZJU1	0.091	18677.61	26.00
124	HLA-A0201	DVKAEEVLAL	DVAEEVLAL	0	0	0	2	1	DVKAEEVLAL	tr_C9ZJU1	0.091	18680.65	26.00
876	HLA-A0201	AGSAVGLCI	AGSAVGLCI	0	0	0	0	0	AGSAVGLCI	tr_C9ZJU1	0.091	18695.21	26.00
809	HLA-A0201	EDLSIGGNIAL	DLIGGNIAL	1	0	0	2	1	DLSIGGNIAL	tr_C9ZJU1	0.091	18697.63	26.00
481	HLA-A0201	RPLGYMWDLLTQ	RLGYMWDLL	0	0	0	1	1	RPLGYMWDLL	tr_C9ZJU1	0.091	18714.64	26.00
75	HLA-A0201	VFAVHCGLM	VFAVHCGLM	0	0	0	0	0	VFAVHCGLM	tr_C9ZJU1	0.091	18757.41	26.00
480	HLA-A0201	GRPLGYMWDL	RPLGYMWDL	1	0	0	0	0	RPLGYMWDL	tr_C9ZJU1	0.091	18767.36	26.00
593	HLA-A0201	QCGRDVLHGL	QCGDVLHGL	0	0	0	3	1	QCGRDVLHGL	tr_C9ZJU1	0.091	18770.00	26.00
437	HLA-A0201	IKCMLSSTDNKM	MLSSTDNKM	3	0	0	0	0	MLSSTDNKM	tr_C9ZJU1	0.091	18771.62	26.00
527	HLA-A0201	RRLGWIKDVIQSS	RLGWVIQSS	1	0	0	4	3	RLGWIKDVIQSS	tr_C9ZJU1	0.091	18776.09	26.00
763	HLA-A0201	RLLNRTYAPTEG	RLLNRTYPT	0	0	0	7	1	RLLNRTYAPT	tr_C9ZJU1	0.091	18779.75	26.00
830	HLA-A0201	ICAALSASRA	ICAALSASA	0	0	0	8	1	ICAALSASRA	tr_C9ZJU1	0.090	18797.23	26.00
391	HLA-A0201	SVTNIATSTYA	SVIATSTYA	0	0	0	2	2	SVTNIATSTYA	tr_C9ZJU1	0.090	18798.04	26.00
554	HLA-A0201	CRQDYLQGFAA	RQDYLQFAA	1	0	0	6	1	RQDYLQGFAA	tr_C9ZJU1	0.090	18805.78	26.00
531	HLA-A0201	WIKDVIQSS	WIKDVIQSS	0	0	0	0	0	WIKDVIQSS	tr_C9ZJU1	0.090	18818.19	26.00
421	HLA-A0201	LTSSPLTLLR	LTSSPLTLL	0	0	0	0	0	LTSSPLTLL	tr_C9ZJU1	0.090	18819.21	26.00
369	HLA-A0201	NFLYFLNES	NFLYFLNES	0	0	0	0	0	NFLYFLNES	tr_C9ZJU1	0.090	18828.78	26.00
315	HLA-A0201	LSRCVSQALWQA	LVSQALWQA	0	0	0	1	3	LSRCVSQALWQA	tr_C9ZJU1	0.090	18831.23	26.00
645	HLA-A0201	HWCPLYHAFLGSF	WLYHAFLGS	1	0	0	1	2	WCPLYHAFLGS	tr_C9ZJU1	0.090	18837.95	26.00
63	HLA-A0201	LVFNHNIMQ	LVFNHNIMQ	0	0	0	0	0	LVFNHNIMQ	tr_C9ZJU1	0.090	18852.63	26.00
711	HLA-A0201	SLDGPWSVEFR	SLDGWSVEF	0	0	0	4	1	SLDGPWSVEF	tr_C9ZJU1	0.090	18868.55	26.00
263	HLA-A0201	SMAVASTFAFE	SMVASTFAF	0	0	0	2	1	SMAVASTFAF	tr_C9ZJU1	0.090	18883.86	26.00
257	HLA-A0201	LPATFVSMA	LPATFVSMA	0	0	0	0	0	LPATFVSMA	tr_C9ZJU1	0.090	18938.29	26.00
483	HLA-A0201	LGYMWDLLTQ	LGYMWLLTQ	0	0	0	5	1	LGYMWDLLTQ	tr_C9ZJU1	0.090	18943.83	26.00
762	HLA-A0201	LRLLNRTYAPTE	RLLNRTYPT	1	0	0	7	1	RLLNRTYAPT	tr_C9ZJU1	0.090	18959.83	26.00
929	HLA-A0201	KHADKIVVL	KHADKIVVL	0	0	0	0	0	KHADKIVVL	tr_C9ZJU1	0.090	18984.86	26.00
772	HLA-A0201	TEGDIFVNGV	TGDIFVNGV	0	0	0	1	1	TEGDIFVNGV	tr_C9ZJU1	0.089	18986.71	26.00
688	HLA-A0201	QVLEGLQSVQR	QVLEGLQSV	0	0	0	0	0	QVLEGLQSV	tr_C9ZJU1	0.089	19008.50	26.00
556	HLA-A0201	QDYLQGFAAL	QDYLGFAAL	0	0	0	4	1	QDYLQGFAAL	tr_C9ZJU1	0.089	19016.11	26.00
755	HLA-A0201	GSGKTTLLRL	GSGTTLLRL	0	0	0	3	1	GSGKTTLLRL	tr_C9ZJU1	0.089	19028.25	26.00
772	HLA-A0201	TEGDIFVNGVHI	TIFVNGVHI	0	0	0	1	3	TEGDIFVNGVHI	tr_C9ZJU1	0.089	19047.21	26.00
318	HLA-A0201	CVSQALWQAS	CVSQALWQA	0	0	0	0	0	CVSQALWQA	tr_C9ZJU1	0.089	19047.21	26.00
436	HLA-A0201	GIKCMLSSTDNKM	MLSSTDNKM	4	0	0	0	0	MLSSTDNKM	tr_C9ZJU1	0.089	19050.30	26.00
498	HLA-A0201	LVPSVFSGCVVD	LVPSVFCVV	0	0	0	6	2	LVPSVFSGCVV	tr_C9ZJU1	0.089	19050.71	26.00
81	HLA-A0201	GLMKNSHPD	GLMKNSHPD	0	0	0	0	0	GLMKNSHPD	tr_C9ZJU1	0.089	19060.20	26.00
428	HLA-A0201	LLRKQKDDGI	LLRQKDDGI	0	0	0	3	1	LLRKQKDDGI	tr_C9ZJU1	0.089	19073.60	26.00
486	HLA-A0201	MWDLLTQHVIWP	MLTQHVIWP	0	0	0	1	3	MWDLLTQHVIWP	tr_C9ZJU1	0.089	19077.12	26.00
118	HLA-A0201	ALCILDDVKAE	ALCILDVKA	0	0	0	5	1	ALCILDDVKA	tr_C9ZJU1	0.089	19081.66	26.00
176	HLA-A0201	SLQSGITSFWK	SLGITSFWK	0	0	0	2	2	SLQSGITSFWK	tr_C9ZJU1	0.089	19090.74	26.00
513	HLA-A0201	RKLQAGGHTCA	KLAGGHTCA	1	0	0	2	1	KLQAGGHTCA	tr_C9ZJU1	0.089	19119.68	26.00
616	HLA-A0201	AVALRLSPNF	AVALRLSPF	0	0	0	8	1	AVALRLSPNF	tr_C9ZJU1	0.089	19135.41	26.00
711	HLA-A0201	SLDGPWSVEFRE	SLWSVEFRE	0	0	0	2	3	SLDGPWSVEFRE	tr_C9ZJU1	0.089	19140.18	26.00
58	HLA-A0201	IHVRTLVFNHNIM	ILVFNHNIM	0	0	0	1	4	IHVRTLVFNHNIM	tr_C9ZJU1	0.089	19169.18	26.00
798	HLA-A0201	VWQEENNLRFIEDL	WQLRFIEDL	1	0	0	2	4	WQEENNLRFIEDL	tr_C9ZJU1	0.089	19188.49	26.00

573	HLA-A0201	MAATAAAAF	MAATAAAAF	0	0	0	0	0	MAATAAAAF	tr_C9ZJU1	0.088	19195.75	26.00
644	HLA-A0201	QHWCPLYHAFLGSF	WLYHAFLGS	2	0	0	1	2	WCPLYHAFLGS	tr_C9ZJU1	0.088	19205.51	26.00
244	HLA-A0201	LSPFRFIIKQLM	SPFRFIILM	1	0	0	7	2	SPFRFIIKQLM	tr_C9ZJU1	0.088	19206.35	26.00
647	HLA-A0201	CPLYHAFLGSF	CLYHAFLGS	0	0	0	1	1	CPLYHAFLGS	tr_C9ZJU1	0.088	19208.63	26.00
779	HLA-A0201	NGVHISQYPA	GVHISQYPA	1	0	0	0	0	GVHISQYPA	tr_C9ZJU1	0.088	19218.20	26.00
435	HLA-A0201	DGIKCMLSSTDNKM	MLSSTDNKM	5	0	0	0	0	MLSSTDNKM	tr_C9ZJU1	0.088	19223.18	26.00
859	HLA-A0201	FSGGEVERLCI	FSGGEVLCI	0	0	0	6	2	FSGGEVERLCI	tr_C9ZJU1	0.088	19235.26	26.00
672	HLA-A0201	HLRRHLAFSG	HLRHLAFSG	0	0	0	2	1	HLRRHLAFSG	tr_C9ZJU1	0.088	19235.26	26.00
492	HLA-A0201	QHVIWPLVPS	HVIWPLVPS	1	0	0	0	0	HVIWPLVPS	tr_C9ZJU1	0.088	19240.46	26.00
682	HLA-A0201	SSLGCYQVLEG	SLGCYVLEG	1	0	0	5	1	SLGCYQVLEG	tr_C9ZJU1	0.088	19254.41	26.00
212	HLA-A0201	LKCILGMHAF	LCILGMHAF	0	0	0	1	1	LKCILGMHAF	tr_C9ZJU1	0.088	19255.24	26.00
117	HLA-A0201	VALCILDDVKAE	VALCILVKA	0	0	0	6	2	VALCILDDVKA	tr_C9ZJU1	0.088	19257.54	26.00
525	HLA-A0201	SVRRLGWIKDVI	SLGWIKDVI	0	0	0	1	3	SVRRLGWIKDVI	tr_C9ZJU1	0.088	19257.95	26.00
522	HLA-A0201	CAVSVRRLGWIKDV	AVLGWIKDV	1	0	0	2	4	AVSVRRLGWIKDV	tr_C9ZJU1	0.088	19258.16	26.00
708	HLA-A0201	APPSLDGPWSVEFR	ASLDGPWSV	0	0	0	1	2	APPSLDGPWSV	tr_C9ZJU1	0.088	19268.37	26.00
446	HLA-A0201	NKMLFQQTFSH	MLFQQTFSH	2	0	0	0	0	MLFQQTFSH	tr_C9ZJU1	0.088	19268.58	26.00
76	HLA-A0201	FAVHCGLMK	FAVHCGLMK	0	0	0	0	0	FAVHCGLMK	tr_C9ZJU1	0.088	19289.23	26.00
209	HLA-A0201	EVILKCILG	EVILKCILG	0	0	0	0	0	EVILKCILG	tr_C9ZJU1	0.088	19295.71	26.00
940	HLA-A0201	GMVAEVGSW	GMVAEVGSW	0	0	0	0	0	GMVAEVGSW	tr_C9ZJU1	0.088	19297.58	26.00
510	HLA-A0201	ALKRKLQAGGHTCA	ALAGGHTCA	0	0	0	2	5	ALKRKLQAGGHTCA	tr_C9ZJU1	0.088	19297.79	26.00
773	HLA-A0201	EGDIFVNGVHI	GIFVNGVHI	1	0	0	1	1	GDIFVNGVHI	tr_C9ZJU1	0.088	19305.53	26.00
54	HLA-A0201	GVESIHVRTLV	GVIHVRTLV	0	0	0	2	2	GVESIHVRTLV	tr_C9ZJU1	0.088	19310.74	26.00
184	HLA-A0201	FWKLMCSSC	FWKLMCSSC	0	0	0	0	0	FWKLMCSSC	tr_C9ZJU1	0.088	19313.88	26.00
877	HLA-A0201	GSAVGLCIF	GSAVGLCIF	0	0	0	0	0	GSAVGLCIF	tr_C9ZJU1	0.088	19320.57	26.00
804	HLA-A0201	NLRFIEDLS	NLRFIEDLS	0	0	0	0	0	NLRFIEDLS	tr_C9ZJU1	0.088	19327.46	26.00
859	HLA-A0201	FSGGEVERLCIA	FSVERLCIA	0	0	0	2	3	FSGGEVERLCIA	tr_C9ZJU1	0.088	19327.68	26.00
889	HLA-A0201	TSAIDTITEGEI	AIDTITEEI	2	0	0	7	1	AIDTITEGEI	tr_C9ZJU1	0.088	19345.03	26.00
773	HLA-A0201	EGDIFVNGV	EGDIFVNGV	0	0	0	0	0	EGDIFVNGV	tr_C9ZJU1	0.088	19345.67	26.00
879	HLA-A0201	AVGLCIFDE	AVGLCIFDE	0	0	0	0	0	AVGLCIFDE	tr_C9ZJU1	0.088	19374.15	26.00
846	HLA-A0201	RSSGIHAPLNV	RSSGIPLNV	0	0	0	5	2	RSSGIHAPLNV	tr_C9ZJU1	0.088	19375.62	26.00
661	HLA-A0201	CGMSEAQVDEAHLR	GMSEAQVHL	1	0	0	7	3	GMSEAQVDEAHL	tr_C9ZJU1	0.088	19399.74	27.00
22	HLA-A0201	MLTRATIKTD	MLTRATIKT	0	0	0	0	0	MLTRATIKT	tr_C9ZJU1	0.087	19401.01	27.00
74	HLA-A0201	SVFAVHCGLMKNS	SVFAVCGLM	0	0	0	5	1	SVFAVHCGLM	tr_C9ZJU1	0.087	19414.02	27.00
524	HLA-A0201	VSVRRLGWIKDV	SVLGWIKDV	1	0	0	2	2	SVRRLGWIKDV	tr_C9ZJU1	0.087	19440.51	27.00
549	HLA-A0201	TCVWQCRQDYLQ	CVWQCQDYL	1	0	0	5	1	CVWQCRQDYL	tr_C9ZJU1	0.087	19469.98	27.00
282	HLA-A0201	VIQSILSDIITS	IQSILIITS	1	0	0	5	2	IQSILSDIITS	tr_C9ZJU1	0.087	19471.45	27.00
898	HLA-A0201	GEIFDTLRL	GEIFDTLRL	0	0	0	0	0	GEIFDTLRL	tr_C9ZJU1	0.087	19479.04	27.00
480	HLA-A0201	GRPLGYMWDLLTQ	GLGYMWDLL	0	0	0	1	2	GRPLGYMWDLL	tr_C9ZJU1	0.087	19481.99	27.00
136	HLA-A0201	LLRGKYSIARYP	LLYSIARYP	0	0	0	2	3	LLRGKYSIARYP	tr_C9ZJU1	0.087	19509.84	27.00
17	HLA-A0201	YEILEMLTR	YEILEMLTR	0	0	0	0	0	YEILEMLTR	tr_C9ZJU1	0.087	19518.07	27.00
180	HLA-A0201	GITSFWKLMCS	GIFWKLMCS	0	0	0	2	2	GITSFWKLMCS	tr_C9ZJU1	0.087	19535.18	27.00
812	HLA-A0201	SIGGNIALGNLW	SIGGNIALL	0	0	0	8	2	SIGGNIALGNL	tr_C9ZJU1	0.087	19547.44	27.00
166	HLA-A0201	GLITAYNRKRSLQ	GLITAYNSL	0	0	0	7	3	GLITAYNRKRSL	tr_C9ZJU1	0.087	19550.62	27.00
726	HLA-A0201	RYSDRHPYVLRRV	YSDYVLRRV	1	0	0	3	3	YSDRHPYVLRRV	tr_C9ZJU1	0.087	19553.16	27.00
909	HLA-A0201	GNNLLRDSTII	GLLRDSTII	0	0	0	1	2	GNNLLRDSTII	tr_C9ZJU1	0.087	19555.49	27.00
839	HLA-A0201	ALNFVQKRSSGIH	ALNFVSSGI	0	0	0	5	3	ALNFVQKRSSGI	tr_C9ZJU1	0.087	19573.90	27.00
445	HLA-A0201	DNKMLFQQTFSH	MLFQQTFSH	3	0	0	0	0	MLFQQTFSH	tr_C9ZJU1	0.087	19605.27	27.00
553	HLA-A0201	QCRQDYLQGFAA	RQDYLQFAA	2	0	0	6	1	RQDYLQGFAA	tr_C9ZJU1	0.086	19639.24	27.00
629	HLA-A0201	SALDQTEIVN	SALDQTEIV	0	0	0	0	0	SALDQTEIV	tr_C9ZJU1	0.086	19657.73	27.00
57	HLA-A0201	SIHVRTLVFN	SIHVRTLVF	0	0	0	0	0	SIHVRTLVF	tr_C9ZJU1	0.086	19664.76	27.00
914	HLA-A0201	RDSTIIIVA	RDSTIIIVA	0	0	0	0	0	RDSTIIIVA	tr_C9ZJU1	0.086	19678.17	27.00
491	HLA-A0201	TQHVIWPLVPS	TQHVIWPLV	0	0	0	0	0	TQHVIWPLV	tr_C9ZJU1	0.086	19683.28	27.00
368	HLA-A0201	SNFLYFLNES	SNFLYFLES	0	0	0	7	1	SNFLYFLNES	tr_C9ZJU1	0.086	19683.92	27.00
301	HLA-A0201	ALATADYDP	ALATADYDP	0	0	0	0	0	ALATADYDP	tr_C9ZJU1	0.086	19686.90	27.00

515	HLA-A0201	LQAGGHTCAVS	LQAGGTCAV	0	0	0	5	1	LQAGGHTCAV	tr_C9ZJU1	0.086	19709.70	27.00
523	HLA-A0201	AVSVRRLGWIKDVI	ALGWIKDVI	0	0	0	1	5	AVSVRRLGWIKDVI	tr_C9ZJU1	0.086	19712.47	27.00
526	HLA-A0201	VRRLGWIKDVIQSS	RLGWVIQSS	2	0	0	4	3	RLGWIKDVIQSS	tr_C9ZJU1	0.086	19720.79	27.00
275	HLA-A0201	LYCIRGFVIQS	YCIGFVIQS	1	0	0	3	1	YCIRGFVIQS	tr_C9ZJU1	0.086	19735.52	27.00
413	HLA-A0201	CSFPVKNSLTSSPL	CSFLTSSPL	0	0	0	3	5	CSFPVKNSLTSSPL	tr_C9ZJU1	0.086	19747.06	27.00
136	HLA-A0201	LLRGKYSIAR	LLRGYSIAR	0	0	0	4	1	LLRGKYSIAR	tr_C9ZJU1	0.086	19751.34	27.00
932	HLA-A0201	DKIVVLNEGMVA	KIVVLNEMV	1	0	0	7	1	KIVVLNEGMV	tr_C9ZJU1	0.086	19754.53	27.00
835	HLA-A0201	SASRALNFVQ	SASRALNFV	0	0	0	0	0	SASRALNFV	tr_C9ZJU1	0.086	19756.02	27.00
761	HLA-A0201	LLRLLNRTYAPTEG	LLRLLYAPT	0	0	0	5	3	LLRLLNRTYAPT	tr_C9ZJU1	0.086	19765.01	27.00
444	HLA-A0201	TDNKMLFQQTFSH	MLFQQTFSH	4	0	0	0	0	MLFQQTFSH	tr_C9ZJU1	0.086	19823.27	27.00
131	HLA-A0201	LALCMLLRGKY	ALCMLLRGY	1	0	0	8	1	ALCMLLRGKY	tr_C9ZJU1	0.086	19824.13	27.00
550	HLA-A0201	CVWQCRQDYLQG	CVWQCQDYL	0	0	0	5	1	CVWQCRQDYL	tr_C9ZJU1	0.085	19846.44	27.00
388	HLA-A0201	QLASVTNIATST	QLASVTTST	0	0	0	6	3	QLASVTNIATST	tr_C9ZJU1	0.085	19863.63	27.00
476	HLA-A0201	FVESGRPLGYM	FVESGLGYM	0	0	0	5	2	FVESGRPLGYM	tr_C9ZJU1	0.085	19877.38	27.00
812	HLA-A0201	SIGGNIALGNLWDC	SILGNLWDC	0	0	0	2	5	SIGGNIALGNLWDC	tr_C9ZJU1	0.085	19882.33	27.00
299	HLA-A0201	YLALATADYDPSD	YLALATAPS	0	0	0	7	3	YLALATADYDPS	tr_C9ZJU1	0.085	19885.56	27.00
262	HLA-A0201	VSMAVASTFAFE	SMVASTFAF	1	0	0	2	1	SMAVASTFAF	tr_C9ZJU1	0.085	19898.69	27.00
71	HLA-A0201	QLPSVFAVHCG	QLSVFAVHC	0	0	0	2	1	QLPSVFAVHC	tr_C9ZJU1	0.085	19901.06	27.00
627	HLA-A0201	TQSALDQTEIV	TQSALTEIV	0	0	0	5	2	TQSALDQTEIV	tr_C9ZJU1	0.085	19912.69	27.00
316	HLA-A0201	SRCVSQALWQA	SVSQALWQA	0	0	0	1	2	SRCVSQALWQA	tr_C9ZJU1	0.085	19913.55	27.00
526	HLA-A0201	VRRLGWIKDVI	VLGWIKDVI	0	0	0	1	2	VRRLGWIKDVI	tr_C9ZJU1	0.085	19921.73	27.00
549	HLA-A0201	TCVWQCRQDYLQGF	CVWQCLQGF	1	0	0	5	4	CVWQCRQDYLQGF	tr_C9ZJU1	0.085	19940.28	27.00
899	HLA-A0201	EIFDTLRLREGNNL	EIFDTLRLL	0	0	0	8	5	EIFDTLRLREGNNL	tr_C9ZJU1	0.085	19940.94	27.00
443	HLA-A0201	STDNKMLFQQTFSH	MLFQQTFSH	5	0	0	0	0	MLFQQTFSH	tr_C9ZJU1	0.085	19948.49	27.00
74	HLA-A0201	SVFAVHCGLMKNSH	SVFAVCGLM	0	0	0	5	1	SVFAVHCGLM	tr_C9ZJU1	0.085	19954.54	27.00
926	HLA-A0201	ATLKHADKIV	ATLKHADIV	0	0	0	7	1	ATLKHADKIV	tr_C9ZJU1	0.085	19966.62	27.00
784	HLA-A0201	SQYPARMLRRRI	SQYPARMLI	0	0	0	8	3	SQYPARMLRRRI	tr_C9ZJU1	0.085	19969.43	27.00
329	HLA-A0201	SVMDELQSR	SVMDELQSR	0	0	0	0	0	SVMDELQSR	tr_C9ZJU1	0.085	19993.64	27.00
103	HLA-A0201	LVNCSIRDILLDD	LVNCSIILL	0	0	0	6	2	LVNCSIRDILL	tr_C9ZJU1	0.085	20018.33	27.00
280	HLA-A0201	GFVIQSILSD	FVIQSILSD	1	0	0	0	0	FVIQSILSD	tr_C9ZJU1	0.085	20021.79	27.00
699	HLA-A0201	KIDRPVRGVA	KIDRPVRGV	0	0	0	0	0	KIDRPVRGV	tr_C9ZJU1	0.084	20041.73	27.00
346	HLA-A0201	HGCRVVFSF	HGCRVVFSF	0	0	0	0	0	HGCRVVFSF	tr_C9ZJU1	0.084	20042.17	27.00
771	HLA-A0201	PTEGDIFVNGVHI	GIFVNGVHI	3	0	0	1	1	GDIFVNGVHI	tr_C9ZJU1	0.084	20044.77	27.00
182	HLA-A0201	TSFWKLMCSS	TSFWLMCSS	0	0	0	4	1	TSFWKLMCSS	tr_C9ZJU1	0.084	20055.62	27.00
758	HLA-A0201	KTTLLRLLNRTY	KLLRLLNRT	0	0	0	1	2	KTTLLRLLNRT	tr_C9ZJU1	0.084	20056.48	27.00
644	HLA-A0201	QHWCPLYHAFL	QHWCPLAFL	0	0	0	6	2	QHWCPLYHAFL	tr_C9ZJU1	0.084	20061.26	27.00
174	HLA-A0201	KRSLQSGITSF	KLQSGITSF	0	0	0	1	2	KRSLQSGITSF	tr_C9ZJU1	0.084	20061.91	27.00
86	HLA-A0201	SHPDHLLVCDIWN	SLLVCDIWN	0	0	0	1	4	SHPDHLLVCDIWN	tr_C9ZJU1	0.084	20064.52	27.00
565	HLA-A0201	LRLSGWELM	LRLSGWELM	0	0	0	0	0	LRLSGWELM	tr_C9ZJU1	0.084	20071.46	27.00
212	HLA-A0201	LKCILGMHA	LKCILGMHA	0	0	0	0	0	LKCILGMHA	tr_C9ZJU1	0.084	20088.85	28.00
747	HLA-A0201	FLGIAGYSGSG	FLGIAGYSG	0	0	0	7	2	FLGIAGYSGSG	tr_C9ZJU1	0.084	20090.14	28.00
35	HLA-A0201	SKQTECKDDASI	KQTECDASI	1	0	0	5	2	KQTECKDDASI	tr_C9ZJU1	0.084	20094.93	28.00
520	HLA-A0201	HTCAVSVRRLGWI	HTCAVSVWI	0	0	0	7	4	HTCAVSVRRLGWI	tr_C9ZJU1	0.084	20102.97	28.00
548	HLA-A0201	STCVWQCRQDYLQ	CVWQCQDYL	2	0	0	5	1	CVWQCRQDYL	tr_C9ZJU1	0.084	20133.23	28.00
574	HLA-A0201	AATAAAAFAT	ATAAAAFAT	0	0	0	1	1	AATAAAAFAT	tr_C9ZJU1	0.084	20157.64	28.00
251	HLA-A0201	IKQLMGLPA	IKQLMGLPA	0	0	0	0	0	IKQLMGLPA	tr_C9ZJU1	0.084	20165.28	28.00
279	HLA-A0201	RGFVIQSILS	RGFVISILS	0	0	0	5	1	RGFVIQSILS	tr_C9ZJU1	0.084	20174.00	28.00
678	HLA-A0201	AFSGSSLGCYQVLE	FLGCYQVLE	1	0	0	1	4	FSGSSLGCYQVLE	tr_C9ZJU1	0.084	20175.76	28.00
646	HLA-A0201	WCPLYHAFLGSFY	WLYHAFLGS	0	0	0	1	2	WCPLYHAFLGS	tr_C9ZJU1	0.084	20202.84	28.00
524	HLA-A0201	VSVRRLGWIKDVI	VLGWIKDVI	0	0	0	1	4	VSVRRLGWIKDVI	tr_C9ZJU1	0.084	20213.55	28.00
945	HLA-A0201	VGSWETLSRT	VSWETLSRT	0	0	0	1	1	VGSWETLSRT	tr_C9ZJU1	0.084	20215.30	28.00
681	HLA-A0201	GSSLGCYQVLEG	SLGCYVLEG	2	0	0	5	1	SLGCYQVLEG	tr_C9ZJU1	0.084	20226.69	28.00
157	HLA-A0201	QEFPEELYLGLI	QEFLYLGLI	0	0	0	3	3	QEFPEELYLGLI	tr_C9ZJU1	0.084	20227.99	28.00
759	HLA-A0201	TTLLRLLNR	TTLLRLLNR	0	0	0	0	0	TTLLRLLNR	tr_C9ZJU1	0.084	20227.99	28.00

864	HLA-A0201	VERLCIARA	VERLCIARA	0	0	0	0	0	VERLCIARA	tr_C9ZJU1	0.084	20243.32	28.00
963	HLA-A0201	MRQSQQLPHFVK	RQSQQLPFV	1	0	0	7	1	RQSQQLPHFV	tr_C9ZJU1	0.083	20278.62	28.00
190	HLA-A0201	SSCSIPQLVT	SSCSIPQLV	0	0	0	0	0	SSCSIPQLV	tr_C9ZJU1	0.083	20306.73	28.00
920	HLA-A0201	IVAHRLATLKHA	IVAHRLATA	0	0	0	8	3	IVAHRLATLKHA	tr_C9ZJU1	0.083	20311.99	28.00
931	HLA-A0201	ADKIVVLNEGMVA	KIVVLNMVA	2	0	0	6	2	KIVVLNEGMVA	tr_C9ZJU1	0.083	20314.19	28.00
824	HLA-A0201	DCSDENICAAL	CSDEICAAL	1	0	0	4	1	CSDENICAAL	tr_C9ZJU1	0.083	20326.29	28.00
642	HLA-A0201	SIQHWCPLYH	SIHWCPLYH	0	0	0	2	1	SIQHWCPLYH	tr_C9ZJU1	0.083	20327.17	28.00
512	HLA-A0201	KRKLQAGGHTCA	KLAGGHTCA	2	0	0	2	1	KLQAGGHTCA	tr_C9ZJU1	0.083	20383.55	28.00
680	HLA-A0201	SGSSLGCYQVLEG	SLGCYVLEG	3	0	0	5	1	SLGCYQVLEG	tr_C9ZJU1	0.083	20384.43	28.00
888	HLA-A0201	PTSAIDTITEGEI	AIDTITEEI	3	0	0	7	1	AIDTITEGEI	tr_C9ZJU1	0.083	20393.03	28.00
513	HLA-A0201	RKLQAGGHTC	KLQAGGHTC	1	0	0	0	0	KLQAGGHTC	tr_C9ZJU1	0.083	20393.25	28.00
630	HLA-A0201	ALDQTEIVN	ALDQTEIVN	0	0	0	0	0	ALDQTEIVN	tr_C9ZJU1	0.083	20395.69	28.00
380	HLA-A0201	MESQFTKSQLA	MQFTKSQLA	0	0	0	1	2	MESQFTKSQLA	tr_C9ZJU1	0.083	20398.11	28.00
547	HLA-A0201	PSTCVWQCRQDYLQ	CVWQCQDYL	3	0	0	5	1	CVWQCRQDYL	tr_C9ZJU1	0.083	20406.71	28.00
296	HLA-A0201	IDAYLALATADYDP	IAYLALATA	0	0	0	1	1	IDAYLALATA	tr_C9ZJU1	0.083	20442.30	28.00
930	HLA-A0201	HADKIVVLNEGMVA	KIVVLNMVA	3	0	0	6	2	KIVVLNEGMVA	tr_C9ZJU1	0.083	20448.49	28.00
759	HLA-A0201	TTLLRLLNRTY	TTLLRLLTY	0	0	0	7	2	TTLLRLLNRTY	tr_C9ZJU1	0.083	20451.14	28.00
487	HLA-A0201	WDLLTQHVIW	WLLTQHVIW	0	0	0	1	1	WDLLTQHVIW	tr_C9ZJU1	0.083	20452.69	28.00
72	HLA-A0201	LPSVFAVHCGLMKN	SVFAVCGLM	2	0	0	5	1	SVFAVHCGLM	tr_C9ZJU1	0.083	20455.12	28.00
367	HLA-A0201	WSNFLYFLNE	WSFLYFLNE	0	0	0	2	1	WSNFLYFLNE	tr_C9ZJU1	0.083	20473.07	28.00
59	HLA-A0201	HVRTLVFNHNI	HVLVFNHNI	0	0	0	2	2	HVRTLVFNHNI	tr_C9ZJU1	0.082	20485.91	28.00
40	HLA-A0201	CKDDASIAIFLNC	CSIAIFLNC	0	0	0	1	4	CKDDASIAIFLNC	tr_C9ZJU1	0.082	20486.36	28.00
682	HLA-A0201	SSLGCYQVLE	SSLGCYQVL	0	0	0	0	0	SSLGCYQVL	tr_C9ZJU1	0.082	20487.02	28.00
224	HLA-A0201	WRPIYAAEI	WRPIYAAEI	0	0	0	0	0	WRPIYAAEI	tr_C9ZJU1	0.082	20499.21	28.00
913	HLA-A0201	LRDSTIIIVA	LRDSTIIIV	0	0	0	0	0	LRDSTIIIV	tr_C9ZJU1	0.082	20511.87	28.00
36	HLA-A0201	KQTECKDDASIAIF	KQTECSIAI	0	0	0	5	4	KQTECKDDASIAI	tr_C9ZJU1	0.082	20531.84	28.00
752	HLA-A0201	GYSGSGKTTLLRL	YSGSTLLRL	1	0	0	4	3	YSGSGKTTLLRL	tr_C9ZJU1	0.082	20538.95	28.00
73	HLA-A0201	PSVFAVHCGLMKN	SVFAVCGLM	1	0	0	5	1	SVFAVHCGLM	tr_C9ZJU1	0.082	20558.08	28.00
125	HLA-A0201	VKAEEVLALCM	KAEEVLALM	1	0	0	8	1	KAEEVLALCM	tr_C9ZJU1	0.082	20581.00	28.00
64	HLA-A0201	VFNHNIMQLPSVF	FIMQLPSVF	1	0	0	1	3	FNHNIMQLPSVF	tr_C9ZJU1	0.082	20608.85	28.00
337	HLA-A0201	RVPALLEVMH	RVPALLEVM	0	0	0	0	0	RVPALLEVM	tr_C9ZJU1	0.082	20616.21	28.00
756	HLA-A0201	SGKTTLLRLLNRTY	SLLRLLNRT	0	0	0	1	4	SGKTTLLRLLNRT	tr_C9ZJU1	0.082	20622.01	28.00
56	HLA-A0201	ESIHVRTLVF	SIHVRTLVF	1	0	0	0	0	SIHVRTLVF	tr_C9ZJU1	0.082	20635.85	28.00
646	HLA-A0201	WCPLYHAFLG	WCPLYHAFL	0	0	0	0	0	WCPLYHAFL	tr_C9ZJU1	0.082	20636.53	28.00
763	HLA-A0201	RLLNRTYAPTEGD	RLLNRTYPT	0	0	0	7	1	RLLNRTYAPT	tr_C9ZJU1	0.082	20644.12	28.00
364	HLA-A0201	GTQWSNFLY	GTQWSNFLY	0	0	0	0	0	GTQWSNFLY	tr_C9ZJU1	0.082	20649.71	28.00
887	HLA-A0201	EPTSAIDTITEGEI	AIDTITEEI	4	0	0	7	1	AIDTITEGEI	tr_C9ZJU1	0.082	20658.64	28.00
527	HLA-A0201	RRLGWIKDV	RRLGWIKDV	0	0	0	0	0	RRLGWIKDV	tr_C9ZJU1	0.082	20660.21	28.00
495	HLA-A0201	IWPLVPSVFSGC	ILVPSVFSG	0	0	0	1	2	IWPLVPSVFSG	tr_C9ZJU1	0.082	20664.46	28.00
878	HLA-A0201	SAVGLCIFDE	SAVLCIFDE	0	0	0	3	1	SAVGLCIFDE	tr_C9ZJU1	0.082	20664.68	28.00
829	HLA-A0201	NICAALSAS	NICAALSAS	0	0	0	0	0	NICAALSAS	tr_C9ZJU1	0.082	20672.73	28.00
376	HLA-A0201	ESLYMESQFTKS	SLYMESQFS	1	0	0	8	2	SLYMESQFTKS	tr_C9ZJU1	0.082	20675.87	28.00
871	HLA-A0201	RAFVKAGSA	RAFVKAGSA	0	0	0	0	0	RAFVKAGSA	tr_C9ZJU1	0.081	20716.16	28.00
751	HLA-A0201	AGYSGSGKTTLLRL	YSGSTLLRL	2	0	0	4	3	YSGSGKTTLLRL	tr_C9ZJU1	0.081	20740.84	28.00
374	HLA-A0201	LNESLYMESQFTKS	SLYMESQFS	3	0	0	8	2	SLYMESQFTKS	tr_C9ZJU1	0.081	20743.31	28.00
499	HLA-A0201	VPSVFSGCVV	VSVFSGCVV	0	0	0	1	1	VPSVFSGCVV	tr_C9ZJU1	0.081	20744.42	28.00
872	HLA-A0201	AFVKAGSAVGLC	FVAGSAVGL	1	0	0	2	1	FVKAGSAVGL	tr_C9ZJU1	0.081	20754.75	28.00
39	HLA-A0201	ECKDDASIAIFLNC	ASIAIFLNC	5	0	0	0	0	ASIAIFLNC	tr_C9ZJU1	0.081	20769.80	29.00
871	HLA-A0201	RAFVKAGSAVGLC	FVAGSAVGL	2	0	0	2	1	FVKAGSAVGL	tr_C9ZJU1	0.081	20772.28	29.00
662	HLA-A0201	GMSEAQVDEAHLRR	GMSEAQVHL	0	0	0	7	3	GMSEAQVDEAHL	tr_C9ZJU1	0.081	20779.02	29.00
823	HLA-A0201	WDCSDENICAAL	CSDEICAAL	2	0	0	4	1	CSDENICAAL	tr_C9ZJU1	0.081	20785.09	29.00
616	HLA-A0201	AVALRLSPNFF	AVALRLNFF	0	0	0	6	2	AVALRLSPNFF	tr_C9ZJU1	0.081	20795.66	29.00
688	HLA-A0201	QVLEGLQSVQRK	QVLEGLQSV	0	0	0	0	0	QVLEGLQSV	tr_C9ZJU1	0.081	20799.94	29.00
141	HLA-A0201	YSIARYPPAAQA	YIARYPPAA	0	0	0	1	1	YSIARYPPAA	tr_C9ZJU1	0.081	20803.76	29.00

615	HLA-A0201	SAVALRLSPNFF	SLRLSPNFF	0	0	0	1	3	SAVALRLSPNFF	tr_C9ZJU1	0.081	20804.22	29.00
272	HLA-A0201	FESLYCIRG	FESLYCIRG	0	0	0	0	0	FESLYCIRG	tr_C9ZJU1	0.081	20804.67	29.00
511	HLA-A0201	LKRKLQAGGHTCA	KLAGGHTCA	3	0	0	2	1	KLQAGGHTCA	tr_C9ZJU1	0.081	20805.80	29.00
412	HLA-A0201	TCSFPVKNSL	CSFPVKNSL	1	0	0	0	0	CSFPVKNSL	tr_C9ZJU1	0.081	20809.17	29.00
829	HLA-A0201	NICAALSASRAL	NICAALSAL	0	0	0	7	3	NICAALSASRAL	tr_C9ZJU1	0.081	20820.20	29.00
34	HLA-A0201	DSKQTECKDDASI	KQTECDASI	2	0	0	5	2	KQTECKDDASI	tr_C9ZJU1	0.081	20830.34	29.00
389	HLA-A0201	LASVTNIAT	LASVTNIAT	0	0	0	0	0	LASVTNIAT	tr_C9ZJU1	0.081	20831.70	29.00
175	HLA-A0201	RSLQSGITSFWK	SLGITSFWK	1	0	0	2	2	SLQSGITSFWK	tr_C9ZJU1	0.081	20833.73	29.00
745	HLA-A0201	GEFLGIAGY	GEFLGIAGY	0	0	0	0	0	GEFLGIAGY	tr_C9ZJU1	0.081	20838.24	29.00
567	HLA-A0201	LSGWELMAATAA	SGWELMAAA	1	0	0	7	2	SGWELMAATAA	tr_C9ZJU1	0.081	20838.46	29.00
757	HLA-A0201	GKTTLLRLLNRTY	TLLRLLNTY	3	0	0	7	1	TLLRLLNRTY	tr_C9ZJU1	0.081	20850.41	29.00
672	HLA-A0201	HLRRHLAFSGSSLG	HLRRHLAFL	0	0	0	8	4	HLRRHLAFSGSSL	tr_C9ZJU1	0.081	20883.82	29.00
839	HLA-A0201	ALNFVQKRS	ALNFVQKRS	0	0	0	0	0	ALNFVQKRS	tr_C9ZJU1	0.081	20898.06	29.00
502	HLA-A0201	VFSGCVVDALK	FSGCVVDAL	1	0	0	0	0	FSGCVVDAL	tr_C9ZJU1	0.081	20904.63	29.00
141	HLA-A0201	YSIARYPPAAQ	YIARYPPAA	0	0	0	1	1	YSIARYPPAA	tr_C9ZJU1	0.081	20923.19	29.00
264	HLA-A0201	MAVASTFAFE	MAVASTFAF	0	0	0	0	0	MAVASTFAF	tr_C9ZJU1	0.080	20931.55	29.00
890	HLA-A0201	SAIDTITEG	SAIDTITEG	0	0	0	0	0	SAIDTITEG	tr_C9ZJU1	0.080	20931.78	29.00
570	HLA-A0201	WELMAATAA	WELMAATAA	0	0	0	0	0	WELMAATAA	tr_C9ZJU1	0.080	20936.99	29.00
748	HLA-A0201	LGIAGYSGSGKTTL	GIAGYSTTL	1	0	0	6	4	GIAGYSGSGKTTL	tr_C9ZJU1	0.080	20940.84	29.00
677	HLA-A0201	LAFSGSSLGC	LAFGSSLGC	0	0	0	3	1	LAFSGSSLGC	tr_C9ZJU1	0.080	20940.84	29.00
641	HLA-A0201	RSIQHWCPLY	RSIQHWCPL	0	0	0	0	0	RSIQHWCPL	tr_C9ZJU1	0.080	20944.48	29.00
257	HLA-A0201	LPATFVSMAVAS	LTFVSMAVA	0	0	0	1	2	LPATFVSMAVA	tr_C9ZJU1	0.080	20945.83	29.00
173	HLA-A0201	RKRSLQSGITSF	KLQSGITSF	1	0	0	1	2	KRSLQSGITSF	tr_C9ZJU1	0.080	20953.08	29.00
693	HLA-A0201	LQSVQRKIDRPV	LQSVIDRPV	0	0	0	4	3	LQSVQRKIDRPV	tr_C9ZJU1	0.080	20954.45	29.00
85	HLA-A0201	NSHPDHLLVCDIWN	SLLVCDIWN	1	0	0	1	4	SHPDHLLVCDIWN	tr_C9ZJU1	0.080	20957.62	29.00
122	HLA-A0201	LDDVKAEEVLALCM	KAEEVLALM	4	0	0	8	1	KAEEVLALCM	tr_C9ZJU1	0.080	20968.74	29.00
614	HLA-A0201	VSAVALRLSPNFF	ALRLSPNFF	4	0	0	0	0	ALRLSPNFF	tr_C9ZJU1	0.080	20972.59	29.00
244	HLA-A0201	LSPFRFIIKQL	SPFRFIIQL	1	0	0	7	1	SPFRFIIKQL	tr_C9ZJU1	0.080	21004.16	29.00
103	HLA-A0201	LVNCSIRDILLDDG	LVNCSIILL	0	0	0	6	2	LVNCSIRDILL	tr_C9ZJU1	0.080	21010.75	29.00
124	HLA-A0201	DVKAEEVLALCM	KAEEVLALM	2	0	0	8	1	KAEEVLALCM	tr_C9ZJU1	0.080	21014.62	29.00
158	HLA-A0201	EFPEELYLGLI	FPEELYLLI	1	0	0	7	1	FPEELYLGLI	tr_C9ZJU1	0.080	21044.66	29.00
507	HLA-A0201	VVDALKRKL	VVDALKRKL	0	0	0	0	0	VVDALKRKL	tr_C9ZJU1	0.080	21057.17	29.00
146	HLA-A0201	YPPAAQADVVV	YAAQADVVV	0	0	0	1	2	YPPAAQADVVV	tr_C9ZJU1	0.080	21061.27	29.00
645	HLA-A0201	HWCPLYHAFLGSFY	WLYHAFLGS	1	0	0	1	2	WCPLYHAFLGS	tr_C9ZJU1	0.080	21075.19	29.00
179	HLA-A0201	SGITSFWKLMCS	GIFWKLMCS	1	0	0	2	2	GITSFWKLMCS	tr_C9ZJU1	0.080	21081.35	29.00
647	HLA-A0201	CPLYHAFLGSFY	CLYHAFLGS	0	0	0	1	1	CPLYHAFLGS	tr_C9ZJU1	0.080	21087.51	29.00
163	HLA-A0201	LYLGLITAY	LYLGLITAY	0	0	0	0	0	LYLGLITAY	tr_C9ZJU1	0.080	21109.19	29.00
472	HLA-A0201	FSMVFVESGRP	FSMVFVESP	0	0	0	8	2	FSMVFVESGRP	tr_C9ZJU1	0.080	21129.06	29.00
662	HLA-A0201	GMSEAQVDEAH	GMSEAQVEA	0	0	0	7	1	GMSEAQVDEA	tr_C9ZJU1	0.080	21138.44	29.00
282	HLA-A0201	VIQSILSDIIT	VIQSILIIT	0	0	0	6	2	VIQSILSDIIT	tr_C9ZJU1	0.080	21150.80	29.00
810	HLA-A0201	DLSIGGNIALGNLW	SIGGNIALL	2	0	0	8	2	SIGGNIALGNL	tr_C9ZJU1	0.079	21181.48	29.00
689	HLA-A0201	VLEGLQSVQRKI	VLEGLQSVI	0	0	0	8	3	VLEGLQSVQRKI	tr_C9ZJU1	0.079	21197.76	29.00
648	HLA-A0201	PLYHAFLGSFY	PLYHAFLFY	0	0	0	7	2	PLYHAFLGSFY	tr_C9ZJU1	0.079	21208.78	29.00
307	HLA-A0201	YDPSDDWLLSRCV	YDWLLSRCV	0	0	0	1	4	YDPSDDWLLSRCV	tr_C9ZJU1	0.079	21211.06	29.00
375	HLA-A0201	NESLYMESQFTKS	SLYMESQFS	2	0	0	8	2	SLYMESQFTKS	tr_C9ZJU1	0.079	21213.13	29.00
382	HLA-A0201	SQFTKSQLAS	SQFTKSQLA	0	0	0	0	0	SQFTKSQLA	tr_C9ZJU1	0.079	21214.28	29.00
684	HLA-A0201	LGCYQVLEGLQ	LCYQVLEGL	0	0	0	1	1	LGCYQVLEGL	tr_C9ZJU1	0.079	21233.80	29.00
807	HLA-A0201	FIEDLSIGGN	FIEDLSIGG	0	0	0	0	0	FIEDLSIGG	tr_C9ZJU1	0.079	21242.99	29.00
33	HLA-A0201	HDSKQTECKDDASI	KQTECDASI	3	0	0	5	2	KQTECKDDASI	tr_C9ZJU1	0.079	21249.89	29.00
753	HLA-A0201	YSGSGKTTLLRLL	YSTTLLRLL	0	0	0	2	4	YSGSGKTTLLRLL	tr_C9ZJU1	0.079	21257.47	29.00
573	HLA-A0201	MAATAAAAFATA	MAAAAFATA	0	0	0	1	3	MAATAAAAFATA	tr_C9ZJU1	0.079	21265.76	29.00
878	HLA-A0201	SAVGLCIFD	SAVGLCIFD	0	0	0	0	0	SAVGLCIFD	tr_C9ZJU1	0.079	21268.53	29.00
123	HLA-A0201	DDVKAEEVLALCM	KAEEVLALM	3	0	0	8	1	KAEEVLALCM	tr_C9ZJU1	0.079	21271.52	29.00
53	HLA-A0201	NGVESIHVRTLV	GVIHVRTLV	1	0	0	2	2	GVESIHVRTLV	tr_C9ZJU1	0.079	21289.25	29.00

626	HLA-A0201	FTQSALDQTEIVNQ	FTLDQTEIV	0	0	0	2	3	FTQSALDQTEIV	tr_C9ZJU1	0.079	21311.83	29.00
143	HLA-A0201	IARYPPAAQA	IAYPPAAQA	0	0	0	2	1	IARYPPAAQA	tr_C9ZJU1	0.079	21312.99	29.00
117	HLA-A0201	VALCILDDVKAEE	VALCILVKA	0	0	0	6	2	VALCILDDVKA	tr_C9ZJU1	0.079	21320.82	29.00
869	HLA-A0201	IARAFVKAGSAV	IARAFVSAV	0	0	0	6	3	IARAFVKAGSAV	tr_C9ZJU1	0.079	21321.74	29.00
265	HLA-A0201	AVASTFAFE	AVASTFAFE	0	0	0	0	0	AVASTFAFE	tr_C9ZJU1	0.079	21325.90	29.00
4	HLA-A0201	ARPAVLLDCERKA	ALLDCERKA	0	0	0	1	4	ARPAVLLDCERKA	tr_C9ZJU1	0.079	21326.36	29.00
73	HLA-A0201	PSVFAVHCGLMKNS	SVFAVCGLM	1	0	0	5	1	SVFAVHCGLM	tr_C9ZJU1	0.079	21339.05	29.00
36	HLA-A0201	KQTECKDDA	KQTECKDDA	0	0	0	0	0	KQTECKDDA	tr_C9ZJU1	0.079	21341.13	29.00
156	HLA-A0201	VQEFPEELYLGLIT	VQFPEELYL	0	0	0	2	1	VQEFPEELYL	tr_C9ZJU1	0.079	21353.83	29.00
60	HLA-A0201	VRTLVFNHNIM	VLVFNHNIM	0	0	0	1	2	VRTLVFNHNIM	tr_C9ZJU1	0.078	21389.90	29.00
607	HLA-A0201	DTLPLLLVS	DTLPLLLVS	0	0	0	0	0	DTLPLLLVS	tr_C9ZJU1	0.078	21400.79	29.00
964	HLA-A0201	RQSQQLPHFVKK	RQSQQLPFV	0	0	0	7	1	RQSQQLPHFV	tr_C9ZJU1	0.078	21401.03	29.00
745	HLA-A0201	GEFLGIAGYSG	FLGIAGYSG	2	0	0	0	0	FLGIAGYSG	tr_C9ZJU1	0.078	21404.27	29.00
651	HLA-A0201	HAFLGSFYD	HAFLGSFYD	0	0	0	0	0	HAFLGSFYD	tr_C9ZJU1	0.078	21405.65	29.00
858	HLA-A0201	EFSGGEVERLCIA	FSVERLCIA	1	0	0	2	3	FSGGEVERLCIA	tr_C9ZJU1	0.078	21407.28	29.00
533	HLA-A0201	KDVIQSSPRA	KVIQSSPRA	0	0	0	1	1	KDVIQSSPRA	tr_C9ZJU1	0.078	21410.29	29.00
482	HLA-A0201	PLGYMWDLLTQ	PLYMWDLLT	0	0	0	2	1	PLGYMWDLLT	tr_C9ZJU1	0.078	21417.00	29.00
475	HLA-A0201	VFVESGRPL	VFVESGRPL	0	0	0	0	0	VFVESGRPL	tr_C9ZJU1	0.078	21419.55	29.00
151	HLA-A0201	QADVVVQEF	QADVVVQEF	0	0	0	0	0	QADVVVQEF	tr_C9ZJU1	0.078	21451.10	30.00
174	HLA-A0201	KRSLQSGITSFWK	SLGITSFWK	2	0	0	2	2	SLQSGITSFWK	tr_C9ZJU1	0.078	21451.56	30.00
554	HLA-A0201	CRQDYLQGFA	RQDYLQGFA	1	0	0	0	0	RQDYLQGFA	tr_C9ZJU1	0.078	21457.82	30.00
859	HLA-A0201	FSGGEVERLC	FSGGEVERL	0	0	0	0	0	FSGGEVERL	tr_C9ZJU1	0.078	21458.75	30.00
870	HLA-A0201	ARAFVKAGSAVGLC	FVAGSAVGL	3	0	0	2	1	FVKAGSAVGL	tr_C9ZJU1	0.078	21460.15	30.00
549	HLA-A0201	TCVWQCRQDYLQG	CVWQCQDYL	1	0	0	5	1	CVWQCRQDYL	tr_C9ZJU1	0.078	21472.69	30.00
178	HLA-A0201	QSGITSFWKLM	SGITSFWLM	1	0	0	7	1	SGITSFWKLM	tr_C9ZJU1	0.078	21480.13	30.00
245	HLA-A0201	SPFRFIIKQLMG	SPFRFIILM	0	0	0	7	2	SPFRFIIKQLM	tr_C9ZJU1	0.078	21486.63	30.00
933	HLA-A0201	KIVVLNEGMVAE	KIVVLNEMV	0	0	0	7	1	KIVVLNEGMV	tr_C9ZJU1	0.078	21496.63	30.00
59	HLA-A0201	HVRTLVFNHNIM	HVRTLVFIM	0	0	0	7	3	HVRTLVFNHNIM	tr_C9ZJU1	0.078	21500.82	30.00
524	HLA-A0201	VSVRRLGWI	VSVRRLGWI	0	0	0	0	0	VSVRRLGWI	tr_C9ZJU1	0.078	21516.41	30.00
228	HLA-A0201	YAAEIGKSLNGDV	YAAEIGKSV	0	0	0	8	4	YAAEIGKSLNGDV	tr_C9ZJU1	0.078	21518.97	30.00
182	HLA-A0201	TSFWKLMCSSC	SFWLMCSSC	1	0	0	3	1	SFWKLMCSSC	tr_C9ZJU1	0.078	21520.13	30.00
320	HLA-A0201	SQALWQASG	SQALWQASG	0	0	0	0	0	SQALWQASG	tr_C9ZJU1	0.078	21529.68	30.00
723	HLA-A0201	VSFRYSDRHPYVL	VSFRYSPYV	0	0	0	6	3	VSFRYSDRHPYV	tr_C9ZJU1	0.078	21538.31	30.00
5	HLA-A0201	RPAVLLDCERKA	RLLDCERKA	0	0	0	1	3	RPAVLLDCERKA	tr_C9ZJU1	0.078	21570.49	30.00
838	HLA-A0201	RALNFVQKRSSGIH	ALNFVSSGI	1	0	0	5	3	ALNFVQKRSSGI	tr_C9ZJU1	0.078	21580.99	30.00
56	HLA-A0201	ESIHVRTLV	ESIHVRTLV	0	0	0	0	0	ESIHVRTLV	tr_C9ZJU1	0.078	21593.61	30.00
118	HLA-A0201	ALCILDDVKAEE	ALCILDVKA	0	0	0	5	1	ALCILDDVKA	tr_C9ZJU1	0.077	21618.16	30.00
569	HLA-A0201	GWELMAATA	GWELMAATA	0	0	0	0	0	GWELMAATA	tr_C9ZJU1	0.077	21618.85	30.00
710	HLA-A0201	PSLDGPWSVEFR	SLDGWSVEF	1	0	0	4	1	SLDGPWSVEF	tr_C9ZJU1	0.077	21626.34	30.00
178	HLA-A0201	QSGITSFWKLMCS	GIFWKLMCS	2	0	0	2	2	GITSFWKLMCS	tr_C9ZJU1	0.077	21635.46	30.00
634	HLA-A0201	TEIVNQLRSI	TIVNQLRSI	0	0	0	1	1	TEIVNQLRSI	tr_C9ZJU1	0.077	21641.56	30.00
72	HLA-A0201	LPSVFAVHC	LPSVFAVHC	0	0	0	0	0	LPSVFAVHC	tr_C9ZJU1	0.077	21646.71	30.00
646	HLA-A0201	WCPLYHAFLGSFYD	WLYHAFLGS	0	0	0	1	2	WCPLYHAFLGS	tr_C9ZJU1	0.077	21671.79	30.00
943	HLA-A0201	AEVGSWETL	AEVGSWETL	0	0	0	0	0	AEVGSWETL	tr_C9ZJU1	0.077	21680.47	30.00
519	HLA-A0201	GHTCAVSVRRLGWI	HTCAVSVWI	1	0	0	7	4	HTCAVSVRRLGWI	tr_C9ZJU1	0.077	21690.09	30.00
745	HLA-A0201	GEFLGIAGYS	GEFLGIAYS	0	0	0	7	1	GEFLGIAGYS	tr_C9ZJU1	0.077	21704.64	30.00
858	HLA-A0201	EFSGGEVERLCI	FSGGEVLCI	1	0	0	6	2	FSGGEVERLCI	tr_C9ZJU1	0.077	21709.57	30.00
710	HLA-A0201	PSLDGPWSVEFRE	SLWSVEFRE	1	0	0	2	3	SLDGPWSVEFRE	tr_C9ZJU1	0.077	21711.21	30.00
298	HLA-A0201	AYLALATADYD	YLALATADY	1	0	0	0	0	YLALATADY	tr_C9ZJU1	0.077	21729.78	30.00
744	HLA-A0201	KGEFLGIAGYSG	FLGIAGYSG	3	0	0	0	0	FLGIAGYSG	tr_C9ZJU1	0.077	21738.95	30.00
387	HLA-A0201	SQLASVTNIATST	QLASVTTST	1	0	0	6	3	QLASVTNIATST	tr_C9ZJU1	0.077	21742.48	30.00
891	HLA-A0201	AIDTITEGEIFDT	AIDTIIFDT	0	0	0	5	4	AIDTITEGEIFDT	tr_C9ZJU1	0.077	21743.42	30.00
713	HLA-A0201	DGPWSVEFREV	GPWSVEFEV	1	0	0	7	1	GPWSVEFREV	tr_C9ZJU1	0.077	21754.25	30.00
173	HLA-A0201	RKRSLQSGITSFWK	SLGITSFWK	3	0	0	2	2	SLQSGITSFWK	tr_C9ZJU1	0.077	21759.43	30.00

367	HLA-A0201	WSNFLYFLN	WSNFLYFLN	0	0	0	0	0	WSNFLYFLN	tr_C9ZJU1	0.077	21762.49	30.00
496	HLA-A0201	WPLVPSVFSGC	WLVPSVFSG	0	0	0	1	1	WPLVPSVFSG	tr_C9ZJU1	0.077	21789.11	30.00
818	HLA-A0201	ALGNLWDCSD	ALGNLWCSD	0	0	0	6	1	ALGNLWDCSD	tr_C9ZJU1	0.077	21799.72	30.00
368	HLA-A0201	SNFLYFLNE	SNFLYFLNE	0	0	0	0	0	SNFLYFLNE	tr_C9ZJU1	0.077	21820.96	30.00
896	HLA-A0201	TEGEIFDTL	TEGEIFDTL	0	0	0	0	0	TEGEIFDTL	tr_C9ZJU1	0.077	21821.43	30.00
506	HLA-A0201	CVVDALKRKLQA	CVVDALLQA	0	0	0	6	3	CVVDALKRKLQA	tr_C9ZJU1	0.077	21832.76	30.00
377	HLA-A0201	SLYMESQFTKSQ	SLYMFTKSQ	0	0	0	4	3	SLYMESQFTKSQ	tr_C9ZJU1	0.077	21851.20	30.00
762	HLA-A0201	LRLLNRTYAPTEG	RLLNRTYPT	1	0	0	7	1	RLLNRTYAPT	tr_C9ZJU1	0.076	21872.49	30.00
3	HLA-A0201	RARPAVLLDCERKA	RLLDCERKA	0	0	0	1	5	RARPAVLLDCERKA	tr_C9ZJU1	0.076	21877.23	30.00
813	HLA-A0201	IGGNIALGNL	IGGNIALGL	0	0	0	8	1	IGGNIALGNL	tr_C9ZJU1	0.076	21895.93	30.00
14	HLA-A0201	RKAYEILEM	RKAYEILEM	0	0	0	0	0	RKAYEILEM	tr_C9ZJU1	0.076	21899.01	30.00
473	HLA-A0201	SMVFVESGR	SMVFVESGR	0	0	0	0	0	SMVFVESGR	tr_C9ZJU1	0.076	21899.96	30.00
811	HLA-A0201	LSIGGNIALGNLW	SIGGNIALL	1	0	0	8	2	SIGGNIALGNL	tr_C9ZJU1	0.076	21912.05	30.00
119	HLA-A0201	LCILDDVKA	LCILDDVKA	0	0	0	0	0	LCILDDVKA	tr_C9ZJU1	0.076	21928.18	30.00
409	HLA-A0201	VSPTCSFPVKNSL	VSPTCSFPL	0	0	0	8	4	VSPTCSFPVKNSL	tr_C9ZJU1	0.076	21940.51	30.00
743	HLA-A0201	NKGEFLGIAGYSG	FLGIAGYSG	4	0	0	0	0	FLGIAGYSG	tr_C9ZJU1	0.076	21947.63	30.00
845	HLA-A0201	KRSSGIHAPLNV	RSSGIPLNV	1	0	0	5	2	RSSGIHAPLNV	tr_C9ZJU1	0.076	21947.63	30.00
333	HLA-A0201	ELQSRVPALLE	ELQSVPALL	0	0	0	4	1	ELQSRVPALL	tr_C9ZJU1	0.076	21952.39	30.00
165	HLA-A0201	LGLITAYNRKRSLQ	GLITAYNSL	1	0	0	7	3	GLITAYNRKRSL	tr_C9ZJU1	0.076	21967.36	30.00
172	HLA-A0201	NRKRSLQSGITSF	KLQSGITSF	2	0	0	1	2	KRSLQSGITSF	tr_C9ZJU1	0.076	21979.25	30.00
52	HLA-A0201	CNGVESIHVRTLV	GVIHVRTLV	2	0	0	2	2	GVESIHVRTLV	tr_C9ZJU1	0.076	21982.34	30.00
742	HLA-A0201	VNKGEFLGIAGYSG	FLGIAGYSG	5	0	0	0	0	FLGIAGYSG	tr_C9ZJU1	0.076	21992.80	30.00
342	HLA-A0201	LEVMHGCRVVF	LMHGCRVVF	0	0	0	1	2	LEVMHGCRVVF	tr_C9ZJU1	0.076	22007.09	30.00
824	HLA-A0201	DCSDENICAA	CSDENICAA	1	0	0	0	0	CSDENICAA	tr_C9ZJU1	0.076	22012.57	30.00
528	HLA-A0201	RLGWIKDVIQSSP	RLGWIVIQS	0	0	0	5	2	RLGWIKDVIQS	tr_C9ZJU1	0.076	22017.81	30.00
216	HLA-A0201	LGMHAFVHWRP	GMHAFVWRP	1	0	0	6	1	GMHAFVHWRP	tr_C9ZJU1	0.076	22036.63	30.00
41	HLA-A0201	KDDASIAIFLNC	ASIAIFLNC	3	0	0	0	0	ASIAIFLNC	tr_C9ZJU1	0.076	22038.06	30.00
450	HLA-A0201	FQQTFSHPSR	FQQTFSHPS	0	0	0	0	0	FQQTFSHPS	tr_C9ZJU1	0.076	22044.73	30.00
594	HLA-A0201	CGRDVLHGL	CGRDVLHGL	0	0	0	0	0	CGRDVLHGL	tr_C9ZJU1	0.076	22055.95	30.00
857	HLA-A0201	REFSGGEVERLCIA	FSVERLCIA	2	0	0	2	3	FSGGEVERLCIA	tr_C9ZJU1	0.076	22057.62	30.00
894	HLA-A0201	TITEGEIFDTLR	ITEGEIFTL	1	0	0	7	1	ITEGEIFDTL	tr_C9ZJU1	0.076	22062.40	30.00
495	HLA-A0201	IWPLVPSVF	IWPLVPSVF	0	0	0	0	0	IWPLVPSVF	tr_C9ZJU1	0.076	22069.80	30.00
109	HLA-A0201	RDILLDDGV	RDILLDDGV	0	0	0	0	0	RDILLDDGV	tr_C9ZJU1	0.076	22079.35	31.00
548	HLA-A0201	STCVWQCRQDYLQG	CVWQCQDYL	2	0	0	5	1	CVWQCRQDYL	tr_C9ZJU1	0.076	22080.79	31.00
386	HLA-A0201	KSQLASVTNIATST	KLASVTNIA	0	0	0	1	2	KSQLASVTNIA	tr_C9ZJU1	0.076	22081.26	31.00
244	HLA-A0201	LSPFRFIIKQLMG	SPFRFIILM	1	0	0	7	2	SPFRFIIKQLM	tr_C9ZJU1	0.076	22086.51	31.00
42	HLA-A0201	DDASIAIFLNC	ASIAIFLNC	2	0	0	0	0	ASIAIFLNC	tr_C9ZJU1	0.075	22093.45	31.00
342	HLA-A0201	LEVMHGCRV	LEVMHGCRV	0	0	0	0	0	LEVMHGCRV	tr_C9ZJU1	0.075	22094.16	31.00
297	HLA-A0201	DAYLALATADYD	YLALATADY	2	0	0	0	0	YLALATADY	tr_C9ZJU1	0.075	22098.71	31.00
663	HLA-A0201	MSEAQVDEA	MSEAQVDEA	0	0	0	0	0	MSEAQVDEA	tr_C9ZJU1	0.075	22114.73	31.00
166	HLA-A0201	GLITAYNRKRSLQS	GLITAYNSL	0	0	0	7	3	GLITAYNRKRSL	tr_C9ZJU1	0.075	22123.35	31.00
398	HLA-A0201	STYAGNGEENQV	STYAGNNQV	0	0	0	6	3	STYAGNGEENQV	tr_C9ZJU1	0.075	22126.94	31.00
716	HLA-A0201	WSVEFREVSF	WSVEFEVSF	0	0	0	5	1	WSVEFREVSF	tr_C9ZJU1	0.075	22129.81	31.00
862	HLA-A0201	GEVERLCIA	GEVERLCIA	0	0	0	0	0	GEVERLCIA	tr_C9ZJU1	0.075	22142.03	31.00
829	HLA-A0201	NICAALSASRALNF	ALSASALNF	4	0	0	5	1	ALSASRALNF	tr_C9ZJU1	0.075	22153.53	31.00
207	HLA-A0201	SPEVILKCIL	SPEVILCIL	0	0	0	6	1	SPEVILKCIL	tr_C9ZJU1	0.075	22163.12	31.00
868	HLA-A0201	CIARAFVKAGSAVG	CIARAFVAV	0	0	0	7	4	CIARAFVKAGSAV	tr_C9ZJU1	0.075	22174.16	31.00
522	HLA-A0201	CAVSVRRLGWI	CAVSVLGWI	0	0	0	5	2	CAVSVRRLGWI	tr_C9ZJU1	0.075	22200.80	31.00
810	HLA-A0201	DLSIGGNIA	DLSIGGNIA	0	0	0	0	0	DLSIGGNIA	tr_C9ZJU1	0.075	22202.96	31.00
297	HLA-A0201	DAYLALATADY	YLALATADY	2	0	0	0	0	YLALATADY	tr_C9ZJU1	0.075	22204.16	31.00
276	HLA-A0201	YCIRGFVIQ	YCIRGFVIQ	0	0	0	0	0	YCIRGFVIQ	tr_C9ZJU1	0.075	22211.38	31.00
133	HLA-A0201	LCMLLRGKYS	LMLLRGKYS	0	0	0	1	1	LCMLLRGKYS	tr_C9ZJU1	0.075	22231.09	31.00
863	HLA-A0201	EVERLCIARA	EVELCIARA	0	0	0	3	1	EVERLCIARA	tr_C9ZJU1	0.075	22233.01	31.00
51	HLA-A0201	NCNGVESIHVRTLV	GVIHVRTLV	3	0	0	2	2	GVESIHVRTLV	tr_C9ZJU1	0.075	22236.62	31.00

832	HLA-A0201	AALSASRALNF	ALSASALNF	1	0	0	5	1	ALSASRALNF	tr_C9ZJU1	0.075	22273.95	31.00
181	HLA-A0201	ITSFWKLMCSSC	SFWLMCSSC	2	0	0	3	1	SFWKLMCSSC	tr_C9ZJU1	0.075	22279.97	31.00
390	HLA-A0201	ASVTNIATSTYA	SVIATSTYA	1	0	0	2	2	SVTNIATSTYA	tr_C9ZJU1	0.075	22285.52	31.00
826	HLA-A0201	SDENICAAL	SDENICAAL	0	0	0	0	0	SDENICAAL	tr_C9ZJU1	0.075	22288.41	31.00
506	HLA-A0201	CVVDALKRKL	CVVDALKRL	0	0	0	8	1	CVVDALKRKL	tr_C9ZJU1	0.075	22290.34	31.00
180	HLA-A0201	GITSFWKLMCSSC	SFWLMCSSC	3	0	0	3	1	SFWKLMCSSC	tr_C9ZJU1	0.075	22320.75	31.00
526	HLA-A0201	VRRLGWIKDVIQ	RLWIKDVIQ	2	0	0	2	1	RLGWIKDVIQ	tr_C9ZJU1	0.075	22321.23	31.00
613	HLA-A0201	LVSAVALRLSPNF	LVSAVALNF	0	0	0	7	4	LVSAVALRLSPNF	tr_C9ZJU1	0.075	22323.16	31.00
290	HLA-A0201	IITSIHIDAY	IITSIHIDA	0	0	0	0	0	IITSIHIDA	tr_C9ZJU1	0.075	22328.48	31.00
649	HLA-A0201	LYHAFLGSF	LYHAFLGSF	0	0	0	0	0	LYHAFLGSF	tr_C9ZJU1	0.074	22348.54	31.00
475	HLA-A0201	VFVESGRPLGYM	FVESGLGYM	1	0	0	5	2	FVESGRPLGYM	tr_C9ZJU1	0.074	22378.05	31.00
462	HLA-A0201	KSSVQYGLVLF	SSVQYLVLF	1	0	0	5	1	SSVQYGLVLF	tr_C9ZJU1	0.074	22380.72	31.00
774	HLA-A0201	GDIFVNGVHISQYP	GIFVNGVHI	0	0	0	1	1	GDIFVNGVHI	tr_C9ZJU1	0.074	22392.11	31.00
525	HLA-A0201	SVRRLGWIKDVIQ	RLWIKDVIQ	3	0	0	2	1	RLGWIKDVIQ	tr_C9ZJU1	0.074	22395.49	31.00
963	HLA-A0201	MRQSQQLPHF	MQSQQLPHF	0	0	0	1	1	MRQSQQLPHF	tr_C9ZJU1	0.074	22397.69	31.00
799	HLA-A0201	WQEENNLRF	WQEENNLRF	0	0	0	0	0	WQEENNLRF	tr_C9ZJU1	0.074	22426.29	31.00
794	HLA-A0201	RIANVWQEENNLRF	RIANVWLRF	0	0	0	6	5	RIANVWQEENNLRF	tr_C9ZJU1	0.074	22463.94	31.00
310	HLA-A0201	SDDWLLSRCVSQ	WLLSRCVSQ	3	0	0	0	0	WLLSRCVSQ	tr_C9ZJU1	0.074	22489.47	31.00
200	HLA-A0201	LAATVLFSPE	LAATVLFPE	0	0	0	7	1	LAATVLFSPE	tr_C9ZJU1	0.074	22497.50	31.00
883	HLA-A0201	CIFDEPTSAID	CIFDEPTSI	0	0	0	8	1	CIFDEPTSAI	tr_C9ZJU1	0.074	22498.23	31.00
688	HLA-A0201	QVLEGLQSVQRKID	QVLEGLQSV	0	0	0	0	0	QVLEGLQSV	tr_C9ZJU1	0.074	22512.35	31.00
271	HLA-A0201	AFESLYCIRGF	FSLYCIRGF	1	0	0	1	1	FESLYCIRGF	tr_C9ZJU1	0.074	22518.93	31.00
142	HLA-A0201	SIARYPPAAQA	SIYPPAAQA	0	0	0	2	2	SIARYPPAAQA	tr_C9ZJU1	0.074	22521.12	31.00
857	HLA-A0201	REFSGGEVERLCI	FSGGEVLCI	2	0	0	6	2	FSGGEVERLCI	tr_C9ZJU1	0.074	22523.07	31.00
474	HLA-A0201	MVFVESGRPLGY	MVFGRPLGY	0	0	0	3	3	MVFVESGRPLGY	tr_C9ZJU1	0.074	22523.56	31.00
298	HLA-A0201	AYLALATADYDPSD	YLALATAPS	1	0	0	7	3	YLALATADYDPS	tr_C9ZJU1	0.074	22536.73	31.00
524	HLA-A0201	VSVRRLGWIKDVIQ	RLWIKDVIQ	4	0	0	2	1	RLGWIKDVIQ	tr_C9ZJU1	0.074	22538.92	31.00
484	HLA-A0201	GYMWDLLTQ	GYMWDLLTQ	0	0	0	0	0	GYMWDLLTQ	tr_C9ZJU1	0.074	22539.17	31.00
828	HLA-A0201	ENICAALSASRAL	NICAALSAL	1	0	0	7	3	NICAALSASRAL	tr_C9ZJU1	0.074	22541.12	31.00
527	HLA-A0201	RRLGWIKDVIQ	RLWIKDVIQ	1	0	0	2	1	RLGWIKDVIQ	tr_C9ZJU1	0.074	22554.04	31.00
474	HLA-A0201	MVFVESGRPLG	MVFESGRPL	0	0	0	3	1	MVFVESGRPL	tr_C9ZJU1	0.074	22555.02	31.00
529	HLA-A0201	LGWIKDVIQS	LGWIKVIQS	0	0	0	5	1	LGWIKDVIQS	tr_C9ZJU1	0.074	22568.20	31.00
545	HLA-A0201	ASPSTCVWQC	ASSTCVWQC	0	0	0	2	1	ASPSTCVWQC	tr_C9ZJU1	0.073	22573.57	31.00
311	HLA-A0201	DDWLLSRCVSQ	WLLSRCVSQ	2	0	0	0	0	WLLSRCVSQ	tr_C9ZJU1	0.073	22581.39	31.00
830	HLA-A0201	ICAALSASRALNF	ALSASALNF	3	0	0	5	1	ALSASRALNF	tr_C9ZJU1	0.073	22587.74	31.00
127	HLA-A0201	AEEVLALCM	AEEVLALCM	0	0	0	0	0	AEEVLALCM	tr_C9ZJU1	0.073	22591.41	31.00
908	HLA-A0201	EGNNLLRDSTII	GLLRDSTII	1	0	0	1	2	GNNLLRDSTII	tr_C9ZJU1	0.073	22592.39	31.00
830	HLA-A0201	ICAALSASRAL	ICAALSAAL	0	0	0	7	2	ICAALSASRAL	tr_C9ZJU1	0.073	22607.79	31.00
487	HLA-A0201	WDLLTQHVIWP	WLTQHVIWP	0	0	0	1	2	WDLLTQHVIWP	tr_C9ZJU1	0.073	22622.73	31.00
831	HLA-A0201	CAALSASRALNF	ALSASALNF	2	0	0	5	1	ALSASRALNF	tr_C9ZJU1	0.073	22629.34	31.00
777	HLA-A0201	FVNGVHISQYP	FVNGVHIYP	0	0	0	7	2	FVNGVHISQYP	tr_C9ZJU1	0.073	22630.80	31.00
175	HLA-A0201	RSLQSGITSFW	SLSGITSFW	1	0	0	2	1	SLQSGITSFW	tr_C9ZJU1	0.073	22633.50	31.00
684	HLA-A0201	LGCYQVLEGLQS	LCYQVLEGL	0	0	0	1	1	LGCYQVLEGL	tr_C9ZJU1	0.073	22633.75	31.00
635	HLA-A0201	EIVNQLRSI	EIVNQLRSI	0	0	0	0	0	EIVNQLRSI	tr_C9ZJU1	0.073	22636.93	31.00
877	HLA-A0201	GSAVGLCIFD	GSAVGLCIF	0	0	0	0	0	GSAVGLCIF	tr_C9ZJU1	0.073	22637.41	31.00
827	HLA-A0201	DENICAALSASRAL	NICAALSAL	2	0	0	7	3	NICAALSASRAL	tr_C9ZJU1	0.073	22638.16	31.00
291	HLA-A0201	ITSIHIDAY	ITSIHIDAY	0	0	0	0	0	ITSIHIDAY	tr_C9ZJU1	0.073	22639.13	31.00
87	HLA-A0201	HPDHLLVCDIWN	HLLVCDIWN	0	0	0	1	3	HPDHLLVCDIWN	tr_C9ZJU1	0.073	22667.56	31.00
487	HLA-A0201	WDLLTQHVI	WDLLTQHVI	0	0	0	0	0	WDLLTQHVI	tr_C9ZJU1	0.073	22670.50	31.00
895	HLA-A0201	ITEGEIFDTLR	ITEGEIFTL	0	0	0	7	1	ITEGEIFDTL	tr_C9ZJU1	0.073	22671.00	31.00
66	HLA-A0201	NHNIMQLPSV	HNIMQLPSV	1	0	0	0	0	HNIMQLPSV	tr_C9ZJU1	0.073	22685.96	32.00
179	HLA-A0201	SGITSFWKLMC	GITSFWLMC	1	0	0	6	1	GITSFWKLMC	tr_C9ZJU1	0.073	22690.88	32.00
306	HLA-A0201	DYDPSDDWLLSRCV	YDWLLSRCV	1	0	0	1	4	YDPSDDWLLSRCV	tr_C9ZJU1	0.073	22691.61	32.00
872	HLA-A0201	AFVKAGSAV	AFVKAGSAV	0	0	0	0	0	AFVKAGSAV	tr_C9ZJU1	0.073	22691.61	32.00

431	HLA-A0201	KQKDDGIKCML	KQDGIKCML	0	0	0	2	2	KQKDDGIKCML	tr_C9ZJU1	0.073	22695.78	32.00
179	HLA-A0201	SGITSFWKLMCSSC	SFWLMCSSC	4	0	0	3	1	SFWKLMCSSC	tr_C9ZJU1	0.073	22699.72	32.00
461	HLA-A0201	DKSSVQYGLVLF	SSVQYLVLF	2	0	0	5	1	SSVQYGLVLF	tr_C9ZJU1	0.073	22712.49	32.00
177	HLA-A0201	LQSGITSFWK	LQSGITFWK	0	0	0	6	1	LQSGITSFWK	tr_C9ZJU1	0.073	22719.37	32.00
122	HLA-A0201	LDDVKAEEVLALC	LAEEVLALC	0	0	0	1	4	LDDVKAEEVLALC	tr_C9ZJU1	0.073	22737.33	32.00
822	HLA-A0201	LWDCSDENI	LWDCSDENI	0	0	0	0	0	LWDCSDENI	tr_C9ZJU1	0.073	22740.78	32.00
486	HLA-A0201	MWDLLTQHVIW	MLLTQHVIW	0	0	0	1	2	MWDLLTQHVIW	tr_C9ZJU1	0.073	22741.01	32.00
746	HLA-A0201	EFLGIAGYSGSG	FLGIAGYSG	1	0	0	7	2	FLGIAGYSGSG	tr_C9ZJU1	0.073	22744.21	32.00
932	HLA-A0201	DKIVVLNEGMVAE	KIVVLNEMV	1	0	0	7	1	KIVVLNEGMV	tr_C9ZJU1	0.073	22747.40	32.00
40	HLA-A0201	CKDDASIAI	CKDDASIAI	0	0	0	0	0	CKDDASIAI	tr_C9ZJU1	0.073	22760.21	32.00
724	HLA-A0201	SFRYSDRHPYV	FRYDRHPYV	1	0	0	3	1	FRYSDRHPYV	tr_C9ZJU1	0.073	22770.07	32.00
427	HLA-A0201	TLLRKQKDDGI	TLLQKDDGI	0	0	0	3	2	TLLRKQKDDGI	tr_C9ZJU1	0.073	22776.22	32.00
499	HLA-A0201	VPSVFSGCV	VPSVFSGCV	0	0	0	0	0	VPSVFSGCV	tr_C9ZJU1	0.073	22776.47	32.00
647	HLA-A0201	CPLYHAFLGSFYD	CLYHAFLGS	0	0	0	1	1	CPLYHAFLGS	tr_C9ZJU1	0.073	22781.16	32.00
657	HLA-A0201	FYDECGMSEAQVD	FYDECGMSV	0	0	0	8	3	FYDECGMSEAQV	tr_C9ZJU1	0.073	22781.89	32.00
741	HLA-A0201	VVNKGEFLGIAGY	VVFLGIAGY	0	0	0	2	4	VVNKGEFLGIAGY	tr_C9ZJU1	0.073	22796.19	32.00
459	HLA-A0201	RHDKSSVQYGLVLF	SSVQYLVLF	4	0	0	5	1	SSVQYGLVLF	tr_C9ZJU1	0.073	22811.48	32.00
255	HLA-A0201	MGLPATFVS	MGLPATFVS	0	0	0	0	0	MGLPATFVS	tr_C9ZJU1	0.073	22818.17	32.00
299	HLA-A0201	YLALATADYDP	YLALATYDP	0	0	0	6	2	YLALATADYDP	tr_C9ZJU1	0.072	22830.50	32.00
844	HLA-A0201	QKRSSGIHAPLNV	RSSGIPLNV	2	0	0	5	2	RSSGIHAPLNV	tr_C9ZJU1	0.072	22843.61	32.00
812	HLA-A0201	SIGGNIALGNLWD	SIGGNIALL	0	0	0	8	2	SIGGNIALGNL	tr_C9ZJU1	0.072	22847.05	32.00
783	HLA-A0201	ISQYPARMLRRRI	SQYPARMLI	1	0	0	8	3	SQYPARMLRRRI	tr_C9ZJU1	0.072	22848.54	32.00
803	HLA-A0201	NNLRFIEDL	NNLRFIEDL	0	0	0	0	0	NNLRFIEDL	tr_C9ZJU1	0.072	22876.75	32.00
856	HLA-A0201	VREFSGGEVERLCI	FSGGEVLCI	3	0	0	6	2	FSGGEVERLCI	tr_C9ZJU1	0.072	22883.18	32.00
460	HLA-A0201	HDKSSVQYGLVLF	SSVQYLVLF	3	0	0	5	1	SSVQYGLVLF	tr_C9ZJU1	0.072	22883.18	32.00
471	HLA-A0201	LFSMVFVESGRP	FSMVFVESP	1	0	0	8	2	FSMVFVESGRP	tr_C9ZJU1	0.072	22898.54	32.00
780	HLA-A0201	GVHISQYPARML	GVHISQYPL	0	0	0	8	3	GVHISQYPARML	tr_C9ZJU1	0.072	22900.28	32.00
146	HLA-A0201	YPPAAQADV	YPPAAQADV	0	0	0	0	0	YPPAAQADV	tr_C9ZJU1	0.072	22926.30	32.00
503	HLA-A0201	FSGCVVDALKR	FSGCVVDAL	0	0	0	0	0	FSGCVVDAL	tr_C9ZJU1	0.072	22932.51	32.00
893	HLA-A0201	DTITEGEIFDTLR	ITEGEIFTL	2	0	0	7	1	ITEGEIFDTL	tr_C9ZJU1	0.072	22944.92	32.00
311	HLA-A0201	DDWLLSRCV	DDWLLSRCV	0	0	0	0	0	DDWLLSRCV	tr_C9ZJU1	0.072	22959.81	32.00
817	HLA-A0201	IALGNLWDCSDEN	ILWDCSDEN	0	0	0	1	4	IALGNLWDCSDEN	tr_C9ZJU1	0.072	22960.55	32.00
709	HLA-A0201	PPSLDGPWSVEFR	SLDGWSVEF	2	0	0	4	1	SLDGPWSVEF	tr_C9ZJU1	0.072	22963.78	32.00
712	HLA-A0201	LDGPWSVEFREV	GPWSVEFEV	2	0	0	7	1	GPWSVEFREV	tr_C9ZJU1	0.072	22991.14	32.00
557	HLA-A0201	DYLQGFAAL	DYLQGFAAL	0	0	0	0	0	DYLQGFAAL	tr_C9ZJU1	0.072	22993.38	32.00
709	HLA-A0201	PPSLDGPWSVEFRE	SLWSVEFRE	2	0	0	2	3	SLDGPWSVEFRE	tr_C9ZJU1	0.072	23002.82	32.00
942	HLA-A0201	VAEVGSWET	VAEVGSWET	0	0	0	0	0	VAEVGSWET	tr_C9ZJU1	0.072	23010.79	32.00
935	HLA-A0201	VVLNEGMVAE	VLNEGMVAE	0	0	0	1	1	VVLNEGMVAE	tr_C9ZJU1	0.072	23015.78	32.00
309	HLA-A0201	PSDDWLLSRCVSQ	WLLSRCVSQ	4	0	0	0	0	WLLSRCVSQ	tr_C9ZJU1	0.072	23018.27	32.00
528	HLA-A0201	RLGWIKDVIQSSPR	RLGWIVIQS	0	0	0	5	2	RLGWIKDVIQS	tr_C9ZJU1	0.072	23028.98	32.00
876	HLA-A0201	AGSAVGLCIF	AGSAVLCIF	0	0	0	5	1	AGSAVGLCIF	tr_C9ZJU1	0.072	23051.67	32.00
931	HLA-A0201	ADKIVVLNEGMVAE	KIVVLNEMV	2	0	0	7	1	KIVVLNEGMV	tr_C9ZJU1	0.072	23063.89	32.00
516	HLA-A0201	QAGGHTCAVSV	QAGGCAVSV	0	0	0	4	2	QAGGHTCAVSV	tr_C9ZJU1	0.071	23083.37	32.00
808	HLA-A0201	IEDLSIGGNIA	ILSIGGNIA	0	0	0	1	2	IEDLSIGGNIA	tr_C9ZJU1	0.071	23088.36	32.00
503	HLA-A0201	FSGCVVDALKRKLQ	FSGCVVDAL	0	0	0	8	4	FSGCVVDALKRKL	tr_C9ZJU1	0.071	23102.36	32.00
556	HLA-A0201	QDYLQGFAA	QDYLQGFAA	0	0	0	0	0	QDYLQGFAA	tr_C9ZJU1	0.071	23118.11	32.00
747	HLA-A0201	FLGIAGYSGSGK	FLGIAGYSK	0	0	0	8	3	FLGIAGYSGSGK	tr_C9ZJU1	0.071	23130.86	32.00
308	HLA-A0201	DPSDDWLLSRCVSQ	WLLSRCVSQ	5	0	0	0	0	WLLSRCVSQ	tr_C9ZJU1	0.071	23154.65	32.00
621	HLA-A0201	LSPNFFTQSAL	LSPNFFTAL	0	0	0	7	2	LSPNFFTQSAL	tr_C9ZJU1	0.071	23155.65	32.00
950	HLA-A0201	TLSRTGPHSC	TLSTGPHSC	0	0	0	3	1	TLSRTGPHSC	tr_C9ZJU1	0.071	23164.42	32.00
730	HLA-A0201	RHPYVLRRV	RHPYVLRRV	0	0	0	0	0	RHPYVLRRV	tr_C9ZJU1	0.071	23167.69	32.00
389	HLA-A0201	LASVTNIATSTYA	SVIATSTYA	2	0	0	2	2	SVTNIATSTYA	tr_C9ZJU1	0.071	23179.47	32.00
782	HLA-A0201	HISQYPARMLRRRI	SQYPARMLI	2	0	0	8	3	SQYPARMLRRRI	tr_C9ZJU1	0.071	23183.24	32.00
798	HLA-A0201	VWQEENNLRFI	WQEENNLFI	1	0	0	7	1	WQEENNLRFI	tr_C9ZJU1	0.071	23183.47	32.00

150	HLA-A0201	AQADVVVQE	AQADVVVQE	0	0	0	0	0	AQADVVVQE	tr_C9ZJU1	0.071	23190.01	32.00
3	HLA-A0201	RARPAVLLDC	RAPAVLLDC	0	0	0	2	1	RARPAVLLDC	tr_C9ZJU1	0.071	23196.28	32.00
174	HLA-A0201	KRSLQSGITSFW	SLSGITSFW	2	0	0	2	1	SLQSGITSFW	tr_C9ZJU1	0.071	23202.31	32.00
883	HLA-A0201	CIFDEPTSAIDT	CIFDSAIDT	0	0	0	4	3	CIFDEPTSAIDT	tr_C9ZJU1	0.071	23204.57	32.00
297	HLA-A0201	DAYLALATAD	DAYLALATA	0	0	0	0	0	DAYLALATA	tr_C9ZJU1	0.071	23208.58	32.00
711	HLA-A0201	SLDGPWSVE	SLDGPWSVE	0	0	0	0	0	SLDGPWSVE	tr_C9ZJU1	0.071	23220.39	32.00
501	HLA-A0201	SVFSGCVVD	SVFSGCVVD	0	0	0	0	0	SVFSGCVVD	tr_C9ZJU1	0.071	23235.46	32.00
178	HLA-A0201	QSGITSFWKLMC	GITSFWLMC	2	0	0	6	1	GITSFWKLMC	tr_C9ZJU1	0.071	23240.99	32.00
756	HLA-A0201	SGKTTLLRLL	SGKTTLLLL	0	0	0	7	1	SGKTTLLRLL	tr_C9ZJU1	0.071	23259.36	32.00
154	HLA-A0201	VVVQEFPEELY	VVVEFPEEL	0	0	0	3	1	VVVQEFPEEL	tr_C9ZJU1	0.071	23265.40	32.00
913	HLA-A0201	LRDSTIIIVAHR	LTIIIVAHR	0	0	0	1	3	LRDSTIIIVAHR	tr_C9ZJU1	0.071	23282.02	33.00
752	HLA-A0201	GYSGSGKTTLLRLL	YSTTLLRLL	1	0	0	2	4	YSGSGKTTLLRLL	tr_C9ZJU1	0.071	23287.06	33.00
50	HLA-A0201	LNCNGVESI	LNCNGVESI	0	0	0	0	0	LNCNGVESI	tr_C9ZJU1	0.071	23292.35	33.00
42	HLA-A0201	DDASIAIFL	DDASIAIFL	0	0	0	0	0	DDASIAIFL	tr_C9ZJU1	0.071	23296.38	33.00
892	HLA-A0201	IDTITEGEIFDTLR	ITEGEIFTL	3	0	0	7	1	ITEGEIFDTL	tr_C9ZJU1	0.071	23298.15	33.00
228	HLA-A0201	YAAEIGKSLN	YAAEIGKSL	0	0	0	0	0	YAAEIGKSL	tr_C9ZJU1	0.071	23298.90	33.00
335	HLA-A0201	QSRVPALLEVM	SVPALLEVM	1	0	0	1	1	SRVPALLEVM	tr_C9ZJU1	0.070	23319.07	33.00
799	HLA-A0201	WQEENNLRFIEDLS	WQLRFIEDL	0	0	0	2	4	WQEENNLRFIEDL	tr_C9ZJU1	0.070	23327.66	33.00
266	HLA-A0201	VASTFAFESLY	VASTFAFEL	0	0	0	8	1	VASTFAFESL	tr_C9ZJU1	0.070	23335.23	33.00
40	HLA-A0201	CKDDASIAIFLNCN	CSIAIFLNC	0	0	0	1	4	CKDDASIAIFLNC	tr_C9ZJU1	0.070	23344.58	33.00
852	HLA-A0201	APLNVREFSG	ALNVREFSG	0	0	0	1	1	APLNVREFSG	tr_C9ZJU1	0.070	23357.71	33.00
776	HLA-A0201	IFVNGVHISQ	FVNGVHISQ	1	0	0	0	0	FVNGVHISQ	tr_C9ZJU1	0.070	23371.88	33.00
661	HLA-A0201	CGMSEAQVDEAH	GMSEAQVEA	1	0	0	7	1	GMSEAQVDEA	tr_C9ZJU1	0.070	23379.46	33.00
83	HLA-A0201	MKNSHPDHLLV	MSHPDHLLV	0	0	0	1	2	MKNSHPDHLLV	tr_C9ZJU1	0.070	23386.29	33.00
745	HLA-A0201	GEFLGIAGYSGSG	FLGIAGYSG	2	0	0	7	2	FLGIAGYSGSG	tr_C9ZJU1	0.070	23398.69	33.00
890	HLA-A0201	SAIDTITEGEIFDT	AIDTIIFDT	1	0	0	5	4	AIDTITEGEIFDT	tr_C9ZJU1	0.070	23408.32	33.00
950	HLA-A0201	TLSRTGPHS	TLSRTGPHS	0	0	0	0	0	TLSRTGPHS	tr_C9ZJU1	0.070	23411.35	33.00
231	HLA-A0201	EIGKSLNGDV	EIGKSLNGV	0	0	0	8	1	EIGKSLNGDV	tr_C9ZJU1	0.070	23432.14	33.00
387	HLA-A0201	SQLASVTNIATSTY	SLASVTNIA	0	0	0	1	1	SQLASVTNIA	tr_C9ZJU1	0.070	23435.18	33.00
267	HLA-A0201	ASTFAFESLY	ASTFAFESL	0	0	0	0	0	ASTFAFESL	tr_C9ZJU1	0.070	23437.97	33.00
831	HLA-A0201	CAALSASRAL	CAALSASAL	0	0	0	7	1	CAALSASRAL	tr_C9ZJU1	0.070	23441.77	33.00
666	HLA-A0201	AQVDEAHLRRHL	AQVDEAHLL	0	0	0	8	3	AQVDEAHLRRHL	tr_C9ZJU1	0.070	23448.87	33.00
347	HLA-A0201	GCRVVFSFP	GCRVVFSFP	0	0	0	0	0	GCRVVFSFP	tr_C9ZJU1	0.070	23453.70	33.00
140	HLA-A0201	KYSIARYPPAAQA	YIARYPPAA	1	0	0	1	1	YSIARYPPAA	tr_C9ZJU1	0.070	23470.45	33.00
173	HLA-A0201	RKRSLQSGITSFW	SLSGITSFW	3	0	0	2	1	SLQSGITSFW	tr_C9ZJU1	0.070	23484.42	33.00
648	HLA-A0201	PLYHAFLGSFYD	PLYHAFLFY	0	0	0	7	2	PLYHAFLGSFY	tr_C9ZJU1	0.070	23487.72	33.00
521	HLA-A0201	TCAVSVRRLGWI	CAVSVLGWI	1	0	0	5	2	CAVSVRRLGWI	tr_C9ZJU1	0.070	23487.72	33.00
894	HLA-A0201	TITEGEIFDTLRLR	TITEGEIFL	0	0	0	8	4	TITEGEIFDTLRL	tr_C9ZJU1	0.070	23496.36	33.00
476	HLA-A0201	FVESGRPLGY	FVSGRPLGY	0	0	0	2	1	FVESGRPLGY	tr_C9ZJU1	0.070	23501.95	33.00
180	HLA-A0201	GITSFWKLMCSS	GIWKLMCSS	0	0	0	2	3	GITSFWKLMCSS	tr_C9ZJU1	0.070	23508.06	33.00
6	HLA-A0201	PAVLLDCERKA	VLLDCERKA	2	0	0	0	0	VLLDCERKA	tr_C9ZJU1	0.070	23520.03	33.00
647	HLA-A0201	CPLYHAFLGSFYDE	CLYHAFLGS	0	0	0	1	1	CPLYHAFLGS	tr_C9ZJU1	0.070	23529.43	33.00
67	HLA-A0201	HNIMQLPSVF	HIMQLPSVF	0	0	0	1	1	HNIMQLPSVF	tr_C9ZJU1	0.070	23530.96	33.00
388	HLA-A0201	QLASVTNIATSTY	QLASVTIAT	0	0	0	6	1	QLASVTNIAT	tr_C9ZJU1	0.070	23533.00	33.00
963	HLA-A0201	MRQSQQLPHFVKK	RQSQQLPFV	1	0	0	7	1	RQSQQLPHFV	tr_C9ZJU1	0.070	23536.56	33.00
591	HLA-A0201	YEQCGRDVLHGL	YEQCVLHGL	0	0	0	4	3	YEQCGRDVLHGL	tr_C9ZJU1	0.070	23539.87	33.00
680	HLA-A0201	SGSSLGCYQVL	SSLGCYQVL	0	0	0	1	2	SGSSLGCYQVL	tr_C9ZJU1	0.070	23545.23	33.00
120	HLA-A0201	CILDDVKAEE	ILDDVKAEE	1	0	0	0	0	ILDDVKAEE	tr_C9ZJU1	0.070	23550.58	33.00
54	HLA-A0201	GVESIHVRTLVF	GVIHVRTLV	0	0	0	2	2	GVESIHVRTLV	tr_C9ZJU1	0.070	23558.48	33.00
304	HLA-A0201	TADYDPSDDWLL	TADYDPWLL	0	0	0	6	3	TADYDPSDDWLL	tr_C9ZJU1	0.070	23558.74	33.00
52	HLA-A0201	CNGVESIHV	CNGVESIHV	0	0	0	0	0	CNGVESIHV	tr_C9ZJU1	0.070	23563.83	33.00
810	HLA-A0201	DLSIGGNIALG	DLIGGNIAL	0	0	0	2	1	DLSIGGNIAL	tr_C9ZJU1	0.070	23569.43	33.00
287	HLA-A0201	LSDIITSIHID	LSIITSIHI	0	0	0	2	1	LSDIITSIHI	tr_C9ZJU1	0.069	23573.78	33.00
816	HLA-A0201	NIALGNLWDCSDEN	ILWDCSDEN	1	0	0	1	4	IALGNLWDCSDEN	tr_C9ZJU1	0.069	23574.80	33.00

188	HLA-A0201	MCSSCSIPQ	MCSSCSIPQ	0	0	0	0	0	MCSSCSIPQ	tr_C9ZJU1	0.069	23580.15	33.00
613	HLA-A0201	LVSAVALRLSP	LVSAVALRL	0	0	0	0	0	LVSAVALRL	tr_C9ZJU1	0.069	23583.21	33.00
577	HLA-A0201	AAAAFATAS	AAAAFATAS	0	0	0	0	0	AAAAFATAS	tr_C9ZJU1	0.069	23583.73	33.00
878	HLA-A0201	SAVGLCIFDEP	AVGLCIFEP	1	0	0	7	1	AVGLCIFDEP	tr_C9ZJU1	0.069	23583.99	33.00
75	HLA-A0201	VFAVHCGLMK	FAVHCGLMK	1	0	0	0	0	FAVHCGLMK	tr_C9ZJU1	0.069	23596.75	33.00
471	HLA-A0201	LFSMVFVESGR	FSMVFVESR	1	0	0	8	1	FSMVFVESGR	tr_C9ZJU1	0.069	23605.68	33.00
762	HLA-A0201	LRLLNRTYAPTEGD	RLLNRTYPT	1	0	0	7	1	RLLNRTYAPT	tr_C9ZJU1	0.069	23609.76	33.00
502	HLA-A0201	VFSGCVVDALKR	FSGCVVDAL	1	0	0	0	0	FSGCVVDAL	tr_C9ZJU1	0.069	23610.28	33.00
592	HLA-A0201	EQCGRDVLHGL	EQCGVLHGL	0	0	0	4	2	EQCGRDVLHGL	tr_C9ZJU1	0.069	23611.04	33.00
727	HLA-A0201	YSDRHPYVLR	YSDRHPYVL	0	0	0	0	0	YSDRHPYVL	tr_C9ZJU1	0.069	23615.38	33.00
527	HLA-A0201	RRLGWIKDVIQSSP	RLGWIVIQS	1	0	0	5	2	RLGWIKDVIQS	tr_C9ZJU1	0.069	23620.50	33.00
306	HLA-A0201	DYDPSDDWLL	YDPSDDWLL	1	0	0	0	0	YDPSDDWLL	tr_C9ZJU1	0.069	23641.21	33.00
145	HLA-A0201	RYPPAAQADVVV	YAAQADVVV	1	0	0	1	2	YPPAAQADVVV	tr_C9ZJU1	0.069	23654.01	33.00
541	HLA-A0201	RAATASPST	RAATASPST	0	0	0	0	0	RAATASPST	tr_C9ZJU1	0.069	23665.53	33.00
756	HLA-A0201	SGKTTLLRL	SGKTTLLRL	0	0	0	0	0	SGKTTLLRL	tr_C9ZJU1	0.069	23681.15	33.00
222	HLA-A0201	VHWRPIYAAEI	VHWIYAAEI	0	0	0	3	2	VHWRPIYAAEI	tr_C9ZJU1	0.069	23686.28	33.00
332	HLA-A0201	DELQSRVPALLE	ELQSVPALL	1	0	0	4	1	ELQSRVPALL	tr_C9ZJU1	0.069	23692.42	33.00
774	HLA-A0201	GDIFVNGVHISQY	GIFVNGVHI	0	0	0	1	1	GDIFVNGVHI	tr_C9ZJU1	0.069	23692.68	33.00
299	HLA-A0201	YLALATADYDPSDD	YLALATAPS	0	0	0	7	3	YLALATADYDPS	tr_C9ZJU1	0.069	23719.36	33.00
744	HLA-A0201	KGEFLGIAGYSGSG	FLGIAGYSG	3	0	0	7	2	FLGIAGYSGSG	tr_C9ZJU1	0.069	23721.15	33.00
216	HLA-A0201	LGMHAFVHW	LGMHAFVHW	0	0	0	0	0	LGMHAFVHW	tr_C9ZJU1	0.069	23722.43	33.00
172	HLA-A0201	NRKRSLQSGITSFW	SLSGITSFW	4	0	0	2	1	SLQSGITSFW	tr_C9ZJU1	0.069	23730.65	33.00
246	HLA-A0201	PFRFIIKQLMG	FRFIIQLMG	1	0	0	5	1	FRFIIKQLMG	tr_C9ZJU1	0.069	23740.66	33.00
258	HLA-A0201	PATFVSMAVAS	ATFVSMAVA	1	0	0	0	0	ATFVSMAVA	tr_C9ZJU1	0.069	23741.18	33.00
101	HLA-A0201	MALVNCSIR	MALVNCSIR	0	0	0	0	0	MALVNCSIR	tr_C9ZJU1	0.069	23748.88	33.00
52	HLA-A0201	CNGVESIHVRTLVF	CIHVRTLVF	0	0	0	1	5	CNGVESIHVRTLVF	tr_C9ZJU1	0.069	23750.17	33.00
625	HLA-A0201	FFTQSALDQT	FTQSALDQT	0	0	0	1	1	FFTQSALDQT	tr_C9ZJU1	0.069	23757.88	33.00
376	HLA-A0201	ESLYMESQFTKSQ	SLYMFTKSQ	1	0	0	4	3	SLYMESQFTKSQ	tr_C9ZJU1	0.069	23765.08	33.00
504	HLA-A0201	SGCVVDALKRKLQA	CVVDALLQA	2	0	0	6	3	CVVDALKRKLQA	tr_C9ZJU1	0.069	23783.09	33.00
157	HLA-A0201	QEFPEELYLGL	QEFELYLGL	0	0	0	3	2	QEFPEELYLGL	tr_C9ZJU1	0.069	23783.09	33.00
505	HLA-A0201	GCVVDALKRKLQA	CVVDALLQA	1	0	0	6	3	CVVDALKRKLQA	tr_C9ZJU1	0.069	23801.11	33.00
813	HLA-A0201	IGGNIALGNLWDCS	ILGNLWDCS	0	0	0	1	5	IGGNIALGNLWDCS	tr_C9ZJU1	0.069	23803.16	33.00
140	HLA-A0201	KYSIARYPPAAQ	YIARYPPAA	1	0	0	1	1	YSIARYPPAA	tr_C9ZJU1	0.069	23819.92	33.00
928	HLA-A0201	LKHADKIVVLNEGM	LIVVLNEGM	0	0	0	1	5	LKHADKIVVLNEGM	tr_C9ZJU1	0.069	23821.97	33.00
846	HLA-A0201	RSSGIHAPLN	RSSGIHAPL	0	0	0	0	0	RSSGIHAPL	tr_C9ZJU1	0.068	23835.38	33.00
379	HLA-A0201	YMESQFTKSQ	YMSQFTKSQ	0	0	0	2	1	YMESQFTKSQ	tr_C9ZJU1	0.068	23844.92	34.00
741	HLA-A0201	VVNKGEFLGIA	VVGEFLGIA	0	0	0	2	2	VVNKGEFLGIA	tr_C9ZJU1	0.068	23852.41	34.00
722	HLA-A0201	EVSFRYSDRHPYVL	VSFRYSYVL	1	0	0	6	4	VSFRYSDRHPYVL	tr_C9ZJU1	0.068	23857.57	34.00
58	HLA-A0201	IHVRTLVFNHNIMQ	ILVFNHNIM	0	0	0	1	4	IHVRTLVFNHNIM	tr_C9ZJU1	0.068	23872.80	34.00
867	HLA-A0201	LCIARAFVKAGSA	LCIARAFSA	0	0	0	7	4	LCIARAFVKAGSA	tr_C9ZJU1	0.068	23896.59	34.00
55	HLA-A0201	VESIHVRTL	VESIHVRTL	0	0	0	0	0	VESIHVRTL	tr_C9ZJU1	0.068	23914.42	34.00
638	HLA-A0201	NQLRSIQHWC	QLRSIQHWC	1	0	0	0	0	QLRSIQHWC	tr_C9ZJU1	0.068	23917.79	34.00
685	HLA-A0201	GCYQVLEGLQS	GCYQVLEGL	0	0	0	0	0	GCYQVLEGL	tr_C9ZJU1	0.068	23924.78	34.00
479	HLA-A0201	SGRPLGYMWDL	SGLGYMWDL	0	0	0	2	2	SGRPLGYMWDL	tr_C9ZJU1	0.068	23929.44	34.00
166	HLA-A0201	GLITAYNRK	GLITAYNRK	0	0	0	0	0	GLITAYNRK	tr_C9ZJU1	0.068	23940.32	34.00
757	HLA-A0201	GKTTLLRLL	GKTTLLRLL	0	0	0	0	0	GKTTLLRLL	tr_C9ZJU1	0.068	23942.91	34.00
867	HLA-A0201	LCIARAFVK	LCIARAFVK	0	0	0	0	0	LCIARAFVK	tr_C9ZJU1	0.068	23943.42	34.00
418	HLA-A0201	KNSLTSSPL	KNSLTSSPL	0	0	0	0	0	KNSLTSSPL	tr_C9ZJU1	0.068	23974.01	34.00
779	HLA-A0201	NGVHISQYPARML	HISQYPAML	3	0	0	7	1	HISQYPARML	tr_C9ZJU1	0.068	23974.54	34.00
774	HLA-A0201	GDIFVNGVHIS	GIFVNGVHI	0	0	0	1	1	GDIFVNGVHI	tr_C9ZJU1	0.068	23994.51	34.00
656	HLA-A0201	SFYDECGMSEAQVD	FYDECGMSV	1	0	0	8	3	FYDECGMSEAQV	tr_C9ZJU1	0.068	24013.21	34.00
842	HLA-A0201	FVQKRSSGIHA	FVQSSGIHA	0	0	0	3	2	FVQKRSSGIHA	tr_C9ZJU1	0.068	24041.81	34.00
324	HLA-A0201	WQASGSVMDELQ	WQASVMDEL	0	0	0	3	2	WQASGSVMDEL	tr_C9ZJU1	0.068	24049.88	34.00
551	HLA-A0201	VWQCRQDYL	VWQCRQDYL	0	0	0	0	0	VWQCRQDYL	tr_C9ZJU1	0.068	24056.90	34.00

366	HLA-A0201	QWSNFLYFLNE	QWSNFLYFL	0	0	0	0	0	QWSNFLYFL	tr_C9ZJU1	0.068	24075.92	34.00
864	HLA-A0201	VERLCIARAF	VERLCIAAF	0	0	0	7	1	VERLCIARAF	tr_C9ZJU1	0.068	24078.00	34.00
495	HLA-A0201	IWPLVPSVFS	IWPLVSVFS	0	0	0	5	1	IWPLVPSVFS	tr_C9ZJU1	0.068	24081.91	34.00
536	HLA-A0201	IQSSPRAAT	IQSSPRAAT	0	0	0	0	0	IQSSPRAAT	tr_C9ZJU1	0.068	24082.16	34.00
853	HLA-A0201	PLNVREFSGGEV	PLNVREFSV	0	0	0	8	3	PLNVREFSGGEV	tr_C9ZJU1	0.068	24087.12	34.00
874	HLA-A0201	VKAGSAVGL	VKAGSAVGL	0	0	0	0	0	VKAGSAVGL	tr_C9ZJU1	0.067	24119.46	34.00
781	HLA-A0201	VHISQYPARML	HISQYPAML	1	0	0	7	1	HISQYPARML	tr_C9ZJU1	0.067	24141.65	34.00
394	HLA-A0201	NIATSTYAG	NIATSTYAG	0	0	0	0	0	NIATSTYAG	tr_C9ZJU1	0.067	24142.43	34.00
159	HLA-A0201	FPEELYLGLIT	FPEELYLLI	0	0	0	7	1	FPEELYLGLI	tr_C9ZJU1	0.067	24149.49	34.00
503	HLA-A0201	FSGCVVDALKRK	FSGCVVDAL	0	0	0	0	0	FSGCVVDAL	tr_C9ZJU1	0.067	24155.50	34.00
88	HLA-A0201	PDHLLVCDIWN	HLLVCDIWN	2	0	0	0	0	HLLVCDIWN	tr_C9ZJU1	0.067	24157.06	34.00
546	HLA-A0201	SPSTCVWQC	SPSTCVWQC	0	0	0	0	0	SPSTCVWQC	tr_C9ZJU1	0.067	24165.43	34.00
275	HLA-A0201	LYCIRGFVIQ	LYCIRGFVI	0	0	0	0	0	LYCIRGFVI	tr_C9ZJU1	0.067	24172.75	34.00
207	HLA-A0201	SPEVILKCILG	SVILKCILG	0	0	0	1	2	SPEVILKCILG	tr_C9ZJU1	0.067	24180.34	34.00
136	HLA-A0201	LLRGKYSIARY	LLRYSIARY	0	0	0	3	2	LLRGKYSIARY	tr_C9ZJU1	0.067	24191.06	34.00
920	HLA-A0201	IVAHRLATLKH	IVAHRLATL	0	0	0	0	0	IVAHRLATL	tr_C9ZJU1	0.067	24191.86	34.00
375	HLA-A0201	NESLYMESQFTKSQ	SLYMFTKSQ	2	0	0	4	3	SLYMESQFTKSQ	tr_C9ZJU1	0.067	24207.04	34.00
773	HLA-A0201	EGDIFVNGVHISQY	FVNGVHISY	4	0	0	8	1	FVNGVHISQY	tr_C9ZJU1	0.067	24208.35	34.00
758	HLA-A0201	KTTLLRLLNR	KTLLRLLNR	0	0	0	1	1	KTTLLRLLNR	tr_C9ZJU1	0.067	24233.77	34.00
904	HLA-A0201	LRLREGNNLLR	LLREGNNLL	0	0	0	1	1	LRLREGNNLL	tr_C9ZJU1	0.067	24239.02	34.00
660	HLA-A0201	ECGMSEAQVDEAH	GMSEAQVEA	2	0	0	7	1	GMSEAQVDEA	tr_C9ZJU1	0.067	24268.41	34.00
264	HLA-A0201	MAVASTFAFES	MAVAFAFES	0	0	0	4	2	MAVASTFAFES	tr_C9ZJU1	0.067	24272.34	34.00
797	HLA-A0201	NVWQEENNLRFIE	NVWNNLRFI	0	0	0	3	3	NVWQEENNLRFI	tr_C9ZJU1	0.067	24278.65	34.00
378	HLA-A0201	LYMESQFTKS	YMESQFTKS	1	0	0	0	0	YMESQFTKS	tr_C9ZJU1	0.067	24290.74	34.00
139	HLA-A0201	GKYSIARYPPAAQA	YIARYPPAA	2	0	0	1	1	YSIARYPPAA	tr_C9ZJU1	0.067	24298.35	34.00
816	HLA-A0201	NIALGNLWDCS	ILGNLWDCS	1	0	0	1	1	IALGNLWDCS	tr_C9ZJU1	0.067	24299.67	34.00
184	HLA-A0201	FWKLMCSSCS	FWLMCSSCS	0	0	0	2	1	FWKLMCSSCS	tr_C9ZJU1	0.067	24318.09	34.00
895	HLA-A0201	ITEGEIFDTLRLR	ITIFDTLRL	0	0	0	2	3	ITEGEIFDTLRL	tr_C9ZJU1	0.067	24349.14	34.00
116	HLA-A0201	GVALCILDD	GVALCILDD	0	0	0	0	0	GVALCILDD	tr_C9ZJU1	0.066	24376.29	34.00
778	HLA-A0201	VNGVHISQYPARML	HISQYPAML	4	0	0	7	1	HISQYPARML	tr_C9ZJU1	0.066	24383.42	34.00
8	HLA-A0201	VLLDCERKAYE	VLLDCEAYE	0	0	0	6	2	VLLDCERKAYE	tr_C9ZJU1	0.066	24393.72	34.00
53	HLA-A0201	NGVESIHVRTLVF	GVIHVRTLV	1	0	0	2	2	GVESIHVRTLV	tr_C9ZJU1	0.066	24401.11	34.00
452	HLA-A0201	QTFSHPSRHDKSSV	QTFSHPSSV	0	0	0	6	5	QTFSHPSRHDKSSV	tr_C9ZJU1	0.066	24411.41	35.00
9	HLA-A0201	LLDCERKAYE	LLCERKAYE	0	0	0	2	1	LLDCERKAYE	tr_C9ZJU1	0.066	24417.74	35.00
882	HLA-A0201	LCIFDEPTSAID	LIFDEPTSA	0	0	0	1	1	LCIFDEPTSA	tr_C9ZJU1	0.066	24432.01	35.00
822	HLA-A0201	LWDCSDENICAALS	LWDCICAAL	0	0	0	4	4	LWDCSDENICAAL	tr_C9ZJU1	0.066	24437.84	35.00
443	HLA-A0201	STDNKMLFQQTF	STMLFQQTF	0	0	0	2	3	STDNKMLFQQTF	tr_C9ZJU1	0.066	24443.39	35.00
860	HLA-A0201	SGGEVERLCI	SGGEVELCI	0	0	0	6	1	SGGEVERLCI	tr_C9ZJU1	0.066	24450.80	35.00
488	HLA-A0201	DLLTQHVIW	DLLTQHVIW	0	0	0	0	0	DLLTQHVIW	tr_C9ZJU1	0.066	24460.58	35.00
676	HLA-A0201	HLAFSGSSLGC	HLAFSSLGC	0	0	0	4	2	HLAFSGSSLGC	tr_C9ZJU1	0.066	24480.18	35.00
55	HLA-A0201	VESIHVRTLVF	VIHVRTLVF	0	0	0	1	2	VESIHVRTLVF	tr_C9ZJU1	0.066	24516.23	35.00
859	HLA-A0201	FSGGEVERLCIAR	FSVERLCIA	0	0	0	2	3	FSGGEVERLCIA	tr_C9ZJU1	0.066	24525.78	35.00
149	HLA-A0201	AAQADVVVQEF	AQADVVVEF	1	0	0	7	1	AQADVVVQEF	tr_C9ZJU1	0.066	24534.80	35.00
644	HLA-A0201	QHWCPLYHAF	QHWCPLYAF	0	0	0	7	1	QHWCPLYHAF	tr_C9ZJU1	0.066	24539.85	35.00
236	HLA-A0201	LNGDVSPKL	LNGDVSPKL	0	0	0	0	0	LNGDVSPKL	tr_C9ZJU1	0.066	24540.64	35.00
819	HLA-A0201	LGNLWDCSDEN	LLWDCSDEN	0	0	0	1	2	LGNLWDCSDEN	tr_C9ZJU1	0.066	24547.81	35.00
659	HLA-A0201	DECGMSEAQVDEAH	GMSEAQVEA	3	0	0	7	1	GMSEAQVDEA	tr_C9ZJU1	0.066	24561.88	35.00
244	HLA-A0201	LSPFRFIIK	LSPFRFIIK	0	0	0	0	0	LSPFRFIIK	tr_C9ZJU1	0.066	24562.42	35.00
815	HLA-A0201	GNIALGNLWDC	IALGNLWDC	2	0	0	0	0	IALGNLWDC	tr_C9ZJU1	0.066	24564.55	35.00
157	HLA-A0201	QEFPEELYLGLIT	QEFLYLGLI	0	0	0	3	3	QEFPEELYLGLI	tr_C9ZJU1	0.066	24565.09	35.00
89	HLA-A0201	DHLLVCDIW	DHLLVCDIW	0	0	0	0	0	DHLLVCDIW	tr_C9ZJU1	0.066	24586.09	35.00
521	HLA-A0201	TCAVSVRRL	TCAVSVRRL	0	0	0	0	0	TCAVSVRRL	tr_C9ZJU1	0.066	24590.34	35.00
692	HLA-A0201	GLQSVQRKID	GLQSVQRKI	0	0	0	0	0	GLQSVQRKI	tr_C9ZJU1	0.066	24591.41	35.00
737	HLA-A0201	RVSFVVNKG	RVSFVVNKG	0	0	0	0	0	RVSFVVNKG	tr_C9ZJU1	0.066	24598.58	35.00

227	HLA-A0201	IYAAEIGKSLNGDV	YAAEIGKSV	1	0	0	8	4	YAAEIGKSLNGDV	tr_C9ZJU1	0.066	24600.46	35.00
502	HLA-A0201	VFSGCVVDALKRK	FSGCVVDAL	1	0	0	0	0	FSGCVVDAL	tr_C9ZJU1	0.065	24620.17	35.00
181	HLA-A0201	ITSFWKLMCSS	ITWKLMCSS	0	0	0	2	2	ITSFWKLMCSS	tr_C9ZJU1	0.065	24621.21	35.00
179	HLA-A0201	SGITSFWKLMCSS	GIWKLMCSS	1	0	0	2	3	GITSFWKLMCSS	tr_C9ZJU1	0.065	24628.15	35.00
775	HLA-A0201	DIFVNGVHISQYP	FVNGVHIYP	2	0	0	7	2	FVNGVHISQYP	tr_C9ZJU1	0.065	24629.22	35.00
877	HLA-A0201	GSAVGLCIFDEP	AVGLCIFEP	2	0	0	7	1	AVGLCIFDEP	tr_C9ZJU1	0.065	24630.01	35.00
839	HLA-A0201	ALNFVQKRSS	ALNFVQRSS	0	0	0	6	1	ALNFVQKRSS	tr_C9ZJU1	0.065	24631.35	35.00
813	HLA-A0201	IGGNIALGNLWDC	IALGNLWDC	4	0	0	0	0	IALGNLWDC	tr_C9ZJU1	0.065	24642.01	35.00
775	HLA-A0201	DIFVNGVHIS	DIFVNGVHI	0	0	0	0	0	DIFVNGVHI	tr_C9ZJU1	0.065	24647.87	35.00
770	HLA-A0201	APTEGDIFVNGV	AGDIFVNGV	0	0	0	1	3	APTEGDIFVNGV	tr_C9ZJU1	0.065	24655.89	35.00
446	HLA-A0201	NKMLFQQTF	NKMLFQQTF	0	0	0	0	0	NKMLFQQTF	tr_C9ZJU1	0.065	24662.55	35.00
914	HLA-A0201	RDSTIIIVAHR	STIIIVAHR	2	0	0	0	0	STIIIVAHR	tr_C9ZJU1	0.065	24663.35	35.00
876	HLA-A0201	AGSAVGLCIFDEP	AVGLCIFEP	3	0	0	7	1	AVGLCIFDEP	tr_C9ZJU1	0.065	24669.49	35.00
244	HLA-A0201	LSPFRFIIKQ	LSFRFIIKQ	0	0	0	2	1	LSPFRFIIKQ	tr_C9ZJU1	0.065	24671.36	35.00
613	HLA-A0201	LVSAVALRLSPN	LVSAVALRL	0	0	0	0	0	LVSAVALRL	tr_C9ZJU1	0.065	24692.72	35.00
59	HLA-A0201	HVRTLVFNHNIMQ	TLFNHNIMQ	3	0	0	2	1	TLVFNHNIMQ	tr_C9ZJU1	0.065	24693.25	35.00
433	HLA-A0201	KDDGIKCML	KDDGIKCML	0	0	0	0	0	KDDGIKCML	tr_C9ZJU1	0.065	24694.06	35.00
423	HLA-A0201	SSPLTLLRK	SSPLTLLRK	0	0	0	0	0	SSPLTLLRK	tr_C9ZJU1	0.065	24698.87	35.00
543	HLA-A0201	ATASPSTCVW	ATASPSTCV	0	0	0	0	0	ATASPSTCV	tr_C9ZJU1	0.065	24702.34	35.00
929	HLA-A0201	KHADKIVVLNEGM	KIVVLNEGM	0	0	0	1	4	KHADKIVVLNEGM	tr_C9ZJU1	0.065	24706.09	35.00
928	HLA-A0201	LKHADKIVVL	LKHAKIVVL	0	0	0	4	1	LKHADKIVVL	tr_C9ZJU1	0.065	24709.02	35.00
590	HLA-A0201	RYEQCGRDVLHGL	YEQCVLHGL	1	0	0	4	3	YEQCGRDVLHGL	tr_C9ZJU1	0.065	24720.26	35.00
808	HLA-A0201	IEDLSIGGNIALG	IEDLSIIAL	0	0	0	6	3	IEDLSIGGNIAL	tr_C9ZJU1	0.065	24724.53	35.00
366	HLA-A0201	QWSNFLYFLNES	QWSNFLYFL	0	0	0	0	0	QWSNFLYFL	tr_C9ZJU1	0.065	24726.94	35.00
146	HLA-A0201	YPPAAQADVVVQEF	YADVVVQEF	0	0	0	1	5	YPPAAQADVVVQEF	tr_C9ZJU1	0.065	24728.02	35.00
814	HLA-A0201	GGNIALGNLWDC	IALGNLWDC	3	0	0	0	0	IALGNLWDC	tr_C9ZJU1	0.065	24740.32	35.00
287	HLA-A0201	LSDIITSIHIDA	LSIITSIHI	0	0	0	2	1	LSDIITSIHI	tr_C9ZJU1	0.065	24740.86	35.00
811	HLA-A0201	LSIGGNIALGNLWD	SIGGNIALL	1	0	0	8	2	SIGGNIALGNL	tr_C9ZJU1	0.065	24741.66	35.00
875	HLA-A0201	KAGSAVGLCIFDEP	AVGLCIFEP	4	0	0	7	1	AVGLCIFDEP	tr_C9ZJU1	0.065	24742.73	35.00
931	HLA-A0201	ADKIVVLNEGM	AIVVLNEGM	0	0	0	1	2	ADKIVVLNEGM	tr_C9ZJU1	0.065	24756.39	35.00
144	HLA-A0201	ARYPPAAQADVVV	YAAQADVVV	2	0	0	1	2	YPPAAQADVVV	tr_C9ZJU1	0.065	24758.53	35.00
574	HLA-A0201	AATAAAAFATA	ATAAAAFTA	1	0	0	7	1	ATAAAAFATA	tr_C9ZJU1	0.065	24758.80	35.00
16	HLA-A0201	AYEILEMLTR	YEILEMLTR	1	0	0	0	0	YEILEMLTR	tr_C9ZJU1	0.065	24762.56	35.00
631	HLA-A0201	LDQTEIVNQLRSI	LIVNQLRSI	0	0	0	1	4	LDQTEIVNQLRSI	tr_C9ZJU1	0.065	24776.21	35.00
774	HLA-A0201	GDIFVNGVHISQ	GIFVNGVHI	0	0	0	1	1	GDIFVNGVHI	tr_C9ZJU1	0.065	24793.65	35.00
930	HLA-A0201	HADKIVVLNEGM	HADKIVVLM	0	0	0	8	3	HADKIVVLNEGM	tr_C9ZJU1	0.065	24806.26	35.00
60	HLA-A0201	VRTLVFNHNIMQ	TLFNHNIMQ	2	0	0	2	1	TLVFNHNIMQ	tr_C9ZJU1	0.065	24817.01	35.00
794	HLA-A0201	RIANVWQEENNLR	RIANVWQEL	0	0	0	8	3	RIANVWQEENNL	tr_C9ZJU1	0.065	24823.99	35.00
868	HLA-A0201	CIARAFVKAGSA	CIARAFVSA	0	0	0	7	3	CIARAFVKAGSA	tr_C9ZJU1	0.065	24831.51	35.00
783	HLA-A0201	ISQYPARML	ISQYPARML	0	0	0	0	0	ISQYPARML	tr_C9ZJU1	0.065	24836.88	35.00
297	HLA-A0201	DAYLALATADYDP	YLALATYDP	2	0	0	6	2	YLALATADYDP	tr_C9ZJU1	0.065	24849.25	35.00
4	HLA-A0201	ARPAVLLDC	ARPAVLLDC	0	0	0	0	0	ARPAVLLDC	tr_C9ZJU1	0.065	24853.54	35.00
776	HLA-A0201	IFVNGVHISQYP	FVNGVHIYP	1	0	0	7	2	FVNGVHISQYP	tr_C9ZJU1	0.065	24858.38	35.00
744	HLA-A0201	KGEFLGIAGY	KGFLGIAGY	0	0	0	2	1	KGEFLGIAGY	tr_C9ZJU1	0.065	24876.96	35.00
298	HLA-A0201	AYLALATADYDP	YLALATYDP	1	0	0	6	2	YLALATADYDP	tr_C9ZJU1	0.064	24882.60	35.00
143	HLA-A0201	IARYPPAAQADVVV	YAAQADVVV	3	0	0	1	2	YPPAAQADVVV	tr_C9ZJU1	0.064	24891.22	35.00
367	HLA-A0201	WSNFLYFLNES	WSFLYFLNE	0	0	0	2	1	WSNFLYFLNE	tr_C9ZJU1	0.064	24892.30	35.00
717	HLA-A0201	SVEFREVSF	SVEFREVSF	0	0	0	0	0	SVEFREVSF	tr_C9ZJU1	0.064	24916.28	35.00
43	HLA-A0201	DASIAIFLNCN	ASIAIFLNC	1	0	0	0	0	ASIAIFLNC	tr_C9ZJU1	0.064	24920.33	35.00
903	HLA-A0201	TLRLREGNNLLR	LLREGNNLL	1	0	0	1	1	LRLREGNNLL	tr_C9ZJU1	0.064	24922.75	35.00
153	HLA-A0201	DVVVQEFPEELY	VVVEFPEEL	1	0	0	3	1	VVVQEFPEEL	tr_C9ZJU1	0.064	24933.27	36.00
882	HLA-A0201	LCIFDEPTSAIDT	LIFDEPTSA	0	0	0	1	1	LCIFDEPTSA	tr_C9ZJU1	0.064	24942.18	36.00
639	HLA-A0201	QLRSIQHWCP	QLRSIQWCP	0	0	0	6	1	QLRSIQHWCP	tr_C9ZJU1	0.064	24943.80	36.00
139	HLA-A0201	GKYSIARYPPAAQ	YIARYPPAA	2	0	0	1	1	YSIARYPPAA	tr_C9ZJU1	0.064	24944.33	36.00

962	HLA-A0201	RMRQSQQLPHF	RMSQQLPHF	0	0	0	2	2	RMRQSQQLPHF	tr_C9ZJU1	0.064	24944.33	36.00
531	HLA-A0201	WIKDVIQSSPRA	WIKDVIPRA	0	0	0	6	3	WIKDVIQSSPRA	tr_C9ZJU1	0.064	24959.18	36.00
543	HLA-A0201	ATASPSTCVWQC	ATATCVWQC	0	0	0	3	3	ATASPSTCVWQC	tr_C9ZJU1	0.064	24968.63	36.00
477	HLA-A0201	VESGRPLGYMWDL	VELGYMWDL	0	0	0	2	4	VESGRPLGYMWDL	tr_C9ZJU1	0.064	24975.66	36.00
754	HLA-A0201	SGSGKTTLL	SGSGKTTLL	0	0	0	0	0	SGSGKTTLL	tr_C9ZJU1	0.064	24981.34	36.00
223	HLA-A0201	HWRPIYAAEI	HWPIYAAEI	0	0	0	2	1	HWRPIYAAEI	tr_C9ZJU1	0.064	24998.10	36.00
207	HLA-A0201	SPEVILKCI	SPEVILKCI	0	0	0	0	0	SPEVILKCI	tr_C9ZJU1	0.064	25001.07	36.00
430	HLA-A0201	RKQKDDGIKCML	KQDGIKCML	1	0	0	2	2	KQKDDGIKCML	tr_C9ZJU1	0.064	25033.02	36.00
727	HLA-A0201	YSDRHPYVLRRVS	YSDYVLRRV	0	0	0	3	3	YSDRHPYVLRRV	tr_C9ZJU1	0.064	25044.93	36.00
504	HLA-A0201	SGCVVDALKRKL	SGCVVDALL	0	0	0	8	3	SGCVVDALKRKL	tr_C9ZJU1	0.064	25050.62	36.00
589	HLA-A0201	DRYEQCGRDVLHGL	YEQCVLHGL	2	0	0	4	3	YEQCGRDVLHGL	tr_C9ZJU1	0.064	25051.97	36.00
178	HLA-A0201	QSGITSFWKLMCSS	GIWKLMCSS	2	0	0	2	3	GITSFWKLMCSS	tr_C9ZJU1	0.064	25072.05	36.00
825	HLA-A0201	CSDENICAALSA	CSDENILSA	0	0	0	6	3	CSDENICAALSA	tr_C9ZJU1	0.064	25072.32	36.00
44	HLA-A0201	ASIAIFLNCNG	SIAIFLCNG	1	0	0	6	1	SIAIFLNCNG	tr_C9ZJU1	0.064	25072.32	36.00
784	HLA-A0201	SQYPARMLRRRIA	SQYPARMLA	0	0	0	8	4	SQYPARMLRRRIA	tr_C9ZJU1	0.064	25082.09	36.00
219	HLA-A0201	HAFVHWRPIY	HAFVHWRPI	0	0	0	0	0	HAFVHWRPI	tr_C9ZJU1	0.064	25084.26	36.00
413	HLA-A0201	CSFPVKNSLT	CSFPVNSLT	0	0	0	5	1	CSFPVKNSLT	tr_C9ZJU1	0.064	25097.84	36.00
650	HLA-A0201	YHAFLGSFY	YHAFLGSFY	0	0	0	0	0	YHAFLGSFY	tr_C9ZJU1	0.064	25109.51	36.00
561	HLA-A0201	GFAALRLSGW	FAALRLSGW	1	0	0	0	0	FAALRLSGW	tr_C9ZJU1	0.064	25110.33	36.00
772	HLA-A0201	TEGDIFVNGVHIS	TIFVNGVHI	0	0	0	1	3	TEGDIFVNGVHI	tr_C9ZJU1	0.064	25128.27	36.00
151	HLA-A0201	QADVVVQEFPEELY	VVVEFPEEL	3	0	0	3	1	VVVQEFPEEL	tr_C9ZJU1	0.064	25129.35	36.00
397	HLA-A0201	TSTYAGNGEENQV	STYAGNNQV	1	0	0	6	3	STYAGNGEENQV	tr_C9ZJU1	0.064	25129.90	36.00
40	HLA-A0201	CKDDASIAIFL	CKASIAIFL	0	0	0	2	2	CKDDASIAIFL	tr_C9ZJU1	0.064	25130.71	36.00
41	HLA-A0201	KDDASIAIFLNCN	ASIAIFLNC	3	0	0	0	0	ASIAIFLNC	tr_C9ZJU1	0.064	25140.23	36.00
383	HLA-A0201	QFTKSQLASVT	FTKSQLASV	1	0	0	0	0	FTKSQLASV	tr_C9ZJU1	0.063	25154.11	36.00
825	HLA-A0201	CSDENICAALS	CSDEICAAL	0	0	0	4	1	CSDENICAAL	tr_C9ZJU1	0.063	25166.09	36.00
746	HLA-A0201	EFLGIAGYSGSGK	FLGIAGYSK	1	0	0	8	3	FLGIAGYSGSGK	tr_C9ZJU1	0.063	25169.35	36.00
690	HLA-A0201	LEGLQSVQRKI	LLQSVQRKI	0	0	0	1	2	LEGLQSVQRKI	tr_C9ZJU1	0.063	25170.99	36.00
124	HLA-A0201	DVKAEEVLALC	KAEEVLALC	2	0	0	0	0	KAEEVLALC	tr_C9ZJU1	0.063	25176.43	36.00
287	HLA-A0201	LSDIITSIHIDAY	LSIITSIHI	0	0	0	2	1	LSDIITSIHI	tr_C9ZJU1	0.063	25195.51	36.00
898	HLA-A0201	GEIFDTLRLR	GIFDTLRLR	0	0	0	1	1	GEIFDTLRLR	tr_C9ZJU1	0.063	25196.05	36.00
795	HLA-A0201	IANVWQEENNL	IVWQEENNL	0	0	0	1	2	IANVWQEENNL	tr_C9ZJU1	0.063	25203.69	36.00
409	HLA-A0201	VSPTCSFPVKN	VSPTCSFPV	0	0	0	0	0	VSPTCSFPV	tr_C9ZJU1	0.063	25205.33	36.00
745	HLA-A0201	GEFLGIAGYSGSGK	FLGIAGYSK	2	0	0	8	3	FLGIAGYSGSGK	tr_C9ZJU1	0.063	25207.51	36.00
152	HLA-A0201	ADVVVQEFPEELY	AVQEFPEEL	0	0	0	1	3	ADVVVQEFPEEL	tr_C9ZJU1	0.063	25210.78	36.00
278	HLA-A0201	IRGFVIQSILS	RGFVISILS	1	0	0	5	1	RGFVIQSILS	tr_C9ZJU1	0.063	25211.33	36.00
389	HLA-A0201	LASVTNIATS	LASVTIATS	0	0	0	5	1	LASVTNIATS	tr_C9ZJU1	0.063	25213.51	36.00
814	HLA-A0201	GGNIALGNLWDCS	ILGNLWDCS	3	0	0	1	1	IALGNLWDCS	tr_C9ZJU1	0.063	25214.32	36.00
815	HLA-A0201	GNIALGNLWDCS	ILGNLWDCS	2	0	0	1	1	IALGNLWDCS	tr_C9ZJU1	0.063	25219.78	36.00
182	HLA-A0201	TSFWKLMCSSCS	TSFWKLMCS	0	0	0	7	3	TSFWKLMCSSCS	tr_C9ZJU1	0.063	25221.96	36.00
42	HLA-A0201	DDASIAIFLNCN	ASIAIFLNC	2	0	0	0	0	ASIAIFLNC	tr_C9ZJU1	0.063	25230.42	36.00
946	HLA-A0201	GSWETLSRTG	GSWETLSTG	0	0	0	7	1	GSWETLSRTG	tr_C9ZJU1	0.063	25239.16	36.00
478	HLA-A0201	ESGRPLGYMWDL	SGLGYMWDL	1	0	0	2	2	SGRPLGYMWDL	tr_C9ZJU1	0.063	25254.74	36.00
644	HLA-A0201	QHWCPLYHAFLG	QHWCPLAFL	0	0	0	6	2	QHWCPLYHAFL	tr_C9ZJU1	0.063	25256.93	36.00
772	HLA-A0201	TEGDIFVNGVHISQ	FVNGVHISQ	5	0	0	0	0	FVNGVHISQ	tr_C9ZJU1	0.063	25268.95	36.00
834	HLA-A0201	LSASRALNF	LSASRALNF	0	0	0	0	0	LSASRALNF	tr_C9ZJU1	0.063	25287.27	36.00
65	HLA-A0201	FNHNIMQLPS	FNHNIMLPS	0	0	0	6	1	FNHNIMQLPS	tr_C9ZJU1	0.063	25294.94	36.00
550	HLA-A0201	CVWQCRQDY	CVWQCRQDY	0	0	0	0	0	CVWQCRQDY	tr_C9ZJU1	0.063	25296.57	36.00
138	HLA-A0201	RGKYSIARYPPAAQ	YIARYPPAA	3	0	0	1	1	YSIARYPPAA	tr_C9ZJU1	0.063	25305.61	36.00
315	HLA-A0201	LSRCVSQALWQAS	LVSQALWQA	0	0	0	1	3	LSRCVSQALWQA	tr_C9ZJU1	0.063	25322.87	36.00
773	HLA-A0201	EGDIFVNGVHIS	GIFVNGVHI	1	0	0	1	1	GDIFVNGVHI	tr_C9ZJU1	0.063	25327.80	36.00
180	HLA-A0201	GITSFWKLMCSSCS	SFWLMCSSC	3	0	0	3	1	SFWKLMCSSC	tr_C9ZJU1	0.063	25336.02	36.00
899	HLA-A0201	EIFDTLRLR	EIFDTLRLR	0	0	0	0	0	EIFDTLRLR	tr_C9ZJU1	0.063	25351.66	36.00
569	HLA-A0201	GWELMAATAA	GWELMAATA	0	0	0	8	1	GWELMAATAA	tr_C9ZJU1	0.063	25370.86	36.00

773	HLA-A0201	EGDIFVNGVHISQ	GIFVNGVHI	1	0	0	1	1	GDIFVNGVHI	tr_C9ZJU1	0.063	25378.83	36.00
136	HLA-A0201	LLRGKYSIARYPP	LLYSIARYP	0	0	0	2	3	LLRGKYSIARYP	tr_C9ZJU1	0.063	25381.84	36.00
218	HLA-A0201	MHAFVHWRPIY	MAFVHWRPI	0	0	0	1	1	MHAFVHWRPI	tr_C9ZJU1	0.063	25382.40	36.00
526	HLA-A0201	VRRLGWIKDV	VRRLGWIKV	0	0	0	8	1	VRRLGWIKDV	tr_C9ZJU1	0.063	25385.41	36.00
38	HLA-A0201	TECKDDASIAIFL	TECSIAIFL	0	0	0	3	4	TECKDDASIAIFL	tr_C9ZJU1	0.063	25387.60	36.00
54	HLA-A0201	GVESIHVRTLVFN	VIHVRTLVF	1	0	0	1	2	VESIHVRTLVF	tr_C9ZJU1	0.063	25390.08	36.00
269	HLA-A0201	TFAFESLYC	TFAFESLYC	0	0	0	0	0	TFAFESLYC	tr_C9ZJU1	0.063	25398.05	36.00
784	HLA-A0201	SQYPARMLR	SQYPARMLR	0	0	0	0	0	SQYPARMLR	tr_C9ZJU1	0.063	25405.47	36.00
7	HLA-A0201	AVLLDCERK	AVLLDCERK	0	0	0	0	0	AVLLDCERK	tr_C9ZJU1	0.062	25428.57	36.00
890	HLA-A0201	SAIDTITEGEIF	SAIDTITEI	0	0	0	7	2	SAIDTITEGEI	tr_C9ZJU1	0.062	25430.77	36.00
278	HLA-A0201	IRGFVIQSILSD	FVIQSILSD	3	0	0	0	0	FVIQSILSD	tr_C9ZJU1	0.062	25434.08	36.00
280	HLA-A0201	GFVIQSILS	GFVIQSILS	0	0	0	0	0	GFVIQSILS	tr_C9ZJU1	0.062	25436.56	36.00
831	HLA-A0201	CAALSASRA	CAALSASRA	0	0	0	0	0	CAALSASRA	tr_C9ZJU1	0.062	25438.75	36.00
965	HLA-A0201	QSQQLPHFVK	QSQQLPHFV	0	0	0	0	0	QSQQLPHFV	tr_C9ZJU1	0.062	25440.41	36.00
648	HLA-A0201	PLYHAFLGSFYDE	PLYHAFLFY	0	0	0	7	2	PLYHAFLGSFY	tr_C9ZJU1	0.062	25441.51	36.00
284	HLA-A0201	QSILSDIIT	QSILSDIIT	0	0	0	0	0	QSILSDIIT	tr_C9ZJU1	0.062	25445.91	37.00
122	HLA-A0201	LDDVKAEEV	LDDVKAEEV	0	0	0	0	0	LDDVKAEEV	tr_C9ZJU1	0.062	25445.91	37.00
123	HLA-A0201	DDVKAEEVLALC	KAEEVLALC	3	0	0	0	0	KAEEVLALC	tr_C9ZJU1	0.062	25447.57	37.00
309	HLA-A0201	PSDDWLLSRCV	PSDDWLLSV	0	0	0	8	2	PSDDWLLSRCV	tr_C9ZJU1	0.062	25457.48	37.00
673	HLA-A0201	LRRHLAFSGSSLG	LLAFSGSSL	0	0	0	1	3	LRRHLAFSGSSL	tr_C9ZJU1	0.062	25485.87	37.00
158	HLA-A0201	EFPEELYLGLIT	FPEELYLLI	1	0	0	7	1	FPEELYLGLI	tr_C9ZJU1	0.062	25485.87	37.00
649	HLA-A0201	LYHAFLGSFY	LYHAFLSFY	0	0	0	6	1	LYHAFLGSFY	tr_C9ZJU1	0.062	25493.59	37.00
46	HLA-A0201	IAIFLNCNG	IAIFLNCNG	0	0	0	0	0	IAIFLNCNG	tr_C9ZJU1	0.062	25518.69	37.00
371	HLA-A0201	LYFLNESLY	LYFLNESLY	0	0	0	0	0	LYFLNESLY	tr_C9ZJU1	0.062	25559.59	37.00
43	HLA-A0201	DASIAIFLNCNG	SIAIFLCNG	2	0	0	6	1	SIAIFLNCNG	tr_C9ZJU1	0.062	25559.87	37.00
50	HLA-A0201	LNCNGVESIHV	NCNGVSIHV	1	0	0	5	1	NCNGVESIHV	tr_C9ZJU1	0.062	25568.72	37.00
76	HLA-A0201	FAVHCGLMKNSHPD	FLMKNSHPD	0	0	0	1	5	FAVHCGLMKNSHPD	tr_C9ZJU1	0.062	25579.25	37.00
902	HLA-A0201	DTLRLREGNNLLR	LLREGNNLL	2	0	0	1	1	LRLREGNNLL	tr_C9ZJU1	0.062	25587.54	37.00
628	HLA-A0201	QSALDQTEI	QSALDQTEI	0	0	0	0	0	QSALDQTEI	tr_C9ZJU1	0.062	25591.42	37.00
696	HLA-A0201	VQRKIDRPVRGV	KIDRPVRGV	3	0	0	0	0	KIDRPVRGV	tr_C9ZJU1	0.062	25591.97	37.00
363	HLA-A0201	LGTQWSNFLY	LTQWSNFLY	0	0	0	1	1	LGTQWSNFLY	tr_C9ZJU1	0.062	25612.75	37.00
741	HLA-A0201	VVNKGEFLGIAGYS	VVFLGIAGY	0	0	0	2	4	VVNKGEFLGIAGY	tr_C9ZJU1	0.062	25618.57	37.00
573	HLA-A0201	MAATAAAAFAT	MTAAAAFAT	0	0	0	1	2	MAATAAAAFAT	tr_C9ZJU1	0.062	25623.00	37.00
183	HLA-A0201	SFWKLMCSSCS	SFWLMCSSC	0	0	0	3	1	SFWKLMCSSC	tr_C9ZJU1	0.062	25632.15	37.00
39	HLA-A0201	ECKDDASIAIFL	CKASIAIFL	1	0	0	2	2	CKDDASIAIFL	tr_C9ZJU1	0.062	25635.48	37.00
673	HLA-A0201	LRRHLAFSGSSLGC	LLAFSGSSL	0	0	0	1	3	LRRHLAFSGSSL	tr_C9ZJU1	0.062	25637.71	37.00
407	HLA-A0201	NQVSPTCSF	NQVSPTCSF	0	0	0	0	0	NQVSPTCSF	tr_C9ZJU1	0.062	25638.25	37.00
818	HLA-A0201	ALGNLWDCSDEN	ALWDCSDEN	0	0	0	1	3	ALGNLWDCSDEN	tr_C9ZJU1	0.062	25652.69	37.00
855	HLA-A0201	NVREFSGGEVERL	NVREFVERL	0	0	0	5	4	NVREFSGGEVERL	tr_C9ZJU1	0.062	25661.02	37.00
657	HLA-A0201	FYDECGMSEAQVDE	FYDECGMSV	0	0	0	8	3	FYDECGMSEAQV	tr_C9ZJU1	0.062	25663.23	37.00
296	HLA-A0201	IDAYLALAT	IDAYLALAT	0	0	0	0	0	IDAYLALAT	tr_C9ZJU1	0.062	25679.62	37.00
833	HLA-A0201	ALSASRALN	ALSASRALN	0	0	0	0	0	ALSASRALN	tr_C9ZJU1	0.062	25682.67	37.00
181	HLA-A0201	ITSFWKLMCSSCS	SFWLMCSSC	2	0	0	3	1	SFWKLMCSSC	tr_C9ZJU1	0.062	25687.13	37.00
810	HLA-A0201	DLSIGGNIALGN	DLSIGGIAL	0	0	0	6	1	DLSIGGNIAL	tr_C9ZJU1	0.062	25692.96	37.00
76	HLA-A0201	FAVHCGLMKN	FVHCGLMKN	0	0	0	1	1	FAVHCGLMKN	tr_C9ZJU1	0.061	25706.32	37.00
148	HLA-A0201	PAAQADVVVQEF	AQADVVVEF	2	0	0	7	1	AQADVVVQEF	tr_C9ZJU1	0.061	25719.38	37.00
150	HLA-A0201	AQADVVVQEFP	AQADVVVFP	0	0	0	7	2	AQADVVVQEFP	tr_C9ZJU1	0.061	25723.28	37.00
695	HLA-A0201	SVQRKIDRPVRGV	KIDRPVRGV	4	0	0	0	0	KIDRPVRGV	tr_C9ZJU1	0.061	25725.79	37.00
809	HLA-A0201	EDLSIGGNIALG	DLIGGNIAL	1	0	0	2	1	DLSIGGNIAL	tr_C9ZJU1	0.061	25754.75	37.00
443	HLA-A0201	STDNKMLFQ	STDNKMLFQ	0	0	0	0	0	STDNKMLFQ	tr_C9ZJU1	0.061	25768.41	37.00
775	HLA-A0201	DIFVNGVHISQY	FVNGVHISY	2	0	0	8	1	FVNGVHISQY	tr_C9ZJU1	0.061	25772.32	37.00
686	HLA-A0201	CYQVLEGLQS	YQVLEGLQS	1	0	0	0	0	YQVLEGLQS	tr_C9ZJU1	0.061	25774.83	37.00
645	HLA-A0201	HWCPLYHAF	HWCPLYHAF	0	0	0	0	0	HWCPLYHAF	tr_C9ZJU1	0.061	25801.34	37.00
7	HLA-A0201	AVLLDCERKAYE	VLLDCEAYE	1	0	0	6	2	VLLDCERKAYE	tr_C9ZJU1	0.061	25808.03	37.00

834	HLA-A0201	LSASRALNFVQ	LSASALNFV	0	0	0	4	1	LSASRALNFV	tr_C9ZJU1	0.061	25810.54	37.00
234	HLA-A0201	KSLNGDVSPK	KLNGDVSPK	0	0	0	1	1	KSLNGDVSPK	tr_C9ZJU1	0.061	25813.90	37.00
273	HLA-A0201	ESLYCIRGF	ESLYCIRGF	0	0	0	0	0	ESLYCIRGF	tr_C9ZJU1	0.061	25823.11	37.00
519	HLA-A0201	GHTCAVSVRRL	HTCAVSVRL	1	0	0	7	1	HTCAVSVRRL	tr_C9ZJU1	0.061	25827.86	37.00
42	HLA-A0201	DDASIAIFLNCNG	SIAIFLCNG	3	0	0	6	1	SIAIFLNCNG	tr_C9ZJU1	0.061	25828.97	37.00
531	HLA-A0201	WIKDVIQSSPRAA	WIKDVIQAA	0	0	0	7	4	WIKDVIQSSPRAA	tr_C9ZJU1	0.061	25831.51	37.00
520	HLA-A0201	HTCAVSVRRLGWIK	HTCAVSVWI	0	0	0	7	4	HTCAVSVRRLGWI	tr_C9ZJU1	0.061	25845.20	37.00
725	HLA-A0201	FRYSDRHPYVL	FSDRHPYVL	0	0	0	1	2	FRYSDRHPYVL	tr_C9ZJU1	0.061	25852.47	37.00
41	HLA-A0201	KDDASIAIFLNCNG	SIAIFLCNG	4	0	0	6	1	SIAIFLNCNG	tr_C9ZJU1	0.061	25857.51	37.00
656	HLA-A0201	SFYDECGMSEA	FYDECGMSA	1	0	0	8	1	FYDECGMSEA	tr_C9ZJU1	0.061	25859.74	37.00
139	HLA-A0201	GKYSIARYPPA	YSIARYPPA	2	0	0	0	0	YSIARYPPA	tr_C9ZJU1	0.061	25865.89	37.00
675	HLA-A0201	RHLAFSGSSLGC	HLAFSSLGC	1	0	0	4	2	HLAFSGSSLGC	tr_C9ZJU1	0.061	25867.30	37.00
413	HLA-A0201	CSFPVKNSLTS	CSFPVSLTS	0	0	0	5	2	CSFPVKNSLTS	tr_C9ZJU1	0.061	25876.26	37.00
771	HLA-A0201	PTEGDIFVNGVHIS	GIFVNGVHI	3	0	0	1	1	GDIFVNGVHI	tr_C9ZJU1	0.061	25892.21	37.00
384	HLA-A0201	FTKSQLASVTN	FTKSQLASV	0	0	0	0	0	FTKSQLASV	tr_C9ZJU1	0.061	25892.50	37.00
279	HLA-A0201	RGFVIQSILSD	RGFVISILS	0	0	0	5	1	RGFVIQSILS	tr_C9ZJU1	0.061	25899.23	37.00
396	HLA-A0201	ATSTYAGNGEENQV	STYAGNNQV	2	0	0	6	3	STYAGNGEENQV	tr_C9ZJU1	0.061	25902.87	37.00
622	HLA-A0201	SPNFFTQSA	SPNFFTQSA	0	0	0	0	0	SPNFFTQSA	tr_C9ZJU1	0.061	25905.68	37.00
147	HLA-A0201	PPAAQADVVVQEF	AQADVVVEF	3	0	0	7	1	AQADVVVQEF	tr_C9ZJU1	0.061	25916.04	37.00
808	HLA-A0201	IEDLSIGGNIALGN	IEDLSIIAL	0	0	0	6	3	IEDLSIGGNIAL	tr_C9ZJU1	0.061	25932.30	37.00
617	HLA-A0201	VALRLSPNF	VALRLSPNF	0	0	0	0	0	VALRLSPNF	tr_C9ZJU1	0.061	25939.88	37.00
689	HLA-A0201	VLEGLQSVQR	VLEGLSVQR	0	0	0	5	1	VLEGLQSVQR	tr_C9ZJU1	0.061	25944.10	37.00
141	HLA-A0201	YSIARYPPAAQAD	YIARYPPAA	0	0	0	1	1	YSIARYPPAA	tr_C9ZJU1	0.061	25960.95	38.00
895	HLA-A0201	ITEGEIFDTLRLRE	ITEGEIFTL	0	0	0	7	1	ITEGEIFDTL	tr_C9ZJU1	0.060	25987.08	38.00
53	HLA-A0201	NGVESIHVRTLVFN	VIHVRTLVF	2	0	0	1	2	VESIHVRTLVF	tr_C9ZJU1	0.060	25992.14	38.00
245	HLA-A0201	SPFRFIIKQ	SPFRFIIKQ	0	0	0	0	0	SPFRFIIKQ	tr_C9ZJU1	0.060	26000.29	38.00
476	HLA-A0201	FVESGRPLGYMW	FVESGLGYM	0	0	0	5	2	FVESGRPLGYM	tr_C9ZJU1	0.060	26008.75	38.00
723	HLA-A0201	VSFRYSDRHPYVLR	VSFRYSPYV	0	0	0	6	3	VSFRYSDRHPYV	tr_C9ZJU1	0.060	26009.87	38.00
55	HLA-A0201	VESIHVRTLVFN	VIHVRTLVF	0	0	0	1	2	VESIHVRTLVF	tr_C9ZJU1	0.060	26010.15	38.00
891	HLA-A0201	AIDTITEGEIF	AIDTITEEI	0	0	0	7	1	AIDTITEGEI	tr_C9ZJU1	0.060	26018.59	38.00
16	HLA-A0201	AYEILEMLT	AYEILEMLT	0	0	0	0	0	AYEILEMLT	tr_C9ZJU1	0.060	26035.21	38.00
315	HLA-A0201	LSRCVSQALW	LSRCVSALW	0	0	0	6	1	LSRCVSQALW	tr_C9ZJU1	0.060	26045.35	38.00
87	HLA-A0201	HPDHLLVCDI	HPDHLLVCI	0	0	0	8	1	HPDHLLVCDI	tr_C9ZJU1	0.060	26070.44	38.00
842	HLA-A0201	FVQKRSSGIH	FVQKRSSGI	0	0	0	0	0	FVQKRSSGI	tr_C9ZJU1	0.060	26071.57	38.00
615	HLA-A0201	SAVALRLSPNF	AVALRLSPF	1	0	0	8	1	AVALRLSPNF	tr_C9ZJU1	0.060	26082.30	38.00
77	HLA-A0201	AVHCGLMKNS	AVHCGLMNS	0	0	0	7	1	AVHCGLMKNS	tr_C9ZJU1	0.060	26096.97	38.00
61	HLA-A0201	RTLVFNHNIMQ	TLFNHNIMQ	1	0	0	2	1	TLVFNHNIMQ	tr_C9ZJU1	0.060	26103.18	38.00
796	HLA-A0201	ANVWQEENNLRFIE	NVWNNLRFI	1	0	0	3	3	NVWQEENNLRFI	tr_C9ZJU1	0.060	26104.60	38.00
946	HLA-A0201	GSWETLSRTGPHSC	GSWETLHSC	0	0	0	6	5	GSWETLSRTGPHSC	tr_C9ZJU1	0.060	26106.01	38.00
647	HLA-A0201	CPLYHAFLG	CPLYHAFLG	0	0	0	0	0	CPLYHAFLG	tr_C9ZJU1	0.060	26106.57	38.00
529	HLA-A0201	LGWIKDVIQSSPRA	LGWIKDVIA	0	0	0	8	5	LGWIKDVIQSSPRA	tr_C9ZJU1	0.060	26109.11	38.00
345	HLA-A0201	MHGCRVVFS	MHGCRVVFS	0	0	0	0	0	MHGCRVVFS	tr_C9ZJU1	0.060	26121.82	38.00
442	HLA-A0201	SSTDNKMLFQQTF	STMLFQQTF	1	0	0	2	3	STDNKMLFQQTF	tr_C9ZJU1	0.060	26132.00	38.00
674	HLA-A0201	RRHLAFSGSSLGC	HLAFSSLGC	2	0	0	4	2	HLAFSGSSLGC	tr_C9ZJU1	0.060	26143.88	38.00
858	HLA-A0201	EFSGGEVERLCIAR	FSVERLCIA	1	0	0	2	3	FSGGEVERLCIA	tr_C9ZJU1	0.060	26145.02	38.00
651	HLA-A0201	HAFLGSFYDE	HAFLGSFYE	0	0	0	8	1	HAFLGSFYDE	tr_C9ZJU1	0.060	26147.00	38.00
429	HLA-A0201	LRKQKDDGIKCML	KQDGIKCML	2	0	0	2	2	KQKDDGIKCML	tr_C9ZJU1	0.060	26152.94	38.00
323	HLA-A0201	LWQASGSVMDELQ	WQASVMDEL	1	0	0	3	2	WQASGSVMDEL	tr_C9ZJU1	0.060	26157.46	38.00
542	HLA-A0201	AATASPSTCVWQC	ATATCVWQC	1	0	0	3	3	ATASPSTCVWQC	tr_C9ZJU1	0.060	26166.52	38.00
529	HLA-A0201	LGWIKDVIQ	LGWIKDVIQ	0	0	0	0	0	LGWIKDVIQ	tr_C9ZJU1	0.060	26172.75	38.00
841	HLA-A0201	NFVQKRSSGIHA	FVQSSGIHA	1	0	0	3	2	FVQKRSSGIHA	tr_C9ZJU1	0.060	26191.73	38.00
814	HLA-A0201	GGNIALGNL	GGNIALGNL	0	0	0	0	0	GGNIALGNL	tr_C9ZJU1	0.060	26197.69	38.00
697	HLA-A0201	QRKIDRPVRGV	KIDRPVRGV	2	0	0	0	0	KIDRPVRGV	tr_C9ZJU1	0.060	26200.24	38.00
208	HLA-A0201	PEVILKCILG	EVILKCILG	1	0	0	0	0	EVILKCILG	tr_C9ZJU1	0.060	26213.27	38.00

870	HLA-A0201	ARAFVKAGSAV	RAFVKASAV	1	0	0	6	1	RAFVKAGSAV	tr_C9ZJU1	0.060	26228.32	38.00
718	HLA-A0201	VEFREVSFRY	VEFEVSFRY	0	0	0	3	1	VEFREVSFRY	tr_C9ZJU1	0.060	26231.15	38.00
665	HLA-A0201	EAQVDEAHLRRHL	AQVDEAHLL	1	0	0	8	3	AQVDEAHLRRHL	tr_C9ZJU1	0.060	26233.14	38.00
622	HLA-A0201	SPNFFTQSAL	SPNFFTQAL	0	0	0	7	1	SPNFFTQSAL	tr_C9ZJU1	0.060	26235.99	38.00
426	HLA-A0201	LTLLRKQKDDGI	TLLQKDDGI	1	0	0	3	2	TLLRKQKDDGI	tr_C9ZJU1	0.060	26253.01	38.00
619	HLA-A0201	LRLSPNFFT	LRLSPNFFT	0	0	0	0	0	LRLSPNFFT	tr_C9ZJU1	0.060	26260.40	38.00
631	HLA-A0201	LDQTEIVNQLR	LQTEIVNQL	0	0	0	1	1	LDQTEIVNQL	tr_C9ZJU1	0.059	26268.36	38.00
868	HLA-A0201	CIARAFVKAG	CIARAFVKA	0	0	0	0	0	CIARAFVKA	tr_C9ZJU1	0.059	26277.74	38.00
459	HLA-A0201	RHDKSSVQYGLVL	SSVQYGLVL	4	0	0	0	0	SSVQYGLVL	tr_C9ZJU1	0.059	26279.17	38.00
960	HLA-A0201	FSRMRQSQQLPHF	FQSQQLPHF	0	0	0	1	4	FSRMRQSQQLPHF	tr_C9ZJU1	0.059	26282.86	38.00
541	HLA-A0201	RAATASPSTCVWQC	ATATCVWQC	2	0	0	3	3	ATASPSTCVWQC	tr_C9ZJU1	0.059	26287.69	38.00
920	HLA-A0201	IVAHRLATLKHAD	IVAHRLATL	0	0	0	0	0	IVAHRLATL	tr_C9ZJU1	0.059	26313.59	38.00
288	HLA-A0201	SDIITSIHID	SDIITSIHI	0	0	0	0	0	SDIITSIHI	tr_C9ZJU1	0.059	26316.16	38.00
138	HLA-A0201	RGKYSIARYPPA	YSIARYPPA	3	0	0	0	0	YSIARYPPA	tr_C9ZJU1	0.059	26317.01	38.00
461	HLA-A0201	DKSSVQYGLVL	SSVQYGLVL	2	0	0	0	0	SSVQYGLVL	tr_C9ZJU1	0.059	26324.70	38.00
441	HLA-A0201	LSSTDNKMLFQQTF	LSMLFQQTF	0	0	0	2	5	LSSTDNKMLFQQTF	tr_C9ZJU1	0.059	26332.68	38.00
667	HLA-A0201	QVDEAHLRRHL	QVDEAHLHL	0	0	0	7	2	QVDEAHLRRHL	tr_C9ZJU1	0.059	26336.38	38.00
737	HLA-A0201	RVSFVVNKGEF	RVSFVVNEF	0	0	0	7	2	RVSFVVNKGEF	tr_C9ZJU1	0.059	26361.19	38.00
308	HLA-A0201	DPSDDWLLSRCV	PSDDWLLSV	1	0	0	8	2	PSDDWLLSRCV	tr_C9ZJU1	0.059	26368.60	38.00
753	HLA-A0201	YSGSGKTTLLRLLN	YSTTLLRLL	0	0	0	2	4	YSGSGKTTLLRLL	tr_C9ZJU1	0.059	26368.89	38.00
776	HLA-A0201	IFVNGVHISQY	FVNGVHISY	1	0	0	8	1	FVNGVHISQY	tr_C9ZJU1	0.059	26393.42	38.00
529	HLA-A0201	LGWIKDVIQSS	LGWIVIQSS	0	0	0	4	2	LGWIKDVIQSS	tr_C9ZJU1	0.059	26394.00	38.00
439	HLA-A0201	CMLSSTDNK	CMLSSTDNK	0	0	0	0	0	CMLSSTDNK	tr_C9ZJU1	0.059	26395.14	38.00
854	HLA-A0201	LNVREFSGGEVERL	FSGGEVERL	5	0	0	0	0	FSGGEVERL	tr_C9ZJU1	0.059	26416.00	38.00
941	HLA-A0201	MVAEVGSWE	MVAEVGSWE	0	0	0	0	0	MVAEVGSWE	tr_C9ZJU1	0.059	26421.71	38.00
614	HLA-A0201	VSAVALRLSPNF	AVALRLSPF	2	0	0	8	1	AVALRLSPNF	tr_C9ZJU1	0.059	26436.59	38.00
460	HLA-A0201	HDKSSVQYGLVL	SSVQYGLVL	3	0	0	0	0	SSVQYGLVL	tr_C9ZJU1	0.059	26452.33	39.00
676	HLA-A0201	HLAFSGSSLGCYQ	HLAFLGCYQ	0	0	0	4	4	HLAFSGSSLGCYQ	tr_C9ZJU1	0.059	26453.75	39.00
518	HLA-A0201	GGHTCAVSVRRL	HTCAVSVRL	2	0	0	7	1	HTCAVSVRRL	tr_C9ZJU1	0.059	26453.75	39.00
928	HLA-A0201	LKHADKIVV	LKHADKIVV	0	0	0	0	0	LKHADKIVV	tr_C9ZJU1	0.059	26455.76	39.00
797	HLA-A0201	NVWQEENNLRFIED	NVWQNLRFI	0	0	0	4	3	NVWQEENNLRFI	tr_C9ZJU1	0.059	26466.07	39.00
137	HLA-A0201	LRGKYSIARYPPA	YSIARYPPA	4	0	0	0	0	YSIARYPPA	tr_C9ZJU1	0.059	26477.52	39.00
409	HLA-A0201	VSPTCSFPVKNS	VSPTCSFPV	0	0	0	0	0	VSPTCSFPV	tr_C9ZJU1	0.059	26484.40	39.00
710	HLA-A0201	PSLDGPWSVE	PSLDGPWSV	0	0	0	0	0	PSLDGPWSV	tr_C9ZJU1	0.059	26485.55	39.00
458	HLA-A0201	SRHDKSSVQYGLVL	SSVQYGLVL	5	0	0	0	0	SSVQYGLVL	tr_C9ZJU1	0.059	26490.71	39.00
517	HLA-A0201	AGGHTCAVSVRRL	HTCAVSVRL	3	0	0	7	1	HTCAVSVRRL	tr_C9ZJU1	0.059	26494.71	39.00
649	HLA-A0201	LYHAFLGSFYD	YAFLGSFYD	1	0	0	1	1	YHAFLGSFYD	tr_C9ZJU1	0.059	26502.17	39.00
852	HLA-A0201	APLNVREFSGGEV	PLNVREFSV	1	0	0	8	3	PLNVREFSGGEV	tr_C9ZJU1	0.059	26509.05	39.00
694	HLA-A0201	QSVQRKIDRPVRGV	KIDRPVRGV	5	0	0	0	0	KIDRPVRGV	tr_C9ZJU1	0.059	26527.71	39.00
614	HLA-A0201	VSAVALRLS	VSAVALRLS	0	0	0	0	0	VSAVALRLS	tr_C9ZJU1	0.059	26534.60	39.00
56	HLA-A0201	ESIHVRTLVFN	SIHVRTLVF	1	0	0	0	0	SIHVRTLVF	tr_C9ZJU1	0.058	26557.28	39.00
824	HLA-A0201	DCSDENICAALSA	CSDENILSA	1	0	0	6	3	CSDENICAALSA	tr_C9ZJU1	0.058	26562.46	39.00
694	HLA-A0201	QSVQRKIDRPV	SVQRKIDPV	1	0	0	7	1	SVQRKIDRPV	tr_C9ZJU1	0.058	26563.60	39.00
475	HLA-A0201	VFVESGRPLGYMW	VFVELGYMW	0	0	0	4	4	VFVESGRPLGYMW	tr_C9ZJU1	0.058	26579.13	39.00
876	HLA-A0201	AGSAVGLCIFD	AGSAVLCIF	0	0	0	5	1	AGSAVGLCIF	tr_C9ZJU1	0.058	26585.75	39.00
324	HLA-A0201	WQASGSVMDELQS	WQASVMDEL	0	0	0	3	2	WQASGSVMDEL	tr_C9ZJU1	0.058	26592.07	39.00
824	HLA-A0201	DCSDENICAALS	CSDEICAAL	1	0	0	4	1	CSDENICAAL	tr_C9ZJU1	0.058	26594.08	39.00
743	HLA-A0201	NKGEFLGIA	NKGEFLGIA	0	0	0	0	0	NKGEFLGIA	tr_C9ZJU1	0.058	26614.24	39.00
784	HLA-A0201	SQYPARMLRR	SQYARMLRR	0	0	0	3	1	SQYPARMLRR	tr_C9ZJU1	0.058	26617.69	39.00
655	HLA-A0201	GSFYDECGMSEA	FYDECMSEA	2	0	0	5	1	FYDECGMSEA	tr_C9ZJU1	0.058	26647.96	39.00
165	HLA-A0201	LGLITAYNR	LGLITAYNR	0	0	0	0	0	LGLITAYNR	tr_C9ZJU1	0.058	26653.14	39.00
823	HLA-A0201	WDCSDENICAALS	CSDEICAAL	2	0	0	4	1	CSDENICAAL	tr_C9ZJU1	0.058	26656.60	39.00
645	HLA-A0201	HWCPLYHAFLG	HWCPLYAFL	0	0	0	6	1	HWCPLYHAFL	tr_C9ZJU1	0.058	26669.02	39.00
83	HLA-A0201	MKNSHPDHL	MKNSHPDHL	0	0	0	0	0	MKNSHPDHL	tr_C9ZJU1	0.058	26686.32	39.00

920	HLA-A0201	IVAHRLATLKHADK	IVAHRLATL	0	0	0	0	0	IVAHRLATL	tr_C9ZJU1	0.058	26713.18	39.00
560	HLA-A0201	QGFAALRLS	QGFAALRLS	0	0	0	0	0	QGFAALRLS	tr_C9ZJU1	0.058	26718.11	39.00
875	HLA-A0201	KAGSAVGLCIFD	AGSAVLCIF	1	0	0	5	1	AGSAVGLCIF	tr_C9ZJU1	0.058	26736.90	39.00
516	HLA-A0201	QAGGHTCAVSVRRL	HTCAVSVRL	4	0	0	7	1	HTCAVSVRRL	tr_C9ZJU1	0.058	26743.85	39.00
903	HLA-A0201	TLRLREGNNLLRD	LLREGNNLL	1	0	0	1	1	LRLREGNNLL	tr_C9ZJU1	0.058	26757.74	39.00
393	HLA-A0201	TNIATSTYA	TNIATSTYA	0	0	0	0	0	TNIATSTYA	tr_C9ZJU1	0.058	26770.47	39.00
738	HLA-A0201	VSFVVNKGEF	VSFVVNGEF	0	0	0	6	1	VSFVVNKGEF	tr_C9ZJU1	0.058	26785.55	39.00
840	HLA-A0201	LNFVQKRSSGIHA	FVQSSGIHA	2	0	0	3	2	FVQKRSSGIHA	tr_C9ZJU1	0.058	26789.02	39.00
889	HLA-A0201	TSAIDTITEGEIF	AIDTITEEI	2	0	0	7	1	AIDTITEGEI	tr_C9ZJU1	0.058	26794.54	39.00
114	HLA-A0201	DDGVALCIL	DDGVALCIL	0	0	0	0	0	DDGVALCIL	tr_C9ZJU1	0.058	26809.90	39.00
640	HLA-A0201	LRSIQHWCPLYH	IQHWCPLYH	3	0	0	0	0	IQHWCPLYH	tr_C9ZJU1	0.058	26815.12	39.00
522	HLA-A0201	CAVSVRRLGWIK	CAVSVLGWI	0	0	0	5	2	CAVSVRRLGWI	tr_C9ZJU1	0.058	26820.64	39.00
54	HLA-A0201	GVESIHVRTLVFNH	GVIHVRTLV	0	0	0	2	2	GVESIHVRTLV	tr_C9ZJU1	0.058	26822.67	39.00
205	HLA-A0201	LFSPEVILK	LFSPEVILK	0	0	0	0	0	LFSPEVILK	tr_C9ZJU1	0.058	26824.12	39.00
793	HLA-A0201	RRIANVWQEENNLR	RIANVWQEL	1	0	0	8	3	RIANVWQEENNL	tr_C9ZJU1	0.058	26825.28	39.00
780	HLA-A0201	GVHISQYPAR	GVHISQYPA	0	0	0	0	0	GVHISQYPA	tr_C9ZJU1	0.058	26825.87	39.00
54	HLA-A0201	GVESIHVRT	GVESIHVRT	0	0	0	0	0	GVESIHVRT	tr_C9ZJU1	0.058	26827.32	39.00
874	HLA-A0201	VKAGSAVGLCIFD	SAVGLCIFD	4	0	0	0	0	SAVGLCIFD	tr_C9ZJU1	0.058	26834.00	39.00
409	HLA-A0201	VSPTCSFPVKNSLT	VSPTCSFPV	0	0	0	0	0	VSPTCSFPV	tr_C9ZJU1	0.058	26836.89	39.00
934	HLA-A0201	IVVLNEGMVAE	ILNEGMVAE	0	0	0	1	2	IVVLNEGMVAE	tr_C9ZJU1	0.057	26856.94	39.00
875	HLA-A0201	KAGSAVGLCIF	AGSAVLCIF	1	0	0	5	1	AGSAVGLCIF	tr_C9ZJU1	0.057	26872.63	39.00
726	HLA-A0201	RYSDRHPYVLRRVS	YSDYVLRRV	1	0	0	3	3	YSDRHPYVLRRV	tr_C9ZJU1	0.057	26885.13	39.00
727	HLA-A0201	YSDRHPYVLRRVSF	YSDYVLRRV	0	0	0	3	3	YSDRHPYVLRRV	tr_C9ZJU1	0.057	26904.35	39.00
85	HLA-A0201	NSHPDHLLVCDIW	NSLLVCDIW	0	0	0	2	4	NSHPDHLLVCDIW	tr_C9ZJU1	0.057	26906.38	39.00
346	HLA-A0201	HGCRVVFSFP	HGCRVVFFP	0	0	0	7	1	HGCRVVFSFP	tr_C9ZJU1	0.057	26906.97	39.00
473	HLA-A0201	SMVFVESGRP	SMVFVESGP	0	0	0	8	1	SMVFVESGRP	tr_C9ZJU1	0.057	26907.25	39.00
9	HLA-A0201	LLDCERKAY	LLDCERKAY	0	0	0	0	0	LLDCERKAY	tr_C9ZJU1	0.057	26920.94	39.00
823	HLA-A0201	WDCSDENICAALSA	CSDENILSA	2	0	0	6	3	CSDENICAALSA	tr_C9ZJU1	0.057	26942.50	40.00
897	HLA-A0201	EGEIFDTLRL	GEIFDTLRL	1	0	0	0	0	GEIFDTLRL	tr_C9ZJU1	0.057	26961.46	40.00
915	HLA-A0201	DSTIIIVAH	DSTIIIVAH	0	0	0	0	0	DSTIIIVAH	tr_C9ZJU1	0.057	26962.03	40.00
37	HLA-A0201	QTECKDDASIAI	QTDDASIAI	0	0	0	2	3	QTECKDDASIAI	tr_C9ZJU1	0.057	26992.98	40.00
677	HLA-A0201	LAFSGSSLG	LAFSGSSLG	0	0	0	0	0	LAFSGSSLG	tr_C9ZJU1	0.057	27003.50	40.00
860	HLA-A0201	SGGEVERLCIARA	GEVLCIARA	2	0	0	3	2	GEVERLCIARA	tr_C9ZJU1	0.057	27010.80	40.00
390	HLA-A0201	ASVTNIATS	ASVTNIATS	0	0	0	0	0	ASVTNIATS	tr_C9ZJU1	0.057	27021.92	40.00
739	HLA-A0201	SFVVNKGEFLG	FVVNKGEFL	1	0	0	0	0	FVVNKGEFL	tr_C9ZJU1	0.057	27023.08	40.00
7	HLA-A0201	AVLLDCERKAY	AVLLDCEAY	0	0	0	7	2	AVLLDCERKAY	tr_C9ZJU1	0.057	27031.85	40.00
621	HLA-A0201	LSPNFFTQS	LSPNFFTQS	0	0	0	0	0	LSPNFFTQS	tr_C9ZJU1	0.057	27045.90	40.00
510	HLA-A0201	ALKRKLQAG	ALKRKLQAG	0	0	0	0	0	ALKRKLQAG	tr_C9ZJU1	0.057	27055.26	40.00
874	HLA-A0201	VKAGSAVGLCI	KASAVGLCI	1	0	0	2	1	KAGSAVGLCI	tr_C9ZJU1	0.057	27060.53	40.00
900	HLA-A0201	IFDTLRLREGNNL	TLLREGNNL	3	0	0	2	1	TLRLREGNNL	tr_C9ZJU1	0.057	27066.98	40.00
250	HLA-A0201	IIKQLMGLP	IIKQLMGLP	0	0	0	0	0	IIKQLMGLP	tr_C9ZJU1	0.057	27084.85	40.00
672	HLA-A0201	HLRRHLAFSGSS	HLLAFSGSS	0	0	0	2	3	HLRRHLAFSGSS	tr_C9ZJU1	0.057	27087.48	40.00
961	HLA-A0201	SRMRQSQQLPHF	RMSQQLPHF	1	0	0	2	2	RMRQSQQLPHF	tr_C9ZJU1	0.057	27095.70	40.00
902	HLA-A0201	DTLRLREGNNL	TLLREGNNL	1	0	0	2	1	TLRLREGNNL	tr_C9ZJU1	0.057	27097.44	40.00
654	HLA-A0201	LGSFYDECGM	LSFYDECGM	0	0	0	1	1	LGSFYDECGM	tr_C9ZJU1	0.057	27109.47	40.00
537	HLA-A0201	QSSPRAATA	QSSPRAATA	0	0	0	0	0	QSSPRAATA	tr_C9ZJU1	0.057	27115.05	40.00
654	HLA-A0201	LGSFYDECGMSEA	FYDECGMSA	3	0	0	8	1	FYDECGMSEA	tr_C9ZJU1	0.057	27116.52	40.00
805	HLA-A0201	LRFIEDLSIG	LRFIEDLSI	0	0	0	0	0	LRFIEDLSI	tr_C9ZJU1	0.057	27128.55	40.00
771	HLA-A0201	PTEGDIFVNGV	PTDIFVNGV	0	0	0	2	2	PTEGDIFVNGV	tr_C9ZJU1	0.056	27132.36	40.00
189	HLA-A0201	CSSCSIPQLVT	CSSCSIPLV	0	0	0	7	1	CSSCSIPQLV	tr_C9ZJU1	0.056	27141.46	40.00
664	HLA-A0201	SEAQVDEAHLRRHL	AQVDEAHLL	2	0	0	8	3	AQVDEAHLRRHL	tr_C9ZJU1	0.056	27141.76	40.00
41	HLA-A0201	KDDASIAIF	KDDASIAIF	0	0	0	0	0	KDDASIAIF	tr_C9ZJU1	0.056	27149.39	40.00
412	HLA-A0201	TCSFPVKNSLTS	CSFPVSLTS	1	0	0	5	2	CSFPVKNSLTS	tr_C9ZJU1	0.056	27158.80	40.00
84	HLA-A0201	KNSHPDHLLVCDIW	NSLLVCDIW	1	0	0	2	4	NSHPDHLLVCDIW	tr_C9ZJU1	0.056	27159.38	40.00

675	HLA-A0201	RHLAFSGSSLG	RLAFSGSSL	0	0	0	1	1	RHLAFSGSSL	tr_C9ZJU1	0.056	27161.74	40.00
317	HLA-A0201	RCVSQALWQAS	RVSQALWQA	0	0	0	1	1	RCVSQALWQA	tr_C9ZJU1	0.056	27167.61	40.00
530	HLA-A0201	GWIKDVIQSSPRA	WIKDVIPRA	1	0	0	6	3	WIKDVIQSSPRA	tr_C9ZJU1	0.056	27170.55	40.00
794	HLA-A0201	RIANVWQEE	RIANVWQEE	0	0	0	0	0	RIANVWQEE	tr_C9ZJU1	0.056	27177.02	40.00
679	HLA-A0201	FSGSSLGCY	FSGSSLGCY	0	0	0	0	0	FSGSSLGCY	tr_C9ZJU1	0.056	27191.43	40.00
641	HLA-A0201	RSIQHWCPLYH	IQHWCPLYH	2	0	0	0	0	IQHWCPLYH	tr_C9ZJU1	0.056	27202.32	40.00
884	HLA-A0201	IFDEPTSAIDTI	IFDEAIDTI	0	0	0	4	3	IFDEPTSAIDTI	tr_C9ZJU1	0.056	27203.80	40.00
107	HLA-A0201	SIRDILLDD	SIRDILLDD	0	0	0	0	0	SIRDILLDD	tr_C9ZJU1	0.056	27213.50	40.00
315	HLA-A0201	LSRCVSQALWQASG	LSRCVLWQA	0	0	0	5	3	LSRCVSQALWQA	tr_C9ZJU1	0.056	27218.22	40.00
316	HLA-A0201	SRCVSQALWQAS	SVSQALWQA	0	0	0	1	2	SRCVSQALWQA	tr_C9ZJU1	0.056	27218.22	40.00
880	HLA-A0201	VGLCIFDEP	VGLCIFDEP	0	0	0	0	0	VGLCIFDEP	tr_C9ZJU1	0.056	27226.18	40.00
633	HLA-A0201	QTEIVNQLRSI	QTEIVLRSI	0	0	0	5	2	QTEIVNQLRSI	tr_C9ZJU1	0.056	27237.08	40.00
735	HLA-A0201	LRRVSFVVNKGEF	RVSFVVNEF	2	0	0	7	2	RVSFVVNKGEF	tr_C9ZJU1	0.056	27242.97	40.00
149	HLA-A0201	AAQADVVVQEFP	AQADVVVFP	1	0	0	7	2	AQADVVVQEFP	tr_C9ZJU1	0.056	27243.26	40.00
903	HLA-A0201	TLRLREGNNLLRDS	LLREGNNLL	1	0	0	1	1	LRLREGNNLL	tr_C9ZJU1	0.056	27247.39	40.00
335	HLA-A0201	QSRVPALLEVMH	QSVPALLEV	0	0	0	2	1	QSRVPALLEV	tr_C9ZJU1	0.056	27252.70	40.00
959	HLA-A0201	CFSRMRQSQQLPHF	RMSQQLPHF	3	0	0	2	2	RMRQSQQLPHF	tr_C9ZJU1	0.056	27258.59	40.00
307	HLA-A0201	YDPSDDWLLSRCVS	YDWLLSRCV	0	0	0	1	4	YDPSDDWLLSRCV	tr_C9ZJU1	0.056	27267.73	40.00
531	HLA-A0201	WIKDVIQSSPRAAT	WIKDVIAAT	0	0	0	6	5	WIKDVIQSSPRAAT	tr_C9ZJU1	0.056	27278.95	40.00
904	HLA-A0201	LRLREGNNLLRD	LLREGNNLL	0	0	0	1	1	LRLREGNNLL	tr_C9ZJU1	0.056	27284.57	40.00
846	HLA-A0201	RSSGIHAPLNVR	RSSGIPLNV	0	0	0	5	2	RSSGIHAPLNV	tr_C9ZJU1	0.056	27289.58	40.00
535	HLA-A0201	VIQSSPRAAT	VIQSSPAAT	0	0	0	6	1	VIQSSPRAAT	tr_C9ZJU1	0.056	27296.08	40.00
874	HLA-A0201	VKAGSAVGLCIF	AGSAVLCIF	2	0	0	5	1	AGSAVGLCIF	tr_C9ZJU1	0.056	27296.68	40.00
107	HLA-A0201	SIRDILLDDG	SIRDILLDG	0	0	0	7	1	SIRDILLDDG	tr_C9ZJU1	0.056	27309.67	40.00
271	HLA-A0201	AFESLYCIRG	FESLYCIRG	1	0	0	0	0	FESLYCIRG	tr_C9ZJU1	0.056	27314.09	40.00
829	HLA-A0201	NICAALSASRA	NICAALSAA	0	0	0	8	2	NICAALSASRA	tr_C9ZJU1	0.056	27320.01	40.00
6	HLA-A0201	PAVLLDCERKAYE	VLLDCEAYE	2	0	0	6	2	VLLDCERKAYE	tr_C9ZJU1	0.056	27332.43	40.00
851	HLA-A0201	HAPLNVREFSGGEV	PLNVREFSV	2	0	0	8	3	PLNVREFSGGEV	tr_C9ZJU1	0.056	27344.55	40.00
901	HLA-A0201	FDTLRLREGNNL	TLLREGNNL	2	0	0	2	1	TLRLREGNNL	tr_C9ZJU1	0.056	27348.99	40.00
905	HLA-A0201	RLREGNNLLRD	RLREGNNLL	0	0	0	0	0	RLREGNNLL	tr_C9ZJU1	0.056	27351.07	40.00
4	HLA-A0201	ARPAVLLDCERKAY	ALLDCERKA	0	0	0	1	4	ARPAVLLDCERKA	tr_C9ZJU1	0.056	27352.54	40.00
57	HLA-A0201	SIHVRTLVFNH	SIHVLVFNH	0	0	0	4	2	SIHVRTLVFNH	tr_C9ZJU1	0.056	27354.02	40.00
431	HLA-A0201	KQKDDGIKCMLSS	KQIKCMLSS	0	0	0	2	4	KQKDDGIKCMLSS	tr_C9ZJU1	0.056	27371.19	40.00
638	HLA-A0201	NQLRSIQHW	NQLRSIQHW	0	0	0	0	0	NQLRSIQHW	tr_C9ZJU1	0.056	27373.85	40.00
676	HLA-A0201	HLAFSGSSLGCY	HLAFSLGCY	0	0	0	4	3	HLAFSGSSLGCY	tr_C9ZJU1	0.056	27378.30	40.00
822	HLA-A0201	LWDCSDENICAA	LSDENICAA	0	0	0	1	3	LWDCSDENICAA	tr_C9ZJU1	0.056	27400.52	40.00
10	HLA-A0201	LDCERKAYEI	LDCERAYEI	0	0	0	5	1	LDCERKAYEI	tr_C9ZJU1	0.056	27407.65	41.00
160	HLA-A0201	PEELYLGLI	PEELYLGLI	0	0	0	0	0	PEELYLGLI	tr_C9ZJU1	0.056	27412.68	41.00
680	HLA-A0201	SGSSLGCYQVLE	GSSLGCYVL	1	0	0	7	1	GSSLGCYQVL	tr_C9ZJU1	0.056	27413.87	41.00
664	HLA-A0201	SEAQVDEAHL	SEAQVDAHL	0	0	0	6	1	SEAQVDEAHL	tr_C9ZJU1	0.056	27421.58	41.00
809	HLA-A0201	EDLSIGGNIALGN	DLSIGGIAL	1	0	0	6	1	DLSIGGNIAL	tr_C9ZJU1	0.056	27423.66	41.00
736	HLA-A0201	RRVSFVVNKGEF	RVSFVVNEF	1	0	0	7	2	RVSFVVNKGEF	tr_C9ZJU1	0.055	27430.49	41.00
829	HLA-A0201	NICAALSASRALN	NICAALSAL	0	0	0	7	3	NICAALSASRAL	tr_C9ZJU1	0.055	27441.18	41.00
443	HLA-A0201	STDNKMLFQQTFS	KMLFQQTFS	4	0	0	0	0	KMLFQQTFS	tr_C9ZJU1	0.055	27442.67	41.00
803	HLA-A0201	NNLRFIEDLS	NLRFIEDLS	1	0	0	0	0	NLRFIEDLS	tr_C9ZJU1	0.055	27447.11	41.00
888	HLA-A0201	PTSAIDTITEGEIF	AIDTITEEI	3	0	0	7	1	AIDTITEGEI	tr_C9ZJU1	0.055	27456.92	41.00
875	HLA-A0201	KAGSAVGLCIFDE	SAVGLCIFE	3	0	0	8	1	SAVGLCIFDE	tr_C9ZJU1	0.055	27462.57	41.00
876	HLA-A0201	AGSAVGLCIFDE	SAVGLCIFE	2	0	0	8	1	SAVGLCIFDE	tr_C9ZJU1	0.055	27496.17	41.00
20	HLA-A0201	LEMLTRATIKT	MLTRATIKT	2	0	0	0	0	MLTRATIKT	tr_C9ZJU1	0.055	27500.03	41.00
869	HLA-A0201	IARAFVKAGS	IARAFVAGS	0	0	0	6	1	IARAFVKAGS	tr_C9ZJU1	0.055	27508.65	41.00
869	HLA-A0201	IARAFVKAGSAVG	IARAFVSAV	0	0	0	6	3	IARAFVKAGSAV	tr_C9ZJU1	0.055	27517.29	41.00
902	HLA-A0201	DTLRLREGNNLLRD	LLREGNNLL	2	0	0	1	1	LRLREGNNLL	tr_C9ZJU1	0.055	27519.37	41.00
83	HLA-A0201	MKNSHPDHLLVCDI	MKHLLVCDI	0	0	0	2	5	MKNSHPDHLLVCDI	tr_C9ZJU1	0.055	27551.55	41.00
752	HLA-A0201	GYSGSGKTTLL	YSGSGTTLL	1	0	0	5	1	YSGSGKTTLL	tr_C9ZJU1	0.055	27553.04	41.00

636	HLA-A0201	IVNQLRSIQHWC	ILRSIQHWC	0	0	0	1	3	IVNQLRSIQHWC	tr_C9ZJU1	0.055	27553.34	41.00
844	HLA-A0201	QKRSSGIHAPL	KSSGIHAPL	1	0	0	1	1	KRSSGIHAPL	tr_C9ZJU1	0.055	27564.38	41.00
783	HLA-A0201	ISQYPARMLRRRIA	SQYPARMLA	1	0	0	8	4	SQYPARMLRRRIA	tr_C9ZJU1	0.055	27564.66	41.00
390	HLA-A0201	ASVTNIATST	ASVTNIATT	0	0	0	8	1	ASVTNIATST	tr_C9ZJU1	0.055	27564.66	41.00
36	HLA-A0201	KQTECKDDASIA	KQTECDASI	0	0	0	5	2	KQTECKDDASI	tr_C9ZJU1	0.055	27566.76	41.00
5	HLA-A0201	RPAVLLDCERKAYE	VLLDCEAYE	3	0	0	6	2	VLLDCERKAYE	tr_C9ZJU1	0.055	27604.07	41.00
674	HLA-A0201	RRHLAFSGSSLG	RLAFSGSSL	0	0	0	1	2	RRHLAFSGSSL	tr_C9ZJU1	0.055	27614.82	41.00
874	HLA-A0201	VKAGSAVGLCIFDE	SAVGLCIFE	4	0	0	8	1	SAVGLCIFDE	tr_C9ZJU1	0.055	27619.01	41.00
338	HLA-A0201	VPALLEVMHG	VALLEVMHG	0	0	0	1	1	VPALLEVMHG	tr_C9ZJU1	0.055	27636.34	41.00
151	HLA-A0201	QADVVVQEFP	QADVVVEFP	0	0	0	6	1	QADVVVQEFP	tr_C9ZJU1	0.055	27643.81	41.00
57	HLA-A0201	SIHVRTLVFNHN	SIHLVFNHN	0	0	0	3	3	SIHVRTLVFNHN	tr_C9ZJU1	0.055	27647.70	41.00
930	HLA-A0201	HADKIVVLN	HADKIVVLN	0	0	0	0	0	HADKIVVLN	tr_C9ZJU1	0.055	27652.19	41.00
20	HLA-A0201	LEMLTRATIK	LEMLTRATI	0	0	0	0	0	LEMLTRATI	tr_C9ZJU1	0.055	27662.96	41.00
914	HLA-A0201	RDSTIIIVAH	RSTIIIVAH	0	0	0	1	1	RDSTIIIVAH	tr_C9ZJU1	0.055	27669.25	41.00
714	HLA-A0201	GPWSVEFREVS	GPWSVEFEV	0	0	0	7	1	GPWSVEFREV	tr_C9ZJU1	0.055	27670.16	41.00
825	HLA-A0201	CSDENICAALSAS	CSDEICAAL	0	0	0	4	1	CSDENICAAL	tr_C9ZJU1	0.055	27673.44	41.00
530	HLA-A0201	GWIKDVIQSSPRAA	WIKDVIQAA	1	0	0	7	4	WIKDVIQSSPRAA	tr_C9ZJU1	0.055	27675.24	41.00
55	HLA-A0201	VESIHVRTLVFNH	VSIHVRTLV	0	0	0	1	1	VESIHVRTLV	tr_C9ZJU1	0.055	27685.13	41.00
741	HLA-A0201	VVNKGEFLG	VVNKGEFLG	0	0	0	0	0	VVNKGEFLG	tr_C9ZJU1	0.055	27685.72	41.00
442	HLA-A0201	SSTDNKMLFQQTFS	SMLFQQTFS	0	0	0	1	5	SSTDNKMLFQQTFS	tr_C9ZJU1	0.055	27686.33	41.00
843	HLA-A0201	VQKRSSGIHAPLN	VQSGIHAPL	0	0	0	2	3	VQKRSSGIHAPL	tr_C9ZJU1	0.055	27688.72	41.00
679	HLA-A0201	FSGSSLGCYQ	FSGSSLCYQ	0	0	0	6	1	FSGSSLGCYQ	tr_C9ZJU1	0.055	27698.31	41.00
675	HLA-A0201	RHLAFSGSSLGCYQ	HLAFLGCYQ	1	0	0	4	4	HLAFSGSSLGCYQ	tr_C9ZJU1	0.055	27702.21	41.00
752	HLA-A0201	GYSGSGKTTL	YSGSGKTTL	1	0	0	0	0	YSGSGKTTL	tr_C9ZJU1	0.055	27702.79	41.00
877	HLA-A0201	GSAVGLCIFDE	SAVGLCIFE	1	0	0	8	1	SAVGLCIFDE	tr_C9ZJU1	0.055	27721.10	41.00
627	HLA-A0201	TQSALDQTEIVN	TQSALTEIV	0	0	0	5	2	TQSALDQTEIV	tr_C9ZJU1	0.054	27752.01	41.00
640	HLA-A0201	LRSIQHWCPLY	LSIQHWCPL	0	0	0	1	1	LRSIQHWCPL	tr_C9ZJU1	0.054	27758.91	41.00
862	HLA-A0201	GEVERLCIARA	GEVLCIARA	0	0	0	3	2	GEVERLCIARA	tr_C9ZJU1	0.054	27759.22	41.00
939	HLA-A0201	EGMVAEVGSW	GMVAEVGSW	1	0	0	0	0	GMVAEVGSW	tr_C9ZJU1	0.054	27768.22	41.00
576	HLA-A0201	TAAAAFATAS	TAAAAFATA	0	0	0	0	0	TAAAAFATA	tr_C9ZJU1	0.054	27773.94	41.00
812	HLA-A0201	SIGGNIALGN	SIGGNIALG	0	0	0	0	0	SIGGNIALG	tr_C9ZJU1	0.054	27776.34	41.00
658	HLA-A0201	YDECGMSEA	YDECGMSEA	0	0	0	0	0	YDECGMSEA	tr_C9ZJU1	0.054	27779.94	41.00
55	HLA-A0201	VESIHVRTLVFNHN	VIHVRTLVF	0	0	0	1	2	VESIHVRTLVF	tr_C9ZJU1	0.054	27780.55	41.00
65	HLA-A0201	FNHNIMQLP	FNHNIMQLP	0	0	0	0	0	FNHNIMQLP	tr_C9ZJU1	0.054	27797.99	41.00
324	HLA-A0201	WQASGSVMDELQSR	WQASVMDEL	0	0	0	3	2	WQASGSVMDEL	tr_C9ZJU1	0.054	27800.08	41.00
345	HLA-A0201	MHGCRVVFSFP	MHGCVVFSF	0	0	0	4	1	MHGCRVVFSF	tr_C9ZJU1	0.054	27803.10	41.00
521	HLA-A0201	TCAVSVRRLGWIK	CAVSVLGWI	1	0	0	5	2	CAVSVRRLGWI	tr_C9ZJU1	0.054	27824.46	41.00
689	HLA-A0201	VLEGLQSVQ	VLEGLQSVQ	0	0	0	0	0	VLEGLQSVQ	tr_C9ZJU1	0.054	27827.78	41.00
780	HLA-A0201	GVHISQYPARM	GVHISQYPM	0	0	0	8	2	GVHISQYPARM	tr_C9ZJU1	0.054	27828.08	41.00
755	HLA-A0201	GSGKTTLLRLL	SGKTTLLLL	1	0	0	7	1	SGKTTLLRLL	tr_C9ZJU1	0.054	27828.98	41.00
415	HLA-A0201	FPVKNSLTSSPL	FPVLTSSPL	0	0	0	3	3	FPVKNSLTSSPL	tr_C9ZJU1	0.054	27829.29	41.00
675	HLA-A0201	RHLAFSGSSLGCY	HLAFSLGCY	1	0	0	4	3	HLAFSGSSLGCY	tr_C9ZJU1	0.054	27833.51	41.00
672	HLA-A0201	HLRRHLAFSGS	HLHLAFSGS	0	0	0	2	2	HLRRHLAFSGS	tr_C9ZJU1	0.054	27841.93	41.00
857	HLA-A0201	REFSGGEVERL	FSGGEVERL	2	0	0	0	0	FSGGEVERL	tr_C9ZJU1	0.054	27844.64	41.00
809	HLA-A0201	EDLSIGGNIA	ELSIGGNIA	0	0	0	1	1	EDLSIGGNIA	tr_C9ZJU1	0.054	27891.08	42.00
43	HLA-A0201	DASIAIFLN	DASIAIFLN	0	0	0	0	0	DASIAIFLN	tr_C9ZJU1	0.054	27899.53	42.00
535	HLA-A0201	VIQSSPRAATA	VIQSSPATA	0	0	0	6	2	VIQSSPRAATA	tr_C9ZJU1	0.054	27916.74	42.00
681	HLA-A0201	GSSLGCYQVLE	GSSLGCYVL	0	0	0	7	1	GSSLGCYQVL	tr_C9ZJU1	0.054	27917.04	42.00
51	HLA-A0201	NCNGVESIHVRTL	GVESIHVTL	3	0	0	7	1	GVESIHVRTL	tr_C9ZJU1	0.054	27936.39	42.00
319	HLA-A0201	VSQALWQAS	VSQALWQAS	0	0	0	0	0	VSQALWQAS	tr_C9ZJU1	0.054	27936.98	42.00
246	HLA-A0201	PFRFIIKQL	PFRFIIKQL	0	0	0	0	0	PFRFIIKQL	tr_C9ZJU1	0.054	27937.59	42.00
905	HLA-A0201	RLREGNNLLRDS	RLREGNNLL	0	0	0	0	0	RLREGNNLL	tr_C9ZJU1	0.054	27942.12	42.00
559	HLA-A0201	LQGFAALRLSGW	LQGFAALRL	0	0	0	0	0	LQGFAALRL	tr_C9ZJU1	0.054	27943.32	42.00
541	HLA-A0201	RAATASPSTCV	RAATASPSV	0	0	0	8	2	RAATASPSTCV	tr_C9ZJU1	0.054	27945.14	42.00

504	HLA-A0201	SGCVVDALK	SGCVVDALK	0	0	0	0	0	SGCVVDALK	tr_C9ZJU1	0.054	27955.13	42.00
238	HLA-A0201	GDVSPKLSPF	GVSPKLSPF	0	0	0	1	1	GDVSPKLSPF	tr_C9ZJU1	0.054	27962.38	42.00
736	HLA-A0201	RRVSFVVNK	RRVSFVVNK	0	0	0	0	0	RRVSFVVNK	tr_C9ZJU1	0.054	27970.86	42.00
336	HLA-A0201	SRVPALLEVMH	SVPALLEVM	0	0	0	1	1	SRVPALLEVM	tr_C9ZJU1	0.054	27971.78	42.00
552	HLA-A0201	WQCRQDYLQGF	WQCDYLQGF	0	0	0	3	2	WQCRQDYLQGF	tr_C9ZJU1	0.054	27987.20	42.00
724	HLA-A0201	SFRYSDRHPYVL	FSDRHPYVL	1	0	0	1	2	FRYSDRHPYVL	tr_C9ZJU1	0.054	27989.03	42.00
53	HLA-A0201	NGVESIHVRTL	GVESIHVTL	1	0	0	7	1	GVESIHVRTL	tr_C9ZJU1	0.054	27994.48	42.00
562	HLA-A0201	FAALRLSGWE	FAALRLSGW	0	0	0	0	0	FAALRLSGW	tr_C9ZJU1	0.054	28003.87	42.00
627	HLA-A0201	TQSALDQTEIVNQ	ALDQTIVNQ	3	0	0	5	1	ALDQTEIVNQ	tr_C9ZJU1	0.054	28005.08	42.00
323	HLA-A0201	LWQASGSVMDELQS	WQASVMDEL	1	0	0	3	2	WQASGSVMDEL	tr_C9ZJU1	0.054	28020.23	42.00
109	HLA-A0201	RDILLDDGVA	RILLDDGVA	0	0	0	1	1	RDILLDDGVA	tr_C9ZJU1	0.053	28038.42	42.00
335	HLA-A0201	QSRVPALLEVMHG	QSVPALLEV	0	0	0	2	1	QSRVPALLEV	tr_C9ZJU1	0.053	28039.34	42.00
146	HLA-A0201	YPPAAQADVV	YPPAAQAVV	0	0	0	7	1	YPPAAQADVV	tr_C9ZJU1	0.053	28050.86	42.00
616	HLA-A0201	AVALRLSPN	AVALRLSPN	0	0	0	0	0	AVALRLSPN	tr_C9ZJU1	0.053	28055.73	42.00
148	HLA-A0201	PAAQADVVVQEFP	AQADVVVFP	2	0	0	7	2	AQADVVVQEFP	tr_C9ZJU1	0.053	28077.90	42.00
300	HLA-A0201	LALATADYDPS	ALATADYPS	1	0	0	7	1	ALATADYDPS	tr_C9ZJU1	0.053	28093.39	42.00
904	HLA-A0201	LRLREGNNLLRDS	LLREGNNLL	0	0	0	1	1	LRLREGNNLL	tr_C9ZJU1	0.053	28097.96	42.00
445	HLA-A0201	DNKMLFQQTFS	KMLFQQTFS	2	0	0	0	0	KMLFQQTFS	tr_C9ZJU1	0.053	28102.21	42.00
754	HLA-A0201	SGSGKTTLLRL	SGSGTLLRL	0	0	0	4	2	SGSGKTTLLRL	tr_C9ZJU1	0.053	28104.33	42.00
411	HLA-A0201	PTCSFPVKNSLTS	CSFPVSLTS	2	0	0	5	2	CSFPVKNSLTS	tr_C9ZJU1	0.053	28110.71	42.00
863	HLA-A0201	EVERLCIARAF	VERLCIAAF	1	0	0	7	1	VERLCIARAF	tr_C9ZJU1	0.053	28125.33	42.00
629	HLA-A0201	SALDQTEIVNQ	ALDQTIVNQ	1	0	0	5	1	ALDQTEIVNQ	tr_C9ZJU1	0.053	28128.67	42.00
410	HLA-A0201	SPTCSFPVKNSL	SSFPVKNSL	0	0	0	1	3	SPTCSFPVKNSL	tr_C9ZJU1	0.053	28131.12	42.00
632	HLA-A0201	DQTEIVNQLRSI	QTEIVLRSI	1	0	0	5	2	QTEIVNQLRSI	tr_C9ZJU1	0.053	28135.36	42.00
816	HLA-A0201	NIALGNLWDCSD	ALGNLWCSD	2	0	0	6	1	ALGNLWDCSD	tr_C9ZJU1	0.053	28136.60	42.00
413	HLA-A0201	CSFPVKNSLTSS	CSFPVLTSS	0	0	0	5	3	CSFPVKNSLTSS	tr_C9ZJU1	0.053	28146.94	42.00
450	HLA-A0201	FQQTFSHPSRHDKS	FQQTFSHPS	0	0	0	8	5	FQQTFSHPSRHDKS	tr_C9ZJU1	0.053	28151.81	42.00
802	HLA-A0201	ENNLRFIEDL	NNLRFIEDL	1	0	0	0	0	NNLRFIEDL	tr_C9ZJU1	0.053	28160.03	42.00
150	HLA-A0201	AQADVVVQEFPE	AQADVVFPE	0	0	0	6	3	AQADVVVQEFPE	tr_C9ZJU1	0.053	28165.52	42.00
668	HLA-A0201	VDEAHLRRHLAFS	VLRRHLAFS	0	0	0	1	4	VDEAHLRRHLAFS	tr_C9ZJU1	0.053	28167.66	42.00
178	HLA-A0201	QSGITSFWK	QSGITSFWK	0	0	0	0	0	QSGITSFWK	tr_C9ZJU1	0.053	28202.12	42.00
50	HLA-A0201	LNCNGVESIHVRTL	GVSIHVRTL	4	0	0	2	1	GVESIHVRTL	tr_C9ZJU1	0.053	28215.24	42.00
425	HLA-A0201	PLTLLRKQKDDGI	TLLQKDDGI	2	0	0	3	2	TLLRKQKDDGI	tr_C9ZJU1	0.053	28216.77	42.00
778	HLA-A0201	VNGVHISQYPARM	VISQYPARM	0	0	0	1	4	VNGVHISQYPARM	tr_C9ZJU1	0.053	28219.52	42.00
147	HLA-A0201	PPAAQADVVVQEFP	AQADVVVFP	3	0	0	7	2	AQADVVVQEFP	tr_C9ZJU1	0.053	28225.62	42.00
552	HLA-A0201	WQCRQDYLQGFA	WQCRQDYFA	0	0	0	7	3	WQCRQDYLQGFA	tr_C9ZJU1	0.053	28231.11	42.00
913	HLA-A0201	LRDSTIIIVAH	LRDSTIIIV	0	0	0	0	0	LRDSTIIIV	tr_C9ZJU1	0.053	28231.42	42.00
392	HLA-A0201	VTNIATSTY	VTNIATSTY	0	0	0	0	0	VTNIATSTY	tr_C9ZJU1	0.053	28237.85	42.00
140	HLA-A0201	KYSIARYPPAAQAD	YIARYPPAA	1	0	0	1	1	YSIARYPPAA	tr_C9ZJU1	0.053	28242.11	42.00
942	HLA-A0201	VAEVGSWETLSRT	VAWETLSRT	0	0	0	2	4	VAEVGSWETLSRT	tr_C9ZJU1	0.053	28249.45	42.00
814	HLA-A0201	GGNIALGNLWDCSD	ALGNLWCSD	4	0	0	6	1	ALGNLWDCSD	tr_C9ZJU1	0.053	28250.67	42.00
410	HLA-A0201	SPTCSFPVKNSLTS	CSFPVSLTS	3	0	0	5	2	CSFPVKNSLTS	tr_C9ZJU1	0.053	28254.35	42.00
337	HLA-A0201	RVPALLEVMHG	RVPALLEVM	0	0	0	0	0	RVPALLEVM	tr_C9ZJU1	0.053	28257.40	42.00
520	HLA-A0201	HTCAVSVRR	HTCAVSVRR	0	0	0	0	0	HTCAVSVRR	tr_C9ZJU1	0.053	28262.59	42.00
241	HLA-A0201	SPKLSPFRFI	SPKLSPFFI	0	0	0	7	1	SPKLSPFRFI	tr_C9ZJU1	0.053	28265.65	42.00
937	HLA-A0201	LNEGMVAEVG	LNEGMVAEV	0	0	0	0	0	LNEGMVAEV	tr_C9ZJU1	0.053	28276.97	42.00
890	HLA-A0201	SAIDTITEGEIFD	SAIDTIIFD	0	0	0	6	4	SAIDTITEGEIFD	tr_C9ZJU1	0.053	28291.06	42.00
805	HLA-A0201	LRFIEDLSIGG	FIEDLSIGG	2	0	0	0	0	FIEDLSIGG	tr_C9ZJU1	0.053	28291.37	42.00
248	HLA-A0201	RFIIKQLMG	RFIIKQLMG	0	0	0	0	0	RFIIKQLMG	tr_C9ZJU1	0.053	28295.04	43.00
856	HLA-A0201	VREFSGGEVERL	FSGGEVERL	3	0	0	0	0	FSGGEVERL	tr_C9ZJU1	0.053	28299.93	43.00
867	HLA-A0201	LCIARAFVKAG	LCIARAFVA	0	0	0	8	1	LCIARAFVKA	tr_C9ZJU1	0.053	28314.93	43.00
424	HLA-A0201	SPLTLLRKQKDDGI	TLLQKDDGI	3	0	0	3	2	TLLRKQKDDGI	tr_C9ZJU1	0.053	28315.55	43.00
834	HLA-A0201	LSASRALNFVQK	LSASALNFV	0	0	0	4	1	LSASRALNFV	tr_C9ZJU1	0.053	28315.55	43.00
674	HLA-A0201	RRHLAFSGSSLGCY	HLAFSLGCY	2	0	0	4	3	HLAFSGSSLGCY	tr_C9ZJU1	0.053	28316.16	43.00

716	HLA-A0201	WSVEFREVSFRY	WSVEVSFRY	0	0	0	3	3	WSVEFREVSFRY	tr_C9ZJU1	0.053	28330.88	43.00
5	HLA-A0201	RPAVLLDCERKAY	RLLDCERKA	0	0	0	1	3	RPAVLLDCERKA	tr_C9ZJU1	0.052	28332.71	43.00
300	HLA-A0201	LALATADYDP	LLATADYDP	0	0	0	1	1	LALATADYDP	tr_C9ZJU1	0.052	28339.47	43.00
383	HLA-A0201	QFTKSQLASVTN	FTKSQLASV	1	0	0	0	0	FTKSQLASV	tr_C9ZJU1	0.052	28340.38	43.00
953	HLA-A0201	RTGPHSCFS	RTGPHSCFS	0	0	0	0	0	RTGPHSCFS	tr_C9ZJU1	0.052	28343.45	43.00
444	HLA-A0201	TDNKMLFQQTFS	KMLFQQTFS	3	0	0	0	0	KMLFQQTFS	tr_C9ZJU1	0.052	28370.75	43.00
52	HLA-A0201	CNGVESIHVRTL	GVESIHVTL	2	0	0	7	1	GVESIHVRTL	tr_C9ZJU1	0.052	28376.28	43.00
828	HLA-A0201	ENICAALSASRALN	NICAALSAL	1	0	0	7	3	NICAALSASRAL	tr_C9ZJU1	0.052	28379.65	43.00
862	HLA-A0201	GEVERLCIARAF	VERLCIAAF	2	0	0	7	1	VERLCIARAF	tr_C9ZJU1	0.052	28398.08	43.00
506	HLA-A0201	CVVDALKRKLQAG	CVVDALLQA	0	0	0	6	3	CVVDALKRKLQA	tr_C9ZJU1	0.052	28411.00	43.00
287	HLA-A0201	LSDIITSIH	LSDIITSIH	0	0	0	0	0	LSDIITSIH	tr_C9ZJU1	0.052	28416.53	43.00
97	HLA-A0201	WNCLMALVN	WNCLMALVN	0	0	0	0	0	WNCLMALVN	tr_C9ZJU1	0.052	28416.53	43.00
825	HLA-A0201	CSDENICAALSASR	CSDEICAAL	0	0	0	4	1	CSDENICAAL	tr_C9ZJU1	0.052	28423.90	43.00
86	HLA-A0201	SHPDHLLVCDIW	SHLLVCDIW	0	0	0	1	3	SHPDHLLVCDIW	tr_C9ZJU1	0.052	28427.60	43.00
85	HLA-A0201	NSHPDHLLVC	NSHPDHLLV	0	0	0	0	0	NSHPDHLLV	tr_C9ZJU1	0.052	28436.52	43.00
6	HLA-A0201	PAVLLDCERKAY	AVLLDCEAY	1	0	0	7	2	AVLLDCERKAY	tr_C9ZJU1	0.052	28451.59	43.00
56	HLA-A0201	ESIHVRTLVFNH	SIHVLVFNH	1	0	0	4	2	SIHVRTLVFNH	tr_C9ZJU1	0.052	28453.45	43.00
108	HLA-A0201	IRDILLDDGVA	IILLDDGVA	0	0	0	1	2	IRDILLDDGVA	tr_C9ZJU1	0.052	28456.53	43.00
891	HLA-A0201	AIDTITEGE	AIDTITEGE	0	0	0	0	0	AIDTITEGE	tr_C9ZJU1	0.052	28462.37	43.00
699	HLA-A0201	KIDRPVRGVAPPS	KIDGVAPPS	0	0	0	3	4	KIDRPVRGVAPPS	tr_C9ZJU1	0.052	28462.99	43.00
310	HLA-A0201	SDDWLLSRC	SDDWLLSRC	0	0	0	0	0	SDDWLLSRC	tr_C9ZJU1	0.052	28478.71	43.00
823	HLA-A0201	WDCSDENICAA	WSDENICAA	0	0	0	1	2	WDCSDENICAA	tr_C9ZJU1	0.052	28492.27	43.00
860	HLA-A0201	SGGEVERLCIARAF	VERLCIAAF	4	0	0	7	1	VERLCIARAF	tr_C9ZJU1	0.052	28512.01	43.00
631	HLA-A0201	LDQTEIVNQLRS	LQTEIVNQL	0	0	0	1	1	LDQTEIVNQL	tr_C9ZJU1	0.052	28512.93	43.00
165	HLA-A0201	LGLITAYNRK	LLITAYNRK	0	0	0	1	1	LGLITAYNRK	tr_C9ZJU1	0.052	28515.70	43.00
714	HLA-A0201	GPWSVEFREVSF	GPWSVEFEV	0	0	0	7	1	GPWSVEFREV	tr_C9ZJU1	0.052	28524.35	43.00
751	HLA-A0201	AGYSGSGKTTLL	YSGSGTTLL	2	0	0	5	1	YSGSGKTTLL	tr_C9ZJU1	0.052	28524.96	43.00
84	HLA-A0201	KNSHPDHLL	KNSHPDHLL	0	0	0	0	0	KNSHPDHLL	tr_C9ZJU1	0.052	28525.89	43.00
766	HLA-A0201	NRTYAPTEGDI	RTYAPTEGI	1	0	0	8	1	RTYAPTEGDI	tr_C9ZJU1	0.052	28536.69	43.00
958	HLA-A0201	SCFSRMRQSQQL	SCFSRMQQL	0	0	0	6	3	SCFSRMRQSQQL	tr_C9ZJU1	0.052	28546.27	43.00
869	HLA-A0201	IARAFVKAG	IARAFVKAG	0	0	0	0	0	IARAFVKAG	tr_C9ZJU1	0.052	28555.21	43.00
714	HLA-A0201	GPWSVEFREVSFRY	GPWSVEFEV	0	0	0	7	1	GPWSVEFREV	tr_C9ZJU1	0.052	28557.39	43.00
797	HLA-A0201	NVWQEENNLRF	NVWENNLRF	0	0	0	3	2	NVWQEENNLRF	tr_C9ZJU1	0.052	28560.78	43.00
775	HLA-A0201	DIFVNGVHISQ	FVNGVHISQ	2	0	0	0	0	FVNGVHISQ	tr_C9ZJU1	0.052	28564.18	43.00
37	HLA-A0201	QTECKDDASI	QTCKDDASI	0	0	0	2	1	QTECKDDASI	tr_C9ZJU1	0.052	28565.42	43.00
750	HLA-A0201	IAGYSGSGKTTLL	YSGSGTTLL	3	0	0	5	1	YSGSGKTTLL	tr_C9ZJU1	0.052	28567.59	43.00
815	HLA-A0201	GNIALGNLWDCSD	NIALGLWDC	1	0	0	5	1	NIALGNLWDC	tr_C9ZJU1	0.052	28568.20	43.00
518	HLA-A0201	GGHTCAVSVR	GGHTCAVSV	0	0	0	0	0	GGHTCAVSV	tr_C9ZJU1	0.052	28598.50	43.00
846	HLA-A0201	RSSGIHAPLNVREF	RSSGIPLNV	0	0	0	5	2	RSSGIHAPLNV	tr_C9ZJU1	0.052	28609.33	43.00
754	HLA-A0201	SGSGKTTLLRLL	SGKTTLLLL	2	0	0	7	1	SGKTTLLRLL	tr_C9ZJU1	0.052	28609.96	43.00
441	HLA-A0201	LSSTDNKML	LSSTDNKML	0	0	0	0	0	LSSTDNKML	tr_C9ZJU1	0.052	28613.37	43.00
56	HLA-A0201	ESIHVRTLVFNHN	SIHLVFNHN	1	0	0	3	3	SIHVRTLVFNHN	tr_C9ZJU1	0.052	28618.63	43.00
861	HLA-A0201	GGEVERLCIARA	GEVLCIARA	1	0	0	3	2	GEVERLCIARA	tr_C9ZJU1	0.052	28621.42	43.00
716	HLA-A0201	WSVEFREVSFRYS	WSVEFFRYS	0	0	0	5	4	WSVEFREVSFRYS	tr_C9ZJU1	0.052	28622.66	43.00
105	HLA-A0201	NCSIRDILLD	NCSIRDILL	0	0	0	0	0	NCSIRDILL	tr_C9ZJU1	0.052	28626.68	43.00
76	HLA-A0201	FAVHCGLMKNS	FAVHCGLMS	0	0	0	8	2	FAVHCGLMKNS	tr_C9ZJU1	0.052	28633.81	43.00
719	HLA-A0201	EFREVSFRY	EFREVSFRY	0	0	0	0	0	EFREVSFRY	tr_C9ZJU1	0.052	28638.76	43.00
201	HLA-A0201	AATVLFSPE	AATVLFSPE	0	0	0	0	0	AATVLFSPE	tr_C9ZJU1	0.051	28647.12	43.00
421	HLA-A0201	LTSSPLTLLRK	LTSSPLTLL	0	0	0	0	0	LTSSPLTLL	tr_C9ZJU1	0.051	28648.68	43.00
591	HLA-A0201	YEQCGRDVLHGLF	YEQCVLHGL	0	0	0	4	3	YEQCGRDVLHGL	tr_C9ZJU1	0.051	28652.39	43.00
341	HLA-A0201	LLEVMHGCR	LLEVMHGCR	0	0	0	0	0	LLEVMHGCR	tr_C9ZJU1	0.051	28657.97	43.00
122	HLA-A0201	LDDVKAEEVLAL	LDDEEVLAL	0	0	0	3	3	LDDVKAEEVLAL	tr_C9ZJU1	0.051	28662.32	43.00
59	HLA-A0201	HVRTLVFNH	HVRTLVFNH	0	0	0	0	0	HVRTLVFNH	tr_C9ZJU1	0.051	28680.62	43.00
522	HLA-A0201	CAVSVRRLGWIKD	CAVSVLGWI	0	0	0	5	2	CAVSVRRLGWI	tr_C9ZJU1	0.051	28684.97	43.00

336	HLA-A0201	SRVPALLEVMHG	SVPALLEVM	0	0	0	1	1	SRVPALLEVM	tr_C9ZJU1	0.051	28687.45	43.00
663	HLA-A0201	MSEAQVDEAHL	MSEAQVAHL	0	0	0	6	2	MSEAQVDEAHL	tr_C9ZJU1	0.051	28688.69	43.00
288	HLA-A0201	SDIITSIHIDA	IITSIHIDA	2	0	0	0	0	IITSIHIDA	tr_C9ZJU1	0.051	28695.22	43.00
667	HLA-A0201	QVDEAHLRRHLAFS	ALRRHLAFS	4	0	0	1	1	AHLRRHLAFS	tr_C9ZJU1	0.051	28695.52	43.00
744	HLA-A0201	KGEFLGIAGYS	GEFLGIAYS	1	0	0	7	1	GEFLGIAGYS	tr_C9ZJU1	0.051	28695.52	43.00
838	HLA-A0201	RALNFVQKRS	RALNFVQRS	0	0	0	7	1	RALNFVQKRS	tr_C9ZJU1	0.051	28706.08	43.00
799	HLA-A0201	WQEENNLRFIE	WQEENNLFI	0	0	0	7	1	WQEENNLRFI	tr_C9ZJU1	0.051	28717.88	43.00
717	HLA-A0201	SVEFREVSFRY	SVEFVSFRY	0	0	0	4	2	SVEFREVSFRY	tr_C9ZJU1	0.051	28731.87	44.00
861	HLA-A0201	GGEVERLCIARAF	VERLCIAAF	3	0	0	7	1	VERLCIARAF	tr_C9ZJU1	0.051	28734.37	44.00
500	HLA-A0201	PSVFSGCVV	PSVFSGCVV	0	0	0	0	0	PSVFSGCVV	tr_C9ZJU1	0.051	28742.76	44.00
797	HLA-A0201	NVWQEENNLR	NVWQEENNL	0	0	0	0	0	NVWQEENNL	tr_C9ZJU1	0.051	28744.30	44.00
476	HLA-A0201	FVESGRPLGYMWD	FVESGLGYM	0	0	0	5	2	FVESGRPLGYM	tr_C9ZJU1	0.051	28746.49	44.00
288	HLA-A0201	SDIITSIHIDAY	IITSIHIAY	2	0	0	7	1	IITSIHIDAY	tr_C9ZJU1	0.051	28747.73	44.00
422	HLA-A0201	TSSPLTLLRK	SSPLTLLRK	1	0	0	0	0	SSPLTLLRK	tr_C9ZJU1	0.051	28778.54	44.00
667	HLA-A0201	QVDEAHLRRHLA	QVDEAHLLA	0	0	0	7	3	QVDEAHLRRHLA	tr_C9ZJU1	0.051	28784.15	44.00
175	HLA-A0201	RSLQSGITS	RSLQSGITS	0	0	0	0	0	RSLQSGITS	tr_C9ZJU1	0.051	28795.99	44.00
628	HLA-A0201	QSALDQTEIVNQ	ALDQTIVNQ	2	0	0	5	1	ALDQTEIVNQ	tr_C9ZJU1	0.051	28796.29	44.00
218	HLA-A0201	MHAFVHWRP	MHAFVHWRP	0	0	0	0	0	MHAFVHWRP	tr_C9ZJU1	0.051	28800.34	44.00
824	HLA-A0201	DCSDENICAALSAS	CSDEICAAL	1	0	0	4	1	CSDENICAAL	tr_C9ZJU1	0.051	28825.29	44.00
113	HLA-A0201	LDDGVALCILD	LDDGVALIL	0	0	0	7	1	LDDGVALCIL	tr_C9ZJU1	0.051	28826.84	44.00
391	HLA-A0201	SVTNIATSTYAG	SVIATSTYA	0	0	0	2	2	SVTNIATSTYA	tr_C9ZJU1	0.051	28829.03	44.00
693	HLA-A0201	LQSVQRKIDRPVR	LQSVIDRPV	0	0	0	4	3	LQSVQRKIDRPV	tr_C9ZJU1	0.051	28859.30	44.00
851	HLA-A0201	HAPLNVREF	HAPLNVREF	0	0	0	0	0	HAPLNVREF	tr_C9ZJU1	0.051	28862.11	44.00
815	HLA-A0201	GNIALGNLW	GNIALGNLW	0	0	0	0	0	GNIALGNLW	tr_C9ZJU1	0.051	28878.36	44.00
594	HLA-A0201	CGRDVLHGLF	CGRDVLHLF	0	0	0	7	1	CGRDVLHGLF	tr_C9ZJU1	0.051	28880.22	44.00
670	HLA-A0201	EAHLRRHLAFS	ALRRHLAFS	1	0	0	1	1	AHLRRHLAFS	tr_C9ZJU1	0.051	28881.79	44.00
289	HLA-A0201	DIITSIHIDAY	IITSIHIAY	1	0	0	7	1	IITSIHIDAY	tr_C9ZJU1	0.051	28885.23	44.00
830	HLA-A0201	ICAALSASRALN	ILSASRALN	0	0	0	1	3	ICAALSASRALN	tr_C9ZJU1	0.051	28890.86	44.00
431	HLA-A0201	KQKDDGIKCMLS	KQDGIKCML	0	0	0	2	2	KQKDDGIKCML	tr_C9ZJU1	0.051	28893.99	44.00
891	HLA-A0201	AIDTITEGEIFD	AIDTITEEI	0	0	0	7	1	AIDTITEGEI	tr_C9ZJU1	0.051	28894.29	44.00
510	HLA-A0201	ALKRKLQAGG	ALRKLQAGG	0	0	0	2	1	ALKRKLQAGG	tr_C9ZJU1	0.051	28902.73	44.00
156	HLA-A0201	VQEFPEELY	VQEFPEELY	0	0	0	0	0	VQEFPEELY	tr_C9ZJU1	0.051	28907.11	44.00
326	HLA-A0201	ASGSVMDELQ	ASGSVMDEL	0	0	0	0	0	ASGSVMDEL	tr_C9ZJU1	0.050	28955.01	44.00
741	HLA-A0201	VVNKGEFLGIAG	VVGEFLGIA	0	0	0	2	2	VVNKGEFLGIA	tr_C9ZJU1	0.050	28955.96	44.00
742	HLA-A0201	VNKGEFLGIAGYS	GEFLGIAYS	3	0	0	7	1	GEFLGIAGYS	tr_C9ZJU1	0.050	28960.65	44.00
496	HLA-A0201	WPLVPSVFS	WPLVPSVFS	0	0	0	0	0	WPLVPSVFS	tr_C9ZJU1	0.050	28966.92	44.00
592	HLA-A0201	EQCGRDVLHGLF	EQCGVLHGL	0	0	0	4	2	EQCGRDVLHGL	tr_C9ZJU1	0.050	28970.36	44.00
905	HLA-A0201	RLREGNNLLRDST	RLREGNNLL	0	0	0	0	0	RLREGNNLL	tr_C9ZJU1	0.050	28974.43	44.00
848	HLA-A0201	SGIHAPLNVR	SGIHAPLNV	0	0	0	0	0	SGIHAPLNV	tr_C9ZJU1	0.050	28979.78	44.00
967	HLA-A0201	QQLPHFVKK	QQLPHFVKK	0	0	0	0	0	QQLPHFVKK	tr_C9ZJU1	0.050	28981.65	44.00
320	HLA-A0201	SQALWQASGS	SQALWQASS	0	0	0	8	1	SQALWQASGS	tr_C9ZJU1	0.050	28984.15	44.00
742	HLA-A0201	VNKGEFLGIAGY	VEFLGIAGY	0	0	0	1	3	VNKGEFLGIAGY	tr_C9ZJU1	0.050	28991.05	44.00
756	HLA-A0201	SGKTTLLRLLNR	STLLRLLNR	0	0	0	1	3	SGKTTLLRLLNR	tr_C9ZJU1	0.050	29026.22	44.00
861	HLA-A0201	GGEVERLCIA	GGVERLCIA	0	0	0	2	1	GGEVERLCIA	tr_C9ZJU1	0.050	29029.98	44.00
522	HLA-A0201	CAVSVRRLGW	CAVSVRLGW	0	0	0	5	1	CAVSVRRLGW	tr_C9ZJU1	0.050	29030.30	44.00
743	HLA-A0201	NKGEFLGIAGYS	GEFLGIAYS	2	0	0	7	1	GEFLGIAGYS	tr_C9ZJU1	0.050	29033.75	44.00
87	HLA-A0201	HPDHLLVCDIW	HPLLVCDIW	0	0	0	2	2	HPDHLLVCDIW	tr_C9ZJU1	0.050	29056.38	44.00
532	HLA-A0201	IKDVIQSSPRAATA	IKDVIAATA	0	0	0	5	5	IKDVIQSSPRAATA	tr_C9ZJU1	0.050	29057.63	44.00
559	HLA-A0201	LQGFAALRLSG	LQGFAALRL	0	0	0	0	0	LQGFAALRL	tr_C9ZJU1	0.050	29060.46	44.00
845	HLA-A0201	KRSSGIHAPLNVR	RSSGIPLNV	1	0	0	5	2	RSSGIHAPLNV	tr_C9ZJU1	0.050	29067.08	44.00
778	HLA-A0201	VNGVHISQYPA	VVHISQYPA	0	0	0	1	2	VNGVHISQYPA	tr_C9ZJU1	0.050	29077.76	44.00
957	HLA-A0201	HSCFSRMRQSQQL	SCFSRMQQL	1	0	0	6	3	SCFSRMRQSQQL	tr_C9ZJU1	0.050	29085.00	44.00
873	HLA-A0201	FVKAGSAVG	FVKAGSAVG	0	0	0	0	0	FVKAGSAVG	tr_C9ZJU1	0.050	29087.20	44.00
58	HLA-A0201	IHVRTLVFNH	IVRTLVFNH	0	0	0	1	1	IHVRTLVFNH	tr_C9ZJU1	0.050	29104.84	44.00

443	HLA-A0201	STDNKMLFQQ	STDNMLFQQ	0	0	0	4	1	STDNKMLFQQ	tr_C9ZJU1	0.050	29110.82	44.00
940	HLA-A0201	GMVAEVGSWE	GMVEVGSWE	0	0	0	3	1	GMVAEVGSWE	tr_C9ZJU1	0.050	29115.54	44.00
742	HLA-A0201	VNKGEFLGI	VNKGEFLGI	0	0	0	0	0	VNKGEFLGI	tr_C9ZJU1	0.050	29117.75	44.00
729	HLA-A0201	DRHPYVLRRV	RHPYVLRRV	1	0	0	0	0	RHPYVLRRV	tr_C9ZJU1	0.050	29126.88	44.00
828	HLA-A0201	ENICAALSASRA	NICAALSAA	1	0	0	8	2	NICAALSASRA	tr_C9ZJU1	0.050	29130.36	45.00
415	HLA-A0201	FPVKNSLTSS	FVKNSLTSS	0	0	0	1	1	FPVKNSLTSS	tr_C9ZJU1	0.050	29131.61	45.00
419	HLA-A0201	NSLTSSPLT	NSLTSSPLT	0	0	0	0	0	NSLTSSPLT	tr_C9ZJU1	0.050	29147.70	45.00
430	HLA-A0201	RKQKDDGIKCMLSS	KQIKCMLSS	1	0	0	2	4	KQKDDGIKCMLSS	tr_C9ZJU1	0.050	29156.52	45.00
662	HLA-A0201	GMSEAQVDE	GMSEAQVDE	0	0	0	0	0	GMSEAQVDE	tr_C9ZJU1	0.050	29160.32	45.00
475	HLA-A0201	VFVESGRPLGYMWD	FVESGLGYM	1	0	0	5	2	FVESGRPLGYM	tr_C9ZJU1	0.050	29165.04	45.00
113	HLA-A0201	LDDGVALCILDD	LVALCILDD	0	0	0	1	3	LDDGVALCILDD	tr_C9ZJU1	0.050	29168.84	45.00
399	HLA-A0201	TYAGNGEENQV	YAGNGEENV	1	0	0	8	1	YAGNGEENQV	tr_C9ZJU1	0.050	29170.72	45.00
713	HLA-A0201	DGPWSVEFREVS	GPWSVEFEV	1	0	0	7	1	GPWSVEFREV	tr_C9ZJU1	0.050	29171.04	45.00
904	HLA-A0201	LRLREGNNLLRDST	LLREGNNLL	0	0	0	1	1	LRLREGNNLL	tr_C9ZJU1	0.050	29176.10	45.00
631	HLA-A0201	LDQTEIVNQLRSIQ	LQTEIVNQL	0	0	0	1	1	LDQTEIVNQL	tr_C9ZJU1	0.050	29182.72	45.00
658	HLA-A0201	YDECGMSEAQV	YCGMSEAQV	0	0	0	1	2	YDECGMSEAQV	tr_C9ZJU1	0.050	29188.73	45.00
474	HLA-A0201	MVFVESGRP	MVFVESGRP	0	0	0	0	0	MVFVESGRP	tr_C9ZJU1	0.050	29195.97	45.00
635	HLA-A0201	EIVNQLRSIQHWC	ILRSIQHWC	1	0	0	1	3	IVNQLRSIQHWC	tr_C9ZJU1	0.050	29210.19	45.00
149	HLA-A0201	AAQADVVVQEFPE	AQADVVFPE	1	0	0	6	3	AQADVVVQEFPE	tr_C9ZJU1	0.050	29226.64	45.00
870	HLA-A0201	ARAFVKAGSA	AAFVKAGSA	0	0	0	1	1	ARAFVKAGSA	tr_C9ZJU1	0.050	29227.91	45.00
827	HLA-A0201	DENICAALSASRA	NICAALSAA	2	0	0	8	2	NICAALSASRA	tr_C9ZJU1	0.050	29228.23	45.00
85	HLA-A0201	NSHPDHLLVCDI	NSHLLVCDI	0	0	0	2	3	NSHPDHLLVCDI	tr_C9ZJU1	0.050	29232.66	45.00
714	HLA-A0201	GPWSVEFREVSFR	GPWSVEFEV	0	0	0	7	1	GPWSVEFREV	tr_C9ZJU1	0.050	29236.13	45.00
834	HLA-A0201	LSASRALNFVQKR	LSASRALFV	0	0	0	7	1	LSASRALNFV	tr_C9ZJU1	0.050	29237.09	45.00
317	HLA-A0201	RCVSQALWQASG	RVSQALWQA	0	0	0	1	1	RCVSQALWQA	tr_C9ZJU1	0.050	29245.61	45.00
754	HLA-A0201	SGSGKTTLLRLLNR	STLLRLLNR	0	0	0	1	5	SGSGKTTLLRLLNR	tr_C9ZJU1	0.050	29250.69	45.00
758	HLA-A0201	KTTLLRLLN	KTTLLRLLN	0	0	0	0	0	KTTLLRLLN	tr_C9ZJU1	0.050	29253.86	45.00
779	HLA-A0201	NGVHISQYPARM	GVHISQYPM	1	0	0	8	2	GVHISQYPARM	tr_C9ZJU1	0.050	29256.38	45.00
671	HLA-A0201	AHLRRHLAFSGSS	HLLAFSGSS	1	0	0	2	3	HLRRHLAFSGSS	tr_C9ZJU1	0.050	29260.82	45.00
867	HLA-A0201	LCIARAFVKAGS	LCIAAFVKA	0	0	0	4	1	LCIARAFVKA	tr_C9ZJU1	0.050	29263.03	45.00
712	HLA-A0201	LDGPWSVEF	LDGPWSVEF	0	0	0	0	0	LDGPWSVEF	tr_C9ZJU1	0.050	29263.99	45.00
40	HLA-A0201	CKDDASIAIFLN	CASIAIFLN	0	0	0	1	3	CKDDASIAIFLN	tr_C9ZJU1	0.050	29265.25	45.00
226	HLA-A0201	PIYAAEIGKSL	YAAEIGKSL	2	0	0	0	0	YAAEIGKSL	tr_C9ZJU1	0.049	29266.84	45.00
666	HLA-A0201	AQVDEAHLRRHLA	AQVDEAHLA	0	0	0	6	4	AQVDEAHLRRHLA	tr_C9ZJU1	0.049	29267.78	45.00
713	HLA-A0201	DGPWSVEFREVSF	GPWSVEFEV	1	0	0	7	1	GPWSVEFREV	tr_C9ZJU1	0.049	29269.99	45.00
865	HLA-A0201	ERLCIARAF	ERLCIARAF	0	0	0	0	0	ERLCIARAF	tr_C9ZJU1	0.049	29271.58	45.00
461	HLA-A0201	DKSSVQYGLV	KSSVQYGLV	1	0	0	0	0	KSSVQYGLV	tr_C9ZJU1	0.049	29289.65	45.00
770	HLA-A0201	APTEGDIFVN	APTEGDIFV	0	0	0	0	0	APTEGDIFV	tr_C9ZJU1	0.049	29290.91	45.00
559	HLA-A0201	LQGFAALRLSGWE	LQGFAALRL	0	0	0	0	0	LQGFAALRL	tr_C9ZJU1	0.049	29308.98	45.00
590	HLA-A0201	RYEQCGRDVLHGLF	YEQCVLHGL	1	0	0	4	3	YEQCGRDVLHGL	tr_C9ZJU1	0.049	29310.89	45.00
148	HLA-A0201	PAAQADVVV	PAAQADVVV	0	0	0	0	0	PAAQADVVV	tr_C9ZJU1	0.049	29314.69	45.00
239	HLA-A0201	DVSPKLSPF	DVSPKLSPF	0	0	0	0	0	DVSPKLSPF	tr_C9ZJU1	0.049	29324.84	45.00
77	HLA-A0201	AVHCGLMKN	AVHCGLMKN	0	0	0	0	0	AVHCGLMKN	tr_C9ZJU1	0.049	29328.32	45.00
316	HLA-A0201	SRCVSQALWQASG	SVSQALWQA	0	0	0	1	2	SRCVSQALWQA	tr_C9ZJU1	0.049	29357.22	45.00
305	HLA-A0201	ADYDPSDDWLL	ADYDPSWLL	0	0	0	6	2	ADYDPSDDWLL	tr_C9ZJU1	0.049	29357.86	45.00
222	HLA-A0201	VHWRPIYAAEIG	VHWIYAAEI	0	0	0	3	2	VHWRPIYAAEI	tr_C9ZJU1	0.049	29362.29	45.00
860	HLA-A0201	SGGEVERLCIA	SGGEVLCIA	0	0	0	5	2	SGGEVERLCIA	tr_C9ZJU1	0.049	29371.84	45.00
966	HLA-A0201	SQQLPHFVK	SQQLPHFVK	0	0	0	0	0	SQQLPHFVK	tr_C9ZJU1	0.049	29395.35	45.00
523	HLA-A0201	AVSVRRLGW	AVSVRRLGW	0	0	0	0	0	AVSVRRLGW	tr_C9ZJU1	0.049	29397.27	45.00
560	HLA-A0201	QGFAALRLSGW	FAALRLSGW	2	0	0	0	0	FAALRLSGW	tr_C9ZJU1	0.049	29405.21	45.00
669	HLA-A0201	DEAHLRRHLAFS	ALRRHLAFS	2	0	0	1	1	AHLRRHLAFS	tr_C9ZJU1	0.049	29409.37	45.00
923	HLA-A0201	HRLATLKHA	HRLATLKHA	0	0	0	0	0	HRLATLKHA	tr_C9ZJU1	0.049	29413.18	45.00
208	HLA-A0201	PEVILKCIL	PEVILKCIL	0	0	0	0	0	PEVILKCIL	tr_C9ZJU1	0.049	29419.86	45.00
414	HLA-A0201	SFPVKNSLTSSPL	FPVLTSSPL	1	0	0	3	3	FPVKNSLTSSPL	tr_C9ZJU1	0.049	29421.77	45.00

826	HLA-A0201	SDENICAALSASRA	NICAALSAA	3	0	0	8	2	NICAALSASRA	tr_C9ZJU1	0.049	29422.40	45.00
412	HLA-A0201	TCSFPVKNSLTSS	CSFPVLTSS	1	0	0	5	3	CSFPVKNSLTSS	tr_C9ZJU1	0.049	29430.69	45.00
260	HLA-A0201	TFVSMAVAS	TFVSMAVAS	0	0	0	0	0	TFVSMAVAS	tr_C9ZJU1	0.049	29439.59	45.00
889	HLA-A0201	TSAIDTITEGEIFD	SAIDTIIFD	1	0	0	6	4	SAIDTITEGEIFD	tr_C9ZJU1	0.049	29449.16	45.00
530	HLA-A0201	GWIKDVIQSS	WIKDVIQSS	1	0	0	0	0	WIKDVIQSS	tr_C9ZJU1	0.049	29449.79	45.00
408	HLA-A0201	QVSPTCSFP	QVSPTCSFP	0	0	0	0	0	QVSPTCSFP	tr_C9ZJU1	0.049	29453.63	45.00
657	HLA-A0201	FYDECGMSE	FYDECGMSE	0	0	0	0	0	FYDECGMSE	tr_C9ZJU1	0.049	29466.38	45.00
380	HLA-A0201	MESQFTKSQL	MESQFTSQL	0	0	0	6	1	MESQFTKSQL	tr_C9ZJU1	0.049	29467.65	45.00
520	HLA-A0201	HTCAVSVRRLGW	HTCAVSVRL	0	0	0	7	1	HTCAVSVRRL	tr_C9ZJU1	0.049	29477.21	45.00
832	HLA-A0201	AALSASRALN	AALSASRAL	0	0	0	0	0	AALSASRAL	tr_C9ZJU1	0.049	29477.86	45.00
743	HLA-A0201	NKGEFLGIAGY	KGFLGIAGY	1	0	0	2	1	KGEFLGIAGY	tr_C9ZJU1	0.049	29485.82	45.00
318	HLA-A0201	CVSQALWQASG	CVSQALWQA	0	0	0	0	0	CVSQALWQA	tr_C9ZJU1	0.049	29498.26	45.00
715	HLA-A0201	PWSVEFREVSFRY	WSVEFSFRY	1	0	0	5	3	WSVEFREVSFRY	tr_C9ZJU1	0.049	29505.61	45.00
808	HLA-A0201	IEDLSIGGNI	IEDLSIGGI	0	0	0	8	1	IEDLSIGGNI	tr_C9ZJU1	0.049	29510.08	45.00
950	HLA-A0201	TLSRTGPHSCFSR	TLSRTCFSR	0	0	0	5	4	TLSRTGPHSCFSR	tr_C9ZJU1	0.049	29516.15	45.00
443	HLA-A0201	STDNKMLFQQT	STDNKMLFT	0	0	0	8	2	STDNKMLFQQT	tr_C9ZJU1	0.049	29517.10	45.00
551	HLA-A0201	VWQCRQDYLQGF	WQCDYLQGF	1	0	0	3	2	WQCRQDYLQGF	tr_C9ZJU1	0.049	29523.50	46.00
240	HLA-A0201	VSPKLSPFRF	VSPLSPFRF	0	0	0	3	1	VSPKLSPFRF	tr_C9ZJU1	0.049	29528.28	46.00
106	HLA-A0201	CSIRDILLD	CSIRDILLD	0	0	0	0	0	CSIRDILLD	tr_C9ZJU1	0.049	29529.25	46.00
123	HLA-A0201	DDVKAEEVLAL	DVAEEVLAL	1	0	0	2	1	DVKAEEVLAL	tr_C9ZJU1	0.049	29535.95	46.00
789	HLA-A0201	RMLRRRIAN	RMLRRRIAN	0	0	0	0	0	RMLRRRIAN	tr_C9ZJU1	0.049	29537.88	46.00
516	HLA-A0201	QAGGHTCAVS	QAGGHTCAV	0	0	0	0	0	QAGGHTCAV	tr_C9ZJU1	0.049	29540.11	46.00
477	HLA-A0201	VESGRPLGYM	VESGRPLYM	0	0	0	7	1	VESGRPLGYM	tr_C9ZJU1	0.049	29542.03	46.00
776	HLA-A0201	IFVNGVHIS	IFVNGVHIS	0	0	0	0	0	IFVNGVHIS	tr_C9ZJU1	0.049	29543.95	46.00
144	HLA-A0201	ARYPPAAQA	ARYPPAAQA	0	0	0	0	0	ARYPPAAQA	tr_C9ZJU1	0.049	29554.17	46.00
782	HLA-A0201	HISQYPARMLR	HISQYPAML	0	0	0	7	1	HISQYPARML	tr_C9ZJU1	0.049	29580.08	46.00
634	HLA-A0201	TEIVNQLRSIQHWC	ILRSIQHWC	2	0	0	1	3	IVNQLRSIQHWC	tr_C9ZJU1	0.049	29582.96	46.00
521	HLA-A0201	TCAVSVRRLGWIKD	CAVSVLGWI	1	0	0	5	2	CAVSVRRLGWI	tr_C9ZJU1	0.048	29596.09	46.00
75	HLA-A0201	VFAVHCGLMKNS	FAVHCGLMS	1	0	0	8	2	FAVHCGLMKNS	tr_C9ZJU1	0.048	29598.02	46.00
417	HLA-A0201	VKNSLTSSPL	VKNSLTSPL	0	0	0	6	1	VKNSLTSSPL	tr_C9ZJU1	0.048	29610.83	46.00
838	HLA-A0201	RALNFVQKR	RALNFVQKR	0	0	0	0	0	RALNFVQKR	tr_C9ZJU1	0.048	29611.15	46.00
671	HLA-A0201	AHLRRHLAFSGS	HLHLAFSGS	1	0	0	2	2	HLRRHLAFSGS	tr_C9ZJU1	0.048	29611.48	46.00
689	HLA-A0201	VLEGLQSVQRKID	VLEGLQSVI	0	0	0	8	3	VLEGLQSVQRKI	tr_C9ZJU1	0.048	29614.34	46.00
591	HLA-A0201	YEQCGRDVLHGLFQ	YEQCVLHGL	0	0	0	4	3	YEQCGRDVLHGL	tr_C9ZJU1	0.048	29620.44	46.00
780	HLA-A0201	GVHISQYPARMLR	GVHISQYPL	0	0	0	8	3	GVHISQYPARML	tr_C9ZJU1	0.048	29624.93	46.00
875	HLA-A0201	KAGSAVGLC	KAGSAVGLC	0	0	0	0	0	KAGSAVGLC	tr_C9ZJU1	0.048	29629.42	46.00
844	HLA-A0201	QKRSSGIHAPLNVR	RSSGIPLNV	2	0	0	5	2	RSSGIHAPLNV	tr_C9ZJU1	0.048	29651.55	46.00
529	HLA-A0201	LGWIKDVIQSSP	LGWIVIQSS	0	0	0	4	2	LGWIKDVIQSS	tr_C9ZJU1	0.048	29655.72	46.00
432	HLA-A0201	QKDDGIKCML	QKDDGICML	0	0	0	6	1	QKDDGIKCML	tr_C9ZJU1	0.048	29663.41	46.00
755	HLA-A0201	GSGKTTLLRLLNR	GTLLRLLNR	0	0	0	1	4	GSGKTTLLRLLNR	tr_C9ZJU1	0.048	29664.71	46.00
505	HLA-A0201	GCVVDALKRKLQAG	CVVDALLQA	1	0	0	6	3	CVVDALKRKLQA	tr_C9ZJU1	0.048	29665.02	46.00
435	HLA-A0201	DGIKCMLSST	GIKCMLSST	1	0	0	0	0	GIKCMLSST	tr_C9ZJU1	0.048	29678.83	46.00
846	HLA-A0201	RSSGIHAPLNVRE	RSSGIPLNV	0	0	0	5	2	RSSGIHAPLNV	tr_C9ZJU1	0.048	29681.07	46.00
534	HLA-A0201	DVIQSSPRAATA	VIQSSPATA	1	0	0	6	2	VIQSSPRAATA	tr_C9ZJU1	0.048	29690.72	46.00
765	HLA-A0201	LNRTYAPTEGDI	RTYAPTEGI	2	0	0	8	1	RTYAPTEGDI	tr_C9ZJU1	0.048	29701.96	46.00
862	HLA-A0201	GEVERLCIAR	GVERLCIAR	0	0	0	1	1	GEVERLCIAR	tr_C9ZJU1	0.048	29708.07	46.00
712	HLA-A0201	LDGPWSVEFREVSF	GPWSVEFEV	2	0	0	7	1	GPWSVEFREV	tr_C9ZJU1	0.048	29709.36	46.00
784	HLA-A0201	SQYPARMLRRRIAN	SQYPARMLI	0	0	0	8	3	SQYPARMLRRRI	tr_C9ZJU1	0.048	29711.28	46.00
539	HLA-A0201	SPRAATASPSTCV	STASPSTCV	0	0	0	1	4	SPRAATASPSTCV	tr_C9ZJU1	0.048	29718.67	46.00
157	HLA-A0201	QEFPEELYLG	QEFPEELYL	0	0	0	0	0	QEFPEELYL	tr_C9ZJU1	0.048	29720.93	46.00
38	HLA-A0201	TECKDDASIAIFLN	TECSIAIFL	0	0	0	3	4	TECKDDASIAIFL	tr_C9ZJU1	0.048	29722.52	46.00
88	HLA-A0201	PDHLLVCDIW	DHLLVCDIW	1	0	0	0	0	DHLLVCDIW	tr_C9ZJU1	0.048	29722.52	46.00
849	HLA-A0201	GIHAPLNVR	GIHAPLNVR	0	0	0	0	0	GIHAPLNVR	tr_C9ZJU1	0.048	29734.43	46.00
764	HLA-A0201	LLNRTYAPTEG	LLNRTYAPG	0	0	0	8	2	LLNRTYAPTEG	tr_C9ZJU1	0.048	29736.04	46.00

168	HLA-A0201	ITAYNRKRSL	ITYNRKRSL	0	0	0	2	1	ITAYNRKRSL	tr_C9ZJU1	0.048	29740.88	46.00
551	HLA-A0201	VWQCRQDYLQGFA	RQDYLQGFA	4	0	0	0	0	RQDYLQGFA	tr_C9ZJU1	0.048	29746.67	46.00
560	HLA-A0201	QGFAALRLSG	QGFAALLSG	0	0	0	6	1	QGFAALRLSG	tr_C9ZJU1	0.048	29753.09	46.00
10	HLA-A0201	LDCERKAYEILEM	LDCERILEM	0	0	0	5	4	LDCERKAYEILEM	tr_C9ZJU1	0.048	29753.75	46.00
757	HLA-A0201	GKTTLLRLLNR	GTLLRLLNR	0	0	0	1	2	GKTTLLRLLNR	tr_C9ZJU1	0.048	29754.07	46.00
35	HLA-A0201	SKQTECKDDASIA	KQTECDASI	1	0	0	5	2	KQTECKDDASI	tr_C9ZJU1	0.048	29754.38	46.00
84	HLA-A0201	KNSHPDHLLVCDI	NSHLLVCDI	1	0	0	2	3	NSHPDHLLVCDI	tr_C9ZJU1	0.048	29769.85	46.00
411	HLA-A0201	PTCSFPVKNSL	CSFPVKNSL	2	0	0	0	0	CSFPVKNSL	tr_C9ZJU1	0.048	29780.79	46.00
716	HLA-A0201	WSVEFREVS	WSVEFREVS	0	0	0	0	0	WSVEFREVS	tr_C9ZJU1	0.048	29802.39	46.00
817	HLA-A0201	IALGNLWDCSD	IALGNLWDC	0	0	0	0	0	IALGNLWDC	tr_C9ZJU1	0.048	29804.01	46.00
720	HLA-A0201	FREVSFRYS	FREVSFRYS	0	0	0	0	0	FREVSFRYS	tr_C9ZJU1	0.048	29804.33	46.00
712	HLA-A0201	LDGPWSVEFREVS	GPWSVEFEV	2	0	0	7	1	GPWSVEFREV	tr_C9ZJU1	0.048	29806.91	46.00
224	HLA-A0201	WRPIYAAEIGKSL	YAAEIGKSL	4	0	0	0	0	YAAEIGKSL	tr_C9ZJU1	0.048	29810.14	46.00
223	HLA-A0201	HWRPIYAAEIGKSL	YAAEIGKSL	5	0	0	0	0	YAAEIGKSL	tr_C9ZJU1	0.048	29816.91	46.00
671	HLA-A0201	AHLRRHLAFSG	HLRHLAFSG	1	0	0	2	1	HLRRHLAFSG	tr_C9ZJU1	0.048	29818.19	46.00
88	HLA-A0201	PDHLLVCDI	PDHLLVCDI	0	0	0	0	0	PDHLLVCDI	tr_C9ZJU1	0.048	29819.17	46.00
2	HLA-A0201	ERARPAVLLDC	RAPAVLLDC	1	0	0	2	1	RARPAVLLDC	tr_C9ZJU1	0.048	29821.10	46.00
161	HLA-A0201	EELYLGLIT	EELYLGLIT	0	0	0	0	0	EELYLGLIT	tr_C9ZJU1	0.048	29822.08	46.00
148	HLA-A0201	PAAQADVVVQEFPE	AQADVVFPE	2	0	0	6	3	AQADVVVQEFPE	tr_C9ZJU1	0.048	29832.41	46.00
773	HLA-A0201	EGDIFVNGVH	EGDIFVNGV	0	0	0	0	0	EGDIFVNGV	tr_C9ZJU1	0.048	29832.41	46.00
955	HLA-A0201	GPHSCFSRM	GPHSCFSRM	0	0	0	0	0	GPHSCFSRM	tr_C9ZJU1	0.048	29837.56	46.00
795	HLA-A0201	IANVWQEENNLRF	NVWENNLRF	2	0	0	3	2	NVWQEENNLRF	tr_C9ZJU1	0.048	29844.34	46.00
834	HLA-A0201	LSASRALNFVQKRS	LSASALNFV	0	0	0	4	1	LSASRALNFV	tr_C9ZJU1	0.048	29851.77	46.00
770	HLA-A0201	APTEGDIFVNGVH	AGDIFVNGV	0	0	0	1	3	APTEGDIFVNGV	tr_C9ZJU1	0.048	29857.26	46.00
742	HLA-A0201	VNKGEFLGIA	VNGEFLGIA	0	0	0	2	1	VNKGEFLGIA	tr_C9ZJU1	0.048	29858.87	46.00
573	HLA-A0201	MAATAAAAFATAS	MAAAAFATA	0	0	0	1	3	MAATAAAAFATA	tr_C9ZJU1	0.048	29864.36	46.00
64	HLA-A0201	VFNHNIMQLP	VFNHNIMQL	0	0	0	0	0	VFNHNIMQL	tr_C9ZJU1	0.048	29867.93	46.00
850	HLA-A0201	IHAPLNVREFSG	ILNVREFSG	0	0	0	1	3	IHAPLNVREFSG	tr_C9ZJU1	0.048	29868.56	46.00
150	HLA-A0201	AQADVVVQEFPEE	AQADVVVEF	0	0	0	7	1	AQADVVVQEF	tr_C9ZJU1	0.048	29874.07	46.00
423	HLA-A0201	SSPLTLLRKQ	SSPLTLLRQ	0	0	0	8	1	SSPLTLLRKQ	tr_C9ZJU1	0.048	29887.33	46.00
538	HLA-A0201	SSPRAATASPSTCV	STASPSTCV	0	0	0	1	5	SSPRAATASPSTCV	tr_C9ZJU1	0.048	29893.46	46.00
225	HLA-A0201	RPIYAAEIGKSL	YAAEIGKSL	3	0	0	0	0	YAAEIGKSL	tr_C9ZJU1	0.048	29897.03	46.00
896	HLA-A0201	TEGEIFDTLRL	TEGEIFLRL	0	0	0	6	2	TEGEIFDTLRL	tr_C9ZJU1	0.048	29904.80	46.00
520	HLA-A0201	HTCAVSVRRLG	HTCAVSVRL	0	0	0	7	1	HTCAVSVRRL	tr_C9ZJU1	0.047	29912.22	46.00
378	HLA-A0201	LYMESQFTK	LYMESQFTK	0	0	0	0	0	LYMESQFTK	tr_C9ZJU1	0.047	29918.71	46.00
670	HLA-A0201	EAHLRRHLAFSGSS	HLLAFSGSS	2	0	0	2	3	HLRRHLAFSGSS	tr_C9ZJU1	0.047	29922.28	46.00
829	HLA-A0201	NICAALSASR	NICAALASR	0	0	0	6	1	NICAALSASR	tr_C9ZJU1	0.047	29942.02	47.00
39	HLA-A0201	ECKDDASIAIFLN	CKASIAIFL	1	0	0	2	2	CKDDASIAIFL	tr_C9ZJU1	0.047	29946.89	47.00
422	HLA-A0201	TSSPLTLLR	TSSPLTLLR	0	0	0	0	0	TSSPLTLLR	tr_C9ZJU1	0.047	29950.44	47.00
855	HLA-A0201	NVREFSGGEVERLC	NVREFVERL	0	0	0	5	4	NVREFSGGEVERL	tr_C9ZJU1	0.047	29955.64	47.00
58	HLA-A0201	IHVRTLVFN	IHVRTLVFN	0	0	0	0	0	IHVRTLVFN	tr_C9ZJU1	0.047	29959.85	47.00
492	HLA-A0201	QHVIWPLVP	QHVIWPLVP	0	0	0	0	0	QHVIWPLVP	tr_C9ZJU1	0.047	29971.20	47.00
956	HLA-A0201	PHSCFSRMRQSQQL	SCFSRMQQL	2	0	0	6	3	SCFSRMRQSQQL	tr_C9ZJU1	0.047	29987.09	47.00
83	HLA-A0201	MKNSHPDHLL	MNSHPDHLL	0	0	0	1	1	MKNSHPDHLL	tr_C9ZJU1	0.047	29991.30	47.00
924	HLA-A0201	RLATLKHADK	RLATLHADK	0	0	0	5	1	RLATLKHADK	tr_C9ZJU1	0.047	30011.75	47.00
649	HLA-A0201	LYHAFLGSFYDE	LYHAFLSFY	0	0	0	6	1	LYHAFLGSFY	tr_C9ZJU1	0.047	30014.04	47.00
37	HLA-A0201	QTECKDDASIAIF	QTDDASIAI	0	0	0	2	3	QTECKDDASIAI	tr_C9ZJU1	0.047	30016.63	47.00
637	HLA-A0201	VNQLRSIQHWC	VLRSIQHWC	0	0	0	1	2	VNQLRSIQHWC	tr_C9ZJU1	0.047	30033.19	47.00
525	HLA-A0201	SVRRLGWIK	SVRRLGWIK	0	0	0	0	0	SVRRLGWIK	tr_C9ZJU1	0.047	30033.85	47.00
780	HLA-A0201	GVHISQYPARMLRR	GVHISQYPL	0	0	0	8	3	GVHISQYPARML	tr_C9ZJU1	0.047	30059.85	47.00
762	HLA-A0201	LRLLNRTYA	LRLLNRTYA	0	0	0	0	0	LRLLNRTYA	tr_C9ZJU1	0.047	30060.51	47.00
899	HLA-A0201	EIFDTLRLRE	EIFDTLLRE	0	0	0	6	1	EIFDTLRLRE	tr_C9ZJU1	0.047	30062.78	47.00
317	HLA-A0201	RCVSQALWQASGS	RVSQALWQA	0	0	0	1	1	RCVSQALWQA	tr_C9ZJU1	0.047	30067.97	47.00
543	HLA-A0201	ATASPSTCVWQCR	ATATCVWQC	0	0	0	3	3	ATASPSTCVWQC	tr_C9ZJU1	0.047	30069.93	47.00

943	HLA-A0201	AEVGSWETLSRT	ASWETLSRT	0	0	0	1	3	AEVGSWETLSRT	tr_C9ZJU1	0.047	30073.83	47.00
318	HLA-A0201	CVSQALWQASGS	CVSQALWQA	0	0	0	0	0	CVSQALWQA	tr_C9ZJU1	0.047	30090.11	47.00
133	HLA-A0201	LCMLLRGKY	LCMLLRGKY	0	0	0	0	0	LCMLLRGKY	tr_C9ZJU1	0.047	30101.52	47.00
696	HLA-A0201	VQRKIDRPVR	VQRKIDRPV	0	0	0	0	0	VQRKIDRPV	tr_C9ZJU1	0.047	30114.55	47.00
169	HLA-A0201	TAYNRKRSL	TAYNRKRSL	0	0	0	0	0	TAYNRKRSL	tr_C9ZJU1	0.047	30117.79	47.00
816	HLA-A0201	NIALGNLWDCSDE	NIALGLWDC	0	0	0	5	1	NIALGNLWDC	tr_C9ZJU1	0.047	30118.12	47.00
506	HLA-A0201	CVVDALKRKLQAGG	CVVDALLQA	0	0	0	6	3	CVVDALKRKLQA	tr_C9ZJU1	0.047	30118.45	47.00
516	HLA-A0201	QAGGHTCAVSVR	QAGGCAVSV	0	0	0	4	2	QAGGHTCAVSV	tr_C9ZJU1	0.047	30119.75	47.00
51	HLA-A0201	NCNGVESIHVR	NCNGVSIHV	0	0	0	5	1	NCNGVESIHV	tr_C9ZJU1	0.047	30127.58	47.00
146	HLA-A0201	YPPAAQADVVVQ	YAAQADVVV	0	0	0	1	2	YPPAAQADVVV	tr_C9ZJU1	0.047	30131.16	47.00
813	HLA-A0201	IGGNIALGNLW	IGGNIALLW	0	0	0	7	2	IGGNIALGNLW	tr_C9ZJU1	0.047	30131.82	47.00
115	HLA-A0201	DGVALCILDD	GVALCILDD	1	0	0	0	0	GVALCILDD	tr_C9ZJU1	0.047	30134.75	47.00
76	HLA-A0201	FAVHCGLMKNSHP	FAVHCGLMP	0	0	0	8	4	FAVHCGLMKNSHP	tr_C9ZJU1	0.047	30149.76	47.00
240	HLA-A0201	VSPKLSPFRFI	VSLSPFRFI	0	0	0	2	2	VSPKLSPFRFI	tr_C9ZJU1	0.047	30170.96	47.00
715	HLA-A0201	PWSVEFREVSFRYS	WSVEFFRYS	1	0	0	5	4	WSVEFREVSFRYS	tr_C9ZJU1	0.047	30172.27	47.00
1	HLA-A0201	RERARPAVLLDC	RAPAVLLDC	2	0	0	2	1	RARPAVLLDC	tr_C9ZJU1	0.047	30174.23	47.00
626	HLA-A0201	FTQSALDQTE	FTQSALDQT	0	0	0	0	0	FTQSALDQT	tr_C9ZJU1	0.047	30174.56	47.00
614	HLA-A0201	VSAVALRLSP	VSAVALLSP	0	0	0	6	1	VSAVALRLSP	tr_C9ZJU1	0.047	30178.80	47.00
670	HLA-A0201	EAHLRRHLAFSGS	HLHLAFSGS	2	0	0	2	2	HLRRHLAFSGS	tr_C9ZJU1	0.047	30183.05	47.00
715	HLA-A0201	PWSVEFREVSF	WSVEFEVSF	1	0	0	5	1	WSVEFREVSF	tr_C9ZJU1	0.047	30185.32	47.00
713	HLA-A0201	DGPWSVEFREVSFR	GPWSVEFEV	1	0	0	7	1	GPWSVEFREV	tr_C9ZJU1	0.047	30185.66	47.00
81	HLA-A0201	GLMKNSHPDH	GLMKNSHPH	0	0	0	8	1	GLMKNSHPDH	tr_C9ZJU1	0.047	30196.44	47.00
782	HLA-A0201	HISQYPARMLRR	HISQYPAML	0	0	0	7	1	HISQYPARML	tr_C9ZJU1	0.047	30201.99	47.00
338	HLA-A0201	VPALLEVMH	VPALLEVMH	0	0	0	0	0	VPALLEVMH	tr_C9ZJU1	0.047	30209.84	47.00
316	HLA-A0201	SRCVSQALWQASGS	SVSQALWQA	0	0	0	1	2	SRCVSQALWQA	tr_C9ZJU1	0.047	30215.07	47.00
848	HLA-A0201	SGIHAPLNVREFSG	SLNVREFSG	0	0	0	1	5	SGIHAPLNVREFSG	tr_C9ZJU1	0.047	30215.73	47.00
454	HLA-A0201	FSHPSRHDKSSV	FSHPSRHSV	0	0	0	7	3	FSHPSRHDKSSV	tr_C9ZJU1	0.047	30226.51	47.00
72	HLA-A0201	LPSVFAVHCG	LPSVFAVHC	0	0	0	0	0	LPSVFAVHC	tr_C9ZJU1	0.046	30240.58	47.00
34	HLA-A0201	DSKQTECKDDASIA	KQTECDASI	2	0	0	5	2	KQTECKDDASI	tr_C9ZJU1	0.046	30241.56	47.00
847	HLA-A0201	SSGIHAPLNVREF	GIHLNVREF	2	0	0	3	2	GIHAPLNVREF	tr_C9ZJU1	0.046	30247.12	47.00
621	HLA-A0201	LSPNFFTQSALD	LSPNFFTAL	0	0	0	7	2	LSPNFFTQSAL	tr_C9ZJU1	0.046	30250.39	47.00
716	HLA-A0201	WSVEFREVSFR	WSVEFVSFR	0	0	0	5	2	WSVEFREVSFR	tr_C9ZJU1	0.046	30255.31	47.00
849	HLA-A0201	GIHAPLNVREFSG	ILNVREFSG	1	0	0	1	3	IHAPLNVREFSG	tr_C9ZJU1	0.046	30260.87	47.00
86	HLA-A0201	SHPDHLLVCDI	HPDHLLVCI	1	0	0	8	1	HPDHLLVCDI	tr_C9ZJU1	0.046	30261.21	47.00
445	HLA-A0201	DNKMLFQQTF	NKMLFQQTF	1	0	0	0	0	NKMLFQQTF	tr_C9ZJU1	0.046	30263.49	47.00
925	HLA-A0201	LATLKHADKI	LTLKHADKI	0	0	0	1	1	LATLKHADKI	tr_C9ZJU1	0.046	30273.31	47.00
670	HLA-A0201	EAHLRRHLAFSG	HLRHLAFSG	2	0	0	2	1	HLRRHLAFSG	tr_C9ZJU1	0.046	30274.62	47.00
893	HLA-A0201	DTITEGEIFDT	TITEGIFDT	1	0	0	5	1	TITEGEIFDT	tr_C9ZJU1	0.046	30275.94	47.00
411	HLA-A0201	PTCSFPVKNSLTSS	CSFPVLTSS	2	0	0	5	3	CSFPVKNSLTSS	tr_C9ZJU1	0.046	30297.56	47.00
0	HLA-A0201	LRERARPAVLLDC	RAPAVLLDC	3	0	0	2	1	RARPAVLLDC	tr_C9ZJU1	0.046	30301.82	47.00
796	HLA-A0201	ANVWQEENNLRF	NVWENNLRF	1	0	0	3	2	NVWQEENNLRF	tr_C9ZJU1	0.046	30302.16	47.00
307	HLA-A0201	YDPSDDWLLS	YDPSDDWLL	0	0	0	0	0	YDPSDDWLL	tr_C9ZJU1	0.046	30310.35	47.00
660	HLA-A0201	ECGMSEAQV	ECGMSEAQV	0	0	0	0	0	ECGMSEAQV	tr_C9ZJU1	0.046	30311.01	47.00
380	HLA-A0201	MESQFTKSQLAS	MQFTKSQLA	0	0	0	1	2	MESQFTKSQLA	tr_C9ZJU1	0.046	30314.29	47.00
826	HLA-A0201	SDENICAALS	SDENICAAL	0	0	0	0	0	SDENICAAL	tr_C9ZJU1	0.046	30338.57	48.00
850	HLA-A0201	IHAPLNVREF	IAPLNVREF	0	0	0	1	1	IHAPLNVREF	tr_C9ZJU1	0.046	30346.45	48.00
391	HLA-A0201	SVTNIATSTY	SVTNIATTY	0	0	0	7	1	SVTNIATSTY	tr_C9ZJU1	0.046	30376.66	48.00
779	HLA-A0201	NGVHISQYPARMLR	GVHISQYPL	1	0	0	8	3	GVHISQYPARML	tr_C9ZJU1	0.046	30378.98	48.00
430	HLA-A0201	RKQKDDGIKCMLS	KQGIKCMLS	1	0	0	2	3	KQKDDGIKCMLS	tr_C9ZJU1	0.046	30392.77	48.00
633	HLA-A0201	QTEIVNQLR	QTEIVNQLR	0	0	0	0	0	QTEIVNQLR	tr_C9ZJU1	0.046	30397.38	48.00
669	HLA-A0201	DEAHLRRHLAFSGS	HLHLAFSGS	3	0	0	2	2	HLRRHLAFSGS	tr_C9ZJU1	0.046	30402.65	48.00
678	HLA-A0201	AFSGSSLGC	AFSGSSLGC	0	0	0	0	0	AFSGSSLGC	tr_C9ZJU1	0.046	30408.24	48.00
592	HLA-A0201	EQCGRDVLHGLFQ	EQCGVLHGL	0	0	0	4	2	EQCGRDVLHGL	tr_C9ZJU1	0.046	30410.87	48.00
13	HLA-A0201	ERKAYEILEM	RKAYEILEM	1	0	0	0	0	RKAYEILEM	tr_C9ZJU1	0.046	30413.16	48.00

533	HLA-A0201	KDVIQSSPRAATA	VIQSSPATA	2	0	0	6	2	VIQSSPRAATA	tr_C9ZJU1	0.046	30420.42	48.00
241	HLA-A0201	SPKLSPFRF	SPKLSPFRF	0	0	0	0	0	SPKLSPFRF	tr_C9ZJU1	0.046	30431.61	48.00
725	HLA-A0201	FRYSDRHPYVLRR	FRYDRHPYV	0	0	0	3	1	FRYSDRHPYV	tr_C9ZJU1	0.046	30439.52	48.00
806	HLA-A0201	RFIEDLSIG	RFIEDLSIG	0	0	0	0	0	RFIEDLSIG	tr_C9ZJU1	0.046	30448.08	48.00
519	HLA-A0201	GHTCAVSVRRLGW	HTCAVSVRL	1	0	0	7	1	HTCAVSVRRL	tr_C9ZJU1	0.046	30449.39	48.00
689	HLA-A0201	VLEGLQSVQRK	VLEGLVQRK	0	0	0	5	2	VLEGLQSVQRK	tr_C9ZJU1	0.046	30451.04	48.00
222	HLA-A0201	VHWRPIYAAEIGK	VHWIYAAEI	0	0	0	3	2	VHWRPIYAAEI	tr_C9ZJU1	0.046	30452.02	48.00
666	HLA-A0201	AQVDEAHLRRHLAF	AQVDEALAF	0	0	0	6	5	AQVDEAHLRRHLAF	tr_C9ZJU1	0.046	30474.11	48.00
529	HLA-A0201	LGWIKDVIQSSPR	LGWIVIQSS	0	0	0	4	2	LGWIKDVIQSS	tr_C9ZJU1	0.046	30474.11	48.00
847	HLA-A0201	SSGIHAPLNVR	SSIHAPLNV	0	0	0	2	1	SSGIHAPLNV	tr_C9ZJU1	0.046	30474.77	48.00
731	HLA-A0201	HPYVLRRVSF	HPYVLRVSF	0	0	0	5	1	HPYVLRRVSF	tr_C9ZJU1	0.046	30476.74	48.00
40	HLA-A0201	CKDDASIAIF	CDDASIAIF	0	0	0	1	1	CKDDASIAIF	tr_C9ZJU1	0.046	30477.41	48.00
892	HLA-A0201	IDTITEGEIFDT	TITEGIFDT	2	0	0	5	1	TITEGEIFDT	tr_C9ZJU1	0.046	30487.96	48.00
668	HLA-A0201	VDEAHLRRHLAFSG	HLRHLAFSG	4	0	0	2	1	HLRRHLAFSG	tr_C9ZJU1	0.046	30489.28	48.00
814	HLA-A0201	GGNIALGNLW	GGNIALNLW	0	0	0	6	1	GGNIALGNLW	tr_C9ZJU1	0.046	30493.90	48.00
615	HLA-A0201	SAVALRLSP	SAVALRLSP	0	0	0	0	0	SAVALRLSP	tr_C9ZJU1	0.046	30493.90	48.00
798	HLA-A0201	VWQEENNLRFIE	WQEENNLFI	1	0	0	7	1	WQEENNLRFI	tr_C9ZJU1	0.046	30514.70	48.00
544	HLA-A0201	TASPSTCVWQC	ASSTCVWQC	1	0	0	2	1	ASPSTCVWQC	tr_C9ZJU1	0.046	30524.26	48.00
953	HLA-A0201	RTGPHSCFSRM	RTGPCFSRM	0	0	0	4	2	RTGPHSCFSRM	tr_C9ZJU1	0.046	30538.14	48.00
669	HLA-A0201	DEAHLRRHLAFSG	HLRHLAFSG	3	0	0	2	1	HLRRHLAFSG	tr_C9ZJU1	0.046	30543.44	48.00
549	HLA-A0201	TCVWQCRQDY	CVWQCRQDY	1	0	0	0	0	CVWQCRQDY	tr_C9ZJU1	0.046	30556.66	48.00
830	HLA-A0201	ICAALSASR	ICAALSASR	0	0	0	0	0	ICAALSASR	tr_C9ZJU1	0.046	30559.95	48.00
228	HLA-A0201	YAAEIGKSLNGDVS	YAAEIGKSV	0	0	0	8	4	YAAEIGKSLNGDV	tr_C9ZJU1	0.045	30561.94	48.00
146	HLA-A0201	YPPAAQADVVVQE	YAAQADVVV	0	0	0	1	2	YPPAAQADVVV	tr_C9ZJU1	0.045	30569.89	48.00
636	HLA-A0201	IVNQLRSIQHWCP	ILRSIQHWC	0	0	0	1	3	IVNQLRSIQHWC	tr_C9ZJU1	0.045	30578.48	48.00
937	HLA-A0201	LNEGMVAEVGSW	LMVAEVGSW	0	0	0	1	3	LNEGMVAEVGSW	tr_C9ZJU1	0.045	30584.44	48.00
427	HLA-A0201	TLLRKQKDDGIKC	TLLDDGIKC	0	0	0	3	4	TLLRKQKDDGIKC	tr_C9ZJU1	0.045	30596.69	48.00
225	HLA-A0201	RPIYAAEIGK	RIYAAEIGK	0	0	0	1	1	RPIYAAEIGK	tr_C9ZJU1	0.045	30606.95	48.00
504	HLA-A0201	SGCVVDALKRKLQ	SGCVVDALL	0	0	0	8	3	SGCVVDALKRKL	tr_C9ZJU1	0.045	30607.61	48.00
930	HLA-A0201	HADKIVVLNE	HADIVVLNE	0	0	0	3	1	HADKIVVLNE	tr_C9ZJU1	0.045	30609.93	48.00
523	HLA-A0201	AVSVRRLGWIK	AVSVRLGWI	0	0	0	4	1	AVSVRRLGWI	tr_C9ZJU1	0.045	30619.19	48.00
942	HLA-A0201	VAEVGSWETLS	VAVGSWETL	0	0	0	2	1	VAEVGSWETL	tr_C9ZJU1	0.045	30630.81	48.00
75	HLA-A0201	VFAVHCGLMKNSHP	FAVHCGLMP	1	0	0	8	4	FAVHCGLMKNSHP	tr_C9ZJU1	0.045	30636.77	48.00
540	HLA-A0201	PRAATASPSTCV	ATASPSTCV	3	0	0	0	0	ATASPSTCV	tr_C9ZJU1	0.045	30641.08	48.00
104	HLA-A0201	VNCSIRDIL	VNCSIRDIL	0	0	0	0	0	VNCSIRDIL	tr_C9ZJU1	0.045	30645.05	48.00
518	HLA-A0201	GGHTCAVSVRRLGW	HTCAVSVRL	2	0	0	7	1	HTCAVSVRRL	tr_C9ZJU1	0.045	30648.37	48.00
479	HLA-A0201	SGRPLGYMW	SGRPLGYMW	0	0	0	0	0	SGRPLGYMW	tr_C9ZJU1	0.045	30648.71	48.00
689	HLA-A0201	VLEGLQSVQRKIDR	VLEGLQSVI	0	0	0	8	3	VLEGLQSVQRKI	tr_C9ZJU1	0.045	30653.67	48.00
595	HLA-A0201	GRDVLHGLF	GRDVLHGLF	0	0	0	0	0	GRDVLHGLF	tr_C9ZJU1	0.045	30663.95	48.00
849	HLA-A0201	GIHAPLNVREF	GIHLNVREF	0	0	0	3	2	GIHAPLNVREF	tr_C9ZJU1	0.045	30669.93	48.00
815	HLA-A0201	GNIALGNLWDCSDE	NIALGLWDC	1	0	0	5	1	NIALGNLWDC	tr_C9ZJU1	0.045	30676.91	48.00
929	HLA-A0201	KHADKIVVLN	KHADKIVVL	0	0	0	0	0	KHADKIVVL	tr_C9ZJU1	0.045	30680.88	48.00
693	HLA-A0201	LQSVQRKIDRPVRG	LQSVIDRPV	0	0	0	4	3	LQSVQRKIDRPV	tr_C9ZJU1	0.045	30681.22	48.00
310	HLA-A0201	SDDWLLSRCVS	SDWLLSRCV	0	0	0	1	1	SDDWLLSRCV	tr_C9ZJU1	0.045	30682.88	48.00
798	HLA-A0201	VWQEENNLRF	WQEENNLRF	1	0	0	0	0	WQEENNLRF	tr_C9ZJU1	0.045	30706.80	49.00
12	HLA-A0201	CERKAYEIL	CERKAYEIL	0	0	0	0	0	CERKAYEIL	tr_C9ZJU1	0.045	30710.10	49.00
828	HLA-A0201	ENICAALSA	ENICAALSA	0	0	0	0	0	ENICAALSA	tr_C9ZJU1	0.045	30713.77	49.00
115	HLA-A0201	DGVALCILD	DGVALCILD	0	0	0	0	0	DGVALCILD	tr_C9ZJU1	0.045	30726.39	49.00
925	HLA-A0201	LATLKHADKIV	LLKHADKIV	0	0	0	1	2	LATLKHADKIV	tr_C9ZJU1	0.045	30747.34	49.00
315	HLA-A0201	LSRCVSQALWQ	LSRCVALWQ	0	0	0	5	2	LSRCVSQALWQ	tr_C9ZJU1	0.045	30752.34	49.00
83	HLA-A0201	MKNSHPDHLLVC	MSHPDHLLV	0	0	0	1	2	MKNSHPDHLLV	tr_C9ZJU1	0.045	30754.00	49.00
698	HLA-A0201	RKIDRPVRGVAPPS	KIDGVAPPS	1	0	0	3	4	KIDRPVRGVAPPS	tr_C9ZJU1	0.045	30757.34	49.00
149	HLA-A0201	AAQADVVVQEFPEE	AQADVVVEF	1	0	0	7	1	AQADVVVQEF	tr_C9ZJU1	0.045	30757.67	49.00
1	HLA-A0201	RERARPAVLL	RERARAVLL	0	0	0	5	1	RERARPAVLL	tr_C9ZJU1	0.045	30761.66	49.00

885	HLA-A0201	FDEPTSAIDTIT	FTSAIDTIT	0	0	0	1	3	FDEPTSAIDTIT	tr_C9ZJU1	0.045	30764.99	49.00
542	HLA-A0201	AATASPSTCVWQCR	ATATCVWQC	1	0	0	3	3	ATASPSTCVWQC	tr_C9ZJU1	0.045	30766.32	49.00
80	HLA-A0201	CGLMKNSHPD	CLMKNSHPD	0	0	0	1	1	CGLMKNSHPD	tr_C9ZJU1	0.045	30769.66	49.00
871	HLA-A0201	RAFVKAGSAVG	RAFVKASAV	0	0	0	6	1	RAFVKAGSAV	tr_C9ZJU1	0.045	30793.64	49.00
757	HLA-A0201	GKTTLLRLLN	GKTTLLRLL	0	0	0	0	0	GKTTLLRLL	tr_C9ZJU1	0.045	30798.62	49.00
534	HLA-A0201	DVIQSSPRAA	VIQSSPRAA	1	0	0	0	0	VIQSSPRAA	tr_C9ZJU1	0.045	30829.30	49.00
849	HLA-A0201	GIHAPLNVREFS	GILNVREFS	0	0	0	2	3	GIHAPLNVREFS	tr_C9ZJU1	0.045	30836.64	49.00
843	HLA-A0201	VQKRSSGIHA	VQRSSGIHA	0	0	0	2	1	VQKRSSGIHA	tr_C9ZJU1	0.045	30836.98	49.00
3	HLA-A0201	RARPAVLLD	RARPAVLLD	0	0	0	0	0	RARPAVLLD	tr_C9ZJU1	0.045	30838.31	49.00
599	HLA-A0201	LHGLFQMADT	LHGLFMADT	0	0	0	5	1	LHGLFQMADT	tr_C9ZJU1	0.045	30838.97	49.00
390	HLA-A0201	ASVTNIATSTYAG	SVIATSTYA	1	0	0	2	2	SVTNIATSTYA	tr_C9ZJU1	0.045	30840.30	49.00
415	HLA-A0201	FPVKNSLTS	FPVKNSLTS	0	0	0	0	0	FPVKNSLTS	tr_C9ZJU1	0.045	30843.30	49.00
59	HLA-A0201	HVRTLVFNHN	HVRTLVFNH	0	0	0	0	0	HVRTLVFNH	tr_C9ZJU1	0.045	30844.32	49.00
41	HLA-A0201	KDDASIAIFLN	KDDAIAIFL	0	0	0	4	1	KDDASIAIFL	tr_C9ZJU1	0.045	30847.98	49.00
309	HLA-A0201	PSDDWLLSRCVS	PSDDWLLSV	0	0	0	8	2	PSDDWLLSRCV	tr_C9ZJU1	0.045	30863.34	49.00
108	HLA-A0201	IRDILLDDG	IRDILLDDG	0	0	0	0	0	IRDILLDDG	tr_C9ZJU1	0.045	30863.34	49.00
323	HLA-A0201	LWQASGSVM	LWQASGSVM	0	0	0	0	0	LWQASGSVM	tr_C9ZJU1	0.045	30864.02	49.00
725	HLA-A0201	FRYSDRHPYVLR	FRYDRHPYV	0	0	0	3	1	FRYSDRHPYV	tr_C9ZJU1	0.045	30869.02	49.00
650	HLA-A0201	YHAFLGSFYDE	YAFLGSFYD	0	0	0	1	1	YHAFLGSFYD	tr_C9ZJU1	0.045	30879.37	49.00
[truncated: 708,221 more chars]
