# Supplementary material for: Immunoinformatic design of a putative multi-epitope vaccine candidate against Trypanosoma brucei gambiense
Source: Comput Struct Biotechnol J. 2022 Oct 7;20:5574–85. doi: 10.1016/j.csbj.2022.10.002 (PMC9576565; doi:10.1016/j.csbj.2022.10.002)
Supplement: Supplementary data 5 [file mmc5.pdf]

jobid=60CF5C66000048EBC9BE1C9B Server Output - DTU Health Tech

---

```
# Tmpdir made /scratch/netMHCIIktrZRf
# Input is in FSA format

# Peptide length 15
        NetMHCII version 2.3.

Strong binder threshold   0.50. Weak binder threshold  10.00.
```

Allele	pos	peptide	core	Of	1-log50k(aff)	affinity(nM)	%Rank	Relia	Identity	Bind Level
DRB1_0101	1	QSSMKCFTSMIPPGG	FTSMIPPGG	6	0.7513	14.7	5.00	0.87	tr_C9ZMD3_	WB
DRB1_0101	2	DEGLKGKNALPANEG	LKGKNALPA	3	0.4831	268.4	45.00	0.63	tr_C9ZMD3_	
DRB1_0101	3	EGLKGKNALPANEGE	LKGKNALPA	2	0.4802	276.9	45.00	0.64	tr_C9ZMD3_	
DRB1_0101	4	EDEGLKGKNALPANE	LKGKNALPA	4	0.4616	338.7	49.00	0.60	tr_C9ZMD3_	
DRB1_0101	5	GLKGKNALPANEGEG	LKGKNALPA	1	0.4573	355.0	50.00	0.57	tr_C9ZMD3_	
DRB1_0101	6	GEDEGLKGKNALPAN	LKGKNALPA	5	0.4346	453.9	55.00	0.57	tr_C9ZMD3_	
DRB1_0101	7	LKGKNALPANEGEGE	KNALPANEG	3	0.3773	843.8	70.00	0.40	tr_C9ZMD3_	
DRB1_0101	8	EQSSMKCFTSMIPPG	SMKCFTSMI	3	0.3673	940.0	70.00	0.45	tr_C9ZMD3_	
DRB1_0101	9	SGEDEGLKGKNALPA	DEGLKGKNA	3	0.3608	1008.5	70.00	0.41	tr_C9ZMD3_	
DRB1_0101	10	EGEQSSMKCFTSMIP	SMKCFTSMI	5	0.3603	1013.4	70.00	0.41	tr_C9ZMD3_	
DRB1_0101	11	GEQSSMKCFTSMIPP	SMKCFTSMI	4	0.3523	1105.1	75.00	0.43	tr_C9ZMD3_	
DRB1_0101	12	GEGEQSSMKCFTSMI	QSSMKCFTS	4	0.3420	1235.6	75.00	0.33	tr_C9ZMD3_	
DRB1_0101	13	DVTSPFGSCEVISGE	FGSCEVISG	5	0.3249	1486.2	80.00	0.45	tr_C9ZMD3_	
DRB1_0101	14	ADVTSPFGSCEVISG	TSPFGSCEV	3	0.3102	1742.9	80.00	0.39	tr_C9ZMD3_	
DRB1_0101	15	VTSPFGSCEVISGED	FGSCEVISG	4	0.3081	1783.5	80.00	0.48	tr_C9ZMD3_	
DRB1_0101	16	VISGEDEGLKGKNAL	DEGLKGKNA	5	0.2947	2062.5	85.00	0.49	tr_C9ZMD3_	
DRB1_0101	17	SCEVISGEDEGLKGK	VISGEDEGL	3	0.2875	2228.3	85.00	0.41	tr_C9ZMD3_	
DRB1_0101	18	TSPFGSCEVISGEDE	FGSCEVISG	3	0.2844	2304.9	85.00	0.57	tr_C9ZMD3_	
DRB1_0101	19	FGSCEVISGEDEGLK	CEVISGEDE	3	0.2818	2369.7	85.00	0.32	tr_C9ZMD3_	
DRB1_0101	20	CEVISGEDEGLKGKN	ISGEDEGLK	3	0.2683	2742.1	90.00	0.39	tr_C9ZMD3_	
DRB1_0101	21	KGKNALPANEGEGEQ	KNALPANEG	2	0.2679	2754.6	90.00	0.69	tr_C9ZMD3_	
DRB1_0101	22	GSCEVISGEDEGLKG	VISGEDEGL	4	0.2655	2827.3	90.00	0.35	tr_C9ZMD3_	
DRB1_0101	23	KADVTSPFGSCEVIS	TSPFGSCEV	4	0.2647	2850.9	90.00	0.47	tr_C9ZMD3_	
DRB1_0101	24	SPFGSCEVISGEDEG	FGSCEVISG	2	0.2626	2916.1	90.00	0.54	tr_C9ZMD3_	
DRB1_0101	25	EVISGEDEGLKGKNA	ISGEDEGLK	2	0.2615	2951.3	90.00	0.37	tr_C9ZMD3_	
DRB1_0101	26	ISGEDEGLKGKNALP	DEGLKGKNA	4	0.2613	2958.0	90.00	0.61	tr_C9ZMD3_	
DRB1_0101	27	PFGSCEVISGEDEGL	FGSCEVISG	1	0.2588	3039.9	90.00	0.37	tr_C9ZMD3_	
DRB1_0101	28	EGEGEQSSMKCFTSM	QSSMKCFTS	5	0.2565	3115.7	90.00	0.43	tr_C9ZMD3_	
DRB1_0101	29	NKADVTSPFGSCEVI	TSPFGSCEV	5	0.2383	3794.3	95.00	0.39	tr_C9ZMD3_	
DRB1_0101	30	VPTTNREQVNKADVT	TNREQVNKA	3	0.2283	4229.5	95.00	0.74	tr_C9ZMD3_	
DRB1_0101	31	PTTNREQVNKADVTS	TNREQVNKA	2	0.2138	4944.4	95.00	0.66	tr_C9ZMD3_	
DRB1_0101	32	GKNALPANEGEGEQS	KNALPANEG	1	0.2085	5237.5	95.00	0.66	tr_C9ZMD3_	
DRB1_0101	33	VNKADVTSPFGSCEV	TSPFGSCEV	6	0.1990	5806.1	95.00	0.26	tr_C9ZMD3_	
DRB1_0101	34	TTNREQVNKADVTSP	TNREQVNKA	1	0.1979	5873.7	95.00	0.54	tr_C9ZMD3_	

DRB1_0101	35	REQVNKADVTSPFGS	VNKADVTSP	3	0.1888	6485.2	100.00	0.49	tr_C9ZMD3_	
DRB1_0101	36	NEGEGEQSSMKCFTS	QSSMKCFTS	6	0.1878	6553.5	100.00	0.35	tr_C9ZMD3_	
DRB1_0101	37	EQVNKADVTSPFGSC	VNKADVTSP	2	0.1831	6897.1	100.00	0.38	tr_C9ZMD3_	
DRB1_0101	38	QVNKADVTSPFGSCE	VNKADVTSP	1	0.1774	7332.8	100.00	0.28	tr_C9ZMD3_	
DRB1_0101	39	NREQVNKADVTSPFG	VNKADVTSP	4	0.1711	7847.8	100.00	0.48	tr_C9ZMD3_	
DRB1_0101	40	TNREQVNKADVTSPF	VNKADVTSP	5	0.1710	7858.4	100.00	0.34	tr_C9ZMD3_	
DRB1_0101	41	KNALPANEGEGEQSS	KNALPANEG	0	0.1598	8875.3	100.00	0.56	tr_C9ZMD3_	
DRB1_0101	42	ANEGEGEQSSMKCFT	GEGEQSSMK	3	0.1496	9903.8	100.00	0.28	tr_C9ZMD3_	
DRB1_0101	43	PANEGEGEQSSMKCF	GEGEQSSMK	4	0.1288	12402.6	100.00	0.29	tr_C9ZMD3_	
DRB1_0101	44	LPANEGEGEQSSMKC	GEGEQSSMK	5	0.1039	16250.8	100.00	0.24	tr_C9ZMD3_	
DRB1_0101	45	ALPANEGEGEQSSMK	ANEGEGEQS	3	0.1024	16508.4	100.00	0.20	tr_C9ZMD3_	
DRB1_0101	46	NALPANEGEGEQSSM	LPANEGEGE	2	0.0878	19329.6	100.00	0.34	tr_C9ZMD3_	

Allele: DRB1_0101. Number of high binders 0. Number of weak binders 1. Number of peptides 46

Allele	pos	peptide	core	Of	1-log50k(aff)	affinity(nM)	%Rank	Relia	Identity	Bind Level
DRB1_0101	1	WSDVAFSVGGVPLRV	FSVGGVPLR	5	0.6601	39.6	14.00	0.47	tr_D0A7J0_	
DRB1_0101	2	FWSDVAFSVGGVPLR	FSVGGVPLR	6	0.5996	76.1	23.00	0.35	tr_D0A7J0_	
DRB1_0101	3	WVTGFWSDVAFSVGG	FWSDVAFSV	4	0.5574	120.2	30.00	0.71	tr_D0A7J0_	
DRB1_0101	4	QWVTGFWSDVAFSVG	FWSDVAFSV	5	0.5574	120.2	30.00	0.60	tr_D0A7J0_	
DRB1_0101	5	VTGFWSDVAFSVGGV	FWSDVAFSV	3	0.5545	124.0	30.00	0.76	tr_D0A7J0_	
DRB1_0101	6	SQWVTGFWSDVAFSV	FWSDVAFSV	6	0.5337	155.3	34.00	0.43	tr_D0A7J0_	
DRB1_0101	7	TGFWSDVAFSVGGVP	FWSDVAFSV	2	0.5279	165.4	35.00	0.77	tr_D0A7J0_	
DRB1_0101	8	GFWSDVAFSVGGVPL	FWSDVAFSV	1	0.5229	174.4	36.00	0.57	tr_D0A7J0_	
DRB1_0101	9	ASIREGLPDLSQWVT	IREGLPDLS	2	0.5062	209.0	40.00	0.69	tr_D0A7J0_	
DRB1_0101	10	GASIREGLPDLSQWV	IREGLPDLS	3	0.5034	215.5	40.00	0.79	tr_D0A7J0_	
DRB1_0101	11	SIREGLPDLSQWVTG	IREGLPDLS	1	0.4726	300.9	47.00	0.62	tr_D0A7J0_	
DRB1_0101	12	LSQWVTGFWSDVAFS	WVTGFWSDV	3	0.4430	414.2	55.00	0.43	tr_D0A7J0_	
DRB1_0101	13	DLSQWVTGFWSDVAF	WVTGFWSDV	4	0.4119	579.8	60.00	0.47	tr_D0A7J0_	
DRB1_0101	14	IREGLPDLSQWVTGF	GLPDLSQWV	3	0.3871	758.8	65.00	0.39	tr_D0A7J0_	
DRB1_0101	15	PDLSQWVTGFWSDVA	WVTGFWSDV	5	0.3748	866.2	70.00	0.41	tr_D0A7J0_	
DRB1_0101	16	LPDLSQWVTGFWSDV	SQWVTGFWS	4	0.3184	1595.3	80.00	0.36	tr_D0A7J0_	
DRB1_0101	17	EGLPDLSQWVTGFWS	GLPDLSQWV	1	0.3054	1836.4	85.00	0.40	tr_D0A7J0_	
DRB1_0101	18	REGLPDLSQWVTGFW	GLPDLSQWV	2	0.3051	1842.7	85.00	0.55	tr_D0A7J0_	
DRB1_0101	19	GLPDLSQWVTGFWSD	SQWVTGFWS	5	0.2930	2100.7	85.00	0.32	tr_D0A7J0_	

Allele: DRB1_0101. Number of high binders 0. Number of weak binders 0. Number of peptides 19

Allele	pos	peptide	core	Of	1-log50k(aff)	affinity(nM)	%Rank	Relia	Identity	Bind Level
DRB1_0101	1	VSFVRYGLAGLVVNE	VRYGLAGLV	3	0.7261	19.4	6.50	0.52	tr_D0A3G8_	WB
DRB1_0101	2	YVSFVRYGLAGLVVN	VRYGLAGLV	4	0.7254	19.5	6.50	0.52	tr_D0A3G8_	WB
DRB1_0101	3	SFVRYGLAGLVVNEF	VRYGLAGLV	2	0.7135	22.2	8.00	0.50	tr_D0A3G8_	WB

DRB1_0101	4	KYVSFVRYGLAGLVV	VRYGLAGLV	5	0.7000	25.7	9.00	0.49	tr_D0A3G8_	WB
DRB1_0101	5	FVRYGLAGLVVNEFD	VRYGLAGLV	1	0.6627	38.4	13.00	0.47	tr_D0A3G8_	
DRB1_0101	6	LKYVSFVRYGLAGLV	VRYGLAGLV	6	0.6485	44.9	15.00	0.40	tr_D0A3G8_	
DRB1_0101	7	GTTYARLQGYFPEEL	YARLQGYFP	3	0.6200	61.0	19.00	0.84	tr_D0A3G8_	
DRB1_0101	8	TTYARLQGYFPEELP	YARLQGYFP	2	0.6096	68.3	21.00	0.81	tr_D0A3G8_	
DRB1_0101	9	DGTTYARLQGYFPEE	YARLQGYFP	4	0.5752	99.1	27.00	0.87	tr_D0A3G8_	
DRB1_0101	10	PFFWRWLKYVSFVRY	WRWLKYVSF	3	0.5718	102.8	27.00	0.34	tr_D0A3G8_	
DRB1_0101	11	FFWRWLKYVSFVRYG	WRWLKYVSF	2	0.5659	109.6	28.00	0.32	tr_D0A3G8_	
DRB1_0101	12	VRYGLAGLVVNEFDG	VRYGLAGLV	0	0.5659	109.7	28.00	0.29	tr_D0A3G8_	
DRB1_0101	13	TYARLQGYFPEELPE	YARLQGYFP	1	0.5650	110.7	28.00	0.77	tr_D0A3G8_	
DRB1_0101	14	PDGTTYARLQGYFPE	YARLQGYFP	5	0.5622	114.1	29.00	0.81	tr_D0A3G8_	
DRB1_0101	15	WLKYVSFVRYGLAGL	FVRYGLAGL	6	0.5577	119.8	30.00	0.22	tr_D0A3G8_	
DRB1_0101	16	FWRWLKYVSFVRYGL	WLKYVSFVR	3	0.5570	120.6	30.00	0.30	tr_D0A3G8_	
DRB1_0101	17	WRWLKYVSFVRYGLA	WLKYVSFVR	2	0.5480	133.0	32.00	0.26	tr_D0A3G8_	
DRB1_0101	18	DGLKFVCEPKVPFCF	LKFVCEPKV	2	0.5383	147.8	33.00	0.47	tr_D0A3G8_	
DRB1_0101	19	LPFFWRWLKYVSFVR	WRWLKYVSF	4	0.5360	151.5	34.00	0.39	tr_D0A3G8_	
DRB1_0101	20	FDGLKFVCEPKVPFC	LKFVCEPKV	3	0.5340	154.7	34.00	0.57	tr_D0A3G8_	
DRB1_0101	21	RWLKYVSFVRYGLAG	WLKYVSFVR	1	0.5292	163.1	35.00	0.25	tr_D0A3G8_	
DRB1_0101	22	GLKFVCEPKVPFCFP	LKFVCEPKV	1	0.5284	164.5	35.00	0.44	tr_D0A3G8_	
DRB1_0101	23	EFDGLKFVCEPKVPF	LKFVCEPKV	4	0.5260	168.8	36.00	0.62	tr_D0A3G8_	
DRB1_0101	24	FPDGTTYARLQGYFP	YARLQGYFP	6	0.4999	223.9	41.00	0.69	tr_D0A3G8_	
DRB1_0101	25	SLPFFWRWLKYVSFV	WRWLKYVSF	5	0.4977	229.1	41.00	0.43	tr_D0A3G8_	
DRB1_0101	26	QSLPFFWRWLKYVSF	FFWRWLKYV	4	0.4764	288.6	46.00	0.39	tr_D0A3G8_	
DRB1_0101	27	LKFVCEPKVPFCFPD	FVCEPKVPF	2	0.4737	297.2	46.00	0.44	tr_D0A3G8_	
DRB1_0101	28	YARLQGYFPEELPEY	YARLQGYFP	0	0.4633	332.6	49.00	0.65	tr_D0A3G8_	
DRB1_0101	29	RYGLAGLVVNEFDGL	YGLAGLVVN	1	0.4608	341.6	49.00	0.31	tr_D0A3G8_	
DRB1_0101	30	GTQSLPFFWRWLKYV	TQSLPFFWR	1	0.4591	348.0	50.00	0.34	tr_D0A3G8_	
DRB1_0101	31	NEFDGLKFVCEPKVP	LKFVCEPKV	5	0.4513	378.8	55.00	0.65	tr_D0A3G8_	
DRB1_0101	32	TQSLPFFWRWLKYVS	FFWRWLKYV	5	0.4458	401.9	55.00	0.41	tr_D0A3G8_	
DRB1_0101	33	VNEFDGLKFVCEPKV	LKFVCEPKV	6	0.4166	551.1	60.00	0.50	tr_D0A3G8_	
DRB1_0101	34	YGLAGLVVNEFDGLK	LAGLVVNEF	2	0.3786	831.9	70.00	0.37	tr_D0A3G8_	
DRB1_0101	35	KFVCEPKVPFCFPDG	FVCEPKVPF	1	0.3645	969.1	70.00	0.56	tr_D0A3G8_	
DRB1_0101	36	GLVVNEFDGLKFVCE	VNEFDGLKF	3	0.3585	1033.5	75.00	0.31	tr_D0A3G8_	
DRB1_0101	37	LAGLVVNEFDGLKFV	VNEFDGLKF	5	0.3550	1073.9	75.00	0.27	tr_D0A3G8_	
DRB1_0101	38	VPFCFPDGTTYARLQ	FCFPDGTTY	2	0.3525	1102.8	75.00	0.31	tr_D0A3G8_	
DRB1_0101	39	AGLVVNEFDGLKFVC	VNEFDGLKF	4	0.3479	1158.9	75.00	0.33	tr_D0A3G8_	
DRB1_0101	40	PFCFPDGTTYARLQG	PDGTTYARL	4	0.3458	1185.3	75.00	0.26	tr_D0A3G8_	
DRB1_0101	41	KVPFCFPDGTTYARL	FCFPDGTTY	3	0.3409	1250.8	75.00	0.38	tr_D0A3G8_	
DRB1_0101	42	GLAGLVVNEFDGLKF	LAGLVVNEF	1	0.3328	1364.5	80.00	0.23	tr_D0A3G8_	
DRB1_0101	43	FCFPDGTTYARLQGY	PDGTTYARL	3	0.3294	1416.1	80.00	0.34	tr_D0A3G8_	
DRB1_0101	44	LVVNEFDGLKFVCEP	VNEFDGLKF	2	0.3202	1563.7	80.00	0.37	tr_D0A3G8_	
DRB1_0101	45	CFPDGTTYARLQGYF	PDGTTYARL	2	0.3069	1806.2	80.00	0.33	tr_D0A3G8_	
DRB1_0101	46	VVNEFDGLKFVCEPK	FDGLKFVCE	4	0.2958	2036.5	85.00	0.41	tr_D0A3G8_	
DRB1_0101	47	PKVPFCFPDGTTYAR	FCFPDGTTY	4	0.2882	2210.6	85.00	0.39	tr_D0A3G8_	
DRB1_0101	48	FVCEPKVPFCFPDGT	FVCEPKVPF	0	0.2767	2504.7	85.00	0.38	tr_D0A3G8_	
DRB1_0101	49	EPKVPFCFPDGTTYA	FCFPDGTTY	5	0.2393	3755.5	95.00	0.44	tr_D0A3G8_	
DRB1_0101	50	CEPKVPFCFPDGTTY	FCFPDGTTY	6	0.2098	5166.4	95.00	0.28	tr_D0A3G8_	
DRB1_0101	51	VCEPKVPFCFPDGTT	CEPKVPFCF	1	0.1958	6009.3	95.00	0.43	tr_D0A3G8_	

Allele: DRB1_0101. Number of high binders 0. Number of weak binders 4. Number of peptides 51

Allele	pos	peptide	core	Of	1-log50k(aff)	affinity(nM)	%Rank	Relia	Identity	Bind Level
DRB1_0101	1	AQTVGGGAGFPMRST	VGGGAGFPM	3	0.5001	223.3	41.00	0.78	tr_D0AAR0_	
DRB1_0101	2	QTVGGGAGFPMRSTP	VGGGAGFPM	2	0.4738	296.8	46.00	0.76	tr_D0AAR0_	
DRB1_0101	3	EAQTVGGGAGFPMRS	VGGGAGFPM	4	0.4711	305.7	47.00	0.79	tr_D0AAR0_	
DRB1_0101	4	YLERRKRGMLRTYLH	ERRKRGMLR	2	0.4652	325.7	48.00	0.38	tr_D0AAR0_	
DRB1_0101	5	GAGFPMRSTPSGVSW	MRSTPSGVS	5	0.4619	337.6	49.00	0.31	tr_D0AAR0_	
DRB1_0101	6	EYLERRKRGMLRTYL	ERRKRGMLR	3	0.4514	378.3	55.00	0.39	tr_D0AAR0_	
DRB1_0101	7	LERRKRGMLRTYLHE	ERRKRGMLR	1	0.4449	405.7	55.00	0.35	tr_D0AAR0_	
DRB1_0101	8	TVGGGAGFPMRSTPS	VGGGAGFPM	1	0.4413	422.2	55.00	0.68	tr_D0AAR0_	
DRB1_0101	9	GGAGFPMRSTPSGVS	GFPMRSTPS	3	0.4227	516.2	60.00	0.32	tr_D0AAR0_	
DRB1_0101	10	TEAQTVGGGAGFPMR	VGGGAGFPM	5	0.4224	517.5	60.00	0.75	tr_D0AAR0_	
DRB1_0101	11	ERRKRGMLRTYLHET	KRGMLRTYL	3	0.4207	527.4	60.00	0.34	tr_D0AAR0_	
DRB1_0101	12	RRKRGMLRTYLHETH	KRGMLRTYL	2	0.4142	565.7	60.00	0.34	tr_D0AAR0_	
DRB1_0101	13	RKRGMLRTYLHETHG	RGMLRTYLH	2	0.3932	710.4	65.00	0.28	tr_D0AAR0_	
DRB1_0101	14	VGGGAGFPMRSTPSG	VGGGAGFPM	0	0.3920	719.5	65.00	0.41	tr_D0AAR0_	
DRB1_0101	15	KRGMLRTYLHETHGR	MLRTYLHET	3	0.3662	951.2	70.00	0.31	tr_D0AAR0_	
DRB1_0101	16	GGGAGFPMRSTPSGV	GFPMRSTPS	4	0.3538	1087.6	75.00	0.44	tr_D0AAR0_	
DRB1_0101	17	RGMLRTYLHETHGRG	MLRTYLHET	2	0.3370	1305.0	75.00	0.30	tr_D0AAR0_	
DRB1_0101	18	MLRTYLHETHGRGGT	YLHETHGRG	4	0.3315	1384.4	80.00	0.44	tr_D0AAR0_	
DRB1_0101	19	GMLRTYLHETHGRGG	YLHETHGRG	5	0.3211	1548.6	80.00	0.38	tr_D0AAR0_	
DRB1_0101	20	LRTYLHETHGRGGTE	YLHETHGRG	3	0.3160	1637.2	80.00	0.52	tr_D0AAR0_	
DRB1_0101	21	GTEAQTVGGGAGFPM	VGGGAGFPM	6	0.3072	1801.4	80.00	0.58	tr_D0AAR0_	
DRB1_0101	22	RTYLHETHGRGGTEA	YLHETHGRG	2	0.2924	2112.7	85.00	0.52	tr_D0AAR0_	
DRB1_0101	23	TYLHETHGRGGTEAQ	YLHETHGRG	1	0.2377	3820.2	95.00	0.48	tr_D0AAR0_	
DRB1_0101	24	YLHETHGRGGTEAQT	YLHETHGRG	0	0.1728	7711.8	100.00	0.30	tr_D0AAR0_	
DRB1_0101	25	RGGTEAQTVGGGAGF	EAQTVGGGA	4	0.1721	7767.6	100.00	0.32	tr_D0AAR0_	
DRB1_0101	26	GRGGTEAQTVGGGAG	RGGTEAQTV	1	0.1664	8259.9	100.00	0.37	tr_D0AAR0_	
DRB1_0101	27	THGRGGTEAQTVGGG	RGGTEAQTV	3	0.1651	8376.5	100.00	0.55	tr_D0AAR0_	
DRB1_0101	28	GGTEAQTVGGGAGFP	EAQTVGGGA	3	0.1611	8747.2	100.00	0.41	tr_D0AAR0_	
DRB1_0101	29	HGRGGTEAQTVGGGA	RGGTEAQTV	2	0.1606	8795.8	100.00	0.47	tr_D0AAR0_	
DRB1_0101	30	ETHGRGGTEAQTVGG	RGGTEAQTV	4	0.1591	8936.9	100.00	0.50	tr_D0AAR0_	
DRB1_0101	31	HETHGRGGTEAQTVG	RGGTEAQTV	5	0.1464	10252.6	100.00	0.41	tr_D0AAR0_	
DRB1_0101	32	LHETHGRGGTEAQTV	THGRGGTEA	3	0.1415	10820.1	100.00	0.34	tr_D0AAR0_	

Allele: DRB1_0101. Number of high binders 0. Number of weak binders 0. Number of peptides 32

Allele	pos	peptide	core	Of	1-log50k(aff)	affinity(nM)	%Rank	Relia	Identity	Bind Level
DRB1_0101	1	GPYNSIRVLFSRHTT	IRVLFSRHT	5	0.7787	11.0	3.00	0.58	tr_D0AAR0_	WB
DRB1_0101	2	PYNSIRVLFSRHTTG	IRVLFSRHT	4	0.7739	11.5	3.50	0.70	tr_D0AAR0_	WB
DRB1_0101	3	YNSIRVLFSRHTTGL	IRVLFSRHT	3	0.7709	11.9	3.50	0.85	tr_D0AAR0_	WB
DRB1_0101	4	MGPYNSIRVLFSRHT	IRVLFSRHT	6	0.7539	14.3	4.50	0.45	tr_D0AAR0_	WB

DRB1_0101	5	RENTFNPLATLALMG	FNPLATLAL	4	0.7426	16.2	5.50	0.78	tr_D0AAR0_	WB
DRB1_0101	6	ATLALMGPYNSIRVL	LALMGPYNS	2	0.7357	17.4	6.00	0.58	tr_D0AAR0_	WB
DRB1_0101	7	LATLALMGPYNSIRV	LALMGPYNS	3	0.7316	18.2	6.00	0.63	tr_D0AAR0_	WB
DRB1_0101	8	ENTFNPLATLALMGP	FNPLATLAL	3	0.7285	18.9	6.50	0.80	tr_D0AAR0_	WB
DRB1_0101	9	TLALMGPYNSIRVLF	LALMGPYNS	1	0.7176	21.2	7.50	0.56	tr_D0AAR0_	WB
DRB1_0101	10	DRENTFNPLATLALM	FNPLATLAL	5	0.7152	21.8	7.50	0.78	tr_D0AAR0_	WB
DRB1_0101	11	PLATLALMGPYNSIR	LALMGPYNS	4	0.7136	22.2	7.50	0.62	tr_D0AAR0_	WB
DRB1_0101	12	NTFNPLATLALMGPY	FNPLATLAL	2	0.7108	22.8	8.00	0.80	tr_D0AAR0_	WB
DRB1_0101	13	LALMGPYNSIRVLFS	YNSIRVLFS	6	0.6959	26.9	9.50	0.29	tr_D0AAR0_	WB
DRB1_0101	14	NPLATLALMGPYNSI	LALMGPYNS	5	0.6697	35.7	13.00	0.58	tr_D0AAR0_	
DRB1_0101	15	ALMGPYNSIRVLFSR	YNSIRVLFS	5	0.6680	36.3	13.00	0.56	tr_D0AAR0_	
DRB1_0101	16	LMGPYNSIRVLFSRH	YNSIRVLFS	4	0.6658	37.2	13.00	0.68	tr_D0AAR0_	
DRB1_0101	17	TFNPLATLALMGPYN	FNPLATLAL	1	0.6629	38.4	13.00	0.74	tr_D0AAR0_	
DRB1_0101	18	FNPLATLALMGPYNS	LALMGPYNS	6	0.6534	42.5	15.00	0.31	tr_D0AAR0_	
DRB1_0101	19	ADRENTFNPLATLAL	FNPLATLAL	6	0.6152	64.3	20.00	0.69	tr_D0AAR0_	
DRB1_0101	20	CADRENTFNPLATLA	NTFNPLATL	5	0.3556	1066.6	75.00	0.39	tr_D0AAR0_	
DRB1_0101	21	KDHKAEMKVELFTEF	KAEMKVELF	3	0.3479	1158.7	75.00	0.40	tr_D0AAR0_	
DRB1_0101	22	YVKDHKAEMKVELFT	KAEMKVELF	5	0.3456	1189.1	75.00	0.29	tr_D0AAR0_	
DRB1_0101	23	HKAEMKVELFTEFCA	KAEMKVELF	1	0.3361	1317.3	75.00	0.29	tr_D0AAR0_	
DRB1_0101	24	PYVKDHKAEMKVELF	VKDHKAEMK	2	0.3327	1366.2	80.00	0.22	tr_D0AAR0_	
DRB1_0101	25	VKDHKAEMKVELFTE	KAEMKVELF	4	0.3317	1381.6	80.00	0.34	tr_D0AAR0_	
DRB1_0101	26	DHKAEMKVELFTEFC	KAEMKVELF	2	0.3211	1550.0	80.00	0.31	tr_D0AAR0_	
DRB1_0101	27	FCADRENTFNPLATL	NTFNPLATL	6	0.3132	1687.6	80.00	0.31	tr_D0AAR0_	
DRB1_0101	28	KAEMKVELFTEFCAD	MKVELFTEF	3	0.3091	1764.3	80.00	0.33	tr_D0AAR0_	
DRB1_0101	29	VELFTEFCADRENTF	FTEFCADRE	3	0.2908	2151.0	85.00	0.47	tr_D0AAR0_	
DRB1_0101	30	MKVELFTEFCADREN	FTEFCADRE	5	0.2895	2179.7	85.00	0.32	tr_D0AAR0_	
DRB1_0101	31	EMKVELFTEFCADRE	KVELFTEFC	2	0.2879	2217.8	85.00	0.24	tr_D0AAR0_	
DRB1_0101	32	ELFTEFCADRENTFN	FTEFCADRE	2	0.2872	2235.4	85.00	0.42	tr_D0AAR0_	
DRB1_0101	33	PPYVKDHKAEMKVEL	VKDHKAEMK	3	0.2870	2240.9	85.00	0.33	tr_D0AAR0_	
DRB1_0101	34	AEMKVELFTEFCADR	KVELFTEFC	3	0.2848	2294.9	85.00	0.25	tr_D0AAR0_	
DRB1_0101	35	KVELFTEFCADRENT	FTEFCADRE	4	0.2811	2388.7	85.00	0.47	tr_D0AAR0_	
DRB1_0101	36	IPPYVKDHKAEMKVE	VKDHKAEMK	4	0.2618	2944.3	90.00	0.35	tr_D0AAR0_	
DRB1_0101	37	LFTEFCADRENTFNP	FTEFCADRE	1	0.2610	2969.2	90.00	0.41	tr_D0AAR0_	
DRB1_0101	38	FTEFCADRENTFNPL	FCADRENTF	3	0.2514	3295.0	90.00	0.48	tr_D0AAR0_	
DRB1_0101	39	EFCADRENTFNPLAT	DRENTFNPL	4	0.2425	3626.3	90.00	0.36	tr_D0AAR0_	
DRB1_0101	40	EIPPYVKDHKAEMKV	VKDHKAEMK	5	0.2413	3673.6	90.00	0.37	tr_D0AAR0_	
DRB1_0101	41	TEFCADRENTFNPLA	FCADRENTF	2	0.2348	3940.3	95.00	0.45	tr_D0AAR0_	

Allele: DRB1_0101. Number of high binders 0. Number of weak binders 13. Number of peptides 41

Allele	pos	peptide	core	Of	1-log50k(aff)	affinity(nM)	%Rank	Relia	Identity	Bind Level
DRB1_0101	1	ARPTFHSVPSLAADG	FHSVPSLAA	4	0.8207	7.0	1.30	0.82	tr_D0A893_	WB
DRB1_0101	2	RPTFHSVPSLAADGP	FHSVPSLAA	3	0.8138	7.5	1.50	0.83	tr_D0A893_	WB
DRB1_0101	3	YARPTFHSVPSLAAD	FHSVPSLAA	5	0.7978	8.9	2.50	0.79	tr_D0A893_	WB
DRB1_0101	4	IFMLFLPLCGSLWAT	FLPLCGSLW	4	0.7952	9.2	2.50	0.56	tr_D0A893_	WB
DRB1_0101	5	LIFMLFLPLCGSLWA	FLPLCGSLW	5	0.7900	9.7	2.50	0.53	tr_D0A893_	WB

DRB1_0101	6	FMLFLPLCGSLWATV	FLPLCGSLW	3	0.7894	9.8	2.50	0.59	tr_D0A893_	WB
DRB1_0101	7	PTFHSVPSLAADGPS	FHSVPSLAA	2	0.7842	10.3	3.00	0.83	tr_D0A893_	WB
DRB1_0101	8	KLIFMLFLPLCGSLW	FLPLCGSLW	6	0.7599	13.4	4.50	0.47	tr_D0A893_	WB
DRB1_0101	9	MLFLPLCGSLWATVY	FLPLCGSLW	2	0.7533	14.4	4.50	0.62	tr_D0A893_	WB
DRB1_0101	10	GYARPTFHSVPSLAA	FHSVPSLAA	6	0.7398	16.7	5.50	0.71	tr_D0A893_	WB
DRB1_0101	11	TFHSVPSLAADGPSP	FHSVPSLAA	1	0.7346	17.7	6.00	0.86	tr_D0A893_	WB
DRB1_0101	12	LFLPLCGSLWATVYS	FLPLCGSLW	1	0.6805	31.7	11.00	0.69	tr_D0A893_	
DRB1_0101	13	RRWCSACGIHRPLRA	WCSACGIHR	2	0.6378	50.3	17.00	0.60	tr_D0A893_	
DRB1_0101	14	MKLIFMLFLPLCGSL	MLFLPLCGS	5	0.6243	58.3	19.00	0.54	tr_D0A893_	
DRB1_0101	15	FHSVPSLAADGPSPR	FHSVPSLAA	0	0.6211	60.3	19.00	0.79	tr_D0A893_	
DRB1_0101	16	RWCSACGIHRPLRAA	WCSACGIHR	1	0.6194	61.5	19.00	0.44	tr_D0A893_	
DRB1_0101	17	ERRWCSACGIHRPLR	WCSACGIHR	3	0.6190	61.7	19.00	0.73	tr_D0A893_	
DRB1_0101	18	SACGIHRPLRAAHCY	CGIHRPLRA	2	0.6011	74.9	22.00	0.43	tr_D0A893_	
DRB1_0101	19	ACGIHRPLRAAHCYF	HRPLRAAHC	4	0.5962	79.0	23.00	0.37	tr_D0A893_	
DRB1_0101	20	FLPLCGSLWATVYSD	FLPLCGSLW	0	0.5941	80.8	23.00	0.52	tr_D0A893_	
DRB1_0101	21	PHLWHNMKLIFMLFL	WHNMKLIFM	3	0.5920	82.6	24.00	0.47	tr_D0A893_	
DRB1_0101	22	WCSACGIHRPLRAAH	CGIHRPLRA	4	0.5887	85.7	24.00	0.46	tr_D0A893_	
DRB1_0101	23	VERRWCSACGIHRPL	WCSACGIHR	4	0.5885	85.9	24.00	0.72	tr_D0A893_	
DRB1_0101	24	EPHLWHNMKLIFMLF	WHNMKLIFM	4	0.5833	90.8	25.00	0.46	tr_D0A893_	
DRB1_0101	25	CSACGIHRPLRAAHC	CGIHRPLRA	3	0.5802	93.9	26.00	0.48	tr_D0A893_	
DRB1_0101	26	CGIHRPLRAAHCYFC	HRPLRAAHC	3	0.5639	112.0	29.00	0.44	tr_D0A893_	
DRB1_0101	27	NMKLIFMLFLPLCGS	MLFLPLCGS	6	0.5624	113.8	29.00	0.46	tr_D0A893_	
DRB1_0101	28	HLWHNMKLIFMLFLP	WHNMKLIFM	2	0.5576	120.0	30.00	0.44	tr_D0A893_	
DRB1_0101	29	GEPHLWHNMKLIFML	WHNMKLIFM	5	0.5573	120.3	30.00	0.46	tr_D0A893_	
DRB1_0101	30	QDHHCGVIGVCVGRR	HCGVIGVCV	3	0.5567	121.0	30.00	0.59	tr_D0A893_	
DRB1_0101	31	PGYARPTFHSVPSLA	YARPTFHSV	2	0.5512	128.5	31.00	0.41	tr_D0A893_	
DRB1_0101	32	DHHCGVIGVCVGRRN	HCGVIGVCV	2	0.5471	134.4	32.00	0.54	tr_D0A893_	
DRB1_0101	33	SVERRWCSACGIHRP	WCSACGIHR	5	0.5311	159.6	35.00	0.68	tr_D0A893_	
DRB1_0101	34	RAAHCYFCGMCVNEQ	HCYFCGMCV	3	0.5276	165.8	36.00	0.62	tr_D0A893_	
DRB1_0101	35	AAHCYFCGMCVNEQD	HCYFCGMCV	2	0.5157	188.7	38.00	0.56	tr_D0A893_	
DRB1_0101	36	EQDHHCGVIGVCVGR	HCGVIGVCV	4	0.5151	189.9	38.00	0.61	tr_D0A893_	
DRB1_0101	37	AHCYFCGMCVNEQDH	HCYFCGMCV	1	0.5081	204.8	39.00	0.46	tr_D0A893_	
DRB1_0101	38	LWHNMKLIFMLFLPL	WHNMKLIFM	1	0.5044	213.1	40.00	0.40	tr_D0A893_	
DRB1_0101	39	DPGYARPTFHSVPSL	YARPTFHSV	3	0.5036	215.1	40.00	0.54	tr_D0A893_	
DRB1_0101	40	VGEPHLWHNMKLIFM	WHNMKLIFM	6	0.5016	219.7	41.00	0.40	tr_D0A893_	
DRB1_0101	41	LRAAHCYFCGMCVNE	HCYFCGMCV	4	0.4986	227.1	41.00	0.68	tr_D0A893_	
DRB1_0101	42	GIHRPLRAAHCYFCG	HRPLRAAHC	2	0.4985	227.3	41.00	0.55	tr_D0A893_	
DRB1_0101	43	PLRAAHCYFCGMCVN	HCYFCGMCV	5	0.4938	239.0	42.00	0.66	tr_D0A893_	
DRB1_0101	44	QSVERRWCSACGIHR	WCSACGIHR	6	0.4817	272.6	45.00	0.55	tr_D0A893_	
DRB1_0101	45	HCYFCGMCVNEQDHH	FCGMCVNEQ	3	0.4722	302.1	47.00	0.48	tr_D0A893_	
DRB1_0101	46	SDPGYARPTFHSVPS	YARPTFHSV	4	0.4676	317.5	48.00	0.50	tr_D0A893_	
DRB1_0101	47	IHRPLRAAHCYFCGM	HRPLRAAHC	1	0.4664	321.6	48.00	0.49	tr_D0A893_	
DRB1_0101	48	NEQDHHCGVIGVCVG	HCGVIGVCV	5	0.4654	325.1	48.00	0.60	tr_D0A893_	
DRB1_0101	49	WHNMKLIFMLFLPLC	HNMKLIFML	1	0.4638	330.8	49.00	0.26	tr_D0A893_	
DRB1_0101	50	RPLRAAHCYFCGMCV	HCYFCGMCV	6	0.4569	356.6	50.00	0.52	tr_D0A893_	
DRB1_0101	51	SSWEDVNGQSVERRW	WEDVNGQSV	2	0.4358	447.8	55.00	0.64	tr_D0A893_	
DRB1_0101	52	YSDPGYARPTFHSVP	YARPTFHSV	5	0.4352	450.7	55.00	0.48	tr_D0A893_	
DRB1_0101	53	ESSWEDVNGQSVERR	WEDVNGQSV	3	0.4287	483.4	60.00	0.73	tr_D0A893_	
DRB1_0101	54	HVVGEPHLWHNMKLI	HLWHNMKLI	6	0.4262	496.9	60.00	0.22	tr_D0A893_	
DRB1_0101	55	HNMKLIFMLFLPLCG	LIFMLFLPL	4	0.4203	529.5	60.00	0.23	tr_D0A893_	

DRB1_0101	56	SWEDVNGQSVERRWC	WEDVNGQSV	1	0.4107	587.5	60.00	0.56	tr_D0A893_
DRB1_0101	57	VYSDPGYARPTFHSV	YARPTFHSV	6	0.4080	605.1	65.00	0.35	tr_D0A893_
DRB1_0101	58	VVGEPHLWHNMKLIF	HLWHNMKLI	5	0.4058	619.6	65.00	0.32	tr_D0A893_
DRB1_0101	59	GSLWATVYSDPGYAR	WATVYSDPG	3	0.3946	699.6	65.00	0.55	tr_D0A893_
DRB1_0101	60	SLWATVYSDPGYARP	WATVYSDPG	2	0.3897	737.6	65.00	0.52	tr_D0A893_
DRB1_0101	61	HRPLRAAHCYFCGMC	LRAAHCYFC	3	0.3895	738.8	65.00	0.37	tr_D0A893_
DRB1_0101	62	RESSWEDVNGQSVER	WEDVNGQSV	4	0.3877	753.8	65.00	0.77	tr_D0A893_
DRB1_0101	63	LPLCGSLWATVYSDP	CGSLWATVY	3	0.3864	764.7	65.00	0.39	tr_D0A893_
DRB1_0101	64	CYFCGMCVNEQDHHC	FCGMCVNEQ	2	0.3855	772.1	65.00	0.65	tr_D0A893_
DRB1_0101	65	VNEQDHHCGVIGVCV	HCGVIGVCV	6	0.3840	784.6	70.00	0.48	tr_D0A893_
DRB1_0101	66	LWATVYSDPGYARPT	WATVYSDPG	1	0.3735	879.2	70.00	0.41	tr_D0A893_
DRB1_0101	67	CGSLWATVYSDPGYA	WATVYSDPG	4	0.3609	1006.7	70.00	0.56	tr_D0A893_
DRB1_0101	68	PLCGSLWATVYSDPG	CGSLWATVY	2	0.3602	1014.6	70.00	0.37	tr_D0A893_
DRB1_0101	69	YFCGMCVNEQDHHCG	FCGMCVNEQ	1	0.3578	1042.1	75.00	0.62	tr_D0A893_
DRB1_0101	70	WEDVNGQSVERRWCS	WEDVNGQSV	0	0.3551	1072.7	75.00	0.40	tr_D0A893_
DRB1_0101	71	LCGSLWATVYSDPGY	WATVYSDPG	5	0.3472	1168.3	75.00	0.40	tr_D0A893_
DRB1_0101	72	ERESSWEDVNGQSVE	WEDVNGQSV	5	0.3387	1280.2	75.00	0.73	tr_D0A893_
DRB1_0101	73	WATVYSDPGYARPTF	TVYSDPGYA	2	0.3313	1387.2	80.00	0.32	tr_D0A893_
DRB1_0101	74	FCGMCVNEQDHHCGV	FCGMCVNEQ	0	0.3038	1868.3	85.00	0.45	tr_D0A893_
DRB1_0101	75	TVYSDPGYARPTFHS	GYARPTFHS	6	0.2955	2043.4	85.00	0.26	tr_D0A893_
DRB1_0101	76	GQSVERRWCSACGIH	RRWCSACGI	5	0.2945	2065.6	85.00	0.46	tr_D0A893_
DRB1_0101	77	ATVYSDPGYARPTFH	TVYSDPGYA	1	0.2895	2180.6	85.00	0.31	tr_D0A893_
DRB1_0101	78	EDVNGQSVERRWCSA	VNGQSVERR	2	0.2745	2565.5	90.00	0.54	tr_D0A893_
DRB1_0101	79	QERESSWEDVNGQSV	WEDVNGQSV	6	0.2738	2583.5	90.00	0.63	tr_D0A893_
DRB1_0101	80	MCVNEQDHHCGVIGV	CVNEQDHHC	1	0.2662	2805.8	90.00	0.31	tr_D0A893_
DRB1_0101	81	HSVPSLAADGPSPRE	LAADGPSPR	5	0.2658	2819.0	90.00	0.35	tr_D0A893_
DRB1_0101	82	CVNEQDHHCGVIGVC	DHHCGVIGV	5	0.2560	3135.0	90.00	0.28	tr_D0A893_
DRB1_0101	83	SVPSLAADGPSPREV	LAADGPSPR	4	0.2558	3141.2	90.00	0.47	tr_D0A893_
DRB1_0101	84	DVNGQSVERRWCSAC	VNGQSVERR	1	0.2535	3220.8	90.00	0.47	tr_D0A893_
DRB1_0101	85	NGQSVERRWCSACGI	RRWCSACGI	6	0.2476	3432.4	90.00	0.38	tr_D0A893_
DRB1_0101	86	VPSLAADGPSPREVE	LAADGPSPR	3	0.2444	3554.3	90.00	0.54	tr_D0A893_
DRB1_0101	87	GMCVNEQDHHCGVIG	CVNEQDHHC	2	0.2423	3636.1	90.00	0.40	tr_D0A893_
DRB1_0101	88	CGMCVNEQDHHCGVI	CVNEQDHHC	3	0.2322	4052.4	95.00	0.45	tr_D0A893_
DRB1_0101	89	PSLAADGPSPREVEG	LAADGPSPR	2	0.2256	4351.6	95.00	0.56	tr_D0A893_
DRB1_0101	90	VNGQSVERRWCSACG	VNGQSVERR	0	0.2219	4532.7	95.00	0.31	tr_D0A893_
DRB1_0101	91	SLAADGPSPREVEGG	LAADGPSPR	1	0.1949	6066.9	95.00	0.56	tr_D0A893_
DRB1_0101	92	GGVQQERESSWEDVN	VQQERESSW	2	0.1590	8953.9	100.00	0.44	tr_D0A893_
DRB1_0101	93	EGGVQQERESSWEDV	VQQERESSW	3	0.1519	9664.9	100.00	0.50	tr_D0A893_
DRB1_0101	94	GVQQERESSWEDVNG	VQQERESSW	1	0.1506	9802.3	100.00	0.38	tr_D0A893_
DRB1_0101	95	VQQERESSWEDVNGQ	ERESSWEDV	3	0.1439	10536.6	100.00	0.34	tr_D0A893_
DRB1_0101	96	EVEGGVQQERESSWE	VQQERESSW	5	0.1370	11356.4	100.00	0.44	tr_D0A893_
DRB1_0101	97	REVEGGVQQERESSW	VQQERESSW	6	0.1370	11356.5	100.00	0.28	tr_D0A893_
DRB1_0101	98	QQERESSWEDVNGQS	SSWEDVNGQ	5	0.1347	11644.8	100.00	0.30	tr_D0A893_
DRB1_0101	99	VEGGVQQERESSWED	VQQERESSW	4	0.1345	11663.3	100.00	0.47	tr_D0A893_
DRB1_0101	100	SPREVEGGVQQERES	REVEGGVQQ	2	0.1314	12068.0	100.00	0.33	tr_D0A893_
DRB1_0101	101	PSPREVEGGVQQERE	REVEGGVQQ	3	0.1295	12317.4	100.00	0.36	tr_D0A893_
DRB1_0101	102	PREVEGGVQQERESS	VEGGVQQER	3	0.1255	12865.1	100.00	0.31	tr_D0A893_
DRB1_0101	103	GPSPREVEGGVQQER	REVEGGVQQ	4	0.1246	12983.1	100.00	0.38	tr_D0A893_
DRB1_0101	104	LAADGPSPREVEGGV	LAADGPSPR	0	0.1162	14227.5	100.00	0.44	tr_D0A893_
DRB1_0101	105	DGPSPREVEGGVQQE	REVEGGVQQ	5	0.0982	17282.3	100.00	0.39	tr_D0A893_

DRB1_0101	106	ADGPSPREVEGGVQQ	PREVEGGVQ	5	0.0875	19407.5	100.00	0.29	tr_D0A893_
DRB1_0101	107	AADGPSPREVEGGVQ	ADGPSPREV	1	0.0771	21708.2	100.00	0.27	tr_D0A893_

Allele: DRB1_0101. Number of high binders 0. Number of weak binders 11. Number of peptides 107

Allele	pos	peptide	core	Of	1-log50k(aff)	affinity(nM)	%Rank	Relia	Identity	Bind Level
DRB1_0101	1	PDVMAYLNGVHVPKV	MAYLNGVHV	3	0.7997	8.7	2.50	0.80	tr_C9ZY92_	WB
DRB1_0101	2	DVMAYLNGVHVPKVM	MAYLNGVHV	2	0.7956	9.1	2.50	0.76	tr_C9ZY92_	WB
DRB1_0101	3	IPDVMAYLNGVHVPK	MAYLNGVHV	4	0.7812	10.7	3.00	0.81	tr_C9ZY92_	WB
DRB1_0101	4	VMAYLNGVHVPKVMT	MAYLNGVHV	1	0.7785	11.0	3.00	0.71	tr_C9ZY92_	WB
DRB1_0101	5	GIPDVMAYLNGVHVP	MAYLNGVHV	5	0.7445	15.9	5.50	0.79	tr_C9ZY92_	WB
DRB1_0101	6	MAYLNGVHVPKVMTL	MAYLNGVHV	0	0.7315	18.3	6.00	0.60	tr_C9ZY92_	WB
DRB1_0101	7	GGIPDVMAYLNGVHV	MAYLNGVHV	6	0.7003	25.6	9.00	0.75	tr_C9ZY92_	WB
DRB1_0101	8	HVPKVMTLRTFVVKS	VMTLRTFVV	4	0.6503	44.0	15.00	0.76	tr_C9ZY92_	
DRB1_0101	9	VPKVMTLRTFVVKSI	VMTLRTFVV	3	0.6480	45.1	15.00	0.77	tr_C9ZY92_	
DRB1_0101	10	VHVPKVMTLRTFVVK	VMTLRTFVV	5	0.6334	52.8	17.00	0.69	tr_C9ZY92_	
DRB1_0101	11	GVHVPKVMTLRTFVV	VMTLRTFVV	6	0.5802	93.8	26.00	0.56	tr_C9ZY92_	
DRB1_0101	12	AYLNGVHVPKVMTLR	LNGVHVPKV	2	0.5300	161.6	35.00	0.41	tr_C9ZY92_	
DRB1_0101	13	YLNGVHVPKVMTLRT	LNGVHVPKV	1	0.4655	324.9	48.00	0.36	tr_C9ZY92_	
DRB1_0101	14	NGVHVPKVMTLRTFV	HVPKVMTLR	3	0.4401	427.5	55.00	0.25	tr_C9ZY92_	
DRB1_0101	15	LNGVHVPKVMTLRTF	LNGVHVPKV	0	0.4354	449.6	55.00	0.22	tr_C9ZY92_	
DRB1_0101	16	GGGIPDVMAYLNGVH	DVMAYLNGV	5	0.4275	490.1	60.00	0.41	tr_C9ZY92_	
DRB1_0101	17	AGGGIPDVMAYLNGV	GIPDVMAYL	3	0.3823	799.0	70.00	0.35	tr_C9ZY92_	
DRB1_0101	18	AAGGGIPDVMAYLNG	GIPDVMAYL	4	0.3123	1704.7	80.00	0.45	tr_C9ZY92_	
DRB1_0101	19	GAAGGGIPDVMAYLN	GIPDVMAYL	5	0.2844	2305.4	85.00	0.44	tr_C9ZY92_	
DRB1_0101	20	YFEPGAAGGGIPDVM	FEPGAAGGG	1	0.2686	2734.3	90.00	0.64	tr_C9ZY92_	
DRB1_0101	21	PGAAGGGIPDVMAYL	GIPDVMAYL	6	0.2414	3668.1	90.00	0.33	tr_C9ZY92_	
DRB1_0101	22	FEPGAAGGGIPDVMA	FEPGAAGGG	0	0.2010	5683.6	95.00	0.37	tr_C9ZY92_	
DRB1_0101	23	EPGAAGGGIPDVMAY	GGGIPDVMA	5	0.1758	7461.3	100.00	0.22	tr_C9ZY92_	

Allele: DRB1_0101. Number of high binders 0. Number of weak binders 7. Number of peptides 23

Allele	pos	peptide	core	Of	1-log50k(aff)	affinity(nM)	%Rank	Relia	Identity	Bind Level
DRB1_0101	1	NNTFSPLATLVLGSE	FSPLATLVL	3	0.7233	20.0	7.00	0.78	tr_C9ZY92_	WB
DRB1_0101	2	ENNTFSPLATLVLGS	FSPLATLVL	4	0.7208	20.5	7.00	0.77	tr_C9ZY92_	WB
DRB1_0101	3	SENNTFSPLATLVLG	FSPLATLVL	5	0.7004	25.6	9.00	0.73	tr_C9ZY92_	WB
DRB1_0101	4	NTFSPLATLVLGSER	FSPLATLVL	2	0.6923	27.9	10.00	0.81	tr_C9ZY92_	
DRB1_0101	5	RDTIRHLFSRQTIWQ	IRHLFSRQT	3	0.6753	33.6	12.00	0.81	tr_C9ZY92_	
DRB1_0101	6	DTIRHLFSRQTIWQY	IRHLFSRQT	2	0.6525	42.9	15.00	0.75	tr_C9ZY92_	
DRB1_0101	7	ERDTIRHLFSRQTIW	IRHLFSRQT	4	0.6504	43.9	15.00	0.82	tr_C9ZY92_	
DRB1_0101	8	TIRHLFSRQTIWQYH	IRHLFSRQT	1	0.6441	47.0	16.00	0.69	tr_C9ZY92_	
DRB1_0101	9	CSENNTFSPLATLVL	FSPLATLVL	6	0.6421	48.0	16.00	0.64	tr_C9ZY92_	

DRB1_0101	10	TFSPLATLVLGSERD	FSPLATLVL	1	0.6125	66.2	20.00	0.79	tr_C9ZY92_
DRB1_0101	11	SERDTIRHLFSRQTI	IRHLFSRQT	5	0.6099	68.1	21.00	0.81	tr_C9ZY92_
DRB1_0101	12	IRHLFSRQTIWQYHP	IRHLFSRQT	0	0.5516	127.9	31.00	0.50	tr_C9ZY92_
DRB1_0101	13	FSPLATLVLGSERDT	FSPLATLVL	0	0.5252	170.2	36.00	0.55	tr_C9ZY92_
DRB1_0101	14	GSERDTIRHLFSRQT	IRHLFSRQT	6	0.5157	188.7	38.00	0.70	tr_C9ZY92_
DRB1_0101	15	LATLVLGSERDTIRH	TLVLGSERD	2	0.4922	243.4	43.00	0.44	tr_C9ZY92_
DRB1_0101	16	PLATLVLGSERDTIR	TLVLGSERD	3	0.4817	272.7	45.00	0.48	tr_C9ZY92_
DRB1_0101	17	TSLFTATCSENNTFS	FTATCSENN	3	0.4769	287.0	46.00	0.85	tr_C9ZY92_
DRB1_0101	18	ATLVLGSERDTIRHL	TLVLGSERD	1	0.4705	307.6	47.00	0.42	tr_C9ZY92_
DRB1_0101	19	TTSLFTATCSENNTF	FTATCSENN	4	0.4571	355.5	50.00	0.81	tr_C9ZY92_
DRB1_0101	20	SLFTATCSENNTFSP	FTATCSENN	2	0.4463	399.7	55.00	0.86	tr_C9ZY92_
DRB1_0101	21	SPLATLVLGSERDTI	TLVLGSERD	4	0.4264	496.0	60.00	0.49	tr_C9ZY92_
DRB1_0101	22	TLVLGSERDTIRHLF	LVLGSERDT	1	0.4175	546.0	60.00	0.30	tr_C9ZY92_
DRB1_0101	23	NTTSLFTATCSENNT	FTATCSENN	5	0.4120	579.6	60.00	0.69	tr_C9ZY92_
DRB1_0101	24	TCSENNTFSPLATLV	NTFSPLATL	5	0.4030	638.5	65.00	0.40	tr_C9ZY92_
DRB1_0101	25	LFTATCSENNTFSPL	FTATCSENN	1	0.4008	654.2	65.00	0.79	tr_C9ZY92_
DRB1_0101	26	RHLFSRQTIWQYHPV	FSRQTIWQY	3	0.3962	687.3	65.00	0.46	tr_C9ZY92_
DRB1_0101	27	ENTTSLFTATCSENN	FTATCSENN	6	0.3312	1388.3	80.00	0.47	tr_C9ZY92_
DRB1_0101	28	VLVWGTENTTSLFTA	WGTENTTSL	3	0.3255	1477.2	80.00	0.38	tr_C9ZY92_
DRB1_0101	29	ATCSENNTFSPLATL	NTFSPLATL	6	0.3199	1569.9	80.00	0.35	tr_C9ZY92_
DRB1_0101	30	LVLGSERDTIRHLFS	LGSERDTIR	2	0.3185	1592.9	80.00	0.22	tr_C9ZY92_
DRB1_0101	31	DVLVWGTENTTSLFT	WGTENTTSL	4	0.3114	1720.4	80.00	0.34	tr_C9ZY92_
DRB1_0101	32	LGSERDTIRHLFSRQ	SERDTIRHL	2	0.3101	1744.7	80.00	0.29	tr_C9ZY92_
DRB1_0101	33	FTATCSENNTFSPLA	FTATCSENN	0	0.3027	1889.8	85.00	0.53	tr_C9ZY92_
DRB1_0101	34	GDVLVWGTENTTSLF	LVWGTENTT	3	0.3012	1921.7	85.00	0.40	tr_C9ZY92_
DRB1_0101	35	LVWGTENTTSLFTAT	WGTENTTSL	2	0.3004	1937.6	85.00	0.38	tr_C9ZY92_
DRB1_0101	36	VLGSERDTIRHLFSR	SERDTIRHL	3	0.2957	2038.6	85.00	0.34	tr_C9ZY92_
DRB1_0101	37	VWGTENTTSLFTATC	WGTENTTSL	1	0.2622	2930.4	90.00	0.34	tr_C9ZY92_
DRB1_0101	38	RGDVLVWGTENTTSL	LVWGTENTT	4	0.2573	3090.6	90.00	0.40	tr_C9ZY92_
DRB1_0101	39	PFQDMEDRGDVLVWG	DMEDRGDVL	3	0.2554	3155.0	90.00	0.36	tr_C9ZY92_
DRB1_0101	40	WGTENTTSLFTATCS	TTSLFTATC	5	0.2517	3284.4	90.00	0.23	tr_C9ZY92_
DRB1_0101	41	FQDMEDRGDVLVWGT	DMEDRGDVL	2	0.2508	3316.0	90.00	0.33	tr_C9ZY92_
DRB1_0101	42	APFQDMEDRGDVLVW	DMEDRGDVL	4	0.2459	3496.1	90.00	0.36	tr_C9ZY92_
DRB1_0101	43	TENTTSLFTATCSEN	TTSLFTATC	3	0.2348	3939.8	95.00	0.34	tr_C9ZY92_
DRB1_0101	44	GTENTTSLFTATCSE	TTSLFTATC	4	0.2302	4144.6	95.00	0.32	tr_C9ZY92_
DRB1_0101	45	CAPFQDMEDRGDVLV	FQDMEDRGD	3	0.2197	4639.7	95.00	0.33	tr_C9ZY92_
DRB1_0101	46	QDMEDRGDVLVWGTE	EDRGDVLVW	3	0.2190	4674.5	95.00	0.25	tr_C9ZY92_
DRB1_0101	47	TATCSENNTFSPLAT	SENNTFSPL	4	0.2013	5660.3	95.00	0.29	tr_C9ZY92_
DRB1_0101	48	DRGDVLVWGTENTTS	LVWGTENTT	5	0.1972	5918.9	95.00	0.42	tr_C9ZY92_
DRB1_0101	49	DMEDRGDVLVWGTEN	EDRGDVLVW	2	0.1899	6405.2	100.00	0.28	tr_C9ZY92_
DRB1_0101	50	GCAPFQDMEDRGDVL	FQDMEDRGD	4	0.1731	7681.1	100.00	0.41	tr_C9ZY92_
DRB1_0101	51	EDRGDVLVWGTENTT	DVLVWGTEN	4	0.1701	7935.8	100.00	0.26	tr_C9ZY92_
DRB1_0101	52	MEDRGDVLVWGTENT	EDRGDVLVW	1	0.1669	8217.9	100.00	0.25	tr_C9ZY92_
DRB1_0101	53	SGCAPFQDMEDRGDV	FQDMEDRGD	5	0.1433	10602.9	100.00	0.40	tr_C9ZY92_

Allele: DRB1_0101. Number of high binders 0. Number of weak binders 3. Number of peptides 53

Allele	pos	peptide	core	Of	1-log50k(aff)	affinity(nM)	%Rank	Relia	Identity	Bind Level
DRB1_0101	1	STAFVRIMGASISVT	FVRIMGASI	3	0.8925	3.2	0.12	0.60	tr_D0A9V0_	SB
DRB1_0101	2	TAFVRIMGASISVTW	FVRIMGASI	2	0.8909	3.3	0.15	0.52	tr_D0A9V0_	SB
DRB1_0101	3	SSTAFVRIMGASISV	FVRIMGASI	4	0.8829	3.6	0.20	0.63	tr_D0A9V0_	SB
DRB1_0101	4	AFVRIMGASISVTWN	FVRIMGASI	1	0.8743	3.9	0.30	0.49	tr_D0A9V0_	SB
DRB1_0101	5	ESSTAFVRIMGASIS	FVRIMGASI	5	0.8524	4.9	0.60	0.72	tr_D0A9V0_	WB
DRB1_0101	6	FVRIMGASISVTWNA	VRIMGASIS	1	0.8355	5.9	0.90	0.52	tr_D0A9V0_	WB
DRB1_0101	7	LADGVMLRGTALYMA	GVMLRGTAL	3	0.8326	6.1	1.00	0.61	tr_D0A9V0_	WB
DRB1_0101	8	ADGVMLRGTALYMAP	GVMLRGTAL	2	0.8294	6.3	1.10	0.59	tr_D0A9V0_	WB
DRB1_0101	9	IVEVRKIQGHYAKLR	VRKIQGHYA	3	0.8229	6.8	1.30	0.88	tr_D0A9V0_	WB
DRB1_0101	10	DGVMLRGTALYMAPE	GVMLRGTAL	1	0.8217	6.9	1.30	0.52	tr_D0A9V0_	WB
DRB1_0101	11	VEVRKIQGHYAKLRR	VRKIQGHYA	2	0.8206	7.0	1.30	0.80	tr_D0A9V0_	WB
DRB1_0101	12	RALISFGAHAALPMH	FGAHAALPM	5	0.8116	7.7	1.60	0.54	tr_D0A9V0_	WB
DRB1_0101	13	ESLFKMYAPSSSSSL	FKMYAPSSS	3	0.8081	8.0	1.70	0.46	tr_D0A9V0_	WB
DRB1_0101	14	SKQFRLGFAAMRQGR	FRLGFAAMR	3	0.8064	8.1	1.80	0.79	tr_D0A9V0_	WB
DRB1_0101	15	DRALISFGAHAALPM	LISFGAHAA	3	0.8052	8.2	1.90	0.35	tr_D0A9V0_	WB
DRB1_0101	16	ISKQFRLGFAAMRQG	FRLGFAAMR	4	0.8026	8.5	1.90	0.73	tr_D0A9V0_	WB
DRB1_0101	17	HYAKLRRQLAVLLKF	LRRQLAVLL	4	0.8025	8.5	2.00	0.81	tr_D0A9V0_	WB
DRB1_0101	18	EVRKIQGHYAKLRRQ	VRKIQGHYA	1	0.8019	8.5	2.00	0.76	tr_D0A9V0_	WB
DRB1_0101	19	YAKLRRQLAVLLKFI	LRRQLAVLL	3	0.8003	8.7	2.00	0.85	tr_D0A9V0_	WB
DRB1_0101	20	SLFKMYAPSSSSSLD	FKMYAPSSS	2	0.8000	8.7	2.00	0.44	tr_D0A9V0_	WB
DRB1_0101	21	ALISFGAHAALPMHC	FGAHAALPM	4	0.7996	8.7	2.50	0.62	tr_D0A9V0_	WB
DRB1_0101	22	SESLFKMYAPSSSSS	LFKMYAPSS	3	0.7994	8.8	2.50	0.57	tr_D0A9V0_	WB
DRB1_0101	23	LQGLNYLHNLGIVHC	LNYLHNLGI	3	0.7990	8.8	2.50	0.51	tr_D0A9V0_	WB
DRB1_0101	24	LISFGAHAALPMHCR	FGAHAALPM	3	0.7983	8.9	2.50	0.70	tr_D0A9V0_	WB
DRB1_0101	25	DPDRALISFGAHAAL	DRALISFGA	2	0.7964	9.1	2.50	0.48	tr_D0A9V0_	WB
DRB1_0101	26	PDRALISFGAHAALP	DRALISFGA	1	0.7963	9.1	2.50	0.44	tr_D0A9V0_	WB
DRB1_0101	27	GVMLRGTALYMAPEV	MLRGTALYM	2	0.7963	9.1	2.50	0.42	tr_D0A9V0_	WB
DRB1_0101	28	RIVEVRKIQGHYAKL	VRKIQGHYA	4	0.7958	9.1	2.50	0.90	tr_D0A9V0_	WB
DRB1_0101	29	TLADGVMLRGTALYM	GVMLRGTAL	4	0.7957	9.1	2.50	0.70	tr_D0A9V0_	WB
DRB1_0101	30	QGLNYLHNLGIVHCD	YLHNLGIVH	4	0.7943	9.3	2.50	0.44	tr_D0A9V0_	WB
DRB1_0101	31	SISKQFRLGFAAMRQ	FRLGFAAMR	5	0.7939	9.3	2.50	0.72	tr_D0A9V0_	WB
DRB1_0101	32	GHYAKLRRQLAVLLK	LRRQLAVLL	5	0.7915	9.5	2.50	0.79	tr_D0A9V0_	WB
DRB1_0101	33	KQFRLGFAAMRQGRY	FRLGFAAMR	2	0.7844	10.3	3.00	0.77	tr_D0A9V0_	WB
DRB1_0101	34	GLNYLHNLGIVHCDV	YLHNLGIVH	3	0.7829	10.5	3.00	0.50	tr_D0A9V0_	WB
DRB1_0101	35	ILQGLNYLHNLGIVH	LNYLHNLGI	4	0.7805	10.7	3.00	0.57	tr_D0A9V0_	WB
DRB1_0101	36	GESSTAFVRIMGASI	FVRIMGASI	6	0.7764	11.2	3.50	0.89	tr_D0A9V0_	WB
DRB1_0101	37	GSESLFKMYAPSSSS	LFKMYAPSS	4	0.7754	11.4	3.50	0.61	tr_D0A9V0_	WB
DRB1_0101	38	LDPDRALISFGAHAA	DRALISFGA	3	0.7702	12.0	3.50	0.63	tr_D0A9V0_	WB
DRB1_0101	39	RDLLFVQSLALGHLE	FVQSLALGH	4	0.7678	12.3	4.00	0.56	tr_D0A9V0_	WB
DRB1_0101	40	ISFGAHAALPMHCRR	FGAHAALPM	2	0.7660	12.6	4.00	0.75	tr_D0A9V0_	WB
DRB1_0101	41	AKLRRQLAVLLKFIP	LRRQLAVLL	2	0.7657	12.6	4.00	0.88	tr_D0A9V0_	WB
DRB1_0101	42	NRDLLFVQSLALGHL	FVQSLALGH	5	0.7612	13.2	4.00	0.50	tr_D0A9V0_	WB
DRB1_0101	43	LSISKQFRLGFAAMR	FRLGFAAMR	6	0.7496	15.0	5.00	0.64	tr_D0A9V0_	WB
DRB1_0101	44	AVLLKFIPEAILARV	LKFIPEAIL	3	0.7492	15.1	5.00	0.57	tr_D0A9V0_	WB
DRB1_0101	45	LNYLHNLGIVHCDVK	YLHNLGIVH	2	0.7488	15.1	5.00	0.58	tr_D0A9V0_	WB
DRB1_0101	46	MLLVSALKSASNLEI	VSALKSASN	3	0.7488	15.2	5.00	0.64	tr_D0A9V0_	WB
DRB1_0101	47	LFKMYAPSSSSSLDG	FKMYAPSSS	1	0.7454	15.7	5.00	0.40	tr_D0A9V0_	WB
DRB1_0101	48	ASHMQGFLAFPVDCV	FLAFPVDCV	6	0.7439	16.0	5.50	0.35	tr_D0A9V0_	WB

DRB1_0101	49	DLLFVQSLALGHLEP	FVQSLALGH	3	0.7438	16.0	5.50	0.58	tr_D0A9V0_	WB
DRB1_0101	50	RTLADGVMLRGTALY	GVMLRGTAL	5	0.7436	16.0	5.50	0.77	tr_D0A9V0_	WB
DRB1_0101	51	SRIVEVRKIQGHYAK	VRKIQGHYA	5	0.7430	16.1	5.50	0.89	tr_D0A9V0_	WB
DRB1_0101	52	VRKIQGHYAKLRRQL	VRKIQGHYA	0	0.7428	16.2	5.50	0.64	tr_D0A9V0_	WB
DRB1_0101	53	QFRLGFAAMRQGRYG	FRLGFAAMR	1	0.7406	16.6	5.50	0.71	tr_D0A9V0_	WB
DRB1_0101	54	HMQGFLAFPVDCVLL	FLAFPVDCV	4	0.7391	16.8	5.50	0.65	tr_D0A9V0_	WB
DRB1_0101	55	IMEYVPGGSLHSVIK	YVPGGSLHS	3	0.7388	16.9	5.50	0.76	tr_D0A9V0_	WB
DRB1_0101	56	IIMEYVPGGSLHSVI	YVPGGSLHS	4	0.7359	17.4	6.00	0.65	tr_D0A9V0_	WB
DRB1_0101	57	LMLLVSALKSASNLE	VSALKSASN	4	0.7356	17.5	6.00	0.59	tr_D0A9V0_	WB
DRB1_0101	58	VLLKFIPEAILARVE	LKFIPEAIL	2	0.7350	17.6	6.00	0.57	tr_D0A9V0_	WB
DRB1_0101	59	DILQGLNYLHNLGIV	LNYLHNLGI	5	0.7341	17.8	6.00	0.64	tr_D0A9V0_	WB
DRB1_0101	60	LLFVQSLALGHLEPV	FVQSLALGH	2	0.7334	17.9	6.00	0.61	tr_D0A9V0_	WB
DRB1_0101	61	SHMQGFLAFPVDCVL	FLAFPVDCV	5	0.7323	18.1	6.00	0.56	tr_D0A9V0_	WB
DRB1_0101	62	LAVLLKFIPEAILAR	LKFIPEAIL	4	0.7313	18.3	6.00	0.56	tr_D0A9V0_	WB
DRB1_0101	63	NADIFLGRSRIVEVR	IFLGRSRIV	3	0.7311	18.4	6.50	0.77	tr_D0A9V0_	WB
DRB1_0101	64	SFGAHAALPMHCRRC	FGAHAALPM	1	0.7310	18.4	6.50	0.72	tr_D0A9V0_	WB
DRB1_0101	65	GGASHMQGFLAFPVD	ASHMQGFLA	2	0.7305	18.5	6.50	0.53	tr_D0A9V0_	WB
DRB1_0101	66	QGHYAKLRRQLAVLL	LRRQLAVLL	6	0.7297	18.6	6.50	0.65	tr_D0A9V0_	WB
DRB1_0101	67	GADHLFQLSYAVRDG	FQLSYAVRD	5	0.7280	19.0	6.50	0.41	tr_D0A9V0_	WB
DRB1_0101	68	SGGASHMQGFLAFPV	ASHMQGFLA	3	0.7272	19.1	6.50	0.62	tr_D0A9V0_	WB
DRB1_0101	69	PLMLLVSALKSASNL	VSALKSASN	5	0.7234	19.9	7.00	0.50	tr_D0A9V0_	WB
DRB1_0101	70	SIIMEYVPGGSLHSV	YVPGGSLHS	5	0.7232	20.0	7.00	0.50	tr_D0A9V0_	WB
DRB1_0101	71	QGRYGEAMAHYLPMV	YGEAMAHYL	3	0.7230	20.0	7.00	0.86	tr_D0A9V0_	WB
DRB1_0101	72	KLRRQLAVLLKFIPE	LRRQLAVLL	1	0.7230	20.0	7.00	0.86	tr_D0A9V0_	WB
DRB1_0101	73	GASHMQGFLAFPVDC	ASHMQGFLA	1	0.7194	20.8	7.50	0.49	tr_D0A9V0_	WB
DRB1_0101	74	ADHLFQLSYAVRDGP	FQLSYAVRD	4	0.7182	21.1	7.50	0.41	tr_D0A9V0_	WB
DRB1_0101	75	RYGEAMAHYLPMVKM	YGEAMAHYL	1	0.7173	21.3	7.50	0.62	tr_D0A9V0_	WB
DRB1_0101	76	SLALGHLEPVIPASV	GHLEPVIPA	4	0.7156	21.7	7.50	0.56	tr_D0A9V0_	WB
DRB1_0101	77	ATRLRSLFNRDLLFV	LRSLFNRDL	3	0.7153	21.8	7.50	0.77	tr_D0A9V0_	WB
DRB1_0101	78	QGSESLFKMYAPSSS	LFKMYAPSS	5	0.7146	21.9	7.50	0.69	tr_D0A9V0_	WB
DRB1_0101	79	LALGHLEPVIPASVD	GHLEPVIPA	3	0.7129	22.3	8.00	0.52	tr_D0A9V0_	WB
DRB1_0101	80	LLVSALKSASNLEIP	VSALKSASN	2	0.7098	23.1	8.00	0.65	tr_D0A9V0_	WB
DRB1_0101	81	MQGFLAFPVDCVLLP	FLAFPVDCV	3	0.7090	23.3	8.00	0.70	tr_D0A9V0_	WB
DRB1_0101	82	LLKFIPEAILARVES	LKFIPEAIL	1	0.7088	23.4	8.00	0.55	tr_D0A9V0_	WB
DRB1_0101	83	TRLRSLFNRDLLFVQ	LRSLFNRDL	2	0.7069	23.8	8.50	0.72	tr_D0A9V0_	WB
DRB1_0101	84	ADIFLGRSRIVEVRK	IFLGRSRIV	2	0.7063	24.0	8.50	0.77	tr_D0A9V0_	WB
DRB1_0101	85	MEYVPGGSLHSVIKN	YVPGGSLHS	2	0.7062	24.0	8.50	0.81	tr_D0A9V0_	WB
DRB1_0101	86	STKYHAALIEVATEH	YHAALIEVA	3	0.7048	24.4	8.50	0.83	tr_D0A9V0_	WB
DRB1_0101	87	GRYGEAMAHYLPMVK	YGEAMAHYL	2	0.7038	24.6	8.50	0.81	tr_D0A9V0_	WB
DRB1_0101	88	DNADIFLGRSRIVEV	IFLGRSRIV	4	0.7036	24.7	8.50	0.76	tr_D0A9V0_	WB
DRB1_0101	89	FNRDLLFVQSLALGH	FVQSLALGH	6	0.7010	25.4	9.00	0.40	tr_D0A9V0_	WB
DRB1_0101	90	ALDPDRALISFGAHA	DRALISFGA	4	0.7000	25.7	9.00	0.75	tr_D0A9V0_	WB
DRB1_0101	91	LFVQSLALGHLEPVI	FVQSLALGH	1	0.6988	26.0	9.00	0.56	tr_D0A9V0_	WB
DRB1_0101	92	RQGRYGEAMAHYLPM	YGEAMAHYL	4	0.6963	26.7	9.50	0.85	tr_D0A9V0_	WB
DRB1_0101	93	VDILQGLNYLHNLGI	LNYLHNLGI	6	0.6959	26.8	9.50	0.41	tr_D0A9V0_	WB
DRB1_0101	94	ALGHLEPVIPASVDH	GHLEPVIPA	2	0.6956	26.9	9.50	0.50	tr_D0A9V0_	WB
DRB1_0101	95	SSTKYHAALIEVATE	YHAALIEVA	4	0.6947	27.2	9.50	0.77	tr_D0A9V0_	WB
DRB1_0101	96	DHLFQLSYAVRDGPC	FQLSYAVRD	3	0.6929	27.7	9.50	0.48	tr_D0A9V0_	WB
DRB1_0101	97	NPSMRPSASSLLSYA	MRPSASSLL	3	0.6923	27.9	10.00	0.91	tr_D0A9V0_	
DRB1_0101	98	FGADHLFQLSYAVRD	HLFQLSYAV	4	0.6908	28.4	10.00	0.49	tr_D0A9V0_	

DRB1_0101	99	AMAHYLPMVKMDMQA	AHYLPMVKM	2	0.6901	28.6	10.00	0.46	tr_D0A9V0_
DRB1_0101	100	DATRLRSLFNRDLLF	LRSLFNRDL	4	0.6875	29.4	11.00	0.81	tr_D0A9V0_
DRB1_0101	101	ISLLEMLLGRPPWQW	LEMLLGRPP	3	0.6873	29.5	11.00	0.71	tr_D0A9V0_
DRB1_0101	102	KSSTKYHAALIEVAT	YHAALIEVA	5	0.6870	29.6	11.00	0.72	tr_D0A9V0_
DRB1_0101	103	QLAVLLKFIPEAILA	LKFIPEAIL	5	0.6853	30.1	11.00	0.61	tr_D0A9V0_
DRB1_0101	104	ARTLADGVMLRGTAL	GVMLRGTAL	6	0.6846	30.3	11.00	0.64	tr_D0A9V0_
DRB1_0101	105	SLLEMLLGRPPWQWS	LEMLLGRPP	2	0.6843	30.4	11.00	0.68	tr_D0A9V0_
DRB1_0101	106	NCGIFKEKPNVFNRR	IFKEKPNVF	3	0.6841	30.5	11.00	0.49	tr_D0A9V0_
DRB1_0101	107	EAMAHYLPMVKMDMQ	AHYLPMVKM	3	0.6815	31.4	11.00	0.50	tr_D0A9V0_
DRB1_0101	108	RLRSLFNRDLLFVQS	LRSLFNRDL	1	0.6802	31.8	11.00	0.67	tr_D0A9V0_
DRB1_0101	109	MAHYLPMVKMDMQAK	YLPMVKMDM	3	0.6781	32.5	12.00	0.47	tr_D0A9V0_
DRB1_0101	110	CGIFKEKPNVFNRRH	FKEKPNVFN	3	0.6769	33.0	12.00	0.48	tr_D0A9V0_
DRB1_0101	111	TKYHAALIEVATEHS	YHAALIEVA	2	0.6730	34.4	12.00	0.84	tr_D0A9V0_
DRB1_0101	112	VRIMGASISVTWNAG	VRIMGASIS	0	0.6725	34.6	12.00	0.70	tr_D0A9V0_
DRB1_0101	113	GTALYMAPEVAAGGR	LYMAPEVAA	3	0.6705	35.3	12.00	0.50	tr_D0A9V0_
DRB1_0101	114	PSMRPSASSLLSYAF	MRPSASSLL	2	0.6701	35.5	13.00	0.86	tr_D0A9V0_
DRB1_0101	115	QSLALGHLEPVIPAS	GHLEPVIPA	5	0.6685	36.1	13.00	0.54	tr_D0A9V0_
DRB1_0101	116	PDNADIFLGRSRIVE	IFLGRSRIV	5	0.6668	36.8	13.00	0.74	tr_D0A9V0_
DRB1_0101	117	LSIIMEYVPGGSLHS	IIMEYVPGG	2	0.6649	37.5	13.00	0.36	tr_D0A9V0_
DRB1_0101	118	DIFLGRSRIVEVRKI	IFLGRSRIV	1	0.6648	37.6	13.00	0.71	tr_D0A9V0_
DRB1_0101	119	TSGGASHMQGFLAFP	ASHMQGFLA	4	0.6646	37.7	13.00	0.75	tr_D0A9V0_
DRB1_0101	120	KPLMLLVSALKSASN	MLLVSALKS	3	0.6646	37.7	13.00	0.35	tr_D0A9V0_
DRB1_0101	121	LLEMLLGRPPWQWSS	LEMLLGRPP	1	0.6627	38.5	13.00	0.62	tr_D0A9V0_
DRB1_0101	122	IKNFGCVSPYVARRF	FGCVSPYVA	3	0.6619	38.8	14.00	0.75	tr_D0A9V0_
DRB1_0101	123	GISLLEMLLGRPPWQ	LEMLLGRPP	4	0.6611	39.1	14.00	0.71	tr_D0A9V0_
DRB1_0101	124	ATSGGASHMQGFLAF	ASHMQGFLA	5	0.6603	39.5	14.00	0.76	tr_D0A9V0_
DRB1_0101	125	MNPSMRPSASSLLSY	MRPSASSLL	4	0.6602	39.5	14.00	0.90	tr_D0A9V0_
DRB1_0101	126	RGTALYMAPEVAAGG	LYMAPEVAA	4	0.6580	40.4	14.00	0.44	tr_D0A9V0_
DRB1_0101	127	VMLRGTALYMAPEVA	MLRGTALYM	1	0.6577	40.6	14.00	0.64	tr_D0A9V0_
DRB1_0101	128	NYLHNLGIVHCDVKP	YLHNLGIVH	1	0.6552	41.7	14.00	0.75	tr_D0A9V0_
DRB1_0101	129	MRQGRYGEAMAHYLP	YGEAMAHYL	5	0.6550	41.8	14.00	0.85	tr_D0A9V0_
DRB1_0101	130	YGEAMAHYLPMVKMD	AHYLPMVKM	5	0.6549	41.8	14.00	0.44	tr_D0A9V0_
DRB1_0101	131	LGHLEPVIPASVDHE	GHLEPVIPA	1	0.6547	42.0	14.00	0.47	tr_D0A9V0_
DRB1_0101	132	TALYMAPEVAAGGRC	LYMAPEVAA	2	0.6545	42.0	14.00	0.49	tr_D0A9V0_
DRB1_0101	133	RPPWQWSSTAPAGSD	WQWSSTAPA	3	0.6542	42.1	14.00	0.83	tr_D0A9V0_
DRB1_0101	134	QGFLAFPVDCVLLPC	FLAFPVDCV	2	0.6541	42.2	15.00	0.80	tr_D0A9V0_
DRB1_0101	135	LFGADHLFQLSYAVR	HLFQLSYAV	5	0.6541	42.2	15.00	0.53	tr_D0A9V0_
DRB1_0101	136	FTVDILQGLNYLHNL	ILQGLNYLH	4	0.6529	42.8	15.00	0.50	tr_D0A9V0_
DRB1_0101	137	GEAMAHYLPMVKMDM	AHYLPMVKM	4	0.6529	42.8	15.00	0.51	tr_D0A9V0_
DRB1_0101	138	VIKNFGCVSPYVARR	FGCVSPYVA	4	0.6500	44.1	15.00	0.71	tr_D0A9V0_
DRB1_0101	139	PMVKMDMQAKRLMRL	KMDMQAKRL	3	0.6500	44.1	15.00	0.46	tr_D0A9V0_
DRB1_0101	140	LVSALKSASNLEIPD	VSALKSASN	1	0.6490	44.6	15.00	0.67	tr_D0A9V0_
DRB1_0101	141	YVSCSITPSHLSIIM	CSITPSHLS	3	0.6489	44.7	15.00	0.56	tr_D0A9V0_
DRB1_0101	142	QFAFDLFAKLPLDHR	FDLFAKLPL	3	0.6488	44.7	15.00	0.40	tr_D0A9V0_
DRB1_0101	143	FRLGFAAMRQGRYGE	FRLGFAAMR	0	0.6487	44.8	15.00	0.54	tr_D0A9V0_
DRB1_0101	144	KNFGCVSPYVARRFT	FGCVSPYVA	2	0.6473	45.4	15.00	0.74	tr_D0A9V0_
DRB1_0101	145	GRPPWQWSSTAPAGS	WQWSSTAPA	4	0.6459	46.1	16.00	0.88	tr_D0A9V0_
DRB1_0101	146	SDATRLRSLFNRDLL	LRSLFNRDL	5	0.6457	46.2	16.00	0.81	tr_D0A9V0_
DRB1_0101	147	HLFQLSYAVRDGPCR	FQLSYAVRD	2	0.6452	46.5	16.00	0.58	tr_D0A9V0_
DRB1_0101	148	RYVSCSITPSHLSII	CSITPSHLS	4	0.6448	46.7	16.00	0.45	tr_D0A9V0_

DRB1_0101	149	FGISLLEMLLGRPPW	LEMLLGRPP	5	0.6434	47.4	16.00	0.63	tr_D0A9V0_
DRB1_0101	150	EYVPGGSLHSVIKNF	YVPGGSLHS	1	0.6429	47.7	16.00	0.84	tr_D0A9V0_
DRB1_0101	151	TVDILQGLNYLHNLG	ILQGLNYLH	3	0.6426	47.8	16.00	0.55	tr_D0A9V0_
DRB1_0101	152	LKFIPEAILARVESK	FIPEAILAR	2	0.6425	47.9	16.00	0.37	tr_D0A9V0_
DRB1_0101	153	LNCGIFKEKPNVFNR	IFKEKPNVF	4	0.6421	48.0	16.00	0.49	tr_D0A9V0_
DRB1_0101	154	FKMYAPSSSSSLDGS	YAPSSSSSL	3	0.6411	48.6	16.00	0.56	tr_D0A9V0_
DRB1_0101	155	GIFKEKPNVFNRRHC	FKEKPNVFN	2	0.6404	49.0	16.00	0.52	tr_D0A9V0_
DRB1_0101	156	SMRPSASSLLSYAFL	MRPSASSLL	1	0.6402	49.1	16.00	0.75	tr_D0A9V0_
DRB1_0101	157	EALDPDRALISFGAH	DRALISFGA	5	0.6396	49.4	16.00	0.75	tr_D0A9V0_
DRB1_0101	158	RFTVDILQGLNYLHN	ILQGLNYLH	5	0.6395	49.4	16.00	0.40	tr_D0A9V0_
DRB1_0101	159	LRGTALYMAPEVAAG	LYMAPEVAA	5	0.6379	50.3	17.00	0.45	tr_D0A9V0_
DRB1_0101	160	AHYLPMVKMDMQAKR	YLPMVKMDM	2	0.6379	50.3	17.00	0.52	tr_D0A9V0_
DRB1_0101	161	PPWQWSSTAPAGSDA	WQWSSTAPA	2	0.6364	51.1	17.00	0.74	tr_D0A9V0_
DRB1_0101	162	FGAHAALPMHCRRCC	FGAHAALPM	0	0.6334	52.8	17.00	0.61	tr_D0A9V0_
DRB1_0101	163	MLRGTALYMAPEVAA	ALYMAPEVA	5	0.6310	54.2	18.00	0.28	tr_D0A9V0_
DRB1_0101	164	VRYVSCSITPSHLSI	CSITPSHLS	5	0.6306	54.4	18.00	0.38	tr_D0A9V0_
DRB1_0101	165	RSRIVEVRKIQGHYA	VRKIQGHYA	6	0.6306	54.4	18.00	0.74	tr_D0A9V0_
DRB1_0101	166	LPMVKMDMQAKRLMR	KMDMQAKRL	4	0.6297	54.9	18.00	0.44	tr_D0A9V0_
DRB1_0101	167	DCVLLPCSTLPIVLF	LLPCSTLPI	3	0.6275	56.3	18.00	0.36	tr_D0A9V0_
DRB1_0101	168	CQFAFDLFAKLPLDH	FAFDLFAKL	2	0.6260	57.2	18.00	0.35	tr_D0A9V0_
DRB1_0101	169	MVKMDMQAKRLMRLC	KMDMQAKRL	2	0.6255	57.5	19.00	0.50	tr_D0A9V0_
DRB1_0101	170	YLPMVKMDMQAKRLM	KMDMQAKRL	5	0.6246	58.1	19.00	0.38	tr_D0A9V0_
DRB1_0101	171	KYHAALIEVATEHSG	YHAALIEVA	1	0.6239	58.5	19.00	0.79	tr_D0A9V0_
DRB1_0101	172	SDIFSFGISLLEMLL	DIFSFGISL	1	0.6236	58.7	19.00	0.33	tr_D0A9V0_
DRB1_0101	173	PWQWSSTAPAGSDAT	WQWSSTAPA	1	0.6218	59.8	19.00	0.67	tr_D0A9V0_
DRB1_0101	174	SVIKNFGCVSPYVAR	FGCVSPYVA	5	0.6213	60.2	19.00	0.64	tr_D0A9V0_
DRB1_0101	175	FAFDLFAKLPLDHRD	FDLFAKLPL	2	0.6213	60.2	19.00	0.38	tr_D0A9V0_
DRB1_0101	176	VSCSITPSHLSIIME	CSITPSHLS	2	0.6183	62.2	20.00	0.57	tr_D0A9V0_
DRB1_0101	177	RQLAVLLKFIPEAIL	LKFIPEAIL	6	0.6168	63.2	20.00	0.52	tr_D0A9V0_
DRB1_0101	178	HLSIIMEYVPGGSLH	IIMEYVPGG	3	0.6162	63.6	20.00	0.52	tr_D0A9V0_
DRB1_0101	179	QSDIFSFGISLLEML	DIFSFGISL	2	0.6160	63.7	20.00	0.40	tr_D0A9V0_
DRB1_0101	180	FVQSLALGHLEPVIP	FVQSLALGH	0	0.6154	64.2	20.00	0.40	tr_D0A9V0_
DRB1_0101	181	WKSSTKYHAALIEVA	YHAALIEVA	6	0.6151	64.4	20.00	0.59	tr_D0A9V0_
DRB1_0101	182	VQSLALGHLEPVIPA	GHLEPVIPA	6	0.6135	65.5	20.00	0.33	tr_D0A9V0_
DRB1_0101	183	HYLPMVKMDMQAKRL	YLPMVKMDM	1	0.6124	66.2	20.00	0.45	tr_D0A9V0_
DRB1_0101	184	CCQFAFDLFAKLPLD	FAFDLFAKL	3	0.6121	66.5	21.00	0.41	tr_D0A9V0_
DRB1_0101	185	SFGISLLEMLLGRPP	LEMLLGRPP	6	0.6111	67.2	21.00	0.47	tr_D0A9V0_
DRB1_0101	186	DIFSFGISLLEMLLG	FGISLLEML	4	0.6105	67.6	21.00	0.31	tr_D0A9V0_
DRB1_0101	187	VDCVLLPCSTLPIVL	CVLLPCSTL	2	0.6091	68.7	21.00	0.41	tr_D0A9V0_
DRB1_0101	188	NFGCVSPYVARRFTV	FGCVSPYVA	1	0.6088	68.9	21.00	0.67	tr_D0A9V0_
DRB1_0101	189	ASVMARTLADGVMLR	MARTLADGV	3	0.6085	69.1	21.00	0.41	tr_D0A9V0_
DRB1_0101	190	GFLAFPVDCVLLPCS	FLAFPVDCV	1	0.6073	70.0	21.00	0.79	tr_D0A9V0_
DRB1_0101	191	AMRQGRYGEAMAHYL	YGEAMAHYL	6	0.6053	71.5	22.00	0.75	tr_D0A9V0_
DRB1_0101	192	SLSISKQFRLGFAAM	LSISKQFRL	1	0.6040	72.6	22.00	0.31	tr_D0A9V0_
DRB1_0101	193	ALYMAPEVAAGGRCT	LYMAPEVAA	1	0.6034	73.1	22.00	0.45	tr_D0A9V0_
DRB1_0101	194	RCCQFAFDLFAKLPL	FAFDLFAKL	4	0.6032	73.2	22.00	0.45	tr_D0A9V0_
DRB1_0101	195	CVLLPCSTLPIVLFE	LLPCSTLPI	2	0.6030	73.3	22.00	0.40	tr_D0A9V0_
DRB1_0101	196	HMNPSMRPSASSLLS	MRPSASSLL	5	0.6025	73.7	22.00	0.86	tr_D0A9V0_
DRB1_0101	197	SVMARTLADGVMLRG	RTLADGVML	4	0.5995	76.2	23.00	0.43	tr_D0A9V0_
DRB1_0101	198	RRFTVDILQGLNYLH	VDILQGLNY	4	0.5991	76.6	23.00	0.37	tr_D0A9V0_

DRB1_0101	199	IPDNADIFLGRSRIV	IFLGRSRIV	6	0.5975	77.8	23.00	0.71	tr_D0A9V0_
DRB1_0101	200	PVDCVLLPCSTLPIV	CVLLPCSTL	3	0.5945	80.4	23.00	0.46	tr_D0A9V0_
DRB1_0101	201	MRPSASSLLSYAFLL	MRPSASSLL	0	0.5942	80.7	23.00	0.32	tr_D0A9V0_
DRB1_0101	202	SHLSIIMEYVPGGSL	IIMEYVPGG	4	0.5932	81.6	24.00	0.52	tr_D0A9V0_
DRB1_0101	203	KPHNVLLGTDGVCKL	NVLLGTDGV	3	0.5926	82.1	24.00	0.45	tr_D0A9V0_
DRB1_0101	204	IVRYVSCSITPSHLS	YVSCSITPS	3	0.5916	83.0	24.00	0.34	tr_D0A9V0_
DRB1_0101	205	LEMLLGRPPWQWSST	LEMLLGRPP	0	0.5912	83.4	24.00	0.49	tr_D0A9V0_
DRB1_0101	206	LGRPPWQWSSTAPAG	WQWSSTAPA	5	0.5894	85.0	24.00	0.92	tr_D0A9V0_
DRB1_0101	207	VPLFLTDSNRNVWTR	FLTDSNRNV	3	0.5893	85.0	24.00	0.70	tr_D0A9V0_
DRB1_0101	208	VLFGADHLFQLSYAV	HLFQLSYAV	6	0.5877	86.6	25.00	0.43	tr_D0A9V0_
DRB1_0101	209	PHNVLLGTDGVCKLS	NVLLGTDGV	2	0.5863	87.9	25.00	0.37	tr_D0A9V0_
DRB1_0101	210	IFSFGISLLEMLLGR	FGISLLEML	3	0.5844	89.7	25.00	0.40	tr_D0A9V0_
DRB1_0101	211	LRSLFNRDLLFVQSL	LRSLFNRDL	0	0.5837	90.4	25.00	0.54	tr_D0A9V0_
DRB1_0101	212	SCSITPSHLSIIMEY	CSITPSHLS	1	0.5836	90.5	25.00	0.53	tr_D0A9V0_
DRB1_0101	213	LRRQLAVLLKFIPEA	LRRQLAVLL	0	0.5833	90.8	25.00	0.66	tr_D0A9V0_
DRB1_0101	214	VMARTLADGVMLRGT	RTLADGVML	3	0.5810	93.1	26.00	0.51	tr_D0A9V0_
DRB1_0101	215	DQGSESLFKMYAPSS	LFKMYAPSS	6	0.5809	93.2	26.00	0.79	tr_D0A9V0_
DRB1_0101	216	LFNRDLLFVQSLALG	LLFVQSLAL	5	0.5809	93.2	26.00	0.61	tr_D0A9V0_
DRB1_0101	217	AFDLFAKLPLDHRDS	FDLFAKLPL	1	0.5802	93.8	26.00	0.38	tr_D0A9V0_
DRB1_0101	218	GHLEPVIPASVDHEA	LEPVIPASV	2	0.5775	96.7	26.00	0.55	tr_D0A9V0_
DRB1_0101	219	IFLGRSRIVEVRKIQ	IFLGRSRIV	0	0.5753	99.0	27.00	0.53	tr_D0A9V0_
DRB1_0101	220	TIFLGVAEDGAQVAI	FLGVAEDGA	2	0.5747	99.6	27.00	0.38	tr_D0A9V0_
DRB1_0101	221	VKMDMQAKRLMRLCR	KMDMQAKRL	1	0.5716	103.0	27.00	0.44	tr_D0A9V0_
DRB1_0101	222	SLKKISEGAFSTIFL	KISEGAFST	3	0.5714	103.2	27.00	0.59	tr_D0A9V0_
DRB1_0101	223	PQSDIFSFGISLLEM	DIFSFGISL	3	0.5703	104.5	28.00	0.47	tr_D0A9V0_
DRB1_0101	224	PASVDHEAAAFVRAC	VDHEAAAFV	3	0.5700	104.9	28.00	0.65	tr_D0A9V0_
DRB1_0101	225	STIFLGVAEDGAQVA	FLGVAEDGA	3	0.5697	105.1	28.00	0.41	tr_D0A9V0_
DRB1_0101	226	IPASVDHEAAAFVRA	VDHEAAAFV	4	0.5694	105.6	28.00	0.65	tr_D0A9V0_
DRB1_0101	227	PLFLTDSNRNVWTRS	FLTDSNRNV	2	0.5667	108.7	28.00	0.69	tr_D0A9V0_
DRB1_0101	228	VEALDPDRALISFGA	DRALISFGA	6	0.5657	109.9	28.00	0.52	tr_D0A9V0_
DRB1_0101	229	IFLGVAEDGAQVAIK	GVAEDGAQV	3	0.5628	113.3	29.00	0.41	tr_D0A9V0_
DRB1_0101	230	RSLKKISEGAFSTIF	KISEGAFST	4	0.5614	115.0	29.00	0.47	tr_D0A9V0_
DRB1_0101	231	HNVLLGTDGVCKLSD	LLGTDGVCK	3	0.5609	115.7	29.00	0.41	tr_D0A9V0_
DRB1_0101	232	VSLSISKQFRLGFAA	LSISKQFRL	2	0.5608	115.8	29.00	0.46	tr_D0A9V0_
DRB1_0101	233	EVSLSISKQFRLGFA	LSISKQFRL	3	0.5605	116.2	29.00	0.56	tr_D0A9V0_
DRB1_0101	234	SEVSLSISKQFRLGF	LSISKQFRL	4	0.5593	117.7	30.00	0.48	tr_D0A9V0_
DRB1_0101	235	VSALKSASNLEIPDN	VSALKSASN	0	0.5587	118.5	30.00	0.43	tr_D0A9V0_
DRB1_0101	236	VVPLFLTDSNRNVWT	FLTDSNRNV	4	0.5587	118.5	30.00	0.63	tr_D0A9V0_
DRB1_0101	237	VASKLHHPNIVRYVS	SKLHHPNIV	2	0.5561	121.9	30.00	0.31	tr_D0A9V0_
DRB1_0101	238	ALNCGIFKEKPNVFN	IFKEKPNVF	5	0.5548	123.5	30.00	0.54	tr_D0A9V0_
DRB1_0101	239	ASVDHEAAAFVRACC	VDHEAAAFV	2	0.5543	124.3	30.00	0.63	tr_D0A9V0_
DRB1_0101	240	FPVDCVLLPCSTLPI	CVLLPCSTL	4	0.5536	125.1	31.00	0.54	tr_D0A9V0_
DRB1_0101	241	HSVIKNFGCVSPYVA	FGCVSPYVA	6	0.5533	125.6	31.00	0.47	tr_D0A9V0_
DRB1_0101	242	EASVMARTLADGVML	MARTLADGV	4	0.5507	129.1	31.00	0.44	tr_D0A9V0_
DRB1_0101	243	MARTLADGVMLRGTA	RTLADGVML	2	0.5507	129.2	31.00	0.56	tr_D0A9V0_
DRB1_0101	244	FSFGISLLEMLLGRP	FGISLLEML	2	0.5507	129.2	31.00	0.41	tr_D0A9V0_
DRB1_0101	245	SSEVSLSISKQFRLG	LSISKQFRL	5	0.5505	129.5	31.00	0.37	tr_D0A9V0_
DRB1_0101	246	FSTIFLGVAEDGAQV	IFLGVAEDG	3	0.5502	129.9	31.00	0.38	tr_D0A9V0_
DRB1_0101	247	FDLFAKLPLDHRDSV	FAKLPLDHR	3	0.5500	130.2	31.00	0.43	tr_D0A9V0_
DRB1_0101	248	IFKEKPNVFNRRHCT	FKEKPNVFN	1	0.5496	130.7	31.00	0.64	tr_D0A9V0_

DRB1_0101	249	LKKISEGAFSTIFLG	KISEGAFST	2	0.5466	135.0	32.00	0.52	tr_D0A9V0_
DRB1_0101	250	AAFVRACCHMNPSMR	FVRACCHMN	2	0.5466	135.1	32.00	0.63	tr_D0A9V0_
DRB1_0101	251	NEFLVGTCGAFSRNA	LVGTCGAFS	3	0.5457	136.4	32.00	0.53	tr_D0A9V0_
DRB1_0101	252	GSDATRLRSLFNRDL	LRSLFNRDL	6	0.5455	136.7	32.00	0.73	tr_D0A9V0_
DRB1_0101	253	FGCVSPYVARRFTVD	FGCVSPYVA	0	0.5451	137.3	32.00	0.51	tr_D0A9V0_
DRB1_0101	254	CSITPSHLSIIMEYV	CSITPSHLS	0	0.5448	137.7	32.00	0.31	tr_D0A9V0_
DRB1_0101	255	AATSGGASHMQGFLA	ASHMQGFLA	6	0.5442	138.6	32.00	0.71	tr_D0A9V0_
DRB1_0101	256	WQWSSTAPAGSDATR	WQWSSTAPA	0	0.5441	138.8	32.00	0.54	tr_D0A9V0_
DRB1_0101	257	TNEFLVGTCGAFSRN	LVGTCGAFS	4	0.5441	138.8	32.00	0.47	tr_D0A9V0_
DRB1_0101	258	PSHLSIIMEYVPGGS	IIMEYVPGG	5	0.5437	139.4	32.00	0.48	tr_D0A9V0_
DRB1_0101	259	ARRFTVDILQGLNYL	VDILQGLNY	5	0.5409	143.7	33.00	0.43	tr_D0A9V0_
DRB1_0101	260	RKIQGHYAKLRRQLA	IQGHYAKLR	2	0.5406	144.1	33.00	0.35	tr_D0A9V0_
DRB1_0101	261	IQGHYAKLRRQLAVL	YAKLRRQLA	4	0.5402	144.7	33.00	0.44	tr_D0A9V0_
DRB1_0101	262	ASKLHHPNIVRYVSC	HHPNIVRYV	4	0.5397	145.5	33.00	0.34	tr_D0A9V0_
DRB1_0101	263	FGSTISEASVMARTL	TISEASVMA	3	0.5393	146.2	33.00	0.53	tr_D0A9V0_
DRB1_0101	264	TPQSDIFSFGISLLE	DIFSFGISL	4	0.5388	146.9	33.00	0.44	tr_D0A9V0_
DRB1_0101	265	VKPHNVLLGTDGVCK	NVLLGTDGV	4	0.5384	147.6	33.00	0.44	tr_D0A9V0_
DRB1_0101	266	VPYVRVLDNHYCVNS	VRVLDNHYC	3	0.5355	152.4	34.00	0.59	tr_D0A9V0_
DRB1_0101	267	DFGSTISEASVMART	TISEASVMA	4	0.5349	153.3	34.00	0.48	tr_D0A9V0_
DRB1_0101	268	KIQGHYAKLRRQLAV	YAKLRRQLA	5	0.5349	153.3	34.00	0.35	tr_D0A9V0_
DRB1_0101	269	AFVRACCHMNPSMRP	FVRACCHMN	1	0.5341	154.7	34.00	0.53	tr_D0A9V0_
DRB1_0101	270	FLAFPVDCVLLPCST	FLAFPVDCV	0	0.5302	161.3	35.00	0.66	tr_D0A9V0_
DRB1_0101	271	YVPGGSLHSVIKNFG	YVPGGSLHS	0	0.5297	162.2	35.00	0.63	tr_D0A9V0_
DRB1_0101	272	SDFGSTISEASVMAR	TISEASVMA	5	0.5283	164.7	35.00	0.38	tr_D0A9V0_
DRB1_0101	273	RRCCQFAFDLFAKLP	FAFDLFAKL	5	0.5278	165.5	35.00	0.61	tr_D0A9V0_
DRB1_0101	274	TRSLKKISEGAFSTI	KISEGAFST	5	0.5273	166.4	36.00	0.41	tr_D0A9V0_
DRB1_0101	275	TVPYVRVLDNHYCVN	VRVLDNHYC	4	0.5272	166.5	36.00	0.51	tr_D0A9V0_
DRB1_0101	276	LFLTDSNRNVWTRSL	FLTDSNRNV	1	0.5265	167.8	36.00	0.73	tr_D0A9V0_
DRB1_0101	277	VLLPCSTLPIVLFEL	LLPCSTLPI	1	0.5256	169.5	36.00	0.45	tr_D0A9V0_
DRB1_0101	278	GSTISEASVMARTLA	TISEASVMA	2	0.5253	170.0	36.00	0.50	tr_D0A9V0_
DRB1_0101	279	NIVRYVSCSITPSHL	YVSCSITPS	4	0.5239	172.6	36.00	0.42	tr_D0A9V0_
DRB1_0101	280	EVASKLHHPNIVRYV	SKLHHPNIV	3	0.5238	172.8	36.00	0.38	tr_D0A9V0_
DRB1_0101	281	TCGAFSRNARVLFGA	GAFSRNARV	2	0.5234	173.5	36.00	0.46	tr_D0A9V0_
DRB1_0101	282	ESLEKEMEVASKLHH	KEMEVASKL	4	0.5232	174.0	36.00	0.75	tr_D0A9V0_
DRB1_0101	283	LFQLSYAVRDGPCRI	FQLSYAVRD	1	0.5218	176.7	37.00	0.76	tr_D0A9V0_
DRB1_0101	284	CRRCCQFAFDLFAKL	FAFDLFAKL	6	0.5217	176.9	37.00	0.50	tr_D0A9V0_
DRB1_0101	285	AFSTIFLGVAEDGAQ	IFLGVAEDG	4	0.5212	177.9	37.00	0.35	tr_D0A9V0_
DRB1_0101	286	AAAFVRACCHMNPSM	FVRACCHMN	3	0.5211	178.0	37.00	0.80	tr_D0A9V0_
DRB1_0101	287	RVLFGADHLFQLSYA	FGADHLFQL	3	0.5186	182.9	37.00	0.37	tr_D0A9V0_
DRB1_0101	288	DVVPLFLTDSNRNVW	FLTDSNRNV	5	0.5184	183.2	37.00	0.58	tr_D0A9V0_
DRB1_0101	289	SGCVEALDPDRALIS	VEALDPDRA	3	0.5184	183.3	37.00	0.77	tr_D0A9V0_
DRB1_0101	290	KMYAPSSSSSLDGSG	YAPSSSSSL	2	0.5173	185.4	38.00	0.88	tr_D0A9V0_
DRB1_0101	291	PYVRVLDNHYCVNSS	VRVLDNHYC	2	0.5167	186.7	38.00	0.60	tr_D0A9V0_
DRB1_0101	292	DTNEFLVGTCGAFSR	LVGTCGAFS	5	0.5154	189.2	38.00	0.43	tr_D0A9V0_
DRB1_0101	293	FLGVAEDGAQVAIKC	GVAEDGAQV	2	0.5154	189.4	38.00	0.50	tr_D0A9V0_
DRB1_0101	294	GCVEALDPDRALISF	VEALDPDRA	2	0.5137	192.9	38.00	0.69	tr_D0A9V0_
DRB1_0101	295	KMDMQAKRLMRLCRQ	KMDMQAKRL	0	0.5135	193.2	38.00	0.29	tr_D0A9V0_
DRB1_0101	296	SLFNRDLLFVQSLAL	LLFVQSLAL	6	0.5132	193.8	38.00	0.59	tr_D0A9V0_
DRB1_0101	297	YLHNLGIVHCDVKPH	YLHNLGIVH	0	0.5122	196.0	39.00	0.60	tr_D0A9V0_
DRB1_0101	298	KKISEGAFSTIFLGV	KISEGAFST	1	0.5119	196.6	39.00	0.45	tr_D0A9V0_

DRB1_0101	299	SSSEVSLSISKQFRL	VSLSISKQF	4	0.5114	197.6	39.00	0.41	tr_D0A9V0_
DRB1_0101	300	CHMNPSMRPSASSLL	MRPSASSLL	6	0.5091	202.7	39.00	0.60	tr_D0A9V0_
DRB1_0101	301	ARVLFGADHLFQLSY	FGADHLFQL	4	0.5083	204.3	39.00	0.33	tr_D0A9V0_
DRB1_0101	302	SVDHEAAAFVRACCH	VDHEAAAFV	1	0.5083	204.5	39.00	0.56	tr_D0A9V0_
DRB1_0101	303	SLEKEMEVASKLHHP	KEMEVASKL	3	0.5082	204.6	39.00	0.74	tr_D0A9V0_
DRB1_0101	304	GTCGAFSRNARVLFG	GAFSRNARV	3	0.5077	205.8	39.00	0.51	tr_D0A9V0_
DRB1_0101	305	NARVLFGADHLFQLS	FGADHLFQL	5	0.5062	209.1	40.00	0.26	tr_D0A9V0_
DRB1_0101	306	TPSHLSIIMEYVPGG	HLSIIMEYV	3	0.5061	209.3	40.00	0.50	tr_D0A9V0_
DRB1_0101	307	SKLHHPNIVRYVSCS	HHPNIVRYV	3	0.5053	211.3	40.00	0.46	tr_D0A9V0_
DRB1_0101	308	FNRRHCTVIAIQVYH	HCTVIAIQV	4	0.5050	211.8	40.00	0.44	tr_D0A9V0_
DRB1_0101	309	FVRACCHMNPSMRPS	CCHMNPSMR	4	0.5048	212.2	40.00	0.32	tr_D0A9V0_
DRB1_0101	310	AFPVDCVLLPCSTLP	CVLLPCSTL	5	0.5047	212.6	40.00	0.63	tr_D0A9V0_
DRB1_0101	311	KISEGAFSTIFLGVA	FSTIFLGVA	6	0.5034	215.5	40.00	0.25	tr_D0A9V0_
DRB1_0101	312	NVLLGTDGVCKLSDF	LLGTDGVCK	2	0.5033	215.7	40.00	0.50	tr_D0A9V0_
DRB1_0101	313	CTPQSDIFSFGISLL	DIFSFGISL	5	0.5008	221.6	41.00	0.55	tr_D0A9V0_
DRB1_0101	314	GAFSTIFLGVAEDGA	IFLGVAEDG	5	0.5002	223.1	41.00	0.33	tr_D0A9V0_
DRB1_0101	315	VIPASVDHEAAAFVR	VDHEAAAFV	5	0.4990	226.1	41.00	0.71	tr_D0A9V0_
DRB1_0101	316	LYMAPEVAAGGRCTP	LYMAPEVAA	0	0.4989	226.2	41.00	0.36	tr_D0A9V0_
DRB1_0101	317	VFNRRHCTVIAIQVY	HCTVIAIQV	5	0.4983	227.9	41.00	0.37	tr_D0A9V0_
DRB1_0101	318	NRRHCTVIAIQVYHP	HCTVIAIQV	3	0.4965	232.3	42.00	0.44	tr_D0A9V0_
DRB1_0101	319	RLGFAAMRQGRYGEA	FAAMRQGRY	3	0.4963	232.7	42.00	0.46	tr_D0A9V0_
DRB1_0101	320	LPIVLFELRPGESSS	FELRPGESS	5	0.4960	233.5	42.00	0.39	tr_D0A9V0_
DRB1_0101	321	EFLVGTCGAFSRNAR	LVGTCGAFS	2	0.4938	239.2	42.00	0.57	tr_D0A9V0_
DRB1_0101	322	KFIPEAILARVESKN	FIPEAILAR	1	0.4935	240.0	42.00	0.62	tr_D0A9V0_
DRB1_0101	323	CGAFSRNARVLFGAD	GAFSRNARV	1	0.4927	242.0	43.00	0.40	tr_D0A9V0_
DRB1_0101	324	IVLFELRPGESSSEV	FELRPGESS	3	0.4920	243.7	43.00	0.50	tr_D0A9V0_
DRB1_0101	325	PIVLFELRPGESSSE	FELRPGESS	4	0.4920	243.9	43.00	0.44	tr_D0A9V0_
DRB1_0101	326	CVEALDPDRALISFG	VEALDPDRA	1	0.4905	247.8	43.00	0.61	tr_D0A9V0_
DRB1_0101	327	RRHCTVIAIQVYHPD	HCTVIAIQV	2	0.4904	248.0	43.00	0.38	tr_D0A9V0_
DRB1_0101	328	DTVPYVRVLDNHYCV	VRVLDNHYC	5	0.4902	248.6	43.00	0.48	tr_D0A9V0_
DRB1_0101	329	RACCHMNPSMRPSAS	CHMNPSMRP	3	0.4870	257.3	44.00	0.50	tr_D0A9V0_
DRB1_0101	330	STISEASVMARTLAD	TISEASVMA	1	0.4869	257.7	44.00	0.39	tr_D0A9V0_
DRB1_0101	331	PCRIAATSGGASHMQ	IAATSGGAS	3	0.4865	258.8	44.00	0.49	tr_D0A9V0_
DRB1_0101	332	EAAAFVRACCHMNPS	FVRACCHMN	4	0.4864	259.1	44.00	0.79	tr_D0A9V0_
DRB1_0101	333	RHCTVIAIQVYHPDV	VIAIQVYHP	4	0.4862	259.6	44.00	0.31	tr_D0A9V0_
DRB1_0101	334	RNARVLFGADHLFQL	RVLFGADHL	3	0.4836	267.0	44.00	0.23	tr_D0A9V0_
DRB1_0101	335	SEASVMARTLADGVM	MARTLADGV	5	0.4835	267.4	44.00	0.41	tr_D0A9V0_
DRB1_0101	336	LSDFGSTISEASVMA	FGSTISEAS	3	0.4828	269.4	45.00	0.62	tr_D0A9V0_
DRB1_0101	337	LEKEMEVASKLHHPN	KEMEVASKL	2	0.4825	270.2	45.00	0.73	tr_D0A9V0_
DRB1_0101	338	VRACCHMNPSMRPSA	CHMNPSMRP	4	0.4810	274.6	45.00	0.47	tr_D0A9V0_
DRB1_0101	339	VGTCGAFSRNARVLF	GAFSRNARV	4	0.4800	277.6	45.00	0.56	tr_D0A9V0_
DRB1_0101	340	LAFPVDCVLLPCSTL	CVLLPCSTL	6	0.4800	277.7	45.00	0.58	tr_D0A9V0_
DRB1_0101	341	HSGCVEALDPDRALI	VEALDPDRA	4	0.4788	281.2	45.00	0.81	tr_D0A9V0_
DRB1_0101	342	LLPCSTLPIVLFELR	CSTLPIVLF	3	0.4784	282.6	45.00	0.46	tr_D0A9V0_
DRB1_0101	343	PNIVRYVSCSITPSH	YVSCSITPS	5	0.4784	282.6	46.00	0.38	tr_D0A9V0_
DRB1_0101	344	FLVGTCGAFSRNARV	LVGTCGAFS	1	0.4772	286.3	46.00	0.49	tr_D0A9V0_
DRB1_0101	345	AESLEKEMEVASKLH	KEMEVASKL	5	0.4766	288.0	46.00	0.73	tr_D0A9V0_
DRB1_0101	346	YHAALIEVATEHSGC	YHAALIEVA	0	0.4761	289.6	46.00	0.61	tr_D0A9V0_
DRB1_0101	347	YYRVLDNHYCVNSSD	VRVLDNHYC	1	0.4749	293.2	46.00	0.64	tr_D0A9V0_
DRB1_0101	348	LLGRPPWQWSSTAPA	WQWSSTAPA	6	0.4730	299.6	47.00	0.78	tr_D0A9V0_

DRB1_0101	349	CRIAATSGGASHMQG	IAATSGGAS	2	0.4725	301.1	47.00	0.49	tr_D0A9V0_
DRB1_0101	350	HCTVIAIQVYHPDVW	VIAIQVYHP	3	0.4706	307.4	47.00	0.37	tr_D0A9V0_
DRB1_0101	351	LVGTCGAFSRNARVL	GAFSRNARV	5	0.4705	307.5	47.00	0.44	tr_D0A9V0_
DRB1_0101	352	MEVASKLHHPNIVRY	SKLHHPNIV	4	0.4689	313.0	47.00	0.45	tr_D0A9V0_
DRB1_0101	353	VARRFTVDILQGLNY	FTVDILQGL	4	0.4676	317.4	48.00	0.40	tr_D0A9V0_
DRB1_0101	354	EGAFSTIFLGVAEDG	FSTIFLGVA	3	0.4648	327.3	48.00	0.43	tr_D0A9V0_
DRB1_0101	355	GVCKLSDFGSTISEA	KLSDFGSTI	3	0.4639	330.5	49.00	0.49	tr_D0A9V0_
DRB1_0101	356	GPCRIAATSGGASHM	IAATSGGAS	4	0.4621	337.0	49.00	0.49	tr_D0A9V0_
DRB1_0101	357	DVKPHNVLLGTDGVC	NVLLGTDGV	5	0.4616	338.7	49.00	0.46	tr_D0A9V0_
DRB1_0101	358	LGVAEDGAQVAIKCI	GVAEDGAQV	1	0.4592	347.7	50.00	0.49	tr_D0A9V0_
DRB1_0101	359	ISEGAFSTIFLGVAE	FSTIFLGVA	5	0.4567	357.3	50.00	0.41	tr_D0A9V0_
DRB1_0101	360	ITPSHLSIIMEYVPG	HLSIIMEYV	4	0.4561	359.7	50.00	0.57	tr_D0A9V0_
DRB1_0101	361	SVTMLVDTNEFLVGT	MLVDTNEFL	3	0.4558	360.8	50.00	0.54	tr_D0A9V0_
DRB1_0101	362	VLFELRPGESSSEVS	FELRPGESS	2	0.4556	361.6	50.00	0.56	tr_D0A9V0_
DRB1_0101	363	WTRSLKKISEGAFST	KISEGAFST	6	0.4546	365.3	55.00	0.25	tr_D0A9V0_
DRB1_0101	364	TLPIVLFELRPGESS	LFELRPGES	5	0.4545	365.7	55.00	0.26	tr_D0A9V0_
DRB1_0101	365	HEAAAFVRACCHMNP	FVRACCHMN	5	0.4545	365.7	55.00	0.71	tr_D0A9V0_
DRB1_0101	366	SITPSHLSIIMEYVP	HLSIIMEYV	5	0.4537	368.9	55.00	0.50	tr_D0A9V0_
DRB1_0101	367	DLFAKLPLDHRDSVT	FAKLPLDHR	2	0.4532	371.1	55.00	0.57	tr_D0A9V0_
DRB1_0101	368	VDTNEFLVGTCGAFS	NEFLVGTCG	3	0.4519	376.2	55.00	0.35	tr_D0A9V0_
DRB1_0101	369	KLHHPNIVRYVSCSI	HHPNIVRYV	2	0.4519	376.4	55.00	0.51	tr_D0A9V0_
DRB1_0101	370	CKLSDFGSTISEASV	FGSTISEAS	5	0.4511	379.4	55.00	0.40	tr_D0A9V0_
DRB1_0101	371	EKEMEVASKLHHPNI	KEMEVASKL	1	0.4505	381.9	55.00	0.64	tr_D0A9V0_
DRB1_0101	372	DGVCKLSDFGSTISE	KLSDFGSTI	4	0.4491	387.7	55.00	0.41	tr_D0A9V0_
DRB1_0101	373	VCKLSDFGSTISEAS	KLSDFGSTI	2	0.4490	388.4	55.00	0.49	tr_D0A9V0_
DRB1_0101	374	MYAPSSSSSLDGSGC	YAPSSSSSL	1	0.4480	392.6	55.00	0.87	tr_D0A9V0_
DRB1_0101	375	LGFAAMRQGRYGEAM	FAAMRQGRY	2	0.4478	393.4	55.00	0.43	tr_D0A9V0_
DRB1_0101	376	GAFSRNARVLFGADH	FSRNARVLF	2	0.4473	395.4	55.00	0.29	tr_D0A9V0_
DRB1_0101	377	ACCHMNPSMRPSASS	CHMNPSMRP	2	0.4464	399.3	55.00	0.50	tr_D0A9V0_
DRB1_0101	378	ADVVPLFLTDSNRNV	FLTDSNRNV	6	0.4451	405.2	55.00	0.34	tr_D0A9V0_
DRB1_0101	379	AQVAIKCIPRLRRDV	IKCIPRLRR	4	0.4444	408.3	55.00	0.34	tr_D0A9V0_
DRB1_0101	380	LHSVIKNFGCVSPYV	VIKNFGCVS	3	0.4436	411.8	55.00	0.48	tr_D0A9V0_
DRB1_0101	381	LHHPNIVRYVSCSIT	HHPNIVRYV	1	0.4435	412.0	55.00	0.36	tr_D0A9V0_
DRB1_0101	382	TDGVCKLSDFGSTIS	KLSDFGSTI	5	0.4427	415.4	55.00	0.40	tr_D0A9V0_
DRB1_0101	383	RRQLAVLLKFIPEAI	LLKFIPEAI	6	0.4426	416.1	55.00	0.33	tr_D0A9V0_
DRB1_0101	384	KLSDFGSTISEASVM	FGSTISEAS	4	0.4424	416.9	55.00	0.51	tr_D0A9V0_
DRB1_0101	385	SEGAFSTIFLGVAED	FSTIFLGVA	4	0.4419	419.4	55.00	0.47	tr_D0A9V0_
DRB1_0101	386	VSPYVARRFTVDILQ	PYVARRFTV	2	0.4403	426.6	55.00	0.31	tr_D0A9V0_
DRB1_0101	387	NVFNRRHCTVIAIQV	HCTVIAIQV	6	0.4398	428.8	55.00	0.26	tr_D0A9V0_
DRB1_0101	388	HLEPVIPASVDHEAA	LEPVIPASV	1	0.4391	432.4	55.00	0.82	tr_D0A9V0_
DRB1_0101	389	VWKSSTKYHAALIEV	STKYHAALI	4	0.4385	435.0	55.00	0.41	tr_D0A9V0_
DRB1_0101	390	QVAIKCIPRLRRDVV	IKCIPRLRR	3	0.4383	436.1	55.00	0.37	tr_D0A9V0_
DRB1_0101	391	SLHSVIKNFGCVSPY	VIKNFGCVS	4	0.4377	438.9	55.00	0.44	tr_D0A9V0_
DRB1_0101	392	RSLFNRDLLFVQSLA	FNRDLLFVQ	3	0.4366	444.2	55.00	0.25	tr_D0A9V0_
DRB1_0101	393	CVSPYVARRFTVDIL	PYVARRFTV	3	0.4352	451.0	55.00	0.35	tr_D0A9V0_
DRB1_0101	394	CTVIAIQVYHPDVWK	VIAIQVYHP	2	0.4345	454.4	55.00	0.37	tr_D0A9V0_
DRB1_0101	395	NDTVPYVRVLDNHYC	VRVLDNHYC	6	0.4338	457.4	55.00	0.31	tr_D0A9V0_
DRB1_0101	396	HPNIVRYVSCSITPS	VRYVSCSIT	4	0.4336	458.5	55.00	0.32	tr_D0A9V0_
DRB1_0101	397	DSVTMLVDTNEFLVG	MLVDTNEFL	4	0.4327	463.2	55.00	0.54	tr_D0A9V0_
DRB1_0101	398	CDVKPHNVLLGTDGV	HNVLLGTDG	5	0.4312	470.7	60.00	0.34	tr_D0A9V0_

DRB1_0101	399	VTMLVDTNEFLVGTC	MLVDTNEFL	2	0.4310	471.7	60.00	0.50	tr_D0A9V0_
DRB1_0101	400	DGAQVAIKCIPRLRR	VAIKCIPRL	4	0.4302	475.6	60.00	0.26	tr_D0A9V0_
DRB1_0101	401	KEMEVASKLHHPNIV	KEMEVASKL	0	0.4296	479.1	60.00	0.37	tr_D0A9V0_
DRB1_0101	402	DHEAAAFVRACCHMN	FVRACCHMN	6	0.4295	479.2	60.00	0.51	tr_D0A9V0_
DRB1_0101	403	ISEASVMARTLADGV	SVMARTLAD	4	0.4290	482.2	60.00	0.32	tr_D0A9V0_
DRB1_0101	404	PYVARRFTVDILQGL	FTVDILQGL	6	0.4285	484.5	60.00	0.20	tr_D0A9V0_
DRB1_0101	405	GAQVAIKCIPRLRRD	VAIKCIPRL	3	0.4277	488.7	60.00	0.28	tr_D0A9V0_
DRB1_0101	406	RIAATSGGASHMQGF	IAATSGGAS	1	0.4275	490.1	60.00	0.48	tr_D0A9V0_
DRB1_0101	407	TISEASVMARTLADG	EASVMARTL	3	0.4270	492.8	60.00	0.32	tr_D0A9V0_
DRB1_0101	408	SPYVARRFTVDILQG	YVARRFTVD	2	0.4268	493.7	60.00	0.28	tr_D0A9V0_
DRB1_0101	409	VAIKCIPRLRRDVVA	IKCIPRLRR	2	0.4265	495.2	60.00	0.35	tr_D0A9V0_
DRB1_0101	410	ESSSEVSLSISKQFR	VSLSISKQF	5	0.4263	496.4	60.00	0.41	tr_D0A9V0_
DRB1_0101	411	VALNCGIFKEKPNVF	IFKEKPNVF	6	0.4258	499.1	60.00	0.56	tr_D0A9V0_
DRB1_0101	412	SRNARVLFGADHLFQ	RVLFGADHL	4	0.4253	501.9	60.00	0.32	tr_D0A9V0_
DRB1_0101	413	FLGRSRIVEVRKIQG	SRIVEVRKI	4	0.4237	510.8	60.00	0.63	tr_D0A9V0_
DRB1_0101	414	DVWKSSTKYHAALIE	STKYHAALI	5	0.4236	511.3	60.00	0.36	tr_D0A9V0_
DRB1_0101	415	RCTPQSDIFSFGISL	DIFSFGISL	6	0.4222	518.8	60.00	0.48	tr_D0A9V0_
DRB1_0101	416	LFAKLPLDHRDSVTM	FAKLPLDHR	1	0.4211	525.2	60.00	0.56	tr_D0A9V0_
DRB1_0101	417	GGSLHSVIKNFGCVS	LHSVIKNFG	3	0.4209	526.0	60.00	0.26	tr_D0A9V0_
DRB1_0101	418	GSLHSVIKNFGCVSP	VIKNFGCVS	5	0.4200	531.3	60.00	0.36	tr_D0A9V0_
DRB1_0101	419	SALKSASNLEIPDNA	LKSASNLEI	2	0.4189	537.4	60.00	0.71	tr_D0A9V0_
DRB1_0101	420	EMEVASKLHHPNIVR	SKLHHPNIV	5	0.4185	540.3	60.00	0.44	tr_D0A9V0_
DRB1_0101	421	FQLSYAVRDGPCRIA	FQLSYAVRD	0	0.4178	543.9	60.00	0.50	tr_D0A9V0_
DRB1_0101	422	LGRSRIVEVRKIQGH	SRIVEVRKI	3	0.4149	561.6	60.00	0.80	tr_D0A9V0_
DRB1_0101	423	LPCSTLPIVLFELRP	CSTLPIVLF	2	0.4148	561.9	60.00	0.52	tr_D0A9V0_
DRB1_0101	424	PCSTLPIVLFELRPG	CSTLPIVLF	1	0.4116	582.2	60.00	0.43	tr_D0A9V0_
DRB1_0101	425	MDMQAKRLMRLCRQR	KRLMRLCRQ	5	0.4112	584.6	60.00	0.28	tr_D0A9V0_
DRB1_0101	426	VDHEAAAFVRACCHM	VDHEAAAFV	0	0.4105	589.0	60.00	0.35	tr_D0A9V0_
DRB1_0101	427	QRCRRNDTVPYVRVL	CRRNDTVPY	2	0.4093	596.5	60.00	0.41	tr_D0A9V0_
DRB1_0101	428	PDVWKSSTKYHAALI	WKSSTKYHA	3	0.4088	599.7	60.00	0.38	tr_D0A9V0_
DRB1_0101	429	EHSGCVEALDPDRAL	VEALDPDRA	5	0.4088	600.0	60.00	0.78	tr_D0A9V0_
DRB1_0101	430	CCHMNPSMRPSASSL	CHMNPSMRP	1	0.4087	600.5	60.00	0.43	tr_D0A9V0_
DRB1_0101	431	TVIAIQVYHPDVWKS	VIAIQVYHP	1	0.4076	607.7	65.00	0.29	tr_D0A9V0_
DRB1_0101	432	LFELRPGESSSEVSL	FELRPGESS	1	0.4072	610.2	65.00	0.56	tr_D0A9V0_
DRB1_0101	433	TMLVDTNEFLVGTCG	MLVDTNEFL	1	0.4067	613.4	65.00	0.41	tr_D0A9V0_
DRB1_0101	434	AIKCIPRLRRDVVAE	IKCIPRLRR	1	0.4063	616.1	65.00	0.29	tr_D0A9V0_
DRB1_0101	435	YVARRFTVDILQGLN	FTVDILQGL	5	0.4060	618.3	65.00	0.43	tr_D0A9V0_
DRB1_0101	436	HHPNIVRYVSCSITP	IVRYVSCSI	4	0.4044	629.3	65.00	0.29	tr_D0A9V0_
DRB1_0101	437	GCVSPYVARRFTVDI	PYVARRFTV	4	0.4042	630.4	65.00	0.41	tr_D0A9V0_
DRB1_0101	438	AFSRNARVLFGADHL	SRNARVLFG	2	0.4025	641.9	65.00	0.24	tr_D0A9V0_
DRB1_0101	439	EMLLGRPPWQWSSTA	MLLGRPPWQ	1	0.4017	647.5	65.00	0.50	tr_D0A9V0_
DRB1_0101	440	GFAAMRQGRYGEAMA	FAAMRQGRY	1	0.4006	655.4	65.00	0.40	tr_D0A9V0_
DRB1_0101	441	DGPCRIAATSGGASH	IAATSGGAS	5	0.3993	664.6	65.00	0.44	tr_D0A9V0_
DRB1_0101	442	GVAEDGAQVAIKCIP	GVAEDGAQV	0	0.3983	671.7	65.00	0.37	tr_D0A9V0_
DRB1_0101	443	RCRRNDTVPYVRVLD	NDTVPYVRV	4	0.3980	674.4	65.00	0.31	tr_D0A9V0_
DRB1_0101	444	SNRNVWTRSLKKISE	NVWTRSLKK	3	0.3970	681.2	65.00	0.40	tr_D0A9V0_
DRB1_0101	445	FLTDSNRNVWTRSLK	FLTDSNRNV	0	0.3968	682.8	65.00	0.64	tr_D0A9V0_
DRB1_0101	446	PGESSTAFVRIMGAS	GESSTAFVR	1	0.3964	686.1	65.00	0.31	tr_D0A9V0_
DRB1_0101	447	GRSRIVEVRKIQGHY	SRIVEVRKI	2	0.3948	697.9	65.00	0.76	tr_D0A9V0_
DRB1_0101	448	MQAKRLMRLCRQRCR	KRLMRLCRQ	3	0.3932	710.1	65.00	0.41	tr_D0A9V0_

DRB1_0101	449	QWSSTAPAGSDATRL	WSSTAPAGS	1	0.3928	713.5	65.00	0.62	tr_D0A9V0_
DRB1_0101	450	HCRRCCQFAFDLFAK	RCCQFAFDL	3	0.3909	728.0	65.00	0.31	tr_D0A9V0_
DRB1_0101	451	QQRDVALNCGIFKEK	VALNCGIFK	4	0.3907	729.3	65.00	0.50	tr_D0A9V0_
DRB1_0101	452	FSRNARVLFGADHLF	RVLFGADHL	5	0.3907	729.9	65.00	0.32	tr_D0A9V0_
DRB1_0101	453	STLPIVLFELRPGES	IVLFELRPG	4	0.3901	734.7	65.00	0.35	tr_D0A9V0_
DRB1_0101	454	QAKRLMRLCRQRCRR	KRLMRLCRQ	2	0.3900	734.9	65.00	0.38	tr_D0A9V0_
DRB1_0101	455	IVHCDVKPHNVLLGT	DVKPHNVLL	4	0.3894	740.2	65.00	0.43	tr_D0A9V0_
DRB1_0101	456	CSTLPIVLFELRPGE	IVLFELRPG	5	0.3883	748.4	65.00	0.31	tr_D0A9V0_
DRB1_0101	457	RDSVTMLVDTNEFLV	MLVDTNEFL	5	0.3879	751.7	65.00	0.56	tr_D0A9V0_
DRB1_0101	458	LVDTNEFLVGTCGAF	NEFLVGTCG	4	0.3873	757.3	65.00	0.51	tr_D0A9V0_
DRB1_0101	459	KLPLDHRDSVTMLVD	LDHRDSVTM	3	0.3868	761.0	65.00	0.38	tr_D0A9V0_
DRB1_0101	460	AKRLMRLCRQRCRRN	LMRLCRQRC	3	0.3845	780.1	65.00	0.29	tr_D0A9V0_
DRB1_0101	461	IKCIPRLRRDVVAES	PRLRRDVVA	4	0.3843	781.8	70.00	0.32	tr_D0A9V0_
DRB1_0101	462	NRNVWTRSLKKISEG	NVWTRSLKK	2	0.3840	784.5	70.00	0.38	tr_D0A9V0_
DRB1_0101	463	VWTRSLKKISEGAFS	LKKISEGAF	5	0.3839	785.7	70.00	0.26	tr_D0A9V0_
DRB1_0101	464	DMQAKRLMRLCRQRC	KRLMRLCRQ	4	0.3834	789.2	70.00	0.34	tr_D0A9V0_
DRB1_0101	465	LPLDHRDSVTMLVDT	HRDSVTMLV	4	0.3832	791.5	70.00	0.38	tr_D0A9V0_
DRB1_0101	466	GIVHCDVKPHNVLLG	DVKPHNVLL	5	0.3824	797.9	70.00	0.40	tr_D0A9V0_
DRB1_0101	467	VLLGTDGVCKLSDFG	LLGTDGVCK	1	0.3800	819.4	70.00	0.63	tr_D0A9V0_
DRB1_0101	468	PVIPASVDHEAAAFV	VDHEAAAFV	6	0.3797	821.8	70.00	0.63	tr_D0A9V0_
DRB1_0101	469	RNDTVPYVRVLDNHY	YVRVLDNHY	6	0.3791	826.9	70.00	0.27	tr_D0A9V0_
DRB1_0101	470	VAESLEKEMEVASKL	KEMEVASKL	6	0.3787	830.3	70.00	0.57	tr_D0A9V0_
DRB1_0101	471	DSNRNVWTRSLKKIS	NVWTRSLKK	4	0.3783	834.6	70.00	0.41	tr_D0A9V0_
DRB1_0101	472	RVESKNEAGGHIRAN	SKNEAGGHI	3	0.3782	835.2	70.00	0.63	tr_D0A9V0_
DRB1_0101	473	HCDVKPHNVLLGTDG	DVKPHNVLL	2	0.3779	837.9	70.00	0.40	tr_D0A9V0_
DRB1_0101	474	TPGESSTAFVRIMGA	GESSTAFVR	2	0.3776	840.3	70.00	0.38	tr_D0A9V0_
DRB1_0101	475	RNVWTRSLKKISEGA	WTRSLKKIS	3	0.3770	846.4	70.00	0.37	tr_D0A9V0_
DRB1_0101	476	GAHAALPMHCRRCCQ	HAALPMHCR	2	0.3767	849.3	70.00	0.54	tr_D0A9V0_
DRB1_0101	477	KCIPRLRRDVVAESL	PRLRRDVVA	3	0.3766	849.9	70.00	0.37	tr_D0A9V0_
DRB1_0101	478	MHCRRCCQFAFDLFA	RCCQFAFDL	4	0.3765	850.7	70.00	0.28	tr_D0A9V0_
DRB1_0101	479	QRDVALNCGIFKEKP	VALNCGIFK	3	0.3745	869.3	70.00	0.51	tr_D0A9V0_
DRB1_0101	480	CRRNDTVPYVRVLDN	NDTVPYVRV	3	0.3745	869.4	70.00	0.41	tr_D0A9V0_
DRB1_0101	481	PNVFNRRHCTVIAIQ	FNRRHCTVI	3	0.3744	870.7	70.00	0.38	tr_D0A9V0_
DRB1_0101	482	MLVDTNEFLVGTCGA	NEFLVGTCG	5	0.3738	875.6	70.00	0.38	tr_D0A9V0_
DRB1_0101	483	RQRCRRNDTVPYVRV	CRRNDTVPY	3	0.3736	878.1	70.00	0.52	tr_D0A9V0_
DRB1_0101	484	GESSSEVSLSISKQF	SSSEVSLSI	2	0.3731	882.4	70.00	0.32	tr_D0A9V0_
DRB1_0101	485	GTDGVCKLSDFGSTI	VCKLSDFGS	4	0.3729	884.2	70.00	0.44	tr_D0A9V0_
DRB1_0101	486	YMAPEVAAGGRCTPQ	MAPEVAAGG	1	0.3728	885.2	70.00	0.42	tr_D0A9V0_
DRB1_0101	487	VIAIQVYHPDVWKSS	IAIQVYHPD	1	0.3715	898.3	70.00	0.23	tr_D0A9V0_
DRB1_0101	488	VHCDVKPHNVLLGTD	DVKPHNVLL	3	0.3693	919.2	70.00	0.47	tr_D0A9V0_
DRB1_0101	489	AKLPLDHRDSVTMLV	LDHRDSVTM	4	0.3689	923.4	70.00	0.45	tr_D0A9V0_
DRB1_0101	490	PGGSLHSVIKNFGCV	LHSVIKNFG	4	0.3679	933.3	70.00	0.34	tr_D0A9V0_
DRB1_0101	491	PLDHRDSVTMLVDTN	HRDSVTMLV	3	0.3672	940.9	70.00	0.43	tr_D0A9V0_
DRB1_0101	492	KPNVFNRRHCTVIAI	FNRRHCTVI	4	0.3665	947.5	70.00	0.38	tr_D0A9V0_
DRB1_0101	493	EDGAQVAIKCIPRLR	VAIKCIPRL	5	0.3665	947.8	70.00	0.29	tr_D0A9V0_
DRB1_0101	494	FAKLPLDHRDSVTML	FAKLPLDHR	0	0.3663	949.9	70.00	0.36	tr_D0A9V0_
DRB1_0101	495	RIMGASISVTWNAGD	IMGASISVT	1	0.3663	950.3	70.00	0.37	tr_D0A9V0_
DRB1_0101	496	FIPEAILARVESKNE	FIPEAILAR	0	0.3660	953.1	70.00	0.34	tr_D0A9V0_
DRB1_0101	497	NVWTRSLKKISEGAF	WTRSLKKIS	2	0.3610	1005.9	70.00	0.29	tr_D0A9V0_
DRB1_0101	498	FAAMRQGRYGEAMAH	AAMRQGRYG	1	0.3593	1024.9	75.00	0.31	tr_D0A9V0_

DRB1_0101	499	AADVVPLFLTDSNRN	VVPLFLTDS	3	0.3579	1040.2	75.00	0.41	tr_D0A9V0_
DRB1_0101	500	IAATSGGASHMQGFL	ATSGGASHM	2	0.3568	1053.2	75.00	0.34	tr_D0A9V0_
DRB1_0101	501	ETPGESSTAFVRIMG	GESSTAFVR	3	0.3567	1053.6	75.00	0.43	tr_D0A9V0_
DRB1_0101	502	PMHCRRCCQFAFDLF	CRRCCQFAF	3	0.3567	1054.4	75.00	0.33	tr_D0A9V0_
DRB1_0101	503	ARVESKNEAGGHIRA	SKNEAGGHI	4	0.3564	1056.9	75.00	0.62	tr_D0A9V0_
DRB1_0101	504	KRLMRLCRQRCRRND	LMRLCRQRC	2	0.3533	1092.9	75.00	0.29	tr_D0A9V0_
DRB1_0101	505	WSSTAPAGSDATRLR	PAGSDATRL	5	0.3528	1098.9	75.00	0.38	tr_D0A9V0_
DRB1_0101	506	RRNDTVPYVRVLDNH	NDTVPYVRV	2	0.3519	1109.8	75.00	0.47	tr_D0A9V0_
DRB1_0101	507	RDVALNCGIFKEKPN	VALNCGIFK	2	0.3518	1111.0	75.00	0.50	tr_D0A9V0_
DRB1_0101	508	AALIEVATEHSGCVE	VATEHSGCV	5	0.3516	1114.2	75.00	0.34	tr_D0A9V0_
DRB1_0101	509	LGIVHCDVKPHNVLL	DVKPHNVLL	6	0.3511	1120.1	75.00	0.27	tr_D0A9V0_
DRB1_0101	510	ALIEVATEHSGCVEA	VATEHSGCV	4	0.3503	1129.3	75.00	0.44	tr_D0A9V0_
DRB1_0101	511	FKEKPNVFNRRHCTV	FKEKPNVFN	0	0.3503	1129.6	75.00	0.62	tr_D0A9V0_
DRB1_0101	512	TETPGESSTAFVRIM	GESSTAFVR	4	0.3492	1143.1	75.00	0.43	tr_D0A9V0_
DRB1_0101	513	VESKNEAGGHIRANG	SKNEAGGHI	2	0.3492	1143.6	75.00	0.62	tr_D0A9V0_
DRB1_0101	514	LIEVATEHSGCVEAL	VATEHSGCV	3	0.3490	1145.2	75.00	0.62	tr_D0A9V0_
DRB1_0101	515	EAGGHIRANGSATET	IRANGSATE	5	0.3490	1145.8	75.00	0.55	tr_D0A9V0_
DRB1_0101	516	IPRLRRDVVAESLEK	LRRDVVAES	3	0.3475	1164.8	75.00	0.46	tr_D0A9V0_
DRB1_0101	517	CIPRLRRDVVAESLE	PRLRRDVVA	2	0.3475	1164.9	75.00	0.35	tr_D0A9V0_
DRB1_0101	518	AGGHIRANGSATETP	IRANGSATE	4	0.3472	1167.7	75.00	0.58	tr_D0A9V0_
DRB1_0101	519	GGHIRANGSATETPG	IRANGSATE	3	0.3472	1168.4	75.00	0.63	tr_D0A9V0_
DRB1_0101	520	STAPAGSDATRLRSL	PAGSDATRL	3	0.3448	1199.1	75.00	0.61	tr_D0A9V0_
DRB1_0101	521	LSYAVRDGPCRIAAT	YAVRDGPCR	2	0.3445	1202.6	75.00	0.35	tr_D0A9V0_
DRB1_0101	522	DQQRDVALNCGIFKE	VALNCGIFK	5	0.3441	1207.6	75.00	0.48	tr_D0A9V0_
DRB1_0101	523	HAALIEVATEHSGCV	LIEVATEHS	3	0.3413	1245.5	75.00	0.37	tr_D0A9V0_
DRB1_0101	524	QLSYAVRDGPCRIAA	YAVRDGPCR	3	0.3407	1253.2	75.00	0.41	tr_D0A9V0_
DRB1_0101	525	VRVLDNHYCVNSSDC	VRVLDNHYC	0	0.3404	1257.2	75.00	0.56	tr_D0A9V0_
DRB1_0101	526	NAADVVPLFLTDSNR	VVPLFLTDS	4	0.3380	1290.4	75.00	0.43	tr_D0A9V0_
DRB1_0101	527	RDGPCRIAATSGGAS	CRIAATSGG	4	0.3368	1307.8	75.00	0.44	tr_D0A9V0_
DRB1_0101	528	ALKSASNLEIPDNAD	LKSASNLEI	1	0.3338	1350.4	80.00	0.76	tr_D0A9V0_
DRB1_0101	529	AEDGAQVAIKCIPRL	DGAQVAIKC	2	0.3321	1375.9	80.00	0.31	tr_D0A9V0_
DRB1_0101	530	IAIQVYHPDVWKSST	YHPDVWKSS	5	0.3310	1391.7	80.00	0.22	tr_D0A9V0_
DRB1_0101	531	SYAVRDGPCRIAATS	YAVRDGPCR	1	0.3292	1419.6	80.00	0.29	tr_D0A9V0_
DRB1_0101	532	PAGSDATRLRSLFNR	ATRLRSLFN	5	0.3277	1443.1	80.00	0.32	tr_D0A9V0_
DRB1_0101	533	LEPVIPASVDHEAAA	LEPVIPASV	0	0.3274	1446.5	80.00	0.58	tr_D0A9V0_
DRB1_0101	534	TAPAGSDATRLRSLF	PAGSDATRL	2	0.3270	1453.9	80.00	0.54	tr_D0A9V0_
DRB1_0101	535	LDHRDSVTMLVDTNE	HRDSVTMLV	2	0.3264	1463.5	80.00	0.47	tr_D0A9V0_
DRB1_0101	536	ANAADVVPLFLTDSN	VVPLFLTDS	5	0.3263	1464.7	80.00	0.37	tr_D0A9V0_
DRB1_0101	537	DSSDQGSESLFKMYA	QGSESLFKM	4	0.3262	1466.3	80.00	0.33	tr_D0A9V0_
DRB1_0101	538	YAPSSSSSLDGSGCA	YAPSSSSSL	0	0.3259	1470.8	80.00	0.65	tr_D0A9V0_
DRB1_0101	539	TDSNRNVWTRSLKKI	NVWTRSLKK	5	0.3258	1472.3	80.00	0.43	tr_D0A9V0_
DRB1_0101	540	VPGGSLHSVIKNFGC	LHSVIKNFG	5	0.3249	1487.2	80.00	0.27	tr_D0A9V0_
DRB1_0101	541	GHIRANGSATETPGE	IRANGSATE	2	0.3236	1507.4	80.00	0.71	tr_D0A9V0_
DRB1_0101	542	APAGSDATRLRSLFN	PAGSDATRL	1	0.3228	1521.7	80.00	0.42	tr_D0A9V0_
DRB1_0101	543	EKPNVFNRRHCTVIA	FNRRHCTVI	5	0.3223	1528.7	80.00	0.41	tr_D0A9V0_
DRB1_0101	544	FELRPGESSSEVSLS	FELRPGESS	0	0.3220	1534.9	80.00	0.43	tr_D0A9V0_
DRB1_0101	545	SSTAPAGSDATRLRS	PAGSDATRL	4	0.3219	1536.0	80.00	0.59	tr_D0A9V0_
DRB1_0101	546	PGESSSEVSLSISKQ	SSSEVSLSI	3	0.3215	1542.3	80.00	0.50	tr_D0A9V0_
DRB1_0101	547	LPMHCRRCCQFAFDL	CRRCCQFAF	4	0.3197	1573.6	80.00	0.38	tr_D0A9V0_
DRB1_0101	548	HPDVWKSSTKYHAAL	WKSSTKYHA	4	0.3186	1592.3	80.00	0.47	tr_D0A9V0_

DRB1_0101	549	MLLGRPPWQWSSTAP	MLLGRPPWQ	0	0.3166	1625.9	80.00	0.41	tr_D0A9V0_
DRB1_0101	550	VAEDGAQVAIKCIPR	DGAQVAIKC	3	0.3164	1630.1	80.00	0.35	tr_D0A9V0_
DRB1_0101	551	AHAALPMHCRRCCQF	HAALPMHCR	1	0.3151	1653.9	80.00	0.53	tr_D0A9V0_
DRB1_0101	552	NLGIVHCDVKPHNVL	IVHCDVKPH	3	0.3143	1667.9	80.00	0.40	tr_D0A9V0_
DRB1_0101	553	ESKNEAGGHIRANGS	SKNEAGGHI	1	0.3126	1699.2	80.00	0.57	tr_D0A9V0_
DRB1_0101	554	HRDSVTMLVDTNEFL	MLVDTNEFL	6	0.3101	1744.2	80.00	0.45	tr_D0A9V0_
DRB1_0101	555	ATETPGESSTAFVRI	GESSTAFVR	5	0.3097	1752.5	80.00	0.41	tr_D0A9V0_
DRB1_0101	556	AIQVYHPDVWKSSTK	YHPDVWKSS	4	0.3097	1753.3	80.00	0.35	tr_D0A9V0_
DRB1_0101	557	SDSSDQGSESLFKMY	QGSESLFKM	5	0.3081	1782.7	80.00	0.29	tr_D0A9V0_
DRB1_0101	558	AAMRQGRYGEAMAHY	MRQGRYGEA	2	0.3078	1788.2	80.00	0.30	tr_D0A9V0_
DRB1_0101	559	IEVATEHSGCVEALD	VATEHSGCV	2	0.3070	1804.0	80.00	0.68	tr_D0A9V0_
DRB1_0101	560	GDQQRDVALNCGIFK	VALNCGIFK	6	0.3058	1828.2	85.00	0.37	tr_D0A9V0_
DRB1_0101	561	GANAADVVPLFLTDS	ADVVPLFLT	4	0.3055	1833.4	85.00	0.32	tr_D0A9V0_
DRB1_0101	562	SSDQGSESLFKMYAP	QGSESLFKM	3	0.3049	1847.0	85.00	0.31	tr_D0A9V0_
DRB1_0101	563	MAPEVAAGGRCTPQS	EVAAGGRCT	3	0.3036	1873.2	85.00	0.50	tr_D0A9V0_
DRB1_0101	564	DVALNCGIFKEKPNV	VALNCGIFK	1	0.3029	1885.7	85.00	0.44	tr_D0A9V0_
DRB1_0101	565	PRLRRDVVAESLEKE	LRRDVVAES	2	0.3029	1886.6	85.00	0.43	tr_D0A9V0_
DRB1_0101	566	CRQRCRRNDTVPYVR	CRRNDTVPY	4	0.3022	1900.3	85.00	0.67	tr_D0A9V0_
DRB1_0101	567	AGSDATRLRSLFNRD	ATRLRSLFN	4	0.3017	1910.9	85.00	0.42	tr_D0A9V0_
DRB1_0101	568	SDQGSESLFKMYAPS	QGSESLFKM	2	0.3012	1920.7	85.00	0.33	tr_D0A9V0_
DRB1_0101	569	LARVESKNEAGGHIR	SKNEAGGHI	5	0.3012	1922.4	85.00	0.60	tr_D0A9V0_
DRB1_0101	570	EIPDNADIFLGRSRI	DIFLGRSRI	6	0.3009	1927.9	85.00	0.41	tr_D0A9V0_
DRB1_0101	571	LLGTDGVCKLSDFGS	LLGTDGVCK	0	0.3004	1939.2	85.00	0.37	tr_D0A9V0_
DRB1_0101	572	RPGESSSEVSLSISK	SSSEVSLSI	4	0.2993	1960.6	85.00	0.52	tr_D0A9V0_
DRB1_0101	573	IPEAILARVESKNEA	AILARVESK	3	0.2968	2014.8	85.00	0.38	tr_D0A9V0_
DRB1_0101	574	VTWNAGDQQRDVALN	WNAGDQQRD	2	0.2965	2021.4	85.00	0.44	tr_D0A9V0_
DRB1_0101	575	IQVYHPDVWKSSTKY	YHPDVWKSS	3	0.2961	2031.3	85.00	0.44	tr_D0A9V0_
DRB1_0101	576	YHPDVWKSSTKYHAA	WKSSTKYHA	5	0.2957	2039.0	85.00	0.43	tr_D0A9V0_
DRB1_0101	577	GRCTPQSDIFSFGIS	CTPQSDIFS	2	0.2930	2099.6	85.00	0.35	tr_D0A9V0_
DRB1_0101	578	LTDSNRNVWTRSLKK	NRNVWTRSL	4	0.2927	2107.3	85.00	0.27	tr_D0A9V0_
DRB1_0101	579	SVTWNAGDQQRDVAL	WNAGDQQRD	3	0.2898	2173.5	85.00	0.47	tr_D0A9V0_
DRB1_0101	580	VRDGPCRIAATSGGA	CRIAATSGG	5	0.2887	2200.0	85.00	0.49	tr_D0A9V0_
DRB1_0101	581	AEGANAADVVPLFLT	ANAADVVPL	3	0.2886	2202.8	85.00	0.37	tr_D0A9V0_
DRB1_0101	582	LHNLGIVHCDVKPHN	IVHCDVKPH	5	0.2835	2327.1	85.00	0.24	tr_D0A9V0_
DRB1_0101	583	LGTDGVCKLSDFGST	VCKLSDFGS	5	0.2833	2332.8	85.00	0.51	tr_D0A9V0_
DRB1_0101	584	RLMRLCRQRCRRNDT	LMRLCRQRC	1	0.2831	2337.2	85.00	0.33	tr_D0A9V0_
DRB1_0101	585	YAVRDGPCRIAATSG	VRDGPCRIA	2	0.2831	2337.5	85.00	0.26	tr_D0A9V0_
DRB1_0101	586	ALPMHCRRCCQFAFD	CRRCCQFAF	5	0.2825	2352.1	85.00	0.38	tr_D0A9V0_
DRB1_0101	587	DGSGCAEGANAADVV	GCAEGANAA	3	0.2824	2353.7	85.00	0.38	tr_D0A9V0_
DRB1_0101	588	GSGCAEGANAADVVP	GCAEGANAA	2	0.2801	2413.1	85.00	0.38	tr_D0A9V0_
DRB1_0101	589	TWNAGDQQRDVALNC	WNAGDQQRD	1	0.2800	2417.8	85.00	0.40	tr_D0A9V0_
DRB1_0101	590	GGRCTPQSDIFSFGI	CTPQSDIFS	3	0.2792	2437.7	85.00	0.44	tr_D0A9V0_
DRB1_0101	591	HIRANGSATETPGES	IRANGSATE	1	0.2787	2450.4	85.00	0.74	tr_D0A9V0_
DRB1_0101	592	EGANAADVVPLFLTD	ADVVPLFLT	5	0.2787	2451.6	85.00	0.33	tr_D0A9V0_
DRB1_0101	593	HAALPMHCRRCCQFA	HAALPMHCR	0	0.2782	2465.3	85.00	0.37	tr_D0A9V0_
DRB1_0101	594	LRPGESSSEVSLSIS	SSSEVSLSI	5	0.2781	2468.4	85.00	0.41	tr_D0A9V0_
DRB1_0101	595	NEAGGHIRANGSATE	IRANGSATE	6	0.2770	2495.8	85.00	0.40	tr_D0A9V0_
DRB1_0101	596	TEHSGCVEALDPDRA	VEALDPDRA	6	0.2750	2550.0	90.00	0.66	tr_D0A9V0_
DRB1_0101	597	PEAILARVESKNEAG	AILARVESK	2	0.2742	2574.3	90.00	0.37	tr_D0A9V0_
DRB1_0101	598	RLRRDVVAESLEKEM	LRRDVVAES	1	0.2740	2578.3	90.00	0.35	tr_D0A9V0_

DRB1_0101	599	SGCAEGANAADVVPL	GCAEGANAA	1	0.2737	2588.6	90.00	0.32	tr_D0A9V0_
DRB1_0101	600	QVYHPDVWKSSTKYH	YHPDVWKSS	2	0.2712	2657.6	90.00	0.45	tr_D0A9V0_
DRB1_0101	601	VYHPDVWKSSTKYHA	YHPDVWKSS	1	0.2705	2677.9	90.00	0.33	tr_D0A9V0_
DRB1_0101	602	IMGASISVTWNAGDQ	MGASISVTW	1	0.2703	2683.9	90.00	0.22	tr_D0A9V0_
DRB1_0101	603	LCRQRCRRNDTVPYV	CRRNDTVPY	5	0.2690	2723.3	90.00	0.58	tr_D0A9V0_
DRB1_0101	604	AALPMHCRRCCQFAF	CRRCCQFAF	6	0.2678	2757.6	90.00	0.31	tr_D0A9V0_
DRB1_0101	605	ESDSSDQGSESLFKM	SSDQGSESL	3	0.2668	2788.5	90.00	0.31	tr_D0A9V0_
DRB1_0101	606	DHRDSVTMLVDTNEF	HRDSVTMLV	1	0.2665	2797.6	90.00	0.47	tr_D0A9V0_
DRB1_0101	607	HNLGIVHCDVKPHNV	IVHCDVKPH	4	0.2651	2839.4	90.00	0.37	tr_D0A9V0_
DRB1_0101	608	LDGSGCAEGANAADV	GCAEGANAA	4	0.2641	2870.6	90.00	0.34	tr_D0A9V0_
DRB1_0101	609	ISVTWNAGDQQRDVA	WNAGDQQRD	4	0.2612	2963.4	90.00	0.47	tr_D0A9V0_
DRB1_0101	610	SKNEAGGHIRANGSA	SKNEAGGHI	0	0.2590	3034.8	90.00	0.38	tr_D0A9V0_
DRB1_0101	611	EVATEHSGCVEALDP	VATEHSGCV	1	0.2536	3215.0	90.00	0.68	tr_D0A9V0_
DRB1_0101	612	EAILARVESKNEAGG	AILARVESK	1	0.2518	3280.6	90.00	0.34	tr_D0A9V0_
DRB1_0101	613	AVRDGPCRIAATSGG	VRDGPCRIA	1	0.2515	3290.5	90.00	0.28	tr_D0A9V0_
DRB1_0101	614	ELRPGESSSEVSLSI	LRPGESSSE	1	0.2513	3298.2	90.00	0.24	tr_D0A9V0_
DRB1_0101	615	SATETPGESSTAFVR	GESSTAFVR	6	0.2501	3341.6	90.00	0.31	tr_D0A9V0_
DRB1_0101	616	GCAEGANAADVVPLF	ANAADVVPL	5	0.2494	3364.6	90.00	0.31	tr_D0A9V0_
DRB1_0101	617	LKSASNLEIPDNADI	LKSASNLEI	0	0.2487	3392.5	90.00	0.49	tr_D0A9V0_
DRB1_0101	618	AGGRCTPQSDIFSFG	CTPQSDIFS	4	0.2477	3429.1	90.00	0.46	tr_D0A9V0_
DRB1_0101	619	WNAGDQQRDVALNCG	GDQQRDVAL	3	0.2469	3456.9	90.00	0.25	tr_D0A9V0_
DRB1_0101	620	APEVAAGGRCTPQSD	EVAAGGRCT	2	0.2467	3466.2	90.00	0.63	tr_D0A9V0_
DRB1_0101	621	SISVTWNAGDQQRDV	WNAGDQQRD	5	0.2460	3491.0	90.00	0.41	tr_D0A9V0_
DRB1_0101	622	KEKPNVFNRRHCTVI	FNRRHCTVI	6	0.2449	3534.2	90.00	0.36	tr_D0A9V0_
DRB1_0101	623	LRRDVVAESLEKEME	VVAESLEKE	4	0.2416	3663.3	90.00	0.41	tr_D0A9V0_
DRB1_0101	624	LMRLCRQRCRRNDTV	LCRQRCRRN	3	0.2402	3716.4	95.00	0.28	tr_D0A9V0_
DRB1_0101	625	CAEGANAADVVPLFL	ANAADVVPL	4	0.2388	3776.0	95.00	0.47	tr_D0A9V0_
DRB1_0101	626	RRDVVAESLEKEMEV	VVAESLEKE	3	0.2373	3836.6	95.00	0.47	tr_D0A9V0_
DRB1_0101	627	ILARVESKNEAGGHI	SKNEAGGHI	6	0.2360	3889.0	95.00	0.41	tr_D0A9V0_
DRB1_0101	628	SLDGSGCAEGANAAD	SGCAEGANA	4	0.2345	3953.5	95.00	0.31	tr_D0A9V0_
DRB1_0101	629	ASNLEIPDNADIFLG	LEIPDNADI	3	0.2334	4001.6	95.00	0.39	tr_D0A9V0_
DRB1_0101	630	AAGGRCTPQSDIFSF	CTPQSDIFS	5	0.2323	4050.8	95.00	0.42	tr_D0A9V0_
DRB1_0101	631	SNLEIPDNADIFLGR	LEIPDNADI	2	0.2316	4080.4	95.00	0.32	tr_D0A9V0_
DRB1_0101	632	VVAESLEKEMEVASK	LEKEMEVAS	5	0.2279	4245.0	95.00	0.29	tr_D0A9V0_
DRB1_0101	633	EPVIPASVDHEAAAF	VIPASVDHE	2	0.2254	4361.2	95.00	0.25	tr_D0A9V0_
DRB1_0101	634	AGDQQRDVALNCGIF	GDQQRDVAL	1	0.2249	4386.9	95.00	0.20	tr_D0A9V0_
DRB1_0101	635	PEVAAGGRCTPQSDI	EVAAGGRCT	1	0.2234	4458.3	95.00	0.55	tr_D0A9V0_
DRB1_0101	636	SSLDGSGCAEGANAA	SGCAEGANA	5	0.2226	4498.4	95.00	0.31	tr_D0A9V0_
DRB1_0101	637	SASNLEIPDNADIFL	LEIPDNADI	4	0.2220	4527.1	95.00	0.34	tr_D0A9V0_
DRB1_0101	638	NLEIPDNADIFLGRS	PDNADIFLG	4	0.2219	4530.9	95.00	0.31	tr_D0A9V0_
DRB1_0101	639	MGASISVTWNAGDQQ	ASISVTWNA	2	0.2198	4633.7	95.00	0.26	tr_D0A9V0_
DRB1_0101	640	SSSSSLDGSGCAEGA	SSSLDGSGC	2	0.2191	4673.3	95.00	0.52	tr_D0A9V0_
DRB1_0101	641	RDVVAESLEKEMEVA	VVAESLEKE	2	0.2185	4699.4	95.00	0.38	tr_D0A9V0_
DRB1_0101	642	KNEAGGHIRANGSAT	GGHIRANGS	4	0.2166	4799.0	95.00	0.28	tr_D0A9V0_
DRB1_0101	643	KSASNLEIPDNADIF	SNLEIPDNA	3	0.2134	4967.5	95.00	0.53	tr_D0A9V0_
DRB1_0101	644	RLCRQRCRRNDTVPY	CRRNDTVPY	6	0.2127	5006.6	95.00	0.38	tr_D0A9V0_
DRB1_0101	645	ASISVTWNAGDQQRD	ISVTWNAGD	2	0.2109	5102.6	95.00	0.22	tr_D0A9V0_
DRB1_0101	646	SSSSLDGSGCAEGAN	SSSLDGSGC	1	0.2099	5159.9	95.00	0.41	tr_D0A9V0_
DRB1_0101	647	LEIPDNADIFLGRSR	PDNADIFLG	3	0.2093	5191.0	95.00	0.35	tr_D0A9V0_
DRB1_0101	648	GASISVTWNAGDQQR	ISVTWNAGD	3	0.2093	5195.7	95.00	0.28	tr_D0A9V0_

DRB1_0101	649	AILARVESKNEAGGH	LARVESKNE	2	0.2091	5205.0	95.00	0.27	tr_D0A9V0_
DRB1_0101	650	NAGDQQRDVALNCGI	GDQQRDVAL	2	0.2067	5342.9	95.00	0.35	tr_D0A9V0_
DRB1_0101	651	SESDSSDQGSESLFK	SSDQGSESL	4	0.2050	5442.1	95.00	0.41	tr_D0A9V0_
DRB1_0101	652	DVVAESLEKEMEVAS	VVAESLEKE	1	0.2047	5457.9	95.00	0.25	tr_D0A9V0_
DRB1_0101	653	SSSLDGSGCAEGANA	LDGSGCAEG	3	0.2042	5487.9	95.00	0.29	tr_D0A9V0_
DRB1_0101	654	PSSSSSLDGSGCAEG	SSSLDGSGC	3	0.2014	5655.4	95.00	0.65	tr_D0A9V0_
DRB1_0101	655	DESDIKDRVPETIDS	IKDRVPETI	4	0.1989	5810.5	95.00	0.75	tr_D0A9V0_
DRB1_0101	656	VAAGGRCTPQSDIFS	CTPQSDIFS	6	0.1887	6490.5	100.00	0.31	tr_D0A9V0_
DRB1_0101	657	EVAAGGRCTPQSDIF	EVAAGGRCT	0	0.1886	6497.7	100.00	0.42	tr_D0A9V0_
DRB1_0101	658	ESDIKDRVPETIDSP	IKDRVPETI	3	0.1876	6568.2	100.00	0.75	tr_D0A9V0_
DRB1_0101	659	VATEHSGCVEALDPD	VATEHSGCV	0	0.1864	6656.7	100.00	0.46	tr_D0A9V0_
DRB1_0101	660	MRLCRQRCRRNDTVP	LCRQRCRRN	2	0.1851	6750.5	100.00	0.34	tr_D0A9V0_
DRB1_0101	661	APSSSSSLDGSGCAE	SSSLDGSGC	4	0.1843	6807.7	100.00	0.67	tr_D0A9V0_
DRB1_0101	662	RVLDNHYCVNSSDCK	RVLDNHYCV	0	0.1831	6896.1	100.00	0.27	tr_D0A9V0_
DRB1_0101	663	SDIKDRVPETIDSPR	IKDRVPETI	2	0.1787	7235.5	100.00	0.69	tr_D0A9V0_
DRB1_0101	664	QDESDIKDRVPETID	IKDRVPETI	5	0.1708	7879.6	100.00	0.69	tr_D0A9V0_
DRB1_0101	665	GSATETPGESSTAFV	PGESSTAFV	6	0.1701	7935.5	100.00	0.23	tr_D0A9V0_
DRB1_0101	666	NHYCVNSSDCKPEEQ	YCVNSSDCK	2	0.1700	7943.1	100.00	0.44	tr_D0A9V0_
DRB1_0101	667	DNHYCVNSSDCKPEE	YCVNSSDCK	3	0.1663	8274.8	100.00	0.47	tr_D0A9V0_
DRB1_0101	668	LDNHYCVNSSDCKPE	YCVNSSDCK	4	0.1640	8481.7	100.00	0.44	tr_D0A9V0_
DRB1_0101	669	IRANGSATETPGESS	IRANGSATE	0	0.1618	8679.6	100.00	0.60	tr_D0A9V0_
DRB1_0101	670	DIKDRVPETIDSPRR	IKDRVPETI	1	0.1572	9130.3	100.00	0.56	tr_D0A9V0_
DRB1_0101	671	VLDNHYCVNSSDCKP	YCVNSSDCK	5	0.1559	9253.1	100.00	0.41	tr_D0A9V0_
DRB1_0101	672	HYCVNSSDCKPEEQD	YCVNSSDCK	1	0.1498	9891.4	100.00	0.47	tr_D0A9V0_
DRB1_0101	673	GSESDSSDQGSESLF	SSDQGSESL	5	0.1424	10716.0	100.00	0.50	tr_D0A9V0_
DRB1_0101	674	ATEHSGCVEALDPDR	EHSGCVEAL	2	0.1411	10866.2	100.00	0.25	tr_D0A9V0_
DRB1_0101	675	NGSATETPGESSTAF	ATETPGESS	3	0.1291	12363.7	100.00	0.32	tr_D0A9V0_
DRB1_0101	676	EQDESDIKDRVPETI	IKDRVPETI	6	0.1280	12516.2	100.00	0.60	tr_D0A9V0_
DRB1_0101	677	IKDRVPETIDSPRRP	IKDRVPETI	0	0.1203	13607.9	100.00	0.38	tr_D0A9V0_
DRB1_0101	678	YCVNSSDCKPEEQDE	YCVNSSDCK	0	0.1138	14589.8	100.00	0.36	tr_D0A9V0_
DRB1_0101	679	ANGSATETPGESSTA	SATETPGES	3	0.1066	15772.7	100.00	0.31	tr_D0A9V0_
DRB1_0101	680	DRVPETIDSPRRPDG	ETIDSPRRP	4	0.0930	18273.2	100.00	0.37	tr_D0A9V0_
DRB1_0101	681	KDRVPETIDSPRRPD	ETIDSPRRP	5	0.0911	18659.2	100.00	0.31	tr_D0A9V0_
DRB1_0101	682	RVPETIDSPRRPDGE	ETIDSPRRP	3	0.0911	18665.5	100.00	0.44	tr_D0A9V0_
DRB1_0101	683	EGSESDSSDQGSESL	SSDQGSESL	6	0.0865	19610.1	100.00	0.46	tr_D0A9V0_
DRB1_0101	684	RANGSATETPGESST	SATETPGES	4	0.0857	19792.4	100.00	0.32	tr_D0A9V0_
DRB1_0101	685	VPETIDSPRRPDGEG	ETIDSPRRP	2	0.0799	21053.5	100.00	0.41	tr_D0A9V0_
DRB1_0101	686	PETIDSPRRPDGEGS	ETIDSPRRP	1	0.0720	22936.0	100.00	0.33	tr_D0A9V0_
DRB1_0101	687	CVNSSDCKPEEQDES	SSDCKPEEQ	3	0.0719	22958.6	100.00	0.23	tr_D0A9V0_
DRB1_0101	688	CKPEEQDESDIKDRV	PEEQDESDI	2	0.0624	25455.3	100.00	0.32	tr_D0A9V0_
DRB1_0101	689	KPEEQDESDIKDRVP	PEEQDESDI	1	0.0599	26152.9	100.00	0.28	tr_D0A9V0_
DRB1_0101	690	ETIDSPRRPDGEGSE	ETIDSPRRP	0	0.0588	26465.5	100.00	0.26	tr_D0A9V0_
DRB1_0101	691	EEQDESDIKDRVPET	DESDIKDRV	3	0.0575	26838.3	100.00	0.34	tr_D0A9V0_
DRB1_0101	692	DCKPEEQDESDIKDR	PEEQDESDI	3	0.0569	27028.6	100.00	0.40	tr_D0A9V0_
DRB1_0101	693	SSDCKPEEQDESDIK	CKPEEQDES	3	0.0565	27141.5	100.00	0.26	tr_D0A9V0_
DRB1_0101	694	VNSSDCKPEEQDESD	SSDCKPEEQ	2	0.0536	27981.4	100.00	0.29	tr_D0A9V0_
DRB1_0101	695	SDCKPEEQDESDIKD	PEEQDESDI	4	0.0535	28014.2	100.00	0.35	tr_D0A9V0_
DRB1_0101	696	NSSDCKPEEQDESDI	SSDCKPEEQ	1	0.0528	28247.9	100.00	0.24	tr_D0A9V0_
DRB1_0101	697	PEEQDESDIKDRVPE	DESDIKDRV	4	0.0518	28550.9	100.00	0.28	tr_D0A9V0_
DRB1_0101	698	SPRRPDGEGSESDSS	PDGEGSESD	4	0.0504	28986.4	100.00	0.37	tr_D0A9V0_

DRB1_0101	699	IDSPRRPDGEGSESD	PRRPDGEGS	3	0.0474	29928.7	100.00	0.31	tr_D0A9V0_
DRB1_0101	700	DSPRRPDGEGSESDS	PDGEGSESD	5	0.0470	30072.9	100.00	0.28	tr_D0A9V0_
DRB1_0101	701	PRRPDGEGSESDSSD	PDGEGSESD	3	0.0465	30218.0	100.00	0.35	tr_D0A9V0_
DRB1_0101	702	TIDSPRRPDGEGSES	PRRPDGEGS	4	0.0464	30264.8	100.00	0.31	tr_D0A9V0_
DRB1_0101	703	RRPDGEGSESDSSDQ	PDGEGSESD	2	0.0446	30854.7	100.00	0.34	tr_D0A9V0_
DRB1_0101	704	GEGSESDSSDQGSES	GSESDSSDQ	2	0.0444	30917.2	100.00	0.20	tr_D0A9V0_
DRB1_0101	705	RPDGEGSESDSSDQG	PDGEGSESD	1	0.0436	31201.8	100.00	0.23	tr_D0A9V0_
DRB1_0101	706	PDGEGSESDSSDQGS	GSESDSSDQ	4	0.0426	31540.2	100.00	0.28	tr_D0A9V0_
DRB1_0101	707	DGEGSESDSSDQGSE	GSESDSSDQ	3	0.0401	32412.0	100.00	0.30	tr_D0A9V0_

Allele: DRB1_0101. Number of high binders 4. Number of weak binders 92. Number of peptides 707

Allele	pos	peptide	core	Of	1-log50k(aff)	affinity(nM)	%Rank	Relia	Identity	Bind Level
DRB1_0101	1	KIIDFLRTQSSLAQF	FLRTQSSLA	4	0.8470	5.2	0.70	0.57	tr_C9ZP82_	WB
DRB1_0101	2	IIDFLRTQSSLAQFG	FLRTQSSLA	3	0.8442	5.4	0.70	0.62	tr_C9ZP82_	WB
DRB1_0101	3	HKIIDFLRTQSSLAQ	FLRTQSSLA	5	0.8268	6.5	1.10	0.50	tr_C9ZP82_	WB
DRB1_0101	4	IDFLRTQSSLAQFGG	FLRTQSSLA	2	0.8190	7.1	1.40	0.67	tr_C9ZP82_	WB
DRB1_0101	5	DTCIISLNPSYIDEE	IISLNPSYI	3	0.7911	9.6	2.50	0.88	tr_C9ZP82_	WB
DRB1_0101	6	DDTCIISLNPSYIDE	IISLNPSYI	4	0.7879	9.9	3.00	0.88	tr_C9ZP82_	WB
DRB1_0101	7	TCIISLNPSYIDEEE	IISLNPSYI	2	0.7779	11.1	3.00	0.89	tr_C9ZP82_	WB
DRB1_0101	8	IDDTCIISLNPSYID	IISLNPSYI	5	0.7729	11.7	3.50	0.87	tr_C9ZP82_	WB
DRB1_0101	9	DFLRTQSSLAQFGGK	FLRTQSSLA	1	0.7714	11.9	3.50	0.68	tr_C9ZP82_	WB
DRB1_0101	10	EHKIIDFLRTQSSLA	IDFLRTQSS	4	0.7698	12.1	3.50	0.50	tr_C9ZP82_	WB
DRB1_0101	11	ADLIFLALSLHITQV	FLALSLHIT	4	0.7678	12.3	4.00	0.56	tr_C9ZP82_	WB
DRB1_0101	12	NHVIVGLDADLIFLA	IVGLDADLI	3	0.7634	12.9	4.00	0.75	tr_C9ZP82_	WB
DRB1_0101	13	DLIFLALSLHITQVF	FLALSLHIT	3	0.7631	13.0	4.00	0.57	tr_C9ZP82_	WB
DRB1_0101	14	DADLIFLALSLHITQ	FLALSLHIT	5	0.7573	13.8	4.50	0.54	tr_C9ZP82_	WB
DRB1_0101	15	VQLLSILPPTSHALL	LLSILPPTS	2	0.7564	13.9	4.50	0.50	tr_C9ZP82_	WB
DRB1_0101	16	ANHVIVGLDADLIFL	IVGLDADLI	4	0.7516	14.7	5.00	0.71	tr_C9ZP82_	WB
DRB1_0101	17	LVQLLSILPPTSHAL	LLSILPPTS	3	0.7491	15.1	5.00	0.57	tr_C9ZP82_	WB
DRB1_0101	18	YYPFYHAPPVIDLAI	FYHAPPVID	3	0.7476	15.3	5.00	0.70	tr_C9ZP82_	WB
DRB1_0101	19	LIFLALSLHITQVFI	FLALSLHIT	2	0.7451	15.8	5.00	0.57	tr_C9ZP82_	WB
DRB1_0101	20	RDTYLKSTGLSNSVQ	YLKSTGLSN	3	0.7363	17.3	6.00	0.60	tr_C9ZP82_	WB
DRB1_0101	21	HVIVGLDADLIFLAL	IVGLDADLI	2	0.7347	17.6	6.00	0.79	tr_C9ZP82_	WB
DRB1_0101	22	WYYPFYHAPPVIDLA	FYHAPPVID	4	0.7342	17.7	6.00	0.68	tr_C9ZP82_	WB
DRB1_0101	23	QLLSILPPTSHALLP	LLSILPPTS	1	0.7293	18.7	6.50	0.50	tr_C9ZP82_	WB
DRB1_0101	24	LDADLIFLALSLHIT	FLALSLHIT	6	0.7275	19.1	6.50	0.47	tr_C9ZP82_	WB
DRB1_0101	25	KANHVIVGLDADLIF	IVGLDADLI	5	0.7264	19.3	6.50	0.65	tr_C9ZP82_	WB
DRB1_0101	26	YPFYHAPPVIDLAIY	FYHAPPVID	2	0.7261	19.4	6.50	0.70	tr_C9ZP82_	WB
DRB1_0101	27	TLVQLLSILPPTSHA	LLSILPPTS	4	0.7251	19.6	7.00	0.57	tr_C9ZP82_	WB
DRB1_0101	28	WRDTYLKSTGLSNSV	YLKSTGLSN	4	0.7248	19.6	7.00	0.58	tr_C9ZP82_	WB
DRB1_0101	29	CIISLNPSYIDEEES	IISLNPSYI	1	0.7206	20.5	7.00	0.92	tr_C9ZP82_	WB
DRB1_0101	30	IFLALSLHITQVFIL	FLALSLHIT	1	0.7181	21.1	7.50	0.50	tr_C9ZP82_	WB
DRB1_0101	31	EANIKRMPLPVVKST	IKRMPLPVV	3	0.7147	21.9	7.50	0.67	tr_C9ZP82_	WB
DRB1_0101	32	FIDDTCIISLNPSYI	IISLNPSYI	6	0.7143	22.0	7.50	0.84	tr_C9ZP82_	WB
DRB1_0101	33	FLRTQSSLAQFGGKA	FLRTQSSLA	0	0.7048	24.4	8.50	0.51	tr_C9ZP82_	WB

DRB1_0101	34	FEYFDISVVGGSLIS	ISVVGGSLI	5	0.7015	25.3	9.00	0.47	tr_C9ZP82_	WB
DRB1_0101	35	SWYYPFYHAPPVIDL	FYHAPPVID	5	0.6995	25.8	9.00	0.64	tr_C9ZP82_	WB
DRB1_0101	36	KEANIKRMPLPVVKS	IKRMPLPVV	4	0.6994	25.8	9.00	0.65	tr_C9ZP82_	WB
DRB1_0101	37	ANIKRMPLPVVKSTP	IKRMPLPVV	2	0.6988	26.0	9.00	0.68	tr_C9ZP82_	WB
DRB1_0101	38	SDAVLNMLNEKMSGN	VLNMLNEKM	3	0.6967	26.6	9.50	0.69	tr_C9ZP82_	WB
DRB1_0101	39	RWRDTYLKSTGLSNS	YLKSTGLSN	5	0.6962	26.8	9.50	0.48	tr_C9ZP82_	WB
DRB1_0101	40	YFDISVVGGSLISEV	ISVVGGSLI	3	0.6933	27.6	9.50	0.66	tr_C9ZP82_	WB
DRB1_0101	41	EYFDISVVGGSLISE	ISVVGGSLI	4	0.6915	28.2	10.00	0.59	tr_C9ZP82_	
DRB1_0101	42	VIVGLDADLIFLALS	IVGLDADLI	1	0.6902	28.6	10.00	0.79	tr_C9ZP82_	
DRB1_0101	43	LDFHLVITSNKTSFV	FHLVITSNK	2	0.6860	29.9	11.00	0.38	tr_C9ZP82_	
DRB1_0101	44	PFYHAPPVIDLAIYL	FYHAPPVID	1	0.6842	30.5	11.00	0.70	tr_C9ZP82_	
DRB1_0101	45	DTLVQLLSILPPTSH	LLSILPPTS	5	0.6814	31.4	11.00	0.54	tr_C9ZP82_	
DRB1_0101	46	GFEYFDISVVGGSLI	ISVVGGSLI	6	0.6798	32.0	11.00	0.32	tr_C9ZP82_	
DRB1_0101	47	KLDFHLVITSNKTSF	FHLVITSNK	3	0.6795	32.1	11.00	0.49	tr_C9ZP82_	
DRB1_0101	48	DTYLKSTGLSNSVQE	YLKSTGLSN	2	0.6763	33.2	12.00	0.70	tr_C9ZP82_	
DRB1_0101	49	DAVLNMLNEKMSGND	VLNMLNEKM	2	0.6741	34.0	12.00	0.64	tr_C9ZP82_	
DRB1_0101	50	LAIYLKEANIKRMPL	YLKEANIKR	3	0.6737	34.2	12.00	0.57	tr_C9ZP82_	
DRB1_0101	51	FEAYAKIEAVKFRRF	YAKIEAVKF	3	0.6723	34.7	12.00	0.70	tr_C9ZP82_	
DRB1_0101	52	VSDAVLNMLNEKMSG	VLNMLNEKM	4	0.6708	35.2	12.00	0.70	tr_C9ZP82_	
DRB1_0101	53	FDISVVGGSLISEVS	ISVVGGSLI	2	0.6695	35.7	13.00	0.69	tr_C9ZP82_	
DRB1_0101	54	FLALSLHITQVFILR	FLALSLHIT	0	0.6642	37.8	13.00	0.38	tr_C9ZP82_	
DRB1_0101	55	LLSILPPTSHALLPM	LSILPPTSH	1	0.6621	38.7	14.00	0.35	tr_C9ZP82_	
DRB1_0101	56	GKANHVIVGLDADLI	IVGLDADLI	6	0.6570	40.9	14.00	0.56	tr_C9ZP82_	
DRB1_0101	57	EAYAKIEAVKFRRFL	YAKIEAVKF	2	0.6569	40.9	14.00	0.64	tr_C9ZP82_	
DRB1_0101	58	DFHLVITSNKTSFVP	FHLVITSNK	1	0.6553	41.7	14.00	0.34	tr_C9ZP82_	
DRB1_0101	59	EVMYIAVDGVAPRAK	YIAVDGVAP	3	0.6536	42.4	15.00	0.71	tr_C9ZP82_	
DRB1_0101	60	GVEIRQLYDVPLERY	IRQLYDVPL	3	0.6531	42.6	15.00	0.87	tr_C9ZP82_	
DRB1_0101	61	NIKRMPLPVVKSTPP	IKRMPLPVV	1	0.6531	42.7	15.00	0.67	tr_C9ZP82_	
DRB1_0101	62	AVLNMLNEKMSGNDP	VLNMLNEKM	1	0.6512	43.5	15.00	0.60	tr_C9ZP82_	
DRB1_0101	63	DLAIYLKEANIKRMP	YLKEANIKR	4	0.6487	44.7	15.00	0.50	tr_C9ZP82_	
DRB1_0101	64	LKEANIKRMPLPVVK	IKRMPLPVV	5	0.6486	44.8	15.00	0.67	tr_C9ZP82_	
DRB1_0101	65	AGYYFFGQPNHIEDG	YFFGQPNHI	3	0.6486	44.8	15.00	0.58	tr_C9ZP82_	
DRB1_0101	66	LFEAYAKIEAVKFRR	YAKIEAVKF	4	0.6473	45.4	15.00	0.74	tr_C9ZP82_	
DRB1_0101	67	IDLAIYLKEANIKRM	YLKEANIKR	5	0.6469	45.6	15.00	0.49	tr_C9ZP82_	
DRB1_0101	68	VMYIAVDGVAPRAKM	YIAVDGVAP	2	0.6436	47.3	16.00	0.74	tr_C9ZP82_	
DRB1_0101	69	VEIRQLYDVPLERYR	IRQLYDVPL	2	0.6423	48.0	16.00	0.86	tr_C9ZP82_	
DRB1_0101	70	GEHKIIDFLRTQSSL	IDFLRTQSS	5	0.6389	49.8	17.00	0.76	tr_C9ZP82_	
DRB1_0101	71	ASWLRNKYPAIVTKN	LRNKYPAIV	3	0.6387	49.8	17.00	0.65	tr_C9ZP82_	
DRB1_0101	72	FASWLRNKYPAIVTK	LRNKYPAIV	4	0.6383	50.1	17.00	0.55	tr_C9ZP82_	
DRB1_0101	73	SAGYYFFGQPNHIED	YFFGQPNHI	4	0.6380	50.3	17.00	0.57	tr_C9ZP82_	
DRB1_0101	74	QKLDFHLVITSNKTS	FHLVITSNK	4	0.6379	50.3	17.00	0.50	tr_C9ZP82_	
DRB1_0101	75	AYAKIEAVKFRRFLL	YAKIEAVKF	1	0.6348	52.0	17.00	0.62	tr_C9ZP82_	
DRB1_0101	76	DISVVGGSLISEVSA	ISVVGGSLI	1	0.6346	52.1	17.00	0.66	tr_C9ZP82_	
DRB1_0101	77	IVLSILLGNDFLPHA	SILLGNDFL	3	0.6336	52.7	17.00	0.46	tr_C9ZP82_	
DRB1_0101	78	KEVMYIAVDGVAPRA	YIAVDGVAP	4	0.6334	52.8	17.00	0.61	tr_C9ZP82_	
DRB1_0101	79	AIYLKEANIKRMPLP	YLKEANIKR	2	0.6315	53.9	18.00	0.61	tr_C9ZP82_	
DRB1_0101	80	GYYFFGQPNHIEDGT	YFFGQPNHI	2	0.6309	54.3	18.00	0.58	tr_C9ZP82_	
DRB1_0101	81	FHLVITSNKTSFVPK	LVITSNKTS	2	0.6302	54.6	18.00	0.31	tr_C9ZP82_	
DRB1_0101	82	RRYMGRAAPAQTGEV	MGRAAPAQT	3	0.6298	54.9	18.00	0.44	tr_C9ZP82_	
DRB1_0101	83	KKTLVSSAGYYFFGQ	LVSSAGYYF	3	0.6288	55.5	18.00	0.75	tr_C9ZP82_	

DRB1_0101	84	EGVEIRQLYDVPLER	IRQLYDVPL	4	0.6261	57.2	18.00	0.86	tr_C9ZP82_
DRB1_0101	85	DRWRDTYLKSTGLSN	DTYLKSTGL	4	0.6233	58.9	19.00	0.58	tr_C9ZP82_
DRB1_0101	86	RLFEAYAKIEAVKFR	YAKIEAVKF	5	0.6224	59.5	19.00	0.60	tr_C9ZP82_
DRB1_0101	87	DKKTLVSSAGYYFFG	LVSSAGYYF	4	0.6209	60.5	19.00	0.73	tr_C9ZP82_
DRB1_0101	88	WSWYYPFYHAPPVID	FYHAPPVID	6	0.6198	61.2	19.00	0.58	tr_C9ZP82_
DRB1_0101	89	ARRYMGRAAPAQTGE	MGRAAPAQT	4	0.6197	61.2	19.00	0.37	tr_C9ZP82_
DRB1_0101	90	KVSDAVLNMLNEKMS	VLNMLNEKM	5	0.6188	61.8	19.00	0.74	tr_C9ZP82_
DRB1_0101	91	IVGLDADLIFLALSL	IVGLDADLI	0	0.6176	62.7	20.00	0.58	tr_C9ZP82_
DRB1_0101	92	PTSHALLPMVLGEVM	HALLPMVLG	3	0.6172	62.9	20.00	0.44	tr_C9ZP82_
DRB1_0101	93	SSAGYYFFGQPNHIE	YFFGQPNHI	5	0.6169	63.1	20.00	0.57	tr_C9ZP82_
DRB1_0101	94	RARRYMGRAAPAQTG	RYMGRAAPA	3	0.6159	63.8	20.00	0.32	tr_C9ZP82_
DRB1_0101	95	VLSILLGNDFLPHAP	SILLGNDFL	2	0.6153	64.2	20.00	0.47	tr_C9ZP82_
DRB1_0101	96	TYLKSTGLSNSVQEA	YLKSTGLSN	1	0.6124	66.3	20.00	0.78	tr_C9ZP82_
DRB1_0101	97	IIVLSILLGNDFLPH	SILLGNDFL	4	0.6121	66.5	21.00	0.42	tr_C9ZP82_
DRB1_0101	98	PPTSHALLPMVLGEV	HALLPMVLG	4	0.6106	67.6	21.00	0.41	tr_C9ZP82_
DRB1_0101	99	TGFEYFDISVVGGSL	FEYFDISVV	2	0.6065	70.7	21.00	0.44	tr_C9ZP82_
DRB1_0101	100	SWLRNKYPAIVTKNC	LRNKYPAIV	2	0.6059	71.1	22.00	0.61	tr_C9ZP82_
DRB1_0101	101	EIRQLYDVPLERYRP	IRQLYDVPL	1	0.6047	72.0	22.00	0.82	tr_C9ZP82_
DRB1_0101	102	ELLVQTVQPKEVMYI	VQTVQPKEV	3	0.6045	72.2	22.00	0.58	tr_C9ZP82_
DRB1_0101	103	ELYVSEVLPYGFLTK	EVLPYGFLT	5	0.6037	72.8	22.00	0.25	tr_C9ZP82_
DRB1_0101	104	KFRRFLLFKSTNKEN	FLLFKSTNK	4	0.6026	73.7	22.00	0.52	tr_C9ZP82_
DRB1_0101	105	MYIAVDGVAPRAKMN	YIAVDGVAP	1	0.6023	73.9	22.00	0.72	tr_C9ZP82_
DRB1_0101	106	TSHALLPMVLGEVMV	HALLPMVLG	2	0.6010	75.0	22.00	0.44	tr_C9ZP82_
DRB1_0101	107	ISVVGGSLISEVSAL	ISVVGGSLI	0	0.6000	75.8	22.00	0.56	tr_C9ZP82_
DRB1_0101	108	FDKKTLVSSAGYYFF	LVSSAGYYF	5	0.5990	76.6	23.00	0.64	tr_C9ZP82_
DRB1_0101	109	VKFRRFLLFKSTNKE	FLLFKSTNK	5	0.5975	77.9	23.00	0.43	tr_C9ZP82_
DRB1_0101	110	FYHAPPVIDLAIYLK	FYHAPPVID	0	0.5965	78.7	23.00	0.49	tr_C9ZP82_
DRB1_0101	111	PKEVMYIAVDGVAPR	YIAVDGVAP	5	0.5957	79.4	23.00	0.56	tr_C9ZP82_
DRB1_0101	112	KFASWLRNKYPAIVT	LRNKYPAIV	5	0.5927	82.0	24.00	0.50	tr_C9ZP82_
DRB1_0101	113	LELYVSEVLPYGFLT	LYVSEVLPY	2	0.5903	84.2	24.00	0.29	tr_C9ZP82_
DRB1_0101	114	TQKLDFHLVITSNKT	FHLVITSNK	5	0.5898	84.6	24.00	0.52	tr_C9ZP82_
DRB1_0101	115	YYFFGQPNHIEDGTL	YFFGQPNHI	1	0.5895	84.9	24.00	0.57	tr_C9ZP82_
DRB1_0101	116	IISLNPSYIDEEESD	IISLNPSYI	0	0.5890	85.3	24.00	0.87	tr_C9ZP82_
DRB1_0101	117	NVDYHDSNVVHLATV	YHDSNVVHL	3	0.5888	85.5	24.00	0.72	tr_C9ZP82_
DRB1_0101	118	LELLVQTVQPKEVMY	VQTVQPKEV	4	0.5887	85.7	24.00	0.56	tr_C9ZP82_
DRB1_0101	119	NLPQLQRLFEAYAKI	LQRLFEAYA	4	0.5884	85.9	24.00	0.47	tr_C9ZP82_
DRB1_0101	120	LPQLQRLFEAYAKIE	LQRLFEAYA	3	0.5872	87.1	25.00	0.47	tr_C9ZP82_
DRB1_0101	121	AVKFRRFLLFKSTNK	FRRFLLFKS	3	0.5871	87.2	25.00	0.46	tr_C9ZP82_
DRB1_0101	122	PDTLVQLLSILPPTS	LLSILPPTS	6	0.5870	87.2	25.00	0.50	tr_C9ZP82_
DRB1_0101	123	SHALLPMVLGEVMVR	HALLPMVLG	1	0.5869	87.3	25.00	0.37	tr_C9ZP82_
DRB1_0101	124	LPPTSHALLPMVLGE	HALLPMVLG	5	0.5859	88.3	25.00	0.41	tr_C9ZP82_
DRB1_0101	125	LLVQTVQPKEVMYIA	VQTVQPKEV	2	0.5858	88.4	25.00	0.62	tr_C9ZP82_
DRB1_0101	126	ASTYMPNKSGMAIVE	YMPNKSGMA	3	0.5855	88.7	25.00	0.74	tr_C9ZP82_
DRB1_0101	127	REGVEIRQLYDVPLE	IRQLYDVPL	5	0.5848	89.4	25.00	0.86	tr_C9ZP82_
DRB1_0101	128	SSLAQFGGKANHVIV	FGGKANHVI	5	0.5840	90.1	25.00	0.41	tr_C9ZP82_
DRB1_0101	129	ILPPTSHALLPMVLG	HALLPMVLG	6	0.5832	90.9	25.00	0.34	tr_C9ZP82_
DRB1_0101	130	LSILLGNDFLPHAPS	SILLGNDFL	1	0.5823	91.8	25.00	0.47	tr_C9ZP82_
DRB1_0101	131	DYHDSNVVHLATVML	YHDSNVVHL	1	0.5822	91.9	25.00	0.49	tr_C9ZP82_
DRB1_0101	132	FRRFLLFKSTNKENS	FLLFKSTNK	3	0.5817	92.4	26.00	0.58	tr_C9ZP82_
DRB1_0101	133	KLECICSELELLVQT	CICSELELL	3	0.5809	93.2	26.00	0.56	tr_C9ZP82_

DRB1_0101	134	DIIVLSILLGNDFLP	SILLGNDFL	5	0.5805	93.6	26.00	0.40	tr_C9ZP82_
DRB1_0101	135	NTGFEYFDISVVGGS	FEYFDISVV	3	0.5802	93.9	26.00	0.61	tr_C9ZP82_
DRB1_0101	136	VASTYMPNKSGMAIV	YMPNKSGMA	4	0.5795	94.6	26.00	0.74	tr_C9ZP82_
DRB1_0101	137	GLDADLIFLALSLHI	IFLALSLHI	6	0.5784	95.7	26.00	0.49	tr_C9ZP82_
DRB1_0101	138	INLPQLQRLFEAYAK	PQLQRLFEA	3	0.5778	96.3	26.00	0.43	tr_C9ZP82_
DRB1_0101	139	YLKEANIKRMPLPVV	IKRMPLPVV	6	0.5753	99.0	27.00	0.50	tr_C9ZP82_
DRB1_0101	140	LYVSEVLPYGFLTKG	EVLPYGFLT	4	0.5745	99.8	27.00	0.32	tr_C9ZP82_
DRB1_0101	141	VDYHDSNVVHLATVM	YHDSNVVHL	2	0.5724	102.1	27.00	0.66	tr_C9ZP82_
DRB1_0101	142	YAKIEAVKFRRFLLF	YAKIEAVKF	0	0.5700	104.9	28.00	0.43	tr_C9ZP82_
DRB1_0101	143	KTLVSSAGYYFFGQP	LVSSAGYYF	2	0.5668	108.5	28.00	0.80	tr_C9ZP82_
DRB1_0101	144	QRLFEAYAKIEAVKF	YAKIEAVKF	6	0.5668	108.6	28.00	0.37	tr_C9ZP82_
DRB1_0101	145	LSILPPTSHALLPMV	LSILPPTSH	0	0.5665	108.9	28.00	0.35	tr_C9ZP82_
DRB1_0101	146	LLELYVSEVLPYGFL	LYVSEVLPY	3	0.5660	109.5	28.00	0.38	tr_C9ZP82_
DRB1_0101	147	RYMGRAAPAQTGEVA	MGRAAPAQT	2	0.5658	109.7	28.00	0.49	tr_C9ZP82_
DRB1_0101	148	EKLECICSELELLVQ	CICSELELL	4	0.5656	110.0	28.00	0.54	tr_C9ZP82_
DRB1_0101	149	PQLQRLFEAYAKIEA	LQRLFEAYA	2	0.5655	110.1	28.00	0.43	tr_C9ZP82_
DRB1_0101	150	WNVDYHDSNVVHLAT	YHDSNVVHL	4	0.5654	110.2	28.00	0.71	tr_C9ZP82_
DRB1_0101	151	SLAQFGGKANHVIVG	FGGKANHVI	4	0.5648	110.9	29.00	0.52	tr_C9ZP82_
DRB1_0101	152	LECICSELELLVQTV	CICSELELL	2	0.5648	111.0	29.00	0.49	tr_C9ZP82_
DRB1_0101	153	WKNFRPCSSPANSKI	FRPCSSPAN	3	0.5637	112.3	29.00	0.76	tr_C9ZP82_
DRB1_0101	154	GNDFLPHAPSAFSGE	FLPHAPSAF	3	0.5623	114.0	29.00	0.56	tr_C9ZP82_
DRB1_0101	155	QSSLAQFGGKANHVI	SLAQFGGKA	2	0.5607	115.9	29.00	0.41	tr_C9ZP82_
DRB1_0101	156	LALSLHITQVFILRD	LHITQVFIL	4	0.5604	116.3	29.00	0.53	tr_C9ZP82_
DRB1_0101	157	LGNDFLPHAPSAFSG	FLPHAPSAF	4	0.5600	116.8	29.00	0.57	tr_C9ZP82_
DRB1_0101	158	SWKNFRPCSSPANSK	FRPCSSPAN	4	0.5579	119.6	30.00	0.71	tr_C9ZP82_
DRB1_0101	159	STYMPNKSGMAIVEE	YMPNKSGMA	2	0.5559	122.1	30.00	0.74	tr_C9ZP82_
DRB1_0101	160	VIDLAIYLKEANIKR	AIYLKEANI	4	0.5555	122.7	30.00	0.49	tr_C9ZP82_
DRB1_0101	161	KNFRPCSSPANSKII	FRPCSSPAN	2	0.5536	125.2	31.00	0.79	tr_C9ZP82_
DRB1_0101	162	ELELLVQTVQPKEVM	VQTVQPKEV	5	0.5535	125.3	31.00	0.48	tr_C9ZP82_
DRB1_0101	163	ECICSELELLVQTVQ	CICSELELL	1	0.5515	128.1	31.00	0.44	tr_C9ZP82_
DRB1_0101	164	NLLELYVSEVLPYGF	LYVSEVLPY	4	0.5510	128.7	31.00	0.35	tr_C9ZP82_
DRB1_0101	165	NDFLPHAPSAFSGES	FLPHAPSAF	2	0.5509	128.9	31.00	0.51	tr_C9ZP82_
DRB1_0101	166	RRFLLFKSTNKENSN	FLLFKSTNK	2	0.5508	129.0	31.00	0.58	tr_C9ZP82_
DRB1_0101	167	PNTGFEYFDISVVGG	FEYFDISVV	4	0.5502	129.9	31.00	0.70	tr_C9ZP82_
DRB1_0101	168	SVHGLYIDLNGIIHP	YIDLNGIIH	5	0.5486	132.2	31.00	0.44	tr_C9ZP82_
DRB1_0101	169	LAQFGGKANHVIVGL	FGGKANHVI	3	0.5479	133.2	32.00	0.61	tr_C9ZP82_
DRB1_0101	170	SSVHGLYIDLNGIIH	YIDLNGIIH	6	0.5476	133.5	32.00	0.33	tr_C9ZP82_
DRB1_0101	171	LVQTVQPKEVMYIAV	VQTVQPKEV	1	0.5476	133.7	32.00	0.53	tr_C9ZP82_
DRB1_0101	172	HALLPMVLGEVMVRS	LLPMVLGEV	2	0.5467	134.9	32.00	0.25	tr_C9ZP82_
DRB1_0101	173	EINLPQLQRLFEAYA	PQLQRLFEA	4	0.5452	137.1	32.00	0.43	tr_C9ZP82_
DRB1_0101	174	QRARRYMGRAAPAQT	RYMGRAAPA	4	0.5444	138.3	32.00	0.40	tr_C9ZP82_
DRB1_0101	175	RTQKLDFHLVITSNK	FHLVITSNK	6	0.5441	138.8	32.00	0.36	tr_C9ZP82_
DRB1_0101	176	QLQRLFEAYAKIEAV	LQRLFEAYA	1	0.5438	139.2	32.00	0.39	tr_C9ZP82_
DRB1_0101	177	IYLKEANIKRMPLPV	YLKEANIKR	1	0.5434	139.8	32.00	0.67	tr_C9ZP82_
DRB1_0101	178	DDIIVLSILLGNDFL	LSILLGNDF	5	0.5432	140.1	32.00	0.42	tr_C9ZP82_
DRB1_0101	179	IKRMPLPVVKSTPPD	IKRMPLPVV	0	0.5429	140.6	33.00	0.60	tr_C9ZP82_
DRB1_0101	180	VLNMLNEKMSGNDPT	LNMLNEKMS	1	0.5392	146.4	33.00	0.44	tr_C9ZP82_
DRB1_0101	181	APSAFSGESALDNLL	FSGESALDN	4	0.5391	146.4	33.00	0.71	tr_C9ZP82_
DRB1_0101	182	DNLLELYVSEVLPYG	LLELYVSEV	2	0.5385	147.5	33.00	0.31	tr_C9ZP82_
DRB1_0101	183	ALSLHITQVFILRDT	LHITQVFIL	3	0.5376	148.8	34.00	0.61	tr_C9ZP82_

DRB1_0101	184	RAKMNQQRARRYMGR	MNQQRARRY	3	0.5373	149.4	34.00	0.82	tr_C9ZP82_
DRB1_0101	185	PSAFSGESALDNLLE	FSGESALDN	3	0.5371	149.7	34.00	0.75	tr_C9ZP82_
DRB1_0101	186	HLVITSNKTSFVPKG	LVITSNKTS	1	0.5359	151.6	34.00	0.35	tr_C9ZP82_
DRB1_0101	187	EEKLECICSELELLV	CICSELELL	5	0.5357	151.9	34.00	0.51	tr_C9ZP82_
DRB1_0101	188	DSNVVHLATVMLPFA	VVHLATVML	3	0.5338	155.1	34.00	0.51	tr_C9ZP82_
DRB1_0101	189	QWNVDYHDSNVVHLA	YHDSNVVHL	5	0.5333	155.9	34.00	0.70	tr_C9ZP82_
DRB1_0101	190	EGEHKIIDFLRTQSS	IDFLRTQSS	6	0.5318	158.5	35.00	0.67	tr_C9ZP82_
DRB1_0101	191	RSWKNFRPCSSPANS	FRPCSSPAN	5	0.5317	158.7	35.00	0.57	tr_C9ZP82_
DRB1_0101	192	VHGLYIDLNGIIHPC	YIDLNGIIH	4	0.5303	161.1	35.00	0.46	tr_C9ZP82_
DRB1_0101	193	WLRNKYPAIVTKNCP	LRNKYPAIV	1	0.5273	166.5	36.00	0.60	tr_C9ZP82_
DRB1_0101	194	QFGGKANHVIVGLDA	FGGKANHVI	1	0.5272	166.6	36.00	0.50	tr_C9ZP82_
DRB1_0101	195	LQRLFEAYAKIEAVK	LFEAYAKIE	3	0.5268	167.3	36.00	0.25	tr_C9ZP82_
DRB1_0101	196	YHDSNVVHLATVMLP	VVHLATVML	5	0.5264	168.0	36.00	0.38	tr_C9ZP82_
DRB1_0101	197	SNVVHLATVMLPFAD	VVHLATVML	2	0.5264	168.1	36.00	0.48	tr_C9ZP82_
DRB1_0101	198	DDRWRDTYLKSTGLS	DTYLKSTGL	5	0.5232	173.9	36.00	0.72	tr_C9ZP82_
DRB1_0101	199	EAVKFRRFLLFKSTN	FRRFLLFKS	4	0.5232	174.0	36.00	0.54	tr_C9ZP82_
DRB1_0101	200	AFDKKTLVSSAGYYF	LVSSAGYYF	6	0.5222	175.9	37.00	0.47	tr_C9ZP82_
DRB1_0101	201	LLGNDFLPHAPSAFS	FLPHAPSAF	5	0.5220	176.3	37.00	0.56	tr_C9ZP82_
DRB1_0101	202	VVREFTSTEMAQAND	FTSTEMAQA	4	0.5218	176.6	37.00	0.72	tr_C9ZP82_
DRB1_0101	203	TDFMAKVSDAVLNML	FMAKVSDAV	2	0.5213	177.6	37.00	0.52	tr_C9ZP82_
DRB1_0101	204	VSSAGYYFFGQPNHI	YFFGQPNHI	6	0.5213	177.6	37.00	0.60	tr_C9ZP82_
DRB1_0101	205	LRTQSSLAQFGGKAN	SLAQFGGKA	5	0.5210	178.2	37.00	0.34	tr_C9ZP82_
DRB1_0101	206	PKFASWLRNKYPAIV	LRNKYPAIV	6	0.5202	179.8	37.00	0.42	tr_C9ZP82_
DRB1_0101	207	YIAVDGVAPRAKMNQ	YIAVDGVAP	0	0.5199	180.2	37.00	0.58	tr_C9ZP82_
DRB1_0101	208	SAFSGESALDNLLEL	FSGESALDN	2	0.5196	181.0	37.00	0.78	tr_C9ZP82_
DRB1_0101	209	NVVHLATVMLPFADM	VVHLATVML	1	0.5191	181.9	37.00	0.35	tr_C9ZP82_
DRB1_0101	210	LDNLLELYVSEVLPY	LLELYVSEV	3	0.5187	182.6	37.00	0.41	tr_C9ZP82_
DRB1_0101	211	IRQLYDVPLERYRPR	IRQLYDVPL	0	0.5184	183.3	37.00	0.65	tr_C9ZP82_
DRB1_0101	212	EVVREFTSTEMAQAN	FTSTEMAQA	5	0.5183	183.5	37.00	0.68	tr_C9ZP82_
DRB1_0101	213	AQFGGKANHVIVGLD	FGGKANHVI	2	0.5179	184.3	37.00	0.62	tr_C9ZP82_
DRB1_0101	214	VCFVWRYYTTTTTDS	WRYYTTTTT	4	0.5167	186.6	38.00	0.70	tr_C9ZP82_
DRB1_0101	215	TYMPNKSGMAIVEEV	YMPNKSGMA	1	0.5165	187.0	38.00	0.65	tr_C9ZP82_
DRB1_0101	216	NVTVADVAVALHSTI	VTVADVAVA	1	0.5161	187.8	38.00	0.31	tr_C9ZP82_
DRB1_0101	217	HGLYIDLNGIIHPCC	YIDLNGIIH	3	0.5145	191.0	38.00	0.47	tr_C9ZP82_
DRB1_0101	218	SREGVEIRQLYDVPL	IRQLYDVPL	6	0.5145	191.1	38.00	0.81	tr_C9ZP82_
DRB1_0101	219	EVASTYMPNKSGMAI	YMPNKSGMA	5	0.5139	192.4	38.00	0.70	tr_C9ZP82_
DRB1_0101	220	HEINLPQLQRLFEAY	PQLQRLFEA	5	0.5136	193.0	38.00	0.40	tr_C9ZP82_
DRB1_0101	221	GTDFMAKVSDAVLNM	FMAKVSDAV	3	0.5129	194.4	38.00	0.61	tr_C9ZP82_
DRB1_0101	222	VPNTGFEYFDISVVG	FEYFDISVV	5	0.5127	195.0	38.00	0.74	tr_C9ZP82_
DRB1_0101	223	CFVWRYYTTTTTDSD	WRYYTTTTT	3	0.5116	197.2	39.00	0.67	tr_C9ZP82_
DRB1_0101	224	NFRPCSSPANSKIID	FRPCSSPAN	1	0.5098	201.1	39.00	0.69	tr_C9ZP82_
DRB1_0101	225	VREFTSTEMAQANDD	FTSTEMAQA	3	0.5090	202.9	39.00	0.79	tr_C9ZP82_
DRB1_0101	226	SILPPTSHALLPMVL	ILPPTSHAL	1	0.5077	205.8	39.00	0.22	tr_C9ZP82_
DRB1_0101	227	YVSEVLPYGFLTKGP	EVLPYGFLT	3	0.5075	206.1	39.00	0.43	tr_C9ZP82_
DRB1_0101	228	HDSNVVHLATVMLPF	VVHLATVML	4	0.5059	209.8	40.00	0.52	tr_C9ZP82_
DRB1_0101	229	KIEAVKFRRFLLFKS	FRRFLLFKS	6	0.5059	209.8	40.00	0.31	tr_C9ZP82_
DRB1_0101	230	PRAKMNQQRARRYMG	MNQQRARRY	4	0.5055	210.7	40.00	0.79	tr_C9ZP82_
DRB1_0101	231	IEAVKFRRFLLFKST	FRRFLLFKS	5	0.5048	212.3	40.00	0.50	tr_C9ZP82_
DRB1_0101	232	DNVTVADVAVALHST	VTVADVAVA	2	0.5047	212.4	40.00	0.40	tr_C9ZP82_
DRB1_0101	233	GGSLISEVSALCDIK	LISEVSALC	3	0.5042	213.7	40.00	0.50	tr_C9ZP82_

DRB1_0101	234	TQSSLAQFGGKANHV	SLAQFGGKA	3	0.5037	214.9	40.00	0.61	tr_C9ZP82_
DRB1_0101	235	DFLPHAPSAFSGESA	FLPHAPSAF	1	0.5003	222.8	41.00	0.47	tr_C9ZP82_
DRB1_0101	236	LSLHITQVFILRDTT	LHITQVFIL	2	0.4998	224.2	41.00	0.65	tr_C9ZP82_
DRB1_0101	237	LPYGFLTKGPHEINL	FLTKGPHEI	4	0.4996	224.6	41.00	0.57	tr_C9ZP82_
DRB1_0101	238	RWRTQKLDFHLVITS	TQKLDFHLV	3	0.4986	227.1	41.00	0.56	tr_C9ZP82_
DRB1_0101	239	SALDNLLELYVSEVL	LLELYVSEV	5	0.4981	228.3	41.00	0.44	tr_C9ZP82_
DRB1_0101	240	FVWRYYTTTTTDSDW	WRYYTTTTT	2	0.4977	229.4	42.00	0.62	tr_C9ZP82_
DRB1_0101	241	RQTLLEDPLYSGALY	TLLEDPLYS	2	0.4968	231.5	42.00	0.65	tr_C9ZP82_
DRB1_0101	242	PHEINLPQLQRLFEA	INLPQLQRL	3	0.4967	231.7	42.00	0.31	tr_C9ZP82_
DRB1_0101	243	FGGKANHVIVGLDAD	NHVIVGLDA	5	0.4967	231.8	42.00	0.42	tr_C9ZP82_
DRB1_0101	244	IKKFTPATESAFDKK	FTPATESAF	3	0.4962	232.9	42.00	0.56	tr_C9ZP82_
DRB1_0101	245	QPKEVMYIAVDGVAP	YIAVDGVAP	6	0.4959	233.7	42.00	0.43	tr_C9ZP82_
DRB1_0101	246	RFLLFKSTNKENSNS	FLLFKSTNK	1	0.4955	234.8	42.00	0.52	tr_C9ZP82_
DRB1_0101	247	PRLSYGADGQVIKRQ	SYGADGQVI	3	0.4950	236.1	42.00	0.55	tr_C9ZP82_
DRB1_0101	248	VRQTLLEDPLYSGAL	TLLEDPLYS	3	0.4939	238.9	42.00	0.74	tr_C9ZP82_
DRB1_0101	249	REFTSTEMAQANDDL	FTSTEMAQA	2	0.4928	241.6	42.00	0.74	tr_C9ZP82_
DRB1_0101	250	SELELLVQTVQPKEV	LLVQTVQPK	4	0.4918	244.4	43.00	0.39	tr_C9ZP82_
DRB1_0101	251	VGGSLISEVSALCDI	LISEVSALC	4	0.4914	245.3	43.00	0.45	tr_C9ZP82_
DRB1_0101	252	AKMNQQRARRYMGRA	MNQQRARRY	2	0.4902	248.5	43.00	0.80	tr_C9ZP82_
DRB1_0101	253	DFMAKVSDAVLNMLN	FMAKVSDAV	1	0.4902	248.7	43.00	0.41	tr_C9ZP82_
DRB1_0101	254	AKVSDAVLNMLNEKM	VLNMLNEKM	6	0.4900	249.2	43.00	0.69	tr_C9ZP82_
DRB1_0101	255	SILLGNDFLPHAPSA	SILLGNDFL	0	0.4899	249.4	43.00	0.41	tr_C9ZP82_
DRB1_0101	256	CICSELELLVQTVQP	ELELLVQTV	4	0.4890	251.9	43.00	0.30	tr_C9ZP82_
DRB1_0101	257	VPRLSYGADGQVIKR	SYGADGQVI	4	0.4883	253.9	43.00	0.50	tr_C9ZP82_
DRB1_0101	258	GSLISEVSALCDIKK	LISEVSALC	2	0.4881	254.3	43.00	0.45	tr_C9ZP82_
DRB1_0101	259	SLHITQVFILRDTTR	LHITQVFIL	1	0.4874	256.3	44.00	0.61	tr_C9ZP82_
DRB1_0101	260	GLYIDLNGIIHPCCH	YIDLNGIIH	2	0.4872	256.7	44.00	0.49	tr_C9ZP82_
DRB1_0101	261	VGLDADLIFLALSLH	LIFLALSLH	6	0.4859	260.5	44.00	0.22	tr_C9ZP82_
DRB1_0101	262	DLRKLSDALDTILRR	LRKLSDALD	1	0.4858	260.8	44.00	0.37	tr_C9ZP82_
DRB1_0101	263	NDNVTVADVAVALHS	VTVADVAVA	3	0.4851	262.7	44.00	0.52	tr_C9ZP82_
DRB1_0101	264	GVCFVWRYYTTTTTD	WRYYTTTTT	5	0.4842	265.3	44.00	0.68	tr_C9ZP82_
DRB1_0101	265	RTQSSLAQFGGKANH	SLAQFGGKA	4	0.4840	265.9	44.00	0.54	tr_C9ZP82_
DRB1_0101	266	HAPSAFSGESALDNL	FSGESALDN	5	0.4838	266.5	44.00	0.63	tr_C9ZP82_
DRB1_0101	267	RLSYGADGQVIKRQR	SYGADGQVI	2	0.4827	269.7	45.00	0.55	tr_C9ZP82_
DRB1_0101	268	ALDNLLELYVSEVLP	LLELYVSEV	4	0.4825	270.2	45.00	0.44	tr_C9ZP82_
DRB1_0101	269	PYGFLTKGPHEINLP	FLTKGPHEI	3	0.4814	273.4	45.00	0.57	tr_C9ZP82_
DRB1_0101	270	PAIVTKNCPSSVHGL	VTKNCPSSV	3	0.4808	275.4	45.00	0.74	tr_C9ZP82_
DRB1_0101	271	AIVTKNCPSSVHGLY	VTKNCPSSV	2	0.4802	277.1	45.00	0.67	tr_C9ZP82_
DRB1_0101	272	VVHLATVMLPFADMD	TVMLPFADM	5	0.4785	282.1	45.00	0.31	tr_C9ZP82_
DRB1_0101	273	IVTKNCPSSVHGLYI	VTKNCPSSV	1	0.4780	283.8	46.00	0.49	tr_C9ZP82_
DRB1_0101	274	GGKANHVIVGLDADL	NHVIVGLDA	4	0.4776	285.0	46.00	0.68	tr_C9ZP82_
DRB1_0101	275	AKIEAVKFRRFLLFK	IEAVKFRRF	2	0.4774	285.7	46.00	0.42	tr_C9ZP82_
DRB1_0101	276	VTVADVAVALHSTID	ADVAVALHS	3	0.4771	286.5	46.00	0.26	tr_C9ZP82_
DRB1_0101	277	PGTDFMAKVSDAVLN	FMAKVSDAV	4	0.4767	287.6	46.00	0.62	tr_C9ZP82_
DRB1_0101	278	LRKLSDALDTILRRV	SDALDTILR	4	0.4762	289.3	46.00	0.29	tr_C9ZP82_
DRB1_0101	279	ERWRTQKLDFHLVIT	TQKLDFHLV	4	0.4754	291.9	46.00	0.56	tr_C9ZP82_
DRB1_0101	280	GDLRKLSDALDTILR	LRKLSDALD	2	0.4752	292.6	46.00	0.46	tr_C9ZP82_
DRB1_0101	281	EESDGVIGVPVCESS	SDGVIGVPV	2	0.4734	298.0	47.00	0.46	tr_C9ZP82_
DRB1_0101	282	DIKKFTPATESAFDK	FTPATESAF	4	0.4727	300.5	47.00	0.53	tr_C9ZP82_
DRB1_0101	283	VSEVLPYGFLTKGPH	EVLPYGFLT	2	0.4725	301.1	47.00	0.47	tr_C9ZP82_

DRB1_0101	284	SVVGGSLISEVSALC	LISEVSALC	6	0.4721	302.5	47.00	0.25	tr_C9ZP82_
DRB1_0101	285	TLVSSAGYYFFGQPN	LVSSAGYYF	1	0.4713	305.2	47.00	0.80	tr_C9ZP82_
DRB1_0101	286	YFFGQPNHIEDGTLP	YFFGQPNHI	0	0.4711	305.7	47.00	0.47	tr_C9ZP82_
DRB1_0101	287	YLKSTGLSNSVQEAC	YLKSTGLSN	0	0.4710	306.0	47.00	0.53	tr_C9ZP82_
DRB1_0101	288	WRTQKLDFHLVITSN	TQKLDFHLV	2	0.4704	308.0	47.00	0.50	tr_C9ZP82_
DRB1_0101	289	KGDLRKLSDALDTIL	LRKLSDALD	3	0.4686	314.0	48.00	0.59	tr_C9ZP82_
DRB1_0101	290	VQTVQPKEVMYIAVD	VQPKEVMYI	3	0.4682	315.3	48.00	0.35	tr_C9ZP82_
DRB1_0101	291	APRAKMNQQRARRYM	MNQQRARRY	5	0.4680	316.1	48.00	0.74	tr_C9ZP82_
DRB1_0101	292	ESDGVIGVPVCESSE	SDGVIGVPV	1	0.4680	316.2	48.00	0.38	tr_C9ZP82_
DRB1_0101	293	PSSVHGLYIDLNGII	GLYIDLNGI	5	0.4679	316.3	48.00	0.32	tr_C9ZP82_
DRB1_0101	294	EEEKLECICSELELL	CICSELELL	6	0.4678	316.7	48.00	0.50	tr_C9ZP82_
DRB1_0101	295	PVIDLAIYLKEANIK	AIYLKEANI	5	0.4678	316.9	48.00	0.44	tr_C9ZP82_
DRB1_0101	296	VVGGSLISEVSALCD	LISEVSALC	5	0.4674	318.2	48.00	0.43	tr_C9ZP82_
DRB1_0101	297	TQVFILRDTTRNPYK	FILRDTTRN	3	0.4673	318.6	48.00	0.73	tr_C9ZP82_
DRB1_0101	298	YMGRAAPAQTGEVAS	MGRAAPAQT	1	0.4661	322.8	48.00	0.47	tr_C9ZP82_
DRB1_0101	299	CDIKKFTPATESAFD	FTPATESAF	5	0.4660	322.9	48.00	0.43	tr_C9ZP82_
DRB1_0101	300	KKFTPATESAFDKKT	FTPATESAF	2	0.4656	324.3	48.00	0.60	tr_C9ZP82_
DRB1_0101	301	LCDIKKFTPATESAF	IKKFTPATE	3	0.4635	331.9	49.00	0.31	tr_C9ZP82_
DRB1_0101	302	LQQVVDAAWPQLTEE	VDAAWPQLT	4	0.4631	333.3	49.00	0.68	tr_C9ZP82_
DRB1_0101	303	ERSWKNFRPCSSPAN	WKNFRPCSS	3	0.4629	333.9	49.00	0.45	tr_C9ZP82_
DRB1_0101	304	ALLPMVLGEVMVRSP	LLPMVLGEV	1	0.4628	334.5	49.00	0.29	tr_C9ZP82_
DRB1_0101	305	SLISEVSALCDIKKF	LISEVSALC	1	0.4613	339.7	49.00	0.41	tr_C9ZP82_
DRB1_0101	306	YGFLTKGPHEINLPQ	FLTKGPHEI	2	0.4609	341.2	49.00	0.62	tr_C9ZP82_
DRB1_0101	307	QTLLEDPLYSGALYN	TLLEDPLYS	1	0.4604	343.2	49.00	0.52	tr_C9ZP82_
DRB1_0101	308	TVPNTGFEYFDISVV	FEYFDISVV	6	0.4593	347.3	50.00	0.63	tr_C9ZP82_
DRB1_0101	309	DWSWYYPFYHAPPVI	YPFYHAPPV	5	0.4592	347.6	50.00	0.38	tr_C9ZP82_
DRB1_0101	310	PTAFEFDSNCISPGT	FEFDSNCIS	3	0.4580	352.3	50.00	0.61	tr_C9ZP82_
DRB1_0101	311	ALDTILRRVPSREGV	ILRRVPSRE	4	0.4568	356.9	50.00	0.32	tr_C9ZP82_
DRB1_0101	312	VLPYGFLTKGPHEIN	FLTKGPHEI	5	0.4553	362.6	50.00	0.53	tr_C9ZP82_
DRB1_0101	313	EVRQTLLEDPLYSGA	TLLEDPLYS	4	0.4551	363.3	50.00	0.76	tr_C9ZP82_
DRB1_0101	314	VLQQVVDAAWPQLTE	VDAAWPQLT	5	0.4548	364.6	50.00	0.61	tr_C9ZP82_
DRB1_0101	315	QQVVDAAWPQLTEEE	VDAAWPQLT	3	0.4532	371.2	55.00	0.74	tr_C9ZP82_
DRB1_0101	316	EERWRTQKLDFHLVI	TQKLDFHLV	5	0.4529	372.4	55.00	0.51	tr_C9ZP82_
DRB1_0101	317	HLATVMLPFADMDVL	TVMLPFADM	3	0.4519	376.2	55.00	0.48	tr_C9ZP82_
DRB1_0101	318	VWRYYTTTTTDSDWS	WRYYTTTTT	1	0.4508	380.6	55.00	0.57	tr_C9ZP82_
DRB1_0101	319	TAFEFDSNCISPGTD	FEFDSNCIS	2	0.4507	381.1	55.00	0.63	tr_C9ZP82_
DRB1_0101	320	QVFILRDTTRNPYKR	FILRDTTRN	2	0.4507	381.4	55.00	0.68	tr_C9ZP82_
DRB1_0101	321	SEVLPYGFLTKGPHE	EVLPYGFLT	1	0.4506	381.7	55.00	0.44	tr_C9ZP82_
DRB1_0101	322	LDTILRRVPSREGVE	ILRRVPSRE	3	0.4505	381.8	55.00	0.36	tr_C9ZP82_
DRB1_0101	323	EEESDGVIGVPVCES	SDGVIGVPV	3	0.4505	381.9	55.00	0.54	tr_C9ZP82_
DRB1_0101	324	QQRARRYMGRAAPAQ	RYMGRAAPA	5	0.4504	382.3	55.00	0.44	tr_C9ZP82_
DRB1_0101	325	YMPNKSGMAIVEEVV	YMPNKSGMA	0	0.4500	384.1	55.00	0.38	tr_C9ZP82_
DRB1_0101	326	VHLATVMLPFADMDV	TVMLPFADM	4	0.4497	385.2	55.00	0.44	tr_C9ZP82_
DRB1_0101	327	KGPHEINLPQLQRLF	HEINLPQLQ	3	0.4491	387.9	55.00	0.36	tr_C9ZP82_
DRB1_0101	328	LPVVKSTPPDTLVQL	VVKSTPPDT	2	0.4485	390.3	55.00	0.49	tr_C9ZP82_
DRB1_0101	329	FLPHAPSAFSGESAL	LPHAPSAFS	1	0.4479	393.0	55.00	0.32	tr_C9ZP82_
DRB1_0101	330	NNDNVTVADVAVALH	VTVADVAVA	4	0.4473	395.5	55.00	0.52	tr_C9ZP82_
DRB1_0101	331	ESALDNLLELYVSEV	LLELYVSEV	6	0.4455	403.4	55.00	0.30	tr_C9ZP82_
DRB1_0101	332	KMNQQRARRYMGRAA	MNQQRARRY	1	0.4452	404.5	55.00	0.68	tr_C9ZP82_
DRB1_0101	333	ILLGNDFLPHAPSAF	FLPHAPSAF	6	0.4437	411.4	55.00	0.40	tr_C9ZP82_

DRB1_0101	334	LHITQVFILRDTTRN	LHITQVFIL	0	0.4432	413.3	55.00	0.41	tr_C9ZP82_
DRB1_0101	335	RNPYKRRAVARQQQR	YKRRAVARQ	3	0.4430	414.4	55.00	0.56	tr_C9ZP82_
DRB1_0101	336	PFADMDVLQQVVDAA	FADMDVLQQ	1	0.4428	415.0	55.00	0.34	tr_C9ZP82_
DRB1_0101	337	AFSGESALDNLLELY	FSGESALDN	1	0.4422	418.0	55.00	0.68	tr_C9ZP82_
DRB1_0101	338	ATVMLPFADMDVLQQ	TVMLPFADM	1	0.4419	419.4	55.00	0.44	tr_C9ZP82_
DRB1_0101	339	GPHEINLPQLQRLFE	INLPQLQRL	4	0.4412	422.3	55.00	0.39	tr_C9ZP82_
DRB1_0101	340	CPSSVHGLYIDLNGI	PSSVHGLYI	1	0.4410	423.6	55.00	0.37	tr_C9ZP82_
DRB1_0101	341	TVMLPFADMDVLQQV	FADMDVLQQ	5	0.4406	425.0	55.00	0.41	tr_C9ZP82_
DRB1_0101	342	LATVMLPFADMDVLQ	TVMLPFADM	2	0.4404	426.2	55.00	0.49	tr_C9ZP82_
DRB1_0101	343	YPAIVTKNCPSSVHG	VTKNCPSSV	4	0.4399	428.6	55.00	0.73	tr_C9ZP82_
DRB1_0101	344	FADMDVLQQVVDAAW	DVLQQVVDA	4	0.4399	428.6	55.00	0.44	tr_C9ZP82_
DRB1_0101	345	PLPVVKSTPPDTLVQ	VVKSTPPDT	3	0.4386	434.6	55.00	0.54	tr_C9ZP82_
DRB1_0101	346	CSELELLVQTVQPKE	ELELLVQTV	2	0.4374	440.4	55.00	0.41	tr_C9ZP82_
DRB1_0101	347	FMAKVSDAVLNMLNE	AKVSDAVLN	2	0.4371	441.6	55.00	0.34	tr_C9ZP82_
DRB1_0101	348	SDGVIGVPVCESSEN	VIGVPVCES	3	0.4365	444.3	55.00	0.40	tr_C9ZP82_
DRB1_0101	349	KNCPSSVHGLYIDLN	PSSVHGLYI	3	0.4349	452.4	55.00	0.61	tr_C9ZP82_
DRB1_0101	350	NKTSFVPKGDLRKLS	FVPKGDLRK	4	0.4348	452.9	55.00	0.62	tr_C9ZP82_
DRB1_0101	351	EGVCFVWRYYTTTTT	WRYYTTTTT	6	0.4347	453.4	55.00	0.54	tr_C9ZP82_
DRB1_0101	352	PPDTLVQLLSILPPT	VQLLSILPP	5	0.4345	454.5	55.00	0.32	tr_C9ZP82_
DRB1_0101	353	ICSELELLVQTVQPK	ELELLVQTV	3	0.4328	462.4	55.00	0.44	tr_C9ZP82_
DRB1_0101	354	KTSFVPKGDLRKLSD	FVPKGDLRK	3	0.4322	465.4	55.00	0.62	tr_C9ZP82_
DRB1_0101	355	EVPRLSYGADGQVIK	SYGADGQVI	5	0.4314	469.5	55.00	0.47	tr_C9ZP82_
DRB1_0101	356	LQWNVDYHDSNVVHL	YHDSNVVHL	6	0.4313	470.5	60.00	0.57	tr_C9ZP82_
DRB1_0101	357	EEVVREFTSTEMAQA	FTSTEMAQA	6	0.4292	481.3	60.00	0.50	tr_C9ZP82_
DRB1_0101	358	DVLQQVVDAAWPQLT	VDAAWPQLT	6	0.4290	482.1	60.00	0.34	tr_C9ZP82_
DRB1_0101	359	DPTAFEFDSNCISPG	FEFDSNCIS	4	0.4289	482.7	60.00	0.66	tr_C9ZP82_
DRB1_0101	360	ITQVFILRDTTRNPY	FILRDTTRN	4	0.4288	483.4	60.00	0.70	tr_C9ZP82_
DRB1_0101	361	RTYSCTVEVPRLSYG	CTVEVPRLS	4	0.4279	487.6	60.00	0.51	tr_C9ZP82_
DRB1_0101	362	LLPMVLGEVMVRSPQ	MVLGEVMVR	3	0.4275	490.2	60.00	0.34	tr_C9ZP82_
DRB1_0101	363	VMLPFADMDVLQQVV	FADMDVLQQ	4	0.4265	495.3	60.00	0.61	tr_C9ZP82_
DRB1_0101	364	DTILRRVPSREGVEI	LRRVPSREG	3	0.4264	495.7	60.00	0.32	tr_C9ZP82_
DRB1_0101	365	EVLPYGFLTKGPHEI	FLTKGPHEI	6	0.4260	497.9	60.00	0.31	tr_C9ZP82_
DRB1_0101	366	LPFADMDVLQQVVDA	FADMDVLQQ	2	0.4251	502.7	60.00	0.52	tr_C9ZP82_
DRB1_0101	367	TRNPYKRRAVARQQQ	YKRRAVARQ	4	0.4245	506.1	60.00	0.52	tr_C9ZP82_
DRB1_0101	368	VQPKEVMYIAVDGVA	VMYIAVDGV	5	0.4243	507.0	60.00	0.50	tr_C9ZP82_
DRB1_0101	369	LYIDLNGIIHPCCHN	YIDLNGIIH	1	0.4243	507.3	60.00	0.50	tr_C9ZP82_
DRB1_0101	370	TYSCTVEVPRLSYGA	CTVEVPRLS	3	0.4237	510.6	60.00	0.60	tr_C9ZP82_
DRB1_0101	371	IDDIIVLSILLGNDF	LSILLGNDF	6	0.4235	511.4	60.00	0.56	tr_C9ZP82_
DRB1_0101	372	AFEFDSNCISPGTDF	FEFDSNCIS	1	0.4234	511.9	60.00	0.54	tr_C9ZP82_
DRB1_0101	373	QQAELESTFPLQWNV	AELESTFPL	2	0.4234	512.0	60.00	0.55	tr_C9ZP82_
DRB1_0101	374	FRPCSSPANSKIIDD	FRPCSSPAN	0	0.4232	513.2	60.00	0.57	tr_C9ZP82_
DRB1_0101	375	PTWTRLCVIFSGSNT	WTRLCVIFS	2	0.4231	514.1	60.00	0.51	tr_C9ZP82_
DRB1_0101	376	SPGTDFMAKVSDAVL	FMAKVSDAV	5	0.4214	523.6	60.00	0.64	tr_C9ZP82_
DRB1_0101	377	VTKNCPSSVHGLYID	PSSVHGLYI	5	0.4193	535.2	60.00	0.43	tr_C9ZP82_
DRB1_0101	378	TVADVAVALHSTIDD	ADVAVALHS	2	0.4184	540.6	60.00	0.32	tr_C9ZP82_
DRB1_0101	379	LSYGADGQVIKRQRR	SYGADGQVI	1	0.4182	542.0	60.00	0.50	tr_C9ZP82_
DRB1_0101	380	PPVIDLAIYLKEANI	PVIDLAIYL	1	0.4162	553.9	60.00	0.35	tr_C9ZP82_
DRB1_0101	381	NCPSSVHGLYIDLNG	PSSVHGLYI	2	0.4157	556.9	60.00	0.56	tr_C9ZP82_
DRB1_0101	382	EFTSTEMAQANDDLD	FTSTEMAQA	1	0.4154	558.4	60.00	0.68	tr_C9ZP82_
DRB1_0101	383	PRTYSCTVEVPRLSY	CTVEVPRLS	5	0.4154	558.5	60.00	0.46	tr_C9ZP82_

DRB1_0101	384	VVDDVFGHRGEVIVD	VFGHRGEVI	4	0.4144	564.7	60.00	0.50	tr_C9ZP82_
DRB1_0101	385	PKGDLRKLSDALDTI	LRKLSDALD	4	0.4135	570.3	60.00	0.62	tr_C9ZP82_
DRB1_0101	386	DALDTILRRVPSREG	DTILRRVPS	3	0.4133	571.4	60.00	0.40	tr_C9ZP82_
DRB1_0101	387	MLPFADMDVLQQVVD	FADMDVLQQ	3	0.4129	573.9	60.00	0.65	tr_C9ZP82_
DRB1_0101	388	KFTPATESAFDKKTL	FTPATESAF	1	0.4111	585.3	60.00	0.54	tr_C9ZP82_
DRB1_0101	389	PVVKSTPPDTLVQLL	VKSTPPDTL	2	0.4108	586.8	60.00	0.44	tr_C9ZP82_
DRB1_0101	390	PQQAELESTFPLQWN	AELESTFPL	3	0.4106	588.2	60.00	0.60	tr_C9ZP82_
DRB1_0101	391	VDDVFGHRGEVIVDD	VFGHRGEVI	3	0.4106	588.2	60.00	0.50	tr_C9ZP82_
DRB1_0101	392	DPTWTRLCVIFSGSN	WTRLCVIFS	3	0.4097	594.0	60.00	0.63	tr_C9ZP82_
DRB1_0101	393	YHAPPVIDLAIYLKE	PVIDLAIYL	4	0.4094	595.9	60.00	0.47	tr_C9ZP82_
DRB1_0101	394	ACEKYVEGVCFVWRY	EKYVEGVCF	2	0.4091	597.6	60.00	0.41	tr_C9ZP82_
DRB1_0101	395	MPNKSGMAIVEEVVR	SGMAIVEEV	4	0.4083	603.3	60.00	0.43	tr_C9ZP82_
DRB1_0101	396	QVVDAAWPQLTEEER	VDAAWPQLT	2	0.4077	607.3	65.00	0.77	tr_C9ZP82_
DRB1_0101	397	ADMDVLQQVVDAAWP	DVLQQVVDA	3	0.4073	609.6	65.00	0.46	tr_C9ZP82_
DRB1_0101	398	VFILRDTTRNPYKRR	FILRDTTRN	1	0.4061	617.6	65.00	0.61	tr_C9ZP82_
DRB1_0101	399	RKLSDALDTILRRVP	SDALDTILR	3	0.4060	618.2	65.00	0.35	tr_C9ZP82_
DRB1_0101	400	IDDRWRDTYLKSTGL	DTYLKSTGL	6	0.4046	627.7	65.00	0.68	tr_C9ZP82_
DRB1_0101	401	MPLPVVKSTPPDTLV	VVKSTPPDT	4	0.4038	633.0	65.00	0.54	tr_C9ZP82_
DRB1_0101	402	ALCDIKKFTPATESA	IKKFTPATE	4	0.4037	634.1	65.00	0.39	tr_C9ZP82_
DRB1_0101	403	TSFVPKGDLRKLSDA	FVPKGDLRK	2	0.4025	641.9	65.00	0.58	tr_C9ZP82_
DRB1_0101	404	CEKYVEGVCFVWRYY	YVEGVCFVW	3	0.4024	642.9	65.00	0.34	tr_C9ZP82_
DRB1_0101	405	MDVLQQVVDAAWPQL	DVLQQVVDA	1	0.4024	643.0	65.00	0.37	tr_C9ZP82_
DRB1_0101	406	PNKSGMAIVEEVVRE	SGMAIVEEV	3	0.4017	647.9	65.00	0.46	tr_C9ZP82_
DRB1_0101	407	DMDVLQQVVDAAWPQ	DVLQQVVDA	2	0.4015	649.3	65.00	0.41	tr_C9ZP82_
DRB1_0101	408	DEVRQTLLEDPLYSG	TLLEDPLYS	5	0.4012	651.0	65.00	0.71	tr_C9ZP82_
DRB1_0101	409	KYPAIVTKNCPSSVH	VTKNCPSSV	5	0.4012	651.4	65.00	0.64	tr_C9ZP82_
DRB1_0101	410	GEVASTYMPNKSGMA	YMPNKSGMA	6	0.4001	659.2	65.00	0.56	tr_C9ZP82_
DRB1_0101	411	TKNCPSSVHGLYIDL	PSSVHGLYI	4	0.4001	659.3	65.00	0.64	tr_C9ZP82_
DRB1_0101	412	SDALDTILRRVPSRE	DTILRRVPS	4	0.3993	664.8	65.00	0.36	tr_C9ZP82_
DRB1_0101	413	TWTRLCVIFSGSNTP	WTRLCVIFS	1	0.3989	668.0	65.00	0.46	tr_C9ZP82_
DRB1_0101	414	NKSGMAIVEEVVREF	SGMAIVEEV	2	0.3983	672.0	65.00	0.44	tr_C9ZP82_
DRB1_0101	415	FLLFKSTNKENSNSG	FKSTNKENS	3	0.3977	676.1	65.00	0.33	tr_C9ZP82_
DRB1_0101	416	TLLEDPLYSGALYNE	TLLEDPLYS	0	0.3959	690.0	65.00	0.35	tr_C9ZP82_
DRB1_0101	417	TPPDTLVQLLSILPP	DTLVQLLSI	3	0.3948	697.7	65.00	0.31	tr_C9ZP82_
DRB1_0101	418	QVVDDVFGHRGEVIV	VFGHRGEVI	5	0.3942	702.3	65.00	0.50	tr_C9ZP82_
DRB1_0101	419	LSDALDTILRRVPSR	DTILRRVPS	5	0.3937	706.5	65.00	0.28	tr_C9ZP82_
DRB1_0101	420	QAELESTFPLQWNVD	AELESTFPL	1	0.3927	714.1	65.00	0.47	tr_C9ZP82_
DRB1_0101	421	EACEKYVEGVCFVWR	EKYVEGVCF	3	0.3919	719.8	65.00	0.45	tr_C9ZP82_
DRB1_0101	422	GFLTKGPHEINLPQL	FLTKGPHEI	1	0.3919	720.5	65.00	0.63	tr_C9ZP82_
DRB1_0101	423	YSCTVEVPRLSYGAD	CTVEVPRLS	2	0.3915	723.4	65.00	0.61	tr_C9ZP82_
DRB1_0101	424	LVITSNKTSFVPKGD	ITSNKTSFV	2	0.3914	724.3	65.00	0.61	tr_C9ZP82_
DRB1_0101	425	TTRNPYKRRAVARQQ	YKRRAVARQ	5	0.3909	728.4	65.00	0.45	tr_C9ZP82_
DRB1_0101	426	DEEESDGVIGVPVCE	SDGVIGVPV	4	0.3903	733.0	65.00	0.62	tr_C9ZP82_
DRB1_0101	427	TKGPHEINLPQLQRL	HEINLPQLQ	4	0.3903	733.0	65.00	0.42	tr_C9ZP82_
DRB1_0101	428	HAPPVIDLAIYLKEA	PVIDLAIYL	3	0.3901	734.1	65.00	0.57	tr_C9ZP82_
DRB1_0101	429	VADVAVALHSTIDDR	VAVALHSTI	3	0.3901	734.2	65.00	0.24	tr_C9ZP82_
DRB1_0101	430	NPYKRRAVARQQQRE	YKRRAVARQ	2	0.3901	734.3	65.00	0.58	tr_C9ZP82_
DRB1_0101	431	SAFDKKTLVSSAGYY	KKTLVSSAG	4	0.3893	740.9	65.00	0.25	tr_C9ZP82_
DRB1_0101	432	KLSDALDTILRRVPS	SDALDTILR	2	0.3886	746.0	65.00	0.34	tr_C9ZP82_
DRB1_0101	433	PHAPSAFSGESALDN	FSGESALDN	6	0.3886	746.3	65.00	0.43	tr_C9ZP82_

DRB1_0101	434	NDPTWTRLCVIFSGS	WTRLCVIFS	4	0.3867	761.6	65.00	0.65	tr_C9ZP82_
DRB1_0101	435	SDTAIALLPEEEKLE	IALLPEEEK	4	0.3856	771.1	65.00	0.54	tr_C9ZP82_
DRB1_0101	436	DDVFGHRGEVIVDDP	VFGHRGEVI	2	0.3853	773.5	65.00	0.51	tr_C9ZP82_
DRB1_0101	437	GESALDNLLELYVSE	LDNLLELYV	4	0.3847	778.3	65.00	0.38	tr_C9ZP82_
DRB1_0101	438	QTVQPKEVMYIAVDG	VQPKEVMYI	2	0.3843	782.0	70.00	0.50	tr_C9ZP82_
DRB1_0101	439	SNNDNVTVADVAVAL	VTVADVAVA	5	0.3840	784.3	70.00	0.58	tr_C9ZP82_
DRB1_0101	440	SNKTSFVPKGDLRKL	FVPKGDLRK	5	0.3839	785.0	70.00	0.60	tr_C9ZP82_
DRB1_0101	441	MGVPKFASWLRNKYP	GVPKFASWL	1	0.3833	790.5	70.00	0.57	tr_C9ZP82_
DRB1_0101	442	WTRLCVIFSGSNTPG	CVIFSGSNT	4	0.3833	790.7	70.00	0.28	tr_C9ZP82_
DRB1_0101	443	APPVIDLAIYLKEAN	PVIDLAIYL	2	0.3831	792.2	70.00	0.52	tr_C9ZP82_
DRB1_0101	444	TAIALLPEEEKLECI	IALLPEEEK	2	0.3823	799.1	70.00	0.53	tr_C9ZP82_
DRB1_0101	445	HITQVFILRDTTRNP	FILRDTTRN	5	0.3817	804.1	70.00	0.58	tr_C9ZP82_
DRB1_0101	446	LPMVLGEVMVRSPQQ	MVLGEVMVR	2	0.3798	821.1	70.00	0.40	tr_C9ZP82_
DRB1_0101	447	EEERWRTQKLDFHLV	TQKLDFHLV	6	0.3782	835.1	70.00	0.47	tr_C9ZP82_
DRB1_0101	448	TVQPKEVMYIAVDGV	VMYIAVDGV	6	0.3772	844.4	70.00	0.28	tr_C9ZP82_
DRB1_0101	449	TILRRVPSREGVEIR	LRRVPSREG	2	0.3772	844.8	70.00	0.44	tr_C9ZP82_
DRB1_0101	450	SPQQAELESTFPLQW	AELESTFPL	4	0.3767	848.8	70.00	0.62	tr_C9ZP82_
DRB1_0101	451	LISEVSALCDIKKFT	VSALCDIKK	4	0.3762	853.6	70.00	0.28	tr_C9ZP82_
DRB1_0101	452	DDPTAFEFDSNCISP	FEFDSNCIS	5	0.3754	861.4	70.00	0.72	tr_C9ZP82_
DRB1_0101	453	KSGMAIVEEVVREFT	SGMAIVEEV	1	0.3752	863.2	70.00	0.38	tr_C9ZP82_
DRB1_0101	454	SALCDIKKFTPATES	CDIKKFTPA	3	0.3747	867.9	70.00	0.36	tr_C9ZP82_
DRB1_0101	455	LRNKYPAIVTKNCPS	LRNKYPAIV	0	0.3741	872.9	70.00	0.52	tr_C9ZP82_
DRB1_0101	456	SNCISPGTDFMAKVS	ISPGTDFMA	3	0.3729	884.7	70.00	0.57	tr_C9ZP82_
DRB1_0101	457	AELESTFPLQWNVDY	LESTFPLQW	2	0.3722	891.3	70.00	0.27	tr_C9ZP82_
DRB1_0101	458	LVSSAGYYFFGQPNH	LVSSAGYYF	0	0.3719	894.1	70.00	0.51	tr_C9ZP82_
DRB1_0101	459	DTAIALLPEEEKLEC	IALLPEEEK	3	0.3689	923.2	70.00	0.56	tr_C9ZP82_
DRB1_0101	460	SDWSWYYPFYHAPPV	YPFYHAPPV	6	0.3682	931.0	70.00	0.31	tr_C9ZP82_
DRB1_0101	461	SGESALDNLLELYVS	LDNLLELYV	5	0.3681	931.5	70.00	0.34	tr_C9ZP82_
DRB1_0101	462	DVFGHRGEVIVDDPT	VFGHRGEVI	1	0.3677	935.3	70.00	0.45	tr_C9ZP82_
DRB1_0101	463	EKYVEGVCFVWRYYT	YVEGVCFVW	2	0.3667	945.6	70.00	0.35	tr_C9ZP82_
DRB1_0101	464	FSGESALDNLLELYV	FSGESALDN	0	0.3665	948.4	70.00	0.38	tr_C9ZP82_
DRB1_0101	465	VPKGDLRKLSDALDT	LRKLSDALD	5	0.3662	951.6	70.00	0.58	tr_C9ZP82_
DRB1_0101	466	DSDTAIALLPEEEKL	IALLPEEEK	5	0.3660	953.0	70.00	0.52	tr_C9ZP82_
DRB1_0101	467	KSTPPDTLVQLLSIL	DTLVQLLSI	5	0.3656	956.8	70.00	0.44	tr_C9ZP82_
DRB1_0101	468	WRYYTTTTTDSDWSW	WRYYTTTTT	0	0.3643	970.9	70.00	0.43	tr_C9ZP82_
DRB1_0101	469	GNDPTWTRLCVIFSG	WTRLCVIFS	5	0.3639	975.1	70.00	0.60	tr_C9ZP82_
DRB1_0101	470	GAEFRKGSNNDNVTV	FRKGSNNDN	3	0.3638	976.6	70.00	0.87	tr_C9ZP82_
DRB1_0101	471	RLCVIFSGSNTPGEG	VIFSGSNTP	3	0.3637	977.4	70.00	0.34	tr_C9ZP82_
DRB1_0101	472	STPPDTLVQLLSILP	DTLVQLLSI	4	0.3634	979.8	70.00	0.42	tr_C9ZP82_
DRB1_0101	473	TRLCVIFSGSNTPGE	CVIFSGSNT	3	0.3627	987.9	70.00	0.29	tr_C9ZP82_
DRB1_0101	474	SCTVEVPRLSYGADG	CTVEVPRLS	1	0.3625	989.4	70.00	0.51	tr_C9ZP82_
DRB1_0101	475	ELESTFPLQWNVDYH	FPLQWNVDY	5	0.3623	991.6	70.00	0.50	tr_C9ZP82_
DRB1_0101	476	VAPRAKMNQQRARRY	MNQQRARRY	6	0.3623	992.0	70.00	0.56	tr_C9ZP82_
DRB1_0101	477	VPKFASWLRNKYPAI	WLRNKYPAI	6	0.3622	992.9	70.00	0.44	tr_C9ZP82_
DRB1_0101	478	VEVPRLSYGADGQVI	SYGADGQVI	6	0.3613	1002.6	70.00	0.32	tr_C9ZP82_
DRB1_0101	479	RPRTYSCTVEVPRLS	CTVEVPRLS	6	0.3610	1006.1	70.00	0.31	tr_C9ZP82_
DRB1_0101	480	SFVPKGDLRKLSDAL	FVPKGDLRK	1	0.3600	1017.3	75.00	0.47	tr_C9ZP82_
DRB1_0101	481	FEFDSNCISPGTDFM	FDSNCISPG	2	0.3584	1034.8	75.00	0.36	tr_C9ZP82_
DRB1_0101	482	IDEEESDGVIGVPVC	SDGVIGVPV	5	0.3581	1038.3	75.00	0.57	tr_C9ZP82_
DRB1_0101	483	DSNCISPGTDFMAKV	ISPGTDFMA	4	0.3579	1040.5	75.00	0.54	tr_C9ZP82_

DRB1_0101	484	VSALCDIKKFTPATE	CDIKKFTPA	4	0.3569	1052.2	75.00	0.33	tr_C9ZP82_
DRB1_0101	485	AEFRKGSNNDNVTVA	FRKGSNNDN	2	0.3564	1057.7	75.00	0.85	tr_C9ZP82_
DRB1_0101	486	YIDLNGIIHPCCHND	YIDLNGIIH	0	0.3552	1071.5	75.00	0.37	tr_C9ZP82_
DRB1_0101	487	VVKSTPPDTLVQLLS	VKSTPPDTL	1	0.3539	1086.7	75.00	0.41	tr_C9ZP82_
DRB1_0101	488	MGRAAPAQTGEVAST	GRAAPAQTG	1	0.3523	1104.9	75.00	0.32	tr_C9ZP82_
DRB1_0101	489	MAKVSDAVLNMLNEK	AKVSDAVLN	1	0.3517	1113.2	75.00	0.37	tr_C9ZP82_
DRB1_0101	490	RMPLPVVKSTPPDTL	VVKSTPPDT	5	0.3512	1118.0	75.00	0.44	tr_C9ZP82_
DRB1_0101	491	ADVAVALHSTIDDRW	VAVALHSTI	2	0.3488	1147.9	75.00	0.24	tr_C9ZP82_
DRB1_0101	492	LERYRPRTYSCTVEV	YRPRTYSCT	3	0.3461	1181.6	75.00	0.56	tr_C9ZP82_
DRB1_0101	493	NFIDDTCIISLNPSY	FIDDTCIIS	1	0.3460	1183.6	75.00	0.29	tr_C9ZP82_
DRB1_0101	494	NQQRARRYMGRAAPA	RYMGRAAPA	6	0.3450	1196.4	75.00	0.40	tr_C9ZP82_
DRB1_0101	495	QEACEKYVEGVCFVW	EKYVEGVCF	4	0.3449	1197.3	75.00	0.47	tr_C9ZP82_
DRB1_0101	496	GEGEHKIIDFLRTQS	HKIIDFLRT	4	0.3448	1198.5	75.00	0.47	tr_C9ZP82_
DRB1_0101	497	EVSALCDIKKFTPAT	CDIKKFTPA	5	0.3445	1202.5	75.00	0.34	tr_C9ZP82_
DRB1_0101	498	FVPKGDLRKLSDALD	LRKLSDALD	6	0.3445	1202.7	75.00	0.38	tr_C9ZP82_
DRB1_0101	499	NESPIESREAHVTTV	IESREAHVT	4	0.3439	1210.4	75.00	0.62	tr_C9ZP82_
DRB1_0101	500	SKIIDDIIVLSILLG	IDDIIVLSI	3	0.3438	1212.0	75.00	0.21	tr_C9ZP82_
DRB1_0101	501	AERSWKNFRPCSSPA	WKNFRPCSS	4	0.3429	1223.2	75.00	0.74	tr_C9ZP82_
DRB1_0101	502	LNMLNEKMSGNDPTW	LNMLNEKMS	0	0.3419	1236.5	75.00	0.57	tr_C9ZP82_
DRB1_0101	503	LPHAPSAFSGESALD	PSAFSGESA	4	0.3410	1249.7	75.00	0.33	tr_C9ZP82_
DRB1_0101	504	FDSNCISPGTDFMAK	ISPGTDFMA	5	0.3409	1251.1	75.00	0.41	tr_C9ZP82_
DRB1_0101	505	ALNDGDGVNSFCVRS	GDGVNSFCV	4	0.3407	1253.9	75.00	0.44	tr_C9ZP82_
DRB1_0101	506	PMVLGEVMVRSPQQA	MVLGEVMVR	1	0.3401	1260.7	75.00	0.34	tr_C9ZP82_
DRB1_0101	507	DALNDGDGVNSFCVR	LNDGDGVNS	2	0.3386	1281.8	75.00	0.41	tr_C9ZP82_
DRB1_0101	508	LESTFPLQWNVDYHD	FPLQWNVDY	4	0.3383	1286.0	75.00	0.56	tr_C9ZP82_
DRB1_0101	509	LLEDPLYSGALYNEG	PLYSGALYN	4	0.3376	1296.3	75.00	0.31	tr_C9ZP82_
DRB1_0101	510	VVDAAWPQLTEEERW	VDAAWPQLT	1	0.3371	1303.6	75.00	0.82	tr_C9ZP82_
DRB1_0101	511	DGVIGVPVCESSENS	VIGVPVCES	2	0.3370	1303.9	75.00	0.50	tr_C9ZP82_
DRB1_0101	512	ESTFPLQWNVDYHDS	FPLQWNVDY	3	0.3364	1313.6	75.00	0.62	tr_C9ZP82_
DRB1_0101	513	NSKIIDDIIVLSILL	IDDIIVLSI	4	0.3349	1333.9	75.00	0.20	tr_C9ZP82_
DRB1_0101	514	VKSTPPDTLVQLLSI	DTLVQLLSI	6	0.3349	1334.7	75.00	0.32	tr_C9ZP82_
DRB1_0101	515	NCISPGTDFMAKVSD	ISPGTDFMA	2	0.3347	1337.6	75.00	0.58	tr_C9ZP82_
DRB1_0101	516	VDDPTAFEFDSNCIS	FEFDSNCIS	6	0.3341	1345.9	80.00	0.62	tr_C9ZP82_
DRB1_0101	517	SEVSALCDIKKFTPA	VSALCDIKK	2	0.3336	1353.8	80.00	0.29	tr_C9ZP82_
DRB1_0101	518	RKRNFIDDTCIISLN	FIDDTCIIS	4	0.3334	1356.7	80.00	0.57	tr_C9ZP82_
DRB1_0101	519	LDEVRQTLLEDPLYS	TLLEDPLYS	6	0.3320	1377.6	80.00	0.55	tr_C9ZP82_
DRB1_0101	520	LNDGDGVNSFCVRST	GDGVNSFCV	3	0.3317	1380.7	80.00	0.50	tr_C9ZP82_
DRB1_0101	521	LEDPLYSGALYNEGE	PLYSGALYN	3	0.3306	1398.1	80.00	0.32	tr_C9ZP82_
DRB1_0101	522	LCVIFSGSNTPGEGE	VIFSGSNTP	2	0.3296	1412.6	80.00	0.31	tr_C9ZP82_
DRB1_0101	523	PYKRRAVARQQQRER	YKRRAVARQ	1	0.3294	1415.9	80.00	0.57	tr_C9ZP82_
DRB1_0101	524	ESPIESREAHVTTVP	IESREAHVT	3	0.3289	1424.4	80.00	0.61	tr_C9ZP82_
DRB1_0101	525	AIALLPEEEKLECIC	IALLPEEEK	1	0.3287	1426.9	80.00	0.51	tr_C9ZP82_
DRB1_0101	526	ESAFDKKTLVSSAGY	FDKKTLVSS	3	0.3270	1453.8	80.00	0.32	tr_C9ZP82_
DRB1_0101	527	MNQQRARRYMGRAAP	MNQQRARRY	0	0.3269	1454.8	80.00	0.49	tr_C9ZP82_
DRB1_0101	528	DTTRNPYKRRAVARQ	PYKRRAVAR	5	0.3263	1464.5	80.00	0.37	tr_C9ZP82_
DRB1_0101	529	SGQLKGNGEGVIEGK	LKGNGEGVI	3	0.3254	1478.9	80.00	0.50	tr_C9ZP82_
DRB1_0101	530	KIIDDIIVLSILLGN	IIVLSILLG	5	0.3250	1484.7	80.00	0.25	tr_C9ZP82_
DRB1_0101	531	SGNDPTWTRLCVIFS	WTRLCVIFS	6	0.3246	1492.0	80.00	0.56	tr_C9ZP82_
DRB1_0101	532	KRMPLPVVKSTPPDT	RMPLPVVKS	1	0.3239	1502.3	80.00	0.41	tr_C9ZP82_
DRB1_0101	533	ISPGTDFMAKVSDAV	FMAKVSDAV	6	0.3236	1508.6	80.00	0.47	tr_C9ZP82_

DRB1_0101	534	SGAEFRKGSNNDNVT	FRKGSNNDN	4	0.3236	1508.6	80.00	0.87	tr_C9ZP82_
DRB1_0101	535	PLERYRPRTYSCTVE	YRPRTYSCT	4	0.3224	1527.1	80.00	0.56	tr_C9ZP82_
DRB1_0101	536	FLTKGPHEINLPQLQ	FLTKGPHEI	0	0.3218	1537.1	80.00	0.46	tr_C9ZP82_
DRB1_0101	537	GVPKFASWLRNKYPA	GVPKFASWL	0	0.3211	1548.5	80.00	0.37	tr_C9ZP82_
DRB1_0101	538	KRNFIDDTCIISLNP	FIDDTCIIS	3	0.3204	1560.6	80.00	0.57	tr_C9ZP82_
DRB1_0101	539	VPLERYRPRTYSCTV	YRPRTYSCT	5	0.3204	1561.1	80.00	0.47	tr_C9ZP82_
DRB1_0101	540	HENRYVESNQVVDDV	NRYVESNQV	2	0.3203	1563.4	80.00	0.49	tr_C9ZP82_
DRB1_0101	541	HSGQLKGNGEGVIEG	LKGNGEGVI	4	0.3197	1572.1	80.00	0.43	tr_C9ZP82_
DRB1_0101	542	PGEGEHKIIDFLRTQ	HKIIDFLRT	5	0.3194	1577.9	80.00	0.44	tr_C9ZP82_
DRB1_0101	543	STFPLQWNVDYHDSN	FPLQWNVDY	2	0.3192	1580.9	80.00	0.62	tr_C9ZP82_
DRB1_0101	544	RSPQQAELESTFPLQ	AELESTFPL	5	0.3190	1584.4	80.00	0.62	tr_C9ZP82_
DRB1_0101	545	RYRPRTYSCTVEVPR	YRPRTYSCT	1	0.3189	1586.1	80.00	0.47	tr_C9ZP82_
DRB1_0101	546	ERYRPRTYSCTVEVP	YRPRTYSCT	2	0.3186	1592.0	80.00	0.55	tr_C9ZP82_
DRB1_0101	547	NKYPAIVTKNCPSSV	VTKNCPSSV	6	0.3183	1597.6	80.00	0.48	tr_C9ZP82_
DRB1_0101	548	ANSKIIDDIIVLSIL	KIIDDIIVL	3	0.3176	1609.0	80.00	0.23	tr_C9ZP82_
DRB1_0101	549	SYGADGQVIKRQRRH	SYGADGQVI	0	0.3169	1620.9	80.00	0.41	tr_C9ZP82_
DRB1_0101	550	YRPRTYSCTVEVPRL	YSCTVEVPR	5	0.3168	1623.8	80.00	0.29	tr_C9ZP82_
DRB1_0101	551	FTSTEMAQANDDLDE	FTSTEMAQA	0	0.3164	1630.9	80.00	0.44	tr_C9ZP82_
DRB1_0101	552	EFRKGSNNDNVTVAD	FRKGSNNDN	1	0.3144	1665.5	80.00	0.81	tr_C9ZP82_
DRB1_0101	553	CTVEVPRLSYGADGQ	CTVEVPRLS	0	0.3134	1683.7	80.00	0.34	tr_C9ZP82_
DRB1_0101	554	SGMAIVEEVVREFTS	GMAIVEEVV	1	0.3130	1690.4	80.00	0.25	tr_C9ZP82_
DRB1_0101	555	YIDEEESDGVIGVPV	SDGVIGVPV	6	0.3130	1691.9	80.00	0.45	tr_C9ZP82_
DRB1_0101	556	RRKRNFIDDTCIISL	FIDDTCIIS	5	0.3125	1700.2	80.00	0.55	tr_C9ZP82_
DRB1_0101	557	GDALNDGDGVNSFCV	LNDGDGVNS	3	0.3121	1706.9	80.00	0.54	tr_C9ZP82_
DRB1_0101	558	VEEVVREFTSTEMAQ	VREFTSTEM	4	0.3119	1710.7	80.00	0.46	tr_C9ZP82_
DRB1_0101	559	FILRDTTRNPYKRRA	FILRDTTRN	0	0.3117	1714.8	80.00	0.43	tr_C9ZP82_
DRB1_0101	560	SPIESREAHVTTVPN	IESREAHVT	2	0.3116	1716.4	80.00	0.57	tr_C9ZP82_
DRB1_0101	561	THENRYVESNQVVDD	NRYVESNQV	3	0.3114	1721.5	80.00	0.55	tr_C9ZP82_
DRB1_0101	562	IAVDGVAPRAKMNQQ	AVDGVAPRA	1	0.3113	1722.8	80.00	0.28	tr_C9ZP82_
DRB1_0101	563	ISEVSALCDIKKFTP	VSALCDIKK	3	0.3111	1726.7	80.00	0.35	tr_C9ZP82_
DRB1_0101	564	RNFIDDTCIISLNPS	FIDDTCIIS	2	0.3106	1736.2	80.00	0.50	tr_C9ZP82_
DRB1_0101	565	VFGHRGEVIVDDPTA	VFGHRGEVI	0	0.3101	1745.6	80.00	0.34	tr_C9ZP82_
DRB1_0101	566	IIDDIIVLSILLGND	IIVLSILLG	4	0.3093	1760.2	80.00	0.29	tr_C9ZP82_
DRB1_0101	567	ENRYVESNQVVDDVF	NRYVESNQV	1	0.3089	1768.5	80.00	0.44	tr_C9ZP82_
DRB1_0101	568	NQVVDDVFGHRGEVI	VFGHRGEVI	6	0.3080	1784.7	80.00	0.47	tr_C9ZP82_
DRB1_0101	569	NDSDTAIALLPEEEK	IALLPEEEK	6	0.3064	1817.1	85.00	0.29	tr_C9ZP82_
DRB1_0101	570	KYVEGVCFVWRYYTT	YVEGVCFVW	1	0.3062	1820.7	85.00	0.36	tr_C9ZP82_
DRB1_0101	571	FTPATESAFDKKTLV	PATESAFDK	2	0.3057	1830.6	85.00	0.40	tr_C9ZP82_
DRB1_0101	572	VSKKAEAERSWKNFR	AEAERSWKN	4	0.3054	1835.4	85.00	0.51	tr_C9ZP82_
DRB1_0101	573	RHSGQLKGNGEGVIE	QLKGNGEGV	4	0.3053	1839.0	85.00	0.40	tr_C9ZP82_
DRB1_0101	574	STHENRYVESNQVVD	NRYVESNQV	4	0.3051	1841.9	85.00	0.61	tr_C9ZP82_
DRB1_0101	575	NDGDGVNSFCVRSTD	GDGVNSFCV	2	0.3041	1862.0	85.00	0.52	tr_C9ZP82_
DRB1_0101	576	GVIEGKKSNSRVTVV	IEGKKSNSR	2	0.3034	1875.6	85.00	0.36	tr_C9ZP82_
DRB1_0101	577	GEVMVRSPQQAELES	VRSPQQAEL	4	0.3031	1881.5	85.00	0.23	tr_C9ZP82_
DRB1_0101	578	LGEVMVRSPQQAELE	VMVRSPQQA	3	0.3031	1882.4	85.00	0.30	tr_C9ZP82_
DRB1_0101	579	HVTTVPNTGFEYFDI	TVPNTGFEY	3	0.3018	1909.4	85.00	0.40	tr_C9ZP82_
DRB1_0101	580	DVAVALHSTIDDRWR	VAVALHSTI	1	0.3016	1912.5	85.00	0.25	tr_C9ZP82_
DRB1_0101	581	EDPLYSGALYNEGEE	YSGALYNEG	4	0.3008	1930.0	85.00	0.33	tr_C9ZP82_
DRB1_0101	582	PEEEKLECICSELEL	LECICSELE	5	0.3003	1939.3	85.00	0.50	tr_C9ZP82_
DRB1_0101	583	CCHNDSDTAIALLPE	NDSDTAIAL	3	0.3003	1939.7	85.00	0.41	tr_C9ZP82_

DRB1_0101	584	EFDSNCISPGTDFMA	FDSNCISPG	1	0.3000	1947.2	85.00	0.32	tr_C9ZP82_
DRB1_0101	585	RVSKKAEAERSWKNF	AEAERSWKN	5	0.2988	1972.3	85.00	0.39	tr_C9ZP82_
DRB1_0101	586	GQLKGNGEGVIEGKK	LKGNGEGVI	2	0.2977	1995.0	85.00	0.48	tr_C9ZP82_
DRB1_0101	587	VQEACEKYVEGVCFV	EKYVEGVCF	5	0.2968	2016.2	85.00	0.52	tr_C9ZP82_
DRB1_0101	588	EGVIEGKKSNSRVTV	IEGKKSNSR	3	0.2965	2020.7	85.00	0.44	tr_C9ZP82_
DRB1_0101	589	VLGEVMVRSPQQAEL	EVMVRSPQQ	3	0.2945	2065.2	85.00	0.32	tr_C9ZP82_
DRB1_0101	590	CVIFSGSNTPGEGEH	FSGSNTPGE	3	0.2939	2080.0	85.00	0.34	tr_C9ZP82_
DRB1_0101	591	PCCHNDSDTAIALLP	NDSDTAIAL	4	0.2924	2113.2	85.00	0.43	tr_C9ZP82_
DRB1_0101	592	EAHVTTVPNTGFEYF	VTTVPNTGF	3	0.2923	2116.0	85.00	0.47	tr_C9ZP82_
DRB1_0101	593	LKSTGLSNSVQEACE	STGLSNSVQ	2	0.2919	2123.8	85.00	0.28	tr_C9ZP82_
DRB1_0101	594	DNESPIESREAHVTT	IESREAHVT	5	0.2914	2136.7	85.00	0.56	tr_C9ZP82_
DRB1_0101	595	CISPGTDFMAKVSDA	ISPGTDFMA	1	0.2900	2169.0	85.00	0.53	tr_C9ZP82_
DRB1_0101	596	KRVSKKAEAERSWKN	SKKAEAERS	3	0.2896	2178.8	85.00	0.40	tr_C9ZP82_
DRB1_0101	597	VIEGKKSNSRVTVVN	IEGKKSNSR	1	0.2879	2219.6	85.00	0.28	tr_C9ZP82_
DRB1_0101	598	CHNDSDTAIALLPEE	NDSDTAIAL	2	0.2878	2220.2	85.00	0.34	tr_C9ZP82_
DRB1_0101	599	EVMVRSPQQAELEST	VRSPQQAEL	3	0.2876	2225.7	85.00	0.28	tr_C9ZP82_
DRB1_0101	600	IVEEVVREFTSTEMA	VREFTSTEM	5	0.2876	2226.6	85.00	0.37	tr_C9ZP82_
DRB1_0101	601	TPGEGEHKIIDFLRT	HKIIDFLRT	6	0.2863	2258.3	85.00	0.34	tr_C9ZP82_
DRB1_0101	602	VITSNKTSFVPKGDL	ITSNKTSFV	1	0.2853	2282.1	85.00	0.69	tr_C9ZP82_
DRB1_0101	603	ESTHENRYVESNQVV	NRYVESNQV	5	0.2835	2327.2	85.00	0.64	tr_C9ZP82_
DRB1_0101	604	DGDGVNSFCVRSTDD	GDGVNSFCV	1	0.2833	2333.0	85.00	0.49	tr_C9ZP82_
DRB1_0101	605	EAERSWKNFRPCSSP	WKNFRPCSS	5	0.2833	2333.2	85.00	0.70	tr_C9ZP82_
DRB1_0101	606	TESAFDKKTLVSSAG	FDKKTLVSS	4	0.2828	2345.2	85.00	0.33	tr_C9ZP82_
DRB1_0101	607	VTTVPNTGFEYFDIS	NTGFEYFDI	5	0.2817	2372.2	85.00	0.31	tr_C9ZP82_
DRB1_0101	608	MVLGEVMVRSPQQAE	EVMVRSPQQ	4	0.2815	2378.6	85.00	0.23	tr_C9ZP82_
DRB1_0101	609	LLFKSTNKENSNSGA	FKSTNKENS	2	0.2813	2382.9	85.00	0.53	tr_C9ZP82_
DRB1_0101	610	AHVTTVPNTGFEYFD	VTTVPNTGF	2	0.2810	2390.5	85.00	0.47	tr_C9ZP82_
DRB1_0101	611	ILRRVPSREGVEIRQ	LRRVPSREG	1	0.2803	2409.9	85.00	0.50	tr_C9ZP82_
DRB1_0101	612	IDLNGIIHPCCHNDS	LNGIIHPCC	2	0.2795	2430.6	85.00	0.28	tr_C9ZP82_
DRB1_0101	613	HPCCHNDSDTAIALL	NDSDTAIAL	5	0.2789	2446.6	85.00	0.47	tr_C9ZP82_
DRB1_0101	614	NSGAEFRKGSNNDNV	FRKGSNNDN	5	0.2782	2463.2	85.00	0.86	tr_C9ZP82_
DRB1_0101	615	DPLYSGALYNEGEEE	YSGALYNEG	3	0.2779	2472.4	85.00	0.43	tr_C9ZP82_
DRB1_0101	616	PSYIDEEESDGVIGV	IDEEESDGV	3	0.2756	2534.0	90.00	0.68	tr_C9ZP82_
DRB1_0101	617	PANSKIIDDIIVLSI	KIIDDIIVL	4	0.2754	2540.0	90.00	0.28	tr_C9ZP82_
DRB1_0101	618	LTKGPHEINLPQLQR	HEINLPQLQ	5	0.2752	2544.6	90.00	0.51	tr_C9ZP82_
DRB1_0101	619	GVIGVPVCESSENSS	VIGVPVCES	1	0.2745	2564.3	90.00	0.53	tr_C9ZP82_
DRB1_0101	620	TFPLQWNVDYHDSNV	FPLQWNVDY	1	0.2745	2565.2	90.00	0.52	tr_C9ZP82_
DRB1_0101	621	HNDSDTAIALLPEEE	NDSDTAIAL	1	0.2742	2572.9	90.00	0.33	tr_C9ZP82_
DRB1_0101	622	HRHSGQLKGNGEGVI	QLKGNGEGV	5	0.2731	2603.1	90.00	0.44	tr_C9ZP82_
DRB1_0101	623	GEGVIEGKKSNSRVT	IEGKKSNSR	4	0.2730	2607.2	90.00	0.47	tr_C9ZP82_
DRB1_0101	624	TTVPNTGFEYFDISV	NTGFEYFDI	4	0.2729	2610.3	90.00	0.44	tr_C9ZP82_
DRB1_0101	625	IALLPEEEKLECICS	IALLPEEEK	0	0.2728	2613.1	90.00	0.38	tr_C9ZP82_
DRB1_0101	626	GRAAPAQTGEVASTY	APAQTGEVA	3	0.2722	2628.4	90.00	0.30	tr_C9ZP82_
DRB1_0101	627	NRYVESNQVVDDVFG	NRYVESNQV	0	0.2719	2637.8	90.00	0.34	tr_C9ZP82_
DRB1_0101	628	SKRVSKKAEAERSWK	SKKAEAERS	4	0.2719	2639.6	90.00	0.37	tr_C9ZP82_
DRB1_0101	629	LNEKMSGNDPTWTRL	MSGNDPTWT	4	0.2717	2643.8	90.00	0.34	tr_C9ZP82_
DRB1_0101	630	SKKAEAERSWKNFRP	AEAERSWKN	3	0.2714	2653.7	90.00	0.56	tr_C9ZP82_
DRB1_0101	631	AGDALNDGDGVNSFC	LNDGDGVNS	4	0.2707	2672.6	90.00	0.60	tr_C9ZP82_
DRB1_0101	632	GSNNDNVTVADVAVA	VTVADVAVA	6	0.2702	2686.4	90.00	0.50	tr_C9ZP82_
DRB1_0101	633	REAHVTTVPNTGFEY	VTTVPNTGF	4	0.2700	2693.9	90.00	0.44	tr_C9ZP82_

DRB1_0101	634	TVEVPRLSYGADGQV	PRLSYGADG	4	0.2698	2697.7	90.00	0.35	tr_C9ZP82_
DRB1_0101	635	DSDWSWYYPFYHAPP	WSWYYPFYH	3	0.2698	2699.5	90.00	0.30	tr_C9ZP82_
DRB1_0101	636	IEGKKSNSRVTVVNK	KSNSRVTVV	4	0.2696	2704.5	90.00	0.38	tr_C9ZP82_
DRB1_0101	637	TSNKTSFVPKGDLRK	FVPKGDLRK	6	0.2681	2747.8	90.00	0.46	tr_C9ZP82_
DRB1_0101	638	RDTTRNPYKRRAVAR	PYKRRAVAR	6	0.2663	2803.3	90.00	0.31	tr_C9ZP82_
DRB1_0101	639	TGEVASTYMPNKSGM	STYMPNKSG	5	0.2655	2825.9	90.00	0.41	tr_C9ZP82_
DRB1_0101	640	DVPLERYRPRTYSCT	YRPRTYSCT	6	0.2647	2851.5	90.00	0.38	tr_C9ZP82_
DRB1_0101	641	SYIDEEESDGVIGVP	IDEEESDGV	2	0.2643	2863.5	90.00	0.67	tr_C9ZP82_
DRB1_0101	642	RAAPAQTGEVASTYM	APAQTGEVA	2	0.2626	2916.0	90.00	0.34	tr_C9ZP82_
DRB1_0101	643	KSTGLSNSVQEACEK	GLSNSVQEA	3	0.2617	2946.2	90.00	0.43	tr_C9ZP82_
DRB1_0101	644	YVEGVCFVWRYYTTT	FVWRYYTTT	6	0.2616	2949.6	90.00	0.20	tr_C9ZP82_
DRB1_0101	645	LPEEEKLECICSELE	LECICSELE	6	0.2605	2984.3	90.00	0.39	tr_C9ZP82_
DRB1_0101	646	EAGDALNDGDGVNSF	LNDGDGVNS	5	0.2601	2996.3	90.00	0.57	tr_C9ZP82_
DRB1_0101	647	MLNEKMSGNDPTWTR	NEKMSGNDP	2	0.2598	3008.0	90.00	0.42	tr_C9ZP82_
DRB1_0101	648	VEGVCFVWRYYTTTT	FVWRYYTTT	5	0.2597	3009.6	90.00	0.36	tr_C9ZP82_
DRB1_0101	649	RQLYDVPLERYRPRT	LYDVPLERY	2	0.2597	3011.2	90.00	0.28	tr_C9ZP82_
DRB1_0101	650	TPATESAFDKKTLVS	PATESAFDK	1	0.2578	3072.8	90.00	0.38	tr_C9ZP82_
DRB1_0101	651	QLKGNGEGVIEGKKS	LKGNGEGVI	1	0.2576	3081.1	90.00	0.47	tr_C9ZP82_
DRB1_0101	652	GMAIVEEVVREFTST	IVEEVVREF	3	0.2574	3085.3	90.00	0.34	tr_C9ZP82_
DRB1_0101	653	DLNGIIHPCCHNDSD	LNGIIHPCC	1	0.2565	3118.2	90.00	0.25	tr_C9ZP82_
DRB1_0101	654	ATESAFDKKTLVSSA	FDKKTLVSS	5	0.2563	3122.4	90.00	0.31	tr_C9ZP82_
DRB1_0101	655	PLYSGALYNEGEEEA	YSGALYNEG	2	0.2554	3154.7	90.00	0.44	tr_C9ZP82_
DRB1_0101	656	AEAERSWKNFRPCSS	WKNFRPCSS	6	0.2544	3188.1	90.00	0.57	tr_C9ZP82_
DRB1_0101	657	PIESREAHVTTVPNT	IESREAHVT	1	0.2541	3197.0	90.00	0.51	tr_C9ZP82_
DRB1_0101	658	PAQTGEVASTYMPNK	GEVASTYMP	4	0.2533	3227.9	90.00	0.33	tr_C9ZP82_
DRB1_0101	659	EGKKSNSRVTVVNKG	KSNSRVTVV	3	0.2521	3267.9	90.00	0.44	tr_C9ZP82_
DRB1_0101	660	RYYTTTTTDSDWSWY	YYTTTTTDS	1	0.2514	3292.0	90.00	0.49	tr_C9ZP82_
DRB1_0101	661	AAPAQTGEVASTYMP	APAQTGEVA	1	0.2510	3307.9	90.00	0.27	tr_C9ZP82_
DRB1_0101	662	GSPKGRRSKRVSKKA	PKGRRSKRV	2	0.2501	3338.6	90.00	0.58	tr_C9ZP82_
DRB1_0101	663	CGSPKGRRSKRVSKK	PKGRRSKRV	3	0.2496	3358.3	90.00	0.58	tr_C9ZP82_
DRB1_0101	664	RSKRVSKKAEAERSW	RVSKKAEAE	3	0.2496	3359.0	90.00	0.31	tr_C9ZP82_
DRB1_0101	665	APAQTGEVASTYMPN	TGEVASTYM	4	0.2493	3368.8	90.00	0.27	tr_C9ZP82_
DRB1_0101	666	SRRKRNFIDDTCIIS	FIDDTCIIS	6	0.2480	3417.8	90.00	0.49	tr_C9ZP82_
DRB1_0101	667	QTGEVASTYMPNKSG	GEVASTYMP	2	0.2479	3419.0	90.00	0.34	tr_C9ZP82_
DRB1_0101	668	VMVRSPQQAELESTF	VRSPQQAEL	2	0.2477	3427.1	90.00	0.36	tr_C9ZP82_
DRB1_0101	669	VRSPQQAELESTFPL	AELESTFPL	6	0.2475	3434.4	90.00	0.44	tr_C9ZP82_
DRB1_0101	670	GDGVNSFCVRSTDDD	VNSFCVRST	3	0.2471	3451.6	90.00	0.33	tr_C9ZP82_
DRB1_0101	671	NPSYIDEEESDGVIG	IDEEESDGV	4	0.2469	3458.5	90.00	0.69	tr_C9ZP82_
DRB1_0101	672	AVDGVAPRAKMNQQR	GVAPRAKMN	3	0.2468	3460.5	90.00	0.26	tr_C9ZP82_
DRB1_0101	673	AQTGEVASTYMPNKS	GEVASTYMP	3	0.2457	3503.4	90.00	0.39	tr_C9ZP82_
DRB1_0101	674	PATESAFDKKTLVSS	SAFDKKTLV	4	0.2455	3510.1	90.00	0.28	tr_C9ZP82_
DRB1_0101	675	AIVEEVVREFTSTEM	VVREFTSTE	5	0.2448	3539.1	90.00	0.28	tr_C9ZP82_
DRB1_0101	676	PLQWNVDYHDSNVVH	VDYHDSNVV	5	0.2447	3539.6	90.00	0.51	tr_C9ZP82_
DRB1_0101	677	TDSDWSWYYPFYHAP	WSWYYPFYH	4	0.2447	3541.7	90.00	0.35	tr_C9ZP82_
DRB1_0101	678	QLYDVPLERYRPRTY	DVPLERYRP	3	0.2446	3546.8	90.00	0.25	tr_C9ZP82_
DRB1_0101	679	RNKYPAIVTKNCPSS	YPAIVTKNC	3	0.2439	3573.0	90.00	0.38	tr_C9ZP82_
DRB1_0101	680	RPCSSPANSKIIDDI	SSPANSKII	3	0.2425	3624.7	90.00	0.51	tr_C9ZP82_
DRB1_0101	681	GKKSNSRVTVVNKGP	KSNSRVTVV	2	0.2425	3624.8	90.00	0.44	tr_C9ZP82_
DRB1_0101	682	KMSGNDPTWTRLCVI	MSGNDPTWT	1	0.2424	3631.6	90.00	0.28	tr_C9ZP82_
DRB1_0101	683	SPKGRRSKRVSKKAE	PKGRRSKRV	1	0.2408	3693.3	90.00	0.49	tr_C9ZP82_

DRB1_0101	684	VAVALHSTIDDRWRD	AVALHSTID	1	0.2408	3695.0	90.00	0.37	tr_C9ZP82_
DRB1_0101	685	FFGQPNHIEDGTLPS	FFGQPNHIE	0	0.2401	3719.9	95.00	0.60	tr_C9ZP82_
DRB1_0101	686	NEKMSGNDPTWTRLC	MSGNDPTWT	3	0.2400	3726.4	95.00	0.39	tr_C9ZP82_
DRB1_0101	687	NGEGVIEGKKSNSRV	IEGKKSNSR	5	0.2399	3728.5	95.00	0.41	tr_C9ZP82_
DRB1_0101	688	LNGIIHPCCHNDSDT	IHPCCHNDS	4	0.2392	3759.7	95.00	0.26	tr_C9ZP82_
DRB1_0101	689	RGEVIVDDPTAFEFD	IVDDPTAFE	4	0.2389	3770.9	95.00	0.32	tr_C9ZP82_
DRB1_0101	690	SVQEACEKYVEGVCF	EKYVEGVCF	6	0.2388	3774.6	95.00	0.39	tr_C9ZP82_
DRB1_0101	691	FPLQWNVDYHDSNVV	VDYHDSNVV	6	0.2371	3843.6	95.00	0.30	tr_C9ZP82_
DRB1_0101	692	LYSGALYNEGEEEAG	YSGALYNEG	1	0.2371	3844.0	95.00	0.40	tr_C9ZP82_
DRB1_0101	693	SNSVQEACEKYVEGV	VQEACEKYV	3	0.2369	3852.0	95.00	0.50	tr_C9ZP82_
DRB1_0101	694	GVAPRAKMNQQRARR	RAKMNQQRA	4	0.2365	3869.0	95.00	0.37	tr_C9ZP82_
DRB1_0101	695	YKRRAVARQQQRERR	YKRRAVARQ	0	0.2355	3910.5	95.00	0.38	tr_C9ZP82_
DRB1_0101	696	VIFSGSNTPGEGEHK	FSGSNTPGE	2	0.2350	3934.0	95.00	0.50	tr_C9ZP82_
DRB1_0101	697	GEVIVDDPTAFEFDS	IVDDPTAFE	3	0.2343	3962.7	95.00	0.34	tr_C9ZP82_
DRB1_0101	698	LSNSVQEACEKYVEG	VQEACEKYV	4	0.2340	3977.4	95.00	0.48	tr_C9ZP82_
DRB1_0101	699	IHPCCHNDSDTAIAL	NDSDTAIAL	6	0.2331	4015.0	95.00	0.31	tr_C9ZP82_
DRB1_0101	700	SPANSKIIDDIIVLS	SKIIDDIIV	4	0.2330	4020.5	95.00	0.29	tr_C9ZP82_
DRB1_0101	701	TDNESPIESREAHVT	IESREAHVT	6	0.2327	4033.5	95.00	0.40	tr_C9ZP82_
DRB1_0101	702	LYDVPLERYRPRTYS	LERYRPRTY	5	0.2326	4036.7	95.00	0.20	tr_C9ZP82_
DRB1_0101	703	LRRVPSREGVEIRQL	VPSREGVEI	3	0.2323	4051.3	95.00	0.31	tr_C9ZP82_
DRB1_0101	704	GLSNSVQEACEKYVE	VQEACEKYV	5	0.2321	4057.1	95.00	0.38	tr_C9ZP82_
DRB1_0101	705	FRKGSNNDNVTVADV	FRKGSNNDN	0	0.2308	4114.0	95.00	0.66	tr_C9ZP82_
DRB1_0101	706	NMLNEKMSGNDPTWT	NEKMSGNDP	3	0.2305	4127.7	95.00	0.53	tr_C9ZP82_
DRB1_0101	707	KKAEAERSWKNFRPC	AEAERSWKN	2	0.2305	4130.3	95.00	0.64	tr_C9ZP82_
DRB1_0101	708	DDDYESTHENRYVES	YESTHENRY	3	0.2304	4134.9	95.00	0.48	tr_C9ZP82_
DRB1_0101	709	TTDSDWSWYYPFYHA	WSWYYPFYH	5	0.2302	4144.2	95.00	0.39	tr_C9ZP82_
DRB1_0101	710	DDYESTHENRYVESN	YESTHENRY	2	0.2299	4154.3	95.00	0.47	tr_C9ZP82_
DRB1_0101	711	NTPGEGEHKIIDFLR	GEHKIIDFL	5	0.2296	4168.6	95.00	0.34	tr_C9ZP82_
DRB1_0101	712	SREAHVTTVPNTGFE	VTTVPNTGF	5	0.2296	4170.6	95.00	0.37	tr_C9ZP82_
DRB1_0101	713	HRGEVIVDDPTAFEF	IVDDPTAFE	5	0.2293	4183.8	95.00	0.32	tr_C9ZP82_
DRB1_0101	714	MSGNDPTWTRLCVIF	PTWTRLCVI	5	0.2293	4185.3	95.00	0.34	tr_C9ZP82_
DRB1_0101	715	ITSNKTSFVPKGDLR	ITSNKTSFV	0	0.2289	4199.1	95.00	0.41	tr_C9ZP82_
DRB1_0101	716	LFKSTNKENSNSGAE	FKSTNKENS	1	0.2277	4257.9	95.00	0.63	tr_C9ZP82_
DRB1_0101	717	KKSNSRVTVVNKGPC	KSNSRVTVV	1	0.2260	4336.2	95.00	0.38	tr_C9ZP82_
DRB1_0101	718	ALYNEGEEEAGDALN	YNEGEEEAG	2	0.2258	4345.5	95.00	0.39	tr_C9ZP82_
DRB1_0101	719	VDAAWPQLTEEERWR	VDAAWPQLT	0	0.2247	4395.2	95.00	0.68	tr_C9ZP82_
DRB1_0101	720	YESTHENRYVESNQV	NRYVESNQV	6	0.2245	4405.7	95.00	0.41	tr_C9ZP82_
DRB1_0101	721	ECGSPKGRRSKRVSK	PKGRRSKRV	4	0.2242	4419.0	95.00	0.54	tr_C9ZP82_
DRB1_0101	722	MAIVEEVVREFTSTE	IVEEVVREF	2	0.2241	4425.6	95.00	0.34	tr_C9ZP82_
DRB1_0101	723	VIKRQRRHRHSGQLK	IKRQRRHRH	1	0.2221	4520.0	95.00	0.32	tr_C9ZP82_
DRB1_0101	724	GALYNEGEEEAGDAL	YNEGEEEAG	3	0.2212	4564.4	95.00	0.41	tr_C9ZP82_
DRB1_0101	725	RRSKRVSKKAEAERS	RVSKKAEAE	4	0.2206	4594.4	95.00	0.31	tr_C9ZP82_
DRB1_0101	726	ILRDTTRNPYKRRAV	DTTRNPYKR	3	0.2205	4598.7	95.00	0.28	tr_C9ZP82_
DRB1_0101	727	TEEERWRTQKLDFHL	WRTQKLDFH	5	0.2198	4637.2	95.00	0.58	tr_C9ZP82_
DRB1_0101	728	VIGVPVCESSENSSS	VIGVPVCES	0	0.2195	4649.7	95.00	0.34	tr_C9ZP82_
DRB1_0101	729	YDVPLERYRPRTYSC	LERYRPRTY	4	0.2194	4655.0	95.00	0.28	tr_C9ZP82_
DRB1_0101	730	TGLSNSVQEACEKYV	GLSNSVQEA	1	0.2189	4679.2	95.00	0.27	tr_C9ZP82_
DRB1_0101	731	QVIKRQRRHRHSGQL	IKRQRRHRH	2	0.2171	4774.4	95.00	0.42	tr_C9ZP82_
DRB1_0101	732	LLPEEEKLECICSEL	EEKLECICS	4	0.2171	4774.7	95.00	0.38	tr_C9ZP82_
DRB1_0101	733	EVIVDDPTAFEFDSN	IVDDPTAFE	2	0.2163	4816.5	95.00	0.34	tr_C9ZP82_

DRB1_0101	734	GQVIKRQRRHRHSGQ	IKRQRRHRH	3	0.2162	4821.8	95.00	0.45	tr_C9ZP82_
DRB1_0101	735	EKMSGNDPTWTRLCV	MSGNDPTWT	2	0.2161	4824.3	95.00	0.40	tr_C9ZP82_
DRB1_0101	736	DGVAPRAKMNQQRAR	GVAPRAKMN	1	0.2147	4899.7	95.00	0.28	tr_C9ZP82_
DRB1_0101	737	VDGVAPRAKMNQQRA	GVAPRAKMN	2	0.2145	4911.1	95.00	0.33	tr_C9ZP82_
DRB1_0101	738	IKRQRRHRHSGQLKG	RRHRHSGQL	4	0.2140	4934.4	95.00	0.28	tr_C9ZP82_
DRB1_0101	739	PKGRRSKRVSKKAEA	PKGRRSKRV	0	0.2140	4938.0	95.00	0.33	tr_C9ZP82_
DRB1_0101	740	NGIIHPCCHNDSDTA	IHPCCHNDS	3	0.2140	4938.6	95.00	0.40	tr_C9ZP82_
DRB1_0101	741	SNQVVDDVFGHRGEV	VDDVFGHRG	4	0.2132	4976.7	95.00	0.55	tr_C9ZP82_
DRB1_0101	742	YSGALYNEGEEEAGD	LYNEGEEEA	4	0.2131	4982.2	95.00	0.34	tr_C9ZP82_
DRB1_0101	743	LNPSYIDEEESDGVI	IDEEESDGV	5	0.2129	4993.2	95.00	0.69	tr_C9ZP82_
DRB1_0101	744	DYESTHENRYVESNQ	YESTHENRY	1	0.2121	5041.3	95.00	0.45	tr_C9ZP82_
DRB1_0101	745	DLDEVRQTLLEDPLY	LDEVRQTLL	1	0.2108	5110.2	95.00	0.26	tr_C9ZP82_
DRB1_0101	746	TDDDYESTHENRYVE	YESTHENRY	4	0.2102	5145.9	95.00	0.49	tr_C9ZP82_
DRB1_0101	747	NSVQEACEKYVEGVC	VQEACEKYV	2	0.2099	5160.9	95.00	0.50	tr_C9ZP82_
DRB1_0101	748	LRDTTRNPYKRRAVA	DTTRNPYKR	2	0.2096	5174.8	95.00	0.23	tr_C9ZP82_
DRB1_0101	749	ESREAHVTTVPNTGF	HVTTVPNTG	5	0.2083	5251.8	95.00	0.30	tr_C9ZP82_
DRB1_0101	750	STGLSNSVQEACEKY	GLSNSVQEA	2	0.2082	5258.4	95.00	0.43	tr_C9ZP82_
DRB1_0101	751	SSPANSKIIDDIIVL	SKIIDDIIV	5	0.2080	5268.9	95.00	0.33	tr_C9ZP82_
DRB1_0101	752	DGQVIKRQRRHRHSG	IKRQRRHRH	4	0.2078	5278.7	95.00	0.46	tr_C9ZP82_
DRB1_0101	753	PCSSPANSKIIDDII	SSPANSKII	2	0.2073	5305.6	95.00	0.47	tr_C9ZP82_
DRB1_0101	754	KRQRRHRHSGQLKGN	RHRHSGQLK	4	0.2061	5379.5	95.00	0.34	tr_C9ZP82_
DRB1_0101	755	ALLPEEEKLECICSE	PEEEKLECI	3	0.2048	5454.0	95.00	0.29	tr_C9ZP82_
DRB1_0101	756	ANDDLDEVRQTLLED	LDEVRQTLL	4	0.2046	5465.5	95.00	0.46	tr_C9ZP82_
DRB1_0101	757	QANDDLDEVRQTLLE	LDEVRQTLL	5	0.2045	5470.4	95.00	0.41	tr_C9ZP82_
DRB1_0101	758	FGHRGEVIVDDPTAF	FGHRGEVIV	0	0.2043	5482.6	95.00	0.28	tr_C9ZP82_
DRB1_0101	759	TSTEMAQANDDLDEV	TEMAQANDD	2	0.2040	5498.8	95.00	0.32	tr_C9ZP82_
DRB1_0101	760	CSSPANSKIIDDIIV	SSPANSKII	1	0.2032	5546.4	95.00	0.35	tr_C9ZP82_
DRB1_0101	761	TECGSPKGRRSKRVS	PKGRRSKRV	5	0.2026	5583.4	95.00	0.49	tr_C9ZP82_
DRB1_0101	762	IESREAHVTTVPNTG	SREAHVTTV	2	0.2016	5644.4	95.00	0.38	tr_C9ZP82_
DRB1_0101	763	SGALYNEGEEEAGDA	LYNEGEEEA	3	0.2011	5676.5	95.00	0.46	tr_C9ZP82_
DRB1_0101	764	RRVPSREGVEIRQLY	VPSREGVEI	2	0.2003	5724.3	95.00	0.31	tr_C9ZP82_
DRB1_0101	765	RQRRHRHSGQLKGNG	RHRHSGQLK	3	0.2002	5733.6	95.00	0.43	tr_C9ZP82_
DRB1_0101	766	IFSGSNTPGEGEHKI	FSGSNTPGE	1	0.1994	5782.4	95.00	0.50	tr_C9ZP82_
DRB1_0101	767	LYNEGEEEAGDALND	YNEGEEEAG	1	0.1985	5838.0	95.00	0.42	tr_C9ZP82_
DRB1_0101	768	TIDDRWRDTYLKSTG	DRWRDTYLK	3	0.1979	5878.0	95.00	0.50	tr_C9ZP82_
DRB1_0101	769	ADGQVIKRQRRHRHS	IKRQRRHRH	5	0.1974	5905.5	95.00	0.37	tr_C9ZP82_
DRB1_0101	770	RERRSRRKRNFIDDT	SRRKRNFID	4	0.1968	5943.5	95.00	0.56	tr_C9ZP82_
DRB1_0101	771	MVRSPQQAELESTFP	VRSPQQAEL	1	0.1968	5944.6	95.00	0.34	tr_C9ZP82_
DRB1_0101	772	GIIHPCCHNDSDTAI	IHPCCHNDS	2	0.1965	5968.3	95.00	0.40	tr_C9ZP82_
DRB1_0101	773	IIHPCCHNDSDTAIA	IHPCCHNDS	1	0.1948	6078.7	95.00	0.33	tr_C9ZP82_
DRB1_0101	774	EEAGDALNDGDGVNS	LNDGDGVNS	6	0.1941	6118.8	95.00	0.47	tr_C9ZP82_
DRB1_0101	775	SNTPGEGEHKIIDFL	TPGEGEHKI	2	0.1923	6245.8	100.00	0.29	tr_C9ZP82_
DRB1_0101	776	DGVNSFCVRSTDDDY	VNSFCVRST	2	0.1917	6280.4	100.00	0.42	tr_C9ZP82_
DRB1_0101	777	GHRGEVIVDDPTAFE	EVIVDDPTA	4	0.1917	6282.2	100.00	0.31	tr_C9ZP82_
DRB1_0101	778	TTTDSDWSWYYPFYH	WSWYYPFYH	6	0.1907	6350.1	100.00	0.37	tr_C9ZP82_
DRB1_0101	779	YYTTTTTDSDWSWYY	YTTTTTDSD	1	0.1903	6380.4	100.00	0.34	tr_C9ZP82_
DRB1_0101	780	ESNQVVDDVFGHRGE	VDDVFGHRG	5	0.1897	6417.5	100.00	0.47	tr_C9ZP82_
DRB1_0101	781	STDDDYESTHENRYV	YESTHENRY	5	0.1893	6449.4	100.00	0.47	tr_C9ZP82_
DRB1_0101	782	RYVESNQVVDDVFGH	YVESNQVVD	1	0.1879	6550.3	100.00	0.34	tr_C9ZP82_
DRB1_0101	783	ERRSRRKRNFIDDTC	SRRKRNFID	3	0.1874	6581.6	100.00	0.62	tr_C9ZP82_

DRB1_0101	784	FKSTNKENSNSGAEF	FKSTNKENS	0	0.1874	6581.9	100.00	0.45	tr_C9ZP82_
DRB1_0101	785	GNGEGVIEGKKSNSR	EGVIEGKKS	3	0.1868	6622.9	100.00	0.35	tr_C9ZP82_
DRB1_0101	786	YGADGQVIKRQRRHR	GADGQVIKR	1	0.1866	6637.9	100.00	0.27	tr_C9ZP82_
DRB1_0101	787	KAEAERSWKNFRPCS	AEAERSWKN	1	0.1865	6646.3	100.00	0.62	tr_C9ZP82_
DRB1_0101	788	RHRHSGQLKGNGEGV	QLKGNGEGV	6	0.1862	6670.4	100.00	0.31	tr_C9ZP82_
DRB1_0101	789	RRSRRKRNFIDDTCI	SRRKRNFID	2	0.1852	6741.0	100.00	0.58	tr_C9ZP82_
DRB1_0101	790	GRRSKRVSKKAEAER	KRVSKKAEA	4	0.1851	6748.5	100.00	0.32	tr_C9ZP82_
DRB1_0101	791	VPSREGVEIRQLYDV	VEIRQLYDV	6	0.1841	6823.9	100.00	0.22	tr_C9ZP82_
DRB1_0101	792	NDDLDEVRQTLLEDP	LDEVRQTLL	3	0.1840	6828.3	100.00	0.47	tr_C9ZP82_
DRB1_0101	793	STIDDRWRDTYLKST	DRWRDTYLK	4	0.1838	6841.2	100.00	0.47	tr_C9ZP82_
DRB1_0101	794	KRRAVARQQQRERRS	VARQQQRER	4	0.1836	6858.1	100.00	0.31	tr_C9ZP82_
DRB1_0101	795	SNSGAEFRKGSNNDN	FRKGSNNDN	6	0.1833	6879.4	100.00	0.70	tr_C9ZP82_
DRB1_0101	796	STEMAQANDDLDEVR	TEMAQANDD	1	0.1826	6934.8	100.00	0.28	tr_C9ZP82_
DRB1_0101	797	GADGQVIKRQRRHRH	IKRQRRHRH	6	0.1825	6937.9	100.00	0.26	tr_C9ZP82_
DRB1_0101	798	AQANDDLDEVRQTLL	LDEVRQTLL	6	0.1823	6955.6	100.00	0.35	tr_C9ZP82_
DRB1_0101	799	STECGSPKGRRSKRV	CGSPKGRRS	3	0.1817	7000.3	100.00	0.32	tr_C9ZP82_
DRB1_0101	800	QRERRSRRKRNFIDD	SRRKRNFID	5	0.1813	7029.0	100.00	0.49	tr_C9ZP82_
DRB1_0101	801	QRRHRHSGQLKGNGE	RHRHSGQLK	2	0.1810	7051.8	100.00	0.38	tr_C9ZP82_
DRB1_0101	802	RSRRKRNFIDDTCII	SRRKRNFID	1	0.1797	7155.7	100.00	0.43	tr_C9ZP82_
DRB1_0101	803	IVDDPTAFEFDSNCI	AFEFDSNCI	6	0.1794	7177.5	100.00	0.32	tr_C9ZP82_
DRB1_0101	804	KGRRSKRVSKKAEAE	RRSKRVSKK	2	0.1785	7246.2	100.00	0.23	tr_C9ZP82_
DRB1_0101	805	FSGSNTPGEGEHKII	FSGSNTPGE	0	0.1784	7259.5	100.00	0.33	tr_C9ZP82_
DRB1_0101	806	DDLDEVRQTLLEDPL	LDEVRQTLL	2	0.1782	7268.3	100.00	0.44	tr_C9ZP82_
DRB1_0101	807	PSREGVEIRQLYDVP	VEIRQLYDV	5	0.1740	7613.1	100.00	0.31	tr_C9ZP82_
DRB1_0101	808	RVPSREGVEIRQLYD	PSREGVEIR	2	0.1731	7687.5	100.00	0.26	tr_C9ZP82_
DRB1_0101	809	QPNHIEDGTLPSSTE	NHIEDGTLP	2	0.1720	7773.1	100.00	0.37	tr_C9ZP82_
DRB1_0101	810	RRAVARQQQRERRSR	VARQQQRER	3	0.1717	7801.6	100.00	0.37	tr_C9ZP82_
DRB1_0101	811	LKGNGEGVIEGKKSN	LKGNGEGVI	0	0.1713	7837.5	100.00	0.38	tr_C9ZP82_
DRB1_0101	812	LTEEERWRTQKLDFH	WRTQKLDFH	6	0.1699	7952.9	100.00	0.47	tr_C9ZP82_
DRB1_0101	813	VESNQVVDDVFGHRG	VDDVFGHRG	6	0.1695	7987.6	100.00	0.38	tr_C9ZP82_
DRB1_0101	814	GVNSFCVRSTDDDYE	VNSFCVRST	1	0.1682	8099.9	100.00	0.35	tr_C9ZP82_
DRB1_0101	815	VIVDDPTAFEFDSNC	IVDDPTAFE	1	0.1670	8208.2	100.00	0.41	tr_C9ZP82_
DRB1_0101	816	PNHIEDGTLPSSTEC	NHIEDGTLP	1	0.1650	8387.2	100.00	0.31	tr_C9ZP82_
DRB1_0101	817	RRHRHSGQLKGNGEG	RHRHSGQLK	1	0.1644	8445.6	100.00	0.32	tr_C9ZP82_
DRB1_0101	818	RAVARQQQRERRSRR	ARQQQRERR	3	0.1627	8597.4	100.00	0.27	tr_C9ZP82_
DRB1_0101	819	QQRERRSRRKRNFID	SRRKRNFID	6	0.1624	8629.6	100.00	0.31	tr_C9ZP82_
DRB1_0101	820	SSTECGSPKGRRSKR	CGSPKGRRS	4	0.1618	8686.2	100.00	0.37	tr_C9ZP82_
DRB1_0101	821	GQPNHIEDGTLPSST	NHIEDGTLP	3	0.1617	8690.1	100.00	0.41	tr_C9ZP82_
DRB1_0101	822	AVALHSTIDDRWRDT	VALHSTIDD	1	0.1616	8697.6	100.00	0.33	tr_C9ZP82_
DRB1_0101	823	YNEGEEEAGDALNDG	GEEEAGDAL	3	0.1605	8808.2	100.00	0.45	tr_C9ZP82_
DRB1_0101	824	PSSTECGSPKGRRSK	TECGSPKGR	3	0.1601	8843.4	100.00	0.23	tr_C9ZP82_
DRB1_0101	825	NHIEDGTLPSSTECG	DGTLPSSTE	4	0.1586	8990.7	100.00	0.20	tr_C9ZP82_
DRB1_0101	826	HSTIDDRWRDTYLKS	DRWRDTYLK	5	0.1579	9061.9	100.00	0.50	tr_C9ZP82_
DRB1_0101	827	IGVPVCESSENSSSD	PVCESSENS	3	0.1576	9089.9	100.00	0.39	tr_C9ZP82_
DRB1_0101	828	DTDNESPIESREAHV	PIESREAHV	6	0.1569	9154.6	100.00	0.31	tr_C9ZP82_
DRB1_0101	829	TEMAQANDDLDEVRQ	MAQANDDLD	2	0.1566	9181.6	100.00	0.32	tr_C9ZP82_
DRB1_0101	830	KGSNNDNVTVADVAV	DNVTVADVA	5	0.1564	9201.8	100.00	0.29	tr_C9ZP82_
DRB1_0101	831	GSNTPGEGEHKIIDF	TPGEGEHKI	3	0.1533	9522.2	100.00	0.44	tr_C9ZP82_
DRB1_0101	832	WPQLTEEERWRTQKL	LTEEERWRT	3	0.1530	9545.5	100.00	0.61	tr_C9ZP82_
DRB1_0101	833	LPSSTECGSPKGRRS	SSTECGSPK	2	0.1511	9752.2	100.00	0.26	tr_C9ZP82_

DRB1_0101	834	PQLTEEERWRTQKLD	LTEEERWRT	2	0.1510	9754.9	100.00	0.62	tr_C9ZP82_
DRB1_0101	835	GVPVCESSENSSSDT	PVCESSENS	2	0.1504	9825.7	100.00	0.38	tr_C9ZP82_
DRB1_0101	836	TTTTDSDWSWYYPFY	TDSDWSWYY	3	0.1475	10136.8	100.00	0.43	tr_C9ZP82_
DRB1_0101	837	HIEDGTLPSSTECGS	DGTLPSSTE	3	0.1474	10148.2	100.00	0.32	tr_C9ZP82_
DRB1_0101	838	YVESNQVVDDVFGHR	QVVDDVFGH	5	0.1470	10187.9	100.00	0.23	tr_C9ZP82_
DRB1_0101	839	TNKENSNSGAEFRKG	ENSNSGAEF	3	0.1463	10273.1	100.00	0.33	tr_C9ZP82_
DRB1_0101	840	TLPSSTECGSPKGRR	SSTECGSPK	3	0.1460	10304.8	100.00	0.31	tr_C9ZP82_
DRB1_0101	841	FGQPNHIEDGTLPSS	NHIEDGTLP	4	0.1452	10395.5	100.00	0.44	tr_C9ZP82_
DRB1_0101	842	KGNGEGVIEGKKSNS	EGVIEGKKS	4	0.1450	10411.6	100.00	0.44	tr_C9ZP82_
DRB1_0101	843	SLNPSYIDEEESDGV	IDEEESDGV	6	0.1437	10561.8	100.00	0.58	tr_C9ZP82_
DRB1_0101	844	VNSFCVRSTDDDYES	FCVRSTDDD	3	0.1431	10628.8	100.00	0.21	tr_C9ZP82_
DRB1_0101	845	AVARQQQRERRSRRK	ARQQQRERR	2	0.1431	10633.9	100.00	0.25	tr_C9ZP82_
DRB1_0101	846	SGSNTPGEGEHKIID	TPGEGEHKI	4	0.1429	10658.8	100.00	0.43	tr_C9ZP82_
DRB1_0101	847	MAQANDDLDEVRQTL	NDDLDEVRQ	4	0.1422	10735.2	100.00	0.19	tr_C9ZP82_
DRB1_0101	848	AWPQLTEEERWRTQK	LTEEERWRT	4	0.1419	10771.9	100.00	0.54	tr_C9ZP82_
DRB1_0101	849	QLTEEERWRTQKLDF	LTEEERWRT	1	0.1403	10957.1	100.00	0.56	tr_C9ZP82_
DRB1_0101	850	STNKENSNSGAEFRK	ENSNSGAEF	4	0.1395	11050.7	100.00	0.31	tr_C9ZP82_
DRB1_0101	851	NKENSNSGAEFRKGS	NSNSGAEFR	3	0.1394	11063.5	100.00	0.29	tr_C9ZP82_
DRB1_0101	852	LHSTIDDRWRDTYLK	DRWRDTYLK	6	0.1390	11113.6	100.00	0.35	tr_C9ZP82_
DRB1_0101	853	GTLPSSTECGSPKGR	SSTECGSPK	4	0.1380	11233.2	100.00	0.28	tr_C9ZP82_
DRB1_0101	854	VARQQQRERRSRRKR	ARQQQRERR	1	0.1378	11257.2	100.00	0.23	tr_C9ZP82_
DRB1_0101	855	VPVCESSENSSSDTD	PVCESSENS	1	0.1365	11416.5	100.00	0.38	tr_C9ZP82_
DRB1_0101	856	QQQRERRSRRKRNFI	QRERRSRRK	2	0.1363	11439.7	100.00	0.31	tr_C9ZP82_
DRB1_0101	857	NSFCVRSTDDDYEST	CVRSTDDDY	3	0.1345	11667.5	100.00	0.25	tr_C9ZP82_
DRB1_0101	858	EMAQANDDLDEVRQT	MAQANDDLD	1	0.1336	11784.5	100.00	0.32	tr_C9ZP82_
DRB1_0101	859	YTTTTTDSDWSWYYP	TDSDWSWYY	5	0.1332	11827.1	100.00	0.32	tr_C9ZP82_
DRB1_0101	860	KSTNKENSNSGAEFR	TNKENSNSG	2	0.1327	11894.8	100.00	0.28	tr_C9ZP82_
DRB1_0101	861	TTTTTDSDWSWYYPF	TDSDWSWYY	4	0.1321	11973.9	100.00	0.43	tr_C9ZP82_
DRB1_0101	862	SFCVRSTDDDYESTH	VRSTDDDYE	3	0.1311	12101.9	100.00	0.33	tr_C9ZP82_
DRB1_0101	863	NEGEEEAGDALNDGD	GEEEAGDAL	2	0.1306	12170.8	100.00	0.56	tr_C9ZP82_
DRB1_0101	864	KENSNSGAEFRKGSN	NSNSGAEFR	2	0.1301	12232.7	100.00	0.31	tr_C9ZP82_
DRB1_0101	865	AAWPQLTEEERWRTQ	LTEEERWRT	5	0.1291	12375.2	100.00	0.47	tr_C9ZP82_
DRB1_0101	866	IEDGTLPSSTECGSP	DGTLPSSTE	2	0.1288	12404.9	100.00	0.34	tr_C9ZP82_
DRB1_0101	867	ARQQQRERRSRRKRN	QRERRSRRK	4	0.1279	12533.2	100.00	0.32	tr_C9ZP82_
DRB1_0101	868	RKGSNNDNVTVADVA	NDNVTVADV	5	0.1257	12828.6	100.00	0.28	tr_C9ZP82_
DRB1_0101	869	RQQQRERRSRRKRNF	QRERRSRRK	3	0.1246	12993.0	100.00	0.40	tr_C9ZP82_
DRB1_0101	870	DGTLPSSTECGSPKG	TLPSSTECG	2	0.1245	13004.4	100.00	0.30	tr_C9ZP82_
DRB1_0101	871	RSTDDDYESTHENRY	YESTHENRY	6	0.1222	13327.6	100.00	0.45	tr_C9ZP82_
DRB1_0101	872	EEEAGDALNDGDGVN	DALNDGDGV	5	0.1222	13331.0	100.00	0.50	tr_C9ZP82_
DRB1_0101	873	EDGTLPSSTECGSPK	TLPSSTECG	3	0.1221	13338.7	100.00	0.36	tr_C9ZP82_
DRB1_0101	874	GEEEAGDALNDGDGV	DALNDGDGV	6	0.1217	13405.2	100.00	0.26	tr_C9ZP82_
DRB1_0101	875	VALHSTIDDRWRDTY	LHSTIDDRW	2	0.1215	13430.8	100.00	0.28	tr_C9ZP82_
DRB1_0101	876	EGEEEAGDALNDGDG	GEEEAGDAL	1	0.1193	13754.9	100.00	0.50	tr_C9ZP82_
DRB1_0101	877	SDTDNESPIESREAH	TDNESPIES	2	0.1151	14395.8	100.00	0.37	tr_C9ZP82_
DRB1_0101	878	ISLNPSYIDEEESDG	ISLNPSYID	0	0.1120	14882.3	100.00	0.48	tr_C9ZP82_
DRB1_0101	879	FCVRSTDDDYESTHE	VRSTDDDYE	2	0.1107	15091.0	100.00	0.36	tr_C9ZP82_
DRB1_0101	880	PVCESSENSSSDTDN	CESSENSSS	2	0.1078	15580.5	100.00	0.32	tr_C9ZP82_
DRB1_0101	881	DAAWPQLTEEERWRT	LTEEERWRT	6	0.1076	15608.7	100.00	0.33	tr_C9ZP82_
DRB1_0101	882	ENSNSGAEFRKGSNN	NSNSGAEFR	1	0.1065	15802.2	100.00	0.28	tr_C9ZP82_
DRB1_0101	883	ALHSTIDDRWRDTYL	LHSTIDDRW	1	0.1044	16157.0	100.00	0.28	tr_C9ZP82_

DRB1_0101	884	SSDTDNESPIESREA	TDNESPIES	3	0.1043	16173.6	100.00	0.48	tr_C9ZP82_
DRB1_0101	885	NSNSGAEFRKGSNND	NSGAEFRKG	2	0.0940	18089.5	100.00	0.28	tr_C9ZP82_
DRB1_0101	886	SSSDTDNESPIESRE	TDNESPIES	4	0.0900	18885.9	100.00	0.48	tr_C9ZP82_
DRB1_0101	887	CVRSTDDDYESTHEN	VRSTDDDYE	1	0.0841	20118.2	100.00	0.41	tr_C9ZP82_
DRB1_0101	888	NSSSDTDNESPIESR	TDNESPIES	5	0.0816	20678.3	100.00	0.46	tr_C9ZP82_
DRB1_0101	889	VRSTDDDYESTHENR	DDYESTHEN	5	0.0772	21680.9	100.00	0.27	tr_C9ZP82_
DRB1_0101	890	VCESSENSSSDTDNE	CESSENSSS	1	0.0729	22722.6	100.00	0.35	tr_C9ZP82_
DRB1_0101	891	ENSSSDTDNESPIES	TDNESPIES	6	0.0521	28451.9	100.00	0.35	tr_C9ZP82_
DRB1_0101	892	CESSENSSSDTDNES	CESSENSSS	0	0.0502	29052.3	100.00	0.22	tr_C9ZP82_
DRB1_0101	893	SENSSSDTDNESPIE	SDTDNESPI	5	0.0384	33001.9	100.00	0.28	tr_C9ZP82_
DRB1_0101	894	SSENSSSDTDNESPI	NSSSDTDNE	3	0.0381	33104.9	100.00	0.22	tr_C9ZP82_
DRB1_0101	895	ESSENSSSDTDNESP	SSENSSSDT	1	0.0364	33714.4	100.00	0.28	tr_C9ZP82_

Allele: DRB1_0101. Number of high binders 0. Number of weak binders 40. Number of peptides 895

Allele	pos	peptide	core	Of	1-log50k(aff)	affinity(nM)	%Rank	Relia	Identity	Bind Level
DRB1_0101	1	TECLSLNFSSRTRCF	LSLNFSSRT	3	0.5913	83.3	24.00	0.62	tr_C9ZP82_	
DRB1_0101	2	CTECLSLNFSSRTRC	LSLNFSSRT	4	0.5750	99.3	27.00	0.55	tr_C9ZP82_	
DRB1_0101	3	LCTECLSLNFSSRTR	LSLNFSSRT	5	0.5736	100.8	27.00	0.47	tr_C9ZP82_	
DRB1_0101	4	ECLSLNFSSRTRCFV	LSLNFSSRT	2	0.5569	120.8	30.00	0.62	tr_C9ZP82_	
DRB1_0101	5	WLCTECLSLNFSSRT	LSLNFSSRT	6	0.5079	205.2	39.00	0.31	tr_C9ZP82_	
DRB1_0101	6	CLSLNFSSRTRCFVC	LSLNFSSRT	1	0.5038	214.5	40.00	0.61	tr_C9ZP82_	
DRB1_0101	7	RTRCFVCRAPFDNRR	FVCRAPFDN	4	0.4619	337.8	49.00	0.62	tr_C9ZP82_	
DRB1_0101	8	QTFVDWLCTECLSLN	VDWLCTECL	3	0.4596	346.1	49.00	0.55	tr_C9ZP82_	
DRB1_0101	9	FVDWLCTECLSLNFS	VDWLCTECL	1	0.4538	368.8	55.00	0.35	tr_C9ZP82_	
DRB1_0101	10	TFVDWLCTECLSLNF	VDWLCTECL	2	0.4530	371.8	55.00	0.48	tr_C9ZP82_	
DRB1_0101	11	PFDNRRCLAVFSCTQ	NRRCLAVFS	3	0.4503	383.0	55.00	0.55	tr_C9ZP82_	
DRB1_0101	12	FDNRRCLAVFSCTQS	NRRCLAVFS	2	0.4484	390.9	55.00	0.47	tr_C9ZP82_	
DRB1_0101	13	TRCFVCRAPFDNRRC	FVCRAPFDN	3	0.4442	409.1	55.00	0.68	tr_C9ZP82_	
DRB1_0101	14	DNRRCLAVFSCTQSA	NRRCLAVFS	1	0.4416	420.7	55.00	0.38	tr_C9ZP82_	
DRB1_0101	15	LAVFSCTQSADQSLY	CTQSADQSL	5	0.4340	456.5	55.00	0.30	tr_C9ZP82_	
DRB1_0101	16	APFDNRRCLAVFSCT	NRRCLAVFS	4	0.4319	467.3	55.00	0.54	tr_C9ZP82_	
DRB1_0101	17	DWLCTECLSLNFSSR	WLCTECLSL	1	0.4312	470.8	60.00	0.23	tr_C9ZP82_	
DRB1_0101	18	RQTFVDWLCTECLSL	VDWLCTECL	4	0.4309	472.2	60.00	0.61	tr_C9ZP82_	
DRB1_0101	19	NRRCLAVFSCTQSAD	AVFSCTQSA	5	0.4193	535.2	60.00	0.23	tr_C9ZP82_	
DRB1_0101	20	AVFSCTQSADQSLYD	CTQSADQSL	4	0.4185	539.8	60.00	0.39	tr_C9ZP82_	
DRB1_0101	21	VDWLCTECLSLNFSS	WLCTECLSL	2	0.4166	551.2	60.00	0.34	tr_C9ZP82_	
DRB1_0101	22	SRTRCFVCRAPFDNR	FVCRAPFDN	5	0.4158	556.3	60.00	0.60	tr_C9ZP82_	
DRB1_0101	23	RCFVCRAPFDNRRCL	FVCRAPFDN	2	0.4153	559.1	60.00	0.65	tr_C9ZP82_	
DRB1_0101	24	LSLNFSSRTRCFVCR	LSLNFSSRT	0	0.4134	570.7	60.00	0.38	tr_C9ZP82_	
DRB1_0101	25	RAPFDNRRCLAVFSC	NRRCLAVFS	5	0.4060	618.2	65.00	0.47	tr_C9ZP82_	
DRB1_0101	26	CLAVFSCTQSADQSL	AVFSCTQSA	2	0.3871	758.5	65.00	0.32	tr_C9ZP82_	
DRB1_0101	27	IRQTFVDWLCTECLS	VDWLCTECL	5	0.3795	823.4	70.00	0.68	tr_C9ZP82_	
DRB1_0101	28	RRCLAVFSCTQSADQ	AVFSCTQSA	4	0.3792	825.8	70.00	0.41	tr_C9ZP82_	
DRB1_0101	29	VFSCTQSADQSLYDP	CTQSADQSL	3	0.3738	876.2	70.00	0.47	tr_C9ZP82_	
DRB1_0101	30	RCLAVFSCTQSADQS	AVFSCTQSA	3	0.3720	893.2	70.00	0.46	tr_C9ZP82_	

DRB1_0101	31	QSLYDPDHSPHIEAV	YDPDHSPHI	3	0.3707	906.3	70.00	0.78	tr_C9ZP82_
DRB1_0101	32	SSGCGMSRNNIRQTF	CGMSRNNIR	3	0.3541	1083.8	75.00	0.55	tr_C9ZP82_
DRB1_0101	33	SLYDPDHSPHIEAVK	YDPDHSPHI	2	0.3507	1124.4	75.00	0.76	tr_C9ZP82_
DRB1_0101	34	CRAPFDNRRCLAVFS	NRRCLAVFS	6	0.3471	1169.3	75.00	0.41	tr_C9ZP82_
DRB1_0101	35	NIRQTFVDWLCTECL	VDWLCTECL	6	0.3465	1176.6	75.00	0.50	tr_C9ZP82_
DRB1_0101	36	SGCGMSRNNIRQTFV	CGMSRNNIR	2	0.3462	1180.6	75.00	0.47	tr_C9ZP82_
DRB1_0101	37	DQSLYDPDHSPHIEA	YDPDHSPHI	4	0.3458	1186.3	75.00	0.77	tr_C9ZP82_
DRB1_0101	38	SSRTRCFVCRAPFDN	FVCRAPFDN	6	0.3410	1248.6	75.00	0.44	tr_C9ZP82_
DRB1_0101	39	CFVCRAPFDNRRCLA	FVCRAPFDN	1	0.3372	1301.8	75.00	0.63	tr_C9ZP82_
DRB1_0101	40	PSSGCGMSRNNIRQT	CGMSRNNIR	4	0.3327	1367.0	80.00	0.51	tr_C9ZP82_
DRB1_0101	41	FSCTQSADQSLYDPD	CTQSADQSL	2	0.3306	1397.6	80.00	0.54	tr_C9ZP82_
DRB1_0101	42	LYDPDHSPHIEAVKL	YDPDHSPHI	1	0.3274	1446.6	80.00	0.62	tr_C9ZP82_
DRB1_0101	43	SLNFSSRTRCFVCRA	NFSSRTRCF	2	0.3239	1502.3	80.00	0.22	tr_C9ZP82_
DRB1_0101	44	SRNNIRQTFVDWLCT	IRQTFVDWL	4	0.3232	1514.5	80.00	0.44	tr_C9ZP82_
DRB1_0101	45	GCGMSRNNIRQTFVD	CGMSRNNIR	1	0.3185	1593.5	80.00	0.40	tr_C9ZP82_
DRB1_0101	46	MSRNNIRQTFVDWLC	IRQTFVDWL	5	0.3055	1833.8	85.00	0.37	tr_C9ZP82_
DRB1_0101	47	ADQSLYDPDHSPHIE	YDPDHSPHI	5	0.3037	1869.4	85.00	0.73	tr_C9ZP82_
DRB1_0101	48	RNNIRQTFVDWLCTE	IRQTFVDWL	3	0.3032	1880.2	85.00	0.46	tr_C9ZP82_
DRB1_0101	49	YDPDHSPHIEAVKLQ	YDPDHSPHI	0	0.3023	1898.3	85.00	0.34	tr_C9ZP82_
DRB1_0101	50	DPSSGCGMSRNNIRQ	CGMSRNNIR	5	0.3015	1916.2	85.00	0.52	tr_C9ZP82_
DRB1_0101	51	DPDHSPHIEAVKLQI	PHIEAVKLQ	5	0.2963	2025.1	85.00	0.34	tr_C9ZP82_
DRB1_0101	52	LNFSSRTRCFVCRAP	SSRTRCFVC	3	0.2874	2230.8	85.00	0.25	tr_C9ZP82_
DRB1_0101	53	GMSRNNIRQTFVDWL	IRQTFVDWL	6	0.2842	2310.6	85.00	0.28	tr_C9ZP82_
DRB1_0101	54	CGMSRNNIRQTFVDW	CGMSRNNIR	0	0.2829	2341.1	85.00	0.23	tr_C9ZP82_
DRB1_0101	55	FVCRAPFDNRRCLAV	FVCRAPFDN	0	0.2724	2623.5	90.00	0.44	tr_C9ZP82_
DRB1_0101	56	FSSRTRCFVCRAPFD	CFVCRAPFD	6	0.2669	2784.8	90.00	0.28	tr_C9ZP82_
DRB1_0101	57	SCTQSADQSLYDPDH	CTQSADQSL	1	0.2664	2800.1	90.00	0.54	tr_C9ZP82_
DRB1_0101	58	NNIRQTFVDWLCTEC	IRQTFVDWL	2	0.2596	3012.5	90.00	0.44	tr_C9ZP82_
DRB1_0101	59	NFSSRTRCFVCRAPF	SSRTRCFVC	2	0.2570	3099.0	90.00	0.25	tr_C9ZP82_
DRB1_0101	60	SADQSLYDPDHSPHI	YDPDHSPHI	6	0.2395	3745.8	95.00	0.63	tr_C9ZP82_
DRB1_0101	61	VCRAPFDNRRCLAVF	FDNRRCLAV	5	0.2304	4133.1	95.00	0.60	tr_C9ZP82_
DRB1_0101	62	CTQSADQSLYDPDHS	CTQSADQSL	0	0.1941	6120.3	95.00	0.40	tr_C9ZP82_
DRB1_0101	63	QSADQSLYDPDHSPH	SLYDPDHSP	5	0.1178	13983.6	100.00	0.22	tr_C9ZP82_
DRB1_0101	64	TQSADQSLYDPDHSP	QSADQSLYD	1	0.1141	14540.9	100.00	0.29	tr_C9ZP82_

Allele: DRB1_0101. Number of high binders 0. Number of weak binders 0. Number of peptides 64

Allele	pos	peptide	core	Of	1-log50k(aff)	affinity(nM)	%Rank	Relia	Identity	Bind Level
DRB1_0101	1	ENICAALSASRALNF	CAALSASRA	3	0.9268	2.2	0.01	0.85	tr_C9ZJU1_	SB
DRB1_0101	2	NICAALSASRALNFV	CAALSASRA	2	0.9268	2.2	0.01	0.84	tr_C9ZJU1_	SB
DRB1_0101	3	ICAALSASRALNFVQ	CAALSASRA	1	0.9161	2.5	0.04	0.83	tr_C9ZJU1_	SB
DRB1_0101	4	DENICAALSASRALN	CAALSASRA	4	0.9150	2.5	0.04	0.86	tr_C9ZJU1_	SB
DRB1_0101	5	RFIIKQLMGLPATFV	IKQLMGLPA	3	0.8977	3.0	0.09	0.58	tr_C9ZJU1_	SB
DRB1_0101	6	FIIKQLMGLPATFVS	IKQLMGLPA	2	0.8975	3.0	0.09	0.47	tr_C9ZJU1_	SB
DRB1_0101	7	SDENICAALSASRAL	CAALSASRA	5	0.8957	3.1	0.10	0.86	tr_C9ZJU1_	SB
DRB1_0101	8	IIKQLMGLPATFVSM	LMGLPATFV	4	0.8925	3.2	0.12	0.46	tr_C9ZJU1_	SB

DRB1_0101	9	CAALSASRALNFVQK	CAALSASRA	0	0.8815	3.6	0.20	0.79	tr_C9ZJU1_	SB
DRB1_0101	10	CRQDYLQGFAALRLS	YLQGFAALR	4	0.8770	3.8	0.25	0.34	tr_C9ZJU1_	SB
DRB1_0101	11	RQDYLQGFAALRLSG	YLQGFAALR	3	0.8767	3.8	0.25	0.39	tr_C9ZJU1_	SB
DRB1_0101	12	IKQLMGLPATFVSMA	LMGLPATFV	3	0.8721	4.0	0.30	0.56	tr_C9ZJU1_	SB
DRB1_0101	13	QCRQDYLQGFAALRL	QDYLQGFAA	3	0.8678	4.2	0.40	0.50	tr_C9ZJU1_	SB
DRB1_0101	14	FRFIIKQLMGLPATF	IKQLMGLPA	4	0.8675	4.2	0.40	0.77	tr_C9ZJU1_	SB
DRB1_0101	15	FNHNIMQLPSVFAVH	IMQLPSVFA	4	0.8550	4.8	0.50	0.75	tr_C9ZJU1_	WB
DRB1_0101	16	SNFLYFLNESLYMES	FLYFLNESL	2	0.8535	4.9	0.60	0.44	tr_C9ZJU1_	WB
DRB1_0101	17	PFRFIIKQLMGLPAT	IKQLMGLPA	5	0.8525	4.9	0.60	0.72	tr_C9ZJU1_	WB
DRB1_0101	18	QDYLQGFAALRLSGW	YLQGFAALR	2	0.8481	5.2	0.70	0.36	tr_C9ZJU1_	WB
DRB1_0101	19	HGLFQMADTLPLLLV	FQMADTLPL	3	0.8461	5.3	0.70	0.90	tr_C9ZJU1_	WB
DRB1_0101	20	VFNHNIMQLPSVFAV	IMQLPSVFA	5	0.8446	5.4	0.70	0.68	tr_C9ZJU1_	WB
DRB1_0101	21	WSNFLYFLNESLYME	FLYFLNESL	3	0.8439	5.4	0.80	0.47	tr_C9ZJU1_	WB
DRB1_0101	22	NHNIMQLPSVFAVHC	IMQLPSVFA	3	0.8426	5.5	0.80	0.77	tr_C9ZJU1_	WB
DRB1_0101	23	NFLYFLNESLYMESQ	YFLNESLYM	3	0.8425	5.5	0.80	0.51	tr_C9ZJU1_	WB
DRB1_0101	24	GLFQMADTLPLLLVS	FQMADTLPL	2	0.8360	5.9	0.90	0.88	tr_C9ZJU1_	WB
DRB1_0101	25	KGEFLGIAGYSGSGK	FLGIAGYSG	3	0.8336	6.1	1.00	0.92	tr_C9ZJU1_	WB
DRB1_0101	26	LHGLFQMADTLPLLL	FQMADTLPL	4	0.8327	6.1	1.00	0.89	tr_C9ZJU1_	WB
DRB1_0101	27	RVSFVVNKGEFLGIA	FVVNKGEFL	3	0.8317	6.2	1.00	0.92	tr_C9ZJU1_	WB
DRB1_0101	28	KQLMGLPATFVSMAV	LMGLPATFV	2	0.8301	6.3	1.10	0.79	tr_C9ZJU1_	WB
DRB1_0101	29	QWSNFLYFLNESLYM	FLYFLNESL	4	0.8236	6.7	1.20	0.58	tr_C9ZJU1_	WB
DRB1_0101	30	HNIMQLPSVFAVHCG	IMQLPSVFA	2	0.8211	6.9	1.30	0.79	tr_C9ZJU1_	WB
DRB1_0101	31	LSGWELMAATAAAAF	WELMAATAA	3	0.8210	6.9	1.30	0.81	tr_C9ZJU1_	WB
DRB1_0101	32	SGWELMAATAAAAFA	WELMAATAA	2	0.8204	7.0	1.30	0.76	tr_C9ZJU1_	WB
DRB1_0101	33	WQCRQDYLQGFAALR	QDYLQGFAA	4	0.8190	7.1	1.40	0.60	tr_C9ZJU1_	WB
DRB1_0101	34	CSDENICAALSASRA	CAALSASRA	6	0.8163	7.3	1.50	0.82	tr_C9ZJU1_	WB
DRB1_0101	35	RRVSFVVNKGEFLGI	FVVNKGEFL	4	0.8162	7.3	1.50	0.93	tr_C9ZJU1_	WB
DRB1_0101	36	LFQMADTLPLLLVSA	FQMADTLPL	1	0.8160	7.3	1.50	0.88	tr_C9ZJU1_	WB
DRB1_0101	37	GEFLGIAGYSGSGKT	FLGIAGYSG	2	0.8145	7.4	1.50	0.89	tr_C9ZJU1_	WB
DRB1_0101	38	NKGEFLGIAGYSGSG	FLGIAGYSG	4	0.8128	7.6	1.60	0.92	tr_C9ZJU1_	WB
DRB1_0101	39	GCYQVLEGLQSVQRK	YQVLEGLQS	2	0.8091	7.9	1.70	0.63	tr_C9ZJU1_	WB
DRB1_0101	40	CYQVLEGLQSVQRKI	YQVLEGLQS	1	0.8087	7.9	1.70	0.58	tr_C9ZJU1_	WB
DRB1_0101	41	FLYFLNESLYMESQF	YFLNESLYM	2	0.8083	8.0	1.70	0.60	tr_C9ZJU1_	WB
DRB1_0101	42	LVFNHNIMQLPSVFA	IMQLPSVFA	6	0.8067	8.1	1.80	0.60	tr_C9ZJU1_	WB
DRB1_0101	43	RLSGWELMAATAAAA	WELMAATAA	4	0.8067	8.1	1.80	0.83	tr_C9ZJU1_	WB
DRB1_0101	44	EELYLGLITAYNRKR	YLGLITAYN	3	0.8062	8.1	1.80	0.77	tr_C9ZJU1_	WB
DRB1_0101	45	GWELMAATAAAAFAT	WELMAATAA	1	0.8060	8.2	1.80	0.64	tr_C9ZJU1_	WB
DRB1_0101	46	VSFVVNKGEFLGIAG	FVVNKGEFL	2	0.8035	8.4	1.90	0.92	tr_C9ZJU1_	WB
DRB1_0101	47	PATFVSMAVASTFAF	FVSMAVAST	3	0.8015	8.6	2.00	0.76	tr_C9ZJU1_	WB
DRB1_0101	48	RAFVKAGSAVGLCIF	FVKAGSAVG	2	0.7991	8.8	2.50	0.59	tr_C9ZJU1_	WB
DRB1_0101	49	LGCYQVLEGLQSVQR	YQVLEGLQS	3	0.7986	8.8	2.50	0.75	tr_C9ZJU1_	WB
DRB1_0101	50	VLHGLFQMADTLPLL	FQMADTLPL	5	0.7983	8.9	2.50	0.88	tr_C9ZJU1_	WB
DRB1_0101	51	ELYLGLITAYNRKRS	YLGLITAYN	2	0.7982	8.9	2.50	0.73	tr_C9ZJU1_	WB
DRB1_0101	52	SPFRFIIKQLMGLPA	IKQLMGLPA	6	0.7980	8.9	2.50	0.62	tr_C9ZJU1_	WB
DRB1_0101	53	ARAFVKAGSAVGLCI	FVKAGSAVG	3	0.7979	8.9	2.50	0.67	tr_C9ZJU1_	WB
DRB1_0101	54	ATFVSMAVASTFAFE	FVSMAVAST	2	0.7929	9.4	2.50	0.74	tr_C9ZJU1_	WB
DRB1_0101	55	EFLGIAGYSGSGKTT	FLGIAGYSG	1	0.7927	9.4	2.50	0.87	tr_C9ZJU1_	WB
DRB1_0101	56	LPATFVSMAVASTFA	FVSMAVAST	4	0.7894	9.8	2.50	0.80	tr_C9ZJU1_	WB
DRB1_0101	57	LRRVSFVVNKGEFLG	FVVNKGEFL	5	0.7880	9.9	3.00	0.92	tr_C9ZJU1_	WB
DRB1_0101	58	PEELYLGLITAYNRK	YLGLITAYN	4	0.7857	10.2	3.00	0.75	tr_C9ZJU1_	WB

DRB1_0101	59	VNKGEFLGIAGYSGS	FLGIAGYSG	5	0.7849	10.3	3.00	0.92	tr_C9ZJU1_	WB
DRB1_0101	60	LRLSGWELMAATAAA	WELMAATAA	5	0.7806	10.7	3.00	0.82	tr_C9ZJU1_	WB
DRB1_0101	61	TSFWKLMCSSCSIPQ	WKLMCSSCS	3	0.7792	10.9	3.00	0.81	tr_C9ZJU1_	WB
DRB1_0101	62	YQVLEGLQSVQRKID	YQVLEGLQS	0	0.7768	11.2	3.50	0.53	tr_C9ZJU1_	WB
DRB1_0101	63	LYLGLITAYNRKRSL	YLGLITAYN	1	0.7756	11.3	3.50	0.68	tr_C9ZJU1_	WB
DRB1_0101	64	DYLQGFAALRLSGWE	YLQGFAALR	1	0.7723	11.7	3.50	0.47	tr_C9ZJU1_	WB
DRB1_0101	65	SFVVNKGEFLGIAGY	FVVNKGEFL	1	0.7716	11.8	3.50	0.88	tr_C9ZJU1_	WB
DRB1_0101	66	ALCMLLRGKYSIARY	CMLLRGKYS	2	0.7714	11.9	3.50	0.69	tr_C9ZJU1_	WB
DRB1_0101	67	GITSFWKLMCSSCSI	WKLMCSSCS	5	0.7704	12.0	3.50	0.81	tr_C9ZJU1_	WB
DRB1_0101	68	WELMAATAAAAFATA	WELMAATAA	0	0.7700	12.0	3.50	0.54	tr_C9ZJU1_	WB
DRB1_0101	69	IARAFVKAGSAVGLC	FVKAGSAVG	4	0.7694	12.1	3.50	0.74	tr_C9ZJU1_	WB
DRB1_0101	70	SFWKLMCSSCSIPQL	WKLMCSSCS	2	0.7685	12.2	4.00	0.75	tr_C9ZJU1_	WB
DRB1_0101	71	ITSFWKLMCSSCSIP	WKLMCSSCS	4	0.7679	12.3	4.00	0.81	tr_C9ZJU1_	WB
DRB1_0101	72	QLMGLPATFVSMAVA	LMGLPATFV	1	0.7673	12.4	4.00	0.78	tr_C9ZJU1_	WB
DRB1_0101	73	LALCMLLRGKYSIAR	CMLLRGKYS	3	0.7670	12.4	4.00	0.74	tr_C9ZJU1_	WB
DRB1_0101	74	TFVSMAVASTFAFES	FVSMAVAST	1	0.7625	13.1	4.00	0.71	tr_C9ZJU1_	WB
DRB1_0101	75	SLGCYQVLEGLQSVQ	YQVLEGLQS	4	0.7624	13.1	4.00	0.81	tr_C9ZJU1_	WB
DRB1_0101	76	ALRLSGWELMAATAA	WELMAATAA	6	0.7618	13.2	4.00	0.56	tr_C9ZJU1_	WB
DRB1_0101	77	NIMQLPSVFAVHCGL	IMQLPSVFA	1	0.7596	13.5	4.50	0.83	tr_C9ZJU1_	WB
DRB1_0101	78	AFVKAGSAVGLCIFD	FVKAGSAVG	1	0.7566	13.9	4.50	0.51	tr_C9ZJU1_	WB
DRB1_0101	79	GLPATFVSMAVASTF	FVSMAVAST	5	0.7525	14.6	5.00	0.77	tr_C9ZJU1_	WB
DRB1_0101	80	FPEELYLGLITAYNR	YLGLITAYN	5	0.7523	14.6	5.00	0.71	tr_C9ZJU1_	WB
DRB1_0101	81	AVALRLSPNFFTQSA	LRLSPNFFT	3	0.7460	15.6	5.00	0.82	tr_C9ZJU1_	WB
DRB1_0101	82	LYFLNESLYMESQFT	YFLNESLYM	1	0.7443	15.9	5.50	0.63	tr_C9ZJU1_	WB
DRB1_0101	83	TQWSNFLYFLNESLY	FLYFLNESL	5	0.7410	16.5	5.50	0.83	tr_C9ZJU1_	WB
DRB1_0101	84	PALLEVMHGCRVVFS	LEVMHGCRV	3	0.7404	16.6	5.50	0.77	tr_C9ZJU1_	WB
DRB1_0101	85	CIARAFVKAGSAVGL	FVKAGSAVG	5	0.7402	16.6	5.50	0.77	tr_C9ZJU1_	WB
DRB1_0101	86	LCMLLRGKYSIARYP	CMLLRGKYS	1	0.7397	16.7	5.50	0.68	tr_C9ZJU1_	WB
DRB1_0101	87	FQMADTLPLLLVSAV	FQMADTLPL	0	0.7395	16.8	5.50	0.81	tr_C9ZJU1_	WB
DRB1_0101	88	FWKLMCSSCSIPQLV	WKLMCSSCS	1	0.7372	17.2	6.00	0.71	tr_C9ZJU1_	WB
DRB1_0101	89	DSTIIIVAHRLATLK	TIIIVAHRL	2	0.7366	17.3	6.00	0.51	tr_C9ZJU1_	WB
DRB1_0101	90	STIIIVAHRLATLKH	TIIIVAHRL	1	0.7353	17.5	6.00	0.43	tr_C9ZJU1_	WB
DRB1_0101	91	PLLLVSAVALRLSPN	LVSAVALRL	3	0.7353	17.5	6.00	0.76	tr_C9ZJU1_	WB
DRB1_0101	92	SAVALRLSPNFFTQS	LRLSPNFFT	4	0.7343	17.7	6.00	0.73	tr_C9ZJU1_	WB
DRB1_0101	93	SSLGCYQVLEGLQSV	YQVLEGLQS	5	0.7321	18.1	6.00	0.81	tr_C9ZJU1_	WB
DRB1_0101	94	FLGIAGYSGSGKTTL	FLGIAGYSG	0	0.7317	18.2	6.00	0.81	tr_C9ZJU1_	WB
DRB1_0101	95	YLQGFAALRLSGWEL	LQGFAALRL	1	0.7316	18.3	6.00	0.34	tr_C9ZJU1_	WB
DRB1_0101	96	VLALCMLLRGKYSIA	CMLLRGKYS	4	0.7313	18.3	6.50	0.79	tr_C9ZJU1_	WB
DRB1_0101	97	ALLEVMHGCRVVFSF	LEVMHGCRV	2	0.7295	18.7	6.50	0.75	tr_C9ZJU1_	WB
DRB1_0101	98	LPLLLVSAVALRLSP	LVSAVALRL	4	0.7294	18.7	6.50	0.71	tr_C9ZJU1_	WB
DRB1_0101	99	GCRVVFSFPVVSLVM	CRVVFSFPV	1	0.7253	19.5	6.50	0.42	tr_C9ZJU1_	WB
DRB1_0101	100	HWRPIYAAEIGKSLN	YAAEIGKSL	5	0.7253	19.5	7.00	0.48	tr_C9ZJU1_	WB
DRB1_0101	101	TLPLLLVSAVALRLS	LVSAVALRL	5	0.7239	19.8	7.00	0.70	tr_C9ZJU1_	WB
DRB1_0101	102	VPALLEVMHGCRVVF	LEVMHGCRV	4	0.7238	19.8	7.00	0.78	tr_C9ZJU1_	WB
DRB1_0101	103	RALNFVQKRSSGIHA	FVQKRSSGI	4	0.7237	19.9	7.00	0.82	tr_C9ZJU1_	WB
DRB1_0101	104	RDSTIIIVAHRLATL	TIIIVAHRL	3	0.7236	19.9	7.00	0.59	tr_C9ZJU1_	WB
DRB1_0101	105	WRPIYAAEIGKSLNG	YAAEIGKSL	4	0.7231	20.0	7.00	0.65	tr_C9ZJU1_	WB
DRB1_0101	106	VWQCRQDYLQGFAAL	QDYLQGFAA	5	0.7219	20.3	7.00	0.78	tr_C9ZJU1_	WB
DRB1_0101	107	VALRLSPNFFTQSAL	LRLSPNFFT	2	0.7210	20.5	7.00	0.83	tr_C9ZJU1_	WB
DRB1_0101	108	ALNFVQKRSSGIHAP	FVQKRSSGI	3	0.7189	20.9	7.50	0.81	tr_C9ZJU1_	WB

DRB1_0101	109	YLGLITAYNRKRSLQ	YLGLITAYN	0	0.7180	21.1	7.50	0.50	tr_C9ZJU1_	WB
DRB1_0101	110	VLRRVSFVVNKGEFL	FVVNKGEFL	6	0.7169	21.4	7.50	0.88	tr_C9ZJU1_	WB
DRB1_0101	111	GFAALRLSGWELMAA	ALRLSGWEL	3	0.7165	21.5	7.50	0.65	tr_C9ZJU1_	WB
DRB1_0101	112	HGCRVVFSFPVVSLV	CRVVFSFPV	2	0.7161	21.6	7.50	0.51	tr_C9ZJU1_	WB
DRB1_0101	113	RHPYVLRRVSFVVNK	YVLRRVSFV	3	0.7126	22.4	8.00	0.77	tr_C9ZJU1_	WB
DRB1_0101	114	VSAVALRLSPNFFTQ	LRLSPNFFT	5	0.7124	22.5	8.00	0.69	tr_C9ZJU1_	WB
DRB1_0101	115	CRVVFSFPVVSLVMW	FSFPVVSLV	4	0.7117	22.6	8.00	0.25	tr_C9ZJU1_	WB
DRB1_0101	116	TIIIVAHRLATLKHA	IVAHRLATL	3	0.7110	22.8	8.00	0.37	tr_C9ZJU1_	WB
DRB1_0101	117	LLLVSAVALRLSPNF	LVSAVALRL	2	0.7103	23.0	8.00	0.77	tr_C9ZJU1_	WB
DRB1_0101	118	LNFVQKRSSGIHAPL	FVQKRSSGI	2	0.7102	23.0	8.00	0.77	tr_C9ZJU1_	WB
DRB1_0101	119	SGITSFWKLMCSSCS	WKLMCSSCS	6	0.7101	23.0	8.00	0.71	tr_C9ZJU1_	WB
DRB1_0101	120	LRRHLAFSGSSLGCY	LAFSGSSLG	4	0.7093	23.2	8.00	0.45	tr_C9ZJU1_	WB
DRB1_0101	121	TSIHIDAYLALATAD	SIHIDAYLA	1	0.7081	23.5	8.50	0.47	tr_C9ZJU1_	WB
DRB1_0101	122	SIHIDAYLALATADY	SIHIDAYLA	0	0.7080	23.6	8.50	0.31	tr_C9ZJU1_	WB
DRB1_0101	123	ITSIHIDAYLALATA	SIHIDAYLA	2	0.7054	24.2	8.50	0.55	tr_C9ZJU1_	WB
DRB1_0101	124	HLRRHLAFSGSSLGC	LAFSGSSLG	5	0.7051	24.3	8.50	0.31	tr_C9ZJU1_	WB
DRB1_0101	125	VHWRPIYAAEIGKSL	WRPIYAAEI	2	0.7050	24.3	8.50	0.59	tr_C9ZJU1_	WB
DRB1_0101	126	FAALRLSGWELMAAT	ALRLSGWEL	2	0.7046	24.4	8.50	0.63	tr_C9ZJU1_	WB
DRB1_0101	127	ILKCILGMHAFVHWR	ILGMHAFVH	4	0.7020	25.1	9.00	0.42	tr_C9ZJU1_	WB
DRB1_0101	128	AFVHWRPIYAAEIGK	WRPIYAAEI	4	0.7013	25.3	9.00	0.58	tr_C9ZJU1_	WB
DRB1_0101	129	GVHISQYPARMLRRR	ISQYPARML	3	0.7011	25.4	9.00	0.55	tr_C9ZJU1_	WB
DRB1_0101	130	AHLRRHLAFSGSSLG	LRRHLAFSG	2	0.7011	25.4	9.00	0.39	tr_C9ZJU1_	WB
DRB1_0101	131	HAFVHWRPIYAAEIG	WRPIYAAEI	5	0.7004	25.6	9.00	0.52	tr_C9ZJU1_	WB
DRB1_0101	132	NGVHISQYPARMLRR	ISQYPARML	4	0.6991	25.9	9.00	0.47	tr_C9ZJU1_	WB
DRB1_0101	133	VILKCILGMHAFVHW	LKCILGMHA	2	0.6987	26.1	9.00	0.44	tr_C9ZJU1_	WB
DRB1_0101	134	LMGLPATFVSMAVAS	LMGLPATFV	0	0.6985	26.1	9.00	0.62	tr_C9ZJU1_	WB
DRB1_0101	135	DVLHGLFQMADTLPL	FQMADTLPL	6	0.6984	26.1	9.00	0.75	tr_C9ZJU1_	WB
DRB1_0101	136	VVNKGEFLGIAGYSG	FLGIAGYSG	6	0.6977	26.3	9.50	0.86	tr_C9ZJU1_	WB
DRB1_0101	137	RPIYAAEIGKSLNGD	YAAEIGKSL	3	0.6969	26.6	9.50	0.82	tr_C9ZJU1_	WB
DRB1_0101	138	CMLLRGKYSIARYPP	CMLLRGKYS	0	0.6967	26.6	9.50	0.60	tr_C9ZJU1_	WB
DRB1_0101	139	RVPALLEVMHGCRVV	LEVMHGCRV	5	0.6966	26.6	9.50	0.74	tr_C9ZJU1_	WB
DRB1_0101	140	LQGFAALRLSGWELM	ALRLSGWEL	5	0.6959	26.9	9.50	0.48	tr_C9ZJU1_	WB
DRB1_0101	141	QGFAALRLSGWELMA	ALRLSGWEL	4	0.6950	27.1	9.50	0.64	tr_C9ZJU1_	WB
DRB1_0101	142	RRHLAFSGSSLGCYQ	LAFSGSSLG	3	0.6949	27.1	9.50	0.54	tr_C9ZJU1_	WB
DRB1_0101	143	LVSAVALRLSPNFFT	LRLSPNFFT	6	0.6944	27.3	9.50	0.43	tr_C9ZJU1_	WB
DRB1_0101	144	LLVSAVALRLSPNFF	LVSAVALRL	1	0.6936	27.5	9.50	0.68	tr_C9ZJU1_	WB
DRB1_0101	145	LLEVMHGCRVVFSFP	LEVMHGCRV	1	0.6930	27.7	9.50	0.75	tr_C9ZJU1_	WB
DRB1_0101	146	YFLNESLYMESQFTK	YFLNESLYM	0	0.6929	27.7	9.50	0.38	tr_C9ZJU1_	WB
DRB1_0101	147	FAFESLYCIRGFVIQ	LYCIRGFVI	5	0.6922	27.9	10.00	0.56	tr_C9ZJU1_	
DRB1_0101	148	FVHWRPIYAAEIGKS	WRPIYAAEI	3	0.6900	28.6	10.00	0.67	tr_C9ZJU1_	
DRB1_0101	149	LRDSTIIIVAHRLAT	TIIIVAHRL	4	0.6897	28.7	10.00	0.66	tr_C9ZJU1_	
DRB1_0101	150	EVERLCIARAFVKAG	LCIARAFVK	4	0.6896	28.7	10.00	0.47	tr_C9ZJU1_	
DRB1_0101	151	LEVMHGCRVVFSFPV	LEVMHGCRV	0	0.6895	28.8	10.00	0.40	tr_C9ZJU1_	
DRB1_0101	152	EVLALCMLLRGKYSI	CMLLRGKYS	5	0.6890	28.9	10.00	0.82	tr_C9ZJU1_	
DRB1_0101	153	MGLPATFVSMAVAST	FVSMAVAST	6	0.6872	29.5	11.00	0.69	tr_C9ZJU1_	
DRB1_0101	154	MHGCRVVFSFPVVSL	CRVVFSFPV	3	0.6865	29.7	11.00	0.63	tr_C9ZJU1_	
DRB1_0101	155	VERLCIARAFVKAGS	LCIARAFVK	3	0.6840	30.5	11.00	0.53	tr_C9ZJU1_	
DRB1_0101	156	LRFIEDLSIGGNIAL	FIEDLSIGG	2	0.6833	30.8	11.00	0.44	tr_C9ZJU1_	
DRB1_0101	157	DRHPYVLRRVSFVVN	YVLRRVSFV	4	0.6829	30.9	11.00	0.80	tr_C9ZJU1_	
DRB1_0101	158	EFPEELYLGLITAYN	YLGLITAYN	6	0.6820	31.2	11.00	0.63	tr_C9ZJU1_	

DRB1_0101	159	TFAFESLYCIRGFVI	LYCIRGFVI	6	0.6819	31.2	11.00	0.41	tr_C9ZJU1_
DRB1_0101	160	ALRLSPNFFTQSALD	LRLSPNFFT	1	0.6815	31.4	11.00	0.81	tr_C9ZJU1_
DRB1_0101	161	FVKAGSAVGLCIFDE	VKAGSAVGL	1	0.6812	31.5	11.00	0.40	tr_C9ZJU1_
DRB1_0101	162	FVSMAVASTFAFESL	FVSMAVAST	0	0.6809	31.6	11.00	0.54	tr_C9ZJU1_
DRB1_0101	163	IKDVIQSSPRAATAS	VIQSSPRAA	3	0.6805	31.7	11.00	0.41	tr_C9ZJU1_
DRB1_0101	164	EVILKCILGMHAFVH	LKCILGMHA	3	0.6802	31.8	11.00	0.52	tr_C9ZJU1_
DRB1_0101	165	IITSIHIDAYLALAT	SIHIDAYLA	3	0.6800	31.9	11.00	0.69	tr_C9ZJU1_
DRB1_0101	166	GEVERLCIARAFVKA	LCIARAFVK	5	0.6797	32.0	11.00	0.39	tr_C9ZJU1_
DRB1_0101	167	AFESLYCIRGFVIQS	LYCIRGFVI	4	0.6778	32.6	12.00	0.64	tr_C9ZJU1_
DRB1_0101	168	RFIEDLSIGGNIALG	FIEDLSIGG	1	0.6777	32.7	12.00	0.34	tr_C9ZJU1_
DRB1_0101	169	KAYEILEMLTRATIK	ILEMLTRAT	4	0.6768	33.0	12.00	0.54	tr_C9ZJU1_
DRB1_0101	170	WKLMCSSCSIPQLVT	WKLMCSSCS	0	0.6763	33.2	12.00	0.61	tr_C9ZJU1_
DRB1_0101	171	MHAFVHWRPIYAAEI	FVHWRPIYA	3	0.6759	33.3	12.00	0.40	tr_C9ZJU1_
DRB1_0101	172	FVVNKGEFLGIAGYS	FVVNKGEFL	0	0.6754	33.5	12.00	0.79	tr_C9ZJU1_
DRB1_0101	173	HPYVLRRVSFVVNKG	YVLRRVSFV	2	0.6753	33.6	12.00	0.70	tr_C9ZJU1_
DRB1_0101	174	RGKYSIARYPPAAQA	YSIARYPPA	3	0.6747	33.8	12.00	0.68	tr_C9ZJU1_
DRB1_0101	175	NLRFIEDLSIGGNIA	FIEDLSIGG	3	0.6729	34.4	12.00	0.58	tr_C9ZJU1_
DRB1_0101	176	VHISQYPARMLRRRI	ISQYPARML	2	0.6718	34.9	12.00	0.58	tr_C9ZJU1_
DRB1_0101	177	NFVQKRSSGIHAPLN	FVQKRSSGI	1	0.6710	35.2	12.00	0.71	tr_C9ZJU1_
DRB1_0101	178	SRALNFVQKRSSGIH	FVQKRSSGI	5	0.6709	35.2	12.00	0.83	tr_C9ZJU1_
DRB1_0101	179	AYEILEMLTRATIKT	ILEMLTRAT	3	0.6679	36.4	13.00	0.60	tr_C9ZJU1_
DRB1_0101	180	LFSMVFVESGRPLGY	FSMVFVESG	1	0.6677	36.4	13.00	0.34	tr_C9ZJU1_
DRB1_0101	181	LLRDSTIIIVAHRLA	TIIIVAHRL	5	0.6673	36.6	13.00	0.67	tr_C9ZJU1_
DRB1_0101	182	WIKDVIQSSPRAATA	VIQSSPRAA	4	0.6664	36.9	13.00	0.37	tr_C9ZJU1_
DRB1_0101	183	AALRLSGWELMAATA	ALRLSGWEL	1	0.6647	37.6	13.00	0.63	tr_C9ZJU1_
DRB1_0101	184	FSMVFVESGRPLGYM	VFVESGRPL	3	0.6644	37.7	13.00	0.42	tr_C9ZJU1_
DRB1_0101	185	VNGVHISQYPARMLR	ISQYPARML	5	0.6641	37.9	13.00	0.43	tr_C9ZJU1_
DRB1_0101	186	GTQWSNFLYFLNESL	FLYFLNESL	6	0.6636	38.1	13.00	0.88	tr_C9ZJU1_
DRB1_0101	187	FESLYCIRGFVIQSI	LYCIRGFVI	3	0.6626	38.5	13.00	0.67	tr_C9ZJU1_
DRB1_0101	188	IRGFVIQSILSDIIT	FVIQSILSD	3	0.6621	38.7	14.00	0.51	tr_C9ZJU1_
DRB1_0101	189	DIITSIHIDAYLALA	SIHIDAYLA	4	0.6617	38.9	14.00	0.71	tr_C9ZJU1_
DRB1_0101	190	RGFVIQSILSDIITS	FVIQSILSD	2	0.6613	39.1	14.00	0.44	tr_C9ZJU1_
DRB1_0101	191	IMQLPSVFAVHCGLM	IMQLPSVFA	0	0.6599	39.6	14.00	0.74	tr_C9ZJU1_
DRB1_0101	192	RKAYEILEMLTRATI	ILEMLTRAT	5	0.6596	39.8	14.00	0.40	tr_C9ZJU1_
DRB1_0101	193	EAHLRRHLAFSGSSL	LRRHLAFSG	3	0.6588	40.1	14.00	0.58	tr_C9ZJU1_
DRB1_0101	194	RVVFSFPVVSLVMWL	FSFPVVSLV	3	0.6585	40.2	14.00	0.43	tr_C9ZJU1_
DRB1_0101	195	IHIDAYLALATADYD	YLALATADY	5	0.6583	40.3	14.00	0.34	tr_C9ZJU1_
DRB1_0101	196	LKCILGMHAFVHWRP	ILGMHAFVH	3	0.6575	40.7	14.00	0.46	tr_C9ZJU1_
DRB1_0101	197	RHLAFSGSSLGCYQV	LAFSGSSLG	2	0.6572	40.8	14.00	0.51	tr_C9ZJU1_
DRB1_0101	198	RKRSLQSGITSFWKL	KRSLQSGIT	1	0.6550	41.8	14.00	0.31	tr_C9ZJU1_
DRB1_0101	199	ERLCIARAFVKAGSA	LCIARAFVK	2	0.6539	42.3	15.00	0.56	tr_C9ZJU1_
DRB1_0101	200	LSPFRFIIKQLMGLP	FRFIIKQLM	3	0.6536	42.4	15.00	0.49	tr_C9ZJU1_
DRB1_0101	201	SDRHPYVLRRVSFVV	YVLRRVSFV	5	0.6524	43.0	15.00	0.81	tr_C9ZJU1_
DRB1_0101	202	GGEVERLCIARAFVK	VERLCIARA	3	0.6521	43.1	15.00	0.43	tr_C9ZJU1_
DRB1_0101	203	VMHGCRVVFSFPVVS	CRVVFSFPV	4	0.6515	43.4	15.00	0.67	tr_C9ZJU1_
DRB1_0101	204	TLVFNHNIMQLPSVF	HNIMQLPSV	5	0.6511	43.6	15.00	0.32	tr_C9ZJU1_
DRB1_0101	205	AIFLNCNGVESIHVR	FLNCNGVES	2	0.6510	43.6	15.00	0.48	tr_C9ZJU1_
DRB1_0101	206	NRKRSLQSGITSFWK	KRSLQSGIT	2	0.6484	44.9	15.00	0.41	tr_C9ZJU1_
DRB1_0101	207	GKYSIARYPPAAQAD	YSIARYPPA	2	0.6484	44.9	15.00	0.66	tr_C9ZJU1_
DRB1_0101	208	TLAATVLFSPEVILK	ATVLFSPEV	3	0.6484	44.9	15.00	0.48	tr_C9ZJU1_

DRB1_0101	209	DTLPLLLVSAVALRL	LVSAVALRL	6	0.6475	45.3	15.00	0.53	tr_C9ZJU1_
DRB1_0101	210	ESLYCIRGFVIQSIL	LYCIRGFVI	2	0.6462	46.0	16.00	0.69	tr_C9ZJU1_
DRB1_0101	211	PLGYMWDLLTQHVIW	WDLLTQHVI	5	0.6461	46.0	16.00	0.39	tr_C9ZJU1_
DRB1_0101	212	SLYCIRGFVIQSILS	LYCIRGFVI	1	0.6460	46.1	16.00	0.55	tr_C9ZJU1_
DRB1_0101	213	LCIARAFVKAGSAVG	FVKAGSAVG	6	0.6454	46.4	16.00	0.51	tr_C9ZJU1_
DRB1_0101	214	KDVIQSSPRAATASP	VIQSSPRAA	2	0.6443	46.9	16.00	0.43	tr_C9ZJU1_
DRB1_0101	215	PIYAAEIGKSLNGDV	YAAEIGKSL	2	0.6439	47.1	16.00	0.81	tr_C9ZJU1_
DRB1_0101	216	KLSPFRFIIKQLMGL	FRFIIKQLM	4	0.6439	47.1	16.00	0.52	tr_C9ZJU1_
DRB1_0101	217	HIDAYLALATADYDP	YLALATADY	4	0.6437	47.2	16.00	0.38	tr_C9ZJU1_
DRB1_0101	218	IAIFLNCNGVESIHV	FLNCNGVES	3	0.6430	47.6	16.00	0.57	tr_C9ZJU1_
DRB1_0101	219	VLFSMVFVESGRPLG	FSMVFVESG	2	0.6423	47.9	16.00	0.54	tr_C9ZJU1_
DRB1_0101	220	FIEDLSIGGNIALGN	LSIGGNIAL	4	0.6412	48.5	16.00	0.29	tr_C9ZJU1_
DRB1_0101	221	LAATVLFSPEVILKC	VLFSPEVIL	4	0.6401	49.1	16.00	0.37	tr_C9ZJU1_
DRB1_0101	222	RTLVFNHNIMQLPSV	LVFNHNIMQ	2	0.6397	49.3	16.00	0.41	tr_C9ZJU1_
DRB1_0101	223	NKMLFQQTFSHPSRH	FQQTFSHPS	4	0.6387	49.8	17.00	0.56	tr_C9ZJU1_
DRB1_0101	224	IDAYLALATADYDPS	YLALATADY	3	0.6387	49.9	17.00	0.42	tr_C9ZJU1_
DRB1_0101	225	SPNFFTQSALDQTEI	FFTQSALDQ	3	0.6374	50.6	17.00	0.80	tr_C9ZJU1_
DRB1_0101	226	GSSLGCYQVLEGLQS	YQVLEGLQS	6	0.6353	51.7	17.00	0.73	tr_C9ZJU1_
DRB1_0101	227	CIRGFVIQSILSDII	FVIQSILSD	4	0.6345	52.2	17.00	0.53	tr_C9ZJU1_
DRB1_0101	228	LYCIRGFVIQSILSD	LYCIRGFVI	0	0.6344	52.2	17.00	0.31	tr_C9ZJU1_
DRB1_0101	229	EEVLALCMLLRGKYS	CMLLRGKYS	6	0.6343	52.3	17.00	0.76	tr_C9ZJU1_
DRB1_0101	230	PYVLRRVSFVVNKGE	YVLRRVSFV	1	0.6338	52.6	17.00	0.64	tr_C9ZJU1_
DRB1_0101	231	YNRKRSLQSGITSFW	KRSLQSGIT	3	0.6338	52.6	17.00	0.51	tr_C9ZJU1_
DRB1_0101	232	QVLEGLQSVQRKIDR	LEGLQSVQR	2	0.6336	52.7	17.00	0.78	tr_C9ZJU1_
DRB1_0101	233	DTLRLREGNNLLRDS	LRLREGNNL	2	0.6334	52.8	17.00	0.46	tr_C9ZJU1_
DRB1_0101	234	NESLYMESQFTKSQL	SLYMESQFT	2	0.6333	52.9	17.00	0.63	tr_C9ZJU1_
DRB1_0101	235	KYSIARYPPAAQADV	YSIARYPPA	1	0.6332	52.9	17.00	0.56	tr_C9ZJU1_
DRB1_0101	236	FDTLRLREGNNLLRD	LRLREGNNL	3	0.6331	53.0	17.00	0.51	tr_C9ZJU1_
DRB1_0101	237	NNLRFIEDLSIGGNI	FIEDLSIGG	4	0.6324	53.4	17.00	0.56	tr_C9ZJU1_
DRB1_0101	238	SDIITSIHIDAYLAL	SIHIDAYLA	5	0.6324	53.4	18.00	0.67	tr_C9ZJU1_
DRB1_0101	239	LRGKYSIARYPPAAQ	YSIARYPPA	4	0.6315	53.9	18.00	0.67	tr_C9ZJU1_
DRB1_0101	240	LNESLYMESQFTKSQ	SLYMESQFT	3	0.6313	54.0	18.00	0.70	tr_C9ZJU1_
DRB1_0101	241	LRLSPNFFTQSALDQ	LRLSPNFFT	0	0.6306	54.4	18.00	0.51	tr_C9ZJU1_
DRB1_0101	242	AATVLFSPEVILKCI	VLFSPEVIL	3	0.6300	54.8	18.00	0.38	tr_C9ZJU1_
DRB1_0101	243	YEILEMLTRATIKTD	ILEMLTRAT	2	0.6299	54.8	18.00	0.62	tr_C9ZJU1_
DRB1_0101	244	TLRLREGNNLLRDST	LREGNNLLR	3	0.6297	54.9	18.00	0.39	tr_C9ZJU1_
DRB1_0101	245	RPLGYMWDLLTQHVI	WDLLTQHVI	6	0.6296	55.0	18.00	0.32	tr_C9ZJU1_
DRB1_0101	246	KTTLLRLLNRTYAPT	LLRLLNRTY	3	0.6286	55.6	18.00	0.46	tr_C9ZJU1_
DRB1_0101	247	VKNSLTSSPLTLLRK	LTSSPLTLL	4	0.6282	55.8	18.00	0.23	tr_C9ZJU1_
DRB1_0101	248	VREFSGGEVERLCIA	FSGGEVERL	3	0.6280	56.0	18.00	0.78	tr_C9ZJU1_
DRB1_0101	249	GFVIQSILSDIITSI	IQSILSDII	3	0.6276	56.2	18.00	0.42	tr_C9ZJU1_
DRB1_0101	250	EVMHGCRVVFSFPVV	CRVVFSFPV	5	0.6265	56.9	18.00	0.65	tr_C9ZJU1_
DRB1_0101	251	SMVFVESGRPLGYMW	VFVESGRPL	2	0.6253	57.6	19.00	0.45	tr_C9ZJU1_
DRB1_0101	252	SRVPALLEVMHGCRV	LEVMHGCRV	6	0.6249	57.9	19.00	0.63	tr_C9ZJU1_
DRB1_0101	253	IFLNCNGVESIHVRT	CNGVESIHV	4	0.6237	58.6	19.00	0.41	tr_C9ZJU1_
DRB1_0101	254	PNFFTQSALDQTEIV	FFTQSALDQ	2	0.6232	58.9	19.00	0.73	tr_C9ZJU1_
DRB1_0101	255	AYNRKRSLQSGITSF	KRSLQSGIT	4	0.6216	60.0	19.00	0.46	tr_C9ZJU1_
DRB1_0101	256	LVLFSMVFVESGRPL	FSMVFVESG	3	0.6211	60.3	19.00	0.69	tr_C9ZJU1_
DRB1_0101	257	RLCIARAFVKAGSAV	LCIARAFVK	1	0.6196	61.3	19.00	0.53	tr_C9ZJU1_
DRB1_0101	258	KRSLQSGITSFWKLM	LQSGITSFW	3	0.6195	61.4	19.00	0.28	tr_C9ZJU1_

DRB1_0101	259	IFDTLRLREGNNLLR	LRLREGNNL	4	0.6189	61.8	19.00	0.55	tr_C9ZJU1_
DRB1_0101	260	REFSGGEVERLCIAR	FSGGEVERL	2	0.6176	62.6	20.00	0.75	tr_C9ZJU1_
DRB1_0101	261	PEVILKCILGMHAFV	LKCILGMHA	4	0.6175	62.7	20.00	0.64	tr_C9ZJU1_
DRB1_0101	262	GKTTLLRLLNRTYAP	LLRLLNRTY	4	0.6175	62.7	20.00	0.46	tr_C9ZJU1_
DRB1_0101	263	ESLYMESQFTKSQLA	SLYMESQFT	1	0.6173	62.8	20.00	0.54	tr_C9ZJU1_
DRB1_0101	264	YMWDLLTQHVIWPLV	WDLLTQHVI	2	0.6173	62.9	20.00	0.44	tr_C9ZJU1_
DRB1_0101	265	PVKNSLTSSPLTLLR	KNSLTSSPL	2	0.6169	63.1	20.00	0.37	tr_C9ZJU1_
DRB1_0101	266	LGYMWDLLTQHVIWP	WDLLTQHVI	4	0.6165	63.4	20.00	0.43	tr_C9ZJU1_
DRB1_0101	267	ELMAATAAAAFATAS	LMAATAAAA	1	0.6159	63.8	20.00	0.46	tr_C9ZJU1_
DRB1_0101	268	SGKTTLLRLLNRTYA	LLRLLNRTY	5	0.6154	64.2	20.00	0.44	tr_C9ZJU1_
DRB1_0101	269	VRTLVFNHNIMQLPS	LVFNHNIMQ	3	0.6134	65.6	20.00	0.56	tr_C9ZJU1_
DRB1_0101	270	IEDLSIGGNIALGNL	LSIGGNIAL	3	0.6133	65.6	20.00	0.43	tr_C9ZJU1_
DRB1_0101	271	VVFSFPVVSLVMWLG	FSFPVVSLV	2	0.6128	66.0	20.00	0.44	tr_C9ZJU1_
DRB1_0101	272	KMLFQQTFSHPSRHD	FQQTFSHPS	3	0.6126	66.1	20.00	0.61	tr_C9ZJU1_
DRB1_0101	273	GYMWDLLTQHVIWPL	WDLLTQHVI	3	0.6125	66.2	20.00	0.48	tr_C9ZJU1_
DRB1_0101	274	GWIKDVIQSSPRAAT	DVIQSSPRA	4	0.6119	66.7	21.00	0.46	tr_C9ZJU1_
DRB1_0101	275	FLNESLYMESQFTKS	SLYMESQFT	4	0.6098	68.1	21.00	0.68	tr_C9ZJU1_
DRB1_0101	276	QHWCPLYHAFLGSFY	WCPLYHAFL	2	0.6085	69.1	21.00	0.42	tr_C9ZJU1_
DRB1_0101	277	HISQYPARMLRRRIA	ISQYPARML	1	0.6084	69.2	21.00	0.60	tr_C9ZJU1_
DRB1_0101	278	EFSGGEVERLCIARA	FSGGEVERL	1	0.6083	69.3	21.00	0.56	tr_C9ZJU1_
DRB1_0101	279	LSPNFFTQSALDQTE	FFTQSALDQ	4	0.6082	69.3	21.00	0.81	tr_C9ZJU1_
DRB1_0101	280	DNKMLFQQTFSHPSR	FQQTFSHPS	5	0.6079	69.6	21.00	0.55	tr_C9ZJU1_
DRB1_0101	281	SQFTKSQLASVTNIA	FTKSQLASV	2	0.6073	70.0	21.00	0.38	tr_C9ZJU1_
DRB1_0101	282	LLRGKYSIARYPPAA	YSIARYPPA	5	0.6065	70.6	21.00	0.59	tr_C9ZJU1_
DRB1_0101	283	TTLLRLLNRTYAPTE	LLRLLNRTY	2	0.6062	70.8	21.00	0.39	tr_C9ZJU1_
DRB1_0101	284	YCIRGFVIQSILSDI	FVIQSILSD	5	0.6060	71.1	22.00	0.50	tr_C9ZJU1_
DRB1_0101	285	STFAFESLYCIRGFV	FAFESLYCI	2	0.6057	71.3	22.00	0.46	tr_C9ZJU1_
DRB1_0101	286	SIAIFLNCNGVESIH	FLNCNGVES	4	0.6047	72.0	22.00	0.61	tr_C9ZJU1_
DRB1_0101	287	ERKAYEILEMLTRAT	YEILEMLTR	4	0.6040	72.6	22.00	0.44	tr_C9ZJU1_
DRB1_0101	288	ESQFTKSQLASVTNI	FTKSQLASV	3	0.6034	73.0	22.00	0.46	tr_C9ZJU1_
DRB1_0101	289	HLAFSGSSLGCYQVL	LAFSGSSLG	1	0.6032	73.2	22.00	0.46	tr_C9ZJU1_
DRB1_0101	290	KSSVQYGLVLFSMVF	YGLVLFSMV	5	0.6025	73.8	22.00	0.25	tr_C9ZJU1_
DRB1_0101	291	FLNCNGVESIHVRTL	CNGVESIHV	3	0.6014	74.6	22.00	0.60	tr_C9ZJU1_
DRB1_0101	292	IIIVAHRLATLKHAD	IVAHRLATL	2	0.6014	74.7	22.00	0.46	tr_C9ZJU1_
DRB1_0101	293	ASTFAFESLYCIRGF	FAFESLYCI	3	0.6009	75.1	22.00	0.52	tr_C9ZJU1_
DRB1_0101	294	DIWNCLMALVNCSIR	CLMALVNCS	4	0.6008	75.2	22.00	0.32	tr_C9ZJU1_
DRB1_0101	295	DAYLALATADYDPSD	YLALATADY	2	0.6000	75.8	22.00	0.50	tr_C9ZJU1_
DRB1_0101	296	NFFTQSALDQTEIVN	FFTQSALDQ	1	0.5999	75.9	23.00	0.64	tr_C9ZJU1_
DRB1_0101	297	GMHAFVHWRPIYAAE	FVHWRPIYA	4	0.5985	77.0	23.00	0.54	tr_C9ZJU1_
DRB1_0101	298	SPKLSPFRFIIKQLM	FRFIIKQLM	6	0.5977	77.7	23.00	0.47	tr_C9ZJU1_
DRB1_0101	299	DKSSVQYGLVLFSMV	SSVQYGLVL	2	0.5970	78.3	23.00	0.32	tr_C9ZJU1_
DRB1_0101	300	FVNGVHISQYPARML	HISQYPARM	5	0.5968	78.5	23.00	0.35	tr_C9ZJU1_
DRB1_0101	301	TQHVIWPLVPSVFSG	IWPLVPSVF	4	0.5960	79.1	23.00	0.44	tr_C9ZJU1_
DRB1_0101	302	NVREFSGGEVERLCI	FSGGEVERL	4	0.5959	79.2	23.00	0.79	tr_C9ZJU1_
DRB1_0101	303	IYAAEIGKSLNGDVS	YAAEIGKSL	1	0.5956	79.5	23.00	0.83	tr_C9ZJU1_
DRB1_0101	304	HVRTLVFNHNIMQLP	LVFNHNIMQ	4	0.5949	80.1	23.00	0.55	tr_C9ZJU1_
DRB1_0101	305	NLLRDSTIIIVAHRL	TIIIVAHRL	6	0.5944	80.5	23.00	0.56	tr_C9ZJU1_
DRB1_0101	306	KNSLTSSPLTLLRKQ	LTSSPLTLL	3	0.5940	80.8	23.00	0.36	tr_C9ZJU1_
DRB1_0101	307	FSGGEVERLCIARAF	VERLCIARA	5	0.5938	81.1	24.00	0.35	tr_C9ZJU1_
DRB1_0101	308	MVFVESGRPLGYMWD	VFVESGRPL	1	0.5934	81.4	24.00	0.38	tr_C9ZJU1_

DRB1_0101	309	PKLSPFRFIIKQLMG	FRFIIKQLM	5	0.5931	81.7	24.00	0.60	tr_C9ZJU1_
DRB1_0101	310	HWCPLYHAFLGSFYD	WCPLYHAFL	1	0.5927	82.0	24.00	0.32	tr_C9ZJU1_
DRB1_0101	311	FPVKNSLTSSPLTLL	KNSLTSSPL	3	0.5924	82.3	24.00	0.47	tr_C9ZJU1_
DRB1_0101	312	YSDRHPYVLRRVSFV	YVLRRVSFV	6	0.5918	82.8	24.00	0.71	tr_C9ZJU1_
DRB1_0101	313	IRDILLDDGVALCIL	ILLDDGVAL	3	0.5900	84.5	24.00	0.62	tr_C9ZJU1_
DRB1_0101	314	EDLSIGGNIALGNLW	LSIGGNIAL	2	0.5897	84.7	24.00	0.43	tr_C9ZJU1_
DRB1_0101	315	IHVRTLVFNHNIMQL	LVFNHNIMQ	5	0.5897	84.8	24.00	0.58	tr_C9ZJU1_
DRB1_0101	316	ENNLRFIEDLSIGGN	FIEDLSIGG	5	0.5876	86.7	25.00	0.50	tr_C9ZJU1_
DRB1_0101	317	IWNCLMALVNCSIRD	CLMALVNCS	3	0.5874	86.8	25.00	0.32	tr_C9ZJU1_
DRB1_0101	318	YGLVLFSMVFVESGR	FSMVFVESG	5	0.5867	87.5	25.00	0.69	tr_C9ZJU1_
DRB1_0101	319	LTQHVIWPLVPSVFS	IWPLVPSVF	5	0.5859	88.3	25.00	0.43	tr_C9ZJU1_
DRB1_0101	320	AALSASRALNFVQKR	LSASRALNF	2	0.5855	88.7	25.00	0.63	tr_C9ZJU1_
DRB1_0101	321	LGMHAFVHWRPIYAA	FVHWRPIYA	5	0.5850	89.2	25.00	0.55	tr_C9ZJU1_
DRB1_0101	322	SGGEVERLCIARAFV	VERLCIARA	4	0.5844	89.7	25.00	0.52	tr_C9ZJU1_
DRB1_0101	323	ATVLFSPEVILKCIL	VLFSPEVIL	2	0.5841	90.0	25.00	0.40	tr_C9ZJU1_
DRB1_0101	324	TAYNRKRSLQSGITS	YNRKRSLQS	2	0.5821	92.0	25.00	0.40	tr_C9ZJU1_
DRB1_0101	325	IQHWCPLYHAFLGSF	WCPLYHAFL	3	0.5816	92.4	26.00	0.56	tr_C9ZJU1_
DRB1_0101	326	IKCMLSSTDNKMLFQ	LSSTDNKML	4	0.5811	93.0	26.00	0.44	tr_C9ZJU1_
DRB1_0101	327	VTLAATVLFSPEVIL	ATVLFSPEV	4	0.5808	93.3	26.00	0.54	tr_C9ZJU1_
DRB1_0101	328	TEGDIFVNGVHISQY	DIFVNGVHI	3	0.5807	93.4	26.00	0.56	tr_C9ZJU1_
DRB1_0101	329	SPEVILKCILGMHAF	LKCILGMHA	5	0.5804	93.7	26.00	0.64	tr_C9ZJU1_
DRB1_0101	330	RDILLDDGVALCILD	ILLDDGVAL	2	0.5794	94.7	26.00	0.60	tr_C9ZJU1_
DRB1_0101	331	SLYMESQFTKSQLAS	SLYMESQFT	0	0.5789	95.2	26.00	0.34	tr_C9ZJU1_
DRB1_0101	332	QHVIWPLVPSVFSGC	IWPLVPSVF	3	0.5786	95.5	26.00	0.46	tr_C9ZJU1_
DRB1_0101	333	RLSPNFFTQSALDQT	FFTQSALDQ	5	0.5778	96.4	26.00	0.85	tr_C9ZJU1_
DRB1_0101	334	DEAHLRRHLAFSGSS	LRRHLAFSG	4	0.5778	96.4	26.00	0.80	tr_C9ZJU1_
DRB1_0101	335	MESQFTKSQLASVTN	FTKSQLASV	4	0.5776	96.6	26.00	0.45	tr_C9ZJU1_
DRB1_0101	336	KCILGMHAFVHWRPI	ILGMHAFVH	2	0.5767	97.5	26.00	0.62	tr_C9ZJU1_
DRB1_0101	337	DKIVVLNEGMVAEVG	VVLNEGMVA	3	0.5762	98.1	26.00	0.46	tr_C9ZJU1_
DRB1_0101	338	GIKCMLSSTDNKMLF	LSSTDNKML	5	0.5749	99.5	27.00	0.31	tr_C9ZJU1_
DRB1_0101	339	QFTKSQLASVTNIAT	TKSQLASVT	2	0.5748	99.5	27.00	0.32	tr_C9ZJU1_
DRB1_0101	340	GDIFVNGVHISQYPA	DIFVNGVHI	1	0.5725	102.1	27.00	0.48	tr_C9ZJU1_
DRB1_0101	341	VFSFPVVSLVMWLGT	FSFPVVSLV	1	0.5715	103.2	27.00	0.40	tr_C9ZJU1_
DRB1_0101	342	MWDLLTQHVIWPLVP	WDLLTQHVI	1	0.5713	103.4	27.00	0.45	tr_C9ZJU1_
DRB1_0101	343	ASIAIFLNCNGVESI	FLNCNGVES	5	0.5699	104.9	28.00	0.57	tr_C9ZJU1_
DRB1_0101	344	KIVVLNEGMVAEVGS	VVLNEGMVA	2	0.5696	105.3	28.00	0.45	tr_C9ZJU1_
DRB1_0101	345	ADKIVVLNEGMVAEV	VVLNEGMVA	4	0.5693	105.7	28.00	0.44	tr_C9ZJU1_
DRB1_0101	346	FVIQSILSDIITSIH	IQSILSDII	2	0.5690	106.0	28.00	0.55	tr_C9ZJU1_
DRB1_0101	347	MDELQSRVPALLEVM	LQSRVPALL	3	0.5687	106.3	28.00	0.62	tr_C9ZJU1_
DRB1_0101	348	VASTFAFESLYCIRG	FAFESLYCI	4	0.5678	107.4	28.00	0.59	tr_C9ZJU1_
DRB1_0101	349	WNCLMALVNCSIRDI	CLMALVNCS	2	0.5668	108.5	28.00	0.31	tr_C9ZJU1_
DRB1_0101	350	EGDIFVNGVHISQYP	DIFVNGVHI	2	0.5668	108.5	28.00	0.54	tr_C9ZJU1_
DRB1_0101	351	CDIWNCLMALVNCSI	WNCLMALVN	3	0.5668	108.6	28.00	0.34	tr_C9ZJU1_
DRB1_0101	352	EILEMLTRATIKTDG	ILEMLTRAT	1	0.5667	108.7	28.00	0.54	tr_C9ZJU1_
DRB1_0101	353	WCPLYHAFLGSFYDE	YHAFLGSFY	4	0.5666	108.8	28.00	0.37	tr_C9ZJU1_
DRB1_0101	354	TLLRLLNRTYAPTEG	LRLLNRTYA	2	0.5662	109.2	28.00	0.46	tr_C9ZJU1_
DRB1_0101	355	SSVQYGLVLFSMVFV	YGLVLFSMV	4	0.5655	110.1	28.00	0.37	tr_C9ZJU1_
DRB1_0101	356	CVWQCRQDYLQGFAA	QDYLQGFAA	6	0.5638	112.1	29.00	0.75	tr_C9ZJU1_
DRB1_0101	357	LNVREFSGGEVERLC	FSGGEVERL	5	0.5637	112.2	29.00	0.73	tr_C9ZJU1_
DRB1_0101	358	LRSIQHWCPLYHAFL	WCPLYHAFL	6	0.5627	113.5	29.00	0.29	tr_C9ZJU1_

DRB1_0101	359	HVIWPLVPSVFSGCV	IWPLVPSVF	2	0.5625	113.7	29.00	0.43	tr_C9ZJU1_
DRB1_0101	360	SQALWQASGSVMDEL	LWQASGSVM	3	0.5612	115.4	29.00	0.55	tr_C9ZJU1_
DRB1_0101	361	SIQHWCPLYHAFLGS	WCPLYHAFL	4	0.5605	116.2	29.00	0.55	tr_C9ZJU1_
DRB1_0101	362	LRLREGNNLLRDSTI	LREGNNLLR	2	0.5602	116.6	29.00	0.46	tr_C9ZJU1_
DRB1_0101	363	GLVLFSMVFVESGRP	FSMVFVESG	4	0.5601	116.7	29.00	0.79	tr_C9ZJU1_
DRB1_0101	364	FVQKRSSGIHAPLNV	FVQKRSSGI	0	0.5600	116.8	29.00	0.54	tr_C9ZJU1_
DRB1_0101	365	YMESQFTKSQLASVT	FTKSQLASV	5	0.5597	117.2	29.00	0.39	tr_C9ZJU1_
DRB1_0101	366	KCMLSSTDNKMLFQQ	LSSTDNKML	3	0.5596	117.3	29.00	0.62	tr_C9ZJU1_
DRB1_0101	367	RSIQHWCPLYHAFLG	WCPLYHAFL	5	0.5591	118.0	30.00	0.46	tr_C9ZJU1_
DRB1_0101	368	MLLRGKYSIARYPPA	YSIARYPPA	6	0.5589	118.2	30.00	0.31	tr_C9ZJU1_
DRB1_0101	369	SDDWLLSRCVSQALW	WLLSRCVSQ	3	0.5585	118.8	30.00	0.29	tr_C9ZJU1_
DRB1_0101	370	ILGMHAFVHWRPIYA	FVHWRPIYA	6	0.5572	120.4	30.00	0.37	tr_C9ZJU1_
DRB1_0101	371	GNNLLRDSTIIIVAH	LLRDSTIII	3	0.5551	123.1	30.00	0.46	tr_C9ZJU1_
DRB1_0101	372	LGLITAYNRKRSLQS	LITAYNRKR	2	0.5550	123.3	30.00	0.51	tr_C9ZJU1_
DRB1_0101	373	QYGLVLFSMVFVESG	FSMVFVESG	6	0.5533	125.6	31.00	0.43	tr_C9ZJU1_
DRB1_0101	374	ASRALNFVQKRSSGI	FVQKRSSGI	6	0.5522	127.1	31.00	0.71	tr_C9ZJU1_
DRB1_0101	375	IIVAHRLATLKHADK	IVAHRLATL	1	0.5518	127.7	31.00	0.43	tr_C9ZJU1_
DRB1_0101	376	VMDELQSRVPALLEV	LQSRVPALL	4	0.5508	129.0	31.00	0.58	tr_C9ZJU1_
DRB1_0101	377	LSDIITSIHIDAYLA	SIHIDAYLA	6	0.5505	129.5	31.00	0.49	tr_C9ZJU1_
DRB1_0101	378	VLEGLQSVQRKIDRP	LEGLQSVQR	1	0.5497	130.5	31.00	0.82	tr_C9ZJU1_
DRB1_0101	379	DVIQSSPRAATASPS	VIQSSPRAA	1	0.5489	131.7	31.00	0.44	tr_C9ZJU1_
DRB1_0101	380	SIRDILLDDGVALCI	ILLDDGVAL	4	0.5487	132.0	31.00	0.62	tr_C9ZJU1_
DRB1_0101	381	DELQSRVPALLEVMH	LQSRVPALL	2	0.5485	132.3	32.00	0.57	tr_C9ZJU1_
DRB1_0101	382	QALWQASGSVMDELQ	LWQASGSVM	2	0.5475	133.7	32.00	0.51	tr_C9ZJU1_
DRB1_0101	383	LLTQHVIWPLVPSVF	IWPLVPSVF	6	0.5473	134.0	32.00	0.38	tr_C9ZJU1_
DRB1_0101	384	EGNNLLRDSTIIIVA	LLRDSTIII	4	0.5467	134.9	32.00	0.43	tr_C9ZJU1_
DRB1_0101	385	HDKSSVQYGLVLFSM	SSVQYGLVL	3	0.5465	135.2	32.00	0.50	tr_C9ZJU1_
DRB1_0101	386	CERKAYEILEMLTRA	YEILEMLTR	5	0.5461	135.8	32.00	0.52	tr_C9ZJU1_
DRB1_0101	387	LNCNGVESIHVRTLV	CNGVESIHV	2	0.5456	136.5	32.00	0.63	tr_C9ZJU1_
DRB1_0101	388	MLFQQTFSHPSRHDK	FQQTFSHPS	2	0.5455	136.7	32.00	0.67	tr_C9ZJU1_
DRB1_0101	389	SRMRQSQQLPHFVKK	QSQQLPHFV	4	0.5455	136.7	32.00	0.44	tr_C9ZJU1_
DRB1_0101	390	EIFDTLRLREGNNLL	LRLREGNNL	5	0.5452	137.1	32.00	0.55	tr_C9ZJU1_
DRB1_0101	391	DLSIGGNIALGNLWD	LSIGGNIAL	1	0.5447	137.9	32.00	0.44	tr_C9ZJU1_
DRB1_0101	392	RSLQSGITSFWKLMC	LQSGITSFW	2	0.5444	138.2	32.00	0.26	tr_C9ZJU1_
DRB1_0101	393	VDEAHLRRHLAFSGS	LRRHLAFSG	5	0.5442	138.6	32.00	0.73	tr_C9ZJU1_
DRB1_0101	394	FSRMRQSQQLPHFVK	SRMRQSQQL	1	0.5442	138.6	32.00	0.38	tr_C9ZJU1_
DRB1_0101	395	YSIARYPPAAQADVV	YSIARYPPA	0	0.5436	139.6	32.00	0.34	tr_C9ZJU1_
DRB1_0101	396	DWLLSRCVSQALWQA	LSRCVSQAL	3	0.5433	140.0	32.00	0.32	tr_C9ZJU1_
DRB1_0101	397	SCSIPQLVTLAATVL	SIPQLVTLA	2	0.5430	140.5	33.00	0.28	tr_C9ZJU1_
DRB1_0101	398	DDWLLSRCVSQALWQ	WLLSRCVSQ	2	0.5429	140.6	33.00	0.28	tr_C9ZJU1_
DRB1_0101	399	GRPLGYMWDLLTQHV	LGYMWDLLT	3	0.5424	141.4	33.00	0.52	tr_C9ZJU1_
DRB1_0101	400	WLLSRCVSQALWQAS	CVSQALWQA	5	0.5414	142.9	33.00	0.37	tr_C9ZJU1_
DRB1_0101	401	YVLRRVSFVVNKGEF	YVLRRVSFV	0	0.5404	144.4	33.00	0.51	tr_C9ZJU1_
DRB1_0101	402	GLITAYNRKRSLQSG	LITAYNRKR	1	0.5385	147.5	33.00	0.40	tr_C9ZJU1_
DRB1_0101	403	DIFVNGVHISQYPAR	DIFVNGVHI	0	0.5383	147.7	33.00	0.37	tr_C9ZJU1_
DRB1_0101	404	LGWIKDVIQSSPRAA	DVIQSSPRA	5	0.5382	147.9	33.00	0.49	tr_C9ZJU1_
DRB1_0101	405	FTKSQLASVTNIATS	TKSQLASVT	1	0.5381	148.0	33.00	0.21	tr_C9ZJU1_
DRB1_0101	406	NCLMALVNCSIRDIL	CLMALVNCS	1	0.5376	148.8	34.00	0.34	tr_C9ZJU1_
DRB1_0101	407	ITAYNRKRSLQSGIT	YNRKRSLQS	3	0.5368	150.2	34.00	0.64	tr_C9ZJU1_
DRB1_0101	408	VIWPLVPSVFSGCVV	IWPLVPSVF	1	0.5360	151.5	34.00	0.36	tr_C9ZJU1_

DRB1_0101	409	HADKIVVLNEGMVAE	VVLNEGMVA	5	0.5354	152.4	34.00	0.39	tr_C9ZJU1_
DRB1_0101	410	CSIPQLVTLAATVLF	LVTLAATVL	5	0.5351	152.9	34.00	0.26	tr_C9ZJU1_
DRB1_0101	411	PSVFAVHCGLMKNSH	FAVHCGLMK	3	0.5344	154.1	34.00	0.59	tr_C9ZJU1_
DRB1_0101	412	LPSVFAVHCGLMKNS	FAVHCGLMK	4	0.5340	154.7	34.00	0.56	tr_C9ZJU1_
DRB1_0101	413	AYLALATADYDPSDD	YLALATADY	1	0.5340	154.7	34.00	0.57	tr_C9ZJU1_
DRB1_0101	414	DILLDDGVALCILDD	ILLDDGVAL	1	0.5339	155.0	34.00	0.55	tr_C9ZJU1_
DRB1_0101	415	SIHVRTLVFNHNIMQ	LVFNHNIMQ	6	0.5339	155.0	34.00	0.43	tr_C9ZJU1_
DRB1_0101	416	PSDDWLLSRCVSQAL	WLLSRCVSQ	4	0.5338	155.0	34.00	0.34	tr_C9ZJU1_
DRB1_0101	417	VCDIWNCLMALVNCS	WNCLMALVN	4	0.5335	155.6	34.00	0.44	tr_C9ZJU1_
DRB1_0101	418	PTEGDIFVNGVHISQ	DIFVNGVHI	4	0.5332	156.1	34.00	0.60	tr_C9ZJU1_
DRB1_0101	419	HRLATLKHADKIVVL	LATLKHADK	2	0.5332	156.2	34.00	0.35	tr_C9ZJU1_
DRB1_0101	420	LMAATAAAAFATASE	AATAAAAFA	2	0.5331	156.2	34.00	0.32	tr_C9ZJU1_
DRB1_0101	421	CPLYHAFLGSFYDEC	YHAFLGSFY	3	0.5325	157.2	35.00	0.41	tr_C9ZJU1_
DRB1_0101	422	LYMESQFTKSQLASV	SQFTKSQLA	4	0.5322	157.9	35.00	0.29	tr_C9ZJU1_
DRB1_0101	423	ELQSRVPALLEVMHG	LQSRVPALL	1	0.5313	159.3	35.00	0.47	tr_C9ZJU1_
DRB1_0101	424	VFVESGRPLGYMWDL	VESGRPLGY	2	0.5307	160.4	35.00	0.35	tr_C9ZJU1_
DRB1_0101	425	SSCSIPQLVTLAATV	SIPQLVTLA	3	0.5303	161.1	35.00	0.41	tr_C9ZJU1_
DRB1_0101	426	LLVCDIWNCLMALVN	WNCLMALVN	6	0.5302	161.2	35.00	0.31	tr_C9ZJU1_
DRB1_0101	427	RLATLKHADKIVVLN	LKHADKIVV	4	0.5297	162.1	35.00	0.34	tr_C9ZJU1_
DRB1_0101	428	SVQYGLVLFSMVFVE	YGLVLFSMV	3	0.5292	163.0	35.00	0.47	tr_C9ZJU1_
DRB1_0101	429	ADTLPLLLVSAVALR	PLLLVSAVA	4	0.5288	163.8	35.00	0.28	tr_C9ZJU1_
DRB1_0101	430	NCNGVESIHVRTLVF	CNGVESIHV	1	0.5274	166.2	36.00	0.52	tr_C9ZJU1_
DRB1_0101	431	AVASTFAFESLYCIR	FAFESLYCI	5	0.5268	167.3	36.00	0.62	tr_C9ZJU1_
DRB1_0101	432	CSSCSIPQLVTLAAT	SIPQLVTLA	4	0.5262	168.4	36.00	0.41	tr_C9ZJU1_
DRB1_0101	433	IVAHRLATLKHADKI	LATLKHADK	5	0.5259	169.0	36.00	0.29	tr_C9ZJU1_
DRB1_0101	434	SVMDELQSRVPALLE	LQSRVPALL	5	0.5255	169.7	36.00	0.54	tr_C9ZJU1_
DRB1_0101	435	EIVNQLRSIQHWCPL	VNQLRSIQH	2	0.5254	169.9	36.00	0.44	tr_C9ZJU1_
DRB1_0101	436	NNLLRDSTIIIVAHR	LLRDSTIII	2	0.5252	170.2	36.00	0.46	tr_C9ZJU1_
DRB1_0101	437	LKRKLQAGGHTCAVS	KLQAGGHTC	3	0.5247	171.1	36.00	0.41	tr_C9ZJU1_
DRB1_0101	438	DGIKCMLSSTDNKML	IKCMLSSTD	2	0.5233	173.7	36.00	0.42	tr_C9ZJU1_
DRB1_0101	439	QLPSVFAVHCGLMKN	FAVHCGLMK	5	0.5228	174.8	36.00	0.52	tr_C9ZJU1_
DRB1_0101	440	AHRLATLKHADKIVV	LATLKHADK	3	0.5221	176.1	37.00	0.47	tr_C9ZJU1_
DRB1_0101	441	FSPEVILKCILGMHA	LKCILGMHA	6	0.5218	176.6	37.00	0.63	tr_C9ZJU1_
DRB1_0101	442	SIPQLVTLAATVLFS	LVTLAATVL	4	0.5215	177.2	37.00	0.38	tr_C9ZJU1_
DRB1_0101	443	SFPVKNSLTSSPLTL	KNSLTSSPL	4	0.5211	178.0	37.00	0.57	tr_C9ZJU1_
DRB1_0101	444	VSQALWQASGSVMDE	LWQASGSVM	4	0.5208	178.6	37.00	0.56	tr_C9ZJU1_
DRB1_0101	445	LVMWLGTQWSNFLYF	LGTQWSNFL	4	0.5206	178.9	37.00	0.38	tr_C9ZJU1_
DRB1_0101	446	HCGLMKNSHPDHLLV	MKNSHPDHL	4	0.5205	179.1	37.00	0.49	tr_C9ZJU1_
DRB1_0101	447	CILGMHAFVHWRPIY	ILGMHAFVH	1	0.5205	179.2	37.00	0.51	tr_C9ZJU1_
DRB1_0101	448	IVNQLRSIQHWCPLY	LRSIQHWCP	4	0.5204	179.3	37.00	0.42	tr_C9ZJU1_
DRB1_0101	449	VMWLGTQWSNFLYFL	LGTQWSNFL	3	0.5197	180.6	37.00	0.49	tr_C9ZJU1_
DRB1_0101	450	SLVMWLGTQWSNFLY	VMWLGTQWS	2	0.5192	181.7	37.00	0.42	tr_C9ZJU1_
DRB1_0101	451	YPARMLRRRIANVWQ	MLRRRIANV	4	0.5190	182.0	37.00	0.30	tr_C9ZJU1_
DRB1_0101	452	QLRSIQHWCPLYHAF	LRSIQHWCP	1	0.5190	182.0	37.00	0.41	tr_C9ZJU1_
DRB1_0101	453	SVFAVHCGLMKNSHP	FAVHCGLMK	2	0.5178	184.4	37.00	0.56	tr_C9ZJU1_
DRB1_0101	454	REGNNLLRDSTIIIV	LLRDSTIII	5	0.5173	185.5	38.00	0.38	tr_C9ZJU1_
DRB1_0101	455	KRKLQAGGHTCAVSV	LQAGGHTCA	3	0.5167	186.6	38.00	0.37	tr_C9ZJU1_
DRB1_0101	456	TDNKMLFQQTFSHPS	MLFQQTFSH	4	0.5165	187.1	38.00	0.39	tr_C9ZJU1_
DRB1_0101	457	CFSRMRQSQQLPHFV	SRMRQSQQL	2	0.5158	188.5	38.00	0.55	tr_C9ZJU1_
DRB1_0101	458	WDLLTQHVIWPLVPS	WDLLTQHVI	0	0.5157	188.7	38.00	0.37	tr_C9ZJU1_

DRB1_0101	459	NQLRSIQHWCPLYHA	LRSIQHWCP	2	0.5152	189.7	38.00	0.50	tr_C9ZJU1_
DRB1_0101	460	LVCDIWNCLMALVNC	WNCLMALVN	5	0.5139	192.4	38.00	0.40	tr_C9ZJU1_
DRB1_0101	461	QTEIVNQLRSIQHWC	VNQLRSIQH	4	0.5137	192.9	38.00	0.58	tr_C9ZJU1_
DRB1_0101	462	LRERARPAVLLDCER	ERARPAVLL	2	0.5135	193.3	38.00	0.67	tr_C9ZJU1_
DRB1_0101	463	CVSQALWQASGSVMD	LWQASGSVM	5	0.5129	194.4	38.00	0.49	tr_C9ZJU1_
DRB1_0101	464	PLYHAFLGSFYDECG	YHAFLGSFY	2	0.5122	195.9	39.00	0.41	tr_C9ZJU1_
DRB1_0101	465	IVVLNEGMVAEVGSW	VVLNEGMVA	1	0.5116	197.3	39.00	0.41	tr_C9ZJU1_
DRB1_0101	466	RHDKSSVQYGLVLFS	SSVQYGLVL	4	0.5109	198.8	39.00	0.53	tr_C9ZJU1_
DRB1_0101	467	VSPKLSPFRFIIKQL	PKLSPFRFI	2	0.5108	199.0	39.00	0.34	tr_C9ZJU1_
DRB1_0101	468	NSLTSSPLTLLRKQK	LTSSPLTLL	2	0.5107	199.1	39.00	0.44	tr_C9ZJU1_
DRB1_0101	469	MCSSCSIPQLVTLAA	SIPQLVTLA	5	0.5105	199.6	39.00	0.34	tr_C9ZJU1_
DRB1_0101	470	TEIVNQLRSIQHWCP	VNQLRSIQH	3	0.5105	199.7	39.00	0.54	tr_C9ZJU1_
DRB1_0101	471	PARMLRRRIANVWQE	LRRRIANVW	4	0.5104	199.9	39.00	0.38	tr_C9ZJU1_
DRB1_0101	472	LLSRCVSQALWQASG	CVSQALWQA	4	0.5099	201.0	39.00	0.47	tr_C9ZJU1_
DRB1_0101	473	GSGKTTLLRLLNRTY	LLRLLNRTY	6	0.5096	201.6	39.00	0.46	tr_C9ZJU1_
DRB1_0101	474	TVLFSPEVILKCILG	VLFSPEVIL	1	0.5095	201.8	39.00	0.43	tr_C9ZJU1_
DRB1_0101	475	ENQVSPTCSFPVKNS	VSPTCSFPV	3	0.5094	202.1	39.00	0.75	tr_C9ZJU1_
DRB1_0101	476	LITAYNRKRSLQSGI	YNRKRSLQS	4	0.5093	202.1	39.00	0.62	tr_C9ZJU1_
DRB1_0101	477	ALKRKLQAGGHTCAV	KLQAGGHTC	4	0.5093	202.2	39.00	0.34	tr_C9ZJU1_
DRB1_0101	478	QEFPEELYLGLITAY	ELYLGLITA	5	0.5081	204.9	39.00	0.32	tr_C9ZJU1_
DRB1_0101	479	VSMAVASTFAFESLY	MAVASTFAF	2	0.5080	205.1	39.00	0.35	tr_C9ZJU1_
DRB1_0101	480	TKSQLASVTNIATST	LASVTNIAT	4	0.5077	205.8	39.00	0.39	tr_C9ZJU1_
DRB1_0101	481	LVTLAATVLFSPEVI	ATVLFSPEV	5	0.5072	206.8	40.00	0.51	tr_C9ZJU1_
DRB1_0101	482	CGLMKNSHPDHLLVC	MKNSHPDHL	3	0.5069	207.5	40.00	0.54	tr_C9ZJU1_
DRB1_0101	483	ADVVVQEFPEELYLG	VVQEFPEEL	3	0.5062	209.0	40.00	0.82	tr_C9ZJU1_
DRB1_0101	484	CMLSSTDNKMLFQQT	LSSTDNKML	2	0.5056	210.4	40.00	0.64	tr_C9ZJU1_
DRB1_0101	485	FFTQSALDQTEIVNQ	FFTQSALDQ	0	0.5055	210.8	40.00	0.42	tr_C9ZJU1_
DRB1_0101	486	EENNLRFIEDLSIGG	LRFIEDLSI	4	0.5052	211.4	40.00	0.49	tr_C9ZJU1_
DRB1_0101	487	RCVSQALWQASGSVM	LWQASGSVM	6	0.5051	211.7	40.00	0.34	tr_C9ZJU1_
DRB1_0101	488	KSQLASVTNIATSTY	LASVTNIAT	3	0.5041	214.0	40.00	0.48	tr_C9ZJU1_
DRB1_0101	489	SFRYSDRHPYVLRRV	YSDRHPYVL	3	0.5039	214.4	40.00	0.47	tr_C9ZJU1_
DRB1_0101	490	VAHRLATLKHADKIV	LATLKHADK	4	0.5028	216.9	40.00	0.41	tr_C9ZJU1_
DRB1_0101	491	NQVSPTCSFPVKNSL	VSPTCSFPV	2	0.5013	220.5	41.00	0.75	tr_C9ZJU1_
DRB1_0101	492	DQTEIVNQLRSIQHW	VNQLRSIQH	5	0.5010	221.2	41.00	0.54	tr_C9ZJU1_
DRB1_0101	493	GRDVLHGLFQMADTL	VLHGLFQMA	3	0.5002	223.1	41.00	0.34	tr_C9ZJU1_
DRB1_0101	494	VSFRYSDRHPYVLRR	YSDRHPYVL	4	0.4996	224.5	41.00	0.44	tr_C9ZJU1_
DRB1_0101	495	VIQSILSDIITSIHI	IQSILSDII	1	0.4979	228.7	41.00	0.51	tr_C9ZJU1_
DRB1_0101	496	SGRPLGYMWDLLTQH	LGYMWDLLT	4	0.4974	230.1	42.00	0.57	tr_C9ZJU1_
DRB1_0101	497	IDRPVRGVAPPSLDG	VRGVAPPSL	4	0.4973	230.3	42.00	0.72	tr_C9ZJU1_
DRB1_0101	498	LCILDDVKAEEVLAL	LDDVKAEEV	3	0.4966	231.9	42.00	0.54	tr_C9ZJU1_
DRB1_0101	499	QYPARMLRRRIANVW	MLRRRIANV	5	0.4958	234.0	42.00	0.28	tr_C9ZJU1_
DRB1_0101	500	DVSPKLSPFRFIIKQ	PKLSPFRFI	3	0.4955	234.7	42.00	0.38	tr_C9ZJU1_
DRB1_0101	501	MAVASTFAFESLYCI	FAFESLYCI	6	0.4953	235.3	42.00	0.44	tr_C9ZJU1_
DRB1_0101	502	KHADKIVVLNEGMVA	IVVLNEGMV	5	0.4951	235.8	42.00	0.47	tr_C9ZJU1_
DRB1_0101	503	LMCSSCSIPQLVTLA	SSCSIPQLV	3	0.4948	236.5	42.00	0.39	tr_C9ZJU1_
DRB1_0101	504	DCERKAYEILEMLTR	YEILEMLTR	6	0.4940	238.5	42.00	0.47	tr_C9ZJU1_
DRB1_0101	505	VNQLRSIQHWCPLYH	LRSIQHWCP	3	0.4931	240.9	42.00	0.57	tr_C9ZJU1_
DRB1_0101	506	FSFPVVSLVMWLGTQ	SFPVVSLVM	1	0.4927	241.9	43.00	0.27	tr_C9ZJU1_
DRB1_0101	507	ALWQASGSVMDELQS	WQASGSVMD	2	0.4919	244.1	43.00	0.43	tr_C9ZJU1_
DRB1_0101	508	VSLVMWLGTQWSNFL	VMWLGTQWS	3	0.4913	245.6	43.00	0.52	tr_C9ZJU1_

DRB1_0101	509	HLLVCDIWNCLMALV	LVCDIWNCL	2	0.4911	246.3	43.00	0.39	tr_C9ZJU1_
DRB1_0101	510	VHCGLMKNSHPDHLL	MKNSHPDHL	5	0.4910	246.6	43.00	0.41	tr_C9ZJU1_
DRB1_0101	511	ISQYPARMLRRRIAN	ISQYPARML	0	0.4906	247.5	43.00	0.44	tr_C9ZJU1_
DRB1_0101	512	RDVLHGLFQMADTLP	VLHGLFQMA	2	0.4904	248.1	43.00	0.29	tr_C9ZJU1_
DRB1_0101	513	ALCILDDVKAEEVLA	LDDVKAEEV	4	0.4899	249.3	43.00	0.38	tr_C9ZJU1_
DRB1_0101	514	CNGVESIHVRTLVFN	CNGVESIHV	0	0.4893	251.2	43.00	0.31	tr_C9ZJU1_
DRB1_0101	515	ARMLRRRIANVWQEE	LRRRIANVW	3	0.4891	251.7	43.00	0.44	tr_C9ZJU1_
DRB1_0101	516	PLNVREFSGGEVERL	FSGGEVERL	6	0.4883	253.8	43.00	0.63	tr_C9ZJU1_
DRB1_0101	517	EENQVSPTCSFPVKN	VSPTCSFPV	4	0.4876	255.6	44.00	0.72	tr_C9ZJU1_
DRB1_0101	518	KLMCSSCSIPQLVTL	SSCSIPQLV	4	0.4875	256.0	44.00	0.34	tr_C9ZJU1_
DRB1_0101	519	ILEMLTRATIKTDGH	ILEMLTRAT	0	0.4874	256.2	44.00	0.34	tr_C9ZJU1_
DRB1_0101	520	GSVMDELQSRVPALL	LQSRVPALL	6	0.4874	256.3	44.00	0.42	tr_C9ZJU1_
DRB1_0101	521	IFVNGVHISQYPARM	HISQYPARM	6	0.4872	256.9	44.00	0.26	tr_C9ZJU1_
DRB1_0101	522	MQLPSVFAVHCGLMK	FAVHCGLMK	6	0.4868	258.0	44.00	0.41	tr_C9ZJU1_
DRB1_0101	523	SCFSRMRQSQQLPHF	SRMRQSQQL	3	0.4865	258.7	44.00	0.67	tr_C9ZJU1_
DRB1_0101	524	DHLLVCDIWNCLMAL	LVCDIWNCL	3	0.4856	261.4	44.00	0.49	tr_C9ZJU1_
DRB1_0101	525	VALCILDDVKAEEVL	LCILDDVKA	2	0.4851	262.6	44.00	0.53	tr_C9ZJU1_
DRB1_0101	526	GDVSPKLSPFRFIIK	PKLSPFRFI	4	0.4851	262.7	44.00	0.36	tr_C9ZJU1_
DRB1_0101	527	CLMALVNCSIRDILL	LMALVNCSI	1	0.4850	263.0	44.00	0.37	tr_C9ZJU1_
DRB1_0101	528	LLRLLNRTYAPTEGD	LRLLNRTYA	1	0.4844	264.8	44.00	0.48	tr_C9ZJU1_
DRB1_0101	529	LATLKHADKIVVLNE	LKHADKIVV	3	0.4844	264.8	44.00	0.45	tr_C9ZJU1_
DRB1_0101	530	QADVVVQEFPEELYL	VVQEFPEEL	4	0.4840	265.8	44.00	0.81	tr_C9ZJU1_
DRB1_0101	531	DVVVQEFPEELYLGL	VVQEFPEEL	2	0.4834	267.5	44.00	0.75	tr_C9ZJU1_
DRB1_0101	532	VQYGLVLFSMVFVES	YGLVLFSMV	2	0.4834	267.6	44.00	0.49	tr_C9ZJU1_
DRB1_0101	533	RLREGNNLLRDSTII	LREGNNLLR	1	0.4832	268.1	44.00	0.50	tr_C9ZJU1_
DRB1_0101	534	ALSASRALNFVQKRS	LSASRALNF	1	0.4825	270.1	45.00	0.64	tr_C9ZJU1_
DRB1_0101	535	LSRCVSQALWQASGS	CVSQALWQA	3	0.4822	271.1	45.00	0.57	tr_C9ZJU1_
DRB1_0101	536	CSIRDILLDDGVALC	ILLDDGVAL	5	0.4820	271.6	45.00	0.71	tr_C9ZJU1_
DRB1_0101	537	DPSDDWLLSRCVSQA	DDWLLSRCV	3	0.4820	271.7	45.00	0.46	tr_C9ZJU1_
DRB1_0101	538	AGGHTCAVSVRRLGW	TCAVSVRRL	4	0.4820	271.8	45.00	0.44	tr_C9ZJU1_
DRB1_0101	539	LAFSGSSLGCYQVLE	LAFSGSSLG	0	0.4817	272.5	45.00	0.43	tr_C9ZJU1_
DRB1_0101	540	VFAVHCGLMKNSHPD	FAVHCGLMK	1	0.4816	272.8	45.00	0.54	tr_C9ZJU1_
DRB1_0101	541	IPQLVTLAATVLFSP	LVTLAATVL	3	0.4813	273.9	45.00	0.41	tr_C9ZJU1_
DRB1_0101	542	DRPVRGVAPPSLDGP	VRGVAPPSL	3	0.4806	275.9	45.00	0.75	tr_C9ZJU1_
DRB1_0101	543	GGHTCAVSVRRLGWI	TCAVSVRRL	3	0.4805	276.0	45.00	0.46	tr_C9ZJU1_
DRB1_0101	544	LFQQTFSHPSRHDKS	FQQTFSHPS	1	0.4800	277.5	45.00	0.72	tr_C9ZJU1_
DRB1_0101	545	YDPSDDWLLSRCVSQ	DDWLLSRCV	4	0.4796	278.8	45.00	0.40	tr_C9ZJU1_
DRB1_0101	546	IWPLVPSVFSGCVVD	WPLVPSVFS	1	0.4785	282.1	45.00	0.32	tr_C9ZJU1_
DRB1_0101	547	LQSRVPALLEVMHGC	PALLEVMHG	5	0.4777	284.6	46.00	0.24	tr_C9ZJU1_
DRB1_0101	548	GEIFDTLRLREGNNL	FDTLRLREG	3	0.4773	285.9	46.00	0.40	tr_C9ZJU1_
DRB1_0101	549	GVALCILDDVKAEEV	LCILDDVKA	3	0.4764	288.6	46.00	0.59	tr_C9ZJU1_
DRB1_0101	550	SLQSGITSFWKLMCS	SGITSFWKL	3	0.4764	288.7	46.00	0.40	tr_C9ZJU1_
DRB1_0101	551	PSVFSGCVVDALKRK	FSGCVVDAL	3	0.4743	295.2	46.00	0.50	tr_C9ZJU1_
DRB1_0101	552	RKLQAGGHTCAVSVR	LQAGGHTCA	2	0.4742	295.7	46.00	0.35	tr_C9ZJU1_
DRB1_0101	553	DASIAIFLNCNGVES	FLNCNGVES	6	0.4737	297.2	46.00	0.41	tr_C9ZJU1_
DRB1_0101	554	LYHAFLGSFYDECGM	YHAFLGSFY	1	0.4737	297.3	46.00	0.40	tr_C9ZJU1_
DRB1_0101	555	SVFSGCVVDALKRKL	FSGCVVDAL	2	0.4714	304.8	47.00	0.46	tr_C9ZJU1_
DRB1_0101	556	MWLGTQWSNFLYFLN	LGTQWSNFL	2	0.4712	305.4	47.00	0.56	tr_C9ZJU1_
DRB1_0101	557	DYDPSDDWLLSRCVS	YDPSDDWLL	1	0.4701	308.9	47.00	0.47	tr_C9ZJU1_
DRB1_0101	558	RPVRGVAPPSLDGPW	VRGVAPPSL	2	0.4699	309.5	47.00	0.74	tr_C9ZJU1_

DRB1_0101	559	VKAGSAVGLCIFDEP	VKAGSAVGL	0	0.4697	310.3	47.00	0.44	tr_C9ZJU1_
DRB1_0101	560	SRHDKSSVQYGLVLF	SSVQYGLVL	5	0.4688	313.5	47.00	0.52	tr_C9ZJU1_
DRB1_0101	561	FRYSDRHPYVLRRVS	YSDRHPYVL	2	0.4687	313.8	48.00	0.51	tr_C9ZJU1_
DRB1_0101	562	EVSFRYSDRHPYVLR	YSDRHPYVL	5	0.4679	316.5	48.00	0.40	tr_C9ZJU1_
DRB1_0101	563	ILLDDGVALCILDDV	ILLDDGVAL	0	0.4674	318.2	48.00	0.46	tr_C9ZJU1_
DRB1_0101	564	LREGNNLLRDSTIII	NNLLRDSTI	4	0.4667	320.6	48.00	0.30	tr_C9ZJU1_
DRB1_0101	565	VVVQEFPEELYLGLI	VVQEFPEEL	1	0.4666	321.0	48.00	0.65	tr_C9ZJU1_
DRB1_0101	566	GHTCAVSVRRLGWIK	TCAVSVRRL	2	0.4658	323.8	48.00	0.44	tr_C9ZJU1_
DRB1_0101	567	APTEGDIFVNGVHIS	DIFVNGVHI	5	0.4655	324.9	48.00	0.69	tr_C9ZJU1_
DRB1_0101	568	QVSPTCSFPVKNSLT	VSPTCSFPV	1	0.4653	325.4	48.00	0.69	tr_C9ZJU1_
DRB1_0101	569	ILDDVKAEEVLALCM	LDDVKAEEV	1	0.4649	326.8	48.00	0.35	tr_C9ZJU1_
DRB1_0101	570	KIDRPVRGVAPPSLD	VRGVAPPSL	5	0.4649	326.9	48.00	0.67	tr_C9ZJU1_
DRB1_0101	571	MADTLPLLLVSAVAL	PLLLVSAVA	5	0.4639	330.5	49.00	0.31	tr_C9ZJU1_
DRB1_0101	572	CGRDVLHGLFQMADT	DVLHGLFQM	3	0.4635	332.1	49.00	0.38	tr_C9ZJU1_
DRB1_0101	573	YAAEIGKSLNGDVSP	YAAEIGKSL	0	0.4630	333.7	49.00	0.65	tr_C9ZJU1_
DRB1_0101	574	CILDDVKAEEVLALC	LDDVKAEEV	2	0.4628	334.3	49.00	0.51	tr_C9ZJU1_
DRB1_0101	575	SQLASVTNIATSTYA	LASVTNIAT	2	0.4623	336.1	49.00	0.46	tr_C9ZJU1_
DRB1_0101	576	QVDEAHLRRHLAFSG	LRRHLAFSG	6	0.4618	338.1	49.00	0.53	tr_C9ZJU1_
DRB1_0101	577	QLVTLAATVLFSPEV	LVTLAATVL	1	0.4616	338.8	49.00	0.34	tr_C9ZJU1_
DRB1_0101	578	VFSGCVVDALKRKLQ	FSGCVVDAL	1	0.4616	338.8	49.00	0.35	tr_C9ZJU1_
DRB1_0101	579	SMAVASTFAFESLYC	VASTFAFES	3	0.4590	348.3	50.00	0.41	tr_C9ZJU1_
DRB1_0101	580	SRCVSQALWQASGSV	CVSQALWQA	2	0.4587	349.5	50.00	0.58	tr_C9ZJU1_
DRB1_0101	581	MLSSTDNKMLFQQTF	LSSTDNKML	1	0.4585	350.5	50.00	0.62	tr_C9ZJU1_
DRB1_0101	582	QMADTLPLLLVSAVA	PLLLVSAVA	6	0.4580	352.2	50.00	0.23	tr_C9ZJU1_
DRB1_0101	583	LSIGGNIALGNLWDC	LSIGGNIAL	0	0.4579	352.8	50.00	0.32	tr_C9ZJU1_
DRB1_0101	584	PQLVTLAATVLFSPE	LVTLAATVL	2	0.4577	353.2	50.00	0.47	tr_C9ZJU1_
DRB1_0101	585	VPSVFSGCVVDALKR	FSGCVVDAL	4	0.4577	353.5	50.00	0.45	tr_C9ZJU1_
DRB1_0101	586	FVESGRPLGYMWDLL	VESGRPLGY	1	0.4575	354.0	50.00	0.37	tr_C9ZJU1_
DRB1_0101	587	LVPSVFSGCVVDALK	FSGCVVDAL	5	0.4570	356.0	50.00	0.36	tr_C9ZJU1_
DRB1_0101	588	DLLTQHVIWPLVPSV	LTQHVIWPL	2	0.4567	357.4	50.00	0.23	tr_C9ZJU1_
DRB1_0101	589	PDHLLVCDIWNCLMA	LVCDIWNCL	4	0.4563	358.8	50.00	0.47	tr_C9ZJU1_
DRB1_0101	590	ESGRPLGYMWDLLTQ	LGYMWDLLT	5	0.4549	364.3	50.00	0.54	tr_C9ZJU1_
DRB1_0101	591	QAGGHTCAVSVRRLG	TCAVSVRRL	5	0.4547	364.9	55.00	0.40	tr_C9ZJU1_
DRB1_0101	592	DDGIKCMLSSTDNKM	IKCMLSSTD	3	0.4547	365.0	55.00	0.62	tr_C9ZJU1_
DRB1_0101	593	HSCFSRMRQSQQLPH	SRMRQSQQL	4	0.4534	370.0	55.00	0.66	tr_C9ZJU1_
DRB1_0101	594	RSSGIHAPLNVREFS	GIHAPLNVR	3	0.4520	375.9	55.00	0.41	tr_C9ZJU1_
DRB1_0101	595	AGYSGSGKTTLLRLL	YSGSGKTTL	2	0.4520	375.9	55.00	0.40	tr_C9ZJU1_
DRB1_0101	596	SIARYPPAAQADVVV	IARYPPAAQ	1	0.4519	376.4	55.00	0.34	tr_C9ZJU1_
DRB1_0101	597	IQSILSDIITSIHID	IQSILSDII	0	0.4517	377.0	55.00	0.29	tr_C9ZJU1_
DRB1_0101	598	LDDVKAEEVLALCML	VKAEEVLAL	3	0.4487	389.3	55.00	0.38	tr_C9ZJU1_
DRB1_0101	599	ESIHVRTLVFNHNIM	VRTLVFNHN	4	0.4487	389.4	55.00	0.22	tr_C9ZJU1_
DRB1_0101	600	GLMKNSHPDHLLVCD	MKNSHPDHL	2	0.4481	391.9	55.00	0.65	tr_C9ZJU1_
DRB1_0101	601	VESGRPLGYMWDLLT	LGYMWDLLT	6	0.4476	394.3	55.00	0.37	tr_C9ZJU1_
DRB1_0101	602	RLGWIKDVIQSSPRA	WIKDVIQSS	3	0.4469	397.0	55.00	0.40	tr_C9ZJU1_
DRB1_0101	603	KRSSGIHAPLNVREF	GIHAPLNVR	4	0.4454	403.5	55.00	0.35	tr_C9ZJU1_
DRB1_0101	604	MAATAAAAFATASER	ATAAAAFAT	2	0.4451	405.0	55.00	0.34	tr_C9ZJU1_
DRB1_0101	605	CSFPVKNSLTSSPLT	KNSLTSSPL	5	0.4441	409.5	55.00	0.58	tr_C9ZJU1_
DRB1_0101	606	DALKRKLQAGGHTCA	KLQAGGHTC	5	0.4438	410.7	55.00	0.30	tr_C9ZJU1_
DRB1_0101	607	RERARPAVLLDCERK	ERARPAVLL	1	0.4435	412.3	55.00	0.69	tr_C9ZJU1_
DRB1_0101	608	ADYDPSDDWLLSRCV	YDPSDDWLL	2	0.4431	413.9	55.00	0.64	tr_C9ZJU1_

DRB1_0101	609	GEENQVSPTCSFPVK	VSPTCSFPV	5	0.4425	416.6	55.00	0.71	tr_C9ZJU1_
DRB1_0101	610	VQEFPEELYLGLITA	ELYLGLITA	6	0.4425	416.7	55.00	0.31	tr_C9ZJU1_
DRB1_0101	611	ECKDDASIAIFLNCN	DDASIAIFL	3	0.4416	420.7	55.00	0.60	tr_C9ZJU1_
DRB1_0101	612	LMKNSHPDHLLVCDI	MKNSHPDHL	1	0.4413	422.2	55.00	0.52	tr_C9ZJU1_
DRB1_0101	613	IAGYSGSGKTTLLRL	YSGSGKTTL	3	0.4403	426.8	55.00	0.47	tr_C9ZJU1_
DRB1_0101	614	QSGITSFWKLMCSSC	SGITSFWKL	1	0.4396	429.7	55.00	0.32	tr_C9ZJU1_
DRB1_0101	615	QCGRDVLHGLFQMAD	DVLHGLFQM	4	0.4395	430.2	55.00	0.38	tr_C9ZJU1_
DRB1_0101	616	FAVHCGLMKNSHPDH	FAVHCGLMK	0	0.4388	433.4	55.00	0.35	tr_C9ZJU1_
DRB1_0101	617	FSGCVVDALKRKLQA	CVVDALKRK	3	0.4379	437.6	55.00	0.26	tr_C9ZJU1_
DRB1_0101	618	QLASVTNIATSTYAG	LASVTNIAT	1	0.4379	437.9	55.00	0.34	tr_C9ZJU1_
DRB1_0101	619	QTECKDDASIAIFLN	DDASIAIFL	5	0.4369	442.5	55.00	0.51	tr_C9ZJU1_
DRB1_0101	620	TECKDDASIAIFLNC	DDASIAIFL	4	0.4365	444.6	55.00	0.55	tr_C9ZJU1_
DRB1_0101	621	DGVALCILDDVKAEE	LCILDDVKA	4	0.4364	444.8	55.00	0.66	tr_C9ZJU1_
DRB1_0101	622	SQYPARMLRRRIANV	ARMLRRRIA	4	0.4360	446.8	55.00	0.31	tr_C9ZJU1_
DRB1_0101	623	VQKRSSGIHAPLNVR	QKRSSGIHA	1	0.4349	452.4	55.00	0.38	tr_C9ZJU1_
DRB1_0101	624	KDDASIAIFLNCNGV	DDASIAIFL	1	0.4345	454.3	55.00	0.44	tr_C9ZJU1_
DRB1_0101	625	QSRVPALLEVMHGCR	PALLEVMHG	4	0.4339	457.2	55.00	0.44	tr_C9ZJU1_
DRB1_0101	626	HTCAVSVRRLGWIKD	TCAVSVRRL	1	0.4337	458.4	55.00	0.41	tr_C9ZJU1_
DRB1_0101	627	HPDHLLVCDIWNCLM	LVCDIWNCL	5	0.4335	459.2	55.00	0.42	tr_C9ZJU1_
DRB1_0101	628	KLQAGGHTCAVSVRR	LQAGGHTCA	1	0.4331	461.1	55.00	0.29	tr_C9ZJU1_
DRB1_0101	629	IARYPPAAQADVVVQ	YPPAAQADV	3	0.4316	468.7	55.00	0.36	tr_C9ZJU1_
DRB1_0101	630	LEGLQSVQRKIDRPV	LEGLQSVQR	0	0.4314	469.5	55.00	0.70	tr_C9ZJU1_
DRB1_0101	631	LTLLRKQKDDGIKCM	LRKQKDDGI	3	0.4310	471.5	60.00	0.74	tr_C9ZJU1_
DRB1_0101	632	TEGEIFDTLRLREGN	GEIFDTLRL	2	0.4302	475.8	60.00	0.41	tr_C9ZJU1_
DRB1_0101	633	LQAGGHTCAVSVRRL	GGHTCAVSV	3	0.4294	479.7	60.00	0.25	tr_C9ZJU1_
DRB1_0101	634	AVHCGLMKNSHPDHL	CGLMKNSHP	3	0.4292	481.0	60.00	0.44	tr_C9ZJU1_
DRB1_0101	635	PLVPSVFSGCVVDAL	FSGCVVDAL	6	0.4289	482.5	60.00	0.25	tr_C9ZJU1_
DRB1_0101	636	GYSGSGKTTLLRLLN	YSGSGKTTL	1	0.4287	483.6	60.00	0.31	tr_C9ZJU1_
DRB1_0101	637	VLFSPEVILKCILGM	FSPEVILKC	2	0.4281	486.9	60.00	0.34	tr_C9ZJU1_
DRB1_0101	638	ATLKHADKIVVLNEG	LKHADKIVV	2	0.4279	487.8	60.00	0.53	tr_C9ZJU1_
DRB1_0101	639	RYSDRHPYVLRRVSF	YSDRHPYVL	1	0.4269	493.1	60.00	0.47	tr_C9ZJU1_
DRB1_0101	640	NGDVSPKLSPFRFII	PKLSPFRFI	5	0.4264	495.7	60.00	0.42	tr_C9ZJU1_
DRB1_0101	641	VVLNEGMVAEVGSWE	VVLNEGMVA	0	0.4264	496.0	60.00	0.33	tr_C9ZJU1_
DRB1_0101	642	LDQTEIVNQLRSIQH	VNQLRSIQH	6	0.4251	502.8	60.00	0.40	tr_C9ZJU1_
DRB1_0101	643	SLTSSPLTLLRKQKD	LTSSPLTLL	1	0.4248	504.5	60.00	0.44	tr_C9ZJU1_
DRB1_0101	644	LQSGITSFWKLMCSS	SGITSFWKL	2	0.4248	504.5	60.00	0.44	tr_C9ZJU1_
DRB1_0101	645	CKDDASIAIFLNCNG	DDASIAIFL	2	0.4244	506.4	60.00	0.56	tr_C9ZJU1_
DRB1_0101	646	SSGIHAPLNVREFSG	GIHAPLNVR	2	0.4237	510.5	60.00	0.40	tr_C9ZJU1_
DRB1_0101	647	EGEIFDTLRLREGNN	FDTLRLREG	4	0.4237	510.6	60.00	0.46	tr_C9ZJU1_
DRB1_0101	648	LASVTNIATSTYAGN	TNIATSTYA	4	0.4227	515.9	60.00	0.35	tr_C9ZJU1_
DRB1_0101	649	SILSDIITSIHIDAY	SDIITSIHI	3	0.4224	517.8	60.00	0.50	tr_C9ZJU1_
DRB1_0101	650	QKRSSGIHAPLNVRE	GIHAPLNVR	5	0.4224	518.0	60.00	0.30	tr_C9ZJU1_
DRB1_0101	651	VVSLVMWLGTQWSNF	VMWLGTQWS	4	0.4222	518.7	60.00	0.62	tr_C9ZJU1_
DRB1_0101	652	KDDGIKCMLSSTDNK	IKCMLSSTD	4	0.4221	519.6	60.00	0.63	tr_C9ZJU1_
DRB1_0101	653	DVKAEEVLALCMLLR	VKAEEVLAL	1	0.4219	520.8	60.00	0.32	tr_C9ZJU1_
DRB1_0101	654	YSGSGKTTLLRLLNR	GKTTLLRLL	4	0.4212	524.4	60.00	0.26	tr_C9ZJU1_
DRB1_0101	655	DDGVALCILDDVKAE	LCILDDVKA	5	0.4212	524.4	60.00	0.64	tr_C9ZJU1_
DRB1_0101	656	WLGTQWSNFLYFLNE	LGTQWSNFL	1	0.4212	524.6	60.00	0.50	tr_C9ZJU1_
DRB1_0101	657	DDVKAEEVLALCMLL	VKAEEVLAL	2	0.4201	530.9	60.00	0.44	tr_C9ZJU1_
DRB1_0101	658	ITEGEIFDTLRLREG	GEIFDTLRL	3	0.4199	531.7	60.00	0.50	tr_C9ZJU1_

DRB1_0101	659	EGMVAEVGSWETLSR	VAEVGSWET	3	0.4187	539.0	60.00	0.56	tr_C9ZJU1_
DRB1_0101	660	RMLRRRIANVWQEEN	LRRRIANVW	2	0.4177	544.6	60.00	0.51	tr_C9ZJU1_
DRB1_0101	661	QSILSDIITSIHIDA	SDIITSIHI	4	0.4171	548.5	60.00	0.39	tr_C9ZJU1_
DRB1_0101	662	DCSDENICAALSASR	DENICAALS	3	0.4154	558.3	60.00	0.24	tr_C9ZJU1_
DRB1_0101	663	ILSDIITSIHIDAYL	SDIITSIHI	2	0.4152	560.0	60.00	0.49	tr_C9ZJU1_
DRB1_0101	664	EQCGRDVLHGLFQMA	DVLHGLFQM	5	0.4145	564.0	60.00	0.42	tr_C9ZJU1_
DRB1_0101	665	PLTLLRKQKDDGIKC	LRKQKDDGI	4	0.4140	567.0	60.00	0.64	tr_C9ZJU1_
DRB1_0101	666	SGSGKTTLLRLLNRT	GKTTLLRLL	3	0.4136	569.6	60.00	0.32	tr_C9ZJU1_
DRB1_0101	667	STDNKMLFQQTFSHP	MLFQQTFSH	5	0.4128	574.5	60.00	0.52	tr_C9ZJU1_
DRB1_0101	668	NEGMVAEVGSWETLS	VAEVGSWET	4	0.4124	576.8	60.00	0.46	tr_C9ZJU1_
DRB1_0101	669	GVESIHVRTLVFNHN	VESIHVRTL	1	0.4122	578.2	60.00	0.26	tr_C9ZJU1_
DRB1_0101	670	ASVTNIATSTYAGNG	TNIATSTYA	3	0.4119	579.8	60.00	0.44	tr_C9ZJU1_
DRB1_0101	671	YLALATADYDPSDDW	LALATADYD	1	0.4118	580.9	60.00	0.42	tr_C9ZJU1_
DRB1_0101	672	SPLTLLRKQKDDGIK	LRKQKDDGI	5	0.4110	585.7	60.00	0.58	tr_C9ZJU1_
DRB1_0101	673	NGVESIHVRTLVFNH	VESIHVRTL	2	0.4108	587.1	60.00	0.35	tr_C9ZJU1_
DRB1_0101	674	AQADVVVQEFPEELY	VVQEFPEEL	5	0.4107	587.9	60.00	0.78	tr_C9ZJU1_
DRB1_0101	675	TLLRKQKDDGIKCML	LRKQKDDGI	2	0.4086	601.2	60.00	0.73	tr_C9ZJU1_
DRB1_0101	676	GIAGYSGSGKTTLLR	YSGSGKTTL	4	0.4081	604.2	60.00	0.45	tr_C9ZJU1_
DRB1_0101	677	WPLVPSVFSGCVVDA	PSVFSGCVV	4	0.4080	605.0	65.00	0.23	tr_C9ZJU1_
DRB1_0101	678	NCSIRDILLDDGVAL	ILLDDGVAL	6	0.4076	607.5	65.00	0.64	tr_C9ZJU1_
DRB1_0101	679	VESIHVRTLVFNHNI	SIHVRTLVF	2	0.4058	619.4	65.00	0.25	tr_C9ZJU1_
DRB1_0101	680	PHSCFSRMRQSQQLP	SRMRQSQQL	5	0.4057	620.0	65.00	0.62	tr_C9ZJU1_
DRB1_0101	681	PVVSLVMWLGTQWSN	VMWLGTQWS	5	0.4038	632.8	65.00	0.50	tr_C9ZJU1_
DRB1_0101	682	SHPDHLLVCDIWNCL	LVCDIWNCL	6	0.4036	634.6	65.00	0.34	tr_C9ZJU1_
DRB1_0101	683	LNEGMVAEVGSWETL	VAEVGSWET	5	0.4034	636.0	65.00	0.32	tr_C9ZJU1_
DRB1_0101	684	VIQSSPRAATASPST	VIQSSPRAA	0	0.4030	638.7	65.00	0.46	tr_C9ZJU1_
DRB1_0101	685	TADYDPSDDWLLSRC	YDPSDDWLL	3	0.4025	642.3	65.00	0.87	tr_C9ZJU1_
DRB1_0101	686	PSRHDKSSVQYGLVL	SSVQYGLVL	6	0.4014	649.5	65.00	0.43	tr_C9ZJU1_
DRB1_0101	687	VVQEFPEELYLGLIT	VVQEFPEEL	0	0.4012	651.3	65.00	0.35	tr_C9ZJU1_
DRB1_0101	688	LKHADKIVVLNEGMV	IVVLNEGMV	6	0.4012	651.4	65.00	0.43	tr_C9ZJU1_
DRB1_0101	689	LDDGVALCILDDVKA	LCILDDVKA	6	0.4010	652.4	65.00	0.50	tr_C9ZJU1_
DRB1_0101	690	LWQASGSVMDELQSR	WQASGSVMD	1	0.4008	654.1	65.00	0.48	tr_C9ZJU1_
DRB1_0101	691	ATADYDPSDDWLLSR	YDPSDDWLL	4	0.4005	656.2	65.00	0.88	tr_C9ZJU1_
DRB1_0101	692	RKIDRPVRGVAPPSL	VRGVAPPSL	6	0.3998	660.8	65.00	0.52	tr_C9ZJU1_
DRB1_0101	693	LMALVNCSIRDILLD	LMALVNCSI	0	0.3997	661.9	65.00	0.28	tr_C9ZJU1_
DRB1_0101	694	SGCVVDALKRKLQAG	CVVDALKRK	2	0.3994	664.3	65.00	0.26	tr_C9ZJU1_
DRB1_0101	695	REVSFRYSDRHPYVL	FRYSDRHPY	4	0.3984	671.0	65.00	0.47	tr_C9ZJU1_
DRB1_0101	696	TCAVSVRRLGWIKDV	SVRRLGWIK	4	0.3956	692.0	65.00	0.25	tr_C9ZJU1_
DRB1_0101	697	DDASIAIFLNCNGVE	AIFLNCNGV	5	0.3948	698.3	65.00	0.31	tr_C9ZJU1_
DRB1_0101	698	WSVEFREVSFRYSDR	FREVSFRYS	4	0.3947	698.5	65.00	0.47	tr_C9ZJU1_
DRB1_0101	699	ARYPPAAQADVVVQE	YPPAAQADV	2	0.3945	700.4	65.00	0.36	tr_C9ZJU1_
DRB1_0101	700	PVRGVAPPSLDGPWS	VRGVAPPSL	1	0.3940	704.1	65.00	0.73	tr_C9ZJU1_
DRB1_0101	701	LDCERKAYEILEMLT	KAYEILEML	5	0.3929	712.3	65.00	0.31	tr_C9ZJU1_
DRB1_0101	702	QEENNLRFIEDLSIG	LRFIEDLSI	5	0.3923	717.4	65.00	0.74	tr_C9ZJU1_
DRB1_0101	703	SVEFREVSFRYSDRH	FREVSFRYS	3	0.3911	726.7	65.00	0.55	tr_C9ZJU1_
DRB1_0101	704	FSGSSLGCYQVLEGL	GSSLGCYQV	2	0.3901	734.0	65.00	0.38	tr_C9ZJU1_
DRB1_0101	705	LRLLNRTYAPTEGDI	LRLLNRTYA	0	0.3893	741.0	65.00	0.50	tr_C9ZJU1_
DRB1_0101	706	LSASRALNFVQKRSS	SRALNFVQK	3	0.3878	752.7	65.00	0.26	tr_C9ZJU1_
DRB1_0101	707	LLDCERKAYEILEML	CERKAYEIL	3	0.3875	755.7	65.00	0.30	tr_C9ZJU1_
DRB1_0101	708	LGIAGYSGSGKTTLL	YSGSGKTTL	5	0.3862	765.7	65.00	0.38	tr_C9ZJU1_

DRB1_0101	709	YHAFLGSFYDECGMS	HAFLGSFYD	1	0.3852	774.6	65.00	0.41	tr_C9ZJU1_
DRB1_0101	710	GMVAEVGSWETLSRT	VAEVGSWET	2	0.3843	781.7	70.00	0.56	tr_C9ZJU1_
DRB1_0101	711	VKAEEVLALCMLLRG	AEEVLALCM	2	0.3834	789.6	70.00	0.25	tr_C9ZJU1_
DRB1_0101	712	SSTDNKMLFQQTFSH	MLFQQTFSH	6	0.3831	791.7	70.00	0.42	tr_C9ZJU1_
DRB1_0101	713	TITEGEIFDTLRLRE	GEIFDTLRL	4	0.3829	794.1	70.00	0.55	tr_C9ZJU1_
DRB1_0101	714	LLRKQKDDGIKCMLS	LRKQKDDGI	1	0.3828	795.0	70.00	0.62	tr_C9ZJU1_
DRB1_0101	715	SIGGNIALGNLWDCS	GNIALGNLW	3	0.3825	797.3	70.00	0.41	tr_C9ZJU1_
DRB1_0101	716	SVTNIATSTYAGNGE	TNIATSTYA	2	0.3818	803.0	70.00	0.46	tr_C9ZJU1_
DRB1_0101	717	AATAAAAFATASERN	ATAAAAFAT	1	0.3818	803.1	70.00	0.34	tr_C9ZJU1_
DRB1_0101	718	SGSSLGCYQVLEGLQ	GSSLGCYQV	1	0.3805	814.6	70.00	0.34	tr_C9ZJU1_
DRB1_0101	719	AFSGSSLGCYQVLEG	GSSLGCYQV	3	0.3802	817.4	70.00	0.46	tr_C9ZJU1_
DRB1_0101	720	FPVVSLVMWLGTQWS	VMWLGTQWS	6	0.3795	823.8	70.00	0.31	tr_C9ZJU1_
DRB1_0101	721	AVLLDCERKAYEILE	VLLDCERKA	1	0.3786	831.9	70.00	0.26	tr_C9ZJU1_
DRB1_0101	722	MKNSHPDHLLVCDIW	PDHLLVCDI	5	0.3785	833.0	70.00	0.34	tr_C9ZJU1_
DRB1_0101	723	QKDDGIKCMLSSTDN	IKCMLSSTD	5	0.3784	833.7	70.00	0.56	tr_C9ZJU1_
DRB1_0101	724	KQTECKDDASIAIFL	DDASIAIFL	6	0.3774	842.6	70.00	0.41	tr_C9ZJU1_
DRB1_0101	725	TLKHADKIVVLNEGM	LKHADKIVV	1	0.3769	846.8	70.00	0.49	tr_C9ZJU1_
DRB1_0101	726	VSPTCSFPVKNSLTS	VSPTCSFPV	0	0.3765	850.4	70.00	0.43	tr_C9ZJU1_
DRB1_0101	727	RRLGWIKDVIQSSPR	WIKDVIQSS	4	0.3759	856.4	70.00	0.54	tr_C9ZJU1_
DRB1_0101	728	GCVVDALKRKLQAGG	VDALKRKLQ	3	0.3750	864.3	70.00	0.19	tr_C9ZJU1_
DRB1_0101	729	FTQSALDQTEIVNQL	SALDQTEIV	3	0.3747	867.1	70.00	0.40	tr_C9ZJU1_
DRB1_0101	730	SGIHAPLNVREFSGG	GIHAPLNVR	1	0.3743	871.3	70.00	0.40	tr_C9ZJU1_
DRB1_0101	731	PAVLLDCERKAYEIL	VLLDCERKA	2	0.3741	872.8	70.00	0.37	tr_C9ZJU1_
DRB1_0101	732	LNGDVSPKLSPFRFI	PKLSPFRFI	6	0.3730	883.6	70.00	0.38	tr_C9ZJU1_
DRB1_0101	733	RYPPAAQADVVVQEF	PAAQADVVV	3	0.3726	887.9	70.00	0.33	tr_C9ZJU1_
DRB1_0101	734	PWSVEFREVSFRYSD	FREVSFRYS	5	0.3723	890.5	70.00	0.41	tr_C9ZJU1_
DRB1_0101	735	KAEEVLALCMLLRGK	EVLALCMLL	3	0.3722	890.9	70.00	0.19	tr_C9ZJU1_
DRB1_0101	736	SGSVMDELQSRVPAL	DELQSRVPA	5	0.3720	893.6	70.00	0.20	tr_C9ZJU1_
DRB1_0101	737	SFPVVSLVMWLGTQW	VVSLVMWLG	3	0.3713	900.1	70.00	0.31	tr_C9ZJU1_
DRB1_0101	738	GPHSCFSRMRQSQQL	SRMRQSQQL	6	0.3706	907.2	70.00	0.47	tr_C9ZJU1_
DRB1_0101	739	LSSTDNKMLFQQTFS	LSSTDNKML	0	0.3705	907.8	70.00	0.47	tr_C9ZJU1_
DRB1_0101	740	VLNEGMVAEVGSWET	LNEGMVAEV	1	0.3686	927.1	70.00	0.26	tr_C9ZJU1_
DRB1_0101	741	AEEVLALCMLLRGKY	VLALCMLLR	3	0.3681	932.2	70.00	0.24	tr_C9ZJU1_
DRB1_0101	742	DTITEGEIFDTLRLR	GEIFDTLRL	5	0.3679	934.1	70.00	0.50	tr_C9ZJU1_
DRB1_0101	743	FQQTFSHPSRHDKSS	FQQTFSHPS	0	0.3672	941.1	70.00	0.50	tr_C9ZJU1_
DRB1_0101	744	LEMLTRATIKTDGHD	MLTRATIKT	2	0.3667	946.2	70.00	0.44	tr_C9ZJU1_
DRB1_0101	745	VLLDCERKAYEILEM	CERKAYEIL	4	0.3649	964.2	70.00	0.29	tr_C9ZJU1_
DRB1_0101	746	LGSFYDECGMSEAQV	FYDECGMSE	3	0.3639	974.8	70.00	0.61	tr_C9ZJU1_
DRB1_0101	747	LGTQWSNFLYFLNES	LGTQWSNFL	0	0.3636	977.9	70.00	0.41	tr_C9ZJU1_
DRB1_0101	748	VDALKRKLQAGGHTC	KRKLQAGGH	4	0.3634	980.3	70.00	0.26	tr_C9ZJU1_
DRB1_0101	749	SVRRLGWIKDVIQSS	WIKDVIQSS	6	0.3628	986.5	70.00	0.20	tr_C9ZJU1_
DRB1_0101	750	YAPTEGDIFVNGVHI	DIFVNGVHI	6	0.3621	993.7	70.00	0.65	tr_C9ZJU1_
DRB1_0101	751	AVSVRRLGWIKDVIQ	SVRRLGWIK	2	0.3589	1028.7	75.00	0.38	tr_C9ZJU1_
DRB1_0101	752	TQSALDQTEIVNQLR	SALDQTEIV	2	0.3581	1038.6	75.00	0.38	tr_C9ZJU1_
DRB1_0101	753	GPWSVEFREVSFRYS	FREVSFRYS	6	0.3571	1049.2	75.00	0.29	tr_C9ZJU1_
DRB1_0101	754	YEQCGRDVLHGLFQM	DVLHGLFQM	6	0.3570	1050.6	75.00	0.33	tr_C9ZJU1_
DRB1_0101	755	GSFYDECGMSEAQVD	FYDECGMSE	2	0.3568	1052.6	75.00	0.53	tr_C9ZJU1_
DRB1_0101	756	LATADYDPSDDWLLS	YDPSDDWLL	5	0.3566	1054.7	75.00	0.85	tr_C9ZJU1_
DRB1_0101	757	CAVSVRRLGWIKDVI	SVRRLGWIK	3	0.3564	1057.0	75.00	0.38	tr_C9ZJU1_
DRB1_0101	758	ERARPAVLLDCERKA	ERARPAVLL	0	0.3551	1072.0	75.00	0.50	tr_C9ZJU1_

DRB1_0101	759	LFSPEVILKCILGMH	FSPEVILKC	1	0.3545	1079.9	75.00	0.32	tr_C9ZJU1_
DRB1_0101	760	RPAVLLDCERKAYEI	VLLDCERKA	3	0.3530	1096.6	75.00	0.52	tr_C9ZJU1_
DRB1_0101	761	NDRYEQCGRDVLHGL	YEQCGRDVL	3	0.3530	1097.1	75.00	0.37	tr_C9ZJU1_
DRB1_0101	762	RIANVWQEENNLRFI	NVWQEENNL	3	0.3528	1099.0	75.00	0.34	tr_C9ZJU1_
DRB1_0101	763	IANVWQEENNLRFIE	WQEENNLRF	4	0.3528	1099.3	75.00	0.38	tr_C9ZJU1_
DRB1_0101	764	TCSFPVKNSLTSSPL	KNSLTSSPL	6	0.3527	1100.3	75.00	0.37	tr_C9ZJU1_
DRB1_0101	765	DRYEQCGRDVLHGLF	YEQCGRDVL	2	0.3516	1113.7	75.00	0.35	tr_C9ZJU1_
DRB1_0101	766	SSPLTLLRKQKDDGI	LTLLRKQKD	3	0.3507	1125.1	75.00	0.47	tr_C9ZJU1_
DRB1_0101	767	VSVRRLGWIKDVIQS	VRRLGWIKD	2	0.3496	1138.4	75.00	0.28	tr_C9ZJU1_
DRB1_0101	768	VRRLGWIKDVIQSSP	WIKDVIQSS	5	0.3492	1143.7	75.00	0.40	tr_C9ZJU1_
DRB1_0101	769	FLGSFYDECGMSEAQ	FYDECGMSE	4	0.3462	1180.7	75.00	0.60	tr_C9ZJU1_
DRB1_0101	770	SASRALNFVQKRSSG	SRALNFVQK	2	0.3448	1199.0	75.00	0.39	tr_C9ZJU1_
DRB1_0101	771	KAGSAVGLCIFDEPT	AGSAVGLCI	1	0.3446	1201.2	75.00	0.50	tr_C9ZJU1_
DRB1_0101	772	AQVDEAHLRRHLAFS	EAHLRRHLA	4	0.3439	1211.1	75.00	0.38	tr_C9ZJU1_
DRB1_0101	773	QSALDQTEIVNQLRS	SALDQTEIV	1	0.3436	1214.7	75.00	0.32	tr_C9ZJU1_
DRB1_0101	774	SFYDECGMSEAQVDE	FYDECGMSE	1	0.3427	1227.0	75.00	0.44	tr_C9ZJU1_
DRB1_0101	775	ALDQTEIVNQLRSIQ	EIVNQLRSI	5	0.3424	1229.8	75.00	0.32	tr_C9ZJU1_
DRB1_0101	776	LTSSPLTLLRKQKDD	LTLLRKQKD	5	0.3410	1248.9	75.00	0.34	tr_C9ZJU1_
DRB1_0101	777	EIGKSLNGDVSPKLS	LNGDVSPKL	5	0.3408	1252.4	75.00	0.44	tr_C9ZJU1_
DRB1_0101	778	IGGNIALGNLWDCSD	GNIALGNLW	2	0.3407	1252.7	75.00	0.47	tr_C9ZJU1_
DRB1_0101	779	VEFREVSFRYSDRHP	FREVSFRYS	2	0.3406	1254.6	75.00	0.56	tr_C9ZJU1_
DRB1_0101	780	WDCSDENICAALSAS	DENICAALS	4	0.3402	1259.8	75.00	0.29	tr_C9ZJU1_
DRB1_0101	781	WQEENNLRFIEDLSI	LRFIEDLSI	6	0.3377	1294.8	75.00	0.62	tr_C9ZJU1_
DRB1_0101	782	KQKDDGIKCMLSSTD	IKCMLSSTD	6	0.3377	1294.8	75.00	0.44	tr_C9ZJU1_
DRB1_0101	783	CVVDALKRKLQAGGH	DALKRKLQA	3	0.3375	1297.2	75.00	0.29	tr_C9ZJU1_
DRB1_0101	784	LLDDGVALCILDDVK	LDDGVALCI	1	0.3371	1302.5	75.00	0.45	tr_C9ZJU1_
DRB1_0101	785	RNDRYEQCGRDVLHG	RYEQCGRDV	3	0.3371	1302.8	75.00	0.40	tr_C9ZJU1_
DRB1_0101	786	RYEQCGRDVLHGLFQ	YEQCGRDVL	1	0.3364	1313.4	75.00	0.31	tr_C9ZJU1_
DRB1_0101	787	GLCIFDEPTSAIDTI	IFDEPTSAI	3	0.3354	1326.7	75.00	0.39	tr_C9ZJU1_
DRB1_0101	788	NSHPDHLLVCDIWNC	PDHLLVCDI	3	0.3346	1338.1	75.00	0.47	tr_C9ZJU1_
DRB1_0101	789	VVDALKRKLQAGGHT	KRKLQAGGH	5	0.3332	1358.5	80.00	0.25	tr_C9ZJU1_
DRB1_0101	790	HAFLGSFYDECGMSE	HAFLGSFYD	0	0.3325	1369.2	80.00	0.35	tr_C9ZJU1_
DRB1_0101	791	ANVWQEENNLRFIED	WQEENNLRF	3	0.3323	1372.3	80.00	0.38	tr_C9ZJU1_
DRB1_0101	792	MVAEVGSWETLSRTG	VAEVGSWET	1	0.3317	1381.0	80.00	0.56	tr_C9ZJU1_
DRB1_0101	793	KNSHPDHLLVCDIWN	PDHLLVCDI	4	0.3317	1381.1	80.00	0.50	tr_C9ZJU1_
DRB1_0101	794	EFREVSFRYSDRHPY	FREVSFRYS	1	0.3283	1433.3	80.00	0.43	tr_C9ZJU1_
DRB1_0101	795	VGLCIFDEPTSAIDT	IFDEPTSAI	4	0.3278	1440.9	80.00	0.37	tr_C9ZJU1_
DRB1_0101	796	VTNIATSTYAGNGEE	TNIATSTYA	1	0.3270	1453.7	80.00	0.47	tr_C9ZJU1_
DRB1_0101	797	ARPAVLLDCERKAYE	VLLDCERKA	4	0.3268	1456.7	80.00	0.55	tr_C9ZJU1_
DRB1_0101	798	AAAAFATASERNDRY	FATASERND	4	0.3265	1460.8	80.00	0.55	tr_C9ZJU1_
DRB1_0101	799	SALDQTEIVNQLRSI	DQTEIVNQL	3	0.3249	1487.1	80.00	0.30	tr_C9ZJU1_
DRB1_0101	800	AEIGKSLNGDVSPKL	IGKSLNGDV	2	0.3243	1496.0	80.00	0.41	tr_C9ZJU1_
DRB1_0101	801	ATAAAAFATASERND	AAAAFATAS	2	0.3241	1499.1	80.00	0.22	tr_C9ZJU1_
DRB1_0101	802	AAAFATASERNDRYE	FATASERND	3	0.3217	1539.2	80.00	0.59	tr_C9ZJU1_
DRB1_0101	803	LRKQKDDGIKCMLSS	LRKQKDDGI	0	0.3210	1551.4	80.00	0.39	tr_C9ZJU1_
DRB1_0101	804	IDTITEGEIFDTLRL	GEIFDTLRL	6	0.3202	1563.7	80.00	0.32	tr_C9ZJU1_
DRB1_0101	805	TAAAAFATASERNDR	FATASERND	5	0.3199	1568.9	80.00	0.40	tr_C9ZJU1_
DRB1_0101	806	AVGLCIFDEPTSAID	IFDEPTSAI	5	0.3189	1587.1	80.00	0.31	tr_C9ZJU1_
DRB1_0101	807	AFLGSFYDECGMSEA	FYDECGMSE	5	0.3178	1606.3	80.00	0.58	tr_C9ZJU1_
DRB1_0101	808	LCIFDEPTSAIDTIT	IFDEPTSAI	2	0.3160	1637.8	80.00	0.36	tr_C9ZJU1_

DRB1_0101	809	ERNDRYEQCGRDVLH	RYEQCGRDV	4	0.3156	1644.8	80.00	0.41	tr_C9ZJU1_
DRB1_0101	810	IGKSLNGDVSPKLSP	LNGDVSPKL	4	0.3140	1672.2	80.00	0.51	tr_C9ZJU1_
DRB1_0101	811	NRTYAPTEGDIFVNG	YAPTEGDIF	3	0.3136	1680.7	80.00	0.80	tr_C9ZJU1_
DRB1_0101	812	ASGSVMDELQSRVPA	MDELQSRVP	5	0.3136	1681.0	80.00	0.23	tr_C9ZJU1_
DRB1_0101	813	MLRRRIANVWQEENN	LRRRIANVW	1	0.3134	1684.6	80.00	0.54	tr_C9ZJU1_
DRB1_0101	814	FREVSFRYSDRHPYV	FRYSDRHPY	5	0.3129	1693.6	80.00	0.51	tr_C9ZJU1_
DRB1_0101	815	WQASGSVMDELQSRV	WQASGSVMD	0	0.3123	1703.9	80.00	0.35	tr_C9ZJU1_
DRB1_0101	816	RARPAVLLDCERKAY	VLLDCERKA	5	0.3107	1733.7	80.00	0.50	tr_C9ZJU1_
DRB1_0101	817	EAQVDEAHLRRHLAF	EAHLRRHLA	5	0.3105	1736.8	80.00	0.34	tr_C9ZJU1_
DRB1_0101	818	SWETLSRTGPHSCFS	LSRTGPHSC	4	0.3097	1752.5	80.00	0.32	tr_C9ZJU1_
DRB1_0101	819	LNRTYAPTEGDIFVN	YAPTEGDIF	4	0.3090	1765.9	80.00	0.73	tr_C9ZJU1_
DRB1_0101	820	GGNIALGNLWDCSDE	GNIALGNLW	1	0.3057	1829.5	85.00	0.47	tr_C9ZJU1_
DRB1_0101	821	MALVNCSIRDILLDD	LVNCSIRDI	2	0.3056	1832.5	85.00	0.28	tr_C9ZJU1_
DRB1_0101	822	SAVGLCIFDEPTSAI	IFDEPTSAI	6	0.3051	1841.5	85.00	0.22	tr_C9ZJU1_
DRB1_0101	823	NGEENQVSPTCSFPV	VSPTCSFPV	6	0.3048	1847.4	85.00	0.58	tr_C9ZJU1_
DRB1_0101	824	VRGVAPPSLDGPWSV	VRGVAPPSL	0	0.3046	1852.5	85.00	0.56	tr_C9ZJU1_
DRB1_0101	825	ETLSRTGPHSCFSRM	LSRTGPHSC	2	0.3033	1877.6	85.00	0.41	tr_C9ZJU1_
DRB1_0101	826	WETLSRTGPHSCFSR	LSRTGPHSC	3	0.3009	1928.1	85.00	0.42	tr_C9ZJU1_
DRB1_0101	827	AAEIGKSLNGDVSPK	IGKSLNGDV	3	0.2998	1951.6	85.00	0.64	tr_C9ZJU1_
DRB1_0101	828	NVWQEENNLRFIEDL	WQEENNLRF	2	0.2990	1967.6	85.00	0.37	tr_C9ZJU1_
DRB1_0101	829	FYDECGMSEAQVDEA	FYDECGMSE	0	0.2990	1968.6	85.00	0.29	tr_C9ZJU1_
DRB1_0101	830	LWDCSDENICAALSA	DENICAALS	5	0.2987	1975.1	85.00	0.31	tr_C9ZJU1_
DRB1_0101	831	LLNRTYAPTEGDIFV	YAPTEGDIF	5	0.2981	1987.3	85.00	0.62	tr_C9ZJU1_
DRB1_0101	832	YPPAAQADVVVQEFP	PAAQADVVV	2	0.2980	1989.9	85.00	0.40	tr_C9ZJU1_
DRB1_0101	833	RRIANVWQEENNLRF	NVWQEENNL	4	0.2976	1998.9	85.00	0.38	tr_C9ZJU1_
DRB1_0101	834	GKSLNGDVSPKLSPF	LNGDVSPKL	3	0.2964	2023.3	85.00	0.65	tr_C9ZJU1_
DRB1_0101	835	RTYAPTEGDIFVNGV	YAPTEGDIF	2	0.2962	2027.6	85.00	0.79	tr_C9ZJU1_
DRB1_0101	836	SPTCSFPVKNSLTSS	SFPVKNSLT	4	0.2949	2056.9	85.00	0.38	tr_C9ZJU1_
DRB1_0101	837	AAFATASERNDRYEQ	FATASERND	2	0.2933	2091.9	85.00	0.64	tr_C9ZJU1_
DRB1_0101	838	GSWETLSRTGPHSCF	WETLSRTGP	2	0.2914	2135.7	85.00	0.28	tr_C9ZJU1_
DRB1_0101	839	TLSRTGPHSCFSRMR	LSRTGPHSC	1	0.2894	2184.0	85.00	0.38	tr_C9ZJU1_
DRB1_0101	840	EMLTRATIKTDGHDS	MLTRATIKT	1	0.2892	2186.7	85.00	0.45	tr_C9ZJU1_
DRB1_0101	841	TSSPLTLLRKQKDDG	LTLLRKQKD	4	0.2875	2227.4	85.00	0.60	tr_C9ZJU1_
DRB1_0101	842	DGPWSVEFREVSFRY	WSVEFREVS	3	0.2862	2261.2	85.00	0.35	tr_C9ZJU1_
DRB1_0101	843	AAQADVVVQEFPEEL	VVQEFPEEL	6	0.2847	2296.5	85.00	0.68	tr_C9ZJU1_
DRB1_0101	844	AGSAVGLCIFDEPTS	AGSAVGLCI	0	0.2837	2323.1	85.00	0.30	tr_C9ZJU1_
DRB1_0101	845	ALATADYDPSDDWLL	YDPSDDWLL	6	0.2831	2337.9	85.00	0.75	tr_C9ZJU1_
DRB1_0101	846	RLLNRTYAPTEGDIF	YAPTEGDIF	6	0.2826	2350.8	85.00	0.32	tr_C9ZJU1_
DRB1_0101	847	KSLNGDVSPKLSPFR	LNGDVSPKL	2	0.2790	2442.6	85.00	0.67	tr_C9ZJU1_
DRB1_0101	848	TRATIKTDGHDSKQT	TIKTDGHDS	3	0.2782	2463.8	85.00	0.70	tr_C9ZJU1_
DRB1_0101	849	STCVWQCRQDYLQGF	CVWQCRQDY	2	0.2772	2491.8	85.00	0.37	tr_C9ZJU1_
DRB1_0101	850	SPRAATASPSTCVWQ	ATASPSTCV	4	0.2769	2499.7	85.00	0.52	tr_C9ZJU1_
DRB1_0101	851	TCVWQCRQDYLQGFA	CVWQCRQDY	1	0.2761	2520.4	85.00	0.26	tr_C9ZJU1_
DRB1_0101	852	SLNGDVSPKLSPFRF	LNGDVSPKL	1	0.2760	2523.8	85.00	0.44	tr_C9ZJU1_
DRB1_0101	853	PTCSFPVKNSLTSSP	SFPVKNSLT	3	0.2760	2525.1	85.00	0.37	tr_C9ZJU1_
DRB1_0101	854	GIHAPLNVREFSGGE	IHAPLNVRE	1	0.2752	2545.4	90.00	0.38	tr_C9ZJU1_
DRB1_0101	855	SEAQVDEAHLRRHLA	EAHLRRHLA	6	0.2746	2563.0	90.00	0.23	tr_C9ZJU1_
DRB1_0101	856	APLNVREFSGGEVER	VREFSGGEV	4	0.2721	2633.4	90.00	0.71	tr_C9ZJU1_
DRB1_0101	857	QSVQRKIDRPVRGVA	VQRKIDRPV	2	0.2702	2687.6	90.00	0.35	tr_C9ZJU1_
DRB1_0101	858	CIFDEPTSAIDTITE	IFDEPTSAI	1	0.2687	2732.7	90.00	0.36	tr_C9ZJU1_

DRB1_0101	859	RKQKDDGIKCMLSST	DDGIKCMLS	4	0.2683	2742.0	90.00	0.31	tr_C9ZJU1_
DRB1_0101	860	SKQTECKDDASIAIF	CKDDASIAI	5	0.2682	2744.9	90.00	0.46	tr_C9ZJU1_
DRB1_0101	861	PSTCVWQCRQDYLQG	CVWQCRQDY	3	0.2668	2787.4	90.00	0.44	tr_C9ZJU1_
DRB1_0101	862	HPSRHDKSSVQYGLV	DKSSVQYGL	5	0.2651	2840.8	90.00	0.32	tr_C9ZJU1_
DRB1_0101	863	RATIKTDGHDSKQTE	TIKTDGHDS	2	0.2643	2864.5	90.00	0.67	tr_C9ZJU1_
DRB1_0101	864	SSPRAATASPSTCVW	ATASPSTCV	5	0.2642	2866.4	90.00	0.41	tr_C9ZJU1_
DRB1_0101	865	PRAATASPSTCVWQC	ATASPSTCV	3	0.2636	2887.0	90.00	0.58	tr_C9ZJU1_
DRB1_0101	866	ALVNCSIRDILLDDG	NCSIRDILL	3	0.2618	2943.8	90.00	0.37	tr_C9ZJU1_
DRB1_0101	867	VAEVGSWETLSRTGP	VAEVGSWET	0	0.2614	2956.7	90.00	0.41	tr_C9ZJU1_
DRB1_0101	868	TNIATSTYAGNGEEN	NIATSTYAG	1	0.2614	2957.1	90.00	0.39	tr_C9ZJU1_
DRB1_0101	869	AIDTITEGEIFDTLR	ITEGEIFDT	4	0.2603	2991.4	90.00	0.25	tr_C9ZJU1_
DRB1_0101	870	SERNDRYEQCGRDVL	RYEQCGRDV	5	0.2601	2998.6	90.00	0.43	tr_C9ZJU1_
DRB1_0101	871	GNIALGNLWDCSDEN	GNIALGNLW	0	0.2594	3020.1	90.00	0.40	tr_C9ZJU1_
DRB1_0101	872	ATSTYAGNGEENQVS	YAGNGEENQ	4	0.2590	3032.9	90.00	0.62	tr_C9ZJU1_
DRB1_0101	873	SVQRKIDRPVRGVAP	VQRKIDRPV	1	0.2577	3074.8	90.00	0.27	tr_C9ZJU1_
DRB1_0101	874	QASGSVMDELQSRVP	GSVMDELQS	3	0.2572	3092.6	90.00	0.32	tr_C9ZJU1_
DRB1_0101	875	LTRATIKTDGHDSKQ	TIKTDGHDS	4	0.2556	3147.8	90.00	0.69	tr_C9ZJU1_
DRB1_0101	876	GSAVGLCIFDEPTSA	AVGLCIFDE	2	0.2549	3171.7	90.00	0.22	tr_C9ZJU1_
DRB1_0101	877	VGSWETLSRTGPHSC	WETLSRTGP	3	0.2542	3196.4	90.00	0.37	tr_C9ZJU1_
DRB1_0101	878	NLWDCSDENICAALS	WDCSDENIC	2	0.2537	3214.1	90.00	0.22	tr_C9ZJU1_
DRB1_0101	879	TSTYAGNGEENQVSP	YAGNGEENQ	3	0.2516	3286.9	90.00	0.62	tr_C9ZJU1_
DRB1_0101	880	LQSVQRKIDRPVRGV	VQRKIDRPV	3	0.2506	3320.8	90.00	0.47	tr_C9ZJU1_
DRB1_0101	881	TYAPTEGDIFVNGVH	YAPTEGDIF	1	0.2496	3357.7	90.00	0.69	tr_C9ZJU1_
DRB1_0101	882	QRKIDRPVRGVAPPS	DRPVRGVAP	4	0.2493	3369.6	90.00	0.39	tr_C9ZJU1_
DRB1_0101	883	MLTRATIKTDGHDSK	TIKTDGHDS	5	0.2491	3375.2	90.00	0.49	tr_C9ZJU1_
DRB1_0101	884	RAATASPSTCVWQCR	ATASPSTCV	2	0.2478	3423.3	90.00	0.62	tr_C9ZJU1_
DRB1_0101	885	SAIDTITEGEIFDTL	IDTITEGEI	2	0.2473	3441.5	90.00	0.29	tr_C9ZJU1_
DRB1_0101	886	SPSTCVWQCRQDYLQ	CVWQCRQDY	4	0.2459	3496.0	90.00	0.44	tr_C9ZJU1_
DRB1_0101	887	VWQEENNLRFIEDLS	WQEENNLRF	1	0.2430	3605.9	90.00	0.38	tr_C9ZJU1_
DRB1_0101	888	LDGPWSVEFREVSFR	WSVEFREVS	4	0.2397	3736.2	95.00	0.38	tr_C9ZJU1_
DRB1_0101	889	LSRTGPHSCFSRMRQ	GPHSCFSRM	4	0.2394	3750.3	95.00	0.28	tr_C9ZJU1_
DRB1_0101	890	STYAGNGEENQVSPT	YAGNGEENQ	2	0.2394	3750.6	95.00	0.60	tr_C9ZJU1_
DRB1_0101	891	AFATASERNDRYEQC	FATASERND	1	0.2394	3750.6	95.00	0.66	tr_C9ZJU1_
DRB1_0101	892	VQRKIDRPVRGVAPP	DRPVRGVAP	5	0.2381	3802.5	95.00	0.25	tr_C9ZJU1_
DRB1_0101	893	QQTFSHPSRHDKSSV	TFSHPSRHD	2	0.2374	3830.3	95.00	0.25	tr_C9ZJU1_
DRB1_0101	894	MSEAQVDEAHLRRHL	EAQVDEAHL	2	0.2367	3861.8	95.00	0.31	tr_C9ZJU1_
DRB1_0101	895	PPSLDGPWSVEFREV	LDGPWSVEF	3	0.2363	3877.5	95.00	0.44	tr_C9ZJU1_
DRB1_0101	896	VAPPSLDGPWSVEFR	PSLDGPWSV	3	0.2335	3999.1	95.00	0.37	tr_C9ZJU1_
DRB1_0101	897	PPAAQADVVVQEFPE	PAAQADVVV	1	0.2325	4042.6	95.00	0.43	tr_C9ZJU1_
DRB1_0101	898	ECGMSEAQVDEAHLR	MSEAQVDEA	3	0.2308	4116.0	95.00	0.28	tr_C9ZJU1_
DRB1_0101	899	APPSLDGPWSVEFRE	PSLDGPWSV	2	0.2305	4131.0	95.00	0.34	tr_C9ZJU1_
DRB1_0101	900	PSLDGPWSVEFREVS	LDGPWSVEF	2	0.2302	4144.6	95.00	0.38	tr_C9ZJU1_
DRB1_0101	901	IQSSPRAATASPSTC	IQSSPRAAT	0	0.2300	4151.8	95.00	0.40	tr_C9ZJU1_
DRB1_0101	902	HAPLNVREFSGGEVE	VREFSGGEV	5	0.2291	4193.7	95.00	0.63	tr_C9ZJU1_
DRB1_0101	903	YDECGMSEAQVDEAH	CGMSEAQVD	3	0.2288	4204.3	95.00	0.33	tr_C9ZJU1_
DRB1_0101	904	TGPHSCFSRMRQSQQ	FSRMRQSQQ	6	0.2286	4214.2	95.00	0.30	tr_C9ZJU1_
DRB1_0101	905	RRRIANVWQEENNLR	NVWQEENNL	5	0.2284	4222.4	95.00	0.41	tr_C9ZJU1_
DRB1_0101	906	CGMSEAQVDEAHLRR	EAQVDEAHL	4	0.2283	4227.1	95.00	0.31	tr_C9ZJU1_
DRB1_0101	907	QSSPRAATASPSTCV	PRAATASPS	3	0.2279	4249.0	95.00	0.37	tr_C9ZJU1_
DRB1_0101	908	EVGSWETLSRTGPHS	WETLSRTGP	4	0.2277	4254.5	95.00	0.44	tr_C9ZJU1_

DRB1_0101	909	LALATADYDPSDDWL	LALATADYD	0	0.2272	4278.0	95.00	0.57	tr_C9ZJU1_
DRB1_0101	910	LRRRIANVWQEENNL	LRRRIANVW	0	0.2266	4304.9	95.00	0.35	tr_C9ZJU1_
DRB1_0101	911	GMSEAQVDEAHLRRH	EAQVDEAHL	3	0.2248	4393.6	95.00	0.34	tr_C9ZJU1_
DRB1_0101	912	SLDGPWSVEFREVSF	WSVEFREVS	5	0.2245	4405.3	95.00	0.33	tr_C9ZJU1_
DRB1_0101	913	ATIKTDGHDSKQTEC	TIKTDGHDS	1	0.2218	4538.9	95.00	0.62	tr_C9ZJU1_
DRB1_0101	914	GLQSVQRKIDRPVRG	VQRKIDRPV	4	0.2200	4627.3	95.00	0.50	tr_C9ZJU1_
DRB1_0101	915	EGLQSVQRKIDRPVR	VQRKIDRPV	5	0.2189	4682.3	95.00	0.41	tr_C9ZJU1_
DRB1_0101	916	GNLWDCSDENICAAL	WDCSDENIC	3	0.2185	4702.2	95.00	0.32	tr_C9ZJU1_
DRB1_0101	917	IATSTYAGNGEENQV	YAGNGEENQ	5	0.2181	4723.5	95.00	0.62	tr_C9ZJU1_
DRB1_0101	918	DECGMSEAQVDEAHL	CGMSEAQVD	2	0.2166	4798.4	95.00	0.32	tr_C9ZJU1_
DRB1_0101	919	VNCSIRDILLDDGVA	NCSIRDILL	1	0.2163	4814.1	95.00	0.31	tr_C9ZJU1_
DRB1_0101	920	IHAPLNVREFSGGEV	VREFSGGEV	6	0.2134	4967.2	95.00	0.47	tr_C9ZJU1_
DRB1_0101	921	AEVGSWETLSRTGPH	WETLSRTGP	5	0.2128	5002.3	95.00	0.37	tr_C9ZJU1_
DRB1_0101	922	TSAIDTITEGEIFDT	IDTITEGEI	3	0.2110	5096.5	95.00	0.46	tr_C9ZJU1_
DRB1_0101	923	SRTGPHSCFSRMRQS	GPHSCFSRM	3	0.2105	5127.1	95.00	0.38	tr_C9ZJU1_
DRB1_0101	924	RTGPHSCFSRMRQSQ	GPHSCFSRM	2	0.2101	5151.7	95.00	0.35	tr_C9ZJU1_
DRB1_0101	925	LVNCSIRDILLDDGV	NCSIRDILL	2	0.2099	5161.9	95.00	0.43	tr_C9ZJU1_
DRB1_0101	926	QTFSHPSRHDKSSVQ	FSHPSRHDK	2	0.2090	5207.8	95.00	0.28	tr_C9ZJU1_
DRB1_0101	927	TYAGNGEENQVSPTC	YAGNGEENQ	1	0.2073	5306.4	95.00	0.58	tr_C9ZJU1_
DRB1_0101	928	ASPSTCVWQCRQDYL	CVWQCRQDY	5	0.2053	5422.9	95.00	0.46	tr_C9ZJU1_
DRB1_0101	929	SHPSRHDKSSVQYGL	HDKSSVQYG	5	0.2041	5493.4	95.00	0.28	tr_C9ZJU1_
DRB1_0101	930	DSKQTECKDDASIAI	CKDDASIAI	6	0.2033	5542.4	95.00	0.40	tr_C9ZJU1_
DRB1_0101	931	AATASPSTCVWQCRQ	ATASPSTCV	1	0.2023	5602.6	95.00	0.60	tr_C9ZJU1_
DRB1_0101	932	GVAPPSLDGPWSVEF	PSLDGPWSV	4	0.2019	5626.2	95.00	0.38	tr_C9ZJU1_
DRB1_0101	933	IFDEPTSAIDTITEG	FDEPTSAID	1	0.1991	5798.3	95.00	0.31	tr_C9ZJU1_
DRB1_0101	934	NIATSTYAGNGEENQ	YAGNGEENQ	6	0.1950	6064.3	95.00	0.28	tr_C9ZJU1_
DRB1_0101	935	LGNLWDCSDENICAA	WDCSDENIC	4	0.1904	6369.7	100.00	0.36	tr_C9ZJU1_
DRB1_0101	936	PTSAIDTITEGEIFD	IDTITEGEI	4	0.1890	6468.7	100.00	0.50	tr_C9ZJU1_
DRB1_0101	937	PAAQADVVVQEFPEE	PAAQADVVV	0	0.1827	6925.4	100.00	0.33	tr_C9ZJU1_
DRB1_0101	938	RGVAPPSLDGPWSVE	PSLDGPWSV	5	0.1823	6952.8	100.00	0.38	tr_C9ZJU1_
DRB1_0101	939	NIALGNLWDCSDENI	IALGNLWDC	1	0.1810	7057.7	100.00	0.23	tr_C9ZJU1_
DRB1_0101	940	TFSHPSRHDKSSVQY	FSHPSRHDK	1	0.1706	7890.4	100.00	0.25	tr_C9ZJU1_
DRB1_0101	941	TIKTDGHDSKQTECK	TIKTDGHDS	0	0.1692	8014.5	100.00	0.42	tr_C9ZJU1_
DRB1_0101	942	EPTSAIDTITEGEIF	IDTITEGEI	5	0.1686	8065.4	100.00	0.55	tr_C9ZJU1_
DRB1_0101	943	FSHPSRHDKSSVQYG	SRHDKSSVQ	4	0.1677	8143.2	100.00	0.23	tr_C9ZJU1_
DRB1_0101	944	ALGNLWDCSDENICA	WDCSDENIC	5	0.1627	8603.0	100.00	0.36	tr_C9ZJU1_
DRB1_0101	945	FATASERNDRYEQCG	FATASERND	0	0.1572	9128.1	100.00	0.50	tr_C9ZJU1_
DRB1_0101	946	IALGNLWDCSDENIC	LWDCSDENI	5	0.1554	9305.7	100.00	0.25	tr_C9ZJU1_
DRB1_0101	947	YAGNGEENQVSPTCS	YAGNGEENQ	0	0.1516	9700.2	100.00	0.41	tr_C9ZJU1_
DRB1_0101	948	TASPSTCVWQCRQDY	CVWQCRQDY	6	0.1510	9754.7	100.00	0.42	tr_C9ZJU1_
DRB1_0101	949	GNGEENQVSPTCSFP	NQVSPTCSF	5	0.1488	9992.6	100.00	0.44	tr_C9ZJU1_
DRB1_0101	950	ATASPSTCVWQCRQD	ATASPSTCV	0	0.1486	10017.1	100.00	0.43	tr_C9ZJU1_
DRB1_0101	951	AGNGEENQVSPTCSF	NQVSPTCSF	6	0.1391	11096.8	100.00	0.32	tr_C9ZJU1_
DRB1_0101	952	ASERNDRYEQCGRDV	RYEQCGRDV	6	0.1373	11313.0	100.00	0.47	tr_C9ZJU1_
DRB1_0101	953	FDEPTSAIDTITEGE	DEPTSAIDT	1	0.1288	12411.9	100.00	0.33	tr_C9ZJU1_
DRB1_0101	954	DEPTSAIDTITEGEI	IDTITEGEI	6	0.1175	14024.2	100.00	0.41	tr_C9ZJU1_
DRB1_0101	955	HDSKQTECKDDASIA	ECKDDASIA	6	0.1134	14652.6	100.00	0.31	tr_C9ZJU1_
DRB1_0101	956	IKTDGHDSKQTECKD	TDGHDSKQT	2	0.1022	16555.5	100.00	0.44	tr_C9ZJU1_
DRB1_0101	957	GHDSKQTECKDDASI	HDSKQTECK	1	0.0822	20540.3	100.00	0.26	tr_C9ZJU1_
DRB1_0101	958	KTDGHDSKQTECKDD	TDGHDSKQT	1	0.0808	20866.7	100.00	0.40	tr_C9ZJU1_

DRB1_0101	959	ATASERNDRYEQCGR	TASERNDRY	1	0.0759	21994.7	100.00	0.47	tr_C9ZJU1_
DRB1_0101	960	TDGHDSKQTECKDDA	HDSKQTECK	3	0.0679	23995.8	100.00	0.34	tr_C9ZJU1_
DRB1_0101	961	TASERNDRYEQCGRD	ERNDRYEQC	3	0.0674	24114.2	100.00	0.23	tr_C9ZJU1_
DRB1_0101	962	DGHDSKQTECKDDAS	HDSKQTECK	2	0.0649	24778.6	100.00	0.34	tr_C9ZJU1_

Allele: DRB1_0101. Number of high binders 14. Number of weak binders 132. Number of peptides 962

Allele	pos	peptide	core	Of	1-log50k(aff)	affinity(nM)	%Rank	Relia	Identity	Bind Level
DRB1_0101	1	KRIFFHSLKVNLKNL	FHSLKVNLK	4	0.8810	3.6	0.20	0.76	tr_C9ZQ90_	SB
DRB1_0101	2	RIFFHSLKVNLKNLT	FHSLKVNLK	3	0.8798	3.7	0.25	0.81	tr_C9ZQ90_	SB
DRB1_0101	3	DKRIFFHSLKVNLKN	FHSLKVNLK	5	0.8615	4.5	0.40	0.71	tr_C9ZQ90_	SB
DRB1_0101	4	IFFHSLKVNLKNLTQ	FHSLKVNLK	2	0.8591	4.6	0.50	0.80	tr_C9ZQ90_	WB
DRB1_0101	5	LTFLGFATTRLLQVV	FLGFATTRL	2	0.8261	6.6	1.20	0.49	tr_C9ZQ90_	WB
DRB1_0101	6	FFHSLKVNLKNLTQS	FHSLKVNLK	1	0.8232	6.8	1.30	0.77	tr_C9ZQ90_	WB
DRB1_0101	7	VDKRIFFHSLKVNLK	FHSLKVNLK	6	0.8231	6.8	1.30	0.65	tr_C9ZQ90_	WB
DRB1_0101	8	PLTFLGFATTRLLQV	FLGFATTRL	3	0.8196	7.0	1.40	0.57	tr_C9ZQ90_	WB
DRB1_0101	9	TFLGFATTRLLQVVI	FLGFATTRL	1	0.8076	8.0	1.80	0.41	tr_C9ZQ90_	WB
DRB1_0101	10	YQEFVAAFNASKESI	FVAAFNASK	3	0.8042	8.3	1.90	0.82	tr_C9ZQ90_	WB
DRB1_0101	11	DPLTFLGFATTRLLQ	FLGFATTRL	4	0.7990	8.8	2.50	0.63	tr_C9ZQ90_	WB
DRB1_0101	12	RNVIHLQPLLAQQLY	LQPLLAQQL	5	0.7988	8.8	2.50	0.31	tr_C9ZQ90_	WB
DRB1_0101	13	FYQEFVAAFNASKES	FVAAFNASK	4	0.7961	9.1	2.50	0.76	tr_C9ZQ90_	WB
DRB1_0101	14	VIHLQPLLAQQLYVL	LQPLLAQQL	3	0.7910	9.6	2.50	0.54	tr_C9ZQ90_	WB
DRB1_0101	15	NVIHLQPLLAQQLYV	LQPLLAQQL	4	0.7872	10.0	3.00	0.48	tr_C9ZQ90_	WB
DRB1_0101	16	QEFVAAFNASKESIA	FVAAFNASK	2	0.7851	10.2	3.00	0.79	tr_C9ZQ90_	WB
DRB1_0101	17	LFYQEFVAAFNASKE	FVAAFNASK	5	0.7825	10.5	3.00	0.59	tr_C9ZQ90_	WB
DRB1_0101	18	IHLQPLLAQQLYVLA	LQPLLAQQL	2	0.7772	11.1	3.50	0.56	tr_C9ZQ90_	WB
DRB1_0101	19	WRNVIHLQPLLAQQL	VIHLQPLLA	3	0.7752	11.4	3.50	0.48	tr_C9ZQ90_	WB
DRB1_0101	20	NMTFIGTLPLSPRLN	FIGTLPLSP	3	0.7694	12.1	3.50	0.49	tr_C9ZQ90_	WB
DRB1_0101	21	VDMYLLAPSSFQHFL	YLLAPSSFQ	3	0.7649	12.7	4.00	0.63	tr_C9ZQ90_	WB
DRB1_0101	22	WDPLTFLGFATTRLL	FLGFATTRL	5	0.7645	12.8	4.00	0.68	tr_C9ZQ90_	WB
DRB1_0101	23	HLQPLLAQQLYVLAV	LQPLLAQQL	1	0.7618	13.2	4.00	0.48	tr_C9ZQ90_	WB
DRB1_0101	24	FLGFATTRLLQVVIS	LGFATTRLL	1	0.7604	13.4	4.50	0.34	tr_C9ZQ90_	WB
DRB1_0101	25	PNMTFIGTLPLSPRL	FIGTLPLSP	4	0.7592	13.5	4.50	0.46	tr_C9ZQ90_	WB
DRB1_0101	26	MTFIGTLPLSPRLNK	FIGTLPLSP	2	0.7570	13.9	4.50	0.49	tr_C9ZQ90_	WB
DRB1_0101	27	LLFYQEFVAAFNASK	FVAAFNASK	6	0.7563	14.0	4.50	0.35	tr_C9ZQ90_	WB
DRB1_0101	28	FHSLKVNLKNLTQSL	FHSLKVNLK	0	0.7547	14.2	4.50	0.63	tr_C9ZQ90_	WB
DRB1_0101	29	TVDMYLLAPSSFQHF	YLLAPSSFQ	4	0.7534	14.4	4.50	0.60	tr_C9ZQ90_	WB
DRB1_0101	30	EFVAAFNASKESIAS	FVAAFNASK	1	0.7463	15.6	5.00	0.74	tr_C9ZQ90_	WB
DRB1_0101	31	DMYLLAPSSFQHFLI	YLLAPSSFQ	2	0.7449	15.8	5.50	0.64	tr_C9ZQ90_	WB
DRB1_0101	32	QRASMRWSLGARSIE	MRWSLGARS	4	0.7446	15.9	5.50	0.79	tr_C9ZQ90_	WB
DRB1_0101	33	RASMRWSLGARSIEE	MRWSLGARS	3	0.7415	16.4	5.50	0.81	tr_C9ZQ90_	WB
DRB1_0101	34	ANVQVSAPLIGLYVV	SAPLIGLYV	5	0.7376	17.1	6.00	0.54	tr_C9ZQ90_	WB
DRB1_0101	35	RTVDMYLLAPSSFQH	YLLAPSSFQ	5	0.7350	17.6	6.00	0.49	tr_C9ZQ90_	WB
DRB1_0101	36	NVQVSAPLIGLYVVL	SAPLIGLYV	4	0.7338	17.8	6.00	0.62	tr_C9ZQ90_	WB
DRB1_0101	37	VPNMTFIGTLPLSPR	MTFIGTLPL	3	0.7330	18.0	6.00	0.47	tr_C9ZQ90_	WB
DRB1_0101	38	FWRNVIHLQPLLAQQ	VIHLQPLLA	4	0.7268	19.2	6.50	0.60	tr_C9ZQ90_	WB

DRB1_0101	39	AQRASMRWSLGARSI	MRWSLGARS	5	0.7248	19.6	7.00	0.77	tr_C9ZQ90_	WB
DRB1_0101	40	LQPLLAQQLYVLAVY	LLAQQLYVL	3	0.7240	19.8	7.00	0.38	tr_C9ZQ90_	WB
DRB1_0101	41	ASMRWSLGARSIEEA	MRWSLGARS	2	0.7176	21.2	7.50	0.74	tr_C9ZQ90_	WB
DRB1_0101	42	SANVQVSAPLIGLYV	SAPLIGLYV	6	0.7150	21.8	7.50	0.42	tr_C9ZQ90_	WB
DRB1_0101	43	TFIGTLPLSPRLNKF	FIGTLPLSP	1	0.7133	22.2	8.00	0.47	tr_C9ZQ90_	WB
DRB1_0101	44	VQVSAPLIGLYVVLT	SAPLIGLYV	3	0.7102	23.0	8.00	0.70	tr_C9ZQ90_	WB
DRB1_0101	45	MYLLAPSSFQHFLIK	YLLAPSSFQ	1	0.7073	23.7	8.50	0.66	tr_C9ZQ90_	WB
DRB1_0101	46	ILLFYQEFVAAFNAS	FYQEFVAAF	3	0.7047	24.4	8.50	0.34	tr_C9ZQ90_	WB
DRB1_0101	47	KFWRNVIHLQPLLAQ	VIHLQPLLA	5	0.6848	30.3	11.00	0.62	tr_C9ZQ90_	
DRB1_0101	48	EVLFEALNNAPQLTN	FEALNNAPQ	3	0.6792	32.2	11.00	0.75	tr_C9ZQ90_	
DRB1_0101	49	SMRWSLGARSIEEAD	MRWSLGARS	1	0.6787	32.3	12.00	0.70	tr_C9ZQ90_	
DRB1_0101	50	QVSAPLIGLYVVLTD	SAPLIGLYV	2	0.6787	32.3	12.00	0.77	tr_C9ZQ90_	
DRB1_0101	51	NRTVDMYLLAPSSFQ	YLLAPSSFQ	6	0.6782	32.5	12.00	0.41	tr_C9ZQ90_	
DRB1_0101	52	DVFCIGLTPPDVAAV	CIGLTPPDV	3	0.6719	34.8	12.00	0.60	tr_C9ZQ90_	
DRB1_0101	53	HWDPLTFLGFATTRL	FLGFATTRL	6	0.6707	35.3	12.00	0.72	tr_C9ZQ90_	
DRB1_0101	54	GEVLFEALNNAPQLT	FEALNNAPQ	4	0.6652	37.4	13.00	0.74	tr_C9ZQ90_	
DRB1_0101	55	VLFEALNNAPQLTNR	FEALNNAPQ	2	0.6650	37.5	13.00	0.76	tr_C9ZQ90_	
DRB1_0101	56	DILLFYQEFVAAFNA	LFYQEFVAA	3	0.6643	37.8	13.00	0.34	tr_C9ZQ90_	
DRB1_0101	57	QPLLAQQLYVLAVYL	LLAQQLYVL	2	0.6630	38.3	13.00	0.50	tr_C9ZQ90_	
DRB1_0101	58	GDVFCIGLTPPDVAA	CIGLTPPDV	4	0.6621	38.7	14.00	0.56	tr_C9ZQ90_	
DRB1_0101	59	VFCIGLTPPDVAAVA	CIGLTPPDV	2	0.6619	38.8	14.00	0.60	tr_C9ZQ90_	
DRB1_0101	60	SVPNMTFIGTLPLSP	MTFIGTLPL	4	0.6607	39.3	14.00	0.58	tr_C9ZQ90_	
DRB1_0101	61	QHFLIKTWSDALVSL	LIKTWSDAL	3	0.6587	40.2	14.00	0.38	tr_C9ZQ90_	
DRB1_0101	62	AVYLSNTSSTGVKAL	YLSNTSSTG	2	0.6566	41.1	14.00	0.44	tr_C9ZQ90_	
DRB1_0101	63	FVAAFNASKESIASS	FVAAFNASK	0	0.6565	41.1	14.00	0.48	tr_C9ZQ90_	
DRB1_0101	64	FQHFLIKTWSDALVS	FLIKTWSDA	3	0.6560	41.4	14.00	0.44	tr_C9ZQ90_	
DRB1_0101	65	MMVFGWLAGEVLFEA	FGWLAGEVL	3	0.6559	41.4	14.00	0.69	tr_C9ZQ90_	
DRB1_0101	66	VYLSNTSSTGVKALV	NTSSTGVKA	4	0.6508	43.8	15.00	0.51	tr_C9ZQ90_	
DRB1_0101	67	MVFGWLAGEVLFEAL	FGWLAGEVL	2	0.6499	44.2	15.00	0.68	tr_C9ZQ90_	
DRB1_0101	68	LAVYLSNTSSTGVKA	YLSNTSSTG	3	0.6492	44.5	15.00	0.58	tr_C9ZQ90_	
DRB1_0101	69	EMMVFGWLAGEVLFE	FGWLAGEVL	4	0.6428	47.7	16.00	0.69	tr_C9ZQ90_	
DRB1_0101	70	VSAPLIGLYVVLTDD	SAPLIGLYV	1	0.6415	48.4	16.00	0.74	tr_C9ZQ90_	
DRB1_0101	71	FIGTLPLSPRLNKFW	TLPLSPRLN	3	0.6373	50.6	17.00	0.31	tr_C9ZQ90_	
DRB1_0101	72	YLLAPSSFQHFLIKT	YLLAPSSFQ	0	0.6358	51.4	17.00	0.53	tr_C9ZQ90_	
DRB1_0101	73	HFLIKTWSDALVSLN	LIKTWSDAL	2	0.6346	52.1	17.00	0.34	tr_C9ZQ90_	
DRB1_0101	74	IGLYVVLTDDKVAQR	YVVLTDDKV	3	0.6332	52.9	17.00	0.80	tr_C9ZQ90_	
DRB1_0101	75	VFGWLAGEVLFEALN	FGWLAGEVL	1	0.6327	53.2	17.00	0.62	tr_C9ZQ90_	
DRB1_0101	76	GEMMVFGWLAGEVLF	FGWLAGEVL	5	0.6325	53.3	17.00	0.67	tr_C9ZQ90_	
DRB1_0101	77	VAQRASMRWSLGARS	MRWSLGARS	6	0.6305	54.5	18.00	0.65	tr_C9ZQ90_	
DRB1_0101	78	SFQHFLIKTWSDALV	FLIKTWSDA	4	0.6299	54.8	18.00	0.44	tr_C9ZQ90_	
DRB1_0101	79	YLSNTSSTGVKALVR	NTSSTGVKA	3	0.6290	55.4	18.00	0.64	tr_C9ZQ90_	
DRB1_0101	80	LGFATTRLLQVVISN	FATTRLLQV	2	0.6278	56.1	18.00	0.50	tr_C9ZQ90_	
DRB1_0101	81	GLYVVLTDDKVAQRA	YVVLTDDKV	2	0.6267	56.8	18.00	0.75	tr_C9ZQ90_	
DRB1_0101	82	VLAVYLSNTSSTGVK	YLSNTSSTG	4	0.6237	58.6	19.00	0.49	tr_C9ZQ90_	
DRB1_0101	83	SERLLFATSFPHWAE	LLFATSFPH	3	0.6232	58.9	19.00	0.63	tr_C9ZQ90_	
DRB1_0101	84	NDILLFYQEFVAAFN	LFYQEFVAA	4	0.6232	59.0	19.00	0.41	tr_C9ZQ90_	
DRB1_0101	85	AGEVLFEALNNAPQL	FEALNNAPQ	5	0.6230	59.1	19.00	0.69	tr_C9ZQ90_	
DRB1_0101	86	SSANVQVSAPLIGLY	NVQVSAPLI	3	0.6218	59.8	19.00	0.45	tr_C9ZQ90_	
DRB1_0101	87	NGDVFCIGLTPPDVA	CIGLTPPDV	5	0.6185	62.0	20.00	0.51	tr_C9ZQ90_	
DRB1_0101	88	PGQLITTGTVPLASD	LITTGTVPL	3	0.6170	63.0	20.00	0.65	tr_C9ZQ90_	

DRB1_0101	89	PLLAQQLYVLAVYLS	LLAQQLYVL	1	0.6155	64.1	20.00	0.43	tr_C9ZQ90_
DRB1_0101	90	NMRKVNAELLADRIY	MRKVNAELL	1	0.6139	65.2	20.00	0.43	tr_C9ZQ90_
DRB1_0101	91	ERLLFATSFPHWAEK	LLFATSFPH	2	0.6139	65.2	20.00	0.56	tr_C9ZQ90_
DRB1_0101	92	LFEALNNAPQLTNRT	FEALNNAPQ	1	0.6136	65.4	20.00	0.76	tr_C9ZQ90_
DRB1_0101	93	FCIGLTPPDVAAVAR	CIGLTPPDV	1	0.6131	65.8	20.00	0.61	tr_C9ZQ90_
DRB1_0101	94	SSERLLFATSFPHWA	LLFATSFPH	4	0.6125	66.2	20.00	0.61	tr_C9ZQ90_
DRB1_0101	95	TPGQLITTGTVPLAS	LITTGTVPL	4	0.6118	66.7	21.00	0.69	tr_C9ZQ90_
DRB1_0101	96	GQLITTGTVPLASDN	LITTGTVPL	2	0.6085	69.1	21.00	0.63	tr_C9ZQ90_
DRB1_0101	97	NKFWRNVIHLQPLLA	VIHLQPLLA	6	0.6076	69.8	21.00	0.63	tr_C9ZQ90_
DRB1_0101	98	LYVVLTDDKVAQRAS	YVVLTDDKV	1	0.6049	71.8	22.00	0.69	tr_C9ZQ90_
DRB1_0101	99	LSVPNMTFIGTLPLS	MTFIGTLPL	5	0.6041	72.5	22.00	0.74	tr_C9ZQ90_
DRB1_0101	100	RLLQVVISNMRKVNA	VVISNMRKV	4	0.6015	74.6	22.00	0.50	tr_C9ZQ90_
DRB1_0101	101	YVLAVYLSNTSSTGV	YLSNTSSTG	5	0.5996	76.1	23.00	0.38	tr_C9ZQ90_
DRB1_0101	102	LIGLYVVLTDDKVAQ	YVVLTDDKV	4	0.5992	76.4	23.00	0.76	tr_C9ZQ90_
DRB1_0101	103	MRKVNAELLADRIYT	NAELLADRI	4	0.5981	77.4	23.00	0.59	tr_C9ZQ90_
DRB1_0101	104	DVDKRIFFHSLKVNL	FFHSLKVNL	6	0.5971	78.2	23.00	0.38	tr_C9ZQ90_
DRB1_0101	105	RLLFATSFPHWAEKN	LLFATSFPH	1	0.5963	78.9	23.00	0.50	tr_C9ZQ90_
DRB1_0101	106	TRLLQVVISNMRKVN	VVISNMRKV	5	0.5962	79.0	23.00	0.31	tr_C9ZQ90_
DRB1_0101	107	LGEMMVFGWLAGEVL	FGWLAGEVL	6	0.5928	82.0	24.00	0.53	tr_C9ZQ90_
DRB1_0101	108	VDTLDKSLVTFGVSL	DKSLVTFGV	4	0.5898	84.6	24.00	0.62	tr_C9ZQ90_
DRB1_0101	109	SNMRKVNAELLADRI	MRKVNAELL	2	0.5886	85.8	24.00	0.60	tr_C9ZQ90_
DRB1_0101	110	DTLDKSLVTFGVSLD	DKSLVTFGV	3	0.5865	87.7	25.00	0.62	tr_C9ZQ90_
DRB1_0101	111	NISVWSMARVLNSSL	WSMARVLNS	4	0.5863	87.9	25.00	0.44	tr_C9ZQ90_
DRB1_0101	112	MDYVIPQEGQLTQSQ	VIPQEGQLT	3	0.5863	87.9	25.00	0.86	tr_C9ZQ90_
DRB1_0101	113	TLDKSLVTFGVSLDS	DKSLVTFGV	2	0.5861	88.0	25.00	0.60	tr_C9ZQ90_
DRB1_0101	114	SMDYVIPQEGQLTQS	VIPQEGQLT	4	0.5821	92.0	25.00	0.76	tr_C9ZQ90_
DRB1_0101	115	HSLKVNLKNLTQSLE	LKVNLKNLT	2	0.5820	92.1	25.00	0.52	tr_C9ZQ90_
DRB1_0101	116	CSSANVQVSAPLIGL	NVQVSAPLI	4	0.5803	93.8	26.00	0.45	tr_C9ZQ90_
DRB1_0101	117	SKESIASSERLLFAT	SIASSERLL	3	0.5799	94.2	26.00	0.52	tr_C9ZQ90_
DRB1_0101	118	VAAVLGVVTADILSV	LGVVTADIL	4	0.5791	95.0	26.00	0.56	tr_C9ZQ90_
DRB1_0101	119	FGWLAGEVLFEALNN	FGWLAGEVL	0	0.5785	95.7	26.00	0.47	tr_C9ZQ90_
DRB1_0101	120	ISVWSMARVLNSSLP	WSMARVLNS	3	0.5784	95.7	26.00	0.44	tr_C9ZQ90_
DRB1_0101	121	LLQVVISNMRKVNAE	VVISNMRKV	3	0.5778	96.4	26.00	0.57	tr_C9ZQ90_
DRB1_0101	122	FLIKTWSDALVSLNR	LIKTWSDAL	1	0.5775	96.6	26.00	0.31	tr_C9ZQ90_
DRB1_0101	123	RARVFVPFNDILLFY	FVPFNDILL	4	0.5775	96.7	26.00	0.70	tr_C9ZQ90_
DRB1_0101	124	LSNTSSTGVKALVRG	NTSSTGVKA	2	0.5766	97.6	26.00	0.70	tr_C9ZQ90_
DRB1_0101	125	TTRLLQVVISNMRKV	LQVVISNMR	4	0.5766	97.6	26.00	0.37	tr_C9ZQ90_
DRB1_0101	126	SLKVNLKNLTQSLEQ	LKVNLKNLT	1	0.5766	97.6	26.00	0.41	tr_C9ZQ90_
DRB1_0101	127	SAPLIGLYVVLTDDK	SAPLIGLYV	0	0.5748	99.6	27.00	0.63	tr_C9ZQ90_
DRB1_0101	128	ARVFVPFNDILLFYQ	FVPFNDILL	3	0.5740	100.5	27.00	0.74	tr_C9ZQ90_
DRB1_0101	129	SSFQHFLIKTWSDAL	FLIKTWSDA	5	0.5718	102.8	27.00	0.45	tr_C9ZQ90_
DRB1_0101	130	SVWSMARVLNSSLPR	WSMARVLNS	2	0.5702	104.6	28.00	0.38	tr_C9ZQ90_
DRB1_0101	131	FTPGQLITTGTVPLA	LITTGTVPL	5	0.5701	104.7	28.00	0.63	tr_C9ZQ90_
DRB1_0101	132	VVISNMRKVNAELLA	MRKVNAELL	5	0.5698	105.0	28.00	0.56	tr_C9ZQ90_
DRB1_0101	133	ISNMRKVNAELLADR	MRKVNAELL	3	0.5684	106.7	28.00	0.80	tr_C9ZQ90_
DRB1_0101	134	ASKESIASSERLLFA	SIASSERLL	4	0.5680	107.2	28.00	0.49	tr_C9ZQ90_
DRB1_0101	135	PLIGLYVVLTDDKVA	YVVLTDDKV	5	0.5655	110.0	28.00	0.73	tr_C9ZQ90_
DRB1_0101	136	TNISVWSMARVLNSS	WSMARVLNS	5	0.5643	111.5	29.00	0.43	tr_C9ZQ90_
DRB1_0101	137	AAVLGVVTADILSVP	LGVVTADIL	3	0.5636	112.3	29.00	0.58	tr_C9ZQ90_
DRB1_0101	138	QLITTGTVPLASDNR	LITTGTVPL	1	0.5618	114.6	29.00	0.63	tr_C9ZQ90_

DRB1_0101	139	FNDILLFYQEFVAAF	LFYQEFVAA	5	0.5616	114.8	29.00	0.46	tr_C9ZQ90_
DRB1_0101	140	LDKSLVTFGVSLDSS	DKSLVTFGV	1	0.5608	115.8	29.00	0.55	tr_C9ZQ90_
DRB1_0101	141	MRWSLGARSIEEADD	MRWSLGARS	0	0.5607	115.9	29.00	0.57	tr_C9ZQ90_
DRB1_0101	142	ASSERLLFATSFPHW	LLFATSFPH	5	0.5604	116.4	29.00	0.59	tr_C9ZQ90_
DRB1_0101	143	RRARVFVPFNDILLF	FVPFNDILL	5	0.5591	118.0	30.00	0.65	tr_C9ZQ90_
DRB1_0101	144	TPPDVAAVARHLQTH	DVAAVARHL	3	0.5590	118.1	30.00	0.87	tr_C9ZQ90_
DRB1_0101	145	GFATTRLLQVVISNM	FATTRLLQV	1	0.5579	119.5	30.00	0.52	tr_C9ZQ90_
DRB1_0101	146	QVVISNMRKVNAELL	VVISNMRKV	1	0.5573	120.3	30.00	0.37	tr_C9ZQ90_
DRB1_0101	147	AVAAVLGVVTADILS	LGVVTADIL	5	0.5559	122.1	30.00	0.50	tr_C9ZQ90_
DRB1_0101	148	RKVNAELLADRIYTE	NAELLADRI	3	0.5559	122.2	30.00	0.71	tr_C9ZQ90_
DRB1_0101	149	LKVNLKNLTQSLEQV	NLKNLTQSL	3	0.5546	123.8	30.00	0.46	tr_C9ZQ90_
DRB1_0101	150	KESIASSERLLFATS	SIASSERLL	2	0.5535	125.3	31.00	0.50	tr_C9ZQ90_
DRB1_0101	151	QVRDTKAVAAVLGVV	DTKAVAAVL	3	0.5505	129.5	31.00	0.37	tr_C9ZQ90_
DRB1_0101	152	RDTKAVAAVLGVVTA	VAAVLGVVT	5	0.5501	130.1	31.00	0.34	tr_C9ZQ90_
DRB1_0101	153	VVDTLDKSLVTFGVS	DKSLVTFGV	5	0.5494	131.0	31.00	0.56	tr_C9ZQ90_
DRB1_0101	154	RVFVPFNDILLFYQE	FVPFNDILL	2	0.5484	132.4	32.00	0.73	tr_C9ZQ90_
DRB1_0101	155	PPDVAAVARHLQTHR	DVAAVARHL	2	0.5480	133.1	32.00	0.81	tr_C9ZQ90_
DRB1_0101	156	DYVIPQEGQLTQSQI	VIPQEGQLT	2	0.5458	136.2	32.00	0.88	tr_C9ZQ90_
DRB1_0101	157	VISNMRKVNAELLAD	MRKVNAELL	4	0.5455	136.6	32.00	0.73	tr_C9ZQ90_
DRB1_0101	158	AVLGVVTADILSVPN	LGVVTADIL	2	0.5448	137.8	32.00	0.60	tr_C9ZQ90_
DRB1_0101	159	FATTRLLQVVISNMR	TRLLQVVIS	3	0.5446	138.0	32.00	0.30	tr_C9ZQ90_
DRB1_0101	160	LQVVISNMRKVNAEL	VVISNMRKV	2	0.5441	138.8	32.00	0.56	tr_C9ZQ90_
DRB1_0101	161	PSMDYVIPQEGQLTQ	VIPQEGQLT	5	0.5430	140.4	33.00	0.67	tr_C9ZQ90_
DRB1_0101	162	EQVRDTKAVAAVLGV	VRDTKAVAA	2	0.5427	140.8	33.00	0.39	tr_C9ZQ90_
DRB1_0101	163	ADRIYTESNIRVDDV	IYTESNIRV	3	0.5405	144.3	33.00	0.86	tr_C9ZQ90_
DRB1_0101	164	LYVLAVYLSNTSSTG	AVYLSNTSS	4	0.5399	145.3	33.00	0.31	tr_C9ZQ90_
DRB1_0101	165	VWSMARVLNSSLPRT	MARVLNSSL	3	0.5396	145.7	33.00	0.38	tr_C9ZQ90_
DRB1_0101	166	VRDTKAVAAVLGVVT	DTKAVAAVL	2	0.5388	147.0	33.00	0.31	tr_C9ZQ90_
DRB1_0101	167	PDVAAVARHLQTHRR	DVAAVARHL	1	0.5371	149.7	34.00	0.72	tr_C9ZQ90_
DRB1_0101	168	ATTRLLQVVISNMRK	LQVVISNMR	5	0.5357	151.9	34.00	0.34	tr_C9ZQ90_
DRB1_0101	169	LLAQQLYVLAVYLSN	LLAQQLYVL	0	0.5354	152.4	34.00	0.22	tr_C9ZQ90_
DRB1_0101	170	YVVLTDDKVAQRASM	YVVLTDDKV	0	0.5335	155.6	34.00	0.58	tr_C9ZQ90_
DRB1_0101	171	DNRTVDMYLLAPSSF	DMYLLAPSS	5	0.5334	155.8	34.00	0.54	tr_C9ZQ90_
DRB1_0101	172	KAVAAVLGVVTADIL	VAAVLGVVT	2	0.5289	163.6	35.00	0.31	tr_C9ZQ90_
DRB1_0101	173	LTPPDVAAVARHLQT	DVAAVARHL	4	0.5289	163.6	35.00	0.86	tr_C9ZQ90_
DRB1_0101	174	DEGAALQGAMCNCNQ	GAALQGAMC	2	0.5280	165.1	35.00	0.63	tr_C9ZQ90_
DRB1_0101	175	KVNAELLADRIYTES	NAELLADRI	2	0.5266	167.7	36.00	0.68	tr_C9ZQ90_
DRB1_0101	176	EGAALQGAMCNCNQG	GAALQGAMC	1	0.5264	168.1	36.00	0.61	tr_C9ZQ90_
DRB1_0101	177	ATNISVWSMARVLNS	SVWSMARVL	4	0.5261	168.5	36.00	0.34	tr_C9ZQ90_
DRB1_0101	178	SLKPMKKGSVTWSVS	LKPMKKGSV	1	0.5258	169.2	36.00	0.35	tr_C9ZQ90_
DRB1_0101	179	LEQVRDTKAVAAVLG	VRDTKAVAA	3	0.5252	170.3	36.00	0.50	tr_C9ZQ90_
DRB1_0101	180	LSLKPMKKGSVTWSV	LKPMKKGSV	2	0.5242	172.0	36.00	0.46	tr_C9ZQ90_
DRB1_0101	181	KVNLKNLTQSLEQVR	NLKNLTQSL	2	0.5237	173.0	36.00	0.41	tr_C9ZQ90_
DRB1_0101	182	SNTSSTGVKALVRGG	NTSSTGVKA	1	0.5224	175.4	37.00	0.67	tr_C9ZQ90_
DRB1_0101	183	GNGDVFCIGLTPPDV	CIGLTPPDV	6	0.5198	180.5	37.00	0.45	tr_C9ZQ90_
DRB1_0101	184	DTKAVAAVLGVVTAD	VAAVLGVVT	4	0.5197	180.7	37.00	0.41	tr_C9ZQ90_
DRB1_0101	185	NRTFTPGQLITTGTV	FTPGQLITT	3	0.5196	180.8	37.00	0.54	tr_C9ZQ90_
DRB1_0101	186	CDEGAALQGAMCNCN	GAALQGAMC	3	0.5196	180.9	37.00	0.67	tr_C9ZQ90_
DRB1_0101	187	HRRARVFVPFNDILL	FVPFNDILL	6	0.5178	184.4	37.00	0.56	tr_C9ZQ90_
DRB1_0101	188	QLYVLAVYLSNTSST	YVLAVYLSN	2	0.5177	184.7	37.00	0.26	tr_C9ZQ90_

DRB1_0101	189	SLSLKPMKKGSVTWS	LKPMKKGSV	3	0.5174	185.2	38.00	0.51	tr_C9ZQ90_
DRB1_0101	190	NRTSFMESLYKQRRY	TSFMESLYK	2	0.5174	185.3	38.00	0.31	tr_C9ZQ90_
DRB1_0101	191	ILSVPNMTFIGTLPL	MTFIGTLPL	6	0.5174	185.3	38.00	0.54	tr_C9ZQ90_
DRB1_0101	192	SLNRTFTPGQLITTG	FTPGQLITT	5	0.5171	185.8	38.00	0.33	tr_C9ZQ90_
DRB1_0101	193	LVTFGVSLDSSKTLG	GVSLDSSKT	4	0.5171	185.8	38.00	0.64	tr_C9ZQ90_
DRB1_0101	194	LNRTFTPGQLITTGT	FTPGQLITT	4	0.5170	186.1	38.00	0.49	tr_C9ZQ90_
DRB1_0101	195	FVKKIAQDNRTVDMY	KIAQDNRTV	3	0.5166	186.8	38.00	0.50	tr_C9ZQ90_
DRB1_0101	196	SMARVLNSSLPRTQV	MARVLNSSL	1	0.5158	188.4	38.00	0.35	tr_C9ZQ90_
DRB1_0101	197	VAAFNASKESIASSE	FNASKESIA	3	0.5154	189.2	38.00	0.76	tr_C9ZQ90_
DRB1_0101	198	ESIASSERLLFATSF	SIASSERLL	1	0.5149	190.2	38.00	0.37	tr_C9ZQ90_
DRB1_0101	199	DKSLVTFGVSLDSSK	DKSLVTFGV	0	0.5134	193.4	38.00	0.34	tr_C9ZQ90_
DRB1_0101	200	IGTLPLSPRLNKFWR	TLPLSPRLN	2	0.5127	195.0	38.00	0.45	tr_C9ZQ90_
DRB1_0101	201	DRIYTESNIRVDDVR	IYTESNIRV	2	0.5126	195.1	38.00	0.82	tr_C9ZQ90_
DRB1_0101	202	VTFGVSLDSSKTLGD	GVSLDSSKT	3	0.5109	198.8	39.00	0.73	tr_C9ZQ90_
DRB1_0101	203	RTSFMESLYKQRRYV	FMESLYKQR	3	0.5108	199.1	39.00	0.25	tr_C9ZQ90_
DRB1_0101	204	LADRIYTESNIRVDD	IYTESNIRV	4	0.5097	201.3	39.00	0.85	tr_C9ZQ90_
DRB1_0101	205	WSMARVLNSSLPRTQ	MARVLNSSL	2	0.5095	201.8	39.00	0.40	tr_C9ZQ90_
DRB1_0101	206	VARHLQTHRRARVFV	LQTHRRARV	4	0.5093	202.1	39.00	0.44	tr_C9ZQ90_
DRB1_0101	207	VLGVVTADILSVPNM	LGVVTADIL	1	0.5065	208.4	40.00	0.58	tr_C9ZQ90_
DRB1_0101	208	KALVRGGEASEVVDT	VRGGEASEV	3	0.5061	209.3	40.00	0.73	tr_C9ZQ90_
DRB1_0101	209	QQLYVLAVYLSNTSS	YVLAVYLSN	3	0.5059	209.8	40.00	0.35	tr_C9ZQ90_
DRB1_0101	210	TNRTSFMESLYKQRR	TSFMESLYK	3	0.5057	210.3	40.00	0.38	tr_C9ZQ90_
DRB1_0101	211	VKALVRGGEASEVVD	VRGGEASEV	4	0.5050	211.8	40.00	0.65	tr_C9ZQ90_
DRB1_0101	212	DSLSLKPMKKGSVTW	LKPMKKGSV	4	0.5048	212.3	40.00	0.43	tr_C9ZQ90_
DRB1_0101	213	VRHFQRDMDNYLDTN	FQRDMDNYL	3	0.5039	214.3	40.00	0.80	tr_C9ZQ90_
DRB1_0101	214	ECSSANVQVSAPLIG	NVQVSAPLI	5	0.5027	217.2	40.00	0.47	tr_C9ZQ90_
DRB1_0101	215	FEALNNAPQLTNRTS	FEALNNAPQ	0	0.5021	218.5	41.00	0.61	tr_C9ZQ90_
DRB1_0101	216	DDKLGEMMVFGWLAG	EMMVFGWLA	5	0.5010	221.2	41.00	0.30	tr_C9ZQ90_
DRB1_0101	217	DKVAQRASMRWSLGA	KVAQRASMR	1	0.5005	222.3	41.00	0.41	tr_C9ZQ90_
DRB1_0101	218	NASKESIASSERLLF	SIASSERLL	5	0.5002	223.1	41.00	0.45	tr_C9ZQ90_
DRB1_0101	219	TKAVAAVLGVVTADI	VAAVLGVVT	3	0.4983	227.8	41.00	0.50	tr_C9ZQ90_
DRB1_0101	220	LLFATSFPHWAEKNT	LLFATSFPH	0	0.4982	228.0	41.00	0.41	tr_C9ZQ90_
DRB1_0101	221	PSSFQHFLIKTWSDA	FLIKTWSDA	6	0.4976	229.6	42.00	0.35	tr_C9ZQ90_
DRB1_0101	222	AVARHLQTHRRARVF	LQTHRRARV	5	0.4963	232.7	42.00	0.34	tr_C9ZQ90_
DRB1_0101	223	CIGLTPPDVAAVARH	CIGLTPPDV	0	0.4960	233.5	42.00	0.54	tr_C9ZQ90_
DRB1_0101	224	SLVTFGVSLDSSKTL	GVSLDSSKT	5	0.4958	233.9	42.00	0.55	tr_C9ZQ90_
DRB1_0101	225	KVAQRASMRWSLGAR	QRASMRWSL	3	0.4956	234.4	42.00	0.41	tr_C9ZQ90_
DRB1_0101	226	GLTPPDVAAVARHLQ	DVAAVARHL	5	0.4953	235.3	42.00	0.76	tr_C9ZQ90_
DRB1_0101	227	MVRHFQRDMDNYLDT	FQRDMDNYL	4	0.4945	237.3	42.00	0.77	tr_C9ZQ90_
DRB1_0101	228	GVKALVRGGEASEVV	VRGGEASEV	5	0.4944	237.6	42.00	0.58	tr_C9ZQ90_
DRB1_0101	229	LAGEVLFEALNNAPQ	FEALNNAPQ	6	0.4940	238.6	42.00	0.51	tr_C9ZQ90_
DRB1_0101	230	EVVDTLDKSLVTFGV	DKSLVTFGV	6	0.4936	239.6	42.00	0.34	tr_C9ZQ90_
DRB1_0101	231	DKLGEMMVFGWLAGE	EMMVFGWLA	4	0.4928	241.6	42.00	0.35	tr_C9ZQ90_
DRB1_0101	232	LKPMKKGSVTWSVSE	KKGSVTWSV	4	0.4917	244.6	43.00	0.42	tr_C9ZQ90_
DRB1_0101	233	MARVLNSSLPRTQVG	LNSSLPRTQ	4	0.4914	245.4	43.00	0.31	tr_C9ZQ90_
DRB1_0101	234	AAFNASKESIASSER	FNASKESIA	2	0.4906	247.4	43.00	0.74	tr_C9ZQ90_
DRB1_0101	235	VSLNRTFTPGQLITT	RTFTPGQLI	4	0.4904	248.1	43.00	0.41	tr_C9ZQ90_
DRB1_0101	236	TFTPGQLITTGTVPL	LITTGTVPL	6	0.4900	249.2	43.00	0.41	tr_C9ZQ90_
DRB1_0101	237	TFGVSLDSSKTLGDD	GVSLDSSKT	2	0.4895	250.5	43.00	0.73	tr_C9ZQ90_
DRB1_0101	238	LIKTWSDALVSLNRT	WSDALVSLN	4	0.4895	250.6	43.00	0.32	tr_C9ZQ90_

DRB1_0101	239	VFVPFNDILLFYQEF	FVPFNDILL	1	0.4879	254.8	43.00	0.70	tr_C9ZQ90_
DRB1_0101	240	DDSLSLKPMKKGSVT	LKPMKKGSV	5	0.4879	254.8	43.00	0.34	tr_C9ZQ90_
DRB1_0101	241	LLADRIYTESNIRVD	IYTESNIRV	5	0.4875	256.1	44.00	0.83	tr_C9ZQ90_
DRB1_0101	242	ECDEGAALQGAMCNC	GAALQGAMC	4	0.4874	256.3	44.00	0.70	tr_C9ZQ90_
DRB1_0101	243	DDKVAQRASMRWSLG	KVAQRASMR	2	0.4870	257.4	44.00	0.46	tr_C9ZQ90_
DRB1_0101	244	AQQLYVLAVYLSNTS	YVLAVYLSN	4	0.4864	259.1	44.00	0.34	tr_C9ZQ90_
DRB1_0101	245	KLGEMMVFGWLAGEV	EMMVFGWLA	3	0.4861	259.8	44.00	0.43	tr_C9ZQ90_
DRB1_0101	246	APLIGLYVVLTDDKV	YVVLTDDKV	6	0.4857	261.0	44.00	0.61	tr_C9ZQ90_
DRB1_0101	247	IASSERLLFATSFPH	LLFATSFPH	6	0.4848	263.6	44.00	0.47	tr_C9ZQ90_
DRB1_0101	248	VNAELLADRIYTESN	NAELLADRI	1	0.4847	263.9	44.00	0.60	tr_C9ZQ90_
DRB1_0101	249	KDDKLGEMMVFGWLA	GEMMVFGWL	5	0.4845	264.4	44.00	0.31	tr_C9ZQ90_
DRB1_0101	250	TSFMESLYKQRRYVI	FMESLYKQR	2	0.4843	265.0	44.00	0.22	tr_C9ZQ90_
DRB1_0101	251	LAQQLYVLAVYLSNT	YVLAVYLSN	5	0.4829	269.0	45.00	0.25	tr_C9ZQ90_
DRB1_0101	252	DDVDKRIFFHSLKVN	RIFFHSLKV	5	0.4811	274.4	45.00	0.48	tr_C9ZQ90_
DRB1_0101	253	GAALQGAMCNCNQGG	GAALQGAMC	0	0.4804	276.3	45.00	0.47	tr_C9ZQ90_
DRB1_0101	254	ARHLQTHRRARVFVP	LQTHRRARV	3	0.4799	277.8	45.00	0.43	tr_C9ZQ90_
DRB1_0101	255	TDDKVAQRASMRWSL	KVAQRASMR	3	0.4760	289.9	46.00	0.54	tr_C9ZQ90_
DRB1_0101	256	RIYTESNIRVDDVRF	IYTESNIRV	1	0.4751	292.6	46.00	0.80	tr_C9ZQ90_
DRB1_0101	257	RHFQRDMDNYLDTNS	FQRDMDNYL	2	0.4740	296.2	46.00	0.81	tr_C9ZQ90_
DRB1_0101	258	ARVLNSSLPRTQVGM	LNSSLPRTQ	3	0.4738	296.7	46.00	0.40	tr_C9ZQ90_
DRB1_0101	259	SLEQVRDTKAVAAVL	VRDTKAVAA	4	0.4738	297.0	46.00	0.52	tr_C9ZQ90_
DRB1_0101	260	VNLKNLTQSLEQVRD	NLKNLTQSL	1	0.4735	297.7	47.00	0.37	tr_C9ZQ90_
DRB1_0101	261	DVAAVARHLQTHRRA	DVAAVARHL	0	0.4725	301.0	47.00	0.49	tr_C9ZQ90_
DRB1_0101	262	RHLQTHRRARVFVPF	LQTHRRARV	2	0.4722	302.2	47.00	0.35	tr_C9ZQ90_
DRB1_0101	263	RTFTPGQLITTGTVP	FTPGQLITT	2	0.4722	302.2	47.00	0.55	tr_C9ZQ90_
DRB1_0101	264	GATNISVWSMARVLN	SVWSMARVL	5	0.4697	310.5	47.00	0.40	tr_C9ZQ90_
DRB1_0101	265	SMVRHFQRDMDNYLD	FQRDMDNYL	5	0.4689	313.1	47.00	0.75	tr_C9ZQ90_
DRB1_0101	266	IKTWSDALVSLNRTF	WSDALVSLN	3	0.4667	320.7	48.00	0.45	tr_C9ZQ90_
DRB1_0101	267	TADILSVPNMTFIGT	ILSVPNMTF	3	0.4651	326.1	48.00	0.56	tr_C9ZQ90_
DRB1_0101	268	CNCNQGGSMAHMRVV	GGSMAHMRV	5	0.4647	327.7	48.00	0.22	tr_C9ZQ90_
DRB1_0101	269	NCNQGGSMAHMRVVD	GSMAHMRVV	5	0.4635	331.8	49.00	0.29	tr_C9ZQ90_
DRB1_0101	270	TPSMDYVIPQEGQLT	VIPQEGQLT	6	0.4616	338.7	49.00	0.50	tr_C9ZQ90_
DRB1_0101	271	AFNASKESIASSERL	FNASKESIA	1	0.4604	343.1	49.00	0.62	tr_C9ZQ90_
DRB1_0101	272	SHWDPLTFLGFATTR	LTFLGFATT	5	0.4602	344.0	49.00	0.39	tr_C9ZQ90_
DRB1_0101	273	PFNDILLFYQEFVAA	LFYQEFVAA	6	0.4601	344.5	49.00	0.46	tr_C9ZQ90_
DRB1_0101	274	KSLVTFGVSLDSSKT	SLVTFGVSL	1	0.4581	351.9	50.00	0.28	tr_C9ZQ90_
DRB1_0101	275	LTNRTSFMESLYKQR	TSFMESLYK	4	0.4574	354.5	50.00	0.41	tr_C9ZQ90_
DRB1_0101	276	LLAPSSFQHFLIKTW	PSSFQHFLI	3	0.4571	355.7	50.00	0.42	tr_C9ZQ90_
DRB1_0101	277	QRRYVIDDFVVGDFG	YVIDDFVVG	3	0.4555	361.8	50.00	0.77	tr_C9ZQ90_
DRB1_0101	278	FNASKESIASSERLL	ESIASSERL	5	0.4553	362.8	50.00	0.29	tr_C9ZQ90_
DRB1_0101	279	GECDEGAALQGAMCN	GAALQGAMC	5	0.4551	363.7	50.00	0.69	tr_C9ZQ90_
DRB1_0101	280	KTWSDALVSLNRTFT	WSDALVSLN	2	0.4538	368.6	55.00	0.36	tr_C9ZQ90_
DRB1_0101	281	ADILSVPNMTFIGTL	ILSVPNMTF	2	0.4524	374.2	55.00	0.51	tr_C9ZQ90_
DRB1_0101	282	LITTGTVPLASDNRS	LITTGTVPL	0	0.4512	379.0	55.00	0.43	tr_C9ZQ90_
DRB1_0101	283	KQRRYVIDDFVVGDF	YVIDDFVVG	4	0.4512	379.3	55.00	0.76	tr_C9ZQ90_
DRB1_0101	284	AAVARHLQTHRRARV	ARHLQTHRR	3	0.4509	380.4	55.00	0.38	tr_C9ZQ90_
DRB1_0101	285	VDDSLSLKPMKKGSV	SLSLKPMKK	3	0.4494	386.8	55.00	0.47	tr_C9ZQ90_
DRB1_0101	286	KPMKKGSVTWSVSEC	KKGSVTWSV	3	0.4489	388.9	55.00	0.56	tr_C9ZQ90_
DRB1_0101	287	LVSLNRTFTPGQLIT	RTFTPGQLI	5	0.4488	389.0	55.00	0.39	tr_C9ZQ90_
DRB1_0101	288	TWSVSECSSANVQVS	VSECSSANV	3	0.4475	394.8	55.00	0.44	tr_C9ZQ90_

DRB1_0101	289	SFMESLYKQRRYVID	LYKQRRYVI	5	0.4472	395.7	55.00	0.28	tr_C9ZQ90_
DRB1_0101	290	VTWSVSECSSANVQV	VSECSSANV	4	0.4467	397.9	55.00	0.41	tr_C9ZQ90_
DRB1_0101	291	SIASSERLLFATSFP	ERLLFATSF	5	0.4462	400.3	55.00	0.38	tr_C9ZQ90_
DRB1_0101	292	ALVRGGEASEVVDTL	VRGGEASEV	2	0.4460	401.1	55.00	0.77	tr_C9ZQ90_
DRB1_0101	293	VTADILSVPNMTFIG	ILSVPNMTF	4	0.4453	404.3	55.00	0.56	tr_C9ZQ90_
DRB1_0101	294	RVLNSSLPRTQVGMT	LNSSLPRTQ	2	0.4445	407.8	55.00	0.39	tr_C9ZQ90_
DRB1_0101	295	LNKFWRNVIHLQPLL	FWRNVIHLQ	3	0.4434	412.5	55.00	0.36	tr_C9ZQ90_
DRB1_0101	296	FGATNISVWSMARVL	SVWSMARVL	6	0.4423	417.4	55.00	0.29	tr_C9ZQ90_
DRB1_0101	297	CNQGGSMAHMRVVDD	GSMAHMRVV	4	0.4421	418.2	55.00	0.33	tr_C9ZQ90_
DRB1_0101	298	FGVSLDSSKTLGDDD	GVSLDSSKT	1	0.4402	426.9	55.00	0.71	tr_C9ZQ90_
DRB1_0101	299	HLQTHRRARVFVPFN	HRRARVFVP	4	0.4399	428.3	55.00	0.34	tr_C9ZQ90_
DRB1_0101	300	RLNKFWRNVIHLQPL	FWRNVIHLQ	4	0.4398	428.9	55.00	0.33	tr_C9ZQ90_
DRB1_0101	301	GTLPLSPRLNKFWRN	TLPLSPRLN	1	0.4397	429.6	55.00	0.47	tr_C9ZQ90_
DRB1_0101	302	SVTWSVSECSSANVQ	VSECSSANV	5	0.4382	436.5	55.00	0.33	tr_C9ZQ90_
DRB1_0101	303	WSVSECSSANVQVSA	VSECSSANV	2	0.4369	442.8	55.00	0.37	tr_C9ZQ90_
DRB1_0101	304	NQGGSMAHMRVVDDS	GSMAHMRVV	3	0.4358	447.9	55.00	0.38	tr_C9ZQ90_
DRB1_0101	305	ISSYLSGNGDVFCIG	YLSGNGDVF	3	0.4351	451.1	55.00	0.55	tr_C9ZQ90_
DRB1_0101	306	SPRLNKFWRNVIHLQ	LNKFWRNVI	3	0.4325	464.3	55.00	0.27	tr_C9ZQ90_
DRB1_0101	307	IGLTPPDVAAVARHL	DVAAVARHL	6	0.4307	473.1	60.00	0.55	tr_C9ZQ90_
DRB1_0101	308	LAPSSFQHFLIKTWS	PSSFQHFLI	2	0.4306	473.9	60.00	0.44	tr_C9ZQ90_
DRB1_0101	309	RRYVIDDFVVGDFGG	YVIDDFVVG	2	0.4303	475.4	60.00	0.75	tr_C9ZQ90_
DRB1_0101	310	GWLAGEVLFEALNNA	WLAGEVLFE	1	0.4299	477.5	60.00	0.41	tr_C9ZQ90_
DRB1_0101	311	LKDDKLGEMMVFGWL	GEMMVFGWL	6	0.4280	487.5	60.00	0.26	tr_C9ZQ90_
DRB1_0101	312	YKQRRYVIDDFVVGD	YVIDDFVVG	5	0.4278	488.4	60.00	0.71	tr_C9ZQ90_
DRB1_0101	313	LGVVTADILSVPNMT	LGVVTADIL	0	0.4277	488.7	60.00	0.49	tr_C9ZQ90_
DRB1_0101	314	ESHWDPLTFLGFATT	LTFLGFATT	6	0.4267	494.2	60.00	0.32	tr_C9ZQ90_
DRB1_0101	315	APSSFQHFLIKTWSD	PSSFQHFLI	1	0.4252	502.0	60.00	0.36	tr_C9ZQ90_
DRB1_0101	316	QGGSMAHMRVVDDSL	GSMAHMRVV	2	0.4236	511.0	60.00	0.36	tr_C9ZQ90_
DRB1_0101	317	NTSSTGVKALVRGGE	NTSSTGVKA	0	0.4234	512.4	60.00	0.48	tr_C9ZQ90_
DRB1_0101	318	PISSYLSGNGDVFCI	YLSGNGDVF	4	0.4228	515.6	60.00	0.51	tr_C9ZQ90_
DRB1_0101	319	LTDDKVAQRASMRWS	KVAQRASMR	4	0.4217	521.6	60.00	0.54	tr_C9ZQ90_
DRB1_0101	320	NLKNLTQSLEQVRDT	LKNLTQSLE	1	0.4216	522.3	60.00	0.26	tr_C9ZQ90_
DRB1_0101	321	PRLNKFWRNVIHLQP	FWRNVIHLQ	5	0.4212	524.3	60.00	0.29	tr_C9ZQ90_
DRB1_0101	322	FVPFNDILLFYQEFV	FVPFNDILL	0	0.4199	531.9	60.00	0.50	tr_C9ZQ90_
DRB1_0101	323	QDNRTVDMYLLAPSS	DMYLLAPSS	6	0.4197	532.9	60.00	0.46	tr_C9ZQ90_
DRB1_0101	324	TGVKALVRGGEASEV	VRGGEASEV	6	0.4194	535.0	60.00	0.37	tr_C9ZQ90_
DRB1_0101	325	VVTADILSVPNMTFI	ILSVPNMTF	5	0.4183	541.3	60.00	0.50	tr_C9ZQ90_
DRB1_0101	326	PRTQVGMTPSMDYVI	VGMTPSMDY	4	0.4166	551.4	60.00	0.31	tr_C9ZQ90_
DRB1_0101	327	ALVSLNRTFTPGQLI	RTFTPGQLI	6	0.4153	559.0	60.00	0.28	tr_C9ZQ90_
DRB1_0101	328	RTQVGMTPSMDYVIP	MTPSMDYVI	5	0.4136	569.5	60.00	0.28	tr_C9ZQ90_
DRB1_0101	329	LYKQRRYVIDDFVVG	YVIDDFVVG	6	0.4132	571.9	60.00	0.56	tr_C9ZQ90_
DRB1_0101	330	SSYLSGNGDVFCIGL	YLSGNGDVF	2	0.4105	588.6	60.00	0.55	tr_C9ZQ90_
DRB1_0101	331	SNFGATNISVWSMAR	FGATNISVW	2	0.4101	591.5	60.00	0.25	tr_C9ZQ90_
DRB1_0101	332	DILSVPNMTFIGTLP	ILSVPNMTF	1	0.4098	593.6	60.00	0.49	tr_C9ZQ90_
DRB1_0101	333	NAELLADRIYTESNI	NAELLADRI	0	0.4097	594.1	60.00	0.45	tr_C9ZQ90_
DRB1_0101	334	QSLEQVRDTKAVAAV	VRDTKAVAA	5	0.4093	596.9	60.00	0.49	tr_C9ZQ90_
DRB1_0101	335	VAAVARHLQTHRRAR	ARHLQTHRR	4	0.4091	597.8	60.00	0.40	tr_C9ZQ90_
DRB1_0101	336	ELLADRIYTESNIRV	IYTESNIRV	6	0.4080	605.0	65.00	0.67	tr_C9ZQ90_
DRB1_0101	337	GGSMAHMRVVDDSLS	GSMAHMRVV	1	0.4070	611.9	65.00	0.31	tr_C9ZQ90_
DRB1_0101	338	FMESLYKQRRYVIDD	LYKQRRYVI	4	0.4063	616.2	65.00	0.40	tr_C9ZQ90_

DRB1_0101	339	TWSDALVSLNRTFTP	WSDALVSLN	1	0.4062	617.1	65.00	0.29	tr_C9ZQ90_
DRB1_0101	340	SSMVRHFQRDMDNYL	FQRDMDNYL	6	0.4055	621.6	65.00	0.64	tr_C9ZQ90_
DRB1_0101	341	QLTNRTSFMESLYKQ	TSFMESLYK	5	0.4046	627.5	65.00	0.38	tr_C9ZQ90_
DRB1_0101	342	ASNFGATNISVWSMA	FGATNISVW	3	0.4037	633.7	65.00	0.34	tr_C9ZQ90_
DRB1_0101	343	NFGATNISVWSMARV	ATNISVWSM	3	0.4025	642.3	65.00	0.22	tr_C9ZQ90_
DRB1_0101	344	PMKKGSVTWSVSECS	KKGSVTWSV	2	0.4024	642.6	65.00	0.50	tr_C9ZQ90_
DRB1_0101	345	MCNCNQGGSMAHMRV	CNQGGSMAH	3	0.4016	648.3	65.00	0.25	tr_C9ZQ90_
DRB1_0101	346	VKKIAQDNRTVDMYL	KIAQDNRTV	2	0.3992	665.6	65.00	0.72	tr_C9ZQ90_
DRB1_0101	347	GSVTWSVSECSSANV	VTWSVSECS	2	0.3988	668.7	65.00	0.28	tr_C9ZQ90_
DRB1_0101	348	SECSSANVQVSAPLI	NVQVSAPLI	6	0.3983	672.1	65.00	0.48	tr_C9ZQ90_
DRB1_0101	349	WLAGEVLFEALNNAP	VLFEALNNA	5	0.3953	694.0	65.00	0.31	tr_C9ZQ90_
DRB1_0101	350	HFQRDMDNYLDTNSD	FQRDMDNYL	1	0.3942	702.3	65.00	0.76	tr_C9ZQ90_
DRB1_0101	351	VVDDSLSLKPMKKGS	SLSLKPMKK	4	0.3939	705.0	65.00	0.51	tr_C9ZQ90_
DRB1_0101	352	ADDVDKRIFFHSLKV	RIFFHSLKV	6	0.3934	708.6	65.00	0.44	tr_C9ZQ90_
DRB1_0101	353	KKIAQDNRTVDMYLL	KIAQDNRTV	1	0.3932	710.4	65.00	0.55	tr_C9ZQ90_
DRB1_0101	354	SEVVDTLDKSLVTFG	VDTLDKSLV	3	0.3926	714.7	65.00	0.50	tr_C9ZQ90_
DRB1_0101	355	VLTDDKVAQRASMRW	KVAQRASMR	5	0.3922	717.6	65.00	0.49	tr_C9ZQ90_
DRB1_0101	356	GSMAHMRVVDDSLSL	MRVVDDSLS	5	0.3917	721.8	65.00	0.34	tr_C9ZQ90_
DRB1_0101	357	LQTHRRARVFVPFND	HRRARVFVP	3	0.3907	729.2	65.00	0.41	tr_C9ZQ90_
DRB1_0101	358	SVSECSSANVQVSAP	VSECSSANV	1	0.3896	738.1	65.00	0.40	tr_C9ZQ90_
DRB1_0101	359	SDAECVSGTSVSANE	CVSGTSVSA	4	0.3894	739.7	65.00	0.68	tr_C9ZQ90_
DRB1_0101	360	DMVARFHRHVNESHW	FHRHVNESH	5	0.3877	753.7	65.00	0.55	tr_C9ZQ90_
DRB1_0101	361	LVRGGEASEVVDTLD	VRGGEASEV	1	0.3852	774.6	65.00	0.76	tr_C9ZQ90_
DRB1_0101	362	DAECVSGTSVSANEC	CVSGTSVSA	3	0.3847	778.9	65.00	0.69	tr_C9ZQ90_
DRB1_0101	363	LSPRLNKFWRNVIHL	LNKFWRNVI	4	0.3841	783.8	70.00	0.36	tr_C9ZQ90_
DRB1_0101	364	LPRTQVGMTPSMDYV	VGMTPSMDY	5	0.3833	790.6	70.00	0.28	tr_C9ZQ90_
DRB1_0101	365	CASNFGATNISVWSM	FGATNISVW	4	0.3832	791.0	70.00	0.29	tr_C9ZQ90_
DRB1_0101	366	TQVGMTPSMDYVIPQ	MTPSMDYVI	4	0.3830	792.9	70.00	0.44	tr_C9ZQ90_
DRB1_0101	367	SYLSGNGDVFCIGLT	YLSGNGDVF	1	0.3805	814.4	70.00	0.49	tr_C9ZQ90_
DRB1_0101	368	PQLTNRTSFMESLYK	QLTNRTSFM	1	0.3805	814.5	70.00	0.29	tr_C9ZQ90_
DRB1_0101	369	GVVTADILSVPNMTF	ILSVPNMTF	6	0.3799	819.8	70.00	0.31	tr_C9ZQ90_
DRB1_0101	370	MESLYKQRRYVIDDF	LYKQRRYVI	3	0.3797	822.2	70.00	0.50	tr_C9ZQ90_
DRB1_0101	371	AECVSGTSVSANECA	CVSGTSVSA	2	0.3790	828.0	70.00	0.68	tr_C9ZQ90_
DRB1_0101	372	SDWKGFAKPEHYLKD	GFAKPEHYL	4	0.3786	831.5	70.00	0.53	tr_C9ZQ90_
DRB1_0101	373	SMAHMRVVDDSLSLK	MRVVDDSLS	4	0.3779	838.4	70.00	0.47	tr_C9ZQ90_
DRB1_0101	374	VLNSSLPRTQVGMTP	NSSLPRTQV	2	0.3766	849.8	70.00	0.38	tr_C9ZQ90_
DRB1_0101	375	WSDALVSLNRTFTPG	LVSLNRTFT	4	0.3756	858.7	70.00	0.40	tr_C9ZQ90_
DRB1_0101	376	FSDAECVSGTSVSAN	CVSGTSVSA	5	0.3751	864.1	70.00	0.62	tr_C9ZQ90_
DRB1_0101	377	RYVIDDFVVGDFGGE	YVIDDFVVG	1	0.3722	891.2	70.00	0.69	tr_C9ZQ90_
DRB1_0101	378	RVVDDSLSLKPMKKG	SLSLKPMKK	5	0.3717	896.3	70.00	0.44	tr_C9ZQ90_
DRB1_0101	379	WKGFAKPEHYLKDDK	GFAKPEHYL	2	0.3716	896.9	70.00	0.50	tr_C9ZQ90_
DRB1_0101	380	VVLTDDKVAQRASMR	TDDKVAQRA	3	0.3711	902.1	70.00	0.34	tr_C9ZQ90_
DRB1_0101	381	DWKGFAKPEHYLKDD	GFAKPEHYL	3	0.3710	903.4	70.00	0.51	tr_C9ZQ90_
DRB1_0101	382	MRVVDDSLSLKPMKK	SLSLKPMKK	6	0.3701	911.9	70.00	0.25	tr_C9ZQ90_
DRB1_0101	383	AMCNCNQGGSMAHMR	CNCNQGGSM	2	0.3691	921.3	70.00	0.28	tr_C9ZQ90_
DRB1_0101	384	DPISSYLSGNGDVFC	YLSGNGDVF	5	0.3681	932.2	70.00	0.46	tr_C9ZQ90_
DRB1_0101	385	YLKDDKLGEMMVFGW	DDKLGEMMV	3	0.3674	938.8	70.00	0.31	tr_C9ZQ90_
DRB1_0101	386	DDPISSYLSGNGDVF	YLSGNGDVF	6	0.3663	950.4	70.00	0.26	tr_C9ZQ90_
DRB1_0101	387	MVARFHRHVNESHWD	FHRHVNESH	4	0.3660	953.0	70.00	0.69	tr_C9ZQ90_
DRB1_0101	388	MKKGSVTWSVSECSS	KKGSVTWSV	1	0.3655	958.4	70.00	0.48	tr_C9ZQ90_

DRB1_0101	389	NESHWDPLTFLGFAT	SHWDPLTFL	2	0.3652	961.3	70.00	0.36	tr_C9ZQ90_
DRB1_0101	390	ESLYKQRRYVIDDFV	LYKQRRYVI	2	0.3651	962.4	70.00	0.46	tr_C9ZQ90_
DRB1_0101	391	MTPSMDYVIPQEGQL	MDYVIPQEG	4	0.3642	972.0	70.00	0.58	tr_C9ZQ90_
DRB1_0101	392	ASEVVDTLDKSLVTF	VDTLDKSLV	4	0.3639	975.2	70.00	0.55	tr_C9ZQ90_
DRB1_0101	393	NSDWKGFAKPEHYLK	GFAKPEHYL	5	0.3637	976.7	70.00	0.51	tr_C9ZQ90_
DRB1_0101	394	DALVSLNRTFTPGQL	LVSLNRTFT	2	0.3633	980.9	70.00	0.37	tr_C9ZQ90_
DRB1_0101	395	LKNLTQSLEQVRDTK	LTQSLEQVR	3	0.3633	981.3	70.00	0.35	tr_C9ZQ90_
DRB1_0101	396	NTKSDMVARFHRHVN	MVARFHRHV	5	0.3628	986.4	70.00	0.37	tr_C9ZQ90_
DRB1_0101	397	SDMVARFHRHVNESH	MVARFHRHV	2	0.3625	989.7	70.00	0.38	tr_C9ZQ90_
DRB1_0101	398	TQSLEQVRDTKAVAA	LEQVRDTKA	3	0.3611	1004.9	70.00	0.41	tr_C9ZQ90_
DRB1_0101	399	SGNGDVFCIGLTPPD	FCIGLTPPD	6	0.3607	1009.1	70.00	0.33	tr_C9ZQ90_
DRB1_0101	400	QTHRRARVFVPFNDI	HRRARVFVP	2	0.3603	1013.3	70.00	0.41	tr_C9ZQ90_
DRB1_0101	401	GGECDEGAALQGAMC	GAALQGAMC	6	0.3600	1016.8	75.00	0.55	tr_C9ZQ90_
DRB1_0101	402	TKSDMVARFHRHVNE	MVARFHRHV	4	0.3581	1038.3	75.00	0.46	tr_C9ZQ90_
DRB1_0101	403	ECASNFGATNISVWS	FGATNISVW	5	0.3581	1038.7	75.00	0.31	tr_C9ZQ90_
DRB1_0101	404	SDALVSLNRTFTPGQ	LVSLNRTFT	3	0.3579	1040.2	75.00	0.46	tr_C9ZQ90_
DRB1_0101	405	THRRARVFVPFNDIL	RVFVPFNDI	5	0.3575	1045.4	75.00	0.35	tr_C9ZQ90_
DRB1_0101	406	KSDMVARFHRHVNES	MVARFHRHV	3	0.3543	1081.9	75.00	0.54	tr_C9ZQ90_
DRB1_0101	407	HYLKDDKLGEMMVFG	KDDKLGEMM	3	0.3543	1082.2	75.00	0.25	tr_C9ZQ90_
DRB1_0101	408	KNTKSDMVARFHRHV	DMVARFHRH	5	0.3506	1125.7	75.00	0.24	tr_C9ZQ90_
DRB1_0101	409	KIAQDNRTVDMYLLA	NRTVDMYLL	5	0.3504	1128.9	75.00	0.38	tr_C9ZQ90_
DRB1_0101	410	KGSVTWSVSECSSAN	VTWSVSECS	3	0.3503	1130.2	75.00	0.50	tr_C9ZQ90_
DRB1_0101	411	MAHMRVVDDSLSLKP	MRVVDDSLS	3	0.3502	1131.0	75.00	0.50	tr_C9ZQ90_
DRB1_0101	412	KKGSVTWSVSECSSA	VTWSVSECS	4	0.3491	1144.3	75.00	0.44	tr_C9ZQ90_
DRB1_0101	413	KGFAKPEHYLKDDKL	GFAKPEHYL	1	0.3467	1174.6	75.00	0.51	tr_C9ZQ90_
DRB1_0101	414	APQLTNRTSFMESLY	QLTNRTSFM	2	0.3463	1179.6	75.00	0.51	tr_C9ZQ90_
DRB1_0101	415	LNSSLPRTQVGMTPS	NSSLPRTQV	1	0.3455	1189.6	75.00	0.35	tr_C9ZQ90_
DRB1_0101	416	AHMRVVDDSLSLKPM	MRVVDDSLS	2	0.3454	1190.8	75.00	0.47	tr_C9ZQ90_
DRB1_0101	417	NAPQLTNRTSFMESL	QLTNRTSFM	3	0.3452	1193.9	75.00	0.62	tr_C9ZQ90_
DRB1_0101	418	TLPLSPRLNKFWRNV	TLPLSPRLN	0	0.3446	1202.0	75.00	0.34	tr_C9ZQ90_
DRB1_0101	419	SLPRTQVGMTPSMDY	PRTQVGMTP	2	0.3432	1219.3	75.00	0.33	tr_C9ZQ90_
DRB1_0101	420	HMRVVDDSLSLKPMK	MRVVDDSLS	1	0.3429	1223.4	75.00	0.37	tr_C9ZQ90_
DRB1_0101	421	SLYKQRRYVIDDFVV	LYKQRRYVI	1	0.3429	1223.4	75.00	0.43	tr_C9ZQ90_
DRB1_0101	422	KNLTQSLEQVRDTKA	LTQSLEQVR	2	0.3415	1242.3	75.00	0.32	tr_C9ZQ90_
DRB1_0101	423	PLSPRLNKFWRNVIH	LNKFWRNVI	5	0.3394	1271.3	75.00	0.33	tr_C9ZQ90_
DRB1_0101	424	GAMCNCNQGGSMAHM	CNCNQGGSM	3	0.3390	1276.0	75.00	0.34	tr_C9ZQ90_
DRB1_0101	425	IYTESNIRVDDVRFG	IYTESNIRV	0	0.3390	1276.2	75.00	0.58	tr_C9ZQ90_
DRB1_0101	426	VARFHRHVNESHWDP	FHRHVNESH	3	0.3386	1281.9	75.00	0.71	tr_C9ZQ90_
DRB1_0101	427	QVGMTPSMDYVIPQE	MTPSMDYVI	3	0.3381	1288.9	75.00	0.56	tr_C9ZQ90_
DRB1_0101	428	ARFHRHVNESHWDPL	FHRHVNESH	2	0.3367	1308.3	75.00	0.65	tr_C9ZQ90_
DRB1_0101	429	VNESHWDPLTFLGFA	SHWDPLTFL	3	0.3360	1319.2	75.00	0.44	tr_C9ZQ90_
DRB1_0101	430	SSTGVKALVRGGEAS	KALVRGGEA	5	0.3355	1326.3	75.00	0.38	tr_C9ZQ90_
DRB1_0101	431	NECASNFGATNISVW	ASNFGATNI	3	0.3353	1328.3	75.00	0.22	tr_C9ZQ90_
DRB1_0101	432	STGVKALVRGGEASE	KALVRGGEA	4	0.3353	1328.4	75.00	0.41	tr_C9ZQ90_
DRB1_0101	433	ECVSGTSVSANECAS	CVSGTSVSA	1	0.3350	1332.3	75.00	0.58	tr_C9ZQ90_
DRB1_0101	434	EHYLKDDKLGEMMVF	KDDKLGEMM	4	0.3317	1380.9	80.00	0.26	tr_C9ZQ90_
DRB1_0101	435	NSSLPRTQVGMTPSM	PRTQVGMTP	4	0.3314	1386.2	80.00	0.39	tr_C9ZQ90_
DRB1_0101	436	VPFNDILLFYQEFVA	ILLFYQEFV	5	0.3299	1408.2	80.00	0.26	tr_C9ZQ90_
DRB1_0101	437	YLSGNGDVFCIGLTP	YLSGNGDVF	0	0.3297	1411.3	80.00	0.30	tr_C9ZQ90_
DRB1_0101	438	LFATSFPHWAEKNTK	FATSFPHWA	1	0.3292	1419.1	80.00	0.63	tr_C9ZQ90_

DRB1_0101	439	GMTPSMDYVIPQEGQ	MDYVIPQEG	5	0.3292	1419.8	80.00	0.47	tr_C9ZQ90_
DRB1_0101	440	VSECSSANVQVSAPL	CSSANVQVS	3	0.3284	1431.1	80.00	0.30	tr_C9ZQ90_
DRB1_0101	441	NLTQSLEQVRDTKAV	LEQVRDTKA	5	0.3276	1443.7	80.00	0.31	tr_C9ZQ90_
DRB1_0101	442	NNAPQLTNRTSFMES	QLTNRTSFM	4	0.3249	1487.2	80.00	0.52	tr_C9ZQ90_
DRB1_0101	443	GVSLDSSKTLGDDDP	GVSLDSSKT	0	0.3229	1519.4	80.00	0.57	tr_C9ZQ90_
DRB1_0101	444	LNNAPQLTNRTSFME	QLTNRTSFM	5	0.3176	1609.6	80.00	0.44	tr_C9ZQ90_
DRB1_0101	445	RFHRHVNESHWDPLT	FHRHVNESH	1	0.3156	1644.4	80.00	0.52	tr_C9ZQ90_
DRB1_0101	446	LTQSLEQVRDTKAVA	LEQVRDTKA	4	0.3140	1672.4	80.00	0.49	tr_C9ZQ90_
DRB1_0101	447	HVNESHWDPLTFLGF	SHWDPLTFL	4	0.3139	1674.9	80.00	0.41	tr_C9ZQ90_
DRB1_0101	448	ALNNAPQLTNRTSFM	NAPQLTNRT	3	0.3126	1698.9	80.00	0.40	tr_C9ZQ90_
DRB1_0101	449	DFGGECDEGAALQGA	FGGECDEGA	1	0.3120	1709.7	80.00	0.42	tr_C9ZQ90_
DRB1_0101	450	AALQGAMCNCNQGGS	LQGAMCNCN	2	0.3117	1715.0	80.00	0.47	tr_C9ZQ90_
DRB1_0101	451	VGMTPSMDYVIPQEG	MTPSMDYVI	2	0.3110	1727.3	80.00	0.49	tr_C9ZQ90_
DRB1_0101	452	EASEVVDTLDKSLVT	VDTLDKSLV	5	0.3106	1735.3	80.00	0.57	tr_C9ZQ90_
DRB1_0101	453	EKNTKSDMVARFHRH	TKSDMVARF	3	0.3105	1736.7	80.00	0.31	tr_C9ZQ90_
DRB1_0101	454	SSLPRTQVGMTPSMD	PRTQVGMTP	3	0.3105	1737.3	80.00	0.45	tr_C9ZQ90_
DRB1_0101	455	LPLSPRLNKFWRNVI	PRLNKFWRN	4	0.3075	1795.4	80.00	0.26	tr_C9ZQ90_
DRB1_0101	456	DDDPISSYLSGNGDV	DDPISSYLS	1	0.3070	1804.9	80.00	0.40	tr_C9ZQ90_
DRB1_0101	457	PFSDAECVSGTSVSA	CVSGTSVSA	6	0.3070	1805.1	80.00	0.38	tr_C9ZQ90_
DRB1_0101	458	TSSTGVKALVRGGEA	STGVKALVR	2	0.3040	1864.8	85.00	0.28	tr_C9ZQ90_
DRB1_0101	459	RWSLGARSIEEADDV	RWSLGARSI	0	0.3031	1881.4	85.00	0.38	tr_C9ZQ90_
DRB1_0101	460	LSGNGDVFCIGLTPP	DVFCIGLTP	5	0.3017	1911.1	85.00	0.31	tr_C9ZQ90_
DRB1_0101	461	GDFGGECDEGAALQG	FGGECDEGA	2	0.3004	1937.2	85.00	0.56	tr_C9ZQ90_
DRB1_0101	462	YVIDDFVVGDFGGEC	YVIDDFVVG	0	0.2954	2046.7	85.00	0.45	tr_C9ZQ90_
DRB1_0101	463	KTLGDDDPISSYLSG	DDPISSYLS	5	0.2948	2060.4	85.00	0.41	tr_C9ZQ90_
DRB1_0101	464	QGAMCNCNQGGSMAH	CNCNQGGSM	4	0.2940	2078.2	85.00	0.38	tr_C9ZQ90_
DRB1_0101	465	TLGDDDPISSYLSGN	DDPISSYLS	4	0.2927	2107.7	85.00	0.52	tr_C9ZQ90_
DRB1_0101	466	ANECASNFGATNISV	CASNFGATN	3	0.2924	2112.5	85.00	0.37	tr_C9ZQ90_
DRB1_0101	467	FGGECDEGAALQGAM	FGGECDEGA	0	0.2915	2133.1	85.00	0.31	tr_C9ZQ90_
DRB1_0101	468	SDNRSSMVRHFQRDM	NRSSMVRHF	2	0.2915	2134.6	85.00	0.35	tr_C9ZQ90_
DRB1_0101	469	RHVNESHWDPLTFLG	SHWDPLTFL	5	0.2915	2135.3	85.00	0.41	tr_C9ZQ90_
DRB1_0101	470	LQGAMCNCNQGGSMA	CNCNQGGSM	5	0.2909	2147.7	85.00	0.34	tr_C9ZQ90_
DRB1_0101	471	VGDFGGECDEGAALQ	FGGECDEGA	3	0.2909	2148.7	85.00	0.68	tr_C9ZQ90_
DRB1_0101	472	AEKNTKSDMVARFHR	TKSDMVARF	4	0.2908	2150.2	85.00	0.31	tr_C9ZQ90_
DRB1_0101	473	CVSGTSVSANECASN	CVSGTSVSA	0	0.2895	2180.1	85.00	0.37	tr_C9ZQ90_
DRB1_0101	474	AQDNRTVDMYLLAPS	NRTVDMYLL	3	0.2895	2180.7	85.00	0.61	tr_C9ZQ90_
DRB1_0101	475	ITTGTVPLASDNRSS	TVPLASDNR	4	0.2894	2183.6	85.00	0.33	tr_C9ZQ90_
DRB1_0101	476	LASDNRSSMVRHFQR	NRSSMVRHF	4	0.2894	2183.6	85.00	0.40	tr_C9ZQ90_
DRB1_0101	477	EALNNAPQLTNRTSF	NAPQLTNRT	4	0.2893	2186.3	85.00	0.33	tr_C9ZQ90_
DRB1_0101	478	LGDDDPISSYLSGNG	DDPISSYLS	3	0.2849	2292.0	85.00	0.63	tr_C9ZQ90_
DRB1_0101	479	HRHVNESHWDPLTFL	SHWDPLTFL	6	0.2837	2321.8	85.00	0.32	tr_C9ZQ90_
DRB1_0101	480	DNRSSMVRHFQRDMD	MVRHFQRDM	5	0.2821	2362.9	85.00	0.31	tr_C9ZQ90_
DRB1_0101	481	AELLADRIYTESNIR	LLADRIYTE	2	0.2813	2382.4	85.00	0.38	tr_C9ZQ90_
DRB1_0101	482	ALQGAMCNCNQGGSM	LQGAMCNCN	1	0.2802	2411.4	85.00	0.36	tr_C9ZQ90_
DRB1_0101	483	VSANECASNFGATNI	NECASNFGA	3	0.2781	2466.4	85.00	0.30	tr_C9ZQ90_
DRB1_0101	484	NRSSMVRHFQRDMDN	MVRHFQRDM	4	0.2775	2484.0	85.00	0.41	tr_C9ZQ90_
DRB1_0101	485	EADDVDKRIFFHSLK	DKRIFFHSL	5	0.2764	2512.2	85.00	0.37	tr_C9ZQ90_
DRB1_0101	486	FQRDMDNYLDTNSDW	FQRDMDNYL	0	0.2758	2529.6	85.00	0.60	tr_C9ZQ90_
DRB1_0101	487	PLASDNRSSMVRHFQ	NRSSMVRHF	5	0.2753	2543.0	90.00	0.32	tr_C9ZQ90_
DRB1_0101	488	WAEKNTKSDMVARFH	TKSDMVARF	5	0.2752	2545.0	90.00	0.28	tr_C9ZQ90_

DRB1_0101	489	HWAEKNTKSDMVARF	WAEKNTKSD	1	0.2751	2549.8	90.00	0.28	tr_C9ZQ90_
DRB1_0101	490	SANECASNFGATNIS	CASNFGATN	4	0.2742	2572.3	90.00	0.34	tr_C9ZQ90_
DRB1_0101	491	GDDDPISSYLSGNGD	DDPISSYLS	2	0.2741	2577.2	90.00	0.60	tr_C9ZQ90_
DRB1_0101	492	IAQDNRTVDMYLLAP	NRTVDMYLL	4	0.2738	2584.4	90.00	0.65	tr_C9ZQ90_
DRB1_0101	493	ASDNRSSMVRHFQRD	NRSSMVRHF	3	0.2729	2609.1	90.00	0.42	tr_C9ZQ90_
DRB1_0101	494	SKTLGDDDPISSYLS	LGDDDPISS	3	0.2725	2621.5	90.00	0.50	tr_C9ZQ90_
DRB1_0101	495	GTVPLASDNRSSMVR	LASDNRSSM	4	0.2720	2636.1	90.00	0.26	tr_C9ZQ90_
DRB1_0101	496	TNSDWKGFAKPEHYL	GFAKPEHYL	6	0.2703	2684.3	90.00	0.44	tr_C9ZQ90_
DRB1_0101	497	PEHYLKDDKLGEMMV	KDDKLGEMM	5	0.2689	2725.7	90.00	0.28	tr_C9ZQ90_
DRB1_0101	498	VVGDFGGECDEGAAL	FGGECDEGA	4	0.2689	2726.5	90.00	0.70	tr_C9ZQ90_
DRB1_0101	499	RSSMVRHFQRDMDNY	MVRHFQRDM	3	0.2666	2792.7	90.00	0.49	tr_C9ZQ90_
DRB1_0101	500	TGTVPLASDNRSSMV	TVPLASDNR	2	0.2666	2794.4	90.00	0.37	tr_C9ZQ90_
DRB1_0101	501	VRGGEASEVVDTLDK	VRGGEASEV	0	0.2652	2836.7	90.00	0.55	tr_C9ZQ90_
DRB1_0101	502	VPLASDNRSSMVRHF	LASDNRSSM	2	0.2649	2845.0	90.00	0.25	tr_C9ZQ90_
DRB1_0101	503	GTSVSANECASNFGA	SANECASNF	4	0.2648	2848.5	90.00	0.35	tr_C9ZQ90_
DRB1_0101	504	GFAKPEHYLKDDKLG	GFAKPEHYL	0	0.2643	2863.0	90.00	0.37	tr_C9ZQ90_
DRB1_0101	505	TSVSANECASNFGAT	SANECASNF	3	0.2634	2893.4	90.00	0.41	tr_C9ZQ90_
DRB1_0101	506	SVSANECASNFGATN	SANECASNF	2	0.2606	2982.6	90.00	0.35	tr_C9ZQ90_
DRB1_0101	507	TVPLASDNRSSMVRH	LASDNRSSM	3	0.2576	3081.1	90.00	0.33	tr_C9ZQ90_
DRB1_0101	508	SGTSVSANECASNFG	SANECASNF	5	0.2570	3101.2	90.00	0.26	tr_C9ZQ90_
DRB1_0101	509	TTGTVPLASDNRSSM	TVPLASDNR	3	0.2551	3164.3	90.00	0.49	tr_C9ZQ90_
DRB1_0101	510	FHRHVNESHWDPLTF	FHRHVNESH	0	0.2542	3195.7	90.00	0.28	tr_C9ZQ90_
DRB1_0101	511	DNYLDTNSDWKGFAK	LDTNSDWKG	3	0.2522	3263.8	90.00	0.50	tr_C9ZQ90_
DRB1_0101	512	PHWAEKNTKSDMVAR	WAEKNTKSD	2	0.2500	3342.4	90.00	0.38	tr_C9ZQ90_
DRB1_0101	513	FVVGDFGGECDEGAA	FGGECDEGA	5	0.2469	3458.7	90.00	0.58	tr_C9ZQ90_
DRB1_0101	514	FPHWAEKNTKSDMVA	WAEKNTKSD	3	0.2448	3537.4	90.00	0.50	tr_C9ZQ90_
DRB1_0101	515	MDNYLDTNSDWKGFA	LDTNSDWKG	4	0.2448	3538.1	90.00	0.43	tr_C9ZQ90_
DRB1_0101	516	FATSFPHWAEKNTKS	FATSFPHWA	0	0.2444	3553.2	90.00	0.48	tr_C9ZQ90_
DRB1_0101	517	VSGTSVSANECASNF	TSVSANECA	3	0.2443	3556.7	90.00	0.34	tr_C9ZQ90_
DRB1_0101	518	NYLDTNSDWKGFAKP	LDTNSDWKG	2	0.2371	3845.5	95.00	0.56	tr_C9ZQ90_
DRB1_0101	519	GEASEVVDTLDKSLV	VDTLDKSLV	6	0.2315	4083.3	95.00	0.54	tr_C9ZQ90_
DRB1_0101	520	VDDVRFGPFSDAECV	VRFGPFSDA	3	0.2300	4152.2	95.00	0.41	tr_C9ZQ90_
DRB1_0101	521	DDVRFGPFSDAECVS	VRFGPFSDA	2	0.2282	4234.2	95.00	0.41	tr_C9ZQ90_
DRB1_0101	522	DVRFGPFSDAECVSG	VRFGPFSDA	1	0.2276	4261.6	95.00	0.34	tr_C9ZQ90_
DRB1_0101	523	GPFSDAECVSGTSVS	AECVSGTSV	5	0.2224	4509.1	95.00	0.36	tr_C9ZQ90_
DRB1_0101	524	SFPHWAEKNTKSDMV	WAEKNTKSD	4	0.2215	4551.5	95.00	0.49	tr_C9ZQ90_
DRB1_0101	525	NIRVDDVRFGPFSDA	IRVDDVRFG	1	0.2159	4837.2	95.00	0.26	tr_C9ZQ90_
DRB1_0101	526	IRVDDVRFGPFSDAE	VRFGPFSDA	5	0.2157	4845.8	95.00	0.35	tr_C9ZQ90_
DRB1_0101	527	RVDDVRFGPFSDAEC	VRFGPFSDA	4	0.2142	4922.9	95.00	0.39	tr_C9ZQ90_
DRB1_0101	528	EEADDVDKRIFFHSL	DKRIFFHSL	6	0.2142	4925.6	95.00	0.32	tr_C9ZQ90_
DRB1_0101	529	KPEHYLKDDKLGEMM	YLKDDKLGE	4	0.2138	4947.3	95.00	0.21	tr_C9ZQ90_
DRB1_0101	530	DMDNYLDTNSDWKGF	LDTNSDWKG	5	0.2136	4958.8	95.00	0.37	tr_C9ZQ90_
DRB1_0101	531	VRFGPFSDAECVSGT	FSDAECVSG	5	0.2124	5023.9	95.00	0.20	tr_C9ZQ90_
DRB1_0101	532	DFVVGDFGGECDEGA	FGGECDEGA	6	0.2119	5051.9	95.00	0.36	tr_C9ZQ90_
DRB1_0101	533	SSKTLGDDDPISSYL	LGDDDPISS	4	0.2095	5180.7	95.00	0.65	tr_C9ZQ90_
DRB1_0101	534	FGPFSDAECVSGTSV	FSDAECVSG	3	0.2044	5474.3	95.00	0.36	tr_C9ZQ90_
DRB1_0101	535	ESNIRVDDVRFGPFS	IRVDDVRFG	3	0.2028	5573.8	95.00	0.50	tr_C9ZQ90_
DRB1_0101	536	YLDTNSDWKGFAKPE	LDTNSDWKG	1	0.2001	5738.8	95.00	0.54	tr_C9ZQ90_
DRB1_0101	537	VIDDFVVGDFGGECD	FVVGDFGGE	4	0.1987	5825.8	95.00	0.42	tr_C9ZQ90_
DRB1_0101	538	TESNIRVDDVRFGPF	IRVDDVRFG	4	0.1980	5870.2	95.00	0.46	tr_C9ZQ90_

DRB1_0101	539	SNIRVDDVRFGPFSD	IRVDDVRFG	2	0.1966	5961.3	95.00	0.46	tr_C9ZQ90_
DRB1_0101	540	TSFPHWAEKNTKSDM	WAEKNTKSD	5	0.1959	6002.1	95.00	0.45	tr_C9ZQ90_
DRB1_0101	541	IDDFVVGDFGGECDE	FVVGDFGGE	3	0.1932	6180.7	95.00	0.41	tr_C9ZQ90_
DRB1_0101	542	YTESNIRVDDVRFGP	IRVDDVRFG	5	0.1930	6198.1	100.00	0.37	tr_C9ZQ90_
DRB1_0101	543	RFGPFSDAECVSGTS	FSDAECVSG	4	0.1910	6327.6	100.00	0.35	tr_C9ZQ90_
DRB1_0101	544	RDMDNYLDTNSDWKG	DNYLDTNSD	3	0.1816	7012.0	100.00	0.37	tr_C9ZQ90_
DRB1_0101	545	WSLGARSIEEADDVD	LGARSIEEA	2	0.1803	7111.7	100.00	0.40	tr_C9ZQ90_
DRB1_0101	546	DDFVVGDFGGECDEG	FVVGDFGGE	2	0.1760	7444.0	100.00	0.41	tr_C9ZQ90_
DRB1_0101	547	AKPEHYLKDDKLGEM	EHYLKDDKL	3	0.1713	7837.8	100.00	0.38	tr_C9ZQ90_
DRB1_0101	548	DSSKTLGDDDPISSY	LGDDDPISS	5	0.1678	8140.9	100.00	0.65	tr_C9ZQ90_
DRB1_0101	549	SIEEADDVDKRIFFH	IEEADDVDK	1	0.1652	8368.5	100.00	0.34	tr_C9ZQ90_
DRB1_0101	550	FAKPEHYLKDDKLGE	EHYLKDDKL	4	0.1622	8641.9	100.00	0.35	tr_C9ZQ90_
DRB1_0101	551	ATSFPHWAEKNTKSD	SFPHWAEKN	2	0.1621	8658.0	100.00	0.23	tr_C9ZQ90_
DRB1_0101	552	IEEADDVDKRIFFHS	DVDKRIFFH	5	0.1606	8792.3	100.00	0.26	tr_C9ZQ90_
DRB1_0101	553	QRDMDNYLDTNSDWK	DNYLDTNSD	4	0.1596	8888.3	100.00	0.39	tr_C9ZQ90_
DRB1_0101	554	RSIEEADDVDKRIFF	IEEADDVDK	2	0.1585	8996.5	100.00	0.47	tr_C9ZQ90_
DRB1_0101	555	ARSIEEADDVDKRIF	IEEADDVDK	3	0.1576	9082.8	100.00	0.57	tr_C9ZQ90_
DRB1_0101	556	LDTNSDWKGFAKPEH	LDTNSDWKG	0	0.1555	9300.5	100.00	0.32	tr_C9ZQ90_
DRB1_0101	557	VSLDSSKTLGDDDPI	LDSSKTLGD	2	0.1538	9466.2	100.00	0.37	tr_C9ZQ90_
DRB1_0101	558	DTNSDWKGFAKPEHY	WKGFAKPEH	5	0.1522	9628.9	100.00	0.28	tr_C9ZQ90_
DRB1_0101	559	SLGARSIEEADDVDK	LGARSIEEA	1	0.1501	9856.2	100.00	0.44	tr_C9ZQ90_
DRB1_0101	560	RGGEASEVVDTLDKS	EASEVVDTL	3	0.1482	10063.5	100.00	0.38	tr_C9ZQ90_
DRB1_0101	561	GARSIEEADDVDKRI	IEEADDVDK	4	0.1481	10070.1	100.00	0.55	tr_C9ZQ90_
DRB1_0101	562	LGARSIEEADDVDKR	IEEADDVDK	5	0.1422	10730.7	100.00	0.38	tr_C9ZQ90_
DRB1_0101	563	GGEASEVVDTLDKSL	EASEVVDTL	2	0.1380	11234.0	100.00	0.31	tr_C9ZQ90_
DRB1_0101	564	LDSSKTLGDDDPISS	LGDDDPISS	6	0.1239	13085.8	100.00	0.50	tr_C9ZQ90_
DRB1_0101	565	SLDSSKTLGDDDPIS	LDSSKTLGD	1	0.1000	16948.1	100.00	0.48	tr_C9ZQ90_

Allele: DRB1_0101. Number of high binders 3. Number of weak binders 43. Number of peptides 565

Allele	pos	peptide	core	Of	1-log50k(aff)	affinity(nM)	%Rank	Relia	Identity	Bind Level
DRB1_0101	1	GFSALHMAASAINYH	ALHMAASAI	3	0.8216	6.9	1.30	0.37	tr_D0A254_	WB
DRB1_0101	2	FSALHMAASAINYHI	LHMAASAIN	3	0.8149	7.4	1.50	0.44	tr_D0A254_	WB
DRB1_0101	3	AGFSALHMAASAINY	ALHMAASAI	4	0.8111	7.7	1.60	0.35	tr_D0A254_	WB
DRB1_0101	4	SALHMAASAINYHIV	LHMAASAIN	2	0.7768	11.2	3.50	0.42	tr_D0A254_	WB
DRB1_0101	5	SAGFSALHMAASAIN	FSALHMAAS	3	0.7750	11.4	3.50	0.40	tr_D0A254_	WB
DRB1_0101	6	ALHMAASAINYHIVR	LHMAASAIN	1	0.7328	18.0	6.00	0.42	tr_D0A254_	WB
DRB1_0101	7	NSAGFSALHMAASAI	FSALHMAAS	4	0.7175	21.3	7.50	0.50	tr_D0A254_	WB
DRB1_0101	8	EVQYVLENYPLAQRG	YVLENYPLA	3	0.7152	21.8	7.50	0.66	tr_D0A254_	WB
DRB1_0101	9	GFDIYLPGSDAFYFK	DIYLPGSDA	2	0.7079	23.6	8.50	0.62	tr_D0A254_	WB
DRB1_0101	10	FDIYLPGSDAFYFKR	DIYLPGSDA	1	0.7054	24.2	8.50	0.57	tr_D0A254_	WB
DRB1_0101	11	PEVQYVLENYPLAQR	YVLENYPLA	4	0.7052	24.3	8.50	0.64	tr_D0A254_	WB
DRB1_0101	12	VQYVLENYPLAQRGA	YVLENYPLA	2	0.7033	24.8	8.50	0.67	tr_D0A254_	WB
DRB1_0101	13	YVIMFWAQGQNIKGH	MFWAQGQNI	3	0.7032	24.8	8.50	0.58	tr_D0A254_	WB
DRB1_0101	14	YCVAVQNASLPLLLL	VAVQNASLP	2	0.6994	25.9	9.00	0.29	tr_D0A254_	WB
DRB1_0101	15	GYVIMFWAQGQNIKG	MFWAQGQNI	4	0.6987	26.0	9.00	0.56	tr_D0A254_	WB

DRB1_0101	16	GDTLLMTAAYKGHIH	LLMTAAYKG	3	0.6897	28.7	10.00	0.72	tr_D0A254_
DRB1_0101	17	DTLLMTAAYKGHIHV	LLMTAAYKG	2	0.6897	28.7	10.00	0.65	tr_D0A254_
DRB1_0101	18	DGFDIYLPGSDAFYF	DIYLPGSDA	3	0.6876	29.4	11.00	0.68	tr_D0A254_
DRB1_0101	19	CVAVQNASLPLLLLL	QNASLPLLL	4	0.6849	30.3	11.00	0.22	tr_D0A254_
DRB1_0101	20	LGYVIMFWAQGQNIK	MFWAQGQNI	5	0.6830	30.9	11.00	0.52	tr_D0A254_
DRB1_0101	21	AYCVAVQNASLPLLL	VAVQNASLP	3	0.6810	31.6	11.00	0.40	tr_D0A254_
DRB1_0101	22	TLLMTAAYKGHIHVV	LLMTAAYKG	1	0.6763	33.2	12.00	0.62	tr_D0A254_
DRB1_0101	23	VIMFWAQGQNIKGHE	MFWAQGQNI	2	0.6700	35.5	13.00	0.65	tr_D0A254_
DRB1_0101	24	VAVQNASLPLLLLLA	QNASLPLLL	3	0.6689	36.0	13.00	0.31	tr_D0A254_
DRB1_0101	25	GGDTLLMTAAYKGHI	LLMTAAYKG	4	0.6684	36.2	13.00	0.70	tr_D0A254_
DRB1_0101	26	SAYCVAVQNASLPLL	CVAVQNASL	3	0.6683	36.2	13.00	0.39	tr_D0A254_
DRB1_0101	27	VVEYLLCAGADVDVV	YLLCAGADV	3	0.6593	39.9	14.00	0.60	tr_D0A254_
DRB1_0101	28	QYVLENYPLAQRGAT	YVLENYPLA	1	0.6573	40.7	14.00	0.68	tr_D0A254_
DRB1_0101	29	PLLLLLAEHRPIDIH	LLLAEHRPI	3	0.6568	41.0	14.00	0.44	tr_D0A254_
DRB1_0101	30	HPEVQYVLENYPLAQ	YVLENYPLA	5	0.6560	41.3	14.00	0.65	tr_D0A254_
DRB1_0101	31	DIYLPGSDAFYFKRQ	DIYLPGSDA	0	0.6531	42.7	15.00	0.47	tr_D0A254_
DRB1_0101	32	DVVEYLLCAGADVDV	YLLCAGADV	4	0.6464	45.9	16.00	0.61	tr_D0A254_
DRB1_0101	33	TNSAGFSALHMAASA	FSALHMAAS	5	0.6454	46.4	16.00	0.56	tr_D0A254_
DRB1_0101	34	IVRFLLDHGADPTKK	FLLDHGADP	3	0.6442	47.0	16.00	0.69	tr_D0A254_
DRB1_0101	35	LPLLLLLAEHRPIDI	LLLAEHRPI	4	0.6423	48.0	16.00	0.42	tr_D0A254_
DRB1_0101	36	VEYLLCAGADVDVVD	YLLCAGADV	2	0.6391	49.7	17.00	0.60	tr_D0A254_
DRB1_0101	37	LHMAASAINYHIVRF	AASAINYHI	3	0.6370	50.8	17.00	0.32	tr_D0A254_
DRB1_0101	38	DDGFDIYLPGSDAFY	DIYLPGSDA	4	0.6367	51.0	17.00	0.73	tr_D0A254_
DRB1_0101	39	LLLLLAEHRPIDIHE	LLLAEHRPI	2	0.6366	51.0	17.00	0.47	tr_D0A254_
DRB1_0101	40	VVRFLVDAGADINAT	FLVDAGADI	3	0.6362	51.2	17.00	0.70	tr_D0A254_
DRB1_0101	41	IMFWAQGQNIKGHES	MFWAQGQNI	1	0.6354	51.7	17.00	0.62	tr_D0A254_
DRB1_0101	42	SLGYVIMFWAQGQNI	MFWAQGQNI	6	0.6334	52.8	17.00	0.36	tr_D0A254_
DRB1_0101	43	YSAYCVAVQNASLPL	CVAVQNASL	4	0.6319	53.7	18.00	0.39	tr_D0A254_
DRB1_0101	44	HVVRFLVDAGADINA	FLVDAGADI	4	0.6307	54.4	18.00	0.68	tr_D0A254_
DRB1_0101	45	ADVVEYLLCAGADVD	YLLCAGADV	5	0.6286	55.6	18.00	0.55	tr_D0A254_
DRB1_0101	46	GGGDTLLMTAAYKGH	LLMTAAYKG	5	0.6282	55.9	18.00	0.68	tr_D0A254_
DRB1_0101	47	LLMTAAYKGHIHVVR	LLMTAAYKG	0	0.6278	56.1	18.00	0.41	tr_D0A254_
DRB1_0101	48	AVQNASLPLLLLLAE	QNASLPLLL	2	0.6206	60.7	19.00	0.38	tr_D0A254_
DRB1_0101	49	HIVRFLLDHGADPTK	FLLDHGADP	4	0.6202	60.9	19.00	0.64	tr_D0A254_
DRB1_0101	50	AYKGHIHVVRFLVDA	GHIHVVRFL	3	0.6202	60.9	19.00	0.34	tr_D0A254_
DRB1_0101	51	AAYKGHIHVVRFLVD	YKGHIHVVR	2	0.6178	62.5	20.00	0.27	tr_D0A254_
DRB1_0101	52	VRFLLDHGADPTKKN	FLLDHGADP	2	0.6177	62.6	20.00	0.73	tr_D0A254_
DRB1_0101	53	TAAYKGHIHVVRFLV	YKGHIHVVR	3	0.6160	63.8	20.00	0.32	tr_D0A254_
DRB1_0101	54	VRFLVDAGADINATN	FLVDAGADI	2	0.6125	66.2	20.00	0.74	tr_D0A254_
DRB1_0101	55	SLPLLLLLAEHRPID	LLLAEHRPI	5	0.6122	66.4	21.00	0.35	tr_D0A254_
DRB1_0101	56	IHVVRFLVDAGADIN	FLVDAGADI	5	0.6061	71.0	21.00	0.58	tr_D0A254_
DRB1_0101	57	YHIVRFLLDHGADPT	FLLDHGADP	5	0.6039	72.6	22.00	0.49	tr_D0A254_
DRB1_0101	58	YADVVEYLLCAGADV	YLLCAGADV	6	0.5990	76.6	23.00	0.49	tr_D0A254_
DRB1_0101	59	EYLLCAGADVDVVDT	YLLCAGADV	1	0.5984	77.1	23.00	0.66	tr_D0A254_
DRB1_0101	60	AINYHIVRFLLDHGA	YHIVRFLLD	3	0.5959	79.2	23.00	0.40	tr_D0A254_
DRB1_0101	61	YKGHIHVVRFLVDAG	GHIHVVRFL	2	0.5939	81.0	23.00	0.34	tr_D0A254_
DRB1_0101	62	LLLLAEHRPIDIHER	LLLAEHRPI	1	0.5894	85.0	24.00	0.42	tr_D0A254_
DRB1_0101	63	KGHIHVVRFLVDAGA	HIHVVRFLV	2	0.5889	85.4	24.00	0.28	tr_D0A254_
DRB1_0101	64	ASLPLLLLLAEHRPI	LLLLLAEHR	4	0.5860	88.2	25.00	0.34	tr_D0A254_
DRB1_0101	65	SDAFYFKRQLHYQPY	FYFKRQLHY	3	0.5850	89.1	25.00	0.57	tr_D0A254_

DRB1_0101	66	NHPEVQYVLENYPLA	YVLENYPLA	6	0.5841	90.0	25.00	0.57	tr_D0A254_
DRB1_0101	67	DAFYFKRQLHYQPYT	FYFKRQLHY	2	0.5839	90.2	25.00	0.50	tr_D0A254_
DRB1_0101	68	ATNSAGFSALHMAAS	FSALHMAAS	6	0.5817	92.4	26.00	0.42	tr_D0A254_
DRB1_0101	69	HIHVVRFLVDAGADI	FLVDAGADI	6	0.5802	93.9	26.00	0.43	tr_D0A254_
DRB1_0101	70	LLHWAAYNNSVAICQ	WAAYNNSVA	3	0.5786	95.6	26.00	0.47	tr_D0A254_
DRB1_0101	71	NYHIVRFLLDHGADP	FLLDHGADP	6	0.5779	96.3	26.00	0.25	tr_D0A254_
DRB1_0101	72	SAINYHIVRFLLDHG	YHIVRFLLD	4	0.5759	98.3	26.00	0.41	tr_D0A254_
DRB1_0101	73	HALLHWAAYNNSVAI	WAAYNNSVA	5	0.5752	99.1	27.00	0.35	tr_D0A254_
DRB1_0101	74	ASAINYHIVRFLLDH	YHIVRFLLD	5	0.5747	99.6	27.00	0.40	tr_D0A254_
DRB1_0101	75	LSPIVDMKSVGTDDG	IVDMKSVGT	3	0.5747	99.6	27.00	0.89	tr_D0A254_
DRB1_0101	76	VQNASLPLLLLLAEH	NASLPLLLL	2	0.5745	99.9	27.00	0.41	tr_D0A254_
DRB1_0101	77	INYHIVRFLLDHGAD	YHIVRFLLD	2	0.5743	100.1	27.00	0.42	tr_D0A254_
DRB1_0101	78	LHWAAYNNSVAICQY	WAAYNNSVA	2	0.5739	100.5	27.00	0.41	tr_D0A254_
DRB1_0101	79	RFLLDHGADPTKKNH	FLLDHGADP	1	0.5738	100.7	27.00	0.71	tr_D0A254_
DRB1_0101	80	QNASLPLLLLLAEHR	NASLPLLLL	1	0.5724	102.1	27.00	0.38	tr_D0A254_
DRB1_0101	81	GYSAYCVAVQNASLP	CVAVQNASL	5	0.5724	102.2	27.00	0.41	tr_D0A254_
DRB1_0101	82	ALLHWAAYNNSVAIC	WAAYNNSVA	4	0.5709	103.8	27.00	0.41	tr_D0A254_
DRB1_0101	83	RFLVDAGADINATNS	FLVDAGADI	1	0.5676	107.6	28.00	0.75	tr_D0A254_
DRB1_0101	84	TDDGFDIYLPGSDAF	DIYLPGSDA	5	0.5661	109.3	28.00	0.81	tr_D0A254_
DRB1_0101	85	MLSPIVDMKSVGTDD	IVDMKSVGT	4	0.5652	110.4	28.00	0.84	tr_D0A254_
DRB1_0101	86	AASAINYHIVRFLLD	INYHIVRFL	4	0.5642	111.6	29.00	0.32	tr_D0A254_
DRB1_0101	87	MTAAYKGHIHVVRFL	YKGHIHVVR	4	0.5614	115.1	29.00	0.40	tr_D0A254_
DRB1_0101	88	AFYFKRQLHYQPYTP	FYFKRQLHY	1	0.5599	117.0	29.00	0.46	tr_D0A254_
DRB1_0101	89	YVLENYPLAQRGATG	YVLENYPLA	0	0.5549	123.4	30.00	0.55	tr_D0A254_
DRB1_0101	90	NYPLAQRGATGMAAG	LAQRGATGM	3	0.5547	123.8	30.00	0.41	tr_D0A254_
DRB1_0101	91	SPIVDMKSVGTDDGF	IVDMKSVGT	2	0.5532	125.8	31.00	0.88	tr_D0A254_
DRB1_0101	92	HWAAYNNSVAICQYL	YNNSVAICQ	4	0.5489	131.7	31.00	0.37	tr_D0A254_
DRB1_0101	93	NASLPLLLLLAEHRP	LLLLLAEHR	5	0.5488	131.8	31.00	0.37	tr_D0A254_
DRB1_0101	94	QGYSAYCVAVQNASL	CVAVQNASL	6	0.5488	131.9	31.00	0.32	tr_D0A254_
DRB1_0101	95	GHIHVVRFLVDAGAD	VRFLVDAGA	5	0.5483	132.6	32.00	0.19	tr_D0A254_
DRB1_0101	96	GHALLHWAAYNNSVA	LHWAAYNNS	4	0.5464	135.3	32.00	0.26	tr_D0A254_
DRB1_0101	97	WAAYNNSVAICQYLV	YNNSVAICQ	3	0.5436	139.5	32.00	0.44	tr_D0A254_
DRB1_0101	98	MFWAQGQNIKGHESV	MFWAQGQNI	0	0.5429	140.6	33.00	0.46	tr_D0A254_
DRB1_0101	99	TSVHMRISPSNLEEA	HMRISPSNL	3	0.5427	140.8	33.00	0.43	tr_D0A254_
DRB1_0101	100	VGGGDTLLMTAAYKG	LLMTAAYKG	6	0.5419	142.0	33.00	0.53	tr_D0A254_
DRB1_0101	101	KTSVHMRISPSNLEE	HMRISPSNL	4	0.5416	142.6	33.00	0.35	tr_D0A254_
DRB1_0101	102	HMAASAINYHIVRFL	AASAINYHI	2	0.5385	147.4	33.00	0.41	tr_D0A254_
DRB1_0101	103	GSLGYVIMFWAQGQN	YVIMFWAQG	4	0.5351	153.0	34.00	0.49	tr_D0A254_
DRB1_0101	104	HQGKMHKTSVHMRIS	MHKTSVHMR	4	0.5337	155.3	34.00	0.38	tr_D0A254_
DRB1_0101	105	MAASAINYHIVRFLL	AASAINYHI	1	0.5334	155.7	34.00	0.32	tr_D0A254_
DRB1_0101	106	HKTSVHMRISPSNLE	SVHMRISPS	3	0.5318	158.5	35.00	0.38	tr_D0A254_
DRB1_0101	107	AAYNNSVAICQYLVE	YNNSVAICQ	2	0.5301	161.4	35.00	0.46	tr_D0A254_
DRB1_0101	108	LMTAAYKGHIHVVRF	YKGHIHVVR	5	0.5290	163.4	35.00	0.32	tr_D0A254_
DRB1_0101	109	ENYPLAQRGATGMAA	PLAQRGATG	3	0.5289	163.6	35.00	0.35	tr_D0A254_
DRB1_0101	110	GSDAFYFKRQLHYQP	FYFKRQLHY	4	0.5288	163.8	35.00	0.62	tr_D0A254_
DRB1_0101	111	YLVEVCKMDINVVGK	VCKMDINVV	4	0.5280	165.2	35.00	0.70	tr_D0A254_
DRB1_0101	112	YPLAQRGATGMAAGD	LAQRGATGM	2	0.5275	166.0	36.00	0.40	tr_D0A254_
DRB1_0101	113	MGHALLHWAAYNNSV	GHALLHWAA	1	0.5241	172.3	36.00	0.34	tr_D0A254_
DRB1_0101	114	SGSLGYVIMFWAQGQ	YVIMFWAQG	5	0.5236	173.2	36.00	0.44	tr_D0A254_
DRB1_0101	115	LVEVCKMDINVVGKD	VCKMDINVV	3	0.5229	174.5	36.00	0.70	tr_D0A254_

DRB1_0101	116	RDEMGHALLHWAAYN	GHALLHWAA	4	0.5214	177.4	37.00	0.37	tr_D0A254_
DRB1_0101	117	YLLCAGADVDVVDTD	YLLCAGADV	0	0.5212	177.7	37.00	0.60	tr_D0A254_
DRB1_0101	118	ERDEMGHALLHWAAY	GHALLHWAA	5	0.5205	179.1	37.00	0.31	tr_D0A254_
DRB1_0101	119	AYNNSVAICQYLVEV	YNNSVAICQ	1	0.5164	187.3	38.00	0.35	tr_D0A254_
DRB1_0101	120	EMGHALLHWAAYNNS	GHALLHWAA	2	0.5159	188.3	38.00	0.44	tr_D0A254_
DRB1_0101	121	QGKMHKTSVHMRISP	MHKTSVHMR	3	0.5120	196.4	39.00	0.38	tr_D0A254_
DRB1_0101	122	VEVCKMDINVVGKDG	VCKMDINVV	2	0.5076	205.9	39.00	0.64	tr_D0A254_
DRB1_0101	123	HERDEMGHALLHWAA	ERDEMGHAL	1	0.5064	208.7	40.00	0.29	tr_D0A254_
DRB1_0101	124	KKNHQGYSAYCVAVQ	HQGYSAYCV	3	0.5062	209.1	40.00	0.50	tr_D0A254_
DRB1_0101	125	ASGSLGYVIMFWAQG	YVIMFWAQG	6	0.5036	215.2	40.00	0.36	tr_D0A254_
DRB1_0101	126	GKMHKTSVHMRISPS	MHKTSVHMR	2	0.5031	216.3	40.00	0.31	tr_D0A254_
DRB1_0101	127	FYFKRQLHYQPYTPC	FKRQLHYQP	2	0.5028	216.8	40.00	0.36	tr_D0A254_
DRB1_0101	128	SVHMRISPSNLEEAC	HMRISPSNL	2	0.5023	218.2	41.00	0.46	tr_D0A254_
DRB1_0101	129	DEMGHALLHWAAYNN	GHALLHWAA	3	0.5009	221.4	41.00	0.47	tr_D0A254_
DRB1_0101	130	TDGYTALNYAKHRNH	YTALNYAKH	3	0.5009	221.5	41.00	0.63	tr_D0A254_
DRB1_0101	131	GATGMAAGDGLLGNQ	MAAGDGLLG	4	0.4990	226.0	41.00	0.63	tr_D0A254_
DRB1_0101	132	TKKNHQGYSAYCVAV	HQGYSAYCV	4	0.4983	227.7	41.00	0.50	tr_D0A254_
DRB1_0101	133	MHKTSVHMRISPSNL	SVHMRISPS	4	0.4981	228.3	41.00	0.47	tr_D0A254_
DRB1_0101	134	GHQGKMHKTSVHMRI	MHKTSVHMR	5	0.4980	228.4	41.00	0.35	tr_D0A254_
DRB1_0101	135	PGSDAFYFKRQLHYQ	FYFKRQLHY	5	0.4958	234.1	42.00	0.71	tr_D0A254_
DRB1_0101	136	EEACASGSLGYVIMF	ASGSLGYVI	4	0.4913	245.6	43.00	0.40	tr_D0A254_
DRB1_0101	137	EACASGSLGYVIMFW	ASGSLGYVI	3	0.4913	245.7	43.00	0.48	tr_D0A254_
DRB1_0101	138	KMHKTSVHMRISPSN	SVHMRISPS	5	0.4909	246.8	43.00	0.37	tr_D0A254_
DRB1_0101	139	PIVDMKSVGTDDGFD	IVDMKSVGT	1	0.4907	247.4	43.00	0.88	tr_D0A254_
DRB1_0101	140	IYLPGSDAFYFKRQL	YLPGSDAFY	1	0.4876	255.8	44.00	0.50	tr_D0A254_
DRB1_0101	141	NATNSAGFSALHMAA	SAGFSALHM	4	0.4860	260.2	44.00	0.58	tr_D0A254_
DRB1_0101	142	QYLVEVCKMDINVVG	VCKMDINVV	5	0.4860	260.2	44.00	0.56	tr_D0A254_
DRB1_0101	143	KNHQGYSAYCVAVQN	HQGYSAYCV	2	0.4818	272.1	45.00	0.48	tr_D0A254_
DRB1_0101	144	RGATGMAAGDGLLGN	MAAGDGLLG	5	0.4817	272.5	45.00	0.51	tr_D0A254_
DRB1_0101	145	ACASGSLGYVIMFWA	ASGSLGYVI	2	0.4758	290.4	46.00	0.46	tr_D0A254_
DRB1_0101	146	PTKKNHQGYSAYCVA	HQGYSAYCV	5	0.4749	293.5	46.00	0.41	tr_D0A254_
DRB1_0101	147	LEEACASGSLGYVIM	ASGSLGYVI	5	0.4747	294.1	46.00	0.32	tr_D0A254_
DRB1_0101	148	IHERDEMGHALLHWA	ERDEMGHAL	2	0.4735	297.8	47.00	0.47	tr_D0A254_
DRB1_0101	149	HQGYSAYCVAVQNAS	YSAYCVAVQ	3	0.4729	299.8	47.00	0.20	tr_D0A254_
DRB1_0101	150	DGYTALNYAKHRNHP	YTALNYAKH	2	0.4720	302.8	47.00	0.61	tr_D0A254_
DRB1_0101	151	LENYPLAQRGATGMA	PLAQRGATG	4	0.4716	304.0	47.00	0.38	tr_D0A254_
DRB1_0101	152	PLAQRGATGMAAGDG	QRGATGMAA	3	0.4708	306.8	47.00	0.38	tr_D0A254_
DRB1_0101	153	INATNSAGFSALHMA	SAGFSALHM	5	0.4706	307.5	47.00	0.48	tr_D0A254_
DRB1_0101	154	NHQGYSAYCVAVQNA	HQGYSAYCV	1	0.4704	307.9	47.00	0.33	tr_D0A254_
DRB1_0101	155	CASGSLGYVIMFWAQ	ASGSLGYVI	1	0.4702	308.6	47.00	0.34	tr_D0A254_
DRB1_0101	156	EVCKMDINVVGKDGR	VCKMDINVV	1	0.4694	311.5	47.00	0.60	tr_D0A254_
DRB1_0101	157	DTDGYTALNYAKHRN	YTALNYAKH	4	0.4689	313.2	47.00	0.68	tr_D0A254_
DRB1_0101	158	GYTALNYAKHRNHPE	YTALNYAKH	1	0.4653	325.4	48.00	0.49	tr_D0A254_
DRB1_0101	159	GYADVVEYLLCAGAD	VEYLLCAGA	5	0.4618	338.2	49.00	0.40	tr_D0A254_
DRB1_0101	160	NLEEACASGSLGYVI	LEEACASGS	1	0.4597	345.9	49.00	0.34	tr_D0A254_
DRB1_0101	161	INVVGKDGRTALVWA	VVGKDGRTA	2	0.4595	346.5	49.00	0.70	tr_D0A254_
DRB1_0101	162	FLLDHGADPTKKNHQ	FLLDHGADP	0	0.4571	355.6	50.00	0.58	tr_D0A254_
DRB1_0101	163	DINVVGKDGRTALVW	VVGKDGRTA	3	0.4540	367.9	55.00	0.75	tr_D0A254_
DRB1_0101	164	GTDDGFDIYLPGSDA	DIYLPGSDA	6	0.4532	370.9	55.00	0.74	tr_D0A254_
DRB1_0101	165	LLLAEHRPIDIHERD	LAEHRPIDI	2	0.4519	376.1	55.00	0.51	tr_D0A254_

DRB1_0101	166	SNLEEACASGSLGYV	LEEACASGS	2	0.4512	379.3	55.00	0.52	tr_D0A254_
DRB1_0101	167	NVVGKDGRTALVWAA	VVGKDGRTA	1	0.4510	380.2	55.00	0.50	tr_D0A254_
DRB1_0101	168	DIHERDEMGHALLHW	ERDEMGHAL	3	0.4488	389.1	55.00	0.55	tr_D0A254_
DRB1_0101	169	YNNSVAICQYLVEVC	SVAICQYLV	3	0.4488	389.2	55.00	0.38	tr_D0A254_
DRB1_0101	170	YFKRQLHYQPYTPCA	LHYQPYTPC	5	0.4487	389.4	55.00	0.39	tr_D0A254_
DRB1_0101	171	FKRQLHYQPYTPCAF	LHYQPYTPC	4	0.4481	392.0	55.00	0.50	tr_D0A254_
DRB1_0101	172	FLVDAGADINATNSA	FLVDAGADI	0	0.4461	400.7	55.00	0.64	tr_D0A254_
DRB1_0101	173	VHMRISPSNLEEACA	HMRISPSNL	1	0.4458	402.0	55.00	0.47	tr_D0A254_
DRB1_0101	174	CQYLVEVCKMDINVV	VCKMDINVV	6	0.4448	406.2	55.00	0.35	tr_D0A254_
DRB1_0101	175	YTALNYAKHRNHPEV	YAKHRNHPE	5	0.4440	409.9	55.00	0.34	tr_D0A254_
DRB1_0101	176	DINATNSAGFSALHM	SAGFSALHM	6	0.4405	425.8	55.00	0.32	tr_D0A254_
DRB1_0101	177	PSNLEEACASGSLGY	LEEACASGS	3	0.4391	432.4	55.00	0.62	tr_D0A254_
DRB1_0101	178	EGYADVVEYLLCAGA	VEYLLCAGA	6	0.4377	438.6	55.00	0.29	tr_D0A254_
DRB1_0101	179	RQLHYQPYTPCAFPV	LHYQPYTPC	2	0.4359	447.4	55.00	0.38	tr_D0A254_
DRB1_0101	180	LGHQGKMHKTSVHMR	KMHKTSVHM	5	0.4347	453.2	55.00	0.38	tr_D0A254_
DRB1_0101	181	PVEWLGHQGKMHKTS	WLGHQGKMH	3	0.4334	459.9	55.00	0.60	tr_D0A254_
DRB1_0101	182	VVGKDGRTALVWAAR	GRTALVWAA	5	0.4307	473.3	60.00	0.34	tr_D0A254_
DRB1_0101	183	VVGGGDTLLMTAAYK	GDTLLMTAA	4	0.4306	474.0	60.00	0.25	tr_D0A254_
DRB1_0101	184	QRGATGMAAGDGLLG	MAAGDGLLG	6	0.4298	477.9	60.00	0.30	tr_D0A254_
DRB1_0101	185	NSVAICQYLVEVCKM	SVAICQYLV	1	0.4288	483.1	60.00	0.28	tr_D0A254_
DRB1_0101	186	NNSVAICQYLVEVCK	SVAICQYLV	2	0.4261	497.5	60.00	0.36	tr_D0A254_
DRB1_0101	187	MDINVVGKDGRTALV	VVGKDGRTA	4	0.4258	498.8	60.00	0.70	tr_D0A254_
DRB1_0101	188	QLHYQPYTPCAFPVE	LHYQPYTPC	1	0.4254	501.3	60.00	0.29	tr_D0A254_
DRB1_0101	189	SVVGGGDTLLMTAAY	VVGGGDTLL	1	0.4252	502.4	60.00	0.28	tr_D0A254_
DRB1_0101	190	LPGSDAFYFKRQLHY	FYFKRQLHY	6	0.4245	506.2	60.00	0.63	tr_D0A254_
DRB1_0101	191	VEWLGHQGKMHKTSV	WLGHQGKMH	2	0.4232	513.1	60.00	0.62	tr_D0A254_
DRB1_0101	192	ESVVGGGDTLLMTAA	VVGGGDTLL	2	0.4224	518.0	60.00	0.40	tr_D0A254_
DRB1_0101	193	HESVVGGGDTLLMTA	VVGGGDTLL	3	0.4217	521.9	60.00	0.50	tr_D0A254_
DRB1_0101	194	KRQLHYQPYTPCAFP	LHYQPYTPC	3	0.4211	525.3	60.00	0.51	tr_D0A254_
DRB1_0101	195	VDTDGYTALNYAKHR	YTALNYAKH	5	0.4208	526.6	60.00	0.63	tr_D0A254_
DRB1_0101	196	FPVEWLGHQGKMHKT	WLGHQGKMH	4	0.4197	533.2	60.00	0.56	tr_D0A254_
DRB1_0101	197	IDIHERDEMGHALLH	ERDEMGHAL	4	0.4187	539.1	60.00	0.56	tr_D0A254_
DRB1_0101	198	DPTKKNHQGYSAYCV	HQGYSAYCV	6	0.4113	583.9	60.00	0.32	tr_D0A254_
DRB1_0101	199	AICQYLVEVCKMDIN	YLVEVCKMD	4	0.4092	597.3	60.00	0.33	tr_D0A254_
DRB1_0101	200	QGQNIKGHESVVGGG	IKGHESVVG	4	0.4076	607.7	65.00	0.57	tr_D0A254_
DRB1_0101	201	NYAKHRNHPEVQYVL	YAKHRNHPE	1	0.4071	610.9	65.00	0.41	tr_D0A254_
DRB1_0101	202	SPSNLEEACASGSLG	LEEACASGS	4	0.4051	624.1	65.00	0.60	tr_D0A254_
DRB1_0101	203	ICQYLVEVCKMDINV	YLVEVCKMD	3	0.4049	625.8	65.00	0.41	tr_D0A254_
DRB1_0101	204	VLENYPLAQRGATGM	PLAQRGATG	5	0.4047	627.3	65.00	0.34	tr_D0A254_
DRB1_0101	205	SVAICQYLVEVCKMD	CQYLVEVCK	4	0.4012	651.1	65.00	0.24	tr_D0A254_
DRB1_0101	206	REGYADVVEYLLCAG	GYADVVEYL	2	0.4009	653.6	65.00	0.40	tr_D0A254_
DRB1_0101	207	DGRTALVWAAREGYA	GRTALVWAA	1	0.4002	658.7	65.00	0.32	tr_D0A254_
DRB1_0101	208	GRTALVWAAREGYAD	TALVWAARE	2	0.3989	667.4	65.00	0.25	tr_D0A254_
DRB1_0101	209	LAQRGATGMAAGDGL	QRGATGMAA	2	0.3987	668.8	65.00	0.45	tr_D0A254_
DRB1_0101	210	ALNYAKHRNHPEVQY	YAKHRNHPE	3	0.3984	671.0	65.00	0.67	tr_D0A254_
DRB1_0101	211	AQRGATGMAAGDGLL	QRGATGMAA	1	0.3969	682.0	65.00	0.32	tr_D0A254_
DRB1_0101	212	VAICQYLVEVCKMDI	YLVEVCKMD	5	0.3966	684.4	65.00	0.28	tr_D0A254_
DRB1_0101	213	AREGYADVVEYLLCA	GYADVVEYL	3	0.3961	688.1	65.00	0.45	tr_D0A254_
DRB1_0101	214	GQNIKGHESVVGGGD	IKGHESVVG	3	0.3947	698.7	65.00	0.63	tr_D0A254_
DRB1_0101	215	KDGRTALVWAAREGY	GRTALVWAA	2	0.3936	707.1	65.00	0.47	tr_D0A254_

DRB1_0101	216	GHESVVGGGDTLLMT	VVGGGDTLL	4	0.3924	716.2	65.00	0.47	tr_D0A254_
DRB1_0101	217	AFPVEWLGHQGKMHK	WLGHQGKMH	5	0.3901	734.1	65.00	0.51	tr_D0A254_
DRB1_0101	218	TALNYAKHRNHPEVQ	YAKHRNHPE	4	0.3901	734.6	65.00	0.58	tr_D0A254_
DRB1_0101	219	VGKDGRTALVWAARE	GRTALVWAA	4	0.3898	736.9	65.00	0.49	tr_D0A254_
DRB1_0101	220	KHRNHPEVQYVLENY	NHPEVQYVL	3	0.3893	741.0	65.00	0.64	tr_D0A254_
DRB1_0101	221	YAKHRNHPEVQYVLE	NHPEVQYVL	5	0.3889	743.8	65.00	0.43	tr_D0A254_
DRB1_0101	222	LHYQPYTPCAFPVEW	PYTPCAFPV	4	0.3887	746.0	65.00	0.29	tr_D0A254_
DRB1_0101	223	RTALVWAAREGYADV	VWAAREGYA	4	0.3879	751.9	65.00	0.32	tr_D0A254_
DRB1_0101	224	GKDGRTALVWAAREG	GRTALVWAA	3	0.3879	752.2	65.00	0.51	tr_D0A254_
DRB1_0101	225	TALVWAAREGYADVV	VWAAREGYA	3	0.3877	753.6	65.00	0.39	tr_D0A254_
DRB1_0101	226	LNYAKHRNHPEVQYV	YAKHRNHPE	2	0.3868	760.8	65.00	0.62	tr_D0A254_
DRB1_0101	227	ADINATNSAGFSALH	INATNSAGF	2	0.3862	766.0	65.00	0.40	tr_D0A254_
DRB1_0101	228	KMDINVVGKDGRTAL	VVGKDGRTA	5	0.3847	778.2	65.00	0.65	tr_D0A254_
DRB1_0101	229	AQGQNIKGHESVVGG	IKGHESVVG	5	0.3847	778.5	65.00	0.54	tr_D0A254_
DRB1_0101	230	EWLGHQGKMHKTSVH	WLGHQGKMH	1	0.3835	788.7	70.00	0.57	tr_D0A254_
DRB1_0101	231	YLPGSDAFYFKRQLH	LPGSDAFYF	1	0.3822	799.8	70.00	0.41	tr_D0A254_
DRB1_0101	232	VCKMDINVVGKDGRT	VCKMDINVV	0	0.3819	802.2	70.00	0.44	tr_D0A254_
DRB1_0101	233	HMRISPSNLEEACAS	MRISPSNLE	1	0.3804	815.6	70.00	0.46	tr_D0A254_
DRB1_0101	234	AKHRNHPEVQYVLEN	NHPEVQYVL	4	0.3796	823.0	70.00	0.58	tr_D0A254_
DRB1_0101	235	HYQPYTPCAFPVEWL	PYTPCAFPV	3	0.3770	846.4	70.00	0.38	tr_D0A254_
DRB1_0101	236	RNHPEVQYVLENYPL	NHPEVQYVL	1	0.3749	866.0	70.00	0.39	tr_D0A254_
DRB1_0101	237	IVDMKSVGTDDGFDI	IVDMKSVGT	0	0.3734	879.6	70.00	0.73	tr_D0A254_
DRB1_0101	238	ISPSNLEEACASGSL	LEEACASGS	5	0.3705	907.9	70.00	0.55	tr_D0A254_
DRB1_0101	239	ALVWAAREGYADVVE	VWAAREGYA	2	0.3689	923.4	70.00	0.37	tr_D0A254_
DRB1_0101	240	PIDIHERDEMGHALL	ERDEMGHAL	5	0.3686	927.0	70.00	0.62	tr_D0A254_
DRB1_0101	241	QNIKGHESVVGGGDT	IKGHESVVG	2	0.3678	934.2	70.00	0.67	tr_D0A254_
DRB1_0101	242	WLGHQGKMHKTSVHM	WLGHQGKMH	0	0.3675	937.3	70.00	0.30	tr_D0A254_
DRB1_0101	243	FWAQGQNIKGHESVV	WAQGQNIKG	1	0.3672	941.3	70.00	0.50	tr_D0A254_
DRB1_0101	244	GADINATNSAGFSAL	INATNSAGF	3	0.3671	941.3	70.00	0.52	tr_D0A254_
DRB1_0101	245	WAAREGYADVVEYLL	GYADVVEYL	5	0.3666	946.8	70.00	0.44	tr_D0A254_
DRB1_0101	246	YQPYTPCAFPVEWLG	PYTPCAFPV	2	0.3575	1045.0	75.00	0.37	tr_D0A254_
DRB1_0101	247	AAREGYADVVEYLLC	GYADVVEYL	4	0.3544	1080.2	75.00	0.50	tr_D0A254_
DRB1_0101	248	WAQGQNIKGHESVVG	IKGHESVVG	6	0.3493	1141.5	75.00	0.33	tr_D0A254_
DRB1_0101	249	KGHESVVGGGDTLLM	VVGGGDTLL	5	0.3460	1183.9	75.00	0.44	tr_D0A254_
DRB1_0101	250	LVWAAREGYADVVEY	VWAAREGYA	1	0.3438	1212.1	75.00	0.32	tr_D0A254_
DRB1_0101	251	CKMDINVVGKDGRTA	VVGKDGRTA	6	0.3401	1261.1	75.00	0.35	tr_D0A254_
DRB1_0101	252	QPYTPCAFPVEWLGH	PYTPCAFPV	1	0.3395	1270.2	75.00	0.32	tr_D0A254_
DRB1_0101	253	HRNHPEVQYVLENYP	NHPEVQYVL	2	0.3383	1286.2	75.00	0.61	tr_D0A254_
DRB1_0101	254	VWAAREGYADVVEYL	GYADVVEYL	6	0.3377	1294.1	75.00	0.26	tr_D0A254_
DRB1_0101	255	VVDTDGYTALNYAKH	YTALNYAKH	6	0.3301	1406.0	80.00	0.43	tr_D0A254_
DRB1_0101	256	CAFPVEWLGHQGKMH	WLGHQGKMH	6	0.3258	1472.9	80.00	0.38	tr_D0A254_
DRB1_0101	257	NIKGHESVVGGGDTL	IKGHESVVG	1	0.3206	1557.7	80.00	0.60	tr_D0A254_
DRB1_0101	258	PYTPCAFPVEWLGHQ	PCAFPVEWL	3	0.3074	1796.4	80.00	0.36	tr_D0A254_
DRB1_0101	259	AGADINATNSAGFSA	INATNSAGF	4	0.3050	1844.4	85.00	0.60	tr_D0A254_
DRB1_0101	260	LLAEHRPIDIHERDE	LAEHRPIDI	1	0.2990	1967.4	85.00	0.83	tr_D0A254_
DRB1_0101	261	IKGHESVVGGGDTLL	VVGGGDTLL	6	0.2987	1975.0	85.00	0.31	tr_D0A254_
DRB1_0101	262	RISPSNLEEACASGS	LEEACASGS	6	0.2968	2015.0	85.00	0.35	tr_D0A254_
DRB1_0101	263	ADPTKKNHQGYSAYC	KKNHQGYSA	4	0.2923	2114.9	85.00	0.55	tr_D0A254_
DRB1_0101	264	VDVVDTDGYTALNYA	VVDTDGYTA	2	0.2850	2289.8	85.00	0.61	tr_D0A254_
DRB1_0101	265	DVVDTDGYTALNYAK	VVDTDGYTA	1	0.2798	2421.5	85.00	0.47	tr_D0A254_

DRB1_0101	266	LLCAGADVDVVDTDG	CAGADVDVV	2	0.2784	2460.0	85.00	0.36	tr_D0A254_
DRB1_0101	267	RPIDIHERDEMGHAL	ERDEMGHAL	6	0.2767	2503.4	85.00	0.52	tr_D0A254_
DRB1_0101	268	DVDVVDTDGYTALNY	VVDTDGYTA	3	0.2729	2609.3	90.00	0.68	tr_D0A254_
DRB1_0101	269	MRISPSNLEEACASG	MRISPSNLE	0	0.2716	2647.9	90.00	0.31	tr_D0A254_
DRB1_0101	270	PCAFPVEWLGHQGKM	VEWLGHQGK	5	0.2689	2726.3	90.00	0.37	tr_D0A254_
DRB1_0101	271	YTPCAFPVEWLGHQG	PCAFPVEWL	2	0.2628	2910.2	90.00	0.39	tr_D0A254_
DRB1_0101	272	TPCAFPVEWLGHQGK	PCAFPVEWL	1	0.2581	3062.7	90.00	0.31	tr_D0A254_
DRB1_0101	273	DAGADINATNSAGFS	INATNSAGF	5	0.2532	3230.5	90.00	0.60	tr_D0A254_
DRB1_0101	274	GADPTKKNHQGYSAY	KKNHQGYSA	5	0.2471	3450.0	90.00	0.58	tr_D0A254_
DRB1_0101	275	ADVDVVDTDGYTALN	VVDTDGYTA	4	0.2422	3636.4	90.00	0.62	tr_D0A254_
DRB1_0101	276	MKSVGTDDGFDIYLP	VGTDDGFDI	3	0.2136	4957.0	95.00	0.58	tr_D0A254_
DRB1_0101	277	DMKSVGTDDGFDIYL	VGTDDGFDI	4	0.2134	4969.5	95.00	0.65	tr_D0A254_
DRB1_0101	278	KSVGTDDGFDIYLPG	VGTDDGFDI	2	0.2121	5038.0	95.00	0.52	tr_D0A254_
DRB1_0101	279	LLDHGADPTKKNHQG	DHGADPTKK	2	0.2063	5362.2	95.00	0.31	tr_D0A254_
DRB1_0101	280	GADVDVVDTDGYTAL	VVDTDGYTA	5	0.2054	5415.4	95.00	0.59	tr_D0A254_
DRB1_0101	281	VGTDDGFDIYLPGSD	FDIYLPGSD	6	0.2007	5702.6	95.00	0.21	tr_D0A254_
DRB1_0101	282	SVGTDDGFDIYLPGS	VGTDDGFDI	1	0.2007	5703.0	95.00	0.40	tr_D0A254_
DRB1_0101	283	LAEHRPIDIHERDEM	LAEHRPIDI	0	0.1960	5998.4	95.00	0.62	tr_D0A254_
DRB1_0101	284	VDAGADINATNSAGF	INATNSAGF	6	0.1954	6039.3	95.00	0.37	tr_D0A254_
DRB1_0101	285	LVDAGADINATNSAG	DAGADINAT	2	0.1897	6422.7	100.00	0.31	tr_D0A254_
DRB1_0101	286	LCAGADVDVVDTDGY	CAGADVDVV	1	0.1820	6976.6	100.00	0.46	tr_D0A254_
DRB1_0101	287	VDMKSVGTDDGFDIY	VGTDDGFDI	5	0.1818	6992.6	100.00	0.53	tr_D0A254_
DRB1_0101	288	HGADPTKKNHQGYSA	KKNHQGYSA	6	0.1603	8829.1	100.00	0.48	tr_D0A254_
DRB1_0101	289	HRPIDIHERDEMGHA	IDIHERDEM	3	0.1543	9412.7	100.00	0.36	tr_D0A254_
DRB1_0101	290	LDHGADPTKKNHQGY	DHGADPTKK	1	0.1430	10645.3	100.00	0.44	tr_D0A254_
DRB1_0101	291	AGADVDVVDTDGYTA	VVDTDGYTA	6	0.1251	12912.5	100.00	0.50	tr_D0A254_
DRB1_0101	292	EHRPIDIHERDEMGH	IDIHERDEM	4	0.1250	12929.0	100.00	0.37	tr_D0A254_
DRB1_0101	293	DHGADPTKKNHQGYS	DHGADPTKK	0	0.1147	14456.1	100.00	0.31	tr_D0A254_
DRB1_0101	294	CAGADVDVVDTDGYT	CAGADVDVV	0	0.1132	14685.0	100.00	0.41	tr_D0A254_
DRB1_0101	295	AEHRPIDIHERDEMG	IDIHERDEM	5	0.1097	15259.2	100.00	0.40	tr_D0A254_

Allele: DRB1_0101. Number of high binders 0. Number of weak binders 15. Number of peptides 295

Allele	pos	peptide	core	Of	1-log50k(aff)	affinity(nM)	%Rank	Relia	Identity	Bind Level
DRB1_0101	1	FLPFLSTRNSVVKLN	FLSTRNSVV	3	0.9116	2.6	0.05	0.92	tr_D0A254_	SB
DRB1_0101	2	PFLPFLSTRNSVVKL	FLSTRNSVV	4	0.9092	2.7	0.05	0.87	tr_D0A254_	SB
DRB1_0101	3	LPFLSTRNSVVKLNT	FLSTRNSVV	2	0.8956	3.1	0.10	0.93	tr_D0A254_	SB
DRB1_0101	4	EPFLPFLSTRNSVVK	FLSTRNSVV	5	0.8895	3.3	0.15	0.85	tr_D0A254_	SB
DRB1_0101	5	PFLSTRNSVVKLNTS	FLSTRNSVV	1	0.8643	4.3	0.40	0.92	tr_D0A254_	SB
DRB1_0101	6	FEPFLPFLSTRNSVV	FLSTRNSVV	6	0.8247	6.7	1.20	0.71	tr_D0A254_	WB
DRB1_0101	7	FLSTRNSVVKLNTSL	FLSTRNSVV	0	0.7590	13.6	4.50	0.84	tr_D0A254_	WB
DRB1_0101	8	GFEPFLPFLSTRNSV	FLPFLSTRN	4	0.6484	44.9	15.00	0.54	tr_D0A254_	
DRB1_0101	9	MGFEPFLPFLSTRNS	FLPFLSTRN	5	0.6291	55.3	18.00	0.49	tr_D0A254_	
DRB1_0101	10	RNSVVKLNTSLNTTD	VVKLNTSLN	3	0.6168	63.2	20.00	0.65	tr_D0A254_	
DRB1_0101	11	TRNSVVKLNTSLNTT	VVKLNTSLN	4	0.6146	64.7	20.00	0.67	tr_D0A254_	
DRB1_0101	12	NSVVKLNTSLNTTDK	VVKLNTSLN	2	0.5861	88.1	25.00	0.57	tr_D0A254_	

DRB1_0101	13	STRNSVVKLNTSLNT	VVKLNTSLN	5	0.5605	116.2	29.00	0.70	tr_D0A254_
DRB1_0101	14	SVVKLNTSLNTTDKD	VVKLNTSLN	1	0.5211	177.9	37.00	0.55	tr_D0A254_
DRB1_0101	15	LSTRNSVVKLNTSLN	VVKLNTSLN	6	0.4549	364.2	50.00	0.58	tr_D0A254_
DRB1_0101	16	VVKLNTSLNTTDKDL	VKLNTSLNT	1	0.3913	725.0	65.00	0.41	tr_D0A254_
DRB1_0101	17	VKLNTSLNTTDKDLH	VKLNTSLNT	0	0.2474	3438.2	90.00	0.38	tr_D0A254_
DRB1_0101	18	KLNTSLNTTDKDLHD	LNTSLNTTD	1	0.1702	7932.4	100.00	0.26	tr_D0A254_
DRB1_0101	19	LNTSLNTTDKDLHDG	NTSLNTTDK	1	0.1164	14196.1	100.00	0.25	tr_D0A254_
DRB1_0101	20	NTSLNTTDKDLHDGG	LNTTDKDLH	3	0.0919	18506.8	100.00	0.39	tr_D0A254_
DRB1_0101	21	TSLNTTDKDLHDGGS	LNTTDKDLH	2	0.0793	21189.1	100.00	0.40	tr_D0A254_
DRB1_0101	22	SLNTTDKDLHDGGSR	LNTTDKDLH	1	0.0729	22719.4	100.00	0.31	tr_D0A254_

Allele: DRB1_0101. Number of high binders 5. Number of weak binders 2. Number of peptides 22

Allele	pos	peptide	core	Of	1-log50k(aff)	affinity(nM)	%Rank	Relia	Identity	Bind Level
DRB1_0101	1	FMFRYIIPTTTNRLG	FRYIIPTTT	2	0.9005	2.9	0.08	0.85	tr_D0A254_	SB
DRB1_0101	2	VFMFRYIIPTTTNRL	FRYIIPTTT	3	0.9003	2.9	0.08	0.88	tr_D0A254_	SB
DRB1_0101	3	WVFMFRYIIPTTTNR	FRYIIPTTT	4	0.8888	3.3	0.15	0.92	tr_D0A254_	SB
DRB1_0101	4	FWVFMFRYIIPTTTN	FRYIIPTTT	5	0.8700	4.1	0.40	0.90	tr_D0A254_	SB
DRB1_0101	5	YFWVFMFRYIIPTTT	FRYIIPTTT	6	0.8383	5.7	0.90	0.83	tr_D0A254_	WB
DRB1_0101	6	LGEYISSSAWNLLVH	YISSSAWNL	3	0.7822	10.5	3.00	0.85	tr_D0A254_	WB
DRB1_0101	7	GEYISSSAWNLLVHA	YISSSAWNL	2	0.7721	11.8	3.50	0.81	tr_D0A254_	WB
DRB1_0101	8	SLGEYISSSAWNLLV	YISSSAWNL	4	0.7635	12.9	4.00	0.87	tr_D0A254_	WB
DRB1_0101	9	EYISSSAWNLLVHAL	YISSSAWNL	1	0.7520	14.6	5.00	0.79	tr_D0A254_	WB
DRB1_0101	10	DSLGEYISSSAWNLL	YISSSAWNL	5	0.7198	20.7	7.00	0.84	tr_D0A254_	WB
DRB1_0101	11	YISSSAWNLLVHALP	YISSSAWNL	0	0.6642	37.8	13.00	0.58	tr_D0A254_	
DRB1_0101	12	LQWLIFISGWRQARV	LIFISGWRQ	3	0.6627	38.4	13.00	0.65	tr_D0A254_	
DRB1_0101	13	QWLIFISGWRQARVM	LIFISGWRQ	2	0.6579	40.5	14.00	0.62	tr_D0A254_	
DRB1_0101	14	VLQWLIFISGWRQAR	LIFISGWRQ	4	0.6406	48.9	16.00	0.63	tr_D0A254_	
DRB1_0101	15	WLIFISGWRQARVML	LIFISGWRQ	1	0.6385	50.0	17.00	0.55	tr_D0A254_	
DRB1_0101	16	LIFISGWRQARVMLK	LIFISGWRQ	0	0.6280	56.0	18.00	0.35	tr_D0A254_	
DRB1_0101	17	SDSLGEYISSSAWNL	YISSSAWNL	6	0.5916	83.0	24.00	0.81	tr_D0A254_	
DRB1_0101	18	MVLQWLIFISGWRQA	LIFISGWRQ	5	0.5876	86.7	25.00	0.63	tr_D0A254_	
DRB1_0101	19	SSAWNLLVHALPCRD	WNLLVHALP	3	0.5725	102.0	27.00	0.32	tr_D0A254_	
DRB1_0101	20	AWNLLVHALPCRDPM	LVHALPCRD	4	0.5705	104.3	27.00	0.41	tr_D0A254_	
DRB1_0101	21	SAWNLLVHALPCRDP	WNLLVHALP	2	0.5700	104.8	28.00	0.28	tr_D0A254_	
DRB1_0101	22	SSSAWNLLVHALPCR	WNLLVHALP	4	0.5623	114.0	29.00	0.34	tr_D0A254_	
DRB1_0101	23	WNLLVHALPCRDPMT	LVHALPCRD	3	0.5478	133.4	32.00	0.46	tr_D0A254_	
DRB1_0101	24	SYFWVFMFRYIIPTT	FMFRYIIPT	5	0.5306	160.6	35.00	0.41	tr_D0A254_	
DRB1_0101	25	FIAVFCFMVLQWLIF	FCFMVLQWL	4	0.5288	163.7	35.00	0.51	tr_D0A254_	
DRB1_0101	26	NLLVHALPCRDPMTI	LVHALPCRD	2	0.5216	177.0	37.00	0.52	tr_D0A254_	
DRB1_0101	27	FMVLQWLIFISGWRQ	LIFISGWRQ	6	0.5176	184.7	37.00	0.57	tr_D0A254_	
DRB1_0101	28	IAVFCFMVLQWLIFI	FCFMVLQWL	3	0.5138	192.5	38.00	0.56	tr_D0A254_	
DRB1_0101	29	ISSSAWNLLVHALPC	WNLLVHALP	5	0.5111	198.3	39.00	0.34	tr_D0A254_	
DRB1_0101	30	IFISGWRQARVMLKC	WRQARVMLK	5	0.5091	202.7	39.00	0.34	tr_D0A254_	
DRB1_0101	31	FFIAVFCFMVLQWLI	FCFMVLQWL	5	0.5082	204.6	39.00	0.47	tr_D0A254_	
DRB1_0101	32	AVFCFMVLQWLIFIS	FCFMVLQWL	2	0.5076	205.9	39.00	0.54	tr_D0A254_	

DRB1_0101	33	KSYFWVFMFRYIIPT	FMFRYIIPT	6	0.5057	210.3	40.00	0.29	tr_D0A254_
DRB1_0101	34	VFCFMVLQWLIFISG	FCFMVLQWL	1	0.5056	210.4	40.00	0.47	tr_D0A254_
DRB1_0101	35	WFFIAVFCFMVLQWL	FCFMVLQWL	6	0.4975	229.8	42.00	0.38	tr_D0A254_
DRB1_0101	36	DGYSAVMDCPVAASN	YSAVMDCPV	2	0.4937	239.3	42.00	0.41	tr_D0A254_
DRB1_0101	37	GYSAVMDCPVAASNH	YSAVMDCPV	1	0.4934	240.1	42.00	0.37	tr_D0A254_
DRB1_0101	38	YDGYSAVMDCPVAAS	YSAVMDCPV	3	0.4887	252.8	43.00	0.49	tr_D0A254_
DRB1_0101	39	FCFMVLQWLIFISGW	FCFMVLQWL	0	0.4667	320.5	48.00	0.37	tr_D0A254_
DRB1_0101	40	CPVAASNHAWFFIAV	AASNHAWFF	3	0.4620	337.2	49.00	0.25	tr_D0A254_
DRB1_0101	41	TGKSYFWVFMFRYII	FWVFMFRYI	5	0.4610	341.1	49.00	0.31	tr_D0A254_
DRB1_0101	42	RYDGYSAVMDCPVAA	YSAVMDCPV	4	0.4588	349.2	50.00	0.51	tr_D0A254_
DRB1_0101	43	DCPVAASNHAWFFIA	AASNHAWFF	4	0.4569	356.5	50.00	0.23	tr_D0A254_
DRB1_0101	44	GKSYFWVFMFRYIIP	FWVFMFRYI	4	0.4542	367.0	55.00	0.32	tr_D0A254_
DRB1_0101	45	YSAVMDCPVAASNHA	SAVMDCPVA	1	0.4507	381.0	55.00	0.26	tr_D0A254_
DRB1_0101	46	LLVHALPCRDPMTID	LVHALPCRD	1	0.4461	400.6	55.00	0.55	tr_D0A254_
DRB1_0101	47	NHAWFFIAVFCFMVL	FIAVFCFMV	5	0.4457	402.3	55.00	0.26	tr_D0A254_
DRB1_0101	48	HAWFFIAVFCFMVLQ	FIAVFCFMV	4	0.4434	412.7	55.00	0.32	tr_D0A254_
DRB1_0101	49	SNHAWFFIAVFCFMV	FFIAVFCFM	5	0.4389	433.0	55.00	0.29	tr_D0A254_
DRB1_0101	50	AWFFIAVFCFMVLQW	FIAVFCFMV	3	0.4308	472.7	60.00	0.36	tr_D0A254_
DRB1_0101	51	FISGWRQARVMLKCP	WRQARVMLK	4	0.4299	477.5	60.00	0.49	tr_D0A254_
DRB1_0101	52	PVAASNHAWFFIAVF	SNHAWFFIA	4	0.4279	487.7	60.00	0.29	tr_D0A254_
DRB1_0101	53	MDCPVAASNHAWFFI	PVAASNHAW	3	0.4270	492.7	60.00	0.25	tr_D0A254_
DRB1_0101	54	ASNHAWFFIAVFCFM	WFFIAVFCF	5	0.4155	557.8	60.00	0.28	tr_D0A254_
DRB1_0101	55	VTGKSYFWVFMFRYI	FWVFMFRYI	6	0.4155	558.0	60.00	0.35	tr_D0A254_
DRB1_0101	56	SGWRQARVMLKCPRS	WRQARVMLK	2	0.4071	610.9	65.00	0.62	tr_D0A254_
DRB1_0101	57	ISGWRQARVMLKCPR	WRQARVMLK	3	0.4058	619.4	65.00	0.70	tr_D0A254_
DRB1_0101	58	VMDCPVAASNHAWFF	CPVAASNHA	3	0.4022	644.3	65.00	0.32	tr_D0A254_
DRB1_0101	59	GWRQARVMLKCPRSD	WRQARVMLK	1	0.3904	731.6	65.00	0.54	tr_D0A254_
DRB1_0101	60	SAVMDCPVAASNHAW	AVMDCPVAA	1	0.3890	743.1	65.00	0.25	tr_D0A254_
DRB1_0101	61	AASNHAWFFIAVFCF	WFFIAVFCF	6	0.3881	750.6	65.00	0.30	tr_D0A254_
DRB1_0101	62	CFMVLQWLIFISGWR	FMVLQWLIF	1	0.3863	764.9	65.00	0.29	tr_D0A254_
DRB1_0101	63	VAASNHAWFFIAVFC	SNHAWFFIA	3	0.3720	893.1	70.00	0.34	tr_D0A254_
DRB1_0101	64	MTIDVTGKSYFWVFM	DVTGKSYFW	3	0.3707	905.6	70.00	0.47	tr_D0A254_
DRB1_0101	65	AVMDCPVAASNHAWF	CPVAASNHA	4	0.3644	969.4	70.00	0.31	tr_D0A254_
DRB1_0101	66	PMTIDVTGKSYFWVF	DVTGKSYFW	4	0.3627	988.1	70.00	0.43	tr_D0A254_
DRB1_0101	67	RQARVMLKCPRSDSL	RVMLKCPRS	3	0.3617	998.9	70.00	0.55	tr_D0A254_
DRB1_0101	68	WRQARVMLKCPRSDS	RVMLKCPRS	4	0.3606	1010.5	70.00	0.41	tr_D0A254_
DRB1_0101	69	QARVMLKCPRSDSLG	RVMLKCPRS	2	0.3598	1018.9	75.00	0.49	tr_D0A254_
DRB1_0101	70	TIDVTGKSYFWVFMF	DVTGKSYFW	2	0.3592	1025.5	75.00	0.44	tr_D0A254_
DRB1_0101	71	DVTGKSYFWVFMFRY	YFWVFMFRY	6	0.3506	1125.4	75.00	0.34	tr_D0A254_
DRB1_0101	72	ARVMLKCPRSDSLGE	RVMLKCPRS	1	0.3415	1242.9	75.00	0.43	tr_D0A254_
DRB1_0101	73	IDVTGKSYFWVFMFR	DVTGKSYFW	1	0.3408	1252.3	75.00	0.35	tr_D0A254_
DRB1_0101	74	DPMTIDVTGKSYFWV	DVTGKSYFW	5	0.3384	1284.6	75.00	0.41	tr_D0A254_
DRB1_0101	75	RDPMTIDVTGKSYFW	MTIDVTGKS	3	0.3139	1674.8	80.00	0.38	tr_D0A254_
DRB1_0101	76	LVHALPCRDPMTIDV	LVHALPCRD	0	0.3087	1771.5	80.00	0.38	tr_D0A254_
DRB1_0101	77	RVMLKCPRSDSLGEY	RVMLKCPRS	0	0.3077	1790.5	80.00	0.32	tr_D0A254_
DRB1_0101	78	KCPRSDSLGEYISSS	RSDSLGEYI	3	0.2924	2114.0	85.00	0.48	tr_D0A254_
DRB1_0101	79	RSDSLGEYISSSAWN	GEYISSSAW	5	0.2916	2131.0	85.00	0.24	tr_D0A254_
DRB1_0101	80	PRSDSLGEYISSSAW	RSDSLGEYI	1	0.2911	2142.5	85.00	0.28	tr_D0A254_
DRB1_0101	81	CPRSDSLGEYISSSA	RSDSLGEYI	2	0.2888	2197.0	85.00	0.38	tr_D0A254_
DRB1_0101	82	LKCPRSDSLGEYISS	RSDSLGEYI	4	0.2819	2366.5	85.00	0.47	tr_D0A254_

DRB1_0101	83	MLKCPRSDSLGEYIS	RSDSLGEYI	5	0.2697	2700.7	90.00	0.40	tr_D0A254_	
DRB1_0101	84	VMLKCPRSDSLGEYI	LKCPRSDSL	2	0.2588	3040.4	90.00	0.31	tr_D0A254_	
DRB1_0101	85	CRDPMTIDVTGKSYF	MTIDVTGKS	4	0.2498	3352.3	90.00	0.46	tr_D0A254_	
DRB1_0101	86	VHALPCRDPMTIDVT	LPCRDPMTI	3	0.2172	4767.9	95.00	0.20	tr_D0A254_	
DRB1_0101	87	PCRDPMTIDVTGKSY	MTIDVTGKS	5	0.2060	5383.2	95.00	0.38	tr_D0A254_	
DRB1_0101	88	HALPCRDPMTIDVTG	CRDPMTIDV	4	0.1924	6237.6	100.00	0.29	tr_D0A254_	
DRB1_0101	89	LPCRDPMTIDVTGKS	CRDPMTIDV	2	0.1827	6927.5	100.00	0.29	tr_D0A254_	
DRB1_0101	90	ALPCRDPMTIDVTGK	CRDPMTIDV	3	0.1810	7053.1	100.00	0.35	tr_D0A254_	

Allele: DRB1_0101. Number of high binders 4. Number of weak binders 6. Number of peptides 90

Allele	pos	peptide	core	Of	1-log50k(aff)	affinity(nM)	%Rank	Relia	Identity	Bind Level
DRB1_0101	1	KTLIHKMSGTALGGR	IHKMSGTAL	3	0.8504	5.0	0.60	0.82	tr_C9ZXW1_	WB
DRB1_0101	2	KKTLIHKMSGTALGG	IHKMSGTAL	4	0.8360	5.9	0.90	0.79	tr_C9ZXW1_	WB
DRB1_0101	3	TLIHKMSGTALGGRV	IHKMSGTAL	2	0.8243	6.7	1.20	0.82	tr_C9ZXW1_	WB
DRB1_0101	4	IKKTLIHKMSGTALG	IHKMSGTAL	5	0.8119	7.7	1.60	0.75	tr_C9ZXW1_	WB
DRB1_0101	5	FTDRYRAVVSFVAQD	YRAVVSFVA	4	0.8066	8.1	1.80	0.89	tr_C9ZXW1_	WB
DRB1_0101	6	GRVLAIMGPSGAGKT	LAIMGPSGA	3	0.8028	8.4	1.90	0.63	tr_C9ZXW1_	WB
DRB1_0101	7	IFTDRYRAVVSFVAQ	YRAVVSFVA	5	0.8012	8.6	2.00	0.87	tr_C9ZXW1_	WB
DRB1_0101	8	TDRYRAVVSFVAQDD	YRAVVSFVA	3	0.7998	8.7	2.50	0.93	tr_C9ZXW1_	WB
DRB1_0101	9	LIHKMSGTALGGRVL	IHKMSGTAL	1	0.7983	8.9	2.50	0.73	tr_C9ZXW1_	WB
DRB1_0101	10	GGRVLAIMGPSGAGK	LAIMGPSGA	4	0.7961	9.1	2.50	0.60	tr_C9ZXW1_	WB
DRB1_0101	11	RAWELYLTTPAASTN	YLTTPAAST	5	0.7921	9.5	2.50	0.34	tr_C9ZXW1_	WB
DRB1_0101	12	RVLAIMGPSGAGKTT	LAIMGPSGA	2	0.7905	9.7	2.50	0.64	tr_C9ZXW1_	WB
DRB1_0101	13	DRYRAVVSFVAQDDI	YRAVVSFVA	2	0.7798	10.8	3.00	0.89	tr_C9ZXW1_	WB
DRB1_0101	14	AWELYLTTPAASTNP	YLTTPAAST	4	0.7738	11.6	3.50	0.40	tr_C9ZXW1_	WB
DRB1_0101	15	RYRAVVSFVAQDDIV	YRAVVSFVA	1	0.7578	13.7	4.50	0.86	tr_C9ZXW1_	WB
DRB1_0101	16	LGGRVLAIMGPSGAG	LAIMGPSGA	5	0.7559	14.0	4.50	0.56	tr_C9ZXW1_	WB
DRB1_0101	17	WRAWELYLTTPAAST	WELYLTTPA	3	0.7529	14.5	4.50	0.34	tr_C9ZXW1_	WB
DRB1_0101	18	TGLDSVNALRVGQML	LDSVNALRV	2	0.7506	14.9	5.00	0.73	tr_C9ZXW1_	WB
DRB1_0101	19	TTGLDSVNALRVGQM	LDSVNALRV	3	0.7501	14.9	5.00	0.81	tr_C9ZXW1_	WB
DRB1_0101	20	TAYFASIGHQVPPRT	YFASIGHQV	2	0.7484	15.2	5.00	0.74	tr_C9ZXW1_	WB
DRB1_0101	21	IHKMSGTALGGRVLA	IHKMSGTAL	0	0.7465	15.5	5.00	0.46	tr_C9ZXW1_	WB
DRB1_0101	22	DDLLLLSEGHVVYHG	LLLSEGHVV	3	0.7434	16.1	5.50	0.69	tr_C9ZXW1_	WB
DRB1_0101	23	ATAYFASIGHQVPPR	YFASIGHQV	3	0.7419	16.3	5.50	0.85	tr_C9ZXW1_	WB
DRB1_0101	24	AIFTDRYRAVVSFVA	YRAVVSFVA	6	0.7407	16.5	5.50	0.75	tr_C9ZXW1_	WB
DRB1_0101	25	WELYLTTPAASTNPC	YLTTPAAST	3	0.7404	16.6	5.50	0.56	tr_C9ZXW1_	WB
DRB1_0101	26	AYFASIGHQVPPRTN	YFASIGHQV	1	0.7387	16.9	5.50	0.58	tr_C9ZXW1_	WB
DRB1_0101	27	GLDSVNALRVGQMLQ	LDSVNALRV	1	0.7379	17.1	5.50	0.63	tr_C9ZXW1_	WB
DRB1_0101	28	FDDLLLLSEGHVVYH	LLLSEGHVV	4	0.7348	17.6	6.00	0.67	tr_C9ZXW1_	WB
DRB1_0101	29	PTTGLDSVNALRVGQ	LDSVNALRV	4	0.7334	17.9	6.00	0.76	tr_C9ZXW1_	WB
DRB1_0101	30	DLLLLSEGHVVYHGP	LLLSEGHVV	2	0.7324	18.1	6.00	0.70	tr_C9ZXW1_	WB
DRB1_0101	31	VLAIMGPSGAGKTTL	LAIMGPSGA	1	0.7314	18.3	6.00	0.69	tr_C9ZXW1_	WB
DRB1_0101	32	KIKKTLIHKMSGTAL	IHKMSGTAL	6	0.7292	18.7	6.50	0.66	tr_C9ZXW1_	WB
DRB1_0101	33	EPTTGLDSVNALRVG	LDSVNALRV	5	0.7100	23.0	8.00	0.71	tr_C9ZXW1_	WB
DRB1_0101	34	EATAYFASIGHQVPP	YFASIGHQV	4	0.7092	23.2	8.00	0.86	tr_C9ZXW1_	WB

DRB1_0101	35	YRAVVSFVAQDDIVM	YRAVVSFVA	0	0.7039	24.6	8.50	0.55	tr_C9ZXW1_	WB
DRB1_0101	36	ELYLTTPAASTNPCL	YLTTPAAST	2	0.7031	24.8	8.50	0.67	tr_C9ZXW1_	WB
DRB1_0101	37	KMSGTALGGRVLAIM	GTALGGRVL	3	0.7030	24.9	8.50	0.61	tr_C9ZXW1_	WB
DRB1_0101	38	TFDDLLLLSEGHVVY	LLLSEGHVV	5	0.7006	25.5	9.00	0.61	tr_C9ZXW1_	WB
DRB1_0101	39	LWRAWELYLTTPAAS	WELYLTTPA	4	0.7005	25.5	9.00	0.46	tr_C9ZXW1_	WB
DRB1_0101	40	EEATAYFASIGHQVP	YFASIGHQV	5	0.6999	25.7	9.00	0.87	tr_C9ZXW1_	WB
DRB1_0101	41	LLLLSEGHVVYHGPR	LLLSEGHVV	1	0.6986	26.1	9.00	0.71	tr_C9ZXW1_	WB
DRB1_0101	42	AVVSFVAQDDIVMAM	FVAQDDIVM	4	0.6961	26.8	9.50	0.80	tr_C9ZXW1_	WB
DRB1_0101	43	HKMSGTALGGRVLAI	GTALGGRVL	4	0.6920	28.0	10.00	0.56	tr_C9ZXW1_	
DRB1_0101	44	VVSFVAQDDIVMAMD	FVAQDDIVM	3	0.6916	28.1	10.00	0.83	tr_C9ZXW1_	
DRB1_0101	45	ALGGRVLAIMGPSGA	VLAIMGPSG	5	0.6904	28.5	10.00	0.47	tr_C9ZXW1_	
DRB1_0101	46	LDSVNALRVGQMLQD	LDSVNALRV	0	0.6816	31.3	11.00	0.46	tr_C9ZXW1_	
DRB1_0101	47	KTTLMSAITGKLHGT	LMSAITGKL	3	0.6752	33.6	12.00	0.47	tr_C9ZXW1_	
DRB1_0101	48	VSFVAQDDIVMAMDT	FVAQDDIVM	2	0.6693	35.8	13.00	0.82	tr_C9ZXW1_	
DRB1_0101	49	MSGTALGGRVLAIMG	GTALGGRVL	2	0.6693	35.8	13.00	0.58	tr_C9ZXW1_	
DRB1_0101	50	GKTTLMSAITGKLHG	LMSAITGKL	4	0.6668	36.8	13.00	0.42	tr_C9ZXW1_	
DRB1_0101	51	RAVVSFVAQDDIVMA	FVAQDDIVM	5	0.6629	38.4	13.00	0.76	tr_C9ZXW1_	
DRB1_0101	52	YFASIGHQVPPRTNP	YFASIGHQV	0	0.6622	38.6	13.00	0.46	tr_C9ZXW1_	
DRB1_0101	53	NVATALVANPCVIVL	LVANPCVIV	5	0.6509	43.7	15.00	0.38	tr_C9ZXW1_	
DRB1_0101	54	AGKTTLMSAITGKLH	LMSAITGKL	5	0.6432	47.5	16.00	0.37	tr_C9ZXW1_	
DRB1_0101	55	VATALVANPCVIVLD	LVANPCVIV	4	0.6406	48.8	16.00	0.46	tr_C9ZXW1_	
DRB1_0101	56	DEPTTGLDSVNALRV	LDSVNALRV	6	0.6358	51.5	17.00	0.57	tr_C9ZXW1_	
DRB1_0101	57	RTVIATVHSPSSELV	IATVHSPSS	3	0.6333	52.9	17.00	0.69	tr_C9ZXW1_	
DRB1_0101	58	NPCLLKVSGPITERD	LLKVSGPIT	3	0.6319	53.6	18.00	0.59	tr_C9ZXW1_	
DRB1_0101	59	TTLMSAITGKLHGTE	LMSAITGKL	2	0.6299	54.8	18.00	0.46	tr_C9ZXW1_	
DRB1_0101	60	TNPCLLKVSGPITER	LLKVSGPIT	4	0.6299	54.8	18.00	0.62	tr_C9ZXW1_	
DRB1_0101	61	REEATAYFASIGHQV	YFASIGHQV	6	0.6248	57.9	19.00	0.86	tr_C9ZXW1_	
DRB1_0101	62	VLWRAWELYLTTPAA	WELYLTTPA	5	0.6248	57.9	19.00	0.50	tr_C9ZXW1_	
DRB1_0101	63	ATALVANPCVIVLDE	LVANPCVIV	3	0.6198	61.1	19.00	0.53	tr_C9ZXW1_	
DRB1_0101	64	ANVATALVANPCVIV	TALVANPCV	4	0.6192	61.6	19.00	0.31	tr_C9ZXW1_	
DRB1_0101	65	SGTALGGRVLAIMGP	GTALGGRVL	1	0.6176	62.6	20.00	0.61	tr_C9ZXW1_	
DRB1_0101	66	LAIMGPSGAGKTTLM	LAIMGPSGA	0	0.6172	62.9	20.00	0.76	tr_C9ZXW1_	
DRB1_0101	67	SFVAQDDIVMAMDTP	FVAQDDIVM	1	0.6155	64.1	20.00	0.82	tr_C9ZXW1_	
DRB1_0101	68	ETFDDLLLLSEGHVV	LLLSEGHVV	6	0.6105	67.6	21.00	0.56	tr_C9ZXW1_	
DRB1_0101	69	PCLLKVSGPITERDP	LLKVSGPIT	2	0.6095	68.4	21.00	0.58	tr_C9ZXW1_	
DRB1_0101	70	LYLTTPAASTNPCLL	YLTTPAAST	1	0.6093	68.5	21.00	0.82	tr_C9ZXW1_	
DRB1_0101	71	DHLKKKGSSWIVQLV	KKKGSSWIV	3	0.6060	71.0	22.00	0.60	tr_C9ZXW1_	
DRB1_0101	72	HLKKKGSSWIVQLVE	KKKGSSWIV	2	0.5944	80.5	23.00	0.61	tr_C9ZXW1_	
DRB1_0101	73	TVIATVHSPSSELVE	IATVHSPSS	2	0.5932	81.6	24.00	0.67	tr_C9ZXW1_	
DRB1_0101	74	KRTVIATVHSPSSEL	IATVHSPSS	4	0.5927	82.0	24.00	0.72	tr_C9ZXW1_	
DRB1_0101	75	GSSWIVQLVELTKRS	WIVQLVELT	3	0.5922	82.4	24.00	0.73	tr_C9ZXW1_	
DRB1_0101	76	EDHLKKKGSSWIVQL	KKKGSSWIV	4	0.5865	87.7	25.00	0.58	tr_C9ZXW1_	
DRB1_0101	77	KGSSWIVQLVELTKR	WIVQLVELT	4	0.5865	87.7	25.00	0.71	tr_C9ZXW1_	
DRB1_0101	78	CEYFMELLQLPKEEL	YFMELLQLP	2	0.5862	88.0	25.00	0.35	tr_C9ZXW1_	
DRB1_0101	79	DSVNALRVGQMLQDL	NALRVGQML	3	0.5840	90.2	25.00	0.52	tr_C9ZXW1_	
DRB1_0101	80	LLLSEGHVVYHGPRE	LLLSEGHVV	0	0.5835	90.6	25.00	0.68	tr_C9ZXW1_	
DRB1_0101	81	DIVMAMDTPYEATYF	VMAMDTPYE	2	0.5831	91.0	25.00	0.44	tr_C9ZXW1_	
DRB1_0101	82	DDIVMAMDTPYEATY	VMAMDTPYE	3	0.5791	95.0	26.00	0.44	tr_C9ZXW1_	
DRB1_0101	83	PCEYFMELLQLPKEE	YFMELLQLP	3	0.5782	95.9	26.00	0.43	tr_C9ZXW1_	
DRB1_0101	84	SSWIVQLVELTKRSY	WIVQLVELT	2	0.5778	96.3	26.00	0.69	tr_C9ZXW1_	

DRB1_0101	85	CLLKVSGPITERDPY	LLKVSGPIT	1	0.5774	96.8	26.00	0.58	tr_C9ZXW1_
DRB1_0101	86	SEVLVNEVINRLHLV	EVINRLHLV	6	0.5773	96.9	26.00	0.32	tr_C9ZXW1_
DRB1_0101	87	GAGKTTLMSAITGKL	TTLMSAITG	4	0.5752	99.1	27.00	0.34	tr_C9ZXW1_
DRB1_0101	88	NPCEYFMELLQLPKE	YFMELLQLP	4	0.5716	103.1	27.00	0.44	tr_C9ZXW1_
DRB1_0101	89	SVNALRVGQMLQDLA	NALRVGQML	2	0.5704	104.4	28.00	0.50	tr_C9ZXW1_
DRB1_0101	90	EVLVNEVINRLHLVE	EVINRLHLV	5	0.5645	111.3	29.00	0.50	tr_C9ZXW1_
DRB1_0101	91	STNPCLLKVSGPITE	LLKVSGPIT	5	0.5631	113.0	29.00	0.66	tr_C9ZXW1_
DRB1_0101	92	EYFMELLQLPKEELE	YFMELLQLP	1	0.5621	114.2	29.00	0.31	tr_C9ZXW1_
DRB1_0101	93	RANVATALVANPCVI	TALVANPCV	5	0.5571	120.6	30.00	0.35	tr_C9ZXW1_
DRB1_0101	94	IVMAMDTPYEATYFA	MAMDTPYEA	2	0.5568	120.9	30.00	0.44	tr_C9ZXW1_
DRB1_0101	95	TALVANPCVIVLDEP	LVANPCVIV	2	0.5541	124.6	30.00	0.60	tr_C9ZXW1_
DRB1_0101	96	YTWENLAFQVPAKDS	LAFQVPAKD	5	0.5532	125.8	31.00	0.58	tr_C9ZXW1_
DRB1_0101	97	TLMSAITGKLHGTEK	LMSAITGKL	1	0.5530	126.0	31.00	0.50	tr_C9ZXW1_
DRB1_0101	98	YEATYFACRVRLGLG	YFACRVRLG	4	0.5528	126.2	31.00	0.61	tr_C9ZXW1_
DRB1_0101	99	KKGSSWIVQLVELTK	WIVQLVELT	5	0.5514	128.2	31.00	0.69	tr_C9ZXW1_
DRB1_0101	100	LKKKGSSWIVQLVEL	KKKGSSWIV	1	0.5483	132.6	32.00	0.62	tr_C9ZXW1_
DRB1_0101	101	VIATVHSPSSELVET	IATVHSPSS	1	0.5480	133.0	32.00	0.59	tr_C9ZXW1_
DRB1_0101	102	TNPCEYFMELLQLPK	YFMELLQLP	5	0.5459	136.0	32.00	0.48	tr_C9ZXW1_
DRB1_0101	103	QDTVLGIPGLVKGVS	VLGIPGLVK	3	0.5447	137.8	32.00	0.57	tr_C9ZXW1_
DRB1_0101	104	QDDIVMAMDTPYEAT	VMAMDTPYE	4	0.5444	138.4	32.00	0.41	tr_C9ZXW1_
DRB1_0101	105	GERKRANVATALVAN	KRANVATAL	3	0.5436	139.5	32.00	0.56	tr_C9ZXW1_
DRB1_0101	106	KKKGSSWIVQLVELT	WIVQLVELT	6	0.5422	141.6	33.00	0.47	tr_C9ZXW1_
DRB1_0101	107	WENLAFQVPAKDSDG	LAFQVPAKD	3	0.5408	143.7	33.00	0.73	tr_C9ZXW1_
DRB1_0101	108	SWIVQLVELTKRSYR	WIVQLVELT	1	0.5406	144.0	33.00	0.61	tr_C9ZXW1_
DRB1_0101	109	VLVNEVINRLHLVEC	EVINRLHLV	4	0.5404	144.4	33.00	0.61	tr_C9ZXW1_
DRB1_0101	110	PYEATYFACRVRLGL	YFACRVRLG	5	0.5389	146.8	33.00	0.54	tr_C9ZXW1_
DRB1_0101	111	TWENLAFQVPAKDSD	LAFQVPAKD	4	0.5385	147.4	33.00	0.67	tr_C9ZXW1_
DRB1_0101	112	LEDHLKKKGSSWIVQ	KKKGSSWIV	5	0.5371	149.7	34.00	0.54	tr_C9ZXW1_
DRB1_0101	113	CQDTVLGIPGLVKGV	VLGIPGLVK	4	0.5356	152.1	34.00	0.56	tr_C9ZXW1_
DRB1_0101	114	EGCCFLNNAIFTDRY	CCFLNNAIF	2	0.5355	152.2	34.00	0.43	tr_C9ZXW1_
DRB1_0101	115	ATYFACRVRLGLGPR	YFACRVRLG	2	0.5334	155.8	34.00	0.58	tr_C9ZXW1_
DRB1_0101	116	EATYFACRVRLGLGP	YFACRVRLG	3	0.5330	156.5	34.00	0.61	tr_C9ZXW1_
DRB1_0101	117	KRANVATALVANPCV	ATALVANPC	5	0.5307	160.3	35.00	0.20	tr_C9ZXW1_
DRB1_0101	118	ENLAFQVPAKDSDGR	LAFQVPAKD	2	0.5299	161.7	35.00	0.74	tr_C9ZXW1_
DRB1_0101	119	ERKRANVATALVANP	KRANVATAL	2	0.5289	163.6	35.00	0.57	tr_C9ZXW1_
DRB1_0101	120	LEGCCFLNNAIFTDR	CCFLNNAIF	3	0.5282	164.8	35.00	0.49	tr_C9ZXW1_
DRB1_0101	121	LVNEVINRLHLVECQ	EVINRLHLV	3	0.5276	165.8	36.00	0.70	tr_C9ZXW1_
DRB1_0101	122	TYFACRVRLGLGPRE	YFACRVRLG	1	0.5267	167.6	36.00	0.44	tr_C9ZXW1_
DRB1_0101	123	VNALRVGQMLQDLAK	NALRVGQML	1	0.5236	173.2	36.00	0.48	tr_C9ZXW1_
DRB1_0101	124	GTALGGRVLAIMGPS	GTALGGRVL	0	0.5201	180.0	37.00	0.58	tr_C9ZXW1_
DRB1_0101	125	GCCFLNNAIFTDRYR	CCFLNNAIF	1	0.5183	183.5	37.00	0.39	tr_C9ZXW1_
DRB1_0101	126	RKRANVATALVANPC	KRANVATAL	1	0.5183	183.5	37.00	0.44	tr_C9ZXW1_
DRB1_0101	127	EVLWRAWELYLTTPA	WELYLTTPA	6	0.5183	183.5	37.00	0.49	tr_C9ZXW1_
DRB1_0101	128	EKRTVIATVHSPSSE	IATVHSPSS	5	0.5175	185.0	38.00	0.69	tr_C9ZXW1_
DRB1_0101	129	DTVLGIPGLVKGVSG	VLGIPGLVK	2	0.5166	186.9	38.00	0.54	tr_C9ZXW1_
DRB1_0101	130	ESEVLVNEVINRLHL	LVNEVINRL	4	0.5138	192.7	38.00	0.42	tr_C9ZXW1_
DRB1_0101	131	YFMELLQLPKEELEV	MELLQLPKE	2	0.5136	193.1	38.00	0.28	tr_C9ZXW1_
DRB1_0101	132	GGERKRANVATALVA	KRANVATAL	4	0.5070	207.2	40.00	0.58	tr_C9ZXW1_
DRB1_0101	133	RESEVLVNEVINRLH	SEVLVNEVI	2	0.5068	207.7	40.00	0.44	tr_C9ZXW1_
DRB1_0101	134	RKIKKTLIHKMSGTA	IKKTLIHKM	2	0.5053	211.1	40.00	0.48	tr_C9ZXW1_

DRB1_0101	135	FVAQDDIVMAMDTPY	FVAQDDIVM	0	0.5042	213.7	40.00	0.62	tr_C9ZXW1_
DRB1_0101	136	YFACRVRLGLGPRES	VRLGLGPRE	5	0.5009	221.4	41.00	0.42	tr_C9ZXW1_
DRB1_0101	137	NYTWENLAFQVPAKD	LAFQVPAKD	6	0.5008	221.8	41.00	0.38	tr_C9ZXW1_
DRB1_0101	138	FASIGHQVPPRTNPC	IGHQVPPRT	3	0.5005	222.4	41.00	0.71	tr_C9ZXW1_
DRB1_0101	139	ECQDTVLGIPGLVKG	VLGIPGLVK	5	0.4995	224.9	41.00	0.53	tr_C9ZXW1_
DRB1_0101	140	RAPINLEGELCMERV	PINLEGELC	2	0.4983	227.7	41.00	0.60	tr_C9ZXW1_
DRB1_0101	141	VNEVINRLHLVECQD	EVINRLHLV	2	0.4978	229.0	41.00	0.74	tr_C9ZXW1_
DRB1_0101	142	TALGGRVLAIMGPSG	VLAIMGPSG	6	0.4973	230.3	42.00	0.62	tr_C9ZXW1_
DRB1_0101	143	SGAGKTTLMSAITGK	TTLMSAITG	5	0.4944	237.5	42.00	0.40	tr_C9ZXW1_
DRB1_0101	144	NAIFTDRYRAVVSFV	IFTDRYRAV	2	0.4939	238.9	42.00	0.21	tr_C9ZXW1_
DRB1_0101	145	YLTTPAASTNPCLLK	YLTTPAAST	0	0.4937	239.3	42.00	0.68	tr_C9ZXW1_
DRB1_0101	146	GRAPINLEGELCMER	PINLEGELC	3	0.4912	245.9	43.00	0.65	tr_C9ZXW1_
DRB1_0101	147	LLKVSGPITERDPYL	LLKVSGPIT	0	0.4898	249.8	43.00	0.49	tr_C9ZXW1_
DRB1_0101	148	NLEGCCFLNNAIFTD	CCFLNNAIF	4	0.4897	249.9	43.00	0.52	tr_C9ZXW1_
DRB1_0101	149	NALRVGQMLQDLAKK	NALRVGQML	0	0.4884	253.6	43.00	0.32	tr_C9ZXW1_
DRB1_0101	150	TPYEATYFACRVRLG	YFACRVRLG	6	0.4860	260.1	44.00	0.41	tr_C9ZXW1_
DRB1_0101	151	ASTNPCLLKVSGPIT	LLKVSGPIT	6	0.4831	268.4	45.00	0.65	tr_C9ZXW1_
DRB1_0101	152	NLAFQVPAKDSDGRK	LAFQVPAKD	1	0.4828	269.5	45.00	0.71	tr_C9ZXW1_
DRB1_0101	153	GRKIKKTLIHKMSGT	IKKTLIHKM	3	0.4826	269.8	45.00	0.56	tr_C9ZXW1_
DRB1_0101	154	ACRVRLGLGPRESEV	VRLGLGPRE	3	0.4798	278.3	45.00	0.69	tr_C9ZXW1_
DRB1_0101	155	KNLEGCCFLNNAIFT	CCFLNNAIF	5	0.4783	282.8	46.00	0.51	tr_C9ZXW1_
DRB1_0101	156	AQDDIVMAMDTPYEA	VMAMDTPYE	5	0.4782	283.1	46.00	0.41	tr_C9ZXW1_
DRB1_0101	157	WIVQLVELTKRSYRM	WIVQLVELT	0	0.4759	290.2	46.00	0.36	tr_C9ZXW1_
DRB1_0101	158	PRESEVLVNEVINRL	SEVLVNEVI	3	0.4754	291.9	46.00	0.55	tr_C9ZXW1_
DRB1_0101	159	TVLGIPGLVKGVSGG	VLGIPGLVK	1	0.4753	292.3	46.00	0.54	tr_C9ZXW1_
DRB1_0101	160	DGRKIKKTLIHKMSG	IKKTLIHKM	4	0.4752	292.3	46.00	0.53	tr_C9ZXW1_
DRB1_0101	161	CCFLNNAIFTDRYRA	CFLNNAIFT	1	0.4677	317.0	48.00	0.28	tr_C9ZXW1_
DRB1_0101	162	FACRVRLGLGPRESE	VRLGLGPRE	4	0.4663	321.9	48.00	0.62	tr_C9ZXW1_
DRB1_0101	163	NEVINRLHLVECQDT	EVINRLHLV	1	0.4660	323.2	48.00	0.69	tr_C9ZXW1_
DRB1_0101	164	RTNPCEYFMELLQLP	YFMELLQLP	6	0.4655	324.7	48.00	0.41	tr_C9ZXW1_
DRB1_0101	165	APINLEGELCMERVV	PINLEGELC	1	0.4652	325.9	48.00	0.51	tr_C9ZXW1_
DRB1_0101	166	IATVHSPSSELVETF	IATVHSPSS	0	0.4604	343.2	49.00	0.39	tr_C9ZXW1_
DRB1_0101	167	SDGRKIKKTLIHKMS	IKKTLIHKM	5	0.4601	344.2	49.00	0.44	tr_C9ZXW1_
DRB1_0101	168	YLEDHLKKKGSSWIV	KKKGSSWIV	6	0.4598	345.4	49.00	0.46	tr_C9ZXW1_
DRB1_0101	169	IVQLVELTKRSYRMY	ELTKRSYRM	5	0.4553	362.5	50.00	0.36	tr_C9ZXW1_
DRB1_0101	170	CRVRLGLGPRESEVL	VRLGLGPRE	2	0.4552	363.2	50.00	0.68	tr_C9ZXW1_
DRB1_0101	171	LDEPTTGLDSVNALR	TTGLDSVNA	4	0.4550	363.7	50.00	0.79	tr_C9ZXW1_
DRB1_0101	172	VMAMDTPYEATYFAC	MAMDTPYEA	1	0.4540	367.8	55.00	0.52	tr_C9ZXW1_
DRB1_0101	173	FMELLQLPKEELEVL	LLQLPKEEL	3	0.4496	385.6	55.00	0.34	tr_C9ZXW1_
DRB1_0101	174	VECQDTVLGIPGLVK	VLGIPGLVK	6	0.4484	390.9	55.00	0.39	tr_C9ZXW1_
DRB1_0101	175	SGGERKRANVATALV	KRANVATAL	5	0.4473	395.5	55.00	0.56	tr_C9ZXW1_
DRB1_0101	176	TGRAPINLEGELCME	PINLEGELC	4	0.4466	398.6	55.00	0.64	tr_C9ZXW1_
DRB1_0101	177	ALVANPCVIVLDEPT	LVANPCVIV	1	0.4462	400.2	55.00	0.73	tr_C9ZXW1_
DRB1_0101	178	LTKRSYRMYPRHPSA	YRMYPRHPS	5	0.4433	412.9	55.00	0.64	tr_C9ZXW1_
DRB1_0101	179	RVGQMLQDLAKKEKR	MLQDLAKKE	4	0.4402	427.0	55.00	0.43	tr_C9ZXW1_
DRB1_0101	180	PCVIVLDEPTTGLDS	IVLDEPTTG	3	0.4401	427.4	55.00	0.41	tr_C9ZXW1_
DRB1_0101	181	LMSAITGKLHGTEKN	MSAITGKLH	1	0.4360	446.7	55.00	0.47	tr_C9ZXW1_
DRB1_0101	182	ELEVLWRAWELYLTT	LWRAWELYL	4	0.4343	455.0	55.00	0.38	tr_C9ZXW1_
DRB1_0101	183	NPCVIVLDEPTTGLD	IVLDEPTTG	4	0.4342	455.9	55.00	0.38	tr_C9ZXW1_
DRB1_0101	184	NNAIFTDRYRAVVSF	IFTDRYRAV	3	0.4335	459.0	55.00	0.34	tr_C9ZXW1_

DRB1_0101	185	ASIGHQVPPRTNPCE	IGHQVPPRT	2	0.4330	461.6	55.00	0.79	tr_C9ZXW1_
DRB1_0101	186	EELEVLWRAWELYLT	LWRAWELYL	5	0.4318	467.7	55.00	0.29	tr_C9ZXW1_
DRB1_0101	187	KEKRTVIATVHSPSS	IATVHSPSS	6	0.4316	468.6	55.00	0.46	tr_C9ZXW1_
DRB1_0101	188	VNYTWENLAFQVPAK	TWENLAFQV	3	0.4289	482.6	60.00	0.34	tr_C9ZXW1_
DRB1_0101	189	QLVELTKRSYRMYPR	ELTKRSYRM	3	0.4283	486.0	60.00	0.58	tr_C9ZXW1_
DRB1_0101	190	VVNYTWENLAFQVPA	TWENLAFQV	4	0.4267	494.3	60.00	0.28	tr_C9ZXW1_
DRB1_0101	191	VQLVELTKRSYRMYP	ELTKRSYRM	4	0.4260	497.9	60.00	0.48	tr_C9ZXW1_
DRB1_0101	192	LRVGQMLQDLAKKEK	MLQDLAKKE	5	0.4249	504.1	60.00	0.29	tr_C9ZXW1_
DRB1_0101	193	VGQMLQDLAKKEKRT	MLQDLAKKE	3	0.4239	509.2	60.00	0.59	tr_C9ZXW1_
DRB1_0101	194	CVIVLDEPTTGLDSV	IVLDEPTTG	2	0.4238	509.9	60.00	0.44	tr_C9ZXW1_
DRB1_0101	195	RVVNYTWENLAFQVP	VNYTWENLA	2	0.4231	513.8	60.00	0.34	tr_C9ZXW1_
DRB1_0101	196	NRLHLVECQDTVLGI	VECQDTVLG	5	0.4229	514.9	60.00	0.41	tr_C9ZXW1_
DRB1_0101	197	RVRLGLGPRESEVLV	VRLGLGPRE	1	0.4229	515.1	60.00	0.63	tr_C9ZXW1_
DRB1_0101	198	ANPCVIVLDEPTTGL	VIVLDEPTT	4	0.4228	515.3	60.00	0.34	tr_C9ZXW1_
DRB1_0101	199	ETGRAPINLEGELCM	PINLEGELC	5	0.4187	539.0	60.00	0.63	tr_C9ZXW1_
DRB1_0101	200	PINLEGELCMERVVN	PINLEGELC	0	0.4164	552.2	60.00	0.39	tr_C9ZXW1_
DRB1_0101	201	KEELEVLWRAWELYL	EVLWRAWEL	4	0.4156	557.4	60.00	0.25	tr_C9ZXW1_
DRB1_0101	202	VETFDDLLLLSEGHV	LLLLSEGHV	6	0.4148	561.9	60.00	0.46	tr_C9ZXW1_
DRB1_0101	203	GPRESEVLVNEVINR	SEVLVNEVI	4	0.4143	565.2	60.00	0.67	tr_C9ZXW1_
DRB1_0101	204	EKNLEGCCFLNNAIF	CCFLNNAIF	6	0.4138	568.5	60.00	0.47	tr_C9ZXW1_
DRB1_0101	205	ERVVNYTWENLAFQV	VNYTWENLA	3	0.4114	583.2	60.00	0.42	tr_C9ZXW1_
DRB1_0101	206	ALRVGQMLQDLAKKE	VGQMLQDLA	3	0.4097	594.0	60.00	0.31	tr_C9ZXW1_
DRB1_0101	207	RLHLVECQDTVLGIP	VECQDTVLG	4	0.4073	609.4	65.00	0.44	tr_C9ZXW1_
DRB1_0101	208	LEVLWRAWELYLTTP	LWRAWELYL	3	0.4068	612.8	65.00	0.38	tr_C9ZXW1_
DRB1_0101	209	PSGAGKTTLMSAITG	KTTLMSAIT	5	0.4054	622.0	65.00	0.35	tr_C9ZXW1_
DRB1_0101	210	VLGIPGLVKGVSGGE	VLGIPGLVK	0	0.4018	647.3	65.00	0.36	tr_C9ZXW1_
DRB1_0101	211	EVINRLHLVECQDTV	EVINRLHLV	0	0.4006	655.7	65.00	0.51	tr_C9ZXW1_
DRB1_0101	212	DSDGRKIKKTLIHKM	IKKTLIHKM	6	0.3996	662.8	65.00	0.34	tr_C9ZXW1_
DRB1_0101	213	LVELTKRSYRMYPRH	ELTKRSYRM	2	0.3993	664.8	65.00	0.64	tr_C9ZXW1_
DRB1_0101	214	ELTKRSYRMYPRHPS	YRMYPRHPS	6	0.3982	673.0	65.00	0.37	tr_C9ZXW1_
DRB1_0101	215	GQMLQDLAKKEKRTV	MLQDLAKKE	2	0.3892	741.3	65.00	0.60	tr_C9ZXW1_
DRB1_0101	216	LEGELCMERVVNYTW	LCMERVVNY	4	0.3887	745.6	65.00	0.47	tr_C9ZXW1_
DRB1_0101	217	CFLNNAIFTDRYRAV	FLNNAIFTD	1	0.3868	761.1	65.00	0.31	tr_C9ZXW1_
DRB1_0101	218	DTPYEATYFACRVRL	EATYFACRV	4	0.3857	770.1	65.00	0.37	tr_C9ZXW1_
DRB1_0101	219	MELLQLPKEELEVLW	LLQLPKEEL	2	0.3852	774.0	65.00	0.44	tr_C9ZXW1_
DRB1_0101	220	LVANPCVIVLDEPTT	LVANPCVIV	0	0.3848	777.6	65.00	0.49	tr_C9ZXW1_
DRB1_0101	221	LNNAIFTDRYRAVVS	IFTDRYRAV	4	0.3841	783.9	70.00	0.37	tr_C9ZXW1_
DRB1_0101	222	LHLVECQDTVLGIPG	VECQDTVLG	3	0.3837	787.1	70.00	0.51	tr_C9ZXW1_
DRB1_0101	223	INRLHLVECQDTVLG	VECQDTVLG	6	0.3803	816.1	70.00	0.24	tr_C9ZXW1_
DRB1_0101	224	VSGGERKRANVATAL	KRANVATAL	6	0.3794	824.9	70.00	0.38	tr_C9ZXW1_
DRB1_0101	225	SSELVETFDDLLLLS	LVETFDDLL	3	0.3775	841.2	70.00	0.58	tr_C9ZXW1_
DRB1_0101	226	VLDEPTTGLDSVNAL	TTGLDSVNA	5	0.3765	850.8	70.00	0.75	tr_C9ZXW1_
DRB1_0101	227	VAQDDIVMAMDTPYE	VMAMDTPYE	6	0.3758	857.0	70.00	0.32	tr_C9ZXW1_
DRB1_0101	228	NLEGELCMERVVNYT	LCMERVVNY	5	0.3757	858.0	70.00	0.44	tr_C9ZXW1_
DRB1_0101	229	PKEELEVLWRAWELY	EELEVLWRA	2	0.3756	859.0	70.00	0.30	tr_C9ZXW1_
DRB1_0101	230	LVECQDTVLGIPGLV	VECQDTVLG	1	0.3747	867.2	70.00	0.31	tr_C9ZXW1_
DRB1_0101	231	IPGLVKGVSGGERKR	LVKGVSGGE	3	0.3746	868.0	70.00	0.40	tr_C9ZXW1_
DRB1_0101	232	INLEGELCMERVVNY	LCMERVVNY	6	0.3741	873.3	70.00	0.28	tr_C9ZXW1_
DRB1_0101	233	HLVECQDTVLGIPGL	VECQDTVLG	2	0.3737	876.7	70.00	0.43	tr_C9ZXW1_
DRB1_0101	234	LPKEELEVLWRAWEL	EELEVLWRA	3	0.3729	884.8	70.00	0.38	tr_C9ZXW1_

DRB1_0101	235	EGELCMERVVNYTWE	LCMERVVNY	3	0.3723	890.4	70.00	0.51	tr_C9ZXW1_
DRB1_0101	236	SIGHQVPPRTNPCEY	IGHQVPPRT	1	0.3712	900.7	70.00	0.76	tr_C9ZXW1_
DRB1_0101	237	VELTKRSYRMYPRHP	ELTKRSYRM	1	0.3702	910.6	70.00	0.57	tr_C9ZXW1_
DRB1_0101	238	LAFQVPAKDSDGRKI	LAFQVPAKD	0	0.3630	985.0	70.00	0.57	tr_C9ZXW1_
DRB1_0101	239	FLNNAIFTDRYRAVV	IFTDRYRAV	5	0.3620	995.3	70.00	0.35	tr_C9ZXW1_
DRB1_0101	240	PGLVKGVSGGERKRA	LVKGVSGGE	2	0.3601	1015.9	70.00	0.33	tr_C9ZXW1_
DRB1_0101	241	VIVLDEPTTGLDSVN	IVLDEPTTG	1	0.3580	1039.7	75.00	0.45	tr_C9ZXW1_
DRB1_0101	242	GIPGLVKGVSGGERK	PGLVKGVSG	2	0.3576	1043.5	75.00	0.29	tr_C9ZXW1_
DRB1_0101	243	SELVETFDDLLLLSE	LVETFDDLL	2	0.3569	1052.3	75.00	0.52	tr_C9ZXW1_
DRB1_0101	244	LGIPGLVKGVSGGER	PGLVKGVSG	3	0.3564	1057.1	75.00	0.34	tr_C9ZXW1_
DRB1_0101	245	TEKNLEGCCFLNNAI	EKNLEGCCF	1	0.3506	1125.3	75.00	0.37	tr_C9ZXW1_
DRB1_0101	246	PSSELVETFDDLLLL	LVETFDDLL	4	0.3503	1129.1	75.00	0.58	tr_C9ZXW1_
DRB1_0101	247	LGPRESEVLVNEVIN	SEVLVNEVI	5	0.3494	1141.2	75.00	0.67	tr_C9ZXW1_
DRB1_0101	248	MERVVNYTWENLAFQ	VNYTWENLA	4	0.3482	1155.6	75.00	0.41	tr_C9ZXW1_
DRB1_0101	249	GTEKNLEGCCFLNNA	EKNLEGCCF	2	0.3473	1167.4	75.00	0.46	tr_C9ZXW1_
DRB1_0101	250	PRTNPCEYFMELLQL	EYFMELLQL	6	0.3466	1175.2	75.00	0.49	tr_C9ZXW1_
DRB1_0101	251	GELCMERVVNYTWEN	LCMERVVNY	2	0.3460	1183.0	75.00	0.54	tr_C9ZXW1_
DRB1_0101	252	QMLQDLAKKEKRTVI	MLQDLAKKE	1	0.3428	1224.8	75.00	0.52	tr_C9ZXW1_
DRB1_0101	253	HGTEKNLEGCCFLNN	EKNLEGCCF	3	0.3409	1250.7	75.00	0.52	tr_C9ZXW1_
DRB1_0101	254	LLQLPKEELEVLWRA	LLQLPKEEL	0	0.3360	1318.8	75.00	0.21	tr_C9ZXW1_
DRB1_0101	255	TTPAASTNPCLLKVS	AASTNPCLL	3	0.3338	1350.8	80.00	0.45	tr_C9ZXW1_
DRB1_0101	256	ELLQLPKEELEVLWR	LLQLPKEEL	1	0.3315	1383.7	80.00	0.44	tr_C9ZXW1_
DRB1_0101	257	VANPCVIVLDEPTTG	VIVLDEPTT	5	0.3315	1384.6	80.00	0.41	tr_C9ZXW1_
DRB1_0101	258	ELVETFDDLLLLSEG	LVETFDDLL	1	0.3302	1403.4	80.00	0.41	tr_C9ZXW1_
DRB1_0101	259	VRLGLGPRESEVLVN	VRLGLGPRE	0	0.3301	1405.6	80.00	0.44	tr_C9ZXW1_
DRB1_0101	260	LTTPAASTNPCLLKV	AASTNPCLL	4	0.3281	1435.6	80.00	0.38	tr_C9ZXW1_
DRB1_0101	261	GLVKGVSGGERKRAN	VKGVSGGER	2	0.3274	1447.6	80.00	0.31	tr_C9ZXW1_
DRB1_0101	262	KKEKRTVIATVHSPS	TVIATVHSP	5	0.3260	1468.7	80.00	0.22	tr_C9ZXW1_
DRB1_0101	263	QLPKEELEVLWRAWE	EELEVLWRA	4	0.3243	1497.3	80.00	0.39	tr_C9ZXW1_
DRB1_0101	264	LQLPKEELEVLWRAW	EELEVLWRA	5	0.3241	1499.9	80.00	0.34	tr_C9ZXW1_
DRB1_0101	265	YHGPREEATAYFASI	REEATAYFA	4	0.3231	1516.2	80.00	0.46	tr_C9ZXW1_
DRB1_0101	266	VINRLHLVECQDTVL	LHLVECQDT	4	0.3207	1556.8	80.00	0.26	tr_C9ZXW1_
DRB1_0101	267	MDTPYEATYFACRVR	EATYFACRV	5	0.3201	1566.5	80.00	0.36	tr_C9ZXW1_
DRB1_0101	268	LAKKEKRTVIATVHS	EKRTVIATV	4	0.3199	1569.4	80.00	0.32	tr_C9ZXW1_
DRB1_0101	269	PETGRAPINLEGELC	PINLEGELC	6	0.3190	1585.3	80.00	0.57	tr_C9ZXW1_
DRB1_0101	270	ATVHSPSSELVETFD	TVHSPSSEL	1	0.3181	1599.8	80.00	0.57	tr_C9ZXW1_
DRB1_0101	271	TPAASTNPCLLKVSG	AASTNPCLL	2	0.3169	1621.4	80.00	0.41	tr_C9ZXW1_
DRB1_0101	272	LHGTEKNLEGCCFLN	EKNLEGCCF	4	0.3168	1622.5	80.00	0.53	tr_C9ZXW1_
DRB1_0101	273	MAMDTPYEATYFACR	MAMDTPYEA	0	0.3159	1638.5	80.00	0.43	tr_C9ZXW1_
DRB1_0101	274	HVVYHGPREEATAYF	YHGPREEAT	3	0.3144	1666.0	80.00	0.38	tr_C9ZXW1_
DRB1_0101	275	GHVVYHGPREEATAY	YHGPREEAT	4	0.3142	1670.0	80.00	0.33	tr_C9ZXW1_
DRB1_0101	276	AKKEKRTVIATVHSP	EKRTVIATV	3	0.3115	1718.6	80.00	0.32	tr_C9ZXW1_
DRB1_0101	277	GPSGAGKTTLMSAIT	KTTLMSAIT	6	0.3113	1722.0	80.00	0.34	tr_C9ZXW1_
DRB1_0101	278	DLAKKEKRTVIATVH	KKEKRTVIA	3	0.3108	1732.5	80.00	0.25	tr_C9ZXW1_
DRB1_0101	279	VYHGPREEATAYFAS	REEATAYFA	5	0.3107	1733.5	80.00	0.37	tr_C9ZXW1_
DRB1_0101	280	VVYHGPREEATAYFA	YHGPREEAT	2	0.3104	1739.9	80.00	0.38	tr_C9ZXW1_
DRB1_0101	281	LVETFDDLLLLSEGH	LVETFDDLL	0	0.3082	1782.3	80.00	0.19	tr_C9ZXW1_
DRB1_0101	282	HGPREEATAYFASIG	REEATAYFA	3	0.3077	1791.0	80.00	0.51	tr_C9ZXW1_
DRB1_0101	283	ELCMERVVNYTWENL	LCMERVVNY	1	0.3073	1799.2	80.00	0.45	tr_C9ZXW1_
DRB1_0101	284	IVLDEPTTGLDSVNA	TTGLDSVNA	6	0.3021	1902.0	85.00	0.41	tr_C9ZXW1_

DRB1_0101	285	PYLEDHLKKKGSSWI	LKKKGSSWI	6	0.3001	1945.3	85.00	0.39	tr_C9ZXW1_
DRB1_0101	286	MSAITGKLHGTEKNL	MSAITGKLH	0	0.2987	1975.0	85.00	0.49	tr_C9ZXW1_
DRB1_0101	287	GPREEATAYFASIGH	REEATAYFA	2	0.2983	1982.1	85.00	0.47	tr_C9ZXW1_
DRB1_0101	288	AIMGPSGAGKTTLMS	MGPSGAGKT	2	0.2981	1987.8	85.00	0.50	tr_C9ZXW1_
DRB1_0101	289	KDSDGRKIKKTLIHK	GRKIKKTLI	4	0.2977	1996.7	85.00	0.43	tr_C9ZXW1_
DRB1_0101	290	EGHVVYHGPREEATA	VVYHGPREE	3	0.2949	2056.3	85.00	0.37	tr_C9ZXW1_
DRB1_0101	291	SPSSELVETFDDLLL	LVETFDDLL	5	0.2938	2081.3	85.00	0.58	tr_C9ZXW1_
DRB1_0101	292	GLGPRESEVLVNEVI	SEVLVNEVI	6	0.2934	2091.5	85.00	0.51	tr_C9ZXW1_
DRB1_0101	293	CMERVVNYTWENLAF	VNYTWENLA	5	0.2932	2095.0	85.00	0.44	tr_C9ZXW1_
DRB1_0101	294	PREEATAYFASIGHQ	REEATAYFA	1	0.2921	2119.8	85.00	0.35	tr_C9ZXW1_
DRB1_0101	295	QDLAKKEKRTVIATV	LAKKEKRTV	2	0.2913	2139.8	85.00	0.28	tr_C9ZXW1_
DRB1_0101	296	KLHGTEKNLEGCCFL	EKNLEGCCF	5	0.2845	2302.5	85.00	0.51	tr_C9ZXW1_
DRB1_0101	297	LCMERVVNYTWENLA	MERVVNYTW	2	0.2832	2335.4	85.00	0.26	tr_C9ZXW1_
DRB1_0101	298	LVKGVSGGERKRANV	VSGGERKRA	4	0.2831	2337.9	85.00	0.32	tr_C9ZXW1_
DRB1_0101	299	GVSGGERKRANVATA	GERKRANVA	4	0.2806	2400.5	85.00	0.35	tr_C9ZXW1_
DRB1_0101	300	IMGPSGAGKTTLMSA	MGPSGAGKT	1	0.2800	2416.7	85.00	0.41	tr_C9ZXW1_
DRB1_0101	301	AMDTPYEATYFACRV	YEATYFACR	5	0.2768	2502.7	85.00	0.28	tr_C9ZXW1_
DRB1_0101	302	MLQDLAKKEKRTVIA	LAKKEKRTV	4	0.2717	2644.1	90.00	0.31	tr_C9ZXW1_
DRB1_0101	303	AASTNPCLLKVSGPI	CLLKVSGPI	6	0.2711	2659.8	90.00	0.34	tr_C9ZXW1_
DRB1_0101	304	MGPSGAGKTTLMSAI	SGAGKTTLM	3	0.2679	2753.6	90.00	0.33	tr_C9ZXW1_
DRB1_0101	305	KGVSGGERKRANVAT	VSGGERKRA	2	0.2675	2766.3	90.00	0.43	tr_C9ZXW1_
DRB1_0101	306	AKDSDGRKIKKTLIH	GRKIKKTLI	5	0.2673	2772.6	90.00	0.40	tr_C9ZXW1_
DRB1_0101	307	PAASTNPCLLKVSGP	AASTNPCLL	1	0.2670	2781.0	90.00	0.41	tr_C9ZXW1_
DRB1_0101	308	LKVSGPITERDPYLE	LKVSGPITE	0	0.2653	2833.8	90.00	0.45	tr_C9ZXW1_
DRB1_0101	309	LQDLAKKEKRTVIAT	LAKKEKRTV	3	0.2645	2858.6	90.00	0.38	tr_C9ZXW1_
DRB1_0101	310	LLSEGHVVYHGPREE	LLSEGHVVY	0	0.2603	2991.6	90.00	0.28	tr_C9ZXW1_
DRB1_0101	311	VKGVSGGERKRANVA	VSGGERKRA	3	0.2601	2996.3	90.00	0.48	tr_C9ZXW1_
DRB1_0101	312	SEGHVVYHGPREEAT	VVYHGPREE	4	0.2538	3207.7	90.00	0.35	tr_C9ZXW1_
DRB1_0101	313	RLGLGPRESEVLVNE	LGPRESEVL	3	0.2493	3369.2	90.00	0.54	tr_C9ZXW1_
DRB1_0101	314	PAKDSDGRKIKKTLI	AKDSDGRKI	1	0.2490	3379.5	90.00	0.41	tr_C9ZXW1_
DRB1_0101	315	RDPYLEDHLKKKGSS	PYLEDHLKK	2	0.2420	3645.3	90.00	0.50	tr_C9ZXW1_
DRB1_0101	316	IGHQVPPRTNPCEYF	IGHQVPPRT	0	0.2405	3706.7	90.00	0.63	tr_C9ZXW1_
DRB1_0101	317	ERDPYLEDHLKKKGS	PYLEDHLKK	3	0.2404	3708.0	90.00	0.51	tr_C9ZXW1_
DRB1_0101	318	TVHSPSSELVETFDD	TVHSPSSEL	0	0.2342	3968.9	95.00	0.37	tr_C9ZXW1_
DRB1_0101	319	DPYLEDHLKKKGSSW	PYLEDHLKK	1	0.2319	4068.7	95.00	0.36	tr_C9ZXW1_
DRB1_0101	320	PPRTNPCEYFMELLQ	NPCEYFMEL	4	0.2313	4093.1	95.00	0.25	tr_C9ZXW1_
DRB1_0101	321	LGLGPRESEVLVNEV	LGPRESEVL	2	0.2294	4176.8	95.00	0.46	tr_C9ZXW1_
DRB1_0101	322	QVPAKDSDGRKIKKT	AKDSDGRKI	3	0.2278	4249.8	95.00	0.65	tr_C9ZXW1_
DRB1_0101	323	FQVPAKDSDGRKIKK	AKDSDGRKI	4	0.2275	4267.3	95.00	0.61	tr_C9ZXW1_
DRB1_0101	324	TERDPYLEDHLKKKG	PYLEDHLKK	4	0.2232	4467.9	95.00	0.50	tr_C9ZXW1_
DRB1_0101	325	AFQVPAKDSDGRKIK	AKDSDGRKI	5	0.2215	4549.3	95.00	0.44	tr_C9ZXW1_
DRB1_0101	326	VPAKDSDGRKIKKTL	AKDSDGRKI	2	0.2211	4572.7	95.00	0.62	tr_C9ZXW1_
DRB1_0101	327	LSEGHVVYHGPREEA	VVYHGPREE	5	0.2169	4786.0	95.00	0.36	tr_C9ZXW1_
DRB1_0101	328	HSPSSELVETFDDLL	LVETFDDLL	6	0.2167	4793.8	95.00	0.47	tr_C9ZXW1_
DRB1_0101	329	SAITGKLHGTEKNLE	KLHGTEKNL	5	0.2131	4984.9	95.00	0.19	tr_C9ZXW1_
DRB1_0101	330	AITGKLHGTEKNLEG	KLHGTEKNL	4	0.2122	5032.0	95.00	0.28	tr_C9ZXW1_
DRB1_0101	331	GKLHGTEKNLEGCCF	EKNLEGCCF	6	0.2112	5089.9	95.00	0.39	tr_C9ZXW1_
DRB1_0101	332	SPETGRAPINLEGEL	TGRAPINLE	3	0.2058	5391.5	95.00	0.38	tr_C9ZXW1_
DRB1_0101	333	VPPRTNPCEYFMELL	NPCEYFMEL	5	0.2016	5646.8	95.00	0.24	tr_C9ZXW1_
DRB1_0101	334	ITGKLHGTEKNLEGC	KLHGTEKNL	3	0.1974	5907.4	95.00	0.31	tr_C9ZXW1_

DRB1_0101	335	ITERDPYLEDHLKKK	PYLEDHLKK	5	0.1950	6062.6	95.00	0.50	tr_C9ZXW1_
DRB1_0101	336	DSPETGRAPINLEGE	ETGRAPINL	3	0.1932	6183.5	95.00	0.37	tr_C9ZXW1_
DRB1_0101	337	PDSPETGRAPINLEG	ETGRAPINL	4	0.1889	6474.7	100.00	0.37	tr_C9ZXW1_
DRB1_0101	338	TGKLHGTEKNLEGCC	LHGTEKNLE	3	0.1763	7423.1	100.00	0.34	tr_C9ZXW1_
DRB1_0101	339	KVSGPITERDPYLED	PITERDPYL	4	0.1672	8189.2	100.00	0.46	tr_C9ZXW1_
DRB1_0101	340	QVPPRTNPCEYFMEL	PRTNPCEYF	3	0.1633	8544.8	100.00	0.31	tr_C9ZXW1_
DRB1_0101	341	PITERDPYLEDHLKK	PYLEDHLKK	6	0.1630	8572.8	100.00	0.34	tr_C9ZXW1_
DRB1_0101	342	MPDSPETGRAPINLE	ETGRAPINL	5	0.1610	8762.0	100.00	0.36	tr_C9ZXW1_
DRB1_0101	343	VSGPITERDPYLEDH	PITERDPYL	3	0.1584	9013.4	100.00	0.58	tr_C9ZXW1_
DRB1_0101	344	SGPITERDPYLEDHL	PITERDPYL	2	0.1515	9711.4	100.00	0.53	tr_C9ZXW1_
DRB1_0101	345	VHSPSSELVETFDDL	VHSPSSELV	0	0.1495	9924.0	100.00	0.31	tr_C9ZXW1_
DRB1_0101	346	GPITERDPYLEDHLK	PITERDPYL	1	0.1351	11587.6	100.00	0.41	tr_C9ZXW1_
DRB1_0101	347	HQVPPRTNPCEYFME	PRTNPCEYF	4	0.1291	12369.9	100.00	0.32	tr_C9ZXW1_
DRB1_0101	348	GHQVPPRTNPCEYFM	PRTNPCEYF	5	0.1099	15224.2	100.00	0.29	tr_C9ZXW1_

Allele: DRB1_0101. Number of high binders 0. Number of weak binders 42. Number of peptides 348

Allele	pos	peptide	core	Of	1-log50k(aff)	affinity(nM)	%Rank	Relia	Identity	Bind Level
DRB1_0101	1	SVIEFFNFDSWSWWK	FFNFDSWSW	4	0.3763	852.6	70.00	0.44	tr_C9ZXW1_	
DRB1_0101	2	VIEFFNFDSWSWWKS	FFNFDSWSW	3	0.3721	892.5	70.00	0.49	tr_C9ZXW1_	
DRB1_0101	3	QSVIEFFNFDSWSWW	FFNFDSWSW	5	0.3540	1085.1	75.00	0.39	tr_C9ZXW1_	
DRB1_0101	4	GQSVIEFFNFDSWSW	FFNFDSWSW	6	0.3130	1691.6	80.00	0.35	tr_C9ZXW1_	
DRB1_0101	5	HCRYPDGQSVIEFFN	YPDGQSVIE	3	0.2827	2348.0	85.00	0.38	tr_C9ZXW1_	
DRB1_0101	6	LHCRYPDGQSVIEFF	YPDGQSVIE	4	0.2810	2390.2	85.00	0.34	tr_C9ZXW1_	
DRB1_0101	7	CRYPDGQSVIEFFNF	YPDGQSVIE	2	0.2695	2706.8	90.00	0.35	tr_C9ZXW1_	
DRB1_0101	8	DLHCRYPDGQSVIEF	CRYPDGQSV	3	0.2560	3132.9	90.00	0.36	tr_C9ZXW1_	
DRB1_0101	9	DGQSVIEFFNFDSWS	VIEFFNFDS	4	0.2381	3803.5	95.00	0.43	tr_C9ZXW1_	
DRB1_0101	10	RYPDGQSVIEFFNFD	YPDGQSVIE	1	0.2363	3879.3	95.00	0.32	tr_C9ZXW1_	
DRB1_0101	11	EFSRLEKICDPPDLH	EKICDPPDL	5	0.2362	3880.3	95.00	0.28	tr_C9ZXW1_	
DRB1_0101	12	FSRLEKICDPPDLHC	EKICDPPDL	4	0.2315	4085.5	95.00	0.32	tr_C9ZXW1_	
DRB1_0101	13	SRLEKICDPPDLHCR	EKICDPPDL	3	0.2270	4287.6	95.00	0.40	tr_C9ZXW1_	
DRB1_0101	14	NEFSRLEKICDPPDL	FSRLEKICD	2	0.2244	4412.4	95.00	0.25	tr_C9ZXW1_	
DRB1_0101	15	PDGQSVIEFFNFDSW	VIEFFNFDS	5	0.2201	4621.8	95.00	0.38	tr_C9ZXW1_	
DRB1_0101	16	RLEKICDPPDLHCRY	EKICDPPDL	2	0.2191	4672.0	95.00	0.39	tr_C9ZXW1_	
DRB1_0101	17	PDLHCRYPDGQSVIE	CRYPDGQSV	4	0.2146	4906.2	95.00	0.40	tr_C9ZXW1_	
DRB1_0101	18	YPDGQSVIEFFNFDS	VIEFFNFDS	6	0.2122	5033.7	95.00	0.22	tr_C9ZXW1_	
DRB1_0101	19	LEKICDPPDLHCRYP	EKICDPPDL	1	0.1832	6889.1	100.00	0.37	tr_C9ZXW1_	
DRB1_0101	20	PPDLHCRYPDGQSVI	CRYPDGQSV	5	0.1708	7874.8	100.00	0.52	tr_C9ZXW1_	
DRB1_0101	21	EKICDPPDLHCRYPD	KICDPPDLH	1	0.1452	10388.8	100.00	0.24	tr_C9ZXW1_	
DRB1_0101	22	DPPDLHCRYPDGQSV	CRYPDGQSV	6	0.1259	12809.6	100.00	0.44	tr_C9ZXW1_	
DRB1_0101	23	KICDPPDLHCRYPDG	KICDPPDLH	0	0.1110	15038.5	100.00	0.25	tr_C9ZXW1_	
DRB1_0101	24	CDPPDLHCRYPDGQS	LHCRYPDGQ	5	0.0874	19424.3	100.00	0.33	tr_C9ZXW1_	
DRB1_0101	25	ICDPPDLHCRYPDGQ	CDPPDLHCR	1	0.0861	19704.8	100.00	0.20	tr_C9ZXW1_	

Allele: DRB1_0101. Number of high binders 0. Number of weak binders 0. Number of peptides 25

Allele	pos	peptide	core	Of	1-log50k(aff)	affinity(nM)	%Rank	Relia	Identity	Bind Level
DRB1_0101	1	LSDYLVRDSLLAMFT	YLVRDSLLA	3	0.7630	13.0	4.00	0.87	tr_D0A423_	WB
DRB1_0101	2	LSYFLCKGLSDYLVR	CKGLSDYLV	5	0.7627	13.0	4.00	0.37	tr_D0A423_	WB
DRB1_0101	3	SLLAMFTQRFGVDVS	FTQRFGVDV	5	0.7599	13.4	4.50	0.47	tr_D0A423_	WB
DRB1_0101	4	SYFLCKGLSDYLVRD	CKGLSDYLV	4	0.7572	13.8	4.50	0.39	tr_D0A423_	WB
DRB1_0101	5	SDYLVRDSLLAMFTQ	YLVRDSLLA	2	0.7533	14.4	4.50	0.85	tr_D0A423_	WB
DRB1_0101	6	GLSDYLVRDSLLAMF	YLVRDSLLA	4	0.7514	14.7	5.00	0.85	tr_D0A423_	WB
DRB1_0101	7	DSLLAMFTQRFGVDV	LLAMFTQRF	2	0.7379	17.0	5.50	0.62	tr_D0A423_	WB
DRB1_0101	8	LAMFTQRFGVDVSVY	FTQRFGVDV	3	0.7348	17.6	6.00	0.87	tr_D0A423_	WB
DRB1_0101	9	LLAMFTQRFGVDVSV	FTQRFGVDV	4	0.7326	18.1	6.00	0.74	tr_D0A423_	WB
DRB1_0101	10	YFLCKGLSDYLVRDS	CKGLSDYLV	3	0.7325	18.1	6.00	0.50	tr_D0A423_	WB
DRB1_0101	11	DYLVRDSLLAMFTQR	YLVRDSLLA	1	0.7253	19.5	6.50	0.77	tr_D0A423_	WB
DRB1_0101	12	VRDSLLAMFTQRFGV	LLAMFTQRF	4	0.7252	19.6	7.00	0.82	tr_D0A423_	WB
DRB1_0101	13	GWSVKPLTAVVSDIF	VKPLTAVVS	3	0.7240	19.8	7.00	0.80	tr_D0A423_	WB
DRB1_0101	14	KGLSDYLVRDSLLAM	YLVRDSLLA	5	0.7221	20.2	7.00	0.83	tr_D0A423_	WB
DRB1_0101	15	YLVRDSLLAMFTQRF	LLAMFTQRF	6	0.7206	20.6	7.00	0.38	tr_D0A423_	WB
DRB1_0101	16	SLSYFLCKGLSDYLV	LCKGLSDYL	5	0.7190	20.9	7.50	0.31	tr_D0A423_	WB
DRB1_0101	17	AMFTQRFGVDVSVYQ	FTQRFGVDV	2	0.7182	21.1	7.50	0.85	tr_D0A423_	WB
DRB1_0101	18	VSVYQRLSNVANMGW	YQRLSNVAN	3	0.7177	21.2	7.50	0.67	tr_D0A423_	WB
DRB1_0101	19	RDSLLAMFTQRFGVD	LLAMFTQRF	3	0.7167	21.4	7.50	0.85	tr_D0A423_	WB
DRB1_0101	20	DVSVYQRLSNVANMG	YQRLSNVAN	4	0.7104	22.9	8.00	0.57	tr_D0A423_	WB
DRB1_0101	21	MGWSVKPLTAVVSDI	VKPLTAVVS	4	0.7096	23.1	8.00	0.75	tr_D0A423_	WB
DRB1_0101	22	YQRLSNVANMGWSVK	NVANMGWSV	5	0.7045	24.5	8.50	0.65	tr_D0A423_	WB
DRB1_0101	23	WSVKPLTAVVSDIFP	VKPLTAVVS	2	0.7030	24.9	8.50	0.80	tr_D0A423_	WB
DRB1_0101	24	LVRDSLLAMFTQRFG	LLAMFTQRF	5	0.6999	25.7	9.00	0.78	tr_D0A423_	WB
DRB1_0101	25	RLSNVANMGWSVKPL	NVANMGWSV	3	0.6998	25.7	9.00	0.81	tr_D0A423_	WB
DRB1_0101	26	VDVSVYQRLSNVANM	YQRLSNVAN	5	0.6943	27.3	9.50	0.53	tr_D0A423_	WB
DRB1_0101	27	CKGLSDYLVRDSLLA	YLVRDSLLA	6	0.6939	27.4	9.50	0.57	tr_D0A423_	WB
DRB1_0101	28	NMGWSVKPLTAVVSD	VKPLTAVVS	5	0.6830	30.9	11.00	0.69	tr_D0A423_	
DRB1_0101	29	FLCKGLSDYLVRDSL	CKGLSDYLV	2	0.6828	30.9	11.00	0.61	tr_D0A423_	
DRB1_0101	30	SVYQRLSNVANMGWS	YQRLSNVAN	2	0.6805	31.7	11.00	0.69	tr_D0A423_	
DRB1_0101	31	VPCLRRLPMFGTAAE	LRRLPMFGT	3	0.6785	32.4	12.00	0.67	tr_D0A423_	
DRB1_0101	32	MFTQRFGVDVSVYQR	FTQRFGVDV	1	0.6766	33.1	12.00	0.76	tr_D0A423_	
DRB1_0101	33	VYQRLSNVANMGWSV	YQRLSNVAN	1	0.6764	33.2	12.00	0.53	tr_D0A423_	
DRB1_0101	34	LSNVANMGWSVKPLT	NVANMGWSV	2	0.6721	34.7	12.00	0.81	tr_D0A423_	
DRB1_0101	35	QRLSNVANMGWSVKP	NVANMGWSV	4	0.6721	34.7	12.00	0.79	tr_D0A423_	
DRB1_0101	36	PCLRRLPMFGTAAEG	LRRLPMFGT	2	0.6700	35.6	13.00	0.66	tr_D0A423_	
DRB1_0101	37	KVPCLRRLPMFGTAA	LRRLPMFGT	4	0.6679	36.3	13.00	0.71	tr_D0A423_	
DRB1_0101	38	SVKPLTAVVSDIFPL	VKPLTAVVS	1	0.6671	36.7	13.00	0.77	tr_D0A423_	
DRB1_0101	39	CLRRLPMFGTAAEGY	LRRLPMFGT	1	0.6460	46.1	16.00	0.61	tr_D0A423_	
DRB1_0101	40	SNVANMGWSVKPLTA	NVANMGWSV	1	0.6432	47.5	16.00	0.75	tr_D0A423_	
DRB1_0101	41	ANMGWSVKPLTAVVS	VKPLTAVVS	6	0.6267	56.8	18.00	0.55	tr_D0A423_	
DRB1_0101	42	EATYVHPDARALFNK	YVHPDARAL	3	0.6211	60.3	19.00	0.80	tr_D0A423_	
DRB1_0101	43	ATYVHPDARALFNKV	YVHPDARAL	2	0.6204	60.8	19.00	0.76	tr_D0A423_	
DRB1_0101	44	LCKGLSDYLVRDSLL	CKGLSDYLV	1	0.6192	61.6	19.00	0.64	tr_D0A423_	
DRB1_0101	45	GVDVSVYQRLSNVAN	SVYQRLSNV	4	0.6171	63.0	20.00	0.37	tr_D0A423_	

DRB1_0101	46	ISLSYFLCKGLSDYL	YFLCKGLSD	4	0.6119	66.7	21.00	0.49	tr_D0A423_
DRB1_0101	47	NKVPCLRRLPMFGTA	LRRLPMFGT	5	0.6095	68.4	21.00	0.75	tr_D0A423_
DRB1_0101	48	GEATYVHPDARALFN	YVHPDARAL	4	0.5840	90.1	25.00	0.78	tr_D0A423_
DRB1_0101	49	TYVHPDARALFNKVP	YVHPDARAL	1	0.5788	95.4	26.00	0.73	tr_D0A423_
DRB1_0101	50	NVANMGWSVKPLTAV	NVANMGWSV	0	0.5733	101.2	27.00	0.57	tr_D0A423_
DRB1_0101	51	FTQRFGVDVSVYQRL	FTQRFGVDV	0	0.5703	104.6	28.00	0.46	tr_D0A423_
DRB1_0101	52	LRRLPMFGTAAEGYG	LRRLPMFGT	0	0.5697	105.2	28.00	0.48	tr_D0A423_
DRB1_0101	53	GPKAVMSISLSYFLC	KAVMSISLS	2	0.5649	110.8	29.00	0.28	tr_D0A423_
DRB1_0101	54	YGPKAVMSISLSYFL	KAVMSISLS	3	0.5629	113.2	29.00	0.30	tr_D0A423_
DRB1_0101	55	PKAVMSISLSYFLCK	MSISLSYFL	4	0.5625	113.8	29.00	0.29	tr_D0A423_
DRB1_0101	56	VKPLTAVVSDIFPLF	VKPLTAVVS	0	0.5603	116.4	29.00	0.60	tr_D0A423_
DRB1_0101	57	FGVDVSVYQRLSNVA	SVYQRLSNV	5	0.5453	137.0	32.00	0.50	tr_D0A423_
DRB1_0101	58	SGEATYVHPDARALF	YVHPDARAL	5	0.5323	157.7	35.00	0.77	tr_D0A423_
DRB1_0101	59	FNKVPCLRRLPMFGT	LRRLPMFGT	6	0.5283	164.6	35.00	0.56	tr_D0A423_
DRB1_0101	60	KAVMSISLSYFLCKG	MSISLSYFL	3	0.5283	164.6	35.00	0.40	tr_D0A423_
DRB1_0101	61	GYGPKAVMSISLSYF	KAVMSISLS	4	0.5283	164.7	35.00	0.31	tr_D0A423_
DRB1_0101	62	SISLSYFLCKGLSDY	YFLCKGLSD	5	0.5187	182.7	37.00	0.53	tr_D0A423_
DRB1_0101	63	RFGVDVSVYQRLSNV	VDVSVYQRL	3	0.5077	205.7	39.00	0.35	tr_D0A423_
DRB1_0101	64	EGYGPKAVMSISLSY	PKAVMSISL	4	0.4924	242.8	43.00	0.30	tr_D0A423_
DRB1_0101	65	MSFAPTRSLPNEVNE	FAPTRSLPN	2	0.4801	277.3	45.00	0.51	tr_D0A423_
DRB1_0101	66	YVHPDARALFNKVPC	YVHPDARAL	0	0.4701	308.9	47.00	0.56	tr_D0A423_
DRB1_0101	67	DARALFNKVPCLRRL	FNKVPCLRR	5	0.4672	319.0	48.00	0.34	tr_D0A423_
DRB1_0101	68	MSISLSYFLCKGLSD	YFLCKGLSD	6	0.4639	330.6	49.00	0.42	tr_D0A423_
DRB1_0101	69	TQRFGVDVSVYQRLS	VDVSVYQRL	5	0.4626	335.0	49.00	0.36	tr_D0A423_
DRB1_0101	70	RALFNKVPCLRRLPM	FNKVPCLRR	3	0.4618	338.2	49.00	0.41	tr_D0A423_
DRB1_0101	71	AEGYGPKAVMSISLS	PKAVMSISL	5	0.4545	365.7	55.00	0.31	tr_D0A423_
DRB1_0101	72	ARALFNKVPCLRRLP	FNKVPCLRR	4	0.4513	378.6	55.00	0.38	tr_D0A423_
DRB1_0101	73	VANMGWSVKPLTAVV	GWSVKPLTA	4	0.4511	379.7	55.00	0.31	tr_D0A423_
DRB1_0101	74	QRFGVDVSVYQRLSN	VDVSVYQRL	4	0.4510	379.8	55.00	0.42	tr_D0A423_
DRB1_0101	75	AVMSISLSYFLCKGL	MSISLSYFL	2	0.4476	394.1	55.00	0.45	tr_D0A423_
DRB1_0101	76	AVVSDIFPLFGYSKR	IFPLFGYSK	5	0.4454	403.8	55.00	0.50	tr_D0A423_
DRB1_0101	77	PDARALFNKVPCLRR	LFNKVPCLR	5	0.4327	463.4	55.00	0.22	tr_D0A423_
DRB1_0101	78	VMSISLSYFLCKGLS	MSISLSYFL	1	0.4310	471.5	60.00	0.34	tr_D0A423_
DRB1_0101	79	ALFNKVPCLRRLPMF	FNKVPCLRR	2	0.4307	473.3	60.00	0.34	tr_D0A423_
DRB1_0101	80	LPTLQSEESGEATYV	LQSEESGEA	3	0.4299	477.2	60.00	0.64	tr_D0A423_
DRB1_0101	81	PTLQSEESGEATYVH	LQSEESGEA	2	0.4282	486.4	60.00	0.54	tr_D0A423_
DRB1_0101	82	TAVVSDIFPLFGYSK	IFPLFGYSK	6	0.4074	609.3	65.00	0.36	tr_D0A423_
DRB1_0101	83	SFAPTRSLPNEVNEP	FAPTRSLPN	1	0.4066	614.0	65.00	0.52	tr_D0A423_
DRB1_0101	84	TLQSEESGEATYVHP	LQSEESGEA	1	0.4030	638.5	65.00	0.45	tr_D0A423_
DRB1_0101	85	ESGEATYVHPDARAL	YVHPDARAL	6	0.3979	675.1	65.00	0.71	tr_D0A423_
DRB1_0101	86	AAEGYGPKAVMSISL	YGPKAVMSI	4	0.3937	706.4	65.00	0.35	tr_D0A423_
DRB1_0101	87	LFNKVPCLRRLPMFG	FNKVPCLRR	1	0.3871	758.4	65.00	0.31	tr_D0A423_
DRB1_0101	88	RRLPMFGTAAEGYGP	RLPMFGTAA	1	0.3851	775.1	65.00	0.57	tr_D0A423_
DRB1_0101	89	KPLTAVVSDIFPLFG	LTAVVSDIF	2	0.3818	803.6	70.00	0.40	tr_D0A423_
DRB1_0101	90	HPDARALFNKVPCLR	DARALFNKV	2	0.3800	819.3	70.00	0.40	tr_D0A423_
DRB1_0101	91	PLTAVVSDIFPLFGY	AVVSDIFPL	3	0.3773	843.3	70.00	0.40	tr_D0A423_
DRB1_0101	92	PLPTLQSEESGEATY	LQSEESGEA	4	0.3766	850.1	70.00	0.63	tr_D0A423_
DRB1_0101	93	LTAVVSDIFPLFGYS	AVVSDIFPL	2	0.3532	1094.9	75.00	0.44	tr_D0A423_
DRB1_0101	94	FAPTRSLPNEVNEPL	TRSLPNEVN	3	0.3494	1141.2	75.00	0.45	tr_D0A423_
DRB1_0101	95	VHPDARALFNKVPCL	DARALFNKV	3	0.3392	1273.5	75.00	0.50	tr_D0A423_

DRB1_0101	96	LQSEESGEATYVHPD	EESGEATYV	3	0.3372	1301.8	75.00	0.49	tr_D0A423_	
DRB1_0101	97	EPLPTLQSEESGEAT	LQSEESGEA	5	0.3361	1316.7	75.00	0.49	tr_D0A423_	
DRB1_0101	98	TAAEGYGPKAVMSIS	YGPKAVMSI	5	0.3283	1433.7	80.00	0.35	tr_D0A423_	
DRB1_0101	99	RLPMFGTAAEGYGPK	RLPMFGTAA	0	0.3193	1579.9	80.00	0.35	tr_D0A423_	
DRB1_0101	100	NEPLPTLQSEESGEA	LPTLQSEES	3	0.3115	1718.1	80.00	0.46	tr_D0A423_	
DRB1_0101	101	NEVNEPLPTLQSEES	NEPLPTLQS	3	0.3027	1890.4	85.00	0.49	tr_D0A423_	
DRB1_0101	102	EVNEPLPTLQSEESG	NEPLPTLQS	2	0.2982	1984.1	85.00	0.42	tr_D0A423_	
DRB1_0101	103	GTAAEGYGPKAVMSI	GYGPKAVMS	5	0.2976	1996.8	85.00	0.28	tr_D0A423_	
DRB1_0101	104	LPNEVNEPLPTLQSE	NEPLPTLQS	5	0.2972	2007.5	85.00	0.32	tr_D0A423_	
DRB1_0101	105	PNEVNEPLPTLQSEE	NEPLPTLQS	4	0.2908	2150.3	85.00	0.42	tr_D0A423_	
DRB1_0101	106	SLPNEVNEPLPTLQS	NEVNEPLPT	3	0.2828	2346.0	85.00	0.23	tr_D0A423_	
DRB1_0101	107	VNEPLPTLQSEESGE	LPTLQSEES	4	0.2792	2438.7	85.00	0.44	tr_D0A423_	
DRB1_0101	108	APTRSLPNEVNEPLP	TRSLPNEVN	2	0.2749	2552.8	90.00	0.57	tr_D0A423_	
DRB1_0101	109	MFGTAAEGYGPKAVM	AAEGYGPKA	4	0.2723	2627.1	90.00	0.26	tr_D0A423_	
DRB1_0101	110	FGTAAEGYGPKAVMS	AAEGYGPKA	3	0.2715	2648.6	90.00	0.32	tr_D0A423_	
DRB1_0101	111	PMFGTAAEGYGPKAV	FGTAAEGYG	2	0.2665	2797.4	90.00	0.32	tr_D0A423_	
DRB1_0101	112	LPMFGTAAEGYGPKA	FGTAAEGYG	3	0.2613	2958.3	90.00	0.41	tr_D0A423_	
DRB1_0101	113	QSEESGEATYVHPDA	EESGEATYV	2	0.2551	3163.2	90.00	0.63	tr_D0A423_	
DRB1_0101	114	TRSLPNEVNEPLPTL	LPNEVNEPL	3	0.2541	3197.6	90.00	0.33	tr_D0A423_	
DRB1_0101	115	PTRSLPNEVNEPLPT	TRSLPNEVN	1	0.2474	3440.1	90.00	0.54	tr_D0A423_	
DRB1_0101	116	RSLPNEVNEPLPTLQ	NEVNEPLPT	4	0.2461	3489.0	90.00	0.26	tr_D0A423_	
DRB1_0101	117	SEESGEATYVHPDAR	EESGEATYV	1	0.2058	5395.9	95.00	0.63	tr_D0A423_	
DRB1_0101	118	EESGEATYVHPDARA	EESGEATYV	0	0.1918	6276.4	100.00	0.30	tr_D0A423_	

Allele: DRB1_0101. Number of high binders 0. Number of weak binders 27. Number of peptides 118

Allele	pos	peptide	core	Of	1-log50k(aff)	affinity(nM)	%Rank	Relia	Identity	Bind Level
DRB1_0101	1	REERYGDALLLYKKK	ERYGDALLL	2	0.5443	138.4	32.00	0.65	tr_D0A423_	
DRB1_0101	2	NREERYGDALLLYKK	ERYGDALLL	3	0.5405	144.2	33.00	0.71	tr_D0A423_	
DRB1_0101	3	EERYGDALLLYKKKR	ERYGDALLL	1	0.5235	173.4	36.00	0.52	tr_D0A423_	
DRB1_0101	4	PNREERYGDALLLYK	ERYGDALLL	4	0.5025	217.5	40.00	0.74	tr_D0A423_	
DRB1_0101	5	CGVFEYNKEVVERNG	FEYNKEVVE	3	0.4697	310.2	47.00	0.50	tr_D0A423_	
DRB1_0101	6	ERYGDALLLYKKKRE	ERYGDALLL	0	0.4577	353.5	50.00	0.40	tr_D0A423_	
DRB1_0101	7	LPNREERYGDALLLY	ERYGDALLL	5	0.4526	373.4	55.00	0.74	tr_D0A423_	
DRB1_0101	8	CCGVFEYNKEVVERN	FEYNKEVVE	4	0.4456	403.0	55.00	0.54	tr_D0A423_	
DRB1_0101	9	DRAINSESNPLSTEG	INSESNPLS	3	0.4455	403.2	55.00	0.74	tr_D0A423_	
DRB1_0101	10	RAINSESNPLSTEGP	INSESNPLS	2	0.4355	449.2	55.00	0.79	tr_D0A423_	
DRB1_0101	11	PDRAINSESNPLSTE	INSESNPLS	4	0.4343	455.0	55.00	0.70	tr_D0A423_	
DRB1_0101	12	CCCGVFEYNKEVVER	FEYNKEVVE	5	0.4188	538.5	60.00	0.54	tr_D0A423_	
DRB1_0101	13	QPDRAINSESNPLST	INSESNPLS	5	0.4140	566.9	60.00	0.68	tr_D0A423_	
DRB1_0101	14	SSFHFQEPGSCCCGV	FHFQEPGSC	2	0.4032	637.5	65.00	0.51	tr_D0A423_	
DRB1_0101	15	LSSFHFQEPGSCCCG	FHFQEPGSC	3	0.4022	644.2	65.00	0.56	tr_D0A423_	
DRB1_0101	16	DLSSFHFQEPGSCCC	FHFQEPGSC	4	0.3752	862.9	70.00	0.60	tr_D0A423_	
DRB1_0101	17	SFHFQEPGSCCCGVF	FHFQEPGSC	1	0.3727	886.8	70.00	0.43	tr_D0A423_	
DRB1_0101	18	SNPLSTEGPDAAGLV	LSTEGPDAA	3	0.3723	889.9	70.00	0.65	tr_D0A423_	
DRB1_0101	19	SCCCGVFEYNKEVVE	FEYNKEVVE	6	0.3580	1039.5	75.00	0.43	tr_D0A423_	

DRB1_0101	20	AINSESNPLSTEGPD	INSESNPLS	1	0.3568	1052.5	75.00	0.79	tr_D0A423_
DRB1_0101	21	NPLSTEGPDAAGLVP	LSTEGPDAA	2	0.3518	1111.3	75.00	0.64	tr_D0A423_
DRB1_0101	22	ELPNREERYGDALLL	ERYGDALLL	6	0.3517	1112.7	75.00	0.69	tr_D0A423_
DRB1_0101	23	RYGDALLLYKKKREE	GDALLLYKK	2	0.3485	1152.4	75.00	0.35	tr_D0A423_
DRB1_0101	24	GDLSSFHFQEPGSCC	FHFQEPGSC	5	0.3461	1182.4	75.00	0.57	tr_D0A423_
DRB1_0101	25	ESNPLSTEGPDAAGL	LSTEGPDAA	4	0.3380	1289.8	75.00	0.65	tr_D0A423_
DRB1_0101	26	TQPDRAINSESNPLS	INSESNPLS	6	0.3276	1443.6	80.00	0.56	tr_D0A423_
DRB1_0101	27	PLSTEGPDAAGLVPY	LSTEGPDAA	1	0.3228	1520.7	80.00	0.56	tr_D0A423_
DRB1_0101	28	FHFQEPGSCCCGVFE	HFQEPGSCC	1	0.3164	1629.3	80.00	0.34	tr_D0A423_
DRB1_0101	29	LVPYEGGAPTGDGDL	YEGGAPTGD	3	0.3127	1696.7	80.00	0.61	tr_D0A423_
DRB1_0101	30	PFSDSLTQPDRAINS	SLTQPDRAI	4	0.3121	1707.9	80.00	0.26	tr_D0A423_
DRB1_0101	31	AGLVPYEGGAPTGDG	VPYEGGAPT	3	0.3106	1736.1	80.00	0.41	tr_D0A423_
DRB1_0101	32	GLVPYEGGAPTGDGD	YEGGAPTGD	4	0.3064	1817.1	85.00	0.50	tr_D0A423_
DRB1_0101	33	FSDSLTQPDRAINSE	SLTQPDRAI	3	0.2923	2115.8	85.00	0.31	tr_D0A423_
DRB1_0101	34	DPFSDSLTQPDRAIN	FSDSLTQPD	2	0.2919	2124.7	85.00	0.33	tr_D0A423_
DRB1_0101	35	YGDALLLYKKKREER	GDALLLYKK	1	0.2911	2143.1	85.00	0.34	tr_D0A423_
DRB1_0101	36	DGDLSSFHFQEPGSC	FHFQEPGSC	6	0.2802	2411.7	85.00	0.50	tr_D0A423_
DRB1_0101	37	SESNPLSTEGPDAAG	LSTEGPDAA	5	0.2751	2548.3	90.00	0.65	tr_D0A423_
DRB1_0101	38	AAGLVPYEGGAPTGD	VPYEGGAPT	4	0.2750	2549.9	90.00	0.41	tr_D0A423_
DRB1_0101	39	GSCCCGVFEYNKEVV	GVFEYNKEV	5	0.2699	2697.3	90.00	0.44	tr_D0A423_
DRB1_0101	40	GDALLLYKKKREERE	DALLLYKKK	1	0.2685	2736.9	90.00	0.25	tr_D0A423_
DRB1_0101	41	ALLLYKKKREEREKK	YKKKREERE	4	0.2671	2778.0	90.00	0.64	tr_D0A423_
DRB1_0101	42	CDPFSDSLTQPDRAI	FSDSLTQPD	3	0.2647	2852.9	90.00	0.43	tr_D0A423_
DRB1_0101	43	SDSLTQPDRAINSES	SLTQPDRAI	2	0.2618	2942.0	90.00	0.34	tr_D0A423_
DRB1_0101	44	VPYEGGAPTGDGDLS	YEGGAPTGD	2	0.2617	2946.0	90.00	0.69	tr_D0A423_
DRB1_0101	45	LLLYKKKREEREKKC	YKKKREERE	3	0.2590	3032.5	90.00	0.74	tr_D0A423_
DRB1_0101	46	DALLLYKKKREEREK	YKKKREERE	5	0.2584	3054.0	90.00	0.45	tr_D0A423_
DRB1_0101	47	DAAGLVPYEGGAPTG	VPYEGGAPT	5	0.2542	3196.8	90.00	0.40	tr_D0A423_
DRB1_0101	48	APTGDGDLSSFHFQE	DGDLSSFHF	4	0.2524	3259.3	90.00	0.44	tr_D0A423_
DRB1_0101	49	LSTEGPDAAGLVPYE	LSTEGPDAA	0	0.2508	3315.1	90.00	0.42	tr_D0A423_
DRB1_0101	50	PTGDGDLSSFHFQEP	DGDLSSFHF	3	0.2378	3813.6	95.00	0.46	tr_D0A423_
DRB1_0101	51	INSESNPLSTEGPDA	INSESNPLS	0	0.2378	3817.0	95.00	0.61	tr_D0A423_
DRB1_0101	52	LLYKKKREEREKKCD	YKKKREERE	2	0.2374	3832.7	95.00	0.75	tr_D0A423_
DRB1_0101	53	TGDGDLSSFHFQEPG	DGDLSSFHF	2	0.2336	3993.7	95.00	0.47	tr_D0A423_
DRB1_0101	54	GAPTGDGDLSSFHFQ	DGDLSSFHF	5	0.2332	4011.1	95.00	0.44	tr_D0A423_
DRB1_0101	55	DSLTQPDRAINSESN	SLTQPDRAI	1	0.2313	4094.5	95.00	0.31	tr_D0A423_
DRB1_0101	56	PDAAGLVPYEGGAPT	AAGLVPYEG	2	0.2227	4494.2	95.00	0.33	tr_D0A423_
DRB1_0101	57	GDGDLSSFHFQEPGS	DGDLSSFHF	1	0.2192	4667.0	95.00	0.41	tr_D0A423_
DRB1_0101	58	KCDPFSDSLTQPDRA	FSDSLTQPD	4	0.2184	4705.3	95.00	0.49	tr_D0A423_
DRB1_0101	59	HFQEPGSCCCGVFEY	HFQEPGSCC	0	0.2162	4820.0	95.00	0.31	tr_D0A423_
DRB1_0101	60	PGSCCCGVFEYNKEV	GVFEYNKEV	6	0.2093	5195.0	95.00	0.30	tr_D0A423_
DRB1_0101	61	EGPDAAGLVPYEGGA	AAGLVPYEG	4	0.2026	5585.9	95.00	0.47	tr_D0A423_
DRB1_0101	62	PYEGGAPTGDGDLSS	YEGGAPTGD	1	0.2006	5706.8	95.00	0.80	tr_D0A423_
DRB1_0101	63	LYKKKREEREKKCDP	YKKKREERE	1	0.1994	5779.7	95.00	0.75	tr_D0A423_
DRB1_0101	64	SLTQPDRAINSESNP	TQPDRAINS	2	0.1976	5893.4	95.00	0.31	tr_D0A423_
DRB1_0101	65	NSESNPLSTEGPDAA	LSTEGPDAA	6	0.1970	5934.3	95.00	0.50	tr_D0A423_
DRB1_0101	66	GPDAAGLVPYEGGAP	AAGLVPYEG	3	0.1957	6016.6	95.00	0.46	tr_D0A423_
DRB1_0101	67	KKCDPFSDSLTQPDR	FSDSLTQPD	5	0.1934	6166.2	95.00	0.47	tr_D0A423_
DRB1_0101	68	EREKKCDPFSDSLTQ	CDPFSDSLT	5	0.1895	6435.9	100.00	0.38	tr_D0A423_
DRB1_0101	69	REKKCDPFSDSLTQP	CDPFSDSLT	4	0.1847	6781.0	100.00	0.38	tr_D0A423_

DRB1_0101	70	TEGPDAAGLVPYEGG	AAGLVPYEG	5	0.1819	6989.4	100.00	0.37	tr_D0A423_	
DRB1_0101	71	EKKCDPFSDSLTQPD	CDPFSDSLT	3	0.1781	7278.7	100.00	0.34	tr_D0A423_	
DRB1_0101	72	LTQPDRAINSESNPL	DRAINSESN	4	0.1774	7338.3	100.00	0.29	tr_D0A423_	
DRB1_0101	73	STEGPDAAGLVPYEG	TEGPDAAGL	1	0.1725	7733.4	100.00	0.21	tr_D0A423_	
DRB1_0101	74	EPGSCCCGVFEYNKE	GSCCCGVFE	2	0.1678	8141.0	100.00	0.33	tr_D0A423_	
DRB1_0101	75	FQEPGSCCCGVFEYN	GSCCCGVFE	4	0.1659	8304.7	100.00	0.35	tr_D0A423_	
DRB1_0101	76	QEPGSCCCGVFEYNK	GSCCCGVFE	3	0.1652	8372.0	100.00	0.37	tr_D0A423_	
DRB1_0101	77	EEREKKCDPFSDSLT	EKKCDPFSD	3	0.1623	8636.3	100.00	0.42	tr_D0A423_	
DRB1_0101	78	GGAPTGDGDLSSFHF	DGDLSSFHF	6	0.1502	9839.9	100.00	0.50	tr_D0A423_	
DRB1_0101	79	YKKKREEREKKCDPF	YKKKREERE	0	0.1298	12271.4	100.00	0.60	tr_D0A423_	
DRB1_0101	80	YEGGAPTGDGDLSSF	YEGGAPTGD	0	0.1263	12748.0	100.00	0.61	tr_D0A423_	
DRB1_0101	81	REEREKKCDPFSDSL	EKKCDPFSD	4	0.1180	13945.5	100.00	0.50	tr_D0A423_	
DRB1_0101	82	KREEREKKCDPFSDS	EKKCDPFSD	5	0.0929	18308.1	100.00	0.50	tr_D0A423_	
DRB1_0101	83	KKREEREKKCDPFSD	EKKCDPFSD	6	0.0758	22021.9	100.00	0.38	tr_D0A423_	
DRB1_0101	84	EGGAPTGDGDLSSFH	GDGDLSSFH	6	0.0688	23741.7	100.00	0.19	tr_D0A423_	
DRB1_0101	85	KKKREEREKKCDPFS	KREEREKKC	2	0.0614	25718.8	100.00	0.27	tr_D0A423_	

Allele: DRB1_0101. Number of high binders 0. Number of weak binders 0. Number of peptides 85

Allele	pos	peptide	core	Of	1-log50k(aff)	affinity(nM)	%Rank	Relia	Identity	Bind Level
DRB1_0101	1	ANLFGYLQRATSVSF	GYLQRATSV	4	0.7629	13.0	4.00	0.47	tr_D0A423_	WB
DRB1_0101	2	NLFGYLQRATSVSFP	GYLQRATSV	3	0.7617	13.2	4.00	0.45	tr_D0A423_	WB
DRB1_0101	3	LFGYLQRATSVSFPG	GYLQRATSV	2	0.7516	14.7	5.00	0.42	tr_D0A423_	WB
DRB1_0101	4	LSNFFIAEADCLADG	FFIAEADCL	3	0.7293	18.7	6.50	0.60	tr_D0A423_	WB
DRB1_0101	5	FGYLQRATSVSFPGA	GYLQRATSV	1	0.7204	20.6	7.00	0.38	tr_D0A423_	WB
DRB1_0101	6	KANLFGYLQRATSVS	GYLQRATSV	5	0.7184	21.0	7.50	0.47	tr_D0A423_	WB
DRB1_0101	7	SNFFIAEADCLADGP	FFIAEADCL	2	0.7120	22.6	8.00	0.57	tr_D0A423_	WB
DRB1_0101	8	ALSNFFIAEADCLAD	FFIAEADCL	4	0.7101	23.0	8.00	0.62	tr_D0A423_	WB
DRB1_0101	9	GALSNFFIAEADCLA	FFIAEADCL	5	0.6928	27.8	9.50	0.63	tr_D0A423_	WB
DRB1_0101	10	NFFIAEADCLADGPH	FFIAEADCL	1	0.6689	36.0	13.00	0.57	tr_D0A423_	
DRB1_0101	11	VSFPGALSNFFIAEA	PGALSNFFI	3	0.6493	44.5	15.00	0.57	tr_D0A423_	
DRB1_0101	12	SVSFPGALSNFFIAE	PGALSNFFI	4	0.6395	49.5	16.00	0.52	tr_D0A423_	
DRB1_0101	13	TSVSFPGALSNFFIA	PGALSNFFI	5	0.6342	52.3	17.00	0.47	tr_D0A423_	
DRB1_0101	14	SFPGALSNFFIAEAD	PGALSNFFI	2	0.6287	55.6	18.00	0.56	tr_D0A423_	
DRB1_0101	15	PGALSNFFIAEADCL	FFIAEADCL	6	0.6225	59.4	19.00	0.42	tr_D0A423_	
DRB1_0101	16	GYLQRATSVSFPGAL	LQRATSVSF	2	0.6088	68.9	21.00	0.41	tr_D0A423_	
DRB1_0101	17	FPGALSNFFIAEADC	PGALSNFFI	1	0.5876	86.6	25.00	0.56	tr_D0A423_	
DRB1_0101	18	ATSVSFPGALSNFFI	SVSFPGALS	2	0.5771	97.1	26.00	0.38	tr_D0A423_	
DRB1_0101	19	FFIAEADCLADGPHF	FIAEADCLA	1	0.5659	109.6	28.00	0.47	tr_D0A423_	
DRB1_0101	20	YLQRATSVSFPGALS	LQRATSVSF	1	0.5323	157.7	35.00	0.49	tr_D0A423_	
DRB1_0101	21	RATSVSFPGALSNFF	SVSFPGALS	3	0.5111	198.4	39.00	0.66	tr_D0A423_	
DRB1_0101	22	QRATSVSFPGALSNF	SVSFPGALS	4	0.4902	248.7	43.00	0.65	tr_D0A423_	
DRB1_0101	23	LQRATSVSFPGALSN	SVSFPGALS	5	0.4856	261.3	44.00	0.51	tr_D0A423_	
DRB1_0101	24	EADCLADGPHFSFAF	CLADGPHFS	3	0.3711	902.2	70.00	0.48	tr_D0A423_	
DRB1_0101	25	ADCLADGPHFSFAFY	CLADGPHFS	2	0.3673	940.2	70.00	0.46	tr_D0A423_	
DRB1_0101	26	FIAEADCLADGPHFS	FIAEADCLA	0	0.3642	972.3	70.00	0.52	tr_D0A423_	

DRB1_0101	27	AEADCLADGPHFSFA	CLADGPHFS	4	0.3546	1077.9	75.00	0.47	tr_D0A423_	
DRB1_0101	28	DCLADGPHFSFAFYQ	CLADGPHFS	1	0.3481	1156.9	75.00	0.34	tr_D0A423_	
DRB1_0101	29	IAEADCLADGPHFSF	CLADGPHFS	5	0.3040	1864.2	85.00	0.45	tr_D0A423_	

Allele: DRB1_0101. Number of high binders 0. Number of weak binders 9. Number of peptides 29

Allele	pos	peptide	core	Of	1-log50k(aff)	affinity(nM)	%Rank	Relia	Identity	Bind Level
DRB1_0101	1	FPMFTYTFGADAKLY	FTYTFGADA	3	0.8387	5.7	0.90	0.79	tr_C9ZVF1_	WB
DRB1_0101	2	PMFTYTFGADAKLYQ	FTYTFGADA	2	0.8268	6.5	1.10	0.75	tr_C9ZVF1_	WB
DRB1_0101	3	MFTYTFGADAKLYQR	FTYTFGADA	1	0.8125	7.6	1.60	0.69	tr_C9ZVF1_	WB
DRB1_0101	4	LFPMFTYTFGADAKL	FTYTFGADA	4	0.8053	8.2	1.80	0.83	tr_C9ZVF1_	WB
DRB1_0101	5	SLFPMFTYTFGADAK	FTYTFGADA	5	0.7635	12.9	4.00	0.83	tr_C9ZVF1_	WB
DRB1_0101	6	GFCELFGKGIADNII	FCELFGKGI	1	0.7468	15.5	5.00	0.56	tr_C9ZVF1_	WB
DRB1_0101	7	FTYTFGADAKLYQRM	FTYTFGADA	0	0.7354	17.5	6.00	0.53	tr_C9ZVF1_	WB
DRB1_0101	8	FCELFGKGIADNIIR	FGKGIADNI	4	0.7095	23.2	8.00	0.47	tr_C9ZVF1_	WB
DRB1_0101	9	SSLFPMFTYTFGADA	FTYTFGADA	6	0.6749	33.7	12.00	0.69	tr_C9ZVF1_	
DRB1_0101	10	CELFGKGIADNIIRS	FGKGIADNI	3	0.6305	54.5	18.00	0.81	tr_C9ZVF1_	
DRB1_0101	11	ELFGKGIADNIIRSS	FGKGIADNI	2	0.6116	66.9	21.00	0.80	tr_C9ZVF1_	
DRB1_0101	12	LFGKGIADNIIRSSL	FGKGIADNI	1	0.5773	96.9	26.00	0.75	tr_C9ZVF1_	
DRB1_0101	13	TYTFGADAKLYQRMS	YTFGADAKL	1	0.5617	114.7	29.00	0.44	tr_C9ZVF1_	
DRB1_0101	14	ADNIIRSSLFPMFTY	IIRSSLFPM	3	0.5282	164.8	35.00	0.50	tr_C9ZVF1_	
DRB1_0101	15	NIIRSSLFPMFTYTF	IIRSSLFPM	1	0.5105	199.6	39.00	0.34	tr_C9ZVF1_	
DRB1_0101	16	IADNIIRSSLFPMFT	IIRSSLFPM	4	0.5055	210.6	40.00	0.49	tr_C9ZVF1_	
DRB1_0101	17	DNIIRSSLFPMFTYT	IIRSSLFPM	2	0.5048	212.3	40.00	0.47	tr_C9ZVF1_	
DRB1_0101	18	YTFGADAKLYQRMSS	YTFGADAKL	0	0.5019	219.0	41.00	0.29	tr_C9ZVF1_	
DRB1_0101	19	IIRSSLFPMFTYTFG	LFPMFTYTF	5	0.4831	268.5	45.00	0.44	tr_C9ZVF1_	
DRB1_0101	20	FGKGIADNIIRSSLF	FGKGIADNI	0	0.4821	271.3	45.00	0.47	tr_C9ZVF1_	
DRB1_0101	21	IRSSLFPMFTYTFGA	LFPMFTYTF	4	0.4814	273.5	45.00	0.62	tr_C9ZVF1_	
DRB1_0101	22	GIADNIIRSSLFPMF	IIRSSLFPM	5	0.4781	283.4	46.00	0.45	tr_C9ZVF1_	
DRB1_0101	23	RSSLFPMFTYTFGAD	LFPMFTYTF	3	0.4739	296.4	46.00	0.64	tr_C9ZVF1_	
DRB1_0101	24	KGIADNIIRSSLFPM	DNIIRSSLF	4	0.4204	529.0	60.00	0.33	tr_C9ZVF1_	
DRB1_0101	25	GKGIADNIIRSSLFP	DNIIRSSLF	5	0.3530	1097.6	75.00	0.37	tr_C9ZVF1_	

Allele: DRB1_0101. Number of high binders 0. Number of weak binders 8. Number of peptides 25

Allele	pos	peptide	core	Of	1-log50k(aff)	affinity(nM)	%Rank	Relia	Identity	Bind Level
DRB1_0101	1	RRRCIYATKAALDSS	YATKAALDS	5	0.6743	33.9	12.00	0.42	tr_C9ZRE0_	
DRB1_0101	2	RRCIYATKAALDSSP	YATKAALDS	4	0.6669	36.7	13.00	0.46	tr_C9ZRE0_	
DRB1_0101	3	RCIYATKAALDSSPF	YATKAALDS	3	0.6583	40.4	14.00	0.60	tr_C9ZRE0_	
DRB1_0101	4	CIYATKAALDSSPFV	YATKAALDS	2	0.6007	75.2	22.00	0.59	tr_C9ZRE0_	
DRB1_0101	5	VRRRCIYATKAALDS	IYATKAALD	5	0.5900	84.4	24.00	0.36	tr_C9ZRE0_	
DRB1_0101	6	PEFAEAFFGKLVRRR	FAEAFFGKL	2	0.5656	109.9	28.00	0.27	tr_C9ZRE0_	

DRB1_0101	7	FAEAFFGKLVRRRCI	FFGKLVRRR	4	0.5606	116.1	29.00	0.44	tr_C9ZRE0_	
DRB1_0101	8	EFAEAFFGKLVRRRC	FFGKLVRRR	5	0.5582	119.1	30.00	0.35	tr_C9ZRE0_	
DRB1_0101	9	RPEFAEAFFGKLVRR	FAEAFFGKL	3	0.5489	131.8	31.00	0.43	tr_C9ZRE0_	
DRB1_0101	10	AFFGKLVRRRCIYAT	LVRRRCIYA	5	0.5451	137.2	32.00	0.31	tr_C9ZRE0_	
DRB1_0101	11	EAFFGKLVRRRCIYA	FFGKLVRRR	2	0.5434	139.9	32.00	0.47	tr_C9ZRE0_	
DRB1_0101	12	AEAFFGKLVRRRCIY	FFGKLVRRR	3	0.5397	145.5	33.00	0.61	tr_C9ZRE0_	
DRB1_0101	13	IYATKAALDSSPFVR	YATKAALDS	1	0.5369	149.9	34.00	0.57	tr_C9ZRE0_	
DRB1_0101	14	KLVRRRCIYATKAAL	LVRRRCIYA	1	0.5323	157.6	35.00	0.40	tr_C9ZRE0_	
DRB1_0101	15	LVRRRCIYATKAALD	RRCIYATKA	3	0.5288	163.7	35.00	0.29	tr_C9ZRE0_	
DRB1_0101	16	SRPEFAEAFFGKLVR	FAEAFFGKL	4	0.5257	169.3	36.00	0.39	tr_C9ZRE0_	
DRB1_0101	17	FFGKLVRRRCIYATK	LVRRRCIYA	4	0.5172	185.7	38.00	0.47	tr_C9ZRE0_	
DRB1_0101	18	GKLVRRRCIYATKAA	LVRRRCIYA	2	0.5147	190.7	38.00	0.52	tr_C9ZRE0_	
DRB1_0101	19	FGKLVRRRCIYATKA	LVRRRCIYA	3	0.5120	196.5	39.00	0.62	tr_C9ZRE0_	
DRB1_0101	20	WSRPEFAEAFFGKLV	FAEAFFGKL	5	0.4821	271.3	45.00	0.37	tr_C9ZRE0_	
DRB1_0101	21	SWSRPEFAEAFFGKL	PEFAEAFFG	4	0.4251	502.9	60.00	0.31	tr_C9ZRE0_	
DRB1_0101	22	MVSWSRPEFAEAFFG	VSWSRPEFA	1	0.4052	623.6	65.00	0.47	tr_C9ZRE0_	
DRB1_0101	23	VSWSRPEFAEAFFGK	PEFAEAFFG	5	0.3725	888.0	70.00	0.26	tr_C9ZRE0_	

Allele: DRB1_0101. Number of high binders 0. Number of weak binders 0. Number of peptides 23

Allele	pos	peptide	core	Of	1-log50k(aff)	affinity(nM)	%Rank	Relia	Identity	Bind Level
DRB1_0101	1	LKRFSFVLPQPLRKS	FSFVLPQPL	3	0.8812	3.6	0.20	0.70	tr_C9ZRE0_	SB
DRB1_0101	2	KRFSFVLPQPLRKSP	FSFVLPQPL	2	0.8758	3.8	0.25	0.70	tr_C9ZRE0_	SB
DRB1_0101	3	FLKRFSFVLPQPLRK	FSFVLPQPL	4	0.8671	4.2	0.40	0.75	tr_C9ZRE0_	SB
DRB1_0101	4	RFSFVLPQPLRKSPI	FSFVLPQPL	1	0.8592	4.6	0.50	0.67	tr_C9ZRE0_	WB
DRB1_0101	5	AFLKRFSFVLPQPLR	FSFVLPQPL	5	0.8341	6.0	1.00	0.77	tr_C9ZRE0_	WB
DRB1_0101	6	FSFVLPQPLRKSPID	FSFVLPQPL	0	0.7937	9.3	2.50	0.57	tr_C9ZRE0_	WB
DRB1_0101	7	QAFLKRFSFVLPQPL	FSFVLPQPL	6	0.7851	10.2	3.00	0.64	tr_C9ZRE0_	WB
DRB1_0101	8	TLECLVSGCAVSVSW	LVSGCAVSV	4	0.6787	32.3	12.00	0.47	tr_C9ZRE0_	
DRB1_0101	9	MTLECLVSGCAVSVS	LECLVSGCA	2	0.6724	34.6	12.00	0.38	tr_C9ZRE0_	
DRB1_0101	10	YVQAFLKRFSFVLPQ	FLKRFSFVL	4	0.6658	37.2	13.00	0.71	tr_C9ZRE0_	
DRB1_0101	11	AYVQAFLKRFSFVLP	FLKRFSFVL	5	0.6554	41.6	14.00	0.63	tr_C9ZRE0_	
DRB1_0101	12	LECLVSGCAVSVSWS	LVSGCAVSV	3	0.6553	41.7	14.00	0.59	tr_C9ZRE0_	
DRB1_0101	13	SAYVQAFLKRFSFVL	FLKRFSFVL	6	0.6489	44.7	15.00	0.46	tr_C9ZRE0_	
DRB1_0101	14	VQAFLKRFSFVLPQP	FLKRFSFVL	3	0.6440	47.1	16.00	0.73	tr_C9ZRE0_	
DRB1_0101	15	CMTLECLVSGCAVSV	LECLVSGCA	3	0.6430	47.6	16.00	0.52	tr_C9ZRE0_	
DRB1_0101	16	SAYTHTSVALGEFAS	YTHTSVALG	2	0.6291	55.3	18.00	0.58	tr_C9ZRE0_	
DRB1_0101	17	GSAYTHTSVALGEFA	YTHTSVALG	3	0.6195	61.4	19.00	0.69	tr_C9ZRE0_	
DRB1_0101	18	AYTHTSVALGEFASW	YTHTSVALG	1	0.6111	67.2	21.00	0.47	tr_C9ZRE0_	
DRB1_0101	19	ECLVSGCAVSVSWSA	LVSGCAVSV	2	0.6042	72.4	22.00	0.68	tr_C9ZRE0_	
DRB1_0101	20	VCMTLECLVSGCAVS	LECLVSGCA	4	0.5996	76.1	23.00	0.60	tr_C9ZRE0_	
DRB1_0101	21	SFVLPQPLRKSPIDV	FVLPQPLRK	1	0.5861	88.1	25.00	0.74	tr_C9ZRE0_	
DRB1_0101	22	AVCMTLECLVSGCAV	LECLVSGCA	5	0.5732	101.3	27.00	0.55	tr_C9ZRE0_	
DRB1_0101	23	YTHTSVALGEFASWT	TSVALGEFA	3	0.5714	103.2	27.00	0.50	tr_C9ZRE0_	
DRB1_0101	24	WSAYVQAFLKRFSFV	SAYVQAFLK	1	0.5692	105.7	28.00	0.34	tr_C9ZRE0_	
DRB1_0101	25	AGSAYTHTSVALGEF	YTHTSVALG	4	0.5674	107.8	28.00	0.80	tr_C9ZRE0_	

DRB1_0101	26	CLVSGCAVSVSWSAY	LVSGCAVSV	1	0.5621	114.3	29.00	0.59	tr_C9ZRE0_
DRB1_0101	27	GCAVSVSWSAYVQAF	VSVSWSAYV	3	0.5563	121.7	30.00	0.52	tr_C9ZRE0_
DRB1_0101	28	VSWSAYVQAFLKRFS	SAYVQAFLK	3	0.5554	122.7	30.00	0.47	tr_C9ZRE0_
DRB1_0101	29	HTSVALGEFASWTVA	TSVALGEFA	1	0.5553	122.9	30.00	0.35	tr_C9ZRE0_
DRB1_0101	30	SWSAYVQAFLKRFSF	SAYVQAFLK	2	0.5536	125.3	31.00	0.41	tr_C9ZRE0_
DRB1_0101	31	THTSVALGEFASWTV	TSVALGEFA	2	0.5508	129.0	31.00	0.50	tr_C9ZRE0_
DRB1_0101	32	SVSWSAYVQAFLKRF	SAYVQAFLK	4	0.5487	132.0	31.00	0.44	tr_C9ZRE0_
DRB1_0101	33	AVSVSWSAYVQAFLK	VSVSWSAYV	1	0.5437	139.3	32.00	0.40	tr_C9ZRE0_
DRB1_0101	34	SGCAVSVSWSAYVQA	VSVSWSAYV	4	0.5436	139.6	32.00	0.53	tr_C9ZRE0_
DRB1_0101	35	VAVCMTLECLVSGCA	LECLVSGCA	6	0.5396	145.7	33.00	0.43	tr_C9ZRE0_
DRB1_0101	36	CAVSVSWSAYVQAFL	VSVSWSAYV	2	0.5376	148.9	34.00	0.52	tr_C9ZRE0_
DRB1_0101	37	VSVSWSAYVQAFLKR	SAYVQAFLK	5	0.5374	149.2	34.00	0.35	tr_C9ZRE0_
DRB1_0101	38	TSVALGEFASWTVAV	LGEFASWTV	4	0.5357	152.0	34.00	0.31	tr_C9ZRE0_
DRB1_0101	39	VAGSAYTHTSVALGE	YTHTSVALG	5	0.5214	177.5	37.00	0.74	tr_C9ZRE0_
DRB1_0101	40	LVSGCAVSVSWSAYV	CAVSVSWSA	4	0.5169	186.3	38.00	0.41	tr_C9ZRE0_
DRB1_0101	41	VSGCAVSVSWSAYVQ	CAVSVSWSA	3	0.5140	192.1	38.00	0.43	tr_C9ZRE0_
DRB1_0101	42	SVALGEFASWTVAVC	LGEFASWTV	3	0.4915	245.2	43.00	0.38	tr_C9ZRE0_
DRB1_0101	43	FVLPQPLRKSPIDVV	FVLPQPLRK	0	0.4732	298.8	47.00	0.42	tr_C9ZRE0_
DRB1_0101	44	VALGEFASWTVAVCM	LGEFASWTV	2	0.4729	300.0	47.00	0.35	tr_C9ZRE0_
DRB1_0101	45	ALGEFASWTVAVCMT	LGEFASWTV	1	0.4609	341.2	49.00	0.28	tr_C9ZRE0_
DRB1_0101	46	AMLPVAGSAYTHTSV	LPVAGSAYT	2	0.4590	348.4	50.00	0.41	tr_C9ZRE0_
DRB1_0101	47	SWTVAVCMTLECLVS	AVCMTLECL	4	0.4531	371.5	55.00	0.46	tr_C9ZRE0_
DRB1_0101	48	WTVAVCMTLECLVSG	AVCMTLECL	3	0.4528	372.5	55.00	0.47	tr_C9ZRE0_
DRB1_0101	49	PVAGSAYTHTSVALG	YTHTSVALG	6	0.4526	373.5	55.00	0.60	tr_C9ZRE0_
DRB1_0101	50	TVAVCMTLECLVSGC	AVCMTLECL	2	0.4452	404.5	55.00	0.47	tr_C9ZRE0_
DRB1_0101	51	LGEFASWTVAVCMTL	EFASWTVAV	2	0.4444	408.0	55.00	0.22	tr_C9ZRE0_
DRB1_0101	52	ASWTVAVCMTLECLV	AVCMTLECL	5	0.4250	503.6	60.00	0.44	tr_C9ZRE0_
DRB1_0101	53	MLPVAGSAYTHTSVA	LPVAGSAYT	1	0.4083	603.0	60.00	0.41	tr_C9ZRE0_
DRB1_0101	54	GEFASWTVAVCMTLE	WTVAVCMTL	5	0.3930	711.7	65.00	0.30	tr_C9ZRE0_
DRB1_0101	55	FASWTVAVCMTLECL	WTVAVCMTL	3	0.3886	746.6	65.00	0.37	tr_C9ZRE0_
DRB1_0101	56	PQPLRKSPIDVVGGR	LRKSPIDVV	3	0.3857	769.9	65.00	0.69	tr_C9ZRE0_
DRB1_0101	57	LPQPLRKSPIDVVGG	LRKSPIDVV	4	0.3760	855.5	70.00	0.61	tr_C9ZRE0_
DRB1_0101	58	VLPQPLRKSPIDVVG	LRKSPIDVV	5	0.3623	992.0	70.00	0.50	tr_C9ZRE0_
DRB1_0101	59	LPVAGSAYTHTSVAL	LPVAGSAYT	0	0.3507	1124.9	75.00	0.25	tr_C9ZRE0_
DRB1_0101	60	EFASWTVAVCMTLEC	WTVAVCMTL	4	0.3449	1197.4	75.00	0.39	tr_C9ZRE0_

Allele: DRB1_0101. Number of high binders 3. Number of weak binders 4. Number of peptides 60

Allele	pos	peptide	core	Of	1-log50k(aff)	affinity(nM)	%Rank	Relia	Identity	Bind Level
DRB1_0101	1	AREFSGFLSGHARTL	FLSGHARTL	6	0.8209	6.9	1.30	0.44	tr_C9ZVS2_	WB
DRB1_0101	2	CFFRYASSYAREFSG	FRYASSYAR	2	0.7554	14.1	4.50	0.66	tr_C9ZVS2_	WB
DRB1_0101	3	WCFFRYASSYAREFS	FRYASSYAR	3	0.7526	14.5	5.00	0.69	tr_C9ZVS2_	WB
DRB1_0101	4	TWCFFRYASSYAREF	FRYASSYAR	4	0.7436	16.0	5.50	0.67	tr_C9ZVS2_	WB
DRB1_0101	5	FFRYASSYAREFSGF	FRYASSYAR	1	0.7294	18.7	6.50	0.63	tr_C9ZVS2_	WB
DRB1_0101	6	SYAREFSGFLSGHAR	FSGFLSGHA	5	0.7106	22.9	8.00	0.61	tr_C9ZVS2_	WB
DRB1_0101	7	YAREFSGFLSGHART	FSGFLSGHA	4	0.7066	23.9	8.50	0.70	tr_C9ZVS2_	WB

DRB1_0101	8	ITWCFFRYASSYARE	FRYASSYAR	5	0.7016	25.2	9.00	0.62	tr_C9ZVS2_	WB
DRB1_0101	9	SSYAREFSGFLSGHA	FSGFLSGHA	6	0.6614	39.0	14.00	0.47	tr_C9ZVS2_	
DRB1_0101	10	FRYASSYAREFSGFL	FRYASSYAR	0	0.6569	40.9	14.00	0.50	tr_C9ZVS2_	
DRB1_0101	11	QITWCFFRYASSYAR	FRYASSYAR	6	0.6372	50.7	17.00	0.46	tr_C9ZVS2_	
DRB1_0101	12	RYASSYAREFSGFLS	YAREFSGFL	5	0.5674	107.8	28.00	0.49	tr_C9ZVS2_	
DRB1_0101	13	ASSYAREFSGFLSGH	YAREFSGFL	3	0.5671	108.2	28.00	0.63	tr_C9ZVS2_	
DRB1_0101	14	YASSYAREFSGFLSG	YAREFSGFL	4	0.5624	113.8	29.00	0.60	tr_C9ZVS2_	
DRB1_0101	15	LQITWCFFRYASSYA	FFRYASSYA	6	0.4722	302.2	47.00	0.41	tr_C9ZVS2_	
DRB1_0101	16	PLQITWCFFRYASSY	WCFFRYASS	5	0.3708	904.9	70.00	0.38	tr_C9ZVS2_	
DRB1_0101	17	MPLQITWCFFRYASS	WCFFRYASS	6	0.3571	1049.1	75.00	0.29	tr_C9ZVS2_	

Allele: DRB1_0101. Number of high binders 0. Number of weak binders 8. Number of peptides 17

Allele	pos	peptide	core	Of	1-log50k(aff)	affinity(nM)	%Rank	Relia	Identity	Bind Level
DRB1_0101	1	CKFTTEGACGGAKIY	FTTEGACGG	2	0.5444	138.3	32.00	0.63	tr_D0A3U1_	
DRB1_0101	2	ECKFTTEGACGGAKI	FTTEGACGG	3	0.5237	173.0	36.00	0.76	tr_D0A3U1_	
DRB1_0101	3	KFTTEGACGGAKIYG	FTTEGACGG	1	0.5178	184.5	37.00	0.55	tr_D0A3U1_	
DRB1_0101	4	TECKFTTEGACGGAK	FTTEGACGG	4	0.4788	281.4	45.00	0.83	tr_D0A3U1_	
DRB1_0101	5	FTTEGACGGAKIYGC	EGACGGAKI	3	0.4320	466.8	55.00	0.35	tr_D0A3U1_	
DRB1_0101	6	ATECKFTTEGACGGA	FTTEGACGG	5	0.4107	587.5	60.00	0.79	tr_D0A3U1_	
DRB1_0101	7	TEGACGGAKIYGCKW	EGACGGAKI	1	0.3534	1092.3	75.00	0.33	tr_D0A3U1_	
DRB1_0101	8	TTEGACGGAKIYGCK	EGACGGAKI	2	0.3528	1099.1	75.00	0.41	tr_D0A3U1_	
DRB1_0101	9	EGACGGAKIYGCKWS	GAKIYGCKW	5	0.3313	1387.1	80.00	0.26	tr_D0A3U1_	
DRB1_0101	10	GACGGAKIYGCKWSG	GAKIYGCKW	4	0.2926	2108.9	85.00	0.37	tr_D0A3U1_	
DRB1_0101	11	AKIYGCKWSGTTCKF	IYGCKWSGT	2	0.2827	2348.1	85.00	0.26	tr_D0A3U1_	
DRB1_0101	12	GAKIYGCKWSGTTCK	IYGCKWSGT	3	0.2769	2500.1	85.00	0.34	tr_D0A3U1_	
DRB1_0101	13	CGGAKIYGCKWSGTT	GAKIYGCKW	2	0.2760	2523.4	85.00	0.35	tr_D0A3U1_	
DRB1_0101	14	ACGGAKIYGCKWSGT	GAKIYGCKW	3	0.2703	2684.4	90.00	0.38	tr_D0A3U1_	
DRB1_0101	15	KIYGCKWSGTTCKFE	CKWSGTTCK	4	0.2694	2712.1	90.00	0.33	tr_D0A3U1_	
DRB1_0101	16	GGAKIYGCKWSGTTC	GAKIYGCKW	1	0.2573	3090.0	90.00	0.32	tr_D0A3U1_	
DRB1_0101	17	IYGCKWSGTTCKFEN	CKWSGTTCK	3	0.2444	3551.4	90.00	0.38	tr_D0A3U1_	
DRB1_0101	18	GTTCKFENPKCSEGS	CKFENPKCS	3	0.2382	3797.9	95.00	0.65	tr_D0A3U1_	
DRB1_0101	19	SGTTCKFENPKCSEG	CKFENPKCS	4	0.2275	4263.7	95.00	0.63	tr_D0A3U1_	
DRB1_0101	20	TTCKFENPKCSEGSD	CKFENPKCS	2	0.2236	4450.1	95.00	0.64	tr_D0A3U1_	
DRB1_0101	21	YGCKWSGTTCKFENP	CKWSGTTCK	2	0.2085	5237.5	95.00	0.44	tr_D0A3U1_	
DRB1_0101	22	WSGTTCKFENPKCSE	CKFENPKCS	5	0.2050	5440.1	95.00	0.59	tr_D0A3U1_	
DRB1_0101	23	TCKFENPKCSEGSDP	CKFENPKCS	1	0.1911	6323.7	100.00	0.63	tr_D0A3U1_	
DRB1_0101	24	KWSGTTCKFENPKCS	CKFENPKCS	6	0.1768	7380.9	100.00	0.44	tr_D0A3U1_	
DRB1_0101	25	GCKWSGTTCKFENPK	CKWSGTTCK	1	0.1758	7460.3	100.00	0.44	tr_D0A3U1_	
DRB1_0101	26	CKWSGTTCKFENPKC	CKWSGTTCK	0	0.1534	9507.2	100.00	0.32	tr_D0A3U1_	
DRB1_0101	27	CKFENPKCSEGSDPS	CKFENPKCS	0	0.1451	10399.5	100.00	0.50	tr_D0A3U1_	
DRB1_0101	28	SEGSDPSDSCKNEVA	SDSCKNEVA	6	0.0981	17306.2	100.00	0.33	tr_D0A3U1_	
DRB1_0101	29	PKCSEGSDPSDSCKN	SEGSDPSDS	3	0.0908	18711.0	100.00	0.41	tr_D0A3U1_	
DRB1_0101	30	FENPKCSEGSDPSDS	KCSEGSDPS	4	0.0893	19033.8	100.00	0.17	tr_D0A3U1_	
DRB1_0101	31	NPKCSEGSDPSDSCK	SEGSDPSDS	4	0.0888	19121.3	100.00	0.40	tr_D0A3U1_	
DRB1_0101	32	KCSEGSDPSDSCKNE	SEGSDPSDS	2	0.0854	19842.8	100.00	0.41	tr_D0A3U1_	

DRB1_0101	33	ENPKCSEGSDPSDSC	SEGSDPSDS	5	0.0842	20095.6	100.00	0.32	tr_D0A3U1_	
DRB1_0101	34	KFENPKCSEGSDPSD	ENPKCSEGS	2	0.0836	20242.0	100.00	0.28	tr_D0A3U1_	
DRB1_0101	35	CSEGSDPSDSCKNEV	SEGSDPSDS	1	0.0817	20657.7	100.00	0.30	tr_D0A3U1_	

Allele: DRB1_0101. Number of high binders 0. Number of weak binders 0. Number of peptides 35

Allele	pos	peptide	core	Of	1-log50k(aff)	affinity(nM)	%Rank	Relia	Identity	Bind Level
DRB1_0101	1	LMIFLPSLTFVSITK	FLPSLTFVS	3	0.7975	8.9	2.50	0.82	tr_D0A0N1_	WB
DRB1_0101	2	SLMIFLPSLTFVSIT	FLPSLTFVS	4	0.7911	9.6	2.50	0.77	tr_D0A0N1_	WB
DRB1_0101	3	MIFLPSLTFVSITKF	FLPSLTFVS	2	0.7787	11.0	3.00	0.81	tr_D0A0N1_	WB
DRB1_0101	4	VSLMIFLPSLTFVSI	FLPSLTFVS	5	0.7740	11.5	3.50	0.71	tr_D0A0N1_	WB
DRB1_0101	5	IFLPSLTFVSITKFE	FLPSLTFVS	1	0.7342	17.7	6.00	0.78	tr_D0A0N1_	WB
DRB1_0101	6	DVSLMIFLPSLTFVS	FLPSLTFVS	6	0.7115	22.7	8.00	0.57	tr_D0A0N1_	WB
DRB1_0101	7	FLPSLTFVSITKFEN	FLPSLTFVS	0	0.6143	64.9	20.00	0.56	tr_D0A0N1_	
DRB1_0101	8	ADVSLMIFLPSLTFV	MIFLPSLTF	5	0.5688	106.3	28.00	0.54	tr_D0A0N1_	
DRB1_0101	9	SLTFVSITKFENAER	FVSITKFEN	3	0.5332	156.1	34.00	0.60	tr_D0A0N1_	
DRB1_0101	10	PSLTFVSITKFENAE	FVSITKFEN	4	0.5238	172.9	36.00	0.52	tr_D0A0N1_	
DRB1_0101	11	LPSLTFVSITKFENA	FVSITKFEN	5	0.5213	177.5	37.00	0.45	tr_D0A0N1_	
DRB1_0101	12	FADVSLMIFLPSLTF	MIFLPSLTF	6	0.5149	190.3	38.00	0.47	tr_D0A0N1_	
DRB1_0101	13	KSVGGFADVSLMIFL	GFADVSLMI	4	0.4371	441.5	55.00	0.51	tr_D0A0N1_	
DRB1_0101	14	GFADVSLMIFLPSLT	SLMIFLPSL	5	0.4214	523.4	60.00	0.29	tr_D0A0N1_	
DRB1_0101	15	GGFADVSLMIFLPSL	GFADVSLMI	1	0.4199	531.7	60.00	0.31	tr_D0A0N1_	
DRB1_0101	16	SVGGFADVSLMIFLP	GFADVSLMI	3	0.4116	581.7	60.00	0.53	tr_D0A0N1_	
DRB1_0101	17	EKSVGGFADVSLMIF	GFADVSLMI	5	0.4051	624.1	65.00	0.46	tr_D0A0N1_	
DRB1_0101	18	VGGFADVSLMIFLPS	GFADVSLMI	2	0.3982	672.4	65.00	0.49	tr_D0A0N1_	

Allele: DRB1_0101. Number of high binders 0. Number of weak binders 6. Number of peptides 18

Allele	pos	peptide	core	Of	1-log50k(aff)	affinity(nM)	%Rank	Relia	Identity	Bind Level
DRB1_0101	1	HFLLLGYEAGRTWKV	FLLLGYEAG	1	0.7379	17.0	5.50	0.43	tr_D0A0N1_	WB
DRB1_0101	2	FLLLGYEAGRTWKVH	YEAGRTWKV	5	0.7312	18.3	6.50	0.54	tr_D0A0N1_	WB
DRB1_0101	3	PLHFLLLGYEAGRTW	FLLLGYEAG	3	0.7296	18.6	6.50	0.54	tr_D0A0N1_	WB
DRB1_0101	4	LLGYEAGRTWKVHAT	YEAGRTWKV	3	0.7253	19.5	7.00	0.88	tr_D0A0N1_	WB
DRB1_0101	5	LHFLLLGYEAGRTWK	FLLLGYEAG	2	0.7238	19.9	7.00	0.55	tr_D0A0N1_	WB
DRB1_0101	6	LLLGYEAGRTWKVHA	YEAGRTWKV	4	0.7216	20.3	7.00	0.81	tr_D0A0N1_	WB
DRB1_0101	7	LGYEAGRTWKVHATT	YEAGRTWKV	2	0.7012	25.3	9.00	0.91	tr_D0A0N1_	WB
DRB1_0101	8	GYEAGRTWKVHATTA	YEAGRTWKV	1	0.6576	40.6	14.00	0.88	tr_D0A0N1_	
DRB1_0101	9	YEAGRTWKVHATTAS	YEAGRTWKV	0	0.5788	95.3	26.00	0.68	tr_D0A0N1_	
DRB1_0101	10	GRTWKVHATTASERA	WKVHATTAS	3	0.5153	189.4	38.00	0.67	tr_D0A0N1_	
DRB1_0101	11	RTWKVHATTASERAD	WKVHATTAS	2	0.5126	195.2	39.00	0.62	tr_D0A0N1_	
DRB1_0101	12	AGRTWKVHATTASER	WKVHATTAS	4	0.4775	285.3	46.00	0.65	tr_D0A0N1_	
DRB1_0101	13	TWKVHATTASERADA	WKVHATTAS	1	0.4599	344.9	49.00	0.55	tr_D0A0N1_	

DRB1_0101	14	EAGRTWKVHATTASE	WKVHATTAS	5	0.4084	602.2	60.00	0.64	tr_D0A0N1_
DRB1_0101	15	WKVHATTASERADAA	HATTASERA	3	0.3914	723.8	65.00	0.32	tr_D0A0N1_
DRB1_0101	16	KVHATTASERADAAQ	HATTASERA	2	0.3367	1308.3	75.00	0.44	tr_D0A0N1_
DRB1_0101	17	VHATTASERADAAQG	TASERADAA	4	0.3167	1625.2	80.00	0.47	tr_D0A0N1_
DRB1_0101	18	HATTASERADAAQGD	TASERADAA	3	0.2780	2470.6	85.00	0.55	tr_D0A0N1_
DRB1_0101	19	GRRDSDDTQIKTFVL	DTQIKTFVL	6	0.2620	2936.7	90.00	0.67	tr_D0A0N1_
DRB1_0101	20	ATTASERADAAQGDS	TASERADAA	2	0.2254	4362.1	95.00	0.65	tr_D0A0N1_
DRB1_0101	21	RDSHGEVSGWETSAG	HGEVSGWET	3	0.2121	5040.2	95.00	0.62	tr_D0A0N1_
DRB1_0101	22	GEVSGWETSAGRRDS	WETSAGRRD	5	0.2094	5189.2	95.00	0.63	tr_D0A0N1_
DRB1_0101	23	SHGEVSGWETSAGRR	HGEVSGWET	1	0.2074	5302.8	95.00	0.48	tr_D0A0N1_
DRB1_0101	24	SRDSHGEVSGWETSA	HGEVSGWET	4	0.2033	5542.9	95.00	0.55	tr_D0A0N1_
DRB1_0101	25	EVSGWETSAGRRDSD	WETSAGRRD	4	0.2025	5589.0	95.00	0.70	tr_D0A0N1_
DRB1_0101	26	TTASERADAAQGDSR	TASERADAA	1	0.2001	5738.5	95.00	0.52	tr_D0A0N1_
DRB1_0101	27	DSHGEVSGWETSAGR	HGEVSGWET	2	0.1993	5787.7	95.00	0.51	tr_D0A0N1_
DRB1_0101	28	HGEVSGWETSAGRRD	WETSAGRRD	6	0.1970	5930.3	95.00	0.28	tr_D0A0N1_
DRB1_0101	29	VSGWETSAGRRDSDD	WETSAGRRD	3	0.1953	6040.4	95.00	0.76	tr_D0A0N1_
DRB1_0101	30	SGWETSAGRRDSDDT	WETSAGRRD	2	0.1773	7344.0	100.00	0.79	tr_D0A0N1_
DRB1_0101	31	DSRDSHGEVSGWETS	HGEVSGWET	5	0.1734	7659.6	100.00	0.45	tr_D0A0N1_
DRB1_0101	32	TASERADAAQGDSRD	SERADAAQG	2	0.1506	9806.8	100.00	0.34	tr_D0A0N1_
DRB1_0101	33	GWETSAGRRDSDDTQ	WETSAGRRD	1	0.1452	10392.7	100.00	0.80	tr_D0A0N1_
DRB1_0101	34	GDSRDSHGEVSGWET	SRDSHGEVS	2	0.1374	11308.2	100.00	0.28	tr_D0A0N1_
DRB1_0101	35	AQGDSRDSHGEVSGW	SRDSHGEVS	4	0.1275	12586.0	100.00	0.40	tr_D0A0N1_
DRB1_0101	36	QGDSRDSHGEVSGWE	SRDSHGEVS	3	0.1261	12773.2	100.00	0.44	tr_D0A0N1_
DRB1_0101	37	AAQGDSRDSHGEVSG	SRDSHGEVS	5	0.1185	13870.3	100.00	0.32	tr_D0A0N1_
DRB1_0101	38	ASERADAAQGDSRDS	SERADAAQG	1	0.1087	15417.0	100.00	0.41	tr_D0A0N1_
DRB1_0101	39	DAAQGDSRDSHGEVS	SRDSHGEVS	6	0.1087	15425.8	100.00	0.23	tr_D0A0N1_
DRB1_0101	40	AGRRDSDDTQIKTFV	RDSDDTQIK	3	0.1067	15755.6	100.00	0.24	tr_D0A0N1_
DRB1_0101	41	WETSAGRRDSDDTQI	WETSAGRRD	0	0.1045	16134.3	100.00	0.62	tr_D0A0N1_
DRB1_0101	42	SERADAAQGDSRDSH	AAQGDSRDS	5	0.0979	17333.8	100.00	0.23	tr_D0A0N1_
DRB1_0101	43	ADAAQGDSRDSHGEV	AAQGDSRDS	2	0.0969	17532.6	100.00	0.31	tr_D0A0N1_
DRB1_0101	44	RADAAQGDSRDSHGE	AAQGDSRDS	3	0.0941	18071.9	100.00	0.41	tr_D0A0N1_
DRB1_0101	45	ERADAAQGDSRDSHG	AAQGDSRDS	4	0.0922	18433.9	100.00	0.35	tr_D0A0N1_
DRB1_0101	46	SAGRRDSDDTQIKTF	RRDSDDTQI	3	0.0912	18636.2	100.00	0.29	tr_D0A0N1_
DRB1_0101	47	TSAGRRDSDDTQIKT	RRDSDDTQI	4	0.0785	21377.2	100.00	0.33	tr_D0A0N1_
DRB1_0101	48	ETSAGRRDSDDTQIK	RRDSDDTQI	5	0.0649	24779.2	100.00	0.35	tr_D0A0N1_

Allele: DRB1_0101. Number of high binders 0. Number of weak binders 7. Number of peptides 48

Allele	pos	peptide	core	Of	1-log50k(aff)	affinity(nM)	%Rank	Relia	Identity	Bind Level
DRB1_0101	1	KQVLLPLQGNISPSA	LLPLQGNIS	3	0.8111	7.7	1.60	0.86	tr_C9ZRP4_	WB
DRB1_0101	2	SKQVLLPLQGNISPS	LLPLQGNIS	4	0.7931	9.4	2.50	0.86	tr_C9ZRP4_	WB
DRB1_0101	3	QVLLPLQGNISPSAE	LLPLQGNIS	2	0.7794	10.9	3.00	0.86	tr_C9ZRP4_	WB
DRB1_0101	4	SSKQVLLPLQGNISP	LLPLQGNIS	5	0.7656	12.6	4.00	0.83	tr_C9ZRP4_	WB
DRB1_0101	5	TRNYKDLINRTVFSL	YKDLINRTV	3	0.7492	15.1	5.00	0.70	tr_C9ZRP4_	WB
DRB1_0101	6	RNYKDLINRTVFSLI	YKDLINRTV	2	0.7469	15.5	5.00	0.67	tr_C9ZRP4_	WB
DRB1_0101	7	VLLPLQGNISPSAES	LLPLQGNIS	1	0.7384	16.9	5.50	0.82	tr_C9ZRP4_	WB

DRB1_0101	8	KSSKQVLLPLQGNIS	LLPLQGNIS	6	0.7143	22.0	7.50	0.80	tr_C9ZRP4_	WB
DRB1_0101	9	NYKDLINRTVFSLIM	YKDLINRTV	1	0.7124	22.5	8.00	0.56	tr_C9ZRP4_	WB
DRB1_0101	10	RTRNYKDLINRTVFS	YKDLINRTV	4	0.7088	23.4	8.00	0.77	tr_C9ZRP4_	WB
DRB1_0101	11	GRTRNYKDLINRTVF	YKDLINRTV	5	0.6466	45.8	16.00	0.87	tr_C9ZRP4_	
DRB1_0101	12	LLPLQGNISPSAESP	LLPLQGNIS	0	0.6323	53.4	18.00	0.71	tr_C9ZRP4_	
DRB1_0101	13	EGRTRNYKDLINRTV	YKDLINRTV	6	0.5355	152.2	34.00	0.81	tr_C9ZRP4_	
DRB1_0101	14	NKSSKQVLLPLQGNI	QVLLPLQGN	5	0.4235	511.7	60.00	0.38	tr_C9ZRP4_	
DRB1_0101	15	KNKSSKQVLLPLQGN	NKSSKQVLL	1	0.4164	552.4	60.00	0.28	tr_C9ZRP4_	
DRB1_0101	16	MKNKSSKQVLLPLQG	NKSSKQVLL	2	0.4162	553.7	60.00	0.43	tr_C9ZRP4_	
DRB1_0101	17	LPLQGNISPSAESPR	LQGNISPSA	2	0.3469	1172.2	75.00	0.67	tr_C9ZRP4_	
DRB1_0101	18	PSSLMTTEGRTRNYK	LMTTEGRTR	3	0.2992	1963.1	85.00	0.55	tr_C9ZRP4_	
DRB1_0101	19	RPSSLMTTEGRTRNY	LMTTEGRTR	4	0.2952	2049.4	85.00	0.44	tr_C9ZRP4_	
DRB1_0101	20	PLQGNISPSAESPRP	LQGNISPSA	1	0.2853	2282.5	85.00	0.69	tr_C9ZRP4_	
DRB1_0101	21	SSLMTTEGRTRNYKD	LMTTEGRTR	2	0.2778	2475.6	85.00	0.60	tr_C9ZRP4_	
DRB1_0101	22	PRPSSLMTTEGRTRN	LMTTEGRTR	5	0.2732	2602.0	90.00	0.34	tr_C9ZRP4_	
DRB1_0101	23	SPRPSSLMTTEGRTR	SSLMTTEGR	4	0.2528	3244.0	90.00	0.25	tr_C9ZRP4_	
DRB1_0101	24	SLMTTEGRTRNYKDL	LMTTEGRTR	1	0.2313	4095.7	95.00	0.55	tr_C9ZRP4_	
DRB1_0101	25	ESPRPSSLMTTEGRT	PSSLMTTEG	4	0.2051	5435.6	95.00	0.29	tr_C9ZRP4_	
DRB1_0101	26	LQGNISPSAESPRPS	LQGNISPSA	0	0.2041	5492.7	95.00	0.46	tr_C9ZRP4_	
DRB1_0101	27	TEGRTRNYKDLINRT	NYKDLINRT	6	0.1965	5968.1	95.00	0.23	tr_C9ZRP4_	
DRB1_0101	28	AESPRPSSLMTTEGR	PSSLMTTEG	5	0.1860	6679.4	100.00	0.24	tr_C9ZRP4_	
DRB1_0101	29	SPSAESPRPSSLMTT	ESPRPSSLM	4	0.1827	6924.6	100.00	0.29	tr_C9ZRP4_	
DRB1_0101	30	LMTTEGRTRNYKDLI	LMTTEGRTR	0	0.1801	7123.1	100.00	0.31	tr_C9ZRP4_	
DRB1_0101	31	PSAESPRPSSLMTTE	ESPRPSSLM	3	0.1793	7185.1	100.00	0.32	tr_C9ZRP4_	
DRB1_0101	32	SAESPRPSSLMTTEG	ESPRPSSLM	2	0.1781	7276.3	100.00	0.29	tr_C9ZRP4_	
DRB1_0101	33	ISPSAESPRPSSLMT	ESPRPSSLM	5	0.1661	8289.1	100.00	0.29	tr_C9ZRP4_	
DRB1_0101	34	TTEGRTRNYKDLINR	GRTRNYKDL	3	0.1627	8601.9	100.00	0.35	tr_C9ZRP4_	
DRB1_0101	35	MTTEGRTRNYKDLIN	GRTRNYKDL	4	0.1514	9718.8	100.00	0.28	tr_C9ZRP4_	
DRB1_0101	36	NISPSAESPRPSSLM	AESPRPSSL	5	0.1482	10057.1	100.00	0.26	tr_C9ZRP4_	
DRB1_0101	37	GNISPSAESPRPSSL	PSAESPRPS	4	0.1372	11334.8	100.00	0.22	tr_C9ZRP4_	
DRB1_0101	38	QGNISPSAESPRPSS	GNISPSAES	1	0.1370	11354.2	100.00	0.32	tr_C9ZRP4_	

Allele: DRB1_0101. Number of high binders 0. Number of weak binders 10. Number of peptides 38

Allele	pos	peptide	core	Of	1-log50k(aff)	affinity(nM)	%Rank	Relia	Identity	Bind Level
DRB1_0101	1	QRRSLLSTLNRSLTE	LLSTLNRSL	4	0.7140	22.1	7.50	0.69	tr_C9ZRP4_	WB
DRB1_0101	2	EQRRSLLSTLNRSLT	LLSTLNRSL	5	0.7034	24.7	8.50	0.63	tr_C9ZRP4_	WB
DRB1_0101	3	FFTWVYLQSYVYRDE	WVYLQSYVY	3	0.6469	45.6	15.00	0.69	tr_C9ZRP4_	
DRB1_0101	4	GFFTWVYLQSYVYRD	WVYLQSYVY	4	0.6435	47.3	16.00	0.69	tr_C9ZRP4_	
DRB1_0101	5	MGFFTWVYLQSYVYR	WVYLQSYVY	5	0.6368	50.9	17.00	0.69	tr_C9ZRP4_	
DRB1_0101	6	EEQRRSLLSTLNRSL	LLSTLNRSL	6	0.6306	54.4	18.00	0.49	tr_C9ZRP4_	
DRB1_0101	7	FTWVYLQSYVYRDEN	WVYLQSYVY	2	0.6246	58.0	19.00	0.67	tr_C9ZRP4_	
DRB1_0101	8	TWVYLQSYVYRDENC	WVYLQSYVY	1	0.5771	97.1	26.00	0.68	tr_C9ZRP4_	
DRB1_0101	9	IMGFFTWVYLQSYVY	WVYLQSYVY	6	0.5549	123.5	30.00	0.68	tr_C9ZRP4_	
DRB1_0101	10	CPSWHTISSCALQLP	WHTISSCAL	3	0.5400	145.0	33.00	0.47	tr_C9ZRP4_	
DRB1_0101	11	PSWHTISSCALQLPE	WHTISSCAL	2	0.5394	146.0	33.00	0.46	tr_C9ZRP4_	

DRB1_0101	12	NCPSWHTISSCALQL	WHTISSCAL	4	0.5266	167.7	36.00	0.52	tr_C9ZRP4_
DRB1_0101	13	KDFGDLIPGHGGITD	FGDLIPGHG	2	0.5234	173.6	36.00	0.47	tr_C9ZRP4_
DRB1_0101	14	MKDFGDLIPGHGGIT	FGDLIPGHG	3	0.5222	175.8	37.00	0.54	tr_C9ZRP4_
DRB1_0101	15	RMDCQGIMGFFTWVY	CQGIMGFFT	3	0.5084	204.1	39.00	0.62	tr_C9ZRP4_
DRB1_0101	16	SWHTISSCALQLPEE	WHTISSCAL	1	0.5070	207.4	40.00	0.44	tr_C9ZRP4_
DRB1_0101	17	DFGDLIPGHGGITDR	FGDLIPGHG	1	0.5008	221.6	41.00	0.39	tr_C9ZRP4_
DRB1_0101	18	MDCQGIMGFFTWVYL	CQGIMGFFT	2	0.4969	231.1	42.00	0.52	tr_C9ZRP4_
DRB1_0101	19	KMKDFGDLIPGHGGI	FGDLIPGHG	4	0.4916	244.9	43.00	0.57	tr_C9ZRP4_
DRB1_0101	20	DCQGIMGFFTWVYLQ	CQGIMGFFT	1	0.4846	264.1	44.00	0.46	tr_C9ZRP4_
DRB1_0101	21	FKMKDFGDLIPGHGG	FGDLIPGHG	5	0.4837	266.7	44.00	0.46	tr_C9ZRP4_
DRB1_0101	22	WVYLQSYVYRDENCP	WVYLQSYVY	0	0.4805	276.2	45.00	0.55	tr_C9ZRP4_
DRB1_0101	23	DRMDCQGIMGFFTWV	CQGIMGFFT	4	0.4769	287.2	46.00	0.63	tr_C9ZRP4_
DRB1_0101	24	PEEQRRSLLSTLNRS	QRRSLLSTL	3	0.4757	290.9	46.00	0.31	tr_C9ZRP4_
DRB1_0101	25	FGDLIPGHGGITDRM	LIPGHGGIT	3	0.4699	309.6	47.00	0.41	tr_C9ZRP4_
DRB1_0101	26	AFKMKDFGDLIPGHG	KMKDFGDLI	2	0.4697	310.4	47.00	0.32	tr_C9ZRP4_
DRB1_0101	27	KRAFKMKDFGDLIPG	FKMKDFGDL	3	0.4593	347.4	50.00	0.52	tr_C9ZRP4_
DRB1_0101	28	CQGIMGFFTWVYLQS	CQGIMGFFT	0	0.4474	395.0	55.00	0.34	tr_C9ZRP4_
DRB1_0101	29	RAFKMKDFGDLIPGH	KMKDFGDLI	3	0.4466	398.6	55.00	0.46	tr_C9ZRP4_
DRB1_0101	30	WHTISSCALQLPEEQ	TISSCALQL	2	0.4448	406.2	55.00	0.34	tr_C9ZRP4_
DRB1_0101	31	ENCPSWHTISSCALQ	WHTISSCAL	5	0.4421	418.5	55.00	0.62	tr_C9ZRP4_
DRB1_0101	32	TDRMDCQGIMGFFTW	CQGIMGFFT	5	0.4354	449.6	55.00	0.64	tr_C9ZRP4_
DRB1_0101	33	LPEEQRRSLLSTLNR	QRRSLLSTL	4	0.4307	473.1	60.00	0.36	tr_C9ZRP4_
DRB1_0101	34	ITDRMDCQGIMGFFT	CQGIMGFFT	6	0.3851	775.4	65.00	0.52	tr_C9ZRP4_
DRB1_0101	35	DENCPSWHTISSCAL	WHTISSCAL	6	0.3826	796.5	70.00	0.51	tr_C9ZRP4_
DRB1_0101	36	QLPEEQRRSLLSTLN	QRRSLLSTL	5	0.3824	798.6	70.00	0.31	tr_C9ZRP4_
DRB1_0101	37	QGIMGFFTWVYLQSY	GIMGFFTWV	1	0.3812	808.5	70.00	0.28	tr_C9ZRP4_
DRB1_0101	38	HTISSCALQLPEEQR	TISSCALQL	1	0.3802	817.2	70.00	0.43	tr_C9ZRP4_
DRB1_0101	39	GIMGFFTWVYLQSYV	FTWVYLQSY	5	0.3739	875.5	70.00	0.32	tr_C9ZRP4_
DRB1_0101	40	GDLIPGHGGITDRMD	LIPGHGGIT	2	0.3564	1058.0	75.00	0.64	tr_C9ZRP4_
DRB1_0101	41	TISSCALQLPEEQRR	CALQLPEEQ	4	0.3499	1134.9	75.00	0.49	tr_C9ZRP4_
DRB1_0101	42	ISSCALQLPEEQRRS	CALQLPEEQ	3	0.3446	1201.1	75.00	0.62	tr_C9ZRP4_
DRB1_0101	43	SSCALQLPEEQRRSL	CALQLPEEQ	2	0.3403	1258.2	75.00	0.58	tr_C9ZRP4_
DRB1_0101	44	SCALQLPEEQRRSLL	CALQLPEEQ	1	0.3261	1468.0	80.00	0.46	tr_C9ZRP4_
DRB1_0101	45	LQLPEEQRRSLLSTL	QRRSLLSTL	6	0.3224	1527.3	80.00	0.26	tr_C9ZRP4_
DRB1_0101	46	YVYRDENCPSWHTIS	DENCPSWHT	4	0.3129	1692.8	80.00	0.40	tr_C9ZRP4_
DRB1_0101	47	SYVYRDENCPSWHTI	DENCPSWHT	5	0.3116	1717.2	80.00	0.31	tr_C9ZRP4_
DRB1_0101	48	CALQLPEEQRRSLLS	CALQLPEEQ	0	0.2968	2016.0	85.00	0.26	tr_C9ZRP4_
DRB1_0101	49	VYLQSYVYRDENCPS	YLQSYVYRD	1	0.2956	2041.0	85.00	0.38	tr_C9ZRP4_
DRB1_0101	50	VYRDENCPSWHTISS	DENCPSWHT	3	0.2899	2172.1	85.00	0.49	tr_C9ZRP4_
DRB1_0101	51	QSYVYRDENCPSWHT	YVYRDENCP	2	0.2870	2240.9	85.00	0.34	tr_C9ZRP4_
DRB1_0101	52	DLIPGHGGITDRMDC	LIPGHGGIT	1	0.2858	2269.8	85.00	0.74	tr_C9ZRP4_
DRB1_0101	53	RDENCPSWHTISSCA	DENCPSWHT	1	0.2679	2754.2	90.00	0.43	tr_C9ZRP4_
DRB1_0101	54	YRDENCPSWHTISSC	DENCPSWHT	2	0.2646	2855.1	90.00	0.52	tr_C9ZRP4_
DRB1_0101	55	ALQLPEEQRRSLLST	PEEQRRSLL	4	0.2563	3125.0	90.00	0.33	tr_C9ZRP4_
DRB1_0101	56	YLQSYVYRDENCPSW	YVYRDENCP	4	0.2463	3479.5	90.00	0.48	tr_C9ZRP4_
DRB1_0101	57	LQSYVYRDENCPSWH	YVYRDENCP	3	0.2462	3483.8	90.00	0.51	tr_C9ZRP4_
DRB1_0101	58	GITDRMDCQGIMGFF	TDRMDCQGI	2	0.2363	3876.5	95.00	0.38	tr_C9ZRP4_
DRB1_0101	59	GGITDRMDCQGIMGF	TDRMDCQGI	3	0.2210	4578.0	95.00	0.44	tr_C9ZRP4_
DRB1_0101	60	LIPGHGGITDRMDCQ	LIPGHGGIT	0	0.2134	4968.1	95.00	0.59	tr_C9ZRP4_
DRB1_0101	61	HGGITDRMDCQGIMG	TDRMDCQGI	4	0.1794	7178.8	100.00	0.47	tr_C9ZRP4_

DRB1_0101	62	GHGGITDRMDCQGIM	TDRMDCQGI	5	0.1611	8746.2	100.00	0.44	tr_C9ZRP4_
DRB1_0101	63	PGHGGITDRMDCQGI	TDRMDCQGI	6	0.1112	15008.6	100.00	0.40	tr_C9ZRP4_
DRB1_0101	64	IPGHGGITDRMDCQG	GHGGITDRM	2	0.0899	18906.2	100.00	0.37	tr_C9ZRP4_

Allele: DRB1_0101. Number of high binders 0. Number of weak binders 2. Number of peptides 64

Allele	pos	peptide	core	Of	1-log50k(aff)	affinity(nM)	%Rank	Relia	Identity	Bind Level
DRB1_0101	1	HTHDMTRPPSHHHHH	MTRPPSHHH	4	0.2160	4829.7	95.00	0.37	tr_D0A888_	
DRB1_0101	2	HHTHDMTRPPSHHHH	HDMTRPPSH	3	0.2087	5229.2	95.00	0.29	tr_D0A888_	
DRB1_0101	3	THDMTRPPSHHHHHD	MTRPPSHHH	3	0.2035	5527.5	95.00	0.40	tr_D0A888_	
DRB1_0101	4	HHHTHDMTRPPSHHH	HDMTRPPSH	4	0.1863	6664.1	100.00	0.28	tr_D0A888_	
DRB1_0101	5	SHHHHHDDHTHGVVD	HHDDHTHGV	4	0.1774	7333.2	100.00	0.39	tr_D0A888_	
DRB1_0101	6	GDTHGGCESGHGTYS	CESGHGTYS	6	0.1719	7781.5	100.00	0.34	tr_D0A888_	
DRB1_0101	7	PSHHHHHDDHTHGVV	HHDDHTHGV	5	0.1705	7902.1	100.00	0.36	tr_D0A888_	
DRB1_0101	8	HDMTRPPSHHHHHDD	MTRPPSHHH	2	0.1667	8238.2	100.00	0.43	tr_D0A888_	
DRB1_0101	9	HHHHHDDHTHGVVDD	HHDDHTHGV	3	0.1661	8286.1	100.00	0.41	tr_D0A888_	
DRB1_0101	10	HHHHDDHTHGVVDDG	HHDDHTHGV	2	0.1499	9879.0	100.00	0.43	tr_D0A888_	
DRB1_0101	11	PPSHHHHHDDHTHGV	HHHHHDDHT	3	0.1363	11446.8	100.00	0.25	tr_D0A888_	
DRB1_0101	12	HHHDDHTHGVVDDGI	HHDDHTHGV	1	0.1326	11905.2	100.00	0.37	tr_D0A888_	
DRB1_0101	13	HTHGVVDDGIDGDTH	GVVDDGIDG	3	0.1296	12303.0	100.00	0.65	tr_D0A888_	
DRB1_0101	14	DMTRPPSHHHHHDDH	MTRPPSHHH	1	0.1277	12560.6	100.00	0.44	tr_D0A888_	
DRB1_0101	15	THGVVDDGIDGDTHG	GVVDDGIDG	2	0.1242	13043.7	100.00	0.68	tr_D0A888_	
DRB1_0101	16	DHTHGVVDDGIDGDT	GVVDDGIDG	4	0.1177	13995.4	100.00	0.62	tr_D0A888_	
DRB1_0101	17	DGDTHGGCESGHGTY	GGCESGHGT	5	0.1154	14339.1	100.00	0.44	tr_D0A888_	
DRB1_0101	18	HGVVDDGIDGDTHGG	GVVDDGIDG	1	0.1119	14903.1	100.00	0.65	tr_D0A888_	
DRB1_0101	19	RPPSHHHHHDDHTHG	HHHHHDDHT	4	0.1039	16254.6	100.00	0.30	tr_D0A888_	
DRB1_0101	20	MTRPPSHHHHHDDHT	MTRPPSHHH	0	0.1034	16325.7	100.00	0.29	tr_D0A888_	
DRB1_0101	21	HHDDHTHGVVDDGID	HHDDHTHGV	0	0.1016	16648.0	100.00	0.31	tr_D0A888_	
DRB1_0101	22	DDHTHGVVDDGIDGD	GVVDDGIDG	5	0.1007	16814.7	100.00	0.55	tr_D0A888_	
DRB1_0101	23	GVVDDGIDGDTHGGC	GVVDDGIDG	0	0.0945	17992.7	100.00	0.50	tr_D0A888_	
DRB1_0101	24	TRPPSHHHHHDDHTH	HHHHHDDHT	5	0.0939	18108.5	100.00	0.28	tr_D0A888_	
DRB1_0101	25	IDGDTHGGCESGHGT	GGCESGHGT	6	0.0922	18435.5	100.00	0.28	tr_D0A888_	
DRB1_0101	26	HDDHTHGVVDDGIDG	GVVDDGIDG	6	0.0911	18653.2	100.00	0.38	tr_D0A888_	
DRB1_0101	27	GIDGDTHGGCESGHG	GDTHGGCES	3	0.0787	21334.2	100.00	0.26	tr_D0A888_	
DRB1_0101	28	DGIDGDTHGGCESGH	IDGDTHGGC	2	0.0724	22849.5	100.00	0.25	tr_D0A888_	
DRB1_0101	29	DDGIDGDTHGGCESG	IDGDTHGGC	3	0.0695	23560.8	100.00	0.24	tr_D0A888_	
DRB1_0101	30	VDDGIDGDTHGGCES	IDGDTHGGC	4	0.0665	24345.5	100.00	0.20	tr_D0A888_	
DRB1_0101	31	VVDDGIDGDTHGGCE	IDGDTHGGC	5	0.0651	24732.8	100.00	0.21	tr_D0A888_	

Allele: DRB1_0101. Number of high binders 0. Number of weak binders 0. Number of peptides 31

Allele	pos	peptide	core	Of	1-log50k(aff)	affinity(nM)	%Rank	Relia	Identity	Bind Level

DRB1_0101	1	LSLLAFATGRLMRVI	LAFATGRLM	3	0.8285	6.4	1.10	0.73	tr_D0A0U2_	WB
DRB1_0101	2	PLSLLAFATGRLMRV	LAFATGRLM	4	0.8232	6.8	1.30	0.70	tr_D0A0U2_	WB
DRB1_0101	3	YDLFATALNNTAGAA	FATALNNTA	3	0.8220	6.9	1.30	0.88	tr_D0A0U2_	WB
DRB1_0101	4	SLLAFATGRLMRVIL	LAFATGRLM	2	0.8197	7.0	1.40	0.69	tr_D0A0U2_	WB
DRB1_0101	5	DLFATALNNTAGAAR	FATALNNTA	2	0.8038	8.4	1.90	0.89	tr_D0A0U2_	WB
DRB1_0101	6	TPLSLLAFATGRLMR	LAFATGRLM	5	0.8019	8.5	2.00	0.62	tr_D0A0U2_	WB
DRB1_0101	7	LLAFATGRLMRVILL	LAFATGRLM	1	0.8000	8.7	2.00	0.62	tr_D0A0U2_	WB
DRB1_0101	8	LYDLFATALNNTAGA	FATALNNTA	4	0.7980	8.9	2.50	0.85	tr_D0A0U2_	WB
DRB1_0101	9	LFATALNNTAGAARL	FATALNNTA	1	0.7857	10.2	3.00	0.86	tr_D0A0U2_	WB
DRB1_0101	10	FLYDLFATALNNTAG	FATALNNTA	5	0.7820	10.6	3.00	0.71	tr_D0A0U2_	WB
DRB1_0101	11	TPQLIRKGGAVTTIR	IRKGGAVTT	4	0.7624	13.1	4.00	0.60	tr_D0A0U2_	WB
DRB1_0101	12	PQLIRKGGAVTTIRE	IRKGGAVTT	3	0.7568	13.9	4.50	0.64	tr_D0A0U2_	WB
DRB1_0101	13	SFLYDLFATALNNTA	FATALNNTA	6	0.7535	14.4	4.50	0.38	tr_D0A0U2_	WB
DRB1_0101	14	LVNFFYADSSIISDD	FFYADSSII	3	0.7515	14.7	5.00	0.57	tr_D0A0U2_	WB
DRB1_0101	15	TLVNFFYADSSIISD	FFYADSSII	4	0.7480	15.3	5.00	0.58	tr_D0A0U2_	WB
DRB1_0101	16	VNFFYADSSIISDDM	FFYADSSII	2	0.7458	15.7	5.00	0.50	tr_D0A0U2_	WB
DRB1_0101	17	EFSFLYDLFATALNN	FLYDLFATA	3	0.7447	15.8	5.50	0.48	tr_D0A0U2_	WB
DRB1_0101	18	VGKYLPISGTVFVIG	YLPISGTVF	3	0.7421	16.3	5.50	0.70	tr_D0A0U2_	WB
DRB1_0101	19	WTPLSLLAFATGRLM	LAFATGRLM	6	0.7397	16.7	5.50	0.49	tr_D0A0U2_	WB
DRB1_0101	20	GVGKYLPISGTVFVI	YLPISGTVF	4	0.7387	16.9	5.50	0.67	tr_D0A0U2_	WB
DRB1_0101	21	AEFSFLYDLFATALN	FLYDLFATA	4	0.7383	17.0	5.50	0.48	tr_D0A0U2_	WB
DRB1_0101	22	QLIRKGGAVTTIRED	IRKGGAVTT	2	0.7361	17.4	6.00	0.69	tr_D0A0U2_	WB
DRB1_0101	23	LAFATGRLMRVILLH	LAFATGRLM	0	0.7330	18.0	6.00	0.53	tr_D0A0U2_	WB
DRB1_0101	24	FSFLYDLFATALNNT	FLYDLFATA	2	0.7283	18.9	6.50	0.44	tr_D0A0U2_	WB
DRB1_0101	25	FAEFSFLYDLFATAL	FLYDLFATA	5	0.7204	20.6	7.00	0.48	tr_D0A0U2_	WB
DRB1_0101	26	NTPQLIRKGGAVTTI	IRKGGAVTT	5	0.7152	21.8	7.50	0.54	tr_D0A0U2_	WB
DRB1_0101	27	EGVGKYLPISGTVFV	YLPISGTVF	5	0.7152	21.8	7.50	0.68	tr_D0A0U2_	WB
DRB1_0101	28	NFFYADSSIISDDMR	FFYADSSII	1	0.7146	21.9	7.50	0.47	tr_D0A0U2_	WB
DRB1_0101	29	DTLVNFFYADSSIIS	FFYADSSII	5	0.7138	22.1	7.50	0.67	tr_D0A0U2_	WB
DRB1_0101	30	GKYLPISGTVFVIGL	YLPISGTVF	2	0.7118	22.6	8.00	0.74	tr_D0A0U2_	WB
DRB1_0101	31	FATALNNTAGAARLV	FATALNNTA	0	0.7094	23.2	8.00	0.70	tr_D0A0U2_	WB
DRB1_0101	32	GTVFVIGLSVPDVKE	FVIGLSVPD	3	0.6830	30.9	11.00	0.67	tr_D0A0U2_	
DRB1_0101	33	SGTVFVIGLSVPDVK	FVIGLSVPD	4	0.6776	32.7	12.00	0.69	tr_D0A0U2_	
DRB1_0101	34	TVFVIGLSVPDVKEI	FVIGLSVPD	2	0.6687	36.1	13.00	0.64	tr_D0A0U2_	
DRB1_0101	35	LIRKGGAVTTIREDG	IRKGGAVTT	1	0.6563	41.2	14.00	0.81	tr_D0A0U2_	
DRB1_0101	36	KYLPISGTVFVIGLS	YLPISGTVF	1	0.6557	41.5	14.00	0.80	tr_D0A0U2_	
DRB1_0101	37	LFAEFSFLYDLFATA	FLYDLFATA	6	0.6544	42.1	14.00	0.47	tr_D0A0U2_	
DRB1_0101	38	AARLVFATSLPHWGD	LVFATSLPH	3	0.6466	45.8	16.00	0.69	tr_D0A0U2_	
DRB1_0101	39	GEGVGKYLPISGTVF	YLPISGTVF	6	0.6439	47.1	16.00	0.65	tr_D0A0U2_	
DRB1_0101	40	GAARLVFATSLPHWG	LVFATSLPH	4	0.6381	50.2	17.00	0.70	tr_D0A0U2_	
DRB1_0101	41	ARLVFATSLPHWGDT	LVFATSLPH	2	0.6318	53.7	18.00	0.66	tr_D0A0U2_	
DRB1_0101	42	ALNNTAGAARLVFAT	NTAGAARLV	3	0.6311	54.1	18.00	0.61	tr_D0A0U2_	
DRB1_0101	43	SRLWTPLSLLAFATG	PLSLLAFAT	5	0.6267	56.8	18.00	0.35	tr_D0A0U2_	
DRB1_0101	44	TALNNTAGAARLVFA	NTAGAARLV	4	0.6266	56.9	18.00	0.49	tr_D0A0U2_	
DRB1_0101	45	FFYADSSIISDDMRY	FYADSSIIS	1	0.6252	57.7	19.00	0.51	tr_D0A0U2_	
DRB1_0101	46	RLWTPLSLLAFATGR	PLSLLAFAT	4	0.6240	58.4	19.00	0.53	tr_D0A0U2_	
DRB1_0101	47	LWTPLSLLAFATGRL	PLSLLAFAT	3	0.6225	59.4	19.00	0.54	tr_D0A0U2_	
DRB1_0101	48	DSRLWTPLSLLAFAT	PLSLLAFAT	6	0.6204	60.8	19.00	0.25	tr_D0A0U2_	
DRB1_0101	49	VFVIGLSVPDVKEIA	FVIGLSVPD	1	0.6189	61.7	19.00	0.60	tr_D0A0U2_	
DRB1_0101	50	ISGTVFVIGLSVPDV	FVIGLSVPD	5	0.6165	63.4	20.00	0.69	tr_D0A0U2_	

DRB1_0101	51	SVAFDSNTPQLIRKG	FDSNTPQLI	3	0.6052	71.7	22.00	0.87	tr_D0A0U2_
DRB1_0101	52	SNTPQLIRKGGAVTT	LIRKGGAVT	5	0.6048	71.9	22.00	0.49	tr_D0A0U2_
DRB1_0101	53	LNNTAGAARLVFATS	NTAGAARLV	2	0.6040	72.5	22.00	0.60	tr_D0A0U2_
DRB1_0101	54	KDSRLWTPLSLLAFA	RLWTPLSLL	3	0.6028	73.5	22.00	0.32	tr_D0A0U2_
DRB1_0101	55	AGAARLVFATSLPHW	LVFATSLPH	5	0.5966	78.7	23.00	0.72	tr_D0A0U2_
DRB1_0101	56	ASVAFDSNTPQLIRK	FDSNTPQLI	4	0.5927	82.0	24.00	0.85	tr_D0A0U2_
DRB1_0101	57	PDTLVNFFYADSSII	FFYADSSII	6	0.5895	84.9	24.00	0.71	tr_D0A0U2_
DRB1_0101	58	RLVFATSLPHWGDTE	LVFATSLPH	1	0.5881	86.2	24.00	0.62	tr_D0A0U2_
DRB1_0101	59	EKDSRLWTPLSLLAF	RLWTPLSLL	4	0.5864	87.8	25.00	0.29	tr_D0A0U2_
DRB1_0101	60	ATALNNTAGAARLVF	NTAGAARLV	5	0.5803	93.8	26.00	0.44	tr_D0A0U2_
DRB1_0101	61	VAFDSNTPQLIRKGG	FDSNTPQLI	2	0.5790	95.2	26.00	0.88	tr_D0A0U2_
DRB1_0101	62	PARLIGGASVAFDSN	LIGGASVAF	3	0.5770	97.2	26.00	0.35	tr_D0A0U2_
DRB1_0101	63	AFATGRLMRVILLHV	FATGRLMRV	1	0.5716	103.0	27.00	0.53	tr_D0A0U2_
DRB1_0101	64	NNTAGAARLVFATSL	NTAGAARLV	1	0.5586	118.7	30.00	0.54	tr_D0A0U2_
DRB1_0101	65	VEKDSRLWTPLSLLA	DSRLWTPLS	3	0.5543	124.2	30.00	0.39	tr_D0A0U2_
DRB1_0101	66	GASVAFDSNTPQLIR	FDSNTPQLI	5	0.5456	136.6	32.00	0.81	tr_D0A0U2_
DRB1_0101	67	VLFAEFSFLYDLFAT	FSFLYDLFA	5	0.5410	143.5	33.00	0.57	tr_D0A0U2_
DRB1_0101	68	IVLFAEFSFLYDLFA	FSFLYDLFA	6	0.5392	146.4	33.00	0.40	tr_D0A0U2_
DRB1_0101	69	RVIVLFAEFSFLYDL	LFAEFSFLY	4	0.5384	147.6	33.00	0.28	tr_D0A0U2_
DRB1_0101	70	LRVIVLFAEFSFLYD	VIVLFAEFS	2	0.5327	157.1	35.00	0.40	tr_D0A0U2_
DRB1_0101	71	AFDSNTPQLIRKGGA	FDSNTPQLI	1	0.5316	158.9	35.00	0.85	tr_D0A0U2_
DRB1_0101	72	YLPISGTVFVIGLSV	YLPISGTVF	0	0.5235	173.5	36.00	0.61	tr_D0A0U2_
DRB1_0101	73	DLRVIVLFAEFSFLY	VIVLFAEFS	3	0.5231	174.1	36.00	0.44	tr_D0A0U2_
DRB1_0101	74	PISGTVFVIGLSVPD	FVIGLSVPD	6	0.5216	177.0	37.00	0.62	tr_D0A0U2_
DRB1_0101	75	TAGAARLVFATSLPH	LVFATSLPH	6	0.5158	188.4	38.00	0.54	tr_D0A0U2_
DRB1_0101	76	FVIGLSVPDVKEIAR	FVIGLSVPD	0	0.5141	192.1	38.00	0.43	tr_D0A0U2_
DRB1_0101	77	FATGRLMRVILLHVE	GRLMRVILL	3	0.5119	196.6	39.00	0.49	tr_D0A0U2_
DRB1_0101	78	ARLIGGASVAFDSNT	LIGGASVAF	2	0.5094	202.1	39.00	0.41	tr_D0A0U2_
DRB1_0101	79	DVEKDSRLWTPLSLL	DSRLWTPLS	4	0.4946	237.2	42.00	0.41	tr_D0A0U2_
DRB1_0101	80	LLHVEEMSPDTLVNF	VEEMSPDTL	3	0.4943	237.9	42.00	0.73	tr_D0A0U2_
DRB1_0101	81	GRLMRVILLHVEEMS	VILLHVEEM	5	0.4919	244.1	43.00	0.37	tr_D0A0U2_
DRB1_0101	82	VIVLFAEFSFLYDLF	LFAEFSFLY	3	0.4908	246.9	43.00	0.41	tr_D0A0U2_
DRB1_0101	83	TGRLMRVILLHVEEM	GRLMRVILL	1	0.4848	263.6	44.00	0.48	tr_D0A0U2_
DRB1_0101	84	LHVEEMSPDTLVNFF	VEEMSPDTL	2	0.4778	284.3	46.00	0.66	tr_D0A0U2_
DRB1_0101	85	NTAGAARLVFATSLP	NTAGAARLV	0	0.4755	291.5	46.00	0.37	tr_D0A0U2_
DRB1_0101	86	SVPDVKEIARKLEER	DVKEIARKL	3	0.4726	300.8	47.00	0.89	tr_D0A0U2_
DRB1_0101	87	ILLHVEEMSPDTLVN	VEEMSPDTL	4	0.4706	307.3	47.00	0.65	tr_D0A0U2_
DRB1_0101	88	LVFATSLPHWGDTET	LVFATSLPH	0	0.4699	309.6	47.00	0.48	tr_D0A0U2_
DRB1_0101	89	NDLRVIVLFAEFSFL	VIVLFAEFS	4	0.4566	357.8	50.00	0.55	tr_D0A0U2_
DRB1_0101	90	VILLHVEEMSPDTLV	VEEMSPDTL	5	0.4534	370.1	55.00	0.52	tr_D0A0U2_
DRB1_0101	91	HVEEMSPDTLVNFFY	VEEMSPDTL	1	0.4494	386.5	55.00	0.56	tr_D0A0U2_
DRB1_0101	92	GGASVAFDSNTPQLI	FDSNTPQLI	6	0.4481	391.9	55.00	0.75	tr_D0A0U2_
DRB1_0101	93	ATGRLMRVILLHVEE	GRLMRVILL	2	0.4470	396.7	55.00	0.69	tr_D0A0U2_
DRB1_0101	94	VPDVKEIARKLEERN	DVKEIARKL	2	0.4457	402.3	55.00	0.88	tr_D0A0U2_
DRB1_0101	95	LSVPDVKEIARKLEE	DVKEIARKL	4	0.4449	406.0	55.00	0.85	tr_D0A0U2_
DRB1_0101	96	GLSVPDVKEIARKLE	DVKEIARKL	5	0.4328	462.7	55.00	0.76	tr_D0A0U2_
DRB1_0101	97	IRKGGAVTTIREDGE	IRKGGAVTT	0	0.4318	467.5	55.00	0.82	tr_D0A0U2_
DRB1_0101	98	RLMRVILLHVEEMSP	VILLHVEEM	4	0.4305	474.1	60.00	0.57	tr_D0A0U2_
DRB1_0101	99	RVILLHVEEMSPDTL	VILLHVEEM	1	0.4217	521.5	60.00	0.39	tr_D0A0U2_
DRB1_0101	100	LMRVILLHVEEMSPD	VILLHVEEM	3	0.4125	576.0	60.00	0.59	tr_D0A0U2_

DRB1_0101	101	FYADSSIISDDMRYG	FYADSSIIS	0	0.4123	577.7	60.00	0.61	tr_D0A0U2_
DRB1_0101	102	ETSSKTAQLFHDVEK	KTAQLFHDV	4	0.4072	610.6	65.00	0.49	tr_D0A0U2_
DRB1_0101	103	DGEGVGKYLPISGTV	GKYLPISGT	5	0.4068	613.2	65.00	0.52	tr_D0A0U2_
DRB1_0101	104	MRVILLHVEEMSPDT	VILLHVEEM	2	0.4050	625.0	65.00	0.56	tr_D0A0U2_
DRB1_0101	105	RLIGGASVAFDSNTP	LIGGASVAF	1	0.4042	630.7	65.00	0.45	tr_D0A0U2_
DRB1_0101	106	VEEMSPDTLVNFFYA	VEEMSPDTL	0	0.4011	652.3	65.00	0.32	tr_D0A0U2_
DRB1_0101	107	PDVKEIARKLEERND	DVKEIARKL	1	0.3975	678.0	65.00	0.85	tr_D0A0U2_
DRB1_0101	108	FDSNTPQLIRKGGAV	FDSNTPQLI	0	0.3943	701.4	65.00	0.70	tr_D0A0U2_
DRB1_0101	109	TETSSKTAQLFHDVE	KTAQLFHDV	5	0.3887	745.2	65.00	0.41	tr_D0A0U2_
DRB1_0101	110	DSNTPQLIRKGGAVT	LIRKGGAVT	6	0.3876	754.4	65.00	0.60	tr_D0A0U2_
DRB1_0101	111	TSSKTAQLFHDVEKD	KTAQLFHDV	3	0.3876	754.8	65.00	0.55	tr_D0A0U2_
DRB1_0101	112	RNDLRVIVLFAEFSF	VIVLFAEFS	5	0.3858	769.4	65.00	0.66	tr_D0A0U2_
DRB1_0101	113	LPISGTVFVIGLSVP	TVFVIGLSV	5	0.3788	829.7	70.00	0.49	tr_D0A0U2_
DRB1_0101	114	IGLSVPDVKEIARKL	DVKEIARKL	6	0.3767	849.4	70.00	0.57	tr_D0A0U2_
DRB1_0101	115	AVTTIREDGEGVGKY	TIREDGEGV	3	0.3728	885.8	70.00	0.80	tr_D0A0U2_
DRB1_0101	116	SPDTLVNFFYADSSI	DTLVNFFYA	2	0.3704	908.9	70.00	0.39	tr_D0A0U2_
DRB1_0101	117	VTTIREDGEGVGKYL	TIREDGEGV	2	0.3700	912.8	70.00	0.68	tr_D0A0U2_
DRB1_0101	118	HDVEKDSRLWTPLSL	DSRLWTPLS	5	0.3644	970.0	70.00	0.50	tr_D0A0U2_
DRB1_0101	119	DTETSSKTAQLFHDV	KTAQLFHDV	6	0.3584	1034.3	75.00	0.31	tr_D0A0U2_
DRB1_0101	120	SSKTAQLFHDVEKDS	KTAQLFHDV	2	0.3496	1138.3	75.00	0.58	tr_D0A0U2_
DRB1_0101	121	MSPDTLVNFFYADSS	DTLVNFFYA	3	0.3460	1183.8	75.00	0.47	tr_D0A0U2_
DRB1_0101	122	ERNDLRVIVLFAEFS	VIVLFAEFS	6	0.3453	1192.0	75.00	0.55	tr_D0A0U2_
DRB1_0101	123	RKLEERNDLRVIVLF	LEERNDLRV	2	0.3449	1197.1	75.00	0.38	tr_D0A0U2_
DRB1_0101	124	ARKLEERNDLRVIVL	LEERNDLRV	3	0.3444	1204.1	75.00	0.43	tr_D0A0U2_
DRB1_0101	125	KLEERNDLRVIVLFA	LEERNDLRV	1	0.3443	1205.1	75.00	0.25	tr_D0A0U2_
DRB1_0101	126	TTIREDGEGVGKYLP	TIREDGEGV	1	0.3389	1278.4	75.00	0.62	tr_D0A0U2_
DRB1_0101	127	VIGLSVPDVKEIARK	VIGLSVPDV	0	0.3345	1340.4	75.00	0.40	tr_D0A0U2_
DRB1_0101	128	GAVTTIREDGEGVGK	TIREDGEGV	4	0.3326	1367.7	80.00	0.81	tr_D0A0U2_
DRB1_0101	129	EDGEGVGKYLPISGT	GKYLPISGT	6	0.3284	1431.6	80.00	0.40	tr_D0A0U2_
DRB1_0101	130	EMSPDTLVNFFYADS	DTLVNFFYA	4	0.3225	1526.1	80.00	0.49	tr_D0A0U2_
DRB1_0101	131	EEMSPDTLVNFFYAD	DTLVNFFYA	5	0.3181	1600.7	80.00	0.41	tr_D0A0U2_
DRB1_0101	132	LEERNDLRVIVLFAE	ERNDLRVIV	2	0.3085	1775.1	80.00	0.18	tr_D0A0U2_
DRB1_0101	133	IARKLEERNDLRVIV	LEERNDLRV	4	0.3063	1817.8	85.00	0.42	tr_D0A0U2_
DRB1_0101	134	SKTAQLFHDVEKDSR	KTAQLFHDV	1	0.3035	1873.9	85.00	0.54	tr_D0A0U2_
DRB1_0101	135	DVKEIARKLEERNDL	DVKEIARKL	0	0.2992	1962.6	85.00	0.70	tr_D0A0U2_
DRB1_0101	136	VFATSLPHWGDTETS	FATSLPHWG	1	0.2944	2067.2	85.00	0.64	tr_D0A0U2_
DRB1_0101	137	FHDVEKDSRLWTPLS	DSRLWTPLS	6	0.2935	2088.0	85.00	0.40	tr_D0A0U2_
DRB1_0101	138	ADSSIISDDMRYGVF	IISDDMRYG	4	0.2928	2105.3	85.00	0.39	tr_D0A0U2_
DRB1_0101	139	DSSIISDDMRYGVFD	IISDDMRYG	3	0.2872	2234.8	85.00	0.39	tr_D0A0U2_
DRB1_0101	140	TIREDGEGVGKYLPI	TIREDGEGV	0	0.2870	2240.1	85.00	0.34	tr_D0A0U2_
DRB1_0101	141	LIGGASVAFDSNTPQ	GGASVAFDS	2	0.2790	2443.9	85.00	0.29	tr_D0A0U2_
DRB1_0101	142	YADSSIISDDMRYGV	IISDDMRYG	5	0.2741	2576.3	90.00	0.33	tr_D0A0U2_
DRB1_0101	143	SSIISDDMRYGVFDD	IISDDMRYG	2	0.2735	2593.8	90.00	0.40	tr_D0A0U2_
DRB1_0101	144	EERNDLRVIVLFAEF	DLRVIVLFA	4	0.2710	2663.8	90.00	0.25	tr_D0A0U2_
DRB1_0101	145	GGAVTTIREDGEGVG	TIREDGEGV	5	0.2700	2692.6	90.00	0.77	tr_D0A0U2_
DRB1_0101	146	GDTETSSKTAQLFHD	TSSKTAQLF	4	0.2691	2720.3	90.00	0.42	tr_D0A0U2_
DRB1_0101	147	EIARKLEERNDLRVI	LEERNDLRV	5	0.2644	2862.1	90.00	0.38	tr_D0A0U2_
DRB1_0101	148	WGDTETSSKTAQLFH	TSSKTAQLF	5	0.2532	3230.5	90.00	0.43	tr_D0A0U2_
DRB1_0101	149	REDGEGVGKYLPISG	DGEGVGKYL	2	0.2432	3599.4	90.00	0.33	tr_D0A0U2_
DRB1_0101	150	KTAQLFHDVEKDSRL	KTAQLFHDV	0	0.2417	3655.9	90.00	0.37	tr_D0A0U2_

DRB1_0101	151	IREDGEGVGKYLPIS	DGEGVGKYL	3	0.2399	3729.9	95.00	0.41	tr_D0A0U2_	
DRB1_0101	152	IGGASVAFDSNTPQL	GGASVAFDS	1	0.2346	3948.9	95.00	0.34	tr_D0A0U2_	
DRB1_0101	153	VFDDTKCDTAEKLPK	TKCDTAEKL	4	0.2338	3985.8	95.00	0.64	tr_D0A0U2_	
DRB1_0101	154	SIISDDMRYGVFDDT	IISDDMRYG	1	0.2320	4062.0	95.00	0.34	tr_D0A0U2_	
DRB1_0101	155	LFHDVEKDSRLWTPL	DVEKDSRLW	3	0.2299	4157.9	95.00	0.34	tr_D0A0U2_	
DRB1_0101	156	AQLFHDVEKDSRLWT	DVEKDSRLW	5	0.2286	4214.3	95.00	0.28	tr_D0A0U2_	
DRB1_0101	157	FDDTKCDTAEKLPKD	TKCDTAEKL	3	0.2262	4324.5	95.00	0.68	tr_D0A0U2_	
DRB1_0101	158	QLFHDVEKDSRLWTP	DVEKDSRLW	4	0.2176	4745.3	95.00	0.29	tr_D0A0U2_	
DRB1_0101	159	PKDDCASNYGATQVS	CASNYGATQ	4	0.2170	4779.6	95.00	0.47	tr_D0A0U2_	
DRB1_0101	160	FATSLPHWGDTETSS	FATSLPHWG	0	0.2161	4826.0	95.00	0.52	tr_D0A0U2_	
DRB1_0101	161	KEIARKLEERNDLRV	LEERNDLRV	6	0.2115	5071.9	95.00	0.28	tr_D0A0U2_	
DRB1_0101	162	HWGDTETSSKTAQLF	ETSSKTAQL	5	0.2097	5168.7	95.00	0.29	tr_D0A0U2_	
DRB1_0101	163	TAQLFHDVEKDSRLW	LFHDVEKDS	3	0.2048	5454.5	95.00	0.22	tr_D0A0U2_	
DRB1_0101	164	GVFDDTKCDTAEKLP	TKCDTAEKL	5	0.2027	5580.1	95.00	0.54	tr_D0A0U2_	
DRB1_0101	165	LPKDDCASNYGATQV	CASNYGATQ	5	0.2011	5673.5	95.00	0.41	tr_D0A0U2_	
DRB1_0101	166	IISDDMRYGVFDDTK	MRYGVFDDT	5	0.1960	5995.4	95.00	0.28	tr_D0A0U2_	
DRB1_0101	167	DDTKCDTAEKLPKDD	TKCDTAEKL	2	0.1913	6308.6	100.00	0.71	tr_D0A0U2_	
DRB1_0101	168	YGVFDDTKCDTAEKL	TKCDTAEKL	6	0.1864	6653.8	100.00	0.37	tr_D0A0U2_	
DRB1_0101	169	KGGAVTTIREDGEGV	TIREDGEGV	6	0.1794	7174.6	100.00	0.68	tr_D0A0U2_	
DRB1_0101	170	DMRYGVFDDTKCDTA	YGVFDDTKC	3	0.1768	7380.4	100.00	0.38	tr_D0A0U2_	
DRB1_0101	171	ISDDMRYGVFDDTKC	MRYGVFDDT	4	0.1766	7399.7	100.00	0.37	tr_D0A0U2_	
DRB1_0101	172	SDDMRYGVFDDTKCD	MRYGVFDDT	3	0.1741	7602.1	100.00	0.37	tr_D0A0U2_	
DRB1_0101	173	DTKCDTAEKLPKDDC	TKCDTAEKL	1	0.1730	7690.2	100.00	0.68	tr_D0A0U2_	
DRB1_0101	174	MRYGVFDDTKCDTAE	YGVFDDTKC	2	0.1709	7868.9	100.00	0.38	tr_D0A0U2_	
DRB1_0101	175	KLPKDDCASNYGATQ	DDCASNYGA	4	0.1681	8109.1	100.00	0.32	tr_D0A0U2_	
DRB1_0101	176	VKEIARKLEERNDLR	IARKLEERN	3	0.1675	8164.9	100.00	0.35	tr_D0A0U2_	
DRB1_0101	177	DDMRYGVFDDTKCDT	MRYGVFDDT	2	0.1644	8442.4	100.00	0.35	tr_D0A0U2_	
DRB1_0101	178	PHWGDTETSSKTAQL	GDTETSSKT	3	0.1594	8914.1	100.00	0.26	tr_D0A0U2_	
DRB1_0101	179	RYGVFDDTKCDTAEK	FDDTKCDTA	4	0.1549	9358.0	100.00	0.34	tr_D0A0U2_	
DRB1_0101	180	EKLPKDDCASNYGAT	DDCASNYGA	5	0.1413	10844.5	100.00	0.34	tr_D0A0U2_	
DRB1_0101	181	LPHWGDTETSSKTAQ	GDTETSSKT	4	0.1342	11703.0	100.00	0.25	tr_D0A0U2_	
DRB1_0101	182	TKCDTAEKLPKDDCA	TKCDTAEKL	0	0.1299	12264.6	100.00	0.54	tr_D0A0U2_	
DRB1_0101	183	SLPHWGDTETSSKTA	WGDTETSSK	4	0.1246	12989.2	100.00	0.25	tr_D0A0U2_	
DRB1_0101	184	ATSLPHWGDTETSSK	PHWGDTETS	4	0.1188	13823.9	100.00	0.25	tr_D0A0U2_	
DRB1_0101	185	AEKLPKDDCASNYGA	PKDDCASNY	4	0.1171	14080.8	100.00	0.22	tr_D0A0U2_	
DRB1_0101	186	TSLPHWGDTETSSKT	PHWGDTETS	3	0.1128	14760.0	100.00	0.28	tr_D0A0U2_	
DRB1_0101	187	RKGGAVTTIREDGEG	GGAVTTIRE	2	0.1001	16927.2	100.00	0.32	tr_D0A0U2_	
DRB1_0101	188	TAEKLPKDDCASNYG	PKDDCASNY	5	0.0896	18966.0	100.00	0.20	tr_D0A0U2_	
DRB1_0101	189	DTAEKLPKDDCASNY	KLPKDDCAS	4	0.0801	21011.0	100.00	0.23	tr_D0A0U2_	
DRB1_0101	190	KCDTAEKLPKDDCAS	DTAEKLPKD	2	0.0736	22542.8	100.00	0.29	tr_D0A0U2_	
DRB1_0101	191	CDTAEKLPKDDCASN	KLPKDDCAS	5	0.0685	23832.8	100.00	0.19	tr_D0A0U2_	

Allele: DRB1_0101. Number of high binders 0. Number of weak binders 31. Number of peptides 191

Allele	pos	peptide	core	Of	1-log50k(aff)	affinity(nM)	%Rank	Relia	Identity	Bind Level
DRB1_0101	1	QCCGFVCALPVPRNS	FVCALPVPR	4	0.7719	11.8	3.50	0.55	tr_C9ZWK4_	WB

DRB1_0101	2	SQCCGFVCALPVPRN	FVCALPVPR	5	0.7596	13.5	4.50	0.51	tr_C9ZWK4_	WB
DRB1_0101	3	CCGFVCALPVPRNSP	FVCALPVPR	3	0.7565	13.9	4.50	0.56	tr_C9ZWK4_	WB
DRB1_0101	4	CGFVCALPVPRNSPL	FVCALPVPR	2	0.7392	16.8	5.50	0.58	tr_C9ZWK4_	WB
DRB1_0101	5	HSQCCGFVCALPVPR	FVCALPVPR	6	0.7178	21.2	7.50	0.47	tr_C9ZWK4_	WB
DRB1_0101	6	GFVCALPVPRNSPLF	FVCALPVPR	1	0.6602	39.5	14.00	0.65	tr_C9ZWK4_	
DRB1_0101	7	PRNSPLFQHLSFSST	FQHLSFSST	6	0.6174	62.8	20.00	0.57	tr_C9ZWK4_	
DRB1_0101	8	NGIVPVCSGMSEKIN	IVPVCSGMS	2	0.5782	95.9	26.00	0.51	tr_C9ZWK4_	
DRB1_0101	9	GIVPVCSGMSEKINK	VCSGMSEKI	4	0.5770	97.2	26.00	0.48	tr_C9ZWK4_	
DRB1_0101	10	AHSQCCGFVCALPVP	CGFVCALPV	5	0.5715	103.2	27.00	0.68	tr_C9ZWK4_	
DRB1_0101	11	MNGIVPVCSGMSEKI	IVPVCSGMS	3	0.5689	106.0	28.00	0.62	tr_C9ZWK4_	
DRB1_0101	12	IVPVCSGMSEKINKK	VCSGMSEKI	3	0.5545	124.0	30.00	0.62	tr_C9ZWK4_	
DRB1_0101	13	FVCALPVPRNSPLFQ	FVCALPVPR	0	0.5342	154.5	34.00	0.51	tr_C9ZWK4_	
DRB1_0101	14	GAHSQCCGFVCALPV	CGFVCALPV	6	0.5034	215.5	40.00	0.60	tr_C9ZWK4_	
DRB1_0101	15	VPVCSGMSEKINKKR	VCSGMSEKI	2	0.4926	242.3	43.00	0.71	tr_C9ZWK4_	
DRB1_0101	16	HSLFKGAHSQCCGFV	FKGAHSQCC	3	0.4907	247.2	43.00	0.57	tr_C9ZWK4_	
DRB1_0101	17	SHSLFKGAHSQCCGF	FKGAHSQCC	4	0.4885	253.2	43.00	0.50	tr_C9ZWK4_	
DRB1_0101	18	HSHSLFKGAHSQCCG	FKGAHSQCC	5	0.4762	289.1	46.00	0.43	tr_C9ZWK4_	
DRB1_0101	19	KINKKRMNATDVHSH	KKRMNATDV	3	0.4543	366.7	55.00	0.65	tr_C9ZWK4_	
DRB1_0101	20	PVCSGMSEKINKKRM	VCSGMSEKI	1	0.4533	370.7	55.00	0.67	tr_C9ZWK4_	
DRB1_0101	21	VHSHSLFKGAHSQCC	HSLFKGAHS	3	0.4521	375.5	55.00	0.37	tr_C9ZWK4_	
DRB1_0101	22	SLFKGAHSQCCGFVC	FKGAHSQCC	2	0.4449	405.8	55.00	0.63	tr_C9ZWK4_	
DRB1_0101	23	EKINKKRMNATDVHS	KKRMNATDV	4	0.4434	412.5	55.00	0.56	tr_C9ZWK4_	
DRB1_0101	24	SEKINKKRMNATDVH	KKRMNATDV	5	0.4178	544.3	60.00	0.50	tr_C9ZWK4_	
DRB1_0101	25	VPRNSPLFQHLSFSS	LFQHLSFSS	6	0.4170	549.0	60.00	0.33	tr_C9ZWK4_	
DRB1_0101	26	LFKGAHSQCCGFVCA	FKGAHSQCC	1	0.4084	602.2	60.00	0.53	tr_C9ZWK4_	
DRB1_0101	27	INKKRMNATDVHSHS	KKRMNATDV	2	0.4038	633.3	65.00	0.67	tr_C9ZWK4_	
DRB1_0101	28	DVHSHSLFKGAHSQC	HSLFKGAHS	4	0.4020	645.4	65.00	0.49	tr_C9ZWK4_	
DRB1_0101	29	TDVHSHSLFKGAHSQ	HSLFKGAHS	5	0.3922	717.9	65.00	0.46	tr_C9ZWK4_	
DRB1_0101	30	NKKRMNATDVHSHSL	KKRMNATDV	1	0.3729	884.2	70.00	0.60	tr_C9ZWK4_	
DRB1_0101	31	FKGAHSQCCGFVCAL	FKGAHSQCC	0	0.3661	951.7	70.00	0.29	tr_C9ZWK4_	
DRB1_0101	32	VCSGMSEKINKKRMN	VCSGMSEKI	0	0.3544	1080.0	75.00	0.50	tr_C9ZWK4_	
DRB1_0101	33	ATDVHSHSLFKGAHS	HSLFKGAHS	6	0.3508	1122.9	75.00	0.35	tr_C9ZWK4_	
DRB1_0101	34	PVPRNSPLFQHLSFS	NSPLFQHLS	4	0.3380	1291.0	75.00	0.26	tr_C9ZWK4_	
DRB1_0101	35	MSEKINKKRMNATDV	INKKRMNAT	4	0.3375	1297.9	75.00	0.47	tr_C9ZWK4_	
DRB1_0101	36	KKRMNATDVHSHSLF	KKRMNATDV	0	0.3347	1337.7	75.00	0.36	tr_C9ZWK4_	
DRB1_0101	37	VCALPVPRNSPLFQH	VCALPVPRN	0	0.3222	1531.5	80.00	0.34	tr_C9ZWK4_	
DRB1_0101	38	LPVPRNSPLFQHLSF	NSPLFQHLS	5	0.3004	1937.6	85.00	0.20	tr_C9ZWK4_	
DRB1_0101	39	KRMNATDVHSHSLFK	ATDVHSHSL	4	0.2976	1998.6	85.00	0.34	tr_C9ZWK4_	
DRB1_0101	40	NATDVHSHSLFKGAH	DVHSHSLFK	3	0.2974	2001.7	85.00	0.25	tr_C9ZWK4_	
DRB1_0101	41	KGAHSQCCGFVCALP	HSQCCGFVC	3	0.2973	2005.3	85.00	0.34	tr_C9ZWK4_	
DRB1_0101	42	RMNATDVHSHSLFKG	ATDVHSHSL	3	0.2938	2080.8	85.00	0.35	tr_C9ZWK4_	
DRB1_0101	43	MNATDVHSHSLFKGA	DVHSHSLFK	4	0.2931	2098.0	85.00	0.24	tr_C9ZWK4_	
DRB1_0101	44	ALPVPRNSPLFQHLS	PRNSPLFQH	4	0.2862	2259.7	85.00	0.19	tr_C9ZWK4_	
DRB1_0101	45	CALPVPRNSPLFQHL	PVPRNSPLF	3	0.2778	2474.2	85.00	0.22	tr_C9ZWK4_	
DRB1_0101	46	GMSEKINKKRMNATD	INKKRMNAT	5	0.2471	3448.5	90.00	0.53	tr_C9ZWK4_	
DRB1_0101	47	SGMSEKINKKRMNAT	INKKRMNAT	6	0.2302	4142.1	95.00	0.33	tr_C9ZWK4_	
DRB1_0101	48	CSGMSEKINKKRMNA	GMSEKINKK	2	0.2042	5488.3	95.00	0.28	tr_C9ZWK4_	

Allele: DRB1_0101. Number of high binders 0. Number of weak binders 5. Number of peptides 48

Allele	pos	peptide	core	Of	1-log50k(aff)	affinity(nM)	%Rank	Relia	Identity	Bind Level
DRB1_0101	1	PYAPVLVELSDIRYL	YAPVLVELS	1	0.6160	63.8	20.00	0.49	tr_D0A5M0_	
DRB1_0101	2	WGPYAPVLVELSDIR	YAPVLVELS	3	0.6138	65.3	20.00	0.71	tr_D0A5M0_	
DRB1_0101	3	GPYAPVLVELSDIRY	YAPVLVELS	2	0.6072	70.1	21.00	0.64	tr_D0A5M0_	
DRB1_0101	4	GWGPYAPVLVELSDI	YAPVLVELS	4	0.6052	71.6	22.00	0.66	tr_D0A5M0_	
DRB1_0101	5	TGWGPYAPVLVELSD	YAPVLVELS	5	0.5968	78.5	23.00	0.55	tr_D0A5M0_	
DRB1_0101	6	YAPVLVELSDIRYLV	YAPVLVELS	0	0.5911	83.4	24.00	0.30	tr_D0A5M0_	
DRB1_0101	7	STGWGPYAPVLVELS	WGPYAPVLV	3	0.5748	99.6	27.00	0.43	tr_D0A5M0_	
DRB1_0101	8	PVLVELSDIRYLVWK	VELSDIRYL	3	0.5549	123.5	30.00	0.43	tr_D0A5M0_	
DRB1_0101	9	APVLVELSDIRYLVW	VELSDIRYL	4	0.5482	132.7	32.00	0.37	tr_D0A5M0_	
DRB1_0101	10	VLVELSDIRYLVWKG	VELSDIRYL	2	0.5217	176.8	37.00	0.43	tr_D0A5M0_	
DRB1_0101	11	CVVSTGWGPYAPVLV	VSTGWGPYA	2	0.4936	239.6	42.00	0.35	tr_D0A5M0_	
DRB1_0101	12	VVSTGWGPYAPVLVE	WGPYAPVLV	5	0.4895	250.6	43.00	0.46	tr_D0A5M0_	
DRB1_0101	13	VSTGWGPYAPVLVEL	WGPYAPVLV	4	0.4883	253.8	43.00	0.58	tr_D0A5M0_	
DRB1_0101	14	SCVVSTGWGPYAPVL	VSTGWGPYA	3	0.4683	315.0	48.00	0.49	tr_D0A5M0_	
DRB1_0101	15	LVELSDIRYLVWKGE	VELSDIRYL	1	0.4650	326.6	48.00	0.41	tr_D0A5M0_	
DRB1_0101	16	VELSDIRYLVWKGEG	VELSDIRYL	0	0.3747	867.1	70.00	0.34	tr_D0A5M0_	
DRB1_0101	17	VWKGEGNTCPAVPAF	GNTCPAVPA	5	0.3511	1119.6	75.00	0.51	tr_D0A5M0_	
DRB1_0101	18	RYLVWKGEGNTCPAV	WKGEGNTCP	4	0.3211	1549.9	80.00	0.40	tr_D0A5M0_	
DRB1_0101	19	IRYLVWKGEGNTCPA	WKGEGNTCP	5	0.3209	1552.8	80.00	0.28	tr_D0A5M0_	
DRB1_0101	20	DIRYLVWKGEGNTCP	IRYLVWKGE	1	0.3058	1829.1	85.00	0.27	tr_D0A5M0_	
DRB1_0101	21	SDIRYLVWKGEGNTC	IRYLVWKGE	2	0.3046	1852.6	85.00	0.35	tr_D0A5M0_	
DRB1_0101	22	LVWKGEGNTCPAVPA	WKGEGNTCP	2	0.3023	1898.4	85.00	0.37	tr_D0A5M0_	
DRB1_0101	23	ELSDIRYLVWKGEGN	IRYLVWKGE	4	0.2929	2102.1	85.00	0.38	tr_D0A5M0_	
DRB1_0101	24	LSDIRYLVWKGEGNT	IRYLVWKGE	3	0.2875	2227.6	85.00	0.43	tr_D0A5M0_	
DRB1_0101	25	YLVWKGEGNTCPAVP	WKGEGNTCP	3	0.2844	2304.2	85.00	0.47	tr_D0A5M0_	

Allele: DRB1_0101. Number of high binders 0. Number of weak binders 0. Number of peptides 25

Allele	pos	peptide	core	Of	1-log50k(aff)	affinity(nM)	%Rank	Relia	Identity	Bind Level
DRB1_0101	1	RRYWMLRGTPQPATF	YWMLRGTPQ	2	0.7430	16.1	5.50	0.56	tr_C9ZJ95_	WB
DRB1_0101	2	ARRYWMLRGTPQPAT	YWMLRGTPQ	3	0.7258	19.4	6.50	0.65	tr_C9ZJ95_	WB
DRB1_0101	3	RYWMLRGTPQPATFG	YWMLRGTPQ	1	0.7219	20.3	7.00	0.48	tr_C9ZJ95_	WB
DRB1_0101	4	YARRYWMLRGTPQPA	YWMLRGTPQ	4	0.6815	31.4	11.00	0.74	tr_C9ZJ95_	
DRB1_0101	5	KHCLNPVDAILGAHE	LNPVDAILG	3	0.6773	32.9	12.00	0.71	tr_C9ZJ95_	
DRB1_0101	6	EKHCLNPVDAILGAH	LNPVDAILG	4	0.6700	35.5	13.00	0.71	tr_C9ZJ95_	
DRB1_0101	7	HCLNPVDAILGAHEG	LNPVDAILG	2	0.6574	40.7	14.00	0.67	tr_C9ZJ95_	
DRB1_0101	8	YWMLRGTPQPATFGS	MLRGTPQPA	2	0.6497	44.3	15.00	0.40	tr_C9ZJ95_	
DRB1_0101	9	LLKYHNGNATSANSK	YHNGNATSA	3	0.6476	45.3	15.00	0.57	tr_C9ZJ95_	
DRB1_0101	10	VEKHCLNPVDAILGA	LNPVDAILG	5	0.6422	48.0	16.00	0.67	tr_C9ZJ95_	
DRB1_0101	11	PLLKYHNGNATSANS	YHNGNATSA	4	0.6389	49.7	17.00	0.47	tr_C9ZJ95_	
DRB1_0101	12	CLNPVDAILGAHEGV	LNPVDAILG	1	0.6357	51.5	17.00	0.57	tr_C9ZJ95_	

DRB1_0101	13	KPLLKYHNGNATSAN	LKYHNGNAT	3	0.6348	52.0	17.00	0.54	tr_C9ZJ95_
DRB1_0101	14	EYARRYWMLRGTPQP	YWMLRGTPQ	5	0.6236	58.7	19.00	0.78	tr_C9ZJ95_
DRB1_0101	15	VEYARRYWMLRGTPQ	YWMLRGTPQ	6	0.5924	82.3	24.00	0.67	tr_C9ZJ95_
DRB1_0101	16	LNPVDAILGAHEGVF	VDAILGAHE	3	0.5910	83.6	24.00	0.34	tr_C9ZJ95_
DRB1_0101	17	LKYHNGNATSANSKP	YHNGNATSA	2	0.5869	87.3	25.00	0.67	tr_C9ZJ95_
DRB1_0101	18	RKPLLKYHNGNATSA	LKYHNGNAT	4	0.5814	92.7	26.00	0.61	tr_C9ZJ95_
DRB1_0101	19	KDIQLLNGQKRKPLL	IQLLNGQKR	2	0.5735	101.0	27.00	0.62	tr_C9ZJ95_
DRB1_0101	20	LKDIQLLNGQKRKPL	IQLLNGQKR	3	0.5717	102.9	27.00	0.75	tr_C9ZJ95_
DRB1_0101	21	VAVVVGVTGDRMFVA	VVGVTGDRM	3	0.5647	111.0	29.00	0.81	tr_C9ZJ95_
DRB1_0101	22	KVEKHCLNPVDAILG	LNPVDAILG	6	0.5643	111.5	29.00	0.47	tr_C9ZJ95_
DRB1_0101	23	TTTYNNVTVAGTSYQ	YNNVTVAGT	3	0.5641	111.7	29.00	0.80	tr_C9ZJ95_
DRB1_0101	24	WDLKDIQLLNGQKRK	IQLLNGQKR	5	0.5565	121.3	30.00	0.70	tr_C9ZJ95_
DRB1_0101	25	DIQLLNGQKRKPLLK	IQLLNGQKR	1	0.5537	125.0	31.00	0.54	tr_C9ZJ95_
DRB1_0101	26	PVDAILGAHEGVFSY	DAILGAHEG	2	0.5513	128.3	31.00	0.38	tr_C9ZJ95_
DRB1_0101	27	AVVVGVTGDRMFVAE	VVGVTGDRM	2	0.5452	137.1	32.00	0.84	tr_C9ZJ95_
DRB1_0101	28	NTTTYNNVTVAGTSY	YNNVTVAGT	4	0.5438	139.3	32.00	0.79	tr_C9ZJ95_
DRB1_0101	29	NPVDAILGAHEGVFS	DAILGAHEG	3	0.5435	139.6	32.00	0.44	tr_C9ZJ95_
DRB1_0101	30	VDAILGAHEGVFSYS	DAILGAHEG	1	0.5399	145.2	33.00	0.33	tr_C9ZJ95_
DRB1_0101	31	TTYNNVTVAGTSYQS	YNNVTVAGT	2	0.5349	153.2	34.00	0.75	tr_C9ZJ95_
DRB1_0101	32	HVAVVVGVTGDRMFV	VVGVTGDRM	4	0.5338	155.2	34.00	0.75	tr_C9ZJ95_
DRB1_0101	33	IQLLNGQKRKPLLKY	LNGQKRKPL	3	0.5305	160.8	35.00	0.41	tr_C9ZJ95_
DRB1_0101	34	DRMFVAEQNWENAAW	FVAEQNWEN	3	0.5263	168.3	36.00	0.81	tr_C9ZJ95_
DRB1_0101	35	RMFVAEQNWENAAWP	FVAEQNWEN	2	0.5213	177.6	37.00	0.85	tr_C9ZJ95_
DRB1_0101	36	DLKDIQLLNGQKRKP	IQLLNGQKR	4	0.5183	183.4	37.00	0.81	tr_C9ZJ95_
DRB1_0101	37	IWDLKDIQLLNGQKR	IQLLNGQKR	6	0.5157	188.6	38.00	0.42	tr_C9ZJ95_
DRB1_0101	38	YQSGLKWQCVEYARR	LKWQCVEYA	4	0.5108	198.9	39.00	0.53	tr_C9ZJ95_
DRB1_0101	39	WMLRGTPQPATFGSV	MLRGTPQPA	1	0.5060	209.7	40.00	0.50	tr_C9ZJ95_
DRB1_0101	40	GPYHNYSRVLNLSCN	YSRVLNLSC	5	0.5036	215.1	40.00	0.34	tr_C9ZJ95_
DRB1_0101	41	QSGLKWQCVEYARRY	LKWQCVEYA	3	0.5033	215.7	40.00	0.65	tr_C9ZJ95_
DRB1_0101	42	SYQSGLKWQCVEYAR	LKWQCVEYA	5	0.5026	217.3	40.00	0.42	tr_C9ZJ95_
DRB1_0101	43	GDRMFVAEQNWENAA	FVAEQNWEN	4	0.5015	220.0	41.00	0.79	tr_C9ZJ95_
DRB1_0101	44	VVVGVTGDRMFVAEQ	VVGVTGDRM	1	0.5011	220.9	41.00	0.80	tr_C9ZJ95_
DRB1_0101	45	DAILGAHEGVFSYSN	LGAHEGVFS	3	0.5001	223.5	41.00	0.41	tr_C9ZJ95_
DRB1_0101	46	GHVAVVVGVTGDRMF	VVGVTGDRM	5	0.4999	224.0	41.00	0.59	tr_C9ZJ95_
DRB1_0101	47	KRKPLLKYHNGNATS	LKYHNGNAT	5	0.4958	234.1	42.00	0.67	tr_C9ZJ95_
DRB1_0101	48	TSYQSGLKWQCVEYA	QSGLKWQCV	3	0.4897	249.9	43.00	0.37	tr_C9ZJ95_
DRB1_0101	49	KYHNGNATSANSKPR	YHNGNATSA	1	0.4863	259.3	44.00	0.84	tr_C9ZJ95_
DRB1_0101	50	AADIWDLKDIQLLNG	WDLKDIQLL	4	0.4837	266.7	44.00	0.51	tr_C9ZJ95_
DRB1_0101	51	TYNNVTVAGTSYQSG	YNNVTVAGT	1	0.4832	268.3	45.00	0.67	tr_C9ZJ95_
DRB1_0101	52	ADIWDLKDIQLLNGQ	WDLKDIQLL	3	0.4824	270.4	45.00	0.52	tr_C9ZJ95_
DRB1_0101	53	PYHNYSRVLNLSCNP	YSRVLNLSC	4	0.4822	271.0	45.00	0.38	tr_C9ZJ95_
DRB1_0101	54	YSRVLNLSCNPNGTA	LNLSCNPNG	4	0.4780	283.7	46.00	0.53	tr_C9ZJ95_
DRB1_0101	55	ENTTTYNNVTVAGTS	YNNVTVAGT	5	0.4780	283.7	46.00	0.75	tr_C9ZJ95_
DRB1_0101	56	YHNYSRVLNLSCNPN	YSRVLNLSC	3	0.4755	291.6	46.00	0.41	tr_C9ZJ95_
DRB1_0101	57	LKWQCVEYARRYWML	LKWQCVEYA	0	0.4752	292.5	46.00	0.25	tr_C9ZJ95_
DRB1_0101	58	QCVEYARRYWMLRGT	EYARRYWML	3	0.4726	300.8	47.00	0.54	tr_C9ZJ95_
DRB1_0101	59	WQCVEYARRYWMLRG	EYARRYWML	4	0.4722	302.2	47.00	0.47	tr_C9ZJ95_
DRB1_0101	60	PGPYHNYSRVLNLSC	HNYSRVLNL	4	0.4700	309.3	47.00	0.33	tr_C9ZJ95_
DRB1_0101	61	SGLKWQCVEYARRYW	LKWQCVEYA	2	0.4679	316.6	48.00	0.68	tr_C9ZJ95_
DRB1_0101	62	KWQCVEYARRYWMLR	EYARRYWML	5	0.4676	317.4	48.00	0.38	tr_C9ZJ95_

DRB1_0101	63	GAADIWDLKDIQLLN	WDLKDIQLL	5	0.4608	341.6	49.00	0.47	tr_C9ZJ95_
DRB1_0101	64	SRVLNLSCNPNGTAC	LNLSCNPNG	3	0.4591	347.9	50.00	0.54	tr_C9ZJ95_
DRB1_0101	65	NYSRVLNLSCNPNGT	LNLSCNPNG	5	0.4562	359.1	50.00	0.38	tr_C9ZJ95_
DRB1_0101	66	DIWDLKDIQLLNGQK	WDLKDIQLL	2	0.4558	360.6	50.00	0.52	tr_C9ZJ95_
DRB1_0101	67	GLKWQCVEYARRYWM	LKWQCVEYA	1	0.4550	364.0	50.00	0.51	tr_C9ZJ95_
DRB1_0101	68	HNYSRVLNLSCNPNG	YSRVLNLSC	2	0.4530	371.7	55.00	0.38	tr_C9ZJ95_
DRB1_0101	69	MFVAEQNWENAAWPG	FVAEQNWEN	1	0.4456	402.8	55.00	0.81	tr_C9ZJ95_
DRB1_0101	70	CVEYARRYWMLRGTP	EYARRYWML	2	0.4411	422.9	55.00	0.52	tr_C9ZJ95_
DRB1_0101	71	GTSYQSGLKWQCVEY	QSGLKWQCV	4	0.4396	429.8	55.00	0.44	tr_C9ZJ95_
DRB1_0101	72	TGDRMFVAEQNWENA	FVAEQNWEN	5	0.4356	448.8	55.00	0.75	tr_C9ZJ95_
DRB1_0101	73	QLLNGQKRKPLLKYH	LNGQKRKPL	2	0.4340	456.7	55.00	0.62	tr_C9ZJ95_
DRB1_0101	74	WPGPYHNYSRVLNLS	HNYSRVLNL	5	0.4315	469.0	55.00	0.38	tr_C9ZJ95_
DRB1_0101	75	RVLNLSCNPNGTACT	LNLSCNPNG	2	0.4306	473.6	60.00	0.51	tr_C9ZJ95_
DRB1_0101	76	ATFGSVDGAADIWDL	FGSVDGAAD	2	0.4273	491.2	60.00	0.73	tr_C9ZJ95_
DRB1_0101	77	NGFPCGHVAVVVGVT	FPCGHVAVV	2	0.4267	494.1	60.00	0.32	tr_C9ZJ95_
DRB1_0101	78	CGHVAVVVGVTGDRM	VVGVTGDRM	6	0.4263	496.5	60.00	0.42	tr_C9ZJ95_
DRB1_0101	79	QPATFGSVDGAADIW	FGSVDGAAD	4	0.4251	503.1	60.00	0.74	tr_C9ZJ95_
DRB1_0101	80	GKVEKHCLNPVDAIL	KHCLNPVDA	4	0.4250	503.6	60.00	0.47	tr_C9ZJ95_
DRB1_0101	81	AGTSYQSGLKWQCVE	QSGLKWQCV	5	0.4241	508.3	60.00	0.39	tr_C9ZJ95_
DRB1_0101	82	PATFGSVDGAADIWD	FGSVDGAAD	3	0.4241	508.5	60.00	0.77	tr_C9ZJ95_
DRB1_0101	83	PNGFPCGHVAVVVGV	FPCGHVAVV	3	0.4189	537.6	60.00	0.38	tr_C9ZJ95_
DRB1_0101	84	AILGAHEGVFSYSNC	LGAHEGVFS	2	0.4179	543.5	60.00	0.58	tr_C9ZJ95_
DRB1_0101	85	GFPCGHVAVVVGVTG	FPCGHVAVV	1	0.4167	550.9	60.00	0.24	tr_C9ZJ95_
DRB1_0101	86	MLRGTPQPATFGSVD	MLRGTPQPA	0	0.4099	592.8	60.00	0.41	tr_C9ZJ95_
DRB1_0101	87	DGAADIWDLKDIQLL	WDLKDIQLL	6	0.4092	597.0	60.00	0.41	tr_C9ZJ95_
DRB1_0101	88	TFGSVDGAADIWDLK	FGSVDGAAD	1	0.4018	647.0	65.00	0.62	tr_C9ZJ95_
DRB1_0101	89	VAGTSYQSGLKWQCV	TSYQSGLKW	3	0.4016	648.2	65.00	0.38	tr_C9ZJ95_
DRB1_0101	90	YNNVTVAGTSYQSGL	YNNVTVAGT	0	0.4008	653.7	65.00	0.37	tr_C9ZJ95_
DRB1_0101	91	QPNGFPCGHVAVVVG	FPCGHVAVV	4	0.3963	687.0	65.00	0.38	tr_C9ZJ95_
DRB1_0101	92	VVGVTGDRMFVAEQN	VVGVTGDRM	0	0.3960	689.3	65.00	0.61	tr_C9ZJ95_
DRB1_0101	93	LLNGQKRKPLLKYHN	LNGQKRKPL	1	0.3956	692.3	65.00	0.57	tr_C9ZJ95_
DRB1_0101	94	FPCGHVAVVVGVTGD	VAVVVGVTG	5	0.3913	724.7	65.00	0.26	tr_C9ZJ95_
DRB1_0101	95	QKRKPLLKYHNGNAT	LKYHNGNAT	6	0.3895	739.2	65.00	0.52	tr_C9ZJ95_
DRB1_0101	96	EGVFSYSNCGATENT	FSYSNCGAT	3	0.3864	764.5	65.00	0.41	tr_C9ZJ95_
DRB1_0101	97	VLNLSCNPNGTACTV	LNLSCNPNG	1	0.3850	775.6	65.00	0.41	tr_C9ZJ95_
DRB1_0101	98	GVFSYSNCGATENTT	FSYSNCGAT	2	0.3843	782.0	70.00	0.40	tr_C9ZJ95_
DRB1_0101	99	RQPNGFPCGHVAVVV	FPCGHVAVV	5	0.3815	805.9	70.00	0.37	tr_C9ZJ95_
DRB1_0101	100	PQPATFGSVDGAADI	FGSVDGAAD	5	0.3802	817.6	70.00	0.70	tr_C9ZJ95_
DRB1_0101	101	SGKVEKHCLNPVDAI	KHCLNPVDA	5	0.3789	829.1	70.00	0.41	tr_C9ZJ95_
DRB1_0101	102	AWPGPYHNYSRVLNL	HNYSRVLNL	6	0.3722	891.7	70.00	0.31	tr_C9ZJ95_
DRB1_0101	103	VFSYSNCGATENTTT	FSYSNCGAT	1	0.3650	963.4	70.00	0.41	tr_C9ZJ95_
DRB1_0101	104	HEGVFSYSNCGATEN	FSYSNCGAT	4	0.3611	1004.7	70.00	0.44	tr_C9ZJ95_
DRB1_0101	105	PCGHVAVVVGVTGDR	VAVVVGVTG	4	0.3567	1054.4	75.00	0.38	tr_C9ZJ95_
DRB1_0101	106	ILGAHEGVFSYSNCG	LGAHEGVFS	1	0.3550	1073.5	75.00	0.52	tr_C9ZJ95_
DRB1_0101	107	TENTTTYNNVTVAGT	YNNVTVAGT	6	0.3538	1087.4	75.00	0.63	tr_C9ZJ95_
DRB1_0101	108	REKDNVTVQGWVRYE	NVTVQGWVR	4	0.3418	1239.0	75.00	0.67	tr_C9ZJ95_
DRB1_0101	109	VREKDNVTVQGWVRY	NVTVQGWVR	5	0.3417	1240.1	75.00	0.57	tr_C9ZJ95_
DRB1_0101	110	PRQPNGFPCGHVAVV	FPCGHVAVV	6	0.3409	1250.6	75.00	0.31	tr_C9ZJ95_
DRB1_0101	111	NNVTVAGTSYQSGLK	TVAGTSYQS	3	0.3405	1256.2	75.00	0.43	tr_C9ZJ95_
DRB1_0101	112	YHNGNATSANSKPRV	YHNGNATSA	0	0.3386	1282.7	75.00	0.70	tr_C9ZJ95_

DRB1_0101	113	VTGDRMFVAEQNWEN	FVAEQNWEN	6	0.3357	1322.4	75.00	0.58	tr_C9ZJ95_
DRB1_0101	114	NSKPRVGDLLIYPRQ	SKPRVGDLL	1	0.3338	1350.1	80.00	0.31	tr_C9ZJ95_
DRB1_0101	115	NVTVAGTSYQSGLKW	TVAGTSYQS	2	0.3327	1366.0	80.00	0.38	tr_C9ZJ95_
DRB1_0101	116	FVAEQNWENAAWPGP	FVAEQNWEN	0	0.3315	1385.0	80.00	0.65	tr_C9ZJ95_
DRB1_0101	117	TSANSKPRVGDLLIY	SKPRVGDLL	4	0.3314	1386.6	80.00	0.60	tr_C9ZJ95_
DRB1_0101	118	LNLSCNPNGTACTVR	LSCNPNGTA	2	0.3275	1445.3	80.00	0.29	tr_C9ZJ95_
DRB1_0101	119	AHEGVFSYSNCGATE	FSYSNCGAT	5	0.3264	1462.9	80.00	0.43	tr_C9ZJ95_
DRB1_0101	120	ACTVREKDNVTVQGW	VREKDNVTV	3	0.3252	1482.6	80.00	0.70	tr_C9ZJ95_
DRB1_0101	121	VTVAGTSYQSGLKWQ	TSYQSGLKW	5	0.3222	1530.5	80.00	0.36	tr_C9ZJ95_
DRB1_0101	122	ANSKPRVGDLLIYPR	SKPRVGDLL	2	0.3211	1549.5	80.00	0.45	tr_C9ZJ95_
DRB1_0101	123	FGSVDGAADIWDLKD	FGSVDGAAD	0	0.3206	1558.2	80.00	0.45	tr_C9ZJ95_
DRB1_0101	124	TVREKDNVTVQGWVR	VREKDNVTV	1	0.3194	1577.6	80.00	0.44	tr_C9ZJ95_
DRB1_0101	125	TVAGTSYQSGLKWQC	TSYQSGLKW	4	0.3184	1594.4	80.00	0.42	tr_C9ZJ95_
DRB1_0101	126	SANSKPRVGDLLIYP	SKPRVGDLL	3	0.3183	1597.3	80.00	0.58	tr_C9ZJ95_
DRB1_0101	127	LNGQKRKPLLKYHNG	LNGQKRKPL	0	0.3154	1648.4	80.00	0.39	tr_C9ZJ95_
DRB1_0101	128	SKPRVGDLLIYPRQP	GDLLIYPRQ	5	0.3124	1702.4	80.00	0.27	tr_C9ZJ95_
DRB1_0101	129	KPRVGDLLIYPRQPN	GDLLIYPRQ	4	0.3121	1707.8	80.00	0.32	tr_C9ZJ95_
DRB1_0101	130	TACTVREKDNVTVQG	VREKDNVTV	4	0.3095	1756.9	80.00	0.68	tr_C9ZJ95_
DRB1_0101	131	FSYSNCGATENTTTY	YSNCGATEN	2	0.3085	1776.5	80.00	0.37	tr_C9ZJ95_
DRB1_0101	132	RVGDLLIYPRQPNGF	GDLLIYPRQ	2	0.3064	1815.5	85.00	0.35	tr_C9ZJ95_
DRB1_0101	133	GSGKVEKHCLNPVDA	KHCLNPVDA	6	0.3026	1893.1	85.00	0.38	tr_C9ZJ95_
DRB1_0101	134	PRVGDLLIYPRQPNG	GDLLIYPRQ	3	0.2999	1948.9	85.00	0.40	tr_C9ZJ95_
DRB1_0101	135	CTVREKDNVTVQGWV	VREKDNVTV	2	0.2978	1992.9	85.00	0.68	tr_C9ZJ95_
DRB1_0101	136	TPQPATFGSVDGAAD	FGSVDGAAD	6	0.2913	2139.4	85.00	0.49	tr_C9ZJ95_
DRB1_0101	137	GAHEGVFSYSNCGAT	FSYSNCGAT	6	0.2877	2222.9	85.00	0.32	tr_C9ZJ95_
DRB1_0101	138	LGAHEGVFSYSNCGA	LGAHEGVFS	0	0.2871	2237.7	85.00	0.33	tr_C9ZJ95_
DRB1_0101	139	LRGTPQPATFGSVDG	GTPQPATFG	2	0.2841	2311.4	85.00	0.38	tr_C9ZJ95_
DRB1_0101	140	VGDLLIYPRQPNGFP	GDLLIYPRQ	1	0.2824	2355.0	85.00	0.27	tr_C9ZJ95_
DRB1_0101	141	GDLLIYPRQPNGFPC	YPRQPNGFP	5	0.2780	2468.9	85.00	0.26	tr_C9ZJ95_
DRB1_0101	142	GTACTVREKDNVTVQ	VREKDNVTV	5	0.2737	2586.2	90.00	0.62	tr_C9ZJ95_
DRB1_0101	143	YPRQPNGFPCGHVAV	PNGFPCGHV	4	0.2722	2629.6	90.00	0.32	tr_C9ZJ95_
DRB1_0101	144	DLLIYPRQPNGFPCG	YPRQPNGFP	4	0.2678	2759.0	90.00	0.34	tr_C9ZJ95_
DRB1_0101	145	ATSANSKPRVGDLLI	SKPRVGDLL	5	0.2668	2786.9	90.00	0.61	tr_C9ZJ95_
DRB1_0101	146	AAWPGPYHNYSRVLN	PYHNYSRVL	5	0.2633	2894.3	90.00	0.31	tr_C9ZJ95_
DRB1_0101	147	IYPRQPNGFPCGHVA	PNGFPCGHV	5	0.2595	3015.5	90.00	0.28	tr_C9ZJ95_
DRB1_0101	148	NLSCNPNGTACTVRE	CNPNGTACT	3	0.2549	3170.3	90.00	0.35	tr_C9ZJ95_
DRB1_0101	149	VDGAADIWDLKDIQL	IWDLKDIQL	6	0.2535	3219.8	90.00	0.48	tr_C9ZJ95_
DRB1_0101	150	LIYPRQPNGFPCGHV	YPRQPNGFP	2	0.2522	3263.3	90.00	0.39	tr_C9ZJ95_
DRB1_0101	151	LLIYPRQPNGFPCGH	YPRQPNGFP	3	0.2502	3334.8	90.00	0.41	tr_C9ZJ95_
DRB1_0101	152	RGTPQPATFGSVDGA	GTPQPATFG	1	0.2484	3400.3	90.00	0.33	tr_C9ZJ95_
DRB1_0101	153	GQKRKPLLKYHNGNA	LLKYHNGNA	6	0.2436	3584.0	90.00	0.32	tr_C9ZJ95_
DRB1_0101	154	GTPQPATFGSVDGAA	PATFGSVDG	4	0.2384	3792.7	95.00	0.39	tr_C9ZJ95_
DRB1_0101	155	VGVTGDRMFVAEQNW	DRMFVAEQN	5	0.2340	3977.5	95.00	0.29	tr_C9ZJ95_
DRB1_0101	156	NGQKRKPLLKYHNGN	GQKRKPLLK	1	0.2310	4106.2	95.00	0.28	tr_C9ZJ95_
DRB1_0101	157	SGIRYPEGSGKVEKH	YPEGSGKVE	4	0.2247	4397.6	95.00	0.41	tr_C9ZJ95_
DRB1_0101	158	GSVDGAADIWDLKDI	DGAADIWDL	3	0.2213	4561.6	95.00	0.45	tr_C9ZJ95_
DRB1_0101	159	LSCNPNGTACTVREK	CNPNGTACT	2	0.2196	4644.8	95.00	0.39	tr_C9ZJ95_
DRB1_0101	160	NAAWPGPYHNYSRVL	PYHNYSRVL	6	0.2185	4702.2	95.00	0.28	tr_C9ZJ95_
DRB1_0101	161	EGSGKVEKHCLNPVD	VEKHCLNPV	5	0.2177	4742.7	95.00	0.37	tr_C9ZJ95_
DRB1_0101	162	NATSANSKPRVGDLL	SKPRVGDLL	6	0.2171	4772.4	95.00	0.37	tr_C9ZJ95_

DRB1_0101	163	GVTGDRMFVAEQNWE	DRMFVAEQN	4	0.2151	4877.3	95.00	0.38	tr_C9ZJ95_
DRB1_0101	164	GIRYPEGSGKVEKHC	YPEGSGKVE	3	0.2128	4999.0	95.00	0.44	tr_C9ZJ95_
DRB1_0101	165	NGTACTVREKDNVTV	VREKDNVTV	6	0.2077	5286.9	95.00	0.43	tr_C9ZJ95_
DRB1_0101	166	SVDGAADIWDLKDIQ	DGAADIWDL	2	0.2003	5727.9	95.00	0.55	tr_C9ZJ95_
DRB1_0101	167	SYSNCGATENTTTYN	YSNCGATEN	1	0.1999	5751.9	95.00	0.49	tr_C9ZJ95_
DRB1_0101	168	ATENTTTYNNVTVAG	TTYNNVTVA	5	0.1980	5871.2	95.00	0.31	tr_C9ZJ95_
DRB1_0101	169	PEGSGKVEKHCLNPV	GKVEKHCLN	4	0.1957	6016.1	95.00	0.36	tr_C9ZJ95_
DRB1_0101	170	IRYPEGSGKVEKHCL	YPEGSGKVE	2	0.1955	6029.5	95.00	0.44	tr_C9ZJ95_
DRB1_0101	171	QNWENAAWPGPYHNY	WENAAWPGP	2	0.1891	6460.9	100.00	0.33	tr_C9ZJ95_
DRB1_0101	172	RYPEGSGKVEKHCLN	YPEGSGKVE	1	0.1880	6541.7	100.00	0.37	tr_C9ZJ95_
DRB1_0101	173	EQNWENAAWPGPYHN	WENAAWPGP	3	0.1821	6969.7	100.00	0.34	tr_C9ZJ95_
DRB1_0101	174	NWENAAWPGPYHNYS	WENAAWPGP	1	0.1809	7063.7	100.00	0.25	tr_C9ZJ95_
DRB1_0101	175	SCNPNGTACTVREKD	CNPNGTACT	1	0.1735	7649.5	100.00	0.40	tr_C9ZJ95_
DRB1_0101	176	AEQNWENAAWPGPYH	WENAAWPGP	4	0.1696	7984.3	100.00	0.34	tr_C9ZJ95_
DRB1_0101	177	WENAAWPGPYHNYSR	AAWPGPYHN	3	0.1689	8039.6	100.00	0.25	tr_C9ZJ95_
DRB1_0101	178	ENAAWPGPYHNYSRV	AAWPGPYHN	2	0.1659	8308.9	100.00	0.21	tr_C9ZJ95_
DRB1_0101	179	HNGNATSANSKPRVG	GNATSANSK	2	0.1635	8520.9	100.00	0.22	tr_C9ZJ95_
DRB1_0101	180	GNATSANSKPRVGDL	TSANSKPRV	3	0.1615	8715.3	100.00	0.35	tr_C9ZJ95_
DRB1_0101	181	YPEGSGKVEKHCLNP	GKVEKHCLN	5	0.1580	9045.8	100.00	0.34	tr_C9ZJ95_
DRB1_0101	182	GATENTTTYNNVTVA	TTTYNNVTV	5	0.1568	9167.2	100.00	0.25	tr_C9ZJ95_
DRB1_0101	183	NGNATSANSKPRVGD	TSANSKPRV	4	0.1567	9179.5	100.00	0.31	tr_C9ZJ95_
DRB1_0101	184	VAEQNWENAAWPGPY	WENAAWPGP	5	0.1553	9315.3	100.00	0.31	tr_C9ZJ95_
DRB1_0101	185	YSNCGATENTTTYNN	CGATENTTT	3	0.1468	10216.6	100.00	0.29	tr_C9ZJ95_
DRB1_0101	186	CNPNGTACTVREKDN	CNPNGTACT	0	0.1395	11054.0	100.00	0.26	tr_C9ZJ95_
DRB1_0101	187	PNGTACTVREKDNVT	TVREKDNVT	6	0.1243	13024.2	100.00	0.33	tr_C9ZJ95_
DRB1_0101	188	SNCGATENTTTYNNV	CGATENTTT	2	0.1157	14293.8	100.00	0.38	tr_C9ZJ95_
DRB1_0101	189	CGATENTTTYNNVTV	ENTTTYNNV	4	0.1112	15008.5	100.00	0.25	tr_C9ZJ95_
DRB1_0101	190	NPNGTACTVREKDNV	GTACTVREK	3	0.1076	15610.7	100.00	0.31	tr_C9ZJ95_
DRB1_0101	191	NCGATENTTTYNNVT	CGATENTTT	1	0.1046	16131.3	100.00	0.25	tr_C9ZJ95_

Allele: DRB1_0101. Number of high binders 0. Number of weak binders 3. Number of peptides 191

Allele	pos	peptide	core	Of	1-log50k(aff)	affinity(nM)	%Rank	Relia	Identity	Bind Level
DRB1_0101	1	GTAWVFAMYPRDSNG	WVFAMYPRD	3	0.5012	220.7	41.00	0.46	tr_C9ZZZ3_	
DRB1_0101	2	DLLKAHGGGELTAFG	LKAHGGGEL	2	0.5010	221.2	41.00	0.67	tr_C9ZZZ3_	
DRB1_0101	3	KGTAWVFAMYPRDSN	WVFAMYPRD	4	0.4842	265.3	44.00	0.46	tr_C9ZZZ3_	
DRB1_0101	4	LLKAHGGGELTAFGE	LKAHGGGEL	1	0.4665	321.1	48.00	0.65	tr_C9ZZZ3_	
DRB1_0101	5	TAFGEKGTAWVFAMY	GEKGTAWVF	3	0.4616	338.8	49.00	0.44	tr_C9ZZZ3_	
DRB1_0101	6	LTAFGEKGTAWVFAM	GEKGTAWVF	4	0.4505	381.9	55.00	0.39	tr_C9ZZZ3_	
DRB1_0101	7	EKGTAWVFAMYPRDS	WVFAMYPRD	5	0.4368	442.9	55.00	0.47	tr_C9ZZZ3_	
DRB1_0101	8	AFGEKGTAWVFAMYP	GEKGTAWVF	2	0.4238	509.8	60.00	0.47	tr_C9ZZZ3_	
DRB1_0101	9	ELTAFGEKGTAWVFA	GEKGTAWVF	5	0.4190	537.3	60.00	0.36	tr_C9ZZZ3_	
DRB1_0101	10	FGEKGTAWVFAMYPR	GEKGTAWVF	1	0.3843	781.7	70.00	0.42	tr_C9ZZZ3_	
DRB1_0101	11	GEKGTAWVFAMYPRD	WVFAMYPRD	6	0.3776	841.0	70.00	0.38	tr_C9ZZZ3_	
DRB1_0101	12	GELTAFGEKGTAWVF	FGEKGTAWV	5	0.3642	972.1	70.00	0.34	tr_C9ZZZ3_	
DRB1_0101	13	LKAHGGGELTAFGEK	LKAHGGGEL	0	0.3470	1170.6	75.00	0.49	tr_C9ZZZ3_	

DRB1_0101	14	GGELTAFGEKGTAWV	LTAFGEKGT	3	0.2956	2041.8	85.00	0.26	tr_C9ZZZ3_	
DRB1_0101	15	GGGELTAFGEKGTAW	ELTAFGEKG	3	0.2592	3025.4	90.00	0.34	tr_C9ZZZ3_	
DRB1_0101	16	HGGGELTAFGEKGTA	ELTAFGEKG	4	0.2449	3532.5	90.00	0.31	tr_C9ZZZ3_	
DRB1_0101	17	AHGGGELTAFGEKGT	GGELTAFGE	3	0.2242	4421.6	95.00	0.40	tr_C9ZZZ3_	
DRB1_0101	18	KAHGGGELTAFGEKG	GGELTAFGE	4	0.2174	4756.4	95.00	0.33	tr_C9ZZZ3_	

Allele: DRB1_0101. Number of high binders 0. Number of weak binders 0. Number of peptides 18

Allele	pos	peptide	core	Of	1-log50k(aff)	affinity(nM)	%Rank	Relia	Identity	Bind Level
DRB1_0101	1	HSVWHGSTVAARDVK	WHGSTVAAR	3	0.5108	199.0	39.00	0.36	tr_D0A148_	
DRB1_0101	2	SVWHGSTVAARDVKR	WHGSTVAAR	2	0.5004	222.6	41.00	0.35	tr_D0A148_	
DRB1_0101	3	THSVWHGSTVAARDV	VWHGSTVAA	3	0.4907	247.2	43.00	0.31	tr_D0A148_	
DRB1_0101	4	YTHSVWHGSTVAARD	VWHGSTVAA	4	0.4778	284.4	46.00	0.29	tr_D0A148_	
DRB1_0101	5	VWHGSTVAARDVKRS	GSTVAARDV	3	0.4620	337.2	49.00	0.32	tr_D0A148_	
DRB1_0101	6	HGSTVAARDVKRSTS	VAARDVKRS	4	0.4379	438.0	55.00	0.41	tr_D0A148_	
DRB1_0101	7	WHGSTVAARDVKRST	GSTVAARDV	2	0.4348	452.8	55.00	0.34	tr_D0A148_	
DRB1_0101	8	GSTVAARDVKRSTSH	VAARDVKRS	3	0.4214	523.3	60.00	0.51	tr_D0A148_	
DRB1_0101	9	ARDVKRSTSHQFCKS	VKRSTSHQF	3	0.4212	524.5	60.00	0.73	tr_D0A148_	
DRB1_0101	10	RDVKRSTSHQFCKSG	VKRSTSHQF	2	0.4014	649.9	65.00	0.66	tr_D0A148_	
DRB1_0101	11	AARDVKRSTSHQFCK	VKRSTSHQF	4	0.3852	774.0	65.00	0.75	tr_D0A148_	
DRB1_0101	12	STVAARDVKRSTSHQ	VAARDVKRS	2	0.3650	963.3	70.00	0.63	tr_D0A148_	
DRB1_0101	13	DVKRSTSHQFCKSGV	VKRSTSHQF	1	0.3470	1171.2	75.00	0.58	tr_D0A148_	
DRB1_0101	14	RSTSHQFCKSGVYSS	FCKSGVYSS	6	0.3411	1247.6	75.00	0.31	tr_D0A148_	
DRB1_0101	15	VAARDVKRSTSHQFC	VKRSTSHQF	5	0.3323	1372.1	80.00	0.62	tr_D0A148_	
DRB1_0101	16	TVAARDVKRSTSHQF	VAARDVKRS	1	0.3322	1374.1	80.00	0.43	tr_D0A148_	
DRB1_0101	17	VKRSTSHQFCKSGVY	VKRSTSHQF	0	0.2915	2133.6	85.00	0.36	tr_D0A148_	
DRB1_0101	18	KRSTSHQFCKSGVYS	STSHQFCKS	2	0.2617	2947.2	90.00	0.22	tr_D0A148_	

Allele: DRB1_0101. Number of high binders 0. Number of weak binders 0. Number of peptides 18

Allele	pos	peptide	core	Of	1-log50k(aff)	affinity(nM)	%Rank	Relia	Identity	Bind Level
DRB1_0101	1	PRFNAIKPTRANILQ	FNAIKPTRA	2	0.8874	3.4	0.17	0.79	tr_D0A6D7_	SB
DRB1_0101	2	RFNAIKPTRANILQH	FNAIKPTRA	1	0.8815	3.6	0.20	0.72	tr_D0A6D7_	SB
DRB1_0101	3	DPRFNAIKPTRANIL	FNAIKPTRA	3	0.8742	3.9	0.30	0.88	tr_D0A6D7_	SB
DRB1_0101	4	DDPRFNAIKPTRANI	FNAIKPTRA	4	0.8378	5.8	0.90	0.93	tr_D0A6D7_	WB
DRB1_0101	5	FNAIKPTRANILQHM	IKPTRANIL	3	0.8351	6.0	0.90	0.40	tr_D0A6D7_	WB
DRB1_0101	6	TDDPRFNAIKPTRAN	FNAIKPTRA	5	0.7918	9.5	2.50	0.94	tr_D0A6D7_	WB
DRB1_0101	7	GTIYSLLSTTRDKLR	YSLLSTTRD	3	0.7640	12.9	4.00	0.75	tr_D0A6D7_	WB
DRB1_0101	8	TQCFISMLLNKTPGT	FISMLLNKT	3	0.7533	14.4	4.50	0.73	tr_D0A6D7_	WB
DRB1_0101	9	TIYSLLSTTRDKLRE	YSLLSTTRD	2	0.7480	15.3	5.00	0.79	tr_D0A6D7_	WB
DRB1_0101	10	PGTIYSLLSTTRDKL	YSLLSTTRD	4	0.7440	16.0	5.50	0.72	tr_D0A6D7_	WB
DRB1_0101	11	ALFLHYSGYGAQVRA	FLHYSGYGA	2	0.7431	16.1	5.50	0.49	tr_D0A6D7_	WB

DRB1_0101	12	QCFISMLLNKTPGTI	FISMLLNKT	2	0.7420	16.3	5.50	0.69	tr_D0A6D7_	WB
DRB1_0101	13	ATQCFISMLLNKTPG	FISMLLNKT	4	0.7388	16.9	5.50	0.76	tr_D0A6D7_	WB
DRB1_0101	14	CFISMLLNKTPGTIY	FISMLLNKT	1	0.7208	20.5	7.00	0.55	tr_D0A6D7_	WB
DRB1_0101	15	LFLHYSGYGAQVRAE	FLHYSGYGA	1	0.7192	20.9	7.50	0.40	tr_D0A6D7_	WB
DRB1_0101	16	DALFLHYSGYGAQVR	FLHYSGYGA	3	0.7190	20.9	7.50	0.54	tr_D0A6D7_	WB
DRB1_0101	17	AATQCFISMLLNKTP	FISMLLNKT	5	0.7106	22.9	8.00	0.80	tr_D0A6D7_	WB
DRB1_0101	18	KKPSKQQRSFRALFI	SKQQRSFRA	3	0.7102	23.0	8.00	0.62	tr_D0A6D7_	WB
DRB1_0101	19	TPGTIYSLLSTTRDK	YSLLSTTRD	5	0.7022	25.1	9.00	0.64	tr_D0A6D7_	WB
DRB1_0101	20	KPSKQQRSFRALFIG	SKQQRSFRA	2	0.7015	25.3	9.00	0.51	tr_D0A6D7_	WB
DRB1_0101	21	HEIISTLPRGVRLTA	STLPRGVRL	4	0.6967	26.6	9.50	0.48	tr_D0A6D7_	WB
DRB1_0101	22	IYSLLSTTRDKLREK	YSLLSTTRD	1	0.6917	28.1	10.00	0.83	tr_D0A6D7_	
DRB1_0101	23	LHEIISTLPRGVRLT	IISTLPRGV	3	0.6897	28.7	10.00	0.45	tr_D0A6D7_	
DRB1_0101	24	EIISTLPRGVRLTAV	STLPRGVRL	3	0.6872	29.5	11.00	0.57	tr_D0A6D7_	
DRB1_0101	25	NAIKPTRANILQHMA	IKPTRANIL	2	0.6791	32.2	11.00	0.88	tr_D0A6D7_	
DRB1_0101	26	PSKQQRSFRALFIGI	SKQQRSFRA	1	0.6791	32.2	11.00	0.41	tr_D0A6D7_	
DRB1_0101	27	DTDDPRFNAIKPTRA	FNAIKPTRA	6	0.6768	33.0	12.00	0.90	tr_D0A6D7_	
DRB1_0101	28	SKQQRSFRALFIGID	QRSFRALFI	3	0.6648	37.6	13.00	0.44	tr_D0A6D7_	
DRB1_0101	29	GAATQCFISMLLNKT	FISMLLNKT	6	0.6648	37.6	13.00	0.75	tr_D0A6D7_	
DRB1_0101	30	GDALFLHYSGYGAQV	FLHYSGYGA	4	0.6634	38.2	13.00	0.58	tr_D0A6D7_	
DRB1_0101	31	FISMLLNKTPGTIYS	FISMLLNKT	0	0.6600	39.6	14.00	0.43	tr_D0A6D7_	
DRB1_0101	32	ELHEIISTLPRGVRL	IISTLPRGV	4	0.6547	41.9	14.00	0.47	tr_D0A6D7_	
DRB1_0101	33	IISTLPRGVRLTAVF	STLPRGVRL	2	0.6502	44.0	15.00	0.65	tr_D0A6D7_	
DRB1_0101	34	FLHYSGYGAQVRAEE	HYSGYGAQV	2	0.6473	45.4	15.00	0.43	tr_D0A6D7_	
DRB1_0101	35	MELLQVAVPYVLVTL	LQVAVPYVL	3	0.6428	47.7	16.00	0.61	tr_D0A6D7_	
DRB1_0101	36	AMELLQVAVPYVLVT	LQVAVPYVL	4	0.6419	48.2	16.00	0.53	tr_D0A6D7_	
DRB1_0101	37	RANILQHMAWLVKDA	ILQHMAWLV	3	0.6380	50.3	17.00	0.60	tr_D0A6D7_	
DRB1_0101	38	IAMELLQVAVPYVLV	LQVAVPYVL	5	0.6375	50.5	17.00	0.47	tr_D0A6D7_	
DRB1_0101	39	KQQRSFRALFIGIDY	QRSFRALFI	2	0.6357	51.5	17.00	0.47	tr_D0A6D7_	
DRB1_0101	40	MKKPSKQQRSFRALF	SKQQRSFRA	4	0.6351	51.9	17.00	0.81	tr_D0A6D7_	
DRB1_0101	41	TRANILQHMAWLVKD	ILQHMAWLV	4	0.6278	56.1	18.00	0.56	tr_D0A6D7_	
DRB1_0101	42	AIKPTRANILQHMAW	IKPTRANIL	1	0.6217	59.9	19.00	0.83	tr_D0A6D7_	
DRB1_0101	43	QQRSFRALFIGIDYK	FRALFIGID	4	0.6216	60.0	19.00	0.46	tr_D0A6D7_	
DRB1_0101	44	ELLQVAVPYVLVTLG	LQVAVPYVL	2	0.6174	62.8	20.00	0.63	tr_D0A6D7_	
DRB1_0101	45	PTRANILQHMAWLVK	ILQHMAWLV	5	0.6162	63.6	20.00	0.56	tr_D0A6D7_	
DRB1_0101	46	KTPGTIYSLLSTTRD	YSLLSTTRD	6	0.6077	69.8	21.00	0.47	tr_D0A6D7_	
DRB1_0101	47	TIAMELLQVAVPYVL	LQVAVPYVL	6	0.6063	70.8	21.00	0.29	tr_D0A6D7_	
DRB1_0101	48	PAELRGCQADAVMMA	LRGCQADAV	3	0.6058	71.2	22.00	0.44	tr_D0A6D7_	
DRB1_0101	49	ANILQHMAWLVKDAK	ILQHMAWLV	2	0.5982	77.3	23.00	0.57	tr_D0A6D7_	
DRB1_0101	50	LLQVAVPYVLVTLGL	LQVAVPYVL	1	0.5978	77.6	23.00	0.53	tr_D0A6D7_	
DRB1_0101	51	QRSFRALFIGIDYKG	FRALFIGID	3	0.5970	78.3	23.00	0.59	tr_D0A6D7_	
DRB1_0101	52	TPAELRGCQADAVMM	LRGCQADAV	4	0.5956	79.5	23.00	0.44	tr_D0A6D7_	
DRB1_0101	53	ISTLPRGVRLTAVFD	STLPRGVRL	1	0.5924	82.3	24.00	0.66	tr_D0A6D7_	
DRB1_0101	54	NELHEIISTLPRGVR	IISTLPRGV	5	0.5870	87.2	25.00	0.50	tr_D0A6D7_	
DRB1_0101	55	PGDALFLHYSGYGAQ	FLHYSGYGA	5	0.5848	89.4	25.00	0.61	tr_D0A6D7_	
DRB1_0101	56	YSLLSTTRDKLREKG	YSLLSTTRD	0	0.5821	92.0	25.00	0.75	tr_D0A6D7_	
DRB1_0101	57	AELRGCQADAVMMAG	LRGCQADAV	2	0.5817	92.3	26.00	0.48	tr_D0A6D7_	
DRB1_0101	58	ELRGCQADAVMMAGT	LRGCQADAV	1	0.5692	105.8	28.00	0.40	tr_D0A6D7_	
DRB1_0101	59	RSFRALFIGIDYKGT	FRALFIGID	2	0.5669	108.5	28.00	0.56	tr_D0A6D7_	
DRB1_0101	60	LTEKFTLTELFSVAE	FTLTELFSV	4	0.5667	108.6	28.00	0.75	tr_D0A6D7_	
DRB1_0101	61	AVPYVLVTLGLQRRP	PYVLVTLGL	2	0.5664	109.0	28.00	0.30	tr_D0A6D7_	

DRB1_0101	62	GTPAELRGCQADAVM	PAELRGCQA	2	0.5642	111.6	29.00	0.45	tr_D0A6D7_
DRB1_0101	63	TELFSVAEPCPNILT	FSVAEPCPN	3	0.5610	115.6	29.00	0.62	tr_D0A6D7_
DRB1_0101	64	LQVAVPYVLVTLGLQ	LQVAVPYVL	0	0.5586	118.6	30.00	0.33	tr_D0A6D7_
DRB1_0101	65	VMKKPSKQQRSFRAL	SKQQRSFRA	5	0.5585	118.7	30.00	0.81	tr_D0A6D7_
DRB1_0101	66	VAVPYVLVTLGLQRR	PYVLVTLGL	3	0.5584	118.8	30.00	0.35	tr_D0A6D7_
DRB1_0101	67	SLTEKFTLTELFSVA	FTLTELFSV	5	0.5565	121.3	30.00	0.74	tr_D0A6D7_
DRB1_0101	68	KGFKQSPQMSASRCL	FKQSPQMSA	2	0.5563	121.6	30.00	0.56	tr_D0A6D7_
DRB1_0101	69	AGTLLDLPFSLICSS	LLDLPFSLI	3	0.5539	124.7	30.00	0.48	tr_D0A6D7_
DRB1_0101	70	HAGTLLDLPFSLICS	LLDLPFSLI	4	0.5529	126.1	31.00	0.49	tr_D0A6D7_
DRB1_0101	71	TEKFTLTELFSVAEP	FTLTELFSV	3	0.5524	126.9	31.00	0.75	tr_D0A6D7_
DRB1_0101	72	ELFSVAEPCPNILTG	FSVAEPCPN	2	0.5517	127.9	31.00	0.57	tr_D0A6D7_
DRB1_0101	73	KPTRANILQHMAWLV	ILQHMAWLV	6	0.5474	133.9	32.00	0.48	tr_D0A6D7_
DRB1_0101	74	VPYVLVTLGLQRRPP	LVTLGLQRR	4	0.5447	137.9	32.00	0.36	tr_D0A6D7_
DRB1_0101	75	REKGFKQSPQMSASR	FKQSPQMSA	4	0.5445	138.2	32.00	0.61	tr_D0A6D7_
DRB1_0101	76	NILQHMAWLVKDAKP	ILQHMAWLV	1	0.5402	144.7	33.00	0.57	tr_D0A6D7_
DRB1_0101	77	SFRALFIGIDYKGTP	FRALFIGID	1	0.5400	145.0	33.00	0.46	tr_D0A6D7_
DRB1_0101	78	EKGFKQSPQMSASRC	FKQSPQMSA	3	0.5394	146.0	33.00	0.66	tr_D0A6D7_
DRB1_0101	79	LRGCQADAVMMAGTM	GCQADAVMM	2	0.5390	146.6	33.00	0.36	tr_D0A6D7_
DRB1_0101	80	SHAGTLLDLPFSLIC	LLDLPFSLI	5	0.5376	148.8	34.00	0.47	tr_D0A6D7_
DRB1_0101	81	ISMLLNKTPGTIYSL	MLLNKTPGT	2	0.5345	154.0	34.00	0.30	tr_D0A6D7_
DRB1_0101	82	GFKQSPQMSASRCLS	FKQSPQMSA	1	0.5340	154.7	34.00	0.48	tr_D0A6D7_
DRB1_0101	83	LPFSLICSSNDCSAV	FSLICSSND	2	0.5337	155.3	34.00	0.68	tr_D0A6D7_
DRB1_0101	84	PYVLVTLGLQRRPPK	LVTLGLQRR	3	0.5299	161.8	35.00	0.42	tr_D0A6D7_
DRB1_0101	85	KGTPAELRGCQADAV	PAELRGCQA	3	0.5285	164.3	35.00	0.57	tr_D0A6D7_
DRB1_0101	86	QADAVMMAGTMEKIG	VMMAGTMEK	4	0.5275	166.1	36.00	0.26	tr_D0A6D7_
DRB1_0101	87	LTELFSVAEPCPNIL	FSVAEPCPN	4	0.5258	169.2	36.00	0.66	tr_D0A6D7_
DRB1_0101	88	GTLLDLPFSLICSSN	LLDLPFSLI	2	0.5253	170.0	36.00	0.47	tr_D0A6D7_
DRB1_0101	89	LFSVAEPCPNILTGE	FSVAEPCPN	1	0.5237	173.0	36.00	0.50	tr_D0A6D7_
DRB1_0101	90	ADAVMMAGTMEKIGI	VMMAGTMEK	3	0.5219	176.5	37.00	0.34	tr_D0A6D7_
DRB1_0101	91	IKPTRANILQHMAWL	IKPTRANIL	0	0.5217	176.8	37.00	0.52	tr_D0A6D7_
DRB1_0101	92	CSHAGTLLDLPFSLI	LLDLPFSLI	6	0.5216	177.0	37.00	0.44	tr_D0A6D7_
DRB1_0101	93	FRALFIGIDYKGTPA	FIGIDYKGT	4	0.5213	177.6	37.00	0.55	tr_D0A6D7_
DRB1_0101	94	CQADAVMMAGTMEKI	DAVMMAGTM	3	0.5205	179.2	37.00	0.34	tr_D0A6D7_
DRB1_0101	95	EKFTLTELFSVAEPC	FTLTELFSV	2	0.5196	180.9	37.00	0.73	tr_D0A6D7_
DRB1_0101	96	DLPFSLICSSNDCSA	FSLICSSND	3	0.5196	180.9	37.00	0.73	tr_D0A6D7_
DRB1_0101	97	SMLLNKTPGTIYSLL	LNKTPGTIY	3	0.5163	187.4	38.00	0.37	tr_D0A6D7_
DRB1_0101	98	TLLDLPFSLICSSND	LLDLPFSLI	1	0.5130	194.3	38.00	0.45	tr_D0A6D7_
DRB1_0101	99	STLPRGVRLTAVFDC	STLPRGVRL	0	0.5128	194.7	38.00	0.53	tr_D0A6D7_
DRB1_0101	100	QVAVPYVLVTLGLQR	PYVLVTLGL	4	0.5083	204.5	39.00	0.43	tr_D0A6D7_
DRB1_0101	101	IDYKGTPAELRGCQA	YKGTPAELR	2	0.5081	204.9	39.00	0.48	tr_D0A6D7_
DRB1_0101	102	DNELHEIISTLPRGV	IISTLPRGV	6	0.5070	207.2	40.00	0.35	tr_D0A6D7_
DRB1_0101	103	PFSLICSSNDCSAVG	FSLICSSND	1	0.5067	208.0	40.00	0.59	tr_D0A6D7_
DRB1_0101	104	LHYSGYGAQVRAEED	HYSGYGAQV	1	0.5029	216.8	40.00	0.56	tr_D0A6D7_
DRB1_0101	105	FIGIDYKGTPAELRG	YKGTPAELR	5	0.5009	221.4	41.00	0.43	tr_D0A6D7_
DRB1_0101	106	KPGDALFLHYSGYGA	FLHYSGYGA	6	0.4990	225.9	41.00	0.54	tr_D0A6D7_
DRB1_0101	107	DYKGTPAELRGCQAD	PAELRGCQA	5	0.4988	226.5	41.00	0.42	tr_D0A6D7_
DRB1_0101	108	AWLVKDAKPGDALFL	WLVKDAKPG	1	0.4985	227.3	41.00	0.37	tr_D0A6D7_
DRB1_0101	109	LLDLPFSLICSSNDC	FSLICSSND	5	0.4953	235.2	42.00	0.54	tr_D0A6D7_
DRB1_0101	110	FKQSPQMSASRCLSL	PQMSASRCL	4	0.4948	236.6	42.00	0.42	tr_D0A6D7_
DRB1_0101	111	TAVFDCSHAGTLLDL	FDCSHAGTL	3	0.4925	242.6	43.00	0.46	tr_D0A6D7_

DRB1_0101	112	GCQADAVMMAGTMEK	DAVMMAGTM	4	0.4922	243.4	43.00	0.31	tr_D0A6D7_
DRB1_0101	113	LFIGIDYKGTPAELR	FIGIDYKGT	1	0.4904	248.0	43.00	0.38	tr_D0A6D7_
DRB1_0101	114	RGCQADAVMMAGTME	GCQADAVMM	1	0.4871	257.1	44.00	0.29	tr_D0A6D7_
DRB1_0101	115	LREKGFKQSPQMSAS	FKQSPQMSA	5	0.4862	259.6	44.00	0.56	tr_D0A6D7_
DRB1_0101	116	GDVNAHVLMFSACGD	HVLMFSACG	5	0.4826	270.0	45.00	0.64	tr_D0A6D7_
DRB1_0101	117	NKTPGTIYSLLSTTR	IYSLLSTTR	6	0.4825	270.2	45.00	0.45	tr_D0A6D7_
DRB1_0101	118	RALFIGIDYKGTPAE	FIGIDYKGT	3	0.4815	273.1	45.00	0.72	tr_D0A6D7_
DRB1_0101	119	LSLTEKFTLTELFSV	FTLTELFSV	6	0.4812	274.2	45.00	0.68	tr_D0A6D7_
DRB1_0101	120	DAVMMAGTMEKIGIP	VMMAGTMEK	2	0.4806	275.9	45.00	0.37	tr_D0A6D7_
DRB1_0101	121	IGIDYKGTPAELRGC	YKGTPAELR	4	0.4801	277.4	45.00	0.56	tr_D0A6D7_
DRB1_0101	122	LDLPFSLICSSNDCS	FSLICSSND	4	0.4800	277.8	45.00	0.69	tr_D0A6D7_
DRB1_0101	123	VNAHVLMFSACGDDE	HVLMFSACG	3	0.4795	279.0	45.00	0.69	tr_D0A6D7_
DRB1_0101	124	YKGTPAELRGCQADA	PAELRGCQA	4	0.4780	283.6	46.00	0.56	tr_D0A6D7_
DRB1_0101	125	DVNAHVLMFSACGDD	HVLMFSACG	4	0.4776	284.9	46.00	0.69	tr_D0A6D7_
DRB1_0101	126	KFTLTELFSVAEPCP	FTLTELFSV	1	0.4771	286.4	46.00	0.73	tr_D0A6D7_
DRB1_0101	127	MAWLVKDAKPGDALF	WLVKDAKPG	2	0.4766	287.9	46.00	0.56	tr_D0A6D7_
DRB1_0101	128	YVLVTLGLQRRPPKG	LVTLGLQRR	2	0.4762	289.4	46.00	0.48	tr_D0A6D7_
DRB1_0101	129	MLLNKTPGTIYSLLS	LNKTPGTIY	2	0.4742	295.6	46.00	0.37	tr_D0A6D7_
DRB1_0101	130	GIDYKGTPAELRGCQ	YKGTPAELR	3	0.4728	300.1	47.00	0.64	tr_D0A6D7_
DRB1_0101	131	WLVKDAKPGDALFLH	AKPGDALFL	5	0.4728	300.2	47.00	0.34	tr_D0A6D7_
DRB1_0101	132	NAHVLMFSACGDDEA	HVLMFSACG	2	0.4725	301.0	47.00	0.67	tr_D0A6D7_
DRB1_0101	133	ASRCLSLTEKFTLTE	CLSLTEKFT	3	0.4640	330.2	49.00	0.50	tr_D0A6D7_
DRB1_0101	134	HMAWLVKDAKPGDAL	WLVKDAKPG	3	0.4640	330.2	49.00	0.68	tr_D0A6D7_
DRB1_0101	135	ALFIGIDYKGTPAEL	FIGIDYKGT	2	0.4635	331.7	49.00	0.57	tr_D0A6D7_
DRB1_0101	136	KRIRTGGDVNAHVLM	IRTGGDVNA	2	0.4621	336.9	49.00	0.55	tr_D0A6D7_
DRB1_0101	137	AVFDCSHAGTLLDLP	FDCSHAGTL	2	0.4613	339.8	49.00	0.47	tr_D0A6D7_
DRB1_0101	138	GGDVNAHVLMFSACG	HVLMFSACG	6	0.4601	344.2	49.00	0.51	tr_D0A6D7_
DRB1_0101	139	MKRIRTGGDVNAHVL	IRTGGDVNA	3	0.4592	347.8	50.00	0.68	tr_D0A6D7_
DRB1_0101	140	AHVLMFSACGDDEAA	HVLMFSACG	1	0.4583	351.1	50.00	0.56	tr_D0A6D7_
DRB1_0101	141	LTAVFDCSHAGTLLD	FDCSHAGTL	4	0.4580	352.2	50.00	0.47	tr_D0A6D7_
DRB1_0101	142	KQSPQMSASRCLSLT	PQMSASRCL	3	0.4539	368.2	55.00	0.57	tr_D0A6D7_
DRB1_0101	143	SRCLSLTEKFTLTEL	CLSLTEKFT	2	0.4532	371.0	55.00	0.44	tr_D0A6D7_
DRB1_0101	144	SASRCLSLTEKFTLT	CLSLTEKFT	4	0.4526	373.3	55.00	0.50	tr_D0A6D7_
DRB1_0101	145	LVKDAKPGDALFLHY	AKPGDALFL	4	0.4514	378.2	55.00	0.51	tr_D0A6D7_
DRB1_0101	146	RIRTGGDVNAHVLMF	IRTGGDVNA	1	0.4501	383.8	55.00	0.37	tr_D0A6D7_
DRB1_0101	147	RLTAVFDCSHAGTLL	FDCSHAGTL	5	0.4443	408.4	55.00	0.49	tr_D0A6D7_
DRB1_0101	148	VDKAMAEAESVEAEV	KAMAEAESV	2	0.4422	417.9	55.00	0.54	tr_D0A6D7_
DRB1_0101	149	AVMMAGTMEKIGIPI	VMMAGTMEK	1	0.4403	426.7	55.00	0.37	tr_D0A6D7_
DRB1_0101	150	GVDKAMAEAESVEAE	KAMAEAESV	3	0.4391	432.2	55.00	0.56	tr_D0A6D7_
DRB1_0101	151	FSLICSSNDCSAVGE	FSLICSSND	0	0.4391	432.2	55.00	0.42	tr_D0A6D7_
DRB1_0101	152	MEKIGIPITERCVLM	GIPITERCV	4	0.4382	436.5	55.00	0.39	tr_D0A6D7_
DRB1_0101	153	VKDAKPGDALFLHYS	AKPGDALFL	3	0.4381	437.1	55.00	0.62	tr_D0A6D7_
DRB1_0101	154	LDNELHEIISTLPRG	LHEIISTLP	4	0.4360	447.1	55.00	0.32	tr_D0A6D7_
DRB1_0101	155	TLTELFSVAEPCPNI	FSVAEPCPN	5	0.4359	447.6	55.00	0.63	tr_D0A6D7_
DRB1_0101	156	SGGAATQCFISMLLN	GAATQCFIS	2	0.4346	453.6	55.00	0.50	tr_D0A6D7_
DRB1_0101	157	MAGTMEKIGIPITER	TMEKIGIPI	3	0.4340	456.9	55.00	0.47	tr_D0A6D7_
DRB1_0101	158	ILQHMAWLVKDAKPG	ILQHMAWLV	0	0.4337	458.0	55.00	0.39	tr_D0A6D7_
DRB1_0101	159	GGAATQCFISMLLNK	GAATQCFIS	1	0.4320	466.6	55.00	0.44	tr_D0A6D7_
DRB1_0101	160	DKAMAEAESVEAEVM	KAMAEAESV	1	0.4318	467.6	55.00	0.44	tr_D0A6D7_
DRB1_0101	161	GSGGAATQCFISMLL	GAATQCFIS	3	0.4309	472.4	60.00	0.54	tr_D0A6D7_

DRB1_0101	162	FTLTELFSVAEPCPN	FTLTELFSV	0	0.4290	481.9	60.00	0.38	tr_D0A6D7_
DRB1_0101	163	RCLSLTEKFTLTELF	SLTEKFTLT	3	0.4262	496.8	60.00	0.35	tr_D0A6D7_
DRB1_0101	164	ILDNELHEIISTLPR	ELHEIISTL	4	0.4259	498.3	60.00	0.29	tr_D0A6D7_
DRB1_0101	165	GTMEKIGIPITERCV	TMEKIGIPI	1	0.4245	506.2	60.00	0.30	tr_D0A6D7_
DRB1_0101	166	VFDCSHAGTLLDLPF	FDCSHAGTL	1	0.4236	510.9	60.00	0.44	tr_D0A6D7_
DRB1_0101	167	LLNKTPGTIYSLLST	LNKTPGTIY	1	0.4226	516.4	60.00	0.35	tr_D0A6D7_
DRB1_0101	168	AGTMEKIGIPITERC	TMEKIGIPI	2	0.4220	519.7	60.00	0.43	tr_D0A6D7_
DRB1_0101	169	FSVAEPCPNILTGEP	FSVAEPCPN	0	0.4219	520.4	60.00	0.35	tr_D0A6D7_
DRB1_0101	170	EKIGIPITERCVLMD	GIPITERCV	3	0.4218	520.9	60.00	0.40	tr_D0A6D7_
DRB1_0101	171	GGVDKAMAEAESVEA	KAMAEAESV	4	0.4205	528.3	60.00	0.60	tr_D0A6D7_
DRB1_0101	172	FDCSHAGTLLDLPFS	GTLLDLPFS	6	0.4181	542.2	60.00	0.27	tr_D0A6D7_
DRB1_0101	173	QSPQMSASRCLSLTE	PQMSASRCL	2	0.4154	558.2	60.00	0.53	tr_D0A6D7_
DRB1_0101	174	VLVTLGLQRRPPKGG	LVTLGLQRR	1	0.4147	562.7	60.00	0.49	tr_D0A6D7_
DRB1_0101	175	KDAKPGDALFLHYSG	AKPGDALFL	2	0.4145	564.0	60.00	0.62	tr_D0A6D7_
DRB1_0101	176	EMKRIRTGGDVNAHV	IRTGGDVNA	4	0.4134	570.7	60.00	0.63	tr_D0A6D7_
DRB1_0101	177	KIGIPITERCVLMDT	PITERCVLM	4	0.4128	574.4	60.00	0.48	tr_D0A6D7_
DRB1_0101	178	HVLMFSACGDDEAAA	HVLMFSACG	0	0.4094	595.7	60.00	0.41	tr_D0A6D7_
DRB1_0101	179	IRTGGDVNAHVLMFS	GDVNAHVLM	4	0.4059	618.7	65.00	0.32	tr_D0A6D7_
DRB1_0101	180	TMEKIGIPITERCVL	GIPITERCV	5	0.4050	624.8	65.00	0.34	tr_D0A6D7_
DRB1_0101	181	MSASRCLSLTEKFTL	CLSLTEKFT	5	0.4047	626.9	65.00	0.55	tr_D0A6D7_
DRB1_0101	182	KLREKGFKQSPQMSA	FKQSPQMSA	6	0.4044	628.8	65.00	0.38	tr_D0A6D7_
DRB1_0101	183	DCSHAGTLLDLPFSL	GTLLDLPFS	5	0.4033	636.7	65.00	0.52	tr_D0A6D7_
DRB1_0101	184	EVMKKPSKQQRSFRA	SKQQRSFRA	6	0.4033	636.8	65.00	0.60	tr_D0A6D7_
DRB1_0101	185	SGSGGAATQCFISML	GAATQCFIS	4	0.3970	681.5	65.00	0.55	tr_D0A6D7_
DRB1_0101	186	KAMAEAESVEAEVMK	MAEAESVEA	2	0.3965	685.3	65.00	0.50	tr_D0A6D7_
DRB1_0101	187	VMMAGTMEKIGIPIT	TMEKIGIPI	5	0.3950	696.3	65.00	0.34	tr_D0A6D7_
DRB1_0101	188	AGDFVEGASGSGGAA	FVEGASGSG	3	0.3943	701.5	65.00	0.64	tr_D0A6D7_
DRB1_0101	189	LNKTPGTIYSLLSTT	PGTIYSLLS	4	0.3929	712.5	65.00	0.24	tr_D0A6D7_
DRB1_0101	190	QHMAWLVKDAKPGDA	WLVKDAKPG	4	0.3908	728.8	65.00	0.71	tr_D0A6D7_
DRB1_0101	191	MMAGTMEKIGIPITE	TMEKIGIPI	4	0.3878	752.7	65.00	0.44	tr_D0A6D7_
DRB1_0101	192	DAKPGDALFLHYSGY	AKPGDALFL	1	0.3872	757.9	65.00	0.49	tr_D0A6D7_
DRB1_0101	193	GDFVEGASGSGGAAT	FVEGASGSG	2	0.3840	784.5	70.00	0.56	tr_D0A6D7_
DRB1_0101	194	PCPNILTGEPRWKTT	LTGEPRWKT	5	0.3831	792.5	70.00	0.41	tr_D0A6D7_
DRB1_0101	195	CILDNELHEIISTLP	ELHEIISTL	5	0.3811	809.3	70.00	0.28	tr_D0A6D7_
DRB1_0101	196	GEMKRIRTGGDVNAH	IRTGGDVNA	5	0.3804	816.0	70.00	0.53	tr_D0A6D7_
DRB1_0101	197	SPQMSASRCLSLTEK	PQMSASRCL	1	0.3800	819.4	70.00	0.46	tr_D0A6D7_
DRB1_0101	198	RTGGDVNAHVLMFSA	GDVNAHVLM	3	0.3781	836.1	70.00	0.39	tr_D0A6D7_
DRB1_0101	199	CPNILTGEPRWKTTP	LTGEPRWKT	4	0.3772	843.9	70.00	0.45	tr_D0A6D7_
DRB1_0101	200	PRGVRLTAVFDCSHA	GVRLTAVFD	2	0.3759	855.9	70.00	0.46	tr_D0A6D7_
DRB1_0101	201	IGIPITERCVLMDTD	PITERCVLM	3	0.3758	856.9	70.00	0.57	tr_D0A6D7_
DRB1_0101	202	LPRGVRLTAVFDCSH	GVRLTAVFD	3	0.3758	857.0	70.00	0.52	tr_D0A6D7_
DRB1_0101	203	GCILDNELHEIISTL	LDNELHEII	3	0.3743	871.3	70.00	0.25	tr_D0A6D7_
DRB1_0101	204	KGGVDKAMAEAESVE	KAMAEAESV	5	0.3723	890.7	70.00	0.52	tr_D0A6D7_
DRB1_0101	205	PNILTGEPRWKTTPL	LTGEPRWKT	3	0.3716	896.8	70.00	0.55	tr_D0A6D7_
DRB1_0101	206	HYSGYGAQVRAEEDK	HYSGYGAQV	0	0.3711	901.9	70.00	0.44	tr_D0A6D7_
DRB1_0101	207	AKPGDALFLHYSGYG	GDALFLHYS	3	0.3684	928.7	70.00	0.28	tr_D0A6D7_
DRB1_0101	208	CLSLTEKFTLTELFS	SLTEKFTLT	2	0.3675	937.8	70.00	0.38	tr_D0A6D7_
DRB1_0101	209	TLPRGVRLTAVFDCS	GVRLTAVFD	4	0.3659	953.7	70.00	0.49	tr_D0A6D7_
DRB1_0101	210	TGGDVNAHVLMFSAC	GDVNAHVLM	2	0.3640	974.2	70.00	0.33	tr_D0A6D7_
DRB1_0101	211	LVTLGLQRRPPKGGV	LVTLGLQRR	0	0.3607	1009.4	70.00	0.31	tr_D0A6D7_

DRB1_0101	212	DFVEGASGSGGAATQ	FVEGASGSG	1	0.3593	1024.9	75.00	0.50	tr_D0A6D7_
DRB1_0101	213	VRLTAVFDCSHAGTL	FDCSHAGTL	6	0.3564	1057.3	75.00	0.45	tr_D0A6D7_
DRB1_0101	214	RGVRLTAVFDCSHAG	GVRLTAVFD	1	0.3556	1066.1	75.00	0.41	tr_D0A6D7_
DRB1_0101	215	LQHMAWLVKDAKPGD	WLVKDAKPG	5	0.3525	1103.0	75.00	0.60	tr_D0A6D7_
DRB1_0101	216	QMSASRCLSLTEKFT	CLSLTEKFT	6	0.3500	1133.4	75.00	0.40	tr_D0A6D7_
DRB1_0101	217	ASGSGGAATQCFISM	GAATQCFIS	5	0.3498	1135.3	75.00	0.49	tr_D0A6D7_
DRB1_0101	218	NAGDFVEGASGSGGA	FVEGASGSG	4	0.3490	1145.0	75.00	0.63	tr_D0A6D7_
DRB1_0101	219	EPCPNILTGEPRWKT	LTGEPRWKT	6	0.3385	1282.9	75.00	0.30	tr_D0A6D7_
DRB1_0101	220	NGCILDNELHEIIST	LDNELHEII	4	0.3365	1311.1	75.00	0.34	tr_D0A6D7_
DRB1_0101	221	NILTGEPRWKTTPLG	LTGEPRWKT	2	0.3252	1481.4	80.00	0.64	tr_D0A6D7_
DRB1_0101	222	VGEMKRIRTGGDVNA	IRTGGDVNA	6	0.3246	1491.3	80.00	0.34	tr_D0A6D7_
DRB1_0101	223	NDCSAVGEMKRIRTG	SAVGEMKRI	3	0.3215	1543.4	80.00	0.40	tr_D0A6D7_
DRB1_0101	224	PQMSASRCLSLTEKF	MSASRCLSL	2	0.3213	1546.2	80.00	0.31	tr_D0A6D7_
DRB1_0101	225	GIPITERCVLMDTDD	PITERCVLM	2	0.3195	1576.7	80.00	0.63	tr_D0A6D7_
DRB1_0101	226	SNDCSAVGEMKRIRT	SAVGEMKRI	4	0.3184	1594.6	80.00	0.38	tr_D0A6D7_
DRB1_0101	227	VEAEVMKKPSKQQRS	EVMKKPSKQ	3	0.3170	1619.0	80.00	0.58	tr_D0A6D7_
DRB1_0101	228	SLICSSNDCSAVGEM	LICSSNDCS	1	0.3133	1685.7	80.00	0.40	tr_D0A6D7_
DRB1_0101	229	PKGGVDKAMAEAESV	VDKAMAEAE	4	0.3115	1718.5	80.00	0.38	tr_D0A6D7_
DRB1_0101	230	EAEVMKKPSKQQRSF	EVMKKPSKQ	2	0.3114	1720.3	80.00	0.53	tr_D0A6D7_
DRB1_0101	231	ENGCILDNELHEIIS	LDNELHEII	5	0.3114	1721.2	80.00	0.34	tr_D0A6D7_
DRB1_0101	232	GVRLTAVFDCSHAGT	GVRLTAVFD	0	0.3105	1737.9	80.00	0.25	tr_D0A6D7_
DRB1_0101	233	IVPCDYEENGCILDN	DYEENGCIL	4	0.3093	1760.0	80.00	0.47	tr_D0A6D7_
DRB1_0101	234	AEVMKKPSKQQRSFR	EVMKKPSKQ	1	0.3091	1764.7	80.00	0.46	tr_D0A6D7_
DRB1_0101	235	VLMFSACGDDEAAAD	FSACGDDEA	3	0.3090	1765.3	80.00	0.56	tr_D0A6D7_
DRB1_0101	236	DCSAVGEMKRIRTGG	SAVGEMKRI	2	0.3088	1770.6	80.00	0.37	tr_D0A6D7_
DRB1_0101	237	VPCDYEENGCILDNE	DYEENGCIL	3	0.3045	1853.9	85.00	0.47	tr_D0A6D7_
DRB1_0101	238	FVEGASGSGGAATQC	VEGASGSGG	1	0.3010	1926.2	85.00	0.35	tr_D0A6D7_
DRB1_0101	239	SSNDCSAVGEMKRIR	SAVGEMKRI	5	0.3004	1937.9	85.00	0.34	tr_D0A6D7_
DRB1_0101	240	SVEAEVMKKPSKQQR	EVMKKPSKQ	4	0.2985	1978.7	85.00	0.58	tr_D0A6D7_
DRB1_0101	241	SVAEPCPNILTGEPR	VAEPCPNIL	1	0.2984	1980.6	85.00	0.41	tr_D0A6D7_
DRB1_0101	242	PCDYEENGCILDNEL	DYEENGCIL	2	0.2934	2089.8	85.00	0.45	tr_D0A6D7_
DRB1_0101	243	LMFSACGDDEAAADL	FSACGDDEA	2	0.2913	2139.3	85.00	0.56	tr_D0A6D7_
DRB1_0101	244	DKLREKGFKQSPQMS	KLREKGFKQ	1	0.2886	2201.1	85.00	0.34	tr_D0A6D7_
DRB1_0101	245	CIVPCDYEENGCILD	DYEENGCIL	5	0.2860	2266.0	85.00	0.45	tr_D0A6D7_
DRB1_0101	246	PNAGDFVEGASGSGG	FVEGASGSG	5	0.2856	2274.3	85.00	0.61	tr_D0A6D7_
DRB1_0101	247	CSAVGEMKRIRTGGD	SAVGEMKRI	1	0.2842	2310.1	85.00	0.39	tr_D0A6D7_
DRB1_0101	248	EENGCILDNELHEII	GCILDNELH	3	0.2822	2358.8	85.00	0.27	tr_D0A6D7_
DRB1_0101	249	AMAEAESVEAEVMKK	MAEAESVEA	1	0.2822	2360.8	85.00	0.54	tr_D0A6D7_
DRB1_0101	250	GASGSGGAATQCFIS	GAATQCFIS	6	0.2774	2485.9	85.00	0.35	tr_D0A6D7_
DRB1_0101	251	RCVLMDTDDPRFNAI	CVLMDTDDP	1	0.2750	2552.3	90.00	0.41	tr_D0A6D7_
DRB1_0101	252	CDYEENGCILDNELH	DYEENGCIL	1	0.2700	2692.5	90.00	0.38	tr_D0A6D7_
DRB1_0101	253	LICSSNDCSAVGEMK	LICSSNDCS	0	0.2684	2740.7	90.00	0.25	tr_D0A6D7_
DRB1_0101	254	ERCVLMDTDDPRFNA	CVLMDTDDP	2	0.2663	2803.1	90.00	0.53	tr_D0A6D7_
DRB1_0101	255	CSSNDCSAVGEMKRI	SAVGEMKRI	6	0.2663	2804.5	90.00	0.22	tr_D0A6D7_
DRB1_0101	256	VTLGLQRRPPKGGVD	LGLQRRPPK	2	0.2662	2806.0	90.00	0.44	tr_D0A6D7_
DRB1_0101	257	IPITERCVLMDTDDP	PITERCVLM	1	0.2659	2815.4	90.00	0.67	tr_D0A6D7_
DRB1_0101	258	SAVGEMKRIRTGGDV	VGEMKRIRT	2	0.2633	2896.4	90.00	0.28	tr_D0A6D7_
DRB1_0101	259	TERCVLMDTDDPRFN	CVLMDTDDP	3	0.2601	2996.0	90.00	0.62	tr_D0A6D7_
DRB1_0101	260	TRDKLREKGFKQSPQ	KLREKGFKQ	3	0.2598	3007.7	90.00	0.67	tr_D0A6D7_
DRB1_0101	261	ESVEAEVMKKPSKQQ	EVMKKPSKQ	5	0.2586	3046.5	90.00	0.47	tr_D0A6D7_

DRB1_0101	262	AEPCPNILTGEPRWK	PNILTGEPR	4	0.2572	3093.1	90.00	0.49	tr_D0A6D7_
DRB1_0101	263	ILTGEPRWKTTPLGG	LTGEPRWKT	1	0.2571	3097.2	90.00	0.68	tr_D0A6D7_
DRB1_0101	264	RDKLREKGFKQSPQM	KLREKGFKQ	2	0.2557	3143.6	90.00	0.59	tr_D0A6D7_
DRB1_0101	265	CVLMDTDDPRFNAIK	MDTDDPRFN	3	0.2555	3152.2	90.00	0.25	tr_D0A6D7_
DRB1_0101	266	MFSACGDDEAAADLP	FSACGDDEA	1	0.2551	3163.5	90.00	0.54	tr_D0A6D7_
DRB1_0101	267	QCIVPCDYEENGCIL	DYEENGCIL	6	0.2530	3236.8	90.00	0.43	tr_D0A6D7_
DRB1_0101	268	VAEPCPNILTGEPRW	PNILTGEPR	5	0.2494	3366.6	90.00	0.33	tr_D0A6D7_
DRB1_0101	269	AVGEMKRIRTGGDVN	MKRIRTGGD	4	0.2488	3389.2	90.00	0.31	tr_D0A6D7_
DRB1_0101	270	AAADLPNAGDFVEGA	DLPNAGDFV	3	0.2458	3500.8	90.00	0.69	tr_D0A6D7_
DRB1_0101	271	TTRDKLREKGFKQSP	KLREKGFKQ	4	0.2455	3509.1	90.00	0.64	tr_D0A6D7_
DRB1_0101	272	STTRDKLREKGFKQS	KLREKGFKQ	5	0.2429	3609.1	90.00	0.61	tr_D0A6D7_
DRB1_0101	273	PITERCVLMDTDDPR	CVLMDTDDP	5	0.2418	3653.6	90.00	0.44	tr_D0A6D7_
DRB1_0101	274	TLGLQRRPPKGGVDK	LGLQRRPPK	1	0.2379	3811.5	95.00	0.41	tr_D0A6D7_
DRB1_0101	275	KEEEFDQCIVPCDYE	FDQCIVPCD	4	0.2356	3907.7	95.00	0.51	tr_D0A6D7_
DRB1_0101	276	EAAADLPNAGDFVEG	DLPNAGDFV	4	0.2295	4175.4	95.00	0.60	tr_D0A6D7_
DRB1_0101	277	AESVEAEVMKKPSKQ	EVMKKPSKQ	6	0.2283	4229.9	95.00	0.28	tr_D0A6D7_
DRB1_0101	278	ITERCVLMDTDDPRF	CVLMDTDDP	4	0.2278	4251.4	95.00	0.69	tr_D0A6D7_
DRB1_0101	279	DKEEEFDQCIVPCDY	FDQCIVPCD	5	0.2278	4253.5	95.00	0.49	tr_D0A6D7_
DRB1_0101	280	SLLSTTRDKLREKGF	LLSTTRDKL	1	0.2274	4268.4	95.00	0.50	tr_D0A6D7_
DRB1_0101	281	EEEFDQCIVPCDYEE	FDQCIVPCD	3	0.2270	4287.8	95.00	0.54	tr_D0A6D7_
DRB1_0101	282	DYEENGCILDNELHE	GCILDNELH	5	0.2270	4288.4	95.00	0.25	tr_D0A6D7_
DRB1_0101	283	YEENGCILDNELHEI	GCILDNELH	4	0.2264	4316.2	95.00	0.31	tr_D0A6D7_
DRB1_0101	284	ICSSNDCSAVGEMKR	CSSNDCSAV	1	0.2240	4428.4	95.00	0.25	tr_D0A6D7_
DRB1_0101	285	AADLPNAGDFVEGAS	DLPNAGDFV	2	0.2231	4474.8	95.00	0.67	tr_D0A6D7_
DRB1_0101	286	EEFDQCIVPCDYEEN	FDQCIVPCD	2	0.2230	4476.6	95.00	0.47	tr_D0A6D7_
DRB1_0101	287	MAEAESVEAEVMKKP	AESVEAEVM	3	0.2174	4755.4	95.00	0.30	tr_D0A6D7_
DRB1_0101	288	LTGEPRWKTTPLGGK	LTGEPRWKT	0	0.2116	5065.3	95.00	0.44	tr_D0A6D7_
DRB1_0101	289	DEAAADLPNAGDFVE	DLPNAGDFV	5	0.2116	5067.1	95.00	0.57	tr_D0A6D7_
DRB1_0101	290	YSGYGAQVRAEEDKE	YSGYGAQVR	0	0.2111	5095.3	95.00	0.28	tr_D0A6D7_
DRB1_0101	291	VLMDTDDPRFNAIKP	MDTDDPRFN	2	0.2099	5161.1	95.00	0.32	tr_D0A6D7_
DRB1_0101	292	LPNAGDFVEGASGSG	FVEGASGSG	6	0.2040	5500.3	95.00	0.44	tr_D0A6D7_
DRB1_0101	293	ADLPNAGDFVEGASG	DLPNAGDFV	1	0.2036	5524.1	95.00	0.56	tr_D0A6D7_
DRB1_0101	294	EFDQCIVPCDYEENG	FDQCIVPCD	1	0.2034	5534.9	95.00	0.42	tr_D0A6D7_
DRB1_0101	295	FSACGDDEAAADLPN	FSACGDDEA	0	0.1991	5800.8	95.00	0.43	tr_D0A6D7_
DRB1_0101	296	EGASGSGGAATQCFI	SGGAATQCF	5	0.1973	5915.5	95.00	0.23	tr_D0A6D7_
DRB1_0101	297	VEGASGSGGAATQCF	GASGSGGAA	2	0.1969	5937.7	95.00	0.26	tr_D0A6D7_
DRB1_0101	298	PPKGGVDKAMAEAES	VDKAMAEAE	5	0.1968	5944.2	95.00	0.56	tr_D0A6D7_
DRB1_0101	299	EAESVEAEVMKKPSK	SVEAEVMKK	3	0.1966	5959.7	95.00	0.28	tr_D0A6D7_
DRB1_0101	300	LGLQRRPPKGGVDKA	LQRRPPKGG	2	0.1922	6246.4	100.00	0.37	tr_D0A6D7_
DRB1_0101	301	LMDTDDPRFNAIKPT	MDTDDPRFN	1	0.1907	6354.7	100.00	0.28	tr_D0A6D7_
DRB1_0101	302	AEAESVEAEVMKKPS	AESVEAEVM	2	0.1899	6409.8	100.00	0.34	tr_D0A6D7_
DRB1_0101	303	LSTTRDKLREKGFKQ	KLREKGFKQ	6	0.1821	6972.5	100.00	0.45	tr_D0A6D7_
DRB1_0101	304	MDTDDPRFNAIKPTR	TDDPRFNAI	2	0.1817	7001.3	100.00	0.25	tr_D0A6D7_
DRB1_0101	305	EDKEEEFDQCIVPCD	FDQCIVPCD	6	0.1813	7028.6	100.00	0.41	tr_D0A6D7_
DRB1_0101	306	DDEAAADLPNAGDFV	DLPNAGDFV	6	0.1798	7144.4	100.00	0.46	tr_D0A6D7_
DRB1_0101	307	DLPNAGDFVEGASGS	DLPNAGDFV	0	0.1770	7366.2	100.00	0.38	tr_D0A6D7_
DRB1_0101	308	LLSTTRDKLREKGFK	LLSTTRDKL	0	0.1747	7550.3	100.00	0.34	tr_D0A6D7_
DRB1_0101	309	FDQCIVPCDYEENGC	FDQCIVPCD	0	0.1618	8684.3	100.00	0.29	tr_D0A6D7_
DRB1_0101	310	DQCIVPCDYEENGCI	CDYEENGCI	6	0.1587	8976.1	100.00	0.34	tr_D0A6D7_
DRB1_0101	311	SGYGAQVRAEEDKEE	YGAQVRAEE	2	0.1536	9488.1	100.00	0.40	tr_D0A6D7_

DRB1_0101	312	SACGDDEAAADLPNA	GDDEAAADL	3	0.1420	10753.9	100.00	0.40	tr_D0A6D7_
DRB1_0101	313	RPPKGGVDKAMAEAE	VDKAMAEAE	6	0.1412	10847.9	100.00	0.39	tr_D0A6D7_
DRB1_0101	314	GLQRRPPKGGVDKAM	LQRRPPKGG	1	0.1348	11633.8	100.00	0.41	tr_D0A6D7_
DRB1_0101	315	ACGDDEAAADLPNAG	GDDEAAADL	2	0.1348	11634.8	100.00	0.38	tr_D0A6D7_
DRB1_0101	316	EEDKEEEFDQCIVPC	KEEEFDQCI	3	0.1339	11742.7	100.00	0.31	tr_D0A6D7_
DRB1_0101	317	GYGAQVRAEEDKEEE	YGAQVRAEE	1	0.1321	11976.0	100.00	0.35	tr_D0A6D7_
DRB1_0101	318	GDDEAAADLPNAGDF	EAAADLPNA	3	0.1300	12243.8	100.00	0.31	tr_D0A6D7_
DRB1_0101	319	CGDDEAAADLPNAGD	GDDEAAADL	1	0.1298	12273.8	100.00	0.29	tr_D0A6D7_
DRB1_0101	320	AEEDKEEEFDQCIVP	KEEEFDQCI	4	0.1253	12887.5	100.00	0.36	tr_D0A6D7_
DRB1_0101	321	RAEEDKEEEFDQCIV	KEEEFDQCI	5	0.1134	14653.7	100.00	0.40	tr_D0A6D7_
DRB1_0101	322	LQRRPPKGGVDKAMA	LQRRPPKGG	0	0.1093	15328.8	100.00	0.23	tr_D0A6D7_
DRB1_0101	323	YGAQVRAEEDKEEEF	VRAEEDKEE	4	0.1082	15503.1	100.00	0.38	tr_D0A6D7_
DRB1_0101	324	RRPPKGGVDKAMAEA	PKGGVDKAM	3	0.1070	15705.2	100.00	0.35	tr_D0A6D7_
DRB1_0101	325	QRRPPKGGVDKAMAE	PKGGVDKAM	4	0.0906	18770.4	100.00	0.36	tr_D0A6D7_
DRB1_0101	326	GAQVRAEEDKEEEFD	VRAEEDKEE	3	0.0861	19691.4	100.00	0.54	tr_D0A6D7_
DRB1_0101	327	AQVRAEEDKEEEFDQ	VRAEEDKEE	2	0.0813	20748.2	100.00	0.55	tr_D0A6D7_
DRB1_0101	328	VRAEEDKEEEFDQCI	KEEEFDQCI	6	0.0786	21360.5	100.00	0.29	tr_D0A6D7_
DRB1_0101	329	QVRAEEDKEEEFDQC	VRAEEDKEE	1	0.0772	21683.3	100.00	0.41	tr_D0A6D7_

Allele: DRB1_0101. Number of high binders 3. Number of weak binders 18. Number of peptides 329

Allele	pos	peptide	core	Of	1-log50k(aff)	affinity(nM)	%Rank	Relia	Identity	Bind Level
DRB1_0101	1	GHSHGGCESGHGTYS	CESGHGTYS	6	0.1829	6909.3	100.00	0.31	tr_D0A886_	
DRB1_0101	2	HHHTHDHGHHHHHHH	HTHDHGHHH	2	0.1508	9778.4	100.00	0.34	tr_D0A886_	
DRB1_0101	3	HHTHDHGHHHHHHHH	HTHDHGHHH	1	0.1452	10386.7	100.00	0.32	tr_D0A886_	
DRB1_0101	4	HGHSHGGCESGHGTY	GGCESGHGT	5	0.1393	11080.1	100.00	0.39	tr_D0A886_	
DRB1_0101	5	HHHHHHHHDDHTHGA	HHHHHDDHT	3	0.1340	11728.8	100.00	0.26	tr_D0A886_	
DRB1_0101	6	HHHHHHHDDHTHGAD	HHHHHDDHT	2	0.1315	12046.0	100.00	0.25	tr_D0A886_	
DRB1_0101	7	HTHDHGHHHHHHHHD	DHGHHHHHH	3	0.1279	12530.6	100.00	0.21	tr_D0A886_	
DRB1_0101	8	GHHHHHHHHDDHTHG	HHHHHDDHT	4	0.1215	13435.4	100.00	0.24	tr_D0A886_	
DRB1_0101	9	HHHHHHDDHTHGADD	HHHHHDDHT	1	0.1205	13582.2	100.00	0.21	tr_D0A886_	
DRB1_0101	10	GHGHSHGGCESGHGT	GHSHGGCES	2	0.1188	13820.1	100.00	0.27	tr_D0A886_	
DRB1_0101	11	HGHHHHHHHHDDHTH	HHHHHDDHT	5	0.1154	14338.3	100.00	0.25	tr_D0A886_	
DRB1_0101	12	HHHHHDDHTHGADDH	HHDDHTHGA	3	0.1096	15269.7	100.00	0.28	tr_D0A886_	
DRB1_0101	13	THDHGHHHHHHHHDD	DHGHHHHHH	2	0.1090	15369.7	100.00	0.23	tr_D0A886_	
DRB1_0101	14	HDHGHHHHHHHHDDH	GHHHHHHHH	3	0.1051	16044.1	100.00	0.22	tr_D0A886_	
DRB1_0101	15	DHGHHHHHHHHDDHT	HHHHHHHHD	3	0.1039	16238.6	100.00	0.17	tr_D0A886_	
DRB1_0101	16	EGHGHSHGGCESGHG	GHSHGGCES	3	0.1027	16451.4	100.00	0.38	tr_D0A886_	
DRB1_0101	17	DHEGHGHSHGGCESG	GHSHGGCES	5	0.0989	17143.2	100.00	0.28	tr_D0A886_	
DRB1_0101	18	HEGHGHSHGGCESGH	GHSHGGCES	4	0.0975	17404.8	100.00	0.38	tr_D0A886_	
DRB1_0101	19	DDHEGHGHSHGGCES	DHEGHGHSH	1	0.0942	18043.2	100.00	0.21	tr_D0A886_	
DRB1_0101	20	ADDHEGHGHSHGGCE	DHEGHGHSH	2	0.0914	18602.6	100.00	0.28	tr_D0A886_	
DRB1_0101	21	HHHHDDHTHGADDHE	HHDDHTHGA	2	0.0897	18937.5	100.00	0.31	tr_D0A886_	
DRB1_0101	22	HGADDHEGHGHSHGG	DHEGHGHSH	4	0.0889	19108.9	100.00	0.30	tr_D0A886_	
DRB1_0101	23	GADDHEGHGHSHGGC	DHEGHGHSH	3	0.0887	19153.0	100.00	0.30	tr_D0A886_	
DRB1_0101	24	THGADDHEGHGHSHG	DHEGHGHSH	5	0.0865	19606.5	100.00	0.26	tr_D0A886_	

DRB1_0101	25	HTHGADDHEGHGHSH	THGADDHEG	1	0.0811	20801.7	100.00	0.27	tr_D0A886_	
DRB1_0101	26	HHHDDHTHGADDHEG	HHDDHTHGA	1	0.0805	20920.5	100.00	0.33	tr_D0A886_	
DRB1_0101	27	DHTHGADDHEGHGHS	THGADDHEG	2	0.0749	22244.1	100.00	0.31	tr_D0A886_	
DRB1_0101	28	HHDDHTHGADDHEGH	THGADDHEG	5	0.0731	22665.4	100.00	0.20	tr_D0A886_	
DRB1_0101	29	DDHTHGADDHEGHGH	THGADDHEG	3	0.0717	23014.5	100.00	0.31	tr_D0A886_	
DRB1_0101	30	HDDHTHGADDHEGHG	THGADDHEG	4	0.0677	24040.0	100.00	0.33	tr_D0A886_	

Allele: DRB1_0101. Number of high binders 0. Number of weak binders 0. Number of peptides 30

Allele	pos	peptide	core	Of	1-log50k(aff)	affinity(nM)	%Rank	Relia	Identity	Bind Level
DRB1_0101	1	GDTHGGCESGHGTYS	CESGHGTYS	6	0.1719	7781.5	100.00	0.34	tr_D0A889_	
DRB1_0101	2	HHHHHDDHTHGVVDD	HHDDHTHGV	3	0.1661	8286.1	100.00	0.41	tr_D0A889_	
DRB1_0101	3	HHHHDDHTHGVVDDG	HHDDHTHGV	2	0.1499	9879.0	100.00	0.43	tr_D0A889_	
DRB1_0101	4	HHHDDHTHGVVDDGI	HHDDHTHGV	1	0.1326	11905.2	100.00	0.37	tr_D0A889_	
DRB1_0101	5	HTHGVVDDGIDGDTH	GVVDDGIDG	3	0.1296	12303.0	100.00	0.65	tr_D0A889_	
DRB1_0101	6	THGVVDDGIDGDTHG	GVVDDGIDG	2	0.1242	13043.7	100.00	0.68	tr_D0A889_	
DRB1_0101	7	DHTHGVVDDGIDGDT	GVVDDGIDG	4	0.1177	13995.4	100.00	0.62	tr_D0A889_	
DRB1_0101	8	DGDTHGGCESGHGTY	GGCESGHGT	5	0.1154	14339.1	100.00	0.44	tr_D0A889_	
DRB1_0101	9	HGVVDDGIDGDTHGG	GVVDDGIDG	1	0.1119	14903.1	100.00	0.65	tr_D0A889_	
DRB1_0101	10	HHDDHTHGVVDDGID	HHDDHTHGV	0	0.1016	16648.0	100.00	0.31	tr_D0A889_	
DRB1_0101	11	DDHTHGVVDDGIDGD	GVVDDGIDG	5	0.1007	16814.7	100.00	0.55	tr_D0A889_	
DRB1_0101	12	GVVDDGIDGDTHGGC	GVVDDGIDG	0	0.0945	17992.7	100.00	0.50	tr_D0A889_	
DRB1_0101	13	IDGDTHGGCESGHGT	GGCESGHGT	6	0.0922	18435.5	100.00	0.28	tr_D0A889_	
DRB1_0101	14	HDDHTHGVVDDGIDG	GVVDDGIDG	6	0.0911	18653.2	100.00	0.38	tr_D0A889_	
DRB1_0101	15	GIDGDTHGGCESGHG	GDTHGGCES	3	0.0787	21334.2	100.00	0.26	tr_D0A889_	
DRB1_0101	16	DGIDGDTHGGCESGH	IDGDTHGGC	2	0.0724	22849.5	100.00	0.25	tr_D0A889_	
DRB1_0101	17	DDGIDGDTHGGCESG	IDGDTHGGC	3	0.0695	23560.8	100.00	0.24	tr_D0A889_	
DRB1_0101	18	VDDGIDGDTHGGCES	IDGDTHGGC	4	0.0665	24345.5	100.00	0.20	tr_D0A889_	
DRB1_0101	19	VVDDGIDGDTHGGCE	IDGDTHGGC	5	0.0651	24732.8	100.00	0.21	tr_D0A889_	

Allele: DRB1_0101. Number of high binders 0. Number of weak binders 0. Number of peptides 19

Allele	pos	peptide	core	Of	1-log50k(aff)	affinity(nM)	%Rank	Relia	Identity	Bind Level
DRB1_0101	1	SNVFNLESATLGAGI	FNLESATLG	3	0.8265	6.5	1.20	0.71	tr_D0AAF8_	WB
DRB1_0101	2	GAGIVMLPSGFLNSG	IVMLPSGFL	3	0.8207	7.0	1.30	0.86	tr_D0AAF8_	WB
DRB1_0101	3	ASNVFNLESATLGAG	FNLESATLG	4	0.8199	7.0	1.40	0.67	tr_D0AAF8_	WB
DRB1_0101	4	LGAGIVMLPSGFLNS	IVMLPSGFL	4	0.8146	7.4	1.50	0.85	tr_D0AAF8_	WB
DRB1_0101	5	AGIVMLPSGFLNSGI	IVMLPSGFL	2	0.8126	7.6	1.60	0.85	tr_D0AAF8_	WB
DRB1_0101	6	NVFNLESATLGAGIV	FNLESATLG	2	0.8039	8.3	1.90	0.73	tr_D0AAF8_	WB
DRB1_0101	7	MASNVFNLESATLGA	FNLESATLG	5	0.7985	8.8	2.50	0.64	tr_D0AAF8_	WB
DRB1_0101	8	TLGAGIVMLPSGFLN	IVMLPSGFL	5	0.7927	9.4	2.50	0.87	tr_D0AAF8_	WB
DRB1_0101	9	VFNLESATLGAGIVM	FNLESATLG	1	0.7649	12.7	4.00	0.70	tr_D0AAF8_	WB

DRB1_0101	10	ATLGAGIVMLPSGFL	IVMLPSGFL	6	0.7344	17.7	6.00	0.83	tr_D0AAF8_	WB
DRB1_0101	11	GMASNVFNLESATLG	FNLESATLG	6	0.7258	19.4	6.50	0.51	tr_D0AAF8_	WB
DRB1_0101	12	FNLESATLGAGIVML	FNLESATLG	0	0.6831	30.8	11.00	0.56	tr_D0AAF8_	
DRB1_0101	13	VFDPIKGVVPSGGMA	FDPIKGVVP	1	0.5922	82.5	24.00	0.41	tr_D0AAF8_	
DRB1_0101	14	TVFDPIKGVVPSGGM	FDPIKGVVP	2	0.5905	84.0	24.00	0.57	tr_D0AAF8_	
DRB1_0101	15	FDPIKGVVPSGGMAS	IKGVVPSGG	3	0.5799	94.2	26.00	0.54	tr_D0AAF8_	
DRB1_0101	16	NTVFDPIKGVVPSGG	FDPIKGVVP	3	0.5749	99.4	27.00	0.73	tr_D0AAF8_	
DRB1_0101	17	LNTVFDPIKGVVPSG	FDPIKGVVP	4	0.5553	122.9	30.00	0.76	tr_D0AAF8_	
DRB1_0101	18	GGMASNVFNLESATL	VFNLESATL	6	0.5454	136.8	32.00	0.53	tr_D0AAF8_	
DRB1_0101	19	PIKGVVPSGGMASNV	IKGVVPSGG	1	0.5420	141.9	33.00	0.44	tr_D0AAF8_	
DRB1_0101	20	CLNTVFDPIKGVVPS	FDPIKGVVP	5	0.5347	153.6	34.00	0.68	tr_D0AAF8_	
DRB1_0101	21	DPIKGVVPSGGMASN	IKGVVPSGG	2	0.5334	155.8	34.00	0.51	tr_D0AAF8_	
DRB1_0101	22	IKGVVPSGGMASNVF	VVPSGGMAS	3	0.5205	179.1	37.00	0.50	tr_D0AAF8_	
DRB1_0101	23	NLESATLGAGIVMLP	ESATLGAGI	2	0.5141	192.0	38.00	0.39	tr_D0AAF8_	
DRB1_0101	24	LESATLGAGIVMLPS	ESATLGAGI	1	0.4954	235.0	42.00	0.32	tr_D0AAF8_	
DRB1_0101	25	GCLNTVFDPIKGVVP	FDPIKGVVP	6	0.4779	284.0	46.00	0.51	tr_D0AAF8_	
DRB1_0101	26	KGVVPSGGMASNVFN	VVPSGGMAS	2	0.4624	336.0	49.00	0.59	tr_D0AAF8_	
DRB1_0101	27	PTSIPSLSQGNVLEL	PSLSQGNVL	4	0.4566	357.7	50.00	0.41	tr_D0AAF8_	
DRB1_0101	28	GVVPSGGMASNVFNL	VVPSGGMAS	1	0.4553	362.7	50.00	0.38	tr_D0AAF8_	
DRB1_0101	29	PSGGMASNVFNLESA	GMASNVFNL	3	0.4497	385.3	55.00	0.57	tr_D0AAF8_	
DRB1_0101	30	ESATLGAGIVMLPSG	SATLGAGIV	1	0.4495	386.0	55.00	0.29	tr_D0AAF8_	
DRB1_0101	31	VVPSGGMASNVFNLE	GMASNVFNL	5	0.4484	390.8	55.00	0.38	tr_D0AAF8_	
DRB1_0101	32	VPSGGMASNVFNLES	GMASNVFNL	4	0.4431	413.9	55.00	0.53	tr_D0AAF8_	
DRB1_0101	33	TSIPSLSQGNVLELP	PSLSQGNVL	3	0.4325	464.1	55.00	0.41	tr_D0AAF8_	
DRB1_0101	34	SGGMASNVFNLESAT	GMASNVFNL	2	0.4290	482.2	60.00	0.56	tr_D0AAF8_	
DRB1_0101	35	HPTSIPSLSQGNVLE	PSLSQGNVL	5	0.4158	556.3	60.00	0.37	tr_D0AAF8_	
DRB1_0101	36	SATLGAGIVMLPSGF	SATLGAGIV	0	0.4141	566.4	60.00	0.22	tr_D0AAF8_	
DRB1_0101	37	SIPSLSQGNVLELPP	PSLSQGNVL	2	0.4017	648.0	65.00	0.44	tr_D0AAF8_	
DRB1_0101	38	RGCLNTVFDPIKGVV	NTVFDPIKG	4	0.3831	792.5	70.00	0.25	tr_D0AAF8_	
DRB1_0101	39	EHPTSIPSLSQGNVL	PSLSQGNVL	6	0.3754	860.6	70.00	0.28	tr_D0AAF8_	
DRB1_0101	40	IPSLSQGNVLELPPD	PSLSQGNVL	1	0.3580	1038.8	75.00	0.44	tr_D0AAF8_	
DRB1_0101	41	HRGCLNTVFDPIKGV	CLNTVFDPI	3	0.3483	1154.1	75.00	0.32	tr_D0AAF8_	
DRB1_0101	42	SQGNVLELPPDSDKS	NVLELPPDS	3	0.3197	1572.4	80.00	0.56	tr_D0AAF8_	
DRB1_0101	43	EHRGCLNTVFDPIKG	CLNTVFDPI	4	0.3123	1704.6	80.00	0.35	tr_D0AAF8_	
DRB1_0101	44	QGNVLELPPDSDKST	NVLELPPDS	2	0.3104	1738.6	80.00	0.52	tr_D0AAF8_	
DRB1_0101	45	PSLSQGNVLELPPDS	PSLSQGNVL	0	0.3082	1781.3	80.00	0.26	tr_D0AAF8_	
DRB1_0101	46	LSQGNVLELPPDSDK	NVLELPPDS	4	0.2949	2056.7	85.00	0.55	tr_D0AAF8_	
DRB1_0101	47	GEHRGCLNTVFDPIK	CLNTVFDPI	5	0.2843	2307.2	85.00	0.37	tr_D0AAF8_	
DRB1_0101	48	DEHPTSIPSLSQGNV	PTSIPSLSQ	3	0.2795	2430.6	85.00	0.43	tr_D0AAF8_	
DRB1_0101	49	GNVLELPPDSDKSTA	NVLELPPDS	1	0.2793	2434.0	85.00	0.47	tr_D0AAF8_	
DRB1_0101	50	SLSQGNVLELPPDSD	NVLELPPDS	5	0.2668	2788.9	90.00	0.43	tr_D0AAF8_	
DRB1_0101	51	MSPRATEPIINEDEH	PRATEPIIN	2	0.2526	3251.7	90.00	0.60	tr_D0AAF8_	
DRB1_0101	52	ATEPIINEDEHPTSI	IINEDEHPT	4	0.2389	3769.0	95.00	0.74	tr_D0AAF8_	
DRB1_0101	53	TEPIINEDEHPTSIP	IINEDEHPT	3	0.2331	4013.6	95.00	0.69	tr_D0AAF8_	
DRB1_0101	54	DGEHRGCLNTVFDPI	HRGCLNTVF	3	0.2276	4262.9	95.00	0.28	tr_D0AAF8_	
DRB1_0101	55	EDEHPTSIPSLSQGN	PTSIPSLSQ	4	0.2269	4295.4	95.00	0.49	tr_D0AAF8_	
DRB1_0101	56	EPIINEDEHPTSIPS	IINEDEHPT	2	0.2265	4311.3	95.00	0.61	tr_D0AAF8_	
DRB1_0101	57	NVLELPPDSDKSTAA	NVLELPPDS	0	0.2209	4579.0	95.00	0.37	tr_D0AAF8_	
DRB1_0101	58	PIINEDEHPTSIPSL	IINEDEHPT	1	0.2194	4656.4	95.00	0.53	tr_D0AAF8_	
DRB1_0101	59	KSTAAADGEHRGCLN	TAAADGEHR	2	0.2160	4830.7	95.00	0.61	tr_D0AAF8_	

DRB1_0101	60	SPRATEPIINEDEHP	PRATEPIIN	1	0.2131	4986.7	95.00	0.62	tr_D0AAF8_
DRB1_0101	61	NEDEHPTSIPSLSQG	PTSIPSLSQ	5	0.2056	5406.2	95.00	0.47	tr_D0AAF8_
DRB1_0101	62	DKSTAAADGEHRGCL	TAAADGEHR	3	0.2056	5408.5	95.00	0.63	tr_D0AAF8_
DRB1_0101	63	STAAADGEHRGCLNT	TAAADGEHR	1	0.1952	6050.4	95.00	0.52	tr_D0AAF8_
DRB1_0101	64	RATEPIINEDEHPTS	IINEDEHPT	5	0.1902	6383.9	100.00	0.71	tr_D0AAF8_
DRB1_0101	65	AADGEHRGCLNTVFD	HRGCLNTVF	5	0.1893	6447.4	100.00	0.36	tr_D0AAF8_
DRB1_0101	66	SDKSTAAADGEHRGC	TAAADGEHR	4	0.1844	6796.2	100.00	0.55	tr_D0AAF8_
DRB1_0101	67	INEDEHPTSIPSLSQ	PTSIPSLSQ	6	0.1833	6880.7	100.00	0.30	tr_D0AAF8_
DRB1_0101	68	IINEDEHPTSIPSLS	IINEDEHPT	0	0.1824	6946.0	100.00	0.34	tr_D0AAF8_
DRB1_0101	69	PRATEPIINEDEHPT	PRATEPIIN	0	0.1817	7002.3	100.00	0.32	tr_D0AAF8_
DRB1_0101	70	DSDKSTAAADGEHRG	TAAADGEHR	5	0.1794	7176.4	100.00	0.48	tr_D0AAF8_
DRB1_0101	71	ADGEHRGCLNTVFDP	HRGCLNTVF	4	0.1793	7185.7	100.00	0.39	tr_D0AAF8_
DRB1_0101	72	AAADGEHRGCLNTVF	HRGCLNTVF	6	0.1786	7243.8	100.00	0.25	tr_D0AAF8_
DRB1_0101	73	TAAADGEHRGCLNTV	TAAADGEHR	0	0.1740	7605.8	100.00	0.28	tr_D0AAF8_
DRB1_0101	74	PDSDKSTAAADGEHR	DKSTAAADG	3	0.1499	9881.5	100.00	0.28	tr_D0AAF8_
DRB1_0101	75	VLELPPDSDKSTAAA	VLELPPDSD	0	0.1389	11124.3	100.00	0.32	tr_D0AAF8_
DRB1_0101	76	PPDSDKSTAAADGEH	DKSTAAADG	4	0.1170	14102.3	100.00	0.36	tr_D0AAF8_
DRB1_0101	77	LPPDSDKSTAAADGE	DKSTAAADG	5	0.1138	14599.1	100.00	0.28	tr_D0AAF8_
DRB1_0101	78	ELPPDSDKSTAAADG	SDKSTAAAD	5	0.1078	15570.9	100.00	0.21	tr_D0AAF8_
DRB1_0101	79	LELPPDSDKSTAAAD	LPPDSDKST	2	0.1013	16714.2	100.00	0.22	tr_D0AAF8_

Allele: DRB1_0101. Number of high binders 0. Number of weak binders 11. Number of peptides 79

Allele	pos	peptide	core	Of	1-log50k(aff)	affinity(nM)	%Rank	Relia	Identity	Bind Level
DRB1_0101	1	GESFQHAGSMGEVSV	FQHAGSMGE	3	0.4424	417.0	55.00	0.67	tr_C9ZL05_	
DRB1_0101	2	ESFQHAGSMGEVSVG	FQHAGSMGE	2	0.4409	423.7	55.00	0.64	tr_C9ZL05_	
DRB1_0101	3	SFQHAGSMGEVSVGV	FQHAGSMGE	1	0.4322	465.5	55.00	0.48	tr_C9ZL05_	
DRB1_0101	4	PGESFQHAGSMGEVS	FQHAGSMGE	4	0.4034	636.0	65.00	0.69	tr_C9ZL05_	
DRB1_0101	5	SMGEVSVGVPRRSTR	VSVGVPRRS	4	0.4009	653.6	65.00	0.48	tr_C9ZL05_	
DRB1_0101	6	GSMGEVSVGVPRRST	VSVGVPRRS	5	0.3973	679.6	65.00	0.35	tr_C9ZL05_	
DRB1_0101	7	MGEVSVGVPRRSTRN	VSVGVPRRS	3	0.3936	707.3	65.00	0.56	tr_C9ZL05_	
DRB1_0101	8	AGSMGEVSVGVPRRS	SMGEVSVGV	2	0.3816	805.3	70.00	0.37	tr_C9ZL05_	
DRB1_0101	9	FQHAGSMGEVSVGVP	FQHAGSMGE	0	0.3699	913.7	70.00	0.27	tr_C9ZL05_	
DRB1_0101	10	HAGSMGEVSVGVPRR	SMGEVSVGV	3	0.3670	942.8	70.00	0.47	tr_C9ZL05_	
DRB1_0101	11	PPGESFQHAGSMGEV	FQHAGSMGE	5	0.3574	1045.6	75.00	0.63	tr_C9ZL05_	
DRB1_0101	12	GEVSVGVPRRSTRNS	VSVGVPRRS	2	0.3534	1092.5	75.00	0.60	tr_C9ZL05_	
DRB1_0101	13	QHAGSMGEVSVGVPR	SMGEVSVGV	4	0.3428	1225.6	75.00	0.41	tr_C9ZL05_	
DRB1_0101	14	EVSVGVPRRSTRNSR	VSVGVPRRS	1	0.3040	1863.2	85.00	0.69	tr_C9ZL05_	
DRB1_0101	15	MDTDRSVRNWGAGHG	DRSVRNWGA	3	0.2908	2149.4	85.00	0.57	tr_C9ZL05_	
DRB1_0101	16	RMDTDRSVRNWGAGH	DRSVRNWGA	4	0.2824	2354.1	85.00	0.58	tr_C9ZL05_	
DRB1_0101	17	SRMDTDRSVRNWGAG	DRSVRNWGA	5	0.2761	2520.1	85.00	0.50	tr_C9ZL05_	
DRB1_0101	18	HPPGESFQHAGSMGE	FQHAGSMGE	6	0.2665	2798.0	90.00	0.50	tr_C9ZL05_	
DRB1_0101	19	NSRMDTDRSVRNWGA	MDTDRSVRN	3	0.2664	2801.0	90.00	0.41	tr_C9ZL05_	
DRB1_0101	20	TRNSRMDTDRSVRNW	SRMDTDRSV	3	0.2549	3172.8	90.00	0.41	tr_C9ZL05_	
DRB1_0101	21	VSVGVPRRSTRNSRM	VSVGVPRRS	0	0.2535	3218.0	90.00	0.41	tr_C9ZL05_	
DRB1_0101	22	RNSRMDTDRSVRNWG	SRMDTDRSV	2	0.2512	3299.5	90.00	0.35	tr_C9ZL05_	

DRB1_0101	23	STRNSRMDTDRSVRN	SRMDTDRSV	4	0.2334	4002.0	95.00	0.44	tr_C9ZL05_	
DRB1_0101	24	RSTRNSRMDTDRSVR	SRMDTDRSV	5	0.2007	5699.7	95.00	0.44	tr_C9ZL05_	
DRB1_0101	25	VGVPRRSTRNSRMDT	RRSTRNSRM	4	0.1930	6197.8	100.00	0.56	tr_C9ZL05_	
DRB1_0101	26	SVGVPRRSTRNSRMD	RRSTRNSRM	5	0.1898	6413.0	100.00	0.43	tr_C9ZL05_	
DRB1_0101	27	DHPPGESFQHAGSMG	PPGESFQHA	2	0.1870	6610.2	100.00	0.29	tr_C9ZL05_	
DRB1_0101	28	GVPRRSTRNSRMDTD	RRSTRNSRM	3	0.1779	7291.9	100.00	0.57	tr_C9ZL05_	
DRB1_0101	29	VPRRSTRNSRMDTDR	RRSTRNSRM	2	0.1669	8214.1	100.00	0.58	tr_C9ZL05_	
DRB1_0101	30	RRSTRNSRMDTDRSV	NSRMDTDRS	5	0.1629	8576.0	100.00	0.29	tr_C9ZL05_	
DRB1_0101	31	NDHPPGESFQHAGSM	PPGESFQHA	3	0.1586	8984.5	100.00	0.38	tr_C9ZL05_	
DRB1_0101	32	PRRSTRNSRMDTDRS	RRSTRNSRM	1	0.1541	9436.6	100.00	0.45	tr_C9ZL05_	
DRB1_0101	33	ANDHPPGESFQHAGS	PPGESFQHA	4	0.1280	12521.2	100.00	0.42	tr_C9ZL05_	
DRB1_0101	34	RANDHPPGESFQHAG	PPGESFQHA	5	0.1200	13647.7	100.00	0.37	tr_C9ZL05_	
DRB1_0101	35	ARANDHPPGESFQHA	PPGESFQHA	6	0.1082	15514.2	100.00	0.25	tr_C9ZL05_	
DRB1_0101	36	EARANDHPPGESFQH	NDHPPGESF	4	0.0842	20105.8	100.00	0.28	tr_C9ZL05_	

Allele: DRB1_0101. Number of high binders 0. Number of weak binders 0. Number of peptides 36

Allele	pos	peptide	core	Of	1-log50k(aff)	affinity(nM)	%Rank	Relia	Identity	Bind Level
DRB1_0101	1	VRDLLYVRPRMLRLG	LLYVRPRML	3	0.7967	9.0	2.50	0.86	tr_C9ZL05_	WB
DRB1_0101	2	NVRDLLYVRPRMLRL	LLYVRPRML	4	0.7886	9.8	2.50	0.88	tr_C9ZL05_	WB
DRB1_0101	3	RDLLYVRPRMLRLGD	LLYVRPRML	2	0.7851	10.2	3.00	0.85	tr_C9ZL05_	WB
DRB1_0101	4	RPRMLRLGDSYLTEK	MLRLGDSYL	3	0.7777	11.1	3.50	0.61	tr_C9ZL05_	WB
DRB1_0101	5	ENVRDLLYVRPRMLR	LLYVRPRML	5	0.7561	14.0	4.50	0.89	tr_C9ZL05_	WB
DRB1_0101	6	PRMLRLGDSYLTEKP	MLRLGDSYL	2	0.7509	14.8	5.00	0.54	tr_C9ZL05_	WB
DRB1_0101	7	VRPRMLRLGDSYLTE	MLRLGDSYL	4	0.7419	16.3	5.50	0.62	tr_C9ZL05_	WB
DRB1_0101	8	DLLYVRPRMLRLGDS	LLYVRPRML	1	0.7391	16.8	5.50	0.81	tr_C9ZL05_	WB
DRB1_0101	9	RMLRLGDSYLTEKPP	MLRLGDSYL	1	0.7288	18.8	6.50	0.48	tr_C9ZL05_	WB
DRB1_0101	10	YVRPRMLRLGDSYLT	MLRLGDSYL	5	0.7092	23.3	8.00	0.68	tr_C9ZL05_	WB
DRB1_0101	11	TENVRDLLYVRPRML	LLYVRPRML	6	0.6839	30.6	11.00	0.86	tr_C9ZL05_	
DRB1_0101	12	LLYVRPRMLRLGDSY	LLYVRPRML	0	0.6673	36.6	13.00	0.64	tr_C9ZL05_	
DRB1_0101	13	PWCLYEVMKTTFFAV	YEVMKTTFF	4	0.6423	48.0	16.00	0.62	tr_C9ZL05_	
DRB1_0101	14	WCLYEVMKTTFFAVT	YEVMKTTFF	3	0.6405	48.9	16.00	0.66	tr_C9ZL05_	
DRB1_0101	15	CLYEVMKTTFFAVTG	YEVMKTTFF	2	0.6293	55.2	18.00	0.71	tr_C9ZL05_	
DRB1_0101	16	MLRLGDSYLTEKPPR	LRLGDSYLT	1	0.6289	55.4	18.00	0.55	tr_C9ZL05_	
DRB1_0101	17	TPWCLYEVMKTTFFA	YEVMKTTFF	5	0.6215	60.1	19.00	0.60	tr_C9ZL05_	
DRB1_0101	18	LGDSYLTEKPPRVGR	YLTEKPPRV	4	0.6096	68.3	21.00	0.89	tr_C9ZL05_	
DRB1_0101	19	GDSYLTEKPPRVGRD	YLTEKPPRV	3	0.6053	71.5	22.00	0.90	tr_C9ZL05_	
DRB1_0101	20	LYVRPRMLRLGDSYL	MLRLGDSYL	6	0.5960	79.1	23.00	0.62	tr_C9ZL05_	
DRB1_0101	21	DSYLTEKPPRVGRDV	YLTEKPPRV	2	0.5927	82.0	24.00	0.90	tr_C9ZL05_	
DRB1_0101	22	SYLTEKPPRVGRDVA	YLTEKPPRV	1	0.5598	117.0	29.00	0.90	tr_C9ZL05_	
DRB1_0101	23	DTPWCLYEVMKTTFF	YEVMKTTFF	6	0.5592	117.8	30.00	0.50	tr_C9ZL05_	
DRB1_0101	24	LRLGDSYLTEKPPRV	YLTEKPPRV	6	0.5540	124.6	30.00	0.45	tr_C9ZL05_	
DRB1_0101	25	RLGDSYLTEKPPRVG	YLTEKPPRV	5	0.5477	133.5	32.00	0.88	tr_C9ZL05_	
DRB1_0101	26	YDTPWCLYEVMKTTF	WCLYEVMKT	4	0.4201	531.0	60.00	0.47	tr_C9ZL05_	
DRB1_0101	27	YLTEKPPRVGRDVAL	YLTEKPPRV	0	0.4140	566.7	60.00	0.71	tr_C9ZL05_	
DRB1_0101	28	DVALSYDTPWCLYEV	LSYDTPWCL	3	0.3906	730.5	65.00	0.64	tr_C9ZL05_	

DRB1_0101	29	VALSYDTPWCLYEVM	LSYDTPWCL	2	0.3859	768.8	65.00	0.64	tr_C9ZL05_	
DRB1_0101	30	SYDTPWCLYEVMKTT	WCLYEVMKT	5	0.3839	785.5	70.00	0.47	tr_C9ZL05_	
DRB1_0101	31	RDVALSYDTPWCLYE	LSYDTPWCL	4	0.3740	873.8	70.00	0.60	tr_C9ZL05_	
DRB1_0101	32	ALSYDTPWCLYEVMK	LSYDTPWCL	1	0.3634	980.1	70.00	0.52	tr_C9ZL05_	
DRB1_0101	33	LSYDTPWCLYEVMKT	WCLYEVMKT	6	0.3633	981.8	70.00	0.37	tr_C9ZL05_	
DRB1_0101	34	GRDVALSYDTPWCLY	LSYDTPWCL	5	0.3565	1056.1	75.00	0.57	tr_C9ZL05_	
DRB1_0101	35	VGRDVALSYDTPWCL	LSYDTPWCL	6	0.2785	2455.2	85.00	0.44	tr_C9ZL05_	
DRB1_0101	36	KPPRVGRDVALSYDT	PRVGRDVAL	2	0.2600	3001.7	90.00	0.37	tr_C9ZL05_	
DRB1_0101	37	EKPPRVGRDVALSYD	PRVGRDVAL	3	0.2466	3468.7	90.00	0.41	tr_C9ZL05_	
DRB1_0101	38	PPRVGRDVALSYDTP	VGRDVALSY	3	0.2415	3663.9	90.00	0.27	tr_C9ZL05_	
DRB1_0101	39	TEKPPRVGRDVALSY	PRVGRDVAL	4	0.2385	3788.1	95.00	0.44	tr_C9ZL05_	
DRB1_0101	40	PRVGRDVALSYDTPW	RDVALSYDT	4	0.2312	4100.2	95.00	0.23	tr_C9ZL05_	
DRB1_0101	41	LTEKPPRVGRDVALS	PRVGRDVAL	5	0.2092	5197.2	95.00	0.41	tr_C9ZL05_	
DRB1_0101	42	RVGRDVALSYDTPWC	RDVALSYDT	3	0.2009	5689.8	95.00	0.29	tr_C9ZL05_	

Allele: DRB1_0101. Number of high binders 0. Number of weak binders 10. Number of peptides 42

Allele	pos	peptide	core	Of	1-log50k(aff)	affinity(nM)	%Rank	Relia	Identity	Bind Level
DRB1_0101	1	TGGNKKTEEAEKANS	KKTEEAEKA	4	0.1655	8343.7	100.00	0.67	tr_D0A070_	
DRB1_0101	2	ETGGNKKTEEAEKAN	KKTEEAEKA	5	0.1364	11434.5	100.00	0.64	tr_D0A070_	
DRB1_0101	3	REASAPHSNADSEGS	SAPHSNADS	3	0.1061	15858.9	100.00	0.35	tr_D0A070_	
DRB1_0101	4	SETGGNKKTEEAEKA	KKTEEAEKA	6	0.1019	16594.8	100.00	0.44	tr_D0A070_	
DRB1_0101	5	EASAPHSNADSEGSE	SAPHSNADS	2	0.0837	20212.2	100.00	0.37	tr_D0A070_	
DRB1_0101	6	SAPHSNADSEGSETG	HSNADSEGS	3	0.0792	21227.1	100.00	0.44	tr_D0A070_	
DRB1_0101	7	ASAPHSNADSEGSET	HSNADSEGS	4	0.0780	21509.9	100.00	0.41	tr_D0A070_	
DRB1_0101	8	HSNADSEGSETGGNK	ADSEGSETG	3	0.0755	22088.9	100.00	0.35	tr_D0A070_	
DRB1_0101	9	APHSNADSEGSETGG	HSNADSEGS	2	0.0752	22166.5	100.00	0.40	tr_D0A070_	
DRB1_0101	10	PHSNADSEGSETGGN	ADSEGSETG	4	0.0748	22263.6	100.00	0.24	tr_D0A070_	
DRB1_0101	11	SEGSETGGNKKTEEA	SETGGNKKT	3	0.0709	23207.3	100.00	0.43	tr_D0A070_	
DRB1_0101	12	GSETGGNKKTEEAEK	GGNKKTEEA	4	0.0704	23349.1	100.00	0.26	tr_D0A070_	
DRB1_0101	13	EGSETGGNKKTEEAE	SETGGNKKT	2	0.0693	23612.3	100.00	0.38	tr_D0A070_	
DRB1_0101	14	NADSEGSETGGNKKT	ADSEGSETG	1	0.0684	23850.1	100.00	0.30	tr_D0A070_	
DRB1_0101	15	ADSEGSETGGNKKTE	SETGGNKKT	5	0.0684	23863.0	100.00	0.32	tr_D0A070_	
DRB1_0101	16	SNADSEGSETGGNKK	ADSEGSETG	2	0.0681	23927.9	100.00	0.38	tr_D0A070_	
DRB1_0101	17	DSEGSETGGNKKTEE	SETGGNKKT	4	0.0619	25592.5	100.00	0.41	tr_D0A070_	

Allele: DRB1_0101. Number of high binders 0. Number of weak binders 0. Number of peptides 17

Allele	pos	peptide	core	Of	1-log50k(aff)	affinity(nM)	%Rank	Relia	Identity	Bind Level
DRB1_0101	1	ISLLSCLGAQAVQTL	LSCLGAQAV	3	0.8181	7.2	1.40	0.56	tr_D0A3E0_	WB
DRB1_0101	2	DTTIAPLAATLGDST	IAPLAATLG	3	0.8144	7.5	1.50	0.88	tr_D0A3E0_	WB
DRB1_0101	3	HDTTIAPLAATLGDS	IAPLAATLG	4	0.8096	7.8	1.70	0.86	tr_D0A3E0_	WB

DRB1_0101	4	TTIAPLAATLGDSTT	IAPLAATLG	2	0.8067	8.1	1.80	0.87	tr_D0A3E0_	WB
DRB1_0101	5	SLLSCLGAQAVQTLH	LSCLGAQAV	2	0.8063	8.1	1.80	0.56	tr_D0A3E0_	WB
DRB1_0101	6	LISLLSCLGAQAVQT	LSCLGAQAV	4	0.8044	8.3	1.90	0.55	tr_D0A3E0_	WB
DRB1_0101	7	ENHMNGLTSYKLYHY	MNGLTSYKL	3	0.7938	9.3	2.50	0.88	tr_D0A3E0_	WB
DRB1_0101	8	LLISLLSCLGAQAVQ	LSCLGAQAV	5	0.7872	10.0	3.00	0.51	tr_D0A3E0_	WB
DRB1_0101	9	AHDTTIAPLAATLGD	IAPLAATLG	5	0.7853	10.2	3.00	0.81	tr_D0A3E0_	WB
DRB1_0101	10	NHMNGLTSYKLYHYS	MNGLTSYKL	2	0.7822	10.6	3.00	0.90	tr_D0A3E0_	WB
DRB1_0101	11	PCGYLNAAGKMMLIN	YLNAAGKMM	3	0.7802	10.8	3.00	0.63	tr_D0A3E0_	WB
DRB1_0101	12	LLSCLGAQAVQTLHL	LSCLGAQAV	1	0.7802	10.8	3.00	0.56	tr_D0A3E0_	WB
DRB1_0101	13	AENHMNGLTSYKLYH	MNGLTSYKL	4	0.7765	11.2	3.50	0.85	tr_D0A3E0_	WB
DRB1_0101	14	CGYLNAAGKMMLINA	YLNAAGKMM	2	0.7699	12.1	3.50	0.63	tr_D0A3E0_	WB
DRB1_0101	15	VPCGYLNAAGKMMLI	YLNAAGKMM	4	0.7673	12.4	4.00	0.59	tr_D0A3E0_	WB
DRB1_0101	16	CLLISLLSCLGAQAV	LSCLGAQAV	6	0.7533	14.4	4.50	0.38	tr_D0A3E0_	WB
DRB1_0101	17	TIAPLAATLGDSTTT	IAPLAATLG	1	0.7446	15.9	5.50	0.85	tr_D0A3E0_	WB
DRB1_0101	18	NAENHMNGLTSYKLY	MNGLTSYKL	5	0.7445	15.9	5.50	0.81	tr_D0A3E0_	WB
DRB1_0101	19	HMNGLTSYKLYHYSA	MNGLTSYKL	1	0.7352	17.6	6.00	0.88	tr_D0A3E0_	WB
DRB1_0101	20	LSCLGAQAVQTLHLV	LSCLGAQAV	0	0.7348	17.6	6.00	0.41	tr_D0A3E0_	WB
DRB1_0101	21	TPPYGQLYAFELLYD	YGQLYAFEL	3	0.7345	17.7	6.00	0.88	tr_D0A3E0_	WB
DRB1_0101	22	ITPPYGQLYAFELLY	YGQLYAFEL	4	0.7337	17.8	6.00	0.89	tr_D0A3E0_	WB
DRB1_0101	23	ATLFDIGVAMQSDGR	FDIGVAMQS	3	0.7278	19.0	6.50	0.84	tr_D0A3E0_	WB
DRB1_0101	24	SAHDTTIAPLAATLG	IAPLAATLG	6	0.7260	19.4	6.50	0.73	tr_D0A3E0_	WB
DRB1_0101	25	GYLNAAGKMMLINAG	YLNAAGKMM	1	0.7257	19.5	6.50	0.67	tr_D0A3E0_	WB
DRB1_0101	26	EVPCGYLNAAGKMML	YLNAAGKMM	5	0.7247	19.7	7.00	0.56	tr_D0A3E0_	WB
DRB1_0101	27	PPYGQLYAFELLYDH	YGQLYAFEL	2	0.7200	20.7	7.00	0.82	tr_D0A3E0_	WB
DRB1_0101	28	MPYACLHGGSLCLLI	CLHGGSLCL	4	0.7153	21.8	7.50	0.41	tr_D0A3E0_	WB
DRB1_0101	29	PYACLHGGSLCLLIS	CLHGGSLCL	3	0.7124	22.5	8.00	0.46	tr_D0A3E0_	WB
DRB1_0101	30	TLFDIGVAMQSDGRI	FDIGVAMQS	2	0.7070	23.8	8.50	0.81	tr_D0A3E0_	WB
DRB1_0101	31	LLRYDVIPQAYAFSH	YDVIPQAYA	3	0.7059	24.1	8.50	0.76	tr_D0A3E0_	WB
DRB1_0101	32	AATLFDIGVAMQSDG	FDIGVAMQS	4	0.7058	24.1	8.50	0.79	tr_D0A3E0_	WB
DRB1_0101	33	LRYDVIPQAYAFSHL	YDVIPQAYA	2	0.7011	25.4	9.00	0.74	tr_D0A3E0_	WB
DRB1_0101	34	AMPYACLHGGSLCLL	YACLHGGSL	3	0.7008	25.5	9.00	0.53	tr_D0A3E0_	WB
DRB1_0101	35	RTHAKMGSLGQHLAQ	KMGSLGQHL	4	0.6997	25.8	9.00	0.67	tr_D0A3E0_	WB
DRB1_0101	36	THAKMGSLGQHLAQE	KMGSLGQHL	3	0.6973	26.4	9.50	0.67	tr_D0A3E0_	WB
DRB1_0101	37	GITPPYGQLYAFELL	YGQLYAFEL	5	0.6961	26.8	9.50	0.85	tr_D0A3E0_	WB
DRB1_0101	38	PYGQLYAFELLYDHE	YGQLYAFEL	1	0.6942	27.3	9.50	0.76	tr_D0A3E0_	WB
DRB1_0101	39	GCLLRGMFPNASEFF	LRGMFPNAS	3	0.6935	27.6	9.50	0.75	tr_D0A3E0_	WB
DRB1_0101	40	HAKMGSLGQHLAQEI	KMGSLGQHL	2	0.6905	28.5	10.00	0.60	tr_D0A3E0_	
DRB1_0101	41	WLLRYDVIPQAYAFS	YDVIPQAYA	4	0.6891	28.9	10.00	0.74	tr_D0A3E0_	
DRB1_0101	42	AGCLLRGMFPNASEF	LRGMFPNAS	4	0.6847	30.3	11.00	0.71	tr_D0A3E0_	
DRB1_0101	43	CAATLFDIGVAMQSD	FDIGVAMQS	5	0.6837	30.7	11.00	0.75	tr_D0A3E0_	
DRB1_0101	44	YACLHGGSLCLLISL	CLHGGSLCL	2	0.6818	31.3	11.00	0.51	tr_D0A3E0_	
DRB1_0101	45	AKMGSLGQHLAQEIL	KMGSLGQHL	1	0.6748	33.7	12.00	0.50	tr_D0A3E0_	
DRB1_0101	46	RYDVIPQAYAFSHLD	YDVIPQAYA	1	0.6726	34.5	12.00	0.71	tr_D0A3E0_	
DRB1_0101	47	GKMMLINAGEFLRNH	MLINAGEFL	3	0.6716	34.9	12.00	0.55	tr_D0A3E0_	
DRB1_0101	48	DWLLRYDVIPQAYAF	YDVIPQAYA	5	0.6690	35.9	13.00	0.74	tr_D0A3E0_	
DRB1_0101	49	LFDIGVAMQSDGRID	FDIGVAMQS	1	0.6674	36.5	13.00	0.78	tr_D0A3E0_	
DRB1_0101	50	YGQLYAFELLYDHEV	YGQLYAFEL	0	0.6659	37.1	13.00	0.46	tr_D0A3E0_	
DRB1_0101	51	LLYDHEVKGYIIRIR	DHEVKGYII	3	0.6658	37.2	13.00	0.56	tr_D0A3E0_	
DRB1_0101	52	CKVFRRVCPAWSCEE	FRRVCPAWS	3	0.6638	38.0	13.00	0.80	tr_D0A3E0_	
DRB1_0101	53	CLLRGMFPNASEFFP	LRGMFPNAS	2	0.6621	38.7	14.00	0.82	tr_D0A3E0_	

DRB1_0101	54	QCKVFRRVCPAWSCE	FRRVCPAWS	4	0.6616	38.9	14.00	0.79	tr_D0A3E0_
DRB1_0101	55	KMMLINAGEFLRNHY	MLINAGEFL	2	0.6615	38.9	14.00	0.50	tr_D0A3E0_
DRB1_0101	56	SAGCLLRGMFPNASE	LRGMFPNAS	5	0.6599	39.6	14.00	0.59	tr_D0A3E0_
DRB1_0101	57	LYAFELLYDHEVKGY	FELLYDHEV	3	0.6594	39.8	14.00	0.82	tr_D0A3E0_
DRB1_0101	58	FELLYDHEVKGYIIR	DHEVKGYII	5	0.6575	40.7	14.00	0.37	tr_D0A3E0_
DRB1_0101	59	AFELLYDHEVKGYII	FELLYDHEV	1	0.6566	41.1	14.00	0.57	tr_D0A3E0_
DRB1_0101	60	LCLLISLLSCLGAQA	LLSCLGAQA	6	0.6561	41.3	14.00	0.32	tr_D0A3E0_
DRB1_0101	61	AGKMMLINAGEFLRN	MLINAGEFL	4	0.6543	42.1	14.00	0.52	tr_D0A3E0_
DRB1_0101	62	DRTHAKMGSLGQHLA	KMGSLGQHL	5	0.6537	42.4	15.00	0.64	tr_D0A3E0_
DRB1_0101	63	KGYIIRIRRGAPGQK	IRIRRGAPG	4	0.6514	43.5	15.00	0.68	tr_D0A3E0_
DRB1_0101	64	KVFRRVCPAWSCEEG	FRRVCPAWS	2	0.6505	43.9	15.00	0.81	tr_D0A3E0_
DRB1_0101	65	ELLYDHEVKGYIIRI	DHEVKGYII	4	0.6466	45.8	16.00	0.54	tr_D0A3E0_
DRB1_0101	66	LYDHEVKGYIIRIRR	DHEVKGYII	2	0.6458	46.2	16.00	0.56	tr_D0A3E0_
DRB1_0101	67	KQCKVFRRVCPAWSC	FRRVCPAWS	5	0.6451	46.5	16.00	0.76	tr_D0A3E0_
DRB1_0101	68	KNAENHMNGLTSYKL	MNGLTSYKL	6	0.6445	46.8	16.00	0.63	tr_D0A3E0_
DRB1_0101	69	MNGLTSYKLYHYSAH	MNGLTSYKL	0	0.6440	47.1	16.00	0.73	tr_D0A3E0_
DRB1_0101	70	YAFELLYDHEVKGYI	FELLYDHEV	2	0.6434	47.4	16.00	0.77	tr_D0A3E0_
DRB1_0101	71	QLYAFELLYDHEVKG	FELLYDHEV	4	0.6410	48.7	16.00	0.81	tr_D0A3E0_
DRB1_0101	72	EAMPYACLHGGSLCL	YACLHGGSL	4	0.6400	49.2	16.00	0.61	tr_D0A3E0_
DRB1_0101	73	IAPLAATLGDSTTTG	IAPLAATLG	0	0.6396	49.4	16.00	0.74	tr_D0A3E0_
DRB1_0101	74	SCLGAQAVQTLHLVQ	CLGAQAVQT	1	0.6367	51.0	17.00	0.35	tr_D0A3E0_
DRB1_0101	75	GQLYAFELLYDHEVK	FELLYDHEV	5	0.6334	52.8	17.00	0.76	tr_D0A3E0_
DRB1_0101	76	VPRTLQSAGCLLRGM	TLQSAGCLL	3	0.6324	53.4	17.00	0.46	tr_D0A3E0_
DRB1_0101	77	GYIIRIRRGAPGQKP	IRIRRGAPG	3	0.6299	54.9	18.00	0.69	tr_D0A3E0_
DRB1_0101	78	HCAATLFDIGVAMQS	FDIGVAMQS	6	0.6284	55.7	18.00	0.65	tr_D0A3E0_
DRB1_0101	79	LLRGMFPNASEFFPA	LRGMFPNAS	1	0.6246	58.0	19.00	0.80	tr_D0A3E0_
DRB1_0101	80	GEVPCGYLNAAGKMM	YLNAAGKMM	6	0.6235	58.8	19.00	0.46	tr_D0A3E0_
DRB1_0101	81	YLNAAGKMMLINAGE	YLNAAGKMM	0	0.6217	59.9	19.00	0.55	tr_D0A3E0_
DRB1_0101	82	KMGSLGQHLAQEILK	LGQHLAQEI	4	0.6167	63.2	20.00	0.29	tr_D0A3E0_
DRB1_0101	83	QSAGCLLRGMFPNAS	LRGMFPNAS	6	0.6163	63.5	20.00	0.44	tr_D0A3E0_
DRB1_0101	84	PRTLQSAGCLLRGMF	TLQSAGCLL	2	0.6155	64.1	20.00	0.47	tr_D0A3E0_
DRB1_0101	85	MMLINAGEFLRNHYN	MLINAGEFL	1	0.6136	65.4	20.00	0.47	tr_D0A3E0_
DRB1_0101	86	YIIRIRRGAPGQKPE	IRIRRGAPG	2	0.6131	65.8	20.00	0.70	tr_D0A3E0_
DRB1_0101	87	DVPRTLQSAGCLLRG	TLQSAGCLL	4	0.6127	66.1	20.00	0.49	tr_D0A3E0_
DRB1_0101	88	AAGKMMLINAGEFLR	MLINAGEFL	5	0.6118	66.7	21.00	0.52	tr_D0A3E0_
DRB1_0101	89	YDHEVKGYIIRIRRG	DHEVKGYII	1	0.6115	66.9	21.00	0.53	tr_D0A3E0_
DRB1_0101	90	CLGAQAVQTLHLVQL	AQAVQTLHL	3	0.6094	68.5	21.00	0.24	tr_D0A3E0_
DRB1_0101	91	TDWLLRYDVIPQAYA	YDVIPQAYA	6	0.6070	70.2	21.00	0.64	tr_D0A3E0_
DRB1_0101	92	VKGYIIRIRRGAPGQ	IRIRRGAPG	5	0.6058	71.2	22.00	0.62	tr_D0A3E0_
DRB1_0101	93	SLCLLISLLSCLGAQ	LLISLLSCL	3	0.6042	72.5	22.00	0.28	tr_D0A3E0_
DRB1_0101	94	HRFVYNASDRTHAKM	FVYNASDRT	2	0.6028	73.6	22.00	0.60	tr_D0A3E0_
DRB1_0101	95	SYKLYHYSAHDTTIA	LYHYSAHDT	3	0.6028	73.6	22.00	0.34	tr_D0A3E0_
DRB1_0101	96	SHRFVYNASDRTHAK	FVYNASDRT	3	0.5997	76.0	23.00	0.67	tr_D0A3E0_
DRB1_0101	97	ACLHGGSLCLLISLL	CLHGGSLCL	1	0.5968	78.5	23.00	0.57	tr_D0A3E0_
DRB1_0101	98	ASEFFPAIHTADVST	FPAIHTADV	4	0.5949	80.1	23.00	0.33	tr_D0A3E0_
DRB1_0101	99	YKLYHYSAHDTTIAP	LYHYSAHDT	2	0.5947	80.3	23.00	0.31	tr_D0A3E0_
DRB1_0101	100	FDIGVAMQSDGRIDK	FDIGVAMQS	0	0.5945	80.4	23.00	0.58	tr_D0A3E0_
DRB1_0101	101	AQAVQTLHLVQLVHR	VQTLHLVQL	3	0.5902	84.3	24.00	0.28	tr_D0A3E0_
DRB1_0101	102	NKQCKVFRRVCPAWS	FRRVCPAWS	6	0.5866	87.6	25.00	0.61	tr_D0A3E0_
DRB1_0101	103	KLYHYSAHDTTIAPL	YSAHDTTIA	4	0.5866	87.6	25.00	0.36	tr_D0A3E0_

DRB1_0101	104	NASEFFPAIHTADVS	SEFFPAIHT	2	0.5859	88.2	25.00	0.38	tr_D0A3E0_
DRB1_0101	105	TGITPPYGQLYAFEL	YGQLYAFEL	6	0.5853	88.9	25.00	0.82	tr_D0A3E0_
DRB1_0101	106	RTLQSAGCLLRGMFP	TLQSAGCLL	1	0.5849	89.2	25.00	0.42	tr_D0A3E0_
DRB1_0101	107	QAVQTLHLVQLVHRH	LHLVQLVHR	5	0.5833	90.8	25.00	0.31	tr_D0A3E0_
DRB1_0101	108	VFRRVCPAWSCEEGY	FRRVCPAWS	1	0.5817	92.3	26.00	0.80	tr_D0A3E0_
DRB1_0101	109	RFVYNASDRTHAKMG	FVYNASDRT	1	0.5816	92.5	26.00	0.51	tr_D0A3E0_
DRB1_0101	110	YDVIPQAYAFSHLDE	YDVIPQAYA	0	0.5809	93.2	26.00	0.60	tr_D0A3E0_
DRB1_0101	111	TDVPRTLQSAGCLLR	TLQSAGCLL	5	0.5786	95.6	26.00	0.47	tr_D0A3E0_
DRB1_0101	112	LGAQAVQTLHLVQLV	AQAVQTLHL	2	0.5751	99.2	27.00	0.31	tr_D0A3E0_
DRB1_0101	113	QTLHLVQLVHRHGAR	LHLVQLVHR	2	0.5717	103.0	27.00	0.31	tr_D0A3E0_
DRB1_0101	114	GSLCLLISLLSCLGA	LLISLLSCL	4	0.5713	103.4	27.00	0.31	tr_D0A3E0_
DRB1_0101	115	VQTLHLVQLVHRHGA	LHLVQLVHR	3	0.5700	104.8	28.00	0.36	tr_D0A3E0_
DRB1_0101	116	TLHLVQLVHRHGARS	LVQLVHRHG	3	0.5682	106.9	28.00	0.28	tr_D0A3E0_
DRB1_0101	117	EHWWRNVCNPKLDTL	WRNVCNPKL	3	0.5661	109.4	28.00	0.86	tr_D0A3E0_
DRB1_0101	118	IIRIRRGAPGQKPEE	IRIRRGAPG	1	0.5639	112.0	29.00	0.66	tr_D0A3E0_
DRB1_0101	119	DHEVKGYIIRIRRGA	DHEVKGYII	0	0.5634	112.6	29.00	0.35	tr_D0A3E0_
DRB1_0101	120	GAQAVQTLHLVQLVH	AQAVQTLHL	1	0.5631	113.0	29.00	0.27	tr_D0A3E0_
DRB1_0101	121	AVQTLHLVQLVHRHG	LHLVQLVHR	4	0.5630	113.1	29.00	0.32	tr_D0A3E0_
DRB1_0101	122	SEFFPAIHTADVSTD	FPAIHTADV	3	0.5615	114.9	29.00	0.38	tr_D0A3E0_
DRB1_0101	123	HWWRNVCNPKLDTLI	WRNVCNPKL	2	0.5606	116.1	29.00	0.83	tr_D0A3E0_
DRB1_0101	124	LYHYSAHDTTIAPLA	YSAHDTTIA	3	0.5587	118.5	30.00	0.48	tr_D0A3E0_
DRB1_0101	125	PNASEFFPAIHTADV	SEFFPAIHT	3	0.5563	121.6	30.00	0.46	tr_D0A3E0_
DRB1_0101	126	EFQMKCMNENHTVYT	MKCMNENHT	3	0.5545	124.0	30.00	0.50	tr_D0A3E0_
DRB1_0101	127	FQMKCMNENHTVYTV	MKCMNENHT	2	0.5501	130.1	31.00	0.46	tr_D0A3E0_
DRB1_0101	128	TSYKLYHYSAHDTTI	LYHYSAHDT	4	0.5481	132.9	32.00	0.37	tr_D0A3E0_
DRB1_0101	129	LHLVQLVHRHGARSP	LVHRHGARS	5	0.5466	135.1	32.00	0.31	tr_D0A3E0_
DRB1_0101	130	AEAMPYACLHGGSLC	YACLHGGSL	5	0.5462	135.6	32.00	0.73	tr_D0A3E0_
DRB1_0101	131	MLINAGEFLRNHYNS	LINAGEFLR	1	0.5448	137.7	32.00	0.37	tr_D0A3E0_
DRB1_0101	132	GEFQMKCMNENHTVY	MKCMNENHT	4	0.5381	148.0	33.00	0.50	tr_D0A3E0_
DRB1_0101	133	DSHRFVYNASDRTHA	FVYNASDRT	4	0.5346	153.7	34.00	0.71	tr_D0A3E0_
DRB1_0101	134	MGSLGQHLAQEILKN	LGQHLAQEI	3	0.5329	156.7	34.00	0.53	tr_D0A3E0_
DRB1_0101	135	GSLGQHLAQEILKNA	LGQHLAQEI	2	0.5317	158.7	35.00	0.47	tr_D0A3E0_
DRB1_0101	136	CLHGGSLCLLISLLS	CLHGGSLCL	0	0.5315	159.1	35.00	0.34	tr_D0A3E0_
DRB1_0101	137	LVQLVHRHGARSPKV	LVHRHGARS	3	0.5303	161.1	35.00	0.68	tr_D0A3E0_
DRB1_0101	138	HLVQLVHRHGARSPK	LVHRHGARS	4	0.5293	162.8	35.00	0.54	tr_D0A3E0_
DRB1_0101	139	DEHWWRNVCNPKLDT	WRNVCNPKL	4	0.5273	166.4	36.00	0.83	tr_D0A3E0_
DRB1_0101	140	EVKGYIIRIRRGAPG	IRIRRGAPG	6	0.5272	166.7	36.00	0.34	tr_D0A3E0_
DRB1_0101	141	EEGYTLNSVTLECVC	GYTLNSVTL	2	0.5240	172.5	36.00	0.35	tr_D0A3E0_
DRB1_0101	142	EGYTLNSVTLECVCS	YTLNSVTLE	2	0.5238	172.8	36.00	0.32	tr_D0A3E0_
DRB1_0101	143	SDRTHAKMGSLGQHL	KMGSLGQHL	6	0.5222	175.8	37.00	0.58	tr_D0A3E0_
DRB1_0101	144	GGSLCLLISLLSCLG	LLISLLSCL	5	0.5208	178.5	37.00	0.31	tr_D0A3E0_
DRB1_0101	145	YHYSAHDTTIAPLAA	YSAHDTTIA	2	0.5197	180.6	37.00	0.49	tr_D0A3E0_
DRB1_0101	146	WWRNVCNPKLDTLID	WRNVCNPKL	1	0.5187	182.6	37.00	0.76	tr_D0A3E0_
DRB1_0101	147	LRGMFPNASEFFPAI	LRGMFPNAS	0	0.5175	185.0	38.00	0.62	tr_D0A3E0_
DRB1_0101	148	NAAGKMMLINAGEFL	MLINAGEFL	6	0.5169	186.3	38.00	0.41	tr_D0A3E0_
DRB1_0101	149	SLGQHLAQEILKNAE	LGQHLAQEI	1	0.5147	190.7	38.00	0.40	tr_D0A3E0_
DRB1_0101	150	CEEGYTLNSVTLECV	GYTLNSVTL	3	0.5139	192.3	38.00	0.39	tr_D0A3E0_
DRB1_0101	151	TLQSAGCLLRGMFPN	LQSAGCLLR	1	0.5103	200.0	39.00	0.31	tr_D0A3E0_
DRB1_0101	152	HPLLRENLGRLRDIK	LRENLGRLR	3	0.5063	208.9	40.00	0.67	tr_D0A3E0_
DRB1_0101	153	VQLVHRHGARSPKVK	LVHRHGARS	2	0.5060	209.5	40.00	0.68	tr_D0A3E0_

DRB1_0101	154	FPNASEFFPAIHTAD	SEFFPAIHT	4	0.5054	211.0	40.00	0.54	tr_D0A3E0_
DRB1_0101	155	GAEAMPYACLHGGSL	YACLHGGSL	6	0.5039	214.3	40.00	0.63	tr_D0A3E0_
DRB1_0101	156	PLLRENLGRLRDIKF	LRENLGRLR	2	0.5017	219.4	41.00	0.57	tr_D0A3E0_
DRB1_0101	157	LLRENLGRLRDIKFF	LRENLGRLR	1	0.4992	225.5	41.00	0.46	tr_D0A3E0_
DRB1_0101	158	YSAHDTTIAPLAATL	HDTTIAPLA	3	0.4990	225.9	41.00	0.27	tr_D0A3E0_
DRB1_0101	159	HYSAHDTTIAPLAAT	YSAHDTTIA	1	0.4988	226.4	41.00	0.39	tr_D0A3E0_
DRB1_0101	160	KHPLLRENLGRLRDI	LRENLGRLR	4	0.4985	227.4	41.00	0.55	tr_D0A3E0_
DRB1_0101	161	GYTLNSVTLECVCSS	LNSVTLECV	3	0.4970	230.9	42.00	0.31	tr_D0A3E0_
DRB1_0101	162	TNTLLSVSREVFSEG	TLLSVSREV	2	0.4961	233.4	42.00	0.41	tr_D0A3E0_
DRB1_0101	163	DTNTLLSVSREVFSE	TLLSVSREV	3	0.4940	238.6	42.00	0.43	tr_D0A3E0_
DRB1_0101	164	WGEFQMKCMNENHTV	MKCMNENHT	5	0.4924	242.8	43.00	0.43	tr_D0A3E0_
DRB1_0101	165	QMKCMNENHTVYTVE	MKCMNENHT	1	0.4910	246.4	43.00	0.41	tr_D0A3E0_
DRB1_0101	166	RRYRELFHCPGNVGK	YRELFHCPG	2	0.4905	247.7	43.00	0.50	tr_D0A3E0_
DRB1_0101	167	FVYNASDRTHAKMGS	YNASDRTHA	2	0.4881	254.3	43.00	0.49	tr_D0A3E0_
DRB1_0101	168	IDTNTLLSVSREVFS	TLLSVSREV	4	0.4861	259.9	44.00	0.42	tr_D0A3E0_
DRB1_0101	169	LDEHWWRNVCNPKLD	WRNVCNPKL	5	0.4857	260.9	44.00	0.82	tr_D0A3E0_
DRB1_0101	170	LTSYKLYHYSAHDTT	LYHYSAHDT	5	0.4853	262.3	44.00	0.31	tr_D0A3E0_
DRB1_0101	171	LQSAGCLLRGMFPNA	GCLLRGMFP	4	0.4840	265.8	44.00	0.51	tr_D0A3E0_
DRB1_0101	172	AGLCYLNRRYRELFH	YLNRRYREL	4	0.4826	270.0	45.00	0.42	tr_D0A3E0_
DRB1_0101	173	MFPNASEFFPAIHTA	SEFFPAIHT	5	0.4819	272.0	45.00	0.50	tr_D0A3E0_
DRB1_0101	174	HGGSLCLLISLLSCL	LLISLLSCL	6	0.4804	276.5	45.00	0.31	tr_D0A3E0_
DRB1_0101	175	EFFPAIHTADVSTDW	FPAIHTADV	2	0.4776	284.9	46.00	0.51	tr_D0A3E0_
DRB1_0101	176	AGEFLRNHYNSNASE	LRNHYNSNA	4	0.4771	286.6	46.00	0.49	tr_D0A3E0_
DRB1_0101	177	CGEVPCGYLNAAGKM	CGYLNAAGK	5	0.4769	287.0	46.00	0.38	tr_D0A3E0_
DRB1_0101	178	NAGEFLRNHYNSNAS	LRNHYNSNA	5	0.4746	294.4	46.00	0.44	tr_D0A3E0_
DRB1_0101	179	WSCEEGYTLNSVTLE	GYTLNSVTL	5	0.4734	298.1	47.00	0.35	tr_D0A3E0_
DRB1_0101	180	HEVKGYIIRIRRGAP	GYIIRIRRG	4	0.4734	298.2	47.00	0.41	tr_D0A3E0_
DRB1_0101	181	GLCYLNRRYRELFHC	YLNRRYREL	3	0.4681	315.9	48.00	0.46	tr_D0A3E0_
DRB1_0101	182	FRRVCPAWSCEEGYT	FRRVCPAWS	0	0.4672	318.8	48.00	0.65	tr_D0A3E0_
DRB1_0101	183	NRRYRELFHCPGNVG	YRELFHCPG	3	0.4655	324.7	48.00	0.61	tr_D0A3E0_
DRB1_0101	184	NTLLSVSREVFSEGF	LSVSREVFS	3	0.4637	331.3	49.00	0.38	tr_D0A3E0_
DRB1_0101	185	PAGLCYLNRRYRELF	YLNRRYREL	5	0.4632	333.1	49.00	0.37	tr_D0A3E0_
DRB1_0101	186	STDVPRTLQSAGCLL	TLQSAGCLL	6	0.4619	337.5	49.00	0.49	tr_D0A3E0_
DRB1_0101	187	LGQHLAQEILKNAEN	HLAQEILKN	3	0.4586	350.0	50.00	0.25	tr_D0A3E0_
DRB1_0101	188	QLVHRHGARSPKVKH	LVHRHGARS	1	0.4584	350.6	50.00	0.60	tr_D0A3E0_
DRB1_0101	189	DKHPLLRENLGRLRD	LRENLGRLR	5	0.4562	359.0	50.00	0.52	tr_D0A3E0_
DRB1_0101	190	EDSHRFVYNASDRTH	FVYNASDRT	5	0.4560	359.9	50.00	0.74	tr_D0A3E0_
DRB1_0101	191	KLDTLIDTNTLLSVS	DTLIDTNTL	2	0.4560	360.0	50.00	0.31	tr_D0A3E0_
DRB1_0101	192	SCEEGYTLNSVTLEC	GYTLNSVTL	4	0.4548	364.7	50.00	0.46	tr_D0A3E0_
DRB1_0101	193	AQEILKNAENHMNGL	ILKNAENHM	3	0.4527	373.0	55.00	0.56	tr_D0A3E0_
DRB1_0101	194	RYRELFHCPGNVGKP	YRELFHCPG	1	0.4517	377.1	55.00	0.41	tr_D0A3E0_
DRB1_0101	195	AWSCEEGYTLNSVTL	WSCEEGYTL	1	0.4514	378.3	55.00	0.54	tr_D0A3E0_
DRB1_0101	196	IRIRRGAPGQKPEEG	IRIRRGAPG	0	0.4504	382.4	55.00	0.52	tr_D0A3E0_
DRB1_0101	197	PKLDTLIDTNTLLSV	DTLIDTNTL	3	0.4486	390.0	55.00	0.34	tr_D0A3E0_
DRB1_0101	198	MSVFYPPLIPSEHYF	YPPLIPSEH	4	0.4475	394.7	55.00	0.23	tr_D0A3E0_
DRB1_0101	199	DIKFFEDSHRFVYNA	FFEDSHRFV	3	0.4470	396.7	55.00	0.57	tr_D0A3E0_
DRB1_0101	200	QEILKNAENHMNGLT	ILKNAENHM	2	0.4457	402.3	55.00	0.52	tr_D0A3E0_
DRB1_0101	201	IKFFEDSHRFVYNAS	FFEDSHRFV	2	0.4420	418.7	55.00	0.53	tr_D0A3E0_
DRB1_0101	202	LNAAGKMMLINAGEF	GKMMLINAG	4	0.4390	432.7	55.00	0.37	tr_D0A3E0_
DRB1_0101	203	GEFLRNHYNSNASEP	LRNHYNSNA	3	0.4379	437.8	55.00	0.50	tr_D0A3E0_

DRB1_0101	204	LHGGSLCLLISLLSC	LCLLISLLS	5	0.4372	441.0	55.00	0.32	tr_D0A3E0_
DRB1_0101	205	WRNVCNPKLDTLIDT	WRNVCNPKL	0	0.4356	448.6	55.00	0.63	tr_D0A3E0_
DRB1_0101	206	LRENLGRLRDIKFFE	LGRLRDIKF	4	0.4345	454.1	55.00	0.47	tr_D0A3E0_
DRB1_0101	207	DIGVAMQSDGRIDKH	VAMQSDGRI	3	0.4328	462.4	55.00	0.69	tr_D0A3E0_
DRB1_0101	208	INAGEFLRNHYNSNA	GEFLRNHYN	3	0.4312	470.6	60.00	0.48	tr_D0A3E0_
DRB1_0101	209	RDIKFFEDSHRFVYN	FFEDSHRFV	4	0.4311	471.0	60.00	0.55	tr_D0A3E0_
DRB1_0101	210	CPAWSCEEGYTLNSV	WSCEEGYTL	3	0.4308	473.0	60.00	0.86	tr_D0A3E0_
DRB1_0101	211	LCYLNRRYRELFHCP	YLNRRYREL	2	0.4296	478.7	60.00	0.54	tr_D0A3E0_
DRB1_0101	212	SQICGEVPCGYLNAA	CGEVPCGYL	3	0.4266	494.8	60.00	0.37	tr_D0A3E0_
DRB1_0101	213	CYLNRRYRELFHCPG	YLNRRYREL	1	0.4257	499.5	60.00	0.43	tr_D0A3E0_
DRB1_0101	214	LIDTNTLLSVSREVF	TLLSVSREV	5	0.4255	500.8	60.00	0.46	tr_D0A3E0_
DRB1_0101	215	PQNRCHCAATLFDIG	NRCHCAATL	2	0.4254	501.2	60.00	0.55	tr_D0A3E0_
DRB1_0101	216	YTLNSVTLECVCSSR	LNSVTLECV	2	0.4250	503.4	60.00	0.40	tr_D0A3E0_
DRB1_0101	217	STDWLLRYDVIPQAY	WLLRYDVIP	3	0.4249	504.0	60.00	0.23	tr_D0A3E0_
DRB1_0101	218	CHCAATLFDIGVAMQ	TLFDIGVAM	5	0.4248	504.4	60.00	0.58	tr_D0A3E0_
DRB1_0101	219	LDTLIDTNTLLSVSR	TLIDTNTLL	2	0.4247	505.1	60.00	0.32	tr_D0A3E0_
DRB1_0101	220	MKCMNENHTVYTVED	MNENHTVYT	3	0.4221	519.4	60.00	0.26	tr_D0A3E0_
DRB1_0101	221	GLTSYKLYHYSAHDT	SYKLYHYSA	3	0.4213	523.9	60.00	0.38	tr_D0A3E0_
DRB1_0101	222	TLNSVTLECVCSSRK	LNSVTLECV	1	0.4210	525.6	60.00	0.30	tr_D0A3E0_
DRB1_0101	223	NPKLDTLIDTNTLLS	DTLIDTNTL	4	0.4191	536.5	60.00	0.34	tr_D0A3E0_
DRB1_0101	224	EILKNAENHMNGLTS	ILKNAENHM	1	0.4191	536.9	60.00	0.40	tr_D0A3E0_
DRB1_0101	225	IGVAMQSDGRIDKHP	VAMQSDGRI	2	0.4186	539.4	60.00	0.72	tr_D0A3E0_
DRB1_0101	226	SWGEFQMKCMNENHT	FQMKCMNEN	4	0.4186	539.5	60.00	0.46	tr_D0A3E0_
DRB1_0101	227	LINAGEFLRNHYNSN	GEFLRNHYN	4	0.4183	541.4	60.00	0.41	tr_D0A3E0_
DRB1_0101	228	PAWSCEEGYTLNSVT	WSCEEGYTL	2	0.4169	549.4	60.00	0.82	tr_D0A3E0_
DRB1_0101	229	FFPAIHTADVSTDWL	FPAIHTADV	1	0.4164	552.3	60.00	0.47	tr_D0A3E0_
DRB1_0101	230	QSQICGEVPCGYLNA	CGEVPCGYL	4	0.4147	562.9	60.00	0.35	tr_D0A3E0_
DRB1_0101	231	DPQNRCHCAATLFDI	NRCHCAATL	3	0.4135	570.3	60.00	0.58	tr_D0A3E0_
DRB1_0101	232	LNRRYRELFHCPGNV	YRELFHCPG	4	0.4131	572.4	60.00	0.67	tr_D0A3E0_
DRB1_0101	233	QNRCHCAATLFDIGV	NRCHCAATL	1	0.4117	581.3	60.00	0.44	tr_D0A3E0_
DRB1_0101	234	ICGEVPCGYLNAAGK	CGEVPCGYL	1	0.4114	583.4	60.00	0.25	tr_D0A3E0_
DRB1_0101	235	IDKHPLLRENLGRLR	LRENLGRLR	6	0.4108	587.2	60.00	0.41	tr_D0A3E0_
DRB1_0101	236	GMFPNASEFFPAIHT	SEFFPAIHT	6	0.4091	598.1	60.00	0.40	tr_D0A3E0_
DRB1_0101	237	HLDEHWWRNVCNPKL	WRNVCNPKL	6	0.4085	602.1	60.00	0.71	tr_D0A3E0_
DRB1_0101	238	NSVTLECVCSSRKCM	LECVCSSRK	4	0.4071	611.2	65.00	0.42	tr_D0A3E0_
DRB1_0101	239	QICGEVPCGYLNAAG	CGEVPCGYL	2	0.4067	613.6	65.00	0.35	tr_D0A3E0_
DRB1_0101	240	PNKQCKVFRRVCPAW	KVFRRVCPA	5	0.4066	614.0	65.00	0.23	tr_D0A3E0_
DRB1_0101	241	DPAGLCYLNRRYREL	YLNRRYREL	6	0.4063	616.4	65.00	0.25	tr_D0A3E0_
DRB1_0101	242	VCPAWSCEEGYTLNS	WSCEEGYTL	4	0.4062	617.2	65.00	0.87	tr_D0A3E0_
DRB1_0101	243	LKNAENHMNGLTSYK	ENHMNGLTS	4	0.4055	621.5	65.00	0.52	tr_D0A3E0_
DRB1_0101	244	FLRNHYNSNASEPFF	YNSNASEPF	5	0.4047	626.9	65.00	0.44	tr_D0A3E0_
DRB1_0101	245	YLNRRYRELFHCPGN	YRELFHCPG	5	0.4039	632.3	65.00	0.49	tr_D0A3E0_
DRB1_0101	246	VSTDWLLRYDVIPQA	DWLLRYDVI	3	0.4038	633.0	65.00	0.31	tr_D0A3E0_
DRB1_0101	247	LAQEILKNAENHMNG	ILKNAENHM	4	0.4025	642.2	65.00	0.55	tr_D0A3E0_
DRB1_0101	248	SVFYPPLIPSEHYFQ	YPPLIPSEH	3	0.4021	644.7	65.00	0.31	tr_D0A3E0_
DRB1_0101	249	ILKNAENHMNGLTSY	ENHMNGLTS	5	0.4021	644.7	65.00	0.31	tr_D0A3E0_
DRB1_0101	250	GQHLAQEILKNAENH	HLAQEILKN	2	0.4011	652.1	65.00	0.26	tr_D0A3E0_
DRB1_0101	251	SSRKCMESRDLSIST	CMESRDLSI	4	0.3999	660.4	65.00	0.44	tr_D0A3E0_
DRB1_0101	252	LRDIKFFEDSHRFVY	FFEDSHRFV	5	0.3991	666.1	65.00	0.54	tr_D0A3E0_
DRB1_0101	253	SVTLECVCSSRKCME	LECVCSSRK	3	0.3984	671.1	65.00	0.45	tr_D0A3E0_

DRB1_0101	254	KFFEDSHRFVYNASD	FFEDSHRFV	1	0.3955	692.6	65.00	0.51	tr_D0A3E0_
DRB1_0101	255	RCHCAATLFDIGVAM	TLFDIGVAM	6	0.3937	706.5	65.00	0.41	tr_D0A3E0_
DRB1_0101	256	VSREVFSEGFCADPQ	EVFSEGFCA	3	0.3925	715.6	65.00	0.66	tr_D0A3E0_
DRB1_0101	257	DTLIDTNTLLSVSRE	LIDTNTLLS	2	0.3922	717.7	65.00	0.28	tr_D0A3E0_
DRB1_0101	258	SCVTYSRSTDVPRTL	VTYSRSTDV	2	0.3916	722.8	65.00	0.58	tr_D0A3E0_
DRB1_0101	259	LNSVTLECVCSSRKC	LECVCSSRK	5	0.3908	728.8	65.00	0.31	tr_D0A3E0_
DRB1_0101	260	EFLRNHYNSNASEPF	LRNHYNSNA	2	0.3903	732.6	65.00	0.50	tr_D0A3E0_
DRB1_0101	261	RRFVKSTKPHDPAGL	VKSTKPHDP	3	0.3893	740.9	65.00	0.55	tr_D0A3E0_
DRB1_0101	262	YRELFHCPGNVGKPP	LFHCPGNVG	3	0.3871	758.9	65.00	0.40	tr_D0A3E0_
DRB1_0101	263	SRKCMESRDLSISTD	CMESRDLSI	3	0.3859	768.9	65.00	0.46	tr_D0A3E0_
DRB1_0101	264	SREVFSEGFCADPQN	EVFSEGFCA	2	0.3857	770.3	65.00	0.65	tr_D0A3E0_
DRB1_0101	265	KTGAEAMPYACLHGG	AEAMPYACL	3	0.3854	772.5	65.00	0.55	tr_D0A3E0_
DRB1_0101	266	NGLTSYKLYHYSAHD	SYKLYHYSA	4	0.3837	786.8	70.00	0.39	tr_D0A3E0_
DRB1_0101	267	KCPYHDFRRFVKSTK	FRRFVKSTK	6	0.3832	791.2	70.00	0.20	tr_D0A3E0_
DRB1_0101	268	HLAQEILKNAENHMN	ILKNAENHM	5	0.3827	795.2	70.00	0.44	tr_D0A3E0_
DRB1_0101	269	CNPKLDTLIDTNTLL	LDTLIDTNT	4	0.3821	800.9	70.00	0.35	tr_D0A3E0_
DRB1_0101	270	NQSQICGEVPCGYLN	ICGEVPCGY	4	0.3814	807.1	70.00	0.28	tr_D0A3E0_
DRB1_0101	271	FRRFVKSTKPHDPAG	VKSTKPHDP	4	0.3810	810.0	70.00	0.46	tr_D0A3E0_
DRB1_0101	272	NSCVTYSRSTDVPRT	VTYSRSTDV	3	0.3800	818.9	70.00	0.73	tr_D0A3E0_
DRB1_0101	273	CVTYSRSTDVPRTLQ	VTYSRSTDV	1	0.3791	827.2	70.00	0.45	tr_D0A3E0_
DRB1_0101	274	CSSRKCMESRDLSIS	CMESRDLSI	5	0.3785	832.7	70.00	0.38	tr_D0A3E0_
DRB1_0101	275	TLLSVSREVFSEGFC	LSVSREVFS	2	0.3773	843.2	70.00	0.47	tr_D0A3E0_
DRB1_0101	276	RENLGRLRDIKFFED	LGRLRDIKF	3	0.3768	847.9	70.00	0.63	tr_D0A3E0_
DRB1_0101	277	PPNKQCKVFRRVCPA	KQCKVFRRV	3	0.3768	848.3	70.00	0.28	tr_D0A3E0_
DRB1_0101	278	DFRRFVKSTKPHDPA	VKSTKPHDP	5	0.3753	861.9	70.00	0.36	tr_D0A3E0_
DRB1_0101	279	FSWGEFQMKCMNENH	FQMKCMNEN	5	0.3748	866.6	70.00	0.50	tr_D0A3E0_
DRB1_0101	280	ADPQNRCHCAATLFD	NRCHCAATL	4	0.3742	872.1	70.00	0.62	tr_D0A3E0_
DRB1_0101	281	STKPHDPAGLCYLNR	HDPAGLCYL	4	0.3740	873.6	70.00	0.42	tr_D0A3E0_
DRB1_0101	282	VFYPPLIPSEHYFQE	YPPLIPSEH	2	0.3740	874.2	70.00	0.31	tr_D0A3E0_
DRB1_0101	283	REVFSEGFCADPQNR	EVFSEGFCA	1	0.3734	879.8	70.00	0.54	tr_D0A3E0_
DRB1_0101	284	LSVSREVFSEGFCAD	EVFSEGFCA	5	0.3727	886.8	70.00	0.59	tr_D0A3E0_
DRB1_0101	285	FPAIHTADVSTDWLL	IHTADVSTD	3	0.3727	886.8	70.00	0.34	tr_D0A3E0_
DRB1_0101	286	TKPHDPAGLCYLNRR	HDPAGLCYL	3	0.3721	892.1	70.00	0.44	tr_D0A3E0_
DRB1_0101	287	CPYHDFRRFVKSTKP	FRRFVKSTK	5	0.3711	901.9	70.00	0.31	tr_D0A3E0_
DRB1_0101	288	DVSTDWLLRYDVIPQ	DWLLRYDVI	4	0.3705	907.7	70.00	0.33	tr_D0A3E0_
DRB1_0101	289	EGYAFSWGEFQMKCM	FSWGEFQMK	4	0.3705	908.2	70.00	0.37	tr_D0A3E0_
DRB1_0101	290	GYAFSWGEFQMKCMN	FSWGEFQMK	3	0.3704	909.2	70.00	0.40	tr_D0A3E0_
DRB1_0101	291	RVCPAWSCEEGYTLN	WSCEEGYTL	5	0.3702	910.6	70.00	0.82	tr_D0A3E0_
DRB1_0101	292	QETIKDKTGAEAMPY	IKDKTGAEA	3	0.3700	912.7	70.00	0.74	tr_D0A3E0_
DRB1_0101	293	DKTGAEAMPYACLHG	AEAMPYACL	4	0.3700	913.1	70.00	0.56	tr_D0A3E0_
DRB1_0101	294	VTLECVCSSRKCMES	LECVCSSRK	2	0.3693	919.3	70.00	0.40	tr_D0A3E0_
DRB1_0101	295	NRCHCAATLFDIGVA	HCAATLFDI	3	0.3689	923.2	70.00	0.28	tr_D0A3E0_
DRB1_0101	296	TLIDTNTLLSVSREV	TLLSVSREV	6	0.3686	927.1	70.00	0.26	tr_D0A3E0_
DRB1_0101	297	LLSVSREVFSEGFCA	EVFSEGFCA	6	0.3685	927.9	70.00	0.38	tr_D0A3E0_
DRB1_0101	298	PYHDFRRFVKSTKPH	FRRFVKSTK	4	0.3684	928.3	70.00	0.38	tr_D0A3E0_
DRB1_0101	299	FEDSHRFVYNASDRT	FVYNASDRT	6	0.3683	929.8	70.00	0.54	tr_D0A3E0_
DRB1_0101	300	SVSREVFSEGFCADP	EVFSEGFCA	4	0.3669	944.3	70.00	0.65	tr_D0A3E0_
DRB1_0101	301	QHLAQEILKNAENHM	HLAQEILKN	1	0.3668	944.8	70.00	0.25	tr_D0A3E0_
DRB1_0101	302	YHDFRRFVKSTKPHD	FRRFVKSTK	3	0.3667	945.8	70.00	0.34	tr_D0A3E0_
DRB1_0101	303	PHDPAGLCYLNRRYR	HDPAGLCYL	1	0.3657	956.7	70.00	0.32	tr_D0A3E0_

DRB1_0101	304	RFVKSTKPHDPAGLC	VKSTKPHDP	2	0.3638	976.2	70.00	0.50	tr_D0A3E0_
DRB1_0101	305	GVAMQSDGRIDKHPL	VAMQSDGRI	1	0.3632	982.0	70.00	0.74	tr_D0A3E0_
DRB1_0101	306	TLECVCSSRKCMESR	CVCSSRKCM	3	0.3613	1002.6	70.00	0.45	tr_D0A3E0_
DRB1_0101	307	ADVSTDWLLRYDVIP	DWLLRYDVI	5	0.3595	1022.0	75.00	0.28	tr_D0A3E0_
DRB1_0101	308	KSTKPHDPAGLCYLN	HDPAGLCYL	5	0.3591	1027.0	75.00	0.36	tr_D0A3E0_
DRB1_0101	309	LVHRHGARSPKVKHN	LVHRHGARS	0	0.3573	1046.9	75.00	0.44	tr_D0A3E0_
DRB1_0101	310	ETIKDKTGAEAMPYA	IKDKTGAEA	2	0.3572	1048.3	75.00	0.64	tr_D0A3E0_
DRB1_0101	311	YNSCVTYSRSTDVPR	VTYSRSTDV	4	0.3569	1052.2	75.00	0.72	tr_D0A3E0_
DRB1_0101	312	AFSWGEFQMKCMNEN	FSWGEFQMK	1	0.3561	1061.2	75.00	0.29	tr_D0A3E0_
DRB1_0101	313	HDPAGLCYLNRRYRE	LCYLNRRYR	5	0.3561	1061.4	75.00	0.27	tr_D0A3E0_
DRB1_0101	314	KDKTGAEAMPYACLH	AEAMPYACL	5	0.3560	1062.4	75.00	0.46	tr_D0A3E0_
DRB1_0101	315	TGAEAMPYACLHGGS	AEAMPYACL	2	0.3554	1069.3	75.00	0.56	tr_D0A3E0_
DRB1_0101	316	HDFRRFVKSTKPHDP	FRRFVKSTK	2	0.3532	1094.1	75.00	0.34	tr_D0A3E0_
DRB1_0101	317	VKSTKPHDPAGLCYL	TKPHDPAGL	3	0.3532	1094.5	75.00	0.42	tr_D0A3E0_
DRB1_0101	318	KPHDPAGLCYLNRRY	HDPAGLCYL	2	0.3528	1099.4	75.00	0.44	tr_D0A3E0_
DRB1_0101	319	RKCMESRDLSISTDT	CMESRDLSI	2	0.3524	1104.4	75.00	0.46	tr_D0A3E0_
DRB1_0101	320	TADVSTDWLLRYDVI	DWLLRYDVI	6	0.3512	1118.2	75.00	0.26	tr_D0A3E0_
DRB1_0101	321	APLAATLGDSTTTGI	LAATLGDST	2	0.3483	1153.8	75.00	0.81	tr_D0A3E0_
DRB1_0101	322	VYNASDRTHAKMGSL	YNASDRTHA	1	0.3474	1165.8	75.00	0.69	tr_D0A3E0_
DRB1_0101	323	CADPQNRCHCAATLF	NRCHCAATL	5	0.3473	1166.7	75.00	0.64	tr_D0A3E0_
DRB1_0101	324	DVIPQAYAFSHLDEH	QAYAFSHLD	4	0.3473	1166.7	75.00	0.23	tr_D0A3E0_
DRB1_0101	325	EEGYAFSWGEFQMKC	YAFSWGEFQ	3	0.3451	1194.5	75.00	0.47	tr_D0A3E0_
DRB1_0101	326	YAFSWGEFQMKCMNE	FSWGEFQMK	2	0.3445	1202.7	75.00	0.41	tr_D0A3E0_
DRB1_0101	327	ESRDLSISTDTLGRN	DLSISTDTL	3	0.3437	1213.8	75.00	0.38	tr_D0A3E0_
DRB1_0101	328	AIHTADVSTDWLLRY	IHTADVSTD	1	0.3428	1224.6	75.00	0.26	tr_D0A3E0_
DRB1_0101	329	PAIHTADVSTDWLLR	IHTADVSTD	2	0.3385	1283.4	75.00	0.31	tr_D0A3E0_
DRB1_0101	330	YFQETIKDKTGAEAM	IKDKTGAEA	5	0.3378	1292.5	75.00	0.62	tr_D0A3E0_
DRB1_0101	331	IKDKTGAEAMPYACL	AEAMPYACL	6	0.3372	1301.9	75.00	0.28	tr_D0A3E0_
DRB1_0101	332	ENLGRLRDIKFFEDS	LGRLRDIKF	2	0.3358	1322.1	75.00	0.63	tr_D0A3E0_
DRB1_0101	333	AYAFSHLDEHWWRNV	FSHLDEHWW	3	0.3357	1322.5	75.00	0.55	tr_D0A3E0_
DRB1_0101	334	KPPNKQCKVFRRVCP	NKQCKVFRR	3	0.3336	1353.2	80.00	0.31	tr_D0A3E0_
DRB1_0101	335	SEHYFQETIKDKTGA	HYFQETIKD	2	0.3333	1357.1	80.00	0.29	tr_D0A3E0_
DRB1_0101	336	RNHYNSNASEPFFPE	YNSNASEPF	3	0.3329	1363.9	80.00	0.62	tr_D0A3E0_
DRB1_0101	337	FQETIKDKTGAEAMP	IKDKTGAEA	4	0.3314	1385.7	80.00	0.70	tr_D0A3E0_
DRB1_0101	338	PEEGYAFSWGEFQMK	YAFSWGEFQ	4	0.3313	1387.2	80.00	0.51	tr_D0A3E0_
DRB1_0101	339	FYPPLIPSEHYFQET	LIPSEHYFQ	4	0.3313	1387.9	80.00	0.34	tr_D0A3E0_
DRB1_0101	340	KCMNENHTVYTVEDN	MNENHTVYT	2	0.3307	1396.2	80.00	0.35	tr_D0A3E0_
DRB1_0101	341	TIKDKTGAEAMPYAC	IKDKTGAEA	1	0.3307	1397.1	80.00	0.51	tr_D0A3E0_
DRB1_0101	342	FVKSTKPHDPAGLCY	VKSTKPHDP	1	0.3291	1420.6	80.00	0.39	tr_D0A3E0_
DRB1_0101	343	QAYAFSHLDEHWWRN	FSHLDEHWW	4	0.3285	1429.8	80.00	0.50	tr_D0A3E0_
DRB1_0101	344	VTYSRSTDVPRTLQS	YSRSTDVPR	2	0.3283	1433.3	80.00	0.33	tr_D0A3E0_
DRB1_0101	345	LRNHYNSNASEPFFP	YNSNASEPF	4	0.3275	1445.9	80.00	0.60	tr_D0A3E0_
DRB1_0101	346	RGMFPNASEFFPAIH	MFPNASEFF	2	0.3270	1452.8	80.00	0.34	tr_D0A3E0_
DRB1_0101	347	RRVCPAWSCEEGYTL	WSCEEGYTL	6	0.3270	1453.4	80.00	0.62	tr_D0A3E0_
DRB1_0101	348	PQAYAFSHLDEHWWR	FSHLDEHWW	5	0.3246	1491.8	80.00	0.41	tr_D0A3E0_
DRB1_0101	349	HNQSQICGEVPCGYL	ICGEVPCGY	5	0.3243	1497.2	80.00	0.29	tr_D0A3E0_
DRB1_0101	350	SYNSCVTYSRSTDVP	VTYSRSTDV	5	0.3239	1502.4	80.00	0.51	tr_D0A3E0_
DRB1_0101	351	RLRDIKFFEDSHRFV	FFEDSHRFV	6	0.3229	1519.1	80.00	0.50	tr_D0A3E0_
DRB1_0101	352	VCSSRKCMESRDLSI	RKCMESRDL	4	0.3228	1520.5	80.00	0.42	tr_D0A3E0_
DRB1_0101	353	VCNPKLDTLIDTNTL	LDTLIDTNT	5	0.3206	1557.1	80.00	0.35	tr_D0A3E0_

DRB1_0101	354	MESRDLSISTDTLGR	DLSISTDTL	4	0.3188	1588.6	80.00	0.41	tr_D0A3E0_
DRB1_0101	355	RELFHCPGNVGKPPN	LFHCPGNVG	2	0.3185	1594.1	80.00	0.50	tr_D0A3E0_
DRB1_0101	356	PSEHYFQETIKDKTG	HYFQETIKD	3	0.3181	1600.6	80.00	0.36	tr_D0A3E0_
DRB1_0101	357	GKPPNKQCKVFRRVC	NKQCKVFRR	4	0.3160	1636.3	80.00	0.35	tr_D0A3E0_
DRB1_0101	358	YAFSHLDEHWWRNVC	FSHLDEHWW	2	0.3160	1637.1	80.00	0.50	tr_D0A3E0_
DRB1_0101	359	EVFSEGFCADPQNRC	EVFSEGFCA	0	0.3158	1640.2	80.00	0.30	tr_D0A3E0_
DRB1_0101	360	ESYNSCVTYSRSTDV	YNSCVTYSR	2	0.3155	1645.3	80.00	0.43	tr_D0A3E0_
DRB1_0101	361	SEPFFPEESYNSCVT	FFPEESYNS	3	0.3151	1654.0	80.00	0.75	tr_D0A3E0_
DRB1_0101	362	NKCPYHDFRRFVKST	HDFRRFVKS	5	0.3146	1661.5	80.00	0.25	tr_D0A3E0_
DRB1_0101	363	NHYNSNASEPFFPEE	YNSNASEPF	2	0.3132	1688.4	80.00	0.55	tr_D0A3E0_
DRB1_0101	364	PLAATLGDSTTTGIT	LAATLGDST	1	0.3119	1711.3	80.00	0.69	tr_D0A3E0_
DRB1_0101	365	ASDRTHAKMGSLGQH	THAKMGSLG	4	0.3110	1727.8	80.00	0.44	tr_D0A3E0_
DRB1_0101	366	KCMESRDLSISTDTL	CMESRDLSI	1	0.3094	1759.3	80.00	0.40	tr_D0A3E0_
DRB1_0101	367	LECVCSSRKCMESRD	CVCSSRKCM	2	0.3091	1764.1	80.00	0.47	tr_D0A3E0_
DRB1_0101	368	RIDKHPLLRENLGRL	LLRENLGRL	6	0.3090	1766.4	80.00	0.29	tr_D0A3E0_
DRB1_0101	369	HYFQETIKDKTGAEA	IKDKTGAEA	6	0.3073	1799.3	80.00	0.28	tr_D0A3E0_
DRB1_0101	370	EPFFPEESYNSCVTY	FFPEESYNS	2	0.3068	1808.2	80.00	0.66	tr_D0A3E0_
DRB1_0101	371	TTTGITPPYGQLYAF	ITPPYGQLY	4	0.3062	1820.9	85.00	0.32	tr_D0A3E0_
DRB1_0101	372	STTTGITPPYGQLYA	TTGITPPYG	2	0.3059	1825.9	85.00	0.40	tr_D0A3E0_
DRB1_0101	373	FFEDSHRFVYNASDR	FFEDSHRFV	0	0.3058	1828.2	85.00	0.37	tr_D0A3E0_
DRB1_0101	374	EHYFQETIKDKTGAE	HYFQETIKD	1	0.3054	1836.5	85.00	0.26	tr_D0A3E0_
DRB1_0101	375	IHTADVSTDWLLRYD	ADVSTDWLL	3	0.3054	1836.5	85.00	0.34	tr_D0A3E0_
DRB1_0101	376	IPQAYAFSHLDEHWW	QAYAFSHLD	2	0.3039	1865.3	85.00	0.29	tr_D0A3E0_
DRB1_0101	377	RSTDVPRTLQSAGCL	PRTLQSAGC	5	0.3035	1874.6	85.00	0.38	tr_D0A3E0_
DRB1_0101	378	NLGRLRDIKFFEDSH	LGRLRDIKF	1	0.3019	1907.7	85.00	0.59	tr_D0A3E0_
DRB1_0101	379	SPKVKHNQSQICGEV	VKHNQSQIC	3	0.3016	1912.4	85.00	0.59	tr_D0A3E0_
DRB1_0101	380	HTADVSTDWLLRYDV	DVSTDWLLR	3	0.2979	1990.7	85.00	0.32	tr_D0A3E0_
DRB1_0101	381	HYNSNASEPFFPEES	YNSNASEPF	1	0.2970	2011.5	85.00	0.45	tr_D0A3E0_
DRB1_0101	382	CMESRDLSISTDTLG	DLSISTDTL	5	0.2968	2014.5	85.00	0.35	tr_D0A3E0_
DRB1_0101	383	PFFPEESYNSCVTYS	FFPEESYNS	1	0.2957	2039.8	85.00	0.54	tr_D0A3E0_
DRB1_0101	384	IPSEHYFQETIKDKT	HYFQETIKD	4	0.2931	2096.9	85.00	0.40	tr_D0A3E0_
DRB1_0101	385	VIPQAYAFSHLDEHW	QAYAFSHLD	3	0.2930	2099.2	85.00	0.34	tr_D0A3E0_
DRB1_0101	386	VGKPPNKQCKVFRRV	NKQCKVFRR	5	0.2924	2114.0	85.00	0.37	tr_D0A3E0_
DRB1_0101	387	DNKCPYHDFRRFVKS	YHDFRRFVK	5	0.2912	2140.3	85.00	0.26	tr_D0A3E0_
DRB1_0101	388	ASEPFFPEESYNSCV	FFPEESYNS	4	0.2890	2191.7	85.00	0.75	tr_D0A3E0_
DRB1_0101	389	YPPLIPSEHYFQETI	LIPSEHYFQ	3	0.2870	2241.1	85.00	0.50	tr_D0A3E0_
DRB1_0101	390	PKVKHNQSQICGEVP	VKHNQSQIC	2	0.2865	2252.8	85.00	0.55	tr_D0A3E0_
DRB1_0101	391	TTGITPPYGQLYAFE	ITPPYGQLY	3	0.2862	2260.9	85.00	0.35	tr_D0A3E0_
DRB1_0101	392	RSPKVKHNQSQICGE	VKHNQSQIC	4	0.2849	2292.7	85.00	0.55	tr_D0A3E0_
DRB1_0101	393	VAMQSDGRIDKHPLL	VAMQSDGRI	0	0.2837	2323.0	85.00	0.54	tr_D0A3E0_
DRB1_0101	394	YNASDRTHAKMGSLG	YNASDRTHA	0	0.2822	2359.7	85.00	0.43	tr_D0A3E0_
DRB1_0101	395	ECVCSSRKCMESRDL	CVCSSRKCM	1	0.2821	2363.3	85.00	0.44	tr_D0A3E0_
DRB1_0101	396	FFPEESYNSCVTYSR	FFPEESYNS	0	0.2798	2421.5	85.00	0.28	tr_D0A3E0_
DRB1_0101	397	AFSHLDEHWWRNVCN	FSHLDEHWW	1	0.2785	2455.8	85.00	0.49	tr_D0A3E0_
DRB1_0101	398	EESYNSCVTYSRSTD	YNSCVTYSR	3	0.2781	2466.4	85.00	0.62	tr_D0A3E0_
DRB1_0101	399	PEESYNSCVTYSRST	YNSCVTYSR	4	0.2774	2485.9	85.00	0.60	tr_D0A3E0_
DRB1_0101	400	FCADPQNRCHCAATL	NRCHCAATL	6	0.2773	2489.4	85.00	0.56	tr_D0A3E0_
DRB1_0101	401	DSTTTGITPPYGQLY	TTGITPPYG	3	0.2771	2494.4	85.00	0.48	tr_D0A3E0_
DRB1_0101	402	TYSRSTDVPRTLQSA	YSRSTDVPR	1	0.2769	2500.6	85.00	0.30	tr_D0A3E0_
DRB1_0101	403	CVCSSRKCMESRDLS	RKCMESRDL	5	0.2755	2537.3	90.00	0.34	tr_D0A3E0_

DRB1_0101	404	RIRRGAPGQKPEEGY	IRRGAPGQK	1	0.2707	2672.9	90.00	0.58	tr_D0A3E0_
DRB1_0101	405	ARSPKVKHNQSQICG	VKHNQSQIC	5	0.2692	2717.1	90.00	0.54	tr_D0A3E0_
DRB1_0101	406	GRIDKHPLLRENLGR	KHPLLRENL	4	0.2682	2744.9	90.00	0.29	tr_D0A3E0_
DRB1_0101	407	PPLIPSEHYFQETIK	LIPSEHYFQ	2	0.2682	2746.0	90.00	0.51	tr_D0A3E0_
DRB1_0101	408	DGRIDKHPLLRENLG	GRIDKHPLL	1	0.2664	2798.8	90.00	0.25	tr_D0A3E0_
DRB1_0101	409	FPEESYNSCVTYSRS	YNSCVTYSR	5	0.2659	2814.3	90.00	0.50	tr_D0A3E0_
DRB1_0101	410	RNVCNPKLDTLIDTN	CNPKLDTLI	3	0.2643	2864.8	90.00	0.52	tr_D0A3E0_
DRB1_0101	411	ELFHCPGNVGKPPNK	LFHCPGNVG	1	0.2640	2874.6	90.00	0.47	tr_D0A3E0_
DRB1_0101	412	VFSEGFCADPQNRCH	FSEGFCADP	1	0.2636	2885.4	90.00	0.32	tr_D0A3E0_
DRB1_0101	413	LIPSEHYFQETIKDK	HYFQETIKD	5	0.2633	2896.7	90.00	0.36	tr_D0A3E0_
DRB1_0101	414	NASDRTHAKMGSLGQ	THAKMGSLG	5	0.2626	2918.1	90.00	0.43	tr_D0A3E0_
DRB1_0101	415	KVKHNQSQICGEVPC	VKHNQSQIC	1	0.2626	2918.9	90.00	0.48	tr_D0A3E0_
DRB1_0101	416	NVCNPKLDTLIDTNT	CNPKLDTLI	2	0.2603	2990.3	90.00	0.39	tr_D0A3E0_
DRB1_0101	417	YNSNASEPFFPEESY	NASEPFFPE	3	0.2601	2996.0	90.00	0.33	tr_D0A3E0_
DRB1_0101	418	PLIPSEHYFQETIKD	LIPSEHYFQ	1	0.2575	3082.5	90.00	0.47	tr_D0A3E0_
DRB1_0101	419	NHTVYTVEDNKCPYH	YTVEDNKCP	4	0.2562	3127.6	90.00	0.70	tr_D0A3E0_
DRB1_0101	420	SDGRIDKHPLLRENL	GRIDKHPLL	2	0.2556	3148.5	90.00	0.37	tr_D0A3E0_
DRB1_0101	421	NASEPFFPEESYNSC	FFPEESYNS	5	0.2533	3224.8	90.00	0.69	tr_D0A3E0_
DRB1_0101	422	HTVYTVEDNKCPYHD	YTVEDNKCP	3	0.2530	3237.2	90.00	0.71	tr_D0A3E0_
DRB1_0101	423	TVYTVEDNKCPYHDF	YTVEDNKCP	2	0.2480	3415.6	90.00	0.65	tr_D0A3E0_
DRB1_0101	424	KPEEGYAFSWGEFQM	YAFSWGEFQ	5	0.2467	3465.5	90.00	0.62	tr_D0A3E0_
DRB1_0101	425	LGRLRDIKFFEDSHR	LGRLRDIKF	0	0.2457	3503.5	90.00	0.38	tr_D0A3E0_
DRB1_0101	426	LAATLGDSTTTGITP	LAATLGDST	0	0.2432	3599.7	90.00	0.53	tr_D0A3E0_
DRB1_0101	427	QSDGRIDKHPLLREN	GRIDKHPLL	3	0.2428	3614.8	90.00	0.47	tr_D0A3E0_
DRB1_0101	428	EDNKCPYHDFRRFVK	PYHDFRRFV	5	0.2410	3686.7	90.00	0.23	tr_D0A3E0_
DRB1_0101	429	LFHCPGNVGKPPNKQ	LFHCPGNVG	0	0.2346	3949.5	95.00	0.33	tr_D0A3E0_
DRB1_0101	430	FSEGFCADPQNRCHC	GFCADPQNR	3	0.2330	4017.2	95.00	0.31	tr_D0A3E0_
DRB1_0101	431	YSRSTDVPRTLQSAG	RSTDVPRTL	2	0.2325	4040.0	95.00	0.30	tr_D0A3E0_
DRB1_0101	432	KHNQSQICGEVPCGY	ICGEVPCGY	6	0.2310	4106.3	95.00	0.28	tr_D0A3E0_
DRB1_0101	433	SNASEPFFPEESYNS	FFPEESYNS	6	0.2310	4107.2	95.00	0.46	tr_D0A3E0_
DRB1_0101	434	VYTVEDNKCPYHDFR	YTVEDNKCP	1	0.2301	4148.6	95.00	0.56	tr_D0A3E0_
DRB1_0101	435	MQSDGRIDKHPLLRE	GRIDKHPLL	4	0.2292	4186.6	95.00	0.47	tr_D0A3E0_
DRB1_0101	436	FSHLDEHWWRNVCNP	FSHLDEHWW	0	0.2282	4231.5	95.00	0.40	tr_D0A3E0_
DRB1_0101	437	SRSTDVPRTLQSAGC	PRTLQSAGC	6	0.2277	4256.2	95.00	0.22	tr_D0A3E0_
DRB1_0101	438	CMNENHTVYTVEDNK	MNENHTVYT	1	0.2267	4303.6	95.00	0.40	tr_D0A3E0_
DRB1_0101	439	NVGKPPNKQCKVFRR	NKQCKVFRR	6	0.2255	4360.3	95.00	0.31	tr_D0A3E0_
DRB1_0101	440	GDSTTTGITPPYGQL	TTGITPPYG	4	0.2248	4392.2	95.00	0.56	tr_D0A3E0_
DRB1_0101	441	ENHTVYTVEDNKCPY	YTVEDNKCP	5	0.2235	4453.4	95.00	0.69	tr_D0A3E0_
DRB1_0101	442	GARSPKVKHNQSQIC	VKHNQSQIC	6	0.2177	4741.7	95.00	0.44	tr_D0A3E0_
DRB1_0101	443	AMQSDGRIDKHPLLR	GRIDKHPLL	5	0.2153	4868.5	95.00	0.49	tr_D0A3E0_
DRB1_0101	444	SEGFCADPQNRCHCA	FCADPQNRC	3	0.2144	4916.7	95.00	0.42	tr_D0A3E0_
DRB1_0101	445	VHRHGARSPKVKHNQ	RHGARSPKV	2	0.2132	4977.0	95.00	0.38	tr_D0A3E0_
DRB1_0101	446	QKPEEGYAFSWGEFQ	YAFSWGEFQ	6	0.2093	5193.7	95.00	0.41	tr_D0A3E0_
DRB1_0101	447	EGFCADPQNRCHCAA	FCADPQNRC	2	0.2036	5523.1	95.00	0.41	tr_D0A3E0_
DRB1_0101	448	VKHNQSQICGEVPCG	VKHNQSQIC	0	0.1998	5758.2	95.00	0.21	tr_D0A3E0_
DRB1_0101	449	GRLRDIKFFEDSHRF	IKFFEDSHR	5	0.1991	5802.5	95.00	0.26	tr_D0A3E0_
DRB1_0101	450	NSNASEPFFPEESYN	NASEPFFPE	2	0.1984	5842.0	95.00	0.46	tr_D0A3E0_
DRB1_0101	451	VEDNKCPYHDFRRFV	DNKCPYHDF	2	0.1970	5930.5	95.00	0.26	tr_D0A3E0_
DRB1_0101	452	CPGNVGKPPNKQCKV	NVGKPPNKQ	3	0.1947	6081.6	95.00	0.62	tr_D0A3E0_
DRB1_0101	453	GFCADPQNRCHCAAT	FCADPQNRC	1	0.1932	6182.3	95.00	0.34	tr_D0A3E0_

DRB1_0101	454	GNVGKPPNKQCKVFR	NVGKPPNKQ	1	0.1925	6226.4	100.00	0.47	tr_D0A3E0_	
DRB1_0101	455	PGNVGKPPNKQCKVF	NVGKPPNKQ	2	0.1923	6244.2	100.00	0.61	tr_D0A3E0_	
DRB1_0101	456	YTVEDNKCPYHDFRR	YTVEDNKCP	0	0.1899	6405.4	100.00	0.36	tr_D0A3E0_	
DRB1_0101	457	HCPGNVGKPPNKQCK	NVGKPPNKQ	4	0.1828	6916.5	100.00	0.57	tr_D0A3E0_	
DRB1_0101	458	FHCPGNVGKPPNKQC	NVGKPPNKQ	5	0.1820	6980.9	100.00	0.43	tr_D0A3E0_	
DRB1_0101	459	IRRGAPGQKPEEGYA	IRRGAPGQK	0	0.1784	7255.1	100.00	0.41	tr_D0A3E0_	
DRB1_0101	460	LGDSTTTGITPPYGQ	TTGITPPYG	5	0.1771	7355.7	100.00	0.51	tr_D0A3E0_	
DRB1_0101	461	SHLDEHWWRNVCNPK	LDEHWWRNV	2	0.1770	7366.7	100.00	0.28	tr_D0A3E0_	
DRB1_0101	462	GQKPEEGYAFSWGEF	QKPEEGYAF	1	0.1724	7746.3	100.00	0.38	tr_D0A3E0_	
DRB1_0101	463	APGQKPEEGYAFSWG	QKPEEGYAF	3	0.1701	7939.9	100.00	0.50	tr_D0A3E0_	
DRB1_0101	464	NENHTVYTVEDNKCP	YTVEDNKCP	6	0.1689	8043.4	100.00	0.51	tr_D0A3E0_	
DRB1_0101	465	TVEDNKCPYHDFRRF	DNKCPYHDF	3	0.1656	8335.0	100.00	0.38	tr_D0A3E0_	
DRB1_0101	466	PGQKPEEGYAFSWGE	QKPEEGYAF	2	0.1652	8369.3	100.00	0.46	tr_D0A3E0_	
DRB1_0101	467	HRHGARSPKVKHNQS	RHGARSPKV	1	0.1646	8424.5	100.00	0.46	tr_D0A3E0_	
DRB1_0101	468	MNENHTVYTVEDNKC	NENHTVYTV	1	0.1645	8434.8	100.00	0.44	tr_D0A3E0_	
DRB1_0101	469	GAPGQKPEEGYAFSW	QKPEEGYAF	4	0.1519	9661.8	100.00	0.50	tr_D0A3E0_	
DRB1_0101	470	HGARSPKVKHNQSQI	KVKHNQSQI	6	0.1484	10033.7	100.00	0.26	tr_D0A3E0_	
DRB1_0101	471	AATLGDSTTTGITPP	LGDSTTTGI	3	0.1468	10213.2	100.00	0.47	tr_D0A3E0_	
DRB1_0101	472	TLGDSTTTGITPPYG	TTGITPPYG	6	0.1465	10243.1	100.00	0.33	tr_D0A3E0_	
DRB1_0101	473	RHGARSPKVKHNQSQ	RHGARSPKV	0	0.1360	11473.7	100.00	0.28	tr_D0A3E0_	
DRB1_0101	474	ATLGDSTTTGITPPY	LGDSTTTGI	2	0.1299	12257.7	100.00	0.46	tr_D0A3E0_	
DRB1_0101	475	RGAPGQKPEEGYAFS	QKPEEGYAF	5	0.1277	12551.6	100.00	0.53	tr_D0A3E0_	
DRB1_0101	476	RRGAPGQKPEEGYAF	QKPEEGYAF	6	0.1170	14091.9	100.00	0.26	tr_D0A3E0_	

Allele: DRB1_0101. Number of high binders 0. Number of weak binders 39. Number of peptides 476

Allele	pos	peptide	core	Of	1-log50k(aff)	affinity(nM)	%Rank	Relia	Identity	Bind Level
DRB1_0101	1	CSLFKTTEACKAVGS	FKTTEACKA	3	0.7529	14.5	4.50	0.83	tr_D0A3U3_	WB
DRB1_0101	2	NCSLFKTTEACKAVG	FKTTEACKA	4	0.7455	15.7	5.00	0.79	tr_D0A3U3_	WB
DRB1_0101	3	SLFKTTEACKAVGSY	FKTTEACKA	2	0.7376	17.1	6.00	0.87	tr_D0A3U3_	WB
DRB1_0101	4	TNCSLFKTTEACKAV	FKTTEACKA	5	0.7164	21.5	7.50	0.74	tr_D0A3U3_	WB
DRB1_0101	5	LFKTTEACKAVGSYG	FKTTEACKA	1	0.6880	29.3	11.00	0.81	tr_D0A3U3_	
DRB1_0101	6	ATNCSLFKTTEACKA	FKTTEACKA	6	0.6139	65.2	20.00	0.55	tr_D0A3U3_	
DRB1_0101	7	FKTTEACKAVGSYGC	FKTTEACKA	0	0.5939	80.9	23.00	0.59	tr_D0A3U3_	
DRB1_0101	8	VATNCSLFKTTEACK	CSLFKTTEA	4	0.4341	456.4	55.00	0.67	tr_D0A3U3_	
DRB1_0101	9	TEACKAVGSYGCEWK	CKAVGSYGC	3	0.4239	509.5	60.00	0.61	tr_D0A3U3_	
DRB1_0101	10	TTEACKAVGSYGCEW	CKAVGSYGC	4	0.4119	580.1	60.00	0.53	tr_D0A3U3_	
DRB1_0101	11	KTTEACKAVGSYGCE	CKAVGSYGC	5	0.4008	654.1	65.00	0.41	tr_D0A3U3_	
DRB1_0101	12	EACKAVGSYGCEWKD	CKAVGSYGC	2	0.3887	745.2	65.00	0.66	tr_D0A3U3_	
DRB1_0101	13	EVATNCSLFKTTEAC	CSLFKTTEA	5	0.3805	815.2	70.00	0.60	tr_D0A3U3_	
DRB1_0101	14	ACKAVGSYGCEWKDT	CKAVGSYGC	1	0.3487	1149.7	75.00	0.67	tr_D0A3U3_	
DRB1_0101	15	CKAVGSYGCEWKDTE	CKAVGSYGC	0	0.2661	2810.2	90.00	0.57	tr_D0A3U3_	
DRB1_0101	16	VCSWKKECDSDSDGV	WKKECDSDS	3	0.2426	3622.7	90.00	0.74	tr_D0A3U3_	
DRB1_0101	17	CSWKKECDSDSDGVN	WKKECDSDS	2	0.2314	4088.8	95.00	0.71	tr_D0A3U3_	
DRB1_0101	18	EVCSWKKECDSDSDG	WKKECDSDS	4	0.2176	4747.0	95.00	0.75	tr_D0A3U3_	
DRB1_0101	19	SWKKECDSDSDGVNP	WKKECDSDS	1	0.2051	5437.5	95.00	0.70	tr_D0A3U3_	

DRB1_0101	20	TEVCSWKKECDSDSD	WKKECDSDS	5	0.1938	6142.7	95.00	0.68	tr_D0A3U3_
DRB1_0101	21	GCEWKDTEVCSWKKE	DTEVCSWKK	5	0.1720	7774.7	100.00	0.34	tr_D0A3U3_
DRB1_0101	22	CEWKDTEVCSWKKEC	DTEVCSWKK	4	0.1666	8242.3	100.00	0.37	tr_D0A3U3_
DRB1_0101	23	DSDSDGVNPCESLIG	DGVNPCESL	4	0.1665	8255.7	100.00	0.28	tr_D0A3U3_
DRB1_0101	24	DTEVCSWKKECDSDS	WKKECDSDS	6	0.1602	8835.5	100.00	0.54	tr_D0A3U3_
DRB1_0101	25	YGCEWKDTEVCSWKK	CEWKDTEVC	2	0.1590	8950.7	100.00	0.26	tr_D0A3U3_
DRB1_0101	26	WKKECDSDSDGVNPC	WKKECDSDS	0	0.1536	9488.6	100.00	0.54	tr_D0A3U3_
DRB1_0101	27	EWKDTEVCSWKKECD	DTEVCSWKK	3	0.1427	10676.5	100.00	0.45	tr_D0A3U3_
DRB1_0101	28	SYGCEWKDTEVCSWK	CEWKDTEVC	3	0.1405	10937.1	100.00	0.33	tr_D0A3U3_
DRB1_0101	29	WKDTEVCSWKKECDS	DTEVCSWKK	2	0.1337	11766.8	100.00	0.44	tr_D0A3U3_
DRB1_0101	30	GSYGCEWKDTEVCSW	CEWKDTEVC	4	0.1311	12100.9	100.00	0.34	tr_D0A3U3_
DRB1_0101	31	KDTEVCSWKKECDSD	DTEVCSWKK	1	0.1254	12876.3	100.00	0.41	tr_D0A3U3_
DRB1_0101	32	CDSDSDGVNPCESLI	DGVNPCESL	5	0.1240	13075.3	100.00	0.34	tr_D0A3U3_
DRB1_0101	33	VGSYGCEWKDTEVCS	CEWKDTEVC	5	0.1220	13362.2	100.00	0.34	tr_D0A3U3_
DRB1_0101	34	KAVGSYGCEWKDTEV	AVGSYGCEW	1	0.1177	13993.5	100.00	0.29	tr_D0A3U3_
DRB1_0101	35	AVGSYGCEWKDTEVC	CEWKDTEVC	6	0.1142	14534.5	100.00	0.25	tr_D0A3U3_
DRB1_0101	36	ECDSDSDGVNPCESL	DGVNPCESL	6	0.0832	20315.9	100.00	0.27	tr_D0A3U3_
DRB1_0101	37	KKECDSDSDGVNPCE	ECDSDSDGV	2	0.0803	20967.8	100.00	0.34	tr_D0A3U3_
DRB1_0101	38	KECDSDSDGVNPCES	DSDSDGVNP	3	0.0752	22155.9	100.00	0.25	tr_D0A3U3_

Allele: DRB1_0101. Number of high binders 0. Number of weak binders 4. Number of peptides 38

Allele	pos	peptide	core	Of	1-log50k(aff)	affinity(nM)	%Rank	Relia	Identity	Bind Level
DRB1_0101	1	ARPYLQMHPTRSTLK	YLQMHPTRS	3	0.8456	5.3	0.70	0.75	tr_C9ZPP9_	WB
DRB1_0101	2	RPYLQMHPTRSTLKN	YLQMHPTRS	2	0.8404	5.6	0.80	0.69	tr_C9ZPP9_	WB
DRB1_0101	3	PYLQMHPTRSTLKNI	YLQMHPTRS	1	0.8042	8.3	1.90	0.67	tr_C9ZPP9_	WB
DRB1_0101	4	YLQMHPTRSTLKNII	YLQMHPTRS	0	0.7289	18.8	6.50	0.52	tr_C9ZPP9_	WB
DRB1_0101	5	REQDYEAVASVLHGA	YEAVASVLH	4	0.5931	81.7	24.00	0.60	tr_C9ZPP9_	
DRB1_0101	6	EQDYEAVASVLHGAG	YEAVASVLH	3	0.5848	89.3	25.00	0.64	tr_C9ZPP9_	
DRB1_0101	7	QDYEAVASVLHGAGL	YEAVASVLH	2	0.5721	102.5	27.00	0.61	tr_C9ZPP9_	
DRB1_0101	8	LREQDYEAVASVLHG	YEAVASVLH	5	0.5698	105.0	28.00	0.53	tr_C9ZPP9_	
DRB1_0101	9	RSTLKNIISLCHHNE	LKNIISLCH	3	0.5643	111.5	29.00	0.63	tr_C9ZPP9_	
DRB1_0101	10	TRSTLKNIISLCHHN	LKNIISLCH	4	0.5510	128.7	31.00	0.69	tr_C9ZPP9_	
DRB1_0101	11	STLKNIISLCHHNEH	LKNIISLCH	2	0.5488	131.8	31.00	0.52	tr_C9ZPP9_	
DRB1_0101	12	DYEAVASVLHGAGLH	YEAVASVLH	1	0.5383	147.7	33.00	0.52	tr_C9ZPP9_	
DRB1_0101	13	ELREQDYEAVASVLH	YEAVASVLH	6	0.5331	156.3	34.00	0.38	tr_C9ZPP9_	
DRB1_0101	14	TFSLNLEGSRGKTQA	LNLEGSRGK	3	0.5237	173.1	36.00	0.35	tr_C9ZPP9_	
DRB1_0101	15	PTRSTLKNIISLCHH	LKNIISLCH	5	0.5194	181.2	37.00	0.66	tr_C9ZPP9_	
DRB1_0101	16	DTFSLNLEGSRGKTQ	SLNLEGSRG	3	0.5093	202.2	39.00	0.38	tr_C9ZPP9_	
DRB1_0101	17	YEAVASVLHGAGLHN	YEAVASVLH	0	0.5069	207.6	40.00	0.38	tr_C9ZPP9_	
DRB1_0101	18	KDTFSLNLEGSRGKT	FSLNLEGSR	3	0.5058	210.0	40.00	0.38	tr_C9ZPP9_	
DRB1_0101	19	TLKNIISLCHHNEHP	LKNIISLCH	1	0.5006	222.1	41.00	0.47	tr_C9ZPP9_	
DRB1_0101	20	LQMHPTRSTLKNIIS	LQMHPTRST	0	0.4814	273.4	45.00	0.47	tr_C9ZPP9_	
DRB1_0101	21	LKDTFSLNLEGSRGK	FSLNLEGSR	4	0.4795	279.1	45.00	0.37	tr_C9ZPP9_	
DRB1_0101	22	FSLNLEGSRGKTQAV	LNLEGSRGK	2	0.4766	288.0	46.00	0.38	tr_C9ZPP9_	
DRB1_0101	23	IEQFLKDTFSLNLEG	LKDTFSLNL	4	0.4715	304.5	47.00	0.37	tr_C9ZPP9_	

DRB1_0101	24	RIEQFLKDTFSLNLE	FLKDTFSLN	4	0.4663	321.8	48.00	0.35	tr_C9ZPP9_
DRB1_0101	25	EQFLKDTFSLNLEGS	LKDTFSLNL	3	0.4617	338.5	49.00	0.41	tr_C9ZPP9_
DRB1_0101	26	HPTRSTLKNIISLCH	LKNIISLCH	6	0.4587	349.4	50.00	0.53	tr_C9ZPP9_
DRB1_0101	27	QFLKDTFSLNLEGSR	LKDTFSLNL	2	0.4530	371.9	55.00	0.34	tr_C9ZPP9_
DRB1_0101	28	FLKDTFSLNLEGSRG	FSLNLEGSR	5	0.4491	388.0	55.00	0.30	tr_C9ZPP9_
DRB1_0101	29	LKNIISLCHHNEHPN	IISLCHHNE	3	0.4407	424.6	55.00	0.35	tr_C9ZPP9_
DRB1_0101	30	RRIEQFLKDTFSLNL	FLKDTFSLN	5	0.4381	436.8	55.00	0.38	tr_C9ZPP9_
DRB1_0101	31	VELREQDYEAVASVL	LREQDYEAV	2	0.4333	460.3	55.00	0.38	tr_C9ZPP9_
DRB1_0101	32	SLNLEGSRGKTQAVE	LNLEGSRGK	1	0.3926	714.5	65.00	0.35	tr_C9ZPP9_
DRB1_0101	33	IGDTSQQRGGTVRRI	TSQQRGGTV	3	0.3922	718.0	65.00	0.69	tr_C9ZPP9_
DRB1_0101	34	GDTSQQRGGTVRRIE	TSQQRGGTV	2	0.3903	732.7	65.00	0.60	tr_C9ZPP9_
DRB1_0101	35	AVELREQDYEAVASV	LREQDYEAV	3	0.3800	819.0	70.00	0.62	tr_C9ZPP9_
DRB1_0101	36	DTSQQRGGTVRRIEQ	TSQQRGGTV	1	0.3778	838.7	70.00	0.53	tr_C9ZPP9_
DRB1_0101	37	QMHPTRSTLKNIISL	MHPTRSTLK	1	0.3769	847.4	70.00	0.38	tr_C9ZPP9_
DRB1_0101	38	KNIISLCHHNEHPNL	IISLCHHNE	2	0.3746	868.3	70.00	0.43	tr_C9ZPP9_
DRB1_0101	39	VRRIEQFLKDTFSLN	FLKDTFSLN	6	0.3526	1101.3	75.00	0.30	tr_C9ZPP9_
DRB1_0101	40	NIISLCHHNEHPNLK	IISLCHHNE	1	0.3501	1131.9	75.00	0.31	tr_C9ZPP9_
DRB1_0101	41	GIGDTSQQRGGTVRR	TSQQRGGTV	4	0.3459	1184.5	75.00	0.75	tr_C9ZPP9_
DRB1_0101	42	RGGTVRRIEQFLKDT	VRRIEQFLK	4	0.3445	1202.9	75.00	0.46	tr_C9ZPP9_
DRB1_0101	43	MHPTRSTLKNIISLC	TRSTLKNII	3	0.3430	1222.7	75.00	0.23	tr_C9ZPP9_
DRB1_0101	44	GGTVRRIEQFLKDTF	VRRIEQFLK	3	0.3387	1281.2	75.00	0.52	tr_C9ZPP9_
DRB1_0101	45	TSQQRGGTVRRIEQF	QRGGTVRRI	3	0.3358	1321.8	75.00	0.41	tr_C9ZPP9_
DRB1_0101	46	QQRGGTVRRIEQFLK	VRRIEQFLK	6	0.3356	1324.5	75.00	0.29	tr_C9ZPP9_
DRB1_0101	47	QRGGTVRRIEQFLKD	VRRIEQFLK	5	0.3316	1382.3	80.00	0.41	tr_C9ZPP9_
DRB1_0101	48	QAVELREQDYEAVAS	LREQDYEAV	4	0.3305	1400.2	80.00	0.68	tr_C9ZPP9_
DRB1_0101	49	GTVRRIEQFLKDTFS	VRRIEQFLK	2	0.3268	1456.4	80.00	0.49	tr_C9ZPP9_
DRB1_0101	50	TVRRIEQFLKDTFSL	VRRIEQFLK	1	0.3135	1682.5	80.00	0.35	tr_C9ZPP9_
DRB1_0101	51	IISLCHHNEHPNLKG	CHHNEHPNL	4	0.3091	1763.9	80.00	0.23	tr_C9ZPP9_
DRB1_0101	52	LNLEGSRGKTQAVEL	LEGSRGKTQ	2	0.3080	1785.7	80.00	0.43	tr_C9ZPP9_
DRB1_0101	53	KGIGDTSQQRGGTVR	TSQQRGGTV	5	0.2984	1981.4	85.00	0.63	tr_C9ZPP9_
DRB1_0101	54	SQQRGGTVRRIEQFL	QRGGTVRRI	2	0.2954	2045.4	85.00	0.52	tr_C9ZPP9_
DRB1_0101	55	TQAVELREQDYEAVA	LREQDYEAV	5	0.2948	2058.2	85.00	0.65	tr_C9ZPP9_
DRB1_0101	56	NLEGSRGKTQAVELR	LEGSRGKTQ	1	0.2898	2173.0	85.00	0.31	tr_C9ZPP9_
DRB1_0101	57	ISLCHHNEHPNLKGI	CHHNEHPNL	3	0.2863	2257.6	85.00	0.30	tr_C9ZPP9_
DRB1_0101	58	EGSRGKTQAVELREQ	GKTQAVELR	4	0.2812	2384.5	85.00	0.40	tr_C9ZPP9_
DRB1_0101	59	LEGSRGKTQAVELRE	GKTQAVELR	5	0.2800	2416.9	85.00	0.31	tr_C9ZPP9_
DRB1_0101	60	SLCHHNEHPNLKGIG	CHHNEHPNL	2	0.2636	2886.2	90.00	0.31	tr_C9ZPP9_
DRB1_0101	61	GSRGKTQAVELREQD	GKTQAVELR	3	0.2635	2890.4	90.00	0.43	tr_C9ZPP9_
DRB1_0101	62	SRGKTQAVELREQDY	GKTQAVELR	2	0.2428	3614.8	90.00	0.46	tr_C9ZPP9_
DRB1_0101	63	LKGIGDTSQQRGGTV	TSQQRGGTV	6	0.2389	3770.8	95.00	0.40	tr_C9ZPP9_
DRB1_0101	64	LCHHNEHPNLKGIGD	HHNEHPNLK	2	0.2258	4345.6	95.00	0.34	tr_C9ZPP9_
DRB1_0101	65	KTQAVELREQDYEAV	LREQDYEAV	6	0.2210	4576.0	95.00	0.56	tr_C9ZPP9_
DRB1_0101	66	HPNLKGIGDTSQQRG	LKGIGDTSQ	3	0.2184	4704.6	95.00	0.38	tr_C9ZPP9_
DRB1_0101	67	PNLKGIGDTSQQRGG	LKGIGDTSQ	2	0.2173	4764.0	95.00	0.35	tr_C9ZPP9_
DRB1_0101	68	NLKGIGDTSQQRGGT	GIGDTSQQR	3	0.2052	5429.2	95.00	0.28	tr_C9ZPP9_
DRB1_0101	69	RGKTQAVELREQDYE	GKTQAVELR	1	0.2048	5451.8	95.00	0.44	tr_C9ZPP9_
DRB1_0101	70	EHPNLKGIGDTSQQR	LKGIGDTSQ	4	0.2028	5570.0	95.00	0.40	tr_C9ZPP9_
DRB1_0101	71	CHHNEHPNLKGIGDT	HHNEHPNLK	1	0.1782	7274.8	100.00	0.40	tr_C9ZPP9_
DRB1_0101	72	NEHPNLKGIGDTSQQ	LKGIGDTSQ	5	0.1742	7591.2	100.00	0.38	tr_C9ZPP9_
DRB1_0101	73	GKTQAVELREQDYEA	GKTQAVELR	0	0.1510	9757.8	100.00	0.29	tr_C9ZPP9_

DRB1_0101	74	HNEHPNLKGIGDTSQ	NLKGIGDTS	5	0.1487	10004.0	100.00	0.32	tr_C9ZPP9_	
DRB1_0101	75	HHNEHPNLKGIGDTS	HHNEHPNLK	0	0.1382	11210.5	100.00	0.24	tr_C9ZPP9_	

Allele: DRB1_0101. Number of high binders 0. Number of weak binders 4. Number of peptides 75

Allele	pos	peptide	core	Of	1-log50k(aff)	affinity(nM)	%Rank	Relia	Identity	Bind Level
DRB1_0101	1	STTFNLGSATLGAGV	FNLGSATLG	3	0.8341	6.0	1.00	0.80	tr_C9ZVS3_	WB
DRB1_0101	2	LSTTFNLGSATLGAG	FNLGSATLG	4	0.8266	6.5	1.20	0.78	tr_C9ZVS3_	WB
DRB1_0101	3	TTFNLGSATLGAGVI	FNLGSATLG	2	0.8146	7.4	1.50	0.80	tr_C9ZVS3_	WB
DRB1_0101	4	ALSTTFNLGSATLGA	FNLGSATLG	5	0.8060	8.2	1.80	0.75	tr_C9ZVS3_	WB
DRB1_0101	5	TFNLGSATLGAGVIS	FNLGSATLG	1	0.7805	10.8	3.00	0.79	tr_C9ZVS3_	WB
DRB1_0101	6	QTHLLNMLSPTEPLG	LLNMLSPTE	3	0.7471	15.4	5.00	0.46	tr_C9ZVS3_	WB
DRB1_0101	7	THLLNMLSPTEPLGS	LLNMLSPTE	2	0.7374	17.1	6.00	0.42	tr_C9ZVS3_	WB
DRB1_0101	8	QQTHLLNMLSPTEPL	LLNMLSPTE	4	0.7307	18.4	6.50	0.50	tr_C9ZVS3_	WB
DRB1_0101	9	GALSTTFNLGSATLG	FNLGSATLG	6	0.7219	20.3	7.00	0.69	tr_C9ZVS3_	WB
DRB1_0101	10	CINAIIPHGGALSTT	IIPHGGALS	4	0.7045	24.5	8.50	0.68	tr_C9ZVS3_	WB
DRB1_0101	11	FNLGSATLGAGVISL	FNLGSATLG	0	0.7035	24.7	8.50	0.57	tr_C9ZVS3_	WB
DRB1_0101	12	INAIIPHGGALSTTF	IIPHGGALS	3	0.7026	25.0	9.00	0.77	tr_C9ZVS3_	WB
DRB1_0101	13	HLLNMLSPTEPLGSG	LLNMLSPTE	1	0.6998	25.7	9.00	0.38	tr_C9ZVS3_	WB
DRB1_0101	14	MQVAMPVSLVDSCLG	QVAMPVSLV	1	0.6961	26.8	9.50	0.55	tr_C9ZVS3_	WB
DRB1_0101	15	QCINAIIPHGGALST	IIPHGGALS	5	0.6893	28.8	10.00	0.58	tr_C9ZVS3_	
DRB1_0101	16	SKFMQCINAIIPHGG	FMQCINAII	2	0.6731	34.4	12.00	0.61	tr_C9ZVS3_	
DRB1_0101	17	TQQTHLLNMLSPTEP	LLNMLSPTE	5	0.6624	38.6	13.00	0.51	tr_C9ZVS3_	
DRB1_0101	18	DSKFMQCINAIIPHG	FMQCINAII	3	0.6592	39.9	14.00	0.69	tr_C9ZVS3_	
DRB1_0101	19	TDSKFMQCINAIIPH	FMQCINAII	4	0.6576	40.6	14.00	0.65	tr_C9ZVS3_	
DRB1_0101	20	KFMQCINAIIPHGGA	FMQCINAII	1	0.6552	41.7	14.00	0.50	tr_C9ZVS3_	
DRB1_0101	21	QVAMPVSLVDSCLGM	QVAMPVSLV	0	0.6522	43.1	15.00	0.40	tr_C9ZVS3_	
DRB1_0101	22	NAIIPHGGALSTTFN	IIPHGGALS	2	0.6518	43.3	15.00	0.85	tr_C9ZVS3_	
DRB1_0101	23	MQCINAIIPHGGALS	INAIIPHGG	3	0.6360	51.4	17.00	0.48	tr_C9ZVS3_	
DRB1_0101	24	TTDSKFMQCINAIIP	FMQCINAII	5	0.6332	52.9	17.00	0.64	tr_C9ZVS3_	
DRB1_0101	25	HTHVHTQQTHLLNML	VHTQQTHLL	3	0.6286	55.6	18.00	0.77	tr_C9ZVS3_	
DRB1_0101	26	LLNMLSPTEPLGSGK	LNMLSPTEP	1	0.6186	62.0	20.00	0.42	tr_C9ZVS3_	
DRB1_0101	27	AIIPHGGALSTTFNL	IIPHGGALS	1	0.6119	66.7	21.00	0.74	tr_C9ZVS3_	
DRB1_0101	28	YSCVWQSLLPLYELA	WQSLLPLYE	4	0.6110	67.3	21.00	0.32	tr_C9ZVS3_	
DRB1_0101	29	THVHTQQTHLLNMLS	VHTQQTHLL	2	0.6092	68.6	21.00	0.70	tr_C9ZVS3_	
DRB1_0101	30	HTQQTHLLNMLSPTE	LLNMLSPTE	6	0.6052	71.7	22.00	0.50	tr_C9ZVS3_	
DRB1_0101	31	SCVWQSLLPLYELAR	WQSLLPLYE	3	0.6041	72.5	22.00	0.37	tr_C9ZVS3_	
DRB1_0101	32	VYSCVWQSLLPLYEL	CVWQSLLPL	3	0.5986	77.0	23.00	0.32	tr_C9ZVS3_	
DRB1_0101	33	VAMPVSLVDSCLGMQ	PVSLVDSCL	3	0.5984	77.1	23.00	0.50	tr_C9ZVS3_	
DRB1_0101	34	THTHVHTQQTHLLNM	VHTQQTHLL	4	0.5984	77.1	23.00	0.73	tr_C9ZVS3_	
DRB1_0101	35	PTTDSKFMQCINAII	FMQCINAII	6	0.5932	81.6	24.00	0.56	tr_C9ZVS3_	
DRB1_0101	36	AMPVSLVDSCLGMQE	PVSLVDSCL	2	0.5922	82.4	24.00	0.46	tr_C9ZVS3_	
DRB1_0101	37	FMQCINAIIPHGGAL	INAIIPHGG	4	0.5891	85.3	24.00	0.52	tr_C9ZVS3_	
DRB1_0101	38	GEGYGAMSAAEEKSH	YGAMSAAEE	3	0.5839	90.3	25.00	0.83	tr_C9ZVS3_	
DRB1_0101	39	TLGAGVISLAIAFQM	ISLAIAFQM	6	0.5821	92.0	25.00	0.55	tr_C9ZVS3_	
DRB1_0101	40	VDSCLGMQELLVLVY	CLGMQELLV	3	0.5820	92.1	25.00	0.57	tr_C9ZVS3_	

DRB1_0101	41	CVWQSLLPLYELARF	WQSLLPLYE	2	0.5801	94.0	26.00	0.34	tr_C9ZVS3_
DRB1_0101	42	LVYSCVWQSLLPLYE	CVWQSLLPL	4	0.5799	94.2	26.00	0.33	tr_C9ZVS3_
DRB1_0101	43	EGYGAMSAAEEKSHH	YGAMSAAEE	2	0.5759	98.3	26.00	0.82	tr_C9ZVS3_
DRB1_0101	44	LYELARFFSFPFQSS	LARFFSFPF	3	0.5752	99.1	27.00	0.36	tr_C9ZVS3_
DRB1_0101	45	YELARFFSFPFQSSS	LARFFSFPF	2	0.5735	101.0	27.00	0.35	tr_C9ZVS3_
DRB1_0101	46	DSCLGMQELLVLVYS	CLGMQELLV	2	0.5731	101.4	27.00	0.54	tr_C9ZVS3_
DRB1_0101	47	HVHTQQTHLLNMLSP	VHTQQTHLL	1	0.5667	108.6	28.00	0.60	tr_C9ZVS3_
DRB1_0101	48	MPVSLVDSCLGMQEL	VSLVDSCLG	2	0.5661	109.4	28.00	0.43	tr_C9ZVS3_
DRB1_0101	49	LVDSCLGMQELLVLV	CLGMQELLV	4	0.5656	110.0	28.00	0.54	tr_C9ZVS3_
DRB1_0101	50	LGSATLGAGVISLAI	LGAGVISLA	5	0.5600	116.8	29.00	0.25	tr_C9ZVS3_
DRB1_0101	51	VLVYSCVWQSLLPLY	YSCVWQSLL	3	0.5599	116.9	29.00	0.34	tr_C9ZVS3_
DRB1_0101	52	EGEGYGAMSAAEEKS	YGAMSAAEE	4	0.5544	124.1	30.00	0.82	tr_C9ZVS3_
DRB1_0101	53	GSATLGAGVISLAIA	LGAGVISLA	4	0.5539	124.8	30.00	0.29	tr_C9ZVS3_
DRB1_0101	54	NLGSATLGAGVISLA	GSATLGAGV	2	0.5521	127.3	31.00	0.35	tr_C9ZVS3_
DRB1_0101	55	VWQSLLPLYELARFF	WQSLLPLYE	1	0.5503	129.8	31.00	0.28	tr_C9ZVS3_
DRB1_0101	56	HTHTHVHTQQTHLLN	VHTQQTHLL	5	0.5480	133.1	32.00	0.74	tr_C9ZVS3_
DRB1_0101	57	RFFSFPFQSSSYQAA	FFSFPFQSS	1	0.5474	133.9	32.00	0.38	tr_C9ZVS3_
DRB1_0101	58	ELARFFSFPFQSSSY	FFSFPFQSS	4	0.5471	134.3	32.00	0.47	tr_C9ZVS3_
DRB1_0101	59	SFPFQSSSYQAAHTH	FQSSSYQAA	3	0.5471	134.4	32.00	0.50	tr_C9ZVS3_
DRB1_0101	60	FFSFPFQSSSYQAAH	FQSSSYQAA	5	0.5461	135.8	32.00	0.35	tr_C9ZVS3_
DRB1_0101	61	SLVDSCLGMQELLVL	CLGMQELLV	5	0.5416	142.6	33.00	0.54	tr_C9ZVS3_
DRB1_0101	62	PLYELARFFSFPFQS	YELARFFSF	2	0.5409	143.6	33.00	0.41	tr_C9ZVS3_
DRB1_0101	63	SCLGMQELLVLVYSC	CLGMQELLV	1	0.5385	147.4	33.00	0.50	tr_C9ZVS3_
DRB1_0101	64	LARFFSFPFQSSSYQ	FFSFPFQSS	3	0.5381	148.1	33.00	0.54	tr_C9ZVS3_
DRB1_0101	65	GYGAMSAAEEKSHHK	YGAMSAAEE	1	0.5370	149.8	34.00	0.82	tr_C9ZVS3_
DRB1_0101	66	LPLYELARFFSFPFQ	YELARFFSF	3	0.5363	151.0	34.00	0.46	tr_C9ZVS3_
DRB1_0101	67	FSFPFQSSSYQAAHT	FQSSSYQAA	4	0.5353	152.7	34.00	0.47	tr_C9ZVS3_
DRB1_0101	68	QSLLPLYELARFFSF	YELARFFSF	6	0.5351	152.9	34.00	0.29	tr_C9ZVS3_
DRB1_0101	69	SATLGAGVISLAIAF	LGAGVISLA	3	0.5339	155.0	34.00	0.35	tr_C9ZVS3_
DRB1_0101	70	ARFFSFPFQSSSYQA	FFSFPFQSS	2	0.5318	158.4	35.00	0.50	tr_C9ZVS3_
DRB1_0101	71	FPFQSSSYQAAHTHT	FQSSSYQAA	2	0.5277	165.7	35.00	0.49	tr_C9ZVS3_
DRB1_0101	72	VHTQQTHLLNMLSPT	VHTQQTHLL	0	0.5263	168.2	36.00	0.38	tr_C9ZVS3_
DRB1_0101	73	IIPHGGALSTTFNLG	IIPHGGALS	0	0.5244	171.8	36.00	0.50	tr_C9ZVS3_
DRB1_0101	74	GGALSTTFNLGSATL	GALSTTFNL	1	0.5221	176.0	37.00	0.37	tr_C9ZVS3_
DRB1_0101	75	VSLVDSCLGMQELLV	CLGMQELLV	6	0.5203	179.6	37.00	0.42	tr_C9ZVS3_
DRB1_0101	76	WQSLLPLYELARFFS	LLPLYELAR	3	0.5175	185.0	38.00	0.32	tr_C9ZVS3_
DRB1_0101	77	PFQSSSYQAAHTHTH	FQSSSYQAA	1	0.5145	191.2	38.00	0.38	tr_C9ZVS3_
DRB1_0101	78	LLPLYELARFFSFPF	YELARFFSF	4	0.5132	193.9	38.00	0.45	tr_C9ZVS3_
DRB1_0101	79	LVLVYSCVWQSLLPL	YSCVWQSLL	4	0.5122	195.9	39.00	0.36	tr_C9ZVS3_
DRB1_0101	80	PVSLVDSCLGMQELL	VSLVDSCLG	1	0.5093	202.2	39.00	0.44	tr_C9ZVS3_
DRB1_0101	81	FQSSSYQAAHTHTHT	YQAAHTHTH	5	0.5083	204.3	39.00	0.31	tr_C9ZVS3_
DRB1_0101	82	SLLPLYELARFFSFP	YELARFFSF	5	0.5045	212.9	40.00	0.46	tr_C9ZVS3_
DRB1_0101	83	LNMLSPTEPLGSGKA	NMLSPTEPL	1	0.4996	224.5	41.00	0.41	tr_C9ZVS3_
DRB1_0101	84	DEGEGYGAMSAAEEK	YGAMSAAEE	5	0.4988	226.5	41.00	0.78	tr_C9ZVS3_
DRB1_0101	85	CLGMQELLVLVYSCV	CLGMQELLV	0	0.4879	255.0	44.00	0.33	tr_C9ZVS3_
DRB1_0101	86	ATLGAGVISLAIAFQ	LGAGVISLA	2	0.4852	262.3	44.00	0.35	tr_C9ZVS3_
DRB1_0101	87	QSSSYQAAHTHTHTH	YQAAHTHTH	4	0.4747	294.0	46.00	0.49	tr_C9ZVS3_
DRB1_0101	88	HGGALSTTFNLGSAT	GALSTTFNL	2	0.4668	320.4	48.00	0.56	tr_C9ZVS3_
DRB1_0101	89	PHGGALSTTFNLGSA	GALSTTFNL	3	0.4652	326.0	48.00	0.62	tr_C9ZVS3_
DRB1_0101	90	ELLVLVYSCVWQSLL	LVYSCVWQS	4	0.4647	327.7	48.00	0.28	tr_C9ZVS3_

DRB1_0101	91	THTHTHVHTQQTHLL	VHTQQTHLL	6	0.4554	362.4	50.00	0.60	tr_C9ZVS3_
DRB1_0101	92	LLVLVYSCVWQSLLP	YSCVWQSLL	5	0.4543	366.5	55.00	0.38	tr_C9ZVS3_
DRB1_0101	93	SSSYQAAHTHTHTHT	YQAAHTHTH	3	0.4528	372.4	55.00	0.59	tr_C9ZVS3_
DRB1_0101	94	IPHGGALSTTFNLGS	GALSTTFNL	4	0.4415	421.0	55.00	0.57	tr_C9ZVS3_
DRB1_0101	95	YGAMSAAEEKSHHKN	YGAMSAAEE	0	0.4347	453.4	55.00	0.65	tr_C9ZVS3_
DRB1_0101	96	QELLVLVYSCVWQSL	LLVLVYSCV	2	0.4084	602.3	60.00	0.43	tr_C9ZVS3_
DRB1_0101	97	NMLSPTEPLGSGKAH	TEPLGSGKA	5	0.3952	694.6	65.00	0.36	tr_C9ZVS3_
DRB1_0101	98	SSYQAAHTHTHTHTH	YQAAHTHTH	2	0.3933	709.5	65.00	0.67	tr_C9ZVS3_
DRB1_0101	99	LGMQELLVLVYSCVW	LLVLVYSCV	5	0.3932	710.1	65.00	0.35	tr_C9ZVS3_
DRB1_0101	100	MQELLVLVYSCVWQS	LLVLVYSCV	3	0.3905	731.6	65.00	0.49	tr_C9ZVS3_
DRB1_0101	101	TPTTDSKFMQCINAI	SKFMQCINA	5	0.3862	766.3	65.00	0.47	tr_C9ZVS3_
DRB1_0101	102	GMQELLVLVYSCVWQ	LLVLVYSCV	4	0.3727	886.4	70.00	0.49	tr_C9ZVS3_
DRB1_0101	103	TDEGEGYGAMSAAEE	YGAMSAAEE	6	0.3705	907.9	70.00	0.62	tr_C9ZVS3_
DRB1_0101	104	MLSPTEPLGSGKAHT	TEPLGSGKA	4	0.3372	1301.0	75.00	0.69	tr_C9ZVS3_
DRB1_0101	105	LSPTEPLGSGKAHTE	TEPLGSGKA	3	0.3329	1363.4	80.00	0.72	tr_C9ZVS3_
DRB1_0101	106	SPTEPLGSGKAHTEV	TEPLGSGKA	2	0.3293	1417.6	80.00	0.62	tr_C9ZVS3_
DRB1_0101	107	PTEPLGSGKAHTEVV	TEPLGSGKA	1	0.3264	1463.2	80.00	0.49	tr_C9ZVS3_
DRB1_0101	108	SYQAAHTHTHTHTHV	YQAAHTHTH	1	0.3178	1606.2	80.00	0.73	tr_C9ZVS3_
DRB1_0101	109	TEPLGSGKAHTEVVT	LGSGKAHTE	3	0.3064	1816.0	85.00	0.38	tr_C9ZVS3_
DRB1_0101	110	EPLGSGKAHTEVVTD	LGSGKAHTE	2	0.2566	3113.4	90.00	0.46	tr_C9ZVS3_
DRB1_0101	111	VTDEGEGYGAMSAAE	GEGYGAMSA	4	0.2535	3219.3	90.00	0.35	tr_C9ZVS3_
DRB1_0101	112	VVTDEGEGYGAMSAA	GEGYGAMSA	5	0.2424	3628.5	90.00	0.34	tr_C9ZVS3_
DRB1_0101	113	DTPTTDSKFMQCINA	SKFMQCINA	6	0.2407	3698.3	90.00	0.55	tr_C9ZVS3_
DRB1_0101	114	PLGSGKAHTEVVTDE	LGSGKAHTE	1	0.2394	3751.9	95.00	0.38	tr_C9ZVS3_
DRB1_0101	115	HTHTHTHVHTQQTHL	HVHTQQTHL	6	0.2390	3765.9	95.00	0.28	tr_C9ZVS3_
DRB1_0101	116	YQAAHTHTHTHTHVH	YQAAHTHTH	0	0.2315	4083.2	95.00	0.47	tr_C9ZVS3_
DRB1_0101	117	EVVTDEGEGYGAMSA	TDEGEGYGA	3	0.2079	5271.4	95.00	0.25	tr_C9ZVS3_
DRB1_0101	118	GAMSAAEEKSHHKNG	AAEEKSHHK	4	0.2007	5701.1	95.00	0.32	tr_C9ZVS3_
DRB1_0101	119	LGSGKAHTEVVTDEG	SGKAHTEVV	2	0.1975	5900.0	95.00	0.38	tr_C9ZVS3_
DRB1_0101	120	THTHTHTHVHTQQTH	HTHTHVHTQ	3	0.1805	7094.4	100.00	0.29	tr_C9ZVS3_
DRB1_0101	121	TEVVTDEGEGYGAMS	TDEGEGYGA	4	0.1717	7797.9	100.00	0.28	tr_C9ZVS3_
DRB1_0101	122	HTHTHTHTHVHTQQT	THTHTHVHT	3	0.1704	7915.0	100.00	0.22	tr_C9ZVS3_
DRB1_0101	123	AAHTHTHTHTHVHTQ	THTHTHTHV	3	0.1678	8137.7	100.00	0.41	tr_C9ZVS3_
DRB1_0101	124	AHTHTHTHTHVHTQQ	THTHTHTHV	2	0.1671	8202.9	100.00	0.34	tr_C9ZVS3_
DRB1_0101	125	AMSAAEEKSHHKNGD	AAEEKSHHK	3	0.1580	9048.1	100.00	0.44	tr_C9ZVS3_
DRB1_0101	126	QAAHTHTHTHTHVHT	THTHTHTHV	4	0.1561	9238.7	100.00	0.44	tr_C9ZVS3_
DRB1_0101	127	HTEVVTDEGEGYGAM	VVTDEGEGY	3	0.1503	9831.4	100.00	0.36	tr_C9ZVS3_
DRB1_0101	128	GSGKAHTEVVTDEGE	SGKAHTEVV	1	0.1395	11058.4	100.00	0.50	tr_C9ZVS3_
DRB1_0101	129	MSAAEEKSHHKNGDT	AAEEKSHHK	2	0.1317	12031.1	100.00	0.49	tr_C9ZVS3_
DRB1_0101	130	GDTPTTDSKFMQCIN	TDSKFMQCI	5	0.1311	12101.0	100.00	0.41	tr_C9ZVS3_
DRB1_0101	131	NGDTPTTDSKFMQCI	TDSKFMQCI	6	0.1186	13849.6	100.00	0.31	tr_C9ZVS3_
DRB1_0101	132	KSHHKNGDTPTTDSK	HKNGDTPTT	3	0.1069	15730.6	100.00	0.47	tr_C9ZVS3_
DRB1_0101	133	AHTEVVTDEGEGYGA	VVTDEGEGY	4	0.1051	16036.3	100.00	0.52	tr_C9ZVS3_
DRB1_0101	134	SAAEEKSHHKNGDTP	AAEEKSHHK	1	0.1033	16345.1	100.00	0.50	tr_C9ZVS3_
DRB1_0101	135	HHKNGDTPTTDSKFM	HKNGDTPTT	1	0.1026	16478.8	100.00	0.38	tr_C9ZVS3_
DRB1_0101	136	SGKAHTEVVTDEGEG	SGKAHTEVV	0	0.1007	16816.0	100.00	0.37	tr_C9ZVS3_
DRB1_0101	137	SHHKNGDTPTTDSKF	HKNGDTPTT	2	0.0991	17104.1	100.00	0.43	tr_C9ZVS3_
DRB1_0101	138	HKNGDTPTTDSKFMQ	TPTTDSKFM	5	0.0991	17109.1	100.00	0.25	tr_C9ZVS3_
DRB1_0101	139	EKSHHKNGDTPTTDS	HKNGDTPTT	4	0.0980	17315.6	100.00	0.44	tr_C9ZVS3_
DRB1_0101	140	KNGDTPTTDSKFMQC	TPTTDSKFM	4	0.0916	18558.8	100.00	0.31	tr_C9ZVS3_

DRB1_0101	141	EEKSHHKNGDTPTTD	HKNGDTPTT	5	0.0886	19180.6	100.00	0.39	tr_C9ZVS3_	
DRB1_0101	142	KAHTEVVTDEGEGYG	VVTDEGEGY	5	0.0824	20502.3	100.00	0.53	tr_C9ZVS3_	
DRB1_0101	143	AAEEKSHHKNGDTPT	AAEEKSHHK	0	0.0808	20864.0	100.00	0.31	tr_C9ZVS3_	
DRB1_0101	144	AEEKSHHKNGDTPTT	HKNGDTPTT	6	0.0807	20886.3	100.00	0.28	tr_C9ZVS3_	
DRB1_0101	145	GKAHTEVVTDEGEGY	VVTDEGEGY	6	0.0698	23492.8	100.00	0.32	tr_C9ZVS3_	

Allele: DRB1_0101. Number of high binders 0. Number of weak binders 14. Number of peptides 145

Allele	pos	peptide	core	Of	1-log50k(aff)	affinity(nM)	%Rank	Relia	Identity	Bind Level
DRB1_0101	1	PLAYIKLPTRRLSYL	YIKLPTRRL	3	0.8621	4.4	0.40	0.89	tr_D0A1S7_	SB
DRB1_0101	2	LAYIKLPTRRLSYLN	YIKLPTRRL	2	0.8556	4.8	0.50	0.91	tr_D0A1S7_	WB
DRB1_0101	3	LSHLYHMTPNLLDNI	LYHMTPNLL	3	0.8541	4.8	0.60	0.81	tr_D0A1S7_	WB
DRB1_0101	4	IPLAYIKLPTRRLSY	YIKLPTRRL	4	0.8466	5.3	0.70	0.85	tr_D0A1S7_	WB
DRB1_0101	5	VLSHLYHMTPNLLDN	LYHMTPNLL	4	0.8464	5.3	0.70	0.82	tr_D0A1S7_	WB
DRB1_0101	6	SHLYHMTPNLLDNIF	LYHMTPNLL	2	0.8432	5.5	0.80	0.77	tr_D0A1S7_	WB
DRB1_0101	7	YVLSHLYHMTPNLLD	LYHMTPNLL	5	0.8317	6.2	1.00	0.77	tr_D0A1S7_	WB
DRB1_0101	8	SIPLAYIKLPTRRLS	YIKLPTRRL	5	0.8191	7.1	1.40	0.77	tr_D0A1S7_	WB
DRB1_0101	9	AYIKLPTRRLSYLNF	YIKLPTRRL	1	0.8171	7.2	1.40	0.89	tr_D0A1S7_	WB
DRB1_0101	10	HLYHMTPNLLDNIFM	LYHMTPNLL	1	0.8133	7.5	1.60	0.74	tr_D0A1S7_	WB
DRB1_0101	11	QDIYYLIQAFNGDAH	YYLIQAFNG	3	0.8071	8.1	1.80	0.71	tr_D0A1S7_	WB
DRB1_0101	12	IYVLSHLYHMTPNLL	LYHMTPNLL	6	0.8070	8.1	1.80	0.69	tr_D0A1S7_	WB
DRB1_0101	13	DIYYLIQAFNGDAHI	YYLIQAFNG	2	0.8049	8.3	1.90	0.71	tr_D0A1S7_	WB
DRB1_0101	14	IYYLIQAFNGDAHIN	YYLIQAFNG	1	0.7975	8.9	2.50	0.67	tr_D0A1S7_	WB
DRB1_0101	15	EAMFFAGGPLILHCL	FFAGGPLIL	3	0.7908	9.6	2.50	0.71	tr_D0A1S7_	WB
DRB1_0101	16	NIAFYAAQDIYYLIQ	FYAAQDIYY	3	0.7887	9.8	2.50	0.50	tr_D0A1S7_	WB
DRB1_0101	17	YNIAFYAAQDIYYLI	FYAAQDIYY	4	0.7821	10.6	3.00	0.50	tr_D0A1S7_	WB
DRB1_0101	18	AQDIYYLIQAFNGDA	YYLIQAFNG	4	0.7820	10.6	3.00	0.77	tr_D0A1S7_	WB
DRB1_0101	19	REAMFFAGGPLILHC	FFAGGPLIL	4	0.7800	10.8	3.00	0.71	tr_D0A1S7_	WB
DRB1_0101	20	RVHMMKIQHAHDVLL	VHMMKIQHA	1	0.7798	10.8	3.00	0.46	tr_D0A1S7_	WB
DRB1_0101	21	AMFFAGGPLILHCLQ	FFAGGPLIL	2	0.7790	10.9	3.00	0.73	tr_D0A1S7_	WB
DRB1_0101	22	IAFYAAQDIYYLIQA	FYAAQDIYY	2	0.7771	11.1	3.50	0.44	tr_D0A1S7_	WB
DRB1_0101	23	NRMLTTLRPPLEHIL	LTTLRPPLE	3	0.7740	11.5	3.50	0.82	tr_D0A1S7_	WB
DRB1_0101	24	YREAMFFAGGPLILH	FFAGGPLIL	5	0.7704	12.0	3.50	0.70	tr_D0A1S7_	WB
DRB1_0101	25	VHMMKIQHAHDVLLA	MKIQHAHDV	3	0.7655	12.7	4.00	0.47	tr_D0A1S7_	WB
DRB1_0101	26	AFEFFLQLAFGVIYV	FFLQLAFGV	3	0.7619	13.1	4.00	0.51	tr_D0A1S7_	WB
DRB1_0101	27	CRVHMMKIQHAHDVL	VHMMKIQHA	2	0.7600	13.4	4.50	0.60	tr_D0A1S7_	WB
DRB1_0101	28	ANRMLTTLRPPLEHI	LTTLRPPLE	4	0.7572	13.8	4.50	0.78	tr_D0A1S7_	WB
DRB1_0101	29	FEFFLQLAFGVIYVL	FFLQLAFGV	2	0.7568	13.9	4.50	0.47	tr_D0A1S7_	WB
DRB1_0101	30	CSIPLAYIKLPTRRL	YIKLPTRRL	6	0.7529	14.5	4.50	0.64	tr_D0A1S7_	WB
DRB1_0101	31	LYNIAFYAAQDIYYL	FYAAQDIYY	5	0.7516	14.7	5.00	0.47	tr_D0A1S7_	WB
DRB1_0101	32	DAFEFFLQLAFGVIY	FFLQLAFGV	4	0.7507	14.8	5.00	0.54	tr_D0A1S7_	WB
DRB1_0101	33	RMLTTLRPPLEHILG	LTTLRPPLE	2	0.7482	15.3	5.00	0.82	tr_D0A1S7_	WB
DRB1_0101	34	HMMKIQHAHDVLLAK	MKIQHAHDV	2	0.7455	15.7	5.00	0.48	tr_D0A1S7_	WB
DRB1_0101	35	AFYAAQDIYYLIQAF	YAAQDIYYL	2	0.7439	16.0	5.50	0.49	tr_D0A1S7_	WB
DRB1_0101	36	SCRVHMMKIQHAHDV	VHMMKIQHA	3	0.7383	17.0	5.50	0.70	tr_D0A1S7_	WB
DRB1_0101	37	RAYINFDLVPTVNSQ	FDLVPTVNS	5	0.7372	17.2	6.00	0.67	tr_D0A1S7_	WB

DRB1_0101	38	MFFAGGPLILHCLQM	FFAGGPLIL	1	0.7366	17.3	6.00	0.71	tr_D0A1S7_	WB
DRB1_0101	39	EFFLQLAFGVIYVLS	FFLQLAFGV	1	0.7360	17.4	6.00	0.43	tr_D0A1S7_	WB
DRB1_0101	40	FANRMLTTLRPPLEH	LTTLRPPLE	5	0.7359	17.4	6.00	0.67	tr_D0A1S7_	WB
DRB1_0101	41	YYLIQAFNGDAHING	YYLIQAFNG	0	0.7324	18.1	6.00	0.58	tr_D0A1S7_	WB
DRB1_0101	42	AAQDIYYLIQAFNGD	YYLIQAFNG	5	0.7304	18.5	6.50	0.82	tr_D0A1S7_	WB
DRB1_0101	43	LYHMTPNLLDNIFMK	LYHMTPNLL	0	0.7290	18.8	6.50	0.58	tr_D0A1S7_	WB
DRB1_0101	44	MMKIQHAHDVLLAKG	IQHAHDVLL	3	0.7283	18.9	6.50	0.41	tr_D0A1S7_	WB
DRB1_0101	45	YINFDLVPTVNSQEV	FDLVPTVNS	3	0.7267	19.2	6.50	0.86	tr_D0A1S7_	WB
DRB1_0101	46	AYINFDLVPTVNSQE	FDLVPTVNS	4	0.7258	19.4	6.50	0.75	tr_D0A1S7_	WB
DRB1_0101	47	RPPLEHILGVRLTDA	LEHILGVRL	3	0.7253	19.5	7.00	0.82	tr_D0A1S7_	WB
DRB1_0101	48	DNIFMKKCSIPLAYI	FMKKCSIPL	3	0.7228	20.1	7.00	0.74	tr_D0A1S7_	WB
DRB1_0101	49	NIFMKKCSIPLAYIK	FMKKCSIPL	2	0.7197	20.8	7.00	0.70	tr_D0A1S7_	WB
DRB1_0101	50	YIKLPTRRLSYLNFI	YIKLPTRRL	0	0.7151	21.8	7.50	0.77	tr_D0A1S7_	WB
DRB1_0101	51	LHCLQMYQSGRILCW	LQMYQSGRI	3	0.7143	22.0	7.50	0.38	tr_D0A1S7_	WB
DRB1_0101	52	YDTLFISGFRVIVLL	TLFISGFRV	2	0.7122	22.5	8.00	0.53	tr_D0A1S7_	WB
DRB1_0101	53	HCLQMYQSGRILCWA	QMYQSGRIL	3	0.7109	22.8	8.00	0.46	tr_D0A1S7_	WB
DRB1_0101	54	VYDTLFISGFRVIVL	TLFISGFRV	3	0.7092	23.3	8.00	0.57	tr_D0A1S7_	WB
DRB1_0101	55	SLVFFRLYNIAFYAA	FFRLYNIAF	3	0.7086	23.4	8.00	0.71	tr_D0A1S7_	WB
DRB1_0101	56	LDNIFMKKCSIPLAY	FMKKCSIPL	4	0.7039	24.6	8.50	0.71	tr_D0A1S7_	WB
DRB1_0101	57	YAAQDIYYLIQAFNG	YYLIQAFNG	6	0.7011	25.4	9.00	0.62	tr_D0A1S7_	WB
DRB1_0101	58	LVFFRLYNIAFYAAQ	FFRLYNIAF	2	0.7007	25.5	9.00	0.62	tr_D0A1S7_	WB
DRB1_0101	59	LRPPLEHILGVRLTD	LEHILGVRL	4	0.6989	26.0	9.00	0.81	tr_D0A1S7_	WB
DRB1_0101	60	PPLEHILGVRLTDAT	LEHILGVRL	2	0.6981	26.2	9.00	0.74	tr_D0A1S7_	WB
DRB1_0101	61	ILHCLQMYQSGRILC	LQMYQSGRI	4	0.6978	26.3	9.00	0.39	tr_D0A1S7_	WB
DRB1_0101	62	INFDLVPTVNSQEVA	FDLVPTVNS	2	0.6975	26.4	9.50	0.88	tr_D0A1S7_	WB
DRB1_0101	63	RRAYINFDLVPTVNS	FDLVPTVNS	6	0.6971	26.5	9.50	0.48	tr_D0A1S7_	WB
DRB1_0101	64	DTLFISGFRVIVLLQ	TLFISGFRV	1	0.6970	26.5	9.50	0.49	tr_D0A1S7_	WB
DRB1_0101	65	IFMKKCSIPLAYIKL	FMKKCSIPL	1	0.6958	26.9	9.50	0.58	tr_D0A1S7_	WB
DRB1_0101	66	LYREAMFFAGGPLIL	FFAGGPLIL	6	0.6939	27.4	9.50	0.68	tr_D0A1S7_	WB
DRB1_0101	67	ADAFEFFLQLAFGVI	FFLQLAFGV	5	0.6920	28.0	10.00	0.62	tr_D0A1S7_	
DRB1_0101	68	MLTTLRPPLEHILGV	LTTLRPPLE	1	0.6916	28.1	10.00	0.78	tr_D0A1S7_	
DRB1_0101	69	TLRPPLEHILGVRLT	LEHILGVRL	5	0.6905	28.5	10.00	0.79	tr_D0A1S7_	
DRB1_0101	70	DSCRVHMMKIQHAHD	VHMMKIQHA	4	0.6885	29.1	10.00	0.84	tr_D0A1S7_	
DRB1_0101	71	KVSFDLPMLSATVIS	DLPMLSATV	4	0.6871	29.5	11.00	0.49	tr_D0A1S7_	
DRB1_0101	72	VSFDLPMLSATVIST	DLPMLSATV	3	0.6863	29.8	11.00	0.56	tr_D0A1S7_	
DRB1_0101	73	FFLQLAFGVIYVLSH	LQLAFGVIY	2	0.6839	30.6	11.00	0.35	tr_D0A1S7_	
DRB1_0101	74	TVYDTLFISGFRVIV	TLFISGFRV	4	0.6834	30.7	11.00	0.60	tr_D0A1S7_	
DRB1_0101	75	RSLVFFRLYNIAFYA	FFRLYNIAF	4	0.6824	31.1	11.00	0.73	tr_D0A1S7_	
DRB1_0101	76	EFANRMLTTLRPPLE	LTTLRPPLE	6	0.6809	31.6	11.00	0.52	tr_D0A1S7_	
DRB1_0101	77	CDSCRVHMMKIQHAH	VHMMKIQHA	5	0.6801	31.9	11.00	0.81	tr_D0A1S7_	
DRB1_0101	78	LILHCLQMYQSGRIL	LQMYQSGRI	5	0.6791	32.2	11.00	0.38	tr_D0A1S7_	
DRB1_0101	79	CLQMYQSGRILCWAD	QMYQSGRIL	2	0.6790	32.2	11.00	0.49	tr_D0A1S7_	
DRB1_0101	80	VFFRLYNIAFYAAQD	FFRLYNIAF	1	0.6778	32.7	12.00	0.54	tr_D0A1S7_	
DRB1_0101	81	MKIQHAHDVLLAKGS	IQHAHDVLL	2	0.6766	33.1	12.00	0.47	tr_D0A1S7_	
DRB1_0101	82	QVLFYCVSCAKSSAE	YCVSCAKSS	4	0.6765	33.1	12.00	0.46	tr_D0A1S7_	
DRB1_0101	83	PLEHILGVRLTDATH	LEHILGVRL	1	0.6762	33.2	12.00	0.69	tr_D0A1S7_	
DRB1_0101	84	SFDLPMLSATVISTI	DLPMLSATV	2	0.6748	33.7	12.00	0.53	tr_D0A1S7_	
DRB1_0101	85	TLFISGFRVIVLLQV	TLFISGFRV	0	0.6745	33.8	12.00	0.37	tr_D0A1S7_	
DRB1_0101	86	RLYNIAFYAAQDIYY	FYAAQDIYY	6	0.6722	34.7	12.00	0.38	tr_D0A1S7_	
DRB1_0101	87	FYAAQDIYYLIQAFN	YAAQDIYYL	1	0.6699	35.6	13.00	0.53	tr_D0A1S7_	

DRB1_0101	88	EEMTLLKGVDNEAVN	MTLLKGVDN	2	0.6670	36.7	13.00	0.66	tr_D0A1S7_
DRB1_0101	89	LQVLFYCVSCAKSSA	YCVSCAKSS	5	0.6670	36.7	13.00	0.41	tr_D0A1S7_
DRB1_0101	90	EMTLLKGVDNEAVNW	MTLLKGVDN	1	0.6661	37.1	13.00	0.58	tr_D0A1S7_
DRB1_0101	91	VLFYCVSCAKSSAEE	YCVSCAKSS	3	0.6652	37.4	13.00	0.50	tr_D0A1S7_
DRB1_0101	92	GEELAQLRSLVFFRL	LAQLRSLVF	3	0.6641	37.9	13.00	0.60	tr_D0A1S7_
DRB1_0101	93	LLDNIFMKKCSIPLA	FMKKCSIPL	5	0.6625	38.6	13.00	0.71	tr_D0A1S7_
DRB1_0101	94	NFDLVPTVNSQEVAF	FDLVPTVNS	1	0.6614	39.0	14.00	0.85	tr_D0A1S7_
DRB1_0101	95	SEEMTLLKGVDNEAV	MTLLKGVDN	3	0.6597	39.7	14.00	0.79	tr_D0A1S7_
DRB1_0101	96	VKVSFDLPMLSATVI	DLPMLSATV	5	0.6595	39.8	14.00	0.46	tr_D0A1S7_
DRB1_0101	97	SYLNFICTEFGLLLD	LNFICTEFG	2	0.6566	41.1	14.00	0.32	tr_D0A1S7_
DRB1_0101	98	FFAGGPLILHCLQMY	FFAGGPLIL	0	0.6562	41.3	14.00	0.50	tr_D0A1S7_
DRB1_0101	99	LRSLVFFRLYNIAFY	FFRLYNIAF	5	0.6536	42.4	15.00	0.70	tr_D0A1S7_
DRB1_0101	100	ELHYSILGIPQGSDA	YSILGIPQG	3	0.6527	42.8	15.00	0.69	tr_D0A1S7_
DRB1_0101	101	YGEELAQLRSLVFFR	LAQLRSLVF	4	0.6520	43.2	15.00	0.61	tr_D0A1S7_
DRB1_0101	102	EELAQLRSLVFFRLY	LAQLRSLVF	2	0.6510	43.6	15.00	0.55	tr_D0A1S7_
DRB1_0101	103	LSYLNFICTEFGLLL	LNFICTEFG	3	0.6496	44.3	15.00	0.38	tr_D0A1S7_
DRB1_0101	104	AFGVIYVLSHLYHMT	YVLSHLYHM	5	0.6484	44.9	15.00	0.44	tr_D0A1S7_
DRB1_0101	105	FTVYDTLFISGFRVI	TLFISGFRV	5	0.6481	45.1	15.00	0.60	tr_D0A1S7_
DRB1_0101	106	LHYSILGIPQGSDAR	YSILGIPQG	2	0.6432	47.5	16.00	0.69	tr_D0A1S7_
DRB1_0101	107	VNWFDVVSTRFAKQM	FDVVSTRFA	3	0.6431	47.6	16.00	0.75	tr_D0A1S7_
DRB1_0101	108	FFRLYNIAFYAAQDI	FFRLYNIAF	0	0.6419	48.2	16.00	0.31	tr_D0A1S7_
DRB1_0101	109	YLNFICTEFGLLLDD	ICTEFGLLL	4	0.6413	48.5	16.00	0.35	tr_D0A1S7_
DRB1_0101	110	FDLPMLSATVISTIV	DLPMLSATV	1	0.6402	49.1	16.00	0.50	tr_D0A1S7_
DRB1_0101	111	LQMYQSGRILCWADA	QMYQSGRIL	1	0.6393	49.6	17.00	0.45	tr_D0A1S7_
DRB1_0101	112	SELHYSILGIPQGSD	YSILGIPQG	4	0.6368	50.9	17.00	0.63	tr_D0A1S7_
DRB1_0101	113	LFYCVSCAKSSAEEW	YCVSCAKSS	2	0.6360	51.3	17.00	0.52	tr_D0A1S7_
DRB1_0101	114	FGVIYVLSHLYHMTP	YVLSHLYHM	4	0.6346	52.1	17.00	0.47	tr_D0A1S7_
DRB1_0101	115	MTLLKGVDNEAVNWF	LKGVDNEAV	3	0.6341	52.4	17.00	0.43	tr_D0A1S7_
DRB1_0101	116	RYGEELAQLRSLVFF	LAQLRSLVF	5	0.6332	52.9	17.00	0.50	tr_D0A1S7_
DRB1_0101	117	DLFAFSCRVPSVYRL	FAFSCRVPS	2	0.6290	55.4	18.00	0.50	tr_D0A1S7_
DRB1_0101	118	AVNWFDVVSTRFAKQ	FDVVSTRFA	4	0.6289	55.5	18.00	0.71	tr_D0A1S7_
DRB1_0101	119	ESELHYSILGIPQGS	YSILGIPQG	5	0.6277	56.2	18.00	0.61	tr_D0A1S7_
DRB1_0101	120	NWFDVVSTRFAKQMR	FDVVSTRFA	2	0.6272	56.5	18.00	0.74	tr_D0A1S7_
DRB1_0101	121	GVIYVLSHLYHMTPN	YVLSHLYHM	3	0.6252	57.7	19.00	0.53	tr_D0A1S7_
DRB1_0101	122	VIYVLSHLYHMTPNL	YVLSHLYHM	2	0.6244	58.2	19.00	0.48	tr_D0A1S7_
DRB1_0101	123	LFAFSCRVPSVYRLT	FAFSCRVPS	1	0.6244	58.2	19.00	0.43	tr_D0A1S7_
DRB1_0101	124	FMKKCSIPLAYIKLP	FMKKCSIPL	0	0.6209	60.4	19.00	0.40	tr_D0A1S7_
DRB1_0101	125	WADAFEFFLQLAFGV	FFLQLAFGV	6	0.6187	61.9	20.00	0.66	tr_D0A1S7_
DRB1_0101	126	SSEEMTLLKGVDNEA	MTLLKGVDN	4	0.6167	63.3	20.00	0.88	tr_D0A1S7_
DRB1_0101	127	TTLRPPLEHILGVRL	LEHILGVRL	6	0.6159	63.8	20.00	0.74	tr_D0A1S7_
DRB1_0101	128	FSWFLQVMCRTLTMG	FLQVMCRTL	3	0.6159	63.8	20.00	0.35	tr_D0A1S7_
DRB1_0101	129	LEHILGVRLTDATHS	LEHILGVRL	0	0.6158	63.9	20.00	0.50	tr_D0A1S7_
DRB1_0101	130	LAFGVIYVLSHLYHM	YVLSHLYHM	6	0.6136	65.4	20.00	0.31	tr_D0A1S7_
DRB1_0101	131	LNFICTEFGLLLDDL	ICTEFGLLL	3	0.6136	65.4	20.00	0.47	tr_D0A1S7_
DRB1_0101	132	ELAQLRSLVFFRLYN	LAQLRSLVF	1	0.6126	66.1	20.00	0.51	tr_D0A1S7_
DRB1_0101	133	TRFAKQMRSAIHRCA	FAKQMRSAI	2	0.6098	68.2	21.00	0.40	tr_D0A1S7_
DRB1_0101	134	PLILHCLQMYQSGRI	LHCLQMYQS	3	0.6096	68.3	21.00	0.41	tr_D0A1S7_
DRB1_0101	135	SWFLQVMCRTLTMGV	FLQVMCRTL	2	0.6093	68.5	21.00	0.32	tr_D0A1S7_
DRB1_0101	136	RFAKQMRSAIHRCAQ	MRSAIHRCA	5	0.6091	68.7	21.00	0.29	tr_D0A1S7_
DRB1_0101	137	CELLWFPWEPPILSV	FPWEPPILS	5	0.6083	69.3	21.00	0.63	tr_D0A1S7_

DRB1_0101	138	VSTRFAKQMRSAIHR	FAKQMRSAI	4	0.6068	70.4	21.00	0.52	tr_D0A1S7_
DRB1_0101	139	NCDSCRVHMMKIQHA	VHMMKIQHA	6	0.6067	70.5	21.00	0.69	tr_D0A1S7_
DRB1_0101	140	LTTLRPPLEHILGVR	LTTLRPPLE	0	0.6058	71.1	22.00	0.63	tr_D0A1S7_
DRB1_0101	141	EFSWFLQVMCRTLTM	FLQVMCRTL	4	0.6058	71.2	22.00	0.34	tr_D0A1S7_
DRB1_0101	142	QLRSLVFFRLYNIAF	FFRLYNIAF	6	0.6057	71.2	22.00	0.60	tr_D0A1S7_
DRB1_0101	143	STRFAKQMRSAIHRC	FAKQMRSAI	3	0.6034	73.1	22.00	0.54	tr_D0A1S7_
DRB1_0101	144	EAVNWFDVVSTRFAK	FDVVSTRFA	5	0.6029	73.4	22.00	0.69	tr_D0A1S7_
DRB1_0101	145	HYSILGIPQGSDARA	YSILGIPQG	1	0.6007	75.3	22.00	0.63	tr_D0A1S7_
DRB1_0101	146	DDLFAFSCRVPSVYR	FAFSCRVPS	3	0.5984	77.1	23.00	0.58	tr_D0A1S7_
DRB1_0101	147	LLQVLFYCVSCAKSS	FYCVSCAKS	5	0.5980	77.4	23.00	0.42	tr_D0A1S7_
DRB1_0101	148	ELLWFPWEPPILSVP	FPWEPPILS	4	0.5971	78.2	23.00	0.69	tr_D0A1S7_
DRB1_0101	149	DRYGEELAQLRSLVF	LAQLRSLVF	6	0.5969	78.4	23.00	0.42	tr_D0A1S7_
DRB1_0101	150	KIQHAHDVLLAKGSE	IQHAHDVLL	1	0.5954	79.6	23.00	0.53	tr_D0A1S7_
DRB1_0101	151	FAFSCRVPSVYRLTV	CRVPSVYRL	4	0.5942	80.7	23.00	0.38	tr_D0A1S7_
DRB1_0101	152	FRLYNIAFYAAQDIY	IAFYAAQDI	5	0.5926	82.1	24.00	0.37	tr_D0A1S7_
DRB1_0101	153	AHDVLLAKGSERYEL	VLLAKGSER	3	0.5924	82.2	24.00	0.46	tr_D0A1S7_
DRB1_0101	154	WFDVVSTRFAKQMRS	FDVVSTRFA	1	0.5908	83.7	24.00	0.68	tr_D0A1S7_
DRB1_0101	155	EVKVSFDLPMLSATV	DLPMLSATV	6	0.5889	85.5	24.00	0.37	tr_D0A1S7_
DRB1_0101	156	KCSIPLAYIKLPTRR	LAYIKLPTR	5	0.5875	86.8	25.00	0.42	tr_D0A1S7_
DRB1_0101	157	WFLQVMCRTLTMGVF	LQVMCRTLT	2	0.5851	89.1	25.00	0.32	tr_D0A1S7_
DRB1_0101	158	LLWFPWEPPILSVPD	FPWEPPILS	3	0.5831	91.0	25.00	0.73	tr_D0A1S7_
DRB1_0101	159	QRRAYINFDLVPTVN	YINFDLVPT	4	0.5826	91.5	25.00	0.62	tr_D0A1S7_
DRB1_0101	160	NFICTEFGLLLDDLF	ICTEFGLLL	2	0.5811	93.0	26.00	0.47	tr_D0A1S7_
DRB1_0101	161	HDVLLAKGSERYELV	VLLAKGSER	2	0.5810	93.1	26.00	0.43	tr_D0A1S7_
DRB1_0101	162	WEIIGMVKRSYVDLY	IGMVKRSYV	3	0.5803	93.8	26.00	0.54	tr_D0A1S7_
DRB1_0101	163	RLSYLNFICTEFGLL	LNFICTEFG	4	0.5795	94.6	26.00	0.46	tr_D0A1S7_
DRB1_0101	164	KEFSWFLQVMCRTLT	FLQVMCRTL	5	0.5789	95.3	26.00	0.34	tr_D0A1S7_
DRB1_0101	165	FYCVSCAKSSAEEWE	YCVSCAKSS	1	0.5772	97.0	26.00	0.52	tr_D0A1S7_
DRB1_0101	166	FLQLAFGVIYVLSHL	LQLAFGVIY	1	0.5765	97.8	26.00	0.43	tr_D0A1S7_
DRB1_0101	167	DLPMLSATVISTIVL	DLPMLSATV	0	0.5755	98.8	27.00	0.32	tr_D0A1S7_
DRB1_0101	168	FDLVPTVNSQEVAFV	FDLVPTVNS	0	0.5745	99.8	27.00	0.70	tr_D0A1S7_
DRB1_0101	169	EWEIIGMVKRSYVDL	IGMVKRSYV	4	0.5745	99.9	27.00	0.48	tr_D0A1S7_
DRB1_0101	170	ISSEEMTLLKGVDNE	MTLLKGVDN	5	0.5728	101.7	27.00	0.87	tr_D0A1S7_
DRB1_0101	171	VVSTRFAKQMRSAIH	FAKQMRSAI	5	0.5713	103.4	27.00	0.46	tr_D0A1S7_
DRB1_0101	172	LDDLFAFSCRVPSVY	FAFSCRVPS	4	0.5712	103.4	27.00	0.56	tr_D0A1S7_
DRB1_0101	173	NLLDNIFMKKCSIPL	FMKKCSIPL	6	0.5687	106.4	28.00	0.58	tr_D0A1S7_
DRB1_0101	174	KKCSIPLAYIKLPTR	SIPLAYIKL	3	0.5674	107.9	28.00	0.33	tr_D0A1S7_
DRB1_0101	175	VFTVYDTLFISGFRV	TLFISGFRV	6	0.5657	109.8	28.00	0.57	tr_D0A1S7_
DRB1_0101	176	FAKQMRSAIHRCAQQ	MRSAIHRCA	4	0.5640	111.8	29.00	0.45	tr_D0A1S7_
DRB1_0101	177	HAHDVLLAKGSERYE	VLLAKGSER	4	0.5625	113.7	29.00	0.46	tr_D0A1S7_
DRB1_0101	178	FLQVMCRTLTMGVFT	LQVMCRTLT	1	0.5610	115.6	29.00	0.30	tr_D0A1S7_
DRB1_0101	179	EIIGMVKRSYVDLYR	IGMVKRSYV	2	0.5602	116.6	29.00	0.58	tr_D0A1S7_
DRB1_0101	180	LFISGFRVIVLLQVL	FRVIVLLQV	5	0.5588	118.4	30.00	0.38	tr_D0A1S7_
DRB1_0101	181	SVYRLTVIVVHTVFL	YRLTVIVVH	2	0.5571	120.5	30.00	0.32	tr_D0A1S7_
DRB1_0101	182	QHAHDVLLAKGSERY	VLLAKGSER	5	0.5546	123.9	30.00	0.38	tr_D0A1S7_
DRB1_0101	183	FICTEFGLLLDDLFA	ICTEFGLLL	1	0.5545	124.0	30.00	0.36	tr_D0A1S7_
DRB1_0101	184	PSVYRLTVIVVHTVF	YRLTVIVVH	3	0.5515	128.1	31.00	0.38	tr_D0A1S7_
DRB1_0101	185	VESELHYSILGIPQG	YSILGIPQG	6	0.5515	128.1	31.00	0.46	tr_D0A1S7_
DRB1_0101	186	CRVPSVYRLTVIVVH	YRLTVIVVH	6	0.5489	131.7	31.00	0.26	tr_D0A1S7_
DRB1_0101	187	IQHAHDVLLAKGSER	HDVLLAKGS	4	0.5469	134.6	32.00	0.28	tr_D0A1S7_

DRB1_0101	188	LWFPWEPPILSVPDR	FPWEPPILS	2	0.5468	134.8	32.00	0.75	tr_D0A1S7_
DRB1_0101	189	GPLILHCLQMYQSGR	LHCLQMYQS	4	0.5465	135.1	32.00	0.56	tr_D0A1S7_
DRB1_0101	190	VPSVYRLTVIVVHTV	YRLTVIVVH	4	0.5461	135.8	32.00	0.38	tr_D0A1S7_
DRB1_0101	191	QMYQSGRILCWADAF	QMYQSGRIL	0	0.5461	135.8	32.00	0.43	tr_D0A1S7_
DRB1_0101	192	LAQLRSLVFFRLYNI	LAQLRSLVF	0	0.5459	136.0	32.00	0.32	tr_D0A1S7_
DRB1_0101	193	QQRRAYINFDLVPTV	YINFDLVPT	5	0.5446	138.0	32.00	0.61	tr_D0A1S7_
DRB1_0101	194	EEWEIIGMVKRSYVD	IGMVKRSYV	5	0.5417	142.4	33.00	0.44	tr_D0A1S7_
DRB1_0101	195	LCELLWFPWEPPILS	FPWEPPILS	6	0.5411	143.3	33.00	0.52	tr_D0A1S7_
DRB1_0101	196	FDVVSTRFAKQMRSA	FDVVSTRFA	0	0.5402	144.7	33.00	0.47	tr_D0A1S7_
DRB1_0101	197	DVLLAKGSERYELVD	VLLAKGSER	1	0.5381	148.0	33.00	0.38	tr_D0A1S7_
DRB1_0101	198	LLDDLFAFSCRVPSV	FAFSCRVPS	5	0.5381	148.0	33.00	0.46	tr_D0A1S7_
DRB1_0101	199	AFSCRVPSVYRLTVI	CRVPSVYRL	3	0.5330	156.5	34.00	0.50	tr_D0A1S7_
DRB1_0101	200	GRILCWADAFEFFLQ	LCWADAFEF	3	0.5309	160.0	35.00	0.60	tr_D0A1S7_
DRB1_0101	201	LQLAFGVIYVLSHLY	LQLAFGVIY	0	0.5303	161.0	35.00	0.24	tr_D0A1S7_
DRB1_0101	202	SGRILCWADAFEFFL	LCWADAFEF	4	0.5299	161.8	35.00	0.58	tr_D0A1S7_
DRB1_0101	203	GGPLILHCLQMYQSG	LHCLQMYQS	5	0.5292	163.1	35.00	0.44	tr_D0A1S7_
DRB1_0101	204	NEAVNWFDVVSTRFA	FDVVSTRFA	6	0.5283	164.5	35.00	0.56	tr_D0A1S7_
DRB1_0101	205	AKQMRSAIHRCAQQR	MRSAIHRCA	3	0.5273	166.5	36.00	0.64	tr_D0A1S7_
DRB1_0101	206	QLAFGVIYVLSHLYH	VIYVLSHLY	5	0.5266	167.8	36.00	0.32	tr_D0A1S7_
DRB1_0101	207	LQVMCRTLTMGVFTV	MCRTLTMGV	3	0.5260	168.7	36.00	0.30	tr_D0A1S7_
DRB1_0101	208	SKEFSWFLQVMCRTL	FLQVMCRTL	6	0.5256	169.6	36.00	0.29	tr_D0A1S7_
DRB1_0101	209	IIGMVKRSYVDLYRE	IGMVKRSYV	1	0.5253	170.1	36.00	0.56	tr_D0A1S7_
DRB1_0101	210	AIHRCAQQRRAYINF	CAQQRRAYI	4	0.5250	170.7	36.00	0.55	tr_D0A1S7_
DRB1_0101	211	DEFANRMLTTLRPPL	NRMLTTLRP	4	0.5240	172.4	36.00	0.56	tr_D0A1S7_
DRB1_0101	212	SAIHRCAQQRRAYIN	CAQQRRAYI	5	0.5240	172.5	36.00	0.46	tr_D0A1S7_
DRB1_0101	213	YSILGIPQGSDARAI	YSILGIPQG	0	0.5238	172.8	36.00	0.47	tr_D0A1S7_
DRB1_0101	214	RVPSVYRLTVIVVHT	YRLTVIVVH	5	0.5226	175.1	36.00	0.38	tr_D0A1S7_
DRB1_0101	215	AISSEEMTLLKGVDN	MTLLKGVDN	6	0.5215	177.2	37.00	0.69	tr_D0A1S7_
DRB1_0101	216	TLLKGVDNEAVNWFD	LKGVDNEAV	2	0.5195	181.0	37.00	0.74	tr_D0A1S7_
DRB1_0101	217	FSCRVPSVYRLTVIV	CRVPSVYRL	2	0.5167	186.6	38.00	0.52	tr_D0A1S7_
DRB1_0101	218	RRLSYLNFICTEFGL	LNFICTEFG	5	0.5166	186.8	38.00	0.47	tr_D0A1S7_
DRB1_0101	219	MKKCSIPLAYIKLPT	SIPLAYIKL	4	0.5153	189.5	38.00	0.34	tr_D0A1S7_
DRB1_0101	220	VYRLTVIVVHTVFLC	YRLTVIVVH	1	0.5147	190.8	38.00	0.28	tr_D0A1S7_
DRB1_0101	221	KQMRSAIHRCAQQRR	MRSAIHRCA	2	0.5145	191.1	38.00	0.62	tr_D0A1S7_
DRB1_0101	222	SCRVPSVYRLTVIVV	CRVPSVYRL	1	0.5145	191.2	38.00	0.42	tr_D0A1S7_
DRB1_0101	223	SEVKVSFDLPMLSAT	VSFDLPMLS	4	0.5138	192.7	38.00	0.28	tr_D0A1S7_
DRB1_0101	224	RILCWADAFEFFLQL	LCWADAFEF	2	0.5133	193.5	38.00	0.61	tr_D0A1S7_
DRB1_0101	225	QVMCRTLTMGVFTVY	MCRTLTMGV	2	0.5114	197.6	39.00	0.26	tr_D0A1S7_
DRB1_0101	226	AEEWEIIGMVKRSYV	IGMVKRSYV	6	0.5085	204.0	39.00	0.38	tr_D0A1S7_
DRB1_0101	227	DVVSTRFAKQMRSAI	TRFAKQMRS	4	0.5067	207.9	40.00	0.30	tr_D0A1S7_
DRB1_0101	228	ICTEFGLLLDDLFAF	FGLLLDDLF	4	0.5066	208.2	40.00	0.37	tr_D0A1S7_
DRB1_0101	229	VLFRVRDEFANRMLT	VRDEFANRM	4	0.5065	208.4	40.00	0.41	tr_D0A1S7_
DRB1_0101	230	IHRCAQQRRAYINFD	CAQQRRAYI	3	0.5063	208.9	40.00	0.60	tr_D0A1S7_
DRB1_0101	231	RSAIHRCAQQRRAYI	CAQQRRAYI	6	0.5056	210.5	40.00	0.32	tr_D0A1S7_
DRB1_0101	232	CTEFGLLLDDLFAFS	FGLLLDDLF	3	0.5055	210.7	40.00	0.42	tr_D0A1S7_
DRB1_0101	233	TEFGLLLDDLFAFSC	FGLLLDDLF	2	0.5051	211.5	40.00	0.38	tr_D0A1S7_
DRB1_0101	234	VDRYGEELAQLRSLV	YGEELAQLR	3	0.5016	219.9	41.00	0.51	tr_D0A1S7_
DRB1_0101	235	EFGLLLDDLFAFSCR	GLLLDDLFA	2	0.5000	223.5	41.00	0.34	tr_D0A1S7_
DRB1_0101	236	WFPWEPPILSVPDRK	FPWEPPILS	1	0.4998	224.0	41.00	0.77	tr_D0A1S7_
DRB1_0101	237	YHMTPNLLDNIFMKK	PNLLDNIFM	4	0.4989	226.3	41.00	0.34	tr_D0A1S7_

DRB1_0101	238	VLLQVLFYCVSCAKS	FYCVSCAKS	6	0.4983	227.7	41.00	0.46	tr_D0A1S7_
DRB1_0101	239	YLIQAFNGDAHINGS	LIQAFNGDA	1	0.4979	228.8	41.00	0.56	tr_D0A1S7_
DRB1_0101	240	ILGVRLTDATHSEIV	VRLTDATHS	3	0.4977	229.3	42.00	0.65	tr_D0A1S7_
DRB1_0101	241	QSGRILCWADAFEFF	LCWADAFEF	5	0.4971	230.7	42.00	0.46	tr_D0A1S7_
DRB1_0101	242	DVLFRVRDEFANRML	VRDEFANRM	5	0.4970	230.9	42.00	0.36	tr_D0A1S7_
DRB1_0101	243	PTVNSQEVAFVAVTR	NSQEVAFVA	3	0.4969	231.3	42.00	0.52	tr_D0A1S7_
DRB1_0101	244	AGGPLILHCLQMYQS	LHCLQMYQS	6	0.4964	232.6	42.00	0.31	tr_D0A1S7_
DRB1_0101	245	VLLAKGSERYELVDR	LLAKGSERY	1	0.4942	238.1	42.00	0.34	tr_D0A1S7_
DRB1_0101	246	TRRLSYLNFICTEFG	LSYLNFICT	3	0.4938	239.1	42.00	0.38	tr_D0A1S7_
DRB1_0101	247	VPTVNSQEVAFVAVT	VNSQEVAFV	3	0.4922	243.4	43.00	0.40	tr_D0A1S7_
DRB1_0101	248	QMRSAIHRCAQQRRA	MRSAIHRCA	1	0.4921	243.6	43.00	0.48	tr_D0A1S7_
DRB1_0101	249	KRSYVDLYREAMFFA	YVDLYREAM	3	0.4905	247.7	43.00	0.41	tr_D0A1S7_
DRB1_0101	250	HRCAQQRRAYINFDL	CAQQRRAYI	2	0.4900	249.0	43.00	0.56	tr_D0A1S7_
DRB1_0101	251	EHILGVRLTDATHSE	VRLTDATHS	5	0.4892	251.4	43.00	0.41	tr_D0A1S7_
DRB1_0101	252	FGLLLDDLFAFSCRV	GLLLDDLFA	1	0.4887	252.6	43.00	0.26	tr_D0A1S7_
DRB1_0101	253	HILGVRLTDATHSEI	VRLTDATHS	4	0.4882	254.0	43.00	0.57	tr_D0A1S7_
DRB1_0101	254	RCAQQRRAYINFDLV	CAQQRRAYI	1	0.4866	258.6	44.00	0.47	tr_D0A1S7_
DRB1_0101	255	RSYVDLYREAMFFAG	YVDLYREAM	2	0.4855	261.6	44.00	0.31	tr_D0A1S7_
DRB1_0101	256	ILCWADAFEFFLQLA	LCWADAFEF	1	0.4839	266.1	44.00	0.48	tr_D0A1S7_
DRB1_0101	257	LLLDDLFAFSCRVPS	DLFAFSCRV	4	0.4832	268.2	44.00	0.34	tr_D0A1S7_
DRB1_0101	258	LPMLSATVISTIVLL	MLSATVIST	2	0.4819	272.1	45.00	0.25	tr_D0A1S7_
DRB1_0101	259	FAGGPLILHCLQMYQ	GGPLILHCL	2	0.4795	279.2	45.00	0.28	tr_D0A1S7_
DRB1_0101	260	VMCRTLTMGVFTVYD	TLTMGVFTV	4	0.4792	280.0	45.00	0.28	tr_D0A1S7_
DRB1_0101	261	FISGFRVIVLLQVLF	FRVIVLLQV	4	0.4783	282.7	46.00	0.57	tr_D0A1S7_
DRB1_0101	262	LFRVRDEFANRMLTT	VRDEFANRM	3	0.4757	290.7	46.00	0.54	tr_D0A1S7_
DRB1_0101	263	AQLRSLVFFRLYNIA	LRSLVFFRL	2	0.4745	294.6	46.00	0.43	tr_D0A1S7_
DRB1_0101	264	LGVRLTDATHSEIVS	VRLTDATHS	2	0.4741	295.9	46.00	0.63	tr_D0A1S7_
DRB1_0101	265	LVPTVNSQEVAFVAV	VNSQEVAFV	4	0.4737	297.3	46.00	0.43	tr_D0A1S7_
DRB1_0101	266	LCWADAFEFFLQLAF	LCWADAFEF	0	0.4732	298.9	47.00	0.25	tr_D0A1S7_
DRB1_0101	267	TVNSQEVAFVAVTRE	NSQEVAFVA	2	0.4687	313.7	48.00	0.50	tr_D0A1S7_
DRB1_0101	268	YRLTVIVVHTVFLCE	VIVVHTVFL	4	0.4680	316.2	48.00	0.34	tr_D0A1S7_
DRB1_0101	269	SYVDLYREAMFFAGG	LYREAMFFA	4	0.4667	320.7	48.00	0.25	tr_D0A1S7_
DRB1_0101	270	LLKGVDNEAVNWFDV	LKGVDNEAV	1	0.4665	321.2	48.00	0.73	tr_D0A1S7_
DRB1_0101	271	AQQRRAYINFDLVPT	YINFDLVPT	6	0.4635	331.8	49.00	0.51	tr_D0A1S7_
DRB1_0101	272	ARAIKGAYREAVRRW	IKGAYREAV	3	0.4629	334.1	49.00	0.55	tr_D0A1S7_
DRB1_0101	273	ATHSEIVSRHARLWE	EIVSRHARL	4	0.4622	336.5	49.00	0.34	tr_D0A1S7_
DRB1_0101	274	ISGFRVIVLLQVLFY	FRVIVLLQV	3	0.4617	338.5	49.00	0.62	tr_D0A1S7_
DRB1_0101	275	RDEFANRMLTTLRPP	NRMLTTLRP	5	0.4616	338.6	49.00	0.50	tr_D0A1S7_
DRB1_0101	276	DARAIKGAYREAVRR	IKGAYREAV	4	0.4604	343.4	49.00	0.51	tr_D0A1S7_
DRB1_0101	277	THSEIVSRHARLWEE	IVSRHARLW	4	0.4598	345.3	49.00	0.40	tr_D0A1S7_
DRB1_0101	278	MRSAIHRCAQQRRAY	IHRCAQQRR	4	0.4593	347.3	50.00	0.25	tr_D0A1S7_
DRB1_0101	279	VDVLFRVRDEFANRM	LFRVRDEFA	3	0.4582	351.4	50.00	0.49	tr_D0A1S7_
DRB1_0101	280	DSEVKVSFDLPMLSA	VSFDLPMLS	5	0.4569	356.6	50.00	0.31	tr_D0A1S7_
DRB1_0101	281	IGMVKRSYVDLYREA	IGMVKRSYV	0	0.4535	369.8	55.00	0.41	tr_D0A1S7_
DRB1_0101	282	GSKEFSWFLQVMCRT	WFLQVMCRT	6	0.4519	376.1	55.00	0.20	tr_D0A1S7_
DRB1_0101	283	VKRSYVDLYREAMFF	YVDLYREAM	4	0.4516	377.5	55.00	0.47	tr_D0A1S7_
DRB1_0101	284	GLLLDDLFAFSCRVP	DLFAFSCRV	5	0.4512	379.0	55.00	0.35	tr_D0A1S7_
DRB1_0101	285	YQSGRILCWADAFEF	GRILCWADA	3	0.4490	388.3	55.00	0.45	tr_D0A1S7_
DRB1_0101	286	SGFRVIVLLQVLFYC	FRVIVLLQV	2	0.4461	400.6	55.00	0.62	tr_D0A1S7_
DRB1_0101	287	LVDRYGEELAQLRSL	YGEELAQLR	4	0.4445	407.6	55.00	0.56	tr_D0A1S7_

DRB1_0101	288	MCRTLTMGVFTVYDT	TLTMGVFTV	3	0.4444	407.9	55.00	0.37	tr_D0A1S7_
DRB1_0101	289	HSEIVSRHARLWEEN	IVSRHARLW	3	0.4428	415.2	55.00	0.49	tr_D0A1S7_
DRB1_0101	290	SDARAIKGAYREAVR	IKGAYREAV	5	0.4409	423.7	55.00	0.42	tr_D0A1S7_
DRB1_0101	291	TPNLLDNIFMKKCSI	PNLLDNIFM	1	0.4408	424.3	55.00	0.34	tr_D0A1S7_
DRB1_0101	292	DLYREAMFFAGGPLI	MFFAGGPLI	6	0.4393	431.1	55.00	0.35	tr_D0A1S7_
DRB1_0101	293	GVRLTDATHSEIVSR	VRLTDATHS	1	0.4391	432.4	55.00	0.56	tr_D0A1S7_
DRB1_0101	294	NGSKEFSWFLQVMCR	GSKEFSWFL	1	0.4387	434.0	55.00	0.31	tr_D0A1S7_
DRB1_0101	295	DATHSEIVSRHARLW	EIVSRHARL	5	0.4371	441.5	55.00	0.35	tr_D0A1S7_
DRB1_0101	296	CWADAFEFFLQLAFG	FEFFLQLAF	5	0.4341	456.4	55.00	0.29	tr_D0A1S7_
DRB1_0101	297	VRDEFANRMLTTLRP	NRMLTTLRP	6	0.4317	468.4	55.00	0.34	tr_D0A1S7_
DRB1_0101	298	LPTRRLSYLNFICTE	LSYLNFICT	5	0.4312	471.0	60.00	0.46	tr_D0A1S7_
DRB1_0101	299	PTRRLSYLNFICTEF	LSYLNFICT	4	0.4295	479.3	60.00	0.51	tr_D0A1S7_
DRB1_0101	300	GFRVIVLLQVLFYCV	FRVIVLLQV	1	0.4288	483.4	60.00	0.51	tr_D0A1S7_
DRB1_0101	301	QEVAFVAVTREHPRA	FVAVTREHP	4	0.4286	484.1	60.00	0.65	tr_D0A1S7_
DRB1_0101	302	RAIKGAYREAVRRWH	IKGAYREAV	2	0.4275	489.9	60.00	0.57	tr_D0A1S7_
DRB1_0101	303	KLPTRRLSYLNFICT	LSYLNFICT	6	0.4274	490.3	60.00	0.31	tr_D0A1S7_
DRB1_0101	304	FRVRDEFANRMLTTL	VRDEFANRM	2	0.4258	498.9	60.00	0.53	tr_D0A1S7_
DRB1_0101	305	VAFVAVTREHPRAPS	FVAVTREHP	2	0.4257	499.5	60.00	0.54	tr_D0A1S7_
DRB1_0101	306	PNLLDNIFMKKCSIP	NIFMKKCSI	5	0.4252	502.5	60.00	0.38	tr_D0A1S7_
DRB1_0101	307	FRVIVLLQVLFYCVS	FRVIVLLQV	0	0.4189	537.8	60.00	0.33	tr_D0A1S7_
DRB1_0101	308	RVRDEFANRMLTTLR	VRDEFANRM	1	0.4182	541.6	60.00	0.40	tr_D0A1S7_
DRB1_0101	309	YCVSCAKSSAEEWEI	YCVSCAKSS	0	0.4178	543.9	60.00	0.52	tr_D0A1S7_
DRB1_0101	310	CRTLTMGVFTVYDTL	TLTMGVFTV	2	0.4175	545.9	60.00	0.40	tr_D0A1S7_
DRB1_0101	311	EVAFVAVTREHPRAP	FVAVTREHP	3	0.4175	546.0	60.00	0.62	tr_D0A1S7_
DRB1_0101	312	HMTPNLLDNIFMKKC	PNLLDNIFM	3	0.4168	549.9	60.00	0.50	tr_D0A1S7_
DRB1_0101	313	RLTVIVVHTVFLCEL	VIVVHTVFL	3	0.4164	552.4	60.00	0.47	tr_D0A1S7_
DRB1_0101	314	CAQQRRAYINFDLVP	CAQQRRAYI	0	0.4163	553.2	60.00	0.31	tr_D0A1S7_
DRB1_0101	315	DLVPTVNSQEVAFVA	VNSQEVAFV	5	0.4158	555.8	60.00	0.45	tr_D0A1S7_
DRB1_0101	316	HINGSKEFSWFLQVM	GSKEFSWFL	3	0.4157	556.8	60.00	0.49	tr_D0A1S7_
DRB1_0101	317	INGSKEFSWFLQVMC	GSKEFSWFL	2	0.4156	557.4	60.00	0.44	tr_D0A1S7_
DRB1_0101	318	MVKRSYVDLYREAMF	YVDLYREAM	5	0.4149	561.7	60.00	0.46	tr_D0A1S7_
DRB1_0101	319	AFVAVTREHPRAPSK	FVAVTREHP	1	0.4130	573.2	60.00	0.43	tr_D0A1S7_
DRB1_0101	320	YVDLYREAMFFAGGP	LYREAMFFA	3	0.4120	579.7	60.00	0.29	tr_D0A1S7_
DRB1_0101	321	PMLSATVISTIVLLY	SATVISTIV	3	0.4119	579.9	60.00	0.22	tr_D0A1S7_
DRB1_0101	322	MGVFTVYDTLFISGF	FTVYDTLFI	3	0.4104	589.5	60.00	0.56	tr_D0A1S7_
DRB1_0101	323	GVFTVYDTLFISGFR	FTVYDTLFI	2	0.4095	595.2	60.00	0.50	tr_D0A1S7_
DRB1_0101	324	VNSQEVAFVAVTREH	NSQEVAFVA	1	0.4095	595.3	60.00	0.49	tr_D0A1S7_
DRB1_0101	325	SEIVSRHARLWEENK	IVSRHARLW	2	0.4045	628.1	65.00	0.50	tr_D0A1S7_
DRB1_0101	326	MYQSGRILCWADAFE	GRILCWADA	4	0.4042	630.6	65.00	0.47	tr_D0A1S7_
DRB1_0101	327	MTPNLLDNIFMKKCS	PNLLDNIFM	2	0.3985	670.6	65.00	0.48	tr_D0A1S7_
DRB1_0101	328	TMGVFTVYDTLFISG	FTVYDTLFI	4	0.3984	671.5	65.00	0.57	tr_D0A1S7_
DRB1_0101	329	FPWEPPILSVPDRKD	FPWEPPILS	0	0.3978	675.3	65.00	0.62	tr_D0A1S7_
DRB1_0101	330	FLCELLWFPWEPPIL	LLWFPWEPP	4	0.3972	679.9	65.00	0.24	tr_D0A1S7_
DRB1_0101	331	ELVDRYGEELAQLRS	YGEELAQLR	5	0.3964	686.3	65.00	0.51	tr_D0A1S7_
DRB1_0101	332	HREVTGEPPRAISSE	VTGEPPRAI	3	0.3962	687.6	65.00	0.75	tr_D0A1S7_
DRB1_0101	333	VHTVFLCELLWFPWE	FLCELLWFP	4	0.3954	693.3	65.00	0.33	tr_D0A1S7_
DRB1_0101	334	GSDARAIKGAYREAV	ARAIKGAYR	3	0.3952	694.8	65.00	0.51	tr_D0A1S7_
DRB1_0101	335	PRAISSEEMTLLKGV	ISSEEMTLL	3	0.3949	697.1	65.00	0.63	tr_D0A1S7_
DRB1_0101	336	PHREVTGEPPRAISS	VTGEPPRAI	4	0.3944	700.9	65.00	0.74	tr_D0A1S7_
DRB1_0101	337	GMVKRSYVDLYREAM	YVDLYREAM	6	0.3941	703.2	65.00	0.28	tr_D0A1S7_

DRB1_0101	338	LTMGVFTVYDTLFIS	FTVYDTLFI	5	0.3934	708.2	65.00	0.50	tr_D0A1S7_
DRB1_0101	339	DVESELHYSILGIPQ	HYSILGIPQ	6	0.3927	714.3	65.00	0.31	tr_D0A1S7_
DRB1_0101	340	TLTMGVFTVYDTLFI	FTVYDTLFI	6	0.3923	717.1	65.00	0.32	tr_D0A1S7_
DRB1_0101	341	TDSEVKVSFDLPMLS	VKVSFDLPM	4	0.3916	722.6	65.00	0.26	tr_D0A1S7_
DRB1_0101	342	PPRAISSEEMTLLKG	ISSEEMTLL	4	0.3909	728.1	65.00	0.57	tr_D0A1S7_
DRB1_0101	343	LTVIVVHTVFLCELL	VIVVHTVFL	2	0.3883	748.9	65.00	0.41	tr_D0A1S7_
DRB1_0101	344	AHINGSKEFSWFLQV	GSKEFSWFL	4	0.3867	761.6	65.00	0.49	tr_D0A1S7_
DRB1_0101	345	IVLLQVLFYCVSCAK	LQVLFYCVS	3	0.3860	767.8	65.00	0.26	tr_D0A1S7_
DRB1_0101	346	SAEEWEIIGMVKRSY	WEIIGMVKR	4	0.3856	771.3	65.00	0.57	tr_D0A1S7_
DRB1_0101	347	RTLTMGVFTVYDTLF	TLTMGVFTV	1	0.3853	773.3	65.00	0.38	tr_D0A1S7_
DRB1_0101	348	IKLPTRRLSYLNFIC	RRLSYLNFI	5	0.3842	782.9	70.00	0.27	tr_D0A1S7_
DRB1_0101	349	KVDVLFRVRDEFANR	LFRVRDEFA	4	0.3834	789.6	70.00	0.60	tr_D0A1S7_
DRB1_0101	350	SQEVAFVAVTREHPR	FVAVTREHP	5	0.3803	816.1	70.00	0.55	tr_D0A1S7_
DRB1_0101	351	VDLYREAMFFAGGPL	YREAMFFAG	3	0.3803	816.1	70.00	0.30	tr_D0A1S7_
DRB1_0101	352	VRLTDATHSEIVSRH	VRLTDATHS	0	0.3770	846.3	70.00	0.34	tr_D0A1S7_
DRB1_0101	353	LKGVDNEAVNWFDVV	LKGVDNEAV	0	0.3764	852.1	70.00	0.56	tr_D0A1S7_
DRB1_0101	354	SILGIPQGSDARAIK	IPQGSDARA	4	0.3761	854.3	70.00	0.31	tr_D0A1S7_
DRB1_0101	355	AIKGAYREAVRRWHP	IKGAYREAV	1	0.3754	861.4	70.00	0.52	tr_D0A1S7_
DRB1_0101	356	HTVFLCELLWFPWEP	FLCELLWFP	3	0.3744	870.6	70.00	0.31	tr_D0A1S7_
DRB1_0101	357	VVHTVFLCELLWFPW	FLCELLWFP	5	0.3733	881.1	70.00	0.33	tr_D0A1S7_
DRB1_0101	358	EPPRAISSEEMTLLK	ISSEEMTLL	5	0.3730	884.0	70.00	0.53	tr_D0A1S7_
DRB1_0101	359	TVIVVHTVFLCELLW	VIVVHTVFL	1	0.3726	887.9	70.00	0.38	tr_D0A1S7_
DRB1_0101	360	NPNCDSCRVHMMKIQ	CDSCRVHMM	3	0.3703	909.6	70.00	0.65	tr_D0A1S7_
DRB1_0101	361	LLAKGSERYELVDRY	KGSERYELV	3	0.3701	911.9	70.00	0.35	tr_D0A1S7_
DRB1_0101	362	YELVDRYGEELAQLR	LVDRYGEEL	2	0.3695	917.4	70.00	0.45	tr_D0A1S7_
DRB1_0101	363	EVLIDVESELHYSIL	IDVESELHY	3	0.3673	939.5	70.00	0.31	tr_D0A1S7_
DRB1_0101	364	VIVLLQVLFYCVSCA	LLQVLFYCV	3	0.3670	942.8	70.00	0.34	tr_D0A1S7_
DRB1_0101	365	REVTGEPPRAISSEE	VTGEPPRAI	2	0.3669	944.1	70.00	0.79	tr_D0A1S7_
DRB1_0101	366	NSQEVAFVAVTREHP	FVAVTREHP	6	0.3666	946.6	70.00	0.26	tr_D0A1S7_
DRB1_0101	367	VLIDVESELHYSILG	IDVESELHY	2	0.3660	952.9	70.00	0.27	tr_D0A1S7_
DRB1_0101	368	LIDVESELHYSILGI	VESELHYSI	3	0.3658	954.9	70.00	0.28	tr_D0A1S7_
DRB1_0101	369	RAISSEEMTLLKGVD	ISSEEMTLL	2	0.3652	961.0	70.00	0.67	tr_D0A1S7_
DRB1_0101	370	FVAVTREHPRAPSKV	VTREHPRAP	3	0.3641	973.1	70.00	0.37	tr_D0A1S7_
DRB1_0101	371	LIQAFNGDAHINGSK	LIQAFNGDA	0	0.3635	978.9	70.00	0.40	tr_D0A1S7_
DRB1_0101	372	ILGIPQGSDARAIKG	IPQGSDARA	3	0.3613	1002.5	70.00	0.38	tr_D0A1S7_
DRB1_0101	373	RNPNCDSCRVHMMKI	CDSCRVHMM	4	0.3604	1012.4	70.00	0.65	tr_D0A1S7_
DRB1_0101	374	SSAEEWEIIGMVKRS	WEIIGMVKR	5	0.3591	1027.2	75.00	0.54	tr_D0A1S7_
DRB1_0101	375	TDATHSEIVSRHARL	EIVSRHARL	6	0.3580	1038.9	75.00	0.28	tr_D0A1S7_
DRB1_0101	376	IVVHTVFLCELLWFP	HTVFLCELL	3	0.3574	1046.2	75.00	0.30	tr_D0A1S7_
DRB1_0101	377	TVFLCELLWFPWEPP	FLCELLWFP	2	0.3550	1073.5	75.00	0.33	tr_D0A1S7_
DRB1_0101	378	APHREVTGEPPRAIS	VTGEPPRAI	5	0.3548	1076.1	75.00	0.71	tr_D0A1S7_
DRB1_0101	379	PNCDSCRVHMMKIQH	CDSCRVHMM	2	0.3547	1077.4	75.00	0.56	tr_D0A1S7_
DRB1_0101	380	VFLCELLWFPWEPPI	FLCELLWFP	1	0.3544	1080.4	75.00	0.31	tr_D0A1S7_
DRB1_0101	381	VIVVHTVFLCELLWF	HTVFLCELL	4	0.3491	1144.0	75.00	0.30	tr_D0A1S7_
DRB1_0101	382	RYELVDRYGEELAQL	LVDRYGEEL	3	0.3489	1146.8	75.00	0.68	tr_D0A1S7_
DRB1_0101	383	WEVLIDVESELHYSI	IDVESELHY	4	0.3485	1152.4	75.00	0.29	tr_D0A1S7_
DRB1_0101	384	DNEAVNWFDVVSTRF	VNWFDVVST	4	0.3483	1154.0	75.00	0.37	tr_D0A1S7_
DRB1_0101	385	LGIPQGSDARAIKGA	PQGSDARAI	3	0.3466	1176.1	75.00	0.38	tr_D0A1S7_
DRB1_0101	386	RVIVLLQVLFYCVSC	LLQVLFYCV	4	0.3451	1195.2	75.00	0.33	tr_D0A1S7_
DRB1_0101	387	IDVESELHYSILGIP	VESELHYSI	2	0.3374	1298.6	75.00	0.25	tr_D0A1S7_

DRB1_0101	388	KSSAEEWEIIGMVKR	WEIIGMVKR	6	0.3365	1311.7	75.00	0.44	tr_D0A1S7_
DRB1_0101	389	DRNPNCDSCRVHMMK	CDSCRVHMM	5	0.3325	1369.6	80.00	0.62	tr_D0A1S7_
DRB1_0101	390	DAHINGSKEFSWFLQ	GSKEFSWFL	5	0.3324	1371.4	80.00	0.52	tr_D0A1S7_
DRB1_0101	391	LWEVLIDVESELHYS	WEVLIDVES	1	0.3311	1390.1	80.00	0.29	tr_D0A1S7_
DRB1_0101	392	CTDSEVKVSFDLPML	SEVKVSFDL	3	0.3307	1396.9	80.00	0.40	tr_D0A1S7_
DRB1_0101	393	SKVDVLFRVRDEFAN	LFRVRDEFA	5	0.3305	1399.6	80.00	0.56	tr_D0A1S7_
DRB1_0101	394	MLSATVISTIVLLYH	SATVISTIV	2	0.3255	1477.6	80.00	0.31	tr_D0A1S7_
DRB1_0101	395	EIVSRHARLWEENKR	IVSRHARLW	1	0.3220	1533.5	80.00	0.50	tr_D0A1S7_
DRB1_0101	396	GEPPRAISSEEMTLL	ISSEEMTLL	6	0.3192	1580.7	80.00	0.39	tr_D0A1S7_
DRB1_0101	397	RLTDATHSEIVSRHA	TDATHSEIV	2	0.3177	1607.3	80.00	0.37	tr_D0A1S7_
DRB1_0101	398	PLWEVLIDVESELHY	WEVLIDVES	2	0.3159	1639.3	80.00	0.43	tr_D0A1S7_
DRB1_0101	399	LTDATHSEIVSRHAR	TDATHSEIV	1	0.3142	1670.0	80.00	0.27	tr_D0A1S7_
DRB1_0101	400	ERYELVDRYGEELAQ	LVDRYGEEL	4	0.3131	1689.0	80.00	0.69	tr_D0A1S7_
DRB1_0101	401	CVSCAKSSAEEWEII	CAKSSAEEW	3	0.3128	1694.2	80.00	0.45	tr_D0A1S7_
DRB1_0101	402	QGSDARAIKGAYREA	ARAIKGAYR	4	0.3125	1701.1	80.00	0.66	tr_D0A1S7_
DRB1_0101	403	VSCAKSSAEEWEIIG	CAKSSAEEW	2	0.3114	1720.9	80.00	0.42	tr_D0A1S7_
DRB1_0101	404	EVTGEPPRAISSEEM	VTGEPPRAI	1	0.3066	1812.5	85.00	0.71	tr_D0A1S7_
DRB1_0101	405	SCAKSSAEEWEIIGM	CAKSSAEEW	1	0.3062	1820.4	85.00	0.33	tr_D0A1S7_
DRB1_0101	406	VDNEAVNWFDVVSTR	VNWFDVVST	5	0.2985	1979.4	85.00	0.41	tr_D0A1S7_
DRB1_0101	407	GIPQGSDARAIKGAY	PQGSDARAI	2	0.2978	1994.5	85.00	0.42	tr_D0A1S7_
DRB1_0101	408	HPRAPSKVDVLFRVR	SKVDVLFRV	5	0.2974	2001.7	85.00	0.40	tr_D0A1S7_
DRB1_0101	409	IKGAYREAVRRWHPD	IKGAYREAV	0	0.2968	2016.3	85.00	0.38	tr_D0A1S7_
DRB1_0101	410	PRAPSKVDVLFRVRD	SKVDVLFRV	4	0.2964	2024.5	85.00	0.44	tr_D0A1S7_
DRB1_0101	411	IPQGSDARAIKGAYR	PQGSDARAI	1	0.2927	2106.5	85.00	0.30	tr_D0A1S7_
DRB1_0101	412	PQGSDARAIKGAYRE	ARAIKGAYR	5	0.2850	2289.0	85.00	0.51	tr_D0A1S7_
DRB1_0101	413	LAKGSERYELVDRYG	KGSERYELV	2	0.2849	2292.8	85.00	0.45	tr_D0A1S7_
DRB1_0101	414	RAPSKVDVLFRVRDE	SKVDVLFRV	3	0.2837	2321.4	85.00	0.48	tr_D0A1S7_
DRB1_0101	415	CAKSSAEEWEIIGMV	SSAEEWEII	3	0.2824	2354.4	85.00	0.38	tr_D0A1S7_
DRB1_0101	416	LSATVISTIVLLYHF	SATVISTIV	1	0.2813	2383.9	85.00	0.28	tr_D0A1S7_
DRB1_0101	417	PSKVDVLFRVRDEFA	LFRVRDEFA	6	0.2810	2391.6	85.00	0.38	tr_D0A1S7_
DRB1_0101	418	SERYELVDRYGEELA	LVDRYGEEL	5	0.2806	2402.0	85.00	0.61	tr_D0A1S7_
DRB1_0101	419	PDRNPNCDSCRVHMM	CDSCRVHMM	6	0.2777	2476.7	85.00	0.58	tr_D0A1S7_
DRB1_0101	420	GDAHINGSKEFSWFL	GSKEFSWFL	6	0.2775	2482.8	85.00	0.46	tr_D0A1S7_
DRB1_0101	421	GVDNEAVNWFDVVST	NEAVNWFDV	3	0.2726	2617.8	90.00	0.31	tr_D0A1S7_
DRB1_0101	422	VAVTREHPRAPSKVD	VTREHPRAP	2	0.2725	2621.6	90.00	0.49	tr_D0A1S7_
DRB1_0101	423	MAPHREVTGEPPRAI	VTGEPPRAI	6	0.2718	2640.2	90.00	0.56	tr_D0A1S7_
DRB1_0101	424	SATVISTIVLLYHFL	ATVISTIVL	1	0.2623	2926.3	90.00	0.19	tr_D0A1S7_
DRB1_0101	425	EHPRAPSKVDVLFRV	SKVDVLFRV	6	0.2573	3090.4	90.00	0.29	tr_D0A1S7_
DRB1_0101	426	TWRRRCTDSEVKVSF	WRRRCTDSE	1	0.2569	3103.4	90.00	0.43	tr_D0A1S7_
DRB1_0101	427	DTWRRRCTDSEVKVS	WRRRCTDSE	2	0.2552	3160.4	90.00	0.51	tr_D0A1S7_
DRB1_0101	428	AKSSAEEWEIIGMVK	SSAEEWEII	2	0.2538	3208.3	90.00	0.39	tr_D0A1S7_
DRB1_0101	429	RCTDSEVKVSFDLPM	SEVKVSFDL	4	0.2526	3250.5	90.00	0.54	tr_D0A1S7_
DRB1_0101	430	SDTWRRRCTDSEVKV	WRRRCTDSE	3	0.2522	3265.0	90.00	0.58	tr_D0A1S7_
DRB1_0101	431	KGVDNEAVNWFDVVS	NEAVNWFDV	4	0.2472	3448.0	90.00	0.33	tr_D0A1S7_
DRB1_0101	432	APSKVDVLFRVRDEF	SKVDVLFRV	2	0.2469	3456.1	90.00	0.50	tr_D0A1S7_
DRB1_0101	433	AVTREHPRAPSKVDV	VTREHPRAP	1	0.2457	3503.1	90.00	0.46	tr_D0A1S7_
DRB1_0101	434	VTGEPPRAISSEEMT	VTGEPPRAI	0	0.2443	3557.7	90.00	0.49	tr_D0A1S7_
DRB1_0101	435	IQAFNGDAHINGSKE	FNGDAHING	3	0.2440	3567.7	90.00	0.50	tr_D0A1S7_
DRB1_0101	436	SVPDRKDKGKNVHRE	DRKDKGKNV	3	0.2406	3703.5	90.00	0.67	tr_D0A1S7_
DRB1_0101	437	GKHTDMAPHREVTGE	TDMAPHREV	3	0.2380	3809.3	95.00	0.52	tr_D0A1S7_

DRB1_0101	438	EGKHTDMAPHREVTG	TDMAPHREV	4	0.2359	3894.1	95.00	0.51	tr_D0A1S7_
DRB1_0101	439	AKGSERYELVDRYGE	KGSERYELV	1	0.2342	3965.5	95.00	0.44	tr_D0A1S7_
DRB1_0101	440	VPDRKDKGKNVHREE	DRKDKGKNV	2	0.2334	4002.2	95.00	0.57	tr_D0A1S7_
DRB1_0101	441	VTREHPRAPSKVDVL	EHPRAPSKV	3	0.2326	4036.0	95.00	0.34	tr_D0A1S7_
DRB1_0101	442	IVSRHARLWEENKRK	IVSRHARLW	0	0.2324	4046.9	95.00	0.41	tr_D0A1S7_
DRB1_0101	443	LSVPDRKDKGKNVHR	DRKDKGKNV	4	0.2293	4181.9	95.00	0.70	tr_D0A1S7_
DRB1_0101	444	PWEPPILSVPDRKDK	PILSVPDRK	4	0.2275	4265.3	95.00	0.28	tr_D0A1S7_
DRB1_0101	445	REHPRAPSKVDVLFR	RAPSKVDVL	4	0.2268	4298.0	95.00	0.32	tr_D0A1S7_
DRB1_0101	446	WEPPILSVPDRKDKG	PILSVPDRK	3	0.2218	4538.5	95.00	0.35	tr_D0A1S7_
DRB1_0101	447	QAFNGDAHINGSKEF	FNGDAHING	2	0.2216	4548.6	95.00	0.52	tr_D0A1S7_
DRB1_0101	448	EPPILSVPDRKDKGK	ILSVPDRKD	3	0.2209	4580.5	95.00	0.46	tr_D0A1S7_
DRB1_0101	449	KHTDMAPHREVTGEP	TDMAPHREV	2	0.2204	4606.0	95.00	0.54	tr_D0A1S7_
DRB1_0101	450	PSDTWRRRCTDSEVK	WRRRCTDSE	4	0.2197	4639.3	95.00	0.65	tr_D0A1S7_
DRB1_0101	451	KGAYREAVRRWHPDR	GAYREAVRR	1	0.2195	4650.6	95.00	0.34	tr_D0A1S7_
DRB1_0101	452	TGEPPRAISSEEMTL	PRAISSEEM	4	0.2155	4854.5	95.00	0.62	tr_D0A1S7_
DRB1_0101	453	EEGKHTDMAPHREVT	TDMAPHREV	5	0.2143	4918.3	95.00	0.45	tr_D0A1S7_
DRB1_0101	454	PDRKDKGKNVHREEE	DRKDKGKNV	1	0.2138	4946.7	95.00	0.51	tr_D0A1S7_
DRB1_0101	455	ILSVPDRKDKGKNVH	DRKDKGKNV	5	0.2102	5143.2	95.00	0.57	tr_D0A1S7_
DRB1_0101	456	WRRRCTDSEVKVSFD	WRRRCTDSE	0	0.2095	5184.8	95.00	0.25	tr_D0A1S7_
DRB1_0101	457	GSERYELVDRYGEEL	LVDRYGEEL	6	0.2082	5257.6	95.00	0.45	tr_D0A1S7_
DRB1_0101	458	KNVHREEEGKHTDMA	HREEEGKHT	3	0.2053	5423.0	95.00	0.62	tr_D0A1S7_
DRB1_0101	459	RRCTDSEVKVSFDLP	SEVKVSFDL	5	0.2047	5456.9	95.00	0.52	tr_D0A1S7_
DRB1_0101	460	TREHPRAPSKVDVLF	EHPRAPSKV	2	0.2025	5587.8	95.00	0.32	tr_D0A1S7_
DRB1_0101	461	PPILSVPDRKDKGKN	ILSVPDRKD	2	0.2005	5712.4	95.00	0.48	tr_D0A1S7_
DRB1_0101	462	PILSVPDRKDKGKNV	ILSVPDRKD	1	0.1985	5835.1	95.00	0.44	tr_D0A1S7_
DRB1_0101	463	RRRCTDSEVKVSFDL	SEVKVSFDL	6	0.1953	6044.1	95.00	0.31	tr_D0A1S7_
DRB1_0101	464	ENKRKNPSDTWRRRC	KNPSDTWRR	4	0.1952	6051.0	95.00	0.38	tr_D0A1S7_
DRB1_0101	465	AFNGDAHINGSKEFS	FNGDAHING	1	0.1931	6186.3	100.00	0.43	tr_D0A1S7_
DRB1_0101	466	NKRKNPSDTWRRRCT	KNPSDTWRR	3	0.1916	6288.3	100.00	0.44	tr_D0A1S7_
DRB1_0101	467	EENKRKNPSDTWRRR	KNPSDTWRR	5	0.1914	6302.5	100.00	0.34	tr_D0A1S7_
DRB1_0101	468	KGSERYELVDRYGEE	KGSERYELV	0	0.1887	6489.9	100.00	0.39	tr_D0A1S7_
DRB1_0101	469	GKNVHREEEGKHTDM	HREEEGKHT	4	0.1836	6858.2	100.00	0.62	tr_D0A1S7_
DRB1_0101	470	GAYREAVRRWHPDRN	YREAVRRWH	2	0.1821	6969.3	100.00	0.32	tr_D0A1S7_
DRB1_0101	471	NVHREEEGKHTDMAP	HREEEGKHT	2	0.1781	7279.7	100.00	0.64	tr_D0A1S7_
DRB1_0101	472	FNGDAHINGSKEFSW	DAHINGSKE	3	0.1774	7335.6	100.00	0.31	tr_D0A1S7_
DRB1_0101	473	NGDAHINGSKEFSWF	INGSKEFSW	5	0.1767	7391.1	100.00	0.31	tr_D0A1S7_
DRB1_0101	474	NPSDTWRRRCTDSEV	WRRRCTDSE	5	0.1740	7611.6	100.00	0.64	tr_D0A1S7_
DRB1_0101	475	DRKDKGKNVHREEEG	DRKDKGKNV	0	0.1729	7701.7	100.00	0.40	tr_D0A1S7_
DRB1_0101	476	HTDMAPHREVTGEPP	TDMAPHREV	1	0.1685	8079.3	100.00	0.54	tr_D0A1S7_
DRB1_0101	477	KRKNPSDTWRRRCTD	KNPSDTWRR	2	0.1683	8096.7	100.00	0.49	tr_D0A1S7_
DRB1_0101	478	EEEGKHTDMAPHREV	HTDMAPHRE	5	0.1657	8324.2	100.00	0.33	tr_D0A1S7_
DRB1_0101	479	WEENKRKNPSDTWRR	KRKNPSDTW	4	0.1632	8556.2	100.00	0.35	tr_D0A1S7_
DRB1_0101	480	KGKNVHREEEGKHTD	HREEEGKHT	5	0.1552	9327.3	100.00	0.57	tr_D0A1S7_
DRB1_0101	481	VSRHARLWEENKRKN	SRHARLWEE	1	0.1540	9447.1	100.00	0.34	tr_D0A1S7_
DRB1_0101	482	VHREEEGKHTDMAPH	HREEEGKHT	1	0.1507	9795.8	100.00	0.65	tr_D0A1S7_
DRB1_0101	483	HPDRNPNCDSCRVHM	NPNCDSCRV	4	0.1503	9829.4	100.00	0.43	tr_D0A1S7_
DRB1_0101	484	AYREAVRRWHPDRNP	YREAVRRWH	1	0.1453	10377.3	100.00	0.38	tr_D0A1S7_
DRB1_0101	485	AVRRWHPDRNPNCDS	WHPDRNPNC	4	0.1440	10530.2	100.00	0.49	tr_D0A1S7_
DRB1_0101	486	SRHARLWEENKRKNP	HARLWEENK	2	0.1434	10592.0	100.00	0.22	tr_D0A1S7_
DRB1_0101	487	DMAPHREVTGEPPRA	HREVTGEPP	4	0.1417	10798.2	100.00	0.43	tr_D0A1S7_

DRB1_0101	488	WHPDRNPNCDSCRVH	NPNCDSCRV	5	0.1390	11117.9	100.00	0.38	tr_D0A1S7_
DRB1_0101	489	RHARLWEENKRKNPS	WEENKRKNP	5	0.1381	11216.1	100.00	0.26	tr_D0A1S7_
DRB1_0101	490	KNPSDTWRRRCTDSE	WRRRCTDSE	6	0.1380	11237.4	100.00	0.47	tr_D0A1S7_
DRB1_0101	491	VRRWHPDRNPNCDSC	WHPDRNPNC	3	0.1377	11269.8	100.00	0.53	tr_D0A1S7_
DRB1_0101	492	EAVRRWHPDRNPNCD	WHPDRNPNC	5	0.1363	11443.4	100.00	0.44	tr_D0A1S7_
DRB1_0101	493	RWHPDRNPNCDSCRV	WHPDRNPNC	1	0.1358	11501.9	100.00	0.44	tr_D0A1S7_
DRB1_0101	494	DKGKNVHREEEGKHT	HREEEGKHT	6	0.1334	11809.1	100.00	0.37	tr_D0A1S7_
DRB1_0101	495	LWEENKRKNPSDTWR	KRKNPSDTW	5	0.1326	11907.1	100.00	0.34	tr_D0A1S7_
DRB1_0101	496	REAVRRWHPDRNPNC	WHPDRNPNC	6	0.1289	12397.0	100.00	0.31	tr_D0A1S7_
DRB1_0101	497	TDMAPHREVTGEPPR	TDMAPHREV	0	0.1276	12568.9	100.00	0.40	tr_D0A1S7_
DRB1_0101	498	RKNPSDTWRRRCTDS	KNPSDTWRR	1	0.1276	12570.1	100.00	0.52	tr_D0A1S7_
DRB1_0101	499	RLWEENKRKNPSDTW	WEENKRKNP	2	0.1267	12690.6	100.00	0.34	tr_D0A1S7_
DRB1_0101	500	YREAVRRWHPDRNPN	YREAVRRWH	0	0.1254	12867.8	100.00	0.23	tr_D0A1S7_
DRB1_0101	501	RRWHPDRNPNCDSCR	WHPDRNPNC	2	0.1245	12994.5	100.00	0.55	tr_D0A1S7_
DRB1_0101	502	RKDKGKNVHREEEGK	DKGKNVHRE	2	0.1225	13286.8	100.00	0.46	tr_D0A1S7_
DRB1_0101	503	HARLWEENKRKNPSD	WEENKRKNP	4	0.1206	13567.5	100.00	0.34	tr_D0A1S7_
DRB1_0101	504	ARLWEENKRKNPSDT	WEENKRKNP	3	0.1158	14285.6	100.00	0.38	tr_D0A1S7_
DRB1_0101	505	HREEEGKHTDMAPHR	HREEEGKHT	0	0.1139	14587.6	100.00	0.43	tr_D0A1S7_
DRB1_0101	506	REEEGKHTDMAPHRE	HTDMAPHRE	6	0.1083	15488.4	100.00	0.31	tr_D0A1S7_
DRB1_0101	507	KDKGKNVHREEEGKH	DKGKNVHRE	1	0.1019	16598.5	100.00	0.34	tr_D0A1S7_

Allele: DRB1_0101. Number of high binders 1. Number of weak binders 65. Number of peptides 507

Allele	pos	peptide	core	Of	1-log50k(aff)	affinity(nM)	%Rank	Relia	Identity	Bind Level
DRB1_0101	1	CSAYTGNGPVSGYCF	YTGNGPVSG	3	0.4281	487.0	60.00	0.74	tr_C9ZKG2_	
DRB1_0101	2	DEELSEIGAPSSGVN	LSEIGAPSS	3	0.4232	513.2	60.00	0.53	tr_C9ZKG2_	
DRB1_0101	3	SAYTGNGPVSGYCFD	YTGNGPVSG	2	0.4229	515.1	60.00	0.73	tr_C9ZKG2_	
DRB1_0101	4	EELSEIGAPSSGVNP	LSEIGAPSS	2	0.4206	527.8	60.00	0.55	tr_C9ZKG2_	
DRB1_0101	5	EDEELSEIGAPSSGV	LSEIGAPSS	4	0.3985	670.7	65.00	0.53	tr_C9ZKG2_	
DRB1_0101	6	ELSEIGAPSSGVNPA	LSEIGAPSS	1	0.3908	728.6	65.00	0.58	tr_C9ZKG2_	
DRB1_0101	7	DCSAYTGNGPVSGYC	YTGNGPVSG	4	0.3846	779.6	65.00	0.76	tr_C9ZKG2_	
DRB1_0101	8	AYTGNGPVSGYCFDK	YTGNGPVSG	1	0.3820	801.8	70.00	0.65	tr_C9ZKG2_	
DRB1_0101	9	DDCSAYTGNGPVSGY	YTGNGPVSG	5	0.3537	1088.6	75.00	0.74	tr_C9ZKG2_	
DRB1_0101	10	QEDEELSEIGAPSSG	LSEIGAPSS	5	0.3356	1324.9	75.00	0.52	tr_C9ZKG2_	
DRB1_0101	11	SGVNPAATSLPQRKK	VNPAATSLP	2	0.3348	1335.4	75.00	0.37	tr_C9ZKG2_	
DRB1_0101	12	SSGVNPAATSLPQRK	VNPAATSLP	3	0.3343	1343.3	80.00	0.44	tr_C9ZKG2_	
DRB1_0101	13	LSEIGAPSSGVNPAA	LSEIGAPSS	0	0.3257	1474.2	80.00	0.44	tr_C9ZKG2_	
DRB1_0101	14	GVNPAATSLPQRKKN	VNPAATSLP	1	0.3083	1778.9	80.00	0.34	tr_C9ZKG2_	
DRB1_0101	15	PSSGVNPAATSLPQR	VNPAATSLP	4	0.3024	1897.1	85.00	0.43	tr_C9ZKG2_	
DRB1_0101	16	VNPAATSLPQRKKNL	AATSLPQRK	3	0.2928	2105.0	85.00	0.39	tr_C9ZKG2_	
DRB1_0101	17	NPAATSLPQRKKNLV	AATSLPQRK	2	0.2876	2226.1	85.00	0.34	tr_C9ZKG2_	
DRB1_0101	18	YTGNGPVSGYCFDKG	YTGNGPVSG	0	0.2733	2600.0	90.00	0.47	tr_C9ZKG2_	
DRB1_0101	19	APSSGVNPAATSLPQ	VNPAATSLP	5	0.2660	2811.9	90.00	0.34	tr_C9ZKG2_	
DRB1_0101	20	VSGYCFDKGLCDEHM	YCFDKGLCD	3	0.2566	3112.4	90.00	0.49	tr_C9ZKG2_	
DRB1_0101	21	SQEDEELSEIGAPSS	LSEIGAPSS	6	0.2560	3133.0	90.00	0.37	tr_C9ZKG2_	
DRB1_0101	22	SGYCFDKGLCDEHMP	YCFDKGLCD	2	0.2547	3178.2	90.00	0.47	tr_C9ZKG2_	

DRB1_0101	23	GYCFDKGLCDEHMPS	YCFDKGLCD	1	0.2500	3344.2	90.00	0.41	tr_C9ZKG2_
DRB1_0101	24	EDDCSAYTGNGPVSG	YTGNGPVSG	6	0.2471	3450.5	90.00	0.58	tr_C9ZKG2_
DRB1_0101	25	YCFDKGLCDEHMPSQ	DKGLCDEHM	3	0.2313	4094.8	95.00	0.35	tr_C9ZKG2_
DRB1_0101	26	GAPSSGVNPAATSLP	SGVNPAATS	4	0.2299	4157.7	95.00	0.24	tr_C9ZKG2_
DRB1_0101	27	IGAPSSGVNPAATSL	SGVNPAATS	5	0.2283	4230.7	95.00	0.28	tr_C9ZKG2_
DRB1_0101	28	PVSGYCFDKGLCDEH	YCFDKGLCD	4	0.2097	5171.0	95.00	0.56	tr_C9ZKG2_
DRB1_0101	29	SEIGAPSSGVNPAAT	GAPSSGVNP	3	0.2076	5292.8	95.00	0.28	tr_C9ZKG2_
DRB1_0101	30	NGPVSGYCFDKGLCD	YCFDKGLCD	6	0.1985	5835.0	95.00	0.32	tr_C9ZKG2_
DRB1_0101	31	EIGAPSSGVNPAATS	GAPSSGVNP	2	0.1950	6063.7	95.00	0.28	tr_C9ZKG2_
DRB1_0101	32	GPVSGYCFDKGLCDE	YCFDKGLCD	5	0.1882	6528.7	100.00	0.54	tr_C9ZKG2_
DRB1_0101	33	TGNGPVSGYCFDKGL	NGPVSGYCF	2	0.1876	6568.4	100.00	0.55	tr_C9ZKG2_
DRB1_0101	34	CFDKGLCDEHMPSQE	DKGLCDEHM	2	0.1872	6599.5	100.00	0.46	tr_C9ZKG2_
DRB1_0101	35	GNGPVSGYCFDKGLC	NGPVSGYCF	1	0.1836	6859.2	100.00	0.44	tr_C9ZKG2_
DRB1_0101	36	FDKGLCDEHMPSQED	DKGLCDEHM	1	0.1662	8276.7	100.00	0.41	tr_C9ZKG2_
DRB1_0101	37	REDDCSAYTGNGPVS	SAYTGNGPV	5	0.1541	9440.0	100.00	0.28	tr_C9ZKG2_
DRB1_0101	38	DKGLCDEHMPSQEDE	DKGLCDEHM	0	0.1364	11429.5	100.00	0.25	tr_C9ZKG2_
DRB1_0101	39	PSQEDEELSEIGAPS	ELSEIGAPS	6	0.1345	11661.1	100.00	0.44	tr_C9ZKG2_
DRB1_0101	40	LCDEHMPSQEDEELS	DEHMPSQED	2	0.1277	12562.0	100.00	0.41	tr_C9ZKG2_
DRB1_0101	41	DEHMPSQEDEELSEI	MPSQEDEEL	3	0.1191	13789.2	100.00	0.37	tr_C9ZKG2_
DRB1_0101	42	CDEHMPSQEDEELSE	DEHMPSQED	1	0.1185	13866.4	100.00	0.32	tr_C9ZKG2_
DRB1_0101	43	GLCDEHMPSQEDEEL	DEHMPSQED	3	0.1181	13930.3	100.00	0.45	tr_C9ZKG2_
DRB1_0101	44	HMPSQEDEELSEIGA	MPSQEDEEL	1	0.1109	15063.0	100.00	0.25	tr_C9ZKG2_
DRB1_0101	45	KGLCDEHMPSQEDEE	DEHMPSQED	4	0.1108	15078.6	100.00	0.34	tr_C9ZKG2_
DRB1_0101	46	EHMPSQEDEELSEIG	MPSQEDEEL	2	0.1051	16028.6	100.00	0.39	tr_C9ZKG2_
DRB1_0101	47	EREDDCSAYTGNGPV	EDDCSAYTG	2	0.1038	16266.8	100.00	0.25	tr_C9ZKG2_
DRB1_0101	48	MPSQEDEELSEIGAP	DEELSEIGA	5	0.1032	16362.6	100.00	0.28	tr_C9ZKG2_
DRB1_0101	49	GDEREDDCSAYTGNG	EDDCSAYTG	4	0.0906	18751.3	100.00	0.31	tr_C9ZKG2_
DRB1_0101	50	DEREDDCSAYTGNGP	EDDCSAYTG	3	0.0863	19645.2	100.00	0.29	tr_C9ZKG2_
DRB1_0101	51	CGDEREDDCSAYTGN	EDDCSAYTG	5	0.0852	19895.9	100.00	0.25	tr_C9ZKG2_
DRB1_0101	52	LCGDEREDDCSAYTG	DEREDDCSA	3	0.0804	20952.9	100.00	0.29	tr_C9ZKG2_
DRB1_0101	53	MHLCGDEREDDCSAY	DEREDDCSA	5	0.0768	21784.9	100.00	0.25	tr_C9ZKG2_
DRB1_0101	54	HLCGDEREDDCSAYT	DEREDDCSA	4	0.0723	22871.1	100.00	0.32	tr_C9ZKG2_

Allele: DRB1_0101. Number of high binders 0. Number of weak binders 0. Number of peptides 54

Allele	pos	peptide	core	Of	1-log50k(aff)	affinity(nM)	%Rank	Relia	Identity	Bind Level
DRB1_0101	1	RACIYLRLPRWVRRS	YLRLPRWVR	4	0.7106	22.9	8.00	0.42	tr_C9ZKG2_	WB
DRB1_0101	2	YRLLQLLLRGSKFGW	QLLLRGSKF	4	0.7096	23.2	8.00	0.57	tr_C9ZKG2_	WB
DRB1_0101	3	HRACIYLRLPRWVRR	YLRLPRWVR	5	0.7087	23.4	8.00	0.37	tr_C9ZKG2_	WB
DRB1_0101	4	AYRLLQLLLRGSKFG	QLLLRGSKF	5	0.7004	25.6	9.00	0.52	tr_C9ZKG2_	WB
DRB1_0101	5	RLLQLLLRGSKFGWT	QLLLRGSKF	3	0.6982	26.2	9.00	0.65	tr_C9ZKG2_	WB
DRB1_0101	6	LLQLLLRGSKFGWTK	QLLLRGSKF	2	0.6833	30.8	11.00	0.55	tr_C9ZKG2_	
DRB1_0101	7	ACIYLRLPRWVRRSP	YLRLPRWVR	3	0.6792	32.2	11.00	0.44	tr_C9ZKG2_	
DRB1_0101	8	THRACIYLRLPRWVR	CIYLRLPRW	4	0.6727	34.5	12.00	0.40	tr_C9ZKG2_	
DRB1_0101	9	LQLLLRGSKFGWTKF	QLLLRGSKF	1	0.6560	41.3	14.00	0.53	tr_C9ZKG2_	
DRB1_0101	10	TKFWLVTHRACIYLR	FWLVTHRAC	2	0.6519	43.2	15.00	0.26	tr_C9ZKG2_	

DRB1_0101	11	CIYLRLPRWVRRSPT	YLRLPRWVR	2	0.6381	50.2	17.00	0.49	tr_C9ZKG2_
DRB1_0101	12	WTKFWLVTHRACIYL	FWLVTHRAC	3	0.6343	52.3	17.00	0.26	tr_C9ZKG2_
DRB1_0101	13	KFWLVTHRACIYLRL	LVTHRACIY	3	0.6302	54.7	18.00	0.22	tr_C9ZKG2_
DRB1_0101	14	GWTKFWLVTHRACIY	KFWLVTHRA	3	0.6158	63.9	20.00	0.34	tr_C9ZKG2_
DRB1_0101	15	VTHRACIYLRLPRWV	CIYLRLPRW	5	0.5992	76.4	23.00	0.45	tr_C9ZKG2_
DRB1_0101	16	QLLLRGSKFGWTKFW	QLLLRGSKF	0	0.5861	88.1	25.00	0.46	tr_C9ZKG2_
DRB1_0101	17	FGWTKFWLVTHRACI	KFWLVTHRA	4	0.5843	89.9	25.00	0.34	tr_C9ZKG2_
DRB1_0101	18	KFGWTKFWLVTHRAC	KFWLVTHRA	5	0.5658	109.7	28.00	0.32	tr_C9ZKG2_
DRB1_0101	19	LVTHRACIYLRLPRW	CIYLRLPRW	6	0.5557	122.5	30.00	0.37	tr_C9ZKG2_
DRB1_0101	20	FWLVTHRACIYLRLP	LVTHRACIY	2	0.5499	130.4	31.00	0.28	tr_C9ZKG2_
DRB1_0101	21	SKFGWTKFWLVTHRA	FGWTKFWLV	2	0.5337	155.2	34.00	0.47	tr_C9ZKG2_
DRB1_0101	22	IYLRLPRWVRRSPTR	YLRLPRWVR	1	0.5321	158.0	35.00	0.62	tr_C9ZKG2_
DRB1_0101	23	WLVTHRACIYLRLPR	HRACIYLRL	4	0.5019	219.0	41.00	0.28	tr_C9ZKG2_
DRB1_0101	24	GSKFGWTKFWLVTHR	FGWTKFWLV	3	0.4665	321.2	48.00	0.70	tr_C9ZKG2_
DRB1_0101	25	RGSKFGWTKFWLVTH	FGWTKFWLV	4	0.4457	402.3	55.00	0.68	tr_C9ZKG2_
DRB1_0101	26	YLRLPRWVRRSPTRS	YLRLPRWVR	0	0.4317	468.0	55.00	0.42	tr_C9ZKG2_
DRB1_0101	27	LRGSKFGWTKFWLVT	FGWTKFWLV	5	0.4297	478.3	60.00	0.63	tr_C9ZKG2_
DRB1_0101	28	LLRGSKFGWTKFWLV	FGWTKFWLV	6	0.4219	520.5	60.00	0.47	tr_C9ZKG2_
DRB1_0101	29	LLLRGSKFGWTKFWL	LLLRGSKFG	0	0.3855	771.8	65.00	0.29	tr_C9ZKG2_
DRB1_0101	30	LPRWVRRSPTRSDDN	WVRRSPTRS	3	0.3577	1043.1	75.00	0.70	tr_C9ZKG2_
DRB1_0101	31	LRLPRWVRRSPTRSD	WVRRSPTRS	5	0.3448	1199.2	75.00	0.54	tr_C9ZKG2_
DRB1_0101	32	RLPRWVRRSPTRSDD	WVRRSPTRS	4	0.3396	1268.7	75.00	0.68	tr_C9ZKG2_
DRB1_0101	33	PRWVRRSPTRSDDNC	WVRRSPTRS	2	0.3303	1401.8	80.00	0.69	tr_C9ZKG2_
DRB1_0101	34	RWVRRSPTRSDDNCD	WVRRSPTRS	1	0.2936	2086.6	85.00	0.69	tr_C9ZKG2_
DRB1_0101	35	WVRRSPTRSDDNCDE	WVRRSPTRS	0	0.1817	7001.6	100.00	0.54	tr_C9ZKG2_
DRB1_0101	36	SPTRSDDNCDEPWNR	DNCDEPWNR	6	0.0794	21166.4	100.00	0.31	tr_C9ZKG2_
DRB1_0101	37	VRRSPTRSDDNCDEP	VRRSPTRSD	0	0.0672	24166.5	100.00	0.47	tr_C9ZKG2_
DRB1_0101	38	RSPTRSDDNCDEPWN	TRSDDNCDE	3	0.0610	25854.7	100.00	0.35	tr_C9ZKG2_
DRB1_0101	39	RRSPTRSDDNCDEPW	TRSDDNCDE	4	0.0510	28787.6	100.00	0.38	tr_C9ZKG2_

Allele: DRB1_0101. Number of high binders 0. Number of weak binders 5. Number of peptides 39

Allele	pos	peptide	core	Of	1-log50k(aff)	affinity(nM)	%Rank	Relia	Identity	Bind Level
DRB1_0101	1	AMRLLLERGAVITQR	LLLERGAVI	3	0.8246	6.7	1.20	0.81	tr_C9ZY45_	WB
DRB1_0101	2	TAMRLLLERGAVITQ	LLLERGAVI	4	0.8133	7.5	1.60	0.75	tr_C9ZY45_	WB
DRB1_0101	3	MRLLLERGAVITQRD	LLLERGAVI	2	0.8088	7.9	1.70	0.83	tr_C9ZY45_	WB
DRB1_0101	4	ITAMRLLLERGAVIT	LLLERGAVI	5	0.8001	8.7	2.00	0.74	tr_C9ZY45_	WB
DRB1_0101	5	RLLLERGAVITQRDD	LLLERGAVI	1	0.7780	11.0	3.00	0.83	tr_C9ZY45_	WB
DRB1_0101	6	NITAMRLLLERGAVI	LLLERGAVI	6	0.7625	13.1	4.00	0.69	tr_C9ZY45_	WB
DRB1_0101	7	SVVTFLLGGADVNAR	FLLGGADVN	4	0.7391	16.8	5.50	0.57	tr_C9ZY45_	WB
DRB1_0101	8	LTALQLAANNAGVSA	LQLAANNAG	3	0.7337	17.8	6.00	0.50	tr_C9ZY45_	WB
DRB1_0101	9	GLTALQLAANNAGVS	LQLAANNAG	4	0.7291	18.7	6.50	0.46	tr_C9ZY45_	WB
DRB1_0101	10	VVTFLLGGADVNARD	FLLGGADVN	3	0.7287	18.8	6.50	0.58	tr_C9ZY45_	WB
DRB1_0101	11	PLMVAAAKGNITAMR	VAAAKGNIT	3	0.7266	19.3	6.50	0.75	tr_C9ZY45_	WB
DRB1_0101	12	QRFVNIRSLWCPTAA	IRSLWCPTA	5	0.7242	19.8	7.00	0.48	tr_C9ZY45_	WB
DRB1_0101	13	ILFLVEQLGMDVNAR	LVEQLGMDV	3	0.7241	19.8	7.00	0.66	tr_C9ZY45_	WB

DRB1_0101	14	VILFLVEQLGMDVNA	LVEQLGMDV	4	0.7204	20.6	7.00	0.58	tr_C9ZY45_	WB
DRB1_0101	15	IGLTALQLAANNAGV	LQLAANNAG	5	0.7158	21.7	7.50	0.36	tr_C9ZY45_	WB
DRB1_0101	16	TPLMVAAAKGNITAM	VAAAKGNIT	4	0.7148	21.9	7.50	0.60	tr_C9ZY45_	WB
DRB1_0101	17	TDVIGLTALQLAANN	VIGLTALQL	2	0.7144	22.0	7.50	0.52	tr_C9ZY45_	WB
DRB1_0101	18	DVIGLTALQLAANNA	VIGLTALQL	1	0.7128	22.4	8.00	0.44	tr_C9ZY45_	WB
DRB1_0101	19	QTDVIGLTALQLAAN	VIGLTALQL	3	0.7112	22.8	8.00	0.63	tr_C9ZY45_	WB
DRB1_0101	20	VIGLTALQLAANNAG	LTALQLAAN	3	0.7111	22.8	8.00	0.34	tr_C9ZY45_	WB
DRB1_0101	21	REPFLGECSNEMIIR	FLGECSNEM	3	0.7107	22.9	8.00	0.85	tr_C9ZY45_	WB
DRB1_0101	22	RFVNIRSLWCPTAAE	IRSLWCPTA	4	0.7087	23.4	8.00	0.56	tr_C9ZY45_	WB
DRB1_0101	23	VTFLLGGADVNARDK	FLLGGADVN	2	0.7081	23.5	8.50	0.57	tr_C9ZY45_	WB
DRB1_0101	24	LMVAAAKGNITAMRL	VAAAKGNIT	2	0.7056	24.2	8.50	0.79	tr_C9ZY45_	WB
DRB1_0101	25	LLHFVCQKGGKGANF	FVCQKGGKG	3	0.7048	24.4	8.50	0.75	tr_C9ZY45_	WB
DRB1_0101	26	ETSVMSAVAVRSSYT	VMSAVAVRS	3	0.7040	24.6	8.50	0.67	tr_C9ZY45_	WB
DRB1_0101	27	PSVVTFLLGGADVNA	FLLGGADVN	5	0.7024	25.0	9.00	0.51	tr_C9ZY45_	WB
DRB1_0101	28	TALQLAANNAGVSAV	LQLAANNAG	2	0.6967	26.6	9.50	0.54	tr_C9ZY45_	WB
DRB1_0101	29	LQRFVNIRSLWCPTA	FVNIRSLWC	3	0.6950	27.1	9.50	0.56	tr_C9ZY45_	WB
DRB1_0101	30	FVILFLVEQLGMDVN	LVEQLGMDV	5	0.6934	27.6	9.50	0.52	tr_C9ZY45_	WB
DRB1_0101	31	TSVMSAVAVRSSYTP	VMSAVAVRS	2	0.6891	28.9	10.00	0.64	tr_C9ZY45_	
DRB1_0101	32	LHFVCQKGGKGANFV	FVCQKGGKG	2	0.6886	29.1	10.00	0.74	tr_C9ZY45_	
DRB1_0101	33	MVAAAKGNITAMRLL	VAAAKGNIT	1	0.6878	29.3	11.00	0.62	tr_C9ZY45_	
DRB1_0101	34	LFLVEQLGMDVNARE	LVEQLGMDV	2	0.6876	29.4	11.00	0.72	tr_C9ZY45_	
DRB1_0101	35	ETPLMVAAAKGNITA	VAAAKGNIT	5	0.6870	29.6	11.00	0.48	tr_C9ZY45_	
DRB1_0101	36	EETSVMSAVAVRSSY	VMSAVAVRS	4	0.6863	29.8	11.00	0.70	tr_C9ZY45_	
DRB1_0101	37	FVNIRSLWCPTAAER	IRSLWCPTA	3	0.6842	30.5	11.00	0.69	tr_C9ZY45_	
DRB1_0101	38	NQTDVIGLTALQLAA	VIGLTALQL	4	0.6834	30.7	11.00	0.72	tr_C9ZY45_	
DRB1_0101	39	EPFLGECSNEMIIRF	FLGECSNEM	2	0.6789	32.3	12.00	0.77	tr_C9ZY45_	
DRB1_0101	40	DLLHFVCQKGGKGAN	FVCQKGGKG	4	0.6763	33.2	12.00	0.74	tr_C9ZY45_	
DRB1_0101	41	GREPFLGECSNEMII	FLGECSNEM	4	0.6756	33.4	12.00	0.85	tr_C9ZY45_	
DRB1_0101	42	IIRFLIDNGADINAR	FLIDNGADI	3	0.6670	36.7	13.00	0.60	tr_C9ZY45_	
DRB1_0101	43	LLLERGAVITQRDDA	LLLERGAVI	0	0.6604	39.4	14.00	0.76	tr_C9ZY45_	
DRB1_0101	44	MIIRFLIDNGADINA	FLIDNGADI	4	0.6585	40.2	14.00	0.54	tr_C9ZY45_	
DRB1_0101	45	TFLLGGADVNARDKD	FLLGGADVN	1	0.6559	41.4	14.00	0.61	tr_C9ZY45_	
DRB1_0101	46	YCKETSLSGREALLQ	ETSLSGREA	3	0.6533	42.6	15.00	0.65	tr_C9ZY45_	
DRB1_0101	47	EGALVFADGLIVVHA	LVFADGLIV	3	0.6521	43.2	15.00	0.70	tr_C9ZY45_	
DRB1_0101	48	GALVFADGLIVVHAL	LVFADGLIV	2	0.6515	43.4	15.00	0.67	tr_C9ZY45_	
DRB1_0101	49	CKETSLSGREALLQR	ETSLSGREA	2	0.6498	44.2	15.00	0.58	tr_C9ZY45_	
DRB1_0101	50	HFVCQKGGKGANFVI	FVCQKGGKG	1	0.6481	45.0	15.00	0.71	tr_C9ZY45_	
DRB1_0101	51	VAAAKGNITAMRLLL	VAAAKGNIT	0	0.6480	45.1	15.00	0.33	tr_C9ZY45_	
DRB1_0101	52	PFLGECSNEMIIRFL	FLGECSNEM	1	0.6469	45.6	15.00	0.75	tr_C9ZY45_	
DRB1_0101	53	SVMSAVAVRSSYTPL	VMSAVAVRS	1	0.6468	45.7	15.00	0.55	tr_C9ZY45_	
DRB1_0101	54	PEETSVMSAVAVRSS	VMSAVAVRS	5	0.6451	46.5	16.00	0.62	tr_C9ZY45_	
DRB1_0101	55	ALQLAANNAGVSAVG	LQLAANNAG	1	0.6443	46.9	16.00	0.56	tr_C9ZY45_	
DRB1_0101	56	KETSLSGREALLQRF	ETSLSGREA	1	0.6418	48.2	16.00	0.52	tr_C9ZY45_	
DRB1_0101	57	VNIRSLWCPTAAERR	IRSLWCPTA	2	0.6418	48.2	16.00	0.74	tr_C9ZY45_	
DRB1_0101	58	NFVILFLVEQLGMDV	LVEQLGMDV	6	0.6413	48.5	16.00	0.38	tr_C9ZY45_	
DRB1_0101	59	FEGALVFADGLIVVH	LVFADGLIV	4	0.6385	50.0	17.00	0.72	tr_C9ZY45_	
DRB1_0101	60	AYCKETSLSGREALL	ETSLSGREA	4	0.6381	50.2	17.00	0.61	tr_C9ZY45_	
DRB1_0101	61	ALVFADGLIVVHALM	LVFADGLIV	1	0.6378	50.3	17.00	0.60	tr_C9ZY45_	
DRB1_0101	62	EMIIRFLIDNGADIN	FLIDNGADI	5	0.6377	50.4	17.00	0.44	tr_C9ZY45_	
DRB1_0101	63	VNQTDVIGLTALQLA	VIGLTALQL	5	0.6356	51.5	17.00	0.69	tr_C9ZY45_	

DRB1_0101	64	EDLLHFVCQKGGKGA	FVCQKGGKG	5	0.6355	51.6	17.00	0.73	tr_C9ZY45_
DRB1_0101	65	AAKGNITAMRLLLER	GNITAMRLL	3	0.6347	52.1	17.00	0.31	tr_C9ZY45_
DRB1_0101	66	IRFLIDNGADINARN	FLIDNGADI	2	0.6343	52.3	17.00	0.64	tr_C9ZY45_
DRB1_0101	67	FLVEQLGMDVNAREG	LVEQLGMDV	1	0.6325	53.3	17.00	0.70	tr_C9ZY45_
DRB1_0101	68	GSWLQRFVNIRSLWC	FVNIRSLWC	6	0.6313	54.0	18.00	0.38	tr_C9ZY45_
DRB1_0101	69	KEVYNLLKAYCKETS	YNLLKAYCK	3	0.6292	55.3	18.00	0.82	tr_C9ZY45_
DRB1_0101	70	FPSVVTFLLGGADVN	FLLGGADVN	6	0.6292	55.3	18.00	0.38	tr_C9ZY45_
DRB1_0101	71	SWLQRFVNIRSLWCP	FVNIRSLWC	5	0.6289	55.4	18.00	0.56	tr_C9ZY45_
DRB1_0101	72	SQNLLSFEGALVFAD	LLSFEGALV	3	0.6214	60.1	19.00	0.44	tr_C9ZY45_
DRB1_0101	73	WLQRFVNIRSLWCPT	FVNIRSLWC	4	0.6198	61.2	19.00	0.68	tr_C9ZY45_
DRB1_0101	74	LGREPFLGECSNEMI	FLGECSNEM	5	0.6197	61.3	19.00	0.85	tr_C9ZY45_
DRB1_0101	75	PSQNLLSFEGALVFA	LLSFEGALV	4	0.6194	61.5	19.00	0.47	tr_C9ZY45_
DRB1_0101	76	AKGNITAMRLLLERG	GNITAMRLL	2	0.6187	61.9	20.00	0.29	tr_C9ZY45_
DRB1_0101	77	PLHRAVLGGDVALVC	HRAVLGGDV	2	0.6185	62.0	20.00	0.51	tr_C9ZY45_
DRB1_0101	78	TPLHRAVLGGDVALV	HRAVLGGDV	3	0.6182	62.2	20.00	0.58	tr_C9ZY45_
DRB1_0101	79	KGNITAMRLLLERGA	ITAMRLLLE	3	0.6172	62.9	20.00	0.31	tr_C9ZY45_
DRB1_0101	80	NIRSLWCPTAAERRV	IRSLWCPTA	1	0.6171	63.0	20.00	0.69	tr_C9ZY45_
DRB1_0101	81	LHRAVLGGDVALVCA	HRAVLGGDV	1	0.6165	63.4	20.00	0.47	tr_C9ZY45_
DRB1_0101	82	SFEGALVFADGLIVV	LVFADGLIV	5	0.6161	63.6	20.00	0.69	tr_C9ZY45_
DRB1_0101	83	AAAKGNITAMRLLLE	KGNITAMRL	3	0.6155	64.1	20.00	0.41	tr_C9ZY45_
DRB1_0101	84	SKEVYNLLKAYCKET	YNLLKAYCK	4	0.6099	68.1	21.00	0.83	tr_C9ZY45_
DRB1_0101	85	QNLLSFEGALVFADG	LLSFEGALV	2	0.6091	68.7	21.00	0.41	tr_C9ZY45_
DRB1_0101	86	GETPLMVAAAKGNIT	LMVAAAKGN	4	0.6085	69.1	21.00	0.38	tr_C9ZY45_
DRB1_0101	87	EVYNLLKAYCKETSL	YNLLKAYCK	2	0.6011	74.9	22.00	0.79	tr_C9ZY45_
DRB1_0101	88	KAYCKETSLSGREAL	ETSLSGREA	5	0.5937	81.1	24.00	0.49	tr_C9ZY45_
DRB1_0101	89	YTPLHRAVLGGDVAL	HRAVLGGDV	4	0.5920	82.6	24.00	0.55	tr_C9ZY45_
DRB1_0101	90	RSKEVYNLLKAYCKE	YNLLKAYCK	5	0.5915	83.1	24.00	0.74	tr_C9ZY45_
DRB1_0101	91	LVFADGLIVVHALMV	LVFADGLIV	0	0.5877	86.6	25.00	0.35	tr_C9ZY45_
DRB1_0101	92	PHMLLLCGRTFHESL	MLLLCGRTF	2	0.5859	88.3	25.00	0.47	tr_C9ZY45_
DRB1_0101	93	LSFEGALVFADGLIV	LVFADGLIV	6	0.5854	88.7	25.00	0.48	tr_C9ZY45_
DRB1_0101	94	NEMIIRFLIDNGADI	IRFLIDNGA	4	0.5842	89.9	25.00	0.54	tr_C9ZY45_
DRB1_0101	95	HRAVLGGDVALVCAL	VLGGDVALV	3	0.5842	89.9	25.00	0.34	tr_C9ZY45_
DRB1_0101	96	GNITAMRLLLERGAV	MRLLLERGA	5	0.5837	90.4	25.00	0.40	tr_C9ZY45_
DRB1_0101	97	EPHMLLLCGRTFHES	MLLLCGRTF	3	0.5837	90.4	25.00	0.49	tr_C9ZY45_
DRB1_0101	98	NLLSFEGALVFADGL	LLSFEGALV	1	0.5826	91.5	25.00	0.34	tr_C9ZY45_
DRB1_0101	99	SPSQNLLSFEGALVF	LLSFEGALV	5	0.5822	91.9	25.00	0.47	tr_C9ZY45_
DRB1_0101	100	VRSSYTPLHRAVLGG	YTPLHRAVL	4	0.5820	92.0	25.00	0.57	tr_C9ZY45_
DRB1_0101	101	AVRSSYTPLHRAVLG	YTPLHRAVL	5	0.5810	93.0	26.00	0.47	tr_C9ZY45_
DRB1_0101	102	RFLIDNGADINARNA	FLIDNGADI	1	0.5787	95.4	26.00	0.75	tr_C9ZY45_
DRB1_0101	103	GGSWLQRFVNIRSLW	SWLQRFVNI	2	0.5787	95.4	26.00	0.25	tr_C9ZY45_
DRB1_0101	104	RMMERGGQSFPLAHY	ERGGQSFPL	3	0.5781	96.1	26.00	0.74	tr_C9ZY45_
DRB1_0101	105	SYTPLHRAVLGGDVA	HRAVLGGDV	5	0.5764	97.8	26.00	0.44	tr_C9ZY45_
DRB1_0101	106	VYNLLKAYCKETSLS	YNLLKAYCK	1	0.5717	102.9	27.00	0.75	tr_C9ZY45_
DRB1_0101	107	RSSYTPLHRAVLGGD	YTPLHRAVL	3	0.5702	104.6	28.00	0.62	tr_C9ZY45_
DRB1_0101	108	FVCQKGGKGANFVIL	FVCQKGGKG	0	0.5689	106.1	28.00	0.51	tr_C9ZY45_
DRB1_0101	109	MMERGGQSFPLAHYL	ERGGQSFPL	2	0.5663	109.1	28.00	0.66	tr_C9ZY45_
DRB1_0101	110	GGDWLFPSVVTFLLG	LFPSVVTFL	4	0.5659	109.6	28.00	0.36	tr_C9ZY45_
DRB1_0101	111	VMSAVAVRSSYTPLH	AVAVRSSYT	3	0.5659	109.6	28.00	0.50	tr_C9ZY45_
DRB1_0101	112	PEPHMLLLCGRTFHE	MLLLCGRTF	4	0.5647	111.1	29.00	0.51	tr_C9ZY45_
DRB1_0101	113	ETSLSGREALLQRFA	SLSGREALL	2	0.5642	111.6	29.00	0.38	tr_C9ZY45_

DRB1_0101	114	HMLLLCGRTFHESLG	MLLLCGRTF	1	0.5618	114.6	29.00	0.44	tr_C9ZY45_
DRB1_0101	115	KPEETSVMSAVAVRS	VMSAVAVRS	6	0.5613	115.1	29.00	0.44	tr_C9ZY45_
DRB1_0101	116	VRSKEVYNLLKAYCK	YNLLKAYCK	6	0.5612	115.4	29.00	0.62	tr_C9ZY45_
DRB1_0101	117	SSYTPLHRAVLGGDV	YTPLHRAVL	2	0.5601	116.6	29.00	0.49	tr_C9ZY45_
DRB1_0101	118	VALVCALLSCGADVN	VCALLSCGA	3	0.5570	120.6	30.00	0.28	tr_C9ZY45_
DRB1_0101	119	MERGGQSFPLAHYLQ	ERGGQSFPL	1	0.5542	124.4	30.00	0.52	tr_C9ZY45_
DRB1_0101	120	LGGDWLFPSVVTFLL	LFPSVVTFL	5	0.5540	124.7	30.00	0.34	tr_C9ZY45_
DRB1_0101	121	VGGSWLQRFVNIRSL	SWLQRFVNI	3	0.5539	124.7	30.00	0.28	tr_C9ZY45_
DRB1_0101	122	GDWLFPSVVTFLLGG	LFPSVVTFL	3	0.5538	125.0	31.00	0.37	tr_C9ZY45_
DRB1_0101	123	ALVCALLSCGADVNQ	ALLSCGADV	4	0.5534	125.5	31.00	0.31	tr_C9ZY45_
DRB1_0101	124	DRMMERGGQSFPLAH	ERGGQSFPL	4	0.5473	134.0	32.00	0.72	tr_C9ZY45_
DRB1_0101	125	SGETPLMVAAAKGNI	LMVAAAKGN	5	0.5460	135.9	32.00	0.38	tr_C9ZY45_
DRB1_0101	126	VAVRSSYTPLHRAVL	YTPLHRAVL	6	0.5459	136.1	32.00	0.35	tr_C9ZY45_
DRB1_0101	127	LPEPHMLLLCGRTFH	MLLLCGRTF	5	0.5457	136.4	32.00	0.55	tr_C9ZY45_
DRB1_0101	128	LLSFEGALVFADGLI	FEGALVFAD	3	0.5456	136.6	32.00	0.31	tr_C9ZY45_
DRB1_0101	129	LQRFACGNSPSQNLL	FACGNSPSQ	3	0.5452	137.1	32.00	0.57	tr_C9ZY45_
DRB1_0101	130	GYTVLHHASRSPFSL	TVLHHASRS	2	0.5442	138.6	32.00	0.30	tr_C9ZY45_
DRB1_0101	131	AGYTVLHHASRSPFS	TVLHHASRS	3	0.5412	143.2	33.00	0.38	tr_C9ZY45_
DRB1_0101	132	FLGECSNEMIIRFLI	FLGECSNEM	0	0.5392	146.4	33.00	0.57	tr_C9ZY45_
DRB1_0101	133	IRSLWCPTAAERRVR	IRSLWCPTA	0	0.5389	146.9	33.00	0.52	tr_C9ZY45_
DRB1_0101	134	DVNQTDVIGLTALQL	VIGLTALQL	6	0.5368	150.1	34.00	0.62	tr_C9ZY45_
DRB1_0101	135	LIVVHALMVLCATLT	VHALMVLCA	3	0.5358	151.8	34.00	0.40	tr_C9ZY45_
DRB1_0101	136	DVALVCALLSCGADV	VCALLSCGA	4	0.5338	155.1	34.00	0.26	tr_C9ZY45_
DRB1_0101	137	LVCALLSCGADVNQT	ALLSCGADV	3	0.5324	157.5	35.00	0.38	tr_C9ZY45_
DRB1_0101	138	LQLAANNAGVSAVGG	LQLAANNAG	0	0.5317	158.6	35.00	0.44	tr_C9ZY45_
DRB1_0101	139	LFPSVVTFLLGGADV	VTFLLGGAD	5	0.5304	160.9	35.00	0.38	tr_C9ZY45_
DRB1_0101	140	DGLIVVHALMVLCAT	VHALMVLCA	5	0.5300	161.6	35.00	0.35	tr_C9ZY45_
DRB1_0101	141	ANFVILFLVEQLGMD	ILFLVEQLG	4	0.5298	161.9	35.00	0.49	tr_C9ZY45_
DRB1_0101	142	IVVHALMVLCATLTN	VHALMVLCA	2	0.5298	162.0	35.00	0.41	tr_C9ZY45_
DRB1_0101	143	QRFACGNSPSQNLLS	FACGNSPSQ	2	0.5293	162.8	35.00	0.54	tr_C9ZY45_
DRB1_0101	144	PLPEPHMLLLCGRTF	MLLLCGRTF	6	0.5286	164.0	35.00	0.44	tr_C9ZY45_
DRB1_0101	145	LKAYCKETSLSGREA	YCKETSLSG	3	0.5253	170.1	36.00	0.58	tr_C9ZY45_
DRB1_0101	146	SAVAVRSSYTPLHRA	AVAVRSSYT	1	0.5244	171.8	36.00	0.41	tr_C9ZY45_
DRB1_0101	147	YTVLHHASRSPFSLQ	TVLHHASRS	1	0.5219	176.4	37.00	0.25	tr_C9ZY45_
DRB1_0101	148	FLLGGADVNARDKDG	FLLGGADVN	0	0.5217	176.9	37.00	0.62	tr_C9ZY45_
DRB1_0101	149	GLIVVHALMVLCATL	VHALMVLCA	4	0.5208	178.5	37.00	0.40	tr_C9ZY45_
DRB1_0101	150	YNLLKAYCKETSLSG	YNLLKAYCK	0	0.5199	180.3	37.00	0.55	tr_C9ZY45_
DRB1_0101	151	LLQCFVDSLLHQVVS	FVDSLLHQV	4	0.5193	181.6	37.00	0.51	tr_C9ZY45_
DRB1_0101	152	SEDLLHFVCQKGGKG	FVCQKGGKG	6	0.5192	181.6	37.00	0.62	tr_C9ZY45_
DRB1_0101	153	RDRMMERGGQSFPLA	ERGGQSFPL	5	0.5178	184.3	37.00	0.64	tr_C9ZY45_
DRB1_0101	154	NSPSQNLLSFEGALV	LLSFEGALV	6	0.5177	184.6	37.00	0.47	tr_C9ZY45_
DRB1_0101	155	RAVLGGDVALVCALL	VLGGDVALV	2	0.5164	187.3	38.00	0.46	tr_C9ZY45_
DRB1_0101	156	ADGLIVVHALMVLCA	LIVVHALMV	3	0.5163	187.4	38.00	0.34	tr_C9ZY45_
DRB1_0101	157	VFADGLIVVHALMVL	LIVVHALMV	5	0.5153	189.5	38.00	0.31	tr_C9ZY45_
DRB1_0101	158	ERGGQSFPLAHYLQH	ERGGQSFPL	0	0.5152	189.6	38.00	0.34	tr_C9ZY45_
DRB1_0101	159	LQCFVDSLLHQVVSE	FVDSLLHQV	3	0.5131	194.0	38.00	0.53	tr_C9ZY45_
DRB1_0101	160	MSAVAVRSSYTPLHR	AVAVRSSYT	2	0.5128	194.7	38.00	0.58	tr_C9ZY45_
DRB1_0101	161	LLQRFACGNSPSQNL	FACGNSPSQ	4	0.5119	196.7	39.00	0.54	tr_C9ZY45_
DRB1_0101	162	DWLFPSVVTFLLGGA	LFPSVVTFL	2	0.5118	196.8	39.00	0.35	tr_C9ZY45_
DRB1_0101	163	VVHALMVLCATLTNE	VHALMVLCA	1	0.5116	197.2	39.00	0.41	tr_C9ZY45_

DRB1_0101	164	GDVALVCALLSCGAD	LVCALLSCG	4	0.5109	198.8	39.00	0.29	tr_C9ZY45_
DRB1_0101	165	DAGYTVLHHASRSPF	YTVLHHASR	3	0.5096	201.5	39.00	0.43	tr_C9ZY45_
DRB1_0101	166	RFACGNSPSQNLLSF	FACGNSPSQ	1	0.5092	202.5	39.00	0.45	tr_C9ZY45_
DRB1_0101	167	GGDVALVCALLSCGA	LVCALLSCG	5	0.5071	207.0	40.00	0.26	tr_C9ZY45_
DRB1_0101	168	QLLQCFVDSLLHQVV	FVDSLLHQV	5	0.5055	210.7	40.00	0.41	tr_C9ZY45_
DRB1_0101	169	AVAVRSSYTPLHRAV	VRSSYTPLH	3	0.5025	217.6	41.00	0.35	tr_C9ZY45_
DRB1_0101	170	ASGETPLMVAAAKGN	ETPLMVAAA	3	0.5020	218.8	41.00	0.37	tr_C9ZY45_
DRB1_0101	171	SLGGDWLFPSVVTFL	WLFPSVVTF	5	0.5005	222.5	41.00	0.28	tr_C9ZY45_
DRB1_0101	172	AVGGSWLQRFVNIRS	SWLQRFVNI	4	0.4974	229.9	42.00	0.37	tr_C9ZY45_
DRB1_0101	173	HASRSPFSLQLLQCF	FSLQLLQCF	6	0.4955	234.9	42.00	0.28	tr_C9ZY45_
DRB1_0101	174	ASRSPFSLQLLQCFV	FSLQLLQCF	5	0.4951	235.7	42.00	0.38	tr_C9ZY45_
DRB1_0101	175	QCFVDSLLHQVVSED	FVDSLLHQV	2	0.4940	238.5	42.00	0.50	tr_C9ZY45_
DRB1_0101	176	WLFPSVVTFLLGGAD	LFPSVVTFL	1	0.4880	254.7	43.00	0.26	tr_C9ZY45_
DRB1_0101	177	ALHVAVTQGNDIATV	VAVTQGNDI	3	0.4871	257.1	44.00	0.65	tr_C9ZY45_
DRB1_0101	178	VLHHASRSPFSLQLL	HASRSPFSL	3	0.4866	258.4	44.00	0.37	tr_C9ZY45_
DRB1_0101	179	TVLHHASRSPFSLQL	HASRSPFSL	4	0.4864	258.9	44.00	0.30	tr_C9ZY45_
DRB1_0101	180	FADGLIVVHALMVLC	LIVVHALMV	4	0.4862	259.6	44.00	0.37	tr_C9ZY45_
DRB1_0101	181	RSPFSLQLLQCFVDS	FSLQLLQCF	3	0.4855	261.6	44.00	0.43	tr_C9ZY45_
DRB1_0101	182	AVLGGDVALVCALLS	VLGGDVALV	1	0.4843	265.0	44.00	0.35	tr_C9ZY45_
DRB1_0101	183	SRSPFSLQLLQCFVD	FSLQLLQCF	4	0.4842	265.4	44.00	0.41	tr_C9ZY45_
DRB1_0101	184	LVEQLGMDVNAREGE	LVEQLGMDV	0	0.4840	265.9	44.00	0.61	tr_C9ZY45_
DRB1_0101	185	ALLQRFACGNSPSQN	FACGNSPSQ	5	0.4835	267.2	44.00	0.42	tr_C9ZY45_
DRB1_0101	186	MLLLCGRTFHESLGG	LLCGRTFHE	2	0.4833	267.8	44.00	0.40	tr_C9ZY45_
DRB1_0101	187	QNDVPLPEPHMLLLC	PLPEPHMLL	4	0.4820	271.7	45.00	0.44	tr_C9ZY45_
DRB1_0101	188	VCALLSCGADVNQTD	ALLSCGADV	2	0.4820	271.8	45.00	0.43	tr_C9ZY45_
DRB1_0101	189	SPFSLQLLQCFVDSL	FSLQLLQCF	2	0.4814	273.6	45.00	0.29	tr_C9ZY45_
DRB1_0101	190	PFSLQLLQCFVDSLL	LQLLQCFVD	3	0.4811	274.5	45.00	0.27	tr_C9ZY45_
DRB1_0101	191	TLGREPFLGECSNEM	FLGECSNEM	6	0.4806	275.8	45.00	0.82	tr_C9ZY45_
DRB1_0101	192	NAGVSAVGGSWLQRF	VSAVGGSWL	3	0.4785	282.2	45.00	0.47	tr_C9ZY45_
DRB1_0101	193	EQNDVPLPEPHMLLL	PLPEPHMLL	5	0.4779	284.0	46.00	0.41	tr_C9ZY45_
DRB1_0101	194	GANFVILFLVEQLGM	ILFLVEQLG	5	0.4762	289.4	46.00	0.62	tr_C9ZY45_
DRB1_0101	195	LQLLQCFVDSLLHQV	FVDSLLHQV	6	0.4747	294.1	46.00	0.29	tr_C9ZY45_
DRB1_0101	196	SNEMIIRFLIDNGAD	IRFLIDNGA	5	0.4734	298.3	47.00	0.67	tr_C9ZY45_
DRB1_0101	197	NDVPLPEPHMLLLCG	PLPEPHMLL	3	0.4730	299.6	47.00	0.48	tr_C9ZY45_
DRB1_0101	198	EALLQRFACGNSPSQ	FACGNSPSQ	6	0.4716	304.0	47.00	0.26	tr_C9ZY45_
DRB1_0101	199	LHHASRSPFSLQLLQ	HASRSPFSL	2	0.4710	306.1	47.00	0.35	tr_C9ZY45_
DRB1_0101	200	CFVDSLLHQVVSEDL	FVDSLLHQV	1	0.4703	308.3	47.00	0.37	tr_C9ZY45_
DRB1_0101	201	FLIDNGADINARNAS	FLIDNGADI	0	0.4686	314.2	48.00	0.63	tr_C9ZY45_
DRB1_0101	202	RRVRSKEVYNLLKAY	VRSKEVYNL	2	0.4670	319.4	48.00	0.38	tr_C9ZY45_
DRB1_0101	203	FVDSLLHQVVSEDLL	LHQVVSEDL	5	0.4669	319.7	48.00	0.40	tr_C9ZY45_
DRB1_0101	204	AGVSAVGGSWLQRFV	VSAVGGSWL	2	0.4647	327.6	48.00	0.44	tr_C9ZY45_
DRB1_0101	205	NNAGVSAVGGSWLQR	VSAVGGSWL	4	0.4637	331.1	49.00	0.44	tr_C9ZY45_
DRB1_0101	206	HHASRSPFSLQLLQC	SRSPFSLQL	3	0.4606	342.3	49.00	0.32	tr_C9ZY45_
DRB1_0101	207	TSLSGREALLQRFAC	SLSGREALL	1	0.4604	343.1	49.00	0.46	tr_C9ZY45_
DRB1_0101	208	ARDKDGATALHVAVT	KDGATALHV	3	0.4600	344.7	49.00	0.64	tr_C9ZY45_
DRB1_0101	209	ERRVRSKEVYNLLKA	VRSKEVYNL	3	0.4588	349.3	50.00	0.43	tr_C9ZY45_
DRB1_0101	210	LGGDVALVCALLSCG	DVALVCALL	3	0.4585	350.4	50.00	0.31	tr_C9ZY45_
DRB1_0101	211	SAVGGSWLQRFVNIR	SWLQRFVNI	5	0.4581	351.8	50.00	0.39	tr_C9ZY45_
DRB1_0101	212	NASGETPLMVAAAKG	ETPLMVAAA	4	0.4580	352.3	50.00	0.48	tr_C9ZY45_
DRB1_0101	213	TALHVAVTQGNDIAT	VAVTQGNDI	4	0.4565	358.1	50.00	0.62	tr_C9ZY45_

DRB1_0101	214	DDAGYTVLHHASRSP	YTVLHHASR	4	0.4555	361.9	50.00	0.49	tr_C9ZY45_
DRB1_0101	215	VPLPEPHMLLLCGRT	PLPEPHMLL	1	0.4536	369.5	55.00	0.41	tr_C9ZY45_
DRB1_0101	216	DVPLPEPHMLLLCGR	PLPEPHMLL	2	0.4529	372.1	55.00	0.47	tr_C9ZY45_
DRB1_0101	217	RDKDGATALHVAVTQ	KDGATALHV	2	0.4528	372.6	55.00	0.56	tr_C9ZY45_
DRB1_0101	218	LHVAVTQGNDIATVC	VAVTQGNDI	2	0.4521	375.4	55.00	0.62	tr_C9ZY45_
DRB1_0101	219	LLKAYCKETSLSGRE	YCKETSLSG	4	0.4505	382.1	55.00	0.75	tr_C9ZY45_
DRB1_0101	220	SLLHQVVSEDLLHFV	LHQVVSEDL	2	0.4504	382.2	55.00	0.50	tr_C9ZY45_
DRB1_0101	221	FSLQLLQCFVDSLLH	LLQCFVDSL	4	0.4467	398.2	55.00	0.29	tr_C9ZY45_
DRB1_0101	222	DSLLHQVVSEDLLHF	LHQVVSEDL	3	0.4463	399.6	55.00	0.56	tr_C9ZY45_
DRB1_0101	223	LKPEETSVMSAVAVR	TSVMSAVAV	5	0.4458	402.1	55.00	0.53	tr_C9ZY45_
DRB1_0101	224	VSAVGGSWLQRFVNI	SWLQRFVNI	6	0.4453	404.2	55.00	0.28	tr_C9ZY45_
DRB1_0101	225	VLGGDVALVCALLSC	DVALVCALL	4	0.4411	422.9	55.00	0.31	tr_C9ZY45_
DRB1_0101	226	REALLQRFACGNSPS	ALLQRFACG	2	0.4391	432.3	55.00	0.28	tr_C9ZY45_
DRB1_0101	227	RDDAGYTVLHHASRS	YTVLHHASR	5	0.4387	433.9	55.00	0.53	tr_C9ZY45_
DRB1_0101	228	NLLKAYCKETSLSGR	YCKETSLSG	5	0.4386	434.6	55.00	0.65	tr_C9ZY45_
DRB1_0101	229	DIATVCTLGREPFLG	CTLGREPFL	5	0.4381	437.0	55.00	0.44	tr_C9ZY45_
DRB1_0101	230	IATVCTLGREPFLGE	CTLGREPFL	4	0.4357	448.4	55.00	0.54	tr_C9ZY45_
DRB1_0101	231	FACGNSPSQNLLSFE	CGNSPSQNL	2	0.4347	453.2	55.00	0.43	tr_C9ZY45_
DRB1_0101	232	GVSAVGGSWLQRFVN	VSAVGGSWL	1	0.4339	457.2	55.00	0.44	tr_C9ZY45_
DRB1_0101	233	NARDKDGATALHVAV	KDGATALHV	4	0.4337	458.1	55.00	0.69	tr_C9ZY45_
DRB1_0101	234	RGGQSFPLAHYLQHE	QSFPLAHYL	3	0.4336	458.4	55.00	0.44	tr_C9ZY45_
DRB1_0101	235	SGREALLQRFACGNS	ALLQRFACG	4	0.4324	464.5	55.00	0.37	tr_C9ZY45_
DRB1_0101	236	CSNEMIIRFLIDNGA	IRFLIDNGA	6	0.4311	471.3	60.00	0.58	tr_C9ZY45_
DRB1_0101	237	AERRVRSKEVYNLLK	VRSKEVYNL	4	0.4287	483.4	60.00	0.42	tr_C9ZY45_
DRB1_0101	238	RVRSKEVYNLLKAYC	RSKEVYNLL	2	0.4275	490.0	60.00	0.30	tr_C9ZY45_
DRB1_0101	239	SLQLLQCFVDSLLHQ	LLQCFVDSL	3	0.4271	491.9	60.00	0.33	tr_C9ZY45_
DRB1_0101	240	VDSLLHQVVSEDLLH	LHQVVSEDL	4	0.4271	492.0	60.00	0.56	tr_C9ZY45_
DRB1_0101	241	NDIATVCTLGREPFL	CTLGREPFL	6	0.4269	493.3	60.00	0.31	tr_C9ZY45_
DRB1_0101	242	GREALLQRFACGNSP	ALLQRFACG	3	0.4263	496.6	60.00	0.34	tr_C9ZY45_
DRB1_0101	243	ATALHVAVTQGNDIA	VAVTQGNDI	5	0.4245	506.1	60.00	0.56	tr_C9ZY45_
DRB1_0101	244	FRDRMMERGGQSFPL	ERGGQSFPL	6	0.4240	508.8	60.00	0.51	tr_C9ZY45_
DRB1_0101	245	RNASGETPLMVAAAK	ETPLMVAAA	5	0.4211	525.1	60.00	0.44	tr_C9ZY45_
DRB1_0101	246	CALLSCGADVNQTDV	ALLSCGADV	1	0.4195	534.0	60.00	0.49	tr_C9ZY45_
DRB1_0101	247	ANNAGVSAVGGSWLQ	VSAVGGSWL	5	0.4185	540.2	60.00	0.38	tr_C9ZY45_
DRB1_0101	248	VITQRDDAGYTVLHH	QRDDAGYTV	3	0.4181	542.6	60.00	0.61	tr_C9ZY45_
DRB1_0101	249	SLSGREALLQRFACG	SLSGREALL	0	0.4168	549.8	60.00	0.23	tr_C9ZY45_
DRB1_0101	250	DKDGATALHVAVTQG	KDGATALHV	1	0.4139	567.5	60.00	0.49	tr_C9ZY45_
DRB1_0101	251	ATVCTLGREPFLGEC	CTLGREPFL	3	0.4129	573.8	60.00	0.56	tr_C9ZY45_
DRB1_0101	252	LLHQVVSEDLLHFVC	LHQVVSEDL	1	0.4123	577.8	60.00	0.46	tr_C9ZY45_
DRB1_0101	253	AVITQRDDAGYTVLH	QRDDAGYTV	4	0.4103	590.3	60.00	0.56	tr_C9ZY45_
DRB1_0101	254	HVAVTQGNDIATVCT	VAVTQGNDI	1	0.4097	593.9	60.00	0.62	tr_C9ZY45_
DRB1_0101	255	KGANFVILFLVEQLG	ILFLVEQLG	6	0.4091	598.2	60.00	0.57	tr_C9ZY45_
DRB1_0101	256	LSGREALLQRFACGN	ALLQRFACG	5	0.4087	600.4	60.00	0.34	tr_C9ZY45_
DRB1_0101	257	CGRTFHESLGGDWLF	FHESLGGDW	4	0.4073	609.3	65.00	0.52	tr_C9ZY45_
DRB1_0101	258	GGQSFPLAHYLQHEE	QSFPLAHYL	2	0.4073	609.7	65.00	0.40	tr_C9ZY45_
DRB1_0101	259	ESLGGDWLFPSVVTF	WLFPSVVTF	6	0.4055	621.8	65.00	0.28	tr_C9ZY45_
DRB1_0101	260	EEQNDVPLPEPHMLL	PLPEPHMLL	6	0.4041	631.3	65.00	0.35	tr_C9ZY45_
DRB1_0101	261	RTFHESLGGDWLFPS	FHESLGGDW	2	0.4006	655.8	65.00	0.44	tr_C9ZY45_
DRB1_0101	262	GRTFHESLGGDWLFP	FHESLGGDW	3	0.3988	668.5	65.00	0.50	tr_C9ZY45_
DRB1_0101	263	QLAANNAGVSAVGGS	LAANNAGVS	1	0.3973	679.6	65.00	0.54	tr_C9ZY45_

DRB1_0101	264	TVCTLGREPFLGECS	CTLGREPFL	2	0.3925	715.6	65.00	0.56	tr_C9ZY45_
DRB1_0101	265	GQSFPLAHYLQHEEQ	QSFPLAHYL	1	0.3912	725.6	65.00	0.37	tr_C9ZY45_
DRB1_0101	266	ELKPEETSVMSAVAV	LKPEETSVM	1	0.3891	742.3	65.00	0.39	tr_C9ZY45_
DRB1_0101	267	ITQRDDAGYTVLHHA	QRDDAGYTV	2	0.3887	745.5	65.00	0.61	tr_C9ZY45_
DRB1_0101	268	LAHYLQHEEQNDVPL	LQHEEQNDV	4	0.3854	772.3	65.00	0.51	tr_C9ZY45_
DRB1_0101	269	VNARDKDGATALHVA	KDGATALHV	5	0.3849	776.4	65.00	0.64	tr_C9ZY45_
DRB1_0101	270	QRDDAGYTVLHHASR	YTVLHHASR	6	0.3795	823.5	70.00	0.44	tr_C9ZY45_
DRB1_0101	271	AAERRVRSKEVYNLL	VRSKEVYNL	5	0.3785	832.7	70.00	0.38	tr_C9ZY45_
DRB1_0101	272	KDGATALHVAVTQGN	ATALHVAVT	3	0.3780	837.3	70.00	0.28	tr_C9ZY45_
DRB1_0101	273	GAVITQRDDAGYTVL	QRDDAGYTV	5	0.3769	847.0	70.00	0.54	tr_C9ZY45_
DRB1_0101	274	VCQKGGKGANFVILF	GGKGANFVI	4	0.3749	866.1	70.00	0.25	tr_C9ZY45_
DRB1_0101	275	ARNASGETPLMVAAA	ASGETPLMV	3	0.3682	930.9	70.00	0.32	tr_C9ZY45_
DRB1_0101	276	AHYLQHEEQNDVPLP	LQHEEQNDV	3	0.3660	953.4	70.00	0.56	tr_C9ZY45_
DRB1_0101	277	GNDIATVCTLGREPF	IATVCTLGR	3	0.3657	955.9	70.00	0.41	tr_C9ZY45_
DRB1_0101	278	GECVKASTGEELKPE	VKASTGEEL	3	0.3640	973.9	70.00	0.85	tr_C9ZY45_
DRB1_0101	279	GNSPSQNLLSFEGAL	SQNLLSFEG	4	0.3638	975.9	70.00	0.40	tr_C9ZY45_
DRB1_0101	280	CQKGGKGANFVILFL	GGKGANFVI	3	0.3635	979.6	70.00	0.34	tr_C9ZY45_
DRB1_0101	281	AANNAGVSAVGGSWL	VSAVGGSWL	6	0.3609	1006.8	70.00	0.29	tr_C9ZY45_
DRB1_0101	282	RSLWCPTAAERRVRS	WCPTAAERR	3	0.3574	1046.5	75.00	0.46	tr_C9ZY45_
DRB1_0101	283	TFHESLGGDWLFPSV	FHESLGGDW	1	0.3564	1057.6	75.00	0.38	tr_C9ZY45_
DRB1_0101	284	TQGNDIATVCTLGRE	IATVCTLGR	5	0.3561	1061.2	75.00	0.36	tr_C9ZY45_
DRB1_0101	285	QGNDIATVCTLGREP	IATVCTLGR	4	0.3561	1061.4	75.00	0.38	tr_C9ZY45_
DRB1_0101	286	EGECVKASTGEELKP	VKASTGEEL	4	0.3545	1079.4	75.00	0.85	tr_C9ZY45_
DRB1_0101	287	LHQVVSEDLLHFVCQ	VVSEDLLHF	3	0.3540	1084.9	75.00	0.41	tr_C9ZY45_
DRB1_0101	288	ACGNSPSQNLLSFEG	CGNSPSQNL	1	0.3539	1086.2	75.00	0.46	tr_C9ZY45_
DRB1_0101	289	EELKPEETSVMSAVA	LKPEETSVM	2	0.3539	1087.0	75.00	0.68	tr_C9ZY45_
DRB1_0101	290	GEELKPEETSVMSAV	LKPEETSVM	3	0.3531	1096.5	75.00	0.79	tr_C9ZY45_
DRB1_0101	291	TQRDDAGYTVLHHAS	QRDDAGYTV	1	0.3530	1097.0	75.00	0.52	tr_C9ZY45_
DRB1_0101	292	VAVTQGNDIATVCTL	VAVTQGNDI	0	0.3525	1102.6	75.00	0.41	tr_C9ZY45_
DRB1_0101	293	VCTLGREPFLGECSN	CTLGREPFL	1	0.3523	1105.6	75.00	0.54	tr_C9ZY45_
DRB1_0101	294	QKGGKGANFVILFLV	GGKGANFVI	2	0.3515	1115.1	75.00	0.32	tr_C9ZY45_
DRB1_0101	295	ECVKASTGEELKPEE	VKASTGEEL	2	0.3504	1128.5	75.00	0.86	tr_C9ZY45_
DRB1_0101	296	LCGRTFHESLGGDWL	FHESLGGDW	5	0.3491	1144.7	75.00	0.55	tr_C9ZY45_
DRB1_0101	297	REGECVKASTGEELK	VKASTGEEL	5	0.3459	1184.4	75.00	0.81	tr_C9ZY45_
DRB1_0101	298	QSFPLAHYLQHEEQN	QSFPLAHYL	0	0.3453	1192.4	75.00	0.31	tr_C9ZY45_
DRB1_0101	299	LAANNAGVSAVGGSW	LAANNAGVS	0	0.3453	1192.8	75.00	0.31	tr_C9ZY45_
DRB1_0101	300	CGNSPSQNLLSFEGA	SQNLLSFEG	5	0.3441	1208.2	75.00	0.29	tr_C9ZY45_
DRB1_0101	301	GATALHVAVTQGNDI	LHVAVTQGN	4	0.3430	1222.5	75.00	0.35	tr_C9ZY45_
DRB1_0101	302	ADVNQTDVIGLTALQ	VNQTDVIGL	2	0.3427	1226.7	75.00	0.31	tr_C9ZY45_
DRB1_0101	303	PLAHYLQHEEQNDVP	LQHEEQNDV	5	0.3421	1234.4	75.00	0.50	tr_C9ZY45_
DRB1_0101	304	HESLGGDWLFPSVVT	GDWLFPSVV	5	0.3387	1280.2	75.00	0.34	tr_C9ZY45_
DRB1_0101	305	LLLCGRTFHESLGGD	LLCGRTFHE	1	0.3362	1315.1	75.00	0.51	tr_C9ZY45_
DRB1_0101	306	VTQGNDIATVCTLGR	NDIATVCTL	4	0.3362	1315.6	75.00	0.28	tr_C9ZY45_
DRB1_0101	307	LGECSNEMIIRFLID	SNEMIIRFL	4	0.3339	1349.2	80.00	0.23	tr_C9ZY45_
DRB1_0101	308	HYLQHEEQNDVPLPE	LQHEEQNDV	2	0.3334	1356.2	80.00	0.57	tr_C9ZY45_
DRB1_0101	309	FHESLGGDWLFPSVV	HESLGGDWL	1	0.3305	1399.6	80.00	0.32	tr_C9ZY45_
DRB1_0101	310	ALLSCGADVNQTDVI	ALLSCGADV	0	0.3287	1427.4	80.00	0.40	tr_C9ZY45_
DRB1_0101	311	TGEELKPEETSVMSA	LKPEETSVM	4	0.3269	1455.5	80.00	0.74	tr_C9ZY45_
DRB1_0101	312	VSEDLLHFVCQKGGK	LHFVCQKGG	5	0.3262	1465.9	80.00	0.34	tr_C9ZY45_
DRB1_0101	313	SLWCPTAAERRVRSK	WCPTAAERR	2	0.3256	1476.2	80.00	0.41	tr_C9ZY45_

DRB1_0101	314	FPLAHYLQHEEQNDV	LQHEEQNDV	6	0.3184	1594.5	80.00	0.36	tr_C9ZY45_
DRB1_0101	315	KGGKGANFVILFLVE	GGKGANFVI	1	0.3127	1696.4	80.00	0.33	tr_C9ZY45_
DRB1_0101	316	CVKASTGEELKPEET	VKASTGEEL	1	0.3116	1716.5	80.00	0.81	tr_C9ZY45_
DRB1_0101	317	GADVNQTDVIGLTAL	VNQTDVIGL	3	0.3115	1719.5	80.00	0.41	tr_C9ZY45_
DRB1_0101	318	LLCGRTFHESLGGDW	FHESLGGDW	6	0.3110	1728.7	80.00	0.34	tr_C9ZY45_
DRB1_0101	319	NARNASGETPLMVAA	ASGETPLMV	4	0.3083	1779.0	80.00	0.41	tr_C9ZY45_
DRB1_0101	320	TAAERRVRSKEVYNL	ERRVRSKEV	3	0.3022	1901.5	85.00	0.49	tr_C9ZY45_
DRB1_0101	321	DGATALHVAVTQGND	ATALHVAVT	2	0.2969	2013.4	85.00	0.35	tr_C9ZY45_
DRB1_0101	322	RGAVITQRDDAGYTV	QRDDAGYTV	6	0.2943	2071.3	85.00	0.36	tr_C9ZY45_
DRB1_0101	323	AVTQGNDIATVCTLG	NDIATVCTL	5	0.2926	2108.3	85.00	0.26	tr_C9ZY45_
DRB1_0101	324	DVNARDKDGATALHV	KDGATALHV	6	0.2906	2154.6	85.00	0.47	tr_C9ZY45_
DRB1_0101	325	HQVVSEDLLHFVCQK	VVSEDLLHF	2	0.2905	2158.5	85.00	0.44	tr_C9ZY45_
DRB1_0101	326	CTLGREPFLGECSNE	CTLGREPFL	0	0.2897	2176.9	85.00	0.46	tr_C9ZY45_
DRB1_0101	327	GECSNEMIIRFLIDN	SNEMIIRFL	3	0.2878	2221.7	85.00	0.33	tr_C9ZY45_
DRB1_0101	328	STGEELKPEETSVMS	LKPEETSVM	5	0.2864	2254.6	85.00	0.65	tr_C9ZY45_
DRB1_0101	329	ECSNEMIIRFLIDNG	SNEMIIRFL	2	0.2850	2290.4	85.00	0.28	tr_C9ZY45_
DRB1_0101	330	LWCPTAAERRVRSKE	WCPTAAERR	1	0.2826	2349.0	85.00	0.44	tr_C9ZY45_
DRB1_0101	331	VVSEDLLHFVCQKGG	LLHFVCQKG	5	0.2823	2357.3	85.00	0.25	tr_C9ZY45_
DRB1_0101	332	GGKGANFVILFLVEQ	FVILFLVEQ	6	0.2822	2359.5	85.00	0.25	tr_C9ZY45_
DRB1_0101	333	QVVSEDLLHFVCQKG	VVSEDLLHF	1	0.2742	2574.7	90.00	0.38	tr_C9ZY45_
DRB1_0101	334	INARNASGETPLMVA	ASGETPLMV	5	0.2735	2591.6	90.00	0.34	tr_C9ZY45_
DRB1_0101	335	SFPLAHYLQHEEQND	FPLAHYLQH	1	0.2703	2685.6	90.00	0.20	tr_C9ZY45_
DRB1_0101	336	VEQLGMDVNAREGEC	QLGMDVNAR	2	0.2692	2717.5	90.00	0.38	tr_C9ZY45_
DRB1_0101	337	GKGANFVILFLVEQL	FVILFLVEQ	5	0.2672	2774.5	90.00	0.35	tr_C9ZY45_
DRB1_0101	338	HEEQNDVPLPEPHML	DVPLPEPHM	5	0.2663	2804.1	90.00	0.57	tr_C9ZY45_
DRB1_0101	339	WVFRDRMMERGGQSF	RMMERGGQS	5	0.2601	2998.0	90.00	0.26	tr_C9ZY45_
DRB1_0101	340	VFRDRMMERGGQSFP	MMERGGQSF	5	0.2599	3005.5	90.00	0.27	tr_C9ZY45_
DRB1_0101	341	YLQHEEQNDVPLPEP	LQHEEQNDV	1	0.2598	3007.5	90.00	0.57	tr_C9ZY45_
DRB1_0101	342	CGADVNQTDVIGLTA	VNQTDVIGL	4	0.2487	3391.6	90.00	0.53	tr_C9ZY45_
DRB1_0101	343	PTAAERRVRSKEVYN	ERRVRSKEV	4	0.2472	3447.6	90.00	0.51	tr_C9ZY45_
DRB1_0101	344	MWVFRDRMMERGGQS	FRDRMMERG	3	0.2433	3596.5	90.00	0.34	tr_C9ZY45_
DRB1_0101	345	LLERGAVITQRDDAG	LLERGAVIT	0	0.2425	3625.6	90.00	0.52	tr_C9ZY45_
DRB1_0101	346	WCPTAAERRVRSKEV	CPTAAERRV	1	0.2404	3711.3	95.00	0.26	tr_C9ZY45_
DRB1_0101	347	EQLGMDVNAREGECV	LGMDVNARE	2	0.2309	4109.5	95.00	0.34	tr_C9ZY45_
DRB1_0101	348	CPTAAERRVRSKEVY	ERRVRSKEV	5	0.2307	4122.3	95.00	0.40	tr_C9ZY45_
DRB1_0101	349	AREGECVKASTGEEL	VKASTGEEL	6	0.2294	4180.1	95.00	0.62	tr_C9ZY45_
DRB1_0101	350	ASTGEELKPEETSVM	LKPEETSVM	6	0.2237	4444.0	95.00	0.50	tr_C9ZY45_
DRB1_0101	351	MDVNAREGECVKAST	VNAREGECV	2	0.2203	4609.0	95.00	0.55	tr_C9ZY45_
DRB1_0101	352	LIDNGADINARNASG	IDNGADINA	1	0.2198	4633.7	95.00	0.31	tr_C9ZY45_
DRB1_0101	353	GMDVNAREGECVKAS	VNAREGECV	3	0.2185	4703.2	95.00	0.57	tr_C9ZY45_
DRB1_0101	354	QLGMDVNAREGECVK	VNAREGECV	5	0.2177	4743.1	95.00	0.34	tr_C9ZY45_
DRB1_0101	355	VKASTGEELKPEETS	VKASTGEEL	0	0.2148	4891.8	95.00	0.63	tr_C9ZY45_
DRB1_0101	356	LLGGADVNARDKDGA	LGGADVNAR	1	0.2127	5008.2	95.00	0.56	tr_C9ZY45_
DRB1_0101	357	DINARNASGETPLMV	ASGETPLMV	6	0.2120	5044.2	95.00	0.23	tr_C9ZY45_
DRB1_0101	358	DVNAREGECVKASTG	VNAREGECV	1	0.2106	5118.4	95.00	0.40	tr_C9ZY45_
DRB1_0101	359	SCGADVNQTDVIGLT	VNQTDVIGL	5	0.2026	5584.4	95.00	0.57	tr_C9ZY45_
DRB1_0101	360	LGMDVNAREGECVKA	VNAREGECV	4	0.2012	5666.8	95.00	0.52	tr_C9ZY45_
DRB1_0101	361	VNAREGECVKASTGE	AREGECVKA	2	0.1908	6342.0	100.00	0.28	tr_C9ZY45_
DRB1_0101	362	ADVNARDKDGATALH	ARDKDGATA	4	0.1873	6590.9	100.00	0.41	tr_C9ZY45_
DRB1_0101	363	IDNGADINARNASGE	GADINARNA	3	0.1859	6688.1	100.00	0.31	tr_C9ZY45_

DRB1_0101	364	ERGAVITQRDDAGYT	ITQRDDAGY	5	0.1836	6857.2	100.00	0.28	tr_C9ZY45_
DRB1_0101	365	ADINARNASGETPLM	ARNASGETP	4	0.1821	6973.6	100.00	0.23	tr_C9ZY45_
DRB1_0101	366	QHEEQNDVPLPEPHM	DVPLPEPHM	6	0.1801	7125.4	100.00	0.54	tr_C9ZY45_
DRB1_0101	367	LLSCGADVNQTDVIG	LLSCGADVN	0	0.1775	7323.6	100.00	0.40	tr_C9ZY45_
DRB1_0101	368	LQHEEQNDVPLPEPH	LQHEEQNDV	0	0.1743	7584.0	100.00	0.46	tr_C9ZY45_
DRB1_0101	369	DNGADINARNASGET	DINARNASG	4	0.1738	7627.6	100.00	0.28	tr_C9ZY45_
DRB1_0101	370	GADINARNASGETPL	DINARNASG	2	0.1665	8251.5	100.00	0.22	tr_C9ZY45_
DRB1_0101	371	NGADINARNASGETP	DINARNASG	3	0.1655	8339.6	100.00	0.28	tr_C9ZY45_
DRB1_0101	372	LSCGADVNQTDVIGL	VNQTDVIGL	6	0.1603	8827.7	100.00	0.43	tr_C9ZY45_
DRB1_0101	373	NAREGECVKASTGEE	GECVKASTG	4	0.1507	9787.6	100.00	0.40	tr_C9ZY45_
DRB1_0101	374	LERGAVITQRDDAGY	VITQRDDAG	5	0.1503	9828.8	100.00	0.31	tr_C9ZY45_
DRB1_0101	375	GADVNARDKDGATAL	ARDKDGATA	5	0.1501	9852.8	100.00	0.42	tr_C9ZY45_
DRB1_0101	376	KASTGEELKPEETSV	GEELKPEET	4	0.1269	12669.7	100.00	0.51	tr_C9ZY45_
DRB1_0101	377	LGGADVNARDKDGAT	LGGADVNAR	0	0.1171	14084.9	100.00	0.47	tr_C9ZY45_
DRB1_0101	378	GGADVNARDKDGATA	ARDKDGATA	6	0.0923	18421.3	100.00	0.38	tr_C9ZY45_

Allele: DRB1_0101. Number of high binders 0. Number of weak binders 30. Number of peptides 378

Allele	pos	peptide	core	Of	1-log50k(aff)	affinity(nM)	%Rank	Relia	Identity	Bind Level
DRB1_0101	1	AVLLGIQLANALIGW	LGIQLANAL	3	0.7959	9.1	2.50	0.36	tr_D0A564_	WB
DRB1_0101	2	VLLGIQLANALIGWY	IQLANALIG	4	0.7955	9.1	2.50	0.39	tr_D0A564_	WB
DRB1_0101	3	LLGIQLANALIGWYE	IQLANALIG	3	0.7905	9.6	2.50	0.46	tr_D0A564_	WB
DRB1_0101	4	WYETIKAGDAVAALK	IKAGDAVAA	4	0.7866	10.1	3.00	0.64	tr_D0A564_	WB
DRB1_0101	5	LIFLRNLWGPMPIVL	LRNLWGPMP	3	0.7834	10.4	3.00	0.57	tr_D0A564_	WB
DRB1_0101	6	WLIFLRNLWGPMPIV	LRNLWGPMP	4	0.7828	10.5	3.00	0.50	tr_D0A564_	WB
DRB1_0101	7	YETIKAGDAVAALKN	IKAGDAVAA	3	0.7811	10.7	3.00	0.78	tr_D0A564_	WB
DRB1_0101	8	GDLVKLGSGSAVPAD	LVKLGSGSA	2	0.7796	10.9	3.00	0.51	tr_D0A564_	WB
DRB1_0101	9	DLVKLGSGSAVPADC	LGSGSAVPA	4	0.7738	11.6	3.50	0.43	tr_D0A564_	WB
DRB1_0101	10	GAVLLGIQLANALIG	LGIQLANAL	4	0.7704	12.0	3.50	0.37	tr_D0A564_	WB
DRB1_0101	11	SWLIFLRNLWGPMPI	LRNLWGPMP	5	0.7677	12.3	4.00	0.44	tr_D0A564_	WB
DRB1_0101	12	GWYETIKAGDAVAAL	IKAGDAVAA	5	0.7627	13.0	4.00	0.51	tr_D0A564_	WB
DRB1_0101	13	PGDLVKLGSGSAVPA	LVKLGSGSA	3	0.7565	13.9	4.50	0.63	tr_D0A564_	WB
DRB1_0101	14	LGIQLANALIGWYET	IQLANALIG	2	0.7537	14.4	4.50	0.50	tr_D0A564_	WB
DRB1_0101	15	LVKLGSGSAVPADCT	LGSGSAVPA	3	0.7491	15.1	5.00	0.59	tr_D0A564_	WB
DRB1_0101	16	IFLRNLWGPMPIVLW	LRNLWGPMP	2	0.7455	15.7	5.00	0.61	tr_D0A564_	WB
DRB1_0101	17	QSTFFGKTATLLQSV	FFGKTATLL	3	0.7406	16.6	5.50	0.87	tr_D0A564_	WB
DRB1_0101	18	ETIKAGDAVAALKNS	IKAGDAVAA	2	0.7318	18.2	6.00	0.89	tr_D0A564_	WB
DRB1_0101	19	PSWLIFLRNLWGPMP	LIFLRNLWG	3	0.7268	19.2	6.50	0.46	tr_D0A564_	WB
DRB1_0101	20	DGAVLLGIQLANALI	LGIQLANAL	5	0.7265	19.3	6.50	0.43	tr_D0A564_	WB
DRB1_0101	21	GQSTFFGKTATLLQS	FFGKTATLL	4	0.7241	19.8	7.00	0.88	tr_D0A564_	WB
DRB1_0101	22	IGWYETIKAGDAVAA	YETIKAGDA	3	0.7154	21.7	7.50	0.55	tr_D0A564_	WB
DRB1_0101	23	STFFGKTATLLQSVE	FFGKTATLL	2	0.7133	22.3	8.00	0.85	tr_D0A564_	WB
DRB1_0101	24	FLRNLWGPMPIVLWI	LRNLWGPMP	1	0.7112	22.8	8.00	0.56	tr_D0A564_	WB
DRB1_0101	25	TIKAGDAVAALKNSL	IKAGDAVAA	1	0.6988	26.0	9.00	0.87	tr_D0A564_	WB
DRB1_0101	26	VPGDLVKLGSGSAVP	LVKLGSGSA	4	0.6919	28.0	10.00	0.79	tr_D0A564_	
DRB1_0101	27	TGQSTFFGKTATLLQ	FFGKTATLL	5	0.6898	28.7	10.00	0.87	tr_D0A564_	

DRB1_0101	28	GIQLANALIGWYETI	IQLANALIG	1	0.6818	31.3	11.00	0.61	tr_D0A564_
DRB1_0101	29	TFFGKTATLLQSVEA	FFGKTATLL	1	0.6810	31.5	11.00	0.73	tr_D0A564_
DRB1_0101	30	LVPGDLVKLGSGSAV	LVKLGSGSA	5	0.6738	34.1	12.00	0.81	tr_D0A564_
DRB1_0101	31	ADGAVLLGIQLANAL	LGIQLANAL	6	0.6702	35.5	13.00	0.38	tr_D0A564_
DRB1_0101	32	DGTWQQIDAALLVPG	WQQIDAALL	3	0.6678	36.4	13.00	0.74	tr_D0A564_
DRB1_0101	33	TPSWLIFLRNLWGPM	LIFLRNLWG	4	0.6649	37.5	13.00	0.68	tr_D0A564_
DRB1_0101	34	VKLGSGSAVPADCTI	LGSGSAVPA	2	0.6619	38.8	14.00	0.81	tr_D0A564_
DRB1_0101	35	RDGTWQQIDAALLVP	WQQIDAALL	4	0.6610	39.2	14.00	0.73	tr_D0A564_
DRB1_0101	36	YRDGTWQQIDAALLV	WQQIDAALL	5	0.6596	39.8	14.00	0.70	tr_D0A564_
DRB1_0101	37	LRNLWGPMPIVLWIV	LRNLWGPMP	0	0.6578	40.5	14.00	0.38	tr_D0A564_
DRB1_0101	38	GTWQQIDAALLVPGD	WQQIDAALL	2	0.6570	40.9	14.00	0.71	tr_D0A564_
DRB1_0101	39	AVAALKNSLKPIATA	VAALKNSLK	1	0.6459	46.1	16.00	0.62	tr_D0A564_
DRB1_0101	40	LIGWYETIKAGDAVA	YETIKAGDA	4	0.6333	52.9	17.00	0.73	tr_D0A564_
DRB1_0101	41	QFALQHFADGAVLLG	FALQHFADG	1	0.6322	53.5	18.00	0.36	tr_D0A564_
DRB1_0101	42	YTGQSTFFGKTATLL	FFGKTATLL	6	0.6313	54.0	18.00	0.69	tr_D0A564_
DRB1_0101	43	SKGLTSAEAEELLLK	LTSAEAEEL	3	0.6304	54.5	18.00	0.75	tr_D0A564_
DRB1_0101	44	TWQQIDAALLVPGDL	WQQIDAALL	1	0.6294	55.1	18.00	0.69	tr_D0A564_
DRB1_0101	45	DAVAALKNSLKPIAT	VAALKNSLK	2	0.6267	56.8	18.00	0.76	tr_D0A564_
DRB1_0101	46	FALQHFADGAVLLGI	HFADGAVLL	4	0.6260	57.2	18.00	0.39	tr_D0A564_
DRB1_0101	47	GDAVAALKNSLKPIA	VAALKNSLK	3	0.6259	57.3	18.00	0.83	tr_D0A564_
DRB1_0101	48	ELPEKKTPSWLIFLR	EKKTPSWLI	3	0.6239	58.5	19.00	0.79	tr_D0A564_
DRB1_0101	49	LPEKKTPSWLIFLRN	EKKTPSWLI	2	0.6213	60.2	19.00	0.75	tr_D0A564_
DRB1_0101	50	IKAGDAVAALKNSLK	IKAGDAVAA	0	0.6203	60.8	19.00	0.51	tr_D0A564_
DRB1_0101	51	IQFALQHFADGAVLL	FALQHFADG	2	0.6182	62.2	20.00	0.45	tr_D0A564_
DRB1_0101	52	ALKNSLKPIATAYRD	KNSLKPIAT	2	0.6135	65.5	20.00	0.41	tr_D0A564_
DRB1_0101	53	AALKNSLKPIATAYR	KNSLKPIAT	3	0.6127	66.1	20.00	0.51	tr_D0A564_
DRB1_0101	54	PSKGLTSAEAEELLL	LTSAEAEEL	4	0.6116	66.8	21.00	0.63	tr_D0A564_
DRB1_0101	55	FFGKTATLLQSVEAD	ATLLQSVEA	5	0.6059	71.1	22.00	0.36	tr_D0A564_
DRB1_0101	56	LKNSLKPIATAYRDG	LKPIATAYR	4	0.6044	72.3	22.00	0.37	tr_D0A564_
DRB1_0101	57	DAALLVPGDLVKLGS	ALLVPGDLV	2	0.6028	73.5	22.00	0.50	tr_D0A564_
DRB1_0101	58	VAALKNSLKPIATAY	KNSLKPIAT	4	0.6028	73.6	22.00	0.34	tr_D0A564_
DRB1_0101	59	IIQFALQHFADGAVL	FALQHFADG	3	0.6026	73.6	22.00	0.55	tr_D0A564_
DRB1_0101	60	IDAALLVPGDLVKLG	ALLVPGDLV	3	0.6010	74.9	22.00	0.52	tr_D0A564_
DRB1_0101	61	KTPSWLIFLRNLWGP	LIFLRNLWG	5	0.6005	75.4	22.00	0.70	tr_D0A564_
DRB1_0101	62	IIIQFALQHFADGAV	FALQHFADG	4	0.5973	78.0	23.00	0.48	tr_D0A564_
DRB1_0101	63	WQQIDAALLVPGDLV	WQQIDAALL	0	0.5968	78.5	23.00	0.44	tr_D0A564_
DRB1_0101	64	VIIIQFALQHFADGA	FALQHFADG	5	0.5963	78.8	23.00	0.37	tr_D0A564_
DRB1_0101	65	AALLVPGDLVKLGSG	ALLVPGDLV	1	0.5954	79.7	23.00	0.47	tr_D0A564_
DRB1_0101	66	NELPEKKTPSWLIFL	EKKTPSWLI	4	0.5948	80.2	23.00	0.78	tr_D0A564_
DRB1_0101	67	ALQHFADGAVLLGIQ	HFADGAVLL	3	0.5921	82.6	24.00	0.50	tr_D0A564_
DRB1_0101	68	AGDAVAALKNSLKPI	VAALKNSLK	4	0.5917	82.9	24.00	0.81	tr_D0A564_
DRB1_0101	69	PEKKTPSWLIFLRNL	EKKTPSWLI	1	0.5908	83.8	24.00	0.65	tr_D0A564_
DRB1_0101	70	AYRDGTWQQIDAALL	WQQIDAALL	6	0.5896	84.8	24.00	0.61	tr_D0A564_
DRB1_0101	71	KGLTSAEAEELLLKY	LTSAEAEEL	2	0.5871	87.2	25.00	0.82	tr_D0A564_
DRB1_0101	72	KLGSGSAVPADCTIN	LGSGSAVPA	1	0.5869	87.3	25.00	0.87	tr_D0A564_
DRB1_0101	73	VDEAALTGESLPVTM	EAALTGESL	2	0.5866	87.6	25.00	0.73	tr_D0A564_
DRB1_0101	74	KTATLLQSVEADIGS	ATLLQSVEA	2	0.5833	90.8	25.00	0.59	tr_D0A564_
DRB1_0101	75	QIDAALLVPGDLVKL	ALLVPGDLV	4	0.5832	90.9	25.00	0.55	tr_D0A564_
DRB1_0101	76	ALIGWYETIKAGDAV	YETIKAGDA	5	0.5808	93.3	26.00	0.66	tr_D0A564_
DRB1_0101	77	IQLANALIGWYETIK	IQLANALIG	0	0.5806	93.5	26.00	0.56	tr_D0A564_

DRB1_0101	78	GKTATLLQSVEADIG	ATLLQSVEA	3	0.5767	97.5	26.00	0.64	tr_D0A564_
DRB1_0101	79	DEAALTGESLPVTMG	EAALTGESL	1	0.5767	97.5	26.00	0.63	tr_D0A564_
DRB1_0101	80	QHFADGAVLLGIQLA	HFADGAVLL	1	0.5764	97.8	26.00	0.35	tr_D0A564_
DRB1_0101	81	LQHFADGAVLLGIQL	HFADGAVLL	2	0.5758	98.5	27.00	0.44	tr_D0A564_
DRB1_0101	82	TVQYTGQSTFFGKTA	YTGQSTFFG	3	0.5751	99.2	27.00	0.80	tr_D0A564_
DRB1_0101	83	LLVPGDLVKLGSGSA	LVKLGSGSA	6	0.5678	107.4	28.00	0.63	tr_D0A564_
DRB1_0101	84	DVDEAALTGESLPVT	EAALTGESL	3	0.5677	107.4	28.00	0.75	tr_D0A564_
DRB1_0101	85	KKTPSWLIFLRNLWG	LIFLRNLWG	6	0.5674	107.8	28.00	0.65	tr_D0A564_
DRB1_0101	86	RNLWGPMPIVLWIVI	WGPMPIVLW	3	0.5656	109.9	28.00	0.41	tr_D0A564_
DRB1_0101	87	KNSLKPIATAYRDGT	LKPIATAYR	3	0.5655	110.1	28.00	0.47	tr_D0A564_
DRB1_0101	88	ATVQYTGQSTFFGKT	YTGQSTFFG	4	0.5646	111.2	29.00	0.69	tr_D0A564_
DRB1_0101	89	TATLLQSVEADIGSI	ATLLQSVEA	1	0.5641	111.8	29.00	0.46	tr_D0A564_
DRB1_0101	90	PPSKGLTSAEAEELL	LTSAEAEEL	5	0.5624	113.8	29.00	0.54	tr_D0A564_
DRB1_0101	91	IVIIIQFALQHFADG	IIIQFALQH	2	0.5587	118.5	30.00	0.31	tr_D0A564_
DRB1_0101	92	FADGAVLLGIQLANA	GAVLLGIQL	3	0.5580	119.3	30.00	0.44	tr_D0A564_
DRB1_0101	93	FGKTATLLQSVEADI	ATLLQSVEA	4	0.5550	123.3	30.00	0.63	tr_D0A564_
DRB1_0101	94	HFADGAVLLGIQLAN	GAVLLGIQL	4	0.5501	130.1	31.00	0.37	tr_D0A564_
DRB1_0101	95	QQIDAALLVPGDLVK	ALLVPGDLV	5	0.5450	137.4	32.00	0.51	tr_D0A564_
DRB1_0101	96	KAGDAVAALKNSLKP	VAALKNSLK	5	0.5445	138.1	32.00	0.74	tr_D0A564_
DRB1_0101	97	EAALTGESLPVTMGT	EAALTGESL	0	0.5394	146.1	33.00	0.50	tr_D0A564_
DRB1_0101	98	VQYTGQSTFFGKTAT	YTGQSTFFG	2	0.5377	148.8	34.00	0.81	tr_D0A564_
DRB1_0101	99	DATVQYTGQSTFFGK	YTGQSTFFG	5	0.5371	149.6	34.00	0.62	tr_D0A564_
DRB1_0101	100	ALLVPGDLVKLGSGS	LVPGDLVKL	2	0.5354	152.5	34.00	0.49	tr_D0A564_
DRB1_0101	101	ATLLQSVEADIGSIR	LQSVEADIG	3	0.5321	157.9	35.00	0.37	tr_D0A564_
DRB1_0101	102	RNELPEKKTPSWLIF	EKKTPSWLI	5	0.5281	164.9	35.00	0.81	tr_D0A564_
DRB1_0101	103	IDVDEAALTGESLPV	EAALTGESL	4	0.5252	170.2	36.00	0.79	tr_D0A564_
DRB1_0101	104	GSVPLLPPSKGLTSA	VPLLPPSKG	2	0.5249	170.8	36.00	0.54	tr_D0A564_
DRB1_0101	105	DGSVPLLPPSKGLTS	VPLLPPSKG	3	0.5228	174.8	36.00	0.54	tr_D0A564_
DRB1_0101	106	NLWGPMPIVLWIVII	WGPMPIVLW	2	0.5224	175.6	37.00	0.35	tr_D0A564_
DRB1_0101	107	EKKTPSWLIFLRNLW	EKKTPSWLI	0	0.5223	175.7	37.00	0.50	tr_D0A564_
DRB1_0101	108	WIVIIIQFALQHFAD	IIIQFALQH	3	0.5066	208.3	40.00	0.40	tr_D0A564_
DRB1_0101	109	GTEHMPKMGSNVVRG	MPKMGSNVV	4	0.5062	209.1	40.00	0.47	tr_D0A564_
DRB1_0101	110	GLTSAEAEELLLKYG	LTSAEAEEL	1	0.5056	210.6	40.00	0.77	tr_D0A564_
DRB1_0101	111	TEHMPKMGSNVVRGE	MPKMGSNVV	3	0.5052	211.4	40.00	0.51	tr_D0A564_
DRB1_0101	112	QYTGQSTFFGKTATL	YTGQSTFFG	1	0.5005	222.3	41.00	0.67	tr_D0A564_
DRB1_0101	113	DDGSVPLLPPSKGLT	VPLLPPSKG	4	0.4996	224.5	41.00	0.51	tr_D0A564_
DRB1_0101	114	LPPSKGLTSAEAEEL	SKGLTSAEA	3	0.4929	241.5	42.00	0.64	tr_D0A564_
DRB1_0101	115	LWIVIIIQFALQHFA	IIIQFALQH	4	0.4928	241.8	42.00	0.44	tr_D0A564_
DRB1_0101	116	ELLLKYGRNELPEKK	LKYGRNELP	3	0.4920	243.8	43.00	0.75	tr_D0A564_
DRB1_0101	117	NALIGWYETIKAGDA	YETIKAGDA	6	0.4898	249.8	43.00	0.45	tr_D0A564_
DRB1_0101	118	VIDVDEAALTGESLP	EAALTGESL	5	0.4755	291.6	46.00	0.64	tr_D0A564_
DRB1_0101	119	EHMPKMGSNVVRGEV	MPKMGSNVV	2	0.4703	308.2	47.00	0.53	tr_D0A564_
DRB1_0101	120	MGTEHMPKMGSNVVR	MPKMGSNVV	5	0.4698	310.1	47.00	0.43	tr_D0A564_
DRB1_0101	121	LLLKYGRNELPEKKT	LKYGRNELP	2	0.4694	311.5	47.00	0.80	tr_D0A564_
DRB1_0101	122	EELLLKYGRNELPEK	LKYGRNELP	4	0.4693	311.6	47.00	0.68	tr_D0A564_
DRB1_0101	123	SVPLLPPSKGLTSAE	VPLLPPSKG	1	0.4592	347.7	50.00	0.56	tr_D0A564_
DRB1_0101	124	GDDGSVPLLPPSKGL	VPLLPPSKG	5	0.4581	351.8	50.00	0.52	tr_D0A564_
DRB1_0101	125	NSLKPIATAYRDGTW	LKPIATAYR	2	0.4550	363.7	50.00	0.57	tr_D0A564_
DRB1_0101	126	LANALIGWYETIKAG	IGWYETIKA	5	0.4541	367.5	55.00	0.32	tr_D0A564_
DRB1_0101	127	RRQSVLSKAISEHRE	SVLSKAISE	3	0.4519	376.2	55.00	0.50	tr_D0A564_

DRB1_0101	128	QLANALIGWYETIKA	ANALIGWYE	2	0.4515	377.7	55.00	0.46	tr_D0A564_
DRB1_0101	129	VDATVQYTGQSTFFG	YTGQSTFFG	6	0.4502	383.1	55.00	0.49	tr_D0A564_
DRB1_0101	130	TLLQSVEADIGSIRI	LQSVEADIG	2	0.4501	383.5	55.00	0.52	tr_D0A564_
DRB1_0101	131	LWGPMPIVLWIVIII	PMPIVLWIV	3	0.4499	384.7	55.00	0.35	tr_D0A564_
DRB1_0101	132	AEELLLKYGRNELPE	LKYGRNELP	5	0.4388	433.7	55.00	0.60	tr_D0A564_
DRB1_0101	133	QRRQSVLSKAISEHR	SVLSKAISE	4	0.4370	442.0	55.00	0.50	tr_D0A564_
DRB1_0101	134	RQSVLSKAISEHREG	SVLSKAISE	2	0.4352	450.8	55.00	0.47	tr_D0A564_
DRB1_0101	135	GVIDVDEAALTGESL	EAALTGESL	6	0.4237	510.8	60.00	0.46	tr_D0A564_
DRB1_0101	136	VLWIVIIIQFALQHF	IIIQFALQH	5	0.4234	512.1	60.00	0.49	tr_D0A564_
DRB1_0101	137	ANALIGWYETIKAGD	IGWYETIKA	4	0.4193	535.2	60.00	0.40	tr_D0A564_
DRB1_0101	138	SVEADIGSIRIILMR	IGSIRIILM	5	0.4176	545.2	60.00	0.38	tr_D0A564_
DRB1_0101	139	LGSGSAVPADCTINE	LGSGSAVPA	0	0.4173	547.1	60.00	0.70	tr_D0A564_
DRB1_0101	140	HMPKMGSNVVRGEVD	MPKMGSNVV	1	0.4167	550.7	60.00	0.53	tr_D0A564_
DRB1_0101	141	LLKYGRNELPEKKTP	LKYGRNELP	1	0.4134	570.6	60.00	0.78	tr_D0A564_
DRB1_0101	142	GRNELPEKKTPSWLI	EKKTPSWLI	6	0.4121	578.5	60.00	0.71	tr_D0A564_
DRB1_0101	143	LLPPSKGLTSAEAEE	SKGLTSAEA	4	0.4047	626.8	65.00	0.80	tr_D0A564_
DRB1_0101	144	LLQSVEADIGSIRII	LQSVEADIG	1	0.3984	671.6	65.00	0.44	tr_D0A564_
DRB1_0101	145	VPLLPPSKGLTSAEA	VPLLPPSKG	0	0.3947	698.8	65.00	0.38	tr_D0A564_
DRB1_0101	146	AALTGESLPVTMGTE	GESLPVTMG	4	0.3921	718.9	65.00	0.40	tr_D0A564_
DRB1_0101	147	QSVEADIGSIRIILM	IGSIRIILM	6	0.3907	729.4	65.00	0.28	tr_D0A564_
DRB1_0101	148	ESLPVTMGTEHMPKM	PVTMGTEHM	3	0.3905	731.6	65.00	0.67	tr_D0A564_
DRB1_0101	149	PQRRQSVLSKAISEH	SVLSKAISE	5	0.3899	736.3	65.00	0.50	tr_D0A564_
DRB1_0101	150	QSVLSKAISEHREGD	LSKAISEHR	3	0.3863	765.5	65.00	0.43	tr_D0A564_
DRB1_0101	151	PLLPPSKGLTSAEAE	SKGLTSAEA	5	0.3862	766.0	65.00	0.69	tr_D0A564_
DRB1_0101	152	LTSAEAEELLLKYGR	LTSAEAEEL	0	0.3833	790.2	70.00	0.50	tr_D0A564_
DRB1_0101	153	SLPVTMGTEHMPKMG	PVTMGTEHM	2	0.3833	790.6	70.00	0.62	tr_D0A564_
DRB1_0101	154	EAEELLLKYGRNELP	LKYGRNELP	6	0.3809	811.4	70.00	0.50	tr_D0A564_
DRB1_0101	155	GESLPVTMGTEHMPK	PVTMGTEHM	4	0.3805	814.3	70.00	0.55	tr_D0A564_
DRB1_0101	156	TMGTEHMPKMGSNVV	HMPKMGSNV	5	0.3791	827.2	70.00	0.44	tr_D0A564_
DRB1_0101	157	KPQRRQSVLSKAISE	SVLSKAISE	6	0.3783	834.4	70.00	0.25	tr_D0A564_
DRB1_0101	158	WGPMPIVLWIVIIIQ	PMPIVLWIV	2	0.3781	835.9	70.00	0.44	tr_D0A564_
DRB1_0101	159	TAYRDGTWQQIDAAL	YRDGTWQQI	2	0.3759	856.7	70.00	0.52	tr_D0A564_
DRB1_0101	160	QKPQRRQSVLSKAIS	PQRRQSVLS	2	0.3751	863.7	70.00	0.43	tr_D0A564_
DRB1_0101	161	IVLWIVIIIQFALQH	IIIQFALQH	6	0.3741	873.5	70.00	0.40	tr_D0A564_
DRB1_0101	162	LQSVEADIGSIRIIL	VEADIGSIR	3	0.3715	898.1	70.00	0.25	tr_D0A564_
DRB1_0101	163	LTGESLPVTMGTEHM	GESLPVTMG	2	0.3673	940.0	70.00	0.45	tr_D0A564_
DRB1_0101	164	PQKPQRRQSVLSKAI	PQRRQSVLS	3	0.3670	942.6	70.00	0.48	tr_D0A564_
DRB1_0101	165	TGESLPVTMGTEHMP	PVTMGTEHM	5	0.3658	954.7	70.00	0.43	tr_D0A564_
DRB1_0101	166	SLKPIATAYRDGTWQ	LKPIATAYR	1	0.3595	1022.2	75.00	0.61	tr_D0A564_
DRB1_0101	167	EGDDGSVPLLPPSKG	SVPLLPPSK	5	0.3569	1051.4	75.00	0.38	tr_D0A564_
DRB1_0101	168	ALTGESLPVTMGTEH	GESLPVTMG	3	0.3568	1052.6	75.00	0.52	tr_D0A564_
DRB1_0101	169	LPVTMGTEHMPKMGS	PVTMGTEHM	1	0.3521	1107.3	75.00	0.56	tr_D0A564_
DRB1_0101	170	KPQKPQRRQSVLSKA	PQRRQSVLS	4	0.3441	1207.9	75.00	0.44	tr_D0A564_
DRB1_0101	171	ATAYRDGTWQQIDAA	YRDGTWQQI	3	0.3431	1220.5	75.00	0.67	tr_D0A564_
DRB1_0101	172	SNVVRGEVDATVQYT	VRGEVDATV	3	0.3418	1238.2	75.00	0.52	tr_D0A564_
DRB1_0101	173	GSNVVRGEVDATVQY	VRGEVDATV	4	0.3357	1322.9	75.00	0.47	tr_D0A564_
DRB1_0101	174	HKPQKPQRRQSVLSK	PQRRQSVLS	5	0.3240	1502.1	80.00	0.37	tr_D0A564_
DRB1_0101	175	PMPIVLWIVIIIQFA	WIVIIIQFA	6	0.3190	1584.1	80.00	0.38	tr_D0A564_
DRB1_0101	176	GPMPIVLWIVIIIQF	PMPIVLWIV	1	0.3181	1600.9	80.00	0.50	tr_D0A564_
DRB1_0101	177	LKYGRNELPEKKTPS	LKYGRNELP	0	0.3153	1650.1	80.00	0.64	tr_D0A564_

DRB1_0101	178	EGVIDVDEAALTGES	IDVDEAALT	3	0.3126	1698.1	80.00	0.61	tr_D0A564_
DRB1_0101	179	MPKMGSNVVRGEVDA	MPKMGSNVV	0	0.3104	1739.2	80.00	0.40	tr_D0A564_
DRB1_0101	180	MGSNVVRGEVDATVQ	VRGEVDATV	5	0.3089	1767.3	80.00	0.41	tr_D0A564_
DRB1_0101	181	SVLSKAISEHREGDD	LSKAISEHR	2	0.3052	1840.9	85.00	0.52	tr_D0A564_
DRB1_0101	182	NVVRGEVDATVQYTG	VRGEVDATV	2	0.3049	1845.2	85.00	0.52	tr_D0A564_
DRB1_0101	183	NEGVIDVDEAALTGE	IDVDEAALT	4	0.3015	1915.3	85.00	0.57	tr_D0A564_
DRB1_0101	184	EVDATVQYTGQSTFF	TVQYTGQST	4	0.2980	1988.5	85.00	0.47	tr_D0A564_
DRB1_0101	185	IATAYRDGTWQQIDA	YRDGTWQQI	4	0.2971	2008.4	85.00	0.69	tr_D0A564_
DRB1_0101	186	PIVLWIVIIIQFALQ	WIVIIIQFA	4	0.2950	2053.8	85.00	0.51	tr_D0A564_
DRB1_0101	187	PVTMGTEHMPKMGSN	PVTMGTEHM	0	0.2896	2178.7	85.00	0.42	tr_D0A564_
DRB1_0101	188	VHKPQKPQRRQSVLS	HKPQKPQRR	1	0.2889	2195.2	85.00	0.29	tr_D0A564_
DRB1_0101	189	INEGVIDVDEAALTG	IDVDEAALT	5	0.2870	2240.3	85.00	0.57	tr_D0A564_
DRB1_0101	190	AEAEELLLKYGRNEL	LLLKYGRNE	5	0.2860	2265.4	85.00	0.49	tr_D0A564_
DRB1_0101	191	MPIVLWIVIIIQFAL	WIVIIIQFA	5	0.2803	2408.8	85.00	0.52	tr_D0A564_
DRB1_0101	192	PIATAYRDGTWQQID	YRDGTWQQI	5	0.2741	2576.0	90.00	0.60	tr_D0A564_
DRB1_0101	193	KMGSNVVRGEVDATV	SNVVRGEVD	3	0.2711	2661.8	90.00	0.25	tr_D0A564_
DRB1_0101	194	VVRGEVDATVQYTGQ	VRGEVDATV	1	0.2661	2809.0	90.00	0.44	tr_D0A564_
DRB1_0101	195	LKPIATAYRDGTWQQ	LKPIATAYR	0	0.2580	3064.9	90.00	0.44	tr_D0A564_
DRB1_0101	196	TSAEAEELLLKYGRN	SAEAEELLL	1	0.2577	3077.5	90.00	0.25	tr_D0A564_
DRB1_0101	197	VTMGTEHMPKMGSNV	HMPKMGSNV	6	0.2562	3125.6	90.00	0.35	tr_D0A564_
DRB1_0101	198	KPIATAYRDGTWQQI	YRDGTWQQI	6	0.2554	3155.4	90.00	0.44	tr_D0A564_
DRB1_0101	199	REGDDGSVPLLPPSK	SVPLLPPSK	6	0.2514	3292.8	90.00	0.43	tr_D0A564_
DRB1_0101	200	SAEAEELLLKYGRNE	LLLKYGRNE	6	0.2427	3616.8	90.00	0.28	tr_D0A564_
DRB1_0101	201	EVHKPQKPQRRQSVL	HKPQKPQRR	2	0.2395	3744.9	95.00	0.41	tr_D0A564_
DRB1_0101	202	TINEGVIDVDEAALT	IDVDEAALT	6	0.2352	3925.3	95.00	0.44	tr_D0A564_
DRB1_0101	203	GEVDATVQYTGQSTF	TVQYTGQST	5	0.2331	4012.7	95.00	0.44	tr_D0A564_
DRB1_0101	204	PKMGSNVVRGEVDAT	SNVVRGEVD	4	0.2281	4237.7	95.00	0.34	tr_D0A564_
DRB1_0101	205	GEVHKPQKPQRRQSV	HKPQKPQRR	3	0.2216	4544.9	95.00	0.46	tr_D0A564_
DRB1_0101	206	VLSKAISEHREGDDG	LSKAISEHR	1	0.2099	5159.1	95.00	0.73	tr_D0A564_
DRB1_0101	207	VRGEVDATVQYTGQS	EVDATVQYT	3	0.2094	5186.7	95.00	0.30	tr_D0A564_
DRB1_0101	208	AGEVHKPQKPQRRQS	HKPQKPQRR	4	0.2004	5717.0	95.00	0.47	tr_D0A564_
DRB1_0101	209	ADCTINEGVIDVDEA	CTINEGVID	2	0.1978	5883.2	95.00	0.36	tr_D0A564_
DRB1_0101	210	VPADCTINEGVIDVD	CTINEGVID	4	0.1964	5972.7	95.00	0.35	tr_D0A564_
DRB1_0101	211	PADCTINEGVIDVDE	CTINEGVID	3	0.1955	6029.5	95.00	0.38	tr_D0A564_
DRB1_0101	212	RGEVDATVQYTGQST	EVDATVQYT	2	0.1912	6319.1	100.00	0.25	tr_D0A564_
DRB1_0101	213	CTINEGVIDVDEAAL	TINEGVIDV	1	0.1909	6338.6	100.00	0.20	tr_D0A564_
DRB1_0101	214	AVPADCTINEGVIDV	CTINEGVID	5	0.1890	6468.1	100.00	0.38	tr_D0A564_
DRB1_0101	215	DCTINEGVIDVDEAA	CTINEGVID	1	0.1881	6532.1	100.00	0.28	tr_D0A564_
DRB1_0101	216	DAGEVHKPQKPQRRQ	HKPQKPQRR	5	0.1877	6563.7	100.00	0.41	tr_D0A564_
DRB1_0101	217	HREGDDGSVPLLPPS	DDGSVPLLP	4	0.1799	7136.4	100.00	0.22	tr_D0A564_
DRB1_0101	218	SEHREGDDGSVPLLP	HREGDDGSV	2	0.1778	7304.8	100.00	0.34	tr_D0A564_
DRB1_0101	219	SGSAVPADCTINEGV	GSAVPADCT	1	0.1778	7305.3	100.00	0.45	tr_D0A564_
DRB1_0101	220	GSGSAVPADCTINEG	GSAVPADCT	2	0.1772	7349.9	100.00	0.48	tr_D0A564_
DRB1_0101	221	YGRNELPEKKTPSWL	PEKKTPSWL	6	0.1739	7616.8	100.00	0.25	tr_D0A564_
DRB1_0101	222	ISEHREGDDGSVPLL	HREGDDGSV	3	0.1731	7682.8	100.00	0.41	tr_D0A564_
DRB1_0101	223	EHREGDDGSVPLLPP	HREGDDGSV	1	0.1725	7736.2	100.00	0.28	tr_D0A564_
DRB1_0101	224	NDAGEVHKPQKPQRR	GEVHKPQKP	3	0.1688	8051.3	100.00	0.34	tr_D0A564_
DRB1_0101	225	SAVPADCTINEGVID	CTINEGVID	6	0.1608	8776.8	100.00	0.30	tr_D0A564_
DRB1_0101	226	GSAVPADCTINEGVI	AVPADCTIN	2	0.1588	8971.5	100.00	0.17	tr_D0A564_
DRB1_0101	227	KYGRNELPEKKTPSW	GRNELPEKK	2	0.1560	9243.6	100.00	0.37	tr_D0A564_

DRB1_0101	228	LSKAISEHREGDDGS	LSKAISEHR	0	0.1305	12188.4	100.00	0.51	tr_D0A564_
DRB1_0101	229	AISEHREGDDGSVPL	HREGDDGSV	4	0.1293	12341.2	100.00	0.46	tr_D0A564_
DRB1_0101	230	TNDAGEVHKPQKPQR	GEVHKPQKP	4	0.1272	12632.4	100.00	0.41	tr_D0A564_
DRB1_0101	231	GTNDAGEVHKPQKPQ	GEVHKPQKP	5	0.1221	13337.8	100.00	0.33	tr_D0A564_
DRB1_0101	232	PGTNDAGEVHKPQKP	NDAGEVHKP	3	0.1151	14393.2	100.00	0.28	tr_D0A564_
DRB1_0101	233	MGDTGPKGVPGTNDA	MGDTGPKGV	0	0.1127	14772.6	100.00	0.34	tr_D0A564_
DRB1_0101	234	VPGTNDAGEVHKPQK	NDAGEVHKP	4	0.1114	14981.4	100.00	0.34	tr_D0A564_
DRB1_0101	235	KAISEHREGDDGSVP	HREGDDGSV	5	0.1003	16889.1	100.00	0.46	tr_D0A564_
DRB1_0101	236	GVPGTNDAGEVHKPQ	NDAGEVHKP	5	0.0991	17120.0	100.00	0.28	tr_D0A564_
DRB1_0101	237	SKAISEHREGDDGSV	HREGDDGSV	6	0.0987	17185.7	100.00	0.33	tr_D0A564_
DRB1_0101	238	PKGVPGTNDAGEVHK	GVPGTNDAG	2	0.0909	18695.2	100.00	0.19	tr_D0A564_
DRB1_0101	239	KGVPGTNDAGEVHKP	PGTNDAGEV	3	0.0894	19010.4	100.00	0.22	tr_D0A564_
DRB1_0101	240	GDTGPKGVPGTNDAG	PKGVPGTND	4	0.0853	19860.6	100.00	0.31	tr_D0A564_
DRB1_0101	241	TGPKGVPGTNDAGEV	PKGVPGTND	2	0.0837	20215.1	100.00	0.28	tr_D0A564_
DRB1_0101	242	GPKGVPGTNDAGEVH	GVPGTNDAG	3	0.0820	20597.9	100.00	0.25	tr_D0A564_
DRB1_0101	243	DTGPKGVPGTNDAGE	PKGVPGTND	3	0.0819	20602.8	100.00	0.37	tr_D0A564_

Allele: DRB1_0101. Number of high binders 0. Number of weak binders 25. Number of peptides 243

Allele	pos	peptide	core	Of	1-log50k(aff)	affinity(nM)	%Rank	Relia	Identity	Bind Level
DRB1_0101	1	CFTFEKGHDLRSLLV	FTFEKGHDL	1	0.7382	17.0	5.50	0.61	tr_D0A564_	WB
DRB1_0101	2	EQCFTFEKGHDLRSL	FTFEKGHDL	3	0.7301	18.5	6.50	0.88	tr_D0A564_	WB
DRB1_0101	3	QCFTFEKGHDLRSLL	FTFEKGHDL	2	0.7298	18.6	6.50	0.85	tr_D0A564_	WB
DRB1_0101	4	RSLLVLSALAAKWRE	LLVLSALAA	2	0.7287	18.8	6.50	0.34	tr_D0A564_	WB
DRB1_0101	5	EHKFLIVEALRQRGY	FLIVEALRQ	3	0.7281	19.0	6.50	0.57	tr_D0A564_	WB
DRB1_0101	6	LRSLLVLSALAAKWR	LLVLSALAA	3	0.7267	19.3	6.50	0.35	tr_D0A564_	WB
DRB1_0101	7	HKFLIVEALRQRGYT	FLIVEALRQ	2	0.7178	21.2	7.50	0.56	tr_D0A564_	WB
DRB1_0101	8	PEHKFLIVEALRQRG	FLIVEALRQ	4	0.7145	22.0	7.50	0.57	tr_D0A564_	WB
DRB1_0101	9	VDIIDKLASRGIRCL	IDKLASRGI	3	0.7131	22.3	8.00	0.69	tr_D0A564_	WB
DRB1_0101	10	DIIDKLASRGIRCLS	IDKLASRGI	2	0.7046	24.4	8.50	0.65	tr_D0A564_	WB
DRB1_0101	11	FTFEKGHDLRSLLVL	GHDLRSLLV	5	0.7039	24.6	8.50	0.38	tr_D0A564_	WB
DRB1_0101	12	SRGIRCLSVAKTDSA	IRCLSVAKT	3	0.7033	24.8	8.50	0.85	tr_D0A564_	WB
DRB1_0101	13	TRLTAIETMSGVNML	IETMSGVNM	5	0.6980	26.3	9.00	0.54	tr_D0A564_	WB
DRB1_0101	14	FPEHKFLIVEALRQR	FLIVEALRQ	5	0.6973	26.5	9.50	0.55	tr_D0A564_	WB
DRB1_0101	15	QEQCFTFEKGHDLRS	FTFEKGHDL	4	0.6914	28.2	10.00	0.89	tr_D0A564_	
DRB1_0101	16	RLTAIETMSGVNMLC	IETMSGVNM	4	0.6913	28.2	10.00	0.61	tr_D0A564_	
DRB1_0101	17	IIDKLASRGIRCLSV	IDKLASRGI	1	0.6912	28.2	10.00	0.57	tr_D0A564_	
DRB1_0101	18	SLLVLSALAAKWREP	LLVLSALAA	1	0.6895	28.8	10.00	0.30	tr_D0A564_	
DRB1_0101	19	DLRSLLVLSALAAKW	LLVLSALAA	4	0.6894	28.8	10.00	0.35	tr_D0A564_	
DRB1_0101	20	KIIVTRLTAIETMSG	VTRLTAIET	3	0.6868	29.6	11.00	0.68	tr_D0A564_	
DRB1_0101	21	VTRLTAIETMSGVNM	LTAIETMSG	3	0.6853	30.1	11.00	0.38	tr_D0A564_	
DRB1_0101	22	KHKIIVTRLTAIETM	VTRLTAIET	5	0.6844	30.4	11.00	0.51	tr_D0A564_	
DRB1_0101	23	KGHDLRSLLVLSALA	GHDLRSLLV	1	0.6837	30.6	11.00	0.47	tr_D0A564_	
DRB1_0101	24	RGIRCLSVAKTDSAG	IRCLSVAKT	2	0.6823	31.1	11.00	0.85	tr_D0A564_	
DRB1_0101	25	LTAIETMSGVNMLCS	IETMSGVNM	3	0.6822	31.1	11.00	0.74	tr_D0A564_	
DRB1_0101	26	ASRGIRCLSVAKTDS	IRCLSVAKT	4	0.6818	31.3	11.00	0.85	tr_D0A564_	

DRB1_0101	27	HKIIVTRLTAIETMS	VTRLTAIET	4	0.6789	32.3	12.00	0.63	tr_D0A564_
DRB1_0101	28	FEKGHDLRSLLVLSA	GHDLRSLLV	3	0.6773	32.8	12.00	0.60	tr_D0A564_
DRB1_0101	29	GHDLRSLLVLSALAA	LRSLLVLSA	3	0.6767	33.1	12.00	0.35	tr_D0A564_
DRB1_0101	30	EKGHDLRSLLVLSAL	GHDLRSLLV	2	0.6760	33.3	12.00	0.53	tr_D0A564_
DRB1_0101	31	VVDIIDKLASRGIRC	IDKLASRGI	4	0.6740	34.0	12.00	0.73	tr_D0A564_
DRB1_0101	32	KFLIVEALRQRGYTC	FLIVEALRQ	1	0.6738	34.1	12.00	0.58	tr_D0A564_
DRB1_0101	33	IIVTRLTAIETMSGV	VTRLTAIET	2	0.6718	34.9	12.00	0.51	tr_D0A564_
DRB1_0101	34	IVTRLTAIETMSGVN	VTRLTAIET	1	0.6694	35.8	13.00	0.44	tr_D0A564_
DRB1_0101	35	LASRGIRCLSVAKTD	IRCLSVAKT	5	0.6644	37.8	13.00	0.77	tr_D0A564_
DRB1_0101	36	HDLRSLLVLSALAAK	LRSLLVLSA	2	0.6579	40.5	14.00	0.34	tr_D0A564_
DRB1_0101	37	MCRMLDLDPNILTAE	MLDLDPNIL	3	0.6572	40.8	14.00	0.49	tr_D0A564_
DRB1_0101	38	SVVDIIDKLASRGIR	IDKLASRGI	5	0.6551	41.8	14.00	0.70	tr_D0A564_
DRB1_0101	39	CRMLDLDPNILTAEK	MLDLDPNIL	2	0.6540	42.2	15.00	0.44	tr_D0A564_
DRB1_0101	40	RMLDLDPNILTAEKL	LDLDPNILT	2	0.6538	42.3	15.00	0.39	tr_D0A564_
DRB1_0101	41	KLASRGIRCLSVAKT	IRCLSVAKT	6	0.6520	43.2	15.00	0.62	tr_D0A564_
DRB1_0101	42	SKHKIIVTRLTAIET	HKIIVTRLT	2	0.6513	43.5	15.00	0.29	tr_D0A564_
DRB1_0101	43	KFSVTKGAPHVIIEM	VTKGAPHVI	3	0.6499	44.2	15.00	0.48	tr_D0A564_
DRB1_0101	44	VDVKMITGDHVLIAK	VKMITGDHV	2	0.6491	44.5	15.00	0.69	tr_D0A564_
DRB1_0101	45	EMCRMLDLDPNILTA	MLDLDPNIL	4	0.6491	44.6	15.00	0.49	tr_D0A564_
DRB1_0101	46	EKFSVTKGAPHVIIE	VTKGAPHVI	4	0.6461	46.0	16.00	0.38	tr_D0A564_
DRB1_0101	47	TAIETMSGVNMLCSD	IETMSGVNM	2	0.6455	46.3	16.00	0.81	tr_D0A564_
DRB1_0101	48	IQEQCFTFEKGHDLR	FTFEKGHDL	5	0.6443	46.9	16.00	0.90	tr_D0A564_
DRB1_0101	49	GVDVKMITGDHVLIA	VKMITGDHV	3	0.6423	47.9	16.00	0.73	tr_D0A564_
DRB1_0101	50	TFEKGHDLRSLLVLS	GHDLRSLLV	4	0.6407	48.8	16.00	0.64	tr_D0A564_
DRB1_0101	51	LLVLSALAAKWREPP	VLSALAAKW	2	0.6400	49.2	16.00	0.34	tr_D0A564_
DRB1_0101	52	GEKFSVTKGAPHVII	FSVTKGAPH	3	0.6342	52.4	17.00	0.46	tr_D0A564_
DRB1_0101	53	GIRCLSVAKTDSAGR	IRCLSVAKT	1	0.6335	52.8	17.00	0.76	tr_D0A564_
DRB1_0101	54	VFPEHKFLIVEALRQ	FLIVEALRQ	6	0.6284	55.7	18.00	0.50	tr_D0A564_
DRB1_0101	55	DVKMITGDHVLIAKE	VKMITGDHV	1	0.6274	56.3	18.00	0.62	tr_D0A564_
DRB1_0101	56	IDKLASRGIRCLSVA	IDKLASRGI	0	0.6196	61.3	19.00	0.34	tr_D0A564_
DRB1_0101	57	KLSKHKIIVTRLTAI	HKIIVTRLT	4	0.6150	64.5	20.00	0.44	tr_D0A564_
DRB1_0101	58	MLDLDPNILTAEKLP	PNILTAEKL	5	0.6126	66.2	20.00	0.44	tr_D0A564_
DRB1_0101	59	VKMITGDHVLIAKEM	VKMITGDHV	0	0.6118	66.7	21.00	0.43	tr_D0A564_
DRB1_0101	60	LDPNILTAEKLPKVD	PNILTAEKL	2	0.6098	68.2	21.00	0.75	tr_D0A564_
DRB1_0101	61	LSKHKIIVTRLTAIE	HKIIVTRLT	3	0.6084	69.2	21.00	0.44	tr_D0A564_
DRB1_0101	62	DLDPNILTAEKLPKV	PNILTAEKL	3	0.6047	72.0	22.00	0.79	tr_D0A564_
DRB1_0101	63	KEMCRMLDLDPNILT	MLDLDPNIL	5	0.6010	74.9	22.00	0.50	tr_D0A564_
DRB1_0101	64	YGVDVKMITGDHVLI	VKMITGDHV	4	0.6006	75.3	22.00	0.75	tr_D0A564_
DRB1_0101	65	AIETMSGVNMLCSDK	IETMSGVNM	1	0.5957	79.4	23.00	0.78	tr_D0A564_
DRB1_0101	66	LDLDPNILTAEKLPK	PNILTAEKL	4	0.5857	88.5	25.00	0.68	tr_D0A564_
DRB1_0101	67	CGILTFLDPPRPDTK	LTFLDPPRP	3	0.5841	90.1	25.00	0.85	tr_D0A564_
DRB1_0101	68	DPNILTAEKLPKVDV	PNILTAEKL	1	0.5821	91.9	25.00	0.70	tr_D0A564_
DRB1_0101	69	FSVTKGAPHVIIEMV	VTKGAPHVI	2	0.5817	92.4	26.00	0.50	tr_D0A564_
DRB1_0101	70	FLIVEALRQRGYTCA	FLIVEALRQ	0	0.5813	92.8	26.00	0.46	tr_D0A564_
DRB1_0101	71	TGDHVLIAKEMCRML	DHVLIAKEM	2	0.5811	93.0	26.00	0.43	tr_D0A564_
DRB1_0101	72	GDHVLIAKEMCRMLD	DHVLIAKEM	1	0.5804	93.7	26.00	0.33	tr_D0A564_
DRB1_0101	73	KKLSKHKIIVTRLTA	HKIIVTRLT	5	0.5780	96.2	26.00	0.40	tr_D0A564_
DRB1_0101	74	GILTFLDPPRPDTKE	LTFLDPPRP	2	0.5722	102.4	27.00	0.86	tr_D0A564_
DRB1_0101	75	DHVLIAKEMCRMLDL	IAKEMCRML	4	0.5700	104.8	28.00	0.33	tr_D0A564_
DRB1_0101	76	IRCLSVAKTDSAGRW	IRCLSVAKT	0	0.5695	105.4	28.00	0.65	tr_D0A564_

DRB1_0101	77	YGDMMLSVGGFAQVF	MLSVGGFAQ	4	0.5578	119.7	30.00	0.28	tr_D0A564_
DRB1_0101	78	SGEKFSVTKGAPHVI	FSVTKGAPH	4	0.5560	121.9	30.00	0.58	tr_D0A564_
DRB1_0101	79	SKKLSKHKIIVTRLT	KHKIIVTRL	5	0.5545	124.1	30.00	0.26	tr_D0A564_
DRB1_0101	80	LCGILTFLDPPRPDT	LTFLDPPRP	4	0.5519	127.5	31.00	0.81	tr_D0A564_
DRB1_0101	81	QYGVDVKMITGDHVL	VKMITGDHV	5	0.5417	142.5	33.00	0.76	tr_D0A564_
DRB1_0101	82	ITGDHVLIAKEMCRM	DHVLIAKEM	3	0.5407	143.9	33.00	0.56	tr_D0A564_
DRB1_0101	83	LVLSALAAKWREPPR	VLSALAAKW	1	0.5401	144.9	33.00	0.44	tr_D0A564_
DRB1_0101	84	KYGDMMLSVGGFAQV	MMLSVGGFA	4	0.5394	146.0	33.00	0.26	tr_D0A564_
DRB1_0101	85	ILTFLDPPRPDTKET	LTFLDPPRP	1	0.5377	148.7	34.00	0.85	tr_D0A564_
DRB1_0101	86	DPTTKRTAATLVDKR	TKRTAATLV	3	0.5372	149.5	34.00	0.80	tr_D0A564_
DRB1_0101	87	GDMMLSVGGFAQVFP	MLSVGGFAQ	3	0.5364	150.8	34.00	0.31	tr_D0A564_
DRB1_0101	88	DSVVDIIDKLASRGI	IDKLASRGI	6	0.5364	150.9	34.00	0.52	tr_D0A564_
DRB1_0101	89	IETMSGVNMLCSDKT	IETMSGVNM	0	0.5332	156.2	34.00	0.52	tr_D0A564_
DRB1_0101	90	MSGVNMLCSDKTGTL	VNMLCSDKT	3	0.5328	156.8	34.00	0.86	tr_D0A564_
DRB1_0101	91	HLCGILTFLDPPRPD	LTFLDPPRP	5	0.5300	161.6	35.00	0.75	tr_D0A564_
DRB1_0101	92	PTTKRTAATLVDKRS	TKRTAATLV	2	0.5299	161.8	35.00	0.81	tr_D0A564_
DRB1_0101	93	DKLASRGIRCLSVAK	KLASRGIRC	1	0.5291	163.2	35.00	0.41	tr_D0A564_
DRB1_0101	94	SGVNMLCSDKTGTLT	VNMLCSDKT	2	0.5253	170.1	36.00	0.81	tr_D0A564_
DRB1_0101	95	KMITGDHVLIAKEMC	DHVLIAKEM	5	0.5216	177.0	37.00	0.43	tr_D0A564_
DRB1_0101	96	HVLIAKEMCRMLDLD	IAKEMCRML	3	0.5168	186.5	38.00	0.43	tr_D0A564_
DRB1_0101	97	PNILTAEKLPKVDVN	PNILTAEKL	0	0.5156	188.8	38.00	0.62	tr_D0A564_
DRB1_0101	98	EIQEQCFTFEKGHDL	FTFEKGHDL	6	0.5131	194.0	38.00	0.85	tr_D0A564_
DRB1_0101	99	QVFPEHKFLIVEALR	HKFLIVEAL	5	0.5129	194.4	38.00	0.60	tr_D0A564_
DRB1_0101	100	MITGDHVLIAKEMCR	DHVLIAKEM	4	0.5111	198.3	39.00	0.56	tr_D0A564_
DRB1_0101	101	FDPTTKRTAATLVDK	TKRTAATLV	4	0.5085	204.0	39.00	0.79	tr_D0A564_
DRB1_0101	102	AKEMCRMLDLDPNIL	MLDLDPNIL	6	0.5079	205.2	39.00	0.49	tr_D0A564_
DRB1_0101	103	EKYGDMMLSVGGFAQ	MMLSVGGFA	5	0.5046	212.9	40.00	0.26	tr_D0A564_
DRB1_0101	104	DMMLSVGGFAQVFPE	MLSVGGFAQ	2	0.5023	218.1	41.00	0.34	tr_D0A564_
DRB1_0101	105	GVNMLCSDKTGTLTL	VNMLCSDKT	1	0.4959	233.8	42.00	0.74	tr_D0A564_
DRB1_0101	106	VGGFAQVFPEHKFLI	FAQVFPEHK	3	0.4938	239.1	42.00	0.69	tr_D0A564_
DRB1_0101	107	TMSGVNMLCSDKTGT	VNMLCSDKT	4	0.4902	248.5	43.00	0.82	tr_D0A564_
DRB1_0101	108	GGFAQVFPEHKFLIV	FAQVFPEHK	2	0.4898	249.6	43.00	0.61	tr_D0A564_
DRB1_0101	109	SVTKGAPHVIIEMVH	VTKGAPHVI	1	0.4855	261.6	44.00	0.55	tr_D0A564_
DRB1_0101	110	TTKRTAATLVDKRSG	TKRTAATLV	1	0.4833	268.0	44.00	0.82	tr_D0A564_
DRB1_0101	111	WHLCGILTFLDPPRP	LTFLDPPRP	6	0.4798	278.4	45.00	0.62	tr_D0A564_
DRB1_0101	112	RGYTCAMTGDGVNDA	TCAMTGDGV	3	0.4793	279.8	45.00	0.54	tr_D0A564_
DRB1_0101	113	ETMSGVNMLCSDKTG	VNMLCSDKT	5	0.4740	296.2	46.00	0.70	tr_D0A564_
DRB1_0101	114	SVGGFAQVFPEHKFL	FAQVFPEHK	4	0.4680	316.1	48.00	0.67	tr_D0A564_
DRB1_0101	115	MMLSVGGFAQVFPEH	MLSVGGFAQ	1	0.4677	317.2	48.00	0.37	tr_D0A564_
DRB1_0101	116	GEKYGDMMLSVGGFA	GDMMLSVGG	4	0.4596	346.1	49.00	0.31	tr_D0A564_
DRB1_0101	117	QRGYTCAMTGDGVND	TCAMTGDGV	4	0.4572	355.3	50.00	0.51	tr_D0A564_
DRB1_0101	118	YEQLEFVPFDPTTKR	FVPFDPTTK	5	0.4537	369.1	55.00	0.38	tr_D0A564_
DRB1_0101	119	QLEFVPFDPTTKRTA	FVPFDPTTK	3	0.4533	370.5	55.00	0.56	tr_D0A564_
DRB1_0101	120	PFDPTTKRTAATLVD	TKRTAATLV	5	0.4511	379.5	55.00	0.72	tr_D0A564_
DRB1_0101	121	RQRGYTCAMTGDGVN	TCAMTGDGV	5	0.4506	381.8	55.00	0.47	tr_D0A564_
DRB1_0101	122	GFAQVFPEHKFLIVE	FAQVFPEHK	1	0.4501	383.7	55.00	0.56	tr_D0A564_
DRB1_0101	123	VLIAKEMCRMLDLDP	IAKEMCRML	2	0.4493	387.0	55.00	0.52	tr_D0A564_
DRB1_0101	124	KETIRRSRQYGVDVK	IRRSRQYGV	3	0.4488	389.0	55.00	0.69	tr_D0A564_
DRB1_0101	125	MLSVGGFAQVFPEHK	FAQVFPEHK	6	0.4435	411.9	55.00	0.24	tr_D0A564_
DRB1_0101	126	RSGEKFSVTKGAPHV	FSVTKGAPH	5	0.4422	417.8	55.00	0.72	tr_D0A564_

DRB1_0101	127	LSVGGFAQVFPEHKF	FAQVFPEHK	5	0.4417	420.1	55.00	0.50	tr_D0A564_
DRB1_0101	128	RQYGVDVKMITGDHV	VKMITGDHV	6	0.4397	429.6	55.00	0.58	tr_D0A564_
DRB1_0101	129	NYEQLEFVPFDPTTK	YEQLEFVPF	1	0.4382	436.6	55.00	0.38	tr_D0A564_
DRB1_0101	130	EQLEFVPFDPTTKRT	FVPFDPTTK	4	0.4378	438.2	55.00	0.52	tr_D0A564_
DRB1_0101	131	LIVEALRQRGYTCAM	ALRQRGYTC	4	0.4367	443.4	55.00	0.30	tr_D0A564_
DRB1_0101	132	GYTCAMTGDGVNDAP	TCAMTGDGV	2	0.4362	445.7	55.00	0.56	tr_D0A564_
DRB1_0101	133	ETIRRSRQYGVDVKM	IRRSRQYGV	2	0.4323	465.3	55.00	0.62	tr_D0A564_
DRB1_0101	134	AQVFPEHKFLIVEAL	HKFLIVEAL	6	0.4320	466.7	55.00	0.54	tr_D0A564_
DRB1_0101	135	TIRRSRQYGVDVKMI	IRRSRQYGV	1	0.4316	468.6	55.00	0.44	tr_D0A564_
DRB1_0101	136	VNMLCSDKTGTLTLN	VNMLCSDKT	0	0.4289	482.5	60.00	0.57	tr_D0A564_
DRB1_0101	137	RDALDTMVLGAADLD	LDTMVLGAA	3	0.4288	483.3	60.00	0.31	tr_D0A564_
DRB1_0101	138	DALDTMVLGAADLDE	TMVLGAADL	4	0.4220	519.8	60.00	0.27	tr_D0A564_
DRB1_0101	139	AGRWHLCGILTFLDP	HLCGILTFL	4	0.4191	536.4	60.00	0.22	tr_D0A564_
DRB1_0101	140	LTFLDPPRPDTKETI	LTFLDPPRP	0	0.4177	544.5	60.00	0.75	tr_D0A564_
DRB1_0101	141	LIAKEMCRMLDLDPN	IAKEMCRML	1	0.4172	547.4	60.00	0.48	tr_D0A564_
DRB1_0101	142	LEFVPFDPTTKRTAA	FVPFDPTTK	2	0.4172	547.9	60.00	0.52	tr_D0A564_
DRB1_0101	143	SAGRWHLCGILTFLD	WHLCGILTF	4	0.4150	560.7	60.00	0.27	tr_D0A564_
DRB1_0101	144	DNYEQLEFVPFDPTT	YEQLEFVPF	2	0.4146	563.1	60.00	0.53	tr_D0A564_
DRB1_0101	145	ALDTMVLGAADLDEC	TMVLGAADL	3	0.4139	567.4	60.00	0.27	tr_D0A564_
DRB1_0101	146	CDNYEQLEFVPFDPT	YEQLEFVPF	3	0.4114	583.1	60.00	0.62	tr_D0A564_
DRB1_0101	147	PRDALDTMVLGAADL	LDTMVLGAA	4	0.4066	614.3	65.00	0.40	tr_D0A564_
DRB1_0101	148	VLSALAAKWREPPRD	LSALAAKWR	1	0.4037	633.6	65.00	0.50	tr_D0A564_
DRB1_0101	149	IRRSRQYGVDVKMIT	QYGVDVKMI	5	0.4029	639.4	65.00	0.29	tr_D0A564_
DRB1_0101	150	DSAGRWHLCGILTFL	WHLCGILTF	5	0.3993	664.5	65.00	0.31	tr_D0A564_
DRB1_0101	151	YTCAMTGDGVNDAPA	TCAMTGDGV	1	0.3992	665.8	65.00	0.56	tr_D0A564_
DRB1_0101	152	TKETIRRSRQYGVDV	IRRSRQYGV	4	0.3988	668.4	65.00	0.76	tr_D0A564_
DRB1_0101	153	IAKEMCRMLDLDPNI	CRMLDLDPN	5	0.3987	669.1	65.00	0.44	tr_D0A564_
DRB1_0101	154	GRWHLCGILTFLDPP	HLCGILTFL	3	0.3983	671.7	65.00	0.22	tr_D0A564_
DRB1_0101	155	IVEALRQRGYTCAMT	ALRQRGYTC	3	0.3973	679.2	65.00	0.38	tr_D0A564_
DRB1_0101	156	VPFDPTTKRTAATLV	TKRTAATLV	6	0.3918	720.6	65.00	0.54	tr_D0A564_
DRB1_0101	157	LGEKYGDMMLSVGGF	GDMMLSVGG	5	0.3877	753.8	65.00	0.31	tr_D0A564_
DRB1_0101	158	RWHLCGILTFLDPPR	LCGILTFLD	3	0.3854	772.9	65.00	0.21	tr_D0A564_
DRB1_0101	159	VEALRQRGYTCAMTG	ALRQRGYTC	2	0.3824	798.5	70.00	0.40	tr_D0A564_
DRB1_0101	160	ECDNYEQLEFVPFDP	YEQLEFVPF	4	0.3807	813.4	70.00	0.67	tr_D0A564_
DRB1_0101	161	DECDNYEQLEFVPFD	YEQLEFVPF	5	0.3805	814.5	70.00	0.65	tr_D0A564_
DRB1_0101	162	VTKGAPHVIIEMVHN	VTKGAPHVI	0	0.3786	832.1	70.00	0.37	tr_D0A564_
DRB1_0101	163	LDTMVLGAADLDECD	MVLGAADLD	3	0.3784	833.4	70.00	0.35	tr_D0A564_
DRB1_0101	164	LRQRGYTCAMTGDGV	TCAMTGDGV	6	0.3782	835.5	70.00	0.34	tr_D0A564_
DRB1_0101	165	FAQVFPEHKFLIVEA	FAQVFPEHK	0	0.3756	858.7	70.00	0.34	tr_D0A564_
DRB1_0101	166	RCLSVAKTDSAGRWH	LSVAKTDSA	2	0.3710	902.7	70.00	0.55	tr_D0A564_
DRB1_0101	167	RRSRQYGVDVKMITG	QYGVDVKMI	4	0.3701	911.5	70.00	0.44	tr_D0A564_
DRB1_0101	168	CLSVAKTDSAGRWHL	VAKTDSAGR	3	0.3658	955.4	70.00	0.34	tr_D0A564_
DRB1_0101	169	EFVPFDPTTKRTAAT	FVPFDPTTK	1	0.3619	995.9	70.00	0.50	tr_D0A564_
DRB1_0101	170	LSVAKTDSAGRWHLC	VAKTDSAGR	2	0.3547	1077.3	75.00	0.32	tr_D0A564_
DRB1_0101	171	TDSAGRWHLCGILTF	RWHLCGILT	5	0.3508	1123.7	75.00	0.25	tr_D0A564_
DRB1_0101	172	EALRQRGYTCAMTGD	ALRQRGYTC	1	0.3507	1125.0	75.00	0.37	tr_D0A564_
DRB1_0101	173	LDECDNYEQLEFVPF	YEQLEFVPF	6	0.3453	1192.5	75.00	0.51	tr_D0A564_
DRB1_0101	174	DLGEKYGDMMLSVGG	LGEKYGDMM	1	0.3437	1213.0	75.00	0.19	tr_D0A564_
DRB1_0101	175	RSRQYGVDVKMITGD	QYGVDVKMI	3	0.3437	1213.4	75.00	0.45	tr_D0A564_
DRB1_0101	176	PPRDALDTMVLGAAD	LDTMVLGAA	5	0.3437	1213.9	75.00	0.43	tr_D0A564_

DRB1_0101	177	NDSVVDIIDKLASRG	VVDIIDKLA	3	0.3429	1223.6	75.00	0.29	tr_D0A564_
DRB1_0101	178	DTKETIRRSRQYGVD	IRRSRQYGV	5	0.3392	1273.2	75.00	0.75	tr_D0A564_
DRB1_0101	179	TKRTAATLVDKRSGE	TKRTAATLV	0	0.3347	1336.7	75.00	0.64	tr_D0A564_
DRB1_0101	180	TCAMTGDGVNDAPAL	TCAMTGDGV	0	0.3342	1345.2	80.00	0.43	tr_D0A564_
DRB1_0101	181	DTMVLGAADLDECDN	MVLGAADLD	2	0.3341	1345.8	80.00	0.43	tr_D0A564_
DRB1_0101	182	KRSGEKFSVTKGAPH	FSVTKGAPH	6	0.3324	1370.5	80.00	0.62	tr_D0A564_
DRB1_0101	183	FVPFDPTTKRTAATL	FDPTTKRTA	3	0.3306	1398.4	80.00	0.45	tr_D0A564_
DRB1_0101	184	LNKMEIQEQCFTFEK	MEIQEQCFT	3	0.3287	1427.2	80.00	0.40	tr_D0A564_
DRB1_0101	185	APHVIIEMVHNQDEI	VIIEMVHNQ	3	0.3268	1457.3	80.00	0.34	tr_D0A564_
DRB1_0101	186	TKGAPHVIIEMVHNQ	HVIIEMVHN	5	0.3266	1459.5	80.00	0.34	tr_D0A564_
DRB1_0101	187	ALRQRGYTCAMTGDG	LRQRGYTCA	1	0.3259	1471.5	80.00	0.25	tr_D0A564_
DRB1_0101	188	SDLGEKYGDMMLSVG	LGEKYGDMM	2	0.3220	1534.7	80.00	0.31	tr_D0A564_
DRB1_0101	189	KGAPHVIIEMVHNQD	HVIIEMVHN	4	0.3200	1567.5	80.00	0.36	tr_D0A564_
DRB1_0101	190	GAPHVIIEMVHNQDE	HVIIEMVHN	3	0.3197	1572.6	80.00	0.34	tr_D0A564_
DRB1_0101	191	NKMEIQEQCFTFEKG	EIQEQCFTF	3	0.3163	1631.7	80.00	0.35	tr_D0A564_
DRB1_0101	192	KTDSAGRWHLCGILT	RWHLCGILT	6	0.3163	1632.6	80.00	0.20	tr_D0A564_
DRB1_0101	193	KTGTLTLNKMEIQEQ	LTLNKMEIQ	4	0.3148	1658.7	80.00	0.31	tr_D0A564_
DRB1_0101	194	TLNKMEIQEQCFTFE	MEIQEQCFT	4	0.3148	1659.4	80.00	0.31	tr_D0A564_
DRB1_0101	195	SVAKTDSAGRWHLCG	TDSAGRWHL	4	0.3135	1681.4	80.00	0.29	tr_D0A564_
DRB1_0101	196	SRQYGVDVKMITGDH	QYGVDVKMI	2	0.3127	1695.9	80.00	0.44	tr_D0A564_
DRB1_0101	197	PHVIIEMVHNQDEIN	VIIEMVHNQ	2	0.3119	1711.5	80.00	0.26	tr_D0A564_
DRB1_0101	198	LTLNKMEIQEQCFTF	MEIQEQCFT	5	0.3105	1738.0	80.00	0.26	tr_D0A564_
DRB1_0101	199	EPPRDALDTMVLGAA	LDTMVLGAA	6	0.3067	1809.9	80.00	0.36	tr_D0A564_
DRB1_0101	200	SDKTGTLTLNKMEIQ	TGTLTLNKM	3	0.3019	1906.3	85.00	0.44	tr_D0A564_
DRB1_0101	201	DKTGTLTLNKMEIQE	TGTLTLNKM	2	0.2978	1992.5	85.00	0.37	tr_D0A564_
DRB1_0101	202	INDSVVDIIDKLASR	VVDIIDKLA	4	0.2976	1998.1	85.00	0.38	tr_D0A564_
DRB1_0101	203	TLTLNKMEIQEQCFT	LNKMEIQEQ	3	0.2975	2000.2	85.00	0.30	tr_D0A564_
DRB1_0101	204	PSDLGEKYGDMMLSV	LGEKYGDMM	3	0.2965	2022.6	85.00	0.41	tr_D0A564_
DRB1_0101	205	AKTDSAGRWHLCGIL	TDSAGRWHL	2	0.2917	2129.7	85.00	0.37	tr_D0A564_
DRB1_0101	206	TLVDKRSGEKFSVTK	VDKRSGEKF	2	0.2916	2131.6	85.00	0.38	tr_D0A564_
DRB1_0101	207	VAKTDSAGRWHLCGI	TDSAGRWHL	3	0.2905	2157.3	85.00	0.41	tr_D0A564_
DRB1_0101	208	TGTLTLNKMEIQEQC	LTLNKMEIQ	3	0.2901	2166.6	85.00	0.35	tr_D0A564_
DRB1_0101	209	NILTAEKLPKVDVND	LTAEKLPKV	2	0.2881	2214.6	85.00	0.54	tr_D0A564_
DRB1_0101	210	TMVLGAADLDECDNY	MVLGAADLD	1	0.2865	2253.2	85.00	0.44	tr_D0A564_
DRB1_0101	211	MLCSDKTGTLTLNKM	CSDKTGTLT	2	0.2848	2295.7	85.00	0.25	tr_D0A564_
DRB1_0101	212	GTLTLNKMEIQEQCF	LTLNKMEIQ	2	0.2839	2316.8	85.00	0.37	tr_D0A564_
DRB1_0101	213	ATLVDKRSGEKFSVT	VDKRSGEKF	3	0.2826	2349.0	85.00	0.47	tr_D0A564_
DRB1_0101	214	KMEIQEQCFTFEKGH	EIQEQCFTF	2	0.2803	2409.7	85.00	0.35	tr_D0A564_
DRB1_0101	215	AMTGDGVNDAPALKR	VNDAPALKR	6	0.2800	2418.2	85.00	0.31	tr_D0A564_
DRB1_0101	216	HVIIEMVHNQDEIND	IEMVHNQDE	3	0.2790	2443.8	85.00	0.28	tr_D0A564_
DRB1_0101	217	LVDKRSGEKFSVTKG	KRSGEKFSV	3	0.2774	2486.3	85.00	0.39	tr_D0A564_
DRB1_0101	218	LCSDKTGTLTLNKME	TGTLTLNKM	5	0.2749	2555.3	90.00	0.38	tr_D0A564_
DRB1_0101	219	NMLCSDKTGTLTLNK	CSDKTGTLT	3	0.2743	2571.8	90.00	0.28	tr_D0A564_
DRB1_0101	220	PDTKETIRRSRQYGV	IRRSRQYGV	6	0.2731	2604.0	90.00	0.56	tr_D0A564_
DRB1_0101	221	AAKWREPPRDALDTM	WREPPRDAL	3	0.2703	2685.1	90.00	0.61	tr_D0A564_
DRB1_0101	222	LAAKWREPPRDALDT	WREPPRDAL	4	0.2688	2728.8	90.00	0.55	tr_D0A564_
DRB1_0101	223	CSDKTGTLTLNKMEI	TGTLTLNKM	4	0.2681	2749.9	90.00	0.52	tr_D0A564_
DRB1_0101	224	KVDVNDMPSDLGEKY	VNDMPSDLG	3	0.2644	2860.5	90.00	0.56	tr_D0A564_
DRB1_0101	225	MPSDLGEKYGDMMLS	LGEKYGDMM	4	0.2625	2921.9	90.00	0.44	tr_D0A564_
DRB1_0101	226	AKWREPPRDALDTMV	WREPPRDAL	2	0.2537	3213.2	90.00	0.56	tr_D0A564_

DRB1_0101	227	ALAAKWREPPRDALD	WREPPRDAL	5	0.2530	3236.4	90.00	0.50	tr_D0A564_
DRB1_0101	228	LSALAAKWREPPRDA	LSALAAKWR	0	0.2515	3288.6	90.00	0.49	tr_D0A564_
DRB1_0101	229	KWREPPRDALDTMVL	WREPPRDAL	1	0.2508	3316.2	90.00	0.44	tr_D0A564_
DRB1_0101	230	EINDSVVDIIDKLAS	VVDIIDKLA	5	0.2500	3342.9	90.00	0.34	tr_D0A564_
DRB1_0101	231	VDKRSGEKFSVTKGA	KRSGEKFSV	2	0.2487	3390.8	90.00	0.34	tr_D0A564_
DRB1_0101	232	PKVDVNDMPSDLGEK	VNDMPSDLG	4	0.2478	3422.8	90.00	0.51	tr_D0A564_
DRB1_0101	233	AATLVDKRSGEKFSV	VDKRSGEKF	4	0.2440	3568.6	90.00	0.58	tr_D0A564_
DRB1_0101	234	VDVNDMPSDLGEKYG	VNDMPSDLG	2	0.2371	3843.7	95.00	0.52	tr_D0A564_
DRB1_0101	235	CAMTGDGVNDAPALK	MTGDGVNDA	2	0.2357	3901.6	95.00	0.33	tr_D0A564_
DRB1_0101	236	SALAAKWREPPRDAL	WREPPRDAL	6	0.2357	3905.1	95.00	0.38	tr_D0A564_
DRB1_0101	237	ILTAEKLPKVDVNDM	LTAEKLPKV	1	0.2342	3969.2	95.00	0.55	tr_D0A564_
DRB1_0101	238	REPPRDALDTMVLGA	RDALDTMVL	4	0.2329	4024.2	95.00	0.42	tr_D0A564_
DRB1_0101	239	VIIEMVHNQDEINDS	IEMVHNQDE	2	0.2298	4162.4	95.00	0.34	tr_D0A564_
DRB1_0101	240	WREPPRDALDTMVLG	RDALDTMVL	5	0.2281	4235.7	95.00	0.34	tr_D0A564_
DRB1_0101	241	DMPSDLGEKYGDMML	LGEKYGDMM	5	0.2224	4506.5	95.00	0.52	tr_D0A564_
DRB1_0101	242	DKRSGEKFSVTKGAP	KRSGEKFSV	1	0.2224	4508.0	95.00	0.35	tr_D0A564_
DRB1_0101	243	DEINDSVVDIIDKLA	SVVDIIDKL	5	0.2224	4509.4	95.00	0.31	tr_D0A564_
DRB1_0101	244	DLDECDNYEQLEFVP	DNYEQLEFV	5	0.2201	4623.1	95.00	0.44	tr_D0A564_
DRB1_0101	245	MEIQEQCFTFEKGHD	EIQEQCFTF	1	0.2199	4631.3	95.00	0.40	tr_D0A564_
DRB1_0101	246	LPKVDVNDMPSDLGE	VNDMPSDLG	5	0.2165	4804.9	95.00	0.44	tr_D0A564_
DRB1_0101	247	IIEMVHNQDEINDSV	MVHNQDEIN	3	0.2073	5309.9	95.00	0.29	tr_D0A564_
DRB1_0101	248	ADLDECDNYEQLEFV	DNYEQLEFV	6	0.2051	5437.1	95.00	0.41	tr_D0A564_
DRB1_0101	249	DVNDMPSDLGEKYGD	VNDMPSDLG	1	0.2023	5602.0	95.00	0.54	tr_D0A564_
DRB1_0101	250	TAATLVDKRSGEKFS	VDKRSGEKF	5	0.2013	5664.3	95.00	0.51	tr_D0A564_
DRB1_0101	251	QDEINDSVVDIIDKL	INDSVVDII	3	0.2002	5731.0	95.00	0.32	tr_D0A564_
DRB1_0101	252	IEMVHNQDEINDSVV	VHNQDEIND	3	0.1935	6159.6	95.00	0.27	tr_D0A564_
DRB1_0101	253	KLPKVDVNDMPSDLG	DVNDMPSDL	5	0.1922	6247.2	100.00	0.35	tr_D0A564_
DRB1_0101	254	NQDEINDSVVDIIDK	INDSVVDII	4	0.1833	6877.8	100.00	0.32	tr_D0A564_
DRB1_0101	255	VHNQDEINDSVVDII	DEINDSVVD	4	0.1816	7006.6	100.00	0.21	tr_D0A564_
DRB1_0101	256	MVHNQDEINDSVVDI	VHNQDEIND	1	0.1799	7142.5	100.00	0.20	tr_D0A564_
DRB1_0101	257	EMVHNQDEINDSVVD	VHNQDEIND	2	0.1796	7164.0	100.00	0.30	tr_D0A564_
DRB1_0101	258	RTAATLVDKRSGEKF	VDKRSGEKF	6	0.1784	7251.9	100.00	0.27	tr_D0A564_
DRB1_0101	259	HNQDEINDSVVDIID	INDSVVDII	5	0.1743	7584.8	100.00	0.31	tr_D0A564_
DRB1_0101	260	KRTAATLVDKRSGEK	TAATLVDKR	2	0.1719	7784.3	100.00	0.32	tr_D0A564_
DRB1_0101	261	NDMPSDLGEKYGDMM	LGEKYGDMM	6	0.1705	7902.1	100.00	0.41	tr_D0A564_
DRB1_0101	262	VNDMPSDLGEKYGDM	VNDMPSDLG	0	0.1698	7965.2	100.00	0.37	tr_D0A564_
DRB1_0101	263	LTAEKLPKVDVNDMP	LTAEKLPKV	0	0.1658	8311.5	100.00	0.38	tr_D0A564_
DRB1_0101	264	RPDTKETIRRSRQYG	DTKETIRRS	2	0.1609	8767.3	100.00	0.41	tr_D0A564_
DRB1_0101	265	MVLGAADLDECDNYE	MVLGAADLD	0	0.1591	8940.7	100.00	0.42	tr_D0A564_
DRB1_0101	266	PRPDTKETIRRSRQY	DTKETIRRS	3	0.1567	9175.2	100.00	0.44	tr_D0A564_
DRB1_0101	267	PPRPDTKETIRRSRQ	DTKETIRRS	4	0.1420	10759.1	100.00	0.46	tr_D0A564_
DRB1_0101	268	TFLDPPRPDTKETIR	FLDPPRPDT	1	0.1394	11062.9	100.00	0.36	tr_D0A564_
DRB1_0101	269	AADLDECDNYEQLEF	DECDNYEQL	4	0.1378	11261.7	100.00	0.38	tr_D0A564_
DRB1_0101	270	DPPRPDTKETIRRSR	DTKETIRRS	5	0.1295	12317.8	100.00	0.44	tr_D0A564_
DRB1_0101	271	EKLPKVDVNDMPSDL	DVNDMPSDL	6	0.1275	12580.0	100.00	0.31	tr_D0A564_
DRB1_0101	272	GAADLDECDNYEQLE	DECDNYEQL	5	0.1195	13719.8	100.00	0.37	tr_D0A564_
DRB1_0101	273	FLDPPRPDTKETIRR	LDPPRPDTK	1	0.1152	14371.4	100.00	0.25	tr_D0A564_
DRB1_0101	274	TAEKLPKVDVNDMPS	AEKLPKVDV	1	0.1136	14621.2	100.00	0.42	tr_D0A564_
DRB1_0101	275	LGAADLDECDNYEQL	LDECDNYEQ	5	0.1134	14652.9	100.00	0.31	tr_D0A564_
DRB1_0101	276	LDPPRPDTKETIRRS	PRPDTKETI	3	0.1114	14979.6	100.00	0.32	tr_D0A564_

DRB1_0101	277	AEKLPKVDVNDMPSD	LPKVDVNDM	3	0.0954	17814.1	100.00	0.34	tr_D0A564_
DRB1_0101	278	VLGAADLDECDNYEQ	LGAADLDEC	1	0.0914	18589.9	100.00	0.32	tr_D0A564_

Allele: DRB1_0101. Number of high binders 0. Number of weak binders 14. Number of peptides 278

Allele	pos	peptide	core	Of	1-log50k(aff)	affinity(nM)	%Rank	Relia	Identity	Bind Level
DRB1_0101	1	VDTFEPATTVYKDHL	FEPATTVYK	3	0.6542	42.2	15.00	0.72	tr_D0A4W6_	
DRB1_0101	2	SVDTFEPATTVYKDH	FEPATTVYK	4	0.6451	46.5	16.00	0.62	tr_D0A4W6_	
DRB1_0101	3	ESVDTFEPATTVYKD	FEPATTVYK	5	0.6225	59.4	19.00	0.56	tr_D0A4W6_	
DRB1_0101	4	DTFEPATTVYKDHLA	FEPATTVYK	2	0.6130	65.9	20.00	0.80	tr_D0A4W6_	
DRB1_0101	5	LSLRQKLQATIPTRH	QKLQATIPT	4	0.5936	81.3	24.00	0.44	tr_D0A4W6_	
DRB1_0101	6	SLRQKLQATIPTRHP	QKLQATIPT	3	0.5857	88.4	25.00	0.44	tr_D0A4W6_	
DRB1_0101	7	YESVDTFEPATTVYK	FEPATTVYK	6	0.5823	91.8	25.00	0.42	tr_D0A4W6_	
DRB1_0101	8	LRQKLQATIPTRHPR	LQATIPTRH	4	0.5766	97.6	26.00	0.39	tr_D0A4W6_	
DRB1_0101	9	TNRFSTNMPLLEERH	FSTNMPLLE	3	0.5686	106.4	28.00	0.60	tr_D0A4W6_	
DRB1_0101	10	CTNRFSTNMPLLEER	FSTNMPLLE	4	0.5663	109.2	28.00	0.56	tr_D0A4W6_	
DRB1_0101	11	RQKLQATIPTRHPRF	LQATIPTRH	3	0.5538	124.9	31.00	0.47	tr_D0A4W6_	
DRB1_0101	12	RCTNRFSTNMPLLEE	NRFSTNMPL	3	0.5493	131.2	31.00	0.44	tr_D0A4W6_	
DRB1_0101	13	TFEPATTVYKDHLAG	FEPATTVYK	1	0.5448	137.6	32.00	0.87	tr_D0A4W6_	
DRB1_0101	14	DLSLRQKLQATIPTR	QKLQATIPT	5	0.5411	143.3	33.00	0.44	tr_D0A4W6_	
DRB1_0101	15	NRFSTNMPLLEERHD	FSTNMPLLE	2	0.5206	178.9	37.00	0.62	tr_D0A4W6_	
DRB1_0101	16	RRCTNRFSTNMPLLE	NRFSTNMPL	4	0.5132	193.9	38.00	0.51	tr_D0A4W6_	
DRB1_0101	17	ERHDLSLRQKLQATI	HDLSLRQKL	2	0.5033	215.8	40.00	0.44	tr_D0A4W6_	
DRB1_0101	18	RYESVDTFEPATTVY	DTFEPATTV	5	0.5008	221.8	41.00	0.39	tr_D0A4W6_	
DRB1_0101	19	RHDLSLRQKLQATIP	HDLSLRQKL	1	0.4988	226.6	41.00	0.37	tr_D0A4W6_	
DRB1_0101	20	HDLSLRQKLQATIPT	LRQKLQATI	4	0.4908	247.1	43.00	0.25	tr_D0A4W6_	
DRB1_0101	21	QKLQATIPTRHPRFY	LQATIPTRH	2	0.4757	291.0	46.00	0.51	tr_D0A4W6_	
DRB1_0101	22	EERHDLSLRQKLQAT	HDLSLRQKL	3	0.4729	299.9	47.00	0.52	tr_D0A4W6_	
DRB1_0101	23	ERYESVDTFEPATTV	YESVDTFEP	2	0.4557	361.1	50.00	0.51	tr_D0A4W6_	
DRB1_0101	24	LEERHDLSLRQKLQA	HDLSLRQKL	4	0.4440	410.0	55.00	0.54	tr_D0A4W6_	
DRB1_0101	25	VRRCTNRFSTNMPLL	NRFSTNMPL	5	0.4417	420.4	55.00	0.60	tr_D0A4W6_	
DRB1_0101	26	ATTVYKDHLAGRSQE	YKDHLAGRS	4	0.4406	425.0	55.00	0.68	tr_D0A4W6_	
DRB1_0101	27	RFSTNMPLLEERHDL	FSTNMPLLE	1	0.4255	500.8	60.00	0.63	tr_D0A4W6_	
DRB1_0101	28	PATTVYKDHLAGRSQ	YKDHLAGRS	5	0.4214	523.2	60.00	0.66	tr_D0A4W6_	
DRB1_0101	29	LLEERHDLSLRQKLQ	HDLSLRQKL	5	0.4165	552.0	60.00	0.46	tr_D0A4W6_	
DRB1_0101	30	TTVYKDHLAGRSQEP	YKDHLAGRS	3	0.4163	553.3	60.00	0.74	tr_D0A4W6_	
DRB1_0101	31	PRFYSSEERRKMERY	YSSEERRKM	3	0.4141	566.1	60.00	0.58	tr_D0A4W6_	
DRB1_0101	32	HPRFYSSEERRKMER	YSSEERRKM	4	0.4089	599.1	60.00	0.51	tr_D0A4W6_	
DRB1_0101	33	SSQCRVPLLGAGSSD	RVPLLGAGS	4	0.4061	617.9	65.00	0.41	tr_D0A4W6_	
DRB1_0101	34	MERYESVDTFEPATT	YESVDTFEP	3	0.4035	635.3	65.00	0.77	tr_D0A4W6_	
DRB1_0101	35	KLQATIPTRHPRFYS	LQATIPTRH	1	0.3957	691.4	65.00	0.54	tr_D0A4W6_	
DRB1_0101	36	QSSQCRVPLLGAGSS	RVPLLGAGS	5	0.3953	693.8	65.00	0.41	tr_D0A4W6_	
DRB1_0101	37	SQCRVPLLGAGSSDD	RVPLLGAGS	3	0.3937	706.6	65.00	0.44	tr_D0A4W6_	
DRB1_0101	38	RFYSSEERRKMERYE	YSSEERRKM	2	0.3917	721.7	65.00	0.63	tr_D0A4W6_	
DRB1_0101	39	TVYKDHLAGRSQEPR	YKDHLAGRS	2	0.3891	742.0	65.00	0.72	tr_D0A4W6_	
DRB1_0101	40	PLLEERHDLSLRQKL	HDLSLRQKL	6	0.3876	754.3	65.00	0.32	tr_D0A4W6_	

DRB1_0101	41	FEPATTVYKDHLAGR	FEPATTVYK	0	0.3817	804.0	70.00	0.67	tr_D0A4W6_
DRB1_0101	42	QCRVPLLGAGSSDDC	RVPLLGAGS	2	0.3791	827.0	70.00	0.42	tr_D0A4W6_
DRB1_0101	43	RHPRFYSSEERRKME	YSSEERRKM	5	0.3712	900.9	70.00	0.47	tr_D0A4W6_
DRB1_0101	44	KMERYESVDTFEPAT	YESVDTFEP	4	0.3688	924.2	70.00	0.82	tr_D0A4W6_
DRB1_0101	45	AVRRCTNRFSTNMPL	NRFSTNMPL	6	0.3653	959.9	70.00	0.38	tr_D0A4W6_
DRB1_0101	46	CRVPLLGAGSSDDCD	RVPLLGAGS	1	0.3440	1208.7	75.00	0.38	tr_D0A4W6_
DRB1_0101	47	MPLLEERHDLSLRQK	LLEERHDLS	2	0.3390	1276.9	75.00	0.32	tr_D0A4W6_
DRB1_0101	48	RKMERYESVDTFEPA	YESVDTFEP	5	0.3375	1296.9	75.00	0.69	tr_D0A4W6_
DRB1_0101	49	NMPLLEERHDLSLRQ	LLEERHDLS	3	0.3373	1300.3	75.00	0.37	tr_D0A4W6_
DRB1_0101	50	EPATTVYKDHLAGRS	YKDHLAGRS	6	0.3368	1306.9	75.00	0.55	tr_D0A4W6_
DRB1_0101	51	GQSSQCRVPLLGAGS	RVPLLGAGS	6	0.3317	1382.0	80.00	0.31	tr_D0A4W6_
DRB1_0101	52	GDNNANEPVAAVRRC	NANEPVAAV	3	0.3309	1394.1	80.00	0.50	tr_D0A4W6_
DRB1_0101	53	TRHPRFYSSEERRKM	FYSSEERRK	5	0.3274	1446.9	80.00	0.47	tr_D0A4W6_
DRB1_0101	54	FSTNMPLLEERHDLS	FSTNMPLLE	0	0.3270	1453.8	80.00	0.31	tr_D0A4W6_
DRB1_0101	55	TNMPLLEERHDLSLR	LLEERHDLS	4	0.3263	1464.2	80.00	0.35	tr_D0A4W6_
DRB1_0101	56	DGDNNANEPVAAVRR	NANEPVAAV	4	0.3253	1479.7	80.00	0.50	tr_D0A4W6_
DRB1_0101	57	VAAVRRCTNRFSTNM	VRRCTNRFS	3	0.3231	1515.9	80.00	0.64	tr_D0A4W6_
DRB1_0101	58	DNNANEPVAAVRRCT	NANEPVAAV	2	0.3228	1520.7	80.00	0.49	tr_D0A4W6_
DRB1_0101	59	MKGQSSQCRVPLLGA	SSQCRVPLL	4	0.3222	1530.5	80.00	0.29	tr_D0A4W6_
DRB1_0101	60	LQATIPTRHPRFYSS	TIPTRHPRF	3	0.3090	1765.3	80.00	0.26	tr_D0A4W6_
DRB1_0101	61	PVAAVRRCTNRFSTN	VRRCTNRFS	4	0.3084	1777.6	80.00	0.61	tr_D0A4W6_
DRB1_0101	62	NNANEPVAAVRRCTN	NANEPVAAV	1	0.3082	1780.4	80.00	0.41	tr_D0A4W6_
DRB1_0101	63	AAVRRCTNRFSTNMP	VRRCTNRFS	2	0.3057	1829.4	85.00	0.62	tr_D0A4W6_
DRB1_0101	64	KGQSSQCRVPLLGAG	SSQCRVPLL	3	0.3026	1892.6	85.00	0.42	tr_D0A4W6_
DRB1_0101	65	FYSSEERRKMERYES	YSSEERRKM	1	0.2996	1954.5	85.00	0.64	tr_D0A4W6_
DRB1_0101	66	RVPLLGAGSSDDCDG	VPLLGAGSS	1	0.2951	2052.9	85.00	0.40	tr_D0A4W6_
DRB1_0101	67	STNMPLLEERHDLSL	LLEERHDLS	5	0.2921	2119.4	85.00	0.28	tr_D0A4W6_
DRB1_0101	68	EPVAAVRRCTNRFST	VRRCTNRFS	5	0.2903	2161.0	85.00	0.50	tr_D0A4W6_
DRB1_0101	69	CDGDNNANEPVAAVR	NANEPVAAV	5	0.2761	2520.7	85.00	0.51	tr_D0A4W6_
DRB1_0101	70	RRKMERYESVDTFEP	YESVDTFEP	6	0.2755	2538.0	90.00	0.35	tr_D0A4W6_
DRB1_0101	71	NEPVAAVRRCTNRFS	VRRCTNRFS	6	0.2601	2997.1	90.00	0.27	tr_D0A4W6_
DRB1_0101	72	NANEPVAAVRRCTNR	NANEPVAAV	0	0.2599	3004.5	90.00	0.26	tr_D0A4W6_
DRB1_0101	73	QATIPTRHPRFYSSE	TIPTRHPRF	2	0.2591	3030.1	90.00	0.32	tr_D0A4W6_
DRB1_0101	74	ERRKMERYESVDTFE	RKMERYESV	2	0.2465	3471.3	90.00	0.44	tr_D0A4W6_
DRB1_0101	75	ANEPVAAVRRCTNRF	PVAAVRRCT	3	0.2415	3666.4	90.00	0.34	tr_D0A4W6_
DRB1_0101	76	EERRKMERYESVDTF	RKMERYESV	3	0.2353	3921.4	95.00	0.49	tr_D0A4W6_
DRB1_0101	77	ATIPTRHPRFYSSEE	TIPTRHPRF	1	0.2300	4151.7	95.00	0.31	tr_D0A4W6_
DRB1_0101	78	YSSEERRKMERYESV	YSSEERRKM	0	0.2198	4636.9	95.00	0.48	tr_D0A4W6_
DRB1_0101	79	PTRHPRFYSSEERRK	FYSSEERRK	6	0.2158	4839.4	95.00	0.49	tr_D0A4W6_
DRB1_0101	80	DCDGDNNANEPVAAV	NANEPVAAV	6	0.2079	5271.0	95.00	0.42	tr_D0A4W6_
DRB1_0101	81	VPLLGAGSSDDCDGD	LLGAGSSDD	2	0.1955	6029.7	95.00	0.38	tr_D0A4W6_
DRB1_0101	82	SEERRKMERYESVDT	RKMERYESV	4	0.1910	6328.0	100.00	0.56	tr_D0A4W6_
DRB1_0101	83	TIPTRHPRFYSSEER	PTRHPRFYS	2	0.1851	6744.7	100.00	0.31	tr_D0A4W6_
DRB1_0101	84	IPTRHPRFYSSEERR	PTRHPRFYS	1	0.1701	7941.0	100.00	0.31	tr_D0A4W6_
DRB1_0101	85	SSEERRKMERYESVD	RKMERYESV	5	0.1611	8749.3	100.00	0.59	tr_D0A4W6_
DRB1_0101	86	PLLGAGSSDDCDGDN	LLGAGSSDD	1	0.1277	12551.2	100.00	0.57	tr_D0A4W6_
DRB1_0101	87	DDCDGDNNANEPVAA	NNANEPVAA	6	0.1094	15300.2	100.00	0.34	tr_D0A4W6_
DRB1_0101	88	LLGAGSSDDCDGDNN	LLGAGSSDD	0	0.0819	20610.2	100.00	0.41	tr_D0A4W6_
DRB1_0101	89	SDDCDGDNNANEPVA	GDNNANEPV	5	0.0734	22598.5	100.00	0.34	tr_D0A4W6_
DRB1_0101	90	SSDDCDGDNNANEPV	CDGDNNANE	4	0.0598	26177.8	100.00	0.22	tr_D0A4W6_

DRB1_0101	91	GSSDDCDGDNNANEP	CDGDNNANE	5	0.0478	29803.4	100.00	0.25	tr_D0A4W6_
DRB1_0101	92	AGSSDDCDGDNNANE	DCDGDNNAN	5	0.0466	30200.4	100.00	0.22	tr_D0A4W6_
DRB1_0101	93	LGAGSSDDCDGDNNA	LGAGSSDDC	0	0.0460	30391.8	100.00	0.30	tr_D0A4W6_
DRB1_0101	94	GAGSSDDCDGDNNAN	SDDCDGDNN	4	0.0426	31523.5	100.00	0.26	tr_D0A4W6_

Allele: DRB1_0101. Number of high binders 0. Number of weak binders 0. Number of peptides 94

Allele	pos	peptide	core	Of	1-log50k(aff)	affinity(nM)	%Rank	Relia	Identity	Bind Level
DRB1_0101	1	QVWIVLHSYASLAFA	VLHSYASLA	4	0.8242	6.7	1.20	0.44	tr_D0A4W6_	WB
DRB1_0101	2	VWIVLHSYASLAFAN	VLHSYASLA	3	0.8213	6.9	1.30	0.51	tr_D0A4W6_	WB
DRB1_0101	3	DSALQTLLSLRTETM	LQTLLSLRT	3	0.8197	7.0	1.40	0.55	tr_D0A4W6_	WB
DRB1_0101	4	QQVWIVLHSYASLAF	WIVLHSYAS	3	0.8067	8.1	1.80	0.53	tr_D0A4W6_	WB
DRB1_0101	5	WIVLHSYASLAFANA	VLHSYASLA	2	0.7966	9.0	2.50	0.49	tr_D0A4W6_	WB
DRB1_0101	6	ADSALQTLLSLRTET	LQTLLSLRT	4	0.7865	10.1	3.00	0.69	tr_D0A4W6_	WB
DRB1_0101	7	EQQVWIVLHSYASLA	WIVLHSYAS	4	0.7666	12.5	4.00	0.63	tr_D0A4W6_	WB
DRB1_0101	8	NADSALQTLLSLRTE	LQTLLSLRT	5	0.7643	12.8	4.00	0.65	tr_D0A4W6_	WB
DRB1_0101	9	IVLHSYASLAFANAD	VLHSYASLA	1	0.7484	15.2	5.00	0.51	tr_D0A4W6_	WB
DRB1_0101	10	ANADSALQTLLSLRT	LQTLLSLRT	6	0.7186	21.0	7.50	0.55	tr_D0A4W6_	WB
DRB1_0101	11	VLHSYASLAFANADS	YASLAFANA	4	0.6954	27.0	9.50	0.38	tr_D0A4W6_	WB
DRB1_0101	12	HEQQVWIVLHSYASL	WIVLHSYAS	5	0.6895	28.8	10.00	0.78	tr_D0A4W6_	
DRB1_0101	13	HSYASLAFANADSAL	YASLAFANA	2	0.6813	31.4	11.00	0.46	tr_D0A4W6_	
DRB1_0101	14	YASLAFANADSALQT	LAFANADSA	3	0.6787	32.3	12.00	0.50	tr_D0A4W6_	
DRB1_0101	15	LHSYASLAFANADSA	YASLAFANA	3	0.6776	32.7	12.00	0.53	tr_D0A4W6_	
DRB1_0101	16	SYASLAFANADSALQ	LAFANADSA	4	0.6765	33.1	12.00	0.44	tr_D0A4W6_	
DRB1_0101	17	ASLAFANADSALQTL	FANADSALQ	4	0.6705	35.3	12.00	0.45	tr_D0A4W6_	
DRB1_0101	18	SLAFANADSALQTLL	FANADSALQ	3	0.6651	37.5	13.00	0.56	tr_D0A4W6_	
DRB1_0101	19	GANGFFCAAPNNTVI	FFCAAPNNT	4	0.6584	40.3	14.00	0.56	tr_D0A4W6_	
DRB1_0101	20	ANGFFCAAPNNTVIP	FFCAAPNNT	3	0.6562	41.3	14.00	0.52	tr_D0A4W6_	
DRB1_0101	21	NGFFCAAPNNTVIPV	FCAAPNNTV	3	0.6546	42.0	14.00	0.45	tr_D0A4W6_	
DRB1_0101	22	SVISFYGANGFFCAA	FYGANGFFC	4	0.6538	42.4	15.00	0.63	tr_D0A4W6_	
DRB1_0101	23	LAFANADSALQTLLS	FANADSALQ	2	0.6488	44.7	15.00	0.53	tr_D0A4W6_	
DRB1_0101	24	AFANADSALQTLLSL	FANADSALQ	1	0.6368	50.9	17.00	0.44	tr_D0A4W6_	
DRB1_0101	25	VSVISFYGANGFFCA	FYGANGFFC	5	0.6320	53.6	18.00	0.53	tr_D0A4W6_	
DRB1_0101	26	FANADSALQTLLSLR	DSALQTLLS	4	0.6240	58.5	19.00	0.55	tr_D0A4W6_	
DRB1_0101	27	GFFCAAPNNTVIPVT	FCAAPNNTV	2	0.6202	60.9	19.00	0.50	tr_D0A4W6_	
DRB1_0101	28	VISFYGANGFFCAAP	FYGANGFFC	3	0.6140	65.1	20.00	0.69	tr_D0A4W6_	
DRB1_0101	29	VHEQQVWIVLHSYAS	WIVLHSYAS	6	0.6101	68.0	21.00	0.71	tr_D0A4W6_	
DRB1_0101	30	YGANGFFCAAPNNTV	FFCAAPNNT	5	0.5830	91.1	25.00	0.57	tr_D0A4W6_	
DRB1_0101	31	ISFYGANGFFCAAPN	FYGANGFFC	2	0.5751	99.2	27.00	0.71	tr_D0A4W6_	
DRB1_0101	32	DVSVISFYGANGFFC	FYGANGFFC	6	0.5672	108.0	28.00	0.38	tr_D0A4W6_	
DRB1_0101	33	LTTLRWGNSSAVSPH	LRWGNSSAV	3	0.5668	108.6	28.00	0.71	tr_D0A4W6_	
DRB1_0101	34	GGLTTLRWGNSSAVS	LRWGNSSAV	5	0.5646	111.2	29.00	0.67	tr_D0A4W6_	
DRB1_0101	35	GLTTLRWGNSSAVSP	LRWGNSSAV	4	0.5616	114.8	29.00	0.68	tr_D0A4W6_	
DRB1_0101	36	FFCAAPNNTVIPVTA	FCAAPNNTV	1	0.5549	123.5	30.00	0.57	tr_D0A4W6_	
DRB1_0101	37	TTLRWGNSSAVSPHF	LRWGNSSAV	2	0.5355	152.2	34.00	0.78	tr_D0A4W6_	
DRB1_0101	38	TATVHEQQVWIVLHS	VHEQQVWIV	3	0.5246	171.3	36.00	0.61	tr_D0A4W6_	

DRB1_0101	39	SFYGANGFFCAAPNN	FYGANGFFC	1	0.5227	175.0	36.00	0.73	tr_D0A4W6_
DRB1_0101	40	VTATVHEQQVWIVLH	VHEQQVWIV	4	0.5184	183.3	37.00	0.57	tr_D0A4W6_
DRB1_0101	41	FYGANGFFCAAPNNT	FFCAAPNNT	6	0.5184	183.3	37.00	0.42	tr_D0A4W6_
DRB1_0101	42	RDVSVISFYGANGFF	VISFYGANG	4	0.5150	190.2	38.00	0.42	tr_D0A4W6_
DRB1_0101	43	TLRWGNSSAVSPHFV	LRWGNSSAV	1	0.5093	202.3	39.00	0.64	tr_D0A4W6_
DRB1_0101	44	SPHFVDAQSGPCVAV	VDAQSGPCV	4	0.5041	213.9	40.00	0.49	tr_D0A4W6_
DRB1_0101	45	ATVHEQQVWIVLHSY	VHEQQVWIV	2	0.5014	220.4	41.00	0.55	tr_D0A4W6_
DRB1_0101	46	AGGLTTLRWGNSSAV	LRWGNSSAV	6	0.4914	245.5	43.00	0.45	tr_D0A4W6_
DRB1_0101	47	PVTATVHEQQVWIVL	VHEQQVWIV	5	0.4883	253.7	43.00	0.55	tr_D0A4W6_
DRB1_0101	48	PHFVDAQSGPCVAVP	VDAQSGPCV	3	0.4852	262.3	44.00	0.51	tr_D0A4W6_
DRB1_0101	49	SRDVSVISFYGANGF	VISFYGANG	5	0.4802	277.0	45.00	0.41	tr_D0A4W6_
DRB1_0101	50	HFVDAQSGPCVAVPQ	VDAQSGPCV	2	0.4631	333.5	49.00	0.50	tr_D0A4W6_
DRB1_0101	51	TVHEQQVWIVLHSYA	VHEQQVWIV	1	0.4568	356.8	50.00	0.49	tr_D0A4W6_
DRB1_0101	52	VSPHFVDAQSGPCVA	VDAQSGPCV	5	0.4500	384.2	55.00	0.50	tr_D0A4W6_
DRB1_0101	53	LRWGNSSAVSPHFVD	LRWGNSSAV	0	0.4358	448.1	55.00	0.40	tr_D0A4W6_
DRB1_0101	54	FCAAPNNTVIPVTAT	FCAAPNNTV	0	0.4210	525.5	60.00	0.56	tr_D0A4W6_
DRB1_0101	55	SGPCVAVPQTLSDSR	CVAVPQTLS	3	0.4137	568.6	60.00	0.61	tr_D0A4W6_
DRB1_0101	56	DSRDVSVISFYGANG	DVSVISFYG	3	0.4104	589.7	60.00	0.36	tr_D0A4W6_
DRB1_0101	57	FVDAQSGPCVAVPQT	VDAQSGPCV	1	0.4093	596.8	60.00	0.52	tr_D0A4W6_
DRB1_0101	58	QSGPCVAVPQTLSDS	CVAVPQTLS	4	0.4076	607.4	65.00	0.51	tr_D0A4W6_
DRB1_0101	59	IPVTATVHEQQVWIV	VHEQQVWIV	6	0.4024	642.6	65.00	0.47	tr_D0A4W6_
DRB1_0101	60	PNNTVIPVTATVHEQ	VIPVTATVH	4	0.3914	724.2	65.00	0.34	tr_D0A4W6_
DRB1_0101	61	APNNTVIPVTATVHE	NTVIPVTAT	3	0.3891	742.3	65.00	0.38	tr_D0A4W6_
DRB1_0101	62	AQSGPCVAVPQTLSD	CVAVPQTLS	5	0.3857	770.6	65.00	0.47	tr_D0A4W6_
DRB1_0101	63	NNTVIPVTATVHEQQ	VIPVTATVH	3	0.3841	783.8	70.00	0.40	tr_D0A4W6_
DRB1_0101	64	GPCVAVPQTLSDSRD	CVAVPQTLS	2	0.3755	860.0	70.00	0.62	tr_D0A4W6_
DRB1_0101	65	DAQSGPCVAVPQTLS	CVAVPQTLS	6	0.3709	904.4	70.00	0.36	tr_D0A4W6_
DRB1_0101	66	RWGNSSAVSPHFVDA	SSAVSPHFV	4	0.3698	914.4	70.00	0.51	tr_D0A4W6_
DRB1_0101	67	AAPNNTVIPVTATVH	NTVIPVTAT	4	0.3675	937.3	70.00	0.41	tr_D0A4W6_
DRB1_0101	68	TAGGLTTLRWGNSSA	LTTLRWGNS	4	0.3631	983.9	70.00	0.50	tr_D0A4W6_
DRB1_0101	69	WGNSSAVSPHFVDAQ	SSAVSPHFV	3	0.3572	1048.7	75.00	0.61	tr_D0A4W6_
DRB1_0101	70	SDSRDVSVISFYGAN	DVSVISFYG	4	0.3546	1078.3	75.00	0.44	tr_D0A4W6_
DRB1_0101	71	AVSPHFVDAQSGPCV	VDAQSGPCV	6	0.3515	1114.7	75.00	0.46	tr_D0A4W6_
DRB1_0101	72	GNSSAVSPHFVDAQS	SSAVSPHFV	2	0.3490	1145.9	75.00	0.62	tr_D0A4W6_
DRB1_0101	73	NTVIPVTATVHEQQV	VIPVTATVH	2	0.3434	1216.7	75.00	0.46	tr_D0A4W6_
DRB1_0101	74	TTAGGLTTLRWGNSS	LTTLRWGNS	5	0.3421	1234.2	75.00	0.43	tr_D0A4W6_
DRB1_0101	75	PCVAVPQTLSDSRDV	CVAVPQTLS	1	0.3417	1239.3	75.00	0.57	tr_D0A4W6_
DRB1_0101	76	VDAQSGPCVAVPQTL	VDAQSGPCV	0	0.3369	1305.7	75.00	0.32	tr_D0A4W6_
DRB1_0101	77	NTTAGGLTTLRWGNS	AGGLTTLRW	3	0.3364	1313.3	75.00	0.25	tr_D0A4W6_
DRB1_0101	78	NSSAVSPHFVDAQSG	SSAVSPHFV	1	0.3339	1349.2	80.00	0.56	tr_D0A4W6_
DRB1_0101	79	TDNTTAGGLTTLRWG	NTTAGGLTT	2	0.3298	1410.0	80.00	0.36	tr_D0A4W6_
DRB1_0101	80	LSDSRDVSVISFYGA	DVSVISFYG	5	0.3206	1557.6	80.00	0.42	tr_D0A4W6_
DRB1_0101	81	PTDNTTAGGLTTLRW	NTTAGGLTT	3	0.3180	1601.9	80.00	0.37	tr_D0A4W6_
DRB1_0101	82	VPQTLSDSRDVSVIS	QTLSDSRDV	2	0.3147	1660.6	80.00	0.46	tr_D0A4W6_
DRB1_0101	83	DNTTAGGLTTLRWGN	NTTAGGLTT	1	0.3144	1665.8	80.00	0.32	tr_D0A4W6_
DRB1_0101	84	AVPQTLSDSRDVSVI	QTLSDSRDV	3	0.3108	1732.4	80.00	0.52	tr_D0A4W6_
DRB1_0101	85	PQTLSDSRDVSVISF	QTLSDSRDV	1	0.3091	1764.6	80.00	0.34	tr_D0A4W6_
DRB1_0101	86	CAAPNNTVIPVTATV	NTVIPVTAT	5	0.3060	1823.6	85.00	0.44	tr_D0A4W6_
DRB1_0101	87	CVAVPQTLSDSRDVS	CVAVPQTLS	0	0.2910	2146.4	85.00	0.43	tr_D0A4W6_
DRB1_0101	88	NPTDNTTAGGLTTLR	NTTAGGLTT	4	0.2851	2286.5	85.00	0.39	tr_D0A4W6_

DRB1_0101	89	TLSDSRDVSVISFYG	DVSVISFYG	6	0.2849	2292.1	85.00	0.28	tr_D0A4W6_
DRB1_0101	90	QTLSDSRDVSVISFY	LSDSRDVSV	2	0.2840	2313.4	85.00	0.31	tr_D0A4W6_
DRB1_0101	91	SSAVSPHFVDAQSGP	SSAVSPHFV	0	0.2835	2326.4	85.00	0.43	tr_D0A4W6_
DRB1_0101	92	AFGSAGWSPPSSALN	FGSAGWSPP	1	0.2752	2544.7	90.00	0.31	tr_D0A4W6_
DRB1_0101	93	TVIPVTATVHEQQVW	VIPVTATVH	1	0.2748	2556.1	90.00	0.46	tr_D0A4W6_
DRB1_0101	94	VIPVTATVHEQQVWI	TVHEQQVWI	6	0.2679	2754.1	90.00	0.25	tr_D0A4W6_
DRB1_0101	95	VAVPQTLSDSRDVSV	QTLSDSRDV	4	0.2614	2957.0	90.00	0.56	tr_D0A4W6_
DRB1_0101	96	FGSAGWSPPSSALNP	WSPPSSALN	5	0.2576	3080.6	90.00	0.33	tr_D0A4W6_
DRB1_0101	97	LNPTDNTTAGGLTTL	NTTAGGLTT	5	0.2544	3188.5	90.00	0.28	tr_D0A4W6_
DRB1_0101	98	SSALNPTDNTTAGGL	LNPTDNTTA	3	0.2518	3278.4	90.00	0.66	tr_D0A4W6_
DRB1_0101	99	GSAGWSPPSSALNPT	WSPPSSALN	4	0.2441	3565.8	90.00	0.45	tr_D0A4W6_
DRB1_0101	100	SAVSPHFVDAQSGPC	PHFVDAQSG	4	0.2382	3799.1	95.00	0.35	tr_D0A4W6_
DRB1_0101	101	SALNPTDNTTAGGLT	LNPTDNTTA	2	0.2346	3949.0	95.00	0.63	tr_D0A4W6_
DRB1_0101	102	ALNPTDNTTAGGLTT	LNPTDNTTA	1	0.2290	4195.9	95.00	0.45	tr_D0A4W6_
DRB1_0101	103	SAGWSPPSSALNPTD	WSPPSSALN	3	0.2290	4196.2	95.00	0.47	tr_D0A4W6_
DRB1_0101	104	PSSALNPTDNTTAGG	LNPTDNTTA	4	0.2275	4263.5	95.00	0.63	tr_D0A4W6_
DRB1_0101	105	PPSSALNPTDNTTAG	LNPTDNTTA	5	0.2070	5322.9	95.00	0.56	tr_D0A4W6_
DRB1_0101	106	AGWSPPSSALNPTDN	WSPPSSALN	2	0.1993	5785.3	95.00	0.47	tr_D0A4W6_
DRB1_0101	107	GWSPPSSALNPTDNT	WSPPSSALN	1	0.1719	7786.5	100.00	0.41	tr_D0A4W6_
DRB1_0101	108	SPPSSALNPTDNTTA	SSALNPTDN	3	0.1677	8143.8	100.00	0.29	tr_D0A4W6_
DRB1_0101	109	WSPPSSALNPTDNTT	SSALNPTDN	4	0.1490	9969.5	100.00	0.31	tr_D0A4W6_

Allele: DRB1_0101. Number of high binders 0. Number of weak binders 11. Number of peptides 109

Allele	pos	peptide	core	Of	1-log50k(aff)	affinity(nM)	%Rank	Relia	Identity	Bind Level
DRB1_0101	1	HGFSWHDLTTYTACS	WHDLTTYTA	4	0.5814	92.6	26.00	0.74	tr_C9ZPL1_	
DRB1_0101	2	GFSWHDLTTYTACSD	WHDLTTYTA	3	0.5686	106.5	28.00	0.76	tr_C9ZPL1_	
DRB1_0101	3	RHGFSWHDLTTYTAC	WHDLTTYTA	5	0.5503	129.8	31.00	0.69	tr_C9ZPL1_	
DRB1_0101	4	FSWHDLTTYTACSDM	WHDLTTYTA	2	0.5479	133.1	32.00	0.78	tr_C9ZPL1_	
DRB1_0101	5	LRHGFSWHDLTTYTA	WHDLTTYTA	6	0.4995	224.7	41.00	0.51	tr_C9ZPL1_	
DRB1_0101	6	SWHDLTTYTACSDMT	WHDLTTYTA	1	0.4878	255.3	44.00	0.74	tr_C9ZPL1_	
DRB1_0101	7	WFLRHGFSWHDLTTY	LRHGFSWHD	2	0.4361	446.5	55.00	0.41	tr_C9ZPL1_	
DRB1_0101	8	FLRHGFSWHDLTTYT	LRHGFSWHD	1	0.4038	633.2	65.00	0.46	tr_C9ZPL1_	
DRB1_0101	9	ACSDMTNGTCLLLAN	DMTNGTCLL	3	0.3980	674.0	65.00	0.38	tr_C9ZPL1_	
DRB1_0101	10	TACSDMTNGTCLLLA	SDMTNGTCL	3	0.3930	711.3	65.00	0.37	tr_C9ZPL1_	
DRB1_0101	11	WHDLTTYTACSDMTN	WHDLTTYTA	0	0.3872	757.9	65.00	0.57	tr_C9ZPL1_	
DRB1_0101	12	CSDMTNGTCLLLANP	DMTNGTCLL	2	0.3678	935.0	70.00	0.34	tr_C9ZPL1_	
DRB1_0101	13	YTACSDMTNGTCLLL	SDMTNGTCL	4	0.3490	1145.4	75.00	0.37	tr_C9ZPL1_	
DRB1_0101	14	SDMTNGTCLLLANPQ	DMTNGTCLL	1	0.3464	1178.0	75.00	0.35	tr_C9ZPL1_	
DRB1_0101	15	TYTACSDMTNGTCLL	SDMTNGTCL	5	0.2859	2267.8	85.00	0.39	tr_C9ZPL1_	
DRB1_0101	16	HDLTTYTACSDMTNG	YTACSDMTN	5	0.2420	3644.5	90.00	0.34	tr_C9ZPL1_	
DRB1_0101	17	TTYTACSDMTNGTCL	YTACSDMTN	2	0.2325	4040.6	95.00	0.34	tr_C9ZPL1_	
DRB1_0101	18	DLTTYTACSDMTNGT	YTACSDMTN	4	0.2294	4179.9	95.00	0.43	tr_C9ZPL1_	
DRB1_0101	19	LTTYTACSDMTNGTC	YTACSDMTN	3	0.2232	4466.1	95.00	0.44	tr_C9ZPL1_	

Allele: DRB1_0101. Number of high binders 0. Number of weak binders 0. Number of peptides 19

Allele	pos	peptide	core	Of	1-log50k(aff)	affinity(nM)	%Rank	Relia	Identity	Bind Level
DRB1_0101	1	DPHVVQQAQPWSILT	VQQAQPWSI	4	0.6529	42.8	15.00	0.69	tr_C9ZPL1_	
DRB1_0101	2	PHVVQQAQPWSILTP	VQQAQPWSI	3	0.6454	46.4	16.00	0.69	tr_C9ZPL1_	
DRB1_0101	3	HVVQQAQPWSILTPT	VQQAQPWSI	2	0.6282	55.8	18.00	0.70	tr_C9ZPL1_	
DRB1_0101	4	VDPHVVQQAQPWSIL	VQQAQPWSI	5	0.6163	63.5	20.00	0.64	tr_C9ZPL1_	
DRB1_0101	5	VVQQAQPWSILTPTN	VQQAQPWSI	1	0.5550	123.3	30.00	0.75	tr_C9ZPL1_	
DRB1_0101	6	FAKLFNIVPLGVDPH	LFNIVPLGV	3	0.5473	134.1	32.00	0.41	tr_C9ZPL1_	
DRB1_0101	7	AKLFNIVPLGVDPHV	LFNIVPLGV	2	0.5207	178.7	37.00	0.40	tr_C9ZPL1_	
DRB1_0101	8	KLFNIVPLGVDPHVV	IVPLGVDPH	4	0.5092	202.4	39.00	0.34	tr_C9ZPL1_	
DRB1_0101	9	VQQAQPWSILTPTNF	VQQAQPWSI	0	0.4929	241.3	42.00	0.41	tr_C9ZPL1_	
DRB1_0101	10	GVDPHVVQQAQPWSI	VQQAQPWSI	6	0.4894	250.9	43.00	0.55	tr_C9ZPL1_	
DRB1_0101	11	LFNIVPLGVDPHVVQ	IVPLGVDPH	3	0.4796	278.7	45.00	0.41	tr_C9ZPL1_	
DRB1_0101	12	FNIVPLGVDPHVVQQ	IVPLGVDPH	2	0.4453	404.3	55.00	0.39	tr_C9ZPL1_	
DRB1_0101	13	NIVPLGVDPHVVQQA	IVPLGVDPH	1	0.4238	509.9	60.00	0.33	tr_C9ZPL1_	
DRB1_0101	14	AQPWSILTPTNFDDW	WSILTPTNF	3	0.4232	513.2	60.00	0.77	tr_C9ZPL1_	
DRB1_0101	15	QQAQPWSILTPTNFD	WSILTPTNF	5	0.4138	568.3	60.00	0.70	tr_C9ZPL1_	
DRB1_0101	16	QPWSILTPTNFDDWK	WSILTPTNF	2	0.4095	595.0	60.00	0.71	tr_C9ZPL1_	
DRB1_0101	17	QAQPWSILTPTNFDD	WSILTPTNF	4	0.4081	604.5	60.00	0.77	tr_C9ZPL1_	
DRB1_0101	18	IVPLGVDPHVVQQAQ	PLGVDPHVV	2	0.3772	844.0	70.00	0.34	tr_C9ZPL1_	
DRB1_0101	19	LGVDPHVVQQAQPWS	HVVQQAQPW	5	0.3221	1532.0	80.00	0.49	tr_C9ZPL1_	
DRB1_0101	20	PLGVDPHVVQQAQPW	VDPHVVQQA	3	0.3199	1570.2	80.00	0.31	tr_C9ZPL1_	
DRB1_0101	21	VPLGVDPHVVQQAQP	PLGVDPHVV	1	0.3114	1721.0	80.00	0.43	tr_C9ZPL1_	

Allele: DRB1_0101. Number of high binders 0. Number of weak binders 0. Number of peptides 21

Allele	pos	peptide	core	Of	1-log50k(aff)	affinity(nM)	%Rank	Relia	Identity	Bind Level
DRB1_0101	1	AFATYHSIQTIVSVL	YHSIQTIVS	4	0.8427	5.5	0.80	0.76	tr_C9ZXD7_	WB
DRB1_0101	2	FATYHSIQTIVSVLC	YHSIQTIVS	3	0.8356	5.9	0.90	0.81	tr_C9ZXD7_	WB
DRB1_0101	3	QAFATYHSIQTIVSV	YHSIQTIVS	5	0.8160	7.3	1.50	0.74	tr_C9ZXD7_	WB
DRB1_0101	4	ATYHSIQTIVSVLCR	YHSIQTIVS	2	0.8152	7.4	1.50	0.81	tr_C9ZXD7_	WB
DRB1_0101	5	TYHSIQTIVSVLCRV	YHSIQTIVS	1	0.7770	11.2	3.50	0.78	tr_C9ZXD7_	WB
DRB1_0101	6	CDRIAKMLPHPILAS	IAKMLPHPI	3	0.7668	12.5	4.00	0.78	tr_C9ZXD7_	WB
DRB1_0101	7	KQAFATYHSIQTIVS	YHSIQTIVS	6	0.7642	12.8	4.00	0.57	tr_C9ZXD7_	WB
DRB1_0101	8	DRIAKMLPHPILASD	IAKMLPHPI	2	0.7611	13.3	4.00	0.74	tr_C9ZXD7_	WB
DRB1_0101	9	TCDRIAKMLPHPILA	IAKMLPHPI	4	0.7463	15.6	5.00	0.80	tr_C9ZXD7_	WB
DRB1_0101	10	RIAKMLPHPILASDD	IAKMLPHPI	1	0.7439	16.0	5.50	0.70	tr_C9ZXD7_	WB
DRB1_0101	11	PTCDRIAKMLPHPIL	IAKMLPHPI	5	0.7005	25.6	9.00	0.80	tr_C9ZXD7_	WB
DRB1_0101	12	YHSIQTIVSVLCRVP	YHSIQTIVS	0	0.6698	35.6	13.00	0.63	tr_C9ZXD7_	
DRB1_0101	13	IAKMLPHPILASDDD	IAKMLPHPI	0	0.6559	41.4	14.00	0.57	tr_C9ZXD7_	
DRB1_0101	14	AKQAFATYHSIQTIV	FATYHSIQT	4	0.6375	50.5	17.00	0.62	tr_C9ZXD7_	
DRB1_0101	15	ENFFTSLERVYTDGK	FTSLERVYT	3	0.6137	65.4	20.00	0.76	tr_C9ZXD7_	

DRB1_0101	16	FPFMSVLLREAVSYS	FMSVLLREA	2	0.5921	82.6	24.00	0.40	tr_C9ZXD7_
DRB1_0101	17	VENFFTSLERVYTDG	FTSLERVYT	4	0.5910	83.6	24.00	0.69	tr_C9ZXD7_
DRB1_0101	18	GPTCDRIAKMLPHPI	IAKMLPHPI	6	0.5874	86.8	25.00	0.77	tr_C9ZXD7_
DRB1_0101	19	EFPFMSVLLREAVSY	FMSVLLREA	3	0.5859	88.3	25.00	0.47	tr_C9ZXD7_
DRB1_0101	20	NFFTSLERVYTDGKC	FTSLERVYT	2	0.5837	90.4	25.00	0.78	tr_C9ZXD7_
DRB1_0101	21	EAKQAFATYHSIQTI	FATYHSIQT	5	0.5782	95.9	26.00	0.62	tr_C9ZXD7_
DRB1_0101	22	RCAYVEFPFMSVLLR	YVEFPFMSV	3	0.5732	101.3	27.00	0.44	tr_C9ZXD7_
DRB1_0101	23	KRMGHLFLGDIVVVG	HLFLGDIVV	4	0.5731	101.4	27.00	0.29	tr_C9ZXD7_
DRB1_0101	24	MVENFFTSLERVYTD	FTSLERVYT	5	0.5726	101.9	27.00	0.62	tr_C9ZXD7_
DRB1_0101	25	VEFPFMSVLLREAVS	FMSVLLREA	4	0.5706	104.2	27.00	0.49	tr_C9ZXD7_
DRB1_0101	26	RMGHLFLGDIVVVGP	FLGDIVVVG	5	0.5687	106.4	28.00	0.31	tr_C9ZXD7_
DRB1_0101	27	YVEFPFMSVLLREAV	FMSVLLREA	5	0.5637	112.2	29.00	0.43	tr_C9ZXD7_
DRB1_0101	28	LGDIVVVGPQSLNWR	IVVVGPQSL	3	0.5626	113.6	29.00	0.71	tr_C9ZXD7_
DRB1_0101	29	LFLGDIVVVGPQSLN	IVVVGPQSL	5	0.5609	115.7	29.00	0.46	tr_C9ZXD7_
DRB1_0101	30	GDIVVVGPQSLNWRV	IVVVGPQSL	2	0.5608	115.8	29.00	0.64	tr_C9ZXD7_
DRB1_0101	31	FLGDIVVVGPQSLNW	IVVVGPQSL	4	0.5605	116.2	29.00	0.61	tr_C9ZXD7_
DRB1_0101	32	MGHLFLGDIVVVGPQ	FLGDIVVVG	4	0.5587	118.5	30.00	0.38	tr_C9ZXD7_
DRB1_0101	33	HLFLGDIVVVGPQSL	FLGDIVVVG	2	0.5537	125.0	31.00	0.38	tr_C9ZXD7_
DRB1_0101	34	YERIKRMGHLFLGDI	IKRMGHLFL	3	0.5529	126.2	31.00	0.51	tr_C9ZXD7_
DRB1_0101	35	AYVEFPFMSVLLREA	FMSVLLREA	6	0.5515	128.1	31.00	0.27	tr_C9ZXD7_
DRB1_0101	36	RIKRMGHLFLGDIVV	IKRMGHLFL	1	0.5504	129.6	31.00	0.35	tr_C9ZXD7_
DRB1_0101	37	ERIKRMGHLFLGDIV	IKRMGHLFL	2	0.5492	131.4	31.00	0.44	tr_C9ZXD7_
DRB1_0101	38	IKRMGHLFLGDIVVV	HLFLGDIVV	5	0.5471	134.3	32.00	0.31	tr_C9ZXD7_
DRB1_0101	39	VPVMRLAEEVAQRMR	MRLAEEVAQ	3	0.5466	135.0	32.00	0.27	tr_C9ZXD7_
DRB1_0101	40	RVPVMRLAEEVAQRM	PVMRLAEEV	2	0.5462	135.6	32.00	0.29	tr_C9ZXD7_
DRB1_0101	41	MRCAYVEFPFMSVLL	YVEFPFMSV	4	0.5453	136.9	32.00	0.46	tr_C9ZXD7_
DRB1_0101	42	GHLFLGDIVVVGPQS	FLGDIVVVG	3	0.5417	142.4	33.00	0.44	tr_C9ZXD7_
DRB1_0101	43	IYERIKRMGHLFLGD	IKRMGHLFL	4	0.5399	145.2	33.00	0.55	tr_C9ZXD7_
DRB1_0101	44	CAYVEFPFMSVLLRE	YVEFPFMSV	2	0.5353	152.6	34.00	0.42	tr_C9ZXD7_
DRB1_0101	45	DMVENFFTSLERVYT	FTSLERVYT	6	0.5345	154.0	34.00	0.51	tr_C9ZXD7_
DRB1_0101	46	DIVVVGPQSLNWRVP	IVVVGPQSL	1	0.5343	154.3	34.00	0.61	tr_C9ZXD7_
DRB1_0101	47	EIYERIKRMGHLFLG	IKRMGHLFL	5	0.5313	159.4	35.00	0.51	tr_C9ZXD7_
DRB1_0101	48	RMRCAYVEFPFMSVL	YVEFPFMSV	5	0.5309	160.1	35.00	0.41	tr_C9ZXD7_
DRB1_0101	49	PQSLNWRVPVMRLAE	WRVPVMRLA	5	0.5307	160.5	35.00	0.38	tr_C9ZXD7_
DRB1_0101	50	VSVLCRVPASAYRTN	LCRVPASAY	3	0.5287	163.9	35.00	0.33	tr_C9ZXD7_
DRB1_0101	51	SLNWRVPVMRLAEEV	WRVPVMRLA	3	0.5263	168.1	36.00	0.43	tr_C9ZXD7_
DRB1_0101	52	WRVPVMRLAEEVAQR	PVMRLAEEV	3	0.5262	168.5	36.00	0.37	tr_C9ZXD7_
DRB1_0101	53	IVSVLCRVPASAYRT	LCRVPASAY	4	0.5243	171.9	36.00	0.29	tr_C9ZXD7_
DRB1_0101	54	QSLNWRVPVMRLAEE	WRVPVMRLA	4	0.5238	172.8	36.00	0.41	tr_C9ZXD7_
DRB1_0101	55	DRQYVQESILSLPDM	YVQESILSL	3	0.5171	185.8	38.00	0.57	tr_C9ZXD7_
DRB1_0101	56	PVMRLAEEVAQRMRC	LAEEVAQRM	4	0.5171	185.9	38.00	0.35	tr_C9ZXD7_
DRB1_0101	57	GPQSLNWRVPVMRLA	LNWRVPVMR	4	0.5126	195.0	38.00	0.28	tr_C9ZXD7_
DRB1_0101	58	NWRVPVMRLAEEVAQ	PVMRLAEEV	4	0.5098	201.0	39.00	0.31	tr_C9ZXD7_
DRB1_0101	59	LNWRVPVMRLAEEVA	WRVPVMRLA	2	0.5082	204.6	39.00	0.38	tr_C9ZXD7_
DRB1_0101	60	RQYVQESILSLPDMV	YVQESILSL	2	0.5077	205.6	39.00	0.52	tr_C9ZXD7_
DRB1_0101	61	TIVSVLCRVPASAYR	VSVLCRVPA	2	0.5073	206.7	40.00	0.31	tr_C9ZXD7_
DRB1_0101	62	VMRLAEEVAQRMRCA	LAEEVAQRM	3	0.5001	223.4	41.00	0.46	tr_C9ZXD7_
DRB1_0101	63	FFTSLERVYTDGKCE	FTSLERVYT	1	0.4990	225.9	41.00	0.81	tr_C9ZXD7_
DRB1_0101	64	DDRQYVQESILSLPD	YVQESILSL	4	0.4971	230.8	42.00	0.57	tr_C9ZXD7_
DRB1_0101	65	EEAKQAFATYHSIQT	FATYHSIQT	6	0.4921	243.6	43.00	0.45	tr_C9ZXD7_

DRB1_0101	66	QRMRCAYVEFPFMSV	YVEFPFMSV	6	0.4866	258.4	44.00	0.34	tr_C9ZXD7_
DRB1_0101	67	DDDRQYVQESILSLP	YVQESILSL	5	0.4837	266.9	44.00	0.54	tr_C9ZXD7_
DRB1_0101	68	WEIYERIKRMGHLFL	IKRMGHLFL	6	0.4792	280.1	45.00	0.38	tr_C9ZXD7_
DRB1_0101	69	QTIVSVLCRVPASAY	VSVLCRVPA	3	0.4765	288.3	46.00	0.38	tr_C9ZXD7_
DRB1_0101	70	SVLCRVPASAYRTND	RVPASAYRT	4	0.4736	297.7	47.00	0.31	tr_C9ZXD7_
DRB1_0101	71	MRLAEEVAQRMRCAY	LAEEVAQRM	2	0.4553	362.7	50.00	0.46	tr_C9ZXD7_
DRB1_0101	72	RLAEEVAQRMRCAYV	EVAQRMRCA	4	0.4512	379.2	55.00	0.35	tr_C9ZXD7_
DRB1_0101	73	IVVVGPQSLNWRVPV	IVVVGPQSL	0	0.4510	379.9	55.00	0.51	tr_C9ZXD7_
DRB1_0101	74	QYVQESILSLPDMVE	YVQESILSL	1	0.4484	390.9	55.00	0.41	tr_C9ZXD7_
DRB1_0101	75	AEEVAQRMRCAYVEF	AQRMRCAYV	4	0.4404	426.0	55.00	0.50	tr_C9ZXD7_
DRB1_0101	76	LAEEVAQRMRCAYVE	EVAQRMRCA	3	0.4404	426.0	55.00	0.34	tr_C9ZXD7_
DRB1_0101	77	IQTIVSVLCRVPASA	VSVLCRVPA	4	0.4358	448.0	55.00	0.38	tr_C9ZXD7_
DRB1_0101	78	SDDDRQYVQESILSL	QYVQESILS	5	0.4348	452.8	55.00	0.43	tr_C9ZXD7_
DRB1_0101	79	HSIQTIVSVLCRVPA	IQTIVSVLC	2	0.4327	463.1	55.00	0.26	tr_C9ZXD7_
DRB1_0101	80	KCEEAKQAFATYHSI	EEAKQAFAT	2	0.4313	470.4	60.00	0.43	tr_C9ZXD7_
DRB1_0101	81	AKMLPHPILASDDDR	MLPHPILAS	2	0.4291	481.5	60.00	0.43	tr_C9ZXD7_
DRB1_0101	82	VGPQSLNWRVPVMRL	LNWRVPVMR	5	0.4268	493.7	60.00	0.31	tr_C9ZXD7_
DRB1_0101	83	EEVAQRMRCAYVEFP	AQRMRCAYV	3	0.4224	517.8	60.00	0.49	tr_C9ZXD7_
DRB1_0101	84	PDMVENFFTSLERVY	NFFTSLERV	5	0.4201	530.8	60.00	0.20	tr_C9ZXD7_
DRB1_0101	85	VLCRVPASAYRTNDE	RVPASAYRT	3	0.4200	531.5	60.00	0.45	tr_C9ZXD7_
DRB1_0101	86	AQRMRCAYVEFPFMS	CAYVEFPFM	5	0.4178	544.4	60.00	0.40	tr_C9ZXD7_
DRB1_0101	87	VAQRMRCAYVEFPFM	AQRMRCAYV	1	0.4172	547.8	60.00	0.33	tr_C9ZXD7_
DRB1_0101	88	CEEAKQAFATYHSIQ	EEAKQAFAT	1	0.4152	560.0	60.00	0.33	tr_C9ZXD7_
DRB1_0101	89	SIQTIVSVLCRVPAS	VSVLCRVPA	5	0.4093	596.7	60.00	0.33	tr_C9ZXD7_
DRB1_0101	90	EVAQRMRCAYVEFPF	AQRMRCAYV	2	0.4047	626.9	65.00	0.47	tr_C9ZXD7_
DRB1_0101	91	GKCEEAKQAFATYHS	EEAKQAFAT	3	0.4027	640.7	65.00	0.55	tr_C9ZXD7_
DRB1_0101	92	YVQESILSLPDMVEN	SILSLPDMV	4	0.3968	683.2	65.00	0.36	tr_C9ZXD7_
DRB1_0101	93	QESILSLPDMVENFF	ILSLPDMVE	3	0.3873	757.2	65.00	0.41	tr_C9ZXD7_
DRB1_0101	94	VQESILSLPDMVENF	SILSLPDMV	3	0.3871	758.9	65.00	0.43	tr_C9ZXD7_
DRB1_0101	95	LPDMVENFFTSLERV	DMVENFFTS	2	0.3849	777.2	65.00	0.31	tr_C9ZXD7_
DRB1_0101	96	DGKCEEAKQAFATYH	EEAKQAFAT	4	0.3779	837.8	70.00	0.54	tr_C9ZXD7_
DRB1_0101	97	ESILSLPDMVENFFT	ILSLPDMVE	2	0.3730	883.2	70.00	0.36	tr_C9ZXD7_
DRB1_0101	98	FTSLERVYTDGKCEE	FTSLERVYT	0	0.3697	915.3	70.00	0.73	tr_C9ZXD7_
DRB1_0101	99	SLPDMVENFFTSLER	DMVENFFTS	3	0.3693	919.9	70.00	0.38	tr_C9ZXD7_
DRB1_0101	100	KRSIDWEIYERIKRM	IDWEIYERI	3	0.3621	994.3	70.00	0.63	tr_C9ZXD7_
DRB1_0101	101	SILSLPDMVENFFTS	ILSLPDMVE	1	0.3564	1058.0	75.00	0.29	tr_C9ZXD7_
DRB1_0101	102	VVGPQSLNWRVPVMR	GPQSLNWRV	2	0.3544	1080.6	75.00	0.28	tr_C9ZXD7_
DRB1_0101	103	DWEIYERIKRMGHLF	RIKRMGHLF	6	0.3538	1087.1	75.00	0.34	tr_C9ZXD7_
DRB1_0101	104	LCRVPASAYRTNDEK	RVPASAYRT	2	0.3516	1113.4	75.00	0.59	tr_C9ZXD7_
DRB1_0101	105	LSLPDMVENFFTSLE	PDMVENFFT	3	0.3502	1130.7	75.00	0.36	tr_C9ZXD7_
DRB1_0101	106	RSIDWEIYERIKRMG	IDWEIYERI	2	0.3493	1141.6	75.00	0.54	tr_C9ZXD7_
DRB1_0101	107	ILSLPDMVENFFTSL	PDMVENFFT	4	0.3480	1158.7	75.00	0.34	tr_C9ZXD7_
DRB1_0101	108	TDGKCEEAKQAFATY	EEAKQAFAT	5	0.3431	1220.5	75.00	0.44	tr_C9ZXD7_
DRB1_0101	109	EKRSIDWEIYERIKR	IDWEIYERI	4	0.3380	1289.7	75.00	0.66	tr_C9ZXD7_
DRB1_0101	110	SIDWEIYERIKRMGH	IDWEIYERI	1	0.3329	1363.0	80.00	0.41	tr_C9ZXD7_
DRB1_0101	111	VVVGPQSLNWRVPVM	GPQSLNWRV	3	0.3319	1379.1	80.00	0.38	tr_C9ZXD7_
DRB1_0101	112	KMLPHPILASDDDRQ	MLPHPILAS	1	0.3212	1548.2	80.00	0.51	tr_C9ZXD7_
DRB1_0101	113	IDWEIYERIKRMGHL	EIYERIKRM	3	0.3182	1597.8	80.00	0.44	tr_C9ZXD7_
DRB1_0101	114	ASAYRTNDEKRSIDW	YRTNDEKRS	3	0.3132	1687.0	80.00	0.82	tr_C9ZXD7_
DRB1_0101	115	DEKRSIDWEIYERIK	IDWEIYERI	5	0.3127	1696.3	80.00	0.60	tr_C9ZXD7_

DRB1_0101	116	SAYRTNDEKRSIDWE	YRTNDEKRS	2	0.2967	2018.1	85.00	0.80	tr_C9ZXD7_	
DRB1_0101	117	YTDGKCEEAKQAFAT	GKCEEAKQA	3	0.2946	2064.0	85.00	0.29	tr_C9ZXD7_	
DRB1_0101	118	PASAYRTNDEKRSID	YRTNDEKRS	4	0.2871	2238.6	85.00	0.82	tr_C9ZXD7_	
DRB1_0101	119	NDEKRSIDWEIYERI	IDWEIYERI	6	0.2745	2565.2	90.00	0.47	tr_C9ZXD7_	
DRB1_0101	120	AYRTNDEKRSIDWEI	YRTNDEKRS	1	0.2690	2721.1	90.00	0.70	tr_C9ZXD7_	
DRB1_0101	121	CRVPASAYRTNDEKR	RVPASAYRT	1	0.2674	2770.7	90.00	0.71	tr_C9ZXD7_	
DRB1_0101	122	VPASAYRTNDEKRSI	YRTNDEKRS	5	0.2659	2814.3	90.00	0.83	tr_C9ZXD7_	
DRB1_0101	123	ASDDDRQYVQESILS	QYVQESILS	6	0.2638	2881.2	90.00	0.47	tr_C9ZXD7_	
DRB1_0101	124	VYTDGKCEEAKQAFA	YTDGKCEEA	1	0.2607	2978.7	90.00	0.32	tr_C9ZXD7_	
DRB1_0101	125	RVYTDGKCEEAKQAF	YTDGKCEEA	2	0.2602	2995.8	90.00	0.47	tr_C9ZXD7_	
DRB1_0101	126	ERVYTDGKCEEAKQA	YTDGKCEEA	3	0.2480	3416.7	90.00	0.57	tr_C9ZXD7_	
DRB1_0101	127	MGPTCDRIAKMLPHP	DRIAKMLPH	5	0.2469	3456.0	90.00	0.38	tr_C9ZXD7_	
DRB1_0101	128	RVPASAYRTNDEKRS	YRTNDEKRS	6	0.2280	4243.8	95.00	0.47	tr_C9ZXD7_	
DRB1_0101	129	YRTNDEKRSIDWEIY	YRTNDEKRS	0	0.2262	4324.9	95.00	0.41	tr_C9ZXD7_	
DRB1_0101	130	LERVYTDGKCEEAKQ	YTDGKCEEA	4	0.2136	4958.3	95.00	0.58	tr_C9ZXD7_	
DRB1_0101	131	HPILASDDDRQYVQE	ILASDDDRQ	2	0.2106	5122.5	95.00	0.33	tr_C9ZXD7_	
DRB1_0101	132	PHPILASDDDRQYVQ	ILASDDDRQ	3	0.2105	5124.1	95.00	0.34	tr_C9ZXD7_	
DRB1_0101	133	PILASDDDRQYVQES	LASDDDRQY	2	0.2002	5730.6	95.00	0.31	tr_C9ZXD7_	
DRB1_0101	134	MLPHPILASDDDRQY	MLPHPILAS	0	0.1968	5944.7	95.00	0.34	tr_C9ZXD7_	
DRB1_0101	135	LPHPILASDDDRQYV	ILASDDDRQ	4	0.1929	6198.8	100.00	0.38	tr_C9ZXD7_	
DRB1_0101	136	SLERVYTDGKCEEAK	YTDGKCEEA	5	0.1913	6309.7	100.00	0.56	tr_C9ZXD7_	
DRB1_0101	137	TNDEKRSIDWEIYER	EKRSIDWEI	3	0.1790	7206.9	100.00	0.50	tr_C9ZXD7_	
DRB1_0101	138	ILASDDDRQYVQESI	LASDDDRQY	1	0.1725	7735.9	100.00	0.31	tr_C9ZXD7_	
DRB1_0101	139	LASDDDRQYVQESIL	RQYVQESIL	6	0.1670	8212.5	100.00	0.19	tr_C9ZXD7_	
DRB1_0101	140	RTNDEKRSIDWEIYE	EKRSIDWEI	4	0.1652	8368.3	100.00	0.45	tr_C9ZXD7_	
DRB1_0101	141	TSLERVYTDGKCEEA	YTDGKCEEA	6	0.1556	9280.7	100.00	0.35	tr_C9ZXD7_	

Allele: DRB1_0101. Number of high binders 0. Number of weak binders 11. Number of peptides 141

Allele	pos	peptide	core	Of	1-log50k(aff)	affinity(nM)	%Rank	Relia	Identity	Bind Level
DRB1_0101	1	LTALTYLHARGVAHR	LTYLHARGV	3	0.8687	4.1	0.40	0.63	tr_C9ZXD7_	SB
DRB1_0101	2	LEGFQYVINTPISST	FQYVINTPI	3	0.8655	4.3	0.40	0.75	tr_C9ZXD7_	SB
DRB1_0101	3	EGFQYVINTPISSTE	FQYVINTPI	2	0.8619	4.5	0.40	0.71	tr_C9ZXD7_	SB
DRB1_0101	4	TALTYLHARGVAHRD	LTYLHARGV	2	0.8606	4.5	0.50	0.63	tr_C9ZXD7_	WB
DRB1_0101	5	GFQYVINTPISSTEV	FQYVINTPI	1	0.8562	4.7	0.50	0.64	tr_C9ZXD7_	WB
DRB1_0101	6	ALTYLHARGVAHRDV	LTYLHARGV	1	0.8494	5.1	0.60	0.54	tr_C9ZXD7_	WB
DRB1_0101	7	ALEGFQYVINTPISS	FQYVINTPI	4	0.8433	5.4	0.80	0.80	tr_C9ZXD7_	WB
DRB1_0101	8	LLRTMFVSLTSVRER	FVSLTSVRE	5	0.8389	5.7	0.90	0.71	tr_C9ZXD7_	WB
DRB1_0101	9	LLTALTYLHARGVAH	LTYLHARGV	4	0.8386	5.7	0.90	0.73	tr_C9ZXD7_	WB
DRB1_0101	10	LRTMFVSLTSVRERC	FVSLTSVRE	4	0.8306	6.2	1.10	0.82	tr_C9ZXD7_	WB
DRB1_0101	11	ERMYTVLLSSASSEG	YTVLLSSAS	3	0.8306	6.3	1.10	0.83	tr_C9ZXD7_	WB
DRB1_0101	12	RTMFVSLTSVRERCE	FVSLTSVRE	3	0.8261	6.6	1.20	0.88	tr_C9ZXD7_	WB
DRB1_0101	13	RMYTVLLSSASSEGD	YTVLLSSAS	2	0.8222	6.8	1.30	0.83	tr_C9ZXD7_	WB
DRB1_0101	14	FQYVINTPISSTEVR	YVINTPISS	2	0.8206	7.0	1.30	0.37	tr_C9ZXD7_	WB
DRB1_0101	15	EERMYTVLLSSASSE	YTVLLSSAS	4	0.8206	7.0	1.30	0.85	tr_C9ZXD7_	WB
DRB1_0101	16	QLLTALTYLHARGVA	LTYLHARGV	5	0.8144	7.4	1.50	0.76	tr_C9ZXD7_	WB

DRB1_0101	17	KLKLRAFATVPVTLF	LRAFATVPV	3	0.8143	7.5	1.50	0.58	tr_C9ZXD7_	WB
DRB1_0101	18	LEERMYTVLLSSASS	YTVLLSSAS	5	0.8101	7.8	1.70	0.85	tr_C9ZXD7_	WB
DRB1_0101	19	LALEGFQYVINTPIS	FQYVINTPI	5	0.8077	8.0	1.80	0.87	tr_C9ZXD7_	WB
DRB1_0101	20	LTYLHARGVAHRDVK	YLHARGVAH	2	0.8034	8.4	1.90	0.46	tr_C9ZXD7_	WB
DRB1_0101	21	IKVLRQLLTALTYLH	LRQLLTALT	3	0.8012	8.6	2.00	0.63	tr_C9ZXD7_	WB
DRB1_0101	22	LKLKLRAFATVPVTL	LRAFATVPV	4	0.8006	8.6	2.00	0.56	tr_C9ZXD7_	WB
DRB1_0101	23	RKVLMAIGTAVSLRK	LMAIGTAVS	3	0.7987	8.8	2.50	0.41	tr_C9ZXD7_	WB
DRB1_0101	24	VIKVLRQLLTALTYL	LRQLLTALT	4	0.7964	9.1	2.50	0.62	tr_C9ZXD7_	WB
DRB1_0101	25	TMFVSLTSVRERCEL	FVSLTSVRE	2	0.7949	9.2	2.50	0.88	tr_C9ZXD7_	WB
DRB1_0101	26	LKLRAFATVPVTLFT	LRAFATVPV	2	0.7939	9.3	2.50	0.55	tr_C9ZXD7_	WB
DRB1_0101	27	VLKLKLRAFATVPVT	LRAFATVPV	5	0.7928	9.4	2.50	0.44	tr_C9ZXD7_	WB
DRB1_0101	28	ILLRTMFVSLTSVRE	FVSLTSVRE	6	0.7885	9.9	2.50	0.51	tr_C9ZXD7_	WB
DRB1_0101	29	TFALQIVATPVAQAA	LQIVATPVA	3	0.7876	10.0	3.00	0.28	tr_C9ZXD7_	WB
DRB1_0101	30	KVLRQLLTALTYLHA	LRQLLTALT	2	0.7870	10.0	3.00	0.69	tr_C9ZXD7_	WB
DRB1_0101	31	VRKVLMAIGTAVSLR	VLMAIGTAV	3	0.7853	10.2	3.00	0.43	tr_C9ZXD7_	WB
DRB1_0101	32	MYTVLLSSASSEGDL	YTVLLSSAS	1	0.7838	10.4	3.00	0.82	tr_C9ZXD7_	WB
DRB1_0101	33	TVIKVLRQLLTALTY	LRQLLTALT	5	0.7823	10.5	3.00	0.56	tr_C9ZXD7_	WB
DRB1_0101	34	NTFALQIVATPVAQA	FALQIVATP	2	0.7802	10.8	3.00	0.39	tr_C9ZXD7_	WB
DRB1_0101	35	RNTFALQIVATPVAQ	FALQIVATP	3	0.7801	10.8	3.00	0.50	tr_C9ZXD7_	WB
DRB1_0101	36	KLRAFATVPVTLFTV	LRAFATVPV	1	0.7782	11.0	3.00	0.50	tr_C9ZXD7_	WB
DRB1_0101	37	RQLLTALTYLHARGV	LTYLHARGV	6	0.7755	11.3	3.50	0.59	tr_C9ZXD7_	WB
DRB1_0101	38	KVLMAIGTAVSLRKN	LMAIGTAVS	2	0.7749	11.4	3.50	0.38	tr_C9ZXD7_	WB
DRB1_0101	39	HLRLQILNSSRNTFA	LQILNSSRN	3	0.7705	12.0	3.50	0.65	tr_C9ZXD7_	WB
DRB1_0101	40	EVLKLKLRAFATVPV	KLKLRAFAT	3	0.7691	12.2	3.50	0.51	tr_C9ZXD7_	WB
DRB1_0101	41	IRDYVVSEVNLSLSE	YVVSEVNLS	3	0.7645	12.8	4.00	0.79	tr_C9ZXD7_	WB
DRB1_0101	42	LRLQILNSSRNTFAL	LQILNSSRN	2	0.7641	12.8	4.00	0.62	tr_C9ZXD7_	WB
DRB1_0101	43	FALQIVATPVAQAAP	IVATPVAQA	4	0.7641	12.8	4.00	0.35	tr_C9ZXD7_	WB
DRB1_0101	44	RLALEGFQYVINTPI	FQYVINTPI	6	0.7589	13.6	4.50	0.76	tr_C9ZXD7_	WB
DRB1_0101	45	VLRQLLTALTYLHAR	LRQLLTALT	1	0.7575	13.8	4.50	0.60	tr_C9ZXD7_	WB
DRB1_0101	46	YIRDYVVSEVNLSLS	YVVSEVNLS	4	0.7567	13.9	4.50	0.80	tr_C9ZXD7_	WB
DRB1_0101	47	SRNTFALQIVATPVA	FALQIVATP	4	0.7540	14.3	4.50	0.54	tr_C9ZXD7_	WB
DRB1_0101	48	RDYVVSEVNLSLSEI	YVVSEVNLS	2	0.7522	14.6	5.00	0.75	tr_C9ZXD7_	WB
DRB1_0101	49	KYRITRWNMAAEVVT	YRITRWNMA	1	0.7513	14.7	5.00	0.62	tr_C9ZXD7_	WB
DRB1_0101	50	KPQNILIKGDVVKIT	NILIKGDVV	3	0.7513	14.7	5.00	0.69	tr_C9ZXD7_	WB
DRB1_0101	51	EVRKVLMAIGTAVSL	VLMAIGTAV	4	0.7490	15.1	5.00	0.48	tr_C9ZXD7_	WB
DRB1_0101	52	CHLRLQILNSSRNTF	LQILNSSRN	4	0.7478	15.3	5.00	0.64	tr_C9ZXD7_	WB
DRB1_0101	53	LTVIKVLRQLLTALT	LRQLLTALT	6	0.7458	15.6	5.00	0.42	tr_C9ZXD7_	WB
DRB1_0101	54	ILEERMYTVLLSSAS	YTVLLSSAS	6	0.7401	16.6	5.50	0.82	tr_C9ZXD7_	WB
DRB1_0101	55	PQNILIKGDVVKITD	NILIKGDVV	2	0.7400	16.7	5.50	0.62	tr_C9ZXD7_	WB
DRB1_0101	56	MFVSLTSVRERCELE	FVSLTSVRE	1	0.7384	17.0	5.50	0.88	tr_C9ZXD7_	WB
DRB1_0101	57	LRAFATVPVTLFTVA	FATVPVTLF	3	0.7370	17.2	6.00	0.57	tr_C9ZXD7_	WB
DRB1_0101	58	QNILIKGDVVKITDF	NILIKGDVV	1	0.7370	17.2	6.00	0.56	tr_C9ZXD7_	WB
DRB1_0101	59	RLQILNSSRNTFALQ	LQILNSSRN	1	0.7364	17.3	6.00	0.56	tr_C9ZXD7_	WB
DRB1_0101	60	AVPILNQEGSLIGRI	ILNQEGSLI	3	0.7342	17.7	6.00	0.79	tr_C9ZXD7_	WB
DRB1_0101	61	EKQYVSSLTITTQPH	YVSSLTITT	3	0.7336	17.9	6.00	0.79	tr_C9ZXD7_	WB
DRB1_0101	62	VPILNQEGSLIGRIL	ILNQEGSLI	2	0.7327	18.0	6.00	0.74	tr_C9ZXD7_	WB
DRB1_0101	63	DKYRITRWNMAAEVV	YRITRWNMA	2	0.7306	18.5	6.50	0.70	tr_C9ZXD7_	WB
DRB1_0101	64	VTLFTVAVPILNQEG	FTVAVPILN	3	0.7300	18.6	6.50	0.88	tr_C9ZXD7_	WB
DRB1_0101	65	ALQIVATPVAQAAPS	IVATPVAQA	3	0.7289	18.8	6.50	0.51	tr_C9ZXD7_	WB
DRB1_0101	66	REKQYVSSLTITTQP	YVSSLTITT	4	0.7255	19.5	6.50	0.75	tr_C9ZXD7_	WB

DRB1_0101	67	PVTLFTVAVPILNQE	FTVAVPILN	4	0.7249	19.6	7.00	0.88	tr_C9ZXD7_	WB
DRB1_0101	68	AVATLLNSLRVPCHL	ATLLNSLRV	2	0.7229	20.1	7.00	0.55	tr_C9ZXD7_	WB
DRB1_0101	69	VATLLNSLRVPCHLR	ATLLNSLRV	1	0.7225	20.1	7.00	0.46	tr_C9ZXD7_	WB
DRB1_0101	70	SREKQYVSSLTITTQ	YVSSLTITT	5	0.7217	20.3	7.00	0.70	tr_C9ZXD7_	WB
DRB1_0101	71	SGEVLKLKLRAFATV	KLKLRAFAT	5	0.7206	20.6	7.00	0.58	tr_C9ZXD7_	WB
DRB1_0101	72	VKPQNILIKGDVVKI	NILIKGDVV	4	0.7180	21.1	7.50	0.74	tr_C9ZXD7_	WB
DRB1_0101	73	PCHLRLQILNSSRNT	LQILNSSRN	5	0.7180	21.1	7.50	0.53	tr_C9ZXD7_	WB
DRB1_0101	74	TKGVLLGRGSFGAVY	VLLGRGSFG	3	0.7176	21.2	7.50	0.62	tr_C9ZXD7_	WB
DRB1_0101	75	VPVTLFTVAVPILNQ	FTVAVPILN	5	0.7172	21.3	7.50	0.82	tr_C9ZXD7_	WB
DRB1_0101	76	PILNQEGSLIGRILI	ILNQEGSLI	1	0.7162	21.6	7.50	0.56	tr_C9ZXD7_	WB
DRB1_0101	77	TLFTVAVPILNQEGS	FTVAVPILN	2	0.7159	21.6	7.50	0.87	tr_C9ZXD7_	WB
DRB1_0101	78	VLMAIGTAVSLRKNA	LMAIGTAVS	1	0.7153	21.8	7.50	0.37	tr_C9ZXD7_	WB
DRB1_0101	79	TYIRDYVVSEVNLSL	YVVSEVNLS	5	0.7145	22.0	7.50	0.81	tr_C9ZXD7_	WB
DRB1_0101	80	YTVLLSSASSEGDLS	YTVLLSSAS	0	0.7143	22.0	7.50	0.72	tr_C9ZXD7_	WB
DRB1_0101	81	VDYYVDGLTSNPVLC	VDGLTSNPV	4	0.7142	22.0	7.50	0.76	tr_C9ZXD7_	WB
DRB1_0101	82	GEVLKLKLRAFATVP	KLKLRAFAT	4	0.7120	22.5	8.00	0.62	tr_C9ZXD7_	WB
DRB1_0101	83	EAVATLLNSLRVPCH	ATLLNSLRV	3	0.7110	22.8	8.00	0.62	tr_C9ZXD7_	WB
DRB1_0101	84	VAVPILNQEGSLIGR	ILNQEGSLI	4	0.7100	23.0	8.00	0.81	tr_C9ZXD7_	WB
DRB1_0101	85	YRITRWNMAAEVVTG	YRITRWNMA	0	0.7098	23.1	8.00	0.38	tr_C9ZXD7_	WB
DRB1_0101	86	CPPLFMETPHMLADM	LFMETPHML	3	0.7093	23.2	8.00	0.62	tr_C9ZXD7_	WB
DRB1_0101	87	DDKYRITRWNMAAEV	YRITRWNMA	3	0.7084	23.4	8.00	0.79	tr_C9ZXD7_	WB
DRB1_0101	88	ATLLNSLRVPCHLRL	LNSLRVPCH	3	0.7081	23.5	8.50	0.47	tr_C9ZXD7_	WB
DRB1_0101	89	DYYVDGLTSNPVLCD	VDGLTSNPV	3	0.7079	23.6	8.50	0.77	tr_C9ZXD7_	WB
DRB1_0101	90	KQYVSSLTITTQPHS	YVSSLTITT	2	0.7077	23.6	8.50	0.79	tr_C9ZXD7_	WB
DRB1_0101	91	LRQLLTALTYLHARG	LRQLLTALT	0	0.7073	23.7	8.50	0.42	tr_C9ZXD7_	WB
DRB1_0101	92	HRLFSSSCSAEVESL	FSSSCSAEV	3	0.7069	23.8	8.50	0.82	tr_C9ZXD7_	WB
DRB1_0101	93	LLHRLFSSSCSAEVE	FSSSCSAEV	5	0.7047	24.4	8.50	0.56	tr_C9ZXD7_	WB
DRB1_0101	94	KGVLLGRGSFGAVYE	VLLGRGSFG	2	0.7015	25.3	9.00	0.56	tr_C9ZXD7_	WB
DRB1_0101	95	PPLFMETPHMLADMT	LFMETPHML	2	0.7011	25.4	9.00	0.62	tr_C9ZXD7_	WB
DRB1_0101	96	YYVDGLTSNPVLCDF	VDGLTSNPV	2	0.6991	25.9	9.00	0.81	tr_C9ZXD7_	WB
DRB1_0101	97	SAKALKVLHTWILEE	LKVLHTWIL	4	0.6985	26.1	9.00	0.64	tr_C9ZXD7_	WB
DRB1_0101	98	TSAKALKVLHTWILE	LKVLHTWIL	5	0.6982	26.2	9.00	0.60	tr_C9ZXD7_	WB
DRB1_0101	99	TEVRKVLMAIGTAVS	VLMAIGTAV	5	0.6972	26.5	9.50	0.56	tr_C9ZXD7_	WB
DRB1_0101	100	AKALKVLHTWILEER	LKVLHTWIL	3	0.6958	26.9	9.50	0.75	tr_C9ZXD7_	WB
DRB1_0101	101	DVDYYVDGLTSNPVL	VDGLTSNPV	5	0.6956	26.9	9.50	0.75	tr_C9ZXD7_	WB
DRB1_0101	102	LHRLFSSSCSAEVES	FSSSCSAEV	4	0.6952	27.1	9.50	0.73	tr_C9ZXD7_	WB
DRB1_0101	103	DYVVSEVNLSLSEIL	YVVSEVNLS	1	0.6952	27.1	9.50	0.70	tr_C9ZXD7_	WB
DRB1_0101	104	MILLRTMFVSLTSVR	LRTMFVSLT	3	0.6943	27.3	9.50	0.66	tr_C9ZXD7_	WB
DRB1_0101	105	LQILNSSRNTFALQI	LNSSRNTFA	3	0.6927	27.8	9.50	0.52	tr_C9ZXD7_	WB
DRB1_0101	106	SSEQLLGQMLRLSPL	SEQLLGQML	1	0.6922	27.9	10.00	0.48	tr_C9ZXD7_	
DRB1_0101	107	WTSSEQLLGQMLRLS	SEQLLGQML	3	0.6913	28.2	10.00	0.62	tr_C9ZXD7_	
DRB1_0101	108	TSSEQLLGQMLRLSP	SEQLLGQML	2	0.6907	28.4	10.00	0.63	tr_C9ZXD7_	
DRB1_0101	109	SSRNTFALQIVATPV	FALQIVATP	5	0.6896	28.7	10.00	0.62	tr_C9ZXD7_	
DRB1_0101	110	DCPPLFMETPHMLAD	LFMETPHML	4	0.6871	29.5	11.00	0.62	tr_C9ZXD7_	
DRB1_0101	111	LFTVAVPILNQEGSL	FTVAVPILN	1	0.6840	30.5	11.00	0.79	tr_C9ZXD7_	
DRB1_0101	112	NILIKGDVVKITDFG	LIKGDVVKI	2	0.6833	30.8	11.00	0.38	tr_C9ZXD7_	
DRB1_0101	113	WTKGVLLGRGSFGAV	VLLGRGSFG	4	0.6827	31.0	11.00	0.62	tr_C9ZXD7_	
DRB1_0101	114	QYVINTPISSTEVRK	YVINTPISS	1	0.6822	31.1	11.00	0.71	tr_C9ZXD7_	
DRB1_0101	115	LQIVATPVAQAAPST	IVATPVAQA	2	0.6814	31.4	11.00	0.57	tr_C9ZXD7_	
DRB1_0101	116	RLFSSSCSAEVESLP	FSSSCSAEV	2	0.6808	31.6	11.00	0.85	tr_C9ZXD7_	

DRB1_0101	117	LLLHRLFSSSCSAEV	LHRLFSSSC	2	0.6779	32.6	12.00	0.40	tr_C9ZXD7_
DRB1_0101	118	FDDKYRITRWNMAAE	YRITRWNMA	4	0.6771	32.9	12.00	0.87	tr_C9ZXD7_
DRB1_0101	119	IIVVPISEPGVRLAL	PISEPGVRL	4	0.6736	34.2	12.00	0.63	tr_C9ZXD7_
DRB1_0101	120	FTVAVPILNQEGSLI	FTVAVPILN	0	0.6730	34.4	12.00	0.44	tr_C9ZXD7_
DRB1_0101	121	TVPVTLFTVAVPILN	FTVAVPILN	6	0.6718	34.8	12.00	0.75	tr_C9ZXD7_
DRB1_0101	122	PEAVATLLNSLRVPC	ATLLNSLRV	4	0.6713	35.0	12.00	0.71	tr_C9ZXD7_
DRB1_0101	123	DLTVIKVLRQLLTAL	IKVLRQLLT	4	0.6706	35.3	12.00	0.68	tr_C9ZXD7_
DRB1_0101	124	NALGYIEGKHVDAQV	LGYIEGKHV	2	0.6696	35.7	13.00	0.74	tr_C9ZXD7_
DRB1_0101	125	RAFATVPVTLFTVAV	FATVPVTLF	2	0.6694	35.8	13.00	0.72	tr_C9ZXD7_
DRB1_0101	126	KQVEELARGMLAEWE	EELARGMLA	3	0.6670	36.7	13.00	0.79	tr_C9ZXD7_
DRB1_0101	127	FSGEVLKLKLRAFAT	KLKLRAFAT	6	0.6669	36.8	13.00	0.38	tr_C9ZXD7_
DRB1_0101	128	GKQVEELARGMLAEW	EELARGMLA	4	0.6662	37.0	13.00	0.79	tr_C9ZXD7_
DRB1_0101	129	KALKVLHTWILEERM	LKVLHTWIL	2	0.6661	37.1	13.00	0.78	tr_C9ZXD7_
DRB1_0101	130	DVKPQNILIKGDVVK	NILIKGDVV	5	0.6651	37.5	13.00	0.81	tr_C9ZXD7_
DRB1_0101	131	LMAIGTAVSLRKNAA	IGTAVSLRK	3	0.6644	37.8	13.00	0.34	tr_C9ZXD7_
DRB1_0101	132	FDCPPLFMETPHMLA	LFMETPHML	5	0.6639	38.0	13.00	0.65	tr_C9ZXD7_
DRB1_0101	133	PLFMETPHMLADMTD	LFMETPHML	1	0.6636	38.1	13.00	0.58	tr_C9ZXD7_
DRB1_0101	134	VPCHLRLQILNSSRN	LRLQILNSS	4	0.6628	38.4	13.00	0.41	tr_C9ZXD7_
DRB1_0101	135	SMILLRTMFVSLTSV	LRTMFVSLT	4	0.6621	38.7	14.00	0.62	tr_C9ZXD7_
DRB1_0101	136	GFDDKYRITRWNMAA	YRITRWNMA	5	0.6604	39.4	14.00	0.86	tr_C9ZXD7_
DRB1_0101	137	IVVPISEPGVRLALE	PISEPGVRL	3	0.6600	39.6	14.00	0.72	tr_C9ZXD7_
DRB1_0101	138	GNALGYIEGKHVDAQ	LGYIEGKHV	3	0.6598	39.7	14.00	0.78	tr_C9ZXD7_
DRB1_0101	139	EWTSSEQLLGQMLRL	SEQLLGQML	4	0.6596	39.8	14.00	0.69	tr_C9ZXD7_
DRB1_0101	140	IPEAVATLLNSLRVP	ATLLNSLRV	5	0.6585	40.3	14.00	0.65	tr_C9ZXD7_
DRB1_0101	141	TYLHARGVAHRDVKP	YLHARGVAH	1	0.6577	40.6	14.00	0.76	tr_C9ZXD7_
DRB1_0101	142	TVAVPILNQEGSLIG	ILNQEGSLI	5	0.6576	40.6	14.00	0.79	tr_C9ZXD7_
DRB1_0101	143	RGLSVIGPLQSFLEY	LSVIGPLQS	2	0.6555	41.6	14.00	0.61	tr_C9ZXD7_
DRB1_0101	144	ALGYIEGKHVDAQVV	LGYIEGKHV	1	0.6536	42.4	15.00	0.65	tr_C9ZXD7_
DRB1_0101	145	QGTLRYMAPEVYRGE	LRYMAPEVY	3	0.6512	43.5	15.00	0.69	tr_C9ZXD7_
DRB1_0101	146	PLQVFSGEVLKLKLR	VFSGEVLKL	3	0.6481	45.0	15.00	0.43	tr_C9ZXD7_
DRB1_0101	147	PRGLSVIGPLQSFLE	LSVIGPLQS	3	0.6481	45.0	15.00	0.63	tr_C9ZXD7_
DRB1_0101	148	SEQLLGQMLRLSPLD	SEQLLGQML	0	0.6476	45.3	15.00	0.31	tr_C9ZXD7_
DRB1_0101	149	ILNQEGSLIGRILIC	EGSLIGRIL	4	0.6462	46.0	16.00	0.34	tr_C9ZXD7_
DRB1_0101	150	TLLNSLRVPCHLRLQ	LNSLRVPCH	2	0.6461	46.0	16.00	0.60	tr_C9ZXD7_
DRB1_0101	151	YGNALGYIEGKHVDA	LGYIEGKHV	4	0.6449	46.6	16.00	0.79	tr_C9ZXD7_
DRB1_0101	152	YGKQVEELARGMLAE	EELARGMLA	5	0.6439	47.1	16.00	0.71	tr_C9ZXD7_
DRB1_0101	153	LQVFSGEVLKLKLRA	VFSGEVLKL	2	0.6435	47.3	16.00	0.38	tr_C9ZXD7_
DRB1_0101	154	ISMILLRTMFVSLTS	LRTMFVSLT	5	0.6399	49.2	16.00	0.61	tr_C9ZXD7_
DRB1_0101	155	PDLTVIKVLRQLLTA	IKVLRQLLT	5	0.6395	49.4	16.00	0.65	tr_C9ZXD7_
DRB1_0101	156	VQGTLRYMAPEVYRG	LRYMAPEVY	4	0.6395	49.4	16.00	0.73	tr_C9ZXD7_
DRB1_0101	157	EIKIMCSLNHPNIVH	IMCSLNHPN	3	0.6385	50.0	17.00	0.25	tr_C9ZXD7_
DRB1_0101	158	FSREKQYVSSLTITT	YVSSLTITT	6	0.6374	50.6	17.00	0.56	tr_C9ZXD7_
DRB1_0101	159	IKIMCSLNHPNIVHY	MCSLNHPNI	3	0.6372	50.7	17.00	0.28	tr_C9ZXD7_
DRB1_0101	160	TPRGLSVIGPLQSFL	LSVIGPLQS	4	0.6367	50.9	17.00	0.64	tr_C9ZXD7_
DRB1_0101	161	DIIVVPISEPGVRLA	PISEPGVRL	5	0.6366	51.0	17.00	0.52	tr_C9ZXD7_
DRB1_0101	162	GVLLGRGSFGAVYEA	VLLGRGSFG	1	0.6360	51.3	17.00	0.53	tr_C9ZXD7_
DRB1_0101	163	YVDGLTSNPVLCDFL	VDGLTSNPV	1	0.6356	51.6	17.00	0.79	tr_C9ZXD7_
DRB1_0101	164	NGYYTIPYGNALGYI	YTIPYGNAL	3	0.6344	52.2	17.00	0.47	tr_C9ZXD7_
DRB1_0101	165	QVEELARGMLAEWEW	EELARGMLA	2	0.6339	52.5	17.00	0.85	tr_C9ZXD7_
DRB1_0101	166	FVSLTSVRERCELEK	FVSLTSVRE	0	0.6321	53.5	18.00	0.82	tr_C9ZXD7_

DRB1_0101	167	RITRWNMAAEVVTGF	WNMAAEVVT	4	0.6318	53.7	18.00	0.47	tr_C9ZXD7_
DRB1_0101	168	QIVATPVAQAAPSTA	IVATPVAQA	1	0.6312	54.1	18.00	0.50	tr_C9ZXD7_
DRB1_0101	169	ATPVAQAAPSTAQIS	VAQAAPSTA	3	0.6308	54.3	18.00	0.71	tr_C9ZXD7_
DRB1_0101	170	GTLRYMAPEVYRGED	LRYMAPEVY	2	0.6299	54.8	18.00	0.68	tr_C9ZXD7_
DRB1_0101	171	ADLLLHRLFSSSCSA	LHRLFSSSC	4	0.6285	55.7	18.00	0.52	tr_C9ZXD7_
DRB1_0101	172	ITRWNMAAEVVTGFL	WNMAAEVVT	3	0.6266	56.8	18.00	0.50	tr_C9ZXD7_
DRB1_0101	173	KNGYYTIPYGNALGY	YYTIPYGNA	3	0.6265	56.9	18.00	0.40	tr_C9ZXD7_
DRB1_0101	174	VVPISEPGVRLALEG	PISEPGVRL	2	0.6263	57.0	18.00	0.80	tr_C9ZXD7_
DRB1_0101	175	PWTKGVLLGRGSFGA	VLLGRGSFG	5	0.6241	58.4	19.00	0.67	tr_C9ZXD7_
DRB1_0101	176	QVFSGEVLKLKLRAF	VFSGEVLKL	1	0.6239	58.5	19.00	0.32	tr_C9ZXD7_
DRB1_0101	177	APLQVFSGEVLKLKL	VFSGEVLKL	4	0.6237	58.7	19.00	0.41	tr_C9ZXD7_
DRB1_0101	178	NEIKIMCSLNHPNIV	IKIMCSLNH	2	0.6236	58.7	19.00	0.40	tr_C9ZXD7_
DRB1_0101	179	IVATPVAQAAPSTAQ	VAQAAPSTA	5	0.6219	59.8	19.00	0.39	tr_C9ZXD7_
DRB1_0101	180	VQDLVHTLSYDIIVV	LVHTLSYDI	3	0.6218	59.9	19.00	0.73	tr_C9ZXD7_
DRB1_0101	181	WEWTSSEQLLGQMLR	SEQLLGQML	5	0.6212	60.3	19.00	0.69	tr_C9ZXD7_
DRB1_0101	182	GYYTIPYGNALGYIE	YTIPYGNAL	2	0.6204	60.8	19.00	0.42	tr_C9ZXD7_
DRB1_0101	183	NSSRNTFALQIVATP	FALQIVATP	6	0.6202	60.9	19.00	0.56	tr_C9ZXD7_
DRB1_0101	184	QILNSSRNTFALQIV	LNSSRNTFA	2	0.6199	61.1	19.00	0.64	tr_C9ZXD7_
DRB1_0101	185	ALKVLHTWILEERMY	LKVLHTWIL	1	0.6196	61.3	19.00	0.71	tr_C9ZXD7_
DRB1_0101	186	DLLLHRLFSSSCSAE	LHRLFSSSC	3	0.6188	61.8	19.00	0.56	tr_C9ZXD7_
DRB1_0101	187	YVINTPISSTEVRKV	YVINTPISS	0	0.6184	62.1	20.00	0.43	tr_C9ZXD7_
DRB1_0101	188	LFSSSCSAEVESLPD	FSSSCSAEV	1	0.6173	62.8	20.00	0.83	tr_C9ZXD7_
DRB1_0101	189	VATPVAQAAPSTAQI	VAQAAPSTA	4	0.6150	64.5	20.00	0.63	tr_C9ZXD7_
DRB1_0101	190	KIMCSLNHPNIVHYF	CSLNHPNIV	3	0.6149	64.5	20.00	0.32	tr_C9ZXD7_
DRB1_0101	191	GLSVIGPLQSFLEYG	LSVIGPLQS	1	0.6140	65.1	20.00	0.54	tr_C9ZXD7_
DRB1_0101	192	LAPLQVFSGEVLKLK	LQVFSGEVL	3	0.6135	65.5	20.00	0.34	tr_C9ZXD7_
DRB1_0101	193	YVVSEVNLSLSEILE	YVVSEVNLS	0	0.6134	65.6	20.00	0.50	tr_C9ZXD7_
DRB1_0101	194	PYGNALGYIEGKHVD	LGYIEGKHV	5	0.6133	65.6	20.00	0.71	tr_C9ZXD7_
DRB1_0101	195	VFSGEVLKLKLRAFA	VLKLKLRAF	5	0.6129	65.9	20.00	0.31	tr_C9ZXD7_
DRB1_0101	196	NISMILLRTMFVSLT	LRTMFVSLT	6	0.6124	66.3	20.00	0.48	tr_C9ZXD7_
DRB1_0101	197	CIPEAVATLLNSLRV	ATLLNSLRV	6	0.6121	66.5	21.00	0.48	tr_C9ZXD7_
DRB1_0101	198	VEELARGMLAEWEWT	EELARGMLA	1	0.6094	68.5	21.00	0.81	tr_C9ZXD7_
DRB1_0101	199	QDLVHTLSYDIIVVP	LVHTLSYDI	2	0.6062	70.8	21.00	0.71	tr_C9ZXD7_
DRB1_0101	200	YDIIVVPISEPGVRL	PISEPGVRL	6	0.6056	71.3	22.00	0.29	tr_C9ZXD7_
DRB1_0101	201	TLRYMAPEVYRGEDH	LRYMAPEVY	1	0.6039	72.7	22.00	0.63	tr_C9ZXD7_
DRB1_0101	202	SVQDLVHTLSYDIIV	LVHTLSYDI	4	0.6005	75.4	22.00	0.69	tr_C9ZXD7_
DRB1_0101	203	GYGKQVEELARGMLA	EELARGMLA	6	0.5996	76.1	23.00	0.59	tr_C9ZXD7_
DRB1_0101	204	TPVAQAAPSTAQISE	VAQAAPSTA	2	0.5993	76.3	23.00	0.69	tr_C9ZXD7_
DRB1_0101	205	RMCFCLQPEGRATAA	FCLQPEGRA	3	0.5986	76.9	23.00	0.55	tr_C9ZXD7_
DRB1_0101	206	DVQGTLRYMAPEVYR	LRYMAPEVY	5	0.5976	77.8	23.00	0.74	tr_C9ZXD7_
DRB1_0101	207	STEVRKVLMAIGTAV	VLMAIGTAV	6	0.5975	77.8	23.00	0.49	tr_C9ZXD7_
DRB1_0101	208	LLNSLRVPCHLRLQI	LNSLRVPCH	1	0.5969	78.3	23.00	0.51	tr_C9ZXD7_
DRB1_0101	209	RTYIRDYVVSEVNLS	YVVSEVNLS	6	0.5934	81.4	24.00	0.74	tr_C9ZXD7_
DRB1_0101	210	PPDLTVIKVLRQLLT	IKVLRQLLT	6	0.5932	81.6	24.00	0.54	tr_C9ZXD7_
DRB1_0101	211	QLLGQMLRLSPLDHK	GQMLRLSPL	3	0.5929	81.8	24.00	0.48	tr_C9ZXD7_
DRB1_0101	212	LGYIEGKHVDAQVVM	LGYIEGKHV	0	0.5912	83.4	24.00	0.49	tr_C9ZXD7_
DRB1_0101	213	AADLLLHRLFSSSCS	LHRLFSSSC	5	0.5901	84.3	24.00	0.47	tr_C9ZXD7_
DRB1_0101	214	YYTIPYGNALGYIEG	YTIPYGNAL	1	0.5901	84.3	24.00	0.43	tr_C9ZXD7_
DRB1_0101	215	LNEIKIMCSLNHPNI	IKIMCSLNH	3	0.5897	84.8	24.00	0.53	tr_C9ZXD7_
DRB1_0101	216	AFATVPVTLFTVAVP	FATVPVTLF	1	0.5891	85.2	24.00	0.69	tr_C9ZXD7_

DRB1_0101	217	RYVIDQTRAIVNCTV	YVIDQTRAI	1	0.5884	85.9	24.00	0.43	tr_C9ZXD7_
DRB1_0101	218	TRWNMAAEVVTGFLE	WNMAAEVVT	2	0.5876	86.6	25.00	0.56	tr_C9ZXD7_
DRB1_0101	219	LVHTLSYDIIVVPIS	YDIIVVPIS	6	0.5869	87.3	25.00	0.45	tr_C9ZXD7_
DRB1_0101	220	ATFRPADALNERYVI	FRPADALNE	2	0.5861	88.1	25.00	0.70	tr_C9ZXD7_
DRB1_0101	221	RVPCHLRLQILNSSR	LRLQILNSS	5	0.5854	88.7	25.00	0.50	tr_C9ZXD7_
DRB1_0101	222	ILNSSRNTFALQIVA	LNSSRNTFA	1	0.5836	90.5	25.00	0.55	tr_C9ZXD7_
DRB1_0101	223	ERYVIDQTRAIVNCT	YVIDQTRAI	2	0.5836	90.5	25.00	0.55	tr_C9ZXD7_
DRB1_0101	224	VPISEPGVRLALEGF	PISEPGVRL	1	0.5832	90.9	25.00	0.70	tr_C9ZXD7_
DRB1_0101	225	NTPISSTEVRKVLMA	ISSTEVRKV	3	0.5831	91.0	25.00	0.73	tr_C9ZXD7_
DRB1_0101	226	TVVFRFPCQITAALE	FRFPCQITA	3	0.5825	91.6	25.00	0.51	tr_C9ZXD7_
DRB1_0101	227	GTVVFRFPCQITAAL	FRFPCQITA	4	0.5821	92.0	25.00	0.51	tr_C9ZXD7_
DRB1_0101	228	VTPRGLSVIGPLQSF	LSVIGPLQS	5	0.5809	93.2	26.00	0.63	tr_C9ZXD7_
DRB1_0101	229	LEESINALLNEIKIM	INALLNEIK	4	0.5808	93.3	26.00	0.47	tr_C9ZXD7_
DRB1_0101	230	EQLLGQMLRLSPLDH	GQMLRLSPL	4	0.5799	94.2	26.00	0.41	tr_C9ZXD7_
DRB1_0101	231	DLAPLQVFSGEVLKL	LQVFSGEVL	4	0.5783	95.9	26.00	0.31	tr_C9ZXD7_
DRB1_0101	232	NERYVIDQTRAIVNC	YVIDQTRAI	3	0.5782	96.0	26.00	0.62	tr_C9ZXD7_
DRB1_0101	233	ESINALLNEIKIMCS	INALLNEIK	2	0.5781	96.1	26.00	0.43	tr_C9ZXD7_
DRB1_0101	234	LLGQMLRLSPLDHKT	GQMLRLSPL	2	0.5780	96.1	26.00	0.49	tr_C9ZXD7_
DRB1_0101	235	LGQMLRLSPLDHKTS	MLRLSPLDH	3	0.5758	98.5	27.00	0.36	tr_C9ZXD7_
DRB1_0101	236	TKNGYYTIPYGNALG	YYTIPYGNA	4	0.5752	99.2	27.00	0.42	tr_C9ZXD7_
DRB1_0101	237	LRMCFCLQPEGRATA	FCLQPEGRA	4	0.5745	99.9	27.00	0.50	tr_C9ZXD7_
DRB1_0101	238	SINALLNEIKIMCSL	INALLNEIK	1	0.5723	102.2	27.00	0.37	tr_C9ZXD7_
DRB1_0101	239	TDVDYYVDGLTSNPV	VDGLTSNPV	6	0.5711	103.7	27.00	0.56	tr_C9ZXD7_
DRB1_0101	240	LSVIGPLQSFLEYGY	LSVIGPLQS	0	0.5707	104.1	27.00	0.40	tr_C9ZXD7_
DRB1_0101	241	RDLAPLQVFSGEVLK	LAPLQVFSG	2	0.5698	105.1	28.00	0.54	tr_C9ZXD7_
DRB1_0101	242	INTPISSTEVRKVLM	ISSTEVRKV	4	0.5696	105.3	28.00	0.65	tr_C9ZXD7_
DRB1_0101	243	NSVQDLVHTLSYDII	LVHTLSYDI	5	0.5688	106.2	28.00	0.58	tr_C9ZXD7_
DRB1_0101	244	RDVKPQNILIKGDVV	NILIKGDVV	6	0.5679	107.2	28.00	0.68	tr_C9ZXD7_
DRB1_0101	245	IPYGNALGYIEGKHV	LGYIEGKHV	6	0.5678	107.4	28.00	0.57	tr_C9ZXD7_
DRB1_0101	246	LSEILEGKAVTPRGL	ILEGKAVTP	3	0.5667	108.6	28.00	0.47	tr_C9ZXD7_
DRB1_0101	247	LKVLHTWILEERMYT	LKVLHTWIL	0	0.5665	108.9	28.00	0.51	tr_C9ZXD7_
DRB1_0101	248	DLEESINALLNEIKI	LEESINALL	1	0.5659	109.5	28.00	0.37	tr_C9ZXD7_
DRB1_0101	249	LFMETPHMLADMTDV	FMETPHMLA	1	0.5651	110.6	28.00	0.41	tr_C9ZXD7_
DRB1_0101	250	VHTLSYDIIVVPISE	YDIIVVPIS	5	0.5642	111.6	29.00	0.64	tr_C9ZXD7_
DRB1_0101	251	LNSLRVPCHLRLQIL	LRVPCHLRL	3	0.5642	111.6	29.00	0.33	tr_C9ZXD7_
DRB1_0101	252	IGFDDKYRITRWNMA	YRITRWNMA	6	0.5640	111.9	29.00	0.82	tr_C9ZXD7_
DRB1_0101	253	SYDIIVVPISEPGVR	YDIIVVPIS	1	0.5634	112.6	29.00	0.44	tr_C9ZXD7_
DRB1_0101	254	EESINALLNEIKIMC	INALLNEIK	3	0.5630	113.0	29.00	0.47	tr_C9ZXD7_
DRB1_0101	255	EWEWTSSEQLLGQML	SEQLLGQML	6	0.5626	113.6	29.00	0.51	tr_C9ZXD7_
DRB1_0101	256	LNERYVIDQTRAIVN	YVIDQTRAI	4	0.5623	114.0	29.00	0.68	tr_C9ZXD7_
DRB1_0101	257	FLRMCFCLQPEGRAT	FCLQPEGRA	5	0.5622	114.1	29.00	0.40	tr_C9ZXD7_
DRB1_0101	258	DLVHTLSYDIIVVPI	LVHTLSYDI	1	0.5619	114.4	29.00	0.62	tr_C9ZXD7_
DRB1_0101	259	LNQEGSLIGRILICA	EGSLIGRIL	3	0.5619	114.5	29.00	0.65	tr_C9ZXD7_
DRB1_0101	260	ERDLAPLQVFSGEVL	LAPLQVFSG	3	0.5614	115.0	29.00	0.58	tr_C9ZXD7_
DRB1_0101	261	MCFCLQPEGRATAAD	FCLQPEGRA	2	0.5604	116.4	29.00	0.55	tr_C9ZXD7_
DRB1_0101	262	GQMLRLSPLDHKTSV	MLRLSPLDH	2	0.5585	118.8	30.00	0.34	tr_C9ZXD7_
DRB1_0101	263	QHNISMILLRTMFVS	MILLRTMFV	5	0.5580	119.4	30.00	0.38	tr_C9ZXD7_
DRB1_0101	264	DATFRPADALNERYV	FRPADALNE	3	0.5577	119.8	30.00	0.77	tr_C9ZXD7_
DRB1_0101	265	MAIGTAVSLRKNAAT	IGTAVSLRK	2	0.5571	120.5	30.00	0.51	tr_C9ZXD7_
DRB1_0101	266	SLSEILEGKAVTPRG	SEILEGKAV	2	0.5566	121.3	30.00	0.47	tr_C9ZXD7_

DRB1_0101	267	IMCSLNHPNIVHYFH	CSLNHPNIV	2	0.5564	121.4	30.00	0.34	tr_C9ZXD7_
DRB1_0101	268	DQHNISMILLRTMFV	MILLRTMFV	6	0.5562	121.8	30.00	0.30	tr_C9ZXD7_
DRB1_0101	269	VVFRFPCQITAALED	FRFPCQITA	2	0.5559	122.2	30.00	0.50	tr_C9ZXD7_
DRB1_0101	270	EELARGMLAEWEWTS	EELARGMLA	0	0.5541	124.5	30.00	0.65	tr_C9ZXD7_
DRB1_0101	271	LFDCPPLFMETPHML	LFMETPHML	6	0.5513	128.4	31.00	0.71	tr_C9ZXD7_
DRB1_0101	272	TFRPADALNERYVID	FRPADALNE	1	0.5512	128.6	31.00	0.63	tr_C9ZXD7_
DRB1_0101	273	PGVRLALEGFQYVIN	LALEGFQYV	4	0.5510	128.7	31.00	0.39	tr_C9ZXD7_
DRB1_0101	274	LSYDIIVVPISEPGV	YDIIVVPIS	2	0.5504	129.7	31.00	0.56	tr_C9ZXD7_
DRB1_0101	275	VINTPISSTEVRKVL	ISSTEVRKV	5	0.5499	130.3	31.00	0.48	tr_C9ZXD7_
DRB1_0101	276	LSLSEILEGKAVTPR	SEILEGKAV	3	0.5492	131.3	31.00	0.55	tr_C9ZXD7_
DRB1_0101	277	YFTEDLEESINALLN	LEESINALL	5	0.5489	131.8	31.00	0.61	tr_C9ZXD7_
DRB1_0101	278	TEDLEESINALLNEI	LEESINALL	3	0.5467	134.9	32.00	0.82	tr_C9ZXD7_
DRB1_0101	279	TPISSTEVRKVLMAI	ISSTEVRKV	2	0.5462	135.6	32.00	0.73	tr_C9ZXD7_
DRB1_0101	280	NQEGSLIGRILICAN	EGSLIGRIL	2	0.5447	137.8	32.00	0.60	tr_C9ZXD7_
DRB1_0101	281	VLLGRGSFGAVYEAT	VLLGRGSFG	0	0.5442	138.6	32.00	0.38	tr_C9ZXD7_
DRB1_0101	282	LLNEIKIMCSLNHPN	IKIMCSLNH	4	0.5440	138.8	32.00	0.60	tr_C9ZXD7_
DRB1_0101	283	EDLEESINALLNEIK	LEESINALL	2	0.5426	141.1	33.00	0.67	tr_C9ZXD7_
DRB1_0101	284	FTEDLEESINALLNE	LEESINALL	4	0.5425	141.1	33.00	0.72	tr_C9ZXD7_
DRB1_0101	285	TLSYDIIVVPISEPG	YDIIVVPIS	3	0.5423	141.5	33.00	0.69	tr_C9ZXD7_
DRB1_0101	286	LHTWILEERMYTVLL	WILEERMYT	3	0.5392	146.3	33.00	0.28	tr_C9ZXD7_
DRB1_0101	287	LRVPCHLRLQILNSS	PCHLRLQIL	3	0.5391	146.5	33.00	0.31	tr_C9ZXD7_
DRB1_0101	288	EPGVRLALEGFQYVI	LALEGFQYV	5	0.5390	146.6	33.00	0.35	tr_C9ZXD7_
DRB1_0101	289	HTWILEERMYTVLLS	ILEERMYTV	3	0.5385	147.4	33.00	0.31	tr_C9ZXD7_
DRB1_0101	290	HTLSYDIIVVPISEP	YDIIVVPIS	4	0.5380	148.2	33.00	0.69	tr_C9ZXD7_
DRB1_0101	291	GVRLALEGFQYVINT	LALEGFQYV	3	0.5380	148.2	33.00	0.45	tr_C9ZXD7_
DRB1_0101	292	PVAQAAPSTAQISEL	VAQAAPSTA	1	0.5379	148.3	33.00	0.65	tr_C9ZXD7_
DRB1_0101	293	YLHARGVAHRDVKPQ	YLHARGVAH	0	0.5355	152.2	34.00	0.56	tr_C9ZXD7_
DRB1_0101	294	YVIDQTRAIVNCTVN	IDQTRAIVN	2	0.5352	152.8	34.00	0.29	tr_C9ZXD7_
DRB1_0101	295	SPWTKGVLLGRGSFG	VLLGRGSFG	6	0.5351	153.0	34.00	0.56	tr_C9ZXD7_
DRB1_0101	296	CDIWSVGCVAAELFD	VGCVAAELF	5	0.5347	153.6	34.00	0.32	tr_C9ZXD7_
DRB1_0101	297	DFLRMCFCLQPEGRA	FCLQPEGRA	6	0.5346	153.8	34.00	0.26	tr_C9ZXD7_
DRB1_0101	298	RGWLNSVQDLVHTLS	WLNSVQDLV	2	0.5338	155.1	34.00	0.40	tr_C9ZXD7_
DRB1_0101	299	VFRFPCQITAALEDI	FRFPCQITA	1	0.5336	155.4	34.00	0.46	tr_C9ZXD7_
DRB1_0101	300	QEGSLIGRILICANA	EGSLIGRIL	1	0.5316	158.8	35.00	0.49	tr_C9ZXD7_
DRB1_0101	301	HNISMILLRTMFVSL	MILLRTMFV	4	0.5296	162.3	35.00	0.45	tr_C9ZXD7_
DRB1_0101	302	SEILEGKAVTPRGLS	ILEGKAVTP	2	0.5285	164.2	35.00	0.46	tr_C9ZXD7_
DRB1_0101	303	DIWSVGCVAAELFDC	VGCVAAELF	4	0.5265	167.8	36.00	0.35	tr_C9ZXD7_
DRB1_0101	304	CFCLQPEGRATAADL	FCLQPEGRA	1	0.5263	168.3	36.00	0.54	tr_C9ZXD7_
DRB1_0101	305	IERDLAPLQVFSGEV	LAPLQVFSG	4	0.5262	168.3	36.00	0.55	tr_C9ZXD7_
DRB1_0101	306	NALLNEIKIMCSLNH	IKIMCSLNH	6	0.5261	168.6	36.00	0.34	tr_C9ZXD7_
DRB1_0101	307	DGTVVFRFPCQITAA	FRFPCQITA	5	0.5256	169.4	36.00	0.53	tr_C9ZXD7_
DRB1_0101	308	LNSSRNTFALQIVAT	SRNTFALQI	3	0.5243	171.8	36.00	0.34	tr_C9ZXD7_
DRB1_0101	309	QSFLEYGYGKQVEEL	LEYGYGKQV	3	0.5223	175.6	37.00	0.80	tr_C9ZXD7_
DRB1_0101	310	DIERDLAPLQVFSGE	LAPLQVFSG	5	0.5214	177.5	37.00	0.38	tr_C9ZXD7_
DRB1_0101	311	PCDIWSVGCVAAELF	VGCVAAELF	6	0.5206	179.0	37.00	0.23	tr_C9ZXD7_
DRB1_0101	312	KTSVDSLFPGTLCLH	VDSLFPGTL	3	0.5172	185.6	38.00	0.44	tr_C9ZXD7_
DRB1_0101	313	IFSREKQYVSSLTIT	KQYVSSLTI	5	0.5165	187.1	38.00	0.37	tr_C9ZXD7_
DRB1_0101	314	TNLVDQHNISMILLR	LVDQHNISM	2	0.5158	188.5	38.00	0.33	tr_C9ZXD7_
DRB1_0101	315	LNSVQDLVHTLSYDI	LVHTLSYDI	6	0.5147	190.7	38.00	0.38	tr_C9ZXD7_
DRB1_0101	316	TAADLLLHRLFSSSC	LHRLFSSSC	6	0.5142	191.7	38.00	0.32	tr_C9ZXD7_

DRB1_0101	317	VDGLTSNPVLCDFLR	VDGLTSNPV	0	0.5141	191.9	38.00	0.67	tr_C9ZXD7_
DRB1_0101	318	GWLNSVQDLVHTLSY	SVQDLVHTL	4	0.5138	192.6	38.00	0.31	tr_C9ZXD7_
DRB1_0101	319	DDATFRPADALNERY	FRPADALNE	4	0.5136	193.0	38.00	0.81	tr_C9ZXD7_
DRB1_0101	320	FATVPVTLFTVAVPI	FATVPVTLF	0	0.5135	193.2	38.00	0.47	tr_C9ZXD7_
DRB1_0101	321	RWNMAAEVVTGFLES	WNMAAEVVT	1	0.5132	193.9	38.00	0.61	tr_C9ZXD7_
DRB1_0101	322	SFLEYGYGKQVEELA	LEYGYGKQV	2	0.5131	194.1	38.00	0.66	tr_C9ZXD7_
DRB1_0101	323	INALLNEIKIMCSLN	NALLNEIKI	1	0.5129	194.5	38.00	0.35	tr_C9ZXD7_
DRB1_0101	324	VDQHNISMILLRTMF	HNISMILLR	3	0.5128	194.6	38.00	0.25	tr_C9ZXD7_
DRB1_0101	325	LVDQHNISMILLRTM	DQHNISMIL	2	0.5125	195.4	39.00	0.21	tr_C9ZXD7_
DRB1_0101	326	NLVDQHNISMILLRT	DQHNISMIL	3	0.5113	197.8	39.00	0.22	tr_C9ZXD7_
DRB1_0101	327	TWILEERMYTVLLSS	EERMYTVLL	4	0.5112	198.2	39.00	0.31	tr_C9ZXD7_
DRB1_0101	328	EKLLPHLRSTCGSIR	LPHLRSTCG	3	0.5110	198.4	39.00	0.34	tr_C9ZXD7_
DRB1_0101	329	CLHPCIPEAVATLLN	CIPEAVATL	4	0.5105	199.5	39.00	0.47	tr_C9ZXD7_
DRB1_0101	330	GDIERDLAPLQVFSG	ERDLAPLQV	3	0.5104	199.7	39.00	0.34	tr_C9ZXD7_
DRB1_0101	331	LHPCIPEAVATLLNS	CIPEAVATL	3	0.5104	199.8	39.00	0.53	tr_C9ZXD7_
DRB1_0101	332	VLHTWILEERMYTVL	WILEERMYT	4	0.5095	201.7	39.00	0.30	tr_C9ZXD7_
DRB1_0101	333	FYFTEDLEESINALL	FTEDLEESI	2	0.5093	202.3	39.00	0.37	tr_C9ZXD7_
DRB1_0101	334	IGRILICANAKDNLG	LICANAKDN	4	0.5091	202.6	39.00	0.40	tr_C9ZXD7_
DRB1_0101	335	EGSLIGRILICANAK	EGSLIGRIL	0	0.5089	203.1	39.00	0.31	tr_C9ZXD7_
DRB1_0101	336	FLEYGYGKQVEELAR	LEYGYGKQV	1	0.5076	206.0	39.00	0.49	tr_C9ZXD7_
DRB1_0101	337	LRYMAPEVYRGEDHC	LRYMAPEVY	0	0.5070	207.2	40.00	0.51	tr_C9ZXD7_
DRB1_0101	338	DRGWLNSVQDLVHTL	WLNSVQDLV	3	0.5065	208.4	40.00	0.49	tr_C9ZXD7_
DRB1_0101	339	ALLNEIKIMCSLNHP	IKIMCSLNH	5	0.5061	209.4	40.00	0.56	tr_C9ZXD7_
DRB1_0101	340	NTKNGYYTIPYGNAL	YYTIPYGNA	5	0.5060	209.7	40.00	0.52	tr_C9ZXD7_
DRB1_0101	341	WILEERMYTVLLSSA	EERMYTVLL	3	0.5059	209.7	40.00	0.38	tr_C9ZXD7_
DRB1_0101	342	PCIPEAVATLLNSLR	CIPEAVATL	1	0.5040	214.2	40.00	0.35	tr_C9ZXD7_
DRB1_0101	343	GRILICANAKDNLGE	LICANAKDN	3	0.5027	217.2	40.00	0.44	tr_C9ZXD7_
DRB1_0101	344	LIGRILICANAKDNL	LICANAKDN	5	0.5021	218.6	41.00	0.28	tr_C9ZXD7_
DRB1_0101	345	VRLALEGFQYVINTP	LALEGFQYV	2	0.5014	220.2	41.00	0.50	tr_C9ZXD7_
DRB1_0101	346	EDVQGTLRYMAPEVY	LRYMAPEVY	6	0.5007	222.0	41.00	0.66	tr_C9ZXD7_
DRB1_0101	347	PISSTEVRKVLMAIG	ISSTEVRKV	1	0.5001	223.4	41.00	0.60	tr_C9ZXD7_
DRB1_0101	348	ILIKGDVVKITDFGT	LIKGDVVKI	1	0.4996	224.6	41.00	0.68	tr_C9ZXD7_
DRB1_0101	349	PISEPGVRLALEGFQ	PISEPGVRL	0	0.4995	224.9	41.00	0.52	tr_C9ZXD7_
DRB1_0101	350	NSLRVPCHLRLQILN	LRVPCHLRL	2	0.4976	229.5	42.00	0.37	tr_C9ZXD7_
DRB1_0101	351	REKLLPHLRSTCGSI	EKLLPHLRS	1	0.4971	230.7	42.00	0.38	tr_C9ZXD7_
DRB1_0101	352	AVTPRGLSVIGPLQS	LSVIGPLQS	6	0.4968	231.5	42.00	0.51	tr_C9ZXD7_
DRB1_0101	353	HPCIPEAVATLLNSL	CIPEAVATL	2	0.4967	231.7	42.00	0.50	tr_C9ZXD7_
DRB1_0101	354	AIGTAVSLRKNAATA	IGTAVSLRK	1	0.4965	232.1	42.00	0.44	tr_C9ZXD7_
DRB1_0101	355	SLRVPCHLRLQILNS	PCHLRLQIL	4	0.4953	235.2	42.00	0.38	tr_C9ZXD7_
DRB1_0101	356	SEPGVRLALEGFQYV	LALEGFQYV	6	0.4953	235.4	42.00	0.25	tr_C9ZXD7_
DRB1_0101	357	ATSDLTGGKMAVKMF	SDLTGGKMA	2	0.4953	235.4	42.00	0.27	tr_C9ZXD7_
DRB1_0101	358	YTIPYGNALGYIEGK	YGNALGYIE	4	0.4941	238.3	42.00	0.31	tr_C9ZXD7_
DRB1_0101	359	LREKLLPHLRSTCGS	EKLLPHLRS	2	0.4932	240.6	42.00	0.44	tr_C9ZXD7_
DRB1_0101	360	LCLHPCIPEAVATLL	CIPEAVATL	5	0.4925	242.4	43.00	0.40	tr_C9ZXD7_
DRB1_0101	361	TSVDSLFPGTLCLHP	VDSLFPGTL	2	0.4920	243.9	43.00	0.41	tr_C9ZXD7_
DRB1_0101	362	GYIEGKHVDAQVVMI	YIEGKHVDA	1	0.4916	244.8	43.00	0.27	tr_C9ZXD7_
DRB1_0101	363	VIDQTRAIVNCTVNV	TRAIVNCTV	4	0.4912	245.8	43.00	0.35	tr_C9ZXD7_
DRB1_0101	364	NLSLSEILEGKAVTP	SEILEGKAV	4	0.4911	246.2	43.00	0.55	tr_C9ZXD7_
DRB1_0101	365	VNLSLSEILEGKAVT	SEILEGKAV	5	0.4908	247.1	43.00	0.55	tr_C9ZXD7_
DRB1_0101	366	ALNERYVIDQTRAIV	YVIDQTRAI	5	0.4900	249.1	43.00	0.79	tr_C9ZXD7_

DRB1_0101	367	KLLPHLRSTCGSIRE	LPHLRSTCG	2	0.4899	249.3	43.00	0.36	tr_C9ZXD7_
DRB1_0101	368	IWSVGCVAAELFDCP	VGCVAAELF	3	0.4899	249.5	43.00	0.37	tr_C9ZXD7_
DRB1_0101	369	YIEGKHVDAQVVMIG	HVDAQVVMI	5	0.4895	250.5	43.00	0.32	tr_C9ZXD7_
DRB1_0101	370	ITNLVDQHNISMILL	LVDQHNISM	3	0.4894	250.9	43.00	0.46	tr_C9ZXD7_
DRB1_0101	371	TSDLTGGKMAVKMFY	TGGKMAVKM	4	0.4893	251.1	43.00	0.27	tr_C9ZXD7_
DRB1_0101	372	SDLTGGKMAVKMFYF	TGGKMAVKM	3	0.4887	252.7	43.00	0.32	tr_C9ZXD7_
DRB1_0101	373	VTGFLESGCVGKPLS	FLESGCVGK	3	0.4875	256.0	44.00	0.38	tr_C9ZXD7_
DRB1_0101	374	IDQTRAIVNCTVNVL	TRAIVNCTV	3	0.4871	257.0	44.00	0.41	tr_C9ZXD7_
DRB1_0101	375	MCSLNHPNIVHYFHC	CSLNHPNIV	1	0.4850	263.1	44.00	0.34	tr_C9ZXD7_
DRB1_0101	376	DQTRAIVNCTVNVLT	TRAIVNCTV	2	0.4834	267.5	44.00	0.35	tr_C9ZXD7_
DRB1_0101	377	FSSSCSAEVESLPDI	FSSSCSAEV	0	0.4831	268.5	45.00	0.71	tr_C9ZXD7_
DRB1_0101	378	QTRAIVNCTVNVLTL	TRAIVNCTV	1	0.4811	274.4	45.00	0.34	tr_C9ZXD7_
DRB1_0101	379	LQSFLEYGYGKQVEE	LEYGYGKQV	4	0.4799	277.9	45.00	0.75	tr_C9ZXD7_
DRB1_0101	380	EVNLSLSEILEGKAV	VNLSLSEIL	1	0.4791	280.2	45.00	0.34	tr_C9ZXD7_
DRB1_0101	381	SLIGRILICANAKDN	ILICANAKD	5	0.4791	280.2	45.00	0.25	tr_C9ZXD7_
DRB1_0101	382	ELREKLLPHLRSTCG	EKLLPHLRS	3	0.4788	281.3	45.00	0.50	tr_C9ZXD7_
DRB1_0101	383	QMLRLSPLDHKTSVD	MLRLSPLDH	1	0.4775	285.3	46.00	0.40	tr_C9ZXD7_
DRB1_0101	384	DLTGGKMAVKMFYFT	TGGKMAVKM	2	0.4763	289.0	46.00	0.30	tr_C9ZXD7_
DRB1_0101	385	VSEVNLSLSEILEGK	VNLSLSEIL	3	0.4756	291.1	46.00	0.62	tr_C9ZXD7_
DRB1_0101	386	HKTSVDSLFPGTLCL	VDSLFPGTL	4	0.4739	296.7	46.00	0.49	tr_C9ZXD7_
DRB1_0101	387	SAEVESLPDIFSREK	VESLPDIFS	3	0.4738	297.0	46.00	0.77	tr_C9ZXD7_
DRB1_0101	388	EATSDLTGGKMAVKM	TSDLTGGKM	2	0.4735	297.8	47.00	0.40	tr_C9ZXD7_
DRB1_0101	389	IGTAVSLRKNAATAQ	VSLRKNAAT	4	0.4735	298.0	47.00	0.23	tr_C9ZXD7_
DRB1_0101	390	TAVSLRKNAATAQEE	LRKNAATAQ	4	0.4731	299.2	47.00	0.42	tr_C9ZXD7_
DRB1_0101	391	IDDATFRPADALNER	FRPADALNE	5	0.4726	300.8	47.00	0.69	tr_C9ZXD7_
DRB1_0101	392	SSTEVRKVLMAIGTA	VRKVLMAIG	4	0.4723	301.7	47.00	0.29	tr_C9ZXD7_
DRB1_0101	393	GTAVSLRKNAATAQE	LRKNAATAQ	5	0.4718	303.3	47.00	0.37	tr_C9ZXD7_
DRB1_0101	394	IDVIVCDRGWLNSVQ	IVCDRGWLN	3	0.4717	303.8	47.00	0.67	tr_C9ZXD7_
DRB1_0101	395	NLFMELCCCSLGDII	FMELCCCSL	2	0.4715	304.5	47.00	0.46	tr_C9ZXD7_
DRB1_0101	396	MFYFTEDLEESINAL	FTEDLEESI	3	0.4713	305.2	47.00	0.56	tr_C9ZXD7_
DRB1_0101	397	CDFLRMCFCLQPEGR	FLRMCFCLQ	2	0.4708	306.5	47.00	0.39	tr_C9ZXD7_
DRB1_0101	398	TVNVLTLITNLVDQH	VLTLITNLV	3	0.4708	306.8	47.00	0.68	tr_C9ZXD7_
DRB1_0101	399	IEGKHVDAQVVMIGF	HVDAQVVMI	4	0.4699	309.8	47.00	0.41	tr_C9ZXD7_
DRB1_0101	400	SVDSLFPGTLCLHPC	VDSLFPGTL	1	0.4694	311.3	47.00	0.34	tr_C9ZXD7_
DRB1_0101	401	SEVNLSLSEILEGKA	VNLSLSEIL	2	0.4694	311.4	47.00	0.57	tr_C9ZXD7_
DRB1_0101	402	VVTGFLESGCVGKPL	FLESGCVGK	4	0.4689	313.1	47.00	0.38	tr_C9ZXD7_
DRB1_0101	403	RILICANAKDNLGEY	LICANAKDN	2	0.4682	315.3	48.00	0.49	tr_C9ZXD7_
DRB1_0101	404	FRFPCQITAALEDID	FRFPCQITA	0	0.4679	316.6	48.00	0.37	tr_C9ZXD7_
DRB1_0101	405	AVSLRKNAATAQEER	LRKNAATAQ	3	0.4678	316.8	48.00	0.56	tr_C9ZXD7_
DRB1_0101	406	KVLHTWILEERMYTV	WILEERMYT	5	0.4678	316.9	48.00	0.31	tr_C9ZXD7_
DRB1_0101	407	CDRGWLNSVQDLVHT	WLNSVQDLV	4	0.4668	320.1	48.00	0.54	tr_C9ZXD7_
DRB1_0101	408	WLNSVQDLVHTLSYD	SVQDLVHTL	3	0.4658	323.7	48.00	0.38	tr_C9ZXD7_
DRB1_0101	409	VNLFMELCCCSLGDI	FMELCCCSL	3	0.4655	324.7	48.00	0.51	tr_C9ZXD7_
DRB1_0101	410	LITNLVDQHNISMIL	LVDQHNISM	4	0.4650	326.4	48.00	0.45	tr_C9ZXD7_
DRB1_0101	411	TGDIERDLAPLQVFS	IERDLAPLQ	3	0.4647	327.8	48.00	0.41	tr_C9ZXD7_
DRB1_0101	412	LGDIIYGRSQKPPDL	IIYGRSQKP	3	0.4638	330.9	49.00	0.82	tr_C9ZXD7_
DRB1_0101	413	TGFLESGCVGKPLSD	FLESGCVGK	2	0.4637	331.2	49.00	0.37	tr_C9ZXD7_
DRB1_0101	414	LAEWEWTSSEQLLGQ	WEWTSSEQL	3	0.4627	334.8	49.00	0.77	tr_C9ZXD7_
DRB1_0101	415	NSVNLFMELCCCSLG	FMELCCCSL	5	0.4619	337.7	49.00	0.46	tr_C9ZXD7_
DRB1_0101	416	YEATSDLTGGKMAVK	TSDLTGGKM	3	0.4611	340.7	49.00	0.51	tr_C9ZXD7_

DRB1_0101	417	FRPADALNERYVIDQ	FRPADALNE	0	0.4610	340.9	49.00	0.35	tr_C9ZXD7_
DRB1_0101	418	DIFSREKQYVSSLTI	FSREKQYVS	2	0.4607	342.2	49.00	0.32	tr_C9ZXD7_
DRB1_0101	419	LFMELCCCSLGDIIY	FMELCCCSL	1	0.4607	342.2	49.00	0.37	tr_C9ZXD7_
DRB1_0101	420	SVNLFMELCCCSLGD	FMELCCCSL	4	0.4606	342.4	49.00	0.49	tr_C9ZXD7_
DRB1_0101	421	LTGGKMAVKMFYFTE	KMAVKMFYF	4	0.4606	342.4	49.00	0.28	tr_C9ZXD7_
DRB1_0101	422	DIDVIVCDRGWLNSV	IVCDRGWLN	4	0.4603	343.6	49.00	0.66	tr_C9ZXD7_
DRB1_0101	423	FCLQPEGRATAADLL	FCLQPEGRA	0	0.4603	343.7	49.00	0.37	tr_C9ZXD7_
DRB1_0101	424	KMFYFTEDLEESINA	FTEDLEESI	4	0.4598	345.5	49.00	0.50	tr_C9ZXD7_
DRB1_0101	425	CTVNVLTLITNLVDQ	VLTLITNLV	4	0.4596	346.0	49.00	0.66	tr_C9ZXD7_
DRB1_0101	426	SELREKLLPHLRSTC	EKLLPHLRS	4	0.4590	348.6	50.00	0.49	tr_C9ZXD7_
DRB1_0101	427	ATAADLLLHRLFSSS	LLLHRLFSS	5	0.4572	355.3	50.00	0.34	tr_C9ZXD7_
DRB1_0101	428	IVCDRGWLNSVQDLV	WLNSVQDLV	6	0.4569	356.5	50.00	0.32	tr_C9ZXD7_
DRB1_0101	429	PLQSFLEYGYGKQVE	LEYGYGKQV	5	0.4564	358.4	50.00	0.74	tr_C9ZXD7_
DRB1_0101	430	GSLIGRILICANAKD	IGRILICAN	3	0.4556	361.4	50.00	0.19	tr_C9ZXD7_
DRB1_0101	431	LLPHLRSTCGSIRED	LRSTCGSIR	4	0.4548	364.6	50.00	0.43	tr_C9ZXD7_
DRB1_0101	432	VVSEVNLSLSEILEG	VNLSLSEIL	4	0.4546	365.5	55.00	0.51	tr_C9ZXD7_
DRB1_0101	433	TLCLHPCIPEAVATL	LHPCIPEAV	3	0.4543	366.6	55.00	0.46	tr_C9ZXD7_
DRB1_0101	434	ISSTEVRKVLMAIGT	ISSTEVRKV	0	0.4537	368.9	55.00	0.26	tr_C9ZXD7_
DRB1_0101	435	YGYGKQVEELARGML	YGKQVEELA	2	0.4537	368.9	55.00	0.50	tr_C9ZXD7_
DRB1_0101	436	VNVLTLITNLVDQHN	VLTLITNLV	2	0.4536	369.6	55.00	0.64	tr_C9ZXD7_
DRB1_0101	437	AEWEWTSSEQLLGQM	WEWTSSEQL	2	0.4535	369.7	55.00	0.71	tr_C9ZXD7_
DRB1_0101	438	LEYGYGKQVEELARG	YGKQVEELA	4	0.4532	370.9	55.00	0.44	tr_C9ZXD7_
DRB1_0101	439	GDIIYGRSQKPPDLT	IIYGRSQKP	2	0.4511	379.7	55.00	0.73	tr_C9ZXD7_
DRB1_0101	440	DDGTVVFRFPCQITA	FRFPCQITA	6	0.4509	380.2	55.00	0.46	tr_C9ZXD7_
DRB1_0101	441	KPPDLTVIKVLRQLL	LTVIKVLRQ	4	0.4496	385.7	55.00	0.61	tr_C9ZXD7_
DRB1_0101	442	PVLCDFLRMCFCLQP	FLRMCFCLQ	5	0.4480	392.6	55.00	0.44	tr_C9ZXD7_
DRB1_0101	443	MLAEWEWTSSEQLLG	WEWTSSEQL	4	0.4473	395.4	55.00	0.75	tr_C9ZXD7_
DRB1_0101	444	NPVLCDFLRMCFCLQ	FLRMCFCLQ	6	0.4468	397.6	55.00	0.35	tr_C9ZXD7_
DRB1_0101	445	NCTVNVLTLITNLVD	VLTLITNLV	5	0.4458	401.8	55.00	0.59	tr_C9ZXD7_
DRB1_0101	446	ISELREKLLPHLRST	EKLLPHLRS	5	0.4451	405.0	55.00	0.47	tr_C9ZXD7_
DRB1_0101	447	EYGYGKQVEELARGM	YGKQVEELA	3	0.4440	409.7	55.00	0.57	tr_C9ZXD7_
DRB1_0101	448	EGKHVDAQVVMIGFD	HVDAQVVMI	3	0.4419	419.3	55.00	0.46	tr_C9ZXD7_
DRB1_0101	449	VCDRGWLNSVQDLVH	WLNSVQDLV	5	0.4417	420.1	55.00	0.53	tr_C9ZXD7_
DRB1_0101	450	KGDVVKITDFGTARQ	VVKITDFGT	3	0.4413	422.1	55.00	0.74	tr_C9ZXD7_
DRB1_0101	451	WSVGCVAAELFDCPP	VGCVAAELF	2	0.4406	425.2	55.00	0.41	tr_C9ZXD7_
DRB1_0101	452	CSAEVESLPDIFSRE	VESLPDIFS	4	0.4403	426.8	55.00	0.76	tr_C9ZXD7_
DRB1_0101	453	TLITNLVDQHNISMI	LVDQHNISM	5	0.4391	432.0	55.00	0.40	tr_C9ZXD7_
DRB1_0101	454	LCDFLRMCFCLQPEG	FLRMCFCLQ	3	0.4387	434.0	55.00	0.47	tr_C9ZXD7_
DRB1_0101	455	FMELCCCSLGDIIYG	CCCSLGDII	4	0.4385	435.2	55.00	0.28	tr_C9ZXD7_
DRB1_0101	456	AEVESLPDIFSREKQ	VESLPDIFS	2	0.4382	436.4	55.00	0.77	tr_C9ZXD7_
DRB1_0101	457	DIIYGRSQKPPDLTV	IIYGRSQKP	1	0.4378	438.2	55.00	0.64	tr_C9ZXD7_
DRB1_0101	458	TRAIVNCTVNVLTLI	IVNCTVNVL	3	0.4363	445.4	55.00	0.28	tr_C9ZXD7_
DRB1_0101	459	DVIVCDRGWLNSVQD	IVCDRGWLN	2	0.4356	448.8	55.00	0.70	tr_C9ZXD7_
DRB1_0101	460	EILEGKAVTPRGLSV	ILEGKAVTP	1	0.4354	450.0	55.00	0.44	tr_C9ZXD7_
DRB1_0101	461	NVLTLITNLVDQHNI	VLTLITNLV	1	0.4354	450.0	55.00	0.50	tr_C9ZXD7_
DRB1_0101	462	VLCDFLRMCFCLQPE	FLRMCFCLQ	4	0.4349	452.2	55.00	0.47	tr_C9ZXD7_
DRB1_0101	463	SPCDIWSVGCVAAEL	DIWSVGCVA	3	0.4345	454.2	55.00	0.28	tr_C9ZXD7_
DRB1_0101	464	GKHVDAQVVMIGFDD	HVDAQVVMI	2	0.4345	454.3	55.00	0.41	tr_C9ZXD7_
DRB1_0101	465	VIVCDRGWLNSVQDL	IVCDRGWLN	1	0.4333	460.0	55.00	0.63	tr_C9ZXD7_
DRB1_0101	466	RATAADLLLHRLFSS	LLLHRLFSS	6	0.4328	462.7	55.00	0.32	tr_C9ZXD7_

DRB1_0101	467	SVIGPLQSFLEYGYG	IGPLQSFLE	2	0.4320	466.7	55.00	0.65	tr_C9ZXD7_
DRB1_0101	468	VDSLFPGTLCLHPCI	DSLFPGTLC	1	0.4317	468.4	55.00	0.28	tr_C9ZXD7_
DRB1_0101	469	MLRLSPLDHKTSVDS	LSPLDHKTS	3	0.4277	488.9	60.00	0.40	tr_C9ZXD7_
DRB1_0101	470	GFLESGCVGKPLSDL	FLESGCVGK	1	0.4274	490.6	60.00	0.32	tr_C9ZXD7_
DRB1_0101	471	ATVPVTLFTVAVPIL	PVTLFTVAV	3	0.4269	493.4	60.00	0.50	tr_C9ZXD7_
DRB1_0101	472	MELCCCSLGDIIYGR	CCCSLGDII	3	0.4264	495.6	60.00	0.38	tr_C9ZXD7_
DRB1_0101	473	VKMFYFTEDLEESIN	FTEDLEESI	5	0.4263	496.2	60.00	0.44	tr_C9ZXD7_
DRB1_0101	474	PTGDIERDLAPLQVF	IERDLAPLQ	4	0.4257	499.4	60.00	0.41	tr_C9ZXD7_
DRB1_0101	475	CSLGDIIYGRSQKPP	IIYGRSQKP	5	0.4257	499.5	60.00	0.72	tr_C9ZXD7_
DRB1_0101	476	TVLLSSASSEGDLST	TVLLSSASS	0	0.4255	500.6	60.00	0.31	tr_C9ZXD7_
DRB1_0101	477	SDIDVIVCDRGWLNS	IVCDRGWLN	5	0.4245	505.9	60.00	0.63	tr_C9ZXD7_
DRB1_0101	478	RILTLRQDSPWTKGV	LRQDSPWTK	4	0.4240	509.0	60.00	0.57	tr_C9ZXD7_
DRB1_0101	479	SCSAEVESLPDIFSR	VESLPDIFS	5	0.4229	514.7	60.00	0.74	tr_C9ZXD7_
DRB1_0101	480	ENSVNLFMELCCCSL	FMELCCCSL	6	0.4227	516.1	60.00	0.43	tr_C9ZXD7_
DRB1_0101	481	VNCTVNVLTLITNLV	VLTLITNLV	6	0.4217	521.6	60.00	0.51	tr_C9ZXD7_
DRB1_0101	482	GMLAEWEWTSSEQLL	WEWTSSEQL	5	0.4206	527.9	60.00	0.67	tr_C9ZXD7_
DRB1_0101	483	LIKGDVVKITDFGTA	VVKITDFGT	5	0.4200	531.2	60.00	0.44	tr_C9ZXD7_
DRB1_0101	484	ILTLRQDSPWTKGVL	LRQDSPWTK	3	0.4193	535.6	60.00	0.70	tr_C9ZXD7_
DRB1_0101	485	SLGDIIYGRSQKPPD	IIYGRSQKP	4	0.4177	544.6	60.00	0.84	tr_C9ZXD7_
DRB1_0101	486	GDVVKITDFGTARQG	VVKITDFGT	2	0.4171	548.5	60.00	0.63	tr_C9ZXD7_
DRB1_0101	487	VAQAAPSTAQISELR	VAQAAPSTA	0	0.4158	556.3	60.00	0.47	tr_C9ZXD7_
DRB1_0101	488	VAHRDVKPQNILIKG	DVKPQNILI	4	0.4131	572.3	60.00	0.51	tr_C9ZXD7_
DRB1_0101	489	TGGKMAVKMFYFTED	KMAVKMFYF	3	0.4127	575.2	60.00	0.35	tr_C9ZXD7_
DRB1_0101	490	EVVTGFLESGCVGKP	FLESGCVGK	5	0.4118	580.7	60.00	0.33	tr_C9ZXD7_
DRB1_0101	491	KHVDAQVVMIGFDDK	HVDAQVVMI	1	0.4115	582.2	60.00	0.32	tr_C9ZXD7_
DRB1_0101	492	QKPPDLTVIKVLRQL	LTVIKVLRQ	5	0.4114	582.9	60.00	0.60	tr_C9ZXD7_
DRB1_0101	493	IKGDVVKITDFGTAR	VVKITDFGT	4	0.4107	587.7	60.00	0.72	tr_C9ZXD7_
DRB1_0101	494	EGRATAADLLLHRLF	GRATAADLL	1	0.4106	588.1	60.00	0.43	tr_C9ZXD7_
DRB1_0101	495	LTLRQDSPWTKGVLL	LRQDSPWTK	2	0.4104	589.7	60.00	0.58	tr_C9ZXD7_
DRB1_0101	496	VSLRKNAATAQEERE	LRKNAATAQ	2	0.4103	590.0	60.00	0.65	tr_C9ZXD7_
DRB1_0101	497	VYEATSDLTGGKMAV	TSDLTGGKM	4	0.4094	595.9	60.00	0.50	tr_C9ZXD7_
DRB1_0101	498	TIPYGNALGYIEGKH	YGNALGYIE	3	0.4085	602.0	60.00	0.60	tr_C9ZXD7_
DRB1_0101	499	ELCCCSLGDIIYGRS	CCCSLGDII	2	0.4081	604.5	60.00	0.36	tr_C9ZXD7_
DRB1_0101	500	LPHLRSTCGSIREDD	LRSTCGSIR	3	0.4057	620.5	65.00	0.55	tr_C9ZXD7_
DRB1_0101	501	PEGRATAADLLLHRL	GRATAADLL	2	0.4052	624.0	65.00	0.47	tr_C9ZXD7_
DRB1_0101	502	SVGCVAAELFDCPPL	VGCVAAELF	1	0.4042	630.4	65.00	0.43	tr_C9ZXD7_
DRB1_0101	503	DVVKITDFGTARQGV	VVKITDFGT	1	0.4036	634.7	65.00	0.51	tr_C9ZXD7_
DRB1_0101	504	GTLCLHPCIPEAVAT	LHPCIPEAV	4	0.4034	635.6	65.00	0.55	tr_C9ZXD7_
DRB1_0101	505	LTLITNLVDQHNISM	ITNLVDQHN	3	0.4028	640.3	65.00	0.36	tr_C9ZXD7_
DRB1_0101	506	LCCCSLGDIIYGRSQ	CCCSLGDII	1	0.4018	646.9	65.00	0.29	tr_C9ZXD7_
DRB1_0101	507	AHRDVKPQNILIKGD	DVKPQNILI	3	0.4015	649.4	65.00	0.55	tr_C9ZXD7_
DRB1_0101	508	ISEPGVRLALEGFQY	GVRLALEGF	4	0.3998	661.2	65.00	0.27	tr_C9ZXD7_
DRB1_0101	509	QISELREKLLPHLRS	EKLLPHLRS	6	0.3997	662.0	65.00	0.31	tr_C9ZXD7_
DRB1_0101	510	GRATAADLLLHRLFS	GRATAADLL	0	0.3991	666.6	65.00	0.24	tr_C9ZXD7_
DRB1_0101	511	GVAHRDVKPQNILIK	DVKPQNILI	5	0.3990	666.8	65.00	0.46	tr_C9ZXD7_
DRB1_0101	512	DHKTSVDSLFPGTLC	VDSLFPGTL	5	0.3988	668.7	65.00	0.54	tr_C9ZXD7_
DRB1_0101	513	AVYEATSDLTGGKMA	TSDLTGGKM	5	0.3978	675.9	65.00	0.37	tr_C9ZXD7_
DRB1_0101	514	DSPWTKGVLLGRGSF	SPWTKGVLL	1	0.3976	677.4	65.00	0.30	tr_C9ZXD7_
DRB1_0101	515	QPEGRATAADLLLHR	GRATAADLL	3	0.3970	681.6	65.00	0.56	tr_C9ZXD7_
DRB1_0101	516	ILEGKAVTPRGLSVI	AVTPRGLSV	5	0.3967	683.8	65.00	0.23	tr_C9ZXD7_

DRB1_0101	517	ERILTLRQDSPWTKG	LRQDSPWTK	5	0.3964	686.1	65.00	0.47	tr_C9ZXD7_
DRB1_0101	518	LEKRLQSSPSDIDVI	RLQSSPSDI	3	0.3956	692.1	65.00	0.35	tr_C9ZXD7_
DRB1_0101	519	CCSLGDIIYGRSQKP	IIYGRSQKP	6	0.3954	693.4	65.00	0.47	tr_C9ZXD7_
DRB1_0101	520	FMETPHMLADMTDVD	FMETPHMLA	0	0.3947	698.8	65.00	0.44	tr_C9ZXD7_
DRB1_0101	521	PGTLCLHPCIPEAVA	LHPCIPEAV	5	0.3938	705.6	65.00	0.46	tr_C9ZXD7_
DRB1_0101	522	VLTLITNLVDQHNIS	VLTLITNLV	0	0.3926	714.8	65.00	0.26	tr_C9ZXD7_
DRB1_0101	523	TLRQDSPWTKGVLLG	LRQDSPWTK	1	0.3919	720.1	65.00	0.47	tr_C9ZXD7_
DRB1_0101	524	LLGRGSFGAVYEATS	LLGRGSFGA	0	0.3914	724.1	65.00	0.28	tr_C9ZXD7_
DRB1_0101	525	SFGAVYEATSDLTGG	VYEATSDLT	4	0.3912	725.3	65.00	0.36	tr_C9ZXD7_
DRB1_0101	526	CSLNHPNIVHYFHCE	CSLNHPNIV	0	0.3909	728.2	65.00	0.33	tr_C9ZXD7_
DRB1_0101	527	RGSFGAVYEATSDLT	FGAVYEATS	3	0.3898	736.5	65.00	0.55	tr_C9ZXD7_
DRB1_0101	528	FLESGCVGKPLSDLV	ESGCVGKPL	2	0.3894	739.9	65.00	0.27	tr_C9ZXD7_
DRB1_0101	529	EYRTYIRDYVVSEVN	RTYIRDYVV	2	0.3873	756.9	65.00	0.44	tr_C9ZXD7_
DRB1_0101	530	GEYRTYIRDYVVSEV	RTYIRDYVV	3	0.3871	758.9	65.00	0.49	tr_C9ZXD7_
DRB1_0101	531	PDIFSREKQYVSSLT	FSREKQYVS	3	0.3870	759.5	65.00	0.52	tr_C9ZXD7_
DRB1_0101	532	RAIVNCTVNVLTLIT	VNCTVNVLT	3	0.3864	764.5	65.00	0.28	tr_C9ZXD7_
DRB1_0101	533	IIYGRSQKPPDLTVI	IIYGRSQKP	0	0.3863	764.9	65.00	0.47	tr_C9ZXD7_
DRB1_0101	534	AQVVMIGFDDKYRIT	QVVMIGFDD	1	0.3852	774.7	65.00	0.46	tr_C9ZXD7_
DRB1_0101	535	AEVVTGFLESGCVGK	TGFLESGCV	4	0.3851	775.2	65.00	0.36	tr_C9ZXD7_
DRB1_0101	536	YRTYIRDYVVSEVNL	RTYIRDYVV	1	0.3850	776.3	65.00	0.36	tr_C9ZXD7_
DRB1_0101	537	WNMAAEVVTGFLESG	WNMAAEVVT	0	0.3845	779.8	65.00	0.46	tr_C9ZXD7_
DRB1_0101	538	GSFGAVYEATSDLTG	FGAVYEATS	2	0.3843	781.9	70.00	0.41	tr_C9ZXD7_
DRB1_0101	539	DAQVVMIGFDDKYRI	QVVMIGFDD	2	0.3840	784.5	70.00	0.53	tr_C9ZXD7_
DRB1_0101	540	ELEKRLQSSPSDIDV	EKRLQSSPS	2	0.3839	785.7	70.00	0.31	tr_C9ZXD7_
DRB1_0101	541	DSLFPGTLCLHPCIP	DSLFPGTLC	0	0.3834	789.5	70.00	0.24	tr_C9ZXD7_
DRB1_0101	542	HRDVKPQNILIKGDV	DVKPQNILI	2	0.3829	793.8	70.00	0.52	tr_C9ZXD7_
DRB1_0101	543	FGAVYEATSDLTGGK	VYEATSDLT	3	0.3826	796.3	70.00	0.47	tr_C9ZXD7_
DRB1_0101	544	EVESLPDIFSREKQY	VESLPDIFS	1	0.3825	797.7	70.00	0.75	tr_C9ZXD7_
DRB1_0101	545	GPLQSFLEYGYGKQV	LEYGYGKQV	6	0.3814	807.2	70.00	0.59	tr_C9ZXD7_
DRB1_0101	546	DALNERYVIDQTRAI	YVIDQTRAI	6	0.3806	813.4	70.00	0.62	tr_C9ZXD7_
DRB1_0101	547	CGSIREDDGTVVFRF	IREDDGTVV	3	0.3782	834.9	70.00	0.67	tr_C9ZXD7_
DRB1_0101	548	ESGCVGKPLSDLVIT	CVGKPLSDL	3	0.3776	840.4	70.00	0.40	tr_C9ZXD7_
DRB1_0101	549	CLQPEGRATAADLLL	GRATAADLL	5	0.3770	846.2	70.00	0.35	tr_C9ZXD7_
DRB1_0101	550	LQPEGRATAADLLLH	GRATAADLL	4	0.3757	858.6	70.00	0.49	tr_C9ZXD7_
DRB1_0101	551	HVDAQVVMIGFDDKY	QVVMIGFDD	4	0.3755	859.9	70.00	0.49	tr_C9ZXD7_
DRB1_0101	552	VDAQVVMIGFDDKYR	QVVMIGFDD	3	0.3751	863.7	70.00	0.60	tr_C9ZXD7_
DRB1_0101	553	SNPVLCDFLRMCFCL	PVLCDFLRM	2	0.3743	871.0	70.00	0.29	tr_C9ZXD7_
DRB1_0101	554	HNTKNGYYTIPYGNA	YYTIPYGNA	6	0.3740	874.4	70.00	0.47	tr_C9ZXD7_
DRB1_0101	555	DIDDATFRPADALNE	FRPADALNE	6	0.3733	880.7	70.00	0.54	tr_C9ZXD7_
DRB1_0101	556	AVKMFYFTEDLEESI	FTEDLEESI	6	0.3723	890.6	70.00	0.31	tr_C9ZXD7_
DRB1_0101	557	RPADALNERYVIDQT	ADALNERYV	2	0.3696	917.2	70.00	0.42	tr_C9ZXD7_
DRB1_0101	558	PSDIDVIVCDRGWLN	IVCDRGWLN	6	0.3690	922.6	70.00	0.46	tr_C9ZXD7_
DRB1_0101	559	GAVYEATSDLTGGKM	VYEATSDLT	2	0.3686	926.9	70.00	0.37	tr_C9ZXD7_
DRB1_0101	560	GSIREDDGTVVFRFP	IREDDGTVV	2	0.3682	930.6	70.00	0.63	tr_C9ZXD7_
DRB1_0101	561	LESGCVGKPLSDLVI	CVGKPLSDL	4	0.3660	953.2	70.00	0.41	tr_C9ZXD7_
DRB1_0101	562	CSPCDIWSVGCVAAE	DIWSVGCVA	4	0.3655	958.4	70.00	0.31	tr_C9ZXD7_
DRB1_0101	563	VVKITDFGTARQGVG	VVKITDFGT	0	0.3653	960.5	70.00	0.32	tr_C9ZXD7_
DRB1_0101	564	ILICANAKDNLGEYR	LICANAKDN	1	0.3637	976.7	70.00	0.50	tr_C9ZXD7_
DRB1_0101	565	CCCSLGDIIYGRSQK	CSLGDIIYG	2	0.3632	982.2	70.00	0.35	tr_C9ZXD7_
DRB1_0101	566	LRQDSPWTKGVLLGR	SPWTKGVLL	4	0.3625	990.4	70.00	0.47	tr_C9ZXD7_

DRB1_0101	567	KAVTPRGLSVIGPLQ	GLSVIGPLQ	6	0.3614	1001.4	70.00	0.28	tr_C9ZXD7_
DRB1_0101	568	CELEKRLQSSPSDID	EKRLQSSPS	3	0.3605	1012.1	70.00	0.42	tr_C9ZXD7_
DRB1_0101	569	APSTAQISELREKLL	STAQISELR	2	0.3597	1020.4	75.00	0.38	tr_C9ZXD7_
DRB1_0101	570	MTDVDYYVDGLTSNP	DYYVDGLTS	4	0.3593	1025.0	75.00	0.57	tr_C9ZXD7_
DRB1_0101	571	TAQISELREKLLPHL	ISELREKLL	3	0.3592	1025.9	75.00	0.60	tr_C9ZXD7_
DRB1_0101	572	FPGTLCLHPCIPEAV	LHPCIPEAV	6	0.3592	1026.1	75.00	0.29	tr_C9ZXD7_
DRB1_0101	573	ELARGMLAEWEWTSS	MLAEWEWTS	5	0.3582	1036.8	75.00	0.31	tr_C9ZXD7_
DRB1_0101	574	RGMLAEWEWTSSEQL	WEWTSSEQL	6	0.3582	1037.5	75.00	0.43	tr_C9ZXD7_
DRB1_0101	575	PSTAQISELREKLLP	ISELREKLL	5	0.3579	1040.4	75.00	0.41	tr_C9ZXD7_
DRB1_0101	576	LRLSPLDHKTSVDSL	LSPLDHKTS	2	0.3577	1042.9	75.00	0.44	tr_C9ZXD7_
DRB1_0101	577	RERILTLRQDSPWTK	ILTLRQDSP	3	0.3575	1044.7	75.00	0.37	tr_C9ZXD7_
DRB1_0101	578	VIGPLQSFLEYGYGK	IGPLQSFLE	1	0.3572	1047.9	75.00	0.63	tr_C9ZXD7_
DRB1_0101	579	YFHCERKENSVNLFM	ERKENSVNL	4	0.3568	1053.0	75.00	0.35	tr_C9ZXD7_
DRB1_0101	580	GGKMAVKMFYFTEDL	KMAVKMFYF	2	0.3567	1053.7	75.00	0.39	tr_C9ZXD7_
DRB1_0101	581	SGCVGKPLSDLVITP	CVGKPLSDL	2	0.3562	1060.0	75.00	0.41	tr_C9ZXD7_
DRB1_0101	582	RCELEKRLQSSPSDI	EKRLQSSPS	4	0.3558	1063.8	75.00	0.41	tr_C9ZXD7_
DRB1_0101	583	GRGSFGAVYEATSDL	FGAVYEATS	4	0.3552	1070.8	75.00	0.58	tr_C9ZXD7_
DRB1_0101	584	LGEYRTYIRDYVVSE	RTYIRDYVV	4	0.3550	1073.4	75.00	0.50	tr_C9ZXD7_
DRB1_0101	585	STAQISELREKLLPH	ISELREKLL	4	0.3545	1079.9	75.00	0.50	tr_C9ZXD7_
DRB1_0101	586	SQKPPDLTVIKVLRQ	LTVIKVLRQ	6	0.3542	1082.5	75.00	0.54	tr_C9ZXD7_
DRB1_0101	587	QDSPWTKGVLLGRGS	SPWTKGVLL	2	0.3539	1086.4	75.00	0.40	tr_C9ZXD7_
DRB1_0101	588	AQISELREKLLPHLR	ISELREKLL	2	0.3538	1087.1	75.00	0.48	tr_C9ZXD7_
DRB1_0101	589	LARGMLAEWEWTSSE	MLAEWEWTS	4	0.3536	1089.5	75.00	0.36	tr_C9ZXD7_
DRB1_0101	590	LPDIFSREKQYVSSL	FSREKQYVS	4	0.3529	1098.1	75.00	0.58	tr_C9ZXD7_
DRB1_0101	591	LEGKAVTPRGLSVIG	AVTPRGLSV	4	0.3526	1101.7	75.00	0.41	tr_C9ZXD7_
DRB1_0101	592	EKRLQSSPSDIDVIV	RLQSSPSDI	2	0.3519	1110.5	75.00	0.35	tr_C9ZXD7_
DRB1_0101	593	TSNPVLCDFLRMCFC	PVLCDFLRM	3	0.3513	1117.7	75.00	0.38	tr_C9ZXD7_
DRB1_0101	594	DGLTSNPVLCDFLRM	PVLCDFLRM	6	0.3512	1118.6	75.00	0.28	tr_C9ZXD7_
DRB1_0101	595	HCERKENSVNLFMEL	ERKENSVNL	2	0.3503	1129.1	75.00	0.31	tr_C9ZXD7_
DRB1_0101	596	QVVMIGFDDKYRITR	QVVMIGFDD	0	0.3495	1139.9	75.00	0.35	tr_C9ZXD7_
DRB1_0101	597	HYFHCERKENSVNLF	FHCERKENS	2	0.3494	1140.3	75.00	0.40	tr_C9ZXD7_
DRB1_0101	598	PLSDLVITPTGDIER	LVITPTGDI	4	0.3475	1163.8	75.00	0.58	tr_C9ZXD7_
DRB1_0101	599	CERKENSVNLFMELC	ERKENSVNL	1	0.3467	1174.0	75.00	0.22	tr_C9ZXD7_
DRB1_0101	600	RQDSPWTKGVLLGRG	SPWTKGVLL	3	0.3456	1188.0	75.00	0.56	tr_C9ZXD7_
DRB1_0101	601	RFPCQITAALEDIDD	FPCQITAAL	1	0.3452	1194.3	75.00	0.41	tr_C9ZXD7_
DRB1_0101	602	PHLRSTCGSIREDDG	LRSTCGSIR	2	0.3448	1198.7	75.00	0.69	tr_C9ZXD7_
DRB1_0101	603	FHCERKENSVNLFME	ERKENSVNL	3	0.3432	1220.2	75.00	0.43	tr_C9ZXD7_
DRB1_0101	604	GCVGKPLSDLVITPT	GKPLSDLVI	3	0.3427	1226.9	75.00	0.29	tr_C9ZXD7_
DRB1_0101	605	GDLSTTGPSAGAAVP	LSTTGPSAG	2	0.3426	1228.3	75.00	0.35	tr_C9ZXD7_
DRB1_0101	606	TRGHNTKNGYYTIPY	HNTKNGYYT	3	0.3422	1233.5	75.00	0.60	tr_C9ZXD7_
DRB1_0101	607	GKAVTPRGLSVIGPL	AVTPRGLSV	2	0.3419	1236.8	75.00	0.37	tr_C9ZXD7_
DRB1_0101	608	EGDLSTTGPSAGAAV	LSTTGPSAG	3	0.3409	1250.4	75.00	0.41	tr_C9ZXD7_
DRB1_0101	609	RGVAHRDVKPQNILI	DVKPQNILI	6	0.3406	1254.3	75.00	0.34	tr_C9ZXD7_
DRB1_0101	610	EGKAVTPRGLSVIGP	AVTPRGLSV	3	0.3400	1262.2	75.00	0.44	tr_C9ZXD7_
DRB1_0101	611	AIVNCTVNVLTLITN	VNCTVNVLT	2	0.3398	1265.6	75.00	0.23	tr_C9ZXD7_
DRB1_0101	612	TPTGDIERDLAPLQV	IERDLAPLQ	5	0.3398	1266.1	75.00	0.49	tr_C9ZXD7_
DRB1_0101	613	TCGSIREDDGTVVFR	IREDDGTVV	4	0.3390	1276.8	75.00	0.75	tr_C9ZXD7_
DRB1_0101	614	NLGEYRTYIRDYVVS	RTYIRDYVV	5	0.3389	1278.0	75.00	0.45	tr_C9ZXD7_
DRB1_0101	615	LGRGSFGAVYEATSD	FGAVYEATS	5	0.3389	1278.3	75.00	0.43	tr_C9ZXD7_
DRB1_0101	616	SIREDDGTVVFRFPC	IREDDGTVV	1	0.3380	1289.9	75.00	0.58	tr_C9ZXD7_

DRB1_0101	617	KPLSDLVITPTGDIE	LVITPTGDI	5	0.3373	1300.4	75.00	0.46	tr_C9ZXD7_
DRB1_0101	618	RLSPLDHKTSVDSLF	LDHKTSVDS	4	0.3368	1306.7	75.00	0.37	tr_C9ZXD7_
DRB1_0101	619	GLTSNPVLCDFLRMC	PVLCDFLRM	5	0.3365	1311.2	75.00	0.36	tr_C9ZXD7_
DRB1_0101	620	SLFPGTLCLHPCIPE	GTLCLHPCI	4	0.3347	1338.0	75.00	0.24	tr_C9ZXD7_
DRB1_0101	621	SSCSAEVESLPDIFS	VESLPDIFS	6	0.3346	1339.0	75.00	0.59	tr_C9ZXD7_
DRB1_0101	622	SLRKNAATAQEERER	LRKNAATAQ	1	0.3340	1347.8	80.00	0.69	tr_C9ZXD7_
DRB1_0101	623	DLSTTGPSAGAAVPQ	TGPSAGAAV	4	0.3306	1398.6	80.00	0.38	tr_C9ZXD7_
DRB1_0101	624	ETPHMLADMTDVDYY	HMLADMTDV	3	0.3303	1403.0	80.00	0.58	tr_C9ZXD7_
DRB1_0101	625	KENSVNLFMELCCCS	NLFMELCCC	5	0.3299	1408.3	80.00	0.25	tr_C9ZXD7_
DRB1_0101	626	LSDLVITPTGDIERD	LVITPTGDI	3	0.3285	1430.8	80.00	0.65	tr_C9ZXD7_
DRB1_0101	627	ERKENSVNLFMELCC	NSVNLFMEL	4	0.3284	1431.2	80.00	0.25	tr_C9ZXD7_
DRB1_0101	628	ARGMLAEWEWTSSEQ	MLAEWEWTS	3	0.3276	1444.0	80.00	0.44	tr_C9ZXD7_
DRB1_0101	629	PADALNERYVIDQTR	DALNERYVI	2	0.3272	1450.1	80.00	0.46	tr_C9ZXD7_
DRB1_0101	630	HCSPCDIWSVGCVAA	DIWSVGCVA	5	0.3269	1455.2	80.00	0.32	tr_C9ZXD7_
DRB1_0101	631	RGHNTKNGYYTIPYG	HNTKNGYYT	2	0.3259	1470.8	80.00	0.55	tr_C9ZXD7_
DRB1_0101	632	CVGKPLSDLVITPTG	GKPLSDLVI	2	0.3256	1475.3	80.00	0.30	tr_C9ZXD7_
DRB1_0101	633	LTSNPVLCDFLRMCF	PVLCDFLRM	4	0.3255	1477.0	80.00	0.47	tr_C9ZXD7_
DRB1_0101	634	VHYFHCERKENSVNL	FHCERKENS	3	0.3245	1493.4	80.00	0.48	tr_C9ZXD7_
DRB1_0101	635	AAPSTAQISELREKL	STAQISELR	3	0.3235	1509.6	80.00	0.62	tr_C9ZXD7_
DRB1_0101	636	RKENSVNLFMELCCC	NSVNLFMEL	3	0.3233	1512.3	80.00	0.28	tr_C9ZXD7_
DRB1_0101	637	TPHMLADMTDVDYYV	HMLADMTDV	2	0.3230	1518.5	80.00	0.58	tr_C9ZXD7_
DRB1_0101	638	DMTDVDYYVDGLTSN	DYYVDGLTS	5	0.3226	1523.8	80.00	0.66	tr_C9ZXD7_
DRB1_0101	639	LFPGTLCLHPCIPEA	GTLCLHPCI	3	0.3206	1558.1	80.00	0.29	tr_C9ZXD7_
DRB1_0101	640	GKPLSDLVITPTGDI	LSDLVITPT	3	0.3205	1559.0	80.00	0.29	tr_C9ZXD7_
DRB1_0101	641	LSTTGPSAGAAVPQV	TGPSAGAAV	3	0.3186	1591.2	80.00	0.52	tr_C9ZXD7_
DRB1_0101	642	METPHMLADMTDVDY	HMLADMTDV	4	0.3181	1600.5	80.00	0.55	tr_C9ZXD7_
DRB1_0101	643	GVGSEDVQGTLRYMA	DVQGTLRYM	5	0.3180	1602.0	80.00	0.28	tr_C9ZXD7_
DRB1_0101	644	QVTRGHNTKNGYYTI	HNTKNGYYT	5	0.3171	1617.8	80.00	0.58	tr_C9ZXD7_
DRB1_0101	645	LDHKTSVDSLFPGTL	VDSLFPGTL	6	0.3163	1631.6	80.00	0.53	tr_C9ZXD7_
DRB1_0101	646	IVNCTVNVLTLITNL	CTVNVLTLI	3	0.3161	1634.5	80.00	0.29	tr_C9ZXD7_
DRB1_0101	647	AQAAPSTAQISELRE	AAPSTAQIS	2	0.3146	1662.3	80.00	0.50	tr_C9ZXD7_
DRB1_0101	648	VGCVAAELFDCPPLF	CVAAELFDC	2	0.3131	1689.7	80.00	0.28	tr_C9ZXD7_
DRB1_0101	649	CVAAELFDCPPLFME	LFDCPPLFM	5	0.3116	1716.5	80.00	0.41	tr_C9ZXD7_
DRB1_0101	650	VTRGHNTKNGYYTIP	HNTKNGYYT	4	0.3115	1718.4	80.00	0.57	tr_C9ZXD7_
DRB1_0101	651	QAAPSTAQISELREK	STAQISELR	4	0.3113	1721.9	80.00	0.46	tr_C9ZXD7_
DRB1_0101	652	VLLSSASSEGDLSTT	LSSASSEGD	2	0.3076	1793.9	80.00	0.35	tr_C9ZXD7_
DRB1_0101	653	VKITDFGTARQGVGS	FGTARQGVG	5	0.3069	1806.7	80.00	0.25	tr_C9ZXD7_
DRB1_0101	654	SEGDLSTTGPSAGAA	DLSTTGPSA	3	0.3037	1870.3	85.00	0.41	tr_C9ZXD7_
DRB1_0101	655	AAEVVTGFLESGCVG	TGFLESGCV	5	0.3033	1878.2	85.00	0.43	tr_C9ZXD7_
DRB1_0101	656	GKMAVKMFYFTEDLE	KMAVKMFYF	1	0.3032	1880.4	85.00	0.36	tr_C9ZXD7_
DRB1_0101	657	SDLVITPTGDIERDL	LVITPTGDI	2	0.3031	1882.4	85.00	0.70	tr_C9ZXD7_
DRB1_0101	658	VAAELFDCPPLFMET	LFDCPPLFM	4	0.3028	1889.0	85.00	0.47	tr_C9ZXD7_
DRB1_0101	659	IGPLQSFLEYGYGKQ	IGPLQSFLE	0	0.3011	1923.0	85.00	0.40	tr_C9ZXD7_
DRB1_0101	660	EDDGTVVFRFPCQIT	VVFRFPCQI	5	0.2997	1952.7	85.00	0.25	tr_C9ZXD7_
DRB1_0101	661	DNLGEYRTYIRDYVV	RTYIRDYVV	6	0.2992	1962.9	85.00	0.34	tr_C9ZXD7_
DRB1_0101	662	ELFDCPPLFMETPHM	PLFMETPHM	6	0.2990	1966.8	85.00	0.32	tr_C9ZXD7_
DRB1_0101	663	KRLQSSPSDIDVIVC	RLQSSPSDI	1	0.2983	1982.1	85.00	0.39	tr_C9ZXD7_
DRB1_0101	664	GCVAAELFDCPPLFM	LFDCPPLFM	6	0.2965	2021.6	85.00	0.34	tr_C9ZXD7_
DRB1_0101	665	SLPDIFSREKQYVSS	FSREKQYVS	5	0.2963	2025.5	85.00	0.56	tr_C9ZXD7_
DRB1_0101	666	ERCELEKRLQSSPSD	EKRLQSSPS	5	0.2959	2034.6	85.00	0.35	tr_C9ZXD7_

DRB1_0101	667	HLRSTCGSIREDDGT	LRSTCGSIR	1	0.2955	2043.6	85.00	0.74	tr_C9ZXD7_
DRB1_0101	668	PHMLADMTDVDYYVD	HMLADMTDV	1	0.2940	2077.5	85.00	0.54	tr_C9ZXD7_
DRB1_0101	669	RYMAPEVYRGEDHCS	YMAPEVYRG	1	0.2931	2098.1	85.00	0.59	tr_C9ZXD7_
DRB1_0101	670	QGVGSEDVQGTLRYM	VGSEDVQGT	2	0.2930	2099.1	85.00	0.45	tr_C9ZXD7_
DRB1_0101	671	SEDVQGTLRYMAPEV	DVQGTLRYM	2	0.2925	2110.6	85.00	0.28	tr_C9ZXD7_
DRB1_0101	672	TTGPSAGAAVPQVTR	TGPSAGAAV	1	0.2918	2126.8	85.00	0.36	tr_C9ZXD7_
DRB1_0101	673	STTGPSAGAAVPQVT	TGPSAGAAV	2	0.2917	2129.0	85.00	0.51	tr_C9ZXD7_
DRB1_0101	674	KITDFGTARQGVGSE	FGTARQGVG	4	0.2909	2147.6	85.00	0.31	tr_C9ZXD7_
DRB1_0101	675	ADALNERYVIDQTRA	DALNERYVI	1	0.2908	2151.2	85.00	0.44	tr_C9ZXD7_
DRB1_0101	676	STCGSIREDDGTVVF	IREDDGTVV	5	0.2905	2158.4	85.00	0.73	tr_C9ZXD7_
DRB1_0101	677	VESLPDIFSREKQYV	VESLPDIFS	0	0.2903	2161.4	85.00	0.60	tr_C9ZXD7_
DRB1_0101	678	SLNHPNIVHYFHCER	NHPNIVHYF	2	0.2901	2166.2	85.00	0.34	tr_C9ZXD7_
DRB1_0101	679	EVYRGEDHCSPCDIW	YRGEDHCSP	2	0.2896	2178.2	85.00	0.54	tr_C9ZXD7_
DRB1_0101	680	VGKPLSDLVITPTGD	LSDLVITPT	4	0.2864	2256.0	85.00	0.35	tr_C9ZXD7_
DRB1_0101	681	AAELFDCPPLFMETP	LFDCPPLFM	3	0.2861	2262.7	85.00	0.47	tr_C9ZXD7_
DRB1_0101	682	RERCELEKRLQSSPS	CELEKRLQS	3	0.2853	2281.3	85.00	0.38	tr_C9ZXD7_
DRB1_0101	683	IREDDGTVVFRFPCQ	DDGTVVFRF	3	0.2842	2309.8	85.00	0.37	tr_C9ZXD7_
DRB1_0101	684	LSPLDHKTSVDSLFP	LDHKTSVDS	3	0.2830	2339.0	85.00	0.45	tr_C9ZXD7_
DRB1_0101	685	FPCQITAALEDIDDA	CQITAALED	2	0.2816	2375.7	85.00	0.28	tr_C9ZXD7_
DRB1_0101	686	IVHYFHCERKENSVN	FHCERKENS	4	0.2815	2377.5	85.00	0.56	tr_C9ZXD7_
DRB1_0101	687	VGSEDVQGTLRYMAP	DVQGTLRYM	4	0.2808	2395.7	85.00	0.33	tr_C9ZXD7_
DRB1_0101	688	GHNTKNGYYTIPYGN	HNTKNGYYT	1	0.2803	2408.2	85.00	0.46	tr_C9ZXD7_
DRB1_0101	689	PEVYRGEDHCSPCDI	YRGEDHCSP	3	0.2803	2408.8	85.00	0.60	tr_C9ZXD7_
DRB1_0101	690	EDIDDATFRPADALN	DATFRPADA	4	0.2801	2414.8	85.00	0.44	tr_C9ZXD7_
DRB1_0101	691	RQGVGSEDVQGTLRY	VGSEDVQGT	3	0.2794	2433.5	85.00	0.59	tr_C9ZXD7_
DRB1_0101	692	TGPSAGAAVPQVTRG	AGAAVPQVT	4	0.2781	2466.7	85.00	0.25	tr_C9ZXD7_
DRB1_0101	693	ANAKDNLGEYRTYIR	NLGEYRTYI	5	0.2765	2510.6	85.00	0.37	tr_C9ZXD7_
DRB1_0101	694	SVRERCELEKRLQSS	CELEKRLQS	5	0.2760	2524.2	85.00	0.39	tr_C9ZXD7_
DRB1_0101	695	AKDNLGEYRTYIRDY	NLGEYRTYI	3	0.2759	2527.6	85.00	0.48	tr_C9ZXD7_
DRB1_0101	696	MAVKMFYFTEDLEES	FYFTEDLEE	5	0.2756	2535.8	90.00	0.22	tr_C9ZXD7_
DRB1_0101	697	NAKDNLGEYRTYIRD	NLGEYRTYI	4	0.2734	2596.6	90.00	0.41	tr_C9ZXD7_
DRB1_0101	698	VYRGEDHCSPCDIWS	YRGEDHCSP	1	0.2732	2600.3	90.00	0.49	tr_C9ZXD7_
DRB1_0101	699	RSQKPPDLTVIKVLR	PDLTVIKVL	5	0.2713	2656.3	90.00	0.31	tr_C9ZXD7_
DRB1_0101	700	ARGVAHRDVKPQNIL	HRDVKPQNI	5	0.2703	2683.3	90.00	0.31	tr_C9ZXD7_
DRB1_0101	701	DLVITPTGDIERDLA	LVITPTGDI	1	0.2699	2697.3	90.00	0.68	tr_C9ZXD7_
DRB1_0101	702	KMAVKMFYFTEDLEE	KMAVKMFYF	0	0.2697	2702.6	90.00	0.24	tr_C9ZXD7_
DRB1_0101	703	AELFDCPPLFMETPH	LFDCPPLFM	2	0.2692	2715.8	90.00	0.49	tr_C9ZXD7_
DRB1_0101	704	NIVHYFHCERKENSV	FHCERKENS	5	0.2685	2737.2	90.00	0.47	tr_C9ZXD7_
DRB1_0101	705	LHARGVAHRDVKPQN	HARGVAHRD	1	0.2681	2749.8	90.00	0.25	tr_C9ZXD7_
DRB1_0101	706	ITDFGTARQGVGSED	FGTARQGVG	3	0.2672	2774.7	90.00	0.39	tr_C9ZXD7_
DRB1_0101	707	KDNLGEYRTYIRDYV	NLGEYRTYI	2	0.2656	2825.2	90.00	0.43	tr_C9ZXD7_
DRB1_0101	708	IYGRSQKPPDLTVIK	GRSQKPPDL	2	0.2650	2843.9	90.00	0.47	tr_C9ZXD7_
DRB1_0101	709	GPSAGAAVPQVTRGH	AGAAVPQVT	3	0.2647	2853.5	90.00	0.36	tr_C9ZXD7_
DRB1_0101	710	REDDGTVVFRFPCQI	DDGTVVFRF	2	0.2617	2945.3	90.00	0.35	tr_C9ZXD7_
DRB1_0101	711	GSEDVQGTLRYMAPE	DVQGTLRYM	3	0.2616	2949.7	90.00	0.35	tr_C9ZXD7_
DRB1_0101	712	LICANAKDNLGEYRT	LICANAKDN	0	0.2603	2989.9	90.00	0.34	tr_C9ZXD7_
DRB1_0101	713	TDFGTARQGVGSEDV	FGTARQGVG	2	0.2592	3027.3	90.00	0.38	tr_C9ZXD7_
DRB1_0101	714	DHCSPCDIWSVGCVA	DIWSVGCVA	6	0.2581	3063.6	90.00	0.35	tr_C9ZXD7_
DRB1_0101	715	ERERILTLRQDSPWT	ILTLRQDSP	4	0.2566	3111.8	90.00	0.49	tr_C9ZXD7_
DRB1_0101	716	GRSQKPPDLTVIKVL	PDLTVIKVL	6	0.2562	3125.3	90.00	0.22	tr_C9ZXD7_

DRB1_0101	717	LNHPNIVHYFHCERK	NHPNIVHYF	1	0.2561	3129.5	90.00	0.28	tr_C9ZXD7_
DRB1_0101	718	ARQGVGSEDVQGTLR	VGSEDVQGT	4	0.2544	3189.3	90.00	0.56	tr_C9ZXD7_
DRB1_0101	719	PQVTRGHNTKNGYYT	HNTKNGYYT	6	0.2543	3191.1	90.00	0.40	tr_C9ZXD7_
DRB1_0101	720	YRGEDHCSPCDIWSV	EDHCSPCDI	3	0.2528	3242.2	90.00	0.30	tr_C9ZXD7_
DRB1_0101	721	VRERCELEKRLQSSP	CELEKRLQS	4	0.2527	3248.5	90.00	0.44	tr_C9ZXD7_
DRB1_0101	722	VVMIGFDDKYRITRW	IGFDDKYRI	3	0.2526	3250.4	90.00	0.30	tr_C9ZXD7_
DRB1_0101	723	APEVYRGEDHCSPCD	YRGEDHCSP	4	0.2521	3267.6	90.00	0.65	tr_C9ZXD7_
DRB1_0101	724	TSVRERCELEKRLQS	ERCELEKRL	4	0.2513	3295.4	90.00	0.34	tr_C9ZXD7_
DRB1_0101	725	PSAGAAVPQVTRGHN	AGAAVPQVT	2	0.2511	3304.8	90.00	0.34	tr_C9ZXD7_
DRB1_0101	726	YGRSQKPPDLTVIKV	GRSQKPPDL	1	0.2493	3368.4	90.00	0.35	tr_C9ZXD7_
DRB1_0101	727	AATAQEERERILTLR	TAQEERERI	2	0.2473	3444.5	90.00	0.33	tr_C9ZXD7_
DRB1_0101	728	SPLDHKTSVDSLFPG	LDHKTSVDS	2	0.2471	3450.3	90.00	0.47	tr_C9ZXD7_
DRB1_0101	729	SSEGDLSTTGPSAGA	DLSTTGPSA	4	0.2470	3454.2	90.00	0.48	tr_C9ZXD7_
DRB1_0101	730	HMLADMTDVDYYVDG	HMLADMTDV	0	0.2469	3457.3	90.00	0.44	tr_C9ZXD7_
DRB1_0101	731	SPSDIDVIVCDRGWL	IDVIVCDRG	4	0.2462	3484.5	90.00	0.52	tr_C9ZXD7_
DRB1_0101	732	SAGAAVPQVTRGHNT	AGAAVPQVT	1	0.2441	3564.2	90.00	0.27	tr_C9ZXD7_
DRB1_0101	733	CANAKDNLGEYRTYI	KDNLGEYRT	4	0.2440	3568.1	90.00	0.32	tr_C9ZXD7_
DRB1_0101	734	LEDIDDATFRPADAL	DATFRPADA	5	0.2425	3625.4	90.00	0.38	tr_C9ZXD7_
DRB1_0101	735	ESLPDIFSREKQYVS	FSREKQYVS	6	0.2413	3675.3	90.00	0.44	tr_C9ZXD7_
DRB1_0101	736	MAPEVYRGEDHCSPC	YRGEDHCSP	5	0.2393	3753.8	95.00	0.63	tr_C9ZXD7_
DRB1_0101	737	ADMTDVDYYVDGLTS	DYYVDGLTS	6	0.2390	3766.3	95.00	0.54	tr_C9ZXD7_
DRB1_0101	738	YMAPEVYRGEDHCSP	YRGEDHCSP	6	0.2386	3781.5	95.00	0.38	tr_C9ZXD7_
DRB1_0101	739	ATAQEERERILTLRQ	EERERILTL	4	0.2384	3791.9	95.00	0.28	tr_C9ZXD7_
DRB1_0101	740	MAAEVVTGFLESGCV	TGFLESGCV	6	0.2377	3818.8	95.00	0.34	tr_C9ZXD7_
DRB1_0101	741	TARQGVGSEDVQGTL	VGSEDVQGT	5	0.2370	3848.7	95.00	0.54	tr_C9ZXD7_
DRB1_0101	742	DFGTARQGVGSEDVQ	FGTARQGVG	1	0.2368	3857.3	95.00	0.33	tr_C9ZXD7_
DRB1_0101	743	AGAAVPQVTRGHNTK	PQVTRGHNT	5	0.2367	3860.3	95.00	0.31	tr_C9ZXD7_
DRB1_0101	744	LLSSASSEGDLSTTG	LSSASSEGD	1	0.2359	3893.3	95.00	0.43	tr_C9ZXD7_
DRB1_0101	745	PNIVHYFHCERKENS	FHCERKENS	6	0.2347	3946.0	95.00	0.29	tr_C9ZXD7_
DRB1_0101	746	HARGVAHRDVKPQNI	VAHRDVKPQ	4	0.2303	4138.5	95.00	0.32	tr_C9ZXD7_
DRB1_0101	747	NAATAQEERERILTL	TAQEERERI	3	0.2289	4201.6	95.00	0.43	tr_C9ZXD7_
DRB1_0101	748	NHPNIVHYFHCERKE	PNIVHYFHC	2	0.2263	4322.7	95.00	0.22	tr_C9ZXD7_
DRB1_0101	749	LTSVRERCELEKRLQ	SVRERCELE	2	0.2262	4328.1	95.00	0.34	tr_C9ZXD7_
DRB1_0101	750	MIGFDDKYRITRWNM	KYRITRWNM	6	0.2257	4351.3	95.00	0.20	tr_C9ZXD7_
DRB1_0101	751	EERERILTLRQDSPW	ILTLRQDSP	5	0.2243	4415.7	95.00	0.44	tr_C9ZXD7_
DRB1_0101	752	PLDHKTSVDSLFPGT	LDHKTSVDS	1	0.2241	4426.8	95.00	0.38	tr_C9ZXD7_
DRB1_0101	753	NMAAEVVTGFLESGC	MAAEVVTGF	1	0.2205	4599.4	95.00	0.24	tr_C9ZXD7_
DRB1_0101	754	VPQVTRGHNTKNGYY	VTRGHNTKN	3	0.2204	4605.2	95.00	0.35	tr_C9ZXD7_
DRB1_0101	755	SLTSVRERCELEKRL	SVRERCELE	3	0.2204	4606.0	95.00	0.41	tr_C9ZXD7_
DRB1_0101	756	GAAVPQVTRGHNTKN	PQVTRGHNT	4	0.2195	4649.7	95.00	0.41	tr_C9ZXD7_
DRB1_0101	757	PCQITAALEDIDDAT	CQITAALED	1	0.2195	4653.4	95.00	0.32	tr_C9ZXD7_
DRB1_0101	758	ITPTGDIERDLAPLQ	IERDLAPLQ	6	0.2185	4701.6	95.00	0.53	tr_C9ZXD7_
DRB1_0101	759	FGTARQGVGSEDVQG	GTARQGVGS	1	0.2161	4824.2	95.00	0.29	tr_C9ZXD7_
DRB1_0101	760	RSTCGSIREDDGTVV	IREDDGTVV	6	0.2150	4884.5	95.00	0.65	tr_C9ZXD7_
DRB1_0101	761	TAQEERERILTLRQD	EERERILTL	3	0.2149	4887.9	95.00	0.34	tr_C9ZXD7_
DRB1_0101	762	AAVPQVTRGHNTKNG	PQVTRGHNT	3	0.2145	4911.4	95.00	0.45	tr_C9ZXD7_
DRB1_0101	763	LVITPTGDIERDLAP	LVITPTGDI	0	0.2135	4963.9	95.00	0.55	tr_C9ZXD7_
DRB1_0101	764	VMIGFDDKYRITRWN	IGFDDKYRI	2	0.2126	5012.4	95.00	0.36	tr_C9ZXD7_
DRB1_0101	765	LRKNAATAQEERERI	LRKNAATAQ	0	0.2124	5023.0	95.00	0.44	tr_C9ZXD7_
DRB1_0101	766	RGEDHCSPCDIWSVG	CSPCDIWSV	5	0.2100	5155.7	95.00	0.40	tr_C9ZXD7_

DRB1_0101	767	AVPQVTRGHNTKNGY	PQVTRGHNT	2	0.2097	5169.1	95.00	0.43	tr_C9ZXD7_	
DRB1_0101	768	HPNIVHYFHCERKEN	NIVHYFHCE	2	0.2090	5210.6	95.00	0.23	tr_C9ZXD7_	
DRB1_0101	769	EDHCSPCDIWSVGCV	CSPCDIWSV	3	0.2090	5213.2	95.00	0.43	tr_C9ZXD7_	
DRB1_0101	770	ICANAKDNLGEYRTY	KDNLGEYRT	5	0.2072	5313.5	95.00	0.28	tr_C9ZXD7_	
DRB1_0101	771	ALEDIDDATFRPADA	IDDATFRPA	4	0.2068	5338.6	95.00	0.25	tr_C9ZXD7_	
DRB1_0101	772	RLQSSPSDIDVIVCD	LQSSPSDID	1	0.2057	5401.6	95.00	0.37	tr_C9ZXD7_	
DRB1_0101	773	SSSCSAEVESLPDIF	SCSAEVESL	2	0.2055	5409.7	95.00	0.41	tr_C9ZXD7_	
DRB1_0101	774	GTARQGVGSEDVQGT	VGSEDVQGT	6	0.2039	5506.7	95.00	0.29	tr_C9ZXD7_	
DRB1_0101	775	VSLTSVRERCELEKR	SVRERCELE	4	0.2019	5628.3	95.00	0.38	tr_C9ZXD7_	
DRB1_0101	776	GEDHCSPCDIWSVGC	CSPCDIWSV	4	0.1978	5883.1	95.00	0.44	tr_C9ZXD7_	
DRB1_0101	777	CQITAALEDIDDATF	ITAALEDID	2	0.1961	5992.9	95.00	0.21	tr_C9ZXD7_	
DRB1_0101	778	KNAATAQEERERILT	TAQEERERI	4	0.1937	6151.4	95.00	0.46	tr_C9ZXD7_	
DRB1_0101	779	QEERERILTLRQDSP	ILTLRQDSP	6	0.1923	6244.5	100.00	0.27	tr_C9ZXD7_	
DRB1_0101	780	SSPSDIDVIVCDRGW	IDVIVCDRG	5	0.1914	6304.5	100.00	0.50	tr_C9ZXD7_	
DRB1_0101	781	AQEERERILTLRQDS	EERERILTL	2	0.1909	6336.1	100.00	0.35	tr_C9ZXD7_	
DRB1_0101	782	TAALEDIDDATFRPA	LEDIDDATF	3	0.1854	6728.8	100.00	0.31	tr_C9ZXD7_	
DRB1_0101	783	LRSTCGSIREDDGTV	LRSTCGSIR	0	0.1840	6832.6	100.00	0.62	tr_C9ZXD7_	
DRB1_0101	784	QITAALEDIDDATFR	AALEDIDDA	3	0.1833	6878.6	100.00	0.23	tr_C9ZXD7_	
DRB1_0101	785	RKNAATAQEERERIL	TAQEERERI	5	0.1786	7242.0	100.00	0.43	tr_C9ZXD7_	
DRB1_0101	786	AALEDIDDATFRPAD	LEDIDDATF	2	0.1770	7368.0	100.00	0.28	tr_C9ZXD7_	
DRB1_0101	787	ASSEGDLSTTGPSAG	DLSTTGPSA	5	0.1758	7461.2	100.00	0.52	tr_C9ZXD7_	
DRB1_0101	788	ITAALEDIDDATFRP	LEDIDDATF	4	0.1698	7966.5	100.00	0.31	tr_C9ZXD7_	
DRB1_0101	789	LSSASSEGDLSTTGP	SASSEGDLS	2	0.1641	8466.6	100.00	0.37	tr_C9ZXD7_	
DRB1_0101	790	QSSPSDIDVIVCDRG	IDVIVCDRG	6	0.1538	9470.4	100.00	0.37	tr_C9ZXD7_	
DRB1_0101	791	MLADMTDVDYYVDGL	DMTDVDYYV	3	0.1533	9516.2	100.00	0.49	tr_C9ZXD7_	
DRB1_0101	792	LADMTDVDYYVDGLT	DMTDVDYYV	2	0.1384	11182.6	100.00	0.51	tr_C9ZXD7_	
DRB1_0101	793	VITPTGDIERDLAPL	ITPTGDIER	1	0.1344	11685.9	100.00	0.23	tr_C9ZXD7_	
DRB1_0101	794	LQSSPSDIDVIVCDR	SSPSDIDVI	2	0.1327	11894.0	100.00	0.32	tr_C9ZXD7_	
DRB1_0101	795	SASSEGDLSTTGPSA	DLSTTGPSA	6	0.1276	12573.6	100.00	0.41	tr_C9ZXD7_	
DRB1_0101	796	SSASSEGDLSTTGPS	SASSEGDLS	1	0.1034	16330.3	100.00	0.41	tr_C9ZXD7_	

Allele: DRB1_0101. Number of high binders 3. Number of weak binders 102. Number of peptides 796

Allele	pos	peptide	core	Of	1-log50k(aff)	affinity(nM)	%Rank	Relia	Identity	Bind Level
DRB1_0101	1	AEFFFSRSPAAETVA	FFFSRSPAA	2	0.8085	7.9	1.70	0.61	tr_C9ZR23_	WB
DRB1_0101	2	EFFFSRSPAAETVAA	FFFSRSPAA	1	0.8028	8.4	1.90	0.47	tr_C9ZR23_	WB
DRB1_0101	3	VAEFFFSRSPAAETV	FFFSRSPAA	3	0.8006	8.6	2.00	0.73	tr_C9ZR23_	WB
DRB1_0101	4	LTFSLTAIKAARDDI	LTAIKAARD	4	0.7806	10.7	3.00	0.49	tr_C9ZR23_	WB
DRB1_0101	5	ALTFSLTAIKAARDD	LTAIKAARD	5	0.7786	11.0	3.00	0.34	tr_C9ZR23_	WB
DRB1_0101	6	TFSLTAIKAARDDIK	LTAIKAARD	3	0.7744	11.5	3.50	0.67	tr_C9ZR23_	WB
DRB1_0101	7	LALTFSLTAIKAARD	FSLTAIKAA	4	0.7696	12.1	3.50	0.33	tr_C9ZR23_	WB
DRB1_0101	8	FFFSRSPAAETVAAN	FSRSPAAET	2	0.7615	13.2	4.00	0.52	tr_C9ZR23_	WB
DRB1_0101	9	GVAEFFFSRSPAAET	FFFSRSPAA	4	0.7610	13.3	4.00	0.80	tr_C9ZR23_	WB
DRB1_0101	10	PLALTFSLTAIKAAR	FSLTAIKAA	5	0.7551	14.2	4.50	0.36	tr_C9ZR23_	WB
DRB1_0101	11	FSLTAIKAARDDIKR	LTAIKAARD	2	0.7318	18.2	6.00	0.81	tr_C9ZR23_	WB
DRB1_0101	12	DGVAEFFFSRSPAAE	FFFSRSPAA	5	0.7095	23.2	8.00	0.81	tr_C9ZR23_	WB

DRB1_0101	13	DVVVLAATNPIVYIR	VLAATNPIV	3	0.7018	25.2	9.00	0.34	tr_C9ZR23_	WB
DRB1_0101	14	SCASIVGKLGQMRVT	IVGKLGQMR	4	0.6994	25.8	9.00	0.48	tr_C9ZR23_	WB
DRB1_0101	15	LPLALTFSLTAIKAA	TFSLTAIKA	5	0.6979	26.3	9.00	0.29	tr_C9ZR23_	WB
DRB1_0101	16	VVVLAATNPIVYIRT	VLAATNPIV	2	0.6951	27.1	9.50	0.36	tr_C9ZR23_	WB
DRB1_0101	17	CDVVVLAATNPIVYI	VVLAATNPI	3	0.6765	33.1	12.00	0.38	tr_C9ZR23_	
DRB1_0101	18	CASIVGKLGQMRVTC	IVGKLGQMR	3	0.6758	33.4	12.00	0.50	tr_C9ZR23_	
DRB1_0101	19	DSCASIVGKLGQMRV	IVGKLGQMR	5	0.6710	35.1	12.00	0.42	tr_C9ZR23_	
DRB1_0101	20	ASIVGKLGQMRVTCS	IVGKLGQMR	2	0.6632	38.3	13.00	0.52	tr_C9ZR23_	
DRB1_0101	21	SIVGKLGQMRVTCSD	IVGKLGQMR	1	0.6440	47.1	16.00	0.44	tr_C9ZR23_	
DRB1_0101	22	VVLAATNPIVYIRTD	LAATNPIVY	2	0.6397	49.3	16.00	0.38	tr_C9ZR23_	
DRB1_0101	23	SLTAIKAARDDIKRH	LTAIKAARD	1	0.6382	50.1	17.00	0.85	tr_C9ZR23_	
DRB1_0101	24	TNPIVYIRTDNLDGE	IVYIRTDNL	3	0.6339	52.5	17.00	0.86	tr_C9ZR23_	
DRB1_0101	25	FFSRSPAAETVAANN	FSRSPAAET	1	0.6298	54.9	18.00	0.75	tr_C9ZR23_	
DRB1_0101	26	FDGVAEFFFSRSPAA	FFFSRSPAA	6	0.6283	55.8	18.00	0.75	tr_C9ZR23_	
DRB1_0101	27	NPIVYIRTDNLDGEL	IVYIRTDNL	2	0.6254	57.6	19.00	0.82	tr_C9ZR23_	
DRB1_0101	28	PCDVVVLAATNPIVY	VVLAATNPI	4	0.6229	59.1	19.00	0.44	tr_C9ZR23_	
DRB1_0101	29	ATNPIVYIRTDNLDG	IVYIRTDNL	4	0.6219	59.8	19.00	0.88	tr_C9ZR23_	
DRB1_0101	30	LLPLALTFSLTAIKA	LTFSLTAIK	5	0.6198	61.2	19.00	0.25	tr_C9ZR23_	
DRB1_0101	31	DDSCASIVGKLGQMR	CASIVGKLG	3	0.6172	62.9	20.00	0.55	tr_C9ZR23_	
DRB1_0101	32	STLLPLALTFSLTAI	LPLALTFSL	3	0.5896	84.8	24.00	0.25	tr_C9ZR23_	
DRB1_0101	33	PIVYIRTDNLDGELD	IVYIRTDNL	1	0.5849	89.2	25.00	0.76	tr_C9ZR23_	
DRB1_0101	34	TLLPLALTFSLTAIK	PLALTFSLT	3	0.5845	89.6	25.00	0.26	tr_C9ZR23_	
DRB1_0101	35	AATNPIVYIRTDNLD	IVYIRTDNL	5	0.5824	91.7	25.00	0.88	tr_C9ZR23_	
DRB1_0101	36	ENNILPQSCVLKNTK	ILPQSCVLK	3	0.5716	103.1	27.00	0.52	tr_C9ZR23_	
DRB1_0101	37	SENNILPQSCVLKNT	ILPQSCVLK	4	0.5711	103.6	27.00	0.48	tr_C9ZR23_	
DRB1_0101	38	LSENNILPQSCVLKN	ILPQSCVLK	5	0.5597	117.1	29.00	0.44	tr_C9ZR23_	
DRB1_0101	39	IVGKLGQMRVTCSDP	LGQMRVTCS	4	0.5570	120.6	30.00	0.35	tr_C9ZR23_	
DRB1_0101	40	QDAIYNKKERKVLNR	YNKKERKVL	4	0.5538	125.0	31.00	0.54	tr_C9ZR23_	
DRB1_0101	41	DAIYNKKERKVLNRE	YNKKERKVL	3	0.5455	136.6	32.00	0.56	tr_C9ZR23_	
DRB1_0101	42	LAATNPIVYIRTDNL	IVYIRTDNL	6	0.5380	148.2	33.00	0.65	tr_C9ZR23_	
DRB1_0101	43	NNILPQSCVLKNTKT	ILPQSCVLK	2	0.5379	148.3	33.00	0.55	tr_C9ZR23_	
DRB1_0101	44	VDDSCASIVGKLGQM	CASIVGKLG	4	0.5324	157.5	35.00	0.69	tr_C9ZR23_	
DRB1_0101	45	AIYNKKERKVLNREA	YNKKERKVL	2	0.5312	159.6	35.00	0.54	tr_C9ZR23_	
DRB1_0101	46	IPCDVVVLAATNPIV	VVLAATNPI	5	0.5183	183.3	37.00	0.55	tr_C9ZR23_	
DRB1_0101	47	NHSIRVGDVILLREG	IRVGDVILL	3	0.5167	186.7	38.00	0.71	tr_C9ZR23_	
DRB1_0101	48	TNHSIRVGDVILLRE	IRVGDVILL	4	0.5153	189.4	38.00	0.67	tr_C9ZR23_	
DRB1_0101	49	LTAIKAARDDIKRHK	LTAIKAARD	0	0.5151	189.9	38.00	0.67	tr_C9ZR23_	
DRB1_0101	50	QSCVLKNTKTAICLA	CVLKNTKTA	2	0.5136	193.0	38.00	0.48	tr_C9ZR23_	
DRB1_0101	51	RTNHSIRVGDVILLR	IRVGDVILL	5	0.5127	195.0	38.00	0.61	tr_C9ZR23_	
DRB1_0101	52	SLSENNILPQSCVLK	NNILPQSCV	4	0.5124	195.6	39.00	0.46	tr_C9ZR23_	
DRB1_0101	53	TVAANNSAPMRVSLS	VAANNSAPM	1	0.5108	199.0	39.00	0.34	tr_C9ZR23_	
DRB1_0101	54	NSAPMRVSLSENNIL	SAPMRVSLS	1	0.5086	203.8	39.00	0.41	tr_C9ZR23_	
DRB1_0101	55	KQDAIYNKKERKVLN	YNKKERKVL	5	0.5050	211.9	40.00	0.50	tr_C9ZR23_	
DRB1_0101	56	SCVLKNTKTAICLAV	CVLKNTKTA	1	0.5022	218.3	41.00	0.37	tr_C9ZR23_	
DRB1_0101	57	SVDDSCASIVGKLGQ	CASIVGKLG	5	0.5020	218.8	41.00	0.61	tr_C9ZR23_	
DRB1_0101	58	VAANNSAPMRVSLSE	SAPMRVSLS	5	0.5002	223.0	41.00	0.50	tr_C9ZR23_	
DRB1_0101	59	ANNSAPMRVSLSENN	SAPMRVSLS	3	0.4991	225.8	41.00	0.77	tr_C9ZR23_	
DRB1_0101	60	VLAATNPIVYIRTDN	LAATNPIVY	1	0.4980	228.5	41.00	0.49	tr_C9ZR23_	
DRB1_0101	61	IVYIRTDNLDGELDL	IVYIRTDNL	0	0.4975	229.7	42.00	0.61	tr_C9ZR23_	
DRB1_0101	62	NILPQSCVLKNTKTA	ILPQSCVLK	1	0.4956	234.6	42.00	0.48	tr_C9ZR23_	

DRB1_0101	63	PQSCVLKNTKTAICL	CVLKNTKTA	3	0.4941	238.4	42.00	0.56	tr_C9ZR23_
DRB1_0101	64	AANNSAPMRVSLSEN	SAPMRVSLS	4	0.4918	244.4	43.00	0.70	tr_C9ZR23_
DRB1_0101	65	SAPMRVSLSENNILP	VSLSENNIL	5	0.4905	247.8	43.00	0.34	tr_C9ZR23_
DRB1_0101	66	ILPQSCVLKNTKTAI	CVLKNTKTA	5	0.4902	248.7	43.00	0.41	tr_C9ZR23_
DRB1_0101	67	HSIRVGDVILLREGE	IRVGDVILL	2	0.4846	264.0	44.00	0.73	tr_C9ZR23_
DRB1_0101	68	NNSAPMRVSLSENNI	SAPMRVSLS	2	0.4835	267.3	44.00	0.65	tr_C9ZR23_
DRB1_0101	69	CVLKNTKTAICLAVY	LKNTKTAIC	2	0.4747	294.0	46.00	0.22	tr_C9ZR23_
DRB1_0101	70	AHQLLSVDDSCASIV	LLSVDDSCA	3	0.4728	300.0	47.00	0.67	tr_C9ZR23_
DRB1_0101	71	PMRVSLSENNILPQS	VSLSENNIL	3	0.4723	301.6	47.00	0.56	tr_C9ZR23_
DRB1_0101	72	FSRSPAAETVAANNS	FSRSPAAET	0	0.4715	304.2	47.00	0.63	tr_C9ZR23_
DRB1_0101	73	VGKLGQMRVTCSDPS	LGQMRVTCS	3	0.4685	314.4	48.00	0.64	tr_C9ZR23_
DRB1_0101	74	IYNKKERKVLNREAM	YNKKERKVL	1	0.4685	314.6	48.00	0.53	tr_C9ZR23_
DRB1_0101	75	HQLLSVDDSCASIVG	LLSVDDSCA	2	0.4671	319.2	48.00	0.63	tr_C9ZR23_
DRB1_0101	76	AMTWETRTNHSIRVG	WETRTNHSI	3	0.4667	320.7	48.00	0.61	tr_C9ZR23_
DRB1_0101	77	APMRVSLSENNILPQ	VSLSENNIL	4	0.4656	324.3	48.00	0.51	tr_C9ZR23_
DRB1_0101	78	ETVAANNSAPMRVSL	VAANNSAPM	2	0.4577	353.5	50.00	0.57	tr_C9ZR23_
DRB1_0101	79	AETVAANNSAPMRVS	VAANNSAPM	3	0.4548	364.7	50.00	0.62	tr_C9ZR23_
DRB1_0101	80	LSVDDSCASIVGKLG	CASIVGKLG	6	0.4542	367.1	55.00	0.42	tr_C9ZR23_
DRB1_0101	81	QLLSVDDSCASIVGK	LLSVDDSCA	1	0.4522	375.0	55.00	0.52	tr_C9ZR23_
DRB1_0101	82	LPQSCVLKNTKTAIC	CVLKNTKTA	4	0.4522	375.1	55.00	0.54	tr_C9ZR23_
DRB1_0101	83	MTWETRTNHSIRVGD	WETRTNHSI	2	0.4511	379.4	55.00	0.57	tr_C9ZR23_
DRB1_0101	84	MRVSLSENNILPQSC	VSLSENNIL	2	0.4469	397.2	55.00	0.64	tr_C9ZR23_
DRB1_0101	85	TRTNHSIRVGDVILL	IRVGDVILL	6	0.4450	405.3	55.00	0.50	tr_C9ZR23_
DRB1_0101	86	VLKNTKTAICLAVYT	NTKTAICLA	3	0.4408	424.2	55.00	0.25	tr_C9ZR23_
DRB1_0101	87	TKCCLNKRNPKVKWA	CLNKRNPKV	3	0.4368	443.2	55.00	0.58	tr_C9ZR23_
DRB1_0101	88	LKNTKTAICLAVYTG	TAICLAVYT	5	0.4367	443.5	55.00	0.23	tr_C9ZR23_
DRB1_0101	89	IAHQLLSVDDSCASI	LLSVDDSCA	4	0.4352	451.0	55.00	0.69	tr_C9ZR23_
DRB1_0101	90	SIRVGDVILLREGED	IRVGDVILL	1	0.4343	455.2	55.00	0.78	tr_C9ZR23_
DRB1_0101	91	TWETRTNHSIRVGDV	WETRTNHSI	1	0.4270	492.7	60.00	0.49	tr_C9ZR23_
DRB1_0101	92	RVSLSENNILPQSCV	VSLSENNIL	1	0.4267	494.0	60.00	0.50	tr_C9ZR23_
DRB1_0101	93	KCCLNKRNPKVKWAQ	CLNKRNPKV	2	0.4265	495.1	60.00	0.51	tr_C9ZR23_
DRB1_0101	94	EAMTWETRTNHSIRV	WETRTNHSI	4	0.4229	515.2	60.00	0.67	tr_C9ZR23_
DRB1_0101	95	HKQDAIYNKKERKVL	IYNKKERKV	5	0.4228	515.6	60.00	0.48	tr_C9ZR23_
DRB1_0101	96	LLSVDDSCASIVGKL	VDDSCASIV	3	0.4172	547.9	60.00	0.40	tr_C9ZR23_
DRB1_0101	97	GKLGQMRVTCSDPSP	LGQMRVTCS	2	0.4171	548.5	60.00	0.71	tr_C9ZR23_
DRB1_0101	98	VSLSENNILPQSCVL	NNILPQSCV	5	0.4166	551.4	60.00	0.47	tr_C9ZR23_
DRB1_0101	99	INCFDGVAEFFFSRS	GVAEFFFSR	5	0.4158	556.3	60.00	0.22	tr_C9ZR23_
DRB1_0101	100	DVPNAIAHQLLSVDD	PNAIAHQLL	2	0.4153	559.2	60.00	0.23	tr_C9ZR23_
DRB1_0101	101	KNTKTAICLAVYTGE	TAICLAVYT	4	0.4142	565.9	60.00	0.31	tr_C9ZR23_
DRB1_0101	102	DDVPNAIAHQLLSVD	VPNAIAHQL	2	0.4133	571.1	60.00	0.23	tr_C9ZR23_
DRB1_0101	103	AIAHQLLSVDDSCAS	LLSVDDSCA	5	0.4124	577.2	60.00	0.54	tr_C9ZR23_
DRB1_0101	104	AAETVAANNSAPMRV	VAANNSAPM	4	0.4097	593.8	60.00	0.65	tr_C9ZR23_
DRB1_0101	105	MINCFDGVAEFFFSR	FDGVAEFFF	4	0.4082	603.6	60.00	0.24	tr_C9ZR23_
DRB1_0101	106	VPNAIAHQLLSVDDS	AIAHQLLSV	3	0.4078	606.1	65.00	0.22	tr_C9ZR23_
DRB1_0101	107	DDIKRHKQDAIYNKK	RHKQDAIYN	4	0.4053	623.0	65.00	0.40	tr_C9ZR23_
DRB1_0101	108	DIPCDVVVLAATNPI	VVLAATNPI	6	0.4031	637.6	65.00	0.44	tr_C9ZR23_
DRB1_0101	109	GDDVPNAIAHQLLSV	VPNAIAHQL	3	0.3989	667.5	65.00	0.32	tr_C9ZR23_
DRB1_0101	110	DIKRHKQDAIYNKKE	RHKQDAIYN	3	0.3971	680.5	65.00	0.41	tr_C9ZR23_
DRB1_0101	111	RDDIKRHKQDAIYNK	IKRHKQDAI	3	0.3970	681.5	65.00	0.51	tr_C9ZR23_
DRB1_0101	112	DTKCCLNKRNPKVKW	CLNKRNPKV	4	0.3906	730.7	65.00	0.59	tr_C9ZR23_

DRB1_0101	113	KLGQMRVTCSDPSPM	LGQMRVTCS	1	0.3892	741.3	65.00	0.57	tr_C9ZR23_
DRB1_0101	114	NAIAHQLLSVDDSCA	LLSVDDSCA	6	0.3839	785.3	70.00	0.30	tr_C9ZR23_
DRB1_0101	115	CCLNKRNPKVKWAQI	CLNKRNPKV	1	0.3826	796.7	70.00	0.46	tr_C9ZR23_
DRB1_0101	116	NCFDGVAEFFFSRSP	GVAEFFFSR	4	0.3825	796.9	70.00	0.31	tr_C9ZR23_
DRB1_0101	117	NTKTAICLAVYTGED	TAICLAVYT	3	0.3825	797.5	70.00	0.37	tr_C9ZR23_
DRB1_0101	118	CFDGVAEFFFSRSPA	GVAEFFFSR	3	0.3701	911.9	70.00	0.36	tr_C9ZR23_
DRB1_0101	119	PNAIAHQLLSVDDSC	AIAHQLLSV	2	0.3662	951.4	70.00	0.24	tr_C9ZR23_
DRB1_0101	120	PMINCFDGVAEFFFS	FDGVAEFFF	5	0.3634	980.6	70.00	0.22	tr_C9ZR23_
DRB1_0101	121	LGQMRVTCSDPSPMI	MRVTCSDPS	3	0.3614	1001.3	70.00	0.31	tr_C9ZR23_
DRB1_0101	122	WETRTNHSIRVGDVI	TRTNHSIRV	2	0.3601	1016.4	75.00	0.44	tr_C9ZR23_
DRB1_0101	123	IKRHKQDAIYNKKER	RHKQDAIYN	2	0.3588	1030.0	75.00	0.46	tr_C9ZR23_
DRB1_0101	124	IRVGDVILLREGEDI	IRVGDVILL	0	0.3576	1043.6	75.00	0.55	tr_C9ZR23_
DRB1_0101	125	REAMTWETRTNHSIR	WETRTNHSI	5	0.3561	1060.6	75.00	0.70	tr_C9ZR23_
DRB1_0101	126	TKTAICLAVYTGEDT	TAICLAVYT	2	0.3561	1060.7	75.00	0.32	tr_C9ZR23_
DRB1_0101	127	RKVLNREAMTWETRT	NREAMTWET	4	0.3477	1161.6	75.00	0.31	tr_C9ZR23_
DRB1_0101	128	PAAETVAANNSAPMR	VAANNSAPM	5	0.3468	1172.9	75.00	0.59	tr_C9ZR23_
DRB1_0101	129	KTAICLAVYTGEDTK	TAICLAVYT	1	0.3468	1173.4	75.00	0.24	tr_C9ZR23_
DRB1_0101	130	YNKKERKVLNREAMT	YNKKERKVL	0	0.3451	1194.6	75.00	0.48	tr_C9ZR23_
DRB1_0101	131	ERKVLNREAMTWETR	KVLNREAMT	2	0.3442	1206.7	75.00	0.29	tr_C9ZR23_
DRB1_0101	132	GGDDVPNAIAHQLLS	VPNAIAHQL	4	0.3420	1235.8	75.00	0.38	tr_C9ZR23_
DRB1_0101	133	ETRTNHSIRVGDVIL	TRTNHSIRV	1	0.3394	1271.1	75.00	0.41	tr_C9ZR23_
DRB1_0101	134	KERKVLNREAMTWET	KVLNREAMT	3	0.3376	1296.1	75.00	0.38	tr_C9ZR23_
DRB1_0101	135	ARDDIKRHKQDAIYN	IKRHKQDAI	4	0.3372	1301.2	75.00	0.57	tr_C9ZR23_
DRB1_0101	136	GQMRVTCSDPSPMIN	VTCSDPSPM	4	0.3342	1344.9	80.00	0.47	tr_C9ZR23_
DRB1_0101	137	EDTKCCLNKRNPKVK	CLNKRNPKV	5	0.3341	1345.7	80.00	0.58	tr_C9ZR23_
DRB1_0101	138	SPMINCFDGVAEFFF	INCFDGVAE	3	0.3300	1406.9	80.00	0.31	tr_C9ZR23_
DRB1_0101	139	QMRVTCSDPSPMINC	VTCSDPSPM	3	0.3287	1426.4	80.00	0.49	tr_C9ZR23_
DRB1_0101	140	NPKVKWAQIDRDISK	VKWAQIDRD	3	0.3228	1521.7	80.00	0.40	tr_C9ZR23_
DRB1_0101	141	CLNKRNPKVKWAQID	CLNKRNPKV	0	0.3161	1634.6	80.00	0.37	tr_C9ZR23_
DRB1_0101	142	KKERKVLNREAMTWE	KVLNREAMT	4	0.3149	1656.9	80.00	0.41	tr_C9ZR23_
DRB1_0101	143	LAVYTGEDTKCCLNK	YTGEDTKCC	3	0.3142	1670.1	80.00	0.65	tr_C9ZR23_
DRB1_0101	144	KPRDVVAPQLSSDHT	DVVAPQLSS	3	0.3132	1688.3	80.00	0.40	tr_C9ZR23_
DRB1_0101	145	CLAVYTGEDTKCCLN	YTGEDTKCC	4	0.3106	1735.6	80.00	0.54	tr_C9ZR23_
DRB1_0101	146	KVLNREAMTWETRTN	NREAMTWET	3	0.3087	1771.6	80.00	0.44	tr_C9ZR23_
DRB1_0101	147	NKKERKVLNREAMTW	KVLNREAMT	5	0.3030	1883.7	85.00	0.41	tr_C9ZR23_
DRB1_0101	148	ICLAVYTGEDTKCCL	YTGEDTKCC	5	0.3029	1886.5	85.00	0.43	tr_C9ZR23_
DRB1_0101	149	TAICLAVYTGEDTKC	LAVYTGEDT	4	0.3019	1906.8	85.00	0.39	tr_C9ZR23_
DRB1_0101	150	VGDVILLREGEDIPC	ILLREGEDI	4	0.3006	1934.5	85.00	0.57	tr_C9ZR23_
DRB1_0101	151	LKPRDVVAPQLSSDH	DVVAPQLSS	4	0.3003	1940.5	85.00	0.39	tr_C9ZR23_
DRB1_0101	152	MRVTCSDPSPMINCF	VTCSDPSPM	2	0.2990	1968.5	85.00	0.47	tr_C9ZR23_
DRB1_0101	153	GDVILLREGEDIPCD	ILLREGEDI	3	0.2945	2066.2	85.00	0.60	tr_C9ZR23_
DRB1_0101	154	PRDVVAPQLSSDHTG	DVVAPQLSS	2	0.2941	2074.7	85.00	0.34	tr_C9ZR23_
DRB1_0101	155	RVGDVILLREGEDIP	ILLREGEDI	5	0.2939	2078.9	85.00	0.52	tr_C9ZR23_
DRB1_0101	156	GEDIPCDVVVLAATN	DIPCDVVVL	2	0.2918	2127.1	85.00	0.38	tr_C9ZR23_
DRB1_0101	157	AICLAVYTGEDTKCC	LAVYTGEDT	3	0.2877	2224.0	85.00	0.40	tr_C9ZR23_
DRB1_0101	158	TGGDDVPNAIAHQLL	VPNAIAHQL	5	0.2861	2263.0	85.00	0.43	tr_C9ZR23_
DRB1_0101	159	VYIRTDNLDGELDLK	YIRTDNLDG	1	0.2853	2281.5	85.00	0.44	tr_C9ZR23_
DRB1_0101	160	EDIPCDVVVLAATNP	DIPCDVVVL	1	0.2852	2283.7	85.00	0.29	tr_C9ZR23_
DRB1_0101	161	PSPMINCFDGVAEFF	INCFDGVAE	4	0.2848	2294.9	85.00	0.37	tr_C9ZR23_
DRB1_0101	162	RNPKVKWAQIDRDIS	VKWAQIDRD	4	0.2848	2295.4	85.00	0.40	tr_C9ZR23_

DRB1_0101	163	LDLKPRDVVAPQLSS	PRDVVAPQL	4	0.2842	2310.0	85.00	0.26	tr_C9ZR23_
DRB1_0101	164	DVILLREGEDIPCDV	ILLREGEDI	2	0.2829	2341.2	85.00	0.62	tr_C9ZR23_
DRB1_0101	165	GELDLKPRDVVAPQL	ELDLKPRDV	1	0.2823	2357.0	85.00	0.28	tr_C9ZR23_
DRB1_0101	166	EGEDIPCDVVVLAAT	DIPCDVVVL	3	0.2816	2375.5	85.00	0.44	tr_C9ZR23_
DRB1_0101	167	NLDGELDLKPRDVVA	ELDLKPRDV	4	0.2809	2393.8	85.00	0.41	tr_C9ZR23_
DRB1_0101	168	ELDLKPRDVVAPQLS	LKPRDVVAP	3	0.2791	2439.7	85.00	0.26	tr_C9ZR23_
DRB1_0101	169	DLKPRDVVAPQLSSD	DVVAPQLSS	5	0.2785	2457.2	85.00	0.32	tr_C9ZR23_
DRB1_0101	170	RDVVAPQLSSDHTGG	VAPQLSSDH	3	0.2779	2471.4	85.00	0.46	tr_C9ZR23_
DRB1_0101	171	AVYTGEDTKCCLNKR	YTGEDTKCC	2	0.2728	2613.4	90.00	0.70	tr_C9ZR23_
DRB1_0101	172	REGEDIPCDVVVLAA	DIPCDVVVL	4	0.2717	2642.7	90.00	0.48	tr_C9ZR23_
DRB1_0101	173	RHKQDAIYNKKERKV	IYNKKERKV	6	0.2707	2673.8	90.00	0.51	tr_C9ZR23_
DRB1_0101	174	TAIKAARDDIKRHKQ	IKAARDDIK	2	0.2703	2684.0	90.00	0.74	tr_C9ZR23_
DRB1_0101	175	VILLREGEDIPCDVV	ILLREGEDI	1	0.2687	2731.9	90.00	0.56	tr_C9ZR23_
DRB1_0101	176	NREAMTWETRTNHSI	WETRTNHSI	6	0.2681	2749.2	90.00	0.47	tr_C9ZR23_
DRB1_0101	177	LDGELDLKPRDVVAP	ELDLKPRDV	3	0.2658	2818.5	90.00	0.43	tr_C9ZR23_
DRB1_0101	178	KRHKQDAIYNKKERK	RHKQDAIYN	1	0.2649	2845.1	90.00	0.52	tr_C9ZR23_
DRB1_0101	179	AARDDIKRHKQDAIY	IKRHKQDAI	5	0.2636	2885.9	90.00	0.68	tr_C9ZR23_
DRB1_0101	180	DGELDLKPRDVVAPQ	ELDLKPRDV	2	0.2632	2900.2	90.00	0.38	tr_C9ZR23_
DRB1_0101	181	RVTCSDPSPMINCFD	VTCSDPSPM	1	0.2631	2901.6	90.00	0.44	tr_C9ZR23_
DRB1_0101	182	SPAAETVAANNSAPM	VAANNSAPM	6	0.2595	3017.8	90.00	0.42	tr_C9ZR23_
DRB1_0101	183	LREGEDIPCDVVVLA	DIPCDVVVL	5	0.2527	3247.7	90.00	0.43	tr_C9ZR23_
DRB1_0101	184	VLNREAMTWETRTNH	NREAMTWET	2	0.2527	3247.8	90.00	0.51	tr_C9ZR23_
DRB1_0101	185	KRNPKVKWAQIDRDI	VKWAQIDRD	5	0.2468	3461.7	90.00	0.40	tr_C9ZR23_
DRB1_0101	186	GEDTKCCLNKRNPKV	CLNKRNPKV	6	0.2458	3497.6	90.00	0.51	tr_C9ZR23_
DRB1_0101	187	DPSPMINCFDGVAEF	INCFDGVAE	5	0.2455	3510.4	90.00	0.37	tr_C9ZR23_
DRB1_0101	188	DNLDGELDLKPRDVV	ELDLKPRDV	5	0.2401	3723.7	95.00	0.40	tr_C9ZR23_
DRB1_0101	189	SRSPAAETVAANNSA	PAAETVAAN	3	0.2380	3808.2	95.00	0.32	tr_C9ZR23_
DRB1_0101	190	VYTGEDTKCCLNKRN	YTGEDTKCC	1	0.2374	3833.7	95.00	0.62	tr_C9ZR23_
DRB1_0101	191	LNREAMTWETRTNHS	NREAMTWET	1	0.2293	4184.2	95.00	0.45	tr_C9ZR23_
DRB1_0101	192	AIKAARDDIKRHKQD	IKAARDDIK	1	0.2289	4200.5	95.00	0.73	tr_C9ZR23_
DRB1_0101	193	YIRTDNLDGELDLKP	TDNLDGELD	3	0.2272	4279.5	95.00	0.39	tr_C9ZR23_
DRB1_0101	194	SDPSPMINCFDGVAE	PSPMINCFD	2	0.2235	4452.0	95.00	0.27	tr_C9ZR23_
DRB1_0101	195	LLREGEDIPCDVVVL	DIPCDVVVL	6	0.2202	4618.1	95.00	0.37	tr_C9ZR23_
DRB1_0101	196	ILLREGEDIPCDVVV	ILLREGEDI	0	0.2169	4784.9	95.00	0.34	tr_C9ZR23_
DRB1_0101	197	LNKRNPKVKWAQIDR	PKVKWAQID	5	0.2148	4894.0	95.00	0.20	tr_C9ZR23_
DRB1_0101	198	DVVAPQLSSDHTGGD	VAPQLSSDH	2	0.2107	5115.1	95.00	0.48	tr_C9ZR23_
DRB1_0101	199	VTCSDPSPMINCFDG	TCSDPSPMI	1	0.2089	5214.8	95.00	0.25	tr_C9ZR23_
DRB1_0101	200	IRTDNLDGELDLKPR	TDNLDGELD	2	0.2069	5327.5	95.00	0.43	tr_C9ZR23_
DRB1_0101	201	NKRNPKVKWAQIDRD	VKWAQIDRD	6	0.2060	5384.7	95.00	0.25	tr_C9ZR23_
DRB1_0101	202	CSDPSPMINCFDGVA	PSPMINCFD	3	0.2041	5493.3	95.00	0.32	tr_C9ZR23_
DRB1_0101	203	TDNLDGELDLKPRDV	LDGELDLKP	3	0.1991	5797.6	95.00	0.36	tr_C9ZR23_
DRB1_0101	204	HTGGDDVPNAIAHQL	VPNAIAHQL	6	0.1937	6148.2	95.00	0.34	tr_C9ZR23_
DRB1_0101	205	RSPAAETVAANNSAP	PAAETVAAN	2	0.1936	6154.7	95.00	0.38	tr_C9ZR23_
DRB1_0101	206	RTDNLDGELDLKPRD	LDGELDLKP	4	0.1917	6280.3	100.00	0.30	tr_C9ZR23_
DRB1_0101	207	TCSDPSPMINCFDGV	PSPMINCFD	4	0.1870	6613.8	100.00	0.31	tr_C9ZR23_
DRB1_0101	208	YTGEDTKCCLNKRNP	YTGEDTKCC	0	0.1804	7103.2	100.00	0.47	tr_C9ZR23_
DRB1_0101	209	KAARDDIKRHKQDAI	IKRHKQDAI	6	0.1749	7537.2	100.00	0.60	tr_C9ZR23_
DRB1_0101	210	IKAARDDIKRHKQDA	IKAARDDIK	0	0.1556	9283.7	100.00	0.56	tr_C9ZR23_
DRB1_0101	211	VVAPQLSSDHTGGDD	VAPQLSSDH	1	0.1485	10027.0	100.00	0.58	tr_C9ZR23_
DRB1_0101	212	TGEDTKCCLNKRNPK	TKCCLNKRN	4	0.1437	10564.9	100.00	0.34	tr_C9ZR23_

DRB1_0101	213	DHTGGDDVPNAIAHQ	GDDVPNAIA	4	0.1333	11823.8	100.00	0.27	tr_C9ZR23_
DRB1_0101	214	SDHTGGDDVPNAIAH	GDDVPNAIA	5	0.1246	12992.4	100.00	0.25	tr_C9ZR23_
DRB1_0101	215	SSDHTGGDDVPNAIA	SDHTGGDDV	1	0.1174	14045.5	100.00	0.24	tr_C9ZR23_
DRB1_0101	216	VAPQLSSDHTGGDDV	VAPQLSSDH	0	0.1052	16013.2	100.00	0.26	tr_C9ZR23_
DRB1_0101	217	LSSDHTGGDDVPNAI	SDHTGGDDV	2	0.0995	17035.2	100.00	0.38	tr_C9ZR23_
DRB1_0101	218	QLSSDHTGGDDVPNA	SDHTGGDDV	3	0.0944	18012.0	100.00	0.40	tr_C9ZR23_
DRB1_0101	219	APQLSSDHTGGDDVP	SDHTGGDDV	5	0.0884	19203.4	100.00	0.31	tr_C9ZR23_
DRB1_0101	220	PQLSSDHTGGDDVPN	SDHTGGDDV	4	0.0883	19236.3	100.00	0.40	tr_C9ZR23_

Allele: DRB1_0101. Number of high binders 0. Number of weak binders 16. Number of peptides 220

Allele	pos	peptide	core	Of	1-log50k(aff)	affinity(nM)	%Rank	Relia	Identity	Bind Level
DRB1_0101	1	ESLLQFSKGVMADVQ	LQFSKGVMA	3	0.8183	7.1	1.40	0.82	tr_C9ZR23_	WB
DRB1_0101	2	SLLQFSKGVMADVQY	LQFSKGVMA	2	0.8140	7.5	1.50	0.76	tr_C9ZR23_	WB
DRB1_0101	3	HYVFAFASEFKTMGV	FAFASEFKT	3	0.8101	7.8	1.70	0.69	tr_C9ZR23_	WB
DRB1_0101	4	RSLVFVHGQLAYNRT	LVFVHGQLA	2	0.8090	7.9	1.70	0.63	tr_C9ZR23_	WB
DRB1_0101	5	VHYVFAFASEFKTMG	FAFASEFKT	4	0.8056	8.2	1.80	0.60	tr_C9ZR23_	WB
DRB1_0101	6	MESLLQFSKGVMADV	LQFSKGVMA	4	0.8039	8.3	1.90	0.80	tr_C9ZR23_	WB
DRB1_0101	7	LRSLVFVHGQLAYNR	LVFVHGQLA	3	0.8033	8.4	1.90	0.69	tr_C9ZR23_	WB
DRB1_0101	8	FVHYVFAFASEFKTM	FAFASEFKT	5	0.7979	8.9	2.50	0.50	tr_C9ZR23_	WB
DRB1_0101	9	SLVFVHGQLAYNRTA	LVFVHGQLA	1	0.7976	8.9	2.50	0.47	tr_C9ZR23_	WB
DRB1_0101	10	LLQFSKGVMADVQYG	LQFSKGVMA	1	0.7916	9.5	2.50	0.73	tr_C9ZR23_	WB
DRB1_0101	11	SKYYFALVIENDVAM	FALVIENDV	4	0.7888	9.8	2.50	0.45	tr_C9ZR23_	WB
DRB1_0101	12	YMESLLQFSKGVMAD	LQFSKGVMA	5	0.7836	10.4	3.00	0.75	tr_C9ZR23_	WB
DRB1_0101	13	MSKYYFALVIENDVA	YYFALVIEN	3	0.7829	10.5	3.00	0.41	tr_C9ZR23_	WB
DRB1_0101	14	VTRLVRSRGFITLAV	LVRSRGFIT	3	0.7827	10.5	3.00	0.82	tr_C9ZR23_	WB
DRB1_0101	15	KYYFALVIENDVAMH	FALVIENDV	3	0.7787	11.0	3.00	0.52	tr_C9ZR23_	WB
DRB1_0101	16	YVFAFASEFKTMGVI	FAFASEFKT	2	0.7767	11.2	3.50	0.73	tr_C9ZR23_	WB
DRB1_0101	17	TRLVRSRGFITLAVG	LVRSRGFIT	2	0.7719	11.8	3.50	0.80	tr_C9ZR23_	WB
DRB1_0101	18	AVTRLVRSRGFITLA	LVRSRGFIT	4	0.7717	11.8	3.50	0.77	tr_C9ZR23_	WB
DRB1_0101	19	DLRSLVFVHGQLAYN	LVFVHGQLA	4	0.7663	12.5	4.00	0.74	tr_C9ZR23_	WB
DRB1_0101	20	LVFVHGQLAYNRTAF	LVFVHGQLA	0	0.7546	14.2	4.50	0.37	tr_C9ZR23_	WB
DRB1_0101	21	GYMESLLQFSKGVMA	LQFSKGVMA	6	0.7545	14.2	4.50	0.68	tr_C9ZR23_	WB
DRB1_0101	22	VEHYAVFGLRTLLVA	VFGLRTLLV	5	0.7532	14.4	4.50	0.44	tr_C9ZR23_	WB
DRB1_0101	23	EHYAVFGLRTLLVAE	VFGLRTLLV	4	0.7489	15.1	5.00	0.47	tr_C9ZR23_	WB
DRB1_0101	24	HYAVFGLRTLLVAEK	VFGLRTLLV	3	0.7452	15.7	5.00	0.60	tr_C9ZR23_	WB
DRB1_0101	25	AAVTRLVRSRGFITL	LVRSRGFIT	5	0.7416	16.4	5.50	0.70	tr_C9ZR23_	WB
DRB1_0101	26	IKYSFYKSVLIGIIQ	YSFYKSVLI	2	0.7399	16.7	5.50	0.50	tr_C9ZR23_	WB
DRB1_0101	27	DMSKYYFALVIENDV	YYFALVIEN	4	0.7389	16.9	5.50	0.48	tr_C9ZR23_	WB
DRB1_0101	28	VFAFASEFKTMGVIV	FAFASEFKT	1	0.7383	17.0	5.50	0.73	tr_C9ZR23_	WB
DRB1_0101	29	WFVHYVFAFASEFKT	YVFAFASEF	4	0.7382	17.0	5.50	0.40	tr_C9ZR23_	WB
DRB1_0101	30	VIKYSFYKSVLIGII	YSFYKSVLI	3	0.7379	17.0	5.50	0.54	tr_C9ZR23_	WB
DRB1_0101	31	TIRSFLQAGIKVWML	SFLQAGIKV	3	0.7354	17.5	6.00	0.62	tr_C9ZR23_	WB
DRB1_0101	32	IRSFLQAGIKVWMLT	SFLQAGIKV	2	0.7295	18.7	6.50	0.54	tr_C9ZR23_	WB
DRB1_0101	33	VLERILTTPSLLKLL	ILTTPSLLK	4	0.7295	18.7	6.50	0.52	tr_C9ZR23_	WB
DRB1_0101	34	KYSFYKSVLIGIIQL	YSFYKSVLI	1	0.7262	19.3	6.50	0.43	tr_C9ZR23_	WB

DRB1_0101	35	YYFALVIENDVAMHH	FALVIENDV	2	0.7257	19.5	6.50	0.60	tr_C9ZR23_	WB
DRB1_0101	36	EVEHYAVFGLRTLLV	HYAVFGLRT	3	0.7246	19.7	7.00	0.45	tr_C9ZR23_	WB
DRB1_0101	37	LERILTTPSLLKLLT	ILTTPSLLK	3	0.7244	19.7	7.00	0.58	tr_C9ZR23_	WB
DRB1_0101	38	RLVRSRGFITLAVGD	LVRSRGFIT	1	0.7241	19.8	7.00	0.80	tr_C9ZR23_	WB
DRB1_0101	39	LQFSKGVMADVQYGD	LQFSKGVMA	0	0.7214	20.4	7.00	0.55	tr_C9ZR23_	WB
DRB1_0101	40	FVIKYSFYKSVLIGI	YSFYKSVLI	4	0.7198	20.7	7.00	0.58	tr_C9ZR23_	WB
DRB1_0101	41	QVLERILTTPSLLKL	ILTTPSLLK	5	0.7192	20.9	7.50	0.44	tr_C9ZR23_	WB
DRB1_0101	42	SDLRSLVFVHGQLAY	LVFVHGQLA	5	0.7191	20.9	7.50	0.81	tr_C9ZR23_	WB
DRB1_0101	43	AFVIKYSFYKSVLIG	YSFYKSVLI	5	0.7123	22.5	8.00	0.58	tr_C9ZR23_	WB
DRB1_0101	44	ETIRSFLQAGIKVWM	SFLQAGIKV	4	0.7101	23.0	8.00	0.63	tr_C9ZR23_	WB
DRB1_0101	45	RSFLQAGIKVWMLTG	SFLQAGIKV	1	0.7095	23.2	8.00	0.50	tr_C9ZR23_	WB
DRB1_0101	46	SYVLVIEGGQVLERI	LVIEGGQVL	3	0.7060	24.1	8.50	0.76	tr_C9ZR23_	WB
DRB1_0101	47	TAFVIKYSFYKSVLI	YSFYKSVLI	6	0.6995	25.8	9.00	0.46	tr_C9ZR23_	WB
DRB1_0101	48	PSLLKLLTELSENCV	LLKLLTELS	2	0.6987	26.0	9.00	0.68	tr_C9ZR23_	WB
DRB1_0101	49	SSYVLVIEGGQVLER	LVIEGGQVL	4	0.6981	26.2	9.00	0.71	tr_C9ZR23_	WB
DRB1_0101	50	AIYYFVKGADDRILE	YYFVKGADD	2	0.6978	26.3	9.00	0.59	tr_C9ZR23_	WB
DRB1_0101	51	YAVFGLRTLLVAEKR	VFGLRTLLV	2	0.6973	26.4	9.50	0.67	tr_C9ZR23_	WB
DRB1_0101	52	NAIYYFVKGADDRIL	YYFVKGADD	3	0.6964	26.7	9.50	0.64	tr_C9ZR23_	WB
DRB1_0101	53	PRRVLEQTPALYKVT	VLEQTPALY	3	0.6960	26.8	9.50	0.47	tr_C9ZR23_	WB
DRB1_0101	54	SLLKLLTELSENCVS	LLKLLTELS	1	0.6955	27.0	9.50	0.55	tr_C9ZR23_	WB
DRB1_0101	55	RRVLEQTPALYKVTR	LEQTPALYK	3	0.6946	27.2	9.50	0.43	tr_C9ZR23_	WB
DRB1_0101	56	ERILTTPSLLKLLTE	ILTTPSLLK	2	0.6940	27.4	9.50	0.63	tr_C9ZR23_	WB
DRB1_0101	57	NGCEKLNVRLVARDA	CEKLNVRLV	2	0.6903	28.5	10.00	0.70	tr_C9ZR23_	
DRB1_0101	58	IYYFVKGADDRILEM	YYFVKGADD	1	0.6897	28.7	10.00	0.50	tr_C9ZR23_	
DRB1_0101	59	GCEKLNVRLVARDAT	CEKLNVRLV	1	0.6883	29.1	10.00	0.56	tr_C9ZR23_	
DRB1_0101	60	TTPSLLKLLTELSEN	LLKLLTELS	4	0.6879	29.3	11.00	0.87	tr_C9ZR23_	
DRB1_0101	61	FAFASEFKTMGVIVE	FAFASEFKT	0	0.6839	30.6	11.00	0.45	tr_C9ZR23_	
DRB1_0101	62	YVLVIEGGQVLERIL	LVIEGGQVL	2	0.6836	30.7	11.00	0.75	tr_C9ZR23_	
DRB1_0101	63	TPSLLKLLTELSENC	LLKLLTELS	3	0.6824	31.1	11.00	0.84	tr_C9ZR23_	
DRB1_0101	64	LCNGCEKLNVRLVAR	CEKLNVRLV	4	0.6811	31.5	11.00	0.82	tr_C9ZR23_	
DRB1_0101	65	EHVLHFGRVLSLCNT	LHFGRVLSL	3	0.6803	31.8	11.00	0.44	tr_C9ZR23_	
DRB1_0101	66	GQVLERILTTPSLLK	LERILTTPS	3	0.6791	32.2	11.00	0.33	tr_C9ZR23_	
DRB1_0101	67	VFVHGQLAYNRTAFV	FVHGQLAYN	1	0.6780	32.6	12.00	0.37	tr_C9ZR23_	
DRB1_0101	68	CNGCEKLNVRLVARD	CEKLNVRLV	3	0.6767	33.0	12.00	0.83	tr_C9ZR23_	
DRB1_0101	69	VPRRVLEQTPALYKV	VLEQTPALY	4	0.6757	33.4	12.00	0.47	tr_C9ZR23_	
DRB1_0101	70	EWFVHYVFAFASEFK	YVFAFASEF	5	0.6756	33.4	12.00	0.50	tr_C9ZR23_	
DRB1_0101	71	PETIRSFLQAGIKVW	SFLQAGIKV	5	0.6734	34.3	12.00	0.61	tr_C9ZR23_	
DRB1_0101	72	GEHVLHFGRVLSLCN	LHFGRVLSL	4	0.6716	34.9	12.00	0.44	tr_C9ZR23_	
DRB1_0101	73	RSRGFITLAVGDGGN	FITLAVGDG	4	0.6712	35.1	12.00	0.54	tr_C9ZR23_	
DRB1_0101	74	YFALVIENDVAMHHD	FALVIENDV	1	0.6687	36.0	13.00	0.63	tr_C9ZR23_	
DRB1_0101	75	HGQLAYNRTAFVIKY	LAYNRTAFV	3	0.6676	36.5	13.00	0.81	tr_C9ZR23_	
DRB1_0101	76	HVLHFGRVLSLCNTV	LHFGRVLSL	2	0.6666	36.9	13.00	0.42	tr_C9ZR23_	
DRB1_0101	77	SRGFITLAVGDGGND	FITLAVGDG	3	0.6660	37.1	13.00	0.54	tr_C9ZR23_	
DRB1_0101	78	YSFYKSVLIGIIQLV	FYKSVLIGI	2	0.6658	37.2	13.00	0.39	tr_C9ZR23_	
DRB1_0101	79	RGFITLAVGDGGNDV	FITLAVGDG	2	0.6631	38.3	13.00	0.48	tr_C9ZR23_	
DRB1_0101	80	LTTPSLLKLLTELSE	LLKLLTELS	5	0.6625	38.6	13.00	0.83	tr_C9ZR23_	
DRB1_0101	81	FSDLRSLVFVHGQLA	LVFVHGQLA	6	0.6624	38.6	13.00	0.73	tr_C9ZR23_	
DRB1_0101	82	KAAVTRLVRSRGFIT	LVRSRGFIT	6	0.6614	39.0	14.00	0.52	tr_C9ZR23_	
DRB1_0101	83	FVHGQLAYNRTAFVI	LAYNRTAFV	5	0.6599	39.6	14.00	0.52	tr_C9ZR23_	
DRB1_0101	84	MEFLFATINGERRCL	FATINGERR	4	0.6595	39.8	14.00	0.44	tr_C9ZR23_	

DRB1_0101	85	AVFGLRTLLVAEKRL	VFGLRTLLV	1	0.6574	40.7	14.00	0.58	tr_C9ZR23_
DRB1_0101	86	EFLFATINGERRCLA	FATINGERR	3	0.6553	41.7	14.00	0.56	tr_C9ZR23_
DRB1_0101	87	LVRSRGFITLAVGDG	LVRSRGFIT	0	0.6553	41.7	14.00	0.41	tr_C9ZR23_
DRB1_0101	88	TLWNGLYSLPQTMLY	LYSLPQTML	5	0.6540	42.3	15.00	0.47	tr_C9ZR23_
DRB1_0101	89	GSSYVLVIEGGQVLE	LVIEGGQVL	5	0.6536	42.4	15.00	0.69	tr_C9ZR23_
DRB1_0101	90	CEKLNVRLVARDATT	NVRLVARDA	4	0.6531	42.7	15.00	0.43	tr_C9ZR23_
DRB1_0101	91	VMEFLFATINGERRC	FLFATINGE	3	0.6529	42.8	15.00	0.41	tr_C9ZR23_
DRB1_0101	92	LLKLLTELSENCVSV	LTELSENCV	4	0.6521	43.1	15.00	0.43	tr_C9ZR23_
DRB1_0101	93	VHGQLAYNRTAFVIK	LAYNRTAFV	4	0.6517	43.3	15.00	0.75	tr_C9ZR23_
DRB1_0101	94	TDMSKYYFALVIEND	YYFALVIEN	5	0.6494	44.4	15.00	0.62	tr_C9ZR23_
DRB1_0101	95	CGEHVLHFGRVLSLC	LHFGRVLSL	5	0.6486	44.8	15.00	0.43	tr_C9ZR23_
DRB1_0101	96	IYKLRLEMENSVTIL	LRLEMENSV	3	0.6482	45.0	15.00	0.50	tr_C9ZR23_
DRB1_0101	97	GQLAYNRTAFVIKYS	LAYNRTAFV	2	0.6479	45.1	15.00	0.77	tr_C9ZR23_
DRB1_0101	98	YKLRLEMENSVTILG	LEMENSVTI	4	0.6468	45.7	15.00	0.43	tr_C9ZR23_
DRB1_0101	99	VLVIEGGQVLERILT	LVIEGGQVL	1	0.6426	47.8	16.00	0.75	tr_C9ZR23_
DRB1_0101	100	LWNGLYSLPQTMLYC	LYSLPQTML	4	0.6415	48.4	16.00	0.50	tr_C9ZR23_
DRB1_0101	101	ALCNGCEKLNVRLVA	CEKLNVRLV	5	0.6412	48.5	16.00	0.80	tr_C9ZR23_
DRB1_0101	102	ILTTPSLLKLLTELS	LLKLLTELS	6	0.6399	49.2	16.00	0.57	tr_C9ZR23_
DRB1_0101	103	RILTTPSLLKLLTEL	ILTTPSLLK	1	0.6385	49.9	17.00	0.63	tr_C9ZR23_
DRB1_0101	104	NVMEFLFATINGERR	FLFATINGE	4	0.6377	50.4	17.00	0.46	tr_C9ZR23_
DRB1_0101	105	WNGLYSLPQTMLYCL	LYSLPQTML	3	0.6362	51.2	17.00	0.57	tr_C9ZR23_
DRB1_0101	106	YYFVKGADDRILEMA	FVKGADDRI	2	0.6346	52.1	17.00	0.44	tr_C9ZR23_
DRB1_0101	107	VLHFGRVLSLCNTVE	LHFGRVLSL	1	0.6338	52.6	17.00	0.40	tr_C9ZR23_
DRB1_0101	108	KLRLEMENSVTILGV	LEMENSVTI	3	0.6336	52.7	17.00	0.55	tr_C9ZR23_
DRB1_0101	109	GDCVLRVLADKLDAF	VLRVLADKL	3	0.6304	54.5	18.00	0.78	tr_C9ZR23_
DRB1_0101	110	RTAFVIKYSFYKSVL	FVIKYSFYK	3	0.6292	55.3	18.00	0.65	tr_C9ZR23_
DRB1_0101	111	VFGLRTLLVAEKRLT	VFGLRTLLV	0	0.6289	55.4	18.00	0.37	tr_C9ZR23_
DRB1_0101	112	SCGEHVLHFGRVLSL	HVLHFGRVL	4	0.6285	55.7	18.00	0.52	tr_C9ZR23_
DRB1_0101	113	AEVEHYAVFGLRTLL	HYAVFGLRT	4	0.6276	56.2	18.00	0.59	tr_C9ZR23_
DRB1_0101	114	GIKVWMLTGDKVQTA	VWMLTGDKV	3	0.6270	56.6	18.00	0.66	tr_C9ZR23_
DRB1_0101	115	RVLEQTPALYKVTRS	LEQTPALYK	2	0.6256	57.5	18.00	0.43	tr_C9ZR23_
DRB1_0101	116	TNAIYYFVKGADDRI	YYFVKGADD	4	0.6254	57.6	19.00	0.74	tr_C9ZR23_
DRB1_0101	117	KLNVRLVARDATTAA	NVRLVARDA	2	0.6245	58.1	19.00	0.55	tr_C9ZR23_
DRB1_0101	118	FLFATINGERRCLAP	FATINGERR	2	0.6244	58.2	19.00	0.65	tr_C9ZR23_
DRB1_0101	119	IKVWMLTGDKVQTAE	VWMLTGDKV	2	0.6238	58.6	19.00	0.66	tr_C9ZR23_
DRB1_0101	120	SFLQAGIKVWMLTGD	SFLQAGIKV	0	0.6229	59.1	19.00	0.40	tr_C9ZR23_
DRB1_0101	121	VRSRGFITLAVGDGG	FITLAVGDG	5	0.6192	61.6	19.00	0.55	tr_C9ZR23_
DRB1_0101	122	LKLLTELSENCVSVI	LTELSENCV	3	0.6185	62.1	20.00	0.69	tr_C9ZR23_
DRB1_0101	123	DCVLRVLADKLDAFE	VLRVLADKL	2	0.6179	62.5	20.00	0.77	tr_C9ZR23_
DRB1_0101	124	QLAYNRTAFVIKYSF	LAYNRTAFV	1	0.6145	64.8	20.00	0.73	tr_C9ZR23_
DRB1_0101	125	LNVRLVARDATTAAV	LVARDATTA	4	0.6128	66.0	20.00	0.47	tr_C9ZR23_
DRB1_0101	126	LHFGRVLSLCNTVEV	FGRVLSLCN	2	0.6119	66.6	21.00	0.34	tr_C9ZR23_
DRB1_0101	127	NGLYSLPQTMLYCLD	LYSLPQTML	2	0.6108	67.4	21.00	0.63	tr_C9ZR23_
DRB1_0101	128	LTLWNGLYSLPQTML	LYSLPQTML	6	0.6090	68.8	21.00	0.37	tr_C9ZR23_
DRB1_0101	129	GLRTLLVAEKRLTRN	LLVAEKRLT	4	0.6085	69.1	21.00	0.44	tr_C9ZR23_
DRB1_0101	130	EEWFVHYVFAFASEF	VHYVFAFAS	4	0.6079	69.6	21.00	0.45	tr_C9ZR23_
DRB1_0101	131	GFGLTLWNGLYSLPQ	FGLTLWNGL	1	0.6066	70.6	21.00	0.40	tr_C9ZR23_
DRB1_0101	132	PGDCVLRVLADKLDA	VLRVLADKL	4	0.6064	70.7	21.00	0.77	tr_C9ZR23_
DRB1_0101	133	EIYKLRLEMENSVTI	LRLEMENSV	4	0.6048	71.9	22.00	0.54	tr_C9ZR23_
DRB1_0101	134	GFITLAVGDGGNDVA	ITLAVGDGG	2	0.6038	72.7	22.00	0.46	tr_C9ZR23_

DRB1_0101	135	KVPRRVLEQTPALYK	VLEQTPALY	5	0.6031	73.3	22.00	0.50	tr_C9ZR23_
DRB1_0101	136	FGLRTLLVAEKRLTR	LLVAEKRLT	5	0.6018	74.4	22.00	0.34	tr_C9ZR23_
DRB1_0101	137	EKLNVRLVARDATTA	NVRLVARDA	3	0.5997	76.0	23.00	0.67	tr_C9ZR23_
DRB1_0101	138	DGFGLTLWNGLYSLP	FGLTLWNGL	2	0.5995	76.2	23.00	0.48	tr_C9ZR23_
DRB1_0101	139	KVWMLTGDKVQTAEQ	VWMLTGDKV	1	0.5993	76.4	23.00	0.62	tr_C9ZR23_
DRB1_0101	140	VTDMSKYYFALVIEN	YYFALVIEN	6	0.5974	77.9	23.00	0.58	tr_C9ZR23_
DRB1_0101	141	WDGFGLTLWNGLYSL	FGLTLWNGL	3	0.5954	79.7	23.00	0.47	tr_C9ZR23_
DRB1_0101	142	NCVSVICARTTPKQK	VSVICARTT	2	0.5953	79.7	23.00	0.61	tr_C9ZR23_
DRB1_0101	143	CVLRVLADKLDAFES	VLRVLADKL	1	0.5953	79.7	23.00	0.71	tr_C9ZR23_
DRB1_0101	144	GTMRYQAASPDEVAL	YQAASPDEV	4	0.5930	81.8	24.00	0.63	tr_C9ZR23_
DRB1_0101	145	ENCVSVICARTTPKQ	VSVICARTT	3	0.5927	82.0	24.00	0.63	tr_C9ZR23_
DRB1_0101	146	VPETIRSFLQAGIKV	SFLQAGIKV	6	0.5920	82.7	24.00	0.51	tr_C9ZR23_
DRB1_0101	147	SFWDGFGLTLWNGLY	FWDGFGLTL	1	0.5916	83.0	24.00	0.35	tr_C9ZR23_
DRB1_0101	148	AGIKVWMLTGDKVQT	VWMLTGDKV	4	0.5901	84.4	24.00	0.69	tr_C9ZR23_
DRB1_0101	149	DFSISRFSDLRSLVF	FSISRFSDL	1	0.5899	84.5	24.00	0.37	tr_C9ZR23_
DRB1_0101	150	KLLTELSENCVSVIC	LTELSENCV	2	0.5894	85.0	24.00	0.67	tr_C9ZR23_
DRB1_0101	151	LRTLLVAEKRLTRNE	LLVAEKRLT	3	0.5893	85.1	24.00	0.47	tr_C9ZR23_
DRB1_0101	152	FASEFKTMGVIVEEK	FKTMGVIVE	4	0.5883	86.0	24.00	0.53	tr_C9ZR23_
DRB1_0101	153	IGIIQLVHNIFHTHY	IQLVHNIFH	3	0.5875	86.8	25.00	0.37	tr_C9ZR23_
DRB1_0101	154	LIGIIQLVHNIFHTH	IQLVHNIFH	4	0.5862	88.0	25.00	0.29	tr_C9ZR23_
DRB1_0101	155	TMRYQAASPDEVALC	YQAASPDEV	3	0.5852	88.9	25.00	0.70	tr_C9ZR23_
DRB1_0101	156	FSISRFSDLRSLVFV	FSDLRSLVF	5	0.5851	89.0	25.00	0.43	tr_C9ZR23_
DRB1_0101	157	FALVIENDVAMHHDG	FALVIENDV	0	0.5850	89.1	25.00	0.52	tr_C9ZR23_
DRB1_0101	158	NRTAFVIKYSFYKSV	FVIKYSFYK	4	0.5849	89.2	25.00	0.73	tr_C9ZR23_
DRB1_0101	159	QNVMEFLFATINGER	FLFATINGE	5	0.5824	91.7	25.00	0.46	tr_C9ZR23_
DRB1_0101	160	TGSSYVLVIEGGQVL	LVIEGGQVL	6	0.5814	92.7	26.00	0.58	tr_C9ZR23_
DRB1_0101	161	HFGRVLSLCNTVEVV	FGRVLSLCN	1	0.5790	95.1	26.00	0.33	tr_C9ZR23_
DRB1_0101	162	FGLTLWNGLYSLPQT	WNGLYSLPQ	5	0.5784	95.8	26.00	0.30	tr_C9ZR23_
DRB1_0101	163	NDVAMIQEAQVGVGI	VAMIQEAQV	2	0.5778	96.4	26.00	0.58	tr_C9ZR23_
DRB1_0101	164	DVAMIQEAQVGVGIT	VAMIQEAQV	1	0.5776	96.5	26.00	0.47	tr_C9ZR23_
DRB1_0101	165	ASEFKTMGVIVEEKS	FKTMGVIVE	3	0.5771	97.1	26.00	0.56	tr_C9ZR23_
DRB1_0101	166	CVSVICARTTPKQKA	VSVICARTT	1	0.5769	97.3	26.00	0.55	tr_C9ZR23_
DRB1_0101	167	LAEVEHYAVFGLRTL	HYAVFGLRT	5	0.5765	97.7	26.00	0.57	tr_C9ZR23_
DRB1_0101	168	GGQVLERILTTPSLL	LERILTTPS	4	0.5760	98.3	26.00	0.47	tr_C9ZR23_
DRB1_0101	169	GSFWDGFGLTLWNGL	FWDGFGLTL	2	0.5730	101.5	27.00	0.47	tr_C9ZR23_
DRB1_0101	170	AFASEFKTMGVIVEE	FKTMGVIVE	5	0.5726	102.0	27.00	0.45	tr_C9ZR23_
DRB1_0101	171	QFSKGVMADVQYGDA	GVMADVQYG	4	0.5725	102.0	27.00	0.48	tr_C9ZR23_
DRB1_0101	172	FWDGFGLTLWNGLYS	FGLTLWNGL	4	0.5722	102.4	27.00	0.45	tr_C9ZR23_
DRB1_0101	173	VLIGIIQLVHNIFHT	GIIQLVHNI	3	0.5711	103.6	27.00	0.28	tr_C9ZR23_
DRB1_0101	174	SENCVSVICARTTPK	VSVICARTT	4	0.5710	103.7	27.00	0.63	tr_C9ZR23_
DRB1_0101	175	SFYKSVLIGIIQLVH	FYKSVLIGI	1	0.5705	104.2	27.00	0.41	tr_C9ZR23_
DRB1_0101	176	QAGIKVWMLTGDKVQ	VWMLTGDKV	5	0.5702	104.6	28.00	0.65	tr_C9ZR23_
DRB1_0101	177	EGYMESLLQFSKGVM	SLLQFSKGV	5	0.5679	107.2	28.00	0.38	tr_C9ZR23_
DRB1_0101	178	NVRLVARDATTAAVE	LVARDATTA	3	0.5677	107.5	28.00	0.57	tr_C9ZR23_
DRB1_0101	179	TMLYCLDRKVPRRVL	LDRKVPRRV	5	0.5676	107.6	28.00	0.47	tr_C9ZR23_
DRB1_0101	180	GNDVAMIQEAQVGVG	VAMIQEAQV	3	0.5647	111.1	29.00	0.63	tr_C9ZR23_
DRB1_0101	181	KAAILAEVEHYAVFG	ILAEVEHYA	3	0.5633	112.8	29.00	0.74	tr_C9ZR23_
DRB1_0101	182	KGERMSSKAAILAEV	RMSSKAAIL	3	0.5631	113.0	29.00	0.41	tr_C9ZR23_
DRB1_0101	183	VGTMRYQAASPDEVA	YQAASPDEV	5	0.5602	116.5	29.00	0.49	tr_C9ZR23_
DRB1_0101	184	EEIYKLRLEMENSVT	LRLEMENSV	5	0.5601	116.6	29.00	0.51	tr_C9ZR23_

DRB1_0101	185	MLYCLDRKVPRRVLE	LDRKVPRRV	4	0.5597	117.2	29.00	0.62	tr_C9ZR23_
DRB1_0101	186	VALCNGCEKLNVRLV	CEKLNVRLV	6	0.5594	117.6	29.00	0.71	tr_C9ZR23_
DRB1_0101	187	STNAIYYFVKGADDR	YYFVKGADD	5	0.5593	117.7	30.00	0.81	tr_C9ZR23_
DRB1_0101	188	FGRVLSLCNTVEVVY	LSLCNTVEV	4	0.5587	118.4	30.00	0.25	tr_C9ZR23_
DRB1_0101	189	GIIQLVHNIFHTHYS	IQLVHNIFH	2	0.5587	118.5	30.00	0.38	tr_C9ZR23_
DRB1_0101	190	GLYSLPQTMLYCLDR	LYSLPQTML	1	0.5573	120.4	30.00	0.57	tr_C9ZR23_
DRB1_0101	191	LRLEMENSVTILGVT	LEMENSVTI	2	0.5572	120.4	30.00	0.65	tr_C9ZR23_
DRB1_0101	192	SPGDCVLRVLADKLD	VLRVLADKL	5	0.5565	121.4	30.00	0.76	tr_C9ZR23_
DRB1_0101	193	SKAAILAEVEHYAVF	ILAEVEHYA	4	0.5564	121.4	30.00	0.71	tr_C9ZR23_
DRB1_0101	194	LYCLDRKVPRRVLEQ	LDRKVPRRV	3	0.5533	125.6	31.00	0.69	tr_C9ZR23_
DRB1_0101	195	RTLLVAEKRLTRNEL	LLVAEKRLT	2	0.5532	125.8	31.00	0.54	tr_C9ZR23_
DRB1_0101	196	VAMIQEAQVGVGITG	QEAQVGVGI	4	0.5519	127.6	31.00	0.29	tr_C9ZR23_
DRB1_0101	197	SVLIGIIQLVHNIFH	GIIQLVHNI	4	0.5508	129.0	31.00	0.29	tr_C9ZR23_
DRB1_0101	198	SISRFSDLRSLVFVH	FSDLRSLVF	4	0.5493	131.2	31.00	0.61	tr_C9ZR23_
DRB1_0101	199	GKGERMSSKAAILAE	RMSSKAAIL	4	0.5491	131.5	31.00	0.42	tr_C9ZR23_
DRB1_0101	200	ADFSISRFSDLRSLV	FSISRFSDL	2	0.5489	131.7	31.00	0.60	tr_C9ZR23_
DRB1_0101	201	AADFSISRFSDLRSL	FSISRFSDL	3	0.5488	131.9	31.00	0.69	tr_C9ZR23_
DRB1_0101	202	ISRFSDLRSLVFVHG	FSDLRSLVF	3	0.5479	133.1	32.00	0.58	tr_C9ZR23_
DRB1_0101	203	LFATINGERRCLAPV	FATINGERR	1	0.5468	134.8	32.00	0.69	tr_C9ZR23_
DRB1_0101	204	FITLAVGDGGNDVAM	ITLAVGDGG	1	0.5464	135.4	32.00	0.43	tr_C9ZR23_
DRB1_0101	205	GGNDVAMIQEAQVGV	VAMIQEAQV	4	0.5425	141.2	33.00	0.64	tr_C9ZR23_
DRB1_0101	206	SEFKTMGVIVEEKST	FKTMGVIVE	2	0.5407	143.9	33.00	0.59	tr_C9ZR23_
DRB1_0101	207	LVIEGGQVLERILTT	LVIEGGQVL	0	0.5387	147.1	33.00	0.62	tr_C9ZR23_
DRB1_0101	208	MRYQAASPDEVALCN	YQAASPDEV	2	0.5376	148.8	34.00	0.78	tr_C9ZR23_
DRB1_0101	209	YNRTAFVIKYSFYKS	FVIKYSFYK	5	0.5361	151.3	34.00	0.69	tr_C9ZR23_
DRB1_0101	210	YFVKGADDRILEMAL	FVKGADDRI	1	0.5326	157.1	35.00	0.61	tr_C9ZR23_
DRB1_0101	211	VLRVLADKLDAFESW	VLRVLADKL	0	0.5295	162.5	35.00	0.49	tr_C9ZR23_
DRB1_0101	212	FSKGVMADVQYGDAA	GVMADVQYG	3	0.5268	167.3	36.00	0.68	tr_C9ZR23_
DRB1_0101	213	GSCGEHVLHFGRVLS	HVLHFGRVL	5	0.5264	168.0	36.00	0.70	tr_C9ZR23_
DRB1_0101	214	GLTLWNGLYSLPQTM	WNGLYSLPQ	4	0.5233	173.9	36.00	0.43	tr_C9ZR23_
DRB1_0101	215	WGKGERMSSKAAILA	RMSSKAAIL	5	0.5229	174.5	36.00	0.41	tr_C9ZR23_
DRB1_0101	216	VSVICARTTPKQKAA	VSVICARTT	0	0.5224	175.4	37.00	0.44	tr_C9ZR23_
DRB1_0101	217	LSENCVSVICARTTP	VSVICARTT	5	0.5222	175.8	37.00	0.61	tr_C9ZR23_
DRB1_0101	218	QTMLYCLDRKVPRRV	MLYCLDRKV	2	0.5184	183.2	37.00	0.43	tr_C9ZR23_
DRB1_0101	219	LLTELSENCVSVICA	LTELSENCV	1	0.5169	186.3	38.00	0.60	tr_C9ZR23_
DRB1_0101	220	SRFSDLRSLVFVHGQ	FSDLRSLVF	2	0.5167	186.7	38.00	0.61	tr_C9ZR23_
DRB1_0101	221	KEEWFVHYVFAFASE	VHYVFAFAS	5	0.5165	187.1	38.00	0.52	tr_C9ZR23_
DRB1_0101	222	AAILAEVEHYAVFGL	ILAEVEHYA	2	0.5159	188.3	38.00	0.69	tr_C9ZR23_
DRB1_0101	223	SSKAAILAEVEHYAV	ILAEVEHYA	5	0.5153	189.4	38.00	0.67	tr_C9ZR23_
DRB1_0101	224	YCLDRKVPRRVLEQT	LDRKVPRRV	2	0.5152	189.7	38.00	0.72	tr_C9ZR23_
DRB1_0101	225	TQNVMEFLFATINGE	MEFLFATIN	4	0.5147	190.7	38.00	0.43	tr_C9ZR23_
DRB1_0101	226	GERMSSKAAILAEVE	RMSSKAAIL	2	0.5135	193.2	38.00	0.38	tr_C9ZR23_
DRB1_0101	227	VWMLTGDKVQTAEQI	VWMLTGDKV	0	0.5133	193.7	38.00	0.46	tr_C9ZR23_
DRB1_0101	228	KEEIYKLRLEMENSV	LRLEMENSV	6	0.5130	194.3	38.00	0.32	tr_C9ZR23_
DRB1_0101	229	ILAEVEHYAVFGLRT	HYAVFGLRT	6	0.5102	200.2	39.00	0.42	tr_C9ZR23_
DRB1_0101	230	IIQLVHNIFHTHYSG	IQLVHNIFH	1	0.5096	201.6	39.00	0.35	tr_C9ZR23_
DRB1_0101	231	WEGYMESLLQFSKGV	GYMESLLQF	2	0.5092	202.4	39.00	0.23	tr_C9ZR23_
DRB1_0101	232	FYKSVLIGIIQLVHN	YKSVLIGII	1	0.5071	207.0	40.00	0.29	tr_C9ZR23_
DRB1_0101	233	QKAAVTRLVRSRGFI	TRLVRSRGF	5	0.5068	207.6	40.00	0.34	tr_C9ZR23_
DRB1_0101	234	LAYNRTAFVIKYSFY	LAYNRTAFV	0	0.5051	211.5	40.00	0.49	tr_C9ZR23_

DRB1_0101	235	DVNPLSTGSSYVLVI	LSTGSSYVL	4	0.5044	213.3	40.00	0.46	tr_C9ZR23_
DRB1_0101	236	KFVTDMSKYYFALVI	FVTDMSKYY	1	0.5030	216.5	40.00	0.36	tr_C9ZR23_
DRB1_0101	237	KQKAAVTRLVRSRGF	QKAAVTRLV	1	0.5029	216.7	40.00	0.38	tr_C9ZR23_
DRB1_0101	238	SGLSTGCGAVGTMRY	LSTGCGAVG	2	0.5018	219.2	41.00	0.51	tr_C9ZR23_
DRB1_0101	239	RAADFSISRFSDLRS	FSISRFSDL	4	0.5008	221.7	41.00	0.75	tr_C9ZR23_
DRB1_0101	240	GGSFWDGFGLTLWNG	FWDGFGLTL	3	0.4994	225.1	41.00	0.69	tr_C9ZR23_
DRB1_0101	241	GRVLSLCNTVEVVYD	LSLCNTVEV	3	0.4990	225.9	41.00	0.32	tr_C9ZR23_
DRB1_0101	242	MGVIVEEKSTNAIYY	IVEEKSTNA	3	0.4988	226.6	41.00	0.67	tr_C9ZR23_
DRB1_0101	243	KSVLIGIIQLVHNIF	GIIQLVHNI	5	0.4981	228.2	41.00	0.31	tr_C9ZR23_
DRB1_0101	244	VNPLSTGSSYVLVIE	LSTGSSYVL	3	0.4980	228.4	41.00	0.53	tr_C9ZR23_
DRB1_0101	245	GLSTGCGAVGTMRYQ	LSTGCGAVG	1	0.4978	229.0	41.00	0.38	tr_C9ZR23_
DRB1_0101	246	GVIVEEKSTNAIYYF	IVEEKSTNA	2	0.4933	240.5	42.00	0.58	tr_C9ZR23_
DRB1_0101	247	LQAGIKVWMLTGDKV	VWMLTGDKV	6	0.4923	243.1	43.00	0.53	tr_C9ZR23_
DRB1_0101	248	QSGLSTGCGAVGTMR	LSTGCGAVG	3	0.4904	248.1	43.00	0.67	tr_C9ZR23_
DRB1_0101	249	TPKQKAAVTRLVRSR	QKAAVTRLV	3	0.4900	249.1	43.00	0.56	tr_C9ZR23_
DRB1_0101	250	AMIQEAQVGVGITGR	QEAQVGVGI	3	0.4896	250.3	43.00	0.60	tr_C9ZR23_
DRB1_0101	251	ELSENCVSVICARTT	VSVICARTT	6	0.4894	250.7	43.00	0.51	tr_C9ZR23_
DRB1_0101	252	RLEMENSVTILGVTA	LEMENSVTI	1	0.4885	253.3	43.00	0.64	tr_C9ZR23_
DRB1_0101	253	DGGNDVAMIQEAQVG	VAMIQEAQV	5	0.4874	256.3	44.00	0.64	tr_C9ZR23_
DRB1_0101	254	AYNRTAFVIKYSFYK	FVIKYSFYK	6	0.4862	259.6	44.00	0.53	tr_C9ZR23_
DRB1_0101	255	TTPKQKAAVTRLVRS	QKAAVTRLV	4	0.4850	263.0	44.00	0.42	tr_C9ZR23_
DRB1_0101	256	VRLVARDATTAAVEV	LVARDATTA	2	0.4843	265.1	44.00	0.70	tr_C9ZR23_
DRB1_0101	257	VLEQTPALYKVTRSG	LEQTPALYK	1	0.4829	269.1	45.00	0.52	tr_C9ZR23_
DRB1_0101	258	YKSVLIGIIQLVHNI	IGIIQLVHN	5	0.4818	272.3	45.00	0.28	tr_C9ZR23_
DRB1_0101	259	SGGSFWDGFGLTLWN	FWDGFGLTL	4	0.4816	272.8	45.00	0.74	tr_C9ZR23_
DRB1_0101	260	CSPGDCVLRVLADKL	VLRVLADKL	6	0.4807	275.4	45.00	0.69	tr_C9ZR23_
DRB1_0101	261	RTTPKQKAAVTRLVR	QKAAVTRLV	5	0.4799	277.8	45.00	0.34	tr_C9ZR23_
DRB1_0101	262	ADVNPLSTGSSYVLV	LSTGSSYVL	5	0.4790	280.6	45.00	0.39	tr_C9ZR23_
DRB1_0101	263	EGGQVLERILTTPSL	LERILTTPS	5	0.4789	280.9	45.00	0.46	tr_C9ZR23_
DRB1_0101	264	VIVEEKSTNAIYYFV	IVEEKSTNA	1	0.4787	281.6	45.00	0.47	tr_C9ZR23_
DRB1_0101	265	VGEKACAMGVMQKRN	CAMGVMQKR	5	0.4784	282.4	45.00	0.29	tr_C9ZR23_
DRB1_0101	266	PKQKAAVTRLVRSRG	QKAAVTRLV	2	0.4774	285.6	46.00	0.50	tr_C9ZR23_
DRB1_0101	267	FVTDMSKYYFALVIE	DMSKYYFAL	3	0.4772	286.1	46.00	0.26	tr_C9ZR23_
DRB1_0101	268	AVGTMRYQAASPDEV	MRYQAASPD	4	0.4764	288.6	46.00	0.44	tr_C9ZR23_
DRB1_0101	269	LSTGCGAVGTMRYQA	CGAVGTMRY	4	0.4757	290.7	46.00	0.46	tr_C9ZR23_
DRB1_0101	270	LYSLPQTMLYCLDRK	LYSLPQTML	0	0.4749	293.4	46.00	0.45	tr_C9ZR23_
DRB1_0101	271	QVDYVLSDKTGTLTQ	YVLSDKTGT	3	0.4720	302.6	47.00	0.45	tr_C9ZR23_
DRB1_0101	272	RFSDLRSLVFVHGQL	FSDLRSLVF	1	0.4718	303.4	47.00	0.54	tr_C9ZR23_
DRB1_0101	273	FATINGERRCLAPVE	FATINGERR	0	0.4715	304.5	47.00	0.49	tr_C9ZR23_
DRB1_0101	274	AILAEVEHYAVFGLR	ILAEVEHYA	1	0.4705	307.6	47.00	0.58	tr_C9ZR23_
DRB1_0101	275	ERMSSKAAILAEVEH	RMSSKAAIL	1	0.4701	309.1	47.00	0.38	tr_C9ZR23_
DRB1_0101	276	KSTNAIYYFVKGADD	YYFVKGADD	6	0.4700	309.3	47.00	0.67	tr_C9ZR23_
DRB1_0101	277	RYQAASPDEVALCNG	YQAASPDEV	1	0.4689	313.2	47.00	0.85	tr_C9ZR23_
DRB1_0101	278	FLQAGIKVWMLTGDK	QAGIKVWML	2	0.4682	315.3	48.00	0.44	tr_C9ZR23_
DRB1_0101	279	PQTMLYCLDRKVPRR	MLYCLDRKV	3	0.4681	315.9	48.00	0.58	tr_C9ZR23_
DRB1_0101	280	ARAADFSISRFSDLR	FSISRFSDL	5	0.4679	316.3	48.00	0.73	tr_C9ZR23_
DRB1_0101	281	SKGVMADVQYGDAAF	GVMADVQYG	2	0.4676	317.5	48.00	0.79	tr_C9ZR23_
DRB1_0101	282	GQVDYVLSDKTGTLT	VDYVLSDKT	2	0.4668	320.3	48.00	0.40	tr_C9ZR23_
DRB1_0101	283	AVGEKACAMGVMQKR	GEKACAMGV	2	0.4666	321.0	48.00	0.35	tr_C9ZR23_
DRB1_0101	284	SWEGYMESLLQFSKG	GYMESLLQF	3	0.4664	321.8	48.00	0.34	tr_C9ZR23_

DRB1_0101	285	IKEEWFVHYVFAFAS	VHYVFAFAS	6	0.4642	329.5	48.00	0.45	tr_C9ZR23_
DRB1_0101	286	YSGGSFWDGFGLTLW	FWDGFGLTL	5	0.4638	330.9	49.00	0.68	tr_C9ZR23_
DRB1_0101	287	TLLVAEKRLTRNELD	LLVAEKRLT	1	0.4637	331.2	49.00	0.50	tr_C9ZR23_
DRB1_0101	288	NPLSTGSSYVLVIEG	LSTGSSYVL	2	0.4598	345.6	49.00	0.58	tr_C9ZR23_
DRB1_0101	289	GEKACAMGVMQKRNG	CAMGVMQKR	4	0.4597	346.0	49.00	0.38	tr_C9ZR23_
DRB1_0101	290	RVLSLCNTVEVVYDD	LSLCNTVEV	2	0.4592	347.7	50.00	0.37	tr_C9ZR23_
DRB1_0101	291	LGQVDYVLSDKTGTL	VDYVLSDKT	3	0.4585	350.3	50.00	0.54	tr_C9ZR23_
DRB1_0101	292	STGCGAVGTMRYQAA	CGAVGTMRY	3	0.4564	358.2	50.00	0.55	tr_C9ZR23_
DRB1_0101	293	RRCLAPVEAEEVQGS	LAPVEAEEV	3	0.4563	358.6	50.00	0.71	tr_C9ZR23_
DRB1_0101	294	IQLVHNIFHTHYSGG	LVHNIFHTH	2	0.4559	360.4	50.00	0.35	tr_C9ZR23_
DRB1_0101	295	EFKTMGVIVEEKSTN	FKTMGVIVE	1	0.4557	361.2	50.00	0.61	tr_C9ZR23_
DRB1_0101	296	TQSGLSTGCGAVGTM	LSTGCGAVG	4	0.4553	362.7	50.00	0.62	tr_C9ZR23_
DRB1_0101	297	ESWEGYMESLLQFSK	GYMESLLQF	4	0.4547	365.0	55.00	0.35	tr_C9ZR23_
DRB1_0101	298	TGCGAVGTMRYQAAS	CGAVGTMRY	2	0.4545	365.7	55.00	0.49	tr_C9ZR23_
DRB1_0101	299	QGSCGEHVLHFGRVL	HVLHFGRVL	6	0.4539	368.4	55.00	0.62	tr_C9ZR23_
DRB1_0101	300	PALYKVTRSGVDLGV	YKVTRSGVD	3	0.4530	371.8	55.00	0.38	tr_C9ZR23_
DRB1_0101	301	ERRCLAPVEAEEVQG	LAPVEAEEV	4	0.4517	376.9	55.00	0.64	tr_C9ZR23_
DRB1_0101	302	RKVPRRVLEQTPALY	VLEQTPALY	6	0.4502	383.1	55.00	0.52	tr_C9ZR23_
DRB1_0101	303	LTQNVMEFLFATING	MEFLFATIN	5	0.4491	388.0	55.00	0.50	tr_C9ZR23_
DRB1_0101	304	HVPETIRSFLQAGIK	IRSFLQAGI	5	0.4490	388.2	55.00	0.37	tr_C9ZR23_
DRB1_0101	305	CLDRKVPRRVLEQTP	LDRKVPRRV	1	0.4443	408.7	55.00	0.73	tr_C9ZR23_
DRB1_0101	306	KGVMADVQYGDAAFP	GVMADVQYG	1	0.4437	411.1	55.00	0.62	tr_C9ZR23_
DRB1_0101	307	TMGVIVEEKSTNAIY	IVEEKSTNA	4	0.4436	411.5	55.00	0.67	tr_C9ZR23_
DRB1_0101	308	EKACAMGVMQKRNGS	CAMGVMQKR	3	0.4435	412.1	55.00	0.46	tr_C9ZR23_
DRB1_0101	309	LSMNNRKEEIYKLRL	NRKEEIYKL	4	0.4426	416.0	55.00	0.74	tr_C9ZR23_
DRB1_0101	310	LTGDKVQTAEQIALT	DKVQTAEQI	3	0.4409	423.9	55.00	0.54	tr_C9ZR23_
DRB1_0101	311	SMNNRKEEIYKLRLE	NRKEEIYKL	3	0.4405	425.5	55.00	0.76	tr_C9ZR23_
DRB1_0101	312	GERRCLAPVEAEEVQ	LAPVEAEEV	5	0.4388	433.4	55.00	0.55	tr_C9ZR23_
DRB1_0101	313	TGDKVQTAEQIALTC	DKVQTAEQI	2	0.4371	441.5	55.00	0.47	tr_C9ZR23_
DRB1_0101	314	RLVARDATTAAVEVN	LVARDATTA	1	0.4369	442.6	55.00	0.61	tr_C9ZR23_
DRB1_0101	315	AEEVQGSCGEHVLHF	VQGSCGEHV	3	0.4364	444.9	55.00	0.77	tr_C9ZR23_
DRB1_0101	316	ALYKVTRSGVDLGVC	YKVTRSGVD	2	0.4355	449.3	55.00	0.40	tr_C9ZR23_
DRB1_0101	317	MIQEAQVGVGITGRE	QEAQVGVGI	2	0.4343	455.4	55.00	0.69	tr_C9ZR23_
DRB1_0101	318	TPALYKVTRSGVDLG	YKVTRSGVD	4	0.4339	457.2	55.00	0.36	tr_C9ZR23_
DRB1_0101	319	FVKGADDRILEMALD	FVKGADDRI	0	0.4335	459.3	55.00	0.44	tr_C9ZR23_
DRB1_0101	320	LTELSENCVSVICAR	LTELSENCV	0	0.4334	459.7	55.00	0.41	tr_C9ZR23_
DRB1_0101	321	IVEEKSTNAIYYFVK	EKSTNAIYY	3	0.4327	462.9	55.00	0.25	tr_C9ZR23_
DRB1_0101	322	MSSKAAILAEVEHYA	ILAEVEHYA	6	0.4315	469.2	55.00	0.44	tr_C9ZR23_
DRB1_0101	323	LPQTMLYCLDRKVPR	MLYCLDRKV	4	0.4314	469.8	60.00	0.59	tr_C9ZR23_
DRB1_0101	324	GDKVQTAEQIALTCS	VQTAEQIAL	3	0.4314	469.8	60.00	0.35	tr_C9ZR23_
DRB1_0101	325	ARTTPKQKAAVTRLV	TPKQKAAVT	3	0.4296	479.1	60.00	0.26	tr_C9ZR23_
DRB1_0101	326	ENSVTILGVTAIEDK	VTILGVTAI	3	0.4294	479.8	60.00	0.59	tr_C9ZR23_
DRB1_0101	327	NSVTILGVTAIEDKL	VTILGVTAI	2	0.4294	480.0	60.00	0.51	tr_C9ZR23_
DRB1_0101	328	GCGAVGTMRYQAASP	CGAVGTMRY	1	0.4280	487.2	60.00	0.46	tr_C9ZR23_
DRB1_0101	329	KACAMGVMQKRNGSV	CAMGVMQKR	2	0.4278	488.3	60.00	0.43	tr_C9ZR23_
DRB1_0101	330	PAVGEKACAMGVMQK	GEKACAMGV	3	0.4273	491.0	60.00	0.41	tr_C9ZR23_
DRB1_0101	331	QWGKGERMSSKAAIL	GERMSSKAA	4	0.4252	502.3	60.00	0.38	tr_C9ZR23_
DRB1_0101	332	SADVNPLSTGSSYVL	VNPLSTGSS	3	0.4245	506.2	60.00	0.47	tr_C9ZR23_
DRB1_0101	333	FESWEGYMESLLQFS	WEGYMESLL	3	0.4239	509.4	60.00	0.34	tr_C9ZR23_
DRB1_0101	334	EEVQGSCGEHVLHFG	VQGSCGEHV	2	0.4221	519.7	60.00	0.74	tr_C9ZR23_

DRB1_0101	335	TAEQIALTCSLCSPG	IALTCSLCS	4	0.4210	525.6	60.00	0.43	tr_C9ZR23_
DRB1_0101	336	VDYVLSDKTGTLTQN	YVLSDKTGT	2	0.4199	531.8	60.00	0.50	tr_C9ZR23_
DRB1_0101	337	MNNRKEEIYKLRLEM	NRKEEIYKL	2	0.4198	532.7	60.00	0.73	tr_C9ZR23_
DRB1_0101	338	SSIVEDLGQVDYVLS	IVEDLGQVD	2	0.4197	533.0	60.00	0.38	tr_C9ZR23_
DRB1_0101	339	LEMENSVTILGVTAI	LEMENSVTI	0	0.4192	536.2	60.00	0.33	tr_C9ZR23_
DRB1_0101	340	NNRKEEIYKLRLEME	NRKEEIYKL	1	0.4188	538.0	60.00	0.52	tr_C9ZR23_
DRB1_0101	341	CGAVGTMRYQAASPD	VGTMRYQAA	3	0.4141	566.7	60.00	0.35	tr_C9ZR23_
DRB1_0101	342	TLTQNVMEFLFATIN	MEFLFATIN	6	0.4131	572.3	60.00	0.43	tr_C9ZR23_
DRB1_0101	343	MTQSGLSTGCGAVGT	LSTGCGAVG	5	0.4124	576.8	60.00	0.56	tr_C9ZR23_
DRB1_0101	344	AEQIALTCSLCSPGD	IALTCSLCS	3	0.4124	577.2	60.00	0.44	tr_C9ZR23_
DRB1_0101	345	CAMGVMQKRNGSVTT	VMQKRNGSV	4	0.4112	584.5	60.00	0.38	tr_C9ZR23_
DRB1_0101	346	RKEEIYKLRLEMENS	IYKLRLEME	4	0.4092	597.3	60.00	0.55	tr_C9ZR23_
DRB1_0101	347	LTCSLCSPGDCVLRV	SLCSPGDCV	3	0.4091	597.9	60.00	0.44	tr_C9ZR23_
DRB1_0101	348	DKVQTAEQIALTCSL	VQTAEQIAL	2	0.4090	598.7	60.00	0.36	tr_C9ZR23_
DRB1_0101	349	QTAEQIALTCSLCSP	IALTCSLCS	5	0.4086	601.3	60.00	0.36	tr_C9ZR23_
DRB1_0101	350	VLADKLDAFESWEGY	ADKLDAFES	2	0.4079	605.9	65.00	0.47	tr_C9ZR23_
DRB1_0101	351	LADKLDAFESWEGYM	LDAFESWEG	4	0.4075	608.3	65.00	0.38	tr_C9ZR23_
DRB1_0101	352	ACAMGVMQKRNGSVT	CAMGVMQKR	1	0.4074	608.9	65.00	0.33	tr_C9ZR23_
DRB1_0101	353	VQTAEQIALTCSLCS	IALTCSLCS	6	0.4067	613.5	65.00	0.20	tr_C9ZR23_
DRB1_0101	354	GVMADVQYGDAAFPT	VQYGDAAFP	5	0.4063	616.5	65.00	0.44	tr_C9ZR23_
DRB1_0101	355	SVTILGVTAIEDKLQ	VTILGVTAI	1	0.4057	620.2	65.00	0.47	tr_C9ZR23_
DRB1_0101	356	NRKEEIYKLRLEMEN	IYKLRLEME	5	0.4053	623.0	65.00	0.40	tr_C9ZR23_
DRB1_0101	357	DLGQVDYVLSDKTGT	VDYVLSDKT	4	0.4037	633.9	65.00	0.50	tr_C9ZR23_
DRB1_0101	358	RVLADKLDAFESWEG	ADKLDAFES	3	0.4037	634.2	65.00	0.56	tr_C9ZR23_
DRB1_0101	359	SIVEDLGQVDYVLSD	IVEDLGQVD	1	0.4030	638.7	65.00	0.29	tr_C9ZR23_
DRB1_0101	360	HYSGGSFWDGFGLTL	FWDGFGLTL	6	0.4015	648.9	65.00	0.61	tr_C9ZR23_
DRB1_0101	361	VNGIKEEWFVHYVFA	IKEEWFVHY	3	0.4010	652.4	65.00	0.31	tr_C9ZR23_
DRB1_0101	362	EVQGSCGEHVLHFGR	VQGSCGEHV	1	0.4010	652.4	65.00	0.63	tr_C9ZR23_
DRB1_0101	363	TCSLCSPGDCVLRVL	SLCSPGDCV	2	0.4008	654.0	65.00	0.41	tr_C9ZR23_
DRB1_0101	364	NSSIVEDLGQVDYVL	IVEDLGQVD	3	0.4008	654.2	65.00	0.52	tr_C9ZR23_
DRB1_0101	365	RMSSKAAILAEVEHY	MSSKAAILA	1	0.4002	658.1	65.00	0.38	tr_C9ZR23_
DRB1_0101	366	QTPALYKVTRSGVDL	YKVTRSGVD	5	0.3997	661.6	65.00	0.35	tr_C9ZR23_
DRB1_0101	367	DPAVGEKACAMGVMQ	GEKACAMGV	4	0.3973	679.1	65.00	0.36	tr_C9ZR23_
DRB1_0101	368	AMGVMQKRNGSVTTE	VMQKRNGSV	3	0.3973	679.5	65.00	0.43	tr_C9ZR23_
DRB1_0101	369	GDGGNDVAMIQEAQV	VAMIQEAQV	6	0.3973	679.6	65.00	0.54	tr_C9ZR23_
DRB1_0101	370	EVNGIKEEWFVHYVF	IKEEWFVHY	4	0.3972	680.0	65.00	0.29	tr_C9ZR23_
DRB1_0101	371	GEWCNVRNSSIVEDL	WCNVRNSSI	2	0.3964	686.0	65.00	0.60	tr_C9ZR23_
DRB1_0101	372	DHVPETIRSFLQAGI	PETIRSFLQ	3	0.3960	689.0	65.00	0.42	tr_C9ZR23_
DRB1_0101	373	MLTGDKVQTAEQIAL	DKVQTAEQI	4	0.3959	689.5	65.00	0.57	tr_C9ZR23_
DRB1_0101	374	LRVLADKLDAFESWE	ADKLDAFES	4	0.3940	704.1	65.00	0.50	tr_C9ZR23_
DRB1_0101	375	ALTCSLCSPGDCVLR	SLCSPGDCV	4	0.3939	704.9	65.00	0.41	tr_C9ZR23_
DRB1_0101	376	MENSVTILGVTAIED	VTILGVTAI	4	0.3938	705.5	65.00	0.60	tr_C9ZR23_
DRB1_0101	377	QIALTCSLCSPGDCV	IALTCSLCS	1	0.3935	707.7	65.00	0.32	tr_C9ZR23_
DRB1_0101	378	ELSMNNRKEEIYKLR	NRKEEIYKL	5	0.3934	708.2	65.00	0.70	tr_C9ZR23_
DRB1_0101	379	EAEEVQGSCGEHVLH	VQGSCGEHV	4	0.3932	710.1	65.00	0.81	tr_C9ZR23_
DRB1_0101	380	ITLAVGDGGNDVAMI	ITLAVGDGG	0	0.3931	711.1	65.00	0.45	tr_C9ZR23_
DRB1_0101	381	IQEAQVGVGITGREG	QEAQVGVGI	1	0.3930	711.8	65.00	0.62	tr_C9ZR23_
DRB1_0101	382	PLSTGSSYVLVIEGG	LSTGSSYVL	1	0.3927	714.0	65.00	0.52	tr_C9ZR23_
DRB1_0101	383	YSLPQTMLYCLDRKV	MLYCLDRKV	6	0.3925	715.4	65.00	0.37	tr_C9ZR23_
DRB1_0101	384	EDLGQVDYVLSDKTG	VDYVLSDKT	5	0.3924	716.7	65.00	0.43	tr_C9ZR23_

DRB1_0101	385	NGIKEEWFVHYVFAF	IKEEWFVHY	2	0.3912	726.0	65.00	0.28	tr_C9ZR23_
DRB1_0101	386	KVQTAEQIALTCSLC	VQTAEQIAL	1	0.3909	728.4	65.00	0.31	tr_C9ZR23_
DRB1_0101	387	VEVNGIKEEWFVHYV	IKEEWFVHY	5	0.3906	730.2	65.00	0.25	tr_C9ZR23_
DRB1_0101	388	GIKEEWFVHYVFAFA	WFVHYVFAF	5	0.3896	738.5	65.00	0.27	tr_C9ZR23_
DRB1_0101	389	QLVHNIFHTHYSGGS	LVHNIFHTH	1	0.3892	741.6	65.00	0.37	tr_C9ZR23_
DRB1_0101	390	GAVGTMRYQAASPDE	MRYQAASPD	5	0.3891	742.0	65.00	0.47	tr_C9ZR23_
DRB1_0101	391	IALTCSLCSPGDCVL	SLCSPGDCV	5	0.3884	748.3	65.00	0.31	tr_C9ZR23_
DRB1_0101	392	EQIALTCSLCSPGDC	IALTCSLCS	2	0.3884	748.3	65.00	0.44	tr_C9ZR23_
DRB1_0101	393	RCLAPVEAEEVQGSC	LAPVEAEEV	2	0.3881	750.5	65.00	0.79	tr_C9ZR23_
DRB1_0101	394	LEQTPALYKVTRSGV	LEQTPALYK	0	0.3876	754.8	65.00	0.31	tr_C9ZR23_
DRB1_0101	395	AARAADFSISRFSDL	FSISRFSDL	6	0.3873	756.7	65.00	0.60	tr_C9ZR23_
DRB1_0101	396	HDGEWCNVRNSSIVE	WCNVRNSSI	4	0.3868	760.6	65.00	0.62	tr_C9ZR23_
DRB1_0101	397	MGVMQKRNGSVTTET	VMQKRNGSV	2	0.3858	769.1	65.00	0.40	tr_C9ZR23_
DRB1_0101	398	NGERRCLAPVEAEEV	RRCLAPVEA	3	0.3851	775.1	65.00	0.38	tr_C9ZR23_
DRB1_0101	399	FKTMGVIVEEKSTNA	FKTMGVIVE	0	0.3851	775.5	65.00	0.38	tr_C9ZR23_
DRB1_0101	400	DGEWCNVRNSSIVED	WCNVRNSSI	3	0.3842	782.8	70.00	0.64	tr_C9ZR23_
DRB1_0101	401	SDPAVGEKACAMGVM	AVGEKACAM	3	0.3840	784.5	70.00	0.43	tr_C9ZR23_
DRB1_0101	402	MADVQYGDAAFPTGS	VQYGDAAFP	3	0.3837	786.9	70.00	0.61	tr_C9ZR23_
DRB1_0101	403	CSLCSPGDCVLRVLA	SLCSPGDCV	1	0.3835	789.0	70.00	0.35	tr_C9ZR23_
DRB1_0101	404	WMLTGDKVQTAEQIA	DKVQTAEQI	5	0.3832	791.7	70.00	0.38	tr_C9ZR23_
DRB1_0101	405	EMENSVTILGVTAIE	VTILGVTAI	5	0.3830	792.8	70.00	0.58	tr_C9ZR23_
DRB1_0101	406	SLPQTMLYCLDRKVP	MLYCLDRKV	5	0.3829	793.6	70.00	0.54	tr_C9ZR23_
DRB1_0101	407	LYKVTRSGVDLGVCQ	YKVTRSGVD	1	0.3825	797.5	70.00	0.39	tr_C9ZR23_
DRB1_0101	408	ALVIENDVAMHHDGE	LVIENDVAM	1	0.3823	799.3	70.00	0.44	tr_C9ZR23_
DRB1_0101	409	VEEKSTNAIYYFVKG	EKSTNAIYY	2	0.3799	819.9	70.00	0.31	tr_C9ZR23_
DRB1_0101	410	SVICARTTPKQKAAV	VICARTTPK	1	0.3791	826.8	70.00	0.39	tr_C9ZR23_
DRB1_0101	411	VSQEMTQSGLSTGCG	EMTQSGLST	3	0.3782	835.5	70.00	0.56	tr_C9ZR23_
DRB1_0101	412	IVEDLGQVDYVLSDK	DLGQVDYVL	3	0.3781	836.6	70.00	0.36	tr_C9ZR23_
DRB1_0101	413	ADVQYGDAAFPTGSS	VQYGDAAFP	2	0.3762	853.2	70.00	0.56	tr_C9ZR23_
DRB1_0101	414	KTMGVIVEEKSTNAI	IVEEKSTNA	5	0.3755	860.2	70.00	0.63	tr_C9ZR23_
DRB1_0101	415	AFESWEGYMESLLQF	WEGYMESLL	4	0.3734	880.2	70.00	0.34	tr_C9ZR23_
DRB1_0101	416	EWCNVRNSSIVEDLG	WCNVRNSSI	1	0.3717	895.9	70.00	0.54	tr_C9ZR23_
DRB1_0101	417	DVSQEMTQSGLSTGC	EMTQSGLST	4	0.3699	913.4	70.00	0.51	tr_C9ZR23_
DRB1_0101	418	HHDGEWCNVRNSSIV	WCNVRNSSI	5	0.3693	919.6	70.00	0.60	tr_C9ZR23_
DRB1_0101	419	ADKLDAFESWEGYME	LDAFESWEG	3	0.3688	925.2	70.00	0.49	tr_C9ZR23_
DRB1_0101	420	VEDLGQVDYVLSDKT	DLGQVDYVL	2	0.3680	932.5	70.00	0.34	tr_C9ZR23_
DRB1_0101	421	EEKSTNAIYYFVKGA	STNAIYYFV	3	0.3672	941.0	70.00	0.32	tr_C9ZR23_
DRB1_0101	422	DYVLSDKTGTLTQNV	YVLSDKTGT	1	0.3668	944.6	70.00	0.46	tr_C9ZR23_
DRB1_0101	423	LVHNIFHTHYSGGSF	FHTHYSGGS	5	0.3656	957.6	70.00	0.34	tr_C9ZR23_
DRB1_0101	424	AVEVNGIKEEWFVHY	VNGIKEEWF	3	0.3642	972.3	70.00	0.27	tr_C9ZR23_
DRB1_0101	425	CARTTPKQKAAVTRL	TPKQKAAVT	4	0.3631	983.5	70.00	0.32	tr_C9ZR23_
DRB1_0101	426	IEGGQVLERILTTPS	LERILTTPS	6	0.3628	986.6	70.00	0.37	tr_C9ZR23_
DRB1_0101	427	TINGERRCLAPVEAE	RRCLAPVEA	5	0.3616	999.5	70.00	0.43	tr_C9ZR23_
DRB1_0101	428	TELSENCVSVICART	CVSVICART	6	0.3611	1005.2	70.00	0.24	tr_C9ZR23_
DRB1_0101	429	VMADVQYGDAAFPTG	VQYGDAAFP	4	0.3589	1029.4	75.00	0.62	tr_C9ZR23_
DRB1_0101	430	VHNIFHTHYSGGSFW	FHTHYSGGS	4	0.3588	1030.3	75.00	0.44	tr_C9ZR23_
DRB1_0101	431	EMTQSGLSTGCGAVG	LSTGCGAVG	6	0.3588	1030.8	75.00	0.35	tr_C9ZR23_
DRB1_0101	432	VLSLCNTVEVVYDDV	LSLCNTVEV	1	0.3585	1033.3	75.00	0.37	tr_C9ZR23_
DRB1_0101	433	ATINGERRCLAPVEA	INGERRCLA	2	0.3579	1040.4	75.00	0.34	tr_C9ZR23_
DRB1_0101	434	VQGSCGEHVLHFGRV	VQGSCGEHV	0	0.3577	1043.0	75.00	0.37	tr_C9ZR23_

DRB1_0101	435	DDVSQEMTQSGLSTG	EMTQSGLST	5	0.3563	1058.1	75.00	0.43	tr_C9ZR23_
DRB1_0101	436	STGSSYVLVIEGGQV	VLVIEGGQV	6	0.3553	1070.4	75.00	0.38	tr_C9ZR23_
DRB1_0101	437	HNIFHTHYSGGSFWD	FHTHYSGGS	3	0.3516	1113.8	75.00	0.50	tr_C9ZR23_
DRB1_0101	438	VEAEEVQGSCGEHVL	VQGSCGEHV	5	0.3512	1118.8	75.00	0.78	tr_C9ZR23_
DRB1_0101	439	EKSTNAIYYFVKGAD	STNAIYYFV	2	0.3507	1124.4	75.00	0.31	tr_C9ZR23_
DRB1_0101	440	SQEMTQSGLSTGCGA	EMTQSGLST	2	0.3506	1125.7	75.00	0.56	tr_C9ZR23_
DRB1_0101	441	EQTPALYKVTRSGVD	LYKVTRSGV	5	0.3495	1139.4	75.00	0.34	tr_C9ZR23_
DRB1_0101	442	RNSSIVEDLGQVDYV	IVEDLGQVD	4	0.3488	1148.6	75.00	0.56	tr_C9ZR23_
DRB1_0101	443	VGITGREGKQAARAA	ITGREGKQA	2	0.3477	1161.9	75.00	0.57	tr_C9ZR23_
DRB1_0101	444	GVGITGREGKQAARA	ITGREGKQA	3	0.3444	1203.7	75.00	0.57	tr_C9ZR23_
DRB1_0101	445	SLCSPGDCVLRVLAD	CSPGDCVLR	2	0.3438	1212.4	75.00	0.26	tr_C9ZR23_
DRB1_0101	446	VGVGITGREGKQAAR	ITGREGKQA	4	0.3432	1220.2	75.00	0.51	tr_C9ZR23_
DRB1_0101	447	LLVAEKRLTRNELDE	LLVAEKRLT	0	0.3422	1233.5	75.00	0.41	tr_C9ZR23_
DRB1_0101	448	VICARTTPKQKAAVT	ARTTPKQKA	3	0.3420	1235.3	75.00	0.23	tr_C9ZR23_
DRB1_0101	449	GITGREGKQAARAAD	ITGREGKQA	1	0.3415	1242.3	75.00	0.44	tr_C9ZR23_
DRB1_0101	450	LVARDATTAAVEVNG	DATTAAVEV	4	0.3407	1253.2	75.00	0.35	tr_C9ZR23_
DRB1_0101	451	DRILEMALDENSSTG	LEMALDENS	3	0.3405	1255.8	75.00	0.52	tr_C9ZR23_
DRB1_0101	452	ITGREGKQAARAADF	REGKQAARA	3	0.3397	1267.5	75.00	0.34	tr_C9ZR23_
DRB1_0101	453	QVGVGITGREGKQAA	GVGITGREG	2	0.3396	1268.7	75.00	0.50	tr_C9ZR23_
DRB1_0101	454	YVLSDKTGTLTQNVM	LSDKTGTLT	2	0.3391	1274.7	75.00	0.30	tr_C9ZR23_
DRB1_0101	455	REGKQAARAADFSIS	KQAARAADF	3	0.3389	1278.4	75.00	0.33	tr_C9ZR23_
DRB1_0101	456	SSDPAVGEKACAMGV	AVGEKACAM	4	0.3388	1279.9	75.00	0.43	tr_C9ZR23_
DRB1_0101	457	DVQYGDAAFPTGSSS	VQYGDAAFP	1	0.3387	1280.3	75.00	0.49	tr_C9ZR23_
DRB1_0101	458	NIFHTHYSGGSFWDG	FHTHYSGGS	2	0.3383	1285.8	75.00	0.42	tr_C9ZR23_
DRB1_0101	459	QEAQVGVGITGREGK	QEAQVGVGI	0	0.3380	1290.0	75.00	0.37	tr_C9ZR23_
DRB1_0101	460	VTILGVTAIEDKLQD	ILGVTAIED	2	0.3365	1310.8	75.00	0.37	tr_C9ZR23_
DRB1_0101	461	GVMQKRNGSVTTETN	MQKRNGSVT	2	0.3364	1312.4	75.00	0.35	tr_C9ZR23_
DRB1_0101	462	INGERRCLAPVEAEE	RRCLAPVEA	4	0.3358	1321.1	75.00	0.51	tr_C9ZR23_
DRB1_0101	463	YKVTRSGVDLGVCQF	YKVTRSGVD	0	0.3356	1324.8	75.00	0.25	tr_C9ZR23_
DRB1_0101	464	YQAASPDEVALCNGC	YQAASPDEV	0	0.3352	1330.0	75.00	0.72	tr_C9ZR23_
DRB1_0101	465	GREGKQAARAADFSI	KQAARAADF	4	0.3351	1331.0	75.00	0.31	tr_C9ZR23_
DRB1_0101	466	DDRILEMALDENSST	LEMALDENS	4	0.3349	1334.3	75.00	0.50	tr_C9ZR23_
DRB1_0101	467	KLQDHVPETIRSFLQ	LQDHVPETI	1	0.3338	1350.5	80.00	0.42	tr_C9ZR23_
DRB1_0101	468	TGREGKQAARAADFS	EGKQAARAA	3	0.3303	1403.1	80.00	0.34	tr_C9ZR23_
DRB1_0101	469	EVALCNGCEKLNVRL	ALCNGCEKL	2	0.3302	1404.3	80.00	0.29	tr_C9ZR23_
DRB1_0101	470	TGTLTQNVMEFLFAT	QNVMEFLFA	5	0.3300	1406.6	80.00	0.29	tr_C9ZR23_
DRB1_0101	471	QDHVPETIRSFLQAG	PETIRSFLQ	4	0.3300	1407.4	80.00	0.53	tr_C9ZR23_
DRB1_0101	472	KTGTLTQNVMEFLFA	GTLTQNVME	2	0.3298	1409.4	80.00	0.22	tr_C9ZR23_
DRB1_0101	473	DEVALCNGCEKLNVR	ALCNGCEKL	3	0.3294	1416.1	80.00	0.29	tr_C9ZR23_
DRB1_0101	474	CLAPVEAEEVQGSCG	LAPVEAEEV	1	0.3294	1416.5	80.00	0.71	tr_C9ZR23_
DRB1_0101	475	ICARTTPKQKAAVTR	TPKQKAAVT	5	0.3276	1443.5	80.00	0.30	tr_C9ZR23_
DRB1_0101	476	EDKLQDHVPETIRSF	LQDHVPETI	3	0.3276	1444.4	80.00	0.67	tr_C9ZR23_
DRB1_0101	477	LDRKVPRRVLEQTPA	LDRKVPRRV	0	0.3272	1450.8	80.00	0.55	tr_C9ZR23_
DRB1_0101	478	GTLTQNVMEFLFATI	QNVMEFLFA	4	0.3271	1451.4	80.00	0.37	tr_C9ZR23_
DRB1_0101	479	IFHTHYSGGSFWDGF	FHTHYSGGS	1	0.3251	1484.0	80.00	0.32	tr_C9ZR23_
DRB1_0101	480	RILEMALDENSSTGG	LEMALDENS	2	0.3242	1497.5	80.00	0.60	tr_C9ZR23_
DRB1_0101	481	LSTGSSYVLVIEGGQ	LSTGSSYVL	0	0.3239	1503.0	80.00	0.31	tr_C9ZR23_
DRB1_0101	482	QEMTQSGLSTGCGAV	EMTQSGLST	1	0.3237	1505.7	80.00	0.43	tr_C9ZR23_
DRB1_0101	483	LQDHVPETIRSFLQA	PETIRSFLQ	5	0.3236	1508.7	80.00	0.45	tr_C9ZR23_
DRB1_0101	484	AQVGVGITGREGKQA	GVGITGREG	3	0.3229	1520.1	80.00	0.59	tr_C9ZR23_

DRB1_0101	485	EFLEKVREAELSMNN	LEKVREAEL	2	0.3228	1521.1	80.00	0.39	tr_C9ZR23_
DRB1_0101	486	VIEGGQVLERILTTP	IEGGQVLER	1	0.3226	1524.7	80.00	0.28	tr_C9ZR23_
DRB1_0101	487	DKLQDHVPETIRSFL	LQDHVPETI	2	0.3221	1533.3	80.00	0.61	tr_C9ZR23_
DRB1_0101	488	SDKTGTLTQNVMEFL	KTGTLTQNV	2	0.3167	1625.7	80.00	0.37	tr_C9ZR23_
DRB1_0101	489	DEFLEKVREAELSMN	LEKVREAEL	3	0.3161	1635.2	80.00	0.38	tr_C9ZR23_
DRB1_0101	490	GKQAARAADFSISRF	AARAADFSI	3	0.3160	1636.5	80.00	0.29	tr_C9ZR23_
DRB1_0101	491	FLEKVREAELSMNNR	LEKVREAEL	1	0.3155	1646.5	80.00	0.25	tr_C9ZR23_
DRB1_0101	492	EGKQAARAADFSISR	KQAARAADF	2	0.3147	1660.7	80.00	0.31	tr_C9ZR23_
DRB1_0101	493	ADDRILEMALDENSS	LEMALDENS	5	0.3141	1671.3	80.00	0.44	tr_C9ZR23_
DRB1_0101	494	PDEVALCNGCEKLNV	ALCNGCEKL	4	0.3140	1672.3	80.00	0.28	tr_C9ZR23_
DRB1_0101	495	LSDKTGTLTQNVMEF	KTGTLTQNV	3	0.3139	1675.6	80.00	0.46	tr_C9ZR23_
DRB1_0101	496	AELSMNNRKEEIYKL	NRKEEIYKL	6	0.3130	1691.1	80.00	0.50	tr_C9ZR23_
DRB1_0101	497	WCNVRNSSIVEDLGQ	WCNVRNSSI	0	0.3124	1701.8	80.00	0.43	tr_C9ZR23_
DRB1_0101	498	YDDVSQEMTQSGLST	EMTQSGLST	6	0.3061	1821.5	85.00	0.25	tr_C9ZR23_
DRB1_0101	499	IEDKLQDHVPETIRS	LQDHVPETI	4	0.3057	1830.4	85.00	0.62	tr_C9ZR23_
DRB1_0101	500	KQAARAADFSISRFS	ARAADFSIS	3	0.3055	1833.7	85.00	0.30	tr_C9ZR23_
DRB1_0101	501	DAFESWEGYMESLLQ	WEGYMESLL	5	0.3045	1853.6	85.00	0.45	tr_C9ZR23_
DRB1_0101	502	DKLDAFESWEGYMES	LDAFESWEG	2	0.3043	1857.9	85.00	0.62	tr_C9ZR23_
DRB1_0101	503	DKTGTLTQNVMEFLF	GTLTQNVME	3	0.3032	1881.4	85.00	0.26	tr_C9ZR23_
DRB1_0101	504	ESADVNPLSTGSSYV	VNPLSTGSS	4	0.3018	1909.0	85.00	0.65	tr_C9ZR23_
DRB1_0101	505	ARDATTAAVEVNGIK	DATTAAVEV	2	0.2992	1963.4	85.00	0.50	tr_C9ZR23_
DRB1_0101	506	LDAFESWEGYMESLL	WEGYMESLL	6	0.2990	1968.0	85.00	0.33	tr_C9ZR23_
DRB1_0101	507	LDEFLEKVREAELSM	LEKVREAEL	4	0.2981	1986.0	85.00	0.39	tr_C9ZR23_
DRB1_0101	508	AAVEVNGIKEEWFVH	VNGIKEEWF	4	0.2976	1998.4	85.00	0.34	tr_C9ZR23_
DRB1_0101	509	LVIENDVAMHHDGEW	IENDVAMHH	2	0.2971	2009.0	85.00	0.36	tr_C9ZR23_
DRB1_0101	510	VLSDKTGTLTQNVME	KTGTLTQNV	4	0.2964	2024.0	85.00	0.41	tr_C9ZR23_
DRB1_0101	511	MHHDGEWCNVRNSSI	WCNVRNSSI	6	0.2959	2034.8	85.00	0.47	tr_C9ZR23_
DRB1_0101	512	VARDATTAAVEVNGI	DATTAAVEV	3	0.2956	2041.8	85.00	0.52	tr_C9ZR23_
DRB1_0101	513	LSLCNTVEVVYDDVS	SLCNTVEVV	1	0.2953	2048.6	85.00	0.34	tr_C9ZR23_
DRB1_0101	514	PQWGKGERMSSKAAI	GERMSSKAA	5	0.2951	2053.1	85.00	0.53	tr_C9ZR23_
DRB1_0101	515	VRNSSIVEDLGQVDY	IVEDLGQVD	5	0.2928	2104.5	85.00	0.57	tr_C9ZR23_
DRB1_0101	516	EAQVGVGITGREGKQ	GVGITGREG	4	0.2927	2105.5	85.00	0.55	tr_C9ZR23_
DRB1_0101	517	LCSPGDCVLRVLADK	CSPGDCVLR	1	0.2921	2120.3	85.00	0.28	tr_C9ZR23_
DRB1_0101	518	SSSDPAVGEKACAMG	AVGEKACAM	5	0.2894	2184.2	85.00	0.44	tr_C9ZR23_
DRB1_0101	519	FHTHYSGGSFWDGFG	THYSGGSFW	2	0.2878	2222.4	85.00	0.28	tr_C9ZR23_
DRB1_0101	520	KLDAFESWEGYMESL	LDAFESWEG	1	0.2874	2230.8	85.00	0.55	tr_C9ZR23_
DRB1_0101	521	KVTRSGVDLGVCQFF	GVDLGVCQF	5	0.2859	2267.8	85.00	0.26	tr_C9ZR23_
DRB1_0101	522	RDATTAAVEVNGIKE	DATTAAVEV	1	0.2857	2272.7	85.00	0.44	tr_C9ZR23_
DRB1_0101	523	VQYGDAAFPTGSSSD	VQYGDAAFP	0	0.2850	2289.2	85.00	0.32	tr_C9ZR23_
DRB1_0101	524	RSGVDLGVCQFFGSF	GVDLGVCQF	2	0.2839	2316.1	85.00	0.31	tr_C9ZR23_
DRB1_0101	525	LEKVREAELSMNNRK	VREAELSMN	3	0.2831	2337.6	85.00	0.34	tr_C9ZR23_
DRB1_0101	526	AIEDKLQDHVPETIR	LQDHVPETI	5	0.2823	2357.3	85.00	0.50	tr_C9ZR23_
DRB1_0101	527	TRSGVDLGVCQFFGS	GVDLGVCQF	3	0.2818	2370.6	85.00	0.37	tr_C9ZR23_
DRB1_0101	528	SPDEVALCNGCEKLN	EVALCNGCE	3	0.2799	2420.4	85.00	0.31	tr_C9ZR23_
DRB1_0101	529	TLAVGDGGNDVAMIQ	AVGDGGNDV	2	0.2773	2489.5	85.00	0.31	tr_C9ZR23_
DRB1_0101	530	VTRSGVDLGVCQFFG	GVDLGVCQF	4	0.2762	2519.3	85.00	0.37	tr_C9ZR23_
DRB1_0101	531	ELDEFLEKVREAELS	LEKVREAEL	5	0.2747	2559.5	90.00	0.28	tr_C9ZR23_
DRB1_0101	532	GADDRILEMALDENS	DRILEMALD	3	0.2728	2612.7	90.00	0.44	tr_C9ZR23_
DRB1_0101	533	VEVVYDDVSQEMTQS	YDDVSQEMT	4	0.2722	2628.7	90.00	0.38	tr_C9ZR23_
DRB1_0101	534	VVYDDVSQEMTQSGL	YDDVSQEMT	2	0.2705	2677.6	90.00	0.34	tr_C9ZR23_

DRB1_0101	535	EVVYDDVSQEMTQSG	YDDVSQEMT	3	0.2704	2681.4	90.00	0.41	tr_C9ZR23_
DRB1_0101	536	VYDDVSQEMTQSGLS	YDDVSQEMT	1	0.2674	2769.1	90.00	0.26	tr_C9ZR23_
DRB1_0101	537	ILEMALDENSSTGGP	LEMALDENS	1	0.2670	2780.4	90.00	0.54	tr_C9ZR23_
DRB1_0101	538	DRKVPRRVLEQTPAL	RRVLEQTPA	5	0.2660	2812.2	90.00	0.31	tr_C9ZR23_
DRB1_0101	539	NGSVTTETNESADVN	VTTETNESA	3	0.2639	2876.3	90.00	0.74	tr_C9ZR23_
DRB1_0101	540	GSSSDPAVGEKACAM	SDPAVGEKA	3	0.2635	2890.8	90.00	0.54	tr_C9ZR23_
DRB1_0101	541	TILGVTAIEDKLQDH	ILGVTAIED	1	0.2628	2910.3	90.00	0.47	tr_C9ZR23_
DRB1_0101	542	PVEAEEVQGSCGEHV	VQGSCGEHV	6	0.2624	2922.4	90.00	0.59	tr_C9ZR23_
DRB1_0101	543	QAARAADFSISRFSD	ARAADFSIS	2	0.2600	3002.4	90.00	0.34	tr_C9ZR23_
DRB1_0101	544	VKGADDRILEMALDE	DRILEMALD	5	0.2589	3037.2	90.00	0.37	tr_C9ZR23_
DRB1_0101	545	EKVREAELSMNNRKE	VREAELSMN	2	0.2574	3087.4	90.00	0.37	tr_C9ZR23_
DRB1_0101	546	KRLTRNELDEFLEKV	TRNELDEFL	3	0.2569	3103.5	90.00	0.50	tr_C9ZR23_
DRB1_0101	547	VMQKRNGSVTTETNE	MQKRNGSVT	1	0.2557	3142.5	90.00	0.47	tr_C9ZR23_
DRB1_0101	548	EKRLTRNELDEFLEK	TRNELDEFL	4	0.2538	3208.8	90.00	0.46	tr_C9ZR23_
DRB1_0101	549	HTHYSGGSFWDGFGL	YSGGSFWDG	3	0.2532	3230.0	90.00	0.38	tr_C9ZR23_
DRB1_0101	550	TVEVVYDDVSQEMTQ	VYDDVSQEM	4	0.2529	3239.9	90.00	0.40	tr_C9ZR23_
DRB1_0101	551	LAPVEAEEVQGSCGE	LAPVEAEEV	0	0.2499	3347.4	90.00	0.53	tr_C9ZR23_
DRB1_0101	552	TAAVEVNGIKEEWFV	VNGIKEEWF	5	0.2495	3361.7	90.00	0.34	tr_C9ZR23_
DRB1_0101	553	AEKRLTRNELDEFLE	TRNELDEFL	5	0.2495	3362.8	90.00	0.34	tr_C9ZR23_
DRB1_0101	554	VAEKRLTRNELDEFL	KRLTRNELD	3	0.2461	3487.5	90.00	0.34	tr_C9ZR23_
DRB1_0101	555	NELDEFLEKVREAEL	EFLEKVREA	4	0.2435	3585.7	90.00	0.33	tr_C9ZR23_
DRB1_0101	556	GSVTTETNESADVNP	VTTETNESA	2	0.2433	3593.7	90.00	0.73	tr_C9ZR23_
DRB1_0101	557	LVAEKRLTRNELDEF	EKRLTRNEL	3	0.2427	3617.6	90.00	0.36	tr_C9ZR23_
DRB1_0101	558	ASPDEVALCNGCEKL	EVALCNGCE	4	0.2421	3642.8	90.00	0.31	tr_C9ZR23_
DRB1_0101	559	THYSGGSFWDGFGLT	YSGGSFWDG	2	0.2416	3660.9	90.00	0.40	tr_C9ZR23_
DRB1_0101	560	DATTAAVEVNGIKEE	TAAVEVNGI	3	0.2397	3738.1	95.00	0.38	tr_C9ZR23_
DRB1_0101	561	LAVGDGGNDVAMIQE	AVGDGGNDV	1	0.2392	3758.9	95.00	0.31	tr_C9ZR23_
DRB1_0101	562	RNGSVTTETNESADV	VTTETNESA	4	0.2387	3779.4	95.00	0.76	tr_C9ZR23_
DRB1_0101	563	NESADVNPLSTGSSY	VNPLSTGSS	5	0.2383	3797.0	95.00	0.64	tr_C9ZR23_
DRB1_0101	564	TAIEDKLQDHVPETI	LQDHVPETI	6	0.2344	3957.8	95.00	0.38	tr_C9ZR23_
DRB1_0101	565	KVREAELSMNNRKEE	VREAELSMN	1	0.2337	3989.7	95.00	0.32	tr_C9ZR23_
DRB1_0101	566	VGDGGNDVAMIQEAQ	NDVAMIQEA	5	0.2317	4076.9	95.00	0.34	tr_C9ZR23_
DRB1_0101	567	RLTRNELDEFLEKVR	TRNELDEFL	2	0.2311	4103.1	95.00	0.49	tr_C9ZR23_
DRB1_0101	568	SVTTETNESADVNPL	VTTETNESA	1	0.2301	4146.1	95.00	0.56	tr_C9ZR23_
DRB1_0101	569	KGADDRILEMALDEN	DRILEMALD	4	0.2284	4224.1	95.00	0.54	tr_C9ZR23_
DRB1_0101	570	AVGDGGNDVAMIQEA	DGGNDVAMI	3	0.2280	4240.8	95.00	0.34	tr_C9ZR23_
DRB1_0101	571	ILGVTAIEDKLQDHV	VTAIEDKLQ	3	0.2276	4262.8	95.00	0.31	tr_C9ZR23_
DRB1_0101	572	NVRNSSIVEDLGQVD	IVEDLGQVD	6	0.2271	4282.3	95.00	0.40	tr_C9ZR23_
DRB1_0101	573	TGSSSDPAVGEKACA	SDPAVGEKA	4	0.2260	4334.8	95.00	0.57	tr_C9ZR23_
DRB1_0101	574	NTVEVVYDDVSQEMT	VYDDVSQEM	5	0.2188	4684.2	95.00	0.37	tr_C9ZR23_
DRB1_0101	575	VAMHHDGEWCNVRNS	HHDGEWCNV	3	0.2168	4787.9	95.00	0.39	tr_C9ZR23_
DRB1_0101	576	REAELSMNNRKEEIY	ELSMNNRKE	3	0.2163	4813.3	95.00	0.39	tr_C9ZR23_
DRB1_0101	577	CNVRNSSIVEDLGQV	NVRNSSIVE	1	0.2160	4829.4	95.00	0.19	tr_C9ZR23_
DRB1_0101	578	TTAAVEVNGIKEEWF	TAAVEVNGI	1	0.2149	4885.8	95.00	0.32	tr_C9ZR23_
DRB1_0101	579	GPQWGKGERMSSKAA	GERMSSKAA	6	0.2119	5052.3	95.00	0.41	tr_C9ZR23_
DRB1_0101	580	DVAMHHDGEWCNVRN	HHDGEWCNV	4	0.2111	5091.0	95.00	0.38	tr_C9ZR23_
DRB1_0101	581	EAELSMNNRKEEIYK	ELSMNNRKE	2	0.2107	5116.0	95.00	0.34	tr_C9ZR23_
DRB1_0101	582	PTGSSSDPAVGEKAC	SDPAVGEKA	5	0.2097	5172.1	95.00	0.46	tr_C9ZR23_
DRB1_0101	583	AMHHDGEWCNVRNSS	HHDGEWCNV	2	0.2088	5222.8	95.00	0.38	tr_C9ZR23_
DRB1_0101	584	VREAELSMNNRKEEI	ELSMNNRKE	4	0.2066	5348.2	95.00	0.31	tr_C9ZR23_

DRB1_0101	585	RNELDEFLEKVREAE	EFLEKVREA	5	0.2054	5416.8	95.00	0.29	tr_C9ZR23_
DRB1_0101	586	LTRNELDEFLEKVRE	TRNELDEFL	1	0.2045	5468.4	95.00	0.36	tr_C9ZR23_
DRB1_0101	587	ATTAAVEVNGIKEEW	TAAVEVNGI	2	0.2045	5468.9	95.00	0.49	tr_C9ZR23_
DRB1_0101	588	FPTGSSSDPAVGEKA	SDPAVGEKA	6	0.2040	5501.5	95.00	0.23	tr_C9ZR23_
DRB1_0101	589	AAFPTGSSSDPAVGE	FPTGSSSDP	2	0.2036	5525.3	95.00	0.47	tr_C9ZR23_
DRB1_0101	590	DAAFPTGSSSDPAVG	FPTGSSSDP	3	0.2021	5615.0	95.00	0.50	tr_C9ZR23_
DRB1_0101	591	AFPTGSSSDPAVGEK	FPTGSSSDP	1	0.2009	5687.2	95.00	0.38	tr_C9ZR23_
DRB1_0101	592	TRNELDEFLEKVREA	ELDEFLEKV	3	0.2005	5713.1	95.00	0.17	tr_C9ZR23_
DRB1_0101	593	NDVAMHHDGEWCNVR	HHDGEWCNV	5	0.2001	5739.0	95.00	0.34	tr_C9ZR23_
DRB1_0101	594	LEMALDENSSTGGPQ	LEMALDENS	0	0.1981	5864.1	95.00	0.35	tr_C9ZR23_
DRB1_0101	595	SLCNTVEVVYDDVSQ	CNTVEVVYD	2	0.1944	6104.2	95.00	0.28	tr_C9ZR23_
DRB1_0101	596	LGVTAIEDKLQDHVP	VTAIEDKLQ	2	0.1941	6123.6	95.00	0.37	tr_C9ZR23_
DRB1_0101	597	GVTAIEDKLQDHVPE	VTAIEDKLQ	1	0.1940	6126.8	95.00	0.28	tr_C9ZR23_
DRB1_0101	598	QYGDAAFPTGSSSDP	QYGDAAFPT	0	0.1940	6128.5	95.00	0.24	tr_C9ZR23_
DRB1_0101	599	TNESADVNPLSTGSS	NESADVNPL	1	0.1897	6420.0	100.00	0.38	tr_C9ZR23_
DRB1_0101	600	VTAIEDKLQDHVPET	IEDKLQDHV	3	0.1880	6537.9	100.00	0.33	tr_C9ZR23_
DRB1_0101	601	VTTETNESADVNPLS	NESADVNPL	5	0.1877	6563.3	100.00	0.34	tr_C9ZR23_
DRB1_0101	602	KRNGSVTTETNESAD	VTTETNESA	5	0.1876	6566.7	100.00	0.73	tr_C9ZR23_
DRB1_0101	603	VIENDVAMHHDGEWC	IENDVAMHH	1	0.1849	6766.4	100.00	0.51	tr_C9ZR23_
DRB1_0101	604	GDAAFPTGSSSDPAV	FPTGSSSDP	4	0.1836	6855.0	100.00	0.46	tr_C9ZR23_
DRB1_0101	605	YGDAAFPTGSSSDPA	FPTGSSSDP	5	0.1748	7540.0	100.00	0.41	tr_C9ZR23_
DRB1_0101	606	CNTVEVVYDDVSQEM	VYDDVSQEM	6	0.1746	7559.2	100.00	0.26	tr_C9ZR23_
DRB1_0101	607	AASPDEVALCNGCEK	EVALCNGCE	5	0.1745	7570.2	100.00	0.35	tr_C9ZR23_
DRB1_0101	608	TETNESADVNPLSTG	NESADVNPL	3	0.1723	7750.0	100.00	0.61	tr_C9ZR23_
DRB1_0101	609	TTETNESADVNPLST	NESADVNPL	4	0.1661	8286.1	100.00	0.52	tr_C9ZR23_
DRB1_0101	610	ENDVAMHHDGEWCNV	HHDGEWCNV	6	0.1643	8447.5	100.00	0.25	tr_C9ZR23_
DRB1_0101	611	ETNESADVNPLSTGS	NESADVNPL	2	0.1635	8529.5	100.00	0.58	tr_C9ZR23_
DRB1_0101	612	MQKRNGSVTTETNES	QKRNGSVTT	1	0.1539	9461.0	100.00	0.34	tr_C9ZR23_
DRB1_0101	613	APVEAEEVQGSCGEH	VEAEEVQGS	2	0.1511	9746.4	100.00	0.73	tr_C9ZR23_
DRB1_0101	614	GGPQWGKGERMSSKA	PQWGKGERM	2	0.1485	10030.1	100.00	0.43	tr_C9ZR23_
DRB1_0101	615	QKRNGSVTTETNESA	VTTETNESA	6	0.1470	10191.7	100.00	0.50	tr_C9ZR23_
DRB1_0101	616	LCNTVEVVYDDVSQE	CNTVEVVYD	1	0.1455	10360.3	100.00	0.28	tr_C9ZR23_
DRB1_0101	617	TGGPQWGKGERMSSK	PQWGKGERM	3	0.1433	10607.0	100.00	0.52	tr_C9ZR23_
DRB1_0101	618	QAASPDEVALCNGCE	EVALCNGCE	6	0.1417	10792.6	100.00	0.18	tr_C9ZR23_
DRB1_0101	619	IENDVAMHHDGEWCN	IENDVAMHH	0	0.1359	11487.5	100.00	0.31	tr_C9ZR23_
DRB1_0101	620	STGGPQWGKGERMSS	PQWGKGERM	4	0.1310	12120.3	100.00	0.53	tr_C9ZR23_
DRB1_0101	621	EMALDENSSTGGPQW	MALDENSST	1	0.1309	12131.6	100.00	0.34	tr_C9ZR23_
DRB1_0101	622	SSTGGPQWGKGERMS	PQWGKGERM	5	0.1207	13542.3	100.00	0.49	tr_C9ZR23_
DRB1_0101	623	NSSTGGPQWGKGERM	PQWGKGERM	6	0.1121	14866.9	100.00	0.34	tr_C9ZR23_
DRB1_0101	624	MALDENSSTGGPQWG	LDENSSTGG	2	0.1065	15787.7	100.00	0.39	tr_C9ZR23_
DRB1_0101	625	LDENSSTGGPQWGKG	NSSTGGPQW	3	0.0991	17110.9	100.00	0.27	tr_C9ZR23_
DRB1_0101	626	ALDENSSTGGPQWGK	LDENSSTGG	1	0.0977	17376.1	100.00	0.34	tr_C9ZR23_
DRB1_0101	627	ENSSTGGPQWGKGER	NSSTGGPQW	1	0.0950	17897.0	100.00	0.28	tr_C9ZR23_
DRB1_0101	628	DENSSTGGPQWGKGE	NSSTGGPQW	2	0.0899	18902.1	100.00	0.32	tr_C9ZR23_

Allele: DRB1_0101. Number of high binders 0. Number of weak binders 56. Number of peptides 628


```
         Allele  pos            peptide      core Of 1-log50k(aff) affinity(nM)    %Rank Relia    Identity Bind Level
--------------------------------------------------------------------------------------------------------------
      DRB1_0101    1    KRASVSRLSRCLWCV  SVSRLSRCL  3        0.4976        229.5    42.00  0.63   tr_C9ZR23_
      DRB1_0101    2    SAELRRQANDPLAVE  LRRQANDPL  3        0.4938        239.2    42.00  0.76   tr_C9ZR23_
      DRB1_0101    3    RSAELRRQANDPLAV  LRRQANDPL  4        0.4801        277.3    45.00  0.79   tr_C9ZR23_
      DRB1_0101    4    AELRRQANDPLAVEK  LRRQANDPL  2        0.4790        280.6    45.00  0.69   tr_C9ZR23_
      DRB1_0101    5    PRDVMRSAELRRQAN  DVMRSAELR  2        0.4767        287.7    46.00  0.37   tr_C9ZR23_
      DRB1_0101    6    YVTVVLADDELTIRN  VVLADDELT  3        0.4761        289.6    46.00  0.73   tr_C9ZR23_
      DRB1_0101    7    DPRDVMRSAELRRQA  DVMRSAELR  3        0.4715        304.3    47.00  0.38   tr_C9ZR23_
      DRB1_0101    8    RASVSRLSRCLWCVP  SVSRLSRCL  2        0.4638        330.7    49.00  0.62   tr_C9ZR23_
      DRB1_0101    9    EKRASVSRLSRCLWC  SVSRLSRCL  4        0.4615        339.1    49.00  0.62   tr_C9ZR23_
      DRB1_0101   10    TYVTVVLADDELTIR  VVLADDELT  4        0.4610        340.9    49.00  0.66   tr_C9ZR23_
      DRB1_0101   11    VEKRASVSRLSRCLW  SVSRLSRCL  5        0.4552        362.9    50.00  0.54   tr_C9ZR23_
      DRB1_0101   12    VTVVLADDELTIRNA  VVLADDELT  2        0.4502        383.2    55.00  0.69   tr_C9ZR23_
      DRB1_0101   13    ELRRQANDPLAVEKR  LRRQANDPL  1        0.4465        398.8    55.00  0.60   tr_C9ZR23_
      DRB1_0101   14    RDVMRSAELRRQAND  DVMRSAELR  1        0.4405        425.9    55.00  0.40   tr_C9ZR23_
      DRB1_0101   15    PDPRDVMRSAELRRQ  DVMRSAELR  4        0.4364        445.1    55.00  0.36   tr_C9ZR23_
      DRB1_0101   16    ASVSRLSRCLWCVPE  SVSRLSRCL  1        0.4218        521.0    60.00  0.50   tr_C9ZR23_
      DRB1_0101   17    AVEKRASVSRLSRCL  SVSRLSRCL  6        0.4151        560.0    60.00  0.31   tr_C9ZR23_
      DRB1_0101   18    STYVTVVLADDELTI  VVLADDELT  5        0.4111        584.8    60.00  0.56   tr_C9ZR23_
      DRB1_0101   19    MRSAELRRQANDPLA  LRRQANDPL  5        0.4109        586.2    60.00  0.78   tr_C9ZR23_
      DRB1_0101   20    TVVLADDELTIRNAN  VVLADDELT  1        0.4084        602.6    60.00  0.68   tr_C9ZR23_
      DRB1_0101   21    LIKIWRPDPRDVMRS  WRPDPRDVM  4        0.3897        737.5    65.00  0.44   tr_C9ZR23_
      DRB1_0101   22    PLAVEKRASVSRLSR  EKRASVSRL  4        0.3896        738.3    65.00  0.28   tr_C9ZR23_
      DRB1_0101   23    RPDPRDVMRSAELRR  RDVMRSAEL  4        0.3841        784.0    70.00  0.44   tr_C9ZR23_
      DRB1_0101   24    IKIWRPDPRDVMRSA  WRPDPRDVM  3        0.3810        810.6    70.00  0.54   tr_C9ZR23_
      DRB1_0101   25    LAVEKRASVSRLSRC  KRASVSRLS  4        0.3736        877.4    70.00  0.30   tr_C9ZR23_
      DRB1_0101   26    LRRQANDPLAVEKRA  LRRQANDPL  0        0.3705        907.4    70.00  0.41   tr_C9ZR23_
      DRB1_0101   27    RCLWCVPEEPSTYVT  LWCVPEEPS  2        0.3623        992.1    70.00  0.31   tr_C9ZR23_
      DRB1_0101   28    SRCLWCVPEEPSTYV  LWCVPEEPS  3        0.3585       1034.1    75.00  0.38   tr_C9ZR23_
      DRB1_0101   29    CLWCVPEEPSTYVTV  VPEEPSTYV  4        0.3584       1035.3    75.00  0.37   tr_C9ZR23_
      DRB1_0101   30    DPLAVEKRASVSRLS  LAVEKRASV  2        0.3482       1155.6    75.00  0.35   tr_C9ZR23_
      DRB1_0101   31    WRPDPRDVMRSAELR  RDVMRSAEL  5        0.3476       1163.3    75.00  0.37   tr_C9ZR23_
      DRB1_0101   32    KIWRPDPRDVMRSAE  WRPDPRDVM  2        0.3472       1168.0    75.00  0.58   tr_C9ZR23_
      DRB1_0101   33    PSTYVTVVLADDELT  VVLADDELT  6        0.3467       1173.9    75.00  0.32   tr_C9ZR23_
      DRB1_0101   34    SVSRLSRCLWCVPEE  SRLSRCLWC  2        0.3433       1217.9    75.00  0.28   tr_C9ZR23_
      DRB1_0101   35    LWCVPEEPSTYVTVV  VPEEPSTYV  3        0.3393       1272.6    75.00  0.44   tr_C9ZR23_
      DRB1_0101   36    VMRSAELRRQANDPL  LRRQANDPL  6        0.3248       1488.5    80.00  0.55   tr_C9ZR23_
      DRB1_0101   37    IWRPDPRDVMRSAEL  WRPDPRDVM  1        0.3241       1499.0    80.00  0.47   tr_C9ZR23_
      DRB1_0101   38    DVMRSAELRRQANDP  DVMRSAELR  0        0.3230       1517.3    80.00  0.37   tr_C9ZR23_
      DRB1_0101   39    NDPLAVEKRASVSRL  LAVEKRASV  3        0.3223       1529.3    80.00  0.49   tr_C9ZR23_
      DRB1_0101   40    LSRCLWCVPEEPSTY  LWCVPEEPS  4        0.3157       1642.6    80.00  0.41   tr_C9ZR23_
      DRB1_0101   41    CVPEEPSTYVTVVLA  VPEEPSTYV  1        0.3058       1827.4    85.00  0.30   tr_C9ZR23_
      DRB1_0101   42    WCVPEEPSTYVTVVL  VPEEPSTYV  2        0.3039       1866.7    85.00  0.44   tr_C9ZR23_
      DRB1_0101   43    VVLADDELTIRNANS  VVLADDELT  0        0.2989       1970.2    85.00  0.51   tr_C9ZR23_
      DRB1_0101   44    SRLSRCLWCVPEEPS  LWCVPEEPS  6        0.2841       2311.1    85.00  0.20   tr_C9ZR23_
      DRB1_0101   45    ANDPLAVEKRASVSR  LAVEKRASV  4        0.2833       2332.4    85.00  0.47   tr_C9ZR23_
      DRB1_0101   46    RLSRCLWCVPEEPST  LWCVPEEPS  5        0.2815       2378.2    85.00  0.40   tr_C9ZR23_
      DRB1_0101   47    VSRLSRCLWCVPEEP  SRLSRCLWC  1        0.2783       2462.1    85.00  0.41   tr_C9ZR23_
      DRB1_0101   48    RRQANDPLAVEKRAS  RQANDPLAV  1        0.2757       2532.9    90.00  0.39   tr_C9ZR23_
```

DRB1_0101	49	RQANDPLAVEKRASV	NDPLAVEKR	3	0.2742	2572.1	90.00	0.28	tr_C9ZR23_	
DRB1_0101	50	VPEEPSTYVTVVLAD	STYVTVVLA	5	0.2680	2750.6	90.00	0.31	tr_C9ZR23_	
DRB1_0101	51	EPSTYVTVVLADDEL	YVTVVLADD	4	0.2670	2782.7	90.00	0.31	tr_C9ZR23_	
DRB1_0101	52	QANDPLAVEKRASVS	LAVEKRASV	5	0.2629	2908.7	90.00	0.41	tr_C9ZR23_	
DRB1_0101	53	PEEPSTYVTVVLADD	STYVTVVLA	4	0.2529	3240.8	90.00	0.38	tr_C9ZR23_	
DRB1_0101	54	VLADDELTIRNANSV	LTIRNANSV	6	0.2512	3300.6	90.00	0.38	tr_C9ZR23_	
DRB1_0101	55	EEPSTYVTVVLADDE	STYVTVVLA	3	0.2479	3420.8	90.00	0.35	tr_C9ZR23_	

Allele: DRB1_0101. Number of high binders 0. Number of weak binders 0. Number of peptides 55

Allele	pos	peptide	core	Of	1-log50k(aff)	affinity(nM)	%Rank	Relia	Identity	Bind Level
DRB1_0101	1	RLFISMIPSPSNATY	FISMIPSPS	2	0.8873	3.4	0.17	0.73	tr_C9ZN43_	SB
DRB1_0101	2	DRLFISMIPSPSNAT	FISMIPSPS	3	0.8858	3.4	0.17	0.77	tr_C9ZN43_	SB
DRB1_0101	3	SDRLFISMIPSPSNA	FISMIPSPS	4	0.8754	3.8	0.25	0.80	tr_C9ZN43_	SB
DRB1_0101	4	NATYLALQSELDTRS	YLALQSELD	3	0.8704	4.1	0.30	0.88	tr_C9ZN43_	SB
DRB1_0101	5	ATYLALQSELDTRSV	YLALQSELD	2	0.8594	4.6	0.50	0.88	tr_C9ZN43_	WB
DRB1_0101	6	SNATYLALQSELDTR	YLALQSELD	4	0.8586	4.6	0.50	0.88	tr_C9ZN43_	WB
DRB1_0101	7	LFISMIPSPSNATYL	FISMIPSPS	1	0.8546	4.8	0.50	0.69	tr_C9ZN43_	WB
DRB1_0101	8	RVYSLLYNPLIPDNL	YSLLYNPLI	2	0.8528	4.9	0.60	0.77	tr_C9ZN43_	WB
DRB1_0101	9	VRVYSLLYNPLIPDN	YSLLYNPLI	3	0.8496	5.1	0.60	0.86	tr_C9ZN43_	WB
DRB1_0101	10	TVRVYSLLYNPLIPD	YSLLYNPLI	4	0.8434	5.4	0.80	0.84	tr_C9ZN43_	WB
DRB1_0101	11	QSDRLFISMIPSPSN	FISMIPSPS	5	0.8385	5.7	0.90	0.81	tr_C9ZN43_	WB
DRB1_0101	12	MTVRVYSLLYNPLIP	YSLLYNPLI	5	0.8361	5.9	0.90	0.83	tr_C9ZN43_	WB
DRB1_0101	13	VYSLLYNPLIPDNLC	YSLLYNPLI	1	0.8304	6.3	1.10	0.67	tr_C9ZN43_	WB
DRB1_0101	14	TYLALQSELDTRSVT	YLALQSELD	1	0.8288	6.4	1.10	0.87	tr_C9ZN43_	WB
DRB1_0101	15	PSNATYLALQSELDT	YLALQSELD	5	0.8215	6.9	1.30	0.87	tr_C9ZN43_	WB
DRB1_0101	16	SHVYFLTASPNAELL	YFLTASPNA	3	0.8134	7.5	1.60	0.38	tr_C9ZN43_	WB
DRB1_0101	17	VMTVRVYSLLYNPLI	YSLLYNPLI	6	0.8053	8.2	1.80	0.78	tr_C9ZN43_	WB
DRB1_0101	18	HVYFLTASPNAELLT	YFLTASPNA	2	0.8004	8.7	2.00	0.37	tr_C9ZN43_	WB
DRB1_0101	19	ISMWSLARALNASVP	WSLARALNA	3	0.7960	9.1	2.50	0.55	tr_C9ZN43_	WB
DRB1_0101	20	NSHVYFLTASPNAEL	YFLTASPNA	4	0.7923	9.5	2.50	0.38	tr_C9ZN43_	WB
DRB1_0101	21	RISMWSLARALNASV	WSLARALNA	4	0.7907	9.6	2.50	0.62	tr_C9ZN43_	WB
DRB1_0101	22	DTEYKMTTSLMSSVG	YKMTTSLMS	3	0.7883	9.9	2.50	0.60	tr_C9ZN43_	WB
DRB1_0101	23	TEYKMTTSLMSSVGR	YKMTTSLMS	2	0.7812	10.7	3.00	0.65	tr_C9ZN43_	WB
DRB1_0101	24	SMWSLARALNASVPP	WSLARALNA	2	0.7806	10.7	3.00	0.55	tr_C9ZN43_	WB
DRB1_0101	25	GDTEYKMTTSLMSSV	YKMTTSLMS	4	0.7803	10.8	3.00	0.59	tr_C9ZN43_	WB
DRB1_0101	26	FISMIPSPSNATYLA	FISMIPSPS	0	0.7800	10.8	3.00	0.47	tr_C9ZN43_	WB
DRB1_0101	27	YSLLYNPLIPDNLCD	YSLLYNPLI	0	0.7749	11.4	3.50	0.50	tr_C9ZN43_	WB
DRB1_0101	28	TLVRYAVSQLRLLRI	YAVSQLRLL	4	0.7721	11.8	3.50	0.44	tr_C9ZN43_	WB
DRB1_0101	29	KGEFFVVLGPVGDGQ	FFVVLGPVG	3	0.7719	11.8	3.50	0.52	tr_C9ZN43_	WB
DRB1_0101	30	VYFLTASPNAELLTL	FLTASPNAE	2	0.7659	12.6	4.00	0.32	tr_C9ZN43_	WB
DRB1_0101	31	ESVRLYSPLNTLMFL	YSPLNTLMF	5	0.7658	12.6	4.00	0.32	tr_C9ZN43_	WB
DRB1_0101	32	LTLVRYAVSQLRLLR	YAVSQLRLL	5	0.7657	12.6	4.00	0.39	tr_C9ZN43_	WB
DRB1_0101	33	MWSLARALNASVPPL	WSLARALNA	1	0.7657	12.6	4.00	0.55	tr_C9ZN43_	WB
DRB1_0101	34	AFVIGLAPGDPSLLA	FVIGLAPGD	1	0.7656	12.6	4.00	0.41	tr_C9ZN43_	WB
DRB1_0101	35	GEFFVVLGPVGDGQA	FVVLGPVGD	3	0.7645	12.8	4.00	0.48	tr_C9ZN43_	WB

DRB1_0101	36	LVRYAVSQLRLLRIG	YAVSQLRLL	3	0.7641	12.8	4.00	0.50	tr_C9ZN43_	WB
DRB1_0101	37	SVRLYSPLNTLMFLI	YSPLNTLMF	4	0.7636	12.9	4.00	0.38	tr_C9ZN43_	WB
DRB1_0101	38	GQSDRLFISMIPSPS	FISMIPSPS	6	0.7634	12.9	4.00	0.81	tr_C9ZN43_	WB
DRB1_0101	39	RKIVWMHGGTLQTVA	IVWMHGGTL	2	0.7602	13.4	4.50	0.65	tr_C9ZN43_	WB
DRB1_0101	40	NAFVIGLAPGDPSLL	FVIGLAPGD	2	0.7584	13.7	4.50	0.55	tr_C9ZN43_	WB
DRB1_0101	41	PLLGYAAARFAQAVL	LGYAAARFA	2	0.7579	13.7	4.50	0.47	tr_C9ZN43_	WB
DRB1_0101	42	LRKIVWMHGGTLQTV	IVWMHGGTL	3	0.7557	14.1	4.50	0.75	tr_C9ZN43_	WB
DRB1_0101	43	TRISMWSLARALNAS	WSLARALNA	5	0.7552	14.1	4.50	0.67	tr_C9ZN43_	WB
DRB1_0101	44	HKGEFFVVLGPVGDG	FFVVLGPVG	4	0.7538	14.4	4.50	0.53	tr_C9ZN43_	WB
DRB1_0101	45	LPLLGYAAARFAQAV	LGYAAARFA	3	0.7511	14.8	5.00	0.57	tr_C9ZN43_	WB
DRB1_0101	46	FGDTEYKMTTSLMSS	YKMTTSLMS	5	0.7463	15.6	5.00	0.56	tr_C9ZN43_	WB
DRB1_0101	47	EFFVVLGPVGDGQAL	FVVLGPVGD	2	0.7450	15.8	5.50	0.50	tr_C9ZN43_	WB
DRB1_0101	48	ADPNAIKLSSAQLAG	IKLSSAQLA	5	0.7446	15.9	5.50	0.58	tr_C9ZN43_	WB
DRB1_0101	49	LLGYAAARFAQAVLP	YAAARFAQA	3	0.7442	15.9	5.50	0.37	tr_C9ZN43_	WB
DRB1_0101	50	VRLYSPLNTLMFLIL	YSPLNTLMF	3	0.7421	16.3	5.50	0.53	tr_C9ZN43_	WB
DRB1_0101	51	LMFLILDRELAQVSS	FLILDRELA	2	0.7409	16.5	5.50	0.64	tr_C9ZN43_	WB
DRB1_0101	52	TLMFLILDRELAQVS	FLILDRELA	3	0.7403	16.6	5.50	0.73	tr_C9ZN43_	WB
DRB1_0101	53	PLPLLGYAAARFAQA	LGYAAARFA	4	0.7400	16.7	5.50	0.51	tr_C9ZN43_	WB
DRB1_0101	54	GNAFVIGLAPGDPSL	FVIGLAPGD	3	0.7391	16.8	5.50	0.63	tr_C9ZN43_	WB
DRB1_0101	55	FVIGLAPGDPSLLAA	LAPGDPSLL	4	0.7390	16.8	5.50	0.47	tr_C9ZN43_	WB
DRB1_0101	56	SPSNATYLALQSELD	YLALQSELD	6	0.7383	17.0	5.50	0.85	tr_C9ZN43_	WB
DRB1_0101	57	KPLPLLGYAAARFAQ	LGYAAARFA	5	0.7369	17.2	6.00	0.42	tr_C9ZN43_	WB
DRB1_0101	58	AESVRLYSPLNTLMF	SVRLYSPLN	2	0.7366	17.3	6.00	0.28	tr_C9ZN43_	WB
DRB1_0101	59	HDAYLLSSSSLEFAL	YLLSSSSLE	3	0.7357	17.5	6.00	0.66	tr_C9ZN43_	WB
DRB1_0101	60	VRYAVSQLRLLRIGF	YAVSQLRLL	2	0.7357	17.5	6.00	0.56	tr_C9ZN43_	WB
DRB1_0101	61	KIVWMHGGTLQTVAV	IVWMHGGTL	1	0.7353	17.5	6.00	0.55	tr_C9ZN43_	WB
DRB1_0101	62	YLALQSELDTRSVTA	YLALQSELD	0	0.7343	17.7	6.00	0.75	tr_C9ZN43_	WB
DRB1_0101	63	WNSHVYFLTASPNAE	VYFLTASPN	4	0.7335	17.9	6.00	0.41	tr_C9ZN43_	WB
DRB1_0101	64	RIGFMYLRGISFGDT	FMYLRGISF	3	0.7335	17.9	6.00	0.70	tr_C9ZN43_	WB
DRB1_0101	65	EYKMTTSLMSSVGRE	YKMTTSLMS	1	0.7330	18.0	6.00	0.67	tr_C9ZN43_	WB
DRB1_0101	66	ALRKIVWMHGGTLQT	IVWMHGGTL	4	0.7321	18.2	6.00	0.86	tr_C9ZN43_	WB
DRB1_0101	67	NTLMFLILDRELAQV	FLILDRELA	4	0.7278	19.0	6.50	0.76	tr_C9ZN43_	WB
DRB1_0101	68	MFLILDRELAQVSSE	FLILDRELA	1	0.7266	19.3	6.50	0.63	tr_C9ZN43_	WB
DRB1_0101	69	WSLARALNASVPPLT	LARALNASV	2	0.7257	19.4	6.50	0.41	tr_C9ZN43_	WB
DRB1_0101	70	LLTLVRYAVSQLRLL	VRYAVSQLR	4	0.7245	19.7	7.00	0.42	tr_C9ZN43_	WB
DRB1_0101	71	MRSYLSANPGVTVFN	YLSANPGVT	3	0.7183	21.1	7.50	0.56	tr_C9ZN43_	WB
DRB1_0101	72	THDAYLLSSSSLEFA	YLLSSSSLE	4	0.7182	21.1	7.50	0.59	tr_C9ZN43_	WB
DRB1_0101	73	RHKGEFFVVLGPVGD	FFVVLGPVG	5	0.7180	21.1	7.50	0.57	tr_C9ZN43_	WB
DRB1_0101	74	LRIGFMYLRGISFGD	FMYLRGISF	4	0.7171	21.3	7.50	0.75	tr_C9ZN43_	WB
DRB1_0101	75	DAYLLSSSSLEFALD	YLLSSSSLE	2	0.7162	21.6	7.50	0.69	tr_C9ZN43_	WB
DRB1_0101	76	QGVIAFAPPAKDLIK	IAFAPPAKD	3	0.7141	22.1	7.50	0.64	tr_C9ZN43_	WB
DRB1_0101	77	LLRIGFMYLRGISFG	FMYLRGISF	5	0.7133	22.2	8.00	0.77	tr_C9ZN43_	WB
DRB1_0101	78	WKPLPLLGYAAARFA	PLPLLGYAA	2	0.7125	22.4	8.00	0.51	tr_C9ZN43_	WB
DRB1_0101	79	RYAVSQLRLLRIGFM	YAVSQLRLL	1	0.7094	23.2	8.00	0.54	tr_C9ZN43_	WB
DRB1_0101	80	FLGQLMLTRTGPLAR	LMLTRTGPL	4	0.7081	23.5	8.50	0.60	tr_C9ZN43_	WB
DRB1_0101	81	LGQLMLTRTGPLARD	LMLTRTGPL	3	0.7076	23.7	8.50	0.64	tr_C9ZN43_	WB
DRB1_0101	82	LNTLMFLILDRELAQ	FLILDRELA	5	0.7068	23.9	8.50	0.75	tr_C9ZN43_	WB
DRB1_0101	83	IGFMYLRGISFGDTE	FMYLRGISF	2	0.7053	24.2	8.50	0.68	tr_C9ZN43_	WB
DRB1_0101	84	HMRSYLSANPGVTVF	YLSANPGVT	4	0.7051	24.3	8.50	0.56	tr_C9ZN43_	WB
DRB1_0101	85	RLYSPLNTLMFLILD	YSPLNTLMF	2	0.7049	24.3	8.50	0.58	tr_C9ZN43_	WB

DRB1_0101	86	VALRKIVWMHGGTLQ	IVWMHGGTL	5	0.7039	24.6	8.50	0.87	tr_C9ZN43_	WB
DRB1_0101	87	GQLMLTRTGPLARDT	LMLTRTGPL	2	0.7032	24.8	8.50	0.57	tr_C9ZN43_	WB
DRB1_0101	88	ATRISMWSLARALNA	WSLARALNA	6	0.7025	25.0	9.00	0.52	tr_C9ZN43_	WB
DRB1_0101	89	CDAINAGLNASLASR	INAGLNASL	3	0.7020	25.1	9.00	0.37	tr_C9ZN43_	WB
DRB1_0101	90	RSYLSANPGVTVFNG	YLSANPGVT	2	0.7015	25.3	9.00	0.54	tr_C9ZN43_	WB
DRB1_0101	91	RLLRIGFMYLRGISF	FMYLRGISF	6	0.6999	25.7	9.00	0.71	tr_C9ZN43_	WB
DRB1_0101	92	LGYAAARFAQAVLPR	YAAARFAQA	2	0.6993	25.9	9.00	0.44	tr_C9ZN43_	WB
DRB1_0101	93	RGNAFVIGLAPGDPS	FVIGLAPGD	4	0.6983	26.2	9.00	0.64	tr_C9ZN43_	WB
DRB1_0101	94	GVIAFAPPAKDLIKF	IAFAPPAKD	2	0.6963	26.7	9.50	0.64	tr_C9ZN43_	WB
DRB1_0101	95	GFMYLRGISFGDTEY	FMYLRGISF	1	0.6956	26.9	9.50	0.62	tr_C9ZN43_	WB
DRB1_0101	96	DAINAGLNASLASRK	NAGLNASLA	3	0.6942	27.3	9.50	0.40	tr_C9ZN43_	WB
DRB1_0101	97	GVEFFLGQLMLTRTG	FFLGQLMLT	3	0.6931	27.7	9.50	0.49	tr_C9ZN43_	WB
DRB1_0101	98	LCDAINAGLNASLAS	INAGLNASL	4	0.6928	27.8	9.50	0.33	tr_C9ZN43_	WB
DRB1_0101	99	YFLTASPNAELLTLV	FLTASPNAE	1	0.6906	28.4	10.00	0.40	tr_C9ZN43_	
DRB1_0101	100	FFLGQLMLTRTGPLA	LMLTRTGPL	5	0.6905	28.5	10.00	0.45	tr_C9ZN43_	
DRB1_0101	101	AYLLSSSSLEFALDT	YLLSSSSLE	1	0.6902	28.6	10.00	0.70	tr_C9ZN43_	
DRB1_0101	102	GDGQALASLPLLERE	GQALASLPL	2	0.6895	28.8	10.00	0.55	tr_C9ZN43_	
DRB1_0101	103	SAWKPLPLLGYAAAR	PLPLLGYAA	4	0.6893	28.9	10.00	0.63	tr_C9ZN43_	
DRB1_0101	104	FLILDRELAQVSSEA	FLILDRELA	0	0.6892	28.9	10.00	0.50	tr_C9ZN43_	
DRB1_0101	105	AINAGLNASLASRKW	NAGLNASLA	2	0.6875	29.4	11.00	0.38	tr_C9ZN43_	
DRB1_0101	106	VGDGQALASLPLLER	GQALASLPL	3	0.6874	29.4	11.00	0.61	tr_C9ZN43_	
DRB1_0101	107	AWKPLPLLGYAAARF	PLPLLGYAA	3	0.6872	29.5	11.00	0.69	tr_C9ZN43_	
DRB1_0101	108	EFFLGQLMLTRTGPL	FFLGQLMLT	1	0.6869	29.6	11.00	0.34	tr_C9ZN43_	
DRB1_0101	109	TPLIRYADPNAIKLS	IRYADPNAI	3	0.6861	29.9	11.00	0.73	tr_C9ZN43_	
DRB1_0101	110	DGVEFFLGQLMLTRT	FFLGQLMLT	4	0.6834	30.7	11.00	0.51	tr_C9ZN43_	
DRB1_0101	111	PQGVIAFAPPAKDLI	IAFAPPAKD	4	0.6831	30.8	11.00	0.61	tr_C9ZN43_	
DRB1_0101	112	DGQALASLPLLEREN	GQALASLPL	1	0.6830	30.9	11.00	0.50	tr_C9ZN43_	
DRB1_0101	113	RTHDAYLLSSSSLEF	YLLSSSSLE	5	0.6821	31.2	11.00	0.55	tr_C9ZN43_	
DRB1_0101	114	FFVVLGPVGDGQALA	FVVLGPVGD	1	0.6805	31.7	11.00	0.58	tr_C9ZN43_	
DRB1_0101	115	QLMLTRTGPLARDTN	LMLTRTGPL	1	0.6795	32.1	11.00	0.52	tr_C9ZN43_	
DRB1_0101	116	VIGLAPGDPSLLAAH	LAPGDPSLL	3	0.6793	32.1	11.00	0.69	tr_C9ZN43_	
DRB1_0101	117	YKMTTSLMSSVGREL	YKMTTSLMS	0	0.6782	32.5	12.00	0.47	tr_C9ZN43_	
DRB1_0101	118	SEALYYGANVLTGNG	LYYGANVLT	3	0.6774	32.8	12.00	0.59	tr_C9ZN43_	
DRB1_0101	119	LAESVRLYSPLNTLM	SVRLYSPLN	3	0.6751	33.6	12.00	0.42	tr_C9ZN43_	
DRB1_0101	120	SFGDTEYKMTTSLMS	YKMTTSLMS	6	0.6751	33.6	12.00	0.44	tr_C9ZN43_	
DRB1_0101	121	SLLYNPLIPDNLCDA	YNPLIPDNL	3	0.6749	33.7	12.00	0.83	tr_C9ZN43_	
DRB1_0101	122	SLARALNASVPPLTS	LARALNASV	1	0.6748	33.7	12.00	0.43	tr_C9ZN43_	
DRB1_0101	123	LVGFAPSTGSNAMRG	FAPSTGSNA	3	0.6746	33.8	12.00	0.81	tr_C9ZN43_	
DRB1_0101	124	PLIRYADPNAIKLSS	IRYADPNAI	2	0.6745	33.9	12.00	0.68	tr_C9ZN43_	
DRB1_0101	125	GWNSHVYFLTASPNA	VYFLTASPN	5	0.6729	34.4	12.00	0.51	tr_C9ZN43_	
DRB1_0101	126	VEFFLGQLMLTRTGP	FFLGQLMLT	2	0.6714	35.0	12.00	0.46	tr_C9ZN43_	
DRB1_0101	127	TSYKIKPVEVLQVAL	IKPVEVLQV	4	0.6713	35.0	12.00	0.47	tr_C9ZN43_	
DRB1_0101	128	NLVGFAPSTGSNAMR	FAPSTGSNA	4	0.6700	35.5	13.00	0.71	tr_C9ZN43_	
DRB1_0101	129	IVWMHGGTLQTVAVL	IVWMHGGTL	0	0.6689	36.0	13.00	0.41	tr_C9ZN43_	
DRB1_0101	130	NLCDAINAGLNASLA	DAINAGLNA	3	0.6684	36.2	13.00	0.41	tr_C9ZN43_	
DRB1_0101	131	SSEALYYGANVLTGN	LYYGANVLT	4	0.6683	36.2	13.00	0.60	tr_C9ZN43_	
DRB1_0101	132	INAGLNASLASRKWT	LNASLASRK	4	0.6644	37.8	13.00	0.37	tr_C9ZN43_	
DRB1_0101	133	SSAWKPLPLLGYAAA	PLPLLGYAA	5	0.6641	37.9	13.00	0.56	tr_C9ZN43_	
DRB1_0101	134	PLNTLMFLILDRELA	FLILDRELA	6	0.6620	38.7	14.00	0.69	tr_C9ZN43_	
DRB1_0101	135	PTSYKIKPVEVLQVA	IKPVEVLQV	5	0.6601	39.5	14.00	0.35	tr_C9ZN43_	

DRB1_0101	136	GRNVIQLSPTLEQQL	NVIQLSPTL	2	0.6599	39.6	14.00	0.38	tr_C9ZN43_
DRB1_0101	137	VVGYVTNTSASAPMS	YVTNTSASA	3	0.6597	39.7	14.00	0.81	tr_C9ZN43_
DRB1_0101	138	YADPNAIKLSSAQLA	IKLSSAQLA	6	0.6594	39.9	14.00	0.50	tr_C9ZN43_
DRB1_0101	139	GQALASLPLLERENL	GQALASLPL	0	0.6589	40.1	14.00	0.40	tr_C9ZN43_
DRB1_0101	140	SYKIKPVEVLQVALE	IKPVEVLQV	3	0.6571	40.9	14.00	0.51	tr_C9ZN43_
DRB1_0101	141	STGSNAMRGWNSHVY	SNAMRGWNS	3	0.6569	41.0	14.00	0.80	tr_C9ZN43_
DRB1_0101	142	EALYYGANVLTGNGR	LYYGANVLT	2	0.6557	41.5	14.00	0.56	tr_C9ZN43_
DRB1_0101	143	RNVIQLSPTLEQQLF	IQLSPTLEQ	3	0.6545	42.0	14.00	0.32	tr_C9ZN43_
DRB1_0101	144	VTPLIRYADPNAIKL	IRYADPNAI	4	0.6544	42.0	14.00	0.75	tr_C9ZN43_
DRB1_0101	145	RPQGVIAFAPPAKDL	IAFAPPAKD	5	0.6544	42.1	14.00	0.53	tr_C9ZN43_
DRB1_0101	146	RGRNVIQLSPTLEQQ	NVIQLSPTL	3	0.6518	43.3	15.00	0.44	tr_C9ZN43_
DRB1_0101	147	ADGVEFFLGQLMLTR	FFLGQLMLT	5	0.6518	43.3	15.00	0.52	tr_C9ZN43_
DRB1_0101	148	DHMRSYLSANPGVTV	YLSANPGVT	5	0.6514	43.5	15.00	0.52	tr_C9ZN43_
DRB1_0101	149	ERVQFATSLPHWADA	VQFATSLPH	2	0.6514	43.5	15.00	0.43	tr_C9ZN43_
DRB1_0101	150	VREFHTALPDSSAWK	FHTALPDSS	3	0.6509	43.7	15.00	0.84	tr_C9ZN43_
DRB1_0101	151	VIAFAPPAKDLIKFV	IAFAPPAKD	1	0.6503	44.0	15.00	0.64	tr_C9ZN43_
DRB1_0101	152	LIRYADPNAIKLSSA	IRYADPNAI	1	0.6502	44.0	15.00	0.59	tr_C9ZN43_
DRB1_0101	153	AERVQFATSLPHWAD	VQFATSLPH	3	0.6493	44.5	15.00	0.47	tr_C9ZN43_
DRB1_0101	154	VGYVTNTSASAPMSA	YVTNTSASA	2	0.6480	45.1	15.00	0.80	tr_C9ZN43_
DRB1_0101	155	ERFHTVQGGTTVFEP	FHTVQGGTT	2	0.6472	45.5	15.00	0.66	tr_C9ZN43_
DRB1_0101	156	NAGLNASLASRKWTV	LNASLASRK	3	0.6472	45.5	15.00	0.47	tr_C9ZN43_
DRB1_0101	157	TGSNAMRGWNSHVYF	SNAMRGWNS	2	0.6444	46.9	16.00	0.76	tr_C9ZN43_
DRB1_0101	158	DERFHTVQGGTTVFE	FHTVQGGTT	3	0.6438	47.2	16.00	0.66	tr_C9ZN43_
DRB1_0101	159	LLYNPLIPDNLCDAI	YNPLIPDNL	2	0.6427	47.7	16.00	0.83	tr_C9ZN43_
DRB1_0101	160	GYAAARFAQAVLPRM	YAAARFAQA	1	0.6421	48.1	16.00	0.44	tr_C9ZN43_
DRB1_0101	161	EVALRKIVWMHGGTL	IVWMHGGTL	6	0.6417	48.3	16.00	0.83	tr_C9ZN43_
DRB1_0101	162	DVALMYDELVSAFNG	VALMYDELV	1	0.6412	48.5	16.00	0.37	tr_C9ZN43_
DRB1_0101	163	ERHKGEFFVVLGPVG	FFVVLGPVG	6	0.6407	48.8	16.00	0.68	tr_C9ZN43_
DRB1_0101	164	IGLAPGDPSLLAAHL	LAPGDPSLL	2	0.6402	49.1	16.00	0.72	tr_C9ZN43_
DRB1_0101	165	FMYLRGISFGDTEYK	YLRGISFGD	2	0.6393	49.6	17.00	0.41	tr_C9ZN43_
DRB1_0101	166	ENLVGFAPSTGSNAM	FAPSTGSNA	5	0.6372	50.6	17.00	0.57	tr_C9ZN43_
DRB1_0101	167	RVQFATSLPHWADAN	FATSLPHWA	3	0.6372	50.7	17.00	0.49	tr_C9ZN43_
DRB1_0101	168	FFDVALMYDELVSAF	VALMYDELV	3	0.6366	51.0	17.00	0.61	tr_C9ZN43_
DRB1_0101	169	VGFAPSTGSNAMRGW	FAPSTGSNA	2	0.6365	51.1	17.00	0.86	tr_C9ZN43_
DRB1_0101	170	NRGNAFVIGLAPGDP	FVIGLAPGD	5	0.6362	51.2	17.00	0.71	tr_C9ZN43_
DRB1_0101	171	FDVALMYDELVSAFN	VALMYDELV	2	0.6344	52.2	17.00	0.49	tr_C9ZN43_
DRB1_0101	172	MSAIVRGADATIIEV	VRGADATII	4	0.6344	52.2	17.00	0.49	tr_C9ZN43_
DRB1_0101	173	SYLSANPGVTVFNGT	YLSANPGVT	1	0.6334	52.8	17.00	0.55	tr_C9ZN43_
DRB1_0101	174	YAVSQLRLLRIGFMY	YAVSQLRLL	0	0.6321	53.6	18.00	0.37	tr_C9ZN43_
DRB1_0101	175	VSSEALYYGANVLTG	LYYGANVLT	5	0.6319	53.7	18.00	0.57	tr_C9ZN43_
DRB1_0101	176	NAERVQFATSLPHWA	VQFATSLPH	4	0.6313	54.0	18.00	0.54	tr_C9ZN43_
DRB1_0101	177	SRTHDAYLLSSSSLE	YLLSSSSLE	6	0.6308	54.3	18.00	0.40	tr_C9ZN43_
DRB1_0101	178	PVGDGQALASLPLLE	GQALASLPL	4	0.6304	54.5	18.00	0.67	tr_C9ZN43_
DRB1_0101	179	EQFAETRPQGVIAFA	FAETRPQGV	2	0.6302	54.7	18.00	0.80	tr_C9ZN43_
DRB1_0101	180	VVVGYVTNTSASAPM	YVTNTSASA	4	0.6299	54.8	18.00	0.80	tr_C9ZN43_
DRB1_0101	181	SAIVRGADATIIEVA	VRGADATII	3	0.6289	55.4	18.00	0.58	tr_C9ZN43_
DRB1_0101	182	RFHTVQGGTTVFEPS	FHTVQGGTT	1	0.6283	55.8	18.00	0.65	tr_C9ZN43_
DRB1_0101	183	REFHTALPDSSAWKP	FHTALPDSS	2	0.6276	56.2	18.00	0.83	tr_C9ZN43_
DRB1_0101	184	GSNAMRGWNSHVYFL	SNAMRGWNS	1	0.6272	56.5	18.00	0.68	tr_C9ZN43_
DRB1_0101	185	NDERFHTVQGGTTVF	FHTVQGGTT	4	0.6258	57.3	18.00	0.69	tr_C9ZN43_

DRB1_0101	186	WEQFAETRPQGVIAF	FAETRPQGV	3	0.6251	57.7	19.00	0.85	tr_C9ZN43_
DRB1_0101	187	VALMYDELVSAFNGN	YDELVSAFN	4	0.6240	58.4	19.00	0.37	tr_C9ZN43_
DRB1_0101	188	ELLTLVRYAVSQLRL	VRYAVSQLR	5	0.6223	59.6	19.00	0.49	tr_C9ZN43_
DRB1_0101	189	YKIKPVEVLQVALER	IKPVEVLQV	2	0.6217	60.0	19.00	0.56	tr_C9ZN43_
DRB1_0101	190	IVREFHTALPDSSAW	FHTALPDSS	4	0.6196	61.3	19.00	0.77	tr_C9ZN43_
DRB1_0101	191	LARALNASVPPLTSP	LNASVPPLT	4	0.6185	62.0	20.00	0.55	tr_C9ZN43_
DRB1_0101	192	IRRFQDHMRSYLSAN	QDHMRSYLS	4	0.6166	63.3	20.00	0.53	tr_C9ZN43_
DRB1_0101	193	PFFDVALMYDELVSA	VALMYDELV	4	0.6155	64.1	20.00	0.62	tr_C9ZN43_
DRB1_0101	194	PPTSYKIKPVEVLQV	SYKIKPVEV	3	0.6143	64.9	20.00	0.40	tr_C9ZN43_
DRB1_0101	195	RKAFMESLYNQRRYV	MESLYNQRR	4	0.6142	65.0	20.00	0.32	tr_C9ZN43_
DRB1_0101	196	CLAESVRLYSPLNTL	SVRLYSPLN	4	0.6129	65.9	20.00	0.50	tr_C9ZN43_
DRB1_0101	197	FEPSRCLAESVRLYS	CLAESVRLY	5	0.6125	66.2	20.00	0.49	tr_C9ZN43_
DRB1_0101	198	GELMVYGWIAGEVLS	YGWIAGEVL	5	0.6121	66.5	21.00	0.40	tr_C9ZN43_
DRB1_0101	199	GYVTNTSASAPMSAI	YVTNTSASA	1	0.6120	66.5	21.00	0.68	tr_C9ZN43_
DRB1_0101	200	AGLNASLASRKWTVA	LNASLASRK	2	0.6086	69.1	21.00	0.51	tr_C9ZN43_
DRB1_0101	201	KMTTSLMSSVGRELC	LMSSVGREL	5	0.6083	69.3	21.00	0.52	tr_C9ZN43_
DRB1_0101	202	YAAARFAQAVLPRME	ARFAQAVLP	3	0.6082	69.4	21.00	0.39	tr_C9ZN43_
DRB1_0101	203	VYGWIAGEVLSQALS	YGWIAGEVL	1	0.6066	70.5	21.00	0.41	tr_C9ZN43_
DRB1_0101	204	PSTGSNAMRGWNSHV	SNAMRGWNS	4	0.6063	70.8	21.00	0.79	tr_C9ZN43_
DRB1_0101	205	ALYYGANVLTGNGRF	LYYGANVLT	1	0.6063	70.8	21.00	0.55	tr_C9ZN43_
DRB1_0101	206	SRKAFMESLYNQRRY	KAFMESLYN	2	0.6061	70.9	21.00	0.37	tr_C9ZN43_
DRB1_0101	207	ISMIPSPSNATYLAL	ISMIPSPSN	0	0.6058	71.2	22.00	0.47	tr_C9ZN43_
DRB1_0101	208	RCLAESVRLYSPLNT	SVRLYSPLN	5	0.6057	71.3	22.00	0.43	tr_C9ZN43_
DRB1_0101	209	PMSAIVRGADATIIE	VRGADATII	5	0.6051	71.7	22.00	0.44	tr_C9ZN43_
DRB1_0101	210	RELAQVSSEALYYGA	LAQVSSEAL	2	0.6042	72.4	22.00	0.61	tr_C9ZN43_
DRB1_0101	211	ELMVYGWIAGEVLSQ	YGWIAGEVL	4	0.6035	73.0	22.00	0.52	tr_C9ZN43_
DRB1_0101	212	MVYGWIAGEVLSQAL	YGWIAGEVL	2	0.6030	73.4	22.00	0.51	tr_C9ZN43_
DRB1_0101	213	KAFMESLYNQRRYVI	MESLYNQRR	3	0.6024	73.9	22.00	0.35	tr_C9ZN43_
DRB1_0101	214	HRGRNVIQLSPTLEQ	NVIQLSPTL	4	0.6023	73.9	22.00	0.50	tr_C9ZN43_
DRB1_0101	215	DNLCDAINAGLNASL	DAINAGLNA	4	0.6013	74.7	22.00	0.49	tr_C9ZN43_
DRB1_0101	216	SRCLAESVRLYSPLN	CLAESVRLY	2	0.6011	74.9	22.00	0.49	tr_C9ZN43_
DRB1_0101	217	QFAETRPQGVIAFAP	FAETRPQGV	1	0.6009	75.1	22.00	0.77	tr_C9ZN43_
DRB1_0101	218	RRFQDHMRSYLSANP	QDHMRSYLS	3	0.6005	75.4	22.00	0.53	tr_C9ZN43_
DRB1_0101	219	DSSAWKPLPLLGYAA	PLPLLGYAA	6	0.5994	76.3	23.00	0.48	tr_C9ZN43_
DRB1_0101	220	TTSLMSSVGRELCGV	LMSSVGREL	3	0.5984	77.1	23.00	0.81	tr_C9ZN43_
DRB1_0101	221	MTTSLMSSVGRELCG	LMSSVGREL	4	0.5964	78.8	23.00	0.67	tr_C9ZN43_
DRB1_0101	222	LMVYGWIAGEVLSQA	YGWIAGEVL	3	0.5960	79.2	23.00	0.60	tr_C9ZN43_
DRB1_0101	223	GPVGDGQALASLPLL	GQALASLPL	5	0.5952	79.8	23.00	0.65	tr_C9ZN43_
DRB1_0101	224	DGELMVYGWIAGEVL	ELMVYGWIA	2	0.5946	80.4	23.00	0.44	tr_C9ZN43_
DRB1_0101	225	YGWIAGEVLSQALSS	YGWIAGEVL	0	0.5941	80.8	23.00	0.28	tr_C9ZN43_
DRB1_0101	226	GLAPGDPSLLAAHLD	LAPGDPSLL	1	0.5923	82.3	24.00	0.72	tr_C9ZN43_
DRB1_0101	227	EPSRCLAESVRLYSP	CLAESVRLY	4	0.5916	83.0	24.00	0.51	tr_C9ZN43_
DRB1_0101	228	PSRCLAESVRLYSPL	CLAESVRLY	3	0.5911	83.4	24.00	0.62	tr_C9ZN43_
DRB1_0101	229	DRELAQVSSEALYYG	LAQVSSEAL	3	0.5905	84.0	24.00	0.62	tr_C9ZN43_
DRB1_0101	230	TAVFGVVDDAMLSIA	FGVVDDAML	3	0.5895	84.9	24.00	0.57	tr_C9ZN43_
DRB1_0101	231	LYNPLIPDNLCDAIN	YNPLIPDNL	1	0.5879	86.4	24.00	0.84	tr_C9ZN43_
DRB1_0101	232	FVVVGYVTNTSASAP	YVTNTSASA	5	0.5869	87.4	25.00	0.63	tr_C9ZN43_
DRB1_0101	233	ARALNASVPPLTSPV	LNASVPPLT	3	0.5868	87.4	25.00	0.75	tr_C9ZN43_
DRB1_0101	234	DVKVELIPPTSYKIK	VELIPPTSY	3	0.5864	87.8	25.00	0.36	tr_C9ZN43_
DRB1_0101	235	AWEQFAETRPQGVIA	FAETRPQGV	4	0.5859	88.3	25.00	0.87	tr_C9ZN43_

DRB1_0101	236	LMLTRTGPLARDTNY	LMLTRTGPL	0	0.5849	89.2	25.00	0.44	tr_C9ZN43_
DRB1_0101	237	VKVELIPPTSYKIKP	VELIPPTSY	2	0.5848	89.3	25.00	0.37	tr_C9ZN43_
DRB1_0101	238	EIVREFHTALPDSSA	FHTALPDSS	5	0.5844	89.7	25.00	0.68	tr_C9ZN43_
DRB1_0101	239	TSRKAFMESLYNQRR	KAFMESLYN	3	0.5840	90.1	25.00	0.42	tr_C9ZN43_
DRB1_0101	240	PRMEYVTPKTLLDTI	MEYVTPKTL	2	0.5833	90.7	25.00	0.50	tr_C9ZN43_
DRB1_0101	241	NVIQLSPTLEQQLFV	IQLSPTLEQ	2	0.5829	91.2	25.00	0.36	tr_C9ZN43_
DRB1_0101	242	QDHMRSYLSANPGVT	YLSANPGVT	6	0.5790	95.1	26.00	0.38	tr_C9ZN43_
DRB1_0101	243	AARFAQAVLPRMEYV	FAQAVLPRM	3	0.5789	95.2	26.00	0.45	tr_C9ZN43_
DRB1_0101	244	AAARFAQAVLPRMEY	FAQAVLPRM	4	0.5787	95.4	26.00	0.40	tr_C9ZN43_
DRB1_0101	245	AVFGVVDDAMLSIAE	FGVVDDAML	2	0.5779	96.3	26.00	0.53	tr_C9ZN43_
DRB1_0101	246	AIRRFQDHMRSYLSA	QDHMRSYLS	5	0.5773	96.9	26.00	0.49	tr_C9ZN43_
DRB1_0101	247	GFAPSTGSNAMRGWN	FAPSTGSNA	1	0.5771	97.1	26.00	0.85	tr_C9ZN43_
DRB1_0101	248	LPRMEYVTPKTLLDT	MEYVTPKTL	3	0.5767	97.5	26.00	0.56	tr_C9ZN43_
DRB1_0101	249	LDRELAQVSSEALYY	LAQVSSEAL	4	0.5752	99.2	27.00	0.57	tr_C9ZN43_
DRB1_0101	250	LIKFVTRLLSDSRTH	VTRLLSDSR	4	0.5751	99.2	27.00	0.29	tr_C9ZN43_
DRB1_0101	251	RMEYVTPKTLLDTIY	YVTPKTLLD	3	0.5747	99.6	27.00	0.46	tr_C9ZN43_
DRB1_0101	252	RENLVGFAPSTGSNA	LVGFAPSTG	3	0.5743	100.1	27.00	0.37	tr_C9ZN43_
DRB1_0101	253	RALNASVPPLTSPVT	LNASVPPLT	2	0.5740	100.5	27.00	0.68	tr_C9ZN43_
DRB1_0101	254	GATRISMWSLARALN	SMWSLARAL	5	0.5723	102.3	27.00	0.43	tr_C9ZN43_
DRB1_0101	255	IPFFDVALMYDELVS	VALMYDELV	5	0.5722	102.4	27.00	0.62	tr_C9ZN43_
DRB1_0101	256	PVTPLIRYADPNAIK	IRYADPNAI	5	0.5722	102.4	27.00	0.79	tr_C9ZN43_
DRB1_0101	257	VFEPSRCLAESVRLY	CLAESVRLY	6	0.5717	102.9	27.00	0.35	tr_C9ZN43_
DRB1_0101	258	SNAMRGWNSHVYFLT	SNAMRGWNS	0	0.5714	103.2	27.00	0.49	tr_C9ZN43_
DRB1_0101	259	KIKPVEVLQVALERH	IKPVEVLQV	1	0.5714	103.3	27.00	0.51	tr_C9ZN43_
DRB1_0101	260	AIVRGADATIIEVAL	VRGADATII	2	0.5712	103.5	27.00	0.67	tr_C9ZN43_
DRB1_0101	261	RFQDHMRSYLSANPG	QDHMRSYLS	2	0.5697	105.2	28.00	0.48	tr_C9ZN43_
DRB1_0101	262	PNRGNAFVIGLAPGD	FVIGLAPGD	6	0.5696	105.2	28.00	0.61	tr_C9ZN43_
DRB1_0101	263	KDLIKFVTRLLSDSR	LIKFVTRLL	2	0.5690	106.0	28.00	0.26	tr_C9ZN43_
DRB1_0101	264	YLLSSSSLEFALDTW	YLLSSSSLE	0	0.5686	106.4	28.00	0.59	tr_C9ZN43_
DRB1_0101	265	TRPQGVIAFAPPAKD	IAFAPPAKD	6	0.5684	106.6	28.00	0.37	tr_C9ZN43_
DRB1_0101	266	EFHTALPDSSAWKPL	FHTALPDSS	1	0.5681	107.0	28.00	0.75	tr_C9ZN43_
DRB1_0101	267	QALASLPLLERENLV	LASLPLLER	2	0.5677	107.4	28.00	0.42	tr_C9ZN43_
DRB1_0101	268	LYSPLNTLMFLILDR	YSPLNTLMF	1	0.5672	108.1	28.00	0.67	tr_C9ZN43_
DRB1_0101	269	QVSSEALYYGANVLT	LYYGANVLT	6	0.5667	108.6	28.00	0.46	tr_C9ZN43_
DRB1_0101	270	ELAQVSSEALYYGAN	LAQVSSEAL	1	0.5648	110.9	29.00	0.54	tr_C9ZN43_
DRB1_0101	271	KVELIPPTSYKIKPV	LIPPTSYKI	3	0.5645	111.3	29.00	0.49	tr_C9ZN43_
DRB1_0101	272	DLIKFVTRLLSDSRT	FVTRLLSDS	4	0.5645	111.3	29.00	0.27	tr_C9ZN43_
DRB1_0101	273	VFGVVDDAMLSIAEV	FGVVDDAML	1	0.5643	111.5	29.00	0.47	tr_C9ZN43_
DRB1_0101	274	APMSAIVRGADATII	SAIVRGADA	3	0.5641	111.7	29.00	0.32	tr_C9ZN43_
DRB1_0101	275	GLNASLASRKWTVAP	LNASLASRK	1	0.5614	115.0	29.00	0.41	tr_C9ZN43_
DRB1_0101	276	AELLTLVRYAVSQLR	LTLVRYAVS	3	0.5609	115.7	29.00	0.31	tr_C9ZN43_
DRB1_0101	277	ALMYDELVSAFNGNP	YDELVSAFN	3	0.5599	116.9	29.00	0.47	tr_C9ZN43_
DRB1_0101	278	LLDTIYMQSIITADE	TIYMQSIIT	3	0.5589	118.2	30.00	0.38	tr_C9ZN43_
DRB1_0101	279	ILDRELAQVSSEALY	LAQVSSEAL	5	0.5577	119.8	30.00	0.44	tr_C9ZN43_
DRB1_0101	280	DSRTHDAYLLSSSSL	DAYLLSSSS	5	0.5572	120.4	30.00	0.34	tr_C9ZN43_
DRB1_0101	281	LDTIYMQSIITADEM	IYMQSIITA	3	0.5567	121.0	30.00	0.38	tr_C9ZN43_
DRB1_0101	282	GNDERFHTVQGGTTV	FHTVQGGTT	5	0.5563	121.6	30.00	0.70	tr_C9ZN43_
DRB1_0101	283	FLTASPNAELLTLVR	LTASPNAEL	1	0.5551	123.2	30.00	0.35	tr_C9ZN43_
DRB1_0101	284	GEVLSQALSSREWLT	LSQALSSRE	3	0.5549	123.4	30.00	0.27	tr_C9ZN43_
DRB1_0101	285	TSLMSSVGRELCGVF	LMSSVGREL	2	0.5548	123.5	30.00	0.84	tr_C9ZN43_

DRB1_0101	286	AGEVLSQALSSREWL	EVLSQALSS	2	0.5542	124.4	30.00	0.31	tr_C9ZN43_
DRB1_0101	287	SREWLTSRKAFMESL	WLTSRKAFM	3	0.5528	126.2	31.00	0.60	tr_C9ZN43_
DRB1_0101	288	VAYYDPAYVPPALSE	YDPAYVPPA	3	0.5528	126.3	31.00	0.44	tr_C9ZN43_
DRB1_0101	289	VTAVFGVVDDAMLSI	FGVVDDAML	4	0.5524	126.8	31.00	0.58	tr_C9ZN43_
DRB1_0101	290	IRYADPNAIKLSSAQ	YADPNAIKL	2	0.5522	127.2	31.00	0.44	tr_C9ZN43_
DRB1_0101	291	TLLDTIYMQSIITAD	TIYMQSIIT	4	0.5520	127.4	31.00	0.37	tr_C9ZN43_
DRB1_0101	292	VSQLRLLRIGFMYLR	LRLLRIGFM	3	0.5499	130.4	31.00	0.47	tr_C9ZN43_
DRB1_0101	293	IPPTSYKIKPVEVLQ	SYKIKPVEV	4	0.5497	130.6	31.00	0.46	tr_C9ZN43_
DRB1_0101	294	AFMESLYNQRRYVID	MESLYNQRR	2	0.5496	130.7	31.00	0.38	tr_C9ZN43_
DRB1_0101	295	EADGVEFFLGQLMLT	FFLGQLMLT	6	0.5494	131.0	31.00	0.59	tr_C9ZN43_
DRB1_0101	296	DVAYYDPAYVPPALS	YDPAYVPPA	4	0.5488	131.8	31.00	0.47	tr_C9ZN43_
DRB1_0101	297	DTIYMQSIITADEMR	IYMQSIITA	2	0.5482	132.8	32.00	0.38	tr_C9ZN43_
DRB1_0101	298	VDDAMLSIAEVAFVD	LSIAEVAFV	5	0.5468	134.8	32.00	0.39	tr_C9ZN43_
DRB1_0101	299	QLRLLRIGFMYLRGI	LRLLRIGFM	1	0.5463	135.5	32.00	0.35	tr_C9ZN43_
DRB1_0101	300	NATLVGRLPNRGNAF	LVGRLPNRG	3	0.5453	137.0	32.00	0.67	tr_C9ZN43_
DRB1_0101	301	IAFAPPAKDLIKFVT	IAFAPPAKD	0	0.5452	137.0	32.00	0.54	tr_C9ZN43_
DRB1_0101	302	LRLLRIGFMYLRGIS	IGFMYLRGI	5	0.5450	137.5	32.00	0.25	tr_C9ZN43_
DRB1_0101	303	VQFATSLPHWADANT	FATSLPHWA	2	0.5448	137.7	32.00	0.56	tr_C9ZN43_
DRB1_0101	304	LFVVVGYVTNTSASA	YVTNTSASA	6	0.5442	138.5	32.00	0.36	tr_C9ZN43_
DRB1_0101	305	IKFVTRLLSDSRTHD	VTRLLSDSR	3	0.5441	138.8	32.00	0.34	tr_C9ZN43_
DRB1_0101	306	AKDLIKFVTRLLSDS	LIKFVTRLL	3	0.5433	139.9	32.00	0.40	tr_C9ZN43_
DRB1_0101	307	PDNLCDAINAGLNAS	DAINAGLNA	5	0.5431	140.2	33.00	0.42	tr_C9ZN43_
DRB1_0101	308	AVSQLRLLRIGFMYL	LRLLRIGFM	4	0.5431	140.2	33.00	0.43	tr_C9ZN43_
DRB1_0101	309	KTLLDTIYMQSIITA	TIYMQSIIT	5	0.5429	140.6	33.00	0.37	tr_C9ZN43_
DRB1_0101	310	YGATRISMWSLARAL	SMWSLARAL	6	0.5425	141.2	33.00	0.29	tr_C9ZN43_
DRB1_0101	311	EVCVVNYGATRISMW	VVNYGATRI	3	0.5413	143.1	33.00	0.47	tr_C9ZN43_
DRB1_0101	312	GWIAGEVLSQALSSR	EVLSQALSS	5	0.5399	145.3	33.00	0.25	tr_C9ZN43_
DRB1_0101	313	SSREWLTSRKAFMES	WLTSRKAFM	4	0.5385	147.4	33.00	0.58	tr_C9ZN43_
DRB1_0101	314	VCVVNYGATRISMWS	VVNYGATRI	2	0.5384	147.5	33.00	0.43	tr_C9ZN43_
DRB1_0101	315	PNAERVQFATSLPHW	VQFATSLPH	5	0.5380	148.3	33.00	0.57	tr_C9ZN43_
DRB1_0101	316	DDAMLSIAEVAFVDP	LSIAEVAFV	4	0.5378	148.6	34.00	0.41	tr_C9ZN43_
DRB1_0101	317	FQDHMRSYLSANPGV	QDHMRSYLS	1	0.5373	149.3	34.00	0.38	tr_C9ZN43_
DRB1_0101	318	SLMSSVGRELCGVFT	LMSSVGREL	1	0.5364	150.8	34.00	0.57	tr_C9ZN43_
DRB1_0101	319	FGVVDDAMLSIAEVA	GVVDDAMLS	1	0.5354	152.5	34.00	0.31	tr_C9ZN43_
DRB1_0101	320	RGWNSHVYFLTASPN	VYFLTASPN	6	0.5342	154.5	34.00	0.54	tr_C9ZN43_
DRB1_0101	321	ARFAQAVLPRMEYVT	FAQAVLPRM	2	0.5341	154.6	34.00	0.47	tr_C9ZN43_
DRB1_0101	322	TPRLHHRGRNVIQLS	RLHHRGRNV	2	0.5336	155.4	34.00	0.41	tr_C9ZN43_
DRB1_0101	323	VWMHGGTLQTVAVLD	WMHGGTLQT	1	0.5336	155.5	34.00	0.46	tr_C9ZN43_
DRB1_0101	324	LMYDELVSAFNGNPN	YDELVSAFN	2	0.5335	155.6	34.00	0.41	tr_C9ZN43_
DRB1_0101	325	APSTGSNAMRGWNSH	SNAMRGWNS	5	0.5334	155.8	34.00	0.80	tr_C9ZN43_
DRB1_0101	326	IAGEVLSQALSSREW	EVLSQALSS	3	0.5328	156.7	34.00	0.38	tr_C9ZN43_
DRB1_0101	327	REWLTSRKAFMESLY	WLTSRKAFM	2	0.5322	157.8	35.00	0.52	tr_C9ZN43_
DRB1_0101	328	EVAFVDPVMLTPRLH	VAFVDPVML	1	0.5320	158.2	35.00	0.50	tr_C9ZN43_
DRB1_0101	329	LAPGDPSLLAAHLDR	LAPGDPSLL	0	0.5319	158.4	35.00	0.44	tr_C9ZN43_
DRB1_0101	330	LILDRELAQVSSEAL	LDRELAQVS	2	0.5311	159.8	35.00	0.56	tr_C9ZN43_
DRB1_0101	331	GTTVFEPSRCLAESV	TVFEPSRCL	2	0.5310	159.8	35.00	0.72	tr_C9ZN43_
DRB1_0101	332	LTPRLHHRGRNVIQL	RLHHRGRNV	3	0.5308	160.2	35.00	0.47	tr_C9ZN43_
DRB1_0101	333	PDVKVELIPPTSYKI	VELIPPTSY	4	0.5299	161.7	35.00	0.42	tr_C9ZN43_
DRB1_0101	334	DAMLSIAEVAFVDPV	LSIAEVAFV	3	0.5293	162.8	35.00	0.51	tr_C9ZN43_
DRB1_0101	335	IKPVEVLQVALERHK	VEVLQVALE	3	0.5292	163.0	35.00	0.43	tr_C9ZN43_

DRB1_0101	336	HHRGRNVIQLSPTLE	NVIQLSPTL	5	0.5291	163.2	35.00	0.59	tr_C9ZN43_
DRB1_0101	337	VDGELMVYGWIAGEV	ELMVYGWIA	3	0.5290	163.4	35.00	0.64	tr_C9ZN43_
DRB1_0101	338	LAQVSSEALYYGANV	QVSSEALYY	2	0.5287	164.0	35.00	0.41	tr_C9ZN43_
DRB1_0101	339	FAPSTGSNAMRGWNS	FAPSTGSNA	0	0.5284	164.4	35.00	0.47	tr_C9ZN43_
DRB1_0101	340	VLPRMEYVTPKTLLD	MEYVTPKTL	4	0.5277	165.8	36.00	0.62	tr_C9ZN43_
DRB1_0101	341	TIYMQSIITADEMRY	IYMQSIITA	1	0.5272	166.5	36.00	0.28	tr_C9ZN43_
DRB1_0101	342	SLEFALDTWREALEA	FALDTWREA	3	0.5271	166.8	36.00	0.66	tr_C9ZN43_
DRB1_0101	343	SQLRLLRIGFMYLRG	LRLLRIGFM	2	0.5269	167.1	36.00	0.50	tr_C9ZN43_
DRB1_0101	344	GGTTVFEPSRCLAES	TVFEPSRCL	3	0.5265	167.9	36.00	0.80	tr_C9ZN43_
DRB1_0101	345	EVLSQALSSREWLTS	LSQALSSRE	2	0.5248	171.0	36.00	0.30	tr_C9ZN43_
DRB1_0101	346	VVDDAMLSIAEVAFV	LSIAEVAFV	6	0.5240	172.4	36.00	0.28	tr_C9ZN43_
DRB1_0101	347	ATLVGRLPNRGNAFV	LVGRLPNRG	2	0.5240	172.5	36.00	0.67	tr_C9ZN43_
DRB1_0101	348	DNATLVGRLPNRGNA	LVGRLPNRG	4	0.5223	175.6	37.00	0.62	tr_C9ZN43_
DRB1_0101	349	LSIAEVAFVDPVMLT	VAFVDPVML	5	0.5209	178.4	37.00	0.56	tr_C9ZN43_
DRB1_0101	350	FHTVQGGTTVFEPSR	FHTVQGGTT	0	0.5205	179.1	37.00	0.56	tr_C9ZN43_
DRB1_0101	351	LMSSVGRELCGVFTV	GRELCGVFT	5	0.5205	179.1	37.00	0.50	tr_C9ZN43_
DRB1_0101	352	VAFVDPVMLTPRLHH	PVMLTPRLH	5	0.5194	181.3	37.00	0.28	tr_C9ZN43_
DRB1_0101	353	MLSIAEVAFVDPVML	VAFVDPVML	6	0.5188	182.4	37.00	0.35	tr_C9ZN43_
DRB1_0101	354	LIPFFDVALMYDELV	VALMYDELV	6	0.5185	183.1	37.00	0.49	tr_C9ZN43_
DRB1_0101	355	LTSRKAFMESLYNQR	KAFMESLYN	4	0.5185	183.1	37.00	0.47	tr_C9ZN43_
DRB1_0101	356	TTVFEPSRCLAESVR	TVFEPSRCL	1	0.5184	183.2	37.00	0.60	tr_C9ZN43_
DRB1_0101	357	ALASLPLLERENLVG	PLLERENLV	5	0.5183	183.4	37.00	0.48	tr_C9ZN43_
DRB1_0101	358	TAWEQFAETRPQGVI	FAETRPQGV	5	0.5178	184.5	37.00	0.87	tr_C9ZN43_
DRB1_0101	359	WIAGEVLSQALSSRE	EVLSQALSS	4	0.5177	184.7	37.00	0.34	tr_C9ZN43_
DRB1_0101	360	PAKDLIKFVTRLLSD	LIKFVTRLL	4	0.5159	188.2	38.00	0.40	tr_C9ZN43_
DRB1_0101	361	DVDGELMVYGWIAGE	ELMVYGWIA	4	0.5156	188.8	38.00	0.64	tr_C9ZN43_
DRB1_0101	362	LSSREWLTSRKAFME	WLTSRKAFM	5	0.5156	188.9	38.00	0.55	tr_C9ZN43_
DRB1_0101	363	ALNASVPPLTSPVTP	LNASVPPLT	1	0.5152	189.7	38.00	0.56	tr_C9ZN43_
DRB1_0101	364	FVVLGPVGDGQALAS	FVVLGPVGD	0	0.5149	190.4	38.00	0.64	tr_C9ZN43_
DRB1_0101	365	AYYDPAYVPPALSED	YDPAYVPPA	2	0.5144	191.3	38.00	0.46	tr_C9ZN43_
DRB1_0101	366	AEVAFVDPVMLTPRL	VAFVDPVML	2	0.5143	191.5	38.00	0.68	tr_C9ZN43_
DRB1_0101	367	YLSANPGVTVFNGTD	LSANPGVTV	1	0.5143	191.6	38.00	0.48	tr_C9ZN43_
DRB1_0101	368	SSSLEFALDTWREAL	FALDTWREA	5	0.5142	191.7	38.00	0.50	tr_C9ZN43_
DRB1_0101	369	SSLEFALDTWREALE	FALDTWREA	4	0.5142	191.8	38.00	0.58	tr_C9ZN43_
DRB1_0101	370	GEVCVVNYGATRISM	VVNYGATRI	4	0.5142	191.8	38.00	0.49	tr_C9ZN43_
DRB1_0101	371	YVTNTSASAPMSAIV	YVTNTSASA	0	0.5142	191.8	38.00	0.45	tr_C9ZN43_
DRB1_0101	372	KFVTRLLSDSRTHDA	VTRLLSDSR	2	0.5122	195.9	39.00	0.34	tr_C9ZN43_
DRB1_0101	373	IAEVAFVDPVMLTPR	VAFVDPVML	3	0.5121	196.1	39.00	0.74	tr_C9ZN43_
DRB1_0101	374	LIPPTSYKIKPVEVL	SYKIKPVEV	5	0.5117	196.9	39.00	0.46	tr_C9ZN43_
DRB1_0101	375	QRRYVIDDLVIGDFG	YVIDDLVIG	3	0.5106	199.4	39.00	0.80	tr_C9ZN43_
DRB1_0101	376	YDVAYYDPAYVPPAL	YDPAYVPPA	5	0.5076	206.0	39.00	0.44	tr_C9ZN43_
DRB1_0101	377	CVVNYGATRISMWSL	VVNYGATRI	1	0.5068	207.9	40.00	0.38	tr_C9ZN43_
DRB1_0101	378	DVMTVRVYSLLYNPL	VYSLLYNPL	6	0.5066	208.3	40.00	0.49	tr_C9ZN43_
DRB1_0101	379	SEIVREFHTALPDSS	FHTALPDSS	6	0.5065	208.3	40.00	0.39	tr_C9ZN43_
DRB1_0101	380	MYDELVSAFNGNPNA	YDELVSAFN	1	0.5065	208.5	40.00	0.38	tr_C9ZN43_
DRB1_0101	381	MEYVTPKTLLDTIYM	YVTPKTLLD	2	0.5057	210.2	40.00	0.44	tr_C9ZN43_
DRB1_0101	382	VIQLSPTLEQQLFVV	IQLSPTLEQ	1	0.5047	212.5	40.00	0.38	tr_C9ZN43_
DRB1_0101	383	PPAKDLIKFVTRLLS	LIKFVTRLL	5	0.5034	215.5	40.00	0.38	tr_C9ZN43_
DRB1_0101	384	DDVDGELMVYGWIAG	ELMVYGWIA	5	0.5027	217.2	40.00	0.63	tr_C9ZN43_
DRB1_0101	385	LASLPLLERENLVGF	PLLERENLV	4	0.5023	218.0	41.00	0.62	tr_C9ZN43_

DRB1_0101	386	FVDPVMLTPRLHHRG	PVMLTPRLH	3	0.5017	219.4	41.00	0.44	tr_C9ZN43_
DRB1_0101	387	CNCNQGGNVLYINVV	CNQGGNVLY	2	0.5016	219.7	41.00	0.35	tr_C9ZN43_
DRB1_0101	388	QGGTTVFEPSRCLAE	TVFEPSRCL	4	0.5013	220.4	41.00	0.81	tr_C9ZN43_
DRB1_0101	389	GNVLYINVVGNDERF	YINVVGNDE	4	0.4999	223.9	41.00	0.44	tr_C9ZN43_
DRB1_0101	390	LYYGANVLTGNGRFG	YYGANVLTG	1	0.4999	223.9	41.00	0.38	tr_C9ZN43_
DRB1_0101	391	YYDPAYVPPALSEDV	PAYVPPALS	3	0.4998	224.0	41.00	0.45	tr_C9ZN43_
DRB1_0101	392	NQRRYVIDDLVIGDF	YVIDDLVIG	4	0.4990	226.1	41.00	0.79	tr_C9ZN43_
DRB1_0101	393	KPVEVLQVALERHKG	VEVLQVALE	2	0.4987	226.8	41.00	0.50	tr_C9ZN43_
DRB1_0101	394	SVTAVFGVVDDAMLS	FGVVDDAML	5	0.4981	228.3	41.00	0.64	tr_C9ZN43_
DRB1_0101	395	SDSRTHDAYLLSSSS	SRTHDAYLL	2	0.4979	228.8	41.00	0.63	tr_C9ZN43_
DRB1_0101	396	ASLPLLERENLVGFA	PLLERENLV	3	0.4976	229.6	42.00	0.75	tr_C9ZN43_
DRB1_0101	397	AFVDPVMLTPRLHHR	PVMLTPRLH	4	0.4973	230.2	42.00	0.41	tr_C9ZN43_
DRB1_0101	398	PRLHHRGRNVIQLSP	RLHHRGRNV	1	0.4969	231.3	42.00	0.35	tr_C9ZN43_
DRB1_0101	399	AQVSSEALYYGANVL	QVSSEALYY	1	0.4968	231.4	42.00	0.37	tr_C9ZN43_
DRB1_0101	400	AVLPRMEYVTPKTLL	MEYVTPKTL	5	0.4966	231.9	42.00	0.70	tr_C9ZN43_
DRB1_0101	401	GGNVLYINVVGNDER	YINVVGNDE	5	0.4960	233.4	42.00	0.34	tr_C9ZN43_
DRB1_0101	402	AMLSIAEVAFVDPVM	LSIAEVAFV	2	0.4936	239.5	42.00	0.62	tr_C9ZN43_
DRB1_0101	403	FGQSDRLFISMIPSP	SDRLFISMI	3	0.4934	240.1	42.00	0.41	tr_C9ZN43_
DRB1_0101	404	VDPVMLTPRLHHRGR	PVMLTPRLH	2	0.4934	240.1	42.00	0.39	tr_C9ZN43_
DRB1_0101	405	SSVGRELCGVFTVDG	GRELCGVFT	3	0.4933	240.4	42.00	0.68	tr_C9ZN43_
DRB1_0101	406	EWLTSRKAFMESLYN	WLTSRKAFM	1	0.4932	240.6	42.00	0.42	tr_C9ZN43_
DRB1_0101	407	SRKWTVAPDVKVELI	WTVAPDVKV	3	0.4921	243.4	43.00	0.74	tr_C9ZN43_
DRB1_0101	408	ALSSREWLTSRKAFM	WLTSRKAFM	6	0.4899	249.5	43.00	0.44	tr_C9ZN43_
DRB1_0101	409	LEFALDTWREALEAD	FALDTWREA	2	0.4898	249.6	43.00	0.66	tr_C9ZN43_
DRB1_0101	410	FMESLYNQRRYVIDD	MESLYNQRR	1	0.4896	250.1	43.00	0.37	tr_C9ZN43_
DRB1_0101	411	SIAEVAFVDPVMLTP	VAFVDPVML	4	0.4894	250.7	43.00	0.70	tr_C9ZN43_
DRB1_0101	412	RAIRRFQDHMRSYLS	QDHMRSYLS	6	0.4885	253.1	43.00	0.38	tr_C9ZN43_
DRB1_0101	413	NCNQGGNVLYINVVG	GNVLYINVV	5	0.4885	253.4	43.00	0.42	tr_C9ZN43_
DRB1_0101	414	IVRGADATIIEVALR	VRGADATII	1	0.4883	253.7	43.00	0.70	tr_C9ZN43_
DRB1_0101	415	IPDNLCDAINAGLNA	DNLCDAINA	2	0.4883	253.8	43.00	0.31	tr_C9ZN43_
DRB1_0101	416	MLTPRLHHRGRNVIQ	RLHHRGRNV	4	0.4877	255.4	44.00	0.47	tr_C9ZN43_
DRB1_0101	417	ACNCNQGGNVLYINV	CNQGGNVLY	3	0.4876	255.8	44.00	0.43	tr_C9ZN43_
DRB1_0101	418	TLVGRLPNRGNAFVI	LVGRLPNRG	1	0.4875	256.0	44.00	0.53	tr_C9ZN43_
DRB1_0101	419	RYADPNAIKLSSAQL	YADPNAIKL	1	0.4872	256.7	44.00	0.43	tr_C9ZN43_
DRB1_0101	420	NYGATRISMWSLARA	TRISMWSLA	4	0.4872	256.9	44.00	0.32	tr_C9ZN43_
DRB1_0101	421	DATIIEVALRKIVWM	TIIEVALRK	2	0.4871	257.1	44.00	0.29	tr_C9ZN43_
DRB1_0101	422	RRYVIDDLVIGDFGG	YVIDDLVIG	2	0.4865	258.7	44.00	0.74	tr_C9ZN43_
DRB1_0101	423	MSSVGRELCGVFTVD	GRELCGVFT	4	0.4860	260.2	44.00	0.67	tr_C9ZN43_
DRB1_0101	424	ISFGDTEYKMTTSLM	EYKMTTSLM	6	0.4860	260.3	44.00	0.57	tr_C9ZN43_
DRB1_0101	425	ATIIEVALRKIVWMH	IEVALRKIV	3	0.4854	261.7	44.00	0.29	tr_C9ZN43_
DRB1_0101	426	TGPLARDTNYRAIRR	LARDTNYRA	3	0.4844	264.8	44.00	0.75	tr_C9ZN43_
DRB1_0101	427	ERENLVGFAPSTGSN	LVGFAPSTG	4	0.4839	266.1	44.00	0.47	tr_C9ZN43_
DRB1_0101	428	VLSQALSSREWLTSR	ALSSREWLT	4	0.4834	267.6	44.00	0.29	tr_C9ZN43_
DRB1_0101	429	LSDSRTHDAYLLSSS	SRTHDAYLL	3	0.4832	268.1	44.00	0.75	tr_C9ZN43_
DRB1_0101	430	NVLYINVVGNDERFH	YINVVGNDE	3	0.4830	268.8	45.00	0.55	tr_C9ZN43_
DRB1_0101	431	TVFEPSRCLAESVRL	PSRCLAESV	4	0.4828	269.2	45.00	0.31	tr_C9ZN43_
DRB1_0101	432	FAETRPQGVIAFAPP	FAETRPQGV	0	0.4825	270.3	45.00	0.58	tr_C9ZN43_
DRB1_0101	433	AACNCNQGGNVLYIN	CNQGGNVLY	4	0.4816	272.9	45.00	0.38	tr_C9ZN43_
DRB1_0101	434	YNPLIPDNLCDAINA	YNPLIPDNL	0	0.4813	273.7	45.00	0.65	tr_C9ZN43_
DRB1_0101	435	SVGRELCGVFTVDGL	GRELCGVFT	2	0.4813	273.7	45.00	0.67	tr_C9ZN43_

DRB1_0101	436	QLFVVVGYVTNTSAS	FVVVGYVTN	2	0.4812	274.0	45.00	0.41	tr_C9ZN43_
DRB1_0101	437	VVNYGATRISMWSLA	YGATRISMW	3	0.4805	276.1	45.00	0.31	tr_C9ZN43_
DRB1_0101	438	VELIPPTSYKIKPVE	LIPPTSYKI	2	0.4775	285.3	46.00	0.56	tr_C9ZN43_
DRB1_0101	439	VQGGTTVFEPSRCLA	TVFEPSRCL	5	0.4768	287.4	46.00	0.82	tr_C9ZN43_
DRB1_0101	440	LLSDSRTHDAYLLSS	SRTHDAYLL	4	0.4768	287.4	46.00	0.71	tr_C9ZN43_
DRB1_0101	441	VNYGATRISMWSLAR	YGATRISMW	2	0.4755	291.3	46.00	0.30	tr_C9ZN43_
DRB1_0101	442	GPLARDTNYRAIRRF	LARDTNYRA	2	0.4754	291.8	46.00	0.70	tr_C9ZN43_
DRB1_0101	443	ASRKWTVAPDVKVEL	WTVAPDVKV	4	0.4750	292.9	46.00	0.75	tr_C9ZN43_
DRB1_0101	444	RFGQSDRLFISMIPS	SDRLFISMI	4	0.4734	298.2	47.00	0.43	tr_C9ZN43_
DRB1_0101	445	SLPLLERENLVGFAP	PLLERENLV	2	0.4734	298.2	47.00	0.76	tr_C9ZN43_
DRB1_0101	446	WLTSRKAFMESLYNQ	KAFMESLYN	5	0.4725	301.1	47.00	0.38	tr_C9ZN43_
DRB1_0101	447	VALERHKGEFFVVLG	LERHKGEFF	2	0.4725	301.2	47.00	0.31	tr_C9ZN43_
DRB1_0101	448	TNTSASAPMSAIVRG	SAPMSAIVR	5	0.4724	301.3	47.00	0.37	tr_C9ZN43_
DRB1_0101	449	ADATIIEVALRKIVW	TIIEVALRK	3	0.4718	303.4	47.00	0.35	tr_C9ZN43_
DRB1_0101	450	QGEVCVVNYGATRIS	VVNYGATRI	5	0.4715	304.4	47.00	0.43	tr_C9ZN43_
DRB1_0101	451	YNQRRYVIDDLVIGD	YVIDDLVIG	5	0.4708	306.8	47.00	0.71	tr_C9ZN43_
DRB1_0101	452	IQLSPTLEQQLFVVV	PTLEQQLFV	4	0.4706	307.5	47.00	0.23	tr_C9ZN43_
DRB1_0101	453	DDNATLVGRLPNRGN	LVGRLPNRG	5	0.4703	308.4	47.00	0.54	tr_C9ZN43_
DRB1_0101	454	APDVKVELIPPTSYK	VELIPPTSY	5	0.4700	309.5	47.00	0.40	tr_C9ZN43_
DRB1_0101	455	QVALERHKGEFFVVL	LERHKGEFF	3	0.4698	310.1	47.00	0.35	tr_C9ZN43_
DRB1_0101	456	NAELLTLVRYAVSQL	LTLVRYAVS	4	0.4696	310.7	47.00	0.45	tr_C9ZN43_
DRB1_0101	457	CNQGGNVLYINVVGN	GNVLYINVV	4	0.4693	311.8	47.00	0.53	tr_C9ZN43_
DRB1_0101	458	PDSSAWKPLPLLGYA	WKPLPLLGY	5	0.4690	312.7	47.00	0.38	tr_C9ZN43_
DRB1_0101	459	FHTALPDSSAWKPLP	FHTALPDSS	0	0.4686	314.1	48.00	0.60	tr_C9ZN43_
DRB1_0101	460	NTSASAPMSAIVRGA	SAPMSAIVR	4	0.4683	315.1	48.00	0.49	tr_C9ZN43_
DRB1_0101	461	ETRPQGVIAFAPPAK	GVIAFAPPA	5	0.4681	315.7	48.00	0.47	tr_C9ZN43_
DRB1_0101	462	DVRVLIPFFDVALMY	LIPFFDVAL	4	0.4679	316.6	48.00	0.31	tr_C9ZN43_
DRB1_0101	463	IYMQSIITADEMRYG	IITADEMRY	5	0.4673	318.7	48.00	0.22	tr_C9ZN43_
DRB1_0101	464	RLLSDSRTHDAYLLS	SRTHDAYLL	5	0.4663	322.0	48.00	0.58	tr_C9ZN43_
DRB1_0101	465	SAPMSAIVRGADATI	SAIVRGADA	4	0.4660	323.0	48.00	0.48	tr_C9ZN43_
DRB1_0101	466	MYDVAYYDPAYVPPA	VAYYDPAYV	3	0.4653	325.4	48.00	0.49	tr_C9ZN43_
DRB1_0101	467	PKTLLDTIYMQSIIT	TIYMQSIIT	6	0.4653	325.6	48.00	0.35	tr_C9ZN43_
DRB1_0101	468	VTNTSASAPMSAIVR	NTSASAPMS	2	0.4646	328.1	48.00	0.23	tr_C9ZN43_
DRB1_0101	469	NPNAERVQFATSLPH	VQFATSLPH	6	0.4644	328.7	48.00	0.35	tr_C9ZN43_
DRB1_0101	470	QQLFVVVGYVTNTSA	FVVVGYVTN	3	0.4640	330.0	49.00	0.50	tr_C9ZN43_
DRB1_0101	471	MYLRGISFGDTEYKM	YLRGISFGD	1	0.4639	330.6	49.00	0.64	tr_C9ZN43_
DRB1_0101	472	DPVMLTPRLHHRGRN	PVMLTPRLH	1	0.4635	331.9	49.00	0.38	tr_C9ZN43_
DRB1_0101	473	WMHGGTLQTVAVLDD	GGTLQTVAV	3	0.4631	333.4	49.00	0.35	tr_C9ZN43_
DRB1_0101	474	QGGNVLYINVVGNDE	GNVLYINVV	2	0.4629	333.9	49.00	0.44	tr_C9ZN43_
DRB1_0101	475	GVVDDAMLSIAEVAF	DAMLSIAEV	4	0.4620	337.5	49.00	0.35	tr_C9ZN43_
DRB1_0101	476	LNASLASRKWTVAPD	SLASRKWTV	3	0.4616	339.0	49.00	0.35	tr_C9ZN43_
DRB1_0101	477	VGRELCGVFTVDGLM	GRELCGVFT	1	0.4610	341.0	49.00	0.60	tr_C9ZN43_
DRB1_0101	478	PDVRVLIPFFDVALM	RVLIPFFDV	3	0.4597	345.8	49.00	0.33	tr_C9ZN43_
DRB1_0101	479	RKWTVAPDVKVELIP	WTVAPDVKV	2	0.4583	351.1	50.00	0.76	tr_C9ZN43_
DRB1_0101	480	RFAQAVLPRMEYVTP	FAQAVLPRM	1	0.4570	356.0	50.00	0.51	tr_C9ZN43_
DRB1_0101	481	YDELVSAFNGNPNAE	DELVSAFNG	1	0.4565	358.1	50.00	0.29	tr_C9ZN43_
DRB1_0101	482	LNASVPPLTSPVTPL	VPPLTSPVT	4	0.4562	359.1	50.00	0.38	tr_C9ZN43_
DRB1_0101	483	EYVTPKTLLDTIYMQ	YVTPKTLLD	1	0.4554	362.2	50.00	0.42	tr_C9ZN43_
DRB1_0101	484	PVEVLQVALERHKGE	VEVLQVALE	1	0.4549	364.1	50.00	0.41	tr_C9ZN43_
DRB1_0101	485	GAACNCNQGGNVLYI	CNQGGNVLY	5	0.4544	366.3	55.00	0.34	tr_C9ZN43_

DRB1_0101	486	YSPLNTLMFLILDRE	YSPLNTLMF	0	0.4542	367.1	55.00	0.45	tr_C9ZN43_
DRB1_0101	487	PLARDTNYRAIRRFQ	LARDTNYRA	1	0.4534	370.2	55.00	0.63	tr_C9ZN43_
DRB1_0101	488	NQGGNVLYINVVGND	GNVLYINVV	3	0.4526	373.6	55.00	0.55	tr_C9ZN43_
DRB1_0101	489	SSSSLEFALDTWREA	FALDTWREA	6	0.4524	374.3	55.00	0.38	tr_C9ZN43_
DRB1_0101	490	LGPVGDGQALASLPL	GQALASLPL	6	0.4524	374.3	55.00	0.56	tr_C9ZN43_
DRB1_0101	491	VRVLIPFFDVALMYD	LIPFFDVAL	3	0.4513	378.7	55.00	0.34	tr_C9ZN43_
DRB1_0101	492	SMIPSPSNATYLALQ	MIPSPSNAT	1	0.4512	379.0	55.00	0.43	tr_C9ZN43_
DRB1_0101	493	LHHRGRNVIQLSPTL	NVIQLSPTL	6	0.4510	380.0	55.00	0.45	tr_C9ZN43_
DRB1_0101	494	YDPAYVPPALSEDVM	PAYVPPALS	2	0.4509	380.2	55.00	0.47	tr_C9ZN43_
DRB1_0101	495	TIIEVALRKIVWMHG	IEVALRKIV	2	0.4509	380.6	55.00	0.27	tr_C9ZN43_
DRB1_0101	496	PVMLTPRLHHRGRNV	PVMLTPRLH	0	0.4501	383.8	55.00	0.28	tr_C9ZN43_
DRB1_0101	497	CGVFTVDGLMRGRVN	VDGLMRGRV	5	0.4499	384.4	55.00	0.29	tr_C9ZN43_
DRB1_0101	498	VMLTPRLHHRGRNVI	RLHHRGRNV	5	0.4486	390.1	55.00	0.44	tr_C9ZN43_
DRB1_0101	499	GVFTVDGLMRGRVND	VDGLMRGRV	4	0.4474	395.2	55.00	0.32	tr_C9ZN43_
DRB1_0101	500	GRFGQSDRLFISMIP	SDRLFISMI	5	0.4473	395.7	55.00	0.40	tr_C9ZN43_
DRB1_0101	501	LYNQRRYVIDDLVIG	YVIDDLVIG	6	0.4468	397.8	55.00	0.56	tr_C9ZN43_
DRB1_0101	502	APGDPSLLAAHLDRN	PSLLAAHLD	4	0.4465	398.9	55.00	0.41	tr_C9ZN43_
DRB1_0101	503	FVTRLLSDSRTHDAY	TRLLSDSRT	2	0.4458	401.8	55.00	0.34	tr_C9ZN43_
DRB1_0101	504	ALERHKGEFFVVLGP	RHKGEFFVV	3	0.4457	402.4	55.00	0.31	tr_C9ZN43_
DRB1_0101	505	SSEIVREFHTALPDS	IVREFHTAL	3	0.4453	404.3	55.00	0.55	tr_C9ZN43_
DRB1_0101	506	SPVTPLIRYADPNAI	IRYADPNAI	6	0.4439	410.1	55.00	0.70	tr_C9ZN43_
DRB1_0101	507	VLYINVVGNDERFHT	YINVVGNDE	2	0.4436	411.8	55.00	0.58	tr_C9ZN43_
DRB1_0101	508	HDDVDGELMVYGWIA	ELMVYGWIA	6	0.4429	414.9	55.00	0.50	tr_C9ZN43_
DRB1_0101	509	LPLLERENLVGFAPS	PLLERENLV	1	0.4427	415.7	55.00	0.68	tr_C9ZN43_
DRB1_0101	510	LERHKGEFFVVLGPV	RHKGEFFVV	2	0.4426	416.2	55.00	0.29	tr_C9ZN43_
DRB1_0101	511	LERENLVGFAPSTGS	LVGFAPSTG	5	0.4424	416.9	55.00	0.43	tr_C9ZN43_
DRB1_0101	512	APPAKDLIKFVTRLL	LIKFVTRLL	6	0.4421	418.4	55.00	0.34	tr_C9ZN43_
DRB1_0101	513	QFATSLPHWADANTS	FATSLPHWA	1	0.4404	426.2	55.00	0.71	tr_C9ZN43_
DRB1_0101	514	RLHHRGRNVIQLSPT	LHHRGRNVI	1	0.4398	428.8	55.00	0.31	tr_C9ZN43_
DRB1_0101	515	QLSPTLEQQLFVVVG	TLEQQLFVV	4	0.4394	430.9	55.00	0.31	tr_C9ZN43_
DRB1_0101	516	LSQALSSREWLTSRK	ALSSREWLT	3	0.4388	433.5	55.00	0.42	tr_C9ZN43_
DRB1_0101	517	EFALDTWREALEADG	FALDTWREA	1	0.4388	433.8	55.00	0.64	tr_C9ZN43_
DRB1_0101	518	NPDVRVLIPFFDVAL	RVLIPFFDV	4	0.4369	442.6	55.00	0.37	tr_C9ZN43_
DRB1_0101	519	ELIPPTSYKIKPVEV	LIPPTSYKI	1	0.4353	450.5	55.00	0.44	tr_C9ZN43_
DRB1_0101	520	NAMRGWNSHVYFLTA	GWNSHVYFL	4	0.4342	455.8	55.00	0.38	tr_C9ZN43_
DRB1_0101	521	TRLLSDSRTHDAYLL	SRTHDAYLL	6	0.4340	456.5	55.00	0.35	tr_C9ZN43_
DRB1_0101	522	TSSEIVREFHTALPD	IVREFHTAL	4	0.4337	458.3	55.00	0.56	tr_C9ZN43_
DRB1_0101	523	GRELCGVFTVDGLMR	GRELCGVFT	0	0.4334	459.7	55.00	0.40	tr_C9ZN43_
DRB1_0101	524	LSPTLEQQLFVVVGY	TLEQQLFVV	3	0.4333	460.4	55.00	0.36	tr_C9ZN43_
DRB1_0101	525	EQQLFVVVGYVTNTS	FVVVGYVTN	4	0.4332	460.7	55.00	0.51	tr_C9ZN43_
DRB1_0101	526	GADATIIEVALRKIV	TIIEVALRK	4	0.4327	463.2	55.00	0.38	tr_C9ZN43_
DRB1_0101	527	LPDSSAWKPLPLLGY	WKPLPLLGY	6	0.4325	464.0	55.00	0.27	tr_C9ZN43_
DRB1_0101	528	LASRKWTVAPDVKVE	WTVAPDVKV	5	0.4324	464.7	55.00	0.67	tr_C9ZN43_
DRB1_0101	529	YRAIRRFQDHMRSYL	IRRFQDHMR	3	0.4324	464.8	55.00	0.29	tr_C9ZN43_
DRB1_0101	530	SVPPLTSPVTPLIRY	LTSPVTPLI	4	0.4317	468.0	55.00	0.28	tr_C9ZN43_
DRB1_0101	531	RTGPLARDTNYRAIR	LARDTNYRA	4	0.4312	470.8	60.00	0.78	tr_C9ZN43_
DRB1_0101	532	VGNDERFHTVQGGTT	FHTVQGGTT	6	0.4301	476.4	60.00	0.60	tr_C9ZN43_
DRB1_0101	533	PNAELLTLVRYAVSQ	LTLVRYAVS	5	0.4294	480.1	60.00	0.46	tr_C9ZN43_
DRB1_0101	534	RYVIDDLVIGDFGGD	YVIDDLVIG	1	0.4271	491.9	60.00	0.67	tr_C9ZN43_
DRB1_0101	535	SPNAELLTLVRYAVS	LTLVRYAVS	6	0.4271	492.1	60.00	0.31	tr_C9ZN43_

DRB1_0101	536	ASAPMSAIVRGADAT	SAIVRGADA	5	0.4270	492.7	60.00	0.41	tr_C9ZN43_
DRB1_0101	537	PGDPSLLAAHLDRNP	PSLLAAHLD	3	0.4269	493.3	60.00	0.43	tr_C9ZN43_
DRB1_0101	538	ASVPPLTSPVTPLIR	VPPLTSPVT	2	0.4266	494.8	60.00	0.41	tr_C9ZN43_
DRB1_0101	539	LQVALERHKGEFFVV	LERHKGEFF	4	0.4255	500.9	60.00	0.40	tr_C9ZN43_
DRB1_0101	540	NASVPPLTSPVTPLI	VPPLTSPVT	3	0.4244	506.8	60.00	0.50	tr_C9ZN43_
DRB1_0101	541	LEQQLFVVVGYVTNT	FVVVGYVTN	5	0.4223	518.4	60.00	0.44	tr_C9ZN43_
DRB1_0101	542	SPTLEQQLFVVVGYV	TLEQQLFVV	2	0.4222	519.1	60.00	0.29	tr_C9ZN43_
DRB1_0101	543	LIPDNLCDAINAGLN	DNLCDAINA	3	0.4206	527.7	60.00	0.49	tr_C9ZN43_
DRB1_0101	544	NGRFGQSDRLFISMI	SDRLFISMI	6	0.4206	527.9	60.00	0.36	tr_C9ZN43_
DRB1_0101	545	LPNRGNAFVIGLAPG	NAFVIGLAP	5	0.4205	528.4	60.00	0.31	tr_C9ZN43_
DRB1_0101	546	LYINVVGNDERFHTV	YINVVGNDE	1	0.4205	528.8	60.00	0.54	tr_C9ZN43_
DRB1_0101	547	TSASAPMSAIVRGAD	SAPMSAIVR	3	0.4203	529.7	60.00	0.58	tr_C9ZN43_
DRB1_0101	548	VFTVDGLMRGRVNDD	VDGLMRGRV	3	0.4201	530.7	60.00	0.38	tr_C9ZN43_
DRB1_0101	549	TLEQQLFVVVGYVTN	FVVVGYVTN	6	0.4194	535.0	60.00	0.30	tr_C9ZN43_
DRB1_0101	550	NTSSEIVREFHTALP	IVREFHTAL	5	0.4191	536.8	60.00	0.56	tr_C9ZN43_
DRB1_0101	551	SASAPMSAIVRGADA	SAPMSAIVR	2	0.4170	548.7	60.00	0.51	tr_C9ZN43_
DRB1_0101	552	TPKTLLDTIYMQSII	KTLLDTIYM	2	0.4169	549.3	60.00	0.50	tr_C9ZN43_
DRB1_0101	553	RVLIPFFDVALMYDE	LIPFFDVAL	2	0.4166	551.3	60.00	0.37	tr_C9ZN43_
DRB1_0101	554	LTASPNAELLTLVRY	PNAELLTLV	4	0.4165	552.1	60.00	0.27	tr_C9ZN43_
DRB1_0101	555	VPPLTSPVTPLIRYA	LTSPVTPLI	3	0.4158	556.3	60.00	0.41	tr_C9ZN43_
DRB1_0101	556	VEVLQVALERHKGEF	LQVALERHK	3	0.4154	558.6	60.00	0.25	tr_C9ZN43_
DRB1_0101	557	ELDTRSVTAVFGVVD	TRSVTAVFG	3	0.4137	568.9	60.00	0.58	tr_C9ZN43_
DRB1_0101	558	DELVSAFNGNPNAER	LVSAFNGNP	2	0.4106	588.1	60.00	0.25	tr_C9ZN43_
DRB1_0101	559	KECFTANDPVPEQGE	FTANDPVPE	3	0.4105	588.8	60.00	0.86	tr_C9ZN43_
DRB1_0101	560	QALSSREWLTSRKAF	REWLTSRKA	5	0.4105	588.8	60.00	0.38	tr_C9ZN43_
DRB1_0101	561	YVTPKTLLDTIYMQS	KTLLDTIYM	4	0.4101	591.7	60.00	0.54	tr_C9ZN43_
DRB1_0101	562	LALQSELDTRSVTAV	QSELDTRSV	3	0.4092	597.0	60.00	0.35	tr_C9ZN43_
DRB1_0101	563	MHGGTLQTVAVLDDN	GGTLQTVAV	2	0.4088	600.0	60.00	0.51	tr_C9ZN43_
DRB1_0101	564	LVGRLPNRGNAFVIG	LVGRLPNRG	0	0.4087	600.4	60.00	0.28	tr_C9ZN43_
DRB1_0101	565	RSVTAVFGVVDDAML	FGVVDDAML	6	0.4083	603.0	60.00	0.52	tr_C9ZN43_
DRB1_0101	566	SELDTRSVTAVFGVV	TRSVTAVFG	4	0.4080	605.1	65.00	0.62	tr_C9ZN43_
DRB1_0101	567	AMRGWNSHVYFLTAS	GWNSHVYFL	3	0.4067	613.4	65.00	0.41	tr_C9ZN43_
DRB1_0101	568	SQALSSREWLTSRKA	ALSSREWLT	2	0.4066	614.2	65.00	0.43	tr_C9ZN43_
DRB1_0101	569	DPAYVPPALSEDVMT	PAYVPPALS	1	0.4066	614.3	65.00	0.47	tr_C9ZN43_
DRB1_0101	570	TNYRAIRRFQDHMRS	YRAIRRFQD	2	0.4063	616.5	65.00	0.61	tr_C9ZN43_
DRB1_0101	571	LCGVFTVDGLMRGRV	FTVDGLMRG	4	0.4062	616.7	65.00	0.32	tr_C9ZN43_
DRB1_0101	572	RLPNRGNAFVIGLAP	PNRGNAFVI	2	0.4062	616.9	65.00	0.35	tr_C9ZN43_
DRB1_0101	573	YMQSIITADEMRYGP	IITADEMRY	4	0.4041	631.1	65.00	0.35	tr_C9ZN43_
DRB1_0101	574	SLASRKWTVAPDVKV	WTVAPDVKV	6	0.4041	631.3	65.00	0.47	tr_C9ZN43_
DRB1_0101	575	PAYVPPALSEDVMTV	PAYVPPALS	0	0.4038	633.0	65.00	0.34	tr_C9ZN43_
DRB1_0101	576	GDPSLLAAHLDRNPD	PSLLAAHLD	2	0.4038	633.0	65.00	0.46	tr_C9ZN43_
DRB1_0101	577	SEDVMTVRVYSLLYN	VMTVRVYSL	3	0.4027	640.8	65.00	0.47	tr_C9ZN43_
DRB1_0101	578	LDDNATLVGRLPNRG	LVGRLPNRG	6	0.4023	643.6	65.00	0.38	tr_C9ZN43_
DRB1_0101	579	LDTRSVTAVFGVVDD	TRSVTAVFG	2	0.4018	647.2	65.00	0.55	tr_C9ZN43_
DRB1_0101	580	QAVLPRMEYVTPKTL	MEYVTPKTL	6	0.4012	651.5	65.00	0.50	tr_C9ZN43_
DRB1_0101	581	EKECFTANDPVPEQG	FTANDPVPE	4	0.3999	660.3	65.00	0.85	tr_C9ZN43_
DRB1_0101	582	MLTRTGPLARDTNYR	LTRTGPLAR	1	0.3989	667.7	65.00	0.42	tr_C9ZN43_
DRB1_0101	583	VTPKTLLDTIYMQSI	KTLLDTIYM	3	0.3988	668.1	65.00	0.57	tr_C9ZN43_
DRB1_0101	584	NYRAIRRFQDHMRSY	YRAIRRFQD	1	0.3987	669.2	65.00	0.50	tr_C9ZN43_
DRB1_0101	585	NTAWEQFAETRPQGV	FAETRPQGV	6	0.3986	669.6	65.00	0.72	tr_C9ZN43_

DRB1_0101	586	SPLNTLMFLILDREL	LMFLILDRE	5	0.3970	681.8	65.00	0.27	tr_C9ZN43_
DRB1_0101	587	MIPSPSNATYLALQS	PSPSNATYL	2	0.3963	686.6	65.00	0.35	tr_C9ZN43_
DRB1_0101	588	IIEVALRKIVWMHGG	VALRKIVWM	3	0.3961	687.8	65.00	0.28	tr_C9ZN43_
DRB1_0101	589	PTLEQQLFVVVGYVT	LEQQLFVVV	2	0.3961	688.6	65.00	0.28	tr_C9ZN43_
DRB1_0101	590	VLIPFFDVALMYDEL	FDVALMYDE	5	0.3958	690.4	65.00	0.26	tr_C9ZN43_
DRB1_0101	591	DTNYRAIRRFQDHMR	YRAIRRFQD	3	0.3953	694.0	65.00	0.67	tr_C9ZN43_
DRB1_0101	592	EQGEVCVVNYGATRI	CVVNYGATR	5	0.3947	698.4	65.00	0.30	tr_C9ZN43_
DRB1_0101	593	GRLPNRGNAFVIGLA	PNRGNAFVI	3	0.3925	715.2	65.00	0.44	tr_C9ZN43_
DRB1_0101	594	PSPSNATYLALQSEL	TYLALQSEL	6	0.3923	716.7	65.00	0.15	tr_C9ZN43_
DRB1_0101	595	KWTVAPDVKVELIPP	WTVAPDVKV	1	0.3920	719.7	65.00	0.79	tr_C9ZN43_
DRB1_0101	596	RNPDVRVLIPFFDVA	RVLIPFFDV	5	0.3913	724.8	65.00	0.40	tr_C9ZN43_
DRB1_0101	597	LSEDVMTVRVYSLLY	VMTVRVYSL	4	0.3912	725.4	65.00	0.43	tr_C9ZN43_
DRB1_0101	598	LARDTNYRAIRRFQD	LARDTNYRA	0	0.3906	730.2	65.00	0.36	tr_C9ZN43_
DRB1_0101	599	ELVSAFNGNPNAERV	FNGNPNAER	5	0.3904	732.3	65.00	0.28	tr_C9ZN43_
DRB1_0101	600	PLLERENLVGFAPST	PLLERENLV	0	0.3898	736.4	65.00	0.47	tr_C9ZN43_
DRB1_0101	601	QSELDTRSVTAVFGV	TRSVTAVFG	5	0.3897	737.6	65.00	0.50	tr_C9ZN43_
DRB1_0101	602	VTRLLSDSRTHDAYL	TRLLSDSRT	1	0.3881	750.4	65.00	0.37	tr_C9ZN43_
DRB1_0101	603	TASPNAELLTLVRYA	PNAELLTLV	3	0.3868	760.7	65.00	0.38	tr_C9ZN43_
DRB1_0101	604	YYGANVLTGNGRFGQ	VLTGNGRFG	5	0.3864	764.4	65.00	0.30	tr_C9ZN43_
DRB1_0101	605	FAQAVLPRMEYVTPK	AVLPRMEYV	3	0.3857	770.0	65.00	0.42	tr_C9ZN43_
DRB1_0101	606	MRGWNSHVYFLTASP	GWNSHVYFL	2	0.3847	778.4	65.00	0.38	tr_C9ZN43_
DRB1_0101	607	MESLYNQRRYVIDDL	LYNQRRYVI	3	0.3837	786.8	70.00	0.38	tr_C9ZN43_
DRB1_0101	608	FAPPAKDLIKFVTRL	PAKDLIKFV	3	0.3834	789.5	70.00	0.26	tr_C9ZN43_
DRB1_0101	609	TVAVLDDNATLVGRL	VLDDNATLV	3	0.3833	790.1	70.00	0.50	tr_C9ZN43_
DRB1_0101	610	FALDTWREALEADGV	FALDTWREA	0	0.3832	791.2	70.00	0.39	tr_C9ZN43_
DRB1_0101	611	VAPDVKVELIPPTSY	VELIPPTSY	6	0.3829	793.8	70.00	0.28	tr_C9ZN43_
DRB1_0101	612	DDDFNTAWEQFAETR	FNTAWEQFA	3	0.3806	813.5	70.00	0.68	tr_C9ZN43_
DRB1_0101	613	ANTSSEIVREFHTAL	IVREFHTAL	6	0.3803	816.5	70.00	0.44	tr_C9ZN43_
DRB1_0101	614	HGGTLQTVAVLDDNA	GGTLQTVAV	1	0.3802	817.6	70.00	0.49	tr_C9ZN43_
DRB1_0101	615	ASPNAELLTLVRYAV	PNAELLTLV	2	0.3800	818.8	70.00	0.38	tr_C9ZN43_
DRB1_0101	616	AETRPQGVIAFAPPA	GVIAFAPPA	6	0.3797	821.8	70.00	0.34	tr_C9ZN43_
DRB1_0101	617	DTRSVTAVFGVVDDA	TRSVTAVFG	1	0.3792	826.1	70.00	0.46	tr_C9ZN43_
DRB1_0101	618	VLQVALERHKGEFFV	LERHKGEFF	5	0.3790	828.4	70.00	0.38	tr_C9ZN43_
DRB1_0101	619	NASLASRKWTVAPDV	SLASRKWTV	2	0.3779	838.0	70.00	0.43	tr_C9ZN43_
DRB1_0101	620	MQSIITADEMRYGPF	IITADEMRY	3	0.3778	839.1	70.00	0.49	tr_C9ZN43_
DRB1_0101	621	PPLTSPVTPLIRYAD	LTSPVTPLI	2	0.3772	844.1	70.00	0.41	tr_C9ZN43_
DRB1_0101	622	GNPNAERVQFATSLP	PNAERVQFA	2	0.3770	846.4	70.00	0.44	tr_C9ZN43_
DRB1_0101	623	RGAACNCNQGGNVLY	CNCNQGGNV	4	0.3769	847.4	70.00	0.34	tr_C9ZN43_
DRB1_0101	624	YLRGISFGDTEYKMT	YLRGISFGD	0	0.3756	858.7	70.00	0.34	tr_C9ZN43_
DRB1_0101	625	ECFTANDPVPEQGEV	FTANDPVPE	2	0.3744	870.2	70.00	0.88	tr_C9ZN43_
DRB1_0101	626	EVLQVALERHKGEFF	LQVALERHK	2	0.3744	870.6	70.00	0.22	tr_C9ZN43_
DRB1_0101	627	EDVMTVRVYSLLYNP	VMTVRVYSL	2	0.3736	878.1	70.00	0.45	tr_C9ZN43_
DRB1_0101	628	DDFNTAWEQFAETRP	FNTAWEQFA	2	0.3733	881.2	70.00	0.67	tr_C9ZN43_
DRB1_0101	629	YINVVGNDERFHTVQ	GNDERFHTV	5	0.3729	885.0	70.00	0.34	tr_C9ZN43_
DRB1_0101	630	VGRLPNRGNAFVIGL	PNRGNAFVI	4	0.3729	885.0	70.00	0.42	tr_C9ZN43_
DRB1_0101	631	FTVDGLMRGRVNDDD	VDGLMRGRV	2	0.3717	895.9	70.00	0.42	tr_C9ZN43_
DRB1_0101	632	ALSEDVMTVRVYSLL	VMTVRVYSL	5	0.3709	903.9	70.00	0.41	tr_C9ZN43_
DRB1_0101	633	AFAPPAKDLIKFVTR	FAPPAKDLI	1	0.3707	906.1	70.00	0.41	tr_C9ZN43_
DRB1_0101	634	PLIPDNLCDAINAGL	DNLCDAINA	4	0.3706	906.5	70.00	0.56	tr_C9ZN43_
DRB1_0101	635	TVQGGTTVFEPSRCL	TVFEPSRCL	6	0.3694	919.1	70.00	0.67	tr_C9ZN43_

DRB1_0101	636	VRGADATIIEVALRK	VRGADATII	0	0.3693	920.1	70.00	0.40	tr_C9ZN43_
DRB1_0101	637	LAAHLDRNPDVRVLI	LDRNPDVRV	4	0.3691	921.7	70.00	0.72	tr_C9ZN43_
DRB1_0101	638	LLERENLVGFAPSTG	LVGFAPSTG	6	0.3682	930.9	70.00	0.34	tr_C9ZN43_
DRB1_0101	639	LEADGVEFFLGQLML	VEFFLGQLM	5	0.3681	931.7	70.00	0.48	tr_C9ZN43_
DRB1_0101	640	AFNGNPNAERVQFAT	PNAERVQFA	5	0.3672	940.5	70.00	0.44	tr_C9ZN43_
DRB1_0101	641	SAFNGNPNAERVQFA	FNGNPNAER	2	0.3670	942.8	70.00	0.44	tr_C9ZN43_
DRB1_0101	642	LVSAFNGNPNAERVQ	FNGNPNAER	4	0.3659	954.3	70.00	0.41	tr_C9ZN43_
DRB1_0101	643	ALPDSSAWKPLPLLG	SAWKPLPLL	5	0.3653	960.7	70.00	0.32	tr_C9ZN43_
DRB1_0101	644	DPSLLAAHLDRNPDV	PSLLAAHLD	1	0.3652	961.0	70.00	0.47	tr_C9ZN43_
DRB1_0101	645	YGANVLTGNGRFGQS	VLTGNGRFG	4	0.3649	964.0	70.00	0.52	tr_C9ZN43_
DRB1_0101	646	RDTNYRAIRRFQDHM	YRAIRRFQD	4	0.3631	983.8	70.00	0.63	tr_C9ZN43_
DRB1_0101	647	QTVAVLDDNATLVGR	VLDDNATLV	4	0.3625	990.2	70.00	0.45	tr_C9ZN43_
DRB1_0101	648	VAVLDDNATLVGRLP	VLDDNATLV	2	0.3624	990.5	70.00	0.45	tr_C9ZN43_
DRB1_0101	649	TRTGPLARDTNYRAI	LARDTNYRA	5	0.3623	992.1	70.00	0.71	tr_C9ZN43_
DRB1_0101	650	ELCGVFTVDGLMRGR	FTVDGLMRG	5	0.3607	1009.1	70.00	0.32	tr_C9ZN43_
DRB1_0101	651	NGNPNAERVQFATSL	PNAERVQFA	3	0.3568	1053.1	75.00	0.55	tr_C9ZN43_
DRB1_0101	652	IEVALRKIVWMHGGT	VALRKIVWM	2	0.3556	1066.6	75.00	0.35	tr_C9ZN43_
DRB1_0101	653	LSSSSLEFALDTWRE	SSLEFALDT	3	0.3547	1077.1	75.00	0.34	tr_C9ZN43_
DRB1_0101	654	GANVLTGNGRFGQSD	VLTGNGRFG	3	0.3544	1080.4	75.00	0.56	tr_C9ZN43_
DRB1_0101	655	EEKECFTANDPVPEQ	FTANDPVPE	5	0.3543	1082.1	75.00	0.83	tr_C9ZN43_
DRB1_0101	656	AAHLDRNPDVRVLIP	LDRNPDVRV	3	0.3539	1086.4	75.00	0.73	tr_C9ZN43_
DRB1_0101	657	RGADATIIEVALRKI	TIIEVALRK	5	0.3538	1087.6	75.00	0.44	tr_C9ZN43_
DRB1_0101	658	RELCGVFTVDGLMRG	VFTVDGLMR	5	0.3534	1092.1	75.00	0.22	tr_C9ZN43_
DRB1_0101	659	PALSEDVMTVRVYSL	VMTVRVYSL	6	0.3532	1094.2	75.00	0.29	tr_C9ZN43_
DRB1_0101	660	DFNTAWEQFAETRPQ	FNTAWEQFA	1	0.3521	1107.8	75.00	0.64	tr_C9ZN43_
DRB1_0101	661	LQSELDTRSVTAVFG	TRSVTAVFG	6	0.3498	1135.6	75.00	0.29	tr_C9ZN43_
DRB1_0101	662	ESLYNQRRYVIDDLV	LYNQRRYVI	2	0.3487	1149.5	75.00	0.41	tr_C9ZN43_
DRB1_0101	663	ARDTNYRAIRRFQDH	YRAIRRFQD	5	0.3476	1162.5	75.00	0.50	tr_C9ZN43_
DRB1_0101	664	CFTANDPVPEQGEVC	FTANDPVPE	1	0.3473	1166.5	75.00	0.81	tr_C9ZN43_
DRB1_0101	665	IPSPSNATYLALQSE	PSPSNATYL	1	0.3451	1195.2	75.00	0.34	tr_C9ZN43_
DRB1_0101	666	AHLDRNPDVRVLIPF	LDRNPDVRV	2	0.3436	1214.7	75.00	0.65	tr_C9ZN43_
DRB1_0101	667	VLDDNATLVGRLPNR	NATLVGRLP	4	0.3434	1216.5	75.00	0.35	tr_C9ZN43_
DRB1_0101	668	LLSSSSLEFALDTWR	SSLEFALDT	4	0.3434	1217.1	75.00	0.35	tr_C9ZN43_
DRB1_0101	669	QSIITADEMRYGPFE	IITADEMRY	2	0.3429	1223.7	75.00	0.50	tr_C9ZN43_
DRB1_0101	670	NDDDFNTAWEQFAET	FNTAWEQFA	4	0.3417	1239.8	75.00	0.69	tr_C9ZN43_
DRB1_0101	671	LQTVAVLDDNATLVG	VAVLDDNAT	3	0.3417	1239.9	75.00	0.38	tr_C9ZN43_
DRB1_0101	672	AYVPPALSEDVMTVR	VPPALSEDV	2	0.3414	1243.8	75.00	0.31	tr_C9ZN43_
DRB1_0101	673	YVPPALSEDVMTVRV	ALSEDVMTV	4	0.3414	1244.3	75.00	0.34	tr_C9ZN43_
DRB1_0101	674	ALQSELDTRSVTAVF	QSELDTRSV	2	0.3412	1247.1	75.00	0.35	tr_C9ZN43_
DRB1_0101	675	VSAFNGNPNAERVQF	FNGNPNAER	3	0.3410	1248.5	75.00	0.55	tr_C9ZN43_
DRB1_0101	676	AVLDDNATLVGRLPN	VLDDNATLV	1	0.3409	1250.8	75.00	0.38	tr_C9ZN43_
DRB1_0101	677	VPPALSEDVMTVRVY	ALSEDVMTV	3	0.3386	1282.3	75.00	0.38	tr_C9ZN43_
DRB1_0101	678	GGTLQTVAVLDDNAT	GGTLQTVAV	0	0.3382	1286.9	75.00	0.36	tr_C9ZN43_
DRB1_0101	679	TRSVTAVFGVVDDAM	VTAVFGVVD	3	0.3373	1300.1	75.00	0.39	tr_C9ZN43_
DRB1_0101	680	ANVLTGNGRFGQSDR	VLTGNGRFG	2	0.3349	1334.1	75.00	0.56	tr_C9ZN43_
DRB1_0101	681	FATSLPHWADANTSS	FATSLPHWA	0	0.3341	1346.1	80.00	0.53	tr_C9ZN43_
DRB1_0101	682	SLYNQRRYVIDDLVI	LYNQRRYVI	1	0.3334	1356.5	80.00	0.34	tr_C9ZN43_
DRB1_0101	683	TWREALEADGVEFFL	REALEADGV	2	0.3329	1363.3	80.00	0.32	tr_C9ZN43_
DRB1_0101	684	DRNPDVRVLIPFFDV	VRVLIPFFD	5	0.3311	1390.0	80.00	0.38	tr_C9ZN43_
DRB1_0101	685	YVIDDLVIGDFGGDC	YVIDDLVIG	0	0.3301	1406.3	80.00	0.49	tr_C9ZN43_

DRB1_0101	686	TALPDSSAWKPLPLL	SSAWKPLPL	5	0.3294	1415.7	80.00	0.29	tr_C9ZN43_
DRB1_0101	687	PPALSEDVMTVRVYS	ALSEDVMTV	2	0.3280	1438.6	80.00	0.38	tr_C9ZN43_
DRB1_0101	688	ASLASRKWTVAPDVK	SLASRKWTV	1	0.3277	1442.8	80.00	0.49	tr_C9ZN43_
DRB1_0101	689	TVDGLMRGRVNDDDF	VDGLMRGRV	1	0.3275	1446.1	80.00	0.44	tr_C9ZN43_
DRB1_0101	690	INVVGNDERFHTVQG	GNDERFHTV	4	0.3272	1449.6	80.00	0.47	tr_C9ZN43_
DRB1_0101	691	DTWREALEADGVEFF	WREALEADG	2	0.3264	1463.3	80.00	0.34	tr_C9ZN43_
DRB1_0101	692	LLAAHLDRNPDVRVL	LDRNPDVRV	5	0.3246	1491.9	80.00	0.69	tr_C9ZN43_
DRB1_0101	693	FNGNPNAERVQFATS	PNAERVQFA	4	0.3240	1501.2	80.00	0.53	tr_C9ZN43_
DRB1_0101	694	PLTSPVTPLIRYADP	LTSPVTPLI	1	0.3237	1506.1	80.00	0.39	tr_C9ZN43_
DRB1_0101	695	GISFGDTEYKMTTSL	SFGDTEYKM	2	0.3229	1520.0	80.00	0.27	tr_C9ZN43_
DRB1_0101	696	NPLIPDNLCDAINAG	DNLCDAINA	5	0.3217	1540.1	80.00	0.56	tr_C9ZN43_
DRB1_0101	697	LDTWREALEADGVEF	WREALEADG	3	0.3204	1561.7	80.00	0.43	tr_C9ZN43_
DRB1_0101	698	ALEADGVEFFLGQLM	VEFFLGQLM	6	0.3186	1591.0	80.00	0.43	tr_C9ZN43_
DRB1_0101	699	LSANPGVTVFNGTDN	LSANPGVTV	0	0.3183	1597.1	80.00	0.67	tr_C9ZN43_
DRB1_0101	700	PEQGEVCVVNYGATR	CVVNYGATR	6	0.3170	1619.7	80.00	0.27	tr_C9ZN43_
DRB1_0101	701	HLDRNPDVRVLIPFF	LDRNPDVRV	1	0.3169	1621.7	80.00	0.56	tr_C9ZN43_
DRB1_0101	702	NVVGNDERFHTVQGG	GNDERFHTV	3	0.3161	1635.2	80.00	0.52	tr_C9ZN43_
DRB1_0101	703	GNGRFGQSDRLFISM	GQSDRLFIS	5	0.3156	1644.9	80.00	0.35	tr_C9ZN43_
DRB1_0101	704	WREALEADGVEFFLG	LEADGVEFF	4	0.3131	1690.0	80.00	0.27	tr_C9ZN43_
DRB1_0101	705	AQAVLPRMEYVTPKT	AVLPRMEYV	2	0.3089	1767.4	80.00	0.51	tr_C9ZN43_
DRB1_0101	706	TLQTVAVLDDNATLV	VAVLDDNAT	4	0.3085	1775.1	80.00	0.41	tr_C9ZN43_
DRB1_0101	707	LRGISFGDTEYKMTT	SFGDTEYKM	4	0.3085	1775.4	80.00	0.31	tr_C9ZN43_
DRB1_0101	708	RGISFGDTEYKMTTS	SFGDTEYKM	3	0.3084	1776.6	80.00	0.35	tr_C9ZN43_
DRB1_0101	709	ALDTWREALEADGVE	WREALEADG	4	0.3047	1849.7	85.00	0.41	tr_C9ZN43_
DRB1_0101	710	VVLGPVGDGQALASL	VGDGQALAS	5	0.3025	1895.2	85.00	0.26	tr_C9ZN43_
DRB1_0101	711	PSLLAAHLDRNPDVR	PSLLAAHLD	0	0.2996	1955.1	85.00	0.49	tr_C9ZN43_
DRB1_0101	712	VNDDDFNTAWEQFAE	FNTAWEQFA	5	0.2972	2006.3	85.00	0.71	tr_C9ZN43_
DRB1_0101	713	WTVAPDVKVELIPPT	WTVAPDVKV	0	0.2951	2052.2	85.00	0.58	tr_C9ZN43_
DRB1_0101	714	LTRTGPLARDTNYRA	LARDTNYRA	6	0.2918	2128.3	85.00	0.39	tr_C9ZN43_
DRB1_0101	715	REALEADGVEFFLGQ	LEADGVEFF	3	0.2871	2238.9	85.00	0.35	tr_C9ZN43_
DRB1_0101	716	VVGNDERFHTVQGGT	GNDERFHTV	2	0.2868	2245.1	85.00	0.56	tr_C9ZN43_
DRB1_0101	717	FNTAWEQFAETRPQG	FNTAWEQFA	0	0.2836	2323.8	85.00	0.46	tr_C9ZN43_
DRB1_0101	718	NVLTGNGRFGQSDRL	VLTGNGRFG	1	0.2831	2336.2	85.00	0.54	tr_C9ZN43_
DRB1_0101	719	HTALPDSSAWKPLPL	HTALPDSSA	0	0.2829	2342.7	85.00	0.23	tr_C9ZN43_
DRB1_0101	720	GTLQTVAVLDDNATL	VAVLDDNAT	5	0.2820	2364.8	85.00	0.34	tr_C9ZN43_
DRB1_0101	721	HTVQGGTTVFEPSRC	HTVQGGTTV	0	0.2803	2408.2	85.00	0.47	tr_C9ZN43_
DRB1_0101	722	LDRNPDVRVLIPFFD	VRVLIPFFD	6	0.2789	2444.7	85.00	0.26	tr_C9ZN43_
DRB1_0101	723	TGNGRFGQSDRLFIS	FGQSDRLFI	5	0.2783	2462.4	85.00	0.28	tr_C9ZN43_
DRB1_0101	724	VPEQGEVCVVNYGAT	PEQGEVCVV	1	0.2777	2478.3	85.00	0.26	tr_C9ZN43_
DRB1_0101	725	PVPEQGEVCVVNYGA	PEQGEVCVV	2	0.2770	2497.8	85.00	0.33	tr_C9ZN43_
DRB1_0101	726	VDGLMRGRVNDDDFN	DGLMRGRVN	1	0.2753	2542.9	90.00	0.38	tr_C9ZN43_
DRB1_0101	727	DHDDVDGELMVYGWI	GELMVYGWI	6	0.2721	2632.2	90.00	0.34	tr_C9ZN43_
DRB1_0101	728	VLGPVGDGQALASLP	VGDGQALAS	4	0.2714	2653.3	90.00	0.31	tr_C9ZN43_
DRB1_0101	729	SIITADEMRYGPFEE	IITADEMRY	1	0.2690	2722.6	90.00	0.57	tr_C9ZN43_
DRB1_0101	730	LPHWADANTSSEIVR	DANTSSEIV	5	0.2664	2800.5	90.00	0.32	tr_C9ZN43_
DRB1_0101	731	HWADANTSSEIVREF	DANTSSEIV	3	0.2654	2829.6	90.00	0.47	tr_C9ZN43_
DRB1_0101	732	DANTSSEIVREFHTA	SEIVREFHT	5	0.2623	2927.3	90.00	0.30	tr_C9ZN43_
DRB1_0101	733	ERGAACNCNQGGNVL	CNCNQGGNV	5	0.2614	2957.2	90.00	0.45	tr_C9ZN43_
DRB1_0101	734	PHWADANTSSEIVRE	DANTSSEIV	4	0.2599	3004.4	90.00	0.38	tr_C9ZN43_
DRB1_0101	735	SLLAAHLDRNPDVRV	LDRNPDVRV	6	0.2593	3024.1	90.00	0.47	tr_C9ZN43_

DRB1_0101	736	DPVPEQGEVCVVNYG	PEQGEVCVV	3	0.2590	3032.0	90.00	0.39	tr_C9ZN43_
DRB1_0101	737	EALEADGVEFFLGQL	LEADGVEFF	2	0.2547	3177.3	90.00	0.34	tr_C9ZN43_
DRB1_0101	738	TVAPDVKVELIPPTS	VKVELIPPT	5	0.2535	3221.1	90.00	0.47	tr_C9ZN43_
DRB1_0101	739	EEEKECFTANDPVPE	FTANDPVPE	6	0.2530	3237.2	90.00	0.69	tr_C9ZN43_
DRB1_0101	740	ADANTSSEIVREFHT	DANTSSEIV	1	0.2525	3255.4	90.00	0.40	tr_C9ZN43_
DRB1_0101	741	NDPVPEQGEVCVVNY	PEQGEVCVV	4	0.2491	3375.2	90.00	0.38	tr_C9ZN43_
DRB1_0101	742	WADANTSSEIVREFH	DANTSSEIV	2	0.2455	3509.4	90.00	0.50	tr_C9ZN43_
DRB1_0101	743	RVNDDDFNTAWEQFA	FNTAWEQFA	6	0.2449	3535.1	90.00	0.55	tr_C9ZN43_
DRB1_0101	744	LTSPVTPLIRYADPN	TSPVTPLIR	1	0.2410	3687.2	90.00	0.26	tr_C9ZN43_
DRB1_0101	745	FTANDPVPEQGEVCV	FTANDPVPE	0	0.2395	3747.2	95.00	0.54	tr_C9ZN43_
DRB1_0101	746	ANDPVPEQGEVCVVN	PEQGEVCVV	5	0.2366	3863.7	95.00	0.34	tr_C9ZN43_
DRB1_0101	747	SLPHWADANTSSEIV	WADANTSSE	4	0.2307	4121.6	95.00	0.28	tr_C9ZN43_
DRB1_0101	748	VIDDLVIGDFGGDCK	DDLVIGDFG	2	0.2279	4247.3	95.00	0.31	tr_C9ZN43_
DRB1_0101	749	NFDHDDVDGELMVYG	FDHDDVDGE	1	0.2254	4361.9	95.00	0.27	tr_C9ZN43_
DRB1_0101	750	TSPVTPLIRYADPNA	PVTPLIRYA	2	0.2227	4492.3	95.00	0.28	tr_C9ZN43_
DRB1_0101	751	IDDLVIGDFGGDCKG	DDLVIGDFG	1	0.2208	4585.4	95.00	0.26	tr_C9ZN43_
DRB1_0101	752	VLTGNGRFGQSDRLF	VLTGNGRFG	0	0.2182	4714.8	95.00	0.37	tr_C9ZN43_
DRB1_0101	753	GDCKGGAGERGAACN	CKGGAGERG	2	0.2170	4779.8	95.00	0.50	tr_C9ZN43_
DRB1_0101	754	FDHDDVDGELMVYGW	HDDVDGELM	2	0.2165	4805.7	95.00	0.25	tr_C9ZN43_
DRB1_0101	755	LTGNGRFGQSDRLFI	GRFGQSDRL	4	0.2140	4936.5	95.00	0.33	tr_C9ZN43_
DRB1_0101	756	DCKGGAGERGAACNC	CKGGAGERG	1	0.2137	4953.9	95.00	0.44	tr_C9ZN43_
DRB1_0101	757	DNFDHDDVDGELMVY	FDHDDVDGE	2	0.2134	4966.4	95.00	0.32	tr_C9ZN43_
DRB1_0101	758	TANDPVPEQGEVCVV	PVPEQGEVC	4	0.2091	5207.5	95.00	0.37	tr_C9ZN43_
DRB1_0101	759	CKGGAGERGAACNCN	GAGERGAAC	3	0.2052	5431.7	95.00	0.40	tr_C9ZN43_
DRB1_0101	760	GGDCKGGAGERGAAC	CKGGAGERG	3	0.2048	5453.2	95.00	0.58	tr_C9ZN43_
DRB1_0101	761	DFGGDCKGGAGERGA	FGGDCKGGA	1	0.2037	5515.7	95.00	0.34	tr_C9ZN43_
DRB1_0101	762	TSLPHWADANTSSEI	PHWADANTS	3	0.2028	5574.1	95.00	0.44	tr_C9ZN43_
DRB1_0101	763	FGGDCKGGAGERGAA	CKGGAGERG	4	0.2023	5605.0	95.00	0.52	tr_C9ZN43_
DRB1_0101	764	DGLMRGRVNDDDFNT	DGLMRGRVN	0	0.2006	5704.5	95.00	0.41	tr_C9ZN43_
DRB1_0101	765	DDLVIGDFGGDCKGG	VIGDFGGDC	3	0.2005	5714.2	95.00	0.25	tr_C9ZN43_
DRB1_0101	766	LVIGDFGGDCKGGAG	FGGDCKGGA	5	0.1996	5767.8	95.00	0.46	tr_C9ZN43_
DRB1_0101	767	DLVIGDFGGDCKGGA	FGGDCKGGA	6	0.1994	5783.0	95.00	0.25	tr_C9ZN43_
DRB1_0101	768	TDNFDHDDVDGELMV	FDHDDVDGE	3	0.1910	6334.3	100.00	0.43	tr_C9ZN43_
DRB1_0101	769	GDFGGDCKGGAGERG	FGGDCKGGA	2	0.1902	6382.8	100.00	0.49	tr_C9ZN43_
DRB1_0101	770	IGDFGGDCKGGAGER	FGGDCKGGA	3	0.1863	6663.7	100.00	0.63	tr_C9ZN43_
DRB1_0101	771	IITADEMRYGPFEEE	IITADEMRY	0	0.1849	6760.0	100.00	0.34	tr_C9ZN43_
DRB1_0101	772	VIGDFGGDCKGGAGE	FGGDCKGGA	4	0.1829	6912.3	100.00	0.56	tr_C9ZN43_
DRB1_0101	773	ATSLPHWADANTSSE	PHWADANTS	4	0.1824	6945.4	100.00	0.44	tr_C9ZN43_
DRB1_0101	774	PGVTVFNGTDNFDHD	FNGTDNFDH	5	0.1816	7011.1	100.00	0.50	tr_C9ZN43_
DRB1_0101	775	VTVFNGTDNFDHDDV	FNGTDNFDH	3	0.1806	7082.1	100.00	0.69	tr_C9ZN43_
DRB1_0101	776	NPGVTVFNGTDNFDH	FNGTDNFDH	6	0.1768	7379.4	100.00	0.35	tr_C9ZN43_
DRB1_0101	777	GVTVFNGTDNFDHDD	FNGTDNFDH	4	0.1756	7480.0	100.00	0.60	tr_C9ZN43_
DRB1_0101	778	GERGAACNCNQGGNV	CNCNQGGNV	6	0.1724	7739.4	100.00	0.46	tr_C9ZN43_
DRB1_0101	779	KGGAGERGAACNCNQ	GAGERGAAC	2	0.1679	8126.7	100.00	0.54	tr_C9ZN43_
DRB1_0101	780	SANPGVTVFNGTDNF	VTVFNGTDN	5	0.1626	8613.0	100.00	0.28	tr_C9ZN43_
DRB1_0101	781	GLMRGRVNDDDFNTA	LMRGRVNDD	1	0.1624	8624.4	100.00	0.32	tr_C9ZN43_
DRB1_0101	782	TVFNGTDNFDHDDVD	FNGTDNFDH	2	0.1606	8794.6	100.00	0.74	tr_C9ZN43_
DRB1_0101	783	GTDNFDHDDVDGELM	FDHDDVDGE	4	0.1581	9035.8	100.00	0.55	tr_C9ZN43_
DRB1_0101	784	GRVNDDDFNTAWEQF	VNDDDFNTA	2	0.1566	9181.4	100.00	0.31	tr_C9ZN43_
DRB1_0101	785	LMRGRVNDDDFNTAW	VNDDDFNTA	5	0.1566	9188.1	100.00	0.31	tr_C9ZN43_

DRB1_0101	786	RGRVNDDDFNTAWEQ	VNDDDFNTA	3	0.1548	9362.5	100.00	0.37	tr_C9ZN43_
DRB1_0101	787	GGAGERGAACNCNQG	GAGERGAAC	1	0.1546	9385.4	100.00	0.41	tr_C9ZN43_
DRB1_0101	788	ANPGVTVFNGTDNFD	VTVFNGTDN	4	0.1446	10460.5	100.00	0.36	tr_C9ZN43_
DRB1_0101	789	MRGRVNDDDFNTAWE	VNDDDFNTA	4	0.1413	10840.5	100.00	0.43	tr_C9ZN43_
DRB1_0101	790	GAGERGAACNCNQGG	GAGERGAAC	0	0.1403	10952.6	100.00	0.29	tr_C9ZN43_
DRB1_0101	791	VFNGTDNFDHDDVDG	FNGTDNFDH	1	0.1358	11502.3	100.00	0.68	tr_C9ZN43_
DRB1_0101	792	FNGTDNFDHDDVDGE	FDHDDVDGE	6	0.1337	11764.3	100.00	0.34	tr_C9ZN43_
DRB1_0101	793	NGTDNFDHDDVDGEL	FDHDDVDGE	5	0.1315	12054.8	100.00	0.60	tr_C9ZN43_
DRB1_0101	794	EEEEKECFTANDPVP	KECFTANDP	4	0.1228	13236.2	100.00	0.22	tr_C9ZN43_
DRB1_0101	795	ITADEMRYGPFEEEE	MRYGPFEEE	5	0.1209	13510.1	100.00	0.28	tr_C9ZN43_
DRB1_0101	796	AGERGAACNCNQGGN	GERGAACNC	1	0.1138	14598.1	100.00	0.22	tr_C9ZN43_
DRB1_0101	797	GPFEEEEEKECFTAN	FEEEEEKEC	2	0.1108	15083.5	100.00	0.35	tr_C9ZN43_
DRB1_0101	798	TADEMRYGPFEEEEE	MRYGPFEEE	4	0.1080	15547.5	100.00	0.34	tr_C9ZN43_
DRB1_0101	799	ADEMRYGPFEEEEEK	MRYGPFEEE	3	0.1074	15649.6	100.00	0.44	tr_C9ZN43_
DRB1_0101	800	PFEEEEEKECFTAND	FEEEEEKEC	1	0.1065	15789.0	100.00	0.31	tr_C9ZN43_
DRB1_0101	801	YGPFEEEEEKECFTA	FEEEEEKEC	3	0.1044	16154.4	100.00	0.45	tr_C9ZN43_
DRB1_0101	802	FEEEEEKECFTANDP	EEEKECFTA	3	0.0986	17212.3	100.00	0.20	tr_C9ZN43_
DRB1_0101	803	EEEEEKECFTANDPV	KECFTANDP	5	0.0965	17594.9	100.00	0.25	tr_C9ZN43_
DRB1_0101	804	EMRYGPFEEEEEKEC	MRYGPFEEE	1	0.0947	17946.0	100.00	0.37	tr_C9ZN43_
DRB1_0101	805	DEMRYGPFEEEEEKE	MRYGPFEEE	2	0.0936	18170.5	100.00	0.44	tr_C9ZN43_
DRB1_0101	806	RYGPFEEEEEKECFT	FEEEEEKEC	4	0.0918	18512.8	100.00	0.47	tr_C9ZN43_
DRB1_0101	807	MRYGPFEEEEEKECF	FEEEEEKEC	5	0.0917	18531.3	100.00	0.37	tr_C9ZN43_

Allele: DRB1_0101. Number of high binders 4. Number of weak binders 94. Number of peptides 807

Allele	pos	peptide	core	Of	1-log50k(aff)	affinity(nM)	%Rank	Relia	Identity	Bind Level
DRB1_0101	1	DPLLRFRSLVINLAS	FRSLVINLA	5	0.8259	6.6	1.20	0.81	tr_C9ZL71_	WB
DRB1_0101	2	PLLRFRSLVINLASP	FRSLVINLA	4	0.8181	7.2	1.40	0.83	tr_C9ZL71_	WB
DRB1_0101	3	LLRFRSLVINLASPD	FRSLVINLA	3	0.8103	7.8	1.70	0.83	tr_C9ZL71_	WB
DRB1_0101	4	GDPLLRFRSLVINLA	FRSLVINLA	6	0.7987	8.8	2.50	0.75	tr_C9ZL71_	WB
DRB1_0101	5	LRFRSLVINLASPDD	FRSLVINLA	2	0.7948	9.2	2.50	0.85	tr_C9ZL71_	WB
DRB1_0101	6	RFRSLVINLASPDDW	FRSLVINLA	1	0.7688	12.2	3.50	0.75	tr_C9ZL71_	WB
DRB1_0101	7	RCVYINLPPGAVLND	YINLPPGAV	3	0.7578	13.7	4.50	0.69	tr_C9ZL71_	WB
DRB1_0101	8	DRCVYINLPPGAVLN	YINLPPGAV	4	0.7555	14.1	4.50	0.70	tr_C9ZL71_	WB
DRB1_0101	9	DDRCVYINLPPGAVL	YINLPPGAV	5	0.7234	19.9	7.00	0.68	tr_C9ZL71_	WB
DRB1_0101	10	CVYINLPPGAVLNDT	YINLPPGAV	2	0.7214	20.4	7.00	0.73	tr_C9ZL71_	WB
DRB1_0101	11	AVGILFQGRAYALTV	LFQGRAYAL	4	0.7209	20.5	7.00	0.57	tr_C9ZL71_	WB
DRB1_0101	12	FRSLVINLASPDDWE	FRSLVINLA	0	0.6906	28.4	10.00	0.54	tr_C9ZL71_	
DRB1_0101	13	SAVGILFQGRAYALT	LFQGRAYAL	5	0.6860	29.9	11.00	0.51	tr_C9ZL71_	
DRB1_0101	14	VYINLPPGAVLNDTG	YINLPPGAV	1	0.6749	33.7	12.00	0.77	tr_C9ZL71_	
DRB1_0101	15	ERRFTLRVAIPFMEE	FTLRVAIPF	3	0.6647	37.6	13.00	0.58	tr_C9ZL71_	
DRB1_0101	16	RRFTLRVAIPFMEEN	FTLRVAIPF	2	0.6624	38.6	13.00	0.57	tr_C9ZL71_	
DRB1_0101	17	DERRFTLRVAIPFME	FTLRVAIPF	4	0.6422	48.0	16.00	0.65	tr_C9ZL71_	
DRB1_0101	18	RFTLRVAIPFMEENS	FTLRVAIPF	1	0.6199	61.1	19.00	0.47	tr_C9ZL71_	
DRB1_0101	19	GDDRCVYINLPPGAV	YINLPPGAV	6	0.6169	63.1	20.00	0.64	tr_C9ZL71_	
DRB1_0101	20	NSAVGILFQGRAYAL	LFQGRAYAL	6	0.6133	65.6	20.00	0.44	tr_C9ZL71_	

DRB1_0101	21	PDERRFTLRVAIPFM	FTLRVAIPF	5	0.5949	80.1	23.00	0.77	tr_C9ZL71_
DRB1_0101	22	NSNWSTAPLPIVCRR	WSTAPLPIV	3	0.5718	102.8	27.00	0.38	tr_C9ZL71_
DRB1_0101	23	FGDPLLRFRSLVINL	LLRFRSLVI	4	0.5692	105.7	28.00	0.61	tr_C9ZL71_
DRB1_0101	24	YINLPPGAVLNDTGG	YINLPPGAV	0	0.5653	110.3	28.00	0.71	tr_C9ZL71_
DRB1_0101	25	SNWSTAPLPIVCRRG	WSTAPLPIV	2	0.5583	118.9	30.00	0.35	tr_C9ZL71_
DRB1_0101	26	DFGDPLLRFRSLVIN	LLRFRSLVI	5	0.5441	138.8	32.00	0.61	tr_C9ZL71_
DRB1_0101	27	SIARNINNSAVGILF	IARNINNSA	1	0.5427	140.8	33.00	0.34	tr_C9ZL71_
DRB1_0101	28	ENSNWSTAPLPIVCR	WSTAPLPIV	4	0.5409	143.7	33.00	0.39	tr_C9ZL71_
DRB1_0101	29	RSLVINLASPDDWEV	LVINLASPD	2	0.5347	153.7	34.00	0.41	tr_C9ZL71_
DRB1_0101	30	GSIARNINNSAVGIL	IARNINNSA	2	0.5273	166.4	36.00	0.49	tr_C9ZL71_
DRB1_0101	31	HPDERRFTLRVAIPF	FTLRVAIPF	6	0.5202	179.7	37.00	0.73	tr_C9ZL71_
DRB1_0101	32	FTLRVAIPFMEENSN	LRVAIPFME	2	0.5178	184.4	37.00	0.54	tr_C9ZL71_
DRB1_0101	33	IARNINNSAVGILFQ	NINNSAVGI	3	0.5128	194.7	38.00	0.32	tr_C9ZL71_
DRB1_0101	34	TDTYRVQWNGRPYSV	YRVQWNGRP	3	0.5095	201.8	39.00	0.73	tr_C9ZL71_
DRB1_0101	35	NWSTAPLPIVCRRGD	TAPLPIVCR	3	0.5076	205.9	39.00	0.38	tr_C9ZL71_
DRB1_0101	36	VDFGDPLLRFRSLVI	LLRFRSLVI	6	0.5048	212.2	40.00	0.52	tr_C9ZL71_
DRB1_0101	37	DTYRVQWNGRPYSVD	YRVQWNGRP	2	0.5028	217.0	40.00	0.69	tr_C9ZL71_
DRB1_0101	38	PGSIARNINNSAVGI	IARNINNSA	3	0.4974	230.0	42.00	0.64	tr_C9ZL71_
DRB1_0101	39	TYRVQWNGRPYSVDF	YRVQWNGRP	1	0.4967	231.9	42.00	0.63	tr_C9ZL71_
DRB1_0101	40	SLVINLASPDDWEVF	VINLASPDD	2	0.4877	255.5	44.00	0.34	tr_C9ZL71_
DRB1_0101	41	SLSLSDVPGSIARNI	LSDVPGSIA	3	0.4832	268.2	44.00	0.56	tr_C9ZL71_
DRB1_0101	42	RVSLSLSDVPGSIAR	LSDVPGSIA	5	0.4830	268.9	45.00	0.35	tr_C9ZL71_
DRB1_0101	43	RPYSVDFGDPLLRFR	YSVDFGDPL	2	0.4782	283.2	46.00	0.49	tr_C9ZL71_
DRB1_0101	44	VSLSLSDVPGSIARN	LSDVPGSIA	4	0.4744	294.9	46.00	0.50	tr_C9ZL71_
DRB1_0101	45	ARNINNSAVGILFQG	NINNSAVGI	2	0.4696	310.8	47.00	0.33	tr_C9ZL71_
DRB1_0101	46	NNSAVGILFQGRAYA	ILFQGRAYA	6	0.4657	324.0	48.00	0.31	tr_C9ZL71_
DRB1_0101	47	HTDTYRVQWNGRPYS	YRVQWNGRP	4	0.4643	328.9	48.00	0.82	tr_C9ZL71_
DRB1_0101	48	EENSNWSTAPLPIVC	WSTAPLPIV	5	0.4639	330.6	49.00	0.44	tr_C9ZL71_
DRB1_0101	49	RNINNSAVGILFQGR	NNSAVGILF	3	0.4616	338.9	49.00	0.43	tr_C9ZL71_
DRB1_0101	50	GRPYSVDFGDPLLRF	YSVDFGDPL	3	0.4604	343.1	49.00	0.57	tr_C9ZL71_
DRB1_0101	51	LSLSDVPGSIARNIN	LSDVPGSIA	2	0.4573	355.0	50.00	0.58	tr_C9ZL71_
DRB1_0101	52	PYSVDFGDPLLRFRS	YSVDFGDPL	1	0.4556	361.4	50.00	0.37	tr_C9ZL71_
DRB1_0101	53	TWRVLDICGRDAPDM	VLDICGRDA	3	0.4495	386.1	55.00	0.54	tr_C9ZL71_
DRB1_0101	54	YSVDFGDPLLRFRSL	VDFGDPLLR	2	0.4479	393.1	55.00	0.22	tr_C9ZL71_
DRB1_0101	55	VPGSIARNINNSAVG	IARNINNSA	4	0.4445	407.5	55.00	0.69	tr_C9ZL71_
DRB1_0101	56	SITWRVLDICGRDAP	VLDICGRDA	5	0.4402	427.0	55.00	0.47	tr_C9ZL71_
DRB1_0101	57	ITWRVLDICGRDAPD	VLDICGRDA	4	0.4398	428.7	55.00	0.51	tr_C9ZL71_
DRB1_0101	58	GRVSLSLSDVPGSIA	LSLSDVPGS	4	0.4364	445.2	55.00	0.31	tr_C9ZL71_
DRB1_0101	59	SVDFGDPLLRFRSLV	VDFGDPLLR	1	0.4232	513.0	60.00	0.19	tr_C9ZL71_
DRB1_0101	60	MEENSNWSTAPLPIV	NWSTAPLPI	5	0.4205	528.8	60.00	0.44	tr_C9ZL71_
DRB1_0101	61	RHTDTYRVQWNGRPY	YRVQWNGRP	5	0.4203	529.5	60.00	0.82	tr_C9ZL71_
DRB1_0101	62	SLSDVPGSIARNINN	LSDVPGSIA	1	0.4154	558.8	60.00	0.55	tr_C9ZL71_
DRB1_0101	63	WSTAPLPIVCRRGDD	TAPLPIVCR	2	0.4135	569.8	60.00	0.50	tr_C9ZL71_
DRB1_0101	64	LVINLASPDDWEVFH	VINLASPDD	1	0.4124	577.2	60.00	0.35	tr_C9ZL71_
DRB1_0101	65	NINNSAVGILFQGRA	NNSAVGILF	2	0.4100	592.1	60.00	0.38	tr_C9ZL71_
DRB1_0101	66	WRVLDICGRDAPDMW	VLDICGRDA	2	0.4098	593.6	60.00	0.56	tr_C9ZL71_
DRB1_0101	67	DVPGSIARNINNSAV	IARNINNSA	5	0.4061	617.7	65.00	0.62	tr_C9ZL71_
DRB1_0101	68	YRVQWNGRPYSVDFG	YRVQWNGRP	0	0.4042	630.7	65.00	0.44	tr_C9ZL71_
DRB1_0101	69	PSITWRVLDICGRDA	WRVLDICGR	4	0.4030	638.7	65.00	0.50	tr_C9ZL71_
DRB1_0101	70	INNSAVGILFQGRAY	VGILFQGRA	5	0.4016	648.6	65.00	0.27	tr_C9ZL71_

DRB1_0101	71	NGRPYSVDFGDPLLR	YSVDFGDPL	4	0.4005	656.4	65.00	0.64	tr_C9ZL71_
DRB1_0101	72	TLRVAIPFMEENSNW	LRVAIPFME	1	0.3933	709.3	65.00	0.70	tr_C9ZL71_
DRB1_0101	73	GAVLNDTGGGRVSLS	LNDTGGGRV	3	0.3900	735.1	65.00	0.76	tr_C9ZL71_
DRB1_0101	74	AVLNDTGGGRVSLSL	LNDTGGGRV	2	0.3851	775.5	65.00	0.70	tr_C9ZL71_
DRB1_0101	75	VFHPDERRFTLRVAI	FHPDERRFT	1	0.3791	826.9	70.00	0.27	tr_C9ZL71_
DRB1_0101	76	RVLDICGRDAPDMWK	VLDICGRDA	1	0.3783	834.4	70.00	0.56	tr_C9ZL71_
DRB1_0101	77	GGRVSLSLSDVPGSI	LSLSDVPGS	5	0.3728	885.8	70.00	0.31	tr_C9ZL71_
DRB1_0101	78	EVFHPDERRFTLRVA	FHPDERRFT	2	0.3673	939.4	70.00	0.37	tr_C9ZL71_
DRB1_0101	79	PGAVLNDTGGGRVSL	LNDTGGGRV	4	0.3668	944.8	70.00	0.75	tr_C9ZL71_
DRB1_0101	80	WKYGTWNSNCATIEL	WNSNCATIE	5	0.3632	982.3	70.00	0.47	tr_C9ZL71_
DRB1_0101	81	RGDDRCVYINLPPGA	CVYINLPPG	5	0.3585	1034.1	75.00	0.48	tr_C9ZL71_
DRB1_0101	82	VLNDTGGGRVSLSLS	LNDTGGGRV	1	0.3545	1078.9	75.00	0.58	tr_C9ZL71_
DRB1_0101	83	WEVFHPDERRFTLRV	FHPDERRFT	3	0.3531	1095.3	75.00	0.41	tr_C9ZL71_
DRB1_0101	84	FHPDERRFTLRVAIP	DERRFTLRV	3	0.3512	1119.2	75.00	0.25	tr_C9ZL71_
DRB1_0101	85	LSDVPGSIARNINNS	VPGSIARNI	3	0.3475	1163.9	75.00	0.40	tr_C9ZL71_
DRB1_0101	86	KYGTWNSNCATIELE	WNSNCATIE	4	0.3446	1201.9	75.00	0.57	tr_C9ZL71_
DRB1_0101	87	MWKYGTWNSNCATIE	WKYGTWNSN	1	0.3432	1220.3	75.00	0.29	tr_C9ZL71_
DRB1_0101	88	DRHTDTYRVQWNGRP	YRVQWNGRP	6	0.3425	1229.0	75.00	0.57	tr_C9ZL71_
DRB1_0101	89	GGGRVSLSLSDVPGS	RVSLSLSDV	3	0.3358	1321.7	75.00	0.32	tr_C9ZL71_
DRB1_0101	90	DWEVFHPDERRFTLR	FHPDERRFT	4	0.3269	1455.4	80.00	0.43	tr_C9ZL71_
DRB1_0101	91	WNGRPYSVDFGDPLL	YSVDFGDPL	5	0.3254	1478.8	80.00	0.69	tr_C9ZL71_
DRB1_0101	92	SDVPGSIARNINNSA	VPGSIARNI	2	0.3252	1482.7	80.00	0.40	tr_C9ZL71_
DRB1_0101	93	LRVAIPFMEENSNWS	LRVAIPFME	0	0.3235	1510.3	80.00	0.48	tr_C9ZL71_
DRB1_0101	94	YGTWNSNCATIELED	WNSNCATIE	3	0.3218	1537.0	80.00	0.64	tr_C9ZL71_
DRB1_0101	95	STAPLPIVCRRGDDR	TAPLPIVCR	1	0.3200	1568.0	80.00	0.62	tr_C9ZL71_
DRB1_0101	96	VAIPFMEENSNWSTA	FMEENSNWS	4	0.3148	1658.5	80.00	0.43	tr_C9ZL71_
DRB1_0101	97	VLDICGRDAPDMWKY	VLDICGRDA	0	0.3126	1699.0	80.00	0.34	tr_C9ZL71_
DRB1_0101	98	PPGAVLNDTGGGRVS	LNDTGGGRV	5	0.3072	1801.4	80.00	0.73	tr_C9ZL71_
DRB1_0101	99	VINLASPDDWEVFHP	INLASPDDW	1	0.3055	1833.2	85.00	0.37	tr_C9ZL71_
DRB1_0101	100	DMWKYGTWNSNCATI	WKYGTWNSN	2	0.3055	1833.6	85.00	0.38	tr_C9ZL71_
DRB1_0101	101	TGGGRVSLSLSDVPG	RVSLSLSDV	4	0.3050	1844.8	85.00	0.31	tr_C9ZL71_
DRB1_0101	102	AIPFMEENSNWSTAP	FMEENSNWS	3	0.3047	1850.4	85.00	0.41	tr_C9ZL71_
DRB1_0101	103	IPFMEENSNWSTAPL	MEENSNWST	3	0.3004	1939.2	85.00	0.44	tr_C9ZL71_
DRB1_0101	104	GTWNSNCATIELEDR	WNSNCATIE	2	0.2966	2020.0	85.00	0.57	tr_C9ZL71_
DRB1_0101	105	DDWEVFHPDERRFTL	FHPDERRFT	5	0.2946	2063.3	85.00	0.41	tr_C9ZL71_
DRB1_0101	106	PIVCRRGDDRCVYIN	CRRGDDRCV	3	0.2923	2116.1	85.00	0.50	tr_C9ZL71_
DRB1_0101	107	FMEENSNWSTAPLPI	NWSTAPLPI	6	0.2921	2119.9	85.00	0.35	tr_C9ZL71_
DRB1_0101	108	PDMWKYGTWNSNCAT	WKYGTWNSN	3	0.2906	2154.1	85.00	0.44	tr_C9ZL71_
DRB1_0101	109	IVCRRGDDRCVYINL	CRRGDDRCV	2	0.2906	2155.5	85.00	0.48	tr_C9ZL71_
DRB1_0101	110	LPIVCRRGDDRCVYI	CRRGDDRCV	4	0.2850	2289.1	85.00	0.49	tr_C9ZL71_
DRB1_0101	111	LNDTGGGRVSLSLSD	LNDTGGGRV	0	0.2823	2357.8	85.00	0.37	tr_C9ZL71_
DRB1_0101	112	DTGGGRVSLSLSDVP	RVSLSLSDV	5	0.2781	2466.6	85.00	0.30	tr_C9ZL71_
DRB1_0101	113	ELEDRHTDTYRVQWN	DRHTDTYRV	3	0.2773	2489.3	85.00	0.75	tr_C9ZL71_
DRB1_0101	114	LEDRHTDTYRVQWNG	DRHTDTYRV	2	0.2721	2632.7	90.00	0.73	tr_C9ZL71_
DRB1_0101	115	NDTGGGRVSLSLSDV	GRVSLSLSD	5	0.2711	2661.1	90.00	0.25	tr_C9ZL71_
DRB1_0101	116	RVAIPFMEENSNWST	FMEENSNWS	5	0.2686	2733.6	90.00	0.38	tr_C9ZL71_
DRB1_0101	117	RVQWNGRPYSVDFGD	QWNGRPYSV	2	0.2682	2745.4	90.00	0.53	tr_C9ZL71_
DRB1_0101	118	IELEDRHTDTYRVQW	DRHTDTYRV	4	0.2674	2770.5	90.00	0.68	tr_C9ZL71_
DRB1_0101	119	PFMEENSNWSTAPLP	MEENSNWST	2	0.2673	2774.2	90.00	0.47	tr_C9ZL71_
DRB1_0101	120	APDMWKYGTWNSNCA	WKYGTWNSN	4	0.2644	2860.7	90.00	0.45	tr_C9ZL71_

DRB1_0101	121	PLPIVCRRGDDRCVY	CRRGDDRCV	5	0.2636	2887.3	90.00	0.43	tr_C9ZL71_
DRB1_0101	122	EDRHTDTYRVQWNGR	DRHTDTYRV	1	0.2626	2918.2	90.00	0.63	tr_C9ZL71_
DRB1_0101	123	TWNSNCATIELEDRH	WNSNCATIE	1	0.2625	2921.6	90.00	0.53	tr_C9ZL71_
DRB1_0101	124	VCRRGDDRCVYINLP	CRRGDDRCV	1	0.2614	2955.1	90.00	0.45	tr_C9ZL71_
DRB1_0101	125	INLPPGAVLNDTGGG	INLPPGAVL	0	0.2576	3079.9	90.00	0.49	tr_C9ZL71_
DRB1_0101	126	RRGDDRCVYINLPPG	CVYINLPPG	6	0.2522	3266.4	90.00	0.44	tr_C9ZL71_
DRB1_0101	127	TIELEDRHTDTYRVQ	DRHTDTYRV	5	0.2508	3316.1	90.00	0.58	tr_C9ZL71_
DRB1_0101	128	QWNGRPYSVDFGDPL	YSVDFGDPL	6	0.2399	3731.4	95.00	0.52	tr_C9ZL71_
DRB1_0101	129	TAPLPIVCRRGDDRC	TAPLPIVCR	0	0.2367	3861.0	95.00	0.43	tr_C9ZL71_
DRB1_0101	130	PDDWEVFHPDERRFT	FHPDERRFT	6	0.2356	3908.7	95.00	0.35	tr_C9ZL71_
DRB1_0101	131	LDICGRDAPDMWKYG	ICGRDAPDM	2	0.2342	3968.3	95.00	0.47	tr_C9ZL71_
DRB1_0101	132	APLPIVCRRGDDRCV	CRRGDDRCV	6	0.2200	4626.7	95.00	0.34	tr_C9ZL71_
DRB1_0101	133	DAPDMWKYGTWNSNC	WKYGTWNSN	5	0.2185	4701.4	95.00	0.49	tr_C9ZL71_
DRB1_0101	134	LPPGAVLNDTGGGRV	LNDTGGGRV	6	0.2184	4708.6	95.00	0.64	tr_C9ZL71_
DRB1_0101	135	CRRGDDRCVYINLPP	CRRGDDRCV	0	0.2163	4814.9	95.00	0.34	tr_C9ZL71_
DRB1_0101	136	VQWNGRPYSVDFGDP	QWNGRPYSV	1	0.2136	4956.7	95.00	0.50	tr_C9ZL71_
DRB1_0101	137	ATIELEDRHTDTYRV	DRHTDTYRV	6	0.2093	5192.7	95.00	0.43	tr_C9ZL71_
DRB1_0101	138	WNSNCATIELEDRHT	WNSNCATIE	0	0.2017	5638.6	95.00	0.37	tr_C9ZL71_
DRB1_0101	139	DICGRDAPDMWKYGT	ICGRDAPDM	1	0.2015	5651.0	95.00	0.41	tr_C9ZL71_
DRB1_0101	140	INLASPDDWEVFHPD	INLASPDDW	0	0.1949	6068.7	95.00	0.35	tr_C9ZL71_
DRB1_0101	141	RDAPDMWKYGTWNSN	WKYGTWNSN	6	0.1886	6497.0	100.00	0.40	tr_C9ZL71_
DRB1_0101	142	ICGRDAPDMWKYGTW	ICGRDAPDM	0	0.1731	7681.2	100.00	0.25	tr_C9ZL71_
DRB1_0101	143	NCATIELEDRHTDTY	TIELEDRHT	3	0.1629	8583.8	100.00	0.27	tr_C9ZL71_
DRB1_0101	144	CATIELEDRHTDTYR	IELEDRHTD	3	0.1628	8589.4	100.00	0.34	tr_C9ZL71_
DRB1_0101	145	SNCATIELEDRHTDT	CATIELEDR	2	0.1582	9031.2	100.00	0.30	tr_C9ZL71_
DRB1_0101	146	SPDDWEVFHPDERRF	WEVFHPDER	4	0.1576	9085.7	100.00	0.41	tr_C9ZL71_
DRB1_0101	147	NSNCATIELEDRHTD	CATIELEDR	3	0.1567	9171.2	100.00	0.35	tr_C9ZL71_
DRB1_0101	148	CGRDAPDMWKYGTWN	RDAPDMWKY	2	0.1326	11903.4	100.00	0.23	tr_C9ZL71_
DRB1_0101	149	NLPPGAVLNDTGGGR	NLPPGAVLN	0	0.1296	12300.4	100.00	0.26	tr_C9ZL71_
DRB1_0101	150	ASPDDWEVFHPDERR	WEVFHPDER	5	0.1228	13238.2	100.00	0.44	tr_C9ZL71_
DRB1_0101	151	GRDAPDMWKYGTWNS	RDAPDMWKY	1	0.1166	14165.9	100.00	0.22	tr_C9ZL71_
DRB1_0101	152	NLASPDDWEVFHPDE	LASPDDWEV	1	0.1147	14455.0	100.00	0.51	tr_C9ZL71_
DRB1_0101	153	LASPDDWEVFHPDER	LASPDDWEV	0	0.1098	15237.5	100.00	0.31	tr_C9ZL71_

Allele: DRB1_0101. Number of high binders 0. Number of weak binders 11. Number of peptides 153

Allele	pos	peptide	core	Of	1-log50k(aff)	affinity(nM)	%Rank	Relia	Identity	Bind Level
DRB1_0101	1	VKSYARSLNALALDT	YARSLNALA	3	0.8890	3.3	0.15	0.86	tr_C9ZL71_	SB
DRB1_0101	2	QVKSYARSLNALALD	YARSLNALA	4	0.8793	3.7	0.25	0.85	tr_C9ZL71_	SB
DRB1_0101	3	KSYARSLNALALDTS	YARSLNALA	2	0.8789	3.7	0.25	0.87	tr_C9ZL71_	SB
DRB1_0101	4	HQVKSYARSLNALAL	YARSLNALA	5	0.8691	4.1	0.40	0.82	tr_C9ZL71_	SB
DRB1_0101	5	SINYTIMCSVHSRPQ	YTIMCSVHS	3	0.8420	5.5	0.80	0.88	tr_C9ZL71_	WB
DRB1_0101	6	KSVWQALRGVISASV	WQALRGVIS	3	0.8390	5.7	0.80	0.77	tr_C9ZL71_	WB
DRB1_0101	7	SVWQALRGVISASVR	WQALRGVIS	2	0.8387	5.7	0.90	0.68	tr_C9ZL71_	WB
DRB1_0101	8	SYARSLNALALDTSC	YARSLNALA	1	0.8374	5.8	0.90	0.86	tr_C9ZL71_	WB
DRB1_0101	9	PSINYTIMCSVHSRP	YTIMCSVHS	4	0.8346	6.0	1.00	0.89	tr_C9ZL71_	WB

DRB1_0101	10	DPSINYTIMCSVHSR	YTIMCSVHS	5	0.8330	6.1	1.00	0.88	tr_C9ZL71_	WB
DRB1_0101	11	VWQALRGVISASVRG	WQALRGVIS	1	0.8281	6.4	1.10	0.61	tr_C9ZL71_	WB
DRB1_0101	12	INYTIMCSVHSRPQD	YTIMCSVHS	2	0.8254	6.6	1.20	0.89	tr_C9ZL71_	WB
DRB1_0101	13	PKSVWQALRGVISAS	WQALRGVIS	4	0.8173	7.2	1.40	0.83	tr_C9ZL71_	WB
DRB1_0101	14	WQALRGVISASVRGA	LRGVISASV	3	0.8133	7.5	1.60	0.33	tr_C9ZL71_	WB
DRB1_0101	15	NYTIMCSVHSRPQDI	YTIMCSVHS	1	0.8089	7.9	1.70	0.88	tr_C9ZL71_	WB
DRB1_0101	16	LAWQPLIQLPALLVW	LIQLPALLV	5	0.8003	8.7	2.00	0.48	tr_C9ZL71_	WB
DRB1_0101	17	NPKSVWQALRGVISA	WQALRGVIS	5	0.7985	8.9	2.50	0.83	tr_C9ZL71_	WB
DRB1_0101	18	MLAWQPLIQLPALLV	LIQLPALLV	6	0.7908	9.6	2.50	0.35	tr_C9ZL71_	WB
DRB1_0101	19	GHQVKSYARSLNALA	YARSLNALA	6	0.7866	10.1	3.00	0.68	tr_C9ZL71_	WB
DRB1_0101	20	AWQPLIQLPALLVWP	LIQLPALLV	4	0.7830	10.5	3.00	0.55	tr_C9ZL71_	WB
DRB1_0101	21	WQPLIQLPALLVWPV	LIQLPALLV	3	0.7636	12.9	4.00	0.63	tr_C9ZL71_	WB
DRB1_0101	22	SDPSINYTIMCSVHS	YTIMCSVHS	6	0.7627	13.0	4.00	0.86	tr_C9ZL71_	WB
DRB1_0101	23	QPLIQLPALLVWPVL	LIQLPALLV	2	0.7532	14.4	4.50	0.68	tr_C9ZL71_	WB
DRB1_0101	24	NNPKSVWQALRGVIS	WQALRGVIS	6	0.7435	16.0	5.50	0.81	tr_C9ZL71_	WB
DRB1_0101	25	QALRGVISASVRGAG	LRGVISASV	2	0.7371	17.2	6.00	0.59	tr_C9ZL71_	WB
DRB1_0101	26	YTIMCSVHSRPQDIE	YTIMCSVHS	0	0.7360	17.4	6.00	0.76	tr_C9ZL71_	WB
DRB1_0101	27	LGGFLKENSSTVYRV	FLKENSSTV	3	0.7292	18.7	6.50	0.78	tr_C9ZL71_	WB
DRB1_0101	28	GMLAWQPLIQLPALL	WQPLIQLPA	4	0.7275	19.1	6.50	0.50	tr_C9ZL71_	WB
DRB1_0101	29	ALRGVISASVRGAGR	LRGVISASV	1	0.7230	20.0	7.00	0.47	tr_C9ZL71_	WB
DRB1_0101	30	PLIQLPALLVWPVLV	LIQLPALLV	1	0.7222	20.2	7.00	0.62	tr_C9ZL71_	WB
DRB1_0101	31	YARSLNALALDTSCK	YARSLNALA	0	0.7207	20.5	7.00	0.66	tr_C9ZL71_	WB
DRB1_0101	32	GGFLKENSSTVYRVF	FLKENSSTV	2	0.7182	21.1	7.50	0.71	tr_C9ZL71_	WB
DRB1_0101	33	PGMLAWQPLIQLPAL	WQPLIQLPA	5	0.7116	22.7	8.00	0.46	tr_C9ZL71_	WB
DRB1_0101	34	IAVFEALDVAAVCAV	FEALDVAAV	3	0.7027	24.9	9.00	0.73	tr_C9ZL71_	WB
DRB1_0101	35	LDFRIPGMLAWQPLI	DFRIPGMLA	1	0.6978	26.3	9.00	0.42	tr_C9ZL71_	WB
DRB1_0101	36	AHAFERAETYLLDAV	FERAETYLL	3	0.6958	26.9	9.50	0.92	tr_C9ZL71_	WB
DRB1_0101	37	ATFRLYGTQVGTEVD	TFRLYGTQV	1	0.6944	27.3	9.50	0.37	tr_C9ZL71_	WB
DRB1_0101	38	KLGGFLKENSSTVYR	FLKENSSTV	4	0.6937	27.5	9.50	0.81	tr_C9ZL71_	WB
DRB1_0101	39	IATFRLYGTQVGTEV	TFRLYGTQV	2	0.6932	27.6	9.50	0.44	tr_C9ZL71_	WB
DRB1_0101	40	PIAVFEALDVAAVCA	FEALDVAAV	4	0.6925	27.9	10.00	0.68	tr_C9ZL71_	
DRB1_0101	41	VLDFRIPGMLAWQPL	DFRIPGMLA	2	0.6904	28.5	10.00	0.48	tr_C9ZL71_	
DRB1_0101	42	GGALNGSVALRLSST	LNGSVALRL	3	0.6886	29.0	10.00	0.66	tr_C9ZL71_	
DRB1_0101	43	AVACLCAYSVRRMLR	VACLCAYSV	1	0.6884	29.1	10.00	0.47	tr_C9ZL71_	
DRB1_0101	44	GAVACLCAYSVRRML	VACLCAYSV	2	0.6875	29.4	11.00	0.58	tr_C9ZL71_	
DRB1_0101	45	IPGMLAWQPLIQLPA	LAWQPLIQL	4	0.6874	29.4	11.00	0.34	tr_C9ZL71_	
DRB1_0101	46	GFLKENSSTVYRVFC	FLKENSSTV	1	0.6860	29.9	11.00	0.64	tr_C9ZL71_	
DRB1_0101	47	YKPIAVFEALDVAAV	FEALDVAAV	6	0.6854	30.1	11.00	0.35	tr_C9ZL71_	
DRB1_0101	48	DGGALNGSVALRLSS	LNGSVALRL	4	0.6833	30.8	11.00	0.62	tr_C9ZL71_	
DRB1_0101	49	QYGYKPIAVFEALDV	YKPIAVFEA	3	0.6823	31.1	11.00	0.64	tr_C9ZL71_	
DRB1_0101	50	AVFEALDVAAVCAVM	FEALDVAAV	2	0.6818	31.3	11.00	0.75	tr_C9ZL71_	
DRB1_0101	51	LRGVISASVRGAGRN	GVISASVRG	2	0.6814	31.4	11.00	0.38	tr_C9ZL71_	
DRB1_0101	52	YGYKPIAVFEALDVA	YKPIAVFEA	2	0.6810	31.6	11.00	0.58	tr_C9ZL71_	
DRB1_0101	53	GGAVACLCAYSVRRM	VACLCAYSV	3	0.6759	33.4	12.00	0.64	tr_C9ZL71_	
DRB1_0101	54	PAHAFERAETYLLDA	FERAETYLL	4	0.6754	33.5	12.00	0.92	tr_C9ZL71_	
DRB1_0101	55	KPIAVFEALDVAAVC	FEALDVAAV	5	0.6743	33.9	12.00	0.57	tr_C9ZL71_	
DRB1_0101	56	TFRLYGTQVGTEVDI	YGTQVGTEV	4	0.6725	34.6	12.00	0.49	tr_C9ZL71_	
DRB1_0101	57	DEIATFRLYGTQVGT	TFRLYGTQV	4	0.6709	35.2	12.00	0.57	tr_C9ZL71_	
DRB1_0101	58	EIATFRLYGTQVGTE	TFRLYGTQV	3	0.6706	35.3	12.00	0.59	tr_C9ZL71_	
DRB1_0101	59	HAFERAETYLLDAVQ	FERAETYLL	2	0.6703	35.4	13.00	0.93	tr_C9ZL71_	

DRB1_0101	60	DFRIPGMLAWQPLIQ	DFRIPGMLA	0	0.6691	35.9	13.00	0.29	tr_C9ZL71_
DRB1_0101	61	GYKPIAVFEALDVAA	YKPIAVFEA	1	0.6682	36.2	13.00	0.47	tr_C9ZL71_
DRB1_0101	62	NDGGALNGSVALRLS	LNGSVALRL	5	0.6649	37.6	13.00	0.54	tr_C9ZL71_
DRB1_0101	63	YVLDFRIPGMLAWQP	DFRIPGMLA	3	0.6606	39.4	14.00	0.59	tr_C9ZL71_
DRB1_0101	64	KQYGYKPIAVFEALD	YKPIAVFEA	4	0.6597	39.7	14.00	0.68	tr_C9ZL71_
DRB1_0101	65	LIQLPALLVWPVLVN	LIQLPALLV	0	0.6588	40.1	14.00	0.50	tr_C9ZL71_
DRB1_0101	66	FRIPGMLAWQPLIQL	LAWQPLIQL	6	0.6582	40.4	14.00	0.24	tr_C9ZL71_
DRB1_0101	67	VKQYGYKPIAVFEAL	YKPIAVFEA	5	0.6581	40.4	14.00	0.62	tr_C9ZL71_
DRB1_0101	68	PLYLPFFNLETAIDC	YLPFFNLET	2	0.6568	41.0	14.00	0.34	tr_C9ZL71_
DRB1_0101	69	LYLPFFNLETAIDCF	FNLETAIDC	5	0.6541	42.2	15.00	0.35	tr_C9ZL71_
DRB1_0101	70	WYRWLARHGGSMYIF	YRWLARHGG	1	0.6538	42.4	15.00	0.36	tr_C9ZL71_
DRB1_0101	71	GALNGSVALRLSSTT	LNGSVALRL	2	0.6528	42.8	15.00	0.69	tr_C9ZL71_
DRB1_0101	72	AAVCAVMDTEFLHHR	CAVMDTEFL	3	0.6521	43.1	15.00	0.82	tr_C9ZL71_
DRB1_0101	73	YLPFFNLETAIDCFN	FNLETAIDC	4	0.6483	44.9	15.00	0.48	tr_C9ZL71_
DRB1_0101	74	MGGAVACLCAYSVRR	VACLCAYSV	4	0.6482	45.0	15.00	0.67	tr_C9ZL71_
DRB1_0101	75	RIPGMLAWQPLIQLP	LAWQPLIQL	5	0.6476	45.3	15.00	0.34	tr_C9ZL71_
DRB1_0101	76	VTVYTFGQPPMGNAA	YTFGQPPMG	3	0.6433	47.4	16.00	0.72	tr_C9ZL71_
DRB1_0101	77	VFEALDVAAVCAVMD	FEALDVAAV	1	0.6424	47.9	16.00	0.70	tr_C9ZL71_
DRB1_0101	78	LPFFNLETAIDCFNI	FNLETAIDC	3	0.6411	48.6	16.00	0.56	tr_C9ZL71_
DRB1_0101	79	EPLYLPFFNLETAID	YLPFFNLET	3	0.6402	49.0	16.00	0.41	tr_C9ZL71_
DRB1_0101	80	SDEIATFRLYGTQVG	TFRLYGTQV	5	0.6401	49.1	16.00	0.55	tr_C9ZL71_
DRB1_0101	81	QTAYDKAIPRTFRVV	YDKAIPRTF	3	0.6391	49.6	17.00	0.60	tr_C9ZL71_
DRB1_0101	82	VACLCAYSVRRMLRE	CLCAYSVRR	2	0.6387	49.9	17.00	0.32	tr_C9ZL71_
DRB1_0101	83	TEFLHHRGKAPRIVI	FLHHRGKAP	2	0.6363	51.2	17.00	0.70	tr_C9ZL71_
DRB1_0101	84	YRWLARHGGSMYIFH	LARHGGSMY	3	0.6353	51.7	17.00	0.34	tr_C9ZL71_
DRB1_0101	85	AVCAVMDTEFLHHRG	CAVMDTEFL	2	0.6352	51.8	17.00	0.82	tr_C9ZL71_
DRB1_0101	86	DWYRWLARHGGSMYI	YRWLARHGG	2	0.6346	52.1	17.00	0.42	tr_C9ZL71_
DRB1_0101	87	AFERAETYLLDAVQQ	FERAETYLL	1	0.6275	56.3	18.00	0.86	tr_C9ZL71_
DRB1_0101	88	RPAHAFERAETYLLD	FERAETYLL	5	0.6266	56.8	18.00	0.89	tr_C9ZL71_
DRB1_0101	89	HEPLYLPFFNLETAI	YLPFFNLET	4	0.6256	57.5	19.00	0.44	tr_C9ZL71_
DRB1_0101	90	TAYDKAIPRTFRVVN	YDKAIPRTF	2	0.6255	57.5	19.00	0.54	tr_C9ZL71_
DRB1_0101	91	QLEYRVRRYLVRLKR	YRVRRYLVR	3	0.6235	58.8	19.00	0.40	tr_C9ZL71_
DRB1_0101	92	ALNGSVALRLSSTTG	LNGSVALRL	1	0.6231	59.1	19.00	0.60	tr_C9ZL71_
DRB1_0101	93	EFLHHRGKAPRIVIA	FLHHRGKAP	1	0.6221	59.7	19.00	0.54	tr_C9ZL71_
DRB1_0101	94	VAAVCAVMDTEFLHH	CAVMDTEFL	4	0.6220	59.7	19.00	0.80	tr_C9ZL71_
DRB1_0101	95	FRLYGTQVGTEVDIN	YGTQVGTEV	3	0.6202	60.9	19.00	0.71	tr_C9ZL71_
DRB1_0101	96	NLSLLLQSIPNDSSS	LLQSIPNDS	4	0.6200	61.0	19.00	0.40	tr_C9ZL71_
DRB1_0101	97	FQTAYDKAIPRTFRV	YDKAIPRTF	4	0.6197	61.3	19.00	0.60	tr_C9ZL71_
DRB1_0101	98	HNLSLLLQSIPNDSS	LLQSIPNDS	5	0.6194	61.4	19.00	0.28	tr_C9ZL71_
DRB1_0101	99	EKLGGFLKENSSTVY	FLKENSSTV	5	0.6187	61.9	20.00	0.81	tr_C9ZL71_
DRB1_0101	100	PRIVIAFRGTANMSN	PRIVIAFRG	0	0.6185	62.0	20.00	0.30	tr_C9ZL71_
DRB1_0101	101	DTEFLHHRGKAPRIV	FLHHRGKAP	3	0.6180	62.4	20.00	0.79	tr_C9ZL71_
DRB1_0101	102	KAPRIVIAFRGTANM	PRIVIAFRG	2	0.6175	62.7	20.00	0.57	tr_C9ZL71_
DRB1_0101	103	APRIVIAFRGTANMS	PRIVIAFRG	1	0.6170	63.1	20.00	0.47	tr_C9ZL71_
DRB1_0101	104	RRGGVHNLSLLLQSI	GVHNLSLLL	3	0.6151	64.4	20.00	0.48	tr_C9ZL71_
DRB1_0101	105	TVYTFGQPPMGNAAF	YTFGQPPMG	2	0.6150	64.4	20.00	0.73	tr_C9ZL71_
DRB1_0101	106	RGVISASVRGAGRNL	GVISASVRG	1	0.6149	64.5	20.00	0.38	tr_C9ZL71_
DRB1_0101	107	EVTVYTFGQPPMGNA	YTFGQPPMG	4	0.6146	64.7	20.00	0.69	tr_C9ZL71_
DRB1_0101	108	KQLEYRVRRYLVRLK	YRVRRYLVR	4	0.6134	65.5	20.00	0.41	tr_C9ZL71_
DRB1_0101	109	PFFNLETAIDCFNIA	FNLETAIDC	2	0.6132	65.7	20.00	0.56	tr_C9ZL71_

DRB1_0101	110	RIVIAFRGTANMSNV	FRGTANMSN	5	0.6108	67.5	21.00	0.41	tr_C9ZL71_
DRB1_0101	111	IVIAFRGTANMSNVR	FRGTANMSN	4	0.6094	68.4	21.00	0.56	tr_C9ZL71_
DRB1_0101	112	MHEPLYLPFFNLETA	PLYLPFFNL	3	0.6051	71.7	22.00	0.47	tr_C9ZL71_
DRB1_0101	113	LEYRVRRYLVRLKRR	YRVRRYLVR	2	0.6024	73.8	22.00	0.38	tr_C9ZL71_
DRB1_0101	114	AFQTAYDKAIPRTFR	YDKAIPRTF	5	0.6012	74.8	22.00	0.48	tr_C9ZL71_
DRB1_0101	115	GKAPRIVIAFRGTAN	PRIVIAFRG	3	0.6005	75.4	22.00	0.63	tr_C9ZL71_
DRB1_0101	116	FLKENSSTVYRVFCT	FLKENSSTV	0	0.5986	77.0	23.00	0.51	tr_C9ZL71_
DRB1_0101	117	RGGVHNLSLLLQSIP	GVHNLSLLL	2	0.5984	77.1	23.00	0.41	tr_C9ZL71_
DRB1_0101	118	VCAVMDTEFLHHRGK	CAVMDTEFL	1	0.5981	77.4	23.00	0.79	tr_C9ZL71_
DRB1_0101	119	RAETYLLDAVQQRMH	YLLDAVQQR	4	0.5976	77.8	23.00	0.54	tr_C9ZL71_
DRB1_0101	120	ENDGGALNGSVALRL	LNGSVALRL	6	0.5969	78.4	23.00	0.40	tr_C9ZL71_
DRB1_0101	121	DVAAVCAVMDTEFLH	CAVMDTEFL	5	0.5931	81.7	24.00	0.77	tr_C9ZL71_
DRB1_0101	122	YRVFCTGHSMGGAVA	FCTGHSMGG	3	0.5930	81.8	24.00	0.25	tr_C9ZL71_
DRB1_0101	123	GGVHNLSLLLQSIPN	NLSLLLQSI	4	0.5927	82.0	24.00	0.27	tr_C9ZL71_
DRB1_0101	124	VFCTGHSMGGAVACL	GHSMGGAVA	4	0.5910	83.6	24.00	0.45	tr_C9ZL71_
DRB1_0101	125	VHNLSLLLQSIPNDS	SLLLQSIPN	4	0.5907	83.8	24.00	0.27	tr_C9ZL71_
DRB1_0101	126	SMGGAVACLCAYSVR	VACLCAYSV	5	0.5907	83.8	24.00	0.69	tr_C9ZL71_
DRB1_0101	127	AYDKAIPRTFRVVNE	YDKAIPRTF	1	0.5901	84.3	24.00	0.50	tr_C9ZL71_
DRB1_0101	128	TVYRVFCTGHSMGGA	YRVFCTGHS	2	0.5897	84.8	24.00	0.52	tr_C9ZL71_
DRB1_0101	129	GVISASVRGAGRNLF	SVRGAGRNL	5	0.5884	85.9	24.00	0.35	tr_C9ZL71_
DRB1_0101	130	DDWYRWLARHGGSMY	YRWLARHGG	3	0.5880	86.3	24.00	0.52	tr_C9ZL71_
DRB1_0101	131	STVYRVFCTGHSMGG	YRVFCTGHS	3	0.5868	87.5	25.00	0.62	tr_C9ZL71_
DRB1_0101	132	AETYLLDAVQQRMHE	YLLDAVQQR	3	0.5860	88.2	25.00	0.57	tr_C9ZL71_
DRB1_0101	133	VIAFRGTANMSNVRE	FRGTANMSN	3	0.5849	89.2	25.00	0.69	tr_C9ZL71_
DRB1_0101	134	LSLLLQSIPNDSSSP	LLQSIPNDS	3	0.5844	89.7	25.00	0.44	tr_C9ZL71_
DRB1_0101	135	GVHNLSLLLQSIPND	NLSLLLQSI	3	0.5842	89.9	25.00	0.28	tr_C9ZL71_
DRB1_0101	136	FLHHRGKAPRIVIAF	FLHHRGKAP	0	0.5822	91.8	25.00	0.35	tr_C9ZL71_
DRB1_0101	137	DVKQYGYKPIAVFEA	YKPIAVFEA	6	0.5798	94.3	26.00	0.46	tr_C9ZL71_
DRB1_0101	138	VYRVFCTGHSMGGAV	YRVFCTGHS	1	0.5785	95.7	26.00	0.41	tr_C9ZL71_
DRB1_0101	139	KRRGGVHNLSLLLQS	GVHNLSLLL	4	0.5775	96.7	26.00	0.52	tr_C9ZL71_
DRB1_0101	140	RGKAPRIVIAFRGTA	PRIVIAFRG	4	0.5763	97.9	26.00	0.62	tr_C9ZL71_
DRB1_0101	141	FERAETYLLDAVQQR	FERAETYLL	0	0.5762	98.0	26.00	0.46	tr_C9ZL71_
DRB1_0101	142	FCTGHSMGGAVACLC	GHSMGGAVA	3	0.5761	98.1	26.00	0.47	tr_C9ZL71_
DRB1_0101	143	WKQLEYRVRRYLVRL	YRVRRYLVR	5	0.5753	99.0	27.00	0.40	tr_C9ZL71_
DRB1_0101	144	FEALDVAAVCAVMDT	FEALDVAAV	0	0.5750	99.3	27.00	0.52	tr_C9ZL71_
DRB1_0101	145	QQRMHEPLYLPFFNL	QRMHEPLYL	1	0.5709	103.8	27.00	0.35	tr_C9ZL71_
DRB1_0101	146	MDTEFLHHRGKAPRI	FLHHRGKAP	4	0.5697	105.2	28.00	0.83	tr_C9ZL71_
DRB1_0101	147	SYNWKGQSHARAQER	WKGQSHARA	3	0.5688	106.2	28.00	0.62	tr_C9ZL71_
DRB1_0101	148	EGHQVKSYARSLNAL	QVKSYARSL	3	0.5669	108.4	28.00	0.56	tr_C9ZL71_
DRB1_0101	149	ISASVRGAGRNLFER	SVRGAGRNL	3	0.5667	108.7	28.00	0.62	tr_C9ZL71_
DRB1_0101	150	EYRVRRYLVRLKRRG	YRVRRYLVR	1	0.5652	110.4	28.00	0.37	tr_C9ZL71_
DRB1_0101	151	SSTVYRVFCTGHSMG	YRVFCTGHS	4	0.5651	110.5	28.00	0.65	tr_C9ZL71_
DRB1_0101	152	ESDEIATFRLYGTQV	TFRLYGTQV	6	0.5648	110.9	29.00	0.47	tr_C9ZL71_
DRB1_0101	153	RMHEPLYLPFFNLET	PLYLPFFNL	4	0.5634	112.6	29.00	0.51	tr_C9ZL71_
DRB1_0101	154	RVFCTGHSMGGAVAC	GHSMGGAVA	5	0.5628	113.4	29.00	0.34	tr_C9ZL71_
DRB1_0101	155	AAFQTAYDKAIPRTF	FQTAYDKAI	2	0.5613	115.2	29.00	0.47	tr_C9ZL71_
DRB1_0101	156	KNKAFGIEGHQVKSY	AFGIEGHQV	3	0.5608	115.8	29.00	0.56	tr_C9ZL71_
DRB1_0101	157	RWLARHGGSMYIFHT	LARHGGSMY	2	0.5607	115.9	29.00	0.44	tr_C9ZL71_
DRB1_0101	158	FFNLETAIDCFNIAF	FNLETAIDC	1	0.5584	118.8	30.00	0.57	tr_C9ZL71_
DRB1_0101	159	QRMHEPLYLPFFNLE	PLYLPFFNL	5	0.5574	120.1	30.00	0.50	tr_C9ZL71_

DRB1_0101	160	ESYNWKGQSHARAQE	WKGQSHARA	4	0.5574	120.1	30.00	0.56	tr_C9ZL71_
DRB1_0101	161	DEVTVYTFGQPPMGN	YTFGQPPMG	5	0.5555	122.6	30.00	0.64	tr_C9ZL71_
DRB1_0101	162	ERAETYLLDAVQQRM	YLLDAVQQR	5	0.5554	122.8	30.00	0.55	tr_C9ZL71_
DRB1_0101	163	NKAFGIEGHQVKSYA	AFGIEGHQV	2	0.5553	122.9	30.00	0.48	tr_C9ZL71_
DRB1_0101	164	CTGHSMGGAVACLCA	GHSMGGAVA	2	0.5552	123.1	30.00	0.44	tr_C9ZL71_
DRB1_0101	165	ETYLLDAVQQRMHEP	YLLDAVQQR	2	0.5545	124.0	30.00	0.58	tr_C9ZL71_
DRB1_0101	166	VYTFGQPPMGNAAFQ	YTFGQPPMG	1	0.5525	126.7	31.00	0.74	tr_C9ZL71_
DRB1_0101	167	FESYNWKGQSHARAQ	WKGQSHARA	5	0.5520	127.4	31.00	0.49	tr_C9ZL71_
DRB1_0101	168	LKRRGGVHNLSLLLQ	GVHNLSLLL	5	0.5517	127.8	31.00	0.47	tr_C9ZL71_
DRB1_0101	169	HRGKAPRIVIAFRGT	PRIVIAFRG	5	0.5499	130.4	31.00	0.57	tr_C9ZL71_
DRB1_0101	170	RESICNRGAAEIVNM	CNRGAAEIV	4	0.5446	138.1	32.00	0.55	tr_C9ZL71_
DRB1_0101	171	ERPAHAFERAETYLL	FERAETYLL	6	0.5432	140.1	32.00	0.82	tr_C9ZL71_
DRB1_0101	172	HSMGGAVACLCAYSV	VACLCAYSV	6	0.5426	141.0	33.00	0.53	tr_C9ZL71_
DRB1_0101	173	IAFRGTANMSNVRED	FRGTANMSN	2	0.5416	142.6	33.00	0.74	tr_C9ZL71_
DRB1_0101	174	VISASVRGAGRNLFE	SVRGAGRNL	4	0.5404	144.4	33.00	0.56	tr_C9ZL71_
DRB1_0101	175	NWKQLEYRVRRYLVR	YRVRRYLVR	6	0.5374	149.2	34.00	0.28	tr_C9ZL71_
DRB1_0101	176	KAFGIEGHQVKSYAR	AFGIEGHQV	1	0.5373	149.4	34.00	0.41	tr_C9ZL71_
DRB1_0101	177	ACLCAYSVRRMLREI	CLCAYSVRR	1	0.5370	149.8	34.00	0.38	tr_C9ZL71_
DRB1_0101	178	AVQQRMHEPLYLPFF	QRMHEPLYL	3	0.5370	149.8	34.00	0.58	tr_C9ZL71_
DRB1_0101	179	SASVRGAGRNLFERP	SVRGAGRNL	2	0.5360	151.5	34.00	0.61	tr_C9ZL71_
DRB1_0101	180	SLLLQSIPNDSSSPP	LLQSIPNDS	2	0.5352	152.9	34.00	0.48	tr_C9ZL71_
DRB1_0101	181	TGHSMGGAVACLCAY	GHSMGGAVA	1	0.5342	154.4	34.00	0.42	tr_C9ZL71_
DRB1_0101	182	LNGSVALRLSSTTGD	LNGSVALRL	0	0.5304	160.9	35.00	0.35	tr_C9ZL71_
DRB1_0101	183	HHRGKAPRIVIAFRG	PRIVIAFRG	6	0.5304	161.0	35.00	0.41	tr_C9ZL71_
DRB1_0101	184	RLYGTQVGTEVDINR	YGTQVGTEV	2	0.5301	161.5	35.00	0.85	tr_C9ZL71_
DRB1_0101	185	NAAFQTAYDKAIPRT	FQTAYDKAI	3	0.5299	161.7	35.00	0.69	tr_C9ZL71_
DRB1_0101	186	LDVAAVCAVMDTEFL	CAVMDTEFL	6	0.5283	164.6	35.00	0.69	tr_C9ZL71_
DRB1_0101	187	QRCHPMKNKAFGIEG	CHPMKNKAF	2	0.5278	165.5	35.00	0.43	tr_C9ZL71_
DRB1_0101	188	CAVMDTEFLHHRGKA	CAVMDTEFL	0	0.5254	170.0	36.00	0.74	tr_C9ZL71_
DRB1_0101	189	NSSTVYRVFCTGHSM	YRVFCTGHS	5	0.5253	170.0	36.00	0.67	tr_C9ZL71_
DRB1_0101	190	ESICNRGAAEIVNMD	CNRGAAEIV	3	0.5239	172.6	36.00	0.60	tr_C9ZL71_
DRB1_0101	191	RLKRRGGVHNLSLLL	GVHNLSLLL	6	0.5236	173.2	36.00	0.35	tr_C9ZL71_
DRB1_0101	192	TYLLDAVQQRMHEPL	YLLDAVQQR	1	0.5235	173.3	36.00	0.58	tr_C9ZL71_
DRB1_0101	193	VQQRMHEPLYLPFFN	QRMHEPLYL	2	0.5231	174.2	36.00	0.56	tr_C9ZL71_
DRB1_0101	194	GRNLFERPAHAFERA	NLFERPAHA	2	0.5228	174.7	36.00	0.44	tr_C9ZL71_
DRB1_0101	195	QLPALLVWPVLVNVM	LPALLVWPV	1	0.5228	174.8	36.00	0.41	tr_C9ZL71_
DRB1_0101	196	SFNWKQLEYRVRRYL	WKQLEYRVR	3	0.5225	175.4	37.00	0.34	tr_C9ZL71_
DRB1_0101	197	ASFNWKQLEYRVRRY	FNWKQLEYR	2	0.5221	176.1	37.00	0.34	tr_C9ZL71_
DRB1_0101	198	RCHPMKNKAFGIEGH	PMKNKAFGI	3	0.5198	180.6	37.00	0.51	tr_C9ZL71_
DRB1_0101	199	PDDWYRWLARHGGSM	YRWLARHGG	4	0.5197	180.7	37.00	0.64	tr_C9ZL71_
DRB1_0101	200	IQLPALLVWPVLVNV	LPALLVWPV	2	0.5197	180.7	37.00	0.49	tr_C9ZL71_
DRB1_0101	201	MKNKAFGIEGHQVKS	AFGIEGHQV	4	0.5172	185.6	38.00	0.61	tr_C9ZL71_
DRB1_0101	202	AGRNLFERPAHAFER	NLFERPAHA	3	0.5166	186.9	38.00	0.49	tr_C9ZL71_
DRB1_0101	203	QTSIEEKLGGFLKEN	IEEKLGGFL	3	0.5162	187.6	38.00	0.57	tr_C9ZL71_
DRB1_0101	204	LNALALDTSCKIRAS	LALDTSCKI	3	0.5152	189.6	38.00	0.62	tr_C9ZL71_
DRB1_0101	205	YRVRRYLVRLKRRGG	YRVRRYLVR	0	0.5150	190.1	38.00	0.25	tr_C9ZL71_
DRB1_0101	206	YNWKGQSHARAQERK	WKGQSHARA	2	0.5144	191.3	38.00	0.68	tr_C9ZL71_
DRB1_0101	207	FNWKQLEYRVRRYLV	WKQLEYRVR	2	0.5139	192.4	38.00	0.26	tr_C9ZL71_
DRB1_0101	208	EQRCHPMKNKAFGIE	CHPMKNKAF	3	0.5129	194.4	38.00	0.49	tr_C9ZL71_
DRB1_0101	209	YDKAIPRTFRVVNES	KAIPRTFRV	2	0.5116	197.2	39.00	0.38	tr_C9ZL71_

DRB1_0101	210	EASYVAEPGREPDAM	YVAEPGREP	3	0.5114	197.6	39.00	0.89	tr_C9ZL71_
DRB1_0101	211	SLNALALDTSCKIRA	LALDTSCKI	4	0.5099	200.9	39.00	0.50	tr_C9ZL71_
DRB1_0101	212	GNAAFQTAYDKAIPR	FQTAYDKAI	4	0.5086	203.7	39.00	0.73	tr_C9ZL71_
DRB1_0101	213	DAVQQRMHEPLYLPF	QRMHEPLYL	4	0.5056	210.5	40.00	0.59	tr_C9ZL71_
DRB1_0101	214	KPTYMEQRCHPMKNK	YMEQRCHPM	3	0.5055	210.7	40.00	0.77	tr_C9ZL71_
DRB1_0101	215	LRESICNRGAAEIVN	CNRGAAEIV	5	0.5031	216.3	40.00	0.48	tr_C9ZL71_
DRB1_0101	216	TSIEEKLGGFLKENS	IEEKLGGFL	2	0.5019	219.1	41.00	0.50	tr_C9ZL71_
DRB1_0101	217	EASFNWKQLEYRVRR	FNWKQLEYR	3	0.5003	222.8	41.00	0.38	tr_C9ZL71_
DRB1_0101	218	ARSLNALALDTSCKI	LNALALDTS	3	0.5001	223.3	41.00	0.34	tr_C9ZL71_
DRB1_0101	219	VMDTEFLHHRGKAPR	FLHHRGKAP	5	0.4985	227.1	41.00	0.82	tr_C9ZL71_
DRB1_0101	220	RNLFERPAHAFERAE	NLFERPAHA	1	0.4975	229.8	42.00	0.41	tr_C9ZL71_
DRB1_0101	221	RSLNALALDTSCKIR	LALDTSCKI	5	0.4970	230.9	42.00	0.35	tr_C9ZL71_
DRB1_0101	222	SICNRGAAEIVNMDM	CNRGAAEIV	2	0.4961	233.1	42.00	0.62	tr_C9ZL71_
DRB1_0101	223	PMKNKAFGIEGHQVK	AFGIEGHQV	5	0.4937	239.3	42.00	0.52	tr_C9ZL71_
DRB1_0101	224	LPALLVWPVLVNVMT	LPALLVWPV	0	0.4927	241.9	43.00	0.20	tr_C9ZL71_
DRB1_0101	225	ASVRGAGRNLFERPA	SVRGAGRNL	1	0.4912	246.0	43.00	0.58	tr_C9ZL71_
DRB1_0101	226	HQTSIEEKLGGFLKE	IEEKLGGFL	4	0.4905	247.8	43.00	0.58	tr_C9ZL71_
DRB1_0101	227	IEGHQVKSYARSLNA	QVKSYARSL	4	0.4894	250.8	43.00	0.61	tr_C9ZL71_
DRB1_0101	228	TYMEQRCHPMKNKAF	YMEQRCHPM	1	0.4893	250.9	43.00	0.52	tr_C9ZL71_
DRB1_0101	229	WLARHGGSMYIFHTE	LARHGGSMY	1	0.4889	252.0	43.00	0.40	tr_C9ZL71_
DRB1_0101	230	RHGNYICKPTYMEQR	YICKPTYME	4	0.4866	258.6	44.00	0.47	tr_C9ZL71_
DRB1_0101	231	NALALDTSCKIRASG	LALDTSCKI	2	0.4838	266.6	44.00	0.62	tr_C9ZL71_
DRB1_0101	232	EEKLGGFLKENSSTV	FLKENSSTV	6	0.4833	267.8	44.00	0.71	tr_C9ZL71_
DRB1_0101	233	LVRLKRRGGVHNLSL	LKRRGGVHN	3	0.4818	272.3	45.00	0.57	tr_C9ZL71_
DRB1_0101	234	AFRGTANMSNVREDI	FRGTANMSN	1	0.4810	274.5	45.00	0.73	tr_C9ZL71_
DRB1_0101	235	RAPLLENDGGALNGS	LLENDGGAL	3	0.4794	279.3	45.00	0.69	tr_C9ZL71_
DRB1_0101	236	IDVKQYGYKPIAVFE	YGYKPIAVF	5	0.4783	282.8	46.00	0.34	tr_C9ZL71_
DRB1_0101	237	AFESYNWKGQSHARA	YNWKGQSHA	4	0.4776	284.8	46.00	0.52	tr_C9ZL71_
DRB1_0101	238	MEQRCHPMKNKAFGI	CHPMKNKAF	4	0.4769	287.0	46.00	0.60	tr_C9ZL71_
DRB1_0101	239	VRLKRRGGVHNLSLL	LKRRGGVHN	2	0.4769	287.1	46.00	0.46	tr_C9ZL71_
DRB1_0101	240	PEASYVAEPGREPDA	YVAEPGREP	4	0.4762	289.2	46.00	0.88	tr_C9ZL71_
DRB1_0101	241	GHSMGGAVACLCAYS	GHSMGGAVA	0	0.4754	291.9	46.00	0.31	tr_C9ZL71_
DRB1_0101	242	AFGIEGHQVKSYARS	FGIEGHQVK	1	0.4749	293.5	46.00	0.28	tr_C9ZL71_
DRB1_0101	243	PTYMEQRCHPMKNKA	YMEQRCHPM	2	0.4737	297.1	46.00	0.75	tr_C9ZL71_
DRB1_0101	244	IEFWKGLRESICNRG	WKGLRESIC	3	0.4718	303.5	47.00	0.49	tr_C9ZL71_
DRB1_0101	245	CLCAYSVRRMLREIE	LCAYSVRRM	1	0.4705	307.8	47.00	0.24	tr_C9ZL71_
DRB1_0101	246	DIEFWKGLRESICNR	WKGLRESIC	4	0.4696	310.6	47.00	0.47	tr_C9ZL71_
DRB1_0101	247	NGSVALRLSSTTGDA	SVALRLSST	2	0.4695	311.2	47.00	0.36	tr_C9ZL71_
DRB1_0101	248	CKPTYMEQRCHPMKN	YMEQRCHPM	4	0.4695	311.2	47.00	0.76	tr_C9ZL71_
DRB1_0101	249	LHHRGKAPRIVIAFR	HRGKAPRIV	2	0.4692	311.9	47.00	0.31	tr_C9ZL71_
DRB1_0101	250	MGNAAFQTAYDKAIP	FQTAYDKAI	5	0.4678	316.9	48.00	0.63	tr_C9ZL71_
DRB1_0101	251	LDAVQQRMHEPLYLP	QRMHEPLYL	5	0.4669	320.1	48.00	0.61	tr_C9ZL71_
DRB1_0101	252	YLVRLKRRGGVHNLS	LKRRGGVHN	4	0.4654	325.0	48.00	0.47	tr_C9ZL71_
DRB1_0101	253	RVRRYLVRLKRRGGV	YLVRLKRRG	4	0.4642	329.4	48.00	0.32	tr_C9ZL71_
DRB1_0101	254	PALLVWPVLVNVMTL	VWPVLVNVM	4	0.4639	330.4	49.00	0.27	tr_C9ZL71_
DRB1_0101	255	VNESDEIATFRLYGT	SDEIATFRL	3	0.4638	330.8	49.00	0.58	tr_C9ZL71_
DRB1_0101	256	TIDVKQYGYKPIAVF	DVKQYGYKP	2	0.4638	330.9	49.00	0.33	tr_C9ZL71_
DRB1_0101	257	GAGRNLFERPAHAFE	NLFERPAHA	4	0.4635	332.0	49.00	0.52	tr_C9ZL71_
DRB1_0101	258	AAEIVNMDMTSTCFL	IVNMDMTST	3	0.4635	332.1	49.00	0.65	tr_C9ZL71_
DRB1_0101	259	RYLVRLKRRGGVHNL	LKRRGGVHN	5	0.4621	336.8	49.00	0.33	tr_C9ZL71_

DRB1_0101	260	YMEQRCHPMKNKAFG	CHPMKNKAF	5	0.4621	337.0	49.00	0.54	tr_C9ZL71_
DRB1_0101	261	HGNYICKPTYMEQRC	YICKPTYME	3	0.4605	343.0	49.00	0.54	tr_C9ZL71_
DRB1_0101	262	VRRYLVRLKRRGGVH	YLVRLKRRG	3	0.4603	343.7	49.00	0.38	tr_C9ZL71_
DRB1_0101	263	WPDDWYRWLARHGGS	YRWLARHGG	5	0.4569	356.4	50.00	0.69	tr_C9ZL71_
DRB1_0101	264	LDEVTVYTFGQPPMG	YTFGQPPMG	6	0.4568	356.8	50.00	0.54	tr_C9ZL71_
DRB1_0101	265	SIEEKLGGFLKENSS	EEKLGGFLK	2	0.4557	361.0	50.00	0.48	tr_C9ZL71_
DRB1_0101	266	APLLENDGGALNGSV	LLENDGGAL	2	0.4555	362.1	50.00	0.65	tr_C9ZL71_
DRB1_0101	267	LENDGGALNGSVALR	GGALNGSVA	4	0.4550	363.7	50.00	0.50	tr_C9ZL71_
DRB1_0101	268	NESDEIATFRLYGTQ	SDEIATFRL	2	0.4544	366.2	55.00	0.56	tr_C9ZL71_
DRB1_0101	269	LYGTQVGTEVDINRH	YGTQVGTEV	1	0.4544	366.3	55.00	0.82	tr_C9ZL71_
DRB1_0101	270	RVVNESDEIATFRLY	SDEIATFRL	5	0.4534	370.1	55.00	0.43	tr_C9ZL71_
DRB1_0101	271	SGCCRGSSNYRSNAR	CRGSSNYRS	3	0.4523	374.5	55.00	0.79	tr_C9ZL71_
DRB1_0101	272	ALLVWPVLVNVMTLI	VWPVLVNVM	3	0.4523	374.7	55.00	0.31	tr_C9ZL71_
DRB1_0101	273	IDCFNIAFESYNWKG	FNIAFESYN	3	0.4517	377.2	55.00	0.44	tr_C9ZL71_
DRB1_0101	274	VVNESDEIATFRLYG	SDEIATFRL	4	0.4513	378.5	55.00	0.56	tr_C9ZL71_
DRB1_0101	275	VRRMLREIEYPLDEV	MLREIEYPL	3	0.4511	379.7	55.00	0.64	tr_C9ZL71_
DRB1_0101	276	PLLENDGGALNGSVA	LLENDGGAL	1	0.4510	379.8	55.00	0.49	tr_C9ZL71_
DRB1_0101	277	RRYLVRLKRRGGVHN	YLVRLKRRG	2	0.4490	388.1	55.00	0.32	tr_C9ZL71_
DRB1_0101	278	AHQTSIEEKLGGFLK	IEEKLGGFL	5	0.4490	388.2	55.00	0.60	tr_C9ZL71_
DRB1_0101	279	LLENDGGALNGSVAL	GGALNGSVA	5	0.4487	389.4	55.00	0.34	tr_C9ZL71_
DRB1_0101	280	SRAPLLENDGGALNG	LLENDGGAL	4	0.4468	397.5	55.00	0.69	tr_C9ZL71_
DRB1_0101	281	NSSCCWEPTVHSGFL	CCWEPTVHS	3	0.4450	405.6	55.00	0.68	tr_C9ZL71_
DRB1_0101	282	AIDCFNIAFESYNWK	FNIAFESYN	4	0.4439	410.2	55.00	0.43	tr_C9ZL71_
DRB1_0101	283	EFWKGLRESICNRGA	WKGLRESIC	2	0.4430	414.1	55.00	0.48	tr_C9ZL71_
DRB1_0101	284	LLVWPVLVNVMTLIY	WPVLVNVMT	3	0.4430	414.3	55.00	0.31	tr_C9ZL71_
DRB1_0101	285	HPMKNKAFGIEGHQV	PMKNKAFGI	1	0.4420	418.8	55.00	0.49	tr_C9ZL71_
DRB1_0101	286	AEIVNMDMTSTCFLP	IVNMDMTST	2	0.4414	421.5	55.00	0.60	tr_C9ZL71_
DRB1_0101	287	PMGNAAFQTAYDKAI	FQTAYDKAI	6	0.4411	422.9	55.00	0.47	tr_C9ZL71_
DRB1_0101	288	SSCCWEPTVHSGFLE	CCWEPTVHS	2	0.4407	424.8	55.00	0.62	tr_C9ZL71_
DRB1_0101	289	EEASFNWKQLEYRVR	FNWKQLEYR	4	0.4405	425.6	55.00	0.43	tr_C9ZL71_
DRB1_0101	290	CHPMKNKAFGIEGHQ	PMKNKAFGI	2	0.4403	426.7	55.00	0.57	tr_C9ZL71_
DRB1_0101	291	NRHGNYICKPTYMEQ	NYICKPTYM	4	0.4395	430.3	55.00	0.45	tr_C9ZL71_
DRB1_0101	292	GSVALRLSSTTGDAP	SVALRLSST	1	0.4389	433.3	55.00	0.32	tr_C9ZL71_
DRB1_0101	293	TNNPKSVWQALRGVI	SVWQALRGV	5	0.4362	446.2	55.00	0.37	tr_C9ZL71_
DRB1_0101	294	SGFLEIWEAHQTSIE	FLEIWEAHQ	2	0.4359	447.3	55.00	0.38	tr_C9ZL71_
DRB1_0101	295	SVRRMLREIEYPLDE	MLREIEYPL	4	0.4359	447.4	55.00	0.62	tr_C9ZL71_
DRB1_0101	296	NWKGQSHARAQERKV	WKGQSHARA	1	0.4348	452.9	55.00	0.76	tr_C9ZL71_
DRB1_0101	297	GLRESICNRGAAEIV	CNRGAAEIV	6	0.4346	453.5	55.00	0.34	tr_C9ZL71_
DRB1_0101	298	LLLQSIPNDSSSPPV	LLQSIPNDS	1	0.4346	453.8	55.00	0.58	tr_C9ZL71_
DRB1_0101	299	HSGFLEIWEAHQTSI	FLEIWEAHQ	3	0.4337	458.3	55.00	0.44	tr_C9ZL71_
DRB1_0101	300	QDIEFWKGLRESICN	WKGLRESIC	5	0.4317	468.1	55.00	0.41	tr_C9ZL71_
DRB1_0101	301	DCFNIAFESYNWKGQ	FNIAFESYN	2	0.4316	468.8	55.00	0.44	tr_C9ZL71_
DRB1_0101	302	IMCSVHSRPQDIEFW	SVHSRPQDI	3	0.4310	471.8	60.00	0.76	tr_C9ZL71_
DRB1_0101	303	GSSNYRSNARESAAY	YRSNARESA	4	0.4308	472.7	60.00	0.80	tr_C9ZL71_
DRB1_0101	304	MYIFHTEKEEASFNW	FHTEKEEAS	3	0.4304	474.6	60.00	0.68	tr_C9ZL71_
DRB1_0101	305	ICNRGAAEIVNMDMT	CNRGAAEIV	1	0.4303	475.3	60.00	0.62	tr_C9ZL71_
DRB1_0101	306	ALALDTSCKIRASGD	LALDTSCKI	1	0.4301	476.2	60.00	0.58	tr_C9ZL71_
DRB1_0101	307	GIEGHQVKSYARSLN	QVKSYARSL	5	0.4299	477.3	60.00	0.56	tr_C9ZL71_
DRB1_0101	308	SNYRSNARESAAYPR	YRSNARESA	2	0.4297	478.4	60.00	0.63	tr_C9ZL71_
DRB1_0101	309	GNYICKPTYMEQRCH	YICKPTYME	2	0.4288	483.1	60.00	0.54	tr_C9ZL71_

DRB1_0101	310	SSNYRSNARESAAYP	YRSNARESA	3	0.4288	483.1	60.00	0.76	tr_C9ZL71_
DRB1_0101	311	RRMLREIEYPLDEVT	MLREIEYPL	2	0.4285	484.5	60.00	0.63	tr_C9ZL71_
DRB1_0101	312	ICKPTYMEQRCHPMK	YMEQRCHPM	5	0.4284	485.3	60.00	0.75	tr_C9ZL71_
DRB1_0101	313	TIMCSVHSRPQDIEF	SVHSRPQDI	4	0.4282	486.2	60.00	0.69	tr_C9ZL71_
DRB1_0101	314	YTFGQPPMGNAAFQT	YTFGQPPMG	0	0.4265	495.4	60.00	0.58	tr_C9ZL71_
DRB1_0101	315	ENSSTVYRVFCTGHS	YRVFCTGHS	6	0.4264	495.6	60.00	0.62	tr_C9ZL71_
DRB1_0101	316	LLDAVQQRMHEPLYL	QRMHEPLYL	6	0.4262	496.8	60.00	0.50	tr_C9ZL71_
DRB1_0101	317	FRVVNESDEIATFRL	VVNESDEIA	2	0.4254	501.0	60.00	0.46	tr_C9ZL71_
DRB1_0101	318	YSVRRMLREIEYPLD	MLREIEYPL	5	0.4250	503.3	60.00	0.54	tr_C9ZL71_
DRB1_0101	319	TSGCCRGSSNYRSNA	CRGSSNYRS	4	0.4250	503.4	60.00	0.72	tr_C9ZL71_
DRB1_0101	320	GCCRGSSNYRSNARE	CRGSSNYRS	2	0.4242	507.7	60.00	0.77	tr_C9ZL71_
DRB1_0101	321	YIFHTEKEEASFNWK	FHTEKEEAS	2	0.4239	509.5	60.00	0.61	tr_C9ZL71_
DRB1_0101	322	LVWPVLVNVMTLIYT	WPVLVNVMT	2	0.4234	512.5	60.00	0.32	tr_C9ZL71_
DRB1_0101	323	SCCWEPTVHSGFLEI	CCWEPTVHS	1	0.4226	516.4	60.00	0.52	tr_C9ZL71_
DRB1_0101	324	YLLDAVQQRMHEPLY	YLLDAVQQR	0	0.4218	521.2	60.00	0.44	tr_C9ZL71_
DRB1_0101	325	NYRSNARESAAYPRE	YRSNARESA	1	0.4211	525.3	60.00	0.50	tr_C9ZL71_
DRB1_0101	326	EIVNMDMTSTCFLPY	IVNMDMTST	1	0.4178	544.1	60.00	0.47	tr_C9ZL71_
DRB1_0101	327	FNLETAIDCFNIAFE	FNLETAIDC	0	0.4178	544.1	60.00	0.50	tr_C9ZL71_
DRB1_0101	328	PSDPSINYTIMCSVH	SDPSINYTI	1	0.4156	557.5	60.00	0.43	tr_C9ZL71_
DRB1_0101	329	VMTLIYTTRHRPETV	LIYTTRHRP	3	0.4132	572.2	60.00	0.30	tr_C9ZL71_
DRB1_0101	330	GFLEIWEAHQTSIEE	FLEIWEAHQ	1	0.4126	575.6	60.00	0.28	tr_C9ZL71_
DRB1_0101	331	DPEASYVAEPGREPD	YVAEPGREP	5	0.4125	576.3	60.00	0.86	tr_C9ZL71_
DRB1_0101	332	FGIEGHQVKSYARSL	QVKSYARSL	6	0.4117	581.6	60.00	0.26	tr_C9ZL71_
DRB1_0101	333	SMYIFHTEKEEASFN	FHTEKEEAS	4	0.4112	584.3	60.00	0.65	tr_C9ZL71_
DRB1_0101	334	TFRVVNESDEIATFR	VVNESDEIA	3	0.4112	584.4	60.00	0.60	tr_C9ZL71_
DRB1_0101	335	RGAGRNLFERPAHAF	NLFERPAHA	5	0.4100	592.3	60.00	0.59	tr_C9ZL71_
DRB1_0101	336	VHSGFLEIWEAHQTS	FLEIWEAHQ	4	0.4090	598.8	60.00	0.47	tr_C9ZL71_
DRB1_0101	337	GAAEIVNMDMTSTCF	IVNMDMTST	4	0.4079	605.6	65.00	0.72	tr_C9ZL71_
DRB1_0101	338	NLFERPAHAFERAET	LFERPAHAF	1	0.4075	608.3	65.00	0.32	tr_C9ZL71_
DRB1_0101	339	EALDVAAVCAVMDTE	LDVAAVCAV	2	0.4054	622.1	65.00	0.41	tr_C9ZL71_
DRB1_0101	340	CFNIAFESYNWKGQS	FNIAFESYN	1	0.4045	628.2	65.00	0.43	tr_C9ZL71_
DRB1_0101	341	MCSVHSRPQDIEFWK	SVHSRPQDI	2	0.4021	645.1	65.00	0.78	tr_C9ZL71_
DRB1_0101	342	DKAIPRTFRVVNESD	KAIPRTFRV	1	0.4017	648.0	65.00	0.52	tr_C9ZL71_
DRB1_0101	343	VNVMTLIYTTRHRPE	MTLIYTTRH	3	0.4010	652.8	65.00	0.44	tr_C9ZL71_
DRB1_0101	344	VLVNVMTLIYTTRHR	MTLIYTTRH	5	0.3999	660.8	65.00	0.46	tr_C9ZL71_
DRB1_0101	345	LCAYSVRRMLREIEY	YSVRRMLRE	3	0.3989	667.6	65.00	0.25	tr_C9ZL71_
DRB1_0101	346	LNSSCCWEPTVHSGF	CCWEPTVHS	4	0.3981	673.2	65.00	0.74	tr_C9ZL71_
DRB1_0101	347	LARHGGSMYIFHTEK	GGSMYIFHT	4	0.3977	676.5	65.00	0.28	tr_C9ZL71_
DRB1_0101	348	VWPVLVNVMTLIYTT	WPVLVNVMT	1	0.3944	701.3	65.00	0.25	tr_C9ZL71_
DRB1_0101	349	FWKGLRESICNRGAA	WKGLRESIC	1	0.3939	704.4	65.00	0.44	tr_C9ZL71_
DRB1_0101	350	LVNVMTLIYTTRHRP	MTLIYTTRH	4	0.3938	705.4	65.00	0.47	tr_C9ZL71_
DRB1_0101	351	TAIDCFNIAFESYNW	FNIAFESYN	5	0.3937	706.6	65.00	0.38	tr_C9ZL71_
DRB1_0101	352	IFHTEKEEASFNWKQ	FHTEKEEAS	1	0.3926	715.0	65.00	0.56	tr_C9ZL71_
DRB1_0101	353	VWPDDWYRWLARHGG	YRWLARHGG	6	0.3924	716.1	65.00	0.56	tr_C9ZL71_
DRB1_0101	354	PQDIEFWKGLRESIC	FWKGLRESI	5	0.3923	716.7	65.00	0.41	tr_C9ZL71_
DRB1_0101	355	FLEIWEAHQTSIEEK	WEAHQTSIE	4	0.3920	719.3	65.00	0.31	tr_C9ZL71_
DRB1_0101	356	SVRGAGRNLFERPAH	SVRGAGRNL	0	0.3916	722.5	65.00	0.47	tr_C9ZL71_
DRB1_0101	357	GSRAPLLENDGGALN	LLENDGGAL	5	0.3913	725.1	65.00	0.75	tr_C9ZL71_
DRB1_0101	358	MTLIYTTRHRPETVP	TTRHRPETV	5	0.3911	726.7	65.00	0.25	tr_C9ZL71_
DRB1_0101	359	RTFRVVNESDEIATF	VVNESDEIA	4	0.3907	729.6	65.00	0.52	tr_C9ZL71_

DRB1_0101	360	INRHGNYICKPTYME	NYICKPTYM	5	0.3903	733.0	65.00	0.48	tr_C9ZL71_
DRB1_0101	361	NVMTLIYTTRHRPET	MTLIYTTRH	2	0.3900	735.4	65.00	0.45	tr_C9ZL71_
DRB1_0101	362	SVALRLSSTTGDAPP	ALRLSSTTG	2	0.3888	744.4	65.00	0.28	tr_C9ZL71_
DRB1_0101	363	TLIYTTRHRPETVPI	TTRHRPETV	4	0.3888	745.0	65.00	0.41	tr_C9ZL71_
DRB1_0101	364	CCRGSSNYRSNARES	CRGSSNYRS	1	0.3887	745.9	65.00	0.74	tr_C9ZL71_
DRB1_0101	365	AYSVRRMLREIEYPL	MLREIEYPL	6	0.3886	746.5	65.00	0.37	tr_C9ZL71_
DRB1_0101	366	AVMDTEFLHHRGKAP	FLHHRGKAP	6	0.3870	759.0	65.00	0.63	tr_C9ZL71_
DRB1_0101	367	GPSDPSINYTIMCSV	SDPSINYTI	2	0.3867	762.1	65.00	0.54	tr_C9ZL71_
DRB1_0101	368	TVHSGFLEIWEAHQT	FLEIWEAHQ	5	0.3861	766.8	65.00	0.43	tr_C9ZL71_
DRB1_0101	369	PTIDVKQYGYKPIAV	DVKQYGYKP	3	0.3858	769.7	65.00	0.59	tr_C9ZL71_
DRB1_0101	370	PVLVNVMTLIYTTRH	MTLIYTTRH	6	0.3850	775.9	65.00	0.27	tr_C9ZL71_
DRB1_0101	371	LKENSSTVYRVFCTG	ENSSTVYRV	2	0.3838	786.2	70.00	0.44	tr_C9ZL71_
DRB1_0101	372	YRSNARESAAYPRET	YRSNARESA	0	0.3820	801.5	70.00	0.31	tr_C9ZL71_
DRB1_0101	373	RMLREIEYPLDEVTV	MLREIEYPL	1	0.3814	807.0	70.00	0.56	tr_C9ZL71_
DRB1_0101	374	WPVLVNVMTLIYTTR	VLVNVMTLI	2	0.3750	864.3	70.00	0.22	tr_C9ZL71_
DRB1_0101	375	RGSSNYRSNARESAA	YRSNARESA	5	0.3748	866.2	70.00	0.78	tr_C9ZL71_
DRB1_0101	376	STNNPKSVWQALRGV	SVWQALRGV	6	0.3746	868.8	70.00	0.28	tr_C9ZL71_
DRB1_0101	377	LIYTTRHRPETVPIH	TTRHRPETV	3	0.3744	870.8	70.00	0.51	tr_C9ZL71_
DRB1_0101	378	GSMYIFHTEKEEASF	FHTEKEEAS	5	0.3739	874.9	70.00	0.57	tr_C9ZL71_
DRB1_0101	379	FRGTANMSNVREDIK	FRGTANMSN	0	0.3739	875.3	70.00	0.50	tr_C9ZL71_
DRB1_0101	380	CRGSSNYRSNARESA	CRGSSNYRS	0	0.3720	893.4	70.00	0.40	tr_C9ZL71_
DRB1_0101	381	KIRASGDPEASYVAE	GDPEASYVA	5	0.3720	893.4	70.00	0.36	tr_C9ZL71_
DRB1_0101	382	ALDVAAVCAVMDTEF	LDVAAVCAV	1	0.3708	905.3	70.00	0.38	tr_C9ZL71_
DRB1_0101	383	CCWEPTVHSGFLEIW	CCWEPTVHS	0	0.3671	941.6	70.00	0.38	tr_C9ZL71_
DRB1_0101	384	IEEKLGGFLKENSST	EEKLGGFLK	1	0.3667	945.6	70.00	0.55	tr_C9ZL71_
DRB1_0101	385	GTSGCCRGSSNYRSN	CRGSSNYRS	5	0.3659	953.8	70.00	0.68	tr_C9ZL71_
DRB1_0101	386	CKIRASGDPEASYVA	IRASGDPEA	2	0.3645	968.4	70.00	0.42	tr_C9ZL71_
DRB1_0101	387	EGPSDPSINYTIMCS	SDPSINYTI	3	0.3610	1006.1	70.00	0.66	tr_C9ZL71_
DRB1_0101	388	YICKPTYMEQRCHPM	YMEQRCHPM	6	0.3599	1018.5	75.00	0.50	tr_C9ZL71_
DRB1_0101	389	CSVHSRPQDIEFWKG	SVHSRPQDI	1	0.3593	1024.7	75.00	0.72	tr_C9ZL71_
DRB1_0101	390	IVNMDMTSTCFLPYV	MDMTSTCFL	3	0.3590	1028.1	75.00	0.31	tr_C9ZL71_
DRB1_0101	391	PRTFRVVNESDEIAT	VVNESDEIA	5	0.3583	1035.6	75.00	0.47	tr_C9ZL71_
DRB1_0101	392	RKFCPSTEQQSVAAT	FCPSTEQQS	2	0.3582	1037.5	75.00	0.44	tr_C9ZL71_
DRB1_0101	393	SLNSSCCWEPTVHSG	CCWEPTVHS	5	0.3580	1039.1	75.00	0.71	tr_C9ZL71_
DRB1_0101	394	IAFESYNWKGQSHAR	YNWKGQSHA	5	0.3578	1041.7	75.00	0.69	tr_C9ZL71_
DRB1_0101	395	TSTCFLPYVEAGREN	TCFLPYVEA	2	0.3566	1054.6	75.00	0.40	tr_C9ZL71_
DRB1_0101	396	KEEASFNWKQLEYRV	FNWKQLEYR	5	0.3558	1064.8	75.00	0.56	tr_C9ZL71_
DRB1_0101	397	PTVHSGFLEIWEAHQ	FLEIWEAHQ	6	0.3554	1069.2	75.00	0.31	tr_C9ZL71_
DRB1_0101	398	RGAAEIVNMDMTSTC	IVNMDMTST	5	0.3514	1116.6	75.00	0.71	tr_C9ZL71_
DRB1_0101	399	CAYSVRRMLREIEYP	YSVRRMLRE	2	0.3511	1119.9	75.00	0.29	tr_C9ZL71_
DRB1_0101	400	NYICKPTYMEQRCHP	YICKPTYME	1	0.3511	1120.2	75.00	0.59	tr_C9ZL71_
DRB1_0101	401	MTSTCFLPYVEAGRE	TCFLPYVEA	3	0.3507	1124.8	75.00	0.41	tr_C9ZL71_
DRB1_0101	402	MRKFCPSTEQQSVAA	FCPSTEQQS	3	0.3499	1134.3	75.00	0.50	tr_C9ZL71_
DRB1_0101	403	ETAIDCFNIAFESYN	FNIAFESYN	6	0.3498	1135.5	75.00	0.22	tr_C9ZL71_
DRB1_0101	404	KAIPRTFRVVNESDE	KAIPRTFRV	0	0.3415	1242.6	75.00	0.46	tr_C9ZL71_
DRB1_0101	405	VEMRKFCPSTEQQSV	MRKFCPSTE	2	0.3414	1243.7	75.00	0.38	tr_C9ZL71_
DRB1_0101	406	GDPEASYVAEPGREP	YVAEPGREP	6	0.3410	1248.6	75.00	0.67	tr_C9ZL71_
DRB1_0101	407	LEIWEAHQTSIEEKL	WEAHQTSIE	3	0.3405	1255.7	75.00	0.47	tr_C9ZL71_
DRB1_0101	408	EMRKFCPSTEQQSVA	FCPSTEQQS	4	0.3404	1257.1	75.00	0.47	tr_C9ZL71_
DRB1_0101	409	VEGPSDPSINYTIMC	SDPSINYTI	4	0.3395	1269.6	75.00	0.68	tr_C9ZL71_

DRB1_0101	410	PPMGNAAFQTAYDKA	AAFQTAYDK	5	0.3384	1284.7	75.00	0.23	tr_C9ZL71_
DRB1_0101	411	WKGQSHARAQERKVG	WKGQSHARA	0	0.3382	1288.1	75.00	0.57	tr_C9ZL71_
DRB1_0101	412	STCFLPYVEAGREND	FLPYVEAGR	3	0.3362	1315.9	75.00	0.28	tr_C9ZL71_
DRB1_0101	413	FHTEKEEASFNWKQL	FHTEKEEAS	0	0.3361	1316.8	75.00	0.38	tr_C9ZL71_
DRB1_0101	414	MLREIEYPLDEVTVY	EYPLDEVTV	5	0.3355	1325.7	75.00	0.34	tr_C9ZL71_
DRB1_0101	415	KFCPSTEQQSVAATP	FCPSTEQQS	1	0.3339	1348.4	80.00	0.33	tr_C9ZL71_
DRB1_0101	416	DINRHGNYICKPTYM	NYICKPTYM	6	0.3338	1350.4	80.00	0.43	tr_C9ZL71_
DRB1_0101	417	DMTSTCFLPYVEAGR	TCFLPYVEA	4	0.3337	1351.2	80.00	0.44	tr_C9ZL71_
DRB1_0101	418	REIEYPLDEVTVYTF	EYPLDEVTV	3	0.3336	1353.0	80.00	0.54	tr_C9ZL71_
DRB1_0101	419	GTEVDINRHGNYICK	DINRHGNYI	4	0.3332	1359.0	80.00	0.60	tr_C9ZL71_
DRB1_0101	420	FNIAFESYNWKGQSH	FNIAFESYN	0	0.3268	1457.3	80.00	0.34	tr_C9ZL71_
DRB1_0101	421	SCKIRASGDPEASYV	IRASGDPEA	3	0.3264	1463.0	80.00	0.58	tr_C9ZL71_
DRB1_0101	422	EPTVHSGFLEIWEAH	SGFLEIWEA	5	0.3250	1485.1	80.00	0.27	tr_C9ZL71_
DRB1_0101	423	KGLRESICNRGAAEI	LRESICNRG	2	0.3244	1495.7	80.00	0.31	tr_C9ZL71_
DRB1_0101	424	TCFLPYVEAGRENDL	FLPYVEAGR	2	0.3241	1500.6	80.00	0.25	tr_C9ZL71_
DRB1_0101	425	CNRGAAEIVNMDMTS	CNRGAAEIV	0	0.3229	1518.6	80.00	0.50	tr_C9ZL71_
DRB1_0101	426	ASQVMRQNVGREGGG	MRQNVGREG	4	0.3227	1522.3	80.00	0.46	tr_C9ZL71_
DRB1_0101	427	IYTTRHRPETVPIHP	TTRHRPETV	2	0.3225	1525.5	80.00	0.60	tr_C9ZL71_
DRB1_0101	428	PVEGPSDPSINYTIM	SDPSINYTI	5	0.3219	1536.2	80.00	0.65	tr_C9ZL71_
DRB1_0101	429	DVEMRKFCPSTEQQS	MRKFCPSTE	3	0.3213	1546.0	80.00	0.41	tr_C9ZL71_
DRB1_0101	430	LALDTSCKIRASGDP	LALDTSCKI	0	0.3207	1555.9	80.00	0.51	tr_C9ZL71_
DRB1_0101	431	KENSSTVYRVFCTGH	ENSSTVYRV	1	0.3191	1583.0	80.00	0.44	tr_C9ZL71_
DRB1_0101	432	TPTIDVKQYGYKPIA	DVKQYGYKP	4	0.3188	1588.6	80.00	0.65	tr_C9ZL71_
DRB1_0101	433	WKGLRESICNRGAAE	LRESICNRG	3	0.3181	1600.1	80.00	0.30	tr_C9ZL71_
DRB1_0101	434	TEVDINRHGNYICKP	DINRHGNYI	3	0.3170	1619.1	80.00	0.59	tr_C9ZL71_
DRB1_0101	435	EIEYPLDEVTVYTFG	EYPLDEVTV	2	0.3162	1634.1	80.00	0.47	tr_C9ZL71_
DRB1_0101	436	NIAFESYNWKGQSHA	YNWKGQSHA	6	0.3150	1655.1	80.00	0.41	tr_C9ZL71_
DRB1_0101	437	LREIEYPLDEVTVYT	EYPLDEVTV	4	0.3145	1664.7	80.00	0.50	tr_C9ZL71_
DRB1_0101	438	YGTQVGTEVDINRHG	YGTQVGTEV	0	0.3142	1668.9	80.00	0.68	tr_C9ZL71_
DRB1_0101	439	RSNARESAAYPRETN	ARESAAYPR	3	0.3139	1674.6	80.00	0.44	tr_C9ZL71_
DRB1_0101	440	VRGAGRNLFERPAHA	NLFERPAHA	6	0.3125	1700.3	80.00	0.47	tr_C9ZL71_
DRB1_0101	441	EAHQTSIEEKLGGFL	IEEKLGGFL	6	0.3116	1716.5	80.00	0.61	tr_C9ZL71_
DRB1_0101	442	SQVMRQNVGREGGGS	MRQNVGREG	3	0.3116	1717.4	80.00	0.57	tr_C9ZL71_
DRB1_0101	443	IRASGDPEASYVAEP	GDPEASYVA	4	0.3111	1725.4	80.00	0.46	tr_C9ZL71_
DRB1_0101	444	QPPMGNAAFQTAYDK	PMGNAAFQT	2	0.3109	1730.9	80.00	0.34	tr_C9ZL71_
DRB1_0101	445	GASQVMRQNVGREGG	MRQNVGREG	5	0.3093	1761.1	80.00	0.38	tr_C9ZL71_
DRB1_0101	446	GGSMYIFHTEKEEAS	FHTEKEEAS	6	0.3088	1769.0	80.00	0.37	tr_C9ZL71_
DRB1_0101	447	RPQDIEFWKGLRESI	FWKGLRESI	6	0.3081	1782.9	80.00	0.44	tr_C9ZL71_
DRB1_0101	448	LETAIDCFNIAFESY	ETAIDCFNI	1	0.3074	1796.8	80.00	0.29	tr_C9ZL71_
DRB1_0101	449	IPRTFRVVNESDEIA	FRVVNESDE	4	0.3064	1815.5	85.00	0.50	tr_C9ZL71_
DRB1_0101	450	LFERPAHAFERAETY	LFERPAHAF	0	0.3061	1822.3	85.00	0.31	tr_C9ZL71_
DRB1_0101	451	EVDINRHGNYICKPT	DINRHGNYI	2	0.3032	1880.1	85.00	0.56	tr_C9ZL71_
DRB1_0101	452	IEYPLDEVTVYTFGQ	EYPLDEVTV	1	0.3027	1891.3	85.00	0.41	tr_C9ZL71_
DRB1_0101	453	GDVEMRKFCPSTEQQ	MRKFCPSTE	4	0.3025	1895.2	85.00	0.39	tr_C9ZL71_
DRB1_0101	454	TSCKIRASGDPEASY	IRASGDPEA	4	0.3004	1938.7	85.00	0.57	tr_C9ZL71_
DRB1_0101	455	EIWEAHQTSIEEKLG	WEAHQTSIE	2	0.2994	1959.0	85.00	0.52	tr_C9ZL71_
DRB1_0101	456	VALRLSSTTGDAPPG	ALRLSSTTG	1	0.2965	2021.1	85.00	0.37	tr_C9ZL71_
DRB1_0101	457	EKEEASFNWKQLEYR	FNWKQLEYR	6	0.2962	2027.4	85.00	0.52	tr_C9ZL71_
DRB1_0101	458	ARHGGSMYIFHTEKE	GGSMYIFHT	3	0.2960	2033.7	85.00	0.49	tr_C9ZL71_
DRB1_0101	459	CFLPYVEAGRENDLG	VEAGRENDL	5	0.2955	2044.1	85.00	0.35	tr_C9ZL71_

DRB1_0101	460	NMDMTSTCFLPYVEA	MDMTSTCFL	1	0.2955	2044.6	85.00	0.31	tr_C9ZL71_
DRB1_0101	461	QGDVEMRKFCPSTEQ	VEMRKFCPS	3	0.2947	2060.8	85.00	0.38	tr_C9ZL71_
DRB1_0101	462	SVHSRPQDIEFWKGL	SVHSRPQDI	0	0.2946	2064.8	85.00	0.47	tr_C9ZL71_
DRB1_0101	463	VDINRHGNYICKPTY	DINRHGNYI	1	0.2931	2096.6	85.00	0.49	tr_C9ZL71_
DRB1_0101	464	NRGAAEIVNMDMTST	IVNMDMTST	6	0.2925	2110.3	85.00	0.58	tr_C9ZL71_
DRB1_0101	465	GSTNNPKSVWQALRG	NNPKSVWQA	3	0.2924	2113.1	85.00	0.38	tr_C9ZL71_
DRB1_0101	466	MDMTSTCFLPYVEAG	TCFLPYVEA	5	0.2924	2113.8	85.00	0.36	tr_C9ZL71_
DRB1_0101	467	ASLNSSCCWEPTVHS	CCWEPTVHS	6	0.2922	2117.7	85.00	0.58	tr_C9ZL71_
DRB1_0101	468	FGQPPMGNAAFQTAY	PMGNAAFQT	4	0.2906	2154.6	85.00	0.44	tr_C9ZL71_
DRB1_0101	469	VNMDMTSTCFLPYVE	MDMTSTCFL	2	0.2890	2193.5	85.00	0.40	tr_C9ZL71_
DRB1_0101	470	PLDEVTVYTFGQPPM	TVYTFGQPP	5	0.2888	2198.1	85.00	0.43	tr_C9ZL71_
DRB1_0101	471	NLETAIDCFNIAFES	ETAIDCFNI	2	0.2886	2201.3	85.00	0.39	tr_C9ZL71_
DRB1_0101	472	YTTRHRPETVPIHPR	TTRHRPETV	1	0.2886	2202.4	85.00	0.51	tr_C9ZL71_
DRB1_0101	473	WEPTVHSGFLEIWEA	PTVHSGFLE	2	0.2884	2207.7	85.00	0.28	tr_C9ZL71_
DRB1_0101	474	LLQSIPNDSSSPPVE	LLQSIPNDS	0	0.2874	2231.2	85.00	0.44	tr_C9ZL71_
DRB1_0101	475	FCPSTEQQSVAATPD	STEQQSVAA	3	0.2854	2280.8	85.00	0.37	tr_C9ZL71_
DRB1_0101	476	SGASQVMRQNVGREG	SQVMRQNVG	3	0.2803	2410.1	85.00	0.37	tr_C9ZL71_
DRB1_0101	477	TFGQPPMGNAAFQTA	PMGNAAFQT	5	0.2794	2433.1	85.00	0.36	tr_C9ZL71_
DRB1_0101	478	VGTEVDINRHGNYIC	DINRHGNYI	5	0.2775	2482.0	85.00	0.60	tr_C9ZL71_
DRB1_0101	479	GQPPMGNAAFQTAYD	PMGNAAFQT	3	0.2757	2530.7	85.00	0.47	tr_C9ZL71_
DRB1_0101	480	GGSTNNPKSVWQALR	NNPKSVWQA	4	0.2756	2535.6	90.00	0.35	tr_C9ZL71_
DRB1_0101	481	GGSRAPLLENDGGAL	LLENDGGAL	6	0.2742	2572.1	90.00	0.77	tr_C9ZL71_
DRB1_0101	482	MSNVREDIKMRRRAW	NVREDIKMR	2	0.2729	2611.2	90.00	0.42	tr_C9ZL71_
DRB1_0101	483	VGTSGCCRGSSNYRS	CRGSSNYRS	6	0.2726	2619.6	90.00	0.52	tr_C9ZL71_
DRB1_0101	484	NMSNVREDIKMRRRA	NVREDIKMR	3	0.2698	2698.6	90.00	0.49	tr_C9ZL71_
DRB1_0101	485	CWEPTVHSGFLEIWE	PTVHSGFLE	3	0.2696	2705.0	90.00	0.34	tr_C9ZL71_
DRB1_0101	486	RASGDPEASYVAEPG	GDPEASYVA	3	0.2681	2749.4	90.00	0.68	tr_C9ZL71_
DRB1_0101	487	SNVREDIKMRRRAWD	NVREDIKMR	1	0.2660	2810.9	90.00	0.32	tr_C9ZL71_
DRB1_0101	488	SNARESAAYPRETNP	ARESAAYPR	2	0.2643	2864.0	90.00	0.46	tr_C9ZL71_
DRB1_0101	489	FERPAHAFERAETYL	HAFERAETY	5	0.2611	2965.9	90.00	0.38	tr_C9ZL71_
DRB1_0101	490	FLPYVEAGRENDLGP	VEAGRENDL	4	0.2609	2971.0	90.00	0.46	tr_C9ZL71_
DRB1_0101	491	SQGDVEMRKFCPSTE	VEMRKFCPS	4	0.2597	3011.8	90.00	0.40	tr_C9ZL71_
DRB1_0101	492	RHGGSMYIFHTEKEE	GGSMYIFHT	2	0.2594	3019.5	90.00	0.51	tr_C9ZL71_
DRB1_0101	493	GTPTIDVKQYGYKPI	DVKQYGYKP	5	0.2562	3125.5	90.00	0.65	tr_C9ZL71_
DRB1_0101	494	HGGSMYIFHTEKEEA	GGSMYIFHT	1	0.2556	3146.7	90.00	0.34	tr_C9ZL71_
DRB1_0101	495	QVMRQNVGREGGGSR	MRQNVGREG	2	0.2555	3149.3	90.00	0.67	tr_C9ZL71_
DRB1_0101	496	DTSCKIRASGDPEAS	IRASGDPEA	5	0.2549	3172.5	90.00	0.53	tr_C9ZL71_
DRB1_0101	497	CPSTEQQSVAATPDH	STEQQSVAA	2	0.2516	3288.0	90.00	0.37	tr_C9ZL71_
DRB1_0101	498	PSGASQVMRQNVGRE	SQVMRQNVG	4	0.2513	3297.2	90.00	0.41	tr_C9ZL71_
DRB1_0101	499	REDIKMRRRAWDEMK	IKMRRRAWD	3	0.2497	3354.6	90.00	0.34	tr_C9ZL71_
DRB1_0101	500	ANMSNVREDIKMRRR	NVREDIKMR	4	0.2495	3362.0	90.00	0.45	tr_C9ZL71_
DRB1_0101	501	IWEAHQTSIEEKLGG	WEAHQTSIE	1	0.2493	3368.2	90.00	0.47	tr_C9ZL71_
DRB1_0101	502	AIPRTFRVVNESDEI	FRVVNESDE	5	0.2481	3413.7	90.00	0.50	tr_C9ZL71_
DRB1_0101	503	ASGDPEASYVAEPGR	GDPEASYVA	2	0.2457	3502.1	90.00	0.65	tr_C9ZL71_
DRB1_0101	504	SRPQDIEFWKGLRES	QDIEFWKGL	3	0.2435	3587.7	90.00	0.31	tr_C9ZL71_
DRB1_0101	505	RPSGASQVMRQNVGR	SQVMRQNVG	5	0.2418	3653.5	90.00	0.38	tr_C9ZL71_
DRB1_0101	506	SGDPEASYVAEPGRE	GDPEASYVA	1	0.2404	3711.3	95.00	0.58	tr_C9ZL71_
DRB1_0101	507	NVREDIKMRRRAWDE	IKMRRRAWD	5	0.2400	3726.6	95.00	0.21	tr_C9ZL71_
DRB1_0101	508	RGTANMSNVREDIKM	TANMSNVRE	2	0.2397	3736.2	95.00	0.54	tr_C9ZL71_
DRB1_0101	509	RRAWDEMKNDRDNAS	WDEMKNDRD	3	0.2397	3736.7	95.00	0.75	tr_C9ZL71_

DRB1_0101	510	DLGPERPSGASQVMR	LGPERPSGA	1	0.2393	3753.3	95.00	0.40	tr_C9ZL71_
DRB1_0101	511	PSTEQQSVAATPDHN	STEQQSVAA	1	0.2391	3762.7	95.00	0.31	tr_C9ZL71_
DRB1_0101	512	LGPERPSGASQVMRQ	ERPSGASQV	3	0.2387	3776.5	95.00	0.44	tr_C9ZL71_
DRB1_0101	513	EYPLDEVTVYTFGQP	EYPLDEVTV	0	0.2371	3844.3	95.00	0.29	tr_C9ZL71_
DRB1_0101	514	RPETVPIHPRDTRWK	TVPIHPRDT	3	0.2363	3876.1	95.00	0.56	tr_C9ZL71_
DRB1_0101	515	TANMSNVREDIKMRR	NVREDIKMR	5	0.2362	3880.9	95.00	0.33	tr_C9ZL71_
DRB1_0101	516	HSRPQDIEFWKGLRE	QDIEFWKGL	4	0.2351	3927.5	95.00	0.28	tr_C9ZL71_
DRB1_0101	517	NDLGPERPSGASQVM	LGPERPSGA	2	0.2346	3950.0	95.00	0.58	tr_C9ZL71_
DRB1_0101	518	RAWDEMKNDRDNASL	WDEMKNDRD	2	0.2346	3950.5	95.00	0.62	tr_C9ZL71_
DRB1_0101	519	VREDIKMRRRAWDEM	IKMRRRAWD	4	0.2344	3958.6	95.00	0.32	tr_C9ZL71_
DRB1_0101	520	GTANMSNVREDIKMR	TANMSNVRE	1	0.2330	4020.8	95.00	0.38	tr_C9ZL71_
DRB1_0101	521	PPVEGPSDPSINYTI	SDPSINYTI	6	0.2324	4043.5	95.00	0.58	tr_C9ZL71_
DRB1_0101	522	HTEKEEASFNWKQLE	EKEEASFNW	2	0.2320	4061.5	95.00	0.40	tr_C9ZL71_
DRB1_0101	523	LPYVEAGRENDLGPE	VEAGRENDL	3	0.2305	4127.0	95.00	0.58	tr_C9ZL71_
DRB1_0101	524	DGGSTNNPKSVWQAL	NNPKSVWQA	5	0.2300	4149.9	95.00	0.30	tr_C9ZL71_
DRB1_0101	525	EDIKMRRRAWDEMKN	IKMRRRAWD	2	0.2289	4203.2	95.00	0.31	tr_C9ZL71_
DRB1_0101	526	ALRLSSTTGDAPPGD	LRLSSTTGD	1	0.2273	4274.9	95.00	0.33	tr_C9ZL71_
DRB1_0101	527	ENDLGPERPSGASQV	LGPERPSGA	3	0.2264	4318.0	95.00	0.67	tr_C9ZL71_
DRB1_0101	528	TSQGDVEMRKFCPST	VEMRKFCPS	5	0.2244	4410.0	95.00	0.47	tr_C9ZL71_
DRB1_0101	529	VHSRPQDIEFWKGLR	SRPQDIEFW	2	0.2243	4413.4	95.00	0.38	tr_C9ZL71_
DRB1_0101	530	RRRAWDEMKNDRDNA	WDEMKNDRD	4	0.2239	4436.6	95.00	0.77	tr_C9ZL71_
DRB1_0101	531	VMRQNVGREGGGSRA	MRQNVGREG	1	0.2233	4462.6	95.00	0.56	tr_C9ZL71_
DRB1_0101	532	NVGREGGGSRAPLLE	GGGSRAPLL	5	0.2211	4571.7	95.00	0.35	tr_C9ZL71_
DRB1_0101	533	TTRHRPETVPIHPRD	TTRHRPETV	0	0.2205	4599.5	95.00	0.36	tr_C9ZL71_
DRB1_0101	534	VGREGGGSRAPLLEN	GGGSRAPLL	4	0.2194	4656.7	95.00	0.41	tr_C9ZL71_
DRB1_0101	535	QVGTEVDINRHGNYI	DINRHGNYI	6	0.2193	4660.6	95.00	0.52	tr_C9ZL71_
DRB1_0101	536	SVWPDDWYRWLARHG	WYRWLARHG	6	0.2174	4756.2	95.00	0.25	tr_C9ZL71_
DRB1_0101	537	ERPSGASQVMRQNVG	SGASQVMRQ	3	0.2172	4768.0	95.00	0.29	tr_C9ZL71_
DRB1_0101	538	AWDEMKNDRDNASLN	WDEMKNDRD	1	0.2158	4842.3	95.00	0.52	tr_C9ZL71_
DRB1_0101	539	STEQQSVAATPDHNG	TEQQSVAAT	1	0.2154	4861.6	95.00	0.23	tr_C9ZL71_
DRB1_0101	540	GPERPSGASQVMRQN	ERPSGASQV	2	0.2144	4912.9	95.00	0.42	tr_C9ZL71_
DRB1_0101	541	QNVGREGGGSRAPLL	GREGGGSRA	3	0.2137	4952.0	95.00	0.38	tr_C9ZL71_
DRB1_0101	542	PERPSGASQVMRQNV	SGASQVMRQ	4	0.2130	4990.8	95.00	0.31	tr_C9ZL71_
DRB1_0101	543	PETVPIHPRDTRWKE	TVPIHPRDT	2	0.2124	5019.7	95.00	0.52	tr_C9ZL71_
DRB1_0101	544	RDNASLNSSCCWEPT	NASLNSSCC	2	0.2117	5063.1	95.00	0.54	tr_C9ZL71_
DRB1_0101	545	RENDLGPERPSGASQ	LGPERPSGA	4	0.2111	5095.6	95.00	0.67	tr_C9ZL71_
DRB1_0101	546	GQSHARAQERKVGTS	HARAQERKV	3	0.2104	5133.1	95.00	0.49	tr_C9ZL71_
DRB1_0101	547	HRPETVPIHPRDTRW	TVPIHPRDT	4	0.2103	5138.3	95.00	0.51	tr_C9ZL71_
DRB1_0101	548	LDTSCKIRASGDPEA	IRASGDPEA	6	0.2102	5145.4	95.00	0.32	tr_C9ZL71_
DRB1_0101	549	TEKEEASFNWKQLEY	EKEEASFNW	1	0.2101	5146.2	95.00	0.37	tr_C9ZL71_
DRB1_0101	550	RHRPETVPIHPRDTR	TVPIHPRDT	5	0.2095	5179.9	95.00	0.42	tr_C9ZL71_
DRB1_0101	551	YPLDEVTVYTFGQPP	TVYTFGQPP	6	0.2088	5221.2	95.00	0.31	tr_C9ZL71_
DRB1_0101	552	KGQSHARAQERKVGT	HARAQERKV	4	0.2070	5325.6	95.00	0.44	tr_C9ZL71_
DRB1_0101	553	ALDTSCKIRASGDPE	LDTSCKIRA	1	0.2042	5490.4	95.00	0.51	tr_C9ZL71_
DRB1_0101	554	DNASLNSSCCWEPTV	NASLNSSCC	1	0.2040	5501.8	95.00	0.41	tr_C9ZL71_
DRB1_0101	555	DIKMRRRAWDEMKND	IKMRRRAWD	1	0.2029	5568.5	95.00	0.28	tr_C9ZL71_
DRB1_0101	556	NARESAAYPRETNPD	ARESAAYPR	1	0.2019	5623.8	95.00	0.44	tr_C9ZL71_
DRB1_0101	557	NDRDNASLNSSCCWE	NASLNSSCC	4	0.2018	5635.6	95.00	0.58	tr_C9ZL71_
DRB1_0101	558	SAAYPRETNPDACTT	YPRETNPDA	3	0.1996	5769.0	95.00	0.70	tr_C9ZL71_
DRB1_0101	559	TEQQSVAATPDHNGT	VAATPDHNG	5	0.1991	5797.8	95.00	0.31	tr_C9ZL71_

DRB1_0101	560	MRRRAWDEMKNDRDN	WDEMKNDRD	5	0.1983	5849.4	95.00	0.68	tr_C9ZL71_
DRB1_0101	561	QSHARAQERKVGTSG	HARAQERKV	2	0.1980	5869.7	95.00	0.50	tr_C9ZL71_
DRB1_0101	562	PYVEAGRENDLGPER	VEAGRENDL	2	0.1975	5900.9	95.00	0.62	tr_C9ZL71_
DRB1_0101	563	WEAHQTSIEEKLGGF	HQTSIEEKL	3	0.1972	5923.1	95.00	0.44	tr_C9ZL71_
DRB1_0101	564	NASLNSSCCWEPTVH	LNSSCCWEP	3	0.1964	5972.2	95.00	0.34	tr_C9ZL71_
DRB1_0101	565	QERKVGTSGCCRGSS	KVGTSGCCR	3	0.1958	6010.2	95.00	0.38	tr_C9ZL71_
DRB1_0101	566	KNDRDNASLNSSCCW	NASLNSSCC	5	0.1957	6017.0	95.00	0.54	tr_C9ZL71_
DRB1_0101	567	DRDNASLNSSCCWEP	NASLNSSCC	3	0.1953	6040.4	95.00	0.57	tr_C9ZL71_
DRB1_0101	568	GRENDLGPERPSGAS	LGPERPSGA	5	0.1938	6141.7	95.00	0.58	tr_C9ZL71_
DRB1_0101	569	ETVPIHPRDTRWKES	TVPIHPRDT	1	0.1934	6167.2	95.00	0.50	tr_C9ZL71_
DRB1_0101	570	TTSQGDVEMRKFCPS	VEMRKFCPS	6	0.1914	6305.3	100.00	0.32	tr_C9ZL71_
DRB1_0101	571	WDEMKNDRDNASLNS	WDEMKNDRD	0	0.1893	6448.0	100.00	0.34	tr_C9ZL71_
DRB1_0101	572	GREGGGSRAPLLEND	GGGSRAPLL	3	0.1887	6491.6	100.00	0.50	tr_C9ZL71_
DRB1_0101	573	ERKVGTSGCCRGSSN	KVGTSGCCR	2	0.1876	6568.7	100.00	0.34	tr_C9ZL71_
DRB1_0101	574	TRHRPETVPIHPRDT	HRPETVPIH	2	0.1849	6763.3	100.00	0.26	tr_C9ZL71_
DRB1_0101	575	AAYPRETNPDACTTS	YPRETNPDA	2	0.1846	6784.3	100.00	0.67	tr_C9ZL71_
DRB1_0101	576	ESVWPDDWYRWLARH	WPDDWYRWL	3	0.1840	6826.3	100.00	0.28	tr_C9ZL71_
DRB1_0101	577	AQERKVGTSGCCRGS	KVGTSGCCR	4	0.1823	6954.2	100.00	0.38	tr_C9ZL71_
DRB1_0101	578	ESAAYPRETNPDACT	YPRETNPDA	4	0.1810	7057.3	100.00	0.69	tr_C9ZL71_
DRB1_0101	579	RKVGTSGCCRGSSNY	KVGTSGCCR	1	0.1792	7193.7	100.00	0.34	tr_C9ZL71_
DRB1_0101	580	KESVWPDDWYRWLAR	VWPDDWYRW	3	0.1786	7236.5	100.00	0.23	tr_C9ZL71_
DRB1_0101	581	KVGTSGCCRGSSNYR	CCRGSSNYR	6	0.1765	7402.9	100.00	0.25	tr_C9ZL71_
DRB1_0101	582	DACTTSQGDVEMRKF	TSQGDVEMR	4	0.1765	7403.6	100.00	0.34	tr_C9ZL71_
DRB1_0101	583	SHARAQERKVGTSGC	HARAQERKV	1	0.1761	7435.0	100.00	0.46	tr_C9ZL71_
DRB1_0101	584	ACTTSQGDVEMRKFC	TSQGDVEMR	3	0.1760	7446.0	100.00	0.34	tr_C9ZL71_
DRB1_0101	585	REGGGSRAPLLENDG	GGGSRAPLL	2	0.1730	7695.3	100.00	0.58	tr_C9ZL71_
DRB1_0101	586	MRQNVGREGGGSRAP	MRQNVGREG	0	0.1713	7837.5	100.00	0.29	tr_C9ZL71_
DRB1_0101	587	NGTPTIDVKQYGYKP	DVKQYGYKP	6	0.1705	7899.3	100.00	0.50	tr_C9ZL71_
DRB1_0101	588	GERRLGSVDDHSGGN	LGSVDDHSG	4	0.1701	7934.1	100.00	0.51	tr_C9ZL71_
DRB1_0101	589	EQQSVAATPDHNGTP	VAATPDHNG	4	0.1694	8001.3	100.00	0.37	tr_C9ZL71_
DRB1_0101	590	KMRRRAWDEMKNDRD	WDEMKNDRD	6	0.1688	8050.7	100.00	0.47	tr_C9ZL71_
DRB1_0101	591	RQNVGREGGGSRAPL	GREGGGSRA	4	0.1678	8141.1	100.00	0.46	tr_C9ZL71_
DRB1_0101	592	MKNDRDNASLNSSCC	NASLNSSCC	6	0.1671	8200.8	100.00	0.34	tr_C9ZL71_
DRB1_0101	593	DDGGSTNNPKSVWQA	GSTNNPKSV	3	0.1670	8205.7	100.00	0.31	tr_C9ZL71_
DRB1_0101	594	ERRLGSVDDHSGGNG	LGSVDDHSG	3	0.1658	8312.0	100.00	0.55	tr_C9ZL71_
DRB1_0101	595	CTTSQGDVEMRKFCP	TSQGDVEMR	2	0.1658	8317.2	100.00	0.29	tr_C9ZL71_
DRB1_0101	596	YVEAGRENDLGPERP	VEAGRENDL	1	0.1654	8352.1	100.00	0.65	tr_C9ZL71_
DRB1_0101	597	QQSVAATPDHNGTPT	VAATPDHNG	3	0.1652	8369.9	100.00	0.46	tr_C9ZL71_
DRB1_0101	598	DGERRLGSVDDHSGG	LGSVDDHSG	5	0.1641	8468.0	100.00	0.41	tr_C9ZL71_
DRB1_0101	599	IKMRRRAWDEMKNDR	KMRRRAWDE	1	0.1620	8661.6	100.00	0.23	tr_C9ZL71_
DRB1_0101	600	WKESVWPDDWYRWLA	VWPDDWYRW	4	0.1620	8668.3	100.00	0.26	tr_C9ZL71_
DRB1_0101	601	RESAAYPRETNPDAC	YPRETNPDA	5	0.1589	8958.1	100.00	0.62	tr_C9ZL71_
DRB1_0101	602	AYPRETNPDACTTSQ	YPRETNPDA	1	0.1585	8998.2	100.00	0.60	tr_C9ZL71_
DRB1_0101	603	PDACTTSQGDVEMRK	TSQGDVEMR	5	0.1584	9009.5	100.00	0.31	tr_C9ZL71_
DRB1_0101	604	TVPIHPRDTRWKESV	IHPRDTRWK	3	0.1569	9153.7	100.00	0.32	tr_C9ZL71_
DRB1_0101	605	RWKESVWPDDWYRWL	VWPDDWYRW	5	0.1565	9195.8	100.00	0.23	tr_C9ZL71_
DRB1_0101	606	RAQERKVGTSGCCRG	KVGTSGCCR	5	0.1560	9241.2	100.00	0.34	tr_C9ZL71_
DRB1_0101	607	TPDHNGTPTIDVKQY	HNGTPTIDV	3	0.1559	9254.5	100.00	0.56	tr_C9ZL71_
DRB1_0101	608	LQSIPNDSSSPPVEG	LQSIPNDSS	0	0.1556	9284.5	100.00	0.41	tr_C9ZL71_
DRB1_0101	609	ARESAAYPRETNPDA	YPRETNPDA	6	0.1550	9342.6	100.00	0.33	tr_C9ZL71_

DRB1_0101	610	LRLSSTTGDAPPGDG	LSSTTGDAP	2	0.1528	9573.4	100.00	0.47	tr_C9ZL71_
DRB1_0101	611	AGRENDLGPERPSGA	LGPERPSGA	6	0.1525	9606.7	100.00	0.44	tr_C9ZL71_
DRB1_0101	612	GDGERRLGSVDDHSG	ERRLGSVDD	3	0.1515	9705.4	100.00	0.46	tr_C9ZL71_
DRB1_0101	613	DEMKNDRDNASLNSS	MKNDRDNAS	2	0.1508	9777.5	100.00	0.36	tr_C9ZL71_
DRB1_0101	614	RRLGSVDDHSGGNGD	LGSVDDHSG	2	0.1501	9856.0	100.00	0.63	tr_C9ZL71_
DRB1_0101	615	QSVAATPDHNGTPTI	VAATPDHNG	2	0.1482	10055.4	100.00	0.45	tr_C9ZL71_
DRB1_0101	616	EGGGSRAPLLENDGG	GGGSRAPLL	1	0.1474	10149.0	100.00	0.54	tr_C9ZL71_
DRB1_0101	617	EMKNDRDNASLNSSC	MKNDRDNAS	1	0.1469	10202.4	100.00	0.31	tr_C9ZL71_
DRB1_0101	618	TRWKESVWPDDWYRW	WKESVWPDD	2	0.1458	10325.3	100.00	0.29	tr_C9ZL71_
DRB1_0101	619	PDHNGTPTIDVKQYG	HNGTPTIDV	2	0.1450	10417.4	100.00	0.59	tr_C9ZL71_
DRB1_0101	620	ATPDHNGTPTIDVKQ	HNGTPTIDV	4	0.1435	10580.3	100.00	0.52	tr_C9ZL71_
DRB1_0101	621	HARAQERKVGTSGCC	HARAQERKV	0	0.1403	10959.2	100.00	0.37	tr_C9ZL71_
DRB1_0101	622	DHNGTPTIDVKQYGY	HNGTPTIDV	1	0.1358	11499.5	100.00	0.52	tr_C9ZL71_
DRB1_0101	623	HNGTPTIDVKQYGYK	HNGTPTIDV	0	0.1348	11635.3	100.00	0.33	tr_C9ZL71_
DRB1_0101	624	DTRWKESVWPDDWYR	WKESVWPDD	3	0.1341	11720.0	100.00	0.31	tr_C9ZL71_
DRB1_0101	625	AATPDHNGTPTIDVK	HNGTPTIDV	5	0.1336	11780.1	100.00	0.47	tr_C9ZL71_
DRB1_0101	626	RDTRWKESVWPDDWY	TRWKESVWP	2	0.1303	12205.2	100.00	0.39	tr_C9ZL71_
DRB1_0101	627	ARAQERKVGTSGCCR	KVGTSGCCR	6	0.1294	12323.8	100.00	0.25	tr_C9ZL71_
DRB1_0101	628	GGGSRAPLLENDGGA	GGGSRAPLL	0	0.1294	12334.6	100.00	0.35	tr_C9ZL71_
DRB1_0101	629	PGDGERRLGSVDDHS	ERRLGSVDD	4	0.1269	12669.9	100.00	0.51	tr_C9ZL71_
DRB1_0101	630	RLGSVDDHSGGNGDD	LGSVDDHSG	1	0.1265	12726.7	100.00	0.57	tr_C9ZL71_
DRB1_0101	631	GDDGGSTNNPKSVWQ	GSTNNPKSV	4	0.1242	13037.9	100.00	0.39	tr_C9ZL71_
DRB1_0101	632	NPDACTTSQGDVEMR	TTSQGDVEM	5	0.1230	13209.9	100.00	0.26	tr_C9ZL71_
DRB1_0101	633	SVAATPDHNGTPTID	VAATPDHNG	1	0.1210	13503.5	100.00	0.44	tr_C9ZL71_
DRB1_0101	634	PRDTRWKESVWPDDW	TRWKESVWP	3	0.1202	13613.1	100.00	0.46	tr_C9ZL71_
DRB1_0101	635	GTQVGTEVDINRHGN	TQVGTEVDI	1	0.1191	13787.7	100.00	0.28	tr_C9ZL71_
DRB1_0101	636	VAATPDHNGTPTIDV	HNGTPTIDV	6	0.1187	13835.1	100.00	0.26	tr_C9ZL71_
DRB1_0101	637	VEAGRENDLGPERPS	VEAGRENDL	0	0.1178	13981.5	100.00	0.43	tr_C9ZL71_
DRB1_0101	638	VPIHPRDTRWKESVW	IHPRDTRWK	2	0.1152	14374.3	100.00	0.43	tr_C9ZL71_
DRB1_0101	639	PIHPRDTRWKESVWP	IHPRDTRWK	1	0.1133	14673.7	100.00	0.31	tr_C9ZL71_
DRB1_0101	640	QSIPNDSSSPPVEGP	DSSSPPVEG	5	0.1132	14689.1	100.00	0.26	tr_C9ZL71_
DRB1_0101	641	IHPRDTRWKESVWPD	TRWKESVWP	5	0.1128	14753.0	100.00	0.39	tr_C9ZL71_
DRB1_0101	642	SPPVEGPSDPSINYT	VEGPSDPSI	3	0.1125	14800.0	100.00	0.22	tr_C9ZL71_
DRB1_0101	643	TQVGTEVDINRHGNY	TEVDINRHG	4	0.1116	14940.9	100.00	0.21	tr_C9ZL71_
DRB1_0101	644	YPRETNPDACTTSQG	YPRETNPDA	0	0.1110	15050.8	100.00	0.40	tr_C9ZL71_
DRB1_0101	645	PPGDGERRLGSVDDH	ERRLGSVDD	5	0.1105	15130.1	100.00	0.43	tr_C9ZL71_
DRB1_0101	646	SIPNDSSSPPVEGPS	DSSSPPVEG	4	0.1073	15659.9	100.00	0.37	tr_C9ZL71_
DRB1_0101	647	RLSSTTGDAPPGDGE	LSSTTGDAP	1	0.1067	15764.6	100.00	0.54	tr_C9ZL71_
DRB1_0101	648	HPRDTRWKESVWPDD	TRWKESVWP	4	0.1054	15987.8	100.00	0.48	tr_C9ZL71_
DRB1_0101	649	NGDDGGSTNNPKSVW	GSTNNPKSV	5	0.1018	16613.5	100.00	0.38	tr_C9ZL71_
DRB1_0101	650	LGSVDDHSGGNGDDG	LGSVDDHSG	0	0.0967	17557.3	100.00	0.38	tr_C9ZL71_
DRB1_0101	651	IPNDSSSPPVEGPSD	DSSSPPVEG	3	0.0932	18249.3	100.00	0.41	tr_C9ZL71_
DRB1_0101	652	SSPPVEGPSDPSINY	VEGPSDPSI	4	0.0914	18607.6	100.00	0.28	tr_C9ZL71_
DRB1_0101	653	EAGRENDLGPERPSG	ENDLGPERP	4	0.0904	18803.7	100.00	0.42	tr_C9ZL71_
DRB1_0101	654	APPGDGERRLGSVDD	ERRLGSVDD	6	0.0900	18887.5	100.00	0.29	tr_C9ZL71_
DRB1_0101	655	TNPDACTTSQGDVEM	CTTSQGDVE	5	0.0876	19387.8	100.00	0.24	tr_C9ZL71_
DRB1_0101	656	LSSTTGDAPPGDGER	LSSTTGDAP	0	0.0779	21516.2	100.00	0.32	tr_C9ZL71_
DRB1_0101	657	PNDSSSPPVEGPSDP	DSSSPPVEG	2	0.0765	21859.5	100.00	0.44	tr_C9ZL71_
DRB1_0101	658	GNGDDGGSTNNPKSV	GSTNNPKSV	6	0.0756	22075.8	100.00	0.29	tr_C9ZL71_
DRB1_0101	659	DAPPGDGERRLGSVD	PPGDGERRL	2	0.0753	22142.0	100.00	0.28	tr_C9ZL71_

DRB1_0101	660	GDAPPGDGERRLGSV	PPGDGERRL	3	0.0725	22830.3	100.00	0.32	tr_C9ZL71_	
DRB1_0101	661	ETNPDACTTSQGDVE	DACTTSQGD	4	0.0690	23700.1	100.00	0.30	tr_C9ZL71_	
DRB1_0101	662	SSSPPVEGPSDPSIN	VEGPSDPSI	5	0.0652	24704.8	100.00	0.29	tr_C9ZL71_	
DRB1_0101	663	NDSSSPPVEGPSDPS	DSSSPPVEG	1	0.0638	25071.5	100.00	0.46	tr_C9ZL71_	
DRB1_0101	664	RETNPDACTTSQGDV	DACTTSQGD	5	0.0636	25120.1	100.00	0.26	tr_C9ZL71_	
DRB1_0101	665	GSVDDHSGGNGDDGG	VDDHSGGNG	2	0.0629	25325.6	100.00	0.44	tr_C9ZL71_	
DRB1_0101	666	PRETNPDACTTSQGD	ETNPDACTT	2	0.0611	25801.1	100.00	0.23	tr_C9ZL71_	
DRB1_0101	667	SVDDHSGGNGDDGGS	VDDHSGGNG	1	0.0609	25856.7	100.00	0.35	tr_C9ZL71_	
DRB1_0101	668	VDDHSGGNGDDGGST	DDHSGGNGD	1	0.0596	26227.2	100.00	0.17	tr_C9ZL71_	
DRB1_0101	669	TGDAPPGDGERRLGS	PPGDGERRL	4	0.0586	26517.1	100.00	0.32	tr_C9ZL71_	
DRB1_0101	670	STTGDAPPGDGERRL	TGDAPPGDG	2	0.0585	26559.9	100.00	0.22	tr_C9ZL71_	
DRB1_0101	671	SSTTGDAPPGDGERR	TGDAPPGDG	3	0.0582	26639.3	100.00	0.23	tr_C9ZL71_	
DRB1_0101	672	DSSSPPVEGPSDPSI	PVEGPSDPS	5	0.0579	26715.5	100.00	0.21	tr_C9ZL71_	
DRB1_0101	673	HSGGNGDDGGSTNNP	GNGDDGGST	3	0.0567	27080.7	100.00	0.38	tr_C9ZL71_	
DRB1_0101	674	DHSGGNGDDGGSTNN	GNGDDGGST	4	0.0566	27097.7	100.00	0.45	tr_C9ZL71_	
DRB1_0101	675	TTGDAPPGDGERRLG	PPGDGERRL	5	0.0561	27255.1	100.00	0.27	tr_C9ZL71_	
DRB1_0101	676	SGGNGDDGGSTNNPK	GNGDDGGST	2	0.0549	27616.3	100.00	0.37	tr_C9ZL71_	
DRB1_0101	677	DDHSGGNGDDGGSTN	GNGDDGGST	5	0.0541	27841.3	100.00	0.41	tr_C9ZL71_	
DRB1_0101	678	GGNGDDGGSTNNPKS	GNGDDGGST	1	0.0540	27876.9	100.00	0.28	tr_C9ZL71_	

Allele: DRB1_0101. Number of high binders 4. Number of weak binders 35. Number of peptides 678

Allele	pos	peptide	core	Of	1-log50k(aff)	affinity(nM)	%Rank	Relia	Identity	Bind Level
DRB1_0101	1	RGYRLLDAPDGIMMF	YRLLDAPDG	2	0.8458	5.3	0.70	0.87	tr_D0A0U3_	WB
DRB1_0101	2	GRGYRLLDAPDGIMM	YRLLDAPDG	3	0.8453	5.3	0.70	0.89	tr_D0A0U3_	WB
DRB1_0101	3	LIRYALGRLRTIRLG	LGRLRTIRL	5	0.8404	5.6	0.80	0.38	tr_D0A0U3_	WB
DRB1_0101	4	IRYALGRLRTIRLGF	LGRLRTIRL	4	0.8318	6.2	1.00	0.49	tr_D0A0U3_	WB
DRB1_0101	5	IGRGYRLLDAPDGIM	YRLLDAPDG	4	0.8255	6.6	1.20	0.91	tr_D0A0U3_	WB
DRB1_0101	6	ALIRYALGRLRTIRL	IRYALGRLR	2	0.8222	6.8	1.30	0.38	tr_D0A0U3_	WB
DRB1_0101	7	RYALGRLRTIRLGFM	LGRLRTIRL	3	0.8194	7.1	1.40	0.64	tr_D0A0U3_	WB
DRB1_0101	8	GYRLLDAPDGIMMFD	YRLLDAPDG	1	0.8173	7.2	1.40	0.87	tr_D0A0U3_	WB
DRB1_0101	9	RVIISPMKAMNATAL	ISPMKAMNA	3	0.8149	7.4	1.50	0.78	tr_D0A0U3_	WB
DRB1_0101	10	VVAFAPLTGSSGGRG	FAPLTGSSG	3	0.8111	7.7	1.60	0.89	tr_D0A0U3_	WB
DRB1_0101	11	VIISPMKAMNATALV	ISPMKAMNA	2	0.8095	7.9	1.70	0.63	tr_D0A0U3_	WB
DRB1_0101	12	DGCFLKGVPRLTGCA	FLKGVPRLT	3	0.8065	8.1	1.80	0.85	tr_D0A0U3_	WB
DRB1_0101	13	SRRWKTAPSVHVKVM	WKTAPSVHV	3	0.8024	8.5	2.00	0.92	tr_D0A0U3_	WB
DRB1_0101	14	DVVAFAPLTGSSGGR	FAPLTGSSG	4	0.8020	8.5	2.00	0.88	tr_D0A0U3_	WB
DRB1_0101	15	IISPMKAMNATALVD	ISPMKAMNA	1	0.8007	8.6	2.00	0.54	tr_D0A0U3_	WB
DRB1_0101	16	GCFLKGVPRLTGCAV	FLKGVPRLT	2	0.8006	8.7	2.00	0.85	tr_D0A0U3_	WB
DRB1_0101	17	VAFAPLTGSSGGRGW	FAPLTGSSG	2	0.7994	8.8	2.50	0.89	tr_D0A0U3_	WB
DRB1_0101	18	WNTFVESRPQAVIVF	FVESRPQAV	3	0.7955	9.1	2.50	0.89	tr_D0A0U3_	WB
DRB1_0101	19	NTFVESRPQAVIVFA	FVESRPQAV	2	0.7925	9.4	2.50	0.88	tr_D0A0U3_	WB
DRB1_0101	20	LALIRYALGRLRTIR	IRYALGRLR	3	0.7916	9.5	2.50	0.54	tr_D0A0U3_	WB
DRB1_0101	21	DDGCFLKGVPRLTGC	FLKGVPRLT	4	0.7873	10.0	3.00	0.82	tr_D0A0U3_	WB
DRB1_0101	22	TARFIRNLVADSRTR	FIRNLVADS	3	0.7868	10.0	3.00	0.50	tr_D0A0U3_	WB
DRB1_0101	23	RLFFDTIVSPFSGAA	FDTIVSPFS	3	0.7868	10.0	3.00	0.81	tr_D0A0U3_	WB

DRB1_0101	24	NLYFTRASPSAELLA	FTRASPSAE	3	0.7865	10.1	3.00	0.43	tr_D0A0U3_	WB
DRB1_0101	25	MRVIISPMKAMNATA	ISPMKAMNA	4	0.7852	10.2	3.00	0.79	tr_D0A0U3_	WB
DRB1_0101	26	RRWKTAPSVHVKVMH	WKTAPSVHV	2	0.7850	10.2	3.00	0.91	tr_D0A0U3_	WB
DRB1_0101	27	VIGRGYRLLDAPDGI	YRLLDAPDG	5	0.7834	10.4	3.00	0.93	tr_D0A0U3_	WB
DRB1_0101	28	TSRRWKTAPSVHVKV	WKTAPSVHV	4	0.7817	10.6	3.00	0.92	tr_D0A0U3_	WB
DRB1_0101	29	PNLYFTRASPSAELL	LYFTRASPS	2	0.7796	10.9	3.00	0.37	tr_D0A0U3_	WB
DRB1_0101	30	ADDGCFLKGVPRLTG	FLKGVPRLT	5	0.7794	10.9	3.00	0.81	tr_D0A0U3_	WB
DRB1_0101	31	AWNTFVESRPQAVIV	FVESRPQAV	4	0.7744	11.5	3.50	0.91	tr_D0A0U3_	WB
DRB1_0101	32	QVSVWSMARALNASI	WSMARALNA	4	0.7707	11.9	3.50	0.64	tr_D0A0U3_	WB
DRB1_0101	33	YALGRLRTIRLGFMY	LGRLRTIRL	2	0.7695	12.1	3.50	0.68	tr_D0A0U3_	WB
DRB1_0101	34	ISPMKAMNATALVDS	MKAMNATAL	3	0.7694	12.1	3.50	0.56	tr_D0A0U3_	WB
DRB1_0101	35	ARFIRNLVADSRTRS	FIRNLVADS	2	0.7693	12.1	3.50	0.46	tr_D0A0U3_	WB
DRB1_0101	36	YRLLDAPDGIMMFDS	YRLLDAPDG	0	0.7675	12.4	4.00	0.78	tr_D0A0U3_	WB
DRB1_0101	37	FMRVIISPMKAMNAT	ISPMKAMNA	5	0.7673	12.4	4.00	0.64	tr_D0A0U3_	WB
DRB1_0101	38	VSVWSMARALNASIP	WSMARALNA	3	0.7629	13.0	4.00	0.66	tr_D0A0U3_	WB
DRB1_0101	39	QDVVAFAPLTGSSGG	FAPLTGSSG	5	0.7624	13.1	4.00	0.82	tr_D0A0U3_	WB
DRB1_0101	40	DTARFIRNLVADSRT	FIRNLVADS	4	0.7613	13.2	4.00	0.50	tr_D0A0U3_	WB
DRB1_0101	41	AFAPLTGSSGGRGWN	FAPLTGSSG	1	0.7612	13.2	4.00	0.89	tr_D0A0U3_	WB
DRB1_0101	42	LLALIRYALGRLRTI	IRYALGRLR	4	0.7607	13.3	4.00	0.54	tr_D0A0U3_	WB
DRB1_0101	43	SRLFFDTIVSPFSGA	FDTIVSPFS	4	0.7593	13.5	4.50	0.80	tr_D0A0U3_	WB
DRB1_0101	44	VYVIGLTAADPVVIA	IGLTAADPV	3	0.7574	13.8	4.50	0.47	tr_D0A0U3_	WB
DRB1_0101	45	LFFDTIVSPFSGAAE	FDTIVSPFS	2	0.7558	14.1	4.50	0.79	tr_D0A0U3_	WB
DRB1_0101	46	TFVESRPQAVIVFAP	FVESRPQAV	1	0.7557	14.1	4.50	0.85	tr_D0A0U3_	WB
DRB1_0101	47	NMWREALAFAASPFV	WREALAFAA	2	0.7554	14.1	4.50	0.67	tr_D0A0U3_	WB
DRB1_0101	48	LYFTRASPSAELLAL	FTRASPSAE	2	0.7534	14.4	4.50	0.47	tr_D0A0U3_	WB
DRB1_0101	49	YVIGLTAADPVVIAA	IGLTAADPV	2	0.7511	14.8	5.00	0.44	tr_D0A0U3_	WB
DRB1_0101	50	CFLKGVPRLTGCAVN	FLKGVPRLT	1	0.7476	15.3	5.00	0.85	tr_D0A0U3_	WB
DRB1_0101	51	NPNLYFTRASPSAEL	LYFTRASPS	3	0.7472	15.4	5.00	0.54	tr_D0A0U3_	WB
DRB1_0101	52	TQVSVWSMARALNAS	WSMARALNA	5	0.7455	15.7	5.00	0.61	tr_D0A0U3_	WB
DRB1_0101	53	SVWSMARALNASIPP	WSMARALNA	2	0.7436	16.0	5.50	0.67	tr_D0A0U3_	WB
DRB1_0101	54	YFMRVIISPMKAMNA	ISPMKAMNA	6	0.7435	16.0	5.50	0.35	tr_D0A0U3_	WB
DRB1_0101	55	MTSRRWKTAPSVHVK	WKTAPSVHV	5	0.7429	16.1	5.50	0.86	tr_D0A0U3_	WB
DRB1_0101	56	DVYVIGLTAADPVVI	IGLTAADPV	4	0.7416	16.4	5.50	0.41	tr_D0A0U3_	WB
DRB1_0101	57	MWREALAFAASPFVD	WREALAFAA	1	0.7388	16.9	5.50	0.55	tr_D0A0U3_	WB
DRB1_0101	58	RVIGRGYRLLDAPDG	YRLLDAPDG	6	0.7384	17.0	5.50	0.64	tr_D0A0U3_	WB
DRB1_0101	59	SSLITLLAEQDVVAF	ITLLAEQDV	3	0.7365	17.3	6.00	0.36	tr_D0A0U3_	WB
DRB1_0101	60	SLITLLAEQDVVAFA	LLAEQDVVA	4	0.7356	17.5	6.00	0.38	tr_D0A0U3_	WB
DRB1_0101	61	ENMWREALAFAASPF	WREALAFAA	3	0.7336	17.8	6.00	0.85	tr_D0A0U3_	WB
DRB1_0101	62	KRFVEAVNAGFNASM	FVEAVNAGF	2	0.7335	17.9	6.00	0.55	tr_D0A0U3_	WB
DRB1_0101	63	RWKTAPSVHVKVMHP	WKTAPSVHV	1	0.7329	18.0	6.00	0.90	tr_D0A0U3_	WB
DRB1_0101	64	SAWNTFVESRPQAVI	FVESRPQAV	5	0.7322	18.1	6.00	0.88	tr_D0A0U3_	WB
DRB1_0101	65	CGVFTLSSSLTGVAS	FTLSSSLTG	3	0.7303	18.5	6.50	0.67	tr_D0A0U3_	WB
DRB1_0101	66	GVFTLSSSLTGVAST	FTLSSSLTG	2	0.7297	18.6	6.50	0.58	tr_D0A0U3_	WB
DRB1_0101	67	GDTARFIRNLVADSR	FIRNLVADS	5	0.7284	18.9	6.50	0.46	tr_D0A0U3_	WB
DRB1_0101	68	RLGFMYLHNVFFGVE	FMYLHNVFF	3	0.7254	19.5	6.50	0.60	tr_D0A0U3_	WB
DRB1_0101	69	EKRFVEAVNAGFNAS	FVEAVNAGF	3	0.7253	19.5	7.00	0.56	tr_D0A0U3_	WB
DRB1_0101	70	FCGVFTLSSSLTGVA	FTLSSSLTG	4	0.7252	19.6	7.00	0.62	tr_D0A0U3_	WB
DRB1_0101	71	VIGLTAADPVVIAAH	LTAADPVVI	3	0.7239	19.8	7.00	0.42	tr_D0A0U3_	WB
DRB1_0101	72	RTPLSLLGFATAYFM	PLSLLGFAT	2	0.7235	19.9	7.00	0.50	tr_D0A0U3_	WB
DRB1_0101	73	RRTPLSLLGFATAYF	PLSLLGFAT	3	0.7190	20.9	7.50	0.62	tr_D0A0U3_	WB

DRB1_0101	74	TPLSLLGFATAYFMR	PLSLLGFAT	1	0.7181	21.1	7.50	0.38	tr_D0A0U3_	WB
DRB1_0101	75	IENMWREALAFAASP	WREALAFAA	4	0.7180	21.1	7.50	0.84	tr_D0A0U3_	WB
DRB1_0101	76	AIENMWREALAFAAS	WREALAFAA	5	0.7174	21.3	7.50	0.81	tr_D0A0U3_	WB
DRB1_0101	77	FFDTIVSPFSGAAEE	FDTIVSPFS	1	0.7174	21.3	7.50	0.79	tr_D0A0U3_	WB
DRB1_0101	78	VFTLSSSLTGVASTR	FTLSSSLTG	1	0.7171	21.3	7.50	0.50	tr_D0A0U3_	WB
DRB1_0101	79	IRLGFMYLHNVFFGV	FMYLHNVFF	4	0.7170	21.4	7.50	0.63	tr_D0A0U3_	WB
DRB1_0101	80	SPMKAMNATALVDSI	MKAMNATAL	2	0.7170	21.4	7.50	0.73	tr_D0A0U3_	WB
DRB1_0101	81	DSRLFFDTIVSPFSG	FDTIVSPFS	5	0.7149	21.8	7.50	0.81	tr_D0A0U3_	WB
DRB1_0101	82	VSSLITLLAEQDVVA	ITLLAEQDV	4	0.7145	22.0	7.50	0.41	tr_D0A0U3_	WB
DRB1_0101	83	GDVYVIGLTAADPVV	IGLTAADPV	5	0.7143	22.0	7.50	0.32	tr_D0A0U3_	WB
DRB1_0101	84	TAYFMRVIISPMKAM	FMRVIISPM	3	0.7138	22.1	7.50	0.52	tr_D0A0U3_	WB
DRB1_0101	85	VWSMARALNASIPPL	WSMARALNA	1	0.7127	22.4	8.00	0.62	tr_D0A0U3_	WB
DRB1_0101	86	RFIRNLVADSRTRSA	FIRNLVADS	1	0.7101	23.0	8.00	0.46	tr_D0A0U3_	WB
DRB1_0101	87	SRRTPLSLLGFATAY	PLSLLGFAT	4	0.7090	23.3	8.00	0.63	tr_D0A0U3_	WB
DRB1_0101	88	AYFMRVIISPMKAMN	FMRVIISPM	2	0.7083	23.5	8.00	0.44	tr_D0A0U3_	WB
DRB1_0101	89	RSYLKSNPGVTVFNA	LKSNPGVTV	3	0.7067	23.9	8.50	0.57	tr_D0A0U3_	WB
DRB1_0101	90	PLSLLGFATAYFMRV	LGFATAYFM	4	0.7046	24.4	8.50	0.41	tr_D0A0U3_	WB
DRB1_0101	91	ELLALIRYALGRLRT	IRYALGRLR	5	0.7018	25.2	9.00	0.60	tr_D0A0U3_	WB
DRB1_0101	92	RFVEAVNAGFNASMT	FVEAVNAGF	1	0.6975	26.4	9.50	0.43	tr_D0A0U3_	WB
DRB1_0101	93	FADDGCFLKGVPRLT	FLKGVPRLT	6	0.6974	26.4	9.50	0.69	tr_D0A0U3_	WB
DRB1_0101	94	VRSYLKSNPGVTVFN	LKSNPGVTV	4	0.6970	26.5	9.50	0.49	tr_D0A0U3_	WB
DRB1_0101	95	LGDFGTVSSLITLLA	FGTVSSLIT	3	0.6956	26.9	9.50	0.73	tr_D0A0U3_	WB
DRB1_0101	96	LITLLAEQDVVAFAP	LLAEQDVVA	3	0.6952	27.0	9.50	0.43	tr_D0A0U3_	WB
DRB1_0101	97	ALGRLRTIRLGFMYL	LGRLRTIRL	1	0.6943	27.3	9.50	0.74	tr_D0A0U3_	WB
DRB1_0101	98	LGFMYLHNVFFGVEE	FMYLHNVFF	2	0.6932	27.6	9.50	0.56	tr_D0A0U3_	WB
DRB1_0101	99	GFCGVFTLSSSLTGV	FTLSSSLTG	5	0.6928	27.8	9.50	0.56	tr_D0A0U3_	WB
DRB1_0101	100	ATAYFMRVIISPMKA	FMRVIISPM	4	0.6926	27.8	10.00	0.55	tr_D0A0U3_	
DRB1_0101	101	ATQVSVWSMARALNA	WSMARALNA	6	0.6920	28.0	10.00	0.50	tr_D0A0U3_	
DRB1_0101	102	RSAYVLVPSMLQFAI	YVLVPSMLQ	3	0.6899	28.7	10.00	0.67	tr_D0A0U3_	
DRB1_0101	103	WREALAFAASPFVDG	LAFAASPFV	4	0.6897	28.7	10.00	0.49	tr_D0A0U3_	
DRB1_0101	104	IEKRFVEAVNAGFNA	FVEAVNAGF	4	0.6862	29.8	11.00	0.62	tr_D0A0U3_	
DRB1_0101	105	WSMARALNASIPPLT	WSMARALNA	0	0.6834	30.7	11.00	0.40	tr_D0A0U3_	
DRB1_0101	106	ASRRTPLSLLGFATA	PLSLLGFAT	5	0.6833	30.8	11.00	0.59	tr_D0A0U3_	
DRB1_0101	107	QAVIVFAPPVGDTAR	IVFAPPVGD	3	0.6827	31.0	11.00	0.69	tr_D0A0U3_	
DRB1_0101	108	GDFGTVSSLITLLAE	FGTVSSLIT	2	0.6816	31.3	11.00	0.71	tr_D0A0U3_	
DRB1_0101	109	LSLLGFATAYFMRVI	LGFATAYFM	3	0.6815	31.4	11.00	0.48	tr_D0A0U3_	
DRB1_0101	110	FFNAFEGVSSADRVL	FEGVSSADR	4	0.6784	32.5	12.00	0.38	tr_D0A0U3_	
DRB1_0101	111	TRSAYVLVPSMLQFA	YVLVPSMLQ	4	0.6782	32.5	12.00	0.69	tr_D0A0U3_	
DRB1_0101	112	VHVRVIGRGYRLLDA	RVIGRGYRL	3	0.6775	32.8	12.00	0.73	tr_D0A0U3_	
DRB1_0101	113	TIRLGFMYLHNVFFG	FMYLHNVFF	5	0.6774	32.8	12.00	0.72	tr_D0A0U3_	
DRB1_0101	114	PMKAMNATALVDSIF	MKAMNATAL	1	0.6768	33.0	12.00	0.69	tr_D0A0U3_	
DRB1_0101	115	YFTRASPSAELLALI	FTRASPSAE	1	0.6759	33.3	12.00	0.49	tr_D0A0U3_	
DRB1_0101	116	WNPNLYFTRASPSAE	LYFTRASPS	4	0.6741	34.0	12.00	0.67	tr_D0A0U3_	
DRB1_0101	117	TPGVAFIDPVPLAPR	VAFIDPVPL	3	0.6728	34.5	12.00	0.79	tr_D0A0U3_	
DRB1_0101	118	EQDVVAFAPLTGSSG	FAPLTGSSG	6	0.6726	34.6	12.00	0.69	tr_D0A0U3_	
DRB1_0101	119	PGVAFIDPVPLAPRL	VAFIDPVPL	2	0.6719	34.8	12.00	0.73	tr_D0A0U3_	
DRB1_0101	120	TALVDSIFAQSVVNV	VDSIFAQSV	3	0.6716	34.9	12.00	0.77	tr_D0A0U3_	
DRB1_0101	121	ALVDSIFAQSVVNVG	VDSIFAQSV	2	0.6697	35.6	13.00	0.65	tr_D0A0U3_	
DRB1_0101	122	PLGDFGTVSSLITLL	FGTVSSLIT	4	0.6696	35.7	13.00	0.73	tr_D0A0U3_	
DRB1_0101	123	IEVFSLLHHQKIEKR	FSLLHHQKI	3	0.6680	36.3	13.00	0.75	tr_D0A0U3_	

DRB1_0101	124	AELLALIRYALGRLR	IRYALGRLR	6	0.6680	36.3	13.00	0.43	tr_D0A0U3_
DRB1_0101	125	GFMYLHNVFFGVEEY	FMYLHNVFF	1	0.6680	36.3	13.00	0.51	tr_D0A0U3_
DRB1_0101	126	FTLSSSLTGVASTRH	SSSLTGVAS	3	0.6661	37.1	13.00	0.38	tr_D0A0U3_
DRB1_0101	127	TVSSLITLLAEQDVV	LITLLAEQD	4	0.6648	37.6	13.00	0.40	tr_D0A0U3_
DRB1_0101	128	EFFNAFEGVSSADRV	FNAFEGVSS	2	0.6648	37.6	13.00	0.47	tr_D0A0U3_
DRB1_0101	129	DVRSYLKSNPGVTVF	LKSNPGVTV	5	0.6619	38.8	14.00	0.47	tr_D0A0U3_
DRB1_0101	130	EVFSLLHHQKIEKRF	FSLLHHQKI	2	0.6617	38.9	14.00	0.69	tr_D0A0U3_
DRB1_0101	131	GVAFIDPVPLAPRLN	VAFIDPVPL	1	0.6604	39.4	14.00	0.63	tr_D0A0U3_
DRB1_0101	132	IGLTAADPVVIAAHL	LTAADPVVI	2	0.6594	39.9	14.00	0.52	tr_D0A0U3_
DRB1_0101	133	SGDVYVIGLTAADPV	YVIGLTAAD	4	0.6592	39.9	14.00	0.28	tr_D0A0U3_
DRB1_0101	134	LVYGWITGEVLAQAL	YGWITGEVL	2	0.6589	40.1	14.00	0.65	tr_D0A0U3_
DRB1_0101	135	LLVYGWITGEVLAQA	YGWITGEVL	3	0.6589	40.1	14.00	0.70	tr_D0A0U3_
DRB1_0101	136	SAYVLVPSMLQFAIE	YVLVPSMLQ	2	0.6587	40.1	14.00	0.61	tr_D0A0U3_
DRB1_0101	137	FATAYFMRVIISPMK	FMRVIISPM	5	0.6578	40.5	14.00	0.56	tr_D0A0U3_
DRB1_0101	138	AVIVFAPPVGDTARF	IVFAPPVGD	2	0.6576	40.6	14.00	0.69	tr_D0A0U3_
DRB1_0101	139	SYLKSNPGVTVFNAS	LKSNPGVTV	2	0.6575	40.7	14.00	0.63	tr_D0A0U3_
DRB1_0101	140	PQAVIVFAPPVGDTA	IVFAPPVGD	4	0.6574	40.7	14.00	0.63	tr_D0A0U3_
DRB1_0101	141	LVDSIFAQSVVNVGE	VDSIFAQSV	1	0.6571	40.8	14.00	0.53	tr_D0A0U3_
DRB1_0101	142	DFGTVSSLITLLAEQ	FGTVSSLIT	1	0.6568	41.0	14.00	0.68	tr_D0A0U3_
DRB1_0101	143	SLLGFATAYFMRVII	LGFATAYFM	2	0.6558	41.4	14.00	0.50	tr_D0A0U3_
DRB1_0101	144	RTIRLGFMYLHNVFF	FMYLHNVFF	6	0.6549	41.8	14.00	0.59	tr_D0A0U3_
DRB1_0101	145	HVRVIGRGYRLLDAP	RVIGRGYRL	2	0.6546	42.0	14.00	0.74	tr_D0A0U3_
DRB1_0101	146	TVHVRVIGRGYRLLD	RVIGRGYRL	4	0.6521	43.1	15.00	0.66	tr_D0A0U3_
DRB1_0101	147	VYGWITGEVLAQALS	YGWITGEVL	1	0.6515	43.4	15.00	0.56	tr_D0A0U3_
DRB1_0101	148	TRHFDSAWNTFVESR	FDSAWNTFV	3	0.6492	44.5	15.00	0.90	tr_D0A0U3_
DRB1_0101	149	AMCWCNQGGNTVHVR	WCNQGGNTV	3	0.6472	45.5	15.00	0.77	tr_D0A0U3_
DRB1_0101	150	REALAFAASPFVDGQ	LAFAASPFV	3	0.6472	45.5	15.00	0.68	tr_D0A0U3_
DRB1_0101	151	QLLVYGWITGEVLAQ	YGWITGEVL	4	0.6470	45.6	15.00	0.68	tr_D0A0U3_
DRB1_0101	152	VFSLLHHQKIEKRFV	FSLLHHQKI	1	0.6465	45.8	16.00	0.62	tr_D0A0U3_
DRB1_0101	153	ATALVDSIFAQSVVN	VDSIFAQSV	4	0.6464	45.9	16.00	0.73	tr_D0A0U3_
DRB1_0101	154	FAPLTGSSGGRGWNP	FAPLTGSSG	0	0.6459	46.1	16.00	0.76	tr_D0A0U3_
DRB1_0101	155	FNAFEGVSSADRVLF	FEGVSSADR	3	0.6429	47.6	16.00	0.51	tr_D0A0U3_
DRB1_0101	156	FLKGVPRLTGCAVNY	FLKGVPRLT	0	0.6422	48.0	16.00	0.64	tr_D0A0U3_
DRB1_0101	157	VDEAMLKTPGVAFID	EAMLKTPGV	2	0.6413	48.5	16.00	0.49	tr_D0A0U3_
DRB1_0101	158	NTVHVRVIGRGYRLL	RVIGRGYRL	5	0.6398	49.3	16.00	0.64	tr_D0A0U3_
DRB1_0101	159	RTRSAYVLVPSMLQF	YVLVPSMLQ	5	0.6387	49.9	17.00	0.69	tr_D0A0U3_
DRB1_0101	160	VVDEAMLKTPGVAFI	EAMLKTPGV	3	0.6384	50.0	17.00	0.55	tr_D0A0U3_
DRB1_0101	161	IEDVLKKTLATFSVE	LKKTLATFS	4	0.6382	50.2	17.00	0.41	tr_D0A0U3_
DRB1_0101	162	SMTSRRWKTAPSVHV	WKTAPSVHV	6	0.6377	50.4	17.00	0.76	tr_D0A0U3_
DRB1_0101	163	GQLLVYGWITGEVLA	YGWITGEVL	5	0.6375	50.5	17.00	0.58	tr_D0A0U3_
DRB1_0101	164	EEFFNAFEGVSSADR	FNAFEGVSS	3	0.6368	50.9	17.00	0.59	tr_D0A0U3_
DRB1_0101	165	ERNVIHLSPTLEQQF	VIHLSPTLE	3	0.6366	51.0	17.00	0.48	tr_D0A0U3_
DRB1_0101	166	LLGFATAYFMRVIIS	LGFATAYFM	1	0.6360	51.3	17.00	0.41	tr_D0A0U3_
DRB1_0101	167	SMARALNASIPPLTN	LNASIPPLT	5	0.6355	51.6	17.00	0.55	tr_D0A0U3_
DRB1_0101	168	FAIENMWREALAFAA	WREALAFAA	6	0.6349	52.0	17.00	0.69	tr_D0A0U3_
DRB1_0101	169	KIEKRFVEAVNAGFN	FVEAVNAGF	5	0.6344	52.3	17.00	0.57	tr_D0A0U3_
DRB1_0101	170	QDSRLFFDTIVSPFS	FDTIVSPFS	6	0.6343	52.3	17.00	0.75	tr_D0A0U3_
DRB1_0101	171	RNVIHLSPTLEQQFY	VIHLSPTLE	2	0.6340	52.5	17.00	0.44	tr_D0A0U3_
DRB1_0101	172	KTPGVAFIDPVPLAP	VAFIDPVPL	4	0.6337	52.6	17.00	0.81	tr_D0A0U3_
DRB1_0101	173	AYVLVPSMLQFAIEN	YVLVPSMLQ	1	0.6326	53.3	17.00	0.55	tr_D0A0U3_

DRB1_0101	174	MCWCNQGGNTVHVRV	WCNQGGNTV	2	0.6322	53.5	18.00	0.73	tr_D0A0U3_
DRB1_0101	175	EIEVFSLLHHQKIEK	FSLLHHQKI	4	0.6312	54.1	18.00	0.78	tr_D0A0U3_
DRB1_0101	176	FVESRPQAVIVFAPP	FVESRPQAV	0	0.6301	54.7	18.00	0.76	tr_D0A0U3_
DRB1_0101	177	YGWITGEVLAQALSV	YGWITGEVL	0	0.6301	54.7	18.00	0.37	tr_D0A0U3_
DRB1_0101	178	YGFCGVFTLSSSLTG	FTLSSSLTG	6	0.6280	56.0	18.00	0.31	tr_D0A0U3_
DRB1_0101	179	RGKLFVVVGPLGDFG	FVVVGPLGD	4	0.6264	57.0	18.00	0.58	tr_D0A0U3_
DRB1_0101	180	VGDTARFIRNLVADS	ARFIRNLVA	4	0.6262	57.1	18.00	0.44	tr_D0A0U3_
DRB1_0101	181	STRHFDSAWNTFVES	FDSAWNTFV	4	0.6254	57.5	19.00	0.88	tr_D0A0U3_
DRB1_0101	182	EDVLKKTLATFSVEP	LKKTLATFS	3	0.6251	57.8	19.00	0.39	tr_D0A0U3_
DRB1_0101	183	GPLGDFGTVSSLITL	FGTVSSLIT	5	0.6243	58.2	19.00	0.70	tr_D0A0U3_
DRB1_0101	184	GKLFVVVGPLGDFGT	FVVVGPLGD	3	0.6238	58.6	19.00	0.62	tr_D0A0U3_
DRB1_0101	185	ITLLAEQDVVAFAPL	LLAEQDVVA	2	0.6229	59.1	19.00	0.52	tr_D0A0U3_
DRB1_0101	186	VAFIDPVPLAPRLNK	VAFIDPVPL	0	0.6220	59.7	19.00	0.46	tr_D0A0U3_
DRB1_0101	187	DEAMLKTPGVAFIDP	EAMLKTPGV	1	0.6218	59.8	19.00	0.46	tr_D0A0U3_
DRB1_0101	188	RLHIVIRSTDAAAIE	IRSTDAAAI	5	0.6218	59.9	19.00	0.49	tr_D0A0U3_
DRB1_0101	189	RPQAVIVFAPPVGDT	IVFAPPVGD	5	0.6203	60.8	19.00	0.56	tr_D0A0U3_
DRB1_0101	190	MERNVIHLSPTLEQQ	VIHLSPTLE	4	0.6200	61.0	19.00	0.47	tr_D0A0U3_
DRB1_0101	191	DVLKKTLATFSVEPQ	KKTLATFSV	3	0.6200	61.0	19.00	0.44	tr_D0A0U3_
DRB1_0101	192	LGRLRTIRLGFMYLH	LGRLRTIRL	0	0.6184	62.1	20.00	0.58	tr_D0A0U3_
DRB1_0101	193	RHFDSAWNTFVESRP	FDSAWNTFV	2	0.6172	62.9	20.00	0.89	tr_D0A0U3_
DRB1_0101	194	VRVIGRGYRLLDAPD	RVIGRGYRL	1	0.6172	62.9	20.00	0.76	tr_D0A0U3_
DRB1_0101	195	LEQQFYVCTSYLSRN	FYVCTSYLS	4	0.6163	63.5	20.00	0.54	tr_D0A0U3_
DRB1_0101	196	NATALVDSIFAQSVV	VDSIFAQSV	5	0.6159	63.8	20.00	0.68	tr_D0A0U3_
DRB1_0101	197	LHIVIRSTDAAAIED	IRSTDAAAI	4	0.6157	64.0	20.00	0.57	tr_D0A0U3_
DRB1_0101	198	EALAFAASPFVDGQV	LAFAASPFV	2	0.6156	64.0	20.00	0.62	tr_D0A0U3_
DRB1_0101	199	DSAWNTFVESRPQAV	FVESRPQAV	6	0.6145	64.8	20.00	0.87	tr_D0A0U3_
DRB1_0101	200	GWNPNLYFTRASPSA	LYFTRASPS	5	0.6132	65.7	20.00	0.74	tr_D0A0U3_
DRB1_0101	201	GLTAADPVVIAAHLN	LTAADPVVI	1	0.6127	66.0	20.00	0.47	tr_D0A0U3_
DRB1_0101	202	KLFVVVGPLGDFGTV	FVVVGPLGD	2	0.6118	66.7	21.00	0.56	tr_D0A0U3_
DRB1_0101	203	LKTPGVAFIDPVPLA	VAFIDPVPL	5	0.6113	67.1	21.00	0.84	tr_D0A0U3_
DRB1_0101	204	GAMCWCNQGGNTVHV	WCNQGGNTV	4	0.6113	67.1	21.00	0.75	tr_D0A0U3_
DRB1_0101	205	MKAMNATALVDSIFA	MKAMNATAL	0	0.6096	68.3	21.00	0.54	tr_D0A0U3_
DRB1_0101	206	HIVIRSTDAAAIEDV	IRSTDAAAI	3	0.6078	69.6	21.00	0.67	tr_D0A0U3_
DRB1_0101	207	FGTVSSLITLLAEQD	FGTVSSLIT	0	0.6078	69.7	21.00	0.50	tr_D0A0U3_
DRB1_0101	208	EQQFYVCTSYLSRND	FYVCTSYLS	3	0.6077	69.7	21.00	0.55	tr_D0A0U3_
DRB1_0101	209	VDSIFAQSVVNVGEM	FAQSVVNVG	4	0.6076	69.8	21.00	0.31	tr_D0A0U3_
DRB1_0101	210	VIVFAPPVGDTARFI	IVFAPPVGD	1	0.6068	70.4	21.00	0.62	tr_D0A0U3_
DRB1_0101	211	VQKFYTAERNASRRT	YTAERNASR	4	0.6067	70.5	21.00	0.46	tr_D0A0U3_
DRB1_0101	212	NRGKLFVVVGPLGDF	FVVVGPLGD	5	0.6057	71.3	22.00	0.54	tr_D0A0U3_
DRB1_0101	213	FVEAVNAGFNASMTS	EAVNAGFNA	2	0.6054	71.5	22.00	0.41	tr_D0A0U3_
DRB1_0101	214	CWCNQGGNTVHVRVI	WCNQGGNTV	1	0.6054	71.5	22.00	0.69	tr_D0A0U3_
DRB1_0101	215	GEVLAQALSVPERLT	LAQALSVPE	3	0.6054	71.5	22.00	0.31	tr_D0A0U3_
DRB1_0101	216	FDTIVSPFSGAAEEL	FDTIVSPFS	0	0.6047	72.0	22.00	0.63	tr_D0A0U3_
DRB1_0101	217	ADRVLFATNLPHWAD	DRVLFATNL	1	0.6026	73.7	22.00	0.28	tr_D0A0U3_
DRB1_0101	218	AIEDVLKKTLATFSV	LKKTLATFS	5	0.6020	74.1	22.00	0.41	tr_D0A0U3_
DRB1_0101	219	MHPFTPSASPISGFQ	FTPSASPIS	3	0.6009	75.1	22.00	0.91	tr_D0A0U3_
DRB1_0101	220	GTVSSLITLLAEQDV	LITLLAEQD	5	0.5997	76.0	23.00	0.41	tr_D0A0U3_
DRB1_0101	221	GFATAYFMRVIISPM	FMRVIISPM	6	0.5969	78.4	23.00	0.47	tr_D0A0U3_
DRB1_0101	222	QQFYVCTSYLSRNDK	FYVCTSYLS	2	0.5965	78.7	23.00	0.54	tr_D0A0U3_
DRB1_0101	223	ALAFAASPFVDGQVV	LAFAASPFV	1	0.5965	78.7	23.00	0.55	tr_D0A0U3_

DRB1_0101	224	SRTRSAYVLVPSMLQ	YVLVPSMLQ	6	0.5962	79.0	23.00	0.60	tr_D0A0U3_
DRB1_0101	225	VLKKTLATFSVEPQS	KKTLATFSV	2	0.5953	79.8	23.00	0.51	tr_D0A0U3_
DRB1_0101	226	NVIHLSPTLEQQFYV	VIHLSPTLE	1	0.5951	79.9	23.00	0.38	tr_D0A0U3_
DRB1_0101	227	NASRRTPLSLLGFAT	PLSLLGFAT	6	0.5941	80.8	23.00	0.52	tr_D0A0U3_
DRB1_0101	228	ASTRHFDSAWNTFVE	FDSAWNTFV	5	0.5939	81.0	23.00	0.83	tr_D0A0U3_
DRB1_0101	229	ARALNASIPPLTNPM	LNASIPPLT	3	0.5921	82.6	24.00	0.80	tr_D0A0U3_
DRB1_0101	230	IEIEVFSLLHHQKIE	FSLLHHQKI	5	0.5919	82.7	24.00	0.69	tr_D0A0U3_
DRB1_0101	231	KRLHIVIRSTDAAAI	HIVIRSTDA	3	0.5909	83.6	24.00	0.37	tr_D0A0U3_
DRB1_0101	232	SADRVLFATNLPHWA	DRVLFATNL	2	0.5909	83.6	24.00	0.43	tr_D0A0U3_
DRB1_0101	233	DSGDVYVIGLTAADP	DVYVIGLTA	3	0.5907	83.8	24.00	0.47	tr_D0A0U3_
DRB1_0101	234	MARALNASIPPLTNP	LNASIPPLT	4	0.5897	84.7	24.00	0.67	tr_D0A0U3_
DRB1_0101	235	WKTAPSVHVKVMHPF	WKTAPSVHV	0	0.5895	84.9	24.00	0.76	tr_D0A0U3_
DRB1_0101	236	VMHPFTPSASPISGF	FTPSASPIS	4	0.5893	85.1	24.00	0.86	tr_D0A0U3_
DRB1_0101	237	TLEQQFYVCTSYLSR	FYVCTSYLS	5	0.5893	85.1	24.00	0.50	tr_D0A0U3_
DRB1_0101	238	TGEVLAQALSVPERL	EVLAQALSV	2	0.5891	85.3	24.00	0.38	tr_D0A0U3_
DRB1_0101	239	CAGWAAGQGAMCWCN	WAAGQGAMC	3	0.5862	88.0	25.00	0.80	tr_D0A0U3_
DRB1_0101	240	EAMLKTPGVAFIDPV	MLKTPGVAF	2	0.5861	88.1	25.00	0.46	tr_D0A0U3_
DRB1_0101	241	TLATFSVEPQSVVVL	FSVEPQSVV	4	0.5857	88.4	25.00	0.41	tr_D0A0U3_
DRB1_0101	242	FSLLHHQKIEKRFVE	FSLLHHQKI	0	0.5857	88.4	25.00	0.53	tr_D0A0U3_
DRB1_0101	243	RALNASIPPLTNPMT	LNASIPPLT	2	0.5854	88.8	25.00	0.71	tr_D0A0U3_
DRB1_0101	244	DGQLLVYGWITGEVL	YGWITGEVL	6	0.5853	88.8	25.00	0.46	tr_D0A0U3_
DRB1_0101	245	FIRNLVADSRTRSAY	IRNLVADSR	1	0.5853	88.9	25.00	0.55	tr_D0A0U3_
DRB1_0101	246	QKFYTAERNASRRTP	YTAERNASR	3	0.5852	89.0	25.00	0.47	tr_D0A0U3_
DRB1_0101	247	LATFSVEPQSVVVLD	FSVEPQSVV	3	0.5851	89.1	25.00	0.42	tr_D0A0U3_
DRB1_0101	248	KMERNVIHLSPTLEQ	VIHLSPTLE	5	0.5836	90.5	25.00	0.49	tr_D0A0U3_
DRB1_0101	249	HEEFFNAFEGVSSAD	FNAFEGVSS	4	0.5833	90.8	25.00	0.65	tr_D0A0U3_
DRB1_0101	250	FMYLHNVFFGVEEYD	YLHNVFFGV	2	0.5833	90.8	25.00	0.52	tr_D0A0U3_
DRB1_0101	251	AGFNASMTSRRWKTA	NASMTSRRW	3	0.5820	92.1	25.00	0.47	tr_D0A0U3_
DRB1_0101	252	DRVLFATNLPHWADE	FATNLPHWA	4	0.5808	93.3	26.00	0.38	tr_D0A0U3_
DRB1_0101	253	FTRASPSAELLALIR	TRASPSAEL	1	0.5796	94.5	26.00	0.38	tr_D0A0U3_
DRB1_0101	254	TLSSSLTGVASTRHF	SSSLTGVAS	2	0.5793	94.8	26.00	0.54	tr_D0A0U3_
DRB1_0101	255	NAFEGVSSADRVLFA	FEGVSSADR	2	0.5791	95.0	26.00	0.61	tr_D0A0U3_
DRB1_0101	256	VVQKFYTAERNASRR	FYTAERNAS	4	0.5781	96.1	26.00	0.47	tr_D0A0U3_
DRB1_0101	257	NAGFNASMTSRRWKT	FNASMTSRR	3	0.5776	96.6	26.00	0.52	tr_D0A0U3_
DRB1_0101	258	LGFATAYFMRVIISP	FATAYFMRV	2	0.5768	97.4	26.00	0.37	tr_D0A0U3_
DRB1_0101	259	GVVDEAMLKTPGVAF	EAMLKTPGV	4	0.5754	98.9	27.00	0.62	tr_D0A0U3_
DRB1_0101	260	YLKSNPGVTVFNASS	LKSNPGVTV	1	0.5741	100.3	27.00	0.70	tr_D0A0U3_
DRB1_0101	261	EVLAQALSVPERLTD	QALSVPERL	4	0.5739	100.5	27.00	0.38	tr_D0A0U3_
DRB1_0101	262	AGWAAGQGAMCWCNQ	WAAGQGAMC	2	0.5729	101.6	27.00	0.81	tr_D0A0U3_
DRB1_0101	263	AEELQREWSTKITTA	LQREWSTKI	3	0.5729	101.6	27.00	0.89	tr_D0A0U3_
DRB1_0101	264	ATFSVEPQSVVVLDG	FSVEPQSVV	2	0.5729	101.7	27.00	0.38	tr_D0A0U3_
DRB1_0101	265	HPFTPSASPISGFQQ	FTPSASPIS	2	0.5707	104.1	27.00	0.94	tr_D0A0U3_
DRB1_0101	266	YVLVPSMLQFAIENM	YVLVPSMLQ	0	0.5697	105.2	28.00	0.43	tr_D0A0U3_
DRB1_0101	267	LHEEFFNAFEGVSSA	FNAFEGVSS	5	0.5683	106.8	28.00	0.60	tr_D0A0U3_
DRB1_0101	268	ECAGWAAGQGAMCWC	WAAGQGAMC	4	0.5677	107.5	28.00	0.79	tr_D0A0U3_
DRB1_0101	269	RDVRSYLKSNPGVTV	YLKSNPGVT	5	0.5671	108.2	28.00	0.52	tr_D0A0U3_
DRB1_0101	270	EELQREWSTKITTAV	LQREWSTKI	2	0.5664	109.0	28.00	0.77	tr_D0A0U3_
DRB1_0101	271	IAAHLNSHDSARVFV	LNSHDSARV	4	0.5660	109.5	28.00	0.69	tr_D0A0U3_
DRB1_0101	272	DSIFAQSVVNVGEMR	FAQSVVNVG	3	0.5627	113.5	29.00	0.51	tr_D0A0U3_
DRB1_0101	273	LTAADPVVIAAHLNS	PVVIAAHLN	5	0.5626	113.6	29.00	0.44	tr_D0A0U3_

DRB1_0101	274	GQVVVTGTNPLARDT	VVTGTNPLA	3	0.5619	114.4	29.00	0.55	tr_D0A0U3_
DRB1_0101	275	DIEIEVFSLLHHQKI	FSLLHHQKI	6	0.5612	115.3	29.00	0.55	tr_D0A0U3_
DRB1_0101	276	PDSGDVYVIGLTAAD	DVYVIGLTA	4	0.5612	115.4	29.00	0.53	tr_D0A0U3_
DRB1_0101	277	IVIRSTDAAAIEDVL	IRSTDAAAI	2	0.5602	116.6	29.00	0.76	tr_D0A0U3_
DRB1_0101	278	KVMHPFTPSASPISG	FTPSASPIS	5	0.5602	116.6	29.00	0.65	tr_D0A0U3_
DRB1_0101	279	VNAGFNASMTSRRWK	FNASMTSRR	4	0.5592	117.9	30.00	0.50	tr_D0A0U3_
DRB1_0101	280	ELQREWSTKITTAVL	LQREWSTKI	1	0.5588	118.3	30.00	0.58	tr_D0A0U3_
DRB1_0101	281	GYGFCGVFTLSSSLT	FCGVFTLSS	3	0.5582	119.1	30.00	0.40	tr_D0A0U3_
DRB1_0101	282	QKIEKRFVEAVNAGF	FVEAVNAGF	6	0.5580	119.3	30.00	0.41	tr_D0A0U3_
DRB1_0101	283	LSSSLTGVASTRHFD	SSSLTGVAS	1	0.5566	121.2	30.00	0.48	tr_D0A0U3_
DRB1_0101	284	SIFAQSVVNVGEMRY	FAQSVVNVG	2	0.5556	122.6	30.00	0.50	tr_D0A0U3_
DRB1_0101	285	QGAMCWCNQGGNTVH	WCNQGGNTV	5	0.5555	122.6	30.00	0.74	tr_D0A0U3_
DRB1_0101	286	ITGEVLAQALSVPER	EVLAQALSV	3	0.5550	123.3	30.00	0.50	tr_D0A0U3_
DRB1_0101	287	HAAVEMEEGASLLEV	VEMEEGASL	3	0.5543	124.3	30.00	0.74	tr_D0A0U3_
DRB1_0101	288	LFVVVGPLGDFGTVS	FVVVGPLGD	1	0.5532	125.7	31.00	0.53	tr_D0A0U3_
DRB1_0101	289	GECAGWAAGQGAMCW	WAAGQGAMC	5	0.5521	127.2	31.00	0.78	tr_D0A0U3_
DRB1_0101	290	APRLNKMERNVIHLS	LNKMERNVI	3	0.5505	129.4	31.00	0.55	tr_D0A0U3_
DRB1_0101	291	AAVEMEEGASLLEVR	VEMEEGASL	2	0.5494	131.0	31.00	0.65	tr_D0A0U3_
DRB1_0101	292	VEAVNAGFNASMTSR	EAVNAGFNA	1	0.5490	131.6	31.00	0.47	tr_D0A0U3_
DRB1_0101	293	LKKTLATFSVEPQSV	KKTLATFSV	1	0.5490	131.6	31.00	0.56	tr_D0A0U3_
DRB1_0101	294	RKTFMQSLYDQRRYV	FMQSLYDQR	3	0.5489	131.7	31.00	0.39	tr_D0A0U3_
DRB1_0101	295	SAELLALIRYALGRL	LALIRYALG	4	0.5474	133.9	32.00	0.54	tr_D0A0U3_
DRB1_0101	296	RVLFATNLPHWADEI	FATNLPHWA	3	0.5470	134.4	32.00	0.52	tr_D0A0U3_
DRB1_0101	297	RNRGKLFVVVGPLGD	FVVVGPLGD	6	0.5460	135.9	32.00	0.41	tr_D0A0U3_
DRB1_0101	298	SRPQAVIVFAPPVGD	IVFAPPVGD	6	0.5448	137.7	32.00	0.42	tr_D0A0U3_
DRB1_0101	299	VEEYDVAQRILRRMG	YDVAQRILR	3	0.5446	138.0	32.00	0.44	tr_D0A0U3_
DRB1_0101	300	DGQVVVTGTNPLARD	VVTGTNPLA	4	0.5446	138.0	32.00	0.53	tr_D0A0U3_
DRB1_0101	301	HFDSAWNTFVESRPQ	FDSAWNTFV	1	0.5440	138.9	32.00	0.83	tr_D0A0U3_
DRB1_0101	302	KFYTAERNASRRTPL	YTAERNASR	2	0.5433	140.0	32.00	0.50	tr_D0A0U3_
DRB1_0101	303	PSMRYLDPDAGKLSR	MRYLDPDAG	2	0.5425	141.1	33.00	0.52	tr_D0A0U3_
DRB1_0101	304	EEYDVAQRILRRMGY	DVAQRILRR	3	0.5419	142.1	33.00	0.39	tr_D0A0U3_
DRB1_0101	305	VLAQALSVPERLTDR	QALSVPERL	3	0.5417	142.5	33.00	0.58	tr_D0A0U3_
DRB1_0101	306	VIAAHLNSHDSARVF	LNSHDSARV	5	0.5412	143.2	33.00	0.60	tr_D0A0U3_
DRB1_0101	307	AFEGVSSADRVLFAT	FEGVSSADR	1	0.5405	144.2	33.00	0.56	tr_D0A0U3_
DRB1_0101	308	AAHLNSHDSARVFVP	LNSHDSARV	3	0.5395	145.8	33.00	0.73	tr_D0A0U3_
DRB1_0101	309	QVVVTGTNPLARDTQ	VVTGTNPLA	2	0.5395	145.9	33.00	0.56	tr_D0A0U3_
DRB1_0101	310	MNATALVDSIFAQSV	VDSIFAQSV	6	0.5394	146.0	33.00	0.56	tr_D0A0U3_
DRB1_0101	311	SMRYLDPDAGKLSRG	MRYLDPDAG	1	0.5387	147.1	33.00	0.46	tr_D0A0U3_
DRB1_0101	312	AAEELQREWSTKITT	LQREWSTKI	4	0.5384	147.6	33.00	0.84	tr_D0A0U3_
DRB1_0101	313	NKMERNVIHLSPTLE	VIHLSPTLE	6	0.5382	147.9	33.00	0.37	tr_D0A0U3_
DRB1_0101	314	PRLNKMERNVIHLSP	LNKMERNVI	2	0.5380	148.2	33.00	0.47	tr_D0A0U3_
DRB1_0101	315	PTLEQQFYVCTSYLS	FYVCTSYLS	6	0.5378	148.5	34.00	0.41	tr_D0A0U3_
DRB1_0101	316	REWSTKITTAVLGVV	WSTKITTAV	2	0.5359	151.6	34.00	0.34	tr_D0A0U3_
DRB1_0101	317	TLLAEQDVVAFAPLT	LLAEQDVVA	1	0.5359	151.7	34.00	0.58	tr_D0A0U3_
DRB1_0101	318	QFYVCTSYLSRNDKK	FYVCTSYLS	1	0.5355	152.2	34.00	0.52	tr_D0A0U3_
DRB1_0101	319	VSVLIPDRPTALHAA	LIPDRPTAL	3	0.5348	153.4	34.00	0.72	tr_D0A0U3_
DRB1_0101	320	GWITGEVLAQALSVP	EVLAQALSV	5	0.5347	153.6	34.00	0.32	tr_D0A0U3_
DRB1_0101	321	SSADRVLFATNLPHW	DRVLFATNL	3	0.5347	153.7	34.00	0.60	tr_D0A0U3_
DRB1_0101	322	GNTVHVRVIGRGYRL	RVIGRGYRL	6	0.5342	154.4	34.00	0.51	tr_D0A0U3_
DRB1_0101	323	TFSVEPQSVVVLDGN	FSVEPQSVV	1	0.5336	155.4	34.00	0.37	tr_D0A0U3_

DRB1_0101	324	GFNASMTSRRWKTAP	NASMTSRRW	2	0.5330	156.5	34.00	0.51	tr_D0A0U3_
DRB1_0101	325	DPVVIAAHLNSHDSA	PVVIAAHLN	1	0.5329	156.6	34.00	0.41	tr_D0A0U3_
DRB1_0101	326	GVEEYDVAQRILRRM	YDVAQRILR	4	0.5329	156.7	34.00	0.45	tr_D0A0U3_
DRB1_0101	327	GATQVSVWSMARALN	SVWSMARAL	5	0.5321	158.0	35.00	0.55	tr_D0A0U3_
DRB1_0101	328	TPSMRYLDPDAGKLS	MRYLDPDAG	3	0.5321	158.0	35.00	0.66	tr_D0A0U3_
DRB1_0101	329	ALNASIPPLTNPMTP	LNASIPPLT	1	0.5310	160.0	35.00	0.57	tr_D0A0U3_
DRB1_0101	330	QSVVVLDGNATVEGH	VVVLDGNAT	2	0.5309	160.0	35.00	0.71	tr_D0A0U3_
DRB1_0101	331	MLKTPGVAFIDPVPL	VAFIDPVPL	6	0.5309	160.1	35.00	0.70	tr_D0A0U3_
DRB1_0101	332	VGPLGDFGTVSSLIT	FGTVSSLIT	6	0.5301	161.4	35.00	0.63	tr_D0A0U3_
DRB1_0101	333	VKVMHPFTPSASPIS	FTPSASPIS	6	0.5299	161.7	35.00	0.37	tr_D0A0U3_
DRB1_0101	334	VDGQVVVTGTNPLAR	VVTGTNPLA	5	0.5292	163.1	35.00	0.51	tr_D0A0U3_
DRB1_0101	335	RLNKMERNVIHLSPT	LNKMERNVI	1	0.5286	164.1	35.00	0.41	tr_D0A0U3_
DRB1_0101	336	PQSVVVLDGNATVEG	VVVLDGNAT	3	0.5286	164.1	35.00	0.77	tr_D0A0U3_
DRB1_0101	337	QRILRRMGYGFCGVF	ILRRMGYGF	2	0.5278	165.6	35.00	0.41	tr_D0A0U3_
DRB1_0101	338	PSAELLALIRYALGR	LALIRYALG	5	0.5274	166.2	36.00	0.51	tr_D0A0U3_
DRB1_0101	339	WITGEVLAQALSVPE	EVLAQALSV	4	0.5266	167.6	36.00	0.43	tr_D0A0U3_
DRB1_0101	340	AQRILRRMGYGFCGV	ILRRMGYGF	3	0.5255	169.7	36.00	0.49	tr_D0A0U3_
DRB1_0101	341	ADPVVIAAHLNSHDS	PVVIAAHLN	2	0.5250	170.5	36.00	0.51	tr_D0A0U3_
DRB1_0101	342	KKTLATFSVEPQSVV	KKTLATFSV	0	0.5245	171.5	36.00	0.38	tr_D0A0U3_
DRB1_0101	343	KTLATFSVEPQSVVV	FSVEPQSVV	5	0.5245	171.6	36.00	0.43	tr_D0A0U3_
DRB1_0101	344	QREWSTKITTAVLGV	WSTKITTAV	3	0.5240	172.4	36.00	0.43	tr_D0A0U3_
DRB1_0101	345	DRKTFMQSLYDQRRY	FMQSLYDQR	4	0.5240	172.4	36.00	0.36	tr_D0A0U3_
DRB1_0101	346	LGVVDEAMLKTPGVA	EAMLKTPGV	5	0.5236	173.3	36.00	0.59	tr_D0A0U3_
DRB1_0101	347	WCNQGGNTVHVRVIG	WCNQGGNTV	0	0.5232	174.0	36.00	0.56	tr_D0A0U3_
DRB1_0101	348	VIHLSPTLEQQFYVC	IHLSPTLEQ	1	0.5230	174.3	36.00	0.28	tr_D0A0U3_
DRB1_0101	349	GWAAGQGAMCWCNQG	WAAGQGAMC	1	0.5225	175.2	36.00	0.80	tr_D0A0U3_
DRB1_0101	350	PFTPSASPISGFQQA	FTPSASPIS	1	0.5216	177.0	37.00	0.87	tr_D0A0U3_
DRB1_0101	351	PVVIAAHLNSHDSAR	IAAHLNSHD	3	0.5213	177.6	37.00	0.29	tr_D0A0U3_
DRB1_0101	352	RILRRMGYGFCGVFT	ILRRMGYGF	1	0.5209	178.3	37.00	0.34	tr_D0A0U3_
DRB1_0101	353	YDVAQRILRRMGYGF	DVAQRILRR	1	0.5209	178.3	37.00	0.35	tr_D0A0U3_
DRB1_0101	354	EVVQKFYTAERNASR	FYTAERNAS	5	0.5208	178.5	37.00	0.41	tr_D0A0U3_
DRB1_0101	355	VASTRHFDSAWNTFV	FDSAWNTFV	6	0.5198	180.5	37.00	0.75	tr_D0A0U3_
DRB1_0101	356	LQREWSTKITTAVLG	WSTKITTAV	4	0.5194	181.3	37.00	0.37	tr_D0A0U3_
DRB1_0101	357	LAFAASPFVDGQVVV	FAASPFVDG	2	0.5192	181.7	37.00	0.43	tr_D0A0U3_
DRB1_0101	358	AHLNSHDSARVFVPF	LNSHDSARV	2	0.5170	186.0	38.00	0.69	tr_D0A0U3_
DRB1_0101	359	TTAVLGVVDEAMLKT	LGVVDEAML	4	0.5169	186.2	38.00	0.51	tr_D0A0U3_
DRB1_0101	360	ASPFVDGQVVVTGTN	FVDGQVVVT	3	0.5164	187.2	38.00	0.81	tr_D0A0U3_
DRB1_0101	361	SSSLTGVASTRHFDS	LTGVASTRH	3	0.5159	188.3	38.00	0.34	tr_D0A0U3_
DRB1_0101	362	AVNAGFNASMTSRRW	FNASMTSRR	5	0.5153	189.5	38.00	0.44	tr_D0A0U3_
DRB1_0101	363	TAADPVVIAAHLNSH	PVVIAAHLN	4	0.5148	190.5	38.00	0.61	tr_D0A0U3_
DRB1_0101	364	LHAAVEMEEGASLLE	VEMEEGASL	4	0.5144	191.3	38.00	0.77	tr_D0A0U3_
DRB1_0101	365	SEVEMQAPFNGVSVL	VEMQAPFNG	2	0.5138	192.7	38.00	0.41	tr_D0A0U3_
DRB1_0101	366	SVHVKVMHPFTPSAS	VKVMHPFTP	3	0.5136	193.1	38.00	0.44	tr_D0A0U3_
DRB1_0101	367	GVSVLIPDRPTALHA	LIPDRPTAL	4	0.5128	194.6	38.00	0.64	tr_D0A0U3_
DRB1_0101	368	AADPVVIAAHLNSHD	PVVIAAHLN	3	0.5124	195.5	39.00	0.59	tr_D0A0U3_
DRB1_0101	369	KTFMQSLYDQRRYVI	FMQSLYDQR	2	0.5123	195.8	39.00	0.33	tr_D0A0U3_
DRB1_0101	370	EYDVAQRILRRMGYG	DVAQRILRR	2	0.5119	196.6	39.00	0.46	tr_D0A0U3_
DRB1_0101	371	QRRYVIDDLVIGDYG	YVIDDLVIG	3	0.5117	197.0	39.00	0.80	tr_D0A0U3_
DRB1_0101	372	IVFAPPVGDTARFIR	IVFAPPVGD	0	0.5112	198.1	39.00	0.44	tr_D0A0U3_
DRB1_0101	373	EAVNAGFNASMTSRR	VNAGFNASM	2	0.5099	201.0	39.00	0.30	tr_D0A0U3_

DRB1_0101	374	LNKMERNVIHLSPTL	MERNVIHLS	3	0.5093	202.2	39.00	0.28	tr_D0A0U3_
DRB1_0101	375	LAPRLNKMERNVIHL	LNKMERNVI	4	0.5093	202.2	39.00	0.61	tr_D0A0U3_
DRB1_0101	376	LPDSGDVYVIGLTAA	DVYVIGLTA	5	0.5093	202.3	39.00	0.63	tr_D0A0U3_
DRB1_0101	377	NVFFGVEEYDVAQRI	FFGVEEYDV	2	0.5074	206.4	40.00	0.60	tr_D0A0U3_
DRB1_0101	378	HNVFFGVEEYDVAQR	FFGVEEYDV	3	0.5053	211.1	40.00	0.68	tr_D0A0U3_
DRB1_0101	379	TDRKTFMQSLYDQRR	KTFMQSLYD	3	0.5049	212.0	40.00	0.38	tr_D0A0U3_
DRB1_0101	380	FGVEEYDVAQRILRR	YDVAQRILR	5	0.5038	214.6	40.00	0.43	tr_D0A0U3_
DRB1_0101	381	EVEMQAPFNGVSVLI	VEMQAPFNG	1	0.5036	215.0	40.00	0.31	tr_D0A0U3_
DRB1_0101	382	AVEMEEGASLLEVRE	VEMEEGASL	1	0.5035	215.2	40.00	0.63	tr_D0A0U3_
DRB1_0101	383	PVGDTARFIRNLVAD	ARFIRNLVA	5	0.5033	215.8	40.00	0.62	tr_D0A0U3_
DRB1_0101	384	AASPFVDGQVVVTGT	FVDGQVVVT	4	0.5032	216.1	40.00	0.78	tr_D0A0U3_
DRB1_0101	385	MGYGFCGVFTLSSSL	FCGVFTLSS	4	0.5024	217.9	41.00	0.44	tr_D0A0U3_
DRB1_0101	386	SVLIPDRPTALHAAV	LIPDRPTAL	2	0.5022	218.3	41.00	0.74	tr_D0A0U3_
DRB1_0101	387	RNASRRTPLSLLGFA	SRRTPLSLL	3	0.5018	219.2	41.00	0.71	tr_D0A0U3_
DRB1_0101	388	FAASPFVDGQVVVTG	FVDGQVVVT	5	0.5010	221.1	41.00	0.66	tr_D0A0U3_
DRB1_0101	389	KKRLHIVIRSTDAAA	HIVIRSTDA	4	0.5009	221.4	41.00	0.50	tr_D0A0U3_
DRB1_0101	390	EPQSVVVLDGNATVE	VVVLDGNAT	4	0.5009	221.4	41.00	0.80	tr_D0A0U3_
DRB1_0101	391	DQRRYVIDDLVIGDY	YVIDDLVIG	4	0.4999	223.8	41.00	0.81	tr_D0A0U3_
DRB1_0101	392	NLVADSRTRSAYVLV	DSRTRSAYV	4	0.4997	224.3	41.00	0.44	tr_D0A0U3_
DRB1_0101	393	IRRFQRDVRSYLKSN	FQRDVRSYL	3	0.4993	225.3	41.00	0.74	tr_D0A0U3_
DRB1_0101	394	TAVLGVVDEAMLKTP	LGVVDEAML	3	0.4988	226.6	41.00	0.51	tr_D0A0U3_
DRB1_0101	395	SPSAELLALIRYALG	LALIRYALG	6	0.4985	227.4	41.00	0.41	tr_D0A0U3_
DRB1_0101	396	SVVVLDGNATVEGHL	VVVLDGNAT	1	0.4971	230.6	42.00	0.65	tr_D0A0U3_
DRB1_0101	397	VVIAAHLNSHDSARV	LNSHDSARV	6	0.4965	232.3	42.00	0.32	tr_D0A0U3_
DRB1_0101	398	YLHNVFFGVEEYDVA	FFGVEEYDV	5	0.4961	233.1	42.00	0.47	tr_D0A0U3_
DRB1_0101	399	FFGVEEYDVAQRILR	YDVAQRILR	6	0.4957	234.3	42.00	0.25	tr_D0A0U3_
DRB1_0101	400	VHVKVMHPFTPSASP	VKVMHPFTP	2	0.4955	234.7	42.00	0.41	tr_D0A0U3_
DRB1_0101	401	MYLHNVFFGVEEYDV	YLHNVFFGV	1	0.4953	235.2	42.00	0.57	tr_D0A0U3_
DRB1_0101	402	VSSADRVLFATNLPH	DRVLFATNL	4	0.4949	236.2	42.00	0.64	tr_D0A0U3_
DRB1_0101	403	PSEVEMQAPFNGVSV	VEMQAPFNG	3	0.4948	236.6	42.00	0.49	tr_D0A0U3_
DRB1_0101	404	IFAQSVVNVGEMRYG	FAQSVVNVG	1	0.4943	237.8	42.00	0.44	tr_D0A0U3_
DRB1_0101	405	EWSTKITTAVLGVVD	TKITTAVLG	3	0.4943	237.9	42.00	0.31	tr_D0A0U3_
DRB1_0101	406	VIRSTDAAAIEDVLK	IRSTDAAAI	1	0.4935	240.0	42.00	0.75	tr_D0A0U3_
DRB1_0101	407	VFFGVEEYDVAQRIL	FFGVEEYDV	1	0.4928	241.8	42.00	0.49	tr_D0A0U3_
DRB1_0101	408	GAAEELQREWSTKIT	LQREWSTKI	5	0.4919	244.1	43.00	0.80	tr_D0A0U3_
DRB1_0101	409	AIRRFQRDVRSYLKS	FQRDVRSYL	4	0.4908	247.1	43.00	0.69	tr_D0A0U3_
DRB1_0101	410	SARVFVPFFDVVLLH	FVPFFDVVL	4	0.4903	248.4	43.00	0.49	tr_D0A0U3_
DRB1_0101	411	RRYVIDDLVIGDYGG	YVIDDLVIG	2	0.4892	251.4	43.00	0.74	tr_D0A0U3_
DRB1_0101	412	AAIEDVLKKTLATFS	DVLKKTLAT	4	0.4878	255.1	44.00	0.38	tr_D0A0U3_
DRB1_0101	413	HVKVMHPFTPSASPI	VKVMHPFTP	1	0.4865	258.7	44.00	0.37	tr_D0A0U3_
DRB1_0101	414	LRTIRLGFMYLHNVF	LGFMYLHNV	5	0.4862	259.5	44.00	0.35	tr_D0A0U3_
DRB1_0101	415	ITTAVLGVVDEAMLK	LGVVDEAML	5	0.4857	261.1	44.00	0.48	tr_D0A0U3_
DRB1_0101	416	DVAQRILRRMGYGFC	ILRRMGYGF	5	0.4851	262.6	44.00	0.49	tr_D0A0U3_
DRB1_0101	417	VAQRILRRMGYGFCG	ILRRMGYGF	4	0.4849	263.4	44.00	0.56	tr_D0A0U3_
DRB1_0101	418	HLNSHDSARVFVPFF	LNSHDSARV	1	0.4844	264.7	44.00	0.59	tr_D0A0U3_
DRB1_0101	419	MQAPFNGVSVLIPDR	FNGVSVLIP	4	0.4835	267.4	44.00	0.38	tr_D0A0U3_
DRB1_0101	420	LLHEEFFNAFEGVSS	FNAFEGVSS	6	0.4835	267.4	44.00	0.41	tr_D0A0U3_
DRB1_0101	421	LVADSRTRSAYVLVP	DSRTRSAYV	3	0.4831	268.4	45.00	0.44	tr_D0A0U3_
DRB1_0101	422	MRYLDPDAGKLSRGQ	YLDPDAGKL	2	0.4830	268.7	45.00	0.30	tr_D0A0U3_
DRB1_0101	423	VEMQAPFNGVSVLIP	QAPFNGVSV	3	0.4828	269.4	45.00	0.26	tr_D0A0U3_

DRB1_0101	424	SMLQFAIENMWREAL	FAIENMWRE	4	0.4828	269.4	45.00	0.36	tr_D0A0U3_
DRB1_0101	425	AEQDVVAFAPLTGSS	DVVAFAPLT	3	0.4826	270.0	45.00	0.41	tr_D0A0U3_
DRB1_0101	426	MTPSMRYLDPDAGKL	MRYLDPDAG	4	0.4814	273.6	45.00	0.71	tr_D0A0U3_
DRB1_0101	427	DSARVFVPFFDVVLL	FVPFFDVVL	5	0.4812	274.1	45.00	0.45	tr_D0A0U3_
DRB1_0101	428	RLRTIRLGFMYLHNV	LGFMYLHNV	6	0.4801	277.5	45.00	0.21	tr_D0A0U3_
DRB1_0101	429	VADSRTRSAYVLVPS	DSRTRSAYV	2	0.4798	278.3	45.00	0.37	tr_D0A0U3_
DRB1_0101	430	PSMLQFAIENMWREA	LQFAIENMW	3	0.4790	280.6	45.00	0.40	tr_D0A0U3_
DRB1_0101	431	RMGYGFCGVFTLSSS	FCGVFTLSS	5	0.4784	282.5	45.00	0.37	tr_D0A0U3_
DRB1_0101	432	QQAVERNRGKLFVVV	VERNRGKLF	3	0.4783	282.8	46.00	0.75	tr_D0A0U3_
DRB1_0101	433	ARVFVPFFDVVLLHE	FVPFFDVVL	3	0.4781	283.4	46.00	0.56	tr_D0A0U3_
DRB1_0101	434	IDGQLLVYGWITGEV	QLLVYGWIT	3	0.4774	285.7	46.00	0.54	tr_D0A0U3_
DRB1_0101	435	PSVHVKVMHPFTPSA	VKVMHPFTP	4	0.4773	285.7	46.00	0.48	tr_D0A0U3_
DRB1_0101	436	ILRRMGYGFCGVFTL	RRMGYGFCG	2	0.4770	286.6	46.00	0.23	tr_D0A0U3_
DRB1_0101	437	MLQFAIENMWREALA	FAIENMWRE	3	0.4765	288.4	46.00	0.42	tr_D0A0U3_
DRB1_0101	438	FFDVVLLHEEFFNAF	VVLLHEEFF	3	0.4763	288.9	46.00	0.34	tr_D0A0U3_
DRB1_0101	439	SPFVDGQVVVTGTNP	FVDGQVVVT	2	0.4762	289.3	46.00	0.83	tr_D0A0U3_
DRB1_0101	440	AVLGVVDEAMLKTPG	LGVVDEAML	2	0.4749	293.3	46.00	0.51	tr_D0A0U3_
DRB1_0101	441	EITASEVVQKFYTAE	EVVQKFYTA	5	0.4746	294.5	46.00	0.41	tr_D0A0U3_
DRB1_0101	442	AFIDPVPLAPRLNKM	PVPLAPRLN	4	0.4741	296.0	46.00	0.35	tr_D0A0U3_
DRB1_0101	443	NGVSVLIPDRPTALH	LIPDRPTAL	5	0.4737	297.3	46.00	0.53	tr_D0A0U3_
DRB1_0101	444	ITASEVVQKFYTAER	EVVQKFYTA	4	0.4736	297.7	47.00	0.60	tr_D0A0U3_
DRB1_0101	445	AFAASPFVDGQVVVT	FVDGQVVVT	6	0.4735	297.7	47.00	0.49	tr_D0A0U3_
DRB1_0101	446	TASEVVQKFYTAERN	EVVQKFYTA	3	0.4735	297.8	47.00	0.66	tr_D0A0U3_
DRB1_0101	447	VLGVVDEAMLKTPGV	LGVVDEAML	1	0.4730	299.6	47.00	0.36	tr_D0A0U3_
DRB1_0101	448	VLFATNLPHWADEIT	FATNLPHWA	2	0.4711	305.8	47.00	0.56	tr_D0A0U3_
DRB1_0101	449	FDVVLLHEEFFNAFE	LLHEEFFNA	4	0.4708	306.6	47.00	0.38	tr_D0A0U3_
DRB1_0101	450	LAQALSVPERLTDRK	QALSVPERL	2	0.4707	307.0	47.00	0.64	tr_D0A0U3_
DRB1_0101	451	SEVVQKFYTAERNAS	EVVQKFYTA	1	0.4705	307.8	47.00	0.50	tr_D0A0U3_
DRB1_0101	452	DDIDGQLLVYGWITG	QLLVYGWIT	5	0.4702	308.6	47.00	0.49	tr_D0A0U3_
DRB1_0101	453	GVSSADRVLFATNLP	DRVLFATNL	5	0.4696	310.8	47.00	0.62	tr_D0A0U3_
DRB1_0101	454	DIDGQLLVYGWITGE	QLLVYGWIT	4	0.4694	311.4	47.00	0.50	tr_D0A0U3_
DRB1_0101	455	KGVPRLTGCAVNYGA	VPRLTGCAV	2	0.4683	315.0	48.00	0.44	tr_D0A0U3_
DRB1_0101	456	YPSEVEMQAPFNGVS	VEMQAPFNG	4	0.4683	315.2	48.00	0.54	tr_D0A0U3_
DRB1_0101	457	DEITASEVVQKFYTA	ITASEVVQK	2	0.4678	316.9	48.00	0.37	tr_D0A0U3_
DRB1_0101	458	YGATQVSVWSMARAL	SVWSMARAL	6	0.4677	317.1	48.00	0.42	tr_D0A0U3_
DRB1_0101	459	RRMGYGFCGVFTLSS	GYGFCGVFT	3	0.4676	317.6	48.00	0.26	tr_D0A0U3_
DRB1_0101	460	ALHAAVEMEEGASLL	VEMEEGASL	5	0.4669	319.9	48.00	0.81	tr_D0A0U3_
DRB1_0101	461	EMQAPFNGVSVLIPD	QAPFNGVSV	2	0.4668	320.3	48.00	0.28	tr_D0A0U3_
DRB1_0101	462	LHNVFFGVEEYDVAQ	FFGVEEYDV	4	0.4667	320.7	48.00	0.64	tr_D0A0U3_
DRB1_0101	463	ADSRTRSAYVLVPSM	SRTRSAYVL	2	0.4665	321.2	48.00	0.38	tr_D0A0U3_
DRB1_0101	464	VEPQSVVVLDGNATV	VVVLDGNAT	5	0.4660	323.2	48.00	0.79	tr_D0A0U3_
DRB1_0101	465	YDQRRYVIDDLVIGD	YVIDDLVIG	5	0.4646	328.1	48.00	0.74	tr_D0A0U3_
DRB1_0101	466	IRNLVADSRTRSAYV	IRNLVADSR	0	0.4645	328.3	48.00	0.37	tr_D0A0U3_
DRB1_0101	467	LLAEQDVVAFAPLTG	DVVAFAPLT	5	0.4645	328.4	48.00	0.35	tr_D0A0U3_
DRB1_0101	468	DVVLLHEEFFNAFEG	LLHEEFFNA	3	0.4644	328.6	48.00	0.38	tr_D0A0U3_
DRB1_0101	469	ERNASRRTPLSLLGF	SRRTPLSLL	4	0.4644	328.8	48.00	0.69	tr_D0A0U3_
DRB1_0101	470	GGECAGWAAGQGAMC	WAAGQGAMC	6	0.4637	331.1	49.00	0.64	tr_D0A0U3_
DRB1_0101	471	QAVERNRGKLFVVVG	VERNRGKLF	2	0.4633	332.5	49.00	0.65	tr_D0A0U3_
DRB1_0101	472	HQKIEKRFVEAVNAG	KRFVEAVNA	5	0.4633	332.6	49.00	0.45	tr_D0A0U3_
DRB1_0101	473	LQFAIENMWREALAF	FAIENMWRE	2	0.4625	335.5	49.00	0.40	tr_D0A0U3_

DRB1_0101	474	FVVVGPLGDFGTVSS	FVVVGPLGD	0	0.4623	336.2	49.00	0.46	tr_D0A0U3_
DRB1_0101	475	VLVPSMLQFAIENMW	LVPSMLQFA	1	0.4619	337.6	49.00	0.29	tr_D0A0U3_
DRB1_0101	476	LKGVPRLTGCAVNYG	VPRLTGCAV	3	0.4607	342.1	49.00	0.49	tr_D0A0U3_
DRB1_0101	477	ASEVVQKFYTAERNA	EVVQKFYTA	2	0.4603	343.6	49.00	0.62	tr_D0A0U3_
DRB1_0101	478	LVPSMLQFAIENMWR	LQFAIENMW	5	0.4595	346.7	49.00	0.35	tr_D0A0U3_
DRB1_0101	479	LNASIPPLTNPMTPS	IPPLTNPMT	4	0.4594	346.9	50.00	0.47	tr_D0A0U3_
DRB1_0101	480	FEGVSSADRVLFATN	FEGVSSADR	0	0.4591	348.1	50.00	0.37	tr_D0A0U3_
DRB1_0101	481	VPSMLQFAIENMWRE	LQFAIENMW	4	0.4584	350.5	50.00	0.38	tr_D0A0U3_
DRB1_0101	482	GRLRTIRLGFMYLHN	LRTIRLGFM	2	0.4582	351.4	50.00	0.37	tr_D0A0U3_
DRB1_0101	483	RRFQRDVRSYLKSNP	FQRDVRSYL	2	0.4574	354.7	50.00	0.76	tr_D0A0U3_
DRB1_0101	484	DSRTRSAYVLVPSML	SRTRSAYVL	1	0.4568	356.7	50.00	0.28	tr_D0A0U3_
DRB1_0101	485	HAIRRFQRDVRSYLK	FQRDVRSYL	5	0.4568	357.0	50.00	0.65	tr_D0A0U3_
DRB1_0101	486	SPTLEQQFYVCTSYL	PTLEQQFYV	1	0.4565	358.0	50.00	0.40	tr_D0A0U3_
DRB1_0101	487	PPVGDTARFIRNLVA	ARFIRNLVA	6	0.4562	359.0	50.00	0.49	tr_D0A0U3_
DRB1_0101	488	FVDGQVVVTGTNPLA	VVVTGTNPL	5	0.4562	359.3	50.00	0.37	tr_D0A0U3_
DRB1_0101	489	RGWNPNLYFTRASPS	LYFTRASPS	6	0.4561	359.4	50.00	0.68	tr_D0A0U3_
DRB1_0101	490	ESRPQAVIVFAPPVG	AVIVFAPPV	5	0.4555	362.1	50.00	0.56	tr_D0A0U3_
DRB1_0101	491	VVVTGTNPLARDTQY	VVTGTNPLA	1	0.4550	363.7	50.00	0.56	tr_D0A0U3_
DRB1_0101	492	SSLTGVASTRHFDSA	LTGVASTRH	2	0.4548	364.5	50.00	0.40	tr_D0A0U3_
DRB1_0101	493	VVLLHEEFFNAFEGV	LLHEEFFNA	2	0.4545	366.0	55.00	0.38	tr_D0A0U3_
DRB1_0101	494	EGVSSADRVLFATNL	DRVLFATNL	6	0.4530	371.9	55.00	0.51	tr_D0A0U3_
DRB1_0101	495	RNLVADSRTRSAYVL	DSRTRSAYV	5	0.4528	372.7	55.00	0.35	tr_D0A0U3_
DRB1_0101	496	IHLSPTLEQQFYVCT	PTLEQQFYV	4	0.4524	374.1	55.00	0.50	tr_D0A0U3_
DRB1_0101	497	QAPFNGVSVLIPDRP	FNGVSVLIP	3	0.4513	378.8	55.00	0.40	tr_D0A0U3_
DRB1_0101	498	KADIEIEVFSLLHHQ	IEIEVFSLL	3	0.4507	381.1	55.00	0.48	tr_D0A0U3_
DRB1_0101	499	LRRMGYGFCGVFTLS	GYGFCGVFT	4	0.4488	389.0	55.00	0.25	tr_D0A0U3_
DRB1_0101	500	TRASPSAELLALIRY	TRASPSAEL	0	0.4486	390.0	55.00	0.25	tr_D0A0U3_
DRB1_0101	501	HDDIDGQLLVYGWIT	QLLVYGWIT	6	0.4486	390.1	55.00	0.34	tr_D0A0U3_
DRB1_0101	502	HDSARVFVPFFDVVL	FVPFFDVVL	6	0.4482	391.7	55.00	0.38	tr_D0A0U3_
DRB1_0101	503	WSTKITTAVLGVVDE	TKITTAVLG	2	0.4481	392.1	55.00	0.35	tr_D0A0U3_
DRB1_0101	504	FQQAVERNRGKLFVV	VERNRGKLF	4	0.4478	393.4	55.00	0.73	tr_D0A0U3_
DRB1_0101	505	DKKRLHIVIRSTDAA	HIVIRSTDA	5	0.4471	396.5	55.00	0.44	tr_D0A0U3_
DRB1_0101	506	KAMNATALVDSIFAQ	TALVDSIFA	5	0.4470	396.6	55.00	0.44	tr_D0A0U3_
DRB1_0101	507	GQGAMCWCNQGGNTV	WCNQGGNTV	6	0.4463	399.7	55.00	0.64	tr_D0A0U3_
DRB1_0101	508	RVFVPFFDVVLLHEE	FVPFFDVVL	2	0.4432	413.4	55.00	0.50	tr_D0A0U3_
DRB1_0101	509	AMLKTPGVAFIDPVP	MLKTPGVAF	1	0.4430	414.4	55.00	0.64	tr_D0A0U3_
DRB1_0101	510	FNASMTSRRWKTAPS	NASMTSRRW	1	0.4427	415.8	55.00	0.55	tr_D0A0U3_
DRB1_0101	511	GVPRLTGCAVNYGAT	LTGCAVNYG	4	0.4423	417.6	55.00	0.35	tr_D0A0U3_
DRB1_0101	512	QFAIENMWREALAFA	IENMWREAL	3	0.4419	419.1	55.00	0.34	tr_D0A0U3_
DRB1_0101	513	VLIPDRPTALHAAVE	LIPDRPTAL	1	0.4410	423.6	55.00	0.66	tr_D0A0U3_
DRB1_0101	514	LAEQDVVAFAPLTGS	DVVAFAPLT	4	0.4406	425.0	55.00	0.44	tr_D0A0U3_
DRB1_0101	515	ERNRGKLFVVVGPLG	LFVVVGPLG	6	0.4396	429.8	55.00	0.44	tr_D0A0U3_
DRB1_0101	516	PLAPRLNKMERNVIH	LNKMERNVI	5	0.4390	432.5	55.00	0.62	tr_D0A0U3_
DRB1_0101	517	FSVEPQSVVVLDGNA	VEPQSVVVL	2	0.4386	434.4	55.00	0.43	tr_D0A0U3_
DRB1_0101	518	PFVDGQVVVTGTNPL	FVDGQVVVT	1	0.4385	435.1	55.00	0.66	tr_D0A0U3_
DRB1_0101	519	YHAIRRFQRDVRSYL	FQRDVRSYL	6	0.4382	436.5	55.00	0.35	tr_D0A0U3_
DRB1_0101	520	PFFDVVLLHEEFFNA	VVLLHEEFF	4	0.4375	439.8	55.00	0.35	tr_D0A0U3_
DRB1_0101	521	PFADDGCFLKGVPRL	GCFLKGVPR	5	0.4359	447.3	55.00	0.43	tr_D0A0U3_
DRB1_0101	522	YLDPDAGKLSRGQLA	GKLSRGQLA	6	0.4355	449.4	55.00	0.54	tr_D0A0U3_
DRB1_0101	523	FYTAERNASRRTPLS	YTAERNASR	1	0.4347	453.0	55.00	0.58	tr_D0A0U3_

DRB1_0101	524	LTDRKTFMQSLYDQR	KTFMQSLYD	4	0.4345	454.4	55.00	0.44	tr_D0A0U3_
DRB1_0101	525	TFMQSLYDQRRYVID	FMQSLYDQR	1	0.4340	456.8	55.00	0.32	tr_D0A0U3_
DRB1_0101	526	ADEITASEVVQKFYT	ITASEVVQK	3	0.4335	459.0	55.00	0.56	tr_D0A0U3_
DRB1_0101	527	RYVIDDLVIGDYGGE	YVIDDLVIG	1	0.4300	476.9	60.00	0.71	tr_D0A0U3_
DRB1_0101	528	HLSPTLEQQFYVCTS	PTLEQQFYV	3	0.4288	483.3	60.00	0.60	tr_D0A0U3_
DRB1_0101	529	FNGVSVLIPDRPTAL	LIPDRPTAL	6	0.4271	492.3	60.00	0.35	tr_D0A0U3_
DRB1_0101	530	GFQQAVERNRGKLFV	VERNRGKLF	5	0.4258	499.0	60.00	0.64	tr_D0A0U3_
DRB1_0101	531	FYVCTSYLSRNDKKR	FYVCTSYLS	0	0.4256	500.0	60.00	0.40	tr_D0A0U3_
DRB1_0101	532	CYPSEVEMQAPFNGV	VEMQAPFNG	5	0.4232	513.0	60.00	0.51	tr_D0A0U3_
DRB1_0101	533	WAAGQGAMCWCNQGG	WAAGQGAMC	0	0.4230	514.6	60.00	0.68	tr_D0A0U3_
DRB1_0101	534	WADEITASEVVQKFY	ITASEVVQK	4	0.4225	516.9	60.00	0.51	tr_D0A0U3_
DRB1_0101	535	LSPTLEQQFYVCTSY	PTLEQQFYV	2	0.4222	519.1	60.00	0.53	tr_D0A0U3_
DRB1_0101	536	TQYHAIRRFQRDVRS	YHAIRRFQR	2	0.4221	519.3	60.00	0.69	tr_D0A0U3_
DRB1_0101	537	LYDQRRYVIDDLVIG	YVIDDLVIG	6	0.4219	520.3	60.00	0.62	tr_D0A0U3_
DRB1_0101	538	FDSAWNTFVESRPQA	FDSAWNTFV	0	0.4214	523.2	60.00	0.67	tr_D0A0U3_
DRB1_0101	539	AERNASRRTPLSLLG	SRRTPLSLL	5	0.4211	524.9	60.00	0.67	tr_D0A0U3_
DRB1_0101	540	VLLHEEFFNAFEGVS	LLHEEFFNA	1	0.4202	530.1	60.00	0.34	tr_D0A0U3_
DRB1_0101	541	ADIEIEVFSLLHHQK	IEIEVFSLL	2	0.4198	532.4	60.00	0.41	tr_D0A0U3_
DRB1_0101	542	APSVHVKVMHPFTPS	VKVMHPFTP	5	0.4188	538.3	60.00	0.44	tr_D0A0U3_
DRB1_0101	543	LKSNPGVTVFNASSN	LKSNPGVTV	0	0.4186	539.4	60.00	0.66	tr_D0A0U3_
DRB1_0101	544	AVERNRGKLFVVVGP	VERNRGKLF	1	0.4175	546.1	60.00	0.53	tr_D0A0U3_
DRB1_0101	545	DTQYHAIRRFQRDVR	YHAIRRFQR	3	0.4169	549.3	60.00	0.68	tr_D0A0U3_
DRB1_0101	546	AVNYGATQVSVWSMA	YGATQVSVW	3	0.4143	565.2	60.00	0.22	tr_D0A0U3_
DRB1_0101	547	VVVLDGNATVEGHLP	VVVLDGNAT	0	0.4140	566.8	60.00	0.55	tr_D0A0U3_
DRB1_0101	548	CAVNYGATQVSVWSM	VNYGATQVS	2	0.4134	570.5	60.00	0.28	tr_D0A0U3_
DRB1_0101	549	FVPFFDVVLLHEEFF	VVLLHEEFF	6	0.4122	578.2	60.00	0.23	tr_D0A0U3_
DRB1_0101	550	HHQKIEKRFVEAVNA	IEKRFVEAV	4	0.4120	579.4	60.00	0.34	tr_D0A0U3_
DRB1_0101	551	RLLDAPDGIMMFDSS	LDAPDGIMM	2	0.4115	582.4	60.00	0.55	tr_D0A0U3_
DRB1_0101	552	FIDPVPLAPRLNKME	PVPLAPRLN	3	0.4113	583.7	60.00	0.44	tr_D0A0U3_
DRB1_0101	553	RASPSAELLALIRYA	PSAELLALI	3	0.4111	585.2	60.00	0.33	tr_D0A0U3_
DRB1_0101	554	VPRLTGCAVNYGATQ	LTGCAVNYG	3	0.4109	586.2	60.00	0.46	tr_D0A0U3_
DRB1_0101	555	IPPLTNPMTPSMRYL	TNPMTPSMR	4	0.4108	586.9	60.00	0.44	tr_D0A0U3_
DRB1_0101	556	NASIPPLTNPMTPSM	IPPLTNPMT	3	0.4107	587.9	60.00	0.68	tr_D0A0U3_
DRB1_0101	557	APFNGVSVLIPDRPT	FNGVSVLIP	2	0.4103	590.0	60.00	0.37	tr_D0A0U3_
DRB1_0101	558	ASPSAELLALIRYAL	SAELLALIR	3	0.4096	594.4	60.00	0.25	tr_D0A0U3_
DRB1_0101	559	SVEPQSVVVLDGNAT	VVVLDGNAT	6	0.4078	606.4	65.00	0.51	tr_D0A0U3_
DRB1_0101	560	KITTAVLGVVDEAML	LGVVDEAML	6	0.4068	612.9	65.00	0.39	tr_D0A0U3_
DRB1_0101	561	LFATNLPHWADEITA	FATNLPHWA	1	0.4065	614.9	65.00	0.61	tr_D0A0U3_
DRB1_0101	562	SIPPLTNPMTPSMRY	IPPLTNPMT	1	0.4063	616.5	65.00	0.38	tr_D0A0U3_
DRB1_0101	563	FTPSASPISGFQQAV	FTPSASPIS	0	0.4045	628.1	65.00	0.66	tr_D0A0U3_
DRB1_0101	564	SLLHHQKIEKRFVEA	LHHQKIEKR	2	0.4044	628.8	65.00	0.56	tr_D0A0U3_
DRB1_0101	565	HWADEITASEVVQKF	ITASEVVQK	5	0.4040	631.5	65.00	0.39	tr_D0A0U3_
DRB1_0101	566	FAQSVVNVGEMRYGP	SVVNVGEMR	3	0.4035	635.1	65.00	0.48	tr_D0A0U3_
DRB1_0101	567	GCAVNYGATQVSVWS	VNYGATQVS	3	0.4028	640.1	65.00	0.30	tr_D0A0U3_
DRB1_0101	568	ASIPPLTNPMTPSMR	IPPLTNPMT	2	0.4010	652.7	65.00	0.61	tr_D0A0U3_
DRB1_0101	569	STKITTAVLGVVDEA	TKITTAVLG	1	0.4007	654.9	65.00	0.38	tr_D0A0U3_
DRB1_0101	570	QYHAIRRFQRDVRSY	YHAIRRFQR	1	0.3984	671.3	65.00	0.57	tr_D0A0U3_
DRB1_0101	571	LLHHQKIEKRFVEAV	LHHQKIEKR	1	0.3966	684.5	65.00	0.40	tr_D0A0U3_
DRB1_0101	572	VESRPQAVIVFAPPV	AVIVFAPPV	6	0.3962	687.8	65.00	0.41	tr_D0A0U3_
DRB1_0101	573	VEMEEGASLLEVREH	VEMEEGASL	0	0.3955	692.5	65.00	0.45	tr_D0A0U3_

DRB1_0101	574	VFAPPVGDTARFIRN	FAPPVGDTA	1	0.3945	700.0	65.00	0.51	tr_D0A0U3_
DRB1_0101	575	HLPDSGDVYVIGLTA	DVYVIGLTA	6	0.3945	700.2	65.00	0.62	tr_D0A0U3_
DRB1_0101	576	VFVPFFDVVLLHEEF	FVPFFDVVL	1	0.3944	701.2	65.00	0.46	tr_D0A0U3_
DRB1_0101	577	NDKKRLHIVIRSTDA	KRLHIVIRS	3	0.3934	708.2	65.00	0.28	tr_D0A0U3_
DRB1_0101	578	AQALSVPERLTDRKT	QALSVPERL	1	0.3934	708.3	65.00	0.74	tr_D0A0U3_
DRB1_0101	579	SLTGVASTRHFDSAW	LTGVASTRH	1	0.3926	714.9	65.00	0.37	tr_D0A0U3_
DRB1_0101	580	SGFQQAVERNRGKLF	VERNRGKLF	6	0.3921	718.8	65.00	0.39	tr_D0A0U3_
DRB1_0101	581	VPFFDVVLLHEEFFN	VVLLHEEFF	5	0.3919	720.0	65.00	0.38	tr_D0A0U3_
DRB1_0101	582	AMNATALVDSIFAQS	TALVDSIFA	4	0.3917	721.4	65.00	0.57	tr_D0A0U3_
DRB1_0101	583	RDTQYHAIRRFQRDV	YHAIRRFQR	4	0.3917	722.0	65.00	0.62	tr_D0A0U3_
DRB1_0101	584	VNYGATQVSVWSMAR	GATQVSVWS	3	0.3909	728.1	65.00	0.28	tr_D0A0U3_
DRB1_0101	585	PMTPSMRYLDPDAGK	MRYLDPDAG	5	0.3890	743.2	65.00	0.78	tr_D0A0U3_
DRB1_0101	586	QRDVRSYLKSNPGVT	YLKSNPGVT	6	0.3890	743.5	65.00	0.60	tr_D0A0U3_
DRB1_0101	587	AAAIEDVLKKTLATF	DVLKKTLAT	5	0.3889	743.8	65.00	0.46	tr_D0A0U3_
DRB1_0101	588	PFNGVSVLIPDRPTA	VSVLIPDRP	4	0.3885	746.9	65.00	0.32	tr_D0A0U3_
DRB1_0101	589	LARDTQYHAIRRFQR	YHAIRRFQR	6	0.3874	756.0	65.00	0.34	tr_D0A0U3_
DRB1_0101	590	LNSHDSARVFVPFFD	LNSHDSARV	0	0.3872	757.5	65.00	0.39	tr_D0A0U3_
DRB1_0101	591	LIPDRPTALHAAVEM	LIPDRPTAL	0	0.3850	775.7	65.00	0.30	tr_D0A0U3_
DRB1_0101	592	TGCAVNYGATQVSVW	VNYGATQVS	4	0.3837	787.2	70.00	0.31	tr_D0A0U3_
DRB1_0101	593	ARDTQYHAIRRFQRD	YHAIRRFQR	5	0.3806	813.8	70.00	0.47	tr_D0A0U3_
DRB1_0101	594	RFQRDVRSYLKSNPG	FQRDVRSYL	1	0.3800	818.9	70.00	0.71	tr_D0A0U3_
DRB1_0101	595	PPLTNPMTPSMRYLD	TNPMTPSMR	3	0.3773	843.7	70.00	0.51	tr_D0A0U3_
DRB1_0101	596	PRLTGCAVNYGATQV	LTGCAVNYG	2	0.3768	848.2	70.00	0.49	tr_D0A0U3_
DRB1_0101	597	PHWADEITASEVVQK	DEITASEVV	4	0.3767	848.5	70.00	0.27	tr_D0A0U3_
DRB1_0101	598	DTIVSPFSGAAEELQ	IVSPFSGAA	2	0.3744	870.6	70.00	0.28	tr_D0A0U3_
DRB1_0101	599	RYLDPDAGKLSRGQL	YLDPDAGKL	1	0.3740	874.4	70.00	0.44	tr_D0A0U3_
DRB1_0101	600	NYGATQVSVWSMARA	GATQVSVWS	2	0.3730	883.7	70.00	0.25	tr_D0A0U3_
DRB1_0101	601	SRNDKKRLHIVIRST	KRLHIVIRS	5	0.3721	892.7	70.00	0.28	tr_D0A0U3_
DRB1_0101	602	IRSTDAAAIEDVLKK	IRSTDAAAI	0	0.3709	903.6	70.00	0.52	tr_D0A0U3_
DRB1_0101	603	TIVSPFSGAAEELQR	FSGAAEELQ	5	0.3708	905.3	70.00	0.41	tr_D0A0U3_
DRB1_0101	604	LHHQKIEKRFVEAVN	IEKRFVEAV	5	0.3706	907.2	70.00	0.31	tr_D0A0U3_
DRB1_0101	605	RLTGCAVNYGATQVS	LTGCAVNYG	1	0.3704	909.0	70.00	0.36	tr_D0A0U3_
DRB1_0101	606	RLTDRKTFMQSLYDQ	KTFMQSLYD	5	0.3703	909.6	70.00	0.41	tr_D0A0U3_
DRB1_0101	607	LTGCAVNYGATQVSV	VNYGATQVS	5	0.3701	911.3	70.00	0.26	tr_D0A0U3_
DRB1_0101	608	SGAAEELQREWSTKI	LQREWSTKI	6	0.3679	933.9	70.00	0.67	tr_D0A0U3_
DRB1_0101	609	SHDSARVFVPFFDVV	RVFVPFFDV	5	0.3660	952.7	70.00	0.40	tr_D0A0U3_
DRB1_0101	610	GIMMFDSSHCYPSEV	IMMFDSSHC	1	0.3658	954.8	70.00	0.28	tr_D0A0U3_
DRB1_0101	611	RNDKKRLHIVIRSTD	KRLHIVIRS	4	0.3644	969.3	70.00	0.31	tr_D0A0U3_
DRB1_0101	612	FAPPVGDTARFIRNL	PVGDTARFI	3	0.3644	969.3	70.00	0.52	tr_D0A0U3_
DRB1_0101	613	VERNRGKLFVVVGPL	VERNRGKLF	0	0.3620	994.7	70.00	0.29	tr_D0A0U3_
DRB1_0101	614	QSVVNVGEMRYGPFA	SVVNVGEMR	1	0.3613	1002.8	70.00	0.41	tr_D0A0U3_
DRB1_0101	615	TSYLSRNDKKRLHIV	LSRNDKKRL	3	0.3606	1010.8	70.00	0.38	tr_D0A0U3_
DRB1_0101	616	DHDDIDGQLLVYGWI	HDDIDGQLL	1	0.3605	1011.6	70.00	0.27	tr_D0A0U3_
DRB1_0101	617	NPLARDTQYHAIRRF	ARDTQYHAI	3	0.3583	1036.1	75.00	0.38	tr_D0A0U3_
DRB1_0101	618	LSRNDKKRLHIVIRS	NDKKRLHIV	3	0.3579	1040.8	75.00	0.35	tr_D0A0U3_
DRB1_0101	619	TAERNASRRTPLSLL	SRRTPLSLL	6	0.3570	1051.0	75.00	0.55	tr_D0A0U3_
DRB1_0101	620	IDPVPLAPRLNKMER	PVPLAPRLN	2	0.3562	1059.8	75.00	0.46	tr_D0A0U3_
DRB1_0101	621	DGIMMFDSSHCYPSE	IMMFDSSHC	2	0.3561	1061.3	75.00	0.36	tr_D0A0U3_
DRB1_0101	622	FMQSLYDQRRYVIDD	MQSLYDQRR	1	0.3556	1066.8	75.00	0.23	tr_D0A0U3_
DRB1_0101	623	TKITTAVLGVVDEAM	TKITTAVLG	0	0.3551	1072.0	75.00	0.29	tr_D0A0U3_

DRB1_0101	624	IPDRPTALHAAVEME	TALHAAVEM	5	0.3544	1080.1	75.00	0.37	tr_D0A0U3_
DRB1_0101	625	GGNTVHVRVIGRGYR	HVRVIGRGY	5	0.3544	1080.4	75.00	0.41	tr_D0A0U3_
DRB1_0101	626	ISGFQQAVERNRGKL	FQQAVERNR	3	0.3543	1081.6	75.00	0.57	tr_D0A0U3_
DRB1_0101	627	PLTNPMTPSMRYLDP	TNPMTPSMR	2	0.3534	1092.4	75.00	0.52	tr_D0A0U3_
DRB1_0101	628	LLDAPDGIMMFDSSH	LDAPDGIMM	1	0.3524	1104.7	75.00	0.51	tr_D0A0U3_
DRB1_0101	629	PDGIMMFDSSHCYPS	IMMFDSSHC	3	0.3511	1120.2	75.00	0.40	tr_D0A0U3_
DRB1_0101	630	PDRPTALHAAVEMEE	TALHAAVEM	4	0.3507	1124.4	75.00	0.41	tr_D0A0U3_
DRB1_0101	631	APPVGDTARFIRNLV	PVGDTARFI	2	0.3491	1144.9	75.00	0.51	tr_D0A0U3_
DRB1_0101	632	VPLAPRLNKMERNVI	LNKMERNVI	6	0.3489	1146.8	75.00	0.55	tr_D0A0U3_
DRB1_0101	633	NQDSRLFFDTIVSPF	FFDTIVSPF	6	0.3485	1151.5	75.00	0.28	tr_D0A0U3_
DRB1_0101	634	VVGPLGDFGTVSSLI	VGPLGDFGT	1	0.3472	1168.0	75.00	0.31	tr_D0A0U3_
DRB1_0101	635	PLARDTQYHAIRRFQ	ARDTQYHAI	2	0.3471	1169.0	75.00	0.35	tr_D0A0U3_
DRB1_0101	636	TNPLARDTQYHAIRR	ARDTQYHAI	4	0.3458	1186.4	75.00	0.36	tr_D0A0U3_
DRB1_0101	637	LPHWADEITASEVVQ	WADEITASE	3	0.3448	1199.4	75.00	0.23	tr_D0A0U3_
DRB1_0101	638	SYLSRNDKKRLHIVI	LSRNDKKRL	2	0.3444	1203.6	75.00	0.29	tr_D0A0U3_
DRB1_0101	639	IMMFDSSHCYPSEVE	FDSSHCYPS	3	0.3439	1211.1	75.00	0.33	tr_D0A0U3_
DRB1_0101	640	DRPTALHAAVEMEEG	TALHAAVEM	3	0.3431	1220.9	75.00	0.45	tr_D0A0U3_
DRB1_0101	641	HCYPSEVEMQAPFNG	VEMQAPFNG	6	0.3429	1223.2	75.00	0.31	tr_D0A0U3_
DRB1_0101	642	IGDYGGECAGWAAGQ	YGGECAGWA	3	0.3419	1237.7	75.00	0.50	tr_D0A0U3_
DRB1_0101	643	NSHDSARVFVPFFDV	RVFVPFFDV	6	0.3412	1246.8	75.00	0.26	tr_D0A0U3_
DRB1_0101	644	PGVTVFNASSNFDHD	FNASSNFDH	5	0.3407	1253.3	75.00	0.49	tr_D0A0U3_
DRB1_0101	645	VTVFNASSNFDHDDI	FNASSNFDH	3	0.3405	1255.6	75.00	0.65	tr_D0A0U3_
DRB1_0101	646	YLSRNDKKRLHIVIR	NDKKRLHIV	4	0.3394	1270.9	75.00	0.31	tr_D0A0U3_
DRB1_0101	647	AQSVVNVGEMRYGPF	SVVNVGEMR	2	0.3388	1278.6	75.00	0.56	tr_D0A0U3_
DRB1_0101	648	VSPFSGAAEELQREW	FSGAAEELQ	3	0.3386	1282.1	75.00	0.63	tr_D0A0U3_
DRB1_0101	649	IVSPFSGAAEELQRE	FSGAAEELQ	4	0.3386	1282.4	75.00	0.56	tr_D0A0U3_
DRB1_0101	650	VVVGPLGDFGTVSSL	VGPLGDFGT	2	0.3379	1291.8	75.00	0.46	tr_D0A0U3_
DRB1_0101	651	CTSYLSRNDKKRLHI	YLSRNDKKR	3	0.3376	1296.0	75.00	0.38	tr_D0A0U3_
DRB1_0101	652	FATNLPHWADEITAS	FATNLPHWA	0	0.3374	1299.2	75.00	0.46	tr_D0A0U3_
DRB1_0101	653	NFDHDDIDGQLLVYG	HDDIDGQLL	3	0.3366	1309.6	75.00	0.42	tr_D0A0U3_
DRB1_0101	654	GVTVFNASSNFDHDD	FNASSNFDH	4	0.3365	1311.8	75.00	0.52	tr_D0A0U3_
DRB1_0101	655	TAPSVHVKVMHPFTP	VKVMHPFTP	6	0.3348	1336.5	75.00	0.37	tr_D0A0U3_
DRB1_0101	656	RPTALHAAVEMEEGA	TALHAAVEM	2	0.3346	1338.1	75.00	0.47	tr_D0A0U3_
DRB1_0101	657	PISGFQQAVERNRGK	FQQAVERNR	4	0.3346	1338.6	75.00	0.50	tr_D0A0U3_
DRB1_0101	658	NASMTSRRWKTAPSV	NASMTSRRW	0	0.3346	1338.7	75.00	0.37	tr_D0A0U3_
DRB1_0101	659	SVVNVGEMRYGPFAD	GEMRYGPFA	5	0.3330	1362.4	80.00	0.31	tr_D0A0U3_
DRB1_0101	660	TALHAAVEMEEGASL	VEMEEGASL	6	0.3324	1371.6	80.00	0.61	tr_D0A0U3_
DRB1_0101	661	GDYGGECAGWAAGQG	YGGECAGWA	2	0.3304	1400.8	80.00	0.47	tr_D0A0U3_
DRB1_0101	662	GPFADDGCFLKGVPR	GCFLKGVPR	6	0.3303	1402.1	80.00	0.37	tr_D0A0U3_
DRB1_0101	663	FDHDDIDGQLLVYGW	HDDIDGQLL	2	0.3294	1415.8	80.00	0.39	tr_D0A0U3_
DRB1_0101	664	VIGDYGGECAGWAAG	YGGECAGWA	4	0.3275	1445.8	80.00	0.48	tr_D0A0U3_
DRB1_0101	665	CNQGGNTVHVRVIGR	NQGGNTVHV	1	0.3273	1448.4	80.00	0.28	tr_D0A0U3_
DRB1_0101	666	LTGVASTRHFDSAWN	TGVASTRHF	1	0.3259	1470.7	80.00	0.32	tr_D0A0U3_
DRB1_0101	667	SNFDHDDIDGQLLVY	HDDIDGQLL	4	0.3254	1479.4	80.00	0.41	tr_D0A0U3_
DRB1_0101	668	YVIDDLVIGDYGGEC	YVIDDLVIG	0	0.3247	1490.7	80.00	0.54	tr_D0A0U3_
DRB1_0101	669	SPISGFQQAVERNRG	FQQAVERNR	5	0.3236	1507.1	80.00	0.34	tr_D0A0U3_
DRB1_0101	670	DPVPLAPRLNKMERN	PVPLAPRLN	1	0.3225	1525.6	80.00	0.42	tr_D0A0U3_
DRB1_0101	671	SPFSGAAEELQREWS	FSGAAEELQ	2	0.3209	1551.8	80.00	0.63	tr_D0A0U3_
DRB1_0101	672	ERLTDRKTFMQSLYD	KTFMQSLYD	6	0.3190	1584.9	80.00	0.35	tr_D0A0U3_
DRB1_0101	673	DAAAIEDVLKKTLAT	EDVLKKTLA	5	0.3186	1592.3	80.00	0.35	tr_D0A0U3_

DRB1_0101	674	APDGIMMFDSSHCYP	IMMFDSSHC	4	0.3183	1596.2	80.00	0.44	tr_D0A0U3_
DRB1_0101	675	YVCTSYLSRNDKKRL	YLSRNDKKR	5	0.3181	1600.8	80.00	0.26	tr_D0A0U3_
DRB1_0101	676	DYGGECAGWAAGQGA	YGGECAGWA	1	0.3177	1606.8	80.00	0.41	tr_D0A0U3_
DRB1_0101	677	VCTSYLSRNDKKRLH	YLSRNDKKR	4	0.3166	1627.4	80.00	0.34	tr_D0A0U3_
DRB1_0101	678	NLPHWADEITASEVV	WADEITASE	4	0.3159	1638.7	80.00	0.23	tr_D0A0U3_
DRB1_0101	679	MMFDSSHCYPSEVEM	FDSSHCYPS	2	0.3124	1701.7	80.00	0.40	tr_D0A0U3_
DRB1_0101	680	NPGVTVFNASSNFDH	FNASSNFDH	6	0.3122	1705.7	80.00	0.31	tr_D0A0U3_
DRB1_0101	681	DAPDGIMMFDSSHCY	IMMFDSSHC	5	0.3108	1732.5	80.00	0.43	tr_D0A0U3_
DRB1_0101	682	LTNPMTPSMRYLDPD	TNPMTPSMR	1	0.3102	1743.5	80.00	0.49	tr_D0A0U3_
DRB1_0101	683	TVFNASSNFDHDDID	FNASSNFDH	2	0.3077	1791.0	80.00	0.76	tr_D0A0U3_
DRB1_0101	684	GTNPLARDTQYHAIR	LARDTQYHA	4	0.3050	1843.6	85.00	0.38	tr_D0A0U3_
DRB1_0101	685	ASPISGFQQAVERNR	SGFQQAVER	4	0.3047	1850.1	85.00	0.30	tr_D0A0U3_
DRB1_0101	686	NPMTPSMRYLDPDAG	MRYLDPDAG	6	0.3030	1885.0	85.00	0.56	tr_D0A0U3_
DRB1_0101	687	QGGNTVHVRVIGRGY	GNTVHVRVI	2	0.3026	1893.3	85.00	0.31	tr_D0A0U3_
DRB1_0101	688	VVTGTNPLARDTQYH	VVTGTNPLA	0	0.3024	1895.8	85.00	0.52	tr_D0A0U3_
DRB1_0101	689	YGGECAGWAAGQGAM	GGECAGWAA	1	0.3023	1898.1	85.00	0.29	tr_D0A0U3_
DRB1_0101	690	QALSVPERLTDRKTF	QALSVPERL	0	0.3005	1935.3	85.00	0.59	tr_D0A0U3_
DRB1_0101	691	FQRDVRSYLKSNPGV	FQRDVRSYL	0	0.3004	1938.0	85.00	0.41	tr_D0A0U3_
DRB1_0101	692	RYGPFADDGCFLKGV	FADDGCFLK	4	0.2979	1991.4	85.00	0.43	tr_D0A0U3_
DRB1_0101	693	NQGGNTVHVRVIGRG	GNTVHVRVI	3	0.2975	1999.3	85.00	0.35	tr_D0A0U3_
DRB1_0101	694	LVIGDYGGECAGWAA	YGGECAGWA	5	0.2974	2002.3	85.00	0.44	tr_D0A0U3_
DRB1_0101	695	MFDSSHCYPSEVEMQ	SSHCYPSEV	3	0.2970	2011.3	85.00	0.34	tr_D0A0U3_
DRB1_0101	696	ASMTSRRWKTAPSVH	RRWKTAPSV	5	0.2966	2020.2	85.00	0.40	tr_D0A0U3_
DRB1_0101	697	KTAPSVHVKVMHPFT	TAPSVHVKV	1	0.2964	2024.0	85.00	0.25	tr_D0A0U3_
DRB1_0101	698	LDAPDGIMMFDSSHC	LDAPDGIMM	0	0.2951	2052.8	85.00	0.34	tr_D0A0U3_
DRB1_0101	699	PFSGAAEELQREWST	FSGAAEELQ	1	0.2950	2055.7	85.00	0.56	tr_D0A0U3_
DRB1_0101	700	SSNFDHDDIDGQLLV	HDDIDGQLL	5	0.2932	2095.9	85.00	0.44	tr_D0A0U3_
DRB1_0101	701	FDSSHCYPSEVEMQA	SSHCYPSEV	2	0.2906	2155.0	85.00	0.27	tr_D0A0U3_
DRB1_0101	702	APLTGSSGGRGWNPN	LTGSSGGRG	2	0.2882	2210.9	85.00	0.71	tr_D0A0U3_
DRB1_0101	703	MQSLYDQRRYVIDDL	LYDQRRYVI	3	0.2871	2239.2	85.00	0.40	tr_D0A0U3_
DRB1_0101	704	VVNVGEMRYGPFADD	GEMRYGPFA	4	0.2857	2272.0	85.00	0.47	tr_D0A0U3_
DRB1_0101	705	PVPLAPRLNKMERNV	LAPRLNKME	3	0.2849	2291.6	85.00	0.32	tr_D0A0U3_
DRB1_0101	706	VNVGEMRYGPFADDG	GEMRYGPFA	3	0.2849	2292.6	85.00	0.55	tr_D0A0U3_
DRB1_0101	707	GGRGWNPNLYFTRAS	WNPNLYFTR	4	0.2822	2360.6	85.00	0.38	tr_D0A0U3_
DRB1_0101	708	SHCYPSEVEMQAPFN	EVEMQAPFN	6	0.2816	2375.3	85.00	0.25	tr_D0A0U3_
DRB1_0101	709	TNLPHWADEITASEV	PHWADEITA	3	0.2785	2457.2	85.00	0.36	tr_D0A0U3_
DRB1_0101	710	HNQDSRLFFDTIVSP	RLFFDTIVS	5	0.2782	2464.8	85.00	0.26	tr_D0A0U3_
DRB1_0101	711	TGVASTRHFDSAWNT	VASTRHFDS	2	0.2748	2555.5	90.00	0.27	tr_D0A0U3_
DRB1_0101	712	PERLTDRKTFMQSLY	DRKTFMQSL	5	0.2746	2563.2	90.00	0.37	tr_D0A0U3_
DRB1_0101	713	PLTGSSGGRGWNPNL	LTGSSGGRG	1	0.2744	2567.7	90.00	0.60	tr_D0A0U3_
DRB1_0101	714	YTAERNASRRTPLSL	YTAERNASR	0	0.2738	2584.7	90.00	0.45	tr_D0A0U3_
DRB1_0101	715	SGGRGWNPNLYFTRA	WNPNLYFTR	5	0.2730	2607.7	90.00	0.34	tr_D0A0U3_
DRB1_0101	716	MRYGPFADDGCFLKG	FADDGCFLK	5	0.2719	2639.0	90.00	0.37	tr_D0A0U3_
DRB1_0101	717	YGPFADDGCFLKGVP	FADDGCFLK	3	0.2713	2654.4	90.00	0.45	tr_D0A0U3_
DRB1_0101	718	QSLYDQRRYVIDDLV	LYDQRRYVI	2	0.2705	2679.2	90.00	0.39	tr_D0A0U3_
DRB1_0101	719	KSNPGVTVFNASSNF	VTVFNASSN	5	0.2701	2689.9	90.00	0.22	tr_D0A0U3_
DRB1_0101	720	EHNQDSRLFFDTIVS	DSRLFFDTI	4	0.2688	2726.8	90.00	0.26	tr_D0A0U3_
DRB1_0101	721	PTALHAAVEMEEGAS	TALHAAVEM	1	0.2673	2771.6	90.00	0.46	tr_D0A0U3_
DRB1_0101	722	SASPISGFQQAVERN	SGFQQAVER	5	0.2642	2868.8	90.00	0.29	tr_D0A0U3_
DRB1_0101	723	NVGEMRYGPFADDGC	GEMRYGPFA	2	0.2636	2885.4	90.00	0.53	tr_D0A0U3_

DRB1_0101	724	SLYDQRRYVIDDLVI	LYDQRRYVI	1	0.2627	2915.8	90.00	0.27	tr_D0A0U3_
DRB1_0101	725	GRGWNPNLYFTRASP	WNPNLYFTR	3	0.2626	2919.0	90.00	0.39	tr_D0A0U3_
DRB1_0101	726	TDAAAIEDVLKKTLA	AAIEDVLKK	3	0.2605	2983.4	90.00	0.41	tr_D0A0U3_
DRB1_0101	727	SNPGVTVFNASSNFD	VTVFNASSN	4	0.2587	3044.5	90.00	0.28	tr_D0A0U3_
DRB1_0101	728	DSSHCYPSEVEMQAP	SSHCYPSEV	1	0.2572	3091.6	90.00	0.26	tr_D0A0U3_
DRB1_0101	729	FSGAAEELQREWSTK	FSGAAEELQ	0	0.2563	3124.2	90.00	0.42	tr_D0A0U3_
DRB1_0101	730	SSHCYPSEVEMQAPF	CYPSEVEMQ	3	0.2548	3175.7	90.00	0.34	tr_D0A0U3_
DRB1_0101	731	RSTDAAAIEDVLKKT	TDAAAIEDV	2	0.2541	3198.6	90.00	0.40	tr_D0A0U3_
DRB1_0101	732	TGTNPLARDTQYHAI	LARDTQYHA	5	0.2530	3238.0	90.00	0.34	tr_D0A0U3_
DRB1_0101	733	REHNQDSRLFFDTIV	DSRLFFDTI	5	0.2512	3300.0	90.00	0.24	tr_D0A0U3_
DRB1_0101	734	SSGGRGWNPNLYFTR	RGWNPNLYF	4	0.2500	3345.1	90.00	0.27	tr_D0A0U3_
DRB1_0101	735	VFNASSNFDHDDIDG	FNASSNFDH	1	0.2492	3372.7	90.00	0.78	tr_D0A0U3_
DRB1_0101	736	GVASTRHFDSAWNTF	TRHFDSAWN	4	0.2491	3375.6	90.00	0.29	tr_D0A0U3_
DRB1_0101	737	STDAAAIEDVLKKTL	AAIEDVLKK	4	0.2459	3495.6	90.00	0.39	tr_D0A0U3_
DRB1_0101	738	LTGSSGGRGWNPNLY	SSGGRGWNP	3	0.2450	3529.6	90.00	0.31	tr_D0A0U3_
DRB1_0101	739	VREHNQDSRLFFDTI	EHNQDSRLF	2	0.2445	3549.5	90.00	0.32	tr_D0A0U3_
DRB1_0101	740	EMRYGPFADDGCFLK	PFADDGCFL	5	0.2444	3553.6	90.00	0.38	tr_D0A0U3_
DRB1_0101	741	VPERLTDRKTFMQSL	LTDRKTFMQ	4	0.2427	3620.4	90.00	0.29	tr_D0A0U3_
DRB1_0101	742	PSASPISGFQQAVER	ASPISGFQQ	2	0.2423	3635.1	90.00	0.23	tr_D0A0U3_
DRB1_0101	743	ATNLPHWADEITASE	PHWADEITA	4	0.2395	3745.9	95.00	0.40	tr_D0A0U3_
DRB1_0101	744	DLVIGDYGGECAGWA	YGGECAGWA	6	0.2384	3792.6	95.00	0.31	tr_D0A0U3_
DRB1_0101	745	VGEMRYGPFADDGCF	GEMRYGPFA	1	0.2355	3911.3	95.00	0.46	tr_D0A0U3_
DRB1_0101	746	LEVREHNQDSRLFFD	EHNQDSRLF	4	0.2339	3980.9	95.00	0.31	tr_D0A0U3_
DRB1_0101	747	EVREHNQDSRLFFDT	EHNQDSRLF	3	0.2337	3989.7	95.00	0.35	tr_D0A0U3_
DRB1_0101	748	LLEVREHNQDSRLFF	EHNQDSRLF	5	0.2326	4034.3	95.00	0.29	tr_D0A0U3_
DRB1_0101	749	TNPMTPSMRYLDPDA	TNPMTPSMR	0	0.2315	4085.3	95.00	0.30	tr_D0A0U3_
DRB1_0101	750	GEMRYGPFADDGCFL	GEMRYGPFA	0	0.2260	4334.8	95.00	0.26	tr_D0A0U3_
DRB1_0101	751	VVLDGNATVEGHLPD	VVLDGNATV	0	0.2258	4344.6	95.00	0.32	tr_D0A0U3_
DRB1_0101	752	EMEEGASLLEVREHN	EMEEGASLL	0	0.2253	4369.5	95.00	0.35	tr_D0A0U3_
DRB1_0101	753	ASSNFDHDDIDGQLL	FDHDDIDGQ	4	0.2244	4411.9	95.00	0.42	tr_D0A0U3_
DRB1_0101	754	GSSGGRGWNPNLYFT	SSGGRGWNP	1	0.2167	4796.6	95.00	0.27	tr_D0A0U3_
DRB1_0101	755	GHLPDSGDVYVIGLT	PDSGDVYVI	3	0.2133	4973.3	95.00	0.41	tr_D0A0U3_
DRB1_0101	756	TPSASPISGFQQAVE	ASPISGFQQ	3	0.2126	5013.1	95.00	0.31	tr_D0A0U3_
DRB1_0101	757	VIDDLVIGDYGGECA	DDLVIGDYG	2	0.2113	5083.3	95.00	0.37	tr_D0A0U3_
DRB1_0101	758	IDDLVIGDYGGECAG	DDLVIGDYG	1	0.2105	5126.2	95.00	0.32	tr_D0A0U3_
DRB1_0101	759	FNASSNFDHDDIDGQ	FNASSNFDH	0	0.2056	5403.6	95.00	0.51	tr_D0A0U3_
DRB1_0101	760	DDLVIGDYGGECAGW	IGDYGGECA	4	0.2041	5494.4	95.00	0.25	tr_D0A0U3_
DRB1_0101	761	TGSSGGRGWNPNLYF	SSGGRGWNP	2	0.2016	5642.6	95.00	0.35	tr_D0A0U3_
DRB1_0101	762	EGHLPDSGDVYVIGL	PDSGDVYVI	4	0.2003	5725.4	95.00	0.39	tr_D0A0U3_
DRB1_0101	763	SVPERLTDRKTFMQS	LTDRKTFMQ	5	0.1987	5825.8	95.00	0.33	tr_D0A0U3_
DRB1_0101	764	SLLEVREHNQDSRLF	EVREHNQDS	3	0.1940	6126.9	95.00	0.22	tr_D0A0U3_
DRB1_0101	765	TVEGHLPDSGDVYVI	VEGHLPDSG	1	0.1887	6491.4	100.00	0.26	tr_D0A0U3_
DRB1_0101	766	LSVPERLTDRKTFMQ	PERLTDRKT	3	0.1882	6528.7	100.00	0.28	tr_D0A0U3_
DRB1_0101	767	AGQGAMCWCNQGGNT	GQGAMCWCN	1	0.1869	6616.8	100.00	0.17	tr_D0A0U3_
DRB1_0101	768	VEGHLPDSGDVYVIG	PDSGDVYVI	5	0.1867	6631.2	100.00	0.35	tr_D0A0U3_
DRB1_0101	769	VTGTNPLARDTQYHA	LARDTQYHA	6	0.1835	6867.1	100.00	0.24	tr_D0A0U3_
DRB1_0101	770	ALSVPERLTDRKTFM	PERLTDRKT	4	0.1744	7574.9	100.00	0.28	tr_D0A0U3_
DRB1_0101	771	NATVEGHLPDSGDVY	VEGHLPDSG	3	0.1722	7757.1	100.00	0.45	tr_D0A0U3_
DRB1_0101	772	AAGQGAMCWCNQGGN	GQGAMCWCN	2	0.1720	7773.1	100.00	0.30	tr_D0A0U3_
DRB1_0101	773	ATVEGHLPDSGDVYV	VEGHLPDSG	2	0.1675	8162.0	100.00	0.42	tr_D0A0U3_

DRB1_0101	774	ASLLEVREHNQDSRL	EVREHNQDS	4	0.1672	8193.4	100.00	0.22	tr_D0A0U3_	
DRB1_0101	775	VLDGNATVEGHLPDS	NATVEGHLP	4	0.1656	8333.3	100.00	0.33	tr_D0A0U3_	
DRB1_0101	776	MEEGASLLEVREHNQ	GASLLEVRE	3	0.1646	8425.1	100.00	0.25	tr_D0A0U3_	
DRB1_0101	777	GNATVEGHLPDSGDV	VEGHLPDSG	4	0.1644	8446.1	100.00	0.40	tr_D0A0U3_	
DRB1_0101	778	LDGNATVEGHLPDSG	NATVEGHLP	3	0.1587	8977.1	100.00	0.41	tr_D0A0U3_	
DRB1_0101	779	GASLLEVREHNQDSR	LLEVREHNQ	3	0.1567	9173.2	100.00	0.23	tr_D0A0U3_	
DRB1_0101	780	NASSNFDHDDIDGQL	FDHDDIDGQ	5	0.1561	9240.1	100.00	0.63	tr_D0A0U3_	
DRB1_0101	781	DGNATVEGHLPDSGD	NATVEGHLP	2	0.1507	9786.6	100.00	0.38	tr_D0A0U3_	
DRB1_0101	782	EGASLLEVREHNQDS	LLEVREHNQ	4	0.1469	10205.9	100.00	0.21	tr_D0A0U3_	
DRB1_0101	783	EEGASLLEVREHNQD	GASLLEVRE	2	0.1410	10879.2	100.00	0.28	tr_D0A0U3_	

Allele: DRB1_0101. Number of high binders 0. Number of weak binders 99. Number of peptides 783

Allele	pos	peptide	core	Of	1-log50k(aff)	affinity(nM)	%Rank	Relia	Identity	Bind Level
DRB1_0101	1	PHFYFLTPAPNAELI	FYFLTPAPN	2	0.8906	3.3	0.15	0.61	tr_C9ZWY7_	SB
DRB1_0101	2	VKVYNLLFTNRAPRR	YNLLFTNRA	3	0.8847	3.5	0.17	0.93	tr_C9ZWY7_	SB
DRB1_0101	3	CGVFTKMHAATVMRA	FTKMHAATV	3	0.8841	3.5	0.17	0.83	tr_C9ZWY7_	SB
DRB1_0101	4	HFYFLTPAPNAELIA	FYFLTPAPN	1	0.8835	3.5	0.20	0.55	tr_C9ZWY7_	SB
DRB1_0101	5	DPHFYFLTPAPNAEL	FYFLTPAPN	3	0.8824	3.6	0.20	0.64	tr_C9ZWY7_	SB
DRB1_0101	6	LVAFAPLTGSSAVRG	FAPLTGSSA	3	0.8804	3.6	0.20	0.90	tr_C9ZWY7_	SB
DRB1_0101	7	KVYNLLFTNRAPRRS	YNLLFTNRA	2	0.8764	3.8	0.25	0.90	tr_C9ZWY7_	SB
DRB1_0101	8	VAFAPLTGSSAVRGW	FAPLTGSSA	2	0.8749	3.9	0.25	0.86	tr_C9ZWY7_	SB
DRB1_0101	9	GVFTKMHAATVMRAD	FTKMHAATV	2	0.8749	3.9	0.25	0.82	tr_C9ZWY7_	SB
DRB1_0101	10	KVKVYNLLFTNRAPR	YNLLFTNRA	4	0.8723	4.0	0.30	0.92	tr_C9ZWY7_	SB
DRB1_0101	11	DLVAFAPLTGSSAVR	FAPLTGSSA	4	0.8700	4.1	0.40	0.89	tr_C9ZWY7_	SB
DRB1_0101	12	LCGVFTKMHAATVMR	FTKMHAATV	4	0.8697	4.1	0.40	0.80	tr_C9ZWY7_	SB
DRB1_0101	13	WDPHFYFLTPAPNAE	FYFLTPAPN	4	0.8611	4.5	0.50	0.66	tr_C9ZWY7_	WB
DRB1_0101	14	AFAPLTGSSAVRGWD	FAPLTGSSA	1	0.8514	5.0	0.60	0.82	tr_C9ZWY7_	WB
DRB1_0101	15	VFTKMHAATVMRADE	FTKMHAATV	1	0.8497	5.1	0.60	0.81	tr_C9ZWY7_	WB
DRB1_0101	16	FYFLTPAPNAELIAL	YFLTPAPNA	1	0.8482	5.2	0.70	0.41	tr_C9ZWY7_	WB
DRB1_0101	17	VYNLLFTNRAPRRSI	YNLLFTNRA	1	0.8460	5.3	0.70	0.86	tr_C9ZWY7_	WB
DRB1_0101	18	IKVKVYNLLFTNRAP	YNLLFTNRA	5	0.8448	5.4	0.70	0.90	tr_C9ZWY7_	WB
DRB1_0101	19	RGVYVLAPSAMQFVI	YVLAPSAMQ	3	0.8444	5.4	0.70	0.75	tr_C9ZWY7_	WB
DRB1_0101	20	RLCGVFTKMHAATVM	FTKMHAATV	5	0.8440	5.4	0.70	0.79	tr_C9ZWY7_	WB
DRB1_0101	21	TRGVYVLAPSAMQFV	YVLAPSAMQ	4	0.8394	5.7	0.80	0.72	tr_C9ZWY7_	WB
DRB1_0101	22	GWDPHFYFLTPAPNA	FYFLTPAPN	5	0.8380	5.8	0.90	0.72	tr_C9ZWY7_	WB
DRB1_0101	23	EDLVAFAPLTGSSAV	FAPLTGSSA	5	0.8370	5.8	0.90	0.89	tr_C9ZWY7_	WB
DRB1_0101	24	RSYLRYHPGVTAFNA	LRYHPGVTA	3	0.8260	6.6	1.20	0.73	tr_C9ZWY7_	WB
DRB1_0101	25	RTRGVYVLAPSAMQF	YVLAPSAMQ	5	0.8237	6.7	1.20	0.69	tr_C9ZWY7_	WB
DRB1_0101	26	ALLRYAINRLRLLRV	LRYAINRLR	2	0.8226	6.8	1.30	0.50	tr_C9ZWY7_	WB
DRB1_0101	27	LLRYAINRLRLLRVG	YAINRLRLL	3	0.8185	7.1	1.40	0.35	tr_C9ZWY7_	WB
DRB1_0101	28	GVYVLAPSAMQFVIG	YVLAPSAMQ	2	0.8167	7.3	1.40	0.76	tr_C9ZWY7_	WB
DRB1_0101	29	PHIVRPLAAKKSIKV	VRPLAAKKS	3	0.8130	7.6	1.60	0.60	tr_C9ZWY7_	WB
DRB1_0101	30	LPHIVRPLAAKKSIK	VRPLAAKKS	4	0.8115	7.7	1.60	0.52	tr_C9ZWY7_	WB
DRB1_0101	31	IALLRYAINRLRLLR	LRYAINRLR	3	0.8094	7.9	1.70	0.56	tr_C9ZWY7_	WB
DRB1_0101	32	SIKVKVYNLLFTNRA	YNLLFTNRA	6	0.8092	7.9	1.70	0.85	tr_C9ZWY7_	WB

DRB1_0101	33	SYLRYHPGVTAFNAS	LRYHPGVTA	2	0.8085	7.9	1.70	0.68	tr_C9ZWY7_	WB
DRB1_0101	34	ARSYLRYHPGVTAFN	LRYHPGVTA	4	0.8014	8.6	2.00	0.76	tr_C9ZWY7_	WB
DRB1_0101	35	HIVRPLAAKKSIKVK	VRPLAAKKS	2	0.8010	8.6	2.00	0.60	tr_C9ZWY7_	WB
DRB1_0101	36	ALLGYATARAMESVV	YATARAMES	4	0.7981	8.9	2.50	0.50	tr_C9ZWY7_	WB
DRB1_0101	37	AMQFVIGATWRTAVE	FVIGATWRT	3	0.7980	8.9	2.50	0.87	tr_C9ZWY7_	WB
DRB1_0101	38	LLGYATARAMESVVS	YATARAMES	3	0.7932	9.4	2.50	0.57	tr_C9ZWY7_	WB
DRB1_0101	39	SAMQFVIGATWRTAV	FVIGATWRT	4	0.7924	9.5	2.50	0.87	tr_C9ZWY7_	WB
DRB1_0101	40	LALLGYATARAMESV	LGYATARAM	3	0.7888	9.8	2.50	0.47	tr_C9ZWY7_	WB
DRB1_0101	41	LRYAINRLRLLRVGF	YAINRLRLL	2	0.7887	9.8	2.50	0.39	tr_C9ZWY7_	WB
DRB1_0101	42	VLPHIVRPLAAKKSI	VRPLAAKKS	5	0.7861	10.1	3.00	0.44	tr_C9ZWY7_	WB
DRB1_0101	43	FTKMHAATVMRADEE	FTKMHAATV	0	0.7845	10.3	3.00	0.70	tr_C9ZWY7_	WB
DRB1_0101	44	LIALLRYAINRLRLL	LRYAINRLR	4	0.7842	10.3	3.00	0.57	tr_C9ZWY7_	WB
DRB1_0101	45	EEFVMTHPQGVIVFT	FVMTHPQGV	2	0.7831	10.5	3.00	0.79	tr_C9ZWY7_	WB
DRB1_0101	46	FAPLTGSSAVRGWDP	FAPLTGSSA	0	0.7827	10.5	3.00	0.70	tr_C9ZWY7_	WB
DRB1_0101	47	VYVLAPSAMQFVIGA	YVLAPSAMQ	1	0.7819	10.6	3.00	0.79	tr_C9ZWY7_	WB
DRB1_0101	48	YLRYHPGVTAFNASD	LRYHPGVTA	1	0.7814	10.6	3.00	0.65	tr_C9ZWY7_	WB
DRB1_0101	49	MQFVIGATWRTAVEE	FVIGATWRT	2	0.7811	10.7	3.00	0.87	tr_C9ZWY7_	WB
DRB1_0101	50	WEEFVMTHPQGVIVF	FVMTHPQGV	3	0.7803	10.8	3.00	0.82	tr_C9ZWY7_	WB
DRB1_0101	51	RRLCGVFTKMHAATV	FTKMHAATV	6	0.7787	11.0	3.00	0.71	tr_C9ZWY7_	WB
DRB1_0101	52	YNLLFTNRAPRRSIE	YNLLFTNRA	0	0.7775	11.1	3.50	0.76	tr_C9ZWY7_	WB
DRB1_0101	53	DRVIVAAPNPLASET	IVAAPNPLA	3	0.7692	12.1	3.50	0.75	tr_C9ZWY7_	WB
DRB1_0101	54	LGYATARAMESVVSR	YATARAMES	2	0.7666	12.5	4.00	0.63	tr_C9ZWY7_	WB
DRB1_0101	55	PLALLGYATARAMES	LGYATARAM	4	0.7661	12.6	4.00	0.47	tr_C9ZWY7_	WB
DRB1_0101	56	TPLALLGYATARAME	LGYATARAM	5	0.7634	12.9	4.00	0.32	tr_C9ZWY7_	WB
DRB1_0101	57	DARSYLRYHPGVTAF	LRYHPGVTA	5	0.7618	13.2	4.00	0.77	tr_C9ZWY7_	WB
DRB1_0101	58	RVIVAAPNPLASETK	IVAAPNPLA	2	0.7615	13.2	4.00	0.79	tr_C9ZWY7_	WB
DRB1_0101	59	RYAINRLRLLRVGFM	INRLRLLRV	3	0.7584	13.7	4.50	0.44	tr_C9ZWY7_	WB
DRB1_0101	60	ARTRGVYVLAPSAMQ	YVLAPSAMQ	6	0.7578	13.7	4.50	0.58	tr_C9ZWY7_	WB
DRB1_0101	61	PSAMQFVIGATWRTA	FVIGATWRT	5	0.7572	13.8	4.50	0.81	tr_C9ZWY7_	WB
DRB1_0101	62	PDRVIVAAPNPLASE	IVAAPNPLA	4	0.7556	14.1	4.50	0.73	tr_C9ZWY7_	WB
DRB1_0101	63	IVRPLAAKKSIKVKV	VRPLAAKKS	1	0.7555	14.1	4.50	0.57	tr_C9ZWY7_	WB
DRB1_0101	64	EFVMTHPQGVIVFTP	FVMTHPQGV	1	0.7542	14.3	4.50	0.76	tr_C9ZWY7_	WB
DRB1_0101	65	LWEEFVMTHPQGVIV	FVMTHPQGV	4	0.7528	14.5	4.50	0.86	tr_C9ZWY7_	WB
DRB1_0101	66	QEDLVAFAPLTGSSA	FAPLTGSSA	6	0.7459	15.6	5.00	0.79	tr_C9ZWY7_	WB
DRB1_0101	67	LVLPHIVRPLAAKKS	IVRPLAAKK	5	0.7453	15.7	5.00	0.28	tr_C9ZWY7_	WB
DRB1_0101	68	WTPLALLGYATARAM	PLALLGYAT	2	0.7437	16.0	5.50	0.44	tr_C9ZWY7_	WB
DRB1_0101	69	QFVIGATWRTAVEEA	FVIGATWRT	1	0.7405	16.6	5.50	0.88	tr_C9ZWY7_	WB
DRB1_0101	70	RGWDPHFYFLTPAPN	FYFLTPAPN	6	0.7398	16.7	5.50	0.85	tr_C9ZWY7_	WB
DRB1_0101	71	IPDRVIVAAPNPLAS	IVAAPNPLA	5	0.7344	17.7	6.00	0.67	tr_C9ZWY7_	WB
DRB1_0101	72	ALKFVRRVAMDARTR	FVRRVAMDA	3	0.7325	18.1	6.00	0.75	tr_C9ZWY7_	WB
DRB1_0101	73	EELINGIFSQSVIVA	INGIFSQSV	3	0.7283	18.9	6.50	0.68	tr_C9ZWY7_	WB
DRB1_0101	74	ELIALLRYAINRLRL	LRYAINRLR	5	0.7264	19.3	6.50	0.59	tr_C9ZWY7_	WB
DRB1_0101	75	ETMGVGLNASFAARH	GVGLNASFA	3	0.7262	19.3	6.50	0.69	tr_C9ZWY7_	WB
DRB1_0101	76	GYATARAMESVVSRM	YATARAMES	1	0.7235	19.9	7.00	0.57	tr_C9ZWY7_	WB
DRB1_0101	77	TMGVGLNASFAARHW	GVGLNASFA	2	0.7225	20.1	7.00	0.68	tr_C9ZWY7_	WB
DRB1_0101	78	ELINGIFSQSVIVAD	INGIFSQSV	2	0.7207	20.5	7.00	0.65	tr_C9ZWY7_	WB
DRB1_0101	79	KWTPLALLGYATARA	PLALLGYAT	3	0.7181	21.1	7.50	0.58	tr_C9ZWY7_	WB
DRB1_0101	80	EALKFVRRVAMDART	FVRRVAMDA	4	0.7159	21.6	7.50	0.69	tr_C9ZWY7_	WB
DRB1_0101	81	KGEFFVVVGPMGDTR	FFVVVGPMG	3	0.7147	21.9	7.50	0.62	tr_C9ZWY7_	WB
DRB1_0101	82	IETMGVGLNASFAAR	GVGLNASFA	4	0.7129	22.3	8.00	0.64	tr_C9ZWY7_	WB

DRB1_0101	83	IGEVLSQALSSRTWL	LSQALSSRT	4	0.7087	23.4	8.00	0.61	tr_C9ZWY7_	WB
DRB1_0101	84	AERLLFATSLPHWAD	LLFATSLPH	3	0.7086	23.4	8.00	0.49	tr_C9ZWY7_	WB
DRB1_0101	85	REALKFVRRVAMDAR	FVRRVAMDA	5	0.7084	23.5	8.00	0.56	tr_C9ZWY7_	WB
DRB1_0101	86	REAFIKSLYNQRRYV	FIKSLYNQR	3	0.7069	23.8	8.50	0.40	tr_C9ZWY7_	WB
DRB1_0101	87	GEVLSQALSSRTWLE	LSQALSSRT	3	0.7067	23.9	8.50	0.62	tr_C9ZWY7_	WB
DRB1_0101	88	QKTIESLGGSMNGIV	IESLGGSMN	3	0.7054	24.2	8.50	0.74	tr_C9ZWY7_	WB
DRB1_0101	89	MGVGLNASFAARHWA	GVGLNASFA	1	0.7044	24.5	8.50	0.62	tr_C9ZWY7_	WB
DRB1_0101	90	LKFVRRVAMDARTRG	FVRRVAMDA	2	0.7044	24.5	8.50	0.76	tr_C9ZWY7_	WB
DRB1_0101	91	LRYHPGVTAFNASDD	LRYHPGVTA	0	0.7032	24.8	8.50	0.55	tr_C9ZWY7_	WB
DRB1_0101	92	SLWEEFVMTHPQGVI	FVMTHPQGV	5	0.7026	25.0	9.00	0.91	tr_C9ZWY7_	WB
DRB1_0101	93	AAERLLFATSLPHWA	LLFATSLPH	4	0.7026	25.0	9.00	0.50	tr_C9ZWY7_	WB
DRB1_0101	94	EAFIKSLYNQRRYVV	IKSLYNQRR	3	0.7007	25.5	9.00	0.45	tr_C9ZWY7_	WB
DRB1_0101	95	SIETMGVGLNASFAA	GVGLNASFA	5	0.6990	26.0	9.00	0.51	tr_C9ZWY7_	WB
DRB1_0101	96	YAINRLRLLRVGFMY	INRLRLLRV	2	0.6989	26.0	9.00	0.43	tr_C9ZWY7_	WB
DRB1_0101	97	GEFFVVVGPMGDTRT	FFVVVGPMG	2	0.6986	26.1	9.00	0.54	tr_C9ZWY7_	WB
DRB1_0101	98	YVLAPSAMQFVIGAT	YVLAPSAMQ	0	0.6978	26.3	9.00	0.66	tr_C9ZWY7_	WB
DRB1_0101	99	YFLTPAPNAELIALL	YFLTPAPNA	0	0.6975	26.4	9.50	0.54	tr_C9ZWY7_	WB
DRB1_0101	100	LINGIFSQSVIVADD	INGIFSQSV	1	0.6964	26.7	9.50	0.60	tr_C9ZWY7_	WB
DRB1_0101	101	VIVAAPNPLASETKY	IVAAPNPLA	1	0.6963	26.7	9.50	0.82	tr_C9ZWY7_	WB
DRB1_0101	102	SKWTPLALLGYATAR	PLALLGYAT	4	0.6962	26.8	9.50	0.54	tr_C9ZWY7_	WB
DRB1_0101	103	RVAFHSVVATSRNTY	FHSVVATSR	3	0.6953	27.0	9.50	0.82	tr_C9ZWY7_	WB
DRB1_0101	104	SEELINGIFSQSVIV	INGIFSQSV	4	0.6933	27.6	9.50	0.69	tr_C9ZWY7_	WB
DRB1_0101	105	FQKTIESLGGSMNGI	IESLGGSMN	4	0.6933	27.6	9.50	0.67	tr_C9ZWY7_	WB
DRB1_0101	106	FFDVALLYSEFVKVF	VALLYSEFV	3	0.6919	28.0	10.00	0.58	tr_C9ZWY7_	
DRB1_0101	107	RSIETMGVGLNASFA	ETMGVGLNA	3	0.6910	28.3	10.00	0.28	tr_C9ZWY7_	
DRB1_0101	108	ERLLFATSLPHWADN	LLFATSLPH	2	0.6904	28.5	10.00	0.46	tr_C9ZWY7_	
DRB1_0101	109	VRPLAAKKSIKVKVY	LAAKKSIKV	3	0.6898	28.7	10.00	0.51	tr_C9ZWY7_	
DRB1_0101	110	LVVGITNADAIRSIT	ITNADAIRS	4	0.6882	29.2	10.00	0.72	tr_C9ZWY7_	
DRB1_0101	111	LLVLPHIVRPLAAKK	PHIVRPLAA	4	0.6882	29.2	10.00	0.29	tr_C9ZWY7_	
DRB1_0101	112	FDVALLYSEFVKVFK	VALLYSEFV	2	0.6879	29.3	11.00	0.50	tr_C9ZWY7_	
DRB1_0101	113	SREAFIKSLYNQRRY	FIKSLYNQR	4	0.6874	29.4	11.00	0.38	tr_C9ZWY7_	
DRB1_0101	114	RVGFMYLQGVHFGDR	FMYLQGVHF	3	0.6858	29.9	11.00	0.75	tr_C9ZWY7_	
DRB1_0101	115	VVDLAMLSVGRVAFI	LAMLSVGRV	3	0.6855	30.0	11.00	0.63	tr_C9ZWY7_	
DRB1_0101	116	VVGITNADAIRSITN	ITNADAIRS	3	0.6852	30.1	11.00	0.83	tr_C9ZWY7_	
DRB1_0101	117	KTIESLGGSMNGIVV	IESLGGSMN	2	0.6836	30.7	11.00	0.71	tr_C9ZWY7_	
DRB1_0101	118	DVALLYSEFVKVFKK	LLYSEFVKV	3	0.6826	31.0	11.00	0.47	tr_C9ZWY7_	
DRB1_0101	119	VDLAMLSVGRVAFIE	LAMLSVGRV	2	0.6824	31.1	11.00	0.58	tr_C9ZWY7_	
DRB1_0101	120	AELIALLRYAINRLR	LRYAINRLR	6	0.6824	31.1	11.00	0.38	tr_C9ZWY7_	
DRB1_0101	121	EVLSQALSSRTWLES	LSQALSSRT	2	0.6812	31.5	11.00	0.62	tr_C9ZWY7_	
DRB1_0101	122	EFFVVVGPMGDTRTL	FFVVVGPMG	1	0.6807	31.6	11.00	0.50	tr_C9ZWY7_	
DRB1_0101	123	NKGEFFVVVGPMGDT	FFVVVGPMG	4	0.6780	32.6	12.00	0.63	tr_C9ZWY7_	
DRB1_0101	124	DRVAFHSVVATSRNT	FHSVVATSR	4	0.6770	32.9	12.00	0.80	tr_C9ZWY7_	
DRB1_0101	125	LLRVGFMYLQGVHFG	FMYLQGVHF	5	0.6732	34.3	12.00	0.72	tr_C9ZWY7_	
DRB1_0101	126	SAFFSLVVGITNADA	FSLVVGITN	3	0.6712	35.1	12.00	0.44	tr_C9ZWY7_	
DRB1_0101	127	LRVGFMYLQGVHFGD	FMYLQGVHF	4	0.6710	35.2	12.00	0.73	tr_C9ZWY7_	
DRB1_0101	128	AFIKSLYNQRRYVVD	IKSLYNQRR	2	0.6706	35.3	12.00	0.48	tr_C9ZWY7_	
DRB1_0101	129	TLKLMTRMGRRLCGV	LMTRMGRRL	3	0.6697	35.7	13.00	0.76	tr_C9ZWY7_	
DRB1_0101	130	FGVVDLAMLSVGRVA	LAMLSVGRV	5	0.6680	36.3	13.00	0.48	tr_C9ZWY7_	
DRB1_0101	131	SLVVGITNADAIRSI	ITNADAIRS	5	0.6656	37.3	13.00	0.62	tr_C9ZWY7_	
DRB1_0101	132	SFFDVALLYSEFVKV	VALLYSEFV	4	0.6654	37.4	13.00	0.62	tr_C9ZWY7_	

DRB1_0101	133	VIGEVLSQALSSRTW	LSQALSSRT	5	0.6629	38.4	13.00	0.51	tr_C9ZWY7_
DRB1_0101	134	FVMTHPQGVIVFTPP	FVMTHPQGV	0	0.6625	38.5	13.00	0.63	tr_C9ZWY7_
DRB1_0101	135	ESKWTPLALLGYATA	PLALLGYAT	5	0.6610	39.2	14.00	0.51	tr_C9ZWY7_
DRB1_0101	136	YATARAMESVVSRMG	ARAMESVVS	3	0.6607	39.3	14.00	0.46	tr_C9ZWY7_
DRB1_0101	137	AVSAFFSLVVGITNA	FFSLVVGIT	4	0.6606	39.3	14.00	0.45	tr_C9ZWY7_
DRB1_0101	138	KLMTRMGRRLCGVFT	LMTRMGRRL	1	0.6600	39.6	14.00	0.45	tr_C9ZWY7_
DRB1_0101	139	QAAERLLFATSLPHW	LLFATSLPH	5	0.6592	39.9	14.00	0.47	tr_C9ZWY7_
DRB1_0101	140	VSAFFSLVVGITNAD	FFSLVVGIT	3	0.6592	39.9	14.00	0.44	tr_C9ZWY7_
DRB1_0101	141	EDARSYLRYHPGVTA	LRYHPGVTA	6	0.6586	40.2	14.00	0.73	tr_C9ZWY7_
DRB1_0101	142	YIPDRVIVAAPNPLA	IVAAPNPLA	6	0.6584	40.3	14.00	0.49	tr_C9ZWY7_
DRB1_0101	143	PDRVAFHSVVATSRN	FHSVVATSR	5	0.6584	40.3	14.00	0.75	tr_C9ZWY7_
DRB1_0101	144	KFVRRVAMDARTRGV	FVRRVAMDA	1	0.6572	40.8	14.00	0.75	tr_C9ZWY7_
DRB1_0101	145	RRSIETMGVGLNASF	IETMGVGLN	3	0.6564	41.2	14.00	0.40	tr_C9ZWY7_
DRB1_0101	146	TIGVKVPTPLIVVVV	GVKVPTPLI	2	0.6555	41.6	14.00	0.34	tr_C9ZWY7_
DRB1_0101	147	RVAMDARTRGVYVLA	DARTRGVYV	4	0.6551	41.7	14.00	0.75	tr_C9ZWY7_
DRB1_0101	148	VFQKTIESLGGSMNG	IESLGGSMN	5	0.6549	41.8	14.00	0.60	tr_C9ZWY7_
DRB1_0101	149	LLLVLPHIVRPLAAK	VLPHIVRPL	3	0.6548	41.9	14.00	0.41	tr_C9ZWY7_
DRB1_0101	150	DLAMLSVGRVAFIEP	LAMLSVGRV	1	0.6539	42.3	15.00	0.57	tr_C9ZWY7_
DRB1_0101	151	AFFSLVVGITNADAI	FSLVVGITN	2	0.6539	42.3	15.00	0.40	tr_C9ZWY7_
DRB1_0101	152	ETLKLMTRMGRRLCG	LMTRMGRRL	4	0.6526	42.9	15.00	0.73	tr_C9ZWY7_
DRB1_0101	153	GVVDLAMLSVGRVAF	LAMLSVGRV	4	0.6519	43.2	15.00	0.68	tr_C9ZWY7_
DRB1_0101	154	VGFMYLQGVHFGDRE	FMYLQGVHF	2	0.6518	43.3	15.00	0.73	tr_C9ZWY7_
DRB1_0101	155	QGVIVFTPPIREALK	IVFTPPIRE	3	0.6512	43.6	15.00	0.64	tr_C9ZWY7_
DRB1_0101	156	RLLFATSLPHWADNN	LLFATSLPH	1	0.6511	43.6	15.00	0.49	tr_C9ZWY7_
DRB1_0101	157	APSAMQFVIGATWRT	FVIGATWRT	6	0.6508	43.8	15.00	0.68	tr_C9ZWY7_
DRB1_0101	158	VAFHSVVATSRNTYG	FHSVVATSR	2	0.6501	44.1	15.00	0.80	tr_C9ZWY7_
DRB1_0101	159	NSEELINGIFSQSVI	INGIFSQSV	5	0.6499	44.2	15.00	0.65	tr_C9ZWY7_
DRB1_0101	160	LMTRMGRRLCGVFTK	GRRLCGVFT	5	0.6499	44.2	15.00	0.57	tr_C9ZWY7_
DRB1_0101	161	AINRLRLLRVGFMYL	INRLRLLRV	1	0.6496	44.3	15.00	0.45	tr_C9ZWY7_
DRB1_0101	162	IREALKFVRRVAMDA	FVRRVAMDA	6	0.6496	44.3	15.00	0.42	tr_C9ZWY7_
DRB1_0101	163	ASCLCNQGGNVVYMN	LCNQGGNVV	3	0.6476	45.3	15.00	0.57	tr_C9ZWY7_
DRB1_0101	164	RLLRVGFMYLQGVHF	FMYLQGVHF	6	0.6439	47.2	16.00	0.68	tr_C9ZWY7_
DRB1_0101	165	IGVKVPTPLIVVVVL	GVKVPTPLI	1	0.6417	48.3	16.00	0.31	tr_C9ZWY7_
DRB1_0101	166	LKLMTRMGRRLCGVF	LMTRMGRRL	2	0.6411	48.6	16.00	0.72	tr_C9ZWY7_
DRB1_0101	167	GVGLNASFAARHWAT	GVGLNASFA	0	0.6405	48.9	16.00	0.51	tr_C9ZWY7_
DRB1_0101	168	VALLYSEFVKVFKKH	LLYSEFVKV	2	0.6404	48.9	16.00	0.55	tr_C9ZWY7_
DRB1_0101	169	VGITNADAIRSITNP	ITNADAIRS	2	0.6396	49.4	16.00	0.91	tr_C9ZWY7_
DRB1_0101	170	QNKGEFFVVVGPMGD	FFVVVGPMG	5	0.6389	49.8	17.00	0.66	tr_C9ZWY7_
DRB1_0101	171	VFGVVDLAMLSVGRV	LAMLSVGRV	6	0.6381	50.2	17.00	0.32	tr_C9ZWY7_
DRB1_0101	172	DARTRGVYVLAPSAM	GVYVLAPSA	5	0.6364	51.1	17.00	0.44	tr_C9ZWY7_
DRB1_0101	173	PRRSIETMGVGLNAS	IETMGVGLN	4	0.6364	51.1	17.00	0.36	tr_C9ZWY7_
DRB1_0101	174	ESREAFIKSLYNQRR	FIKSLYNQR	5	0.6360	51.4	17.00	0.37	tr_C9ZWY7_
DRB1_0101	175	QETLKLMTRMGRRLC	LMTRMGRRL	5	0.6349	52.0	17.00	0.65	tr_C9ZWY7_
DRB1_0101	176	FSLVVGITNADAIRS	ITNADAIRS	6	0.6323	53.5	18.00	0.29	tr_C9ZWY7_
DRB1_0101	177	STIGVKVPTPLIVVV	GVKVPTPLI	3	0.6320	53.6	18.00	0.40	tr_C9ZWY7_
DRB1_0101	178	ADGALMVSGWVIGEV	ALMVSGWVI	3	0.6319	53.6	18.00	0.46	tr_C9ZWY7_
DRB1_0101	179	GASCLCNQGGNVVYM	LCNQGGNVV	4	0.6316	53.8	18.00	0.54	tr_C9ZWY7_
DRB1_0101	180	SRTWLESREAFIKSL	WLESREAFI	3	0.6312	54.1	18.00	0.71	tr_C9ZWY7_
DRB1_0101	181	FFSLVVGITNADAIR	FSLVVGITN	1	0.6312	54.1	18.00	0.38	tr_C9ZWY7_
DRB1_0101	182	MGRRLCGVFTKMHAA	GRRLCGVFT	1	0.6303	54.6	18.00	0.55	tr_C9ZWY7_

DRB1_0101	183	AAVSAFFSLVVGITN	FFSLVVGIT	5	0.6303	54.6	18.00	0.45	tr_C9ZWY7_
DRB1_0101	184	RRVAMDARTRGVYVL	DARTRGVYV	5	0.6294	55.2	18.00	0.65	tr_C9ZWY7_
DRB1_0101	185	TRMGRRLCGVFTKMH	GRRLCGVFT	3	0.6284	55.8	18.00	0.77	tr_C9ZWY7_
DRB1_0101	186	INGIFSQSVIVADDM	INGIFSQSV	0	0.6283	55.8	18.00	0.40	tr_C9ZWY7_
DRB1_0101	187	GVIVFTPPIREALKF	IVFTPPIRE	2	0.6273	56.4	18.00	0.62	tr_C9ZWY7_
DRB1_0101	188	VLSQALSSRTWLESR	LSQALSSRT	1	0.6267	56.8	18.00	0.63	tr_C9ZWY7_
DRB1_0101	189	TIESLGGSMNGIVVP	IESLGGSMN	1	0.6263	57.0	18.00	0.65	tr_C9ZWY7_
DRB1_0101	190	VAMDARTRGVYVLAP	DARTRGVYV	3	0.6261	57.2	18.00	0.81	tr_C9ZWY7_
DRB1_0101	191	FFVVVGPMGDTRTLA	FVVVGPMGD	1	0.6258	57.3	18.00	0.39	tr_C9ZWY7_
DRB1_0101	192	PLLLVLPHIVRPLAA	VLPHIVRPL	4	0.6258	57.4	18.00	0.38	tr_C9ZWY7_
DRB1_0101	193	SCLCNQGGNVVYMNS	LCNQGGNVV	2	0.6246	58.1	19.00	0.56	tr_C9ZWY7_
DRB1_0101	194	VSFFDVALLYSEFVK	VALLYSEFV	5	0.6222	59.6	19.00	0.61	tr_C9ZWY7_
DRB1_0101	195	DGALMVSGWVIGEVL	ALMVSGWVI	2	0.6217	60.0	19.00	0.41	tr_C9ZWY7_
DRB1_0101	196	GGFAGVITGRHITTY	AGVITGRHI	3	0.6215	60.1	19.00	0.52	tr_C9ZWY7_
DRB1_0101	197	GFMYLQGVHFGDREY	FMYLQGVHF	1	0.6208	60.5	19.00	0.71	tr_C9ZWY7_
DRB1_0101	198	GRRLCGVFTKMHAAT	GVFTKMHAA	5	0.6207	60.6	19.00	0.30	tr_C9ZWY7_
DRB1_0101	199	PQGVIVFTPPIREAL	IVFTPPIRE	4	0.6200	61.0	19.00	0.61	tr_C9ZWY7_
DRB1_0101	200	AFHSVVATSRNTYGR	FHSVVATSR	1	0.6193	61.5	19.00	0.74	tr_C9ZWY7_
DRB1_0101	201	DADGALMVSGWVIGE	ALMVSGWVI	4	0.6187	61.9	20.00	0.46	tr_C9ZWY7_
DRB1_0101	202	MDARTRGVYVLAPSA	DARTRGVYV	1	0.6184	62.1	20.00	0.56	tr_C9ZWY7_
DRB1_0101	203	MTRMGRRLCGVFTKM	GRRLCGVFT	4	0.6159	63.8	20.00	0.77	tr_C9ZWY7_
DRB1_0101	204	RMGRRLCGVFTKMHA	GRRLCGVFT	2	0.6150	64.5	20.00	0.75	tr_C9ZWY7_
DRB1_0101	205	ITFFLIPLLLVLPHI	FFLIPLLLV	2	0.6148	64.5	20.00	0.63	tr_C9ZWY7_
DRB1_0101	206	FVIGATWRTAVEEAA	FVIGATWRT	0	0.6146	64.7	20.00	0.70	tr_C9ZWY7_
DRB1_0101	207	PPAITFFLIPLLLVL	FFLIPLLLV	5	0.6136	65.4	20.00	0.68	tr_C9ZWY7_
DRB1_0101	208	GFAGVITGRHITTYS	AGVITGRHI	2	0.6129	65.9	20.00	0.54	tr_C9ZWY7_
DRB1_0101	209	FGGFAGVITGRHITT	AGVITGRHI	4	0.6125	66.2	20.00	0.44	tr_C9ZWY7_
DRB1_0101	210	INRLRLLRVGFMYLQ	LRLLRVGFM	3	0.6124	66.2	20.00	0.52	tr_C9ZWY7_
DRB1_0101	211	TAVFGVVDLAMLSVG	FGVVDLAML	3	0.6101	67.9	21.00	0.65	tr_C9ZWY7_
DRB1_0101	212	PQAAERLLFATSLPH	ERLLFATSL	4	0.6088	68.9	21.00	0.51	tr_C9ZWY7_
DRB1_0101	213	TFFLIPLLLVLPHIV	FFLIPLLLV	1	0.6086	69.0	21.00	0.56	tr_C9ZWY7_
DRB1_0101	214	SSRTWLESREAFIKS	WLESREAFI	4	0.6067	70.5	21.00	0.73	tr_C9ZWY7_
DRB1_0101	215	RTWLESREAFIKSLY	WLESREAFI	2	0.6066	70.5	21.00	0.68	tr_C9ZWY7_
DRB1_0101	216	AITFFLIPLLLVLPH	FFLIPLLLV	3	0.6061	70.9	21.00	0.65	tr_C9ZWY7_
DRB1_0101	217	VIVFTPPIREALKFV	IVFTPPIRE	1	0.6057	71.2	22.00	0.55	tr_C9ZWY7_
DRB1_0101	218	AVFGVVDLAMLSVGR	FGVVDLAML	2	0.6047	72.0	22.00	0.57	tr_C9ZWY7_
DRB1_0101	219	PAITFFLIPLLLVLP	FFLIPLLLV	4	0.6039	72.6	22.00	0.67	tr_C9ZWY7_
DRB1_0101	220	DDADGALMVSGWVIG	ALMVSGWVI	5	0.6019	74.2	22.00	0.44	tr_C9ZWY7_
DRB1_0101	221	GTMRRRPPAITFFLI	MRRRPPAIT	2	0.6012	74.8	22.00	0.57	tr_C9ZWY7_
DRB1_0101	222	FIKSLYNQRRYVVDD	IKSLYNQRR	1	0.6011	74.9	22.00	0.50	tr_C9ZWY7_
DRB1_0101	223	CSTIGVKVPTPLIVV	GVKVPTPLI	4	0.6010	75.0	22.00	0.39	tr_C9ZWY7_
DRB1_0101	224	AMDARTRGVYVLAPS	DARTRGVYV	2	0.6004	75.5	22.00	0.83	tr_C9ZWY7_
DRB1_0101	225	NPDRVAFHSVVATSR	FHSVVATSR	6	0.6002	75.6	22.00	0.57	tr_C9ZWY7_
DRB1_0101	226	VTAVFGVVDLAMLSV	FGVVDLAML	4	0.5992	76.4	23.00	0.67	tr_C9ZWY7_
DRB1_0101	227	LAMLSVGRVAFIEPI	LAMLSVGRV	0	0.5989	76.7	23.00	0.45	tr_C9ZWY7_
DRB1_0101	228	GFGGFAGVITGRHIT	AGVITGRHI	5	0.5969	78.4	23.00	0.38	tr_C9ZWY7_
DRB1_0101	229	VSTLKLLETAADQNK	LKLLETAAD	3	0.5957	79.4	23.00	0.74	tr_C9ZWY7_
DRB1_0101	230	GVKVPTPLIVVVVLV	PTPLIVVVV	4	0.5949	80.1	23.00	0.34	tr_C9ZWY7_
DRB1_0101	231	RPPAITFFLIPLLLV	FFLIPLLLV	6	0.5945	80.4	23.00	0.68	tr_C9ZWY7_
DRB1_0101	232	RPLAAKKSIKVKVYN	LAAKKSIKV	2	0.5942	80.7	23.00	0.86	tr_C9ZWY7_

DRB1_0101	233	FFLIPLLLVLPHIVR	FFLIPLLLV	0	0.5936	81.2	24.00	0.40	tr_C9ZWY7_
DRB1_0101	234	WVIGEVLSQALSSRT	LSQALSSRT	6	0.5933	81.5	24.00	0.27	tr_C9ZWY7_
DRB1_0101	235	GGTMRRRPPAITFFL	MRRRPPAIT	3	0.5926	82.1	24.00	0.68	tr_C9ZWY7_
DRB1_0101	236	YQETLKLMTRMGRRL	LMTRMGRRL	6	0.5924	82.3	24.00	0.51	tr_C9ZWY7_
DRB1_0101	237	SVFQKTIESLGGSMN	IESLGGSMN	6	0.5919	82.7	24.00	0.37	tr_C9ZWY7_
DRB1_0101	238	QQLFLLVGYIARDKK	FLLVGYIAR	3	0.5910	83.5	24.00	0.44	tr_C9ZWY7_
DRB1_0101	239	GDTRTLAVLPLLRQE	LAVLPLLRQ	5	0.5895	84.9	24.00	0.44	tr_C9ZWY7_
DRB1_0101	240	GALMVSGWVIGEVLS	ALMVSGWVI	1	0.5888	85.6	24.00	0.41	tr_C9ZWY7_
DRB1_0101	241	RNVIHFSPTIEQQLF	IHFSPTIEQ	3	0.5873	87.0	25.00	0.44	tr_C9ZWY7_
DRB1_0101	242	ATARAMESVVSRMGR	ARAMESVVS	2	0.5871	87.1	25.00	0.62	tr_C9ZWY7_
DRB1_0101	243	RSVSTLKLLETAADQ	LKLLETAAD	5	0.5865	87.7	25.00	0.45	tr_C9ZWY7_
DRB1_0101	244	NAELIALLRYAINRL	IALLRYAIN	4	0.5857	88.5	25.00	0.55	tr_C9ZWY7_
DRB1_0101	245	DSLWEEFVMTHPQGV	FVMTHPQGV	6	0.5856	88.5	25.00	0.84	tr_C9ZWY7_
DRB1_0101	246	SVSTLKLLETAADQN	LKLLETAAD	4	0.5846	89.5	25.00	0.58	tr_C9ZWY7_
DRB1_0101	247	FAGVITGRHITTYST	AGVITGRHI	1	0.5839	90.2	25.00	0.54	tr_C9ZWY7_
DRB1_0101	248	APRRSIETMGVGLNA	IETMGVGLN	5	0.5822	91.9	25.00	0.38	tr_C9ZWY7_
DRB1_0101	249	AVPYIPDRVIVAAPN	YIPDRVIVA	3	0.5821	91.9	25.00	0.77	tr_C9ZWY7_
DRB1_0101	250	SAAVSAFFSLVVGIT	FFSLVVGIT	6	0.5808	93.3	26.00	0.38	tr_C9ZWY7_
DRB1_0101	251	RVAFIEPITLSPKLS	VAFIEPITL	1	0.5801	94.0	26.00	0.33	tr_C9ZWY7_
DRB1_0101	252	IPLLLVLPHIVRPLA	VLPHIVRPL	5	0.5795	94.6	26.00	0.33	tr_C9ZWY7_
DRB1_0101	253	GITNADAIRSITNPD	ITNADAIRS	1	0.5788	95.3	26.00	0.92	tr_C9ZWY7_
DRB1_0101	254	MGDTRTLAVLPLLRQ	LAVLPLLRQ	6	0.5780	96.2	26.00	0.38	tr_C9ZWY7_
DRB1_0101	255	LSSRTWLESREAFIK	WLESREAFI	5	0.5779	96.3	26.00	0.70	tr_C9ZWY7_
DRB1_0101	256	DTRTLAVLPLLRQED	LAVLPLLRQ	4	0.5765	97.8	26.00	0.47	tr_C9ZWY7_
DRB1_0101	257	RGASCLCNQGGNVVY	LCNQGGNVV	5	0.5738	100.6	27.00	0.53	tr_C9ZWY7_
DRB1_0101	258	QLFLLVGYIARDKKT	FLLVGYIAR	2	0.5734	101.1	27.00	0.41	tr_C9ZWY7_
DRB1_0101	259	CLCNQGGNVVYMNSL	LCNQGGNVV	1	0.5732	101.3	27.00	0.51	tr_C9ZWY7_
DRB1_0101	260	EAAVPYIPDRVIVAA	YIPDRVIVA	5	0.5724	102.2	27.00	0.75	tr_C9ZWY7_
DRB1_0101	261	TARAMESVVSRMGRV	ARAMESVVS	1	0.5716	103.0	27.00	0.50	tr_C9ZWY7_
DRB1_0101	262	HPQGVIVFTPPIREA	IVFTPPIRE	5	0.5711	103.6	27.00	0.60	tr_C9ZWY7_
DRB1_0101	263	STLKLLETAADQNKG	LKLLETAAD	2	0.5707	104.1	27.00	0.76	tr_C9ZWY7_
DRB1_0101	264	DESKWTPLALLGYAT	PLALLGYAT	6	0.5704	104.3	28.00	0.49	tr_C9ZWY7_
DRB1_0101	265	ISVWSMARVLNPSVP	MARVLNPSV	5	0.5695	105.4	28.00	0.46	tr_C9ZWY7_
DRB1_0101	266	SVTAVFGVVDLAMLS	FGVVDLAML	5	0.5690	106.0	28.00	0.64	tr_C9ZWY7_
DRB1_0101	267	AAVPYIPDRVIVAAP	YIPDRVIVA	4	0.5688	106.2	28.00	0.76	tr_C9ZWY7_
DRB1_0101	268	VNSEELINGIFSQSV	INGIFSQSV	6	0.5683	106.8	28.00	0.54	tr_C9ZWY7_
DRB1_0101	269	HLDNHRGVRVFVSFF	LDNHRGVRV	1	0.5682	106.9	28.00	0.41	tr_C9ZWY7_
DRB1_0101	270	TMRRRPPAITFFLIP	MRRRPPAIT	1	0.5676	107.6	28.00	0.54	tr_C9ZWY7_
DRB1_0101	271	FVSFFDVALLYSEFV	VALLYSEFV	6	0.5670	108.3	28.00	0.44	tr_C9ZWY7_
DRB1_0101	272	NVIHFSPTIEQQLFL	IHFSPTIEQ	2	0.5665	108.9	28.00	0.42	tr_C9ZWY7_
DRB1_0101	273	VKVFKKHPQAAERLL	FKKHPQAAE	3	0.5659	109.6	28.00	0.41	tr_C9ZWY7_
DRB1_0101	274	GRVAFIEPITLSPKL	VAFIEPITL	2	0.5658	109.8	28.00	0.47	tr_C9ZWY7_
DRB1_0101	275	GRNVIHFSPTIEQQL	IHFSPTIEQ	4	0.5656	110.0	28.00	0.44	tr_C9ZWY7_
DRB1_0101	276	SMARVLNPSVPPVTK	MARVLNPSV	1	0.5654	110.3	28.00	0.43	tr_C9ZWY7_
DRB1_0101	277	PNAELIALLRYAINR	IALLRYAIN	5	0.5653	110.3	28.00	0.52	tr_C9ZWY7_
DRB1_0101	278	TRTLAVLPLLRQEDL	LAVLPLLRQ	3	0.5653	110.3	28.00	0.55	tr_C9ZWY7_
DRB1_0101	279	GWVIGEVLSQALSSR	IGEVLSQAL	3	0.5644	111.4	29.00	0.24	tr_C9ZWY7_
DRB1_0101	280	EHLDNHRGVRVFVSF	LDNHRGVRV	2	0.5638	112.1	29.00	0.52	tr_C9ZWY7_
DRB1_0101	281	DQNKGEFFVVVGPMG	FFVVVGPMG	6	0.5635	112.5	29.00	0.69	tr_C9ZWY7_
DRB1_0101	282	FVKVFKKHPQAAERL	VFKKHPQAA	3	0.5614	115.1	29.00	0.44	tr_C9ZWY7_

DRB1_0101	283	EQQLFLLVGYIARDK	FLLVGYIAR	4	0.5611	115.5	29.00	0.41	tr_C9ZWY7_
DRB1_0101	284	VIHFSPTIEQQLFLL	FSPTIEQQL	3	0.5596	117.4	29.00	0.41	tr_C9ZWY7_
DRB1_0101	285	PIREALKFVRRVAMD	LKFVRRVAM	5	0.5579	119.5	30.00	0.46	tr_C9ZWY7_
DRB1_0101	286	AEHLDNHRGVRVFVS	LDNHRGVRV	3	0.5572	120.4	30.00	0.62	tr_C9ZWY7_
DRB1_0101	287	VPYIPDRVIVAAPNP	YIPDRVIVA	2	0.5557	122.4	30.00	0.75	tr_C9ZWY7_
DRB1_0101	288	ARHWATAENVTVEII	WATAENVTV	3	0.5548	123.6	30.00	0.69	tr_C9ZWY7_
DRB1_0101	289	VWSMARVLNPSVPPV	MARVLNPSV	3	0.5546	123.9	30.00	0.62	tr_C9ZWY7_
DRB1_0101	290	PPIREALKFVRRVAM	LKFVRRVAM	6	0.5544	124.1	30.00	0.35	tr_C9ZWY7_
DRB1_0101	291	VGRVAFIEPITLSPK	VAFIEPITL	3	0.5542	124.4	30.00	0.55	tr_C9ZWY7_
DRB1_0101	292	LLFATSLPHWADNNT	FATSLPHWA	2	0.5540	124.7	30.00	0.50	tr_C9ZWY7_
DRB1_0101	293	TWLESREAFIKSLYN	WLESREAFI	1	0.5534	125.4	31.00	0.57	tr_C9ZWY7_
DRB1_0101	294	ALLYSEFVKVFKKHP	LLYSEFVKV	1	0.5532	125.7	31.00	0.65	tr_C9ZWY7_
DRB1_0101	295	KVFKKHPQAAERLLF	FKKHPQAAE	2	0.5528	126.3	31.00	0.39	tr_C9ZWY7_
DRB1_0101	296	SGWVIGEVLSQALSS	WVIGEVLSQ	2	0.5525	126.7	31.00	0.35	tr_C9ZWY7_
DRB1_0101	297	VAFIEPITLSPKLSH	FIEPITLSP	2	0.5523	127.0	31.00	0.25	tr_C9ZWY7_
DRB1_0101	298	SVWSMARVLNPSVPP	MARVLNPSV	4	0.5514	128.2	31.00	0.50	tr_C9ZWY7_
DRB1_0101	299	PYIPDRVIVAAPNPL	YIPDRVIVA	1	0.5514	128.2	31.00	0.54	tr_C9ZWY7_
DRB1_0101	300	FVRRVAMDARTRGVY	FVRRVAMDA	0	0.5508	129.0	31.00	0.51	tr_C9ZWY7_
DRB1_0101	301	NRLRLLRVGFMYLQG	LRLLRVGFM	2	0.5504	129.6	31.00	0.60	tr_C9ZWY7_
DRB1_0101	302	VDDSAAVSAFFSLVV	SAAVSAFFS	3	0.5490	131.5	31.00	0.65	tr_C9ZWY7_
DRB1_0101	303	HISVWSMARVLNPSV	MARVLNPSV	6	0.5488	131.9	31.00	0.29	tr_C9ZWY7_
DRB1_0101	304	TGGTMRRRPPAITFF	MRRRPPAIT	4	0.5477	133.5	32.00	0.69	tr_C9ZWY7_
DRB1_0101	305	AGFGGFAGVITGRHI	FGGFAGVIT	2	0.5476	133.5	32.00	0.36	tr_C9ZWY7_
DRB1_0101	306	AARHWATAENVTVEI	WATAENVTV	4	0.5466	135.0	32.00	0.67	tr_C9ZWY7_
DRB1_0101	307	PRSVSTLKLLETAAD	VSTLKLLET	3	0.5465	135.2	32.00	0.42	tr_C9ZWY7_
DRB1_0101	308	DDSAAVSAFFSLVVG	SAAVSAFFS	2	0.5452	137.2	32.00	0.61	tr_C9ZWY7_
DRB1_0101	309	PLAAKKSIKVKVYNL	LAAKKSIKV	1	0.5449	137.5	32.00	0.67	tr_C9ZWY7_
DRB1_0101	310	RLRLLRVGFMYLQGV	LRLLRVGFM	1	0.5438	139.2	32.00	0.50	tr_C9ZWY7_
DRB1_0101	311	RCSTIGVKVPTPLIV	GVKVPTPLI	5	0.5435	139.7	32.00	0.43	tr_C9ZWY7_
DRB1_0101	312	HRMIPLRDGITVFNI	MIPLRDGIT	2	0.5427	140.8	33.00	0.65	tr_C9ZWY7_
DRB1_0101	313	LIPLLLVLPHIVRPL	LLVLPHIVR	4	0.5427	140.9	33.00	0.21	tr_C9ZWY7_
DRB1_0101	314	IVFTPPIREALKFVR	IVFTPPIRE	0	0.5418	142.3	33.00	0.37	tr_C9ZWY7_
DRB1_0101	315	IHFSPTIEQQLFLLV	FSPTIEQQL	2	0.5418	142.3	33.00	0.41	tr_C9ZWY7_
DRB1_0101	316	IVAAPNPLASETKYH	IVAAPNPLA	0	0.5417	142.3	33.00	0.79	tr_C9ZWY7_
DRB1_0101	317	APNAELIALLRYAIN	IALLRYAIN	6	0.5411	143.3	33.00	0.40	tr_C9ZWY7_
DRB1_0101	318	WSMARVLNPSVPPVT	MARVLNPSV	2	0.5404	144.4	33.00	0.54	tr_C9ZWY7_
DRB1_0101	319	HDDADGALMVSGWVI	ALMVSGWVI	6	0.5399	145.2	33.00	0.38	tr_C9ZWY7_
DRB1_0101	320	VRRVAMDARTRGVYV	DARTRGVYV	6	0.5397	145.5	33.00	0.50	tr_C9ZWY7_
DRB1_0101	321	DHRMIPLRDGITVFN	MIPLRDGIT	3	0.5389	146.8	33.00	0.69	tr_C9ZWY7_
DRB1_0101	322	ALMVSGWVIGEVLSQ	ALMVSGWVI	0	0.5387	147.1	33.00	0.36	tr_C9ZWY7_
DRB1_0101	323	DSAAVSAFFSLVVGI	SAAVSAFFS	1	0.5383	147.7	33.00	0.55	tr_C9ZWY7_
DRB1_0101	324	FMYLQGVHFGDREYQ	FMYLQGVHF	0	0.5381	148.0	33.00	0.60	tr_C9ZWY7_
DRB1_0101	325	LSPKLSHPGRNVIHF	KLSHPGRNV	3	0.5375	149.0	34.00	0.56	tr_C9ZWY7_
DRB1_0101	326	FHSVVATSRNTYGRM	FHSVVATSR	0	0.5350	153.0	34.00	0.41	tr_C9ZWY7_
DRB1_0101	327	KKSIKVKVYNLLFTN	IKVKVYNLL	3	0.5345	154.0	34.00	0.37	tr_C9ZWY7_
DRB1_0101	328	LAVLPLLRQEDLVAF	LLRQEDLVA	5	0.5340	154.8	34.00	0.47	tr_C9ZWY7_
DRB1_0101	329	LFLLVGYIARDKKTT	FLLVGYIAR	1	0.5336	155.4	34.00	0.35	tr_C9ZWY7_
DRB1_0101	330	MARVLNPSVPPVTKA	LNPSVPPVT	4	0.5335	155.6	34.00	0.49	tr_C9ZWY7_
DRB1_0101	331	VSGWVIGEVLSQALS	WVIGEVLSQ	3	0.5334	155.7	34.00	0.38	tr_C9ZWY7_
DRB1_0101	332	HPQAAERLLFATSLP	ERLLFATSL	5	0.5334	155.8	34.00	0.62	tr_C9ZWY7_

DRB1_0101	333	LESREAFIKSLYNQR	EAFIKSLYN	4	0.5327	157.0	34.00	0.41	tr_C9ZWY7_
DRB1_0101	334	IESLGGSMNGIVVPD	IESLGGSMN	0	0.5321	158.1	35.00	0.43	tr_C9ZWY7_
DRB1_0101	335	AKKSIKVKVYNLLFT	SIKVKVYNL	3	0.5316	158.8	35.00	0.41	tr_C9ZWY7_
DRB1_0101	336	LAAKKSIKVKVYNLL	LAAKKSIKV	0	0.5303	161.0	35.00	0.32	tr_C9ZWY7_
DRB1_0101	337	AVLPLLRQEDLVAFA	LLRQEDLVA	4	0.5285	164.2	35.00	0.64	tr_C9ZWY7_
DRB1_0101	338	ARAMESVVSRMGRVN	MESVVSRMG	3	0.5285	164.3	35.00	0.32	tr_C9ZWY7_
DRB1_0101	339	FAARHWATAENVTVE	WATAENVTV	5	0.5270	167.0	36.00	0.56	tr_C9ZWY7_
DRB1_0101	340	PPRSVSTLKLLETAA	VSTLKLLET	4	0.5255	169.7	36.00	0.53	tr_C9ZWY7_
DRB1_0101	341	KHPQAAERLLFATSL	ERLLFATSL	6	0.5233	173.7	36.00	0.50	tr_C9ZWY7_
DRB1_0101	342	GNVVYMNSLGDDHRM	VVYMNSLGD	2	0.5228	174.7	36.00	0.62	tr_C9ZWY7_
DRB1_0101	343	GSVFQKTIESLGGSM	FQKTIESLG	3	0.5224	175.4	37.00	0.40	tr_C9ZWY7_
DRB1_0101	344	SPKLSHPGRNVIHFS	KLSHPGRNV	2	0.5200	180.0	37.00	0.56	tr_C9ZWY7_
DRB1_0101	345	VFKKHPQAAERLLFA	KHPQAAERL	3	0.5200	180.1	37.00	0.34	tr_C9ZWY7_
DRB1_0101	346	RTLAVLPLLRQEDLV	LAVLPLLRQ	2	0.5187	182.7	37.00	0.60	tr_C9ZWY7_
DRB1_0101	347	VVDDSAAVSAFFSLV	SAAVSAFFS	4	0.5184	183.2	37.00	0.68	tr_C9ZWY7_
DRB1_0101	348	LDNHRGVRVFVSFFD	HRGVRVFVS	3	0.5180	184.1	37.00	0.41	tr_C9ZWY7_
DRB1_0101	349	RNTYGRMQGELDTRS	YGRMQGELD	3	0.5175	185.0	38.00	0.79	tr_C9ZWY7_
DRB1_0101	350	DDHRMIPLRDGITVF	MIPLRDGIT	4	0.5158	188.4	38.00	0.69	tr_C9ZWY7_
DRB1_0101	351	TLSPKLSHPGRNVIH	KLSHPGRNV	4	0.5149	190.2	38.00	0.51	tr_C9ZWY7_
DRB1_0101	352	NGIFSQSVIVADDMW	GIFSQSVIV	1	0.5147	190.7	38.00	0.35	tr_C9ZWY7_
DRB1_0101	353	GGNVVYMNSLGDDHR	VVYMNSLGD	3	0.5146	190.9	38.00	0.68	tr_C9ZWY7_
DRB1_0101	354	NVVYMNSLGDDHRMI	VVYMNSLGD	1	0.5134	193.4	38.00	0.51	tr_C9ZWY7_
DRB1_0101	355	LSQALSSRTWLESRE	LSQALSSRT	0	0.5128	194.7	38.00	0.40	tr_C9ZWY7_
DRB1_0101	356	PGRNVIHFSPTIEQQ	IHFSPTIEQ	5	0.5119	196.6	39.00	0.43	tr_C9ZWY7_
DRB1_0101	357	EFVKVFKKHPQAAER	VFKKHPQAA	4	0.5110	198.5	39.00	0.43	tr_C9ZWY7_
DRB1_0101	358	VGLNASFAARHWATA	LNASFAARH	2	0.5107	199.1	39.00	0.44	tr_C9ZWY7_
DRB1_0101	359	TLAVLPLLRQEDLVA	LAVLPLLRQ	1	0.5092	202.5	39.00	0.38	tr_C9ZWY7_
DRB1_0101	360	TLKLLETAADQNKGE	LKLLETAAD	1	0.5081	204.9	39.00	0.78	tr_C9ZWY7_
DRB1_0101	361	AAKKSIKVKVYNLLF	SIKVKVYNL	4	0.5077	205.7	39.00	0.44	tr_C9ZWY7_
DRB1_0101	362	SVGRVAFIEPITLSP	VAFIEPITL	4	0.5069	207.6	40.00	0.57	tr_C9ZWY7_
DRB1_0101	363	IEQQLFLLVGYIARD	FLLVGYIAR	5	0.5067	207.9	40.00	0.40	tr_C9ZWY7_
DRB1_0101	364	SFAARHWATAENVTV	WATAENVTV	6	0.5067	207.9	40.00	0.34	tr_C9ZWY7_
DRB1_0101	365	VAEHLDNHRGVRVFV	LDNHRGVRV	4	0.5065	208.3	40.00	0.73	tr_C9ZWY7_
DRB1_0101	366	TVFNIDRCSTIGVKV	FNIDRCSTI	2	0.5064	208.7	40.00	0.45	tr_C9ZWY7_
DRB1_0101	367	VKVPTPLIVVVVLVV	PTPLIVVVV	3	0.5063	208.8	40.00	0.48	tr_C9ZWY7_
DRB1_0101	368	FLIPLLLVLPHIVRP	LLVLPHIVR	5	0.5052	211.3	40.00	0.25	tr_C9ZWY7_
DRB1_0101	369	TKMHAATVMRADEEF	MHAATVMRA	2	0.5046	212.8	40.00	0.71	tr_C9ZWY7_
DRB1_0101	370	VLPLLRQEDLVAFAP	LLRQEDLVA	3	0.5041	213.8	40.00	0.64	tr_C9ZWY7_
DRB1_0101	371	GECHGMAGERGASCL	CHGMAGERG	2	0.5026	217.5	40.00	0.68	tr_C9ZWY7_
DRB1_0101	372	LSVGRVAFIEPITLS	VAFIEPITL	5	0.5022	218.3	41.00	0.60	tr_C9ZWY7_
DRB1_0101	373	TLVFGLTEADITTVA	FGLTEADIT	3	0.5022	218.4	41.00	0.49	tr_C9ZWY7_
DRB1_0101	374	APLTGSSAVRGWDPH	LTGSSAVRG	2	0.5020	218.7	41.00	0.79	tr_C9ZWY7_
DRB1_0101	375	TIEQQLFLLVGYIAR	FLLVGYIAR	6	0.5018	219.2	41.00	0.33	tr_C9ZWY7_
DRB1_0101	376	HFSPTIEQQLFLLVG	FSPTIEQQL	1	0.5016	219.7	41.00	0.38	tr_C9ZWY7_
DRB1_0101	377	KSIKVKVYNLLFTNR	IKVKVYNLL	2	0.5014	220.3	41.00	0.33	tr_C9ZWY7_
DRB1_0101	378	NVTVEIIPPPPRSVS	VEIIPPPPR	3	0.5010	221.1	41.00	0.56	tr_C9ZWY7_
DRB1_0101	379	MVSGWVIGEVLSQAL	WVIGEVLSQ	4	0.5003	222.8	41.00	0.46	tr_C9ZWY7_
DRB1_0101	380	RHWATAENVTVEIIP	WATAENVTV	2	0.5001	223.4	41.00	0.75	tr_C9ZWY7_
DRB1_0101	381	RSVTAVFGVVDLAML	FGVVDLAML	6	0.5000	223.7	41.00	0.60	tr_C9ZWY7_
DRB1_0101	382	GATHISVWSMARVLN	SVWSMARVL	5	0.4994	225.0	41.00	0.36	tr_C9ZWY7_

DRB1_0101	383	RMIPLRDGITVFNID	MIPLRDGIT	1	0.4992	225.6	41.00	0.58	tr_C9ZWY7_
DRB1_0101	384	LRLLRVGFMYLQGVH	LRLLRVGFM	0	0.4990	226.1	41.00	0.31	tr_C9ZWY7_
DRB1_0101	385	VRVFVSFFDVALLYS	FVSFFDVAL	3	0.4985	227.4	41.00	0.44	tr_C9ZWY7_
DRB1_0101	386	ATHISVWSMARVLNP	SVWSMARVL	4	0.4974	229.9	42.00	0.33	tr_C9ZWY7_
DRB1_0101	387	NTYGRMQGELDTRSV	YGRMQGELD	2	0.4973	230.2	42.00	0.70	tr_C9ZWY7_
DRB1_0101	388	VTVEIIPPPPRSVST	VEIIPPPPR	2	0.4972	230.6	42.00	0.55	tr_C9ZWY7_
DRB1_0101	389	FLTPAPNAELIALLR	LTPAPNAEL	1	0.4949	236.4	42.00	0.48	tr_C9ZWY7_
DRB1_0101	390	THISVWSMARVLNPS	SVWSMARVL	3	0.4945	237.3	42.00	0.36	tr_C9ZWY7_
DRB1_0101	391	AGVITGRHITTYSTG	AGVITGRHI	0	0.4944	237.7	42.00	0.44	tr_C9ZWY7_
DRB1_0101	392	FGGECHGMAGERGAS	CHGMAGERG	4	0.4943	237.9	42.00	0.75	tr_C9ZWY7_
DRB1_0101	393	RQEDLVAFAPLTGSS	DLVAFAPLT	3	0.4941	238.3	42.00	0.35	tr_C9ZWY7_
DRB1_0101	394	RVFVSFFDVALLYSE	FVSFFDVAL	2	0.4941	238.4	42.00	0.40	tr_C9ZWY7_
DRB1_0101	395	STGGTMRRRPPAITF	MRRRPPAIT	5	0.4927	241.9	43.00	0.62	tr_C9ZWY7_
DRB1_0101	396	VFNIDRCSTIGVKVP	IDRCSTIGV	3	0.4924	242.6	43.00	0.31	tr_C9ZWY7_
DRB1_0101	397	AFIEPITLSPKLSHP	PITLSPKLS	4	0.4923	243.1	43.00	0.34	tr_C9ZWY7_
DRB1_0101	398	ITVFNIDRCSTIGVK	FNIDRCSTI	3	0.4919	244.0	43.00	0.54	tr_C9ZWY7_
DRB1_0101	399	ARVLNPSVPPVTKAA	LNPSVPPVT	3	0.4908	246.9	43.00	0.71	tr_C9ZWY7_
DRB1_0101	400	FLLVGYIARDKKTTS	LVGYIARDK	2	0.4901	248.9	43.00	0.28	tr_C9ZWY7_
DRB1_0101	401	GGECHGMAGERGASC	CHGMAGERG	3	0.4889	252.0	43.00	0.77	tr_C9ZWY7_
DRB1_0101	402	ECHGMAGERGASCLC	CHGMAGERG	1	0.4888	252.4	43.00	0.59	tr_C9ZWY7_
DRB1_0101	403	LPLLRQEDLVAFAPL	LLRQEDLVA	2	0.4877	255.4	44.00	0.60	tr_C9ZWY7_
DRB1_0101	404	WLESREAFIKSLYNQ	EAFIKSLYN	5	0.4876	255.6	44.00	0.32	tr_C9ZWY7_
DRB1_0101	405	SRNTYGRMQGELDTR	YGRMQGELD	4	0.4875	256.1	44.00	0.80	tr_C9ZWY7_
DRB1_0101	406	NLLFTNRAPRRSIET	FTNRAPRRS	3	0.4870	257.5	44.00	0.41	tr_C9ZWY7_
DRB1_0101	407	IGSVFQKTIESLGGS	FQKTIESLG	4	0.4865	258.6	44.00	0.42	tr_C9ZWY7_
DRB1_0101	408	ALSSRTWLESREAFI	WLESREAFI	6	0.4850	263.1	44.00	0.62	tr_C9ZWY7_
DRB1_0101	409	GLNASFAARHWATAE	LNASFAARH	1	0.4846	264.2	44.00	0.38	tr_C9ZWY7_
DRB1_0101	410	TTGFAAEGCAVNYGA	FAAEGCAVN	3	0.4845	264.3	44.00	0.55	tr_C9ZWY7_
DRB1_0101	411	GVRVFVSFFDVALLY	FVSFFDVAL	4	0.4845	264.5	44.00	0.42	tr_C9ZWY7_
DRB1_0101	412	SAGFGGFAGVITGRH	FGGFAGVIT	3	0.4845	264.5	44.00	0.53	tr_C9ZWY7_
DRB1_0101	413	DTLVFGLTEADITTV	FGLTEADIT	4	0.4838	266.5	44.00	0.45	tr_C9ZWY7_
DRB1_0101	414	PPPRSVSTLKLLETA	VSTLKLLET	5	0.4835	267.3	44.00	0.49	tr_C9ZWY7_
DRB1_0101	415	FEDSCVPTTGFAAEG	CVPTTGFAA	4	0.4833	267.9	44.00	0.77	tr_C9ZWY7_
DRB1_0101	416	THPQGVIVFTPPIRE	IVFTPPIRE	6	0.4832	268.2	44.00	0.50	tr_C9ZWY7_
DRB1_0101	417	LMVSGWVIGEVLSQA	WVIGEVLSQ	5	0.4828	269.3	45.00	0.41	tr_C9ZWY7_
DRB1_0101	418	ITLSPKLSHPGRNVI	KLSHPGRNV	5	0.4809	275.1	45.00	0.47	tr_C9ZWY7_
DRB1_0101	419	EEAAVPYIPDRVIVA	YIPDRVIVA	6	0.4798	278.1	45.00	0.64	tr_C9ZWY7_
DRB1_0101	420	GIGSVFQKTIESLGG	SVFQKTIES	3	0.4789	280.8	45.00	0.46	tr_C9ZWY7_
DRB1_0101	421	TVEIIPPPPRSVSTL	VEIIPPPPR	1	0.4786	281.8	45.00	0.49	tr_C9ZWY7_
DRB1_0101	422	RAPRRSIETMGVGLN	SIETMGVGL	5	0.4770	286.9	46.00	0.51	tr_C9ZWY7_
DRB1_0101	423	PKLSHPGRNVIHFSP	KLSHPGRNV	1	0.4762	289.4	46.00	0.52	tr_C9ZWY7_
DRB1_0101	424	RYHPGVTAFNASDDF	YHPGVTAFN	1	0.4759	290.2	46.00	0.68	tr_C9ZWY7_
DRB1_0101	425	PFEDSCVPTTGFAAE	CVPTTGFAA	5	0.4757	290.9	46.00	0.63	tr_C9ZWY7_
DRB1_0101	426	FTPPIREALKFVRRV	PIREALKFV	3	0.4750	293.1	46.00	0.31	tr_C9ZWY7_
DRB1_0101	427	LVVDDSAAVSAFFSL	SAAVSAFFS	5	0.4748	293.6	46.00	0.61	tr_C9ZWY7_
DRB1_0101	428	MLSVGRVAFIEPITL	VAFIEPITL	6	0.4738	296.9	46.00	0.39	tr_C9ZWY7_
DRB1_0101	429	TPPIREALKFVRRVA	IREALKFVR	3	0.4737	297.1	46.00	0.32	tr_C9ZWY7_
DRB1_0101	430	PMGDTRTLAVLPLLR	TRTLAVLPL	4	0.4737	297.3	46.00	0.56	tr_C9ZWY7_
DRB1_0101	431	FKKHPQAAERLLFAT	KHPQAAERL	2	0.4734	298.2	47.00	0.34	tr_C9ZWY7_
DRB1_0101	432	TYGRMQGELDTRSVT	YGRMQGELD	1	0.4728	300.2	47.00	0.64	tr_C9ZWY7_

DRB1_0101	433	ERGASCLCNQGGNVV	LCNQGGNVV	6	0.4723	301.6	47.00	0.48	tr_C9ZWY7_
DRB1_0101	434	VFTPPIREALKFVRR	FTPPIREAL	1	0.4710	305.9	47.00	0.27	tr_C9ZWY7_
DRB1_0101	435	NFGGECHGMAGERGA	CHGMAGERG	5	0.4710	306.0	47.00	0.66	tr_C9ZWY7_
DRB1_0101	436	DRCSTIGVKVPTPLI	IGVKVPTPL	5	0.4708	306.8	47.00	0.33	tr_C9ZWY7_
DRB1_0101	437	PPVTKAATPSMRYAN	TKAATPSMR	3	0.4704	308.0	47.00	0.35	tr_C9ZWY7_
DRB1_0101	438	MRRRPPAITFFLIPL	MRRRPPAIT	0	0.4701	309.0	47.00	0.35	tr_C9ZWY7_
DRB1_0101	439	SCVPTTGFAAEGCAV	CVPTTGFAA	1	0.4693	311.8	47.00	0.68	tr_C9ZWY7_
DRB1_0101	440	LCNQGGNVVYMNSLG	LCNQGGNVV	0	0.4683	315.0	48.00	0.41	tr_C9ZWY7_
DRB1_0101	441	PTTGFAAEGCAVNYG	FAAEGCAVN	4	0.4681	315.7	48.00	0.52	tr_C9ZWY7_
DRB1_0101	442	PLLRQEDLVAFAPLT	LLRQEDLVA	1	0.4672	318.7	48.00	0.46	tr_C9ZWY7_
DRB1_0101	443	PVTKAATPSMRYANR	KAATPSMRY	3	0.4672	318.7	48.00	0.35	tr_C9ZWY7_
DRB1_0101	444	YGATHISVWSMARVL	SVWSMARVL	6	0.4669	319.7	48.00	0.26	tr_C9ZWY7_
DRB1_0101	445	PAPNAELIALLRYAI	NAELIALLR	3	0.4653	325.5	48.00	0.33	tr_C9ZWY7_
DRB1_0101	446	VFVSFFDVALLYSEF	FVSFFDVAL	1	0.4650	326.5	48.00	0.28	tr_C9ZWY7_
DRB1_0101	447	GDDHRMIPLRDGITV	MIPLRDGIT	5	0.4650	326.7	48.00	0.69	tr_C9ZWY7_
DRB1_0101	448	DSCVPTTGFAAEGCA	CVPTTGFAA	2	0.4647	327.7	48.00	0.80	tr_C9ZWY7_
DRB1_0101	449	RAMESVVSRMGRVNS	MESVVSRMG	2	0.4641	329.6	48.00	0.41	tr_C9ZWY7_
DRB1_0101	450	FNIDRCSTIGVKVPT	IDRCSTIGV	2	0.4640	330.0	49.00	0.33	tr_C9ZWY7_
DRB1_0101	451	PLTGSSAVRGWDPHF	LTGSSAVRG	1	0.4639	330.3	49.00	0.65	tr_C9ZWY7_
DRB1_0101	452	RGVRVFVSFFDVALL	FVSFFDVAL	5	0.4639	330.4	49.00	0.39	tr_C9ZWY7_
DRB1_0101	453	LVFGLTEADITTVAE	FGLTEADIT	2	0.4638	330.9	49.00	0.51	tr_C9ZWY7_
DRB1_0101	454	FSPTIEQQLFLLVGY	TIEQQLFLL	3	0.4632	333.1	49.00	0.38	tr_C9ZWY7_
DRB1_0101	455	QGGNVVYMNSLGDDH	VVYMNSLGD	4	0.4623	336.1	49.00	0.72	tr_C9ZWY7_
DRB1_0101	456	ENVTVEIIPPPPRSV	VEIIPPPPR	4	0.4615	339.1	49.00	0.60	tr_C9ZWY7_
DRB1_0101	457	LLYSEFVKVFKKHPQ	LLYSEFVKV	0	0.4603	343.5	49.00	0.44	tr_C9ZWY7_
DRB1_0101	458	VPPVTKAATPSMRYA	TKAATPSMR	4	0.4597	345.8	49.00	0.37	tr_C9ZWY7_
DRB1_0101	459	ITNADAIRSITNPDR	ITNADAIRS	0	0.4597	345.9	49.00	0.65	tr_C9ZWY7_
DRB1_0101	460	EDSCVPTTGFAAEGC	CVPTTGFAA	3	0.4593	347.2	50.00	0.85	tr_C9ZWY7_
DRB1_0101	461	DNHRGVRVFVSFFDV	HRGVRVFVS	2	0.4589	348.9	50.00	0.46	tr_C9ZWY7_
DRB1_0101	462	GDTLVFGLTEADITT	FGLTEADIT	5	0.4586	349.9	50.00	0.36	tr_C9ZWY7_
DRB1_0101	463	IKSLYNQRRYVVDDL	LYNQRRYVV	3	0.4586	350.1	50.00	0.34	tr_C9ZWY7_
DRB1_0101	464	HRGVRVFVSFFDVAL	FVSFFDVAL	6	0.4581	351.8	50.00	0.28	tr_C9ZWY7_
DRB1_0101	465	TVAEHLDNHRGVRVF	LDNHRGVRV	5	0.4580	352.2	50.00	0.65	tr_C9ZWY7_
DRB1_0101	466	TGFAAEGCAVNYGAT	FAAEGCAVN	2	0.4571	355.9	50.00	0.55	tr_C9ZWY7_
DRB1_0101	467	TPAPNAELIALLRYA	PNAELIALL	3	0.4564	358.5	50.00	0.33	tr_C9ZWY7_
DRB1_0101	468	QSAGFGGFAGVITGR	FGGFAGVIT	4	0.4562	359.2	50.00	0.56	tr_C9ZWY7_
DRB1_0101	469	PGIGSVFQKTIESLG	SVFQKTIES	4	0.4560	360.0	50.00	0.47	tr_C9ZWY7_
DRB1_0101	470	ADAIRSITNPDRVAF	IRSITNPDR	3	0.4551	363.4	50.00	0.80	tr_C9ZWY7_
DRB1_0101	471	SEFVKVFKKHPQAAE	VFKKHPQAA	5	0.4543	366.7	55.00	0.38	tr_C9ZWY7_
DRB1_0101	472	LRQEDLVAFAPLTGS	DLVAFAPLT	4	0.4542	367.0	55.00	0.37	tr_C9ZWY7_
DRB1_0101	473	FVVVGPMGDTRTLAV	FVVVGPMGD	0	0.4540	368.0	55.00	0.38	tr_C9ZWY7_
DRB1_0101	474	EYQETLKLMTRMGRR	TLKLMTRMG	4	0.4530	371.7	55.00	0.28	tr_C9ZWY7_
DRB1_0101	475	FIEPITLSPKLSHPG	PITLSPKLS	3	0.4520	376.0	55.00	0.43	tr_C9ZWY7_
DRB1_0101	476	CHGMAGERGASCLCN	CHGMAGERG	0	0.4516	377.5	55.00	0.44	tr_C9ZWY7_
DRB1_0101	477	NHRGVRVFVSFFDVA	HRGVRVFVS	1	0.4514	378.2	55.00	0.38	tr_C9ZWY7_
DRB1_0101	478	RVLNPSVPPVTKAAT	LNPSVPPVT	2	0.4495	386.0	55.00	0.73	tr_C9ZWY7_
DRB1_0101	479	GIFSQSVIVADDMWY	IFSQSVIVA	1	0.4487	389.7	55.00	0.33	tr_C9ZWY7_
DRB1_0101	480	REYQETLKLMTRMGR	YQETLKLMT	2	0.4465	399.0	55.00	0.24	tr_C9ZWY7_
DRB1_0101	481	HPGRNVIHFSPTIEQ	NVIHFSPTI	4	0.4455	403.2	55.00	0.41	tr_C9ZWY7_
DRB1_0101	482	LLRQEDLVAFAPLTG	DLVAFAPLT	5	0.4453	403.9	55.00	0.34	tr_C9ZWY7_

DRB1_0101	483	VRRFQEDARSYLRYH	FQEDARSYL	3	0.4449	405.7	55.00	0.61	tr_C9ZWY7_
DRB1_0101	484	TNPDRVAFHSVVATS	DRVAFHSVV	3	0.4444	407.9	55.00	0.29	tr_C9ZWY7_
DRB1_0101	485	GATWRTAVEEAAVPY	WRTAVEEAA	3	0.4441	409.5	55.00	0.80	tr_C9ZWY7_
DRB1_0101	486	ATWRTAVEEAAVPYI	WRTAVEEAA	2	0.4440	409.9	55.00	0.71	tr_C9ZWY7_
DRB1_0101	487	ESLGGSMNGIVVPDN	GGSMNGIVV	3	0.4430	414.3	55.00	0.67	tr_C9ZWY7_
DRB1_0101	488	RHITTYSTGGTMRRR	YSTGGTMRR	5	0.4429	414.6	55.00	0.35	tr_C9ZWY7_
DRB1_0101	489	VVYMNSLGDDHRMIP	YMNSLGDDH	2	0.4428	415.1	55.00	0.35	tr_C9ZWY7_
DRB1_0101	490	DDESKWTPLALLGYA	SKWTPLALL	3	0.4422	417.9	55.00	0.44	tr_C9ZWY7_
DRB1_0101	491	CVPTTGFAAEGCAVN	CVPTTGFAA	0	0.4418	419.7	55.00	0.38	tr_C9ZWY7_
DRB1_0101	492	LLFTNRAPRRSIETM	FTNRAPRRS	2	0.4412	422.7	55.00	0.45	tr_C9ZWY7_
DRB1_0101	493	VLAPSAMQFVIGATW	PSAMQFVIG	3	0.4411	422.9	55.00	0.31	tr_C9ZWY7_
DRB1_0101	494	DEEFDSLWEEFVMTH	FDSLWEEFV	3	0.4408	424.4	55.00	0.71	tr_C9ZWY7_
DRB1_0101	495	DAIRSITNPDRVAFH	IRSITNPDR	2	0.4390	432.7	55.00	0.74	tr_C9ZWY7_
DRB1_0101	496	NQGGNVVYMNSLGDD	VVYMNSLGD	5	0.4389	433.1	55.00	0.65	tr_C9ZWY7_
DRB1_0101	497	AMLSVGRVAFIEPIT	LSVGRVAFI	2	0.4388	433.4	55.00	0.54	tr_C9ZWY7_
DRB1_0101	498	GITVFNIDRCSTIGV	FNIDRCSTI	4	0.4387	434.0	55.00	0.55	tr_C9ZWY7_
DRB1_0101	499	LAPSAMQFVIGATWR	PSAMQFVIG	2	0.4385	435.1	55.00	0.31	tr_C9ZWY7_
DRB1_0101	500	LNASFAARHWATAEN	FAARHWATA	4	0.4380	437.6	55.00	0.42	tr_C9ZWY7_
DRB1_0101	501	ASFAARHWATAENVT	FAARHWATA	2	0.4371	441.6	55.00	0.46	tr_C9ZWY7_
DRB1_0101	502	VPTTGFAAEGCAVNY	FAAEGCAVN	5	0.4361	446.6	55.00	0.48	tr_C9ZWY7_
DRB1_0101	503	NASFAARHWATAENV	FAARHWATA	3	0.4352	450.7	55.00	0.51	tr_C9ZWY7_
DRB1_0101	504	PITLSPKLSHPGRNV	LSPKLSHPG	3	0.4338	457.5	55.00	0.34	tr_C9ZWY7_
DRB1_0101	505	SPTIEQQLFLLVGYI	TIEQQLFLL	2	0.4328	462.8	55.00	0.32	tr_C9ZWY7_
DRB1_0101	506	GQSAGFGGFAGVITG	FGGFAGVIT	5	0.4305	474.5	60.00	0.51	tr_C9ZWY7_
DRB1_0101	507	HSVVATSRNTYGRMQ	VATSRNTYG	3	0.4302	476.1	60.00	0.55	tr_C9ZWY7_
DRB1_0101	508	AVRRFQEDARSYLRY	FQEDARSYL	4	0.4300	477.1	60.00	0.60	tr_C9ZWY7_
DRB1_0101	509	KVPTPLIVVVVLVVD	PTPLIVVVV	2	0.4299	477.6	60.00	0.63	tr_C9ZWY7_
DRB1_0101	510	SLGGSMNGIVVPDND	GGSMNGIVV	2	0.4296	479.0	60.00	0.69	tr_C9ZWY7_
DRB1_0101	511	EEFDSLWEEFVMTHP	FDSLWEEFV	2	0.4295	479.6	60.00	0.71	tr_C9ZWY7_
DRB1_0101	512	VTKAATPSMRYANRN	KAATPSMRY	2	0.4291	481.4	60.00	0.39	tr_C9ZWY7_
DRB1_0101	513	QRRYVVDDLVFGNFG	YVVDDLVFG	3	0.4286	484.0	60.00	0.70	tr_C9ZWY7_
DRB1_0101	514	LTPAPNAELIALLRY	PNAELIALL	4	0.4279	487.9	60.00	0.26	tr_C9ZWY7_
DRB1_0101	515	PTIEQQLFLLVGYIA	TIEQQLFLL	1	0.4274	490.3	60.00	0.25	tr_C9ZWY7_
DRB1_0101	516	AMESVVSRMGRVNSE	SVVSRMGRV	3	0.4264	495.9	60.00	0.41	tr_C9ZWY7_
DRB1_0101	517	KKHPQAAERLLFATS	QAAERLLFA	4	0.4249	503.8	60.00	0.31	tr_C9ZWY7_
DRB1_0101	518	PPPPRSVSTLKLLET	PRSVSTLKL	3	0.4230	514.2	60.00	0.41	tr_C9ZWY7_
DRB1_0101	519	LDTRSVTAVFGVVDL	TRSVTAVFG	2	0.4227	516.3	60.00	0.51	tr_C9ZWY7_
DRB1_0101	520	ANRNRGSLSGKQLAG	RGSLSGKQL	4	0.4226	516.7	60.00	0.50	tr_C9ZWY7_
DRB1_0101	521	AIRSITNPDRVAFHS	IRSITNPDR	1	0.4219	520.4	60.00	0.68	tr_C9ZWY7_
DRB1_0101	522	AVNYGATHISVWSMA	VNYGATHIS	1	0.4214	523.2	60.00	0.26	tr_C9ZWY7_
DRB1_0101	523	GPMGDTRTLAVLPLL	TRTLAVLPL	5	0.4206	528.2	60.00	0.55	tr_C9ZWY7_
DRB1_0101	524	TSRNTYGRMQGELDT	YGRMQGELD	5	0.4203	529.5	60.00	0.77	tr_C9ZWY7_
DRB1_0101	525	HGDTLVFGLTEADIT	LVFGLTEAD	4	0.4202	530.4	60.00	0.37	tr_C9ZWY7_
DRB1_0101	526	GPFEDSCVPTTGFAA	CVPTTGFAA	6	0.4188	538.2	60.00	0.49	tr_C9ZWY7_
DRB1_0101	527	VLVVDDSAAVSAFFS	SAAVSAFFS	6	0.4180	542.8	60.00	0.38	tr_C9ZWY7_
DRB1_0101	528	KDDESKWTPLALLGY	SKWTPLALL	4	0.4178	544.1	60.00	0.41	tr_C9ZWY7_
DRB1_0101	529	NADAIRSITNPDRVA	IRSITNPDR	4	0.4173	547.3	60.00	0.78	tr_C9ZWY7_
DRB1_0101	530	HITTYSTGGTMRRRP	YSTGGTMRR	4	0.4166	551.6	60.00	0.42	tr_C9ZWY7_
DRB1_0101	531	CAVNYGATHISVWSM	VNYGATHIS	2	0.4160	554.6	60.00	0.35	tr_C9ZWY7_
DRB1_0101	532	DREYQETLKLMTRMG	YQETLKLMT	3	0.4153	558.9	60.00	0.30	tr_C9ZWY7_

DRB1_0101	533	ADEEFDSLWEEFVMT	FDSLWEEFV	4	0.4153	559.3	60.00	0.75	tr_C9ZWY7_
DRB1_0101	534	KMHAATVMRADEEFD	MHAATVMRA	1	0.4144	564.3	60.00	0.74	tr_C9ZWY7_
DRB1_0101	535	VNYGATHISVWSMAR	ATHISVWSM	4	0.4140	566.9	60.00	0.25	tr_C9ZWY7_
DRB1_0101	536	ELDTRSVTAVFGVVD	TRSVTAVFG	3	0.4137	568.9	60.00	0.58	tr_C9ZWY7_
DRB1_0101	537	VFGLTEADITTVAEH	FGLTEADIT	1	0.4134	570.8	60.00	0.52	tr_C9ZWY7_
DRB1_0101	538	VIGATWRTAVEEAAV	WRTAVEEAA	5	0.4129	573.6	60.00	0.75	tr_C9ZWY7_
DRB1_0101	539	NQRRYVVDDLVFGNF	YVVDDLVFG	4	0.4127	574.9	60.00	0.74	tr_C9ZWY7_
DRB1_0101	540	GCAVNYGATHISVWS	VNYGATHIS	3	0.4127	575.3	60.00	0.37	tr_C9ZWY7_
DRB1_0101	541	VGQSAGFGGFAGVIT	FGGFAGVIT	6	0.4121	578.7	60.00	0.39	tr_C9ZWY7_
DRB1_0101	542	RRFQEDARSYLRYHP	FQEDARSYL	2	0.4120	579.3	60.00	0.61	tr_C9ZWY7_
DRB1_0101	543	GFAAEGCAVNYGATH	FAAEGCAVN	1	0.4101	591.3	60.00	0.55	tr_C9ZWY7_
DRB1_0101	544	VPGIGSVFQKTIESL	SVFQKTIES	5	0.4098	593.6	60.00	0.48	tr_C9ZWY7_
DRB1_0101	545	NYGATHISVWSMARV	ATHISVWSM	3	0.4092	597.6	60.00	0.25	tr_C9ZWY7_
DRB1_0101	546	MTHPQGVIVFTPPIR	GVIVFTPPI	5	0.4080	605.1	65.00	0.37	tr_C9ZWY7_
DRB1_0101	547	RRYVVDDLVFGNFGG	YVVDDLVFG	2	0.4079	606.0	65.00	0.65	tr_C9ZWY7_
DRB1_0101	548	TWRTAVEEAAVPYIP	WRTAVEEAA	1	0.4078	606.1	65.00	0.62	tr_C9ZWY7_
DRB1_0101	549	LFATSLPHWADNNTT	FATSLPHWA	1	0.4077	606.8	65.00	0.73	tr_C9ZWY7_
DRB1_0101	550	CNQGGNVVYMNSLGD	VVYMNSLGD	6	0.4068	612.8	65.00	0.41	tr_C9ZWY7_
DRB1_0101	551	GDREYQETLKLMTRM	YQETLKLMT	4	0.4059	619.0	65.00	0.34	tr_C9ZWY7_
DRB1_0101	552	KLSHPGRNVIHFSPT	KLSHPGRNV	0	0.4041	630.9	65.00	0.42	tr_C9ZWY7_
DRB1_0101	553	IEPITLSPKLSHPGR	PITLSPKLS	2	0.4038	633.0	65.00	0.46	tr_C9ZWY7_
DRB1_0101	554	DHDDADGALMVSGWV	GALMVSGWV	6	0.4036	634.6	65.00	0.41	tr_C9ZWY7_
DRB1_0101	555	DTRSVTAVFGVVDLA	TRSVTAVFG	1	0.4035	635.5	65.00	0.44	tr_C9ZWY7_
DRB1_0101	556	VMTHPQGVIVFTPPI	MTHPQGVIV	1	0.4035	635.5	65.00	0.50	tr_C9ZWY7_
DRB1_0101	557	GELDTRSVTAVFGVV	TRSVTAVFG	4	0.4034	636.1	65.00	0.63	tr_C9ZWY7_
DRB1_0101	558	IGATWRTAVEEAAVP	WRTAVEEAA	4	0.4030	638.8	65.00	0.80	tr_C9ZWY7_
DRB1_0101	559	YSEFVKVFKKHPQAA	FVKVFKKHP	3	0.4024	643.1	65.00	0.29	tr_C9ZWY7_
DRB1_0101	560	VEIIPPPPRSVSTLK	IIPPPPRSV	2	0.4017	647.6	65.00	0.40	tr_C9ZWY7_
DRB1_0101	561	YANRNRGSLSGKQLA	RGSLSGKQL	5	0.4017	647.7	65.00	0.49	tr_C9ZWY7_
DRB1_0101	562	EFDSLWEEFVMTHPQ	FDSLWEEFV	1	0.3986	670.0	65.00	0.63	tr_C9ZWY7_
DRB1_0101	563	YGRMQGELDTRSVTA	YGRMQGELD	0	0.3980	674.5	65.00	0.44	tr_C9ZWY7_
DRB1_0101	564	SVVATSRNTYGRMQG	VATSRNTYG	2	0.3978	675.6	65.00	0.57	tr_C9ZWY7_
DRB1_0101	565	RVNSEELINGIFSQS	EELINGIFS	4	0.3972	680.4	65.00	0.45	tr_C9ZWY7_
DRB1_0101	566	ITNPDRVAFHSVVAT	DRVAFHSVV	4	0.3967	683.7	65.00	0.31	tr_C9ZWY7_
DRB1_0101	567	MIPLRDGITVFNIDR	LRDGITVFN	3	0.3963	687.0	65.00	0.36	tr_C9ZWY7_
DRB1_0101	568	LGGSMNGIVVPDNDT	GGSMNGIVV	1	0.3954	693.5	65.00	0.65	tr_C9ZWY7_
DRB1_0101	569	IDRCSTIGVKVPTPL	RCSTIGVKV	2	0.3953	694.4	65.00	0.31	tr_C9ZWY7_
DRB1_0101	570	SVPPVTKAATPSMRY	VTKAATPSM	4	0.3950	696.2	65.00	0.38	tr_C9ZWY7_
DRB1_0101	571	HWATAENVTVEIIPP	WATAENVTV	1	0.3943	701.7	65.00	0.83	tr_C9ZWY7_
DRB1_0101	572	EPITLSPKLSHPGRN	PITLSPKLS	1	0.3918	720.7	65.00	0.37	tr_C9ZWY7_
DRB1_0101	573	MESVVSRMGRVNSEE	SVVSRMGRV	2	0.3913	724.8	65.00	0.44	tr_C9ZWY7_
DRB1_0101	574	YSTGGTMRRRPPAIT	MRRRPPAIT	6	0.3910	727.2	65.00	0.47	tr_C9ZWY7_
DRB1_0101	575	EGCAVNYGATHISVW	VNYGATHIS	4	0.3906	730.8	65.00	0.38	tr_C9ZWY7_
DRB1_0101	576	LKLLETAADQNKGEF	LKLLETAAD	0	0.3902	733.7	65.00	0.70	tr_C9ZWY7_
DRB1_0101	577	YNQRRYVVDDLVFGN	YVVDDLVFG	5	0.3900	735.5	65.00	0.67	tr_C9ZWY7_
DRB1_0101	578	AENVTVEIIPPPPRS	VEIIPPPPR	5	0.3899	736.3	65.00	0.61	tr_C9ZWY7_
DRB1_0101	579	LTGSSAVRGWDPHFY	LTGSSAVRG	0	0.3896	738.5	65.00	0.46	tr_C9ZWY7_
DRB1_0101	580	GRHITTYSTGGTMRR	TTYSTGGTM	4	0.3872	757.6	65.00	0.34	tr_C9ZWY7_
DRB1_0101	581	NIDRCSTIGVKVPTP	RCSTIGVKV	3	0.3864	764.0	65.00	0.40	tr_C9ZWY7_
DRB1_0101	582	KSLYNQRRYVVDDLV	LYNQRRYVV	2	0.3851	774.9	65.00	0.44	tr_C9ZWY7_

DRB1_0101	583	GGDVPGIGSVFQKTI	DVPGIGSVF	2	0.3848	777.6	65.00	0.44	tr_C9ZWY7_
DRB1_0101	584	ITTYSTGGTMRRRPP	YSTGGTMRR	3	0.3848	777.8	65.00	0.46	tr_C9ZWY7_
DRB1_0101	585	HAVRRFQEDARSYLR	FQEDARSYL	5	0.3847	778.2	65.00	0.55	tr_C9ZWY7_
DRB1_0101	586	DFDHDDADGALMVSG	FDHDDADGA	1	0.3844	781.5	70.00	0.35	tr_C9ZWY7_
DRB1_0101	587	FGDREYQETLKLMTR	YQETLKLMT	5	0.3834	789.4	70.00	0.29	tr_C9ZWY7_
DRB1_0101	588	TTVAEHLDNHRGVRV	LDNHRGVRV	6	0.3834	789.6	70.00	0.55	tr_C9ZWY7_
DRB1_0101	589	GNFGGECHGMAGERG	CHGMAGERG	6	0.3821	800.6	70.00	0.47	tr_C9ZWY7_
DRB1_0101	590	RRRPPAITFFLIPLL	PPAITFFLI	3	0.3819	802.7	70.00	0.34	tr_C9ZWY7_
DRB1_0101	591	SQALSSRTWLESREA	ALSSRTWLE	2	0.3798	820.7	70.00	0.41	tr_C9ZWY7_
DRB1_0101	592	LLVGYIARDKKTTSL	LVGYIARDK	1	0.3784	833.1	70.00	0.31	tr_C9ZWY7_
DRB1_0101	593	IARDKKTTSLHMVTR	DKKTTSLHM	3	0.3781	836.0	70.00	0.57	tr_C9ZWY7_
DRB1_0101	594	LGDDHRMIPLRDGIT	MIPLRDGIT	6	0.3778	838.6	70.00	0.47	tr_C9ZWY7_
DRB1_0101	595	QGELDTRSVTAVFGV	TRSVTAVFG	5	0.3773	843.6	70.00	0.51	tr_C9ZWY7_
DRB1_0101	596	DDFDHDDADGALMVS	FDHDDADGA	2	0.3764	851.5	70.00	0.40	tr_C9ZWY7_
DRB1_0101	597	RGGDVPGIGSVFQKT	DVPGIGSVF	3	0.3764	852.0	70.00	0.56	tr_C9ZWY7_
DRB1_0101	598	ARDKKTTSLHMVTRG	DKKTTSLHM	2	0.3755	860.4	70.00	0.48	tr_C9ZWY7_
DRB1_0101	599	RRPPAITFFLIPLLL	ITFFLIPLL	5	0.3752	862.5	70.00	0.25	tr_C9ZWY7_
DRB1_0101	600	RADEEFDSLWEEFVM	FDSLWEEFV	5	0.3737	876.9	70.00	0.79	tr_C9ZWY7_
DRB1_0101	601	GRVNSEELINGIFSQ	EELINGIFS	5	0.3718	894.8	70.00	0.42	tr_C9ZWY7_
DRB1_0101	602	TRSVTAVFGVVDLAM	VTAVFGVVD	3	0.3714	898.7	70.00	0.38	tr_C9ZWY7_
DRB1_0101	603	FDHDDADGALMVSGW	DDADGALMV	3	0.3709	903.6	70.00	0.42	tr_C9ZWY7_
DRB1_0101	604	DGITVFNIDRCSTIG	FNIDRCSTI	5	0.3703	909.4	70.00	0.60	tr_C9ZWY7_
DRB1_0101	605	SMRYANRNRGSLSGK	YANRNRGSL	3	0.3683	930.1	70.00	0.37	tr_C9ZWY7_
DRB1_0101	606	RDKKTTSLHMVTRGG	DKKTTSLHM	1	0.3671	941.5	70.00	0.35	tr_C9ZWY7_
DRB1_0101	607	GDVPGIGSVFQKTIE	DVPGIGSVF	1	0.3670	943.0	70.00	0.35	tr_C9ZWY7_
DRB1_0101	608	NPLASETKYHAVRRF	LASETKYHA	2	0.3665	947.6	70.00	0.47	tr_C9ZWY7_
DRB1_0101	609	EDLSPHGDTLVFGLT	LSPHGDTLV	2	0.3655	958.4	70.00	0.58	tr_C9ZWY7_
DRB1_0101	610	FGLTEADITTVAEHL	LTEADITTV	2	0.3649	964.1	70.00	0.31	tr_C9ZWY7_
DRB1_0101	611	DVPGIGSVFQKTIES	GIGSVFQKT	3	0.3649	964.7	70.00	0.29	tr_C9ZWY7_
DRB1_0101	612	MKDDESKWTPLALLG	SKWTPLALL	5	0.3646	967.2	70.00	0.46	tr_C9ZWY7_
DRB1_0101	613	SSAVRGWDPHFYFLT	GWDPHFYFL	5	0.3640	974.3	70.00	0.17	tr_C9ZWY7_
DRB1_0101	614	NEDLSPHGDTLVFGL	LSPHGDTLV	3	0.3625	990.4	70.00	0.69	tr_C9ZWY7_
DRB1_0101	615	SVGQSAGFGGFAGVI	SAGFGGFAG	4	0.3624	990.9	70.00	0.32	tr_C9ZWY7_
DRB1_0101	616	ESVVSRMGRVNSEEL	SVVSRMGRV	1	0.3623	992.4	70.00	0.38	tr_C9ZWY7_
DRB1_0101	617	PNPLASETKYHAVRR	LASETKYHA	3	0.3617	998.4	70.00	0.57	tr_C9ZWY7_
DRB1_0101	618	LYNQRRYVVDDLVFG	YVVDDLVFG	6	0.3613	1002.7	70.00	0.49	tr_C9ZWY7_
DRB1_0101	619	LYSEFVKVFKKHPQA	FVKVFKKHP	4	0.3605	1011.9	70.00	0.35	tr_C9ZWY7_
DRB1_0101	620	IFSQSVIVADDMWYG	QSVIVADDM	3	0.3597	1020.6	75.00	0.23	tr_C9ZWY7_
DRB1_0101	621	SHPGRNVIHFSPTIE	NVIHFSPTI	5	0.3588	1030.7	75.00	0.52	tr_C9ZWY7_
DRB1_0101	622	RYVVDDLVFGNFGGE	YVVDDLVFG	1	0.3569	1051.4	75.00	0.57	tr_C9ZWY7_
DRB1_0101	623	SVVSRMGRVNSEELI	MGRVNSEEL	5	0.3555	1067.3	75.00	0.22	tr_C9ZWY7_
DRB1_0101	624	VLNPSVPPVTKAATP	LNPSVPPVT	1	0.3543	1082.1	75.00	0.75	tr_C9ZWY7_
DRB1_0101	625	SDDFDHDDADGALMV	FDHDDADGA	3	0.3542	1083.4	75.00	0.54	tr_C9ZWY7_
DRB1_0101	626	TNADAIRSITNPDRV	IRSITNPDR	5	0.3535	1091.0	75.00	0.78	tr_C9ZWY7_
DRB1_0101	627	PLASETKYHAVRRFQ	LASETKYHA	1	0.3529	1097.8	75.00	0.35	tr_C9ZWY7_
DRB1_0101	628	IRSITNPDRVAFHSV	IRSITNPDR	0	0.3526	1101.3	75.00	0.43	tr_C9ZWY7_
DRB1_0101	629	PSMRYANRNRGSLSG	MRYANRNRG	2	0.3525	1102.5	75.00	0.37	tr_C9ZWY7_
DRB1_0101	630	ASVGQSAGFGGFAGV	VGQSAGFGG	2	0.3523	1105.8	75.00	0.34	tr_C9ZWY7_
DRB1_0101	631	RFQEDARSYLRYHPG	FQEDARSYL	1	0.3502	1131.4	75.00	0.56	tr_C9ZWY7_
DRB1_0101	632	VGPMGDTRTLAVLPL	TRTLAVLPL	6	0.3501	1132.3	75.00	0.45	tr_C9ZWY7_

DRB1_0101	633	DLSPHGDTLVFGLTE	LSPHGDTLV	1	0.3498	1135.3	75.00	0.50	tr_C9ZWY7_
DRB1_0101	634	GSSAVRGWDPHFYFL	SSAVRGWDP	1	0.3493	1141.7	75.00	0.34	tr_C9ZWY7_
DRB1_0101	635	AEGCAVNYGATHISV	VNYGATHIS	5	0.3488	1147.6	75.00	0.34	tr_C9ZWY7_
DRB1_0101	636	LSHPGRNVIHFSPTI	NVIHFSPTI	6	0.3467	1173.9	75.00	0.41	tr_C9ZWY7_
DRB1_0101	637	LFTNRAPRRSIETMG	FTNRAPRRS	1	0.3449	1197.2	75.00	0.54	tr_C9ZWY7_
DRB1_0101	638	SITNPDRVAFHSVVA	DRVAFHSVV	5	0.3448	1198.9	75.00	0.31	tr_C9ZWY7_
DRB1_0101	639	MGRVNSEELINGIFS	EELINGIFS	6	0.3442	1206.4	75.00	0.28	tr_C9ZWY7_
DRB1_0101	640	TKAATPSMRYANRNR	KAATPSMRY	1	0.3440	1209.5	75.00	0.49	tr_C9ZWY7_
DRB1_0101	641	PHGDTLVFGLTEADI	LVFGLTEAD	5	0.3439	1211.1	75.00	0.37	tr_C9ZWY7_
DRB1_0101	642	TRGGDVPGIGSVFQK	DVPGIGSVF	4	0.3437	1212.6	75.00	0.61	tr_C9ZWY7_
DRB1_0101	643	VYMNSLGDDHRMIPL	YMNSLGDDH	1	0.3425	1228.7	75.00	0.41	tr_C9ZWY7_
DRB1_0101	644	MASVGQSAGFGGFAG	VGQSAGFGG	3	0.3424	1229.9	75.00	0.41	tr_C9ZWY7_
DRB1_0101	645	YIARDKKTTSLHMVT	DKKTTSLHM	4	0.3398	1265.8	75.00	0.56	tr_C9ZWY7_
DRB1_0101	646	FDSLWEEFVMTHPQG	FDSLWEEFV	0	0.3391	1274.5	75.00	0.49	tr_C9ZWY7_
DRB1_0101	647	RYANRNRGSLSGKQL	YANRNRGSL	1	0.3390	1276.1	75.00	0.25	tr_C9ZWY7_
DRB1_0101	648	FAAEGCAVNYGATHI	FAAEGCAVN	0	0.3375	1296.9	75.00	0.40	tr_C9ZWY7_
DRB1_0101	649	GVITGRHITTYSTGG	GRHITTYST	4	0.3375	1297.6	75.00	0.38	tr_C9ZWY7_
DRB1_0101	650	MRYANRNRGSLSGKQ	YANRNRGSL	2	0.3368	1307.7	75.00	0.35	tr_C9ZWY7_
DRB1_0101	651	APNPLASETKYHAVR	LASETKYHA	4	0.3368	1307.7	75.00	0.50	tr_C9ZWY7_
DRB1_0101	652	IIPPPPRSVSTLKLL	PRSVSTLKL	5	0.3365	1312.0	75.00	0.46	tr_C9ZWY7_
DRB1_0101	653	TPSMRYANRNRGSLS	MRYANRNRG	3	0.3364	1312.9	75.00	0.42	tr_C9ZWY7_
DRB1_0101	654	VVVLVVDDSAAVSAF	LVVDDSAAV	3	0.3363	1314.9	75.00	0.35	tr_C9ZWY7_
DRB1_0101	655	TTYSTGGTMRRRPPA	YSTGGTMRR	2	0.3336	1353.7	80.00	0.52	tr_C9ZWY7_
DRB1_0101	656	VVLVVDDSAAVSAFF	LVVDDSAAV	2	0.3330	1361.4	80.00	0.38	tr_C9ZWY7_
DRB1_0101	657	IPPPPRSVSTLKLLE	PRSVSTLKL	4	0.3330	1362.4	80.00	0.57	tr_C9ZWY7_
DRB1_0101	658	ATSRNTYGRMQGELD	YGRMQGELD	6	0.3329	1362.9	80.00	0.63	tr_C9ZWY7_
DRB1_0101	659	ADQNKGEFFVVVGPM	QNKGEFFVV	2	0.3328	1364.8	80.00	0.31	tr_C9ZWY7_
DRB1_0101	660	YGPFEDSCVPTTGFA	FEDSCVPTT	3	0.3325	1369.7	80.00	0.67	tr_C9ZWY7_
DRB1_0101	661	MQGELDTRSVTAVFG	TRSVTAVFG	6	0.3319	1378.3	80.00	0.32	tr_C9ZWY7_
DRB1_0101	662	TGRHITTYSTGGTMR	TTYSTGGTM	5	0.3318	1379.3	80.00	0.34	tr_C9ZWY7_
DRB1_0101	663	TEADITTVAEHLDNH	DITTVAEHL	3	0.3317	1381.5	80.00	0.75	tr_C9ZWY7_
DRB1_0101	664	VVVGPMGDTRTLAVL	VGPMGDTRT	2	0.3316	1383.6	80.00	0.45	tr_C9ZWY7_
DRB1_0101	665	LASETKYHAVRRFQE	TKYHAVRRF	4	0.3302	1404.6	80.00	0.31	tr_C9ZWY7_
DRB1_0101	666	WRTAVEEAAVPYIPD	WRTAVEEAA	0	0.3298	1409.5	80.00	0.38	tr_C9ZWY7_
DRB1_0101	667	WYGPFEDSCVPTTGF	FEDSCVPTT	4	0.3298	1410.7	80.00	0.60	tr_C9ZWY7_
DRB1_0101	668	EMKDDESKWTPLALL	SKWTPLALL	6	0.3297	1411.1	80.00	0.40	tr_C9ZWY7_
DRB1_0101	669	SLYNQRRYVVDDLVF	LYNQRRYVV	1	0.3295	1415.0	80.00	0.40	tr_C9ZWY7_
DRB1_0101	670	LSPHGDTLVFGLTEA	LSPHGDTLV	0	0.3294	1416.6	80.00	0.28	tr_C9ZWY7_
DRB1_0101	671	YHPGVTAFNASDDFD	YHPGVTAFN	0	0.3291	1420.8	80.00	0.43	tr_C9ZWY7_
DRB1_0101	672	VVATSRNTYGRMQGE	VATSRNTYG	1	0.3290	1422.3	80.00	0.56	tr_C9ZWY7_
DRB1_0101	673	HFGDREYQETLKLMT	REYQETLKL	4	0.3287	1427.3	80.00	0.34	tr_C9ZWY7_
DRB1_0101	674	NRAPRRSIETMGVGL	SIETMGVGL	6	0.3284	1431.7	80.00	0.51	tr_C9ZWY7_
DRB1_0101	675	GYIARDKKTTSLHMV	DKKTTSLHM	5	0.3284	1432.3	80.00	0.41	tr_C9ZWY7_
DRB1_0101	676	GGSMNGIVVPDNDTT	GGSMNGIVV	0	0.3283	1433.4	80.00	0.51	tr_C9ZWY7_
DRB1_0101	677	VVSRMGRVNSEELIN	MGRVNSEEL	4	0.3282	1434.3	80.00	0.39	tr_C9ZWY7_
DRB1_0101	678	LVGYIARDKKTTSLH	YIARDKKTT	3	0.3273	1449.5	80.00	0.50	tr_C9ZWY7_
DRB1_0101	679	VPTPLIVVVVLVVDD	PTPLIVVVV	1	0.3271	1452.0	80.00	0.71	tr_C9ZWY7_
DRB1_0101	680	SRMGRVNSEELINGI	GRVNSEELI	3	0.3270	1453.1	80.00	0.38	tr_C9ZWY7_
DRB1_0101	681	VVVVLVVDDSAAVSA	LVVDDSAAV	4	0.3261	1467.0	80.00	0.38	tr_C9ZWY7_
DRB1_0101	682	LNEDLSPHGDTLVFG	LSPHGDTLV	4	0.3256	1476.2	80.00	0.69	tr_C9ZWY7_

DRB1_0101	683	TAADQNKGEFFVVVG	QNKGEFFVV	4	0.3255	1478.0	80.00	0.33	tr_C9ZWY7_
DRB1_0101	684	VSRMGRVNSEELING	MGRVNSEEL	3	0.3251	1483.7	80.00	0.44	tr_C9ZWY7_
DRB1_0101	685	EADITTVAEHLDNHR	DITTVAEHL	2	0.3251	1484.3	80.00	0.65	tr_C9ZWY7_
DRB1_0101	686	VRGWDPHFYFLTPAP	WDPHFYFLT	3	0.3235	1509.2	80.00	0.27	tr_C9ZWY7_
DRB1_0101	687	QALSSRTWLESREAF	ALSSRTWLE	1	0.3212	1547.0	80.00	0.39	tr_C9ZWY7_
DRB1_0101	688	HGMAGERGASCLCNQ	MAGERGASC	2	0.3203	1563.4	80.00	0.56	tr_C9ZWY7_
DRB1_0101	689	YHAVRRFQEDARSYL	FQEDARSYL	6	0.3201	1565.9	80.00	0.46	tr_C9ZWY7_
DRB1_0101	690	QEDARSYLRYHPGVT	YLRYHPGVT	6	0.3197	1573.8	80.00	0.38	tr_C9ZWY7_
DRB1_0101	691	DKKTTSLHMVTRGGD	SLHMVTRGG	5	0.3181	1599.9	80.00	0.17	tr_C9ZWY7_
DRB1_0101	692	TSLHMVTRGGDVPGI	HMVTRGGDV	3	0.3177	1607.9	80.00	0.50	tr_C9ZWY7_
DRB1_0101	693	KKTTSLHMVTRGGDV	SLHMVTRGG	4	0.3170	1619.2	80.00	0.25	tr_C9ZWY7_
DRB1_0101	694	SAVRGWDPHFYFLTP	WDPHFYFLT	5	0.3153	1648.8	80.00	0.20	tr_C9ZWY7_
DRB1_0101	695	ADITTVAEHLDNHRG	DITTVAEHL	1	0.3150	1655.0	80.00	0.56	tr_C9ZWY7_
DRB1_0101	696	EIIPPPPRSVSTLKL	IIPPPPRSV	1	0.3147	1661.1	80.00	0.49	tr_C9ZWY7_
DRB1_0101	697	RMGRVNSEELINGIF	GRVNSEELI	2	0.3146	1662.3	80.00	0.35	tr_C9ZWY7_
DRB1_0101	698	LTEADITTVAEHLDN	DITTVAEHL	4	0.3135	1681.2	80.00	0.73	tr_C9ZWY7_
DRB1_0101	699	MWYGPFEDSCVPTTG	FEDSCVPTT	5	0.3125	1699.7	80.00	0.56	tr_C9ZWY7_
DRB1_0101	700	RDGITVFNIDRCSTI	FNIDRCSTI	6	0.3117	1715.3	80.00	0.44	tr_C9ZWY7_
DRB1_0101	701	AADQNKGEFFVVVGP	QNKGEFFVV	3	0.3109	1730.4	80.00	0.35	tr_C9ZWY7_
DRB1_0101	702	RMQGELDTRSVTAVF	QGELDTRSV	2	0.3091	1764.1	80.00	0.33	tr_C9ZWY7_
DRB1_0101	703	FSQSVIVADDMWYGP	QSVIVADDM	2	0.3083	1779.0	80.00	0.31	tr_C9ZWY7_
DRB1_0101	704	GLTEADITTVAEHLD	DITTVAEHL	5	0.3077	1790.4	80.00	0.56	tr_C9ZWY7_
DRB1_0101	705	ETAADQNKGEFFVVV	QNKGEFFVV	5	0.3076	1792.5	80.00	0.32	tr_C9ZWY7_
DRB1_0101	706	GRMQGELDTRSVTAV	QGELDTRSV	3	0.3062	1821.0	85.00	0.38	tr_C9ZWY7_
DRB1_0101	707	TGSSAVRGWDPHFYF	SSAVRGWDP	2	0.3059	1826.8	85.00	0.47	tr_C9ZWY7_
DRB1_0101	708	TTSLHMVTRGGDVPG	HMVTRGGDV	4	0.3055	1834.8	85.00	0.46	tr_C9ZWY7_
DRB1_0101	709	FATSLPHWADNNTTS	FATSLPHWA	0	0.3050	1843.8	85.00	0.59	tr_C9ZWY7_
DRB1_0101	710	IVVVVLVVDDSAAVS	LVVDDSAAV	5	0.3046	1852.0	85.00	0.29	tr_C9ZWY7_
DRB1_0101	711	VTRGGDVPGIGSVFQ	DVPGIGSVF	5	0.3043	1857.7	85.00	0.56	tr_C9ZWY7_
DRB1_0101	712	TLNEDLSPHGDTLVF	LSPHGDTLV	5	0.3039	1867.0	85.00	0.57	tr_C9ZWY7_
DRB1_0101	713	KTTSLHMVTRGGDVP	HMVTRGGDV	5	0.3037	1869.7	85.00	0.37	tr_C9ZWY7_
DRB1_0101	714	LVFGNFGGECHGMAG	FGGECHGMA	5	0.3035	1873.6	85.00	0.47	tr_C9ZWY7_
DRB1_0101	715	SPHGDTLVFGLTEAD	GDTLVFGLT	3	0.3016	1912.9	85.00	0.22	tr_C9ZWY7_
DRB1_0101	716	AVRGWDPHFYFLTPA	WDPHFYFLT	4	0.3016	1914.1	85.00	0.25	tr_C9ZWY7_
DRB1_0101	717	ASETKYHAVRRFQED	TKYHAVRRF	3	0.3014	1916.7	85.00	0.38	tr_C9ZWY7_
DRB1_0101	718	SQSVIVADDMWYGPF	IVADDMWYG	4	0.3008	1929.3	85.00	0.40	tr_C9ZWY7_
DRB1_0101	719	ETKYHAVRRFQEDAR	YHAVRRFQE	3	0.2979	1991.9	85.00	0.55	tr_C9ZWY7_
DRB1_0101	720	SLHMVTRGGDVPGIG	HMVTRGGDV	2	0.2979	1992.4	85.00	0.49	tr_C9ZWY7_
DRB1_0101	721	FGNFGGECHGMAGER	FGGECHGMA	3	0.2975	1999.2	85.00	0.63	tr_C9ZWY7_
DRB1_0101	722	AAPNPLASETKYHAV	LASETKYHA	5	0.2972	2006.3	85.00	0.45	tr_C9ZWY7_
DRB1_0101	723	VVGPMGDTRTLAVLP	VGPMGDTRT	1	0.2967	2017.5	85.00	0.40	tr_C9ZWY7_
DRB1_0101	724	RSITNPDRVAFHSVV	PDRVAFHSV	5	0.2962	2028.7	85.00	0.28	tr_C9ZWY7_
DRB1_0101	725	VGYIARDKKTTSLHM	YIARDKKTT	2	0.2948	2058.3	85.00	0.51	tr_C9ZWY7_
DRB1_0101	726	TAENVTVEIIPPPPR	VEIIPPPPR	6	0.2944	2068.7	85.00	0.50	tr_C9ZWY7_
DRB1_0101	727	MRADEEFDSLWEEFV	FDSLWEEFV	6	0.2943	2070.4	85.00	0.69	tr_C9ZWY7_
DRB1_0101	728	AAEGCAVNYGATHIS	CAVNYGATH	4	0.2941	2074.9	85.00	0.28	tr_C9ZWY7_
DRB1_0101	729	SETKYHAVRRFQEDA	YHAVRRFQE	4	0.2936	2087.2	85.00	0.45	tr_C9ZWY7_
DRB1_0101	730	GERGASCLCNQGGNV	CLCNQGGNV	6	0.2925	2111.3	85.00	0.43	tr_C9ZWY7_
DRB1_0101	731	YVVDDLVFGNFGGEC	YVVDDLVFG	0	0.2915	2133.8	85.00	0.31	tr_C9ZWY7_
DRB1_0101	732	ITGRHITTYSTGGTM	GRHITTYST	2	0.2913	2138.5	85.00	0.50	tr_C9ZWY7_

DRB1_0101	733	IPLRDGITVFNIDRC	LRDGITVFN	2	0.2905	2157.3	85.00	0.51	tr_C9ZWY7_
DRB1_0101	734	YMNSLGDDHRMIPLR	SLGDDHRMI	3	0.2901	2166.3	85.00	0.31	tr_C9ZWY7_
DRB1_0101	735	ATPSMRYANRNRGSL	MRYANRNRG	4	0.2900	2168.9	85.00	0.51	tr_C9ZWY7_
DRB1_0101	736	ASDDFDHDDADGALM	FDHDDADGA	4	0.2897	2176.2	85.00	0.66	tr_C9ZWY7_
DRB1_0101	737	VFGNFGGECHGMAGE	FGGECHGMA	4	0.2888	2197.4	85.00	0.56	tr_C9ZWY7_
DRB1_0101	738	TYSTGGTMRRRPPAI	YSTGGTMRR	1	0.2887	2200.4	85.00	0.49	tr_C9ZWY7_
DRB1_0101	739	DLVFGNFGGECHGMA	FGGECHGMA	6	0.2862	2261.3	85.00	0.31	tr_C9ZWY7_
DRB1_0101	740	GMAGERGASCLCNQG	MAGERGASC	1	0.2856	2274.1	85.00	0.52	tr_C9ZWY7_
DRB1_0101	741	FQEDARSYLRYHPGV	FQEDARSYL	0	0.2855	2276.5	85.00	0.36	tr_C9ZWY7_
DRB1_0101	742	VITGRHITTYSTGGT	GRHITTYST	3	0.2854	2280.3	85.00	0.56	tr_C9ZWY7_
DRB1_0101	743	DITTVAEHLDNHRGV	DITTVAEHL	0	0.2845	2301.4	85.00	0.32	tr_C9ZWY7_
DRB1_0101	744	PSVPPVTKAATPSMR	VTKAATPSM	5	0.2844	2305.2	85.00	0.42	tr_C9ZWY7_
DRB1_0101	745	VEEAAVPYIPDRVIV	AVPYIPDRV	4	0.2818	2370.9	85.00	0.28	tr_C9ZWY7_
DRB1_0101	746	LIVVVVLVVDDSAAV	VVVLVVDDS	3	0.2817	2373.6	85.00	0.42	tr_C9ZWY7_
DRB1_0101	747	QSVIVADDMWYGPFE	IVADDMWYG	3	0.2798	2421.3	85.00	0.48	tr_C9ZWY7_
DRB1_0101	748	SLGDDHRMIPLRDGI	DHRMIPLRD	4	0.2788	2447.5	85.00	0.44	tr_C9ZWY7_
DRB1_0101	749	KAATPSMRYANRNRG	KAATPSMRY	0	0.2785	2457.4	85.00	0.33	tr_C9ZWY7_
DRB1_0101	750	WATAENVTVEIIPPP	WATAENVTV	0	0.2775	2484.3	85.00	0.71	tr_C9ZWY7_
DRB1_0101	751	TKYHAVRRFQEDARS	YHAVRRFQE	2	0.2772	2492.5	85.00	0.56	tr_C9ZWY7_
DRB1_0101	752	RTAVEEAAVPYIPDR	VEEAAVPYI	3	0.2755	2537.9	90.00	0.29	tr_C9ZWY7_
DRB1_0101	753	LHMVTRGGDVPGIGS	HMVTRGGDV	1	0.2736	2589.7	90.00	0.47	tr_C9ZWY7_
DRB1_0101	754	VHFGDREYQETLKLM	REYQETLKL	5	0.2711	2661.3	90.00	0.37	tr_C9ZWY7_
DRB1_0101	755	DMWYGPFEDSCVPTT	FEDSCVPTT	6	0.2707	2673.3	90.00	0.41	tr_C9ZWY7_
DRB1_0101	756	ITTVAEHLDNHRGVR	AEHLDNHRG	4	0.2703	2684.8	90.00	0.43	tr_C9ZWY7_
DRB1_0101	757	MHAATVMRADEEFDS	MHAATVMRA	0	0.2696	2704.1	90.00	0.57	tr_C9ZWY7_
DRB1_0101	758	PTPLIVVVVLVVDDS	PTPLIVVVV	0	0.2686	2733.9	90.00	0.49	tr_C9ZWY7_
DRB1_0101	759	LETAADQNKGEFFVV	QNKGEFFVV	6	0.2636	2887.4	90.00	0.28	tr_C9ZWY7_
DRB1_0101	760	PLRDGITVFNIDRCS	LRDGITVFN	1	0.2606	2981.1	90.00	0.46	tr_C9ZWY7_
DRB1_0101	761	NSLGDDHRMIPLRDG	DHRMIPLRD	5	0.2571	3096.3	90.00	0.37	tr_C9ZWY7_
DRB1_0101	762	KYHAVRRFQEDARSY	YHAVRRFQE	1	0.2552	3162.3	90.00	0.49	tr_C9ZWY7_
DRB1_0101	763	VDDLVFGNFGGECHG	DDLVFGNFG	1	0.2550	3167.5	90.00	0.28	tr_C9ZWY7_
DRB1_0101	764	DDLVFGNFGGECHGM	FGNFGGECH	4	0.2550	3168.3	90.00	0.19	tr_C9ZWY7_
DRB1_0101	765	MNSLGDDHRMIPLRD	SLGDDHRMI	2	0.2547	3178.0	90.00	0.29	tr_C9ZWY7_
DRB1_0101	766	VVDDLVFGNFGGECH	DDLVFGNFG	2	0.2545	3183.5	90.00	0.34	tr_C9ZWY7_
DRB1_0101	767	VATSRNTYGRMQGEL	VATSRNTYG	0	0.2540	3202.3	90.00	0.34	tr_C9ZWY7_
DRB1_0101	768	MYLQGVHFGDREYQE	YLQGVHFGD	1	0.2536	3216.3	90.00	0.39	tr_C9ZWY7_
DRB1_0101	769	MVTRGGDVPGIGSVF	DVPGIGSVF	6	0.2529	3239.4	90.00	0.42	tr_C9ZWY7_
DRB1_0101	770	AVEEAAVPYIPDRVI	EEAAVPYIP	2	0.2454	3514.5	90.00	0.28	tr_C9ZWY7_
DRB1_0101	771	TTLNEDLSPHGDTLV	LSPHGDTLV	6	0.2439	3570.8	90.00	0.40	tr_C9ZWY7_
DRB1_0101	772	VAAPNPLASETKYHA	PNPLASETK	3	0.2432	3599.1	90.00	0.28	tr_C9ZWY7_
DRB1_0101	773	TAVEEAAVPYIPDRV	EEAAVPYIP	3	0.2424	3632.2	90.00	0.30	tr_C9ZWY7_
DRB1_0101	774	LNPSVPPVTKAATPS	LNPSVPPVT	0	0.2413	3672.3	90.00	0.51	tr_C9ZWY7_
DRB1_0101	775	HMVTRGGDVPGIGSV	HMVTRGGDV	0	0.2410	3683.9	90.00	0.34	tr_C9ZWY7_
DRB1_0101	776	MAGERGASCLCNQGG	MAGERGASC	0	0.2409	3689.9	90.00	0.32	tr_C9ZWY7_
DRB1_0101	777	FTNRAPRRSIETMGV	FTNRAPRRS	0	0.2392	3760.1	95.00	0.40	tr_C9ZWY7_
DRB1_0101	778	PLIVVVVLVVDDSAA	VVVLVVDDS	4	0.2349	3939.1	95.00	0.49	tr_C9ZWY7_
DRB1_0101	779	AATPSMRYANRNRGS	MRYANRNRG	5	0.2344	3959.3	95.00	0.54	tr_C9ZWY7_
DRB1_0101	780	SVIVADDMWYGPFED	IVADDMWYG	2	0.2274	4270.7	95.00	0.56	tr_C9ZWY7_
DRB1_0101	781	GSMNGIVVPDNDTTL	MNGIVVPDN	2	0.2240	4427.9	95.00	0.35	tr_C9ZWY7_
DRB1_0101	782	LRDGITVFNIDRCST	ITVFNIDRC	4	0.2235	4452.4	95.00	0.24	tr_C9ZWY7_

DRB1_0101	783	NASDDFDHDDADGAL	FDHDDADGA	5	0.2217	4544.1	95.00	0.83	tr_C9ZWY7_
DRB1_0101	784	HPGVTAFNASDDFDH	VTAFNASDD	3	0.2210	4575.2	95.00	0.31	tr_C9ZWY7_
DRB1_0101	785	DEMKDDESKWTPLAL	DDESKWTPL	4	0.2201	4620.6	95.00	0.28	tr_C9ZWY7_
DRB1_0101	786	WADNNTTSETVKRFH	DNNTTSETV	2	0.2172	4768.6	95.00	0.35	tr_C9ZWY7_
DRB1_0101	787	SMNGIVVPDNDTTLN	MNGIVVPDN	1	0.2148	4892.4	95.00	0.25	tr_C9ZWY7_
DRB1_0101	788	HWADNNTTSETVKRF	DNNTTSETV	3	0.2129	4997.6	95.00	0.45	tr_C9ZWY7_
DRB1_0101	789	GVHFGDREYQETLKL	FGDREYQET	3	0.2102	5143.5	95.00	0.29	tr_C9ZWY7_
DRB1_0101	790	ADNNTTSETVKRFHD	TTSETVKRF	4	0.2046	5465.3	95.00	0.37	tr_C9ZWY7_
DRB1_0101	791	AATVMRADEEFDSLW	MRADEEFDS	4	0.2034	5537.8	95.00	0.29	tr_C9ZWY7_
DRB1_0101	792	LLETAADQNKGEFFV	ADQNKGEFF	5	0.1991	5799.8	95.00	0.38	tr_C9ZWY7_
DRB1_0101	793	ATVMRADEEFDSLWE	MRADEEFDS	3	0.1956	6022.6	95.00	0.32	tr_C9ZWY7_
DRB1_0101	794	TPLIVVVVLVVDDSA	VVVLVVDDS	5	0.1950	6063.0	95.00	0.53	tr_C9ZWY7_
DRB1_0101	795	MNGIVVPDNDTTLNE	GIVVPDNDT	2	0.1920	6264.4	100.00	0.29	tr_C9ZWY7_
DRB1_0101	796	DDMWYGPFEDSCVPT	YGPFEDSCV	4	0.1908	6347.6	100.00	0.56	tr_C9ZWY7_
DRB1_0101	797	KLLETAADQNKGEFF	LETAADQNK	2	0.1895	6431.0	100.00	0.31	tr_C9ZWY7_
DRB1_0101	798	AGERGASCLCNQGGN	ASCLCNQGG	5	0.1886	6499.4	100.00	0.34	tr_C9ZWY7_
DRB1_0101	799	HAATVMRADEEFDSL	MRADEEFDS	5	0.1880	6537.7	100.00	0.23	tr_C9ZWY7_
DRB1_0101	800	NPSVPPVTKAATPSM	VTKAATPSM	6	0.1879	6547.6	100.00	0.38	tr_C9ZWY7_
DRB1_0101	801	HDEMKDDESKWTPLA	MKDDESKWT	3	0.1879	6548.9	100.00	0.34	tr_C9ZWY7_
DRB1_0101	802	VIVADDMWYGPFEDS	IVADDMWYG	1	0.1857	6707.9	100.00	0.55	tr_C9ZWY7_
DRB1_0101	803	TNRAPRRSIETMGVG	RRSIETMGV	5	0.1855	6720.7	100.00	0.48	tr_C9ZWY7_
DRB1_0101	804	PHWADNNTTSETVKR	DNNTTSETV	4	0.1853	6731.2	100.00	0.44	tr_C9ZWY7_
DRB1_0101	805	PGVTAFNASDDFDHD	FNASDDFDH	5	0.1837	6853.7	100.00	0.34	tr_C9ZWY7_
DRB1_0101	806	TVMRADEEFDSLWEE	MRADEEFDS	2	0.1782	7272.7	100.00	0.32	tr_C9ZWY7_
DRB1_0101	807	DNNTTSETVKRFHDE	TTSETVKRF	3	0.1739	7616.1	100.00	0.46	tr_C9ZWY7_
DRB1_0101	808	QGVHFGDREYQETLK	FGDREYQET	4	0.1735	7652.8	100.00	0.34	tr_C9ZWY7_
DRB1_0101	809	YLQGVHFGDREYQET	YLQGVHFGD	0	0.1728	7711.7	100.00	0.22	tr_C9ZWY7_
DRB1_0101	810	ADDMWYGPFEDSCVP	YGPFEDSCV	5	0.1726	7722.7	100.00	0.47	tr_C9ZWY7_
DRB1_0101	811	DTTLNEDLSPHGDTL	LNEDLSPHG	3	0.1697	7970.0	100.00	0.49	tr_C9ZWY7_
DRB1_0101	812	LPHWADNNTTSETVK	DNNTTSETV	5	0.1680	8116.7	100.00	0.35	tr_C9ZWY7_
DRB1_0101	813	NGIVVPDNDTTLNED	VVPDNDTTL	3	0.1648	8402.5	100.00	0.37	tr_C9ZWY7_
DRB1_0101	814	FHDEMKDDESKWTPL	MKDDESKWT	4	0.1647	8415.0	100.00	0.35	tr_C9ZWY7_
DRB1_0101	815	LQGVHFGDREYQETL	FGDREYQET	5	0.1628	8591.1	100.00	0.29	tr_C9ZWY7_
DRB1_0101	816	GVTAFNASDDFDHDD	FNASDDFDH	4	0.1614	8719.0	100.00	0.44	tr_C9ZWY7_
DRB1_0101	817	VADDMWYGPFEDSCV	YGPFEDSCV	6	0.1575	9097.4	100.00	0.42	tr_C9ZWY7_
DRB1_0101	818	FNASDDFDHDDADGA	FDHDDADGA	6	0.1496	9904.4	100.00	0.63	tr_C9ZWY7_
DRB1_0101	819	GIVVPDNDTTLNEDL	VVPDNDTTL	2	0.1492	9951.0	100.00	0.34	tr_C9ZWY7_
DRB1_0101	820	NNTTSETVKRFHDEM	TTSETVKRF	2	0.1486	10011.4	100.00	0.51	tr_C9ZWY7_
DRB1_0101	821	VMRADEEFDSLWEEF	MRADEEFDS	1	0.1478	10102.4	100.00	0.31	tr_C9ZWY7_
DRB1_0101	822	VTAFNASDDFDHDDA	FNASDDFDH	3	0.1475	10140.0	100.00	0.63	tr_C9ZWY7_
DRB1_0101	823	NDTTLNEDLSPHGDT	LNEDLSPHG	4	0.1446	10458.5	100.00	0.49	tr_C9ZWY7_
DRB1_0101	824	KRFHDEMKDDESKWT	FHDEMKDDE	2	0.1421	10750.3	100.00	0.26	tr_C9ZWY7_
DRB1_0101	825	RFHDEMKDDESKWTP	MKDDESKWT	5	0.1414	10826.9	100.00	0.34	tr_C9ZWY7_
DRB1_0101	826	SLPHWADNNTTSETV	WADNNTTSE	4	0.1358	11500.3	100.00	0.41	tr_C9ZWY7_
DRB1_0101	827	ATAENVTVEIIPPPP	VTVEIIPPP	5	0.1356	11531.6	100.00	0.37	tr_C9ZWY7_
DRB1_0101	828	IVADDMWYGPFEDSC	IVADDMWYG	0	0.1352	11575.1	100.00	0.34	tr_C9ZWY7_
DRB1_0101	829	DNDTTLNEDLSPHGD	LNEDLSPHG	5	0.1300	12246.0	100.00	0.42	tr_C9ZWY7_
DRB1_0101	830	ATSLPHWADNNTTSE	PHWADNNTT	4	0.1293	12336.1	100.00	0.28	tr_C9ZWY7_
DRB1_0101	831	NTTSETVKRFHDEMK	TTSETVKRF	1	0.1272	12620.9	100.00	0.34	tr_C9ZWY7_
DRB1_0101	832	VKRFHDEMKDDESKW	FHDEMKDDE	3	0.1244	13014.6	100.00	0.38	tr_C9ZWY7_

DRB1_0101	833	TAFNASDDFDHDDAD	FNASDDFDH	2	0.1238	13098.4	100.00	0.75	tr_C9ZWY7_
DRB1_0101	834	TVKRFHDEMKDDESK	FHDEMKDDE	4	0.1227	13261.8	100.00	0.37	tr_C9ZWY7_
DRB1_0101	835	IVVPDNDTTLNEDLS	VVPDNDTTL	1	0.1203	13611.5	100.00	0.31	tr_C9ZWY7_
DRB1_0101	836	TSLPHWADNNTTSET	WADNNTTSE	5	0.1194	13733.4	100.00	0.36	tr_C9ZWY7_
DRB1_0101	837	ETVKRFHDEMKDDES	FHDEMKDDE	5	0.1161	14230.3	100.00	0.28	tr_C9ZWY7_
DRB1_0101	838	SETVKRFHDEMKDDE	VKRFHDEMK	3	0.1136	14622.5	100.00	0.23	tr_C9ZWY7_
DRB1_0101	839	TTSETVKRFHDEMKD	VKRFHDEMK	5	0.1133	14678.6	100.00	0.24	tr_C9ZWY7_
DRB1_0101	840	PDNDTTLNEDLSPHG	LNEDLSPHG	6	0.1131	14700.2	100.00	0.28	tr_C9ZWY7_
DRB1_0101	841	TSETVKRFHDEMKDD	VKRFHDEMK	4	0.1118	14913.3	100.00	0.28	tr_C9ZWY7_
DRB1_0101	842	AFNASDDFDHDDADG	FNASDDFDH	1	0.1044	16165.4	100.00	0.69	tr_C9ZWY7_
DRB1_0101	843	VPDNDTTLNEDLSPH	NDTTLNEDL	3	0.0971	17479.2	100.00	0.43	tr_C9ZWY7_
DRB1_0101	844	VVPDNDTTLNEDLSP	NDTTLNEDL	4	0.0971	17493.8	100.00	0.38	tr_C9ZWY7_

Allele: DRB1_0101. Number of high binders 12. Number of weak binders 93. Number of peptides 844

Allele	pos	peptide	core	Of	1-log50k(aff)	affinity(nM)	%Rank	Relia	Identity	Bind Level
DRB1_0101	1	GQLFLHAFGTTTSDS	FLHAFGTTT	3	0.8709	4.0	0.30	0.83	tr_C9ZZQ4_	SB
DRB1_0101	2	SGQLFLHAFGTTTSD	FLHAFGTTT	4	0.8636	4.4	0.40	0.81	tr_C9ZZQ4_	SB
DRB1_0101	3	QLFLHAFGTTTSDSA	FLHAFGTTT	2	0.8585	4.6	0.50	0.85	tr_C9ZZQ4_	WB
DRB1_0101	4	YSGQLFLHAFGTTTS	FLHAFGTTT	5	0.8582	4.6	0.50	0.77	tr_C9ZZQ4_	WB
DRB1_0101	5	RELYFLRPSPTIETM	LYFLRPSPT	2	0.8253	6.6	1.20	0.69	tr_C9ZZQ4_	WB
DRB1_0101	6	VRELYFLRPSPTIET	LYFLRPSPT	3	0.8239	6.7	1.20	0.78	tr_C9ZZQ4_	WB
DRB1_0101	7	VLIRHALGHLGVLRL	IRHALGHLG	2	0.8231	6.8	1.30	0.77	tr_C9ZZQ4_	WB
DRB1_0101	8	MVLIRHALGHLGVLR	IRHALGHLG	3	0.8222	6.8	1.30	0.80	tr_C9ZZQ4_	WB
DRB1_0101	9	LFLHAFGTTTSDSAK	FLHAFGTTT	1	0.8168	7.3	1.40	0.88	tr_C9ZZQ4_	WB
DRB1_0101	10	LIRHALGHLGVLRLG	IRHALGHLG	1	0.8124	7.6	1.60	0.62	tr_C9ZZQ4_	WB
DRB1_0101	11	TMVLIRHALGHLGVL	IRHALGHLG	4	0.8111	7.7	1.60	0.73	tr_C9ZZQ4_	WB
DRB1_0101	12	WVRELYFLRPSPTIE	LYFLRPSPT	4	0.8075	8.0	1.80	0.82	tr_C9ZZQ4_	WB
DRB1_0101	13	ELYFLRPSPTIETMV	LYFLRPSPT	1	0.8018	8.5	2.00	0.59	tr_C9ZZQ4_	WB
DRB1_0101	14	GKLLIAGTNPLAKNE	LLIAGTNPL	2	0.7926	9.4	2.50	0.41	tr_C9ZZQ4_	WB
DRB1_0101	15	EYSGQLFLHAFGTTT	FLHAFGTTT	6	0.7913	9.6	2.50	0.68	tr_C9ZZQ4_	WB
DRB1_0101	16	PGKLLIAGTNPLAKN	LLIAGTNPL	3	0.7903	9.7	2.50	0.43	tr_C9ZZQ4_	WB
DRB1_0101	17	LYEKFVTAFNGSAAA	FVTAFNGSA	4	0.7901	9.7	2.50	0.76	tr_C9ZZQ4_	WB
DRB1_0101	18	RWVRELYFLRPSPTI	LYFLRPSPT	5	0.7860	10.1	3.00	0.85	tr_C9ZZQ4_	WB
DRB1_0101	19	ETMVLIRHALGHLGV	IRHALGHLG	5	0.7774	11.1	3.50	0.70	tr_C9ZZQ4_	WB
DRB1_0101	20	YEKFVTAFNGSAAAP	FVTAFNGSA	3	0.7748	11.4	3.50	0.80	tr_C9ZZQ4_	WB
DRB1_0101	21	LRSISLSVPPHALST	ISLSVPPHA	3	0.7733	11.6	3.50	0.79	tr_C9ZZQ4_	WB
DRB1_0101	22	KLLIAGTNPLAKNEQ	LIAGTNPLA	2	0.7733	11.6	3.50	0.47	tr_C9ZZQ4_	WB
DRB1_0101	23	KFVTAFNGSAAAPRL	FVTAFNGSA	1	0.7700	12.0	3.50	0.56	tr_C9ZZQ4_	WB
DRB1_0101	24	EKFVTAFNGSAAAPR	FVTAFNGSA	2	0.7679	12.3	4.00	0.70	tr_C9ZZQ4_	WB
DRB1_0101	25	LYFLRPSPTIETMVL	LYFLRPSPT	0	0.7679	12.3	4.00	0.44	tr_C9ZZQ4_	WB
DRB1_0101	26	CLRSISLSVPPHALS	ISLSVPPHA	4	0.7631	13.0	4.00	0.79	tr_C9ZZQ4_	WB
DRB1_0101	27	FPGKLLIAGTNPLAK	LLIAGTNPL	4	0.7628	13.0	4.00	0.46	tr_C9ZZQ4_	WB
DRB1_0101	28	RSISLSVPPHALSTF	ISLSVPPHA	2	0.7581	13.7	4.50	0.75	tr_C9ZZQ4_	WB
DRB1_0101	29	LLYEKFVTAFNGSAA	FVTAFNGSA	5	0.7570	13.9	4.50	0.62	tr_C9ZZQ4_	WB
DRB1_0101	30	IRHALGHLGVLRLGF	IRHALGHLG	0	0.7565	13.9	4.50	0.43	tr_C9ZZQ4_	WB

DRB1_0101	31	NISLVRRRGAALTGH	LVRRRGAAL	3	0.7463	15.6	5.00	0.65	tr_C9ZZQ4_	WB
DRB1_0101	32	ISLVRRRGAALTGHL	LVRRRGAAL	2	0.7430	16.1	5.50	0.56	tr_C9ZZQ4_	WB
DRB1_0101	33	FERFAATKPQAVLLF	FAATKPQAV	3	0.7424	16.2	5.50	0.81	tr_C9ZZQ4_	WB
DRB1_0101	34	TGRFLRKLVADRRTS	LRKLVADRR	4	0.7405	16.6	5.50	0.40	tr_C9ZZQ4_	WB
DRB1_0101	35	SCLRSISLSVPPHAL	ISLSVPPHA	5	0.7355	17.5	6.00	0.73	tr_C9ZZQ4_	WB
DRB1_0101	36	VFERFAATKPQAVLL	FAATKPQAV	4	0.7293	18.7	6.50	0.75	tr_C9ZZQ4_	WB
DRB1_0101	37	LNISLVRRRGAALTG	LVRRRGAAL	4	0.7285	18.9	6.50	0.62	tr_C9ZZQ4_	WB
DRB1_0101	38	DTGRFLRKLVADRRT	LRKLVADRR	5	0.7235	19.9	7.00	0.32	tr_C9ZZQ4_	WB
DRB1_0101	39	GRFLRKLVADRRTSG	LRKLVADRR	3	0.7231	20.0	7.00	0.43	tr_C9ZZQ4_	WB
DRB1_0101	40	SLVRRRGAALTGHLP	LVRRRGAAL	1	0.7213	20.4	7.00	0.44	tr_C9ZZQ4_	WB
DRB1_0101	41	FVTAFNGSAAAPRLV	FNGSAAAPR	4	0.7213	20.4	7.00	0.45	tr_C9ZZQ4_	WB
DRB1_0101	42	ALLYEKFVTAFNGSA	FVTAFNGSA	6	0.7196	20.8	7.00	0.32	tr_C9ZZQ4_	WB
DRB1_0101	43	TIAVKATASWLVGAL	VKATASWLV	3	0.7187	21.0	7.50	0.71	tr_C9ZZQ4_	WB
DRB1_0101	44	ERFAATKPQAVLLFG	FAATKPQAV	2	0.7159	21.6	7.50	0.79	tr_C9ZZQ4_	WB
DRB1_0101	45	RLGFMYLQGHHYGEK	FMYLQGHHY	3	0.7096	23.1	8.00	0.76	tr_C9ZZQ4_	WB
DRB1_0101	46	NTIAVKATASWLVGA	VKATASWLV	4	0.7095	23.2	8.00	0.63	tr_C9ZZQ4_	WB
DRB1_0101	47	VTAFNGSAAAPRLVF	FNGSAAAPR	3	0.7056	24.2	8.50	0.62	tr_C9ZZQ4_	WB
DRB1_0101	48	IETMVLIRHALGHLG	IRHALGHLG	6	0.7024	25.0	9.00	0.56	tr_C9ZZQ4_	WB
DRB1_0101	49	FLHAFGTTTSDSAKA	FLHAFGTTT	0	0.7012	25.4	9.00	0.69	tr_C9ZZQ4_	WB
DRB1_0101	50	TAFNGSAAAPRLVFA	FNGSAAAPR	2	0.7005	25.5	9.00	0.54	tr_C9ZZQ4_	WB
DRB1_0101	51	RRWVRELYFLRPSPT	LYFLRPSPT	6	0.6998	25.7	9.00	0.80	tr_C9ZZQ4_	WB
DRB1_0101	52	SLNISLVRRRGAALT	LVRRRGAAL	5	0.6995	25.8	9.00	0.58	tr_C9ZZQ4_	WB
DRB1_0101	53	VDVIERHLAAHSELR	IERHLAAHS	3	0.6994	25.8	9.00	0.70	tr_C9ZZQ4_	WB
DRB1_0101	54	SISLSVPPHALSTFS	ISLSVPPHA	1	0.6971	26.5	9.50	0.72	tr_C9ZZQ4_	WB
DRB1_0101	55	FRRHVIHLSPTVEQQ	HVIHLSPTV	3	0.6964	26.7	9.50	0.46	tr_C9ZZQ4_	WB
DRB1_0101	56	RFAATKPQAVLLFGA	FAATKPQAV	1	0.6953	27.0	9.50	0.73	tr_C9ZZQ4_	WB
DRB1_0101	57	AVFERFAATKPQAVL	FAATKPQAV	5	0.6936	27.5	9.50	0.58	tr_C9ZZQ4_	WB
DRB1_0101	58	SDTGRFLRKLVADRR	GRFLRKLVA	3	0.6922	27.9	10.00	0.40	tr_C9ZZQ4_	
DRB1_0101	59	KFRRHVIHLSPTVEQ	HVIHLSPTV	4	0.6893	28.9	10.00	0.43	tr_C9ZZQ4_	
DRB1_0101	60	AVCYCNQGGNVVYVR	YCNQGGNVV	3	0.6881	29.2	10.00	0.62	tr_C9ZZQ4_	
DRB1_0101	61	RRHVIHLSPTVEQQI	HVIHLSPTV	2	0.6873	29.5	11.00	0.41	tr_C9ZZQ4_	
DRB1_0101	62	LLIAGTNPLAKNEQY	LIAGTNPLA	1	0.6871	29.5	11.00	0.52	tr_C9ZZQ4_	
DRB1_0101	63	PFPGKLLIAGTNPLA	LLIAGTNPL	5	0.6851	30.2	11.00	0.51	tr_C9ZZQ4_	
DRB1_0101	64	IAVKATASWLVGALR	VKATASWLV	2	0.6837	30.6	11.00	0.73	tr_C9ZZQ4_	
DRB1_0101	65	VTKALMKMTNTLFID	LMKMTNTLF	4	0.6827	31.0	11.00	0.71	tr_C9ZZQ4_	
DRB1_0101	66	SGAYVLAPSGAQVFL	YVLAPSGAQ	3	0.6808	31.6	11.00	0.52	tr_C9ZZQ4_	
DRB1_0101	67	LRLGFMYLQGHHYGE	FMYLQGHHY	4	0.6803	31.8	11.00	0.81	tr_C9ZZQ4_	
DRB1_0101	68	LGFMYLQGHHYGEKE	FMYLQGHHY	2	0.6798	32.0	11.00	0.74	tr_C9ZZQ4_	
DRB1_0101	69	DVIERHLAAHSELRV	IERHLAAHS	2	0.6788	32.3	12.00	0.62	tr_C9ZZQ4_	
DRB1_0101	70	EVALLYEKFVTAFNG	LLYEKFVTA	3	0.6776	32.7	12.00	0.61	tr_C9ZZQ4_	
DRB1_0101	71	KIFTTAVNAGFNASM	FTTAVNAGF	2	0.6774	32.8	12.00	0.60	tr_C9ZZQ4_	
DRB1_0101	72	MSCLRSISLSVPPHA	ISLSVPPHA	6	0.6763	33.2	12.00	0.40	tr_C9ZZQ4_	
DRB1_0101	73	TAVFGIVTKALMKMT	FGIVTKALM	3	0.6744	33.9	12.00	0.32	tr_C9ZZQ4_	
DRB1_0101	74	VALLYEKFVTAFNGS	LLYEKFVTA	2	0.6734	34.3	12.00	0.57	tr_C9ZZQ4_	
DRB1_0101	75	IVTKALMKMTNTLFI	LMKMTNTLF	5	0.6732	34.3	12.00	0.69	tr_C9ZZQ4_	
DRB1_0101	76	NKFRRHVIHLSPTVE	FRRHVIHLS	2	0.6727	34.5	12.00	0.40	tr_C9ZZQ4_	
DRB1_0101	77	AFNGSAAAPRLVFAT	FNGSAAAPR	1	0.6722	34.7	12.00	0.44	tr_C9ZZQ4_	
DRB1_0101	78	PKIFTTAVNAGFNAS	FTTAVNAGF	3	0.6711	35.1	12.00	0.68	tr_C9ZZQ4_	
DRB1_0101	79	DVDVIERHLAAHSEL	IERHLAAHS	4	0.6700	35.5	13.00	0.67	tr_C9ZZQ4_	
DRB1_0101	80	VTAVFGIVTKALMKM	FGIVTKALM	4	0.6699	35.6	13.00	0.30	tr_C9ZZQ4_	

DRB1_0101	81	DNTIAVKATASWLVG	VKATASWLV	5	0.6689	36.0	13.00	0.59	tr_C9ZZQ4_
DRB1_0101	82	RHALGHLGVLRLGFM	LGHLGVLRL	3	0.6688	36.0	13.00	0.66	tr_C9ZZQ4_
DRB1_0101	83	GAYVLAPSGAQVFLE	YVLAPSGAQ	2	0.6686	36.1	13.00	0.52	tr_C9ZZQ4_
DRB1_0101	84	TKALMKMTNTLFIDP	LMKMTNTLF	3	0.6682	36.2	13.00	0.69	tr_C9ZZQ4_
DRB1_0101	85	VLRLGFMYLQGHHYG	FMYLQGHHY	5	0.6677	36.4	13.00	0.76	tr_C9ZZQ4_
DRB1_0101	86	VIERHLAAHSELRVL	IERHLAAHS	1	0.6674	36.6	13.00	0.54	tr_C9ZZQ4_
DRB1_0101	87	SEVALLYEKFVTAFN	LLYEKFVTA	4	0.6670	36.7	13.00	0.64	tr_C9ZZQ4_
DRB1_0101	88	VCYCNQGGNVVYVRE	YCNQGGNVV	2	0.6646	37.7	13.00	0.63	tr_C9ZZQ4_
DRB1_0101	89	AVFGIVTKALMKMTN	GIVTKALMK	3	0.6629	38.4	13.00	0.35	tr_C9ZZQ4_
DRB1_0101	90	RHVIHLSPTVEQQIY	IHLSPTVEQ	3	0.6625	38.5	13.00	0.32	tr_C9ZZQ4_
DRB1_0101	91	NAIVCAAEAHGIMKV	VCAAEAHGI	3	0.6618	38.8	14.00	0.55	tr_C9ZZQ4_
DRB1_0101	92	AYVLAPSGAQVFLEL	YVLAPSGAQ	1	0.6589	40.1	14.00	0.46	tr_C9ZZQ4_
DRB1_0101	93	ANAIVCAAEAHGIMK	VCAAEAHGI	4	0.6582	40.4	14.00	0.46	tr_C9ZZQ4_
DRB1_0101	94	RFLRKLVADRRTSGA	LRKLVADRR	2	0.6578	40.6	14.00	0.63	tr_C9ZZQ4_
DRB1_0101	95	LVRRRGAALTGHLPT	VRRRGAALT	1	0.6565	41.1	14.00	0.40	tr_C9ZZQ4_
DRB1_0101	96	GAVCYCNQGGNVVYV	YCNQGGNVV	4	0.6561	41.3	14.00	0.63	tr_C9ZZQ4_
DRB1_0101	97	VLLLLMPYPLQVHGA	LLMPYPLQV	3	0.6561	41.3	14.00	0.38	tr_C9ZZQ4_
DRB1_0101	98	YVLLLLMPYPLQVHG	LLLMPYPLQ	3	0.6509	43.7	15.00	0.37	tr_C9ZZQ4_
DRB1_0101	99	GAQVFLELMWKRVLL	FLELMWKRV	4	0.6508	43.8	15.00	0.60	tr_C9ZZQ4_
DRB1_0101	100	FSEVALLYEKFVTAF	LLYEKFVTA	5	0.6464	45.9	16.00	0.68	tr_C9ZZQ4_
DRB1_0101	101	AQVFLELMWKRVLLD	FLELMWKRV	3	0.6460	46.1	16.00	0.60	tr_C9ZZQ4_
DRB1_0101	102	MYVLLLLMPYPLQVH	LLLMPYPLQ	4	0.6448	46.7	16.00	0.36	tr_C9ZZQ4_
DRB1_0101	103	RVKVQVVQSSKSHKT	VQVVQSSKS	3	0.6441	47.0	16.00	0.70	tr_C9ZZQ4_
DRB1_0101	104	GFMYLQGHHYGEKEY	FMYLQGHHY	1	0.6435	47.4	16.00	0.69	tr_C9ZZQ4_
DRB1_0101	105	SVTLHSPLNGLVIFF	HSPLNGLVI	4	0.6429	47.6	16.00	0.38	tr_C9ZZQ4_
DRB1_0101	106	ALRVMEEMGYKLCGV	VMEEMGYKL	3	0.6425	47.9	16.00	0.77	tr_C9ZZQ4_
DRB1_0101	107	TSGAYVLAPSGAQVF	YVLAPSGAQ	4	0.6415	48.3	16.00	0.55	tr_C9ZZQ4_
DRB1_0101	108	KALMKMTNTLFIDPI	LMKMTNTLF	2	0.6412	48.5	16.00	0.67	tr_C9ZZQ4_
DRB1_0101	109	TVTAVFGIVTKALMK	FGIVTKALM	5	0.6409	48.7	16.00	0.30	tr_C9ZZQ4_
DRB1_0101	110	LLLLMPYPLQVHGAP	LLMPYPLQV	2	0.6390	49.7	17.00	0.39	tr_C9ZZQ4_
DRB1_0101	111	RVMEEMGYKLCGVFV	VMEEMGYKL	1	0.6377	50.4	17.00	0.50	tr_C9ZZQ4_
DRB1_0101	112	DSVTLHSPLNGLVIF	HSPLNGLVI	5	0.6375	50.5	17.00	0.31	tr_C9ZZQ4_
DRB1_0101	113	GIVTKALMKMTNTLF	LMKMTNTLF	6	0.6368	50.9	17.00	0.55	tr_C9ZZQ4_
DRB1_0101	114	IFTTAVNAGFNASME	FTTAVNAGF	1	0.6362	51.2	17.00	0.44	tr_C9ZZQ4_
DRB1_0101	115	NAVFERFAATKPQAV	FERFAATKP	3	0.6358	51.5	17.00	0.50	tr_C9ZZQ4_
DRB1_0101	116	HALGHLGVLRLGFMY	LGHLGVLRL	2	0.6334	52.8	17.00	0.64	tr_C9ZZQ4_
DRB1_0101	117	VFGIVTKALMKMTNT	GIVTKALMK	2	0.6332	52.9	17.00	0.38	tr_C9ZZQ4_
DRB1_0101	118	VPKIFTTAVNAGFNA	FTTAVNAGF	4	0.6327	53.2	17.00	0.74	tr_C9ZZQ4_
DRB1_0101	119	LNKFRRHVIHLSPTV	FRRHVIHLS	3	0.6324	53.4	18.00	0.53	tr_C9ZZQ4_
DRB1_0101	120	AVKATASWLVGALRH	VKATASWLV	1	0.6318	53.7	18.00	0.71	tr_C9ZZQ4_
DRB1_0101	121	GAVFVFGISASDVDV	FVFGISASD	3	0.6311	54.1	18.00	0.65	tr_C9ZZQ4_
DRB1_0101	122	GEMMVYGWAAGEVLS	YGWAAGEVL	5	0.6301	54.7	18.00	0.38	tr_C9ZZQ4_
DRB1_0101	123	QVFLELMWKRVLLDS	FLELMWKRV	2	0.6294	55.1	18.00	0.60	tr_C9ZZQ4_
DRB1_0101	124	RVLVLFSEVALLYEK	VLFSEVALL	3	0.6273	56.4	18.00	0.34	tr_C9ZZQ4_
DRB1_0101	125	VALRVMEEMGYKLCG	VMEEMGYKL	4	0.6252	57.7	19.00	0.75	tr_C9ZZQ4_
DRB1_0101	126	SGAQVFLELMWKRVL	FLELMWKRV	5	0.6249	57.9	19.00	0.59	tr_C9ZZQ4_
DRB1_0101	127	GVLRLGFMYLQGHHY	FMYLQGHHY	6	0.6246	58.1	19.00	0.69	tr_C9ZZQ4_
DRB1_0101	128	CYCNQGGNVVYVREV	YCNQGGNVV	1	0.6242	58.3	19.00	0.58	tr_C9ZZQ4_
DRB1_0101	129	ELRVLVLFSEVALLY	VLVLFSEVA	3	0.6228	59.2	19.00	0.50	tr_C9ZZQ4_
DRB1_0101	130	LRVLVLFSEVALLYE	VLVLFSEVA	2	0.6226	59.3	19.00	0.45	tr_C9ZZQ4_

DRB1_0101	131	VMEEMGYKLCGVFVV	GYKLCGVFV	5	0.6218	59.9	19.00	0.49	tr_C9ZZQ4_
DRB1_0101	132	GSLNISLVRRRGAAL	LVRRRGAAL	6	0.6216	60.0	19.00	0.42	tr_C9ZZQ4_
DRB1_0101	133	NQRRYVIADFVIGDF	YVIADFVIG	4	0.6208	60.5	19.00	0.68	tr_C9ZZQ4_
DRB1_0101	134	VKVQVVQSSKSHKTP	VQVVQSSKS	2	0.6204	60.8	19.00	0.64	tr_C9ZZQ4_
DRB1_0101	135	EMMVYGWAAGEVLSQ	YGWAAGEVL	4	0.6201	61.0	19.00	0.52	tr_C9ZZQ4_
DRB1_0101	136	AVFVFGISASDVDVI	FVFGISASD	2	0.6200	61.0	19.00	0.63	tr_C9ZZQ4_
DRB1_0101	137	YPLQVHGAPANINVK	QVHGAPANI	3	0.6197	61.3	19.00	0.32	tr_C9ZZQ4_
DRB1_0101	138	YFLRPSPTIETMVLI	LRPSPTIET	2	0.6194	61.5	19.00	0.46	tr_C9ZZQ4_
DRB1_0101	139	EANAIVCAAEAHGIM	VCAAEAHGI	5	0.6180	62.4	20.00	0.41	tr_C9ZZQ4_
DRB1_0101	140	APRLVFATNLPHWYD	LVFATNLPH	3	0.6180	62.4	20.00	0.61	tr_C9ZZQ4_
DRB1_0101	141	DRVKVQVVQSSKSHK	VQVVQSSKS	4	0.6179	62.4	20.00	0.73	tr_C9ZZQ4_
DRB1_0101	142	SDVDVIERHLAAHSE	IERHLAAHS	5	0.6177	62.6	20.00	0.61	tr_C9ZZQ4_
DRB1_0101	143	EEMGYKLCGVFVVLN	GYKLCGVFV	3	0.6166	63.3	20.00	0.62	tr_C9ZZQ4_
DRB1_0101	144	KSDTGRFLRKLVADR	GRFLRKLVA	4	0.6164	63.5	20.00	0.52	tr_C9ZZQ4_
DRB1_0101	145	AIVCAAEAHGIMKVI	VCAAEAHGI	2	0.6163	63.6	20.00	0.59	tr_C9ZZQ4_
DRB1_0101	146	QRRYVIADFVIGDFG	YVIADFVIG	3	0.6140	65.1	20.00	0.69	tr_C9ZZQ4_
DRB1_0101	147	SGAVFVFGISASDVD	FVFGISASD	4	0.6128	66.0	20.00	0.63	tr_C9ZZQ4_
DRB1_0101	148	YNQRRYVIADFVIGD	YVIADFVIG	5	0.6126	66.2	20.00	0.62	tr_C9ZZQ4_
DRB1_0101	149	ADSVTLHSPLNGLVI	SVTLHSPLN	2	0.6122	66.4	21.00	0.27	tr_C9ZZQ4_
DRB1_0101	150	AAPRLVFATNLPHWY	LVFATNLPH	4	0.6117	66.8	21.00	0.62	tr_C9ZZQ4_
DRB1_0101	151	VTLHSPLNGLVIFFG	HSPLNGLVI	3	0.6109	67.3	21.00	0.54	tr_C9ZZQ4_
DRB1_0101	152	PYPLQVHGAPANINV	LQVHGAPAN	3	0.6104	67.7	21.00	0.31	tr_C9ZZQ4_
DRB1_0101	153	LRVMEEMGYKLCGVF	VMEEMGYKL	2	0.6095	68.4	21.00	0.78	tr_C9ZZQ4_
DRB1_0101	154	EMGYKLCGVFVVLNV	GYKLCGVFV	2	0.6091	68.6	21.00	0.57	tr_C9ZZQ4_
DRB1_0101	155	KLVADRRTSGAYVLA	DRRTSGAYV	4	0.6078	69.6	21.00	0.69	tr_C9ZZQ4_
DRB1_0101	156	IERHLAAHSELRVLV	IERHLAAHS	0	0.6061	71.0	22.00	0.31	tr_C9ZZQ4_
DRB1_0101	157	VMYVLLLLMPYPLQV	LLLMPYPLQ	5	0.6050	71.8	22.00	0.38	tr_C9ZZQ4_
DRB1_0101	158	LFSEVALLYEKFVTA	LLYEKFVTA	6	0.6041	72.5	22.00	0.51	tr_C9ZZQ4_
DRB1_0101	159	MEEMGYKLCGVFVVL	GYKLCGVFV	4	0.6040	72.6	22.00	0.60	tr_C9ZZQ4_
DRB1_0101	160	YVLAPSGAQVFLELM	LAPSGAQVF	2	0.6028	73.6	22.00	0.39	tr_C9ZZQ4_
DRB1_0101	161	LLLMPYPLQVHGAPA	LLMPYPLQV	1	0.6021	74.1	22.00	0.38	tr_C9ZZQ4_
DRB1_0101	162	MMVYGWAAGEVLSQA	YGWAAGEVL	3	0.6017	74.4	22.00	0.67	tr_C9ZZQ4_
DRB1_0101	163	FGIVTKALMKMTNTL	GIVTKALMK	1	0.6006	75.3	22.00	0.40	tr_C9ZZQ4_
DRB1_0101	164	EKWGGSLNISLVRRR	GGSLNISLV	3	0.6000	75.8	23.00	0.41	tr_C9ZZQ4_
DRB1_0101	165	PRLVFATNLPHWYDN	LVFATNLPH	2	0.5991	76.5	23.00	0.57	tr_C9ZZQ4_
DRB1_0101	166	LEYSGQLFLHAFGTT	QLFLHAFGT	5	0.5989	76.7	23.00	0.45	tr_C9ZZQ4_
DRB1_0101	167	KWGGSLNISLVRRRG	GGSLNISLV	2	0.5978	77.6	23.00	0.40	tr_C9ZZQ4_
DRB1_0101	168	TSGAVFVFGISASDV	FVFGISASD	5	0.5977	77.7	23.00	0.63	tr_C9ZZQ4_
DRB1_0101	169	EVALRVMEEMGYKLC	VMEEMGYKL	5	0.5973	78.0	23.00	0.68	tr_C9ZZQ4_
DRB1_0101	170	LYNQRRYVIADFVIG	YVIADFVIG	6	0.5972	78.1	23.00	0.48	tr_C9ZZQ4_
DRB1_0101	171	MPYPLQVHGAPANIN	LQVHGAPAN	4	0.5966	78.6	23.00	0.28	tr_C9ZZQ4_
DRB1_0101	172	QGAVCYCNQGGNVVY	YCNQGGNVV	5	0.5963	78.9	23.00	0.65	tr_C9ZZQ4_
DRB1_0101	173	EDDFSMQTPKDGTVG	FSMQTPKDG	3	0.5950	80.0	23.00	0.90	tr_C9ZZQ4_
DRB1_0101	174	LVADRRTSGAYVLAP	DRRTSGAYV	3	0.5944	80.6	23.00	0.68	tr_C9ZZQ4_
DRB1_0101	175	FNGSAAAPRLVFATN	GSAAAPRLV	2	0.5943	80.6	23.00	0.32	tr_C9ZZQ4_
DRB1_0101	176	NVKVLLCTWNTRVPK	LLCTWNTRV	4	0.5925	82.1	24.00	0.51	tr_C9ZZQ4_
DRB1_0101	177	DGEMMVYGWAAGEVL	EMMVYGWAA	2	0.5922	82.4	24.00	0.41	tr_C9ZZQ4_
DRB1_0101	178	ISLSVPPHALSTFSR	ISLSVPPHA	0	0.5919	82.7	24.00	0.49	tr_C9ZZQ4_
DRB1_0101	179	KVQVVQSSKSHKTPE	VQVVQSSKS	1	0.5909	83.6	24.00	0.54	tr_C9ZZQ4_
DRB1_0101	180	GDNTIAVKATASWLV	VKATASWLV	6	0.5903	84.2	24.00	0.44	tr_C9ZZQ4_

DRB1_0101	181	RTSGAYVLAPSGAQV	YVLAPSGAQ	5	0.5867	87.5	25.00	0.55	tr_C9ZZQ4_
DRB1_0101	182	DDFSMQTPKDGTVGL	FSMQTPKDG	2	0.5852	89.0	25.00	0.90	tr_C9ZZQ4_
DRB1_0101	183	VKVLLCTWNTRVPKI	LLCTWNTRV	3	0.5851	89.0	25.00	0.54	tr_C9ZZQ4_
DRB1_0101	184	MGYKLCGVFVVLNVN	GYKLCGVFV	1	0.5850	89.1	25.00	0.51	tr_C9ZZQ4_
DRB1_0101	185	HVIHLSPTVEQQIYV	IHLSPTVEQ	2	0.5849	89.2	25.00	0.38	tr_C9ZZQ4_
DRB1_0101	186	FAATKPQAVLLFGAP	FAATKPQAV	0	0.5840	90.1	25.00	0.59	tr_C9ZZQ4_
DRB1_0101	187	PLQVHGAPANINVKV	QVHGAPANI	2	0.5837	90.4	25.00	0.31	tr_C9ZZQ4_
DRB1_0101	188	ALGHLGVLRLGFMYL	LGHLGVLRL	1	0.5815	92.6	26.00	0.55	tr_C9ZZQ4_
DRB1_0101	189	KSGITIVFGPVGDDT	ITIVFGPVG	3	0.5810	93.1	26.00	0.77	tr_C9ZZQ4_
DRB1_0101	190	VFLELMWKRVLLDSQ	FLELMWKRV	1	0.5799	94.1	26.00	0.54	tr_C9ZZQ4_
DRB1_0101	191	WGGSLNISLVRRRGA	LNISLVRRR	4	0.5790	95.1	26.00	0.37	tr_C9ZZQ4_
DRB1_0101	192	RVPKIFTTAVNAGFN	FTTAVNAGF	5	0.5775	96.7	26.00	0.69	tr_C9ZZQ4_
DRB1_0101	193	GEVLSQALSVPEWLK	EVLSQALSV	1	0.5763	98.0	26.00	0.34	tr_C9ZZQ4_
DRB1_0101	194	RTVTAVFGIVTKALM	VFGIVTKAL	5	0.5758	98.4	26.00	0.25	tr_C9ZZQ4_
DRB1_0101	195	VEDDFSMQTPKDGTV	FSMQTPKDG	4	0.5754	98.9	27.00	0.89	tr_C9ZZQ4_
DRB1_0101	196	VADRRTSGAYVLAPS	DRRTSGAYV	2	0.5753	99.0	27.00	0.63	tr_C9ZZQ4_
DRB1_0101	197	SLEYSGQLFLHAFGT	YSGQLFLHA	3	0.5739	100.5	27.00	0.42	tr_C9ZZQ4_
DRB1_0101	198	ADRVKVQVVQSSKSH	VQVVQSSKS	5	0.5735	100.9	27.00	0.56	tr_C9ZZQ4_
DRB1_0101	199	RTTFMDSLYNQRRYV	MDSLYNQRR	4	0.5727	101.8	27.00	0.31	tr_C9ZZQ4_
DRB1_0101	200	VFVFGISASDVDVIE	FVFGISASD	1	0.5724	102.2	27.00	0.56	tr_C9ZZQ4_
DRB1_0101	201	VLVLFSEVALLYEKF	VLFSEVALL	2	0.5715	103.1	27.00	0.35	tr_C9ZZQ4_
DRB1_0101	202	RGAALTGHLPTSGAV	GAALTGHLP	1	0.5712	103.5	27.00	0.40	tr_C9ZZQ4_
DRB1_0101	203	INVKVLLCTWNTRVP	LLCTWNTRV	5	0.5705	104.3	27.00	0.45	tr_C9ZZQ4_
DRB1_0101	204	TLHSPLNGLVIFFGD	HSPLNGLVI	2	0.5703	104.5	28.00	0.55	tr_C9ZZQ4_
DRB1_0101	205	GAALTGHLPTSGAVF	LTGHLPTSG	3	0.5692	105.7	28.00	0.34	tr_C9ZZQ4_
DRB1_0101	206	RRYVIADFVIGDFGG	YVIADFVIG	2	0.5683	106.8	28.00	0.74	tr_C9ZZQ4_
DRB1_0101	207	GGSLNISLVRRRGAA	LNISLVRRR	3	0.5668	108.5	28.00	0.43	tr_C9ZZQ4_
DRB1_0101	208	FTTAVNAGFNASMES	TAVNAGFNA	2	0.5665	108.9	28.00	0.38	tr_C9ZZQ4_
DRB1_0101	209	MVYGWAAGEVLSQAL	YGWAAGEVL	2	0.5655	110.1	28.00	0.68	tr_C9ZZQ4_
DRB1_0101	210	DKSGITIVFGPVGDD	ITIVFGPVG	4	0.5649	110.8	29.00	0.80	tr_C9ZZQ4_
DRB1_0101	211	DFSMQTPKDGTVGLA	FSMQTPKDG	1	0.5635	112.5	29.00	0.88	tr_C9ZZQ4_
DRB1_0101	212	VRRRGAALTGHLPTS	GAALTGHLP	4	0.5635	112.5	29.00	0.58	tr_C9ZZQ4_
DRB1_0101	213	QIYVLTRYLSNNSGK	TRYLSNNSG	5	0.5632	112.9	29.00	0.34	tr_C9ZZQ4_
DRB1_0101	214	HYGEKEYEVALRVME	KEYEVALRV	4	0.5622	114.1	29.00	0.62	tr_C9ZZQ4_
DRB1_0101	215	PQAVLLFGAPKSDTG	VLLFGAPKS	3	0.5621	114.2	29.00	0.51	tr_C9ZZQ4_
DRB1_0101	216	SELRVLVLFSEVALL	VLVLFSEVA	4	0.5613	115.2	29.00	0.62	tr_C9ZZQ4_
DRB1_0101	217	LVIFFGDNTIAVKAT	FFGDNTIAV	3	0.5612	115.4	29.00	0.41	tr_C9ZZQ4_
DRB1_0101	218	KPQAVLLFGAPKSDT	VLLFGAPKS	4	0.5606	116.1	29.00	0.46	tr_C9ZZQ4_
DRB1_0101	219	RRGAALTGHLPTSGA	GAALTGHLP	2	0.5605	116.2	29.00	0.60	tr_C9ZZQ4_
DRB1_0101	220	KEANAIVCAAEAHGI	NAIVCAAEA	3	0.5603	116.4	29.00	0.43	tr_C9ZZQ4_
DRB1_0101	221	LCGVFVVLNVNDKPA	FVVLNVNDK	4	0.5599	116.9	29.00	0.80	tr_C9ZZQ4_
DRB1_0101	222	FNAVFERFAATKPQA	FERFAATKP	4	0.5595	117.4	29.00	0.64	tr_C9ZZQ4_
DRB1_0101	223	NINVKVLLCTWNTRV	KVLLCTWNT	4	0.5594	117.5	29.00	0.38	tr_C9ZZQ4_
DRB1_0101	224	LEKWGGSLNISLVRR	GGSLNISLV	4	0.5586	118.6	30.00	0.41	tr_C9ZZQ4_
DRB1_0101	225	ALMKMTNTLFIDPIT	LMKMTNTLF	1	0.5584	118.9	30.00	0.70	tr_C9ZZQ4_
DRB1_0101	226	LMPYPLQVHGAPANI	PLQVHGAPA	4	0.5582	119.1	30.00	0.22	tr_C9ZZQ4_
DRB1_0101	227	IVCAAEAHGIMKVIR	VCAAEAHGI	1	0.5573	120.2	30.00	0.59	tr_C9ZZQ4_
DRB1_0101	228	GLVIFFGDNTIAVKA	FFGDNTIAV	4	0.5569	120.8	30.00	0.37	tr_C9ZZQ4_
DRB1_0101	229	AAAPRLVFATNLPHW	LVFATNLPH	5	0.5567	121.1	30.00	0.60	tr_C9ZZQ4_
DRB1_0101	230	QLNKFRRHVIHLSPT	FRRHVIHLS	4	0.5564	121.4	30.00	0.69	tr_C9ZZQ4_

DRB1_0101	231	AGEVLSQALSVPEWL	EVLSQALSV	2	0.5564	121.5	30.00	0.47	tr_C9ZZQ4_
DRB1_0101	232	PSGAQVFLELMWKRV	FLELMWKRV	6	0.5560	122.0	30.00	0.60	tr_C9ZZQ4_
DRB1_0101	233	TTFMDSLYNQRRYVI	MDSLYNQRR	3	0.5557	122.3	30.00	0.39	tr_C9ZZQ4_
DRB1_0101	234	QAVLLFGAPKSDTGR	VLLFGAPKS	2	0.5557	122.4	30.00	0.52	tr_C9ZZQ4_
DRB1_0101	235	QQIYVLTRYLSNNSG	YVLTRYLSN	3	0.5546	123.8	30.00	0.42	tr_C9ZZQ4_
DRB1_0101	236	TDKSGITIVFGPVGD	ITIVFGPVG	5	0.5534	125.4	31.00	0.81	tr_C9ZZQ4_
DRB1_0101	237	TFMDSLYNQRRYVIA	MDSLYNQRR	2	0.5515	128.1	31.00	0.34	tr_C9ZZQ4_
DRB1_0101	238	RLVFATNLPHWYDNE	LVFATNLPH	1	0.5513	128.4	31.00	0.61	tr_C9ZZQ4_
DRB1_0101	239	LVLFSEVALLYEKFV	LFSEVALLY	2	0.5506	129.3	31.00	0.28	tr_C9ZZQ4_
DRB1_0101	240	AALTGHLPTSGAVFV	TGHLPTSGA	3	0.5503	129.8	31.00	0.38	tr_C9ZZQ4_
DRB1_0101	241	NADSVTLHSPLNGLV	SVTLHSPLN	3	0.5494	131.0	31.00	0.41	tr_C9ZZQ4_
DRB1_0101	242	RRRGAALTGHLPTSG	GAALTGHLP	3	0.5490	131.6	31.00	0.70	tr_C9ZZQ4_
DRB1_0101	243	YGEKEYEVALRVMEE	KEYEVALRV	3	0.5480	133.0	32.00	0.65	tr_C9ZZQ4_
DRB1_0101	244	SGITIVFGPVGDDTT	ITIVFGPVG	2	0.5480	133.0	32.00	0.75	tr_C9ZZQ4_
DRB1_0101	245	CGVFVVLNVNDKPAP	FVVLNVNDK	3	0.5477	133.5	32.00	0.77	tr_C9ZZQ4_
DRB1_0101	246	ASDVDVIERHLAAHS	IERHLAAHS	6	0.5468	134.7	32.00	0.47	tr_C9ZZQ4_
DRB1_0101	247	FMYLQGHHYGEKEYE	FMYLQGHHY	0	0.5462	135.7	32.00	0.64	tr_C9ZZQ4_
DRB1_0101	248	HHYGEKEYEVALRVM	KEYEVALRV	5	0.5459	136.1	32.00	0.58	tr_C9ZZQ4_
DRB1_0101	249	ADRRTSGAYVLAPSG	DRRTSGAYV	1	0.5448	137.7	32.00	0.55	tr_C9ZZQ4_
DRB1_0101	250	TIETMVLIRHALGHL	MVLIRHALG	4	0.5446	138.0	32.00	0.54	tr_C9ZZQ4_
DRB1_0101	251	LLMPYPLQVHGAPAN	PYPLQVHGA	3	0.5445	138.2	32.00	0.27	tr_C9ZZQ4_
DRB1_0101	252	DRTTFMDSLYNQRRY	TTFMDSLYN	2	0.5444	138.3	32.00	0.46	tr_C9ZZQ4_
DRB1_0101	253	ERHLAAHSELRVLVL	LAAHSELRV	3	0.5436	139.5	32.00	0.38	tr_C9ZZQ4_
DRB1_0101	254	KVLLCTWNTRVPKIF	LLCTWNTRV	2	0.5432	140.2	33.00	0.61	tr_C9ZZQ4_
DRB1_0101	255	SPFPGKLLIAGTNPL	LLIAGTNPL	6	0.5431	140.3	33.00	0.50	tr_C9ZZQ4_
DRB1_0101	256	SLEKWGGSLNISLVR	GGSLNISLV	5	0.5412	143.2	33.00	0.34	tr_C9ZZQ4_
DRB1_0101	257	VYGWAAGEVLSQALS	YGWAAGEVL	1	0.5403	144.6	33.00	0.63	tr_C9ZZQ4_
DRB1_0101	258	DSQVSPFPGKLLIAG	VSPFPGKLL	3	0.5395	145.8	33.00	0.62	tr_C9ZZQ4_
DRB1_0101	259	GVFVVLNVNDKPAPD	FVVLNVNDK	2	0.5362	151.2	34.00	0.80	tr_C9ZZQ4_
DRB1_0101	260	FLRKLVADRRTSGAY	LRKLVADRR	1	0.5361	151.2	34.00	0.70	tr_C9ZZQ4_
DRB1_0101	261	VIFFGDNTIAVKATA	FFGDNTIAV	2	0.5361	151.3	34.00	0.40	tr_C9ZZQ4_
DRB1_0101	262	RHLAAHSELRVLVLF	LAAHSELRV	2	0.5356	152.1	34.00	0.38	tr_C9ZZQ4_
DRB1_0101	263	EFNAVFERFAATKPQ	FERFAATKP	5	0.5343	154.2	34.00	0.55	tr_C9ZZQ4_
DRB1_0101	264	ALTGHLPTSGAVFVF	TGHLPTSGA	2	0.5341	154.7	34.00	0.37	tr_C9ZZQ4_
DRB1_0101	265	SQVSPFPGKLLIAGT	VSPFPGKLL	2	0.5340	154.7	34.00	0.56	tr_C9ZZQ4_
DRB1_0101	266	TDGEMMVYGWAAGEV	EMMVYGWAA	3	0.5334	155.7	34.00	0.60	tr_C9ZZQ4_
DRB1_0101	267	RKLVADRRTSGAYVL	DRRTSGAYV	5	0.5332	156.2	34.00	0.67	tr_C9ZZQ4_
DRB1_0101	268	EVLSQALSVPEWLKD	LSQALSVPE	2	0.5331	156.3	34.00	0.28	tr_C9ZZQ4_
DRB1_0101	269	LDSQVSPFPGKLLIA	VSPFPGKLL	4	0.5329	156.7	34.00	0.61	tr_C9ZZQ4_
DRB1_0101	270	YGWAAGEVLSQALSV	YGWAAGEVL	0	0.5328	156.8	34.00	0.34	tr_C9ZZQ4_
DRB1_0101	271	EQQIYVLTRYLSNNS	YVLTRYLSN	4	0.5327	157.0	35.00	0.41	tr_C9ZZQ4_
DRB1_0101	272	IYVLTRYLSNNSGKE	TRYLSNNSG	4	0.5322	157.8	35.00	0.46	tr_C9ZZQ4_
DRB1_0101	273	KDRTTFMDSLYNQRR	TTFMDSLYN	3	0.5319	158.3	35.00	0.56	tr_C9ZZQ4_
DRB1_0101	274	TKPQAVLLFGAPKSD	VLLFGAPKS	5	0.5317	158.7	35.00	0.46	tr_C9ZZQ4_
DRB1_0101	275	AAGEVLSQALSVPEW	EVLSQALSV	3	0.5316	158.8	35.00	0.56	tr_C9ZZQ4_
DRB1_0101	276	PQLNKFRRHVIHLSP	FRRHVIHLS	5	0.5299	161.8	35.00	0.67	tr_C9ZZQ4_
DRB1_0101	277	YVLTRYLSNNSGKEA	TRYLSNNSG	3	0.5292	163.0	35.00	0.59	tr_C9ZZQ4_
DRB1_0101	278	YVMYVLLLLMPYPLQ	LLLMPYPLQ	6	0.5279	165.3	35.00	0.44	tr_C9ZZQ4_
DRB1_0101	279	YKLCGVFVVLNVNDK	FVVLNVNDK	6	0.5273	166.4	36.00	0.54	tr_C9ZZQ4_
DRB1_0101	280	PKSDTGRFLRKLVAD	GRFLRKLVA	5	0.5267	167.5	36.00	0.64	tr_C9ZZQ4_

DRB1_0101	281	VEQQIYVLTRYLSNN	QIYVLTRYL	3	0.5266	167.7	36.00	0.41	tr_C9ZZQ4_
DRB1_0101	282	KRTVTAVFGIVTKAL	VTAVFGIVT	3	0.5253	170.1	36.00	0.48	tr_C9ZZQ4_
DRB1_0101	283	LGHLGVLRLGFMYLQ	LGHLGVLRL	0	0.5249	170.8	36.00	0.34	tr_C9ZZQ4_
DRB1_0101	284	YEVALRVMEEMGYKL	VMEEMGYKL	6	0.5247	171.2	36.00	0.57	tr_C9ZZQ4_
DRB1_0101	285	GEKEYEVALRVMEEM	KEYEVALRV	2	0.5234	173.5	36.00	0.61	tr_C9ZZQ4_
DRB1_0101	286	YCNQGGNVVYVREVE	YCNQGGNVV	0	0.5232	173.9	36.00	0.49	tr_C9ZZQ4_
DRB1_0101	287	VKATASWLVGALRHD	VKATASWLV	0	0.5231	174.1	36.00	0.58	tr_C9ZZQ4_
DRB1_0101	288	FMDSLYNQRRYVIAD	MDSLYNQRR	1	0.5230	174.4	36.00	0.29	tr_C9ZZQ4_
DRB1_0101	289	RRTSGAYVLAPSGAQ	YVLAPSGAQ	6	0.5227	174.8	36.00	0.41	tr_C9ZZQ4_
DRB1_0101	290	KLCGVFVVLNVNDKP	FVVLNVNDK	5	0.5226	175.1	36.00	0.76	tr_C9ZZQ4_
DRB1_0101	291	NEQYVAIKRFQEVMR	YVAIKRFQE	3	0.5223	175.7	37.00	0.47	tr_C9ZZQ4_
DRB1_0101	292	NGLVIFFGDNTIAVK	FFGDNTIAV	5	0.5219	176.4	37.00	0.32	tr_C9ZZQ4_
DRB1_0101	293	TGSLEYSGQLFLHAF	YSGQLFLHA	5	0.5209	178.4	37.00	0.41	tr_C9ZZQ4_
DRB1_0101	294	EQYVAIKRFQEVMRE	YVAIKRFQE	2	0.5187	182.7	37.00	0.45	tr_C9ZZQ4_
DRB1_0101	295	LTRYLSNNSGKEANA	TRYLSNNSG	1	0.5173	185.5	38.00	0.44	tr_C9ZZQ4_
DRB1_0101	296	GSLEYSGQLFLHAFG	YSGQLFLHA	4	0.5171	185.8	38.00	0.49	tr_C9ZZQ4_
DRB1_0101	297	LQVHGAPANINVKVL	VHGAPANIN	2	0.5171	185.9	38.00	0.35	tr_C9ZZQ4_
DRB1_0101	298	SLYNQRRYVIADFVI	RRYVIADFV	5	0.5171	185.9	38.00	0.37	tr_C9ZZQ4_
DRB1_0101	299	QVSPFPGKLLIAGTN	VSPFPGKLL	1	0.5157	188.6	38.00	0.43	tr_C9ZZQ4_
DRB1_0101	300	NGSAAAPRLVFATNL	GSAAAPRLV	1	0.5155	189.1	38.00	0.31	tr_C9ZZQ4_
DRB1_0101	301	VLTRYLSNNSGKEAN	TRYLSNNSG	2	0.5146	190.9	38.00	0.55	tr_C9ZZQ4_
DRB1_0101	302	VQVVQSSKSHKTPEE	VQSSKSHKT	3	0.5140	192.1	38.00	0.55	tr_C9ZZQ4_
DRB1_0101	303	VLFSEVALLYEKFVT	VALLYEKFV	5	0.5138	192.6	38.00	0.29	tr_C9ZZQ4_
DRB1_0101	304	GYKLCGVFVVLNVND	GYKLCGVFV	0	0.5129	194.5	38.00	0.46	tr_C9ZZQ4_
DRB1_0101	305	DRRTSGAYVLAPSGA	GAYVLAPSG	5	0.5126	195.1	38.00	0.30	tr_C9ZZQ4_
DRB1_0101	306	KSLEKWGGSLNISLV	EKWGGSLNI	3	0.5115	197.5	39.00	0.31	tr_C9ZZQ4_
DRB1_0101	307	FLELMWKRVLLDSQV	FLELMWKRV	0	0.5103	200.1	39.00	0.38	tr_C9ZZQ4_
DRB1_0101	308	GITIVFGPVGDDTTL	ITIVFGPVG	1	0.5092	202.4	39.00	0.71	tr_C9ZZQ4_
DRB1_0101	309	QYVAIKRFQEVMREY	YVAIKRFQE	1	0.5080	205.1	39.00	0.38	tr_C9ZZQ4_
DRB1_0101	310	FLRPSPTIETMVLIR	LRPSPTIET	1	0.5059	209.7	40.00	0.56	tr_C9ZZQ4_
DRB1_0101	311	VFVVLNVNDKPAPDK	FVVLNVNDK	1	0.5049	212.0	40.00	0.75	tr_C9ZZQ4_
DRB1_0101	312	AVLLFGAPKSDTGRF	VLLFGAPKS	1	0.5047	212.5	40.00	0.52	tr_C9ZZQ4_
DRB1_0101	313	EKEYEVALRVMEEMG	KEYEVALRV	1	0.5035	215.3	40.00	0.56	tr_C9ZZQ4_
DRB1_0101	314	PTSGAVFVFGISASD	FVFGISASD	6	0.5033	215.8	40.00	0.56	tr_C9ZZQ4_
DRB1_0101	315	KKKRTVTAVFGIVTK	VTAVFGIVT	5	0.5032	215.9	40.00	0.38	tr_C9ZZQ4_
DRB1_0101	316	EVEDDFSMQTPKDGT	FSMQTPKDG	5	0.5032	216.0	40.00	0.89	tr_C9ZZQ4_
DRB1_0101	317	TVEQQIYVLTRYLSN	QIYVLTRYL	4	0.5030	216.5	40.00	0.41	tr_C9ZZQ4_
DRB1_0101	318	DTDGEMMVYGWAAGE	EMMVYGWAA	4	0.5021	218.5	41.00	0.62	tr_C9ZZQ4_
DRB1_0101	319	KKRTVTAVFGIVTKA	VTAVFGIVT	4	0.5013	220.5	41.00	0.47	tr_C9ZZQ4_
DRB1_0101	320	VIHLSPTVEQQIYVL	IHLSPTVEQ	1	0.5010	221.3	41.00	0.39	tr_C9ZZQ4_
DRB1_0101	321	LHSPLNGLVIFFGDN	HSPLNGLVI	1	0.5001	223.3	41.00	0.56	tr_C9ZZQ4_
DRB1_0101	322	ATKPQAVLLFGAPKS	VLLFGAPKS	6	0.4994	225.0	41.00	0.35	tr_C9ZZQ4_
DRB1_0101	323	PTIETMVLIRHALGH	MVLIRHALG	5	0.4993	225.4	41.00	0.55	tr_C9ZZQ4_
DRB1_0101	324	ETDKSGITIVFGPVG	ITIVFGPVG	6	0.4990	226.2	41.00	0.73	tr_C9ZZQ4_
DRB1_0101	325	WAAGEVLSQALSVPE	EVLSQALSV	4	0.4977	229.2	42.00	0.57	tr_C9ZZQ4_
DRB1_0101	326	HSELRVLVLFSEVAL	VLVLFSEVA	5	0.4970	230.9	42.00	0.71	tr_C9ZZQ4_
DRB1_0101	327	VLSQALSVPEWLKDR	QALSVPEWL	3	0.4969	231.3	42.00	0.41	tr_C9ZZQ4_
DRB1_0101	328	KEFNAVFERFAATKP	FNAVFERFA	2	0.4968	231.5	42.00	0.45	tr_C9ZZQ4_
DRB1_0101	329	TRVPKIFTTAVNAGF	FTTAVNAGF	6	0.4952	235.5	42.00	0.50	tr_C9ZZQ4_
DRB1_0101	330	TTAVNAGFNASMESR	VNAGFNASM	3	0.4949	236.3	42.00	0.41	tr_C9ZZQ4_

DRB1_0101	331	APKSDTGRFLRKLVA	GRFLRKLVA	6	0.4948	236.4	42.00	0.54	tr_C9ZZQ4_
DRB1_0101	332	HLAAHSELRVLVLFS	LAAHSELRV	1	0.4933	240.4	42.00	0.29	tr_C9ZZQ4_
DRB1_0101	333	ASWLVGALRHDTGSL	LVGALRHDT	3	0.4923	243.1	43.00	0.52	tr_C9ZZQ4_
DRB1_0101	334	LTGHLPTSGAVFVFG	TGHLPTSGA	1	0.4921	243.5	43.00	0.33	tr_C9ZZQ4_
DRB1_0101	335	LLDSQVSPFPGKLLI	VSPFPGKLL	5	0.4917	244.5	43.00	0.51	tr_C9ZZQ4_
DRB1_0101	336	EPQLNKFRRHVIHLS	FRRHVIHLS	6	0.4910	246.4	43.00	0.56	tr_C9ZZQ4_
DRB1_0101	337	IFFGDNTIAVKATAS	FFGDNTIAV	1	0.4902	248.6	43.00	0.35	tr_C9ZZQ4_
DRB1_0101	338	TSETGITDPNYFLKH	GITDPNYFL	4	0.4892	251.3	43.00	0.75	tr_C9ZZQ4_
DRB1_0101	339	SAAAPRLVFATNLPH	LVFATNLPH	6	0.4877	255.4	44.00	0.41	tr_C9ZZQ4_
DRB1_0101	340	KQGAVCYCNQGGNVV	YCNQGGNVV	6	0.4863	259.4	44.00	0.52	tr_C9ZZQ4_
DRB1_0101	341	FVFGISASDVDVIER	FVFGISASD	0	0.4837	266.7	44.00	0.41	tr_C9ZZQ4_
DRB1_0101	342	GWAAGEVLSQALSVP	EVLSQALSV	5	0.4833	268.0	44.00	0.52	tr_C9ZZQ4_
DRB1_0101	343	IADRVKVQVVQSSKS	VKVQVVQSS	4	0.4811	274.5	45.00	0.41	tr_C9ZZQ4_
DRB1_0101	344	SETGITDPNYFLKHD	GITDPNYFL	3	0.4807	275.7	45.00	0.76	tr_C9ZZQ4_
DRB1_0101	345	MREYLKTHTSETGIT	YLKTHTSET	3	0.4805	276.1	45.00	0.49	tr_C9ZZQ4_
DRB1_0101	346	LSTFSRTGMLYVMYV	FSRTGMLYV	3	0.4800	277.6	45.00	0.43	tr_C9ZZQ4_
DRB1_0101	347	DSLYNQRRYVIADFV	YNQRRYVIA	3	0.4798	278.3	45.00	0.32	tr_C9ZZQ4_
DRB1_0101	348	QKHEVVAFGPMTGSG	EVVAFGPMT	3	0.4791	280.3	45.00	0.50	tr_C9ZZQ4_
DRB1_0101	349	VCAAEAHGIMKVIRK	VCAAEAHGI	0	0.4790	280.7	45.00	0.45	tr_C9ZZQ4_
DRB1_0101	350	TRYLSNNSGKEANAI	LSNNSGKEA	3	0.4785	282.2	45.00	0.41	tr_C9ZZQ4_
DRB1_0101	351	HDTDGEMMVYGWAAG	EMMVYGWAA	5	0.4784	282.3	45.00	0.63	tr_C9ZZQ4_
DRB1_0101	352	YVAIKRFQEVMREYL	IKRFQEVMR	3	0.4783	282.9	46.00	0.30	tr_C9ZZQ4_
DRB1_0101	353	TASWLVGALRHDTGS	LVGALRHDT	4	0.4778	284.2	46.00	0.44	tr_C9ZZQ4_
DRB1_0101	354	PKDGTVGLAALRCNA	TVGLAALRC	4	0.4775	285.3	46.00	0.43	tr_C9ZZQ4_
DRB1_0101	355	DTGSLEYSGQLFLHA	YSGQLFLHA	6	0.4774	285.7	46.00	0.32	tr_C9ZZQ4_
DRB1_0101	356	RYVIADFVIGDFGGD	YVIADFVIG	1	0.4759	290.3	46.00	0.80	tr_C9ZZQ4_
DRB1_0101	357	GHHYGEKEYEVALRV	KEYEVALRV	6	0.4757	290.9	46.00	0.41	tr_C9ZZQ4_
DRB1_0101	358	SPTIETMVLIRHALG	MVLIRHALG	6	0.4740	296.3	46.00	0.43	tr_C9ZZQ4_
DRB1_0101	359	VLAPSGAQVFLELMW	LAPSGAQVF	1	0.4724	301.5	47.00	0.47	tr_C9ZZQ4_
DRB1_0101	360	PTVEQQIYVLTRYLS	QIYVLTRYL	5	0.4723	301.7	47.00	0.43	tr_C9ZZQ4_
DRB1_0101	361	KHEVVAFGPMTGSGE	EVVAFGPMT	2	0.4712	305.5	47.00	0.43	tr_C9ZZQ4_
DRB1_0101	362	SPTVEQQIYVLTRYL	TVEQQIYVL	2	0.4702	308.7	47.00	0.26	tr_C9ZZQ4_
DRB1_0101	363	LIAGTNPLAKNEQYV	LIAGTNPLA	0	0.4694	311.3	47.00	0.62	tr_C9ZZQ4_
DRB1_0101	364	GKEANAIVCAAEAHG	NAIVCAAEA	4	0.4681	315.9	48.00	0.56	tr_C9ZZQ4_
DRB1_0101	365	KDGTVGLAALRCNAD	TVGLAALRC	3	0.4680	316.0	48.00	0.46	tr_C9ZZQ4_
DRB1_0101	366	EKKKRTVTAVFGIVT	KRTVTAVFG	3	0.4680	316.0	48.00	0.45	tr_C9ZZQ4_
DRB1_0101	367	LRKLVADRRTSGAYV	DRRTSGAYV	6	0.4672	318.9	48.00	0.44	tr_C9ZZQ4_
DRB1_0101	368	ALSTFSRTGMLYVMY	FSRTGMLYV	4	0.4672	319.0	48.00	0.41	tr_C9ZZQ4_
DRB1_0101	369	STFSRTGMLYVMYVL	FSRTGMLYV	2	0.4668	320.3	48.00	0.41	tr_C9ZZQ4_
DRB1_0101	370	ANINVKVLLCTWNTR	KVLLCTWNT	5	0.4662	322.4	48.00	0.47	tr_C9ZZQ4_
DRB1_0101	371	LHAFGTTTSDSAKAL	FGTTTSDSA	3	0.4646	328.0	48.00	0.51	tr_C9ZZQ4_
DRB1_0101	372	KRVLLDSQVSPFPGK	RVLLDSQVS	1	0.4637	331.1	49.00	0.41	tr_C9ZZQ4_
DRB1_0101	373	LQKHEVVAFGPMTGS	EVVAFGPMT	4	0.4635	331.9	49.00	0.49	tr_C9ZZQ4_
DRB1_0101	374	VLLCTWNTRVPKIFT	LLCTWNTRV	1	0.4634	332.3	49.00	0.63	tr_C9ZZQ4_
DRB1_0101	375	DSISELQKHEVVAFG	SELQKHEVV	3	0.4633	332.7	49.00	0.32	tr_C9ZZQ4_
DRB1_0101	376	LNGLVIFFGDNTIAV	LVIFFGDNT	3	0.4632	332.8	49.00	0.29	tr_C9ZZQ4_
DRB1_0101	377	KNEQYVAIKRFQEVM	YVAIKRFQE	4	0.4632	332.9	49.00	0.51	tr_C9ZZQ4_
DRB1_0101	378	GSAAAPRLVFATNLP	PRLVFATNL	5	0.4628	334.5	49.00	0.41	tr_C9ZZQ4_
DRB1_0101	379	CNADSVTLHSPLNGL	SVTLHSPLN	4	0.4622	336.6	49.00	0.50	tr_C9ZZQ4_
DRB1_0101	380	SWLVGALRHDTGSLE	LVGALRHDT	2	0.4607	342.1	49.00	0.48	tr_C9ZZQ4_

DRB1_0101	381	PHALSTFSRTGMLYV	STFSRTGML	4	0.4597	345.8	49.00	0.26	tr_C9ZZQ4_
DRB1_0101	382	HALSTFSRTGMLYVM	FSRTGMLYV	5	0.4591	348.2	50.00	0.29	tr_C9ZZQ4_
DRB1_0101	383	IHLSPTVEQQIYVLT	PTVEQQIYV	4	0.4589	348.8	50.00	0.31	tr_C9ZZQ4_
DRB1_0101	384	REYLKTHTSETGITD	YLKTHTSET	2	0.4589	348.8	50.00	0.46	tr_C9ZZQ4_
DRB1_0101	385	SISELQKHEVVAFGP	SELQKHEVV	2	0.4573	354.8	50.00	0.31	tr_C9ZZQ4_
DRB1_0101	386	HEVVAFGPMTGSGEV	EVVAFGPMT	1	0.4572	355.4	50.00	0.29	tr_C9ZZQ4_
DRB1_0101	387	MDSLYNQRRYVIADF	YNQRRYVIA	4	0.4567	357.1	50.00	0.30	tr_C9ZZQ4_
DRB1_0101	388	RKSLEKWGGSLNISL	EKWGGSLNI	4	0.4566	357.6	50.00	0.35	tr_C9ZZQ4_
DRB1_0101	389	HAFGTTTSDSAKALK	FGTTTSDSA	2	0.4566	357.7	50.00	0.46	tr_C9ZZQ4_
DRB1_0101	390	LMWKRVLLDSQVSPF	RVLLDSQVS	4	0.4563	358.8	50.00	0.57	tr_C9ZZQ4_
DRB1_0101	391	AHSELRVLVLFSEVA	VLVLFSEVA	6	0.4563	358.9	50.00	0.60	tr_C9ZZQ4_
DRB1_0101	392	DKEFNAVFERFAATK	FNAVFERFA	3	0.4555	362.1	50.00	0.59	tr_C9ZZQ4_
DRB1_0101	393	PPHALSTFSRTGMLY	PHALSTFSR	1	0.4548	364.5	50.00	0.31	tr_C9ZZQ4_
DRB1_0101	394	FSMQTPKDGTVGLAA	FSMQTPKDG	0	0.4539	368.4	55.00	0.69	tr_C9ZZQ4_
DRB1_0101	395	ATASWLVGALRHDTG	LVGALRHDT	5	0.4517	377.0	55.00	0.40	tr_C9ZZQ4_
DRB1_0101	396	WKRVLLDSQVSPFPG	RVLLDSQVS	2	0.4509	380.4	55.00	0.47	tr_C9ZZQ4_
DRB1_0101	397	VPPHALSTFSRTGML	PHALSTFSR	2	0.4506	381.4	55.00	0.37	tr_C9ZZQ4_
DRB1_0101	398	MWKRVLLDSQVSPFP	RVLLDSQVS	3	0.4502	383.3	55.00	0.53	tr_C9ZZQ4_
DRB1_0101	399	VMREYLKTHTSETGI	YLKTHTSET	4	0.4498	384.8	55.00	0.49	tr_C9ZZQ4_
DRB1_0101	400	HLSPTVEQQIYVLTR	PTVEQQIYV	3	0.4498	385.0	55.00	0.36	tr_C9ZZQ4_
DRB1_0101	401	LSPTVEQQIYVLTRY	TVEQQIYVL	3	0.4497	385.3	55.00	0.37	tr_C9ZZQ4_
DRB1_0101	402	AGFNASMESRNWTIA	SMESRNWTI	5	0.4496	385.8	55.00	0.40	tr_C9ZZQ4_
DRB1_0101	403	ETGITDPNYFLKHDT	GITDPNYFL	2	0.4493	386.9	55.00	0.76	tr_C9ZZQ4_
DRB1_0101	404	HTSETGITDPNYFLK	GITDPNYFL	5	0.4491	387.8	55.00	0.74	tr_C9ZZQ4_
DRB1_0101	405	PITIEPQLNKFRRHV	IEPQLNKFR	3	0.4487	389.4	55.00	0.59	tr_C9ZZQ4_
DRB1_0101	406	VSPFPGKLLIAGTNP	GKLLIAGTN	5	0.4487	389.5	55.00	0.25	tr_C9ZZQ4_
DRB1_0101	407	AFGTTTSDSAKALKE	FGTTTSDSA	1	0.4476	394.3	55.00	0.38	tr_C9ZZQ4_
DRB1_0101	408	KEYEVALRVMEEMGY	YEVALRVME	2	0.4466	398.3	55.00	0.26	tr_C9ZZQ4_
DRB1_0101	409	DGTVGLAALRCNADS	TVGLAALRC	2	0.4460	401.1	55.00	0.42	tr_C9ZZQ4_
DRB1_0101	410	VLLDSQVSPFPGKLL	VSPFPGKLL	6	0.4459	401.6	55.00	0.34	tr_C9ZZQ4_
DRB1_0101	411	TPKDGTVGLAALRCN	TVGLAALRC	5	0.4458	402.1	55.00	0.39	tr_C9ZZQ4_
DRB1_0101	412	LSQALSVPEWLKDRT	QALSVPEWL	2	0.4455	403.2	55.00	0.44	tr_C9ZZQ4_
DRB1_0101	413	LMKMTNTLFIDPITI	LMKMTNTLF	0	0.4450	405.3	55.00	0.43	tr_C9ZZQ4_
DRB1_0101	414	LKDRTTFMDSLYNQR	TTFMDSLYN	4	0.4433	412.8	55.00	0.65	tr_C9ZZQ4_
DRB1_0101	415	DPITIEPQLNKFRRH	IEPQLNKFR	4	0.4419	419.4	55.00	0.52	tr_C9ZZQ4_
DRB1_0101	416	GTVGLAALRCNADSV	TVGLAALRC	1	0.4410	423.5	55.00	0.38	tr_C9ZZQ4_
DRB1_0101	417	GHLGVLRLGFMYLQG	VLRLGFMYL	4	0.4395	430.2	55.00	0.31	tr_C9ZZQ4_
DRB1_0101	418	HSPLNGLVIFFGDNT	HSPLNGLVI	0	0.4389	433.1	55.00	0.46	tr_C9ZZQ4_
DRB1_0101	419	LDSISELQKHEVVAF	ISELQKHEV	3	0.4387	434.0	55.00	0.37	tr_C9ZZQ4_
DRB1_0101	420	AATKPQAVLLFGAPK	ATKPQAVLL	1	0.4382	436.5	55.00	0.28	tr_C9ZZQ4_
DRB1_0101	421	LVFATNLPHWYDNET	LVFATNLPH	0	0.4377	438.8	55.00	0.45	tr_C9ZZQ4_
DRB1_0101	422	TAVNAGFNASMESRN	VNAGFNASM	2	0.4373	440.7	55.00	0.44	tr_C9ZZQ4_
DRB1_0101	423	EVVAFGPMTGSGEVR	FGPMTGSGE	4	0.4366	443.8	55.00	0.45	tr_C9ZZQ4_
DRB1_0101	424	FGDNTIAVKATASWL	AVKATASWL	6	0.4358	447.6	55.00	0.26	tr_C9ZZQ4_
DRB1_0101	425	LAAHSELRVLVLFSE	HSELRVLVL	3	0.4356	449.0	55.00	0.32	tr_C9ZZQ4_
DRB1_0101	426	ELQKHEVVAFGPMTG	EVVAFGPMT	5	0.4355	449.2	55.00	0.43	tr_C9ZZQ4_
DRB1_0101	427	EAHGIMKVIRKSLEK	HGIMKVIRK	2	0.4355	449.3	55.00	0.32	tr_C9ZZQ4_
DRB1_0101	428	AEAHGIMKVIRKSLE	HGIMKVIRK	3	0.4354	449.6	55.00	0.38	tr_C9ZZQ4_
DRB1_0101	429	RVLLDSQVSPFPGKL	LDSQVSPFP	3	0.4350	452.0	55.00	0.34	tr_C9ZZQ4_
DRB1_0101	430	IDPITIEPQLNKFRR	IEPQLNKFR	5	0.4337	458.1	55.00	0.41	tr_C9ZZQ4_

DRB1_0101	431	VLLFGAPKSDTGRFL	VLLFGAPKS	0	0.4328	462.8	55.00	0.40	tr_C9ZZQ4_
DRB1_0101	432	SRNWTIADRVKVQVV	WTIADRVKV	3	0.4322	465.7	55.00	0.62	tr_C9ZZQ4_
DRB1_0101	433	HLGVLRLGFMYLQGH	VLRLGFMYL	3	0.4318	467.7	55.00	0.32	tr_C9ZZQ4_
DRB1_0101	434	LELMWKRVLLDSQVS	RVLLDSQVS	6	0.4317	468.2	55.00	0.31	tr_C9ZZQ4_
DRB1_0101	435	SLSVPPHALSTFSRT	PHALSTFSR	5	0.4311	471.1	60.00	0.34	tr_C9ZZQ4_
DRB1_0101	436	GFNASMESRNWTIAD	SMESRNWTI	4	0.4304	475.0	60.00	0.45	tr_C9ZZQ4_
DRB1_0101	437	TGHLPTSGAVFVFGI	HLPTSGAVF	2	0.4303	475.3	60.00	0.35	tr_C9ZZQ4_
DRB1_0101	438	VAIKRFQEVMREYLK	IKRFQEVMR	2	0.4291	481.3	60.00	0.31	tr_C9ZZQ4_
DRB1_0101	439	SVPPHALSTFSRTGM	PHALSTFSR	3	0.4285	484.8	60.00	0.49	tr_C9ZZQ4_
DRB1_0101	440	AHGIMKVIRKSLEKW	GIMKVIRKS	2	0.4277	489.1	60.00	0.27	tr_C9ZZQ4_
DRB1_0101	441	SGKEANAIVCAAEAH	NAIVCAAEA	5	0.4275	490.1	60.00	0.56	tr_C9ZZQ4_
DRB1_0101	442	ISELQKHEVVAFGPM	LQKHEVVAF	3	0.4272	491.7	60.00	0.37	tr_C9ZZQ4_
DRB1_0101	443	LGVLRLGFMYLQGHH	VLRLGFMYL	2	0.4265	495.4	60.00	0.30	tr_C9ZZQ4_
DRB1_0101	444	WLVGALRHDTGSLEY	LVGALRHDT	1	0.4258	499.2	60.00	0.38	tr_C9ZZQ4_
DRB1_0101	445	AAEAHGIMKVIRKSL	HGIMKVIRK	4	0.4253	501.8	60.00	0.40	tr_C9ZZQ4_
DRB1_0101	446	FFGDNTIAVKATASW	GDNTIAVKA	2	0.4251	502.6	60.00	0.26	tr_C9ZZQ4_
DRB1_0101	447	RNWTIADRVKVQVVQ	WTIADRVKV	2	0.4239	509.2	60.00	0.56	tr_C9ZZQ4_
DRB1_0101	448	SELQKHEVVAFGPMT	LQKHEVVAF	2	0.4235	511.5	60.00	0.27	tr_C9ZZQ4_
DRB1_0101	449	EEKKKRTVTAVFGIV	KRTVTAVFG	4	0.4235	511.8	60.00	0.55	tr_C9ZZQ4_
DRB1_0101	450	FVVLNVNDKPAPDKE	FVVLNVNDK	0	0.4206	527.9	60.00	0.65	tr_C9ZZQ4_
DRB1_0101	451	QVVQSSKSHKTPEEF	VQSSKSHKT	2	0.4202	530.3	60.00	0.82	tr_C9ZZQ4_
DRB1_0101	452	TFSRTGMLYVMYVLL	FSRTGMLYV	1	0.4196	533.4	60.00	0.39	tr_C9ZZQ4_
DRB1_0101	453	ITIVFGPVGDDTTLD	ITIVFGPVG	0	0.4190	537.1	60.00	0.60	tr_C9ZZQ4_
DRB1_0101	454	ELMWKRVLLDSQVSP	RVLLDSQVS	5	0.4181	542.7	60.00	0.49	tr_C9ZZQ4_
DRB1_0101	455	PDKEFNAVFERFAAT	FNAVFERFA	4	0.4170	548.9	60.00	0.59	tr_C9ZZQ4_
DRB1_0101	456	LAPSGAQVFLELMWK	SGAQVFLEL	3	0.4159	555.4	60.00	0.25	tr_C9ZZQ4_
DRB1_0101	457	NAGFNASMESRNWTI	FNASMESRN	3	0.4144	564.4	60.00	0.47	tr_C9ZZQ4_
DRB1_0101	458	ITIEPQLNKFRRHVI	IEPQLNKFR	2	0.4144	564.8	60.00	0.58	tr_C9ZZQ4_
DRB1_0101	459	AKNEQYVAIKRFQEV	YVAIKRFQE	5	0.4141	566.6	60.00	0.48	tr_C9ZZQ4_
DRB1_0101	460	IRKSLEKWGGSLNIS	EKWGGSLNI	5	0.4140	567.3	60.00	0.33	tr_C9ZZQ4_
DRB1_0101	461	AIKRFQEVMREYLKT	FQEVMREYL	4	0.4138	568.2	60.00	0.33	tr_C9ZZQ4_
DRB1_0101	462	SASDVDVIERHLAAH	DVIERHLAA	5	0.4114	583.4	60.00	0.56	tr_C9ZZQ4_
DRB1_0101	463	PTIRKFHATVESREK	IRKFHATVE	2	0.4113	583.9	60.00	0.34	tr_C9ZZQ4_
DRB1_0101	464	TGITDPNYFLKHDTD	GITDPNYFL	1	0.4112	584.4	60.00	0.74	tr_C9ZZQ4_
DRB1_0101	465	KHDTDGEMMVYGWAA	EMMVYGWAA	6	0.4089	599.5	60.00	0.50	tr_C9ZZQ4_
DRB1_0101	466	FIDPITIEPQLNKFR	ITIEPQLNK	4	0.4087	600.6	60.00	0.29	tr_C9ZZQ4_
DRB1_0101	467	KATASWLVGALRHDT	LVGALRHDT	6	0.4083	603.2	60.00	0.28	tr_C9ZZQ4_
DRB1_0101	468	TVGLAALRCNADSVT	LAALRCNAD	3	0.4081	604.2	60.00	0.39	tr_C9ZZQ4_
DRB1_0101	469	LSVPPHALSTFSRTG	PHALSTFSR	4	0.4066	614.4	65.00	0.45	tr_C9ZZQ4_
DRB1_0101	470	VVAFGPMTGSGEVRR	FGPMTGSGE	3	0.4053	623.0	65.00	0.60	tr_C9ZZQ4_
DRB1_0101	471	FGTTTSDSAKALKEE	TTTSDSAKA	2	0.4035	635.3	65.00	0.31	tr_C9ZZQ4_
DRB1_0101	472	QTPKDGTVGLAALRC	TVGLAALRC	6	0.4034	635.8	65.00	0.28	tr_C9ZZQ4_
DRB1_0101	473	QVHGAPANINVKVLL	VHGAPANIN	1	0.4020	645.4	65.00	0.36	tr_C9ZZQ4_
DRB1_0101	474	RYLSNNSGKEANAIV	LSNNSGKEA	2	0.4019	646.6	65.00	0.45	tr_C9ZZQ4_
DRB1_0101	475	SPTIRKFHATVESRE	IRKFHATVE	3	0.4007	654.5	65.00	0.45	tr_C9ZZQ4_
DRB1_0101	476	TNPLAKNEQYVAIKR	LAKNEQYVA	3	0.3999	660.2	65.00	0.38	tr_C9ZZQ4_
DRB1_0101	477	IKRFQEVMREYLKTH	FQEVMREYL	3	0.3998	661.2	65.00	0.38	tr_C9ZZQ4_
DRB1_0101	478	ESRNWTIADRVKVQV	WTIADRVKV	4	0.3998	661.4	65.00	0.64	tr_C9ZZQ4_
DRB1_0101	479	FNASMESRNWTIADR	SMESRNWTI	3	0.3993	664.5	65.00	0.61	tr_C9ZZQ4_
DRB1_0101	480	WLKDRTTFMDSLYNQ	TTFMDSLYN	5	0.3987	669.0	65.00	0.56	tr_C9ZZQ4_

DRB1_0101	481	APSGAQVFLELMWKR	SGAQVFLEL	2	0.3984	671.6	65.00	0.23	tr_C9ZZQ4_
DRB1_0101	482	EWLKDRTTFMDSLYN	WLKDRTTFM	1	0.3970	681.4	65.00	0.47	tr_C9ZZQ4_
DRB1_0101	483	LVGALRHDTGSLEYS	LRHDTGSLE	4	0.3944	700.8	65.00	0.47	tr_C9ZZQ4_
DRB1_0101	484	NPLAKNEQYVAIKRF	LAKNEQYVA	2	0.3936	706.8	65.00	0.32	tr_C9ZZQ4_
DRB1_0101	485	PLNGLVIFFGDNTIA	LVIFFGDNT	4	0.3924	716.3	65.00	0.40	tr_C9ZZQ4_
DRB1_0101	486	CAAEAHGIMKVIRKS	HGIMKVIRK	5	0.3916	722.7	65.00	0.33	tr_C9ZZQ4_
DRB1_0101	487	VAFGPMTGSGEVRRW	FGPMTGSGE	2	0.3912	726.0	65.00	0.57	tr_C9ZZQ4_
DRB1_0101	488	LRPSPTIETMVLIRH	LRPSPTIET	0	0.3904	732.3	65.00	0.35	tr_C9ZZQ4_
DRB1_0101	489	EYLKTHTSETGITDP	YLKTHTSET	1	0.3901	734.0	65.00	0.41	tr_C9ZZQ4_
DRB1_0101	490	HGIMKVIRKSLEKWG	MKVIRKSLE	3	0.3890	743.4	65.00	0.28	tr_C9ZZQ4_
DRB1_0101	491	APDKEFNAVFERFAA	FNAVFERFA	5	0.3886	746.2	65.00	0.54	tr_C9ZZQ4_
DRB1_0101	492	AVNAGFNASMESRNW	VNAGFNASM	1	0.3886	746.6	65.00	0.38	tr_C9ZZQ4_
DRB1_0101	493	QGHHYGEKEYEVALR	HYGEKEYEV	3	0.3885	747.0	65.00	0.44	tr_C9ZZQ4_
DRB1_0101	494	LLCTWNTRVPKIFTT	LLCTWNTRV	0	0.3883	748.7	65.00	0.44	tr_C9ZZQ4_
DRB1_0101	495	PANINVKVLLCTWNT	KVLLCTWNT	6	0.3876	754.2	65.00	0.41	tr_C9ZZQ4_
DRB1_0101	496	HWYDNETSSPTIRKF	YDNETSSPT	2	0.3871	758.7	65.00	0.52	tr_C9ZZQ4_
DRB1_0101	497	VPEWLKDRTTFMDSL	WLKDRTTFM	3	0.3866	762.6	65.00	0.76	tr_C9ZZQ4_
DRB1_0101	498	TIADRVKVQVVQSSK	VKVQVVQSS	5	0.3860	767.9	65.00	0.56	tr_C9ZZQ4_
DRB1_0101	499	KEEKKKRTVTAVFGI	KRTVTAVFG	5	0.3848	778.0	65.00	0.50	tr_C9ZZQ4_
DRB1_0101	500	SSPTIRKFHATVESR	IRKFHATVE	4	0.3840	784.5	70.00	0.46	tr_C9ZZQ4_
DRB1_0101	501	SQALSVPEWLKDRTT	QALSVPEWL	1	0.3835	788.6	70.00	0.47	tr_C9ZZQ4_
DRB1_0101	502	NWTIADRVKVQVVQS	WTIADRVKV	1	0.3829	793.6	70.00	0.52	tr_C9ZZQ4_
DRB1_0101	503	HDTGSLEYSGQLFLH	LEYSGQLFL	5	0.3829	793.6	70.00	0.28	tr_C9ZZQ4_
DRB1_0101	504	KRFQEVMREYLKTHT	FQEVMREYL	2	0.3828	794.6	70.00	0.40	tr_C9ZZQ4_
DRB1_0101	505	PHWYDNETSSPTIRK	YDNETSSPT	3	0.3827	795.4	70.00	0.57	tr_C9ZZQ4_
DRB1_0101	506	TLDSISELQKHEVVA	ISELQKHEV	4	0.3826	796.8	70.00	0.40	tr_C9ZZQ4_
DRB1_0101	507	GHLPTSGAVFVFGIS	HLPTSGAVF	1	0.3818	803.5	70.00	0.32	tr_C9ZZQ4_
DRB1_0101	508	NSGKEANAIVCAAEA	NAIVCAAEA	6	0.3800	818.9	70.00	0.34	tr_C9ZZQ4_
DRB1_0101	509	LPTSGAVFVFGISAS	AVFVFGISA	5	0.3799	820.0	70.00	0.30	tr_C9ZZQ4_
DRB1_0101	510	FSRTGMLYVMYVLLL	TGMLYVMYV	3	0.3790	827.7	70.00	0.31	tr_C9ZZQ4_
DRB1_0101	511	VNAGFNASMESRNWT	FNASMESRN	4	0.3760	855.6	70.00	0.47	tr_C9ZZQ4_
DRB1_0101	512	AFGPMTGSGEVRRWV	FGPMTGSGE	1	0.3759	856.3	70.00	0.48	tr_C9ZZQ4_
DRB1_0101	513	VRRWVRELYFLRPSP	WVRELYFLR	3	0.3751	863.5	70.00	0.50	tr_C9ZZQ4_
DRB1_0101	514	TIEPQLNKFRRHVIH	IEPQLNKFR	1	0.3742	872.6	70.00	0.47	tr_C9ZZQ4_
DRB1_0101	515	EYEVALRVMEEMGYK	LRVMEEMGY	5	0.3733	880.5	70.00	0.36	tr_C9ZZQ4_
DRB1_0101	516	EVRRWVRELYFLRPS	WVRELYFLR	4	0.3730	883.7	70.00	0.52	tr_C9ZZQ4_
DRB1_0101	517	EVMREYLKTHTSETG	YLKTHTSET	5	0.3720	893.4	70.00	0.47	tr_C9ZZQ4_
DRB1_0101	518	PSPTIETMVLIRHAL	PTIETMVLI	2	0.3709	904.3	70.00	0.29	tr_C9ZZQ4_
DRB1_0101	519	GTNPLAKNEQYVAIK	LAKNEQYVA	4	0.3696	916.6	70.00	0.38	tr_C9ZZQ4_
DRB1_0101	520	REVEDDFSMQTPKDG	FSMQTPKDG	6	0.3691	921.5	70.00	0.80	tr_C9ZZQ4_
DRB1_0101	521	HLPTSGAVFVFGISA	LPTSGAVFV	1	0.3691	921.8	70.00	0.25	tr_C9ZZQ4_
DRB1_0101	522	VVQSSKSHKTPEEFI	VQSSKSHKT	1	0.3690	922.3	70.00	0.71	tr_C9ZZQ4_
DRB1_0101	523	PEWLKDRTTFMDSLY	WLKDRTTFM	2	0.3685	927.6	70.00	0.75	tr_C9ZZQ4_
DRB1_0101	524	RCNADSVTLHSPLNG	SVTLHSPLN	5	0.3681	931.4	70.00	0.57	tr_C9ZZQ4_
DRB1_0101	525	LFIDPITIEPQLNKF	ITIEPQLNK	5	0.3677	935.3	70.00	0.28	tr_C9ZZQ4_
DRB1_0101	526	LYVMYVLLLLMPYPL	LLLLMPYPL	6	0.3676	936.3	70.00	0.56	tr_C9ZZQ4_
DRB1_0101	527	NTRVPKIFTTAVNAG	KIFTTAVNA	5	0.3667	946.1	70.00	0.29	tr_C9ZZQ4_
DRB1_0101	528	TLFIDPITIEPQLNK	IDPITIEPQ	3	0.3665	947.7	70.00	0.28	tr_C9ZZQ4_
DRB1_0101	529	AAHSELRVLVLFSEV	HSELRVLVL	2	0.3664	948.5	70.00	0.38	tr_C9ZZQ4_
DRB1_0101	530	YVIADFVIGDFGGDC	YVIADFVIG	0	0.3664	949.1	70.00	0.62	tr_C9ZZQ4_

DRB1_0101	531	VGLAALRCNADSVTL	LAALRCNAD	2	0.3659	953.7	70.00	0.40	tr_C9ZZQ4_
DRB1_0101	532	VIRKSLEKWGGSLNI	SLEKWGGSL	4	0.3644	969.8	70.00	0.35	tr_C9ZZQ4_
DRB1_0101	533	PLAKNEQYVAIKRFQ	AKNEQYVAI	2	0.3640	974.4	70.00	0.26	tr_C9ZZQ4_
DRB1_0101	534	WTIADRVKVQVVQSS	WTIADRVKV	0	0.3638	976.3	70.00	0.31	tr_C9ZZQ4_
DRB1_0101	535	MESRNWTIADRVKVQ	WTIADRVKV	5	0.3628	986.8	70.00	0.62	tr_C9ZZQ4_
DRB1_0101	536	IEPQLNKFRRHVIHL	LNKFRRHVI	4	0.3611	1004.7	70.00	0.25	tr_C9ZZQ4_
DRB1_0101	537	GEVRRWVRELYFLRP	WVRELYFLR	5	0.3595	1022.1	75.00	0.50	tr_C9ZZQ4_
DRB1_0101	538	GLAALRCNADSVTLH	LAALRCNAD	1	0.3587	1031.9	75.00	0.28	tr_C9ZZQ4_
DRB1_0101	539	SVPEWLKDRTTFMDS	WLKDRTTFM	4	0.3584	1034.2	75.00	0.77	tr_C9ZZQ4_
DRB1_0101	540	LAKNEQYVAIKRFQE	YVAIKRFQE	6	0.3580	1039.5	75.00	0.29	tr_C9ZZQ4_
DRB1_0101	541	RPSPTIETMVLIRHA	PTIETMVLI	3	0.3573	1046.7	75.00	0.43	tr_C9ZZQ4_
DRB1_0101	542	YLSNNSGKEANAIVC	LSNNSGKEA	1	0.3561	1060.6	75.00	0.36	tr_C9ZZQ4_
DRB1_0101	543	NYFLKHDTDGEMMVY	LKHDTDGEM	3	0.3561	1061.4	75.00	0.25	tr_C9ZZQ4_
DRB1_0101	544	RFQEVMREYLKTHTS	FQEVMREYL	1	0.3548	1075.7	75.00	0.31	tr_C9ZZQ4_
DRB1_0101	545	NTLFIDPITIEPQLN	IDPITIEPQ	4	0.3546	1077.9	75.00	0.26	tr_C9ZZQ4_
DRB1_0101	546	SPLNGLVIFFGDNTI	LVIFFGDNT	5	0.3517	1112.1	75.00	0.38	tr_C9ZZQ4_
DRB1_0101	547	NASMESRNWTIADRV	SMESRNWTI	2	0.3475	1163.9	75.00	0.63	tr_C9ZZQ4_
DRB1_0101	548	WYDNETSSPTIRKFH	YDNETSSPT	1	0.3475	1164.3	75.00	0.44	tr_C9ZZQ4_
DRB1_0101	549	SNNSGKEANAIVCAA	SGKEANAIV	3	0.3474	1166.1	75.00	0.31	tr_C9ZZQ4_
DRB1_0101	550	SGEVRRWVRELYFLR	WVRELYFLR	6	0.3472	1168.0	75.00	0.43	tr_C9ZZQ4_
DRB1_0101	551	VGALRHDTGSLEYSG	LRHDTGSLE	3	0.3463	1179.5	75.00	0.65	tr_C9ZZQ4_
DRB1_0101	552	LAALRCNADSVTLHS	LRCNADSVT	3	0.3460	1183.5	75.00	0.29	tr_C9ZZQ4_
DRB1_0101	553	MQTPKDGTVGLAALR	KDGTVGLAA	4	0.3457	1186.7	75.00	0.38	tr_C9ZZQ4_
DRB1_0101	554	THTSETGITDPNYFL	GITDPNYFL	6	0.3453	1192.4	75.00	0.62	tr_C9ZZQ4_
DRB1_0101	555	LQGHHYGEKEYEVAL	HYGEKEYEV	4	0.3448	1198.9	75.00	0.45	tr_C9ZZQ4_
DRB1_0101	556	TNTLFIDPITIEPQL	TLFIDPITI	2	0.3430	1222.3	75.00	0.34	tr_C9ZZQ4_
DRB1_0101	557	LPHWYDNETSSPTIR	YDNETSSPT	4	0.3420	1235.3	75.00	0.61	tr_C9ZZQ4_
DRB1_0101	558	LSNNSGKEANAIVCA	NSGKEANAI	3	0.3412	1247.0	75.00	0.31	tr_C9ZZQ4_
DRB1_0101	559	WNTRVPKIFTTAVNA	VPKIFTTAV	4	0.3391	1275.8	75.00	0.28	tr_C9ZZQ4_
DRB1_0101	560	TTLDSISELQKHEVV	ISELQKHEV	5	0.3381	1288.9	75.00	0.35	tr_C9ZZQ4_
DRB1_0101	561	PNYFLKHDTDGEMMV	FLKHDTDGE	3	0.3378	1293.2	75.00	0.31	tr_C9ZZQ4_
DRB1_0101	562	FGAPKSDTGRFLRKL	PKSDTGRFL	3	0.3363	1314.9	75.00	0.55	tr_C9ZZQ4_
DRB1_0101	563	YFLKHDTDGEMMVYG	LKHDTDGEM	2	0.3362	1315.4	75.00	0.26	tr_C9ZZQ4_
DRB1_0101	564	GAPKSDTGRFLRKLV	PKSDTGRFL	2	0.3359	1319.8	75.00	0.46	tr_C9ZZQ4_
DRB1_0101	565	SRTGMLYVMYVLLLL	TGMLYVMYV	2	0.3348	1336.1	75.00	0.31	tr_C9ZZQ4_
DRB1_0101	566	RHDTGSLEYSGQLFL	GSLEYSGQL	4	0.3334	1356.4	80.00	0.28	tr_C9ZZQ4_
DRB1_0101	567	ISASDVDVIERHLAA	DVIERHLAA	6	0.3333	1357.4	80.00	0.40	tr_C9ZZQ4_
DRB1_0101	568	FGPMTGSGEVRRWVR	MTGSGEVRR	3	0.3330	1362.7	80.00	0.31	tr_C9ZZQ4_
DRB1_0101	569	LSVPEWLKDRTTFMD	WLKDRTTFM	5	0.3312	1388.8	80.00	0.75	tr_C9ZZQ4_
DRB1_0101	570	GTTTSDSAKALKEEK	TTTSDSAKA	1	0.3306	1397.8	80.00	0.31	tr_C9ZZQ4_
DRB1_0101	571	LKEEKKKRTVTAVFG	KRTVTAVFG	6	0.3296	1413.4	80.00	0.38	tr_C9ZZQ4_
DRB1_0101	572	TWNTRVPKIFTTAVN	VPKIFTTAV	5	0.3285	1429.8	80.00	0.22	tr_C9ZZQ4_
DRB1_0101	573	TSSPTIRKFHATVES	IRKFHATVE	5	0.3285	1430.6	80.00	0.41	tr_C9ZZQ4_
DRB1_0101	574	CNQGGNVVYVREVED	NQGGNVVYV	1	0.3283	1433.7	80.00	0.34	tr_C9ZZQ4_
DRB1_0101	575	PAPDKEFNAVFERFA	FNAVFERFA	6	0.3281	1436.7	80.00	0.45	tr_C9ZZQ4_
DRB1_0101	576	VHGAPANINVKVLLC	VHGAPANIN	0	0.3280	1437.5	80.00	0.22	tr_C9ZZQ4_
DRB1_0101	577	SMESRNWTIADRVKV	WTIADRVKV	6	0.3277	1441.9	80.00	0.41	tr_C9ZZQ4_
DRB1_0101	578	CTWNTRVPKIFTTAV	WNTRVPKIF	2	0.3267	1458.7	80.00	0.30	tr_C9ZZQ4_
DRB1_0101	579	GIMKVIRKSLEKWGG	MKVIRKSLE	2	0.3235	1509.6	80.00	0.34	tr_C9ZZQ4_
DRB1_0101	580	NNSGKEANAIVCAAE	SGKEANAIV	2	0.3223	1528.7	80.00	0.29	tr_C9ZZQ4_

DRB1_0101	581	YLKTHTSETGITDPN	LKTHTSETG	1	0.3222	1530.2	80.00	0.39	tr_C9ZZQ4_
DRB1_0101	582	MTNTLFIDPITIEPQ	TLFIDPITI	3	0.3222	1531.1	80.00	0.43	tr_C9ZZQ4_
DRB1_0101	583	LLFGAPKSDTGRFLR	PKSDTGRFL	5	0.3219	1536.5	80.00	0.40	tr_C9ZZQ4_
DRB1_0101	584	VQSSKSHKTPEEFIK	VQSSKSHKT	0	0.3205	1559.6	80.00	0.36	tr_C9ZZQ4_
DRB1_0101	585	GALRHDTGSLEYSGQ	LRHDTGSLE	2	0.3199	1569.8	80.00	0.67	tr_C9ZZQ4_
DRB1_0101	586	VFGISASDVDVIERH	VFGISASDV	0	0.3168	1623.1	80.00	0.34	tr_C9ZZQ4_
DRB1_0101	587	ASMESRNWTIADRVK	SMESRNWTI	1	0.3161	1636.0	80.00	0.58	tr_C9ZZQ4_
DRB1_0101	588	LCTWNTRVPKIFTTA	WNTRVPKIF	3	0.3111	1725.5	80.00	0.41	tr_C9ZZQ4_
DRB1_0101	589	ALSVPEWLKDRTTFM	WLKDRTTFM	6	0.3111	1726.5	80.00	0.57	tr_C9ZZQ4_
DRB1_0101	590	LFGAPKSDTGRFLRK	PKSDTGRFL	4	0.3106	1736.5	80.00	0.50	tr_C9ZZQ4_
DRB1_0101	591	AGTNPLAKNEQYVAI	PLAKNEQYV	4	0.3100	1747.8	80.00	0.34	tr_C9ZZQ4_
DRB1_0101	592	FQEVMREYLKTHTSE	QEVMREYLK	1	0.3089	1767.8	80.00	0.27	tr_C9ZZQ4_
DRB1_0101	593	MKMTNTLFIDPITIE	TLFIDPITI	5	0.3086	1773.2	80.00	0.38	tr_C9ZZQ4_
DRB1_0101	594	QALSVPEWLKDRTTF	QALSVPEWL	0	0.3058	1829.0	85.00	0.39	tr_C9ZZQ4_
DRB1_0101	595	GITDPNYFLKHDTDG	GITDPNYFL	0	0.3050	1844.0	85.00	0.63	tr_C9ZZQ4_
DRB1_0101	596	ALRHDTGSLEYSGQL	LRHDTGSLE	1	0.3047	1850.3	85.00	0.55	tr_C9ZZQ4_
DRB1_0101	597	RTGMLYVMYVLLLLM	LYVMYVLLL	4	0.3046	1851.7	85.00	0.35	tr_C9ZZQ4_
DRB1_0101	598	QEVMREYLKTHTSET	REYLKTHTS	4	0.3046	1852.2	85.00	0.28	tr_C9ZZQ4_
DRB1_0101	599	YDNETSSPTIRKFHA	ETSSPTIRK	3	0.3043	1857.5	85.00	0.31	tr_C9ZZQ4_
DRB1_0101	600	APANINVKVLLCTWN	NINVKVLLC	3	0.3040	1864.6	85.00	0.28	tr_C9ZZQ4_
DRB1_0101	601	KMTNTLFIDPITIEP	TLFIDPITI	4	0.3028	1889.1	85.00	0.45	tr_C9ZZQ4_
DRB1_0101	602	AALRCNADSVTLHSP	LRCNADSVT	2	0.3019	1906.4	85.00	0.35	tr_C9ZZQ4_
DRB1_0101	603	LRCNADSVTLHSPLN	SVTLHSPLN	6	0.3016	1912.2	85.00	0.44	tr_C9ZZQ4_
DRB1_0101	604	HGAPANINVKVLLCT	PANINVKVL	3	0.3004	1937.5	85.00	0.28	tr_C9ZZQ4_
DRB1_0101	605	DPNYFLKHDTDGEMM	FLKHDTDGE	4	0.2970	2010.5	85.00	0.34	tr_C9ZZQ4_
DRB1_0101	606	KVIRKSLEKWGGSLN	SLEKWGGSL	5	0.2940	2077.1	85.00	0.34	tr_C9ZZQ4_
DRB1_0101	607	TTTSDSAKALKEEKK	SDSAKALKE	3	0.2929	2101.8	85.00	0.35	tr_C9ZZQ4_
DRB1_0101	608	GAPANINVKVLLCTW	NINVKVLLC	4	0.2928	2105.1	85.00	0.29	tr_C9ZZQ4_
DRB1_0101	609	AKQGAVCYCNQGGNV	CYCNQGGNV	6	0.2910	2144.8	85.00	0.43	tr_C9ZZQ4_
DRB1_0101	610	ETSSPTIRKFHATVE	IRKFHATVE	6	0.2910	2146.6	85.00	0.28	tr_C9ZZQ4_
DRB1_0101	611	FLKHDTDGEMMVYGW	KHDTDGEMM	2	0.2897	2175.8	85.00	0.26	tr_C9ZZQ4_
DRB1_0101	612	VFATNLPHWYDNETS	FATNLPHWY	1	0.2892	2187.9	85.00	0.58	tr_C9ZZQ4_
DRB1_0101	613	IMKVIRKSLEKWGGS	MKVIRKSLE	1	0.2891	2190.0	85.00	0.34	tr_C9ZZQ4_
DRB1_0101	614	YLQGHHYGEKEYEVA	HYGEKEYEV	5	0.2868	2245.8	85.00	0.44	tr_C9ZZQ4_
DRB1_0101	615	CEGEAAKQGAVCYCN	AAKQGAVCY	4	0.2867	2248.4	85.00	0.37	tr_C9ZZQ4_
DRB1_0101	616	LRHDTGSLEYSGQLF	GSLEYSGQL	5	0.2857	2272.3	85.00	0.25	tr_C9ZZQ4_
DRB1_0101	617	NLPHWYDNETSSPTI	YDNETSSPT	5	0.2850	2289.9	85.00	0.60	tr_C9ZZQ4_
DRB1_0101	618	LKHDTDGEMMVYGWA	GEMMVYGWA	6	0.2834	2330.6	85.00	0.21	tr_C9ZZQ4_
DRB1_0101	619	EGEAAKQGAVCYCNQ	AAKQGAVCY	3	0.2826	2350.9	85.00	0.46	tr_C9ZZQ4_
DRB1_0101	620	LSKETDKSGITIVFG	TDKSGITIV	4	0.2810	2389.6	85.00	0.35	tr_C9ZZQ4_
DRB1_0101	621	MKVIRKSLEKWGGSL	IRKSLEKWG	3	0.2799	2419.9	85.00	0.20	tr_C9ZZQ4_
DRB1_0101	622	GPMTGSGEVRRWVRE	MTGSGEVRR	2	0.2766	2507.4	85.00	0.39	tr_C9ZZQ4_
DRB1_0101	623	GEAAKQGAVCYCNQG	AAKQGAVCY	2	0.2744	2568.6	90.00	0.45	tr_C9ZZQ4_
DRB1_0101	624	KETDKSGITIVFGPV	TDKSGITIV	2	0.2743	2569.6	90.00	0.28	tr_C9ZZQ4_
DRB1_0101	625	DTTLDSISELQKHEV	LDSISELQK	3	0.2741	2575.9	90.00	0.25	tr_C9ZZQ4_
DRB1_0101	626	NETSSPTIRKFHATV	ETSSPTIRK	1	0.2729	2610.2	90.00	0.25	tr_C9ZZQ4_
DRB1_0101	627	DCEGEAAKQGAVCYC	AAKQGAVCY	5	0.2720	2636.0	90.00	0.30	tr_C9ZZQ4_
DRB1_0101	628	PMTGSGEVRRWVREL	MTGSGEVRR	1	0.2693	2714.3	90.00	0.34	tr_C9ZZQ4_
DRB1_0101	629	TGMLYVMYVLLLLMP	LYVMYVLLL	3	0.2689	2726.5	90.00	0.41	tr_C9ZZQ4_
DRB1_0101	630	NQGGNVVYVREVEDD	GNVVYVREV	3	0.2675	2768.0	90.00	0.44	tr_C9ZZQ4_

DRB1_0101	631	MYLQGHHYGEKEYEV	MYLQGHHYG	0	0.2669	2784.4	90.00	0.29	tr_C9ZZQ4_
DRB1_0101	632	SMQTPKDGTVGLAAL	KDGTVGLAA	5	0.2650	2843.3	90.00	0.44	tr_C9ZZQ4_
DRB1_0101	633	ALRCNADSVTLHSPL	LRCNADSVT	1	0.2646	2856.3	90.00	0.28	tr_C9ZZQ4_
DRB1_0101	634	IAGTNPLAKNEQYVA	PLAKNEQYV	5	0.2642	2866.1	90.00	0.31	tr_C9ZZQ4_
DRB1_0101	635	ALKEEKKKRTVTAVF	EKKKRTVTA	4	0.2600	3002.0	90.00	0.25	tr_C9ZZQ4_
DRB1_0101	636	ELSKETDKSGITIVF	TDKSGITIV	5	0.2598	3008.9	90.00	0.31	tr_C9ZZQ4_
DRB1_0101	637	SKETDKSGITIVFGP	TDKSGITIV	3	0.2587	3043.6	90.00	0.38	tr_C9ZZQ4_
DRB1_0101	638	DNETSSPTIRKFHAT	ETSSPTIRK	2	0.2580	3067.2	90.00	0.33	tr_C9ZZQ4_
DRB1_0101	639	GDCEGEAAKQGAVCY	CEGEAAKQG	2	0.2561	3130.3	90.00	0.37	tr_C9ZZQ4_
DRB1_0101	640	MLYVMYVLLLLMPYP	YVLLLLMPY	5	0.2524	3258.4	90.00	0.28	tr_C9ZZQ4_
DRB1_0101	641	EAAKQGAVCYCNQGG	AAKQGAVCY	1	0.2501	3338.6	90.00	0.35	tr_C9ZZQ4_
DRB1_0101	642	EEFIKDELSKETDKS	IKDELSKET	3	0.2486	3394.8	90.00	0.52	tr_C9ZZQ4_
DRB1_0101	643	SSKSHKTPEEFIKDE	SHKTPEEFI	3	0.2484	3403.3	90.00	0.60	tr_C9ZZQ4_
DRB1_0101	644	GMLYVMYVLLLLMPY	LYVMYVLLL	2	0.2476	3432.9	90.00	0.38	tr_C9ZZQ4_
DRB1_0101	645	GSGEVRRWVRELYFL	RRWVRELYF	5	0.2474	3440.1	90.00	0.23	tr_C9ZZQ4_
DRB1_0101	646	KALKEEKKKRTVTAV	LKEEKKKRT	2	0.2465	3471.1	90.00	0.38	tr_C9ZZQ4_
DRB1_0101	647	DDTTLDSISELQKHE	LDSISELQK	4	0.2464	3478.2	90.00	0.27	tr_C9ZZQ4_
DRB1_0101	648	QSSKSHKTPEEFIKD	SHKTPEEFI	4	0.2462	3484.4	90.00	0.59	tr_C9ZZQ4_
DRB1_0101	649	GDDTTLDSISELQKH	DTTLDSISE	2	0.2454	3514.6	90.00	0.29	tr_C9ZZQ4_
DRB1_0101	650	MTGSGEVRRWVRELY	GSGEVRRWV	2	0.2404	3711.2	95.00	0.19	tr_C9ZZQ4_
DRB1_0101	651	PEEFIKDELSKETDK	IKDELSKET	4	0.2398	3732.1	95.00	0.49	tr_C9ZZQ4_
DRB1_0101	652	TIVFGPVGDDTTLDS	FGPVGDDTT	3	0.2373	3837.8	95.00	0.51	tr_C9ZZQ4_
DRB1_0101	653	TDPNYFLKHDTDGEM	FLKHDTDGE	5	0.2369	3854.8	95.00	0.38	tr_C9ZZQ4_
DRB1_0101	654	KPAPDKEFNAVFERF	DKEFNAVFE	4	0.2359	3893.5	95.00	0.28	tr_C9ZZQ4_
DRB1_0101	655	GGNVVYVREVEDDFS	GNVVYVREV	1	0.2356	3905.6	95.00	0.34	tr_C9ZZQ4_
DRB1_0101	656	SKSHKTPEEFIKDEL	SHKTPEEFI	2	0.2345	3953.4	95.00	0.56	tr_C9ZZQ4_
DRB1_0101	657	FGGDCEGEAAKQGAV	CEGEAAKQG	4	0.2332	4011.9	95.00	0.42	tr_C9ZZQ4_
DRB1_0101	658	DFGGDCEGEAAKQGA	FGGDCEGEA	1	0.2317	4076.8	95.00	0.40	tr_C9ZZQ4_
DRB1_0101	659	EFIKDELSKETDKSG	IKDELSKET	2	0.2293	4183.5	95.00	0.55	tr_C9ZZQ4_
DRB1_0101	660	AKALKEEKKKRTVTA	LKEEKKKRT	3	0.2292	4186.8	95.00	0.49	tr_C9ZZQ4_
DRB1_0101	661	GISASDVDVIERHLA	VDVIERHLA	6	0.2290	4195.3	95.00	0.42	tr_C9ZZQ4_
DRB1_0101	662	QGGNVVYVREVEDDF	GNVVYVREV	2	0.2288	4205.4	95.00	0.51	tr_C9ZZQ4_
DRB1_0101	663	GNVVYVREVEDDFSM	YVREVEDDF	4	0.2273	4275.1	95.00	0.34	tr_C9ZZQ4_
DRB1_0101	664	TGSGEVRRWVRELYF	EVRRWVREL	4	0.2266	4309.1	95.00	0.23	tr_C9ZZQ4_
DRB1_0101	665	FGISASDVDVIERHL	ISASDVDVI	2	0.2247	4397.1	95.00	0.46	tr_C9ZZQ4_
DRB1_0101	666	VGDDTTLDSISELQK	DTTLDSISE	3	0.2235	4455.0	95.00	0.38	tr_C9ZZQ4_
DRB1_0101	667	IADFVIGDFGGDCEG	FVIGDFGGD	3	0.2207	4592.4	95.00	0.47	tr_C9ZZQ4_
DRB1_0101	668	TTSDSAKALKEEKKK	SDSAKALKE	2	0.2200	4628.0	95.00	0.49	tr_C9ZZQ4_
DRB1_0101	669	LKTHTSETGITDPNY	HTSETGITD	3	0.2196	4644.4	95.00	0.41	tr_C9ZZQ4_
DRB1_0101	670	GGDCEGEAAKQGAVC	CEGEAAKQG	3	0.2191	4673.3	95.00	0.49	tr_C9ZZQ4_
DRB1_0101	671	AAKQGAVCYCNQGGN	KQGAVCYCN	2	0.2187	4689.6	95.00	0.21	tr_C9ZZQ4_
DRB1_0101	672	VIADFVIGDFGGDCE	FVIGDFGGD	4	0.2158	4842.5	95.00	0.44	tr_C9ZZQ4_
DRB1_0101	673	FATNLPHWYDNETSS	FATNLPHWY	0	0.2155	4855.7	95.00	0.45	tr_C9ZZQ4_
DRB1_0101	674	GDFGGDCEGEAAKQG	FGGDCEGEA	2	0.2152	4872.1	95.00	0.47	tr_C9ZZQ4_
DRB1_0101	675	DELSKETDKSGITIV	ETDKSGITI	5	0.2152	4872.4	95.00	0.32	tr_C9ZZQ4_
DRB1_0101	676	SAKALKEEKKKRTVT	LKEEKKKRT	4	0.2109	5103.2	95.00	0.47	tr_C9ZZQ4_
DRB1_0101	677	NVVYVREVEDDFSMQ	YVREVEDDF	3	0.2079	5270.7	95.00	0.44	tr_C9ZZQ4_
DRB1_0101	678	ADFVIGDFGGDCEGE	FVIGDFGGD	2	0.2066	5349.8	95.00	0.50	tr_C9ZZQ4_
DRB1_0101	679	IGDFGGDCEGEAAKQ	FGGDCEGEA	3	0.2066	5350.5	95.00	0.58	tr_C9ZZQ4_
DRB1_0101	680	KSHKTPEEFIKDELS	SHKTPEEFI	1	0.2057	5401.6	95.00	0.52	tr_C9ZZQ4_

DRB1_0101	681	TPEEFIKDELSKETD	FIKDELSKE	4	0.2053	5423.0	95.00	0.38	tr_C9ZZQ4_
DRB1_0101	682	DKPAPDKEFNAVFER	PDKEFNAVF	4	0.2050	5442.2	95.00	0.25	tr_C9ZZQ4_
DRB1_0101	683	IVFGPVGDDTTLDSI	FGPVGDDTT	2	0.2045	5472.6	95.00	0.57	tr_C9ZZQ4_
DRB1_0101	684	TNLPHWYDNETSSPT	YDNETSSPT	6	0.2030	5562.5	95.00	0.49	tr_C9ZZQ4_
DRB1_0101	685	DFVIGDFGGDCEGEA	FVIGDFGGD	1	0.1987	5827.6	95.00	0.42	tr_C9ZZQ4_
DRB1_0101	686	FVIGDFGGDCEGEAA	FGGDCEGEA	5	0.1959	6004.1	95.00	0.35	tr_C9ZZQ4_
DRB1_0101	687	VVYVREVEDDFSMQT	YVREVEDDF	2	0.1953	6044.2	95.00	0.44	tr_C9ZZQ4_
DRB1_0101	688	VVLNVNDKPAPDKEF	VNDKPAPDK	4	0.1947	6081.6	95.00	0.27	tr_C9ZZQ4_
DRB1_0101	689	VIGDFGGDCEGEAAK	FGGDCEGEA	4	0.1927	6214.9	100.00	0.56	tr_C9ZZQ4_
DRB1_0101	690	FIKDELSKETDKSGI	IKDELSKET	1	0.1923	6245.2	100.00	0.51	tr_C9ZZQ4_
DRB1_0101	691	DSAKALKEEKKKRTV	LKEEKKKRT	5	0.1886	6494.6	100.00	0.45	tr_C9ZZQ4_
DRB1_0101	692	KTPEEFIKDELSKET	FIKDELSKE	5	0.1863	6658.1	100.00	0.40	tr_C9ZZQ4_
DRB1_0101	693	KTHTSETGITDPNYF	HTSETGITD	2	0.1819	6987.0	100.00	0.46	tr_C9ZZQ4_
DRB1_0101	694	TSDSAKALKEEKKKR	SDSAKALKE	1	0.1817	6999.1	100.00	0.45	tr_C9ZZQ4_
DRB1_0101	695	PVGDDTTLDSISELQ	DTTLDSISE	4	0.1784	7254.1	100.00	0.44	tr_C9ZZQ4_
DRB1_0101	696	ITDPNYFLKHDTDGE	FLKHDTDGE	6	0.1766	7394.5	100.00	0.27	tr_C9ZZQ4_
DRB1_0101	697	SDSAKALKEEKKKRT	AKALKEEKK	3	0.1735	7652.4	100.00	0.34	tr_C9ZZQ4_
DRB1_0101	698	NDKPAPDKEFNAVFE	PDKEFNAVF	5	0.1686	8069.1	100.00	0.25	tr_C9ZZQ4_
DRB1_0101	699	VYVREVEDDFSMQTP	YVREVEDDF	1	0.1684	8083.0	100.00	0.37	tr_C9ZZQ4_
DRB1_0101	700	KDELSKETDKSGITI	LSKETDKSG	3	0.1674	8173.2	100.00	0.35	tr_C9ZZQ4_
DRB1_0101	701	VFGPVGDDTTLDSIS	FGPVGDDTT	1	0.1672	8193.3	100.00	0.61	tr_C9ZZQ4_
DRB1_0101	702	VLNVNDKPAPDKEFN	VNDKPAPDK	3	0.1633	8546.2	100.00	0.39	tr_C9ZZQ4_
DRB1_0101	703	IKDELSKETDKSGIT	LSKETDKSG	4	0.1487	10008.1	100.00	0.34	tr_C9ZZQ4_
DRB1_0101	704	FGPVGDDTTLDSISE	FGPVGDDTT	0	0.1475	10138.0	100.00	0.41	tr_C9ZZQ4_
DRB1_0101	705	SHKTPEEFIKDELSK	SHKTPEEFI	0	0.1467	10221.5	100.00	0.34	tr_C9ZZQ4_
DRB1_0101	706	YVREVEDDFSMQTPK	VREVEDDFS	1	0.1426	10682.5	100.00	0.25	tr_C9ZZQ4_
DRB1_0101	707	VNDKPAPDKEFNAVF	PAPDKEFNA	4	0.1414	10830.5	100.00	0.30	tr_C9ZZQ4_
DRB1_0101	708	ATNLPHWYDNETSSP	WYDNETSSP	6	0.1399	11002.1	100.00	0.23	tr_C9ZZQ4_
DRB1_0101	709	GPVGDDTTLDSISEL	DTTLDSISE	5	0.1396	11036.0	100.00	0.49	tr_C9ZZQ4_
DRB1_0101	710	HKTPEEFIKDELSKE	FIKDELSKE	6	0.1393	11079.4	100.00	0.39	tr_C9ZZQ4_
DRB1_0101	711	LNVNDKPAPDKEFNA	VNDKPAPDK	2	0.1362	11448.4	100.00	0.42	tr_C9ZZQ4_
DRB1_0101	712	VREVEDDFSMQTPKD	VEDDFSMQT	3	0.1266	12708.0	100.00	0.29	tr_C9ZZQ4_
DRB1_0101	713	NVNDKPAPDKEFNAV	VNDKPAPDK	1	0.1261	12779.2	100.00	0.35	tr_C9ZZQ4_

Allele: DRB1_0101. Number of high binders 2. Number of weak binders 55. Number of peptides 713

Allele	pos	peptide	core	Of	1-log50k(aff)	affinity(nM)	%Rank	Relia	Identity	Bind Level
DRB1_0101	1	QLDVTALGSVPLRGV	VTALGSVPL	3	0.8197	7.0	1.40	0.83	tr_C9ZZQ4_	WB
DRB1_0101	2	VEIYQLDAVPGRTFA	IYQLDAVPG	2	0.8046	8.3	1.90	0.85	tr_C9ZZQ4_	WB
DRB1_0101	3	LDVTALGSVPLRGVP	VTALGSVPL	2	0.8042	8.3	1.90	0.83	tr_C9ZZQ4_	WB
DRB1_0101	4	DSFMIASKSATAAVR	MIASKSATA	3	0.8039	8.3	1.90	0.52	tr_C9ZZQ4_	WB
DRB1_0101	5	PVEIYQLDAVPGRTF	IYQLDAVPG	3	0.8017	8.5	2.00	0.87	tr_C9ZZQ4_	WB
DRB1_0101	6	GYDYYGQTANMAART	YYGQTANMA	3	0.7996	8.7	2.50	0.85	tr_C9ZZQ4_	WB
DRB1_0101	7	EQLDVTALGSVPLRG	VTALGSVPL	4	0.7973	9.0	2.50	0.84	tr_C9ZZQ4_	WB
DRB1_0101	8	SFMIASKSATAAVRL	MIASKSATA	2	0.7957	9.1	2.50	0.53	tr_C9ZZQ4_	WB
DRB1_0101	9	YDYYGQTANMAARTE	YYGQTANMA	2	0.7943	9.3	2.50	0.82	tr_C9ZZQ4_	WB

DRB1_0101	10	GDSFMIASKSATAAV	MIASKSATA	4	0.7892	9.8	2.50	0.42	tr_C9ZZQ4_	WB
DRB1_0101	11	KGYDYYGQTANMAAR	YYGQTANMA	4	0.7820	10.6	3.00	0.83	tr_C9ZZQ4_	WB
DRB1_0101	12	EIYQLDAVPGRTFAP	IYQLDAVPG	1	0.7818	10.6	3.00	0.81	tr_C9ZZQ4_	WB
DRB1_0101	13	DVTALGSVPLRGVPE	VTALGSVPL	1	0.7814	10.6	3.00	0.79	tr_C9ZZQ4_	WB
DRB1_0101	14	EPVEIYQLDAVPGRT	IYQLDAVPG	4	0.7802	10.8	3.00	0.88	tr_C9ZZQ4_	WB
DRB1_0101	15	SNVLHALLGTFTIVQ	LHALLGTFT	3	0.7731	11.6	3.50	0.67	tr_C9ZZQ4_	WB
DRB1_0101	16	VSNVLHALLGTFTIV	LHALLGTFT	4	0.7639	12.9	4.00	0.68	tr_C9ZZQ4_	WB
DRB1_0101	17	REQLDVTALGSVPLR	VTALGSVPL	5	0.7631	13.0	4.00	0.84	tr_C9ZZQ4_	WB
DRB1_0101	18	DYYGQTANMAARTES	YYGQTANMA	1	0.7627	13.0	4.00	0.79	tr_C9ZZQ4_	WB
DRB1_0101	19	FMIASKSATAAVRLA	MIASKSATA	1	0.7589	13.6	4.50	0.50	tr_C9ZZQ4_	WB
DRB1_0101	20	IGDSFMIASKSATAA	MIASKSATA	5	0.7573	13.8	4.50	0.36	tr_C9ZZQ4_	WB
DRB1_0101	21	NVLHALLGTFTIVQR	LHALLGTFT	2	0.7569	13.9	4.50	0.65	tr_C9ZZQ4_	WB
DRB1_0101	22	SSVILISNPAVDQST	LISNPAVDQ	4	0.7439	16.0	5.50	0.38	tr_C9ZZQ4_	WB
DRB1_0101	23	SSSVILISNPAVDQS	VILISNPAV	3	0.7438	16.0	5.50	0.35	tr_C9ZZQ4_	WB
DRB1_0101	24	PEPVEIYQLDAVPGR	IYQLDAVPG	5	0.7422	16.3	5.50	0.88	tr_C9ZZQ4_	WB
DRB1_0101	25	SVSNVLHALLGTFTI	LHALLGTFT	5	0.7388	16.9	5.50	0.69	tr_C9ZZQ4_	WB
DRB1_0101	26	EVYRKLWNGLRIRAG	YRKLWNGLR	2	0.7300	18.6	6.50	0.70	tr_C9ZZQ4_	WB
DRB1_0101	27	TKGYDYYGQTANMAA	YYGQTANMA	5	0.7286	18.9	6.50	0.82	tr_C9ZZQ4_	WB
DRB1_0101	28	PEVYRKLWNGLRIRA	YRKLWNGLR	3	0.7269	19.2	6.50	0.77	tr_C9ZZQ4_	WB
DRB1_0101	29	VTALGSVPLRGVPEP	VTALGSVPL	0	0.7223	20.2	7.00	0.70	tr_C9ZZQ4_	WB
DRB1_0101	30	RATYFSLSTAEREQL	YFSLSTAER	3	0.7201	20.7	7.00	0.63	tr_C9ZZQ4_	WB
DRB1_0101	31	VLHALLGTFTIVQRQ	LHALLGTFT	1	0.7200	20.7	7.00	0.61	tr_C9ZZQ4_	WB
DRB1_0101	32	VYRKLWNGLRIRAGI	YRKLWNGLR	1	0.7199	20.7	7.00	0.64	tr_C9ZZQ4_	WB
DRB1_0101	33	SVILISNPAVDQSTE	LISNPAVDQ	3	0.7191	20.9	7.50	0.45	tr_C9ZZQ4_	WB
DRB1_0101	34	IYQLDAVPGRTFAPL	IYQLDAVPG	0	0.7128	22.4	8.00	0.68	tr_C9ZZQ4_	WB
DRB1_0101	35	QEAIHRLAVKVGHIV	IHRLAVKVG	3	0.7095	23.2	8.00	0.54	tr_C9ZZQ4_	WB
DRB1_0101	36	DSSSVILISNPAVDQ	VILISNPAV	4	0.7075	23.7	8.50	0.38	tr_C9ZZQ4_	WB
DRB1_0101	37	DPEVYRKLWNGLRIR	YRKLWNGLR	4	0.6993	25.9	9.00	0.79	tr_C9ZZQ4_	WB
DRB1_0101	38	EAIHRLAVKVGHIVD	IHRLAVKVG	2	0.6974	26.4	9.50	0.46	tr_C9ZZQ4_	WB
DRB1_0101	39	TIGDSFMIASKSATA	DSFMIASKS	3	0.6933	27.6	9.50	0.41	tr_C9ZZQ4_	WB
DRB1_0101	40	YYGQTANMAARTESI	YYGQTANMA	0	0.6928	27.8	9.50	0.65	tr_C9ZZQ4_	WB
DRB1_0101	41	ATYFSLSTAEREQLD	YFSLSTAER	2	0.6890	28.9	10.00	0.60	tr_C9ZZQ4_	
DRB1_0101	42	EYYQEAIHRLAVKVG	YYQEAIHRL	1	0.6863	29.8	11.00	0.35	tr_C9ZZQ4_	
DRB1_0101	43	TRATYFSLSTAEREQ	YFSLSTAER	4	0.6816	31.4	11.00	0.67	tr_C9ZZQ4_	
DRB1_0101	44	YQEAIHRLAVKVGHI	IHRLAVKVG	4	0.6791	32.2	11.00	0.58	tr_C9ZZQ4_	
DRB1_0101	45	YYQEAIHRLAVKVGH	IHRLAVKVG	5	0.6772	32.9	12.00	0.42	tr_C9ZZQ4_	
DRB1_0101	46	TSQWASHPNVMADAV	WASHPNVMA	3	0.6763	33.2	12.00	0.82	tr_C9ZZQ4_	
DRB1_0101	47	ESVSNVLHALLGTFT	LHALLGTFT	6	0.6761	33.3	12.00	0.64	tr_C9ZZQ4_	
DRB1_0101	48	NDEYYQEAIHRLAVK	YYQEAIHRL	3	0.6729	34.4	12.00	0.61	tr_C9ZZQ4_	
DRB1_0101	49	AIHRLAVKVGHIVDV	IHRLAVKVG	1	0.6722	34.7	12.00	0.41	tr_C9ZZQ4_	
DRB1_0101	50	DEYYQEAIHRLAVKV	YYQEAIHRL	2	0.6685	36.1	13.00	0.50	tr_C9ZZQ4_	
DRB1_0101	51	RTESIANGGQVLLTR	SIANGGQVL	3	0.6659	37.1	13.00	0.71	tr_C9ZZQ4_	
DRB1_0101	52	MIASKSATAAVRLAR	KSATAAVRL	4	0.6615	39.0	14.00	0.25	tr_C9ZZQ4_	
DRB1_0101	53	STSQWASHPNVMADA	WASHPNVMA	4	0.6601	39.6	14.00	0.80	tr_C9ZZQ4_	
DRB1_0101	54	LDPEVYRKLWNGLRI	YRKLWNGLR	5	0.6560	41.4	14.00	0.73	tr_C9ZZQ4_	
DRB1_0101	55	YRKLWNGLRIRAGIH	YRKLWNGLR	0	0.6544	42.1	14.00	0.47	tr_C9ZZQ4_	
DRB1_0101	56	IDNFYRAEEKQVAEL	FYRAEEKQV	3	0.6535	42.5	15.00	0.55	tr_C9ZZQ4_	
DRB1_0101	57	FNDCMSISSQLGTGG	CMSISSQLG	3	0.6529	42.7	15.00	0.69	tr_C9ZZQ4_	
DRB1_0101	58	LHALLGTFTIVQRQK	LHALLGTFT	0	0.6496	44.3	15.00	0.43	tr_C9ZZQ4_	
DRB1_0101	59	HHSLIRTLIGNYDCY	IRTLIGNYD	4	0.6493	44.5	15.00	0.71	tr_C9ZZQ4_	

DRB1_0101	60	SQWASHPNVMADAVA	WASHPNVMA	2	0.6489	44.6	15.00	0.83	tr_C9ZZQ4_
DRB1_0101	61	DNFYRAEEKQVAELN	FYRAEEKQV	2	0.6484	44.9	15.00	0.47	tr_C9ZZQ4_
DRB1_0101	62	TFNDCMSISSQLGTG	CMSISSQLG	4	0.6458	46.2	16.00	0.67	tr_C9ZZQ4_
DRB1_0101	63	AHHSLIRTLIGNYDC	IRTLIGNYD	5	0.6438	47.2	16.00	0.54	tr_C9ZZQ4_
DRB1_0101	64	SDSSSVILISNPAVD	SVILISNPA	4	0.6437	47.3	16.00	0.44	tr_C9ZZQ4_
DRB1_0101	65	TYFSLSTAEREQLDV	YFSLSTAER	1	0.6423	47.9	16.00	0.54	tr_C9ZZQ4_
DRB1_0101	66	TESIANGGQVLLTRA	SIANGGQVL	2	0.6421	48.1	16.00	0.64	tr_C9ZZQ4_
DRB1_0101	67	VILISNPAVDQSTED	LISNPAVDQ	2	0.6386	49.9	17.00	0.62	tr_C9ZZQ4_
DRB1_0101	68	NDCMSISSQLGTGGE	CMSISSQLG	2	0.6372	50.7	17.00	0.65	tr_C9ZZQ4_
DRB1_0101	69	WNDEYYQEAIHRLAV	YYQEAIHRL	4	0.6371	50.7	17.00	0.61	tr_C9ZZQ4_
DRB1_0101	70	EREQLDVTALGSVPL	VTALGSVPL	6	0.6360	51.4	17.00	0.81	tr_C9ZZQ4_
DRB1_0101	71	NFYRAEEKQVAELNS	YRAEEKQVA	2	0.6358	51.5	17.00	0.54	tr_C9ZZQ4_
DRB1_0101	72	PNVMADAVAAHHSLI	NVMADAVAA	1	0.6357	51.5	17.00	0.50	tr_C9ZZQ4_
DRB1_0101	73	HSLIRTLIGNYDCYE	IRTLIGNYD	3	0.6353	51.7	17.00	0.79	tr_C9ZZQ4_
DRB1_0101	74	QWASHPNVMADAVAA	WASHPNVMA	1	0.6345	52.2	17.00	0.69	tr_C9ZZQ4_
DRB1_0101	75	AAHHSLIRTLIGNYD	IRTLIGNYD	6	0.6337	52.6	17.00	0.33	tr_C9ZZQ4_
DRB1_0101	76	LTRATYFSLSTAERE	YFSLSTAER	5	0.6298	54.9	18.00	0.65	tr_C9ZZQ4_
DRB1_0101	77	HPNVMADAVAAHHSL	NVMADAVAA	2	0.6298	54.9	18.00	0.65	tr_C9ZZQ4_
DRB1_0101	78	GGQVLLTRATYFSLS	VLLTRATYF	3	0.6258	57.4	18.00	0.65	tr_C9ZZQ4_
DRB1_0101	79	ARTESIANGGQVLLT	SIANGGQVL	4	0.6234	58.8	19.00	0.69	tr_C9ZZQ4_
DRB1_0101	80	VPEPVEIYQLDAVPG	IYQLDAVPG	6	0.6201	61.0	19.00	0.88	tr_C9ZZQ4_
DRB1_0101	81	IHRLAVKVGHIVDVA	LAVKVGHIV	3	0.6195	61.4	19.00	0.50	tr_C9ZZQ4_
DRB1_0101	82	TTFNDCMSISSQLGT	CMSISSQLG	5	0.6178	62.5	20.00	0.60	tr_C9ZZQ4_
DRB1_0101	83	NVMADAVAAHHSLIR	NVMADAVAA	0	0.6165	63.4	20.00	0.28	tr_C9ZZQ4_
DRB1_0101	84	SHPNVMADAVAAHHS	NVMADAVAA	3	0.6148	64.6	20.00	0.75	tr_C9ZZQ4_
DRB1_0101	85	NSTSQWASHPNVMAD	WASHPNVMA	5	0.6119	66.7	21.00	0.77	tr_C9ZZQ4_
DRB1_0101	86	NGGQVLLTRATYFSL	VLLTRATYF	4	0.6109	67.4	21.00	0.64	tr_C9ZZQ4_
DRB1_0101	87	GQVLLTRATYFSLST	VLLTRATYF	2	0.6107	67.5	21.00	0.63	tr_C9ZZQ4_
DRB1_0101	88	KTIGDSFMIASKSAT	DSFMIASKS	4	0.6085	69.1	21.00	0.50	tr_C9ZZQ4_
DRB1_0101	89	DCMSISSQLGTGGES	CMSISSQLG	1	0.6082	69.3	21.00	0.61	tr_C9ZZQ4_
DRB1_0101	90	VTKGYDYYGQTANMA	YYGQTANMA	6	0.6065	70.7	21.00	0.78	tr_C9ZZQ4_
DRB1_0101	91	ESIANGGQVLLTRAT	SIANGGQVL	1	0.6062	70.9	21.00	0.62	tr_C9ZZQ4_
DRB1_0101	92	SLIRTLIGNYDCYEV	IRTLIGNYD	2	0.6041	72.5	22.00	0.82	tr_C9ZZQ4_
DRB1_0101	93	WASHPNVMADAVAAH	NVMADAVAA	5	0.6011	74.9	22.00	0.54	tr_C9ZZQ4_
DRB1_0101	94	SIDNFYRAEEKQVAE	FYRAEEKQV	4	0.5987	76.9	23.00	0.58	tr_C9ZZQ4_
DRB1_0101	95	LWNGLRIRAGIHTGL	LRIRAGIHT	4	0.5965	78.7	23.00	0.54	tr_C9ZZQ4_
DRB1_0101	96	VAAHHSLIRTLIGNY	HHSLIRTLI	3	0.5920	82.7	24.00	0.43	tr_C9ZZQ4_
DRB1_0101	97	QLDPEVYRKLWNGLR	YRKLWNGLR	6	0.5876	86.7	25.00	0.62	tr_C9ZZQ4_
DRB1_0101	98	AVAAHHSLIRTLIGN	HHSLIRTLI	4	0.5867	87.6	25.00	0.37	tr_C9ZZQ4_
DRB1_0101	99	VWNDEYYQEAIHRLA	YYQEAIHRL	5	0.5854	88.8	25.00	0.63	tr_C9ZZQ4_
DRB1_0101	100	IASKSATAAVRLARD	KSATAAVRL	3	0.5853	88.9	25.00	0.41	tr_C9ZZQ4_
DRB1_0101	101	WNGLRIRAGIHTGLC	LRIRAGIHT	3	0.5848	89.3	25.00	0.56	tr_C9ZZQ4_
DRB1_0101	102	SSDSSSVILISNPAV	SVILISNPA	5	0.5844	89.7	25.00	0.56	tr_C9ZZQ4_
DRB1_0101	103	KLWNGLRIRAGIHTG	LRIRAGIHT	5	0.5831	91.0	25.00	0.46	tr_C9ZZQ4_
DRB1_0101	104	ASHPNVMADAVAAHH	NVMADAVAA	4	0.5798	94.3	26.00	0.79	tr_C9ZZQ4_
DRB1_0101	105	ANGGQVLLTRATYFS	VLLTRATYF	5	0.5791	95.0	26.00	0.63	tr_C9ZZQ4_
DRB1_0101	106	QVLLTRATYFSLSTA	VLLTRATYF	1	0.5774	96.7	26.00	0.55	tr_C9ZZQ4_
DRB1_0101	107	DAVAAHHSLIRTLIG	HHSLIRTLI	5	0.5740	100.4	27.00	0.33	tr_C9ZZQ4_
DRB1_0101	108	RKLWNGLRIRAGIHT	WNGLRIRAG	3	0.5713	103.4	27.00	0.42	tr_C9ZZQ4_
DRB1_0101	109	ADAVAAHHSLIRTLI	HHSLIRTLI	6	0.5674	107.8	28.00	0.19	tr_C9ZZQ4_

DRB1_0101	110	LIRTLIGNYDCYEVK	IRTLIGNYD	1	0.5653	110.3	28.00	0.79	tr_C9ZZQ4_
DRB1_0101	111	AARTESIANGGQVLL	SIANGGQVL	5	0.5620	114.3	29.00	0.72	tr_C9ZZQ4_
DRB1_0101	112	ASKSATAAVRLARDL	KSATAAVRL	2	0.5616	114.8	29.00	0.46	tr_C9ZZQ4_
DRB1_0101	113	CMSISSQLGTGGESV	CMSISSQLG	0	0.5603	116.5	29.00	0.47	tr_C9ZZQ4_
DRB1_0101	114	NGLRIRAGIHTGLCD	LRIRAGIHT	2	0.5599	116.9	29.00	0.63	tr_C9ZZQ4_
DRB1_0101	115	VMADAVAAHHSLIRT	ADAVAAHHS	2	0.5568	120.9	30.00	0.29	tr_C9ZZQ4_
DRB1_0101	116	VKTIGDSFMIASKSA	DSFMIASKS	5	0.5526	126.6	31.00	0.50	tr_C9ZZQ4_
DRB1_0101	117	MADAVAAHHSLIRTL	AVAAHHSLI	3	0.5523	127.0	31.00	0.25	tr_C9ZZQ4_
DRB1_0101	118	DSIDNFYRAEEKQVA	FYRAEEKQV	5	0.5490	131.6	31.00	0.62	tr_C9ZZQ4_
DRB1_0101	119	SIANGGQVLLTRATY	SIANGGQVL	0	0.5486	132.2	31.00	0.47	tr_C9ZZQ4_
DRB1_0101	120	FYRAEEKQVAELNSK	YRAEEKQVA	1	0.5450	137.4	32.00	0.62	tr_C9ZZQ4_
DRB1_0101	121	ENSTSQWASHPNVMA	WASHPNVMA	6	0.5435	139.7	32.00	0.71	tr_C9ZZQ4_
DRB1_0101	122	LLTRATYFSLSTAER	YFSLSTAER	6	0.5411	143.3	33.00	0.57	tr_C9ZZQ4_
DRB1_0101	123	HTTFNDCMSISSQLG	CMSISSQLG	6	0.5409	143.6	33.00	0.54	tr_C9ZZQ4_
DRB1_0101	124	HRLAVKVGHIVDVAA	LAVKVGHIV	2	0.5393	146.2	33.00	0.67	tr_C9ZZQ4_
DRB1_0101	125	YFSLSTAEREQLDVT	FSLSTAERE	1	0.5392	146.3	33.00	0.52	tr_C9ZZQ4_
DRB1_0101	126	GLRIRAGIHTGLCDI	LRIRAGIHT	1	0.5309	160.1	35.00	0.58	tr_C9ZZQ4_
DRB1_0101	127	IANGGQVLLTRATYF	VLLTRATYF	6	0.5304	161.0	35.00	0.58	tr_C9ZZQ4_
DRB1_0101	128	SKSATAAVRLARDLQ	KSATAAVRL	1	0.5195	181.0	37.00	0.44	tr_C9ZZQ4_
DRB1_0101	129	RLAVKVGHIVDVAAV	LAVKVGHIV	1	0.5162	187.7	38.00	0.56	tr_C9ZZQ4_
DRB1_0101	130	VGHIVDVAAVERVGR	VDVAAVERV	4	0.5147	190.8	38.00	0.59	tr_C9ZZQ4_
DRB1_0101	131	ILISNPAVDQSTEDL	LISNPAVDQ	1	0.5052	211.4	40.00	0.78	tr_C9ZZQ4_
DRB1_0101	132	GESVSNVLHALLGTF	SNVLHALLG	4	0.5020	218.9	41.00	0.42	tr_C9ZZQ4_
DRB1_0101	133	HALLGTFTIVQRQKE	HALLGTFTI	0	0.4966	232.0	42.00	0.60	tr_C9ZZQ4_
DRB1_0101	134	EVKTIGDSFMIASKS	KTIGDSFMI	2	0.4940	238.5	42.00	0.33	tr_C9ZZQ4_
DRB1_0101	135	RTFAPLRLDHEAYVA	FAPLRLDHE	2	0.4909	246.8	43.00	0.43	tr_C9ZZQ4_
DRB1_0101	136	VLLTRATYFSLSTAE	VLLTRATYF	0	0.4895	250.5	43.00	0.40	tr_C9ZZQ4_
DRB1_0101	137	GHIVDVAAVERVGRP	VDVAAVERV	3	0.4879	255.0	43.00	0.60	tr_C9ZZQ4_
DRB1_0101	138	KVGHIVDVAAVERVG	VDVAAVERV	5	0.4851	262.7	44.00	0.49	tr_C9ZZQ4_
DRB1_0101	139	GRTFAPLRLDHEAYV	FAPLRLDHE	3	0.4795	279.2	45.00	0.44	tr_C9ZZQ4_
DRB1_0101	140	TALGSVPLRGVPEPV	SVPLRGVPE	4	0.4760	289.8	46.00	0.40	tr_C9ZZQ4_
DRB1_0101	141	GGESVSNVLHALLGT	SNVLHALLG	5	0.4733	298.6	47.00	0.36	tr_C9ZZQ4_
DRB1_0101	142	IRTLIGNYDCYEVKT	IRTLIGNYD	0	0.4715	304.5	47.00	0.72	tr_C9ZZQ4_
DRB1_0101	143	YEVKTIGDSFMIASK	KTIGDSFMI	3	0.4701	309.2	47.00	0.47	tr_C9ZZQ4_
DRB1_0101	144	KSATAAVRLARDLQR	SATAAVRLA	1	0.4698	310.1	47.00	0.34	tr_C9ZZQ4_
DRB1_0101	145	YGQTANMAARTESIA	QTANMAART	2	0.4684	314.7	48.00	0.32	tr_C9ZZQ4_
DRB1_0101	146	AVWNDEYYQEAIHRL	YYQEAIHRL	6	0.4659	323.2	48.00	0.60	tr_C9ZZQ4_
DRB1_0101	147	TFAPLRLDHEAYVAD	LRLDHEAYV	4	0.4654	325.1	48.00	0.46	tr_C9ZZQ4_
DRB1_0101	148	CYEVKTIGDSFMIAS	KTIGDSFMI	4	0.4636	331.4	49.00	0.38	tr_C9ZZQ4_
DRB1_0101	149	LRIRAGIHTGLCDIR	LRIRAGIHT	0	0.4563	358.9	50.00	0.41	tr_C9ZZQ4_
DRB1_0101	150	TGGESVSNVLHALLG	SNVLHALLG	6	0.4500	384.1	55.00	0.28	tr_C9ZZQ4_
DRB1_0101	151	PGRTFAPLRLDHEAY	FAPLRLDHE	4	0.4461	400.8	55.00	0.43	tr_C9ZZQ4_
DRB1_0101	152	GQTANMAARTESIAN	NMAARTESI	4	0.4446	407.1	55.00	0.47	tr_C9ZZQ4_
DRB1_0101	153	VPGRTFAPLRLDHEA	GRTFAPLRL	2	0.4377	438.6	55.00	0.44	tr_C9ZZQ4_
DRB1_0101	154	DCYEVKTIGDSFMIA	KTIGDSFMI	5	0.4370	442.3	55.00	0.34	tr_C9ZZQ4_
DRB1_0101	155	VKVGHIVDVAAVERV	VDVAAVERV	6	0.4366	444.1	55.00	0.31	tr_C9ZZQ4_
DRB1_0101	156	LAVKVGHIVDVAAVE	LAVKVGHIV	0	0.4359	447.4	55.00	0.39	tr_C9ZZQ4_
DRB1_0101	157	HIVDVAAVERVGRPT	VDVAAVERV	2	0.4358	447.9	55.00	0.69	tr_C9ZZQ4_
DRB1_0101	158	MSISSQLGTGGESVS	ISSQLGTGG	2	0.4349	452.4	55.00	0.61	tr_C9ZZQ4_
DRB1_0101	159	TAAVRLARDLQRCFL	VRLARDLQR	3	0.4331	461.2	55.00	0.56	tr_C9ZZQ4_

DRB1_0101	160	PSAQLDPEVYRKLWN	LDPEVYRKL	4	0.4325	464.1	55.00	0.54	tr_C9ZZQ4_
DRB1_0101	161	PPSAQLDPEVYRKLW	LDPEVYRKL	5	0.4281	486.7	60.00	0.49	tr_C9ZZQ4_
DRB1_0101	162	FAPLRLDHEAYVADE	LRLDHEAYV	3	0.4277	488.7	60.00	0.56	tr_C9ZZQ4_
DRB1_0101	163	ALGSVPLRGVPEPVE	SVPLRGVPE	3	0.4272	491.5	60.00	0.51	tr_C9ZZQ4_
DRB1_0101	164	AAVRLARDLQRCFLN	VRLARDLQR	2	0.4260	497.9	60.00	0.52	tr_C9ZZQ4_
DRB1_0101	165	AVPGRTFAPLRLDHE	GRTFAPLRL	3	0.4236	510.8	60.00	0.52	tr_C9ZZQ4_
DRB1_0101	166	YQLDAVPGRTFAPLR	LDAVPGRTF	2	0.4221	519.4	60.00	0.57	tr_C9ZZQ4_
DRB1_0101	167	AVRLARDLQRCFLNY	VRLARDLQR	1	0.4203	529.5	60.00	0.38	tr_C9ZZQ4_
DRB1_0101	168	QTANMAARTESIANG	NMAARTESI	3	0.4175	546.1	60.00	0.58	tr_C9ZZQ4_
DRB1_0101	169	QLDAVPGRTFAPLRL	LDAVPGRTF	1	0.4163	553.2	60.00	0.35	tr_C9ZZQ4_
DRB1_0101	170	SISSQLGTGGESVSN	ISSQLGTGG	1	0.4162	553.9	60.00	0.46	tr_C9ZZQ4_
DRB1_0101	171	SAQLDPEVYRKLWNG	LDPEVYRKL	3	0.4154	558.8	60.00	0.62	tr_C9ZZQ4_
DRB1_0101	172	LGSVPLRGVPEPVEI	SVPLRGVPE	2	0.4083	603.4	60.00	0.51	tr_C9ZZQ4_
DRB1_0101	173	SATAAVRLARDLQRC	VRLARDLQR	5	0.4079	605.8	65.00	0.37	tr_C9ZZQ4_
DRB1_0101	174	AQLDPEVYRKLWNGL	LDPEVYRKL	2	0.4072	610.0	65.00	0.61	tr_C9ZZQ4_
DRB1_0101	175	VRLARDLQRCFLNYR	RDLQRCFLN	4	0.4064	615.9	65.00	0.28	tr_C9ZZQ4_
DRB1_0101	176	ATAAVRLARDLQRCF	VRLARDLQR	4	0.4063	615.9	65.00	0.52	tr_C9ZZQ4_
DRB1_0101	177	LDAVPGRTFAPLRLD	GRTFAPLRL	5	0.4056	621.2	65.00	0.42	tr_C9ZZQ4_
DRB1_0101	178	MAARTESIANGGQVL	SIANGGQVL	6	0.4043	630.0	65.00	0.68	tr_C9ZZQ4_
DRB1_0101	179	RLARDLQRCFLNYRW	RDLQRCFLN	3	0.4023	643.8	65.00	0.32	tr_C9ZZQ4_
DRB1_0101	180	GTFTIVQRQKELTAI	FTIVQRQKE	2	0.4001	658.9	65.00	0.29	tr_C9ZZQ4_
DRB1_0101	181	CERWRVPLPPWKEAV	RVPLPPWKE	4	0.3977	676.2	65.00	0.41	tr_C9ZZQ4_
DRB1_0101	182	GSVPLRGVPEPVEIY	SVPLRGVPE	1	0.3917	721.8	65.00	0.40	tr_C9ZZQ4_
DRB1_0101	183	DAVPGRTFAPLRLDH	GRTFAPLRL	4	0.3898	737.1	65.00	0.57	tr_C9ZZQ4_
DRB1_0101	184	EPPSAQLDPEVYRKL	LDPEVYRKL	6	0.3892	741.5	65.00	0.36	tr_C9ZZQ4_
DRB1_0101	185	TDSIDNFYRAEEKQV	FYRAEEKQV	6	0.3886	746.0	65.00	0.59	tr_C9ZZQ4_
DRB1_0101	186	YDCYEVKTIGDSFMI	VKTIGDSFM	5	0.3859	768.1	65.00	0.26	tr_C9ZZQ4_
DRB1_0101	187	TFTIVQRQKELTAIC	TIVQRQKEL	2	0.3851	775.0	65.00	0.28	tr_C9ZZQ4_
DRB1_0101	188	IVDVAAVERVGRPTE	VDVAAVERV	1	0.3846	779.0	65.00	0.69	tr_C9ZZQ4_
DRB1_0101	189	LGTFTIVQRQKELTA	FTIVQRQKE	3	0.3840	784.6	70.00	0.37	tr_C9ZZQ4_
DRB1_0101	190	TLIFTDIENSTSQWA	FTDIENSTS	3	0.3834	789.3	70.00	0.59	tr_C9ZZQ4_
DRB1_0101	191	HEAYVADESSDVSHT	YVADESSDV	3	0.3831	792.2	70.00	0.71	tr_C9ZZQ4_
DRB1_0101	192	ICERWRVPLPPWKEA	WRVPLPPWK	4	0.3824	798.0	70.00	0.31	tr_C9ZZQ4_
DRB1_0101	193	ERWRVPLPPWKEAVW	RVPLPPWKE	3	0.3822	799.5	70.00	0.47	tr_C9ZZQ4_
DRB1_0101	194	LARDLQRCFLNYRWG	RDLQRCFLN	2	0.3775	841.9	70.00	0.32	tr_C9ZZQ4_
DRB1_0101	195	LIFTDIENSTSQWAS	FTDIENSTS	2	0.3770	845.9	70.00	0.52	tr_C9ZZQ4_
DRB1_0101	196	ESSDSSSVILISNPA	SVILISNPA	6	0.3770	846.3	70.00	0.70	tr_C9ZZQ4_
DRB1_0101	197	EAYVADESSDVSHTT	YVADESSDV	2	0.3754	860.5	70.00	0.64	tr_C9ZZQ4_
DRB1_0101	198	APLRLDHEAYVADES	LRLDHEAYV	2	0.3749	865.6	70.00	0.67	tr_C9ZZQ4_
DRB1_0101	199	TANMAARTESIANGG	NMAARTESI	2	0.3743	871.0	70.00	0.62	tr_C9ZZQ4_
DRB1_0101	200	FTIVQRQKELTAICE	VQRQKELTA	3	0.3727	886.8	70.00	0.22	tr_C9ZZQ4_
DRB1_0101	201	ISSQLGTGGESVSNV	QLGTGGESV	3	0.3718	895.4	70.00	0.44	tr_C9ZZQ4_
DRB1_0101	202	AVKVGHIVDVAAVER	GHIVDVAAV	4	0.3686	926.2	70.00	0.37	tr_C9ZZQ4_
DRB1_0101	203	GTGGESVSNVLHALL	SVSNVLHAL	5	0.3676	936.8	70.00	0.37	tr_C9ZZQ4_
DRB1_0101	204	SVPLRGVPEPVEIYQ	LRGVPEPVE	3	0.3657	956.1	70.00	0.28	tr_C9ZZQ4_
DRB1_0101	205	RIRAGIHTGLCDIRY	IRAGIHTGL	1	0.3629	986.2	70.00	0.33	tr_C9ZZQ4_
DRB1_0101	206	YRAEEKQVAELNSKA	YRAEEKQVA	0	0.3626	989.0	70.00	0.54	tr_C9ZZQ4_
DRB1_0101	207	QRQKELTAICERWRV	QKELTAICE	2	0.3614	1001.7	70.00	0.49	tr_C9ZZQ4_
DRB1_0101	208	ARDLQRCFLNYRWGT	RDLQRCFLN	1	0.3597	1020.2	75.00	0.28	tr_C9ZZQ4_
DRB1_0101	209	IFTDIENSTSQWASH	FTDIENSTS	1	0.3554	1068.9	75.00	0.44	tr_C9ZZQ4_

DRB1_0101	210	AICERWRVPLPPWKE	WRVPLPPWK	5	0.3550	1073.4	75.00	0.30	tr_C9ZZQ4_
DRB1_0101	211	DHEAYVADESSDVSH	YVADESSDV	4	0.3529	1098.7	75.00	0.74	tr_C9ZZQ4_
DRB1_0101	212	TIVQRQKELTAICER	QKELTAICE	5	0.3528	1099.0	75.00	0.33	tr_C9ZZQ4_
DRB1_0101	213	AYVADESSDVSHTTF	YVADESSDV	1	0.3502	1131.1	75.00	0.58	tr_C9ZZQ4_
DRB1_0101	214	VQRQKELTAICERWR	QKELTAICE	3	0.3492	1142.8	75.00	0.57	tr_C9ZZQ4_
DRB1_0101	215	LLGTFTIVQRQKELT	FTIVQRQKE	4	0.3489	1146.3	75.00	0.35	tr_C9ZZQ4_
DRB1_0101	216	NYDCYEVKTIGDSFM	YDCYEVKTI	1	0.3476	1163.6	75.00	0.32	tr_C9ZZQ4_
DRB1_0101	217	VTLIFTDIENSTSQW	FTDIENSTS	4	0.3474	1166.1	75.00	0.57	tr_C9ZZQ4_
DRB1_0101	218	PVTLIFTDIENSTSQ	FTDIENSTS	5	0.3460	1183.7	75.00	0.39	tr_C9ZZQ4_
DRB1_0101	219	ALLGTFTIVQRQKEL	FTIVQRQKE	5	0.3421	1234.1	75.00	0.31	tr_C9ZZQ4_
DRB1_0101	220	RQKELTAICERWRVP	QKELTAICE	1	0.3414	1244.2	75.00	0.37	tr_C9ZZQ4_
DRB1_0101	221	RWRVPLPPWKEAVWN	RVPLPPWKE	2	0.3409	1250.7	75.00	0.53	tr_C9ZZQ4_
DRB1_0101	222	SSQLGTGGESVSNVL	QLGTGGESV	2	0.3371	1303.2	75.00	0.47	tr_C9ZZQ4_
DRB1_0101	223	IVQRQKELTAICERW	QKELTAICE	4	0.3306	1398.2	80.00	0.49	tr_C9ZZQ4_
DRB1_0101	224	SHTTFNDCMSISSQL	FNDCMSISS	4	0.3278	1440.9	80.00	0.43	tr_C9ZZQ4_
DRB1_0101	225	PLRLDHEAYVADESS	LRLDHEAYV	1	0.3273	1448.1	80.00	0.63	tr_C9ZZQ4_
DRB1_0101	226	TAICERWRVPLPPWK	ERWRVPLPP	4	0.3257	1474.3	80.00	0.25	tr_C9ZZQ4_
DRB1_0101	227	GNYDCYEVKTIGDSF	YDCYEVKTI	2	0.3254	1478.5	80.00	0.47	tr_C9ZZQ4_
DRB1_0101	228	RCFLNYRWGTDSIDN	LNYRWGTDS	3	0.3250	1484.6	80.00	0.44	tr_C9ZZQ4_
DRB1_0101	229	IRAGIHTGLCDIRYD	GIHTGLCDI	3	0.3248	1488.1	80.00	0.28	tr_C9ZZQ4_
DRB1_0101	230	ANMAARTESIANGGQ	NMAARTESI	1	0.3247	1489.8	80.00	0.64	tr_C9ZZQ4_
DRB1_0101	231	DPVTLIFTDIENSTS	LIFTDIENS	4	0.3224	1527.0	80.00	0.34	tr_C9ZZQ4_
DRB1_0101	232	FSLSTAEREQLDVTA	FSLSTAERE	0	0.3224	1527.8	80.00	0.53	tr_C9ZZQ4_
DRB1_0101	233	LGTGGESVSNVLHAL	SVSNVLHAL	6	0.3223	1528.7	80.00	0.23	tr_C9ZZQ4_
DRB1_0101	234	LISNPAVDQSTEDLE	LISNPAVDQ	0	0.3220	1533.9	80.00	0.68	tr_C9ZZQ4_
DRB1_0101	235	QRCFLNYRWGTDSID	LNYRWGTDS	4	0.3214	1544.4	80.00	0.40	tr_C9ZZQ4_
DRB1_0101	236	IGNYDCYEVKTIGDS	YDCYEVKTI	3	0.3214	1544.8	80.00	0.58	tr_C9ZZQ4_
DRB1_0101	237	QKELTAICERWRVPL	LTAICERWR	3	0.3198	1571.9	80.00	0.27	tr_C9ZZQ4_
DRB1_0101	238	SQLGTGGESVSNVLH	QLGTGGESV	1	0.3196	1574.2	80.00	0.37	tr_C9ZZQ4_
DRB1_0101	239	LQRCFLNYRWGTDSI	LNYRWGTDS	5	0.3191	1583.8	80.00	0.34	tr_C9ZZQ4_
DRB1_0101	240	RDLQRCFLNYRWGTD	QRCFLNYRW	3	0.3190	1584.6	80.00	0.29	tr_C9ZZQ4_
DRB1_0101	241	CFLNYRWGTDSIDNF	YRWGTDSID	4	0.3137	1678.9	80.00	0.41	tr_C9ZZQ4_
DRB1_0101	242	VPLRGVPEPVEIYQL	GVPEPVEIY	4	0.3134	1684.0	80.00	0.32	tr_C9ZZQ4_
DRB1_0101	243	TLIGNYDCYEVKTIG	YDCYEVKTI	5	0.3123	1704.5	80.00	0.56	tr_C9ZZQ4_
DRB1_0101	244	FLNYRWGTDSIDNFY	YRWGTDSID	3	0.3099	1749.0	80.00	0.49	tr_C9ZZQ4_
DRB1_0101	245	AEEKQVAELNSKAEP	QVAELNSKA	4	0.3067	1809.5	80.00	0.51	tr_C9ZZQ4_
DRB1_0101	246	TAEREQLDVTALGSV	REQLDVTAL	3	0.3043	1858.6	85.00	0.43	tr_C9ZZQ4_
DRB1_0101	247	QLGTGGESVSNVLHA	TGGESVSNV	3	0.3037	1871.2	85.00	0.26	tr_C9ZZQ4_
DRB1_0101	248	NYRWGTDSIDNFYRA	YRWGTDSID	1	0.3036	1871.3	85.00	0.35	tr_C9ZZQ4_
DRB1_0101	249	RAEEKQVAELNSKAE	QVAELNSKA	5	0.3031	1882.7	85.00	0.46	tr_C9ZZQ4_
DRB1_0101	250	AEREQLDVTALGSVP	REQLDVTAL	2	0.3025	1895.5	85.00	0.35	tr_C9ZZQ4_
DRB1_0101	251	LIGNYDCYEVKTIGD	YDCYEVKTI	4	0.3017	1910.2	85.00	0.61	tr_C9ZZQ4_
DRB1_0101	252	LDHEAYVADESSDVS	YVADESSDV	5	0.3003	1941.2	85.00	0.71	tr_C9ZZQ4_
DRB1_0101	253	RAGIHTGLCDIRYDE	GIHTGLCDI	2	0.2996	1955.8	85.00	0.31	tr_C9ZZQ4_
DRB1_0101	254	VAELNSKAEPPSAQL	SKAEPPSAQ	5	0.2968	2014.4	85.00	0.34	tr_C9ZZQ4_
DRB1_0101	255	AELNSKAEPPSAQLD	SKAEPPSAQ	4	0.2959	2034.5	85.00	0.38	tr_C9ZZQ4_
DRB1_0101	256	FTDIENSTSQWASHP	IENSTSQWA	3	0.2944	2068.7	85.00	0.45	tr_C9ZZQ4_
DRB1_0101	257	DLQRCFLNYRWGTDS	QRCFLNYRW	2	0.2932	2096.0	85.00	0.25	tr_C9ZZQ4_
DRB1_0101	258	TDPVTLIFTDIENST	PVTLIFTDI	2	0.2928	2104.7	85.00	0.38	tr_C9ZZQ4_
DRB1_0101	259	YRWGTDSIDNFYRAE	TDSIDNFYR	4	0.2923	2116.0	85.00	0.36	tr_C9ZZQ4_

DRB1_0101	260	VDVAAVERVGRPTES	VDVAAVERV	0	0.2914	2137.4	85.00	0.51	tr_C9ZZQ4_
DRB1_0101	261	RTLIGNYDCYEVKTI	YDCYEVKTI	6	0.2905	2158.3	85.00	0.41	tr_C9ZZQ4_
DRB1_0101	262	EKQVAELNSKAEPPS	QVAELNSKA	2	0.2892	2188.3	85.00	0.49	tr_C9ZZQ4_
DRB1_0101	263	EEKQVAELNSKAEPP	QVAELNSKA	3	0.2875	2228.9	85.00	0.54	tr_C9ZZQ4_
DRB1_0101	264	AGIHTGLCDIRYDEV	HTGLCDIRY	3	0.2872	2236.0	85.00	0.31	tr_C9ZZQ4_
DRB1_0101	265	IENSTSQWASHPNVM	STSQWASHP	3	0.2864	2256.1	85.00	0.23	tr_C9ZZQ4_
DRB1_0101	266	LNYRWGTDSIDNFYR	YRWGTDSID	2	0.2834	2330.1	85.00	0.51	tr_C9ZZQ4_
DRB1_0101	267	PLRGVPEPVEIYQLD	GVPEPVEIY	3	0.2781	2466.5	85.00	0.34	tr_C9ZZQ4_
DRB1_0101	268	KQVAELNSKAEPPSA	QVAELNSKA	1	0.2757	2532.4	85.00	0.42	tr_C9ZZQ4_
DRB1_0101	269	SAPMDPTDPVTLIFT	MDPTDPVTL	3	0.2744	2567.2	90.00	0.62	tr_C9ZZQ4_
DRB1_0101	270	PTDPVTLIFTDIENS	PVTLIFTDI	3	0.2715	2649.8	90.00	0.47	tr_C9ZZQ4_
DRB1_0101	271	RPTESSDSSSVILIS	TESSDSSSV	2	0.2705	2677.6	90.00	0.41	tr_C9ZZQ4_
DRB1_0101	272	ELNSKAEPPSAQLDP	SKAEPPSAQ	3	0.2699	2695.9	90.00	0.39	tr_C9ZZQ4_
DRB1_0101	273	LTAICERWRVPLPPW	ERWRVPLPP	5	0.2699	2696.5	90.00	0.21	tr_C9ZZQ4_
DRB1_0101	274	KELTAICERWRVPLP	LTAICERWR	2	0.2696	2706.1	90.00	0.31	tr_C9ZZQ4_
DRB1_0101	275	PMDPTDPVTLIFTDI	MDPTDPVTL	1	0.2685	2735.8	90.00	0.35	tr_C9ZZQ4_
DRB1_0101	276	YVADESSDVSHTTFN	YVADESSDV	0	0.2678	2759.4	90.00	0.40	tr_C9ZZQ4_
DRB1_0101	277	VSAPMDPTDPVTLIF	MDPTDPVTL	4	0.2668	2787.0	90.00	0.60	tr_C9ZZQ4_
DRB1_0101	278	EVTKGYDYYGQTANM	YDYYGQTAN	5	0.2667	2790.0	90.00	0.22	tr_C9ZZQ4_
DRB1_0101	279	NMAARTESIANGGQV	NMAARTESI	0	0.2662	2805.3	90.00	0.44	tr_C9ZZQ4_
DRB1_0101	280	STAEREQLDVTALGS	REQLDVTAL	4	0.2613	2958.3	90.00	0.50	tr_C9ZZQ4_
DRB1_0101	281	RWGTDSIDNFYRAEE	DSIDNFYRA	4	0.2606	2981.1	90.00	0.38	tr_C9ZZQ4_
DRB1_0101	282	AEPPSAQLDPEVYRK	SAQLDPEVY	4	0.2590	3033.6	90.00	0.56	tr_C9ZZQ4_
DRB1_0101	283	GRPTESSDSSSVILI	TESSDSSSV	3	0.2589	3036.1	90.00	0.56	tr_C9ZZQ4_
DRB1_0101	284	GIHTGLCDIRYDEVT	HTGLCDIRY	2	0.2583	3055.5	90.00	0.34	tr_C9ZZQ4_
DRB1_0101	285	QVAELNSKAEPPSAQ	VAELNSKAE	1	0.2576	3079.1	90.00	0.22	tr_C9ZZQ4_
DRB1_0101	286	EAVWNDEYYQEAIHR	WNDEYYQEA	3	0.2571	3095.7	90.00	0.38	tr_C9ZZQ4_
DRB1_0101	287	APMDPTDPVTLIFTD	MDPTDPVTL	2	0.2531	3232.8	90.00	0.61	tr_C9ZZQ4_
DRB1_0101	288	GTDSIDNFYRAEEKQ	DSIDNFYRA	2	0.2525	3255.2	90.00	0.44	tr_C9ZZQ4_
DRB1_0101	289	WRVPLPPWKEAVWND	RVPLPPWKE	1	0.2518	3279.0	90.00	0.55	tr_C9ZZQ4_
DRB1_0101	290	WGTDSIDNFYRAEEK	DSIDNFYRA	3	0.2510	3307.4	90.00	0.45	tr_C9ZZQ4_
DRB1_0101	291	PTESSDSSSVILISN	TESSDSSSV	1	0.2509	3312.3	90.00	0.28	tr_C9ZZQ4_
DRB1_0101	292	MDPTDPVTLIFTDIE	PVTLIFTDI	5	0.2494	3364.6	90.00	0.43	tr_C9ZZQ4_
DRB1_0101	293	LSTAEREQLDVTALG	REQLDVTAL	5	0.2490	3381.0	90.00	0.40	tr_C9ZZQ4_
DRB1_0101	294	ELTAICERWRVPLPP	TAICERWRV	2	0.2480	3417.7	90.00	0.22	tr_C9ZZQ4_
DRB1_0101	295	LNSKAEPPSAQLDPE	SKAEPPSAQ	2	0.2470	3454.2	90.00	0.44	tr_C9ZZQ4_
DRB1_0101	296	ITLRDARDNVSAPMD	LRDARDNVS	2	0.2467	3464.9	90.00	0.51	tr_C9ZZQ4_
DRB1_0101	297	LRLDHEAYVADESSD	LRLDHEAYV	0	0.2467	3466.6	90.00	0.47	tr_C9ZZQ4_
DRB1_0101	298	LRGVPEPVEIYQLDA	GVPEPVEIY	2	0.2462	3483.4	90.00	0.44	tr_C9ZZQ4_
DRB1_0101	299	VSHTTFNDCMSISSQ	FNDCMSISS	5	0.2440	3568.9	90.00	0.50	tr_C9ZZQ4_
DRB1_0101	300	DPTDPVTLIFTDIEN	PVTLIFTDI	4	0.2428	3613.0	90.00	0.52	tr_C9ZZQ4_
DRB1_0101	301	SLSTAEREQLDVTAL	LSTAEREQL	1	0.2391	3763.4	95.00	0.35	tr_C9ZZQ4_
DRB1_0101	302	RGVPEPVEIYQLDAV	GVPEPVEIY	1	0.2376	3824.7	95.00	0.36	tr_C9ZZQ4_
DRB1_0101	303	TDIENSTSQWASHPN	IENSTSQWA	2	0.2365	3868.4	95.00	0.53	tr_C9ZZQ4_
DRB1_0101	304	CDIRYDEVTKGYDYY	IRYDEVTKG	2	0.2362	3880.7	95.00	0.55	tr_C9ZZQ4_
DRB1_0101	305	NVSAPMDPTDPVTLI	MDPTDPVTL	5	0.2342	3967.0	95.00	0.55	tr_C9ZZQ4_
DRB1_0101	306	LCDIRYDEVTKGYDY	IRYDEVTKG	3	0.2339	3980.6	95.00	0.62	tr_C9ZZQ4_
DRB1_0101	307	DIRYDEVTKGYDYYG	IRYDEVTKG	1	0.2326	4035.4	95.00	0.43	tr_C9ZZQ4_
DRB1_0101	308	DIENSTSQWASHPNV	IENSTSQWA	1	0.2322	4053.0	95.00	0.44	tr_C9ZZQ4_
DRB1_0101	309	IRYDEVTKGYDYYGQ	DEVTKGYDY	3	0.2294	4179.5	95.00	0.35	tr_C9ZZQ4_

DRB1_0101	310	DEVTKGYDYYGQTAN	VTKGYDYYG	2	0.2267	4302.0	95.00	0.34	tr_C9ZZQ4_
DRB1_0101	311	RLDHEAYVADESSDV	YVADESSDV	6	0.2201	4618.4	95.00	0.54	tr_C9ZZQ4_
DRB1_0101	312	GVPEPVEIYQLDAVP	PEPVEIYQL	2	0.2201	4621.6	95.00	0.22	tr_C9ZZQ4_
DRB1_0101	313	IHTGLCDIRYDEVTK	HTGLCDIRY	1	0.2178	4735.7	95.00	0.36	tr_C9ZZQ4_
DRB1_0101	314	YDEVTKGYDYYGQTA	VTKGYDYYG	3	0.2154	4864.5	95.00	0.38	tr_C9ZZQ4_
DRB1_0101	315	VGRPTESSDSSSVIL	TESSDSSSV	4	0.2142	4922.9	95.00	0.62	tr_C9ZZQ4_
DRB1_0101	316	TGLCDIRYDEVTKGY	IRYDEVTKG	5	0.2135	4962.0	95.00	0.53	tr_C9ZZQ4_
DRB1_0101	317	TESSDSSSVILISNP	SSDSSSVIL	2	0.2133	4974.5	95.00	0.27	tr_C9ZZQ4_
DRB1_0101	318	HTGLCDIRYDEVTKG	IRYDEVTKG	6	0.2118	5055.2	95.00	0.32	tr_C9ZZQ4_
DRB1_0101	319	RYDEVTKGYDYYGQT	DEVTKGYDY	2	0.2099	5161.1	95.00	0.38	tr_C9ZZQ4_
DRB1_0101	320	KAEPPSAQLDPEVYR	SAQLDPEVY	5	0.2094	5188.6	95.00	0.54	tr_C9ZZQ4_
DRB1_0101	321	GLCDIRYDEVTKGYD	IRYDEVTKG	4	0.2069	5330.2	95.00	0.60	tr_C9ZZQ4_
DRB1_0101	322	TLRDARDNVSAPMDP	LRDARDNVS	1	0.2064	5357.7	95.00	0.51	tr_C9ZZQ4_
DRB1_0101	323	DVSHTTFNDCMSISS	FNDCMSISS	6	0.2026	5581.6	95.00	0.37	tr_C9ZZQ4_
DRB1_0101	324	VAAVERVGRPTESSD	ERVGRPTES	4	0.2006	5707.3	95.00	0.29	tr_C9ZZQ4_
DRB1_0101	325	DVAAVERVGRPTESS	ERVGRPTES	5	0.1991	5798.9	95.00	0.27	tr_C9ZZQ4_
DRB1_0101	326	NSKAEPPSAQLDPEV	SKAEPPSAQ	1	0.1986	5833.4	95.00	0.47	tr_C9ZZQ4_
DRB1_0101	327	LEGDAFECGWEEKGK	DAFECGWEE	3	0.1985	5836.5	95.00	0.47	tr_C9ZZQ4_
DRB1_0101	328	EGDAFECGWEEKGKQ	FECGWEEKG	4	0.1963	5976.7	95.00	0.46	tr_C9ZZQ4_
DRB1_0101	329	AAVERVGRPTESSDS	ERVGRPTES	3	0.1898	6411.3	100.00	0.36	tr_C9ZZQ4_
DRB1_0101	330	SKAEPPSAQLDPEVY	SAQLDPEVY	6	0.1893	6445.3	100.00	0.32	tr_C9ZZQ4_
DRB1_0101	331	KEAVWNDEYYQEAIH	WNDEYYQEA	4	0.1846	6783.6	100.00	0.47	tr_C9ZZQ4_
DRB1_0101	332	LRDARDNVSAPMDPT	LRDARDNVS	0	0.1786	7243.4	100.00	0.32	tr_C9ZZQ4_
DRB1_0101	333	DLEGDAFECGWEEKG	DAFECGWEE	4	0.1752	7514.0	100.00	0.40	tr_C9ZZQ4_
DRB1_0101	334	WKEAVWNDEYYQEAI	WNDEYYQEA	5	0.1728	7707.7	100.00	0.43	tr_C9ZZQ4_
DRB1_0101	335	DNVSAPMDPTDPVTL	MDPTDPVTL	6	0.1715	7819.6	100.00	0.41	tr_C9ZZQ4_
DRB1_0101	336	VADESSDVSHTTFND	DESSDVSHT	2	0.1674	8176.4	100.00	0.35	tr_C9ZZQ4_
DRB1_0101	337	RVGRPTESSDSSSVI	TESSDSSSV	5	0.1623	8635.2	100.00	0.65	tr_C9ZZQ4_
DRB1_0101	338	AVERVGRPTESSDSS	ERVGRPTES	2	0.1618	8686.3	100.00	0.38	tr_C9ZZQ4_
DRB1_0101	339	PWKEAVWNDEYYQEA	AVWNDEYYQ	4	0.1582	9030.8	100.00	0.31	tr_C9ZZQ4_
DRB1_0101	340	EDLEGDAFECGWEEK	DAFECGWEE	5	0.1576	9086.2	100.00	0.40	tr_C9ZZQ4_
DRB1_0101	341	RVPLPPWKEAVWNDE	RVPLPPWKE	0	0.1565	9190.9	100.00	0.45	tr_C9ZZQ4_
DRB1_0101	342	SDVSHTTFNDCMSIS	TTFNDCMSI	5	0.1565	9199.3	100.00	0.30	tr_C9ZZQ4_
DRB1_0101	343	TEDLEGDAFECGWEE	DLEGDAFEC	2	0.1504	9828.1	100.00	0.29	tr_C9ZZQ4_
DRB1_0101	344	QSTEDLEGDAFECGW	DLEGDAFEC	4	0.1499	9871.6	100.00	0.34	tr_C9ZZQ4_
DRB1_0101	345	STEDLEGDAFECGWE	DLEGDAFEC	3	0.1479	10089.0	100.00	0.35	tr_C9ZZQ4_
DRB1_0101	346	SSDVSHTTFNDCMSI	DVSHTTFND	2	0.1451	10408.4	100.00	0.25	tr_C9ZZQ4_
DRB1_0101	347	RDARDNVSAPMDPTD	ARDNVSAPM	2	0.1346	11652.1	100.00	0.35	tr_C9ZZQ4_
DRB1_0101	348	DQSTEDLEGDAFECG	DLEGDAFEC	5	0.1338	11758.6	100.00	0.32	tr_C9ZZQ4_
DRB1_0101	349	RDNVSAPMDPTDPVT	SAPMDPTDP	4	0.1327	11901.7	100.00	0.44	tr_C9ZZQ4_
DRB1_0101	350	ADESSDVSHTTFNDC	DESSDVSHT	1	0.1323	11946.6	100.00	0.35	tr_C9ZZQ4_
DRB1_0101	351	ARDNVSAPMDPTDPV	SAPMDPTDP	5	0.1308	12145.8	100.00	0.32	tr_C9ZZQ4_
DRB1_0101	352	ISNPAVDQSTEDLEG	AVDQSTEDL	4	0.1293	12343.2	100.00	0.51	tr_C9ZZQ4_
DRB1_0101	353	ESSDVSHTTFNDCMS	DVSHTTFND	3	0.1286	12441.2	100.00	0.35	tr_C9ZZQ4_
DRB1_0101	354	LPPWKEAVWNDEYYQ	WKEAVWNDE	3	0.1285	12445.6	100.00	0.50	tr_C9ZZQ4_
DRB1_0101	355	DESSDVSHTTFNDCM	DVSHTTFND	4	0.1275	12586.4	100.00	0.28	tr_C9ZZQ4_
DRB1_0101	356	PPWKEAVWNDEYYQE	WKEAVWNDE	2	0.1273	12606.5	100.00	0.46	tr_C9ZZQ4_
DRB1_0101	357	VERVGRPTESSDSSS	ERVGRPTES	1	0.1266	12711.8	100.00	0.41	tr_C9ZZQ4_
DRB1_0101	358	ERVGRPTESSDSSSV	TESSDSSSV	6	0.1225	13286.5	100.00	0.38	tr_C9ZZQ4_
DRB1_0101	359	DARDNVSAPMDPTDP	ARDNVSAPM	1	0.1214	13440.8	100.00	0.26	tr_C9ZZQ4_

DRB1_0101	360	SNPAVDQSTEDLEGD	AVDQSTEDL	3	0.1210	13500.6	100.00	0.56	tr_C9ZZQ4_	
DRB1_0101	361	PLPPWKEAVWNDEYY	WKEAVWNDE	4	0.1127	14766.9	100.00	0.54	tr_C9ZZQ4_	
DRB1_0101	362	NPAVDQSTEDLEGDA	AVDQSTEDL	2	0.1121	14862.9	100.00	0.50	tr_C9ZZQ4_	
DRB1_0101	363	PAVDQSTEDLEGDAF	AVDQSTEDL	1	0.1107	15088.9	100.00	0.38	tr_C9ZZQ4_	
DRB1_0101	364	VDQSTEDLEGDAFEC	TEDLEGDAF	4	0.1039	16249.5	100.00	0.43	tr_C9ZZQ4_	
DRB1_0101	365	AVDQSTEDLEGDAFE	TEDLEGDAF	5	0.1005	16855.3	100.00	0.39	tr_C9ZZQ4_	
DRB1_0101	366	VPLPPWKEAVWNDEY	WKEAVWNDE	5	0.1002	16910.0	100.00	0.45	tr_C9ZZQ4_	

Allele: DRB1_0101. Number of high binders 0. Number of weak binders 40. Number of peptides 366

Allele	pos	peptide	core	Of	1-log50k(aff)	affinity(nM)	%Rank	Relia	Identity	Bind Level
DRB1_0101	1	DRFFLHAFNTTIAEA	FLHAFNTTI	3	0.9118	2.6	0.05	0.88	tr_D0A0W7_	SB
DRB1_0101	2	RFFLHAFNTTIAEAA	FLHAFNTTI	2	0.9113	2.6	0.05	0.86	tr_D0A0W7_	SB
DRB1_0101	3	SDRFFLHAFNTTIAE	FLHAFNTTI	4	0.9029	2.9	0.07	0.86	tr_D0A0W7_	SB
DRB1_0101	4	HSDRFFLHAFNTTIA	FLHAFNTTI	5	0.8944	3.1	0.12	0.83	tr_D0A0W7_	SB
DRB1_0101	5	FFLHAFNTTIAEAAN	FLHAFNTTI	1	0.8869	3.4	0.17	0.79	tr_D0A0W7_	SB
DRB1_0101	6	PYLYFLRADPAAETL	LYFLRADPA	2	0.8754	3.8	0.25	0.67	tr_D0A0W7_	SB
DRB1_0101	7	YLYFLRADPAAETLA	LYFLRADPA	1	0.8729	4.0	0.30	0.59	tr_D0A0W7_	SB
DRB1_0101	8	NPYLYFLRADPAAET	LYFLRADPA	3	0.8693	4.1	0.40	0.75	tr_D0A0W7_	SB
DRB1_0101	9	WNPYLYFLRADPAAE	LYFLRADPA	4	0.8512	5.0	0.60	0.80	tr_D0A0W7_	WB
DRB1_0101	10	GSTFIRQWNPYLYFL	FIRQWNPYL	3	0.8501	5.1	0.60	0.89	tr_D0A0W7_	WB
DRB1_0101	11	LPVILRAPVALLLLL	ILRAPVALL	3	0.8463	5.3	0.70	0.64	tr_D0A0W7_	WB
DRB1_0101	12	STFIRQWNPYLYFLR	FIRQWNPYL	2	0.8425	5.5	0.80	0.87	tr_D0A0W7_	WB
DRB1_0101	13	TLPVILRAPVALLLL	ILRAPVALL	4	0.8415	5.6	0.80	0.59	tr_D0A0W7_	WB
DRB1_0101	14	LYFLRADPAAETLAL	LYFLRADPA	0	0.8385	5.7	0.90	0.44	tr_D0A0W7_	WB
DRB1_0101	15	GHSDRFFLHAFNTTI	FLHAFNTTI	6	0.8375	5.8	0.90	0.75	tr_D0A0W7_	WB
DRB1_0101	16	YDLFTTALNNTAGAA	FTTALNNTA	3	0.8367	5.9	0.90	0.90	tr_D0A0W7_	WB
DRB1_0101	17	PVILRAPVALLLLLI	ILRAPVALL	2	0.8347	6.0	1.00	0.67	tr_D0A0W7_	WB
DRB1_0101	18	TGSTFIRQWNPYLYF	FIRQWNPYL	4	0.8295	6.3	1.10	0.92	tr_D0A0W7_	WB
DRB1_0101	19	LSLLAFATGRLMRVI	LAFATGRLM	3	0.8285	6.4	1.10	0.73	tr_D0A0W7_	WB
DRB1_0101	20	QWNPYLYFLRADPAA	LYFLRADPA	5	0.8276	6.5	1.10	0.84	tr_D0A0W7_	WB
DRB1_0101	21	PLSLLAFATGRLMRV	LAFATGRLM	4	0.8232	6.8	1.30	0.70	tr_D0A0W7_	WB
DRB1_0101	22	DTLPVILRAPVALLL	ILRAPVALL	5	0.8220	6.9	1.30	0.55	tr_D0A0W7_	WB
DRB1_0101	23	DLFTTALNNTAGAAR	FTTALNNTA	2	0.8218	6.9	1.30	0.89	tr_D0A0W7_	WB
DRB1_0101	24	SLLAFATGRLMRVIL	LAFATGRLM	2	0.8197	7.0	1.40	0.69	tr_D0A0W7_	WB
DRB1_0101	25	LYDLFTTALNNTAGA	FTTALNNTA	4	0.8145	7.4	1.50	0.88	tr_D0A0W7_	WB
DRB1_0101	26	FLHAFNTTIAEAAND	FLHAFNTTI	0	0.8138	7.5	1.50	0.61	tr_D0A0W7_	WB
DRB1_0101	27	QMWLHALATAGTSFA	WLHALATAG	2	0.8085	7.9	1.70	0.47	tr_D0A0W7_	WB
DRB1_0101	28	TFIRQWNPYLYFLRA	FIRQWNPYL	1	0.8080	8.0	1.70	0.81	tr_D0A0W7_	WB
DRB1_0101	29	LFTTALNNTAGAARL	FTTALNNTA	1	0.8075	8.0	1.80	0.86	tr_D0A0W7_	WB
DRB1_0101	30	IKRFQGVMSEYLKAH	FQGVMSEYL	3	0.8068	8.1	1.80	0.71	tr_D0A0W7_	WB
DRB1_0101	31	AIKRFQGVMSEYLKA	FQGVMSEYL	4	0.8052	8.2	1.80	0.67	tr_D0A0W7_	WB
DRB1_0101	32	AQMWLHALATAGTSF	WLHALATAG	3	0.8038	8.4	1.90	0.57	tr_D0A0W7_	WB
DRB1_0101	33	TPLSLLAFATGRLMR	LAFATGRLM	5	0.8019	8.5	2.00	0.62	tr_D0A0W7_	WB
DRB1_0101	34	LLAFATGRLMRVILL	LAFATGRLM	1	0.8000	8.7	2.00	0.62	tr_D0A0W7_	WB
DRB1_0101	35	FERFAAALPQAIIVF	FAAALPQAI	3	0.7958	9.1	2.50	0.75	tr_D0A0W7_	WB

DRB1_0101	36	MTGSTFIRQWNPYLY	FIRQWNPYL	5	0.7952	9.2	2.50	0.92	tr_D0A0W7_	WB
DRB1_0101	37	KRFQGVMSEYLKAHV	FQGVMSEYL	2	0.7936	9.3	2.50	0.74	tr_D0A0W7_	WB
DRB1_0101	38	VFERFAAALPQAIIV	FAAALPQAI	4	0.7923	9.5	2.50	0.69	tr_D0A0W7_	WB
DRB1_0101	39	IAIKRFQGVMSEYLK	FQGVMSEYL	5	0.7907	9.6	2.50	0.57	tr_D0A0W7_	WB
DRB1_0101	40	VILRAPVALLLLLIV	ILRAPVALL	1	0.7877	9.9	3.00	0.73	tr_D0A0W7_	WB
DRB1_0101	41	LAQMWLHALATAGTS	WLHALATAG	4	0.7844	10.3	3.00	0.61	tr_D0A0W7_	WB
DRB1_0101	42	MWLHALATAGTSFAP	WLHALATAG	1	0.7815	10.6	3.00	0.42	tr_D0A0W7_	WB
DRB1_0101	43	LLYDLFTTALNNTAG	FTTALNNTA	5	0.7782	11.0	3.00	0.81	tr_D0A0W7_	WB
DRB1_0101	44	RFQGVMSEYLKAHVS	FQGVMSEYL	1	0.7727	11.7	3.50	0.56	tr_D0A0W7_	WB
DRB1_0101	45	QVSVWSMARALNASI	WSMARALNA	4	0.7707	11.9	3.50	0.64	tr_D0A0W7_	WB
DRB1_0101	46	YIAIKRFQGVMSEYL	IKRFQGVMS	3	0.7704	12.0	3.50	0.41	tr_D0A0W7_	WB
DRB1_0101	47	DIVTYLALPILMKHN	YLALPILMK	4	0.7701	12.0	3.50	0.70	tr_D0A0W7_	WB
DRB1_0101	48	EVFERFAAALPQAII	FAAALPQAI	5	0.7699	12.1	3.50	0.60	tr_D0A0W7_	WB
DRB1_0101	49	AALWQKGASHLVGKG	WQKGASHLV	3	0.7698	12.1	3.50	0.81	tr_D0A0W7_	WB
DRB1_0101	50	MDTLPVILRAPVALL	ILRAPVALL	6	0.7693	12.1	3.50	0.46	tr_D0A0W7_	WB
DRB1_0101	51	GDIVTYLALPILMKH	YLALPILMK	5	0.7641	12.8	4.00	0.67	tr_D0A0W7_	WB
DRB1_0101	52	ERFAAALPQAIIVFG	FAAALPQAI	2	0.7633	12.9	4.00	0.82	tr_D0A0W7_	WB
DRB1_0101	53	VSVWSMARALNASIP	WSMARALNA	3	0.7629	13.0	4.00	0.66	tr_D0A0W7_	WB
DRB1_0101	54	GTVFVIGLSVPNVRE	FVIGLSVPN	3	0.7618	13.2	4.00	0.75	tr_D0A0W7_	WB
DRB1_0101	55	IVTYLALPILMKHNV	YLALPILMK	3	0.7613	13.2	4.00	0.73	tr_D0A0W7_	WB
DRB1_0101	56	VTYLALPILMKHNVV	YLALPILMK	2	0.7602	13.4	4.50	0.69	tr_D0A0W7_	WB
DRB1_0101	57	YLALPILMKHNVVAF	ILMKHNVVA	5	0.7598	13.4	4.50	0.40	tr_D0A0W7_	WB
DRB1_0101	58	AAALWQKGASHLVGK	WQKGASHLV	4	0.7595	13.5	4.50	0.76	tr_D0A0W7_	WB
DRB1_0101	59	TYLALPILMKHNVVA	YLALPILMK	1	0.7585	13.6	4.50	0.50	tr_D0A0W7_	WB
DRB1_0101	60	SGTVFVIGLSVPNVR	FVIGLSVPN	4	0.7549	14.2	4.50	0.75	tr_D0A0W7_	WB
DRB1_0101	61	LSSYLAGDGVGTVDA	YLAGDGVGT	3	0.7542	14.3	4.50	0.77	tr_D0A0W7_	WB
DRB1_0101	62	SLAQMWLHALATAGT	WLHALATAG	5	0.7539	14.3	4.50	0.58	tr_D0A0W7_	WB
DRB1_0101	63	TVFVIGLSVPNVREI	FVIGLSVPN	2	0.7523	14.6	5.00	0.69	tr_D0A0W7_	WB
DRB1_0101	64	LVNFFYADSSIISDD	FFYADSSII	3	0.7515	14.7	5.00	0.57	tr_D0A0W7_	WB
DRB1_0101	65	RQWNPYLYFLRADPA	LYFLRADPA	6	0.7488	15.1	5.00	0.81	tr_D0A0W7_	WB
DRB1_0101	66	ALWQKGASHLVGKGD	WQKGASHLV	2	0.7488	15.2	5.00	0.82	tr_D0A0W7_	WB
DRB1_0101	67	TLVNFFYADSSIISD	FFYADSSII	4	0.7480	15.3	5.00	0.58	tr_D0A0W7_	WB
DRB1_0101	68	RTTFVRSLYSQRRYV	FVRSLYSQR	3	0.7470	15.4	5.00	0.41	tr_D0A0W7_	WB
DRB1_0101	69	VNFFYADSSIISDDM	FFYADSSII	2	0.7458	15.7	5.00	0.50	tr_D0A0W7_	WB
DRB1_0101	70	TQVSVWSMARALNAS	WSMARALNA	5	0.7455	15.7	5.00	0.61	tr_D0A0W7_	WB
DRB1_0101	71	SVWSMARALNASIPP	WSMARALNA	2	0.7436	16.0	5.50	0.67	tr_D0A0W7_	WB
DRB1_0101	72	LALPILMKHNVVAFS	ILMKHNVVA	4	0.7428	16.2	5.50	0.56	tr_D0A0W7_	WB
DRB1_0101	73	VGKYLPISGTVFVIG	YLPISGTVF	3	0.7421	16.3	5.50	0.70	tr_D0A0W7_	WB
DRB1_0101	74	WTPLSLLAFATGRLM	LAFATGRLM	6	0.7397	16.7	5.50	0.49	tr_D0A0W7_	WB
DRB1_0101	75	GVGKYLPISGTVFVI	YLPISGTVF	4	0.7387	16.9	5.50	0.67	tr_D0A0W7_	WB
DRB1_0101	76	VLSSYLAGDGVGTVD	YLAGDGVGT	4	0.7375	17.1	6.00	0.73	tr_D0A0W7_	WB
DRB1_0101	77	LGDIVTYLALPILMK	YLALPILMK	6	0.7369	17.2	6.00	0.64	tr_D0A0W7_	WB
DRB1_0101	78	TTFVRSLYSQRRYVI	VRSLYSQRR	3	0.7368	17.2	6.00	0.41	tr_D0A0W7_	WB
DRB1_0101	79	YVLSSYLAGDGVGTV	YLAGDGVGT	5	0.7361	17.4	6.00	0.65	tr_D0A0W7_	WB
DRB1_0101	80	LTEFGVSLRSAVIRE	GVSLRSAVI	4	0.7335	17.9	6.00	0.65	tr_D0A0W7_	WB
DRB1_0101	81	LAFATGRLMRVILLH	LAFATGRLM	0	0.7330	18.0	6.00	0.53	tr_D0A0W7_	WB
DRB1_0101	82	WLHALATAGTSFAPG	LHALATAGT	1	0.7326	18.0	6.00	0.35	tr_D0A0W7_	WB
DRB1_0101	83	RFAAALPQAIIVFGA	FAAALPQAI	1	0.7298	18.6	6.50	0.77	tr_D0A0W7_	WB
DRB1_0101	84	REVFERFAAALPQAI	FAAALPQAI	6	0.7296	18.6	6.50	0.36	tr_D0A0W7_	WB
DRB1_0101	85	LIRYALCQLRVLRLG	LCQLRVLRL	5	0.7288	18.8	6.50	0.45	tr_D0A0W7_	WB

DRB1_0101	86	FTTALNNTAGAARLV	FTTALNNTA	0	0.7286	18.8	6.50	0.69	tr_D0A0W7_	WB
DRB1_0101	87	SLTEFGVSLRSAVIR	GVSLRSAVI	5	0.7274	19.1	6.50	0.62	tr_D0A0W7_	WB
DRB1_0101	88	ALPILMKHNVVAFSP	ILMKHNVVA	3	0.7264	19.3	6.50	0.55	tr_D0A0W7_	WB
DRB1_0101	89	TEFGVSLRSAVIRED	GVSLRSAVI	3	0.7249	19.6	7.00	0.70	tr_D0A0W7_	WB
DRB1_0101	90	IRYALCQLRVLRLGF	LCQLRVLRL	4	0.7242	19.8	7.00	0.58	tr_D0A0W7_	WB
DRB1_0101	91	QNKIVIVYGPLGDIV	IVIVYGPLG	3	0.7214	20.4	7.00	0.83	tr_D0A0W7_	WB
DRB1_0101	92	NKIVIVYGPLGDIVT	IVIVYGPLG	2	0.7193	20.8	7.50	0.75	tr_D0A0W7_	WB
DRB1_0101	93	SSYLAGDGVGTVDAV	YLAGDGVGT	2	0.7187	21.0	7.50	0.83	tr_D0A0W7_	WB
DRB1_0101	94	SQYIAIKRFQGVMSE	IKRFQGVMS	5	0.7186	21.0	7.50	0.43	tr_D0A0W7_	WB
DRB1_0101	95	VFVIGLSVPNVREIA	FVIGLSVPN	1	0.7182	21.1	7.50	0.62	tr_D0A0W7_	WB
DRB1_0101	96	FQGVMSEYLKAHVSE	SEYLKAHVS	5	0.7181	21.1	7.50	0.31	tr_D0A0W7_	WB
DRB1_0101	97	DRTTFVRSLYSQRRY	FVRSLYSQR	4	0.7169	21.4	7.50	0.43	tr_D0A0W7_	WB
DRB1_0101	98	KAAALWQKGASHLVG	WQKGASHLV	5	0.7168	21.4	7.50	0.73	tr_D0A0W7_	WB
DRB1_0101	99	FRRHVIHLSPTLEQQ	HVIHLSPTL	3	0.7163	21.5	7.50	0.38	tr_D0A0W7_	WB
DRB1_0101	100	EGVGKYLPISGTVFV	YLPISGTVF	5	0.7152	21.8	7.50	0.68	tr_D0A0W7_	WB
DRB1_0101	101	NFFYADSSIISDDMR	FFYADSSII	1	0.7146	21.9	7.50	0.47	tr_D0A0W7_	WB
DRB1_0101	102	QYIAIKRFQGVMSEY	IKRFQGVMS	4	0.7141	22.0	7.50	0.52	tr_D0A0W7_	WB
DRB1_0101	103	RYALCQLRVLRLGFM	LCQLRVLRL	3	0.7135	22.2	8.00	0.62	tr_D0A0W7_	WB
DRB1_0101	104	KFRRHVIHLSPTLEQ	HVIHLSPTL	4	0.7131	22.3	8.00	0.39	tr_D0A0W7_	WB
DRB1_0101	105	VWSMARALNASIPPL	WSMARALNA	1	0.7127	22.4	8.00	0.62	tr_D0A0W7_	WB
DRB1_0101	106	ETLVNFFYADSSIIS	FFYADSSII	5	0.7123	22.5	8.00	0.67	tr_D0A0W7_	WB
DRB1_0101	107	KIVIVYGPLGDIVTY	IVIVYGPLG	1	0.7119	22.6	8.00	0.65	tr_D0A0W7_	WB
DRB1_0101	108	GKYLPISGTVFVIGL	YLPISGTVF	2	0.7118	22.6	8.00	0.74	tr_D0A0W7_	WB
DRB1_0101	109	ARSYILGPSSVQASL	YILGPSSVQ	3	0.7115	22.7	8.00	0.60	tr_D0A0W7_	WB
DRB1_0101	110	LHAFNTTIAEAANDL	FNTTIAEAA	3	0.7113	22.7	8.00	0.81	tr_D0A0W7_	WB
DRB1_0101	111	ALIRYALCQLRVLRL	LCQLRVLRL	6	0.7108	22.8	8.00	0.33	tr_D0A0W7_	WB
DRB1_0101	112	SLLYDLFTTALNNTA	FTTALNNTA	6	0.7106	22.9	8.00	0.59	tr_D0A0W7_	WB
DRB1_0101	113	LPILMKHNVVAFSPM	ILMKHNVVA	2	0.7090	23.3	8.00	0.54	tr_D0A0W7_	WB
DRB1_0101	114	ISGTVFVIGLSVPNV	FVIGLSVPN	5	0.7079	23.6	8.50	0.78	tr_D0A0W7_	WB
DRB1_0101	115	RRHVIHLSPTLEQQL	VIHLSPTLE	3	0.7067	23.9	8.50	0.46	tr_D0A0W7_	WB
DRB1_0101	116	RSYILGPSSVQASLA	YILGPSSVQ	2	0.7066	23.9	8.50	0.53	tr_D0A0W7_	WB
DRB1_0101	117	YFLRADPAAETLALI	FLRADPAAE	1	0.7034	24.8	8.50	0.54	tr_D0A0W7_	WB
DRB1_0101	118	TFVRSLYSQRRYVIN	VRSLYSQRR	2	0.7000	25.7	9.00	0.40	tr_D0A0W7_	WB
DRB1_0101	119	QEYVNAINAGFNASL	NAINAGFNA	4	0.6987	26.1	9.00	0.33	tr_D0A0W7_	WB
DRB1_0101	120	DQNKIVIVYGPLGDI	IVIVYGPLG	4	0.6968	26.6	9.50	0.88	tr_D0A0W7_	WB
DRB1_0101	121	EFGVSLRSAVIREDG	GVSLRSAVI	2	0.6964	26.7	9.50	0.72	tr_D0A0W7_	WB
DRB1_0101	122	LWQKGASHLVGKGDL	WQKGASHLV	1	0.6958	26.9	9.50	0.83	tr_D0A0W7_	WB
DRB1_0101	123	HAFNTTIAEAANDLR	FNTTIAEAA	2	0.6955	27.0	9.50	0.79	tr_D0A0W7_	WB
DRB1_0101	124	PMTGSTFIRQWNPYL	FIRQWNPYL	6	0.6946	27.2	9.50	0.88	tr_D0A0W7_	WB
DRB1_0101	125	FIRQWNPYLYFLRAD	FIRQWNPYL	0	0.6939	27.4	9.50	0.71	tr_D0A0W7_	WB
DRB1_0101	126	IARSYILGPSSVQAS	YILGPSSVQ	4	0.6937	27.5	9.50	0.54	tr_D0A0W7_	WB
DRB1_0101	127	ASLAQMWLHALATAG	WLHALATAG	6	0.6934	27.6	9.50	0.44	tr_D0A0W7_	WB
DRB1_0101	128	WSMARALNASIPPLA	WSMARALNA	0	0.6931	27.7	9.50	0.38	tr_D0A0W7_	WB
DRB1_0101	129	ATQVSVWSMARALNA	WSMARALNA	6	0.6920	28.0	10.00	0.50	tr_D0A0W7_	
DRB1_0101	130	NKFRRHVIHLSPTLE	FRRHVIHLS	2	0.6918	28.1	10.00	0.38	tr_D0A0W7_	
DRB1_0101	131	ILRAPVALLLLLIVL	ILRAPVALL	0	0.6915	28.2	10.00	0.59	tr_D0A0W7_	
DRB1_0101	132	DSQYIAIKRFQGVMS	YIAIKRFQG	3	0.6872	29.5	11.00	0.44	tr_D0A0W7_	
DRB1_0101	133	VVAFSPMTGSTFIRQ	FSPMTGSTF	3	0.6854	30.1	11.00	0.82	tr_D0A0W7_	
DRB1_0101	134	RSLTEFGVSLRSAVI	GVSLRSAVI	6	0.6848	30.3	11.00	0.45	tr_D0A0W7_	
DRB1_0101	135	NVVAFSPMTGSTFIR	FSPMTGSTF	4	0.6842	30.5	11.00	0.75	tr_D0A0W7_	

DRB1_0101	136	EYVNAINAGFNASLE	NAINAGFNA	3	0.6839	30.6	11.00	0.40	tr_D0A0W7_
DRB1_0101	137	VLRLGFMYLQGVHYG	FMYLQGVHY	5	0.6836	30.7	11.00	0.65	tr_D0A0W7_
DRB1_0101	138	SRQWTVAGSVKVEVV	WTVAGSVKV	3	0.6833	30.8	11.00	0.86	tr_D0A0W7_
DRB1_0101	139	KDRTTFVRSLYSQRR	TTFVRSLYS	3	0.6810	31.6	11.00	0.38	tr_D0A0W7_
DRB1_0101	140	LRLGFMYLQGVHYGD	FMYLQGVHY	4	0.6808	31.6	11.00	0.68	tr_D0A0W7_
DRB1_0101	141	EERIARSYILGPSSV	ERIARSYIL	1	0.6794	32.1	11.00	0.72	tr_D0A0W7_
DRB1_0101	142	VAEERIARSYILGPS	ERIARSYIL	3	0.6790	32.2	11.00	0.91	tr_D0A0W7_
DRB1_0101	143	ERIARSYILGPSSVQ	ERIARSYIL	0	0.6786	32.4	12.00	0.47	tr_D0A0W7_
DRB1_0101	144	RLGFMYLQGVHYGDE	FMYLQGVHY	3	0.6783	32.5	12.00	0.69	tr_D0A0W7_
DRB1_0101	145	RHVIHLSPTLEQQLY	VIHLSPTLE	2	0.6782	32.5	12.00	0.41	tr_D0A0W7_
DRB1_0101	146	VMSEYLKAHVSETNI	SEYLKAHVS	2	0.6771	32.9	12.00	0.60	tr_D0A0W7_
DRB1_0101	147	PILMKHNVVAFSPMT	ILMKHNVVA	1	0.6762	33.2	12.00	0.51	tr_D0A0W7_
DRB1_0101	148	GQLLFTGTNPLAKDS	LLFTGTNPL	2	0.6740	34.0	12.00	0.48	tr_D0A0W7_
DRB1_0101	149	MMVAEERIARSYILG	ERIARSYIL	5	0.6736	34.2	12.00	0.81	tr_D0A0W7_
DRB1_0101	150	MSEYLKAHVSETNIT	SEYLKAHVS	1	0.6728	34.5	12.00	0.49	tr_D0A0W7_
DRB1_0101	151	GVMSEYLKAHVSETN	SEYLKAHVS	3	0.6714	35.0	12.00	0.62	tr_D0A0W7_
DRB1_0101	152	DEEYALTVNVMSQMG	YALTVNVMS	3	0.6711	35.1	12.00	0.74	tr_D0A0W7_
DRB1_0101	153	YALCQLRVLRLGFMY	LCQLRVLRL	2	0.6708	35.2	12.00	0.65	tr_D0A0W7_
DRB1_0101	154	AEERIARSYILGPSS	ERIARSYIL	2	0.6707	35.3	12.00	0.86	tr_D0A0W7_
DRB1_0101	155	AFNTTIAEAANDLRY	FNTTIAEAA	1	0.6691	35.9	13.00	0.66	tr_D0A0W7_
DRB1_0101	156	SMARALNASIPPLAN	LNASIPPLA	5	0.6688	36.0	13.00	0.60	tr_D0A0W7_
DRB1_0101	157	PQEYVNAINAGFNAS	YVNAINAGF	3	0.6666	36.9	13.00	0.38	tr_D0A0W7_
DRB1_0101	158	GDEEYALTVNVMSQM	YALTVNVMS	4	0.6646	37.7	13.00	0.72	tr_D0A0W7_
DRB1_0101	159	PGQLLFTGTNPLAKD	LLFTGTNPL	3	0.6646	37.7	13.00	0.52	tr_D0A0W7_
DRB1_0101	160	LYVLSSYLAGDGVGT	YLAGDGVGT	6	0.6645	37.7	13.00	0.41	tr_D0A0W7_
DRB1_0101	161	MVAEERIARSYILGP	ERIARSYIL	4	0.6644	37.7	13.00	0.88	tr_D0A0W7_
DRB1_0101	162	VAFSPMTGSTFIRQW	FSPMTGSTF	2	0.6629	38.4	13.00	0.82	tr_D0A0W7_
DRB1_0101	163	TDQNKIVIVYGPLGD	IVIVYGPLG	5	0.6629	38.4	13.00	0.88	tr_D0A0W7_
DRB1_0101	164	ESRQWTVAGSVKVEV	WTVAGSVKV	4	0.6616	38.9	14.00	0.84	tr_D0A0W7_
DRB1_0101	165	IVIVYGPLGDIVTYL	IVIVYGPLG	0	0.6614	39.0	14.00	0.41	tr_D0A0W7_
DRB1_0101	166	RIARSYILGPSSVQA	YILGPSSVQ	5	0.6612	39.1	14.00	0.51	tr_D0A0W7_
DRB1_0101	167	AMDTLPVILRAPVAL	PVILRAPVA	5	0.6612	39.1	14.00	0.68	tr_D0A0W7_
DRB1_0101	168	QGVMSEYLKAHVSET	SEYLKAHVS	4	0.6607	39.3	14.00	0.58	tr_D0A0W7_
DRB1_0101	169	EEYALTVNVMSQMGY	YALTVNVMS	2	0.6594	39.8	14.00	0.70	tr_D0A0W7_
DRB1_0101	170	RQWTVAGSVKVEVVY	WTVAGSVKV	2	0.6588	40.1	14.00	0.84	tr_D0A0W7_
DRB1_0101	171	KYLPISGTVFVIGLS	YLPISGTVF	1	0.6557	41.5	14.00	0.80	tr_D0A0W7_
DRB1_0101	172	QLLFTGTNPLAKDSQ	LLFTGTNPL	1	0.6556	41.5	14.00	0.44	tr_D0A0W7_
DRB1_0101	173	RVLRLGFMYLQGVHY	FMYLQGVHY	6	0.6552	41.7	14.00	0.62	tr_D0A0W7_
DRB1_0101	174	HNVVAFSPMTGSTFI	FSPMTGSTF	5	0.6549	41.8	14.00	0.67	tr_D0A0W7_
DRB1_0101	175	LLIVLPQLSVGAEAN	VLPQLSVGA	3	0.6546	42.0	14.00	0.52	tr_D0A0W7_
DRB1_0101	176	SYILGPSSVQASLAQ	YILGPSSVQ	1	0.6540	42.2	15.00	0.52	tr_D0A0W7_
DRB1_0101	177	LLLIVLPQLSVGAEA	VLPQLSVGA	4	0.6529	42.8	15.00	0.44	tr_D0A0W7_
DRB1_0101	178	YVNAINAGFNASLES	NAINAGFNA	2	0.6525	42.9	15.00	0.44	tr_D0A0W7_
DRB1_0101	179	SYLAGDGVGTVDAVI	YLAGDGVGT	1	0.6521	43.1	15.00	0.88	tr_D0A0W7_
DRB1_0101	180	APGQLLFTGTNPLAK	LLFTGTNPL	4	0.6516	43.4	15.00	0.57	tr_D0A0W7_
DRB1_0101	181	TLEQQLYVLSSYLAG	LYVLSSYLA	5	0.6514	43.5	15.00	0.38	tr_D0A0W7_
DRB1_0101	182	FGVSLRSAVIREDGE	GVSLRSAVI	1	0.6501	44.1	15.00	0.78	tr_D0A0W7_
DRB1_0101	183	LEQQLYVLSSYLAGD	LYVLSSYLA	4	0.6481	45.0	15.00	0.43	tr_D0A0W7_
DRB1_0101	184	QQLYVLSSYLAGDGV	LYVLSSYLA	2	0.6475	45.3	15.00	0.40	tr_D0A0W7_
DRB1_0101	185	FREVFERFAAALPQA	FERFAAALP	4	0.6468	45.7	15.00	0.44	tr_D0A0W7_

DRB1_0101	186	EQQLYVLSSYLAGDG	LYVLSSYLA	3	0.6467	45.7	15.00	0.44	tr_D0A0W7_
DRB1_0101	187	AARLVFATSLPHWGD	LVFATSLPH	3	0.6466	45.8	16.00	0.69	tr_D0A0W7_
DRB1_0101	188	QMGYELHGVFTVMSS	YELHGVFTV	3	0.6462	45.9	16.00	0.38	tr_D0A0W7_
DRB1_0101	189	RIVTAVFGPVTEAML	VTAVFGPVT	2	0.6446	46.8	16.00	0.52	tr_D0A0W7_
DRB1_0101	190	SQMGYELHGVFTVMS	GYELHGVFT	3	0.6439	47.1	16.00	0.44	tr_D0A0W7_
DRB1_0101	191	GEGVGKYLPISGTVF	YLPISGTVF	6	0.6439	47.1	16.00	0.65	tr_D0A0W7_
DRB1_0101	192	LALIRYALCQLRVLR	IRYALCQLR	3	0.6439	47.1	16.00	0.37	tr_D0A0W7_
DRB1_0101	193	VNAINAGFNASLESR	INAGFNASL	3	0.6430	47.6	16.00	0.42	tr_D0A0W7_
DRB1_0101	194	IVTAVFGPVTEAMLD	FGPVTEAML	5	0.6415	48.4	16.00	0.52	tr_D0A0W7_
DRB1_0101	195	FVIGLSVPNVREIAR	FVIGLSVPN	0	0.6410	48.6	16.00	0.47	tr_D0A0W7_
DRB1_0101	196	ARALNASIPPLANPM	LNASIPPLA	3	0.6400	49.1	16.00	0.82	tr_D0A0W7_
DRB1_0101	197	LGFMYLQGVHYGDEE	FMYLQGVHY	2	0.6393	49.5	17.00	0.69	tr_D0A0W7_
DRB1_0101	198	GAARLVFATSLPHWG	LVFATSLPH	4	0.6381	50.2	17.00	0.70	tr_D0A0W7_
DRB1_0101	199	LNKFRRHVIHLSPTL	FRRHVIHLS	3	0.6376	50.5	17.00	0.56	tr_D0A0W7_
DRB1_0101	200	QRRYVINDIVIGDYG	YVINDIVIG	3	0.6360	51.3	17.00	0.83	tr_D0A0W7_
DRB1_0101	201	RALNASIPPLANPMT	LNASIPPLA	2	0.6350	51.9	17.00	0.71	tr_D0A0W7_
DRB1_0101	202	YGDEEYALTVNVMSQ	YALTVNVMS	5	0.6347	52.1	17.00	0.64	tr_D0A0W7_
DRB1_0101	203	ALCQLRVLRLGFMYL	LCQLRVLRL	1	0.6346	52.1	17.00	0.56	tr_D0A0W7_
DRB1_0101	204	SQRRYVINDIVIGDY	YVINDIVIG	4	0.6344	52.3	17.00	0.82	tr_D0A0W7_
DRB1_0101	205	SKAAALWQKGASHLV	WQKGASHLV	6	0.6330	53.0	17.00	0.59	tr_D0A0W7_
DRB1_0101	206	ARLVFATSLPHWGDT	LVFATSLPH	2	0.6318	53.7	18.00	0.66	tr_D0A0W7_
DRB1_0101	207	ALNNTAGAARLVFAT	NTAGAARLV	3	0.6311	54.1	18.00	0.61	tr_D0A0W7_
DRB1_0101	208	LHALATAGTSFAPGQ	LATAGTSFA	3	0.6309	54.3	18.00	0.51	tr_D0A0W7_
DRB1_0101	209	QLYVLSSYLAGDGVG	LYVLSSYLA	1	0.6308	54.3	18.00	0.35	tr_D0A0W7_
DRB1_0101	210	FVRSLYSQRRYVIND	VRSLYSQRR	1	0.6302	54.7	18.00	0.42	tr_D0A0W7_
DRB1_0101	211	MARALNASIPPLANP	LNASIPPLA	4	0.6286	55.6	18.00	0.73	tr_D0A0W7_
DRB1_0101	212	PTLEQQLYVLSSYLA	QLYVLSSYL	5	0.6281	55.9	18.00	0.35	tr_D0A0W7_
DRB1_0101	213	LLLLIVLPQLSVGAE	VLPQLSVGA	5	0.6280	56.0	18.00	0.38	tr_D0A0W7_
DRB1_0101	214	SRLWTPLSLLAFATG	PLSLLAFAT	5	0.6267	56.8	18.00	0.35	tr_D0A0W7_
DRB1_0101	215	TALNNTAGAARLVFA	NTAGAARLV	4	0.6266	56.9	18.00	0.49	tr_D0A0W7_
DRB1_0101	216	FFYADSSIISDDMRY	FYADSSIIS	1	0.6252	57.7	19.00	0.51	tr_D0A0W7_
DRB1_0101	217	RLWTPLSLLAFATGR	PLSLLAFAT	4	0.6240	58.4	19.00	0.53	tr_D0A0W7_
DRB1_0101	218	VTAVFGPVTEAMLDT	FGPVTEAML	4	0.6237	58.6	19.00	0.67	tr_D0A0W7_
DRB1_0101	219	LIVLPQLSVGAEANA	VLPQLSVGA	2	0.6233	58.9	19.00	0.50	tr_D0A0W7_
DRB1_0101	220	LWTPLSLLAFATGRL	PLSLLAFAT	3	0.6225	59.4	19.00	0.54	tr_D0A0W7_
DRB1_0101	221	AKFLMMMVAEERIAR	LMMMVAEER	3	0.6210	60.4	19.00	0.34	tr_D0A0W7_
DRB1_0101	222	DSRLWTPLSLLAFAT	PLSLLAFAT	6	0.6204	60.8	19.00	0.25	tr_D0A0W7_
DRB1_0101	223	AFSPMTGSTFIRQWN	FSPMTGSTF	1	0.6189	61.7	19.00	0.79	tr_D0A0W7_
DRB1_0101	224	MGYELHGVFTVMSSD	YELHGVFTV	2	0.6187	61.9	20.00	0.41	tr_D0A0W7_
DRB1_0101	225	LESRQWTVAGSVKVE	WTVAGSVKV	5	0.6175	62.7	20.00	0.81	tr_D0A0W7_
DRB1_0101	226	PISGTVFVIGLSVPN	FVIGLSVPN	6	0.6168	63.2	20.00	0.74	tr_D0A0W7_
DRB1_0101	227	HVIHLSPTLEQQLYV	VIHLSPTLE	1	0.6155	64.1	20.00	0.35	tr_D0A0W7_
DRB1_0101	228	KFLMMMVAEERIARS	LMMMVAEER	2	0.6146	64.7	20.00	0.29	tr_D0A0W7_
DRB1_0101	229	SEYLKAHVSETNITE	YLKAHVSET	2	0.6145	64.8	20.00	0.40	tr_D0A0W7_
DRB1_0101	230	MSQMGYELHGVFTVM	GYELHGVFT	4	0.6140	65.2	20.00	0.44	tr_D0A0W7_
DRB1_0101	231	VAMDTLPVILRAPVA	PVILRAPVA	6	0.6124	66.3	20.00	0.56	tr_D0A0W7_
DRB1_0101	232	FAAALPQAIIVFGAP	FAAALPQAI	0	0.6123	66.3	20.00	0.57	tr_D0A0W7_
DRB1_0101	233	EYALTVNVMSQMGYE	YALTVNVMS	1	0.6121	66.5	21.00	0.70	tr_D0A0W7_
DRB1_0101	234	QASLAQMWLHALATA	LAQMWLHAL	3	0.6112	67.1	21.00	0.38	tr_D0A0W7_
DRB1_0101	235	ERIVTAVFGPVTEAM	VTAVFGPVT	3	0.6106	67.6	21.00	0.76	tr_D0A0W7_

DRB1_0101	236	MPQEYVNAINAGFNA	YVNAINAGF	4	0.6105	67.7	21.00	0.43	tr_D0A0W7_
DRB1_0101	237	MMMVAEERIARSYIL	ERIARSYIL	6	0.6101	68.0	21.00	0.65	tr_D0A0W7_
DRB1_0101	238	EFREVFERFAAALPQ	FERFAAALP	5	0.6089	68.8	21.00	0.41	tr_D0A0W7_
DRB1_0101	239	SLEWLKDRTTFVRSL	WLKDRTTFV	3	0.6089	68.9	21.00	0.64	tr_D0A0W7_
DRB1_0101	240	ESGFTVVKTSQCYTE	FTVVKTSQC	3	0.6077	69.7	21.00	0.84	tr_D0A0W7_
DRB1_0101	241	TLALIRYALCQLRVL	IRYALCQLR	4	0.6070	70.2	21.00	0.39	tr_D0A0W7_
DRB1_0101	242	GQTTTMESGFTVVKT	TTMESGFTV	3	0.6067	70.5	21.00	0.65	tr_D0A0W7_
DRB1_0101	243	SGFTVVKTSQCYTES	FTVVKTSQC	2	0.6066	70.6	21.00	0.82	tr_D0A0W7_
DRB1_0101	244	EFSLLYDLFTTALNN	LYDLFTTAL	4	0.6063	70.8	21.00	0.30	tr_D0A0W7_
DRB1_0101	245	YSQRRYVINDIVIGD	YVINDIVIG	5	0.6046	72.1	22.00	0.74	tr_D0A0W7_
DRB1_0101	246	LNNTAGAARLVFATS	NTAGAARLV	2	0.6040	72.5	22.00	0.60	tr_D0A0W7_
DRB1_0101	247	KDSQYIAIKRFQGVM	YIAIKRFQG	4	0.6031	73.3	22.00	0.63	tr_D0A0W7_
DRB1_0101	248	RRYVINDIVIGDYGG	YVINDIVIG	2	0.6029	73.4	22.00	0.85	tr_D0A0W7_
DRB1_0101	249	KDSRLWTPLSLLAFA	RLWTPLSLL	3	0.6028	73.5	22.00	0.32	tr_D0A0W7_
DRB1_0101	250	RDPSEGKLSGASLVG	GKLSGASLV	5	0.6010	75.0	22.00	0.51	tr_D0A0W7_
DRB1_0101	251	AGTSFAPGQLLFTGT	TSFAPGQLL	2	0.6001	75.7	22.00	0.47	tr_D0A0W7_
DRB1_0101	252	LLLLLIVLPQLSVGA	LIVLPQLSV	4	0.5999	75.8	23.00	0.38	tr_D0A0W7_
DRB1_0101	253	VYGWICGEVLSQALS	YGWICGEVL	1	0.5990	76.6	23.00	0.41	tr_D0A0W7_
DRB1_0101	254	RRSLTEFGVSLRSAV	SLTEFGVSL	2	0.5990	76.6	23.00	0.47	tr_D0A0W7_
DRB1_0101	255	GEFSLLYDLFTTALN	FSLLYDLFT	2	0.5987	76.9	23.00	0.35	tr_D0A0W7_
DRB1_0101	256	GFMYLQGVHYGDEEY	FMYLQGVHY	1	0.5985	77.0	23.00	0.68	tr_D0A0W7_
DRB1_0101	257	TAGTSFAPGQLLFTG	TSFAPGQLL	3	0.5985	77.1	23.00	0.51	tr_D0A0W7_
DRB1_0101	258	KHNVVAFSPMTGSTF	FSPMTGSTF	6	0.5971	78.2	23.00	0.48	tr_D0A0W7_
DRB1_0101	259	ETDQNKIVIVYGPLG	IVIVYGPLG	6	0.5966	78.7	23.00	0.88	tr_D0A0W7_
DRB1_0101	260	AGAARLVFATSLPHW	LVFATSLPH	5	0.5966	78.7	23.00	0.72	tr_D0A0W7_
DRB1_0101	261	FLRRSLTEFGVSLRS	SLTEFGVSL	4	0.5963	78.9	23.00	0.50	tr_D0A0W7_
DRB1_0101	262	QWTVAGSVKVEVVYP	WTVAGSVKV	1	0.5955	79.6	23.00	0.85	tr_D0A0W7_
DRB1_0101	263	CECNQGSKAVYVKEM	NQGSKAVYV	3	0.5953	79.7	23.00	0.79	tr_D0A0W7_
DRB1_0101	264	MESGFTVVKTSQCYT	FTVVKTSQC	4	0.5950	80.0	23.00	0.85	tr_D0A0W7_
DRB1_0101	265	SSLEWLKDRTTFVRS	WLKDRTTFV	4	0.5943	80.6	23.00	0.61	tr_D0A0W7_
DRB1_0101	266	LKDRTTFVRSLYSQR	TTFVRSLYS	4	0.5942	80.7	23.00	0.55	tr_D0A0W7_
DRB1_0101	267	TCECNQGSKAVYVKE	NQGSKAVYV	4	0.5934	81.4	24.00	0.68	tr_D0A0W7_
DRB1_0101	268	QTTTMESGFTVVKTS	TTMESGFTV	2	0.5933	81.5	24.00	0.66	tr_D0A0W7_
DRB1_0101	269	NGQTTTMESGFTVVK	TTMESGFTV	4	0.5924	82.3	24.00	0.62	tr_D0A0W7_
DRB1_0101	270	FSLLYDLFTTALNNT	LYDLFTTAL	3	0.5922	82.4	24.00	0.34	tr_D0A0W7_
DRB1_0101	271	GERIVTAVFGPVTEA	VTAVFGPVT	4	0.5921	82.6	24.00	0.71	tr_D0A0W7_
DRB1_0101	272	LRRSLTEFGVSLRSA	SLTEFGVSL	3	0.5920	82.7	24.00	0.56	tr_D0A0W7_
DRB1_0101	273	TAKFLMMMVAEERIA	LMMMVAEER	4	0.5902	84.3	24.00	0.32	tr_D0A0W7_
DRB1_0101	274	ATCECNQGSKAVYVK	NQGSKAVYV	5	0.5900	84.5	24.00	0.62	tr_D0A0W7_
DRB1_0101	275	SELQGPLNGLAVFME	QGPLNGLAV	3	0.5898	84.6	24.00	0.53	tr_D0A0W7_
DRB1_0101	276	VMSQMGYELHGVFTV	GYELHGVFT	5	0.5894	85.0	24.00	0.41	tr_D0A0W7_
DRB1_0101	277	RLVFATSLPHWGDTE	LVFATSLPH	1	0.5881	86.2	24.00	0.62	tr_D0A0W7_
DRB1_0101	278	SSELQGPLNGLAVFM	QGPLNGLAV	4	0.5877	86.6	25.00	0.48	tr_D0A0W7_
DRB1_0101	279	NATVKVLSATWNSYM	VKVLSATWN	3	0.5876	86.7	25.00	0.49	tr_D0A0W7_
DRB1_0101	280	GTSFAPGQLLFTGTN	FAPGQLLFT	3	0.5872	87.1	25.00	0.48	tr_D0A0W7_
DRB1_0101	281	EKDSRLWTPLSLLAF	RLWTPLSLL	4	0.5864	87.8	25.00	0.29	tr_D0A0W7_
DRB1_0101	282	PETLVNFFYADSSII	FFYADSSII	6	0.5863	87.9	25.00	0.74	tr_D0A0W7_
DRB1_0101	283	FLRADPAAETLALIR	LRADPAAET	1	0.5863	87.9	25.00	0.39	tr_D0A0W7_
DRB1_0101	284	YGWICGEVLSQALSS	YGWICGEVL	0	0.5860	88.2	25.00	0.28	tr_D0A0W7_
DRB1_0101	285	NAINAGFNASLESRQ	INAGFNASL	2	0.5858	88.3	25.00	0.50	tr_D0A0W7_

DRB1_0101	286	VQASLAQMWLHALAT	LAQMWLHAL	4	0.5854	88.8	25.00	0.34	tr_D0A0W7_
DRB1_0101	287	ATVKVLSATWNSYMP	VKVLSATWN	2	0.5835	90.6	25.00	0.44	tr_D0A0W7_
DRB1_0101	288	TTALNNTAGAARLVF	NTAGAARLV	5	0.5832	90.9	25.00	0.43	tr_D0A0W7_
DRB1_0101	289	SSVQASLAQMWLHAL	VQASLAQMW	2	0.5821	91.9	25.00	0.38	tr_D0A0W7_
DRB1_0101	290	ALNASIPPLANPMTP	LNASIPPLA	1	0.5812	92.8	26.00	0.58	tr_D0A0W7_
DRB1_0101	291	LCQLRVLRLGFMYLQ	LCQLRVLRL	0	0.5804	93.7	26.00	0.38	tr_D0A0W7_
DRB1_0101	292	PSSVQASLAQMWLHA	VQASLAQMW	3	0.5803	93.8	26.00	0.46	tr_D0A0W7_
DRB1_0101	293	VCRVAMDTLPVILRA	MDTLPVILR	5	0.5798	94.3	26.00	0.44	tr_D0A0W7_
DRB1_0101	294	VIVYGPLGDIVTYLA	YGPLGDIVT	3	0.5796	94.5	26.00	0.57	tr_D0A0W7_
DRB1_0101	295	LLFTGTNPLAKDSQY	FTGTNPLAK	2	0.5793	94.8	26.00	0.35	tr_D0A0W7_
DRB1_0101	296	MVYGWICGEVLSQAL	YGWICGEVL	2	0.5792	94.9	26.00	0.55	tr_D0A0W7_
DRB1_0101	297	YILGPSSVQASLAQM	YILGPSSVQ	0	0.5790	95.1	26.00	0.38	tr_D0A0W7_
DRB1_0101	298	GEVLSQALSSLEWLK	LSQALSSLE	3	0.5773	96.9	26.00	0.26	tr_D0A0W7_
DRB1_0101	299	CGEVLSQALSSLEWL	EVLSQALSS	2	0.5768	97.4	26.00	0.25	tr_D0A0W7_
DRB1_0101	300	DFLRRSLTEFGVSLR	SLTEFGVSL	5	0.5756	98.7	27.00	0.47	tr_D0A0W7_
DRB1_0101	301	SVQASLAQMWLHALA	LAQMWLHAL	5	0.5756	98.7	27.00	0.25	tr_D0A0W7_
DRB1_0101	302	ILMKHNVVAFSPMTG	ILMKHNVVA	0	0.5753	99.0	27.00	0.40	tr_D0A0W7_
DRB1_0101	303	LSSLEWLKDRTTFVR	WLKDRTTFV	5	0.5748	99.5	27.00	0.51	tr_D0A0W7_
DRB1_0101	304	ICGEVLSQALSSLEW	EVLSQALSS	3	0.5743	100.1	27.00	0.26	tr_D0A0W7_
DRB1_0101	305	HYGDEEYALTVNVMS	YALTVNVMS	6	0.5740	100.4	27.00	0.50	tr_D0A0W7_
DRB1_0101	306	RVIVLFGEFSLLYDL	VIVLFGEFS	1	0.5733	101.2	27.00	0.34	tr_D0A0W7_
DRB1_0101	307	LRVIVLFGEFSLLYD	VIVLFGEFS	2	0.5729	101.6	27.00	0.44	tr_D0A0W7_
DRB1_0101	308	FAPGQLLFTGTNPLA	LLFTGTNPL	5	0.5726	101.9	27.00	0.58	tr_D0A0W7_
DRB1_0101	309	FGEFSLLYDLFTTAL	FSLLYDLFT	3	0.5726	102.0	27.00	0.46	tr_D0A0W7_
DRB1_0101	310	WNSYMPQEYVNAINA	YMPQEYVNA	3	0.5725	102.0	27.00	0.52	tr_D0A0W7_
DRB1_0101	311	LGHSDRFFLHAFNTT	FFLHAFNTT	6	0.5720	102.6	27.00	0.38	tr_D0A0W7_
DRB1_0101	312	LMVYGWICGEVLSQA	YGWICGEVL	3	0.5717	102.9	27.00	0.68	tr_D0A0W7_
DRB1_0101	313	AFATGRLMRVILLHV	FATGRLMRV	1	0.5716	103.0	27.00	0.53	tr_D0A0W7_
DRB1_0101	314	DLRVIVLFGEFSLLY	VIVLFGEFS	3	0.5693	105.6	28.00	0.48	tr_D0A0W7_
DRB1_0101	315	FNTTIAEAANDLRYE	FNTTIAEAA	0	0.5688	106.2	28.00	0.47	tr_D0A0W7_
DRB1_0101	316	ELMVYGWICGEVLSQ	YGWICGEVL	4	0.5683	106.8	28.00	0.66	tr_D0A0W7_
DRB1_0101	317	LEWLKDRTTFVRSLY	WLKDRTTFV	2	0.5683	106.8	28.00	0.68	tr_D0A0W7_
DRB1_0101	318	EWLKDRTTFVRSLYS	WLKDRTTFV	1	0.5679	107.3	28.00	0.47	tr_D0A0W7_
DRB1_0101	319	ELQGPLNGLAVFMED	QGPLNGLAV	2	0.5679	107.3	28.00	0.51	tr_D0A0W7_
DRB1_0101	320	NSYMPQEYVNAINAG	YMPQEYVNA	2	0.5676	107.6	28.00	0.50	tr_D0A0W7_
DRB1_0101	321	GYELHGVFTVMSSDG	YELHGVFTV	1	0.5674	107.8	28.00	0.40	tr_D0A0W7_
DRB1_0101	322	IVYGPLGDIVTYLAL	YGPLGDIVT	2	0.5660	109.5	28.00	0.57	tr_D0A0W7_
DRB1_0101	323	FLMMMVAEERIARSY	LMMMVAEER	1	0.5651	110.5	28.00	0.25	tr_D0A0W7_
DRB1_0101	324	PNITFIDPLELKPRL	ITFIDPLEL	2	0.5650	110.7	28.00	0.71	tr_D0A0W7_
DRB1_0101	325	IVLPQLSVGAEANAT	VLPQLSVGA	1	0.5648	110.9	29.00	0.51	tr_D0A0W7_
DRB1_0101	326	GFTVVKTSQCYTESS	FTVVKTSQC	1	0.5646	111.2	29.00	0.80	tr_D0A0W7_
DRB1_0101	327	TAVFGPVTEAMLDTP	FGPVTEAML	3	0.5640	111.9	29.00	0.82	tr_D0A0W7_
DRB1_0101	328	TWNSYMPQEYVNAIN	YMPQEYVNA	4	0.5624	113.8	29.00	0.46	tr_D0A0W7_
DRB1_0101	329	ECNQGSKAVYVKEML	NQGSKAVYV	2	0.5620	114.3	29.00	0.84	tr_D0A0W7_
DRB1_0101	330	GWICGEVLSQALSSL	CGEVLSQAL	3	0.5620	114.3	29.00	0.28	tr_D0A0W7_
DRB1_0101	331	GELMVYGWICGEVLS	YGWICGEVL	5	0.5611	115.5	29.00	0.56	tr_D0A0W7_
DRB1_0101	332	RLNKFRRHVIHLSPT	FRRHVIHLS	4	0.5607	116.0	29.00	0.68	tr_D0A0W7_
DRB1_0101	333	AVCRVAMDTLPVILR	RVAMDTLPV	3	0.5606	116.0	29.00	0.50	tr_D0A0W7_
DRB1_0101	334	ATAGTSFAPGQLLFT	TSFAPGQLL	4	0.5606	116.1	29.00	0.56	tr_D0A0W7_
DRB1_0101	335	CRVAMDTLPVILRAP	MDTLPVILR	4	0.5605	116.2	29.00	0.46	tr_D0A0W7_

DRB1_0101	336	TPNITFIDPLELKPR	ITFIDPLEL	3	0.5602	116.6	29.00	0.75	tr_D0A0W7_
DRB1_0101	337	TVKVLSATWNSYMPQ	LSATWNSYM	4	0.5597	117.2	29.00	0.40	tr_D0A0W7_
DRB1_0101	338	NNTAGAARLVFATSL	NTAGAARLV	1	0.5586	118.7	30.00	0.54	tr_D0A0W7_
DRB1_0101	339	YMPQEYVNAINAGFN	YVNAINAGF	5	0.5577	119.8	30.00	0.39	tr_D0A0W7_
DRB1_0101	340	WICGEVLSQALSSLE	EVLSQALSS	4	0.5571	120.5	30.00	0.26	tr_D0A0W7_
DRB1_0101	341	LYSQRRYVINDIVIG	YVINDIVIG	6	0.5565	121.3	30.00	0.58	tr_D0A0W7_
DRB1_0101	342	GPSSVQASLAQMWLH	VQASLAQMW	4	0.5565	121.3	30.00	0.44	tr_D0A0W7_
DRB1_0101	343	YALTVNVMSQMGYEL	YALTVNVMS	0	0.5565	121.4	30.00	0.44	tr_D0A0W7_
DRB1_0101	344	VEKDSRLWTPLSLLA	DSRLWTPLS	3	0.5543	124.2	30.00	0.39	tr_D0A0W7_
DRB1_0101	345	TMESGFTVVKTSQCY	FTVVKTSQC	5	0.5539	124.8	30.00	0.82	tr_D0A0W7_
DRB1_0101	346	QGERIVTAVFGPVTE	VTAVFGPVT	5	0.5539	124.9	30.00	0.61	tr_D0A0W7_
DRB1_0101	347	NITFIDPLELKPRLN	ITFIDPLEL	1	0.5534	125.5	31.00	0.64	tr_D0A0W7_
DRB1_0101	348	WQKGASHLVGKGDLG	WQKGASHLV	0	0.5512	128.5	31.00	0.79	tr_D0A0W7_
DRB1_0101	349	ATWNSYMPQEYVNAI	YMPQEYVNA	5	0.5509	128.9	31.00	0.40	tr_D0A0W7_
DRB1_0101	350	DEFREVFERFAAALP	FERFAAALP	6	0.5496	130.8	31.00	0.35	tr_D0A0W7_
DRB1_0101	351	EVLSQALSSLEWLKD	LSQALSSLE	2	0.5468	134.8	32.00	0.31	tr_D0A0W7_
DRB1_0101	352	AKDSQYIAIKRFQGV	YIAIKRFQG	5	0.5464	135.4	32.00	0.64	tr_D0A0W7_
DRB1_0101	353	SPTLEQQLYVLSSYL	QLYVLSSYL	6	0.5451	137.3	32.00	0.32	tr_D0A0W7_
DRB1_0101	354	WLKDRTTFVRSLYSQ	TTFVRSLYS	5	0.5448	137.7	32.00	0.46	tr_D0A0W7_
DRB1_0101	355	SVPNVREIARKLEER	NVREIARKL	3	0.5438	139.3	32.00	0.88	tr_D0A0W7_
DRB1_0101	356	ETLALIRYALCQLRV	IRYALCQLR	5	0.5400	145.1	33.00	0.38	tr_D0A0W7_
DRB1_0101	357	NDLRYEQGERIVTAV	LRYEQGERI	2	0.5398	145.4	33.00	0.57	tr_D0A0W7_
DRB1_0101	358	VIHLSPTLEQQLYVL	IHLSPTLEQ	1	0.5398	145.4	33.00	0.26	tr_D0A0W7_
DRB1_0101	359	SYMPQEYVNAINAGF	YMPQEYVNA	1	0.5390	146.7	33.00	0.41	tr_D0A0W7_
DRB1_0101	360	LLHVEEMSPETLVNF	VEEMSPETL	3	0.5388	147.0	33.00	0.69	tr_D0A0W7_
DRB1_0101	361	LQGPLNGLAVFMEDD	QGPLNGLAV	1	0.5380	148.2	33.00	0.49	tr_D0A0W7_
DRB1_0101	362	RYVINDIVIGDYGGT	YVINDIVIG	1	0.5376	148.9	34.00	0.81	tr_D0A0W7_
DRB1_0101	363	FRDPSEGKLSGASLV	EGKLSGASL	5	0.5375	149.1	34.00	0.44	tr_D0A0W7_
DRB1_0101	364	ALLLLLIVLPQLSVG	LIVLPQLSV	5	0.5368	150.1	34.00	0.44	tr_D0A0W7_
DRB1_0101	365	ANDLRYEQGERIVTA	LRYEQGERI	3	0.5356	152.1	34.00	0.68	tr_D0A0W7_
DRB1_0101	366	LGPSSVQASLAQMWL	VQASLAQMW	5	0.5353	152.7	34.00	0.35	tr_D0A0W7_
DRB1_0101	367	DTAKFLMMMVAEERI	FLMMMVAEE	4	0.5353	152.7	34.00	0.41	tr_D0A0W7_
DRB1_0101	368	NVMSQMGYELHGVFT	VMSQMGYEL	1	0.5350	153.1	34.00	0.38	tr_D0A0W7_
DRB1_0101	369	TTTMESGFTVVKTSQ	TTMESGFTV	1	0.5346	153.9	34.00	0.65	tr_D0A0W7_
DRB1_0101	370	PLGDIVTYLALPILM	IVTYLALPI	4	0.5338	155.0	34.00	0.28	tr_D0A0W7_
DRB1_0101	371	TVNVMSQMGYELHGV	VMSQMGYEL	3	0.5322	157.8	35.00	0.70	tr_D0A0W7_
DRB1_0101	372	GATQVSVWSMARALN	SVWSMARAL	5	0.5321	158.0	35.00	0.55	tr_D0A0W7_
DRB1_0101	373	TSFAPGQLLFTGTNP	FAPGQLLFT	2	0.5320	158.2	35.00	0.53	tr_D0A0W7_
DRB1_0101	374	PRLNKFRRHVIHLSP	FRRHVIHLS	5	0.5319	158.4	35.00	0.64	tr_D0A0W7_
DRB1_0101	375	GLSVPNVREIARKLE	NVREIARKL	5	0.5305	160.7	35.00	0.70	tr_D0A0W7_
DRB1_0101	376	RVAMDTLPVILRAPV	MDTLPVILR	3	0.5302	161.4	35.00	0.50	tr_D0A0W7_
DRB1_0101	377	MSMLHLSDRNASLAP	MLHLSDRNA	2	0.5296	162.4	35.00	0.60	tr_D0A0W7_
DRB1_0101	378	DLRYEQGERIVTAVF	LRYEQGERI	1	0.5294	162.7	35.00	0.49	tr_D0A0W7_
DRB1_0101	379	SLESRQWTVAGSVKV	WTVAGSVKV	6	0.5293	162.9	35.00	0.72	tr_D0A0W7_
DRB1_0101	380	LSVPNVREIARKLEE	NVREIARKL	4	0.5291	163.2	35.00	0.79	tr_D0A0W7_
DRB1_0101	381	ADYFLTHGTEGELMV	YFLTHGTEG	2	0.5287	164.0	35.00	0.41	tr_D0A0W7_
DRB1_0101	382	EGELMVYGWICGEVL	YGWICGEVL	6	0.5268	167.2	36.00	0.44	tr_D0A0W7_
DRB1_0101	383	LTVNVMSQMGYELHG	VMSQMGYEL	4	0.5267	167.5	36.00	0.67	tr_D0A0W7_
DRB1_0101	384	LHVEEMSPETLVNFF	VEEMSPETL	2	0.5258	169.2	36.00	0.65	tr_D0A0W7_
DRB1_0101	385	ANATVKVLSATWNSY	VKVLSATWN	4	0.5257	169.2	36.00	0.56	tr_D0A0W7_

DRB1_0101	386	CNQGSKAVYVKEMLE	NQGSKAVYV	1	0.5243	171.9	36.00	0.82	tr_D0A0W7_
DRB1_0101	387	TTMESGFTVVKTSQC	FTVVKTSQC	6	0.5240	172.4	36.00	0.44	tr_D0A0W7_
DRB1_0101	388	VIVLFGEFSLLYDLF	LFGEFSLLY	3	0.5239	172.6	36.00	0.29	tr_D0A0W7_
DRB1_0101	389	YLPISGTVFVIGLSV	YLPISGTVF	0	0.5235	173.5	36.00	0.61	tr_D0A0W7_
DRB1_0101	390	YLAGDGVGTVDAVIC	YLAGDGVGT	0	0.5228	174.8	36.00	0.69	tr_D0A0W7_
DRB1_0101	391	ALATAGTSFAPGQLL	LATAGTSFA	1	0.5210	178.1	37.00	0.56	tr_D0A0W7_
DRB1_0101	392	DYFLTHGTEGELMVY	YFLTHGTEG	1	0.5210	178.2	37.00	0.32	tr_D0A0W7_
DRB1_0101	393	AVFGPVTEAMLDTPN	FGPVTEAML	2	0.5199	180.4	37.00	0.87	tr_D0A0W7_
DRB1_0101	394	LFGEFSLLYDLFTTA	FSLLYDLFT	4	0.5198	180.4	37.00	0.51	tr_D0A0W7_
DRB1_0101	395	ENGQTTTMESGFTVV	TTMESGFTV	5	0.5193	181.4	37.00	0.62	tr_D0A0W7_
DRB1_0101	396	NDLRVIVLFGEFSLL	VIVLFGEFS	4	0.5191	181.9	37.00	0.58	tr_D0A0W7_
DRB1_0101	397	HALATAGTSFAPGQL	LATAGTSFA	2	0.5181	183.9	37.00	0.79	tr_D0A0W7_
DRB1_0101	398	VYGPLGDIVTYLALP	YGPLGDIVT	1	0.5180	184.1	37.00	0.53	tr_D0A0W7_
DRB1_0101	399	GVSLRSAVIREDGEG	GVSLRSAVI	0	0.5177	184.6	37.00	0.73	tr_D0A0W7_
DRB1_0101	400	IVLFGEFSLLYDLFT	FSLLYDLFT	6	0.5177	184.6	37.00	0.31	tr_D0A0W7_
DRB1_0101	401	ESSELQGPLNGLAVF	QGPLNGLAV	5	0.5176	184.9	38.00	0.43	tr_D0A0W7_
DRB1_0101	402	GATCECNQGSKAVYV	NQGSKAVYV	6	0.5168	186.5	38.00	0.50	tr_D0A0W7_
DRB1_0101	403	TAGAARLVFATSLPH	LVFATSLPH	6	0.5158	188.4	38.00	0.54	tr_D0A0W7_
DRB1_0101	404	VLSQALSSLEWLKDR	LSQALSSLE	1	0.5143	191.5	38.00	0.23	tr_D0A0W7_
DRB1_0101	405	SVKVEVVYPRNLDLM	VEVVYPRNL	3	0.5143	191.6	38.00	0.51	tr_D0A0W7_
DRB1_0101	406	LATAGTSFAPGQLLF	TSFAPGQLL	5	0.5135	193.2	38.00	0.56	tr_D0A0W7_
DRB1_0101	407	ALTVNVMSQMGYELH	VMSQMGYEL	5	0.5135	193.3	38.00	0.58	tr_D0A0W7_
DRB1_0101	408	VPNVREIARKLEERN	NVREIARKL	2	0.5131	194.0	38.00	0.87	tr_D0A0W7_
DRB1_0101	409	FATGRLMRVILLHVE	GRLMRVILL	3	0.5119	196.6	39.00	0.49	tr_D0A0W7_
DRB1_0101	410	LMMMVAEERIARSYI	MMVAEERIA	2	0.5117	197.1	39.00	0.26	tr_D0A0W7_
DRB1_0101	411	VRSLYSQRRYVINDI	LYSQRRYVI	3	0.5116	197.3	39.00	0.37	tr_D0A0W7_
DRB1_0101	412	DTPNITFIDPLELKP	ITFIDPLEL	4	0.5101	200.4	39.00	0.82	tr_D0A0W7_
DRB1_0101	413	VKVLSATWNSYMPQE	LSATWNSYM	3	0.5097	201.2	39.00	0.54	tr_D0A0W7_
DRB1_0101	414	VLFGEFSLLYDLFTT	FSLLYDLFT	5	0.5087	203.4	39.00	0.49	tr_D0A0W7_
DRB1_0101	415	ILLHVEEMSPETLVN	VEEMSPETL	4	0.5085	204.1	39.00	0.69	tr_D0A0W7_
DRB1_0101	416	AINAGFNASLESRQW	INAGFNASL	1	0.5073	206.7	40.00	0.53	tr_D0A0W7_
DRB1_0101	417	ADFLRRSLTEFGVSL	LRRSLTEFG	3	0.5073	206.7	40.00	0.29	tr_D0A0W7_
DRB1_0101	418	ILGPSSVQASLAQMW	PSSVQASLA	3	0.5066	208.2	40.00	0.38	tr_D0A0W7_
DRB1_0101	419	IHLSPTLEQQLYVLS	PTLEQQLYV	4	0.5053	211.1	40.00	0.32	tr_D0A0W7_
DRB1_0101	420	VLPQLSVGAEANATV	PQLSVGAEA	2	0.5052	211.4	40.00	0.37	tr_D0A0W7_
DRB1_0101	421	EQGERIVTAVFGPVT	VTAVFGPVT	6	0.5048	212.3	40.00	0.50	tr_D0A0W7_
DRB1_0101	422	FMYLQGVHYGDEEYA	FMYLQGVHY	0	0.5037	214.8	40.00	0.58	tr_D0A0W7_
DRB1_0101	423	EADYFLTHGTEGELM	YFLTHGTEG	3	0.5029	216.7	40.00	0.51	tr_D0A0W7_
DRB1_0101	424	SFAPGQLLFTGTNPL	FAPGQLLFT	1	0.5028	216.9	40.00	0.42	tr_D0A0W7_
DRB1_0101	425	VNVMSQMGYELHGVF	VMSQMGYEL	2	0.5026	217.3	40.00	0.66	tr_D0A0W7_
DRB1_0101	426	WTVAGSVKVEVVYPR	WTVAGSVKV	0	0.5026	217.5	40.00	0.68	tr_D0A0W7_
DRB1_0101	427	LRYEQGERIVTAVFG	YEQGERIVT	2	0.5021	218.6	41.00	0.34	tr_D0A0W7_
DRB1_0101	428	ALSSLEWLKDRTTFV	LEWLKDRTT	4	0.5018	219.3	41.00	0.41	tr_D0A0W7_
DRB1_0101	429	LNASIPPLANPMTPS	IPPLANPMT	4	0.5016	219.8	41.00	0.36	tr_D0A0W7_
DRB1_0101	430	KPRLNKFRRHVIHLS	FRRHVIHLS	6	0.5012	220.7	41.00	0.51	tr_D0A0W7_
DRB1_0101	431	HVEEMSPETLVNFFY	VEEMSPETL	1	0.5011	221.0	41.00	0.57	tr_D0A0W7_
DRB1_0101	432	VALLLLLIVLPQLSV	LLIVLPQLS	5	0.4997	224.2	41.00	0.47	tr_D0A0W7_
DRB1_0101	433	KDTAKFLMMMVAEER	FLMMMVAEE	5	0.4989	226.3	41.00	0.44	tr_D0A0W7_
DRB1_0101	434	HLSPTLEQQLYVLSS	PTLEQQLYV	3	0.4975	229.8	42.00	0.40	tr_D0A0W7_
DRB1_0101	435	QLRVLRLGFMYLQGV	VLRLGFMYL	3	0.4954	235.0	42.00	0.29	tr_D0A0W7_

DRB1_0101	436	AETLALIRYALCQLR	IRYALCQLR	6	0.4953	235.2	42.00	0.27	tr_D0A0W7_
DRB1_0101	437	LRVLRLGFMYLQGVH	VLRLGFMYL	2	0.4949	236.2	42.00	0.24	tr_D0A0W7_
DRB1_0101	438	SMLHLSDRNASLAPS	MLHLSDRNA	1	0.4949	236.4	42.00	0.52	tr_D0A0W7_
DRB1_0101	439	DVEKDSRLWTPLSLL	DSRLWTPLS	4	0.4946	237.2	42.00	0.41	tr_D0A0W7_
DRB1_0101	440	LSPTLEQQLYVLSSY	PTLEQQLYV	2	0.4937	239.5	42.00	0.38	tr_D0A0W7_
DRB1_0101	441	IGLSVPNVREIARKL	NVREIARKL	6	0.4934	240.2	42.00	0.49	tr_D0A0W7_
DRB1_0101	442	YELHGVFTVMSSDGE	LHGVFTVMS	2	0.4931	241.0	42.00	0.29	tr_D0A0W7_
DRB1_0101	443	ITFIDPLELKPRLNK	ITFIDPLEL	0	0.4923	243.1	43.00	0.49	tr_D0A0W7_
DRB1_0101	444	GRLMRVILLHVEEMS	VILLHVEEM	5	0.4919	244.1	43.00	0.37	tr_D0A0W7_
DRB1_0101	445	LDTPNITFIDPLELK	ITFIDPLEL	5	0.4896	250.1	43.00	0.85	tr_D0A0W7_
DRB1_0101	446	GPLGDIVTYLALPIL	IVTYLALPI	5	0.4895	250.6	43.00	0.30	tr_D0A0W7_
DRB1_0101	447	YGPLGDIVTYLALPI	YGPLGDIVT	0	0.4882	253.9	43.00	0.31	tr_D0A0W7_
DRB1_0101	448	LMKHNVVAFSPMTGS	NVVAFSPMT	4	0.4853	262.2	44.00	0.34	tr_D0A0W7_
DRB1_0101	449	TGRLMRVILLHVEEM	GRLMRVILL	1	0.4848	263.6	44.00	0.48	tr_D0A0W7_
DRB1_0101	450	MKHNVVAFSPMTGST	NVVAFSPMT	3	0.4846	264.3	44.00	0.36	tr_D0A0W7_
DRB1_0101	451	VKVEVVYPRNLDLMP	VEVVYPRNL	2	0.4830	268.7	45.00	0.48	tr_D0A0W7_
DRB1_0101	452	CQLRVLRLGFMYLQG	LRVLRLGFM	2	0.4826	270.0	45.00	0.44	tr_D0A0W7_
DRB1_0101	453	EANATVKVLSATWNS	VKVLSATWN	5	0.4824	270.5	45.00	0.56	tr_D0A0W7_
DRB1_0101	454	VFGPVTEAMLDTPNI	FGPVTEAML	1	0.4801	277.3	45.00	0.75	tr_D0A0W7_
DRB1_0101	455	YFLTHGTEGELMVYG	LTHGTEGEL	2	0.4798	278.2	45.00	0.22	tr_D0A0W7_
DRB1_0101	456	LPQLSVGAEANATVK	PQLSVGAEA	1	0.4792	280.1	45.00	0.34	tr_D0A0W7_
DRB1_0101	457	VILLHVEEMSPETLV	VEEMSPETL	5	0.4791	280.3	45.00	0.59	tr_D0A0W7_
DRB1_0101	458	GSVKVEVVYPRNLDL	VEVVYPRNL	4	0.4791	280.3	45.00	0.53	tr_D0A0W7_
DRB1_0101	459	AANDLRYEQGERIVT	LRYEQGERI	4	0.4782	283.1	46.00	0.75	tr_D0A0W7_
DRB1_0101	460	DDEFREVFERFAAAL	EVFERFAAA	5	0.4769	287.2	46.00	0.43	tr_D0A0W7_
DRB1_0101	461	SATWNSYMPQEYVNA	NSYMPQEYV	4	0.4757	290.7	46.00	0.49	tr_D0A0W7_
DRB1_0101	462	NTAGAARLVFATSLP	NTAGAARLV	0	0.4755	291.5	46.00	0.37	tr_D0A0W7_
DRB1_0101	463	FSPMTGSTFIRQWNP	FSPMTGSTF	0	0.4752	292.4	46.00	0.68	tr_D0A0W7_
DRB1_0101	464	PRNLDLMPQDFIKEQ	LDLMPQDFI	3	0.4742	295.7	46.00	0.65	tr_D0A0W7_
DRB1_0101	465	GAVCRVAMDTLPVIL	RVAMDTLPV	4	0.4708	306.7	47.00	0.65	tr_D0A0W7_
DRB1_0101	466	LVFATSLPHWGDTET	LVFATSLPH	0	0.4699	309.6	47.00	0.48	tr_D0A0W7_
DRB1_0101	467	RNLDLMPQDFIKEQL	LDLMPQDFI	2	0.4698	310.1	47.00	0.63	tr_D0A0W7_
DRB1_0101	468	FTVVKTSQCYTESSE	FTVVKTSQC	0	0.4688	313.3	47.00	0.68	tr_D0A0W7_
DRB1_0101	469	YGATQVSVWSMARAL	SVWSMARAL	6	0.4677	317.1	48.00	0.42	tr_D0A0W7_
DRB1_0101	470	LSQALSSLEWLKDRT	SQALSSLEW	1	0.4663	322.1	48.00	0.29	tr_D0A0W7_
DRB1_0101	471	KVEVVYPRNLDLMPQ	VEVVYPRNL	1	0.4641	329.7	49.00	0.42	tr_D0A0W7_
DRB1_0101	472	PNVREIARKLEERND	NVREIARKL	1	0.4634	332.2	49.00	0.83	tr_D0A0W7_
DRB1_0101	473	VIGLSVPNVREIARK	IGLSVPNVR	1	0.4626	335.1	49.00	0.25	tr_D0A0W7_
DRB1_0101	474	RSLYSQRRYVINDIV	LYSQRRYVI	2	0.4615	339.2	49.00	0.43	tr_D0A0W7_
DRB1_0101	475	YPRNLDLMPQDFIKE	LDLMPQDFI	4	0.4595	346.5	49.00	0.65	tr_D0A0W7_
DRB1_0101	476	VHYGDEEYALTVNVM	EEYALTVNV	5	0.4586	350.1	50.00	0.24	tr_D0A0W7_
DRB1_0101	477	EYLKAHVSETNITEA	YLKAHVSET	1	0.4585	350.5	50.00	0.53	tr_D0A0W7_
DRB1_0101	478	SQALSSLEWLKDRTT	LSSLEWLKD	3	0.4543	366.7	55.00	0.25	tr_D0A0W7_
DRB1_0101	479	RNDLRVIVLFGEFSL	VIVLFGEFS	5	0.4537	369.0	55.00	0.67	tr_D0A0W7_
DRB1_0101	480	LAKDSQYIAIKRFQG	YIAIKRFQG	6	0.4532	371.0	55.00	0.51	tr_D0A0W7_
DRB1_0101	481	TESSELQGPLNGLAV	QGPLNGLAV	6	0.4530	371.8	55.00	0.25	tr_D0A0W7_
DRB1_0101	482	QGPLNGLAVFMEDDD	PLNGLAVFM	2	0.4528	372.6	55.00	0.40	tr_D0A0W7_
DRB1_0101	483	AGFNASLESRQWTVA	FNASLESRQ	2	0.4528	372.8	55.00	0.44	tr_D0A0W7_
DRB1_0101	484	INAGFNASLESRQWT	FNASLESRQ	4	0.4520	375.7	55.00	0.38	tr_D0A0W7_
DRB1_0101	485	NTTIAEAANDLRYEQ	TIAEAANDL	2	0.4489	388.5	55.00	0.49	tr_D0A0W7_

DRB1_0101	486	PQLSVGAEANATVKV	VGAEANATV	4	0.4487	389.4	55.00	0.29	tr_D0A0W7_
DRB1_0101	487	NAGFNASLESRQWTV	FNASLESRQ	3	0.4487	389.5	55.00	0.54	tr_D0A0W7_
DRB1_0101	488	ATGRLMRVILLHVEE	GRLMRVILL	2	0.4470	396.7	55.00	0.69	tr_D0A0W7_
DRB1_0101	489	TEADYFLTHGTEGEL	YFLTHGTEG	4	0.4466	398.4	55.00	0.55	tr_D0A0W7_
DRB1_0101	490	LHGVFTVMSSDGEPA	FTVMSSDGE	4	0.4450	405.5	55.00	0.50	tr_D0A0W7_
DRB1_0101	491	VYPRNLDLMPQDFIK	LDLMPQDFI	5	0.4428	415.2	55.00	0.70	tr_D0A0W7_
DRB1_0101	492	TNPLAKDSQYIAIKR	PLAKDSQYI	2	0.4397	429.6	55.00	0.55	tr_D0A0W7_
DRB1_0101	493	RSAVIREDGEGVGKY	VIREDGEGV	3	0.4375	439.8	55.00	0.86	tr_D0A0W7_
DRB1_0101	494	RVILLHVEEMSPETL	VILLHVEEM	1	0.4359	447.3	55.00	0.37	tr_D0A0W7_
DRB1_0101	495	VEEMSPETLVNFFYA	VEEMSPETL	0	0.4342	455.5	55.00	0.35	tr_D0A0W7_
DRB1_0101	496	KVLSATWNSYMPQEY	LSATWNSYM	2	0.4335	459.2	55.00	0.68	tr_D0A0W7_
DRB1_0101	497	QALSSLEWLKDRTTF	LEWLKDRTT	5	0.4332	460.9	55.00	0.49	tr_D0A0W7_
DRB1_0101	498	LFTGTNPLAKDSQYI	FTGTNPLAK	1	0.4329	462.4	55.00	0.51	tr_D0A0W7_
DRB1_0101	499	AGSVKVEVVYPRNLD	VEVVYPRNL	5	0.4326	463.5	55.00	0.49	tr_D0A0W7_
DRB1_0101	500	GTNPLAKDSQYIAIK	PLAKDSQYI	3	0.4317	468.4	55.00	0.61	tr_D0A0W7_
DRB1_0101	501	NASIPPLANPMTPSM	IPPLANPMT	3	0.4314	469.8	60.00	0.58	tr_D0A0W7_
DRB1_0101	502	PQAIIVFGAPEKDTA	IVFGAPEKD	4	0.4314	469.8	60.00	0.38	tr_D0A0W7_
DRB1_0101	503	HGVFTVMSSDGEPAP	FTVMSSDGE	3	0.4310	471.5	60.00	0.56	tr_D0A0W7_
DRB1_0101	504	QLSVGAEANATVKVL	VGAEANATV	3	0.4309	472.2	60.00	0.46	tr_D0A0W7_
DRB1_0101	505	RLMRVILLHVEEMSP	VILLHVEEM	4	0.4305	474.1	60.00	0.57	tr_D0A0W7_
DRB1_0101	506	QAIIVFGAPEKDTAK	IVFGAPEKD	3	0.4297	478.6	60.00	0.51	tr_D0A0W7_
DRB1_0101	507	AAETLALIRYALCQL	LALIRYALC	4	0.4288	482.9	60.00	0.41	tr_D0A0W7_
DRB1_0101	508	NQGSKAVYVKEMLEN	NQGSKAVYV	0	0.4275	490.0	60.00	0.64	tr_D0A0W7_
DRB1_0101	509	DDTASKAAALWQKGA	DTASKAAAL	1	0.4273	491.1	60.00	0.52	tr_D0A0W7_
DRB1_0101	510	IADFLRRSLTEFGVS	LRRSLTEFG	4	0.4267	494.1	60.00	0.38	tr_D0A0W7_
DRB1_0101	511	NPLAKDSQYIAIKRF	PLAKDSQYI	1	0.4266	494.8	60.00	0.39	tr_D0A0W7_
DRB1_0101	512	GVFTVMSSDGEPAPD	FTVMSSDGE	2	0.4266	494.8	60.00	0.54	tr_D0A0W7_
DRB1_0101	513	FGPVTEAMLDTPNIT	FGPVTEAML	0	0.4265	495.4	60.00	0.44	tr_D0A0W7_
DRB1_0101	514	ELHGVFTVMSSDGEP	FTVMSSDGE	5	0.4261	497.3	60.00	0.41	tr_D0A0W7_
DRB1_0101	515	TEGELMVYGWICGEV	ELMVYGWIC	3	0.4258	499.2	60.00	0.49	tr_D0A0W7_
DRB1_0101	516	AEANATVKVLSATWN	VKVLSATWN	6	0.4249	504.2	60.00	0.39	tr_D0A0W7_
DRB1_0101	517	SLYSQRRYVINDIVI	LYSQRRYVI	1	0.4245	506.3	60.00	0.33	tr_D0A0W7_
DRB1_0101	518	EKDTAKFLMMMVAEE	FLMMMVAEE	6	0.4242	507.9	60.00	0.49	tr_D0A0W7_
DRB1_0101	519	DLGHSDRFFLHAFNT	GHSDRFFLH	2	0.4225	517.4	60.00	0.34	tr_D0A0W7_
DRB1_0101	520	MLHLSDRNASLAPSG	MLHLSDRNA	0	0.4224	517.7	60.00	0.34	tr_D0A0W7_
DRB1_0101	521	PAAETLALIRYALCQ	LALIRYALC	5	0.4220	520.0	60.00	0.34	tr_D0A0W7_
DRB1_0101	522	LRAPVALLLLLIVLP	LRAPVALLL	0	0.4219	520.4	60.00	0.62	tr_D0A0W7_
DRB1_0101	523	GFNASLESRQWTVAG	FNASLESRQ	1	0.4210	525.4	60.00	0.35	tr_D0A0W7_
DRB1_0101	524	DDDTASKAAALWQKG	DTASKAAAL	2	0.4196	533.8	60.00	0.61	tr_D0A0W7_
DRB1_0101	525	GTEGELMVYGWICGE	ELMVYGWIC	4	0.4193	535.4	60.00	0.47	tr_D0A0W7_
DRB1_0101	526	LMRVILLHVEEMSPE	VILLHVEEM	3	0.4186	539.3	60.00	0.58	tr_D0A0W7_
DRB1_0101	527	ASIPPLANPMTPSMS	IPPLANPMT	2	0.4184	540.4	60.00	0.52	tr_D0A0W7_
DRB1_0101	528	NLDLMPQDFIKEQLE	LDLMPQDFI	1	0.4181	542.7	60.00	0.57	tr_D0A0W7_
DRB1_0101	529	PVTEAMLDTPNITFI	EAMLDTPNI	3	0.4180	543.0	60.00	0.74	tr_D0A0W7_
DRB1_0101	530	LPQAIIVFGAPEKDT	IVFGAPEKD	5	0.4177	544.9	60.00	0.28	tr_D0A0W7_
DRB1_0101	531	EAANDLRYEQGERIV	LRYEQGERI	5	0.4173	547.3	60.00	0.84	tr_D0A0W7_
DRB1_0101	532	RYEQGERIVTAVFGP	YEQGERIVT	1	0.4159	555.7	60.00	0.38	tr_D0A0W7_
DRB1_0101	533	SAVIREDGEGVGKYL	VIREDGEGV	2	0.4144	564.5	60.00	0.74	tr_D0A0W7_
DRB1_0101	534	GGAVCRVAMDTLPVI	RVAMDTLPV	5	0.4142	565.7	60.00	0.58	tr_D0A0W7_
DRB1_0101	535	DPAAETLALIRYALC	LALIRYALC	6	0.4141	566.2	60.00	0.28	tr_D0A0W7_

DRB1_0101	536	VTEAMLDTPNITFID	EAMLDTPNI	2	0.4124	577.1	60.00	0.69	tr_D0A0W7_
DRB1_0101	537	FYADSSIISDDMRYG	FYADSSIIS	0	0.4123	577.7	60.00	0.61	tr_D0A0W7_
DRB1_0101	538	HGTEGELMVYGWICG	ELMVYGWIC	5	0.4119	580.2	60.00	0.46	tr_D0A0W7_
DRB1_0101	539	ASKAAALWQKGASHL	LWQKGASHL	6	0.4107	587.9	60.00	0.35	tr_D0A0W7_
DRB1_0101	540	YVKEMLENGQTTTME	EMLENGQTT	3	0.4103	590.3	60.00	0.49	tr_D0A0W7_
DRB1_0101	541	AAALPQAIIVFGAPE	PQAIIVFGA	4	0.4101	591.7	60.00	0.34	tr_D0A0W7_
DRB1_0101	542	LRADPAAETLALIRY	LRADPAAET	0	0.4100	592.3	60.00	0.31	tr_D0A0W7_
DRB1_0101	543	SIPPLANPMTPSMSF	IPPLANPMT	1	0.4088	599.9	60.00	0.42	tr_D0A0W7_
DRB1_0101	544	ETSSKTAQLFHDVEK	KTAQLFHDV	4	0.4072	610.6	65.00	0.49	tr_D0A0W7_
DRB1_0101	545	EDDDTASKAAALWQK	DTASKAAAL	3	0.4068	612.7	65.00	0.69	tr_D0A0W7_
DRB1_0101	546	DGEGVGKYLPISGTV	GKYLPISGT	5	0.4068	613.2	65.00	0.52	tr_D0A0W7_
DRB1_0101	547	MRVILLHVEEMSPET	VILLHVEEM	2	0.4068	613.3	65.00	0.56	tr_D0A0W7_
DRB1_0101	548	VAGSVKVEVVYPRNL	VEVVYPRNL	6	0.4060	618.6	65.00	0.35	tr_D0A0W7_
DRB1_0101	549	GEHSLPTGGAVCRVA	SLPTGGAVC	3	0.4056	621.1	65.00	0.27	tr_D0A0W7_
DRB1_0101	550	TTIAEAANDLRYEQG	TIAEAANDL	1	0.4054	622.1	65.00	0.46	tr_D0A0W7_
DRB1_0101	551	VKEMLENGQTTTMES	EMLENGQTT	2	0.4048	626.5	65.00	0.40	tr_D0A0W7_
DRB1_0101	552	YVINDIVIGDYGGTC	YVINDIVIG	0	0.4044	628.8	65.00	0.68	tr_D0A0W7_
DRB1_0101	553	VFTVMSSDGEPAPDD	FTVMSSDGE	1	0.4035	635.2	65.00	0.51	tr_D0A0W7_
DRB1_0101	554	AIIVFGAPEKDTAKF	IVFGAPEKD	2	0.4033	636.9	65.00	0.52	tr_D0A0W7_
DRB1_0101	555	AALPQAIIVFGAPEK	PQAIIVFGA	3	0.4031	637.9	65.00	0.44	tr_D0A0W7_
DRB1_0101	556	VYVKEMLENGQTTTM	EMLENGQTT	4	0.4016	648.3	65.00	0.46	tr_D0A0W7_
DRB1_0101	557	LRSAVIREDGEGVGK	VIREDGEGV	4	0.3998	661.2	65.00	0.86	tr_D0A0W7_
DRB1_0101	558	EHSLPTGGAVCRVAM	LPTGGAVCR	3	0.3994	663.9	65.00	0.31	tr_D0A0W7_
DRB1_0101	559	VLSATWNSYMPQEYV	LSATWNSYM	1	0.3977	676.1	65.00	0.49	tr_D0A0W7_
DRB1_0101	560	ALPQAIIVFGAPEKD	PQAIIVFGA	2	0.3974	678.6	65.00	0.40	tr_D0A0W7_
DRB1_0101	561	YEQGERIVTAVFGPV	ERIVTAVFG	4	0.3942	702.8	65.00	0.40	tr_D0A0W7_
DRB1_0101	562	LENGQTTTMESGFTV	TTMESGFTV	6	0.3932	710.5	65.00	0.49	tr_D0A0W7_
DRB1_0101	563	DTASKAAALWQKGAS	DTASKAAAL	0	0.3927	714.3	65.00	0.37	tr_D0A0W7_
DRB1_0101	564	VVYPRNLDLMPQDFI	LDLMPQDFI	6	0.3926	714.8	65.00	0.55	tr_D0A0W7_
DRB1_0101	565	GVHYGDEEYALTVNV	YGDEEYALT	3	0.3925	715.2	65.00	0.31	tr_D0A0W7_
DRB1_0101	566	KEMLENGQTTTMESG	LENGQTTTM	3	0.3908	729.1	65.00	0.49	tr_D0A0W7_
DRB1_0101	567	LSATWNSYMPQEYVN	NSYMPQEYV	5	0.3907	729.5	65.00	0.51	tr_D0A0W7_
DRB1_0101	568	IRQWNPYLYFLRADP	YLYFLRADP	6	0.3903	732.6	65.00	0.21	tr_D0A0W7_
DRB1_0101	569	PLAKDSQYIAIKRFQ	PLAKDSQYI	0	0.3902	733.4	65.00	0.23	tr_D0A0W7_
DRB1_0101	570	LSVGAEANATVKVLS	VGAEANATV	2	0.3888	744.5	65.00	0.43	tr_D0A0W7_
DRB1_0101	571	TETSSKTAQLFHDVE	KTAQLFHDV	5	0.3887	745.2	65.00	0.41	tr_D0A0W7_
DRB1_0101	572	TSSKTAQLFHDVEKD	KTAQLFHDV	3	0.3876	754.8	65.00	0.55	tr_D0A0W7_
DRB1_0101	573	GGEHSLPTGGAVCRV	SLPTGGAVC	4	0.3854	772.8	65.00	0.26	tr_D0A0W7_
DRB1_0101	574	GIADFLRRSLTEFGV	LRRSLTEFG	5	0.3849	776.6	65.00	0.38	tr_D0A0W7_
DRB1_0101	575	VEVVYPRNLDLMPQD	VVYPRNLDL	2	0.3845	780.2	65.00	0.44	tr_D0A0W7_
DRB1_0101	576	TEAMLDTPNITFIDP	EAMLDTPNI	1	0.3841	783.7	70.00	0.67	tr_D0A0W7_
DRB1_0101	577	TGTNPLAKDSQYIAI	PLAKDSQYI	4	0.3823	798.8	70.00	0.63	tr_D0A0W7_
DRB1_0101	578	SNYGATQVSVWSMAR	GATQVSVWS	3	0.3817	804.6	70.00	0.29	tr_D0A0W7_
DRB1_0101	579	ADPAAETLALIRYAL	ETLALIRYA	5	0.3809	811.0	70.00	0.28	tr_D0A0W7_
DRB1_0101	580	YTESSELQGPLNGLA	SSELQGPLN	3	0.3805	814.9	70.00	0.43	tr_D0A0W7_
DRB1_0101	581	PDDEFREVFERFAAA	FREVFERFA	4	0.3804	815.5	70.00	0.40	tr_D0A0W7_
DRB1_0101	582	GPVTEAMLDTPNITF	EAMLDTPNI	4	0.3794	824.7	70.00	0.77	tr_D0A0W7_
DRB1_0101	583	LPISGTVFVIGLSVP	TVFVIGLSV	5	0.3788	829.7	70.00	0.49	tr_D0A0W7_
DRB1_0101	584	THGTEGELMVYGWIC	ELMVYGWIC	6	0.3788	829.9	70.00	0.34	tr_D0A0W7_
DRB1_0101	585	SFRDPSEGKLSGASL	FRDPSEGKL	1	0.3767	849.0	70.00	0.44	tr_D0A0W7_

DRB1_0101	586	RADPAAETLALIRYA	PAAETLALI	3	0.3763	852.7	70.00	0.41	tr_D0A0W7_
DRB1_0101	587	AVIREDGEGVGKYLP	VIREDGEGV	1	0.3752	862.5	70.00	0.67	tr_D0A0W7_
DRB1_0101	588	ASNYGATQVSVWSMA	YGATQVSVW	3	0.3737	877.4	70.00	0.32	tr_D0A0W7_
DRB1_0101	589	GDLGHSDRFFLHAFN	GHSDRFFLH	3	0.3735	878.6	70.00	0.47	tr_D0A0W7_
DRB1_0101	590	FTGTNPLAKDSQYIA	PLAKDSQYI	5	0.3733	880.8	70.00	0.51	tr_D0A0W7_
DRB1_0101	591	NYGATQVSVWSMARA	GATQVSVWS	2	0.3730	883.7	70.00	0.25	tr_D0A0W7_
DRB1_0101	592	ITEADYFLTHGTEGE	YFLTHGTEG	5	0.3713	899.7	70.00	0.60	tr_D0A0W7_
DRB1_0101	593	IPPLANPMTPSMSFR	ANPMTPSMS	4	0.3713	899.8	70.00	0.34	tr_D0A0W7_
DRB1_0101	594	QGVHYGDEEYALTVN	HYGDEEYAL	3	0.3710	902.7	70.00	0.26	tr_D0A0W7_
DRB1_0101	595	FLTHGTEGELMVYGW	THGTEGELM	2	0.3694	918.2	70.00	0.32	tr_D0A0W7_
DRB1_0101	596	TVDAVICSNEADGIA	DAVICSNEA	2	0.3683	930.2	70.00	0.54	tr_D0A0W7_
DRB1_0101	597	PVALLLLLIVLPQLS	LLIVLPQLS	6	0.3676	936.7	70.00	0.63	tr_D0A0W7_
DRB1_0101	598	LKPRLNKFRRHVIHL	LNKFRRHVI	4	0.3673	939.7	70.00	0.29	tr_D0A0W7_
DRB1_0101	599	QLELETDQNKIVIVY	LETDQNKIV	3	0.3661	951.7	70.00	0.60	tr_D0A0W7_
DRB1_0101	600	ERNDLRVIVLFGEFS	VIVLFGEFS	6	0.3659	954.1	70.00	0.63	tr_D0A0W7_
DRB1_0101	601	HDVEKDSRLWTPLSL	DSRLWTPLS	5	0.3644	970.0	70.00	0.50	tr_D0A0W7_
DRB1_0101	602	HSLPTGGAVCRVAMD	LPTGGAVCR	2	0.3635	978.8	70.00	0.33	tr_D0A0W7_
DRB1_0101	603	IIVFGAPEKDTAKFL	IVFGAPEKD	1	0.3624	990.9	70.00	0.47	tr_D0A0W7_
DRB1_0101	604	PEKDTAKFLMMMVAE	KDTAKFLMM	2	0.3595	1023.1	75.00	0.25	tr_D0A0W7_
DRB1_0101	605	DTETSSKTAQLFHDV	KTAQLFHDV	6	0.3584	1034.3	75.00	0.31	tr_D0A0W7_
DRB1_0101	606	SMSFRDPSEGKLSGA	FRDPSEGKL	3	0.3583	1035.8	75.00	0.72	tr_D0A0W7_
DRB1_0101	607	FIDPLELKPRLNKFR	PLELKPRLN	3	0.3582	1036.7	75.00	0.29	tr_D0A0W7_
DRB1_0101	608	HGATCECNQGSKAVY	CECNQGSKA	4	0.3577	1042.9	75.00	0.56	tr_D0A0W7_
DRB1_0101	609	TFIDPLELKPRLNKF	IDPLELKPR	2	0.3569	1051.9	75.00	0.28	tr_D0A0W7_
DRB1_0101	610	YLKAHVSETNITEAD	LKAHVSETN	1	0.3566	1054.7	75.00	0.34	tr_D0A0W7_
DRB1_0101	611	MEDDDTASKAAALWQ	DTASKAAAL	4	0.3562	1059.7	75.00	0.68	tr_D0A0W7_
DRB1_0101	612	MLDTPNITFIDPLEL	ITFIDPLEL	6	0.3559	1063.1	75.00	0.86	tr_D0A0W7_
DRB1_0101	613	KGDLGHSDRFFLHAF	GHSDRFFLH	4	0.3558	1064.8	75.00	0.51	tr_D0A0W7_
DRB1_0101	614	NVREIARKLEERNDL	NVREIARKL	0	0.3547	1076.8	75.00	0.69	tr_D0A0W7_
DRB1_0101	615	AVYVKEMLENGQTTT	EMLENGQTT	5	0.3545	1079.6	75.00	0.41	tr_D0A0W7_
DRB1_0101	616	SGGEHSLPTGGAVCR	EHSLPTGGA	3	0.3541	1083.5	75.00	0.28	tr_D0A0W7_
DRB1_0101	617	IDPLELKPRLNKFRR	LKPRLNKFR	5	0.3535	1091.0	75.00	0.32	tr_D0A0W7_
DRB1_0101	618	NASLAPSGGEHSLPT	LAPSGGEHS	3	0.3524	1104.6	75.00	0.76	tr_D0A0W7_
DRB1_0101	619	VDAVICSNEADGIAD	DAVICSNEA	1	0.3521	1108.2	75.00	0.49	tr_D0A0W7_
DRB1_0101	620	SPETLVNFFYADSSI	PETLVNFFY	1	0.3518	1111.4	75.00	0.28	tr_D0A0W7_
DRB1_0101	621	GVGTVDAVICSNEAD	VGTVDAVIC	1	0.3509	1122.6	75.00	0.37	tr_D0A0W7_
DRB1_0101	622	SVGAEANATVKVLSA	VGAEANATV	1	0.3508	1123.1	75.00	0.37	tr_D0A0W7_
DRB1_0101	623	APEKDTAKFLMMMVA	KDTAKFLMM	3	0.3508	1123.3	75.00	0.30	tr_D0A0W7_
DRB1_0101	624	SSKTAQLFHDVEKDS	KTAQLFHDV	2	0.3496	1138.3	75.00	0.58	tr_D0A0W7_
DRB1_0101	625	DPLELKPRLNKFRRH	LKPRLNKFR	4	0.3494	1140.2	75.00	0.41	tr_D0A0W7_
DRB1_0101	626	FNASLESRQWTVAGS	SLESRQWTV	3	0.3493	1142.3	75.00	0.33	tr_D0A0W7_
DRB1_0101	627	PLELKPRLNKFRRHV	LKPRLNKFR	3	0.3491	1144.2	75.00	0.49	tr_D0A0W7_
DRB1_0101	628	NITEADYFLTHGTEG	YFLTHGTEG	6	0.3489	1146.3	75.00	0.41	tr_D0A0W7_
DRB1_0101	629	SLRSAVIREDGEGVG	VIREDGEGV	5	0.3476	1163.7	75.00	0.76	tr_D0A0W7_
DRB1_0101	630	SETNITEADYFLTHG	NITEADYFL	3	0.3459	1184.7	75.00	0.58	tr_D0A0W7_
DRB1_0101	631	RNASLAPSGGEHSLP	LAPSGGEHS	4	0.3458	1185.7	75.00	0.70	tr_D0A0W7_
DRB1_0101	632	LQGVHYGDEEYALTV	HYGDEEYAL	4	0.3452	1193.5	75.00	0.28	tr_D0A0W7_
DRB1_0101	633	RKLEERNDLRVIVLF	LEERNDLRV	2	0.3449	1197.1	75.00	0.38	tr_D0A0W7_
DRB1_0101	634	EQLELETDQNKIVIV	LETDQNKIV	4	0.3449	1198.1	75.00	0.57	tr_D0A0W7_
DRB1_0101	635	ARKLEERNDLRVIVL	LEERNDLRV	3	0.3444	1204.1	75.00	0.43	tr_D0A0W7_

DRB1_0101	636	GTVDAVICSNEADGI	DAVICSNEA	3	0.3436	1214.8	75.00	0.62	tr_D0A0W7_
DRB1_0101	637	DRNASLAPSGGEHSL	LAPSGGEHS	5	0.3431	1221.3	75.00	0.64	tr_D0A0W7_
DRB1_0101	638	PPLANPMTPSMSFRD	ANPMTPSMS	3	0.3429	1224.2	75.00	0.42	tr_D0A0W7_
DRB1_0101	639	PLANPMTPSMSFRDP	ANPMTPSMS	2	0.3422	1232.7	75.00	0.40	tr_D0A0W7_
DRB1_0101	640	LTHGTEGELMVYGWI	THGTEGELM	1	0.3417	1240.1	75.00	0.28	tr_D0A0W7_
DRB1_0101	641	CASNYGATQVSVWSM	YGATQVSVW	4	0.3406	1254.0	75.00	0.32	tr_D0A0W7_
DRB1_0101	642	KAVYVKEMLENGQTT	YVKEMLENG	3	0.3399	1264.8	75.00	0.55	tr_D0A0W7_
DRB1_0101	643	CYTESSELQGPLNGL	SSELQGPLN	4	0.3396	1267.8	75.00	0.41	tr_D0A0W7_
DRB1_0101	644	GAEANATVKVLSATW	NATVKVLSA	4	0.3393	1272.2	75.00	0.47	tr_D0A0W7_
DRB1_0101	645	DGVGTVDAVICSNEA	VGTVDAVIC	2	0.3376	1296.4	75.00	0.52	tr_D0A0W7_
DRB1_0101	646	LELETDQNKIVIVYG	LETDQNKIV	2	0.3375	1297.4	75.00	0.59	tr_D0A0W7_
DRB1_0101	647	LDLMPQDFIKEQLEL	LDLMPQDFI	0	0.3374	1299.0	75.00	0.47	tr_D0A0W7_
DRB1_0101	648	LELKPRLNKFRRHVI	LKPRLNKFR	2	0.3367	1308.2	75.00	0.47	tr_D0A0W7_
DRB1_0101	649	ELKPRLNKFRRHVIH	LKPRLNKFR	1	0.3361	1317.3	75.00	0.30	tr_D0A0W7_
DRB1_0101	650	MSFRDPSEGKLSGAS	FRDPSEGKL	2	0.3353	1328.5	75.00	0.73	tr_D0A0W7_
DRB1_0101	651	ASLAPSGGEHSLPTG	LAPSGGEHS	2	0.3336	1353.9	80.00	0.78	tr_D0A0W7_
DRB1_0101	652	GAPEKDTAKFLMMMV	PEKDTAKFL	2	0.3329	1363.4	80.00	0.31	tr_D0A0W7_
DRB1_0101	653	PSMSFRDPSEGKLSG	FRDPSEGKL	4	0.3324	1371.1	80.00	0.69	tr_D0A0W7_
DRB1_0101	654	MSPETLVNFFYADSS	PETLVNFFY	2	0.3322	1373.3	80.00	0.39	tr_D0A0W7_
DRB1_0101	655	PQDFIKEQLELETDQ	FIKEQLELE	3	0.3322	1373.3	80.00	0.69	tr_D0A0W7_
DRB1_0101	656	FGAPEKDTAKFLMMM	PEKDTAKFL	3	0.3309	1393.5	80.00	0.38	tr_D0A0W7_
DRB1_0101	657	ETNITEADYFLTHGT	NITEADYFL	2	0.3307	1396.7	80.00	0.53	tr_D0A0W7_
DRB1_0101	658	FTVMSSDGEPAPDDE	VMSSDGEPA	2	0.3299	1408.9	80.00	0.50	tr_D0A0W7_
DRB1_0101	659	VGTVDAVICSNEADG	DAVICSNEA	4	0.3296	1413.8	80.00	0.50	tr_D0A0W7_
DRB1_0101	660	VSETNITEADYFLTH	NITEADYFL	4	0.3288	1425.3	80.00	0.59	tr_D0A0W7_
DRB1_0101	661	EDGEGVGKYLPISGT	GKYLPISGT	6	0.3284	1431.6	80.00	0.40	tr_D0A0W7_
DRB1_0101	662	VGAEANATVKVLSAT	NATVKVLSA	5	0.3271	1451.5	80.00	0.31	tr_D0A0W7_
DRB1_0101	663	EEMSPETLVNFFYAD	PETLVNFFY	4	0.3260	1469.1	80.00	0.41	tr_D0A0W7_
DRB1_0101	664	QCYTESSELQGPLNG	CYTESSELQ	1	0.3254	1478.8	80.00	0.35	tr_D0A0W7_
DRB1_0101	665	FMEDDDTASKAAALW	DTASKAAAL	5	0.3251	1484.0	80.00	0.62	tr_D0A0W7_
DRB1_0101	666	TASKAAALWQKGASH	SKAAALWQK	2	0.3244	1494.9	80.00	0.26	tr_D0A0W7_
DRB1_0101	667	QDFIKEQLELETDQN	FIKEQLELE	2	0.3229	1519.1	80.00	0.64	tr_D0A0W7_
DRB1_0101	668	DGIADFLRRSLTEFG	LRRSLTEFG	6	0.3218	1537.4	80.00	0.25	tr_D0A0W7_
DRB1_0101	669	KEQLELETDQNKIVI	LETDQNKIV	5	0.3218	1537.6	80.00	0.50	tr_D0A0W7_
DRB1_0101	670	SQCYTESSELQGPLN	CYTESSELQ	2	0.3218	1537.9	80.00	0.48	tr_D0A0W7_
DRB1_0101	671	TNITEADYFLTHGTE	NITEADYFL	1	0.3205	1560.0	80.00	0.46	tr_D0A0W7_
DRB1_0101	672	LANPMTPSMSFRDPS	MTPSMSFRD	4	0.3204	1560.4	80.00	0.34	tr_D0A0W7_
DRB1_0101	673	GKGDLGHSDRFFLHA	GHSDRFFLH	5	0.3195	1576.1	80.00	0.48	tr_D0A0W7_
DRB1_0101	674	MPQDFIKEQLELETD	FIKEQLELE	4	0.3181	1600.5	80.00	0.68	tr_D0A0W7_
DRB1_0101	675	DAVICSNEADGIADF	ICSNEADGI	3	0.3179	1604.3	80.00	0.39	tr_D0A0W7_
DRB1_0101	676	KLEERNDLRVIVLFG	LEERNDLRV	1	0.3173	1615.2	80.00	0.30	tr_D0A0W7_
DRB1_0101	677	TGGAVCRVAMDTLPV	RVAMDTLPV	6	0.3165	1628.6	80.00	0.39	tr_D0A0W7_
DRB1_0101	678	EAMLDTPNITFIDPL	EAMLDTPNI	0	0.3157	1642.0	80.00	0.56	tr_D0A0W7_
DRB1_0101	679	LMPQDFIKEQLELET	FIKEQLELE	5	0.3155	1645.6	80.00	0.59	tr_D0A0W7_
DRB1_0101	680	EMSPETLVNFFYADS	PETLVNFFY	3	0.3152	1652.1	80.00	0.47	tr_D0A0W7_
DRB1_0101	681	GDGVGTVDAVICSNE	VGTVDAVIC	3	0.3149	1657.0	80.00	0.63	tr_D0A0W7_
DRB1_0101	682	GPLNGLAVFMEDDDT	PLNGLAVFM	1	0.3143	1666.8	80.00	0.55	tr_D0A0W7_
DRB1_0101	683	TIAEAANDLRYEQGE	IAEAANDLR	1	0.3138	1675.9	80.00	0.44	tr_D0A0W7_
DRB1_0101	684	LHLSDRNASLAPSGG	LSDRNASLA	2	0.3132	1688.3	80.00	0.39	tr_D0A0W7_
DRB1_0101	685	AGDGVGTVDAVICSN	VGTVDAVIC	4	0.3131	1689.2	80.00	0.61	tr_D0A0W7_

DRB1_0101	686	EMLENGQTTTMESGF	LENGQTTTM	2	0.3113	1722.9	80.00	0.60	tr_D0A0W7_
DRB1_0101	687	KGASHLVGKGDLGHS	HLVGKGDLG	4	0.3112	1723.5	80.00	0.34	tr_D0A0W7_
DRB1_0101	688	TSQCYTESSELQGPL	CYTESSELQ	3	0.3089	1767.0	80.00	0.58	tr_D0A0W7_
DRB1_0101	689	VIREDGEGVGKYLPI	VIREDGEGV	0	0.3081	1782.8	80.00	0.43	tr_D0A0W7_
DRB1_0101	690	SLPTGGAVCRVAMDT	LPTGGAVCR	1	0.3076	1793.2	80.00	0.33	tr_D0A0W7_
DRB1_0101	691	IARKLEERNDLRVIV	LEERNDLRV	4	0.3063	1817.8	85.00	0.42	tr_D0A0W7_
DRB1_0101	692	EVVYPRNLDLMPQDF	VVYPRNLDL	1	0.3063	1818.2	85.00	0.46	tr_D0A0W7_
DRB1_0101	693	ASHLVGKGDLGHSDR	LVGKGDLGH	3	0.3043	1858.4	85.00	0.39	tr_D0A0W7_
DRB1_0101	694	SKTAQLFHDVEKDSR	KTAQLFHDV	1	0.3035	1873.9	85.00	0.54	tr_D0A0W7_
DRB1_0101	695	DCASNYGATQVSVWS	YGATQVSVW	5	0.3025	1894.0	85.00	0.28	tr_D0A0W7_
DRB1_0101	696	TPSMSFRDPSEGKLS	FRDPSEGKL	5	0.3025	1895.1	85.00	0.66	tr_D0A0W7_
DRB1_0101	697	VSLRSAVIREDGEGV	VIREDGEGV	6	0.3024	1897.3	85.00	0.50	tr_D0A0W7_
DRB1_0101	698	SLAPSGGEHSLPTGG	LAPSGGEHS	1	0.3022	1900.0	85.00	0.72	tr_D0A0W7_
DRB1_0101	699	GASHLVGKGDLGHSD	LVGKGDLGH	4	0.3016	1913.2	85.00	0.31	tr_D0A0W7_
DRB1_0101	700	VFGAPEKDTAKFLMM	PEKDTAKFL	4	0.3012	1921.7	85.00	0.38	tr_D0A0W7_
DRB1_0101	701	AEAANDLRYEQGERI	LRYEQGERI	6	0.3000	1947.0	85.00	0.81	tr_D0A0W7_
DRB1_0101	702	TVAGSVKVEVVYPRN	VKVEVVYPR	5	0.2976	1998.6	85.00	0.46	tr_D0A0W7_
DRB1_0101	703	NASLESRQWTVAGSV	SLESRQWTV	2	0.2960	2031.6	85.00	0.34	tr_D0A0W7_
DRB1_0101	704	SKAVYVKEMLENGQT	YVKEMLENG	4	0.2957	2039.6	85.00	0.62	tr_D0A0W7_
DRB1_0101	705	IVFGAPEKDTAKFLM	PEKDTAKFL	5	0.2952	2051.2	85.00	0.27	tr_D0A0W7_
DRB1_0101	706	LAGDGVGTVDAVICS	VGTVDAVIC	5	0.2947	2061.1	85.00	0.56	tr_D0A0W7_
DRB1_0101	707	VFATSLPHWGDTETS	FATSLPHWG	1	0.2944	2067.2	85.00	0.64	tr_D0A0W7_
DRB1_0101	708	YLQGVHYGDEEYALT	HYGDEEYAL	5	0.2936	2086.3	85.00	0.32	tr_D0A0W7_
DRB1_0101	709	FHDVEKDSRLWTPLS	DSRLWTPLS	6	0.2935	2088.0	85.00	0.40	tr_D0A0W7_
DRB1_0101	710	ELETDQNKIVIVYGP	LETDQNKIV	1	0.2929	2102.0	85.00	0.51	tr_D0A0W7_
DRB1_0101	711	ADSSIISDDMRYGVF	IISDDMRYG	4	0.2928	2105.3	85.00	0.39	tr_D0A0W7_
DRB1_0101	712	KHGATCECNQGSKAV	CECNQGSKA	5	0.2915	2134.0	85.00	0.57	tr_D0A0W7_
DRB1_0101	713	MYLQGVHYGDEEYAL	MYLQGVHYG	0	0.2912	2141.7	85.00	0.30	tr_D0A0W7_
DRB1_0101	714	ADGIADFLRRSLTEF	DGIADFLRR	1	0.2884	2206.0	85.00	0.23	tr_D0A0W7_
DRB1_0101	715	SHLVGKGDLGHSDRF	LVGKGDLGH	2	0.2883	2208.8	85.00	0.37	tr_D0A0W7_
DRB1_0101	716	YGGTCEGEAAKHGAT	CEGEAAKHG	4	0.2882	2211.9	85.00	0.54	tr_D0A0W7_
DRB1_0101	717	DSSIISDDMRYGVFD	IISDDMRYG	3	0.2872	2234.8	85.00	0.39	tr_D0A0W7_
DRB1_0101	718	PSGGEHSLPTGGAVC	EHSLPTGGA	4	0.2870	2239.6	85.00	0.36	tr_D0A0W7_
DRB1_0101	719	VKTSQCYTESSELQG	CYTESSELQ	5	0.2856	2275.2	85.00	0.50	tr_D0A0W7_
DRB1_0101	720	HVSETNITEADYFLT	NITEADYFL	5	0.2855	2277.6	85.00	0.58	tr_D0A0W7_
DRB1_0101	721	DFIKEQLELETDQNK	FIKEQLELE	1	0.2842	2310.5	85.00	0.58	tr_D0A0W7_
DRB1_0101	722	DLMPQDFIKEQLELE	FIKEQLELE	6	0.2828	2343.9	85.00	0.47	tr_D0A0W7_
DRB1_0101	723	VFMEDDDTASKAAAL	DTASKAAAL	6	0.2823	2357.9	85.00	0.41	tr_D0A0W7_
DRB1_0101	724	KTSQCYTESSELQGP	CYTESSELQ	4	0.2822	2360.9	85.00	0.53	tr_D0A0W7_
DRB1_0101	725	DYGGTCEGEAAKHGA	CEGEAAKHG	5	0.2816	2376.0	85.00	0.43	tr_D0A0W7_
DRB1_0101	726	ASLESRQWTVAGSVK	SLESRQWTV	1	0.2776	2480.5	85.00	0.29	tr_D0A0W7_
DRB1_0101	727	LEERNDLRVIVLFGE	ERNDLRVIV	2	0.2747	2559.4	90.00	0.19	tr_D0A0W7_
DRB1_0101	728	YADSSIISDDMRYGV	IISDDMRYG	5	0.2741	2576.3	90.00	0.33	tr_D0A0W7_
DRB1_0101	729	SSIISDDMRYGVFDD	IISDDMRYG	2	0.2735	2593.8	90.00	0.40	tr_D0A0W7_
DRB1_0101	730	SPMTGSTFIRQWNPY	PMTGSTFIR	1	0.2728	2613.6	90.00	0.29	tr_D0A0W7_
DRB1_0101	731	QKGASHLVGKGDLGH	HLVGKGDLG	5	0.2727	2614.6	90.00	0.32	tr_D0A0W7_
DRB1_0101	732	LETDQNKIVIVYGPL	LETDQNKIV	0	0.2723	2625.6	90.00	0.30	tr_D0A0W7_
DRB1_0101	733	GSKAVYVKEMLENGQ	YVKEMLENG	5	0.2715	2650.4	90.00	0.54	tr_D0A0W7_
DRB1_0101	734	GDTETSSKTAQLFHD	TSSKTAQLF	4	0.2691	2720.3	90.00	0.42	tr_D0A0W7_
DRB1_0101	735	MLENGQTTTMESGFT	LENGQTTTM	1	0.2690	2721.3	90.00	0.58	tr_D0A0W7_

DRB1_0101	736	NEADGIADFLRRSLT	DGIADFLRR	3	0.2683	2743.2	90.00	0.39	tr_D0A0W7_
DRB1_0101	737	GGTCEGEAAKHGATC	CEGEAAKHG	3	0.2683	2743.2	90.00	0.62	tr_D0A0W7_
DRB1_0101	738	SDRNASLAPSGGEHS	LAPSGGEHS	6	0.2678	2758.8	90.00	0.38	tr_D0A0W7_
DRB1_0101	739	VGKGDLGHSDRFFLH	GHSDRFFLH	6	0.2671	2778.3	90.00	0.34	tr_D0A0W7_
DRB1_0101	740	DDCASNYGATQVSVW	CASNYGATQ	2	0.2671	2778.9	90.00	0.32	tr_D0A0W7_
DRB1_0101	741	IKEQLELETDQNKIV	LETDQNKIV	6	0.2665	2798.2	90.00	0.41	tr_D0A0W7_
DRB1_0101	742	APDDEFREVFERFAA	FREVFERFA	5	0.2652	2837.1	90.00	0.58	tr_D0A0W7_
DRB1_0101	743	EIARKLEERNDLRVI	LEERNDLRV	5	0.2644	2862.1	90.00	0.38	tr_D0A0W7_
DRB1_0101	744	LPTGGAVCRVAMDTL	GAVCRVAMD	4	0.2640	2873.0	90.00	0.24	tr_D0A0W7_
DRB1_0101	745	AVICSNEADGIADFL	ICSNEADGI	2	0.2636	2884.7	90.00	0.50	tr_D0A0W7_
DRB1_0101	746	EADGIADFLRRSLTE	DGIADFLRR	2	0.2629	2908.5	90.00	0.36	tr_D0A0W7_
DRB1_0101	747	ANPMTPSMSFRDPSE	MTPSMSFRD	3	0.2622	2929.9	90.00	0.50	tr_D0A0W7_
DRB1_0101	748	HLSDRNASLAPSGGE	LSDRNASLA	1	0.2616	2950.3	90.00	0.34	tr_D0A0W7_
DRB1_0101	749	VFDDTKCDTAEKLSK	TKCDTAEKL	4	0.2606	2982.1	90.00	0.65	tr_D0A0W7_
DRB1_0101	750	HLVGKGDLGHSDRFF	LVGKGDLGH	1	0.2563	3122.7	90.00	0.31	tr_D0A0W7_
DRB1_0101	751	WGDTETSSKTAQLFH	TSSKTAQLF	5	0.2532	3230.5	90.00	0.43	tr_D0A0W7_
DRB1_0101	752	SNEADGIADFLRRSL	DGIADFLRR	4	0.2525	3255.7	90.00	0.36	tr_D0A0W7_
DRB1_0101	753	FDDTKCDTAEKLSKD	TKCDTAEKL	3	0.2506	3323.8	90.00	0.70	tr_D0A0W7_
DRB1_0101	754	LAPSGGEHSLPTGGA	LAPSGGEHS	0	0.2505	3325.2	90.00	0.46	tr_D0A0W7_
DRB1_0101	755	KDDCASNYGATQVSV	CASNYGATQ	3	0.2496	3357.6	90.00	0.46	tr_D0A0W7_
DRB1_0101	756	GTCEGEAAKHGATCE	CEGEAAKHG	2	0.2466	3469.4	90.00	0.64	tr_D0A0W7_
DRB1_0101	757	LAVFMEDDDTASKAA	FMEDDDTAS	3	0.2452	3522.6	90.00	0.41	tr_D0A0W7_
DRB1_0101	758	GDYGGTCEGEAAKHG	YGGTCEGEA	2	0.2444	3553.2	90.00	0.31	tr_D0A0W7_
DRB1_0101	759	MTPSMSFRDPSEGKL	FRDPSEGKL	6	0.2439	3573.1	90.00	0.56	tr_D0A0W7_
DRB1_0101	760	REDGEGVGKYLPISG	DGEGVGKYL	2	0.2432	3599.4	90.00	0.33	tr_D0A0W7_
DRB1_0101	761	KTAQLFHDVEKDSRL	KTAQLFHDV	0	0.2417	3655.9	90.00	0.37	tr_D0A0W7_
DRB1_0101	762	VICSNEADGIADFLR	ICSNEADGI	1	0.2415	3665.8	90.00	0.41	tr_D0A0W7_
DRB1_0101	763	APSGGEHSLPTGGAV	EHSLPTGGA	5	0.2407	3698.0	90.00	0.38	tr_D0A0W7_
DRB1_0101	764	VVKTSQCYTESSELQ	CYTESSELQ	6	0.2403	3715.0	95.00	0.34	tr_D0A0W7_
DRB1_0101	765	ICSNEADGIADFLRR	EADGIADFL	4	0.2400	3725.9	95.00	0.27	tr_D0A0W7_
DRB1_0101	766	APVALLLLLIVLPQL	PVALLLLLI	1	0.2400	3726.9	95.00	0.32	tr_D0A0W7_
DRB1_0101	767	IREDGEGVGKYLPIS	DGEGVGKYL	3	0.2399	3729.9	95.00	0.41	tr_D0A0W7_
DRB1_0101	768	PLNGLAVFMEDDDTA	PLNGLAVFM	0	0.2389	3770.2	95.00	0.44	tr_D0A0W7_
DRB1_0101	769	RAPVALLLLLIVLPQ	PVALLLLLI	2	0.2364	3874.5	95.00	0.47	tr_D0A0W7_
DRB1_0101	770	QGSKAVYVKEMLENG	YVKEMLENG	6	0.2350	3933.2	95.00	0.34	tr_D0A0W7_
DRB1_0101	771	FIKEQLELETDQNKI	FIKEQLELE	0	0.2348	3941.9	95.00	0.31	tr_D0A0W7_
DRB1_0101	772	AVFMEDDDTASKAAA	FMEDDDTAS	2	0.2330	4020.1	95.00	0.37	tr_D0A0W7_
DRB1_0101	773	CSNEADGIADFLRRS	EADGIADFL	3	0.2320	4060.5	95.00	0.30	tr_D0A0W7_
DRB1_0101	774	SIISDDMRYGVFDDT	IISDDMRYG	1	0.2320	4062.0	95.00	0.34	tr_D0A0W7_
DRB1_0101	775	LVGKGDLGHSDRFFL	LGHSDRFFL	6	0.2316	4082.4	95.00	0.22	tr_D0A0W7_
DRB1_0101	776	PTGGAVCRVAMDTLP	GAVCRVAMD	3	0.2303	4138.5	95.00	0.25	tr_D0A0W7_
DRB1_0101	777	LFHDVEKDSRLWTPL	DVEKDSRLW	3	0.2299	4157.9	95.00	0.34	tr_D0A0W7_
DRB1_0101	778	GLAVFMEDDDTASKA	FMEDDDTAS	4	0.2293	4181.1	95.00	0.40	tr_D0A0W7_
DRB1_0101	779	EERNDLRVIVLFGEF	RNDLRVIVL	2	0.2292	4188.5	95.00	0.20	tr_D0A0W7_
DRB1_0101	780	AQLFHDVEKDSRLWT	DVEKDSRLW	5	0.2286	4214.3	95.00	0.28	tr_D0A0W7_
DRB1_0101	781	GVFDDTKCDTAEKLS	TKCDTAEKL	5	0.2260	4333.1	95.00	0.60	tr_D0A0W7_
DRB1_0101	782	AHVSETNITEADYFL	NITEADYFL	6	0.2244	4411.6	95.00	0.43	tr_D0A0W7_
DRB1_0101	783	SKDDCASNYGATQVS	CASNYGATQ	4	0.2231	4472.3	95.00	0.47	tr_D0A0W7_
DRB1_0101	784	LKAHVSETNITEADY	HVSETNITE	3	0.2230	4477.5	95.00	0.41	tr_D0A0W7_
DRB1_0101	785	LSDRNASLAPSGGEH	NASLAPSGG	4	0.2221	4520.6	95.00	0.35	tr_D0A0W7_

DRB1_0101	786	QLFHDVEKDSRLWTP	DVEKDSRLW	4	0.2176	4745.3	95.00	0.29	tr_D0A0W7_
DRB1_0101	787	FATSLPHWGDTETSS	FATSLPHWG	0	0.2161	4826.0	95.00	0.52	tr_D0A0W7_
DRB1_0101	788	NPMTPSMSFRDPSEG	MTPSMSFRD	2	0.2155	4858.5	95.00	0.63	tr_D0A0W7_
DRB1_0101	789	DDTKCDTAEKLSKDD	TKCDTAEKL	2	0.2147	4900.1	95.00	0.75	tr_D0A0W7_
DRB1_0101	790	TVVKTSQCYTESSEL	VVKTSQCYT	1	0.2137	4954.4	95.00	0.23	tr_D0A0W7_
DRB1_0101	791	LSKDDCASNYGATQV	DDCASNYGA	3	0.2123	5026.2	95.00	0.30	tr_D0A0W7_
DRB1_0101	792	REIARKLEERNDLRV	LEERNDLRV	6	0.2118	5056.7	95.00	0.27	tr_D0A0W7_
DRB1_0101	793	IGDYGGTCEGEAAKH	YGGTCEGEA	3	0.2112	5088.3	95.00	0.50	tr_D0A0W7_
DRB1_0101	794	HWGDTETSSKTAQLF	ETSSKTAQL	5	0.2097	5168.7	95.00	0.29	tr_D0A0W7_
DRB1_0101	795	TCEGEAAKHGATCEC	CEGEAAKHG	1	0.2083	5249.8	95.00	0.60	tr_D0A0W7_
DRB1_0101	796	TVMSSDGEPAPDDEF	VMSSDGEPA	1	0.2083	5250.6	95.00	0.78	tr_D0A0W7_
DRB1_0101	797	VINDIVIGDYGGTCE	NDIVIGDYG	2	0.2071	5318.4	95.00	0.31	tr_D0A0W7_
DRB1_0101	798	TAQLFHDVEKDSRLW	LFHDVEKDS	3	0.2048	5454.5	95.00	0.22	tr_D0A0W7_
DRB1_0101	799	AKHGATCECNQGSKA	CECNQGSKA	6	0.2016	5644.4	95.00	0.46	tr_D0A0W7_
DRB1_0101	800	PAPDDEFREVFERFA	FREVFERFA	6	0.2013	5663.9	95.00	0.47	tr_D0A0W7_
DRB1_0101	801	INDIVIGDYGGTCEG	IVIGDYGGT	3	0.1994	5779.0	95.00	0.26	tr_D0A0W7_
DRB1_0101	802	IISDDMRYGVFDDTK	MRYGVFDDT	5	0.1960	5995.4	95.00	0.28	tr_D0A0W7_
DRB1_0101	803	NGLAVFMEDDDTASK	FMEDDDTAS	5	0.1959	6001.0	95.00	0.37	tr_D0A0W7_
DRB1_0101	804	DTKCDTAEKLSKDDC	TKCDTAEKL	1	0.1939	6132.2	95.00	0.73	tr_D0A0W7_
DRB1_0101	805	KLSKDDCASNYGATQ	DDCASNYGA	4	0.1868	6623.8	100.00	0.29	tr_D0A0W7_
DRB1_0101	806	IAEAANDLRYEQGER	IAEAANDLR	0	0.1868	6624.9	100.00	0.45	tr_D0A0W7_
DRB1_0101	807	YGVFDDTKCDTAEKL	TKCDTAEKL	6	0.1864	6653.8	100.00	0.37	tr_D0A0W7_
DRB1_0101	808	VIGDYGGTCEGEAAK	YGGTCEGEA	4	0.1854	6728.4	100.00	0.47	tr_D0A0W7_
DRB1_0101	809	PMTPSMSFRDPSEGK	MTPSMSFRD	1	0.1851	6747.8	100.00	0.54	tr_D0A0W7_
DRB1_0101	810	KAHVSETNITEADYF	HVSETNITE	2	0.1813	7029.0	100.00	0.46	tr_D0A0W7_
DRB1_0101	811	NDIVIGDYGGTCEGE	IVIGDYGGT	2	0.1775	7328.3	100.00	0.28	tr_D0A0W7_
DRB1_0101	812	DMRYGVFDDTKCDTA	YGVFDDTKC	3	0.1768	7380.4	100.00	0.38	tr_D0A0W7_
DRB1_0101	813	ISDDMRYGVFDDTKC	MRYGVFDDT	4	0.1766	7399.7	100.00	0.37	tr_D0A0W7_
DRB1_0101	814	AMLDTPNITFIDPLE	MLDTPNITF	1	0.1755	7487.8	100.00	0.34	tr_D0A0W7_
DRB1_0101	815	SDDMRYGVFDDTKCD	MRYGVFDDT	3	0.1741	7602.1	100.00	0.37	tr_D0A0W7_
DRB1_0101	816	MRYGVFDDTKCDTAE	YGVFDDTKC	2	0.1709	7868.9	100.00	0.38	tr_D0A0W7_
DRB1_0101	817	VREIARKLEERNDLR	IARKLEERN	3	0.1692	8019.1	100.00	0.37	tr_D0A0W7_
DRB1_0101	818	IVIGDYGGTCEGEAA	YGGTCEGEA	5	0.1676	8153.7	100.00	0.37	tr_D0A0W7_
DRB1_0101	819	LNGLAVFMEDDDTAS	VFMEDDDTA	5	0.1662	8276.8	100.00	0.22	tr_D0A0W7_
DRB1_0101	820	EKLSKDDCASNYGAT	DDCASNYGA	5	0.1649	8395.7	100.00	0.26	tr_D0A0W7_
DRB1_0101	821	DDMRYGVFDDTKCDT	MRYGVFDDT	2	0.1644	8442.4	100.00	0.35	tr_D0A0W7_
DRB1_0101	822	PHWGDTETSSKTAQL	GDTETSSKT	3	0.1594	8914.1	100.00	0.26	tr_D0A0W7_
DRB1_0101	823	CEGEAAKHGATCECN	CEGEAAKHG	0	0.1563	9214.8	100.00	0.40	tr_D0A0W7_
DRB1_0101	824	RYGVFDDTKCDTAEK	FDDTKCDTA	4	0.1549	9358.0	100.00	0.34	tr_D0A0W7_
DRB1_0101	825	DIVIGDYGGTCEGEA	VIGDYGGTC	2	0.1538	9466.1	100.00	0.25	tr_D0A0W7_
DRB1_0101	826	AEKLSKDDCASNYGA	LSKDDCASN	3	0.1448	10433.6	100.00	0.32	tr_D0A0W7_
DRB1_0101	827	TKCDTAEKLSKDDCA	TKCDTAEKL	0	0.1393	11071.0	100.00	0.59	tr_D0A0W7_
DRB1_0101	828	VMSSDGEPAPDDEFR	VMSSDGEPA	0	0.1356	11528.7	100.00	0.61	tr_D0A0W7_
DRB1_0101	829	LPHWGDTETSSKTAQ	GDTETSSKT	4	0.1342	11703.0	100.00	0.25	tr_D0A0W7_
DRB1_0101	830	SLPHWGDTETSSKTA	WGDTETSSK	4	0.1246	12989.2	100.00	0.25	tr_D0A0W7_
DRB1_0101	831	ATSLPHWGDTETSSK	PHWGDTETS	4	0.1188	13823.9	100.00	0.25	tr_D0A0W7_
DRB1_0101	832	EPAPDDEFREVFERF	DEFREVFER	5	0.1161	14241.2	100.00	0.26	tr_D0A0W7_
DRB1_0101	833	AAKHGATCECNQGSK	ATCECNQGS	5	0.1147	14451.4	100.00	0.43	tr_D0A0W7_
DRB1_0101	834	TSLPHWGDTETSSKT	PHWGDTETS	3	0.1128	14760.0	100.00	0.28	tr_D0A0W7_
DRB1_0101	835	TAEKLSKDDCASNYG	LSKDDCASN	4	0.1114	14975.5	100.00	0.36	tr_D0A0W7_

DRB1_0101	836	EGEAAKHGATCECNQ	EAAKHGATC	2	0.1014	16700.3	100.00	0.40	tr_D0A0W7_
DRB1_0101	837	EAAKHGATCECNQGS	ATCECNQGS	6	0.0994	17061.6	100.00	0.22	tr_D0A0W7_
DRB1_0101	838	GEAAKHGATCECNQG	EAAKHGATC	1	0.0973	17448.6	100.00	0.36	tr_D0A0W7_
DRB1_0101	839	DTAEKLSKDDCASNY	LSKDDCASN	5	0.0939	18096.3	100.00	0.33	tr_D0A0W7_
DRB1_0101	840	GEPAPDDEFREVFER	PAPDDEFRE	2	0.0864	19634.6	100.00	0.20	tr_D0A0W7_
DRB1_0101	841	CDTAEKLSKDDCASN	LSKDDCASN	6	0.0725	22827.3	100.00	0.26	tr_D0A0W7_
DRB1_0101	842	DGEPAPDDEFREVFE	PAPDDEFRE	3	0.0685	23831.0	100.00	0.27	tr_D0A0W7_
DRB1_0101	843	SDGEPAPDDEFREVF	PAPDDEFRE	4	0.0668	24262.1	100.00	0.27	tr_D0A0W7_
DRB1_0101	844	MSSDGEPAPDDEFRE	SDGEPAPDD	2	0.0644	24911.2	100.00	0.34	tr_D0A0W7_
DRB1_0101	845	KCDTAEKLSKDDCAS	DTAEKLSKD	2	0.0642	24959.5	100.00	0.22	tr_D0A0W7_
DRB1_0101	846	SSDGEPAPDDEFREV	PAPDDEFRE	5	0.0607	25921.7	100.00	0.28	tr_D0A0W7_

Allele: DRB1_0101. Number of high binders 8. Number of weak binders 120. Number of peptides 846

Allele	pos	peptide	core	Of	1-log50k(aff)	affinity(nM)	%Rank	Relia	Identity	Bind Level
DRB1_0101	1	PDFHSVANAHAIKGY	FHSVANAHA	2	0.8384	5.7	0.90	0.81	tr_C9ZUI4_	WB
DRB1_0101	2	PPDFHSVANAHAIKG	FHSVANAHA	3	0.8348	6.0	1.00	0.84	tr_C9ZUI4_	WB
DRB1_0101	3	DFHSVANAHAIKGYR	FHSVANAHA	1	0.8276	6.5	1.10	0.75	tr_C9ZUI4_	WB
DRB1_0101	4	TLATLIAQYSEHIAV	LIAQYSEHI	4	0.8179	7.2	1.40	0.64	tr_C9ZUI4_	WB
DRB1_0101	5	IPPDFHSVANAHAIK	FHSVANAHA	4	0.8166	7.3	1.50	0.86	tr_C9ZUI4_	WB
DRB1_0101	6	LATLIAQYSEHIAVW	LIAQYSEHI	3	0.8058	8.2	1.80	0.75	tr_C9ZUI4_	WB
DRB1_0101	7	TTLATLIAQYSEHIA	LIAQYSEHI	5	0.8024	8.5	2.00	0.52	tr_C9ZUI4_	WB
DRB1_0101	8	PPGFKLTVVPPAPSA	FKLTVVPPA	3	0.8006	8.6	2.00	0.64	tr_C9ZUI4_	WB
DRB1_0101	9	PGFKLTVVPPAPSAD	FKLTVVPPA	2	0.8004	8.7	2.00	0.60	tr_C9ZUI4_	WB
DRB1_0101	10	GFKLTVVPPAPSADA	FKLTVVPPA	1	0.7954	9.2	2.50	0.50	tr_C9ZUI4_	WB
DRB1_0101	11	FHSVANAHAIKGYRV	FHSVANAHA	0	0.7938	9.3	2.50	0.62	tr_C9ZUI4_	WB
DRB1_0101	12	MEKASLGALGYRVNI	SLGALGYRV	4	0.7842	10.3	3.00	0.81	tr_C9ZUI4_	WB
DRB1_0101	13	EKASLGALGYRVNID	SLGALGYRV	3	0.7752	11.4	3.50	0.86	tr_C9ZUI4_	WB
DRB1_0101	14	ASDFLMESIFNADDA	FLMESIFNA	3	0.7687	12.2	4.00	0.84	tr_C9ZUI4_	WB
DRB1_0101	15	TTTLATLIAQYSEHI	LATLIAQYS	3	0.7654	12.7	4.00	0.68	tr_C9ZUI4_	WB
DRB1_0101	16	ATLIAQYSEHIAVWA	LIAQYSEHI	2	0.7637	12.9	4.00	0.86	tr_C9ZUI4_	WB
DRB1_0101	17	KASLGALGYRVNIDD	SLGALGYRV	2	0.7608	13.3	4.00	0.89	tr_C9ZUI4_	WB
DRB1_0101	18	CDIVRLGRSTLVTTL	VRLGRSTLV	3	0.7581	13.7	4.50	0.82	tr_C9ZUI4_	WB
DRB1_0101	19	AMEKASLGALGYRVN	SLGALGYRV	5	0.7579	13.7	4.50	0.74	tr_C9ZUI4_	WB
DRB1_0101	20	SIPPDFHSVANAHAI	FHSVANAHA	5	0.7575	13.8	4.50	0.87	tr_C9ZUI4_	WB
DRB1_0101	21	IPPGFKLTVVPPAPS	FKLTVVPPA	4	0.7545	14.2	4.50	0.73	tr_C9ZUI4_	WB
DRB1_0101	22	PASDFLMESIFNADD	FLMESIFNA	4	0.7501	14.9	5.00	0.85	tr_C9ZUI4_	WB
DRB1_0101	23	FGLHLYSMVETVRLV	YSMVETVRL	5	0.7501	14.9	5.00	0.53	tr_C9ZUI4_	WB
DRB1_0101	24	GLHLYSMVETVRLVE	YSMVETVRL	4	0.7470	15.5	5.00	0.62	tr_C9ZUI4_	WB
DRB1_0101	25	SDFLMESIFNADDAD	FLMESIFNA	2	0.7462	15.6	5.00	0.84	tr_C9ZUI4_	WB
DRB1_0101	26	FKLTVVPPAPSADAP	LTVVPPAPS	2	0.7447	15.8	5.50	0.52	tr_C9ZUI4_	WB
DRB1_0101	27	VCDIVRLGRSTLVTT	VRLGRSTLV	4	0.7428	16.2	5.50	0.77	tr_C9ZUI4_	WB
DRB1_0101	28	LHLYSMVETVRLVEE	YSMVETVRL	3	0.7410	16.5	5.50	0.66	tr_C9ZUI4_	WB
DRB1_0101	29	TLIAQYSEHIAVWAR	LIAQYSEHI	1	0.7395	16.7	5.50	0.75	tr_C9ZUI4_	WB
DRB1_0101	30	DIVRLGRSTLVTTLQ	VRLGRSTLV	2	0.7393	16.8	5.50	0.85	tr_C9ZUI4_	WB
DRB1_0101	31	APASDFLMESIFNAD	FLMESIFNA	5	0.7391	16.8	5.50	0.85	tr_C9ZUI4_	WB

DRB1_0101	32	CTIFMQFGLHLYSMV	IFMQFGLHL	2	0.7279	19.0	6.50	0.68	tr_C9ZUI4_	WB
DRB1_0101	33	PMELTLAVNTALLGL	LTLAVNTAL	3	0.7272	19.1	6.50	0.51	tr_C9ZUI4_	WB
DRB1_0101	34	MCTIFMQFGLHLYSM	IFMQFGLHL	3	0.7267	19.2	6.50	0.76	tr_C9ZUI4_	WB
DRB1_0101	35	LALNCLTSAYSMSVL	LNCLTSAYS	2	0.7232	20.0	7.00	0.55	tr_C9ZUI4_	WB
DRB1_0101	36	VANAHAIKGYRVIAL	AHAIKGYRV	3	0.7193	20.8	7.50	0.76	tr_C9ZUI4_	WB
DRB1_0101	37	DVGALKQAHAGIAVL	LKQAHAGIA	4	0.7177	21.2	7.50	0.40	tr_C9ZUI4_	WB
DRB1_0101	38	SVCDIVRLGRSTLVT	VRLGRSTLV	5	0.7171	21.3	7.50	0.73	tr_C9ZUI4_	WB
DRB1_0101	39	MMCTIFMQFGLHLYS	IFMQFGLHL	4	0.7167	21.4	7.50	0.78	tr_C9ZUI4_	WB
DRB1_0101	40	MELTLAVNTALLGLV	LTLAVNTAL	2	0.7165	21.5	7.50	0.50	tr_C9ZUI4_	WB
DRB1_0101	41	ANAHAIKGYRVIALA	AHAIKGYRV	2	0.7163	21.5	7.50	0.73	tr_C9ZUI4_	WB
DRB1_0101	42	VGALKQAHAGIAVLN	LKQAHAGIA	3	0.7142	22.0	7.50	0.44	tr_C9ZUI4_	WB
DRB1_0101	43	ALNCLTSAYSMSVLQ	LTSAYSMSV	4	0.7137	22.1	7.50	0.38	tr_C9ZUI4_	WB
DRB1_0101	44	ILALNCLTSAYSMSV	LNCLTSAYS	3	0.7130	22.3	8.00	0.64	tr_C9ZUI4_	WB
DRB1_0101	45	TIFMQFGLHLYSMVE	IFMQFGLHL	1	0.7126	22.4	8.00	0.62	tr_C9ZUI4_	WB
DRB1_0101	46	HLYSMVETVRLVEEA	YSMVETVRL	2	0.7123	22.5	8.00	0.73	tr_C9ZUI4_	WB
DRB1_0101	47	ASLGALGYRVNIDDT	SLGALGYRV	1	0.7119	22.6	8.00	0.89	tr_C9ZUI4_	WB
DRB1_0101	48	PTTTLATLIAQYSEH	LATLIAQYS	4	0.7087	23.4	8.00	0.88	tr_C9ZUI4_	WB
DRB1_0101	49	NDVGALKQAHAGIAV	VGALKQAHA	2	0.7068	23.9	8.50	0.47	tr_C9ZUI4_	WB
DRB1_0101	50	QFGLHLYSMVETVRL	YSMVETVRL	6	0.7062	24.0	8.50	0.41	tr_C9ZUI4_	WB
DRB1_0101	51	LPMELTLAVNTALLG	LTLAVNTAL	4	0.7058	24.1	8.50	0.50	tr_C9ZUI4_	WB
DRB1_0101	52	YKILALNCLTSAYSM	LNCLTSAYS	5	0.7037	24.7	8.50	0.45	tr_C9ZUI4_	WB
DRB1_0101	53	IVRLGRSTLVTTLQM	VRLGRSTLV	1	0.7036	24.7	8.50	0.85	tr_C9ZUI4_	WB
DRB1_0101	54	KGYRVIALAYRPLKE	VIALAYRPL	4	0.7029	24.9	8.50	0.33	tr_C9ZUI4_	WB
DRB1_0101	55	GKYVVAKGSPEAIAQ	YVVAKGSPE	2	0.7022	25.1	9.00	0.50	tr_C9ZUI4_	WB
DRB1_0101	56	KGLLNTLKTVPPKAE	LNTLKTVPP	3	0.7018	25.2	9.00	0.70	tr_C9ZUI4_	WB
DRB1_0101	57	DFLMESIFNADDADM	FLMESIFNA	1	0.7017	25.2	9.00	0.85	tr_C9ZUI4_	WB
DRB1_0101	58	KILALNCLTSAYSMS	LNCLTSAYS	4	0.7008	25.5	9.00	0.60	tr_C9ZUI4_	WB
DRB1_0101	59	KYVVAKGSPEAIAQL	VAKGSPEAI	3	0.6981	26.2	9.00	0.42	tr_C9ZUI4_	WB
DRB1_0101	60	NAHAIKGYRVIALAY	AHAIKGYRV	1	0.6980	26.2	9.00	0.66	tr_C9ZUI4_	WB
DRB1_0101	61	GKGLLNTLKTVPPKA	LNTLKTVPP	4	0.6965	26.7	9.50	0.68	tr_C9ZUI4_	WB
DRB1_0101	62	ELTLAVNTALLGLVK	LTLAVNTAL	1	0.6957	26.9	9.50	0.40	tr_C9ZUI4_	WB
DRB1_0101	63	DAMEKASLGALGYRV	SLGALGYRV	6	0.6953	27.0	9.50	0.56	tr_C9ZUI4_	WB
DRB1_0101	64	MYKILALNCLTSAYS	YKILALNCL	1	0.6945	27.3	9.50	0.34	tr_C9ZUI4_	WB
DRB1_0101	65	VCFLCMSKSQPMPTL	LCMSKSQPM	3	0.6906	28.4	10.00	0.69	tr_C9ZUI4_	
DRB1_0101	66	KIPPGFKLTVVPPAP	FKLTVVPPA	5	0.6899	28.7	10.00	0.84	tr_C9ZUI4_	
DRB1_0101	67	IKGYRVIALAYRPLK	VIALAYRPL	5	0.6896	28.7	10.00	0.31	tr_C9ZUI4_	
DRB1_0101	68	YMMCTIFMQFGLHLY	IFMQFGLHL	5	0.6882	29.2	10.00	0.78	tr_C9ZUI4_	
DRB1_0101	69	GYRVIALAYRPLKEE	VIALAYRPL	3	0.6856	30.0	11.00	0.37	tr_C9ZUI4_	
DRB1_0101	70	LNCLTSAYSMSVLQM	LTSAYSMSV	3	0.6851	30.2	11.00	0.51	tr_C9ZUI4_	
DRB1_0101	71	SVANAHAIKGYRVIA	AHAIKGYRV	4	0.6848	30.3	11.00	0.72	tr_C9ZUI4_	
DRB1_0101	72	ELPMELTLAVNTALL	LTLAVNTAL	5	0.6810	31.5	11.00	0.50	tr_C9ZUI4_	
DRB1_0101	73	NAPASDFLMESIFNA	FLMESIFNA	6	0.6796	32.0	11.00	0.76	tr_C9ZUI4_	
DRB1_0101	74	LQMYKILALNCLTSA	YKILALNCL	3	0.6776	32.7	12.00	0.54	tr_C9ZUI4_	
DRB1_0101	75	TNDVGALKQAHAGIA	VGALKQAHA	3	0.6768	33.0	12.00	0.60	tr_C9ZUI4_	
DRB1_0101	76	DGKYVVAKGSPEAIA	YVVAKGSPE	3	0.6748	33.7	12.00	0.53	tr_C9ZUI4_	
DRB1_0101	77	CFLCMSKSQPMPTLC	LCMSKSQPM	2	0.6745	33.8	12.00	0.68	tr_C9ZUI4_	
DRB1_0101	78	IPTTTLATLIAQYSE	LATLIAQYS	5	0.6739	34.1	12.00	0.85	tr_C9ZUI4_	
DRB1_0101	79	QMYKILALNCLTSAY	YKILALNCL	2	0.6728	34.5	12.00	0.50	tr_C9ZUI4_	
DRB1_0101	80	AHAIKGYRVIALAYR	AHAIKGYRV	0	0.6707	35.3	12.00	0.38	tr_C9ZUI4_	
DRB1_0101	81	GLLNTLKTVPPKAEL	LNTLKTVPP	2	0.6683	36.2	13.00	0.64	tr_C9ZUI4_	

DRB1_0101	82	CHSLLQLEGTDTVAG	LLQLEGTDT	3	0.6627	38.5	13.00	0.67	tr_C9ZUI4_
DRB1_0101	83	LLNTLKTVPPKAELV	LNTLKTVPP	1	0.6622	38.7	14.00	0.50	tr_C9ZUI4_
DRB1_0101	84	TVCFLCMSKSQPMPT	LCMSKSQPM	4	0.6609	39.2	14.00	0.62	tr_C9ZUI4_
DRB1_0101	85	TLQMYKILALNCLTS	YKILALNCL	4	0.6607	39.3	14.00	0.56	tr_C9ZUI4_
DRB1_0101	86	REHMVLMAGDGTNDV	MVLMAGDGT	3	0.6599	39.7	14.00	0.81	tr_C9ZUI4_
DRB1_0101	87	DGKGLLNTLKTVPPK	LNTLKTVPP	5	0.6582	40.4	14.00	0.57	tr_C9ZUI4_
DRB1_0101	88	HSVANAHAIKGYRVI	AHAIKGYRV	5	0.6573	40.8	14.00	0.56	tr_C9ZUI4_
DRB1_0101	89	YRVIALAYRPLKEEE	VIALAYRPL	2	0.6568	41.0	14.00	0.38	tr_C9ZUI4_
DRB1_0101	90	GALKQAHAGIAVLNA	LKQAHAGIA	2	0.6538	42.4	15.00	0.50	tr_C9ZUI4_
DRB1_0101	91	RCVIITGDSVQTAIS	IITGDSVQT	3	0.6534	42.5	15.00	0.52	tr_C9ZUI4_
DRB1_0101	92	IFMQFGLHLYSMVET	IFMQFGLHL	0	0.6526	42.9	15.00	0.44	tr_C9ZUI4_
DRB1_0101	93	LYSMVETVRLVEEAD	YSMVETVRL	1	0.6523	43.0	15.00	0.77	tr_C9ZUI4_
DRB1_0101	94	HSLLQLEGTDTVAGD	LLQLEGTDT	2	0.6503	44.0	15.00	0.69	tr_C9ZUI4_
DRB1_0101	95	PFLANLRRMPCIVSA	LRRMPCIVS	5	0.6498	44.2	15.00	0.59	tr_C9ZUI4_
DRB1_0101	96	HKIPPGFKLTVVPPA	FKLTVVPPA	6	0.6484	44.9	15.00	0.68	tr_C9ZUI4_
DRB1_0101	97	LTLAVNTALLGLVKQ	LAVNTALLG	2	0.6464	45.8	16.00	0.51	tr_C9ZUI4_
DRB1_0101	98	HRCVIITGDSVQTAI	IITGDSVQT	4	0.6455	46.3	16.00	0.48	tr_C9ZUI4_
DRB1_0101	99	TCHSLLQLEGTDTVA	LLQLEGTDT	4	0.6438	47.2	16.00	0.65	tr_C9ZUI4_
DRB1_0101	100	TSVCDIVRLGRSTLV	VRLGRSTLV	6	0.6418	48.2	16.00	0.62	tr_C9ZUI4_
DRB1_0101	101	FLCMSKSQPMPTLCP	LCMSKSQPM	1	0.6394	49.5	17.00	0.65	tr_C9ZUI4_
DRB1_0101	102	TTLQMYKILALNCLT	YKILALNCL	5	0.6390	49.7	17.00	0.56	tr_C9ZUI4_
DRB1_0101	103	ALKQAHAGIAVLNAT	LKQAHAGIA	1	0.6354	51.7	17.00	0.44	tr_C9ZUI4_
DRB1_0101	104	VTTLQMYKILALNCL	YKILALNCL	6	0.6349	52.0	17.00	0.37	tr_C9ZUI4_
DRB1_0101	105	LIAQYSEHIAVWARC	LIAQYSEHI	0	0.6340	52.4	17.00	0.48	tr_C9ZUI4_
DRB1_0101	106	LNTLKTVPPKAELVL	TVPPKAELV	5	0.6333	52.9	17.00	0.28	tr_C9ZUI4_
DRB1_0101	107	QREHMVLMAGDGTND	MVLMAGDGT	4	0.6309	54.2	18.00	0.83	tr_C9ZUI4_
DRB1_0101	108	HAGIAVLNATSMDAS	IAVLNATSM	3	0.6291	55.3	18.00	0.69	tr_C9ZUI4_
DRB1_0101	109	AIKGYRVIALAYRPL	YRVIALAYR	4	0.6287	55.6	18.00	0.29	tr_C9ZUI4_
DRB1_0101	110	FPFLANLRRMPCIVS	LRRMPCIVS	6	0.6284	55.7	18.00	0.36	tr_C9ZUI4_
DRB1_0101	111	PYMMCTIFMQFGLHL	IFMQFGLHL	6	0.6282	55.8	18.00	0.71	tr_C9ZUI4_
DRB1_0101	112	LTVCFLCMSKSQPMP	LCMSKSQPM	5	0.6273	56.4	18.00	0.55	tr_C9ZUI4_
DRB1_0101	113	TIEMLQSGSHRCVII	MLQSGSHRC	3	0.6271	56.5	18.00	0.32	tr_C9ZUI4_
DRB1_0101	114	PELPMELTLAVNTAL	LTLAVNTAL	6	0.6268	56.7	18.00	0.26	tr_C9ZUI4_
DRB1_0101	115	AGIAVLNATSMDASQ	IAVLNATSM	2	0.6260	57.2	18.00	0.64	tr_C9ZUI4_
DRB1_0101	116	EHMVLMAGDGTNDVG	MVLMAGDGT	2	0.6254	57.5	19.00	0.80	tr_C9ZUI4_
DRB1_0101	117	NCLTSAYSMSVLQMD	LTSAYSMSV	2	0.6198	61.2	19.00	0.64	tr_C9ZUI4_
DRB1_0101	118	SHRCVIITGDSVQTA	CVIITGDSV	3	0.6174	62.8	20.00	0.52	tr_C9ZUI4_
DRB1_0101	119	VRFPFLANLRRMPCI	FLANLRRMP	4	0.6170	63.0	20.00	0.41	tr_C9ZUI4_
DRB1_0101	120	KNLIFAGLAVFQCPL	FAGLAVFQC	4	0.6157	64.0	20.00	0.46	tr_C9ZUI4_
DRB1_0101	121	RFPFLANLRRMPCIV	FLANLRRMP	3	0.6156	64.0	20.00	0.47	tr_C9ZUI4_
DRB1_0101	122	ESIPPDFHSVANAHA	FHSVANAHA	6	0.6154	64.2	20.00	0.86	tr_C9ZUI4_
DRB1_0101	123	STLVTTLQMYKILAL	VTTLQMYKI	3	0.6146	64.7	20.00	0.62	tr_C9ZUI4_
DRB1_0101	124	CIQIITAVVPPELPM	ITAVVPPEL	4	0.6144	64.9	20.00	0.64	tr_C9ZUI4_
DRB1_0101	125	SLLQLEGTDTVAGDA	LLQLEGTDT	1	0.6131	65.8	20.00	0.67	tr_C9ZUI4_
DRB1_0101	126	SLGALGYRVNIDDTV	SLGALGYRV	0	0.6127	66.1	20.00	0.76	tr_C9ZUI4_
DRB1_0101	127	MQFGLHLYSMVETVR	HLYSMVETV	5	0.6124	66.3	20.00	0.36	tr_C9ZUI4_
DRB1_0101	128	SIPTTTLATLIAQYS	LATLIAQYS	6	0.6116	66.9	21.00	0.74	tr_C9ZUI4_
DRB1_0101	129	TLVTTLQMYKILALN	VTTLQMYKI	2	0.6107	67.5	21.00	0.55	tr_C9ZUI4_
DRB1_0101	130	TILKCRQQLVASSMK	CRQQLVASS	4	0.6106	67.6	21.00	0.34	tr_C9ZUI4_
DRB1_0101	131	DTIEMLQSGSHRCVI	IEMLQSGSH	2	0.6105	67.7	21.00	0.38	tr_C9ZUI4_

DRB1_0101	132	FLANLRRMPCIVSAP	LRRMPCIVS	4	0.6100	68.0	21.00	0.68	tr_C9ZUI4_
DRB1_0101	133	DRRSILAKTFVQTRR	SILAKTFVQ	3	0.6083	69.2	21.00	0.66	tr_C9ZUI4_
DRB1_0101	134	KQREHMVLMAGDGTN	MVLMAGDGT	5	0.6076	69.8	21.00	0.83	tr_C9ZUI4_
DRB1_0101	135	RRSILAKTFVQTRRH	SILAKTFVQ	2	0.6075	69.9	21.00	0.62	tr_C9ZUI4_
DRB1_0101	136	SMSVLQMDGVKHGES	VLQMDGVKH	3	0.6074	69.9	21.00	0.71	tr_C9ZUI4_
DRB1_0101	137	IQIITAVVPPELPME	ITAVVPPEL	3	0.6070	70.2	21.00	0.75	tr_C9ZUI4_
DRB1_0101	138	LVRFPFLANLRRMPC	FPFLANLRR	3	0.6067	70.5	21.00	0.36	tr_C9ZUI4_
DRB1_0101	139	SVQTAISVGRDVTIL	TAISVGRDV	3	0.6065	70.7	21.00	0.69	tr_C9ZUI4_
DRB1_0101	140	EKNLIFAGLAVFQCP	FAGLAVFQC	5	0.6063	70.8	21.00	0.42	tr_C9ZUI4_
DRB1_0101	141	YVVAKGSPEAIAQLC	VAKGSPEAI	2	0.6057	71.2	22.00	0.54	tr_C9ZUI4_
DRB1_0101	142	CVIITGDSVQTAISV	IITGDSVQT	2	0.6054	71.5	22.00	0.50	tr_C9ZUI4_
DRB1_0101	143	NLIFAGLAVFQCPLK	FAGLAVFQC	3	0.6035	73.0	22.00	0.58	tr_C9ZUI4_
DRB1_0101	144	ACIQIITAVVPPELP	ITAVVPPEL	5	0.6032	73.2	22.00	0.52	tr_C9ZUI4_
DRB1_0101	145	YSMSVLQMDGVKHGE	VLQMDGVKH	4	0.6025	73.7	22.00	0.65	tr_C9ZUI4_
DRB1_0101	146	HMVLMAGDGTNDVGA	MVLMAGDGT	1	0.6021	74.1	22.00	0.75	tr_C9ZUI4_
DRB1_0101	147	ILKCRQQLVASSMKK	CRQQLVASS	3	0.6013	74.7	22.00	0.41	tr_C9ZUI4_
DRB1_0101	148	GTNDVGALKQAHAGI	VGALKQAHA	4	0.5989	76.7	23.00	0.71	tr_C9ZUI4_
DRB1_0101	149	GIAVLNATSMDASQN	IAVLNATSM	1	0.5984	77.1	23.00	0.56	tr_C9ZUI4_
DRB1_0101	150	AHAGIAVLNATSMDA	IAVLNATSM	4	0.5983	77.2	23.00	0.73	tr_C9ZUI4_
DRB1_0101	151	VTILKCRQQLVASSM	ILKCRQQLV	2	0.5968	78.4	23.00	0.37	tr_C9ZUI4_
DRB1_0101	152	NTALLGLVKQNVFCT	LLGLVKQNV	3	0.5966	78.6	23.00	0.41	tr_C9ZUI4_
DRB1_0101	153	NTLKTVPPKAELVLV	TVPPKAELV	4	0.5958	79.3	23.00	0.51	tr_C9ZUI4_
DRB1_0101	154	AYSMSVLQMDGVKHG	VLQMDGVKH	5	0.5956	79.5	23.00	0.55	tr_C9ZUI4_
DRB1_0101	155	FLMESIFNADDADMG	FLMESIFNA	0	0.5932	81.6	24.00	0.81	tr_C9ZUI4_
DRB1_0101	156	PDGKYVVAKGSPEAI	YVVAKGSPE	4	0.5928	82.0	24.00	0.59	tr_C9ZUI4_
DRB1_0101	157	KLFLACIQIITAVVP	CIQIITAVV	5	0.5919	82.7	24.00	0.24	tr_C9ZUI4_
DRB1_0101	158	FMQFGLHLYSMVETV	FGLHLYSMV	3	0.5912	83.4	24.00	0.32	tr_C9ZUI4_
DRB1_0101	159	LVTTLQMYKILALNC	VTTLQMYKI	1	0.5905	84.0	24.00	0.43	tr_C9ZUI4_
DRB1_0101	160	ILVRFPFLANLRRMP	FPFLANLRR	4	0.5902	84.2	24.00	0.37	tr_C9ZUI4_
DRB1_0101	161	WKLFLACIQIITAVV	LFLACIQII	2	0.5898	84.6	24.00	0.24	tr_C9ZUI4_
DRB1_0101	162	TALLGLVKQNVFCTE	LLGLVKQNV	2	0.5883	86.0	24.00	0.34	tr_C9ZUI4_
DRB1_0101	163	RVIALAYRPLKEEER	VIALAYRPL	1	0.5872	87.0	25.00	0.43	tr_C9ZUI4_
DRB1_0101	164	KDTIEMLQSGSHRCV	IEMLQSGSH	3	0.5865	87.7	25.00	0.55	tr_C9ZUI4_
DRB1_0101	165	LKCRQQLVASSMKKK	CRQQLVASS	2	0.5863	87.9	25.00	0.38	tr_C9ZUI4_
DRB1_0101	166	KLTVVPPAPSADAPF	LTVVPPAPS	1	0.5861	88.1	25.00	0.82	tr_C9ZUI4_
DRB1_0101	167	VRLGRSTLVTTLQMY	VRLGRSTLV	0	0.5852	88.9	25.00	0.66	tr_C9ZUI4_
DRB1_0101	168	CEKNLIFAGLAVFQC	FAGLAVFQC	6	0.5850	89.1	25.00	0.32	tr_C9ZUI4_
DRB1_0101	169	TLAVNTALLGLVKQN	LAVNTALLG	1	0.5838	90.3	25.00	0.51	tr_C9ZUI4_
DRB1_0101	170	TLKTVPPKAELVLVT	TVPPKAELV	3	0.5836	90.5	25.00	0.57	tr_C9ZUI4_
DRB1_0101	171	LKQAHAGIAVLNATS	AHAGIAVLN	3	0.5836	90.5	25.00	0.22	tr_C9ZUI4_
DRB1_0101	172	DSVQTAISVGRDVTI	TAISVGRDV	4	0.5832	90.9	25.00	0.64	tr_C9ZUI4_
DRB1_0101	173	ILTVCFLCMSKSQPM	LCMSKSQPM	6	0.5828	91.2	25.00	0.38	tr_C9ZUI4_
DRB1_0101	174	APQVKLGDASIAAPF	VKLGDASIA	3	0.5827	91.4	25.00	0.64	tr_C9ZUI4_
DRB1_0101	175	VQTAISVGRDVTILK	TAISVGRDV	2	0.5826	91.5	25.00	0.67	tr_C9ZUI4_
DRB1_0101	176	VTCHSLLQLEGTDTV	LLQLEGTDT	5	0.5819	92.1	25.00	0.65	tr_C9ZUI4_
DRB1_0101	177	LANLRRMPCIVSAPD	LRRMPCIVS	3	0.5812	92.9	26.00	0.78	tr_C9ZUI4_
DRB1_0101	178	LACIQIITAVVPPEL	ITAVVPPEL	6	0.5809	93.1	26.00	0.38	tr_C9ZUI4_
DRB1_0101	179	QIITAVVPPELPMEL	ITAVVPPEL	2	0.5808	93.2	26.00	0.75	tr_C9ZUI4_
DRB1_0101	180	IEMLQSGSHRCVIIT	MLQSGSHRC	2	0.5789	95.2	26.00	0.37	tr_C9ZUI4_
DRB1_0101	181	QAHAGIAVLNATSMD	IAVLNATSM	5	0.5777	96.5	26.00	0.62	tr_C9ZUI4_

DRB1_0101	182	KILVRFPFLANLRRM	FPFLANLRR	5	0.5770	97.2	26.00	0.41	tr_C9ZUI4_
DRB1_0101	183	RSTLVTTLQMYKILA	VTTLQMYKI	4	0.5770	97.2	26.00	0.64	tr_C9ZUI4_
DRB1_0101	184	LDRRSILAKTFVQTR	SILAKTFVQ	4	0.5759	98.4	26.00	0.64	tr_C9ZUI4_
DRB1_0101	185	PQVKLGDASIAAPFT	VKLGDASIA	2	0.5741	100.3	27.00	0.57	tr_C9ZUI4_
DRB1_0101	186	PPELPMELTLAVNTA	ELPMELTLA	2	0.5727	101.8	27.00	0.33	tr_C9ZUI4_
DRB1_0101	187	HAIKGYRVIALAYRP	YRVIALAYR	5	0.5726	101.9	27.00	0.30	tr_C9ZUI4_
DRB1_0101	188	KQAHAGIAVLNATSM	IAVLNATSM	6	0.5722	102.4	27.00	0.37	tr_C9ZUI4_
DRB1_0101	189	MSVLQMDGVKHGESQ	VLQMDGVKH	2	0.5720	102.6	27.00	0.73	tr_C9ZUI4_
DRB1_0101	190	KCRQQLVASSMKKKG	RQQLVASSM	2	0.5702	104.6	28.00	0.37	tr_C9ZUI4_
DRB1_0101	191	CTEPFRIPYAGKVDT	FRIPYAGKV	4	0.5699	104.9	28.00	0.77	tr_C9ZUI4_
DRB1_0101	192	RWKLFLACIQIITAV	LFLACIQII	3	0.5680	107.2	28.00	0.35	tr_C9ZUI4_
DRB1_0101	193	ADGKGLLNTLKTVPP	LNTLKTVPP	6	0.5654	110.3	28.00	0.41	tr_C9ZUI4_
DRB1_0101	194	DRWKLFLACIQIITA	LFLACIQII	4	0.5648	110.9	29.00	0.32	tr_C9ZUI4_
DRB1_0101	195	GAPQVKLGDASIAAP	VKLGDASIA	4	0.5640	111.9	29.00	0.63	tr_C9ZUI4_
DRB1_0101	196	AVNTALLGLVKQNVF	LLGLVKQNV	5	0.5640	111.9	29.00	0.44	tr_C9ZUI4_
DRB1_0101	197	LIFAGLAVFQCPLKK	FAGLAVFQC	2	0.5635	112.5	29.00	0.58	tr_C9ZUI4_
DRB1_0101	198	GGAPQVKLGDASIAA	VKLGDASIA	5	0.5624	113.8	29.00	0.63	tr_C9ZUI4_
DRB1_0101	199	GDSVQTAISVGRDVT	TAISVGRDV	5	0.5622	114.1	29.00	0.52	tr_C9ZUI4_
DRB1_0101	200	YSMVETVRLVEEADA	YSMVETVRL	0	0.5617	114.7	29.00	0.64	tr_C9ZUI4_
DRB1_0101	201	ANLRRMPCIVSAPDG	LRRMPCIVS	2	0.5616	114.8	29.00	0.78	tr_C9ZUI4_
DRB1_0101	202	VNTALLGLVKQNVFC	LLGLVKQNV	4	0.5606	116.0	29.00	0.44	tr_C9ZUI4_
DRB1_0101	203	YKILVRFPFLANLRR	FPFLANLRR	6	0.5580	119.4	30.00	0.27	tr_C9ZUI4_
DRB1_0101	204	ALLGLVKQNVFCTEP	LVKQNVFCT	4	0.5571	120.5	30.00	0.41	tr_C9ZUI4_
DRB1_0101	205	TEPFRIPYAGKVDTC	FRIPYAGKV	3	0.5565	121.3	30.00	0.77	tr_C9ZUI4_
DRB1_0101	206	KQYKILVRFPFLANL	YKILVRFPF	2	0.5546	123.8	30.00	0.47	tr_C9ZUI4_
DRB1_0101	207	EKQYKILVRFPFLAN	YKILVRFPF	3	0.5543	124.3	30.00	0.56	tr_C9ZUI4_
DRB1_0101	208	FCTEPFRIPYAGKVD	FRIPYAGKV	5	0.5527	126.4	31.00	0.65	tr_C9ZUI4_
DRB1_0101	209	LAVNTALLGLVKQNV	NTALLGLVK	3	0.5519	127.5	31.00	0.32	tr_C9ZUI4_
DRB1_0101	210	DVTILKCRQQLVASS	ILKCRQQLV	3	0.5516	127.9	31.00	0.49	tr_C9ZUI4_
DRB1_0101	211	RSILAKTFVQTRRHK	SILAKTFVQ	1	0.5493	131.1	31.00	0.56	tr_C9ZUI4_
DRB1_0101	212	CLTSAYSMSVLQMDG	LTSAYSMSV	1	0.5492	131.3	31.00	0.59	tr_C9ZUI4_
DRB1_0101	213	IAVLNATSMDASQNV	VLNATSMDA	2	0.5486	132.1	31.00	0.37	tr_C9ZUI4_
DRB1_0101	214	GRSTLVTTLQMYKIL	VTTLQMYKI	5	0.5485	132.3	32.00	0.59	tr_C9ZUI4_
DRB1_0101	215	QVKLGDASIAAPFTC	VKLGDASIA	1	0.5480	133.1	32.00	0.54	tr_C9ZUI4_
DRB1_0101	216	LFLACIQIITAVVPP	CIQIITAVV	4	0.5471	134.3	32.00	0.31	tr_C9ZUI4_
DRB1_0101	217	MDGVKHGESQMILSG	VKHGESQMI	3	0.5459	136.0	32.00	0.81	tr_C9ZUI4_
DRB1_0101	218	FEKQYKILVRFPFLA	YKILVRFPF	4	0.5455	136.7	32.00	0.55	tr_C9ZUI4_
DRB1_0101	219	VIITGDSVQTAISVG	IITGDSVQT	1	0.5418	142.3	33.00	0.51	tr_C9ZUI4_
DRB1_0101	220	QTAISVGRDVTILKC	TAISVGRDV	1	0.5412	143.1	33.00	0.62	tr_C9ZUI4_
DRB1_0101	221	SAYSMSVLQMDGVKH	VLQMDGVKH	6	0.5401	144.9	33.00	0.38	tr_C9ZUI4_
DRB1_0101	222	LKTVPPKAELVLVTC	TVPPKAELV	2	0.5393	146.2	33.00	0.60	tr_C9ZUI4_
DRB1_0101	223	IAQYSEHIAVWARCA	YSEHIAVWA	3	0.5389	146.8	33.00	0.61	tr_C9ZUI4_
DRB1_0101	224	QYKILVRFPFLANLR	YKILVRFPF	1	0.5361	151.2	34.00	0.38	tr_C9ZUI4_
DRB1_0101	225	VPPELPMELTLAVNT	ELPMELTLA	3	0.5360	151.5	34.00	0.47	tr_C9ZUI4_
DRB1_0101	226	GFEKQYKILVRFPFL	YKILVRFPF	5	0.5356	152.1	34.00	0.49	tr_C9ZUI4_
DRB1_0101	227	GSHRCVIITGDSVQT	CVIITGDSV	4	0.5352	152.8	34.00	0.69	tr_C9ZUI4_
DRB1_0101	228	MEQVRHKMAQARRKA	VRHKMAQAR	3	0.5346	153.8	34.00	0.60	tr_C9ZUI4_
DRB1_0101	229	CIVWTDAATGKEVNL	IVWTDAATG	1	0.5323	157.6	35.00	0.57	tr_C9ZUI4_
DRB1_0101	230	DGVKHGESQMILSGI	VKHGESQMI	2	0.5310	160.0	35.00	0.75	tr_C9ZUI4_
DRB1_0101	231	LAKTFVQTRRHKVPS	FVQTRRHKV	4	0.5309	160.1	35.00	0.83	tr_C9ZUI4_

DRB1_0101	232	FSGVDMADGKGLLNT	VDMADGKGL	3	0.5302	161.2	35.00	0.71	tr_C9ZUI4_
DRB1_0101	233	LLGLVKQNVFCTEPF	LVKQNVFCT	3	0.5292	163.0	35.00	0.55	tr_C9ZUI4_
DRB1_0101	234	NLDRRSILAKTFVQT	SILAKTFVQ	5	0.5291	163.1	35.00	0.59	tr_C9ZUI4_
DRB1_0101	235	GDAMEKASLGALGYR	EKASLGALG	4	0.5287	163.9	35.00	0.34	tr_C9ZUI4_
DRB1_0101	236	SGFEKQYKILVRFPF	YKILVRFPF	6	0.5287	164.0	35.00	0.37	tr_C9ZUI4_
DRB1_0101	237	VFCTEPFRIPYAGKV	FRIPYAGKV	6	0.5279	165.4	35.00	0.45	tr_C9ZUI4_
DRB1_0101	238	AKTFVQTRRHKVPSD	FVQTRRHKV	3	0.5274	166.3	36.00	0.86	tr_C9ZUI4_
DRB1_0101	239	DCEKNLIFAGLAVFQ	NLIFAGLAV	4	0.5273	166.4	36.00	0.34	tr_C9ZUI4_
DRB1_0101	240	LCMSKSQPMPTLCPQ	LCMSKSQPM	0	0.5267	167.6	36.00	0.50	tr_C9ZUI4_
DRB1_0101	241	RDRWKLFLACIQIIT	LFLACIQII	5	0.5266	167.6	36.00	0.34	tr_C9ZUI4_
DRB1_0101	242	LFSGVDMADGKGLLN	VDMADGKGL	4	0.5253	170.0	36.00	0.62	tr_C9ZUI4_
DRB1_0101	243	QMDGVKHGESQMILS	VKHGESQMI	4	0.5246	171.4	36.00	0.80	tr_C9ZUI4_
DRB1_0101	244	EPFRIPYAGKVDTCC	FRIPYAGKV	2	0.5242	172.2	36.00	0.81	tr_C9ZUI4_
DRB1_0101	245	DCIVWTDAATGKEVN	IVWTDAATG	2	0.5239	172.6	36.00	0.70	tr_C9ZUI4_
DRB1_0101	246	NLRRMPCIVSAPDGK	LRRMPCIVS	1	0.5238	172.9	36.00	0.75	tr_C9ZUI4_
DRB1_0101	247	IITAVVPPELPMELT	ITAVVPPEL	1	0.5233	173.8	36.00	0.68	tr_C9ZUI4_
DRB1_0101	248	MLFSGVDMADGKGLL	VDMADGKGL	5	0.5226	175.1	36.00	0.47	tr_C9ZUI4_
DRB1_0101	249	GVKHGESQMILSGII	VKHGESQMI	1	0.5226	175.2	36.00	0.64	tr_C9ZUI4_
DRB1_0101	250	DGTNDVGALKQAHAG	VGALKQAHA	5	0.5207	178.7	37.00	0.73	tr_C9ZUI4_
DRB1_0101	251	CRQQLVASSMKKKGN	RQQLVASSM	1	0.5206	178.9	37.00	0.31	tr_C9ZUI4_
DRB1_0101	252	AQYSEHIAVWARCAP	YSEHIAVWA	2	0.5168	186.4	38.00	0.58	tr_C9ZUI4_
DRB1_0101	253	SILAKTFVQTRRHKV	FVQTRRHKV	6	0.5166	186.9	38.00	0.41	tr_C9ZUI4_
DRB1_0101	254	IFAGLAVFQCPLKKD	FAGLAVFQC	1	0.5156	189.0	38.00	0.57	tr_C9ZUI4_
DRB1_0101	255	FMEQVRHKMAQARRK	VRHKMAQAR	4	0.5149	190.3	38.00	0.60	tr_C9ZUI4_
DRB1_0101	256	WDLCVNAESIPTTTL	CVNAESIPT	3	0.5147	190.7	38.00	0.47	tr_C9ZUI4_
DRB1_0101	257	RDVTILKCRQQLVAS	ILKCRQQLV	4	0.5132	193.9	38.00	0.54	tr_C9ZUI4_
DRB1_0101	258	EQVRHKMAQARRKAE	VRHKMAQAR	2	0.5115	197.4	39.00	0.56	tr_C9ZUI4_
DRB1_0101	259	EWDLCVNAESIPTTT	DLCVNAESI	2	0.5109	198.7	39.00	0.38	tr_C9ZUI4_
DRB1_0101	260	AKDTIEMLQSGSHRC	IEMLQSGSH	4	0.5107	199.1	39.00	0.62	tr_C9ZUI4_
DRB1_0101	261	LLQLEGTDTVAGDAM	LLQLEGTDT	0	0.5101	200.6	39.00	0.47	tr_C9ZUI4_
DRB1_0101	262	FLACIQIITAVVPPE	CIQIITAVV	3	0.5098	201.2	39.00	0.37	tr_C9ZUI4_
DRB1_0101	263	SVLQMDGVKHGESQM	VLQMDGVKH	1	0.5093	202.3	39.00	0.80	tr_C9ZUI4_
DRB1_0101	264	TKVFHPYMMCTIFMQ	FHPYMMCTI	3	0.5091	202.7	39.00	0.39	tr_C9ZUI4_
DRB1_0101	265	TGDSVQTAISVGRDV	DSVQTAISV	2	0.5086	203.8	39.00	0.32	tr_C9ZUI4_
DRB1_0101	266	PKDHKIPPGFKLTVV	HKIPPGFKL	3	0.5084	204.2	39.00	0.62	tr_C9ZUI4_
DRB1_0101	267	MVLMAGDGTNDVGAL	MVLMAGDGT	0	0.5072	206.8	40.00	0.65	tr_C9ZUI4_
DRB1_0101	268	ITKVFHPYMMCTIFM	FHPYMMCTI	4	0.5071	207.0	40.00	0.34	tr_C9ZUI4_
DRB1_0101	269	NNSGFEKQYKILVRF	FEKQYKILV	4	0.5069	207.6	40.00	0.40	tr_C9ZUI4_
DRB1_0101	270	KQNVFCTEPFRIPYA	VFCTEPFRI	3	0.5058	209.9	40.00	0.59	tr_C9ZUI4_
DRB1_0101	271	MADGKGLLNTLKTVP	GLLNTLKTV	5	0.5054	210.9	40.00	0.34	tr_C9ZUI4_
DRB1_0101	272	EMLFSGVDMADGKGL	FSGVDMADG	3	0.5037	214.9	40.00	0.40	tr_C9ZUI4_
DRB1_0101	273	SGVDMADGKGLLNTL	VDMADGKGL	2	0.5036	215.0	40.00	0.71	tr_C9ZUI4_
DRB1_0101	274	VIALAYRPLKEEERS	IALAYRPLK	1	0.5025	217.6	41.00	0.38	tr_C9ZUI4_
DRB1_0101	275	EMLQSGSHRCVIITG	MLQSGSHRC	1	0.5010	221.1	41.00	0.41	tr_C9ZUI4_
DRB1_0101	276	ILAKTFVQTRRHKVP	FVQTRRHKV	5	0.5001	223.3	41.00	0.76	tr_C9ZUI4_
DRB1_0101	277	KTFVQTRRHKVPSDD	FVQTRRHKV	2	0.4993	225.3	41.00	0.85	tr_C9ZUI4_
DRB1_0101	278	QYSEHIAVWARCAPT	YSEHIAVWA	1	0.4977	229.2	42.00	0.46	tr_C9ZUI4_
DRB1_0101	279	IITGDSVQTAISVGR	DSVQTAISV	4	0.4965	232.1	42.00	0.41	tr_C9ZUI4_
DRB1_0101	280	DDCIVWTDAATGKEV	IVWTDAATG	3	0.4964	232.6	42.00	0.80	tr_C9ZUI4_
DRB1_0101	281	PFRIPYAGKVDTCCF	FRIPYAGKV	1	0.4963	232.7	42.00	0.73	tr_C9ZUI4_

DRB1_0101	282	DMADGKGLLNTLKTV	GLLNTLKTV	6	0.4961	233.2	42.00	0.26 tr_C9ZUI4_
DRB1_0101	283	VKLGDASIAAPFTCR	DASIAAPFT	4	0.4953	235.2	42.00	0.33 tr_C9ZUI4_
DRB1_0101	284	IVTDLKQREHMVLMA	DLKQREHMV	3	0.4952	235.5	42.00	0.58 tr_C9ZUI4_
DRB1_0101	285	VVPPELPMELTLAVN	ELPMELTLA	4	0.4951	235.9	42.00	0.54 tr_C9ZUI4_
DRB1_0101	286	QNVFCTEPFRIPYAG	VFCTEPFRI	2	0.4949	236.3	42.00	0.51 tr_C9ZUI4_
DRB1_0101	287	DEWDLCVNAESIPTT	DLCVNAESI	3	0.4934	240.2	42.00	0.41 tr_C9ZUI4_
DRB1_0101	288	LGRSTLVTTLQMYKI	VTTLQMYKI	6	0.4920	243.8	43.00	0.38 tr_C9ZUI4_
DRB1_0101	289	KDHKIPPGFKLTVVP	HKIPPGFKL	2	0.4919	244.0	43.00	0.62 tr_C9ZUI4_
DRB1_0101	290	KVFHPYMMCTIFMQF	FHPYMMCTI	2	0.4917	244.5	43.00	0.35 tr_C9ZUI4_
DRB1_0101	291	GVDMADGKGLLNTLK	VDMADGKGL	1	0.4913	245.7	43.00	0.56 tr_C9ZUI4_
DRB1_0101	292	VNLDRRSILAKTFVQ	SILAKTFVQ	6	0.4902	248.6	43.00	0.43 tr_C9ZUI4_
DRB1_0101	293	LTSAYSMSVLQMDGV	AYSMSVLQM	3	0.4879	254.9	43.00	0.31 tr_C9ZUI4_
DRB1_0101	294	VRLVEEADAEGVATM	VEEADAEGV	3	0.4878	255.1	44.00	0.73 tr_C9ZUI4_
DRB1_0101	295	AGDAMEKASLGALGY	DAMEKASLG	2	0.4878	255.2	44.00	0.37 tr_C9ZUI4_
DRB1_0101	296	KTVPPKAELVLVTCH	TVPPKAELV	1	0.4877	255.4	44.00	0.57 tr_C9ZUI4_
DRB1_0101	297	QVRHKMAQARRKAEI	VRHKMAQAR	1	0.4876	255.7	44.00	0.52 tr_C9ZUI4_
DRB1_0101	298	LKQREHMVLMAGDGT	MVLMAGDGT	6	0.4867	258.1	44.00	0.69 tr_C9ZUI4_
DRB1_0101	299	KNNSGFEKQYKILVR	FEKQYKILV	5	0.4863	259.5	44.00	0.35 tr_C9ZUI4_
DRB1_0101	300	NRDRWKLFLACIQII	LFLACIQII	6	0.4861	260.0	44.00	0.30 tr_C9ZUI4_
DRB1_0101	301	KLGDASIAAPFTCRS	DASIAAPFT	3	0.4860	260.1	44.00	0.45 tr_C9ZUI4_
DRB1_0101	302	TCRSRALTSVCDIVR	SRALTSVCD	3	0.4856	261.3	44.00	0.67 tr_C9ZUI4_
DRB1_0101	303	NSGFEKQYKILVRFP	FEKQYKILV	3	0.4850	263.1	44.00	0.37 tr_C9ZUI4_
DRB1_0101	304	VVAKGSPEAIAQLCE	VAKGSPEAI	1	0.4848	263.5	44.00	0.69 tr_C9ZUI4_
DRB1_0101	305	VTDLKQREHMVLMAG	DLKQREHMV	2	0.4836	267.1	44.00	0.54 tr_C9ZUI4_
DRB1_0101	306	RPITKVFHPYMMCTI	ITKVFHPYM	2	0.4832	268.2	44.00	0.31 tr_C9ZUI4_
DRB1_0101	307	PITKVFHPYMMCTIF	FHPYMMCTI	5	0.4821	271.4	45.00	0.33 tr_C9ZUI4_
DRB1_0101	308	LGDASIAAPFTCRSR	SIAAPFTCR	4	0.4819	272.1	45.00	0.34 tr_C9ZUI4_
DRB1_0101	309	CRSRALTSVCDIVRL	SRALTSVCD	2	0.4807	275.6	45.00	0.56 tr_C9ZUI4_
DRB1_0101	310	DLCVNAESIPTTTLA	CVNAESIPT	2	0.4798	278.1	45.00	0.54 tr_C9ZUI4_
DRB1_0101	311	PFMEQVRHKMAQARR	VRHKMAQAR	5	0.4798	278.3	45.00	0.49 tr_C9ZUI4_
DRB1_0101	312	GRDVTILKCRQQLVA	ILKCRQQLV	5	0.4797	278.5	45.00	0.50 tr_C9ZUI4_
DRB1_0101	313	VPKDHKIPPGFKLTV	HKIPPGFKL	4	0.4788	281.2	45.00	0.62 tr_C9ZUI4_
DRB1_0101	314	APDGKYVVAKGSPEA	YVVAKGSPE	5	0.4779	284.1	46.00	0.72 tr_C9ZUI4_
DRB1_0101	315	VKHGESQMILSGIIL	VKHGESQMI	0	0.4765	288.5	46.00	0.34 tr_C9ZUI4_
DRB1_0101	316	TSAYSMSVLQMDGVK	MSVLQMDGV	5	0.4752	292.4	46.00	0.28 tr_C9ZUI4_
DRB1_0101	317	GDASIAAPFTCRSRA	SIAAPFTCR	3	0.4748	293.8	46.00	0.41 tr_C9ZUI4_
DRB1_0101	318	LQMDGVKHGESQMIL	VKHGESQMI	5	0.4734	298.2	47.00	0.78 tr_C9ZUI4_
DRB1_0101	319	SGSHRCVIITGDSVQ	CVIITGDSV	5	0.4722	302.0	47.00	0.80 tr_C9ZUI4_
DRB1_0101	320	NVFCTEPFRIPYAGK	VFCTEPFRI	1	0.4720	302.7	47.00	0.39 tr_C9ZUI4_
DRB1_0101	321	VLQMDGVKHGESQMI	VLQMDGVKH	0	0.4720	302.8	47.00	0.44 tr_C9ZUI4_
DRB1_0101	322	TDEMLFSGVDMADGK	MLFSGVDMA	3	0.4716	303.9	47.00	0.40 tr_C9ZUI4_
DRB1_0101	323	KAELVLVTCHSLLQL	LVLVTCHSL	3	0.4710	306.2	47.00	0.38 tr_C9ZUI4_
DRB1_0101	324	TVRLVEEADAEGVAT	VEEADAEGV	4	0.4696	310.9	47.00	0.65 tr_C9ZUI4_
DRB1_0101	325	DAKDTIEMLQSGSHR	IEMLQSGSH	5	0.4695	310.9	47.00	0.71 tr_C9ZUI4_
DRB1_0101	326	AVVPPELPMELTLAV	ELPMELTLA	5	0.4679	316.3	48.00	0.52 tr_C9ZUI4_
DRB1_0101	327	DEMLFSGVDMADGKG	FSGVDMADG	4	0.4673	318.5	48.00	0.38 tr_C9ZUI4_
DRB1_0101	328	RSRALTSVCDIVRLG	SRALTSVCD	1	0.4667	320.5	48.00	0.53 tr_C9ZUI4_
DRB1_0101	329	FAGLAVFQCPLKKDA	FAGLAVFQC	0	0.4666	320.9	48.00	0.38 tr_C9ZUI4_
DRB1_0101	330	VKQNVFCTEPFRIPY	VFCTEPFRI	4	0.4666	321.1	48.00	0.62 tr_C9ZUI4_
DRB1_0101	331	GLVKQNVFCTEPFRI	LVKQNVFCT	1	0.4665	321.2	48.00	0.46 tr_C9ZUI4_

DRB1_0101	332	LVTCHSLLQLEGTDT	LLQLEGTDT	6	0.4665	321.3	48.00	0.54	tr_C9ZUI4_
DRB1_0101	333	LTVVPPAPSADAPFM	LTVVPPAPS	0	0.4665	321.4	48.00	0.69	tr_C9ZUI4_
DRB1_0101	334	MGGAPQVKLGDASIA	VKLGDASIA	6	0.4658	323.8	48.00	0.47	tr_C9ZUI4_
DRB1_0101	335	EDCEKNLIFAGLAVF	NLIFAGLAV	5	0.4656	324.4	48.00	0.35	tr_C9ZUI4_
DRB1_0101	336	IVWTDAATGKEVNLD	TDAATGKEV	3	0.4654	325.1	48.00	0.45	tr_C9ZUI4_
DRB1_0101	337	RQQLVASSMKKKGNG	LVASSMKKK	3	0.4644	328.8	48.00	0.40	tr_C9ZUI4_
DRB1_0101	338	GVATMRQAGAEGEFK	VATMRQAGA	1	0.4640	330.0	49.00	0.41	tr_C9ZUI4_
DRB1_0101	339	RLVEEADAEGVATMR	VEEADAEGV	2	0.4638	330.8	49.00	0.76	tr_C9ZUI4_
DRB1_0101	340	FTCRSRALTSVCDIV	SRALTSVCD	4	0.4622	336.7	49.00	0.59	tr_C9ZUI4_
DRB1_0101	341	LGLVKQNVFCTEPFR	LVKQNVFCT	2	0.4616	338.7	49.00	0.63	tr_C9ZUI4_
DRB1_0101	342	VDDCIVWTDAATGKE	IVWTDAATG	4	0.4597	345.7	49.00	0.87	tr_C9ZUI4_
DRB1_0101	343	DIVTDLKQREHMVLM	DLKQREHMV	4	0.4594	346.9	50.00	0.56	tr_C9ZUI4_
DRB1_0101	344	DASIAAPFTCRSRAL	SIAAPFTCR	2	0.4591	347.9	50.00	0.43	tr_C9ZUI4_
DRB1_0101	345	PFTCRSRALTSVCDI	SRALTSVCD	5	0.4582	351.4	50.00	0.47	tr_C9ZUI4_
DRB1_0101	346	EGVATMRQAGAEGEF	VATMRQAGA	2	0.4570	356.1	50.00	0.62	tr_C9ZUI4_
DRB1_0101	347	AELVLVTCHSLLQLE	LVLVTCHSL	2	0.4559	360.4	50.00	0.31	tr_C9ZUI4_
DRB1_0101	348	PKAELVLVTCHSLLQ	LVLVTCHSL	4	0.4545	365.7	55.00	0.38	tr_C9ZUI4_
DRB1_0101	349	VAGDAMEKASLGALG	DAMEKASLG	3	0.4520	375.9	55.00	0.57	tr_C9ZUI4_
DRB1_0101	350	VFHPYMMCTIFMQFG	PYMMCTIFM	3	0.4520	376.1	55.00	0.37	tr_C9ZUI4_
DRB1_0101	351	AEGVATMRQAGAEGE	VATMRQAGA	3	0.4518	376.7	55.00	0.76	tr_C9ZUI4_
DRB1_0101	352	DDEWDLCVNAESIPT	DLCVNAESI	4	0.4496	385.7	55.00	0.47	tr_C9ZUI4_
DRB1_0101	353	YSEHIAVWARCAPTH	HIAVWARCA	3	0.4495	386.2	55.00	0.42	tr_C9ZUI4_
DRB1_0101	354	VDMADGKGLLNTLKT	VDMADGKGL	0	0.4495	386.3	55.00	0.33	tr_C9ZUI4_
DRB1_0101	355	HGESQMILSGIILTV	QMILSGIIL	4	0.4494	386.5	55.00	0.27	tr_C9ZUI4_
DRB1_0101	356	ITGDSVQTAISVGRD	DSVQTAISV	3	0.4487	389.4	55.00	0.50	tr_C9ZUI4_
DRB1_0101	357	EHIAVWARCAPTHKE	WARCAPTHK	5	0.4486	389.9	55.00	0.28	tr_C9ZUI4_
DRB1_0101	358	PPKAELVLVTCHSLL	LVLVTCHSL	5	0.4481	391.9	55.00	0.30	tr_C9ZUI4_
DRB1_0101	359	LRRMPCIVSAPDGKY	LRRMPCIVS	0	0.4480	392.4	55.00	0.54	tr_C9ZUI4_
DRB1_0101	360	LVKQNVFCTEPFRIP	VFCTEPFRI	5	0.4480	392.4	55.00	0.50	tr_C9ZUI4_
DRB1_0101	361	TDLKQREHMVLMAGD	DLKQREHMV	1	0.4468	397.6	55.00	0.50	tr_C9ZUI4_
DRB1_0101	362	ELVLVTCHSLLQLEG	LVLVTCHSL	1	0.4451	404.9	55.00	0.23	tr_C9ZUI4_
DRB1_0101	363	TAISVGRDVTILKCR	TAISVGRDV	0	0.4434	412.4	55.00	0.44	tr_C9ZUI4_
DRB1_0101	364	ASIAAPFTCRSRALT	SIAAPFTCR	1	0.4413	422.1	55.00	0.38	tr_C9ZUI4_
DRB1_0101	365	SEHIAVWARCAPTHK	HIAVWARCA	2	0.4376	439.3	55.00	0.40	tr_C9ZUI4_
DRB1_0101	366	GESQMILSGIILTVC	QMILSGIIL	3	0.4368	443.0	55.00	0.29	tr_C9ZUI4_
DRB1_0101	367	ITAVVPPELPMELTL	ITAVVPPEL	0	0.4364	444.9	55.00	0.48	tr_C9ZUI4_
DRB1_0101	368	IILTVCFLCMSKSQP	VCFLCMSKS	4	0.4351	451.4	55.00	0.39	tr_C9ZUI4_
DRB1_0101	369	QRPITKVFHPYMMCT	ITKVFHPYM	3	0.4349	452.1	55.00	0.46	tr_C9ZUI4_
DRB1_0101	370	AVLNATSMDASQNVG	VLNATSMDA	1	0.4349	452.2	55.00	0.48	tr_C9ZUI4_
DRB1_0101	371	KHGESQMILSGIILT	QMILSGIIL	5	0.4348	452.8	55.00	0.30	tr_C9ZUI4_
DRB1_0101	372	AGKVVSQPEMNAPAS	VVSQPEMNA	3	0.4332	460.6	55.00	0.71	tr_C9ZUI4_
DRB1_0101	373	DHKIPPGFKLTVVPP	HKIPPGFKL	1	0.4331	460.9	55.00	0.66	tr_C9ZUI4_
DRB1_0101	374	TTDEMLFSGVDMADG	MLFSGVDMA	4	0.4326	463.4	55.00	0.41	tr_C9ZUI4_
DRB1_0101	375	GALGYRVNIDDTVVY	YRVNIDDTV	4	0.4308	473.0	60.00	0.71	tr_C9ZUI4_
DRB1_0101	376	ETVRLVEEADAEGVA	VEEADAEGV	5	0.4307	473.5	60.00	0.57	tr_C9ZUI4_
DRB1_0101	377	EVDDCIVWTDAATGK	IVWTDAATG	5	0.4303	475.4	60.00	0.86	tr_C9ZUI4_
DRB1_0101	378	ALGYRVNIDDTVVYD	YRVNIDDTV	3	0.4301	476.3	60.00	0.75	tr_C9ZUI4_
DRB1_0101	379	APFMEQVRHKMAQAR	FMEQVRHKM	2	0.4284	485.1	60.00	0.32	tr_C9ZUI4_
DRB1_0101	380	TVAGDAMEKASLGAL	DAMEKASLG	4	0.4278	488.5	60.00	0.56	tr_C9ZUI4_
DRB1_0101	381	DAEGVATMRQAGAEG	VATMRQAGA	4	0.4276	489.4	60.00	0.83	tr_C9ZUI4_

DRB1_0101	382	TFVQTRRHKVPSDDE	FVQTRRHKV	1	0.4269	493.2	60.00	0.83	tr_C9ZUI4_
DRB1_0101	383	ESQMILSGIILTVCF	QMILSGIIL	2	0.4266	495.0	60.00	0.28	tr_C9ZUI4_
DRB1_0101	384	DVPKDHKIPPGFKLT	HKIPPGFKL	5	0.4265	495.1	60.00	0.60	tr_C9ZUI4_
DRB1_0101	385	FHPYMMCTIFMQFGL	PYMMCTIFM	2	0.4213	523.8	60.00	0.40	tr_C9ZUI4_
DRB1_0101	386	LVLVTCHSLLQLEGT	VLVTCHSLL	1	0.4210	525.4	60.00	0.19	tr_C9ZUI4_
DRB1_0101	387	AGLAVFQCPLKKDAK	AVFQCPLKK	3	0.4200	531.5	60.00	0.38	tr_C9ZUI4_
DRB1_0101	388	TAVVPPELPMELTLA	ELPMELTLA	6	0.4185	539.9	60.00	0.42	tr_C9ZUI4_
DRB1_0101	389	KEVNLDRRSILAKTF	NLDRRSILA	3	0.4182	541.6	60.00	0.38	tr_C9ZUI4_
DRB1_0101	390	LTSVCDIVRLGRSTL	IVRLGRSTL	6	0.4172	547.6	60.00	0.37	tr_C9ZUI4_
DRB1_0101	391	HIAVWARCAPTHKED	WARCAPTHK	4	0.4167	550.8	60.00	0.34	tr_C9ZUI4_
DRB1_0101	392	RKNNSGFEKQYKILV	SGFEKQYKI	4	0.4166	551.4	60.00	0.31	tr_C9ZUI4_
DRB1_0101	393	APFTCRSRALTSVCD	FTCRSRALT	2	0.4155	557.7	60.00	0.31	tr_C9ZUI4_
DRB1_0101	394	PNRDRWKLFLACIQI	WKLFLACIQ	5	0.4155	557.9	60.00	0.26	tr_C9ZUI4_
DRB1_0101	395	SQMILSGIILTVCFL	ILSGIILTV	3	0.4153	559.4	60.00	0.26	tr_C9ZUI4_
DRB1_0101	396	PQRPITKVFHPYMMC	ITKVFHPYM	4	0.4148	562.4	60.00	0.49	tr_C9ZUI4_
DRB1_0101	397	RLGRSTLVTTLQMYK	GRSTLVTTL	2	0.4139	567.3	60.00	0.32	tr_C9ZUI4_
DRB1_0101	398	TVPPKAELVLVTCHS	PKAELVLVT	3	0.4137	569.2	60.00	0.34	tr_C9ZUI4_
DRB1_0101	399	HPYMMCTIFMQFGLH	PYMMCTIFM	1	0.4127	575.3	60.00	0.40	tr_C9ZUI4_
DRB1_0101	400	SIAAPFTCRSRALTS	FTCRSRALT	5	0.4126	575.8	60.00	0.34	tr_C9ZUI4_
DRB1_0101	401	EVNLDRRSILAKTFV	NLDRRSILA	2	0.4121	578.8	60.00	0.34	tr_C9ZUI4_
DRB1_0101	402	ESIPTTTLATLIAQY	PTTTLATLI	3	0.4093	596.8	60.00	0.60	tr_C9ZUI4_
DRB1_0101	403	MLQSGSHRCVIITGD	LQSGSHRCV	1	0.4089	599.4	60.00	0.47	tr_C9ZUI4_
DRB1_0101	404	EDIVTDLKQREHMVL	DLKQREHMV	5	0.4086	600.9	60.00	0.59	tr_C9ZUI4_
DRB1_0101	405	GKEVNLDRRSILAKT	NLDRRSILA	4	0.4083	603.1	60.00	0.38	tr_C9ZUI4_
DRB1_0101	406	MPTLCPQRPITKVFH	LCPQRPITK	3	0.4063	616.2	65.00	0.55	tr_C9ZUI4_
DRB1_0101	407	GKVVSQPEMNAPASD	VVSQPEMNA	2	0.4063	616.3	65.00	0.71	tr_C9ZUI4_
DRB1_0101	408	PCIVSAPDGKYVVAK	VSAPDGKYV	3	0.4058	619.4	65.00	0.38	tr_C9ZUI4_
DRB1_0101	409	MPCIVSAPDGKYVVA	IVSAPDGKY	3	0.4053	623.0	65.00	0.35	tr_C9ZUI4_
DRB1_0101	410	LGYRVNIDDTVVYDP	YRVNIDDTV	2	0.4051	624.2	65.00	0.75	tr_C9ZUI4_
DRB1_0101	411	EAGKVVSQPEMNAPA	VVSQPEMNA	4	0.4041	630.9	65.00	0.74	tr_C9ZUI4_
DRB1_0101	412	LCVNAESIPTTTLAT	CVNAESIPT	1	0.4023	643.2	65.00	0.62	tr_C9ZUI4_
DRB1_0101	413	LVEEADAEGVATMRQ	VEEADAEGV	1	0.4022	644.5	65.00	0.67	tr_C9ZUI4_
DRB1_0101	414	KKSRETAEAGKVVSQ	ETAEAGKVV	4	0.4020	645.7	65.00	0.67	tr_C9ZUI4_
DRB1_0101	415	VLNATSMDASQNVGG	TSMDASQNV	4	0.4007	654.5	65.00	0.44	tr_C9ZUI4_
DRB1_0101	416	VATMRQAGAEGEFKP	MRQAGAEGE	3	0.4005	656.2	65.00	0.44	tr_C9ZUI4_
DRB1_0101	417	SRALTSVCDIVRLGR	SRALTSVCD	0	0.4002	658.1	65.00	0.34	tr_C9ZUI4_
DRB1_0101	418	IALAYRPLKEEERSK	YRPLKEEER	4	0.3995	663.0	65.00	0.37	tr_C9ZUI4_
DRB1_0101	419	IAAPFTCRSRALTSV	FTCRSRALT	4	0.3973	679.1	65.00	0.41	tr_C9ZUI4_
DRB1_0101	420	VRHKMAQARRKAEIV	HKMAQARRK	2	0.3970	681.2	65.00	0.34	tr_C9ZUI4_
DRB1_0101	421	TGKEVNLDRRSILAK	NLDRRSILA	5	0.3968	683.1	65.00	0.34	tr_C9ZUI4_
DRB1_0101	422	GLAVFQCPLKKDAKD	AVFQCPLKK	2	0.3966	684.8	65.00	0.37	tr_C9ZUI4_
DRB1_0101	423	GDGTNDVGALKQAHA	VGALKQAHA	6	0.3959	689.7	65.00	0.69	tr_C9ZUI4_
DRB1_0101	424	VPPKAELVLVTCHSL	PKAELVLVT	2	0.3958	690.7	65.00	0.37	tr_C9ZUI4_
DRB1_0101	425	LGALGYRVNIDDTVV	YRVNIDDTV	5	0.3947	699.0	65.00	0.64	tr_C9ZUI4_
DRB1_0101	426	GIILTVCFLCMSKSQ	VCFLCMSKS	5	0.3945	700.3	65.00	0.47	tr_C9ZUI4_
DRB1_0101	427	PMPTLCPQRPITKVF	LCPQRPITK	4	0.3943	701.5	65.00	0.53	tr_C9ZUI4_
DRB1_0101	428	VGRDVTILKCRQQLV	ILKCRQQLV	6	0.3929	712.4	65.00	0.47	tr_C9ZUI4_
DRB1_0101	429	CPQRPITKVFHPYMM	ITKVFHPYM	5	0.3927	714.1	65.00	0.47	tr_C9ZUI4_
DRB1_0101	430	LTTDEMLFSGVDMAD	MLFSGVDMA	5	0.3917	721.9	65.00	0.43	tr_C9ZUI4_
DRB1_0101	431	IAVWARCAPTHKEDI	WARCAPTHK	3	0.3896	738.4	65.00	0.48	tr_C9ZUI4_

DRB1_0101	432	AAPFTCRSRALTSVC	FTCRSRALT	3	0.3895	739.2	65.00	0.41	tr_C9ZUI4_
DRB1_0101	433	SDDEWDLCVNAESIP	DLCVNAESI	5	0.3890	743.4	65.00	0.47	tr_C9ZUI4_
DRB1_0101	434	FRIPYAGKVDTCCFD	FRIPYAGKV	0	0.3880	751.4	65.00	0.63	tr_C9ZUI4_
DRB1_0101	435	ADAEGVATMRQAGAE	VATMRQAGA	5	0.3872	757.7	65.00	0.77	tr_C9ZUI4_
DRB1_0101	436	REDCEKNLIFAGLAV	NLIFAGLAV	6	0.3871	758.6	65.00	0.34	tr_C9ZUI4_
DRB1_0101	437	QSGSHRCVIITGDSV	CVIITGDSV	6	0.3865	763.4	65.00	0.68	tr_C9ZUI4_
DRB1_0101	438	RKAEIVQIARWNKQL	VQIARWNKQ	5	0.3864	764.5	65.00	0.29	tr_C9ZUI4_
DRB1_0101	439	MNAPASDFLMESIFN	NAPASDFLM	1	0.3860	768.0	65.00	0.49	tr_C9ZUI4_
DRB1_0101	440	RMPCIVSAPDGKYVV	IVSAPDGKY	4	0.3849	777.2	65.00	0.33	tr_C9ZUI4_
DRB1_0101	441	DLKQREHMVLMAGDG	DLKQREHMV	0	0.3846	779.6	65.00	0.38	tr_C9ZUI4_
DRB1_0101	442	KAEIVQIARWNKQLE	VQIARWNKQ	4	0.3835	788.9	70.00	0.36	tr_C9ZUI4_
DRB1_0101	443	KSRETAEAGKVVSQP	ETAEAGKVV	3	0.3812	808.5	70.00	0.70	tr_C9ZUI4_
DRB1_0101	444	LNATSMDASQNVGGS	TSMDASQNV	3	0.3806	813.4	70.00	0.54	tr_C9ZUI4_
DRB1_0101	445	DTVAGDAMEKASLGA	DAMEKASLG	5	0.3803	816.1	70.00	0.49	tr_C9ZUI4_
DRB1_0101	446	CMSKSQPMPTLCPQR	SQPMPTLCP	4	0.3795	823.5	70.00	0.45	tr_C9ZUI4_
DRB1_0101	447	DPNRDRWKLFLACIQ	RWKLFLACI	5	0.3794	824.6	70.00	0.25	tr_C9ZUI4_
DRB1_0101	448	AESIPTTTLATLIAQ	PTTTLATLI	4	0.3788	830.1	70.00	0.56	tr_C9ZUI4_
DRB1_0101	449	CIVSAPDGKYVVAKG	VSAPDGKYV	2	0.3733	880.3	70.00	0.43	tr_C9ZUI4_
DRB1_0101	450	PEMNAPASDFLMESI	NAPASDFLM	3	0.3708	904.9	70.00	0.67	tr_C9ZUI4_
DRB1_0101	451	VLVTCHSLLQLEGTD	HSLLQLEGT	5	0.3705	907.5	70.00	0.25	tr_C9ZUI4_
DRB1_0101	452	TLTTDEMLFSGVDMA	MLFSGVDMA	6	0.3702	910.4	70.00	0.28	tr_C9ZUI4_
DRB1_0101	453	KMAQARRKAEIVQIA	QARRKAEIV	3	0.3702	910.8	70.00	0.55	tr_C9ZUI4_
DRB1_0101	454	QQLVASSMKKKGNGE	LVASSMKKK	2	0.3690	922.5	70.00	0.53	tr_C9ZUI4_
DRB1_0101	455	VAKGSPEAIAQLCES	VAKGSPEAI	0	0.3689	924.0	70.00	0.55	tr_C9ZUI4_
DRB1_0101	456	AQARRKAEIVQIARW	QARRKAEIV	1	0.3688	924.5	70.00	0.34	tr_C9ZUI4_
DRB1_0101	457	EMNAPASDFLMESIF	NAPASDFLM	2	0.3674	938.9	70.00	0.64	tr_C9ZUI4_
DRB1_0101	458	NAESIPTTTLATLIA	PTTTLATLI	5	0.3667	945.7	70.00	0.52	tr_C9ZUI4_
DRB1_0101	459	SGIILTVCFLCMSKS	VCFLCMSKS	6	0.3666	946.6	70.00	0.30	tr_C9ZUI4_
DRB1_0101	460	KVVSQPEMNAPASDF	VVSQPEMNA	1	0.3655	958.8	70.00	0.63	tr_C9ZUI4_
DRB1_0101	461	MAQARRKAEIVQIAR	QARRKAEIV	2	0.3645	968.4	70.00	0.49	tr_C9ZUI4_
DRB1_0101	462	DAPFMEQVRHKMAQA	FMEQVRHKM	3	0.3640	974.5	70.00	0.46	tr_C9ZUI4_
DRB1_0101	463	LAVFQCPLKKDAKDT	AVFQCPLKK	1	0.3639	975.0	70.00	0.38	tr_C9ZUI4_
DRB1_0101	464	SKKSRETAEAGKVVS	ETAEAGKVV	5	0.3638	975.8	70.00	0.60	tr_C9ZUI4_
DRB1_0101	465	QPEMNAPASDFLMES	NAPASDFLM	4	0.3626	989.3	70.00	0.62	tr_C9ZUI4_
DRB1_0101	466	PTLCPQRPITKVFHP	LCPQRPITK	2	0.3624	991.0	70.00	0.59	tr_C9ZUI4_
DRB1_0101	467	QPMPTLCPQRPITKV	LCPQRPITK	5	0.3618	997.8	70.00	0.44	tr_C9ZUI4_
DRB1_0101	468	AVWARCAPTHKEDIV	WARCAPTHK	2	0.3601	1015.9	70.00	0.47	tr_C9ZUI4_
DRB1_0101	469	GYRVNIDDTVVYDPP	YRVNIDDTV	1	0.3589	1029.2	75.00	0.74	tr_C9ZUI4_
DRB1_0101	470	AEIVQIARWNKQLEE	VQIARWNKQ	3	0.3574	1045.6	75.00	0.41	tr_C9ZUI4_
DRB1_0101	471	RRKAEIVQIARWNKQ	AEIVQIARW	3	0.3571	1049.2	75.00	0.20	tr_C9ZUI4_
DRB1_0101	472	KDAKDTIEMLQSGSH	IEMLQSGSH	6	0.3561	1060.4	75.00	0.57	tr_C9ZUI4_
DRB1_0101	473	NATSMDASQNVGGSG	TSMDASQNV	2	0.3534	1092.2	75.00	0.54	tr_C9ZUI4_
DRB1_0101	474	HKMAQARRKAEIVQI	QARRKAEIV	4	0.3531	1096.0	75.00	0.49	tr_C9ZUI4_
DRB1_0101	475	QMILSGIILTVCFLC	ILSGIILTV	2	0.3524	1103.8	75.00	0.29	tr_C9ZUI4_
DRB1_0101	476	RHKMAQARRKAEIVQ	HKMAQARRK	1	0.3518	1111.4	75.00	0.34	tr_C9ZUI4_
DRB1_0101	477	QARRKAEIVQIARWN	RRKAEIVQI	2	0.3477	1161.4	75.00	0.27	tr_C9ZUI4_
DRB1_0101	478	PSDDEWDLCVNAESI	WDLCVNAES	5	0.3476	1163.1	75.00	0.40	tr_C9ZUI4_
DRB1_0101	479	VWTDAATGKEVNLDR	TDAATGKEV	2	0.3461	1182.6	75.00	0.75	tr_C9ZUI4_
DRB1_0101	480	SAPDGKYVVAKGSPE	YVVAKGSPE	6	0.3438	1211.8	75.00	0.61	tr_C9ZUI4_
DRB1_0101	481	VETVRLVEEADAEGV	VRLVEEADA	3	0.3434	1216.6	75.00	0.44	tr_C9ZUI4_

DRB1_0101	482	RRMPCIVSAPDGKYV	IVSAPDGKY	5	0.3407	1253.1	75.00	0.32	tr_C9ZUI4_
DRB1_0101	483	TDTVAGDAMEKASLG	DAMEKASLG	6	0.3400	1263.1	75.00	0.28	tr_C9ZUI4_
DRB1_0101	484	AEAGKVVSQPEMNAP	VVSQPEMNA	5	0.3391	1275.7	75.00	0.71	tr_C9ZUI4_
DRB1_0101	485	IARWNKQLEESKKSR	WNKQLEESK	3	0.3381	1289.0	75.00	0.68	tr_C9ZUI4_
DRB1_0101	486	ARRKAEIVQIARWNK	AEIVQIARW	4	0.3381	1289.1	75.00	0.20	tr_C9ZUI4_
DRB1_0101	487	MSKSQPMPTLCPQRP	SQPMPTLCP	3	0.3369	1305.8	75.00	0.51	tr_C9ZUI4_
DRB1_0101	488	ATGKEVNLDRRSILA	VNLDRRSIL	5	0.3349	1334.4	75.00	0.37	tr_C9ZUI4_
DRB1_0101	489	SRETAEAGKVVSQPE	ETAEAGKVV	2	0.3345	1340.9	75.00	0.69	tr_C9ZUI4_
DRB1_0101	490	SQPEMNAPASDFLME	NAPASDFLM	5	0.3341	1345.9	80.00	0.53	tr_C9ZUI4_
DRB1_0101	491	PDVPKDHKIPPGFKL	HKIPPGFKL	6	0.3327	1367.2	80.00	0.56	tr_C9ZUI4_
DRB1_0101	492	RALTSVCDIVRLGRS	TSVCDIVRL	3	0.3315	1385.1	80.00	0.25	tr_C9ZUI4_
DRB1_0101	493	LCPQRPITKVFHPYM	ITKVFHPYM	6	0.3308	1394.7	80.00	0.38	tr_C9ZUI4_
DRB1_0101	494	CVNAESIPTTTLATL	CVNAESIPT	0	0.3275	1444.9	80.00	0.38	tr_C9ZUI4_
DRB1_0101	495	MILSGIILTVCFLCM	GIILTVCFL	4	0.3264	1463.4	80.00	0.22	tr_C9ZUI4_
DRB1_0101	496	KEDIVTDLKQREHMV	DLKQREHMV	6	0.3231	1515.5	80.00	0.44	tr_C9ZUI4_
DRB1_0101	497	SQPMPTLCPQRPITK	LCPQRPITK	6	0.3228	1521.4	80.00	0.25	tr_C9ZUI4_
DRB1_0101	498	NRKNNSGFEKQYKIL	SGFEKQYKI	5	0.3224	1528.4	80.00	0.41	tr_C9ZUI4_
DRB1_0101	499	ARWNKQLEESKKSRE	WNKQLEESK	2	0.3215	1542.2	80.00	0.63	tr_C9ZUI4_
DRB1_0101	500	ALTSVCDIVRLGRST	CDIVRLGRS	5	0.3206	1557.9	80.00	0.26	tr_C9ZUI4_
DRB1_0101	501	TLCPQRPITKVFHPY	LCPQRPITK	1	0.3203	1563.6	80.00	0.56	tr_C9ZUI4_
DRB1_0101	502	ADAPFMEQVRHKMAQ	FMEQVRHKM	4	0.3201	1566.8	80.00	0.50	tr_C9ZUI4_
DRB1_0101	503	EDPNRDRWKLFLACI	RWKLFLACI	6	0.3197	1572.2	80.00	0.27	tr_C9ZUI4_
DRB1_0101	504	AQLCESIPPDFHSVA	LCESIPPDF	2	0.3182	1597.9	80.00	0.31	tr_C9ZUI4_
DRB1_0101	505	SMVETVRLVEEADAE	VRLVEEADA	5	0.3172	1616.7	80.00	0.41	tr_C9ZUI4_
DRB1_0101	506	VNAESIPTTTLATLI	PTTTLATLI	6	0.3168	1622.9	80.00	0.40	tr_C9ZUI4_
DRB1_0101	507	SKSQPMPTLCPQRPI	SQPMPTLCP	2	0.3151	1652.4	80.00	0.50	tr_C9ZUI4_
DRB1_0101	508	EIVQIARWNKQLEES	VQIARWNKQ	2	0.3151	1653.4	80.00	0.43	tr_C9ZUI4_
DRB1_0101	509	EADAEGVATMRQAGA	VATMRQAGA	6	0.3147	1660.7	80.00	0.57	tr_C9ZUI4_
DRB1_0101	510	DMGGAPQVKLGDASI	MGGAPQVKL	1	0.3131	1689.9	80.00	0.44	tr_C9ZUI4_
DRB1_0101	511	IAQLCESIPPDFHSV	LCESIPPDF	3	0.3126	1698.7	80.00	0.37	tr_C9ZUI4_
DRB1_0101	512	MESIFNADDADMGGA	FNADDADMG	4	0.3121	1707.4	80.00	0.52	tr_C9ZUI4_
DRB1_0101	513	IVSAPDGKYVVAKGS	VSAPDGKYV	1	0.3121	1708.1	80.00	0.43	tr_C9ZUI4_
DRB1_0101	514	LSGIILTVCFLCMSK	ILTVCFLCM	4	0.3116	1717.8	80.00	0.32	tr_C9ZUI4_
DRB1_0101	515	DEVDDCIVWTDAATG	IVWTDAATG	6	0.3114	1720.7	80.00	0.74	tr_C9ZUI4_
DRB1_0101	516	QIARWNKQLEESKKS	WNKQLEESK	4	0.3111	1725.7	80.00	0.67	tr_C9ZUI4_
DRB1_0101	517	VWARCAPTHKEDIVT	WARCAPTHK	1	0.3109	1729.5	80.00	0.49	tr_C9ZUI4_
DRB1_0101	518	ILSGIILTVCFLCMS	GIILTVCFL	3	0.3102	1742.4	80.00	0.29	tr_C9ZUI4_
DRB1_0101	519	TCCFDKTGTLTTDEM	FDKTGTLTT	3	0.3084	1776.9	80.00	0.63	tr_C9ZUI4_
DRB1_0101	520	ATSMDASQNVGGSGE	TSMDASQNV	1	0.3075	1794.4	80.00	0.50	tr_C9ZUI4_
DRB1_0101	521	WTDAATGKEVNLDRR	TDAATGKEV	1	0.3064	1815.6	85.00	0.67	tr_C9ZUI4_
DRB1_0101	522	EAIAQLCESIPPDFH	IAQLCESIP	2	0.3057	1831.1	85.00	0.25	tr_C9ZUI4_
DRB1_0101	523	AIAQLCESIPPDFHS	LCESIPPDF	4	0.3051	1842.3	85.00	0.28	tr_C9ZUI4_
DRB1_0101	524	LQLEGTDTVAGDAME	LEGTDTVAG	2	0.3050	1844.9	85.00	0.44	tr_C9ZUI4_
DRB1_0101	525	VQIARWNKQLEESKK	WNKQLEESK	5	0.3047	1849.4	85.00	0.55	tr_C9ZUI4_
DRB1_0101	526	ALAYRPLKEEERSKE	YRPLKEEER	3	0.3043	1858.9	85.00	0.66	tr_C9ZUI4_
DRB1_0101	527	FVQTRRHKVPSDDEW	FVQTRRHKV	0	0.3026	1892.9	85.00	0.75	tr_C9ZUI4_
DRB1_0101	528	KSQPMPTLCPQRPIT	SQPMPTLCP	1	0.3016	1912.6	85.00	0.38	tr_C9ZUI4_
DRB1_0101	529	IVQIARWNKQLEESK	VQIARWNKQ	1	0.3016	1913.8	85.00	0.35	tr_C9ZUI4_
DRB1_0101	530	KGSPEAIAQLCESIP	AIAQLCESI	5	0.3014	1916.6	85.00	0.31	tr_C9ZUI4_
DRB1_0101	531	QLCESIPPDFHSVAN	SIPPDFHSV	4	0.3005	1935.1	85.00	0.28	tr_C9ZUI4_

DRB1_0101	532	DADMGGAPQVKLGDA	MGGAPQVKL	3	0.3004	1937.2	85.00	0.56	tr_C9ZUI4_
DRB1_0101	533	VEEADAEGVATMRQA	VEEADAEGV	0	0.2994	1958.8	85.00	0.47	tr_C9ZUI4_
DRB1_0101	534	GTLTTDEMLFSGVDM	EMLFSGVDM	6	0.2955	2043.9	85.00	0.27	tr_C9ZUI4_
DRB1_0101	535	GSPEAIAQLCESIPP	AIAQLCESI	4	0.2943	2071.5	85.00	0.34	tr_C9ZUI4_
DRB1_0101	536	ESIFNADDADMGGAP	FNADDADMG	3	0.2932	2094.4	85.00	0.57	tr_C9ZUI4_
DRB1_0101	537	ADDADMGGAPQVKLG	MGGAPQVKL	5	0.2930	2099.4	85.00	0.43	tr_C9ZUI4_
DRB1_0101	538	SADAPFMEQVRHKMA	FMEQVRHKM	5	0.2918	2126.4	85.00	0.50	tr_C9ZUI4_
DRB1_0101	539	CCFDKTGTLTTDEML	FDKTGTLTT	2	0.2910	2146.3	85.00	0.58	tr_C9ZUI4_
DRB1_0101	540	LMESIFNADDADMGG	FNADDADMG	5	0.2901	2165.7	85.00	0.43	tr_C9ZUI4_
DRB1_0101	541	DTVVYDPPAQKEDIK	VVYDPPAQK	2	0.2897	2176.0	85.00	0.63	tr_C9ZUI4_
DRB1_0101	542	PEAIAQLCESIPPDF	AIAQLCESI	2	0.2892	2188.4	85.00	0.31	tr_C9ZUI4_
DRB1_0101	543	QLVASSMKKKGNGED	LVASSMKKK	1	0.2891	2189.9	85.00	0.54	tr_C9ZUI4_
DRB1_0101	544	AISVGRDVTILKCRQ	ISVGRDVTI	1	0.2889	2194.6	85.00	0.44	tr_C9ZUI4_
DRB1_0101	545	DDADMGGAPQVKLGD	MGGAPQVKL	4	0.2884	2206.9	85.00	0.51	tr_C9ZUI4_
DRB1_0101	546	RWNKQLEESKKSRET	WNKQLEESK	1	0.2880	2217.0	85.00	0.55	tr_C9ZUI4_
DRB1_0101	547	VSQPEMNAPASDFLM	NAPASDFLM	6	0.2870	2241.3	85.00	0.38	tr_C9ZUI4_
DRB1_0101	548	GTDTVAGDAMEKASL	TVAGDAMEK	3	0.2869	2242.3	85.00	0.43	tr_C9ZUI4_
DRB1_0101	549	RETAEAGKVVSQPEM	ETAEAGKVV	1	0.2866	2251.0	85.00	0.68	tr_C9ZUI4_
DRB1_0101	550	DTCCFDKTGTLTTDE	FDKTGTLTT	4	0.2862	2261.3	85.00	0.60	tr_C9ZUI4_
DRB1_0101	551	DREDCEKNLIFAGLA	KNLIFAGLA	6	0.2848	2293.9	85.00	0.24	tr_C9ZUI4_
DRB1_0101	552	DDTVVYDPPAQKEDI	VVYDPPAQK	3	0.2847	2298.2	85.00	0.65	tr_C9ZUI4_
DRB1_0101	553	SIFNADDADMGGAPQ	FNADDADMG	2	0.2843	2306.5	85.00	0.58	tr_C9ZUI4_
DRB1_0101	554	ESKKSRETAEAGKVV	ETAEAGKVV	6	0.2818	2369.3	85.00	0.44	tr_C9ZUI4_
DRB1_0101	555	VVSQPEMNAPASDFL	VVSQPEMNA	0	0.2813	2383.3	85.00	0.38	tr_C9ZUI4_
DRB1_0101	556	ADMGGAPQVKLGDAS	MGGAPQVKL	2	0.2804	2405.7	85.00	0.62	tr_C9ZUI4_
DRB1_0101	557	SPEAIAQLCESIPPD	AIAQLCESI	3	0.2804	2407.1	85.00	0.37	tr_C9ZUI4_
DRB1_0101	558	LCESIPPDFHSVANA	SIPPDFHSV	3	0.2792	2439.2	85.00	0.38	tr_C9ZUI4_
DRB1_0101	559	AKGSPEAIAQLCESI	GSPEAIAQL	2	0.2789	2447.0	85.00	0.25	tr_C9ZUI4_
DRB1_0101	560	AVFQCPLKKDAKDTI	FQCPLKKDA	2	0.2768	2500.7	85.00	0.37	tr_C9ZUI4_
DRB1_0101	561	LAYRPLKEEERSKEA	YRPLKEEER	2	0.2754	2541.1	90.00	0.69	tr_C9ZUI4_
DRB1_0101	562	KTGTLTTDEMLFSGV	GTLTTDEML	2	0.2736	2590.7	90.00	0.28	tr_C9ZUI4_
DRB1_0101	563	VDTCCFDKTGTLTTD	FDKTGTLTT	5	0.2733	2598.0	90.00	0.53	tr_C9ZUI4_
DRB1_0101	564	CFDKTGTLTTDEMLF	FDKTGTLTT	1	0.2733	2599.6	90.00	0.42	tr_C9ZUI4_
DRB1_0101	565	TAEAGKVVSQPEMNA	VVSQPEMNA	6	0.2732	2602.6	90.00	0.57	tr_C9ZUI4_
DRB1_0101	566	VLMAGDGTNDVGALK	MAGDGTNDV	2	0.2710	2662.9	90.00	0.37	tr_C9ZUI4_
DRB1_0101	567	TVVYDPPAQKEDIKG	VVYDPPAQK	1	0.2691	2718.5	90.00	0.63	tr_C9ZUI4_
DRB1_0101	568	YRVNIDDTVVYDPPA	YRVNIDDTV	0	0.2674	2768.4	90.00	0.61	tr_C9ZUI4_
DRB1_0101	569	MVETVRLVEEADAEG	VRLVEEADA	4	0.2666	2792.7	90.00	0.69	tr_C9ZUI4_
DRB1_0101	570	SVGRDVTILKCRQQL	VTILKCRQQ	5	0.2649	2846.5	90.00	0.41	tr_C9ZUI4_
DRB1_0101	571	LQSGSHRCVIITGDS	LQSGSHRCV	0	0.2643	2864.6	90.00	0.37	tr_C9ZUI4_
DRB1_0101	572	CESIPPDFHSVANAH	SIPPDFHSV	2	0.2642	2868.5	90.00	0.39	tr_C9ZUI4_
DRB1_0101	573	FDKTGTLTTDEMLFS	GTLTTDEML	4	0.2641	2869.2	90.00	0.25	tr_C9ZUI4_
DRB1_0101	574	IDDTVVYDPPAQKED	VVYDPPAQK	4	0.2621	2933.8	90.00	0.61	tr_C9ZUI4_
DRB1_0101	575	VSAPDGKYVVAKGSP	VSAPDGKYV	0	0.2561	3131.0	90.00	0.27	tr_C9ZUI4_
DRB1_0101	576	PLKKDAKDTIEMLQS	DAKDTIEML	4	0.2561	3131.8	90.00	0.41	tr_C9ZUI4_
DRB1_0101	577	TGTLTTDEMLFSGVD	TDEMLFSGV	5	0.2549	3170.1	90.00	0.28	tr_C9ZUI4_
DRB1_0101	578	ISVGRDVTILKCRQQ	SVGRDVTIL	1	0.2542	3195.4	90.00	0.26	tr_C9ZUI4_
DRB1_0101	579	LKKDAKDTIEMLQSG	DAKDTIEML	3	0.2540	3201.7	90.00	0.44	tr_C9ZUI4_
DRB1_0101	580	DKTGTLTTDEMLFSG	GTLTTDEML	3	0.2518	3278.4	90.00	0.32	tr_C9ZUI4_
DRB1_0101	581	IFNADDADMGGAPQV	FNADDADMG	1	0.2512	3299.7	90.00	0.46	tr_C9ZUI4_

DRB1_0101	582	NIDDTVVYDPPAQKE	VVYDPPAQK	5	0.2489	3382.6	90.00	0.57	tr_C9ZUI4_
DRB1_0101	583	NADDADMGGAPQVKL	MGGAPQVKL	6	0.2478	3426.0	90.00	0.32	tr_C9ZUI4_
DRB1_0101	584	CPLKKDAKDTIEMLQ	DAKDTIEML	5	0.2476	3433.2	90.00	0.37	tr_C9ZUI4_
DRB1_0101	585	AYRPLKEEERSKEAI	YRPLKEEER	1	0.2466	3470.5	90.00	0.56	tr_C9ZUI4_
DRB1_0101	586	KKDAKDTIEMLQSGS	DAKDTIEML	2	0.2460	3491.0	90.00	0.42	tr_C9ZUI4_
DRB1_0101	587	AATGKEVNLDRRSIL	EVNLDRRSI	5	0.2458	3500.7	90.00	0.37	tr_C9ZUI4_
DRB1_0101	588	WARCAPTHKEDIVTD	WARCAPTHK	0	0.2449	3533.9	90.00	0.34	tr_C9ZUI4_
DRB1_0101	589	LEDPNRDRWKLFLAC	DRWKLFLAC	6	0.2435	3588.5	90.00	0.24	tr_C9ZUI4_
DRB1_0101	590	KVDTCCFDKTGTLTT	FDKTGTLTT	6	0.2414	3670.1	90.00	0.38	tr_C9ZUI4_
DRB1_0101	591	PSADAPFMEQVRHKM	FMEQVRHKM	6	0.2365	3870.5	95.00	0.38	tr_C9ZUI4_
DRB1_0101	592	KEDIKGKSTTGSSKN	IKGKSTTGS	3	0.2317	4076.6	95.00	0.61	tr_C9ZUI4_
DRB1_0101	593	KEEERSKEAIHNMDR	ERSKEAIHN	3	0.2308	4115.9	95.00	0.51	tr_C9ZUI4_
DRB1_0101	594	EGTDTVAGDAMEKAS	TVAGDAMEK	4	0.2306	4123.0	95.00	0.46	tr_C9ZUI4_
DRB1_0101	595	QCPLKKDAKDTIEML	LKKDAKDTI	3	0.2304	4134.8	95.00	0.31	tr_C9ZUI4_
DRB1_0101	596	TDAATGKEVNLDRRS	TDAATGKEV	0	0.2279	4247.4	95.00	0.52	tr_C9ZUI4_
DRB1_0101	597	TSMDASQNVGGSGEH	MDASQNVGG	2	0.2263	4322.3	95.00	0.39	tr_C9ZUI4_
DRB1_0101	598	FNADDADMGGAPQVK	FNADDADMG	0	0.2262	4326.0	95.00	0.31	tr_C9ZUI4_
DRB1_0101	599	MDREDCEKNLIFAGL	EDCEKNLIF	3	0.2262	4327.2	95.00	0.25	tr_C9ZUI4_
DRB1_0101	600	QKEDIKGKSTTGSSK	IKGKSTTGS	4	0.2223	4514.1	95.00	0.57	tr_C9ZUI4_
DRB1_0101	601	LKEEERSKEAIHNMD	ERSKEAIHN	4	0.2195	4650.5	95.00	0.46	tr_C9ZUI4_
DRB1_0101	602	TVVPPAPSADAPFME	VPPAPSADA	2	0.2192	4668.5	95.00	0.44	tr_C9ZUI4_
DRB1_0101	603	PLKEEERSKEAIHNM	ERSKEAIHN	5	0.2159	4836.9	95.00	0.44	tr_C9ZUI4_
DRB1_0101	604	WNKQLEESKKSRETA	WNKQLEESK	0	0.2146	4905.2	95.00	0.34	tr_C9ZUI4_
DRB1_0101	605	QLEGTDTVAGDAMEK	LEGTDTVAG	1	0.2127	5007.3	95.00	0.50	tr_C9ZUI4_
DRB1_0101	606	LMAGDGTNDVGALKQ	MAGDGTNDV	1	0.2116	5065.5	95.00	0.38	tr_C9ZUI4_
DRB1_0101	607	GLEDPNRDRWKLFLA	PNRDRWKLF	4	0.2107	5117.2	95.00	0.27	tr_C9ZUI4_
DRB1_0101	608	LEGTDTVAGDAMEKA	TVAGDAMEK	5	0.2070	5322.0	95.00	0.35	tr_C9ZUI4_
DRB1_0101	609	EEADAEGVATMRQAG	DAEGVATMR	3	0.2067	5339.4	95.00	0.26	tr_C9ZUI4_
DRB1_0101	610	RIPYAGKVDTCCFDK	YAGKVDTCC	3	0.2058	5392.3	95.00	0.38	tr_C9ZUI4_
DRB1_0101	611	VFQCPLKKDAKDTIE	FQCPLKKDA	1	0.2056	5404.5	95.00	0.34	tr_C9ZUI4_
DRB1_0101	612	EEERSKEAIHNMDRE	ERSKEAIHN	2	0.2043	5483.2	95.00	0.46	tr_C9ZUI4_
DRB1_0101	613	VPSDDEWDLCVNAES	WDLCVNAES	6	0.2038	5509.7	95.00	0.52	tr_C9ZUI4_
DRB1_0101	614	YRPLKEEERSKEAIH	YRPLKEEER	0	0.2022	5606.8	95.00	0.37	tr_C9ZUI4_
DRB1_0101	615	VNIDDTVVYDPPAQK	VVYDPPAQK	6	0.1995	5775.0	95.00	0.43	tr_C9ZUI4_
DRB1_0101	616	ETAEAGKVVSQPEMN	ETAEAGKVV	0	0.1993	5786.3	95.00	0.50	tr_C9ZUI4_
DRB1_0101	617	APSADAPFMEQVRHK	PSADAPFME	1	0.1990	5802.9	95.00	0.25	tr_C9ZUI4_
DRB1_0101	618	EDIKGKSTTGSSKNE	IKGKSTTGS	2	0.1983	5852.8	95.00	0.61	tr_C9ZUI4_
DRB1_0101	619	FQCPLKKDAKDTIEM	LKKDAKDTI	4	0.1969	5939.2	95.00	0.34	tr_C9ZUI4_
DRB1_0101	620	QNRKNNSGFEKQYKI	SGFEKQYKI	6	0.1964	5971.8	95.00	0.41	tr_C9ZUI4_
DRB1_0101	621	VVYDPPAQKEDIKGK	VVYDPPAQK	0	0.1953	6041.4	95.00	0.49	tr_C9ZUI4_
DRB1_0101	622	IPYAGKVDTCCFDKT	YAGKVDTCC	2	0.1950	6059.6	95.00	0.29	tr_C9ZUI4_
DRB1_0101	623	RGLEDPNRDRWKLFL	PNRDRWKLF	5	0.1933	6174.3	95.00	0.20	tr_C9ZUI4_
DRB1_0101	624	NMDREDCEKNLIFAG	EDCEKNLIF	4	0.1924	6233.7	100.00	0.28	tr_C9ZUI4_
DRB1_0101	625	PAPSADAPFMEQVRH	PSADAPFME	2	0.1904	6373.3	100.00	0.33	tr_C9ZUI4_
DRB1_0101	626	PPAPSADAPFMEQVR	PSADAPFME	3	0.1893	6448.3	100.00	0.35	tr_C9ZUI4_
DRB1_0101	627	GKVDTCCFDKTGTLT	CFDKTGTLT	6	0.1866	6641.9	100.00	0.26	tr_C9ZUI4_
DRB1_0101	628	AQKEDIKGKSTTGSS	IKGKSTTGS	5	0.1862	6668.0	100.00	0.51	tr_C9ZUI4_
DRB1_0101	629	ARCAPTHKEDIVTDL	CAPTHKEDI	2	0.1853	6737.2	100.00	0.22	tr_C9ZUI4_
DRB1_0101	630	HKEDIVTDLKQREHM	IVTDLKQRE	4	0.1836	6856.4	100.00	0.45	tr_C9ZUI4_
DRB1_0101	631	PYAGKVDTCCFDKTG	YAGKVDTCC	1	0.1829	6908.9	100.00	0.25	tr_C9ZUI4_

DRB1_0101	632	KRGLEDPNRDRWKLF	LEDPNRDRW	3	0.1825	6943.5	100.00	0.20	tr_C9ZUI4_
DRB1_0101	633	VVPPAPSADAPFMEQ	VPPAPSADA	1	0.1806	7083.0	100.00	0.37	tr_C9ZUI4_
DRB1_0101	634	RCAPTHKEDIVTDLK	HKEDIVTDL	5	0.1804	7099.6	100.00	0.29	tr_C9ZUI4_
DRB1_0101	635	VPPAPSADAPFMEQV	PSADAPFME	4	0.1804	7103.9	100.00	0.33	tr_C9ZUI4_
DRB1_0101	636	APTHKEDIVTDLKQR	HKEDIVTDL	3	0.1800	7129.2	100.00	0.47	tr_C9ZUI4_
DRB1_0101	637	RPLKEEERSKEAIHN	LKEEERSKE	2	0.1788	7223.6	100.00	0.30	tr_C9ZUI4_
DRB1_0101	638	MAGDGTNDVGALKQA	GTNDVGALK	4	0.1787	7228.5	100.00	0.22	tr_C9ZUI4_
DRB1_0101	639	EERSKEAIHNMDRED	ERSKEAIHN	1	0.1761	7434.4	100.00	0.46	tr_C9ZUI4_
DRB1_0101	640	CAPTHKEDIVTDLKQ	HKEDIVTDL	4	0.1746	7563.9	100.00	0.38	tr_C9ZUI4_
DRB1_0101	641	AGDGTNDVGALKQAH	GTNDVGALK	3	0.1720	7778.7	100.00	0.31	tr_C9ZUI4_
DRB1_0101	642	HNMDREDCEKNLIFA	EDCEKNLIF	5	0.1718	7795.0	100.00	0.31	tr_C9ZUI4_
DRB1_0101	643	DIKGKSTTGSSKNET	IKGKSTTGS	1	0.1696	7977.3	100.00	0.55	tr_C9ZUI4_
DRB1_0101	644	PTHKEDIVTDLKQRE	HKEDIVTDL	2	0.1694	7999.3	100.00	0.41	tr_C9ZUI4_
DRB1_0101	645	EESKKSRETAEAGKV	RETAEAGKV	6	0.1655	8343.1	100.00	0.27	tr_C9ZUI4_
DRB1_0101	646	DAATGKEVNLDRRSI	EVNLDRRSI	6	0.1644	8441.6	100.00	0.36	tr_C9ZUI4_
DRB1_0101	647	LVASSMKKKGNGEDE	LVASSMKKK	0	0.1641	8467.7	100.00	0.47	tr_C9ZUI4_
DRB1_0101	648	THKEDIVTDLKQREH	IVTDLKQRE	5	0.1637	8510.0	100.00	0.35	tr_C9ZUI4_
DRB1_0101	649	YAGKVDTCCFDKTGT	VDTCCFDKT	4	0.1623	8634.0	100.00	0.22	tr_C9ZUI4_
DRB1_0101	650	AGKVDTCCFDKTGTL	VDTCCFDKT	3	0.1607	8787.5	100.00	0.22	tr_C9ZUI4_
DRB1_0101	651	EPDVPKDHKIPPGFK	DHKIPPGFK	6	0.1583	9014.2	100.00	0.44	tr_C9ZUI4_
DRB1_0101	652	IHNMDREDCEKNLIF	EDCEKNLIF	6	0.1581	9040.5	100.00	0.23	tr_C9ZUI4_
DRB1_0101	653	SMDASQNVGGSGEHN	MDASQNVGG	1	0.1523	9622.4	100.00	0.46	tr_C9ZUI4_
DRB1_0101	654	NKQLEESKKSRETAE	LEESKKSRE	3	0.1515	9709.7	100.00	0.29	tr_C9ZUI4_
DRB1_0101	655	EDEVDDCIVWTDAAT	DEVDDCIVW	1	0.1485	10031.3	100.00	0.28	tr_C9ZUI4_
DRB1_0101	656	KQLEESKKSRETAEA	LEESKKSRE	2	0.1439	10538.8	100.00	0.32	tr_C9ZUI4_
DRB1_0101	657	RVNIDDTVVYDPPAQ	DTVVYDPPA	5	0.1434	10594.3	100.00	0.23	tr_C9ZUI4_
DRB1_0101	658	PAQKEDIKGKSTTGS	IKGKSTTGS	6	0.1432	10614.8	100.00	0.30	tr_C9ZUI4_
DRB1_0101	659	SQNRKNNSGFEKQYK	RKNNSGFEK	3	0.1405	10928.3	100.00	0.29	tr_C9ZUI4_
DRB1_0101	660	AIHNMDREDCEKNLI	IHNMDREDC	1	0.1387	11150.7	100.00	0.35	tr_C9ZUI4_
DRB1_0101	661	ERSKEAIHNMDREDC	ERSKEAIHN	0	0.1377	11275.3	100.00	0.28	tr_C9ZUI4_
DRB1_0101	662	KSQNRKNNSGFEKQY	NRKNNSGFE	3	0.1368	11380.6	100.00	0.27	tr_C9ZUI4_
DRB1_0101	663	KVPSDDEWDLCVNAE	DEWDLCVNA	5	0.1322	11958.4	100.00	0.29	tr_C9ZUI4_
DRB1_0101	664	ASQNVGGSGEHNSNE	NVGGSGEHN	3	0.1308	12138.0	100.00	0.38	tr_C9ZUI4_
DRB1_0101	665	EAIHNMDREDCEKNL	IHNMDREDC	2	0.1308	12149.2	100.00	0.43	tr_C9ZUI4_
DRB1_0101	666	GEDEVDDCIVWTDAA	DEVDDCIVW	2	0.1305	12186.4	100.00	0.34	tr_C9ZUI4_
DRB1_0101	667	IKGKSTTGSSKNETS	IKGKSTTGS	0	0.1303	12212.7	100.00	0.37	tr_C9ZUI4_
DRB1_0101	668	QLEESKKSRETAEAG	LEESKKSRE	1	0.1301	12239.2	100.00	0.25	tr_C9ZUI4_
DRB1_0101	669	DASQNVGGSGEHNSN	NVGGSGEHN	4	0.1292	12354.6	100.00	0.38	tr_C9ZUI4_
DRB1_0101	670	KEAIHNMDREDCEKN	IHNMDREDC	3	0.1284	12457.1	100.00	0.56	tr_C9ZUI4_
DRB1_0101	671	MDASQNVGGSGEHNS	NVGGSGEHN	5	0.1279	12532.9	100.00	0.25	tr_C9ZUI4_
DRB1_0101	672	LEESKKSRETAEAGK	KSRETAEAG	5	0.1268	12685.3	100.00	0.23	tr_C9ZUI4_
DRB1_0101	673	NGEDEVDDCIVWTDA	DEVDDCIVW	3	0.1251	12917.7	100.00	0.38	tr_C9ZUI4_
DRB1_0101	674	SKSQNRKNNSGFEKQ	QNRKNNSGF	3	0.1237	13108.9	100.00	0.29	tr_C9ZUI4_
DRB1_0101	675	HKVPSDDEWDLCVNA	VPSDDEWDL	2	0.1230	13211.9	100.00	0.29	tr_C9ZUI4_
DRB1_0101	676	SQNVGGSGEHNSNEP	NVGGSGEHN	2	0.1200	13643.5	100.00	0.38	tr_C9ZUI4_
DRB1_0101	677	SKEAIHNMDREDCEK	IHNMDREDC	4	0.1185	13870.9	100.00	0.60	tr_C9ZUI4_
DRB1_0101	678	SKNETSGSSKSQNRK	SGSSKSQNR	5	0.1158	14280.4	100.00	0.21	tr_C9ZUI4_
DRB1_0101	679	SSKNETSGSSKSQNR	NETSGSSKS	3	0.1136	14627.7	100.00	0.33	tr_C9ZUI4_
DRB1_0101	680	VASSMKKKGNGEDEV	SMKKKGNGE	3	0.1133	14676.7	100.00	0.31	tr_C9ZUI4_
DRB1_0101	681	PPAQKEDIKGKSTTG	KEDIKGKST	4	0.1124	14826.1	100.00	0.31	tr_C9ZUI4_

DRB1_0101	682	RSKEAIHNMDREDCE	IHNMDREDC	5	0.1120	14886.4	100.00	0.41	tr_C9ZUI4_
DRB1_0101	683	VYDPPAQKEDIKGKS	VYDPPAQKE	0	0.1119	14896.2	100.00	0.26	tr_C9ZUI4_
DRB1_0101	684	KNETSGSSKSQNRKN	SGSSKSQNR	4	0.1118	14917.8	100.00	0.31	tr_C9ZUI4_
DRB1_0101	685	NETSGSSKSQNRKNN	SGSSKSQNR	3	0.1115	14962.3	100.00	0.35	tr_C9ZUI4_
DRB1_0101	686	QNVGGSGEHNSNEPH	NVGGSGEHN	1	0.1102	15177.8	100.00	0.33	tr_C9ZUI4_
DRB1_0101	687	ETSGSSKSQNRKNNS	SGSSKSQNR	2	0.1098	15243.2	100.00	0.31	tr_C9ZUI4_
DRB1_0101	688	ASSMKKKGNGEDEVD	SMKKKGNGE	2	0.1096	15266.3	100.00	0.31	tr_C9ZUI4_
DRB1_0101	689	GNGEDEVDDCIVWTD	DEVDDCIVW	4	0.1089	15396.5	100.00	0.41	tr_C9ZUI4_
DRB1_0101	690	KGNGEDEVDDCIVWT	DEVDDCIVW	5	0.1078	15578.8	100.00	0.38	tr_C9ZUI4_
DRB1_0101	691	TSGSSKSQNRKNNSG	SGSSKSQNR	1	0.1052	16012.7	100.00	0.25	tr_C9ZUI4_
DRB1_0101	692	RHKVPSDDEWDLCVN	VPSDDEWDL	3	0.1046	16123.3	100.00	0.42	tr_C9ZUI4_
DRB1_0101	693	SSKSQNRKNNSGFEK	QNRKNNSGF	4	0.1042	16200.9	100.00	0.29	tr_C9ZUI4_
DRB1_0101	694	TGSSKNETSGSSKSQ	SKNETSGSS	3	0.1022	16546.2	100.00	0.35	tr_C9ZUI4_
DRB1_0101	695	DPPAQKEDIKGKSTT	KEDIKGKST	5	0.1020	16574.7	100.00	0.27	tr_C9ZUI4_
DRB1_0101	696	GSSKNETSGSSKSQN	SKNETSGSS	2	0.1018	16625.5	100.00	0.30	tr_C9ZUI4_
DRB1_0101	697	SSMKKKGNGEDEVDD	MKKKGNGED	2	0.1013	16714.1	100.00	0.30	tr_C9ZUI4_
DRB1_0101	698	RRHKVPSDDEWDLCV	VPSDDEWDL	4	0.1003	16884.4	100.00	0.41	tr_C9ZUI4_
DRB1_0101	699	TTGSSKNETSGSSKS	SKNETSGSS	4	0.0998	16980.6	100.00	0.34	tr_C9ZUI4_
DRB1_0101	700	SGSSKSQNRKNNSGF	SSKSQNRKN	2	0.0986	17210.7	100.00	0.25	tr_C9ZUI4_
DRB1_0101	701	STTGSSKNETSGSSK	TGSSKNETS	2	0.0964	17614.2	100.00	0.28	tr_C9ZUI4_
DRB1_0101	702	YDPPAQKEDIKGKST	PAQKEDIKG	3	0.0959	17713.8	100.00	0.20	tr_C9ZUI4_
DRB1_0101	703	QTRRHKVPSDDEWDL	TRRHKVPSD	1	0.0954	17812.9	100.00	0.19	tr_C9ZUI4_
DRB1_0101	704	TRRHKVPSDDEWDLC	VPSDDEWDL	5	0.0944	17998.5	100.00	0.30	tr_C9ZUI4_
DRB1_0101	705	NVGGSGEHNSNEPHN	VGGSGEHNS	1	0.0944	18013.3	100.00	0.32	tr_C9ZUI4_
DRB1_0101	706	KSTTGSSKNETSGSS	TGSSKNETS	3	0.0927	18334.4	100.00	0.41	tr_C9ZUI4_
DRB1_0101	707	GSSKSQNRKNNSGFE	QNRKNNSGF	5	0.0911	18653.6	100.00	0.25	tr_C9ZUI4_
DRB1_0101	708	SGEHNSNEPHNEPDV	HNSNEPHNE	3	0.0905	18778.5	100.00	0.53	tr_C9ZUI4_
DRB1_0101	709	KGKSTTGSSKNETSG	GKSTTGSSK	1	0.0905	18789.9	100.00	0.31	tr_C9ZUI4_
DRB1_0101	710	EHNSNEPHNEPDVPK	HNSNEPHNE	1	0.0880	19302.4	100.00	0.41	tr_C9ZUI4_
DRB1_0101	711	VQTRRHKVPSDDEWD	TRRHKVPSD	2	0.0871	19483.9	100.00	0.24	tr_C9ZUI4_
DRB1_0101	712	VGGSGEHNSNEPHNE	HNSNEPHNE	6	0.0861	19703.9	100.00	0.28	tr_C9ZUI4_
DRB1_0101	713	KKGNGEDEVDDCIVW	DEVDDCIVW	6	0.0859	19742.6	100.00	0.32	tr_C9ZUI4_
DRB1_0101	714	GKSTTGSSKNETSGS	TGSSKNETS	4	0.0853	19875.0	100.00	0.35	tr_C9ZUI4_
DRB1_0101	715	GEHNSNEPHNEPDVP	HNSNEPHNE	2	0.0839	20176.2	100.00	0.51	tr_C9ZUI4_
DRB1_0101	716	GSGEHNSNEPHNEPD	HNSNEPHNE	4	0.0835	20250.5	100.00	0.50	tr_C9ZUI4_
DRB1_0101	717	GGSGEHNSNEPHNEP	HNSNEPHNE	5	0.0828	20408.7	100.00	0.45	tr_C9ZUI4_
DRB1_0101	718	SMKKKGNGEDEVDDC	MKKKGNGED	1	0.0820	20595.0	100.00	0.28	tr_C9ZUI4_
DRB1_0101	719	NEPDVPKDHKIPPGF	KDHKIPPGF	6	0.0783	21433.9	100.00	0.25	tr_C9ZUI4_
DRB1_0101	720	HNSNEPHNEPDVPKD	HNSNEPHNE	0	0.0773	21656.3	100.00	0.30	tr_C9ZUI4_
DRB1_0101	721	SNEPHNEPDVPKDHK	PHNEPDVPK	3	0.0693	23620.0	100.00	0.41	tr_C9ZUI4_
DRB1_0101	722	NEPHNEPDVPKDHKI	PHNEPDVPK	2	0.0688	23760.4	100.00	0.35	tr_C9ZUI4_
DRB1_0101	723	EPHNEPDVPKDHKIP	PHNEPDVPK	1	0.0658	24525.5	100.00	0.25	tr_C9ZUI4_
DRB1_0101	724	NSNEPHNEPDVPKDH	PHNEPDVPK	4	0.0646	24864.3	100.00	0.40	tr_C9ZUI4_
DRB1_0101	725	MKKKGNGEDEVDDCI	KKKGNGEDE	1	0.0622	25508.5	100.00	0.19	tr_C9ZUI4_
DRB1_0101	726	KKKGNGEDEVDDCIV	GEDEVDDCI	5	0.0613	25767.3	100.00	0.23	tr_C9ZUI4_
DRB1_0101	727	HNEPDVPKDHKIPPG	PDVPKDHKI	3	0.0609	25878.8	100.00	0.31	tr_C9ZUI4_
DRB1_0101	728	PHNEPDVPKDHKIPP	PDVPKDHKI	4	0.0602	26064.5	100.00	0.28	tr_C9ZUI4_

Allele: DRB1_0101. Number of high binders 0. Number of weak binders 64. Number of peptides 728

Allele	pos	peptide	core	Of	1-log50k(aff)	affinity(nM)	%Rank	Relia	Identity	Bind Level
DRB1_0101	1	IRAFRGLKSAERLIF	FRGLKSAER	3	0.8792	3.7	0.25	0.78	tr_C9ZNH3_	SB
DRB1_0101	2	FIRAFRGLKSAERLI	FRGLKSAER	4	0.8790	3.7	0.25	0.69	tr_C9ZNH3_	SB
DRB1_0101	3	TLLSFNRSLNSVVSL	FNRSLNSVV	4	0.8753	3.9	0.25	0.79	tr_C9ZNH3_	SB
DRB1_0101	4	LLSFNRSLNSVVSLD	FNRSLNSVV	3	0.8721	4.0	0.30	0.80	tr_C9ZNH3_	SB
DRB1_0101	5	DFIRAFRGLKSAERL	FRGLKSAER	5	0.8645	4.3	0.40	0.54	tr_C9ZNH3_	SB
DRB1_0101	6	LSFNRSLNSVVSLDR	FNRSLNSVV	2	0.8616	4.5	0.40	0.78	tr_C9ZNH3_	SB
DRB1_0101	7	RAFRGLKSAERLIFA	FRGLKSAER	2	0.8589	4.6	0.50	0.83	tr_C9ZNH3_	WB
DRB1_0101	8	RTLLSFNRSLNSVVS	FNRSLNSVV	5	0.8451	5.3	0.70	0.74	tr_C9ZNH3_	WB
DRB1_0101	9	NAYFLHVSPAAELLA	FLHVSPAAE	3	0.8385	5.7	0.90	0.38	tr_C9ZNH3_	WB
DRB1_0101	10	DDFIRAFRGLKSAER	IRAFRGLKS	3	0.8338	6.0	1.00	0.46	tr_C9ZNH3_	WB
DRB1_0101	11	PNAYFLHVSPAAELL	YFLHVSPAA	3	0.8262	6.6	1.20	0.44	tr_C9ZNH3_	WB
DRB1_0101	12	SFNRSLNSVVSLDRD	FNRSLNSVV	1	0.8262	6.6	1.20	0.75	tr_C9ZNH3_	WB
DRB1_0101	13	AYFLHVSPAAELLAL	FLHVSPAAE	2	0.8251	6.6	1.20	0.37	tr_C9ZNH3_	WB
DRB1_0101	14	AFRGLKSAERLIFAT	FRGLKSAER	1	0.8198	7.0	1.40	0.83	tr_C9ZNH3_	WB
DRB1_0101	15	SSAYILSPTPHVTNI	YILSPTPHV	3	0.8089	7.9	1.70	0.87	tr_C9ZNH3_	WB
DRB1_0101	16	ERLFIHSVPSTSGNV	FIHSVPSTS	3	0.8063	8.1	1.80	0.78	tr_C9ZNH3_	WB
DRB1_0101	17	RLFIHSVPSTSGNVM	FIHSVPSTS	2	0.8035	8.4	1.90	0.75	tr_C9ZNH3_	WB
DRB1_0101	18	SAYILSPTPHVTNIE	YILSPTPHV	2	0.7934	9.3	2.50	0.85	tr_C9ZNH3_	WB
DRB1_0101	19	TSSAYILSPTPHVTN	YILSPTPHV	4	0.7917	9.5	2.50	0.87	tr_C9ZNH3_	WB
DRB1_0101	20	SPNAYFLHVSPAAEL	YFLHVSPAA	4	0.7897	9.7	2.50	0.50	tr_C9ZNH3_	WB
DRB1_0101	21	SASFLDAVAQHIVGV	FLDAVAQHI	3	0.7880	9.9	3.00	0.83	tr_C9ZNH3_	WB
DRB1_0101	22	NRTLLSFNRSLNSVV	FNRSLNSVV	6	0.7873	10.0	3.00	0.57	tr_C9ZNH3_	WB
DRB1_0101	23	SERLFIHSVPSTSGN	FIHSVPSTS	4	0.7785	11.0	3.00	0.76	tr_C9ZNH3_	WB
DRB1_0101	24	YFLHVSPAAELLALL	FLHVSPAAE	1	0.7773	11.1	3.50	0.39	tr_C9ZNH3_	WB
DRB1_0101	25	LALLRYAVSQLHLLR	LRYAVSQLH	3	0.7760	11.3	3.50	0.50	tr_C9ZNH3_	WB
DRB1_0101	26	TSASFLDAVAQHIVG	FLDAVAQHI	4	0.7752	11.4	3.50	0.83	tr_C9ZNH3_	WB
DRB1_0101	27	ALLRYAVSQLHLLRI	LRYAVSQLH	2	0.7738	11.6	3.50	0.50	tr_C9ZNH3_	WB
DRB1_0101	28	YDDFIRAFRGLKSAE	IRAFRGLKS	4	0.7706	12.0	3.50	0.59	tr_C9ZNH3_	WB
DRB1_0101	29	ASFLDAVAQHIVGVD	FLDAVAQHI	2	0.7648	12.7	4.00	0.81	tr_C9ZNH3_	WB
DRB1_0101	30	RTSSAYILSPTPHVT	YILSPTPHV	5	0.7614	13.2	4.00	0.86	tr_C9ZNH3_	WB
DRB1_0101	31	RGALFVSGNGHLGKS	FVSGNGHLG	4	0.7597	13.5	4.50	0.83	tr_C9ZNH3_	WB
DRB1_0101	32	LLRYAVSQLHLLRIG	YAVSQLHLL	3	0.7597	13.5	4.50	0.44	tr_C9ZNH3_	WB
DRB1_0101	33	FYDDFIRAFRGLKSA	IRAFRGLKS	5	0.7585	13.6	4.50	0.55	tr_C9ZNH3_	WB
DRB1_0101	34	GALFVSGNGHLGKSE	FVSGNGHLG	3	0.7577	13.8	4.50	0.87	tr_C9ZNH3_	WB
DRB1_0101	35	AYILSPTPHVTNIEI	YILSPTPHV	1	0.7570	13.9	4.50	0.84	tr_C9ZNH3_	WB
DRB1_0101	36	FTSASFLDAVAQHIV	FLDAVAQHI	5	0.7509	14.8	5.00	0.81	tr_C9ZNH3_	WB
DRB1_0101	37	PLARALNAAVPPLTK	LNAAVPPLT	5	0.7469	15.5	5.00	0.61	tr_C9ZNH3_	WB
DRB1_0101	38	LLALLRYAVSQLHLL	LRYAVSQLH	4	0.7462	15.6	5.00	0.55	tr_C9ZNH3_	WB
DRB1_0101	39	ALFVSGNGHLGKSER	FVSGNGHLG	2	0.7458	15.6	5.00	0.86	tr_C9ZNH3_	WB
DRB1_0101	40	KSERLFIHSVPSTSG	FIHSVPSTS	5	0.7425	16.2	5.50	0.76	tr_C9ZNH3_	WB
DRB1_0101	41	LFIHSVPSTSGNVMS	FIHSVPSTS	1	0.7413	16.4	5.50	0.76	tr_C9ZNH3_	WB
DRB1_0101	42	QKVLTTIYTHSVISV	LTTIYTHSV	3	0.7376	17.1	6.00	0.76	tr_C9ZNH3_	WB
DRB1_0101	43	FRGLKSAERLIFATN	FRGLKSAER	0	0.7353	17.5	6.00	0.66	tr_C9ZNH3_	WB
DRB1_0101	44	RIGFMYLQNVHFGDS	FMYLQNVHF	3	0.7297	18.6	6.50	0.77	tr_C9ZNH3_	WB
DRB1_0101	45	LCGVFTLESSFDGEA	FTLESSFDG	4	0.7259	19.4	6.50	0.89	tr_C9ZNH3_	WB

DRB1_0101	46	HRGALFVSGNGHLGK	FVSGNGHLG	5	0.7242	19.8	7.00	0.78	tr_C9ZNH3_	WB
DRB1_0101	47	LRIGFMYLQNVHFGD	FMYLQNVHF	4	0.7228	20.1	7.00	0.79	tr_C9ZNH3_	WB
DRB1_0101	48	CGVFTLESSFDGEAD	FTLESSFDG	3	0.7220	20.2	7.00	0.89	tr_C9ZNH3_	WB
DRB1_0101	49	PQKVLTTIYTHSVIS	LTTIYTHSV	4	0.7220	20.2	7.00	0.74	tr_C9ZNH3_	WB
DRB1_0101	50	LARALNAAVPPLTKP	LNAAVPPLT	4	0.7213	20.4	7.00	0.71	tr_C9ZNH3_	WB
DRB1_0101	51	AFAPFTGSDAVRGWS	FTGSDAVRG	4	0.7213	20.4	7.00	0.59	tr_C9ZNH3_	WB
DRB1_0101	52	LLRIGFMYLQNVHFG	FMYLQNVHF	5	0.7193	20.9	7.50	0.75	tr_C9ZNH3_	WB
DRB1_0101	53	WSPNAYFLHVSPAAE	YFLHVSPAA	5	0.7186	21.0	7.50	0.58	tr_C9ZNH3_	WB
DRB1_0101	54	VAFAPFTGSDAVRGW	FTGSDAVRG	5	0.7177	21.2	7.50	0.49	tr_C9ZNH3_	WB
DRB1_0101	55	KNEFFVVVGPLSDSD	FFVVVGPLS	3	0.7175	21.3	7.50	0.63	tr_C9ZNH3_	WB
DRB1_0101	56	SFLDAVAQHIVGVDP	FLDAVAQHI	1	0.7170	21.4	7.50	0.81	tr_C9ZNH3_	WB
DRB1_0101	57	AVELMSQMGRSLCGV	LMSQMGRSL	3	0.7159	21.6	7.50	0.82	tr_C9ZNH3_	WB
DRB1_0101	58	NKNEFFVVVGPLSDS	FFVVVGPLS	4	0.7155	21.7	7.50	0.66	tr_C9ZNH3_	WB
DRB1_0101	59	VMFGWIVGEVLARAL	FGWIVGEVL	2	0.7155	21.7	7.50	0.49	tr_C9ZNH3_	WB
DRB1_0101	60	MFGWIVGEVLARALE	FGWIVGEVL	1	0.7104	22.9	8.00	0.41	tr_C9ZNH3_	WB
DRB1_0101	61	RTPLALLGFTSASFL	PLALLGFTS	2	0.7093	23.2	8.00	0.44	tr_C9ZNH3_	WB
DRB1_0101	62	KVLTTIYTHSVISVD	LTTIYTHSV	2	0.7068	23.9	8.50	0.75	tr_C9ZNH3_	WB
DRB1_0101	63	LAVELMSQMGRSLCG	LMSQMGRSL	4	0.7065	24.0	8.50	0.75	tr_C9ZNH3_	WB
DRB1_0101	64	GVFTLESSFDGEADD	FTLESSFDG	2	0.7061	24.0	8.50	0.90	tr_C9ZNH3_	WB
DRB1_0101	65	LRYAVSQLHLLRIGF	YAVSQLHLL	2	0.7060	24.1	8.50	0.50	tr_C9ZNH3_	WB
DRB1_0101	66	FNRSLNSVVSLDRDA	FNRSLNSVV	0	0.7056	24.2	8.50	0.59	tr_C9ZNH3_	WB
DRB1_0101	67	LFYDDFIRAFRGLKS	IRAFRGLKS	6	0.7038	24.6	8.50	0.41	tr_C9ZNH3_	WB
DRB1_0101	68	SLLLIGLTASDVGGI	LIGLTASDV	3	0.7009	25.4	9.00	0.71	tr_C9ZNH3_	WB
DRB1_0101	69	GSLLLIGLTASDVGG	LIGLTASDV	4	0.7000	25.7	9.00	0.68	tr_C9ZNH3_	WB
DRB1_0101	70	KSRMTFMDSLYNQRR	MTFMDSLYN	3	0.6993	25.9	9.00	0.72	tr_C9ZNH3_	WB
DRB1_0101	71	QVMFGWIVGEVLARA	FGWIVGEVL	3	0.6991	25.9	9.00	0.58	tr_C9ZNH3_	WB
DRB1_0101	72	LFVSGNGHLGKSERL	FVSGNGHLG	1	0.6988	26.0	9.00	0.86	tr_C9ZNH3_	WB
DRB1_0101	73	AQHIVGVDPQKVLTT	IVGVDPQKV	3	0.6980	26.3	9.00	0.94	tr_C9ZNH3_	WB
DRB1_0101	74	NEFFVVVGPLSDSDT	FFVVVGPLS	2	0.6973	26.5	9.50	0.57	tr_C9ZNH3_	WB
DRB1_0101	75	ASDVGGIAAHLSANR	VGGIAAHLS	3	0.6960	26.8	9.50	0.56	tr_C9ZNH3_	WB
DRB1_0101	76	LYTLLFSSVNDPFAR	YTLLFSSVN	1	0.6958	26.9	9.50	0.45	tr_C9ZNH3_	WB
DRB1_0101	77	DPQKVLTTIYTHSVI	LTTIYTHSV	5	0.6956	26.9	9.50	0.68	tr_C9ZNH3_	WB
DRB1_0101	78	IGFMYLQNVHFGDSE	FMYLQNVHF	2	0.6953	27.0	9.50	0.75	tr_C9ZNH3_	WB
DRB1_0101	79	SGVQMLAPLYTLLFS	VQMLAPLYT	2	0.6944	27.3	9.50	0.23	tr_C9ZNH3_	WB
DRB1_0101	80	SDVGGIAAHLSANRN	VGGIAAHLS	2	0.6940	27.4	9.50	0.50	tr_C9ZNH3_	WB
DRB1_0101	81	QMLAPLYTLLFSSVN	YTLLFSSVN	6	0.6926	27.8	9.50	0.47	tr_C9ZNH3_	WB
DRB1_0101	82	ELAVELMSQMGRSLC	LMSQMGRSL	5	0.6924	27.9	10.00	0.69	tr_C9ZNH3_	
DRB1_0101	83	ARALNAAVPPLTKPE	LNAAVPPLT	3	0.6921	28.0	10.00	0.86	tr_C9ZNH3_	
DRB1_0101	84	LVAFAPFTGSDAVRG	FAPFTGSDA	3	0.6903	28.5	10.00	0.62	tr_C9ZNH3_	
DRB1_0101	85	VELMSQMGRSLCGVF	LMSQMGRSL	2	0.6893	28.8	10.00	0.81	tr_C9ZNH3_	
DRB1_0101	86	SRMTFMDSLYNQRRY	MTFMDSLYN	2	0.6890	28.9	10.00	0.63	tr_C9ZNH3_	
DRB1_0101	87	NRTSSAYILSPTPHV	YILSPTPHV	6	0.6890	28.9	10.00	0.71	tr_C9ZNH3_	
DRB1_0101	88	ESGVQMLAPLYTLLF	VQMLAPLYT	3	0.6887	29.0	10.00	0.31	tr_C9ZNH3_	
DRB1_0101	89	WPLARALNAAVPPLT	LARALNAAV	2	0.6882	29.2	10.00	0.44	tr_C9ZNH3_	
DRB1_0101	90	DRTPLALLGFTSASF	PLALLGFTS	3	0.6867	29.7	11.00	0.51	tr_C9ZNH3_	
DRB1_0101	91	ELLALLRYAVSQLHL	LRYAVSQLH	5	0.6863	29.8	11.00	0.54	tr_C9ZNH3_	
DRB1_0101	92	ELMSQMGRSLCGVFT	LMSQMGRSL	1	0.6856	30.0	11.00	0.59	tr_C9ZNH3_	
DRB1_0101	93	MLAPLYTLLFSSVND	YTLLFSSVN	5	0.6855	30.0	11.00	0.68	tr_C9ZNH3_	
DRB1_0101	94	PLYTLLFSSVNDPFA	YTLLFSSVN	2	0.6845	30.4	11.00	0.62	tr_C9ZNH3_	
DRB1_0101	95	DVGGIAAHLSANRNV	VGGIAAHLS	1	0.6823	31.1	11.00	0.41	tr_C9ZNH3_	

DRB1_0101	96	TPLALLGFTSASFLD	PLALLGFTS	1	0.6815	31.4	11.00	0.40	tr_C9ZNH3_
DRB1_0101	97	HLLRIGFMYLQNVHF	FMYLQNVHF	6	0.6812	31.5	11.00	0.68	tr_C9ZNH3_
DRB1_0101	98	FLHVSPAAELLALLR	LHVSPAAEL	1	0.6812	31.5	11.00	0.26	tr_C9ZNH3_
DRB1_0101	99	QGVMIFAPQVSDAMR	GVMIFAPQV	1	0.6807	31.6	11.00	0.43	tr_C9ZNH3_
DRB1_0101	100	DGSLLLIGLTASDVG	LIGLTASDV	5	0.6805	31.7	11.00	0.62	tr_C9ZNH3_
DRB1_0101	101	APLYTLLFSSVNDPF	YTLLFSSVN	3	0.6804	31.7	11.00	0.76	tr_C9ZNH3_
DRB1_0101	102	GQVMFGWIVGEVLAR	FGWIVGEVL	4	0.6800	31.9	11.00	0.59	tr_C9ZNH3_
DRB1_0101	103	ENKNEFFVVVGPLSD	FFVVVGPLS	5	0.6799	31.9	11.00	0.71	tr_C9ZNH3_
DRB1_0101	104	GFTSASFLDAVAQHI	FLDAVAQHI	6	0.6798	31.9	11.00	0.69	tr_C9ZNH3_
DRB1_0101	105	QHIVGVDPQKVLTTI	IVGVDPQKV	2	0.6795	32.1	11.00	0.92	tr_C9ZNH3_
DRB1_0101	106	IAGYLASENASTLHA	YLASENAST	3	0.6785	32.4	12.00	0.58	tr_C9ZNH3_
DRB1_0101	107	VAQHIVGVDPQKVLT	IVGVDPQKV	4	0.6781	32.5	12.00	0.91	tr_C9ZNH3_
DRB1_0101	108	FGWIVGEVLARALEC	FGWIVGEVL	0	0.6768	33.0	12.00	0.32	tr_C9ZNH3_
DRB1_0101	109	GVQMLAPLYTLLFSS	LAPLYTLLF	4	0.6749	33.7	12.00	0.27	tr_C9ZNH3_
DRB1_0101	110	FAPFTGSDAVRGWSP	FTGSDAVRG	3	0.6742	34.0	12.00	0.70	tr_C9ZNH3_
DRB1_0101	111	VLPLVSDNGFRATRR	LVSDNGFRA	3	0.6731	34.4	12.00	0.83	tr_C9ZNH3_
DRB1_0101	112	SLCGVFTLESSFDGE	FTLESSFDG	5	0.6729	34.4	12.00	0.87	tr_C9ZNH3_
DRB1_0101	113	YELAVELMSQMGRSL	LMSQMGRSL	6	0.6721	34.7	12.00	0.49	tr_C9ZNH3_
DRB1_0101	114	SDRTPLALLGFTSAS	PLALLGFTS	4	0.6709	35.2	12.00	0.47	tr_C9ZNH3_
DRB1_0101	115	AGYLASENASTLHAV	YLASENAST	2	0.6684	36.1	13.00	0.48	tr_C9ZNH3_
DRB1_0101	116	YTLLFSSVNDPFARS	FSSVNDPFA	4	0.6678	36.4	13.00	0.45	tr_C9ZNH3_
DRB1_0101	117	RMTFMDSLYNQRRYV	MTFMDSLYN	1	0.6675	36.5	13.00	0.56	tr_C9ZNH3_
DRB1_0101	118	LLLIGLTASDVGGIA	LIGLTASDV	2	0.6666	36.9	13.00	0.75	tr_C9ZNH3_
DRB1_0101	119	LAPLYTLLFSSVNDP	YTLLFSSVN	4	0.6664	36.9	13.00	0.74	tr_C9ZNH3_
DRB1_0101	120	RALNAAVPPLTKPET	LNAAVPPLT	2	0.6652	37.4	13.00	0.86	tr_C9ZNH3_
DRB1_0101	121	GVMIFAPQVSDAMRF	FAPQVSDAM	4	0.6632	38.2	13.00	0.47	tr_C9ZNH3_
DRB1_0101	122	VGGIAAHLSANRNVR	IAAHLSANR	3	0.6632	38.3	13.00	0.38	tr_C9ZNH3_
DRB1_0101	123	GFMYLQNVHFGDSEY	FMYLQNVHF	1	0.6614	39.0	14.00	0.74	tr_C9ZNH3_
DRB1_0101	124	TASDVGGIAAHLSAN	VGGIAAHLS	4	0.6601	39.5	14.00	0.56	tr_C9ZNH3_
DRB1_0101	125	GECEPLAAAYGATCY	CEPLAAAYG	2	0.6600	39.6	14.00	0.64	tr_C9ZNH3_
DRB1_0101	126	EFFVVVGPLSDSDTL	FFVVVGPLS	1	0.6597	39.7	14.00	0.52	tr_C9ZNH3_
DRB1_0101	127	DESGVQMLAPLYTLL	VQMLAPLYT	4	0.6583	40.3	14.00	0.37	tr_C9ZNH3_
DRB1_0101	128	RYAVSQLHLLRIGFM	YAVSQLHLL	1	0.6565	41.1	14.00	0.50	tr_C9ZNH3_
DRB1_0101	129	GVLPLVSDNGFRATR	LVSDNGFRA	4	0.6560	41.4	14.00	0.73	tr_C9ZNH3_
DRB1_0101	130	VFTLESSFDGEADDA	FTLESSFDG	1	0.6555	41.6	14.00	0.92	tr_C9ZNH3_
DRB1_0101	131	VQMLAPLYTLLFSSV	LAPLYTLLF	3	0.6553	41.7	14.00	0.34	tr_C9ZNH3_
DRB1_0101	132	LMSQMGRSLCGVFTL	GRSLCGVFT	5	0.6550	41.8	14.00	0.46	tr_C9ZNH3_
DRB1_0101	133	ISLWPLARALNAAVP	LARALNAAV	5	0.6533	42.6	15.00	0.50	tr_C9ZNH3_
DRB1_0101	134	PLALLGFTSASFLDA	LGFTSASFL	4	0.6515	43.4	15.00	0.18	tr_C9ZNH3_
DRB1_0101	135	GKSERLFIHSVPSTS	FIHSVPSTS	6	0.6512	43.5	15.00	0.62	tr_C9ZNH3_
DRB1_0101	136	GGQVMFGWIVGEVLA	FGWIVGEVL	5	0.6511	43.6	15.00	0.56	tr_C9ZNH3_
DRB1_0101	137	PQGVMIFAPQVSDAM	GVMIFAPQV	2	0.6508	43.7	15.00	0.56	tr_C9ZNH3_
DRB1_0101	138	AERLIFATNLPHWAD	LIFATNLPH	3	0.6496	44.3	15.00	0.44	tr_C9ZNH3_
DRB1_0101	139	FGGECEPLAAAYGAT	CEPLAAAYG	4	0.6491	44.6	15.00	0.73	tr_C9ZNH3_
DRB1_0101	140	VNAGLHASFAVRQWS	LHASFAVRQ	4	0.6478	45.2	15.00	0.45	tr_C9ZNH3_
DRB1_0101	141	GGECEPLAAAYGATC	CEPLAAAYG	3	0.6476	45.3	15.00	0.73	tr_C9ZNH3_
DRB1_0101	142	LWPLARALNAAVPPL	LARALNAAV	3	0.6472	45.5	15.00	0.68	tr_C9ZNH3_
DRB1_0101	143	VLTTIYTHSVISVDD	LTTIYTHSV	1	0.6471	45.5	15.00	0.68	tr_C9ZNH3_
DRB1_0101	144	GVNAGLHASFAVRQW	LHASFAVRQ	5	0.6470	45.6	15.00	0.34	tr_C9ZNH3_
DRB1_0101	145	LPLVSDNGFRATRRL	LVSDNGFRA	2	0.6468	45.7	15.00	0.86	tr_C9ZNH3_

DRB1_0101	146	YILSPTPHVTNIEIA	YILSPTPHV	0	0.6462	46.0	16.00	0.77	tr_C9ZNH3_
DRB1_0101	147	LKSRMTFMDSLYNQR	MTFMDSLYN	4	0.6455	46.3	16.00	0.77	tr_C9ZNH3_
DRB1_0101	148	NDPFARSVNGAIHRG	FARSVNGAI	3	0.6454	46.4	16.00	0.83	tr_C9ZNH3_
DRB1_0101	149	VIAGYLASENASTLH	YLASENAST	4	0.6448	46.7	16.00	0.53	tr_C9ZNH3_
DRB1_0101	150	AELLALLRYAVSQLH	LALLRYAVS	3	0.6436	47.3	16.00	0.38	tr_C9ZNH3_
DRB1_0101	151	ERLIFATNLPHWADH	LIFATNLPH	2	0.6421	48.1	16.00	0.38	tr_C9ZNH3_
DRB1_0101	152	AMRFLMKLVTDNRTS	FLMKLVTDN	3	0.6410	48.6	16.00	0.49	tr_C9ZNH3_
DRB1_0101	153	SAERLIFATNLPHWA	LIFATNLPH	4	0.6407	48.8	16.00	0.47	tr_C9ZNH3_
DRB1_0101	154	CSEFLKSRMTFMDSL	FLKSRMTFM	3	0.6396	49.4	16.00	0.58	tr_C9ZNH3_
DRB1_0101	155	ASDRTPLALLGFTSA	PLALLGFTS	5	0.6393	49.5	17.00	0.47	tr_C9ZNH3_
DRB1_0101	156	VNRTLLSFNRSLNSV	TLLSFNRSL	3	0.6387	49.9	17.00	0.47	tr_C9ZNH3_
DRB1_0101	157	DLVAFAPFTGSDAVR	FAPFTGSDA	4	0.6375	50.5	17.00	0.77	tr_C9ZNH3_
DRB1_0101	158	ECEPLAAAYGATCYC	CEPLAAAYG	1	0.6372	50.7	17.00	0.59	tr_C9ZNH3_
DRB1_0101	159	SLWPLARALNAAVPP	LARALNAAV	4	0.6361	51.3	17.00	0.57	tr_C9ZNH3_
DRB1_0101	160	GIAAHLSANRNVRVF	LSANRNVRV	5	0.6352	51.8	17.00	0.34	tr_C9ZNH3_
DRB1_0101	161	GWSPNAYFLHVSPAA	YFLHVSPAA	6	0.6346	52.1	17.00	0.56	tr_C9ZNH3_
DRB1_0101	162	HIVGVDPQKVLTTIY	IVGVDPQKV	1	0.6342	52.4	17.00	0.87	tr_C9ZNH3_
DRB1_0101	163	RNVIYLSPTLEQQLF	IYLSPTLEQ	3	0.6337	52.6	17.00	0.40	tr_C9ZNH3_
DRB1_0101	164	DFGGECEPLAAAYGA	CEPLAAAYG	5	0.6331	53.0	17.00	0.67	tr_C9ZNH3_
DRB1_0101	165	HISLWPLARALNAAV	LARALNAAV	6	0.6326	53.3	17.00	0.31	tr_C9ZNH3_
DRB1_0101	166	MRFLMKLVTDNRTSS	FLMKLVTDN	2	0.6320	53.6	18.00	0.42	tr_C9ZNH3_
DRB1_0101	167	NGVNAGLHASFAVRQ	NAGLHASFA	3	0.6309	54.2	18.00	0.44	tr_C9ZNH3_
DRB1_0101	168	ATCYCNEGGRMIYMM	YCNEGGRMI	3	0.6309	54.3	18.00	0.61	tr_C9ZNH3_
DRB1_0101	169	GYLASENASTLHAVM	YLASENAST	1	0.6299	54.8	18.00	0.44	tr_C9ZNH3_
DRB1_0101	170	AYNNGVNAGLHASFA	YNNGVNAGL	1	0.6289	55.4	18.00	0.47	tr_C9ZNH3_
DRB1_0101	171	GRNVIYLSPTLEQQL	VIYLSPTLE	3	0.6286	55.6	18.00	0.46	tr_C9ZNH3_
DRB1_0101	172	GGIAAHLSANRNVRV	IAAHLSANR	2	0.6277	56.2	18.00	0.43	tr_C9ZNH3_
DRB1_0101	173	AVAQHIVGVDPQKVL	IVGVDPQKV	5	0.6275	56.3	18.00	0.88	tr_C9ZNH3_
DRB1_0101	174	TDGSLLLIGLTASDV	LIGLTASDV	6	0.6272	56.4	18.00	0.46	tr_C9ZNH3_
DRB1_0101	175	KVYSLLYDPFLPDAY	YSLLYDPFL	2	0.6266	56.8	18.00	0.71	tr_C9ZNH3_
DRB1_0101	176	DPFARSVNGAIHRGA	FARSVNGAI	2	0.6260	57.2	18.00	0.79	tr_C9ZNH3_
DRB1_0101	177	SGVLPLVSDNGFRAT	LVSDNGFRA	5	0.6251	57.7	19.00	0.61	tr_C9ZNH3_
DRB1_0101	178	ECSEFLKSRMTFMDS	FLKSRMTFM	4	0.6248	58.0	19.00	0.55	tr_C9ZNH3_
DRB1_0101	179	EFLKSRMTFMDSLYN	FLKSRMTFM	1	0.6236	58.7	19.00	0.44	tr_C9ZNH3_
DRB1_0101	180	VDPQKVLTTIYTHSV	LTTIYTHSV	6	0.6233	58.9	19.00	0.56	tr_C9ZNH3_
DRB1_0101	181	VNDPFARSVNGAIHR	FARSVNGAI	4	0.6230	59.1	19.00	0.87	tr_C9ZNH3_
DRB1_0101	182	YGGQVMFGWIVGEVL	GQVMFGWIV	2	0.6212	60.2	19.00	0.42	tr_C9ZNH3_
DRB1_0101	183	RLIFATNLPHWADHR	FATNLPHWA	3	0.6212	60.2	19.00	0.46	tr_C9ZNH3_
DRB1_0101	184	FVIAGYLASENASTL	YLASENAST	5	0.6212	60.3	19.00	0.44	tr_C9ZNH3_
DRB1_0101	185	SEFLKSRMTFMDSLY	FLKSRMTFM	2	0.6206	60.7	19.00	0.60	tr_C9ZNH3_
DRB1_0101	186	SEYELAVELMSQMGR	YELAVELMS	2	0.6204	60.8	19.00	0.44	tr_C9ZNH3_
DRB1_0101	187	FLKSRMTFMDSLYNQ	MTFMDSLYN	5	0.6202	60.9	19.00	0.66	tr_C9ZNH3_
DRB1_0101	188	DAMRFLMKLVTDNRT	FLMKLVTDN	4	0.6201	61.0	19.00	0.53	tr_C9ZNH3_
DRB1_0101	189	LTASDVGGIAAHLSA	VGGIAAHLS	5	0.6196	61.3	19.00	0.50	tr_C9ZNH3_
DRB1_0101	190	WELFAGTHPQGVMIF	FAGTHPQGV	3	0.6195	61.4	19.00	0.71	tr_C9ZNH3_
DRB1_0101	191	PDAYNNGVNAGLHAS	YNNGVNAGL	3	0.6193	61.5	19.00	0.67	tr_C9ZNH3_
DRB1_0101	192	LECSEFLKSRMTFMD	FLKSRMTFM	5	0.6180	62.4	20.00	0.46	tr_C9ZNH3_
DRB1_0101	193	HPQGVMIFAPQVSDA	GVMIFAPQV	3	0.6179	62.4	20.00	0.69	tr_C9ZNH3_
DRB1_0101	194	DAYNNGVNAGLHASF	YNNGVNAGL	2	0.6178	62.5	20.00	0.61	tr_C9ZNH3_
DRB1_0101	195	IAAHLSANRNVRVFI	LSANRNVRV	4	0.6174	62.8	20.00	0.56	tr_C9ZNH3_

DRB1_0101	196	VMIFAPQVSDAMRFL	FAPQVSDAM	3	0.6174	62.8	20.00	0.68	tr_C9ZNH3_
DRB1_0101	197	ELFAGTHPQGVMIFA	FAGTHPQGV	2	0.6164	63.4	20.00	0.69	tr_C9ZNH3_
DRB1_0101	198	NAGLHASFAVRQWST	LHASFAVRQ	3	0.6161	63.6	20.00	0.54	tr_C9ZNH3_
DRB1_0101	199	TLLFSSVNDPFARSV	FSSVNDPFA	3	0.6159	63.8	20.00	0.69	tr_C9ZNH3_
DRB1_0101	200	IRRFHANRPNASDRT	FHANRPNAS	3	0.6139	65.2	20.00	0.83	tr_C9ZNH3_
DRB1_0101	201	ALFYDDFIRAFRGLK	DDFIRAFRG	4	0.6118	66.7	21.00	0.57	tr_C9ZNH3_
DRB1_0101	202	TSGVKMFPGQVVLSG	VKMFPGQVV	3	0.6117	66.7	21.00	0.34	tr_C9ZNH3_
DRB1_0101	203	IHRGALFVSGNGHLG	FVSGNGHLG	6	0.6116	66.8	21.00	0.68	tr_C9ZNH3_
DRB1_0101	204	TCYCNEGGRMIYMMI	YCNEGGRMI	2	0.6114	67.0	21.00	0.59	tr_C9ZNH3_
DRB1_0101	205	YAVSQLHLLRIGFMY	LHLLRIGFM	5	0.6103	67.8	21.00	0.40	tr_C9ZNH3_
DRB1_0101	206	TIRRFHANRPNASDR	FHANRPNAS	4	0.6100	68.0	21.00	0.74	tr_C9ZNH3_
DRB1_0101	207	SGVKMFPGQVVLSGV	VKMFPGQVV	2	0.6098	68.2	21.00	0.28	tr_C9ZNH3_
DRB1_0101	208	LLIGLTASDVGGIAA	LIGLTASDV	1	0.6095	68.4	21.00	0.77	tr_C9ZNH3_
DRB1_0101	209	EENKNEFFVVVGPLS	FFVVVGPLS	6	0.6094	68.5	21.00	0.76	tr_C9ZNH3_
DRB1_0101	210	GATCYCNEGGRMIYM	YCNEGGRMI	4	0.6093	68.5	21.00	0.59	tr_C9ZNH3_
DRB1_0101	211	DSEYELAVELMSQMG	YELAVELMS	3	0.6080	69.5	21.00	0.50	tr_C9ZNH3_
DRB1_0101	212	MTFMDSLYNQRRYVI	MDSLYNQRR	3	0.6072	70.1	21.00	0.31	tr_C9ZNH3_
DRB1_0101	213	PLVSDNGFRATRRLR	LVSDNGFRA	1	0.6056	71.3	22.00	0.76	tr_C9ZNH3_
DRB1_0101	214	RMIYMMILGDDYLPR	YMMILGDDY	3	0.6051	71.7	22.00	0.56	tr_C9ZNH3_
DRB1_0101	215	APFTGSDAVRGWSPN	FTGSDAVRG	2	0.6051	71.7	22.00	0.88	tr_C9ZNH3_
DRB1_0101	216	AMHISLWPLARALNA	MHISLWPLA	1	0.6049	71.9	22.00	0.22	tr_C9ZNH3_
DRB1_0101	217	PFARSVNGAIHRGAL	FARSVNGAI	1	0.6036	72.9	22.00	0.69	tr_C9ZNH3_
DRB1_0101	218	YNNGVNAGLHASFAV	NAGLHASFA	5	0.6035	72.9	22.00	0.32	tr_C9ZNH3_
DRB1_0101	219	CDESGVQMLAPLYTL	VQMLAPLYT	5	0.6029	73.5	22.00	0.37	tr_C9ZNH3_
DRB1_0101	220	QQLFVIAGYLASENA	FVIAGYLAS	3	0.6019	74.3	22.00	0.56	tr_C9ZNH3_
DRB1_0101	221	ALECSEFLKSRMTFM	FLKSRMTFM	6	0.6014	74.6	22.00	0.38	tr_C9ZNH3_
DRB1_0101	222	TTSGVKMFPGQVVLS	VKMFPGQVV	4	0.6000	75.8	22.00	0.40	tr_C9ZNH3_
DRB1_0101	223	GDSEYELAVELMSQM	YELAVELMS	4	0.5999	75.8	23.00	0.50	tr_C9ZNH3_
DRB1_0101	224	RFLMKLVTDNRTSSA	FLMKLVTDN	1	0.5997	76.0	23.00	0.37	tr_C9ZNH3_
DRB1_0101	225	SDLRKMLDTRAVTSV	LRKMLDTRA	2	0.5992	76.5	23.00	0.54	tr_C9ZNH3_
DRB1_0101	226	SDAMRFLMKLVTDNR	FLMKLVTDN	5	0.5979	77.5	23.00	0.47	tr_C9ZNH3_
DRB1_0101	227	MIFAPQVSDAMRFLM	FAPQVSDAM	2	0.5977	77.7	23.00	0.66	tr_C9ZNH3_
DRB1_0101	228	PGRNVIYLSPTLEQQ	VIYLSPTLE	4	0.5975	77.9	23.00	0.49	tr_C9ZNH3_
DRB1_0101	229	MHISLWPLARALNAA	WPLARALNA	5	0.5972	78.1	23.00	0.35	tr_C9ZNH3_
DRB1_0101	230	VVNRTLLSFNRSLNS	TLLSFNRSL	4	0.5967	78.6	23.00	0.46	tr_C9ZNH3_
DRB1_0101	231	YGAMHISLWPLARAL	MHISLWPLA	3	0.5963	78.9	23.00	0.34	tr_C9ZNH3_
DRB1_0101	232	LALLGFTSASFLDAV	LGFTSASFL	3	0.5961	79.1	23.00	0.34	tr_C9ZNH3_
DRB1_0101	233	TWELFAGTHPQGVMI	FAGTHPQGV	4	0.5955	79.6	23.00	0.69	tr_C9ZNH3_
DRB1_0101	234	CEPLAAAYGATCYCN	LAAAYGATC	3	0.5953	79.7	23.00	0.41	tr_C9ZNH3_
DRB1_0101	235	TVVNRTLLSFNRSLN	TLLSFNRSL	5	0.5940	80.8	23.00	0.32	tr_C9ZNH3_
DRB1_0101	236	EYELAVELMSQMGRS	YELAVELMS	1	0.5926	82.1	24.00	0.36	tr_C9ZNH3_
DRB1_0101	237	GVKMFPGQVVLSGVL	MFPGQVVLS	3	0.5923	82.3	24.00	0.35	tr_C9ZNH3_
DRB1_0101	238	GAMHISLWPLARALN	MHISLWPLA	2	0.5921	82.6	24.00	0.26	tr_C9ZNH3_
DRB1_0101	239	MSDLRKMLDTRAVTS	LRKMLDTRA	3	0.5919	82.7	24.00	0.65	tr_C9ZNH3_
DRB1_0101	240	LPDAYNNGVNAGLHA	YNNGVNAGL	4	0.5918	82.8	24.00	0.67	tr_C9ZNH3_
DRB1_0101	241	NNGVNAGLHASFAVR	NAGLHASFA	4	0.5915	83.1	24.00	0.46	tr_C9ZNH3_
DRB1_0101	242	QLFVIAGYLASENAS	FVIAGYLAS	2	0.5911	83.5	24.00	0.52	tr_C9ZNH3_
DRB1_0101	243	GWIVGEVLARALECS	WIVGEVLAR	1	0.5909	83.6	24.00	0.33	tr_C9ZNH3_
DRB1_0101	244	LLFSSVNDPFARSVN	FSSVNDPFA	2	0.5901	84.3	24.00	0.69	tr_C9ZNH3_
DRB1_0101	245	SQMGRSLCGVFTLES	GRSLCGVFT	3	0.5878	86.5	24.00	0.80	tr_C9ZNH3_

DRB1_0101	246	FLDAVAQHIVGVDPQ	FLDAVAQHI	0	0.5873	86.9	25.00	0.63	tr_C9ZNH3_
DRB1_0101	247	VYSLLYDPFLPDAYN	YSLLYDPFL	1	0.5862	88.0	25.00	0.65	tr_C9ZNH3_
DRB1_0101	248	CYCNEGGRMIYMMIL	YCNEGGRMI	1	0.5859	88.3	25.00	0.49	tr_C9ZNH3_
DRB1_0101	249	FVSGNGHLGKSERLF	FVSGNGHLG	0	0.5859	88.3	25.00	0.73	tr_C9ZNH3_
DRB1_0101	250	NVIYLSPTLEQQLFV	IYLSPTLEQ	2	0.5852	89.0	25.00	0.37	tr_C9ZNH3_
DRB1_0101	251	THPQGVMIFAPQVSD	GVMIFAPQV	4	0.5847	89.4	25.00	0.74	tr_C9ZNH3_
DRB1_0101	252	RRFHANRPNASDRTP	FHANRPNAS	2	0.5839	90.2	25.00	0.88	tr_C9ZNH3_
DRB1_0101	253	EQQLFVIAGYLASEN	FVIAGYLAS	4	0.5838	90.3	25.00	0.51	tr_C9ZNH3_
DRB1_0101	254	KSAERLIFATNLPHW	LIFATNLPH	5	0.5836	90.5	25.00	0.48	tr_C9ZNH3_
DRB1_0101	255	EDLVAFAPFTGSDAV	FAPFTGSDA	5	0.5836	90.5	25.00	0.68	tr_C9ZNH3_
DRB1_0101	256	FFVVVGPLSDSDTLS	FVVVGPLSD	1	0.5831	91.0	25.00	0.45	tr_C9ZNH3_
DRB1_0101	257	FIHSVPSTSGNVMSD	FIHSVPSTS	0	0.5829	91.2	25.00	0.62	tr_C9ZNH3_
DRB1_0101	258	LFAGTHPQGVMIFAP	FAGTHPQGV	1	0.5823	91.8	25.00	0.64	tr_C9ZNH3_
DRB1_0101	259	IKAYALSGTGAVEFD	YALSGTGAV	3	0.5821	92.0	25.00	0.58	tr_C9ZNH3_
DRB1_0101	260	DLRKMLDTRAVTSVL	LRKMLDTRA	1	0.5818	92.2	25.00	0.47	tr_C9ZNH3_
DRB1_0101	261	ALNAAVPPLTKPETP	LNAAVPPLT	1	0.5818	92.3	26.00	0.86	tr_C9ZNH3_
DRB1_0101	262	DIKAYALSGTGAVEF	YALSGTGAV	4	0.5805	93.6	26.00	0.58	tr_C9ZNH3_
DRB1_0101	263	GTHPQGVMIFAPQVS	GVMIFAPQV	5	0.5803	93.8	26.00	0.68	tr_C9ZNH3_
DRB1_0101	264	NYGAMHISLWPLARA	MHISLWPLA	4	0.5797	94.4	26.00	0.33	tr_C9ZNH3_
DRB1_0101	265	MSQMGRSLCGVFTLE	GRSLCGVFT	4	0.5793	94.8	26.00	0.71	tr_C9ZNH3_
DRB1_0101	266	LFVIAGYLASENAST	FVIAGYLAS	1	0.5776	96.6	26.00	0.43	tr_C9ZNH3_
DRB1_0101	267	ETVVNRTLLSFNRSL	TVVNRTLLS	1	0.5775	96.7	26.00	0.34	tr_C9ZNH3_
DRB1_0101	268	GRMIYMMILGDDYLP	YMMILGDDY	4	0.5771	97.0	26.00	0.55	tr_C9ZNH3_
DRB1_0101	269	RSLCGVFTLESSFDG	FTLESSFDG	6	0.5765	97.7	26.00	0.79	tr_C9ZNH3_
DRB1_0101	270	FGDSEYELAVELMSQ	YELAVELMS	5	0.5764	97.9	26.00	0.46	tr_C9ZNH3_
DRB1_0101	271	VTNIEIAWTLAGTTS	IEIAWTLAG	3	0.5760	98.3	26.00	0.56	tr_C9ZNH3_
DRB1_0101	272	GGRMIYMMILGDDYL	YMMILGDDY	5	0.5743	100.1	27.00	0.60	tr_C9ZNH3_
DRB1_0101	273	ETIRRFHANRPNASD	FHANRPNAS	5	0.5741	100.2	27.00	0.64	tr_C9ZNH3_
DRB1_0101	274	VSQLHLLRIGFMYLQ	LHLLRIGFM	3	0.5734	101.1	27.00	0.57	tr_C9ZNH3_
DRB1_0101	275	AGLHASFAVRQWSTA	LHASFAVRQ	2	0.5723	102.3	27.00	0.55	tr_C9ZNH3_
DRB1_0101	276	EDCSRIVGNHTDAVD	CSRIVGNHT	2	0.5691	105.9	28.00	0.74	tr_C9ZNH3_
DRB1_0101	277	LEQQLFVIAGYLASE	FVIAGYLAS	5	0.5687	106.4	28.00	0.44	tr_C9ZNH3_
DRB1_0101	278	QMGRSLCGVFTLESS	GRSLCGVFT	2	0.5684	106.6	28.00	0.77	tr_C9ZNH3_
DRB1_0101	279	LHVSPAAELLALLRY	HVSPAAELL	1	0.5674	107.8	28.00	0.29	tr_C9ZNH3_
DRB1_0101	280	LTTIYTHSVISVDDM	LTTIYTHSV	0	0.5660	109.5	28.00	0.47	tr_C9ZNH3_
DRB1_0101	281	IFAPQVSDAMRFLMK	FAPQVSDAM	1	0.5659	109.6	28.00	0.58	tr_C9ZNH3_
DRB1_0101	282	HVTNIEIAWTLAGTT	IEIAWTLAG	4	0.5655	110.1	28.00	0.51	tr_C9ZNH3_
DRB1_0101	283	LSGVLPLVSDNGFRA	VLPLVSDNG	3	0.5651	110.6	28.00	0.46	tr_C9ZNH3_
DRB1_0101	284	VTDNRTSSAYILSPT	NRTSSAYIL	3	0.5648	110.9	29.00	0.72	tr_C9ZNH3_
DRB1_0101	285	NGFRATRRLRADIKA	FRATRRLRA	2	0.5646	111.2	29.00	0.54	tr_C9ZNH3_
DRB1_0101	286	TNIEIAWTLAGTTSG	IEIAWTLAG	2	0.5643	111.6	29.00	0.49	tr_C9ZNH3_
DRB1_0101	287	ALLGFTSASFLDAVA	FTSASFLDA	4	0.5641	111.7	29.00	0.34	tr_C9ZNH3_
DRB1_0101	288	PFTGSDAVRGWSPNA	FTGSDAVRG	1	0.5640	111.9	29.00	0.79	tr_C9ZNH3_
DRB1_0101	289	FMYLQNVHFGDSEYE	FMYLQNVHF	0	0.5637	112.3	29.00	0.61	tr_C9ZNH3_
DRB1_0101	290	SVNDPFARSVNGAIH	FARSVNGAI	5	0.5629	113.2	29.00	0.81	tr_C9ZNH3_
DRB1_0101	291	MIYMMILGDDYLPRP	YMMILGDDY	2	0.5625	113.7	29.00	0.55	tr_C9ZNH3_
DRB1_0101	292	AAHLSANRNVRVFIP	LSANRNVRV	3	0.5608	115.8	29.00	0.69	tr_C9ZNH3_
DRB1_0101	293	SVNGAIHRGALFVSG	GAIHRGALF	3	0.5599	116.9	29.00	0.54	tr_C9ZNH3_
DRB1_0101	294	VALFYDDFIRAFRGL	DDFIRAFRG	5	0.5595	117.4	29.00	0.54	tr_C9ZNH3_
DRB1_0101	295	SQLHLLRIGFMYLQN	LHLLRIGFM	2	0.5592	117.8	30.00	0.52	tr_C9ZNH3_

DRB1_0101	296	AVSQLHLLRIGFMYL	LHLLRIGFM	4	0.5592	117.8	30.00	0.56	tr_C9ZNH3_
DRB1_0101	297	NVRVFIPFFDVALFY	FIPFFDVAL	4	0.5590	118.1	30.00	0.57	tr_C9ZNH3_
DRB1_0101	298	YGATCYCNEGGRMIY	YCNEGGRMI	5	0.5590	118.1	30.00	0.60	tr_C9ZNH3_
DRB1_0101	299	VMSDLRKMLDTRAVT	LRKMLDTRA	4	0.5589	118.2	30.00	0.68	tr_C9ZNH3_
DRB1_0101	300	NIEIAWTLAGTTSGV	WTLAGTTSG	5	0.5588	118.4	30.00	0.32	tr_C9ZNH3_
DRB1_0101	301	DCSRIVGNHTDAVDG	CSRIVGNHT	1	0.5586	118.7	30.00	0.68	tr_C9ZNH3_
DRB1_0101	302	AAELLALLRYAVSQL	LALLRYAVS	4	0.5579	119.5	30.00	0.56	tr_C9ZNH3_
DRB1_0101	303	LVTDNRTSSAYILSP	NRTSSAYIL	4	0.5577	119.7	30.00	0.68	tr_C9ZNH3_
DRB1_0101	304	VNYGAMHISLWPLAR	MHISLWPLA	5	0.5577	119.7	30.00	0.29	tr_C9ZNH3_
DRB1_0101	305	KLVTDNRTSSAYILS	NRTSSAYIL	5	0.5575	120.0	30.00	0.62	tr_C9ZNH3_
DRB1_0101	306	GDFGGECEPLAAAYG	CEPLAAAYG	6	0.5567	121.1	30.00	0.50	tr_C9ZNH3_
DRB1_0101	307	FARSVNGAIHRGALF	FARSVNGAI	0	0.5567	121.1	30.00	0.44	tr_C9ZNH3_
DRB1_0101	308	LLGFTSASFLDAVAQ	FTSASFLDA	3	0.5550	123.4	30.00	0.46	tr_C9ZNH3_
DRB1_0101	309	GTTSGVKMFPGQVVL	GVKMFPGQV	4	0.5547	123.7	30.00	0.42	tr_C9ZNH3_
DRB1_0101	310	ATWELFAGTHPQGVM	FAGTHPQGV	5	0.5545	123.9	30.00	0.62	tr_C9ZNH3_
DRB1_0101	311	KAYALSGTGAVEFDP	YALSGTGAV	2	0.5543	124.2	30.00	0.58	tr_C9ZNH3_
DRB1_0101	312	TRLIETVVNRTLLSF	IETVVNRTL	3	0.5543	124.2	30.00	0.38	tr_C9ZNH3_
DRB1_0101	313	DEDCSRIVGNHTDAV	CSRIVGNHT	3	0.5522	127.2	31.00	0.76	tr_C9ZNH3_
DRB1_0101	314	VNGAIHRGALFVSGN	GAIHRGALF	2	0.5520	127.5	31.00	0.50	tr_C9ZNH3_
DRB1_0101	315	RNVRVFIPFFDVALF	FIPFFDVAL	5	0.5517	127.8	31.00	0.52	tr_C9ZNH3_
DRB1_0101	316	GFRATRRLRADIKAY	TRRLRADIK	4	0.5515	128.0	31.00	0.47	tr_C9ZNH3_
DRB1_0101	317	GLHASFAVRQWSTAS	LHASFAVRQ	1	0.5515	128.1	31.00	0.44	tr_C9ZNH3_
DRB1_0101	318	HPGRNVIYLSPTLEQ	VIYLSPTLE	5	0.5506	129.3	31.00	0.52	tr_C9ZNH3_
DRB1_0101	319	EIAWTLAGTTSGVKM	WTLAGTTSG	3	0.5492	131.4	31.00	0.63	tr_C9ZNH3_
DRB1_0101	320	TLEQQLFVIAGYLAS	FVIAGYLAS	6	0.5488	131.9	31.00	0.32	tr_C9ZNH3_
DRB1_0101	321	FRATRRLRADIKAYA	TRRLRADIK	3	0.5487	132.0	31.00	0.57	tr_C9ZNH3_
DRB1_0101	322	IEIAWTLAGTTSGVK	WTLAGTTSG	4	0.5476	133.7	32.00	0.51	tr_C9ZNH3_
DRB1_0101	323	LRKMLDTRAVTSVLG	RKMLDTRAV	1	0.5467	134.9	32.00	0.25	tr_C9ZNH3_
DRB1_0101	324	RLIETVVNRTLLSFN	TVVNRTLLS	4	0.5457	136.4	32.00	0.45	tr_C9ZNH3_
DRB1_0101	325	PHVTNIEIAWTLAGT	IEIAWTLAG	5	0.5455	136.7	32.00	0.43	tr_C9ZNH3_
DRB1_0101	326	PGQVVLSGVLPLVSD	VVLSGVLPL	3	0.5452	137.1	32.00	0.50	tr_C9ZNH3_
DRB1_0101	327	LVSDNGFRATRRLRA	LVSDNGFRA	0	0.5452	137.1	32.00	0.49	tr_C9ZNH3_
DRB1_0101	328	QLHLLRIGFMYLQNV	LHLLRIGFM	1	0.5450	137.3	32.00	0.41	tr_C9ZNH3_
DRB1_0101	329	VSDAMRFLMKLVTDN	FLMKLVTDN	6	0.5450	137.5	32.00	0.31	tr_C9ZNH3_
DRB1_0101	330	DNGFRATRRLRADIK	FRATRRLRA	3	0.5438	139.1	32.00	0.61	tr_C9ZNH3_
DRB1_0101	331	NVMSDLRKMLDTRAV	LRKMLDTRA	5	0.5434	139.8	32.00	0.58	tr_C9ZNH3_
DRB1_0101	332	NASDRTPLALLGFTS	PLALLGFTS	6	0.5428	140.7	33.00	0.47	tr_C9ZNH3_
DRB1_0101	333	TDNRTSSAYILSPTP	NRTSSAYIL	2	0.5422	141.6	33.00	0.73	tr_C9ZNH3_
DRB1_0101	334	LFSSVNDPFARSVNG	FSSVNDPFA	1	0.5422	141.7	33.00	0.70	tr_C9ZNH3_
DRB1_0101	335	VRVFIPFFDVALFYD	FIPFFDVAL	3	0.5420	141.9	33.00	0.62	tr_C9ZNH3_
DRB1_0101	336	GQVVLSGVLPLVSDN	VVLSGVLPL	2	0.5414	142.9	33.00	0.47	tr_C9ZNH3_
DRB1_0101	337	LIETVVNRTLLSFNR	TVVNRTLLS	3	0.5413	143.0	33.00	0.53	tr_C9ZNH3_
DRB1_0101	338	ADIKAYALSGTGAVE	YALSGTGAV	5	0.5406	144.2	33.00	0.50	tr_C9ZNH3_
DRB1_0101	339	RSVNGAIHRGALFVS	GAIHRGALF	4	0.5396	145.7	33.00	0.58	tr_C9ZNH3_
DRB1_0101	340	FLPDAYNNGVNAGLH	YNNGVNAGL	5	0.5394	146.0	33.00	0.57	tr_C9ZNH3_
DRB1_0101	341	MGRSLCGVFTLESSF	GRSLCGVFT	1	0.5387	147.1	33.00	0.69	tr_C9ZNH3_
DRB1_0101	342	FAVRQWSTASNVNVE	WSTASNVNV	5	0.5373	149.4	34.00	0.49	tr_C9ZNH3_
DRB1_0101	343	APLNAALPTDGSLLL	LNAALPTDG	2	0.5371	149.8	34.00	0.51	tr_C9ZNH3_
DRB1_0101	344	IVGVDPQKVLTTIYT	IVGVDPQKV	0	0.5362	151.1	34.00	0.62	tr_C9ZNH3_
DRB1_0101	345	VRQWSTASNVNVEVI	WSTASNVNV	3	0.5361	151.3	34.00	0.74	tr_C9ZNH3_

DRB1_0101	346	IAWTLAGTTSGVKMF	WTLAGTTSG	2	0.5361	151.3	34.00	0.64	tr_C9ZNH3_
DRB1_0101	347	SFAVRQWSTASNVNV	FAVRQWSTA	1	0.5359	151.7	34.00	0.29	tr_C9ZNH3_
DRB1_0101	348	IYMMILGDDYLPRPA	YMMILGDDY	1	0.5349	153.3	34.00	0.49	tr_C9ZNH3_
DRB1_0101	349	FPGQVVLSGVLPLVS	VVLSGVLPL	4	0.5345	153.9	34.00	0.54	tr_C9ZNH3_
DRB1_0101	350	QRRYVIDDIVIGDFG	YVIDDIVIG	3	0.5343	154.2	34.00	0.86	tr_C9ZNH3_
DRB1_0101	351	LHLLRIGFMYLQNVH	IGFMYLQNV	5	0.5332	156.0	34.00	0.26	tr_C9ZNH3_
DRB1_0101	352	QVVLSGVLPLVSDNG	VVLSGVLPL	1	0.5331	156.2	34.00	0.34	tr_C9ZNH3_
DRB1_0101	353	ATRLIETVVNRTLLS	IETVVNRTL	4	0.5326	157.1	35.00	0.31	tr_C9ZNH3_
DRB1_0101	354	RGLKSAERLIFATNL	ERLIFATNL	6	0.5322	157.8	35.00	0.28	tr_C9ZNH3_
DRB1_0101	355	YLASENASTLHAVMR	LASENASTL	1	0.5311	159.7	35.00	0.49	tr_C9ZNH3_
DRB1_0101	356	VIYLSPTLEQQLFVI	IYLSPTLEQ	1	0.5307	160.4	35.00	0.34	tr_C9ZNH3_
DRB1_0101	357	PLNAALPTDGSLLLI	ALPTDGSLL	4	0.5305	160.7	35.00	0.46	tr_C9ZNH3_
DRB1_0101	358	WIVGEVLARALECSE	IVGEVLARA	1	0.5298	162.0	35.00	0.31	tr_C9ZNH3_
DRB1_0101	359	IETVVNRTLLSFNRS	TVVNRTLLS	2	0.5295	162.4	35.00	0.51	tr_C9ZNH3_
DRB1_0101	360	LIFATNLPHWADHRP	FATNLPHWA	2	0.5295	162.5	35.00	0.56	tr_C9ZNH3_
DRB1_0101	361	PAAELLALLRYAVSQ	LALLRYAVS	5	0.5294	162.7	35.00	0.56	tr_C9ZNH3_
DRB1_0101	362	HFGDSEYELAVELMS	SEYELAVEL	4	0.5283	164.7	35.00	0.34	tr_C9ZNH3_
DRB1_0101	363	LVNYGAMHISLWPLA	YGAMHISLW	3	0.5280	165.2	35.00	0.29	tr_C9ZNH3_
DRB1_0101	364	GLTASDVGGIAAHLS	DVGGIAAHL	5	0.5280	165.2	35.00	0.44	tr_C9ZNH3_
DRB1_0101	365	AYGGQVMFGWIVGEV	GQVMFGWIV	3	0.5277	165.7	35.00	0.80	tr_C9ZNH3_
DRB1_0101	366	NGAIHRGALFVSGNG	GAIHRGALF	1	0.5276	165.9	36.00	0.47	tr_C9ZNH3_
DRB1_0101	367	RATRRLRADIKAYAL	TRRLRADIK	2	0.5262	168.4	36.00	0.54	tr_C9ZNH3_
DRB1_0101	368	LKSAERLIFATNLPH	ERLIFATNL	4	0.5259	169.0	36.00	0.49	tr_C9ZNH3_
DRB1_0101	369	AVRQWSTASNVNVEV	WSTASNVNV	4	0.5241	172.3	36.00	0.69	tr_C9ZNH3_
DRB1_0101	370	VKMFPGQVVLSGVLP	MFPGQVVLS	2	0.5239	172.7	36.00	0.41	tr_C9ZNH3_
DRB1_0101	371	NQRRYVIDDIVIGDF	YVIDDIVIG	4	0.5232	173.9	36.00	0.85	tr_C9ZNH3_
DRB1_0101	372	RFHANRPNASDRTPL	FHANRPNAS	1	0.5231	174.2	36.00	0.89	tr_C9ZNH3_
DRB1_0101	373	DGCLVNYGAMHISLW	LVNYGAMHI	3	0.5226	175.2	36.00	0.37	tr_C9ZNH3_
DRB1_0101	374	DNRTSSAYILSPTPH	NRTSSAYIL	1	0.5222	175.8	37.00	0.69	tr_C9ZNH3_
DRB1_0101	375	AGTHPQGVMIFAPQV	GVMIFAPQV	6	0.5200	180.1	37.00	0.56	tr_C9ZNH3_
DRB1_0101	376	AAYGGQVMFGWIVGE	GQVMFGWIV	4	0.5173	185.4	38.00	0.76	tr_C9ZNH3_
DRB1_0101	377	SPAAELLALLRYAVS	LALLRYAVS	6	0.5160	188.0	38.00	0.42	tr_C9ZNH3_
DRB1_0101	378	SETIRRFHANRPNAS	FHANRPNAS	6	0.5149	190.4	38.00	0.47	tr_C9ZNH3_
DRB1_0101	379	YSLLYDPFLPDAYNN	YSLLYDPFL	0	0.5140	192.1	38.00	0.50	tr_C9ZNH3_
DRB1_0101	380	TFMDSLYNQRRYVID	MDSLYNQRR	2	0.5138	192.7	38.00	0.38	tr_C9ZNH3_
DRB1_0101	381	LGFTSASFLDAVAQH	FTSASFLDA	2	0.5128	194.6	38.00	0.49	tr_C9ZNH3_
DRB1_0101	382	DAAYGGQVMFGWIVG	GQVMFGWIV	5	0.5123	195.7	39.00	0.69	tr_C9ZNH3_
DRB1_0101	383	LIGLTASDVGGIAAH	LIGLTASDV	0	0.5118	196.7	39.00	0.63	tr_C9ZNH3_
DRB1_0101	384	RVFIPFFDVALFYDD	FIPFFDVAL	2	0.5113	197.9	39.00	0.60	tr_C9ZNH3_
DRB1_0101	385	FTLESSFDGEADDAE	FTLESSFDG	0	0.5109	198.7	39.00	0.82	tr_C9ZNH3_
DRB1_0101	386	RKMLDTRAVTSVLGV	TRAVTSVLG	5	0.5108	198.9	39.00	0.37	tr_C9ZNH3_
DRB1_0101	387	CLVNYGAMHISLWPL	LVNYGAMHI	1	0.5098	201.2	39.00	0.28	tr_C9ZNH3_
DRB1_0101	388	NRNVRVFIPFFDVAL	FIPFFDVAL	6	0.5096	201.6	39.00	0.38	tr_C9ZNH3_
DRB1_0101	389	VVLSGVLPLVSDNGF	VLPLVSDNG	5	0.5092	202.5	39.00	0.34	tr_C9ZNH3_
DRB1_0101	390	ATRRLRADIKAYALS	TRRLRADIK	1	0.5083	204.5	39.00	0.41	tr_C9ZNH3_
DRB1_0101	391	YCNEGGRMIYMMILG	YCNEGGRMI	0	0.5076	205.9	39.00	0.38	tr_C9ZNH3_
DRB1_0101	392	ACDESGVQMLAPLYT	GVQMLAPLY	5	0.5070	207.3	40.00	0.34	tr_C9ZNH3_
DRB1_0101	393	RRYVIDDIVIGDFGG	YVIDDIVIG	2	0.5067	207.9	40.00	0.81	tr_C9ZNH3_
DRB1_0101	394	GCLVNYGAMHISLWP	LVNYGAMHI	2	0.5060	209.5	40.00	0.35	tr_C9ZNH3_
DRB1_0101	395	RQWSTASNVNVEVIH	WSTASNVNV	2	0.5038	214.6	40.00	0.79	tr_C9ZNH3_

DRB1_0101	396	ARSVNGAIHRGALFV	GAIHRGALF	5	0.5035	215.4	40.00	0.54	tr_C9ZNH3_
DRB1_0101	397	GNVMSDLRKMLDTRA	NVMSDLRKM	1	0.5025	217.7	41.00	0.41	tr_C9ZNH3_
DRB1_0101	398	VDGCLVNYGAMHISL	LVNYGAMHI	4	0.5020	218.9	41.00	0.40	tr_C9ZNH3_
DRB1_0101	399	PTDGSLLLIGLTASD	SLLLIGLTA	4	0.5012	220.7	41.00	0.31	tr_C9ZNH3_
DRB1_0101	400	DAPLNAALPTDGSLL	LNAALPTDG	3	0.5011	221.0	41.00	0.58	tr_C9ZNH3_
DRB1_0101	401	FAPQVSDAMRFLMKL	FAPQVSDAM	0	0.5003	222.9	41.00	0.31	tr_C9ZNH3_
DRB1_0101	402	AHLSANRNVRVFIPF	LSANRNVRV	2	0.5002	223.1	41.00	0.78	tr_C9ZNH3_
DRB1_0101	403	LHASFAVRQWSTASN	FAVRQWSTA	4	0.4992	225.5	41.00	0.47	tr_C9ZNH3_
DRB1_0101	404	TPHVTNIEIAWTLAG	TNIEIAWTL	4	0.4984	227.6	41.00	0.34	tr_C9ZNH3_
DRB1_0101	405	NPRLRHPGRNVIYLS	RLRHPGRNV	2	0.4983	227.7	41.00	0.33	tr_C9ZNH3_
DRB1_0101	406	LNAALPTDGSLLLIG	ALPTDGSLL	3	0.4967	231.7	42.00	0.60	tr_C9ZNH3_
DRB1_0101	407	KMFPGQVVLSGVLPL	MFPGQVVLS	1	0.4967	231.8	42.00	0.33	tr_C9ZNH3_
DRB1_0101	408	ASFAVRQWSTASNVN	FAVRQWSTA	2	0.4961	233.4	42.00	0.49	tr_C9ZNH3_
DRB1_0101	409	MFPGQVVLSGVLPLV	VVLSGVLPL	5	0.4960	233.4	42.00	0.52	tr_C9ZNH3_
DRB1_0101	410	ADEDCSRIVGNHTDA	CSRIVGNHT	4	0.4957	234.2	42.00	0.86	tr_C9ZNH3_
DRB1_0101	411	VLSGVLPLVSDNGFR	VLPLVSDNG	4	0.4949	236.2	42.00	0.52	tr_C9ZNH3_
DRB1_0101	412	HVSPAAELLALLRYA	PAAELLALL	3	0.4947	236.8	42.00	0.22	tr_C9ZNH3_
DRB1_0101	413	FAGTHPQGVMIFAPQ	FAGTHPQGV	0	0.4945	237.3	42.00	0.51	tr_C9ZNH3_
DRB1_0101	414	EEDLVAFAPFTGSDA	FAPFTGSDA	6	0.4940	238.6	42.00	0.47	tr_C9ZNH3_
DRB1_0101	415	EGGRMIYMMILGDDY	YMMILGDDY	6	0.4939	239.0	42.00	0.52	tr_C9ZNH3_
DRB1_0101	416	CSRIVGNHTDAVDGC	CSRIVGNHT	0	0.4936	239.7	42.00	0.57	tr_C9ZNH3_
DRB1_0101	417	HASFAVRQWSTASNV	FAVRQWSTA	3	0.4929	241.4	42.00	0.56	tr_C9ZNH3_
DRB1_0101	418	DATRLIETVVNRTLL	IETVVNRTL	5	0.4928	241.6	42.00	0.28	tr_C9ZNH3_
DRB1_0101	419	YNQRRYVIDDIVIGD	YVIDDIVIG	5	0.4919	244.1	43.00	0.79	tr_C9ZNH3_
DRB1_0101	420	KMLDTRAVTSVLGVV	TRAVTSVLG	4	0.4903	248.5	43.00	0.51	tr_C9ZNH3_
DRB1_0101	421	SDNGFRATRRLRADI	FRATRRLRA	4	0.4885	253.3	43.00	0.68	tr_C9ZNH3_
DRB1_0101	422	HPLSYEVPPPELLRS	YEVPPPELL	4	0.4884	253.4	43.00	0.46	tr_C9ZNH3_
DRB1_0101	423	AYALSGTGAVEFDPL	YALSGTGAV	1	0.4880	254.7	43.00	0.54	tr_C9ZNH3_
DRB1_0101	424	GLKSAERLIFATNLP	ERLIFATNL	5	0.4869	257.6	44.00	0.48	tr_C9ZNH3_
DRB1_0101	425	PLSYEVPPPELLRSI	YEVPPPELL	3	0.4859	260.5	44.00	0.51	tr_C9ZNH3_
DRB1_0101	426	DVALFYDDFIRAFRG	DDFIRAFRG	6	0.4858	260.7	44.00	0.41	tr_C9ZNH3_
DRB1_0101	427	LNPRLRHPGRNVIYL	RLRHPGRNV	3	0.4853	262.3	44.00	0.43	tr_C9ZNH3_
DRB1_0101	428	PTLEQQLFVIAGYLA	QLFVIAGYL	5	0.4843	264.9	44.00	0.25	tr_C9ZNH3_
DRB1_0101	429	QVSDAMRFLMKLVTD	MRFLMKLVT	5	0.4840	266.0	44.00	0.28	tr_C9ZNH3_
DRB1_0101	430	IYLSPTLEQQLFVIA	PTLEQQLFV	4	0.4827	269.6	45.00	0.23	tr_C9ZNH3_
DRB1_0101	431	AWTLAGTTSGVKMFP	WTLAGTTSG	1	0.4822	271.0	45.00	0.67	tr_C9ZNH3_
DRB1_0101	432	RADIKAYALSGTGAV	YALSGTGAV	6	0.4819	271.9	45.00	0.38	tr_C9ZNH3_
DRB1_0101	433	FLMKLVTDNRTSSAY	MKLVTDNRT	2	0.4819	272.1	45.00	0.35	tr_C9ZNH3_
DRB1_0101	434	GRSLCGVFTLESSFD	GRSLCGVFT	0	0.4818	272.4	45.00	0.59	tr_C9ZNH3_
DRB1_0101	435	FTGSDAVRGWSPNAY	FTGSDAVRG	0	0.4812	274.0	45.00	0.55	tr_C9ZNH3_
DRB1_0101	436	AGTTSGVKMFPGQVV	GVKMFPGQV	5	0.4809	275.0	45.00	0.44	tr_C9ZNH3_
DRB1_0101	437	IHPLSYEVPPPELLR	YEVPPPELL	5	0.4805	276.1	45.00	0.40	tr_C9ZNH3_
DRB1_0101	438	TRRLRADIKAYALSG	LRADIKAYA	3	0.4801	277.3	45.00	0.47	tr_C9ZNH3_
DRB1_0101	439	PLAFDGDAAYGGQVM	FDGDAAYGG	3	0.4778	284.3	46.00	0.62	tr_C9ZNH3_
DRB1_0101	440	PQVSDAMRFLMKLVT	SDAMRFLMK	3	0.4763	289.0	46.00	0.23	tr_C9ZNH3_
DRB1_0101	441	TATWELFAGTHPQGV	FAGTHPQGV	6	0.4754	291.7	46.00	0.46	tr_C9ZNH3_
DRB1_0101	442	MKLVTDNRTSSAYIL	NRTSSAYIL	6	0.4753	292.2	46.00	0.44	tr_C9ZNH3_
DRB1_0101	443	LPTDGSLLLIGLTAS	SLLLIGLTA	5	0.4728	300.0	47.00	0.31	tr_C9ZNH3_
DRB1_0101	444	LAFDGDAAYGGQVMF	FDGDAAYGG	2	0.4728	300.2	47.00	0.60	tr_C9ZNH3_
DRB1_0101	445	RSIIEENKNEFFVVV	IEENKNEFF	3	0.4726	300.6	47.00	0.62	tr_C9ZNH3_

DRB1_0101	446	AVDGCLVNYGAMHIS	LVNYGAMHI	5	0.4721	302.3	47.00	0.38	tr_C9ZNH3_
DRB1_0101	447	PRLRHPGRNVIYLSP	LRHPGRNVI	2	0.4698	310.0	47.00	0.31	tr_C9ZNH3_
DRB1_0101	448	RALECSEFLKSRMTF	SEFLKSRMT	5	0.4692	311.9	47.00	0.57	tr_C9ZNH3_
DRB1_0101	449	SDTLSVLPSLEEEDL	LSVLPSLEE	3	0.4682	315.3	48.00	0.64	tr_C9ZNH3_
DRB1_0101	450	FMDSLYNQRRYVIDD	MDSLYNQRR	1	0.4681	315.6	48.00	0.35	tr_C9ZNH3_
DRB1_0101	451	GDAAYGGQVMFGWIV	GQVMFGWIV	6	0.4679	316.4	48.00	0.57	tr_C9ZNH3_
DRB1_0101	452	RHPGRNVIYLSPTLE	VIYLSPTLE	6	0.4673	318.5	48.00	0.50	tr_C9ZNH3_
DRB1_0101	453	MLDTRAVTSVLGVVD	TRAVTSVLG	3	0.4662	322.4	48.00	0.59	tr_C9ZNH3_
DRB1_0101	454	LGKSERLFIHSVPST	SERLFIHSV	3	0.4645	328.2	48.00	0.38	tr_C9ZNH3_
DRB1_0101	455	YMMILGDDYLPRPAA	MMILGDDYL	1	0.4637	331.2	49.00	0.29	tr_C9ZNH3_
DRB1_0101	456	SSVNDPFARSVNGAI	FARSVNGAI	6	0.4634	332.3	49.00	0.62	tr_C9ZNH3_
DRB1_0101	457	DAVAQHIVGVDPQKV	IVGVDPQKV	6	0.4614	339.4	49.00	0.84	tr_C9ZNH3_
DRB1_0101	458	FADEDCSRIVGNHTD	CSRIVGNHT	5	0.4612	340.3	49.00	0.74	tr_C9ZNH3_
DRB1_0101	459	GVDPQKVLTTIYTHS	QKVLTTIYT	4	0.4608	341.6	49.00	0.64	tr_C9ZNH3_
DRB1_0101	460	GAIHRGALFVSGNGH	GAIHRGALF	0	0.4602	343.9	49.00	0.35	tr_C9ZNH3_
DRB1_0101	461	SDSDTLSVLPSLEEE	LSVLPSLEE	5	0.4594	347.0	50.00	0.54	tr_C9ZNH3_
DRB1_0101	462	TSGNVMSDLRKMLDT	NVMSDLRKM	3	0.4586	349.8	50.00	0.74	tr_C9ZNH3_
DRB1_0101	463	LSYEVPPPELLRSII	YEVPPPELL	2	0.4584	350.8	50.00	0.57	tr_C9ZNH3_
DRB1_0101	464	RDAPLNAALPTDGSL	LNAALPTDG	4	0.4564	358.4	50.00	0.62	tr_C9ZNH3_
DRB1_0101	465	VSDNGFRATRRLRAD	FRATRRLRA	5	0.4563	358.7	50.00	0.60	tr_C9ZNH3_
DRB1_0101	466	AYGATCYCNEGGRMI	YCNEGGRMI	6	0.4563	359.0	50.00	0.55	tr_C9ZNH3_
DRB1_0101	467	APQVSDAMRFLMKLV	SDAMRFLMK	4	0.4559	360.5	50.00	0.29	tr_C9ZNH3_
DRB1_0101	468	DSDTLSVLPSLEEED	LSVLPSLEE	4	0.4553	362.8	50.00	0.59	tr_C9ZNH3_
DRB1_0101	469	EPLAAAYGATCYCNE	LAAAYGATC	2	0.4548	364.8	55.00	0.75	tr_C9ZNH3_
DRB1_0101	470	HLSANRNVRVFIPFF	LSANRNVRV	1	0.4530	371.6	55.00	0.73	tr_C9ZNH3_
DRB1_0101	471	PFLPDAYNNGVNAGL	YNNGVNAGL	6	0.4524	374.1	55.00	0.40	tr_C9ZNH3_
DRB1_0101	472	LYNQRRYVIDDIVIG	YVIDDIVIG	6	0.4522	375.0	55.00	0.64	tr_C9ZNH3_
DRB1_0101	473	NAALPTDGSLLLIGL	ALPTDGSLL	2	0.4516	377.6	55.00	0.64	tr_C9ZNH3_
DRB1_0101	474	RYVIDDIVIGDFGGE	YVIDDIVIG	1	0.4514	378.2	55.00	0.74	tr_C9ZNH3_
DRB1_0101	475	LDTRAVTSVLGVVDD	TRAVTSVLG	2	0.4511	379.7	55.00	0.56	tr_C9ZNH3_
DRB1_0101	476	IGDFGGECEPLAAAY	FGGECEPLA	3	0.4503	382.8	55.00	0.65	tr_C9ZNH3_
DRB1_0101	477	RGWSPNAYFLHVSPA	AYFLHVSPA	6	0.4501	383.6	55.00	0.32	tr_C9ZNH3_
DRB1_0101	478	SPTLEQQLFVIAGYL	TLEQQLFVI	2	0.4500	384.2	55.00	0.25	tr_C9ZNH3_
DRB1_0101	479	SIIEENKNEFFVVVG	IEENKNEFF	2	0.4496	385.6	55.00	0.55	tr_C9ZNH3_
DRB1_0101	480	FSSVNDPFARSVNGA	FSSVNDPFA	0	0.4492	387.5	55.00	0.55	tr_C9ZNH3_
DRB1_0101	481	VVFIDPITLNPRLRH	FIDPITLNP	2	0.4476	394.1	55.00	0.22	tr_C9ZNH3_
DRB1_0101	482	DAVRGWSPNAYFLHV	GWSPNAYFL	4	0.4465	398.8	55.00	0.39	tr_C9ZNH3_
DRB1_0101	483	AVRGWSPNAYFLHVS	GWSPNAYFL	3	0.4463	399.8	55.00	0.41	tr_C9ZNH3_
DRB1_0101	484	IVGEVLARALECSEF	EVLARALEC	3	0.4462	400.2	55.00	0.33	tr_C9ZNH3_
DRB1_0101	485	STSGNVMSDLRKMLD	NVMSDLRKM	4	0.4454	403.9	55.00	0.70	tr_C9ZNH3_
DRB1_0101	486	VIHPLSYEVPPPELL	LSYEVPPPE	4	0.4436	411.7	55.00	0.33	tr_C9ZNH3_
DRB1_0101	487	VFIPFFDVALFYDDF	FIPFFDVAL	1	0.4436	411.8	55.00	0.57	tr_C9ZNH3_
DRB1_0101	488	DVVFIDPITLNPRLR	VVFIDPITL	1	0.4433	413.0	55.00	0.29	tr_C9ZNH3_
DRB1_0101	489	DDATRLIETVVNRTL	TRLIETVVN	3	0.4430	414.2	55.00	0.34	tr_C9ZNH3_
DRB1_0101	490	YLSPTLEQQLFVIAG	TLEQQLFVI	4	0.4423	417.5	55.00	0.28	tr_C9ZNH3_
DRB1_0101	491	SGNVMSDLRKMLDTR	NVMSDLRKM	2	0.4423	417.5	55.00	0.70	tr_C9ZNH3_
DRB1_0101	492	RRLRADIKAYALSGT	LRADIKAYA	2	0.4420	418.8	55.00	0.47	tr_C9ZNH3_
DRB1_0101	493	DPLAFDGDAAYGGQV	FDGDAAYGG	4	0.4419	419.3	55.00	0.64	tr_C9ZNH3_
DRB1_0101	494	LRSIIEENKNEFFVV	IEENKNEFF	4	0.4401	427.7	55.00	0.65	tr_C9ZNH3_
DRB1_0101	495	SDAVRGWSPNAYFLH	GWSPNAYFL	5	0.4399	428.4	55.00	0.29	tr_C9ZNH3_

DRB1_0101	496	VSPAAELLALLRYAV	PAAELLALL	2	0.4370	442.1	55.00	0.31	tr_C9ZNH3_
DRB1_0101	497	VHFGDSEYELAVELM	SEYELAVEL	5	0.4356	449.0	55.00	0.46	tr_C9ZNH3_
DRB1_0101	498	AFDGDAAYGGQVMFG	FDGDAAYGG	1	0.4346	454.0	55.00	0.50	tr_C9ZNH3_
DRB1_0101	499	PSTSGNVMSDLRKML	NVMSDLRKM	5	0.4344	454.6	55.00	0.68	tr_C9ZNH3_
DRB1_0101	500	LSPTLEQQLFVIAGY	TLEQQLFVI	3	0.4336	458.5	55.00	0.33	tr_C9ZNH3_
DRB1_0101	501	NVNVEVIHPLSYEVP	VIHPLSYEV	5	0.4332	460.8	55.00	0.32	tr_C9ZNH3_
DRB1_0101	502	SYEVPPPELLRSIIE	YEVPPPELL	1	0.4329	462.2	55.00	0.47	tr_C9ZNH3_
DRB1_0101	503	NRSLNSVVSLDRDAP	NRSLNSVVS	0	0.4325	464.1	55.00	0.55	tr_C9ZNH3_
DRB1_0101	504	NGHLGKSERLFIHSV	GKSERLFIH	4	0.4305	474.1	60.00	0.28	tr_C9ZNH3_
DRB1_0101	505	AALPTDGSLLLIGLT	ALPTDGSLL	1	0.4283	485.5	60.00	0.56	tr_C9ZNH3_
DRB1_0101	506	PTPHVTNIEIAWTLA	TNIEIAWTL	5	0.4276	489.5	60.00	0.38	tr_C9ZNH3_
DRB1_0101	507	DTLSVLPSLEEEDLV	LSVLPSLEE	2	0.4266	494.5	60.00	0.69	tr_C9ZNH3_
DRB1_0101	508	AFADEDCSRIVGNHT	CSRIVGNHT	6	0.4260	498.1	60.00	0.53	tr_C9ZNH3_
DRB1_0101	509	DTRAVTSVLGVVDDA	TRAVTSVLG	1	0.4256	500.0	60.00	0.50	tr_C9ZNH3_
DRB1_0101	510	VGVDPQKVLTTIYTH	QKVLTTIYT	5	0.4247	505.0	60.00	0.60	tr_C9ZNH3_
DRB1_0101	511	RLRHPGRNVIYLSPT	LRHPGRNVI	1	0.4245	506.3	60.00	0.34	tr_C9ZNH3_
DRB1_0101	512	PDVVFIDPITLNPRL	VVFIDPITL	2	0.4237	510.7	60.00	0.47	tr_C9ZNH3_
DRB1_0101	513	LSDSDTLSVLPSLEE	LSVLPSLEE	6	0.4233	513.0	60.00	0.38	tr_C9ZNH3_
DRB1_0101	514	DAVDGCLVNYGAMHI	CLVNYGAMH	5	0.4227	515.8	60.00	0.26	tr_C9ZNH3_
DRB1_0101	515	ALPTDGSLLLIGLTA	SLLLIGLTA	6	0.4216	522.1	60.00	0.29	tr_C9ZNH3_
DRB1_0101	516	TLNPRLRHPGRNVIY	RLRHPGRNV	4	0.4204	529.3	60.00	0.44	tr_C9ZNH3_
DRB1_0101	517	GHLGKSERLFIHSVP	SERLFIHSV	5	0.4203	529.4	60.00	0.35	tr_C9ZNH3_
DRB1_0101	518	TTIYTHSVISVDDMQ	YTHSVISVD	3	0.4197	533.0	60.00	0.46	tr_C9ZNH3_
DRB1_0101	519	ARALECSEFLKSRMT	SEFLKSRMT	6	0.4183	541.2	60.00	0.34	tr_C9ZNH3_
DRB1_0101	520	VNVEVIHPLSYEVPP	VIHPLSYEV	4	0.4165	551.8	60.00	0.38	tr_C9ZNH3_
DRB1_0101	521	QWSTASNVNVEVIHP	WSTASNVNV	1	0.4165	551.9	60.00	0.80	tr_C9ZNH3_
DRB1_0101	522	PNASDRTPLALLGFT	DRTPLALLG	4	0.4145	563.9	60.00	0.40	tr_C9ZNH3_
DRB1_0101	523	LHAVMRDDATRLIET	VMRDDATRL	3	0.4140	567.2	60.00	0.52	tr_C9ZNH3_
DRB1_0101	524	ANRNVRVFIPFFDVA	RVFIPFFDV	5	0.4139	567.4	60.00	0.47	tr_C9ZNH3_
DRB1_0101	525	SNVNVEVIHPLSYEV	VNVEVIHPL	2	0.4137	568.6	60.00	0.38	tr_C9ZNH3_
DRB1_0101	526	VIGDFGGECEPLAAA	FGGECEPLA	4	0.4136	569.6	60.00	0.67	tr_C9ZNH3_
DRB1_0101	527	HLGKSERLFIHSVPS	SERLFIHSV	4	0.4125	576.1	60.00	0.41	tr_C9ZNH3_
DRB1_0101	528	RLRADIKAYALSGTG	LRADIKAYA	1	0.4123	577.6	60.00	0.38	tr_C9ZNH3_
DRB1_0101	529	AEFTATWELFAGTHP	FTATWELFA	2	0.4119	580.3	60.00	0.57	tr_C9ZNH3_
DRB1_0101	530	IFATNLPHWADHRPS	FATNLPHWA	1	0.4116	581.9	60.00	0.68	tr_C9ZNH3_
DRB1_0101	531	FDPLAFDGDAAYGGQ	FDGDAAYGG	5	0.4111	584.9	60.00	0.58	tr_C9ZNH3_
DRB1_0101	532	IIEENKNEFFVVVGP	IEENKNEFF	1	0.4109	586.6	60.00	0.46	tr_C9ZNH3_
DRB1_0101	533	PLAAAYGATCYCNEG	LAAAYGATC	1	0.4101	591.5	60.00	0.73	tr_C9ZNH3_
DRB1_0101	534	TPDVVFIDPITLNPR	VVFIDPITL	3	0.4095	595.1	60.00	0.58	tr_C9ZNH3_
DRB1_0101	535	LNAAVPPLTKPETPS	LNAAVPPLT	0	0.4095	595.2	60.00	0.68	tr_C9ZNH3_
DRB1_0101	536	SVVSLDRDAPLNAAL	LDRDAPLNA	4	0.4083	603.3	60.00	0.51	tr_C9ZNH3_
DRB1_0101	537	IGLTASDVGGIAAHL	DVGGIAAHL	6	0.4065	615.0	65.00	0.44	tr_C9ZNH3_
DRB1_0101	538	DACDESGVQMLAPLY	GVQMLAPLY	6	0.4063	616.4	65.00	0.43	tr_C9ZNH3_
DRB1_0101	539	EFTATWELFAGTHPQ	FTATWELFA	1	0.4053	623.0	65.00	0.44	tr_C9ZNH3_
DRB1_0101	540	ITFDACDESGVQMLA	FDACDESGV	2	0.4045	628.5	65.00	0.46	tr_C9ZNH3_
DRB1_0101	541	FDVALFYDDFIRAFR	LFYDDFIRA	4	0.4034	636.2	65.00	0.45	tr_C9ZNH3_
DRB1_0101	542	VVSLDRDAPLNAALP	LDRDAPLNA	3	0.4030	638.6	65.00	0.48	tr_C9ZNH3_
DRB1_0101	543	TLHAVMRDDATRLIE	VMRDDATRL	4	0.4028	640.0	65.00	0.50	tr_C9ZNH3_
DRB1_0101	544	WTLAGTTSGVKMFPG	WTLAGTTSG	0	0.4026	641.3	65.00	0.47	tr_C9ZNH3_
DRB1_0101	545	CNEGGRMIYMMILGD	GRMIYMMIL	4	0.4003	657.9	65.00	0.29	tr_C9ZNH3_

DRB1_0101	546	RPNASDRTPLALLGF	SDRTPLALL	4	0.4001	659.3	65.00	0.37	tr_C9ZNH3_
DRB1_0101	547	RDDATRLIETVVNRT	TRLIETVVN	4	0.3998	661.4	65.00	0.40	tr_C9ZNH3_
DRB1_0101	548	TDAVDGCLVNYGAMH	VDGCLVNYG	3	0.3994	663.8	65.00	0.23	tr_C9ZNH3_
DRB1_0101	549	VSLDRDAPLNAALPT	LDRDAPLNA	2	0.3979	674.6	65.00	0.41	tr_C9ZNH3_
DRB1_0101	550	NEGGRMIYMMILGDD	GRMIYMMIL	3	0.3979	675.0	65.00	0.31	tr_C9ZNH3_
DRB1_0101	551	FVVVGPLSDSDTLSV	FVVVGPLSD	0	0.3976	677.2	65.00	0.51	tr_C9ZNH3_
DRB1_0101	552	AIHRGALFVSGNGHL	IHRGALFVS	1	0.3970	681.3	65.00	0.25	tr_C9ZNH3_
DRB1_0101	553	DRDAPLNAALPTDGS	LNAALPTDG	5	0.3970	681.6	65.00	0.49	tr_C9ZNH3_
DRB1_0101	554	GSDAVRGWSPNAYFL	SDAVRGWSP	1	0.3969	682.4	65.00	0.28	tr_C9ZNH3_
DRB1_0101	555	LRADIKAYALSGTGA	DIKAYALSG	3	0.3966	684.2	65.00	0.39	tr_C9ZNH3_
DRB1_0101	556	TLSVLPSLEEEDLVA	LSVLPSLEE	1	0.3963	686.6	65.00	0.65	tr_C9ZNH3_
DRB1_0101	557	FIPFFDVALFYDDFI	FIPFFDVAL	0	0.3962	687.6	65.00	0.30	tr_C9ZNH3_
DRB1_0101	558	VFIDPITLNPRLRHP	PITLNPRLR	4	0.3956	692.1	65.00	0.26	tr_C9ZNH3_
DRB1_0101	559	VRGWSPNAYFLHVSP	GWSPNAYFL	2	0.3946	699.5	65.00	0.44	tr_C9ZNH3_
DRB1_0101	560	FFDVALFYDDFIRAF	LFYDDFIRA	5	0.3945	700.3	65.00	0.38	tr_C9ZNH3_
DRB1_0101	561	HAVMRDDATRLIETV	VMRDDATRL	2	0.3945	700.5	65.00	0.48	tr_C9ZNH3_
DRB1_0101	562	LITFDACDESGVQML	FDACDESGV	3	0.3941	703.3	65.00	0.56	tr_C9ZNH3_
DRB1_0101	563	HTDAVDGCLVNYGAM	TDAVDGCLV	1	0.3935	707.7	65.00	0.43	tr_C9ZNH3_
DRB1_0101	564	LMKLVTDNRTSSAYI	MKLVTDNRT	1	0.3914	723.9	65.00	0.42	tr_C9ZNH3_
DRB1_0101	565	VGEVLARALECSEFL	EVLARALEC	2	0.3907	729.6	65.00	0.37	tr_C9ZNH3_
DRB1_0101	566	YALSGTGAVEFDPLA	YALSGTGAV	0	0.3890	743.2	65.00	0.43	tr_C9ZNH3_
DRB1_0101	567	LLRSIIEENKNEFFV	IEENKNEFF	5	0.3875	755.6	65.00	0.65	tr_C9ZNH3_
DRB1_0101	568	DAEFTATWELFAGTH	FTATWELFA	3	0.3873	757.1	65.00	0.69	tr_C9ZNH3_
DRB1_0101	569	VVDDATLSTPDVVFI	DATLSTPDV	3	0.3856	771.1	65.00	0.60	tr_C9ZNH3_
DRB1_0101	570	NHTDAVDGCLVNYGA	TDAVDGCLV	2	0.3839	785.3	70.00	0.58	tr_C9ZNH3_
DRB1_0101	571	LRHPGRNVIYLSPTL	NVIYLSPTL	6	0.3833	790.6	70.00	0.23	tr_C9ZNH3_
DRB1_0101	572	VISVDDMQYGAFADE	DDMQYGAFA	4	0.3832	790.9	70.00	0.62	tr_C9ZNH3_
DRB1_0101	573	EVIHPLSYEVPPPEL	LSYEVPPPE	5	0.3825	797.5	70.00	0.32	tr_C9ZNH3_
DRB1_0101	574	GNGHLGKSERLFIHS	GHLGKSERL	2	0.3824	798.6	70.00	0.41	tr_C9ZNH3_
DRB1_0101	575	TFDACDESGVQMLAP	FDACDESGV	1	0.3814	806.8	70.00	0.43	tr_C9ZNH3_
DRB1_0101	576	NVEVIHPLSYEVPPP	VIHPLSYEV	3	0.3812	808.8	70.00	0.42	tr_C9ZNH3_
DRB1_0101	577	IEENKNEFFVVVGPL	ENKNEFFVV	2	0.3811	809.5	70.00	0.28	tr_C9ZNH3_
DRB1_0101	578	TRAVTSVLGVVDDAT	TRAVTSVLG	0	0.3803	816.2	70.00	0.37	tr_C9ZNH3_
DRB1_0101	579	LASENASTLHAVMRD	LASENASTL	0	0.3797	821.7	70.00	0.39	tr_C9ZNH3_
DRB1_0101	580	FHANRPNASDRTPLA	FHANRPNAS	0	0.3794	824.5	70.00	0.73	tr_C9ZNH3_
DRB1_0101	581	GEVLARALECSEFLK	EVLARALEC	1	0.3784	833.5	70.00	0.28	tr_C9ZNH3_
DRB1_0101	582	YEVPPPELLRSIIEE	PELLRSIIE	5	0.3781	835.8	70.00	0.41	tr_C9ZNH3_
DRB1_0101	583	FTATWELFAGTHPQG	WELFAGTHP	4	0.3771	845.4	70.00	0.51	tr_C9ZNH3_
DRB1_0101	584	FDGDAAYGGQVMFGW	FDGDAAYGG	0	0.3766	849.6	70.00	0.28	tr_C9ZNH3_
DRB1_0101	585	STLHAVMRDDATRLI	VMRDDATRL	5	0.3764	851.4	70.00	0.47	tr_C9ZNH3_
DRB1_0101	586	VDDATLSTPDVVFID	DATLSTPDV	2	0.3763	852.8	70.00	0.56	tr_C9ZNH3_
DRB1_0101	587	ASNVNVEVIHPLSYE	VNVEVIHPL	3	0.3761	854.6	70.00	0.54	tr_C9ZNH3_
DRB1_0101	588	SLDRDAPLNAALPTD	LDRDAPLNA	1	0.3760	855.6	70.00	0.37	tr_C9ZNH3_
DRB1_0101	589	MRDDATRLIETVVNR	ATRLIETVV	4	0.3756	858.8	70.00	0.31	tr_C9ZNH3_
DRB1_0101	590	SVISVDDMQYGAFAD	DDMQYGAFA	5	0.3755	859.6	70.00	0.60	tr_C9ZNH3_
DRB1_0101	591	NSVVSLDRDAPLNAA	LDRDAPLNA	5	0.3752	862.8	70.00	0.44	tr_C9ZNH3_
DRB1_0101	592	DDAEFTATWELFAGT	FTATWELFA	4	0.3736	877.8	70.00	0.67	tr_C9ZNH3_
DRB1_0101	593	LDRDAPLNAALPTDG	DAPLNAALP	3	0.3725	888.8	70.00	0.34	tr_C9ZNH3_
DRB1_0101	594	MDSLYNQRRYVIDDI	LYNQRRYVI	3	0.3709	904.2	70.00	0.41	tr_C9ZNH3_
DRB1_0101	595	ISVDDMQYGAFADED	DDMQYGAFA	3	0.3690	922.2	70.00	0.69	tr_C9ZNH3_

DRB1_0101	596	AVMRDDATRLIETVV	VMRDDATRL	1	0.3686	926.5	70.00	0.39	tr_C9ZNH3_
DRB1_0101	597	GNHTDAVDGCLVNYG	TDAVDGCLV	3	0.3683	929.4	70.00	0.68	tr_C9ZNH3_
DRB1_0101	598	STPDVVFIDPITLNP	VVFIDPITL	4	0.3683	930.2	70.00	0.65	tr_C9ZNH3_
DRB1_0101	599	EVLARALECSEFLKS	RALECSEFL	4	0.3680	933.1	70.00	0.36	tr_C9ZNH3_
DRB1_0101	600	SGNGHLGKSERLFIH	GHLGKSERL	3	0.3679	933.8	70.00	0.48	tr_C9ZNH3_
DRB1_0101	601	VEVIHPLSYEVPPPE	VIHPLSYEV	2	0.3672	940.9	70.00	0.33	tr_C9ZNH3_
DRB1_0101	602	SANRNVRVFIPFFDV	RVFIPFFDV	6	0.3653	960.1	70.00	0.41	tr_C9ZNH3_
DRB1_0101	603	SSETIRRFHANRPNA	IRRFHANRP	4	0.3651	962.2	70.00	0.70	tr_C9ZNH3_
DRB1_0101	604	GDDYLPRPAADGLIT	YLPRPAADG	3	0.3618	997.9	70.00	0.60	tr_C9ZNH3_
DRB1_0101	605	PFFDVALFYDDFIRA	FDVALFYDD	2	0.3616	999.8	70.00	0.30	tr_C9ZNH3_
DRB1_0101	606	FIDPITLNPRLRHPG	PITLNPRLR	3	0.3615	1000.4	70.00	0.28	tr_C9ZNH3_
DRB1_0101	607	EEEDLVAFAPFTGSD	LVAFAPFTG	4	0.3612	1003.8	70.00	0.34	tr_C9ZNH3_
DRB1_0101	608	NVHFGDSEYELAVEL	SEYELAVEL	6	0.3593	1024.6	75.00	0.40	tr_C9ZNH3_
DRB1_0101	609	TASNVNVEVIHPLSY	VNVEVIHPL	4	0.3577	1042.8	75.00	0.62	tr_C9ZNH3_
DRB1_0101	610	VMRDDATRLIETVVN	ATRLIETVV	5	0.3571	1049.5	75.00	0.24	tr_C9ZNH3_
DRB1_0101	611	DDYLPRPAADGLITF	YLPRPAADG	2	0.3568	1052.3	75.00	0.55	tr_C9ZNH3_
DRB1_0101	612	SPTPHVTNIEIAWTL	VTNIEIAWT	5	0.3567	1053.6	75.00	0.27	tr_C9ZNH3_
DRB1_0101	613	FDACDESGVQMLAPL	CDESGVQML	3	0.3565	1056.1	75.00	0.43	tr_C9ZNH3_
DRB1_0101	614	LARALECSEFLKSRM	RALECSEFL	2	0.3565	1056.8	75.00	0.44	tr_C9ZNH3_
DRB1_0101	615	IVIGDFGGECEPLAA	FGGECEPLA	5	0.3564	1057.9	75.00	0.69	tr_C9ZNH3_
DRB1_0101	616	ITLNPRLRHPGRNVI	RLRHPGRNV	5	0.3564	1057.9	75.00	0.40	tr_C9ZNH3_
DRB1_0101	617	TIYTHSVISVDDMQY	YTHSVISVD	2	0.3563	1058.4	75.00	0.50	tr_C9ZNH3_
DRB1_0101	618	LAGTTSGVKMFPGQV	GVKMFPGQV	6	0.3562	1059.2	75.00	0.43	tr_C9ZNH3_
DRB1_0101	619	EFDPLAFDGDAAYGG	FDGDAAYGG	6	0.3560	1061.7	75.00	0.36	tr_C9ZNH3_
DRB1_0101	620	LSANRNVRVFIPFFD	LSANRNVRV	0	0.3551	1071.9	75.00	0.45	tr_C9ZNH3_
DRB1_0101	621	VLARALECSEFLKSR	RALECSEFL	3	0.3538	1088.0	75.00	0.48	tr_C9ZNH3_
DRB1_0101	622	MMILGDDYLPRPAAD	MILGDDYLP	1	0.3530	1097.6	75.00	0.28	tr_C9ZNH3_
DRB1_0101	623	ADDAEFTATWELFAG	FTATWELFA	5	0.3520	1109.2	75.00	0.62	tr_C9ZNH3_
DRB1_0101	624	DDATLSTPDVVFIDP	DATLSTPDV	1	0.3516	1114.4	75.00	0.51	tr_C9ZNH3_
DRB1_0101	625	LEEEDLVAFAPFTGS	DLVAFAPFT	4	0.3497	1136.6	75.00	0.28	tr_C9ZNH3_
DRB1_0101	626	YVIDDIVIGDFGGEC	YVIDDIVIG	0	0.3487	1148.9	75.00	0.56	tr_C9ZNH3_
DRB1_0101	627	LSTPDVVFIDPITLN	VVFIDPITL	5	0.3483	1154.2	75.00	0.69	tr_C9ZNH3_
DRB1_0101	628	LGDDYLPRPAADGLI	YLPRPAADG	4	0.3475	1164.2	75.00	0.56	tr_C9ZNH3_
DRB1_0101	629	NRPNASDRTPLALLG	SDRTPLALL	5	0.3469	1172.2	75.00	0.38	tr_C9ZNH3_
DRB1_0101	630	PLSDSDTLSVLPSLE	SDTLSVLPS	4	0.3461	1181.6	75.00	0.47	tr_C9ZNH3_
DRB1_0101	631	WSTASNVNVEVIHPL	WSTASNVNV	0	0.3460	1183.0	75.00	0.48	tr_C9ZNH3_
DRB1_0101	632	IPFFDVALFYDDFIR	FDVALFYDD	3	0.3453	1192.7	75.00	0.40	tr_C9ZNH3_
DRB1_0101	633	VGNHTDAVDGCLVNY	TDAVDGCLV	4	0.3450	1195.8	75.00	0.72	tr_C9ZNH3_
DRB1_0101	634	GVVDDATLSTPDVVF	DATLSTPDV	4	0.3431	1221.5	75.00	0.60	tr_C9ZNH3_
DRB1_0101	635	LSVLPSLEEEDLVAF	LSVLPSLEE	0	0.3421	1234.3	75.00	0.43	tr_C9ZNH3_
DRB1_0101	636	SVDDMQYGAFADEDC	DDMQYGAFA	2	0.3381	1289.3	75.00	0.70	tr_C9ZNH3_
DRB1_0101	637	IHSVPSTSGNVMSDL	IHSVPSTSG	0	0.3379	1291.6	75.00	0.37	tr_C9ZNH3_
DRB1_0101	638	GLITFDACDESGVQM	FDACDESGV	4	0.3372	1301.6	75.00	0.63	tr_C9ZNH3_
DRB1_0101	639	RIVGNHTDAVDGCLV	IVGNHTDAV	1	0.3369	1305.2	75.00	0.42	tr_C9ZNH3_
DRB1_0101	640	HSVISVDDMQYGAFA	DDMQYGAFA	6	0.3367	1308.4	75.00	0.48	tr_C9ZNH3_
DRB1_0101	641	TGSDAVRGWSPNAYF	SDAVRGWSP	2	0.3359	1320.1	75.00	0.45	tr_C9ZNH3_
DRB1_0101	642	SLLYDPFLPDAYNNG	YDPFLPDAY	3	0.3353	1328.4	75.00	0.41	tr_C9ZNH3_
DRB1_0101	643	IVGNHTDAVDGCLVN	TDAVDGCLV	5	0.3350	1333.2	75.00	0.58	tr_C9ZNH3_
DRB1_0101	644	DPFLPDAYNNGVNAG	DAYNNGVNA	5	0.3342	1345.2	80.00	0.41	tr_C9ZNH3_
DRB1_0101	645	VPPPELLRSIIEENK	PELLRSIIE	3	0.3329	1363.1	80.00	0.60	tr_C9ZNH3_

DRB1_0101	646	DSLYNQRRYVIDDIV	LYNQRRYVI	2	0.3324	1370.9	80.00	0.41	tr_C9ZNH3_
DRB1_0101	647	PPPELLRSIIEENKN	PELLRSIIE	2	0.3320	1376.7	80.00	0.56	tr_C9ZNH3_
DRB1_0101	648	VPSTSGNVMSDLRKM	NVMSDLRKM	6	0.3288	1424.8	80.00	0.52	tr_C9ZNH3_
DRB1_0101	649	DYLPRPAADGLITFD	YLPRPAADG	1	0.3259	1470.2	80.00	0.56	tr_C9ZNH3_
DRB1_0101	650	VTSVLGVVDDATLST	GVVDDATLS	5	0.3247	1490.4	80.00	0.29	tr_C9ZNH3_
DRB1_0101	651	YLPRPAADGLITFDA	YLPRPAADG	0	0.3231	1515.7	80.00	0.26	tr_C9ZNH3_
DRB1_0101	652	PSSETIRRFHANRPN	IRRFHANRP	5	0.3209	1552.4	80.00	0.60	tr_C9ZNH3_
DRB1_0101	653	IDPITLNPRLRHPGR	ITLNPRLRH	3	0.3207	1556.0	80.00	0.40	tr_C9ZNH3_
DRB1_0101	654	AVTSVLGVVDDATLS	VTSVLGVVD	1	0.3202	1564.6	80.00	0.24	tr_C9ZNH3_
DRB1_0101	655	SLYNQRRYVIDDIVI	LYNQRRYVI	1	0.3195	1576.0	80.00	0.34	tr_C9ZNH3_
DRB1_0101	656	YGAFADEDCSRIVGN	FADEDCSRI	3	0.3189	1586.2	80.00	0.75	tr_C9ZNH3_
DRB1_0101	657	LGVVDDATLSTPDVV	DATLSTPDV	5	0.3181	1601.1	80.00	0.50	tr_C9ZNH3_
DRB1_0101	658	LLYDPFLPDAYNNGV	YDPFLPDAY	2	0.3178	1605.8	80.00	0.34	tr_C9ZNH3_
DRB1_0101	659	FATNLPHWADHRPSS	FATNLPHWA	0	0.3177	1606.6	80.00	0.55	tr_C9ZNH3_
DRB1_0101	660	ELLRSIIEENKNEFF	IEENKNEFF	6	0.3166	1626.4	80.00	0.55	tr_C9ZNH3_
DRB1_0101	661	DGDAAYGGQVMFGWI	DAAYGGQVM	2	0.3142	1669.9	80.00	0.30	tr_C9ZNH3_
DRB1_0101	662	PPELLRSIIEENKNE	PELLRSIIE	1	0.3141	1671.2	80.00	0.52	tr_C9ZNH3_
DRB1_0101	663	RAVTSVLGVVDDATL	VTSVLGVVD	2	0.3121	1707.3	80.00	0.44	tr_C9ZNH3_
DRB1_0101	664	ILGDDYLPRPAADGL	YLPRPAADG	5	0.3112	1724.2	80.00	0.50	tr_C9ZNH3_
DRB1_0101	665	DATLSTPDVVFIDPI	DATLSTPDV	0	0.3109	1730.3	80.00	0.38	tr_C9ZNH3_
DRB1_0101	666	VSGNGHLGKSERLFI	GHLGKSERL	4	0.3097	1753.5	80.00	0.58	tr_C9ZNH3_
DRB1_0101	667	EVPPPELLRSIIEEN	PELLRSIIE	4	0.3077	1790.6	80.00	0.58	tr_C9ZNH3_
DRB1_0101	668	STASNVNVEVIHPLS	VNVEVIHPL	5	0.3072	1800.9	80.00	0.60	tr_C9ZNH3_
DRB1_0101	669	QYGAFADEDCSRIVG	FADEDCSRI	4	0.3056	1832.3	85.00	0.71	tr_C9ZNH3_
DRB1_0101	670	PITLNPRLRHPGRNV	ITLNPRLRH	1	0.3055	1833.9	85.00	0.31	tr_C9ZNH3_
DRB1_0101	671	RPAADGLITFDACDE	ADGLITFDA	3	0.3053	1838.1	85.00	0.58	tr_C9ZNH3_
DRB1_0101	672	YDPFLPDAYNNGVNA	FLPDAYNNG	3	0.3053	1838.2	85.00	0.37	tr_C9ZNH3_
DRB1_0101	673	PRPAADGLITFDACD	ADGLITFDA	4	0.3050	1843.2	85.00	0.59	tr_C9ZNH3_
DRB1_0101	674	LNSVVSLDRDAPLNA	VSLDRDAPL	4	0.3047	1850.0	85.00	0.40	tr_C9ZNH3_
DRB1_0101	675	LPRPAADGLITFDAC	ADGLITFDA	5	0.3041	1862.7	85.00	0.55	tr_C9ZNH3_
DRB1_0101	676	TSVLGVVDDATLSTP	GVVDDATLS	4	0.3032	1879.9	85.00	0.38	tr_C9ZNH3_
DRB1_0101	677	LAAAYGATCYCNEGG	LAAAYGATC	0	0.3028	1888.1	85.00	0.57	tr_C9ZNH3_
DRB1_0101	678	GPLSDSDTLSVLPSL	SDTLSVLPS	5	0.3026	1891.8	85.00	0.50	tr_C9ZNH3_
DRB1_0101	679	ASTLHAVMRDDATRL	LHAVMRDDA	3	0.3022	1901.8	85.00	0.30	tr_C9ZNH3_
DRB1_0101	680	SLEEEDLVAFAPFTG	DLVAFAPFT	5	0.3014	1917.6	85.00	0.33	tr_C9ZNH3_
DRB1_0101	681	SRIVGNHTDAVDGCL	IVGNHTDAV	2	0.3012	1922.0	85.00	0.71	tr_C9ZNH3_
DRB1_0101	682	DGLITFDACDESGVQ	FDACDESGV	5	0.3011	1922.9	85.00	0.60	tr_C9ZNH3_
DRB1_0101	683	ADGLITFDACDESGV	FDACDESGV	6	0.3000	1946.8	85.00	0.34	tr_C9ZNH3_
DRB1_0101	684	EADDAEFTATWELFA	FTATWELFA	6	0.2996	1955.3	85.00	0.54	tr_C9ZNH3_
DRB1_0101	685	GAFADEDCSRIVGNH	FADEDCSRI	2	0.2980	1989.6	85.00	0.71	tr_C9ZNH3_
DRB1_0101	686	LYDPFLPDAYNNGVN	YDPFLPDAY	1	0.2949	2056.9	85.00	0.29	tr_C9ZNH3_
DRB1_0101	687	VDDMQYGAFADEDCS	DDMQYGAFA	1	0.2945	2065.0	85.00	0.69	tr_C9ZNH3_
DRB1_0101	688	ASENASTLHAVMRDD	ASTLHAVMR	4	0.2945	2067.0	85.00	0.26	tr_C9ZNH3_
DRB1_0101	689	TLSTPDVVFIDPITL	VVFIDPITL	6	0.2926	2109.0	85.00	0.58	tr_C9ZNH3_
DRB1_0101	690	IYTHSVISVDDMQYG	YTHSVISVD	1	0.2910	2145.4	85.00	0.46	tr_C9ZNH3_
DRB1_0101	691	DIVIGDFGGECEPLA	FGGECEPLA	6	0.2909	2148.7	85.00	0.53	tr_C9ZNH3_
DRB1_0101	692	MILGDDYLPRPAADG	DDYLPRPAA	4	0.2900	2168.0	85.00	0.28	tr_C9ZNH3_
DRB1_0101	693	PAADGLITFDACDES	ADGLITFDA	2	0.2847	2296.9	85.00	0.56	tr_C9ZNH3_
DRB1_0101	694	QNVHFGDSEYELAVE	FGDSEYELA	4	0.2845	2303.0	85.00	0.43	tr_C9ZNH3_
DRB1_0101	695	PELLRSIIEENKNEF	PELLRSIIE	0	0.2844	2305.1	85.00	0.38	tr_C9ZNH3_

DRB1_0101	696	VEFDPLAFDGDAAYG	FDPLAFDGD	2	0.2837	2321.6	85.00	0.28	tr_C9ZNH3_
DRB1_0101	697	LDAVAQHIVGVDPQK	LDAVAQHIV	0	0.2831	2337.6	85.00	0.39	tr_C9ZNH3_
DRB1_0101	698	DPITLNPRLRHPGRN	ITLNPRLRH	2	0.2828	2343.6	85.00	0.44	tr_C9ZNH3_
DRB1_0101	699	VLGVVDDATLSTPDV	GVVDDATLS	2	0.2823	2356.8	85.00	0.34	tr_C9ZNH3_
DRB1_0101	700	MQYGAFADEDCSRIV	FADEDCSRI	5	0.2816	2376.2	85.00	0.67	tr_C9ZNH3_
DRB1_0101	701	TLAGTTSGVKMFPGQ	AGTTSGVKM	2	0.2811	2389.3	85.00	0.38	tr_C9ZNH3_
DRB1_0101	702	HWADHRPSSETIRRF	WADHRPSSE	1	0.2799	2419.3	85.00	0.35	tr_C9ZNH3_
DRB1_0101	703	LQNVHFGDSEYELAV	FGDSEYELA	5	0.2781	2466.5	85.00	0.41	tr_C9ZNH3_
DRB1_0101	704	AADGLITFDACDESG	ADGLITFDA	1	0.2780	2469.4	85.00	0.50	tr_C9ZNH3_
DRB1_0101	705	SVLGVVDDATLSTPD	GVVDDATLS	3	0.2770	2497.2	85.00	0.44	tr_C9ZNH3_
DRB1_0101	706	RSLNSVVSLDRDAPL	LNSVVSLDR	2	0.2759	2525.9	85.00	0.50	tr_C9ZNH3_
DRB1_0101	707	MYLQNVHFGDSEYEL	YLQNVHFGD	1	0.2757	2533.0	90.00	0.37	tr_C9ZNH3_
DRB1_0101	708	RPSSETIRRFHANRP	IRRFHANRP	6	0.2743	2570.8	90.00	0.38	tr_C9ZNH3_
DRB1_0101	709	ALSGTGAVEFDPLAF	LSGTGAVEF	1	0.2737	2588.0	90.00	0.34	tr_C9ZNH3_
DRB1_0101	710	LPHWADHRPSSETIR	WADHRPSSE	3	0.2727	2615.5	90.00	0.55	tr_C9ZNH3_
DRB1_0101	711	SENASTLHAVMRDDA	ASTLHAVMR	3	0.2721	2632.3	90.00	0.37	tr_C9ZNH3_
DRB1_0101	712	PHWADHRPSSETIRR	WADHRPSSE	2	0.2699	2696.1	90.00	0.47	tr_C9ZNH3_
DRB1_0101	713	SVLPSLEEEDLVAFA	LPSLEEEDL	2	0.2679	2755.3	90.00	0.31	tr_C9ZNH3_
DRB1_0101	714	ANRPNASDRTPLALL	SDRTPLALL	6	0.2679	2755.7	90.00	0.38	tr_C9ZNH3_
DRB1_0101	715	AAYGATCYCNEGGRM	CYCNEGGRM	6	0.2624	2922.9	90.00	0.54	tr_C9ZNH3_
DRB1_0101	716	HSVPSTSGNVMSDLR	SVPSTSGNV	1	0.2620	2936.2	90.00	0.28	tr_C9ZNH3_
DRB1_0101	717	PSLEEEDLVAFAPFT	DLVAFAPFT	6	0.2610	2969.9	90.00	0.23	tr_C9ZNH3_
DRB1_0101	718	NASTLHAVMRDDATR	LHAVMRDDA	4	0.2607	2978.8	90.00	0.34	tr_C9ZNH3_
DRB1_0101	719	SLNSVVSLDRDAPLN	VSLDRDAPL	5	0.2590	3033.1	90.00	0.34	tr_C9ZNH3_
DRB1_0101	720	AVEFDPLAFDGDAAY	FDPLAFDGD	3	0.2547	3178.2	90.00	0.34	tr_C9ZNH3_
DRB1_0101	721	ENASTLHAVMRDDAT	ASTLHAVMR	2	0.2535	3219.2	90.00	0.34	tr_C9ZNH3_
DRB1_0101	722	NLPHWADHRPSSETI	WADHRPSSE	4	0.2513	3295.8	90.00	0.54	tr_C9ZNH3_
DRB1_0101	723	LSGTGAVEFDPLAFD	GAVEFDPLA	4	0.2510	3308.3	90.00	0.31	tr_C9ZNH3_
DRB1_0101	724	LSPTPHVTNIEIAWT	VTNIEIAWT	6	0.2508	3313.6	90.00	0.31	tr_C9ZNH3_
DRB1_0101	725	YLQNVHFGDSEYELA	FGDSEYELA	6	0.2506	3322.6	90.00	0.28	tr_C9ZNH3_
DRB1_0101	726	SGTGAVEFDPLAFDG	GAVEFDPLA	3	0.2504	3328.0	90.00	0.35	tr_C9ZNH3_
DRB1_0101	727	ILSPTPHVTNIEIAW	ILSPTPHVT	0	0.2487	3391.2	90.00	0.23	tr_C9ZNH3_
DRB1_0101	728	PLTKPETPSMHYHNL	TKPETPSMH	2	0.2480	3418.4	90.00	0.38	tr_C9ZNH3_
DRB1_0101	729	VLPSLEEEDLVAFAP	LEEEDLVAF	4	0.2465	3472.5	90.00	0.28	tr_C9ZNH3_
DRB1_0101	730	SVPSTSGNVMSDLRK	PSTSGNVMS	2	0.2459	3494.2	90.00	0.28	tr_C9ZNH3_
DRB1_0101	731	PPLTKPETPSMHYHN	TKPETPSMH	3	0.2457	3501.6	90.00	0.46	tr_C9ZNH3_
DRB1_0101	732	GTGAVEFDPLAFDGD	GAVEFDPLA	2	0.2434	3591.0	90.00	0.30	tr_C9ZNH3_
DRB1_0101	733	LPSLEEEDLVAFAPF	LEEEDLVAF	3	0.2416	3660.0	90.00	0.29	tr_C9ZNH3_
DRB1_0101	734	TGAVEFDPLAFDGDA	GAVEFDPLA	1	0.2398	3734.7	95.00	0.28	tr_C9ZNH3_
DRB1_0101	735	HRPSSETIRRFHANR	SETIRRFHA	4	0.2384	3790.0	95.00	0.35	tr_C9ZNH3_
DRB1_0101	736	VPPLTKPETPSMHYH	TKPETPSMH	4	0.2370	3848.7	95.00	0.43	tr_C9ZNH3_
DRB1_0101	737	VVVGPLSDSDTLSVL	VGPLSDSDT	2	0.2370	3850.2	95.00	0.30	tr_C9ZNH3_
DRB1_0101	738	WADHRPSSETIRRFH	PSSETIRRF	5	0.2346	3949.4	95.00	0.20	tr_C9ZNH3_
DRB1_0101	739	GAVEFDPLAFDGDAA	FDPLAFDGD	4	0.2333	4005.7	95.00	0.34	tr_C9ZNH3_
DRB1_0101	740	TNLPHWADHRPSSET	WADHRPSSE	5	0.2330	4018.1	95.00	0.51	tr_C9ZNH3_
DRB1_0101	741	ATLSTPDVVFIDPIT	LSTPDVVFI	2	0.2304	4132.0	95.00	0.41	tr_C9ZNH3_
DRB1_0101	742	YTHSVISVDDMQYGA	YTHSVISVD	0	0.2276	4260.5	95.00	0.25	tr_C9ZNH3_
DRB1_0101	743	DHRPSSETIRRFHAN	PSSETIRRF	3	0.2258	4345.7	95.00	0.28	tr_C9ZNH3_
DRB1_0101	744	DDMQYGAFADEDCSR	DDMQYGAFA	0	0.2235	4453.6	95.00	0.48	tr_C9ZNH3_
DRB1_0101	745	ADHRPSSETIRRFHA	PSSETIRRF	4	0.2223	4512.3	95.00	0.28	tr_C9ZNH3_

DRB1_0101	746	VGPLSDSDTLSVLPS	LSDSDTLSV	3	0.2212	4564.2	95.00	0.34	tr_C9ZNH3_
DRB1_0101	747	LTKPETPSMHYHNLE	TKPETPSMH	1	0.2197	4639.5	95.00	0.31	tr_C9ZNH3_
DRB1_0101	748	VIDDIVIGDFGGECE	DDIVIGDFG	2	0.2138	4945.3	95.00	0.29	tr_C9ZNH3_
DRB1_0101	749	THSVISVDDMQYGAF	VISVDDMQY	3	0.2137	4950.5	95.00	0.34	tr_C9ZNH3_
DRB1_0101	750	DMQYGAFADEDCSRI	FADEDCSRI	6	0.2128	5000.2	95.00	0.55	tr_C9ZNH3_
DRB1_0101	751	AVPPLTKPETPSMHY	LTKPETPSM	4	0.2064	5359.2	95.00	0.42	tr_C9ZNH3_
DRB1_0101	752	ATNLPHWADHRPSSE	WADHRPSSE	6	0.2046	5464.8	95.00	0.36	tr_C9ZNH3_
DRB1_0101	753	VVGPLSDSDTLSVLP	LSDSDTLSV	4	0.2035	5531.1	95.00	0.42	tr_C9ZNH3_
DRB1_0101	754	IDDIVIGDFGGECEP	DDIVIGDFG	1	0.1987	5827.3	95.00	0.25	tr_C9ZNH3_
DRB1_0101	755	DDIVIGDFGGECEPL	VIGDFGGEC	3	0.1910	6333.4	100.00	0.20	tr_C9ZNH3_
DRB1_0101	756	SSFDGEADDAEFTAT	FDGEADDAE	2	0.1887	6491.8	100.00	0.50	tr_C9ZNH3_
DRB1_0101	757	ESSFDGEADDAEFTA	FDGEADDAE	3	0.1870	6614.4	100.00	0.56	tr_C9ZNH3_
DRB1_0101	758	MHYHNLEEDDHPKSV	YHNLEEDDH	2	0.1807	7075.2	100.00	0.36	tr_C9ZNH3_
DRB1_0101	759	GEADDAEFTATWELF	AEFTATWEL	5	0.1774	7333.7	100.00	0.34	tr_C9ZNH3_
DRB1_0101	760	SFDGEADDAEFTATW	FDGEADDAE	1	0.1765	7407.2	100.00	0.40	tr_C9ZNH3_
DRB1_0101	761	NAAVPPLTKPETPSM	NAAVPPLTK	0	0.1738	7625.3	100.00	0.37	tr_C9ZNH3_
DRB1_0101	762	SMHYHNLEEDDHPKS	YHNLEEDDH	3	0.1705	7902.1	100.00	0.50	tr_C9ZNH3_
DRB1_0101	763	TKPETPSMHYHNLEE	TPSMHYHNL	4	0.1689	8042.2	100.00	0.31	tr_C9ZNH3_
DRB1_0101	764	AAAYGATCYCNEGGR	AYGATCYCN	2	0.1643	8447.8	100.00	0.23	tr_C9ZNH3_
DRB1_0101	765	LESSFDGEADDAEFT	FDGEADDAE	4	0.1638	8495.0	100.00	0.61	tr_C9ZNH3_
DRB1_0101	766	AAVPPLTKPETPSMH	LTKPETPSM	5	0.1595	8905.8	100.00	0.41	tr_C9ZNH3_
DRB1_0101	767	HANRPNASDRTPLAL	NASDRTPLA	5	0.1583	9014.5	100.00	0.44	tr_C9ZNH3_
DRB1_0101	768	KPETPSMHYHNLEED	TPSMHYHNL	3	0.1515	9708.4	100.00	0.38	tr_C9ZNH3_
DRB1_0101	769	PSMHYHNLEEDDHPK	YHNLEEDDH	4	0.1514	9722.5	100.00	0.55	tr_C9ZNH3_
DRB1_0101	770	ETPSMHYHNLEEDDH	YHNLEEDDH	6	0.1461	10291.5	100.00	0.26	tr_C9ZNH3_
DRB1_0101	771	TPSMHYHNLEEDDHP	YHNLEEDDH	5	0.1426	10688.7	100.00	0.38	tr_C9ZNH3_
DRB1_0101	772	FDGEADDAEFTATWE	GEADDAEFT	2	0.1423	10727.6	100.00	0.38	tr_C9ZNH3_
DRB1_0101	773	TLESSFDGEADDAEF	FDGEADDAE	5	0.1415	10821.8	100.00	0.56	tr_C9ZNH3_
DRB1_0101	774	PETPSMHYHNLEEDD	TPSMHYHNL	2	0.1363	11441.0	100.00	0.34	tr_C9ZNH3_
DRB1_0101	775	DGEADDAEFTATWEL	GEADDAEFT	1	0.1325	11927.7	100.00	0.26	tr_C9ZNH3_

Allele: DRB1_0101. Number of high binders 6. Number of weak binders 75. Number of peptides 775

Allele	pos	peptide	core	Of	1-log50k(aff)	affinity(nM)	%Rank	Relia	Identity	Bind Level
DRB1_0101	1	SPRFVNLRDSVVAAS	FVNLRDSVV	3	0.8545	4.8	0.60	0.89	tr_C9ZZ34_	WB
DRB1_0101	2	ASPRFVNLRDSVVAA	FVNLRDSVV	4	0.8417	5.5	0.80	0.91	tr_C9ZZ34_	WB
DRB1_0101	3	PRFVNLRDSVVAASP	FVNLRDSVV	2	0.8411	5.6	0.80	0.89	tr_C9ZZ34_	WB
DRB1_0101	4	RFVNLRDSVVAASPL	FVNLRDSVV	1	0.8239	6.7	1.20	0.88	tr_C9ZZ34_	WB
DRB1_0101	5	LGKQLQGALANPTLW	LQGALANPT	4	0.8160	7.3	1.50	0.56	tr_C9ZZ34_	WB
DRB1_0101	6	VASPRFVNLRDSVVA	FVNLRDSVV	5	0.8105	7.8	1.70	0.92	tr_C9ZZ34_	WB
DRB1_0101	7	GKQLQGALANPTLWC	LQGALANPT	3	0.8035	8.4	1.90	0.59	tr_C9ZZ34_	WB
DRB1_0101	8	ALGKQLQGALANPTL	LQGALANPT	5	0.8003	8.7	2.00	0.51	tr_C9ZZ34_	WB
DRB1_0101	9	KQLQGALANPTLWCS	LQGALANPT	2	0.7810	10.7	3.00	0.69	tr_C9ZZ34_	WB
DRB1_0101	10	KSSFSPLVASPRFVN	FSPLVASPR	3	0.7707	12.0	3.50	0.76	tr_C9ZZ34_	WB
DRB1_0101	11	SSFSPLVASPRFVNL	FSPLVASPR	2	0.7535	14.4	4.50	0.68	tr_C9ZZ34_	WB
DRB1_0101	12	IALGKQLQGALANPT	GKQLQGALA	3	0.7504	14.9	5.00	0.60	tr_C9ZZ34_	WB

DRB1_0101	13	PKSSFSPLVASPRFV	FSPLVASPR	4	0.7503	14.9	5.00	0.79	tr_C9ZZ34_	WB
DRB1_0101	14	LVASPRFVNLRDSVV	FVNLRDSVV	6	0.7365	17.3	6.00	0.88	tr_C9ZZ34_	WB
DRB1_0101	15	QLQGALANPTLWCSK	LQGALANPT	1	0.7332	17.9	6.00	0.61	tr_C9ZZ34_	WB
DRB1_0101	16	FVNLRDSVVAASPLD	FVNLRDSVV	0	0.7311	18.4	6.50	0.80	tr_C9ZZ34_	WB
DRB1_0101	17	SFSPLVASPRFVNLR	FSPLVASPR	1	0.7263	19.3	6.50	0.61	tr_C9ZZ34_	WB
DRB1_0101	18	SPKSSFSPLVASPRF	FSPLVASPR	5	0.7102	23.0	8.00	0.80	tr_C9ZZ34_	WB
DRB1_0101	19	DNIALGKQLQGALAN	GKQLQGALA	5	0.7004	25.6	9.00	0.61	tr_C9ZZ34_	WB
DRB1_0101	20	NIALGKQLQGALANP	GKQLQGALA	4	0.6868	29.6	11.00	0.70	tr_C9ZZ34_	
DRB1_0101	21	SDNIALGKQLQGALA	GKQLQGALA	6	0.6731	34.4	12.00	0.44	tr_C9ZZ34_	
DRB1_0101	22	LQGALANPTLWCSKE	LQGALANPT	0	0.6459	46.1	16.00	0.44	tr_C9ZZ34_	
DRB1_0101	23	FSPLVASPRFVNLRD	FSPLVASPR	0	0.6432	47.5	16.00	0.46	tr_C9ZZ34_	
DRB1_0101	24	GSFCSQFSDNIALGK	FCSQFSDNI	2	0.6319	53.7	18.00	0.62	tr_C9ZZ34_	
DRB1_0101	25	GQSLPFFGMRSRDGT	FFGMRSRDG	5	0.6256	57.5	18.00	0.71	tr_C9ZZ34_	
DRB1_0101	26	GGSFCSQFSDNIALG	FCSQFSDNI	3	0.6212	60.2	19.00	0.71	tr_C9ZZ34_	
DRB1_0101	27	SFCSQFSDNIALGKQ	FCSQFSDNI	1	0.6209	60.5	19.00	0.50	tr_C9ZZ34_	
DRB1_0101	28	GSPKSSFSPLVASPR	FSPLVASPR	6	0.6190	61.7	19.00	0.65	tr_C9ZZ34_	
DRB1_0101	29	SLPFFGMRSRDGTDT	FFGMRSRDG	3	0.6153	64.2	20.00	0.86	tr_C9ZZ34_	
DRB1_0101	30	QFSDNIALGKQLQGA	DNIALGKQL	3	0.6099	68.1	21.00	0.51	tr_C9ZZ34_	
DRB1_0101	31	QSLPFFGMRSRDGTD	FFGMRSRDG	4	0.6043	72.3	22.00	0.82	tr_C9ZZ34_	
DRB1_0101	32	RGGSFCSQFSDNIAL	FCSQFSDNI	4	0.5993	76.3	23.00	0.75	tr_C9ZZ34_	
DRB1_0101	33	FSDNIALGKQLQGAL	DNIALGKQL	2	0.5977	77.7	23.00	0.46	tr_C9ZZ34_	
DRB1_0101	34	SQFSDNIALGKQLQG	DNIALGKQL	4	0.5935	81.3	24.00	0.47	tr_C9ZZ34_	
DRB1_0101	35	FCSQFSDNIALGKQL	FSDNIALGK	4	0.5884	85.9	24.00	0.28	tr_C9ZZ34_	
DRB1_0101	36	LPFFGMRSRDGTDTC	FFGMRSRDG	2	0.5879	86.4	24.00	0.88	tr_C9ZZ34_	
DRB1_0101	37	EGQSLPFFGMRSRDG	FFGMRSRDG	6	0.5730	101.5	27.00	0.50	tr_C9ZZ34_	
DRB1_0101	38	CSQFSDNIALGKQLQ	DNIALGKQL	5	0.5693	105.7	28.00	0.42	tr_C9ZZ34_	
DRB1_0101	39	PLGWEGLEGQSLPFF	WEGLEGQSL	3	0.5645	111.3	29.00	0.83	tr_C9ZZ34_	
DRB1_0101	40	GWEGLEGQSLPFFGM	WEGLEGQSL	1	0.5618	114.6	29.00	0.61	tr_C9ZZ34_	
DRB1_0101	41	LGWEGLEGQSLPFFG	WEGLEGQSL	2	0.5585	118.7	30.00	0.79	tr_C9ZZ34_	
DRB1_0101	42	PFFGMRSRDGTDTCI	FFGMRSRDG	1	0.5401	144.8	33.00	0.81	tr_C9ZZ34_	
DRB1_0101	43	NPLGWEGLEGQSLPF	WEGLEGQSL	4	0.5358	151.7	34.00	0.81	tr_C9ZZ34_	
DRB1_0101	44	WEGLEGQSLPFFGMR	WEGLEGQSL	0	0.5350	153.1	34.00	0.38	tr_C9ZZ34_	
DRB1_0101	45	LRDSVVAASPLDYSE	VVAASPLDY	4	0.5272	166.6	36.00	0.51	tr_C9ZZ34_	
DRB1_0101	46	ERGGSFCSQFSDNIA	FCSQFSDNI	5	0.5266	167.7	36.00	0.81	tr_C9ZZ34_	
DRB1_0101	47	RDSVVAASPLDYSEE	VVAASPLDY	3	0.5188	182.5	37.00	0.58	tr_C9ZZ34_	
DRB1_0101	48	NLRDSVVAASPLDYS	VVAASPLDY	5	0.5149	190.4	38.00	0.45	tr_C9ZZ34_	
DRB1_0101	49	VCPISMERNLRPSSS	ISMERNLRP	3	0.5046	212.8	40.00	0.70	tr_C9ZZ34_	
DRB1_0101	50	CPISMERNLRPSSSV	ISMERNLRP	2	0.4969	231.2	42.00	0.63	tr_C9ZZ34_	
DRB1_0101	51	GNPLGWEGLEGQSLP	WEGLEGQSL	5	0.4967	231.7	42.00	0.78	tr_C9ZZ34_	
DRB1_0101	52	PISMERNLRPSSSVS	ISMERNLRP	1	0.4844	264.7	44.00	0.52	tr_C9ZZ34_	
DRB1_0101	53	HVCPISMERNLRPSS	ISMERNLRP	4	0.4835	267.4	44.00	0.67	tr_C9ZZ34_	
DRB1_0101	54	GLEGQSLPFFGMRSR	GQSLPFFGM	3	0.4832	268.2	44.00	0.57	tr_C9ZZ34_	
DRB1_0101	55	LEGQSLPFFGMRSRD	GQSLPFFGM	2	0.4739	296.5	46.00	0.58	tr_C9ZZ34_	
DRB1_0101	56	SPLVASPRFVNLRDS	PLVASPRFV	1	0.4737	297.2	46.00	0.47	tr_C9ZZ34_	
DRB1_0101	57	SGNCLESPDLKVASA	CLESPDLKV	3	0.4706	307.4	47.00	0.60	tr_C9ZZ34_	
DRB1_0101	58	HDPWKKVLRCLTITT	WKKVLRCLT	3	0.4684	314.8	48.00	0.34	tr_C9ZZ34_	
DRB1_0101	59	QGALANPTLWCSKEE	GALANPTLW	1	0.4616	338.7	49.00	0.55	tr_C9ZZ34_	
DRB1_0101	60	YSGNCLESPDLKVAS	CLESPDLKV	4	0.4611	340.7	49.00	0.51	tr_C9ZZ34_	
DRB1_0101	61	DSVVAASPLDYSEEH	VVAASPLDY	2	0.4586	350.1	50.00	0.59	tr_C9ZZ34_	
DRB1_0101	62	EGLEGQSLPFFGMRS	GQSLPFFGM	4	0.4563	358.7	50.00	0.54	tr_C9ZZ34_	

DRB1_0101	63	FFGMRSRDGTDTCIH	FFGMRSRDG	0	0.4563	358.8	50.00	0.62	tr_C9ZZ34_
DRB1_0101	64	AIRDRVKGGIIVTTG	DRVKGGIIV	3	0.4554	362.2	50.00	0.62	tr_C9ZZ34_
DRB1_0101	65	ISMERNLRPSSSVSW	ERNLRPSSS	3	0.4538	368.5	55.00	0.36	tr_C9ZZ34_
DRB1_0101	66	PHVCPISMERNLRPS	ISMERNLRP	5	0.4519	376.3	55.00	0.60	tr_C9ZZ34_
DRB1_0101	67	DLKVASASQPGPAAP	LKVASASQP	1	0.4501	383.6	55.00	0.47	tr_C9ZZ34_
DRB1_0101	68	VNLRDSVVAASPLDY	DSVVAASPL	4	0.4470	396.7	55.00	0.37	tr_C9ZZ34_
DRB1_0101	69	PDLKVASASQPGPAA	LKVASASQP	2	0.4461	400.5	55.00	0.60	tr_C9ZZ34_
DRB1_0101	70	DAIRDRVKGGIIVTT	DRVKGGIIV	4	0.4440	410.0	55.00	0.62	tr_C9ZZ34_
DRB1_0101	71	SGNPLGWEGLEGQSL	WEGLEGQSL	6	0.4431	413.9	55.00	0.69	tr_C9ZZ34_
DRB1_0101	72	GNCLESPDLKVASAS	CLESPDLKV	2	0.4430	414.1	55.00	0.61	tr_C9ZZ34_
DRB1_0101	73	GYSGNCLESPDLKVA	CLESPDLKV	5	0.4402	427.0	55.00	0.44	tr_C9ZZ34_
DRB1_0101	74	SHDPWKKVLRCLTIT	WKKVLRCLT	4	0.4379	437.6	55.00	0.38	tr_C9ZZ34_
DRB1_0101	75	IRDRVKGGIIVTTGE	DRVKGGIIV	2	0.4351	451.4	55.00	0.62	tr_C9ZZ34_
DRB1_0101	76	LKVASASQPGPAAPS	SASQPGPAA	4	0.4261	497.4	60.00	0.45	tr_C9ZZ34_
DRB1_0101	77	SPRFASGPSERGGSF	FASGPSERG	3	0.4249	504.1	60.00	0.85	tr_C9ZZ34_
DRB1_0101	78	SPDLKVASASQPGPA	LKVASASQP	3	0.4224	517.8	60.00	0.74	tr_C9ZZ34_
DRB1_0101	79	SMERNLRPSSSVSWL	LRPSSSVSW	5	0.4183	541.5	60.00	0.38	tr_C9ZZ34_
DRB1_0101	80	GDAIRDRVKGGIIVT	DRVKGGIIV	5	0.4121	578.5	60.00	0.63	tr_C9ZZ34_
DRB1_0101	81	MERNLRPSSSVSWLC	LRPSSSVSW	4	0.4097	593.8	60.00	0.44	tr_C9ZZ34_
DRB1_0101	82	AGSPKSSFSPLVASP	SSFSPLVAS	5	0.4081	604.2	60.00	0.30	tr_C9ZZ34_
DRB1_0101	83	KGGIIVTTGEGAGSS	IIVTTGEGA	3	0.4065	614.8	65.00	0.75	tr_C9ZZ34_
DRB1_0101	84	PRFASGPSERGGSFC	FASGPSERG	2	0.4035	635.1	65.00	0.86	tr_C9ZZ34_
DRB1_0101	85	SERGGSFCSQFSDNI	FCSQFSDNI	6	0.4031	637.7	65.00	0.69	tr_C9ZZ34_
DRB1_0101	86	RDRVKGGIIVTTGEG	DRVKGGIIV	1	0.4017	647.7	65.00	0.59	tr_C9ZZ34_
DRB1_0101	87	HSPRFASGPSERGGS	FASGPSERG	4	0.4004	656.7	65.00	0.81	tr_C9ZZ34_
DRB1_0101	88	NCLESPDLKVASASQ	CLESPDLKV	1	0.3995	663.3	65.00	0.60	tr_C9ZZ34_
DRB1_0101	89	LRDIASTSPLGDAIR	IASTSPLGD	3	0.3929	712.3	65.00	0.44	tr_C9ZZ34_
DRB1_0101	90	SVVAASPLDYSEEHS	VVAASPLDY	1	0.3894	740.1	65.00	0.68	tr_C9ZZ34_
DRB1_0101	91	LAGSPKSSFSPLVAS	PKSSFSPLV	4	0.3881	750.3	65.00	0.27	tr_C9ZZ34_
DRB1_0101	92	QPHVCPISMERNLRP	ISMERNLRP	6	0.3866	762.9	65.00	0.41	tr_C9ZZ34_
DRB1_0101	93	PASTINSSPLGMGRG	TINSSPLGM	3	0.3861	766.7	65.00	0.50	tr_C9ZZ34_
DRB1_0101	94	PSHDPWKKVLRCLTI	WKKVLRCLT	5	0.3843	781.8	70.00	0.40	tr_C9ZZ34_
DRB1_0101	95	ERNLRPSSSVSWLCD	LRPSSSVSW	3	0.3838	785.9	70.00	0.47	tr_C9ZZ34_
DRB1_0101	96	PLVASPRFVNLRDSV	LVASPRFVN	1	0.3832	791.2	70.00	0.34	tr_C9ZZ34_
DRB1_0101	97	SPASTINSSPLGMGR	TINSSPLGM	4	0.3808	812.0	70.00	0.47	tr_C9ZZ34_
DRB1_0101	98	VGYSGNCLESPDLKV	GNCLESPDL	4	0.3742	872.0	70.00	0.29	tr_C9ZZ34_
DRB1_0101	99	ESPDLKVASASQPGP	LKVASASQP	4	0.3739	874.8	70.00	0.74	tr_C9ZZ34_
DRB1_0101	100	GGIIVTTGEGAGSSP	IIVTTGEGA	2	0.3732	881.9	70.00	0.71	tr_C9ZZ34_
DRB1_0101	101	ALRDIASTSPLGDAI	IASTSPLGD	4	0.3722	891.5	70.00	0.40	tr_C9ZZ34_
DRB1_0101	102	ASTINSSPLGMGRGS	TINSSPLGM	2	0.3707	905.6	70.00	0.52	tr_C9ZZ34_
DRB1_0101	103	RFASGPSERGGSFCS	FASGPSERG	1	0.3706	906.9	70.00	0.86	tr_C9ZZ34_
DRB1_0101	104	KVASASQPGPAAPSH	SASQPGPAA	3	0.3699	913.3	70.00	0.68	tr_C9ZZ34_
DRB1_0101	105	RDIASTSPLGDAIRD	IASTSPLGD	2	0.3699	913.7	70.00	0.44	tr_C9ZZ34_
DRB1_0101	106	VKGGIIVTTGEGAGS	IIVTTGEGA	4	0.3666	946.7	70.00	0.74	tr_C9ZZ34_
DRB1_0101	107	LESPDLKVASASQPG	LKVASASQP	5	0.3646	967.5	70.00	0.70	tr_C9ZZ34_
DRB1_0101	108	CALRDIASTSPLGDA	IASTSPLGD	5	0.3605	1011.4	70.00	0.31	tr_C9ZZ34_
DRB1_0101	109	EHSPRFASGPSERGG	FASGPSERG	5	0.3600	1017.1	75.00	0.74	tr_C9ZZ34_
DRB1_0101	110	GIIVTTGEGAGSSPA	IIVTTGEGA	1	0.3579	1040.9	75.00	0.62	tr_C9ZZ34_
DRB1_0101	111	ETLSEGLAGSPKSSF	LSEGLAGSP	2	0.3563	1058.6	75.00	0.37	tr_C9ZZ34_
DRB1_0101	112	CLESPDLKVASASQP	CLESPDLKV	0	0.3553	1069.8	75.00	0.34	tr_C9ZZ34_

DRB1_0101	113	SVSWLCDETLSEGLA	VSWLCDETL	1	0.3531	1095.9	75.00	0.41	tr_C9ZZ34_
DRB1_0101	114	RVGYSGNCLESPDLK	YSGNCLESP	3	0.3495	1139.7	75.00	0.34	tr_C9ZZ34_
DRB1_0101	115	RNLRPSSSVSWLCDE	LRPSSSVSW	2	0.3465	1176.8	75.00	0.52	tr_C9ZZ34_
DRB1_0101	116	DETLSEGLAGSPKSS	LSEGLAGSP	3	0.3426	1227.4	75.00	0.44	tr_C9ZZ34_
DRB1_0101	117	CDETLSEGLAGSPKS	LSEGLAGSP	4	0.3416	1241.7	75.00	0.34	tr_C9ZZ34_
DRB1_0101	118	GALANPTLWCSKEES	GALANPTLW	0	0.3404	1257.1	75.00	0.52	tr_C9ZZ34_
DRB1_0101	119	TLSEGLAGSPKSSFS	SEGLAGSPK	2	0.3400	1262.9	75.00	0.30	tr_C9ZZ34_
DRB1_0101	120	LCDETLSEGLAGSPK	LSEGLAGSP	5	0.3397	1266.9	75.00	0.25	tr_C9ZZ34_
DRB1_0101	121	SSVSWLCDETLSEGL	VSWLCDETL	2	0.3392	1273.8	75.00	0.58	tr_C9ZZ34_
DRB1_0101	122	MCALRDIASTSPLGD	DIASTSPLG	5	0.3388	1279.9	75.00	0.21	tr_C9ZZ34_
DRB1_0101	123	DRVKGGIIVTTGEGA	DRVKGGIIV	0	0.3375	1297.3	75.00	0.41	tr_C9ZZ34_
DRB1_0101	124	LGDAIRDRVKGGIIV	DRVKGGIIV	6	0.3363	1314.9	75.00	0.50	tr_C9ZZ34_
DRB1_0101	125	SSPASTINSSPLGMG	TINSSPLGM	5	0.3328	1364.9	80.00	0.41	tr_C9ZZ34_
DRB1_0101	126	SSSVSWLCDETLSEG	VSWLCDETL	3	0.3327	1365.9	80.00	0.72	tr_C9ZZ34_
DRB1_0101	127	HSHSYDQPHVCPISM	YDQPHVCPI	4	0.3280	1437.4	80.00	0.54	tr_C9ZZ34_
DRB1_0101	128	VSWLCDETLSEGLAG	CDETLSEGL	4	0.3272	1450.7	80.00	0.28	tr_C9ZZ34_
DRB1_0101	129	PRVGYSGNCLESPDL	YSGNCLESP	4	0.3262	1466.7	80.00	0.31	tr_C9ZZ34_
DRB1_0101	130	SHSYDQPHVCPISME	YDQPHVCPI	3	0.3257	1474.8	80.00	0.54	tr_C9ZZ34_
DRB1_0101	131	RVKGGIIVTTGEGAG	IIVTTGEGA	5	0.3255	1476.8	80.00	0.67	tr_C9ZZ34_
DRB1_0101	132	DIASTSPLGDAIRDR	IASTSPLGD	1	0.3253	1481.0	80.00	0.34	tr_C9ZZ34_
DRB1_0101	133	VASASQPGPAAPSHD	SASQPGPAA	2	0.3244	1494.8	80.00	0.68	tr_C9ZZ34_
DRB1_0101	134	PSSSVSWLCDETLSE	VSWLCDETL	4	0.3233	1512.6	80.00	0.71	tr_C9ZZ34_
DRB1_0101	135	RPSSSVSWLCDETLS	VSWLCDETL	5	0.3179	1603.4	80.00	0.63	tr_C9ZZ34_
DRB1_0101	136	SWLCDETLSEGLAGS	CDETLSEGL	3	0.3164	1630.3	80.00	0.39	tr_C9ZZ34_
DRB1_0101	137	HSYDQPHVCPISMER	YDQPHVCPI	2	0.3121	1707.8	80.00	0.50	tr_C9ZZ34_
DRB1_0101	138	STINSSPLGMGRGSD	TINSSPLGM	1	0.3115	1719.2	80.00	0.50	tr_C9ZZ34_
DRB1_0101	139	PLDYSEEHSPRFASG	YSEEHSPRF	3	0.3088	1770.3	80.00	0.65	tr_C9ZZ34_
DRB1_0101	140	APSHDPWKKVLRCLT	WKKVLRCLT	6	0.3084	1777.6	80.00	0.40	tr_C9ZZ34_
DRB1_0101	141	IHSHSYDQPHVCPIS	YDQPHVCPI	5	0.3082	1781.3	80.00	0.48	tr_C9ZZ34_
DRB1_0101	142	WLCDETLSEGLAGSP	CDETLSEGL	2	0.3055	1833.8	85.00	0.35	tr_C9ZZ34_
DRB1_0101	143	GLAGSPKSSFSPLVA	PKSSFSPLV	5	0.3038	1867.8	85.00	0.30	tr_C9ZZ34_
DRB1_0101	144	SPRVGYSGNCLESPD	RVGYSGNCL	2	0.3036	1872.0	85.00	0.42	tr_C9ZZ34_
DRB1_0101	145	HSPRVGYSGNCLESP	RVGYSGNCL	3	0.2994	1959.5	85.00	0.49	tr_C9ZZ34_
DRB1_0101	146	SPLDYSEEHSPRFAS	YSEEHSPRF	4	0.2970	2011.2	85.00	0.62	tr_C9ZZ34_
DRB1_0101	147	DQPHVCPISMERNLR	HVCPISMER	3	0.2966	2018.7	85.00	0.25	tr_C9ZZ34_
DRB1_0101	148	PHSPRVGYSGNCLES	RVGYSGNCL	4	0.2965	2022.7	85.00	0.59	tr_C9ZZ34_
DRB1_0101	149	LDYSEEHSPRFASGP	YSEEHSPRF	2	0.2947	2061.4	85.00	0.69	tr_C9ZZ34_
DRB1_0101	150	IASTSPLGDAIRDRV	STSPLGDAI	2	0.2939	2080.5	85.00	0.31	tr_C9ZZ34_
DRB1_0101	151	LRPSSSVSWLCDETL	VSWLCDETL	6	0.2929	2102.6	85.00	0.44	tr_C9ZZ34_
DRB1_0101	152	IIVTTGEGAGSSPAS	IIVTTGEGA	0	0.2923	2114.9	85.00	0.47	tr_C9ZZ34_
DRB1_0101	153	LSEGLAGSPKSSFSP	SEGLAGSPK	1	0.2876	2225.1	85.00	0.32	tr_C9ZZ34_
DRB1_0101	154	ASASQPGPAAPSHDP	SASQPGPAA	1	0.2804	2405.9	85.00	0.65	tr_C9ZZ34_
DRB1_0101	155	NLRPSSSVSWLCDET	LRPSSSVSW	1	0.2772	2491.9	85.00	0.44	tr_C9ZZ34_
DRB1_0101	156	CIHSHSYDQPHVCPI	YDQPHVCPI	6	0.2750	2551.6	90.00	0.35	tr_C9ZZ34_
DRB1_0101	157	EEHSPRFASGPSERG	FASGPSERG	6	0.2748	2556.0	90.00	0.62	tr_C9ZZ34_
DRB1_0101	158	SYDQPHVCPISMERN	YDQPHVCPI	1	0.2732	2600.0	90.00	0.41	tr_C9ZZ34_
DRB1_0101	159	GSSPASTINSSPLGM	TINSSPLGM	6	0.2710	2664.1	90.00	0.30	tr_C9ZZ34_
DRB1_0101	160	ASTSPLGDAIRDRVK	STSPLGDAI	1	0.2684	2740.2	90.00	0.25	tr_C9ZZ34_
DRB1_0101	161	YDQPHVCPISMERNL	HVCPISMER	4	0.2669	2785.9	90.00	0.23	tr_C9ZZ34_
DRB1_0101	162	FASGPSERGGSFCSQ	FASGPSERG	0	0.2662	2805.3	90.00	0.67	tr_C9ZZ34_

DRB1_0101	163	ASPLDYSEEHSPRFA	YSEEHSPRF	5	0.2655	2828.3	90.00	0.60	tr_C9ZZ34_
DRB1_0101	164	SPHSPRVGYSGNCLE	RVGYSGNCL	5	0.2620	2936.2	90.00	0.55	tr_C9ZZ34_
DRB1_0101	165	SEGLAGSPKSSFSPL	LAGSPKSSF	3	0.2570	3100.7	90.00	0.34	tr_C9ZZ34_
DRB1_0101	166	EGLAGSPKSSFSPLV	LAGSPKSSF	2	0.2510	3306.2	90.00	0.33	tr_C9ZZ34_
DRB1_0101	167	DYSEEHSPRFASGPS	YSEEHSPRF	1	0.2505	3324.3	90.00	0.67	tr_C9ZZ34_
DRB1_0101	168	STSPLGDAIRDRVKG	LGDAIRDRV	4	0.2398	3732.0	95.00	0.30	tr_C9ZZ34_
DRB1_0101	169	VVAASPLDYSEEHSP	VVAASPLDY	0	0.2367	3861.3	95.00	0.51	tr_C9ZZ34_
DRB1_0101	170	AHSPRSGNPLGWEGL	HSPRSGNPL	1	0.2364	3875.8	95.00	0.31	tr_C9ZZ34_
DRB1_0101	171	HSPRSGNPLGWEGLE	GNPLGWEGL	5	0.2320	4060.8	95.00	0.34	tr_C9ZZ34_
DRB1_0101	172	AAPSHDPWKKVLRCL	PWKKVLRCL	6	0.2319	4065.4	95.00	0.26	tr_C9ZZ34_
DRB1_0101	173	TCIHSHSYDQPHVCP	CIHSHSYDQ	1	0.2289	4202.7	95.00	0.35	tr_C9ZZ34_
DRB1_0101	174	DTCIHSHSYDQPHVC	CIHSHSYDQ	2	0.2283	4229.1	95.00	0.38	tr_C9ZZ34_
DRB1_0101	175	SSPLGMGRGSDDSAH	LGMGRGSDD	3	0.2247	4397.7	95.00	0.56	tr_C9ZZ34_
DRB1_0101	176	PRSGNPLGWEGLEGQ	GNPLGWEGL	3	0.2241	4424.4	95.00	0.50	tr_C9ZZ34_
DRB1_0101	177	TINSSPLGMGRGSDD	INSSPLGMG	1	0.2238	4441.3	95.00	0.26	tr_C9ZZ34_
DRB1_0101	178	RSGNPLGWEGLEGQS	GNPLGWEGL	2	0.2230	4478.3	95.00	0.51	tr_C9ZZ34_
DRB1_0101	179	TDTCIHSHSYDQPHV	CIHSHSYDQ	3	0.2212	4563.8	95.00	0.40	tr_C9ZZ34_
DRB1_0101	180	GEGAGSSPASTINSS	AGSSPASTI	3	0.2207	4590.6	95.00	0.45	tr_C9ZZ34_
DRB1_0101	181	FGMRSRDGTDTCIHS	MRSRDGTDT	2	0.2206	4596.1	95.00	0.55	tr_C9ZZ34_
DRB1_0101	182	SPRSGNPLGWEGLEG	GNPLGWEGL	4	0.2199	4633.3	95.00	0.43	tr_C9ZZ34_
DRB1_0101	183	TGEGAGSSPASTINS	AGSSPASTI	4	0.2192	4665.4	95.00	0.40	tr_C9ZZ34_
DRB1_0101	184	PTLWCSKEESPRSTA	CSKEESPRS	4	0.2179	4732.9	95.00	0.28	tr_C9ZZ34_
DRB1_0101	185	SPLGMGRGSDDSAHS	LGMGRGSDD	2	0.2168	4787.9	95.00	0.53	tr_C9ZZ34_
DRB1_0101	186	NSSPLGMGRGSDDSA	LGMGRGSDD	4	0.2150	4881.8	95.00	0.56	tr_C9ZZ34_
DRB1_0101	187	PSPHSPRVGYSGNCL	RVGYSGNCL	6	0.2134	4969.1	95.00	0.47	tr_C9ZZ34_
DRB1_0101	188	TLWCSKEESPRSTAP	CSKEESPRS	3	0.2089	5214.1	95.00	0.29	tr_C9ZZ34_
DRB1_0101	189	PLGDAIRDRVKGGII	LGDAIRDRV	1	0.2072	5313.1	95.00	0.23	tr_C9ZZ34_
DRB1_0101	190	GTDTCIHSHSYDQPH	CIHSHSYDQ	4	0.2059	5385.4	95.00	0.40	tr_C9ZZ34_
DRB1_0101	191	EGAGSSPASTINSSP	AGSSPASTI	2	0.2059	5386.2	95.00	0.47	tr_C9ZZ34_
DRB1_0101	192	IVTTGEGAGSSPAST	VTTGEGAGS	1	0.2053	5421.5	95.00	0.35	tr_C9ZZ34_
DRB1_0101	193	SASQPGPAAPSHDPW	SASQPGPAA	0	0.2050	5443.3	95.00	0.51	tr_C9ZZ34_
DRB1_0101	194	SAHSPRSGNPLGWEG	HSPRSGNPL	2	0.2050	5443.3	95.00	0.52	tr_C9ZZ34_
DRB1_0101	195	TTGEGAGSSPASTIN	AGSSPASTI	5	0.2044	5478.5	95.00	0.35	tr_C9ZZ34_
DRB1_0101	196	TSPLGDAIRDRVKGG	LGDAIRDRV	3	0.2040	5501.7	95.00	0.43	tr_C9ZZ34_
DRB1_0101	197	VTTGEGAGSSPASTI	GAGSSPAST	5	0.2018	5630.8	95.00	0.20	tr_C9ZZ34_
DRB1_0101	198	INSSPLGMGRGSDDS	LGMGRGSDD	5	0.2002	5733.3	95.00	0.44	tr_C9ZZ34_
DRB1_0101	199	AASPLDYSEEHSPRF	YSEEHSPRF	6	0.1997	5761.9	95.00	0.49	tr_C9ZZ34_
DRB1_0101	200	YSEEHSPRFASGPSE	YSEEHSPRF	0	0.1988	5819.1	95.00	0.51	tr_C9ZZ34_
DRB1_0101	201	AGSSPASTINSSPLG	ASTINSSPL	5	0.1974	5905.2	95.00	0.34	tr_C9ZZ34_
DRB1_0101	202	DSAHSPRSGNPLGWE	HSPRSGNPL	3	0.1973	5911.4	95.00	0.54	tr_C9ZZ34_
DRB1_0101	203	GAGSSPASTINSSPL	AGSSPASTI	1	0.1944	6103.7	95.00	0.43	tr_C9ZZ34_
DRB1_0101	204	PLGMGRGSDDSAHSP	LGMGRGSDD	1	0.1919	6271.8	100.00	0.51	tr_C9ZZ34_
DRB1_0101	205	DGTDTCIHSHSYDQP	CIHSHSYDQ	5	0.1901	6392.6	100.00	0.34	tr_C9ZZ34_
DRB1_0101	206	PSERGGSFCSQFSDN	GGSFCSQFS	4	0.1901	6392.9	100.00	0.35	tr_C9ZZ34_
DRB1_0101	207	NPTLWCSKEESPRST	CSKEESPRS	5	0.1886	6496.8	100.00	0.28	tr_C9ZZ34_
DRB1_0101	208	GMRSRDGTDTCIHSH	MRSRDGTDT	1	0.1864	6651.5	100.00	0.50	tr_C9ZZ34_
DRB1_0101	209	SPLGDAIRDRVKGGI	LGDAIRDRV	2	0.1854	6723.9	100.00	0.40	tr_C9ZZ34_
DRB1_0101	210	LWCSKEESPRSTAPS	CSKEESPRS	2	0.1854	6728.2	100.00	0.28	tr_C9ZZ34_
DRB1_0101	211	RDGTDTCIHSHSYDQ	DTCIHSHSY	4	0.1848	6769.8	100.00	0.35	tr_C9ZZ34_
DRB1_0101	212	DDSAHSPRSGNPLGW	HSPRSGNPL	4	0.1787	7228.6	100.00	0.55	tr_C9ZZ34_

DRB1_0101	213	PAAPSHDPWKKVLRC	PSHDPWKKV	3	0.1785	7246.0	100.00	0.36	tr_C9ZZ34_	
DRB1_0101	214	GPAAPSHDPWKKVLR	PSHDPWKKV	4	0.1746	7556.5	100.00	0.37	tr_C9ZZ34_	
DRB1_0101	215	GPSERGGSFCSQFSD	SERGGSFCS	2	0.1735	7652.1	100.00	0.34	tr_C9ZZ34_	
DRB1_0101	216	RSRDGTDTCIHSHSY	DTCIHSHSY	6	0.1674	8174.5	100.00	0.21	tr_C9ZZ34_	
DRB1_0101	217	LGMGRGSDDSAHSPR	LGMGRGSDD	0	0.1645	8433.5	100.00	0.34	tr_C9ZZ34_	
DRB1_0101	218	PGPAAPSHDPWKKVL	PSHDPWKKV	5	0.1637	8505.9	100.00	0.35	tr_C9ZZ34_	
DRB1_0101	219	SGPSERGGSFCSQFS	SERGGSFCS	3	0.1624	8625.1	100.00	0.41	tr_C9ZZ34_	
DRB1_0101	220	MRSRDGTDTCIHSHS	SRDGTDTCI	2	0.1622	8648.2	100.00	0.25	tr_C9ZZ34_	
DRB1_0101	221	SPRSTAPSPHSPRVG	STAPSPHSP	3	0.1620	8668.6	100.00	0.20	tr_C9ZZ34_	
DRB1_0101	222	SRDGTDTCIHSHSYD	DTCIHSHSY	5	0.1602	8834.8	100.00	0.34	tr_C9ZZ34_	
DRB1_0101	223	PRSTAPSPHSPRVGY	TAPSPHSPR	3	0.1554	9303.0	100.00	0.21	tr_C9ZZ34_	
DRB1_0101	224	SDDSAHSPRSGNPLG	HSPRSGNPL	5	0.1550	9344.2	100.00	0.52	tr_C9ZZ34_	
DRB1_0101	225	ANPTLWCSKEESPRS	WCSKEESPR	5	0.1536	9485.3	100.00	0.28	tr_C9ZZ34_	
DRB1_0101	226	RSTAPSPHSPRVGYS	TAPSPHSPR	2	0.1520	9650.4	100.00	0.22	tr_C9ZZ34_	
DRB1_0101	227	ESPRSTAPSPHSPRV	RSTAPSPHS	3	0.1499	9881.4	100.00	0.29	tr_C9ZZ34_	
DRB1_0101	228	ALANPTLWCSKEESP	LANPTLWCS	1	0.1452	10396.8	100.00	0.35	tr_C9ZZ34_	
DRB1_0101	229	SEEHSPRFASGPSER	PRFASGPSE	5	0.1444	10486.8	100.00	0.28	tr_C9ZZ34_	
DRB1_0101	230	WCSKEESPRSTAPSP	CSKEESPRS	1	0.1443	10492.6	100.00	0.31	tr_C9ZZ34_	
DRB1_0101	231	QPGPAAPSHDPWKKV	PSHDPWKKV	6	0.1436	10568.8	100.00	0.26	tr_C9ZZ34_	
DRB1_0101	232	TAPSPHSPRVGYSGN	HSPRVGYSG	5	0.1400	10991.9	100.00	0.22	tr_C9ZZ34_	
DRB1_0101	233	STAPSPHSPRVGYSG	TAPSPHSPR	1	0.1397	11033.0	100.00	0.17	tr_C9ZZ34_	
DRB1_0101	234	VAASPLDYSEEHSPR	LDYSEEHSP	5	0.1363	11444.8	100.00	0.35	tr_C9ZZ34_	
DRB1_0101	235	MGRGSDDSAHSPRSG	DDSAHSPRS	5	0.1321	11974.6	100.00	0.26	tr_C9ZZ34_	
DRB1_0101	236	GMGRGSDDSAHSPRS	MGRGSDDSA	1	0.1302	12226.2	100.00	0.23	tr_C9ZZ34_	
DRB1_0101	237	ASGPSERGGSFCSQF	SERGGSFCS	4	0.1287	12420.6	100.00	0.49	tr_C9ZZ34_	
DRB1_0101	238	APSPHSPRVGYSGNC	HSPRVGYSG	4	0.1286	12430.3	100.00	0.31	tr_C9ZZ34_	
DRB1_0101	239	GSDDSAHSPRSGNPL	HSPRSGNPL	6	0.1283	12479.3	100.00	0.34	tr_C9ZZ34_	
DRB1_0101	240	LANPTLWCSKEESPR	LWCSKEESP	5	0.1270	12659.2	100.00	0.23	tr_C9ZZ34_	
DRB1_0101	241	GRGSDDSAHSPRSGN	DDSAHSPRS	4	0.1233	13169.3	100.00	0.31	tr_C9ZZ34_	
DRB1_0101	242	EESPRSTAPSPHSPR	PRSTAPSPH	3	0.1229	13222.4	100.00	0.29	tr_C9ZZ34_	
DRB1_0101	243	ASQPGPAAPSHDPWK	SQPGPAAPS	1	0.1208	13525.0	100.00	0.39	tr_C9ZZ34_	
DRB1_0101	244	SQPGPAAPSHDPWKK	GPAAPSHDP	3	0.1175	14025.1	100.00	0.27	tr_C9ZZ34_	
DRB1_0101	245	CSKEESPRSTAPSPH	SKEESPRST	1	0.1138	14602.6	100.00	0.38	tr_C9ZZ34_	
DRB1_0101	246	RGSDDSAHSPRSGNP	DDSAHSPRS	3	0.1106	15111.3	100.00	0.35	tr_C9ZZ34_	
DRB1_0101	247	SKEESPRSTAPSPHS	PRSTAPSPH	5	0.0981	17306.6	100.00	0.23	tr_C9ZZ34_	
DRB1_0101	248	KEESPRSTAPSPHSP	PRSTAPSPH	4	0.0930	18280.0	100.00	0.32	tr_C9ZZ34_	

Allele: DRB1_0101. Number of high binders 0. Number of weak binders 19. Number of peptides 248

Allele	pos	peptide	core	Of	1-log50k(aff)	affinity(nM)	%Rank	Relia	Identity	Bind Level
DRB1_0101	1	SREQYAPLMAAFGLM	YAPLMAAFG	4	0.8477	5.2	0.70	0.87	tr_C9ZZ34_	WB
DRB1_0101	2	REQYAPLMAAFGLMC	YAPLMAAFG	3	0.8450	5.4	0.70	0.87	tr_C9ZZ34_	WB
DRB1_0101	3	NSSFLAQQVTFLVCR	FLAQQVTFL	3	0.8397	5.7	0.80	0.79	tr_C9ZZ34_	WB
DRB1_0101	4	SNSSFLAQQVTFLVC	FLAQQVTFL	4	0.8278	6.4	1.10	0.76	tr_C9ZZ34_	WB
DRB1_0101	5	LSNSSFLAQQVTFLV	FLAQQVTFL	5	0.8254	6.6	1.20	0.75	tr_C9ZZ34_	WB
DRB1_0101	6	SSREQYAPLMAAFGL	YAPLMAAFG	5	0.8239	6.7	1.20	0.81	tr_C9ZZ34_	WB

DRB1_0101	7	SSFLAQQVTFLVCRL	FLAQQVTFL	2	0.8198	7.0	1.40	0.76	tr_C9ZZ34_	WB
DRB1_0101	8	EQYAPLMAAFGLMCE	YAPLMAAFG	2	0.8150	7.4	1.50	0.83	tr_C9ZZ34_	WB
DRB1_0101	9	MLSNSSFLAQQVTFL	FLAQQVTFL	6	0.7939	9.3	2.50	0.63	tr_C9ZZ34_	WB
DRB1_0101	10	KNAMLSNSSFLAQQV	MLSNSSFLA	3	0.7869	10.0	3.00	0.73	tr_C9ZZ34_	WB
DRB1_0101	11	ITEFVSGGSLAALVE	FVSGGSLAA	3	0.7831	10.4	3.00	0.87	tr_C9ZZ34_	WB
DRB1_0101	12	QYAPLMAAFGLMCEK	YAPLMAAFG	1	0.7830	10.5	3.00	0.79	tr_C9ZZ34_	WB
DRB1_0101	13	IITEFVSGGSLAALV	FVSGGSLAA	4	0.7802	10.8	3.00	0.85	tr_C9ZZ34_	WB
DRB1_0101	14	SKNAMLSNSSFLAQQ	MLSNSSFLA	4	0.7769	11.2	3.50	0.69	tr_C9ZZ34_	WB
DRB1_0101	15	LPFVWGIAMHLSQLL	FVWGIAMHL	2	0.7739	11.5	3.50	0.57	tr_C9ZZ34_	WB
DRB1_0101	16	SFLAQQVTFLVCRLF	FLAQQVTFL	1	0.7733	11.6	3.50	0.74	tr_C9ZZ34_	WB
DRB1_0101	17	PFVWGIAMHLSQLLV	FVWGIAMHL	1	0.7709	11.9	3.50	0.46	tr_C9ZZ34_	WB
DRB1_0101	18	LRSFSMGTVSLICRV	FSMGTVSLI	3	0.7678	12.3	4.00	0.75	tr_C9ZZ34_	WB
DRB1_0101	19	TEFVSGGSLAALVET	FVSGGSLAA	2	0.7664	12.5	4.00	0.86	tr_C9ZZ34_	WB
DRB1_0101	20	SLPFVWGIAMHLSQL	FVWGIAMHL	3	0.7638	12.9	4.00	0.73	tr_C9ZZ34_	WB
DRB1_0101	21	GSSREQYAPLMAAFG	YAPLMAAFG	6	0.7618	13.2	4.00	0.71	tr_C9ZZ34_	WB
DRB1_0101	22	TLRSFSMGTVSLICR	FSMGTVSLI	4	0.7587	13.6	4.50	0.74	tr_C9ZZ34_	WB
DRB1_0101	23	AIVYQLQEATSFSRK	YQLQEATSF	3	0.7570	13.9	4.50	0.68	tr_C9ZZ34_	WB
DRB1_0101	24	RSFSMGTVSLICRVA	FSMGTVSLI	2	0.7552	14.1	4.50	0.75	tr_C9ZZ34_	WB
DRB1_0101	25	NAMLSNSSFLAQQVT	MLSNSSFLA	2	0.7534	14.4	4.50	0.69	tr_C9ZZ34_	WB
DRB1_0101	26	LSEIRMLSASHHKNI	IRMLSASHH	3	0.7524	14.6	5.00	0.79	tr_C9ZZ34_	WB
DRB1_0101	27	GSKNAMLSNSSFLAQ	MLSNSSFLA	5	0.7512	14.8	5.00	0.61	tr_C9ZZ34_	WB
DRB1_0101	28	SEIRMLSASHHKNIV	IRMLSASHH	2	0.7486	15.2	5.00	0.69	tr_C9ZZ34_	WB
DRB1_0101	29	YIITEFVSGGSLAAL	FVSGGSLAA	5	0.7446	15.8	5.50	0.79	tr_C9ZZ34_	WB
DRB1_0101	30	FVWGIAMHLSQLLVG	IAMHLSQLL	4	0.7422	16.3	5.50	0.28	tr_C9ZZ34_	WB
DRB1_0101	31	SSLPFVWGIAMHLSQ	FVWGIAMHL	4	0.7363	17.3	6.00	0.81	tr_C9ZZ34_	WB
DRB1_0101	32	DAIVYQLQEATSFSR	YQLQEATSF	4	0.7345	17.7	6.00	0.65	tr_C9ZZ34_	WB
DRB1_0101	33	HTLRSFSMGTVSLIC	FSMGTVSLI	5	0.7337	17.8	6.00	0.69	tr_C9ZZ34_	WB
DRB1_0101	34	TSSLPFVWGIAMHLS	FVWGIAMHL	5	0.7265	19.3	6.50	0.76	tr_C9ZZ34_	WB
DRB1_0101	35	IVYQLQEATSFSRKE	YQLQEATSF	2	0.7264	19.3	6.50	0.75	tr_C9ZZ34_	WB
DRB1_0101	36	TLSEIRMLSASHHKN	IRMLSASHH	4	0.7244	19.7	7.00	0.79	tr_C9ZZ34_	WB
DRB1_0101	37	EFVSGGSLAALVETF	FVSGGSLAA	1	0.7242	19.8	7.00	0.84	tr_C9ZZ34_	WB
DRB1_0101	38	EIRMLSASHHKNIVR	IRMLSASHH	1	0.7207	20.5	7.00	0.64	tr_C9ZZ34_	WB
DRB1_0101	39	AMLSNSSFLAQQVTF	MLSNSSFLA	1	0.7205	20.6	7.00	0.58	tr_C9ZZ34_	WB
DRB1_0101	40	STLSEIRMLSASHHK	IRMLSASHH	5	0.7116	22.7	8.00	0.74	tr_C9ZZ34_	WB
DRB1_0101	41	RTSSLYGEEVRLAYR	LYGEEVRLA	4	0.7094	23.2	8.00	0.47	tr_C9ZZ34_	WB
DRB1_0101	42	SHEIIPVSSSPLLKN	IIPVSSSPL	3	0.7075	23.7	8.50	0.75	tr_C9ZZ34_	WB
DRB1_0101	43	KDAIVYQLQEATSFS	YQLQEATSF	5	0.7074	23.7	8.50	0.58	tr_C9ZZ34_	WB
DRB1_0101	44	GRTSSLYGEEVRLAY	TSSLYGEEV	2	0.7014	25.3	9.00	0.51	tr_C9ZZ34_	WB
DRB1_0101	45	FGSKNAMLSNSSFLA	MLSNSSFLA	6	0.6990	26.0	9.00	0.45	tr_C9ZZ34_	WB
DRB1_0101	46	VCRLFLPALDLEMEG	FLPALDLEM	4	0.6989	26.0	9.00	0.52	tr_C9ZZ34_	WB
DRB1_0101	47	EYRLLHRVANSCAMM	LHRVANSCA	4	0.6983	26.2	9.00	0.53	tr_C9ZZ34_	WB
DRB1_0101	48	CRLFLPALDLEMEGV	FLPALDLEM	3	0.6971	26.5	9.50	0.61	tr_C9ZZ34_	WB
DRB1_0101	49	HEIIPVSSSPLLKNL	IIPVSSSPL	2	0.6959	26.9	9.50	0.71	tr_C9ZZ34_	WB
DRB1_0101	50	LVCRLFLPALDLEME	FLPALDLEM	5	0.6936	27.5	9.50	0.44	tr_C9ZZ34_	WB
DRB1_0101	51	SFSMGTVSLICRVAP	FSMGTVSLI	1	0.6926	27.8	9.50	0.71	tr_C9ZZ34_	WB
DRB1_0101	52	RVPLRAVRSHEIIPV	LRAVRSHEI	3	0.6918	28.1	10.00	0.84	tr_C9ZZ34_	
DRB1_0101	53	VVTTKRLRAVQAMSQ	TKRLRAVQA	3	0.6893	28.8	10.00	0.76	tr_C9ZZ34_	
DRB1_0101	54	CIDAFALPRPRELKV	FALPRPREL	4	0.6878	29.3	11.00	0.83	tr_C9ZZ34_	
DRB1_0101	55	YAPLMAAFGLMCEKR	YAPLMAAFG	0	0.6871	29.5	11.00	0.62	tr_C9ZZ34_	
DRB1_0101	56	VTTKRLRAVQAMSQQ	TKRLRAVQA	2	0.6871	29.5	11.00	0.69	tr_C9ZZ34_	

DRB1_0101	57	RTSSLPFVWGIAMHL	FVWGIAMHL	6	0.6865	29.7	11.00	0.67	tr_C9ZZ34_
DRB1_0101	58	RSHEIIPVSSSPLLK	IIPVSSSPL	4	0.6848	30.3	11.00	0.76	tr_C9ZZ34_
DRB1_0101	59	VWGIAMHLSQLLVGV	IAMHLSQLL	3	0.6828	30.9	11.00	0.43	tr_C9ZZ34_
DRB1_0101	60	YRLLHRVANSCAMMD	LHRVANSCA	3	0.6825	31.1	11.00	0.57	tr_C9ZZ34_
DRB1_0101	61	IDAFALPRPRELKVC	FALPRPREL	3	0.6815	31.4	11.00	0.86	tr_C9ZZ34_
DRB1_0101	62	SGRTSSLYGEEVRLA	TSSLYGEEV	3	0.6813	31.5	11.00	0.69	tr_C9ZZ34_
DRB1_0101	63	QSEYALVTLRQNTLM	YALVTLRQN	3	0.6808	31.6	11.00	0.34	tr_C9ZZ34_
DRB1_0101	64	SEYALVTLRQNTLMN	LVTLRQNTL	4	0.6788	32.3	12.00	0.41	tr_C9ZZ34_
DRB1_0101	65	GGRVPLRAVRSHEII	LRAVRSHEI	5	0.6765	33.1	12.00	0.74	tr_C9ZZ34_
DRB1_0101	66	TSSLYGEEVRLAYRV	LYGEEVRLA	3	0.6753	33.6	12.00	0.56	tr_C9ZZ34_
DRB1_0101	67	TTKRLRAVQAMSQQF	TKRLRAVQA	1	0.6740	34.0	12.00	0.61	tr_C9ZZ34_
DRB1_0101	68	FLVCRLFLPALDLEM	RLFLPALDL	4	0.6733	34.3	12.00	0.38	tr_C9ZZ34_
DRB1_0101	69	VYQLQEATSFSRKEA	YQLQEATSF	1	0.6716	34.9	12.00	0.71	tr_C9ZZ34_
DRB1_0101	70	GRVPLRAVRSHEIIP	LRAVRSHEI	4	0.6700	35.5	13.00	0.78	tr_C9ZZ34_
DRB1_0101	71	GVVTTKRLRAVQAMS	TKRLRAVQA	4	0.6692	35.8	13.00	0.77	tr_C9ZZ34_
DRB1_0101	72	EEYRLLHRVANSCAM	LHRVANSCA	5	0.6669	36.8	13.00	0.46	tr_C9ZZ34_
DRB1_0101	73	EYALVTLRQNTLMNS	LVTLRQNTL	3	0.6654	37.4	13.00	0.47	tr_C9ZZ34_
DRB1_0101	74	RLFLPALDLEMEGVV	FLPALDLEM	2	0.6651	37.5	13.00	0.65	tr_C9ZZ34_
DRB1_0101	75	NEIQSEYALVTLRQN	IQSEYALVT	2	0.6645	37.7	13.00	0.47	tr_C9ZZ34_
DRB1_0101	76	PNEIQSEYALVTLRQ	IQSEYALVT	3	0.6630	38.3	13.00	0.51	tr_C9ZZ34_
DRB1_0101	77	WGIAMHLSQLLVGVI	IAMHLSQLL	2	0.6625	38.6	13.00	0.43	tr_C9ZZ34_
DRB1_0101	78	QSTLSEIRMLSASHH	IRMLSASHH	6	0.6589	40.1	14.00	0.55	tr_C9ZZ34_
DRB1_0101	79	EIIPVSSSPLLKNLD	IIPVSSSPL	1	0.6587	40.1	14.00	0.69	tr_C9ZZ34_
DRB1_0101	80	LYIITEFVSGGSLAA	FVSGGSLAA	6	0.6585	40.3	14.00	0.54	tr_C9ZZ34_
DRB1_0101	81	VPLRAVRSHEIIPVS	LRAVRSHEI	2	0.6585	40.3	14.00	0.81	tr_C9ZZ34_
DRB1_0101	82	DAFALPRPRELKVCC	FALPRPREL	2	0.6550	41.8	14.00	0.89	tr_C9ZZ34_
DRB1_0101	83	ACIDAFALPRPRELK	FALPRPREL	5	0.6541	42.2	15.00	0.77	tr_C9ZZ34_
DRB1_0101	84	IQSEYALVTLRQNTL	YALVTLRQN	4	0.6541	42.2	15.00	0.34	tr_C9ZZ34_
DRB1_0101	85	QPNEIQSEYALVTLR	IQSEYALVT	4	0.6529	42.8	15.00	0.50	tr_C9ZZ34_
DRB1_0101	86	IRMLSASHHKNIVRY	IRMLSASHH	0	0.6528	42.8	15.00	0.50	tr_C9ZZ34_
DRB1_0101	87	FLAQQVTFLVCRLFL	FLAQQVTFL	0	0.6510	43.6	15.00	0.55	tr_C9ZZ34_
DRB1_0101	88	SSLYGEEVRLAYRVT	LYGEEVRLA	2	0.6502	44.0	15.00	0.56	tr_C9ZZ34_
DRB1_0101	89	SELQKLESQWARTSS	LQKLESQWA	2	0.6429	47.7	16.00	0.61	tr_C9ZZ34_
DRB1_0101	90	QLLLETFGSKNAMLS	LETFGSKNA	3	0.6422	48.0	16.00	0.28	tr_C9ZZ34_
DRB1_0101	91	RTIAVVAPPHGENTI	IAVVAPPHG	2	0.6420	48.1	16.00	0.82	tr_C9ZZ34_
DRB1_0101	92	RLLHRVANSCAMMDL	LHRVANSCA	2	0.6404	49.0	16.00	0.59	tr_C9ZZ34_
DRB1_0101	93	EIQSEYALVTLRQNT	IQSEYALVT	1	0.6399	49.2	16.00	0.35	tr_C9ZZ34_
DRB1_0101	94	DRTIAVVAPPHGENT	IAVVAPPHG	3	0.6397	49.3	16.00	0.81	tr_C9ZZ34_
DRB1_0101	95	GIAMHLSQLLVGVIR	IAMHLSQLL	1	0.6373	50.6	17.00	0.39	tr_C9ZZ34_
DRB1_0101	96	ELQKLESQWARTSSL	LQKLESQWA	1	0.6365	51.1	17.00	0.47	tr_C9ZZ34_
DRB1_0101	97	EGVVTTKRLRAVQAM	TKRLRAVQA	5	0.6357	51.5	17.00	0.66	tr_C9ZZ34_
DRB1_0101	98	TKRLRAVQAMSQQFT	TKRLRAVQA	0	0.6354	51.7	17.00	0.40	tr_C9ZZ34_
DRB1_0101	99	DCAFRLVSELQKLES	FRLVSELQK	3	0.6338	52.6	17.00	0.58	tr_C9ZZ34_
DRB1_0101	100	LLLETFGSKNAMLSN	FGSKNAMLS	5	0.6327	53.2	17.00	0.38	tr_C9ZZ34_
DRB1_0101	101	VSELQKLESQWARTS	LQKLESQWA	3	0.6317	53.8	18.00	0.75	tr_C9ZZ34_
DRB1_0101	102	RDCAFRLVSELQKLE	FRLVSELQK	4	0.6317	53.8	18.00	0.54	tr_C9ZZ34_
DRB1_0101	103	YALVTLRQNTLMNSS	LVTLRQNTL	2	0.6314	54.0	18.00	0.47	tr_C9ZZ34_
DRB1_0101	104	DGNIVAIKEYPVPSM	IVAIKEYPV	3	0.6314	54.0	18.00	0.85	tr_C9ZZ34_
DRB1_0101	105	EKDAIVYQLQEATSF	YQLQEATSF	6	0.6308	54.3	18.00	0.45	tr_C9ZZ34_
DRB1_0101	106	PLRAVRSHEIIPVSS	LRAVRSHEI	1	0.6307	54.4	18.00	0.75	tr_C9ZZ34_

DRB1_0101	107	AFALPRPRELKVCCD	FALPRPREL	1	0.6290	55.4	18.00	0.86	tr_C9ZZ34_
DRB1_0101	108	CAFRLVSELQKLESQ	FRLVSELQK	2	0.6286	55.6	18.00	0.58	tr_C9ZZ34_
DRB1_0101	109	GNIVAIKEYPVPSMD	IVAIKEYPV	2	0.6280	56.0	18.00	0.81	tr_C9ZZ34_
DRB1_0101	110	TFLVCRLFLPALDLE	RLFLPALDL	5	0.6278	56.1	18.00	0.40	tr_C9ZZ34_
DRB1_0101	111	VPFDIRLDSVVVTFH	DIRLDSVVV	3	0.6216	60.0	19.00	0.68	tr_C9ZZ34_
DRB1_0101	112	PRDCAFRLVSELQKL	FRLVSELQK	5	0.6207	60.6	19.00	0.50	tr_C9ZZ34_
DRB1_0101	113	FQPNEIQSEYALVTL	IQSEYALVT	5	0.6203	60.8	19.00	0.44	tr_C9ZZ34_
DRB1_0101	114	LLETFGSKNAMLSNS	FGSKNAMLS	4	0.6199	61.1	19.00	0.50	tr_C9ZZ34_
DRB1_0101	115	FVSGGSLAALVETFH	FVSGGSLAA	0	0.6198	61.2	19.00	0.70	tr_C9ZZ34_
DRB1_0101	116	VRSHEIIPVSSSPLL	IIPVSSSPL	5	0.6195	61.4	19.00	0.74	tr_C9ZZ34_
DRB1_0101	117	AKKFIRKKIEKAGMS	FIRKKIEKA	3	0.6191	61.7	19.00	0.85	tr_C9ZZ34_
DRB1_0101	118	GCLYIITEFVSGGSL	YIITEFVSG	3	0.6162	63.6	20.00	0.44	tr_C9ZZ34_
DRB1_0101	119	SDRTIAVVAPPHGEN	IAVVAPPHG	4	0.6153	64.2	20.00	0.81	tr_C9ZZ34_
DRB1_0101	120	SEEYRLLHRVANSCA	YRLLHRVAN	3	0.6127	66.1	20.00	0.36	tr_C9ZZ34_
DRB1_0101	121	NGCLYIITEFVSGGS	CLYIITEFV	2	0.6124	66.3	20.00	0.41	tr_C9ZZ34_
DRB1_0101	122	KKFIRKKIEKAGMSI	FIRKKIEKA	2	0.6120	66.6	21.00	0.75	tr_C9ZZ34_
DRB1_0101	123	SLYGEEVRLAYRVTE	LYGEEVRLA	1	0.6108	67.4	21.00	0.49	tr_C9ZZ34_
DRB1_0101	124	RVYRGLHPDGNIVAI	YRGLHPDGN	2	0.6089	68.8	21.00	0.62	tr_C9ZZ34_
DRB1_0101	125	AGGRVPLRAVRSHEI	LRAVRSHEI	6	0.6081	69.4	21.00	0.52	tr_C9ZZ34_
DRB1_0101	126	PFDIRLDSVVVTFHT	DIRLDSVVV	2	0.6080	69.5	21.00	0.68	tr_C9ZZ34_
DRB1_0101	127	GVPFDIRLDSVVVTF	DIRLDSVVV	4	0.6037	72.8	22.00	0.65	tr_C9ZZ34_
DRB1_0101	128	QNGCLYIITEFVSGG	CLYIITEFV	3	0.6032	73.2	22.00	0.51	tr_C9ZZ34_
DRB1_0101	129	DFAGGRVPLRAVRSH	FAGGRVPLR	1	0.6025	73.8	22.00	0.44	tr_C9ZZ34_
DRB1_0101	130	DQLLLETFGSKNAML	LETFGSKNA	4	0.6018	74.4	22.00	0.26	tr_C9ZZ34_
DRB1_0101	131	FSMGTVSLICRVAPL	FSMGTVSLI	0	0.6012	74.8	22.00	0.57	tr_C9ZZ34_
DRB1_0101	132	PSGRTSSLYGEEVRL	TSSLYGEEV	4	0.6011	74.9	22.00	0.86	tr_C9ZZ34_
DRB1_0101	133	VTFLVCRLFLPALDL	RLFLPALDL	6	0.6009	75.1	22.00	0.35	tr_C9ZZ34_
DRB1_0101	134	DACIDAFALPRPREL	FALPRPREL	6	0.5989	76.7	23.00	0.53	tr_C9ZZ34_
DRB1_0101	135	PDFAGGRVPLRAVRS	FAGGRVPLR	2	0.5989	76.7	23.00	0.57	tr_C9ZZ34_
DRB1_0101	136	CLYIITEFVSGGSLA	YIITEFVSG	2	0.5987	76.8	23.00	0.37	tr_C9ZZ34_
DRB1_0101	137	PDGNIVAIKEYPVPS	IVAIKEYPV	4	0.5982	77.2	23.00	0.83	tr_C9ZZ34_
DRB1_0101	138	YENFHELAGPDFAGG	FHELAGPDF	3	0.5971	78.2	23.00	0.83	tr_C9ZZ34_
DRB1_0101	139	PNNVLVSIDGVCKLS	LVSIDGVCK	4	0.5960	79.2	23.00	0.71	tr_C9ZZ34_
DRB1_0101	140	NIVAIKEYPVPSMDE	IVAIKEYPV	1	0.5946	80.4	23.00	0.76	tr_C9ZZ34_
DRB1_0101	141	TFGSKNAMLSNSSFL	FGSKNAMLS	1	0.5945	80.5	23.00	0.41	tr_C9ZZ34_
DRB1_0101	142	RSNKFSTPTAVRHNY	FSTPTAVRH	4	0.5929	81.8	24.00	0.42	tr_C9ZZ34_
DRB1_0101	143	GRVYRGLHPDGNIVA	YRGLHPDGN	3	0.5917	82.9	24.00	0.67	tr_C9ZZ34_
DRB1_0101	144	STTLKTCFGTPVCMS	LKTCFGTPV	3	0.5911	83.5	24.00	0.68	tr_C9ZZ34_
DRB1_0101	145	AFRLVSELQKLESQW	FRLVSELQK	1	0.5907	83.8	24.00	0.56	tr_C9ZZ34_
DRB1_0101	146	LVSELQKLESQWART	LQKLESQWA	4	0.5901	84.4	24.00	0.76	tr_C9ZZ34_
DRB1_0101	147	NNVLVSIDGVCKLSD	LVSIDGVCK	3	0.5901	84.4	24.00	0.75	tr_C9ZZ34_
DRB1_0101	148	STNFVLDSSCNIPKV	FVLDSSCNI	3	0.5893	85.1	24.00	0.75	tr_C9ZZ34_
DRB1_0101	149	LETFGSKNAMLSNSS	FGSKNAMLS	3	0.5869	87.3	25.00	0.65	tr_C9ZZ34_
DRB1_0101	150	VYRGLHPDGNIVAIK	YRGLHPDGN	1	0.5856	88.6	25.00	0.52	tr_C9ZZ34_
DRB1_0101	151	CPLLMLQPCYDVFRV	LLMLQPCYD	2	0.5851	89.1	25.00	0.38	tr_C9ZZ34_
DRB1_0101	152	EAKKFIRKKIEKAGM	FIRKKIEKA	4	0.5841	90.0	25.00	0.89	tr_C9ZZ34_
DRB1_0101	153	SDQLLLETFGSKNAM	QLLLETFGS	2	0.5814	92.7	26.00	0.34	tr_C9ZZ34_
DRB1_0101	154	RYENFHELAGPDFAG	FHELAGPDF	4	0.5810	93.0	26.00	0.85	tr_C9ZZ34_
DRB1_0101	155	DCPLLMLQPCYDVFR	LLMLQPCYD	3	0.5801	94.0	26.00	0.41	tr_C9ZZ34_
DRB1_0101	156	ALDLEMEGVVTTKRL	LEMEGVVTT	3	0.5780	96.2	26.00	0.46	tr_C9ZZ34_

DRB1_0101	157	LQKLESQWARTSSLP	LESQWARTS	3	0.5777	96.5	26.00	0.47	tr_C9ZZ34_
DRB1_0101	158	ENFHELAGPDFAGGR	FHELAGPDF	2	0.5768	97.4	26.00	0.80	tr_C9ZZ34_
DRB1_0101	159	TNFVLDSSCNIPKVN	FVLDSSCNI	2	0.5768	97.4	26.00	0.70	tr_C9ZZ34_
DRB1_0101	160	FDIRLDSVVVTFHTP	DIRLDSVVV	1	0.5766	97.7	26.00	0.64	tr_C9ZZ34_
DRB1_0101	161	GPDFAGGRVPLRAVR	FAGGRVPLR	3	0.5763	97.9	26.00	0.70	tr_C9ZZ34_
DRB1_0101	162	SNKFSTPTAVRHNYW	FSTPTAVRH	3	0.5754	98.9	27.00	0.57	tr_C9ZZ34_
DRB1_0101	163	TIAVVAPPHGENTIQ	IAVVAPPHG	1	0.5753	99.0	27.00	0.80	tr_C9ZZ34_
DRB1_0101	164	LLHRVANSCAMMDLS	LHRVANSCA	1	0.5752	99.2	27.00	0.58	tr_C9ZZ34_
DRB1_0101	165	APLLQQEKDAIVYQL	LQQEKDAIV	3	0.5745	99.9	27.00	0.75	tr_C9ZZ34_
DRB1_0101	166	VSLICRVAPLLQQEK	CRVAPLLQQ	4	0.5738	100.6	27.00	0.37	tr_C9ZZ34_
DRB1_0101	167	SPNNVLVSIDGVCKL	LVSIDGVCK	5	0.5730	101.5	27.00	0.64	tr_C9ZZ34_
DRB1_0101	168	FQNGCLYIITEFVSG	CLYIITEFV	4	0.5726	101.9	27.00	0.54	tr_C9ZZ34_
DRB1_0101	169	ETFGSKNAMLSNSSF	FGSKNAMLS	2	0.5715	103.2	27.00	0.61	tr_C9ZZ34_
DRB1_0101	170	IIPVSSSPLLKNLDR	IIPVSSSPL	0	0.5710	103.7	27.00	0.57	tr_C9ZZ34_
DRB1_0101	171	IAMHLSQLLVGVIRT	IAMHLSQLL	0	0.5710	103.7	27.00	0.28	tr_C9ZZ34_
DRB1_0101	172	KSDRTIAVVAPPHGE	IAVVAPPHG	5	0.5709	103.8	27.00	0.76	tr_C9ZZ34_
DRB1_0101	173	FGVPFDIRLDSVVVT	DIRLDSVVV	5	0.5708	103.9	27.00	0.65	tr_C9ZZ34_
DRB1_0101	174	GSTTLKTCFGTPVCM	LKTCFGTPV	4	0.5705	104.3	27.00	0.69	tr_C9ZZ34_
DRB1_0101	175	EPPFDLMDVHFADFV	FDLMDVHFA	3	0.5704	104.4	28.00	0.81	tr_C9ZZ34_
DRB1_0101	176	HNYWSVREARASDST	YWSVREARA	2	0.5702	104.6	28.00	0.49	tr_C9ZZ34_
DRB1_0101	177	DRSNKFSTPTAVRHN	FSTPTAVRH	5	0.5688	106.2	28.00	0.38	tr_C9ZZ34_
DRB1_0101	178	KFIRKKIEKAGMSID	FIRKKIEKA	1	0.5687	106.3	28.00	0.56	tr_C9ZZ34_
DRB1_0101	179	PLMAAFGLMCEKRFS	FGLMCEKRF	5	0.5681	107.1	28.00	0.58	tr_C9ZZ34_
DRB1_0101	180	TVSLICRVAPLLQQE	LICRVAPLL	3	0.5671	108.2	28.00	0.28	tr_C9ZZ34_
DRB1_0101	181	LRAVRSHEIIPVSSS	LRAVRSHEI	0	0.5668	108.5	28.00	0.56	tr_C9ZZ34_
DRB1_0101	182	FAGGRVPLRAVRSHE	RVPLRAVRS	4	0.5668	108.5	28.00	0.33	tr_C9ZZ34_
DRB1_0101	183	RHNYWSVREARASDS	YWSVREARA	3	0.5667	108.6	28.00	0.61	tr_C9ZZ34_
DRB1_0101	184	TTLKTCFGTPVCMSP	LKTCFGTPV	2	0.5657	109.8	28.00	0.63	tr_C9ZZ34_
DRB1_0101	185	LFLPALDLEMEGVVT	FLPALDLEM	1	0.5651	110.5	28.00	0.69	tr_C9ZZ34_
DRB1_0101	186	PALDLEMEGVVTTKR	LEMEGVVTT	4	0.5651	110.6	28.00	0.41	tr_C9ZZ34_
DRB1_0101	187	QLLVGVIRTPSGRTS	VGVIRTPSG	3	0.5650	110.6	28.00	0.52	tr_C9ZZ34_
DRB1_0101	188	RLEFDIYGPVGAGSF	FDIYGPVGA	3	0.5632	112.9	29.00	0.70	tr_C9ZZ34_
DRB1_0101	189	PPFDLMDVHFADFVR	FDLMDVHFA	2	0.5628	113.4	29.00	0.75	tr_C9ZZ34_
DRB1_0101	190	GTVSLICRVAPLLQQ	LICRVAPLL	4	0.5618	114.5	29.00	0.28	tr_C9ZZ34_
DRB1_0101	191	MAAFGLMCEKRFSQA	FGLMCEKRF	3	0.5610	115.6	29.00	0.75	tr_C9ZZ34_
DRB1_0101	192	APLMAAFGLMCEKRF	FGLMCEKRF	6	0.5606	116.1	29.00	0.40	tr_C9ZZ34_
DRB1_0101	193	SFQPNEIQSEYALVT	IQSEYALVT	6	0.5601	116.8	29.00	0.31	tr_C9ZZ34_
DRB1_0101	194	ALVTLRQNTLMNSSI	LVTLRQNTL	1	0.5595	117.5	29.00	0.49	tr_C9ZZ34_
DRB1_0101	195	NVLVSIDGVCKLSDF	LVSIDGVCK	2	0.5585	118.7	30.00	0.77	tr_C9ZZ34_
DRB1_0101	196	FRLVSELQKLESQWA	FRLVSELQK	0	0.5581	119.3	30.00	0.36	tr_C9ZZ34_
DRB1_0101	197	GSTNFVLDSSCNIPK	FVLDSSCNI	4	0.5575	120.0	30.00	0.81	tr_C9ZZ34_
DRB1_0101	198	CDIIRRYTGDILRGL	IRRYTGDIL	3	0.5575	120.0	30.00	0.55	tr_C9ZZ34_
DRB1_0101	199	NYWSVREARASDSTS	YWSVREARA	1	0.5574	120.2	30.00	0.44	tr_C9ZZ34_
DRB1_0101	200	IRRYTGDILRGLQYL	GDILRGLQY	5	0.5569	120.9	30.00	0.33	tr_C9ZZ34_
DRB1_0101	201	RLVSELQKLESQWAR	LQKLESQWA	5	0.5560	122.1	30.00	0.71	tr_C9ZZ34_
DRB1_0101	202	VETFHGLPCDIIRRY	FHGLPCDII	3	0.5558	122.3	30.00	0.68	tr_C9ZZ34_
DRB1_0101	203	LRGLQYLHDRQIVHR	LQYLHDRQI	3	0.5557	122.4	30.00	0.61	tr_C9ZZ34_
DRB1_0101	204	IQSTLSEIRMLSASH	STLSEIRML	2	0.5551	123.2	30.00	0.58	tr_C9ZZ34_
DRB1_0101	205	LLVGVIRTPSGRTSS	VGVIRTPSG	2	0.5550	123.3	30.00	0.49	tr_C9ZZ34_
DRB1_0101	206	IIRRYTGDILRGLQY	IRRYTGDIL	1	0.5548	123.5	30.00	0.38	tr_C9ZZ34_

DRB1_0101	207	PQYYAAPGSFQPNEI	YYAAPGSFQ	2	0.5543	124.2	30.00	0.52	tr_C9ZZ34_
DRB1_0101	208	LYGEEVRLAYRVTEL	EEVRLAYRV	3	0.5540	124.7	30.00	0.49	tr_C9ZZ34_
DRB1_0101	209	MEGVVTTKRLRAVQA	TKRLRAVQA	6	0.5531	125.9	31.00	0.47	tr_C9ZZ34_
DRB1_0101	210	FGRVYRGLHPDGNIV	YRGLHPDGN	4	0.5529	126.1	31.00	0.74	tr_C9ZZ34_
DRB1_0101	211	SPQYYAAPGSFQPNE	YYAAPGSFQ	3	0.5525	126.7	31.00	0.61	tr_C9ZZ34_
DRB1_0101	212	LDCPLLMLQPCYDVF	LLMLQPCYD	4	0.5524	126.8	31.00	0.41	tr_C9ZZ34_
DRB1_0101	213	LRQNTLMNSSITRDV	NTLMNSSIT	3	0.5521	127.3	31.00	0.43	tr_C9ZZ34_
DRB1_0101	214	LDLEMEGVVTTKRLR	LEMEGVVTT	2	0.5516	128.0	31.00	0.45	tr_C9ZZ34_
DRB1_0101	215	CFQNGCLYIITEFVS	CLYIITEFV	5	0.5512	128.5	31.00	0.64	tr_C9ZZ34_
DRB1_0101	216	TRYENFHELAGPDFA	FHELAGPDF	5	0.5508	129.0	31.00	0.82	tr_C9ZZ34_
DRB1_0101	217	EIQSTLSEIRMLSAS	STLSEIRML	3	0.5506	129.4	31.00	0.63	tr_C9ZZ34_
DRB1_0101	218	DIIRRYTGDILRGLQ	IRRYTGDIL	2	0.5502	129.8	31.00	0.52	tr_C9ZZ34_
DRB1_0101	219	LMAAFGLMCEKRFSQ	FGLMCEKRF	4	0.5497	130.6	31.00	0.68	tr_C9ZZ34_
DRB1_0101	220	PLLMLQPCYDVFRVC	LLMLQPCYD	1	0.5484	132.5	32.00	0.35	tr_C9ZZ34_
DRB1_0101	221	YQLQEATSFSRKEAS	YQLQEATSF	0	0.5480	133.0	32.00	0.56	tr_C9ZZ34_
DRB1_0101	222	PEPPFDLMDVHFADF	FDLMDVHFA	4	0.5476	133.6	32.00	0.84	tr_C9ZZ34_
DRB1_0101	223	PLLQQEKDAIVYQLQ	LQQEKDAIV	2	0.5473	134.0	32.00	0.70	tr_C9ZZ34_
DRB1_0101	224	PFDLMDVHFADFVRQ	FDLMDVHFA	1	0.5467	135.0	32.00	0.67	tr_C9ZZ34_
DRB1_0101	225	APRLHRLCKYLAQEH	LHRLCKYLA	3	0.5465	135.3	32.00	0.64	tr_C9ZZ34_
DRB1_0101	226	NSDQLLLETFGSKNA	QLLLETFGS	3	0.5462	135.7	32.00	0.45	tr_C9ZZ34_
DRB1_0101	227	AAFGLMCEKRFSQAS	FGLMCEKRF	2	0.5453	136.9	32.00	0.69	tr_C9ZZ34_
DRB1_0101	228	LVGVIRTPSGRTSSL	VIRTPSGRT	3	0.5453	137.0	32.00	0.44	tr_C9ZZ34_
DRB1_0101	229	LVETFHGLPCDIIRR	FHGLPCDII	4	0.5443	138.5	32.00	0.66	tr_C9ZZ34_
DRB1_0101	230	NFVLDSSCNIPKVNT	FVLDSSCNI	1	0.5432	140.1	32.00	0.65	tr_C9ZZ34_
DRB1_0101	231	SLICRVAPLLQQEKD	CRVAPLLQQ	3	0.5426	141.0	33.00	0.40	tr_C9ZZ34_
DRB1_0101	232	NFHELAGPDFAGGRV	FHELAGPDF	1	0.5426	141.0	33.00	0.79	tr_C9ZZ34_
DRB1_0101	233	YDVFRVCVTAVPVDV	FRVCVTAVP	3	0.5425	141.3	33.00	0.44	tr_C9ZZ34_
DRB1_0101	234	HPDGNIVAIKEYPVP	IVAIKEYPV	5	0.5422	141.7	33.00	0.86	tr_C9ZZ34_
DRB1_0101	235	VAPLLQQEKDAIVYQ	LQQEKDAIV	4	0.5419	142.1	33.00	0.71	tr_C9ZZ34_
DRB1_0101	236	ERLEFDIYGPVGAGS	FDIYGPVGA	4	0.5408	143.8	33.00	0.69	tr_C9ZZ34_
DRB1_0101	237	RPRDCAFRLVSELQK	FRLVSELQK	6	0.5402	144.8	33.00	0.44	tr_C9ZZ34_
DRB1_0101	238	PEIQSTLSEIRMLSA	STLSEIRML	4	0.5401	144.9	33.00	0.57	tr_C9ZZ34_
DRB1_0101	239	VCIVTHQRSSSACGV	VTHQRSSSA	3	0.5393	146.2	33.00	0.73	tr_C9ZZ34_
DRB1_0101	240	SQLLVGVIRTPSGRT	VGVIRTPSG	4	0.5383	147.7	33.00	0.50	tr_C9ZZ34_
DRB1_0101	241	TLCFCVACSYPWSQE	FCVACSYPW	3	0.5381	148.1	33.00	0.74	tr_C9ZZ34_
DRB1_0101	242	AGPDFAGGRVPLRAV	FAGGRVPLR	4	0.5375	148.9	34.00	0.69	tr_C9ZZ34_
DRB1_0101	243	RRYTGDILRGLQYLH	GDILRGLQY	4	0.5373	149.3	34.00	0.42	tr_C9ZZ34_
DRB1_0101	244	RQNTLMNSSITRDVC	NTLMNSSIT	2	0.5373	149.4	34.00	0.38	tr_C9ZZ34_
DRB1_0101	245	KRLRAVQAMSQQFTE	LRAVQAMSQ	2	0.5370	149.9	34.00	0.47	tr_C9ZZ34_
DRB1_0101	246	GDILRGLQYLHDRQI	LRGLQYLHD	3	0.5369	150.0	34.00	0.23	tr_C9ZZ34_
DRB1_0101	247	NAPRLHRLCKYLAQE	LHRLCKYLA	4	0.5368	150.1	34.00	0.61	tr_C9ZZ34_
DRB1_0101	248	VGSSREQYAPLMAAF	SREQYAPLM	3	0.5368	150.2	34.00	0.35	tr_C9ZZ34_
DRB1_0101	249	TELCRILDCPLLMLQ	CRILDCPLL	3	0.5356	152.2	34.00	0.50	tr_C9ZZ34_
DRB1_0101	250	ITLCFCVACSYPWSQ	FCVACSYPW	4	0.5354	152.5	34.00	0.72	tr_C9ZZ34_
DRB1_0101	251	VSPQYYAAPGSFQPN	YYAAPGSFQ	4	0.5341	154.6	34.00	0.61	tr_C9ZZ34_
DRB1_0101	252	PCYDVFRVCVTAVPV	FRVCVTAVP	5	0.5340	154.8	34.00	0.41	tr_C9ZZ34_
DRB1_0101	253	RYTGDILRGLQYLHD	GDILRGLQY	3	0.5334	155.8	34.00	0.41	tr_C9ZZ34_
DRB1_0101	254	CYDVFRVCVTAVPVD	FRVCVTAVP	4	0.5333	156.0	34.00	0.43	tr_C9ZZ34_
DRB1_0101	255	ETFHGLPCDIIRRYT	FHGLPCDII	2	0.5332	156.1	34.00	0.70	tr_C9ZZ34_
DRB1_0101	256	QYYAAPGSFQPNEIQ	YYAAPGSFQ	1	0.5329	156.6	34.00	0.47	tr_C9ZZ34_

DRB1_0101	257	ILRGLQYLHDRQIVH	LQYLHDRQI	4	0.5328	156.9	34.00	0.56	tr_C9ZZ34_
DRB1_0101	258	EISEEYRLLHRVANS	YRLLHRVAN	5	0.5328	156.9	34.00	0.49	tr_C9ZZ34_
DRB1_0101	259	YTGDILRGLQYLHDR	GDILRGLQY	2	0.5325	157.4	35.00	0.34	tr_C9ZZ34_
DRB1_0101	260	GMSIDMNATSLSVSQ	IDMNATSLS	3	0.5294	162.6	35.00	0.50	tr_C9ZZ34_
DRB1_0101	261	RCFQNGCLYIITEFV	CLYIITEFV	6	0.5293	162.8	35.00	0.51	tr_C9ZZ34_
DRB1_0101	262	AGMSIDMNATSLSVS	IDMNATSLS	4	0.5289	163.6	35.00	0.43	tr_C9ZZ34_
DRB1_0101	263	LEFDIYGPVGAGSFG	FDIYGPVGA	2	0.5286	164.2	35.00	0.68	tr_C9ZZ34_
DRB1_0101	264	AMSQQFTEVVLRVAR	FTEVVLRVA	5	0.5280	165.1	35.00	0.34	tr_C9ZZ34_
DRB1_0101	265	HNAPRLHRLCKYLAQ	LHRLCKYLA	5	0.5273	166.4	36.00	0.55	tr_C9ZZ34_
DRB1_0101	266	DILRGLQYLHDRQIV	LQYLHDRQI	5	0.5272	166.6	36.00	0.47	tr_C9ZZ34_
DRB1_0101	267	TLKTCFGTPVCMSPE	LKTCFGTPV	1	0.5270	167.0	36.00	0.57	tr_C9ZZ34_
DRB1_0101	268	ISEEYRLLHRVANSC	YRLLHRVAN	4	0.5270	167.0	36.00	0.53	tr_C9ZZ34_
DRB1_0101	269	CIVTHQRSSSACGVM	VTHQRSSSA	2	0.5262	168.4	36.00	0.69	tr_C9ZZ34_
DRB1_0101	270	VRHNYWSVREARASD	YWSVREARA	4	0.5261	168.6	36.00	0.63	tr_C9ZZ34_
DRB1_0101	271	LDRSNKFSTPTAVRH	NKFSTPTAV	4	0.5255	169.8	36.00	0.44	tr_C9ZZ34_
DRB1_0101	272	PRLHRLCKYLAQEHE	LHRLCKYLA	2	0.5238	172.9	36.00	0.63	tr_C9ZZ34_
DRB1_0101	273	VTLRQNTLMNSSITR	NTLMNSSIT	5	0.5235	173.5	36.00	0.42	tr_C9ZZ34_
DRB1_0101	274	LPALDLEMEGVVTTK	DLEMEGVVT	4	0.5231	174.2	36.00	0.40	tr_C9ZZ34_
DRB1_0101	275	RLRAVQAMSQQFTEV	VQAMSQQFT	4	0.5227	174.9	36.00	0.32	tr_C9ZZ34_
DRB1_0101	276	VTELCRILDCPLLML	CRILDCPLL	4	0.5227	175.0	36.00	0.47	tr_C9ZZ34_
DRB1_0101	277	TLRQNTLMNSSITRD	NTLMNSSIT	4	0.5226	175.0	36.00	0.41	tr_C9ZZ34_
DRB1_0101	278	NKFSTPTAVRHNYWS	FSTPTAVRH	2	0.5223	175.7	37.00	0.63	tr_C9ZZ34_
DRB1_0101	279	DVFRVCVTAVPVDVI	FRVCVTAVP	2	0.5198	180.6	37.00	0.44	tr_C9ZZ34_
DRB1_0101	280	TGDILRGLQYLHDRQ	GDILRGLQY	1	0.5191	181.9	37.00	0.35	tr_C9ZZ34_
DRB1_0101	281	TGSTTLKTCFGTPVC	LKTCFGTPV	5	0.5188	182.4	37.00	0.68	tr_C9ZZ34_
DRB1_0101	282	DLEMEGVVTTKRLRA	LEMEGVVTT	1	0.5179	184.3	37.00	0.41	tr_C9ZZ34_
DRB1_0101	283	PCDIIRRYTGDILRG	IRRYTGDIL	4	0.5174	185.2	38.00	0.56	tr_C9ZZ34_
DRB1_0101	284	PECNYKSDRTIAVVA	YKSDRTIAV	4	0.5170	186.0	38.00	0.70	tr_C9ZZ34_
DRB1_0101	285	RGLQYLHDRQIVHRD	LQYLHDRQI	2	0.5170	186.0	38.00	0.62	tr_C9ZZ34_
DRB1_0101	286	YRGLHPDGNIVAIKE	LHPDGNIVA	3	0.5169	186.2	38.00	0.38	tr_C9ZZ34_
DRB1_0101	287	SPEPPFDLMDVHFAD	FDLMDVHFA	5	0.5168	186.5	38.00	0.86	tr_C9ZZ34_
DRB1_0101	288	REAKKFIRKKIEKAG	FIRKKIEKA	5	0.5166	186.9	38.00	0.87	tr_C9ZZ34_
DRB1_0101	289	RMLSASHHKNIVRYV	LSASHHKNI	2	0.5162	187.6	38.00	0.31	tr_C9ZZ34_
DRB1_0101	290	YWSVREARASDSTSL	VREARASDS	3	0.5161	187.8	38.00	0.44	tr_C9ZZ34_
DRB1_0101	291	ECNYKSDRTIAVVAP	YKSDRTIAV	3	0.5148	190.5	38.00	0.69	tr_C9ZZ34_
DRB1_0101	292	FIRKKIEKAGMSIDM	KIEKAGMSI	4	0.5147	190.7	38.00	0.35	tr_C9ZZ34_
DRB1_0101	293	LRAVQAMSQQFTEVV	VQAMSQQFT	3	0.5144	191.3	38.00	0.43	tr_C9ZZ34_
DRB1_0101	294	LVTLRQNTLMNSSIT	VTLRQNTLM	1	0.5138	192.5	38.00	0.22	tr_C9ZZ34_
DRB1_0101	295	ILDCPLLMLQPCYDV	LLMLQPCYD	5	0.5135	193.2	38.00	0.46	tr_C9ZZ34_
DRB1_0101	296	LSQLLVGVIRTPSGR	VGVIRTPSG	5	0.5131	194.1	38.00	0.41	tr_C9ZZ34_
DRB1_0101	297	ELCRILDCPLLMLQP	CRILDCPLL	2	0.5130	194.2	38.00	0.52	tr_C9ZZ34_
DRB1_0101	298	AERLEFDIYGPVGAG	FDIYGPVGA	5	0.5130	194.3	38.00	0.68	tr_C9ZZ34_
DRB1_0101	299	RNDIWGLGITLCFCV	DIWGLGITL	2	0.5116	197.3	39.00	0.47	tr_C9ZZ34_
DRB1_0101	300	TPSGRTSSLYGEEVR	TSSLYGEEV	5	0.5114	197.7	39.00	0.85	tr_C9ZZ34_
DRB1_0101	301	YKSDRTIAVVAPPHG	IAVVAPPHG	6	0.5110	198.5	39.00	0.53	tr_C9ZZ34_
DRB1_0101	302	QAMSQQFTEVVLRVA	MSQQFTEVV	2	0.5108	199.0	39.00	0.34	tr_C9ZZ34_
DRB1_0101	303	LCFCVACSYPWSQED	FCVACSYPW	2	0.5101	200.4	39.00	0.75	tr_C9ZZ34_
DRB1_0101	304	ALVETFHGLPCDIIR	FHGLPCDII	5	0.5092	202.5	39.00	0.63	tr_C9ZZ34_
DRB1_0101	305	AFGLMCEKRFSQASE	FGLMCEKRF	1	0.5092	202.5	39.00	0.69	tr_C9ZZ34_
DRB1_0101	306	EVRLAYRVTELCRIL	YRVTELCRI	5	0.5087	203.4	39.00	0.56	tr_C9ZZ34_

DRB1_0101	307	RVAPLLQQEKDAIVY	LQQEKDAIV	5	0.5087	203.6	39.00	0.68	tr_C9ZZ34_
DRB1_0101	308	NPEIQSTLSEIRMLS	STLSEIRML	5	0.5086	203.8	39.00	0.53	tr_C9ZZ34_
DRB1_0101	309	MSIDMNATSLSVSQY	IDMNATSLS	2	0.5083	204.5	39.00	0.47	tr_C9ZZ34_
DRB1_0101	310	GITLCFCVACSYPWS	FCVACSYPW	5	0.5077	205.6	39.00	0.64	tr_C9ZZ34_
DRB1_0101	311	MGTVSLICRVAPLLQ	LICRVAPLL	5	0.5077	205.8	39.00	0.32	tr_C9ZZ34_
DRB1_0101	312	MSQQFTEVVLRVARE	FTEVVLRVA	4	0.5076	206.0	39.00	0.41	tr_C9ZZ34_
DRB1_0101	313	DVCIVTHQRSSSACG	VTHQRSSSA	4	0.5068	207.8	40.00	0.65	tr_C9ZZ34_
DRB1_0101	314	AVRSHEIIPVSSSPL	IIPVSSSPL	6	0.5066	208.1	40.00	0.55	tr_C9ZZ34_
DRB1_0101	315	VFRVCVTAVPVDVIH	FRVCVTAVP	1	0.5064	208.6	40.00	0.41	tr_C9ZZ34_
DRB1_0101	316	RLHRLCKYLAQEHEG	LHRLCKYLA	1	0.5051	211.6	40.00	0.54	tr_C9ZZ34_
DRB1_0101	317	PRNDIWGLGITLCFC	DIWGLGITL	3	0.5051	211.6	40.00	0.50	tr_C9ZZ34_
DRB1_0101	318	TFHGLPCDIIRRYTG	FHGLPCDII	1	0.5050	211.9	40.00	0.66	tr_C9ZZ34_
DRB1_0101	319	QEISEEYRLLHRVAN	YRLLHRVAN	6	0.5042	213.8	40.00	0.38	tr_C9ZZ34_
DRB1_0101	320	EFDIYGPVGAGSFGR	FDIYGPVGA	1	0.5041	213.9	40.00	0.64	tr_C9ZZ34_
DRB1_0101	321	AVRHNYWSVREARAS	YWSVREARA	5	0.5034	215.4	40.00	0.65	tr_C9ZZ34_
DRB1_0101	322	FALPRPRELKVCCDV	FALPRPREL	0	0.5034	215.6	40.00	0.73	tr_C9ZZ34_
DRB1_0101	323	QVTFLVCRLFLPALD	FLVCRLFLP	3	0.5032	216.0	40.00	0.31	tr_C9ZZ34_
DRB1_0101	324	VRLAYRVTELCRILD	YRVTELCRI	4	0.5028	216.9	40.00	0.61	tr_C9ZZ34_
DRB1_0101	325	FLPALDLEMEGVVTT	DLEMEGVVT	5	0.5024	217.8	41.00	0.29	tr_C9ZZ34_
DRB1_0101	326	DPRNDIWGLGITLCF	DIWGLGITL	4	0.5019	219.1	41.00	0.52	tr_C9ZZ34_
DRB1_0101	327	RAVQAMSQQFTEVVL	VQAMSQQFT	2	0.5017	219.5	41.00	0.38	tr_C9ZZ34_
DRB1_0101	328	LLQQEKDAIVYQLQE	LQQEKDAIV	1	0.5009	221.4	41.00	0.71	tr_C9ZZ34_
DRB1_0101	329	SFGRVYRGLHPDGNI	YRGLHPDGN	5	0.5000	223.6	41.00	0.68	tr_C9ZZ34_
DRB1_0101	330	EFGVPFDIRLDSVVV	DIRLDSVVV	6	0.4986	226.9	41.00	0.52	tr_C9ZZ34_
DRB1_0101	331	KAGMSIDMNATSLSV	SIDMNATSL	4	0.4985	227.2	41.00	0.38	tr_C9ZZ34_
DRB1_0101	332	PDKFDSATTNFSQEV	FDSATTNFS	3	0.4980	228.6	41.00	0.85	tr_C9ZZ34_
DRB1_0101	333	NDIWGLGITLCFCVA	DIWGLGITL	1	0.4972	230.6	42.00	0.34	tr_C9ZZ34_
DRB1_0101	334	VGVIRTPSGRTSSLY	VIRTPSGRT	2	0.4970	230.9	42.00	0.50	tr_C9ZZ34_
DRB1_0101	335	DIRLDSVVVTFHTPS	DIRLDSVVV	0	0.4964	232.5	42.00	0.52	tr_C9ZZ34_
DRB1_0101	336	VLVSIDGVCKLSDFG	LVSIDGVCK	1	0.4959	233.7	42.00	0.80	tr_C9ZZ34_
DRB1_0101	337	ISPNNVLVSIDGVCK	LVSIDGVCK	6	0.4951	235.7	42.00	0.54	tr_C9ZZ34_
DRB1_0101	338	QKLESQWARTSSLPF	LESQWARTS	2	0.4949	236.2	42.00	0.56	tr_C9ZZ34_
DRB1_0101	339	VANSCAMMDLSRFGQ	CAMMDLSRF	4	0.4947	236.9	42.00	0.79	tr_C9ZZ34_
DRB1_0101	340	QPCYDVFRVCVTAVP	YDVFRVCVT	3	0.4930	241.3	42.00	0.33	tr_C9ZZ34_
DRB1_0101	341	ANSCAMMDLSRFGQC	CAMMDLSRF	3	0.4927	241.9	42.00	0.76	tr_C9ZZ34_
DRB1_0101	342	ISPEPPFDLMDVHFA	FDLMDVHFA	6	0.4926	242.1	43.00	0.71	tr_C9ZZ34_
DRB1_0101	343	RDVCIVTHQRSSSAC	VTHQRSSSA	5	0.4925	242.5	43.00	0.54	tr_C9ZZ34_
DRB1_0101	344	QSDACIDAFALPRPR	DACIDAFAL	2	0.4925	242.5	43.00	0.64	tr_C9ZZ34_
DRB1_0101	345	CNYKSDRTIAVVAPP	YKSDRTIAV	2	0.4923	243.1	43.00	0.69	tr_C9ZZ34_
DRB1_0101	346	TQQSDACIDAFALPR	DACIDAFAL	4	0.4923	243.1	43.00	0.71	tr_C9ZZ34_
DRB1_0101	347	CVGSSREQYAPLMAA	SREQYAPLM	4	0.4917	244.6	43.00	0.34	tr_C9ZZ34_
DRB1_0101	348	GGSTNFVLDSSCNIP	FVLDSSCNI	5	0.4910	246.4	43.00	0.81	tr_C9ZZ34_
DRB1_0101	349	LCRILDCPLLMLQPC	CRILDCPLL	1	0.4908	247.0	43.00	0.52	tr_C9ZZ34_
DRB1_0101	350	RLAYRVTELCRILDC	YRVTELCRI	3	0.4905	247.9	43.00	0.64	tr_C9ZZ34_
DRB1_0101	351	SDACIDAFALPRPRE	DACIDAFAL	1	0.4902	248.7	43.00	0.56	tr_C9ZZ34_
DRB1_0101	352	DTADVRSFISKLRRG	DVRSFISKL	3	0.4898	249.7	43.00	0.41	tr_C9ZZ34_
DRB1_0101	353	SMGTVSLICRVAPLL	SLICRVAPL	5	0.4898	249.7	43.00	0.26	tr_C9ZZ34_
DRB1_0101	354	AMHLSQLLVGVIRTP	LSQLLVGVI	3	0.4897	249.9	43.00	0.30	tr_C9ZZ34_
DRB1_0101	355	DVRSFISKLRRGCIS	ISKLRRGCI	5	0.4895	250.5	43.00	0.25	tr_C9ZZ34_
DRB1_0101	356	EEVRLAYRVTELCRI	YRVTELCRI	6	0.4894	250.8	43.00	0.35	tr_C9ZZ34_

DRB1_0101	357	QQSDACIDAFALPRP	DACIDAFAL	3	0.4890	251.8	43.00	0.73	tr_C9ZZ34_
DRB1_0101	358	DKFDSATTNFSQEVT	FDSATTNFS	2	0.4887	252.7	43.00	0.81	tr_C9ZZ34_
DRB1_0101	359	MLSASHHKNIVRYVD	HHKNIVRYV	5	0.4877	255.4	44.00	0.40	tr_C9ZZ34_
DRB1_0101	360	LLMLQPCYDVFRVCV	LMLQPCYDV	1	0.4875	256.1	44.00	0.47	tr_C9ZZ34_
DRB1_0101	361	QNTLMNSSITRDVCI	NTLMNSSIT	1	0.4868	257.9	44.00	0.35	tr_C9ZZ34_
DRB1_0101	362	KLESQWARTSSLPFV	LESQWARTS	1	0.4858	260.8	44.00	0.35	tr_C9ZZ34_
DRB1_0101	363	LPCDIIRRYTGDILR	IRRYTGDIL	5	0.4856	261.4	44.00	0.54	tr_C9ZZ34_
DRB1_0101	364	IVAIKEYPVPSMDEN	IVAIKEYPV	0	0.4851	262.7	44.00	0.66	tr_C9ZZ34_
DRB1_0101	365	SNSDQLLLETFGSKN	QLLLETFGS	4	0.4849	263.3	44.00	0.50	tr_C9ZZ34_
DRB1_0101	366	TADVRSFISKLRRGC	DVRSFISKL	2	0.4835	267.3	44.00	0.44	tr_C9ZZ34_
DRB1_0101	367	LAGPDFAGGRVPLRA	FAGGRVPLR	5	0.4827	269.5	45.00	0.72	tr_C9ZZ34_
DRB1_0101	368	SQQFTEVVLRVAREE	FTEVVLRVA	3	0.4825	270.1	45.00	0.43	tr_C9ZZ34_
DRB1_0101	369	NSCAMMDLSRFGQCE	CAMMDLSRF	2	0.4811	274.3	45.00	0.69	tr_C9ZZ34_
DRB1_0101	370	RVANSCAMMDLSRFG	CAMMDLSRF	5	0.4811	274.3	45.00	0.71	tr_C9ZZ34_
DRB1_0101	371	IRKKIEKAGMSIDMN	KIEKAGMSI	3	0.4801	277.4	45.00	0.49	tr_C9ZZ34_
DRB1_0101	372	YGEEVRLAYRVTELC	EEVRLAYRV	2	0.4801	277.5	45.00	0.58	tr_C9ZZ34_
DRB1_0101	373	GLQYLHDRQIVHRDI	LQYLHDRQI	1	0.4800	277.6	45.00	0.50	tr_C9ZZ34_
DRB1_0101	374	RKKIEKAGMSIDMNA	KIEKAGMSI	2	0.4799	277.8	45.00	0.44	tr_C9ZZ34_
DRB1_0101	375	IVTHQRSSSACGVMP	VTHQRSSSA	1	0.4799	278.1	45.00	0.63	tr_C9ZZ34_
DRB1_0101	376	QQVTFLVCRLFLPAL	FLVCRLFLP	4	0.4795	279.2	45.00	0.38	tr_C9ZZ34_
DRB1_0101	377	AVQAMSQQFTEVVLR	MSQQFTEVV	4	0.4788	281.4	45.00	0.38	tr_C9ZZ34_
DRB1_0101	378	RVTELCRILDCPLLM	CRILDCPLL	5	0.4784	282.5	45.00	0.50	tr_C9ZZ34_
DRB1_0101	379	ARTSSLPFVWGIAMH	LPFVWGIAM	5	0.4784	282.6	46.00	0.34	tr_C9ZZ34_
DRB1_0101	380	LSASHHKNIVRYVDR	HHKNIVRYV	4	0.4769	287.1	46.00	0.61	tr_C9ZZ34_
DRB1_0101	381	HLSQLLVGVIRTPSG	LLVGVIRTP	4	0.4760	290.0	46.00	0.17	tr_C9ZZ34_
DRB1_0101	382	LICRVAPLLQQEKDA	CRVAPLLQQ	2	0.4755	291.4	46.00	0.43	tr_C9ZZ34_
DRB1_0101	383	EDTADVRSFISKLRR	DVRSFISKL	4	0.4753	292.0	46.00	0.40	tr_C9ZZ34_
DRB1_0101	384	ESDYCSTGSTTLKTC	YCSTGSTTL	3	0.4745	294.6	46.00	0.77	tr_C9ZZ34_
DRB1_0101	385	HRVANSCAMMDLSRF	CAMMDLSRF	6	0.4743	295.3	46.00	0.55	tr_C9ZZ34_
DRB1_0101	386	TESDYCSTGSTTLKT	YCSTGSTTL	4	0.4738	296.9	46.00	0.76	tr_C9ZZ34_
DRB1_0101	387	FRVCVTAVPVDVIHR	CVTAVPVDV	3	0.4735	297.9	47.00	0.32	tr_C9ZZ34_
DRB1_0101	388	LHRVANSCAMMDLSR	LHRVANSCA	0	0.4733	298.4	47.00	0.44	tr_C9ZZ34_
DRB1_0101	389	LKVCCDVEGHPLKTN	CCDVEGHPL	3	0.4731	299.3	47.00	0.68	tr_C9ZZ34_
DRB1_0101	390	VQAMSQQFTEVVLRV	MSQQFTEVV	3	0.4726	300.8	47.00	0.47	tr_C9ZZ34_
DRB1_0101	391	ADVRSFISKLRRGCI	DVRSFISKL	1	0.4721	302.4	47.00	0.34	tr_C9ZZ34_
DRB1_0101	392	MHLSQLLVGVIRTPS	LSQLLVGVI	2	0.4719	303.2	47.00	0.29	tr_C9ZZ34_
DRB1_0101	393	FDLMDVHFADFVRQC	FDLMDVHFA	0	0.4712	305.5	47.00	0.56	tr_C9ZZ34_
DRB1_0101	394	LEMEGVVTTKRLRAV	LEMEGVVTT	0	0.4711	305.6	47.00	0.25	tr_C9ZZ34_
DRB1_0101	395	LHPDGNIVAIKEYPV	IVAIKEYPV	6	0.4685	314.3	48.00	0.70	tr_C9ZZ34_
DRB1_0101	396	RSFISKLRRGCISPE	SKLRRGCIS	4	0.4685	314.5	48.00	0.40	tr_C9ZZ34_
DRB1_0101	397	GEEVRLAYRVTELCR	EEVRLAYRV	1	0.4684	314.7	48.00	0.49	tr_C9ZZ34_
DRB1_0101	398	DHNAPRLHRLCKYLA	LHRLCKYLA	6	0.4680	316.2	48.00	0.44	tr_C9ZZ34_
DRB1_0101	399	YVDRCFQNGCLYIIT	CFQNGCLYI	4	0.4679	316.5	48.00	0.31	tr_C9ZZ34_
DRB1_0101	400	GSFQPNEIQSEYALV	NEIQSEYAL	5	0.4679	316.5	48.00	0.26	tr_C9ZZ34_
DRB1_0101	401	VRSFISKLRRGCISP	SKLRRGCIS	5	0.4677	317.1	48.00	0.34	tr_C9ZZ34_
DRB1_0101	402	CFCVACSYPWSQEDT	FCVACSYPW	1	0.4673	318.7	48.00	0.74	tr_C9ZZ34_
DRB1_0101	403	WARTSSLPFVWGIAM	TSSLPFVWG	3	0.4660	323.1	48.00	0.23	tr_C9ZZ34_
DRB1_0101	404	CRVAPLLQQEKDAIV	LQQEKDAIV	6	0.4658	323.6	48.00	0.41	tr_C9ZZ34_
DRB1_0101	405	HVSPQYYAAPGSFQP	YYAAPGSFQ	5	0.4652	325.8	48.00	0.65	tr_C9ZZ34_
DRB1_0101	406	SDYCSTGSTTLKTCF	YCSTGSTTL	2	0.4652	325.8	48.00	0.73	tr_C9ZZ34_

DRB1_0101	407	CRILDCPLLMLQPCY	CRILDCPLL	0	0.4647	327.6	48.00	0.37	tr_C9ZZ34_
DRB1_0101	408	DPDKFDSATTNFSQE	FDSATTNFS	4	0.4643	329.2	48.00	0.85	tr_C9ZZ34_
DRB1_0101	409	KVCCDVEGHPLKTNL	CCDVEGHPL	2	0.4642	329.4	48.00	0.62	tr_C9ZZ34_
DRB1_0101	410	MPECNYKSDRTIAVV	YKSDRTIAV	5	0.4619	337.7	49.00	0.69	tr_C9ZZ34_
DRB1_0101	411	SQWARTSSLPFVWGI	WARTSSLPF	2	0.4593	347.5	50.00	0.44	tr_C9ZZ34_
DRB1_0101	412	QEKDAIVYQLQEATS	IVYQLQEAT	5	0.4591	348.1	50.00	0.62	tr_C9ZZ34_
DRB1_0101	413	AALVETFHGLPCDII	FHGLPCDII	6	0.4579	352.8	50.00	0.49	tr_C9ZZ34_
DRB1_0101	414	RYVDRCFQNGCLYII	DRCFQNGCL	3	0.4575	354.2	50.00	0.34	tr_C9ZZ34_
DRB1_0101	415	VDRCFQNGCLYIITE	CFQNGCLYI	3	0.4573	354.9	50.00	0.34	tr_C9ZZ34_
DRB1_0101	416	IAVVAPPHGENTIQN	IAVVAPPHG	0	0.4573	355.0	50.00	0.70	tr_C9ZZ34_
DRB1_0101	417	CHVSPQYYAAPGSFQ	YYAAPGSFQ	6	0.4571	355.8	50.00	0.51	tr_C9ZZ34_
DRB1_0101	418	STGSTTLKTCFGTPV	LKTCFGTPV	6	0.4568	356.9	50.00	0.58	tr_C9ZZ34_
DRB1_0101	419	ESQWARTSSLPFVWG	WARTSSLPF	3	0.4564	358.6	50.00	0.48	tr_C9ZZ34_
DRB1_0101	420	LAQQVTFLVCRLFLP	FLVCRLFLP	6	0.4558	360.6	50.00	0.31	tr_C9ZZ34_
DRB1_0101	421	KSDFSTSNSDQLLLE	FSTSNSDQL	3	0.4543	366.8	55.00	0.65	tr_C9ZZ34_
DRB1_0101	422	KFDSATTNFSQEVTG	FDSATTNFS	1	0.4535	369.8	55.00	0.77	tr_C9ZZ34_
DRB1_0101	423	NTRYENFHELAGPDF	FHELAGPDF	6	0.4527	373.0	55.00	0.68	tr_C9ZZ34_
DRB1_0101	424	FDIYGPVGAGSFGRV	FDIYGPVGA	0	0.4524	374.4	55.00	0.44	tr_C9ZZ34_
DRB1_0101	425	STQQSDACIDAFALP	DACIDAFAL	5	0.4518	376.7	55.00	0.69	tr_C9ZZ34_
DRB1_0101	426	LESQWARTSSLPFVW	WARTSSLPF	4	0.4511	379.4	55.00	0.46	tr_C9ZZ34_
DRB1_0101	427	VDPRNDIWGLGITLC	DIWGLGITL	5	0.4504	382.3	55.00	0.57	tr_C9ZZ34_
DRB1_0101	428	AQEISEEYRLLHRVA	EISEEYRLL	2	0.4502	383.1	55.00	0.47	tr_C9ZZ34_
DRB1_0101	429	LGITLCFCVACSYPW	FCVACSYPW	6	0.4500	384.1	55.00	0.44	tr_C9ZZ34_
DRB1_0101	430	LQPCYDVFRVCVTAV	YDVFRVCVT	4	0.4496	385.6	55.00	0.46	tr_C9ZZ34_
DRB1_0101	431	ELKVCCDVEGHPLKT	CCDVEGHPL	4	0.4486	390.0	55.00	0.66	tr_C9ZZ34_
DRB1_0101	432	NTLMNSSITRDVCIV	MNSSITRDV	3	0.4476	394.3	55.00	0.41	tr_C9ZZ34_
DRB1_0101	433	QQFTEVVLRVAREEF	FTEVVLRVA	2	0.4473	395.3	55.00	0.44	tr_C9ZZ34_
DRB1_0101	434	DIWGLGITLCFCVAC	WGLGITLCF	2	0.4472	396.1	55.00	0.18	tr_C9ZZ34_
DRB1_0101	435	LMLQPCYDVFRVCVT	LMLQPCYDV	0	0.4468	397.4	55.00	0.37	tr_C9ZZ34_
DRB1_0101	436	SVNLVRPRDCAFRLV	VNLVRPRDC	1	0.4467	397.9	55.00	0.40	tr_C9ZZ34_
DRB1_0101	437	SASHHKNIVRYVDRC	HHKNIVRYV	3	0.4466	398.4	55.00	0.71	tr_C9ZZ34_
DRB1_0101	438	LKTCFGTPVCMSPEA	LKTCFGTPV	0	0.4466	398.6	55.00	0.39	tr_C9ZZ34_
DRB1_0101	439	QWARTSSLPFVWGIA	WARTSSLPF	1	0.4464	399.3	55.00	0.37	tr_C9ZZ34_
DRB1_0101	440	KAERLEFDIYGPVGA	FDIYGPVGA	6	0.4462	400.2	55.00	0.60	tr_C9ZZ34_
DRB1_0101	441	NYKSDRTIAVVAPPH	YKSDRTIAV	1	0.4448	406.3	55.00	0.62	tr_C9ZZ34_
DRB1_0101	442	YRVTELCRILDCPLL	CRILDCPLL	6	0.4447	406.6	55.00	0.32	tr_C9ZZ34_
DRB1_0101	443	NNPEIQSTLSEIRML	STLSEIRML	6	0.4440	409.6	55.00	0.41	tr_C9ZZ34_
DRB1_0101	444	NSVNLVRPRDCAFRL	VNLVRPRDC	2	0.4440	409.7	55.00	0.57	tr_C9ZZ34_
DRB1_0101	445	EKRFSQASEQLEKVL	FSQASEQLE	3	0.4440	410.0	55.00	0.54	tr_C9ZZ34_
DRB1_0101	446	RILDCPLLMLQPCYD	LLMLQPCYD	6	0.4436	411.5	55.00	0.40	tr_C9ZZ34_
DRB1_0101	447	ADPDKFDSATTNFSQ	FDSATTNFS	5	0.4432	413.5	55.00	0.82	tr_C9ZZ34_
DRB1_0101	448	TRDVCIVTHQRSSSA	VTHQRSSSA	6	0.4430	414.4	55.00	0.37	tr_C9ZZ34_
DRB1_0101	449	SCAMMDLSRFGQCEC	CAMMDLSRF	1	0.4428	415.2	55.00	0.69	tr_C9ZZ34_
DRB1_0101	450	KFSTPTAVRHNYWSV	FSTPTAVRH	1	0.4422	417.8	55.00	0.63	tr_C9ZZ34_
DRB1_0101	451	KVLELDHNAPRLHRL	LDHNAPRLH	4	0.4420	418.7	55.00	0.27	tr_C9ZZ34_
DRB1_0101	452	SGGSLAALVETFHGL	SLAALVETF	3	0.4411	422.7	55.00	0.25	tr_C9ZZ34_
DRB1_0101	453	TKSDFSTSNSDQLLL	FSTSNSDQL	4	0.4394	430.7	55.00	0.67	tr_C9ZZ34_
DRB1_0101	454	KKIEKAGMSIDMNAT	EKAGMSIDM	3	0.4391	432.0	55.00	0.34	tr_C9ZZ34_
DRB1_0101	455	SIDMNATSLSVSQYP	IDMNATSLS	1	0.4383	435.9	55.00	0.48	tr_C9ZZ34_
DRB1_0101	456	FVLDSSCNIPKVNTR	FVLDSSCNI	0	0.4382	436.4	55.00	0.53	tr_C9ZZ34_

DRB1_0101	457	KRFSQASEQLEKVLE	FSQASEQLE	2	0.4376	439.2	55.00	0.49	tr_C9ZZ34_
DRB1_0101	458	DYCSTGSTTLKTCFG	YCSTGSTTL	1	0.4373	440.9	55.00	0.58	tr_C9ZZ34_
DRB1_0101	459	YYAAPGSFQPNEIQS	YAAPGSFQP	1	0.4371	441.5	55.00	0.43	tr_C9ZZ34_
DRB1_0101	460	SDFSTSNSDQLLLET	FSTSNSDQL	2	0.4358	447.8	55.00	0.61	tr_C9ZZ34_
DRB1_0101	461	FHGLPCDIIRRYTGD	FHGLPCDII	0	0.4346	453.9	55.00	0.58	tr_C9ZZ34_
DRB1_0101	462	LQYLHDRQIVHRDIS	LQYLHDRQI	0	0.4345	454.2	55.00	0.34	tr_C9ZZ34_
DRB1_0101	463	EKAGMSIDMNATSLS	SIDMNATSL	5	0.4340	456.5	55.00	0.40	tr_C9ZZ34_
DRB1_0101	464	LAYRVTELCRILDCP	YRVTELCRI	2	0.4340	456.6	55.00	0.67	tr_C9ZZ34_
DRB1_0101	465	VCCDVEGHPLKTNLH	CCDVEGHPL	1	0.4331	461.2	55.00	0.56	tr_C9ZZ34_
DRB1_0101	466	LHRLCKYLAQEHEGE	LHRLCKYLA	0	0.4330	461.8	55.00	0.35	tr_C9ZZ34_
DRB1_0101	467	SFISKLRRGCISPEP	SKLRRGCIS	3	0.4325	464.4	55.00	0.42	tr_C9ZZ34_
DRB1_0101	468	EMEGVVTTKRLRAVQ	VVTTKRLRA	4	0.4322	465.5	55.00	0.31	tr_C9ZZ34_
DRB1_0101	469	PNSVNLVRPRDCAFR	VNLVRPRDC	3	0.4315	469.2	55.00	0.65	tr_C9ZZ34_
DRB1_0101	470	ASHHKNIVRYVDRCF	HHKNIVRYV	2	0.4310	471.7	60.00	0.65	tr_C9ZZ34_
DRB1_0101	471	RVCVTAVPVDVIHRR	VTAVPVDVI	3	0.4300	476.8	60.00	0.42	tr_C9ZZ34_
DRB1_0101	472	ATSFSRKEASDAGNK	FSRKEASDA	3	0.4298	477.8	60.00	0.88	tr_C9ZZ34_
DRB1_0101	473	DRCFQNGCLYIITEF	FQNGCLYII	3	0.4298	478.0	60.00	0.34	tr_C9ZZ34_
DRB1_0101	474	RGCISPEPPFDLMDV	ISPEPPFDL	3	0.4296	478.8	60.00	0.83	tr_C9ZZ34_
DRB1_0101	475	AQQVTFLVCRLFLPA	FLVCRLFLP	5	0.4288	483.3	60.00	0.47	tr_C9ZZ34_
DRB1_0101	476	CAQEISEEYRLLHRV	EISEEYRLL	3	0.4287	483.5	60.00	0.50	tr_C9ZZ34_
DRB1_0101	477	KGGSTNFVLDSSCNI	FVLDSSCNI	6	0.4277	489.1	60.00	0.74	tr_C9ZZ34_
DRB1_0101	478	FHELAGPDFAGGRVP	FHELAGPDF	0	0.4266	494.7	60.00	0.67	tr_C9ZZ34_
DRB1_0101	479	LSTQQSDACIDAFAL	DACIDAFAL	6	0.4258	498.8	60.00	0.56	tr_C9ZZ34_
DRB1_0101	480	QEDTADVRSFISKLR	DVRSFISKL	5	0.4254	501.3	60.00	0.35	tr_C9ZZ34_
DRB1_0101	481	GGSLAALVETFHGLP	AALVETFHG	4	0.4244	506.6	60.00	0.25	tr_C9ZZ34_
DRB1_0101	482	RFSQASEQLEKVLEL	FSQASEQLE	1	0.4243	507.0	60.00	0.34	tr_C9ZZ34_
DRB1_0101	483	FGQCECHVSPQYYAA	CHVSPQYYA	5	0.4242	507.8	60.00	0.51	tr_C9ZZ34_
DRB1_0101	484	EKVLELDHNAPRLHR	LELDHNAPR	3	0.4237	510.4	60.00	0.27	tr_C9ZZ34_
DRB1_0101	485	TTESDYCSTGSTTLK	YCSTGSTTL	5	0.4232	513.1	60.00	0.73	tr_C9ZZ34_
DRB1_0101	486	GTPVCMSPEACMGVV	VCMSPEACM	3	0.4213	524.0	60.00	0.42	tr_C9ZZ34_
DRB1_0101	487	QCECHVSPQYYAAPG	CHVSPQYYA	3	0.4213	524.0	60.00	0.54	tr_C9ZZ34_
DRB1_0101	488	FTEVVLRVAREEFGV	LRVAREEFG	5	0.4203	529.6	60.00	0.48	tr_C9ZZ34_
DRB1_0101	489	ICRVAPLLQQEKDAI	CRVAPLLQQ	1	0.4178	544.0	60.00	0.47	tr_C9ZZ34_
DRB1_0101	490	QIVHRDISPNNVLVS	HRDISPNNV	3	0.4176	545.1	60.00	0.50	tr_C9ZZ34_
DRB1_0101	491	QFTEVVLRVAREEFG	FTEVVLRVA	1	0.4174	546.7	60.00	0.31	tr_C9ZZ34_
DRB1_0101	492	NLDRSNKFSTPTAVR	NKFSTPTAV	5	0.4171	548.4	60.00	0.51	tr_C9ZZ34_
DRB1_0101	493	WSVREARASDSTSLS	VREARASDS	2	0.4163	552.8	60.00	0.58	tr_C9ZZ34_
DRB1_0101	494	TSNSDQLLLETFGSK	QLLLETFGS	5	0.4158	556.1	60.00	0.55	tr_C9ZZ34_
DRB1_0101	495	FGTPVCMSPEACMGV	PVCMSPEAC	3	0.4149	561.5	60.00	0.42	tr_C9ZZ34_
DRB1_0101	496	IVHRDISPNNVLVSI	HRDISPNNV	2	0.4145	563.8	60.00	0.44	tr_C9ZZ34_
DRB1_0101	497	GSLAALVETFHGLPC	AALVETFHG	3	0.4139	567.4	60.00	0.26	tr_C9ZZ34_
DRB1_0101	498	AYRVTELCRILDCPL	YRVTELCRI	1	0.4137	568.6	60.00	0.58	tr_C9ZZ34_
DRB1_0101	499	FGLMCEKRFSQASEQ	FGLMCEKRF	0	0.4128	574.7	60.00	0.54	tr_C9ZZ34_
DRB1_0101	500	GQCECHVSPQYYAAP	CHVSPQYYA	4	0.4116	581.9	60.00	0.52	tr_C9ZZ34_
DRB1_0101	501	GSFGRVYRGLHPDGN	YRGLHPDGN	6	0.4115	582.8	60.00	0.50	tr_C9ZZ34_
DRB1_0101	502	RELKVCCDVEGHPLK	CCDVEGHPL	5	0.4112	584.2	60.00	0.64	tr_C9ZZ34_
DRB1_0101	503	VRIYLYDPKAPKERE	YLYDPKAPK	3	0.4105	588.9	60.00	0.36	tr_C9ZZ34_
DRB1_0101	504	SHHKNIVRYVDRCFQ	HHKNIVRYV	1	0.4101	591.5	60.00	0.54	tr_C9ZZ34_
DRB1_0101	505	GCAQEISEEYRLLHR	EISEEYRLL	4	0.4100	592.3	60.00	0.50	tr_C9ZZ34_
DRB1_0101	506	GAVECAMQPPTSEDN	CAMQPPTSE	4	0.4093	596.7	60.00	0.50	tr_C9ZZ34_

DRB1_0101	507	CEKRFSQASEQLEKV	FSQASEQLE	4	0.4089	598.9	60.00	0.51	tr_C9ZZ34_
DRB1_0101	508	ECVGSSREQYAPLMA	SREQYAPLM	5	0.4082	603.9	60.00	0.39	tr_C9ZZ34_
DRB1_0101	509	EVVLRVAREEFGVPF	LRVAREEFG	3	0.4074	608.8	65.00	0.58	tr_C9ZZ34_
DRB1_0101	510	VSGGSLAALVETFHG	SLAALVETF	4	0.4074	608.9	65.00	0.23	tr_C9ZZ34_
DRB1_0101	511	VMPECNYKSDRTIAV	YKSDRTIAV	6	0.4073	609.8	65.00	0.44	tr_C9ZZ34_
DRB1_0101	512	TSFSRKEASDAGNKE	FSRKEASDA	2	0.4070	611.6	65.00	0.83	tr_C9ZZ34_
DRB1_0101	513	GLPCDIIRRYTGDIL	IRRYTGDIL	6	0.4064	615.6	65.00	0.42	tr_C9ZZ34_
DRB1_0101	514	TAVRHNYWSVREARA	YWSVREARA	6	0.4062	616.6	65.00	0.57	tr_C9ZZ34_
DRB1_0101	515	TRYVREGPQWCAVDR	VREGPQWCA	3	0.4059	619.2	65.00	0.52	tr_C9ZZ34_
DRB1_0101	516	LVSIDGVCKLSDFGG	LVSIDGVCK	0	0.4054	622.4	65.00	0.58	tr_C9ZZ34_
DRB1_0101	517	TPVCMSPEACMGVVD	VCMSPEACM	2	0.4053	623.0	65.00	0.41	tr_C9ZZ34_
DRB1_0101	518	RRGCISPEPPFDLMD	ISPEPPFDL	4	0.4044	629.1	65.00	0.79	tr_C9ZZ34_
DRB1_0101	519	ITRYVREGPQWCAVD	VREGPQWCA	4	0.4043	630.0	65.00	0.46	tr_C9ZZ34_
DRB1_0101	520	VLELDHNAPRLHRLC	LDHNAPRLH	3	0.4042	630.5	65.00	0.34	tr_C9ZZ34_
DRB1_0101	521	AVECAMQPPTSEDNV	CAMQPPTSE	3	0.4040	632.0	65.00	0.62	tr_C9ZZ34_
DRB1_0101	522	GVIRTPSGRTSSLYG	VIRTPSGRT	1	0.4033	636.9	65.00	0.58	tr_C9ZZ34_
DRB1_0101	523	MLQPCYDVFRVCVTA	YDVFRVCVT	5	0.4031	638.3	65.00	0.54	tr_C9ZZ34_
DRB1_0101	524	PGSFQPNEIQSEYAL	FQPNEIQSE	3	0.4021	644.8	65.00	0.38	tr_C9ZZ34_
DRB1_0101	525	LEKVLELDHNAPRLH	KVLELDHNA	2	0.4018	647.0	65.00	0.34	tr_C9ZZ34_
DRB1_0101	526	VNLVRPRDCAFRLVS	VNLVRPRDC	0	0.4016	648.1	65.00	0.28	tr_C9ZZ34_
DRB1_0101	527	VVTFHTPSGPNSVNL	FHTPSGPNS	3	0.4014	649.8	65.00	0.46	tr_C9ZZ34_
DRB1_0101	528	DFSTSNSDQLLLETF	FSTSNSDQL	1	0.4013	650.5	65.00	0.56	tr_C9ZZ34_
DRB1_0101	529	FNDVVEPGCAQEISE	VEPGCAQEI	4	0.4003	657.3	65.00	0.58	tr_C9ZZ34_
DRB1_0101	530	RAVRSHEIIPVSSSP	VRSHEIIPV	2	0.4002	658.2	65.00	0.59	tr_C9ZZ34_
DRB1_0101	531	ELAGPDFAGGRVPLR	FAGGRVPLR	6	0.4000	659.7	65.00	0.65	tr_C9ZZ34_
DRB1_0101	532	SVVVTFHTPSGPNSV	VVTFHTPSG	2	0.3998	661.5	65.00	0.35	tr_C9ZZ34_
DRB1_0101	533	VVVTFHTPSGPNSVN	FHTPSGPNS	4	0.3997	661.8	65.00	0.38	tr_C9ZZ34_
DRB1_0101	534	VRYVDRCFQNGCLYI	DRCFQNGCL	4	0.3994	663.8	65.00	0.40	tr_C9ZZ34_
DRB1_0101	535	CECHVSPQYYAAPGS	CHVSPQYYA	2	0.3984	671.0	65.00	0.52	tr_C9ZZ34_
DRB1_0101	536	EKMNSSLSDITRYVR	SLSDITRYV	5	0.3983	672.2	65.00	0.38	tr_C9ZZ34_
DRB1_0101	537	DVRIYLYDPKAPKER	YLYDPKAPK	4	0.3980	673.9	65.00	0.35	tr_C9ZZ34_
DRB1_0101	538	DITRYVREGPQWCAV	VREGPQWCA	5	0.3980	674.1	65.00	0.44	tr_C9ZZ34_
DRB1_0101	539	GGAVECAMQPPTSED	CAMQPPTSE	5	0.3979	674.8	65.00	0.45	tr_C9ZZ34_
DRB1_0101	540	VTFHTPSGPNSVNLV	FHTPSGPNS	2	0.3974	678.5	65.00	0.45	tr_C9ZZ34_
DRB1_0101	541	ECHVSPQYYAAPGSF	CHVSPQYYA	1	0.3971	680.7	65.00	0.47	tr_C9ZZ34_
DRB1_0101	542	PFNDVVEPGCAQEIS	VEPGCAQEI	5	0.3957	691.2	65.00	0.50	tr_C9ZZ34_
DRB1_0101	543	VRPRDCAFRLVSELQ	CAFRLVSEL	5	0.3950	696.5	65.00	0.34	tr_C9ZZ34_
DRB1_0101	544	YCSTGSTTLKTCFGT	YCSTGSTTL	0	0.3933	709.7	65.00	0.36	tr_C9ZZ34_
DRB1_0101	545	GPNSVNLVRPRDCAF	VNLVRPRDC	4	0.3927	714.1	65.00	0.65	tr_C9ZZ34_
DRB1_0101	546	KMNSSLSDITRYVRE	SLSDITRYV	4	0.3924	716.2	65.00	0.46	tr_C9ZZ34_
DRB1_0101	547	RGLHPDGNIVAIKEY	LHPDGNIVA	2	0.3920	719.7	65.00	0.60	tr_C9ZZ34_
DRB1_0101	548	DREAKKFIRKKIEKA	FIRKKIEKA	6	0.3906	730.3	65.00	0.77	tr_C9ZZ34_
DRB1_0101	549	LRRGCISPEPPFDLM	ISPEPPFDL	5	0.3906	730.4	65.00	0.75	tr_C9ZZ34_
DRB1_0101	550	RQIVHRDISPNNVLV	HRDISPNNV	4	0.3900	735.3	65.00	0.47	tr_C9ZZ34_
DRB1_0101	551	FISKLRRGCISPEPP	SKLRRGCIS	2	0.3888	744.5	65.00	0.44	tr_C9ZZ34_
DRB1_0101	552	VVLRVAREEFGVPFD	LRVAREEFG	2	0.3876	754.5	65.00	0.58	tr_C9ZZ34_
DRB1_0101	553	IWGLGITLCFCVACS	WGLGITLCF	1	0.3867	761.6	65.00	0.19	tr_C9ZZ34_
DRB1_0101	554	TEVVLRVAREEFGVP	LRVAREEFG	4	0.3857	770.4	65.00	0.58	tr_C9ZZ34_
DRB1_0101	555	EATSFSRKEASDAGN	FSRKEASDA	4	0.3856	770.7	65.00	0.87	tr_C9ZZ34_
DRB1_0101	556	GVCKLSDFGGAVECA	KLSDFGGAV	3	0.3855	771.7	65.00	0.28	tr_C9ZZ34_

DRB1_0101	557	SLAALVETFHGLPCD	AALVETFHG	2	0.3854	772.7	65.00	0.31	tr_C9ZZ34_
DRB1_0101	558	TLMNSSITRDVCIVT	MNSSITRDV	2	0.3852	774.4	65.00	0.47	tr_C9ZZ34_
DRB1_0101	559	CFGTPVCMSPEACMG	PVCMSPEAC	4	0.3842	782.9	70.00	0.41	tr_C9ZZ34_
DRB1_0101	560	GCISPEPPFDLMDVH	ISPEPPFDL	2	0.3832	791.1	70.00	0.81	tr_C9ZZ34_
DRB1_0101	561	VCVTAVPVDVIHRRT	VTAVPVDVI	2	0.3830	792.9	70.00	0.45	tr_C9ZZ34_
DRB1_0101	562	FCVACSYPWSQEDTA	FCVACSYPW	0	0.3825	797.3	70.00	0.60	tr_C9ZZ34_
DRB1_0101	563	VHRDISPNNVLVSID	HRDISPNNV	1	0.3822	800.3	70.00	0.36	tr_C9ZZ34_
DRB1_0101	564	LQQEKDAIVYQLQEA	LQQEKDAIV	0	0.3816	805.1	70.00	0.50	tr_C9ZZ34_
DRB1_0101	565	VLRVAREEFGVPFDI	LRVAREEFG	1	0.3808	812.2	70.00	0.46	tr_C9ZZ34_
DRB1_0101	566	IDGVCKLSDFGGAVE	VCKLSDFGG	3	0.3807	812.6	70.00	0.41	tr_C9ZZ34_
DRB1_0101	567	VCKLSDFGGAVECAM	KLSDFGGAV	2	0.3805	814.3	70.00	0.31	tr_C9ZZ34_
DRB1_0101	568	VTHQRSSSACGVMPE	QRSSSACGV	3	0.3804	815.4	70.00	0.38	tr_C9ZZ34_
DRB1_0101	569	PGCAQEISEEYRLLH	EISEEYRLL	5	0.3803	816.7	70.00	0.43	tr_C9ZZ34_
DRB1_0101	570	LELDHNAPRLHRLCK	LDHNAPRLH	2	0.3796	822.5	70.00	0.34	tr_C9ZZ34_
DRB1_0101	571	TFHTPSGPNSVNLVR	FHTPSGPNS	1	0.3796	823.1	70.00	0.38	tr_C9ZZ34_
DRB1_0101	572	NDVVEPGCAQEISEE	VEPGCAQEI	3	0.3793	825.5	70.00	0.65	tr_C9ZZ34_
DRB1_0101	573	ACGVMPECNYKSDRT	VMPECNYKS	3	0.3791	827.3	70.00	0.62	tr_C9ZZ34_
DRB1_0101	574	VIHRRTCGGDVRIYL	HRRTCGGDV	2	0.3783	834.6	70.00	0.60	tr_C9ZZ34_
DRB1_0101	575	DVIHRRTCGGDVRIY	HRRTCGGDV	3	0.3782	835.2	70.00	0.62	tr_C9ZZ34_
DRB1_0101	576	CCDVEGHPLKTNLHK	CCDVEGHPL	0	0.3771	845.1	70.00	0.38	tr_C9ZZ34_
DRB1_0101	577	FGGAVECAMQPPTSE	CAMQPPTSE	6	0.3770	846.4	70.00	0.26	tr_C9ZZ34_
DRB1_0101	578	DGVCKLSDFGGAVEC	VCKLSDFGG	2	0.3768	847.6	70.00	0.36	tr_C9ZZ34_
DRB1_0101	579	RIYLYDPKAPKEREC	YLYDPKAPK	2	0.3755	860.1	70.00	0.37	tr_C9ZZ34_
DRB1_0101	580	IEKAGMSIDMNATSL	SIDMNATSL	6	0.3747	867.4	70.00	0.26	tr_C9ZZ34_
DRB1_0101	581	SACGVMPECNYKSDR	VMPECNYKS	4	0.3745	868.9	70.00	0.56	tr_C9ZZ34_
DRB1_0101	582	VECAMQPPTSEDNVT	CAMQPPTSE	2	0.3739	875.4	70.00	0.64	tr_C9ZZ34_
DRB1_0101	583	EEFGVPFDIRLDSVV	FGVPFDIRL	2	0.3733	880.4	70.00	0.40	tr_C9ZZ34_
DRB1_0101	584	WGLGITLCFCVACSY	GITLCFCVA	3	0.3731	882.9	70.00	0.31	tr_C9ZZ34_
DRB1_0101	585	RLCKYLAQEHEGEKM	YLAQEHEGE	4	0.3730	883.1	70.00	0.47	tr_C9ZZ34_
DRB1_0101	586	HRLCKYLAQEHEGEK	YLAQEHEGE	5	0.3726	887.7	70.00	0.32	tr_C9ZZ34_
DRB1_0101	587	MCEKRFSQASEQLEK	FSQASEQLE	5	0.3721	892.2	70.00	0.45	tr_C9ZZ34_
DRB1_0101	588	VVDPRNDIWGLGITL	DIWGLGITL	6	0.3717	896.3	70.00	0.54	tr_C9ZZ34_
DRB1_0101	589	IPVSSSPLLKNLDRS	PVSSSPLLK	1	0.3710	902.9	70.00	0.50	tr_C9ZZ34_
DRB1_0101	590	RDAKDRPSASELLFH	DRPSASELL	4	0.3706	906.8	70.00	0.65	tr_C9ZZ34_
DRB1_0101	591	KIEKAGMSIDMNATS	EKAGMSIDM	2	0.3702	910.7	70.00	0.37	tr_C9ZZ34_
DRB1_0101	592	CAMMDLSRFGQCECH	CAMMDLSRF	0	0.3700	913.2	70.00	0.56	tr_C9ZZ34_
DRB1_0101	593	RYVREGPQWCAVDRE	VREGPQWCA	2	0.3699	913.7	70.00	0.55	tr_C9ZZ34_
DRB1_0101	594	VAREEFGVPFDIRLD	FGVPFDIRL	5	0.3693	920.0	70.00	0.35	tr_C9ZZ34_
DRB1_0101	595	IHRRTCGGDVRIYLY	HRRTCGGDV	1	0.3686	926.9	70.00	0.50	tr_C9ZZ34_
DRB1_0101	596	RVAREEFGVPFDIRL	EEFGVPFDI	4	0.3678	934.5	70.00	0.39	tr_C9ZZ34_
DRB1_0101	597	REEFGVPFDIRLDSV	FGVPFDIRL	3	0.3678	935.2	70.00	0.53	tr_C9ZZ34_
DRB1_0101	598	AKDRPSASELLFHDF	DRPSASELL	2	0.3677	935.8	70.00	0.63	tr_C9ZZ34_
DRB1_0101	599	IRLDSVVVTFHTPSG	DSVVVTFHT	3	0.3673	940.1	70.00	0.31	tr_C9ZZ34_
DRB1_0101	600	LCDHKGGSTNFVLDS	HKGGSTNFV	3	0.3668	944.8	70.00	0.55	tr_C9ZZ34_
DRB1_0101	601	LMDVHFADFVRQCLK	VHFADFVRQ	3	0.3664	949.2	70.00	0.47	tr_C9ZZ34_
DRB1_0101	602	FSQASEQLEKVLELD	QASEQLEKV	2	0.3655	958.8	70.00	0.38	tr_C9ZZ34_
DRB1_0101	603	LCKYLAQEHEGEKMN	YLAQEHEGE	3	0.3654	959.6	70.00	0.50	tr_C9ZZ34_
DRB1_0101	604	MNSSLSDITRYVREG	SLSDITRYV	3	0.3654	959.7	70.00	0.56	tr_C9ZZ34_
DRB1_0101	605	QLEKVLELDHNAPRL	KVLELDHNA	3	0.3648	965.3	70.00	0.46	tr_C9ZZ34_
DRB1_0101	606	CGVMPECNYKSDRTI	VMPECNYKS	2	0.3647	966.7	70.00	0.57	tr_C9ZZ34_

DRB1_0101	607	DSVVVTFHTPSGPNS	VVTFHTPSG	3	0.3644	969.7	70.00	0.44	tr_C9ZZ34_
DRB1_0101	608	TCFGTPVCMSPEACM	PVCMSPEAC	5	0.3636	978.5	70.00	0.38	tr_C9ZZ34_
DRB1_0101	609	AREEFGVPFDIRLDS	FGVPFDIRL	4	0.3634	979.9	70.00	0.43	tr_C9ZZ34_
DRB1_0101	610	RECVGSSREQYAPLM	VGSSREQYA	3	0.3634	980.4	70.00	0.34	tr_C9ZZ34_
DRB1_0101	611	LRVAREEFGVPFDIR	AREEFGVPF	3	0.3631	983.1	70.00	0.31	tr_C9ZZ34_
DRB1_0101	612	RLCDHKGGSTNFVLD	HKGGSTNFV	4	0.3626	988.5	70.00	0.49	tr_C9ZZ34_
DRB1_0101	613	IYGPVGAGSFGRVYR	PVGAGSFGR	3	0.3619	996.7	70.00	0.26	tr_C9ZZ34_
DRB1_0101	614	ELDHNAPRLHRLCKY	HNAPRLHRL	3	0.3609	1007.3	70.00	0.31	tr_C9ZZ34_
DRB1_0101	615	GVMPECNYKSDRTIA	VMPECNYKS	1	0.3604	1012.4	70.00	0.53	tr_C9ZZ34_
DRB1_0101	616	DAKDRPSASELLFHD	DRPSASELL	3	0.3598	1019.5	75.00	0.67	tr_C9ZZ34_
DRB1_0101	617	SFSRKEASDAGNKER	FSRKEASDA	1	0.3592	1026.1	75.00	0.80	tr_C9ZZ34_
DRB1_0101	618	KDRPSASELLFHDFM	DRPSASELL	1	0.3589	1029.1	75.00	0.53	tr_C9ZZ34_
DRB1_0101	619	VHFADFVRQCLKRDA	FVRQCLKRD	5	0.3584	1034.9	75.00	0.33	tr_C9ZZ34_
DRB1_0101	620	LAALVETFHGLPCDI	VETFHGLPC	4	0.3581	1038.0	75.00	0.23	tr_C9ZZ34_
DRB1_0101	621	LDHNAPRLHRLCKYL	HNAPRLHRL	2	0.3579	1040.0	75.00	0.29	tr_C9ZZ34_
DRB1_0101	622	KTCFGTPVCMSPEAC	CFGTPVCMS	2	0.3576	1043.8	75.00	0.31	tr_C9ZZ34_
DRB1_0101	623	MDVHFADFVRQCLKR	VHFADFVRQ	2	0.3576	1044.0	75.00	0.41	tr_C9ZZ34_
DRB1_0101	624	GDVRIYLYDPKAPKE	RIYLYDPKA	3	0.3564	1057.6	75.00	0.31	tr_C9ZZ34_
DRB1_0101	625	KTKSDFSTSNSDQLL	FSTSNSDQL	5	0.3564	1057.9	75.00	0.70	tr_C9ZZ34_
DRB1_0101	626	DRPSASELLFHDFMV	ELLFHDFMV	6	0.3563	1058.2	75.00	0.30	tr_C9ZZ34_
DRB1_0101	627	SVREARASDSTSLST	VREARASDS	1	0.3558	1064.1	75.00	0.61	tr_C9ZZ34_
DRB1_0101	628	HFADFVRQCLKRDAK	FVRQCLKRD	4	0.3557	1065.2	75.00	0.48	tr_C9ZZ34_
DRB1_0101	629	HRDISPNNVLVSIDG	DISPNNVLV	2	0.3557	1065.6	75.00	0.30	tr_C9ZZ34_
DRB1_0101	630	DVHFADFVRQCLKRD	VHFADFVRQ	1	0.3555	1067.7	75.00	0.34	tr_C9ZZ34_
DRB1_0101	631	GLGITLCFCVACSYP	GITLCFCVA	2	0.3551	1071.9	75.00	0.34	tr_C9ZZ34_
DRB1_0101	632	VDVIHRRTCGGDVRI	HRRTCGGDV	4	0.3543	1082.1	75.00	0.56	tr_C9ZZ34_
DRB1_0101	633	FSTPTAVRHNYWSVR	FSTPTAVRH	0	0.3542	1082.8	75.00	0.34	tr_C9ZZ34_
DRB1_0101	634	RPSASELLFHDFMVN	ELLFHDFMV	5	0.3541	1084.6	75.00	0.35	tr_C9ZZ34_
DRB1_0101	635	NRLCDHKGGSTNFVL	HKGGSTNFV	5	0.3538	1087.7	75.00	0.45	tr_C9ZZ34_
DRB1_0101	636	RTPSGRTSSLYGEEV	TSSLYGEEV	6	0.3537	1089.1	75.00	0.75	tr_C9ZZ34_
DRB1_0101	637	RFGQCECHVSPQYYA	CHVSPQYYA	6	0.3531	1095.7	75.00	0.41	tr_C9ZZ34_
DRB1_0101	638	GEKMNSSLSDITRYV	MNSSLSDIT	3	0.3526	1101.9	75.00	0.38	tr_C9ZZ34_
DRB1_0101	639	DIYGPVGAGSFGRVY	YGPVGAGSF	2	0.3522	1106.8	75.00	0.29	tr_C9ZZ34_
DRB1_0101	640	SGPNSVNLVRPRDCA	VNLVRPRDC	5	0.3518	1111.0	75.00	0.60	tr_C9ZZ34_
DRB1_0101	641	HHKNIVRYVDRCFQN	HHKNIVRYV	0	0.3518	1111.2	75.00	0.37	tr_C9ZZ34_
DRB1_0101	642	SYPWSQEDTADVRSF	WSQEDTADV	3	0.3518	1111.3	75.00	0.75	tr_C9ZZ34_
DRB1_0101	643	QQEKDAIVYQLQEAT	IVYQLQEAT	6	0.3516	1113.8	75.00	0.50	tr_C9ZZ34_
DRB1_0101	644	LVRPRDCAFRLVSEL	RPRDCAFRL	2	0.3512	1118.3	75.00	0.28	tr_C9ZZ34_
DRB1_0101	645	ITRDVCIVTHQRSSS	DVCIVTHQR	3	0.3511	1119.4	75.00	0.41	tr_C9ZZ34_
DRB1_0101	646	LMNSSITRDVCIVTH	MNSSITRDV	1	0.3490	1145.8	75.00	0.34	tr_C9ZZ34_
DRB1_0101	647	FSTSNSDQLLLETFG	FSTSNSDQL	0	0.3489	1147.3	75.00	0.41	tr_C9ZZ34_
DRB1_0101	648	SDSTSLSTQQSDACI	STSLSTQQS	2	0.3473	1167.4	75.00	0.62	tr_C9ZZ34_
DRB1_0101	649	SIDGVCKLSDFGGAV	VCKLSDFGG	4	0.3471	1169.0	75.00	0.46	tr_C9ZZ34_
DRB1_0101	650	PVCMSPEACMGVVDP	VCMSPEACM	1	0.3469	1171.4	75.00	0.38	tr_C9ZZ34_
DRB1_0101	651	CISPEPPFDLMDVHF	ISPEPPFDL	1	0.3466	1175.3	75.00	0.76	tr_C9ZZ34_
DRB1_0101	652	RDISPNNVLVSIDGV	NNVLVSIDG	5	0.3462	1181.3	75.00	0.32	tr_C9ZZ34_
DRB1_0101	653	ISKLRRGCISPEPPF	SKLRRGCIS	1	0.3460	1183.9	75.00	0.40	tr_C9ZZ34_
DRB1_0101	654	RLDSVVVTFHTPSGP	VVTFHTPSG	5	0.3436	1214.5	75.00	0.33	tr_C9ZZ34_
DRB1_0101	655	DLMDVHFADFVRQCL	VHFADFVRQ	4	0.3431	1221.2	75.00	0.45	tr_C9ZZ34_
DRB1_0101	656	DVVEPGCAQEISEEY	VEPGCAQEI	2	0.3427	1226.4	75.00	0.68	tr_C9ZZ34_

DRB1_0101	657	IVRYVDRCFQNGCLY	DRCFQNGCL	5	0.3418	1238.2	75.00	0.38	tr_C9ZZ34_
DRB1_0101	658	DSTSLSTQQSDACID	STSLSTQQS	1	0.3416	1240.5	75.00	0.47	tr_C9ZZ34_
DRB1_0101	659	SSPLLKNLDRSNKFS	LLKNLDRSN	3	0.3416	1240.7	75.00	0.37	tr_C9ZZ34_
DRB1_0101	660	CDHKGGSTNFVLDSS	HKGGSTNFV	2	0.3411	1248.2	75.00	0.55	tr_C9ZZ34_
DRB1_0101	661	DISPNNVLVSIDGVC	NNVLVSIDG	4	0.3407	1253.6	75.00	0.44	tr_C9ZZ34_
DRB1_0101	662	QEATSFSRKEASDAG	FSRKEASDA	5	0.3406	1254.0	75.00	0.82	tr_C9ZZ34_
DRB1_0101	663	SSACGVMPECNYKSD	VMPECNYKS	5	0.3403	1258.2	75.00	0.46	tr_C9ZZ34_
DRB1_0101	664	SPLLKNLDRSNKFST	LKNLDRSNK	3	0.3400	1263.4	75.00	0.40	tr_C9ZZ34_
DRB1_0101	665	SITRDVCIVTHQRSS	DVCIVTHQR	4	0.3390	1276.5	75.00	0.38	tr_C9ZZ34_
DRB1_0101	666	STSLSTQQSDACIDA	STQQSDACI	4	0.3385	1284.0	75.00	0.27	tr_C9ZZ34_
DRB1_0101	667	LDSVVVTFHTPSGPN	VVTFHTPSG	4	0.3383	1286.5	75.00	0.38	tr_C9ZZ34_
DRB1_0101	668	EPGCAQEISEEYRLL	EISEEYRLL	6	0.3377	1294.3	75.00	0.34	tr_C9ZZ34_
DRB1_0101	669	HADPDKFDSATTNFS	FDSATTNFS	6	0.3376	1295.7	75.00	0.78	tr_C9ZZ34_
DRB1_0101	670	CKLSDFGGAVECAMQ	KLSDFGGAV	1	0.3373	1300.4	75.00	0.29	tr_C9ZZ34_
DRB1_0101	671	FADFVRQCLKRDAKD	FVRQCLKRD	3	0.3363	1314.5	75.00	0.54	tr_C9ZZ34_
DRB1_0101	672	YGPVGAGSFGRVYRG	VGAGSFGRV	3	0.3358	1321.3	75.00	0.35	tr_C9ZZ34_
DRB1_0101	673	APGSFQPNEIQSEYA	FQPNEIQSE	4	0.3357	1322.7	75.00	0.44	tr_C9ZZ34_
DRB1_0101	674	PRELKVCCDVEGHPL	CCDVEGHPL	6	0.3355	1325.3	75.00	0.47	tr_C9ZZ34_
DRB1_0101	675	YPFNDVVEPGCAQEI	VEPGCAQEI	6	0.3349	1334.1	75.00	0.35	tr_C9ZZ34_
DRB1_0101	676	YPWSQEDTADVRSFI	WSQEDTADV	2	0.3348	1336.4	75.00	0.69	tr_C9ZZ34_
DRB1_0101	677	CKYLAQEHEGEKMNS	YLAQEHEGE	2	0.3343	1343.1	80.00	0.50	tr_C9ZZ34_
DRB1_0101	678	SQEDTADVRSFISKL	TADVRSFIS	4	0.3329	1363.5	80.00	0.38	tr_C9ZZ34_
DRB1_0101	679	NLVRPRDCAFRLVSE	RPRDCAFRL	3	0.3329	1363.8	80.00	0.34	tr_C9ZZ34_
DRB1_0101	680	SSITRDVCIVTHQRS	DVCIVTHQR	5	0.3326	1368.5	80.00	0.29	tr_C9ZZ34_
DRB1_0101	681	ECAMQPPTSEDNVTL	CAMQPPTSE	1	0.3325	1369.0	80.00	0.63	tr_C9ZZ34_
DRB1_0101	682	CSYPWSQEDTADVRS	WSQEDTADV	4	0.3317	1381.0	80.00	0.71	tr_C9ZZ34_
DRB1_0101	683	ASDSTSLSTQQSDAC	STSLSTQQS	3	0.3306	1398.7	80.00	0.77	tr_C9ZZ34_
DRB1_0101	684	KRDAKDRPSASELLF	DRPSASELL	5	0.3304	1401.3	80.00	0.62	tr_C9ZZ34_
DRB1_0101	685	YAAPGSFQPNEIQSE	YAAPGSFQP	0	0.3281	1436.9	80.00	0.31	tr_C9ZZ34_
DRB1_0101	686	GGDVRIYLYDPKAPK	RIYLYDPKA	4	0.3279	1440.0	80.00	0.35	tr_C9ZZ34_
DRB1_0101	687	CVTAVPVDVIHRRTC	VTAVPVDVI	1	0.3271	1451.5	80.00	0.44	tr_C9ZZ34_
DRB1_0101	688	GLHPDGNIVAIKEYP	LHPDGNIVA	1	0.3265	1461.4	80.00	0.54	tr_C9ZZ34_
DRB1_0101	689	NIVRYVDRCFQNGCL	DRCFQNGCL	6	0.3262	1466.3	80.00	0.24	tr_C9ZZ34_
DRB1_0101	690	SDITRYVREGPQWCA	YVREGPQWC	5	0.3240	1501.7	80.00	0.47	tr_C9ZZ34_
DRB1_0101	691	LMCEKRFSQASEQLE	FSQASEQLE	6	0.3237	1506.3	80.00	0.28	tr_C9ZZ34_
DRB1_0101	692	IDMNATSLSVSQYPF	DMNATSLSV	1	0.3233	1513.2	80.00	0.31	tr_C9ZZ34_
DRB1_0101	693	NSSITRDVCIVTHQR	ITRDVCIVT	3	0.3230	1517.6	80.00	0.38	tr_C9ZZ34_
DRB1_0101	694	PVDVIHRRTCGGDVR	HRRTCGGDV	5	0.3228	1520.5	80.00	0.51	tr_C9ZZ34_
DRB1_0101	695	FDSATTNFSQEVTGR	FDSATTNFS	0	0.3222	1530.7	80.00	0.58	tr_C9ZZ34_
DRB1_0101	696	DRQIVHRDISPNNVL	HRDISPNNV	5	0.3209	1552.3	80.00	0.49	tr_C9ZZ34_
DRB1_0101	697	NSSLSDITRYVREGP	SLSDITRYV	2	0.3202	1564.3	80.00	0.59	tr_C9ZZ34_
DRB1_0101	698	ADFVRQCLKRDAKDR	FVRQCLKRD	2	0.3196	1574.9	80.00	0.48	tr_C9ZZ34_
DRB1_0101	699	FHTPSGPNSVNLVRP	HTPSGPNSV	1	0.3190	1585.4	80.00	0.34	tr_C9ZZ34_
DRB1_0101	700	DFGGAVECAMQPPTS	GAVECAMQP	3	0.3172	1616.6	80.00	0.35	tr_C9ZZ34_
DRB1_0101	701	GPVGAGSFGRVYRGL	VGAGSFGRV	2	0.3166	1626.1	80.00	0.32	tr_C9ZZ34_
DRB1_0101	702	IYLYDPKAPKERECV	YDPKAPKER	3	0.3162	1634.1	80.00	0.38	tr_C9ZZ34_
DRB1_0101	703	TSLSTQQSDACIDAF	STQQSDACI	3	0.3159	1639.8	80.00	0.47	tr_C9ZZ34_
DRB1_0101	704	SQASEQLEKVLELDH	EQLEKVLEL	4	0.3154	1647.0	80.00	0.47	tr_C9ZZ34_
DRB1_0101	705	HRRTCGGDVRIYLYD	HRRTCGGDV	0	0.3153	1649.1	80.00	0.33	tr_C9ZZ34_
DRB1_0101	706	RASDSTSLSTQQSDA	STSLSTQQS	4	0.3151	1652.7	80.00	0.79	tr_C9ZZ34_

DRB1_0101	707	VVEPGCAQEISEEYR	VEPGCAQEI	1	0.3144	1666.6	80.00	0.67	tr_C9ZZ34_
DRB1_0101	708	SSSPLLKNLDRSNKF	LLKNLDRSN	4	0.3135	1682.4	80.00	0.38	tr_C9ZZ34_
DRB1_0101	709	EQLEKVLELDHNAPR	KVLELDHNA	4	0.3132	1686.8	80.00	0.43	tr_C9ZZ34_
DRB1_0101	710	VIRTPSGRTSSLYGE	VIRTPSGRT	0	0.3123	1703.3	80.00	0.45	tr_C9ZZ34_
DRB1_0101	711	PWSQEDTADVRSFIS	WSQEDTADV	1	0.3122	1706.0	80.00	0.57	tr_C9ZZ34_
DRB1_0101	712	DHKGGSTNFVLDSSC	HKGGSTNFV	1	0.3120	1709.1	80.00	0.54	tr_C9ZZ34_
DRB1_0101	713	KKTGYAPNRLCDHKG	KTGYAPNRL	1	0.3101	1745.5	80.00	0.35	tr_C9ZZ34_
DRB1_0101	714	HKNIVRYVDRCFQNG	IVRYVDRCF	3	0.3096	1754.3	80.00	0.37	tr_C9ZZ34_
DRB1_0101	715	DKKTGYAPNRLCDHK	KTGYAPNRL	2	0.3086	1774.0	80.00	0.41	tr_C9ZZ34_
DRB1_0101	716	QLQEATSFSRKEASD	LQEATSFSR	1	0.3082	1780.7	80.00	0.70	tr_C9ZZ34_
DRB1_0101	717	STSNSDQLLLETFGS	QLLLETFGS	6	0.3073	1799.3	80.00	0.45	tr_C9ZZ34_
DRB1_0101	718	MNSSITRDVCIVTHQ	ITRDVCIVT	4	0.3072	1800.6	80.00	0.37	tr_C9ZZ34_
DRB1_0101	719	TPTAVRHNYWSVREA	AVRHNYWSV	3	0.3061	1821.4	85.00	0.32	tr_C9ZZ34_
DRB1_0101	720	AAPGSFQPNEIQSEY	FQPNEIQSE	5	0.3061	1823.0	85.00	0.40	tr_C9ZZ34_
DRB1_0101	721	ACSYPWSQEDTADVR	WSQEDTADV	5	0.3058	1828.8	85.00	0.67	tr_C9ZZ34_
DRB1_0101	722	KLSDFGGAVECAMQP	FGGAVECAM	4	0.3057	1830.6	85.00	0.31	tr_C9ZZ34_
DRB1_0101	723	TTNFSQEVTGRKEDA	FSQEVTGRK	3	0.3056	1831.5	85.00	0.76	tr_C9ZZ34_
DRB1_0101	724	KNIVRYVDRCFQNGC	IVRYVDRCF	2	0.3053	1837.3	85.00	0.31	tr_C9ZZ34_
DRB1_0101	725	ATSLSVSQYPFNDVV	SVSQYPFND	4	0.3036	1872.0	85.00	0.28	tr_C9ZZ34_
DRB1_0101	726	WSQEDTADVRSFISK	TADVRSFIS	5	0.3036	1872.2	85.00	0.32	tr_C9ZZ34_
DRB1_0101	727	EGEKMNSSLSDITRY	MNSSLSDIT	4	0.3034	1875.8	85.00	0.41	tr_C9ZZ34_
DRB1_0101	728	PLLKNLDRSNKFSTP	LKNLDRSNK	2	0.3029	1885.5	85.00	0.41	tr_C9ZZ34_
DRB1_0101	729	NDKKTGYAPNRLCDH	KTGYAPNRL	3	0.3029	1886.8	85.00	0.45	tr_C9ZZ34_
DRB1_0101	730	QYLHDRQIVHRDISP	YLHDRQIVH	1	0.3025	1895.4	85.00	0.32	tr_C9ZZ34_
DRB1_0101	731	LTTESDYCSTGSTTL	YCSTGSTTL	6	0.3016	1912.8	85.00	0.58	tr_C9ZZ34_
DRB1_0101	732	KLRRGCISPEPPFDL	ISPEPPFDL	6	0.3006	1934.5	85.00	0.51	tr_C9ZZ34_
DRB1_0101	733	PVGAGSFGRVYRGLH	VGAGSFGRV	1	0.3003	1940.7	85.00	0.22	tr_C9ZZ34_
DRB1_0101	734	PTAVRHNYWSVREAR	VRHNYWSVR	3	0.3002	1942.6	85.00	0.28	tr_C9ZZ34_
DRB1_0101	735	ENNPEIQSTLSEIRM	IQSTLSEIR	5	0.2994	1959.0	85.00	0.46	tr_C9ZZ34_
DRB1_0101	736	SATTNFSQEVTGRKE	FSQEVTGRK	5	0.2987	1974.4	85.00	0.60	tr_C9ZZ34_
DRB1_0101	737	KNLDRSNKFSTPTAV	NKFSTPTAV	6	0.2985	1978.5	85.00	0.42	tr_C9ZZ34_
DRB1_0101	738	SSSACGVMPECNYKS	CGVMPECNY	4	0.2985	1978.8	85.00	0.39	tr_C9ZZ34_
DRB1_0101	739	SLSTQQSDACIDAFA	STQQSDACI	2	0.2963	2025.7	85.00	0.42	tr_C9ZZ34_
DRB1_0101	740	VEGHPLKTNLHKHAD	GHPLKTNLH	2	0.2959	2035.3	85.00	0.43	tr_C9ZZ34_
DRB1_0101	741	STPTAVRHNYWSVRE	AVRHNYWSV	4	0.2957	2039.1	85.00	0.29	tr_C9ZZ34_
DRB1_0101	742	PSGPNSVNLVRPRDC	VNLVRPRDC	6	0.2948	2059.4	85.00	0.36	tr_C9ZZ34_
DRB1_0101	743	AGSFGRVYRGLHPDG	GRVYRGLHP	4	0.2945	2066.1	85.00	0.44	tr_C9ZZ34_
DRB1_0101	744	ATTNFSQEVTGRKED	FSQEVTGRK	4	0.2920	2121.7	85.00	0.68	tr_C9ZZ34_
DRB1_0101	745	VSIDGVCKLSDFGGA	VCKLSDFGG	5	0.2914	2135.8	85.00	0.42	tr_C9ZZ34_
DRB1_0101	746	NATSLSVSQYPFNDV	LSVSQYPFN	4	0.2909	2148.0	85.00	0.21	tr_C9ZZ34_
DRB1_0101	747	SEQLEKVLELDHNAP	EQLEKVLEL	1	0.2896	2178.6	85.00	0.41	tr_C9ZZ34_
DRB1_0101	748	TSLSVSQYPFNDVVE	SVSQYPFND	3	0.2887	2199.5	85.00	0.33	tr_C9ZZ34_
DRB1_0101	749	DMNATSLSVSQYPFN	TSLSVSQYP	4	0.2886	2201.2	85.00	0.27	tr_C9ZZ34_
DRB1_0101	750	ASEQLEKVLELDHNA	EQLEKVLEL	2	0.2881	2214.7	85.00	0.56	tr_C9ZZ34_
DRB1_0101	751	QASEQLEKVLELDHN	EQLEKVLEL	3	0.2877	2224.8	85.00	0.62	tr_C9ZZ34_
DRB1_0101	752	LQEATSFSRKEASDA	FSRKEASDA	6	0.2873	2234.0	85.00	0.50	tr_C9ZZ34_
DRB1_0101	753	GLMCEKRFSQASEQL	CEKRFSQAS	3	0.2862	2260.6	85.00	0.25	tr_C9ZZ34_
DRB1_0101	754	PVSSSPLLKNLDRSN	PVSSSPLLK	0	0.2861	2261.6	85.00	0.32	tr_C9ZZ34_
DRB1_0101	755	YVREGPQWCAVDREA	VREGPQWCA	1	0.2855	2277.2	85.00	0.57	tr_C9ZZ34_
DRB1_0101	756	DVEGHPLKTNLHKHA	GHPLKTNLH	3	0.2854	2279.8	85.00	0.47	tr_C9ZZ34_

DRB1_0101	757	HKGGSTNFVLDSSCN	HKGGSTNFV	0	0.2846	2300.2	85.00	0.38	tr_C9ZZ34_
DRB1_0101	758	HGLPCDIIRRYTGDI	CDIIRRYTG	4	0.2830	2340.5	85.00	0.39	tr_C9ZZ34_
DRB1_0101	759	PNDKKTGYAPNRLCD	KTGYAPNRL	4	0.2830	2340.9	85.00	0.44	tr_C9ZZ34_
DRB1_0101	760	THQRSSSACGVMPEC	QRSSSACGV	2	0.2819	2368.3	85.00	0.53	tr_C9ZZ34_
DRB1_0101	761	CSTGSTTLKTCFGTP	STTLKTCFG	4	0.2811	2389.6	85.00	0.62	tr_C9ZZ34_
DRB1_0101	762	DFVRQCLKRDAKDRP	FVRQCLKRD	1	0.2808	2395.2	85.00	0.43	tr_C9ZZ34_
DRB1_0101	763	KYLAQEHEGEKMNSS	YLAQEHEGE	1	0.2802	2411.6	85.00	0.52	tr_C9ZZ34_
DRB1_0101	764	LSDFGGAVECAMQPP	FGGAVECAM	3	0.2793	2435.6	85.00	0.36	tr_C9ZZ34_
DRB1_0101	765	ERECVGSSREQYAPL	VGSSREQYA	4	0.2788	2448.5	85.00	0.47	tr_C9ZZ34_
DRB1_0101	766	SKLRRGCISPEPPFD	LRRGCISPE	2	0.2788	2449.3	85.00	0.34	tr_C9ZZ34_
DRB1_0101	767	VGAGSFGRVYRGLHP	GSFGRVYRG	3	0.2770	2495.7	85.00	0.25	tr_C9ZZ34_
DRB1_0101	768	MNATSLSVSQYPFND	TSLSVSQYP	3	0.2767	2503.6	85.00	0.27	tr_C9ZZ34_
DRB1_0101	769	VEPGCAQEISEEYRL	CAQEISEEY	4	0.2765	2509.7	85.00	0.40	tr_C9ZZ34_
DRB1_0101	770	PKVNTRYENFHELAG	TRYENFHEL	4	0.2762	2519.0	85.00	0.62	tr_C9ZZ34_
DRB1_0101	771	GAGSFGRVYRGLHPD	GRVYRGLHP	5	0.2760	2524.4	85.00	0.41	tr_C9ZZ34_
DRB1_0101	772	CDVEGHPLKTNLHKH	GHPLKTNLH	4	0.2758	2530.4	85.00	0.43	tr_C9ZZ34_
DRB1_0101	773	EGHPLKTNLHKHADP	GHPLKTNLH	1	0.2754	2538.9	90.00	0.38	tr_C9ZZ34_
DRB1_0101	774	TNFSQEVTGRKEDAQ	FSQEVTGRK	2	0.2737	2586.0	90.00	0.73	tr_C9ZZ34_
DRB1_0101	775	SDFGGAVECAMQPPT	GAVECAMQP	4	0.2737	2587.8	90.00	0.35	tr_C9ZZ34_
DRB1_0101	776	VLDSSCNIPKVNTRY	LDSSCNIPK	1	0.2723	2628.0	90.00	0.41	tr_C9ZZ34_
DRB1_0101	777	QPNDKKTGYAPNRLC	KTGYAPNRL	5	0.2720	2634.1	90.00	0.41	tr_C9ZZ34_
DRB1_0101	778	KTGYAPNRLCDHKGG	YAPNRLCDH	3	0.2720	2636.6	90.00	0.26	tr_C9ZZ34_
DRB1_0101	779	PNRLCDHKGGSTNFV	HKGGSTNFV	6	0.2710	2663.2	90.00	0.34	tr_C9ZZ34_
DRB1_0101	780	VREARASDSTSLSTQ	ARASDSTSL	3	0.2710	2664.1	90.00	0.45	tr_C9ZZ34_
DRB1_0101	781	HEGEKMNSSLSDITR	MNSSLSDIT	5	0.2694	2710.1	90.00	0.37	tr_C9ZZ34_
DRB1_0101	782	HTPSGPNSVNLVRPR	SGPNSVNLV	3	0.2694	2711.4	90.00	0.58	tr_C9ZZ34_
DRB1_0101	783	QYPFNDVVEPGCAQE	DVVEPGCAQ	5	0.2675	2766.8	90.00	0.37	tr_C9ZZ34_
DRB1_0101	784	SLSVSQYPFNDVVEP	SVSQYPFND	2	0.2671	2779.6	90.00	0.29	tr_C9ZZ34_
DRB1_0101	785	NKAERLEFDIYGPVG	LEFDIYGPV	5	0.2653	2834.7	90.00	0.41	tr_C9ZZ34_
DRB1_0101	786	VCMSPEACMGVVDPR	VCMSPEACM	0	0.2648	2849.5	90.00	0.28	tr_C9ZZ34_
DRB1_0101	787	VSSSPLLKNLDRSNK	LLKNLDRSN	5	0.2640	2873.2	90.00	0.32	tr_C9ZZ34_
DRB1_0101	788	GDCYNKAERLEFDIY	DCYNKAERL	1	0.2637	2883.5	90.00	0.37	tr_C9ZZ34_
DRB1_0101	789	YLYDPKAPKERECVG	YDPKAPKER	2	0.2636	2886.6	90.00	0.44	tr_C9ZZ34_
DRB1_0101	790	WCAVDREAKKFIRKK	DREAKKFIR	4	0.2620	2937.3	90.00	0.34	tr_C9ZZ34_
DRB1_0101	791	ARASDSTSLSTQQSD	STSLSTQQS	5	0.2619	2939.6	90.00	0.64	tr_C9ZZ34_
DRB1_0101	792	KVNTRYENFHELAGP	TRYENFHEL	3	0.2616	2948.2	90.00	0.61	tr_C9ZZ34_
DRB1_0101	793	SSLSDITRYVREGPQ	SLSDITRYV	1	0.2593	3024.7	90.00	0.62	tr_C9ZZ34_
DRB1_0101	794	IQNGDCYNKAERLEF	DCYNKAERL	4	0.2588	3040.5	90.00	0.49	tr_C9ZZ34_
DRB1_0101	795	LLKNLDRSNKFSTPT	LKNLDRSNK	1	0.2586	3045.6	90.00	0.44	tr_C9ZZ34_
DRB1_0101	796	KRTMVLTTESDYCST	LTTESDYCS	5	0.2578	3072.7	90.00	0.28	tr_C9ZZ34_
DRB1_0101	797	PRPRELKVCCDVEGH	PRELKVCCD	2	0.2558	3139.6	90.00	0.41	tr_C9ZZ34_
DRB1_0101	798	LKRDAKDRPSASELL	DRPSASELL	6	0.2558	3141.6	90.00	0.49	tr_C9ZZ34_
DRB1_0101	799	CGGDVRIYLYDPKAP	RIYLYDPKA	5	0.2550	3166.9	90.00	0.39	tr_C9ZZ34_
DRB1_0101	800	CAVDREAKKFIRKKI	DREAKKFIR	3	0.2550	3168.0	90.00	0.40	tr_C9ZZ34_
DRB1_0101	801	QWCAVDREAKKFIRK	DREAKKFIR	5	0.2544	3186.5	90.00	0.25	tr_C9ZZ34_
DRB1_0101	802	DSATTNFSQEVTGRK	FSQEVTGRK	6	0.2541	3197.3	90.00	0.42	tr_C9ZZ34_
DRB1_0101	803	HQRSSSACGVMPECN	QRSSSACGV	1	0.2540	3201.9	90.00	0.47	tr_C9ZZ34_
DRB1_0101	804	RTMVLTTESDYCSTG	LTTESDYCS	4	0.2537	3213.4	90.00	0.38	tr_C9ZZ34_
DRB1_0101	805	SQYPFNDVVEPGCAQ	FNDVVEPGC	4	0.2533	3226.0	90.00	0.26	tr_C9ZZ34_
DRB1_0101	806	IPKVNTRYENFHELA	TRYENFHEL	5	0.2533	3226.2	90.00	0.58	tr_C9ZZ34_

DRB1_0101	807	VTAVPVDVIHRRTCG	VTAVPVDVI	0	0.2511	3303.1	90.00	0.41	tr_C9ZZ34_
DRB1_0101	808	LPRPRELKVCCDVEG	PRELKVCCD	3	0.2507	3320.0	90.00	0.49	tr_C9ZZ34_
DRB1_0101	809	DCYNKAERLEFDIYG	DCYNKAERL	0	0.2502	3336.7	90.00	0.28	tr_C9ZZ34_
DRB1_0101	810	KSPTRKRTMVLTTES	PTRKRTMVL	2	0.2502	3337.2	90.00	0.40	tr_C9ZZ34_
DRB1_0101	811	QNGDCYNKAERLEFD	DCYNKAERL	3	0.2497	3353.9	90.00	0.52	tr_C9ZZ34_
DRB1_0101	812	SRFGQCECHVSPQYY	FGQCECHVS	2	0.2490	3380.1	90.00	0.31	tr_C9ZZ34_
DRB1_0101	813	NGDCYNKAERLEFDI	DCYNKAERL	2	0.2485	3397.5	90.00	0.46	tr_C9ZZ34_
DRB1_0101	814	EKSPTRKRTMVLTTE	PTRKRTMVL	3	0.2481	3414.6	90.00	0.47	tr_C9ZZ34_
DRB1_0101	815	HDRQIVHRDISPNNV	HRDISPNNV	6	0.2477	3428.1	90.00	0.35	tr_C9ZZ34_
DRB1_0101	816	RSSSACGVMPECNYK	CGVMPECNY	5	0.2476	3430.7	90.00	0.44	tr_C9ZZ34_
DRB1_0101	817	YLHDRQIVHRDISPN	DRQIVHRDI	3	0.2468	3460.9	90.00	0.25	tr_C9ZZ34_
DRB1_0101	818	VPVDVIHRRTCGGDV	HRRTCGGDV	6	0.2468	3461.0	90.00	0.37	tr_C9ZZ34_
DRB1_0101	819	RPRELKVCCDVEGHP	PRELKVCCD	1	0.2452	3521.5	90.00	0.38	tr_C9ZZ34_
DRB1_0101	820	RKRTMVLTTESDYCS	TMVLTTESD	3	0.2452	3522.3	90.00	0.41	tr_C9ZZ34_
DRB1_0101	821	DEKSPTRKRTMVLTT	PTRKRTMVL	4	0.2450	3528.0	90.00	0.44	tr_C9ZZ34_
DRB1_0101	822	TPSGPNSVNLVRPRD	SGPNSVNLV	2	0.2436	3582.6	90.00	0.62	tr_C9ZZ34_
DRB1_0101	823	FSRKEASDAGNKERS	FSRKEASDA	0	0.2429	3610.7	90.00	0.65	tr_C9ZZ34_
DRB1_0101	824	GHPLKTNLHKHADPD	LKTNLHKHA	3	0.2425	3624.7	90.00	0.44	tr_C9ZZ34_
DRB1_0101	825	FVRQCLKRDAKDRPS	CLKRDAKDR	4	0.2422	3639.2	90.00	0.28	tr_C9ZZ34_
DRB1_0101	826	TIQNGDCYNKAERLE	DCYNKAERL	5	0.2417	3656.5	90.00	0.44	tr_C9ZZ34_
DRB1_0101	827	TCGGDVRIYLYDPKA	RIYLYDPKA	6	0.2409	3690.0	90.00	0.31	tr_C9ZZ34_
DRB1_0101	828	ACMGVVDPRNDIWGL	MGVVDPRND	2	0.2390	3765.2	95.00	0.28	tr_C9ZZ34_
DRB1_0101	829	PSMDENNPEIQSTLS	MDENNPEIQ	2	0.2383	3795.3	95.00	0.55	tr_C9ZZ34_
DRB1_0101	830	QRSSSACGVMPECNY	SSSACGVMP	2	0.2383	3796.7	95.00	0.29	tr_C9ZZ34_
DRB1_0101	831	EHEGEKMNSSLSDIT	GEKMNSSLS	3	0.2379	3809.8	95.00	0.47	tr_C9ZZ34_
DRB1_0101	832	CAMQPPTSEDNVTLT	CAMQPPTSE	0	0.2376	3823.3	95.00	0.52	tr_C9ZZ34_
DRB1_0101	833	LSVSQYPFNDVVEPG	YPFNDVVEP	5	0.2375	3830.0	95.00	0.28	tr_C9ZZ34_
DRB1_0101	834	VNTRYENFHELAGPD	TRYENFHEL	2	0.2368	3858.1	95.00	0.57	tr_C9ZZ34_
DRB1_0101	835	YNKAERLEFDIYGPV	LEFDIYGPV	6	0.2363	3876.8	95.00	0.30	tr_C9ZZ34_
DRB1_0101	836	VPSMDENNPEIQSTL	MDENNPEIQ	3	0.2363	3878.5	95.00	0.59	tr_C9ZZ34_
DRB1_0101	837	NTIQNGDCYNKAERL	DCYNKAERL	6	0.2349	3936.2	95.00	0.27	tr_C9ZZ34_
DRB1_0101	838	CKTKSDFSTSNSDQL	FSTSNSDQL	6	0.2346	3949.8	95.00	0.62	tr_C9ZZ34_
DRB1_0101	839	TMVLTTESDYCSTGS	LTTESDYCS	3	0.2344	3958.1	95.00	0.50	tr_C9ZZ34_
DRB1_0101	840	ALPRPRELKVCCDVE	PRELKVCCD	4	0.2344	3960.0	95.00	0.50	tr_C9ZZ34_
DRB1_0101	841	DENNPEIQSTLSEIR	IQSTLSEIR	6	0.2321	4060.0	95.00	0.38	tr_C9ZZ34_
DRB1_0101	842	LDSSCNIPKVNTRYE	NIPKVNTRY	5	0.2306	4125.2	95.00	0.27	tr_C9ZZ34_
DRB1_0101	843	VACSYPWSQEDTADV	WSQEDTADV	6	0.2303	4138.9	95.00	0.49	tr_C9ZZ34_
DRB1_0101	844	SMDENNPEIQSTLSE	MDENNPEIQ	1	0.2299	4155.9	95.00	0.49	tr_C9ZZ34_
DRB1_0101	845	AVDREAKKFIRKKIE	DREAKKFIR	2	0.2294	4178.5	95.00	0.41	tr_C9ZZ34_
DRB1_0101	846	EACMGVVDPRNDIWG	MGVVDPRND	3	0.2283	4228.7	95.00	0.31	tr_C9ZZ34_
DRB1_0101	847	SDEKSPTRKRTMVLT	PTRKRTMVL	5	0.2281	4236.4	95.00	0.48	tr_C9ZZ34_
DRB1_0101	848	RRTCGGDVRIYLYDP	TCGGDVRIY	2	0.2268	4295.5	95.00	0.25	tr_C9ZZ34_
DRB1_0101	849	VAIKEYPVPSMDENN	IKEYPVPSM	2	0.2266	4307.0	95.00	0.47	tr_C9ZZ34_
DRB1_0101	850	LSRFGQCECHVSPQY	FGQCECHVS	3	0.2264	4314.8	95.00	0.40	tr_C9ZZ34_
DRB1_0101	851	SPTRKRTMVLTTESD	PTRKRTMVL	1	0.2264	4317.7	95.00	0.35	tr_C9ZZ34_
DRB1_0101	852	PTRKRTMVLTTESDY	TMVLTTESD	5	0.2261	4328.5	95.00	0.31	tr_C9ZZ34_
DRB1_0101	853	PQWCAVDREAKKFIR	CAVDREAKK	3	0.2257	4347.5	95.00	0.25	tr_C9ZZ34_
DRB1_0101	854	MGVVDPRNDIWGLGI	GVVDPRNDI	1	0.2248	4392.3	95.00	0.20	tr_C9ZZ34_
DRB1_0101	855	PEACMGVVDPRNDIW	MGVVDPRND	4	0.2245	4403.8	95.00	0.26	tr_C9ZZ34_
DRB1_0101	856	NFSQEVTGRKEDAQE	FSQEVTGRK	1	0.2243	4414.1	95.00	0.70	tr_C9ZZ34_

DRB1_0101	857	CMGVVDPRNDIWGLG	MGVVDPRND	1	0.2238	4438.3	95.00	0.25	tr_C9ZZ34_
DRB1_0101	858	GVVDPRNDIWGLGIT	RNDIWGLGI	5	0.2223	4511.7	95.00	0.31	tr_C9ZZ34_
DRB1_0101	859	MMDLSRFGQCECHVS	MDLSRFGQC	1	0.2221	4523.5	95.00	0.23	tr_C9ZZ34_
DRB1_0101	860	EARASDSTSLSTQQS	STSLSTQQS	6	0.2217	4540.3	95.00	0.37	tr_C9ZZ34_
DRB1_0101	861	SQPNDKKTGYAPNRL	KTGYAPNRL	6	0.2195	4649.3	95.00	0.33	tr_C9ZZ34_
DRB1_0101	862	HELAGPDFAGGRVPL	HELAGPDFA	0	0.2176	4746.2	95.00	0.28	tr_C9ZZ34_
DRB1_0101	863	VSQYPFNDVVEPGCA	YPFNDVVEP	3	0.2173	4761.4	95.00	0.38	tr_C9ZZ34_
DRB1_0101	864	TGYAPNRLCDHKGGS	YAPNRLCDH	2	0.2155	4857.9	95.00	0.29	tr_C9ZZ34_
DRB1_0101	865	TRKRTMVLTTESDYC	TMVLTTESD	4	0.2151	4875.6	95.00	0.41	tr_C9ZZ34_
DRB1_0101	866	LHDRQIVHRDISPNN	DRQIVHRDI	2	0.2136	4955.5	95.00	0.25	tr_C9ZZ34_
DRB1_0101	867	AMMDLSRFGQCECHV	MDLSRFGQC	2	0.2123	5029.3	95.00	0.32	tr_C9ZZ34_
DRB1_0101	868	MDLSRFGQCECHVSP	FGQCECHVS	5	0.2122	5034.6	95.00	0.34	tr_C9ZZ34_
DRB1_0101	869	RTCGGDVRIYLYDPK	GGDVRIYLY	3	0.2122	5035.2	95.00	0.20	tr_C9ZZ34_
DRB1_0101	870	MDENNPEIQSTLSEI	NNPEIQSTL	3	0.2112	5089.1	95.00	0.31	tr_C9ZZ34_
DRB1_0101	871	QEHEGEKMNSSLSDI	GEKMNSSLS	4	0.2105	5126.2	95.00	0.50	tr_C9ZZ34_
DRB1_0101	872	REARASDSTSLSTQQ	ARASDSTSL	2	0.2100	5155.9	95.00	0.60	tr_C9ZZ34_
DRB1_0101	873	KERECVGSSREQYAP	VGSSREQYA	5	0.2097	5171.1	95.00	0.47	tr_C9ZZ34_
DRB1_0101	874	NIPKVNTRYENFHEL	TRYENFHEL	6	0.2081	5262.9	95.00	0.44	tr_C9ZZ34_
DRB1_0101	875	YLAQEHEGEKMNSSL	YLAQEHEGE	0	0.2070	5323.6	95.00	0.34	tr_C9ZZ34_
DRB1_0101	876	SPEACMGVVDPRNDI	MGVVDPRND	5	0.2066	5348.9	95.00	0.20	tr_C9ZZ34_
DRB1_0101	877	SSCNIPKVNTRYENF	NIPKVNTRY	3	0.2057	5400.1	95.00	0.44	tr_C9ZZ34_
DRB1_0101	878	LKNLDRSNKFSTPTA	KNLDRSNKF	1	0.2050	5442.8	95.00	0.25	tr_C9ZZ34_
DRB1_0101	879	VDREAKKFIRKKIEK	DREAKKFIR	1	0.2048	5451.1	95.00	0.35	tr_C9ZZ34_
DRB1_0101	880	CMSPEACMGVVDPRN	PEACMGVVD	3	0.2044	5478.2	95.00	0.26	tr_C9ZZ34_
DRB1_0101	881	APNRLCDHKGGSTNF	LCDHKGGST	4	0.2034	5534.7	95.00	0.34	tr_C9ZZ34_
DRB1_0101	882	DSSCNIPKVNTRYEN	NIPKVNTRY	4	0.2030	5562.9	95.00	0.40	tr_C9ZZ34_
DRB1_0101	883	GYAPNRLCDHKGGST	PNRLCDHKG	3	0.2019	5628.9	95.00	0.37	tr_C9ZZ34_
DRB1_0101	884	YAPNRLCDHKGGSTN	PNRLCDHKG	2	0.2010	5684.6	95.00	0.35	tr_C9ZZ34_
DRB1_0101	885	PVPSMDENNPEIQST	MDENNPEIQ	4	0.2001	5739.1	95.00	0.62	tr_C9ZZ34_
DRB1_0101	886	SCNIPKVNTRYENFH	NIPKVNTRY	2	0.1991	5801.7	95.00	0.40	tr_C9ZZ34_
DRB1_0101	887	SVSQYPFNDVVEPGC	YPFNDVVEP	4	0.1990	5807.8	95.00	0.35	tr_C9ZZ34_
DRB1_0101	888	MVLTTESDYCSTGST	LTTESDYCS	2	0.1986	5830.7	95.00	0.56	tr_C9ZZ34_
DRB1_0101	889	TSDEKSPTRKRTMVL	PTRKRTMVL	6	0.1979	5873.3	95.00	0.39	tr_C9ZZ34_
DRB1_0101	890	TAVPVDVIHRRTCGG	AVPVDVIHR	1	0.1972	5917.1	95.00	0.29	tr_C9ZZ34_
DRB1_0101	891	DLSRFGQCECHVSPQ	FGQCECHVS	4	0.1959	6005.3	95.00	0.41	tr_C9ZZ34_
DRB1_0101	892	GPQWCAVDREAKKFI	WCAVDREAK	3	0.1954	6038.8	95.00	0.32	tr_C9ZZ34_
DRB1_0101	893	IRTPSGRTSSLYGEE	IRTPSGRTS	0	0.1950	6060.2	95.00	0.30	tr_C9ZZ34_
DRB1_0101	894	MSPEACMGVVDPRND	PEACMGVVD	2	0.1947	6080.1	95.00	0.25	tr_C9ZZ34_
DRB1_0101	895	CYNKAERLEFDIYGP	YNKAERLEF	1	0.1943	6111.6	95.00	0.25	tr_C9ZZ34_
DRB1_0101	896	VREGPQWCAVDREAK	VREGPQWCA	0	0.1926	6224.1	100.00	0.47	tr_C9ZZ34_
DRB1_0101	897	ENTIQNGDCYNKAER	IQNGDCYNK	3	0.1912	6317.4	100.00	0.35	tr_C9ZZ34_
DRB1_0101	898	PKERECVGSSREQYA	VGSSREQYA	6	0.1907	6351.4	100.00	0.38	tr_C9ZZ34_
DRB1_0101	899	VRQCLKRDAKDRPSA	CLKRDAKDR	3	0.1903	6377.7	100.00	0.50	tr_C9ZZ34_
DRB1_0101	900	LYDPKAPKERECVGS	YDPKAPKER	1	0.1903	6380.0	100.00	0.48	tr_C9ZZ34_
DRB1_0101	901	LSDITRYVREGPQWC	YVREGPQWC	6	0.1893	6451.2	100.00	0.56	tr_C9ZZ34_
DRB1_0101	902	HPLKTNLHKHADPDK	LKTNLHKHA	2	0.1869	6619.6	100.00	0.54	tr_C9ZZ34_
DRB1_0101	903	VLTTESDYCSTGSTT	LTTESDYCS	1	0.1838	6846.2	100.00	0.45	tr_C9ZZ34_
DRB1_0101	904	RQCLKRDAKDRPSAS	CLKRDAKDR	2	0.1834	6874.6	100.00	0.45	tr_C9ZZ34_
DRB1_0101	905	AVPVDVIHRRTCGGD	VIHRRTCGG	5	0.1822	6963.6	100.00	0.27	tr_C9ZZ34_
DRB1_0101	906	PLKTNLHKHADPDKF	LKTNLHKHA	1	0.1819	6988.5	100.00	0.40	tr_C9ZZ34_

DRB1_0101	907	SSSCKTKSDFSTSNS	CKTKSDFST	3	0.1810	7056.7	100.00	0.53	tr_C9ZZ34_
DRB1_0101	908	AQEHEGEKMNSSLSD	GEKMNSSLS	5	0.1804	7103.4	100.00	0.40	tr_C9ZZ34_
DRB1_0101	909	YPVPSMDENNPEIQS	MDENNPEIQ	5	0.1794	7181.4	100.00	0.56	tr_C9ZZ34_
DRB1_0101	910	SLSDITRYVREGPQW	SLSDITRYV	0	0.1775	7324.1	100.00	0.48	tr_C9ZZ34_
DRB1_0101	911	GENTIQNGDCYNKAE	IQNGDCYNK	4	0.1752	7514.4	100.00	0.34	tr_C9ZZ34_
DRB1_0101	912	CNIPKVNTRYENFHE	NIPKVNTRY	1	0.1746	7562.4	100.00	0.34	tr_C9ZZ34_
DRB1_0101	913	AIKEYPVPSMDENNP	IKEYPVPSM	1	0.1744	7579.5	100.00	0.46	tr_C9ZZ34_
DRB1_0101	914	LAQEHEGEKMNSSLS	GEKMNSSLS	6	0.1726	7726.0	100.00	0.23	tr_C9ZZ34_
DRB1_0101	915	NVTLTSDEKSPTRKR	VTLTSDEKS	1	0.1694	7998.0	100.00	0.31	tr_C9ZZ34_
DRB1_0101	916	QCLKRDAKDRPSASE	CLKRDAKDR	1	0.1681	8111.9	100.00	0.36	tr_C9ZZ34_
DRB1_0101	917	CVACSYPWSQEDTAD	YPWSQEDTA	5	0.1675	8164.1	100.00	0.30	tr_C9ZZ34_
DRB1_0101	918	SSCKTKSDFSTSNSD	CKTKSDFST	2	0.1671	8197.5	100.00	0.51	tr_C9ZZ34_
DRB1_0101	919	ESSSCKTKSDFSTSN	CKTKSDFST	4	0.1670	8210.7	100.00	0.52	tr_C9ZZ34_
DRB1_0101	920	EGPQWCAVDREAKKF	WCAVDREAK	4	0.1646	8419.5	100.00	0.34	tr_C9ZZ34_
DRB1_0101	921	CLKRDAKDRPSASEL	DAKDRPSAS	4	0.1642	8457.4	100.00	0.28	tr_C9ZZ34_
DRB1_0101	922	LKTNLHKHADPDKFD	LHKHADPDK	4	0.1631	8566.2	100.00	0.28	tr_C9ZZ34_
DRB1_0101	923	DNVTLTSDEKSPTRK	VTLTSDEKS	2	0.1630	8571.3	100.00	0.38	tr_C9ZZ34_
DRB1_0101	924	FSQEVTGRKEDAQES	FSQEVTGRK	0	0.1599	8861.6	100.00	0.46	tr_C9ZZ34_
DRB1_0101	925	AVVAPPHGENTIQNG	AVVAPPHGE	0	0.1593	8925.1	100.00	0.31	tr_C9ZZ34_
DRB1_0101	926	KTNLHKHADPDKFDS	HKHADPDKF	4	0.1588	8965.1	100.00	0.36	tr_C9ZZ34_
DRB1_0101	927	HGENTIQNGDCYNKA	IQNGDCYNK	5	0.1579	9061.0	100.00	0.32	tr_C9ZZ34_
DRB1_0101	928	VTLTSDEKSPTRKRT	DEKSPTRKR	5	0.1577	9072.4	100.00	0.20	tr_C9ZZ34_
DRB1_0101	929	YDPKAPKERECVGSS	KAPKERECV	3	0.1557	9280.1	100.00	0.38	tr_C9ZZ34_
DRB1_0101	930	TNLHKHADPDKFDSA	HKHADPDKF	3	0.1547	9377.1	100.00	0.41	tr_C9ZZ34_
DRB1_0101	931	EDNVTLTSDEKSPTR	VTLTSDEKS	3	0.1546	9384.4	100.00	0.41	tr_C9ZZ34_
DRB1_0101	932	GRKEDAQESSSCKTK	DAQESSSCK	4	0.1534	9508.4	100.00	0.32	tr_C9ZZ34_
DRB1_0101	933	RKEDAQESSSCKTKS	DAQESSSCK	3	0.1528	9570.7	100.00	0.38	tr_C9ZZ34_
DRB1_0101	934	SCKTKSDFSTSNSDQ	CKTKSDFST	1	0.1505	9813.9	100.00	0.42	tr_C9ZZ34_
DRB1_0101	935	TLTSDEKSPTRKRTM	DEKSPTRKR	4	0.1495	9920.5	100.00	0.31	tr_C9ZZ34_
DRB1_0101	936	LTSDEKSPTRKRTMV	DEKSPTRKR	3	0.1487	10004.6	100.00	0.31	tr_C9ZZ34_
DRB1_0101	937	TGRKEDAQESSSCKT	GRKEDAQES	1	0.1462	10276.4	100.00	0.37	tr_C9ZZ34_
DRB1_0101	938	QESSSCKTKSDFSTS	CKTKSDFST	5	0.1449	10423.2	100.00	0.45	tr_C9ZZ34_
DRB1_0101	939	AMQPPTSEDNVTLTS	PTSEDNVTL	4	0.1435	10586.7	100.00	0.63	tr_C9ZZ34_
DRB1_0101	940	EYPVPSMDENNPEIQ	MDENNPEIQ	6	0.1433	10607.1	100.00	0.42	tr_C9ZZ34_
DRB1_0101	941	PKAPKERECVGSSRE	KAPKERECV	1	0.1433	10607.5	100.00	0.38	tr_C9ZZ34_
DRB1_0101	942	REGPQWCAVDREAKK	WCAVDREAK	5	0.1419	10771.6	100.00	0.36	tr_C9ZZ34_
DRB1_0101	943	SEDNVTLTSDEKSPT	NVTLTSDEK	3	0.1405	10930.8	100.00	0.43	tr_C9ZZ34_
DRB1_0101	944	VTGRKEDAQESSSCK	GRKEDAQES	2	0.1402	10966.8	100.00	0.44	tr_C9ZZ34_
DRB1_0101	945	KEDAQESSSCKTKSD	DAQESSSCK	2	0.1396	11041.8	100.00	0.36	tr_C9ZZ34_
DRB1_0101	946	NLHKHADPDKFDSAT	HKHADPDKF	2	0.1394	11060.7	100.00	0.46	tr_C9ZZ34_
DRB1_0101	947	KAPKERECVGSSREQ	ERECVGSSR	4	0.1387	11146.9	100.00	0.28	tr_C9ZZ34_
DRB1_0101	948	MQPPTSEDNVTLTSD	PTSEDNVTL	3	0.1371	11340.9	100.00	0.65	tr_C9ZZ34_
DRB1_0101	949	TSQPNDKKTGYAPNR	NDKKTGYAP	4	0.1358	11506.5	100.00	0.43	tr_C9ZZ34_
DRB1_0101	950	DPKAPKERECVGSSR	KAPKERECV	2	0.1357	11514.0	100.00	0.39	tr_C9ZZ34_
DRB1_0101	951	QEVTGRKEDAQESSS	GRKEDAQES	4	0.1335	11794.3	100.00	0.51	tr_C9ZZ34_
DRB1_0101	952	EVTGRKEDAQESSSC	GRKEDAQES	3	0.1323	11953.8	100.00	0.53	tr_C9ZZ34_
DRB1_0101	953	APKERECVGSSREQY	ERECVGSSR	3	0.1320	11990.1	100.00	0.33	tr_C9ZZ34_
DRB1_0101	954	PTSEDNVTLTSDEKS	NVTLTSDEK	5	0.1265	12722.1	100.00	0.41	tr_C9ZZ34_
DRB1_0101	955	KHADPDKFDSATTNF	PDKFDSATT	4	0.1260	12790.9	100.00	0.44	tr_C9ZZ34_
DRB1_0101	956	LHKHADPDKFDSATT	HKHADPDKF	1	0.1251	12920.6	100.00	0.41	tr_C9ZZ34_

DRB1_0101	957	IKEYPVPSMDENNPE	IKEYPVPSM	0	0.1246	12987.5	100.00	0.35	tr_C9ZZ34_
DRB1_0101	958	SQEVTGRKEDAQESS	GRKEDAQES	5	0.1234	13150.7	100.00	0.41	tr_C9ZZ34_
DRB1_0101	959	NKERSETSQPNDKKT	SETSQPNDK	4	0.1226	13277.1	100.00	0.33	tr_C9ZZ34_
DRB1_0101	960	KERSETSQPNDKKTG	SETSQPNDK	3	0.1217	13401.8	100.00	0.35	tr_C9ZZ34_
DRB1_0101	961	EDAQESSSCKTKSDF	DAQESSSCK	1	0.1213	13460.9	100.00	0.29	tr_C9ZZ34_
DRB1_0101	962	TSEDNVTLTSDEKSP	NVTLTSDEK	4	0.1205	13570.1	100.00	0.47	tr_C9ZZ34_
DRB1_0101	963	QPPTSEDNVTLTSDE	PTSEDNVTL	2	0.1198	13673.5	100.00	0.66	tr_C9ZZ34_
DRB1_0101	964	GNKERSETSQPNDKK	ERSETSQPN	3	0.1175	14019.5	100.00	0.37	tr_C9ZZ34_
DRB1_0101	965	AQESSSCKTKSDFST	CKTKSDFST	6	0.1166	14161.6	100.00	0.26	tr_C9ZZ34_
DRB1_0101	966	PPTSEDNVTLTSDEK	PTSEDNVTL	1	0.1163	14211.0	100.00	0.45	tr_C9ZZ34_
DRB1_0101	967	ETSQPNDKKTGYAPN	NDKKTGYAP	5	0.1143	14520.6	100.00	0.32	tr_C9ZZ34_
DRB1_0101	968	PHGENTIQNGDCYNK	NTIQNGDCY	4	0.1110	15044.7	100.00	0.35	tr_C9ZZ34_
DRB1_0101	969	HKHADPDKFDSATTN	PDKFDSATT	5	0.1109	15055.3	100.00	0.26	tr_C9ZZ34_
DRB1_0101	970	RSETSQPNDKKTGYA	ETSQPNDKK	2	0.1077	15590.9	100.00	0.29	tr_C9ZZ34_
DRB1_0101	971	SRKEASDAGNKERSE	EASDAGNKE	3	0.1070	15708.6	100.00	0.31	tr_C9ZZ34_
DRB1_0101	972	ERSETSQPNDKKTGY	SETSQPNDK	2	0.1063	15826.5	100.00	0.32	tr_C9ZZ34_
DRB1_0101	973	AGNKERSETSQPNDK	ERSETSQPN	4	0.1052	16027.4	100.00	0.36	tr_C9ZZ34_
DRB1_0101	974	DAQESSSCKTKSDFS	AQESSSCKT	1	0.1048	16083.7	100.00	0.31	tr_C9ZZ34_
DRB1_0101	975	SETSQPNDKKTGYAP	PNDKKTGYA	5	0.1018	16614.9	100.00	0.25	tr_C9ZZ34_
DRB1_0101	976	VVAPPHGENTIQNGD	VAPPHGENT	1	0.1001	16931.7	100.00	0.41	tr_C9ZZ34_
DRB1_0101	977	KEYPVPSMDENNPEI	EYPVPSMDE	1	0.0953	17837.4	100.00	0.31	tr_C9ZZ34_
DRB1_0101	978	RKEASDAGNKERSET	EASDAGNKE	2	0.0941	18057.4	100.00	0.34	tr_C9ZZ34_
DRB1_0101	979	KEASDAGNKERSETS	EASDAGNKE	1	0.0893	19034.4	100.00	0.22	tr_C9ZZ34_
DRB1_0101	980	EASDAGNKERSETSQ	GNKERSETS	5	0.0873	19432.3	100.00	0.41	tr_C9ZZ34_
DRB1_0101	981	SDAGNKERSETSQPN	GNKERSETS	3	0.0866	19579.6	100.00	0.46	tr_C9ZZ34_
DRB1_0101	982	DAGNKERSETSQPND	GNKERSETS	2	0.0855	19820.7	100.00	0.45	tr_C9ZZ34_
DRB1_0101	983	PPHGENTIQNGDCYN	NTIQNGDCY	5	0.0841	20130.8	100.00	0.34	tr_C9ZZ34_
DRB1_0101	984	ASDAGNKERSETSQP	GNKERSETS	4	0.0799	21063.3	100.00	0.47	tr_C9ZZ34_
DRB1_0101	985	VAPPHGENTIQNGDC	PHGENTIQN	3	0.0794	21179.0	100.00	0.27	tr_C9ZZ34_
DRB1_0101	986	APPHGENTIQNGDCY	PHGENTIQN	2	0.0736	22546.0	100.00	0.26	tr_C9ZZ34_

Allele: DRB1_0101. Number of high binders 0. Number of weak binders 51. Number of peptides 986

Allele	pos	peptide	core	Of	1-log50k(aff)	affinity(nM)	%Rank	Relia	Identity	Bind Level
DRB1_0101	1	SSFNDLWALPLQLSL	FNDLWALPL	2	0.8291	6.4	1.10	0.71	tr_C9ZMP2_	WB
DRB1_0101	2	ASSFNDLWALPLQLS	FNDLWALPL	3	0.8274	6.5	1.10	0.79	tr_C9ZMP2_	WB
DRB1_0101	3	SFNDLWALPLQLSLA	FNDLWALPL	1	0.8189	7.1	1.40	0.60	tr_C9ZMP2_	WB
DRB1_0101	4	AASSFNDLWALPLQL	FNDLWALPL	4	0.8017	8.5	2.00	0.86	tr_C9ZMP2_	WB
DRB1_0101	5	IVLYIRGLLTSELFR	IRGLLTSEL	4	0.7982	8.9	2.50	0.65	tr_C9ZMP2_	WB
DRB1_0101	6	KIVLYIRGLLTSELF	IRGLLTSEL	5	0.7869	10.0	3.00	0.51	tr_C9ZMP2_	WB
DRB1_0101	7	VLYIRGLLTSELFRC	IRGLLTSEL	3	0.7855	10.2	3.00	0.68	tr_C9ZMP2_	WB
DRB1_0101	8	LYIRGLLTSELFRCT	IRGLLTSEL	2	0.7661	12.6	4.00	0.73	tr_C9ZMP2_	WB
DRB1_0101	9	DAASSFNDLWALPLQ	FNDLWALPL	5	0.7611	13.3	4.00	0.91	tr_C9ZMP2_	WB
DRB1_0101	10	QKIVLYIRGLLTSEL	VLYIRGLLT	3	0.7565	13.9	4.50	0.44	tr_C9ZMP2_	WB
DRB1_0101	11	IMNHLSLDVARVADA	HLSLDVARV	3	0.7469	15.5	5.00	0.54	tr_C9ZMP2_	WB
DRB1_0101	12	DIMNHLSLDVARVAD	HLSLDVARV	4	0.7410	16.5	5.50	0.47	tr_C9ZMP2_	WB

DRB1_0101	13	YIRGLLTSELFRCTL	IRGLLTSEL	1	0.7409	16.5	5.50	0.69	tr_C9ZMP2_	WB
DRB1_0101	14	GDIMNHLSLDVARVA	HLSLDVARV	5	0.7379	17.1	6.00	0.46	tr_C9ZMP2_	WB
DRB1_0101	15	MNHLSLDVARVADAA	HLSLDVARV	2	0.7308	18.4	6.50	0.51	tr_C9ZMP2_	WB
DRB1_0101	16	NHLSLDVARVADAAS	HLSLDVARV	1	0.7079	23.6	8.50	0.43	tr_C9ZMP2_	WB
DRB1_0101	17	QGDIMNHLSLDVARV	HLSLDVARV	6	0.6843	30.4	11.00	0.37	tr_C9ZMP2_	
DRB1_0101	18	ADAASSFNDLWALPL	FNDLWALPL	6	0.6810	31.5	11.00	0.86	tr_C9ZMP2_	
DRB1_0101	19	LQKIVLYIRGLLTSE	VLYIRGLLT	4	0.6756	33.4	12.00	0.74	tr_C9ZMP2_	
DRB1_0101	20	HYRVGLQKIVLYIRG	YRVGLQKIV	1	0.6603	39.5	14.00	0.55	tr_C9ZMP2_	
DRB1_0101	21	HLSLDVARVADAASS	LDVARVADA	3	0.6602	39.5	14.00	0.40	tr_C9ZMP2_	
DRB1_0101	22	GLQKIVLYIRGLLTS	VLYIRGLLT	5	0.6584	40.3	14.00	0.68	tr_C9ZMP2_	
DRB1_0101	23	IRGLLTSELFRCTLC	IRGLLTSEL	0	0.6470	45.6	15.00	0.51	tr_C9ZMP2_	
DRB1_0101	24	VGLQKIVLYIRGLLT	VLYIRGLLT	6	0.6312	54.1	18.00	0.60	tr_C9ZMP2_	
DRB1_0101	25	YRVGLQKIVLYIRGL	YRVGLQKIV	0	0.5945	80.4	23.00	0.34	tr_C9ZMP2_	
DRB1_0101	26	TQGDIMNHLSLDVAR	IMNHLSLDV	4	0.5875	86.8	25.00	0.44	tr_C9ZMP2_	
DRB1_0101	27	LSLDVARVADAASSF	LDVARVADA	2	0.5470	134.5	32.00	0.60	tr_C9ZMP2_	
DRB1_0101	28	RTQGDIMNHLSLDVA	IMNHLSLDV	5	0.5300	161.6	35.00	0.41	tr_C9ZMP2_	
DRB1_0101	29	RVGLQKIVLYIRGLL	KIVLYIRGL	5	0.5162	187.7	38.00	0.25	tr_C9ZMP2_	
DRB1_0101	30	RGLLTSELFRCTLCR	LTSELFRCT	3	0.5094	201.9	39.00	0.35	tr_C9ZMP2_	
DRB1_0101	31	SELFRCTLCRRKHLF	FRCTLCRRK	3	0.4760	289.8	46.00	0.40	tr_C9ZMP2_	
DRB1_0101	32	SLDVARVADAASSFN	LDVARVADA	1	0.4756	291.1	46.00	0.60	tr_C9ZMP2_	
DRB1_0101	33	TSELFRCTLCRRKHL	FRCTLCRRK	4	0.4489	388.7	55.00	0.41	tr_C9ZMP2_	
DRB1_0101	34	ELFRCTLCRRKHLFD	FRCTLCRRK	2	0.4477	394.0	55.00	0.39	tr_C9ZMP2_	
DRB1_0101	35	GLLTSELFRCTLCRR	LTSELFRCT	2	0.4402	427.0	55.00	0.35	tr_C9ZMP2_	
DRB1_0101	36	LFRCTLCRRKHLFDG	CTLCRRKHL	3	0.4292	480.8	60.00	0.39	tr_C9ZMP2_	
DRB1_0101	37	GRTQGDIMNHLSLDV	IMNHLSLDV	6	0.4234	512.2	60.00	0.35	tr_C9ZMP2_	
DRB1_0101	38	LLTSELFRCTLCRRK	LTSELFRCT	1	0.4183	541.5	60.00	0.28	tr_C9ZMP2_	
DRB1_0101	39	LTSELFRCTLCRRKH	FRCTLCRRK	5	0.4069	612.3	65.00	0.37	tr_C9ZMP2_	
DRB1_0101	40	FRCTLCRRKHLFDGR	CTLCRRKHL	2	0.3954	693.1	65.00	0.43	tr_C9ZMP2_	
DRB1_0101	41	LDVARVADAASSFND	LDVARVADA	0	0.3720	892.8	70.00	0.44	tr_C9ZMP2_	
DRB1_0101	42	RCTLCRRKHLFDGRT	CTLCRRKHL	1	0.3340	1347.7	80.00	0.47	tr_C9ZMP2_	
DRB1_0101	43	FDGRTQGDIMNHLSL	TQGDIMNHL	4	0.3210	1550.6	80.00	0.29	tr_C9ZMP2_	
DRB1_0101	44	DGRTQGDIMNHLSLD	TQGDIMNHL	3	0.3206	1557.8	80.00	0.29	tr_C9ZMP2_	
DRB1_0101	45	VARVADAASSFNDLW	DAASSFNDL	5	0.3019	1906.3	85.00	0.29	tr_C9ZMP2_	
DRB1_0101	46	RVADAASSFNDLWAL	DAASSFNDL	3	0.2963	2026.2	85.00	0.47	tr_C9ZMP2_	
DRB1_0101	47	LFDGRTQGDIMNHLS	FDGRTQGDI	1	0.2941	2074.1	85.00	0.32	tr_C9ZMP2_	
DRB1_0101	48	VADAASSFNDLWALP	DAASSFNDL	2	0.2928	2103.7	85.00	0.45	tr_C9ZMP2_	
DRB1_0101	49	DVARVADAASSFNDL	ARVADAASS	2	0.2914	2137.5	85.00	0.28	tr_C9ZMP2_	
DRB1_0101	50	HLFDGRTQGDIMNHL	FDGRTQGDI	2	0.2909	2148.7	85.00	0.46	tr_C9ZMP2_	
DRB1_0101	51	ARVADAASSFNDLWA	DAASSFNDL	4	0.2893	2186.0	85.00	0.40	tr_C9ZMP2_	
DRB1_0101	52	KHLFDGRTQGDIMNH	FDGRTQGDI	3	0.2847	2297.9	85.00	0.55	tr_C9ZMP2_	
DRB1_0101	53	CTLCRRKHLFDGRTQ	CTLCRRKHL	0	0.2736	2589.8	90.00	0.36	tr_C9ZMP2_	
DRB1_0101	54	RKHLFDGRTQGDIMN	FDGRTQGDI	4	0.2720	2634.1	90.00	0.50	tr_C9ZMP2_	
DRB1_0101	55	RRKHLFDGRTQGDIM	FDGRTQGDI	5	0.2547	3178.9	90.00	0.49	tr_C9ZMP2_	
DRB1_0101	56	CRRKHLFDGRTQGDI	FDGRTQGDI	6	0.2116	5063.5	95.00	0.34	tr_C9ZMP2_	
DRB1_0101	57	TLCRRKHLFDGRTQG	CRRKHLFDG	2	0.1894	6439.3	100.00	0.22	tr_C9ZMP2_	
DRB1_0101	58	LCRRKHLFDGRTQGD	KHLFDGRTQ	4	0.1644	8440.1	100.00	0.22	tr_C9ZMP2_	

Allele: DRB1_0101. Number of high binders 0. Number of weak binders 16. Number of peptides 58

Allele	pos	peptide	core	Of	1-log50k(aff)	affinity(nM)	%Rank	Relia	Identity	Bind Level
DRB1_0101	1	QRARVALARALYAGK	VALARALYA	4	0.8762	3.8	0.25	0.84	tr_C9ZMP2_	SB
DRB1_0101	2	RARVALARALYAGKE	VALARALYA	3	0.8747	3.9	0.30	0.88	tr_C9ZMP2_	SB
DRB1_0101	3	GQRARVALARALYAG	VALARALYA	5	0.8573	4.7	0.50	0.77	tr_C9ZMP2_	WB
DRB1_0101	4	ARVALARALYAGKEL	VALARALYA	2	0.8465	5.3	0.70	0.89	tr_C9ZMP2_	WB
DRB1_0101	5	GGQRARVALARALYA	VALARALYA	6	0.8207	7.0	1.30	0.64	tr_C9ZMP2_	WB
DRB1_0101	6	SYVSWCRLSPFLVMR	WCRLSPFLV	4	0.8124	7.6	1.60	0.75	tr_C9ZMP2_	WB
DRB1_0101	7	RVALARALYAGKELY	VALARALYA	1	0.8116	7.7	1.60	0.90	tr_C9ZMP2_	WB
DRB1_0101	8	YVSWCRLSPFLVMRP	WCRLSPFLV	3	0.8032	8.4	1.90	0.75	tr_C9ZMP2_	WB
DRB1_0101	9	ALALFNSLTMPLNAY	FNSLTMPLN	4	0.7925	9.4	2.50	0.65	tr_C9ZMP2_	WB
DRB1_0101	10	VSWCRLSPFLVMRPD	WCRLSPFLV	2	0.7861	10.1	3.00	0.74	tr_C9ZMP2_	WB
DRB1_0101	11	ESYVSWCRLSPFLVM	WCRLSPFLV	5	0.7850	10.2	3.00	0.74	tr_C9ZMP2_	WB
DRB1_0101	12	LALFNSLTMPLNAYP	FNSLTMPLN	3	0.7828	10.5	3.00	0.66	tr_C9ZMP2_	WB
DRB1_0101	13	TALALFNSLTMPLNA	FNSLTMPLN	5	0.7739	11.5	3.50	0.60	tr_C9ZMP2_	WB
DRB1_0101	14	STAYVEQQPFLMSGT	YVEQQPFLM	3	0.7703	12.0	3.50	0.89	tr_C9ZMP2_	WB
DRB1_0101	15	ALFNSLTMPLNAYPC	FNSLTMPLN	2	0.7680	12.3	4.00	0.64	tr_C9ZMP2_	WB
DRB1_0101	16	LACYIGRVGWKLSAF	YIGRVGWKL	3	0.7667	12.5	4.00	0.87	tr_C9ZMP2_	WB
DRB1_0101	17	SWCRLSPFLVMRPDA	WCRLSPFLV	1	0.7634	12.9	4.00	0.70	tr_C9ZMP2_	WB
DRB1_0101	18	FSTAYVEQQPFLMSG	YVEQQPFLM	4	0.7621	13.1	4.00	0.90	tr_C9ZMP2_	WB
DRB1_0101	19	KSTFLDGLAGEHLID	LDGLAGEHL	4	0.7576	13.8	4.50	0.66	tr_C9ZMP2_	WB
DRB1_0101	20	RGIRLSGGQRARVAL	IRLSGGQRA	2	0.7554	14.1	4.50	0.68	tr_C9ZMP2_	WB
DRB1_0101	21	TLACYIGRVGWKLSA	YIGRVGWKL	4	0.7549	14.2	4.50	0.85	tr_C9ZMP2_	WB
DRB1_0101	22	GKSTFLDGLAGEHLI	LDGLAGEHL	5	0.7525	14.5	5.00	0.62	tr_C9ZMP2_	WB
DRB1_0101	23	ACYIGRVGWKLSAFI	YIGRVGWKL	2	0.7516	14.7	5.00	0.86	tr_C9ZMP2_	WB
DRB1_0101	24	PSFMLHIPTFQATSS	MLHIPTFQA	3	0.7505	14.9	5.00	0.71	tr_C9ZMP2_	WB
DRB1_0101	25	RPSFMLHIPTFQATS	MLHIPTFQA	4	0.7480	15.3	5.00	0.64	tr_C9ZMP2_	WB
DRB1_0101	26	TAYVEQQPFLMSGTL	YVEQQPFLM	2	0.7479	15.3	5.00	0.87	tr_C9ZMP2_	WB
DRB1_0101	27	CYIGRVGWKLSAFII	YIGRVGWKL	1	0.7451	15.8	5.00	0.70	tr_C9ZMP2_	WB
DRB1_0101	28	WCRLSPFLVMRPDAC	WCRLSPFLV	0	0.7451	15.8	5.00	0.46	tr_C9ZMP2_	WB
DRB1_0101	29	DRGIRLSGGQRARVA	IRLSGGQRA	3	0.7440	16.0	5.50	0.73	tr_C9ZMP2_	WB
DRB1_0101	30	SVRYEAVINAVALTQ	YEAVINAVA	3	0.7410	16.5	5.50	0.77	tr_C9ZMP2_	WB
DRB1_0101	31	VALARALYAGKELYL	VALARALYA	0	0.7355	17.5	6.00	0.63	tr_C9ZMP2_	WB
DRB1_0101	32	RFSTAYVEQQPFLMS	YVEQQPFLM	5	0.7331	17.9	6.00	0.90	tr_C9ZMP2_	WB
DRB1_0101	33	SFMLHIPTFQATSSQ	MLHIPTFQA	2	0.7325	18.1	6.00	0.75	tr_C9ZMP2_	WB
DRB1_0101	34	LFNSLTMPLNAYPCV	FNSLTMPLN	1	0.7318	18.2	6.00	0.59	tr_C9ZMP2_	WB
DRB1_0101	35	DSVRYEAVINAVALT	YEAVINAVA	4	0.7300	18.6	6.50	0.74	tr_C9ZMP2_	WB
DRB1_0101	36	STFLDGLAGEHLIDP	LDGLAGEHL	3	0.7291	18.8	6.50	0.73	tr_C9ZMP2_	WB
DRB1_0101	37	GIRLSGGQRARVALA	IRLSGGQRA	1	0.7229	20.1	7.00	0.60	tr_C9ZMP2_	WB
DRB1_0101	38	AYVEQQPFLMSGTLR	YVEQQPFLM	1	0.7201	20.7	7.00	0.76	tr_C9ZMP2_	WB
DRB1_0101	39	VTALALFNSLTMPLN	FNSLTMPLN	6	0.7200	20.7	7.00	0.44	tr_C9ZMP2_	WB
DRB1_0101	40	PRPSFMLHIPTFQAT	MLHIPTFQA	5	0.7196	20.8	7.00	0.59	tr_C9ZMP2_	WB
DRB1_0101	41	ETLACYIGRVGWKLS	YIGRVGWKL	5	0.7187	21.0	7.50	0.81	tr_C9ZMP2_	WB
DRB1_0101	42	VRYEAVINAVALTQN	YEAVINAVA	2	0.7129	22.3	8.00	0.76	tr_C9ZMP2_	WB
DRB1_0101	43	YDSVRYEAVINAVAL	YEAVINAVA	5	0.7088	23.4	8.00	0.67	tr_C9ZMP2_	WB
DRB1_0101	44	GDRGIRLSGGQRARV	IRLSGGQRA	4	0.7083	23.5	8.00	0.75	tr_C9ZMP2_	WB
DRB1_0101	45	VESYVSWCRLSPFLV	WCRLSPFLV	6	0.7078	23.6	8.50	0.78	tr_C9ZMP2_	WB
DRB1_0101	46	QQPFLMSGTLRENIL	FLMSGTLRE	3	0.7076	23.7	8.50	0.79	tr_C9ZMP2_	WB

DRB1_0101	47	RLSPFLVMRPDACHG	FLVMRPDAC	4	0.6984	26.1	9.00	0.84	tr_C9ZMP2_	WB
DRB1_0101	48	YIGRVGWKLSAFIII	YIGRVGWKL	0	0.6968	26.6	9.50	0.49	tr_C9ZMP2_	WB
DRB1_0101	49	LSPFLVMRPDACHGD	FLVMRPDAC	3	0.6918	28.1	10.00	0.85	tr_C9ZMP2_	
DRB1_0101	50	CRLSPFLVMRPDACH	FLVMRPDAC	5	0.6914	28.2	10.00	0.73	tr_C9ZMP2_	
DRB1_0101	51	QPFLMSGTLRENILM	FLMSGTLRE	2	0.6899	28.7	10.00	0.75	tr_C9ZMP2_	
DRB1_0101	52	SGKSTFLDGLAGEHL	LDGLAGEHL	6	0.6882	29.2	10.00	0.49	tr_C9ZMP2_	
DRB1_0101	53	EGQLLFSQRLVKTAA	LFSQRLVKT	4	0.6845	30.4	11.00	0.31	tr_C9ZMP2_	
DRB1_0101	54	GQLLFSQRLVKTAAT	LFSQRLVKT	3	0.6814	31.4	11.00	0.38	tr_C9ZMP2_	
DRB1_0101	55	TFLDGLAGEHLIDPN	LDGLAGEHL	2	0.6803	31.8	11.00	0.83	tr_C9ZMP2_	
DRB1_0101	56	DTFLVGDRGIRLSGG	LVGDRGIRL	3	0.6797	32.0	11.00	0.73	tr_C9ZMP2_	
DRB1_0101	57	GDTFLVGDRGIRLSG	LVGDRGIRL	4	0.6795	32.1	11.00	0.64	tr_C9ZMP2_	
DRB1_0101	58	EQQPFLMSGTLRENI	FLMSGTLRE	4	0.6791	32.2	11.00	0.73	tr_C9ZMP2_	
DRB1_0101	59	RYEAVINAVALTQNF	YEAVINAVA	1	0.6777	32.7	12.00	0.74	tr_C9ZMP2_	
DRB1_0101	60	SPFLVMRPDACHGDL	FLVMRPDAC	2	0.6755	33.5	12.00	0.81	tr_C9ZMP2_	
DRB1_0101	61	YVEQQPFLMSGTLRE	YVEQQPFLM	0	0.6749	33.7	12.00	0.38	tr_C9ZMP2_	
DRB1_0101	62	FMLHIPTFQATSSQL	MLHIPTFQA	1	0.6708	35.2	12.00	0.74	tr_C9ZMP2_	
DRB1_0101	63	IRLSGGQRARVALAR	IRLSGGQRA	0	0.6707	35.3	12.00	0.40	tr_C9ZMP2_	
DRB1_0101	64	ILGCLDPTVARHIVI	LGCLDPTVA	1	0.6677	36.4	13.00	0.45	tr_C9ZMP2_	
DRB1_0101	65	DILGCLDPTVARHIV	LGCLDPTVA	2	0.6651	37.5	13.00	0.53	tr_C9ZMP2_	
DRB1_0101	66	IPTFQATSSQLIAVV	FQATSSQLI	3	0.6651	37.5	13.00	0.82	tr_C9ZMP2_	
DRB1_0101	67	FNSLTMPLNAYPCVI	FNSLTMPLN	0	0.6616	38.9	14.00	0.39	tr_C9ZMP2_	
DRB1_0101	68	VEGQLLFSQRLVKTA	GQLLFSQRL	2	0.6579	40.5	14.00	0.41	tr_C9ZMP2_	
DRB1_0101	69	LARALYAGKELYLID	LYAGKELYL	4	0.6540	42.3	15.00	0.67	tr_C9ZMP2_	
DRB1_0101	70	QLLFSQRLVKTAATT	FSQRLVKTA	3	0.6536	42.4	15.00	0.38	tr_C9ZMP2_	
DRB1_0101	71	LPRPSFMLHIPTFQA	MLHIPTFQA	6	0.6523	43.0	15.00	0.44	tr_C9ZMP2_	
DRB1_0101	72	DGDTFLVGDRGIRLS	LVGDRGIRL	5	0.6499	44.2	15.00	0.60	tr_C9ZMP2_	
DRB1_0101	73	GGELTPGKAVTALAL	LTPGKAVTA	3	0.6485	44.8	15.00	0.69	tr_C9ZMP2_	
DRB1_0101	74	GELTPGKAVTALALF	LTPGKAVTA	2	0.6482	45.0	15.00	0.65	tr_C9ZMP2_	
DRB1_0101	75	SAFIIISAALMQISR	FIIISAALM	2	0.6479	45.1	15.00	0.56	tr_C9ZMP2_	
DRB1_0101	76	VGDRGIRLSGGQRAR	IRLSGGQRA	5	0.6469	45.6	15.00	0.76	tr_C9ZMP2_	
DRB1_0101	77	LSAFIIISAALMQIS	FIIISAALM	3	0.6464	45.9	16.00	0.64	tr_C9ZMP2_	
DRB1_0101	78	ALARALYAGKELYLI	LYAGKELYL	5	0.6462	46.0	16.00	0.65	tr_C9ZMP2_	
DRB1_0101	79	VEQQPFLMSGTLREN	FLMSGTLRE	5	0.6460	46.0	16.00	0.67	tr_C9ZMP2_	
DRB1_0101	80	PFLMSGTLRENILMG	FLMSGTLRE	1	0.6452	46.5	16.00	0.72	tr_C9ZMP2_	
DRB1_0101	81	HIPTFQATSSQLIAV	FQATSSQLI	4	0.6450	46.6	16.00	0.79	tr_C9ZMP2_	
DRB1_0101	82	ARALYAGKELYLIDD	LYAGKELYL	3	0.6445	46.8	16.00	0.68	tr_C9ZMP2_	
DRB1_0101	83	SGGQRARVALARALY	ARVALARAL	5	0.6434	47.4	16.00	0.41	tr_C9ZMP2_	
DRB1_0101	84	WETLACYIGRVGWKL	YIGRVGWKL	6	0.6431	47.5	16.00	0.70	tr_C9ZMP2_	
DRB1_0101	85	PTFQATSSQLIAVVG	FQATSSQLI	2	0.6416	48.3	16.00	0.82	tr_C9ZMP2_	
DRB1_0101	86	SRFSTAYVEQQPFLM	YVEQQPFLM	6	0.6416	48.3	16.00	0.80	tr_C9ZMP2_	
DRB1_0101	87	GWKLSAFIIISAALM	FIIISAALM	6	0.6380	50.3	17.00	0.45	tr_C9ZMP2_	
DRB1_0101	88	DDILGCLDPTVARHI	LGCLDPTVA	3	0.6374	50.5	17.00	0.61	tr_C9ZMP2_	
DRB1_0101	89	KLSAFIIISAALMQI	FIIISAALM	4	0.6366	51.0	17.00	0.65	tr_C9ZMP2_	
DRB1_0101	90	LNEIQPLLQAKGCVG	IQPLLQAKG	3	0.6353	51.7	17.00	0.41	tr_C9ZMP2_	
DRB1_0101	91	YRCVEGQLLFSQRLV	GQLLFSQRL	5	0.6347	52.1	17.00	0.35	tr_C9ZMP2_	
DRB1_0101	92	TFLVGDRGIRLSGGQ	LVGDRGIRL	2	0.6344	52.2	17.00	0.83	tr_C9ZMP2_	
DRB1_0101	93	VYRCVEGQLLFSQRL	YRCVEGQLL	1	0.6334	52.8	17.00	0.57	tr_C9ZMP2_	
DRB1_0101	94	MYDSVRYEAVINAVA	YEAVINAVA	6	0.6324	53.4	17.00	0.47	tr_C9ZMP2_	
DRB1_0101	95	PFLVMRPDACHGDLY	FLVMRPDAC	1	0.6313	54.0	18.00	0.77	tr_C9ZMP2_	
DRB1_0101	96	WKLSAFIIISAALMQ	FIIISAALM	5	0.6295	55.1	18.00	0.66	tr_C9ZMP2_	

DRB1_0101	97	SLNEIQPLLQAKGCV	IQPLLQAKG	4	0.6292	55.2	18.00	0.41	tr_C9ZMP2_
DRB1_0101	98	ASSLNEIQPLLQAKG	LNEIQPLLQ	3	0.6292	55.3	18.00	0.41	tr_C9ZMP2_
DRB1_0101	99	SSLNEIQPLLQAKGC	LNEIQPLLQ	2	0.6274	56.4	18.00	0.38	tr_C9ZMP2_
DRB1_0101	100	AFIIISAALMQISRN	FIIISAALM	1	0.6251	57.7	19.00	0.47	tr_C9ZMP2_
DRB1_0101	101	ELTPGKAVTALALFN	LTPGKAVTA	1	0.6244	58.2	19.00	0.55	tr_C9ZMP2_
DRB1_0101	102	IQPLLQAKGCVGEMR	LLQAKGCVG	3	0.6241	58.4	19.00	0.70	tr_C9ZMP2_
DRB1_0101	103	DVVYRCVEGQLLFSQ	YRCVEGQLL	3	0.6237	58.7	19.00	0.80	tr_C9ZMP2_
DRB1_0101	104	LGCLDPTVARHIVIN	LDPTVARHI	3	0.6231	59.0	19.00	0.56	tr_C9ZMP2_
DRB1_0101	105	MLHIPTFQATSSQLI	MLHIPTFQA	0	0.6213	60.2	19.00	0.48	tr_C9ZMP2_
DRB1_0101	106	RALYAGKELYLIDDI	LYAGKELYL	2	0.6210	60.4	19.00	0.69	tr_C9ZMP2_
DRB1_0101	107	VVYRCVEGQLLFSQR	YRCVEGQLL	2	0.6206	60.7	19.00	0.79	tr_C9ZMP2_
DRB1_0101	108	NEIQPLLQAKGCVGE	LLQAKGCVG	5	0.6192	61.6	19.00	0.43	tr_C9ZMP2_
DRB1_0101	109	AVTALALFNSLTMPL	ALALFNSLT	3	0.6179	62.4	20.00	0.43	tr_C9ZMP2_
DRB1_0101	110	SCVIVATHNQELVER	IVATHNQEL	3	0.6178	62.5	20.00	0.73	tr_C9ZMP2_
DRB1_0101	111	RHIVINALVGSAKSG	INALVGSAK	4	0.6175	62.7	20.00	0.38	tr_C9ZMP2_
DRB1_0101	112	EIQPLLQAKGCVGEM	LLQAKGCVG	4	0.6171	63.0	20.00	0.57	tr_C9ZMP2_
DRB1_0101	113	GKAVTALALFNSLTM	ALALFNSLT	5	0.6168	63.2	20.00	0.32	tr_C9ZMP2_
DRB1_0101	114	CVEGQLLFSQRLVKT	GQLLFSQRL	3	0.6164	63.5	20.00	0.56	tr_C9ZMP2_
DRB1_0101	115	LLFSQRLVKTAATTV	FSQRLVKTA	2	0.6162	63.6	20.00	0.31	tr_C9ZMP2_
DRB1_0101	116	LFSQRLVKTAATTVA	LVKTAATTV	5	0.6152	64.3	20.00	0.40	tr_C9ZMP2_
DRB1_0101	117	MGGELTPGKAVTALA	LTPGKAVTA	4	0.6150	64.4	20.00	0.70	tr_C9ZMP2_
DRB1_0101	118	FLDGLAGEHLIDPND	LDGLAGEHL	1	0.6140	65.2	20.00	0.87	tr_C9ZMP2_
DRB1_0101	119	ARHIVINALVGSAKS	HIVINALVG	2	0.6135	65.5	20.00	0.33	tr_C9ZMP2_
DRB1_0101	120	TFQATSSQLIAVVGV	FQATSSQLI	1	0.6109	67.4	21.00	0.77	tr_C9ZMP2_
DRB1_0101	121	LSGGQRARVALARAL	GQRARVALA	3	0.6100	68.0	21.00	0.36	tr_C9ZMP2_
DRB1_0101	122	ADVVYRCVEGQLLFS	YRCVEGQLL	4	0.6074	69.9	21.00	0.80	tr_C9ZMP2_
DRB1_0101	123	LHIPTFQATSSQLIA	FQATSSQLI	5	0.6026	73.7	22.00	0.77	tr_C9ZMP2_
DRB1_0101	124	FIIISAALMQISRNA	FIIISAALM	0	0.6024	73.9	22.00	0.29	tr_C9ZMP2_
DRB1_0101	125	HDLLIDIRSGVIRLN	LIDIRSGVI	3	0.5986	77.0	23.00	0.53	tr_C9ZMP2_
DRB1_0101	126	IDDILGCLDPTVARH	LGCLDPTVA	4	0.5981	77.4	23.00	0.68	tr_C9ZMP2_
DRB1_0101	127	RSGVIRLNTQGGAGL	VIRLNTQGG	3	0.5957	79.4	23.00	0.65	tr_C9ZMP2_
DRB1_0101	128	GRVGWKLSAFIIISA	GWKLSAFII	3	0.5952	79.8	23.00	0.54	tr_C9ZMP2_
DRB1_0101	129	RVGWKLSAFIIISAA	GWKLSAFII	2	0.5949	80.1	23.00	0.51	tr_C9ZMP2_
DRB1_0101	130	HIVINALVGSAKSGS	INALVGSAK	3	0.5939	80.9	23.00	0.47	tr_C9ZMP2_
DRB1_0101	131	FSQRLVKTAATTVAE	LVKTAATTV	4	0.5936	81.2	24.00	0.52	tr_C9ZMP2_
DRB1_0101	132	KAVTALALFNSLTMP	ALALFNSLT	4	0.5916	83.0	24.00	0.41	tr_C9ZMP2_
DRB1_0101	133	CVIVATHNQELVERA	IVATHNQEL	2	0.5899	84.5	24.00	0.70	tr_C9ZMP2_
DRB1_0101	134	CASSLNEIQPLLQAK	LNEIQPLLQ	4	0.5893	85.1	24.00	0.47	tr_C9ZMP2_
DRB1_0101	135	VARHIVINALVGSAK	HIVINALVG	3	0.5885	85.8	24.00	0.41	tr_C9ZMP2_
DRB1_0101	136	SGVIRLNTQGGAGLP	VIRLNTQGG	2	0.5885	85.8	24.00	0.54	tr_C9ZMP2_
DRB1_0101	137	RLSGGQRARVALARA	GQRARVALA	4	0.5884	85.9	24.00	0.34	tr_C9ZMP2_
DRB1_0101	138	GVIRLNTQGGAGLPR	LNTQGGAGL	4	0.5873	86.9	25.00	0.43	tr_C9ZMP2_
DRB1_0101	139	DLLIDIRSGVIRLNT	LIDIRSGVI	2	0.5869	87.4	25.00	0.52	tr_C9ZMP2_
DRB1_0101	140	IGRVGWKLSAFIIIS	GWKLSAFII	4	0.5850	89.1	25.00	0.54	tr_C9ZMP2_
DRB1_0101	141	GSCVIVATHNQELVE	IVATHNQEL	4	0.5836	90.5	25.00	0.69	tr_C9ZMP2_
DRB1_0101	142	SQLIAVVGVSGSGKS	IAVVGVSGS	3	0.5778	96.4	26.00	0.44	tr_C9ZMP2_
DRB1_0101	143	NHDLLIDIRSGVIRL	LIDIRSGVI	4	0.5774	96.8	26.00	0.58	tr_C9ZMP2_
DRB1_0101	144	RADVVYRCVEGQLLF	YRCVEGQLL	5	0.5763	97.9	26.00	0.79	tr_C9ZMP2_
DRB1_0101	145	RCVEGQLLFSQRLVK	GQLLFSQRL	4	0.5762	98.0	26.00	0.67	tr_C9ZMP2_
DRB1_0101	146	LVGDRGIRLSGGQRA	IRLSGGQRA	6	0.5760	98.3	26.00	0.47	tr_C9ZMP2_

DRB1_0101	147	QPLLQAKGCVGEMRR	LLQAKGCVG	2	0.5759	98.3	26.00	0.75	tr_C9ZMP2_
DRB1_0101	148	FLVGDRGIRLSGGQR	LVGDRGIRL	1	0.5758	98.4	26.00	0.76	tr_C9ZMP2_
DRB1_0101	149	PDGDTFLVGDRGIRL	LVGDRGIRL	6	0.5748	99.6	27.00	0.46	tr_C9ZMP2_
DRB1_0101	150	SSQLIAVVGVSGSGK	LIAVVGVSG	3	0.5737	100.7	27.00	0.43	tr_C9ZMP2_
DRB1_0101	151	LIDDILGCLDPTVAR	LGCLDPTVA	5	0.5724	102.1	27.00	0.64	tr_C9ZMP2_
DRB1_0101	152	VGWKLSAFIIISAAL	GWKLSAFII	1	0.5720	102.6	27.00	0.43	tr_C9ZMP2_
DRB1_0101	153	YEAVINAVALTQNFQ	YEAVINAVA	0	0.5716	103.1	27.00	0.55	tr_C9ZMP2_
DRB1_0101	154	PGKAVTALALFNSLT	GKAVTALAL	1	0.5710	103.7	27.00	0.24	tr_C9ZMP2_
DRB1_0101	155	LTPGKAVTALALFNS	GKAVTALAL	3	0.5708	103.9	27.00	0.37	tr_C9ZMP2_
DRB1_0101	156	SQRLVKTAATTVAEP	LVKTAATTV	3	0.5670	108.3	28.00	0.56	tr_C9ZMP2_
DRB1_0101	157	LLIDIRSGVIRLNTQ	LIDIRSGVI	1	0.5583	118.9	30.00	0.46	tr_C9ZMP2_
DRB1_0101	158	SGSCVIVATHNQELV	IVATHNQEL	5	0.5561	121.9	30.00	0.63	tr_C9ZMP2_
DRB1_0101	159	ALYAGKELYLIDDIL	LYAGKELYL	1	0.5550	123.4	30.00	0.59	tr_C9ZMP2_
DRB1_0101	160	GLPRPSFMLHIPTFQ	FMLHIPTFQ	6	0.5540	124.6	30.00	0.32	tr_C9ZMP2_
DRB1_0101	161	NSLTMPLNAYPCVIN	TMPLNAYPC	3	0.5536	125.2	31.00	0.41	tr_C9ZMP2_
DRB1_0101	162	QLIAVVGVSGSGKST	IAVVGVSGS	2	0.5534	125.5	31.00	0.40	tr_C9ZMP2_
DRB1_0101	163	TPGKAVTALALFNSL	GKAVTALAL	2	0.5478	133.3	32.00	0.38	tr_C9ZMP2_
DRB1_0101	164	QRLVKTAATTVAEPS	LVKTAATTV	2	0.5456	136.5	32.00	0.50	tr_C9ZMP2_
DRB1_0101	165	VIVATHNQELVERAD	IVATHNQEL	1	0.5431	140.3	33.00	0.71	tr_C9ZMP2_
DRB1_0101	166	AGLPRPSFMLHIPTF	GLPRPSFML	1	0.5427	140.8	33.00	0.47	tr_C9ZMP2_
DRB1_0101	167	TVARHIVINALVGSA	HIVINALVG	4	0.5410	143.5	33.00	0.48	tr_C9ZMP2_
DRB1_0101	168	IIISAALMQISRNAG	ISAALMQIS	2	0.5402	144.8	33.00	0.28	tr_C9ZMP2_
DRB1_0101	169	GCLDPTVARHIVINA	LDPTVARHI	2	0.5376	148.9	34.00	0.72	tr_C9ZMP2_
DRB1_0101	170	IVINALVGSAKSGSC	INALVGSAK	2	0.5373	149.4	34.00	0.56	tr_C9ZMP2_
DRB1_0101	171	RNHDLLIDIRSGVIR	LIDIRSGVI	5	0.5359	151.6	34.00	0.54	tr_C9ZMP2_
DRB1_0101	172	FLMSGTLRENILMGL	FLMSGTLRE	0	0.5357	151.9	34.00	0.56	tr_C9ZMP2_
DRB1_0101	173	FLVMRPDACHGDLYG	FLVMRPDAC	0	0.5352	152.8	34.00	0.67	tr_C9ZMP2_
DRB1_0101	174	QRLAGIHGLPHPFRR	IHGLPHPFR	5	0.5324	157.5	35.00	0.35	tr_C9ZMP2_
DRB1_0101	175	VIRLNTQGGAGLPRP	LNTQGGAGL	3	0.5316	158.9	35.00	0.56	tr_C9ZMP2_
DRB1_0101	176	TSSQLIAVVGVSGSG	LIAVVGVSG	4	0.5285	164.3	35.00	0.41	tr_C9ZMP2_
DRB1_0101	177	PCASSLNEIQPLLQA	LNEIQPLLQ	5	0.5282	164.9	35.00	0.47	tr_C9ZMP2_
DRB1_0101	178	LMYDSVRYEAVINAV	VRYEAVINA	5	0.5273	166.4	36.00	0.38	tr_C9ZMP2_
DRB1_0101	179	RQRLAGIHGLPHPFR	LAGIHGLPH	3	0.5251	170.4	36.00	0.44	tr_C9ZMP2_
DRB1_0101	180	QGGAGLPRPSFMLHI	GLPRPSFML	4	0.5249	170.8	36.00	0.63	tr_C9ZMP2_
DRB1_0101	181	CLDPTVARHIVINAL	LDPTVARHI	1	0.5214	177.4	37.00	0.57	tr_C9ZMP2_
DRB1_0101	182	RLAGIHGLPHPFRRN	IHGLPHPFR	4	0.5214	177.4	37.00	0.43	tr_C9ZMP2_
DRB1_0101	183	PTVARHIVINALVGS	HIVINALVG	5	0.5213	177.6	37.00	0.44	tr_C9ZMP2_
DRB1_0101	184	SLTMPLNAYPCVING	TMPLNAYPC	2	0.5206	179.0	37.00	0.42	tr_C9ZMP2_
DRB1_0101	185	GAGLPRPSFMLHIPT	GLPRPSFML	2	0.5184	183.3	37.00	0.60	tr_C9ZMP2_
DRB1_0101	186	IISAALMQISRNAGD	ALMQISRNA	4	0.5177	184.6	37.00	0.33	tr_C9ZMP2_
DRB1_0101	187	FQATSSQLIAVVGVS	FQATSSQLI	0	0.5174	185.3	38.00	0.56	tr_C9ZMP2_
DRB1_0101	188	LIAVVGVSGSGKSTF	IAVVGVSGS	1	0.5170	186.0	38.00	0.36	tr_C9ZMP2_
DRB1_0101	189	INAVALTQNFQTCFY	VALTQNFQT	3	0.5168	186.4	38.00	0.67	tr_C9ZMP2_
DRB1_0101	190	IRSGVIRLNTQGGAG	VIRLNTQGG	4	0.5154	189.3	38.00	0.70	tr_C9ZMP2_
DRB1_0101	191	GGAGLPRPSFMLHIP	GLPRPSFML	3	0.5147	190.7	38.00	0.64	tr_C9ZMP2_
DRB1_0101	192	PLLQAKGCVGEMRRQ	LLQAKGCVG	1	0.5146	191.0	38.00	0.73	tr_C9ZMP2_
DRB1_0101	193	YLIDDILGCLDPTVA	LGCLDPTVA	6	0.5074	206.5	40.00	0.49	tr_C9ZMP2_
DRB1_0101	194	GSGKSTFLDGLAGEH	STFLDGLAG	4	0.5061	209.3	40.00	0.67	tr_C9ZMP2_
DRB1_0101	195	HAAVSRFSTAYVEQQ	VSRFSTAYV	3	0.5053	211.0	40.00	0.59	tr_C9ZMP2_
DRB1_0101	196	GKELYLIDDILGCLD	YLIDDILGC	4	0.5046	212.8	40.00	0.32	tr_C9ZMP2_

DRB1_0101	197	ERADVVYRCVEGQLL	YRCVEGQLL	6	0.5026	217.5	40.00	0.72	tr_C9ZMP2_
DRB1_0101	198	YPCVINGLVESYVSW	INGLVESYV	4	0.5017	219.6	41.00	0.56	tr_C9ZMP2_
DRB1_0101	199	ISAALMQISRNAGDQ	ALMQISRNA	3	0.5003	222.8	41.00	0.43	tr_C9ZMP2_
DRB1_0101	200	VINALVGSAKSGSCV	INALVGSAK	1	0.5003	223.0	41.00	0.56	tr_C9ZMP2_
DRB1_0101	201	AGKELYLIDDILGCL	YLIDDILGC	5	0.5001	223.4	41.00	0.31	tr_C9ZMP2_
DRB1_0101	202	ESGSIEAAEVLETSK	IEAAEVLET	4	0.4990	226.2	41.00	0.44	tr_C9ZMP2_
DRB1_0101	203	GLMYDSVRYEAVINA	YDSVRYEAV	3	0.4948	236.5	42.00	0.52	tr_C9ZMP2_
DRB1_0101	204	DIRSGVIRLNTQGGA	VIRLNTQGG	5	0.4938	239.2	42.00	0.60	tr_C9ZMP2_
DRB1_0101	205	LAGIHGLPHPFRRNH	IHGLPHPFR	3	0.4937	239.5	42.00	0.52	tr_C9ZMP2_
DRB1_0101	206	GHAAVSRFSTAYVEQ	VSRFSTAYV	4	0.4936	239.7	42.00	0.55	tr_C9ZMP2_
DRB1_0101	207	DPTVARHIVINALVG	HIVINALVG	6	0.4909	246.7	43.00	0.35	tr_C9ZMP2_
DRB1_0101	208	NQELVERADVVYRCV	VERADVVYR	4	0.4896	250.2	43.00	0.52	tr_C9ZMP2_
DRB1_0101	209	AYPCVINGLVESYVS	INGLVESYV	5	0.4885	253.2	43.00	0.44	tr_C9ZMP2_
DRB1_0101	210	SGSIEAAEVLETSKH	IEAAEVLET	3	0.4880	254.5	43.00	0.52	tr_C9ZMP2_
DRB1_0101	211	NAVALTQNFQTCFYP	VALTQNFQT	2	0.4873	256.6	44.00	0.69	tr_C9ZMP2_
DRB1_0101	212	KELYLIDDILGCLDP	YLIDDILGC	3	0.4849	263.4	44.00	0.34	tr_C9ZMP2_
DRB1_0101	213	ATSSQLIAVVGVSGS	LIAVVGVSG	5	0.4826	269.8	45.00	0.41	tr_C9ZMP2_
DRB1_0101	214	LIDIRSGVIRLNTQG	DIRSGVIRL	2	0.4819	271.9	45.00	0.26	tr_C9ZMP2_
DRB1_0101	215	LYAGKELYLIDDILG	LYAGKELYL	0	0.4803	276.8	45.00	0.31	tr_C9ZMP2_
DRB1_0101	216	PCVINGLVESYVSWC	INGLVESYV	3	0.4799	277.8	45.00	0.56	tr_C9ZMP2_
DRB1_0101	217	VINAVALTQNFQTCF	VALTQNFQT	4	0.4793	279.8	45.00	0.62	tr_C9ZMP2_
DRB1_0101	218	TQGGAGLPRPSFMLH	GLPRPSFML	5	0.4789	281.1	45.00	0.61	tr_C9ZMP2_
DRB1_0101	219	PESGSIEAAEVLETS	IEAAEVLET	5	0.4783	282.8	46.00	0.34	tr_C9ZMP2_
DRB1_0101	220	PHPFRRNHDLLIDIR	FRRNHDLLI	3	0.4748	293.6	46.00	0.81	tr_C9ZMP2_
DRB1_0101	221	HPFRRNHDLLIDIRS	FRRNHDLLI	2	0.4747	293.9	46.00	0.72	tr_C9ZMP2_
DRB1_0101	222	LDPTVARHIVINALV	LDPTVARHI	0	0.4747	294.2	46.00	0.28	tr_C9ZMP2_
DRB1_0101	223	LDGLAGEHLIDPNDA	LDGLAGEHL	0	0.4746	294.2	46.00	0.72	tr_C9ZMP2_
DRB1_0101	224	RLVKTAATTVAEPSS	LVKTAATTV	1	0.4744	295.0	46.00	0.50	tr_C9ZMP2_
DRB1_0101	225	RENILMGLMYDSVRY	LMGLMYDSV	4	0.4743	295.4	46.00	0.34	tr_C9ZMP2_
DRB1_0101	226	QELVERADVVYRCVE	VERADVVYR	3	0.4734	298.3	47.00	0.55	tr_C9ZMP2_
DRB1_0101	227	SAALMQISRNAGDQY	ALMQISRNA	2	0.4701	309.2	47.00	0.46	tr_C9ZMP2_
DRB1_0101	228	TGHAAVSRFSTAYVE	VSRFSTAYV	5	0.4699	309.6	47.00	0.49	tr_C9ZMP2_
DRB1_0101	229	GDQYVVTWAKSGDGN	YVVTWAKSG	3	0.4699	309.8	47.00	0.74	tr_C9ZMP2_
DRB1_0101	230	HNQELVERADVVYRC	VERADVVYR	5	0.4691	312.4	47.00	0.44	tr_C9ZMP2_
DRB1_0101	231	DQYVVTWAKSGDGNT	YVVTWAKSG	2	0.4686	314.0	48.00	0.68	tr_C9ZMP2_
DRB1_0101	232	QYVVTWAKSGDGNTT	YVVTWAKSG	1	0.4681	315.7	48.00	0.62	tr_C9ZMP2_
DRB1_0101	233	AAVSRFSTAYVEQQP	VSRFSTAYV	2	0.4668	320.1	48.00	0.58	tr_C9ZMP2_
DRB1_0101	234	ENILMGLMYDSVRYE	LMGLMYDSV	3	0.4639	330.5	49.00	0.34	tr_C9ZMP2_
DRB1_0101	235	THNQELVERADVVYR	ELVERADVV	4	0.4639	330.6	49.00	0.40	tr_C9ZMP2_
DRB1_0101	236	LMGLMYDSVRYEAVI	YDSVRYEAV	5	0.4636	331.4	49.00	0.42	tr_C9ZMP2_
DRB1_0101	237	CVINGLVESYVSWCR	INGLVESYV	2	0.4636	331.6	49.00	0.48	tr_C9ZMP2_
DRB1_0101	238	ILMGLMYDSVRYEAV	YDSVRYEAV	6	0.4627	334.8	49.00	0.22	tr_C9ZMP2_
DRB1_0101	239	IRLNTQGGAGLPRPS	LNTQGGAGL	2	0.4597	345.7	49.00	0.71	tr_C9ZMP2_
DRB1_0101	240	RRNHDLLIDIRSGVI	LIDIRSGVI	6	0.4597	345.8	49.00	0.42	tr_C9ZMP2_
DRB1_0101	241	AVINAVALTQNFQTC	VALTQNFQT	5	0.4569	356.4	50.00	0.50	tr_C9ZMP2_
DRB1_0101	242	RRQRLAGIHGLPHPF	LAGIHGLPH	4	0.4568	357.0	50.00	0.50	tr_C9ZMP2_
DRB1_0101	243	IAVVGVSGSGKSTFL	VVGVSGSGK	2	0.4566	357.4	50.00	0.31	tr_C9ZMP2_
DRB1_0101	244	INALVGSAKSGSCVI	INALVGSAK	0	0.4554	362.3	50.00	0.31	tr_C9ZMP2_
DRB1_0101	245	NILMGLMYDSVRYEA	LMGLMYDSV	2	0.4553	362.5	50.00	0.35	tr_C9ZMP2_
DRB1_0101	246	PFRRNHDLLIDIRSG	FRRNHDLLI	1	0.4552	363.2	50.00	0.60	tr_C9ZMP2_

DRB1_0101	247	MGLMYDSVRYEAVIN	YDSVRYEAV	4	0.4533	370.6	55.00	0.56	tr_C9ZMP2_
DRB1_0101	248	NAYPCVINGLVESYV	INGLVESYV	6	0.4499	384.4	55.00	0.32	tr_C9ZMP2_
DRB1_0101	249	AGIHGLPHPFRRNHD	IHGLPHPFR	2	0.4488	389.3	55.00	0.62	tr_C9ZMP2_
DRB1_0101	250	LRENILMGLMYDSVR	LMGLMYDSV	5	0.4474	395.1	55.00	0.31	tr_C9ZMP2_
DRB1_0101	251	LTMPLNAYPCVINGL	TMPLNAYPC	1	0.4471	396.2	55.00	0.40	tr_C9ZMP2_
DRB1_0101	252	AGDQYVVTWAKSGDG	YVVTWAKSG	4	0.4457	402.5	55.00	0.77	tr_C9ZMP2_
DRB1_0101	253	TPESGSIEAAEVLET	SGSIEAAEV	3	0.4440	409.8	55.00	0.44	tr_C9ZMP2_
DRB1_0101	254	VINGLVESYVSWCRL	INGLVESYV	1	0.4438	410.8	55.00	0.41	tr_C9ZMP2_
DRB1_0101	255	LPHPFRRNHDLLIDI	FRRNHDLLI	4	0.4433	412.8	55.00	0.81	tr_C9ZMP2_
DRB1_0101	256	ATGHAAVSRFSTAYV	VSRFSTAYV	6	0.4428	415.4	55.00	0.32	tr_C9ZMP2_
DRB1_0101	257	IVATHNQELVERADV	IVATHNQEL	0	0.4427	415.5	55.00	0.51	tr_C9ZMP2_
DRB1_0101	258	YAGKELYLIDDILGC	LYLIDDILG	5	0.4412	422.4	55.00	0.29	tr_C9ZMP2_
DRB1_0101	259	SGSGKSTFLDGLAGE	STFLDGLAG	5	0.4382	436.2	55.00	0.69	tr_C9ZMP2_
DRB1_0101	260	EAVINAVALTQNFQT	VINAVALTQ	2	0.4379	438.0	55.00	0.20	tr_C9ZMP2_
DRB1_0101	261	AVALTQNFQTCFYPD	VALTQNFQT	1	0.4373	440.6	55.00	0.67	tr_C9ZMP2_
DRB1_0101	262	KSGSCVIVATHNQEL	IVATHNQEL	6	0.4366	444.2	55.00	0.47	tr_C9ZMP2_
DRB1_0101	263	IDIRSGVIRLNTQGG	VIRLNTQGG	6	0.4353	450.4	55.00	0.33	tr_C9ZMP2_
DRB1_0101	264	YPDGDTFLVGDRGIR	DTFLVGDRG	4	0.4351	451.2	55.00	0.50	tr_C9ZMP2_
DRB1_0101	265	MRRQRLAGIHGLPHP	LAGIHGLPH	5	0.4314	469.7	60.00	0.44	tr_C9ZMP2_
DRB1_0101	266	AVSRFSTAYVEQQPF	VSRFSTAYV	1	0.4308	472.8	60.00	0.48	tr_C9ZMP2_
DRB1_0101	267	GSIEAAEVLETSKHG	IEAAEVLET	2	0.4297	478.6	60.00	0.62	tr_C9ZMP2_
DRB1_0101	268	ELYLIDDILGCLDPT	YLIDDILGC	2	0.4281	487.0	60.00	0.34	tr_C9ZMP2_
DRB1_0101	269	EMRRQRLAGIHGLPH	LAGIHGLPH	6	0.4241	508.6	60.00	0.29	tr_C9ZMP2_
DRB1_0101	270	MPCASSLNEIQPLLQ	LNEIQPLLQ	6	0.4219	520.4	60.00	0.41	tr_C9ZMP2_
DRB1_0101	271	RLNTQGGAGLPRPSF	LNTQGGAGL	1	0.4210	525.8	60.00	0.64	tr_C9ZMP2_
DRB1_0101	272	AKPHATQQHVALANK	HATQQHVAL	3	0.4193	535.3	60.00	0.43	tr_C9ZMP2_
DRB1_0101	273	TMPLNAYPCVINGLV	PLNAYPCVI	2	0.4188	538.5	60.00	0.26	tr_C9ZMP2_
DRB1_0101	274	ELVERADVVYRCVEG	VERADVVYR	2	0.4174	546.7	60.00	0.56	tr_C9ZMP2_
DRB1_0101	275	ALVGSAKSGSCVIVA	SAKSGSCVI	4	0.4166	551.5	60.00	0.41	tr_C9ZMP2_
DRB1_0101	276	LVGSAKSGSCVIVAT	SAKSGSCVI	3	0.4134	570.9	60.00	0.46	tr_C9ZMP2_
DRB1_0101	277	NAGDQYVVTWAKSGD	YVVTWAKSG	5	0.4131	572.5	60.00	0.69	tr_C9ZMP2_
DRB1_0101	278	AALMQISRNAGDQYV	ALMQISRNA	1	0.4129	574.1	60.00	0.40	tr_C9ZMP2_
DRB1_0101	279	NALVGSAKSGSCVIV	SAKSGSCVI	5	0.4115	582.5	60.00	0.31	tr_C9ZMP2_
DRB1_0101	280	FRRNHDLLIDIRSGV	FRRNHDLLI	0	0.4102	591.0	60.00	0.35	tr_C9ZMP2_
DRB1_0101	281	FQTCFYPDGDTFLVG	FYPDGDTFL	4	0.4097	593.7	60.00	0.47	tr_C9ZMP2_
DRB1_0101	282	QATSSQLIAVVGVSG	LIAVVGVSG	6	0.4086	601.1	60.00	0.35	tr_C9ZMP2_
DRB1_0101	283	FYPDGDTFLVGDRGI	DTFLVGDRG	5	0.4065	614.7	65.00	0.43	tr_C9ZMP2_
DRB1_0101	284	INGLVESYVSWCRLS	INGLVESYV	0	0.4051	624.0	65.00	0.25	tr_C9ZMP2_
DRB1_0101	285	TLRENILMGLMYDSV	ILMGLMYDS	5	0.4050	625.2	65.00	0.28	tr_C9ZMP2_
DRB1_0101	286	KPHATQQHVALANKE	HATQQHVAL	2	0.4035	635.0	65.00	0.41	tr_C9ZMP2_
DRB1_0101	287	NTQGGAGLPRPSFML	GLPRPSFML	6	0.4033	636.3	65.00	0.50	tr_C9ZMP2_
DRB1_0101	288	GLPHPFRRNHDLLID	FRRNHDLLI	5	0.4026	641.2	65.00	0.78	tr_C9ZMP2_
DRB1_0101	289	QTCFYPDGDTFLVGD	FYPDGDTFL	3	0.4017	647.8	65.00	0.47	tr_C9ZMP2_
DRB1_0101	290	VSRFSTAYVEQQPFL	FSTAYVEQQ	3	0.4006	655.6	65.00	0.44	tr_C9ZMP2_
DRB1_0101	291	AWETLACYIGRVGWK	TLACYIGRV	3	0.3995	663.0	65.00	0.26	tr_C9ZMP2_
DRB1_0101	292	TAATTVAEPSSPAAK	TVAEPSSPA	4	0.3994	664.2	65.00	0.68	tr_C9ZMP2_
DRB1_0101	293	TLAWETLACYIGRVG	WETLACYIG	3	0.3990	666.8	65.00	0.31	tr_C9ZMP2_
DRB1_0101	294	SKHGTLAWETLACYI	GTLAWETLA	3	0.3989	667.3	65.00	0.28	tr_C9ZMP2_
DRB1_0101	295	LYLIDDILGCLDPTV	YLIDDILGC	1	0.3978	675.6	65.00	0.27	tr_C9ZMP2_
DRB1_0101	296	TCFYPDGDTFLVGDR	YPDGDTFLV	3	0.3974	678.7	65.00	0.48	tr_C9ZMP2_

DRB1_0101	297	LLQAKGCVGEMRRQR	LLQAKGCVG	0	0.3966	684.7	65.00	0.58	tr_C9ZMP2_
DRB1_0101	298	KHGTLAWETLACYIG	GTLAWETLA	2	0.3960	689.0	65.00	0.25	tr_C9ZMP2_
DRB1_0101	299	CFYPDGDTFLVGDRG	YPDGDTFLV	2	0.3943	701.7	65.00	0.43	tr_C9ZMP2_
DRB1_0101	300	AATTVAEPSSPAAKP	TVAEPSSPA	3	0.3935	708.1	65.00	0.64	tr_C9ZMP2_
DRB1_0101	301	YVVTWAKSGDGNTTR	YVVTWAKSG	0	0.3920	719.5	65.00	0.44	tr_C9ZMP2_
DRB1_0101	302	GIHGLPHPFRRNHDL	IHGLPHPFR	1	0.3912	725.9	65.00	0.63	tr_C9ZMP2_
DRB1_0101	303	ATHNQELVERADVVY	ELVERADVV	5	0.3901	734.4	65.00	0.43	tr_C9ZMP2_
DRB1_0101	304	HGTLAWETLACYIGR	LAWETLACY	3	0.3899	736.2	65.00	0.23	tr_C9ZMP2_
DRB1_0101	305	TSKHGTLAWETLACY	GTLAWETLA	4	0.3891	742.2	65.00	0.29	tr_C9ZMP2_
DRB1_0101	306	AAKPHATQQHVALAN	HATQQHVAL	4	0.3885	747.4	65.00	0.40	tr_C9ZMP2_
DRB1_0101	307	GTLAWETLACYIGRV	WETLACYIG	4	0.3862	766.0	65.00	0.31	tr_C9ZMP2_
DRB1_0101	308	LVESYVSWCRLSPFL	ESYVSWCRL	2	0.3846	779.0	65.00	0.29	tr_C9ZMP2_
DRB1_0101	309	MSGTLRENILMGLMY	GTLRENILM	2	0.3846	779.6	65.00	0.24	tr_C9ZMP2_
DRB1_0101	310	VGSAKSGSCVIVATH	SAKSGSCVI	2	0.3843	781.9	70.00	0.44	tr_C9ZMP2_
DRB1_0101	311	ATPESGSIEAAEVLE	SGSIEAAEV	4	0.3841	783.4	70.00	0.51	tr_C9ZMP2_
DRB1_0101	312	LAWETLACYIGRVGW	WETLACYIG	2	0.3836	788.2	70.00	0.30	tr_C9ZMP2_
DRB1_0101	313	ATTVAEPSSPAAKPH	TVAEPSSPA	2	0.3828	794.7	70.00	0.60	tr_C9ZMP2_
DRB1_0101	314	AVVGVSGSGKSTFLD	VVGVSGSGK	1	0.3825	796.9	70.00	0.28	tr_C9ZMP2_
DRB1_0101	315	PHATQQHVALANKEK	HATQQHVAL	1	0.3823	798.8	70.00	0.33	tr_C9ZMP2_
DRB1_0101	316	LNAYPCVINGLVESY	YPCVINGLV	3	0.3822	799.9	70.00	0.44	tr_C9ZMP2_
DRB1_0101	317	NFQTCFYPDGDTFLV	FYPDGDTFL	5	0.3764	852.1	70.00	0.46	tr_C9ZMP2_
DRB1_0101	318	PLNAYPCVINGLVES	YPCVINGLV	4	0.3741	872.7	70.00	0.40	tr_C9ZMP2_
DRB1_0101	319	VSGSGKSTFLDGLAG	STFLDGLAG	6	0.3741	873.5	70.00	0.50	tr_C9ZMP2_
DRB1_0101	320	LMSGTLRENILMGLM	GTLRENILM	3	0.3715	897.9	70.00	0.29	tr_C9ZMP2_
DRB1_0101	321	MPLNAYPCVINGLVE	YPCVINGLV	5	0.3690	922.2	70.00	0.34	tr_C9ZMP2_
DRB1_0101	322	LETSKHGTLAWETLA	KHGTLAWET	4	0.3685	928.2	70.00	0.31	tr_C9ZMP2_
DRB1_0101	323	HATQQHVALANKEKG	QHVALANKE	4	0.3683	930.1	70.00	0.19	tr_C9ZMP2_
DRB1_0101	324	LYGFASGESDSGPKM	FASGESDSG	3	0.3681	931.8	70.00	0.47	tr_C9ZMP2_
DRB1_0101	325	VGEMRRQRLAGIHGL	MRRQRLAGI	3	0.3663	949.8	70.00	0.42	tr_C9ZMP2_
DRB1_0101	326	VATHNQELVERADVV	HNQELVERA	3	0.3663	949.9	70.00	0.41	tr_C9ZMP2_
DRB1_0101	327	DATGHAAVSRFSTAY	GHAAVSRFS	3	0.3661	952.0	70.00	0.35	tr_C9ZMP2_
DRB1_0101	328	RNAGDQYVVTWAKSG	YVVTWAKSG	6	0.3643	971.1	70.00	0.54	tr_C9ZMP2_
DRB1_0101	329	DLYGFASGESDSGPK	YGFASGESD	2	0.3635	978.9	70.00	0.45	tr_C9ZMP2_
DRB1_0101	330	GEMRRQRLAGIHGLP	MRRQRLAGI	2	0.3631	983.3	70.00	0.37	tr_C9ZMP2_
DRB1_0101	331	VALTQNFQTCFYPDG	VALTQNFQT	0	0.3594	1024.0	75.00	0.44	tr_C9ZMP2_
DRB1_0101	332	SGTLRENILMGLMYD	ENILMGLMY	5	0.3591	1027.5	75.00	0.26	tr_C9ZMP2_
DRB1_0101	333	ETSKHGTLAWETLAC	KHGTLAWET	3	0.3575	1045.0	75.00	0.34	tr_C9ZMP2_
DRB1_0101	334	HGLPHPFRRNHDLLI	FRRNHDLLI	6	0.3564	1057.6	75.00	0.67	tr_C9ZMP2_
DRB1_0101	335	ITEEYQDMVVSSEEM	YQDMVVSSE	4	0.3557	1065.2	75.00	0.47	tr_C9ZMP2_
DRB1_0101	336	GTLRENILMGLMYDS	ENILMGLMY	4	0.3527	1100.8	75.00	0.28	tr_C9ZMP2_
DRB1_0101	337	SIEAAEVLETSKHGT	IEAAEVLET	1	0.3520	1109.1	75.00	0.61	tr_C9ZMP2_
DRB1_0101	338	ATQQHVALANKEKGH	QHVALANKE	3	0.3520	1109.5	75.00	0.28	tr_C9ZMP2_
DRB1_0101	339	HGDLYGFASGESDSG	YGFASGESD	4	0.3516	1114.1	75.00	0.52	tr_C9ZMP2_
DRB1_0101	340	AAEVLETSKHGTLAW	EVLETSKHG	2	0.3510	1121.3	75.00	0.36	tr_C9ZMP2_
DRB1_0101	341	GDLYGFASGESDSGP	YGFASGESD	3	0.3503	1129.9	75.00	0.49	tr_C9ZMP2_
DRB1_0101	342	GLVESYVSWCRLSPF	ESYVSWCRL	3	0.3490	1145.0	75.00	0.28	tr_C9ZMP2_
DRB1_0101	343	LVKTAATTVAEPSSP	VKTAATTVA	1	0.3481	1156.5	75.00	0.50	tr_C9ZMP2_
DRB1_0101	344	NDATGHAAVSRFSTA	TGHAAVSRF	3	0.3479	1158.8	75.00	0.25	tr_C9ZMP2_
DRB1_0101	345	TTVAEPSSPAAKPHA	TVAEPSSPA	1	0.3459	1185.2	75.00	0.54	tr_C9ZMP2_
DRB1_0101	346	ALMQISRNAGDQYVV	MQISRNAGD	2	0.3458	1186.4	75.00	0.31	tr_C9ZMP2_

DRB1_0101	347	LVERADVVYRCVEGQ	VERADVVYR	1	0.3451	1194.6	75.00	0.60	tr_C9ZMP2_
DRB1_0101	348	PAAKPHATQQHVALA	HATQQHVAL	5	0.3449	1197.2	75.00	0.41	tr_C9ZMP2_
DRB1_0101	349	VVGVSGSGKSTFLDG	SGSGKSTFL	4	0.3438	1211.9	75.00	0.28	tr_C9ZMP2_
DRB1_0101	350	LNTQGGAGLPRPSFM	LNTQGGAGL	0	0.3428	1225.1	75.00	0.43	tr_C9ZMP2_
DRB1_0101	351	NGLVESYVSWCRLSP	ESYVSWCRL	4	0.3411	1247.9	75.00	0.25	tr_C9ZMP2_
DRB1_0101	352	TEEYQDMVVSSEEMP	YQDMVVSSE	3	0.3409	1250.4	75.00	0.45	tr_C9ZMP2_
DRB1_0101	353	GSAKSGSCVIVATHN	SAKSGSCVI	1	0.3401	1262.0	75.00	0.40	tr_C9ZMP2_
DRB1_0101	354	GCVGEMRRQRLAGIH	MRRQRLAGI	5	0.3400	1262.2	75.00	0.37	tr_C9ZMP2_
DRB1_0101	355	TQQHVALANKEKGHG	HVALANKEK	3	0.3400	1262.4	75.00	0.34	tr_C9ZMP2_
DRB1_0101	356	EITEEYQDMVVSSEE	YQDMVVSSE	5	0.3376	1295.7	75.00	0.41	tr_C9ZMP2_
DRB1_0101	357	AEVLETSKHGTLAWE	LETSKHGTL	3	0.3373	1300.0	75.00	0.38	tr_C9ZMP2_
DRB1_0101	358	CVGEMRRQRLAGIHG	MRRQRLAGI	4	0.3365	1311.7	75.00	0.40	tr_C9ZMP2_
DRB1_0101	359	EAAEVLETSKHGTLA	EVLETSKHG	3	0.3361	1317.4	75.00	0.47	tr_C9ZMP2_
DRB1_0101	360	TEITEEYQDMVVSSE	EEYQDMVVS	4	0.3330	1361.7	80.00	0.25	tr_C9ZMP2_
DRB1_0101	361	VLETSKHGTLAWETL	KHGTLAWET	5	0.3325	1369.2	80.00	0.30	tr_C9ZMP2_
DRB1_0101	362	QQHVALANKEKGHGK	VALANKEKG	3	0.3313	1387.9	80.00	0.44	tr_C9ZMP2_
DRB1_0101	363	AATPESGSIEAAEVL	SGSIEAAEV	5	0.3297	1411.4	80.00	0.55	tr_C9ZMP2_
DRB1_0101	364	PNDATGHAAVSRFST	TGHAAVSRF	4	0.3295	1415.0	80.00	0.23	tr_C9ZMP2_
DRB1_0101	365	KGCVGEMRRQRLAGI	VGEMRRQRL	3	0.3285	1430.7	80.00	0.35	tr_C9ZMP2_
DRB1_0101	366	KTAATTVAEPSSPAA	TVAEPSSPA	5	0.3240	1501.6	80.00	0.61	tr_C9ZMP2_
DRB1_0101	367	IHGLPHPFRRNHDLL	IHGLPHPFR	0	0.3220	1534.6	80.00	0.52	tr_C9ZMP2_
DRB1_0101	368	FTEITEEYQDMVVSS	EEYQDMVVS	5	0.3218	1537.4	80.00	0.28	tr_C9ZMP2_
DRB1_0101	369	EVLETSKHGTLAWET	LETSKHGTL	2	0.3216	1540.8	80.00	0.35	tr_C9ZMP2_
DRB1_0101	370	CHGDLYGFASGESDS	YGFASGESD	5	0.3175	1610.6	80.00	0.50	tr_C9ZMP2_
DRB1_0101	371	IEAAEVLETSKHGTL	EVLETSKHG	4	0.3165	1629.0	80.00	0.38	tr_C9ZMP2_
DRB1_0101	372	SAKSGSCVIVATHNQ	CVIVATHNQ	6	0.3152	1650.7	80.00	0.29	tr_C9ZMP2_
DRB1_0101	373	EMPCASSLNEIQPLL	SSLNEIQPL	5	0.3146	1662.0	80.00	0.41	tr_C9ZMP2_
DRB1_0101	374	MFTEITEEYQDMVVS	ITEEYQDMV	4	0.3142	1668.6	80.00	0.29	tr_C9ZMP2_
DRB1_0101	375	VGVSGSGKSTFLDGL	SGSGKSTFL	3	0.3118	1714.1	80.00	0.43	tr_C9ZMP2_
DRB1_0101	376	EEYQDMVVSSEEMPC	YQDMVVSSE	2	0.3114	1720.8	80.00	0.43	tr_C9ZMP2_
DRB1_0101	377	YGFASGESDSGPKMF	FASGESDSG	2	0.3107	1733.7	80.00	0.50	tr_C9ZMP2_
DRB1_0101	378	LMQISRNAGDQYVVT	MQISRNAGD	1	0.3082	1781.2	80.00	0.27	tr_C9ZMP2_
DRB1_0101	379	EYQDMVVSSEEMPCA	DMVVSSEEM	3	0.3070	1803.7	80.00	0.31	tr_C9ZMP2_
DRB1_0101	380	DPNDATGHAAVSRFS	TGHAAVSRF	5	0.3059	1826.8	85.00	0.24	tr_C9ZMP2_
DRB1_0101	381	AKGCVGEMRRQRLAG	VGEMRRQRL	4	0.3011	1924.1	85.00	0.40	tr_C9ZMP2_
DRB1_0101	382	QNFQTCFYPDGDTFL	FYPDGDTFL	6	0.2995	1957.6	85.00	0.41	tr_C9ZMP2_
DRB1_0101	383	YQDMVVSSEEMPCAS	DMVVSSEEM	2	0.2954	2047.0	85.00	0.33	tr_C9ZMP2_
DRB1_0101	384	KMFTEITEEYQDMVV	FTEITEEYQ	2	0.2940	2077.1	85.00	0.38	tr_C9ZMP2_
DRB1_0101	385	QHVALANKEKGHGKV	VALANKEKG	2	0.2935	2088.6	85.00	0.45	tr_C9ZMP2_
DRB1_0101	386	MQISRNAGDQYVVTW	SRNAGDQYV	3	0.2898	2173.5	85.00	0.29	tr_C9ZMP2_
DRB1_0101	387	KEKGHGKVGNVLGDV	GHGKVGNVL	3	0.2895	2181.7	85.00	0.61	tr_C9ZMP2_
DRB1_0101	388	QAKGCVGEMRRQRLA	VGEMRRQRL	5	0.2880	2216.2	85.00	0.34	tr_C9ZMP2_
DRB1_0101	389	VGNVLGDVPWPSSDS	VLGDVPWPS	3	0.2872	2234.7	85.00	0.59	tr_C9ZMP2_
DRB1_0101	390	AKSGSCVIVATHNQE	CVIVATHNQ	5	0.2867	2247.6	85.00	0.47	tr_C9ZMP2_
DRB1_0101	391	EHLIDPNDATGHAAV	IDPNDATGH	3	0.2863	2257.1	85.00	0.28	tr_C9ZMP2_
DRB1_0101	392	IDPNDATGHAAVSRF	PNDATGHAA	2	0.2845	2302.5	85.00	0.32	tr_C9ZMP2_
DRB1_0101	393	KVGNVLGDVPWPSSD	VLGDVPWPS	4	0.2833	2333.3	85.00	0.55	tr_C9ZMP2_
DRB1_0101	394	LIDPNDATGHAAVSR	PNDATGHAA	3	0.2832	2335.6	85.00	0.40	tr_C9ZMP2_
DRB1_0101	395	QISRNAGDQYVVTWA	SRNAGDQYV	2	0.2776	2481.0	85.00	0.26	tr_C9ZMP2_
DRB1_0101	396	DMVVSSEEMPCASSL	VSSEEMPCA	3	0.2762	2518.0	85.00	0.25	tr_C9ZMP2_

DRB1_0101	397	QDMVVSSEEMPCASS	DMVVSSEEM	1	0.2759	2525.4	85.00	0.31	tr_C9ZMP2_
DRB1_0101	398	TVAEPSSPAAKPHAT	TVAEPSSPA	0	0.2759	2526.4	85.00	0.38	tr_C9ZMP2_
DRB1_0101	399	VERADVVYRCVEGQL	VERADVVYR	0	0.2758	2527.9	85.00	0.28	tr_C9ZMP2_
DRB1_0101	400	GKVGNVLGDVPWPSS	VLGDVPWPS	5	0.2730	2607.6	90.00	0.50	tr_C9ZMP2_
DRB1_0101	401	GEHLIDPNDATGHAA	EHLIDPNDA	1	0.2723	2626.2	90.00	0.40	tr_C9ZMP2_
DRB1_0101	402	HLIDPNDATGHAAVS	PNDATGHAA	4	0.2714	2653.2	90.00	0.34	tr_C9ZMP2_
DRB1_0101	403	PKMFTEITEEYQDMV	FTEITEEYQ	3	0.2696	2703.8	90.00	0.44	tr_C9ZMP2_
DRB1_0101	404	GVSGSGKSTFLDGLA	SGSGKSTFL	2	0.2688	2728.0	90.00	0.50	tr_C9ZMP2_
DRB1_0101	405	ACHGDLYGFASGESD	YGFASGESD	6	0.2679	2755.1	90.00	0.33	tr_C9ZMP2_
DRB1_0101	406	AGEHLIDPNDATGHA	EHLIDPNDA	2	0.2674	2770.0	90.00	0.51	tr_C9ZMP2_
DRB1_0101	407	ALTQNFQTCFYPDGD	FQTCFYPDG	5	0.2670	2783.1	90.00	0.34	tr_C9ZMP2_
DRB1_0101	408	MVVSSEEMPCASSLN	VSSEEMPCA	2	0.2660	2811.1	90.00	0.26	tr_C9ZMP2_
DRB1_0101	409	SPAAKPHATQQHVAL	HATQQHVAL	6	0.2655	2826.5	90.00	0.31	tr_C9ZMP2_
DRB1_0101	410	NKEKGHGKVGNVLGD	GHGKVGNVL	4	0.2654	2831.7	90.00	0.60	tr_C9ZMP2_
DRB1_0101	411	ANKEKGHGKVGNVLG	GHGKVGNVL	5	0.2652	2835.7	90.00	0.52	tr_C9ZMP2_
DRB1_0101	412	GNVLGDVPWPSSDSS	VLGDVPWPS	2	0.2635	2889.4	90.00	0.61	tr_C9ZMP2_
DRB1_0101	413	ISRNAGDQYVVTWAK	NAGDQYVVT	3	0.2629	2908.7	90.00	0.23	tr_C9ZMP2_
DRB1_0101	414	SRNAGDQYVVTWAKS	DQYVVTWAK	5	0.2600	2999.2	90.00	0.24	tr_C9ZMP2_
DRB1_0101	415	EKGHGKVGNVLGDVP	GHGKVGNVL	2	0.2600	2999.7	90.00	0.57	tr_C9ZMP2_
DRB1_0101	416	LAGEHLIDPNDATGH	EHLIDPNDA	3	0.2592	3025.8	90.00	0.59	tr_C9ZMP2_
DRB1_0101	417	VKTAATTVAEPSSPA	TVAEPSSPA	6	0.2587	3044.5	90.00	0.35	tr_C9ZMP2_
DRB1_0101	418	LQAKGCVGEMRRQRL	CVGEMRRQR	5	0.2571	3097.3	90.00	0.24	tr_C9ZMP2_
DRB1_0101	419	EEMPCASSLNEIQPL	CASSLNEIQ	4	0.2549	3170.8	90.00	0.28	tr_C9ZMP2_
DRB1_0101	420	SSEEMPCASSLNEIQ	EEMPCASSL	2	0.2504	3329.9	90.00	0.34	tr_C9ZMP2_
DRB1_0101	421	VSSEEMPCASSLNEI	EEMPCASSL	3	0.2476	3433.2	90.00	0.32	tr_C9ZMP2_
DRB1_0101	422	VVSSEEMPCASSLNE	EEMPCASSL	4	0.2468	3460.6	90.00	0.31	tr_C9ZMP2_
DRB1_0101	423	GFASGESDSGPKMFT	FASGESDSG	1	0.2459	3497.0	90.00	0.57	tr_C9ZMP2_
DRB1_0101	424	KGHGKVGNVLGDVPW	GHGKVGNVL	1	0.2450	3531.4	90.00	0.49	tr_C9ZMP2_
DRB1_0101	425	HVALANKEKGHGKVG	VALANKEKG	1	0.2431	3604.2	90.00	0.43	tr_C9ZMP2_
DRB1_0101	426	GLAGEHLIDPNDATG	EHLIDPNDA	4	0.2375	3826.4	95.00	0.58	tr_C9ZMP2_
DRB1_0101	427	GPKMFTEITEEYQDM	FTEITEEYQ	4	0.2344	3960.1	95.00	0.49	tr_C9ZMP2_
DRB1_0101	428	LTQNFQTCFYPDGDT	FQTCFYPDG	4	0.2338	3984.4	95.00	0.45	tr_C9ZMP2_
DRB1_0101	429	SEEMPCASSLNEIQP	EEMPCASSL	1	0.2330	4018.3	95.00	0.29	tr_C9ZMP2_
DRB1_0101	430	DGLAGEHLIDPNDAT	EHLIDPNDA	5	0.2330	4018.4	95.00	0.47	tr_C9ZMP2_
DRB1_0101	431	PWPSSDSSPARAPAA	SSDSSPARA	3	0.2322	4055.0	95.00	0.31	tr_C9ZMP2_
DRB1_0101	432	TQNFQTCFYPDGDTF	FQTCFYPDG	3	0.2310	4105.4	95.00	0.55	tr_C9ZMP2_
DRB1_0101	433	LVMRPDACHGDLYGF	LVMRPDACH	0	0.2302	4144.5	95.00	0.32	tr_C9ZMP2_
DRB1_0101	434	WPSSDSSPARAPAAT	SSDSSPARA	2	0.2299	4158.1	95.00	0.29	tr_C9ZMP2_
DRB1_0101	435	LANKEKGHGKVGNVL	GHGKVGNVL	6	0.2293	4184.8	95.00	0.41	tr_C9ZMP2_
DRB1_0101	436	NVLGDVPWPSSDSSP	VLGDVPWPS	1	0.2266	4307.6	95.00	0.57	tr_C9ZMP2_
DRB1_0101	437	DACHGDLYGFASGES	HGDLYGFAS	3	0.2193	4660.5	95.00	0.38	tr_C9ZMP2_
DRB1_0101	438	SGPKMFTEITEEYQD	FTEITEEYQ	5	0.2183	4710.4	95.00	0.41	tr_C9ZMP2_
DRB1_0101	439	RPDACHGDLYGFASG	HGDLYGFAS	5	0.2155	4855.2	95.00	0.35	tr_C9ZMP2_
DRB1_0101	440	VPWPSSDSSPARAPA	WPSSDSSPA	2	0.2147	4901.5	95.00	0.41	tr_C9ZMP2_
DRB1_0101	441	DSGPKMFTEITEEYQ	FTEITEEYQ	6	0.2135	4963.3	95.00	0.26	tr_C9ZMP2_
DRB1_0101	442	HGKVGNVLGDVPWPS	VLGDVPWPS	6	0.2130	4989.2	95.00	0.34	tr_C9ZMP2_
DRB1_0101	443	PSSDSSPARAPAATP	SSDSSPARA	1	0.2121	5040.3	95.00	0.30	tr_C9ZMP2_
DRB1_0101	444	PDACHGDLYGFASGE	HGDLYGFAS	4	0.2077	5286.6	95.00	0.38	tr_C9ZMP2_
DRB1_0101	445	SSPAAKPHATQQHVA	SPAAKPHAT	1	0.2038	5514.2	95.00	0.28	tr_C9ZMP2_
DRB1_0101	446	GESDSGPKMFTEITE	SGPKMFTEI	4	0.2006	5709.0	95.00	0.25	tr_C9ZMP2_

DRB1_0101	447	FASGESDSGPKMFTE	FASGESDSG	0	0.1998	5757.0	95.00	0.39	tr_C9ZMP2_
DRB1_0101	448	SSDSSPARAPAATPE	SSPARAPAA	3	0.1986	5831.7	95.00	0.30	tr_C9ZMP2_
DRB1_0101	449	VAEPSSPAAKPHATQ	SPAAKPHAT	5	0.1950	6065.5	95.00	0.25	tr_C9ZMP2_
DRB1_0101	450	PAATPESGSIEAAEV	SGSIEAAEV	6	0.1923	6242.5	100.00	0.50	tr_C9ZMP2_
DRB1_0101	451	SDSGPKMFTEITEEY	SGPKMFTEI	2	0.1922	6246.9	100.00	0.28	tr_C9ZMP2_
DRB1_0101	452	SGESDSGPKMFTEIT	SGPKMFTEI	5	0.1922	6250.5	100.00	0.22	tr_C9ZMP2_
DRB1_0101	453	ESDSGPKMFTEITEE	SGPKMFTEI	3	0.1911	6323.1	100.00	0.31	tr_C9ZMP2_
DRB1_0101	454	DVPWPSSDSSPARAP	WPSSDSSPA	3	0.1905	6362.8	100.00	0.47	tr_C9ZMP2_
DRB1_0101	455	MRPDACHGDLYGFAS	HGDLYGFAS	6	0.1893	6446.2	100.00	0.26	tr_C9ZMP2_
DRB1_0101	456	GHGKVGNVLGDVPWP	GHGKVGNVL	0	0.1892	6453.0	100.00	0.26	tr_C9ZMP2_
DRB1_0101	457	VALANKEKGHGKVGN	VALANKEKG	0	0.1890	6466.5	100.00	0.33	tr_C9ZMP2_
DRB1_0101	458	PSSPAAKPHATQQHV	SPAAKPHAT	2	0.1870	6609.6	100.00	0.38	tr_C9ZMP2_
DRB1_0101	459	VLGDVPWPSSDSSPA	VLGDVPWPS	0	0.1826	6933.3	100.00	0.35	tr_C9ZMP2_
DRB1_0101	460	GDVPWPSSDSSPARA	WPSSDSSPA	4	0.1814	7026.7	100.00	0.58	tr_C9ZMP2_
DRB1_0101	461	SDSSPARAPAATPES	PARAPAATP	4	0.1791	7203.0	100.00	0.41	tr_C9ZMP2_
DRB1_0101	462	EPSSPAAKPHATQQH	SPAAKPHAT	3	0.1760	7449.7	100.00	0.43	tr_C9ZMP2_
DRB1_0101	463	AEPSSPAAKPHATQQ	SPAAKPHAT	4	0.1747	7555.7	100.00	0.36	tr_C9ZMP2_
DRB1_0101	464	DSSPARAPAATPESG	PARAPAATP	3	0.1697	7967.8	100.00	0.55	tr_C9ZMP2_
DRB1_0101	465	VMRPDACHGDLYGFA	PDACHGDLY	3	0.1653	8361.0	100.00	0.22	tr_C9ZMP2_
DRB1_0101	466	ASGESDSGPKMFTEI	GESDSGPKM	2	0.1652	8373.6	100.00	0.26	tr_C9ZMP2_
DRB1_0101	467	ALANKEKGHGKVGNV	ANKEKGHGK	2	0.1615	8706.8	100.00	0.25	tr_C9ZMP2_
DRB1_0101	468	SSPARAPAATPESGS	PARAPAATP	2	0.1613	8733.7	100.00	0.52	tr_C9ZMP2_
DRB1_0101	469	LGDVPWPSSDSSPAR	WPSSDSSPA	5	0.1463	10267.1	100.00	0.55	tr_C9ZMP2_
DRB1_0101	470	SPARAPAATPESGSI	PARAPAATP	1	0.1378	11256.4	100.00	0.50	tr_C9ZMP2_
DRB1_0101	471	RAPAATPESGSIEAA	ATPESGSIE	4	0.1330	11854.3	100.00	0.34	tr_C9ZMP2_
DRB1_0101	472	APAATPESGSIEAAE	ATPESGSIE	3	0.1294	12332.9	100.00	0.34	tr_C9ZMP2_
DRB1_0101	473	ARAPAATPESGSIEA	ATPESGSIE	5	0.1147	14447.8	100.00	0.28	tr_C9ZMP2_
DRB1_0101	474	PARAPAATPESGSIE	PARAPAATP	0	0.1143	14523.0	100.00	0.31	tr_C9ZMP2_

Allele: DRB1_0101. Number of high binders 2. Number of weak binders 46. Number of peptides 474

Allele	pos	peptide	core	Of	1-log50k(aff)	affinity(nM)	%Rank	Relia	Identity	Bind Level
DRB1_0101	1	GGVVIRSLGGRVVMS	VIRSLGGRV	3	0.8611	4.5	0.50	0.51	tr_C9ZMP2_	WB
DRB1_0101	2	GVVIRSLGGRVVMSH	VIRSLGGRV	2	0.8603	4.5	0.50	0.48	tr_C9ZMP2_	WB
DRB1_0101	3	DGGVVIRSLGGRVVM	VIRSLGGRV	4	0.8430	5.5	0.80	0.50	tr_C9ZMP2_	WB
DRB1_0101	4	VVIRSLGGRVVMSHL	IRSLGGRVV	2	0.8358	5.9	0.90	0.49	tr_C9ZMP2_	WB
DRB1_0101	5	LDGGVVIRSLGGRVV	VIRSLGGRV	5	0.7979	8.9	2.50	0.54	tr_C9ZMP2_	WB
DRB1_0101	6	VIRSLGGRVVMSHLR	IRSLGGRVV	1	0.7720	11.8	3.50	0.58	tr_C9ZMP2_	WB
DRB1_0101	7	ALDGGVVIRSLGGRV	VIRSLGGRV	6	0.6669	36.8	13.00	0.55	tr_C9ZMP2_	
DRB1_0101	8	IRSLGGRVVMSHLRR	IRSLGGRVV	0	0.6568	41.0	14.00	0.59	tr_C9ZMP2_	
DRB1_0101	9	LGGRVVMSHLRRASR	VVMSHLRRA	4	0.6108	67.4	21.00	0.54	tr_C9ZMP2_	
DRB1_0101	10	GGRVVMSHLRRASRN	VVMSHLRRA	3	0.6094	68.5	21.00	0.59	tr_C9ZMP2_	
DRB1_0101	11	SVLYHRIQCPYRASS	HRIQCPYRA	4	0.6070	70.3	21.00	0.43	tr_C9ZMP2_	
DRB1_0101	12	VLYHRIQCPYRASSR	HRIQCPYRA	3	0.5996	76.1	23.00	0.51	tr_C9ZMP2_	
DRB1_0101	13	VSVLYHRIQCPYRAS	HRIQCPYRA	5	0.5901	84.4	24.00	0.38	tr_C9ZMP2_	
DRB1_0101	14	SLGGRVVMSHLRRAS	VVMSHLRRA	5	0.5859	88.2	25.00	0.43	tr_C9ZMP2_	

DRB1_0101	15	GRVVMSHLRRASRNT	VVMSHLRRA	2	0.5820	92.1	25.00	0.60	tr_C9ZMP2_
DRB1_0101	16	LYHRIQCPYRASSRE	HRIQCPYRA	2	0.5682	106.9	28.00	0.58	tr_C9ZMP2_
DRB1_0101	17	PVSVLYHRIQCPYRA	YHRIQCPYR	5	0.5645	111.3	29.00	0.34	tr_C9ZMP2_
DRB1_0101	18	RSLGGRVVMSHLRRA	VVMSHLRRA	6	0.5518	127.6	31.00	0.29	tr_C9ZMP2_
DRB1_0101	19	TMRDALDGGVVIRSL	DALDGGVVI	3	0.5465	135.3	32.00	0.47	tr_C9ZMP2_
DRB1_0101	20	RELRRLEEAANAPLL	LEEAANAPL	5	0.5335	155.6	34.00	0.42	tr_C9ZMP2_
DRB1_0101	21	MRDALDGGVVIRSLG	DALDGGVVI	2	0.5329	156.6	34.00	0.44	tr_C9ZMP2_
DRB1_0101	22	IQCPYRASSRELRRL	YRASSRELR	4	0.5315	159.0	35.00	0.71	tr_C9ZMP2_
DRB1_0101	23	RDALDGGVVIRSLGG	DALDGGVVI	1	0.5307	160.4	35.00	0.38	tr_C9ZMP2_
DRB1_0101	24	QCPYRASSRELRRLE	YRASSRELR	3	0.5297	162.2	35.00	0.73	tr_C9ZMP2_
DRB1_0101	25	LRRLEEAANAPLLDT	LEEAANAPL	3	0.5280	165.2	35.00	0.56	tr_C9ZMP2_
DRB1_0101	26	ELRRLEEAANAPLLD	LEEAANAPL	4	0.5258	169.2	36.00	0.50	tr_C9ZMP2_
DRB1_0101	27	DALDGGVVIRSLGGR	GVVIRSLGG	5	0.5170	185.9	38.00	0.41	tr_C9ZMP2_
DRB1_0101	28	RVVMSHLRRASRNTD	VVMSHLRRA	1	0.5156	188.9	38.00	0.63	tr_C9ZMP2_
DRB1_0101	29	DTMRDALDGGVVIRS	DALDGGVVI	4	0.5140	192.1	38.00	0.44	tr_C9ZMP2_
DRB1_0101	30	YHRIQCPYRASSREL	HRIQCPYRA	1	0.5136	193.1	38.00	0.60	tr_C9ZMP2_
DRB1_0101	31	CPYRASSRELRRLEE	YRASSRELR	2	0.5020	218.8	41.00	0.73	tr_C9ZMP2_
DRB1_0101	32	LDTMRDALDGGVVIR	DALDGGVVI	5	0.4962	233.0	42.00	0.41	tr_C9ZMP2_
DRB1_0101	33	RRLEEAANAPLLDTM	LEEAANAPL	2	0.4845	264.6	44.00	0.60	tr_C9ZMP2_
DRB1_0101	34	HRIQCPYRASSRELR	HRIQCPYRA	0	0.4813	273.7	45.00	0.38	tr_C9ZMP2_
DRB1_0101	35	RIQCPYRASSRELRR	YRASSRELR	5	0.4759	290.3	46.00	0.69	tr_C9ZMP2_
DRB1_0101	36	SRELRRLEEAANAPL	LRRLEEAAN	3	0.4734	298.2	47.00	0.54	tr_C9ZMP2_
DRB1_0101	37	PYRASSRELRRLEEA	YRASSRELR	1	0.4597	345.7	49.00	0.68	tr_C9ZMP2_
DRB1_0101	38	LLDTMRDALDGGVVI	DALDGGVVI	6	0.4450	405.3	55.00	0.26	tr_C9ZMP2_
DRB1_0101	39	RLEEAANAPLLDTMR	LEEAANAPL	1	0.4200	531.5	60.00	0.57	tr_C9ZMP2_
DRB1_0101	40	SRNTDLLLRVKFNSM	DLLLRVKFN	4	0.4111	584.9	60.00	0.37	tr_C9ZMP2_
DRB1_0101	41	VVMSHLRRASRNTDL	VVMSHLRRA	0	0.3867	761.9	65.00	0.46	tr_C9ZMP2_
DRB1_0101	42	PLLDTMRDALDGGVV	LDTMRDALD	2	0.3850	776.1	65.00	0.38	tr_C9ZMP2_
DRB1_0101	43	SSRELRRLEEAANAP	LRRLEEAAN	4	0.3793	825.1	70.00	0.62	tr_C9ZMP2_
DRB1_0101	44	YRASSRELRRLEEAA	YRASSRELR	0	0.3687	926.1	70.00	0.60	tr_C9ZMP2_
DRB1_0101	45	ASSRELRRLEEAANA	LRRLEEAAN	5	0.3661	951.7	70.00	0.64	tr_C9ZMP2_
DRB1_0101	46	APLLDTMRDALDGGV	LDTMRDALD	3	0.3606	1010.5	70.00	0.55	tr_C9ZMP2_
DRB1_0101	47	LRRASRNTDLLLRVK	SRNTDLLLR	4	0.3465	1177.0	75.00	0.37	tr_C9ZMP2_
DRB1_0101	48	HLRRASRNTDLLLRV	RRASRNTDL	2	0.3445	1202.9	75.00	0.28	tr_C9ZMP2_
DRB1_0101	49	SHLRRASRNTDLLLR	RRASRNTDL	3	0.3397	1267.5	75.00	0.32	tr_C9ZMP2_
DRB1_0101	50	RASRNTDLLLRVKFN	DLLLRVKFN	6	0.3311	1390.2	80.00	0.28	tr_C9ZMP2_
DRB1_0101	51	RRASRNTDLLLRVKF	SRNTDLLLR	3	0.3303	1402.4	80.00	0.46	tr_C9ZMP2_
DRB1_0101	52	NAPLLDTMRDALDGG	LDTMRDALD	4	0.3291	1420.8	80.00	0.57	tr_C9ZMP2_
DRB1_0101	53	ASRNTDLLLRVKFNS	DLLLRVKFN	5	0.3280	1437.6	80.00	0.44	tr_C9ZMP2_
DRB1_0101	54	LEEAANAPLLDTMRD	LEEAANAPL	0	0.3199	1568.8	80.00	0.34	tr_C9ZMP2_
DRB1_0101	55	MSHLRRASRNTDLLL	LRRASRNTD	3	0.3116	1716.2	80.00	0.29	tr_C9ZMP2_
DRB1_0101	56	ANAPLLDTMRDALDG	LDTMRDALD	5	0.3051	1841.2	85.00	0.51	tr_C9ZMP2_
DRB1_0101	57	VMSHLRRASRNTDLL	LRRASRNTD	4	0.2882	2212.0	85.00	0.20	tr_C9ZMP2_
DRB1_0101	58	RASSRELRRLEEAAN	LRRLEEAAN	6	0.2673	2772.5	90.00	0.47	tr_C9ZMP2_
DRB1_0101	59	AANAPLLDTMRDALD	LDTMRDALD	6	0.2594	3021.8	90.00	0.42	tr_C9ZMP2_
DRB1_0101	60	EEAANAPLLDTMRDA	EAANAPLLD	1	0.2430	3604.9	90.00	0.25	tr_C9ZMP2_
DRB1_0101	61	EAANAPLLDTMRDAL	NAPLLDTMR	3	0.2337	3988.7	95.00	0.24	tr_C9ZMP2_

Allele: DRB1_0101. Number of high binders 0. Number of weak binders 6. Number of peptides 61

Allele	pos	peptide	core	Of	1-log50k(aff)	affinity(nM)	%Rank	Relia	Identity	Bind Level
DRB1_0101	1	LARVILQRSPLLLLD	VILQRSPLL	3	0.8769	3.8	0.25	0.76	tr_C9ZMP2_	SB
DRB1_0101	2	ALARVILQRSPLLLL	VILQRSPLL	4	0.8728	4.0	0.30	0.75	tr_C9ZMP2_	SB
DRB1_0101	3	ARVILQRSPLLLLDE	VILQRSPLL	2	0.8707	4.1	0.30	0.74	tr_C9ZMP2_	SB
DRB1_0101	4	IALARVILQRSPLLL	VILQRSPLL	5	0.8446	5.4	0.70	0.79	tr_C9ZMP2_	WB
DRB1_0101	5	RVILQRSPLLLLDEP	VILQRSPLL	1	0.8445	5.4	0.70	0.73	tr_C9ZMP2_	WB
DRB1_0101	6	RRLPLEVLRTRLSVL	LEVLRTRLS	4	0.8111	7.7	1.60	0.88	tr_C9ZMP2_	WB
DRB1_0101	7	RLPLEVLRTRLSVLP	LEVLRTRLS	3	0.7996	8.7	2.50	0.86	tr_C9ZMP2_	WB
DRB1_0101	8	DRVIVIENGHVAGDG	IVIENGHVA	3	0.7963	9.1	2.50	0.85	tr_C9ZMP2_	WB
DRB1_0101	9	RVIVIENGHVAGDGT	IVIENGHVA	2	0.7932	9.4	2.50	0.85	tr_C9ZMP2_	WB
DRB1_0101	10	FDRVIVIENGHVAGD	IVIENGHVA	4	0.7876	10.0	3.00	0.83	tr_C9ZMP2_	WB
DRB1_0101	11	GTVAQLRAASVWPFS	VAQLRAASV	2	0.7843	10.3	3.00	0.79	tr_C9ZMP2_	WB
DRB1_0101	12	DGTVAQLRAASVWPF	VAQLRAASV	3	0.7828	10.5	3.00	0.85	tr_C9ZMP2_	WB
DRB1_0101	13	TRRLPLEVLRTRLSV	LEVLRTRLS	5	0.7779	11.1	3.50	0.85	tr_C9ZMP2_	WB
DRB1_0101	14	FFDRVIVIENGHVAG	IVIENGHVA	5	0.7764	11.2	3.50	0.79	tr_C9ZMP2_	WB
DRB1_0101	15	LPLEVLRTRLSVLPQ	LEVLRTRLS	2	0.7762	11.3	3.50	0.78	tr_C9ZMP2_	WB
DRB1_0101	16	LIALARVILQRSPLL	VILQRSPLL	6	0.7719	11.8	3.50	0.76	tr_C9ZMP2_	WB
DRB1_0101	17	GDGTVAQLRAASVWP	VAQLRAASV	4	0.7633	13.0	4.00	0.86	tr_C9ZMP2_	WB
DRB1_0101	18	GQRHLIALARVILQR	HLIALARVI	3	0.7610	13.3	4.00	0.51	tr_C9ZMP2_	WB
DRB1_0101	19	AGDGTVAQLRAASVW	VAQLRAASV	5	0.7599	13.4	4.50	0.83	tr_C9ZMP2_	WB
DRB1_0101	20	TVAQLRAASVWPFSS	VAQLRAASV	1	0.7567	13.9	4.50	0.75	tr_C9ZMP2_	WB
DRB1_0101	21	PLEVLRTRLSVLPQQ	LEVLRTRLS	1	0.7539	14.3	4.50	0.71	tr_C9ZMP2_	WB
DRB1_0101	22	QRHLIALARVILQRS	HLIALARVI	2	0.7522	14.6	5.00	0.50	tr_C9ZMP2_	WB
DRB1_0101	23	GGQRHLIALARVILQ	HLIALARVI	4	0.7401	16.6	5.50	0.47	tr_C9ZMP2_	WB
DRB1_0101	24	RQYFSLASEEPRTLG	YFSLASEEP	2	0.7345	17.7	6.00	0.47	tr_C9ZMP2_	WB
DRB1_0101	25	VIVIENGHVAGDGTV	IVIENGHVA	1	0.7323	18.1	6.00	0.85	tr_C9ZMP2_	WB
DRB1_0101	26	VILQRSPLLLLDEPT	VILQRSPLL	0	0.7316	18.2	6.00	0.62	tr_C9ZMP2_	WB
DRB1_0101	27	DTRRLPLEVLRTRLS	LEVLRTRLS	6	0.7229	20.1	7.00	0.67	tr_C9ZMP2_	WB
DRB1_0101	28	QYFSLASEEPRTLGM	LASEEPRTL	4	0.7210	20.5	7.00	0.43	tr_C9ZMP2_	WB
DRB1_0101	29	RHLIALARVILQRSP	HLIALARVI	1	0.7160	21.6	7.50	0.53	tr_C9ZMP2_	WB
DRB1_0101	30	VRQYFSLASEEPRTL	YFSLASEEP	3	0.7126	22.4	8.00	0.60	tr_C9ZMP2_	WB
DRB1_0101	31	GPEVLRSLSFHVNAG	VLRSLSFHV	3	0.7107	22.9	8.00	0.75	tr_C9ZMP2_	WB
DRB1_0101	32	SGGQRHLIALARVIL	HLIALARVI	5	0.6992	25.9	9.00	0.52	tr_C9ZMP2_	WB
DRB1_0101	33	SGPEVLRSLSFHVNA	VLRSLSFHV	4	0.6991	25.9	9.00	0.72	tr_C9ZMP2_	WB
DRB1_0101	34	DFFDRVIVIENGHVA	IVIENGHVA	6	0.6942	27.3	9.50	0.67	tr_C9ZMP2_	WB
DRB1_0101	35	YFSLASEEPRTLGMF	LASEEPRTL	3	0.6919	28.0	10.00	0.61	tr_C9ZMP2_	
DRB1_0101	36	PEVLRSLSFHVNAGE	VLRSLSFHV	2	0.6911	28.3	10.00	0.74	tr_C9ZMP2_	
DRB1_0101	37	LEVLRTRLSVLPQQP	LEVLRTRLS	0	0.6734	34.3	12.00	0.60	tr_C9ZMP2_	
DRB1_0101	38	VAQLRAASVWPFSSP	VAQLRAASV	0	0.6696	35.7	13.00	0.62	tr_C9ZMP2_	
DRB1_0101	39	VAGDGTVAQLRAASV	VAQLRAASV	6	0.6674	36.6	13.00	0.76	tr_C9ZMP2_	
DRB1_0101	40	RVRQYFSLASEEPRT	YFSLASEEP	4	0.6620	38.7	14.00	0.70	tr_C9ZMP2_	
DRB1_0101	41	QQPFIFSGSLRQNID	FIFSGSLRQ	3	0.6606	39.4	14.00	0.70	tr_C9ZMP2_	
DRB1_0101	42	SLSFHVNAGEKVAIV	FHVNAGEKV	3	0.6593	39.9	14.00	0.83	tr_C9ZMP2_	
DRB1_0101	43	PSGPEVLRSLSFHVN	VLRSLSFHV	5	0.6564	41.1	14.00	0.67	tr_C9ZMP2_	
DRB1_0101	44	HLIALARVILQRSPL	IALARVILQ	2	0.6556	41.5	14.00	0.34	tr_C9ZMP2_	
DRB1_0101	45	RSPLLLLDEPTAQSS	LLLLDEPTA	3	0.6552	41.7	14.00	0.73	tr_C9ZMP2_	

DRB1_0101	46	VSGGQRHLIALARVI	HLIALARVI	6	0.6483	45.0	15.00	0.41	tr_C9ZMP2_
DRB1_0101	47	PQQPFIFSGSLRQNI	FIFSGSLRQ	4	0.6472	45.5	15.00	0.76	tr_C9ZMP2_
DRB1_0101	48	LSFHVNAGEKVAIVG	FHVNAGEKV	2	0.6439	47.1	16.00	0.81	tr_C9ZMP2_
DRB1_0101	49	GKSSIFSALLRLVEI	IFSALLRLV	4	0.6438	47.2	16.00	0.49	tr_C9ZMP2_
DRB1_0101	50	AGKSSIFSALLRLVE	IFSALLRLV	5	0.6418	48.2	16.00	0.44	tr_C9ZMP2_
DRB1_0101	51	QPFIFSGSLRQNIDP	FIFSGSLRQ	2	0.6403	49.0	16.00	0.67	tr_C9ZMP2_
DRB1_0101	52	SPLLLLDEPTAQSSA	LLLLDEPTA	2	0.6402	49.0	16.00	0.66	tr_C9ZMP2_
DRB1_0101	53	EVLRSLSFHVNAGEK	VLRSLSFHV	1	0.6368	50.9	17.00	0.73	tr_C9ZMP2_
DRB1_0101	54	AEAALWSSLAEHLRS	LWSSLAEHL	4	0.6355	51.6	17.00	0.43	tr_C9ZMP2_
DRB1_0101	55	QRSPLLLLDEPTAQS	LLLLDEPTA	4	0.6317	53.8	18.00	0.74	tr_C9ZMP2_
DRB1_0101	56	VATSVKLDGVALDYN	SVKLDGVAL	3	0.6310	54.2	18.00	0.85	tr_C9ZMP2_
DRB1_0101	57	KSSIFSALLRLVEIE	IFSALLRLV	3	0.6296	55.0	18.00	0.51	tr_C9ZMP2_
DRB1_0101	58	GAGKSSIFSALLRLV	IFSALLRLV	6	0.6281	55.9	18.00	0.37	tr_C9ZMP2_
DRB1_0101	59	ATSVKLDGVALDYNV	SVKLDGVAL	2	0.6266	56.9	18.00	0.81	tr_C9ZMP2_
DRB1_0101	60	EAEAALWSSLAEHLR	EAALWSSLA	2	0.6252	57.7	19.00	0.46	tr_C9ZMP2_
DRB1_0101	61	RSLSFHVNAGEKVAI	FHVNAGEKV	4	0.6246	58.1	19.00	0.85	tr_C9ZMP2_
DRB1_0101	62	ERVRQYFSLASEEPR	YFSLASEEP	5	0.6133	65.6	20.00	0.66	tr_C9ZMP2_
DRB1_0101	63	SFHVNAGEKVAIVGR	FHVNAGEKV	1	0.6126	66.1	20.00	0.75	tr_C9ZMP2_
DRB1_0101	64	IVIENGHVAGDGTVA	IVIENGHVA	0	0.6124	66.3	20.00	0.81	tr_C9ZMP2_
DRB1_0101	65	PLLLLDEPTAQSSAE	LLLLDEPTA	1	0.6118	66.7	21.00	0.64	tr_C9ZMP2_
DRB1_0101	66	FSLASEEPRTLGMFF	LASEEPRTL	2	0.6116	66.8	21.00	0.81	tr_C9ZMP2_
DRB1_0101	67	PFIFSGSLRQNIDPF	FIFSGSLRQ	1	0.6074	69.9	21.00	0.59	tr_C9ZMP2_
DRB1_0101	68	TSVKLDGVALDYNVT	SVKLDGVAL	1	0.6061	71.0	22.00	0.71	tr_C9ZMP2_
DRB1_0101	69	DVATSVKLDGVALDY	SVKLDGVAL	4	0.6054	71.5	22.00	0.86	tr_C9ZMP2_
DRB1_0101	70	EAALWSSLAEHLRST	LWSSLAEHL	3	0.6047	72.0	22.00	0.50	tr_C9ZMP2_
DRB1_0101	71	RTRLSVLPQQPFIFS	VLPQQPFIF	5	0.6027	73.6	22.00	0.43	tr_C9ZMP2_
DRB1_0101	72	TRLSVLPQQPFIFSG	VLPQQPFIF	4	0.5978	77.6	23.00	0.49	tr_C9ZMP2_
DRB1_0101	73	LQRSPLLLLDEPTAQ	LLLLDEPTA	5	0.5948	80.1	23.00	0.71	tr_C9ZMP2_
DRB1_0101	74	AEAEAALWSSLAEHL	EAALWSSLA	3	0.5904	84.1	24.00	0.56	tr_C9ZMP2_
DRB1_0101	75	LRSLSFHVNAGEKVA	FHVNAGEKV	5	0.5886	85.7	24.00	0.74	tr_C9ZMP2_
DRB1_0101	76	TTVLCITHKLSHIDF	CITHKLSHI	4	0.5879	86.3	24.00	0.37	tr_C9ZMP2_
DRB1_0101	77	RLSVLPQQPFIFSGS	VLPQQPFIF	3	0.5867	87.5	25.00	0.59	tr_C9ZMP2_
DRB1_0101	78	STTVLCITHKLSHID	TVLCITHKL	2	0.5839	90.2	25.00	0.41	tr_C9ZMP2_
DRB1_0101	79	RSTTVLCITHKLSHI	TVLCITHKL	3	0.5806	93.5	26.00	0.48	tr_C9ZMP2_
DRB1_0101	80	SSIFSALLRLVEIES	IFSALLRLV	2	0.5780	96.1	26.00	0.48	tr_C9ZMP2_
DRB1_0101	81	LPQQPFIFSGSLRQN	FIFSGSLRQ	5	0.5756	98.7	27.00	0.85	tr_C9ZMP2_
DRB1_0101	82	SGGQVVLSNVSMRYD	VLSNVSMRY	5	0.5744	100.0	27.00	0.38	tr_C9ZMP2_
DRB1_0101	83	VLRSLSFHVNAGEKV	FHVNAGEKV	6	0.5738	100.6	27.00	0.38	tr_C9ZMP2_
DRB1_0101	84	GQVVLSNVSMRYDPS	VLSNVSMRY	3	0.5675	107.7	28.00	0.47	tr_C9ZMP2_
DRB1_0101	85	AALWSSLAEHLRSTT	LWSSLAEHL	2	0.5665	108.9	28.00	0.56	tr_C9ZMP2_
DRB1_0101	86	TVLCITHKLSHIDFF	CITHKLSHI	3	0.5657	109.9	28.00	0.40	tr_C9ZMP2_
DRB1_0101	87	GGQVVLSNVSMRYDP	VLSNVSMRY	4	0.5630	113.1	29.00	0.41	tr_C9ZMP2_
DRB1_0101	88	DPSGPEVLRSLSFHV	VLRSLSFHV	6	0.5622	114.0	29.00	0.56	tr_C9ZMP2_
DRB1_0101	89	LRTRLSVLPQQPFIF	LSVLPQQPF	4	0.5598	117.1	29.00	0.52	tr_C9ZMP2_
DRB1_0101	90	RDVATSVKLDGVALD	SVKLDGVAL	5	0.5532	125.8	31.00	0.80	tr_C9ZMP2_
DRB1_0101	91	SMRYDPSGPEVLRSL	YDPSGPEVL	3	0.5515	128.2	31.00	0.83	tr_C9ZMP2_
DRB1_0101	92	ILQRSPLLLLDEPTA	LLLLDEPTA	6	0.5513	128.4	31.00	0.55	tr_C9ZMP2_
DRB1_0101	93	LLLLDEPTAQSSAEA	LLLLDEPTA	0	0.5490	131.6	31.00	0.53	tr_C9ZMP2_
DRB1_0101	94	YDTRRLPLEVLRTRL	TRRLPLEVL	2	0.5480	133.0	32.00	0.47	tr_C9ZMP2_
DRB1_0101	95	TGAGKSSIFSALLRL	KSSIFSALL	4	0.5409	143.7	33.00	0.24	tr_C9ZMP2_

DRB1_0101	96	SLASEEPRTLGMFFS	LASEEPRTL	1	0.5407	143.9	33.00	0.75	tr_C9ZMP2_
DRB1_0101	97	GYDTRRLPLEVLRTR	TRRLPLEVL	3	0.5407	143.9	33.00	0.51	tr_C9ZMP2_
DRB1_0101	98	ALWSSLAEHLRSTTV	LWSSLAEHL	1	0.5401	144.9	33.00	0.44	tr_C9ZMP2_
DRB1_0101	99	PSGGQVVLSNVSMRY	VVLSNVSMR	5	0.5399	145.2	33.00	0.28	tr_C9ZMP2_
DRB1_0101	100	ALLRLVEIESGSIHI	LVEIESGSI	4	0.5365	150.6	34.00	0.34	tr_C9ZMP2_
DRB1_0101	101	RVSGGQRHLIALARV	QRHLIALAR	5	0.5344	154.1	34.00	0.38	tr_C9ZMP2_
DRB1_0101	102	PFGLHSEDAVRDVAT	LHSEDAVRD	3	0.5343	154.3	34.00	0.60	tr_C9ZMP2_
DRB1_0101	103	DPFGLHSEDAVRDVA	LHSEDAVRD	4	0.5301	161.4	35.00	0.54	tr_C9ZMP2_
DRB1_0101	104	SIFSALLRLVEIESG	IFSALLRLV	1	0.5291	163.2	35.00	0.42	tr_C9ZMP2_
DRB1_0101	105	MRYDPSGPEVLRSLS	YDPSGPEVL	2	0.5288	163.8	35.00	0.81	tr_C9ZMP2_
DRB1_0101	106	LLRLVEIESGSIHID	LVEIESGSI	3	0.5281	165.0	35.00	0.38	tr_C9ZMP2_
DRB1_0101	107	FIFSGSLRQNIDPFG	FSGSLRQNI	2	0.5279	165.3	35.00	0.44	tr_C9ZMP2_
DRB1_0101	108	QVVLSNVSMRYDPSG	VLSNVSMRY	2	0.5272	166.5	36.00	0.49	tr_C9ZMP2_
DRB1_0101	109	FHVNAGEKVAIVGRT	FHVNAGEKV	0	0.5217	176.9	37.00	0.59	tr_C9ZMP2_
DRB1_0101	110	VSMRYDPSGPEVLRS	YDPSGPEVL	4	0.5206	179.0	37.00	0.83	tr_C9ZMP2_
DRB1_0101	111	SVKLDGVALDYNVTD	SVKLDGVAL	0	0.5199	180.4	37.00	0.62	tr_C9ZMP2_
DRB1_0101	112	LSVLPQQPFIFSGSL	VLPQQPFIF	2	0.5186	182.8	37.00	0.66	tr_C9ZMP2_
DRB1_0101	113	HLRSTTVLCITHKLS	TVLCITHKL	5	0.5167	186.7	38.00	0.47	tr_C9ZMP2_
DRB1_0101	114	VLRTRLSVLPQQPFI	LSVLPQQPF	5	0.5151	189.9	38.00	0.47	tr_C9ZMP2_
DRB1_0101	115	SAEAEAALWSSLAEH	EAALWSSLA	4	0.5141	191.9	38.00	0.64	tr_C9ZMP2_
DRB1_0101	116	EVLRTRLSVLPQQPF	VLRTRLSVL	1	0.5138	192.6	38.00	0.34	tr_C9ZMP2_
DRB1_0101	117	LRSTTVLCITHKLSH	TVLCITHKL	4	0.5134	193.4	38.00	0.59	tr_C9ZMP2_
DRB1_0101	118	RYDPSGPEVLRSLSF	YDPSGPEVL	1	0.5134	193.5	38.00	0.69	tr_C9ZMP2_
DRB1_0101	119	SALLRLVEIESGSIH	LRLVEIESG	3	0.5132	193.8	38.00	0.44	tr_C9ZMP2_
DRB1_0101	120	SSLAEHLRSTTVLCI	AEHLRSTTV	3	0.5116	197.3	39.00	0.55	tr_C9ZMP2_
DRB1_0101	121	VERVRQYFSLASEEP	YFSLASEEP	6	0.5109	198.8	39.00	0.44	tr_C9ZMP2_
DRB1_0101	122	DGYDTRRLPLEVLRT	TRRLPLEVL	4	0.5102	200.2	39.00	0.53	tr_C9ZMP2_
DRB1_0101	123	LWSSLAEHLRSTTVL	AEHLRSTTV	5	0.5090	202.8	39.00	0.37	tr_C9ZMP2_
DRB1_0101	124	LRLVEIESGSIHIDG	LVEIESGSI	2	0.5090	202.8	39.00	0.41	tr_C9ZMP2_
DRB1_0101	125	SLAEHLRSTTVLCIT	AEHLRSTTV	2	0.5075	206.3	40.00	0.44	tr_C9ZMP2_
DRB1_0101	126	VLPQQPFIFSGSLRQ	FIFSGSLRQ	6	0.5065	208.5	40.00	0.65	tr_C9ZMP2_
DRB1_0101	127	IDPFGLHSEDAVRDV	LHSEDAVRD	5	0.5031	216.3	40.00	0.47	tr_C9ZMP2_
DRB1_0101	128	IDGYDTRRLPLEVLR	TRRLPLEVL	5	0.5029	216.7	40.00	0.42	tr_C9ZMP2_
DRB1_0101	129	SSAEAEAALWSSLAE	EAALWSSLA	5	0.5028	216.8	40.00	0.54	tr_C9ZMP2_
DRB1_0101	130	LAEHLRSTTVLCITH	LRSTTVLCI	4	0.4986	227.0	41.00	0.40	tr_C9ZMP2_
DRB1_0101	131	SRVSGGQRHLIALAR	VSGGQRHLI	2	0.4947	236.7	42.00	0.31	tr_C9ZMP2_
DRB1_0101	132	TETELISVERVRQYF	TELISVERV	2	0.4925	242.5	43.00	0.56	tr_C9ZMP2_
DRB1_0101	133	RTGAGKSSIFSALLR	GAGKSSIFS	2	0.4911	246.3	43.00	0.25	tr_C9ZMP2_
DRB1_0101	134	STETELISVERVRQY	TELISVERV	3	0.4904	248.0	43.00	0.63	tr_C9ZMP2_
DRB1_0101	135	NVSMRYDPSGPEVLR	YDPSGPEVL	5	0.4899	249.5	43.00	0.74	tr_C9ZMP2_
DRB1_0101	136	FGLHSEDAVRDVATS	LHSEDAVRD	2	0.4891	251.7	43.00	0.69	tr_C9ZMP2_
DRB1_0101	137	VLCITHKLSHIDFFD	CITHKLSHI	2	0.4881	254.2	43.00	0.46	tr_C9ZMP2_
DRB1_0101	138	EHLRSTTVLCITHKL	LRSTTVLCI	2	0.4838	266.5	44.00	0.41	tr_C9ZMP2_
DRB1_0101	139	HIDGYDTRRLPLEVL	IDGYDTRRL	1	0.4802	276.9	45.00	0.34	tr_C9ZMP2_
DRB1_0101	140	WSSLAEHLRSTTVLC	AEHLRSTTV	4	0.4800	277.6	45.00	0.56	tr_C9ZMP2_
DRB1_0101	141	VRDVATSVKLDGVAL	SVKLDGVAL	6	0.4790	280.6	45.00	0.64	tr_C9ZMP2_
DRB1_0101	142	ETELISVERVRQYFS	TELISVERV	1	0.4775	285.3	46.00	0.50	tr_C9ZMP2_
DRB1_0101	143	YDPSGPEVLRSLSFH	PEVLRSLSF	5	0.4745	294.5	46.00	0.38	tr_C9ZMP2_
DRB1_0101	144	QSSAEAEAALWSSLA	EAALWSSLA	6	0.4713	305.0	47.00	0.41	tr_C9ZMP2_
DRB1_0101	145	AEHLRSTTVLCITHK	LRSTTVLCI	3	0.4708	306.6	47.00	0.49	tr_C9ZMP2_

DRB1_0101	146	RLVEIESGSIHIDGY	LVEIESGSI	1	0.4677	317.2	48.00	0.35	tr_C9ZMP2_
DRB1_0101	147	EKVAIVGRTGAGKSS	KVAIVGRTG	1	0.4677	317.2	48.00	0.36	tr_C9ZMP2_
DRB1_0101	148	LISVERVRQYFSLAS	ERVRQYFSL	4	0.4668	320.2	48.00	0.27	tr_C9ZMP2_
DRB1_0101	149	IDFFDRVIVIENGHV	FDRVIVIEN	3	0.4659	323.5	48.00	0.34	tr_C9ZMP2_
DRB1_0101	150	FSALLRLVEIESGSI	LRLVEIESG	4	0.4656	324.4	48.00	0.49	tr_C9ZMP2_
DRB1_0101	151	GEKVAIVGRTGAGKS	KVAIVGRTG	2	0.4653	325.3	48.00	0.41	tr_C9ZMP2_
DRB1_0101	152	LCITHKLSHIDFFDR	CITHKLSHI	1	0.4598	345.4	49.00	0.44	tr_C9ZMP2_
DRB1_0101	153	TELISVERVRQYFSL	ISVERVRQY	3	0.4579	352.7	50.00	0.37	tr_C9ZMP2_
DRB1_0101	154	KVAIVGRTGAGKSSI	IVGRTGAGK	3	0.4539	368.4	55.00	0.40	tr_C9ZMP2_
DRB1_0101	155	HVAGDGTVAQLRAAS	GTVAQLRAA	5	0.4529	372.1	55.00	0.23	tr_C9ZMP2_
DRB1_0101	156	IFSALLRLVEIESGS	LRLVEIESG	5	0.4522	375.1	55.00	0.41	tr_C9ZMP2_
DRB1_0101	157	SSRVSGGQRHLIALA	VSGGQRHLI	3	0.4518	376.7	55.00	0.43	tr_C9ZMP2_
DRB1_0101	158	AGEKVAIVGRTGAGK	KVAIVGRTG	3	0.4517	376.9	55.00	0.54	tr_C9ZMP2_
DRB1_0101	159	GHVAGDGTVAQLRAA	HVAGDGTVA	1	0.4513	378.5	55.00	0.44	tr_C9ZMP2_
DRB1_0101	160	ISVERVRQYFSLASE	RVRQYFSLA	4	0.4505	382.1	55.00	0.27	tr_C9ZMP2_
DRB1_0101	161	SVLPQQPFIFSGSLR	VLPQQPFIF	1	0.4502	383.2	55.00	0.63	tr_C9ZMP2_
DRB1_0101	162	GSIHIDGYDTRRLPL	IDGYDTRRL	4	0.4458	401.8	55.00	0.55	tr_C9ZMP2_
DRB1_0101	163	AQLRAASVWPFSSPN	LRAASVWPF	2	0.4440	409.8	55.00	0.75	tr_C9ZMP2_
DRB1_0101	164	ELISVERVRQYFSLA	ISVERVRQY	2	0.4415	421.1	55.00	0.32	tr_C9ZMP2_
DRB1_0101	165	SIHIDGYDTRRLPLE	IDGYDTRRL	3	0.4390	432.7	55.00	0.60	tr_C9ZMP2_
DRB1_0101	166	SVERVRQYFSLASEE	RVRQYFSLA	3	0.4387	434.0	55.00	0.29	tr_C9ZMP2_
DRB1_0101	167	NGHVAGDGTVAQLRA	HVAGDGTVA	2	0.4361	446.4	55.00	0.60	tr_C9ZMP2_
DRB1_0101	168	PRTLGMFFSPPRSNW	LGMFFSPPR	3	0.4345	454.4	55.00	0.32	tr_C9ZMP2_
DRB1_0101	169	ASTETELISVERVRQ	TELISVERV	4	0.4338	457.7	55.00	0.70	tr_C9ZMP2_
DRB1_0101	170	GRTGAGKSSIFSALL	GAGKSSIFS	3	0.4311	471.2	60.00	0.34	tr_C9ZMP2_
DRB1_0101	171	NIDPFGLHSEDAVRD	FGLHSEDAV	4	0.4286	484.1	60.00	0.47	tr_C9ZMP2_
DRB1_0101	172	WPSGGQVVLSNVSMR	QVVLSNVSM	5	0.4256	500.2	60.00	0.39	tr_C9ZMP2_
DRB1_0101	173	LASEEPRTLGMFFSP	LASEEPRTL	0	0.4232	513.3	60.00	0.52	tr_C9ZMP2_
DRB1_0101	174	VVLSNVSMRYDPSGP	VLSNVSMRY	1	0.4226	516.6	60.00	0.56	tr_C9ZMP2_
DRB1_0101	175	ENGHVAGDGTVAQLR	HVAGDGTVA	3	0.4188	538.1	60.00	0.63	tr_C9ZMP2_
DRB1_0101	176	IFSGSLRQNIDPFGL	FSGSLRQNI	1	0.4184	540.6	60.00	0.52	tr_C9ZMP2_
DRB1_0101	177	HIDFFDRVIVIENGH	FDRVIVIEN	4	0.4179	543.9	60.00	0.43	tr_C9ZMP2_
DRB1_0101	178	SGSIHIDGYDTRRLP	IDGYDTRRL	5	0.4164	552.3	60.00	0.44	tr_C9ZMP2_
DRB1_0101	179	EPRTLGMFFSPPRSN	LGMFFSPPR	4	0.4151	560.5	60.00	0.31	tr_C9ZMP2_
DRB1_0101	180	DSSRVSGGQRHLIAL	VSGGQRHLI	4	0.4107	587.4	60.00	0.50	tr_C9ZMP2_
DRB1_0101	181	ALDYNVTDSSRVSGG	YNVTDSSRV	3	0.4084	602.4	60.00	0.72	tr_C9ZMP2_
DRB1_0101	182	IHIDGYDTRRLPLEV	IDGYDTRRL	2	0.4083	603.0	60.00	0.61	tr_C9ZMP2_
DRB1_0101	183	RTLGMFFSPPRSNWP	LGMFFSPPR	2	0.4068	612.7	65.00	0.34	tr_C9ZMP2_
DRB1_0101	184	SHIDFFDRVIVIENG	FDRVIVIEN	5	0.4066	614.5	65.00	0.37	tr_C9ZMP2_
DRB1_0101	185	NAGEKVAIVGRTGAG	KVAIVGRTG	4	0.4063	616.3	65.00	0.49	tr_C9ZMP2_
DRB1_0101	186	QLRAASVWPFSSPNA	LRAASVWPF	1	0.4062	616.7	65.00	0.58	tr_C9ZMP2_
DRB1_0101	187	CITHKLSHIDFFDRV	CITHKLSHI	0	0.4051	624.0	65.00	0.27	tr_C9ZMP2_
DRB1_0101	188	VALDYNVTDSSRVSG	YNVTDSSRV	4	0.3993	665.0	65.00	0.68	tr_C9ZMP2_
DRB1_0101	189	GLHSEDAVRDVATSV	LHSEDAVRD	1	0.3950	696.6	65.00	0.70	tr_C9ZMP2_
DRB1_0101	190	VAIVGRTGAGKSSIF	IVGRTGAGK	2	0.3942	702.8	65.00	0.44	tr_C9ZMP2_
DRB1_0101	191	SNVSMRYDPSGPEVL	YDPSGPEVL	6	0.3924	716.3	65.00	0.62	tr_C9ZMP2_
DRB1_0101	192	AQSSAEAEAALWSSL	SAEAEAALW	3	0.3919	720.5	65.00	0.42	tr_C9ZMP2_
DRB1_0101	193	TLGMFFSPPRSNWPS	MFFSPPRSN	3	0.3902	733.4	65.00	0.29	tr_C9ZMP2_
DRB1_0101	194	FSGSLRQNIDPFGLH	LRQNIDPFG	4	0.3874	756.0	65.00	0.33	tr_C9ZMP2_
DRB1_0101	195	LSHIDFFDRVIVIEN	DFFDRVIVI	4	0.3864	764.0	65.00	0.36	tr_C9ZMP2_

DRB1_0101	196	GVALDYNVTDSSRVS	YNVTDSSRV	5	0.3854	772.4	65.00	0.55	tr_C9ZMP2_
DRB1_0101	197	ESGSIHIDGYDTRRL	SIHIDGYDT	3	0.3848	778.0	65.00	0.53	tr_C9ZMP2_
DRB1_0101	198	VNAGEKVAIVGRTGA	KVAIVGRTG	5	0.3842	782.6	70.00	0.41	tr_C9ZMP2_
DRB1_0101	199	LDYNVTDSSRVSGGQ	YNVTDSSRV	2	0.3787	830.4	70.00	0.77	tr_C9ZMP2_
DRB1_0101	200	VGRTGAGKSSIFSAL	GAGKSSIFS	4	0.3776	840.5	70.00	0.39	tr_C9ZMP2_
DRB1_0101	201	EEPRTLGMFFSPPRS	PRTLGMFFS	2	0.3769	847.4	70.00	0.40	tr_C9ZMP2_
DRB1_0101	202	LVEIESGSIHIDGYD	EIESGSIHI	2	0.3730	883.3	70.00	0.41	tr_C9ZMP2_
DRB1_0101	203	AIVGRTGAGKSSIFS	IVGRTGAGK	1	0.3700	913.1	70.00	0.35	tr_C9ZMP2_
DRB1_0101	204	IENGHVAGDGTVAQL	HVAGDGTVA	4	0.3669	943.8	70.00	0.68	tr_C9ZMP2_
DRB1_0101	205	TDSSRVSGGQRHLIA	VSGGQRHLI	5	0.3655	957.8	70.00	0.48	tr_C9ZMP2_
DRB1_0101	206	TAQSSAEAEAALWSS	SAEAEAALW	4	0.3655	958.4	70.00	0.38	tr_C9ZMP2_
DRB1_0101	207	HKLSHIDFFDRVIVI	DFFDRVIVI	6	0.3642	971.5	70.00	0.26	tr_C9ZMP2_
DRB1_0101	208	QNIDPFGLHSEDAVR	FGLHSEDAV	5	0.3637	977.6	70.00	0.60	tr_C9ZMP2_
DRB1_0101	209	KLSHIDFFDRVIVIE	DFFDRVIVI	5	0.3610	1006.3	70.00	0.34	tr_C9ZMP2_
DRB1_0101	210	LGMFFSPPRSNWPSG	MFFSPPRSN	2	0.3573	1047.1	75.00	0.25	tr_C9ZMP2_
DRB1_0101	211	IVGRTGAGKSSIFSA	GAGKSSIFS	5	0.3537	1088.3	75.00	0.29	tr_C9ZMP2_
DRB1_0101	212	ASVWPFSSPNADASL	VWPFSSPNA	2	0.3498	1135.6	75.00	0.32	tr_C9ZMP2_
DRB1_0101	213	DGVALDYNVTDSSRV	GVALDYNVT	1	0.3479	1159.0	75.00	0.31	tr_C9ZMP2_
DRB1_0101	214	SVWPFSSPNADASLT	FSSPNADAS	4	0.3478	1161.1	75.00	0.32	tr_C9ZMP2_
DRB1_0101	215	SGSLRQNIDPFGLHS	LRQNIDPFG	3	0.3461	1181.6	75.00	0.43	tr_C9ZMP2_
DRB1_0101	216	PTAQSSAEAEAALWS	SAEAEAALW	5	0.3457	1187.6	75.00	0.34	tr_C9ZMP2_
DRB1_0101	217	KLDGVALDYNVTDSS	GVALDYNVT	3	0.3454	1190.8	75.00	0.54	tr_C9ZMP2_
DRB1_0101	218	VKLDGVALDYNVTDS	GVALDYNVT	4	0.3443	1205.2	75.00	0.51	tr_C9ZMP2_
DRB1_0101	219	HVNAGEKVAIVGRTG	NAGEKVAIV	2	0.3426	1228.0	75.00	0.41	tr_C9ZMP2_
DRB1_0101	220	SEEPRTLGMFFSPPR	PRTLGMFFS	3	0.3423	1231.3	75.00	0.51	tr_C9ZMP2_
DRB1_0101	221	GSLRQNIDPFGLHSE	LRQNIDPFG	2	0.3403	1258.1	75.00	0.37	tr_C9ZMP2_
DRB1_0101	222	ITHKLSHIDFFDRVI	LSHIDFFDR	4	0.3342	1345.2	80.00	0.27	tr_C9ZMP2_
DRB1_0101	223	LRAASVWPFSSPNAD	LRAASVWPF	0	0.3307	1397.0	80.00	0.37	tr_C9ZMP2_
DRB1_0101	224	DYNVTDSSRVSGGQR	YNVTDSSRV	1	0.3305	1398.8	80.00	0.78	tr_C9ZMP2_
DRB1_0101	225	NWPSGGQVVLSNVSM	QVVLSNVSM	6	0.3292	1419.8	80.00	0.33	tr_C9ZMP2_
DRB1_0101	226	RSNWPSGGQVVLSNV	NWPSGGQVV	2	0.3289	1423.7	80.00	0.43	tr_C9ZMP2_
DRB1_0101	227	LHSEDAVRDVATSVK	LHSEDAVRD	0	0.3280	1437.6	80.00	0.43	tr_C9ZMP2_
DRB1_0101	228	LLLDEPTAQSSAEAE	LLDEPTAQS	1	0.3267	1458.9	80.00	0.31	tr_C9ZMP2_
DRB1_0101	229	THKLSHIDFFDRVIV	LSHIDFFDR	3	0.3242	1497.9	80.00	0.28	tr_C9ZMP2_
DRB1_0101	230	PRSNWPSGGQVVLSN	NWPSGGQVV	3	0.3228	1521.7	80.00	0.47	tr_C9ZMP2_
DRB1_0101	231	LDGVALDYNVTDSSR	GVALDYNVT	2	0.3200	1568.3	80.00	0.52	tr_C9ZMP2_
DRB1_0101	232	AASVWPFSSPNADAS	VWPFSSPNA	3	0.3183	1596.2	80.00	0.41	tr_C9ZMP2_
DRB1_0101	233	EPTAQSSAEAEAALW	QSSAEAEAA	4	0.3178	1606.1	80.00	0.24	tr_C9ZMP2_
DRB1_0101	234	RQNIDPFGLHSEDAV	FGLHSEDAV	6	0.3172	1616.4	80.00	0.41	tr_C9ZMP2_
DRB1_0101	235	DAVRDVATSVKLDGV	RDVATSVKL	3	0.3164	1630.3	80.00	0.46	tr_C9ZMP2_
DRB1_0101	236	SLRQNIDPFGLHSED	RQNIDPFGL	2	0.3157	1642.4	80.00	0.43	tr_C9ZMP2_
DRB1_0101	237	EDAVRDVATSVKLDG	RDVATSVKL	4	0.3117	1716.1	80.00	0.40	tr_C9ZMP2_
DRB1_0101	238	VIENGHVAGDGTVAQ	HVAGDGTVA	5	0.3106	1735.4	80.00	0.62	tr_C9ZMP2_
DRB1_0101	239	SNWPSGGQVVLSNVS	NWPSGGQVV	1	0.3053	1837.9	85.00	0.31	tr_C9ZMP2_
DRB1_0101	240	SEDAVRDVATSVKLD	AVRDVATSV	3	0.3052	1839.2	85.00	0.38	tr_C9ZMP2_
DRB1_0101	241	AVRDVATSVKLDGVA	RDVATSVKL	2	0.3050	1844.5	85.00	0.48	tr_C9ZMP2_
DRB1_0101	242	PPRSNWPSGGQVVLS	NWPSGGQVV	4	0.3030	1883.6	85.00	0.50	tr_C9ZMP2_
DRB1_0101	243	RAASVWPFSSPNADA	VWPFSSPNA	4	0.2938	2081.2	85.00	0.46	tr_C9ZMP2_
DRB1_0101	244	IESGSIHIDGYDTRR	SIHIDGYDT	4	0.2910	2146.8	85.00	0.69	tr_C9ZMP2_
DRB1_0101	245	ASEEPRTLGMFFSPP	PRTLGMFFS	4	0.2895	2180.9	85.00	0.56	tr_C9ZMP2_

DRB1_0101	246	VEIESGSIHIDGYDT	EIESGSIHI	1	0.2894	2182.8	85.00	0.44	tr_C9ZMP2_	
DRB1_0101	247	VTDSSRVSGGQRHLI	VSGGQRHLI	6	0.2886	2202.6	85.00	0.43	tr_C9ZMP2_	
DRB1_0101	248	GMFFSPPRSNWPSGG	MFFSPPRSN	1	0.2875	2229.7	85.00	0.29	tr_C9ZMP2_	
DRB1_0101	249	HSEDAVRDVATSVKL	AVRDVATSV	4	0.2846	2300.5	85.00	0.44	tr_C9ZMP2_	
DRB1_0101	250	VLSNVSMRYDPSGPE	VLSNVSMRY	0	0.2805	2403.8	85.00	0.43	tr_C9ZMP2_	
DRB1_0101	251	LRQNIDPFGLHSEDA	RQNIDPFGL	1	0.2785	2455.4	85.00	0.38	tr_C9ZMP2_	
DRB1_0101	252	DEPTAQSSAEAEAAL	PTAQSSAEA	2	0.2771	2494.9	85.00	0.37	tr_C9ZMP2_	
DRB1_0101	253	EIESGSIHIDGYDTR	SIHIDGYDT	5	0.2713	2655.4	90.00	0.49	tr_C9ZMP2_	
DRB1_0101	254	LLDEPTAQSSAEAEA	PTAQSSAEA	4	0.2681	2750.1	90.00	0.30	tr_C9ZMP2_	
DRB1_0101	255	YNVTDSSRVSGGQRH	YNVTDSSRV	0	0.2652	2835.3	90.00	0.63	tr_C9ZMP2_	
DRB1_0101	256	SPPRSNWPSGGQVVL	NWPSGGQVV	5	0.2623	2925.7	90.00	0.52	tr_C9ZMP2_	
DRB1_0101	257	LDEPTAQSSAEAEAA	PTAQSSAEA	3	0.2623	2928.1	90.00	0.47	tr_C9ZMP2_	
DRB1_0101	258	LSNVSMRYDPSGPEV	NVSMRYDPS	2	0.2306	4124.8	95.00	0.28	tr_C9ZMP2_	
DRB1_0101	259	MFFSPPRSNWPSGGQ	FSPPRSNWP	2	0.2263	4321.8	95.00	0.34	tr_C9ZMP2_	
DRB1_0101	260	FSPPRSNWPSGGQVV	NWPSGGQVV	6	0.2027	5578.7	95.00	0.40	tr_C9ZMP2_	
DRB1_0101	261	NVTDSSRVSGGQRHL	SRVSGGQRH	5	0.1786	7239.1	100.00	0.38	tr_C9ZMP2_	
DRB1_0101	262	FFSPPRSNWPSGGQV	FSPPRSNWP	1	0.1668	8228.6	100.00	0.43	tr_C9ZMP2_	

Allele: DRB1_0101. Number of high binders 3. Number of weak binders 31. Number of peptides 262

Allele	pos	peptide	core	Of	1-log50k(aff)	affinity(nM)	%Rank	Relia	Identity	Bind Level
DRB1_0101	1	GSDFVSVPSRDVRAG	FVSVPSRDV	3	0.8029	8.4	1.90	0.89	tr_D0A9H6_	WB
DRB1_0101	2	SDFVSVPSRDVRAGD	FVSVPSRDV	2	0.7920	9.5	2.50	0.89	tr_D0A9H6_	WB
DRB1_0101	3	RGSDFVSVPSRDVRA	FVSVPSRDV	4	0.7913	9.6	2.50	0.90	tr_D0A9H6_	WB
DRB1_0101	4	SNPFAAGTASNIYPR	FAAGTASNI	3	0.7671	12.4	4.00	0.93	tr_D0A9H6_	WB
DRB1_0101	5	DFVSVPSRDVRAGDV	FVSVPSRDV	1	0.7597	13.5	4.50	0.86	tr_D0A9H6_	WB
DRB1_0101	6	NPFAAGTASNIYPRA	FAAGTASNI	2	0.7475	15.4	5.00	0.92	tr_D0A9H6_	WB
DRB1_0101	7	LRGSDFVSVPSRDVR	FVSVPSRDV	5	0.7393	16.8	5.50	0.92	tr_D0A9H6_	WB
DRB1_0101	8	SSNPFAAGTASNIYP	FAAGTASNI	4	0.7346	17.7	6.00	0.92	tr_D0A9H6_	WB
DRB1_0101	9	SSSNPFAAGTASNIY	FAAGTASNI	5	0.7229	20.1	7.00	0.91	tr_D0A9H6_	WB
DRB1_0101	10	PFAAGTASNIYPRAG	FAAGTASNI	1	0.6997	25.8	9.00	0.90	tr_D0A9H6_	WB
DRB1_0101	11	CAHFLNNGSLVGWKR	FLNNGSLVG	3	0.6943	27.3	9.50	0.51	tr_D0A9H6_	WB
DRB1_0101	12	AHFLNNGSLVGWKRC	FLNNGSLVG	2	0.6847	30.3	11.00	0.50	tr_D0A9H6_	
DRB1_0101	13	FVSVPSRDVRAGDVI	FVSVPSRDV	0	0.6822	31.1	11.00	0.81	tr_D0A9H6_	
DRB1_0101	14	TCAHFLNNGSLVGWK	FLNNGSLVG	4	0.6779	32.6	12.00	0.48	tr_D0A9H6_	
DRB1_0101	15	GQLRLRCGSVVSLSI	LRLRCGSVV	2	0.6628	38.4	13.00	0.38	tr_D0A9H6_	
DRB1_0101	16	SDKLVNSVGVQVLRG	LVNSVGVQV	3	0.6606	39.4	14.00	0.62	tr_D0A9H6_	
DRB1_0101	17	KSDKLVNSVGVQVLR	LVNSVGVQV	4	0.6572	40.8	14.00	0.61	tr_D0A9H6_	
DRB1_0101	18	IGQLRLRCGSVVSLS	LRLRCGSVV	3	0.6561	41.3	14.00	0.41	tr_D0A9H6_	
DRB1_0101	19	SKGEKSSLTSRRINL	KSSLTSRRI	4	0.6551	41.8	14.00	0.82	tr_D0A9H6_	
DRB1_0101	20	QLRLRCGSVVSLSID	LRCGSVVSL	3	0.6526	42.9	15.00	0.35	tr_D0A9H6_	
DRB1_0101	21	KPETIKALGTVEDII	IKALGTVED	4	0.6433	47.4	16.00	0.57	tr_D0A9H6_	
DRB1_0101	22	RKSDKLVNSVGVQVL	LVNSVGVQV	5	0.6377	50.4	17.00	0.49	tr_D0A9H6_	
DRB1_0101	23	ATCAHFLNNGSLVGW	HFLNNGSLV	4	0.6326	53.3	17.00	0.50	tr_D0A9H6_	
DRB1_0101	24	LGDVVPADAVVLNTS	VVPADAVVL	3	0.6326	53.3	17.00	0.87	tr_D0A9H6_	
DRB1_0101	25	PETIKALGTVEDIIE	IKALGTVED	3	0.6314	53.9	18.00	0.65	tr_D0A9H6_	

DRB1_0101	26	KNTKMLLNLKSKGEK	TKMLLNLKS	2	0.6310	54.2	18.00	0.34	tr_D0A9H6_
DRB1_0101	27	HFLNNGSLVGWKRCD	FLNNGSLVG	1	0.6302	54.7	18.00	0.46	tr_D0A9H6_
DRB1_0101	28	NTKMLLNLKSKGEKS	LLNLKSKGE	4	0.6286	55.6	18.00	0.36	tr_D0A9H6_
DRB1_0101	29	DKLVNSVGVQVLRGS	LVNSVGVQV	2	0.6264	57.0	18.00	0.68	tr_D0A9H6_
DRB1_0101	30	WIGQLRLRCGSVVSL	LRLRCGSVV	4	0.6250	57.8	19.00	0.47	tr_D0A9H6_
DRB1_0101	31	VLRGSDFVSVPSRDV	FVSVPSRDV	6	0.6233	58.9	19.00	0.81	tr_D0A9H6_
DRB1_0101	32	AVVYTGKNTKMLLNL	YTGKNTKML	3	0.6198	61.2	19.00	0.62	tr_D0A9H6_
DRB1_0101	33	TKMLLNLKSKGEKSS	LLNLKSKGE	3	0.6136	65.4	20.00	0.45	tr_D0A9H6_
DRB1_0101	34	GLGDVVPADAVVLNT	VVPADAVVL	4	0.6119	66.6	21.00	0.81	tr_D0A9H6_
DRB1_0101	35	ETIKALGTVEDIIEG	IKALGTVED	2	0.6085	69.1	21.00	0.71	tr_D0A9H6_
DRB1_0101	36	GDVVPADAVVLNTSL	VVPADAVVL	2	0.6065	70.6	21.00	0.85	tr_D0A9H6_
DRB1_0101	37	CAVVYTGKNTKMLLN	YTGKNTKML	4	0.6048	71.9	22.00	0.58	tr_D0A9H6_
DRB1_0101	38	VVYTGKNTKMLLNLK	YTGKNTKML	2	0.5987	76.9	23.00	0.63	tr_D0A9H6_
DRB1_0101	39	LRLRCGSVVSLSIDQ	LRCGSVVSL	2	0.5987	76.9	23.00	0.41	tr_D0A9H6_
DRB1_0101	40	AKPETIKALGTVEDI	IKALGTVED	5	0.5980	77.5	23.00	0.51	tr_D0A9H6_
DRB1_0101	41	KSKGEKSSLTSRRIN	KSSLTSRRI	5	0.5943	80.6	23.00	0.76	tr_D0A9H6_
DRB1_0101	42	GKNTKMLLNLKSKGE	TKMLLNLKS	3	0.5902	84.2	24.00	0.46	tr_D0A9H6_
DRB1_0101	43	CGLGDVVPADAVVLN	VVPADAVVL	5	0.5883	86.0	24.00	0.69	tr_D0A9H6_
DRB1_0101	44	CSSSNPFAAGTASNI	FAAGTASNI	6	0.5880	86.3	24.00	0.84	tr_D0A9H6_
DRB1_0101	45	DVVPADAVVLNTSLV	VVPADAVVL	1	0.5827	91.4	25.00	0.73	tr_D0A9H6_
DRB1_0101	46	FKGVLLRGATPCPDL	GVLLRGATP	2	0.5779	96.2	26.00	0.41	tr_D0A9H6_
DRB1_0101	47	KLVNSVGVQVLRGSD	LVNSVGVQV	1	0.5743	100.1	27.00	0.67	tr_D0A9H6_
DRB1_0101	48	LCAVVYTGKNTKMLL	YTGKNTKML	5	0.5726	101.9	27.00	0.52	tr_D0A9H6_
DRB1_0101	49	VYTGKNTKMLLNLKS	YTGKNTKML	1	0.5719	102.8	27.00	0.52	tr_D0A9H6_
DRB1_0101	50	KMLLNLKSKGEKSSL	LLNLKSKGE	2	0.5697	105.2	28.00	0.49	tr_D0A9H6_
DRB1_0101	51	YTGKNTKMLLNLKSK	TKMLLNLKS	5	0.5693	105.6	28.00	0.43	tr_D0A9H6_
DRB1_0101	52	DFKGVLLRGATPCPD	GVLLRGATP	3	0.5660	109.5	28.00	0.40	tr_D0A9H6_
DRB1_0101	53	SWIGQLRLRCGSVVS	LRLRCGSVV	5	0.5656	109.9	28.00	0.50	tr_D0A9H6_
DRB1_0101	54	FAAGTASNIYPRAGA	FAAGTASNI	0	0.5643	111.6	29.00	0.81	tr_D0A9H6_
DRB1_0101	55	RRKSDKLVNSVGVQV	DKLVNSVGV	4	0.5621	114.2	29.00	0.43	tr_D0A9H6_
DRB1_0101	56	AATCAHFLNNGSLVG	HFLNNGSLV	5	0.5607	115.9	29.00	0.53	tr_D0A9H6_
DRB1_0101	57	GDFKGVLLRGATPCP	GVLLRGATP	4	0.5599	116.9	29.00	0.44	tr_D0A9H6_
DRB1_0101	58	TGKNTKMLLNLKSKG	TKMLLNLKS	4	0.5563	121.6	30.00	0.52	tr_D0A9H6_
DRB1_0101	59	DGDFKGVLLRGATPC	GVLLRGATP	5	0.5555	122.7	30.00	0.49	tr_D0A9H6_
DRB1_0101	60	KGVLLRGATPCPDLH	GVLLRGATP	1	0.5480	133.1	32.00	0.43	tr_D0A9H6_
DRB1_0101	61	RLRCGSVVSLSIDQF	CGSVVSLSI	3	0.5457	136.4	32.00	0.42	tr_D0A9H6_
DRB1_0101	62	GDVILCGLGDVVPAD	ILCGLGDVV	3	0.5448	137.7	32.00	0.38	tr_D0A9H6_
DRB1_0101	63	GDGDFKGVLLRGATP	FKGVLLRGA	4	0.5439	139.1	32.00	0.38	tr_D0A9H6_
DRB1_0101	64	AGDVILCGLGDVVPA	ILCGLGDVV	4	0.5398	145.4	33.00	0.42	tr_D0A9H6_
DRB1_0101	65	SVGVQVLRGSDFVSV	VQVLRGSDF	3	0.5386	147.2	33.00	0.61	tr_D0A9H6_
DRB1_0101	66	DVILCGLGDVVPADA	ILCGLGDVV	2	0.5380	148.3	33.00	0.38	tr_D0A9H6_
DRB1_0101	67	HSWIGQLRLRCGSVV	LRLRCGSVV	6	0.5378	148.6	34.00	0.36	tr_D0A9H6_
DRB1_0101	68	SIDQFLPRGCIIRNT	FLPRGCIIR	4	0.5350	153.2	34.00	0.48	tr_D0A9H6_
DRB1_0101	69	LCGLGDVVPADAVVL	VVPADAVVL	6	0.5338	155.1	34.00	0.38	tr_D0A9H6_
DRB1_0101	70	TIKALGTVEDIIEGC	IKALGTVED	1	0.5310	159.9	35.00	0.75	tr_D0A9H6_
DRB1_0101	71	LSIDQFLPRGCIIRN	FLPRGCIIR	5	0.5285	164.3	35.00	0.43	tr_D0A9H6_
DRB1_0101	72	NAKTRRAKPETIKAL	TRRAKPETI	3	0.5261	168.5	36.00	0.82	tr_D0A9H6_
DRB1_0101	73	VILCGLGDVVPADAV	CGLGDVVPA	3	0.5249	170.7	36.00	0.43	tr_D0A9H6_
DRB1_0101	74	RAKPETIKALGTVED	PETIKALGT	3	0.5162	187.6	38.00	0.31	tr_D0A9H6_
DRB1_0101	75	IDQFLPRGCIIRNTE	FLPRGCIIR	3	0.5137	192.9	38.00	0.51	tr_D0A9H6_

DRB1_0101	76	AKTRRAKPETIKALG	TRRAKPETI	2	0.5127	194.8	38.00	0.80	tr_D0A9H6_
DRB1_0101	77	VVPADAVVLNTSLVD	AVVLNTSLV	5	0.5122	195.9	39.00	0.44	tr_D0A9H6_
DRB1_0101	78	NSVGVQVLRGSDFVS	VQVLRGSDF	4	0.5090	202.8	39.00	0.64	tr_D0A9H6_
DRB1_0101	79	VGVQVLRGSDFVSVP	VQVLRGSDF	2	0.5077	205.7	39.00	0.61	tr_D0A9H6_
DRB1_0101	80	RGDGDFKGVLLRGAT	FKGVLLRGA	5	0.5074	206.3	40.00	0.50	tr_D0A9H6_
DRB1_0101	81	RGCIIRNTEWVLCAV	IIRNTEWVL	3	0.5058	210.0	40.00	0.54	tr_D0A9H6_
DRB1_0101	82	LKSKGEKSSLTSRRI	KSSLTSRRI	6	0.5009	221.5	41.00	0.60	tr_D0A9H6_
DRB1_0101	83	RAGDVILCGLGDVVP	DVILCGLGD	3	0.5004	222.7	41.00	0.42	tr_D0A9H6_
DRB1_0101	84	LVNSVGVQVLRGSDF	VQVLRGSDF	6	0.4978	229.0	41.00	0.31	tr_D0A9H6_
DRB1_0101	85	LRCGSVVSLSIDQFL	CGSVVSLSI	2	0.4965	232.1	42.00	0.44	tr_D0A9H6_
DRB1_0101	86	SLSIDQFLPRGCIIR	FLPRGCIIR	6	0.4960	233.5	42.00	0.32	tr_D0A9H6_
DRB1_0101	87	GVLLRGATPCPDLHS	LRGATPCPD	3	0.4936	239.5	42.00	0.26	tr_D0A9H6_
DRB1_0101	88	ILCGLGDVVPADAVV	CGLGDVVPA	2	0.4909	246.7	43.00	0.43	tr_D0A9H6_
DRB1_0101	89	GVQVLRGSDFVSVPS	VQVLRGSDF	1	0.4905	247.8	43.00	0.56	tr_D0A9H6_
DRB1_0101	90	PRGCIIRNTEWVLCA	IIRNTEWVL	4	0.4892	251.4	43.00	0.50	tr_D0A9H6_
DRB1_0101	91	FLNNGSLVGWKRCDS	FLNNGSLVG	0	0.4874	256.4	44.00	0.38	tr_D0A9H6_
DRB1_0101	92	KTRRAKPETIKALGT	TRRAKPETI	1	0.4865	258.8	44.00	0.69	tr_D0A9H6_
DRB1_0101	93	VRAGDVILCGLGDVV	DVILCGLGD	4	0.4864	259.0	44.00	0.46	tr_D0A9H6_
DRB1_0101	94	MLLNLKSKGEKSSLT	LLNLKSKGE	1	0.4860	260.1	44.00	0.46	tr_D0A9H6_
DRB1_0101	95	VNSVGVQVLRGSDFV	VQVLRGSDF	5	0.4856	261.2	44.00	0.58	tr_D0A9H6_
DRB1_0101	96	DAATCAHFLNNGSLV	HFLNNGSLV	6	0.4843	265.0	44.00	0.44	tr_D0A9H6_
DRB1_0101	97	DQFLPRGCIIRNTEW	FLPRGCIIR	2	0.4809	274.9	45.00	0.50	tr_D0A9H6_
DRB1_0101	98	PADAVVLNTSLVDGV	AVVLNTSLV	3	0.4743	295.4	46.00	0.69	tr_D0A9H6_
DRB1_0101	99	VLCAVVYTGKNTKML	YTGKNTKML	6	0.4733	298.4	47.00	0.38	tr_D0A9H6_
DRB1_0101	100	TNAKTRRAKPETIKA	TRRAKPETI	4	0.4729	299.8	47.00	0.83	tr_D0A9H6_
DRB1_0101	101	LSTLLPLCIVVGVGM	STLLPLCIV	1	0.4721	302.5	47.00	0.24	tr_D0A9H6_
DRB1_0101	102	ADAVVLNTSLVDGVT	AVVLNTSLV	2	0.4702	308.6	47.00	0.68	tr_D0A9H6_
DRB1_0101	103	SGLCSSSNPFAAGTA	LCSSSNPFA	2	0.4671	319.3	48.00	0.44	tr_D0A9H6_
DRB1_0101	104	GCIIRNTEWVLCAVV	IIRNTEWVL	2	0.4663	322.0	48.00	0.50	tr_D0A9H6_
DRB1_0101	105	PSGLCSSSNPFAAGT	LCSSSNPFA	3	0.4600	344.8	49.00	0.50	tr_D0A9H6_
DRB1_0101	106	GCLPDAATCAHFLNN	DAATCAHFL	4	0.4582	351.3	50.00	0.52	tr_D0A9H6_
DRB1_0101	107	STLLPLCIVVGVGMW	LCIVVGVGM	5	0.4573	354.9	50.00	0.40	tr_D0A9H6_
DRB1_0101	108	DGVTYIETSNLDGET	VTYIETSNL	2	0.4564	358.5	50.00	0.60	tr_D0A9H6_
DRB1_0101	109	ARGDGDFKGVLLRGA	DGDFKGVLL	3	0.4558	360.8	50.00	0.40	tr_D0A9H6_
DRB1_0101	110	LPLCIVVGVGMWKDL	LCIVVGVGM	2	0.4552	362.9	50.00	0.50	tr_D0A9H6_
DRB1_0101	111	QFLPRGCIIRNTEWV	FLPRGCIIR	1	0.4552	363.0	50.00	0.48	tr_D0A9H6_
DRB1_0101	112	SNIYPRAGAPGSAVA	YPRAGAPGS	3	0.4545	365.9	55.00	0.40	tr_D0A9H6_
DRB1_0101	113	GVTYIETSNLDGETN	VTYIETSNL	1	0.4538	368.8	55.00	0.51	tr_D0A9H6_
DRB1_0101	114	VDGVTYIETSNLDGE	VTYIETSNL	3	0.4537	369.1	55.00	0.68	tr_D0A9H6_
DRB1_0101	115	TLLPLCIVVGVGMWK	LCIVVGVGM	4	0.4535	369.8	55.00	0.53	tr_D0A9H6_
DRB1_0101	116	TRRAKPETIKALGTV	PETIKALGT	5	0.4531	371.6	55.00	0.35	tr_D0A9H6_
DRB1_0101	117	EGCLPDAATCAHFLN	DAATCAHFL	5	0.4527	373.2	55.00	0.44	tr_D0A9H6_
DRB1_0101	118	DNGKGAISQNVLTEN	GAISQNVLT	4	0.4521	375.3	55.00	0.56	tr_D0A9H6_
DRB1_0101	119	PLCIVVGVGMWKDLW	LCIVVGVGM	1	0.4521	375.5	55.00	0.41	tr_D0A9H6_
DRB1_0101	120	NGKGAISQNVLTENG	GAISQNVLT	3	0.4520	376.0	55.00	0.57	tr_D0A9H6_
DRB1_0101	121	NIYPRAGAPGSAVAL	YPRAGAPGS	2	0.4518	376.8	55.00	0.35	tr_D0A9H6_
DRB1_0101	122	LHSWIGQLRLRCGSV	IGQLRLRCG	4	0.4517	377.0	55.00	0.29	tr_D0A9H6_
DRB1_0101	123	KRRKSDKLVNSVGVQ	DKLVNSVGV	5	0.4508	380.9	55.00	0.57	tr_D0A9H6_
DRB1_0101	124	VPADAVVLNTSLVDG	AVVLNTSLV	4	0.4505	382.2	55.00	0.76	tr_D0A9H6_
DRB1_0101	125	IYPRAGAPGSAVALA	GAPGSAVAL	5	0.4483	391.2	55.00	0.28	tr_D0A9H6_

DRB1_0101	126	FLPRGCIIRNTEWVL	IIRNTEWVL	6	0.4476	394.2	55.00	0.27	tr_D0A9H6_
DRB1_0101	127	LLPLCIVVGVGMWKD	LCIVVGVGM	3	0.4469	397.2	55.00	0.55	tr_D0A9H6_
DRB1_0101	128	DAVVLNTSLVDGVTY	AVVLNTSLV	1	0.4450	405.2	55.00	0.61	tr_D0A9H6_
DRB1_0101	129	VQVLRGSDFVSVPSR	VQVLRGSDF	0	0.4444	408.3	55.00	0.41	tr_D0A9H6_
DRB1_0101	130	IEGCLPDAATCAHFL	CLPDAATCA	3	0.4410	423.4	55.00	0.48	tr_D0A9H6_
DRB1_0101	131	LPRGCIIRNTEWVLC	IIRNTEWVL	5	0.4404	426.1	55.00	0.47	tr_D0A9H6_
DRB1_0101	132	RRAKPETIKALGTVE	PETIKALGT	4	0.4401	427.5	55.00	0.48	tr_D0A9H6_
DRB1_0101	133	RCGSVVSLSIDQFLP	CGSVVSLSI	1	0.4395	430.1	55.00	0.50	tr_D0A9H6_
DRB1_0101	134	GPSGLCSSSNPFAAG	LCSSSNPFA	4	0.4359	447.4	55.00	0.50	tr_D0A9H6_
DRB1_0101	135	CIIRNTEWVLCAVVY	IIRNTEWVL	1	0.4332	460.8	55.00	0.42	tr_D0A9H6_
DRB1_0101	136	GLCSSSNPFAAGTAS	LCSSSNPFA	1	0.4326	463.8	55.00	0.39	tr_D0A9H6_
DRB1_0101	137	LVDGVTYIETSNLDG	VTYIETSNL	4	0.4324	464.7	55.00	0.79	tr_D0A9H6_
DRB1_0101	138	GDNGKGAISQNVLTE	GAISQNVLT	5	0.4317	468.3	55.00	0.50	tr_D0A9H6_
DRB1_0101	139	NVTQSFAEQTSKKGT	FAEQTSKKG	5	0.4305	474.4	60.00	0.58	tr_D0A9H6_
DRB1_0101	140	DLHSWIGQLRLRCGS	IGQLRLRCG	5	0.4295	479.6	60.00	0.26	tr_D0A9H6_
DRB1_0101	141	TSNLDGETNAKTRRA	LDGETNAKT	3	0.4276	489.3	60.00	0.85	tr_D0A9H6_
DRB1_0101	142	CLPDAATCAHFLNNG	DAATCAHFL	3	0.4274	490.3	60.00	0.62	tr_D0A9H6_
DRB1_0101	143	ASNIYPRAGAPGSAV	IYPRAGAPG	3	0.4250	503.4	60.00	0.44	tr_D0A9H6_
DRB1_0101	144	DVRAGDVILCGLGDV	DVILCGLGD	5	0.4227	515.8	60.00	0.50	tr_D0A9H6_
DRB1_0101	145	SQNVLTENGRTSGGT	VLTENGRTS	3	0.4189	537.8	60.00	0.83	tr_D0A9H6_
DRB1_0101	146	PDLHSWIGQLRLRCG	HSWIGQLRL	3	0.4180	542.8	60.00	0.26	tr_D0A9H6_
DRB1_0101	147	TQSFAEQTSKKGTEG	FAEQTSKKG	3	0.4179	543.6	60.00	0.87	tr_D0A9H6_
DRB1_0101	148	IIEGCLPDAATCAHF	CLPDAATCA	4	0.4156	557.1	60.00	0.40	tr_D0A9H6_
DRB1_0101	149	LLNLKSKGEKSSLTS	LLNLKSKGE	0	0.4134	570.9	60.00	0.25	tr_D0A9H6_
DRB1_0101	150	VTQSFAEQTSKKGTE	FAEQTSKKG	4	0.4117	581.0	60.00	0.75	tr_D0A9H6_
DRB1_0101	151	DIIEGCLPDAATCAH	IEGCLPDAA	2	0.4112	584.7	60.00	0.48	tr_D0A9H6_
DRB1_0101	152	LCIVVGVGMWKDLWE	LCIVVGVGM	0	0.4108	587.2	60.00	0.26	tr_D0A9H6_
DRB1_0101	153	YPRAGAPGSAVALAN	GAPGSAVAL	4	0.4103	590.4	60.00	0.44	tr_D0A9H6_
DRB1_0101	154	GKGAISQNVLTENGR	GAISQNVLT	2	0.4088	599.7	60.00	0.63	tr_D0A9H6_
DRB1_0101	155	ETNAKTRRAKPETIK	TRRAKPETI	5	0.4088	600.0	60.00	0.81	tr_D0A9H6_
DRB1_0101	156	VSLSIDQFLPRGCII	DQFLPRGCI	5	0.4052	623.8	65.00	0.25	tr_D0A9H6_
DRB1_0101	157	CGSVVSLSIDQFLPR	VVSLSIDQF	3	0.4030	638.7	65.00	0.22	tr_D0A9H6_
DRB1_0101	158	TNVTQSFAEQTSKKG	VTQSFAEQT	2	0.3984	671.4	65.00	0.55	tr_D0A9H6_
DRB1_0101	159	ETSNLDGETNAKTRR	LDGETNAKT	4	0.3977	676.1	65.00	0.80	tr_D0A9H6_
DRB1_0101	160	SNLDGETNAKTRRAK	LDGETNAKT	2	0.3976	677.1	65.00	0.85	tr_D0A9H6_
DRB1_0101	161	AVVLNTSLVDGVTYI	AVVLNTSLV	0	0.3965	685.1	65.00	0.43	tr_D0A9H6_
DRB1_0101	162	ISQNVLTENGRTSGG	VLTENGRTS	4	0.3949	696.9	65.00	0.81	tr_D0A9H6_
DRB1_0101	163	SLVDGVTYIETSNLD	VTYIETSNL	5	0.3941	703.2	65.00	0.73	tr_D0A9H6_
DRB1_0101	164	VTYIETSNLDGETNA	YIETSNLDG	2	0.3933	709.4	65.00	0.41	tr_D0A9H6_
DRB1_0101	165	RDVRAGDVILCGLGD	VRAGDVILC	2	0.3929	712.7	65.00	0.41	tr_D0A9H6_
DRB1_0101	166	EDIIEGCLPDAATCA	IEGCLPDAA	3	0.3923	716.8	65.00	0.51	tr_D0A9H6_
DRB1_0101	167	QSFAEQTSKKGTEGG	FAEQTSKKG	2	0.3918	721.0	65.00	0.87	tr_D0A9H6_
DRB1_0101	168	TSGQRARASSAFRDV	ARASSAFRD	5	0.3917	721.7	65.00	0.34	tr_D0A9H6_
DRB1_0101	169	VLLRGATPCPDLHSW	LRGATPCPD	2	0.3913	724.8	65.00	0.44	tr_D0A9H6_
DRB1_0101	170	IKALGTVEDIIEGCL	IKALGTVED	0	0.3890	743.0	65.00	0.60	tr_D0A9H6_
DRB1_0101	171	GGDNGKGAISQNVLT	GKGAISQNV	4	0.3890	743.5	65.00	0.41	tr_D0A9H6_
DRB1_0101	172	SSAFRDVEVTEVEDG	FRDVEVTEV	3	0.3885	746.8	65.00	0.85	tr_D0A9H6_
DRB1_0101	173	LPDAATCAHFLNNGS	DAATCAHFL	2	0.3871	759.0	65.00	0.70	tr_D0A9H6_
DRB1_0101	174	GKRRKSDKLVNSVGV	DKLVNSVGV	6	0.3868	761.4	65.00	0.41	tr_D0A9H6_
DRB1_0101	175	QNVLTENGRTSGGTS	VLTENGRTS	2	0.3845	780.5	65.00	0.83	tr_D0A9H6_

DRB1_0101	176	SGQRARASSAFRDVE	ARASSAFRD	4	0.3837	787.1	70.00	0.42	tr_D0A9H6_
DRB1_0101	177	RASSAFRDVEVTEVE	FRDVEVTEV	5	0.3823	799.3	70.00	0.73	tr_D0A9H6_
DRB1_0101	178	CPDLHSWIGQLRLRC	HSWIGQLRL	4	0.3822	800.0	70.00	0.28	tr_D0A9H6_
DRB1_0101	179	PDAATCAHFLNNGSL	DAATCAHFL	1	0.3820	801.6	70.00	0.59	tr_D0A9H6_
DRB1_0101	180	GGPSGLCSSSNPFAA	LCSSSNPFA	5	0.3819	802.8	70.00	0.52	tr_D0A9H6_
DRB1_0101	181	GQRARASSAFRDVEV	ARASSAFRD	3	0.3812	808.7	70.00	0.50	tr_D0A9H6_
DRB1_0101	182	ASSAFRDVEVTEVED	FRDVEVTEV	4	0.3806	814.1	70.00	0.79	tr_D0A9H6_
DRB1_0101	183	TSGGTSGQRARASSA	TSGQRARAS	4	0.3801	817.9	70.00	0.37	tr_D0A9H6_
DRB1_0101	184	PCPDLHSWIGQLRLR	HSWIGQLRL	5	0.3759	856.5	70.00	0.28	tr_D0A9H6_
DRB1_0101	185	RTSGGTSGQRARASS	SGGTSGQRA	2	0.3740	874.0	70.00	0.48	tr_D0A9H6_
DRB1_0101	186	LNLKSKGEKSSLTSR	SKGEKSSLT	4	0.3736	877.8	70.00	0.34	tr_D0A9H6_
DRB1_0101	187	IIRNTEWVLCAVVYT	IIRNTEWVL	0	0.3722	891.2	70.00	0.22	tr_D0A9H6_
DRB1_0101	188	LNNGSLVGWKRCDSR	NGSLVGWKR	2	0.3711	902.1	70.00	0.67	tr_D0A9H6_
DRB1_0101	189	VCNTLELSSKERTIT	LELSSKERT	4	0.3707	905.6	70.00	0.32	tr_D0A9H6_
DRB1_0101	190	PRAGAPGSAVALAND	GAPGSAVAL	3	0.3679	934.1	70.00	0.55	tr_D0A9H6_
DRB1_0101	191	NLKSKGEKSSLTSRR	SKGEKSSLT	3	0.3678	935.2	70.00	0.47	tr_D0A9H6_
DRB1_0101	192	GSVVSLSIDQFLPRG	VVSLSIDQF	2	0.3673	940.3	70.00	0.31	tr_D0A9H6_
DRB1_0101	193	SGGTSGQRARASSAF	TSGQRARAS	3	0.3658	955.7	70.00	0.41	tr_D0A9H6_
DRB1_0101	194	TASNIYPRAGAPGSA	IYPRAGAPG	4	0.3654	959.4	70.00	0.47	tr_D0A9H6_
DRB1_0101	195	VVSLSIDQFLPRGCI	LSIDQFLPR	3	0.3650	963.1	70.00	0.22	tr_D0A9H6_
DRB1_0101	196	QRARASSAFRDVEVT	ARASSAFRD	2	0.3649	964.3	70.00	0.53	tr_D0A9H6_
DRB1_0101	197	PTNVTQSFAEQTSKK	VTQSFAEQT	3	0.3643	971.2	70.00	0.77	tr_D0A9H6_
DRB1_0101	198	LCSSSNPFAAGTASN	CSSSNPFAA	1	0.3631	983.2	70.00	0.36	tr_D0A9H6_
DRB1_0101	199	RDRVRGGPSGLCSSS	VRGGPSGLC	3	0.3625	990.1	70.00	0.50	tr_D0A9H6_
DRB1_0101	200	IRDRVRGGPSGLCSS	VRGGPSGLC	4	0.3612	1004.4	70.00	0.46	tr_D0A9H6_
DRB1_0101	201	SVCNTLELSSKERTI	LELSSKERT	5	0.3604	1013.1	70.00	0.28	tr_D0A9H6_
DRB1_0101	202	SRDVRAGDVILCGLG	VRAGDVILC	3	0.3583	1035.6	75.00	0.57	tr_D0A9H6_
DRB1_0101	203	SVVSLSIDQFLPRGC	VSLSIDQFL	2	0.3573	1046.7	75.00	0.26	tr_D0A9H6_
DRB1_0101	204	KGAISQNVLTENGRT	GAISQNVLT	1	0.3565	1056.7	75.00	0.70	tr_D0A9H6_
DRB1_0101	205	SAFRDVEVTEVEDGT	FRDVEVTEV	2	0.3554	1069.4	75.00	0.87	tr_D0A9H6_
DRB1_0101	206	ARASSAFRDVEVTEV	FRDVEVTEV	6	0.3553	1070.0	75.00	0.47	tr_D0A9H6_
DRB1_0101	207	CNTLELSSKERTITE	LELSSKERT	3	0.3553	1070.3	75.00	0.38	tr_D0A9H6_
DRB1_0101	208	GTSGQRARASSAFRD	TSGQRARAS	1	0.3532	1094.3	75.00	0.25	tr_D0A9H6_
DRB1_0101	209	RAGAPGSAVALANDG	GAPGSAVAL	2	0.3518	1111.0	75.00	0.51	tr_D0A9H6_
DRB1_0101	210	IETSNLDGETNAKTR	LDGETNAKT	5	0.3503	1129.7	75.00	0.70	tr_D0A9H6_
DRB1_0101	211	AISQNVLTENGRTSG	VLTENGRTS	5	0.3476	1162.8	75.00	0.75	tr_D0A9H6_
DRB1_0101	212	DARGDGDFKGVLLRG	DGDFKGVLL	4	0.3471	1169.0	75.00	0.62	tr_D0A9H6_
DRB1_0101	213	GSAVALANDGNGVLN	LANDGNGVL	5	0.3469	1171.6	75.00	0.34	tr_D0A9H6_
DRB1_0101	214	VEDIIEGCLPDAATC	IEGCLPDAA	4	0.3454	1190.5	75.00	0.56	tr_D0A9H6_
DRB1_0101	215	GRTSGGTSGQRARAS	SGGTSGQRA	3	0.3446	1201.7	75.00	0.55	tr_D0A9H6_
DRB1_0101	216	TSLVDGVTYIETSNL	VTYIETSNL	6	0.3442	1206.7	75.00	0.50	tr_D0A9H6_
DRB1_0101	217	GGTSGQRARASSAFR	TSGQRARAS	2	0.3435	1216.0	75.00	0.41	tr_D0A9H6_
DRB1_0101	218	NLDGETNAKTRRAKP	LDGETNAKT	1	0.3429	1224.2	75.00	0.81	tr_D0A9H6_
DRB1_0101	219	WVLCAVVYTGKNTKM	VYTGKNTKM	6	0.3422	1233.5	75.00	0.31	tr_D0A9H6_
DRB1_0101	220	PSRDVRAGDVILCGL	VRAGDVILC	4	0.3409	1250.2	75.00	0.56	tr_D0A9H6_
DRB1_0101	221	NTEWVLCAVVYTGKN	WVLCAVVYT	3	0.3399	1264.1	75.00	0.35	tr_D0A9H6_
DRB1_0101	222	TVEDIIEGCLPDAAT	IEGCLPDAA	5	0.3384	1285.1	75.00	0.49	tr_D0A9H6_
DRB1_0101	223	NNGSLVGWKRCDSRG	NGSLVGWKR	1	0.3377	1295.2	75.00	0.62	tr_D0A9H6_
DRB1_0101	224	SFAEQTSKKGTEGGH	FAEQTSKKG	1	0.3338	1349.9	80.00	0.88	tr_D0A9H6_
DRB1_0101	225	TEWVLCAVVYTGKNT	WVLCAVVYT	2	0.3334	1356.3	80.00	0.29	tr_D0A9H6_

DRB1_0101	226	RNTEWVLCAVVYTGK	WVLCAVVYT	4	0.3317	1381.7	80.00	0.35	tr_D0A9H6_
DRB1_0101	227	SAVALANDGNGVLND	LANDGNGVL	4	0.3303	1402.9	80.00	0.38	tr_D0A9H6_
DRB1_0101	228	DRVRGGPSGLCSSSN	VRGGPSGLC	2	0.3261	1467.8	80.00	0.47	tr_D0A9H6_
DRB1_0101	229	DIRDRVRGGPSGLCS	VRGGPSGLC	5	0.3257	1473.7	80.00	0.44	tr_D0A9H6_
DRB1_0101	230	TPCPDLHSWIGQLRL	LHSWIGQLR	5	0.3250	1485.8	80.00	0.29	tr_D0A9H6_
DRB1_0101	231	GSVCNTLELSSKERT	CNTLELSSK	3	0.3233	1513.2	80.00	0.25	tr_D0A9H6_
DRB1_0101	232	CIVVGVGMWKDLWED	IVVGVGMWK	1	0.3229	1519.5	80.00	0.33	tr_D0A9H6_
DRB1_0101	233	QVLRGSDFVSVPSRD	LRGSDFVSV	2	0.3224	1527.6	80.00	0.47	tr_D0A9H6_
DRB1_0101	234	PPTNVTQSFAEQTSK	VTQSFAEQT	4	0.3222	1530.2	80.00	0.71	tr_D0A9H6_
DRB1_0101	235	GAISQNVLTENGRTS	VLTENGRTS	6	0.3208	1554.0	80.00	0.40	tr_D0A9H6_
DRB1_0101	236	EWVLCAVVYTGKNTK	WVLCAVVYT	1	0.3202	1565.1	80.00	0.23	tr_D0A9H6_
DRB1_0101	237	IRNTEWVLCAVVYTG	WVLCAVVYT	5	0.3199	1569.4	80.00	0.34	tr_D0A9H6_
DRB1_0101	238	NVLTENGRTSGGTSG	VLTENGRTS	1	0.3194	1577.8	80.00	0.79	tr_D0A9H6_
DRB1_0101	239	NTLELSSKERTITEC	LELSSKERT	2	0.3179	1604.0	80.00	0.41	tr_D0A9H6_
DRB1_0101	240	PGSAVALANDGNGVL	SAVALANDG	2	0.3179	1604.5	80.00	0.28	tr_D0A9H6_
DRB1_0101	241	RARASSAFRDVEVTE	ARASSAFRD	1	0.3164	1629.5	80.00	0.47	tr_D0A9H6_
DRB1_0101	242	NDARGDGDFKGVLLR	DGDFKGVLL	5	0.3160	1637.1	80.00	0.65	tr_D0A9H6_
DRB1_0101	243	LNTSLVDGVTYIETS	SLVDGVTYI	3	0.3142	1668.7	80.00	0.38	tr_D0A9H6_
DRB1_0101	244	TPQSVELPPPTNVTQ	SVELPPPTN	3	0.3137	1678.8	80.00	0.38	tr_D0A9H6_
DRB1_0101	245	VLNTSLVDGVTYIET	SLVDGVTYI	4	0.3131	1689.1	80.00	0.32	tr_D0A9H6_
DRB1_0101	246	LLRGATPCPDLHSWI	LRGATPCPD	1	0.3113	1722.5	80.00	0.49	tr_D0A9H6_
DRB1_0101	247	TPLETRGSVCNTLEL	TRGSVCNTL	4	0.3101	1744.3	80.00	0.37	tr_D0A9H6_
DRB1_0101	248	PLETRGSVCNTLELS	TRGSVCNTL	3	0.3097	1751.8	80.00	0.41	tr_D0A9H6_
DRB1_0101	249	VPSRDVRAGDVILCG	VRAGDVILC	5	0.3087	1770.8	80.00	0.51	tr_D0A9H6_
DRB1_0101	250	ETRGSVCNTLELSSK	TRGSVCNTL	1	0.3077	1791.7	80.00	0.29	tr_D0A9H6_
DRB1_0101	251	AVALANDGNGVLNDA	LANDGNGVL	3	0.3072	1800.3	80.00	0.49	tr_D0A9H6_
DRB1_0101	252	VVLNTSLVDGVTYIE	SLVDGVTYI	5	0.3041	1861.3	85.00	0.26	tr_D0A9H6_
DRB1_0101	253	PQSVELPPPTNVTQS	VELPPPTNV	3	0.3024	1897.1	85.00	0.42	tr_D0A9H6_
DRB1_0101	254	AGAPGSAVALANDGN	GAPGSAVAL	1	0.3016	1913.4	85.00	0.47	tr_D0A9H6_
DRB1_0101	255	AFRDVEVTEVEDGTP	FRDVEVTEV	1	0.3008	1929.3	85.00	0.86	tr_D0A9H6_
DRB1_0101	256	TYIETSNLDGETNAK	YIETSNLDG	1	0.2997	1952.6	85.00	0.55	tr_D0A9H6_
DRB1_0101	257	RGSVCNTLELSSKER	CNTLELSSK	4	0.2989	1969.6	85.00	0.27	tr_D0A9H6_
DRB1_0101	258	NGSLVGWKRCDSRGG	NGSLVGWKR	0	0.2987	1975.0	85.00	0.44	tr_D0A9H6_
DRB1_0101	259	SVPSRDVRAGDVILC	VRAGDVILC	6	0.2985	1978.9	85.00	0.31	tr_D0A9H6_
DRB1_0101	260	GTPQSVELPPPTNVT	SVELPPPTN	4	0.2979	1991.9	85.00	0.38	tr_D0A9H6_
DRB1_0101	261	LETRGSVCNTLELSS	TRGSVCNTL	2	0.2955	2043.1	85.00	0.39	tr_D0A9H6_
DRB1_0101	262	TRGSVCNTLELSSKE	SVCNTLELS	3	0.2947	2062.1	85.00	0.26	tr_D0A9H6_
DRB1_0101	263	GTASNIYPRAGAPGS	IYPRAGAPG	5	0.2941	2075.0	85.00	0.48	tr_D0A9H6_
DRB1_0101	264	YIETSNLDGETNAKT	LDGETNAKT	6	0.2940	2078.1	85.00	0.39	tr_D0A9H6_
DRB1_0101	265	VSVPSRDVRAGDVIL	SVPSRDVRA	1	0.2932	2096.0	85.00	0.37	tr_D0A9H6_
DRB1_0101	266	RVRGGPSGLCSSSNP	VRGGPSGLC	1	0.2931	2097.2	85.00	0.51	tr_D0A9H6_
DRB1_0101	267	PTPLETRGSVCNTLE	LETRGSVCN	3	0.2921	2120.0	85.00	0.31	tr_D0A9H6_
DRB1_0101	268	NTSLVDGVTYIETSN	SLVDGVTYI	2	0.2917	2129.7	85.00	0.39	tr_D0A9H6_
DRB1_0101	269	NGRTSGGTSGQRARA	SGGTSGQRA	4	0.2913	2139.8	85.00	0.64	tr_D0A9H6_
DRB1_0101	270	EVTEVEDGTPQSVEL	VEDGTPQSV	4	0.2895	2181.0	85.00	0.77	tr_D0A9H6_
DRB1_0101	271	RGGDNGKGAISQNVL	GKGAISQNV	5	0.2853	2283.3	85.00	0.56	tr_D0A9H6_
DRB1_0101	272	GETNAKTRRAKPETI	TRRAKPETI	6	0.2846	2300.8	85.00	0.68	tr_D0A9H6_
DRB1_0101	273	VTEVEDGTPQSVELP	VEDGTPQSV	3	0.2829	2341.3	85.00	0.74	tr_D0A9H6_
DRB1_0101	274	GTVEDIIEGCLPDAA	IEGCLPDAA	6	0.2822	2359.1	85.00	0.31	tr_D0A9H6_
DRB1_0101	275	RGGPSGLCSSSNPFA	LCSSSNPFA	6	0.2775	2481.9	85.00	0.41	tr_D0A9H6_

DRB1_0101	276	QSVELPPPTNVTQSF	VELPPPTNV	2	0.2770	2496.7	85.00	0.44	tr_D0A9H6_
DRB1_0101	277	TPTPLETRGSVCNTL	LETRGSVCN	4	0.2757	2533.3	90.00	0.32	tr_D0A9H6_
DRB1_0101	278	VALANDGNGVLNDAR	LANDGNGVL	2	0.2756	2535.9	90.00	0.52	tr_D0A9H6_
DRB1_0101	279	APGSAVALANDGNGV	SAVALANDG	3	0.2746	2562.7	90.00	0.44	tr_D0A9H6_
DRB1_0101	280	DGKRRKSDKLVNSVG	KRRKSDKLV	2	0.2745	2563.9	90.00	0.45	tr_D0A9H6_
DRB1_0101	281	TEVEDGTPQSVELPP	VEDGTPQSV	2	0.2669	2783.5	90.00	0.71	tr_D0A9H6_
DRB1_0101	282	TLELSSKERTITECD	LELSSKERT	1	0.2649	2846.3	90.00	0.39	tr_D0A9H6_
DRB1_0101	283	GAPGSAVALANDGNG	SAVALANDG	4	0.2633	2895.8	90.00	0.32	tr_D0A9H6_
DRB1_0101	284	SDIRDRVRGGPSGLC	VRGGPSGLC	6	0.2632	2898.9	90.00	0.38	tr_D0A9H6_
DRB1_0101	285	ATPCPDLHSWIGQLR	CPDLHSWIG	3	0.2632	2900.1	90.00	0.26	tr_D0A9H6_
DRB1_0101	286	PPPTNVTQSFAEQTS	VTQSFAEQT	5	0.2620	2937.2	90.00	0.68	tr_D0A9H6_
DRB1_0101	287	IVVGVGMWKDLWEDG	VVGVGMWKD	1	0.2595	3017.9	90.00	0.25	tr_D0A9H6_
DRB1_0101	288	DGTPQSVELPPPTNV	SVELPPPTN	5	0.2587	3042.4	90.00	0.38	tr_D0A9H6_
DRB1_0101	289	HGRTSTATPTPLETR	TSTATPTPL	3	0.2567	3108.8	90.00	0.47	tr_D0A9H6_
DRB1_0101	290	LNDARGDGDFKGVLL	DGDFKGVLL	6	0.2514	3294.1	90.00	0.58	tr_D0A9H6_
DRB1_0101	291	GMWKDLWEDGKRRKS	WKDLWEDGK	2	0.2470	3453.5	90.00	0.28	tr_D0A9H6_
DRB1_0101	292	GRTSTATPTPLETRG	TSTATPTPL	2	0.2451	3525.5	90.00	0.48	tr_D0A9H6_
DRB1_0101	293	EDGKRRKSDKLVNSV	KRRKSDKLV	3	0.2439	3571.6	90.00	0.56	tr_D0A9H6_
DRB1_0101	294	MWKDLWEDGKRRKSD	DLWEDGKRR	3	0.2429	3609.6	90.00	0.35	tr_D0A9H6_
DRB1_0101	295	LDGETNAKTRRAKPE	LDGETNAKT	0	0.2426	3623.6	90.00	0.62	tr_D0A9H6_
DRB1_0101	296	VEVTEVEDGTPQSVE	VEDGTPQSV	5	0.2348	3942.2	95.00	0.73	tr_D0A9H6_
DRB1_0101	297	ENGRTSGGTSGQRAR	SGGTSGQRA	5	0.2317	4077.2	95.00	0.64	tr_D0A9H6_
DRB1_0101	298	GHGRTSTATPTPLET	TSTATPTPL	4	0.2300	4152.8	95.00	0.46	tr_D0A9H6_
DRB1_0101	299	AGTASNIYPRAGAPG	IYPRAGAPG	6	0.2288	4204.0	95.00	0.30	tr_D0A9H6_
DRB1_0101	300	RTSTATPTPLETRGS	TSTATPTPL	1	0.2283	4227.7	95.00	0.43	tr_D0A9H6_
DRB1_0101	301	EVEDGTPQSVELPPP	VEDGTPQSV	1	0.2278	4252.5	95.00	0.63	tr_D0A9H6_
DRB1_0101	302	ATPTPLETRGSVCNT	LETRGSVCN	5	0.2269	4292.3	95.00	0.32	tr_D0A9H6_
DRB1_0101	303	LRGATPCPDLHSWIG	LRGATPCPD	0	0.2267	4302.1	95.00	0.31	tr_D0A9H6_
DRB1_0101	304	ALANDGNGVLNDARG	LANDGNGVL	1	0.2265	4311.4	95.00	0.51	tr_D0A9H6_
DRB1_0101	305	WEDGKRRKSDKLVNS	KRRKSDKLV	4	0.2249	4387.5	95.00	0.52	tr_D0A9H6_
DRB1_0101	306	VGMWKDLWEDGKRRK	WKDLWEDGK	3	0.2222	4517.2	95.00	0.36	tr_D0A9H6_
DRB1_0101	307	WKDLWEDGKRRKSDK	WEDGKRRKS	4	0.2219	4530.9	95.00	0.34	tr_D0A9H6_
DRB1_0101	308	VLTENGRTSGGTSGQ	VLTENGRTS	0	0.2200	4624.6	95.00	0.67	tr_D0A9H6_
DRB1_0101	309	LWEDGKRRKSDKLVN	KRRKSDKLV	5	0.2181	4723.1	95.00	0.46	tr_D0A9H6_
DRB1_0101	310	SVELPPPTNVTQSFA	VELPPPTNV	1	0.2161	4823.4	95.00	0.49	tr_D0A9H6_
DRB1_0101	311	GATPCPDLHSWIGQL	CPDLHSWIG	4	0.2137	4951.3	95.00	0.28	tr_D0A9H6_
DRB1_0101	312	FAEQTSKKGTEGGHG	FAEQTSKKG	0	0.2135	4963.9	95.00	0.77	tr_D0A9H6_
DRB1_0101	313	VRGGPSGLCSSSNPF	VRGGPSGLC	0	0.2130	4988.5	95.00	0.34	tr_D0A9H6_
DRB1_0101	314	LGTVEDIIEGCLPDA	EDIIEGCLP	4	0.2118	5054.0	95.00	0.23	tr_D0A9H6_
DRB1_0101	315	GSLVGWKRCDSRGGD	WKRCDSRGG	5	0.2041	5495.5	95.00	0.50	tr_D0A9H6_
DRB1_0101	316	GGHGRTSTATPTPLE	TSTATPTPL	5	0.2038	5510.0	95.00	0.41	tr_D0A9H6_
DRB1_0101	317	KDLWEDGKRRKSDKL	WEDGKRRKS	3	0.2016	5642.9	95.00	0.47	tr_D0A9H6_
DRB1_0101	318	DLWEDGKRRKSDKLV	WEDGKRRKS	2	0.2015	5653.5	95.00	0.31	tr_D0A9H6_
DRB1_0101	319	AAGTASNIYPRAGAP	GTASNIYPR	2	0.2003	5724.1	95.00	0.34	tr_D0A9H6_
DRB1_0101	320	LVGWKRCDSRGGDNG	WKRCDSRGG	3	0.2002	5729.0	95.00	0.71	tr_D0A9H6_
DRB1_0101	321	SRGGDNGKGAISQNV	GKGAISQNV	6	0.2000	5744.1	95.00	0.39	tr_D0A9H6_
DRB1_0101	322	TATPTPLETRGSVCN	PLETRGSVC	5	0.1990	5805.0	95.00	0.32	tr_D0A9H6_
DRB1_0101	323	RGATPCPDLHSWIGQ	CPDLHSWIG	5	0.1981	5863.9	95.00	0.25	tr_D0A9H6_
DRB1_0101	324	LELSSKERTITECDD	ELSSKERTI	1	0.1975	5898.2	95.00	0.31	tr_D0A9H6_
DRB1_0101	325	SLVGWKRCDSRGGDN	WKRCDSRGG	4	0.1972	5923.3	95.00	0.67	tr_D0A9H6_

DRB1_0101	326	VVGVGMWKDLWEDGK	VGVGMWKDL	1	0.1959	6005.5	95.00	0.26	tr_D0A9H6_
DRB1_0101	327	KALGTVEDIIEGCLP	GTVEDIIEG	3	0.1954	6039.9	95.00	0.29	tr_D0A9H6_
DRB1_0101	328	FRDVEVTEVEDGTPQ	FRDVEVTEV	0	0.1947	6080.8	95.00	0.75	tr_D0A9H6_
DRB1_0101	329	TSTATPTPLETRGSV	STATPTPLE	1	0.1918	6279.3	100.00	0.25	tr_D0A9H6_
DRB1_0101	330	GVGMWKDLWEDGKRR	WKDLWEDGK	4	0.1911	6320.8	100.00	0.39	tr_D0A9H6_
DRB1_0101	331	EDGTPQSVELPPPTN	SVELPPPTN	6	0.1895	6436.7	100.00	0.34	tr_D0A9H6_
DRB1_0101	332	VGWKRCDSRGGDNGK	WKRCDSRGG	2	0.1894	6443.1	100.00	0.69	tr_D0A9H6_
DRB1_0101	333	LPPPTNVTQSFAEQT	VTQSFAEQT	6	0.1840	6832.7	100.00	0.49	tr_D0A9H6_
DRB1_0101	334	VGVGMWKDLWEDGKR	WKDLWEDGK	5	0.1837	6851.0	100.00	0.28	tr_D0A9H6_
DRB1_0101	335	VEDGTPQSVELPPPT	VEDGTPQSV	0	0.1813	7031.5	100.00	0.37	tr_D0A9H6_
DRB1_0101	336	GNGVLNDARGDGDFK	GVLNDARGD	2	0.1798	7147.4	100.00	0.31	tr_D0A9H6_
DRB1_0101	337	STATPTPLETRGSVC	PTPLETRGS	4	0.1780	7286.3	100.00	0.25	tr_D0A9H6_
DRB1_0101	338	GWKRCDSRGGDNGKG	WKRCDSRGG	1	0.1773	7342.6	100.00	0.63	tr_D0A9H6_
DRB1_0101	339	NGVLNDARGDGDFKG	LNDARGDGD	3	0.1765	7403.2	100.00	0.42	tr_D0A9H6_
DRB1_0101	340	PSSTVETSDIRDRVR	VETSDIRDR	4	0.1743	7582.8	100.00	0.26	tr_D0A9H6_
DRB1_0101	341	ALGTVEDIIEGCLPD	GTVEDIIEG	2	0.1738	7621.7	100.00	0.26	tr_D0A9H6_
DRB1_0101	342	SSTVETSDIRDRVRG	VETSDIRDR	3	0.1729	7696.6	100.00	0.29	tr_D0A9H6_
DRB1_0101	343	TECDDRVPSSTVETS	DDRVPSSTV	3	0.1680	8121.4	100.00	0.65	tr_D0A9H6_
DRB1_0101	344	RTITECDDRVPSSTV	TITECDDRV	1	0.1674	8171.0	100.00	0.29	tr_D0A9H6_
DRB1_0101	345	TITECDDRVPSSTVE	DDRVPSSTV	5	0.1643	8447.1	100.00	0.47	tr_D0A9H6_
DRB1_0101	346	EGGHGRTSTATPTPL	GRTSTATPT	4	0.1639	8486.0	100.00	0.46	tr_D0A9H6_
DRB1_0101	347	VPSSTVETSDIRDRV	STVETSDIR	3	0.1633	8541.7	100.00	0.24	tr_D0A9H6_
DRB1_0101	348	TENGRTSGGTSGQRA	SGGTSGQRA	6	0.1631	8557.6	100.00	0.46	tr_D0A9H6_
DRB1_0101	349	LANDGNGVLNDARGD	LANDGNGVL	0	0.1626	8607.8	100.00	0.35	tr_D0A9H6_
DRB1_0101	350	DGNGVLNDARGDGDF	GVLNDARGD	3	0.1626	8608.5	100.00	0.34	tr_D0A9H6_
DRB1_0101	351	TSDIRDRVRGGPSGL	RVRGGPSGL	6	0.1625	8621.4	100.00	0.34	tr_D0A9H6_
DRB1_0101	352	GVLNDARGDGDFKGV	LNDARGDGD	2	0.1600	8858.0	100.00	0.41	tr_D0A9H6_
DRB1_0101	353	KERTITECDDRVPSS	TITECDDRV	3	0.1594	8909.5	100.00	0.54	tr_D0A9H6_
DRB1_0101	354	DVEVTEVEDGTPQSV	VEDGTPQSV	6	0.1583	9021.3	100.00	0.62	tr_D0A9H6_
DRB1_0101	355	ITECDDRVPSSTVET	DDRVPSSTV	4	0.1581	9034.4	100.00	0.62	tr_D0A9H6_
DRB1_0101	356	ECDDRVPSSTVETSD	DDRVPSSTV	2	0.1567	9180.0	100.00	0.64	tr_D0A9H6_
DRB1_0101	357	STVETSDIRDRVRGG	ETSDIRDRV	3	0.1566	9186.9	100.00	0.25	tr_D0A9H6_
DRB1_0101	358	CDDRVPSSTVETSDI	DDRVPSSTV	1	0.1520	9653.9	100.00	0.52	tr_D0A9H6_
DRB1_0101	359	ERTITECDDRVPSST	TITECDDRV	2	0.1508	9778.9	100.00	0.46	tr_D0A9H6_
DRB1_0101	360	WKRCDSRGGDNGKGA	WKRCDSRGG	0	0.1443	10494.8	100.00	0.44	tr_D0A9H6_
DRB1_0101	361	SKERTITECDDRVPS	TITECDDRV	4	0.1439	10542.7	100.00	0.55	tr_D0A9H6_
DRB1_0101	362	VLNDARGDGDFKGVL	LNDARGDGD	1	0.1436	10577.9	100.00	0.38	tr_D0A9H6_
DRB1_0101	363	DSRGGDNGKGAISQN	GDNGKGAIS	4	0.1432	10618.4	100.00	0.35	tr_D0A9H6_
DRB1_0101	364	RVPSSTVETSDIRDR	SSTVETSDI	3	0.1417	10795.6	100.00	0.30	tr_D0A9H6_
DRB1_0101	365	NDGNGVLNDARGDGD	GVLNDARGD	4	0.1416	10805.4	100.00	0.32	tr_D0A9H6_
DRB1_0101	366	DDRVPSSTVETSDIR	DDRVPSSTV	0	0.1401	10985.9	100.00	0.31	tr_D0A9H6_
DRB1_0101	367	VELPPPTNVTQSFAE	VELPPPTNV	0	0.1326	11907.9	100.00	0.34	tr_D0A9H6_
DRB1_0101	368	CDSRGGDNGKGAISQ	GDNGKGAIS	5	0.1290	12378.3	100.00	0.32	tr_D0A9H6_
DRB1_0101	369	ANDGNGVLNDARGDG	GVLNDARGD	5	0.1281	12499.4	100.00	0.33	tr_D0A9H6_
DRB1_0101	370	TVETSDIRDRVRGGP	ETSDIRDRV	2	0.1277	12560.2	100.00	0.29	tr_D0A9H6_
DRB1_0101	371	ELSSKERTITECDDR	LSSKERTIT	1	0.1253	12887.1	100.00	0.34	tr_D0A9H6_
DRB1_0101	372	DRVPSSTVETSDIRD	SSTVETSDI	4	0.1234	13156.1	100.00	0.29	tr_D0A9H6_
DRB1_0101	373	SSKERTITECDDRVP	TITECDDRV	5	0.1228	13247.2	100.00	0.56	tr_D0A9H6_
DRB1_0101	374	DGETNAKTRRAKPET	ETNAKTRRA	2	0.1175	14021.9	100.00	0.44	tr_D0A9H6_
DRB1_0101	375	TEGGHGRTSTATPTP	GRTSTATPT	5	0.1173	14060.7	100.00	0.52	tr_D0A9H6_

DRB1_0101	376	RCDSRGGDNGKGAIS	SRGGDNGKG	3	0.1119	14905.2	100.00	0.29	tr_D0A9H6_
DRB1_0101	377	LSSKERTITECDDRV	TITECDDRV	6	0.1114	14974.2	100.00	0.34	tr_D0A9H6_
DRB1_0101	378	KKGTEGGHGRTSTAT	TEGGHGRTS	3	0.1107	15101.0	100.00	0.33	tr_D0A9H6_
DRB1_0101	379	GTEGGHGRTSTATPT	GRTSTATPT	6	0.1099	15222.9	100.00	0.32	tr_D0A9H6_
DRB1_0101	380	ELPPPTNVTQSFAEQ	PTNVTQSFA	4	0.1088	15407.5	100.00	0.24	tr_D0A9H6_
DRB1_0101	381	SKKGTEGGHGRTSTA	TEGGHGRTS	4	0.1072	15683.7	100.00	0.32	tr_D0A9H6_
DRB1_0101	382	VETSDIRDRVRGGPS	ETSDIRDRV	1	0.1071	15690.1	100.00	0.26	tr_D0A9H6_
DRB1_0101	383	ETSDIRDRVRGGPSG	DIRDRVRGG	3	0.1064	15809.9	100.00	0.15	tr_D0A9H6_
DRB1_0101	384	TSKKGTEGGHGRTST	KKGTEGGHG	2	0.1034	16333.8	100.00	0.31	tr_D0A9H6_
DRB1_0101	385	LTENGRTSGGTSGQR	GRTSGGTSG	4	0.1030	16406.8	100.00	0.40	tr_D0A9H6_
DRB1_0101	386	QTSKKGTEGGHGRTS	KKGTEGGHG	3	0.0990	17123.9	100.00	0.34	tr_D0A9H6_
DRB1_0101	387	KRCDSRGGDNGKGAI	SRGGDNGKG	4	0.0969	17521.8	100.00	0.34	tr_D0A9H6_
DRB1_0101	388	KGTEGGHGRTSTATP	TEGGHGRTS	2	0.0951	17859.4	100.00	0.34	tr_D0A9H6_
DRB1_0101	389	EQTSKKGTEGGHGRT	KKGTEGGHG	4	0.0867	19576.0	100.00	0.32	tr_D0A9H6_
DRB1_0101	390	AEQTSKKGTEGGHGR	KKGTEGGHG	5	0.0770	21726.5	100.00	0.30	tr_D0A9H6_
DRB1_0101	391	RDVEVTEVEDGTPQS	TEVEDGTPQ	5	0.0650	24747.5	100.00	0.41	tr_D0A9H6_

Allele: DRB1_0101. Number of high binders 0. Number of weak binders 11. Number of peptides 391

Allele	pos	peptide	core	Of	1-log50k(aff)	affinity(nM)	%Rank	Relia	Identity	Bind Level
DRB1_0101	1	PLFRYLRNLSLCHSV	FRYLRNLSL	2	0.9105	2.6	0.05	0.79	tr_D0A9H6_	SB
DRB1_0101	2	EPLFRYLRNLSLCHS	FRYLRNLSL	3	0.9099	2.6	0.05	0.82	tr_D0A9H6_	SB
DRB1_0101	3	LFRYLRNLSLCHSVV	FRYLRNLSL	1	0.9064	2.8	0.06	0.75	tr_D0A9H6_	SB
DRB1_0101	4	KEPLFRYLRNLSLCH	FRYLRNLSL	4	0.8998	3.0	0.09	0.86	tr_D0A9H6_	SB
DRB1_0101	5	EKEPLFRYLRNLSLC	FRYLRNLSL	5	0.8750	3.9	0.25	0.92	tr_D0A9H6_	SB
DRB1_0101	6	FRYLRNLSLCHSVVC	FRYLRNLSL	0	0.8740	3.9	0.30	0.66	tr_D0A9H6_	SB
DRB1_0101	7	GEAFLRLVEARRLSV	LRLVEARRL	4	0.8550	4.8	0.50	0.73	tr_D0A9H6_	WB
DRB1_0101	8	EAFLRLVEARRLSVP	LRLVEARRL	3	0.8458	5.3	0.70	0.75	tr_D0A9H6_	WB
DRB1_0101	9	MEKEPLFRYLRNLSL	FRYLRNLSL	6	0.8408	5.6	0.80	0.92	tr_D0A9H6_	WB
DRB1_0101	10	LGEAFLRLVEARRLS	LRLVEARRL	5	0.8336	6.1	1.00	0.68	tr_D0A9H6_	WB
DRB1_0101	11	AFLRLVEARRLSVPP	LRLVEARRL	2	0.8320	6.2	1.00	0.76	tr_D0A9H6_	WB
DRB1_0101	12	CLVYSLHKNAFLTSA	YSLHKNAFL	3	0.7889	9.8	2.50	0.70	tr_D0A9H6_	WB
DRB1_0101	13	ALGEAFLRLVEARRL	LRLVEARRL	6	0.7843	10.3	3.00	0.56	tr_D0A9H6_	WB
DRB1_0101	14	FLRLVEARRLSVPPA	LRLVEARRL	1	0.7843	10.3	3.00	0.80	tr_D0A9H6_	WB
DRB1_0101	15	KTSCLNSQLAYVRYV	TSCLNSQLA	1	0.7764	11.2	3.50	0.35	tr_D0A9H6_	WB
DRB1_0101	16	GIGIMGLEGSQAELA	IMGLEGSQA	3	0.7751	11.4	3.50	0.82	tr_D0A9H6_	WB
DRB1_0101	17	HCLVYSLHKNAFLTS	YSLHKNAFL	4	0.7722	11.8	3.50	0.68	tr_D0A9H6_	WB
DRB1_0101	18	IGIMGLEGSQAELAS	IMGLEGSQA	2	0.7685	12.2	4.00	0.79	tr_D0A9H6_	WB
DRB1_0101	19	TVKFLMQADIVVWML	FLMQADIVV	3	0.7647	12.8	4.00	0.58	tr_D0A9H6_	WB
DRB1_0101	20	ETVKFLMQADIVVWM	FLMQADIVV	4	0.7617	13.2	4.00	0.56	tr_D0A9H6_	WB
DRB1_0101	21	LKRLLMVHGRFSLYR	LLMVHGRFS	3	0.7592	13.5	4.50	0.64	tr_D0A9H6_	WB
DRB1_0101	22	RDNLFALQGRTSKHV	LFALQGRTS	3	0.7558	14.0	4.50	0.79	tr_D0A9H6_	WB
DRB1_0101	23	PKTSCLNSQLAYVRY	TSCLNSQLA	2	0.7558	14.1	4.50	0.46	tr_D0A9H6_	WB
DRB1_0101	24	IREVLRSQLSAASNK	LRSQLSAAS	4	0.7526	14.5	5.00	0.80	tr_D0A9H6_	WB
DRB1_0101	25	TSCLNSQLAYVRYVF	LNSQLAYVR	3	0.7510	14.8	5.00	0.31	tr_D0A9H6_	WB
DRB1_0101	26	AHCLVYSLHKNAFLT	YSLHKNAFL	5	0.7508	14.8	5.00	0.65	tr_D0A9H6_	WB

DRB1_0101	27	NLFALQGRTSKHVFV	LFALQGRTS	1	0.7492	15.1	5.00	0.58	tr_D0A9H6_	WB
DRB1_0101	28	VGIGIMGLEGSQAEL	IMGLEGSQA	4	0.7477	15.3	5.00	0.81	tr_D0A9H6_	WB
DRB1_0101	29	RPKTSCLNSQLAYVR	TSCLNSQLA	3	0.7465	15.5	5.00	0.56	tr_D0A9H6_	WB
DRB1_0101	30	REVLRSQLSAASNKC	LRSQLSAAS	3	0.7438	16.0	5.50	0.82	tr_D0A9H6_	WB
DRB1_0101	31	PETVKFLMQADIVVW	FLMQADIVV	5	0.7423	16.3	5.50	0.53	tr_D0A9H6_	WB
DRB1_0101	32	KRLLMVHGRFSLYRD	LLMVHGRFS	2	0.7413	16.4	5.50	0.68	tr_D0A9H6_	WB
DRB1_0101	33	DNLFALQGRTSKHVF	LFALQGRTS	2	0.7401	16.6	5.50	0.68	tr_D0A9H6_	WB
DRB1_0101	34	VKFLMQADIVVWMLT	FLMQADIVV	2	0.7375	17.1	6.00	0.58	tr_D0A9H6_	WB
DRB1_0101	35	FLKRLLMVHGRFSLY	LLMVHGRFS	4	0.7349	17.6	6.00	0.62	tr_D0A9H6_	WB
DRB1_0101	36	GIMGLEGSQAELASD	IMGLEGSQA	1	0.7296	18.7	6.50	0.76	tr_D0A9H6_	WB
DRB1_0101	37	ARDNLFALQGRTSKH	LFALQGRTS	4	0.7200	20.7	7.00	0.81	tr_D0A9H6_	WB
DRB1_0101	38	LFALQGRTSKHVFVK	LQGRTSKHV	3	0.7165	21.5	7.50	0.29	tr_D0A9H6_	WB
DRB1_0101	39	SCLNSQLAYVRYVFT	SQLAYVRYV	4	0.7152	21.8	7.50	0.34	tr_D0A9H6_	WB
DRB1_0101	40	RIREVLRSQLSAASN	LRSQLSAAS	5	0.7134	22.2	8.00	0.70	tr_D0A9H6_	WB
DRB1_0101	41	FLLLVKGADSSMMSI	LLLVKGADS	1	0.7130	22.3	8.00	0.41	tr_D0A9H6_	WB
DRB1_0101	42	RYLRNLSLCHSVVCF	YLRNLSLCH	1	0.7118	22.6	8.00	0.55	tr_D0A9H6_	WB
DRB1_0101	43	PFLLLVKGADSSMMS	LLLVKGADS	2	0.7096	23.2	8.00	0.50	tr_D0A9H6_	WB
DRB1_0101	44	RLLMVHGRFSLYRDA	LLMVHGRFS	1	0.7092	23.2	8.00	0.60	tr_D0A9H6_	WB
DRB1_0101	45	CCRMTPLQKAKIVKL	CRMTPLQKA	1	0.7075	23.7	8.50	0.41	tr_D0A9H6_	WB
DRB1_0101	46	FRFLKRLLMVHGRFS	LLMVHGRFS	6	0.7071	23.8	8.50	0.38	tr_D0A9H6_	WB
DRB1_0101	47	RFLKRLLMVHGRFSL	LLMVHGRFS	5	0.7049	24.4	8.50	0.63	tr_D0A9H6_	WB
DRB1_0101	48	QFTSQRKLMSVLLSR	QRKLMSVLL	4	0.7037	24.7	8.50	0.57	tr_D0A9H6_	WB
DRB1_0101	49	VCCRMTPLQKAKIVK	CRMTPLQKA	2	0.7033	24.8	8.50	0.52	tr_D0A9H6_	WB
DRB1_0101	50	PKFRFLKRLLMVHGR	FRFLKRLLM	2	0.7029	24.9	8.50	0.62	tr_D0A9H6_	WB
DRB1_0101	51	LQFTSQRKLMSVLLS	QRKLMSVLL	5	0.7027	24.9	9.00	0.47	tr_D0A9H6_	WB
DRB1_0101	52	SVGIGIMGLEGSQAE	IMGLEGSQA	5	0.7001	25.7	9.00	0.76	tr_D0A9H6_	WB
DRB1_0101	53	VPETVKFLMQADIVV	FLMQADIVV	6	0.6989	26.0	9.00	0.44	tr_D0A9H6_	WB
DRB1_0101	54	LPFLLLVKGADSSMM	LLLVKGADS	3	0.6975	26.4	9.50	0.58	tr_D0A9H6_	WB
DRB1_0101	55	CRMTPLQKAKIVKLF	MTPLQKAKI	2	0.6961	26.8	9.50	0.25	tr_D0A9H6_	WB
DRB1_0101	56	AVCCRMTPLQKAKIV	CRMTPLQKA	3	0.6959	26.8	9.50	0.54	tr_D0A9H6_	WB
DRB1_0101	57	IPKFRFLKRLLMVHG	FRFLKRLLM	3	0.6944	27.3	9.50	0.69	tr_D0A9H6_	WB
DRB1_0101	58	KFLMQADIVVWMLTG	FLMQADIVV	1	0.6936	27.5	9.50	0.65	tr_D0A9H6_	WB
DRB1_0101	59	DAHCLVYSLHKNAFL	YSLHKNAFL	6	0.6933	27.6	9.50	0.56	tr_D0A9H6_	WB
DRB1_0101	60	TREFFSIGMRCRSAV	FFSIGMRCR	3	0.6892	28.9	10.00	0.51	tr_D0A9H6_	
DRB1_0101	61	KFRFLKRLLMVHGRF	FRFLKRLLM	1	0.6884	29.1	10.00	0.58	tr_D0A9H6_	
DRB1_0101	62	VKLFQENTGGVALAI	FQENTGGVA	3	0.6858	29.9	11.00	0.66	tr_D0A9H6_	
DRB1_0101	63	EVLRSQLSAASNKCD	LRSQLSAAS	2	0.6857	30.0	11.00	0.86	tr_D0A9H6_	
DRB1_0101	64	LQLYLIANDRRMASY	YLIANDRRM	3	0.6855	30.1	11.00	0.47	tr_D0A9H6_	
DRB1_0101	65	SRPKTSCLNSQLAYV	TSCLNSQLA	4	0.6854	30.1	11.00	0.63	tr_D0A9H6_	
DRB1_0101	66	ELQFTSQRKLMSVLL	FTSQRKLMS	3	0.6806	31.7	11.00	0.44	tr_D0A9H6_	
DRB1_0101	67	AIPKFRFLKRLLMVH	FRFLKRLLM	4	0.6790	32.2	11.00	0.74	tr_D0A9H6_	
DRB1_0101	68	KLFQENTGGVALAIG	FQENTGGVA	2	0.6779	32.6	12.00	0.62	tr_D0A9H6_	
DRB1_0101	69	CLNSQLAYVRYVFTD	SQLAYVRYV	3	0.6778	32.6	12.00	0.38	tr_D0A9H6_	
DRB1_0101	70	FTSQRKLMSVLLSRC	QRKLMSVLL	3	0.6724	34.6	12.00	0.69	tr_D0A9H6_	
DRB1_0101	71	REFFSIGMRCRSAVC	FFSIGMRCR	2	0.6723	34.7	12.00	0.49	tr_D0A9H6_	
DRB1_0101	72	AARDNLFALQGRTSK	LFALQGRTS	5	0.6719	34.8	12.00	0.81	tr_D0A9H6_	
DRB1_0101	73	YAIPKFRFLKRLLMV	FRFLKRLLM	5	0.6709	35.2	12.00	0.68	tr_D0A9H6_	
DRB1_0101	74	LLLVKGADSSMMSIM	VKGADSSMM	3	0.6695	35.7	13.00	0.37	tr_D0A9H6_	
DRB1_0101	75	VLQLYLIANDRRMAS	YLIANDRRM	4	0.6691	35.9	13.00	0.44	tr_D0A9H6_	
DRB1_0101	76	DYAIPKFRFLKRLLM	FRFLKRLLM	6	0.6676	36.5	13.00	0.55	tr_D0A9H6_	

DRB1_0101	77	LRLVEARRLSVPPAV	LRLVEARRL	0	0.6669	36.8	13.00	0.77	tr_D0A9H6_
DRB1_0101	78	IVKLFQENTGGVALA	FQENTGGVA	4	0.6649	37.5	13.00	0.67	tr_D0A9H6_
DRB1_0101	79	LTREFFSIGMRCRSA	FFSIGMRCR	4	0.6641	37.9	13.00	0.51	tr_D0A9H6_
DRB1_0101	80	IMGLEGSQAELASDY	IMGLEGSQA	0	0.6626	38.5	13.00	0.62	tr_D0A9H6_
DRB1_0101	81	SAVCCRMTPLQKAKI	CRMTPLQKA	4	0.6587	40.1	14.00	0.62	tr_D0A9H6_
DRB1_0101	82	IDSVAKKGLRTLVLG	SVAKKGLRT	2	0.6562	41.2	14.00	0.58	tr_D0A9H6_
DRB1_0101	83	TSQRKLMSVLLSRCP	QRKLMSVLL	2	0.6560	41.3	14.00	0.62	tr_D0A9H6_
DRB1_0101	84	KLPFLLLVKGADSSM	LLLVKGADS	4	0.6525	42.9	15.00	0.67	tr_D0A9H6_
DRB1_0101	85	RFLHGHTGSASWRMT	FLHGHTGSA	1	0.6519	43.2	15.00	0.37	tr_D0A9H6_
DRB1_0101	86	GRFLHGHTGSASWRM	FLHGHTGSA	2	0.6503	44.0	15.00	0.45	tr_D0A9H6_
DRB1_0101	87	QLYLIANDRRMASYD	YLIANDRRM	2	0.6489	44.6	15.00	0.49	tr_D0A9H6_
DRB1_0101	88	RMTPLQKAKIVKLFQ	LQKAKIVKL	4	0.6469	45.6	15.00	0.46	tr_D0A9H6_
DRB1_0101	89	EIDSVAKKGLRTLVL	SVAKKGLRT	3	0.6467	45.7	15.00	0.62	tr_D0A9H6_
DRB1_0101	90	VGRFLHGHTGSASWR	FLHGHTGSA	3	0.6380	50.2	17.00	0.60	tr_D0A9H6_
DRB1_0101	91	KVLQLYLIANDRRMA	YLIANDRRM	5	0.6372	50.7	17.00	0.43	tr_D0A9H6_
DRB1_0101	92	KIVKLFQENTGGVAL	FQENTGGVA	5	0.6369	50.8	17.00	0.56	tr_D0A9H6_
DRB1_0101	93	DSVAKKGLRTLVLGQ	SVAKKGLRT	1	0.6365	51.1	17.00	0.50	tr_D0A9H6_
DRB1_0101	94	ERIREVLRSQLSAAS	LRSQLSAAS	6	0.6354	51.7	17.00	0.48	tr_D0A9H6_
DRB1_0101	95	GALGEAFLRLVEARR	FLRLVEARR	6	0.6354	51.7	17.00	0.40	tr_D0A9H6_
DRB1_0101	96	VLRSQLSAASNKCDS	LRSQLSAAS	1	0.6328	53.1	17.00	0.83	tr_D0A9H6_
DRB1_0101	97	SQRKLMSVLLSRCPD	QRKLMSVLL	1	0.6290	55.4	18.00	0.56	tr_D0A9H6_
DRB1_0101	98	LFQENTGGVALAIGD	FQENTGGVA	1	0.6246	58.1	19.00	0.58	tr_D0A9H6_
DRB1_0101	99	KGVLRYSRPKTSCLN	LRYSRPKTS	3	0.6225	59.4	19.00	0.76	tr_D0A9H6_
DRB1_0101	100	LLMVHGRFSLYRDAH	LLMVHGRFS	0	0.6217	59.9	19.00	0.50	tr_D0A9H6_
DRB1_0101	101	EFFSIGMRCRSAVCC	FFSIGMRCR	1	0.6209	60.4	19.00	0.44	tr_D0A9H6_
DRB1_0101	102	AELQFTSQRKLMSVL	FTSQRKLMS	4	0.6195	61.4	19.00	0.47	tr_D0A9H6_
DRB1_0101	103	LNSQLAYVRYVFTDK	SQLAYVRYV	2	0.6191	61.7	19.00	0.47	tr_D0A9H6_
DRB1_0101	104	SSVGIGIMGLEGSQA	IMGLEGSQA	6	0.6183	62.2	20.00	0.59	tr_D0A9H6_
DRB1_0101	105	FALQGRTSKHVFVKV	LQGRTSKHV	2	0.6131	65.8	20.00	0.45	tr_D0A9H6_
DRB1_0101	106	NDALMHERSLTMSRK	LMHERSLTM	3	0.6128	66.0	20.00	0.73	tr_D0A9H6_
DRB1_0101	107	DLTREFFSIGMRCRS	FFSIGMRCR	5	0.6126	66.1	20.00	0.52	tr_D0A9H6_
DRB1_0101	108	NARGKKLPFLLLVKG	GKKLPFLLL	3	0.6114	67.0	21.00	0.62	tr_D0A9H6_
DRB1_0101	109	SNARGKKLPFLLLVK	GKKLPFLLL	4	0.6108	67.4	21.00	0.58	tr_D0A9H6_
DRB1_0101	110	KKLPFLLLVKGADSS	LLLVKGADS	5	0.6084	69.2	21.00	0.69	tr_D0A9H6_
DRB1_0101	111	RPEVESAVAAAVEAA	VESAVAAAV	3	0.6037	72.8	22.00	0.65	tr_D0A9H6_
DRB1_0101	112	ARGKKLPFLLLVKGA	GKKLPFLLL	2	0.6033	73.1	22.00	0.56	tr_D0A9H6_
DRB1_0101	113	GKKLPFLLLVKGADS	LLLVKGADS	6	0.6028	73.6	22.00	0.38	tr_D0A9H6_
DRB1_0101	114	GVGRFLHGHTGSASW	FLHGHTGSA	4	0.6022	74.0	22.00	0.56	tr_D0A9H6_
DRB1_0101	115	RSAVCCRMTPLQKAK	CRMTPLQKA	5	0.6011	74.9	22.00	0.63	tr_D0A9H6_
DRB1_0101	116	FKGVLRYSRPKTSCL	LRYSRPKTS	4	0.6005	75.4	22.00	0.75	tr_D0A9H6_
DRB1_0101	117	NVMTYVGGCTATERH	MTYVGGCTA	2	0.5983	77.2	23.00	0.49	tr_D0A9H6_
DRB1_0101	118	RPGALGEAFLRLVEA	GALGEAFLR	2	0.5970	78.2	23.00	0.41	tr_D0A9H6_
DRB1_0101	119	SVAKKGLRTLVLGQR	KKGLRTLVL	3	0.5970	78.3	23.00	0.37	tr_D0A9H6_
DRB1_0101	120	NKVLQLYLIANDRRM	YLIANDRRM	6	0.5962	79.0	23.00	0.23	tr_D0A9H6_
DRB1_0101	121	MTPLQKAKIVKLFQE	LQKAKIVKL	3	0.5955	79.6	23.00	0.56	tr_D0A9H6_
DRB1_0101	122	VAELQFTSQRKLMSV	LQFTSQRKL	3	0.5950	80.0	23.00	0.46	tr_D0A9H6_
DRB1_0101	123	DALMHERSLTMSRKV	LMHERSLTM	2	0.5945	80.4	23.00	0.66	tr_D0A9H6_
DRB1_0101	124	GVLRYSRPKTSCLNS	LRYSRPKTS	2	0.5943	80.6	23.00	0.73	tr_D0A9H6_
DRB1_0101	125	DVSMIQESSVGIGIM	MIQESSVGI	3	0.5943	80.6	23.00	0.67	tr_D0A9H6_
DRB1_0101	126	YSRPKTSCLNSQLAY	TSCLNSQLA	5	0.5923	82.3	24.00	0.73	tr_D0A9H6_

DRB1_0101	127	YLRNLSLCHSVVCFD	YLRNLSLCH	0	0.5906	83.9	24.00	0.35	tr_D0A9H6_
DRB1_0101	128	ALMHERSLTMSRKVR	LMHERSLTM	1	0.5903	84.1	24.00	0.52	tr_D0A9H6_
DRB1_0101	129	HNDALMHERSLTMSR	LMHERSLTM	4	0.5894	85.0	24.00	0.67	tr_D0A9H6_
DRB1_0101	130	FLHGHTGSASWRMTC	HTGSASWRM	4	0.5887	85.6	24.00	0.46	tr_D0A9H6_
DRB1_0101	131	ENVMTYVGGCTATER	MTYVGGCTA	3	0.5816	92.5	26.00	0.56	tr_D0A9H6_
DRB1_0101	132	DSNARGKKLPFLLLV	GKKLPFLLL	5	0.5812	92.9	26.00	0.57	tr_D0A9H6_
DRB1_0101	133	VSMIQESSVGIGIMG	MIQESSVGI	2	0.5800	94.1	26.00	0.62	tr_D0A9H6_
DRB1_0101	134	NDVSMIQESSVGIGI	MIQESSVGI	4	0.5792	94.9	26.00	0.58	tr_D0A9H6_
DRB1_0101	135	RGKKLPFLLLVKGAD	GKKLPFLLL	1	0.5792	94.9	26.00	0.50	tr_D0A9H6_
DRB1_0101	136	VEIDSVAKKGLRTLV	SVAKKGLRT	4	0.5789	95.2	26.00	0.70	tr_D0A9H6_
DRB1_0101	137	PGALGEAFLRLVEAR	GALGEAFLR	1	0.5774	96.8	26.00	0.34	tr_D0A9H6_
DRB1_0101	138	AKIVKLFQENTGGVA	FQENTGGVA	6	0.5760	98.3	26.00	0.38	tr_D0A9H6_
DRB1_0101	139	VMTYVGGCTATERHD	YVGGCTATE	3	0.5756	98.7	27.00	0.47	tr_D0A9H6_
DRB1_0101	140	QRKLMSVLLSRCPDM	QRKLMSVLL	0	0.5754	98.9	27.00	0.38	tr_D0A9H6_
DRB1_0101	141	LYLIANDRRMASYDE	YLIANDRRM	1	0.5731	101.4	27.00	0.55	tr_D0A9H6_
DRB1_0101	142	TGGVALAIGDGANDV	VALAIGDGA	3	0.5705	104.3	27.00	0.77	tr_D0A9H6_
DRB1_0101	143	DRPEVESAVAAAVEA	VESAVAAAV	4	0.5683	106.8	28.00	0.64	tr_D0A9H6_
DRB1_0101	144	NSQLAYVRYVFTDKT	SQLAYVRYV	1	0.5673	108.0	28.00	0.47	tr_D0A9H6_
DRB1_0101	145	KERPGALGEAFLRLV	GALGEAFLR	4	0.5666	108.7	28.00	0.45	tr_D0A9H6_
DRB1_0101	146	VLRYSRPKTSCLNSQ	LRYSRPKTS	1	0.5662	109.2	28.00	0.67	tr_D0A9H6_
[truncated: 566,765 more chars]
