# Supplementary material for: Immunoinformatic design of a putative multi-epitope vaccine candidate against Trypanosoma brucei gambiense
Source: Comput Struct Biotechnol J. 2022 Oct 7;20:5574–85. doi: 10.1016/j.csbj.2022.10.002 (PMC9576565; doi:10.1016/j.csbj.2022.10.002)
Supplement: Supplementary data 6 [file mmc6.pdf]

jobid=60CF4B5C000035F585CA8838 Server Output - DTU Health Tech

```
# Tmpdir made /scratch/netMHCII9g6Tpj
# Input is in FSA format

# Peptide length 15
        NetMHCII version 2.3.

Strong binder threshold   0.50. Weak binder threshold  10.00.
```

Allele	pos	peptide	core	Of	1-log50k(aff)	affinity(nM)	%Rank	Relia	Identity	Bind Level
HLA-DQA10501-DQB10201	1	PFGSCEVISGEDEGL	VISGEDEGL	6	0.4332	460.6	12.00	0.41	tr_C9ZMD3_	
HLA-DQA10501-DQB10201	2	FGSCEVISGEDEGLK	VISGEDEGL	5	0.4028	639.9	16.00	0.43	tr_C9ZMD3_	
HLA-DQA10501-DQB10201	3	GSCEVISGEDEGLKG	VISGEDEGL	4	0.3736	877.9	20.00	0.45	tr_C9ZMD3_	
HLA-DQA10501-DQB10201	4	SPFGSCEVISGEDEG	CEVISGEDE	5	0.3515	1115.1	24.00	0.51	tr_C9ZMD3_	
HLA-DQA10501-DQB10201	5	SCEVISGEDEGLKGK	VISGEDEGL	3	0.3441	1207.6	25.00	0.43	tr_C9ZMD3_	
HLA-DQA10501-DQB10201	6	TSPFGSCEVISGEDE	CEVISGEDE	6	0.3351	1330.9	27.00	0.55	tr_C9ZMD3_	
HLA-DQA10501-DQB10201	7	CEVISGEDEGLKGKN	VISGEDEGL	2	0.3057	1829.2	34.00	0.49	tr_C9ZMD3_	
HLA-DQA10501-DQB10201	8	EVISGEDEGLKGKNA	VISGEDEGL	1	0.2417	3657.4	50.00	0.73	tr_C9ZMD3_	
HLA-DQA10501-DQB10201	9	KADVTSPFGSCEVIS	PFGSCEVIS	6	0.2350	3932.6	55.00	0.35	tr_C9ZMD3_	
HLA-DQA10501-DQB10201	10	EQSSMKCFTSMIPPG	MKCFTSMIP	4	0.2292	4189.8	55.00	0.38	tr_C9ZMD3_	
HLA-DQA10501-DQB10201	11	VTSPFGSCEVISGED	PFGSCEVIS	3	0.2266	4305.2	55.00	0.37	tr_C9ZMD3_	
HLA-DQA10501-DQB10201	12	QSSMKCFTSMIPPGG	MKCFTSMIP	3	0.2237	4445.9	60.00	0.33	tr_C9ZMD3_	
HLA-DQA10501-DQB10201	13	ADVTSPFGSCEVISG	PFGSCEVIS	5	0.2183	4709.2	60.00	0.34	tr_C9ZMD3_	
HLA-DQA10501-DQB10201	14	DVTSPFGSCEVISGE	PFGSCEVIS	4	0.2156	4850.4	60.00	0.34	tr_C9ZMD3_	
HLA-DQA10501-DQB10201	15	NKADVTSPFGSCEVI	TSPFGSCEV	5	0.2121	5038.8	60.00	0.32	tr_C9ZMD3_	
HLA-DQA10501-DQB10201	16	NREQVNKADVTSPFG	KADVTSPFG	6	0.2105	5127.0	60.00	0.40	tr_C9ZMD3_	
HLA-DQA10501-DQB10201	17	VNKADVTSPFGSCEV	TSPFGSCEV	6	0.2056	5407.1	65.00	0.35	tr_C9ZMD3_	
HLA-DQA10501-DQB10201	18	REQVNKADVTSPFGS	KADVTSPFG	5	0.2018	5634.0	65.00	0.41	tr_C9ZMD3_	
HLA-DQA10501-DQB10201	19	GEQSSMKCFTSMIPP	MKCFTSMIP	5	0.2002	5729.2	65.00	0.41	tr_C9ZMD3_	
HLA-DQA10501-DQB10201	20	EQVNKADVTSPFGSC	KADVTSPFG	4	0.1943	6112.0	65.00	0.40	tr_C9ZMD3_	
HLA-DQA10501-DQB10201	21	TNREQVNKADVTSPF	NKADVTSPF	6	0.1866	6639.5	70.00	0.35	tr_C9ZMD3_	
HLA-DQA10501-DQB10201	22	QVNKADVTSPFGSCE	KADVTSPFG	3	0.1829	6909.0	70.00	0.44	tr_C9ZMD3_	
HLA-DQA10501-DQB10201	23	VISGEDEGLKGKNAL	VISGEDEGL	0	0.1756	7478.9	70.00	0.77	tr_C9ZMD3_	
HLA-DQA10501-DQB10201	24	GKNALPANEGEGEQS	LPANEGEGE	4	0.1755	7490.3	70.00	0.31	tr_C9ZMD3_	
HLA-DQA10501-DQB10201	25	NALPANEGEGEQSSM	LPANEGEGE	2	0.1725	7731.3	70.00	0.34	tr_C9ZMD3_	
HLA-DQA10501-DQB10201	26	KNALPANEGEGEQSS	LPANEGEGE	3	0.1718	7789.6	75.00	0.34	tr_C9ZMD3_	
HLA-DQA10501-DQB10201	27	KGKNALPANEGEGEQ	LPANEGEGE	5	0.1707	7887.7	75.00	0.40	tr_C9ZMD3_	
HLA-DQA10501-DQB10201	28	EGEQSSMKCFTSMIP	MKCFTSMIP	6	0.1659	8309.1	75.00	0.48	tr_C9ZMD3_	
HLA-DQA10501-DQB10201	29	ALPANEGEGEQSSMK	LPANEGEGE	1	0.1529	9560.8	80.00	0.35	tr_C9ZMD3_	
HLA-DQA10501-DQB10201	30	LKGKNALPANEGEGE	LPANEGEGE	6	0.1498	9883.9	80.00	0.46	tr_C9ZMD3_	
HLA-DQA10501-DQB10201	31	LPANEGEGEQSSMKC	LPANEGEGE	0	0.1375	11291.3	80.00	0.27	tr_C9ZMD3_	
HLA-DQA10501-DQB10201	32	TTNREQVNKADVTSP	EQVNKADVT	4	0.1325	11921.4	85.00	0.51	tr_C9ZMD3_	
HLA-DQA10501-DQB10201	33	EGLKGKNALPANEGE	KNALPANEG	5	0.1239	13089.1	85.00	0.25	tr_C9ZMD3_	
HLA-DQA10501-DQB10201	34	PANEGEGEQSSMKCF	ANEGEGEQS	1	0.1170	14104.0	85.00	0.22	tr_C9ZMD3_	

HLA-DQA10501-DQB10201	35	VPTTNREQVNKADVT	EQVNKADVT	6	0.1168	14123.2	85.00	0.55	tr_C9ZMD3_	
HLA-DQA10501-DQB10201	36	DEGLKGKNALPANEG	KNALPANEG	6	0.1156	14320.6	85.00	0.25	tr_C9ZMD3_	
HLA-DQA10501-DQB10201	37	PTTNREQVNKADVTS	EQVNKADVT	5	0.1147	14449.6	85.00	0.58	tr_C9ZMD3_	
HLA-DQA10501-DQB10201	38	ANEGEGEQSSMKCFT	EQSSMKCFT	6	0.1130	14719.0	90.00	0.29	tr_C9ZMD3_	
HLA-DQA10501-DQB10201	39	EDEGLKGKNALPANE	LKGKNALPA	4	0.1117	14935.4	90.00	0.33	tr_C9ZMD3_	
HLA-DQA10501-DQB10201	40	GLKGKNALPANEGEG	NALPANEGE	5	0.1107	15096.4	90.00	0.22	tr_C9ZMD3_	
HLA-DQA10501-DQB10201	41	GEGEQSSMKCFTSMI	SMKCFTSMI	6	0.1006	16837.3	90.00	0.35	tr_C9ZMD3_	
HLA-DQA10501-DQB10201	42	NEGEGEQSSMKCFTS	EQSSMKCFT	5	0.0967	17559.0	90.00	0.33	tr_C9ZMD3_	
HLA-DQA10501-DQB10201	43	GEDEGLKGKNALPAN	LKGKNALPA	5	0.0931	18264.7	90.00	0.44	tr_C9ZMD3_	
HLA-DQA10501-DQB10201	44	EGEGEQSSMKCFTSM	EQSSMKCFT	4	0.0912	18637.8	95.00	0.33	tr_C9ZMD3_	
HLA-DQA10501-DQB10201	45	SGEDEGLKGKNALPA	LKGKNALPA	6	0.0898	18923.3	95.00	0.48	tr_C9ZMD3_	
HLA-DQA10501-DQB10201	46	ISGEDEGLKGKNALP	EGLKGKNAL	5	0.0605	25972.2	100.00	0.24	tr_C9ZMD3_	

Allele: HLA-DQA10501-DQB10201. Number of high binders 0. Number of weak binders 0. Number of peptides 46

Allele	pos	peptide	core	Of	1-log50k(aff)	affinity(nM)	%Rank	Relia	Identity	Bind Level
HLA-DQA10501-DQB10201	1	DLSQWVTGFWSDVAF	VTGFWSDVA	5	0.5039	214.3	5.50	0.41	tr_D0A7J0_	WB
HLA-DQA10501-DQB10201	2	SQWVTGFWSDVAFSV	VTGFWSDVA	3	0.4958	234.1	6.00	0.36	tr_D0A7J0_	WB
HLA-DQA10501-DQB10201	3	LSQWVTGFWSDVAFS	VTGFWSDVA	4	0.4856	261.3	6.50	0.42	tr_D0A7J0_	WB
HLA-DQA10501-DQB10201	4	QWVTGFWSDVAFSVG	VTGFWSDVA	2	0.4781	283.4	7.00	0.36	tr_D0A7J0_	WB
HLA-DQA10501-DQB10201	5	PDLSQWVTGFWSDVA	VTGFWSDVA	6	0.4537	369.1	9.50	0.42	tr_D0A7J0_	WB
HLA-DQA10501-DQB10201	6	WVTGFWSDVAFSVGG	VTGFWSDVA	1	0.4462	400.2	10.00	0.34	tr_D0A7J0_	
HLA-DQA10501-DQB10201	7	GFWSDVAFSVGGVPL	AFSVGGVPL	6	0.3992	665.3	16.00	0.25	tr_D0A7J0_	
HLA-DQA10501-DQB10201	8	VTGFWSDVAFSVGGV	FWSDVAFSV	3	0.3958	690.7	17.00	0.27	tr_D0A7J0_	
HLA-DQA10501-DQB10201	9	WSDVAFSVGGVPLRV	SVGGVPLRV	6	0.3825	797.4	19.00	0.34	tr_D0A7J0_	
HLA-DQA10501-DQB10201	10	TGFWSDVAFSVGGVP	FWSDVAFSV	2	0.3806	813.9	19.00	0.28	tr_D0A7J0_	
HLA-DQA10501-DQB10201	11	FWSDVAFSVGGVPLR	AFSVGGVPL	5	0.3660	952.9	21.00	0.22	tr_D0A7J0_	
HLA-DQA10501-DQB10201	12	LPDLSQWVTGFWSDV	WVTGFWSDV	6	0.3625	990.3	22.00	0.29	tr_D0A7J0_	
HLA-DQA10501-DQB10201	13	GASIREGLPDLSQWV	IREGLPDLS	3	0.3213	1545.6	30.00	0.49	tr_D0A7J0_	
HLA-DQA10501-DQB10201	14	ASIREGLPDLSQWVT	IREGLPDLS	2	0.3084	1778.3	33.00	0.52	tr_D0A7J0_	
HLA-DQA10501-DQB10201	15	EGLPDLSQWVTGFWS	LSQWVTGFW	5	0.2992	1964.1	35.00	0.37	tr_D0A7J0_	
HLA-DQA10501-DQB10201	16	GLPDLSQWVTGFWSD	LSQWVTGFW	4	0.2963	2027.0	36.00	0.36	tr_D0A7J0_	
HLA-DQA10501-DQB10201	17	SIREGLPDLSQWVTG	IREGLPDLS	1	0.2767	2506.1	41.00	0.52	tr_D0A7J0_	
HLA-DQA10501-DQB10201	18	REGLPDLSQWVTGFW	LSQWVTGFW	6	0.2717	2642.8	42.00	0.46	tr_D0A7J0_	
HLA-DQA10501-DQB10201	19	IREGLPDLSQWVTGF	IREGLPDLS	0	0.2404	3708.7	55.00	0.35	tr_D0A7J0_	

Allele: HLA-DQA10501-DQB10201. Number of high binders 0. Number of weak binders 5. Number of peptides 19

Allele	pos	peptide	core	Of	1-log50k(aff)	affinity(nM)	%Rank	Relia	Identity	Bind Level
HLA-DQA10501-DQB10201	1	YARLQGYFPEELPEY	LQGYFPEEL	3	0.6873	29.5	0.25	0.57	tr_D0A3G8_	SB
HLA-DQA10501-DQB10201	2	TTYARLQGYFPEELP	LQGYFPEEL	5	0.6727	34.5	0.40	0.68	tr_D0A3G8_	SB
HLA-DQA10501-DQB10201	3	TYARLQGYFPEELPE	LQGYFPEEL	4	0.6656	37.3	0.40	0.67	tr_D0A3G8_	SB

HLA-DQA10501-DQB10201	4	FVRYGLAGLVVNEFD	LAGLVVNEF	5	0.6597	39.7	0.40	0.67	tr_D0A3G8_	SB
HLA-DQA10501-DQB10201	5	RYGLAGLVVNEFDGL	LAGLVVNEF	3	0.6453	46.4	0.60	0.56	tr_D0A3G8_	WB
HLA-DQA10501-DQB10201	6	GTTYARLQGYFPEEL	LQGYFPEEL	6	0.6449	46.6	0.60	0.72	tr_D0A3G8_	WB
HLA-DQA10501-DQB10201	7	VRYGLAGLVVNEFDG	LAGLVVNEF	4	0.6327	53.2	0.70	0.64	tr_D0A3G8_	WB
HLA-DQA10501-DQB10201	8	SFVRYGLAGLVVNEF	LAGLVVNEF	6	0.6265	56.9	0.80	0.75	tr_D0A3G8_	WB
HLA-DQA10501-DQB10201	9	YGLAGLVVNEFDGLK	LAGLVVNEF	2	0.6155	64.1	1.00	0.59	tr_D0A3G8_	WB
HLA-DQA10501-DQB10201	10	GLAGLVVNEFDGLKF	LAGLVVNEF	1	0.5878	86.4	1.60	0.55	tr_D0A3G8_	WB
HLA-DQA10501-DQB10201	11	LAGLVVNEFDGLKFV	LAGLVVNEF	0	0.5460	135.9	3.00	0.49	tr_D0A3G8_	WB
HLA-DQA10501-DQB10201	12	KYVSFVRYGLAGLVV	RYGLAGLVV	6	0.4439	410.1	11.00	0.62	tr_D0A3G8_	
HLA-DQA10501-DQB10201	13	YVSFVRYGLAGLVVN	RYGLAGLVV	5	0.4161	554.3	14.00	0.58	tr_D0A3G8_	
HLA-DQA10501-DQB10201	14	VSFVRYGLAGLVVNE	RYGLAGLVV	4	0.4104	589.8	14.00	0.54	tr_D0A3G8_	
HLA-DQA10501-DQB10201	15	AGLVVNEFDGLKFVC	LVVNEFDGL	2	0.4062	616.7	15.00	0.35	tr_D0A3G8_	
HLA-DQA10501-DQB10201	16	DGTTYARLQGYFPEE	RLQGYFPEE	6	0.3911	726.1	17.00	0.28	tr_D0A3G8_	
HLA-DQA10501-DQB10201	17	GLVVNEFDGLKFVCE	LVVNEFDGL	1	0.3418	1238.4	26.00	0.39	tr_D0A3G8_	
HLA-DQA10501-DQB10201	18	LKYVSFVRYGLAGLV	VRYGLAGLV	6	0.3403	1258.6	26.00	0.46	tr_D0A3G8_	
HLA-DQA10501-DQB10201	19	LVVNEFDGLKFVCEP	LVVNEFDGL	0	0.3304	1400.8	28.00	0.31	tr_D0A3G8_	
HLA-DQA10501-DQB10201	20	PDGTTYARLQGYFPE	YARLQGYFP	5	0.3271	1452.0	29.00	0.34	tr_D0A3G8_	
HLA-DQA10501-DQB10201	21	FPDGTTYARLQGYFP	YARLQGYFP	6	0.3171	1617.0	31.00	0.35	tr_D0A3G8_	
HLA-DQA10501-DQB10201	22	PFFWRWLKYVSFVRY	WRWLKYVSF	3	0.3104	1738.8	33.00	0.33	tr_D0A3G8_	
HLA-DQA10501-DQB10201	23	FFWRWLKYVSFVRYG	WRWLKYVSF	2	0.2870	2239.5	38.00	0.37	tr_D0A3G8_	
HLA-DQA10501-DQB10201	24	WLKYVSFVRYGLAGL	FVRYGLAGL	6	0.2827	2346.2	39.00	0.54	tr_D0A3G8_	
HLA-DQA10501-DQB10201	25	SLPFFWRWLKYVSFV	WRWLKYVSF	5	0.2816	2374.6	40.00	0.54	tr_D0A3G8_	
HLA-DQA10501-DQB10201	26	LPFFWRWLKYVSFVR	WRWLKYVSF	4	0.2811	2389.3	40.00	0.46	tr_D0A3G8_	
HLA-DQA10501-DQB10201	27	FWRWLKYVSFVRYGL	WRWLKYVSF	1	0.2767	2504.9	41.00	0.28	tr_D0A3G8_	
HLA-DQA10501-DQB10201	28	EFDGLKFVCEPKVPF	LKFVCEPKV	4	0.2689	2725.6	43.00	0.43	tr_D0A3G8_	
HLA-DQA10501-DQB10201	29	QSLPFFWRWLKYVSF	WRWLKYVSF	6	0.2669	2786.4	44.00	0.65	tr_D0A3G8_	
HLA-DQA10501-DQB10201	30	WRWLKYVSFVRYGLA	VSFVRYGLA	6	0.2623	2925.7	45.00	0.20	tr_D0A3G8_	
HLA-DQA10501-DQB10201	31	VNEFDGLKFVCEPKV	LKFVCEPKV	6	0.2619	2939.8	45.00	0.38	tr_D0A3G8_	
HLA-DQA10501-DQB10201	32	NEFDGLKFVCEPKVP	LKFVCEPKV	5	0.2560	3132.9	47.00	0.44	tr_D0A3G8_	
HLA-DQA10501-DQB10201	33	FDGLKFVCEPKVPFC	LKFVCEPKV	3	0.2533	3227.4	47.00	0.45	tr_D0A3G8_	
HLA-DQA10501-DQB10201	34	VVNEFDGLKFVCEPK	VNEFDGLKF	1	0.2509	3312.7	48.00	0.28	tr_D0A3G8_	
HLA-DQA10501-DQB10201	35	CFPDGTTYARLQGYF	TYARLQGYF	6	0.2423	3634.8	50.00	0.47	tr_D0A3G8_	
HLA-DQA10501-DQB10201	36	RWLKYVSFVRYGLAG	VSFVRYGLA	5	0.2292	4187.0	55.00	0.26	tr_D0A3G8_	
HLA-DQA10501-DQB10201	37	DGLKFVCEPKVPFCF	LKFVCEPKV	2	0.2251	4375.3	55.00	0.43	tr_D0A3G8_	
HLA-DQA10501-DQB10201	38	GLKFVCEPKVPFCFP	LKFVCEPKV	1	0.2225	4501.0	60.00	0.37	tr_D0A3G8_	
HLA-DQA10501-DQB10201	39	LKFVCEPKVPFCFPD	LKFVCEPKV	0	0.1972	5918.0	65.00	0.31	tr_D0A3G8_	
HLA-DQA10501-DQB10201	40	FVCEPKVPFCFPDGT	VPFCFPDGT	6	0.1885	6507.9	70.00	0.31	tr_D0A3G8_	
HLA-DQA10501-DQB10201	41	GTQSLPFFWRWLKYV	GTQSLPFFW	0	0.1691	8027.1	75.00	0.34	tr_D0A3G8_	
HLA-DQA10501-DQB10201	42	VCEPKVPFCFPDGTT	VPFCFPDGT	5	0.1686	8070.1	75.00	0.40	tr_D0A3G8_	
HLA-DQA10501-DQB10201	43	CEPKVPFCFPDGTTY	VPFCFPDGT	4	0.1683	8093.4	75.00	0.41	tr_D0A3G8_	
HLA-DQA10501-DQB10201	44	KFVCEPKVPFCFPDG	FVCEPKVPF	1	0.1675	8164.7	75.00	0.32	tr_D0A3G8_	
HLA-DQA10501-DQB10201	45	FCFPDGTTYARLQGY	TTYARLQGY	6	0.1658	8314.6	75.00	0.44	tr_D0A3G8_	
HLA-DQA10501-DQB10201	46	EPKVPFCFPDGTTYA	VPFCFPDGT	3	0.1636	8520.3	75.00	0.40	tr_D0A3G8_	
HLA-DQA10501-DQB10201	47	KVPFCFPDGTTYARL	VPFCFPDGT	1	0.1633	8545.7	75.00	0.34	tr_D0A3G8_	
HLA-DQA10501-DQB10201	48	PKVPFCFPDGTTYAR	VPFCFPDGT	2	0.1462	10278.0	80.00	0.44	tr_D0A3G8_	
HLA-DQA10501-DQB10201	49	VPFCFPDGTTYARLQ	PDGTTYARL	5	0.1419	10763.4	80.00	0.31	tr_D0A3G8_	
HLA-DQA10501-DQB10201	50	TQSLPFFWRWLKYVS	FFWRWLKYV	5	0.1351	11597.5	80.00	0.38	tr_D0A3G8_	
HLA-DQA10501-DQB10201	51	PFCFPDGTTYARLQG	PDGTTYARL	4	0.1173	14058.7	85.00	0.34	tr_D0A3G8_	

Allele: HLA-DQA10501-DQB10201. Number of high binders 4. Number of weak binders 7. Number of peptides 51

Allele	pos	peptide	core	Of	1-log50k(aff)	affinity(nM)	%Rank	Relia	Identity	Bind Level
HLA-DQA10501-DQB10201	1	LHETHGRGGTEAQTV	RGGTEAQTV	6	0.1952	6047.2	65.00	0.67	tr_D0AAR0_	
HLA-DQA10501-DQB10201	2	RRKRGMLRTYLHETH	LRTYLHETH	6	0.1832	6886.8	70.00	0.29	tr_D0AAR0_	
HLA-DQA10501-DQB10201	3	GTEAQTVGGGAGFPM	VGGGAGFPM	6	0.1753	7503.3	70.00	0.50	tr_D0AAR0_	
HLA-DQA10501-DQB10201	4	HETHGRGGTEAQTVG	RGGTEAQTV	5	0.1716	7811.7	75.00	0.65	tr_D0AAR0_	
HLA-DQA10501-DQB10201	5	RKRGMLRTYLHETHG	LRTYLHETH	5	0.1694	7999.4	75.00	0.31	tr_D0AAR0_	
HLA-DQA10501-DQB10201	6	ERRKRGMLRTYLHET	MLRTYLHET	6	0.1654	8352.7	75.00	0.32	tr_D0AAR0_	
HLA-DQA10501-DQB10201	7	EAQTVGGGAGFPMRS	VGGGAGFPM	4	0.1613	8732.4	75.00	0.43	tr_D0AAR0_	
HLA-DQA10501-DQB10201	8	TEAQTVGGGAGFPMR	VGGGAGFPM	5	0.1608	8776.6	75.00	0.48	tr_D0AAR0_	
HLA-DQA10501-DQB10201	9	ETHGRGGTEAQTVGG	RGGTEAQTV	4	0.1539	9453.0	75.00	0.66	tr_D0AAR0_	
HLA-DQA10501-DQB10201	10	AQTVGGGAGFPMRST	VGGGAGFPM	3	0.1519	9661.2	80.00	0.42	tr_D0AAR0_	
HLA-DQA10501-DQB10201	11	KRGMLRTYLHETHGR	LRTYLHETH	4	0.1512	9741.1	80.00	0.29	tr_D0AAR0_	
HLA-DQA10501-DQB10201	12	QTVGGGAGFPMRSTP	VGGGAGFPM	2	0.1456	10347.4	80.00	0.41	tr_D0AAR0_	
HLA-DQA10501-DQB10201	13	LERRKRGMLRTYLHE	KRGMLRTYL	4	0.1383	11198.4	80.00	0.47	tr_D0AAR0_	
HLA-DQA10501-DQB10201	14	THGRGGTEAQTVGGG	RGGTEAQTV	3	0.1366	11404.0	80.00	0.64	tr_D0AAR0_	
HLA-DQA10501-DQB10201	15	HGRGGTEAQTVGGGA	RGGTEAQTV	2	0.1308	12147.6	85.00	0.60	tr_D0AAR0_	
HLA-DQA10501-DQB10201	16	RGMLRTYLHETHGRG	LRTYLHETH	3	0.1288	12405.5	85.00	0.34	tr_D0AAR0_	
HLA-DQA10501-DQB10201	17	TVGGGAGFPMRSTPS	VGGGAGFPM	1	0.1278	12543.2	85.00	0.44	tr_D0AAR0_	
HLA-DQA10501-DQB10201	18	EYLERRKRGMLRTYL	KRGMLRTYL	6	0.1274	12597.1	85.00	0.68	tr_D0AAR0_	
HLA-DQA10501-DQB10201	19	YLERRKRGMLRTYLH	KRGMLRTYL	5	0.1244	13009.3	85.00	0.51	tr_D0AAR0_	
HLA-DQA10501-DQB10201	20	GAGFPMRSTPSGVSW	PMRSTPSGV	4	0.1211	13489.9	85.00	0.41	tr_D0AAR0_	
HLA-DQA10501-DQB10201	21	GGTEAQTVGGGAGFP	TVGGGAGFP	6	0.1157	14292.3	85.00	0.40	tr_D0AAR0_	
HLA-DQA10501-DQB10201	22	GRGGTEAQTVGGGAG	RGGTEAQTV	1	0.1136	14628.7	90.00	0.56	tr_D0AAR0_	
HLA-DQA10501-DQB10201	23	GMLRTYLHETHGRGG	LRTYLHETH	2	0.1121	14868.8	90.00	0.38	tr_D0AAR0_	
HLA-DQA10501-DQB10201	24	RGGTEAQTVGGGAGF	RGGTEAQTV	0	0.1100	15210.5	90.00	0.35	tr_D0AAR0_	
HLA-DQA10501-DQB10201	25	GGGAGFPMRSTPSGV	PMRSTPSGV	6	0.1071	15692.8	90.00	0.42	tr_D0AAR0_	
HLA-DQA10501-DQB10201	26	GGAGFPMRSTPSGVS	PMRSTPSGV	5	0.1061	15866.1	90.00	0.41	tr_D0AAR0_	
HLA-DQA10501-DQB10201	27	VGGGAGFPMRSTPSG	VGGGAGFPM	0	0.1054	15979.3	90.00	0.33	tr_D0AAR0_	
HLA-DQA10501-DQB10201	28	YLHETHGRGGTEAQT	GRGGTEAQT	6	0.1004	16869.0	90.00	0.31	tr_D0AAR0_	
HLA-DQA10501-DQB10201	29	MLRTYLHETHGRGGT	LRTYLHETH	1	0.0917	18535.1	90.00	0.44	tr_D0AAR0_	
HLA-DQA10501-DQB10201	30	TYLHETHGRGGTEAQ	THGRGGTEA	5	0.0821	20574.3	95.00	0.55	tr_D0AAR0_	
HLA-DQA10501-DQB10201	31	RTYLHETHGRGGTEA	THGRGGTEA	6	0.0795	21153.8	95.00	0.57	tr_D0AAR0_	
HLA-DQA10501-DQB10201	32	LRTYLHETHGRGGTE	LRTYLHETH	0	0.0707	23278.2	95.00	0.38	tr_D0AAR0_	

Allele: HLA-DQA10501-DQB10201. Number of high binders 0. Number of weak binders 0. Number of peptides 32

Allele	pos	peptide	core	Of	1-log50k(aff)	affinity(nM)	%Rank	Relia	Identity	Bind Level
HLA-DQA10501-DQB10201	1	EMKVELFTEFCADRE	FTEFCADRE	6	0.5788	95.3	1.80	0.60	tr_D0AAR0_	WB
HLA-DQA10501-DQB10201	2	MKVELFTEFCADREN	FTEFCADRE	5	0.5683	106.8	2.50	0.62	tr_D0AAR0_	WB
HLA-DQA10501-DQB10201	3	VELFTEFCADRENTF	FTEFCADRE	3	0.5625	113.7	2.50	0.70	tr_D0AAR0_	WB
HLA-DQA10501-DQB10201	4	KVELFTEFCADRENT	FTEFCADRE	4	0.5582	119.1	2.50	0.65	tr_D0AAR0_	WB

HLA-DQA10501-DQB10201	5	ELFTEFCADRENTFN	FTEFCADRE	2	0.5396	145.6	3.50	0.73	tr_D0AAR0_	WB
HLA-DQA10501-DQB10201	6	LFTEFCADRENTFNP	FTEFCADRE	1	0.5037	214.8	5.50	0.73	tr_D0AAR0_	WB
HLA-DQA10501-DQB10201	7	FTEFCADRENTFNPL	FTEFCADRE	0	0.4448	406.1	10.00	0.63	tr_D0AAR0_	
HLA-DQA10501-DQB10201	8	KAEMKVELFTEFCAD	ELFTEFCAD	6	0.4250	503.4	13.00	0.30	tr_D0AAR0_	
HLA-DQA10501-DQB10201	9	AEMKVELFTEFCADR	ELFTEFCAD	5	0.4003	657.5	16.00	0.29	tr_D0AAR0_	
HLA-DQA10501-DQB10201	10	HKAEMKVELFTEFCA	KAEMKVELF	1	0.3943	701.9	17.00	0.33	tr_D0AAR0_	
HLA-DQA10501-DQB10201	11	DHKAEMKVELFTEFC	KAEMKVELF	2	0.3730	883.8	20.00	0.44	tr_D0AAR0_	
HLA-DQA10501-DQB10201	12	KDHKAEMKVELFTEF	KAEMKVELF	3	0.3509	1121.7	24.00	0.56	tr_D0AAR0_	
HLA-DQA10501-DQB10201	13	PYVKDHKAEMKVELF	KAEMKVELF	6	0.3490	1145.9	24.00	0.79	tr_D0AAR0_	
HLA-DQA10501-DQB10201	14	VKDHKAEMKVELFTE	KAEMKVELF	4	0.3399	1263.6	26.00	0.58	tr_D0AAR0_	
HLA-DQA10501-DQB10201	15	YVKDHKAEMKVELFT	KAEMKVELF	5	0.3356	1324.2	27.00	0.69	tr_D0AAR0_	
HLA-DQA10501-DQB10201	16	NTFNPLATLALMGPY	LATLALMGP	5	0.3063	1818.8	34.00	0.25	tr_D0AAR0_	
HLA-DQA10501-DQB10201	17	LALMGPYNSIRVLFS	YNSIRVLFS	6	0.3028	1888.9	34.00	0.54	tr_D0AAR0_	
HLA-DQA10501-DQB10201	18	TFNPLATLALMGPYN	TLALMGPYN	6	0.2990	1966.9	35.00	0.21	tr_D0AAR0_	
HLA-DQA10501-DQB10201	19	ENTFNPLATLALMGP	LATLALMGP	6	0.2888	2197.6	38.00	0.23	tr_D0AAR0_	
HLA-DQA10501-DQB10201	20	FNPLATLALMGPYNS	TLALMGPYN	5	0.2728	2611.7	42.00	0.26	tr_D0AAR0_	
HLA-DQA10501-DQB10201	21	DRENTFNPLATLALM	FNPLATLAL	5	0.2719	2637.0	42.00	0.32	tr_D0AAR0_	
HLA-DQA10501-DQB10201	22	ALMGPYNSIRVLFSR	YNSIRVLFS	5	0.2716	2646.3	42.00	0.54	tr_D0AAR0_	
HLA-DQA10501-DQB10201	23	ADRENTFNPLATLAL	FNPLATLAL	6	0.2680	2752.3	43.00	0.44	tr_D0AAR0_	
HLA-DQA10501-DQB10201	24	RENTFNPLATLALMG	FNPLATLAL	4	0.2645	2859.1	44.00	0.24	tr_D0AAR0_	
HLA-DQA10501-DQB10201	25	NPLATLALMGPYNSI	TLALMGPYN	4	0.2500	3342.9	48.00	0.31	tr_D0AAR0_	
HLA-DQA10501-DQB10201	26	LMGPYNSIRVLFSRH	YNSIRVLFS	4	0.2494	3364.9	48.00	0.51	tr_D0AAR0_	
HLA-DQA10501-DQB10201	27	TLALMGPYNSIRVLF	PYNSIRVLF	6	0.2446	3546.0	50.00	0.55	tr_D0AAR0_	
HLA-DQA10501-DQB10201	28	MGPYNSIRVLFSRHT	YNSIRVLFS	3	0.2381	3804.2	55.00	0.49	tr_D0AAR0_	
HLA-DQA10501-DQB10201	29	TEFCADRENTFNPLA	FCADRENTF	2	0.2261	4330.5	55.00	0.35	tr_D0AAR0_	
HLA-DQA10501-DQB10201	30	GPYNSIRVLFSRHTT	YNSIRVLFS	2	0.2229	4480.7	60.00	0.50	tr_D0AAR0_	
HLA-DQA10501-DQB10201	31	PLATLALMGPYNSIR	TLALMGPYN	3	0.2218	4537.8	60.00	0.35	tr_D0AAR0_	
HLA-DQA10501-DQB10201	32	LATLALMGPYNSIRV	TLALMGPYN	2	0.2139	4938.9	60.00	0.36	tr_D0AAR0_	
HLA-DQA10501-DQB10201	33	CADRENTFNPLATLA	TFNPLATLA	6	0.2120	5046.5	60.00	0.44	tr_D0AAR0_	
HLA-DQA10501-DQB10201	34	EFCADRENTFNPLAT	FCADRENTF	1	0.2075	5295.7	60.00	0.35	tr_D0AAR0_	
HLA-DQA10501-DQB10201	35	ATLALMGPYNSIRVL	TLALMGPYN	1	0.1959	6002.7	65.00	0.35	tr_D0AAR0_	
HLA-DQA10501-DQB10201	36	PYNSIRVLFSRHTTG	YNSIRVLFS	1	0.1952	6048.9	65.00	0.50	tr_D0AAR0_	
HLA-DQA10501-DQB10201	37	FCADRENTFNPLATL	NTFNPLATL	6	0.1912	6319.1	65.00	0.27	tr_D0AAR0_	
HLA-DQA10501-DQB10201	38	YNSIRVLFSRHTTGL	YNSIRVLFS	0	0.1510	9756.3	80.00	0.48	tr_D0AAR0_	
HLA-DQA10501-DQB10201	39	PPYVKDHKAEMKVEL	HKAEMKVEL	6	0.1299	12264.9	85.00	0.40	tr_D0AAR0_	
HLA-DQA10501-DQB10201	40	IPPYVKDHKAEMKVE	YVKDHKAEM	3	0.1035	16318.6	90.00	0.35	tr_D0AAR0_	
HLA-DQA10501-DQB10201	41	EIPPYVKDHKAEMKV	YVKDHKAEM	4	0.0941	18067.8	90.00	0.42	tr_D0AAR0_	

Allele: HLA-DQA10501-DQB10201. Number of high binders 0. Number of weak binders 6. Number of peptides 41

Allele	pos	peptide	core	Of	1-log50k(aff)	affinity(nM)	%Rank	Relia	Identity	Bind Level
HLA-DQA10501-DQB10201	1	PSPREVEGGVQQERE	EGGVQQERE	6	0.3906	730.6	17.00	0.46	tr_D0A893_	
HLA-DQA10501-DQB10201	2	RPTFHSVPSLAADGP	VPSLAADGP	6	0.3746	868.3	20.00	0.28	tr_D0A893_	
HLA-DQA10501-DQB10201	3	SPREVEGGVQQERES	EGGVQQERE	5	0.3716	897.0	20.00	0.45	tr_D0A893_	
HLA-DQA10501-DQB10201	4	PTFHSVPSLAADGPS	VPSLAADGP	5	0.3615	1000.8	22.00	0.31	tr_D0A893_	
HLA-DQA10501-DQB10201	5	LCGSLWATVYSDPGY	WATVYSDPG	5	0.3604	1012.6	22.00	0.26	tr_D0A893_	

HLA-DQA10501-DQB10201	6	REVEGGVQQERESSW	EGGVQQERE	3	0.3559	1063.0	23.00	0.44	tr_D0A893_
HLA-DQA10501-DQB10201	7	PREVEGGVQQERESS	EGGVQQERE	4	0.3541	1083.6	23.00	0.44	tr_D0A893_
HLA-DQA10501-DQB10201	8	CGSLWATVYSDPGYA	WATVYSDPG	4	0.3485	1151.8	25.00	0.29	tr_D0A893_
HLA-DQA10501-DQB10201	9	YARPTFHSVPSLAAD	TFHSVPSLA	4	0.3447	1200.6	25.00	0.50	tr_D0A893_
HLA-DQA10501-DQB10201	10	TFHSVPSLAADGPSP	VPSLAADGP	4	0.3390	1276.4	26.00	0.39	tr_D0A893_
HLA-DQA10501-DQB10201	11	AAHCYFCGMCVNEQD	FCGMCVNEQ	5	0.3375	1296.7	27.00	0.50	tr_D0A893_
HLA-DQA10501-DQB10201	12	PLCGSLWATVYSDPG	WATVYSDPG	6	0.3367	1308.5	27.00	0.29	tr_D0A893_
HLA-DQA10501-DQB10201	13	ARPTFHSVPSLAADG	TFHSVPSLA	3	0.3338	1350.7	28.00	0.46	tr_D0A893_
HLA-DQA10501-DQB10201	14	GYARPTFHSVPSLAA	TFHSVPSLA	5	0.3302	1403.9	28.00	0.48	tr_D0A893_
HLA-DQA10501-DQB10201	15	EVEGGVQQERESSWE	EGGVQQERE	2	0.3265	1461.5	29.00	0.47	tr_D0A893_
HLA-DQA10501-DQB10201	16	GSLWATVYSDPGYAR	WATVYSDPG	3	0.3265	1461.8	29.00	0.28	tr_D0A893_
HLA-DQA10501-DQB10201	17	AHCYFCGMCVNEQDH	FCGMCVNEQ	4	0.3209	1553.2	30.00	0.49	tr_D0A893_
HLA-DQA10501-DQB10201	18	HLWHNMKLIFMLFLP	WHNMKLIFM	2	0.3170	1620.4	31.00	0.48	tr_D0A893_
HLA-DQA10501-DQB10201	19	KLIFMLFLPLCGSLW	FLPLCGSLW	6	0.3158	1641.5	31.00	0.42	tr_D0A893_
HLA-DQA10501-DQB10201	20	LPLCGSLWATVYSDP	SLWATVYSD	5	0.3151	1653.9	32.00	0.31	tr_D0A893_
HLA-DQA10501-DQB10201	21	SLWATVYSDPGYARP	WATVYSDPG	2	0.3107	1733.0	33.00	0.30	tr_D0A893_
HLA-DQA10501-DQB10201	22	EPHLWHNMKLIFMLF	WHNMKLIFM	4	0.3102	1743.0	33.00	0.60	tr_D0A893_
HLA-DQA10501-DQB10201	23	RAAHCYFCGMCVNEQ	FCGMCVNEQ	6	0.3084	1777.2	33.00	0.50	tr_D0A893_
HLA-DQA10501-DQB10201	24	LWHNMKLIFMLFLPL	WHNMKLIFM	1	0.3057	1830.0	34.00	0.47	tr_D0A893_
HLA-DQA10501-DQB10201	25	VGEPHLWHNMKLIFM	WHNMKLIFM	6	0.3049	1845.8	34.00	0.71	tr_D0A893_
HLA-DQA10501-DQB10201	26	LIFMLFLPLCGSLWA	FLPLCGSLW	5	0.3038	1868.6	34.00	0.39	tr_D0A893_
HLA-DQA10501-DQB10201	27	PHLWHNMKLIFMLFL	WHNMKLIFM	3	0.3031	1882.2	34.00	0.55	tr_D0A893_
HLA-DQA10501-DQB10201	28	HCYFCGMCVNEQDHH	FCGMCVNEQ	3	0.3023	1897.8	35.00	0.44	tr_D0A893_
HLA-DQA10501-DQB10201	29	FHSVPSLAADGPSPR	VPSLAADGP	3	0.3006	1934.9	35.00	0.46	tr_D0A893_
HLA-DQA10501-DQB10201	30	PGYARPTFHSVPSLA	TFHSVPSLA	6	0.2996	1954.9	35.00	0.59	tr_D0A893_
HLA-DQA10501-DQB10201	31	GEPHLWHNMKLIFML	WHNMKLIFM	5	0.2937	2084.2	37.00	0.70	tr_D0A893_
HLA-DQA10501-DQB10201	32	CYFCGMCVNEQDHHC	FCGMCVNEQ	2	0.2914	2137.2	37.00	0.48	tr_D0A893_
HLA-DQA10501-DQB10201	33	SSWEDVNGQSVERRW	WEDVNGQSV	2	0.2889	2193.9	38.00	0.41	tr_D0A893_
HLA-DQA10501-DQB10201	34	ESSWEDVNGQSVERR	WEDVNGQSV	3	0.2881	2214.6	38.00	0.47	tr_D0A893_
HLA-DQA10501-DQB10201	35	IFMLFLPLCGSLWAT	FLPLCGSLW	4	0.2864	2254.3	38.00	0.38	tr_D0A893_
HLA-DQA10501-DQB10201	36	WHNMKLIFMLFLPLC	WHNMKLIFM	0	0.2857	2273.2	39.00	0.37	tr_D0A893_
HLA-DQA10501-DQB10201	37	ERESSWEDVNGQSVE	WEDVNGQSV	5	0.2810	2390.1	40.00	0.62	tr_D0A893_
HLA-DQA10501-DQB10201	38	RESSWEDVNGQSVER	WEDVNGQSV	4	0.2805	2402.7	40.00	0.57	tr_D0A893_
HLA-DQA10501-DQB10201	39	FLPLCGSLWATVYSD	SLWATVYSD	6	0.2792	2437.5	40.00	0.38	tr_D0A893_
HLA-DQA10501-DQB10201	40	GPSPREVEGGVQQER	EVEGGVQQE	5	0.2786	2453.7	40.00	0.55	tr_D0A893_
HLA-DQA10501-DQB10201	41	QERESSWEDVNGQSV	WEDVNGQSV	6	0.2779	2471.3	41.00	0.62	tr_D0A893_
HLA-DQA10501-DQB10201	42	SWEDVNGQSVERRWC	WEDVNGQSV	1	0.2725	2619.8	42.00	0.40	tr_D0A893_
HLA-DQA10501-DQB10201	43	YFCGMCVNEQDHHCG	FCGMCVNEQ	1	0.2716	2647.4	42.00	0.50	tr_D0A893_
HLA-DQA10501-DQB10201	44	HNMKLIFMLFLPLCG	FMLFLPLCG	6	0.2688	2727.7	43.00	0.37	tr_D0A893_
HLA-DQA10501-DQB10201	45	HSVPSLAADGPSPRE	VPSLAADGP	2	0.2656	2824.9	44.00	0.47	tr_D0A893_
HLA-DQA10501-DQB10201	46	LWATVYSDPGYARPT	WATVYSDPG	1	0.2653	2834.8	44.00	0.31	tr_D0A893_
HLA-DQA10501-DQB10201	47	DGPSPREVEGGVQQE	EVEGGVQQE	6	0.2651	2839.5	44.00	0.63	tr_D0A893_
HLA-DQA10501-DQB10201	48	VEGGVQQERESSWED	EGGVQQERE	1	0.2625	2921.0	45.00	0.54	tr_D0A893_
HLA-DQA10501-DQB10201	49	EGGVQQERESSWEDV	EGGVQQERE	0	0.2613	2958.9	45.00	0.32	tr_D0A893_
HLA-DQA10501-DQB10201	50	NMKLIFMLFLPLCGS	FMLFLPLCG	5	0.2604	2987.8	45.00	0.34	tr_D0A893_
HLA-DQA10501-DQB10201	51	FMLFLPLCGSLWATV	FLPLCGSLW	3	0.2595	3016.2	46.00	0.41	tr_D0A893_
HLA-DQA10501-DQB10201	52	MKLIFMLFLPLCGSL	FMLFLPLCG	4	0.2571	3096.9	46.00	0.38	tr_D0A893_
HLA-DQA10501-DQB10201	53	SVPSLAADGPSPREV	VPSLAADGP	1	0.2536	3215.3	47.00	0.45	tr_D0A893_
HLA-DQA10501-DQB10201	54	LFLPLCGSLWATVYS	FLPLCGSLW	1	0.2386	3781.2	55.00	0.28	tr_D0A893_
HLA-DQA10501-DQB10201	55	MLFLPLCGSLWATVY	FLPLCGSLW	2	0.2378	3814.8	55.00	0.35	tr_D0A893_

HLA-DQA10501-DQB10201	56	WEDVNGQSVERRWCS	WEDVNGQSV	0	0.2373	3834.5	55.00	0.29	tr_D0A893_
HLA-DQA10501-DQB10201	57	DPGYARPTFHSVPSL	PTFHSVPSL	6	0.2259	4337.7	55.00	0.58	tr_D0A893_
HLA-DQA10501-DQB10201	58	IHRPLRAAHCYFCGM	PLRAAHCYF	3	0.2255	4357.7	55.00	0.48	tr_D0A893_
HLA-DQA10501-DQB10201	59	WATVYSDPGYARPTF	WATVYSDPG	0	0.2253	4368.9	55.00	0.25	tr_D0A893_
HLA-DQA10501-DQB10201	60	RPLRAAHCYFCGMCV	PLRAAHCYF	1	0.2245	4408.4	55.00	0.42	tr_D0A893_
HLA-DQA10501-DQB10201	61	LRAAHCYFCGMCVNE	YFCGMCVNE	6	0.2231	4471.9	60.00	0.37	tr_D0A893_
HLA-DQA10501-DQB10201	62	GGVQQERESSWEDVN	ERESSWEDV	5	0.2162	4818.0	60.00	0.38	tr_D0A893_
HLA-DQA10501-DQB10201	63	HRPLRAAHCYFCGMC	PLRAAHCYF	2	0.2161	4826.6	60.00	0.47	tr_D0A893_
HLA-DQA10501-DQB10201	64	VPSLAADGPSPREVE	VPSLAADGP	0	0.2137	4951.8	60.00	0.35	tr_D0A893_
HLA-DQA10501-DQB10201	65	ACGIHRPLRAAHCYF	PLRAAHCYF	6	0.2102	5141.9	60.00	0.73	tr_D0A893_
HLA-DQA10501-DQB10201	66	ATVYSDPGYARPTFH	PGYARPTFH	6	0.2093	5193.6	60.00	0.34	tr_D0A893_
HLA-DQA10501-DQB10201	67	GVQQERESSWEDVNG	ERESSWEDV	4	0.2082	5255.0	60.00	0.34	tr_D0A893_
HLA-DQA10501-DQB10201	68	CGIHRPLRAAHCYFC	PLRAAHCYF	5	0.2064	5360.7	65.00	0.67	tr_D0A893_
HLA-DQA10501-DQB10201	69	PLRAAHCYFCGMCVN	PLRAAHCYF	0	0.2059	5388.8	65.00	0.32	tr_D0A893_
HLA-DQA10501-DQB10201	70	FCGMCVNEQDHHCGV	FCGMCVNEQ	0	0.2049	5444.6	65.00	0.50	tr_D0A893_
HLA-DQA10501-DQB10201	71	VQQERESSWEDVNGQ	ERESSWEDV	3	0.1948	6074.3	65.00	0.33	tr_D0A893_
HLA-DQA10501-DQB10201	72	GIHRPLRAAHCYFCG	PLRAAHCYF	4	0.1925	6226.5	65.00	0.66	tr_D0A893_
HLA-DQA10501-DQB10201	73	NGQSVERRWCSACGI	RRWCSACGI	6	0.1806	7084.7	70.00	0.75	tr_D0A893_
HLA-DQA10501-DQB10201	74	EDVNGQSVERRWCSA	VNGQSVERR	2	0.1802	7117.1	70.00	0.46	tr_D0A893_
HLA-DQA10501-DQB10201	75	QQERESSWEDVNGQS	ERESSWEDV	2	0.1793	7184.0	70.00	0.35	tr_D0A893_
HLA-DQA10501-DQB10201	76	TVYSDPGYARPTFHS	PGYARPTFH	5	0.1755	7483.2	70.00	0.41	tr_D0A893_
HLA-DQA10501-DQB10201	77	SVERRWCSACGIHRP	RRWCSACGI	3	0.1752	7512.3	70.00	0.59	tr_D0A893_
HLA-DQA10501-DQB10201	78	VERRWCSACGIHRPL	RRWCSACGI	2	0.1734	7661.5	70.00	0.55	tr_D0A893_
HLA-DQA10501-DQB10201	79	QSVERRWCSACGIHR	RRWCSACGI	4	0.1635	8529.0	75.00	0.62	tr_D0A893_
HLA-DQA10501-DQB10201	80	GQSVERRWCSACGIH	RRWCSACGI	5	0.1612	8741.7	75.00	0.76	tr_D0A893_
HLA-DQA10501-DQB10201	81	ERRWCSACGIHRPLR	RRWCSACGI	1	0.1555	9295.3	75.00	0.51	tr_D0A893_
HLA-DQA10501-DQB10201	82	VYSDPGYARPTFHSV	PGYARPTFH	4	0.1513	9732.5	80.00	0.55	tr_D0A893_
HLA-DQA10501-DQB10201	83	YSDPGYARPTFHSVP	PGYARPTFH	3	0.1503	9831.4	80.00	0.56	tr_D0A893_
HLA-DQA10501-DQB10201	84	HVVGEPHLWHNMKLI	HVVGEPHLW	0	0.1502	9841.0	80.00	0.49	tr_D0A893_
HLA-DQA10501-DQB10201	85	PSLAADGPSPREVEG	SLAADGPSP	1	0.1466	10233.5	80.00	0.34	tr_D0A893_
HLA-DQA10501-DQB10201	86	DVNGQSVERRWCSAC	VNGQSVERR	1	0.1439	10540.7	80.00	0.53	tr_D0A893_
HLA-DQA10501-DQB10201	87	ADGPSPREVEGGVQQ	PREVEGGVQ	5	0.1425	10698.0	80.00	0.46	tr_D0A893_
HLA-DQA10501-DQB10201	88	RRWCSACGIHRPLRA	RRWCSACGI	0	0.1424	10714.2	80.00	0.40	tr_D0A893_
HLA-DQA10501-DQB10201	89	VNEQDHHCGVIGVCV	HCGVIGVCV	6	0.1382	11206.5	80.00	0.41	tr_D0A893_
HLA-DQA10501-DQB10201	90	QDHHCGVIGVCVGRR	VIGVCVGRR	6	0.1372	11325.9	80.00	0.37	tr_D0A893_
HLA-DQA10501-DQB10201	91	VVGEPHLWHNMKLIF	LWHNMKLIF	6	0.1357	11516.6	80.00	0.42	tr_D0A893_
HLA-DQA10501-DQB10201	92	SDPGYARPTFHSVPS	PGYARPTFH	2	0.1342	11701.6	85.00	0.52	tr_D0A893_
HLA-DQA10501-DQB10201	93	AADGPSPREVEGGVQ	PREVEGGVQ	6	0.1308	12139.7	85.00	0.56	tr_D0A893_
HLA-DQA10501-DQB10201	94	DHHCGVIGVCVGRRN	VIGVCVGRR	5	0.1295	12311.9	85.00	0.38	tr_D0A893_
HLA-DQA10501-DQB10201	95	SLAADGPSPREVEGG	SLAADGPSP	0	0.1284	12460.7	85.00	0.29	tr_D0A893_
HLA-DQA10501-DQB10201	96	NEQDHHCGVIGVCVG	HCGVIGVCV	5	0.1261	12783.8	85.00	0.36	tr_D0A893_
HLA-DQA10501-DQB10201	97	EQDHHCGVIGVCVGR	HCGVIGVCV	4	0.1239	13088.6	85.00	0.31	tr_D0A893_
HLA-DQA10501-DQB10201	98	CGMCVNEQDHHCGVI	CGMCVNEQD	0	0.1144	14507.0	85.00	0.28	tr_D0A893_
HLA-DQA10501-DQB10201	99	CVNEQDHHCGVIGVC	DHHCGVIGV	5	0.1054	15983.4	90.00	0.43	tr_D0A893_
HLA-DQA10501-DQB10201	100	VNGQSVERRWCSACG	VNGQSVERR	0	0.1000	16941.3	90.00	0.46	tr_D0A893_
HLA-DQA10501-DQB10201	101	RWCSACGIHRPLRAA	WCSACGIHR	1	0.0980	17315.6	90.00	0.34	tr_D0A893_
HLA-DQA10501-DQB10201	102	LAADGPSPREVEGGV	AADGPSPRE	1	0.0968	17541.0	90.00	0.22	tr_D0A893_
HLA-DQA10501-DQB10201	103	MCVNEQDHHCGVIGV	DHHCGVIGV	6	0.0953	17827.0	90.00	0.52	tr_D0A893_
HLA-DQA10501-DQB10201	104	SACGIHRPLRAAHCY	RPLRAAHCY	6	0.0937	18142.4	90.00	0.31	tr_D0A893_
HLA-DQA10501-DQB10201	105	WCSACGIHRPLRAAH	CGIHRPLRA	4	0.0829	20384.9	95.00	0.28	tr_D0A893_

HLA-DQA10501-DQB10201	106	CSACGIHRPLRAAHC	CGIHRPLRA	3	0.0755	22100.6	95.00	0.31	tr_D0A893_	
HLA-DQA10501-DQB10201	107	GMCVNEQDHHCGVIG	GMCVNEQDH	0	0.0753	22139.9	95.00	0.29	tr_D0A893_	

Allele: HLA-DQA10501-DQB10201. Number of high binders 0. Number of weak binders 0. Number of peptides 107

Allele	pos	peptide	core	Of	1-log50k(aff)	affinity(nM)	%Rank	Relia	Identity	Bind Level
HLA-DQA10501-DQB10201	1	GAAGGGIPDVMAYLN	AGGGIPDVM	2	0.3701	911.5	21.00	0.54	tr_C9ZY92_	
HLA-DQA10501-DQB10201	2	PGAAGGGIPDVMAYL	AGGGIPDVM	3	0.3695	917.9	21.00	0.57	tr_C9ZY92_	
HLA-DQA10501-DQB10201	3	GIPDVMAYLNGVHVP	MAYLNGVHV	5	0.3655	958.1	21.00	0.28	tr_C9ZY92_	
HLA-DQA10501-DQB10201	4	EPGAAGGGIPDVMAY	AGGGIPDVM	4	0.3622	993.6	22.00	0.62	tr_C9ZY92_	
HLA-DQA10501-DQB10201	5	FEPGAAGGGIPDVMA	AGGGIPDVM	5	0.3573	1046.7	23.00	0.63	tr_C9ZY92_	
HLA-DQA10501-DQB10201	6	GGIPDVMAYLNGVHV	MAYLNGVHV	6	0.3497	1137.3	24.00	0.31	tr_C9ZY92_	
HLA-DQA10501-DQB10201	7	YFEPGAAGGGIPDVM	AGGGIPDVM	6	0.3469	1172.4	25.00	0.64	tr_C9ZY92_	
HLA-DQA10501-DQB10201	8	AGGGIPDVMAYLNGV	DVMAYLNGV	6	0.3446	1201.5	25.00	0.40	tr_C9ZY92_	
HLA-DQA10501-DQB10201	9	AAGGGIPDVMAYLNG	AGGGIPDVM	1	0.3429	1224.3	26.00	0.54	tr_C9ZY92_	
HLA-DQA10501-DQB10201	10	IPDVMAYLNGVHVPK	DVMAYLNGV	2	0.3394	1270.8	26.00	0.43	tr_C9ZY92_	
HLA-DQA10501-DQB10201	11	PDVMAYLNGVHVPKV	DVMAYLNGV	1	0.3329	1364.1	28.00	0.35	tr_C9ZY92_	
HLA-DQA10501-DQB10201	12	DVMAYLNGVHVPKVM	MAYLNGVHV	2	0.3271	1452.2	29.00	0.26	tr_C9ZY92_	
HLA-DQA10501-DQB10201	13	GGGIPDVMAYLNGVH	DVMAYLNGV	5	0.3124	1702.2	32.00	0.45	tr_C9ZY92_	
HLA-DQA10501-DQB10201	14	VMAYLNGVHVPKVMT	MAYLNGVHV	1	0.2641	2871.7	44.00	0.31	tr_C9ZY92_	
HLA-DQA10501-DQB10201	15	MAYLNGVHVPKVMTL	MAYLNGVHV	0	0.2449	3533.9	50.00	0.20	tr_C9ZY92_	
HLA-DQA10501-DQB10201	16	AYLNGVHVPKVMTLR	LNGVHVPKV	2	0.1985	5840.2	65.00	0.35	tr_C9ZY92_	
HLA-DQA10501-DQB10201	17	VPKVMTLRTFVVKSI	LRTFVVKSI	6	0.1894	6438.9	70.00	0.44	tr_C9ZY92_	
HLA-DQA10501-DQB10201	18	GVHVPKVMTLRTFVV	VMTLRTFVV	6	0.1877	6563.7	70.00	0.38	tr_C9ZY92_	
HLA-DQA10501-DQB10201	19	YLNGVHVPKVMTLRT	LNGVHVPKV	1	0.1771	7358.2	70.00	0.28	tr_C9ZY92_	
HLA-DQA10501-DQB10201	20	LNGVHVPKVMTLRTF	PKVMTLRTF	6	0.1669	8217.8	75.00	0.23	tr_C9ZY92_	
HLA-DQA10501-DQB10201	21	NGVHVPKVMTLRTFV	KVMTLRTFV	6	0.1638	8496.5	75.00	0.32	tr_C9ZY92_	
HLA-DQA10501-DQB10201	22	VHVPKVMTLRTFVVK	VMTLRTFVV	5	0.1585	8995.1	75.00	0.34	tr_C9ZY92_	
HLA-DQA10501-DQB10201	23	HVPKVMTLRTFVVKS	VMTLRTFVV	4	0.1564	9205.7	75.00	0.32	tr_C9ZY92_	

Allele: HLA-DQA10501-DQB10201. Number of high binders 0. Number of weak binders 0. Number of peptides 23

Allele	pos	peptide	core	Of	1-log50k(aff)	affinity(nM)	%Rank	Relia	Identity	Bind Level
HLA-DQA10501-DQB10201	1	APFQDMEDRGDVLVW	DMEDRGDVL	4	0.3513	1117.6	24.00	0.44	tr_C9ZY92_	
HLA-DQA10501-DQB10201	2	PFQDMEDRGDVLVWG	DMEDRGDVL	3	0.3450	1196.5	25.00	0.43	tr_C9ZY92_	
HLA-DQA10501-DQB10201	3	FQDMEDRGDVLVWGT	DMEDRGDVL	2	0.3276	1443.6	29.00	0.44	tr_C9ZY92_	
HLA-DQA10501-DQB10201	4	CAPFQDMEDRGDVLV	DMEDRGDVL	5	0.3209	1552.1	30.00	0.49	tr_C9ZY92_	
HLA-DQA10501-DQB10201	5	GCAPFQDMEDRGDVL	DMEDRGDVL	6	0.3042	1860.5	34.00	0.51	tr_C9ZY92_	
HLA-DQA10501-DQB10201	6	SPLATLVLGSERDTI	TLVLGSERD	4	0.3032	1879.5	34.00	0.37	tr_C9ZY92_	
HLA-DQA10501-DQB10201	7	TFSPLATLVLGSERD	TLVLGSERD	6	0.2986	1976.6	35.00	0.35	tr_C9ZY92_	
HLA-DQA10501-DQB10201	8	QDMEDRGDVLVWGTE	DMEDRGDVL	1	0.2924	2114.4	37.00	0.39	tr_C9ZY92_	
HLA-DQA10501-DQB10201	9	CSENNTFSPLATLVL	FSPLATLVL	6	0.2884	2206.0	38.00	0.40	tr_C9ZY92_	

HLA-DQA10501-DQB10201	10	FSPLATLVLGSERDT	TLVLGSERD	5	0.2848	2295.4	39.00	0.40	tr_C9ZY92_
HLA-DQA10501-DQB10201	11	DMEDRGDVLVWGTEN	DMEDRGDVL	0	0.2829	2343.1	39.00	0.25	tr_C9ZY92_
HLA-DQA10501-DQB10201	12	PLATLVLGSERDTIR	TLVLGSERD	3	0.2819	2367.2	40.00	0.36	tr_C9ZY92_
HLA-DQA10501-DQB10201	13	NNTFSPLATLVLGSE	LATLVLGSE	6	0.2771	2494.2	41.00	0.40	tr_C9ZY92_
HLA-DQA10501-DQB10201	14	DVLVWGTENTTSLFT	TENTTSLFT	6	0.2681	2748.9	43.00	0.29	tr_C9ZY92_
HLA-DQA10501-DQB10201	15	LATLVLGSERDTIRH	TLVLGSERD	2	0.2651	2839.2	44.00	0.42	tr_C9ZY92_
HLA-DQA10501-DQB10201	16	NTFSPLATLVLGSER	LATLVLGSE	5	0.2616	2949.4	45.00	0.32	tr_C9ZY92_
HLA-DQA10501-DQB10201	17	SENNTFSPLATLVLG	FSPLATLVL	5	0.2589	3037.8	46.00	0.32	tr_C9ZY92_
HLA-DQA10501-DQB10201	18	ENTTSLFTATCSENN	SLFTATCSE	4	0.2561	3129.8	47.00	0.38	tr_C9ZY92_
HLA-DQA10501-DQB10201	19	TTSLFTATCSENNTF	SLFTATCSE	2	0.2532	3230.5	47.00	0.33	tr_C9ZY92_
HLA-DQA10501-DQB10201	20	MEDRGDVLVWGTENT	VLVWGTENT	6	0.2513	3298.7	48.00	0.22	tr_C9ZY92_
HLA-DQA10501-DQB10201	21	NTTSLFTATCSENNT	SLFTATCSE	3	0.2502	3335.4	48.00	0.37	tr_C9ZY92_
HLA-DQA10501-DQB10201	22	ENNTFSPLATLVLGS	FSPLATLVL	4	0.2498	3349.7	48.00	0.26	tr_C9ZY92_
HLA-DQA10501-DQB10201	23	GDVLVWGTENTTSLF	WGTENTTSL	5	0.2469	3456.0	49.00	0.22	tr_C9ZY92_
HLA-DQA10501-DQB10201	24	EDRGDVLVWGTENTT	VLVWGTENT	5	0.2465	3471.4	49.00	0.25	tr_C9ZY92_
HLA-DQA10501-DQB10201	25	TENTTSLFTATCSEN	SLFTATCSE	5	0.2437	3579.5	50.00	0.48	tr_C9ZY92_
HLA-DQA10501-DQB10201	26	ATLVLGSERDTIRHL	TLVLGSERD	1	0.2422	3637.9	50.00	0.47	tr_C9ZY92_
HLA-DQA10501-DQB10201	27	GTENTTSLFTATCSE	SLFTATCSE	6	0.2382	3797.5	55.00	0.48	tr_C9ZY92_
HLA-DQA10501-DQB10201	28	VLVWGTENTTSLFTA	TENTTSLFT	5	0.2379	3811.9	55.00	0.30	tr_C9ZY92_
HLA-DQA10501-DQB10201	29	TSLFTATCSENNTFS	SLFTATCSE	1	0.2349	3935.1	55.00	0.33	tr_C9ZY92_
HLA-DQA10501-DQB10201	30	RGDVLVWGTENTTSL	LVWGTENTT	4	0.2308	4117.5	55.00	0.22	tr_C9ZY92_
HLA-DQA10501-DQB10201	31	TCSENNTFSPLATLV	TFSPLATLV	6	0.2300	4150.2	55.00	0.41	tr_C9ZY92_
HLA-DQA10501-DQB10201	32	TLVLGSERDTIRHLF	TLVLGSERD	0	0.2283	4226.3	55.00	0.37	tr_C9ZY92_
HLA-DQA10501-DQB10201	33	SGCAPFQDMEDRGDV	PFQDMEDRG	4	0.2277	4257.3	55.00	0.32	tr_C9ZY92_
HLA-DQA10501-DQB10201	34	DRGDVLVWGTENTTS	LVWGTENTT	5	0.2213	4560.2	60.00	0.25	tr_C9ZY92_
HLA-DQA10501-DQB10201	35	SLFTATCSENNTFSP	FTATCSENN	2	0.2104	5129.9	60.00	0.34	tr_C9ZY92_
HLA-DQA10501-DQB10201	36	LVWGTENTTSLFTAT	TENTTSLFT	4	0.2038	5509.8	65.00	0.32	tr_C9ZY92_
HLA-DQA10501-DQB10201	37	WGTENTTSLFTATCS	TSLFTATCS	6	0.2032	5550.2	65.00	0.38	tr_C9ZY92_
HLA-DQA10501-DQB10201	38	LVLGSERDTIRHLFS	VLGSERDTI	1	0.1972	5918.9	65.00	0.28	tr_C9ZY92_
HLA-DQA10501-DQB10201	39	DTIRHLFSRQTIWQY	FSRQTIWQY	6	0.1906	6356.7	65.00	0.25	tr_C9ZY92_
HLA-DQA10501-DQB10201	40	ATCSENNTFSPLATL	NTFSPLATL	6	0.1884	6510.0	70.00	0.31	tr_C9ZY92_
HLA-DQA10501-DQB10201	41	VWGTENTTSLFTATC	TENTTSLFT	3	0.1871	6605.3	70.00	0.37	tr_C9ZY92_
HLA-DQA10501-DQB10201	42	ERDTIRHLFSRQTIW	RHLFSRQTI	5	0.1750	7526.7	70.00	0.28	tr_C9ZY92_
HLA-DQA10501-DQB10201	43	TIRHLFSRQTIWQYH	FSRQTIWQY	5	0.1725	7732.8	70.00	0.25	tr_C9ZY92_
HLA-DQA10501-DQB10201	44	SERDTIRHLFSRQTI	RHLFSRQTI	6	0.1718	7789.8	75.00	0.32	tr_C9ZY92_
HLA-DQA10501-DQB10201	45	LFTATCSENNTFSPL	FTATCSENN	1	0.1690	8034.9	75.00	0.35	tr_C9ZY92_
HLA-DQA10501-DQB10201	46	IRHLFSRQTIWQYHP	FSRQTIWQY	4	0.1689	8037.7	75.00	0.27	tr_C9ZY92_
HLA-DQA10501-DQB10201	47	RDTIRHLFSRQTIWQ	RHLFSRQTI	4	0.1686	8065.7	75.00	0.28	tr_C9ZY92_
HLA-DQA10501-DQB10201	48	RHLFSRQTIWQYHPV	FSRQTIWQY	3	0.1679	8129.5	75.00	0.29	tr_C9ZY92_
HLA-DQA10501-DQB10201	49	VLGSERDTIRHLFSR	ERDTIRHLF	4	0.1674	8175.5	75.00	0.25	tr_C9ZY92_
HLA-DQA10501-DQB10201	50	TATCSENNTFSPLAT	ENNTFSPLA	5	0.1544	9403.8	75.00	0.39	tr_C9ZY92_
HLA-DQA10501-DQB10201	51	FTATCSENNTFSPLA	ENNTFSPLA	6	0.1540	9449.0	75.00	0.40	tr_C9ZY92_
HLA-DQA10501-DQB10201	52	GSERDTIRHLFSRQT	IRHLFSRQT	6	0.1447	10453.4	80.00	0.23	tr_C9ZY92_
HLA-DQA10501-DQB10201	53	LGSERDTIRHLFSRQ	ERDTIRHLF	3	0.1372	11333.0	80.00	0.32	tr_C9ZY92_

Allele: HLA-DQA10501-DQB10201. Number of high binders 0. Number of weak binders 0. Number of peptides 53

Allele	pos	peptide	core	Of	1-log50k(aff)	affinity(nM)	%Rank	Relia	Identity	Bind Level
HLA-DQA10501-DQB10201	1	CAEGANAADVVPLFL	AADVVPLFL	6	0.6786	32.4	0.30	0.35	tr_D0A9V0_	SB
HLA-DQA10501-DQB10201	2	AEGANAADVVPLFLT	AADVVPLFL	5	0.6541	42.2	0.50	0.35	tr_D0A9V0_	WB
HLA-DQA10501-DQB10201	3	EGANAADVVPLFLTD	AADVVPLFL	4	0.6387	49.8	0.70	0.38	tr_D0A9V0_	WB
HLA-DQA10501-DQB10201	4	GCAEGANAADVVPLF	NAADVVPLF	6	0.6158	63.9	1.00	0.40	tr_D0A9V0_	WB
HLA-DQA10501-DQB10201	5	GANAADVVPLFLTDS	AADVVPLFL	3	0.5990	76.6	1.30	0.44	tr_D0A9V0_	WB
HLA-DQA10501-DQB10201	6	GAFSTIFLGVAEDGA	IFLGVAEDG	5	0.5949	80.1	1.40	0.41	tr_D0A9V0_	WB
HLA-DQA10501-DQB10201	7	AFSTIFLGVAEDGAQ	FLGVAEDGA	5	0.5907	83.8	1.50	0.42	tr_D0A9V0_	WB
HLA-DQA10501-DQB10201	8	FSTIFLGVAEDGAQV	FLGVAEDGA	4	0.5885	85.8	1.60	0.46	tr_D0A9V0_	WB
HLA-DQA10501-DQB10201	9	SHMQGFLAFPVDCVL	FLAFPVDCV	5	0.5718	102.9	2.00	0.45	tr_D0A9V0_	WB
HLA-DQA10501-DQB10201	10	ANAADVVPLFLTDSN	AADVVPLFL	2	0.5716	103.0	2.00	0.46	tr_D0A9V0_	WB
HLA-DQA10501-DQB10201	11	STIFLGVAEDGAQVA	FLGVAEDGA	3	0.5700	104.9	2.50	0.47	tr_D0A9V0_	WB
HLA-DQA10501-DQB10201	12	HMQGFLAFPVDCVLL	FLAFPVDCV	4	0.5651	110.5	2.50	0.47	tr_D0A9V0_	WB
HLA-DQA10501-DQB10201	13	MQGFLAFPVDCVLLP	FLAFPVDCV	3	0.5647	111.0	2.50	0.51	tr_D0A9V0_	WB
HLA-DQA10501-DQB10201	14	ASHMQGFLAFPVDCV	FLAFPVDCV	6	0.5602	116.5	2.50	0.49	tr_D0A9V0_	WB
HLA-DQA10501-DQB10201	15	TIFLGVAEDGAQVAI	FLGVAEDGA	2	0.5509	129.0	3.00	0.50	tr_D0A9V0_	WB
HLA-DQA10501-DQB10201	16	EGAFSTIFLGVAEDG	IFLGVAEDG	6	0.5476	133.7	3.00	0.49	tr_D0A9V0_	WB
HLA-DQA10501-DQB10201	17	QGFLAFPVDCVLLPC	FLAFPVDCV	2	0.5399	145.2	3.50	0.55	tr_D0A9V0_	WB
HLA-DQA10501-DQB10201	18	SGCAEGANAADVVPL	EGANAADVV	4	0.5244	171.7	4.00	0.50	tr_D0A9V0_	WB
HLA-DQA10501-DQB10201	19	KCIPRLRRDVVAESL	RRDVVAESL	6	0.5151	189.9	4.50	0.82	tr_D0A9V0_	WB
HLA-DQA10501-DQB10201	20	HRDSVTMLVDTNEFL	TMLVDTNEF	5	0.5088	203.3	5.00	0.47	tr_D0A9V0_	WB
HLA-DQA10501-DQB10201	21	NAADVVPLFLTDSNR	AADVVPLFL	1	0.5084	204.2	5.00	0.53	tr_D0A9V0_	WB
HLA-DQA10501-DQB10201	22	CIPRLRRDVVAESLE	RRDVVAESL	5	0.5079	205.4	5.00	0.72	tr_D0A9V0_	WB
HLA-DQA10501-DQB10201	23	SDIFSFGISLLEMLL	SFGISLLEM	4	0.5066	208.2	5.00	0.50	tr_D0A9V0_	WB
HLA-DQA10501-DQB10201	24	RLRRDVVAESLEKEM	RRDVVAESL	2	0.5059	209.8	5.00	0.46	tr_D0A9V0_	WB
HLA-DQA10501-DQB10201	25	RDSVTMLVDTNEFLV	TMLVDTNEF	4	0.5054	210.9	5.00	0.40	tr_D0A9V0_	WB
HLA-DQA10501-DQB10201	26	VMLRGTALYMAPEVA	ALYMAPEVA	6	0.5038	214.6	5.50	0.49	tr_D0A9V0_	WB
HLA-DQA10501-DQB10201	27	EHSGCVEALDPDRAL	VEALDPDRA	5	0.5016	219.8	5.50	0.50	tr_D0A9V0_	WB
HLA-DQA10501-DQB10201	28	QSDIFSFGISLLEML	SFGISLLEM	5	0.5016	219.9	5.50	0.56	tr_D0A9V0_	WB
HLA-DQA10501-DQB10201	29	IFLGVAEDGAQVAIK	FLGVAEDGA	1	0.5010	221.2	5.50	0.54	tr_D0A9V0_	WB
HLA-DQA10501-DQB10201	30	GFLAFPVDCVLLPCS	FLAFPVDCV	1	0.4964	232.4	6.00	0.57	tr_D0A9V0_	WB
HLA-DQA10501-DQB10201	31	TEHSGCVEALDPDRA	VEALDPDRA	6	0.4961	233.2	6.00	0.40	tr_D0A9V0_	WB
HLA-DQA10501-DQB10201	32	SSTKYHAALIEVATE	YHAALIEVA	4	0.4957	234.3	6.00	0.48	tr_D0A9V0_	WB
HLA-DQA10501-DQB10201	33	PRLRRDVVAESLEKE	RRDVVAESL	3	0.4938	239.2	6.00	0.50	tr_D0A9V0_	WB
HLA-DQA10501-DQB10201	34	MLRGTALYMAPEVAA	ALYMAPEVA	5	0.4922	243.3	6.00	0.49	tr_D0A9V0_	WB
HLA-DQA10501-DQB10201	35	LRRDVVAESLEKEME	RRDVVAESL	1	0.4906	247.5	6.00	0.44	tr_D0A9V0_	WB
HLA-DQA10501-DQB10201	36	DSVTMLVDTNEFLVG	TMLVDTNEF	3	0.4860	260.1	6.50	0.39	tr_D0A9V0_	WB
HLA-DQA10501-DQB10201	37	WKSSTKYHAALIEVA	YHAALIEVA	6	0.4844	264.7	6.50	0.63	tr_D0A9V0_	WB
HLA-DQA10501-DQB10201	38	HSGCVEALDPDRALI	VEALDPDRA	4	0.4835	267.4	6.50	0.54	tr_D0A9V0_	WB
HLA-DQA10501-DQB10201	39	IPRLRRDVVAESLEK	RRDVVAESL	4	0.4826	269.8	7.00	0.62	tr_D0A9V0_	WB
HLA-DQA10501-DQB10201	40	PQSDIFSFGISLLEM	SFGISLLEM	6	0.4808	275.2	7.00	0.71	tr_D0A9V0_	WB
HLA-DQA10501-DQB10201	41	STKYHAALIEVATEH	YHAALIEVA	3	0.4771	286.5	7.00	0.47	tr_D0A9V0_	WB
HLA-DQA10501-DQB10201	42	GSGCAEGANAADVVP	EGANAADVV	5	0.4756	291.1	7.50	0.61	tr_D0A9V0_	WB
HLA-DQA10501-DQB10201	43	DIFSFGISLLEMLLG	SFGISLLEM	3	0.4753	292.0	7.50	0.48	tr_D0A9V0_	WB
HLA-DQA10501-DQB10201	44	DHRDSVTMLVDTNEF	TMLVDTNEF	6	0.4704	308.2	8.00	0.64	tr_D0A9V0_	WB
HLA-DQA10501-DQB10201	45	TKYHAALIEVATEHS	YHAALIEVA	2	0.4700	309.3	8.00	0.38	tr_D0A9V0_	WB
HLA-DQA10501-DQB10201	46	KSSTKYHAALIEVAT	YHAALIEVA	5	0.4663	321.8	8.00	0.57	tr_D0A9V0_	WB
HLA-DQA10501-DQB10201	47	RNARVLFGADHLFQL	RVLFGADHL	3	0.4647	327.6	8.50	0.39	tr_D0A9V0_	WB
HLA-DQA10501-DQB10201	48	LRGTALYMAPEVAAG	ALYMAPEVA	4	0.4644	328.8	8.50	0.49	tr_D0A9V0_	WB

HLA-DQA10501-DQB10201	49	SVTMLVDTNEFLVGT	TMLVDTNEF	2	0.4621	337.1	8.50	0.40	tr_D0A9V0_	WB
HLA-DQA10501-DQB10201	50	ARVLFGADHLFQLSY	RVLFGADHL	1	0.4605	342.7	8.50	0.33	tr_D0A9V0_	WB
HLA-DQA10501-DQB10201	51	NARVLFGADHLFQLS	RVLFGADHL	2	0.4597	345.8	8.50	0.38	tr_D0A9V0_	WB
HLA-DQA10501-DQB10201	52	RRDVVAESLEKEMEV	RRDVVAESL	0	0.4587	349.7	9.00	0.27	tr_D0A9V0_	WB
HLA-DQA10501-DQB10201	53	SGCVEALDPDRALIS	VEALDPDRA	3	0.4568	356.7	9.00	0.57	tr_D0A9V0_	WB
HLA-DQA10501-DQB10201	54	GASHMQGFLAFPVDC	QGFLAFPVD	5	0.4541	367.4	9.50	0.30	tr_D0A9V0_	WB
HLA-DQA10501-DQB10201	55	PYVARRFTVDILQGL	FTVDILQGL	6	0.4530	371.9	9.50	0.34	tr_D0A9V0_	WB
HLA-DQA10501-DQB10201	56	SEGAFSTIFLGVAED	TIFLGVAED	6	0.4522	375.0	9.50	0.47	tr_D0A9V0_	WB
HLA-DQA10501-DQB10201	57	GGASHMQGFLAFPVD	MQGFLAFPV	5	0.4509	380.3	9.50	0.44	tr_D0A9V0_	WB
HLA-DQA10501-DQB10201	58	SRNARVLFGADHLFQ	RVLFGADHL	4	0.4474	395.0	10.00	0.44	tr_D0A9V0_	
HLA-DQA10501-DQB10201	59	RVLFGADHLFQLSYA	RVLFGADHL	0	0.4445	407.8	10.00	0.24	tr_D0A9V0_	
HLA-DQA10501-DQB10201	60	FLAFPVDCVLLPCST	FLAFPVDCV	0	0.4439	410.4	11.00	0.54	tr_D0A9V0_	
HLA-DQA10501-DQB10201	61	IFSFGISLLEMLLGR	SFGISLLEM	2	0.4424	417.0	11.00	0.47	tr_D0A9V0_	
HLA-DQA10501-DQB10201	62	GVMLRGTALYMAPEV	TALYMAPEV	6	0.4424	417.1	11.00	0.56	tr_D0A9V0_	
HLA-DQA10501-DQB10201	63	FSRNARVLFGADHLF	RVLFGADHL	5	0.4413	422.0	11.00	0.55	tr_D0A9V0_	
HLA-DQA10501-DQB10201	64	RGTALYMAPEVAAGG	ALYMAPEVA	3	0.4384	435.3	11.00	0.50	tr_D0A9V0_	
HLA-DQA10501-DQB10201	65	KYHAALIEVATEHSG	YHAALIEVA	1	0.4377	438.7	11.00	0.37	tr_D0A9V0_	
HLA-DQA10501-DQB10201	66	DGSGCAEGANAADVV	EGANAADVV	6	0.4363	445.6	11.00	0.61	tr_D0A9V0_	
HLA-DQA10501-DQB10201	67	FVQSLALGHLEPVIP	LALGHLEPV	4	0.4351	451.2	12.00	0.25	tr_D0A9V0_	
HLA-DQA10501-DQB10201	68	VTMLVDTNEFLVGTC	TMLVDTNEF	1	0.4339	457.1	12.00	0.37	tr_D0A9V0_	
HLA-DQA10501-DQB10201	69	YVARRFTVDILQGLN	FTVDILQGL	5	0.4337	458.2	12.00	0.31	tr_D0A9V0_	
HLA-DQA10501-DQB10201	70	GCVEALDPDRALISF	VEALDPDRA	2	0.4322	465.6	12.00	0.61	tr_D0A9V0_	
HLA-DQA10501-DQB10201	71	QGRYGEAMAHYLPMV	YGEAMAHYL	3	0.4319	467.1	12.00	0.34	tr_D0A9V0_	
HLA-DQA10501-DQB10201	72	GTALYMAPEVAAGGR	ALYMAPEVA	2	0.4228	515.7	13.00	0.54	tr_D0A9V0_	
HLA-DQA10501-DQB10201	73	DRALISFGAHAALPM	FGAHAALPM	6	0.4225	517.2	13.00	0.62	tr_D0A9V0_	
HLA-DQA10501-DQB10201	74	VARRFTVDILQGLNY	FTVDILQGL	4	0.4219	520.6	13.00	0.31	tr_D0A9V0_	
HLA-DQA10501-DQB10201	75	LFVQSLALGHLEPVI	LALGHLEPV	5	0.4211	525.2	13.00	0.23	tr_D0A9V0_	
HLA-DQA10501-DQB10201	76	ATEHSGCVEALDPDR	EHSGCVEAL	2	0.4201	531.1	13.00	0.32	tr_D0A9V0_	
HLA-DQA10501-DQB10201	77	FSFGISLLEMLLGRP	SFGISLLEM	1	0.4187	538.7	13.00	0.46	tr_D0A9V0_	
HLA-DQA10501-DQB10201	78	ALISFGAHAALPMHC	FGAHAALPM	4	0.4187	539.1	13.00	0.51	tr_D0A9V0_	
HLA-DQA10501-DQB10201	79	RQLAVLLKFIPEAIL	LLKFIPEAI	5	0.4185	540.2	13.00	0.64	tr_D0A9V0_	
HLA-DQA10501-DQB10201	80	PVIPASVDHEAAAFV	VDHEAAAFV	6	0.4173	547.1	14.00	0.38	tr_D0A9V0_	
HLA-DQA10501-DQB10201	81	QLAVLLKFIPEAILA	LLKFIPEAI	4	0.4141	566.3	14.00	0.59	tr_D0A9V0_	
HLA-DQA10501-DQB10201	82	HCTVIAIQVYHPDVW	IQVYHPDVW	6	0.4141	566.6	14.00	0.47	tr_D0A9V0_	
HLA-DQA10501-DQB10201	83	RRQLAVLLKFIPEAI	LLKFIPEAI	6	0.4134	570.4	14.00	0.69	tr_D0A9V0_	
HLA-DQA10501-DQB10201	84	SLKKISEGAFSTIFL	EGAFSTIFL	6	0.4092	597.1	15.00	0.50	tr_D0A9V0_	
HLA-DQA10501-DQB10201	85	ARRFTVDILQGLNYL	FTVDILQGL	3	0.4087	600.2	15.00	0.32	tr_D0A9V0_	
HLA-DQA10501-DQB10201	86	VQSLALGHLEPVIPA	LALGHLEPV	3	0.4083	602.9	15.00	0.28	tr_D0A9V0_	
HLA-DQA10501-DQB10201	87	VATEHSGCVEALDPD	EHSGCVEAL	3	0.4065	614.8	15.00	0.38	tr_D0A9V0_	
HLA-DQA10501-DQB10201	88	GRYGEAMAHYLPMVK	YGEAMAHYL	2	0.4056	620.9	15.00	0.31	tr_D0A9V0_	
HLA-DQA10501-DQB10201	89	RDVVAESLEKEMEVA	VVAESLEKE	2	0.4051	624.4	15.00	0.44	tr_D0A9V0_	
HLA-DQA10501-DQB10201	90	YHAALIEVATEHSGC	YHAALIEVA	0	0.4050	624.8	15.00	0.26	tr_D0A9V0_	
HLA-DQA10501-DQB10201	91	VLLKFIPEAILARVE	LLKFIPEAI	1	0.4045	628.4	15.00	0.38	tr_D0A9V0_	
HLA-DQA10501-DQB10201	92	SITPSHLSIIMEYVP	HLSIIMEYV	5	0.4031	637.7	15.00	0.41	tr_D0A9V0_	
HLA-DQA10501-DQB10201	93	SPYVARRFTVDILQG	ARRFTVDIL	4	0.4025	641.9	16.00	0.41	tr_D0A9V0_	
HLA-DQA10501-DQB10201	94	LLFVQSLALGHLEPV	LALGHLEPV	6	0.4000	659.7	16.00	0.27	tr_D0A9V0_	
HLA-DQA10501-DQB10201	95	VSPYVARRFTVDILQ	ARRFTVDIL	5	0.3993	664.5	16.00	0.58	tr_D0A9V0_	
HLA-DQA10501-DQB10201	96	CVEALDPDRALISFG	VEALDPDRA	1	0.3960	689.1	17.00	0.63	tr_D0A9V0_	
HLA-DQA10501-DQB10201	97	CVSPYVARRFTVDIL	ARRFTVDIL	6	0.3957	690.9	17.00	0.79	tr_D0A9V0_	
HLA-DQA10501-DQB10201	98	SASNLEIPDNADIFL	EIPDNADIF	5	0.3955	692.9	17.00	0.44	tr_D0A9V0_	

HLA-DQA10501-DQB10201	99	RALISFGAHAALPMH	FGAHAALPM	5	0.3940	703.7	17.00	0.61	tr_D0A9V0_
HLA-DQA10501-DQB10201	100	RYGEAMAHYLPMVKM	YGEAMAHYL	1	0.3938	705.4	17.00	0.28	tr_D0A9V0_
HLA-DQA10501-DQB10201	101	TALYMAPEVAAGGRC	ALYMAPEVA	1	0.3933	709.1	17.00	0.57	tr_D0A9V0_
HLA-DQA10501-DQB10201	102	VIPASVDHEAAAFVR	VDHEAAAFV	5	0.3916	722.2	17.00	0.34	tr_D0A9V0_
HLA-DQA10501-DQB10201	103	LAVLLKFIPEAILAR	LLKFIPEAI	3	0.3906	730.8	17.00	0.57	tr_D0A9V0_
HLA-DQA10501-DQB10201	104	LISFGAHAALPMHCR	FGAHAALPM	3	0.3889	743.7	18.00	0.51	tr_D0A9V0_
HLA-DQA10501-DQB10201	105	RQGRYGEAMAHYLPM	YGEAMAHYL	4	0.3874	756.3	18.00	0.40	tr_D0A9V0_
HLA-DQA10501-DQB10201	106	AFSRNARVLFGADHL	RVLFGADHL	6	0.3853	773.6	18.00	0.65	tr_D0A9V0_
HLA-DQA10501-DQB10201	107	TMLVDTNEFLVGTCG	MLVDTNEFL	1	0.3847	778.3	18.00	0.48	tr_D0A9V0_
HLA-DQA10501-DQB10201	108	CTVIAIQVYHPDVWK	IQVYHPDVW	5	0.3839	785.7	18.00	0.44	tr_D0A9V0_
HLA-DQA10501-DQB10201	109	TPSHLSIIMEYVPGG	HLSIIMEYV	3	0.3838	785.9	18.00	0.31	tr_D0A9V0_
HLA-DQA10501-DQB10201	110	ITPSHLSIIMEYVPG	HLSIIMEYV	4	0.3833	790.5	18.00	0.37	tr_D0A9V0_
HLA-DQA10501-DQB10201	111	AVLLKFIPEAILARV	LLKFIPEAI	2	0.3832	791.2	18.00	0.50	tr_D0A9V0_
HLA-DQA10501-DQB10201	112	FLGVAEDGAQVAIKC	FLGVAEDGA	0	0.3831	792.5	18.00	0.61	tr_D0A9V0_
HLA-DQA10501-DQB10201	113	QSLALGHLEPVIPAS	LALGHLEPV	2	0.3821	800.6	19.00	0.29	tr_D0A9V0_
HLA-DQA10501-DQB10201	114	EVATEHSGCVEALDP	EHSGCVEAL	4	0.3804	815.3	19.00	0.41	tr_D0A9V0_
HLA-DQA10501-DQB10201	115	VLFGADHLFQLSYAV	LFGADHLFQ	1	0.3794	824.8	19.00	0.32	tr_D0A9V0_
HLA-DQA10501-DQB10201	116	ISEGAFSTIFLGVAE	EGAFSTIFL	2	0.3782	835.5	19.00	0.22	tr_D0A9V0_
HLA-DQA10501-DQB10201	117	EPVIPASVDHEAAAF	VIPASVDHE	2	0.3779	838.0	19.00	0.25	tr_D0A9V0_
HLA-DQA10501-DQB10201	118	CSITPSHLSIIMEYV	HLSIIMEYV	6	0.3775	841.8	19.00	0.47	tr_D0A9V0_
HLA-DQA10501-DQB10201	119	SGGASHMQGFLAFPV	MQGFLAFPV	6	0.3744	870.0	20.00	0.61	tr_D0A9V0_
HLA-DQA10501-DQB10201	120	MRQGRYGEAMAHYLP	YGEAMAHYL	5	0.3740	874.4	20.00	0.46	tr_D0A9V0_
HLA-DQA10501-DQB10201	121	ASNLEIPDNADIFLG	EIPDNADIF	4	0.3739	875.2	20.00	0.43	tr_D0A9V0_
HLA-DQA10501-DQB10201	122	VWKSSTKYHAALIEV	KYHAALIEV	6	0.3733	880.5	20.00	0.66	tr_D0A9V0_
HLA-DQA10501-DQB10201	123	EASVMARTLADGVML	MARTLADGV	4	0.3729	884.3	20.00	0.49	tr_D0A9V0_
HLA-DQA10501-DQB10201	124	RRFTVDILQGLNYLH	FTVDILQGL	2	0.3722	891.1	20.00	0.41	tr_D0A9V0_
HLA-DQA10501-DQB10201	125	KISEGAFSTIFLGVA	EGAFSTIFL	3	0.3713	900.1	20.00	0.30	tr_D0A9V0_
HLA-DQA10501-DQB10201	126	TVIAIQVYHPDVWKS	IQVYHPDVW	4	0.3687	925.4	21.00	0.46	tr_D0A9V0_
HLA-DQA10501-DQB10201	127	LLKFIPEAILARVES	PEAILARVE	5	0.3670	943.1	21.00	0.34	tr_D0A9V0_
HLA-DQA10501-DQB10201	128	DVVAESLEKEMEVAS	VVAESLEKE	1	0.3668	945.0	21.00	0.47	tr_D0A9V0_
HLA-DQA10501-DQB10201	129	PSHLSIIMEYVPGGS	HLSIIMEYV	2	0.3666	947.0	21.00	0.31	tr_D0A9V0_
HLA-DQA10501-DQB10201	130	YGEAMAHYLPMVKMD	AMAHYLPMV	3	0.3651	962.9	22.00	0.31	tr_D0A9V0_
HLA-DQA10501-DQB10201	131	SLALGHLEPVIPASV	LALGHLEPV	1	0.3639	974.5	22.00	0.26	tr_D0A9V0_
HLA-DQA10501-DQB10201	132	LKKISEGAFSTIFLG	EGAFSTIFL	5	0.3637	976.8	22.00	0.45	tr_D0A9V0_
HLA-DQA10501-DQB10201	133	KKISEGAFSTIFLGV	EGAFSTIFL	4	0.3627	987.5	22.00	0.40	tr_D0A9V0_
HLA-DQA10501-DQB10201	134	IPASVDHEAAAFVRA	VDHEAAAFV	4	0.3624	990.5	22.00	0.38	tr_D0A9V0_
HLA-DQA10501-DQB10201	135	ISFGAHAALPMHCRR	FGAHAALPM	2	0.3616	999.9	22.00	0.55	tr_D0A9V0_
HLA-DQA10501-DQB10201	136	TAFVRIMGASISVTW	IMGASISVT	5	0.3611	1005.3	22.00	0.23	tr_D0A9V0_
HLA-DQA10501-DQB10201	137	DLLFVQSLALGHLEP	SLALGHLEP	6	0.3600	1016.8	22.00	0.28	tr_D0A9V0_
HLA-DQA10501-DQB10201	138	AADVVPLFLTDSNRN	AADVVPLFL	0	0.3559	1063.7	23.00	0.70	tr_D0A9V0_
HLA-DQA10501-DQB10201	139	AMRQGRYGEAMAHYL	YGEAMAHYL	6	0.3551	1072.4	23.00	0.47	tr_D0A9V0_
HLA-DQA10501-DQB10201	140	CCQFAFDLFAKLPLD	DLFAKLPLD	6	0.3510	1120.8	24.00	0.29	tr_D0A9V0_
HLA-DQA10501-DQB10201	141	SEASVMARTLADGVM	MARTLADGV	5	0.3499	1134.1	24.00	0.57	tr_D0A9V0_
HLA-DQA10501-DQB10201	142	SHLSIIMEYVPGGSL	HLSIIMEYV	1	0.3496	1138.5	24.00	0.29	tr_D0A9V0_
HLA-DQA10501-DQB10201	143	SFGISLLEMLLGRPP	SFGISLLEM	0	0.3485	1151.8	25.00	0.37	tr_D0A9V0_
HLA-DQA10501-DQB10201	144	VIAIQVYHPDVWKSS	IQVYHPDVW	3	0.3470	1171.0	25.00	0.47	tr_D0A9V0_
HLA-DQA10501-DQB10201	145	HLEPVIPASVDHEAA	VIPASVDHE	4	0.3466	1176.3	25.00	0.38	tr_D0A9V0_
HLA-DQA10501-DQB10201	146	LRSLFNRDLLFVQSL	RDLLFVQSL	6	0.3465	1176.7	25.00	0.40	tr_D0A9V0_
HLA-DQA10501-DQB10201	147	SNLEIPDNADIFLGR	EIPDNADIF	3	0.3456	1188.2	25.00	0.44	tr_D0A9V0_
HLA-DQA10501-DQB10201	148	ASVMARTLADGVMLR	MARTLADGV	3	0.3448	1199.5	25.00	0.50	tr_D0A9V0_

HLA-DQA10501-DQB10201	149	CQFAFDLFAKLPLDH	DLFAKLPLD	5	0.3445	1203.4	25.00	0.26	tr_D0A9V0_
HLA-DQA10501-DQB10201	150	HAALIEVATEHSGCV	LIEVATEHS	3	0.3434	1217.5	26.00	0.39	tr_D0A9V0_
HLA-DQA10501-DQB10201	151	DGVMLRGTALYMAPE	MLRGTALYM	3	0.3424	1229.8	26.00	0.31	tr_D0A9V0_
HLA-DQA10501-DQB10201	152	RFTVDILQGLNYLHN	FTVDILQGL	1	0.3424	1231.0	26.00	0.43	tr_D0A9V0_
HLA-DQA10501-DQB10201	153	AFVRIMGASISVTWN	IMGASISVT	4	0.3423	1232.1	26.00	0.25	tr_D0A9V0_
HLA-DQA10501-DQB10201	154	LSDFGSTISEASVMA	DFGSTISEA	2	0.3417	1239.9	26.00	0.28	tr_D0A9V0_
HLA-DQA10501-DQB10201	155	SISKQFRLGFAAMRQ	RLGFAAMRQ	6	0.3416	1241.7	26.00	0.72	tr_D0A9V0_
HLA-DQA10501-DQB10201	156	HSVIKNFGCVSPYVA	FGCVSPYVA	6	0.3391	1275.1	26.00	0.42	tr_D0A9V0_
HLA-DQA10501-DQB10201	157	PASVDHEAAAFVRAC	VDHEAAAFV	3	0.3383	1286.2	27.00	0.41	tr_D0A9V0_
HLA-DQA10501-DQB10201	158	KSASNLEIPDNADIF	EIPDNADIF	6	0.3368	1307.2	27.00	0.54	tr_D0A9V0_
HLA-DQA10501-DQB10201	159	ADGVMLRGTALYMAP	MLRGTALYM	4	0.3358	1321.4	27.00	0.34	tr_D0A9V0_
HLA-DQA10501-DQB10201	160	NDTVPYVRVLDNHYC	YVRVLDNHY	5	0.3352	1330.7	27.00	0.37	tr_D0A9V0_
HLA-DQA10501-DQB10201	161	RSLFNRDLLFVQSLA	RDLLFVQSL	5	0.3350	1332.9	27.00	0.35	tr_D0A9V0_
HLA-DQA10501-DQB10201	162	RNDTVPYVRVLDNHY	YVRVLDNHY	6	0.3346	1339.3	27.00	0.35	tr_D0A9V0_
HLA-DQA10501-DQB10201	163	LEPVIPASVDHEAAA	VIPASVDHE	3	0.3337	1351.2	28.00	0.39	tr_D0A9V0_
HLA-DQA10501-DQB10201	164	LALGHLEPVIPASVD	LEPVIPASV	5	0.3323	1372.9	28.00	0.23	tr_D0A9V0_
HLA-DQA10501-DQB10201	165	GHLEPVIPASVDHEA	VIPASVDHE	5	0.3321	1376.1	28.00	0.44	tr_D0A9V0_
HLA-DQA10501-DQB10201	166	ATRLRSLFNRDLLFV	SLFNRDLLF	5	0.3300	1406.9	28.00	0.37	tr_D0A9V0_
HLA-DQA10501-DQB10201	167	GEAMAHYLPMVKMDM	AMAHYLPMV	2	0.3298	1409.9	28.00	0.31	tr_D0A9V0_
HLA-DQA10501-DQB10201	168	ASVDHEAAAFVRACC	VDHEAAAFV	2	0.3295	1415.3	28.00	0.43	tr_D0A9V0_
HLA-DQA10501-DQB10201	169	DTVPYVRVLDNHYCV	YVRVLDNHY	4	0.3287	1427.6	29.00	0.35	tr_D0A9V0_
HLA-DQA10501-DQB10201	170	QFAFDLFAKLPLDHR	DLFAKLPLD	4	0.3274	1447.6	29.00	0.25	tr_D0A9V0_
HLA-DQA10501-DQB10201	171	RHCTVIAIQVYHPDV	AIQVYHPDV	6	0.3273	1449.2	29.00	0.29	tr_D0A9V0_
HLA-DQA10501-DQB10201	172	KLSDFGSTISEASVM	DFGSTISEA	3	0.3265	1461.8	29.00	0.34	tr_D0A9V0_
HLA-DQA10501-DQB10201	173	SVDHEAAAFVRACCH	VDHEAAAFV	1	0.3263	1463.9	29.00	0.32	tr_D0A9V0_
HLA-DQA10501-DQB10201	174	SLFNRDLLFVQSLAL	RDLLFVQSL	4	0.3262	1466.4	29.00	0.32	tr_D0A9V0_
HLA-DQA10501-DQB10201	175	SDFGSTISEASVMAR	DFGSTISEA	1	0.3246	1491.5	29.00	0.27	tr_D0A9V0_
HLA-DQA10501-DQB10201	176	STAFVRIMGASISVT	FVRIMGASI	3	0.3243	1496.3	30.00	0.30	tr_D0A9V0_
HLA-DQA10501-DQB10201	177	ISEASVMARTLADGV	MARTLADGV	6	0.3243	1496.5	30.00	0.63	tr_D0A9V0_
HLA-DQA10501-DQB10201	178	NLEIPDNADIFLGRS	EIPDNADIF	2	0.3239	1502.4	30.00	0.46	tr_D0A9V0_
HLA-DQA10501-DQB10201	179	FVRIMGASISVTWNA	IMGASISVT	3	0.3237	1506.0	30.00	0.28	tr_D0A9V0_
HLA-DQA10501-DQB10201	180	AALIEVATEHSGCVE	LIEVATEHS	2	0.3230	1518.5	30.00	0.41	tr_D0A9V0_
HLA-DQA10501-DQB10201	181	LDHRDSVTMLVDTNE	VTMLVDTNE	6	0.3217	1539.8	30.00	0.44	tr_D0A9V0_
HLA-DQA10501-DQB10201	182	SFGAHAALPMHCRRC	FGAHAALPM	1	0.3215	1542.1	30.00	0.58	tr_D0A9V0_
HLA-DQA10501-DQB10201	183	SVIKNFGCVSPYVAR	FGCVSPYVA	5	0.3209	1552.8	30.00	0.44	tr_D0A9V0_
HLA-DQA10501-DQB10201	184	FAFDLFAKLPLDHRD	DLFAKLPLD	3	0.3199	1568.8	30.00	0.28	tr_D0A9V0_
HLA-DQA10501-DQB10201	185	TPQSDIFSFGISLLE	FSFGISLLE	6	0.3191	1582.5	31.00	0.49	tr_D0A9V0_
HLA-DQA10501-DQB10201	186	SVMARTLADGVMLRG	MARTLADGV	2	0.3190	1584.1	31.00	0.50	tr_D0A9V0_
HLA-DQA10501-DQB10201	187	SSTAFVRIMGASISV	FVRIMGASI	4	0.3167	1624.1	31.00	0.37	tr_D0A9V0_
HLA-DQA10501-DQB10201	188	RCCQFAFDLFAKLPL	FDLFAKLPL	6	0.3162	1633.8	31.00	0.25	tr_D0A9V0_
HLA-DQA10501-DQB10201	189	SISVTWNAGDQQRDV	VTWNAGDQQ	3	0.3140	1673.4	32.00	0.54	tr_D0A9V0_
HLA-DQA10501-DQB10201	190	VEALDPDRALISFGA	VEALDPDRA	0	0.3122	1706.4	32.00	0.57	tr_D0A9V0_
HLA-DQA10501-DQB10201	191	ISVTWNAGDQQRDVA	VTWNAGDQQ	2	0.3119	1710.5	32.00	0.51	tr_D0A9V0_
HLA-DQA10501-DQB10201	192	ASISVTWNAGDQQRD	VTWNAGDQQ	4	0.3117	1715.1	32.00	0.56	tr_D0A9V0_
HLA-DQA10501-DQB10201	193	EAMAHYLPMVKMDMQ	AMAHYLPMV	1	0.3116	1717.8	32.00	0.31	tr_D0A9V0_
HLA-DQA10501-DQB10201	194	ISKQFRLGFAAMRQG	RLGFAAMRQ	5	0.3112	1724.5	33.00	0.70	tr_D0A9V0_
HLA-DQA10501-DQB10201	195	IEVATEHSGCVEALD	EHSGCVEAL	5	0.3105	1738.4	33.00	0.47	tr_D0A9V0_
HLA-DQA10501-DQB10201	196	SCSITPSHLSIIMEY	SHLSIIMEY	6	0.3101	1744.9	33.00	0.57	tr_D0A9V0_
HLA-DQA10501-DQB10201	197	MRPSASSLLSYAFLL	SLLSYAFLL	6	0.3100	1747.7	33.00	0.27	tr_D0A9V0_
HLA-DQA10501-DQB10201	198	MLVDTNEFLVGTCGA	MLVDTNEFL	0	0.3098	1751.6	33.00	0.46	tr_D0A9V0_

HLA-DQA10501-DQB10201	199	TRLRSLFNRDLLFVQ	SLFNRDLLF	4	0.3087	1771.9	33.00	0.34	tr_D0A9V0_
HLA-DQA10501-DQB10201	200	DATRLRSLFNRDLLF	SLFNRDLLF	6	0.3085	1774.9	33.00	0.44	tr_D0A9V0_
HLA-DQA10501-DQB10201	201	IAIQVYHPDVWKSST	IQVYHPDVW	2	0.3085	1776.0	33.00	0.57	tr_D0A9V0_
HLA-DQA10501-DQB10201	202	LKFIPEAILARVESK	PEAILARVE	4	0.3068	1808.1	33.00	0.41	tr_D0A9V0_
HLA-DQA10501-DQB10201	203	KQFRLGFAAMRQGRY	RLGFAAMRQ	3	0.3052	1839.3	34.00	0.58	tr_D0A9V0_
HLA-DQA10501-DQB10201	204	TVPYVRVLDNHYCVN	YVRVLDNHY	3	0.3052	1839.7	34.00	0.41	tr_D0A9V0_
HLA-DQA10501-DQB10201	205	LGHLEPVIPASVDHE	VIPASVDHE	6	0.3051	1841.9	34.00	0.46	tr_D0A9V0_
HLA-DQA10501-DQB10201	206	RDLLFVQSLALGHLE	VQSLALGHL	5	0.3035	1875.2	34.00	0.28	tr_D0A9V0_
HLA-DQA10501-DQB10201	207	RSLKKISEGAFSTIF	SEGAFSTIF	6	0.3032	1879.4	34.00	0.71	tr_D0A9V0_
HLA-DQA10501-DQB10201	208	CKLSDFGSTISEASV	DFGSTISEA	4	0.3029	1885.6	34.00	0.44	tr_D0A9V0_
HLA-DQA10501-DQB10201	209	LFGADHLFQLSYAVR	LFGADHLFQ	0	0.3018	1908.2	35.00	0.27	tr_D0A9V0_
HLA-DQA10501-DQB10201	210	LIEVATEHSGCVEAL	EHSGCVEAL	6	0.3011	1922.6	35.00	0.43	tr_D0A9V0_
HLA-DQA10501-DQB10201	211	DFGSTISEASVMART	TISEASVMA	4	0.3005	1936.1	35.00	0.21	tr_D0A9V0_
HLA-DQA10501-DQB10201	212	NRDLLFVQSLALGHL	VQSLALGHL	6	0.2996	1954.9	35.00	0.32	tr_D0A9V0_
HLA-DQA10501-DQB10201	213	IKNFGCVSPYVARRF	FGCVSPYVA	3	0.2986	1976.5	35.00	0.41	tr_D0A9V0_
HLA-DQA10501-DQB10201	214	LADGVMLRGTALYMA	MLRGTALYM	5	0.2976	1997.5	36.00	0.42	tr_D0A9V0_
HLA-DQA10501-DQB10201	215	LEIPDNADIFLGRSR	EIPDNADIF	1	0.2974	2001.1	36.00	0.47	tr_D0A9V0_
HLA-DQA10501-DQB10201	216	VIKNFGCVSPYVARR	FGCVSPYVA	4	0.2972	2005.4	36.00	0.45	tr_D0A9V0_
HLA-DQA10501-DQB10201	217	FTVDILQGLNYLHNL	FTVDILQGL	0	0.2963	2026.2	36.00	0.34	tr_D0A9V0_
HLA-DQA10501-DQB10201	218	RLRSLFNRDLLFVQS	SLFNRDLLF	3	0.2961	2029.9	36.00	0.31	tr_D0A9V0_
HLA-DQA10501-DQB10201	219	RRCCQFAFDLFAKLP	FAFDLFAKL	5	0.2961	2030.5	36.00	0.26	tr_D0A9V0_
HLA-DQA10501-DQB10201	220	QFRLGFAAMRQGRYG	RLGFAAMRQ	2	0.2951	2053.6	36.00	0.62	tr_D0A9V0_
HLA-DQA10501-DQB10201	221	VMARTLADGVMLRGT	MARTLADGV	1	0.2944	2067.8	37.00	0.48	tr_D0A9V0_
HLA-DQA10501-DQB10201	222	HLSIIMEYVPGGSLH	HLSIIMEYV	0	0.2939	2078.8	37.00	0.29	tr_D0A9V0_
HLA-DQA10501-DQB10201	223	SVTWNAGDQQRDVAL	VTWNAGDQQ	1	0.2936	2086.7	37.00	0.49	tr_D0A9V0_
HLA-DQA10501-DQB10201	224	FGADHLFQLSYAVRD	FQLSYAVRD	6	0.2935	2087.8	37.00	0.38	tr_D0A9V0_
HLA-DQA10501-DQB10201	225	VVAESLEKEMEVASK	VVAESLEKE	0	0.2919	2125.8	37.00	0.41	tr_D0A9V0_
HLA-DQA10501-DQB10201	226	VCKLSDFGSTISEAS	DFGSTISEA	5	0.2901	2167.1	38.00	0.47	tr_D0A9V0_
HLA-DQA10501-DQB10201	227	MGASISVTWNAGDQQ	VTWNAGDQQ	6	0.2894	2182.5	38.00	0.60	tr_D0A9V0_
HLA-DQA10501-DQB10201	228	FGSTISEASVMARTL	EASVMARTL	6	0.2888	2196.9	38.00	0.25	tr_D0A9V0_
HLA-DQA10501-DQB10201	229	SKQFRLGFAAMRQGR	RLGFAAMRQ	4	0.2881	2214.6	38.00	0.69	tr_D0A9V0_
HLA-DQA10501-DQB10201	230	VDHEAAAFVRACCHM	VDHEAAAFV	0	0.2869	2241.9	38.00	0.20	tr_D0A9V0_
HLA-DQA10501-DQB10201	231	SMRPSASSLLSYAFL	SSLLSYAFL	6	0.2869	2241.9	38.00	0.38	tr_D0A9V0_
HLA-DQA10501-DQB10201	232	RRHCTVIAIQVYHPD	VIAIQVYHP	5	0.2869	2244.0	38.00	0.31	tr_D0A9V0_
HLA-DQA10501-DQB10201	233	TLADGVMLRGTALYM	MLRGTALYM	6	0.2864	2254.1	38.00	0.55	tr_D0A9V0_
HLA-DQA10501-DQB10201	234	FGISLLEMLLGRPPW	FGISLLEML	0	0.2854	2280.0	39.00	0.25	tr_D0A9V0_
HLA-DQA10501-DQB10201	235	LHSVIKNFGCVSPYV	NFGCVSPYV	6	0.2851	2286.6	39.00	0.44	tr_D0A9V0_
HLA-DQA10501-DQB10201	236	RRNDTVPYVRVLDNH	VPYVRVLDN	5	0.2851	2288.1	39.00	0.41	tr_D0A9V0_
HLA-DQA10501-DQB10201	237	KFIPEAILARVESKN	PEAILARVE	3	0.2846	2299.8	39.00	0.41	tr_D0A9V0_
HLA-DQA10501-DQB10201	238	LFNRDLLFVQSLALG	RDLLFVQSL	3	0.2843	2306.0	39.00	0.37	tr_D0A9V0_
HLA-DQA10501-DQB10201	239	AMAHYLPMVKMDMQA	AMAHYLPMV	0	0.2842	2308.7	39.00	0.23	tr_D0A9V0_
HLA-DQA10501-DQB10201	240	GESSTAFVRIMGASI	FVRIMGASI	6	0.2828	2344.8	39.00	0.56	tr_D0A9V0_
HLA-DQA10501-DQB10201	241	FNRDLLFVQSLALGH	RDLLFVQSL	2	0.2821	2361.9	40.00	0.31	tr_D0A9V0_
HLA-DQA10501-DQB10201	242	ESSTAFVRIMGASIS	FVRIMGASI	5	0.2808	2395.0	40.00	0.52	tr_D0A9V0_
HLA-DQA10501-DQB10201	243	ALYMAPEVAAGGRCT	ALYMAPEVA	0	0.2803	2408.6	40.00	0.69	tr_D0A9V0_
HLA-DQA10501-DQB10201	244	AAMRQGRYGEAMAHY	RYGEAMAHY	6	0.2787	2450.8	40.00	0.47	tr_D0A9V0_
HLA-DQA10501-DQB10201	245	VDTNEFLVGTCGAFS	LVGTCGAFS	6	0.2781	2466.0	41.00	0.33	tr_D0A9V0_
HLA-DQA10501-DQB10201	246	LRRQLAVLLKFIPEA	VLLKFIPEA	6	0.2779	2472.9	41.00	0.50	tr_D0A9V0_
HLA-DQA10501-DQB10201	247	ALGHLEPVIPASVDH	LEPVIPASV	4	0.2777	2478.2	41.00	0.31	tr_D0A9V0_
HLA-DQA10501-DQB10201	248	MHCRRCCQFAFDLFA	CQFAFDLFA	6	0.2772	2490.6	41.00	0.34	tr_D0A9V0_

HLA-DQA10501-DQB10201	249	ADHLFQLSYAVRDGP	FQLSYAVRD	4	0.2772	2491.0	41.00	0.38	tr_D0A9V0_
HLA-DQA10501-DQB10201	250	AFDLFAKLPLDHRDS	DLFAKLPLD	2	0.2770	2495.7	41.00	0.31	tr_D0A9V0_
HLA-DQA10501-DQB10201	251	KNFGCVSPYVARRFT	FGCVSPYVA	2	0.2757	2531.9	41.00	0.46	tr_D0A9V0_
HLA-DQA10501-DQB10201	252	GASISVTWNAGDQQR	VTWNAGDQQ	5	0.2756	2533.6	41.00	0.66	tr_D0A9V0_
HLA-DQA10501-DQB10201	253	VPYVRVLDNHYCVNS	YVRVLDNHY	2	0.2756	2535.6	41.00	0.44	tr_D0A9V0_
HLA-DQA10501-DQB10201	254	DHLFQLSYAVRDGPC	FQLSYAVRD	3	0.2737	2587.3	42.00	0.40	tr_D0A9V0_
HLA-DQA10501-DQB10201	255	CRRCCQFAFDLFAKL	FAFDLFAKL	6	0.2731	2604.4	42.00	0.29	tr_D0A9V0_
HLA-DQA10501-DQB10201	256	FIPEAILARVESKNE	PEAILARVE	2	0.2720	2636.8	42.00	0.44	tr_D0A9V0_
HLA-DQA10501-DQB10201	257	HPNIVRYVSCSITPS	IVRYVSCSI	3	0.2719	2639.4	42.00	0.39	tr_D0A9V0_
HLA-DQA10501-DQB10201	258	GLNYLHNLGIVHCDV	NLGIVHCDV	6	0.2714	2653.8	42.00	0.53	tr_D0A9V0_
HLA-DQA10501-DQB10201	259	LAFPVDCVLLPCSTL	LAFPVDCVL	0	0.2713	2654.7	42.00	0.28	tr_D0A9V0_
HLA-DQA10501-DQB10201	260	KLPLDHRDSVTMLVD	RDSVTMLVD	6	0.2706	2674.6	43.00	0.44	tr_D0A9V0_
HLA-DQA10501-DQB10201	261	FRLGFAAMRQGRYGE	RLGFAAMRQ	1	0.2705	2679.6	43.00	0.60	tr_D0A9V0_
HLA-DQA10501-DQB10201	262	VRIMGASISVTWNAG	IMGASISVT	2	0.2697	2703.0	43.00	0.32	tr_D0A9V0_
HLA-DQA10501-DQB10201	263	HHPNIVRYVSCSITP	IVRYVSCSI	4	0.2687	2731.8	43.00	0.44	tr_D0A9V0_
HLA-DQA10501-DQB10201	264	ALIEVATEHSGCVEA	LIEVATEHS	1	0.2681	2750.2	43.00	0.51	tr_D0A9V0_
HLA-DQA10501-DQB10201	265	PDRALISFGAHAALP	ISFGAHAAL	5	0.2675	2766.0	43.00	0.34	tr_D0A9V0_
HLA-DQA10501-DQB10201	266	CRRNDTVPYVRVLDN	VPYVRVLDN	6	0.2675	2767.8	43.00	0.50	tr_D0A9V0_
HLA-DQA10501-DQB10201	267	NRRHCTVIAIQVYHP	VIAIQVYHP	6	0.2669	2785.1	44.00	0.28	tr_D0A9V0_
HLA-DQA10501-DQB10201	268	AIQVYHPDVWKSSTK	IQVYHPDVW	1	0.2653	2832.2	44.00	0.63	tr_D0A9V0_
HLA-DQA10501-DQB10201	269	GADHLFQLSYAVRDG	FQLSYAVRD	5	0.2642	2866.8	44.00	0.35	tr_D0A9V0_
HLA-DQA10501-DQB10201	270	DTNEFLVGTCGAFSR	LVGTCGAFS	5	0.2639	2878.0	44.00	0.34	tr_D0A9V0_
HLA-DQA10501-DQB10201	271	ESDIKDRVPETIDSP	IKDRVPETI	3	0.2607	2978.8	45.00	0.56	tr_D0A9V0_
HLA-DQA10501-DQB10201	272	GVCKLSDFGSTISEA	DFGSTISEA	6	0.2606	2982.9	45.00	0.59	tr_D0A9V0_
HLA-DQA10501-DQB10201	273	PLDHRDSVTMLVDTN	RDSVTMLVD	4	0.2595	3016.4	46.00	0.38	tr_D0A9V0_
HLA-DQA10501-DQB10201	274	STISEASVMARTLAD	EASVMARTL	4	0.2594	3020.9	46.00	0.22	tr_D0A9V0_
HLA-DQA10501-DQB10201	275	NFGCVSPYVARRFTV	FGCVSPYVA	1	0.2589	3037.5	46.00	0.46	tr_D0A9V0_
HLA-DQA10501-DQB10201	276	VFNRRHCTVIAIQVY	CTVIAIQVY	6	0.2584	3054.5	46.00	0.23	tr_D0A9V0_
HLA-DQA10501-DQB10201	277	EIPDNADIFLGRSRI	EIPDNADIF	0	0.2576	3078.8	46.00	0.41	tr_D0A9V0_
HLA-DQA10501-DQB10201	278	STLPIVLFELRPGES	LFELRPGES	6	0.2574	3084.9	46.00	0.27	tr_D0A9V0_
HLA-DQA10501-DQB10201	279	TSGGASHMQGFLAFP	HMQGFLAFP	6	0.2573	3090.7	46.00	0.31	tr_D0A9V0_
HLA-DQA10501-DQB10201	280	FGAHAALPMHCRRCC	FGAHAALPM	0	0.2570	3100.0	46.00	0.51	tr_D0A9V0_
HLA-DQA10501-DQB10201	281	PNIVRYVSCSITPSH	IVRYVSCSI	2	0.2565	3115.1	46.00	0.41	tr_D0A9V0_
HLA-DQA10501-DQB10201	282	LVDTNEFLVGTCGAF	EFLVGTCGA	5	0.2560	3132.2	47.00	0.40	tr_D0A9V0_
HLA-DQA10501-DQB10201	283	VTWNAGDQQRDVALN	VTWNAGDQQ	0	0.2559	3135.8	47.00	0.41	tr_D0A9V0_
HLA-DQA10501-DQB10201	284	LPLDHRDSVTMLVDT	RDSVTMLVD	5	0.2554	3154.3	47.00	0.41	tr_D0A9V0_
HLA-DQA10501-DQB10201	285	GISLLEMLLGRPPWQ	MLLGRPPWQ	6	0.2550	3169.2	47.00	0.31	tr_D0A9V0_
HLA-DQA10501-DQB10201	286	TLPIVLFELRPGESS	LFELRPGES	5	0.2537	3214.0	47.00	0.33	tr_D0A9V0_
HLA-DQA10501-DQB10201	287	VSALKSASNLEIPDN	LKSASNLEI	3	0.2532	3230.5	47.00	0.36	tr_D0A9V0_
HLA-DQA10501-DQB10201	288	LVSALKSASNLEIPD	LKSASNLEI	4	0.2528	3242.4	47.00	0.43	tr_D0A9V0_
HLA-DQA10501-DQB10201	289	CTPQSDIFSFGISLL	IFSFGISLL	6	0.2526	3252.3	47.00	0.38	tr_D0A9V0_
HLA-DQA10501-DQB10201	290	NIVRYVSCSITPSHL	IVRYVSCSI	1	0.2511	3303.4	48.00	0.39	tr_D0A9V0_
HLA-DQA10501-DQB10201	291	TNEFLVGTCGAFSRN	LVGTCGAFS	4	0.2511	3304.0	48.00	0.37	tr_D0A9V0_
HLA-DQA10501-DQB10201	292	GSTISEASVMARTLA	EASVMARTL	5	0.2508	3314.1	48.00	0.26	tr_D0A9V0_
HLA-DQA10501-DQB10201	293	MARTLADGVMLRGTA	MARTLADGV	0	0.2505	3326.8	48.00	0.37	tr_D0A9V0_
HLA-DQA10501-DQB10201	294	RIMGASISVTWNAGD	IMGASISVT	1	0.2496	3359.5	48.00	0.31	tr_D0A9V0_
HLA-DQA10501-DQB10201	295	FDLFAKLPLDHRDSV	DLFAKLPLD	1	0.2488	3388.1	49.00	0.31	tr_D0A9V0_
HLA-DQA10501-DQB10201	296	ISLLEMLLGRPPWQW	MLLGRPPWQ	5	0.2487	3389.6	49.00	0.36	tr_D0A9V0_
HLA-DQA10501-DQB10201	297	LFQLSYAVRDGPCRI	FQLSYAVRD	1	0.2487	3391.6	49.00	0.36	tr_D0A9V0_
HLA-DQA10501-DQB10201	298	SALKSASNLEIPDNA	LKSASNLEI	2	0.2469	3458.1	49.00	0.30	tr_D0A9V0_

HLA-DQA10501-DQB10201	299	LDGSGCAEGANAADV	AEGANAADV	6	0.2466	3468.6	49.00	0.69	tr_D0A9V0_
HLA-DQA10501-DQB10201	300	HCRRCCQFAFDLFAK	CQFAFDLFA	5	0.2463	3480.2	49.00	0.31	tr_D0A9V0_
HLA-DQA10501-DQB10201	301	LHHPNIVRYVSCSIT	IVRYVSCSI	5	0.2461	3487.1	49.00	0.55	tr_D0A9V0_
HLA-DQA10501-DQB10201	302	DVWKSSTKYHAALIE	TKYHAALIE	6	0.2457	3502.0	49.00	0.75	tr_D0A9V0_
HLA-DQA10501-DQB10201	303	QDESDIKDRVPETID	IKDRVPETI	5	0.2452	3521.6	49.00	0.62	tr_D0A9V0_
HLA-DQA10501-DQB10201	304	PMHCRRCCQFAFDLF	CCQFAFDLF	6	0.2452	3522.2	49.00	0.47	tr_D0A9V0_
HLA-DQA10501-DQB10201	305	ALKSASNLEIPDNAD	SNLEIPDNA	5	0.2448	3535.8	50.00	0.23	tr_D0A9V0_
HLA-DQA10501-DQB10201	306	SDIKDRVPETIDSPR	IKDRVPETI	2	0.2447	3540.1	50.00	0.57	tr_D0A9V0_
HLA-DQA10501-DQB10201	307	FNRRHCTVIAIQVYH	RHCTVIAIQ	3	0.2431	3604.0	50.00	0.23	tr_D0A9V0_
HLA-DQA10501-DQB10201	308	KPHNVLLGTDGVCKL	NVLLGTDGV	3	0.2427	3617.8	50.00	0.28	tr_D0A9V0_
HLA-DQA10501-DQB10201	309	PYVRVLDNHYCVNSS	YVRVLDNHY	1	0.2427	3620.0	50.00	0.51	tr_D0A9V0_
HLA-DQA10501-DQB10201	310	LPIVLFELRPGESSS	LFELRPGES	4	0.2424	3630.4	50.00	0.35	tr_D0A9V0_
HLA-DQA10501-DQB10201	311	HLFQLSYAVRDGPCR	FQLSYAVRD	2	0.2412	3677.9	55.00	0.44	tr_D0A9V0_
HLA-DQA10501-DQB10201	312	CSTLPIVLFELRPGE	PIVLFELRP	4	0.2399	3729.6	55.00	0.31	tr_D0A9V0_
HLA-DQA10501-DQB10201	313	LSIIMEYVPGGSLHS	IIMEYVPGG	2	0.2385	3788.4	55.00	0.31	tr_D0A9V0_
HLA-DQA10501-DQB10201	314	GAFSRNARVLFGADH	FSRNARVLF	2	0.2382	3798.4	55.00	0.34	tr_D0A9V0_
HLA-DQA10501-DQB10201	315	LLVSALKSASNLEIP	LKSASNLEI	5	0.2378	3815.2	55.00	0.46	tr_D0A9V0_
HLA-DQA10501-DQB10201	316	NYLHNLGIVHCDVKP	NLGIVHCDV	4	0.2377	3817.7	55.00	0.51	tr_D0A9V0_
HLA-DQA10501-DQB10201	317	DVKPHNVLLGTDGVC	NVLLGTDGV	5	0.2372	3839.1	55.00	0.43	tr_D0A9V0_
HLA-DQA10501-DQB10201	318	KLHHPNIVRYVSCSI	IVRYVSCSI	6	0.2369	3851.4	55.00	0.71	tr_D0A9V0_
HLA-DQA10501-DQB10201	319	PSMRPSASSLLSYAF	ASSLLSYAF	6	0.2364	3874.7	55.00	0.35	tr_D0A9V0_
HLA-DQA10501-DQB10201	320	NEFLVGTCGAFSRNA	LVGTCGAFS	3	0.2363	3879.5	55.00	0.38	tr_D0A9V0_
HLA-DQA10501-DQB10201	321	IPEAILARVESKNEA	PEAILARVE	1	0.2358	3900.3	55.00	0.40	tr_D0A9V0_
HLA-DQA10501-DQB10201	322	LPCSTLPIVLFELRP	PIVLFELRP	6	0.2357	3903.9	55.00	0.40	tr_D0A9V0_
HLA-DQA10501-DQB10201	323	DESDIKDRVPETIDS	IKDRVPETI	4	0.2354	3916.4	55.00	0.62	tr_D0A9V0_
HLA-DQA10501-DQB10201	324	NVFNRRHCTVIAIQV	RHCTVIAIQ	5	0.2350	3934.3	55.00	0.31	tr_D0A9V0_
HLA-DQA10501-DQB10201	325	DHEAAAFVRACCHMN	AAFVRACCH	4	0.2343	3963.6	55.00	0.32	tr_D0A9V0_
HLA-DQA10501-DQB10201	326	EQDESDIKDRVPETI	IKDRVPETI	6	0.2340	3973.9	55.00	0.63	tr_D0A9V0_
HLA-DQA10501-DQB10201	327	TISEASVMARTLADG	EASVMARTL	3	0.2333	4007.5	55.00	0.26	tr_D0A9V0_
HLA-DQA10501-DQB10201	328	SLLEMLLGRPPWQWS	MLLGRPPWQ	4	0.2331	4013.0	55.00	0.38	tr_D0A9V0_
HLA-DQA10501-DQB10201	329	LNYLHNLGIVHCDVK	NLGIVHCDV	5	0.2325	4040.0	55.00	0.50	tr_D0A9V0_
HLA-DQA10501-DQB10201	330	HEAAAFVRACCHMNP	AAFVRACCH	3	0.2312	4097.1	55.00	0.40	tr_D0A9V0_
HLA-DQA10501-DQB10201	331	CGAFSRNARVLFGAD	FSRNARVLF	3	0.2311	4101.6	55.00	0.42	tr_D0A9V0_
HLA-DQA10501-DQB10201	332	VAESLEKEMEVASKL	KEMEVASKL	6	0.2309	4109.8	55.00	0.26	tr_D0A9V0_
HLA-DQA10501-DQB10201	333	PHNVLLGTDGVCKLS	NVLLGTDGV	2	0.2281	4236.0	55.00	0.34	tr_D0A9V0_
HLA-DQA10501-DQB10201	334	VDILQGLNYLHNLGI	DILQGLNYL	1	0.2280	4242.9	55.00	0.28	tr_D0A9V0_
HLA-DQA10501-DQB10201	335	LGVAEDGAQVAIKCI	VAEDGAQVA	2	0.2267	4304.7	55.00	0.49	tr_D0A9V0_
HLA-DQA10501-DQB10201	336	PIVLFELRPGESSSE	LFELRPGES	3	0.2265	4313.6	55.00	0.38	tr_D0A9V0_
HLA-DQA10501-DQB10201	337	DPDRALISFGAHAAL	ISFGAHAAL	6	0.2264	4314.4	55.00	0.41	tr_D0A9V0_
HLA-DQA10501-DQB10201	338	DIKDRVPETIDSPRR	IKDRVPETI	1	0.2264	4317.7	55.00	0.50	tr_D0A9V0_
HLA-DQA10501-DQB10201	339	AFPVDCVLLPCSTLP	VDCVLLPCS	3	0.2263	4323.1	55.00	0.25	tr_D0A9V0_
HLA-DQA10501-DQB10201	340	MLLVSALKSASNLEI	LKSASNLEI	6	0.2260	4335.0	55.00	0.50	tr_D0A9V0_
HLA-DQA10501-DQB10201	341	AGDQQRDVALNCGIF	DVALNCGIF	6	0.2250	4383.4	55.00	0.26	tr_D0A9V0_
HLA-DQA10501-DQB10201	342	IKCIPRLRRDVVAES	LRRDVVAES	6	0.2248	4390.9	55.00	0.47	tr_D0A9V0_
HLA-DQA10501-DQB10201	343	VKPHNVLLGTDGVCK	NVLLGTDGV	4	0.2245	4403.7	55.00	0.35	tr_D0A9V0_
HLA-DQA10501-DQB10201	344	FQLSYAVRDGPCRIA	FQLSYAVRD	0	0.2242	4420.3	55.00	0.26	tr_D0A9V0_
HLA-DQA10501-DQB10201	345	TVDILQGLNYLHNLG	DILQGLNYL	2	0.2223	4509.8	60.00	0.30	tr_D0A9V0_
HLA-DQA10501-DQB10201	346	RTLADGVMLRGTALY	VMLRGTALY	6	0.2223	4511.0	60.00	0.35	tr_D0A9V0_
HLA-DQA10501-DQB10201	347	IVRYVSCSITPSHLS	IVRYVSCSI	0	0.2222	4515.8	60.00	0.34	tr_D0A9V0_
HLA-DQA10501-DQB10201	348	LKSASNLEIPDNADI	SNLEIPDNA	4	0.2215	4552.3	60.00	0.29	tr_D0A9V0_

HLA-DQA10501-DQB10201	349	HNVLLGTDGVCKLSD	NVLLGTDGV	1	0.2211	4573.1	60.00	0.34	tr_D0A9V0_
HLA-DQA10501-DQB10201	350	CVLLPCSTLPIVLFE	STLPIVLFE	6	0.2205	4599.4	60.00	0.23	tr_D0A9V0_
HLA-DQA10501-DQB10201	351	SDATRLRSLFNRDLL	RSLFNRDLL	6	0.2198	4635.3	60.00	0.50	tr_D0A9V0_
HLA-DQA10501-DQB10201	352	PCSTLPIVLFELRPG	PIVLFELRP	5	0.2197	4639.7	60.00	0.37	tr_D0A9V0_
HLA-DQA10501-DQB10201	353	GVAEDGAQVAIKCIP	VAEDGAQVA	1	0.2187	4691.3	60.00	0.46	tr_D0A9V0_
HLA-DQA10501-DQB10201	354	VLLPCSTLPIVLFEL	TLPIVLFEL	6	0.2185	4703.0	60.00	0.18	tr_D0A9V0_
HLA-DQA10501-DQB10201	355	IMGASISVTWNAGDQ	MGASISVTW	1	0.2181	4720.6	60.00	0.34	tr_D0A9V0_
HLA-DQA10501-DQB10201	356	HYAKLRRQLAVLLKF	LRRQLAVLL	4	0.2181	4722.9	60.00	0.37	tr_D0A9V0_
HLA-DQA10501-DQB10201	357	GTCGAFSRNARVLFG	FSRNARVLF	5	0.2171	4772.2	60.00	0.54	tr_D0A9V0_
HLA-DQA10501-DQB10201	358	LLEMLLGRPPWQWSS	MLLGRPPWQ	3	0.2169	4784.7	60.00	0.43	tr_D0A9V0_
HLA-DQA10501-DQB10201	359	YLHNLGIVHCDVKPH	NLGIVHCDV	3	0.2167	4791.9	60.00	0.52	tr_D0A9V0_
HLA-DQA10501-DQB10201	360	FPVDCVLLPCSTLPI	VDCVLLPCS	2	0.2153	4867.2	60.00	0.22	tr_D0A9V0_
HLA-DQA10501-DQB10201	361	MAHYLPMVKMDMQAK	PMVKMDMQA	5	0.2152	4873.6	60.00	0.19	tr_D0A9V0_
HLA-DQA10501-DQB10201	362	TCGAFSRNARVLFGA	FSRNARVLF	4	0.2146	4903.4	60.00	0.49	tr_D0A9V0_
HLA-DQA10501-DQB10201	363	SLHSVIKNFGCVSPY	VIKNFGCVS	4	0.2144	4914.7	60.00	0.38	tr_D0A9V0_
HLA-DQA10501-DQB10201	364	FGCVSPYVARRFTVD	FGCVSPYVA	0	0.2143	4917.6	60.00	0.40	tr_D0A9V0_
HLA-DQA10501-DQB10201	365	NVLLGTDGVCKLSDF	NVLLGTDGV	0	0.2141	4931.5	60.00	0.25	tr_D0A9V0_
HLA-DQA10501-DQB10201	366	TPGESSTAFVRIMGA	TAFVRIMGA	6	0.2138	4945.7	60.00	0.50	tr_D0A9V0_
HLA-DQA10501-DQB10201	367	QGHYAKLRRQLAVLL	LRRQLAVLL	6	0.2135	4964.2	60.00	0.60	tr_D0A9V0_
HLA-DQA10501-DQB10201	368	SIIMEYVPGGSLHSV	IIMEYVPGG	1	0.2128	5003.0	60.00	0.29	tr_D0A9V0_
HLA-DQA10501-DQB10201	369	VDCVLLPCSTLPIVL	PCSTLPIVL	6	0.2121	5040.3	60.00	0.27	tr_D0A9V0_
HLA-DQA10501-DQB10201	370	IVLFELRPGESSSEV	LFELRPGES	2	0.2120	5041.5	60.00	0.43	tr_D0A9V0_
HLA-DQA10501-DQB10201	371	EAAAFVRACCHMNPS	AAFVRACCH	2	0.2120	5045.7	60.00	0.41	tr_D0A9V0_
HLA-DQA10501-DQB10201	372	RLGFAAMRQGRYGEA	RLGFAAMRQ	0	0.2115	5073.3	60.00	0.47	tr_D0A9V0_
HLA-DQA10501-DQB10201	373	GDQQRDVALNCGIFK	QRDVALNCG	3	0.2104	5131.7	60.00	0.22	tr_D0A9V0_
HLA-DQA10501-DQB10201	374	EFLVGTCGAFSRNAR	LVGTCGAFS	2	0.2103	5140.2	60.00	0.44	tr_D0A9V0_
HLA-DQA10501-DQB10201	375	DILQGLNYLHNLGIV	DILQGLNYL	0	0.2101	5151.4	60.00	0.21	tr_D0A9V0_
HLA-DQA10501-DQB10201	376	DCVLLPCSTLPIVLF	PCSTLPIVL	5	0.2096	5177.7	60.00	0.25	tr_D0A9V0_
HLA-DQA10501-DQB10201	377	YVRVLDNHYCVNSSD	YVRVLDNHY	0	0.2095	5182.9	60.00	0.46	tr_D0A9V0_
HLA-DQA10501-DQB10201	378	DNADIFLGRSRIVEV	LGRSRIVEV	6	0.2093	5192.8	60.00	0.58	tr_D0A9V0_
HLA-DQA10501-DQB10201	379	YAKLRRQLAVLLKFI	LRRQLAVLL	3	0.2083	5249.3	60.00	0.37	tr_D0A9V0_
HLA-DQA10501-DQB10201	380	ATSGGASHMQGFLAF	ASHMQGFLA	5	0.2079	5272.6	60.00	0.39	tr_D0A9V0_
HLA-DQA10501-DQB10201	381	DIFLGRSRIVEVRKI	LGRSRIVEV	3	0.2079	5274.0	60.00	0.30	tr_D0A9V0_
HLA-DQA10501-DQB10201	382	RCTPQSDIFSFGISL	DIFSFGISL	6	0.2077	5285.6	60.00	0.54	tr_D0A9V0_
HLA-DQA10501-DQB10201	383	DQQRDVALNCGIFKE	QRDVALNCG	2	0.2066	5348.1	65.00	0.19	tr_D0A9V0_
HLA-DQA10501-DQB10201	384	GFAAMRQGRYGEAMA	RQGRYGEAM	5	0.2061	5374.6	65.00	0.34	tr_D0A9V0_
HLA-DQA10501-DQB10201	385	IIMEYVPGGSLHSVI	MEYVPGGSL	2	0.2061	5377.5	65.00	0.23	tr_D0A9V0_
HLA-DQA10501-DQB10201	386	KLRRQLAVLLKFIPE	RQLAVLLKF	3	0.2059	5391.0	65.00	0.25	tr_D0A9V0_
HLA-DQA10501-DQB10201	387	CDVKPHNVLLGTDGV	NVLLGTDGV	6	0.2053	5421.0	65.00	0.48	tr_D0A9V0_
HLA-DQA10501-DQB10201	388	QGLNYLHNLGIVHCD	YLHNLGIVH	4	0.2047	5459.1	65.00	0.25	tr_D0A9V0_
HLA-DQA10501-DQB10201	389	GCVSPYVARRFTVDI	VARRFTVDI	6	0.2045	5468.3	65.00	0.38	tr_D0A9V0_
HLA-DQA10501-DQB10201	390	AKLRRQLAVLLKFIP	LRRQLAVLL	2	0.2042	5487.3	65.00	0.31	tr_D0A9V0_
HLA-DQA10501-DQB10201	391	AKLPLDHRDSVTMLV	HRDSVTMLV	6	0.2039	5504.9	65.00	0.56	tr_D0A9V0_
HLA-DQA10501-DQB10201	392	PNVFNRRHCTVIAIQ	RHCTVIAIQ	6	0.2035	5531.5	65.00	0.38	tr_D0A9V0_
HLA-DQA10501-DQB10201	393	RCRRNDTVPYVRVLD	RRNDTVPYV	2	0.2028	5573.4	65.00	0.37	tr_D0A9V0_
HLA-DQA10501-DQB10201	394	VSCSITPSHLSIIME	PSHLSIIME	6	0.2024	5597.1	65.00	0.62	tr_D0A9V0_
HLA-DQA10501-DQB10201	395	GSLHSVIKNFGCVSP	VIKNFGCVS	5	0.2016	5643.5	65.00	0.41	tr_D0A9V0_
HLA-DQA10501-DQB10201	396	HYLPMVKMDMQAKRL	KMDMQAKRL	6	0.2016	5646.5	65.00	0.31	tr_D0A9V0_
HLA-DQA10501-DQB10201	397	ARTLADGVMLRGTAL	RTLADGVML	1	0.2011	5675.6	65.00	0.29	tr_D0A9V0_
HLA-DQA10501-DQB10201	398	LLPCSTLPIVLFELR	PCSTLPIVL	2	0.2011	5676.3	65.00	0.20	tr_D0A9V0_

HLA-DQA10501-DQB10201	399	VGTCGAFSRNARVLF	FSRNARVLF	6	0.2005	5711.5	65.00	0.72	tr_D0A9V0_
HLA-DQA10501-DQB10201	400	NADIFLGRSRIVEVR	LGRSRIVEV	5	0.2005	5714.2	65.00	0.51	tr_D0A9V0_
HLA-DQA10501-DQB10201	401	RPPWQWSSTAPAGSD	WQWSSTAPA	3	0.2004	5718.0	65.00	0.50	tr_D0A9V0_
HLA-DQA10501-DQB10201	402	LQGLNYLHNLGIVHC	YLHNLGIVH	5	0.2002	5731.9	65.00	0.26	tr_D0A9V0_
HLA-DQA10501-DQB10201	403	AESLEKEMEVASKLH	KEMEVASKL	5	0.1998	5755.2	65.00	0.27	tr_D0A9V0_
HLA-DQA10501-DQB10201	404	PVDCVLLPCSTLPIV	VDCVLLPCS	1	0.1998	5757.0	65.00	0.20	tr_D0A9V0_
HLA-DQA10501-DQB10201	405	IKDRVPETIDSPRRP	IKDRVPETI	0	0.1998	5757.2	65.00	0.34	tr_D0A9V0_
HLA-DQA10501-DQB10201	406	FAAMRQGRYGEAMAH	RQGRYGEAM	4	0.1989	5811.4	65.00	0.31	tr_D0A9V0_
HLA-DQA10501-DQB10201	407	DLFAKLPLDHRDSVT	LFAKLPLDH	1	0.1986	5832.0	65.00	0.29	tr_D0A9V0_
HLA-DQA10501-DQB10201	408	VLFELRPGESSSEVS	LFELRPGES	1	0.1982	5855.8	65.00	0.34	tr_D0A9V0_
HLA-DQA10501-DQB10201	409	TWNAGDQQRDVALNC	WNAGDQQRD	1	0.1966	5961.7	65.00	0.33	tr_D0A9V0_
HLA-DQA10501-DQB10201	410	QGSESLFKMYAPSSS	LFKMYAPSS	5	0.1964	5970.9	65.00	0.36	tr_D0A9V0_
HLA-DQA10501-DQB10201	411	LEMLLGRPPWQWSST	MLLGRPPWQ	2	0.1960	5998.4	65.00	0.49	tr_D0A9V0_
HLA-DQA10501-DQB10201	412	ILQGLNYLHNLGIVH	YLHNLGIVH	6	0.1955	6028.7	65.00	0.31	tr_D0A9V0_
HLA-DQA10501-DQB10201	413	NAGDQQRDVALNCGI	QRDVALNCG	5	0.1954	6037.6	65.00	0.32	tr_D0A9V0_
HLA-DQA10501-DQB10201	414	PGESSTAFVRIMGAS	TAFVRIMGA	5	0.1952	6052.3	65.00	0.46	tr_D0A9V0_
HLA-DQA10501-DQB10201	415	PEAILARVESKNEAG	PEAILARVE	0	0.1948	6075.8	65.00	0.27	tr_D0A9V0_
HLA-DQA10501-DQB10201	416	PPWQWSSTAPAGSDA	WQWSSTAPA	2	0.1938	6139.3	65.00	0.50	tr_D0A9V0_
HLA-DQA10501-DQB10201	417	PWQWSSTAPAGSDAT	WQWSSTAPA	1	0.1938	6143.7	65.00	0.44	tr_D0A9V0_
HLA-DQA10501-DQB10201	418	DQGSESLFKMYAPSS	LFKMYAPSS	6	0.1923	6239.6	65.00	0.44	tr_D0A9V0_
HLA-DQA10501-DQB10201	419	LGFAAMRQGRYGEAM	RQGRYGEAM	6	0.1921	6259.2	65.00	0.41	tr_D0A9V0_
HLA-DQA10501-DQB10201	420	MNPSMRPSASSLLSY	PSASSLLSY	6	0.1914	6302.8	65.00	0.45	tr_D0A9V0_
HLA-DQA10501-DQB10201	421	VAEDGAQVAIKCIPR	VAEDGAQVA	0	0.1902	6384.4	65.00	0.31	tr_D0A9V0_
HLA-DQA10501-DQB10201	422	IFLGRSRIVEVRKIQ	LGRSRIVEV	2	0.1900	6398.1	65.00	0.31	tr_D0A9V0_
HLA-DQA10501-DQB10201	423	WNAGDQQRDVALNCG	QRDVALNCG	6	0.1897	6417.6	65.00	0.34	tr_D0A9V0_
HLA-DQA10501-DQB10201	424	RSRIVEVRKIQGHYA	VRKIQGHYA	6	0.1897	6421.9	65.00	0.57	tr_D0A9V0_
HLA-DQA10501-DQB10201	425	YLPMVKMDMQAKRLM	KMDMQAKRL	5	0.1896	6426.9	70.00	0.29	tr_D0A9V0_
HLA-DQA10501-DQB10201	426	GHYAKLRRQLAVLLK	LRRQLAVLL	5	0.1892	6452.7	70.00	0.46	tr_D0A9V0_
HLA-DQA10501-DQB10201	427	ADIFLGRSRIVEVRK	LGRSRIVEV	4	0.1892	6458.8	70.00	0.40	tr_D0A9V0_
HLA-DQA10501-DQB10201	428	GRPPWQWSSTAPAGS	WQWSSTAPA	4	0.1877	6561.9	70.00	0.56	tr_D0A9V0_
HLA-DQA10501-DQB10201	429	AATSGGASHMQGFLA	ASHMQGFLA	6	0.1864	6650.5	70.00	0.53	tr_D0A9V0_
HLA-DQA10501-DQB10201	430	AEDGAQVAIKCIPRL	VAIKCIPRL	6	0.1863	6659.2	70.00	0.33	tr_D0A9V0_
HLA-DQA10501-DQB10201	431	GGSLHSVIKNFGCVS	VIKNFGCVS	6	0.1860	6682.1	70.00	0.41	tr_D0A9V0_
HLA-DQA10501-DQB10201	432	IQVYHPDVWKSSTKY	IQVYHPDVW	0	0.1860	6683.7	70.00	0.73	tr_D0A9V0_
HLA-DQA10501-DQB10201	433	GSESLFKMYAPSSSS	LFKMYAPSS	4	0.1856	6711.3	70.00	0.37	tr_D0A9V0_
HLA-DQA10501-DQB10201	434	QWSSTAPAGSDATRL	PAGSDATRL	6	0.1840	6825.4	70.00	0.51	tr_D0A9V0_
HLA-DQA10501-DQB10201	435	AGGHIRANGSATETP	IRANGSATE	4	0.1839	6834.1	70.00	0.31	tr_D0A9V0_
HLA-DQA10501-DQB10201	436	LHNLGIVHCDVKPHN	NLGIVHCDV	2	0.1835	6864.0	70.00	0.56	tr_D0A9V0_
HLA-DQA10501-DQB10201	437	GESSSEVSLSISKQF	VSLSISKQF	6	0.1833	6877.7	70.00	0.43	tr_D0A9V0_
HLA-DQA10501-DQB10201	438	AAAFVRACCHMNPSM	AAFVRACCH	1	0.1831	6892.6	70.00	0.44	tr_D0A9V0_
HLA-DQA10501-DQB10201	439	LGRPPWQWSSTAPAG	WQWSSTAPA	5	0.1827	6924.3	70.00	0.65	tr_D0A9V0_
HLA-DQA10501-DQB10201	440	LMLLVSALKSASNLE	ALKSASNLE	6	0.1824	6949.7	70.00	0.29	tr_D0A9V0_
HLA-DQA10501-DQB10201	441	LSISKQFRLGFAAMR	FRLGFAAMR	6	0.1823	6956.3	70.00	0.34	tr_D0A9V0_
HLA-DQA10501-DQB10201	442	LDPDRALISFGAHAA	LISFGAHAA	6	0.1815	7015.6	70.00	0.34	tr_D0A9V0_
HLA-DQA10501-DQB10201	443	NPSMRPSASSLLSYA	PSASSLLSY	5	0.1814	7022.0	70.00	0.36	tr_D0A9V0_
HLA-DQA10501-DQB10201	444	QQRDVALNCGIFKEK	QRDVALNCG	1	0.1799	7139.9	70.00	0.19	tr_D0A9V0_
HLA-DQA10501-DQB10201	445	FLGRSRIVEVRKIQG	LGRSRIVEV	1	0.1798	7148.5	70.00	0.25	tr_D0A9V0_
HLA-DQA10501-DQB10201	446	SSDQGSESLFKMYAP	SESLFKMYA	5	0.1789	7217.6	70.00	0.28	tr_D0A9V0_
HLA-DQA10501-DQB10201	447	AHYLPMVKMDMQAKR	PMVKMDMQA	4	0.1786	7239.3	70.00	0.24	tr_D0A9V0_
HLA-DQA10501-DQB10201	448	LLGRPPWQWSSTAPA	WQWSSTAPA	6	0.1781	7277.0	70.00	0.69	tr_D0A9V0_

HLA-DQA10501-DQB10201	449	LCRQRCRRNDTVPYV	RRNDTVPYV	6	0.1756	7481.5	70.00	0.71	tr_D0A9V0_
HLA-DQA10501-DQB10201	450	VLLGTDGVCKLSDFG	LGTDGVCKL	2	0.1750	7526.7	70.00	0.32	tr_D0A9V0_
HLA-DQA10501-DQB10201	451	AIKCIPRLRRDVVAE	RLRRDVVAE	6	0.1741	7599.2	70.00	0.40	tr_D0A9V0_
HLA-DQA10501-DQB10201	452	PLMLLVSALKSASNL	MLLVSALKS	2	0.1738	7627.8	70.00	0.37	tr_D0A9V0_
HLA-DQA10501-DQB10201	453	QLSYAVRDGPCRIAA	AVRDGPCRI	4	0.1735	7652.0	70.00	0.39	tr_D0A9V0_
HLA-DQA10501-DQB10201	454	KPLMLLVSALKSASN	MLLVSALKS	3	0.1731	7684.2	70.00	0.39	tr_D0A9V0_
HLA-DQA10501-DQB10201	455	GSATETPGESSTAFV	PGESSTAFV	6	0.1729	7702.9	70.00	0.62	tr_D0A9V0_
HLA-DQA10501-DQB10201	456	FLVGTCGAFSRNARV	LVGTCGAFS	1	0.1724	7739.4	70.00	0.54	tr_D0A9V0_
HLA-DQA10501-DQB10201	457	SESLFKMYAPSSSSS	LFKMYAPSS	3	0.1710	7858.0	75.00	0.37	tr_D0A9V0_
HLA-DQA10501-DQB10201	458	EMLLGRPPWQWSSTA	MLLGRPPWQ	1	0.1705	7901.6	75.00	0.50	tr_D0A9V0_
HLA-DQA10501-DQB10201	459	ALDPDRALISFGAHA	ALISFGAHA	6	0.1704	7908.5	75.00	0.32	tr_D0A9V0_
HLA-DQA10501-DQB10201	460	VALNCGIFKEKPNVF	IFKEKPNVF	6	0.1697	7970.5	75.00	0.57	tr_D0A9V0_
HLA-DQA10501-DQB10201	461	ESLEKEMEVASKLHH	KEMEVASKL	4	0.1693	8003.2	75.00	0.33	tr_D0A9V0_
HLA-DQA10501-DQB10201	462	GGHIRANGSATETPG	IRANGSATE	3	0.1693	8009.8	75.00	0.33	tr_D0A9V0_
HLA-DQA10501-DQB10201	463	KPNVFNRRHCTVIAI	RRHCTVIAI	6	0.1690	8030.3	75.00	0.65	tr_D0A9V0_
HLA-DQA10501-DQB10201	464	EDGAQVAIKCIPRLR	VAIKCIPRL	5	0.1688	8047.9	75.00	0.30	tr_D0A9V0_
HLA-DQA10501-DQB10201	465	RIVEVRKIQGHYAKL	VRKIQGHYA	4	0.1687	8058.7	75.00	0.60	tr_D0A9V0_
HLA-DQA10501-DQB10201	466	DSSDQGSESLFKMYA	SESLFKMYA	6	0.1683	8095.1	75.00	0.28	tr_D0A9V0_
HLA-DQA10501-DQB10201	467	SRIVEVRKIQGHYAK	VRKIQGHYA	5	0.1683	8095.9	75.00	0.58	tr_D0A9V0_
HLA-DQA10501-DQB10201	468	VRYVSCSITPSHLSI	YVSCSITPS	2	0.1682	8099.7	75.00	0.30	tr_D0A9V0_
HLA-DQA10501-DQB10201	469	WQWSSTAPAGSDATR	WQWSSTAPA	0	0.1680	8123.4	75.00	0.35	tr_D0A9V0_
HLA-DQA10501-DQB10201	470	ESLFKMYAPSSSSSL	FKMYAPSSS	3	0.1674	8176.0	75.00	0.34	tr_D0A9V0_
HLA-DQA10501-DQB10201	471	ADVVPLFLTDSNRNV	VPLFLTDSN	3	0.1659	8308.7	75.00	0.31	tr_D0A9V0_
HLA-DQA10501-DQB10201	472	GHIRANGSATETPGE	IRANGSATE	2	0.1654	8350.9	75.00	0.32	tr_D0A9V0_
HLA-DQA10501-DQB10201	473	SLEKEMEVASKLHHP	KEMEVASKL	3	0.1651	8379.0	75.00	0.40	tr_D0A9V0_
HLA-DQA10501-DQB10201	474	LFELRPGESSSEVSL	PGESSSEVS	5	0.1642	8462.1	75.00	0.31	tr_D0A9V0_
HLA-DQA10501-DQB10201	475	YVPGGSLHSVIKNFG	LHSVIKNFG	6	0.1639	8486.4	75.00	0.46	tr_D0A9V0_
HLA-DQA10501-DQB10201	476	PMVKMDMQAKRLMRL	KMDMQAKRL	3	0.1639	8487.6	75.00	0.33	tr_D0A9V0_
HLA-DQA10501-DQB10201	477	TETPGESSTAFVRIM	PGESSTAFV	3	0.1637	8502.4	75.00	0.37	tr_D0A9V0_
HLA-DQA10501-DQB10201	478	HNLGIVHCDVKPHNV	NLGIVHCDV	1	0.1633	8544.3	75.00	0.57	tr_D0A9V0_
HLA-DQA10501-DQB10201	479	CRQRCRRNDTVPYVR	RRNDTVPYV	5	0.1624	8628.1	75.00	0.61	tr_D0A9V0_
HLA-DQA10501-DQB10201	480	QRDVALNCGIFKEKP	VALNCGIFK	3	0.1621	8651.2	75.00	0.27	tr_D0A9V0_
HLA-DQA10501-DQB10201	481	ESSSEVSLSISKQFR	VSLSISKQF	5	0.1620	8662.9	75.00	0.39	tr_D0A9V0_
HLA-DQA10501-DQB10201	482	EALDPDRALISFGAH	DRALISFGA	5	0.1618	8685.1	75.00	0.32	tr_D0A9V0_
HLA-DQA10501-DQB10201	483	SDSSDQGSESLFKMY	SSDQGSESL	2	0.1617	8695.5	75.00	0.29	tr_D0A9V0_
HLA-DQA10501-DQB10201	484	RQRCRRNDTVPYVRV	RRNDTVPYV	4	0.1616	8700.4	75.00	0.55	tr_D0A9V0_
HLA-DQA10501-DQB10201	485	QRCRRNDTVPYVRVL	RRNDTVPYV	3	0.1616	8705.9	75.00	0.52	tr_D0A9V0_
HLA-DQA10501-DQB10201	486	IPDNADIFLGRSRIV	IPDNADIFL	0	0.1614	8725.2	75.00	0.41	tr_D0A9V0_
HLA-DQA10501-DQB10201	487	LPMVKMDMQAKRLMR	KMDMQAKRL	4	0.1603	8824.8	75.00	0.34	tr_D0A9V0_
HLA-DQA10501-DQB10201	488	NSSDCKPEEQDESDI	KPEEQDESD	5	0.1602	8832.3	75.00	0.26	tr_D0A9V0_
HLA-DQA10501-DQB10201	489	KDRVPETIDSPRRPD	RVPETIDSP	2	0.1600	8852.8	75.00	0.41	tr_D0A9V0_
HLA-DQA10501-DQB10201	490	ATETPGESSTAFVRI	PGESSTAFV	4	0.1599	8865.8	75.00	0.44	tr_D0A9V0_
HLA-DQA10501-DQB10201	491	SLSISKQFRLGFAAM	QFRLGFAAM	6	0.1593	8921.5	75.00	0.51	tr_D0A9V0_
HLA-DQA10501-DQB10201	492	ALNCGIFKEKPNVFN	IFKEKPNVF	5	0.1589	8958.0	75.00	0.60	tr_D0A9V0_
HLA-DQA10501-DQB10201	493	GSDATRLRSLFNRDL	LRSLFNRDL	6	0.1586	8985.4	75.00	0.40	tr_D0A9V0_
HLA-DQA10501-DQB10201	494	LPMHCRRCCQFAFDL	RRCCQFAFD	5	0.1586	8988.5	75.00	0.48	tr_D0A9V0_
HLA-DQA10501-DQB10201	495	WSSTAPAGSDATRLR	PAGSDATRL	5	0.1585	9001.5	75.00	0.53	tr_D0A9V0_
HLA-DQA10501-DQB10201	496	ETPGESSTAFVRIMG	PGESSTAFV	2	0.1583	9014.0	75.00	0.30	tr_D0A9V0_
HLA-DQA10501-DQB10201	497	SSSEVSLSISKQFRL	VSLSISKQF	4	0.1579	9061.0	75.00	0.38	tr_D0A9V0_
HLA-DQA10501-DQB10201	498	VRVLDNHYCVNSSDC	HYCVNSSDC	6	0.1576	9090.0	75.00	0.25	tr_D0A9V0_

HLA-DQA10501-DQB10201	499	GGRCTPQSDIFSFGI	QSDIFSFGI	6	0.1575	9094.6	75.00	0.37	tr_D0A9V0_
HLA-DQA10501-DQB10201	500	VNSSDCKPEEQDESD	KPEEQDESD	6	0.1574	9106.6	75.00	0.32	tr_D0A9V0_
HLA-DQA10501-DQB10201	501	DVVPLFLTDSNRNVW	VPLFLTDSN	2	0.1573	9112.1	75.00	0.26	tr_D0A9V0_
HLA-DQA10501-DQB10201	502	SDQGSESLFKMYAPS	SESLFKMYA	4	0.1570	9143.5	75.00	0.32	tr_D0A9V0_
HLA-DQA10501-DQB10201	503	EAGGHIRANGSATET	IRANGSATE	5	0.1570	9149.6	75.00	0.43	tr_D0A9V0_
HLA-DQA10501-DQB10201	504	GSESDSSDQGSESLF	SSDQGSESL	5	0.1570	9150.1	75.00	0.49	tr_D0A9V0_
HLA-DQA10501-DQB10201	505	ELRPGESSSEVSLSI	SSSEVSLSI	6	0.1567	9175.1	75.00	0.35	tr_D0A9V0_
HLA-DQA10501-DQB10201	506	SLFKMYAPSSSSSLD	FKMYAPSSS	2	0.1564	9206.2	75.00	0.30	tr_D0A9V0_
HLA-DQA10501-DQB10201	507	IMEYVPGGSLHSVIK	MEYVPGGSL	1	0.1557	9272.7	75.00	0.33	tr_D0A9V0_
HLA-DQA10501-DQB10201	508	VPGGSLHSVIKNFGC	LHSVIKNFG	5	0.1556	9288.9	75.00	0.50	tr_D0A9V0_
HLA-DQA10501-DQB10201	509	FAKLPLDHRDSVTML	DHRDSVTML	6	0.1553	9318.5	75.00	0.47	tr_D0A9V0_
HLA-DQA10501-DQB10201	510	HIRANGSATETPGES	IRANGSATE	1	0.1551	9331.9	75.00	0.31	tr_D0A9V0_
HLA-DQA10501-DQB10201	511	LSYAVRDGPCRIAAT	AVRDGPCRI	3	0.1542	9431.1	75.00	0.46	tr_D0A9V0_
HLA-DQA10501-DQB10201	512	IVEVRKIQGHYAKLR	VRKIQGHYA	3	0.1534	9513.3	80.00	0.62	tr_D0A9V0_
HLA-DQA10501-DQB10201	513	DGVCKLSDFGSTISE	LSDFGSTIS	5	0.1531	9536.7	80.00	0.34	tr_D0A9V0_
HLA-DQA10501-DQB10201	514	ESDSSDQGSESLFKM	SSDQGSESL	3	0.1510	9760.1	80.00	0.37	tr_D0A9V0_
HLA-DQA10501-DQB10201	515	PRRPDGEGSESDSSD	PDGEGSESD	3	0.1508	9781.1	80.00	0.32	tr_D0A9V0_
HLA-DQA10501-DQB10201	516	KMDMQAKRLMRLCRQ	KRLMRLCRQ	6	0.1506	9805.4	80.00	0.41	tr_D0A9V0_
HLA-DQA10501-DQB10201	517	EAILARVESKNEAGG	AILARVESK	1	0.1501	9850.3	80.00	0.41	tr_D0A9V0_
HLA-DQA10501-DQB10201	518	GRCTPQSDIFSFGIS	QSDIFSFGI	5	0.1499	9876.4	80.00	0.31	tr_D0A9V0_
HLA-DQA10501-DQB10201	519	VHCDVKPHNVLLGTD	PHNVLLGTD	6	0.1497	9901.9	80.00	0.62	tr_D0A9V0_
HLA-DQA10501-DQB10201	520	AAFVRACCHMNPSMR	AAFVRACCH	0	0.1495	9917.0	80.00	0.41	tr_D0A9V0_
HLA-DQA10501-DQB10201	521	MVKMDMQAKRLMRLC	KMDMQAKRL	2	0.1495	9922.3	80.00	0.37	tr_D0A9V0_
HLA-DQA10501-DQB10201	522	NHYCVNSSDCKPEEQ	SSDCKPEEQ	6	0.1495	9924.5	80.00	0.31	tr_D0A9V0_
HLA-DQA10501-DQB10201	523	DRVPETIDSPRRPDG	RVPETIDSP	1	0.1491	9957.3	80.00	0.41	tr_D0A9V0_
HLA-DQA10501-DQB10201	524	LGRSRIVEVRKIQGH	SRIVEVRKI	3	0.1491	9960.0	80.00	0.22	tr_D0A9V0_
HLA-DQA10501-DQB10201	525	DGAQVAIKCIPRLRR	VAIKCIPRL	4	0.1490	9973.7	80.00	0.33	tr_D0A9V0_
HLA-DQA10501-DQB10201	526	VAIKCIPRLRRDVVA	PRLRRDVVA	6	0.1489	9984.7	80.00	0.36	tr_D0A9V0_
HLA-DQA10501-DQB10201	527	PGGSLHSVIKNFGCV	LHSVIKNFG	4	0.1488	9999.5	80.00	0.52	tr_D0A9V0_
HLA-DQA10501-DQB10201	528	HCDVKPHNVLLGTDG	PHNVLLGTD	5	0.1479	10092.4	80.00	0.56	tr_D0A9V0_
HLA-DQA10501-DQB10201	529	SEVSLSISKQFRLGF	VSLSISKQF	2	0.1476	10128.0	80.00	0.31	tr_D0A9V0_
HLA-DQA10501-DQB10201	530	RRPDGEGSESDSSDQ	PDGEGSESD	2	0.1475	10138.2	80.00	0.32	tr_D0A9V0_
HLA-DQA10501-DQB10201	531	LEKEMEVASKLHHPN	KEMEVASKL	2	0.1468	10217.5	80.00	0.44	tr_D0A9V0_
HLA-DQA10501-DQB10201	532	TAPAGSDATRLRSLF	PAGSDATRL	2	0.1467	10223.9	80.00	0.50	tr_D0A9V0_
HLA-DQA10501-DQB10201	533	APAGSDATRLRSLFN	PAGSDATRL	1	0.1465	10243.1	80.00	0.37	tr_D0A9V0_
HLA-DQA10501-DQB10201	534	LRPGESSSEVSLSIS	SSSEVSLSI	5	0.1463	10268.0	80.00	0.31	tr_D0A9V0_
HLA-DQA10501-DQB10201	535	SATETPGESSTAFVR	PGESSTAFV	5	0.1463	10268.1	80.00	0.60	tr_D0A9V0_
HLA-DQA10501-DQB10201	536	HYCVNSSDCKPEEQD	SSDCKPEEQ	5	0.1455	10356.4	80.00	0.36	tr_D0A9V0_
HLA-DQA10501-DQB10201	537	SSEVSLSISKQFRLG	EVSLSISKQ	2	0.1454	10369.7	80.00	0.34	tr_D0A9V0_
HLA-DQA10501-DQB10201	538	LFAKLPLDHRDSVTM	FAKLPLDHR	1	0.1452	10390.1	80.00	0.34	tr_D0A9V0_
HLA-DQA10501-DQB10201	539	FELRPGESSSEVSLS	PGESSSEVS	4	0.1446	10459.2	80.00	0.29	tr_D0A9V0_
HLA-DQA10501-DQB10201	540	YVSCSITPSHLSIIM	TPSHLSIIM	6	0.1439	10544.2	80.00	0.31	tr_D0A9V0_
HLA-DQA10501-DQB10201	541	EGSESDSSDQGSESL	SSDQGSESL	6	0.1434	10592.5	80.00	0.56	tr_D0A9V0_
HLA-DQA10501-DQB10201	542	RPDGEGSESDSSDQG	PDGEGSESD	1	0.1434	10598.5	80.00	0.28	tr_D0A9V0_
HLA-DQA10501-DQB10201	543	LNCGIFKEKPNVFNR	IFKEKPNVF	4	0.1433	10607.8	80.00	0.65	tr_D0A9V0_
HLA-DQA10501-DQB10201	544	MLLGRPPWQWSSTAP	MLLGRPPWQ	0	0.1411	10859.6	80.00	0.46	tr_D0A9V0_
HLA-DQA10501-DQB10201	545	LFKMYAPSSSSSLDG	FKMYAPSSS	1	0.1409	10882.3	80.00	0.31	tr_D0A9V0_
HLA-DQA10501-DQB10201	546	VEVRKIQGHYAKLRR	VRKIQGHYA	2	0.1408	10902.9	80.00	0.63	tr_D0A9V0_
HLA-DQA10501-DQB10201	547	SESDSSDQGSESLFK	SSDQGSESL	4	0.1405	10939.4	80.00	0.43	tr_D0A9V0_
HLA-DQA10501-DQB10201	548	GAHAALPMHCRRCCQ	AHAALPMHC	1	0.1404	10940.9	80.00	0.61	tr_D0A9V0_

HLA-DQA10501-DQB10201	549	VLDNHYCVNSSDCKP	HYCVNSSDC	4	0.1404	10948.9	80.00	0.28	tr_D0A9V0_
HLA-DQA10501-DQB10201	550	EYVPGGSLHSVIKNF	SLHSVIKNF	6	0.1401	10976.3	80.00	0.33	tr_D0A9V0_
HLA-DQA10501-DQB10201	551	SSTAPAGSDATRLRS	PAGSDATRL	4	0.1384	11183.6	80.00	0.63	tr_D0A9V0_
HLA-DQA10501-DQB10201	552	DNHYCVNSSDCKPEE	HYCVNSSDC	2	0.1382	11204.9	80.00	0.25	tr_D0A9V0_
HLA-DQA10501-DQB10201	553	EVSLSISKQFRLGFA	VSLSISKQF	1	0.1381	11219.2	80.00	0.25	tr_D0A9V0_
HLA-DQA10501-DQB10201	554	RVLDNHYCVNSSDCK	HYCVNSSDC	5	0.1379	11247.2	80.00	0.25	tr_D0A9V0_
HLA-DQA10501-DQB10201	555	SSDCKPEEQDESDIK	KPEEQDESD	4	0.1377	11269.2	80.00	0.25	tr_D0A9V0_
HLA-DQA10501-DQB10201	556	GAQVAIKCIPRLRRD	VAIKCIPRL	3	0.1376	11276.2	80.00	0.40	tr_D0A9V0_
HLA-DQA10501-DQB10201	557	VVPLFLTDSNRNVWT	VPLFLTDSN	1	0.1376	11282.1	80.00	0.25	tr_D0A9V0_
HLA-DQA10501-DQB10201	558	LYMAPEVAAGGRCTP	LYMAPEVAA	0	0.1372	11332.7	80.00	0.27	tr_D0A9V0_
HLA-DQA10501-DQB10201	559	TDGVCKLSDFGSTIS	LSDFGSTIS	6	0.1370	11355.4	80.00	0.38	tr_D0A9V0_
HLA-DQA10501-DQB10201	560	RYVSCSITPSHLSII	YVSCSITPS	1	0.1369	11363.1	80.00	0.34	tr_D0A9V0_
HLA-DQA10501-DQB10201	561	EKEMEVASKLHHPNI	EMEVASKLH	2	0.1369	11372.6	80.00	0.41	tr_D0A9V0_
HLA-DQA10501-DQB10201	562	PGESSSEVSLSISKQ	EVSLSISKQ	6	0.1368	11382.4	80.00	0.41	tr_D0A9V0_
HLA-DQA10501-DQB10201	563	NCGIFKEKPNVFNRR	IFKEKPNVF	3	0.1368	11384.5	80.00	0.63	tr_D0A9V0_
HLA-DQA10501-DQB10201	564	STAPAGSDATRLRSL	PAGSDATRL	3	0.1367	11389.1	80.00	0.59	tr_D0A9V0_
HLA-DQA10501-DQB10201	565	IRANGSATETPGESS	RANGSATET	1	0.1360	11475.8	80.00	0.25	tr_D0A9V0_
HLA-DQA10501-DQB10201	566	EEQDESDIKDRVPET	DIKDRVPET	6	0.1356	11526.2	80.00	0.32	tr_D0A9V0_
HLA-DQA10501-DQB10201	567	GRSRIVEVRKIQGHY	IVEVRKIQG	4	0.1355	11538.8	80.00	0.24	tr_D0A9V0_
HLA-DQA10501-DQB10201	568	PDGEGSESDSSDQGS	PDGEGSESD	0	0.1355	11541.4	80.00	0.20	tr_D0A9V0_
HLA-DQA10501-DQB10201	569	LLGTDGVCKLSDFGS	LGTDGVCKL	1	0.1355	11545.3	80.00	0.37	tr_D0A9V0_
HLA-DQA10501-DQB10201	570	VKMDMQAKRLMRLCR	KMDMQAKRL	1	0.1338	11753.6	85.00	0.38	tr_D0A9V0_
HLA-DQA10501-DQB10201	571	SDCKPEEQDESDIKD	KPEEQDESD	3	0.1338	11760.6	85.00	0.24	tr_D0A9V0_
HLA-DQA10501-DQB10201	572	AGGRCTPQSDIFSFG	PQSDIFSFG	6	0.1334	11811.7	85.00	0.32	tr_D0A9V0_
HLA-DQA10501-DQB10201	573	LDNHYCVNSSDCKPE	HYCVNSSDC	3	0.1333	11815.7	85.00	0.28	tr_D0A9V0_
HLA-DQA10501-DQB10201	574	YCVNSSDCKPEEQDE	SSDCKPEEQ	4	0.1331	11847.0	85.00	0.41	tr_D0A9V0_
HLA-DQA10501-DQB10201	575	ALPMHCRRCCQFAFD	RRCCQFAFD	6	0.1330	11857.0	85.00	0.62	tr_D0A9V0_
HLA-DQA10501-DQB10201	576	RDVALNCGIFKEKPN	VALNCGIFK	2	0.1326	11909.7	85.00	0.35	tr_D0A9V0_
HLA-DQA10501-DQB10201	577	KPEEQDESDIKDRVP	DESDIKDRV	5	0.1321	11973.4	85.00	0.29	tr_D0A9V0_
HLA-DQA10501-DQB10201	578	NLGIVHCDVKPHNVL	NLGIVHCDV	0	0.1315	12046.9	85.00	0.50	tr_D0A9V0_
HLA-DQA10501-DQB10201	579	SPRRPDGEGSESDSS	PDGEGSESD	4	0.1314	12064.4	85.00	0.38	tr_D0A9V0_
HLA-DQA10501-DQB10201	580	NEAGGHIRANGSATE	IRANGSATE	6	0.1314	12065.3	85.00	0.56	tr_D0A9V0_
HLA-DQA10501-DQB10201	581	QVAIKCIPRLRRDVV	IPRLRRDVV	6	0.1308	12146.7	85.00	0.34	tr_D0A9V0_
HLA-DQA10501-DQB10201	582	SYAVRDGPCRIAATS	AVRDGPCRI	2	0.1288	12413.7	85.00	0.56	tr_D0A9V0_
HLA-DQA10501-DQB10201	583	AGSDATRLRSLFNRD	RLRSLFNRD	6	0.1287	12427.2	85.00	0.32	tr_D0A9V0_
HLA-DQA10501-DQB10201	584	HMNPSMRPSASSLLS	MRPSASSLL	5	0.1285	12449.8	85.00	0.55	tr_D0A9V0_
HLA-DQA10501-DQB10201	585	TRSLKKISEGAFSTI	ISEGAFSTI	6	0.1282	12484.0	85.00	0.34	tr_D0A9V0_
HLA-DQA10501-DQB10201	586	CVNSSDCKPEEQDES	SSDCKPEEQ	3	0.1276	12564.9	85.00	0.43	tr_D0A9V0_
HLA-DQA10501-DQB10201	587	MEYVPGGSLHSVIKN	PGGSLHSVI	4	0.1276	12571.1	85.00	0.35	tr_D0A9V0_
HLA-DQA10501-DQB10201	588	CGIFKEKPNVFNRRH	IFKEKPNVF	2	0.1275	12579.2	85.00	0.62	tr_D0A9V0_
HLA-DQA10501-DQB10201	589	CKPEEQDESDIKDRV	DESDIKDRV	6	0.1273	12611.7	85.00	0.26	tr_D0A9V0_
HLA-DQA10501-DQB10201	590	PDNADIFLGRSRIVE	FLGRSRIVE	6	0.1272	12629.7	85.00	0.30	tr_D0A9V0_
HLA-DQA10501-DQB10201	591	IDSPRRPDGEGSESD	PDGEGSESD	6	0.1265	12720.1	85.00	0.52	tr_D0A9V0_
HLA-DQA10501-DQB10201	592	IQGHYAKLRRQLAVL	KLRRQLAVL	6	0.1264	12732.9	85.00	0.44	tr_D0A9V0_
HLA-DQA10501-DQB10201	593	DSPRRPDGEGSESDS	PDGEGSESD	5	0.1255	12865.4	85.00	0.50	tr_D0A9V0_
HLA-DQA10501-DQB10201	594	EVRKIQGHYAKLRRQ	VRKIQGHYA	1	0.1252	12907.2	85.00	0.61	tr_D0A9V0_
HLA-DQA10501-DQB10201	595	RVPETIDSPRRPDGE	RVPETIDSP	0	0.1244	13015.9	85.00	0.33	tr_D0A9V0_
HLA-DQA10501-DQB10201	596	RPGESSSEVSLSISK	PGESSSEVS	1	0.1237	13112.6	85.00	0.22	tr_D0A9V0_
HLA-DQA10501-DQB10201	597	IAATSGGASHMQGFL	GASHMQGFL	6	0.1231	13197.1	85.00	0.36	tr_D0A9V0_
HLA-DQA10501-DQB10201	598	AAGGRCTPQSDIFSF	TPQSDIFSF	6	0.1230	13208.1	85.00	0.40	tr_D0A9V0_

HLA-DQA10501-DQB10201	599	PAGSDATRLRLSLFNR	PAGSDATRL	0	0.1227	13250.7	85.00	0.29	tr_D0A9V0_
HLA-DQA10501-DQB10201	600	MDMQAKRLMRLCRQR	KRLMRLCRQ	5	0.1221	13346.2	85.00	0.40	tr_D0A9V0_
HLA-DQA10501-DQB10201	601	AQVAIKCIPRLRRDV	VAIKCIPRL	2	0.1212	13468.8	85.00	0.49	tr_D0A9V0_
HLA-DQA10501-DQB10201	602	LVGTCGAFSRNARVL	LVGTCGAFS	0	0.1212	13469.6	85.00	0.48	tr_D0A9V0_
HLA-DQA10501-DQB10201	603	CHMNPSMRPSASSLL	MRPSASSLL	6	0.1210	13508.2	85.00	0.69	tr_D0A9V0_
HLA-DQA10501-DQB10201	604	VSLSISKQFRLGFAA	SKQFRLGFA	5	0.1209	13522.1	85.00	0.22	tr_D0A9V0_
HLA-DQA10501-DQB10201	605	DCKPEEQDESDIKDR	KPEEQDESD	2	0.1203	13605.9	85.00	0.25	tr_D0A9V0_
HLA-DQA10501-DQB10201	606	PEEQDESDIKDRVPE	DESDIKDRV	4	0.1202	13617.3	85.00	0.36	tr_D0A9V0_
HLA-DQA10501-DQB10201	607	GIFKEKPNVFNRRHC	IFKEKPNVF	1	0.1194	13733.7	85.00	0.62	tr_D0A9V0_
HLA-DQA10501-DQB10201	608	SLDGSGCAEGANAAD	SGCAEGANA	4	0.1182	13920.2	85.00	0.28	tr_D0A9V0_
HLA-DQA10501-DQB10201	609	PDVWKSSTKYHAALI	STKYHAALI	6	0.1182	13922.5	85.00	0.79	tr_D0A9V0_
HLA-DQA10501-DQB10201	610	DMQAKRLMRLCRQRC	KRLMRLCRQ	4	0.1164	14186.6	85.00	0.40	tr_D0A9V0_
HLA-DQA10501-DQB10201	611	DGEGSESDSSDQGSE	EGSESDSSD	2	0.1150	14412.8	85.00	0.22	tr_D0A9V0_
HLA-DQA10501-DQB10201	612	VPLFLTDSNRNVWTR	LFLTDSNRN	2	0.1146	14469.5	85.00	0.28	tr_D0A9V0_
HLA-DQA10501-DQB10201	613	YAVRDGPCRIAATSG	AVRDGPCRI	1	0.1145	14481.7	85.00	0.58	tr_D0A9V0_
HLA-DQA10501-DQB10201	614	KEMEVASKLHHPNIV	EMEVASKLH	1	0.1138	14598.9	90.00	0.40	tr_D0A9V0_
HLA-DQA10501-DQB10201	615	AHAALPMHCRRCCQF	AHAALPMHC	0	0.1130	14729.4	90.00	0.49	tr_D0A9V0_
HLA-DQA10501-DQB10201	616	DVALNCGIFKEKPNV	VALNCGIFK	1	0.1123	14840.1	90.00	0.38	tr_D0A9V0_
HLA-DQA10501-DQB10201	617	SSSLDGSGCAEGANA	SLDGSGCAE	2	0.1117	14927.8	90.00	0.28	tr_D0A9V0_
HLA-DQA10501-DQB10201	618	FKMYAPSSSSSLDGS	YAPSSSSSL	3	0.1104	15141.7	90.00	0.31	tr_D0A9V0_
HLA-DQA10501-DQB10201	619	SSSSSLDGSGCAEGA	SLDGSGCAE	4	0.1088	15401.0	90.00	0.35	tr_D0A9V0_
HLA-DQA10501-DQB10201	620	GEGSESDSSDQGSES	SESDSSDQG	3	0.1074	15637.9	90.00	0.17	tr_D0A9V0_
HLA-DQA10501-DQB10201	621	GTDGVCKLSDFGSTI	KLSDFGSTI	6	0.1071	15688.4	90.00	0.41	tr_D0A9V0_
HLA-DQA10501-DQB10201	622	SSLDGSGCAEGANAA	SLDGSGCAE	1	0.1067	15767.7	90.00	0.26	tr_D0A9V0_
HLA-DQA10501-DQB10201	623	SKLHHPNIVRYVSCS	NIVRYVSCS	6	0.1060	15885.2	90.00	0.21	tr_D0A9V0_
HLA-DQA10501-DQB10201	624	ASKLHHPNIVRYVSC	PNIVRYVSC	6	0.1051	16035.8	90.00	0.28	tr_D0A9V0_
HLA-DQA10501-DQB10201	625	AILARVESKNEAGGH	AILARVESK	0	0.1044	16165.0	90.00	0.47	tr_D0A9V0_
HLA-DQA10501-DQB10201	626	SSSSLDGSGCAEGAN	SLDGSGCAE	3	0.1039	16239.0	90.00	0.35	tr_D0A9V0_
HLA-DQA10501-DQB10201	627	LGTDGVCKLSDFGST	LGTDGVCKL	0	0.1033	16353.4	90.00	0.35	tr_D0A9V0_
HLA-DQA10501-DQB10201	628	VRKIQGHYAKLRRQL	VRKIQGHYA	0	0.1031	16387.8	90.00	0.46	tr_D0A9V0_
HLA-DQA10501-DQB10201	629	RANGSATETPGESST	ANGSATETP	1	0.1024	16514.7	90.00	0.31	tr_D0A9V0_
HLA-DQA10501-DQB10201	630	RIAATSGGASHMQGF	SGGASHMQG	5	0.1015	16665.1	90.00	0.18	tr_D0A9V0_
HLA-DQA10501-DQB10201	631	YMAPEVAAGGRCTPQ	PEVAAGGRC	3	0.1007	16812.7	90.00	0.28	tr_D0A9V0_
HLA-DQA10501-DQB10201	632	MQAKRLMRLCRQRCR	KRLMRLCRQ	3	0.0999	16956.1	90.00	0.41	tr_D0A9V0_
HLA-DQA10501-DQB10201	633	APSSSSSLDGSGCAE	SLDGSGCAE	6	0.0996	17013.8	90.00	0.55	tr_D0A9V0_
HLA-DQA10501-DQB10201	634	NGSATETPGESSTAF	TPGESSTAF	6	0.0994	17061.9	90.00	0.44	tr_D0A9V0_
HLA-DQA10501-DQB10201	635	WTRSLKKISEGAFST	LKKISEGAF	4	0.0967	17556.9	90.00	0.39	tr_D0A9V0_
HLA-DQA10501-DQB10201	636	AFVRACCHMNPSMRP	VRACCHMNP	2	0.0963	17645.3	90.00	0.26	tr_D0A9V0_
HLA-DQA10501-DQB10201	637	PSSSSSLDGSGCAEG	SLDGSGCAE	5	0.0957	17749.4	90.00	0.49	tr_D0A9V0_
HLA-DQA10501-DQB10201	638	LFLTDSNRNVWTRSL	NRNVWTRSL	6	0.0955	17788.1	90.00	0.28	tr_D0A9V0_
HLA-DQA10501-DQB10201	639	CRIAATSGGASHMQG	RIAATSGGA	1	0.0955	17801.0	90.00	0.27	tr_D0A9V0_
HLA-DQA10501-DQB10201	640	PLFLTDSNRNVWTRS	LFLTDSNRN	1	0.0954	17816.0	90.00	0.35	tr_D0A9V0_
HLA-DQA10501-DQB10201	641	EVASKLHHPNIVRYV	LHHPNIVRY	5	0.0952	17850.5	90.00	0.32	tr_D0A9V0_
HLA-DQA10501-DQB10201	642	VWTRSLKKISEGAFS	LKKISEGAF	5	0.0947	17951.1	90.00	0.47	tr_D0A9V0_
HLA-DQA10501-DQB10201	643	VASKLHHPNIVRYVS	LHHPNIVRY	4	0.0943	18024.6	90.00	0.28	tr_D0A9V0_
HLA-DQA10501-DQB10201	644	EVAAGGRCTPQSDIF	RCTPQSDIF	6	0.0937	18139.5	90.00	0.34	tr_D0A9V0_
HLA-DQA10501-DQB10201	645	PCRIAATSGGASHMQ	RIAATSGGA	2	0.0929	18298.8	90.00	0.31	tr_D0A9V0_
HLA-DQA10501-DQB10201	646	VAAGGRCTPQSDIFS	RCTPQSDIF	5	0.0922	18437.3	90.00	0.29	tr_D0A9V0_
HLA-DQA10501-DQB10201	647	VPETIDSPRRPDGEG	PETIDSPRR	1	0.0899	18900.4	95.00	0.35	tr_D0A9V0_
HLA-DQA10501-DQB10201	648	RKIQGHYAKLRRQLA	YAKLRRQLA	6	0.0891	19057.1	95.00	0.48	tr_D0A9V0_

HLA-DQA10501-DQB10201	649	KIQGHYAKLRRQLAV	YAKLRRQLA	5	0.0889	19110.0	95.00	0.44	tr_D0A9V0_
HLA-DQA10501-DQB10201	650	GPCRIAATSGGASHM	RIAATSGGA	3	0.0888	19127.8	95.00	0.38	tr_D0A9V0_
HLA-DQA10501-DQB10201	651	QAKRLMRLCRQRCRR	KRLMRLCRQ	2	0.0887	19153.6	95.00	0.46	tr_D0A9V0_
HLA-DQA10501-DQB10201	652	NVWTRSLKKISEGAF	LKKISEGAF	6	0.0884	19204.7	95.00	0.57	tr_D0A9V0_
HLA-DQA10501-DQB10201	653	IFKEKPNVFNRRHCT	IFKEKPNVF	0	0.0882	19261.3	95.00	0.62	tr_D0A9V0_
HLA-DQA10501-DQB10201	654	KMYAPSSSSSLDGSG	YAPSSSSSL	2	0.0877	19366.4	95.00	0.38	tr_D0A9V0_
HLA-DQA10501-DQB10201	655	APEVAAGGRCTPQSD	PEVAAGGRC	1	0.0858	19752.0	95.00	0.24	tr_D0A9V0_
HLA-DQA10501-DQB10201	656	MYAPSSSSSLDGSGC	YAPSSSSSL	1	0.0858	19753.5	95.00	0.35	tr_D0A9V0_
HLA-DQA10501-DQB10201	657	LGIVHCDVKPHNVLL	GIVHCDVKP	1	0.0858	19759.9	95.00	0.28	tr_D0A9V0_
HLA-DQA10501-DQB10201	658	MAPEVAAGGRCTPQS	PEVAAGGRC	2	0.0856	19797.8	95.00	0.28	tr_D0A9V0_
HLA-DQA10501-DQB10201	659	AVRDGPCRIAATSGG	AVRDGPCRI	0	0.0853	19857.2	95.00	0.50	tr_D0A9V0_
HLA-DQA10501-DQB10201	660	RDGPCRIAATSGGAS	RIAATSGGA	5	0.0846	20022.2	95.00	0.46	tr_D0A9V0_
HLA-DQA10501-DQB10201	661	MEVASKLHHPNIVRY	LHHPNIVRY	6	0.0837	20220.3	95.00	0.38	tr_D0A9V0_
HLA-DQA10501-DQB10201	662	EMEVASKLHHPNIVR	EMEVASKLH	0	0.0836	20233.9	95.00	0.40	tr_D0A9V0_
HLA-DQA10501-DQB10201	663	ANGSATETPGESSTA	ANGSATETP	0	0.0816	20687.3	95.00	0.25	tr_D0A9V0_
HLA-DQA10501-DQB10201	664	DGPCRIAATSGGASH	RIAATSGGA	4	0.0815	20693.3	95.00	0.41	tr_D0A9V0_
HLA-DQA10501-DQB10201	665	PEVAAGGRCTPQSDI	EVAAGGRCT	1	0.0814	20717.3	95.00	0.19	tr_D0A9V0_
HLA-DQA10501-DQB10201	666	YAPSSSSSLDGSGCA	YAPSSSSSL	0	0.0786	21356.4	95.00	0.23	tr_D0A9V0_
HLA-DQA10501-DQB10201	667	IVHCDVKPHNVLLGT	KPHNVLLGT	6	0.0784	21417.5	95.00	0.31	tr_D0A9V0_
HLA-DQA10501-DQB10201	668	AKRLMRLCRQRCRRN	KRLMRLCRQ	1	0.0780	21506.4	95.00	0.46	tr_D0A9V0_
HLA-DQA10501-DQB10201	669	GIVHCDVKPHNVLLG	VKPHNVLLG	6	0.0767	21815.8	95.00	0.19	tr_D0A9V0_
HLA-DQA10501-DQB10201	670	RLCRQRCRRNDTVPY	CRRNDTVPY	6	0.0757	22045.7	95.00	0.50	tr_D0A9V0_
HLA-DQA10501-DQB10201	671	VRDGPCRIAATSGGA	RIAATSGGA	6	0.0757	22047.8	95.00	0.55	tr_D0A9V0_
HLA-DQA10501-DQB10201	672	FLTDSNRNVWTRSLK	NRNVWTRSL	5	0.0749	22231.6	95.00	0.29	tr_D0A9V0_
HLA-DQA10501-DQB10201	673	PETIDSPRRPDGEGS	PETIDSPRR	0	0.0728	22744.2	95.00	0.31	tr_D0A9V0_
HLA-DQA10501-DQB10201	674	FVRACCHMNPSMRPS	VRACCHMNP	1	0.0710	23194.3	95.00	0.28	tr_D0A9V0_
HLA-DQA10501-DQB10201	675	EKPNVFNRRHCTVIA	NRRHCTVIA	6	0.0693	23620.2	95.00	0.39	tr_D0A9V0_
HLA-DQA10501-DQB10201	676	AALPMHCRRCCQFAF	CRRCCQFAF	6	0.0692	23643.5	95.00	0.31	tr_D0A9V0_
HLA-DQA10501-DQB10201	677	ILARVESKNEAGGHI	ARVESKNEA	2	0.0662	24440.2	95.00	0.28	tr_D0A9V0_
HLA-DQA10501-DQB10201	678	KNEAGGHIRANGSAT	HIRANGSAT	6	0.0660	24493.9	95.00	0.34	tr_D0A9V0_
HLA-DQA10501-DQB10201	679	KRLMRLCRQRCRRND	KRLMRLCRQ	0	0.0646	24858.7	95.00	0.42	tr_D0A9V0_
HLA-DQA10501-DQB10201	680	TIDSPRRPDGEGSES	RPDGEGSES	6	0.0629	25314.9	100.00	0.32	tr_D0A9V0_
HLA-DQA10501-DQB10201	681	ETIDSPRRPDGEGSE	RRPDGEGSE	6	0.0616	25685.5	100.00	0.30	tr_D0A9V0_
HLA-DQA10501-DQB10201	682	QVYHPDVWKSSTKYH	QVYHPDVWK	0	0.0589	26433.4	100.00	0.38	tr_D0A9V0_
HLA-DQA10501-DQB10201	683	SKNEAGGHIRANGSA	GGHIRANGS	5	0.0575	26832.0	100.00	0.36	tr_D0A9V0_
HLA-DQA10501-DQB10201	684	KEKPNVFNRRHCTVI	VFNRRHCTV	5	0.0572	26925.9	100.00	0.44	tr_D0A9V0_
HLA-DQA10501-DQB10201	685	LTDSNRNVWTRSLKK	NRNVWTRSL	4	0.0570	26990.1	100.00	0.37	tr_D0A9V0_
HLA-DQA10501-DQB10201	686	HAALPMHCRRCCQFA	MHCRRCCQF	5	0.0565	27123.8	100.00	0.41	tr_D0A9V0_
HLA-DQA10501-DQB10201	687	FKEKPNVFNRRHCTV	VFNRRHCTV	6	0.0553	27475.0	100.00	0.50	tr_D0A9V0_
HLA-DQA10501-DQB10201	688	LARVESKNEAGGHIR	ARVESKNEA	1	0.0549	27611.5	100.00	0.32	tr_D0A9V0_
HLA-DQA10501-DQB10201	689	ESKNEAGGHIRANGS	GGHIRANGS	6	0.0542	27813.6	100.00	0.33	tr_D0A9V0_
HLA-DQA10501-DQB10201	690	VRACCHMNPSMRPSA	VRACCHMNP	0	0.0539	27909.5	100.00	0.24	tr_D0A9V0_
HLA-DQA10501-DQB10201	691	ARVESKNEAGGHIRA	NEAGGHIRA	6	0.0531	28163.4	100.00	0.23	tr_D0A9V0_
HLA-DQA10501-DQB10201	692	TDSNRNVWTRSLKKI	NRNVWTRSL	3	0.0528	28247.0	100.00	0.31	tr_D0A9V0_
HLA-DQA10501-DQB10201	693	SNRNVWTRSLKKISE	TRSLKKISE	6	0.0512	28737.2	100.00	0.29	tr_D0A9V0_
HLA-DQA10501-DQB10201	694	RNVWTRSLKKISEGA	SLKKISEGA	6	0.0500	29117.4	100.00	0.35	tr_D0A9V0_
HLA-DQA10501-DQB10201	695	DSNRNVWTRSLKKIS	NRNVWTRSL	2	0.0492	29372.2	100.00	0.31	tr_D0A9V0_
HLA-DQA10501-DQB10201	696	CCHMNPSMRPSASSL	SMRPSASSL	6	0.0489	29465.1	100.00	0.44	tr_D0A9V0_
HLA-DQA10501-DQB10201	697	HPDVWKSSTKYHAAL	SSTKYHAAL	6	0.0489	29466.1	100.00	0.27	tr_D0A9V0_
HLA-DQA10501-DQB10201	698	NRNVWTRSLKKISEG	TRSLKKISE	5	0.0451	30681.6	100.00	0.25	tr_D0A9V0_

HLA-DQA10501-DQB10201	699	RVESKNEAGGHIRAN	NEAGGHIRA	5	0.0450	30732.1	100.00	0.29	tr_D0A9V0_	
HLA-DQA10501-DQB10201	700	VESKNEAGGHIRANG	NEAGGHIRA	4	0.0441	31030.1	100.00	0.28	tr_D0A9V0_	
HLA-DQA10501-DQB10201	701	VYHPDVWKSSTKYHA	DVWKSSTKY	4	0.0433	31312.4	100.00	0.40	tr_D0A9V0_	
HLA-DQA10501-DQB10201	702	MRLCRQRCRRNDTVP	QRCRRNDTV	5	0.0430	31383.3	100.00	0.47	tr_D0A9V0_	
HLA-DQA10501-DQB10201	703	LMRLCRQRCRRNDTV	QRCRRNDTV	6	0.0425	31561.0	100.00	0.41	tr_D0A9V0_	
HLA-DQA10501-DQB10201	704	RACCHMNPSMRPSAS	CHMNPSMRP	3	0.0425	31566.8	100.00	0.31	tr_D0A9V0_	
HLA-DQA10501-DQB10201	705	YHPDVWKSSTKYHAA	DVWKSSTKY	3	0.0414	31949.6	100.00	0.37	tr_D0A9V0_	
HLA-DQA10501-DQB10201	706	ACCHMNPSMRPSASS	CHMNPSMRP	2	0.0409	32127.4	100.00	0.29	tr_D0A9V0_	
HLA-DQA10501-DQB10201	707	RLMRLCRQRCRRNDT	LMRLCRQRC	1	0.0376	33298.2	100.00	0.41	tr_D0A9V0_	

Allele: HLA-DQA10501-DQB10201. Number of high binders 1. Number of weak binders 56. Number of peptides 707

Allele	pos	peptide	core	Of	1-log50k(aff)	affinity(nM)	%Rank	Relia	Identity	Bind Level
HLA-DQA10501-DQB10201	1	KANHVIVGLDADLIF	IVGLDADLI	5	0.6917	28.1	0.20	0.60	tr_C9ZP82_	SB
HLA-DQA10501-DQB10201	2	ANHVIVGLDADLIFL	IVGLDADLI	4	0.6854	30.1	0.25	0.60	tr_C9ZP82_	SB
HLA-DQA10501-DQB10201	3	NHVIVGLDADLIFLA	IVGLDADLI	3	0.6709	35.2	0.40	0.62	tr_C9ZP82_	SB
HLA-DQA10501-DQB10201	4	HVIVGLDADLIFLAL	IVGLDADLI	2	0.6640	37.9	0.40	0.61	tr_C9ZP82_	SB
HLA-DQA10501-DQB10201	5	GKANHVIVGLDADLI	IVGLDADLI	6	0.6523	43.0	0.50	0.68	tr_C9ZP82_	WB
HLA-DQA10501-DQB10201	6	VIVGLDADLIFLALS	IVGLDADLI	1	0.6350	51.9	0.70	0.63	tr_C9ZP82_	WB
HLA-DQA10501-DQB10201	7	HNDSDTAIALLPEEE	AIALLPEEE	6	0.6313	54.0	0.70	0.68	tr_C9ZP82_	WB
HLA-DQA10501-DQB10201	8	DSDTAIALLPEEEKL	AIALLPEEE	4	0.6103	67.8	1.10	0.71	tr_C9ZP82_	WB
HLA-DQA10501-DQB10201	9	NDSDTAIALLPEEEK	AIALLPEEE	5	0.6101	67.9	1.10	0.67	tr_C9ZP82_	WB
HLA-DQA10501-DQB10201	10	SDTAIALLPEEEKLE	AIALLPEEE	3	0.6014	74.7	1.30	0.74	tr_C9ZP82_	WB
HLA-DQA10501-DQB10201	11	KEVMYIAVDGVAPRA	VMYIAVDGV	2	0.5999	75.9	1.30	0.43	tr_C9ZP82_	WB
HLA-DQA10501-DQB10201	12	ENRYVESNQVVDDVF	SNQVVDDVF	6	0.5990	76.6	1.30	0.50	tr_C9ZP82_	WB
HLA-DQA10501-DQB10201	13	QPKEVMYIAVDGVAP	VMYIAVDGV	4	0.5909	83.7	1.50	0.51	tr_C9ZP82_	WB
HLA-DQA10501-DQB10201	14	PKEVMYIAVDGVAPR	VMYIAVDGV	3	0.5865	87.7	1.60	0.47	tr_C9ZP82_	WB
HLA-DQA10501-DQB10201	15	DTAIALLPEEEKLEC	AIALLPEEE	2	0.5848	89.3	1.70	0.75	tr_C9ZP82_	WB
HLA-DQA10501-DQB10201	16	EVMYIAVDGVAPRAK	VMYIAVDGV	1	0.5813	92.8	1.80	0.44	tr_C9ZP82_	WB
HLA-DQA10501-DQB10201	17	NRYVESNQVVDDVFG	SNQVVDDVF	5	0.5690	106.0	2.50	0.49	tr_C9ZP82_	WB
HLA-DQA10501-DQB10201	18	IVGLDADLIFLALSL	IVGLDADLI	0	0.5620	114.3	2.50	0.62	tr_C9ZP82_	WB
HLA-DQA10501-DQB10201	19	LDNLLELYVSEVLPY	LELYVSEVL	4	0.5610	115.6	2.50	0.33	tr_C9ZP82_	WB
HLA-DQA10501-DQB10201	20	VMLPFADMDVLQQVV	FADMDVLQQ	4	0.5552	123.1	3.00	0.27	tr_C9ZP82_	WB
HLA-DQA10501-DQB10201	21	ALDNLLELYVSEVLP	LELYVSEVL	5	0.5537	125.1	3.00	0.34	tr_C9ZP82_	WB
HLA-DQA10501-DQB10201	22	TVMLPFADMDVLQQV	FADMDVLQQ	5	0.5522	127.1	3.00	0.29	tr_C9ZP82_	WB
HLA-DQA10501-DQB10201	23	ATVMLPFADMDVLQQ	FADMDVLQQ	6	0.5509	129.0	3.00	0.31	tr_C9ZP82_	WB
HLA-DQA10501-DQB10201	24	MLPFADMDVLQQVVD	FADMDVLQQ	3	0.5424	141.3	3.50	0.28	tr_C9ZP82_	WB
HLA-DQA10501-DQB10201	25	TAIALLPEEEKLECI	AIALLPEEE	1	0.5419	142.2	3.50	0.75	tr_C9ZP82_	WB
HLA-DQA10501-DQB10201	26	DNLLELYVSEVLPYG	LELYVSEVL	3	0.5415	142.7	3.50	0.31	tr_C9ZP82_	WB
HLA-DQA10501-DQB10201	27	NLLELYVSEVLPYGF	LELYVSEVL	2	0.5405	144.3	3.50	0.25	tr_C9ZP82_	WB
HLA-DQA10501-DQB10201	28	VMYIAVDGVAPRAKM	VMYIAVDGV	0	0.5386	147.2	3.50	0.41	tr_C9ZP82_	WB
HLA-DQA10501-DQB10201	29	VQPKEVMYIAVDGVA	VMYIAVDGV	5	0.5379	148.3	3.50	0.56	tr_C9ZP82_	WB
HLA-DQA10501-DQB10201	30	AFSGESALDNLLELY	ALDNLLELY	6	0.5362	151.1	3.50	0.29	tr_C9ZP82_	WB
HLA-DQA10501-DQB10201	31	LPFADMDVLQQVVDA	FADMDVLQQ	2	0.5361	151.3	3.50	0.25	tr_C9ZP82_	WB
HLA-DQA10501-DQB10201	32	YSGALYNEGEEEAGD	ALYNEGEEE	3	0.5349	153.2	3.50	0.67	tr_C9ZP82_	WB
HLA-DQA10501-DQB10201	33	PLYSGALYNEGEEEA	ALYNEGEEE	5	0.5339	154.9	3.50	0.65	tr_C9ZP82_	WB

HLA-DQA10501-DQB10201	34	RYVESNQVVDDVFGH	SNQVVDDVF	4	0.5339	155.0	3.50	0.50	tr_C9ZP82_	WB
HLA-DQA10501-DQB10201	35	LATVMLPFADMDVLQ	PFADMDVLQ	6	0.5277	165.7	4.00	0.23	tr_C9ZP82_	WB
HLA-DQA10501-DQB10201	36	LLELYVSEVLPYGFL	ELYVSEVLP	2	0.5245	171.4	4.00	0.29	tr_C9ZP82_	WB
HLA-DQA10501-DQB10201	37	SPQQAELESTFPLQW	LESTFPLQW	6	0.5204	179.3	4.50	0.36	tr_C9ZP82_	WB
HLA-DQA10501-DQB10201	38	IISLNPSYIDEEESD	PSYIDEEES	5	0.5187	182.6	4.50	0.32	tr_C9ZP82_	WB
HLA-DQA10501-DQB10201	39	LYSGALYNEGEEEAG	ALYNEGEEE	4	0.5187	182.7	4.50	0.64	tr_C9ZP82_	WB
HLA-DQA10501-DQB10201	40	TVQPKEVMYIAVDGV	VMYIAVDGV	6	0.5152	189.7	4.50	0.69	tr_C9ZP82_	WB
HLA-DQA10501-DQB10201	41	FSGESALDNLLELYV	ALDNLLELY	5	0.5144	191.4	4.50	0.29	tr_C9ZP82_	WB
HLA-DQA10501-DQB10201	42	YMPNKSGMAIVEEVV	GMAIVEEVV	6	0.5115	197.5	5.00	0.43	tr_C9ZP82_	WB
HLA-DQA10501-DQB10201	43	PFADMDVLQQVVDAA	VLQQVVDAA	6	0.5114	197.7	5.00	0.25	tr_C9ZP82_	WB
HLA-DQA10501-DQB10201	44	SGALYNEGEEEAGDA	ALYNEGEEE	2	0.5100	200.7	5.00	0.64	tr_C9ZP82_	WB
HLA-DQA10501-DQB10201	45	CIISLNPSYIDEEES	PSYIDEEES	6	0.5074	206.4	5.00	0.31	tr_C9ZP82_	WB
HLA-DQA10501-DQB10201	46	HLATVMLPFADMDVL	LPFADMDVL	6	0.5057	210.3	5.00	0.26	tr_C9ZP82_	WB
HLA-DQA10501-DQB10201	47	DDVFGHRGEVIVDDP	RGEVIVDDP	6	0.5045	212.9	5.50	0.39	tr_C9ZP82_	WB
HLA-DQA10501-DQB10201	48	ADMDVLQQVVDAAWP	VLQQVVDAA	4	0.5043	213.3	5.50	0.28	tr_C9ZP82_	WB
HLA-DQA10501-DQB10201	49	SALDNLLELYVSEVL	LELYVSEVL	6	0.5031	216.2	5.50	0.44	tr_C9ZP82_	WB
HLA-DQA10501-DQB10201	50	DVLQQVVDAAWPQLT	LQQVVDAAW	2	0.5011	221.1	5.50	0.19	tr_C9ZP82_	WB
HLA-DQA10501-DQB10201	51	TSTEMAQANDDLDEV	EMAQANDDL	3	0.5000	223.6	5.50	0.38	tr_C9ZP82_	WB
HLA-DQA10501-DQB10201	52	DVFGHRGEVIVDDPT	RGEVIVDDP	5	0.4993	225.4	5.50	0.36	tr_C9ZP82_	WB
HLA-DQA10501-DQB10201	53	FADMDVLQQVVDAAW	VLQQVVDAA	5	0.4991	225.9	5.50	0.25	tr_C9ZP82_	WB
HLA-DQA10501-DQB10201	54	MDVLQQVVDAAWPQL	VLQQVVDAA	2	0.4949	236.2	6.00	0.21	tr_C9ZP82_	WB
HLA-DQA10501-DQB10201	55	PQQAELESTFPLQWN	LESTFPLQW	5	0.4947	236.8	6.00	0.37	tr_C9ZP82_	WB
HLA-DQA10501-DQB10201	56	YVESNQVVDDVFGHR	SNQVVDDVF	3	0.4933	240.4	6.00	0.53	tr_C9ZP82_	WB
HLA-DQA10501-DQB10201	57	SAFSGESALDNLLEL	SALDNLLEL	6	0.4915	245.1	6.00	0.32	tr_C9ZP82_	WB
HLA-DQA10501-DQB10201	58	GALYNEGEEEAGDAL	ALYNEGEEE	1	0.4900	249.2	6.00	0.61	tr_C9ZP82_	WB
HLA-DQA10501-DQB10201	59	LELYVSEVLPYGFLT	ELYVSEVLP	1	0.4894	250.8	6.50	0.28	tr_C9ZP82_	WB
HLA-DQA10501-DQB10201	60	EFTSTEMAQANDDLD	EMAQANDDL	5	0.4884	253.5	6.50	0.47	tr_C9ZP82_	WB
HLA-DQA10501-DQB10201	61	FTSTEMAQANDDLDE	EMAQANDDL	4	0.4871	257.1	6.50	0.45	tr_C9ZP82_	WB
HLA-DQA10501-DQB10201	62	DPLYSGALYNEGEEE	ALYNEGEEE	6	0.4867	258.2	6.50	0.73	tr_C9ZP82_	WB
HLA-DQA10501-DQB10201	63	VFGHRGEVIVDDPTA	RGEVIVDDP	4	0.4858	260.7	6.50	0.34	tr_C9ZP82_	WB
HLA-DQA10501-DQB10201	64	VVDAAWPQLTEEERW	PQLTEEERW	6	0.4841	265.7	6.50	0.32	tr_C9ZP82_	WB
HLA-DQA10501-DQB10201	65	FGHRGEVIVDDPTAF	RGEVIVDDP	3	0.4821	271.3	7.00	0.35	tr_C9ZP82_	WB
HLA-DQA10501-DQB10201	66	DMDVLQQVVDAAWPQ	VLQQVVDAA	3	0.4819	272.0	7.00	0.26	tr_C9ZP82_	WB
HLA-DQA10501-DQB10201	67	NKSGMAIVEEVVREF	GMAIVEEVV	3	0.4818	272.4	7.00	0.38	tr_C9ZP82_	WB
HLA-DQA10501-DQB10201	68	KSGMAIVEEVVREFT	GMAIVEEVV	2	0.4813	273.7	7.00	0.31	tr_C9ZP82_	WB
HLA-DQA10501-DQB10201	69	TCIISLNPSYIDEEE	IISLNPSYI	2	0.4803	276.8	7.00	0.34	tr_C9ZP82_	WB
HLA-DQA10501-DQB10201	70	DLDEVRQTLLEDPLY	EVRQTLLED	3	0.4801	277.5	7.00	0.28	tr_C9ZP82_	WB
HLA-DQA10501-DQB10201	71	ISLNPSYIDEEESDG	PSYIDEEES	4	0.4799	277.9	7.00	0.37	tr_C9ZP82_	WB
HLA-DQA10501-DQB10201	72	HENRYVESNQVVDDV	ESNQVVDDV	6	0.4786	282.0	7.00	0.30	tr_C9ZP82_	WB
HLA-DQA10501-DQB10201	73	VHLATVMLPFADMDV	LATVMLPFA	2	0.4773	285.7	7.00	0.35	tr_C9ZP82_	WB
HLA-DQA10501-DQB10201	74	SGESALDNLLELYVS	ALDNLLELY	4	0.4769	287.1	7.50	0.31	tr_C9ZP82_	WB
HLA-DQA10501-DQB10201	75	HRGEVIVDDPTAFEF	RGEVIVDDP	1	0.4760	289.9	7.50	0.28	tr_C9ZP82_	WB
HLA-DQA10501-DQB10201	76	SLNPSYIDEEESDGV	PSYIDEEES	3	0.4748	293.6	7.50	0.35	tr_C9ZP82_	WB
HLA-DQA10501-DQB10201	77	STEMAQANDDLDEVR	EMAQANDDL	2	0.4741	296.0	7.50	0.39	tr_C9ZP82_	WB
HLA-DQA10501-DQB10201	78	KNCPSSVHGLYIDLN	VHGLYIDLN	6	0.4739	296.4	7.50	0.65	tr_C9ZP82_	WB
HLA-DQA10501-DQB10201	79	MPNKSGMAIVEEVVR	GMAIVEEVV	5	0.4730	299.6	7.50	0.41	tr_C9ZP82_	WB
HLA-DQA10501-DQB10201	80	VLQQVVDAAWPQLTE	VVDAAWPQL	4	0.4711	305.7	7.50	0.28	tr_C9ZP82_	WB
HLA-DQA10501-DQB10201	81	VVHLATVMLPFADMD	LATVMLPFA	3	0.4710	305.9	7.50	0.41	tr_C9ZP82_	WB
HLA-DQA10501-DQB10201	82	QQAELESTFPLQWNV	LESTFPLQW	4	0.4700	309.3	8.00	0.40	tr_C9ZP82_	WB
HLA-DQA10501-DQB10201	83	PNKSGMAIVEEVVRE	GMAIVEEVV	4	0.4695	311.0	8.00	0.40	tr_C9ZP82_	WB

HLA-DQA10501-DQB10201	84	GGKANHVIVGLDADL	VIVGLDADL	6	0.4670	319.7	8.00	0.51	tr_C9ZP82_	WB
HLA-DQA10501-DQB10201	85	GHRGEVIVDDPTAFE	RGEVIVDDP	2	0.4657	324.0	8.00	0.32	tr_C9ZP82_	WB
HLA-DQA10501-DQB10201	86	CHNDSDTAIALLPEE	TAIALLPEE	6	0.4655	324.8	8.00	0.54	tr_C9ZP82_	WB
HLA-DQA10501-DQB10201	87	REFTSTEMAQANDDL	EMAQANDDL	6	0.4645	328.3	8.50	0.49	tr_C9ZP82_	WB
HLA-DQA10501-DQB10201	88	TYMPNKSGMAIVEEV	SGMAIVEEV	6	0.4641	329.6	8.50	0.60	tr_C9ZP82_	WB
HLA-DQA10501-DQB10201	89	GVEIRQLYDVPLERY	QLYDVPLER	5	0.4618	338.2	8.50	0.26	tr_C9ZP82_	WB
HLA-DQA10501-DQB10201	90	ESALDNLLELYVSEV	LLELYVSEV	6	0.4614	339.6	8.50	0.35	tr_C9ZP82_	WB
HLA-DQA10501-DQB10201	91	RGEVIVDDPTAFEFD	VIVDDPTAF	3	0.4606	342.4	8.50	0.22	tr_C9ZP82_	WB
HLA-DQA10501-DQB10201	92	LNPSYIDEEESDGVI	PSYIDEEES	2	0.4585	350.2	9.00	0.34	tr_C9ZP82_	WB
HLA-DQA10501-DQB10201	93	LQQVVDAAWPQLTEE	VVDAAWPQL	3	0.4582	351.3	9.00	0.29	tr_C9ZP82_	WB
HLA-DQA10501-DQB10201	94	LDEVRQTLLEDPLYS	EVRQTLLED	2	0.4566	357.6	9.00	0.28	tr_C9ZP82_	WB
HLA-DQA10501-DQB10201	95	RSPQQAELESTFPLQ	QAELESTFP	4	0.4566	357.7	9.00	0.28	tr_C9ZP82_	WB
HLA-DQA10501-DQB10201	96	DTCIISLNPSYIDEE	IISLNPSYI	3	0.4548	364.8	9.00	0.47	tr_C9ZP82_	WB
HLA-DQA10501-DQB10201	97	GESALDNLLELYVSE	ALDNLLELY	3	0.4547	364.9	9.00	0.31	tr_C9ZP82_	WB
HLA-DQA10501-DQB10201	98	CPSSVHGLYIDLNGI	VHGLYIDLN	4	0.4528	372.7	9.50	0.59	tr_C9ZP82_	WB
HLA-DQA10501-DQB10201	99	PSAFSGESALDNLLE	GESALDNLL	5	0.4527	373.0	9.50	0.18	tr_C9ZP82_	WB
HLA-DQA10501-DQB10201	100	AIALLPEEEKLECIC	AIALLPEEE	0	0.4521	375.5	9.50	0.81	tr_C9ZP82_	WB
HLA-DQA10501-DQB10201	101	DWSWYYPFYHAPPVI	PFYHAPPVI	6	0.4519	376.1	9.50	0.38	tr_C9ZP82_	WB
HLA-DQA10501-DQB10201	102	NCPSSVHGLYIDLNG	VHGLYIDLN	5	0.4518	376.6	9.50	0.62	tr_C9ZP82_	WB
HLA-DQA10501-DQB10201	103	VGGSLISEVSALCDI	ISEVSALCD	5	0.4516	377.5	9.50	0.34	tr_C9ZP82_	WB
HLA-DQA10501-DQB10201	104	DDLDEVRQTLLEDPL	EVRQTLLED	4	0.4512	379.3	9.50	0.34	tr_C9ZP82_	WB
HLA-DQA10501-DQB10201	105	SWYYPFYHAPPVIDL	PFYHAPPVI	4	0.4505	382.1	9.50	0.40	tr_C9ZP82_	WB
HLA-DQA10501-DQB10201	106	PSSVHGLYIDLNGII	VHGLYIDLN	3	0.4493	386.9	9.50	0.55	tr_C9ZP82_	WB
HLA-DQA10501-DQB10201	107	EEKLECICSELELLV	LECICSELE	3	0.4470	396.8	10.00	0.41	tr_C9ZP82_	
HLA-DQA10501-DQB10201	108	SGMAIVEEVVREFTS	GMAIVEEVV	1	0.4462	400.1	10.00	0.34	tr_C9ZP82_	
HLA-DQA10501-DQB10201	109	VESNQVVDDVFGHRG	SNQVVDDVF	2	0.4460	401.0	10.00	0.60	tr_C9ZP82_	
HLA-DQA10501-DQB10201	110	QAELESTFPLQWNVD	LESTFPLQW	3	0.4453	404.0	10.00	0.44	tr_C9ZP82_	
HLA-DQA10501-DQB10201	111	QRLFEAYAKIEAVKF	EAYAKIEAV	4	0.4426	415.9	11.00	0.43	tr_C9ZP82_	
HLA-DQA10501-DQB10201	112	IDDTCIISLNPSYID	IISLNPSYI	5	0.4420	418.8	11.00	0.56	tr_C9ZP82_	
HLA-DQA10501-DQB10201	113	APSAFSGESALDNLL	GESALDNLL	6	0.4417	420.2	11.00	0.19	tr_C9ZP82_	
HLA-DQA10501-DQB10201	114	SSVHGLYIDLNGIIH	VHGLYIDLN	2	0.4400	427.8	11.00	0.52	tr_C9ZP82_	
HLA-DQA10501-DQB10201	115	QQVVDAAWPQLTEEE	VVDAAWPQL	2	0.4399	428.3	11.00	0.28	tr_C9ZP82_	
HLA-DQA10501-DQB10201	116	TEMAQANDDLDEVRQ	EMAQANDDL	1	0.4394	430.9	11.00	0.40	tr_C9ZP82_	
HLA-DQA10501-DQB10201	117	EEEKLECICSELELL	LECICSELE	4	0.4387	433.9	11.00	0.46	tr_C9ZP82_	
HLA-DQA10501-DQB10201	118	SILPPTSHALLPMVL	SHALLPMVL	6	0.4387	434.2	11.00	0.64	tr_C9ZP82_	
HLA-DQA10501-DQB10201	119	WYYPFYHAPPVIDLA	PFYHAPPVI	3	0.4377	438.9	11.00	0.38	tr_C9ZP82_	
HLA-DQA10501-DQB10201	120	VRSPQQAELESTFPL	QAELESTFP	5	0.4375	439.9	11.00	0.34	tr_C9ZP82_	
HLA-DQA10501-DQB10201	121	DDTCIISLNPSYIDE	IISLNPSYI	4	0.4372	441.4	11.00	0.56	tr_C9ZP82_	
HLA-DQA10501-DQB10201	122	SPGTDFMAKVSDAVL	FMAKVSDAV	5	0.4372	441.4	11.00	0.60	tr_C9ZP82_	
HLA-DQA10501-DQB10201	123	VDDVFGHRGEVIVDD	VFGHRGEVI	3	0.4366	444.0	11.00	0.41	tr_C9ZP82_	
HLA-DQA10501-DQB10201	124	WSWYYPFYHAPPVID	PFYHAPPVI	5	0.4358	447.8	11.00	0.40	tr_C9ZP82_	
HLA-DQA10501-DQB10201	125	THENRYVESNQVVDD	NRYVESNQV	3	0.4343	455.4	12.00	0.29	tr_C9ZP82_	
HLA-DQA10501-DQB10201	126	VVGGSLISEVSALCD	ISEVSALCD	6	0.4338	457.7	12.00	0.38	tr_C9ZP82_	
HLA-DQA10501-DQB10201	127	ALYNEGEEEAGDALN	ALYNEGEEE	0	0.4330	461.4	12.00	0.55	tr_C9ZP82_	
HLA-DQA10501-DQB10201	128	EGVEIRQLYDVPLER	QLYDVPLER	6	0.4329	462.1	12.00	0.28	tr_C9ZP82_	
HLA-DQA10501-DQB10201	129	QVVDAAWPQLTEEER	WPQLTEEER	6	0.4325	464.2	12.00	0.22	tr_C9ZP82_	
HLA-DQA10501-DQB10201	130	VEIRQLYDVPLERYR	QLYDVPLER	4	0.4321	466.2	12.00	0.27	tr_C9ZP82_	
HLA-DQA10501-DQB10201	131	ADVAVALHSTIDDRW	LHSTIDDRW	6	0.4320	466.8	12.00	0.51	tr_C9ZP82_	
HLA-DQA10501-DQB10201	132	REGVEIRQLYDVPLE	RQLYDVPLE	6	0.4310	471.6	12.00	0.34	tr_C9ZP82_	
HLA-DQA10501-DQB10201	133	AIVEEVVREFTSTEM	VREFTSTEM	6	0.4262	496.8	13.00	0.58	tr_C9ZP82_	

HLA-DQA10501-DQB10201	134	TVPNTGFEYFDISVV	FEYFDISVV	6	0.4259	498.6	13.00	0.52	tr_C9ZP82_
HLA-DQA10501-DQB10201	135	YYPFYHAPPVIDLAI	PFYHAPPVI	2	0.4254	501.0	13.00	0.34	tr_C9ZP82_
HLA-DQA10501-DQB10201	136	DEVRQTLLEDPLYSG	EVRQTLLED	1	0.4242	507.5	13.00	0.28	tr_C9ZP82_
HLA-DQA10501-DQB10201	137	EIRQLYDVPLERYRP	QLYDVPLER	3	0.4241	508.5	13.00	0.34	tr_C9ZP82_
HLA-DQA10501-DQB10201	138	VDAAWPQLTEEERWR	PQLTEEERW	5	0.4240	509.1	13.00	0.34	tr_C9ZP82_
HLA-DQA10501-DQB10201	139	EKLECICSELELLVQ	LECICSELE	2	0.4230	514.2	13.00	0.41	tr_C9ZP82_
HLA-DQA10501-DQB10201	140	GTDFMAKVSDAVLNM	FMAKVSDAV	3	0.4222	519.1	13.00	0.54	tr_C9ZP82_
HLA-DQA10501-DQB10201	141	NVVHLATVMLPFADM	LATVMLPFA	4	0.4215	522.6	13.00	0.47	tr_C9ZP82_
HLA-DQA10501-DQB10201	142	TDFMAKVSDAVLNML	FMAKVSDAV	2	0.4213	524.2	13.00	0.49	tr_C9ZP82_
HLA-DQA10501-DQB10201	143	SVHGLYIDLNGIIHP	VHGLYIDLN	1	0.4206	527.8	13.00	0.47	tr_C9ZP82_
HLA-DQA10501-DQB10201	144	PGTDFMAKVSDAVLN	FMAKVSDAV	4	0.4205	528.3	13.00	0.62	tr_C9ZP82_
HLA-DQA10501-DQB10201	145	GGSLISEVSALCDIK	ISEVSALCD	4	0.4196	533.8	13.00	0.34	tr_C9ZP82_
HLA-DQA10501-DQB10201	146	FIDDTCIISLNPSYI	IISLNPSYI	6	0.4185	540.0	13.00	0.50	tr_C9ZP82_
HLA-DQA10501-DQB10201	147	GEVIVDDPTAFEFDS	VIVDDPTAF	2	0.4182	541.6	13.00	0.31	tr_C9ZP82_
HLA-DQA10501-DQB10201	148	SNVVHLATVMLPFAD	LATVMLPFA	5	0.4179	543.5	14.00	0.50	tr_C9ZP82_
HLA-DQA10501-DQB10201	149	MYIAVDGVAPRAKMN	YIAVDGVAP	1	0.4169	549.2	14.00	0.36	tr_C9ZP82_
HLA-DQA10501-DQB10201	150	HAPSAFSGESALDNL	PSAFSGESA	2	0.4160	555.1	14.00	0.23	tr_C9ZP82_
HLA-DQA10501-DQB10201	151	DIIVLSILLGNDFLP	SILLGNDFL	5	0.4145	563.8	14.00	0.37	tr_C9ZP82_
HLA-DQA10501-DQB10201	152	NDDLDEVRQTLLEDP	EVRQTLLED	5	0.4136	569.8	14.00	0.43	tr_C9ZP82_
HLA-DQA10501-DQB10201	153	VPNTGFEYFDISVVG	FEYFDISVV	5	0.4135	570.2	14.00	0.46	tr_C9ZP82_
HLA-DQA10501-DQB10201	154	QLQRLFEAYAKIEAV	EAYAKIEAV	6	0.4117	581.1	14.00	0.69	tr_C9ZP82_
HLA-DQA10501-DQB10201	155	VAVALHSTIDDRWRD	LHSTIDDRW	4	0.4097	594.3	15.00	0.60	tr_C9ZP82_
HLA-DQA10501-DQB10201	156	EVIVDDPTAFEFDSN	VIVDDPTAF	1	0.4093	596.3	15.00	0.28	tr_C9ZP82_
HLA-DQA10501-DQB10201	157	NPSYIDEEESDGVIG	PSYIDEEES	1	0.4093	596.5	15.00	0.38	tr_C9ZP82_
HLA-DQA10501-DQB10201	158	DVAVALHSTIDDRWR	LHSTIDDRW	5	0.4087	600.8	15.00	0.56	tr_C9ZP82_
HLA-DQA10501-DQB10201	159	PTSHALLPMVLGEVM	SHALLPMVL	2	0.4086	601.0	15.00	0.40	tr_C9ZP82_
HLA-DQA10501-DQB10201	160	DSNVVHLATVMLPFA	LATVMLPFA	6	0.4082	603.8	15.00	0.48	tr_C9ZP82_
HLA-DQA10501-DQB10201	161	IVEEVVREFTSTEMA	VREFTSTEM	5	0.4074	608.8	15.00	0.51	tr_C9ZP82_
HLA-DQA10501-DQB10201	162	DSDWSWYYPFYHAPP	WSWYYPFYH	3	0.4048	626.5	15.00	0.47	tr_C9ZP82_
HLA-DQA10501-DQB10201	163	YPFYHAPPVIDLAIY	PFYHAPPVI	1	0.4043	629.7	15.00	0.30	tr_C9ZP82_
HLA-DQA10501-DQB10201	164	ELYVSEVLPYGFLTK	ELYVSEVLP	0	0.4041	630.9	15.00	0.27	tr_C9ZP82_
HLA-DQA10501-DQB10201	165	AELESTFPLQWNVDY	LESTFPLQW	2	0.4034	636.0	15.00	0.50	tr_C9ZP82_
HLA-DQA10501-DQB10201	166	PEEEKLECICSELEL	LECICSELE	5	0.4031	638.1	16.00	0.54	tr_C9ZP82_
HLA-DQA10501-DQB10201	167	RLFEAYAKIEAVKFR	EAYAKIEAV	3	0.4025	642.0	16.00	0.44	tr_C9ZP82_
HLA-DQA10501-DQB10201	168	IIVLSILLGNDFLPH	SILLGNDFL	4	0.4013	650.8	16.00	0.34	tr_C9ZP82_
HLA-DQA10501-DQB10201	169	ESNQVVDDVFGHRGE	SNQVVDDVF	1	0.3985	670.4	16.00	0.62	tr_C9ZP82_
HLA-DQA10501-DQB10201	170	DAAWPQLTEEERWRT	PQLTEEERW	4	0.3982	672.8	16.00	0.37	tr_C9ZP82_
HLA-DQA10501-DQB10201	171	TSHALLPMVLGEVMV	SHALLPMVL	1	0.3974	678.9	16.00	0.35	tr_C9ZP82_
HLA-DQA10501-DQB10201	172	EEESDGVIGVPVCES	VIGVPVCES	6	0.3967	683.7	16.00	0.47	tr_C9ZP82_
HLA-DQA10501-DQB10201	173	SNNDNVTVADVAVAL	TVADVAVAL	6	0.3965	684.8	16.00	0.37	tr_C9ZP82_
HLA-DQA10501-DQB10201	174	SDWSWYYPFYHAPPV	WSWYYPFYH	2	0.3962	687.8	16.00	0.42	tr_C9ZP82_
HLA-DQA10501-DQB10201	175	GMAIVEEVVREFTST	VEEVVREFT	4	0.3958	690.7	17.00	0.29	tr_C9ZP82_
HLA-DQA10501-DQB10201	176	TDSDWSWYYPFYHAP	WSWYYPFYH	4	0.3953	694.1	17.00	0.47	tr_C9ZP82_
HLA-DQA10501-DQB10201	177	ISPGTDFMAKVSDAV	FMAKVSDAV	6	0.3949	697.3	17.00	0.75	tr_C9ZP82_
HLA-DQA10501-DQB10201	178	ILPPTSHALLPMVLG	SHALLPMVL	5	0.3937	706.3	17.00	0.62	tr_C9ZP82_
HLA-DQA10501-DQB10201	179	VREFTSTEMAQANDD	FTSTEMAQA	3	0.3929	712.2	17.00	0.31	tr_C9ZP82_
HLA-DQA10501-DQB10201	180	IVLSILLGNDFLPHA	SILLGNDFL	3	0.3929	712.7	17.00	0.34	tr_C9ZP82_
HLA-DQA10501-DQB10201	181	DFMAKVSDAVLNMLN	FMAKVSDAV	1	0.3928	712.9	17.00	0.47	tr_C9ZP82_
HLA-DQA10501-DQB10201	182	KLECICSELELLVQT	LECICSELE	1	0.3925	715.5	17.00	0.43	tr_C9ZP82_
HLA-DQA10501-DQB10201	183	PNTGFEYFDISVVGG	FEYFDISVV	4	0.3920	719.8	17.00	0.42	tr_C9ZP82_

HLA-DQA10501-DQB10201	184	LECICSELELLVQTV	LECICSELE	0	0.3906	730.7	17.00	0.30	tr_C9ZP82_
HLA-DQA10501-DQB10201	185	EEVVREFTSTEMAQA	VREFTSTEM	3	0.3888	744.8	18.00	0.41	tr_C9ZP82_
HLA-DQA10501-DQB10201	186	SREGVEIRQLYDVPL	IRQLYDVPL	6	0.3880	751.4	18.00	0.32	tr_C9ZP82_
HLA-DQA10501-DQB10201	187	GSLISEVSALCDIKK	ISEVSALCD	3	0.3880	751.5	18.00	0.34	tr_C9ZP82_
HLA-DQA10501-DQB10201	188	MVRSPQQAELESTFP	QAELESTFP	6	0.3875	755.5	18.00	0.31	tr_C9ZP82_
HLA-DQA10501-DQB10201	189	AVALHSTIDDRWRDT	LHSTIDDRW	3	0.3863	765.4	18.00	0.63	tr_C9ZP82_
HLA-DQA10501-DQB10201	190	IRQLYDVPLERYRPR	QLYDVPLER	2	0.3861	766.9	18.00	0.39	tr_C9ZP82_
HLA-DQA10501-DQB10201	191	CICSELELLVQTVQP	ELLVQTVQP	6	0.3857	770.0	18.00	0.41	tr_C9ZP82_
HLA-DQA10501-DQB10201	192	LPPTSHALLPMVLGE	SHALLPMVL	4	0.3848	777.9	18.00	0.58	tr_C9ZP82_
HLA-DQA10501-DQB10201	193	NNDNVTVADVAVALH	TVADVAVAL	5	0.3848	777.9	18.00	0.31	tr_C9ZP82_
HLA-DQA10501-DQB10201	194	VEEVVREFTSTEMAQ	VREFTSTEM	4	0.3846	779.0	18.00	0.52	tr_C9ZP82_
HLA-DQA10501-DQB10201	195	PFYHAPPVIDLAIYL	PFYHAPPVI	0	0.3845	780.5	18.00	0.24	tr_C9ZP82_
HLA-DQA10501-DQB10201	196	PPTSHALLPMVLGEV	SHALLPMVL	3	0.3830	792.6	18.00	0.52	tr_C9ZP82_
HLA-DQA10501-DQB10201	197	LQRLFEAYAKIEAVK	EAYAKIEAV	5	0.3824	798.1	19.00	0.62	tr_C9ZP82_
HLA-DQA10501-DQB10201	198	TTTDSDWSWYYPFYH	WSWYYPFYH	6	0.3822	800.0	19.00	0.47	tr_C9ZP82_
HLA-DQA10501-DQB10201	199	EMAQANDDLDEVRQT	EMAQANDDL	0	0.3811	809.6	19.00	0.29	tr_C9ZP82_
HLA-DQA10501-DQB10201	200	VLSILLGNDFLPHAP	SILLGNDFL	2	0.3809	811.2	19.00	0.31	tr_C9ZP82_
HLA-DQA10501-DQB10201	201	LFEAYAKIEAVKFRR	EAYAKIEAV	2	0.3781	835.8	19.00	0.46	tr_C9ZP82_
HLA-DQA10501-DQB10201	202	STHENRYVESNQVVD	NRYVESNQV	4	0.3776	840.8	19.00	0.47	tr_C9ZP82_
HLA-DQA10501-DQB10201	203	LPEEEKLECICSELE	LECICSELE	6	0.3776	841.0	19.00	0.53	tr_C9ZP82_
HLA-DQA10501-DQB10201	204	SLISEVSALCDIKKF	ISEVSALCD	2	0.3773	843.0	19.00	0.34	tr_C9ZP82_
HLA-DQA10501-DQB10201	205	EESDGVIGVPVCESS	VIGVPVCES	5	0.3766	849.6	19.00	0.47	tr_C9ZP82_
HLA-DQA10501-DQB10201	206	PDTLVQLLSILPPTS	LLSILPPTS	6	0.3766	850.1	19.00	0.28	tr_C9ZP82_
HLA-DQA10501-DQB10201	207	VHGLYIDLNGIIHPC	VHGLYIDLN	0	0.3753	861.7	20.00	0.36	tr_C9ZP82_
HLA-DQA10501-DQB10201	208	EVRQTLLEDPLYSGA	QTLLEDPLY	3	0.3741	872.9	20.00	0.28	tr_C9ZP82_
HLA-DQA10501-DQB10201	209	TTDSDWSWYYPFYHA	WSWYYPFYH	5	0.3741	873.0	20.00	0.50	tr_C9ZP82_
HLA-DQA10501-DQB10201	210	FVWRYYTTTTTDSDW	WRYYTTTTT	2	0.3737	877.0	20.00	0.20	tr_C9ZP82_
HLA-DQA10501-DQB10201	211	VNSFCVRSTDDDYES	VRSTDDDYE	5	0.3731	883.0	20.00	0.37	tr_C9ZP82_
HLA-DQA10501-DQB10201	212	PHAPSAFSGESALDN	PSAFSGESA	3	0.3716	897.3	20.00	0.35	tr_C9ZP82_
HLA-DQA10501-DQB10201	213	VVDDVFGHRGEVIVD	VFGHRGEVI	4	0.3713	899.6	20.00	0.57	tr_C9ZP82_
HLA-DQA10501-DQB10201	214	NTGFEYFDISVVGGS	FEYFDISVV	3	0.3713	900.0	20.00	0.44	tr_C9ZP82_
HLA-DQA10501-DQB10201	215	SVVGGSLISEVSALC	VGGSLISEV	2	0.3706	906.5	21.00	0.19	tr_C9ZP82_
HLA-DQA10501-DQB10201	216	EVVREFTSTEMAQAN	VREFTSTEM	2	0.3701	911.9	21.00	0.38	tr_C9ZP82_
HLA-DQA10501-DQB10201	217	TGFEYFDISVVGGSL	FEYFDISVV	2	0.3700	912.8	21.00	0.42	tr_C9ZP82_
HLA-DQA10501-DQB10201	218	PPDTLVQLLSILPPT	VQLLSILPP	5	0.3697	915.3	21.00	0.23	tr_C9ZP82_
HLA-DQA10501-DQB10201	219	STYMPNKSGMAIVEE	KSGMAIVEE	6	0.3669	944.2	21.00	0.87	tr_C9ZP82_
HLA-DQA10501-DQB10201	220	NDNVTVADVAVALHS	TVADVAVAL	4	0.3667	946.0	21.00	0.28	tr_C9ZP82_
HLA-DQA10501-DQB10201	221	FGGKANHVIVGLDAD	NHVIVGLDA	5	0.3659	954.0	21.00	0.38	tr_C9ZP82_
HLA-DQA10501-DQB10201	222	NSFCVRSTDDDYEST	VRSTDDDYE	4	0.3658	955.3	21.00	0.38	tr_C9ZP82_
HLA-DQA10501-DQB10201	223	GVNSFCVRSTDDDYE	VRSTDDDYE	6	0.3641	973.2	22.00	0.35	tr_C9ZP82_
HLA-DQA10501-DQB10201	224	ESDGVIGVPVCESSE	VIGVPVCES	4	0.3631	983.4	22.00	0.50	tr_C9ZP82_
HLA-DQA10501-DQB10201	225	VVREFTSTEMAQAND	FTSTEMAQA	4	0.3617	998.2	22.00	0.32	tr_C9ZP82_
HLA-DQA10501-DQB10201	226	IVDDPTAFEFDSNCI	AFEFDSNCI	6	0.3613	1002.6	22.00	0.33	tr_C9ZP82_
HLA-DQA10501-DQB10201	227	AAWPQLTEEERWRTQ	PQLTEEERW	3	0.3609	1007.0	22.00	0.40	tr_C9ZP82_
HLA-DQA10501-DQB10201	228	VALHSTIDDRWRDTY	LHSTIDDRW	2	0.3603	1013.3	22.00	0.65	tr_C9ZP82_
HLA-DQA10501-DQB10201	229	GFEYFDISVVGGSLI	FEYFDISVV	1	0.3598	1019.4	22.00	0.35	tr_C9ZP82_
HLA-DQA10501-DQB10201	230	ANDDLDEVRQTLLED	EVRQTLLED	6	0.3597	1020.1	22.00	0.56	tr_C9ZP82_
HLA-DQA10501-DQB10201	231	DDIIVLSILLGNDFL	LSILLGNDF	5	0.3591	1026.6	23.00	0.38	tr_C9ZP82_
HLA-DQA10501-DQB10201	232	KGDLRKLSDALDTIL	KLSDALDTI	5	0.3586	1032.1	23.00	0.38	tr_C9ZP82_
HLA-DQA10501-DQB10201	233	DTLVQLLSILPPTSH	LLSILPPTS	5	0.3584	1034.7	23.00	0.31	tr_C9ZP82_

HLA-DQA10501-DQB10201	234	ELESTFPLQWNVDYH	LESTFPLQW	1	0.3581	1037.7	23.00	0.54	tr_C9ZP82_
HLA-DQA10501-DQB10201	235	ISVVGGSLISEVSAL	VGGSLISEV	3	0.3580	1039.8	23.00	0.28	tr_C9ZP82_
HLA-DQA10501-DQB10201	236	CCHNDSDTAIALLPE	DTAIALLPE	6	0.3578	1042.1	23.00	0.50	tr_C9ZP82_
HLA-DQA10501-DQB10201	237	SELELLVQTVQPKEV	ELLVQTVQP	3	0.3576	1043.7	23.00	0.30	tr_C9ZP82_
HLA-DQA10501-DQB10201	238	VIVDDPTAFEFDSNC	VIVDDPTAF	0	0.3553	1070.4	23.00	0.29	tr_C9ZP82_
HLA-DQA10501-DQB10201	239	SAGYYFFGQPNHIED	YFFGQPNHI	4	0.3549	1075.1	23.00	0.63	tr_C9ZP82_
HLA-DQA10501-DQB10201	240	FEAYAKIEAVKFRRF	EAYAKIEAV	1	0.3545	1079.7	23.00	0.41	tr_C9ZP82_
HLA-DQA10501-DQB10201	241	WRYYTTTTTDSDWSW	YTTTTTDSD	3	0.3527	1100.3	24.00	0.30	tr_C9ZP82_
HLA-DQA10501-DQB10201	242	VWRYYTTTTTDSDWS	YTTTTTDSD	4	0.3521	1108.1	24.00	0.26	tr_C9ZP82_
HLA-DQA10501-DQB10201	243	FMAKVSDAVLNMLNE	FMAKVSDAV	0	0.3516	1113.5	24.00	0.27	tr_C9ZP82_
HLA-DQA10501-DQB10201	244	DDPTAFEFDSNCISP	AFEFDSNCI	4	0.3510	1121.2	24.00	0.39	tr_C9ZP82_
HLA-DQA10501-DQB10201	245	MAIVEEVVREFTSTE	VEEVVREFT	3	0.3506	1126.2	24.00	0.44	tr_C9ZP82_
HLA-DQA10501-DQB10201	246	SHALLPMVLGEVMVR	LPMVLGEVM	4	0.3505	1127.6	24.00	0.30	tr_C9ZP82_
HLA-DQA10501-DQB10201	247	TTVPNTGFEYFDISV	VPNTGFEYF	2	0.3492	1142.8	24.00	0.19	tr_C9ZP82_
HLA-DQA10501-DQB10201	248	PSREGVEIRQLYDVP	EIRQLYDVP	6	0.3478	1160.8	25.00	0.28	tr_C9ZP82_
HLA-DQA10501-DQB10201	249	TPPDTLVQLLSILPP	VQLLSILPP	6	0.3477	1162.3	25.00	0.28	tr_C9ZP82_
HLA-DQA10501-DQB10201	250	ELELLVQTVQPKEVM	VQTVQPKEV	5	0.3473	1166.4	25.00	0.32	tr_C9ZP82_
HLA-DQA10501-DQB10201	251	LSILLGNDFLPHAPS	SILLGNDFL	1	0.3471	1169.0	25.00	0.33	tr_C9ZP82_
HLA-DQA10501-DQB10201	252	ESTHENRYVESNQVV	NRYVESNQV	5	0.3467	1174.4	25.00	0.57	tr_C9ZP82_
HLA-DQA10501-DQB10201	253	GSNNDNVTVADVAVA	DNVTVADVA	4	0.3462	1180.3	25.00	0.49	tr_C9ZP82_
HLA-DQA10501-DQB10201	254	QVVDDVFGHRGEVIV	VFGHRGEVI	5	0.3460	1182.7	25.00	0.61	tr_C9ZP82_
HLA-DQA10501-DQB10201	255	VTTVPNTGFEYFDIS	VPNTGFEYF	3	0.3453	1192.7	25.00	0.30	tr_C9ZP82_
HLA-DQA10501-DQB10201	256	CFVWRYYTTTTTDSD	WRYYTTTTT	3	0.3452	1193.1	25.00	0.30	tr_C9ZP82_
HLA-DQA10501-DQB10201	257	SFCVRSTDDDYESTH	VRSTDDDYE	3	0.3447	1200.1	25.00	0.40	tr_C9ZP82_
HLA-DQA10501-DQB10201	258	RQLYDVPLERYRPRT	QLYDVPLER	1	0.3440	1209.2	25.00	0.44	tr_C9ZP82_
HLA-DQA10501-DQB10201	259	HDSNVVHLATVMLPF	HLATVMLPF	6	0.3439	1210.9	25.00	0.34	tr_C9ZP82_
HLA-DQA10501-DQB10201	260	FDISVVGGSLISEVS	VGGSLISEV	5	0.3435	1215.3	26.00	0.35	tr_C9ZP82_
HLA-DQA10501-DQB10201	261	PSYIDEEESDGVIGV	PSYIDEEES	0	0.3429	1223.2	26.00	0.34	tr_C9ZP82_
HLA-DQA10501-DQB10201	262	VGLDADLIFLALSLH	VGLDADLIF	0	0.3415	1242.6	26.00	0.37	tr_C9ZP82_
HLA-DQA10501-DQB10201	263	SDGVIGVPVCESSEN	VIGVPVCES	3	0.3408	1251.3	26.00	0.52	tr_C9ZP82_
HLA-DQA10501-DQB10201	264	YFDISVVGGSLISEV	VGGSLISEV	6	0.3408	1251.3	26.00	0.41	tr_C9ZP82_
HLA-DQA10501-DQB10201	265	VDDPTAFEFDSNCIS	AFEFDSNCI	5	0.3405	1255.4	26.00	0.38	tr_C9ZP82_
HLA-DQA10501-DQB10201	266	AAPAQTGEVASTYMP	TGEVASTYM	5	0.3404	1257.4	26.00	0.60	tr_C9ZP82_
HLA-DQA10501-DQB10201	267	LPHAPSAFSGESALD	PSAFSGESA	4	0.3383	1286.4	27.00	0.48	tr_C9ZP82_
HLA-DQA10501-DQB10201	268	NFIDDTCIISLNPSY	CIISLNPSY	6	0.3372	1300.9	27.00	0.37	tr_C9ZP82_
HLA-DQA10501-DQB10201	269	AGYYFFGQPNHIEDG	YFFGQPNHI	3	0.3370	1304.6	27.00	0.63	tr_C9ZP82_
HLA-DQA10501-DQB10201	270	DNVTVADVAVALHST	TVADVAVAL	3	0.3367	1308.9	27.00	0.32	tr_C9ZP82_
HLA-DQA10501-DQB10201	271	DISVVGGSLISEVSA	VGGSLISEV	4	0.3365	1311.0	27.00	0.34	tr_C9ZP82_
HLA-DQA10501-DQB10201	272	RAAPAQTGEVASTYM	TGEVASTYM	6	0.3363	1314.8	27.00	0.62	tr_C9ZP82_
HLA-DQA10501-DQB10201	273	HVTTVPNTGFEYFDI	VPNTGFEYF	4	0.3346	1339.2	27.00	0.35	tr_C9ZP82_
HLA-DQA10501-DQB10201	274	SSAGYYFFGQPNHIE	YFFGQPNHI	5	0.3334	1355.7	28.00	0.62	tr_C9ZP82_
HLA-DQA10501-DQB10201	275	FEYFDISVVGGSLIS	FEYFDISVV	0	0.3323	1372.4	28.00	0.25	tr_C9ZP82_
HLA-DQA10501-DQB10201	276	LISEVSALCDIKKFT	ISEVSALCD	1	0.3317	1381.0	28.00	0.37	tr_C9ZP82_
HLA-DQA10501-DQB10201	277	TLVQLLSILPPTSHA	LLSILPPTS	4	0.3305	1399.1	28.00	0.35	tr_C9ZP82_
HLA-DQA10501-DQB10201	278	ICSELELLVQTVQPK	ELLVQTVQP	5	0.3304	1401.5	28.00	0.43	tr_C9ZP82_
HLA-DQA10501-DQB10201	279	LELLVQTVQPKEVMY	VQTVQPKEV	4	0.3289	1424.6	29.00	0.36	tr_C9ZP82_
HLA-DQA10501-DQB10201	280	CSELELLVQTVQPKE	ELLVQTVQP	4	0.3286	1428.6	29.00	0.42	tr_C9ZP82_
HLA-DQA10501-DQB10201	281	GDLRKLSDALDTILR	KLSDALDTI	4	0.3286	1428.7	29.00	0.38	tr_C9ZP82_
HLA-DQA10501-DQB10201	282	LESTFPLQWNVDYHD	LESTFPLQW	0	0.3275	1445.3	29.00	0.37	tr_C9ZP82_
HLA-DQA10501-DQB10201	283	DPTAFEFDSNCISPG	AFEFDSNCI	3	0.3274	1447.0	29.00	0.38	tr_C9ZP82_

HLA-DQA10501-DQB10201	284	FCVRSTDDDYESTHE	VRSTDDDYE	2	0.3257	1474.2	29.00	0.44	tr_C9ZP82_
HLA-DQA10501-DQB10201	285	QTVQPKEVMYIAVDG	EVMYIAVDG	6	0.3255	1476.7	29.00	0.62	tr_C9ZP82_
HLA-DQA10501-DQB10201	286	HGLYIDLNGIIHPCC	YIDLNGIIH	3	0.3248	1488.0	29.00	0.20	tr_C9ZP82_
HLA-DQA10501-DQB10201	287	TVADVAVALHSTIDD	VALHSTIDD	6	0.3248	1489.1	29.00	0.29	tr_C9ZP82_
HLA-DQA10501-DQB10201	288	RYYTTTTTDSDWSWY	YTTTTTDSD	2	0.3244	1494.4	30.00	0.30	tr_C9ZP82_
HLA-DQA10501-DQB10201	289	APAQTGEVASTYMPN	TGEVASTYM	4	0.3241	1500.2	30.00	0.59	tr_C9ZP82_
HLA-DQA10501-DQB10201	290	GYYFFGQPNHIEDGT	YFFGQPNHI	2	0.3241	1500.3	30.00	0.65	tr_C9ZP82_
HLA-DQA10501-DQB10201	291	KGSNNDNVTVADVAV	DNVTVADVA	5	0.3232	1515.0	30.00	0.63	tr_C9ZP82_
HLA-DQA10501-DQB10201	292	NQVVDDVFGHRGEVI	VFGHRGEVI	6	0.3229	1520.0	30.00	0.63	tr_C9ZP82_
HLA-DQA10501-DQB10201	293	VSSAGYYFFGQPNHI	YFFGQPNHI	6	0.3228	1521.6	30.00	0.58	tr_C9ZP82_
HLA-DQA10501-DQB10201	294	LIFLALSLHITQVFI	LSLHITQVF	5	0.3217	1538.5	30.00	0.33	tr_C9ZP82_
HLA-DQA10501-DQB10201	295	DLIFLALSLHITQVF	LSLHITQVF	6	0.3217	1539.2	30.00	0.43	tr_C9ZP82_
HLA-DQA10501-DQB10201	296	RKGSNNDNVTVADVA	DNVTVADVA	6	0.3211	1548.5	30.00	0.74	tr_C9ZP82_
HLA-DQA10501-DQB10201	297	HALLPMVLGEVMVRS	LPMVLGEVM	3	0.3210	1551.2	30.00	0.31	tr_C9ZP82_
HLA-DQA10501-DQB10201	298	DEEESDGVIGVPVCE	SDGVIGVPV	4	0.3210	1551.5	30.00	0.34	tr_C9ZP82_
HLA-DQA10501-DQB10201	299	IFLALSLHITQVFIL	LSLHITQVF	4	0.3192	1581.6	31.00	0.32	tr_C9ZP82_
HLA-DQA10501-DQB10201	300	DGVIGVPVCESSENS	VIGVPVCES	2	0.3190	1584.4	31.00	0.54	tr_C9ZP82_
HLA-DQA10501-DQB10201	301	KIIDFLRTQSSLAQF	RTQSSLAQF	6	0.3187	1590.9	31.00	0.28	tr_C9ZP82_
HLA-DQA10501-DQB10201	302	ECICSELELLVQTVQ	ELELLVQTV	5	0.3185	1593.0	31.00	0.31	tr_C9ZP82_
HLA-DQA10501-DQB10201	303	AWPQLTEEERWRTQK	PQLTEEERW	2	0.3183	1597.8	31.00	0.44	tr_C9ZP82_
HLA-DQA10501-DQB10201	304	YYFFGQPNHIEDGTL	YFFGQPNHI	1	0.3182	1599.0	31.00	0.59	tr_C9ZP82_
HLA-DQA10501-DQB10201	305	IDDIIVLSILLGNDF	LSILLGNDF	6	0.3163	1631.8	31.00	0.56	tr_C9ZP82_
HLA-DQA10501-DQB10201	306	QFGGKANHVIVGLDA	NHVIVGLDA	6	0.3163	1632.3	31.00	0.50	tr_C9ZP82_
HLA-DQA10501-DQB10201	307	ALLPMVLGEVMVRSP	LPMVLGEVM	2	0.3163	1632.5	31.00	0.28	tr_C9ZP82_
HLA-DQA10501-DQB10201	308	ALHSTIDDRWRDTYL	LHSTIDDRW	1	0.3162	1632.8	31.00	0.72	tr_C9ZP82_
HLA-DQA10501-DQB10201	309	PKGDLRKLSDALDTI	KLSDALDTI	6	0.3158	1641.0	31.00	0.50	tr_C9ZP82_
HLA-DQA10501-DQB10201	310	YIAVDGVAPRAKMNQ	YIAVDGVAP	0	0.3151	1653.0	32.00	0.43	tr_C9ZP82_
HLA-DQA10501-DQB10201	311	YRPRTYSCTVEVPRL	TYSCTVEVP	4	0.3150	1655.3	32.00	0.35	tr_C9ZP82_
HLA-DQA10501-DQB10201	312	SNQVVDDVFGHRGEV	SNQVVDDVF	0	0.3143	1666.9	32.00	0.58	tr_C9ZP82_
HLA-DQA10501-DQB10201	313	VRQTLLEDPLYSGAL	QTLLEDPLY	2	0.3132	1688.2	32.00	0.40	tr_C9ZP82_
HLA-DQA10501-DQB10201	314	TTTTDSDWSWYYPFY	DWSWYYPFY	6	0.3126	1698.1	32.00	0.47	tr_C9ZP82_
HLA-DQA10501-DQB10201	315	VMVRSPQQAELESTF	PQQAELEST	5	0.3125	1699.8	32.00	0.28	tr_C9ZP82_
HLA-DQA10501-DQB10201	316	LVQLLSILPPTSHAL	LLSILPPTS	3	0.3120	1709.1	32.00	0.43	tr_C9ZP82_
HLA-DQA10501-DQB10201	317	RVPSREGVEIRQLYD	REGVEIRQL	4	0.3120	1709.2	32.00	0.42	tr_C9ZP82_
HLA-DQA10501-DQB10201	318	QEACEKYVEGVCFVW	EKYVEGVCF	4	0.3115	1719.5	32.00	0.41	tr_C9ZP82_
HLA-DQA10501-DQB10201	319	AKVSDAVLNMLNEKM	VLNMLNEKM	6	0.3113	1721.9	32.00	0.43	tr_C9ZP82_
HLA-DQA10501-DQB10201	320	VADVAVALHSTIDDR	VALHSTIDD	5	0.3110	1728.6	33.00	0.28	tr_C9ZP82_
HLA-DQA10501-DQB10201	321	AHVTTVPNTGFEYFD	VPNTGFEYF	5	0.3099	1748.8	33.00	0.41	tr_C9ZP82_
HLA-DQA10501-DQB10201	322	VCFVWRYYTTTTTDS	WRYYTTTTT	4	0.3090	1765.5	33.00	0.37	tr_C9ZP82_
HLA-DQA10501-DQB10201	323	ACEKYVEGVCFVWRY	EKYVEGVCF	2	0.3083	1780.4	33.00	0.34	tr_C9ZP82_
HLA-DQA10501-DQB10201	324	VPSREGVEIRQLYDV	REGVEIRQL	3	0.3081	1783.6	33.00	0.38	tr_C9ZP82_
HLA-DQA10501-DQB10201	325	MAQANDDLDEVRQTL	QANDDLDEV	2	0.3081	1784.2	33.00	0.36	tr_C9ZP82_
HLA-DQA10501-DQB10201	326	EACEKYVEGVCFVWR	EKYVEGVCF	3	0.3075	1795.0	33.00	0.37	tr_C9ZP82_
HLA-DQA10501-DQB10201	327	YYTTTTTDSDWSWYY	YTTTTTDSD	1	0.3075	1795.7	33.00	0.31	tr_C9ZP82_
HLA-DQA10501-DQB10201	328	YHDSNVVHLATVMLP	VHLATVMLP	6	0.3074	1796.8	33.00	0.33	tr_C9ZP82_
HLA-DQA10501-DQB10201	329	IIDFLRTQSSLAQFG	FLRTQSSLA	3	0.3072	1800.8	33.00	0.30	tr_C9ZP82_
HLA-DQA10501-DQB10201	330	VEVPRLSYGADGQVI	SYGADGQVI	6	0.3072	1801.2	33.00	0.38	tr_C9ZP82_
HLA-DQA10501-DQB10201	331	ERYRPRTYSCTVEVP	TYSCTVEVP	6	0.3063	1818.0	34.00	0.42	tr_C9ZP82_
HLA-DQA10501-DQB10201	332	LQWNVDYHDSNVVHL	YHDSNVVHL	6	0.3062	1819.7	34.00	0.40	tr_C9ZP82_
HLA-DQA10501-DQB10201	333	PANSKIIDDIIVLSI	IDDIIVLSI	6	0.3054	1835.3	34.00	0.27	tr_C9ZP82_

HLA-DQA10501-DQB10201	334	DGVNSFCVRSTDDDY	CVRSTDDDY	6	0.3054	1835.4	34.00	0.43	tr_C9ZP82_
HLA-DQA10501-DQB10201	335	FYHAPPVIDLAIYLK	PVIDLAIYL	5	0.3053	1837.4	34.00	0.22	tr_C9ZP82_
HLA-DQA10501-DQB10201	336	NVTVADVAVALHSTI	TVADVAVAL	2	0.3043	1858.2	34.00	0.38	tr_C9ZP82_
HLA-DQA10501-DQB10201	337	DLRKLSDALDTILRR	KLSDALDTI	3	0.3042	1859.5	34.00	0.38	tr_C9ZP82_
HLA-DQA10501-DQB10201	338	LYVSEVLPYGFLTKG	VSEVLPYGF	2	0.3040	1863.5	34.00	0.43	tr_C9ZP82_
HLA-DQA10501-DQB10201	339	YIDEEESDGVIGVPV	SDGVIGVPV	6	0.3035	1873.8	34.00	0.48	tr_C9ZP82_
HLA-DQA10501-DQB10201	340	PTAFEFDSNCISPGT	AFEFDSNCI	2	0.3029	1885.9	34.00	0.40	tr_C9ZP82_
HLA-DQA10501-DQB10201	341	EVMVRSPQQAELEST	PQQAELEST	6	0.3026	1893.5	34.00	0.25	tr_C9ZP82_
HLA-DQA10501-DQB10201	342	ELLVQTVQPKEVMYI	VQTVQPKEV	3	0.3020	1904.7	35.00	0.42	tr_C9ZP82_
HLA-DQA10501-DQB10201	343	EYFDISVVGGSLISE	VVGGSLISE	6	0.3015	1915.6	35.00	0.31	tr_C9ZP82_
HLA-DQA10501-DQB10201	344	GVCFVWRYYTTTTTD	WRYYTTTTT	5	0.3011	1923.4	35.00	0.44	tr_C9ZP82_
HLA-DQA10501-DQB10201	345	SILLGNDFLPHAPSA	SILLGNDFL	0	0.3009	1926.9	35.00	0.36	tr_C9ZP82_
HLA-DQA10501-DQB10201	346	FPLQWNVDYHDSNVV	LQWNVDYHD	2	0.3003	1939.5	35.00	0.26	tr_C9ZP82_
HLA-DQA10501-DQB10201	347	LGEVMVRSPQQAELE	EVMVRSPQQ	2	0.2991	1965.5	35.00	0.25	tr_C9ZP82_
HLA-DQA10501-DQB10201	348	VTVADVAVALHSTID	TVADVAVAL	1	0.2989	1970.3	35.00	0.34	tr_C9ZP82_
HLA-DQA10501-DQB10201	349	RRVPSREGVEIRQLY	REGVEIRQL	5	0.2977	1994.6	36.00	0.48	tr_C9ZP82_
HLA-DQA10501-DQB10201	350	LPMVLGEVMVRSPQQ	EVMVRSPQQ	6	0.2964	2022.9	36.00	0.28	tr_C9ZP82_
HLA-DQA10501-DQB10201	351	IDEEESDGVIGVPVC	SDGVIGVPV	5	0.2961	2030.5	36.00	0.42	tr_C9ZP82_
HLA-DQA10501-DQB10201	352	EDPLYSGALYNEGEE	YSGALYNEG	4	0.2961	2030.9	36.00	0.22	tr_C9ZP82_
HLA-DQA10501-DQB10201	353	TKNCPSSVHGLYIDL	SVHGLYIDL	6	0.2960	2032.0	36.00	0.40	tr_C9ZP82_
HLA-DQA10501-DQB10201	354	CEKYVEGVCFVWRYY	EKYVEGVCF	1	0.2958	2038.2	36.00	0.32	tr_C9ZP82_
HLA-DQA10501-DQB10201	355	ANSKIIDDIIVLSIL	NSKIIDDII	1	0.2955	2044.2	36.00	0.20	tr_C9ZP82_
HLA-DQA10501-DQB10201	356	SPANSKIIDDIIVLS	NSKIIDDII	3	0.2954	2046.8	36.00	0.31	tr_C9ZP82_
HLA-DQA10501-DQB10201	357	GLYIDLNGIIHPCCH	YIDLNGIIH	2	0.2947	2061.5	36.00	0.22	tr_C9ZP82_
HLA-DQA10501-DQB10201	358	FLPHAPSAFSGESAL	PSAFSGESA	5	0.2947	2062.5	36.00	0.51	tr_C9ZP82_
HLA-DQA10501-DQB10201	359	RPRTYSCTVEVPRLS	TYSCTVEVP	3	0.2942	2073.7	37.00	0.38	tr_C9ZP82_
HLA-DQA10501-DQB10201	360	LLPMVLGEVMVRSPQ	LPMVLGEVM	1	0.2931	2097.5	37.00	0.31	tr_C9ZP82_
HLA-DQA10501-DQB10201	361	DFLPHAPSAFSGESA	PSAFSGESA	6	0.2926	2108.0	37.00	0.55	tr_C9ZP82_
HLA-DQA10501-DQB10201	362	LYNEGEEEAGDALND	LYNEGEEEA	0	0.2919	2125.1	37.00	0.34	tr_C9ZP82_
HLA-DQA10501-DQB10201	363	AQANDDLDEVRQTLL	QANDDLDEV	1	0.2915	2133.5	37.00	0.38	tr_C9ZP82_
HLA-DQA10501-DQB10201	364	VQEACEKYVEGVCFV	EKYVEGVCF	5	0.2914	2135.4	37.00	0.45	tr_C9ZP82_
HLA-DQA10501-DQB10201	365	SSPANSKIIDDIIVL	NSKIIDDII	4	0.2913	2138.6	37.00	0.41	tr_C9ZP82_
HLA-DQA10501-DQB10201	366	TFPLQWNVDYHDSNV	LQWNVDYHD	3	0.2904	2159.0	37.00	0.28	tr_C9ZP82_
HLA-DQA10501-DQB10201	367	ILLGNDFLPHAPSAF	FLPHAPSAF	6	0.2903	2161.9	38.00	0.42	tr_C9ZP82_
HLA-DQA10501-DQB10201	368	FLALSLHITQVFILR	LSLHITQVF	3	0.2900	2168.8	38.00	0.32	tr_C9ZP82_
HLA-DQA10501-DQB10201	369	STPPDTLVQLLSILP	LVQLLSILP	6	0.2893	2184.9	38.00	0.26	tr_C9ZP82_
HLA-DQA10501-DQB10201	370	TAFEFDSNCISPGTD	AFEFDSNCI	1	0.2891	2190.3	38.00	0.41	tr_C9ZP82_
HLA-DQA10501-DQB10201	371	LALSLHITQVFILRD	ITQVFILRD	6	0.2883	2208.6	38.00	0.22	tr_C9ZP82_
HLA-DQA10501-DQB10201	372	PRTYSCTVEVPRLSY	TYSCTVEVP	2	0.2883	2210.0	38.00	0.34	tr_C9ZP82_
HLA-DQA10501-DQB10201	373	RYRPRTYSCTVEVPR	TYSCTVEVP	5	0.2881	2214.7	38.00	0.41	tr_C9ZP82_
HLA-DQA10501-DQB10201	374	STFPLQWNVDYHDSN	LQWNVDYHD	4	0.2879	2218.2	38.00	0.30	tr_C9ZP82_
HLA-DQA10501-DQB10201	375	GEVMVRSPQQAELES	RSPQQAELE	5	0.2874	2231.2	38.00	0.29	tr_C9ZP82_
HLA-DQA10501-DQB10201	376	RQTLLEDPLYSGALY	QTLLEDPLY	1	0.2859	2267.6	39.00	0.38	tr_C9ZP82_
HLA-DQA10501-DQB10201	377	MAKVSDAVLNMLNEK	VSDAVLNML	3	0.2849	2291.3	39.00	0.22	tr_C9ZP82_
HLA-DQA10501-DQB10201	378	EAYAKIEAVKFRRFL	YAKIEAVKF	2	0.2848	2295.6	39.00	0.47	tr_C9ZP82_
HLA-DQA10501-DQB10201	379	ESTFPLQWNVDYHDS	LQWNVDYHD	5	0.2834	2328.8	39.00	0.37	tr_C9ZP82_
HLA-DQA10501-DQB10201	380	KVSDAVLNMLNEKMS	VLNMLNEKM	5	0.2832	2334.5	39.00	0.41	tr_C9ZP82_
HLA-DQA10501-DQB10201	381	YESTHENRYVESNQV	NRYVESNQV	6	0.2820	2364.2	40.00	0.73	tr_C9ZP82_
HLA-DQA10501-DQB10201	382	ISEVSALCDIKKFTP	ISEVSALCD	0	0.2818	2369.2	40.00	0.30	tr_C9ZP82_
HLA-DQA10501-DQB10201	383	EHKIIDFLRTQSSLA	FLRTQSSLA	6	0.2794	2433.6	40.00	0.38	tr_C9ZP82_

HLA-DQA10501-DQB10201	384	LRKLSDALDTILRRV	KLSDALDTI	2	0.2785	2455.3	41.00	0.38	tr_C9ZP82_
HLA-DQA10501-DQB10201	385	PMVLGEVMVRSPQQA	EVMVRSPQQ	5	0.2784	2459.2	41.00	0.35	tr_C9ZP82_
HLA-DQA10501-DQB10201	386	NSKIIDDIIVLSILL	IDDIIVLSI	4	0.2784	2460.2	41.00	0.26	tr_C9ZP82_
HLA-DQA10501-DQB10201	387	PAQTGEVASTYMPNK	TGEVASTYM	3	0.2782	2464.1	41.00	0.57	tr_C9ZP82_
HLA-DQA10501-DQB10201	388	EVPRLSYGADGQVIK	SYGADGQVI	5	0.2777	2478.4	41.00	0.35	tr_C9ZP82_
HLA-DQA10501-DQB10201	389	KRNFIDDTCIISLNP	DTCIISLNP	6	0.2776	2480.6	41.00	0.25	tr_C9ZP82_
HLA-DQA10501-DQB10201	390	EKYVEGVCFVWRYYT	KYVEGVCFV	1	0.2759	2525.9	41.00	0.25	tr_C9ZP82_
HLA-DQA10501-DQB10201	391	VQLLSILPPTSHALL	LLSILPPTS	2	0.2758	2530.6	41.00	0.50	tr_C9ZP82_
HLA-DQA10501-DQB10201	392	DYHDSNVVHLATVML	NVVHLATVM	5	0.2753	2544.1	41.00	0.32	tr_C9ZP82_
HLA-DQA10501-DQB10201	393	QANDDLDEVRQTLLE	LDEVRQTLL	5	0.2750	2551.2	41.00	0.29	tr_C9ZP82_
HLA-DQA10501-DQB10201	394	QLYDVPLERYRPRTY	LYDVPLERY	1	0.2748	2556.4	41.00	0.47	tr_C9ZP82_
HLA-DQA10501-DQB10201	395	VLGEVMVRSPQQAEL	EVMVRSPQQ	3	0.2741	2575.6	42.00	0.37	tr_C9ZP82_
HLA-DQA10501-DQB10201	396	LEDPLYSGALYNEGE	YSGALYNEG	5	0.2740	2578.3	42.00	0.28	tr_C9ZP82_
HLA-DQA10501-DQB10201	397	VDYHDSNVVHLATVM	NVVHLATVM	6	0.2738	2584.1	42.00	0.37	tr_C9ZP82_
HLA-DQA10501-DQB10201	398	MVLGEVMVRSPQQAE	EVMVRSPQQ	4	0.2735	2592.8	42.00	0.34	tr_C9ZP82_
HLA-DQA10501-DQB10201	399	EERWRTQKLDFHLVI	WRTQKLDFH	3	0.2731	2603.7	42.00	0.34	tr_C9ZP82_
HLA-DQA10501-DQB10201	400	GVIGVPVCESSENSS	VIGVPVCES	1	0.2729	2610.6	42.00	0.60	tr_C9ZP82_
HLA-DQA10501-DQB10201	401	HKIIDFLRTQSSLAQ	FLRTQSSLA	5	0.2729	2611.0	42.00	0.38	tr_C9ZP82_
HLA-DQA10501-DQB10201	402	RNFIDDTCIISLNPS	DTCIISLNP	5	0.2721	2633.9	42.00	0.22	tr_C9ZP82_
HLA-DQA10501-DQB10201	403	IDFLRTQSSLAQFGG	FLRTQSSLA	2	0.2717	2644.5	42.00	0.32	tr_C9ZP82_
HLA-DQA10501-DQB10201	404	SYIDEEESDGVIGVP	SYIDEEESD	0	0.2715	2649.9	42.00	0.24	tr_C9ZP82_
HLA-DQA10501-DQB10201	405	PQLQRLFEAYAKIEA	FEAYAKIEA	6	0.2704	2680.7	43.00	0.49	tr_C9ZP82_
HLA-DQA10501-DQB10201	406	RRKRNFIDDTCIISL	NFIDDTCII	4	0.2700	2693.8	43.00	0.21	tr_C9ZP82_
HLA-DQA10501-DQB10201	407	PCCHNDSDTAIALLP	DSDTAIALL	5	0.2698	2699.4	43.00	0.46	tr_C9ZP82_
HLA-DQA10501-DQB10201	408	SVQEACEKYVEGVCF	EKYVEGVCF	6	0.2697	2702.9	43.00	0.65	tr_C9ZP82_
HLA-DQA10501-DQB10201	409	PLQWNVDYHDSNVVH	VDYHDSNVV	5	0.2695	2707.7	43.00	0.23	tr_C9ZP82_
HLA-DQA10501-DQB10201	410	QTLLEDPLYSGALYN	QTLLEDPLY	0	0.2693	2712.5	43.00	0.32	tr_C9ZP82_
HLA-DQA10501-DQB10201	411	CVRSTDDDYESTHEN	VRSTDDDYE	1	0.2693	2713.0	43.00	0.52	tr_C9ZP82_
HLA-DQA10501-DQB10201	412	YTTTTTDSDWSWYYP	TTTTTDSDW	1	0.2686	2734.6	43.00	0.30	tr_C9ZP82_
HLA-DQA10501-DQB10201	413	QWNVDYHDSNVVHLA	YHDSNVVHL	5	0.2676	2763.6	43.00	0.36	tr_C9ZP82_
HLA-DQA10501-DQB10201	414	AQTGEVASTYMPNKS	TGEVASTYM	2	0.2672	2775.1	43.00	0.57	tr_C9ZP82_
HLA-DQA10501-DQB10201	415	VKSTPPDTLVQLLSI	DTLVQLLSI	6	0.2665	2798.0	44.00	0.32	tr_C9ZP82_
HLA-DQA10501-DQB10201	416	EAHVTTVPNTGFEYF	VPNTGFEYF	6	0.2654	2831.9	44.00	0.49	tr_C9ZP82_
HLA-DQA10501-DQB10201	417	ALSLHITQVFILRDT	ITQVFILRD	5	0.2653	2833.7	44.00	0.16	tr_C9ZP82_
HLA-DQA10501-DQB10201	418	YFFGQPNHIEDGTLP	YFFGQPNHI	0	0.2628	2910.8	45.00	0.47	tr_C9ZP82_
HLA-DQA10501-DQB10201	419	YHAPPVIDLAIYLKE	PVIDLAIYL	4	0.2614	2955.5	45.00	0.32	tr_C9ZP82_
HLA-DQA10501-DQB10201	420	NEGEEEAGDALNDGD	GEEEAGDAL	2	0.2611	2963.9	45.00	0.28	tr_C9ZP82_
HLA-DQA10501-DQB10201	421	GLDADLIFLALSLHI	IFLALSLHI	6	0.2610	2969.8	45.00	0.28	tr_C9ZP82_
HLA-DQA10501-DQB10201	422	LRRVPSREGVEIRQL	REGVEIRQL	6	0.2604	2988.4	45.00	0.53	tr_C9ZP82_
HLA-DQA10501-DQB10201	423	VVKSTPPDTLVQLLS	PDTLVQLLS	6	0.2590	3033.0	46.00	0.32	tr_C9ZP82_
HLA-DQA10501-DQB10201	424	WNVDYHDSNVVHLAT	YHDSNVVHL	4	0.2587	3042.8	46.00	0.36	tr_C9ZP82_
HLA-DQA10501-DQB10201	425	LSLHITQVFILRDTT	QVFILRDTT	6	0.2585	3048.6	46.00	0.20	tr_C9ZP82_
HLA-DQA10501-DQB10201	426	LLEDPLYSGALYNEG	LYSGALYNE	5	0.2585	3050.7	46.00	0.26	tr_C9ZP82_
HLA-DQA10501-DQB10201	427	VPRLSYGADGQVIKR	SYGADGQVI	4	0.2577	3075.2	46.00	0.33	tr_C9ZP82_
HLA-DQA10501-DQB10201	428	AFEFDSNCISPGTDF	AFEFDSNCI	0	0.2571	3095.8	46.00	0.31	tr_C9ZP82_
HLA-DQA10501-DQB10201	429	LLPEEEKLECICSEL	EKLECICSE	5	0.2563	3121.9	46.00	0.41	tr_C9ZP82_
HLA-DQA10501-DQB10201	430	RKLSDALDTILRRVP	KLSDALDTI	1	0.2551	3163.3	47.00	0.39	tr_C9ZP82_
HLA-DQA10501-DQB10201	431	WPQLTEEERWRTQKL	PQLTEEERW	1	0.2551	3165.4	47.00	0.52	tr_C9ZP82_
HLA-DQA10501-DQB10201	432	RTYSCTVEVPRLSYG	TYSCTVEVP	1	0.2545	3183.9	47.00	0.38	tr_C9ZP82_
HLA-DQA10501-DQB10201	433	RSRRKRNFIDDTCII	NFIDDTCII	6	0.2542	3196.3	47.00	0.33	tr_C9ZP82_

HLA-DQA10501-DQB10201	434	YNEGEEEAGDALNDG	GEEEAGDAL	3	0.2539	3205.6	47.00	0.34	tr_C9ZP82_
HLA-DQA10501-DQB10201	435	ERWRTQKLDFHLVIT	WRTQKLDFH	2	0.2536	3217.1	47.00	0.35	tr_C9ZP82_
HLA-DQA10501-DQB10201	436	SKIIDDIIVLSILLG	IDDIIVLSI	3	0.2534	3223.5	47.00	0.28	tr_C9ZP82_
HLA-DQA10501-DQB10201	437	TVEVPRLSYGADGQV	LSYGADGQV	6	0.2522	3266.5	48.00	0.40	tr_C9ZP82_
HLA-DQA10501-DQB10201	438	VSDAVLNMLNEKMSG	VLNMLNEKM	4	0.2520	3272.8	48.00	0.44	tr_C9ZP82_
HLA-DQA10501-DQB10201	439	LYIDLNGIIHPCCHN	YIDLNGIIH	1	0.2517	3283.6	48.00	0.28	tr_C9ZP82_
HLA-DQA10501-DQB10201	440	GEHKIIDFLRTQSSL	IIDFLRTQS	4	0.2513	3296.6	48.00	0.20	tr_C9ZP82_
HLA-DQA10501-DQB10201	441	KTLVSSAGYYFFGQP	LVSSAGYYF	2	0.2510	3307.3	48.00	0.25	tr_C9ZP82_
HLA-DQA10501-DQB10201	442	GPHEINLPQLQRLFE	PHEINLPQL	1	0.2510	3307.7	48.00	0.31	tr_C9ZP82_
HLA-DQA10501-DQB10201	443	FLTKGPHEINLPQLQ	PHEINLPQL	5	0.2510	3308.5	48.00	0.52	tr_C9ZP82_
HLA-DQA10501-DQB10201	444	TKGPHEINLPQLQRL	PHEINLPQL	3	0.2508	3314.6	48.00	0.40	tr_C9ZP82_
HLA-DQA10501-DQB10201	445	LLVQTVQPKEVMYIA	VQTVQPKEV	2	0.2503	3334.6	48.00	0.57	tr_C9ZP82_
HLA-DQA10501-DQB10201	446	LSILPPTSHALLPMV	TSHALLPMV	6	0.2502	3337.7	48.00	0.57	tr_C9ZP82_
HLA-DQA10501-DQB10201	447	CSSPANSKIIDDIIV	NSKIIDDII	5	0.2499	3346.4	48.00	0.54	tr_C9ZP82_
HLA-DQA10501-DQB10201	448	SRRKRNFIDDTCIIS	NFIDDTCII	5	0.2497	3355.8	48.00	0.28	tr_C9ZP82_
HLA-DQA10501-DQB10201	449	CDIKKFTPATESAFD	FTPATESAF	5	0.2492	3374.6	48.00	0.43	tr_C9ZP82_
HLA-DQA10501-DQB10201	450	LDADLIFLALSLHIT	FLALSLHIT	6	0.2491	3377.8	48.00	0.25	tr_C9ZP82_
HLA-DQA10501-DQB10201	451	RKRNFIDDTCIISLN	NFIDDTCII	3	0.2489	3382.7	48.00	0.22	tr_C9ZP82_
HLA-DQA10501-DQB10201	452	TLLEDPLYSGALYNE	LYSGALYNE	6	0.2484	3402.0	49.00	0.34	tr_C9ZP82_
HLA-DQA10501-DQB10201	453	FDKKTLVSSAGYYFF	LVSSAGYYF	5	0.2484	3402.3	49.00	0.33	tr_C9ZP82_
HLA-DQA10501-DQB10201	454	TLVSSAGYYFFGQPN	LVSSAGYYF	1	0.2483	3404.6	49.00	0.22	tr_C9ZP82_
HLA-DQA10501-DQB10201	455	SGNDPTWTRLCVIFS	WTRLCVIFS	6	0.2478	3423.1	49.00	0.44	tr_C9ZP82_
HLA-DQA10501-DQB10201	456	KGPHEINLPQLQRLF	PHEINLPQL	2	0.2474	3438.8	49.00	0.37	tr_C9ZP82_
HLA-DQA10501-DQB10201	457	PHEINLPQLQRLFEA	PHEINLPQL	0	0.2472	3446.0	49.00	0.22	tr_C9ZP82_
HLA-DQA10501-DQB10201	458	NVDYHDSNVVHLATV	YHDSNVVHL	3	0.2470	3453.6	49.00	0.37	tr_C9ZP82_
HLA-DQA10501-DQB10201	459	KSTPPDTLVQLLSIL	DTLVQLLSI	5	0.2469	3457.7	49.00	0.34	tr_C9ZP82_
HLA-DQA10501-DQB10201	460	GFLTKGPHEINLPQL	PHEINLPQL	6	0.2456	3506.1	49.00	0.60	tr_C9ZP82_
HLA-DQA10501-DQB10201	461	EEERWRTQKLDFHLV	WRTQKLDFH	4	0.2446	3543.3	50.00	0.47	tr_C9ZP82_
HLA-DQA10501-DQB10201	462	YVSEVLPYGFLTKGP	VSEVLPYGF	1	0.2445	3547.7	50.00	0.52	tr_C9ZP82_
HLA-DQA10501-DQB10201	463	GEEEAGDALNDGDGV	DALNDGDGV	6	0.2442	3560.6	50.00	0.20	tr_C9ZP82_
HLA-DQA10501-DQB10201	464	LLGNDFLPHAPSAFS	FLPHAPSAF	5	0.2434	3590.5	50.00	0.53	tr_C9ZP82_
HLA-DQA10501-DQB10201	465	PVVKSTPPDTLVQLL	PPDTLVQLL	6	0.2428	3613.1	50.00	0.37	tr_C9ZP82_
HLA-DQA10501-DQB10201	466	EGEEEAGDALNDGDG	GEEEAGDAL	1	0.2426	3621.6	50.00	0.26	tr_C9ZP82_
HLA-DQA10501-DQB10201	467	ADLIFLALSLHITQV	ALSLHITQV	6	0.2425	3625.2	50.00	0.32	tr_C9ZP82_
HLA-DQA10501-DQB10201	468	LVSSAGYYFFGQPNH	YYFFGQPNH	6	0.2423	3635.1	50.00	0.25	tr_C9ZP82_
HLA-DQA10501-DQB10201	469	VQTVQPKEVMYIAVD	KEVMYIAVD	6	0.2420	3646.7	50.00	0.42	tr_C9ZP82_
HLA-DQA10501-DQB10201	470	KIIDDIIVLSILLGN	IDDIIVLSI	2	0.2407	3698.6	55.00	0.25	tr_C9ZP82_
HLA-DQA10501-DQB10201	471	TEEERWRTQKLDFHL	WRTQKLDFH	5	0.2406	3702.0	55.00	0.50	tr_C9ZP82_
HLA-DQA10501-DQB10201	472	PRLSYGADGQVIKRQ	SYGADGQVI	3	0.2405	3706.6	55.00	0.35	tr_C9ZP82_
HLA-DQA10501-DQB10201	473	HAPPVIDLAIYLKEA	PVIDLAIYL	3	0.2404	3708.9	55.00	0.36	tr_C9ZP82_
HLA-DQA10501-DQB10201	474	QRARRYMGRAAPAQT	YMGRAAPAQ	5	0.2395	3747.4	55.00	0.37	tr_C9ZP82_
HLA-DQA10501-DQB10201	475	PPVIDLAIYLKEANI	PVIDLAIYL	1	0.2394	3750.8	55.00	0.28	tr_C9ZP82_
HLA-DQA10501-DQB10201	476	LVQTVQPKEVMYIAV	VQTVQPKEV	1	0.2387	3780.4	55.00	0.47	tr_C9ZP82_
HLA-DQA10501-DQB10201	477	GNDPTWTRLCVIFSG	WTRLCVIFS	5	0.2383	3794.0	55.00	0.38	tr_C9ZP82_
HLA-DQA10501-DQB10201	478	PCSSPANSKIIDDII	NSKIIDDII	6	0.2375	3827.3	55.00	0.64	tr_C9ZP82_
HLA-DQA10501-DQB10201	479	DFLRTQSSLAQFGGK	FLRTQSSLA	1	0.2362	3881.7	55.00	0.35	tr_C9ZP82_
HLA-DQA10501-DQB10201	480	EGEHKIIDFLRTQSS	IIDFLRTQS	5	0.2355	3910.5	55.00	0.22	tr_C9ZP82_
HLA-DQA10501-DQB10201	481	AQFGGKANHVIVGLD	ANHVIVGLD	6	0.2351	3927.1	55.00	0.54	tr_C9ZP82_
HLA-DQA10501-DQB10201	482	IIDDIIVLSILLGND	VLSILLGND	6	0.2349	3936.6	55.00	0.28	tr_C9ZP82_
HLA-DQA10501-DQB10201	483	EGVCFVWRYYTTTTT	WRYYTTTTT	6	0.2348	3941.2	55.00	0.73	tr_C9ZP82_

HLA-DQA10501-DQB10201	484	HEINLPQLQRLFEAY	QLQRLFEAY	6	0.2346	3948.3	55.00	0.27	tr_C9ZP82_
HLA-DQA10501-DQB10201	485	RYMGRAAPAQTGEVA	APAQTGEVA	6	0.2346	3948.9	55.00	0.28	tr_C9ZP82_
HLA-DQA10501-DQB10201	486	LHSTIDDRWRDTYLK	LHSTIDDRW	0	0.2329	4023.3	55.00	0.74	tr_C9ZP82_
HLA-DQA10501-DQB10201	487	ALLPEEEKLECICSE	EKLECICSE	6	0.2328	4029.6	55.00	0.47	tr_C9ZP82_
HLA-DQA10501-DQB10201	488	DADLIFLALSLHITQ	FLALSLHIT	5	0.2325	4038.9	55.00	0.22	tr_C9ZP82_
HLA-DQA10501-DQB10201	489	APPVIDLAIYLKEAN	PVIDLAIYL	2	0.2325	4040.4	55.00	0.35	tr_C9ZP82_
HLA-DQA10501-DQB10201	490	DIKKFTPATESAFDK	FTPATESAF	4	0.2324	4043.0	55.00	0.41	tr_C9ZP82_
HLA-DQA10501-DQB10201	491	LTEEERWRTQKLDFH	WRTQKLDFH	6	0.2322	4052.8	55.00	0.48	tr_C9ZP82_
HLA-DQA10501-DQB10201	492	QTGEVASTYMPNKSG	TGEVASTYM	1	0.2317	4075.4	55.00	0.62	tr_C9ZP82_
HLA-DQA10501-DQB10201	493	LTKGPHEINLPQLQR	PHEINLPQL	4	0.2314	4088.6	55.00	0.46	tr_C9ZP82_
HLA-DQA10501-DQB10201	494	TTTTTDSDWSWYYPF	TTTTDSDWS	1	0.2305	4128.4	55.00	0.26	tr_C9ZP82_
HLA-DQA10501-DQB10201	495	LERYRPRTYSCTVEV	RTYSCTVEV	6	0.2303	4138.3	55.00	0.63	tr_C9ZP82_
HLA-DQA10501-DQB10201	496	NDPTWTRLCVIFSGS	WTRLCVIFS	4	0.2297	4165.6	55.00	0.35	tr_C9ZP82_
HLA-DQA10501-DQB10201	497	RWRTQKLDFHLVITS	WRTQKLDFH	1	0.2281	4238.0	55.00	0.35	tr_C9ZP82_
HLA-DQA10501-DQB10201	498	RARRYMGRAAPAQTG	YMGRAAPAQ	4	0.2277	4257.2	55.00	0.37	tr_C9ZP82_
HLA-DQA10501-DQB10201	499	RRSRRKRNFIDDTCI	RNFIDDTCI	6	0.2266	4307.7	55.00	0.40	tr_C9ZP82_
HLA-DQA10501-DQB10201	500	SDAVLNMLNEKMSGN	VLNMLNEKM	3	0.2242	4421.4	55.00	0.52	tr_C9ZP82_
HLA-DQA10501-DQB10201	501	ARRYMGRAAPAQTGE	YMGRAAPAQ	3	0.2242	4421.8	55.00	0.36	tr_C9ZP82_
HLA-DQA10501-DQB10201	502	LGNDFLPHAPSAFSG	FLPHAPSAF	4	0.2238	4438.8	60.00	0.56	tr_C9ZP82_
HLA-DQA10501-DQB10201	503	DKKTLVSSAGYYFFG	LVSSAGYYF	4	0.2230	4477.3	60.00	0.32	tr_C9ZP82_
HLA-DQA10501-DQB10201	504	GDGVNSFCVRSTDDD	SFCVRSTDD	5	0.2227	4492.1	60.00	0.41	tr_C9ZP82_
HLA-DQA10501-DQB10201	505	KYVEGVCFVWRYYTT	VEGVCFVWR	2	0.2226	4498.6	60.00	0.26	tr_C9ZP82_
HLA-DQA10501-DQB10201	506	WRTQKLDFHLVITSN	DFHLVITSN	6	0.2224	4506.5	60.00	0.26	tr_C9ZP82_
HLA-DQA10501-DQB10201	507	EINLPQLQRLFEAYA	LQRLFEAYA	6	0.2220	4527.5	60.00	0.26	tr_C9ZP82_
HLA-DQA10501-DQB10201	508	QLLSILPPTSHALLP	LLSILPPTS	1	0.2212	4566.0	60.00	0.59	tr_C9ZP82_
HLA-DQA10501-DQB10201	509	KKTLVSSAGYYFFGQ	LVSSAGYYF	3	0.2200	4624.6	60.00	0.29	tr_C9ZP82_
HLA-DQA10501-DQB10201	510	LPQLQRLFEAYAKIE	LFEAYAKIE	6	0.2192	4665.1	60.00	0.31	tr_C9ZP82_
HLA-DQA10501-DQB10201	511	GEGEHKIIDFLRTQS	GEHKIIDFL	2	0.2189	4682.1	60.00	0.28	tr_C9ZP82_
HLA-DQA10501-DQB10201	512	RLSYGADGQVIKRQR	SYGADGQVI	2	0.2189	4683.3	60.00	0.44	tr_C9ZP82_
HLA-DQA10501-DQB10201	513	RRYMGRAAPAQTGEV	YMGRAAPAQ	2	0.2187	4693.2	60.00	0.32	tr_C9ZP82_
HLA-DQA10501-DQB10201	514	VPKGDLRKLSDALDT	RKLSDALDT	6	0.2185	4699.4	60.00	0.39	tr_C9ZP82_
HLA-DQA10501-DQB10201	515	DPTWTRLCVIFSGSN	WTRLCVIFS	3	0.2180	4725.2	60.00	0.37	tr_C9ZP82_
HLA-DQA10501-DQB10201	516	VTKNCPSSVHGLYID	PSSVHGLYI	5	0.2167	4793.3	60.00	0.62	tr_C9ZP82_
HLA-DQA10501-DQB10201	517	GRAAPAQTGEVASTY	QTGEVASTY	6	0.2164	4807.6	60.00	0.40	tr_C9ZP82_
HLA-DQA10501-DQB10201	518	KLSDALDTILRRVPS	LSDALDTIL	1	0.2161	4823.2	60.00	0.39	tr_C9ZP82_
HLA-DQA10501-DQB10201	519	GNDFLPHAPSAFSGE	FLPHAPSAF	3	0.2161	4823.8	60.00	0.61	tr_C9ZP82_
HLA-DQA10501-DQB10201	520	EEEAGDALNDGDGVN	DALNDGDGV	5	0.2159	4833.2	60.00	0.25	tr_C9ZP82_
HLA-DQA10501-DQB10201	521	VRSTDDDYESTHENR	VRSTDDDYE	0	0.2150	4882.6	60.00	0.50	tr_C9ZP82_
HLA-DQA10501-DQB10201	522	TYSCTVEVPRLSYGA	TYSCTVEVP	0	0.2147	4897.9	60.00	0.35	tr_C9ZP82_
HLA-DQA10501-DQB10201	523	MPLPVVKSTPPDTLV	VKSTPPDTL	5	0.2146	4904.8	60.00	0.54	tr_C9ZP82_
HLA-DQA10501-DQB10201	524	YMGRAAPAQTGEVAS	APAQTGEVA	5	0.2140	4934.8	60.00	0.37	tr_C9ZP82_
HLA-DQA10501-DQB10201	525	SLHITQVFILRDTTR	QVFILRDTT	5	0.2137	4954.8	60.00	0.25	tr_C9ZP82_
HLA-DQA10501-DQB10201	526	CISPGTDFMAKVSDA	DFMAKVSDA	6	0.2128	4999.9	60.00	0.47	tr_C9ZP82_
HLA-DQA10501-DQB10201	527	QQRARRYMGRAAPAQ	YMGRAAPAQ	6	0.2125	5014.7	60.00	0.45	tr_C9ZP82_
HLA-DQA10501-DQB10201	528	GDALNDGDGVNSFCV	DALNDGDGV	1	0.2124	5022.0	60.00	0.25	tr_C9ZP82_
HLA-DQA10501-DQB10201	529	TPGEGEHKIIDFLRT	GEHKIIDFL	4	0.2113	5085.0	60.00	0.37	tr_C9ZP82_
HLA-DQA10501-DQB10201	530	TGLSNSVQEACEKYV	VQEACEKYV	6	0.2110	5098.3	60.00	0.23	tr_C9ZP82_
HLA-DQA10501-DQB10201	531	RMPLPVVKSTPPDTL	VKSTPPDTL	6	0.2104	5132.4	60.00	0.52	tr_C9ZP82_
HLA-DQA10501-DQB10201	532	HPCCHNDSDTAIALL	DSDTAIALL	6	0.2101	5148.4	60.00	0.63	tr_C9ZP82_
HLA-DQA10501-DQB10201	533	IKKFTPATESAFDKK	FTPATESAF	3	0.2096	5175.0	60.00	0.38	tr_C9ZP82_

HLA-DQA10501-DQB10201	534	AGDALNDGDGVNSFC	DALNDGDGV	2	0.2091	5205.4	60.00	0.29	tr_C9ZP82_
HLA-DQA10501-DQB10201	535	PVIDLAIYLKEANIK	PVIDLAIYL	0	0.2087	5227.3	60.00	0.27	tr_C9ZP82_
HLA-DQA10501-DQB10201	536	AFDKKTLVSSAGYYF	LVSSAGYYF	6	0.2085	5237.9	60.00	0.49	tr_C9ZP82_
HLA-DQA10501-DQB10201	537	NDFLPHAPSAFSGES	FLPHAPSAF	2	0.2084	5242.7	60.00	0.60	tr_C9ZP82_
HLA-DQA10501-DQB10201	538	IALLPEEEKLECICS	IALLPEEEK	0	0.2080	5264.7	60.00	0.26	tr_C9ZP82_
HLA-DQA10501-DQB10201	539	LPVVKSTPPDTLVQL	VKSTPPDTL	3	0.2075	5294.5	60.00	0.52	tr_C9ZP82_
HLA-DQA10501-DQB10201	540	NTPGEGEHKIIDFLR	GEHKIIDFL	5	0.2064	5359.1	65.00	0.44	tr_C9ZP82_
HLA-DQA10501-DQB10201	541	NLPQLQRLFEAYAKI	RLFEAYAKI	6	0.2063	5366.3	65.00	0.26	tr_C9ZP82_
HLA-DQA10501-DQB10201	542	AYAKIEAVKFRRFLL	YAKIEAVKF	1	0.2052	5430.8	65.00	0.68	tr_C9ZP82_
HLA-DQA10501-DQB10201	543	DGDGVNSFCVRSTDD	SFCVRSTDD	6	0.2050	5442.3	65.00	0.40	tr_C9ZP82_
HLA-DQA10501-DQB10201	544	PLPVVKSTPPDTLVQ	VKSTPPDTL	4	0.2042	5486.0	65.00	0.54	tr_C9ZP82_
HLA-DQA10501-DQB10201	545	STGLSNSVQEACEKY	SVQEACEKY	6	0.2038	5512.1	65.00	0.25	tr_C9ZP82_
HLA-DQA10501-DQB10201	546	MSGNDPTWTRLCVIF	PTWTRLCVI	5	0.2035	5532.0	65.00	0.50	tr_C9ZP82_
HLA-DQA10501-DQB10201	547	DAVLNMLNEKMSGND	VLNMLNEKM	2	0.2027	5581.1	65.00	0.56	tr_C9ZP82_
HLA-DQA10501-DQB10201	548	YIDLNGIIHPCCHND	LNGIIHPCC	3	0.2024	5595.8	65.00	0.34	tr_C9ZP82_
HLA-DQA10501-DQB10201	549	LKSTGLSNSVQEACE	GLSNSVQEA	4	0.2019	5629.1	65.00	0.31	tr_C9ZP82_
HLA-DQA10501-DQB10201	550	EAGDALNDGDGVNSF	DALNDGDGV	3	0.2015	5652.2	65.00	0.30	tr_C9ZP82_
HLA-DQA10501-DQB10201	551	LLSILPPTSHALLPM	LLSILPPTS	0	0.2014	5656.6	65.00	0.44	tr_C9ZP82_
HLA-DQA10501-DQB10201	552	YVEGVCFVWRYYTTT	VEGVCFVWR	1	0.2011	5674.9	65.00	0.29	tr_C9ZP82_
HLA-DQA10501-DQB10201	553	PGEGEHKIIDFLRTQ	GEHKIIDFL	3	0.2007	5699.9	65.00	0.33	tr_C9ZP82_
HLA-DQA10501-DQB10201	554	INLPQLQRLFEAYAK	LQRLFEAYA	5	0.2002	5730.4	65.00	0.23	tr_C9ZP82_
HLA-DQA10501-DQB10201	555	VIGVPVCESSENSSS	VIGVPVCES	0	0.2001	5734.9	65.00	0.64	tr_C9ZP82_
HLA-DQA10501-DQB10201	556	KMSGNDPTWTRLCVI	PTWTRLCVI	6	0.1995	5772.6	65.00	0.46	tr_C9ZP82_
HLA-DQA10501-DQB10201	557	DALNDGDGVNSFCVR	DALNDGDGV	0	0.1987	5826.0	65.00	0.16	tr_C9ZP82_
HLA-DQA10501-DQB10201	558	SENSSSDTDNESPIE	SSSDTDNES	3	0.1980	5869.5	65.00	0.25	tr_C9ZP82_
HLA-DQA10501-DQB10201	559	CTVEVPRLSYGADGQ	RLSYGADGQ	6	0.1976	5895.5	65.00	0.57	tr_C9ZP82_
HLA-DQA10501-DQB10201	560	SNTPGEGEHKIIDFL	GEHKIIDFL	6	0.1971	5928.6	65.00	0.57	tr_C9ZP82_
HLA-DQA10501-DQB10201	561	FLRTQSSLAQFGGKA	RTQSSLAQF	2	0.1967	5952.8	65.00	0.33	tr_C9ZP82_
HLA-DQA10501-DQB10201	562	FEFDSNCISPGTDFM	FEFDSNCIS	0	0.1951	6055.6	65.00	0.26	tr_C9ZP82_
HLA-DQA10501-DQB10201	563	LCDIKKFTPATESAF	FTPATESAF	6	0.1947	6080.8	65.00	0.51	tr_C9ZP82_
HLA-DQA10501-DQB10201	564	ENSSSDTDNESPIES	SSSDTDNES	2	0.1944	6100.5	65.00	0.25	tr_C9ZP82_
HLA-DQA10501-DQB10201	565	EEAGDALNDGDGVNS	DALNDGDGV	4	0.1941	6121.2	65.00	0.28	tr_C9ZP82_
HLA-DQA10501-DQB10201	566	PTWTRLCVIFSGSNT	WTRLCVIFS	2	0.1941	6125.1	65.00	0.37	tr_C9ZP82_
HLA-DQA10501-DQB10201	567	KKFTPATESAFDKKT	FTPATESAF	2	0.1934	6165.8	65.00	0.39	tr_C9ZP82_
HLA-DQA10501-DQB10201	568	GLSNSVQEACEKYVE	VQEACEKYV	5	0.1930	6194.3	65.00	0.25	tr_C9ZP82_
HLA-DQA10501-DQB10201	569	SEVSALCDIKKFTPA	EVSALCDIK	1	0.1928	6211.4	65.00	0.34	tr_C9ZP82_
HLA-DQA10501-DQB10201	570	KSTGLSNSVQEACEK	GLSNSVQEA	3	0.1925	6226.3	65.00	0.23	tr_C9ZP82_
HLA-DQA10501-DQB10201	571	LHITQVFILRDTTRN	QVFILRDTT	4	0.1920	6263.9	65.00	0.24	tr_C9ZP82_
HLA-DQA10501-DQB10201	572	RTQKLDFHLVITSNK	DFHLVITSN	5	0.1902	6388.8	65.00	0.24	tr_C9ZP82_
HLA-DQA10501-DQB10201	573	RSTDDDYESTHENRY	DYESTHENR	5	0.1897	6420.8	65.00	0.31	tr_C9ZP82_
HLA-DQA10501-DQB10201	574	DDDYESTHENRYVES	DYESTHENR	2	0.1893	6451.7	70.00	0.31	tr_C9ZP82_
HLA-DQA10501-DQB10201	575	KFASWLRNKYPAIVT	WLRNKYPAI	4	0.1880	6537.3	70.00	0.37	tr_C9ZP82_
HLA-DQA10501-DQB10201	576	IAVDGVAPRAKMNQQ	AVDGVAPRA	1	0.1872	6594.6	70.00	0.65	tr_C9ZP82_
HLA-DQA10501-DQB10201	577	STDDDYESTHENRYV	DYESTHENR	4	0.1871	6603.1	70.00	0.34	tr_C9ZP82_
HLA-DQA10501-DQB10201	578	TQKLDFHLVITSNKT	DFHLVITSN	4	0.1863	6658.0	70.00	0.23	tr_C9ZP82_
HLA-DQA10501-DQB10201	579	HITQVFILRDTTRNP	QVFILRDTT	3	0.1850	6754.7	70.00	0.29	tr_C9ZP82_
HLA-DQA10501-DQB10201	580	TWTRLCVIFSGSNTP	WTRLCVIFS	1	0.1842	6813.0	70.00	0.34	tr_C9ZP82_
HLA-DQA10501-DQB10201	581	KFTPATESAFDKKTL	FTPATESAF	1	0.1840	6825.6	70.00	0.38	tr_C9ZP82_
HLA-DQA10501-DQB10201	582	REAHVTTVPNTGFEY	TVPNTGFEY	6	0.1836	6858.4	70.00	0.35	tr_C9ZP82_
HLA-DQA10501-DQB10201	583	LYDVPLERYRPRTYS	LYDVPLERY	0	0.1833	6881.1	70.00	0.60	tr_C9ZP82_

HLA-DQA10501-DQB10201	584	LDFHLVITSNKTSFV	ITSNKTSFV	6	0.1826	6930.5	70.00	0.22	tr_C9ZP82_
HLA-DQA10501-DQB10201	585	LNDGDGVNSFCVRST	VNSFCVRST	6	0.1818	6992.0	70.00	0.26	tr_C9ZP82_
HLA-DQA10501-DQB10201	586	PKFASWLRNKYPAIV	WLRNKYPAI	5	0.1818	6992.6	70.00	0.38	tr_C9ZP82_
HLA-DQA10501-DQB10201	587	TDDDYESTHENRYVE	DYESTHENR	3	0.1796	7159.8	70.00	0.36	tr_C9ZP82_
HLA-DQA10501-DQB10201	588	TGEVASTYMPNKSGM	TGEVASTYM	0	0.1796	7162.3	70.00	0.54	tr_C9ZP82_
HLA-DQA10501-DQB10201	589	DFHLVITSNKTSFVP	ITSNKTSFV	5	0.1791	7197.9	70.00	0.29	tr_C9ZP82_
HLA-DQA10501-DQB10201	590	NSSSDTDNESPIESR	SSSDTDNES	1	0.1791	7198.3	70.00	0.25	tr_C9ZP82_
HLA-DQA10501-DQB10201	591	MGRAAPAQTGEVAST	APAQTGEVA	4	0.1788	7220.4	70.00	0.44	tr_C9ZP82_
HLA-DQA10501-DQB10201	592	ITQVFILRDTTRNPY	QVFILRDTT	2	0.1786	7236.4	70.00	0.32	tr_C9ZP82_
HLA-DQA10501-DQB10201	593	LSYGADGQVIKRQRR	SYGADGQVI	1	0.1784	7253.9	70.00	0.60	tr_C9ZP82_
HLA-DQA10501-DQB10201	594	IVTKNCPSSVHGLYI	PSSVHGLYI	6	0.1774	7331.9	70.00	0.71	tr_C9ZP82_
HLA-DQA10501-DQB10201	595	FRKGSNNDNVTVADV	NDNVTVADV	6	0.1769	7374.8	70.00	0.70	tr_C9ZP82_
HLA-DQA10501-DQB10201	596	SSENSSSDTDNESPI	SSSDTDNES	4	0.1763	7423.9	70.00	0.31	tr_C9ZP82_
HLA-DQA10501-DQB10201	597	YLKSTGLSNSVQEAC	GLSNSVQEA	5	0.1755	7485.9	70.00	0.37	tr_C9ZP82_
HLA-DQA10501-DQB10201	598	DTILRRVPSREGVEI	VPSREGVEI	6	0.1754	7497.9	70.00	0.41	tr_C9ZP82_
HLA-DQA10501-DQB10201	599	FFGQPNHIEDGTLPS	PNHIEDGTL	4	0.1748	7541.9	70.00	0.34	tr_C9ZP82_
HLA-DQA10501-DQB10201	600	ESSENSSSDTDNESP	SSSDTDNES	5	0.1746	7563.5	70.00	0.31	tr_C9ZP82_
HLA-DQA10501-DQB10201	601	NDGDGVNSFCVRSTD	VNSFCVRST	5	0.1739	7616.0	70.00	0.28	tr_C9ZP82_
HLA-DQA10501-DQB10201	602	SPIESREAHVTTVPN	EAHVTTVPN	6	0.1727	7719.1	70.00	0.30	tr_C9ZP82_
HLA-DQA10501-DQB10201	603	LAQFGGKANHVIVGL	KANHVIVGL	6	0.1724	7740.0	70.00	0.65	tr_C9ZP82_
HLA-DQA10501-DQB10201	604	ALNDGDGVNSFCVRS	DGDGVNSFC	3	0.1722	7758.0	70.00	0.24	tr_C9ZP82_
HLA-DQA10501-DQB10201	605	VSEVLPYGFLTKGPH	VSEVLPYGF	0	0.1708	7878.5	75.00	0.54	tr_C9ZP82_
HLA-DQA10501-DQB10201	606	FVPKGDLRKLSDALD	LRKLSDALD	6	0.1707	7882.1	75.00	0.55	tr_C9ZP82_
HLA-DQA10501-DQB10201	607	PIESREAHVTTVPNT	EAHVTTVPN	5	0.1706	7894.6	75.00	0.25	tr_C9ZP82_
HLA-DQA10501-DQB10201	608	NMLNEKMSGNDPTWT	MSGNDPTWT	6	0.1702	7929.8	75.00	0.51	tr_C9ZP82_
HLA-DQA10501-DQB10201	609	ERRSRRKRNFIDDTC	KRNFIDDTC	6	0.1700	7946.1	75.00	0.50	tr_C9ZP82_
HLA-DQA10501-DQB10201	610	QLTEEERWRTQKLDF	RWRTQKLDF	6	0.1699	7957.6	75.00	0.54	tr_C9ZP82_
HLA-DQA10501-DQB10201	611	QKLDFHLVITSNKTS	DFHLVITSN	3	0.1698	7961.9	75.00	0.28	tr_C9ZP82_
HLA-DQA10501-DQB10201	612	LSNSVQEACEKYVEG	VQEACEKYV	4	0.1696	7980.0	75.00	0.26	tr_C9ZP82_
HLA-DQA10501-DQB10201	613	YAKIEAVKFRRFLLF	YAKIEAVKF	0	0.1692	8016.8	75.00	0.53	tr_C9ZP82_
HLA-DQA10501-DQB10201	614	DDYESTHENRYVESN	DYESTHENR	1	0.1679	8127.8	75.00	0.31	tr_C9ZP82_
HLA-DQA10501-DQB10201	615	KLDFHLVITSNKTSF	FHLVITSNK	3	0.1672	8191.9	75.00	0.23	tr_C9ZP82_
HLA-DQA10501-DQB10201	616	VPKFASWLRNKYPAI	WLRNKYPAI	6	0.1670	8205.0	75.00	0.44	tr_C9ZP82_
HLA-DQA10501-DQB10201	617	ILRRVPSREGVEIRQ	VPSREGVEI	4	0.1669	8215.8	75.00	0.35	tr_C9ZP82_
HLA-DQA10501-DQB10201	618	ESPIESREAHVTTVP	REAHVTTVP	6	0.1668	8229.7	75.00	0.28	tr_C9ZP82_
HLA-DQA10501-DQB10201	619	TILRRVPSREGVEIR	VPSREGVEI	5	0.1663	8266.6	75.00	0.39	tr_C9ZP82_
HLA-DQA10501-DQB10201	620	TYLKSTGLSNSVQEA	GLSNSVQEA	6	0.1647	8411.8	75.00	0.41	tr_C9ZP82_
HLA-DQA10501-DQB10201	621	LNEKMSGNDPTWTRL	MSGNDPTWT	4	0.1647	8416.6	75.00	0.53	tr_C9ZP82_
HLA-DQA10501-DQB10201	622	AVLNMLNEKMSGNDP	VLNMLNEKM	1	0.1642	8461.7	75.00	0.64	tr_C9ZP82_
HLA-DQA10501-DQB10201	623	FASWLRNKYPAIVTK	WLRNKYPAI	3	0.1634	8530.5	75.00	0.38	tr_C9ZP82_
HLA-DQA10501-DQB10201	624	YLKEANIKRMPLPVV	IKRMPLPVV	6	0.1630	8567.2	75.00	0.44	tr_C9ZP82_
HLA-DQA10501-DQB10201	625	VIDLAIYLKEANIKR	AIYLKEANI	4	0.1627	8594.7	75.00	0.23	tr_C9ZP82_
HLA-DQA10501-DQB10201	626	NSVQEACEKYVEGVC	VQEACEKYV	2	0.1623	8633.6	75.00	0.28	tr_C9ZP82_
HLA-DQA10501-DQB10201	627	MLNEKMSGNDPTWTR	MSGNDPTWT	5	0.1612	8742.2	75.00	0.51	tr_C9ZP82_
HLA-DQA10501-DQB10201	628	IDLNGIIHPCCHNDS	LNGIIHPCC	2	0.1605	8807.4	75.00	0.44	tr_C9ZP82_
HLA-DQA10501-DQB10201	629	NCISPGTDFMAKVSD	PGTDFMAKV	4	0.1603	8827.2	75.00	0.28	tr_C9ZP82_
HLA-DQA10501-DQB10201	630	PQLTEEERWRTQKLD	PQLTEEERW	0	0.1599	8867.3	75.00	0.62	tr_C9ZP82_
HLA-DQA10501-DQB10201	631	SNSVQEACEKYVEGV	VQEACEKYV	3	0.1593	8919.7	75.00	0.35	tr_C9ZP82_
HLA-DQA10501-DQB10201	632	NEKMSGNDPTWTRLC	MSGNDPTWT	3	0.1591	8941.0	75.00	0.56	tr_C9ZP82_
HLA-DQA10501-DQB10201	633	ESREAHVTTVPNTGF	EAHVTTVPN	3	0.1589	8957.7	75.00	0.20	tr_C9ZP82_

HLA-DQA10501-DQB10201	634	VEGVCFVWRYYTTTT	FVWRYYTTT	5	0.1585	8995.5	75.00	0.23	tr_C9ZP82_
HLA-DQA10501-DQB10201	635	SCTVEVPRLSYGADG	PRLSYGADG	6	0.1583	9017.6	75.00	0.35	tr_C9ZP82_
HLA-DQA10501-DQB10201	636	WTRLCVIFSGSNTPG	WTRLCVIFS	0	0.1579	9062.1	75.00	0.30	tr_C9ZP82_
HLA-DQA10501-DQB10201	637	SSSDTDNESPIESRE	DTDNESPIE	3	0.1566	9186.4	75.00	0.23	tr_C9ZP82_
HLA-DQA10501-DQB10201	638	DYESTHENRYVESNQ	THENRYVES	4	0.1566	9190.3	75.00	0.22	tr_C9ZP82_
HLA-DQA10501-DQB10201	639	FTPATESAFDKKTLV	TPATESAFD	1	0.1558	9265.1	75.00	0.34	tr_C9ZP82_
HLA-DQA10501-DQB10201	640	LSDALDTILRRVPSR	LSDALDTIL	0	0.1554	9310.5	75.00	0.38	tr_C9ZP82_
HLA-DQA10501-DQB10201	641	YSCTVEVPRLSYGAD	SCTVEVPRL	1	0.1551	9331.8	75.00	0.38	tr_C9ZP82_
HLA-DQA10501-DQB10201	642	SAFDKKTLVSSAGYY	TLVSSAGYY	6	0.1550	9349.4	75.00	0.49	tr_C9ZP82_
HLA-DQA10501-DQB10201	643	EFDSNCISPGTDFMA	CISPGTDFM	5	0.1537	9479.7	80.00	0.30	tr_C9ZP82_
HLA-DQA10501-DQB10201	644	EKMSGNDPTWTRLCV	MSGNDPTWT	2	0.1537	9482.7	80.00	0.55	tr_C9ZP82_
HLA-DQA10501-DQB10201	645	PYGFLTKGPHEINLP	FLTKGPHEI	3	0.1533	9515.5	80.00	0.56	tr_C9ZP82_
HLA-DQA10501-DQB10201	646	IESREAHVTTVPNTG	EAHVTTVPN	4	0.1530	9548.8	80.00	0.21	tr_C9ZP82_
HLA-DQA10501-DQB10201	647	RPCSSPANSKIIDDI	ANSKIIDDI	6	0.1522	9637.3	80.00	0.73	tr_C9ZP82_
HLA-DQA10501-DQB10201	648	EVLPYGFLTKGPHEI	FLTKGPHEI	6	0.1516	9691.8	80.00	0.44	tr_C9ZP82_
HLA-DQA10501-DQB10201	649	SREAHVTTVPNTGFE	AHVTTVPNT	3	0.1514	9719.6	80.00	0.22	tr_C9ZP82_
HLA-DQA10501-DQB10201	650	TQVFILRDTTRNPYK	QVFILRDTT	1	0.1513	9723.3	80.00	0.38	tr_C9ZP82_
HLA-DQA10501-DQB10201	651	CESSENSSSDTDNES	SSSDTDNES	6	0.1509	9769.8	80.00	0.31	tr_C9ZP82_
HLA-DQA10501-DQB10201	652	YGFLTKGPHEINLPQ	FLTKGPHEI	2	0.1497	9902.1	80.00	0.57	tr_C9ZP82_
HLA-DQA10501-DQB10201	653	NQQRARRYMGRAAPA	RYMGRAAPA	6	0.1491	9962.9	80.00	0.69	tr_C9ZP82_
HLA-DQA10501-DQB10201	654	NESPIESREAHVTTV	PIESREAHV	3	0.1487	10010.0	80.00	0.36	tr_C9ZP82_
HLA-DQA10501-DQB10201	655	LKEANIKRMPLPVVK	IKRMPLPVV	5	0.1484	10034.2	80.00	0.38	tr_C9ZP82_
HLA-DQA10501-DQB10201	656	DTDNESPIESREAHV	PIESREAHV	6	0.1480	10082.6	80.00	0.34	tr_C9ZP82_
HLA-DQA10501-DQB10201	657	DSNCISPGTDFMAKV	PGTDFMAKV	6	0.1480	10086.0	80.00	0.34	tr_C9ZP82_
HLA-DQA10501-DQB10201	658	HSGQLKGNGEGVIEG	KGNGEGVIE	5	0.1468	10213.4	80.00	0.25	tr_C9ZP82_
HLA-DQA10501-DQB10201	659	FGQPNHIEDGTLPSS	PNHIEDGTL	3	0.1464	10259.1	80.00	0.37	tr_C9ZP82_
HLA-DQA10501-DQB10201	660	LPYGFLTKGPHEINL	FLTKGPHEI	4	0.1460	10297.1	80.00	0.56	tr_C9ZP82_
HLA-DQA10501-DQB10201	661	SDALDTILRRVPSRE	ILRRVPSRE	6	0.1455	10357.5	80.00	0.47	tr_C9ZP82_
HLA-DQA10501-DQB10201	662	VLPYGFLTKGPHEIN	FLTKGPHEI	5	0.1442	10501.6	80.00	0.56	tr_C9ZP82_
HLA-DQA10501-DQB10201	663	SSDTDNESPIESREA	DTDNESPIE	2	0.1442	10508.8	80.00	0.22	tr_C9ZP82_
HLA-DQA10501-DQB10201	664	IDLAIYLKEANIKRM	AIYLKEANI	3	0.1440	10522.1	80.00	0.28	tr_C9ZP82_
HLA-DQA10501-DQB10201	665	KRMPLPVVKSTPPDT	VVKSTPPDT	6	0.1438	10544.9	80.00	0.48	tr_C9ZP82_
HLA-DQA10501-DQB10201	666	SGQLKGNGEGVIEGK	QLKGNGEGV	2	0.1437	10559.3	80.00	0.24	tr_C9ZP82_
HLA-DQA10501-DQB10201	667	RHSGQLKGNGEGVIE	KGNGEGVIE	6	0.1437	10560.1	80.00	0.30	tr_C9ZP82_
HLA-DQA10501-DQB10201	668	KEANIKRMPLPVVKS	IKRMPLPVV	4	0.1432	10615.4	80.00	0.34	tr_C9ZP82_
HLA-DQA10501-DQB10201	669	PLERYRPRTYSCTVE	PRTYSCTVE	6	0.1432	10617.6	80.00	0.60	tr_C9ZP82_
HLA-DQA10501-DQB10201	670	FKSTNKENSNSGAEF	ENSNSGAEF	6	0.1422	10730.7	80.00	0.84	tr_C9ZP82_
HLA-DQA10501-DQB10201	671	DTYLKSTGLSNSVQE	TGLSNSVQE	6	0.1415	10818.6	80.00	0.49	tr_C9ZP82_
HLA-DQA10501-DQB10201	672	ASWLRNKYPAIVTKN	WLRNKYPAI	2	0.1409	10884.4	80.00	0.40	tr_C9ZP82_
HLA-DQA10501-DQB10201	673	FHLVITSNKTSFVPK	ITSNKTSFV	4	0.1409	10891.1	80.00	0.37	tr_C9ZP82_
HLA-DQA10501-DQB10201	674	TRLCVIFSGSNTPGE	VIFSGSNTP	4	0.1408	10902.3	80.00	0.35	tr_C9ZP82_
HLA-DQA10501-DQB10201	675	TDNESPIESREAHVT	PIESREAHV	5	0.1406	10920.9	80.00	0.42	tr_C9ZP82_
HLA-DQA10501-DQB10201	676	MGVPKFASWLRNKYP	FASWLRNKY	5	0.1392	11090.8	80.00	0.40	tr_C9ZP82_
HLA-DQA10501-DQB10201	677	NPYKRRAVARQQQRE	RRAVARQQQ	4	0.1387	11149.6	80.00	0.41	tr_C9ZP82_
HLA-DQA10501-DQB10201	678	DALDTILRRVPSREG	ILRRVPSRE	5	0.1380	11238.2	80.00	0.47	tr_C9ZP82_
HLA-DQA10501-DQB10201	679	KSTNKENSNSGAEFR	ENSNSGAEF	5	0.1373	11322.0	80.00	0.62	tr_C9ZP82_
HLA-DQA10501-DQB10201	680	LRTQSSLAQFGGKAN	RTQSSLAQF	1	0.1370	11355.7	80.00	0.44	tr_C9ZP82_
HLA-DQA10501-DQB10201	681	SWLRNKYPAIVTKNC	WLRNKYPAI	1	0.1366	11403.0	80.00	0.34	tr_C9ZP82_
HLA-DQA10501-DQB10201	682	DNESPIESREAHVTT	PIESREAHV	4	0.1357	11514.7	80.00	0.41	tr_C9ZP82_
HLA-DQA10501-DQB10201	683	SNCISPGTDFMAKVS	PGTDFMAKV	5	0.1355	11540.1	80.00	0.31	tr_C9ZP82_

HLA-DQA10501-DQB10201	684	QVFILRDTTRNPYKR	QVFILRDTT	0	0.1341	11719.2	85.00	0.32	tr_C9ZP82_
HLA-DQA10501-DQB10201	685	EANIKRMPLPVVKST	IKRMPLPVV	3	0.1336	11782.2	85.00	0.37	tr_C9ZP82_
HLA-DQA10501-DQB10201	686	SYGADGQVIKRQRRH	SYGADGQVI	0	0.1312	12093.3	85.00	0.62	tr_C9ZP82_
HLA-DQA10501-DQB10201	687	GVPKFASWLRNKYPA	FASWLRNKY	4	0.1303	12205.8	85.00	0.41	tr_C9ZP82_
HLA-DQA10501-DQB10201	688	ALDTILRRVPSREGV	ILRRVPSRE	4	0.1303	12213.0	85.00	0.54	tr_C9ZP82_
HLA-DQA10501-DQB10201	689	EVSALCDIKKFTPAT	EVSALCDIK	0	0.1303	12215.0	85.00	0.38	tr_C9ZP82_
HLA-DQA10501-DQB10201	690	LDTILRRVPSREGVE	ILRRVPSRE	3	0.1301	12230.0	85.00	0.54	tr_C9ZP82_
HLA-DQA10501-DQB10201	691	AVDGVAPRAKMNQQR	AVDGVAPRA	0	0.1297	12290.6	85.00	0.67	tr_C9ZP82_
HLA-DQA10501-DQB10201	692	ASTYMPNKSGMAIVE	NKSGMAIVE	6	0.1288	12414.1	85.00	0.38	tr_C9ZP82_
HLA-DQA10501-DQB10201	693	GQPNHIEDGTLPSST	PNHIEDGTL	2	0.1280	12523.4	85.00	0.33	tr_C9ZP82_
HLA-DQA10501-DQB10201	694	DRWRDTYLKSTGLSN	YLKSTGLSN	6	0.1278	12544.7	85.00	0.33	tr_C9ZP82_
HLA-DQA10501-DQB10201	695	GQLKGNGEGVIEGKK	QLKGNGEGV	1	0.1275	12583.0	85.00	0.23	tr_C9ZP82_
HLA-DQA10501-DQB10201	696	WLRNKYPAIVTKNCP	WLRNKYPAI	0	0.1268	12679.2	85.00	0.23	tr_C9ZP82_
HLA-DQA10501-DQB10201	697	SEVLPYGFLTKGPHE	GFLTKGPHE	6	0.1263	12745.8	85.00	0.28	tr_C9ZP82_
HLA-DQA10501-DQB10201	698	LCVIFSGSNTPGEGE	VIFSGSNTP	2	0.1257	12830.2	85.00	0.35	tr_C9ZP82_
HLA-DQA10501-DQB10201	699	IDDRWRDTYLKSTGL	DTYLKSTGL	6	0.1257	12835.2	85.00	0.34	tr_C9ZP82_
HLA-DQA10501-DQB10201	700	RLCVIFSGSNTPGEG	VIFSGSNTP	3	0.1254	12879.6	85.00	0.38	tr_C9ZP82_
HLA-DQA10501-DQB10201	701	DLAIYLKEANIKRMP	AIYLKEANI	2	0.1253	12887.4	85.00	0.34	tr_C9ZP82_
HLA-DQA10501-DQB10201	702	DLNGIIHPCCHNDSD	LNGIIHPCC	1	0.1252	12907.8	85.00	0.44	tr_C9ZP82_
HLA-DQA10501-DQB10201	703	ANIKRMPLPVVKSTP	IKRMPLPVV	2	0.1247	12978.1	85.00	0.38	tr_C9ZP82_
HLA-DQA10501-DQB10201	704	SDTDNESPIESREAH	DTDNESPIE	1	0.1246	12986.9	85.00	0.25	tr_C9ZP82_
HLA-DQA10501-DQB10201	705	IYLKEANIKRMPLPV	NIKRMPLPV	6	0.1238	13093.9	85.00	0.31	tr_C9ZP82_
HLA-DQA10501-DQB10201	706	PYKRRAVARQQQRER	RRAVARQQQ	3	0.1236	13120.4	85.00	0.38	tr_C9ZP82_
HLA-DQA10501-DQB10201	707	VCESSENSSSDTDNE	NSSSDTDNE	6	0.1234	13150.2	85.00	0.43	tr_C9ZP82_
HLA-DQA10501-DQB10201	708	FDSNCISPGTDFMAK	CISPGTDFM	4	0.1232	13186.1	85.00	0.35	tr_C9ZP82_
HLA-DQA10501-DQB10201	709	IHPCCHNDSDTAIAL	NDSDTAIAL	6	0.1231	13192.6	85.00	0.35	tr_C9ZP82_
HLA-DQA10501-DQB10201	710	QPNHIEDGTLPSSTE	PNHIEDGTL	1	0.1222	13326.6	85.00	0.32	tr_C9ZP82_
HLA-DQA10501-DQB10201	711	TRNPYKRRAVARQQQ	RRAVARQQQ	6	0.1213	13460.5	85.00	0.64	tr_C9ZP82_
HLA-DQA10501-DQB10201	712	GSNTPGEGEHKIIDF	EGEHKIIDF	6	0.1199	13666.5	85.00	0.58	tr_C9ZP82_
HLA-DQA10501-DQB10201	713	ALCDIKKFTPATESA	IKKFTPATE	4	0.1197	13691.4	85.00	0.40	tr_C9ZP82_
HLA-DQA10501-DQB10201	714	LAIYLKEANIKRMPL	EANIKRMPL	6	0.1195	13723.1	85.00	0.22	tr_C9ZP82_
HLA-DQA10501-DQB10201	715	NIKRMPLPVVKSTPP	IKRMPLPVV	1	0.1195	13723.6	85.00	0.34	tr_C9ZP82_
HLA-DQA10501-DQB10201	716	RWRDTYLKSTGLSNS	YLKSTGLSN	5	0.1193	13753.0	85.00	0.38	tr_C9ZP82_
HLA-DQA10501-DQB10201	717	IKRMPLPVVKSTPPD	LPVVKSTPP	5	0.1192	13772.9	85.00	0.24	tr_C9ZP82_
HLA-DQA10501-DQB10201	718	KIEAVKFRRFLLFKS	FRRFLLFKS	6	0.1190	13794.6	85.00	0.28	tr_C9ZP82_
HLA-DQA10501-DQB10201	719	DDRWRDTYLKSTGLS	DTYLKSTGL	5	0.1189	13808.9	85.00	0.31	tr_C9ZP82_
HLA-DQA10501-DQB10201	720	RNPYKRRAVARQQQR	RRAVARQQQ	5	0.1184	13887.7	85.00	0.56	tr_C9ZP82_
HLA-DQA10501-DQB10201	721	STIDDRWRDTYLKST	WRDTYLKST	6	0.1182	13920.5	85.00	0.38	tr_C9ZP82_
HLA-DQA10501-DQB10201	722	QLKGNGEGVIEGKKS	KGNGEGVIE	2	0.1173	14047.5	85.00	0.22	tr_C9ZP82_
HLA-DQA10501-DQB10201	723	ERSWKNFRPCSSPAN	WKNFRPCSS	3	0.1173	14052.3	85.00	0.57	tr_C9ZP82_
HLA-DQA10501-DQB10201	724	STNKENSNSGAEFRK	ENSNSGAEF	4	0.1173	14055.8	85.00	0.62	tr_C9ZP82_
HLA-DQA10501-DQB10201	725	WRDTYLKSTGLSNSV	YLKSTGLSN	4	0.1165	14183.1	85.00	0.38	tr_C9ZP82_
HLA-DQA10501-DQB10201	726	CVIFSGSNTPGEGEH	VIFSGSNTP	1	0.1158	14282.4	85.00	0.37	tr_C9ZP82_
HLA-DQA10501-DQB10201	727	ESAFDKKTLVSSAGY	KTLVSSAGY	6	0.1157	14300.0	85.00	0.72	tr_C9ZP82_
HLA-DQA10501-DQB10201	728	YKRRAVARQQQRERR	RRAVARQQQ	2	0.1156	14315.2	85.00	0.38	tr_C9ZP82_
HLA-DQA10501-DQB10201	729	IEAVKFRRFLLFKST	FRRFLLFKS	5	0.1153	14357.7	85.00	0.25	tr_C9ZP82_
HLA-DQA10501-DQB10201	730	EAERSWKNFRPCSSP	WKNFRPCSS	5	0.1150	14403.0	85.00	0.68	tr_C9ZP82_
HLA-DQA10501-DQB10201	731	AIYLKEANIKRMPLP	EANIKRMPL	5	0.1149	14429.6	85.00	0.23	tr_C9ZP82_
HLA-DQA10501-DQB10201	732	VLNMLNEKMSGNDPT	VLNMLNEKM	0	0.1146	14471.1	85.00	0.65	tr_C9ZP82_
HLA-DQA10501-DQB10201	733	AERSWKNFRPCSSPA	WKNFRPCSS	4	0.1139	14576.5	90.00	0.65	tr_C9ZP82_

HLA-DQA10501-DQB10201	734	HLVITSNKTSFVPKG	ITSNKTSFV	3	0.1139	14581.3	90.00	0.44	tr_C9ZP82_
HLA-DQA10501-DQB10201	735	RSKRVSKKAEAERSW	SKKAEAERS	5	0.1123	14840.5	90.00	0.35	tr_C9ZP82_
HLA-DQA10501-DQB10201	736	AKIEAVKFRRFLLFK	IEAVKFRRF	2	0.1121	14861.7	90.00	0.21	tr_C9ZP82_
HLA-DQA10501-DQB10201	737	LNMLNEKMSGNDPTW	KMSGNDPTW	6	0.1119	14904.4	90.00	0.51	tr_C9ZP82_
HLA-DQA10501-DQB10201	738	VSALCDIKKFTPATE	IKKFTPATE	6	0.1114	14981.4	90.00	0.53	tr_C9ZP82_
HLA-DQA10501-DQB10201	739	RSWKNFRPCSSPANS	WKNFRPCSS	2	0.1110	15044.7	90.00	0.55	tr_C9ZP82_
HLA-DQA10501-DQB10201	740	HRHSGQLKGNGEGVI	QLKGNGEGV	5	0.1103	15158.6	90.00	0.49	tr_C9ZP82_
HLA-DQA10501-DQB10201	741	AEAERSWKNFRPCSS	WKNFRPCSS	6	0.1095	15293.7	90.00	0.62	tr_C9ZP82_
HLA-DQA10501-DQB10201	742	GEVASTYMPNKSGMA	EVASTYMPN	1	0.1090	15380.3	90.00	0.38	tr_C9ZP82_
HLA-DQA10501-DQB10201	743	RDTYLKSTGLSNSVQ	YLKSTGLSN	3	0.1089	15390.6	90.00	0.38	tr_C9ZP82_
HLA-DQA10501-DQB10201	744	VPVCESSENSSSDTD	SENSSSDTD	6	0.1074	15639.4	90.00	0.44	tr_C9ZP82_
HLA-DQA10501-DQB10201	745	RERRSRRKRNFIDDT	RKRNFIDDT	6	0.1074	15642.5	90.00	0.71	tr_C9ZP82_
HLA-DQA10501-DQB10201	746	TIDDRWRDTYLKSTG	WRDTYLKST	5	0.1073	15661.3	90.00	0.38	tr_C9ZP82_
HLA-DQA10501-DQB10201	747	TNKENSNSGAEFRKG	ENSNSGAEF	3	0.1071	15688.6	90.00	0.61	tr_C9ZP82_
HLA-DQA10501-DQB10201	748	EAVKFRRFLLFKSTN	FRRFLLFKS	4	0.1069	15726.3	90.00	0.26	tr_C9ZP82_
HLA-DQA10501-DQB10201	749	VASTYMPNKSGMAIV	PNKSGMAIV	6	0.1062	15838.3	90.00	0.38	tr_C9ZP82_
HLA-DQA10501-DQB10201	750	SALCDIKKFTPATES	IKKFTPATE	5	0.1057	15937.2	90.00	0.51	tr_C9ZP82_
HLA-DQA10501-DQB10201	751	RTQSSLAQFGGKANH	TQSSLAQFG	1	0.1049	16067.0	90.00	0.38	tr_C9ZP82_
HLA-DQA10501-DQB10201	752	PNHIEDGTLPSSTEC	PNHIEDGTL	0	0.1043	16178.7	90.00	0.24	tr_C9ZP82_
HLA-DQA10501-DQB10201	753	VIFSGSNTPGEGEHK	VIFSGSNTP	0	0.1032	16364.9	90.00	0.31	tr_C9ZP82_
HLA-DQA10501-DQB10201	754	PVCESSENSSSDTDN	SENSSSDTD	5	0.1024	16505.9	90.00	0.43	tr_C9ZP82_
HLA-DQA10501-DQB10201	755	SKRVSKKAEAERSWK	RVSKKAEAE	2	0.1020	16579.7	90.00	0.34	tr_C9ZP82_
HLA-DQA10501-DQB10201	756	LVITSNKTSFVPKGD	ITSNKTSFV	2	0.1017	16636.7	90.00	0.49	tr_C9ZP82_
HLA-DQA10501-DQB10201	757	HSTIDDRWRDTYLKS	RWRDTYLKS	6	0.1014	16687.9	90.00	0.24	tr_C9ZP82_
HLA-DQA10501-DQB10201	758	SWKNFRPCSSPANSK	WKNFRPCSS	1	0.1007	16815.6	90.00	0.54	tr_C9ZP82_
HLA-DQA10501-DQB10201	759	SFVPKGDLRKLSDAL	DLRKLSDAL	6	0.1006	16830.0	90.00	0.56	tr_C9ZP82_
HLA-DQA10501-DQB10201	760	NKENSNSGAEFRKGS	ENSNSGAEF	2	0.0993	17071.0	90.00	0.62	tr_C9ZP82_
HLA-DQA10501-DQB10201	761	KRRAVARQQQRERRS	RRAVARQQQ	1	0.0991	17108.7	90.00	0.35	tr_C9ZP82_
HLA-DQA10501-DQB10201	762	EVASTYMPNKSGMAI	EVASTYMPN	0	0.0990	17133.2	90.00	0.22	tr_C9ZP82_
HLA-DQA10501-DQB10201	763	VITSNKTSFVPKGDL	ITSNKTSFV	1	0.0979	17332.9	90.00	0.41	tr_C9ZP82_
HLA-DQA10501-DQB10201	764	SSLAQFGGKANHVIV	QFGGKANHV	4	0.0976	17392.2	90.00	0.34	tr_C9ZP82_
HLA-DQA10501-DQB10201	765	LRNKYPAIVTKNCPS	PAIVTKNCP	5	0.0968	17534.3	90.00	0.22	tr_C9ZP82_
HLA-DQA10501-DQB10201	766	AVKFRRFLLFKSTNK	FRRFLLFKS	3	0.0968	17539.4	90.00	0.24	tr_C9ZP82_
HLA-DQA10501-DQB10201	767	LKGNGEGVIEGKKSN	KGNGEGVIE	1	0.0956	17767.9	90.00	0.25	tr_C9ZP82_
HLA-DQA10501-DQB10201	768	QSSLAQFGGKANHVI	QFGGKANHV	5	0.0955	17795.4	90.00	0.37	tr_C9ZP82_
HLA-DQA10501-DQB10201	769	TPATESAFDKKTLVS	TPATESAFD	0	0.0949	17902.6	90.00	0.30	tr_C9ZP82_
HLA-DQA10501-DQB10201	770	KRVSKKAEAERSWKN	RVSKKAEAE	1	0.0943	18031.1	90.00	0.32	tr_C9ZP82_
HLA-DQA10501-DQB10201	771	IIHPCCHNDSDTAIA	CHNDSDTAI	5	0.0935	18176.6	90.00	0.36	tr_C9ZP82_
HLA-DQA10501-DQB10201	772	RRSKRVSKKAEAERS	SKKAEAERS	6	0.0929	18291.6	90.00	0.41	tr_C9ZP82_
HLA-DQA10501-DQB10201	773	NHIEDGTLPSSTECG	TLPSSTECG	6	0.0927	18330.9	90.00	0.19	tr_C9ZP82_
HLA-DQA10501-DQB10201	774	VKFRRFLLFKSTNKE	FLLFKSTNK	5	0.0926	18351.9	90.00	0.24	tr_C9ZP82_
HLA-DQA10501-DQB10201	775	EFRKGSNNDNVTVAD	NNDNVTVAD	6	0.0926	18368.2	90.00	0.31	tr_C9ZP82_
HLA-DQA10501-DQB10201	776	NKYPAIVTKNCPSSV	VTKNCPSSV	6	0.0920	18476.0	90.00	0.28	tr_C9ZP82_
HLA-DQA10501-DQB10201	777	IGVPVCESSENSSSD	PVCESSENS	3	0.0917	18537.3	90.00	0.36	tr_C9ZP82_
HLA-DQA10501-DQB10201	778	RVSKKAEAERSWKNF	SKKAEAERS	2	0.0915	18580.9	90.00	0.33	tr_C9ZP82_
HLA-DQA10501-DQB10201	779	KENSNSGAEFRKGSN	ENSNSGAEF	1	0.0912	18629.8	95.00	0.58	tr_C9ZP82_
HLA-DQA10501-DQB10201	780	LNGIIHPCCHNDSDT	LNGIIHPCC	0	0.0888	19129.4	95.00	0.36	tr_C9ZP82_
HLA-DQA10501-DQB10201	781	GIIHPCCHNDSDTAI	CHNDSDTAI	6	0.0880	19301.1	95.00	0.47	tr_C9ZP82_
HLA-DQA10501-DQB10201	782	SLAQFGGKANHVIVG	QFGGKANHV	3	0.0878	19337.9	95.00	0.33	tr_C9ZP82_
HLA-DQA10501-DQB10201	783	KFRRFLLFKSTNKEN	FLLFKSTNK	4	0.0870	19506.2	95.00	0.25	tr_C9ZP82_

HLA-DQA10501-DQB10201	784	VPLERYRPRTYSCTV	RPRTYSCTV	6	0.0854	19836.6	95.00	0.37	tr_C9ZP82_
HLA-DQA10501-DQB10201	785	TQSSLAQFGGKANHV	QFGGKANHV	6	0.0854	19840.7	95.00	0.40	tr_C9ZP82_
HLA-DQA10501-DQB10201	786	FRPCSSPANSKIIDD	PANSKIIDD	6	0.0845	20035.7	95.00	0.63	tr_C9ZP82_
HLA-DQA10501-DQB10201	787	GVPVCESSENSSSDT	PVCESSENS	2	0.0845	20042.0	95.00	0.36	tr_C9ZP82_
HLA-DQA10501-DQB10201	788	HIEDGTLPSSTECGS	TLPSSTECG	5	0.0845	20047.8	95.00	0.19	tr_C9ZP82_
HLA-DQA10501-DQB10201	789	VFILRDTTRNPYKRR	FILRDTTRN	1	0.0841	20126.9	95.00	0.38	tr_C9ZP82_
HLA-DQA10501-DQB10201	790	RNKYPAIVTKNCPSS	PAIVTKNCP	4	0.0837	20220.6	95.00	0.26	tr_C9ZP82_
HLA-DQA10501-DQB10201	791	KYPAIVTKNCPSSVH	VTKNCPSSV	5	0.0829	20383.1	95.00	0.31	tr_C9ZP82_
HLA-DQA10501-DQB10201	792	RRAVARQQQRERRSR	AVARQQQRE	2	0.0827	20435.2	95.00	0.38	tr_C9ZP82_
HLA-DQA10501-DQB10201	793	FSGSNTPGEGEHKII	GSNTPGEGE	2	0.0822	20547.2	95.00	0.22	tr_C9ZP82_
HLA-DQA10501-DQB10201	794	FRRFLLFKSTNKENS	FLLFKSTNK	3	0.0821	20566.1	95.00	0.25	tr_C9ZP82_
HLA-DQA10501-DQB10201	795	IEDGTLPSSTECGSP	TLPSSTECG	4	0.0814	20731.2	95.00	0.28	tr_C9ZP82_
HLA-DQA10501-DQB10201	796	IFSGSNTPGEGEHKI	FSGSNTPGE	1	0.0813	20748.9	95.00	0.34	tr_C9ZP82_
HLA-DQA10501-DQB10201	797	ITSNKTSFVPKGDLR	ITSNKTSFV	0	0.0809	20846.1	95.00	0.35	tr_C9ZP82_
HLA-DQA10501-DQB10201	798	GKKSNSRVTVVNKGP	SRVTVVNKG	5	0.0808	20861.2	95.00	0.32	tr_C9ZP82_
HLA-DQA10501-DQB10201	799	KKSNSRVTVVNKGPC	SRVTVVNKG	4	0.0792	21224.2	95.00	0.29	tr_C9ZP82_
HLA-DQA10501-DQB10201	800	WKNFRPCSSPANSKI	WKNFRPCSS	0	0.0780	21509.4	95.00	0.48	tr_C9ZP82_
HLA-DQA10501-DQB10201	801	ENSNSGAEFRKGSNN	ENSNSGAEF	0	0.0759	21986.9	95.00	0.48	tr_C9ZP82_
HLA-DQA10501-DQB10201	802	YDVPLERYRPRTYSC	LERYRPRTY	4	0.0759	21993.8	95.00	0.47	tr_C9ZP82_
HLA-DQA10501-DQB10201	803	MNQQRARRYMGRAAP	ARRYMGRAA	5	0.0756	22057.6	95.00	0.38	tr_C9ZP82_
HLA-DQA10501-DQB10201	804	VSKKAEAERSWKNFR	SKKAEAERS	1	0.0740	22463.7	95.00	0.41	tr_C9ZP82_
HLA-DQA10501-DQB10201	805	AEFRKGSNNDNVTVA	SNNDNVTVA	6	0.0735	22583.8	95.00	0.31	tr_C9ZP82_
HLA-DQA10501-DQB10201	806	KGNGEGVIEGKKSNS	NGEGVIEGK	2	0.0724	22849.5	95.00	0.23	tr_C9ZP82_
HLA-DQA10501-DQB10201	807	RHRHSGQLKGNGEGV	QLKGNGEGV	6	0.0718	22982.4	95.00	0.63	tr_C9ZP82_
HLA-DQA10501-DQB10201	808	GRRSKRVSKKAEAER	RVSKKAEAE	5	0.0704	23352.2	95.00	0.64	tr_C9ZP82_
HLA-DQA10501-DQB10201	809	SGSNTPGEGEHKIID	PGEGEHKII	5	0.0702	23389.1	95.00	0.25	tr_C9ZP82_
HLA-DQA10501-DQB10201	810	EGKKSNSRVTVVNKG	SRVTVVNKG	6	0.0702	23389.6	95.00	0.34	tr_C9ZP82_
HLA-DQA10501-DQB10201	811	VAPRAKMNQQRARRY	MNQQRARRY	6	0.0701	23414.6	95.00	0.35	tr_C9ZP82_
HLA-DQA10501-DQB10201	812	TTRNPYKRRAVARQQ	KRRAVARQQ	6	0.0701	23426.3	95.00	0.56	tr_C9ZP82_
HLA-DQA10501-DQB10201	813	YPAIVTKNCPSSVHG	VTKNCPSSV	4	0.0699	23468.9	95.00	0.38	tr_C9ZP82_
HLA-DQA10501-DQB10201	814	RRFLLFKSTNKENSN	FLLFKSTNK	2	0.0697	23515.7	95.00	0.27	tr_C9ZP82_
HLA-DQA10501-DQB10201	815	KGRRSKRVSKKAEAE	RVSKKAEAE	6	0.0694	23595.2	95.00	0.75	tr_C9ZP82_
HLA-DQA10501-DQB10201	816	AIVTKNCPSSVHGLY	VTKNCPSSV	2	0.0686	23791.8	95.00	0.39	tr_C9ZP82_
HLA-DQA10501-DQB10201	817	SKKAEAERSWKNFRP	SKKAEAERS	0	0.0682	23915.7	95.00	0.23	tr_C9ZP82_
HLA-DQA10501-DQB10201	818	DVPLERYRPRTYSCT	LERYRPRTY	3	0.0678	24008.5	95.00	0.47	tr_C9ZP82_
HLA-DQA10501-DQB10201	819	KTSFVPKGDLRKLSD	SFVPKGDLR	2	0.0676	24050.7	95.00	0.22	tr_C9ZP82_
HLA-DQA10501-DQB10201	820	SNKTSFVPKGDLRKL	SFVPKGDLR	4	0.0671	24203.6	95.00	0.25	tr_C9ZP82_
HLA-DQA10501-DQB10201	821	EDGTLPSSTECGSPK	TLPSSTECG	3	0.0670	24228.8	95.00	0.30	tr_C9ZP82_
HLA-DQA10501-DQB10201	822	IEGKKSNSRVTVVNK	KSNSRVTVV	4	0.0662	24418.3	95.00	0.44	tr_C9ZP82_
HLA-DQA10501-DQB10201	823	PATESAFDKKTLVSS	ATESAFDKK	1	0.0661	24443.9	95.00	0.22	tr_C9ZP82_
HLA-DQA10501-DQB10201	824	GAEFRKGSNNDNVTV	RKGSNNDNV	4	0.0659	24512.8	95.00	0.31	tr_C9ZP82_
HLA-DQA10501-DQB10201	825	NGIIHPCCHNDSDTA	CCHNDSDTA	6	0.0657	24564.5	95.00	0.22	tr_C9ZP82_
HLA-DQA10501-DQB10201	826	VIEGKKSNSRVTVVN	KSNSRVTVV	5	0.0652	24693.5	95.00	0.62	tr_C9ZP82_
HLA-DQA10501-DQB10201	827	NKTSFVPKGDLRKLS	SFVPKGDLR	3	0.0643	24938.9	95.00	0.25	tr_C9ZP82_
HLA-DQA10501-DQB10201	828	VDGVAPRAKMNQQRA	RAKMNQQRA	6	0.0638	25064.2	95.00	0.49	tr_C9ZP82_
HLA-DQA10501-DQB10201	829	TSNKTSFVPKGDLRK	SFVPKGDLR	5	0.0633	25202.6	100.00	0.25	tr_C9ZP82_
HLA-DQA10501-DQB10201	830	SGAEFRKGSNNDNVT	RKGSNNDNV	5	0.0633	25207.5	100.00	0.39	tr_C9ZP82_
HLA-DQA10501-DQB10201	831	RFLLFKSTNKENSNS	FLLFKSTNK	1	0.0631	25256.1	100.00	0.27	tr_C9ZP82_
HLA-DQA10501-DQB10201	832	TSFVPKGDLRKLSDA	SFVPKGDLR	1	0.0627	25359.6	100.00	0.22	tr_C9ZP82_
HLA-DQA10501-DQB10201	833	APRAKMNQQRARRYM	MNQQRARRY	5	0.0626	25395.6	100.00	0.35	tr_C9ZP82_

HLA-DQA10501-DQB10201	834	RAVARQQQRERRSRR	AVARQQQRE	1	0.0625	25419.5	100.00	0.58	tr_C9ZP82_
HLA-DQA10501-DQB10201	835	ATESAFDKKTLVSSA	DKKTLVSSA	6	0.0621	25544.4	100.00	0.23	tr_C9ZP82_
HLA-DQA10501-DQB10201	836	GVIEGKKSNSRVTVV	KSNSRVTVV	6	0.0611	25821.2	100.00	0.77	tr_C9ZP82_
HLA-DQA10501-DQB10201	837	KMNQQRARRYMGRAA	ARRYMGRAA	6	0.0596	26247.1	100.00	0.50	tr_C9ZP82_
HLA-DQA10501-DQB10201	838	PAIVTKNCPSSVHGL	VTKNCPSSV	3	0.0587	26492.4	100.00	0.51	tr_C9ZP82_
HLA-DQA10501-DQB10201	839	DGTLPSSTECGSPKG	TLPSSTECG	2	0.0577	26789.9	100.00	0.35	tr_C9ZP82_
HLA-DQA10501-DQB10201	840	NSGAEFRKGSNNDNV	RKGSNNDNV	6	0.0571	26954.8	100.00	0.52	tr_C9ZP82_
HLA-DQA10501-DQB10201	841	FILRDTTRNPYKRRA	FILRDTTRN	0	0.0569	27027.2	100.00	0.42	tr_C9ZP82_
HLA-DQA10501-DQB10201	842	GNGEGVIEGKKSNSR	NGEGVIEGK	1	0.0564	27170.3	100.00	0.37	tr_C9ZP82_
HLA-DQA10501-DQB10201	843	FLLFKSTNKENSNSG	LLFKSTNKE	1	0.0547	27652.5	100.00	0.30	tr_C9ZP82_
HLA-DQA10501-DQB10201	844	TESAFDKKTLVSSAG	KKTLVSSAG	6	0.0542	27805.2	100.00	0.28	tr_C9ZP82_
HLA-DQA10501-DQB10201	845	KAEAERSWKNFRPCS	SWKNFRPCS	6	0.0540	27872.1	100.00	0.20	tr_C9ZP82_
HLA-DQA10501-DQB10201	846	PRAKMNQQRARRYMG	MNQQRARRY	4	0.0531	28154.3	100.00	0.36	tr_C9ZP82_
HLA-DQA10501-DQB10201	847	KKAEAERSWKNFRPC	ERSWKNFRP	5	0.0528	28229.9	100.00	0.24	tr_C9ZP82_
HLA-DQA10501-DQB10201	848	DTTRNPYKRRAVARQ	YKRRAVARQ	6	0.0493	29334.7	100.00	0.63	tr_C9ZP82_
HLA-DQA10501-DQB10201	849	AKMNQQRARRYMGRA	MNQQRARRY	2	0.0491	29404.6	100.00	0.37	tr_C9ZP82_
HLA-DQA10501-DQB10201	850	DGVAPRAKMNQQRAR	RAKMNQQRA	5	0.0491	29406.8	100.00	0.54	tr_C9ZP82_
HLA-DQA10501-DQB10201	851	RAKMNQQRARRYMGR	MNQQRARRY	3	0.0486	29546.2	100.00	0.39	tr_C9ZP82_
HLA-DQA10501-DQB10201	852	GVAPRAKMNQQRARR	RAKMNQQRA	4	0.0485	29599.9	100.00	0.50	tr_C9ZP82_
HLA-DQA10501-DQB10201	853	YGADGQVIKRQRRHR	YGADGQVIK	0	0.0482	29681.7	100.00	0.33	tr_C9ZP82_
HLA-DQA10501-DQB10201	854	AVARQQQRERRSRRK	AVARQQQRE	0	0.0481	29727.4	100.00	0.53	tr_C9ZP82_
HLA-DQA10501-DQB10201	855	NFRPCSSPANSKIID	FRPCSSPAN	1	0.0468	30128.9	100.00	0.27	tr_C9ZP82_
HLA-DQA10501-DQB10201	856	GTLPSSTECGSPKGR	TLPSSTECG	1	0.0451	30700.1	100.00	0.41	tr_C9ZP82_
HLA-DQA10501-DQB10201	857	KNFRPCSSPANSKII	FRPCSSPAN	2	0.0451	30709.8	100.00	0.37	tr_C9ZP82_
HLA-DQA10501-DQB10201	858	NSNSGAEFRKGSNND	NSNSGAEFR	0	0.0449	30764.7	100.00	0.45	tr_C9ZP82_
HLA-DQA10501-DQB10201	859	QRERRSRRKRNFIDD	RRKRNFIDD	6	0.0437	31169.7	100.00	0.49	tr_C9ZP82_
HLA-DQA10501-DQB10201	860	NGEGVIEGKKSNSRV	NGEGVIEGK	0	0.0435	31238.3	100.00	0.31	tr_C9ZP82_
HLA-DQA10501-DQB10201	861	QRRHRHSGQLKGNGE	SGQLKGNGE	6	0.0414	31956.2	100.00	0.39	tr_C9ZP82_
HLA-DQA10501-DQB10201	862	LLFKSTNKENSNSGA	FKSTNKENS	2	0.0383	33032.3	100.00	0.28	tr_C9ZP82_
HLA-DQA10501-DQB10201	863	SNSGAEFRKGSNNDN	FRKGSNNDN	6	0.0374	33357.3	100.00	0.37	tr_C9ZP82_
HLA-DQA10501-DQB10201	864	RRHRHSGQLKGNGEG	SGQLKGNGE	5	0.0361	33826.1	100.00	0.41	tr_C9ZP82_
HLA-DQA10501-DQB10201	865	IKRQRRHRHSGQLKG	RHRHSGQLK	5	0.0358	33929.2	100.00	0.43	tr_C9ZP82_
HLA-DQA10501-DQB10201	866	TLPSSTECGSPKGRR	TLPSSTECG	0	0.0348	34319.8	100.00	0.38	tr_C9ZP82_
HLA-DQA10501-DQB10201	867	KRQRRHRHSGQLKGN	RHRHSGQLK	4	0.0342	34548.9	100.00	0.41	tr_C9ZP82_
HLA-DQA10501-DQB10201	868	VIKRQRRHRHSGQLK	RHRHSGQLK	6	0.0341	34570.6	100.00	0.55	tr_C9ZP82_
HLA-DQA10501-DQB10201	869	LFKSTNKENSNSGAE	KENSNSGAE	6	0.0332	34909.3	100.00	0.23	tr_C9ZP82_
HLA-DQA10501-DQB10201	870	ILRDTTRNPYKRRAV	LRDTTRNPY	1	0.0323	35254.3	100.00	0.46	tr_C9ZP82_
HLA-DQA10501-DQB10201	871	RQRRHRHSGQLKGNG	RHRHSGQLK	3	0.0321	35342.9	100.00	0.36	tr_C9ZP82_
HLA-DQA10501-DQB10201	872	GADGQVIKRQRRHRH	ADGQVIKRQ	1	0.0287	36648.6	100.00	0.41	tr_C9ZP82_
HLA-DQA10501-DQB10201	873	GEGVIEGKKSNSRVT	EGVIEGKKS	1	0.0271	37303.9	100.00	0.43	tr_C9ZP82_
HLA-DQA10501-DQB10201	874	QVIKRQRRHRHSGQL	RRHRHSGQL	6	0.0248	38215.4	100.00	0.60	tr_C9ZP82_
HLA-DQA10501-DQB10201	875	RDTTRNPYKRRAVAR	PYKRRAVAR	6	0.0238	38639.0	100.00	0.52	tr_C9ZP82_
HLA-DQA10501-DQB10201	876	LRDTTRNPYKRRAVA	LRDTTRNPY	0	0.0237	38685.9	100.00	0.38	tr_C9ZP82_
HLA-DQA10501-DQB10201	877	EGVIEGKKSNSRVTV	EGVIEGKKS	0	0.0230	38965.7	100.00	0.30	tr_C9ZP82_
HLA-DQA10501-DQB10201	878	QQRERRSRRKRNFID	SRRKRNFID	6	0.0226	39140.2	100.00	0.34	tr_C9ZP82_
HLA-DQA10501-DQB10201	879	VARQQQRERRSRRKR	ARQQQRERR	1	0.0222	39324.4	100.00	0.44	tr_C9ZP82_
HLA-DQA10501-DQB10201	880	PKGRRSKRVSKKAEA	KRVSKKAEA	6	0.0221	39347.8	100.00	0.57	tr_C9ZP82_
HLA-DQA10501-DQB10201	881	ADGQVIKRQRRHRHS	ADGQVIKRQ	0	0.0215	39610.5	100.00	0.39	tr_C9ZP82_
HLA-DQA10501-DQB10201	882	LPSSTECGSPKGRRS	LPSSTECGS	0	0.0211	39789.2	100.00	0.34	tr_C9ZP82_
HLA-DQA10501-DQB10201	883	QQQRERRSRRKRNFI	RSRRKRNFI	6	0.0162	41965.2	100.00	0.49	tr_C9ZP82_

HLA-DQA10501-DQB10201	884	ARQQQRERRSRRKRN	ARQQQRERR	0	0.0153	42359.3	100.00	0.47	tr_C9ZP82_
HLA-DQA10501-DQB10201	885	STECGSPKGRRSKRV	PKGRRSKRV	6	0.0151	42445.6	100.00	0.44	tr_C9ZP82_
HLA-DQA10501-DQB10201	886	PSSTECGSPKGRRSK	PSSTECGSP	0	0.0132	43330.0	100.00	0.28	tr_C9ZP82_
HLA-DQA10501-DQB10201	887	GQVIKRQRRHRHSGQ	QRRHRHSGQ	6	0.0126	43607.9	100.00	0.43	tr_C9ZP82_
HLA-DQA10501-DQB10201	888	DGQVIKRQRRHRHSG	DGQVIKRQR	0	0.0125	43681.1	100.00	0.25	tr_C9ZP82_
HLA-DQA10501-DQB10201	889	TECGSPKGRRSKRVS	PKGRRSKRV	5	0.0123	43765.3	100.00	0.52	tr_C9ZP82_
HLA-DQA10501-DQB10201	890	SPKGRRSKRVSKKAE	SKRVSKKAE	6	0.0121	43874.9	100.00	0.28	tr_C9ZP82_
HLA-DQA10501-DQB10201	891	RQQQRERRSRRKRNF	RRSRRKRNF	6	0.0112	44303.2	100.00	0.42	tr_C9ZP82_
HLA-DQA10501-DQB10201	892	ECGSPKGRRSKRVSK	PKGRRSKRV	4	0.0111	44345.0	100.00	0.52	tr_C9ZP82_
HLA-DQA10501-DQB10201	893	GSPKGRRSKRVSKKA	PKGRRSKRV	2	0.0110	44379.1	100.00	0.34	tr_C9ZP82_
HLA-DQA10501-DQB10201	894	SSTECGSPKGRRSKR	STECGSPKG	1	0.0103	44741.6	100.00	0.34	tr_C9ZP82_
HLA-DQA10501-DQB10201	895	CGSPKGRRSKRVSKK	PKGRRSKRV	3	0.0103	44748.9	100.00	0.40	tr_C9ZP82_

Allele: HLA-DQA10501-DQB10201. Number of high binders 4. Number of weak binders 102. Number of peptides 895

Allele	pos	peptide	core	Of	1-log50k(aff)	affinity(nM)	%Rank	Relia	Identity	Bind Level
HLA-DQA10501-DQB10201	1	NIRQTFVDWLCTECL	VDWLCTECL	6	0.5213	177.6	4.50	0.46	tr_C9ZP82_	WB
HLA-DQA10501-DQB10201	2	RQTFVDWLCTECLSL	VDWLCTECL	4	0.5073	206.7	5.00	0.44	tr_C9ZP82_	WB
HLA-DQA10501-DQB10201	3	IRQTFVDWLCTECLS	VDWLCTECL	5	0.5064	208.7	5.00	0.47	tr_C9ZP82_	WB
HLA-DQA10501-DQB10201	4	QTFVDWLCTECLSLN	VDWLCTECL	3	0.4917	244.7	6.00	0.43	tr_C9ZP82_	WB
HLA-DQA10501-DQB10201	5	TFVDWLCTECLSLNF	VDWLCTECL	2	0.4773	285.8	7.00	0.41	tr_C9ZP82_	WB
HLA-DQA10501-DQB10201	6	FVDWLCTECLSLNFS	VDWLCTECL	1	0.4478	393.5	10.00	0.44	tr_C9ZP82_	
HLA-DQA10501-DQB10201	7	NNIRQTFVDWLCTEC	IRQTFVDWL	2	0.4264	495.7	13.00	0.38	tr_C9ZP82_	
HLA-DQA10501-DQB10201	8	RNNIRQTFVDWLCTE	IRQTFVDWL	3	0.4149	561.5	14.00	0.38	tr_C9ZP82_	
HLA-DQA10501-DQB10201	9	GMSRNNIRQTFVDWL	IRQTFVDWL	6	0.4111	585.2	14.00	0.61	tr_C9ZP82_	
HLA-DQA10501-DQB10201	10	MSRNNIRQTFVDWLC	IRQTFVDWL	5	0.4017	647.6	16.00	0.51	tr_C9ZP82_	
HLA-DQA10501-DQB10201	11	SRNNIRQTFVDWLCT	IRQTFVDWL	4	0.3924	716.6	17.00	0.50	tr_C9ZP82_	
HLA-DQA10501-DQB10201	12	VDWLCTECLSLNFSS	VDWLCTECL	0	0.3566	1055.7	23.00	0.38	tr_C9ZP82_	
HLA-DQA10501-DQB10201	13	CGMSRNNIRQTFVDW	NIRQTFVDW	6	0.2728	2613.6	42.00	0.37	tr_C9ZP82_	
HLA-DQA10501-DQB10201	14	VFSCTQSADQSLYDP	QSADQSLYD	5	0.2416	3662.4	55.00	0.28	tr_C9ZP82_	
HLA-DQA10501-DQB10201	15	FSCTQSADQSLYDPD	QSADQSLYD	4	0.2413	3672.5	55.00	0.31	tr_C9ZP82_	
HLA-DQA10501-DQB10201	16	AVFSCTQSADQSLYD	QSADQSLYD	6	0.2379	3812.6	55.00	0.27	tr_C9ZP82_	
HLA-DQA10501-DQB10201	17	DWLCTECLSLNFSSR	WLCTECLSL	1	0.2335	3997.4	55.00	0.44	tr_C9ZP82_	
HLA-DQA10501-DQB10201	18	DPDHSPHIEAVKLQI	HIEAVKLQI	6	0.2236	4450.6	60.00	0.35	tr_C9ZP82_	
HLA-DQA10501-DQB10201	19	SCTQSADQSLYDPDH	QSADQSLYD	3	0.2207	4593.1	60.00	0.32	tr_C9ZP82_	
HLA-DQA10501-DQB10201	20	CTQSADQSLYDPDHS	QSADQSLYD	2	0.2200	4624.3	60.00	0.25	tr_C9ZP82_	
HLA-DQA10501-DQB10201	21	TQSADQSLYDPDHSP	QSLYDPDHS	5	0.2164	4808.1	60.00	0.28	tr_C9ZP82_	
HLA-DQA10501-DQB10201	22	LAVFSCTQSADQSLY	CTQSADQSL	5	0.2140	4938.3	60.00	0.22	tr_C9ZP82_	
HLA-DQA10501-DQB10201	23	CLAVFSCTQSADQSL	CTQSADQSL	6	0.2085	5240.5	60.00	0.22	tr_C9ZP82_	
HLA-DQA10501-DQB10201	24	GCGMSRNNIRQTFVD	RNNIRQTFV	5	0.2036	5521.1	65.00	0.57	tr_C9ZP82_	
HLA-DQA10501-DQB10201	25	RRCLAVFSCTQSADQ	CLAVFSCTQ	2	0.1903	6377.0	65.00	0.25	tr_C9ZP82_	
HLA-DQA10501-DQB10201	26	RCLAVFSCTQSADQS	FSCTQSADQ	5	0.1896	6426.2	70.00	0.22	tr_C9ZP82_	
HLA-DQA10501-DQB10201	27	PFDNRRCLAVFSCTQ	CLAVFSCTQ	6	0.1894	6440.8	70.00	0.31	tr_C9ZP82_	
HLA-DQA10501-DQB10201	28	NRRCLAVFSCTQSAD	CLAVFSCTQ	3	0.1864	6656.1	70.00	0.28	tr_C9ZP82_	
HLA-DQA10501-DQB10201	29	YDPDHSPHIEAVKLQ	SPHIEAVKL	5	0.1863	6664.8	70.00	0.50	tr_C9ZP82_	
HLA-DQA10501-DQB10201	30	QSADQSLYDPDHSPH	QSLYDPDHS	4	0.1857	6707.7	70.00	0.32	tr_C9ZP82_	

HLA-DQA10501-DQB10201	31	FDNRRCLAVFSCTQS	CLAVFSCTQ	5	0.1856	6713.9	70.00	0.28	tr_C9ZP82_
HLA-DQA10501-DQB10201	32	SGCGMSRNNIRQTFV	RNNIRQTFV	6	0.1844	6796.3	70.00	0.63	tr_C9ZP82_
HLA-DQA10501-DQB10201	33	WLCTECLSLNFSSRT	WLCTECLSL	0	0.1820	6977.6	70.00	0.39	tr_C9ZP82_
HLA-DQA10501-DQB10201	34	RAPFDNRRCLAVFSC	RRCLAVFSC	6	0.1779	7291.3	70.00	0.32	tr_C9ZP82_
HLA-DQA10501-DQB10201	35	DNRRCLAVFSCTQSA	CLAVFSCTQ	4	0.1751	7518.2	70.00	0.28	tr_C9ZP82_
HLA-DQA10501-DQB10201	36	FSSRTRCFVCRAPFD	CFVCRAPFD	6	0.1731	7684.8	70.00	0.35	tr_C9ZP82_
HLA-DQA10501-DQB10201	37	APFDNRRCLAVFSCT	RRCLAVFSC	5	0.1712	7841.6	75.00	0.25	tr_C9ZP82_
HLA-DQA10501-DQB10201	38	SSRTRCFVCRAPFDN	CFVCRAPFD	5	0.1704	7908.8	75.00	0.35	tr_C9ZP82_
HLA-DQA10501-DQB10201	39	SADQSLYDPDHSPHI	QSLYDPDHS	3	0.1674	8175.9	75.00	0.41	tr_C9ZP82_
HLA-DQA10501-DQB10201	40	LYDPDHSPHIEAVKL	SPHIEAVKL	6	0.1671	8200.1	75.00	0.64	tr_C9ZP82_
HLA-DQA10501-DQB10201	41	SRTRCFVCRAPFDNR	CFVCRAPFD	4	0.1616	8700.0	75.00	0.33	tr_C9ZP82_
HLA-DQA10501-DQB10201	42	CRAPFDNRRCLAVFS	NRRCLAVFS	6	0.1611	8747.7	75.00	0.36	tr_C9ZP82_
HLA-DQA10501-DQB10201	43	ADQSLYDPDHSPHIE	QSLYDPDHS	2	0.1557	9272.8	75.00	0.41	tr_C9ZP82_
HLA-DQA10501-DQB10201	44	RTRCFVCRAPFDNRR	CFVCRAPFD	3	0.1485	10024.1	80.00	0.35	tr_C9ZP82_
HLA-DQA10501-DQB10201	45	DQSLYDPDHSPHIEA	QSLYDPDHS	1	0.1448	10436.2	80.00	0.42	tr_C9ZP82_
HLA-DQA10501-DQB10201	46	NFSSRTRCFVCRAPF	RTRCFVCRA	4	0.1409	10887.5	80.00	0.40	tr_C9ZP82_
HLA-DQA10501-DQB10201	47	TRCFVCRAPFDNRRC	CFVCRAPFD	2	0.1381	11223.8	80.00	0.40	tr_C9ZP82_
HLA-DQA10501-DQB10201	48	VCRAPFDNRRCLAVF	DNRRCLAVF	6	0.1371	11347.3	80.00	0.41	tr_C9ZP82_
HLA-DQA10501-DQB10201	49	LNFSSRTRCFVCRAP	RTRCFVCRA	5	0.1325	11923.0	85.00	0.41	tr_C9ZP82_
HLA-DQA10501-DQB10201	50	SLNFSSRTRCFVCRA	RTRCFVCRA	6	0.1305	12188.8	85.00	0.41	tr_C9ZP82_
HLA-DQA10501-DQB10201	51	CFVCRAPFDNRRCLA	CFVCRAPFD	0	0.1302	12227.5	85.00	0.29	tr_C9ZP82_
HLA-DQA10501-DQB10201	52	RCFVCRAPFDNRRCL	CFVCRAPFD	1	0.1299	12257.4	85.00	0.38	tr_C9ZP82_
HLA-DQA10501-DQB10201	53	QSLYDPDHSPHIEAV	QSLYDPDHS	0	0.1228	13242.6	85.00	0.30	tr_C9ZP82_
HLA-DQA10501-DQB10201	54	ECLSLNFSSRTRCFV	FSSRTRCFV	6	0.1218	13383.1	85.00	0.46	tr_C9ZP82_
HLA-DQA10501-DQB10201	55	LCTECLSLNFSSRTR	TECLSLNFS	2	0.1171	14084.3	85.00	0.44	tr_C9ZP82_
HLA-DQA10501-DQB10201	56	CLSLNFSSRTRCFVC	FSSRTRCFV	5	0.1157	14302.3	85.00	0.50	tr_C9ZP82_
HLA-DQA10501-DQB10201	57	CTECLSLNFSSRTRC	TECLSLNFS	1	0.1067	15755.9	90.00	0.40	tr_C9ZP82_
HLA-DQA10501-DQB10201	58	FVCRAPFDNRRCLAV	PFDNRRCLA	5	0.1048	16091.0	90.00	0.31	tr_C9ZP82_
HLA-DQA10501-DQB10201	59	LSLNFSSRTRCFVCR	FSSRTRCFV	4	0.1046	16130.4	90.00	0.47	tr_C9ZP82_
HLA-DQA10501-DQB10201	60	TECLSLNFSSRTRCF	LSLNFSSRT	3	0.0975	17414.6	90.00	0.27	tr_C9ZP82_
HLA-DQA10501-DQB10201	61	SSGCGMSRNNIRQTF	SRNNIRQTF	6	0.0949	17898.3	90.00	0.55	tr_C9ZP82_
HLA-DQA10501-DQB10201	62	SLYDPDHSPHIEAVK	DHSPHIEAV	5	0.0875	19397.0	95.00	0.23	tr_C9ZP82_
HLA-DQA10501-DQB10201	63	PSSGCGMSRNNIRQT	MSRNNIRQT	6	0.0403	32341.9	100.00	0.29	tr_C9ZP82_
HLA-DQA10501-DQB10201	64	DPSSGCGMSRNNIRQ	PSSGCGMSR	1	0.0368	33589.8	100.00	0.27	tr_C9ZP82_

Allele: HLA-DQA10501-DQB10201. Number of high binders 0. Number of weak binders 5. Number of peptides 64

Allele	pos	peptide	core	Of	1-log50k(aff)	affinity(nM)	%Rank	Relia	Identity	Bind Level
HLA-DQA10501-DQB10201	1	AQADVVVQEFPEELY	VVQEFPEEL	5	0.6929	27.7	0.20	0.46	tr_C9ZJU1_	SB
HLA-DQA10501-DQB10201	2	QADVVVQEFPEELYL	VVQEFPEEL	4	0.6851	30.2	0.25	0.50	tr_C9ZJU1_	SB
HLA-DQA10501-DQB10201	3	AAQADVVVQEFPEEL	VVQEFPEEL	6	0.6779	32.6	0.30	0.47	tr_C9ZJU1_	SB
HLA-DQA10501-DQB10201	4	ADVVVQEFPEELYLG	VVQEFPEEL	3	0.6509	43.7	0.50	0.52	tr_C9ZJU1_	WB
HLA-DQA10501-DQB10201	5	DVVVQEFPEELYLGL	VVQEFPEEL	2	0.6391	49.6	0.60	0.51	tr_C9ZJU1_	WB
HLA-DQA10501-DQB10201	6	HIDAYLALATADYDP	LALATADYD	5	0.6085	69.1	1.10	0.44	tr_C9ZJU1_	WB
HLA-DQA10501-DQB10201	7	VVVQEFPEELYLGLI	VVQEFPEEL	1	0.6045	72.2	1.20	0.50	tr_C9ZJU1_	WB
HLA-DQA10501-DQB10201	8	IDAYLALATADYDPS	LALATADYD	4	0.5975	77.8	1.40	0.46	tr_C9ZJU1_	WB

HLA-DQA10501-DQB10201	9	DAYLALATADYDPSD	LALATADYD	3	0.5962	78.9	1.40	0.53	tr_C9ZJU1_	WB
HLA-DQA10501-DQB10201	10	LHGLFQMADTLPLLL	MADTLPLLL	6	0.5928	81.9	1.50	0.23	tr_C9ZJU1_	WB
HLA-DQA10501-DQB10201	11	IHIDAYLALATADYD	YLALATADY	5	0.5871	87.1	1.60	0.40	tr_C9ZJU1_	WB
HLA-DQA10501-DQB10201	12	PAAQADVVVQEFPEE	QADVVVQEF	3	0.5857	88.5	1.60	0.46	tr_C9ZJU1_	WB
HLA-DQA10501-DQB10201	13	CIRGFVIQSILSDII	IQSILSDII	6	0.5828	91.3	1.70	0.61	tr_C9ZJU1_	WB
HLA-DQA10501-DQB10201	14	YPPAAQADVVVQEFP	QADVVVQEF	5	0.5780	96.2	1.80	0.62	tr_C9ZJU1_	WB
HLA-DQA10501-DQB10201	15	RYPPAAQADVVVQEF	QADVVVQEF	6	0.5750	99.3	1.90	0.66	tr_C9ZJU1_	WB
HLA-DQA10501-DQB10201	16	CPLYHAFLGSFYDEC	FLGSFYDEC	6	0.5743	100.1	1.90	0.34	tr_C9ZJU1_	WB
HLA-DQA10501-DQB10201	17	HGLFQMADTLPLLLV	FQMADTLPL	3	0.5737	100.7	2.00	0.25	tr_C9ZJU1_	WB
HLA-DQA10501-DQB10201	18	AYLALATADYDPSDD	LALATADYD	2	0.5704	104.4	2.50	0.54	tr_C9ZJU1_	WB
HLA-DQA10501-DQB10201	19	VLHGLFQMADTLPLL	FQMADTLPL	5	0.5694	105.5	2.50	0.28	tr_C9ZJU1_	WB
HLA-DQA10501-DQB10201	20	PLYHAFLGSFYDECG	FLGSFYDEC	5	0.5657	109.8	2.50	0.32	tr_C9ZJU1_	WB
HLA-DQA10501-DQB10201	21	IRGFVIQSILSDIIT	IQSILSDII	5	0.5600	116.9	2.50	0.62	tr_C9ZJU1_	WB
HLA-DQA10501-DQB10201	22	LYHAFLGSFYDECGM	FLGSFYDEC	4	0.5593	117.7	2.50	0.33	tr_C9ZJU1_	WB
HLA-DQA10501-DQB10201	23	PPAAQADVVVQEFPE	QADVVVQEF	4	0.5580	119.3	2.50	0.61	tr_C9ZJU1_	WB
HLA-DQA10501-DQB10201	24	QWSNFLYFLNESLYM	FLYFLNESL	4	0.5513	128.3	3.00	0.35	tr_C9ZJU1_	WB
HLA-DQA10501-DQB10201	25	WSNFLYFLNESLYME	FLYFLNESL	3	0.5511	128.7	3.00	0.35	tr_C9ZJU1_	WB
HLA-DQA10501-DQB10201	26	GLFQMADTLPLLLVS	FQMADTLPL	2	0.5465	135.2	3.00	0.28	tr_C9ZJU1_	WB
HLA-DQA10501-DQB10201	27	LCILDDVKAEEVLAL	LDDVKAEEV	3	0.5443	138.4	3.00	0.39	tr_C9ZJU1_	WB
HLA-DQA10501-DQB10201	28	FLYFLNESLYMESQF	YFLNESLYM	2	0.5410	143.5	3.50	0.23	tr_C9ZJU1_	WB
HLA-DQA10501-DQB10201	29	DVLHGLFQMADTLPL	FQMADTLPL	6	0.5397	145.5	3.50	0.35	tr_C9ZJU1_	WB
HLA-DQA10501-DQB10201	30	SNFLYFLNESLYMES	FLYFLNESL	2	0.5344	154.2	3.50	0.32	tr_C9ZJU1_	WB
HLA-DQA10501-DQB10201	31	RGFVIQSILSDIITS	IQSILSDII	4	0.5307	160.4	4.00	0.65	tr_C9ZJU1_	WB
HLA-DQA10501-DQB10201	32	YLALATADYDPSDDW	LALATADYD	1	0.5305	160.7	4.00	0.55	tr_C9ZJU1_	WB
HLA-DQA10501-DQB10201	33	ILDDVKAEEVLALCM	LDDVKAEEV	1	0.5296	162.4	4.00	0.29	tr_C9ZJU1_	WB
HLA-DQA10501-DQB10201	34	PLVPSVFSGCVVDAL	FSGCVVDAL	6	0.5267	167.6	4.00	0.64	tr_C9ZJU1_	WB
HLA-DQA10501-DQB10201	35	CILDDVKAEEVLALC	LDDVKAEEV	2	0.5266	167.6	4.00	0.39	tr_C9ZJU1_	WB
HLA-DQA10501-DQB10201	36	ALCILDDVKAEEVLA	LDDVKAEEV	4	0.5250	170.6	4.00	0.54	tr_C9ZJU1_	WB
HLA-DQA10501-DQB10201	37	GFVIQSILSDIITSI	IQSILSDII	3	0.5232	173.9	4.00	0.62	tr_C9ZJU1_	WB
HLA-DQA10501-DQB10201	38	VALCILDDVKAEEVL	LDDVKAEEV	5	0.5200	180.0	4.50	0.59	tr_C9ZJU1_	WB
HLA-DQA10501-DQB10201	39	QALWQASGSVMDELQ	ASGSVMDEL	5	0.5161	187.9	4.50	0.49	tr_C9ZJU1_	WB
HLA-DQA10501-DQB10201	40	NFLYFLNESLYMESQ	YFLNESLYM	3	0.5154	189.4	4.50	0.30	tr_C9ZJU1_	WB
HLA-DQA10501-DQB10201	41	YHAFLGSFYDECGMS	FLGSFYDEC	3	0.5142	191.7	4.50	0.39	tr_C9ZJU1_	WB
HLA-DQA10501-DQB10201	42	SIHIDAYLALATADY	YLALATADY	6	0.5141	191.9	4.50	0.54	tr_C9ZJU1_	WB
HLA-DQA10501-DQB10201	43	SQALWQASGSVMDEL	ASGSVMDEL	6	0.5120	196.4	5.00	0.63	tr_C9ZJU1_	WB
HLA-DQA10501-DQB10201	44	WCPLYHAFLGSFYDE	YHAFLGSFY	4	0.5095	201.7	5.00	0.45	tr_C9ZJU1_	WB
HLA-DQA10501-DQB10201	45	VSMAVASTFAFESLY	ASTFAFESL	5	0.5091	202.6	5.00	0.28	tr_C9ZJU1_	WB
HLA-DQA10501-DQB10201	46	VVQEFPEELYLGLIT	VVQEFPEEL	0	0.5055	210.7	5.00	0.51	tr_C9ZJU1_	WB
HLA-DQA10501-DQB10201	47	PNFFTQSALDQTEIV	FFTQSALDQ	2	0.5053	211.2	5.00	0.35	tr_C9ZJU1_	WB
HLA-DQA10501-DQB10201	48	LFQMADTLPLLLVSA	FQMADTLPL	1	0.5046	212.8	5.50	0.31	tr_C9ZJU1_	WB
HLA-DQA10501-DQB10201	49	GVALCILDDVKAEEV	LDDVKAEEV	6	0.5039	214.4	5.50	0.54	tr_C9ZJU1_	WB
HLA-DQA10501-DQB10201	50	ALWQASGSVMDELQS	ASGSVMDEL	4	0.5017	219.6	5.50	0.50	tr_C9ZJU1_	WB
HLA-DQA10501-DQB10201	51	FVIQSILSDIITSIH	IQSILSDII	2	0.5017	219.6	5.50	0.65	tr_C9ZJU1_	WB
HLA-DQA10501-DQB10201	52	LVPSVFSGCVVDALK	FSGCVVDAL	5	0.4960	233.5	6.00	0.60	tr_C9ZJU1_	WB
HLA-DQA10501-DQB10201	53	HWCPLYHAFLGSFYD	YHAFLGSFY	5	0.4957	234.3	6.00	0.49	tr_C9ZJU1_	WB
HLA-DQA10501-DQB10201	54	TQWSNFLYFLNESLY	FLYFLNESL	5	0.4951	235.9	6.00	0.50	tr_C9ZJU1_	WB
HLA-DQA10501-DQB10201	55	LYFLNESLYMESQFT	YFLNESLYM	1	0.4934	240.2	6.00	0.23	tr_C9ZJU1_	WB
HLA-DQA10501-DQB10201	56	SGWELMAATAAAAFA	MAATAAAAF	5	0.4934	240.3	6.00	0.25	tr_C9ZJU1_	WB
HLA-DQA10501-DQB10201	57	SMAVASTFAFESLYC	ASTFAFESL	4	0.4912	245.9	6.00	0.26	tr_C9ZJU1_	WB
HLA-DQA10501-DQB10201	58	SPNFFTQSALDQTEI	FFTQSALDQ	3	0.4878	255.2	6.50	0.40	tr_C9ZJU1_	WB

HLA-DQA10501-DQB10201	59	GTQWSNFLYFLNESL	FLYFLNESL	6	0.4850	262.9	6.50	0.61	tr_C9ZJU1_	WB
HLA-DQA10501-DQB10201	60	LDDVKAEEVLALCML	LDDVKAEEV	0	0.4848	263.6	6.50	0.19	tr_C9ZJU1_	WB
HLA-DQA10501-DQB10201	61	LSGWELMAATAAAAF	MAATAAAAF	6	0.4846	264.0	6.50	0.35	tr_C9ZJU1_	WB
HLA-DQA10501-DQB10201	62	LDCERKAYEILEMLT	RKAYEILEM	4	0.4842	265.2	6.50	0.34	tr_C9ZJU1_	WB
HLA-DQA10501-DQB10201	63	ILLDDGVALCILDDV	VALCILDDV	6	0.4840	265.9	6.50	0.57	tr_C9ZJU1_	WB
HLA-DQA10501-DQB10201	64	NFFTQSALDQTEIVN	FFTQSALDQ	1	0.4836	267.0	6.50	0.34	tr_C9ZJU1_	WB
HLA-DQA10501-DQB10201	65	FVSMAVASTFAFESL	ASTFAFESL	6	0.4821	271.5	7.00	0.34	tr_C9ZJU1_	WB
HLA-DQA10501-DQB10201	66	QCRQDYLQGFAALRL	LQGFAALRL	6	0.4808	275.2	7.00	0.69	tr_C9ZJU1_	WB
HLA-DQA10501-DQB10201	67	LSPNFFTQSALDQTE	FFTQSALDQ	4	0.4805	276.1	7.00	0.44	tr_C9ZJU1_	WB
HLA-DQA10501-DQB10201	68	MAVASTFAFESLYCI	ASTFAFESL	3	0.4805	276.2	7.00	0.22	tr_C9ZJU1_	WB
HLA-DQA10501-DQB10201	69	HAFLGSFYDECGMSE	FLGSFYDEC	2	0.4798	278.2	7.00	0.41	tr_C9ZJU1_	WB
HLA-DQA10501-DQB10201	70	RLSPNFFTQSALDQT	FFTQSALDQ	5	0.4774	285.6	7.00	0.47	tr_C9ZJU1_	WB
HLA-DQA10501-DQB10201	71	FYDECGMSEAQVDEA	MSEAQVDEA	6	0.4763	288.9	7.50	0.66	tr_C9ZJU1_	WB
HLA-DQA10501-DQB10201	72	GWELMAATAAAAFAT	MAATAAAAF	4	0.4755	291.6	7.50	0.25	tr_C9ZJU1_	WB
HLA-DQA10501-DQB10201	73	RFIIKQLMGLPATFV	LMGLPATFV	6	0.4728	300.1	7.50	0.54	tr_C9ZJU1_	WB
HLA-DQA10501-DQB10201	74	LWQASGSVMDELQSR	ASGSVMDEL	3	0.4709	306.2	7.50	0.50	tr_C9ZJU1_	WB
HLA-DQA10501-DQB10201	75	VPSVFSGCVVDALKR	FSGCVVDAL	4	0.4683	315.2	8.00	0.62	tr_C9ZJU1_	WB
HLA-DQA10501-DQB10201	76	FESLYCIRGFVIQSI	IRGFVIQSI	6	0.4673	318.5	8.00	0.84	tr_C9ZJU1_	WB
HLA-DQA10501-DQB10201	77	SAIDTITEGEIFDTL	TEGEIFDTL	6	0.4670	319.7	8.00	0.22	tr_C9ZJU1_	WB
HLA-DQA10501-DQB10201	78	YCIRGFVIQSILSDI	IRGFVIQSI	2	0.4663	322.0	8.00	0.50	tr_C9ZJU1_	WB
HLA-DQA10501-DQB10201	79	DECGMSEAQVDEAHL	MSEAQVDEA	4	0.4628	334.5	8.50	0.66	tr_C9ZJU1_	WB
HLA-DQA10501-DQB10201	80	RDVLHGLFQMADTLP	GLFQMADTL	5	0.4612	340.2	8.50	0.42	tr_C9ZJU1_	WB
HLA-DQA10501-DQB10201	81	QHWCPLYHAFLGSFY	YHAFLGSFY	6	0.4548	364.8	9.00	0.58	tr_C9ZJU1_	WB
HLA-DQA10501-DQB10201	82	KIDRPVRGVAPPSLD	VRGVAPPSL	5	0.4541	367.5	9.50	0.81	tr_C9ZJU1_	WB
HLA-DQA10501-DQB10201	83	YDECGMSEAQVDEAH	MSEAQVDEA	5	0.4533	370.7	9.50	0.70	tr_C9ZJU1_	WB
HLA-DQA10501-DQB10201	84	WELMAATAAAAFATA	MAATAAAAF	3	0.4529	372.2	9.50	0.25	tr_C9ZJU1_	WB
HLA-DQA10501-DQB10201	85	ATFVSMAVASTFAFE	SMAVASTFA	4	0.4522	375.0	9.50	0.32	tr_C9ZJU1_	WB
HLA-DQA10501-DQB10201	86	ESLYCIRGFVIQSIL	IRGFVIQSI	5	0.4520	376.0	9.50	0.74	tr_C9ZJU1_	WB
HLA-DQA10501-DQB10201	87	PTSAIDTITEGEIFD	DTITEGEIF	5	0.4515	378.0	9.50	0.34	tr_C9ZJU1_	WB
HLA-DQA10501-DQB10201	88	LLDCERKAYEILEML	RKAYEILEM	5	0.4508	380.7	9.50	0.52	tr_C9ZJU1_	WB
HLA-DQA10501-DQB10201	89	FIIKQLMGLPATFVS	LMGLPATFV	5	0.4507	381.2	9.50	0.52	tr_C9ZJU1_	WB
HLA-DQA10501-DQB10201	90	WQASGSVMDELQSRV	ASGSVMDEL	2	0.4489	388.5	10.00	0.49	tr_C9ZJU1_	
HLA-DQA10501-DQB10201	91	LALATADYDPSDDWL	LALATADYD	0	0.4486	390.0	10.00	0.48	tr_C9ZJU1_	
HLA-DQA10501-DQB10201	92	LRLSPNFFTQSALDQ	FFTQSALDQ	6	0.4483	391.3	10.00	0.58	tr_C9ZJU1_	
HLA-DQA10501-DQB10201	93	AVASTFAFESLYCIR	ASTFAFESL	2	0.4452	404.4	10.00	0.21	tr_C9ZJU1_	
HLA-DQA10501-DQB10201	94	LYCIRGFVIQSILSD	IRGFVIQSI	3	0.4449	405.9	10.00	0.63	tr_C9ZJU1_	
HLA-DQA10501-DQB10201	95	TSAIDTITEGEIFDT	DTITEGEIF	4	0.4448	406.4	10.00	0.30	tr_C9ZJU1_	
HLA-DQA10501-DQB10201	96	TFVSMAVASTFAFES	SMAVASTFA	3	0.4446	407.2	10.00	0.26	tr_C9ZJU1_	
HLA-DQA10501-DQB10201	97	YFLNESLYMESQFTK	ESLYMESQF	4	0.4444	408.2	10.00	0.25	tr_C9ZJU1_	
HLA-DQA10501-DQB10201	98	AIDTITEGEIFDTLR	DTITEGEIF	2	0.4431	414.0	11.00	0.26	tr_C9ZJU1_	
HLA-DQA10501-DQB10201	99	DCERKAYEILEMLTR	RKAYEILEM	3	0.4427	415.5	11.00	0.34	tr_C9ZJU1_	
HLA-DQA10501-DQB10201	100	PATFVSMAVASTFAF	SMAVASTFA	5	0.4427	415.8	11.00	0.37	tr_C9ZJU1_	
HLA-DQA10501-DQB10201	101	VIQSILSDIITSIHI	IQSILSDII	1	0.4426	416.0	11.00	0.67	tr_C9ZJU1_	
HLA-DQA10501-DQB10201	102	DRPVRGVAPPSLDGP	VRGVAPPSL	3	0.4416	420.7	11.00	0.81	tr_C9ZJU1_	
HLA-DQA10501-DQB10201	103	LLDDGVALCILDDVK	VALCILDDV	5	0.4407	424.6	11.00	0.54	tr_C9ZJU1_	
HLA-DQA10501-DQB10201	104	FFTQSALDQTEIVNQ	SALDQTEIV	4	0.4406	425.1	11.00	0.25	tr_C9ZJU1_	
HLA-DQA10501-DQB10201	105	CRQDYLQGFAALRLS	LQGFAALRL	5	0.4403	426.6	11.00	0.70	tr_C9ZJU1_	
HLA-DQA10501-DQB10201	106	AFLGSFYDECGMSEA	FLGSFYDEC	1	0.4400	427.8	11.00	0.43	tr_C9ZJU1_	
HLA-DQA10501-DQB10201	107	DIITSIHIDAYLALA	ITSIHIDAY	2	0.4385	435.0	11.00	0.21	tr_C9ZJU1_	
HLA-DQA10501-DQB10201	108	SDIITSIHIDAYLAL	ITSIHIDAY	3	0.4383	436.0	11.00	0.23	tr_C9ZJU1_	

HLA-DQA10501-DQB10201	109	FQMADTLPLLLVSAV	MADTLPLLL	2	0.4367	443.5	11.00	0.38	tr_C9ZJU1_
HLA-DQA10501-DQB10201	110	IIKQLMGLPATFVSM	LMGLPATFV	4	0.4354	449.7	11.00	0.56	tr_C9ZJU1_
HLA-DQA10501-DQB10201	111	IDRPVRGVAPPSLDG	VRGVAPPSL	4	0.4322	465.5	12.00	0.81	tr_C9ZJU1_
HLA-DQA10501-DQB10201	112	PSVFSGCVVDALKRK	FSGCVVDAL	3	0.4320	466.9	12.00	0.60	tr_C9ZJU1_
HLA-DQA10501-DQB10201	113	RLSGWELMAATAAAA	LMAATAAAA	6	0.4316	468.5	12.00	0.35	tr_C9ZJU1_
HLA-DQA10501-DQB10201	114	ECGMSEAQVDEAHLR	MSEAQVDEA	3	0.4314	469.9	12.00	0.67	tr_C9ZJU1_
HLA-DQA10501-DQB10201	115	RKIDRPVRGVAPPSL	VRGVAPPSL	6	0.4307	473.3	12.00	0.82	tr_C9ZJU1_
HLA-DQA10501-DQB10201	116	LLVSAVALRLSPNFF	ALRLSPNFF	6	0.4300	477.0	12.00	0.54	tr_C9ZJU1_
HLA-DQA10501-DQB10201	117	DDVKAEEVLALCMLL	AEEVLALCM	4	0.4295	479.5	12.00	0.22	tr_C9ZJU1_
HLA-DQA10501-DQB10201	118	MHAFVHWRPIYAAEI	WRPIYAAEI	6	0.4280	487.4	12.00	0.57	tr_C9ZJU1_
HLA-DQA10501-DQB10201	119	EPTSAIDTITEGEIF	DTITEGEIF	6	0.4280	487.5	12.00	0.33	tr_C9ZJU1_
HLA-DQA10501-DQB10201	120	LPATFVSMAVASTFA	SMAVASTFA	6	0.4279	488.0	12.00	0.39	tr_C9ZJU1_
HLA-DQA10501-DQB10201	121	PLNVREFSGGEVERL	FSGGEVERL	6	0.4274	490.7	12.00	0.52	tr_C9ZJU1_
HLA-DQA10501-DQB10201	122	LLTQHVIWPLVPSVF	IWPLVPSVF	6	0.4272	491.3	12.00	0.41	tr_C9ZJU1_
HLA-DQA10501-DQB10201	123	LNVREFSGGEVERLC	FSGGEVERL	5	0.4270	492.5	12.00	0.52	tr_C9ZJU1_
HLA-DQA10501-DQB10201	124	CERKAYEILEMLTRA	RKAYEILEM	2	0.4256	500.3	13.00	0.31	tr_C9ZJU1_
HLA-DQA10501-DQB10201	125	SLYCIRGFVIQSILS	IRGFVIQSI	4	0.4247	504.9	13.00	0.71	tr_C9ZJU1_
HLA-DQA10501-DQB10201	126	VVLNEGMVAEVGSWE	MVAEVGSWE	6	0.4243	507.2	13.00	0.30	tr_C9ZJU1_
HLA-DQA10501-DQB10201	127	LSDIITSIHIDAYLA	ITSIHIDAY	4	0.4237	510.4	13.00	0.31	tr_C9ZJU1_
HLA-DQA10501-DQB10201	128	TSIHIDAYLALATAD	AYLALATAD	6	0.4235	511.7	13.00	0.37	tr_C9ZJU1_
HLA-DQA10501-DQB10201	129	NVREFSGGEVERLCI	FSGGEVERL	4	0.4226	516.6	13.00	0.53	tr_C9ZJU1_
HLA-DQA10501-DQB10201	130	LNEGMVAEVGSWETL	MVAEVGSWE	4	0.4221	519.2	13.00	0.37	tr_C9ZJU1_
HLA-DQA10501-DQB10201	131	ALVNCSIRDILLDDG	IRDILLDDG	6	0.4220	520.1	13.00	0.51	tr_C9ZJU1_
HLA-DQA10501-DQB10201	132	VLNEGMVAEVGSWET	MVAEVGSWE	5	0.4213	524.0	13.00	0.36	tr_C9ZJU1_
HLA-DQA10501-DQB10201	133	LDDGVALCILDDVKA	VALCILDDV	4	0.4205	528.3	13.00	0.56	tr_C9ZJU1_
HLA-DQA10501-DQB10201	134	ILSDIITSIHIDAYL	ITSIHIDAY	5	0.4197	533.2	13.00	0.34	tr_C9ZJU1_
HLA-DQA10501-DQB10201	135	NCSIRDILLDDGVAL	IRDILLDDG	3	0.4195	534.5	13.00	0.37	tr_C9ZJU1_
HLA-DQA10501-DQB10201	136	IDTITEGEIFDTLRL	TEGEIFDTL	4	0.4189	537.8	13.00	0.22	tr_C9ZJU1_
HLA-DQA10501-DQB10201	137	RPVRGVAPPSLDGPW	VRGVAPPSL	2	0.4189	538.0	13.00	0.77	tr_C9ZJU1_
HLA-DQA10501-DQB10201	138	LTQHVIWPLVPSVFS	IWPLVPSVF	5	0.4184	540.4	13.00	0.36	tr_C9ZJU1_
HLA-DQA10501-DQB10201	139	VKAGSAVGLCIFDEP	AVGLCIFDE	5	0.4177	544.7	14.00	0.34	tr_C9ZJU1_
HLA-DQA10501-DQB10201	140	LVNCSIRDILLDDGV	IRDILLDDG	5	0.4175	546.2	14.00	0.46	tr_C9ZJU1_
HLA-DQA10501-DQB10201	141	SVFSGCVVDALKRKL	FSGCVVDAL	2	0.4173	546.9	14.00	0.61	tr_C9ZJU1_
HLA-DQA10501-DQB10201	142	ARYPPAAQADVVVQE	AQADVVVQE	6	0.4168	550.0	14.00	0.29	tr_C9ZJU1_
HLA-DQA10501-DQB10201	143	IKQLMGLPATFVSMA	LMGLPATFV	3	0.4157	556.5	14.00	0.57	tr_C9ZJU1_
HLA-DQA10501-DQB10201	144	IITSIHIDAYLALAT	HIDAYLALA	5	0.4152	559.4	14.00	0.21	tr_C9ZJU1_
HLA-DQA10501-DQB10201	145	QDYLQGFAALRLSGW	LQGFAALRL	3	0.4150	561.0	14.00	0.67	tr_C9ZJU1_
HLA-DQA10501-DQB10201	146	HAFVHWRPIYAAEIG	WRPIYAAEI	5	0.4149	561.8	14.00	0.46	tr_C9ZJU1_
HLA-DQA10501-DQB10201	147	KQLMGLPATFVSMAV	LMGLPATFV	2	0.4148	561.9	14.00	0.47	tr_C9ZJU1_
HLA-DQA10501-DQB10201	148	DYLQGFAALRLSGWE	LQGFAALRL	2	0.4132	572.1	14.00	0.63	tr_C9ZJU1_
HLA-DQA10501-DQB10201	149	VTLAATVLFSPEVIL	TVLFSPEVI	5	0.4127	574.9	14.00	0.55	tr_C9ZJU1_
HLA-DQA10501-DQB10201	150	LVTLAATVLFSPEVI	TVLFSPEVI	6	0.4126	575.7	14.00	0.54	tr_C9ZJU1_
HLA-DQA10501-DQB10201	151	DGVALCILDDVKAEE	VALCILDDV	2	0.4118	580.6	14.00	0.49	tr_C9ZJU1_
HLA-DQA10501-DQB10201	152	LVSAVALRLSPNFFT	ALRLSPNFF	5	0.4094	595.7	15.00	0.56	tr_C9ZJU1_
HLA-DQA10501-DQB10201	153	VREFSGGEVERLCIA	FSGGEVERL	3	0.4091	597.8	15.00	0.52	tr_C9ZJU1_
HLA-DQA10501-DQB10201	154	LRLSGWELMAATAAA	WELMAATAA	5	0.4068	612.9	15.00	0.44	tr_C9ZJU1_
HLA-DQA10501-DQB10201	155	VNCSIRDILLDDGVA	IRDILLDDG	4	0.4068	613.2	15.00	0.43	tr_C9ZJU1_
HLA-DQA10501-DQB10201	156	RQDYLQGFAALRLSG	LQGFAALRL	4	0.4068	613.3	15.00	0.70	tr_C9ZJU1_
HLA-DQA10501-DQB10201	157	DDGVALCILDDVKAE	VALCILDDV	3	0.4064	615.3	15.00	0.59	tr_C9ZJU1_
HLA-DQA10501-DQB10201	158	ERKAYEILEMLTRAT	RKAYEILEM	1	0.4060	618.1	15.00	0.31	tr_C9ZJU1_

HLA-DQA10501-DQB10201	159	VASTFAFESLYCIRG	ASTFAFESL	1	0.4054	622.4	15.00	0.24	tr_C9ZJU1_
HLA-DQA10501-DQB10201	160	LMAATAAAAFATASE	AATAAAAFA	2	0.4043	629.7	15.00	0.33	tr_C9ZJU1_
HLA-DQA10501-DQB10201	161	CSIRDILLDDGVALC	IRDILLDDG	2	0.4032	637.6	15.00	0.38	tr_C9ZJU1_
HLA-DQA10501-DQB10201	162	NEGMVAEVGSWETLS	MVAEVGSWE	3	0.4031	638.2	16.00	0.40	tr_C9ZJU1_
HLA-DQA10501-DQB10201	163	KAGSAVGLCIFDEPT	VGLCIFDEP	5	0.4030	638.6	16.00	0.31	tr_C9ZJU1_
HLA-DQA10501-DQB10201	164	ELMAATAAAAFATAS	AATAAAAFA	3	0.4022	644.3	16.00	0.34	tr_C9ZJU1_
HLA-DQA10501-DQB10201	165	CGMSEAQVDEAHLRR	MSEAQVDEA	2	0.4020	645.5	16.00	0.67	tr_C9ZJU1_
HLA-DQA10501-DQB10201	166	ITSIHIDAYLALATA	DAYLALATA	6	0.4009	653.4	16.00	0.20	tr_C9ZJU1_
HLA-DQA10501-DQB10201	167	DKIVVLNEGMVAEVG	NEGMVAEVG	6	0.4008	654.3	16.00	0.46	tr_C9ZJU1_
HLA-DQA10501-DQB10201	168	MDELQSRVPALLEVM	RVPALLEVM	6	0.3986	669.7	16.00	0.34	tr_C9ZJU1_
HLA-DQA10501-DQB10201	169	LVFNHNIMQLPSVFA	NIMQLPSVF	5	0.3982	673.1	16.00	0.30	tr_C9ZJU1_
HLA-DQA10501-DQB10201	170	VLLDCERKAYEILEM	RKAYEILEM	6	0.3978	675.6	16.00	0.78	tr_C9ZJU1_
HLA-DQA10501-DQB10201	171	YMWDLLTQHVIWPLV	WDLLTQHVI	2	0.3960	689.2	17.00	0.31	tr_C9ZJU1_
HLA-DQA10501-DQB10201	172	MWDLLTQHVIWPLVP	WDLLTQHVI	1	0.3959	690.0	17.00	0.26	tr_C9ZJU1_
HLA-DQA10501-DQB10201	173	PVRGVAPPSLDGPWS	VRGVAPPSL	1	0.3955	692.7	17.00	0.75	tr_C9ZJU1_
HLA-DQA10501-DQB10201	174	QASGSVMDELQSRVP	ASGSVMDEL	1	0.3945	700.1	17.00	0.49	tr_C9ZJU1_
HLA-DQA10501-DQB10201	175	SIRDILLDDGVALCI	IRDILLDDG	1	0.3937	706.2	17.00	0.37	tr_C9ZJU1_
HLA-DQA10501-DQB10201	176	YLQGFAALRLSGWEL	LQGFAALRL	1	0.3934	708.8	17.00	0.52	tr_C9ZJU1_
HLA-DQA10501-DQB10201	177	WQEENNLRFIEDLSI	LRFIEDLSI	6	0.3919	720.4	17.00	0.35	tr_C9ZJU1_
HLA-DQA10501-DQB10201	178	IVVLNEGMVAEVGSW	NEGMVAEVG	4	0.3918	721.1	17.00	0.45	tr_C9ZJU1_
HLA-DQA10501-DQB10201	179	LGYMWDLLTQHVIWP	WDLLTQHVI	4	0.3913	724.9	17.00	0.33	tr_C9ZJU1_
HLA-DQA10501-DQB10201	180	PLGYMWDLLTQHVIW	WDLLTQHVI	5	0.3901	734.5	17.00	0.32	tr_C9ZJU1_
HLA-DQA10501-DQB10201	181	GRDVLHGLFQMADTL	GLFQMADTL	6	0.3889	744.2	18.00	0.49	tr_C9ZJU1_
HLA-DQA10501-DQB10201	182	VFNHNIMQLPSVFAV	NIMQLPSVF	4	0.3885	747.1	18.00	0.33	tr_C9ZJU1_
HLA-DQA10501-DQB10201	183	QLMGLPATFVSMAVA	LMGLPATFV	1	0.3877	754.0	18.00	0.47	tr_C9ZJU1_
HLA-DQA10501-DQB10201	184	TQHVIWPLVPSVFSG	IWPLVPSVF	4	0.3876	754.4	18.00	0.36	tr_C9ZJU1_
HLA-DQA10501-DQB10201	185	FLGSFYDECGMSEAQ	FLGSFYDEC	0	0.3869	760.3	18.00	0.37	tr_C9ZJU1_
HLA-DQA10501-DQB10201	186	GLPATFVSMAVASTF	VSMAVASTF	6	0.3859	768.9	18.00	0.47	tr_C9ZJU1_
HLA-DQA10501-DQB10201	187	GYMWDLLTQHVIWPL	WDLLTQHVI	3	0.3854	772.8	18.00	0.37	tr_C9ZJU1_
HLA-DQA10501-DQB10201	188	KIVVLNEGMVAEVGS	NEGMVAEVG	5	0.3853	773.9	18.00	0.44	tr_C9ZJU1_
HLA-DQA10501-DQB10201	189	ASTFAFESLYCIRGF	STFAFESLY	1	0.3850	776.4	18.00	0.27	tr_C9ZJU1_
HLA-DQA10501-DQB10201	190	IRDILLDDGVALCIL	DDGVALCIL	6	0.3841	783.7	18.00	0.26	tr_C9ZJU1_
HLA-DQA10501-DQB10201	191	AGSAVGLCIFDEPTS	VGLCIFDEP	4	0.3832	791.5	18.00	0.31	tr_C9ZJU1_
HLA-DQA10501-DQB10201	192	RPLGYMWDLLTQHVI	WDLLTQHVI	6	0.3824	797.8	19.00	0.30	tr_C9ZJU1_
HLA-DQA10501-DQB10201	193	AFVHWRPIYAAEIGK	WRPIYAAEI	4	0.3792	826.2	19.00	0.44	tr_C9ZJU1_
HLA-DQA10501-DQB10201	194	ALRLSGWELMAATAA	WELMAATAA	6	0.3779	837.8	19.00	0.53	tr_C9ZJU1_
HLA-DQA10501-DQB10201	195	MAATAAAAFATASER	AATAAAAFA	1	0.3779	838.0	19.00	0.33	tr_C9ZJU1_
HLA-DQA10501-DQB10201	196	SIARYPPAAQADVVV	PPAAQADVV	5	0.3771	845.2	19.00	0.42	tr_C9ZJU1_
HLA-DQA10501-DQB10201	197	SILSDIITSIHIDAY	ITSIHIDAY	6	0.3771	845.3	19.00	0.42	tr_C9ZJU1_
HLA-DQA10501-DQB10201	198	TLAATVLFSPEVILK	TVLFSPEVI	4	0.3770	845.8	19.00	0.54	tr_C9ZJU1_
HLA-DQA10501-DQB10201	199	LGTQWSNFLYFLNES	NFLYFLNES	6	0.3765	851.1	20.00	0.38	tr_C9ZJU1_
HLA-DQA10501-DQB10201	200	DTITEGEIFDTLRLR	TEGEIFDTL	3	0.3762	853.8	20.00	0.23	tr_C9ZJU1_
HLA-DQA10501-DQB10201	201	DILLDDGVALCILDD	GVALCILDD	6	0.3753	862.3	20.00	0.26	tr_C9ZJU1_
HLA-DQA10501-DQB10201	202	EGMVAEVGSWETLSR	MVAEVGSWE	2	0.3752	863.0	20.00	0.47	tr_C9ZJU1_
HLA-DQA10501-DQB10201	203	TLVFNHNIMQLPSVF	NIMQLPSVF	6	0.3751	863.8	20.00	0.35	tr_C9ZJU1_
HLA-DQA10501-DQB10201	204	FLNESLYMESQFTKS	ESLYMESQF	3	0.3749	866.0	20.00	0.35	tr_C9ZJU1_
HLA-DQA10501-DQB10201	205	DEPTSAIDTITEGEI	IDTITEGEI	6	0.3744	870.4	20.00	0.32	tr_C9ZJU1_
HLA-DQA10501-DQB10201	206	REFSGGEVERLCIAR	FSGGEVERL	2	0.3741	873.3	20.00	0.58	tr_C9ZJU1_
HLA-DQA10501-DQB10201	207	VSAVALRLSPNFFTQ	ALRLSPNFF	4	0.3738	875.7	20.00	0.67	tr_C9ZJU1_
HLA-DQA10501-DQB10201	208	IQSILSDIITSIHID	IQSILSDII	0	0.3736	877.8	20.00	0.56	tr_C9ZJU1_

HLA-DQA10501-DQB10201	209	VFSGCVVDALKRKLQ	FSGCVVDAL	1	0.3727	886.6	20.00	0.66	tr_C9ZJU1_
HLA-DQA10501-DQB10201	210	IARYPPAAQADVVVQ	PPAAQADVV	4	0.3722	891.2	20.00	0.37	tr_C9ZJU1_
HLA-DQA10501-DQB10201	211	DELQSRVPALLEVMH	RVPALLEVM	5	0.3719	893.8	20.00	0.31	tr_C9ZJU1_
HLA-DQA10501-DQB10201	212	ALRLSPNFFTQSALD	NFFTQSALD	6	0.3695	917.4	21.00	0.49	tr_C9ZJU1_
HLA-DQA10501-DQB10201	213	FRFIIKQLMGLPATF	QLMGLPATF	6	0.3690	922.9	21.00	0.43	tr_C9ZJU1_
HLA-DQA10501-DQB10201	214	VLRRVSFVVNKGEFL	FVVNKGEFL	6	0.3688	924.2	21.00	0.74	tr_C9ZJU1_
HLA-DQA10501-DQB10201	215	FTQSALDQTEIVNQL	SALDQTEIV	3	0.3683	929.9	21.00	0.41	tr_C9ZJU1_
HLA-DQA10501-DQB10201	216	GMSEAQVDEAHLRRH	MSEAQVDEA	1	0.3677	935.7	21.00	0.73	tr_C9ZJU1_
HLA-DQA10501-DQB10201	217	GSAVGLCIFDEPTSA	VGLCIFDEP	3	0.3661	952.4	21.00	0.31	tr_C9ZJU1_
HLA-DQA10501-DQB10201	218	WLGTQWSNFLYFLNE	SNFLYFLNE	6	0.3659	954.2	21.00	0.24	tr_C9ZJU1_
HLA-DQA10501-DQB10201	219	RKAYEILEMLTRATI	AYEILEMLT	2	0.3647	967.1	22.00	0.34	tr_C9ZJU1_
HLA-DQA10501-DQB10201	220	RRVSFVVNKGEFLGI	FVVNKGEFL	4	0.3633	981.4	22.00	0.72	tr_C9ZJU1_
HLA-DQA10501-DQB10201	221	QEENNLRFIEDLSIG	LRFIEDLSI	5	0.3624	991.2	22.00	0.35	tr_C9ZJU1_
HLA-DQA10501-DQB10201	222	LVLFSMVFVESGRPL	FSMVFVESG	3	0.3610	1005.6	22.00	0.37	tr_C9ZJU1_
HLA-DQA10501-DQB10201	223	LAATVLFSPEVILKC	TVLFSPEVI	3	0.3599	1018.7	22.00	0.56	tr_C9ZJU1_
HLA-DQA10501-DQB10201	224	WDLLTQHVIWPLVPS	TQHVIWPLV	4	0.3593	1024.8	22.00	0.28	tr_C9ZJU1_
HLA-DQA10501-DQB10201	225	DVKAEEVLALCMLLR	AEEVLALCM	3	0.3592	1026.4	23.00	0.25	tr_C9ZJU1_
HLA-DQA10501-DQB10201	226	FNHNIMQLPSVFAVH	NIMQLPSVF	3	0.3589	1029.4	23.00	0.34	tr_C9ZJU1_
HLA-DQA10501-DQB10201	227	QHVIWPLVPSVFSGC	IWPLVPSVF	3	0.3589	1029.5	23.00	0.43	tr_C9ZJU1_
HLA-DQA10501-DQB10201	228	LRRVSFVVNKGEFLG	FVVNKGEFL	5	0.3589	1029.5	23.00	0.75	tr_C9ZJU1_
HLA-DQA10501-DQB10201	229	VMWLGTQWSNFLYFL	QWSNFLYFL	6	0.3583	1035.4	23.00	0.22	tr_C9ZJU1_
HLA-DQA10501-DQB10201	230	VQEFPEELYLGLITA	VQEFPEELY	0	0.3580	1038.9	23.00	0.25	tr_C9ZJU1_
HLA-DQA10501-DQB10201	231	VMDELQSRVPALLEV	LQSRVPALL	4	0.3578	1041.1	23.00	0.40	tr_C9ZJU1_
HLA-DQA10501-DQB10201	232	LQGFAALRLSGWELM	LQGFAALRL	0	0.3575	1045.2	23.00	0.31	tr_C9ZJU1_
HLA-DQA10501-DQB10201	233	RVSFVVNKGEFLGIA	FVVNKGEFL	3	0.3573	1047.3	23.00	0.72	tr_C9ZJU1_
HLA-DQA10501-DQB10201	234	MKNSHPDHLLVCDIW	DHLLVCDIW	6	0.3571	1049.6	23.00	0.62	tr_C9ZJU1_
HLA-DQA10501-DQB10201	235	LVMWLGTQWSNFLYF	WLGTQWSNF	3	0.3564	1057.0	23.00	0.25	tr_C9ZJU1_
HLA-DQA10501-DQB10201	236	LRERARPAVLLDCER	RPAVLLDCE	5	0.3558	1064.2	23.00	0.34	tr_C9ZJU1_
HLA-DQA10501-DQB10201	237	SVMDELQSRVPALLE	LQSRVPALL	5	0.3557	1065.1	23.00	0.40	tr_C9ZJU1_
HLA-DQA10501-DQB10201	238	FVKAGSAVGLCIFDE	AVGLCIFDE	6	0.3549	1075.1	23.00	0.41	tr_C9ZJU1_
HLA-DQA10501-DQB10201	239	SAVALRLSPNFFTQS	ALRLSPNFF	3	0.3545	1079.8	23.00	0.69	tr_C9ZJU1_
HLA-DQA10501-DQB10201	240	SLDGPWSVEFREVSF	SVEFREVSF	6	0.3541	1083.9	23.00	0.32	tr_C9ZJU1_
HLA-DQA10501-DQB10201	241	DLLTQHVIWPLVPSV	VIWPLVPSV	6	0.3540	1084.9	23.00	0.28	tr_C9ZJU1_
HLA-DQA10501-DQB10201	242	VWQEENNLRFIEDLS	NLRFIEDLS	6	0.3535	1091.1	24.00	0.35	tr_C9ZJU1_
HLA-DQA10501-DQB10201	243	WPLVPSVFSGCVVDA	VFSGCVVDA	6	0.3530	1097.5	24.00	0.53	tr_C9ZJU1_
HLA-DQA10501-DQB10201	244	LLNRTYAPTEGDIFV	YAPTEGDIF	5	0.3524	1103.7	24.00	0.57	tr_C9ZJU1_
HLA-DQA10501-DQB10201	245	SFVVNKGEFLGIAGY	FVVNKGEFL	1	0.3516	1114.1	24.00	0.62	tr_C9ZJU1_
HLA-DQA10501-DQB10201	246	VSFVVNKGEFLGIAG	FVVNKGEFL	2	0.3515	1114.6	24.00	0.72	tr_C9ZJU1_
HLA-DQA10501-DQB10201	247	NHNIMQLPSVFAVHC	NIMQLPSVF	2	0.3511	1119.9	24.00	0.31	tr_C9ZJU1_
HLA-DQA10501-DQB10201	248	RTYAPTEGDIFVNGV	YAPTEGDIF	2	0.3508	1124.0	24.00	0.41	tr_C9ZJU1_
HLA-DQA10501-DQB10201	249	VLFSMVFVESGRPLG	FSMVFVESG	2	0.3499	1134.4	24.00	0.38	tr_C9ZJU1_
HLA-DQA10501-DQB10201	250	QYGLVLFSMVFVESG	FSMVFVESG	6	0.3498	1136.1	24.00	0.46	tr_C9ZJU1_
HLA-DQA10501-DQB10201	251	RMLRRRIANVWQEEN	RIANVWQEE	5	0.3496	1138.3	24.00	0.46	tr_C9ZJU1_
HLA-DQA10501-DQB10201	252	SLVMWLGTQWSNFLY	WLGTQWSNF	4	0.3494	1140.1	24.00	0.26	tr_C9ZJU1_
HLA-DQA10501-DQB10201	253	SAVGLCIFDEPTSAI	VGLCIFDEP	2	0.3485	1151.3	25.00	0.35	tr_C9ZJU1_
HLA-DQA10501-DQB10201	254	MWLGTQWSNFLYFLN	QWSNFLYFL	5	0.3485	1152.3	25.00	0.20	tr_C9ZJU1_
HLA-DQA10501-DQB10201	255	VSLVMWLGTQWSNFL	WLGTQWSNF	5	0.3482	1155.3	25.00	0.25	tr_C9ZJU1_
HLA-DQA10501-DQB10201	256	ADKIVVLNEGMVAEV	LNEGMVAEV	6	0.3470	1170.8	25.00	0.34	tr_C9ZJU1_
HLA-DQA10501-DQB10201	257	QMADTLPLLLVSAVA	MADTLPLLL	1	0.3468	1172.6	25.00	0.44	tr_C9ZJU1_
HLA-DQA10501-DQB10201	258	GLVLFSMVFVESGRP	FSMVFVESG	4	0.3462	1180.3	25.00	0.43	tr_C9ZJU1_

HLA-DQA10501-DQB10201	259	GMVAEVGSWETLSRT	MVAEVGSWE	1	0.3461	1181.7	25.00	0.50	tr_C9ZJU1_
HLA-DQA10501-DQB10201	260	RDILLDDGVALCILD	DDGVALCIL	5	0.3457	1187.6	25.00	0.35	tr_C9ZJU1_
HLA-DQA10501-DQB10201	261	LFSMVFVESGRPLGY	FSMVFVESG	1	0.3456	1189.1	25.00	0.31	tr_C9ZJU1_
HLA-DQA10501-DQB10201	262	VSQALWQASGSVMDE	QASGSVMDE	6	0.3453	1192.5	25.00	0.38	tr_C9ZJU1_
HLA-DQA10501-DQB10201	263	ELQSRVPALLEVMHG	RVPALLEVM	4	0.3446	1202.1	25.00	0.29	tr_C9ZJU1_
HLA-DQA10501-DQB10201	264	FVHWRPIYAAEIGKS	WRPIYAAEI	3	0.3430	1222.2	26.00	0.49	tr_C9ZJU1_
HLA-DQA10501-DQB10201	265	EENNLRFIEDLSIGG	LRFIEDLSI	4	0.3427	1226.7	26.00	0.34	tr_C9ZJU1_
HLA-DQA10501-DQB10201	266	RRIANVWQEENNLRF	RIANVWQEE	1	0.3422	1232.6	26.00	0.31	tr_C9ZJU1_
HLA-DQA10501-DQB10201	267	GSVMDELQSRVPALL	LQSRVPALL	6	0.3422	1232.8	26.00	0.47	tr_C9ZJU1_
HLA-DQA10501-DQB10201	268	LRRRIANVWQEENNL	RIANVWQEE	3	0.3419	1236.4	26.00	0.41	tr_C9ZJU1_
HLA-DQA10501-DQB10201	269	SSLGCYQVLEGLQSV	YQVLEGLQS	5	0.3415	1242.1	26.00	0.30	tr_C9ZJU1_
HLA-DQA10501-DQB10201	270	EFSGGEVERLCIARA	FSGGEVERL	1	0.3410	1249.2	26.00	0.58	tr_C9ZJU1_
HLA-DQA10501-DQB10201	271	ALATADYDPSDDWLL	DYDPSDDWL	5	0.3409	1250.6	26.00	0.26	tr_C9ZJU1_
HLA-DQA10501-DQB10201	272	AATVLFSPEVILKCI	TVLFSPEVI	2	0.3404	1257.9	26.00	0.57	tr_C9ZJU1_
HLA-DQA10501-DQB10201	273	LNRTYAPTEGDIFVN	YAPTEGDIF	4	0.3389	1278.5	26.00	0.57	tr_C9ZJU1_
HLA-DQA10501-DQB10201	274	AVALRLSPNFFTQSA	ALRLSPNFF	2	0.3382	1288.1	27.00	0.69	tr_C9ZJU1_
HLA-DQA10501-DQB10201	275	FVVNKGEFLGIAGYS	FVVNKGEFL	0	0.3378	1292.8	27.00	0.48	tr_C9ZJU1_
HLA-DQA10501-DQB10201	276	GRPLGYMWDLLTQHV	MWDLLTQHV	6	0.3377	1294.3	27.00	0.34	tr_C9ZJU1_
HLA-DQA10501-DQB10201	277	RVVFSFPVVSLVMWL	VVFSFPVVS	1	0.3371	1303.4	27.00	0.21	tr_C9ZJU1_
HLA-DQA10501-DQB10201	278	AATAAAAFATASERN	AAAFATASE	4	0.3369	1305.4	27.00	0.28	tr_C9ZJU1_
HLA-DQA10501-DQB10201	279	HNIMQLPSVFAVHCG	NIMQLPSVF	1	0.3365	1310.9	27.00	0.31	tr_C9ZJU1_
HLA-DQA10501-DQB10201	280	KNSHPDHLLVCDIWN	DHLLVCDIW	5	0.3362	1315.7	27.00	0.54	tr_C9ZJU1_
HLA-DQA10501-DQB10201	281	MLRRRIANVWQEENN	RIANVWQEE	4	0.3360	1319.2	27.00	0.46	tr_C9ZJU1_
HLA-DQA10501-DQB10201	282	HVIWPLVPSVFSGCV	IWPLVPSVF	2	0.3359	1320.3	27.00	0.44	tr_C9ZJU1_
HLA-DQA10501-DQB10201	283	ARMLRRRIANVWQEE	RIANVWQEE	6	0.3356	1324.3	27.00	0.51	tr_C9ZJU1_
HLA-DQA10501-DQB10201	284	DGPWSVEFREVSFRY	EFREVSFRY	6	0.3353	1328.4	27.00	0.26	tr_C9ZJU1_
HLA-DQA10501-DQB10201	285	ASGSVMDELQSRVPA	ASGSVMDEL	0	0.3345	1340.6	27.00	0.38	tr_C9ZJU1_
HLA-DQA10501-DQB10201	286	FVESGRPLGYMWDLL	PLGYMWDLL	6	0.3339	1348.3	27.00	0.58	tr_C9ZJU1_
HLA-DQA10501-DQB10201	287	FDEPTSAIDTITEGE	AIDTITEGE	6	0.3336	1353.2	28.00	0.27	tr_C9ZJU1_
HLA-DQA10501-DQB10201	288	NVWQEENNLRFIEDL	NNLRFIEDL	6	0.3333	1357.4	28.00	0.41	tr_C9ZJU1_
HLA-DQA10501-DQB10201	289	EFPEELYLGLITAYN	YLGLITAYN	6	0.3328	1365.6	28.00	0.25	tr_C9ZJU1_
HLA-DQA10501-DQB10201	290	ASIAIFLNCNGVESI	FLNCNGVES	5	0.3326	1367.7	28.00	0.43	tr_C9ZJU1_
HLA-DQA10501-DQB10201	291	CRVVFSFPVVSLVMW	VVFSFPVVS	2	0.3324	1371.7	28.00	0.25	tr_C9ZJU1_
HLA-DQA10501-DQB10201	292	RLLNRTYAPTEGDIF	YAPTEGDIF	6	0.3323	1372.2	28.00	0.59	tr_C9ZJU1_
HLA-DQA10501-DQB10201	293	VRGVAPPSLDGPWSV	VRGVAPPSL	0	0.3322	1374.2	28.00	0.71	tr_C9ZJU1_
HLA-DQA10501-DQB10201	294	TITEGEIFDTLRLRE	TEGEIFDTL	2	0.3311	1391.0	28.00	0.28	tr_C9ZJU1_
HLA-DQA10501-DQB10201	295	LNESLYMESQFTKSQ	ESLYMESQF	2	0.3304	1400.8	28.00	0.35	tr_C9ZJU1_
HLA-DQA10501-DQB10201	296	LLLVSAVALRLSPNF	LVSAVALRL	2	0.3301	1405.6	28.00	0.46	tr_C9ZJU1_
HLA-DQA10501-DQB10201	297	VALRLSPNFFTQSAL	ALRLSPNFF	1	0.3298	1409.8	28.00	0.61	tr_C9ZJU1_
HLA-DQA10501-DQB10201	298	STFAFESLYCIRGFV	FAFESLYCI	2	0.3297	1411.9	28.00	0.30	tr_C9ZJU1_
HLA-DQA10501-DQB10201	299	QEFPEELYLGLITAY	PEELYLGLI	3	0.3296	1412.6	28.00	0.28	tr_C9ZJU1_
HLA-DQA10501-DQB10201	300	IAIFLNCNGVESIHV	FLNCNGVES	3	0.3291	1420.4	29.00	0.36	tr_C9ZJU1_
HLA-DQA10501-DQB10201	301	QSILSDIITSIHIDA	IITSIHIDA	6	0.3285	1430.0	29.00	0.45	tr_C9ZJU1_
HLA-DQA10501-DQB10201	302	GFAALRLSGWELMAA	LSGWELMAA	6	0.3285	1430.4	29.00	0.19	tr_C9ZJU1_
HLA-DQA10501-DQB10201	303	GCRVVFSFPVVSLVM	SFPVVSLVM	6	0.3270	1453.7	29.00	0.22	tr_C9ZJU1_
HLA-DQA10501-DQB10201	304	VIWPLVPSVFSGCVV	IWPLVPSVF	1	0.3256	1476.4	29.00	0.32	tr_C9ZJU1_
HLA-DQA10501-DQB10201	305	GSSLGCYQVLEGLQS	YQVLEGLQS	6	0.3243	1496.7	30.00	0.31	tr_C9ZJU1_
HLA-DQA10501-DQB10201	306	ATAAAAFATASERND	AAAFATASE	3	0.3237	1506.0	30.00	0.32	tr_C9ZJU1_
HLA-DQA10501-DQB10201	307	SFYDECGMSEAQVDE	FYDECGMSE	1	0.3232	1513.8	30.00	0.37	tr_C9ZJU1_
HLA-DQA10501-DQB10201	308	GSFYDECGMSEAQVD	FYDECGMSE	2	0.3227	1522.4	30.00	0.38	tr_C9ZJU1_

HLA-DQA10501-DQB10201	309	IQHWCPLYHAFLGSF	LYHAFLGSF	6	0.3226	1524.8	30.00	0.52	tr_C9ZJU1_
HLA-DQA10501-DQB10201	310	MHGCRVVFSFPVVSL	VVFSFPVVS	5	0.3220	1534.5	30.00	0.31	tr_C9ZJU1_
HLA-DQA10501-DQB10201	311	CIFDEPTSAIDTITE	PTSAIDTIT	5	0.3218	1536.8	30.00	0.34	tr_C9ZJU1_
HLA-DQA10501-DQB10201	312	LDGPWSVEFREVSFR	SVEFREVSF	5	0.3214	1543.9	30.00	0.30	tr_C9ZJU1_
HLA-DQA10501-DQB10201	313	HPDHLLVCDIWNCLM	DHLLVCDIW	2	0.3211	1548.6	30.00	0.44	tr_C9ZJU1_
HLA-DQA10501-DQB10201	314	QLVTLAATVLFSPEV	ATVLFSPEV	6	0.3208	1554.6	30.00	0.33	tr_C9ZJU1_
HLA-DQA10501-DQB10201	315	APLNVREFSGGEVER	REFSGGEVE	5	0.3207	1555.8	30.00	0.46	tr_C9ZJU1_
HLA-DQA10501-DQB10201	316	NRTYAPTEGDIFVNG	YAPTEGDIF	3	0.3205	1560.1	30.00	0.56	tr_C9ZJU1_
HLA-DQA10501-DQB10201	317	RERARPAVLLDCERK	RPAVLLDCE	4	0.3200	1567.6	30.00	0.34	tr_C9ZJU1_
HLA-DQA10501-DQB10201	318	QGFAALRLSGWELMA	LRLSGWELM	5	0.3200	1568.6	30.00	0.19	tr_C9ZJU1_
HLA-DQA10501-DQB10201	319	NSHPDHLLVCDIWNC	DHLLVCDIW	4	0.3199	1569.9	30.00	0.52	tr_C9ZJU1_
HLA-DQA10501-DQB10201	320	ATVLFSPEVILKCIL	TVLFSPEVI	1	0.3197	1573.5	31.00	0.55	tr_C9ZJU1_
HLA-DQA10501-DQB10201	321	SLGCYQVLEGLQSVQ	YQVLEGLQS	4	0.3196	1574.0	31.00	0.29	tr_C9ZJU1_
HLA-DQA10501-DQB10201	322	SHPDHLLVCDIWNCL	DHLLVCDIW	3	0.3195	1577.1	31.00	0.50	tr_C9ZJU1_
HLA-DQA10501-DQB10201	323	GPWSVEFREVSFRYS	EFREVSFRY	5	0.3193	1579.7	31.00	0.24	tr_C9ZJU1_
HLA-DQA10501-DQB10201	324	ENNLRFIEDLSIGGN	LRFIEDLSI	3	0.3188	1588.6	31.00	0.39	tr_C9ZJU1_
HLA-DQA10501-DQB10201	325	TQSALDQTEIVNQLR	SALDQTEIV	2	0.3183	1597.4	31.00	0.49	tr_C9ZJU1_
HLA-DQA10501-DQB10201	326	TYAPTEGDIFVNGVH	YAPTEGDIF	1	0.3176	1608.8	31.00	0.43	tr_C9ZJU1_
HLA-DQA10501-DQB10201	327	VESGRPLGYMWDLLT	PLGYMWDLL	5	0.3170	1619.5	31.00	0.52	tr_C9ZJU1_
HLA-DQA10501-DQB10201	328	FSMVFVESGRPLGYM	MVFVESGRP	2	0.3170	1619.6	31.00	0.27	tr_C9ZJU1_
HLA-DQA10501-DQB10201	329	SIAIFLNCNGVESIH	FLNCNGVES	4	0.3164	1630.9	31.00	0.42	tr_C9ZJU1_
HLA-DQA10501-DQB10201	330	FAALRLSGWELMAAT	LSGWELMAA	5	0.3157	1643.2	31.00	0.19	tr_C9ZJU1_
HLA-DQA10501-DQB10201	331	RRRIANVWQEENNLR	RIANVWQEE	2	0.3153	1649.4	32.00	0.43	tr_C9ZJU1_
HLA-DQA10501-DQB10201	332	LATADYDPSDDWLLS	DYDPSDDWL	4	0.3150	1654.9	32.00	0.30	tr_C9ZJU1_
HLA-DQA10501-DQB10201	333	SPFRFIIKQLMGLPA	FRFIIKQLM	2	0.3149	1656.8	32.00	0.37	tr_C9ZJU1_
HLA-DQA10501-DQB10201	334	MGLPATFVSMAVAST	FVSMAVAST	6	0.3140	1672.4	32.00	0.51	tr_C9ZJU1_
HLA-DQA10501-DQB10201	335	NIMQLPSVFAVHCGL	IMQLPSVFA	1	0.3140	1672.7	32.00	0.29	tr_C9ZJU1_
HLA-DQA10501-DQB10201	336	YGLVLFSMVFVESGR	FSMVFVESG	5	0.3136	1679.4	32.00	0.50	tr_C9ZJU1_
HLA-DQA10501-DQB10201	337	HGCRVVFSFPVVSLV	VVFSFPVVS	4	0.3128	1694.4	32.00	0.28	tr_C9ZJU1_
HLA-DQA10501-DQB10201	338	CGRDVLHGLFQMADT	HGLFQMADT	6	0.3124	1701.8	32.00	0.31	tr_C9ZJU1_
HLA-DQA10501-DQB10201	339	RTLVFNHNIMQLPSV	HNIMQLPSV	6	0.3124	1703.0	32.00	0.30	tr_C9ZJU1_
HLA-DQA10501-DQB10201	340	DTLPLLLVSAVALRL	LVSAVALRL	6	0.3122	1705.8	32.00	0.63	tr_C9ZJU1_
HLA-DQA10501-DQB10201	341	VVFSFPVVSLVMWLG	FSFPVVSLV	2	0.3119	1712.3	32.00	0.19	tr_C9ZJU1_
HLA-DQA10501-DQB10201	342	AVGLCIFDEPTSAID	VGLCIFDEP	1	0.3112	1725.0	33.00	0.35	tr_C9ZJU1_
HLA-DQA10501-DQB10201	343	VHWRPIYAAEIGKSL	WRPIYAAEI	2	0.3109	1730.4	33.00	0.47	tr_C9ZJU1_
HLA-DQA10501-DQB10201	344	MALVNCSIRDILLDD	SIRDILLDD	6	0.3101	1745.5	33.00	0.43	tr_C9ZJU1_
HLA-DQA10501-DQB10201	345	VVSLVMWLGTQWSNF	VMWLGTQWS	4	0.3101	1745.9	33.00	0.37	tr_C9ZJU1_
HLA-DQA10501-DQB10201	346	FSGGEVERLCIARAF	FSGGEVERL	0	0.3099	1749.2	33.00	0.38	tr_C9ZJU1_
HLA-DQA10501-DQB10201	347	RIANVWQEENNLRFI	WQEENNLRF	5	0.3097	1753.6	33.00	0.34	tr_C9ZJU1_
HLA-DQA10501-DQB10201	348	PWSVEFREVSFRYSD	EFREVSFRY	4	0.3093	1760.1	33.00	0.27	tr_C9ZJU1_
HLA-DQA10501-DQB10201	349	VWQCRQDYLQGFAAL	DYLQGFAAL	6	0.3089	1767.6	33.00	0.28	tr_C9ZJU1_
HLA-DQA10501-DQB10201	350	HADKIVVLNEGMVAE	VVLNEGMVA	5	0.3088	1769.4	33.00	0.38	tr_C9ZJU1_
HLA-DQA10501-DQB10201	351	LMGLPATFVSMAVAS	LMGLPATFV	0	0.3087	1771.1	33.00	0.41	tr_C9ZJU1_
HLA-DQA10501-DQB10201	352	VQYGLVLFSMVFVES	LFSMVFVES	6	0.3085	1776.0	33.00	0.38	tr_C9ZJU1_
HLA-DQA10501-DQB10201	353	ESGRPLGYMWDLLTQ	PLGYMWDLL	4	0.3083	1780.0	33.00	0.47	tr_C9ZJU1_
HLA-DQA10501-DQB10201	354	VMHGCRVVFSFPVVS	VVFSFPVVS	6	0.3081	1783.6	33.00	0.30	tr_C9ZJU1_
HLA-DQA10501-DQB10201	355	LGCYQVLEGLQSVQR	YQVLEGLQS	3	0.3075	1794.8	33.00	0.25	tr_C9ZJU1_
HLA-DQA10501-DQB10201	356	KAYEILEMLTRATIK	AYEILEMLT	1	0.3075	1795.3	33.00	0.41	tr_C9ZJU1_
HLA-DQA10501-DQB10201	357	SGRPLGYMWDLLTQH	PLGYMWDLL	3	0.3075	1795.4	33.00	0.39	tr_C9ZJU1_
HLA-DQA10501-DQB10201	358	CDIWNCLMALVNCSI	LMALVNCSI	6	0.3069	1806.0	33.00	0.31	tr_C9ZJU1_

HLA-DQA10501-DQB10201	359	FPEELYLGLITAYNR	YLGLITAYN	5	0.3069	1807.5	33.00	0.23	tr_C9ZJU1_
HLA-DQA10501-DQB10201	360	YSIARYPPAAQADVV	PPAAQADVV	6	0.3067	1810.6	33.00	0.68	tr_C9ZJU1_
HLA-DQA10501-DQB10201	361	LGSFYDECGMSEAQV	FYDECGMSE	3	0.3062	1820.8	34.00	0.48	tr_C9ZJU1_
HLA-DQA10501-DQB10201	362	LLVCDIWNCLMALVN	WNCLMALVN	6	0.3062	1821.3	34.00	0.38	tr_C9ZJU1_
HLA-DQA10501-DQB10201	363	VRRLGWIKDVIQSSP	WIKDVIQSS	5	0.3057	1830.8	34.00	0.43	tr_C9ZJU1_
HLA-DQA10501-DQB10201	364	EGDIFVNGVHISQYP	VNGVHISQY	5	0.3050	1844.6	34.00	0.41	tr_C9ZJU1_
HLA-DQA10501-DQB10201	365	IFDEPTSAIDTITEG	PTSAIDTIT	4	0.3048	1848.5	34.00	0.31	tr_C9ZJU1_
HLA-DQA10501-DQB10201	366	MVAEVGSWETLSRTG	MVAEVGSWE	0	0.3046	1851.4	34.00	0.50	tr_C9ZJU1_
HLA-DQA10501-DQB10201	367	AIFLNCNGVESIHVR	FLNCNGVES	2	0.3044	1855.6	34.00	0.40	tr_C9ZJU1_
HLA-DQA10501-DQB10201	368	LPLLLVSAVALRLSP	LVSAVALRL	4	0.3043	1857.2	34.00	0.57	tr_C9ZJU1_
HLA-DQA10501-DQB10201	369	LCIFDEPTSAIDTIT	PTSAIDTIT	6	0.3024	1897.6	35.00	0.41	tr_C9ZJU1_
HLA-DQA10501-DQB10201	370	LQSRVPALLEVMHGC	RVPALLEVM	3	0.3019	1907.7	35.00	0.41	tr_C9ZJU1_
HLA-DQA10501-DQB10201	371	GMHAFVHWRPIYAAE	FVHWRPIYA	4	0.3014	1918.0	35.00	0.45	tr_C9ZJU1_
HLA-DQA10501-DQB10201	372	PFRFIIKQLMGLPAT	FRFIIKQLM	1	0.3008	1929.6	35.00	0.34	tr_C9ZJU1_
HLA-DQA10501-DQB10201	373	HAPLNVREFSGGEVE	REFSGGEVE	6	0.3005	1935.4	35.00	0.50	tr_C9ZJU1_
HLA-DQA10501-DQB10201	374	TAAAAFATASERNDR	AAAFATASE	2	0.3003	1940.7	35.00	0.35	tr_C9ZJU1_
HLA-DQA10501-DQB10201	375	SGSSLGCYQVLEGLQ	LGCYQVLEG	4	0.2998	1950.5	35.00	0.28	tr_C9ZJU1_
HLA-DQA10501-DQB10201	376	MSEAQVDEAHLRRHL	MSEAQVDEA	0	0.2997	1952.5	35.00	0.66	tr_C9ZJU1_
HLA-DQA10501-DQB10201	377	TLPLLLVSAVALRLS	LVSAVALRL	5	0.2992	1964.1	35.00	0.56	tr_C9ZJU1_
HLA-DQA10501-DQB10201	378	YAPTEGDIFVNGVHI	YAPTEGDIF	0	0.2991	1965.6	35.00	0.35	tr_C9ZJU1_
HLA-DQA10501-DQB10201	379	LSPFRFIIKQLMGLP	FRFIIKQLM	3	0.2990	1968.3	35.00	0.46	tr_C9ZJU1_
HLA-DQA10501-DQB10201	380	QSALDQTEIVNQLRS	SALDQTEIV	1	0.2984	1980.5	36.00	0.45	tr_C9ZJU1_
HLA-DQA10501-DQB10201	381	KHADKIVVLNEGMVA	VVLNEGMVA	6	0.2981	1986.4	36.00	0.42	tr_C9ZJU1_
HLA-DQA10501-DQB10201	382	NESLYMESQFTKSQL	SLYMESQFT	2	0.2977	1995.4	36.00	0.37	tr_C9ZJU1_
HLA-DQA10501-DQB10201	383	HDKSSVQYGLVLFSM	QYGLVLFSM	6	0.2974	2001.5	36.00	0.54	tr_C9ZJU1_
HLA-DQA10501-DQB10201	384	ILGMHAFVHWRPIYA	FVHWRPIYA	6	0.2973	2004.1	36.00	0.46	tr_C9ZJU1_
HLA-DQA10501-DQB10201	385	AALRLSGWELMAATA	GWELMAATA	6	0.2973	2004.9	36.00	0.22	tr_C9ZJU1_
HLA-DQA10501-DQB10201	386	ERARPAVLLDCERKA	RPAVLLDCE	3	0.2972	2005.6	36.00	0.38	tr_C9ZJU1_
HLA-DQA10501-DQB10201	387	AAAAFATASERNDRY	AAAFATASE	1	0.2966	2019.0	36.00	0.26	tr_C9ZJU1_
HLA-DQA10501-DQB10201	388	LGMHAFVHWRPIYAA	FVHWRPIYA	5	0.2962	2027.8	36.00	0.46	tr_C9ZJU1_
HLA-DQA10501-DQB10201	389	LVCDIWNCLMALVNC	WNCLMALVN	5	0.2960	2031.9	36.00	0.42	tr_C9ZJU1_
HLA-DQA10501-DQB10201	390	AFVKAGSAVGLCIFD	AGSAVGLCI	4	0.2953	2047.7	36.00	0.31	tr_C9ZJU1_
HLA-DQA10501-DQB10201	391	EVMHGCRVVFSFPVV	MHGCRVVFS	2	0.2952	2051.2	36.00	0.31	tr_C9ZJU1_
HLA-DQA10501-DQB10201	392	HVRTLVFNHNIMQLP	VFNHNIMQL	5	0.2949	2056.8	36.00	0.41	tr_C9ZJU1_
HLA-DQA10501-DQB10201	393	IWPLVPSVFSGCVVD	IWPLVPSVF	0	0.2947	2061.7	36.00	0.25	tr_C9ZJU1_
HLA-DQA10501-DQB10201	394	TEGDIFVNGVHISQY	VNGVHISQY	6	0.2945	2066.7	37.00	0.34	tr_C9ZJU1_
HLA-DQA10501-DQB10201	395	SGSVMDELQSRVPAL	ELQSRVPAL	6	0.2925	2111.8	37.00	0.32	tr_C9ZJU1_
HLA-DQA10501-DQB10201	396	KSSVQYGLVLFSMVF	QYGLVLFSM	4	0.2919	2125.1	37.00	0.41	tr_C9ZJU1_
HLA-DQA10501-DQB10201	397	PDHLLVCDIWNCLMA	DHLLVCDIW	1	0.2918	2127.0	37.00	0.44	tr_C9ZJU1_
HLA-DQA10501-DQB10201	398	PLLLVSAVALRLSPN	LVSAVALRL	3	0.2916	2132.7	37.00	0.55	tr_C9ZJU1_
HLA-DQA10501-DQB10201	399	SVRRLGWIKDVIQSS	WIKDVIQSS	6	0.2915	2133.9	37.00	0.35	tr_C9ZJU1_
HLA-DQA10501-DQB10201	400	NNLRFIEDLSIGGNI	LRFIEDLSI	2	0.2914	2135.7	37.00	0.40	tr_C9ZJU1_
HLA-DQA10501-DQB10201	401	FSGSSLGCYQVLEGL	LGCYQVLEG	5	0.2911	2143.8	37.00	0.29	tr_C9ZJU1_
HLA-DQA10501-DQB10201	402	VKAEEVLALCMLLRG	AEEVLALCM	2	0.2910	2144.9	37.00	0.32	tr_C9ZJU1_
HLA-DQA10501-DQB10201	403	FPVVSLVMWLGTQWS	VMWLGTQWS	6	0.2895	2181.4	38.00	0.38	tr_C9ZJU1_
HLA-DQA10501-DQB10201	404	PQLVTLAATVLFSPE	LVTLAATVL	2	0.2894	2183.9	38.00	0.29	tr_C9ZJU1_
HLA-DQA10501-DQB10201	405	ATADYDPSDDWLLSR	DYDPSDDWL	3	0.2887	2199.2	38.00	0.31	tr_C9ZJU1_
HLA-DQA10501-DQB10201	406	DHLLVCDIWNCLMAL	DIWNCLMAL	6	0.2886	2201.2	38.00	0.29	tr_C9ZJU1_
HLA-DQA10501-DQB10201	407	GDIFVNGVHISQYPA	VNGVHISQY	4	0.2884	2205.8	38.00	0.43	tr_C9ZJU1_
HLA-DQA10501-DQB10201	408	CVWQCRQDYLQGFAA	VWQCRQDYL	1	0.2884	2207.0	38.00	0.22	tr_C9ZJU1_

HLA-DQA10501-DQB10201	409	ANVWQEENNLRFIED	ENNLRFIED	6	0.2875	2228.4	38.00	0.34	tr_C9ZJU1_
HLA-DQA10501-DQB10201	410	RRLGWIKDVIQSSPR	WIKDVIQSS	4	0.2870	2240.7	38.00	0.43	tr_C9ZJU1_
HLA-DQA10501-DQB10201	411	DKSSVQYGLVLFSMV	QYGLVLFSM	5	0.2869	2242.8	38.00	0.46	tr_C9ZJU1_
HLA-DQA10501-DQB10201	412	TFAFESLYCIRGFVI	FAFESLYCI	1	0.2868	2245.5	38.00	0.34	tr_C9ZJU1_
HLA-DQA10501-DQB10201	413	ITEGEIFDTLRLREG	TEGEIFDTL	1	0.2865	2253.9	38.00	0.32	tr_C9ZJU1_
HLA-DQA10501-DQB10201	414	MADTLPLLLVSAVAL	MADTLPLLL	0	0.2852	2284.2	39.00	0.41	tr_C9ZJU1_
HLA-DQA10501-DQB10201	415	DASIAIFLNCNGVES	FLNCNGVES	6	0.2848	2293.9	39.00	0.46	tr_C9ZJU1_
HLA-DQA10501-DQB10201	416	HWRPIYAAEIGKSLN	WRPIYAAEI	1	0.2845	2302.9	39.00	0.46	tr_C9ZJU1_
HLA-DQA10501-DQB10201	417	WSVEFREVSFRYSDR	EFREVSFRY	3	0.2840	2314.4	39.00	0.26	tr_C9ZJU1_
HLA-DQA10501-DQB10201	418	IPQLVTLAATVLFSP	LVTLAATVL	3	0.2835	2327.8	39.00	0.34	tr_C9ZJU1_
HLA-DQA10501-DQB10201	419	RAFVKAGSAVGLCIF	AGSAVGLCI	5	0.2834	2328.7	39.00	0.31	tr_C9ZJU1_
HLA-DQA10501-DQB10201	420	DIWNCLMALVNCSIR	WNCLMALVN	2	0.2834	2329.8	39.00	0.31	tr_C9ZJU1_
HLA-DQA10501-DQB10201	421	VCDIWNCLMALVNCS	WNCLMALVN	4	0.2824	2354.1	40.00	0.43	tr_C9ZJU1_
HLA-DQA10501-DQB10201	422	QCGRDVLHGLFQMAD	LHGLFQMAD	6	0.2823	2358.5	40.00	0.32	tr_C9ZJU1_
HLA-DQA10501-DQB10201	423	VRTLVFNHNIMQLPS	VFNHNIMQL	4	0.2811	2387.3	40.00	0.39	tr_C9ZJU1_
HLA-DQA10501-DQB10201	424	PSLDGPWSVEFREVS	PWSVEFREV	5	0.2809	2393.0	40.00	0.29	tr_C9ZJU1_
HLA-DQA10501-DQB10201	425	SVQYGLVLFSMVFVE	QYGLVLFSM	2	0.2806	2402.0	40.00	0.34	tr_C9ZJU1_
HLA-DQA10501-DQB10201	426	LEVMHGCRVVFSFPV	MHGCRVVFS	3	0.2801	2414.7	40.00	0.37	tr_C9ZJU1_
HLA-DQA10501-DQB10201	427	SSVQYGLVLFSMVFV	QYGLVLFSM	3	0.2793	2435.5	40.00	0.35	tr_C9ZJU1_
HLA-DQA10501-DQB10201	428	RLGWIKDVIQSSPRA	WIKDVIQSS	3	0.2783	2461.2	41.00	0.37	tr_C9ZJU1_
HLA-DQA10501-DQB10201	429	SALDQTEIVNQLRSI	SALDQTEIV	0	0.2783	2461.7	41.00	0.31	tr_C9ZJU1_
HLA-DQA10501-DQB10201	430	FSGCVVDALKRKLQA	FSGCVVDAL	0	0.2780	2470.8	41.00	0.73	tr_C9ZJU1_
HLA-DQA10501-DQB10201	431	VFSFPVVSLVMWLGT	SFPVVSLVM	2	0.2775	2483.0	41.00	0.19	tr_C9ZJU1_
HLA-DQA10501-DQB10201	432	TCVWQCRQDYLQGFA	VWQCRQDYL	2	0.2767	2504.3	41.00	0.29	tr_C9ZJU1_
HLA-DQA10501-DQB10201	433	STCVWQCRQDYLQGF	VWQCRQDYL	3	0.2765	2509.5	41.00	0.35	tr_C9ZJU1_
HLA-DQA10501-DQB10201	434	CSIPQLVTLAATVLF	LVTLAATVL	5	0.2764	2513.5	41.00	0.42	tr_C9ZJU1_
HLA-DQA10501-DQB10201	435	NIALGNLWDCSDENI	LWDCSDENI	6	0.2762	2518.2	41.00	0.31	tr_C9ZJU1_
HLA-DQA10501-DQB10201	436	IFLNCNGVESIHVRT	FLNCNGVES	1	0.2761	2520.4	41.00	0.38	tr_C9ZJU1_
HLA-DQA10501-DQB10201	437	WQCRQDYLQGFAALR	DYLQGFAAL	5	0.2759	2525.8	41.00	0.23	tr_C9ZJU1_
HLA-DQA10501-DQB10201	438	SIPQLVTLAATVLFS	LVTLAATVL	4	0.2759	2527.8	41.00	0.34	tr_C9ZJU1_
HLA-DQA10501-DQB10201	439	KLSPFRFIIKQLMGL	FRFIIKQLM	4	0.2757	2532.1	41.00	0.51	tr_C9ZJU1_
HLA-DQA10501-DQB10201	440	CVSQALWQASGSVMD	WQASGSVMD	6	0.2749	2554.8	41.00	0.41	tr_C9ZJU1_
HLA-DQA10501-DQB10201	441	IFVNGVHISQYPARM	VNGVHISQY	2	0.2746	2562.8	42.00	0.33	tr_C9ZJU1_
HLA-DQA10501-DQB10201	442	GCYQVLEGLQSVQRK	YQVLEGLQS	2	0.2728	2611.7	42.00	0.30	tr_C9ZJU1_
HLA-DQA10501-DQB10201	443	DIFVNGVHISQYPAR	VNGVHISQY	3	0.2727	2614.4	42.00	0.38	tr_C9ZJU1_
HLA-DQA10501-DQB10201	444	SPKLSPFRFIIKQLM	FRFIIKQLM	6	0.2722	2628.8	42.00	0.69	tr_C9ZJU1_
HLA-DQA10501-DQB10201	445	LLEVMHGCRVVFSFP	MHGCRVVFS	4	0.2718	2641.6	42.00	0.37	tr_C9ZJU1_
HLA-DQA10501-DQB10201	446	PPSLDGPWSVEFREV	PWSVEFREV	6	0.2717	2643.6	42.00	0.33	tr_C9ZJU1_
HLA-DQA10501-DQB10201	447	IGGNIALGNLWDCSD	NIALGNLWD	3	0.2716	2648.2	42.00	0.37	tr_C9ZJU1_
HLA-DQA10501-DQB10201	448	RARPAVLLDCERKAY	RPAVLLDCE	2	0.2707	2672.3	43.00	0.41	tr_C9ZJU1_
HLA-DQA10501-DQB10201	449	FVNGVHISQYPARML	HISQYPARM	5	0.2703	2684.6	43.00	0.23	tr_C9ZJU1_
HLA-DQA10501-DQB10201	450	SMVFVESGRPLGYMW	MVFVESGRP	1	0.2702	2686.0	43.00	0.25	tr_C9ZJU1_
HLA-DQA10501-DQB10201	451	HLLVCDIWNCLMALV	IWNCLMALV	6	0.2701	2689.3	43.00	0.32	tr_C9ZJU1_
HLA-DQA10501-DQB10201	452	PVVSLVMWLGTQWSN	VMWLGTQWS	5	0.2697	2700.5	43.00	0.40	tr_C9ZJU1_
HLA-DQA10501-DQB10201	453	AAAFATASERNDRYE	AFATASERN	2	0.2693	2714.9	43.00	0.25	tr_C9ZJU1_
HLA-DQA10501-DQB10201	454	SGGEVERLCIARAFV	VERLCIARA	4	0.2690	2723.4	43.00	0.29	tr_C9ZJU1_
HLA-DQA10501-DQB10201	455	ENICAALSASRALNF	LSASRALNF	6	0.2688	2728.4	43.00	0.33	tr_C9ZJU1_
HLA-DQA10501-DQB10201	456	NICAALSASRALNFV	LSASRALNF	5	0.2679	2756.0	43.00	0.31	tr_C9ZJU1_
HLA-DQA10501-DQB10201	457	ARAFVKAGSAVGLCI	AGSAVGLCI	6	0.2678	2759.2	43.00	0.40	tr_C9ZJU1_
HLA-DQA10501-DQB10201	458	DLSIGGNIALGNLWD	NIALGNLWD	6	0.2668	2787.8	44.00	0.34	tr_C9ZJU1_

HLA-DQA10501-DQB10201	459	SCSIPQLVTLAATVL	LVTLAATVL	6	0.2667	2789.8	44.00	0.50	tr_C9ZJU1_
HLA-DQA10501-DQB10201	460	IMQLPSVFAVHCGLM	SVFAVHCGL	5	0.2666	2794.7	44.00	0.22	tr_C9ZJU1_
HLA-DQA10501-DQB10201	461	TVLFSPEVILKCILG	TVLFSPEVI	0	0.2665	2796.6	44.00	0.54	tr_C9ZJU1_
HLA-DQA10501-DQB10201	462	ALLEVMHGCRVVFSF	MHGCRVVFS	5	0.2635	2890.7	44.00	0.38	tr_C9ZJU1_
HLA-DQA10501-DQB10201	463	TADYDPSDDWLLSRC	DYDPSDDWL	2	0.2634	2893.5	45.00	0.37	tr_C9ZJU1_
HLA-DQA10501-DQB10201	464	SFPVVSLVMWLGTQW	LVMWLGTQW	6	0.2632	2897.9	45.00	0.38	tr_C9ZJU1_
HLA-DQA10501-DQB10201	465	LWDCSDENICAALSA	ENICAALSA	6	0.2630	2905.7	45.00	0.31	tr_C9ZJU1_
HLA-DQA10501-DQB10201	466	QSRVPALLEVMHGCR	RVPALLEVM	2	0.2619	2939.8	45.00	0.50	tr_C9ZJU1_
HLA-DQA10501-DQB10201	467	MVFVESGRPLGYMWD	VESGRPLGY	3	0.2615	2951.2	45.00	0.25	tr_C9ZJU1_
HLA-DQA10501-DQB10201	468	LSIGGNIALGNLWDC	NIALGNLWD	5	0.2607	2977.2	45.00	0.37	tr_C9ZJU1_
HLA-DQA10501-DQB10201	469	GGNIALGNLWDCSDE	NIALGNLWD	2	0.2605	2984.2	45.00	0.33	tr_C9ZJU1_
HLA-DQA10501-DQB10201	470	SIGGNIALGNLWDCS	NIALGNLWD	4	0.2604	2989.0	45.00	0.37	tr_C9ZJU1_
HLA-DQA10501-DQB10201	471	GLCIFDEPTSAIDTI	EPTSAIDTI	6	0.2602	2994.6	45.00	0.48	tr_C9ZJU1_
HLA-DQA10501-DQB10201	472	NLRFIEDLSIGGNIA	LRFIEDLSI	1	0.2594	3020.7	46.00	0.41	tr_C9ZJU1_
HLA-DQA10501-DQB10201	473	IALGNLWDCSDENIC	LWDCSDENI	5	0.2584	3052.7	46.00	0.35	tr_C9ZJU1_
HLA-DQA10501-DQB10201	474	ESLYMESQFTKSQLA	SLYMESQFT	1	0.2582	3058.8	46.00	0.41	tr_C9ZJU1_
HLA-DQA10501-DQB10201	475	IWNCLMALVNCSIRD	LMALVNCSI	4	0.2573	3088.4	46.00	0.34	tr_C9ZJU1_
HLA-DQA10501-DQB10201	476	VVNKGEFLGIAGYSG	FLGIAGYSG	6	0.2564	3120.8	46.00	0.31	tr_C9ZJU1_
HLA-DQA10501-DQB10201	477	GNIALGNLWDCSDEN	NIALGNLWD	1	0.2562	3126.8	46.00	0.28	tr_C9ZJU1_
HLA-DQA10501-DQB10201	478	ARPAVLLDCERKAYE	RPAVLLDCE	1	0.2553	3155.8	47.00	0.43	tr_C9ZJU1_
HLA-DQA10501-DQB10201	479	PEELYLGLITAYNRK	YLGLITAYN	4	0.2553	3158.1	47.00	0.27	tr_C9ZJU1_
HLA-DQA10501-DQB10201	480	SVEFREVSFRYSDRH	EFREVSFRY	2	0.2544	3189.5	47.00	0.28	tr_C9ZJU1_
HLA-DQA10501-DQB10201	481	AVSVRRLGWIKDVIQ	LGWIKDVIQ	6	0.2541	3199.1	47.00	0.44	tr_C9ZJU1_
HLA-DQA10501-DQB10201	482	ICAALSASRALNFVQ	LSASRALNF	4	0.2536	3216.1	47.00	0.31	tr_C9ZJU1_
HLA-DQA10501-DQB10201	483	IATSTYAGNGEENQV	YAGNGEENQ	5	0.2532	3230.0	47.00	0.38	tr_C9ZJU1_
HLA-DQA10501-DQB10201	484	LGWIKDVIQSSPRAA	WIKDVIQSS	2	0.2507	3318.3	48.00	0.44	tr_C9ZJU1_
HLA-DQA10501-DQB10201	485	SRVPALLEVMHGCRV	RVPALLEVM	1	0.2505	3327.0	48.00	0.44	tr_C9ZJU1_
HLA-DQA10501-DQB10201	486	AFSGSSLGCYQVLEG	LGCYQVLEG	6	0.2502	3334.7	48.00	0.37	tr_C9ZJU1_
HLA-DQA10501-DQB10201	487	SDENICAALSASRAL	CAALSASRA	5	0.2494	3363.7	48.00	0.37	tr_C9ZJU1_
HLA-DQA10501-DQB10201	488	CILGMHAFVHWRPIY	MHAFVHWRP	4	0.2492	3371.3	48.00	0.29	tr_C9ZJU1_
HLA-DQA10501-DQB10201	489	PKLSPFRFIIKQLMG	FRFIIKQLM	5	0.2491	3374.8	48.00	0.68	tr_C9ZJU1_
HLA-DQA10501-DQB10201	490	KSQLASVTNIATSTY	VTNIATSTY	6	0.2490	3379.3	48.00	0.28	tr_C9ZJU1_
HLA-DQA10501-DQB10201	491	VFVESGRPLGYMWDL	VESGRPLGY	2	0.2485	3397.8	49.00	0.26	tr_C9ZJU1_
HLA-DQA10501-DQB10201	492	CYQVLEGLQSVQRKI	YQVLEGLQS	1	0.2483	3406.1	49.00	0.31	tr_C9ZJU1_
HLA-DQA10501-DQB10201	493	PALLEVMHGCRVVFS	MHGCRVVFS	6	0.2483	3406.7	49.00	0.41	tr_C9ZJU1_
HLA-DQA10501-DQB10201	494	NLWDCSDENICAALS	DENICAALS	6	0.2483	3406.8	49.00	0.34	tr_C9ZJU1_
HLA-DQA10501-DQB10201	495	FWKLMCSSCSIPQLV	SSCSIPQLV	6	0.2482	3411.3	49.00	0.37	tr_C9ZJU1_
HLA-DQA10501-DQB10201	496	VGLCIFDEPTSAIDT	VGLCIFDEP	0	0.2481	3411.6	49.00	0.35	tr_C9ZJU1_
HLA-DQA10501-DQB10201	497	LKHADKIVVLNEGMV	KIVVLNEGM	5	0.2477	3428.6	49.00	0.43	tr_C9ZJU1_
HLA-DQA10501-DQB10201	498	LRSIQHWCPLYHAFL	WCPLYHAFL	6	0.2467	3463.7	49.00	0.36	tr_C9ZJU1_
HLA-DQA10501-DQB10201	499	TSTYAGNGEENQVSP	YAGNGEENQ	3	0.2463	3481.4	49.00	0.43	tr_C9ZJU1_
HLA-DQA10501-DQB10201	500	WRPIYAAEIGKSLNG	WRPIYAAEI	0	0.2456	3505.9	49.00	0.37	tr_C9ZJU1_
HLA-DQA10501-DQB10201	501	SQLASVTNIATSTYA	VTNIATSTY	5	0.2455	3509.5	49.00	0.25	tr_C9ZJU1_
HLA-DQA10501-DQB10201	502	ALGNLWDCSDENICA	LWDCSDENI	4	0.2455	3510.2	49.00	0.37	tr_C9ZJU1_
HLA-DQA10501-DQB10201	503	IANVWQEENNLRFIE	WQEENNLRF	4	0.2453	3518.9	49.00	0.46	tr_C9ZJU1_
HLA-DQA10501-DQB10201	504	VILKCILGMHAFVHW	LKCILGMHA	2	0.2450	3529.5	50.00	0.31	tr_C9ZJU1_
HLA-DQA10501-DQB10201	505	ATSTYAGNGEENQVS	YAGNGEENQ	4	0.2442	3560.0	50.00	0.40	tr_C9ZJU1_
HLA-DQA10501-DQB10201	506	KAEEVLALCMLLRGK	AEEVLALCM	1	0.2439	3572.7	50.00	0.38	tr_C9ZJU1_
HLA-DQA10501-DQB10201	507	PTEGDIFVNGVHISQ	FVNGVHISQ	6	0.2436	3581.9	50.00	0.34	tr_C9ZJU1_
HLA-DQA10501-DQB10201	508	NCLMALVNCSIRDIL	LMALVNCSI	2	0.2436	3582.3	50.00	0.30	tr_C9ZJU1_

HLA-DQA10501-DQB10201	509	PEVILKCILGMHAFV	LKCILGMHA	4	0.2436	3583.3	50.00	0.44	tr_C9ZJU1_
HLA-DQA10501-DQB10201	510	QFTKSQLASVTNIAT	QLASVTNIA	5	0.2431	3602.9	50.00	0.28	tr_C9ZJU1_
HLA-DQA10501-DQB10201	511	TEGEIFDTLRLREGN	EGEIFDTLR	1	0.2429	3611.7	50.00	0.28	tr_C9ZJU1_
HLA-DQA10501-DQB10201	512	LMALVNCSIRDILLD	VNCSIRDIL	4	0.2425	3624.6	50.00	0.34	tr_C9ZJU1_
HLA-DQA10501-DQB10201	513	VNKGEFLGIAGYSGS	FLGIAGYSG	5	0.2425	3626.1	50.00	0.32	tr_C9ZJU1_
HLA-DQA10501-DQB10201	514	VSVRRLGWIKDVIQS	LGWIKDVIQ	5	0.2422	3638.2	50.00	0.44	tr_C9ZJU1_
HLA-DQA10501-DQB10201	515	FAFESLYCIRGFVIQ	FAFESLYCI	0	0.2419	3651.4	50.00	0.28	tr_C9ZJU1_
HLA-DQA10501-DQB10201	516	WNCLMALVNCSIRDI	LMALVNCSI	3	0.2417	3657.5	50.00	0.38	tr_C9ZJU1_
HLA-DQA10501-DQB10201	517	FSFPVVSLVMWLGTQ	SFPVVSLVM	1	0.2411	3681.7	55.00	0.22	tr_C9ZJU1_
HLA-DQA10501-DQB10201	518	EVILKCILGMHAFVH	LKCILGMHA	3	0.2410	3684.8	55.00	0.37	tr_C9ZJU1_
HLA-DQA10501-DQB10201	519	EQCGRDVLHGLFQMA	VLHGLFQMA	6	0.2405	3705.0	55.00	0.29	tr_C9ZJU1_
HLA-DQA10501-DQB10201	520	CLMALVNCSIRDILL	VNCSIRDIL	5	0.2388	3772.5	55.00	0.29	tr_C9ZJU1_
HLA-DQA10501-DQB10201	521	NIATSTYAGNGEENQ	YAGNGEENQ	6	0.2382	3799.2	55.00	0.41	tr_C9ZJU1_
HLA-DQA10501-DQB10201	522	LKCILGMHAFVHWRP	MHAFVHWRP	6	0.2382	3800.5	55.00	0.29	tr_C9ZJU1_
HLA-DQA10501-DQB10201	523	LMKNSHPDHLLVCDI	PDHLLVCDI	6	0.2372	3838.6	55.00	0.62	tr_C9ZJU1_
HLA-DQA10501-DQB10201	524	LGNLWDCSDENICAA	LWDCSDENI	3	0.2371	3843.7	55.00	0.38	tr_C9ZJU1_
HLA-DQA10501-DQB10201	525	GGEVERLCIARAFVK	VERLCIARA	3	0.2370	3849.4	55.00	0.29	tr_C9ZJU1_
HLA-DQA10501-DQB10201	526	IHVRTLVFNHNIMQL	VFNHNIMQL	6	0.2363	3877.0	55.00	0.55	tr_C9ZJU1_
HLA-DQA10501-DQB10201	527	SDDWLLSRCVSQALW	SRCVSQALW	6	0.2359	3896.3	55.00	0.38	tr_C9ZJU1_
HLA-DQA10501-DQB10201	528	AYEILEMLTRATIKT	AYEILEMLT	0	0.2352	3924.7	55.00	0.37	tr_C9ZJU1_
HLA-DQA10501-DQB10201	529	TLKHADKIVVLNEGM	KIVVLNEGM	6	0.2349	3936.4	55.00	0.50	tr_C9ZJU1_
HLA-DQA10501-DQB10201	530	VEFREVSFRYSDRHP	EFREVSFRY	1	0.2340	3975.8	55.00	0.26	tr_C9ZJU1_
HLA-DQA10501-DQB10201	531	CSDENICAALSASRA	CAALSASRA	6	0.2336	3992.9	55.00	0.37	tr_C9ZJU1_
HLA-DQA10501-DQB10201	532	GNLWDCSDENICAAL	LWDCSDENI	2	0.2332	4011.5	55.00	0.37	tr_C9ZJU1_
HLA-DQA10501-DQB10201	533	SPSTCVWQCRQDYLQ	VWQCRQDYL	5	0.2329	4024.1	55.00	0.52	tr_C9ZJU1_
HLA-DQA10501-DQB10201	534	APPSLDGPWSVEFRE	GPWSVEFRE	6	0.2327	4033.0	55.00	0.22	tr_C9ZJU1_
HLA-DQA10501-DQB10201	535	DDWLLSRCVSQALWQ	SRCVSQALW	5	0.2313	4092.6	55.00	0.35	tr_C9ZJU1_
HLA-DQA10501-DQB10201	536	LRFIEDLSIGGNIAL	DLSIGGNIA	5	0.2304	4135.2	55.00	0.30	tr_C9ZJU1_
HLA-DQA10501-DQB10201	537	DENICAALSASRALN	CAALSASRA	4	0.2301	4148.1	55.00	0.41	tr_C9ZJU1_
HLA-DQA10501-DQB10201	538	MQLPSVFAVHCGLMK	SVFAVHCGL	4	0.2298	4160.2	55.00	0.22	tr_C9ZJU1_
HLA-DQA10501-DQB10201	539	KQTECKDDASIAIFL	DDASIAIFL	6	0.2293	4183.2	55.00	0.60	tr_C9ZJU1_
HLA-DQA10501-DQB10201	540	FTKSQLASVTNIATS	QLASVTNIA	4	0.2292	4188.6	55.00	0.29	tr_C9ZJU1_
HLA-DQA10501-DQB10201	541	DDASIAIFLNCNGVE	IFLNCNGVE	6	0.2287	4210.5	55.00	0.35	tr_C9ZJU1_
HLA-DQA10501-DQB10201	542	ADYDPSDDWLLSRCV	DYDPSDDWL	1	0.2284	4221.8	55.00	0.44	tr_C9ZJU1_
HLA-DQA10501-DQB10201	543	ALDQTEIVNQLRSIQ	EIVNQLRSI	5	0.2284	4222.4	55.00	0.26	tr_C9ZJU1_
HLA-DQA10501-DQB10201	544	FLNCNGVESIHVRTL	FLNCNGVES	0	0.2282	4235.4	55.00	0.28	tr_C9ZJU1_
HLA-DQA10501-DQB10201	545	YQVLEGLQSVQRKID	LEGLQSVQR	3	0.2279	4246.0	55.00	0.31	tr_C9ZJU1_
HLA-DQA10501-DQB10201	546	STYAGNGEENQVSPT	YAGNGEENQ	2	0.2279	4247.6	55.00	0.43	tr_C9ZJU1_
HLA-DQA10501-DQB10201	547	SQFTKSQLASVTNIA	QLASVTNIA	6	0.2272	4281.2	55.00	0.35	tr_C9ZJU1_
HLA-DQA10501-DQB10201	548	RSIQHWCPLYHAFLG	WCPLYHAFL	5	0.2270	4287.5	55.00	0.35	tr_C9ZJU1_
HLA-DQA10501-DQB10201	549	SIQHWCPLYHAFLGS	WCPLYHAFL	4	0.2269	4292.7	55.00	0.34	tr_C9ZJU1_
HLA-DQA10501-DQB10201	550	KCILGMHAFVHWRPI	MHAFVHWRP	5	0.2261	4329.7	55.00	0.34	tr_C9ZJU1_
HLA-DQA10501-DQB10201	551	TKSQLASVTNIATST	QLASVTNIA	3	0.2257	4349.0	55.00	0.27	tr_C9ZJU1_
HLA-DQA10501-DQB10201	552	AAFATASERNDRYEQ	AFATASERN	1	0.2256	4354.3	55.00	0.34	tr_C9ZJU1_
HLA-DQA10501-DQB10201	553	APTEGDIFVNGVHIS	EGDIFVNGV	3	0.2252	4372.6	55.00	0.47	tr_C9ZJU1_
HLA-DQA10501-DQB10201	554	YNRKRSLQSGITSFW	LQSGITSFW	6	0.2245	4404.7	55.00	0.61	tr_C9ZJU1_
HLA-DQA10501-DQB10201	555	YEQCGRDVLHGLFQM	GRDVLHGLF	4	0.2243	4416.5	55.00	0.30	tr_C9ZJU1_
HLA-DQA10501-DQB10201	556	GEVERLCIARAFVKA	VERLCIARA	2	0.2240	4431.6	60.00	0.28	tr_C9ZJU1_
HLA-DQA10501-DQB10201	557	PSTCVWQCRQDYLQG	VWQCRQDYL	4	0.2237	4446.0	60.00	0.49	tr_C9ZJU1_
HLA-DQA10501-DQB10201	558	CSSCSIPQLVTLAAT	PQLVTLAAT	6	0.2237	4446.6	60.00	0.34	tr_C9ZJU1_

HLA-DQA10501-DQB10201	559	CAALSASRALNFVQK	LSASRALNF	3	0.2236	4449.1	60.00	0.31	tr_C9ZJU1_
HLA-DQA10501-DQB10201	560	WDCSDENICAALSAS	ENICAALSA	5	0.2234	4459.1	60.00	0.32	tr_C9ZJU1_
HLA-DQA10501-DQB10201	561	NKGEFLGIAGYSGSG	FLGIAGYSG	4	0.2225	4502.8	60.00	0.34	tr_C9ZJU1_
HLA-DQA10501-DQB10201	562	RVPALLEVMHGCRVV	RVPALLEVM	0	0.2219	4533.6	60.00	0.33	tr_C9ZJU1_
HLA-DQA10501-DQB10201	563	EFREVSFRYSDRHPY	VSFRYSDRH	4	0.2215	4551.5	60.00	0.19	tr_C9ZJU1_
HLA-DQA10501-DQB10201	564	EELYLGLITAYNRKR	YLGLITAYN	3	0.2212	4568.6	60.00	0.29	tr_C9ZJU1_
HLA-DQA10501-DQB10201	565	RPAVLLDCERKAYEI	PAVLLDCER	1	0.2210	4573.8	60.00	0.36	tr_C9ZJU1_
HLA-DQA10501-DQB10201	566	VNQLRSIQHWCPLYH	IQHWCPLYH	6	0.2210	4575.5	60.00	0.27	tr_C9ZJU1_
HLA-DQA10501-DQB10201	567	PARMLRRRIANVWQE	RRIANVWQE	6	0.2206	4597.5	60.00	0.34	tr_C9ZJU1_
HLA-DQA10501-DQB10201	568	QTECKDDASIAIFLN	DDASIAIFL	5	0.2205	4598.9	60.00	0.48	tr_C9ZJU1_
HLA-DQA10501-DQB10201	569	FREVSFRYSDRHPYV	RYSDRHPYV	6	0.2200	4626.0	60.00	0.23	tr_C9ZJU1_
HLA-DQA10501-DQB10201	570	SLQSGITSFWKLMCS	LQSGITSFW	1	0.2196	4647.5	60.00	0.43	tr_C9ZJU1_
HLA-DQA10501-DQB10201	571	SRHDKSSVQYGLVLF	SSVQYGLVL	5	0.2195	4651.5	60.00	0.40	tr_C9ZJU1_
HLA-DQA10501-DQB10201	572	SSCSIPQLVTLAATV	QLVTLAATV	6	0.2194	4653.6	60.00	0.38	tr_C9ZJU1_
HLA-DQA10501-DQB10201	573	NRKRSLQSGITSFWK	LQSGITSFW	5	0.2192	4665.7	60.00	0.51	tr_C9ZJU1_
HLA-DQA10501-DQB10201	574	LAFSGSSLGCYQVLE	SLGCYQVLE	6	0.2191	4669.4	60.00	0.32	tr_C9ZJU1_
HLA-DQA10501-DQB10201	575	RHDKSSVQYGLVLFS	SSVQYGLVL	4	0.2188	4684.6	60.00	0.34	tr_C9ZJU1_
HLA-DQA10501-DQB10201	576	NCNGVESIHVRTLVF	SIHVRTLVF	6	0.2186	4698.6	60.00	0.38	tr_C9ZJU1_
HLA-DQA10501-DQB10201	577	ECKDDASIAIFLNCN	DDASIAIFL	3	0.2182	4715.5	60.00	0.32	tr_C9ZJU1_
HLA-DQA10501-DQB10201	578	GWIKDVIQSSPRAAT	WIKDVIQSS	1	0.2182	4716.7	60.00	0.46	tr_C9ZJU1_
HLA-DQA10501-DQB10201	579	MCSSCSIPQLVTLAA	SSCSIPQLV	2	0.2182	4718.6	60.00	0.32	tr_C9ZJU1_
HLA-DQA10501-DQB10201	580	IHAPLNVREFSGGEV	VREFSGGEV	6	0.2179	4734.6	60.00	0.71	tr_C9ZJU1_
HLA-DQA10501-DQB10201	581	LDQTEIVNQLRSIQH	EIVNQLRSI	4	0.2171	4774.0	60.00	0.24	tr_C9ZJU1_
HLA-DQA10501-DQB10201	582	DWLLSRCVSQALWQA	SRCVSQALW	4	0.2167	4794.2	60.00	0.32	tr_C9ZJU1_
HLA-DQA10501-DQB10201	583	ILKCILGMHAFVHWR	LKCILGMHA	1	0.2163	4815.8	60.00	0.35	tr_C9ZJU1_
HLA-DQA10501-DQB10201	584	RYEQCGRDVLHGLFQ	GRDVLHGLF	5	0.2162	4821.1	60.00	0.35	tr_C9ZJU1_
HLA-DQA10501-DQB10201	585	WKLMCSSCSIPQLVT	SSCSIPQLV	5	0.2158	4841.1	60.00	0.44	tr_C9ZJU1_
HLA-DQA10501-DQB10201	586	VNGVHISQYPARMLR	HISQYPARM	4	0.2148	4894.0	60.00	0.38	tr_C9ZJU1_
HLA-DQA10501-DQB10201	587	ESQFTKSQLASVTNI	SQLASVTNI	6	0.2147	4897.2	60.00	0.37	tr_C9ZJU1_
HLA-DQA10501-DQB10201	588	RKRSLQSGITSFWKL	LQSGITSFW	4	0.2147	4900.5	60.00	0.53	tr_C9ZJU1_
HLA-DQA10501-DQB10201	589	KRSLQSGITSFWKLM	LQSGITSFW	3	0.2140	4936.9	60.00	0.53	tr_C9ZJU1_
HLA-DQA10501-DQB10201	590	KLMCSSCSIPQLVTL	SSCSIPQLV	4	0.2134	4968.6	60.00	0.49	tr_C9ZJU1_
HLA-DQA10501-DQB10201	591	TECKDDASIAIFLNC	DDASIAIFL	4	0.2133	4971.6	60.00	0.44	tr_C9ZJU1_
HLA-DQA10501-DQB10201	592	DQTEIVNQLRSIQHW	EIVNQLRSI	3	0.2127	5004.6	60.00	0.26	tr_C9ZJU1_
HLA-DQA10501-DQB10201	593	ASPSTCVWQCRQDYL	VWQCRQDYL	6	0.2127	5008.5	60.00	0.61	tr_C9ZJU1_
HLA-DQA10501-DQB10201	594	NQLRSIQHWCPLYHA	IQHWCPLYH	5	0.2126	5010.2	60.00	0.25	tr_C9ZJU1_
HLA-DQA10501-DQB10201	595	AEEVLALCMLLRGKY	AEEVLALCM	0	0.2120	5044.8	60.00	0.35	tr_C9ZJU1_
HLA-DQA10501-DQB10201	596	EDLSIGGNIALGNLW	GNIALGNLW	6	0.2119	5047.8	60.00	0.36	tr_C9ZJU1_
HLA-DQA10501-DQB10201	597	IARAFVKAGSAVGLC	VKAGSAVGL	5	0.2115	5071.5	60.00	0.42	tr_C9ZJU1_
HLA-DQA10501-DQB10201	598	VPALLEVMHGCRVVF	LEVMHGCRV	4	0.2114	5074.3	60.00	0.31	tr_C9ZJU1_
HLA-DQA10501-DQB10201	599	DRYEQCGRDVLHGLF	GRDVLHGLF	6	0.2112	5085.8	60.00	0.45	tr_C9ZJU1_
HLA-DQA10501-DQB10201	600	QLRSIQHWCPLYHAF	IQHWCPLYH	4	0.2112	5086.4	60.00	0.33	tr_C9ZJU1_
HLA-DQA10501-DQB10201	601	RSLQSGITSFWKLMC	LQSGITSFW	2	0.2109	5107.3	60.00	0.50	tr_C9ZJU1_
HLA-DQA10501-DQB10201	602	SFWKLMCSSCSIPQL	WKLMCSSCS	2	0.2098	5166.9	60.00	0.26	tr_C9ZJU1_
HLA-DQA10501-DQB10201	603	REVSFRYSDRHPYVL	RYSDRHPYV	5	0.2090	5207.9	60.00	0.26	tr_C9ZJU1_
HLA-DQA10501-DQB10201	604	CFSRMRQSQQLPHFV	SRMRQSQQL	2	0.2088	5222.3	60.00	0.26	tr_C9ZJU1_
HLA-DQA10501-DQB10201	605	QLPSVFAVHCGLMKN	SVFAVHCGL	3	0.2084	5246.9	60.00	0.25	tr_C9ZJU1_
HLA-DQA10501-DQB10201	606	ITSFWKLMCSSCSIP	WKLMCSSCS	4	0.2081	5263.8	60.00	0.31	tr_C9ZJU1_
HLA-DQA10501-DQB10201	607	QLASVTNIATSTYAG	VTNIATSTY	4	0.2071	5317.6	60.00	0.26	tr_C9ZJU1_
HLA-DQA10501-DQB10201	608	CKDDASIAIFLNCNG	DDASIAIFL	2	0.2069	5331.6	65.00	0.34	tr_C9ZJU1_

HLA-DQA10501-DQB10201	609	ADTLPLLLVSAVALR	PLLLVSAVA	4	0.2066	5345.9	65.00	0.45	tr_C9ZJU1_
HLA-DQA10501-DQB10201	610	AFESLYCIRGFVIQS	ESLYCIRGF	2	0.2064	5361.2	65.00	0.25	tr_C9ZJU1_
HLA-DQA10501-DQB10201	611	VAPPSLDGPWSVEFR	LDGPWSVEF	5	0.2056	5403.0	65.00	0.28	tr_C9ZJU1_
HLA-DQA10501-DQB10201	612	IVNQLRSIQHWCPLY	SIQHWCPLY	6	0.2054	5415.2	65.00	0.29	tr_C9ZJU1_
HLA-DQA10501-DQB10201	613	LMCSSCSIPQLVTLA	SSCSIPQLV	3	0.2053	5425.8	65.00	0.46	tr_C9ZJU1_
HLA-DQA10501-DQB10201	614	AALSASRALNFVQKR	LSASRALNF	2	0.2042	5486.5	65.00	0.39	tr_C9ZJU1_
HLA-DQA10501-DQB10201	615	WLLSRCVSQALWQAS	SRCVSQALW	3	0.2040	5499.6	65.00	0.33	tr_C9ZJU1_
HLA-DQA10501-DQB10201	616	KGEFLGIAGYSGSGK	EFLGIAGYS	2	0.2039	5507.2	65.00	0.34	tr_C9ZJU1_
HLA-DQA10501-DQB10201	617	KDDASIAIFLNCNGV	DDASIAIFL	1	0.2037	5517.6	65.00	0.33	tr_C9ZJU1_
HLA-DQA10501-DQB10201	618	YVLRRVSFVVNKGEF	SFVVNKGEF	6	0.2036	5521.4	65.00	0.25	tr_C9ZJU1_
HLA-DQA10501-DQB10201	619	TYAGNGEENQVSPTC	YAGNGEENQ	1	0.2026	5584.9	65.00	0.41	tr_C9ZJU1_
HLA-DQA10501-DQB10201	620	DCSDENICAALSASR	ENICAALSA	4	0.2012	5670.1	65.00	0.34	tr_C9ZJU1_
HLA-DQA10501-DQB10201	621	EVERLCIARAFVKAG	VERLCIARA	1	0.2005	5715.6	65.00	0.31	tr_C9ZJU1_
HLA-DQA10501-DQB10201	622	LSSTDNKMLFQQTFS	KMLFQQTFS	6	0.2002	5733.6	65.00	0.38	tr_C9ZJU1_
HLA-DQA10501-DQB10201	623	CIARAFVKAGSAVGL	VKAGSAVGL	6	0.1995	5772.5	65.00	0.47	tr_C9ZJU1_
HLA-DQA10501-DQB10201	624	SCFSRMRQSQQLPHF	SRMRQSQQL	3	0.1992	5792.3	65.00	0.35	tr_C9ZJU1_
HLA-DQA10501-DQB10201	625	SPEVILKCILGMHAF	LKCILGMHA	5	0.1990	5803.1	65.00	0.47	tr_C9ZJU1_
HLA-DQA10501-DQB10201	626	STDNKMLFQQTFSHP	KMLFQQTFS	4	0.1984	5844.7	65.00	0.34	tr_C9ZJU1_
HLA-DQA10501-DQB10201	627	AVLLDCERKAYEILE	ERKAYEILE	6	0.1975	5899.4	65.00	0.50	tr_C9ZJU1_
HLA-DQA10501-DQB10201	628	CNGVESIHVRTLVFN	SIHVRTLVF	5	0.1969	5937.7	65.00	0.34	tr_C9ZJU1_
HLA-DQA10501-DQB10201	629	TSFWKLMCSSCSIPQ	WKLMCSSCS	3	0.1968	5948.8	65.00	0.30	tr_C9ZJU1_
HLA-DQA10501-DQB10201	630	GITSFWKLMCSSCSI	WKLMCSSCS	5	0.1964	5969.2	65.00	0.25	tr_C9ZJU1_
HLA-DQA10501-DQB10201	631	AFATASERNDRYEQC	TASERNDRY	3	0.1964	5974.2	65.00	0.25	tr_C9ZJU1_
HLA-DQA10501-DQB10201	632	RDSTIIIVAHRLATL	IVAHRLATL	6	0.1958	6010.1	65.00	0.41	tr_C9ZJU1_
HLA-DQA10501-DQB10201	633	GVAPPSLDGPWSVEF	LDGPWSVEF	6	0.1956	6022.2	65.00	0.32	tr_C9ZJU1_
HLA-DQA10501-DQB10201	634	CAVSVRRLGWIKDVI	RLGWIKDVI	6	0.1952	6046.5	65.00	0.52	tr_C9ZJU1_
HLA-DQA10501-DQB10201	635	FSPEVILKCILGMHA	LKCILGMHA	6	0.1948	6073.9	65.00	0.43	tr_C9ZJU1_
HLA-DQA10501-DQB10201	636	RCVSQALWQASGSVM	LWQASGSVM	6	0.1947	6083.2	65.00	0.54	tr_C9ZJU1_
HLA-DQA10501-DQB10201	637	HLAFSGSSLGCYQVL	SSLGCYQVL	6	0.1946	6088.6	65.00	0.25	tr_C9ZJU1_
HLA-DQA10501-DQB10201	638	LNCNGVESIHVRTLV	ESIHVRTLV	6	0.1944	6100.8	65.00	0.25	tr_C9ZJU1_
HLA-DQA10501-DQB10201	639	FDTLRLREGNNLLRD	REGNNLLRD	6	0.1942	6118.0	65.00	0.27	tr_C9ZJU1_
HLA-DQA10501-DQB10201	640	MLSSTDNKMLFQQTF	NKMLFQQTF	6	0.1938	6141.4	65.00	0.51	tr_C9ZJU1_
HLA-DQA10501-DQB10201	641	VAEVGSWETLSRTGP	EVGSWETLS	2	0.1936	6153.7	65.00	0.29	tr_C9ZJU1_
HLA-DQA10501-DQB10201	642	NLLRDSTIIIVAHRL	TIIIVAHRL	6	0.1936	6158.3	65.00	0.34	tr_C9ZJU1_
HLA-DQA10501-DQB10201	643	DRHPYVLRRVSFVVN	YVLRRVSFV	4	0.1920	6263.3	65.00	0.29	tr_C9ZJU1_
HLA-DQA10501-DQB10201	644	LRLLNRTYAPTEGDI	RTYAPTEGD	5	0.1917	6280.2	65.00	0.26	tr_C9ZJU1_
HLA-DQA10501-DQB10201	645	RFIEDLSIGGNIALG	DLSIGGNIA	4	0.1916	6287.7	65.00	0.40	tr_C9ZJU1_
HLA-DQA10501-DQB10201	646	GVESIHVRTLVFNHN	SIHVRTLVF	3	0.1911	6326.7	65.00	0.25	tr_C9ZJU1_
HLA-DQA10501-DQB10201	647	QTEIVNQLRSIQHWC	EIVNQLRSI	2	0.1907	6352.7	65.00	0.31	tr_C9ZJU1_
HLA-DQA10501-DQB10201	648	LYMESQFTKSQLASV	FTKSQLASV	6	0.1905	6363.8	65.00	0.56	tr_C9ZJU1_
HLA-DQA10501-DQB10201	649	EGEIFDTLRLREGNN	IFDTLRLRE	3	0.1900	6401.0	65.00	0.29	tr_C9ZJU1_
HLA-DQA10501-DQB10201	650	EVSFRYSDRHPYVLR	RYSDRHPYV	4	0.1897	6421.0	65.00	0.25	tr_C9ZJU1_
HLA-DQA10501-DQB10201	651	WIKDVIQSSPRAATA	WIKDVIQSS	0	0.1895	6437.6	70.00	0.40	tr_C9ZJU1_
HLA-DQA10501-DQB10201	652	FIEDLSIGGNIALGN	DLSIGGNIA	3	0.1894	6442.0	70.00	0.33	tr_C9ZJU1_
HLA-DQA10501-DQB10201	653	EGNNLLRDSTIIIVA	RDSTIIIVA	6	0.1890	6471.3	70.00	0.25	tr_C9ZJU1_
HLA-DQA10501-DQB10201	654	REGNNLLRDSTIIIV	LRDSTIIIV	6	0.1888	6483.8	70.00	0.40	tr_C9ZJU1_
HLA-DQA10501-DQB10201	655	SDRHPYVLRRVSFVV	YVLRRVSFV	5	0.1872	6594.5	70.00	0.34	tr_C9ZJU1_
HLA-DQA10501-DQB10201	656	ELYLGLITAYNRKRS	YLGLITAYN	2	0.1872	6594.9	70.00	0.38	tr_C9ZJU1_
HLA-DQA10501-DQB10201	657	TDNKMLFQQTFSHPS	KMLFQQTFS	3	0.1872	6595.7	70.00	0.34	tr_C9ZJU1_
HLA-DQA10501-DQB10201	658	DTLRLREGNNLLRDS	RLREGNNLL	3	0.1868	6623.1	70.00	0.22	tr_C9ZJU1_

HLA-DQA10501-DQB10201	659	TNIATSTYAGNGEEN	TYAGNGEEN	6	0.1868	6623.7	70.00	0.41	tr_C9ZJU1_
HLA-DQA10501-DQB10201	660	LQSGITSFWKLMCSS	LQSGITSFW	0	0.1860	6686.0	70.00	0.37	tr_C9ZJU1_
HLA-DQA10501-DQB10201	661	LPSVFAVHCGLMKNS	SVFAVHCGL	2	0.1857	6702.7	70.00	0.28	tr_C9ZJU1_
HLA-DQA10501-DQB10201	662	GEFLGIAGYSGSGKT	EFLGIAGYS	1	0.1856	6710.2	70.00	0.37	tr_C9ZJU1_
HLA-DQA10501-DQB10201	663	LATLKHADKIVVLNE	ADKIVVLNE	6	0.1853	6733.9	70.00	0.35	tr_C9ZJU1_
HLA-DQA10501-DQB10201	664	FSRMRQSQQLPHFVK	SRMRQSQQL	1	0.1852	6741.3	70.00	0.28	tr_C9ZJU1_
HLA-DQA10501-DQB10201	665	SSTDNKMLFQQTFSH	KMLFQQTFS	5	0.1849	6761.5	70.00	0.38	tr_C9ZJU1_
HLA-DQA10501-DQB10201	666	VESIHVRTLVFNHNI	SIHVRTLVF	2	0.1849	6764.8	70.00	0.26	tr_C9ZJU1_
HLA-DQA10501-DQB10201	667	TEIVNQLRSIQHWCP	EIVNQLRSI	1	0.1845	6789.3	70.00	0.28	tr_C9ZJU1_
HLA-DQA10501-DQB10201	668	PSRHDKSSVQYGLVL	SSVQYGLVL	6	0.1841	6822.3	70.00	0.56	tr_C9ZJU1_
HLA-DQA10501-DQB10201	669	PYVLRRVSFVVNKGE	YVLRRVSFV	1	0.1840	6827.0	70.00	0.22	tr_C9ZJU1_
HLA-DQA10501-DQB10201	670	NGVESIHVRTLVFNH	SIHVRTLVF	4	0.1838	6840.6	70.00	0.34	tr_C9ZJU1_
HLA-DQA10501-DQB10201	671	PSDDWLLSRCVSQAL	LSRCVSQAL	6	0.1838	6846.0	70.00	0.40	tr_C9ZJU1_
HLA-DQA10501-DQB10201	672	GNNLLRDSTIIIVAH	LRDSTIIIV	4	0.1827	6926.1	70.00	0.22	tr_C9ZJU1_
HLA-DQA10501-DQB10201	673	ATLKHADKIVVLNEG	ADKIVVLNE	5	0.1814	7025.4	70.00	0.30	tr_C9ZJU1_
HLA-DQA10501-DQB10201	674	LRLREGNNLLRDSTI	NNLLRDSTI	6	0.1811	7048.3	70.00	0.21	tr_C9ZJU1_
HLA-DQA10501-DQB10201	675	RRHLAFSGSSLGCYQ	HLAFSGSSL	2	0.1804	7104.1	70.00	0.31	tr_C9ZJU1_
HLA-DQA10501-DQB10201	676	IEDLSIGGNIALGNL	DLSIGGNIA	2	0.1797	7151.2	70.00	0.30	tr_C9ZJU1_
HLA-DQA10501-DQB10201	677	ESIHVRTLVFNHNIM	TLVFNHNIM	6	0.1796	7159.5	70.00	0.20	tr_C9ZJU1_
HLA-DQA10501-DQB10201	678	DNKMLFQQTFSHPSR	KMLFQQTFS	2	0.1794	7180.9	70.00	0.34	tr_C9ZJU1_
HLA-DQA10501-DQB10201	679	EIVNQLRSIQHWCPL	EIVNQLRSI	0	0.1786	7238.8	70.00	0.20	tr_C9ZJU1_
HLA-DQA10501-DQB10201	680	DYDPSDDWLLSRCVS	DYDPSDDWL	0	0.1784	7255.5	70.00	0.35	tr_C9ZJU1_
HLA-DQA10501-DQB10201	681	SGITSFWKLMCSSCS	WKLMCSSCS	6	0.1777	7307.1	70.00	0.32	tr_C9ZJU1_
HLA-DQA10501-DQB10201	682	YPARMLRRRIANVWQ	LRRRIANVW	5	0.1777	7307.3	70.00	0.48	tr_C9ZJU1_
HLA-DQA10501-DQB10201	683	YMESQFTKSQLASVT	FTKSQLASV	5	0.1777	7307.4	70.00	0.57	tr_C9ZJU1_
HLA-DQA10501-DQB10201	684	RHLAFSGSSLGCYQV	FSGSSLGCY	4	0.1769	7373.6	70.00	0.28	tr_C9ZJU1_
HLA-DQA10501-DQB10201	685	HPYVLRRVSFVVNKG	YVLRRVSFV	2	0.1767	7391.7	70.00	0.26	tr_C9ZJU1_
HLA-DQA10501-DQB10201	686	RSSGIHAPLNVREFS	APLNVREFS	6	0.1766	7398.9	70.00	0.18	tr_C9ZJU1_
HLA-DQA10501-DQB10201	687	RHPYVLRRVSFVVNK	YVLRRVSFV	3	0.1763	7421.1	70.00	0.28	tr_C9ZJU1_
HLA-DQA10501-DQB10201	688	TLRLREGNNLLRDST	REGNNLLRD	4	0.1762	7432.1	70.00	0.23	tr_C9ZJU1_
HLA-DQA10501-DQB10201	689	SLYMESQFTKSQLAS	SLYMESQFT	0	0.1758	7465.8	70.00	0.38	tr_C9ZJU1_
HLA-DQA10501-DQB10201	690	ALSASRALNFVQKRS	LSASRALNF	1	0.1755	7484.2	70.00	0.40	tr_C9ZJU1_
HLA-DQA10501-DQB10201	691	LLSRCVSQALWQASG	SRCVSQALW	2	0.1750	7524.3	70.00	0.34	tr_C9ZJU1_
HLA-DQA10501-DQB10201	692	NGVHISQYPARMLRR	HISQYPARM	3	0.1749	7533.3	70.00	0.47	tr_C9ZJU1_
HLA-DQA10501-DQB10201	693	LRRHLAFSGSSLGCY	HLAFSGSSL	3	0.1748	7540.7	70.00	0.34	tr_C9ZJU1_
HLA-DQA10501-DQB10201	694	LLRDSTIIIVAHRLA	TIIIVAHRL	5	0.1745	7567.3	70.00	0.30	tr_C9ZJU1_
HLA-DQA10501-DQB10201	695	LREGNNLLRDSTIII	LLRDSTIII	6	0.1745	7568.2	70.00	0.38	tr_C9ZJU1_
HLA-DQA10501-DQB10201	696	LASVTNIATSTYAGN	VTNIATSTY	3	0.1745	7572.1	70.00	0.33	tr_C9ZJU1_
HLA-DQA10501-DQB10201	697	RPIYAAEIGKSLNGD	YAAEIGKSL	3	0.1735	7650.0	70.00	0.31	tr_C9ZJU1_
HLA-DQA10501-DQB10201	698	GEIFDTLRLREGNNL	IFDTLRLRE	2	0.1728	7710.9	70.00	0.34	tr_C9ZJU1_
HLA-DQA10501-DQB10201	699	AEIGKSLNGDVSPKL	LNGDVSPKL	6	0.1727	7716.2	70.00	0.46	tr_C9ZJU1_
HLA-DQA10501-DQB10201	700	KYSIARYPPAAQADV	YPPAAQADV	6	0.1724	7740.7	70.00	0.31	tr_C9ZJU1_
HLA-DQA10501-DQB10201	701	EIFDTLRLREGNNLL	LRLREGNNL	5	0.1721	7770.9	70.00	0.30	tr_C9ZJU1_
HLA-DQA10501-DQB10201	702	LLRLLNRTYAPTEGD	RTYAPTEGD	6	0.1713	7832.9	75.00	0.31	tr_C9ZJU1_
HLA-DQA10501-DQB10201	703	QVLEGLQSVQRKIDR	LEGLQSVQR	2	0.1712	7847.5	75.00	0.50	tr_C9ZJU1_
HLA-DQA10501-DQB10201	704	DSTIIIVAHRLATLK	IVAHRLATL	5	0.1699	7953.8	75.00	0.40	tr_C9ZJU1_
HLA-DQA10501-DQB10201	705	YEILEMLTRATIKTD	EILEMLTRA	1	0.1697	7974.5	75.00	0.27	tr_C9ZJU1_
HLA-DQA10501-DQB10201	706	VERLCIARAFVKAGS	ERLCIARAF	1	0.1696	7981.0	75.00	0.25	tr_C9ZJU1_
HLA-DQA10501-DQB10201	707	NNLLRDSTIIIVAHR	LRDSTIIIV	3	0.1695	7986.9	75.00	0.22	tr_C9ZJU1_
HLA-DQA10501-DQB10201	708	RLREGNNLLRDSTII	NNLLRDSTI	5	0.1693	8007.1	75.00	0.23	tr_C9ZJU1_

HLA-DQA10501-DQB10201	709	FPVKNSLTSSPLTLL	SLTSSPLTL	5	0.1693	8010.2	75.00	0.47	tr_C9ZJU1_
HLA-DQA10501-DQB10201	710	PAVLLDCERKAYEIL	PAVLLDCER	0	0.1688	8051.2	75.00	0.37	tr_C9ZJU1_
HLA-DQA10501-DQB10201	711	KRSSGIHAPLNVREF	SGIHAPLNV	3	0.1683	8095.4	75.00	0.28	tr_C9ZJU1_
HLA-DQA10501-DQB10201	712	SERNDRYEQCGRDVL	RYEQCGRDV	5	0.1682	8104.9	75.00	0.35	tr_C9ZJU1_
HLA-DQA10501-DQB10201	713	PHSCFSRMRQSQQLP	SRMRQSQQL	5	0.1677	8143.8	75.00	0.56	tr_C9ZJU1_
HLA-DQA10501-DQB10201	714	QRKIDRPVRGVAPPS	PVRGVAPPS	6	0.1674	8175.5	75.00	0.47	tr_C9ZJU1_
HLA-DQA10501-DQB10201	715	MESQFTKSQLASVTN	FTKSQLASV	4	0.1665	8250.9	75.00	0.57	tr_C9ZJU1_
HLA-DQA10501-DQB10201	716	SSGIHAPLNVREFSG	SGIHAPLNV	1	0.1645	8434.8	75.00	0.25	tr_C9ZJU1_
HLA-DQA10501-DQB10201	717	VLFSPEVILKCILGM	PEVILKCIL	4	0.1642	8456.7	75.00	0.23	tr_C9ZJU1_
HLA-DQA10501-DQB10201	718	IFDTLRLREGNNLLR	LRLREGNNL	4	0.1640	8475.3	75.00	0.33	tr_C9ZJU1_
HLA-DQA10501-DQB10201	719	RGVAPPSLDGPWSVE	PPSLDGPWS	4	0.1638	8494.1	75.00	0.31	tr_C9ZJU1_
HLA-DQA10501-DQB10201	720	HSCFSRMRQSQQLPH	SRMRQSQQL	4	0.1626	8606.7	75.00	0.48	tr_C9ZJU1_
HLA-DQA10501-DQB10201	721	SIHVRTLVFNHNIMQ	TLVFNHNIM	5	0.1622	8642.3	75.00	0.21	tr_C9ZJU1_
HLA-DQA10501-DQB10201	722	EIGKSLNGDVSPKLS	LNGDVSPKL	5	0.1614	8717.0	75.00	0.46	tr_C9ZJU1_
HLA-DQA10501-DQB10201	723	VSFRYSDRHPYVLRR	RYSDRHPYV	3	0.1610	8761.1	75.00	0.33	tr_C9ZJU1_
HLA-DQA10501-DQB10201	724	YSDRHPYVLRRVSFV	YVLRRVSFV	6	0.1608	8782.0	75.00	0.46	tr_C9ZJU1_
HLA-DQA10501-DQB10201	725	QSGITSFWKLMCSSC	TSFWKLMCS	4	0.1607	8789.2	75.00	0.23	tr_C9ZJU1_
HLA-DQA10501-DQB10201	726	YAGNGEENQVSPTCS	YAGNGEENQ	0	0.1597	8883.5	75.00	0.34	tr_C9ZJU1_
HLA-DQA10501-DQB10201	727	SFPVKNSLTSSPLTL	SLTSSPLTL	6	0.1597	8887.1	75.00	0.52	tr_C9ZJU1_
HLA-DQA10501-DQB10201	728	HRLATLKHADKIVVL	KHADKIVVL	6	0.1586	8984.6	75.00	0.56	tr_C9ZJU1_
HLA-DQA10501-DQB10201	729	LRDSTIIIVAHRLAT	TIIIVAHRL	4	0.1586	8986.2	75.00	0.34	tr_C9ZJU1_
HLA-DQA10501-DQB10201	730	IGKSLNGDVSPKLSP	LNGDVSPKL	4	0.1586	8990.2	75.00	0.55	tr_C9ZJU1_
HLA-DQA10501-DQB10201	731	LSRCVSQALWQASGS	SRCVSQALW	1	0.1579	9053.9	75.00	0.32	tr_C9ZJU1_
HLA-DQA10501-DQB10201	732	PSVFAVHCGLMKNSH	SVFAVHCGL	1	0.1578	9066.4	75.00	0.33	tr_C9ZJU1_
HLA-DQA10501-DQB10201	733	GVHISQYPARMLRRR	HISQYPARM	2	0.1573	9118.1	75.00	0.56	tr_C9ZJU1_
HLA-DQA10501-DQB10201	734	YAAEIGKSLNGDVSP	GKSLNGDVS	5	0.1571	9133.9	75.00	0.31	tr_C9ZJU1_
HLA-DQA10501-DQB10201	735	SFRYSDRHPYVLRRV	RYSDRHPYV	2	0.1568	9168.4	75.00	0.29	tr_C9ZJU1_
HLA-DQA10501-DQB10201	736	GKSLNGDVSPKLSPF	LNGDVSPKL	3	0.1562	9221.0	75.00	0.57	tr_C9ZJU1_
HLA-DQA10501-DQB10201	737	FATASERNDRYEQCG	TASERNDRY	2	0.1561	9232.9	75.00	0.38	tr_C9ZJU1_
HLA-DQA10501-DQB10201	738	ERNDRYEQCGRDVLH	RYEQCGRDV	4	0.1559	9258.1	75.00	0.34	tr_C9ZJU1_
HLA-DQA10501-DQB10201	739	NGEENQVSPTCSFPV	NQVSPTCSF	4	0.1559	9259.2	75.00	0.24	tr_C9ZJU1_
HLA-DQA10501-DQB10201	740	ERLCIARAFVKAGSA	RAFVKAGSA	6	0.1555	9298.4	75.00	0.34	tr_C9ZJU1_
HLA-DQA10501-DQB10201	741	RLATLKHADKIVVLN	KHADKIVVL	5	0.1555	9298.8	75.00	0.50	tr_C9ZJU1_
HLA-DQA10501-DQB10201	742	LYLGLITAYNRKRSL	YLGLITAYN	1	0.1540	9447.4	75.00	0.47	tr_C9ZJU1_
HLA-DQA10501-DQB10201	743	STIIIVAHRLATLKH	IVAHRLATL	4	0.1534	9505.9	80.00	0.38	tr_C9ZJU1_
HLA-DQA10501-DQB10201	744	NKMLFQQTFSHPSRH	KMLFQQTFS	1	0.1528	9567.9	80.00	0.29	tr_C9ZJU1_
HLA-DQA10501-DQB10201	745	EFLGIAGYSGSGKTT	FLGIAGYSG	1	0.1528	9572.5	80.00	0.50	tr_C9ZJU1_
HLA-DQA10501-DQB10201	746	SRCVSQALWQASGSV	ALWQASGSV	6	0.1527	9578.9	80.00	0.31	tr_C9ZJU1_
HLA-DQA10501-DQB10201	747	LRGKYSIARYPPAAQ	RGKYSIARY	1	0.1527	9582.1	80.00	0.26	tr_C9ZJU1_
HLA-DQA10501-DQB10201	748	LLRGKYSIARYPPAA	RGKYSIARY	2	0.1526	9590.6	80.00	0.30	tr_C9ZJU1_
HLA-DQA10501-DQB10201	749	SEAQVDEAHLRRHLA	EAQVDEAHL	1	0.1520	9658.7	80.00	0.48	tr_C9ZJU1_
HLA-DQA10501-DQB10201	750	SLNGDVSPKLSPFRF	LNGDVSPKL	1	0.1517	9685.0	80.00	0.47	tr_C9ZJU1_
HLA-DQA10501-DQB10201	751	DPSDDWLLSRCVSQA	WLLSRCVSQ	5	0.1515	9706.7	80.00	0.28	tr_C9ZJU1_
HLA-DQA10501-DQB10201	752	FVQKRSSGIHAPLNV	SGIHAPLNV	6	0.1511	9754.2	80.00	0.33	tr_C9ZJU1_
HLA-DQA10501-DQB10201	753	IYAAEIGKSLNGDVS	YAAEIGKSL	1	0.1509	9766.2	80.00	0.35	tr_C9ZJU1_
HLA-DQA10501-DQB10201	754	ASVTNIATSTYAGNG	VTNIATSTY	2	0.1508	9776.3	80.00	0.40	tr_C9ZJU1_
HLA-DQA10501-DQB10201	755	MLLRGKYSIARYPPA	RGKYSIARY	3	0.1507	9787.9	80.00	0.34	tr_C9ZJU1_
HLA-DQA10501-DQB10201	756	VTNIATSTYAGNGEE	TSTYAGNGE	5	0.1496	9912.3	80.00	0.29	tr_C9ZJU1_
HLA-DQA10501-DQB10201	757	QKRSSGIHAPLNVRE	SGIHAPLNV	4	0.1495	9915.2	80.00	0.28	tr_C9ZJU1_
HLA-DQA10501-DQB10201	758	RLCIARAFVKAGSAV	RAFVKAGSA	5	0.1490	9970.7	80.00	0.34	tr_C9ZJU1_

HLA-DQA10501-DQB10201	759	GKYSIARYPPAAQAD	IARYPPAAQ	4	0.1489	9984.2	80.00	0.21	tr_C9ZJU1_
HLA-DQA10501-DQB10201	760	GPHSCFSRMRQSQQL	SRMRQSQQL	6	0.1488	9992.7	80.00	0.67	tr_C9ZJU1_
HLA-DQA10501-DQB10201	761	PVKNSLTSSPLTLLR	SLTSSPLTL	4	0.1487	10010.0	80.00	0.47	tr_C9ZJU1_
HLA-DQA10501-DQB10201	762	EENQVSPTCSFPVKN	NQVSPTCSF	2	0.1484	10042.8	80.00	0.19	tr_C9ZJU1_
HLA-DQA10501-DQB10201	763	AEVGSWETLSRTGPH	EVGSWETLS	1	0.1481	10066.0	80.00	0.34	tr_C9ZJU1_
HLA-DQA10501-DQB10201	764	SVTNIATSTYAGNGE	VTNIATSTY	1	0.1481	10074.6	80.00	0.35	tr_C9ZJU1_
HLA-DQA10501-DQB10201	765	LSASRALNFVQKRSS	SASRALNFV	1	0.1476	10121.1	80.00	0.28	tr_C9ZJU1_
HLA-DQA10501-DQB10201	766	NDRYEQCGRDVLHGL	RYEQCGRDV	2	0.1473	10155.4	80.00	0.28	tr_C9ZJU1_
HLA-DQA10501-DQB10201	767	RGKYSIARYPPAAQA	YSIARYPPA	3	0.1471	10184.8	80.00	0.25	tr_C9ZJU1_
HLA-DQA10501-DQB10201	768	VSPKLSPFRFIIKQL	PFRFIIKQL	6	0.1467	10222.9	80.00	0.57	tr_C9ZJU1_
HLA-DQA10501-DQB10201	769	SKQTECKDDASIAIF	KDDASIAIF	6	0.1458	10321.5	80.00	0.69	tr_C9ZJU1_
HLA-DQA10501-DQB10201	770	VHISQYPARMLRRRI	HISQYPARM	1	0.1458	10328.8	80.00	0.52	tr_C9ZJU1_
HLA-DQA10501-DQB10201	771	PIYAAEIGKSLNGDV	YAAEIGKSL	2	0.1457	10335.0	80.00	0.40	tr_C9ZJU1_
HLA-DQA10501-DQB10201	772	EEVLALCMLLRGKYS	EEVLALCML	0	0.1457	10340.7	80.00	0.37	tr_C9ZJU1_
HLA-DQA10501-DQB10201	773	QYPARMLRRRIANVW	LRRRIANVW	6	0.1455	10361.1	80.00	0.75	tr_C9ZJU1_
HLA-DQA10501-DQB10201	774	SRMRQSQQLPHFVKK	QSQQLPHFV	4	0.1454	10370.2	80.00	0.28	tr_C9ZJU1_
HLA-DQA10501-DQB10201	775	ASERNDRYEQCGRDV	RYEQCGRDV	6	0.1445	10467.0	80.00	0.46	tr_C9ZJU1_
HLA-DQA10501-DQB10201	776	EAHLRRHLAFSGSSL	HLAFSGSSL	6	0.1437	10565.9	80.00	0.65	tr_C9ZJU1_
HLA-DQA10501-DQB10201	777	KSLNGDVSPKLSPFR	LNGDVSPKL	2	0.1433	10603.8	80.00	0.63	tr_C9ZJU1_
HLA-DQA10501-DQB10201	778	YDPSDDWLLSRCVSQ	WLLSRCVSQ	6	0.1428	10663.2	80.00	0.32	tr_C9ZJU1_
HLA-DQA10501-DQB10201	779	SGIHAPLNVREFSGG	APLNVREFS	4	0.1423	10725.9	80.00	0.21	tr_C9ZJU1_
HLA-DQA10501-DQB10201	780	RNDRYEQCGRDVLHG	RYEQCGRDV	3	0.1417	10795.1	80.00	0.35	tr_C9ZJU1_
HLA-DQA10501-DQB10201	781	LCIARAFVKAGSAVG	RAFVKAGSA	4	0.1415	10819.4	80.00	0.34	tr_C9ZJU1_
HLA-DQA10501-DQB10201	782	GEENQVSPTCSFPVK	NQVSPTCSF	3	0.1412	10846.8	80.00	0.22	tr_C9ZJU1_
HLA-DQA10501-DQB10201	783	HLRRHLAFSGSSLGC	HLAFSGSSL	4	0.1412	10856.0	80.00	0.49	tr_C9ZJU1_
HLA-DQA10501-DQB10201	784	GNGEENQVSPTCSFP	NQVSPTCSF	5	0.1408	10892.8	80.00	0.31	tr_C9ZJU1_
HLA-DQA10501-DQB10201	785	SSPRAATASPSTCVW	RAATASPST	3	0.1406	10925.5	80.00	0.28	tr_C9ZJU1_
HLA-DQA10501-DQB10201	786	GIKCMLSSTDNKMLF	MLSSTDNKM	4	0.1401	10978.7	80.00	0.54	tr_C9ZJU1_
HLA-DQA10501-DQB10201	787	LFSPEVILKCILGMH	PEVILKCIL	3	0.1397	11030.7	80.00	0.31	tr_C9ZJU1_
HLA-DQA10501-DQB10201	788	TIIIVAHRLATLKHA	IVAHRLATL	3	0.1392	11087.3	80.00	0.42	tr_C9ZJU1_
HLA-DQA10501-DQB10201	789	AGNGEENQVSPTCSF	NQVSPTCSF	6	0.1388	11137.2	80.00	0.30	tr_C9ZJU1_
HLA-DQA10501-DQB10201	790	VLEGLQSVQRKIDRP	LEGLQSVQR	1	0.1386	11159.4	80.00	0.51	tr_C9ZJU1_
HLA-DQA10501-DQB10201	791	SVFAVHCGLMKNSHP	SVFAVHCGL	0	0.1386	11161.1	80.00	0.36	tr_C9ZJU1_
HLA-DQA10501-DQB10201	792	EILEMLTRATIKTDG	EILEMLTRA	0	0.1382	11204.3	80.00	0.22	tr_C9ZJU1_
HLA-DQA10501-DQB10201	793	IKCMLSSTDNKMLFQ	MLSSTDNKM	3	0.1374	11301.5	80.00	0.50	tr_C9ZJU1_
HLA-DQA10501-DQB10201	794	TLLRLLNRTYAPTEG	NRTYAPTEG	6	0.1364	11431.3	80.00	0.31	tr_C9ZJU1_
HLA-DQA10501-DQB10201	795	CMLSSTDNKMLFQQT	MLSSTDNKM	1	0.1361	11468.1	80.00	0.41	tr_C9ZJU1_
HLA-DQA10501-DQB10201	796	GLMKNSHPDHLLVCD	MKNSHPDHL	2	0.1359	11489.1	80.00	0.46	tr_C9ZJU1_
HLA-DQA10501-DQB10201	797	TCAVSVRRLGWIKDV	RRLGWIKDV	6	0.1347	11640.3	80.00	0.29	tr_C9ZJU1_
HLA-DQA10501-DQB10201	798	CMLLRGKYSIARYPP	RGKYSIARY	4	0.1347	11644.7	80.00	0.52	tr_C9ZJU1_
HLA-DQA10501-DQB10201	799	VIQSSPRAATASPST	PRAATASPS	5	0.1343	11697.1	85.00	0.38	tr_C9ZJU1_
HLA-DQA10501-DQB10201	800	DDGIKCMLSSTDNKM	MLSSTDNKM	6	0.1334	11800.8	85.00	0.51	tr_C9ZJU1_
HLA-DQA10501-DQB10201	801	FRYSDRHPYVLRRVS	RYSDRHPYV	1	0.1334	11801.0	85.00	0.31	tr_C9ZJU1_
HLA-DQA10501-DQB10201	802	VQKRSSGIHAPLNVR	SGIHAPLNV	5	0.1334	11811.1	85.00	0.36	tr_C9ZJU1_
HLA-DQA10501-DQB10201	803	DGIKCMLSSTDNKML	MLSSTDNKM	5	0.1325	11927.6	85.00	0.55	tr_C9ZJU1_
HLA-DQA10501-DQB10201	804	LNGDVSPKLSPFRFI	SPKLSPFRF	5	0.1323	11943.6	85.00	0.34	tr_C9ZJU1_
HLA-DQA10501-DQB10201	805	AHLRRHLAFSGSSLG	HLAFSGSSL	5	0.1321	11969.1	85.00	0.61	tr_C9ZJU1_
HLA-DQA10501-DQB10201	806	VKNSLTSSPLTLLRK	SLTSSPLTL	3	0.1315	12046.8	85.00	0.44	tr_C9ZJU1_
HLA-DQA10501-DQB10201	807	SPRAATASPSTCVWQ	RAATASPST	2	0.1313	12076.6	85.00	0.28	tr_C9ZJU1_
HLA-DQA10501-DQB10201	808	QSSPRAATASPSTCV	PRAATASPS	3	0.1306	12168.9	85.00	0.34	tr_C9ZJU1_

HLA-DQA10501-DQB10201	809	TASPSTCVWQCRQDY	CVWQCRQDY	6	0.1305	12185.0	85.00	0.29	tr_C9ZJU1_
HLA-DQA10501-DQB10201	810	ATASERNDRYEQCGR	TASERNDRY	1	0.1301	12237.8	85.00	0.39	tr_C9ZJU1_
HLA-DQA10501-DQB10201	811	DVIQSSPRAATASPS	PRAATASPS	6	0.1299	12260.6	85.00	0.41	tr_C9ZJU1_
HLA-DQA10501-DQB10201	812	AAEIGKSLNGDVSPK	GKSLNGDVS	4	0.1298	12271.8	85.00	0.36	tr_C9ZJU1_
HLA-DQA10501-DQB10201	813	GGHTCAVSVRRLGWI	VSVRRLGWI	6	0.1294	12332.3	85.00	0.20	tr_C9ZJU1_
HLA-DQA10501-DQB10201	814	RYSDRHPYVLRRVSF	PYVLRRVSF	6	0.1286	12439.1	85.00	0.34	tr_C9ZJU1_
HLA-DQA10501-DQB10201	815	KCMLSSTDNKMLFQQ	MLSSTDNKM	2	0.1279	12524.5	85.00	0.51	tr_C9ZJU1_
HLA-DQA10501-DQB10201	816	IIIVAHRLATLKHAD	IVAHRLATL	2	0.1278	12547.3	85.00	0.44	tr_C9ZJU1_
HLA-DQA10501-DQB10201	817	IQSSPRAATASPSTC	PRAATASPS	4	0.1274	12602.5	85.00	0.40	tr_C9ZJU1_
HLA-DQA10501-DQB10201	818	AYNRKRSLQSGITSF	SLQSGITSF	6	0.1256	12843.1	85.00	0.47	tr_C9ZJU1_
HLA-DQA10501-DQB10201	819	TTLLRLLNRTYAPTE	LNRTYAPTE	6	0.1252	12908.1	85.00	0.29	tr_C9ZJU1_
HLA-DQA10501-DQB10201	820	HTCAVSVRRLGWIKD	VRRLGWIKD	6	0.1247	12965.5	85.00	0.22	tr_C9ZJU1_
HLA-DQA10501-DQB10201	821	ENQVSPTCSFPVKNS	PTCSFPVKN	5	0.1243	13034.7	85.00	0.23	tr_C9ZJU1_
HLA-DQA10501-DQB10201	822	EAQVDEAHLRRHLAF	EAQVDEAHL	0	0.1233	13170.3	85.00	0.32	tr_C9ZJU1_
HLA-DQA10501-DQB10201	823	KMLFQQTFSHPSRHD	KMLFQQTFS	0	0.1232	13189.6	85.00	0.31	tr_C9ZJU1_
HLA-DQA10501-DQB10201	824	AGGHTCAVSVRRLGW	HTCAVSVRR	3	0.1221	13335.2	85.00	0.26	tr_C9ZJU1_
HLA-DQA10501-DQB10201	825	GIHAPLNVREFSGGE	APLNVREFS	3	0.1220	13352.4	85.00	0.25	tr_C9ZJU1_
HLA-DQA10501-DQB10201	826	LCMLLRGKYSIARYP	RGKYSIARY	5	0.1206	13560.9	85.00	0.61	tr_C9ZJU1_
HLA-DQA10501-DQB10201	827	TASERNDRYEQCGRD	ERNDRYEQC	3	0.1204	13594.1	85.00	0.28	tr_C9ZJU1_
HLA-DQA10501-DQB10201	828	PRAATASPSTCVWQC	RAATASPST	1	0.1197	13698.9	85.00	0.27	tr_C9ZJU1_
HLA-DQA10501-DQB10201	829	LQAGGHTCAVSVRRL	HTCAVSVRR	5	0.1195	13729.3	85.00	0.28	tr_C9ZJU1_
HLA-DQA10501-DQB10201	830	VHCGLMKNSHPDHLL	MKNSHPDHL	5	0.1191	13776.4	85.00	0.60	tr_C9ZJU1_
HLA-DQA10501-DQB10201	831	GKTTLLRLLNRTYAP	LRLLNRTYA	5	0.1189	13811.9	85.00	0.28	tr_C9ZJU1_
HLA-DQA10501-DQB10201	832	NFVQKRSSGIHAPLN	SSGIHAPLN	6	0.1185	13874.0	85.00	0.37	tr_C9ZJU1_
HLA-DQA10501-DQB10201	833	HCGLMKNSHPDHLLV	MKNSHPDHL	4	0.1180	13953.7	85.00	0.60	tr_C9ZJU1_
HLA-DQA10501-DQB10201	834	KNSLTSSPLTLLRKQ	SLTSSPLTL	2	0.1179	13965.8	85.00	0.47	tr_C9ZJU1_
HLA-DQA10501-DQB10201	835	CGLMKNSHPDHLLVC	MKNSHPDHL	3	0.1175	14020.0	85.00	0.56	tr_C9ZJU1_
HLA-DQA10501-DQB10201	836	VQRKIDRPVRGVAPP	DRPVRGVAP	5	0.1166	14160.1	85.00	0.21	tr_C9ZJU1_
HLA-DQA10501-DQB10201	837	EVGSWETLSRTGPHS	EVGSWETLS	0	0.1156	14314.7	85.00	0.29	tr_C9ZJU1_
HLA-DQA10501-DQB10201	838	ALCMLLRGKYSIARY	RGKYSIARY	6	0.1154	14345.9	85.00	0.62	tr_C9ZJU1_
HLA-DQA10501-DQB10201	839	HPSRHDKSSVQYGLV	KSSVQYGLV	6	0.1153	14363.8	85.00	0.46	tr_C9ZJU1_
HLA-DQA10501-DQB10201	840	IIVAHRLATLKHADK	IVAHRLATL	1	0.1151	14393.6	85.00	0.43	tr_C9ZJU1_
HLA-DQA10501-DQB10201	841	GHTCAVSVRRLGWIK	TCAVSVRRL	2	0.1151	14399.1	85.00	0.22	tr_C9ZJU1_
HLA-DQA10501-DQB10201	842	ILEMLTRATIKTDGH	EMLTRATIK	2	0.1147	14456.7	85.00	0.28	tr_C9ZJU1_
HLA-DQA10501-DQB10201	843	IKDVIQSSPRAATAS	DVIQSSPRA	2	0.1146	14470.9	85.00	0.36	tr_C9ZJU1_
HLA-DQA10501-DQB10201	844	HISQYPARMLRRRIA	HISQYPARM	0	0.1143	14522.1	85.00	0.42	tr_C9ZJU1_
HLA-DQA10501-DQB10201	845	KTTLLRLLNRTYAPT	LRLLNRTYA	4	0.1131	14706.1	90.00	0.27	tr_C9ZJU1_
HLA-DQA10501-DQB10201	846	NGDVSPKLSPFRFII	SPKLSPFRF	4	0.1104	15150.4	90.00	0.47	tr_C9ZJU1_
HLA-DQA10501-DQB10201	847	AVHCGLMKNSHPDHL	MKNSHPDHL	6	0.1103	15160.6	90.00	0.63	tr_C9ZJU1_
HLA-DQA10501-DQB10201	848	AATASPSTCVWQCRQ	STCVWQCRQ	6	0.1098	15249.3	90.00	0.27	tr_C9ZJU1_
HLA-DQA10501-DQB10201	849	CSFPVKNSLTSSPLT	KNSLTSSPL	5	0.1093	15320.5	90.00	0.34	tr_C9ZJU1_
HLA-DQA10501-DQB10201	850	SASRALNFVQKRSSG	SASRALNFV	0	0.1084	15470.3	90.00	0.26	tr_C9ZJU1_
HLA-DQA10501-DQB10201	851	LEMLTRATIKTDGHD	EMLTRATIK	1	0.1080	15542.6	90.00	0.23	tr_C9ZJU1_
HLA-DQA10501-DQB10201	852	LEGLQSVQRKIDRPV	LEGLQSVQR	0	0.1078	15574.6	90.00	0.41	tr_C9ZJU1_
HLA-DQA10501-DQB10201	853	QAGGHTCAVSVRRLG	HTCAVSVRR	4	0.1073	15651.3	90.00	0.27	tr_C9ZJU1_
HLA-DQA10501-DQB10201	854	YLGLITAYNRKRSLQ	YLGLITAYN	0	0.1072	15671.4	90.00	0.52	tr_C9ZJU1_
HLA-DQA10501-DQB10201	855	KDVIQSSPRAATASP	DVIQSSPRA	1	0.1072	15675.0	90.00	0.35	tr_C9ZJU1_
HLA-DQA10501-DQB10201	856	QKDDGIKCMLSSTDN	IKCMLSSTD	5	0.1069	15728.7	90.00	0.38	tr_C9ZJU1_
HLA-DQA10501-DQB10201	857	IVAHRLATLKHADKI	IVAHRLATL	0	0.1068	15746.9	90.00	0.31	tr_C9ZJU1_
HLA-DQA10501-DQB10201	858	NSLTSSPLTLLRKQK	SLTSSPLTL	1	0.1065	15793.0	90.00	0.48	tr_C9ZJU1_

HLA-DQA10501-DQB10201	859	KQKDDGIKCMLSSTD	IKCMLSSTD	6	0.1064	15807.8	90.00	0.37	tr_C9ZJU1_
HLA-DQA10501-DQB10201	860	NQVSPTCSFPVKNSL	PTCSFPVKN	4	0.1061	15868.0	90.00	0.26	tr_C9ZJU1_
HLA-DQA10501-DQB10201	861	SVQRKIDRPVRGVAP	RKIDRPVRG	3	0.1051	16031.2	90.00	0.29	tr_C9ZJU1_
HLA-DQA10501-DQB10201	862	SGKTTLLRLLNRTYA	LRLLNRTYA	6	0.1041	16204.2	90.00	0.26	tr_C9ZJU1_
HLA-DQA10501-DQB10201	863	RAATASPSTCVWQCR	TASPSTCVW	3	0.1039	16245.1	90.00	0.21	tr_C9ZJU1_
HLA-DQA10501-DQB10201	864	ATASPSTCVWQCRQD	STCVWQCRQ	5	0.1038	16269.8	90.00	0.28	tr_C9ZJU1_
HLA-DQA10501-DQB10201	865	KLQAGGHTCAVSVRR	HTCAVSVRR	6	0.1027	16450.8	90.00	0.29	tr_C9ZJU1_
HLA-DQA10501-DQB10201	866	KRKLQAGGHTCAVSV	GGHTCAVSV	6	0.1007	16826.0	90.00	0.47	tr_C9ZJU1_
HLA-DQA10501-DQB10201	867	TCSFPVKNSLTSSPL	KNSLTSSPL	6	0.0993	17080.8	90.00	0.48	tr_C9ZJU1_
HLA-DQA10501-DQB10201	868	FLGIAGYSGSGKTTL	FLGIAGYSG	0	0.0990	17130.2	90.00	0.49	tr_C9ZJU1_
HLA-DQA10501-DQB10201	869	VFAVHCGLMKNSHPD	FAVHCGLMK	1	0.0990	17139.7	90.00	0.38	tr_C9ZJU1_
HLA-DQA10501-DQB10201	870	EMLTRATIKTDGHDS	RATIKTDGH	4	0.0985	17229.3	90.00	0.25	tr_C9ZJU1_
HLA-DQA10501-DQB10201	871	EVLALCMLLRGKYSI	EVLALCMLL	0	0.0982	17272.2	90.00	0.32	tr_C9ZJU1_
HLA-DQA10501-DQB10201	872	KDDGIKCMLSSTDNK	IKCMLSSTD	4	0.0976	17396.6	90.00	0.40	tr_C9ZJU1_
HLA-DQA10501-DQB10201	873	GDVSPKLSPFRFIIK	SPKLSPFRF	3	0.0973	17454.4	90.00	0.43	tr_C9ZJU1_
HLA-DQA10501-DQB10201	874	GSGKTTLLRLLNRTY	LLRLLNRTY	6	0.0973	17456.7	90.00	0.23	tr_C9ZJU1_
HLA-DQA10501-DQB10201	875	LNFVQKRSSGIHAPL	RSSGIHAPL	6	0.0964	17622.6	90.00	0.47	tr_C9ZJU1_
HLA-DQA10501-DQB10201	876	DVSPKLSPFRFIIKQ	SPKLSPFRF	2	0.0939	18100.5	90.00	0.38	tr_C9ZJU1_
HLA-DQA10501-DQB10201	877	SGSGKTTLLRLLNRT	TLLRLLNRT	6	0.0927	18330.1	90.00	0.27	tr_C9ZJU1_
HLA-DQA10501-DQB10201	878	LQSVQRKIDRPVRGV	RKIDRPVRG	5	0.0927	18339.4	90.00	0.38	tr_C9ZJU1_
HLA-DQA10501-DQB10201	879	AHRLATLKHADKIVV	LKHADKIVV	6	0.0925	18372.2	90.00	0.28	tr_C9ZJU1_
HLA-DQA10501-DQB10201	880	MLFQQTFSHPSRHDK	LFQQTFSHP	1	0.0925	18378.7	90.00	0.26	tr_C9ZJU1_
HLA-DQA10501-DQB10201	881	QSVQRKIDRPVRGVA	RKIDRPVRG	4	0.0920	18478.6	90.00	0.35	tr_C9ZJU1_
HLA-DQA10501-DQB10201	882	YSGSGKTTLLRLLNR	TTLLRLLNR	6	0.0919	18488.4	90.00	0.22	tr_C9ZJU1_
HLA-DQA10501-DQB10201	883	RKLQAGGHTCAVSVR	GGHTCAVSV	5	0.0904	18809.8	95.00	0.37	tr_C9ZJU1_
HLA-DQA10501-DQB10201	884	GLQSVQRKIDRPVRG	RKIDRPVRG	6	0.0901	18855.1	95.00	0.37	tr_C9ZJU1_
HLA-DQA10501-DQB10201	885	QVSPTCSFPVKNSLT	PTCSFPVKN	3	0.0900	18882.4	95.00	0.29	tr_C9ZJU1_
HLA-DQA10501-DQB10201	886	AQVDEAHLRRHLAFS	AHLRRHLAF	5	0.0887	19154.7	95.00	0.29	tr_C9ZJU1_
HLA-DQA10501-DQB10201	887	GYSGSGKTTLLRLLN	SGKTTLLRL	4	0.0883	19223.4	95.00	0.35	tr_C9ZJU1_
HLA-DQA10501-DQB10201	888	VGSWETLSRTGPHSC	WETLSRTGP	3	0.0882	19254.6	95.00	0.28	tr_C9ZJU1_
HLA-DQA10501-DQB10201	889	VAHRLATLKHADKIV	ATLKHADKI	5	0.0882	19260.7	95.00	0.25	tr_C9ZJU1_
HLA-DQA10501-DQB10201	890	SLTSSPLTLLRKQKD	SLTSSPLTL	0	0.0882	19262.3	95.00	0.40	tr_C9ZJU1_
HLA-DQA10501-DQB10201	891	DEAHLRRHLAFSGSS	RHLAFSGSS	6	0.0874	19431.7	95.00	0.29	tr_C9ZJU1_
HLA-DQA10501-DQB10201	892	LLRKQKDDGIKCMLS	DDGIKCMLS	6	0.0862	19685.0	95.00	0.37	tr_C9ZJU1_
HLA-DQA10501-DQB10201	893	GSWETLSRTGPHSCF	WETLSRTGP	2	0.0856	19804.2	95.00	0.26	tr_C9ZJU1_
HLA-DQA10501-DQB10201	894	MLTRATIKTDGHDSK	RATIKTDGH	3	0.0839	20176.4	95.00	0.23	tr_C9ZJU1_
HLA-DQA10501-DQB10201	895	ASRALNFVQKRSSGI	RALNFVQKR	2	0.0831	20353.1	95.00	0.31	tr_C9ZJU1_
HLA-DQA10501-DQB10201	896	SGCVVDALKRKLQAG	SGCVVDALK	0	0.0826	20466.9	95.00	0.38	tr_C9ZJU1_
HLA-DQA10501-DQB10201	897	LRKQKDDGIKCMLSS	DDGIKCMLS	5	0.0825	20487.0	95.00	0.32	tr_C9ZJU1_
HLA-DQA10501-DQB10201	898	AGYSGSGKTTLLRLL	SGKTTLLRL	5	0.0824	20492.1	95.00	0.44	tr_C9ZJU1_
HLA-DQA10501-DQB10201	899	IAGYSGSGKTTLLRL	SGKTTLLRL	6	0.0817	20657.1	95.00	0.51	tr_C9ZJU1_
HLA-DQA10501-DQB10201	900	QVDEAHLRRHLAFSG	AHLRRHLAF	4	0.0812	20779.5	95.00	0.29	tr_C9ZJU1_
HLA-DQA10501-DQB10201	901	VDEAHLRRHLAFSGS	RRHLAFSGS	6	0.0809	20844.1	95.00	0.29	tr_C9ZJU1_
HLA-DQA10501-DQB10201	902	RKQKDDGIKCMLSST	DDGIKCMLS	4	0.0807	20888.1	95.00	0.34	tr_C9ZJU1_
HLA-DQA10501-DQB10201	903	SHPSRHDKSSVQYGL	RHDKSSVQY	4	0.0807	20889.7	95.00	0.40	tr_C9ZJU1_
HLA-DQA10501-DQB10201	904	SWETLSRTGPHSCFS	WETLSRTGP	1	0.0795	21155.4	95.00	0.24	tr_C9ZJU1_
HLA-DQA10501-DQB10201	905	TAYNRKRSLQSGITS	RSLQSGITS	6	0.0793	21210.6	95.00	0.37	tr_C9ZJU1_
HLA-DQA10501-DQB10201	906	DSKQTECKDDASIAI	CKDDASIAI	6	0.0787	21338.6	95.00	0.34	tr_C9ZJU1_
HLA-DQA10501-DQB10201	907	LTRATIKTDGHDSKQ	RATIKTDGH	2	0.0785	21392.9	95.00	0.27	tr_C9ZJU1_
HLA-DQA10501-DQB10201	908	VSPTCSFPVKNSLTS	PTCSFPVKN	2	0.0782	21463.2	95.00	0.31	tr_C9ZJU1_

HLA-DQA10501-DQB10201	909	LFQQTFSHPSRHDKS	QQTFSHPSR	2	0.0774	21651.6	95.00	0.20	tr_C9ZJU1_
HLA-DQA10501-DQB10201	910	EGLQSVQRKIDRPVR	VQRKIDRPV	5	0.0772	21681.9	95.00	0.22	tr_C9ZJU1_
HLA-DQA10501-DQB10201	911	TLLRKQKDDGIKCML	KDDGIKCML	6	0.0760	21965.9	95.00	0.31	tr_C9ZJU1_
HLA-DQA10501-DQB10201	912	TFSHPSRHDKSSVQY	RHDKSSVQY	6	0.0757	22044.7	95.00	0.53	tr_C9ZJU1_
HLA-DQA10501-DQB10201	913	FAVHCGLMKNSHPDH	FAVHCGLMK	0	0.0750	22215.9	95.00	0.31	tr_C9ZJU1_
HLA-DQA10501-DQB10201	914	LKRKLQAGGHTCAVS	QAGGHTCAV	5	0.0740	22455.4	95.00	0.34	tr_C9ZJU1_
HLA-DQA10501-DQB10201	915	VLALCMLLRGKYSIA	VLALCMLLR	0	0.0737	22514.1	95.00	0.28	tr_C9ZJU1_
HLA-DQA10501-DQB10201	916	TRATIKTDGHDSKQT	RATIKTDGH	1	0.0735	22580.9	95.00	0.23	tr_C9ZJU1_
HLA-DQA10501-DQB10201	917	RATIKTDGHDSKQTE	DGHDSKQTE	6	0.0734	22604.1	95.00	0.17	tr_C9ZJU1_
HLA-DQA10501-DQB10201	918	TGPHSCFSRMRQSQQ	FSRMRQSQQ	6	0.0733	22634.0	95.00	0.43	tr_C9ZJU1_
HLA-DQA10501-DQB10201	919	PTCSFPVKNSLTSSP	PVKNSLTSS	5	0.0726	22789.8	95.00	0.30	tr_C9ZJU1_
HLA-DQA10501-DQB10201	920	SPTCSFPVKNSLTSS	PTCSFPVKN	1	0.0724	22837.9	95.00	0.28	tr_C9ZJU1_
HLA-DQA10501-DQB10201	921	ATIKTDGHDSKQTEC	DGHDSKQTE	5	0.0712	23149.9	95.00	0.18	tr_C9ZJU1_
HLA-DQA10501-DQB10201	922	SSPLTLLRKQKDDGI	LRKQKDDGI	6	0.0708	23238.5	95.00	0.41	tr_C9ZJU1_
HLA-DQA10501-DQB10201	923	ISQYPARMLRRRIAN	ISQYPARML	0	0.0695	23559.2	95.00	0.31	tr_C9ZJU1_
HLA-DQA10501-DQB10201	924	ALKRKLQAGGHTCAV	QAGGHTCAV	6	0.0693	23620.2	95.00	0.41	tr_C9ZJU1_
HLA-DQA10501-DQB10201	925	LSRTGPHSCFSRMRQ	HSCFSRMRQ	6	0.0690	23687.8	95.00	0.31	tr_C9ZJU1_
HLA-DQA10501-DQB10201	926	DGHDSKQTECKDDAS	QTECKDDAS	6	0.0685	23827.1	95.00	0.31	tr_C9ZJU1_
HLA-DQA10501-DQB10201	927	ITAYNRKRSLQSGIT	KRSLQSGIT	6	0.0681	23926.3	95.00	0.50	tr_C9ZJU1_
HLA-DQA10501-DQB10201	928	ETLSRTGPHSCFSRM	GPHSCFSRM	6	0.0678	24017.4	95.00	0.20	tr_C9ZJU1_
HLA-DQA10501-DQB10201	929	GHDSKQTECKDDASI	QTECKDDAS	5	0.0677	24024.4	95.00	0.28	tr_C9ZJU1_
HLA-DQA10501-DQB10201	930	FSHPSRHDKSSVQYG	RHDKSSVQY	5	0.0677	24047.8	95.00	0.50	tr_C9ZJU1_
HLA-DQA10501-DQB10201	931	WETLSRTGPHSCFSR	TLSRTGPHS	2	0.0667	24286.8	95.00	0.25	tr_C9ZJU1_
HLA-DQA10501-DQB10201	932	LGIAGYSGSGKTTLL	YSGSGKTTL	5	0.0662	24419.9	95.00	0.28	tr_C9ZJU1_
HLA-DQA10501-DQB10201	933	HDSKQTECKDDASIA	QTECKDDAS	4	0.0660	24474.9	95.00	0.23	tr_C9ZJU1_
HLA-DQA10501-DQB10201	934	TLSRTGPHSCFSRMR	GPHSCFSRM	5	0.0645	24879.1	95.00	0.20	tr_C9ZJU1_
HLA-DQA10501-DQB10201	935	TDGHDSKQTECKDDA	KQTECKDDA	6	0.0638	25068.2	95.00	0.34	tr_C9ZJU1_
HLA-DQA10501-DQB10201	936	SRTGPHSCFSRMRQS	HSCFSRMRQ	5	0.0635	25155.7	100.00	0.32	tr_C9ZJU1_
HLA-DQA10501-DQB10201	937	LTLLRKQKDDGIKCM	LRKQKDDGI	3	0.0630	25279.9	100.00	0.35	tr_C9ZJU1_
HLA-DQA10501-DQB10201	938	LTSSPLTLLRKQKDD	LTSSPLTLL	0	0.0630	25295.5	100.00	0.34	tr_C9ZJU1_
HLA-DQA10501-DQB10201	939	ALNFVQKRSSGIHAP	FVQKRSSGI	3	0.0629	25303.7	100.00	0.34	tr_C9ZJU1_
HLA-DQA10501-DQB10201	940	SRALNFVQKRSSGIH	RALNFVQKR	1	0.0624	25454.2	100.00	0.37	tr_C9ZJU1_
HLA-DQA10501-DQB10201	941	RTGPHSCFSRMRQSQ	HSCFSRMRQ	4	0.0621	25525.9	100.00	0.32	tr_C9ZJU1_
HLA-DQA10501-DQB10201	942	SPLTLLRKQKDDGIK	LRKQKDDGI	5	0.0619	25589.5	100.00	0.41	tr_C9ZJU1_
HLA-DQA10501-DQB10201	943	IKTDGHDSKQTECKD	DSKQTECKD	6	0.0610	25836.3	100.00	0.27	tr_C9ZJU1_
HLA-DQA10501-DQB10201	944	SQYPARMLRRRIANV	MLRRRIANV	6	0.0608	25886.6	100.00	0.41	tr_C9ZJU1_
HLA-DQA10501-DQB10201	945	RALNFVQKRSSGIHA	QKRSSGIHA	6	0.0602	26053.5	100.00	0.29	tr_C9ZJU1_
HLA-DQA10501-DQB10201	946	PLTLLRKQKDDGIKC	LRKQKDDGI	4	0.0602	26062.3	100.00	0.41	tr_C9ZJU1_
HLA-DQA10501-DQB10201	947	FQQTFSHPSRHDKSS	QQTFSHPSR	1	0.0594	26303.1	100.00	0.27	tr_C9ZJU1_
HLA-DQA10501-DQB10201	948	TIKTDGHDSKQTECK	DGHDSKQTE	4	0.0586	26521.7	100.00	0.20	tr_C9ZJU1_
HLA-DQA10501-DQB10201	949	KTDGHDSKQTECKDD	DSKQTECKD	5	0.0578	26763.2	100.00	0.30	tr_C9ZJU1_
HLA-DQA10501-DQB10201	950	GIAGYSGSGKTTLLR	YSGSGKTTL	4	0.0576	26815.1	100.00	0.28	tr_C9ZJU1_
HLA-DQA10501-DQB10201	951	LALCMLLRGKYSIAR	LLRGKYSIA	5	0.0565	27134.4	100.00	0.22	tr_C9ZJU1_
HLA-DQA10501-DQB10201	952	LGLITAYNRKRSLQS	LGLITAYNR	0	0.0541	27859.1	100.00	0.43	tr_C9ZJU1_
HLA-DQA10501-DQB10201	953	DALKRKLQAGGHTCA	LQAGGHTCA	6	0.0523	28385.8	100.00	0.38	tr_C9ZJU1_
HLA-DQA10501-DQB10201	954	TSSPLTLLRKQKDDG	LTLLRKQKD	4	0.0498	29176.4	100.00	0.40	tr_C9ZJU1_
HLA-DQA10501-DQB10201	955	QQTFSHPSRHDKSSV	QQTFSHPSR	0	0.0485	29582.7	100.00	0.19	tr_C9ZJU1_
HLA-DQA10501-DQB10201	956	LITAYNRKRSLQSGI	RKRSLQSGI	6	0.0456	30514.0	100.00	0.40	tr_C9ZJU1_
HLA-DQA10501-DQB10201	957	QTFSHPSRHDKSSVQ	SRHDKSSVQ	6	0.0436	31203.1	100.00	0.26	tr_C9ZJU1_
HLA-DQA10501-DQB10201	958	GCVVDALKRKLQAGG	VVDALKRKL	2	0.0428	31474.4	100.00	0.36	tr_C9ZJU1_

HLA-DQA10501-DQB10201	959	VDALKRKLQAGGHTC	RKLQAGGHT	5	0.0370	33503.1	100.00	0.35	tr_C9ZJU1_	
HLA-DQA10501-DQB10201	960	GLITAYNRKRSLQSG	YNRKRSLQS	5	0.0353	34113.2	100.00	0.25	tr_C9ZJU1_	
HLA-DQA10501-DQB10201	961	VVDALKRKLQAGGHT	RKLQAGGHT	6	0.0350	34237.5	100.00	0.38	tr_C9ZJU1_	
HLA-DQA10501-DQB10201	962	CVVDALKRKLQAGGH	VVDALKRKL	1	0.0327	35107.0	100.00	0.37	tr_C9ZJU1_	

Allele: HLA-DQA10501-DQB10201. Number of high binders 3. Number of weak binders 86. Number of peptides 962

Allele	pos	peptide	core	Of	1-log50k(aff)	affinity(nM)	%Rank	Relia	Identity	Bind Level
HLA-DQA10501-DQB10201	1	EMMVFGWLAGEVLFE	FGWLAGEVL	4	0.7114	22.7	0.12	0.62	tr_C9ZQ90_	SB
HLA-DQA10501-DQB10201	2	MMVFGWLAGEVLFEA	FGWLAGEVL	3	0.7060	24.1	0.15	0.57	tr_C9ZQ90_	SB
HLA-DQA10501-DQB10201	3	MVFGWLAGEVLFEAL	FGWLAGEVL	2	0.7043	24.5	0.15	0.54	tr_C9ZQ90_	SB
HLA-DQA10501-DQB10201	4	GEMMVFGWLAGEVLF	FGWLAGEVL	5	0.6977	26.3	0.17	0.69	tr_C9ZQ90_	SB
HLA-DQA10501-DQB10201	5	LGEMMVFGWLAGEVL	FGWLAGEVL	6	0.6914	28.2	0.20	0.71	tr_C9ZQ90_	SB
HLA-DQA10501-DQB10201	6	VFGWLAGEVLFEALN	FGWLAGEVL	1	0.6769	33.0	0.30	0.55	tr_C9ZQ90_	SB
HLA-DQA10501-DQB10201	7	FGWLAGEVLFEALNN	FGWLAGEVL	0	0.6181	62.3	1.00	0.47	tr_C9ZQ90_	WB
HLA-DQA10501-DQB10201	8	ESLYKQRRYVIDDFV	RRYVIDDFV	6	0.5829	91.2	1.70	0.57	tr_C9ZQ90_	WB
HLA-DQA10501-DQB10201	9	KAVAAVLGVVTADIL	LGVVTADIL	6	0.5827	91.4	1.70	0.52	tr_C9ZQ90_	WB
HLA-DQA10501-DQB10201	10	SLYKQRRYVIDDFVV	RRYVIDDFV	5	0.5773	96.9	1.90	0.58	tr_C9ZQ90_	WB
HLA-DQA10501-DQB10201	11	AVAAVLGVVTADILS	LGVVTADIL	5	0.5630	113.1	2.50	0.52	tr_C9ZQ90_	WB
HLA-DQA10501-DQB10201	12	AAVLGVVTADILSVP	LGVVTADIL	3	0.5555	122.7	3.00	0.57	tr_C9ZQ90_	WB
HLA-DQA10501-DQB10201	13	VAAVLGVVTADILSV	LGVVTADIL	4	0.5553	122.9	3.00	0.53	tr_C9ZQ90_	WB
HLA-DQA10501-DQB10201	14	YKQRRYVIDDFVVGD	RRYVIDDFV	3	0.5532	125.7	3.00	0.61	tr_C9ZQ90_	WB
HLA-DQA10501-DQB10201	15	LYKQRRYVIDDFVVG	RRYVIDDFV	4	0.5510	128.8	3.00	0.61	tr_C9ZQ90_	WB
HLA-DQA10501-DQB10201	16	KQRRYVIDDFVVGDF	RRYVIDDFV	2	0.5469	134.6	3.00	0.50	tr_C9ZQ90_	WB
HLA-DQA10501-DQB10201	17	AVLGVVTADILSVPN	LGVVTADIL	2	0.5354	152.5	3.50	0.58	tr_C9ZQ90_	WB
HLA-DQA10501-DQB10201	18	QRRYVIDDFVVGDFG	RRYVIDDFV	1	0.5298	162.0	4.00	0.47	tr_C9ZQ90_	WB
HLA-DQA10501-DQB10201	19	FNDILLFYQEFVAAF	FYQEFVAAF	6	0.5250	170.6	4.00	0.43	tr_C9ZQ90_	WB
HLA-DQA10501-DQB10201	20	NDILLFYQEFVAAFN	FYQEFVAAF	5	0.5147	190.7	4.50	0.38	tr_C9ZQ90_	WB
HLA-DQA10501-DQB10201	21	DILLFYQEFVAAFNA	FYQEFVAAF	4	0.5134	193.3	4.50	0.34	tr_C9ZQ90_	WB
HLA-DQA10501-DQB10201	22	LVRGGEASEVVDTLD	EASEVVDTL	5	0.5132	193.8	5.00	0.44	tr_C9ZQ90_	WB
HLA-DQA10501-DQB10201	23	VLGVVTADILSVPNM	LGVVTADIL	1	0.5104	199.9	5.00	0.53	tr_C9ZQ90_	WB
HLA-DQA10501-DQB10201	24	SNMRKVNAELLADRI	NAELLADRI	6	0.5099	201.0	5.00	0.38	tr_C9ZQ90_	WB
HLA-DQA10501-DQB10201	25	NKFWRNVIHLQPLLA	VIHLQPLLA	6	0.5055	210.7	5.00	0.42	tr_C9ZQ90_	WB
HLA-DQA10501-DQB10201	26	WSLGARSIEEADDVD	RSIEEADDV	5	0.5030	216.4	5.50	0.32	tr_C9ZQ90_	WB
HLA-DQA10501-DQB10201	27	NMRKVNAELLADRIY	NAELLADRI	5	0.4928	241.8	6.00	0.38	tr_C9ZQ90_	WB
HLA-DQA10501-DQB10201	28	ALVRGGEASEVVDTL	EASEVVDTL	6	0.4892	251.4	6.50	0.50	tr_C9ZQ90_	WB
HLA-DQA10501-DQB10201	29	TKAVAAVLGVVTADI	VLGVVTADI	6	0.4843	264.9	6.50	0.66	tr_C9ZQ90_	WB
HLA-DQA10501-DQB10201	30	ILLFYQEFVAAFNAS	FYQEFVAAF	3	0.4840	266.0	6.50	0.39	tr_C9ZQ90_	WB
HLA-DQA10501-DQB10201	31	KFWRNVIHLQPLLAQ	VIHLQPLLA	5	0.4822	271.0	7.00	0.41	tr_C9ZQ90_	WB
HLA-DQA10501-DQB10201	32	VISNMRKVNAELLAD	MRKVNAELL	4	0.4811	274.2	7.00	0.62	tr_C9ZQ90_	WB
HLA-DQA10501-DQB10201	33	FVPFNDILLFYQEFV	ILLFYQEFV	6	0.4796	278.8	7.00	0.45	tr_C9ZQ90_	WB
HLA-DQA10501-DQB10201	34	VGMTPSMDYVIPQEG	SMDYVIPQE	5	0.4701	308.9	8.00	0.49	tr_C9ZQ90_	WB
HLA-DQA10501-DQB10201	35	RARVFVPFNDILLFY	PFNDILLFY	6	0.4681	315.9	8.00	0.31	tr_C9ZQ90_	WB
HLA-DQA10501-DQB10201	36	RRYVIDDFVVGDFGG	RRYVIDDFV	0	0.4663	321.8	8.00	0.40	tr_C9ZQ90_	WB
HLA-DQA10501-DQB10201	37	VRGGEASEVVDTLDK	EASEVVDTL	4	0.4650	326.4	8.00	0.46	tr_C9ZQ90_	WB
HLA-DQA10501-DQB10201	38	SLGARSIEEADDVDK	RSIEEADDV	4	0.4650	326.5	8.00	0.34	tr_C9ZQ90_	WB

HLA-DQA10501-DQB10201	39	MESLYKQRRYVIDDF	QRRYVIDDF	6	0.4648	327.2	8.00	0.92	tr_C9ZQ90_	WB
HLA-DQA10501-DQB10201	40	GWLAGEVLFEALNNA	WLAGEVLFE	1	0.4645	328.2	8.50	0.35	tr_C9ZQ90_	WB
HLA-DQA10501-DQB10201	41	FWRNVIHLQPLLAQQ	VIHLQPLLA	4	0.4627	334.7	8.50	0.40	tr_C9ZQ90_	WB
HLA-DQA10501-DQB10201	42	RWSLGARSIEEADDV	RSIEEADDV	6	0.4587	349.7	9.00	0.35	tr_C9ZQ90_	WB
HLA-DQA10501-DQB10201	43	VFVPFNDILLFYQEF	DILLFYQEF	6	0.4577	353.5	9.00	0.31	tr_C9ZQ90_	WB
HLA-DQA10501-DQB10201	44	VVISNMRKVNAELLA	MRKVNAELL	5	0.4544	366.2	9.00	0.71	tr_C9ZQ90_	WB
HLA-DQA10501-DQB10201	45	ISNMRKVNAELLADR	MRKVNAELL	3	0.4542	367.1	9.00	0.62	tr_C9ZQ90_	WB
HLA-DQA10501-DQB10201	46	MRKVNAELLADRIYT	NAELLADRI	4	0.4541	367.6	9.50	0.38	tr_C9ZQ90_	WB
HLA-DQA10501-DQB10201	47	VPFNDILLFYQEFVA	ILLFYQEFV	5	0.4535	369.7	9.50	0.44	tr_C9ZQ90_	WB
HLA-DQA10501-DQB10201	48	MTPSMDYVIPQEGQL	SMDYVIPQE	3	0.4532	370.8	9.50	0.46	tr_C9ZQ90_	WB
HLA-DQA10501-DQB10201	49	APLIGLYVVLTDDKV	YVVLTDDKV	6	0.4528	372.7	9.50	0.50	tr_C9ZQ90_	WB
HLA-DQA10501-DQB10201	50	VIDDFVVGDFGGECD	VGDFGGECD	6	0.4520	375.7	9.50	0.28	tr_C9ZQ90_	WB
HLA-DQA10501-DQB10201	51	KLGEMMVFGWLAGEV	VFGWLAGEV	6	0.4509	380.4	9.50	0.67	tr_C9ZQ90_	WB
HLA-DQA10501-DQB10201	52	GMTPSMDYVIPQEGQ	SMDYVIPQE	4	0.4489	388.7	10.00	0.49	tr_C9ZQ90_	
HLA-DQA10501-DQB10201	53	TPSMDYVIPQEGQLT	SMDYVIPQE	2	0.4474	395.2	10.00	0.42	tr_C9ZQ90_	
HLA-DQA10501-DQB10201	54	WRNVIHLQPLLAQQL	VIHLQPLLA	3	0.4464	399.4	10.00	0.40	tr_C9ZQ90_	
HLA-DQA10501-DQB10201	55	ARVFVPFNDILLFYQ	PFNDILLFY	5	0.4464	399.5	10.00	0.26	tr_C9ZQ90_	
HLA-DQA10501-DQB10201	56	QVVISNMRKVNAELL	MRKVNAELL	6	0.4462	400.2	10.00	0.81	tr_C9ZQ90_	
HLA-DQA10501-DQB10201	57	LGVVTADILSVPNMT	LGVVTADIL	0	0.4460	401.1	10.00	0.47	tr_C9ZQ90_	
HLA-DQA10501-DQB10201	58	QVGMTPSMDYVIPQE	SMDYVIPQE	6	0.4442	409.0	11.00	0.62	tr_C9ZQ90_	
HLA-DQA10501-DQB10201	59	PLIGLYVVLTDDKVA	YVVLTDDKV	5	0.4423	417.7	11.00	0.51	tr_C9ZQ90_	
HLA-DQA10501-DQB10201	60	IDDFVVGDFGGECDE	VGDFGGECD	5	0.4417	420.1	11.00	0.31	tr_C9ZQ90_	
HLA-DQA10501-DQB10201	61	PFNDILLFYQEFVAA	ILLFYQEFV	4	0.4401	427.7	11.00	0.42	tr_C9ZQ90_	
HLA-DQA10501-DQB10201	62	QGGSMAHMRVVDDSL	HMRVVDDSL	6	0.4396	429.8	11.00	0.56	tr_C9ZQ90_	
HLA-DQA10501-DQB10201	63	WLAGEVLFEALNNAP	WLAGEVLFE	0	0.4396	429.8	11.00	0.25	tr_C9ZQ90_	
HLA-DQA10501-DQB10201	64	GSMAHMRVVDDSLSL	HMRVVDDSL	4	0.4378	438.5	11.00	0.50	tr_C9ZQ90_	
HLA-DQA10501-DQB10201	65	LLFYQEFVAAFNASK	FYQEFVAAF	2	0.4364	444.9	11.00	0.45	tr_C9ZQ90_	
HLA-DQA10501-DQB10201	66	LFYQEFVAAFNASKE	FYQEFVAAF	1	0.4354	449.7	11.00	0.40	tr_C9ZQ90_	
HLA-DQA10501-DQB10201	67	LGARSIEEADDVDKR	RSIEEADDV	3	0.4345	454.4	12.00	0.34	tr_C9ZQ90_	
HLA-DQA10501-DQB10201	68	RGGEASEVVDTLDKS	EASEVVDTL	3	0.4336	458.6	12.00	0.47	tr_C9ZQ90_	
HLA-DQA10501-DQB10201	69	RRARVFVPFNDILLF	FVPFNDILL	5	0.4318	467.7	12.00	0.28	tr_C9ZQ90_	
HLA-DQA10501-DQB10201	70	SVPNMTFIGTLPLSP	FIGTLPLSP	6	0.4287	483.4	12.00	0.34	tr_C9ZQ90_	
HLA-DQA10501-DQB10201	71	PSMDYVIPQEGQLTQ	SMDYVIPQE	1	0.4265	495.4	13.00	0.40	tr_C9ZQ90_	
HLA-DQA10501-DQB10201	72	RVFVPFNDILLFYQE	PFNDILLFY	4	0.4250	503.2	13.00	0.26	tr_C9ZQ90_	
HLA-DQA10501-DQB10201	73	LIGLYVVLTDDKVAQ	YVVLTDDKV	4	0.4238	510.0	13.00	0.50	tr_C9ZQ90_	
HLA-DQA10501-DQB10201	74	GGSMAHMRVVDDSLS	HMRVVDDSL	5	0.4233	512.9	13.00	0.56	tr_C9ZQ90_	
HLA-DQA10501-DQB10201	75	GGEASEVVDTLDKSL	EASEVVDTL	2	0.4212	524.4	13.00	0.49	tr_C9ZQ90_	
HLA-DQA10501-DQB10201	76	GARSIEEADDVDKRI	SIEEADDVD	3	0.4196	533.7	13.00	0.35	tr_C9ZQ90_	
HLA-DQA10501-DQB10201	77	LNKFWRNVIHLQPLL	WRNVIHLQP	4	0.4192	536.2	13.00	0.38	tr_C9ZQ90_	
HLA-DQA10501-DQB10201	78	DDFVVGDFGGECDEG	VGDFGGECD	4	0.4178	544.0	14.00	0.35	tr_C9ZQ90_	
HLA-DQA10501-DQB10201	79	HRRARVFVPFNDILL	FVPFNDILL	6	0.4173	547.1	14.00	0.31	tr_C9ZQ90_	
HLA-DQA10501-DQB10201	80	RNVIHLQPLLAQQLY	VIHLQPLLA	2	0.4126	575.8	14.00	0.44	tr_C9ZQ90_	
HLA-DQA10501-DQB10201	81	DFVVGDFGGECDEGA	VGDFGGECD	3	0.4106	588.5	14.00	0.34	tr_C9ZQ90_	
HLA-DQA10501-DQB10201	82	VPNMTFIGTLPLSPR	FIGTLPLSP	5	0.4054	622.1	15.00	0.35	tr_C9ZQ90_	
HLA-DQA10501-DQB10201	83	RKVNAELLADRIYTE	NAELLADRI	3	0.4052	623.8	15.00	0.54	tr_C9ZQ90_	
HLA-DQA10501-DQB10201	84	YVIDDFVVGDFGGEC	DDFVVGDFG	3	0.4031	638.1	16.00	0.31	tr_C9ZQ90_	
HLA-DQA10501-DQB10201	85	FYQEFVAAFNASKES	FYQEFVAAF	0	0.4023	643.7	16.00	0.34	tr_C9ZQ90_	
HLA-DQA10501-DQB10201	86	NVIHLQPLLAQQLYV	VIHLQPLLA	1	0.4021	644.6	16.00	0.40	tr_C9ZQ90_	
HLA-DQA10501-DQB10201	87	RYVIDDFVVGDFGGE	DDFVVGDFG	4	0.4003	657.5	16.00	0.31	tr_C9ZQ90_	
HLA-DQA10501-DQB10201	88	SMAHMRVVDDSLSLK	HMRVVDDSL	3	0.4000	659.4	16.00	0.47	tr_C9ZQ90_	

HLA-DQA10501-DQB10201	89	GEASEVVDTLDKSLV	EASEVVDTL	1	0.3980	674.4	16.00	0.44	tr_C9ZQ90_
HLA-DQA10501-DQB10201	90	MRWSLGARSIEEADD	LGARSIEEA	4	0.3973	679.2	16.00	0.31	tr_C9ZQ90_
HLA-DQA10501-DQB10201	91	IGLYVVLTDDKVAQR	YVVLTDDKV	3	0.3951	696.0	17.00	0.54	tr_C9ZQ90_
HLA-DQA10501-DQB10201	92	ARSIEEADDVDKRIF	SIEEADDVD	2	0.3947	698.7	17.00	0.41	tr_C9ZQ90_
HLA-DQA10501-DQB10201	93	SMDYVIPQEGQLTQS	SMDYVIPQE	0	0.3942	702.7	17.00	0.34	tr_C9ZQ90_
HLA-DQA10501-DQB10201	94	VKALVRGGEASEVVD	VRGGEASEV	4	0.3939	704.7	17.00	0.41	tr_C9ZQ90_
HLA-DQA10501-DQB10201	95	RIYTESNIRVDDVRF	ESNIRVDDV	4	0.3939	705.1	17.00	0.51	tr_C9ZQ90_
HLA-DQA10501-DQB10201	96	SMVRHFQRDMDNYLD	FQRDMDNYL	5	0.3935	708.2	17.00	0.50	tr_C9ZQ90_
HLA-DQA10501-DQB10201	97	HWDPLTFLGFATTRL	FLGFATTRL	6	0.3932	710.5	17.00	0.75	tr_C9ZQ90_
HLA-DQA10501-DQB10201	98	DNRTVDMYLLAPSSF	MYLLAPSSF	6	0.3916	722.2	17.00	0.58	tr_C9ZQ90_
HLA-DQA10501-DQB10201	99	PNMTFIGTLPLSPRL	FIGTLPLSP	4	0.3905	731.3	17.00	0.40	tr_C9ZQ90_
HLA-DQA10501-DQB10201	100	KALVRGGEASEVVDT	RGGEASEVV	4	0.3900	735.0	17.00	0.37	tr_C9ZQ90_
HLA-DQA10501-DQB10201	101	MVRHFQRDMDNYLDT	FQRDMDNYL	4	0.3897	737.8	17.00	0.50	tr_C9ZQ90_
HLA-DQA10501-DQB10201	102	VRHFQRDMDNYLDTN	FQRDMDNYL	3	0.3893	740.5	18.00	0.50	tr_C9ZQ90_
HLA-DQA10501-DQB10201	103	WDPLTFLGFATTRLL	FLGFATTRL	5	0.3858	769.6	18.00	0.73	tr_C9ZQ90_
HLA-DQA10501-DQB10201	104	FVVGDFGGECDEGAA	VGDFGGECD	2	0.3840	784.7	18.00	0.32	tr_C9ZQ90_
HLA-DQA10501-DQB10201	105	ADRIYTESNIRVDDV	ESNIRVDDV	6	0.3819	802.2	19.00	0.65	tr_C9ZQ90_
HLA-DQA10501-DQB10201	106	MAHMRVVDDSLSLKP	HMRVVDDSL	2	0.3818	803.1	19.00	0.50	tr_C9ZQ90_
HLA-DQA10501-DQB10201	107	KVNAELLADRIYTES	NAELLADRI	2	0.3796	822.4	19.00	0.55	tr_C9ZQ90_
HLA-DQA10501-DQB10201	108	ILSVPNMTFIGTLPL	MTFIGTLPL	6	0.3772	844.8	19.00	0.38	tr_C9ZQ90_
HLA-DQA10501-DQB10201	109	PLTFLGFATTRLLQV	FLGFATTRL	3	0.3757	858.3	20.00	0.56	tr_C9ZQ90_
HLA-DQA10501-DQB10201	110	NGDVFCIGLTPPDVA	CIGLTPPDV	5	0.3755	860.1	20.00	0.39	tr_C9ZQ90_
HLA-DQA10501-DQB10201	111	LSVPNMTFIGTLPLS	NMTFIGTLP	4	0.3731	882.5	20.00	0.33	tr_C9ZQ90_
HLA-DQA10501-DQB10201	112	GDVFCIGLTPPDVAA	CIGLTPPDV	4	0.3719	894.4	20.00	0.40	tr_C9ZQ90_
HLA-DQA10501-DQB10201	113	APQLTNRTSFMESLY	RTSFMESLY	6	0.3713	899.6	20.00	0.46	tr_C9ZQ90_
HLA-DQA10501-DQB10201	114	NRTVDMYLLAPSSFQ	MYLLAPSSF	5	0.3706	907.1	21.00	0.58	tr_C9ZQ90_
HLA-DQA10501-DQB10201	115	SAPLIGLYVVLTDDK	LYVVLTDDK	6	0.3696	916.6	21.00	0.37	tr_C9ZQ90_
HLA-DQA10501-DQB10201	116	LTFLGFATTRLLQVV	FLGFATTRL	2	0.3685	927.9	21.00	0.57	tr_C9ZQ90_
HLA-DQA10501-DQB10201	117	RLNKFWRNVIHLQPL	WRNVIHLQP	5	0.3678	934.6	21.00	0.44	tr_C9ZQ90_
HLA-DQA10501-DQB10201	118	GLYVVLTDDKVAQRA	YVVLTDDKV	2	0.3662	951.3	21.00	0.57	tr_C9ZQ90_
HLA-DQA10501-DQB10201	119	LDKSLVTFGVSLDSS	TFGVSLDSS	6	0.3647	966.5	22.00	0.57	tr_C9ZQ90_
HLA-DQA10501-DQB10201	120	DVFCIGLTPPDVAAV	CIGLTPPDV	3	0.3647	966.7	22.00	0.43	tr_C9ZQ90_
HLA-DQA10501-DQB10201	121	AGEVLFEALNNAPQL	EALNNAPQL	6	0.3647	966.7	22.00	0.31	tr_C9ZQ90_
HLA-DQA10501-DQB10201	122	SSMVRHFQRDMDNYL	FQRDMDNYL	6	0.3641	973.4	22.00	0.48	tr_C9ZQ90_
HLA-DQA10501-DQB10201	123	GVVTADILSVPNMTF	VVTADILSV	1	0.3634	980.3	22.00	0.22	tr_C9ZQ90_
HLA-DQA10501-DQB10201	124	SIASSERLLFATSFP	ERLLFATSF	5	0.3629	986.0	22.00	0.57	tr_C9ZQ90_
HLA-DQA10501-DQB10201	125	IYTESNIRVDDVRFG	ESNIRVDDV	3	0.3619	996.7	22.00	0.48	tr_C9ZQ90_
HLA-DQA10501-DQB10201	126	RHFQRDMDNYLDTNS	FQRDMDNYL	2	0.3615	1000.4	22.00	0.52	tr_C9ZQ90_
HLA-DQA10501-DQB10201	127	DTKAVAAVLGVVTAD	VAAVLGVVT	4	0.3600	1016.6	22.00	0.34	tr_C9ZQ90_
HLA-DQA10501-DQB10201	128	ESIASSERLLFATSF	ERLLFATSF	6	0.3599	1017.9	22.00	0.51	tr_C9ZQ90_
HLA-DQA10501-DQB10201	129	THRRARVFVPFNDIL	VFVPFNDIL	6	0.3590	1028.4	23.00	0.40	tr_C9ZQ90_
HLA-DQA10501-DQB10201	130	TFLGFATTRLLQVVI	FLGFATTRL	1	0.3575	1045.1	23.00	0.47	tr_C9ZQ90_
HLA-DQA10501-DQB10201	131	DPLTFLGFATTRLLQ	FLGFATTRL	4	0.3572	1048.8	23.00	0.74	tr_C9ZQ90_
HLA-DQA10501-DQB10201	132	GVKALVRGGEASEVV	VRGGEASEV	5	0.3569	1051.8	23.00	0.45	tr_C9ZQ90_
HLA-DQA10501-DQB10201	133	NMTFIGTLPLSPRLN	FIGTLPLSP	3	0.3566	1055.1	23.00	0.46	tr_C9ZQ90_
HLA-DQA10501-DQB10201	134	SSERLLFATSFPHWA	ERLLFATSF	2	0.3562	1060.2	23.00	0.38	tr_C9ZQ90_
HLA-DQA10501-DQB10201	135	YTESNIRVDDVRFGP	ESNIRVDDV	2	0.3556	1066.1	23.00	0.42	tr_C9ZQ90_
HLA-DQA10501-DQB10201	136	SMRWSLGARSIEEAD	LGARSIEEA	5	0.3555	1068.3	23.00	0.37	tr_C9ZQ90_
HLA-DQA10501-DQB10201	137	GEVLFEALNNAPQLT	EALNNAPQL	5	0.3533	1093.5	24.00	0.29	tr_C9ZQ90_
HLA-DQA10501-DQB10201	138	LAGEVLFEALNNAPQ	AGEVLFEAL	1	0.3526	1101.3	24.00	0.24	tr_C9ZQ90_

HLA-DQA10501-DQB10201	139	VIHLQPLLAQQLYVL	VIHLQPLLA	0	0.3514	1116.5	24.00	0.32	tr_C9ZQ90_
HLA-DQA10501-DQB10201	140	EASEVVDTLDKSLVT	ASEVVDTLD	1	0.3510	1121.6	24.00	0.37	tr_C9ZQ90_
HLA-DQA10501-DQB10201	141	RSIEEADDVDKRIFF	SIEEADDVD	1	0.3495	1138.9	24.00	0.44	tr_C9ZQ90_
HLA-DQA10501-DQB10201	142	DRIYTESNIRVDDVR	ESNIRVDDV	5	0.3493	1141.9	24.00	0.63	tr_C9ZQ90_
HLA-DQA10501-DQB10201	143	ASSERLLFATSFPHW	ERLLFATSF	3	0.3493	1142.0	24.00	0.48	tr_C9ZQ90_
HLA-DQA10501-DQB10201	144	RTVDMYLLAPSSFQH	MYLLAPSSF	4	0.3491	1144.4	24.00	0.57	tr_C9ZQ90_
HLA-DQA10501-DQB10201	145	VSAPLIGLYVVLTDD	GLYVVLTDD	6	0.3490	1146.2	24.00	0.36	tr_C9ZQ90_
HLA-DQA10501-DQB10201	146	ASKESIASSERLLFA	ESIASSERL	3	0.3486	1150.2	25.00	0.39	tr_C9ZQ90_
HLA-DQA10501-DQB10201	147	VNAELLADRIYTESN	NAELLADRI	1	0.3484	1153.1	25.00	0.60	tr_C9ZQ90_
HLA-DQA10501-DQB10201	148	AHMRVVDDSLSLKPM	HMRVVDDSL	1	0.3481	1157.1	25.00	0.51	tr_C9ZQ90_
HLA-DQA10501-DQB10201	149	TVDMYLLAPSSFQHF	MYLLAPSSF	3	0.3480	1157.5	25.00	0.58	tr_C9ZQ90_
HLA-DQA10501-DQB10201	150	SERLLFATSFPHWAE	ERLLFATSF	1	0.3472	1168.4	25.00	0.33	tr_C9ZQ90_
HLA-DQA10501-DQB10201	151	DDVRFGPFSDAECVS	PFSDAECVS	6	0.3465	1177.5	25.00	0.26	tr_C9ZQ90_
HLA-DQA10501-DQB10201	152	NASKESIASSERLLF	ESIASSERL	4	0.3461	1181.9	25.00	0.47	tr_C9ZQ90_
HLA-DQA10501-DQB10201	153	VVTADILSVPNMTFI	DILSVPNMT	4	0.3450	1196.6	25.00	0.27	tr_C9ZQ90_
HLA-DQA10501-DQB10201	154	HFQRDMDNYLDTNSD	FQRDMDNYL	1	0.3448	1198.4	25.00	0.50	tr_C9ZQ90_
HLA-DQA10501-DQB10201	155	DKSLVTFGVSLDSSK	TFGVSLDSS	5	0.3436	1214.8	26.00	0.54	tr_C9ZQ90_
HLA-DQA10501-DQB10201	156	TESNIRVDDVRFGPF	NIRVDDVRF	3	0.3418	1238.8	26.00	0.37	tr_C9ZQ90_
HLA-DQA10501-DQB10201	157	EVLFEALNNAPQLTN	EALNNAPQL	4	0.3410	1249.4	26.00	0.28	tr_C9ZQ90_
HLA-DQA10501-DQB10201	158	GNGDVFCIGLTPPDV	CIGLTPPDV	6	0.3400	1262.3	26.00	0.49	tr_C9ZQ90_
HLA-DQA10501-DQB10201	159	PQLTNRTSFMESLYK	RTSFMESLY	5	0.3388	1279.2	26.00	0.41	tr_C9ZQ90_
HLA-DQA10501-DQB10201	160	VVGDFGGECDEGAAL	VGDFGGECD	1	0.3383	1286.1	27.00	0.31	tr_C9ZQ90_
HLA-DQA10501-DQB10201	161	VFCIGLTPPDVAAVA	CIGLTPPDV	2	0.3381	1289.4	27.00	0.46	tr_C9ZQ90_
HLA-DQA10501-DQB10201	162	IASSERLLFATSFPH	ERLLFATSF	4	0.3369	1306.1	27.00	0.53	tr_C9ZQ90_
HLA-DQA10501-DQB10201	163	SLVTFGVSLDSSKTL	TFGVSLDSS	3	0.3358	1321.0	27.00	0.52	tr_C9ZQ90_
HLA-DQA10501-DQB10201	164	VDMYLLAPSSFQHFL	MYLLAPSSF	2	0.3339	1349.4	27.00	0.58	tr_C9ZQ90_
HLA-DQA10501-DQB10201	165	YQEFVAAFNASKESI	VAAFNASKE	4	0.3334	1356.1	28.00	0.28	tr_C9ZQ90_
HLA-DQA10501-DQB10201	166	KSLVTFGVSLDSSKT	TFGVSLDSS	4	0.3320	1377.0	28.00	0.54	tr_C9ZQ90_
HLA-DQA10501-DQB10201	167	ASMRWSLGARSIEEA	LGARSIEEA	6	0.3315	1384.2	28.00	0.38	tr_C9ZQ90_
HLA-DQA10501-DQB10201	168	DVRFGPFSDAECVSG	PFSDAECVS	5	0.3314	1386.3	28.00	0.29	tr_C9ZQ90_
HLA-DQA10501-DQB10201	169	DILSVPNMTFIGTLP	NMTFIGTLP	6	0.3298	1410.3	28.00	0.44	tr_C9ZQ90_
HLA-DQA10501-DQB10201	170	SKESIASSERLLFAT	ESIASSERL	2	0.3298	1410.5	28.00	0.40	tr_C9ZQ90_
HLA-DQA10501-DQB10201	171	FLGFATTRLLQVVIS	FLGFATTRL	0	0.3286	1428.7	29.00	0.33	tr_C9ZQ90_
HLA-DQA10501-DQB10201	172	FQRDMDNYLDTNSDW	FQRDMDNYL	0	0.3286	1429.2	29.00	0.33	tr_C9ZQ90_
HLA-DQA10501-DQB10201	173	VRDTKAVAAVLGVVT	VAAVLGVVT	6	0.3284	1431.4	29.00	0.40	tr_C9ZQ90_
HLA-DQA10501-DQB10201	174	LYVVLTDDKVAQRAS	YVVLTDDKV	1	0.3256	1475.3	29.00	0.67	tr_C9ZQ90_
HLA-DQA10501-DQB10201	175	VDDVRFGPFSDAECV	FGPFSDAEC	5	0.3255	1476.6	29.00	0.33	tr_C9ZQ90_
HLA-DQA10501-DQB10201	176	DMYLLAPSSFQHFLI	MYLLAPSSF	1	0.3228	1521.7	30.00	0.54	tr_C9ZQ90_
HLA-DQA10501-DQB10201	177	MTFIGTLPLSPRLNK	FIGTLPLSP	2	0.3208	1554.7	30.00	0.62	tr_C9ZQ90_
HLA-DQA10501-DQB10201	178	FCIGLTPPDVAAVAR	CIGLTPPDV	1	0.3199	1569.2	30.00	0.46	tr_C9ZQ90_
HLA-DQA10501-DQB10201	179	LQPLLAQQLYVLAVY	QQLYVLAVY	6	0.3192	1582.3	31.00	0.29	tr_C9ZQ90_
HLA-DQA10501-DQB10201	180	PISSYLSGNGDVFCI	YLSGNGDVF	4	0.3184	1595.4	31.00	0.57	tr_C9ZQ90_
HLA-DQA10501-DQB10201	181	QLTNRTSFMESLYKQ	RTSFMESLY	4	0.3179	1603.5	31.00	0.41	tr_C9ZQ90_
HLA-DQA10501-DQB10201	182	RDTKAVAAVLGVVTA	VAAVLGVVT	5	0.3173	1614.2	31.00	0.40	tr_C9ZQ90_
HLA-DQA10501-DQB10201	183	NQGGSMAHMRVVDDS	MAHMRVVDD	5	0.3172	1615.8	31.00	0.41	tr_C9ZQ90_
HLA-DQA10501-DQB10201	184	QVSAPLIGLYVVLTD	IGLYVVLTD	6	0.3169	1622.2	31.00	0.38	tr_C9ZQ90_
HLA-DQA10501-DQB10201	185	DPISSYLSGNGDVFC	YLSGNGDVF	5	0.3154	1647.0	32.00	0.58	tr_C9ZQ90_
HLA-DQA10501-DQB10201	186	QPLLAQQLYVLAVYL	QLYVLAVYL	6	0.3144	1665.9	32.00	0.25	tr_C9ZQ90_
HLA-DQA10501-DQB10201	187	LVTFGVSLDSSKTLG	TFGVSLDSS	2	0.3128	1694.9	32.00	0.56	tr_C9ZQ90_
HLA-DQA10501-DQB10201	188	HFLIKTWSDALVSLN	TWSDALVSL	5	0.3124	1702.2	32.00	0.31	tr_C9ZQ90_

HLA-DQA10501-DQB10201	189	NAELLADRIYTESNI	NAELLADRI	0	0.3101	1744.3	33.00	0.41	tr_C9ZQ90_
HLA-DQA10501-DQB10201	190	VRFGPFSDAECVSGT	PFSDAECVS	4	0.3099	1748.7	33.00	0.29	tr_C9ZQ90_
HLA-DQA10501-DQB10201	191	ERLLFATSFPHWAEK	FATSFPHWA	4	0.3097	1753.5	33.00	0.27	tr_C9ZQ90_
HLA-DQA10501-DQB10201	192	QTHRRARVFVPFNDI	RVFVPFNDI	6	0.3081	1783.2	33.00	0.25	tr_C9ZQ90_
HLA-DQA10501-DQB10201	193	QHFLIKTWSDALVSL	TWSDALVSL	6	0.3075	1794.9	33.00	0.40	tr_C9ZQ90_
HLA-DQA10501-DQB10201	194	ESNIRVDDVRFGPFS	NIRVDDVRF	2	0.3071	1801.9	33.00	0.41	tr_C9ZQ90_
HLA-DQA10501-DQB10201	195	SANVQVSAPLIGLYV	NVQVSAPLI	2	0.3068	1809.5	33.00	0.34	tr_C9ZQ90_
HLA-DQA10501-DQB10201	196	KESIASSERLLFATS	ESIASSERL	1	0.3067	1809.8	33.00	0.38	tr_C9ZQ90_
HLA-DQA10501-DQB10201	197	IAQDNRTVDMYLLAP	DNRTVDMYL	3	0.3067	1810.0	33.00	0.29	tr_C9ZQ90_
HLA-DQA10501-DQB10201	198	AFNASKESIASSERL	ESIASSERL	6	0.3065	1813.6	34.00	0.62	tr_C9ZQ90_
HLA-DQA10501-DQB10201	199	ISSYLSGNGDVFCIG	YLSGNGDVF	3	0.3062	1820.1	34.00	0.55	tr_C9ZQ90_
HLA-DQA10501-DQB10201	200	FNASKESIASSERLL	ESIASSERL	5	0.3038	1868.9	34.00	0.58	tr_C9ZQ90_
HLA-DQA10501-DQB10201	201	TQVGMTPSMDYVIPQ	QVGMTPSMD	1	0.3037	1870.4	34.00	0.22	tr_C9ZQ90_
HLA-DQA10501-DQB10201	202	SSYLSGNGDVFCIGL	YLSGNGDVF	2	0.3037	1870.9	34.00	0.51	tr_C9ZQ90_
HLA-DQA10501-DQB10201	203	SECSSANVQVSAPLI	NVQVSAPLI	6	0.3016	1913.5	35.00	0.54	tr_C9ZQ90_
HLA-DQA10501-DQB10201	204	SSANVQVSAPLIGLY	NVQVSAPLI	3	0.3011	1923.7	35.00	0.44	tr_C9ZQ90_
HLA-DQA10501-DQB10201	205	HLQPLLAQQLYVLAV	AQQLYVLAV	6	0.3007	1931.6	35.00	0.26	tr_C9ZQ90_
HLA-DQA10501-DQB10201	206	QEFVAAFNASKESIA	VAAFNASKE	3	0.3004	1937.9	35.00	0.29	tr_C9ZQ90_
HLA-DQA10501-DQB10201	207	VLFEALNNAPQLTNR	EALNNAPQL	3	0.3004	1938.4	35.00	0.34	tr_C9ZQ90_
HLA-DQA10501-DQB10201	208	LTNRTSFMESLYKQR	RTSFMESLY	3	0.2997	1952.9	35.00	0.37	tr_C9ZQ90_
HLA-DQA10501-DQB10201	209	PRLNKFWRNVIHLQP	WRNVIHLQP	6	0.2990	1967.4	35.00	0.62	tr_C9ZQ90_
HLA-DQA10501-DQB10201	210	CSSANVQVSAPLIGL	NVQVSAPLI	4	0.2978	1994.2	36.00	0.44	tr_C9ZQ90_
HLA-DQA10501-DQB10201	211	VTADILSVPNMTFIG	DILSVPNMT	3	0.2957	2038.3	36.00	0.31	tr_C9ZQ90_
HLA-DQA10501-DQB10201	212	ECSSANVQVSAPLIG	NVQVSAPLI	5	0.2954	2046.0	36.00	0.47	tr_C9ZQ90_
HLA-DQA10501-DQB10201	213	HMRVVDDSLSLKPMK	HMRVVDDSL	0	0.2937	2083.6	37.00	0.42	tr_C9ZQ90_
HLA-DQA10501-DQB10201	214	CNQGGSMAHMRVVDD	MAHMRVVDD	6	0.2936	2085.3	37.00	0.61	tr_C9ZQ90_
HLA-DQA10501-DQB10201	215	VGDFGGECDEGAALQ	FGGECDEGA	3	0.2936	2087.0	37.00	0.37	tr_C9ZQ90_
HLA-DQA10501-DQB10201	216	AQDNRTVDMYLLAPS	DNRTVDMYL	2	0.2932	2095.9	37.00	0.25	tr_C9ZQ90_
HLA-DQA10501-DQB10201	217	EQVRDTKAVAAVLGV	KAVAAVLGV	6	0.2929	2102.4	37.00	0.31	tr_C9ZQ90_
HLA-DQA10501-DQB10201	218	RVDDVRFGPFSDAEC	FGPFSDAEC	6	0.2924	2113.1	37.00	0.34	tr_C9ZQ90_
HLA-DQA10501-DQB10201	219	LIKTWSDALVSLNRT	WSDALVSLN	4	0.2909	2148.7	37.00	0.21	tr_C9ZQ90_
HLA-DQA10501-DQB10201	220	TGVKALVRGGEASEV	VRGGEASEV	6	0.2890	2192.7	38.00	0.65	tr_C9ZQ90_
HLA-DQA10501-DQB10201	221	RTQVGMTPSMDYVIP	QVGMTPSMD	2	0.2884	2206.5	38.00	0.26	tr_C9ZQ90_
HLA-DQA10501-DQB10201	222	QVRDTKAVAAVLGVV	KAVAAVLGV	5	0.2877	2223.6	38.00	0.27	tr_C9ZQ90_
HLA-DQA10501-DQB10201	223	FLIKTWSDALVSLNR	TWSDALVSL	4	0.2870	2240.5	38.00	0.31	tr_C9ZQ90_
HLA-DQA10501-DQB10201	224	IHLQPLLAQQLYVLA	LQPLLAQQL	2	0.2863	2258.5	39.00	0.29	tr_C9ZQ90_
HLA-DQA10501-DQB10201	225	NAPQLTNRTSFMESL	NRTSFMESL	6	0.2860	2265.8	39.00	0.57	tr_C9ZQ90_
HLA-DQA10501-DQB10201	226	QDNRTVDMYLLAPSS	DMYLLAPSS	6	0.2853	2282.9	39.00	0.22	tr_C9ZQ90_
HLA-DQA10501-DQB10201	227	SYLSGNGDVFCIGLT	YLSGNGDVF	1	0.2846	2299.3	39.00	0.50	tr_C9ZQ90_
HLA-DQA10501-DQB10201	228	ANVQVSAPLIGLYVV	NVQVSAPLI	1	0.2846	2299.5	39.00	0.38	tr_C9ZQ90_
HLA-DQA10501-DQB10201	229	VTFGVSLDSSKTLGD	TFGVSLDSS	1	0.2826	2349.0	39.00	0.57	tr_C9ZQ90_
HLA-DQA10501-DQB10201	230	RASMRWSLGARSIEE	SLGARSIEE	6	0.2820	2365.8	40.00	0.47	tr_C9ZQ90_
HLA-DQA10501-DQB10201	231	RFGPFSDAECVSGTS	PFSDAECVS	3	0.2811	2387.3	40.00	0.31	tr_C9ZQ90_
HLA-DQA10501-DQB10201	232	LAQQLYVLAVYLSNT	VLAVYLSNT	6	0.2804	2406.6	40.00	0.23	tr_C9ZQ90_
HLA-DQA10501-DQB10201	233	LLAQQLYVLAVYLSN	YVLAVYLSN	6	0.2797	2425.5	40.00	0.21	tr_C9ZQ90_
HLA-DQA10501-DQB10201	234	MDYVIPQEGQLTQSQ	MDYVIPQEG	0	0.2794	2431.4	40.00	0.38	tr_C9ZQ90_
HLA-DQA10501-DQB10201	235	PLLAQQLYVLAVYLS	QLYVLAVYL	5	0.2793	2436.2	40.00	0.23	tr_C9ZQ90_
HLA-DQA10501-DQB10201	236	DKLGEMMVFGWLAGE	MVFGWLAGE	6	0.2781	2466.1	41.00	0.32	tr_C9ZQ90_
HLA-DQA10501-DQB10201	237	TFIGTLPLSPRLNKF	FIGTLPLSP	1	0.2781	2467.2	41.00	0.70	tr_C9ZQ90_
HLA-DQA10501-DQB10201	238	IKTWSDALVSLNRTF	WSDALVSLN	3	0.2774	2484.9	41.00	0.26	tr_C9ZQ90_

HLA-DQA10501-DQB10201	239	DDPISSYLSGNGDVF	YLSGNGDVF	6	0.2738	2585.8	42.00	0.73	tr_C9ZQ90_
HLA-DQA10501-DQB10201	240	ADILSVPNMTFIGTL	PNMTFIGTL	6	0.2731	2603.4	42.00	0.31	tr_C9ZQ90_
HLA-DQA10501-DQB10201	241	FGPFSDAECVSGTSV	PFSDAECVS	2	0.2720	2634.6	42.00	0.28	tr_C9ZQ90_
HLA-DQA10501-DQB10201	242	LQTHRRARVFVPFND	RARVFVPFN	5	0.2720	2635.4	42.00	0.37	tr_C9ZQ90_
HLA-DQA10501-DQB10201	243	TWSDALVSLNRTFTP	DALVSLNRT	3	0.2715	2648.6	42.00	0.29	tr_C9ZQ90_
HLA-DQA10501-DQB10201	244	TNRTSFMESLYKQRR	RTSFMESLY	2	0.2706	2675.4	43.00	0.39	tr_C9ZQ90_
HLA-DQA10501-DQB10201	245	EFVAAFNASKESIAS	VAAFNASKE	2	0.2703	2683.6	43.00	0.33	tr_C9ZQ90_
HLA-DQA10501-DQB10201	246	SLPRTQVGMTPSMDY	QVGMTPSMD	5	0.2699	2697.4	43.00	0.32	tr_C9ZQ90_
HLA-DQA10501-DQB10201	247	LPRTQVGMTPSMDYV	QVGMTPSMD	4	0.2698	2698.6	43.00	0.32	tr_C9ZQ90_
HLA-DQA10501-DQB10201	248	KIAQDNRTVDMYLLA	DNRTVDMYL	4	0.2698	2699.8	43.00	0.32	tr_C9ZQ90_
HLA-DQA10501-DQB10201	249	KKIAQDNRTVDMYLL	DNRTVDMYL	5	0.2692	2716.7	43.00	0.41	tr_C9ZQ90_
HLA-DQA10501-DQB10201	250	VQVSAPLIGLYVVLT	LIGLYVVLT	6	0.2686	2733.3	43.00	0.38	tr_C9ZQ90_
HLA-DQA10501-DQB10201	251	CIGLTPPDVAAVARH	IGLTPPDVA	1	0.2680	2752.4	43.00	0.42	tr_C9ZQ90_
HLA-DQA10501-DQB10201	252	KTWSDALVSLNRTFT	WSDALVSLN	2	0.2674	2769.3	43.00	0.25	tr_C9ZQ90_
HLA-DQA10501-DQB10201	253	NVQVSAPLIGLYVVL	NVQVSAPLI	0	0.2664	2800.7	44.00	0.30	tr_C9ZQ90_
HLA-DQA10501-DQB10201	254	YLSGNGDVFCIGLTP	YLSGNGDVF	0	0.2663	2802.6	44.00	0.38	tr_C9ZQ90_
HLA-DQA10501-DQB10201	255	TADILSVPNMTFIGT	DILSVPNMT	2	0.2657	2821.5	44.00	0.37	tr_C9ZQ90_
HLA-DQA10501-DQB10201	256	LEQVRDTKAVAAVLG	TKAVAAVLG	6	0.2652	2836.0	44.00	0.37	tr_C9ZQ90_
HLA-DQA10501-DQB10201	257	PRTQVGMTPSMDYVI	QVGMTPSMD	3	0.2652	2837.8	44.00	0.33	tr_C9ZQ90_
HLA-DQA10501-DQB10201	258	QRDMDNYLDTNSDWK	NYLDTNSDW	5	0.2632	2899.1	45.00	0.37	tr_C9ZQ90_
HLA-DQA10501-DQB10201	259	MYLLAPSSFQHFLIK	MYLLAPSSF	0	0.2629	2907.4	45.00	0.56	tr_C9ZQ90_
HLA-DQA10501-DQB10201	260	GFATTRLLQVVISNM	FATTRLLQV	1	0.2629	2909.1	45.00	0.25	tr_C9ZQ90_
HLA-DQA10501-DQB10201	261	AQQLYVLAVYLSNTS	YVLAVYLSN	4	0.2621	2934.6	45.00	0.20	tr_C9ZQ90_
HLA-DQA10501-DQB10201	262	LGFATTRLLQVVISN	FATTRLLQV	2	0.2619	2939.0	45.00	0.32	tr_C9ZQ90_
HLA-DQA10501-DQB10201	263	SNIRVDDVRFGPFSD	NIRVDDVRF	1	0.2610	2969.8	45.00	0.47	tr_C9ZQ90_
HLA-DQA10501-DQB10201	264	SIEEADDVDKRIFFH	SIEEADDVD	0	0.2597	3009.0	46.00	0.46	tr_C9ZQ90_
HLA-DQA10501-DQB10201	265	KDDKLGEMMVFGWLA	EMMVFGWLA	6	0.2594	3019.4	46.00	0.26	tr_C9ZQ90_
HLA-DQA10501-DQB10201	266	MKKGSVTWSVSECSS	VTWSVSECS	5	0.2587	3044.8	46.00	0.55	tr_C9ZQ90_
HLA-DQA10501-DQB10201	267	FQHFLIKTWSDALVS	LIKTWSDAL	4	0.2579	3070.4	46.00	0.28	tr_C9ZQ90_
HLA-DQA10501-DQB10201	268	LKVNLKNLTQSLEQV	NLTQSLEQV	6	0.2577	3076.8	46.00	0.51	tr_C9ZQ90_
HLA-DQA10501-DQB10201	269	LFEALNNAPQLTNRT	EALNNAPQL	2	0.2576	3081.0	46.00	0.42	tr_C9ZQ90_
HLA-DQA10501-DQB10201	270	HVNESHWDPLTFLGF	WDPLTFLGF	6	0.2566	3112.1	46.00	0.34	tr_C9ZQ90_
HLA-DQA10501-DQB10201	271	ASEVVDTLDKSLVTF	ASEVVDTLD	0	0.2562	3128.0	46.00	0.35	tr_C9ZQ90_
HLA-DQA10501-DQB10201	272	PGQLITTGTVPLASD	ITTGTVPLA	4	0.2561	3128.8	47.00	0.28	tr_C9ZQ90_
HLA-DQA10501-DQB10201	273	PMKKGSVTWSVSECS	VTWSVSECS	6	0.2560	3133.6	47.00	0.63	tr_C9ZQ90_
HLA-DQA10501-DQB10201	274	ISVWSMARVLNSSLP	WSMARVLNS	3	0.2560	3133.8	47.00	0.26	tr_C9ZQ90_
HLA-DQA10501-DQB10201	275	RLLFATSFPHWAEKN	FATSFPHWA	3	0.2556	3147.5	47.00	0.43	tr_C9ZQ90_
HLA-DQA10501-DQB10201	276	TLDKSLVTFGVSLDS	VTFGVSLDS	6	0.2537	3213.0	47.00	0.41	tr_C9ZQ90_
HLA-DQA10501-DQB10201	277	VKKIAQDNRTVDMYL	DNRTVDMYL	6	0.2526	3251.7	47.00	0.55	tr_C9ZQ90_
HLA-DQA10501-DQB10201	278	SLEQVRDTKAVAAVL	DTKAVAAVL	6	0.2525	3256.2	48.00	0.54	tr_C9ZQ90_
HLA-DQA10501-DQB10201	279	NISVWSMARVLNSSL	WSMARVLNS	4	0.2521	3268.1	48.00	0.25	tr_C9ZQ90_
HLA-DQA10501-DQB10201	280	KKGSVTWSVSECSSA	VTWSVSECS	4	0.2516	3285.1	48.00	0.52	tr_C9ZQ90_
HLA-DQA10501-DQB10201	281	RSSMVRHFQRDMDNY	HFQRDMDNY	6	0.2516	3286.1	48.00	0.34	tr_C9ZQ90_
HLA-DQA10501-DQB10201	282	KGFAKPEHYLKDDKL	EHYLKDDKL	6	0.2508	3316.7	48.00	0.75	tr_C9ZQ90_
HLA-DQA10501-DQB10201	283	SFQHFLIKTWSDALV	LIKTWSDAL	5	0.2507	3319.4	48.00	0.32	tr_C9ZQ90_
HLA-DQA10501-DQB10201	284	YVVLTDDKVAQRASM	YVVLTDDKV	0	0.2506	3323.8	48.00	0.71	tr_C9ZQ90_
HLA-DQA10501-DQB10201	285	DDKLGEMMVFGWLAG	EMMVFGWLA	5	0.2502	3336.3	48.00	0.25	tr_C9ZQ90_
HLA-DQA10501-DQB10201	286	VNLKNLTQSLEQVRD	NLTQSLEQV	4	0.2500	3345.4	48.00	0.41	tr_C9ZQ90_
HLA-DQA10501-DQB10201	287	ATNISVWSMARVLNS	WSMARVLNS	6	0.2499	3348.3	48.00	0.32	tr_C9ZQ90_
HLA-DQA10501-DQB10201	288	VNESHWDPLTFLGFA	WDPLTFLGF	5	0.2472	3446.4	49.00	0.31	tr_C9ZQ90_

HLA-DQA10501-DQB10201	289	YLKDDKLGEMMVFGW	LGEMMVFGW	6	0.2470	3453.8	49.00	0.28	tr_C9ZQ90_
HLA-DQA10501-DQB10201	290	FATTRLLQVVISNMR	LLQVVISNM	5	0.2466	3469.6	49.00	0.14	tr_C9ZQ90_
HLA-DQA10501-DQB10201	291	GSVTWSVSECSSANV	VTWSVSECS	2	0.2463	3480.1	49.00	0.47	tr_C9ZQ90_
HLA-DQA10501-DQB10201	292	SGNGDVFCIGLTPPD	VFCIGLTPP	5	0.2457	3502.5	49.00	0.34	tr_C9ZQ90_
HLA-DQA10501-DQB10201	293	EHYLKDDKLGEMMVF	DKLGEMMVF	6	0.2451	3525.0	50.00	0.35	tr_C9ZQ90_
HLA-DQA10501-DQB10201	294	GQLITTGTVPLASDN	ITTGTVPLA	3	0.2445	3549.3	50.00	0.29	tr_C9ZQ90_
HLA-DQA10501-DQB10201	295	LKDDKLGEMMVFGWL	DKLGEMMVF	3	0.2442	3559.6	50.00	0.31	tr_C9ZQ90_
HLA-DQA10501-DQB10201	296	KGSVTWSVSECSSAN	VTWSVSECS	3	0.2432	3599.3	50.00	0.50	tr_C9ZQ90_
HLA-DQA10501-DQB10201	297	NESHWDPLTFLGFAT	WDPLTFLGF	4	0.2420	3646.9	50.00	0.23	tr_C9ZQ90_
HLA-DQA10501-DQB10201	298	QQLYVLAVYLSNTSS	YVLAVYLSN	3	0.2406	3701.8	55.00	0.22	tr_C9ZQ90_
HLA-DQA10501-DQB10201	299	FTPGQLITTGTVPLA	ITTGTVPLA	6	0.2402	3716.8	55.00	0.34	tr_C9ZQ90_
HLA-DQA10501-DQB10201	300	HRHVNESHWDPLTFL	SHWDPLTFL	6	0.2401	3720.7	55.00	0.40	tr_C9ZQ90_
HLA-DQA10501-DQB10201	301	SVWSMARVLNSSLPR	WSMARVLNS	2	0.2400	3725.5	55.00	0.26	tr_C9ZQ90_
HLA-DQA10501-DQB10201	302	NRTSFMESLYKQRRY	RTSFMESLY	1	0.2399	3729.2	55.00	0.41	tr_C9ZQ90_
HLA-DQA10501-DQB10201	303	TPGQLITTGTVPLAS	ITTGTVPLA	5	0.2393	3753.1	55.00	0.29	tr_C9ZQ90_
HLA-DQA10501-DQB10201	304	TFGVSLDSSKTLGDD	TFGVSLDSS	0	0.2380	3808.1	55.00	0.51	tr_C9ZQ90_
HLA-DQA10501-DQB10201	305	TNISVWSMARVLNSS	WSMARVLNS	5	0.2376	3824.0	55.00	0.31	tr_C9ZQ90_
HLA-DQA10501-DQB10201	306	QLITTGTVPLASDNR	ITTGTVPLA	2	0.2369	3851.7	55.00	0.29	tr_C9ZQ90_
HLA-DQA10501-DQB10201	307	RDMDNYLDTNSDWKG	NYLDTNSDW	4	0.2367	3862.9	55.00	0.37	tr_C9ZQ90_
HLA-DQA10501-DQB10201	308	HLQTHRRARVFVPFN	RARVFVPFN	6	0.2364	3875.5	55.00	0.38	tr_C9ZQ90_
HLA-DQA10501-DQB10201	309	WSDALVSLNRTFTPG	DALVSLNRT	2	0.2345	3952.1	55.00	0.35	tr_C9ZQ90_
HLA-DQA10501-DQB10201	310	NLKNLTQSLEQVRDT	NLTQSLEQV	3	0.2335	3995.5	55.00	0.42	tr_C9ZQ90_
HLA-DQA10501-DQB10201	311	FVAAFNASKESIASS	VAAFNASKE	1	0.2333	4007.8	55.00	0.36	tr_C9ZQ90_
HLA-DQA10501-DQB10201	312	LSGNGDVFCIGLTPP	VFCIGLTPP	6	0.2322	4052.1	55.00	0.32	tr_C9ZQ90_
HLA-DQA10501-DQB10201	313	NIRVDDVRFGPFSDA	NIRVDDVRF	0	0.2320	4061.6	55.00	0.38	tr_C9ZQ90_
HLA-DQA10501-DQB10201	314	KVNLKNLTQSLEQVR	NLTQSLEQV	5	0.2316	4082.1	55.00	0.50	tr_C9ZQ90_
HLA-DQA10501-DQB10201	315	DMDNYLDTNSDWKGF	NYLDTNSDW	3	0.2313	4095.1	55.00	0.38	tr_C9ZQ90_
HLA-DQA10501-DQB10201	316	ECDEGAALQGAMCNC	GAALQGAMC	4	0.2307	4119.9	55.00	0.29	tr_C9ZQ90_
HLA-DQA10501-DQB10201	317	GDFGGECDEGAALQG	FGGECDEGA	2	0.2306	4124.9	55.00	0.52	tr_C9ZQ90_
HLA-DQA10501-DQB10201	318	CASNFGATNISVWSM	FGATNISVW	4	0.2302	4141.5	55.00	0.37	tr_C9ZQ90_
HLA-DQA10501-DQB10201	319	IRVDDVRFGPFSDAE	RFGPFSDAE	6	0.2301	4148.6	55.00	0.40	tr_C9ZQ90_
HLA-DQA10501-DQB10201	320	SSLPRTQVGMTPSMD	QVGMTPSMD	6	0.2296	4169.4	55.00	0.46	tr_C9ZQ90_
HLA-DQA10501-DQB10201	321	VWSMARVLNSSLPRT	WSMARVLNS	1	0.2295	4172.1	55.00	0.22	tr_C9ZQ90_
HLA-DQA10501-DQB10201	322	GECDEGAALQGAMCN	GAALQGAMC	5	0.2294	4180.4	55.00	0.34	tr_C9ZQ90_
HLA-DQA10501-DQB10201	323	ESHWDPLTFLGFATT	WDPLTFLGF	3	0.2294	4180.5	55.00	0.23	tr_C9ZQ90_
HLA-DQA10501-DQB10201	324	FGATNISVWSMARVL	ISVWSMARV	5	0.2292	4188.0	55.00	0.28	tr_C9ZQ90_
HLA-DQA10501-DQB10201	325	VSECSSANVQVSAPL	ANVQVSAPL	6	0.2283	4230.5	55.00	0.49	tr_C9ZQ90_
HLA-DQA10501-DQB10201	326	NFGATNISVWSMARV	ISVWSMARV	6	0.2279	4249.0	55.00	0.32	tr_C9ZQ90_
HLA-DQA10501-DQB10201	327	SHWDPLTFLGFATTR	TFLGFATTR	6	0.2276	4259.1	55.00	0.28	tr_C9ZQ90_
HLA-DQA10501-DQB10201	328	QLYVLAVYLSNTSST	YVLAVYLSN	2	0.2273	4272.6	55.00	0.24	tr_C9ZQ90_
HLA-DQA10501-DQB10201	329	SVTWSVSECSSANVQ	VTWSVSECS	1	0.2268	4296.9	55.00	0.46	tr_C9ZQ90_
HLA-DQA10501-DQB10201	330	LADRIYTESNIRVDD	IYTESNIRV	4	0.2258	4342.7	55.00	0.31	tr_C9ZQ90_
HLA-DQA10501-DQB10201	331	DFGGECDEGAALQGA	FGGECDEGA	1	0.2251	4378.9	55.00	0.43	tr_C9ZQ90_
HLA-DQA10501-DQB10201	332	SSFQHFLIKTWSDAL	LIKTWSDAL	6	0.2240	4430.1	60.00	0.40	tr_C9ZQ90_
HLA-DQA10501-DQB10201	333	GFAKPEHYLKDDKLG	EHYLKDDKL	5	0.2235	4455.6	60.00	0.75	tr_C9ZQ90_
HLA-DQA10501-DQB10201	334	LLFATSFPHWAEKNT	FATSFPHWA	2	0.2232	4470.8	60.00	0.54	tr_C9ZQ90_
HLA-DQA10501-DQB10201	335	LLADRIYTESNIRVD	IYTESNIRV	5	0.2221	4520.0	60.00	0.31	tr_C9ZQ90_
HLA-DQA10501-DQB10201	336	FAKPEHYLKDDKLGE	EHYLKDDKL	4	0.2206	4597.5	60.00	0.72	tr_C9ZQ90_
HLA-DQA10501-DQB10201	337	GPFSDAECVSGTSVS	PFSDAECVS	1	0.2205	4601.1	60.00	0.34	tr_C9ZQ90_
HLA-DQA10501-DQB10201	338	ELLADRIYTESNIRV	IYTESNIRV	6	0.2201	4619.6	60.00	0.30	tr_C9ZQ90_

HLA-DQA10501-DQB10201	339	CDEGAALQGAMCNCN	GAALQGAMC	3	0.2199	4628.6	60.00	0.31	tr_C9ZQ90_
HLA-DQA10501-DQB10201	340	AELLADRIYTESNIR	DRIYTESNI	5	0.2180	4726.3	60.00	0.17	tr_C9ZQ90_
HLA-DQA10501-DQB10201	341	DYVIPQEGQLTQSQI	YVIPQEGQL	1	0.2179	4731.3	60.00	0.29	tr_C9ZQ90_
HLA-DQA10501-DQB10201	342	LITTGTVPLASDNRS	ITTGTVPLA	1	0.2179	4733.9	60.00	0.25	tr_C9ZQ90_
HLA-DQA10501-DQB10201	343	ASNFGATNISVWSMA	FGATNISVW	3	0.2177	4741.4	60.00	0.38	tr_C9ZQ90_
HLA-DQA10501-DQB10201	344	FEALNNAPQLTNRTS	EALNNAPQL	1	0.2164	4807.6	60.00	0.47	tr_C9ZQ90_
HLA-DQA10501-DQB10201	345	AKPEHYLKDDKLGEM	EHYLKDDKL	3	0.2164	4811.1	60.00	0.67	tr_C9ZQ90_
HLA-DQA10501-DQB10201	346	KPEHYLKDDKLGEMM	EHYLKDDKL	2	0.2162	4820.2	60.00	0.58	tr_C9ZQ90_
HLA-DQA10501-DQB10201	347	AECVSGTSVSANECA	GTSVSANEC	5	0.2161	4826.2	60.00	0.31	tr_C9ZQ90_
HLA-DQA10501-DQB10201	348	RHVNESHWDPLTFLG	SHWDPLTFL	5	0.2161	4827.8	60.00	0.37	tr_C9ZQ90_
HLA-DQA10501-DQB10201	349	DTLDKSLVTFGVSLD	LVTFGVSLD	6	0.2160	4830.8	60.00	0.44	tr_C9ZQ90_
HLA-DQA10501-DQB10201	350	PEHYLKDDKLGEMMV	EHYLKDDKL	1	0.2146	4902.3	60.00	0.50	tr_C9ZQ90_
HLA-DQA10501-DQB10201	351	TFTPGQLITTGTVPL	LITTGTVPL	6	0.2142	4927.3	60.00	0.51	tr_C9ZQ90_
HLA-DQA10501-DQB10201	352	GATNISVWSMARVLN	ISVWSMARV	4	0.2134	4970.5	60.00	0.29	tr_C9ZQ90_
HLA-DQA10501-DQB10201	353	DEGAALQGAMCNCNQ	GAALQGAMC	2	0.2115	5068.8	60.00	0.28	tr_C9ZQ90_
HLA-DQA10501-DQB10201	354	HYLKDDKLGEMMVFG	DKLGEMMVF	5	0.2114	5076.6	60.00	0.41	tr_C9ZQ90_
HLA-DQA10501-DQB10201	355	MDNYLDTNSDWKGFA	NYLDTNSDW	2	0.2112	5088.1	60.00	0.38	tr_C9ZQ90_
HLA-DQA10501-DQB10201	356	ECASNFGATNISVWS	FGATNISVW	5	0.2111	5092.9	60.00	0.42	tr_C9ZQ90_
HLA-DQA10501-DQB10201	357	FIGTLPLSPRLNKFW	FIGTLPLSP	0	0.2108	5110.9	60.00	0.77	tr_C9ZQ90_
HLA-DQA10501-DQB10201	358	NECASNFGATNISVW	FGATNISVW	6	0.2103	5136.7	60.00	0.46	tr_C9ZQ90_
HLA-DQA10501-DQB10201	359	IGLTPPDVAAVARHL	IGLTPPDVA	0	0.2101	5150.8	60.00	0.35	tr_C9ZQ90_
HLA-DQA10501-DQB10201	360	LKNLTQSLEQVRDTK	NLTQSLEQV	2	0.2099	5159.5	60.00	0.46	tr_C9ZQ90_
HLA-DQA10501-DQB10201	361	RHLQTHRRARVFVPF	RRARVFVPF	6	0.2097	5171.1	60.00	0.56	tr_C9ZQ90_
HLA-DQA10501-DQB10201	362	VSGTSVSANECASNF	GTSVSANEC	2	0.2092	5198.2	60.00	0.28	tr_C9ZQ90_
HLA-DQA10501-DQB10201	363	FHRHVNESHWDPLTF	ESHWDPLTF	6	0.2090	5212.8	60.00	0.28	tr_C9ZQ90_
HLA-DQA10501-DQB10201	364	QRASMRWSLGARSIE	WSLGARSIE	6	0.2086	5235.2	60.00	0.37	tr_C9ZQ90_
HLA-DQA10501-DQB10201	365	VSLDSSKTLGDDDPI	KTLGDDDPI	6	0.2081	5261.6	60.00	0.35	tr_C9ZQ90_
HLA-DQA10501-DQB10201	366	SLDSSKTLGDDDPIS	SKTLGDDDP	4	0.2079	5274.3	60.00	0.28	tr_C9ZQ90_
HLA-DQA10501-DQB10201	367	SNFGATNISVWSMAR	FGATNISVW	2	0.2064	5357.6	65.00	0.40	tr_C9ZQ90_
HLA-DQA10501-DQB10201	368	GGECDEGAALQGAMC	GAALQGAMC	6	0.2064	5360.4	65.00	0.36	tr_C9ZQ90_
HLA-DQA10501-DQB10201	369	LDSSKTLGDDDPISS	SKTLGDDDP	3	0.2055	5414.1	65.00	0.27	tr_C9ZQ90_
HLA-DQA10501-DQB10201	370	DSSKTLGDDDPISSY	KTLGDDDPI	3	0.2051	5433.1	65.00	0.28	tr_C9ZQ90_
HLA-DQA10501-DQB10201	371	ECVSGTSVSANECAS	GTSVSANEC	4	0.2049	5444.7	65.00	0.34	tr_C9ZQ90_
HLA-DQA10501-DQB10201	372	VDKRIFFHSLKVNLK	FFHSLKVNL	5	0.2046	5461.9	65.00	0.38	tr_C9ZQ90_
HLA-DQA10501-DQB10201	373	LYVLAVYLSNTSSTG	YVLAVYLSN	1	0.2040	5500.9	65.00	0.26	tr_C9ZQ90_
HLA-DQA10501-DQB10201	374	DVDKRIFFHSLKVNL	FFHSLKVNL	6	0.2037	5519.6	65.00	0.50	tr_C9ZQ90_
HLA-DQA10501-DQB10201	375	ATTRLLQVVISNMRK	LLQVVISNM	4	0.2027	5578.0	65.00	0.19	tr_C9ZQ90_
HLA-DQA10501-DQB10201	376	FGGECDEGAALQGAM	FGGECDEGA	0	0.2013	5665.9	65.00	0.29	tr_C9ZQ90_
HLA-DQA10501-DQB10201	377	WSMARVLNSSLPRTQ	MARVLNSSL	2	0.2011	5676.5	65.00	0.24	tr_C9ZQ90_
HLA-DQA10501-DQB10201	378	MRVVDDSLSLKPMKK	RVVDDSLSL	1	0.2007	5698.8	65.00	0.49	tr_C9ZQ90_
HLA-DQA10501-DQB10201	379	NRSSMVRHFQRDMDN	VRHFQRDMD	5	0.1997	5759.3	65.00	0.46	tr_C9ZQ90_
HLA-DQA10501-DQB10201	380	KNLTQSLEQVRDTKA	NLTQSLEQV	1	0.1990	5809.1	65.00	0.44	tr_C9ZQ90_
HLA-DQA10501-DQB10201	381	DKRIFFHSLKVNLKN	FFHSLKVNL	4	0.1945	6096.8	65.00	0.37	tr_C9ZQ90_
HLA-DQA10501-DQB10201	382	VLTDDKVAQRASMRW	VAQRASMRW	6	0.1943	6109.8	65.00	0.59	tr_C9ZQ90_
HLA-DQA10501-DQB10201	383	DAECVSGTSVSANEC	GTSVSANEC	6	0.1942	6116.9	65.00	0.30	tr_C9ZQ90_
HLA-DQA10501-DQB10201	384	DALVSLNRTFTPGQL	NRTFTPGQL	6	0.1933	6178.5	65.00	0.28	tr_C9ZQ90_
HLA-DQA10501-DQB10201	385	VAAFNASKESIASSE	AFNASKESI	2	0.1932	6181.5	65.00	0.30	tr_C9ZQ90_
HLA-DQA10501-DQB10201	386	CVSGTSVSANECASN	GTSVSANEC	3	0.1929	6199.6	65.00	0.33	tr_C9ZQ90_
HLA-DQA10501-DQB10201	387	TNSDWKGFAKPEHYL	KGFAKPEHY	5	0.1929	6204.3	65.00	0.42	tr_C9ZQ90_
HLA-DQA10501-DQB10201	388	DNYLDTNSDWKGFAK	NYLDTNSDW	1	0.1924	6238.6	65.00	0.38	tr_C9ZQ90_

HLA-DQA10501-DQB10201	389	SSKTLGDDDPISSYL	KTLGDDDPI	2	0.1923	6239.5	65.00	0.28	tr_C9ZQ90_
HLA-DQA10501-DQB10201	390	SGTSVSANECASNFG	GTSVSANEC	1	0.1912	6319.9	65.00	0.28	tr_C9ZQ90_
HLA-DQA10501-DQB10201	391	KRIFFHSLKVNLKNL	FFHSLKVNL	3	0.1908	6345.2	65.00	0.37	tr_C9ZQ90_
HLA-DQA10501-DQB10201	392	YVLAVYLSNTSSTGV	VLAVYLSNT	1	0.1908	6345.3	65.00	0.25	tr_C9ZQ90_
HLA-DQA10501-DQB10201	393	LFATSFPHWAEKNTK	FATSFPHWA	1	0.1904	6373.7	65.00	0.56	tr_C9ZQ90_
HLA-DQA10501-DQB10201	394	ARFHRHVNESHWDPL	RHVNESHWD	4	0.1899	6405.4	65.00	0.26	tr_C9ZQ90_
HLA-DQA10501-DQB10201	395	ALVSLNRTFTPGQLI	NRTFTPGQL	5	0.1889	6477.3	70.00	0.37	tr_C9ZQ90_
HLA-DQA10501-DQB10201	396	APSSFQHFLIKTWSD	FQHFLIKTW	4	0.1882	6526.0	70.00	0.36	tr_C9ZQ90_
HLA-DQA10501-DQB10201	397	SDALVSLNRTFTPGQ	DALVSLNRT	1	0.1881	6534.6	70.00	0.39	tr_C9ZQ90_
HLA-DQA10501-DQB10201	398	DNRSSMVRHFQRDMD	VRHFQRDMD	6	0.1874	6579.7	70.00	0.52	tr_C9ZQ90_
HLA-DQA10501-DQB10201	399	DTNSDWKGFAKPEHY	KGFAKPEHY	6	0.1864	6655.2	70.00	0.43	tr_C9ZQ90_
HLA-DQA10501-DQB10201	400	PFSDAECVSGTSVSA	AECVSGTSV	4	0.1852	6738.6	70.00	0.37	tr_C9ZQ90_
HLA-DQA10501-DQB10201	401	LLAPSSFQHFLIKTW	FQHFLIKTW	6	0.1849	6765.1	70.00	0.42	tr_C9ZQ90_
HLA-DQA10501-DQB10201	402	PSSFQHFLIKTWSDA	FQHFLIKTW	3	0.1843	6804.6	70.00	0.34	tr_C9ZQ90_
HLA-DQA10501-DQB10201	403	TTRLLQVVISNMRKV	LLQVVISNM	3	0.1836	6859.8	70.00	0.25	tr_C9ZQ90_
HLA-DQA10501-DQB10201	404	VTWSVSECSSANVQV	VTWSVSECS	0	0.1834	6876.0	70.00	0.32	tr_C9ZQ90_
HLA-DQA10501-DQB10201	405	VARFHRHVNESHWDP	RHVNESHWD	5	0.1825	6943.8	70.00	0.29	tr_C9ZQ90_
HLA-DQA10501-DQB10201	406	RIFFHSLKVNLKNLT	FFHSLKVNL	2	0.1816	7005.9	70.00	0.38	tr_C9ZQ90_
HLA-DQA10501-DQB10201	407	LTPPDVAAVARHLQT	DVAAVARHL	4	0.1812	7037.2	70.00	0.34	tr_C9ZQ90_
HLA-DQA10501-DQB10201	408	QSLEQVRDTKAVAAV	VRDTKAVAA	5	0.1811	7049.4	70.00	0.55	tr_C9ZQ90_
HLA-DQA10501-DQB10201	409	SLKVNLKNLTQSLEQ	KNLTQSLEQ	6	0.1801	7123.1	70.00	0.44	tr_C9ZQ90_
HLA-DQA10501-DQB10201	410	ITTGTVPLASDNRSS	ITTGTVPLA	0	0.1800	7127.3	70.00	0.25	tr_C9ZQ90_
HLA-DQA10501-DQB10201	411	RTSFMESLYKQRRYV	RTSFMESLY	0	0.1799	7140.3	70.00	0.38	tr_C9ZQ90_
HLA-DQA10501-DQB10201	412	SEVVDTLDKSLVTFG	EVVDTLDKS	1	0.1798	7145.7	70.00	0.31	tr_C9ZQ90_
HLA-DQA10501-DQB10201	413	LTDDKVAQRASMRWS	VAQRASMRW	5	0.1792	7192.3	70.00	0.57	tr_C9ZQ90_
HLA-DQA10501-DQB10201	414	RFHRHVNESHWDPLT	RHVNESHWD	3	0.1787	7235.9	70.00	0.27	tr_C9ZQ90_
HLA-DQA10501-DQB10201	415	TQSLEQVRDTKAVAA	VRDTKAVAA	6	0.1782	7270.5	70.00	0.54	tr_C9ZQ90_
HLA-DQA10501-DQB10201	416	EGAALQGAMCNCNQG	GAALQGAMC	1	0.1776	7318.4	70.00	0.31	tr_C9ZQ90_
HLA-DQA10501-DQB10201	417	HWAEKNTKSDMVARF	TKSDMVARF	6	0.1774	7332.7	70.00	0.65	tr_C9ZQ90_
HLA-DQA10501-DQB10201	418	GVSLDSSKTLGDDDP	SKTLGDDDP	6	0.1773	7343.2	70.00	0.36	tr_C9ZQ90_
HLA-DQA10501-DQB10201	419	TDDKVAQRASMRWSL	VAQRASMRW	4	0.1767	7390.3	70.00	0.56	tr_C9ZQ90_
HLA-DQA10501-DQB10201	420	WAEKNTKSDMVARFH	TKSDMVARF	5	0.1759	7451.3	70.00	0.50	tr_C9ZQ90_
HLA-DQA10501-DQB10201	421	GLTPPDVAAVARHLQ	DVAAVARHL	5	0.1754	7494.3	70.00	0.41	tr_C9ZQ90_
HLA-DQA10501-DQB10201	422	FVKKIAQDNRTVDMY	QDNRTVDMY	6	0.1743	7588.8	70.00	0.33	tr_C9ZQ90_
HLA-DQA10501-DQB10201	423	LAPSSFQHFLIKTWS	FQHFLIKTW	5	0.1741	7600.7	70.00	0.35	tr_C9ZQ90_
HLA-DQA10501-DQB10201	424	MVARFHRHVNESHWD	RHVNESHWD	6	0.1736	7645.6	70.00	0.31	tr_C9ZQ90_
HLA-DQA10501-DQB10201	425	AAFNASKESIASSER	AFNASKESI	1	0.1723	7754.2	70.00	0.29	tr_C9ZQ90_
HLA-DQA10501-DQB10201	426	NLTQSLEQVRDTKAV	NLTQSLEQV	0	0.1721	7764.6	70.00	0.37	tr_C9ZQ90_
HLA-DQA10501-DQB10201	427	NSDWKGFAKPEHYLK	KGFAKPEHY	4	0.1710	7861.5	75.00	0.41	tr_C9ZQ90_
HLA-DQA10501-DQB10201	428	SDWKGFAKPEHYLKD	KGFAKPEHY	3	0.1707	7883.0	75.00	0.46	tr_C9ZQ90_
HLA-DQA10501-DQB10201	429	LVSLNRTFTPGQLIT	NRTFTPGQL	4	0.1704	7913.8	75.00	0.37	tr_C9ZQ90_
HLA-DQA10501-DQB10201	430	SMARVLNSSLPRTQV	VLNSSLPRT	4	0.1686	8065.7	75.00	0.28	tr_C9ZQ90_
HLA-DQA10501-DQB10201	431	SKTLGDDDPISSYLS	TLGDDDPIS	2	0.1672	8193.4	75.00	0.26	tr_C9ZQ90_
HLA-DQA10501-DQB10201	432	STGVKALVRGGEASE	KALVRGGEA	4	0.1665	8248.3	75.00	0.39	tr_C9ZQ90_
HLA-DQA10501-DQB10201	433	IFFHSLKVNLKNLTQ	FFHSLKVNL	1	0.1662	8277.5	75.00	0.41	tr_C9ZQ90_
HLA-DQA10501-DQB10201	434	TPPDVAAVARHLQTH	DVAAVARHL	3	0.1662	8278.2	75.00	0.34	tr_C9ZQ90_
HLA-DQA10501-DQB10201	435	TRLLQVVISNMRKVN	LLQVVISNM	2	0.1650	8383.4	75.00	0.29	tr_C9ZQ90_
HLA-DQA10501-DQB10201	436	LQVVISNMRKVNAEL	NMRKVNAEL	6	0.1640	8478.8	75.00	0.44	tr_C9ZQ90_
HLA-DQA10501-DQB10201	437	GTSVSANECASNFGA	TSVSANECA	1	0.1636	8512.5	75.00	0.28	tr_C9ZQ90_
HLA-DQA10501-DQB10201	438	AQRASMRWSLGARSI	RWSLGARSI	6	0.1634	8534.8	75.00	0.42	tr_C9ZQ90_

HLA-DQA10501-DQB10201	439	YLLAPSSFQHFLIKT	PSSFQHFLI	4	0.1627	8602.3	75.00	0.24	tr_C9ZQ90_
HLA-DQA10501-DQB10201	440	DDKVAQRASMRWSLG	VAQRASMRW	3	0.1624	8623.4	75.00	0.54	tr_C9ZQ90_
HLA-DQA10501-DQB10201	441	EALNNAPQLTNRTSF	ALNNAPQLT	1	0.1624	8630.7	75.00	0.49	tr_C9ZQ90_
HLA-DQA10501-DQB10201	442	NSSLPRTQVGMTPSM	TQVGMTPSM	6	0.1613	8730.5	75.00	0.34	tr_C9ZQ90_
HLA-DQA10501-DQB10201	443	VSLNRTFTPGQLITT	NRTFTPGQL	3	0.1612	8738.3	75.00	0.37	tr_C9ZQ90_
HLA-DQA10501-DQB10201	444	EVVDTLDKSLVTFGV	VVDTLDKSL	1	0.1595	8900.2	75.00	0.28	tr_C9ZQ90_
HLA-DQA10501-DQB10201	445	NYLDTNSDWKGFAKP	YLDTNSDWK	1	0.1588	8966.8	75.00	0.44	tr_C9ZQ90_
HLA-DQA10501-DQB10201	446	SDAECVSGTSVSANE	AECVSGTSV	2	0.1588	8973.3	75.00	0.33	tr_C9ZQ90_
HLA-DQA10501-DQB10201	447	VDTLDKSLVTFGVSL	SLVTFGVSL	6	0.1583	9020.4	75.00	0.49	tr_C9ZQ90_
HLA-DQA10501-DQB10201	448	SVSECSSANVQVSAP	SANVQVSAP	6	0.1581	9041.1	75.00	0.38	tr_C9ZQ90_
HLA-DQA10501-DQB10201	449	DWKGFAKPEHYLKDD	KGFAKPEHY	2	0.1580	9046.9	75.00	0.46	tr_C9ZQ90_
HLA-DQA10501-DQB10201	450	ANECASNFGATNISV	NFGATNISV	6	0.1580	9051.4	75.00	0.50	tr_C9ZQ90_
HLA-DQA10501-DQB10201	451	AEKNTKSDMVARFHR	TKSDMVARF	4	0.1561	9234.1	75.00	0.49	tr_C9ZQ90_
HLA-DQA10501-DQB10201	452	RVVDDSLSLKPMKKG	RVVDDSLSL	0	0.1555	9296.2	75.00	0.56	tr_C9ZQ90_
HLA-DQA10501-DQB10201	453	RTFTPGQLITTGTVP	QLITTGTVP	6	0.1552	9330.0	75.00	0.38	tr_C9ZQ90_
HLA-DQA10501-DQB10201	454	NNAPQLTNRTSFMES	QLTNRTSFM	4	0.1543	9417.9	75.00	0.43	tr_C9ZQ90_
HLA-DQA10501-DQB10201	455	NCNQGGSMAHMRVVD	GGSMAHMRV	4	0.1541	9440.9	75.00	0.35	tr_C9ZQ90_
HLA-DQA10501-DQB10201	456	IEEADDVDKRIFFHS	EADDVDKRI	2	0.1521	9641.5	80.00	0.38	tr_C9ZQ90_
HLA-DQA10501-DQB10201	457	FGVSLDSSKTLGDDD	VSLDSSKTL	2	0.1514	9719.8	80.00	0.38	tr_C9ZQ90_
HLA-DQA10501-DQB10201	458	TSSTGVKALVRGGEA	KALVRGGEA	6	0.1506	9806.8	80.00	0.46	tr_C9ZQ90_
HLA-DQA10501-DQB10201	459	DKVAQRASMRWSLGA	VAQRASMRW	2	0.1503	9834.1	80.00	0.55	tr_C9ZQ90_
HLA-DQA10501-DQB10201	460	FSDAECVSGTSVSAN	AECVSGTSV	3	0.1501	9858.5	80.00	0.40	tr_C9ZQ90_
HLA-DQA10501-DQB10201	461	PPDVAAVARHLQTHR	DVAAVARHL	2	0.1498	9885.1	80.00	0.38	tr_C9ZQ90_
HLA-DQA10501-DQB10201	462	MARVLNSSLPRTQVG	VLNSSLPRT	3	0.1471	10177.0	80.00	0.32	tr_C9ZQ90_
HLA-DQA10501-DQB10201	463	VAQRASMRWSLGARS	MRWSLGARS	6	0.1465	10242.0	80.00	0.44	tr_C9ZQ90_
HLA-DQA10501-DQB10201	464	SPRLNKFWRNVIHLQ	FWRNVIHLQ	6	0.1458	10319.7	80.00	0.45	tr_C9ZQ90_
HLA-DQA10501-DQB10201	465	TTGTVPLASDNRSSM	TVPLASDNR	3	0.1458	10322.2	80.00	0.28	tr_C9ZQ90_
HLA-DQA10501-DQB10201	466	RLLQVVISNMRKVNA	LLQVVISNM	1	0.1444	10484.9	80.00	0.37	tr_C9ZQ90_
HLA-DQA10501-DQB10201	467	SLNRTFTPGQLITTG	NRTFTPGQL	2	0.1435	10579.6	80.00	0.41	tr_C9ZQ90_
HLA-DQA10501-DQB10201	468	LNRTFTPGQLITTGT	NRTFTPGQL	1	0.1423	10718.0	80.00	0.32	tr_C9ZQ90_
HLA-DQA10501-DQB10201	469	HSLKVNLKNLTQSLE	LKNLTQSLE	6	0.1423	10719.7	80.00	0.47	tr_C9ZQ90_
HLA-DQA10501-DQB10201	470	SSTGVKALVRGGEAS	KALVRGGEA	5	0.1413	10838.2	80.00	0.47	tr_C9ZQ90_
HLA-DQA10501-DQB10201	471	FFHSLKVNLKNLTQS	FHSLKVNLK	1	0.1412	10849.8	80.00	0.42	tr_C9ZQ90_
HLA-DQA10501-DQB10201	472	VLAVYLSNTSSTGVK	LAVYLSNTS	1	0.1398	11020.5	80.00	0.25	tr_C9ZQ90_
HLA-DQA10501-DQB10201	473	EKNTKSDMVARFHRH	TKSDMVARF	3	0.1394	11060.2	80.00	0.50	tr_C9ZQ90_
HLA-DQA10501-DQB10201	474	GAALQGAMCNCNQGG	AALQGAMCN	1	0.1387	11151.9	80.00	0.29	tr_C9ZQ90_
HLA-DQA10501-DQB10201	475	VSANECASNFGATNI	ASNFGATNI	6	0.1386	11163.3	80.00	0.48	tr_C9ZQ90_
HLA-DQA10501-DQB10201	476	ADDVDKRIFFHSLKV	RIFFHSLKV	6	0.1379	11246.2	80.00	0.47	tr_C9ZQ90_
HLA-DQA10501-DQB10201	477	TWSVSECSSANVQVS	VSECSSANV	3	0.1369	11370.0	80.00	0.20	tr_C9ZQ90_
HLA-DQA10501-DQB10201	478	WKGFAKPEHYLKDDK	KGFAKPEHY	1	0.1368	11379.7	80.00	0.44	tr_C9ZQ90_
HLA-DQA10501-DQB10201	479	EEADDVDKRIFFHSL	EADDVDKRI	1	0.1364	11429.5	80.00	0.34	tr_C9ZQ90_
HLA-DQA10501-DQB10201	480	KTLGDDDPISSYLSG	TLGDDDPIS	1	0.1364	11435.2	80.00	0.37	tr_C9ZQ90_
HLA-DQA10501-DQB10201	481	WSVSECSSANVQVSA	SSANVQVSA	6	0.1359	11489.7	80.00	0.25	tr_C9ZQ90_
HLA-DQA10501-DQB10201	482	PDVAAVARHLQTHRR	DVAAVARHL	1	0.1356	11530.8	80.00	0.40	tr_C9ZQ90_
HLA-DQA10501-DQB10201	483	NRTFTPGQLITTGTV	RTFTPGQLI	1	0.1355	11542.4	80.00	0.23	tr_C9ZQ90_
HLA-DQA10501-DQB10201	484	KPMKKGSVTWSVSEC	SVTWSVSEC	6	0.1351	11585.6	80.00	0.50	tr_C9ZQ90_
HLA-DQA10501-DQB10201	485	TGTVPLASDNRSSMV	TVPLASDNR	2	0.1349	11619.4	80.00	0.31	tr_C9ZQ90_
HLA-DQA10501-DQB10201	486	DDVDKRIFFHSLKVN	RIFFHSLKV	5	0.1346	11655.1	80.00	0.41	tr_C9ZQ90_
HLA-DQA10501-DQB10201	487	DMVARFHRHVNESHW	FHRHVNESH	5	0.1344	11684.2	80.00	0.41	tr_C9ZQ90_
HLA-DQA10501-DQB10201	488	LLQVVISNMRKVNAE	LLQVVISNM	0	0.1330	11856.6	85.00	0.32	tr_C9ZQ90_

HLA-DQA10501-DQB10201	489	VVDTLDKSLVTFGVS	VVDTLDKSL	0	0.1314	12068.1	85.00	0.22	tr_C9ZQ90_
HLA-DQA10501-DQB10201	490	LDTNSDWKGFAKPEH	WKGFAKPEH	6	0.1314	12069.5	85.00	0.61	tr_C9ZQ90_
HLA-DQA10501-DQB10201	491	VLNSSLPRTQVGMTP	PRTQVGMTP	6	0.1311	12101.9	85.00	0.55	tr_C9ZQ90_
HLA-DQA10501-DQB10201	492	ALNNAPQLTNRTSFM	QLTNRTSFM	6	0.1307	12161.7	85.00	0.43	tr_C9ZQ90_
HLA-DQA10501-DQB10201	493	LNNAPQLTNRTSFME	QLTNRTSFM	5	0.1305	12187.7	85.00	0.56	tr_C9ZQ90_
HLA-DQA10501-DQB10201	494	FATSFPHWAEKNTKS	FATSFPHWA	0	0.1303	12209.0	85.00	0.62	tr_C9ZQ90_
HLA-DQA10501-DQB10201	495	KVAQRASMRWSLGAR	VAQRASMRW	1	0.1299	12261.3	85.00	0.50	tr_C9ZQ90_
HLA-DQA10501-DQB10201	496	LTQSLEQVRDTKAVA	LEQVRDTKA	4	0.1295	12313.0	85.00	0.32	tr_C9ZQ90_
HLA-DQA10501-DQB10201	497	LNSSLPRTQVGMTPS	PRTQVGMTP	5	0.1294	12331.9	85.00	0.50	tr_C9ZQ90_
HLA-DQA10501-DQB10201	498	KNTKSDMVARFHRHV	TKSDMVARF	2	0.1291	12368.7	85.00	0.50	tr_C9ZQ90_
HLA-DQA10501-DQB10201	499	TSVSANECASNFGAT	SANECASNF	3	0.1280	12520.4	85.00	0.28	tr_C9ZQ90_
HLA-DQA10501-DQB10201	500	DDDPISSYLSGNGDV	SSYLSGNGD	5	0.1268	12677.9	85.00	0.37	tr_C9ZQ90_
HLA-DQA10501-DQB10201	501	SANECASNFGATNIS	ASNFGATNI	5	0.1222	13331.9	85.00	0.48	tr_C9ZQ90_
HLA-DQA10501-DQB10201	502	YLSNTSSTGVKALVR	STGVKALVR	6	0.1221	13338.4	85.00	0.44	tr_C9ZQ90_
HLA-DQA10501-DQB10201	503	DVAAVARHLQTHRRA	DVAAVARHL	0	0.1219	13370.3	85.00	0.34	tr_C9ZQ90_
HLA-DQA10501-DQB10201	504	NTKSDMVARFHRHVN	TKSDMVARF	1	0.1216	13415.5	85.00	0.49	tr_C9ZQ90_
HLA-DQA10501-DQB10201	505	TSFMESLYKQRRYVI	FMESLYKQR	2	0.1216	13421.6	85.00	0.30	tr_C9ZQ90_
HLA-DQA10501-DQB10201	506	CNCNQGGSMAHMRVV	GGSMAHMRV	5	0.1209	13516.9	85.00	0.51	tr_C9ZQ90_
HLA-DQA10501-DQB10201	507	ARHLQTHRRARVFVP	THRRARVFV	5	0.1209	13522.8	85.00	0.40	tr_C9ZQ90_
HLA-DQA10501-DQB10201	508	GTVPLASDNRSSMVR	VPLASDNRS	2	0.1208	13524.8	85.00	0.35	tr_C9ZQ90_
HLA-DQA10501-DQB10201	509	ARVLNSSLPRTQVGM	VLNSSLPRT	2	0.1207	13548.9	85.00	0.38	tr_C9ZQ90_
HLA-DQA10501-DQB10201	510	FHSLKVNLKNLTQSL	NLKNLTQSL	6	0.1206	13555.0	85.00	0.37	tr_C9ZQ90_
HLA-DQA10501-DQB10201	511	MCNCNQGGSMAHMRV	GGSMAHMRV	6	0.1184	13883.7	85.00	0.58	tr_C9ZQ90_
HLA-DQA10501-DQB10201	512	TKSDMVARFHRHVNE	TKSDMVARF	0	0.1178	13979.2	85.00	0.31	tr_C9ZQ90_
HLA-DQA10501-DQB10201	513	SDNRSSMVRHFQRDM	MVRHFQRDM	6	0.1170	14102.0	85.00	0.29	tr_C9ZQ90_
HLA-DQA10501-DQB10201	514	SFMESLYKQRRYVID	FMESLYKQR	1	0.1149	14420.3	85.00	0.25	tr_C9ZQ90_
HLA-DQA10501-DQB10201	515	LSPRLNKFWRNVIHL	KFWRNVIHL	6	0.1147	14460.0	85.00	0.49	tr_C9ZQ90_
HLA-DQA10501-DQB10201	516	LSNTSSTGVKALVRG	STGVKALVR	5	0.1141	14544.2	85.00	0.45	tr_C9ZQ90_
HLA-DQA10501-DQB10201	517	VVLTDDKVAQRASMR	VVLTDDKVA	0	0.1136	14633.1	90.00	0.22	tr_C9ZQ90_
HLA-DQA10501-DQB10201	518	TLGDDDPISSYLSGN	TLGDDDPIS	0	0.1124	14812.9	90.00	0.26	tr_C9ZQ90_
HLA-DQA10501-DQB10201	519	FMESLYKQRRYVIDD	YKQRRYVID	5	0.1123	14831.5	90.00	0.22	tr_C9ZQ90_
HLA-DQA10501-DQB10201	520	EADDVDKRIFFHSLK	DKRIFFHSL	5	0.1112	15009.4	90.00	0.28	tr_C9ZQ90_
HLA-DQA10501-DQB10201	521	SDMVARFHRHVNESH	FHRHVNESH	6	0.1108	15071.6	90.00	0.46	tr_C9ZQ90_
HLA-DQA10501-DQB10201	522	LAVYLSNTSSTGVKA	LAVYLSNTS	0	0.1106	15107.0	90.00	0.20	tr_C9ZQ90_
HLA-DQA10501-DQB10201	523	YLDTNSDWKGFAKPE	YLDTNSDWK	0	0.1093	15328.2	90.00	0.50	tr_C9ZQ90_
HLA-DQA10501-DQB10201	524	TVPLASDNRSSMVRH	VPLASDNRS	1	0.1071	15699.9	90.00	0.34	tr_C9ZQ90_
HLA-DQA10501-DQB10201	525	NTSSTGVKALVRGGE	STGVKALVR	3	0.1067	15765.5	90.00	0.40	tr_C9ZQ90_
HLA-DQA10501-DQB10201	526	VYLSNTSSTGVKALV	SSTGVKALV	6	0.1058	15921.3	90.00	0.34	tr_C9ZQ90_
HLA-DQA10501-DQB10201	527	GDDDPISSYLSGNGD	SSYLSGNGD	6	0.1048	16084.2	90.00	0.43	tr_C9ZQ90_
HLA-DQA10501-DQB10201	528	VARHLQTHRRARVFV	THRRARVFV	6	0.1042	16196.0	90.00	0.48	tr_C9ZQ90_
HLA-DQA10501-DQB10201	529	SVSANECASNFGATN	SANECASNF	2	0.1029	16422.4	90.00	0.34	tr_C9ZQ90_
HLA-DQA10501-DQB10201	530	AALQGAMCNCNQGGS	AALQGAMCN	0	0.1026	16475.6	90.00	0.25	tr_C9ZQ90_
HLA-DQA10501-DQB10201	531	SNTSSTGVKALVRGG	STGVKALVR	4	0.1021	16573.6	90.00	0.44	tr_C9ZQ90_
HLA-DQA10501-DQB10201	532	ASDNRSSMVRHFQRD	NRSSMVRHF	3	0.0998	16974.3	90.00	0.28	tr_C9ZQ90_
HLA-DQA10501-DQB10201	533	RVLNSSLPRTQVGMT	VLNSSLPRT	1	0.0988	17171.2	90.00	0.38	tr_C9ZQ90_
HLA-DQA10501-DQB10201	534	AVYLSNTSSTGVKAL	LSNTSSTGV	3	0.0944	18001.1	90.00	0.20	tr_C9ZQ90_
HLA-DQA10501-DQB10201	535	KSDMVARFHRHVNES	ARFHRHVNE	5	0.0924	18399.6	90.00	0.32	tr_C9ZQ90_
HLA-DQA10501-DQB10201	536	VPLASDNRSSMVRHF	NRSSMVRHF	6	0.0924	18402.2	90.00	0.40	tr_C9ZQ90_
HLA-DQA10501-DQB10201	537	LASDNRSSMVRHFQR	NRSSMVRHF	4	0.0916	18568.4	90.00	0.34	tr_C9ZQ90_
HLA-DQA10501-DQB10201	538	PLASDNRSSMVRHFQ	NRSSMVRHF	5	0.0900	18884.9	95.00	0.38	tr_C9ZQ90_

HLA-DQA10501-DQB10201	539	LGDDDPISSYLSGNG	PISSYLSGN	5	0.0882	19249.6	95.00	0.26	tr_C9ZQ90_
HLA-DQA10501-DQB10201	540	FPHWAEKNTKSDMVA	EKNTKSDMV	5	0.0871	19476.1	95.00	0.43	tr_C9ZQ90_
HLA-DQA10501-DQB10201	541	LKPMKKGSVTWSVSE	GSVTWSVSE	6	0.0829	20396.1	95.00	0.41	tr_C9ZQ90_
HLA-DQA10501-DQB10201	542	SFPHWAEKNTKSDMV	EKNTKSDMV	6	0.0825	20477.9	95.00	0.49	tr_C9ZQ90_
HLA-DQA10501-DQB10201	543	VAAVARHLQTHRRAR	VAAVARHLQ	0	0.0814	20730.7	95.00	0.26	tr_C9ZQ90_
HLA-DQA10501-DQB10201	544	ALQGAMCNCNQGGSM	QGAMCNCNQ	2	0.0776	21600.8	95.00	0.29	tr_C9ZQ90_
HLA-DQA10501-DQB10201	545	VVDDSLSLKPMKKGS	VVDDSLSLK	0	0.0774	21629.4	95.00	0.24	tr_C9ZQ90_
HLA-DQA10501-DQB10201	546	PHWAEKNTKSDMVAR	EKNTKSDMV	4	0.0762	21923.0	95.00	0.44	tr_C9ZQ90_
HLA-DQA10501-DQB10201	547	PLSPRLNKFWRNVIH	LNKFWRNVI	5	0.0758	22019.0	95.00	0.40	tr_C9ZQ90_
HLA-DQA10501-DQB10201	548	LPLSPRLNKFWRNVI	LNKFWRNVI	6	0.0742	22399.1	95.00	0.44	tr_C9ZQ90_
HLA-DQA10501-DQB10201	549	IGTLPLSPRLNKFWR	PLSPRLNKF	4	0.0666	24321.0	95.00	0.22	tr_C9ZQ90_
HLA-DQA10501-DQB10201	550	AVARHLQTHRRARVF	QTHRRARVF	6	0.0666	24325.2	95.00	0.31	tr_C9ZQ90_
HLA-DQA10501-DQB10201	551	GTLPLSPRLNKFWRN	PLSPRLNKF	3	0.0612	25796.0	100.00	0.21	tr_C9ZQ90_
HLA-DQA10501-DQB10201	552	AAVARHLQTHRRARV	RHLQTHRRA	4	0.0606	25945.8	100.00	0.23	tr_C9ZQ90_
HLA-DQA10501-DQB10201	553	ATSFPHWAEKNTKSD	SFPHWAEKN	2	0.0599	26147.6	100.00	0.24	tr_C9ZQ90_
HLA-DQA10501-DQB10201	554	SLKPMKKGSVTWSVS	PMKKGSVTW	3	0.0599	26148.7	100.00	0.25	tr_C9ZQ90_
HLA-DQA10501-DQB10201	555	VDDSLSLKPMKKGSV	SLSLKPMKK	3	0.0592	26346.3	100.00	0.32	tr_C9ZQ90_
HLA-DQA10501-DQB10201	556	TLPLSPRLNKFWRNV	PLSPRLNKF	2	0.0585	26563.0	100.00	0.22	tr_C9ZQ90_
HLA-DQA10501-DQB10201	557	TSFPHWAEKNTKSDM	SFPHWAEKN	1	0.0579	26732.3	100.00	0.19	tr_C9ZQ90_
HLA-DQA10501-DQB10201	558	LSLKPMKKGSVTWSV	PMKKGSVTW	4	0.0576	26803.8	100.00	0.31	tr_C9ZQ90_
HLA-DQA10501-DQB10201	559	AMCNCNQGGSMAHMR	NQGGSMAHM	5	0.0569	27013.1	100.00	0.43	tr_C9ZQ90_
HLA-DQA10501-DQB10201	560	LQGAMCNCNQGGSMA	QGAMCNCNQ	1	0.0551	27546.2	100.00	0.33	tr_C9ZQ90_
HLA-DQA10501-DQB10201	561	GAMCNCNQGGSMAHM	NQGGSMAHM	6	0.0543	27771.8	100.00	0.58	tr_C9ZQ90_
HLA-DQA10501-DQB10201	562	SLSLKPMKKGSVTWS	PMKKGSVTW	5	0.0533	28100.7	100.00	0.43	tr_C9ZQ90_
HLA-DQA10501-DQB10201	563	DSLSLKPMKKGSVTW	PMKKGSVTW	6	0.0505	28950.3	100.00	0.50	tr_C9ZQ90_
HLA-DQA10501-DQB10201	564	DDSLSLKPMKKGSVT	SLSLKPMKK	2	0.0436	31205.8	100.00	0.49	tr_C9ZQ90_
HLA-DQA10501-DQB10201	565	QGAMCNCNQGGSMAH	QGAMCNCNQ	0	0.0395	32622.7	100.00	0.28	tr_C9ZQ90_

Allele: HLA-DQA10501-DQB10201. Number of high binders 6. Number of weak binders 45. Number of peptides 565

Allele	pos	peptide	core	Of	1-log50k(aff)	affinity(nM)	%Rank	Relia	Identity	Bind Level
HLA-DQA10501-DQB10201	1	WAAREGYADVVEYLL	GYADVVEYL	5	0.6046	72.1	1.20	0.29	tr_D0A254_	WB
HLA-DQA10501-DQB10201	2	AAREGYADVVEYLLC	GYADVVEYL	4	0.5769	97.3	1.90	0.31	tr_D0A254_	WB
HLA-DQA10501-DQB10201	3	VWAAREGYADVVEYL	GYADVVEYL	6	0.5582	119.1	2.50	0.40	tr_D0A254_	WB
HLA-DQA10501-DQB10201	4	AREGYADVVEYLLCA	GYADVVEYL	3	0.5519	127.6	3.00	0.32	tr_D0A254_	WB
HLA-DQA10501-DQB10201	5	YLLCAGADVDVVDTD	CAGADVDVV	3	0.5312	159.6	4.00	0.23	tr_D0A254_	WB
HLA-DQA10501-DQB10201	6	RNHPEVQYVLENYPL	EVQYVLENY	4	0.5169	186.2	4.50	0.51	tr_D0A254_	WB
HLA-DQA10501-DQB10201	7	REGYADVVEYLLCAG	GYADVVEYL	2	0.5138	192.6	4.50	0.37	tr_D0A254_	WB
HLA-DQA10501-DQB10201	8	EYLLCAGADVDVVDT	CAGADVDVV	4	0.5115	197.4	5.00	0.25	tr_D0A254_	WB
HLA-DQA10501-DQB10201	9	LCAGADVDVVDTDGY	CAGADVDVV	1	0.5099	201.0	5.00	0.21	tr_D0A254_	WB
HLA-DQA10501-DQB10201	10	HRNHPEVQYVLENYP	EVQYVLENY	5	0.5042	213.6	5.50	0.57	tr_D0A254_	WB
HLA-DQA10501-DQB10201	11	LLCAGADVDVVDTDG	CAGADVDVV	2	0.5040	214.2	5.50	0.23	tr_D0A254_	WB
HLA-DQA10501-DQB10201	12	NHPEVQYVLENYPLA	EVQYVLENY	3	0.5037	214.9	5.50	0.50	tr_D0A254_	WB
HLA-DQA10501-DQB10201	13	VEYLLCAGADVDVVD	CAGADVDVV	5	0.5032	216.1	5.50	0.27	tr_D0A254_	WB
HLA-DQA10501-DQB10201	14	LVWAAREGYADVVEY	EGYADVVEY	6	0.4997	224.3	5.50	0.44	tr_D0A254_	WB
HLA-DQA10501-DQB10201	15	HPEVQYVLENYPLAQ	EVQYVLENY	2	0.4940	238.5	6.00	0.47	tr_D0A254_	WB

HLA-DQA10501-DQB10201	16	CAGADVDVVDTDGYT	ADVDVVDTD	3	0.4853	262.1	6.50	0.20	tr_D0A254_	WB
HLA-DQA10501-DQB10201	17	EGYADVVEYLLCAGA	GYADVVEYL	1	0.4762	289.2	7.50	0.38	tr_D0A254_	WB
HLA-DQA10501-DQB10201	18	KHRNHPEVQYVLENY	EVQYVLENY	6	0.4726	300.8	7.50	0.68	tr_D0A254_	WB
HLA-DQA10501-DQB10201	19	PEVQYVLENYPLAQR	EVQYVLENY	1	0.4660	323.0	8.00	0.48	tr_D0A254_	WB
HLA-DQA10501-DQB10201	20	IHVVRFLVDAGADIN	FLVDAGADI	5	0.4594	347.0	9.00	0.52	tr_D0A254_	WB
HLA-DQA10501-DQB10201	21	HIHVVRFLVDAGADI	FLVDAGADI	6	0.4561	359.5	9.00	0.56	tr_D0A254_	WB
HLA-DQA10501-DQB10201	22	HYQPYTPCAFPVEWL	TPCAFPVEW	5	0.4552	363.2	9.00	0.36	tr_D0A254_	WB
HLA-DQA10501-DQB10201	23	HVVRFLVDAGADINA	FLVDAGADI	4	0.4515	378.0	9.50	0.52	tr_D0A254_	WB
HLA-DQA10501-DQB10201	24	VVEYLLCAGADVDVV	CAGADVDVV	6	0.4492	387.4	9.50	0.28	tr_D0A254_	WB
HLA-DQA10501-DQB10201	25	AGADVDVVDTDGYTA	ADVDVVDTD	2	0.4469	397.1	10.00	0.25	tr_D0A254_	
HLA-DQA10501-DQB10201	26	VVRFLVDAGADINAT	FLVDAGADI	3	0.4424	416.9	11.00	0.52	tr_D0A254_	
HLA-DQA10501-DQB10201	27	ALVWAAREGYADVVE	AREGYADVV	5	0.4326	463.8	12.00	0.34	tr_D0A254_	
HLA-DQA10501-DQB10201	28	GYADVVEYLLCAGAD	YADVVEYLL	1	0.4314	469.8	12.00	0.31	tr_D0A254_	
HLA-DQA10501-DQB10201	29	EVQYVLENYPLAQRG	EVQYVLENY	0	0.4223	518.4	13.00	0.42	tr_D0A254_	
HLA-DQA10501-DQB10201	30	YQPYTPCAFPVEWLG	TPCAFPVEW	4	0.4215	522.6	13.00	0.37	tr_D0A254_	
HLA-DQA10501-DQB10201	31	DVVEYLLCAGADVDV	LLCAGADVD	5	0.4179	543.7	14.00	0.26	tr_D0A254_	
HLA-DQA10501-DQB10201	32	VRFLVDAGADINATN	FLVDAGADI	2	0.4159	555.4	14.00	0.57	tr_D0A254_	
HLA-DQA10501-DQB10201	33	AYCVAVQNASLPLLL	QNASLPLLL	6	0.4100	592.2	15.00	0.40	tr_D0A254_	
HLA-DQA10501-DQB10201	34	ADVVEYLLCAGADVD	EYLLCAGAD	4	0.4076	607.8	15.00	0.34	tr_D0A254_	
HLA-DQA10501-DQB10201	35	DGFDIYLPGSDAFYF	YLPGSDAFY	5	0.4021	645.1	16.00	0.31	tr_D0A254_	
HLA-DQA10501-DQB10201	36	YADVVEYLLCAGADV	EYLLCAGAD	5	0.3984	671.0	16.00	0.28	tr_D0A254_	
HLA-DQA10501-DQB10201	37	LHYQPYTPCAFPVEW	TPCAFPVEW	6	0.3979	674.6	16.00	0.44	tr_D0A254_	
HLA-DQA10501-DQB10201	38	GADVDVVDTDGYTAL	VDVVDTDGY	3	0.3959	689.7	17.00	0.25	tr_D0A254_	
HLA-DQA10501-DQB10201	39	YCVAVQNASLPLLLL	QNASLPLLL	5	0.3868	760.8	18.00	0.40	tr_D0A254_	
HLA-DQA10501-DQB10201	40	TALVWAAREGYADVV	AREGYADVV	6	0.3868	761.0	18.00	0.43	tr_D0A254_	
HLA-DQA10501-DQB10201	41	RFLVDAGADINATNS	FLVDAGADI	1	0.3839	785.5	18.00	0.60	tr_D0A254_	
HLA-DQA10501-DQB10201	42	QPYTPCAFPVEWLGH	TPCAFPVEW	3	0.3832	791.1	18.00	0.41	tr_D0A254_	
HLA-DQA10501-DQB10201	43	SLGYVIMFWAQGQNI	MFWAQGQNI	6	0.3719	894.3	20.00	0.56	tr_D0A254_	
HLA-DQA10501-DQB10201	44	EACASGSLGYVIMFW	SLGYVIMFW	6	0.3708	905.3	21.00	0.64	tr_D0A254_	
HLA-DQA10501-DQB10201	45	CVAVQNASLPLLLLL	QNASLPLLL	4	0.3656	957.5	21.00	0.41	tr_D0A254_	
HLA-DQA10501-DQB10201	46	DDGFDIYLPGSDAFY	YLPGSDAFY	6	0.3635	979.0	22.00	0.32	tr_D0A254_	
HLA-DQA10501-DQB10201	47	GFDIYLPGSDAFYFK	YLPGSDAFY	4	0.3634	979.9	22.00	0.34	tr_D0A254_	
HLA-DQA10501-DQB10201	48	PYTPCAFPVEWLGHQ	TPCAFPVEW	2	0.3592	1026.2	23.00	0.43	tr_D0A254_	
HLA-DQA10501-DQB10201	49	DVDVVDTDGYTALNY	TDGYTALNY	6	0.3586	1032.5	23.00	0.34	tr_D0A254_	
HLA-DQA10501-DQB10201	50	NHQGYSAYCVAVQNA	YSAYCVAVQ	4	0.3570	1050.8	23.00	0.29	tr_D0A254_	
HLA-DQA10501-DQB10201	51	GHIHVVRFLVDAGAD	RFLVDAGAD	6	0.3570	1050.9	23.00	0.33	tr_D0A254_	
HLA-DQA10501-DQB10201	52	SAYCVAVQNASLPLL	VQNASLPLL	6	0.3552	1071.7	23.00	0.36	tr_D0A254_	
HLA-DQA10501-DQB10201	53	ADVDVVDTDGYTALN	VDVVDTDGY	2	0.3536	1089.5	24.00	0.35	tr_D0A254_	
HLA-DQA10501-DQB10201	54	DINATNSAGFSALHM	SAGFSALHM	6	0.3493	1141.5	24.00	0.75	tr_D0A254_	
HLA-DQA10501-DQB10201	55	YSAYCVAVQNASLPL	AYCVAVQNA	2	0.3455	1189.3	25.00	0.26	tr_D0A254_	
HLA-DQA10501-DQB10201	56	LHWAAYNNSVAICQY	NNSVAICQY	6	0.3453	1192.0	25.00	0.46	tr_D0A254_	
HLA-DQA10501-DQB10201	57	VAVQNASLPLLLLLA	QNASLPLLL	3	0.3450	1195.7	25.00	0.41	tr_D0A254_	
HLA-DQA10501-DQB10201	58	AQRGATGMAAGDGLL	TGMAAGDGL	5	0.3443	1205.9	25.00	0.58	tr_D0A254_	
HLA-DQA10501-DQB10201	59	AYNNSVAICQYLVEV	AICQYLVEV	6	0.3441	1207.5	25.00	0.34	tr_D0A254_	
HLA-DQA10501-DQB10201	60	KNHQGYSAYCVAVQN	YSAYCVAVQ	5	0.3407	1252.8	26.00	0.41	tr_D0A254_	
HLA-DQA10501-DQB10201	61	YTPCAFPVEWLGHQG	TPCAFPVEW	1	0.3400	1262.2	26.00	0.41	tr_D0A254_	
HLA-DQA10501-DQB10201	62	NSVAICQYLVEVCKM	QYLVEVCKM	6	0.3356	1325.0	27.00	0.46	tr_D0A254_	
HLA-DQA10501-DQB10201	63	GYSAYCVAVQNASLP	AYCVAVQNA	3	0.3349	1334.5	27.00	0.29	tr_D0A254_	
HLA-DQA10501-DQB10201	64	VDVVDTDGYTALNYA	TDGYTALNY	5	0.3340	1346.8	27.00	0.35	tr_D0A254_	
HLA-DQA10501-DQB10201	65	QGYSAYCVAVQNASL	YSAYCVAVQ	2	0.3339	1349.3	27.00	0.29	tr_D0A254_	

HLA-DQA10501-DQB10201	66	FLVDAGADINATNSA	FLVDAGADI	0	0.3334	1356.9	28.00	0.58	tr_D0A254_
HLA-DQA10501-DQB10201	67	FDIYLPGSDAFYFKR	YLPGSDAFY	3	0.3333	1357.6	28.00	0.34	tr_D0A254_
HLA-DQA10501-DQB10201	68	HWAAYNNSVAICQYL	NNSVAICQY	5	0.3332	1358.6	28.00	0.42	tr_D0A254_
HLA-DQA10501-DQB10201	69	HQGYSAYCVAVQNAS	YSAYCVAVQ	3	0.3326	1367.7	28.00	0.31	tr_D0A254_
HLA-DQA10501-DQB10201	70	ACASGSLGYVIMFWA	SLGYVIMFW	5	0.3325	1368.8	28.00	0.58	tr_D0A254_
HLA-DQA10501-DQB10201	71	KKNHQGYSAYCVAVQ	YSAYCVAVQ	6	0.3319	1377.8	28.00	0.51	tr_D0A254_
HLA-DQA10501-DQB10201	72	YNNSVAICQYLVEVC	AICQYLVEV	5	0.3317	1381.3	28.00	0.31	tr_D0A254_
HLA-DQA10501-DQB10201	73	QRGATGMAAGDGLLG	TGMAAGDGL	4	0.3305	1399.9	28.00	0.56	tr_D0A254_
HLA-DQA10501-DQB10201	74	KGHESVVGGGDTLLM	VVGGGDTLL	5	0.3304	1400.6	28.00	0.46	tr_D0A254_
HLA-DQA10501-DQB10201	75	GRTALVWAAREGYAD	LVWAAREGY	4	0.3304	1401.3	28.00	0.31	tr_D0A254_
HLA-DQA10501-DQB10201	76	RTALVWAAREGYADV	LVWAAREGY	3	0.3303	1402.7	28.00	0.28	tr_D0A254_
HLA-DQA10501-DQB10201	77	VAICQYLVEVCKMDI	QYLVEVCKM	4	0.3282	1435.3	29.00	0.40	tr_D0A254_
HLA-DQA10501-DQB10201	78	INATNSAGFSALHMA	SAGFSALHM	5	0.3265	1461.8	29.00	0.72	tr_D0A254_
HLA-DQA10501-DQB10201	79	MKSVGTDDGFDIYLP	VGTDDGFDI	3	0.3248	1487.7	29.00	0.21	tr_D0A254_
HLA-DQA10501-DQB10201	80	GHESVVGGGDTLLMT	VVGGGDTLL	4	0.3217	1539.6	30.00	0.45	tr_D0A254_
HLA-DQA10501-DQB10201	81	LGYVIMFWAQGQNIK	MFWAQGQNI	5	0.3209	1553.5	30.00	0.63	tr_D0A254_
HLA-DQA10501-DQB10201	82	SVAICQYLVEVCKMD	QYLVEVCKM	5	0.3206	1556.9	30.00	0.49	tr_D0A254_
HLA-DQA10501-DQB10201	83	DMKSVGTDDGFDIYL	KSVGTDDGF	2	0.3206	1557.4	30.00	0.19	tr_D0A254_
HLA-DQA10501-DQB10201	84	RGATGMAAGDGLLGN	TGMAAGDGL	3	0.3203	1563.3	30.00	0.57	tr_D0A254_
HLA-DQA10501-DQB10201	85	TDDGFDIYLPGSDAF	IYLPGSDAF	6	0.3187	1589.8	31.00	0.38	tr_D0A254_
HLA-DQA10501-DQB10201	86	AVQNASLPLLLLLAE	QNASLPLLL	2	0.3172	1615.3	31.00	0.44	tr_D0A254_
HLA-DQA10501-DQB10201	87	IVDMKSVGTDDGFDI	KSVGTDDGF	4	0.3172	1616.5	31.00	0.23	tr_D0A254_
HLA-DQA10501-DQB10201	88	NATNSAGFSALHMAA	SAGFSALHM	4	0.3167	1624.7	31.00	0.63	tr_D0A254_
HLA-DQA10501-DQB10201	89	VDMKSVGTDDGFDIY	KSVGTDDGF	3	0.3155	1645.3	31.00	0.19	tr_D0A254_
HLA-DQA10501-DQB10201	90	DGRTALVWAAREGYA	TALVWAARE	3	0.3149	1656.5	32.00	0.37	tr_D0A254_
HLA-DQA10501-DQB10201	91	LAQRGATGMAAGDGL	TGMAAGDGL	6	0.3142	1669.8	32.00	0.66	tr_D0A254_
HLA-DQA10501-DQB10201	92	HESVVGGGDTLLMTA	VVGGGDTLL	3	0.3133	1685.9	32.00	0.44	tr_D0A254_
HLA-DQA10501-DQB10201	93	KDGRTALVWAAREGY	TALVWAARE	4	0.3128	1694.8	32.00	0.46	tr_D0A254_
HLA-DQA10501-DQB10201	94	SGSLGYVIMFWAQGQ	SLGYVIMFW	2	0.3126	1698.7	32.00	0.44	tr_D0A254_
HLA-DQA10501-DQB10201	95	WAAYNNSVAICQYLV	NNSVAICQY	4	0.3116	1717.0	32.00	0.41	tr_D0A254_
HLA-DQA10501-DQB10201	96	CASGSLGYVIMFWAQ	SLGYVIMFW	4	0.3108	1731.2	33.00	0.59	tr_D0A254_
HLA-DQA10501-DQB10201	97	GATGMAAGDGLLGNQ	TGMAAGDGL	2	0.3108	1732.6	33.00	0.56	tr_D0A254_
HLA-DQA10501-DQB10201	98	KSVGTDDGFDIYLPG	VGTDDGFDI	2	0.3083	1778.5	33.00	0.22	tr_D0A254_
HLA-DQA10501-DQB10201	99	AAYNNSVAICQYLVE	NNSVAICQY	3	0.3078	1788.3	33.00	0.44	tr_D0A254_
HLA-DQA10501-DQB10201	100	KGHIHVVRFLVDAGA	VRFLVDAGA	6	0.3061	1821.8	34.00	0.39	tr_D0A254_
HLA-DQA10501-DQB10201	101	AICQYLVEVCKMDIN	QYLVEVCKM	3	0.3041	1863.0	34.00	0.42	tr_D0A254_
HLA-DQA10501-DQB10201	102	AGFSALHMAASAINY	HMAASAINY	6	0.3035	1875.2	34.00	0.42	tr_D0A254_
HLA-DQA10501-DQB10201	103	GYVIMFWAQGQNIKG	MFWAQGQNI	4	0.3018	1908.1	35.00	0.61	tr_D0A254_
HLA-DQA10501-DQB10201	104	NSAGFSALHMAASAI	SAGFSALHM	1	0.3017	1910.7	35.00	0.36	tr_D0A254_
HLA-DQA10501-DQB10201	105	ESVVGGGDTLLMTAA	VVGGGDTLL	2	0.3017	1911.5	35.00	0.47	tr_D0A254_
HLA-DQA10501-DQB10201	106	ATNSAGFSALHMAAS	SAGFSALHM	3	0.3004	1939.2	35.00	0.60	tr_D0A254_
HLA-DQA10501-DQB10201	107	PIVDMKSVGTDDGFD	KSVGTDDGF	5	0.3001	1943.8	35.00	0.28	tr_D0A254_
HLA-DQA10501-DQB10201	108	YHIVRFLLDHGADPT	IVRFLLDHG	2	0.3000	1946.7	35.00	0.22	tr_D0A254_
HLA-DQA10501-DQB10201	109	LPLLLLLAEHRPIDI	LLLLLAEHR	2	0.2972	2006.2	36.00	0.34	tr_D0A254_
HLA-DQA10501-DQB10201	110	IKGHESVVGGGDTLL	VVGGGDTLL	6	0.2960	2033.3	36.00	0.58	tr_D0A254_
HLA-DQA10501-DQB10201	111	QLHYQPYTPCAFPVE	YQPYTPCAF	3	0.2959	2034.4	36.00	0.41	tr_D0A254_
HLA-DQA10501-DQB10201	112	VQNASLPLLLLLAEH	QNASLPLLL	1	0.2955	2043.6	36.00	0.41	tr_D0A254_
HLA-DQA10501-DQB10201	113	TNSAGFSALHMAASA	SAGFSALHM	2	0.2939	2080.5	37.00	0.55	tr_D0A254_
HLA-DQA10501-DQB10201	114	DIYLPGSDAFYFKRQ	YLPGSDAFY	2	0.2931	2097.2	37.00	0.37	tr_D0A254_
HLA-DQA10501-DQB10201	115	NYHIVRFLLDHGADP	IVRFLLDHG	3	0.2916	2132.3	37.00	0.25	tr_D0A254_

HLA-DQA10501-DQB10201	116	ICQYLVEVCKMDINV	QYLVEVCKM	2	0.2911	2143.6	37.00	0.44	tr_D0A254_
HLA-DQA10501-DQB10201	117	ASGSLGYVIMFWAQG	SLGYVIMFW	3	0.2907	2151.7	37.00	0.57	tr_D0A254_
HLA-DQA10501-DQB10201	118	SVVGGGDTLLMTAAY	VVGGGDTLL	1	0.2893	2185.4	38.00	0.37	tr_D0A254_
HLA-DQA10501-DQB10201	119	GFSALHMAASAINYH	HMAASAINY	5	0.2891	2191.4	38.00	0.38	tr_D0A254_
HLA-DQA10501-DQB10201	120	SVGTDDGFDIYLPGS	VGTDDGFDI	1	0.2873	2232.5	38.00	0.19	tr_D0A254_
HLA-DQA10501-DQB10201	121	GSLGYVIMFWAQGQN	SLGYVIMFW	1	0.2863	2257.3	39.00	0.40	tr_D0A254_
HLA-DQA10501-DQB10201	122	NNSVAICQYLVEVCK	AICQYLVEV	4	0.2857	2272.9	39.00	0.32	tr_D0A254_
HLA-DQA10501-DQB10201	123	FSALHMAASAINYHI	HMAASAINY	4	0.2847	2297.3	39.00	0.38	tr_D0A254_
HLA-DQA10501-DQB10201	124	VGTDDGFDIYLPGSD	FDIYLPGSD	6	0.2843	2307.2	39.00	0.21	tr_D0A254_
HLA-DQA10501-DQB10201	125	YVIMFWAQGQNIKGH	MFWAQGQNI	3	0.2829	2342.8	39.00	0.59	tr_D0A254_
HLA-DQA10501-DQB10201	126	VQYVLENYPLAQRGA	VQYVLENYP	0	0.2824	2354.5	40.00	0.29	tr_D0A254_
HLA-DQA10501-DQB10201	127	PLLLLLAEHRPIDIH	LLLLLAEHR	1	0.2792	2438.0	40.00	0.34	tr_D0A254_
HLA-DQA10501-DQB10201	128	SPIVDMKSVGTDDGF	KSVGTDDGF	6	0.2782	2463.6	41.00	0.28	tr_D0A254_
HLA-DQA10501-DQB10201	129	CQYLVEVCKMDINVV	QYLVEVCKM	1	0.2770	2498.0	41.00	0.41	tr_D0A254_
HLA-DQA10501-DQB10201	130	LHMAASAINYHIVRF	AINYHIVRF	6	0.2758	2529.9	41.00	0.38	tr_D0A254_
HLA-DQA10501-DQB10201	131	HMAASAINYHIVRFL	AINYHIVRF	5	0.2751	2549.4	41.00	0.32	tr_D0A254_
HLA-DQA10501-DQB10201	132	SAGFSALHMAASAIN	ALHMAASAI	5	0.2745	2563.7	42.00	0.26	tr_D0A254_
HLA-DQA10501-DQB10201	133	GTDDGFDIYLPGSDA	FDIYLPGSD	5	0.2743	2571.3	42.00	0.23	tr_D0A254_
HLA-DQA10501-DQB10201	134	LAEHRPIDIHERDEM	IDIHERDEM	6	0.2743	2571.8	42.00	0.32	tr_D0A254_
HLA-DQA10501-DQB10201	135	SAINYHIVRFLLDHG	IVRFLLDHG	6	0.2736	2589.0	42.00	0.24	tr_D0A254_
HLA-DQA10501-DQB10201	136	TPCAFPVEWLGHQGK	PCAFPVEWL	1	0.2724	2625.2	42.00	0.38	tr_D0A254_
HLA-DQA10501-DQB10201	137	AASAINYHIVRFLLD	AINYHIVRF	3	0.2708	2668.6	43.00	0.28	tr_D0A254_
HLA-DQA10501-DQB10201	138	YKGHIHVVRFLVDAG	VVRFLVDAG	6	0.2704	2681.8	43.00	0.48	tr_D0A254_
HLA-DQA10501-DQB10201	139	ALLHWAAYNNSVAIC	WAAYNNSVA	4	0.2700	2692.6	43.00	0.40	tr_D0A254_
HLA-DQA10501-DQB10201	140	VGKDGRTALVWAARE	TALVWAARE	6	0.2696	2704.5	43.00	0.66	tr_D0A254_
HLA-DQA10501-DQB10201	141	DVVDTDGYTALNYAK	TDGYTALNY	4	0.2690	2721.7	43.00	0.40	tr_D0A254_
HLA-DQA10501-DQB10201	142	LLHWAAYNNSVAICQ	WAAYNNSVA	3	0.2686	2732.8	43.00	0.35	tr_D0A254_
HLA-DQA10501-DQB10201	143	RQLHYQPYTPCAFPV	YQPYTPCAF	4	0.2683	2743.4	43.00	0.58	tr_D0A254_
HLA-DQA10501-DQB10201	144	SALHMAASAINYHIV	HMAASAINY	3	0.2682	2746.6	43.00	0.41	tr_D0A254_
HLA-DQA10501-DQB10201	145	LLLLLAEHRPIDIHE	LAEHRPIDI	4	0.2677	2761.8	43.00	0.29	tr_D0A254_
HLA-DQA10501-DQB10201	146	HIVRFLLDHGADPTK	FLLDHGADP	4	0.2676	2763.6	43.00	0.23	tr_D0A254_
HLA-DQA10501-DQB10201	147	AINYHIVRFLLDHGA	IVRFLLDHG	5	0.2650	2842.9	44.00	0.25	tr_D0A254_
HLA-DQA10501-DQB10201	148	INYHIVRFLLDHGAD	IVRFLLDHG	4	0.2645	2858.5	44.00	0.27	tr_D0A254_
HLA-DQA10501-DQB10201	149	QYLVEVCKMDINVVG	QYLVEVCKM	0	0.2642	2866.2	44.00	0.36	tr_D0A254_
HLA-DQA10501-DQB10201	150	ASAINYHIVRFLLDH	AINYHIVRF	2	0.2642	2868.3	44.00	0.26	tr_D0A254_
HLA-DQA10501-DQB10201	151	SLPLLLLLAEHRPID	LLLLLAEHR	3	0.2638	2881.1	44.00	0.45	tr_D0A254_
HLA-DQA10501-DQB10201	152	GKDGRTALVWAAREG	TALVWAARE	5	0.2623	2927.5	45.00	0.58	tr_D0A254_
HLA-DQA10501-DQB10201	153	AEHRPIDIHERDEMG	IDIHERDEM	5	0.2607	2978.4	45.00	0.34	tr_D0A254_
HLA-DQA10501-DQB10201	154	HALLHWAAYNNSVAI	WAAYNNSVA	5	0.2585	3050.1	46.00	0.45	tr_D0A254_
HLA-DQA10501-DQB10201	155	IYLPGSDAFYFKRQL	YLPGSDAFY	1	0.2582	3060.7	46.00	0.36	tr_D0A254_
HLA-DQA10501-DQB10201	156	AKHRNHPEVQYVLEN	HPEVQYVLE	5	0.2568	3107.8	46.00	0.36	tr_D0A254_
HLA-DQA10501-DQB10201	157	ASLPLLLLLAEHRPI	LLLLLAEHR	4	0.2564	3119.7	46.00	0.45	tr_D0A254_
HLA-DQA10501-DQB10201	158	DIHERDEMGHALLHW	EMGHALLHW	6	0.2564	3121.5	46.00	0.56	tr_D0A254_
HLA-DQA10501-DQB10201	159	NASLPLLLLLAEHRP	LLLLLAEHR	5	0.2550	3167.5	47.00	0.50	tr_D0A254_
HLA-DQA10501-DQB10201	160	QNASLPLLLLLAEHR	LLLLLAEHR	6	0.2536	3215.5	47.00	0.41	tr_D0A254_
HLA-DQA10501-DQB10201	161	MAASAINYHIVRFLL	AINYHIVRF	4	0.2534	3224.4	47.00	0.36	tr_D0A254_
HLA-DQA10501-DQB10201	162	VIMFWAQGQNIKGHE	MFWAQGQNI	2	0.2530	3238.5	47.00	0.65	tr_D0A254_
HLA-DQA10501-DQB10201	163	TKKNHQGYSAYCVAV	QGYSAYCVA	5	0.2518	3279.6	48.00	0.38	tr_D0A254_
HLA-DQA10501-DQB10201	164	KRQLHYQPYTPCAFP	YQPYTPCAF	5	0.2499	3345.8	48.00	0.64	tr_D0A254_
HLA-DQA10501-DQB10201	165	ALHMAASAINYHIVR	HMAASAINY	2	0.2495	3361.4	48.00	0.47	tr_D0A254_

HLA-DQA10501-DQB10201	166	IHERDEMGHALLHWA	EMGHALLHW	5	0.2460	3490.1	49.00	0.49	tr_D0A254_
HLA-DQA10501-DQB10201	167	TSVHMRISPSNLEEA	ISPSNLEEA	6	0.2437	3580.3	50.00	0.33	tr_D0A254_
HLA-DQA10501-DQB10201	168	SVHMRISPSNLEEAC	ISPSNLEEA	5	0.2427	3618.0	50.00	0.32	tr_D0A254_
HLA-DQA10501-DQB10201	169	EHRPIDIHERDEMGH	IDIHERDEM	4	0.2414	3669.7	55.00	0.32	tr_D0A254_
HLA-DQA10501-DQB10201	170	IVRFLLDHGADPTKK	FLLDHGADP	3	0.2402	3717.8	55.00	0.23	tr_D0A254_
HLA-DQA10501-DQB10201	171	LLLAEHRPIDIHERD	RPIDIHERD	6	0.2400	3726.6	55.00	0.33	tr_D0A254_
HLA-DQA10501-DQB10201	172	FKRQLHYQPYTPCAF	YQPYTPCAF	6	0.2392	3758.9	55.00	0.60	tr_D0A254_
HLA-DQA10501-DQB10201	173	VHMRISPSNLEEACA	ISPSNLEEA	4	0.2377	3820.6	55.00	0.28	tr_D0A254_
HLA-DQA10501-DQB10201	174	LLAEHRPIDIHERDE	RPIDIHERD	5	0.2373	3836.1	55.00	0.32	tr_D0A254_
HLA-DQA10501-DQB10201	175	VVDTDGYTALNYAKH	TDGYTALNY	3	0.2363	3879.8	55.00	0.43	tr_D0A254_
HLA-DQA10501-DQB10201	176	VVGGGDTLLMTAAYK	VVGGGDTLL	0	0.2330	4019.3	55.00	0.35	tr_D0A254_
HLA-DQA10501-DQB10201	177	ERDEMGHALLHWAAY	EMGHALLHW	3	0.2306	4123.6	55.00	0.49	tr_D0A254_
HLA-DQA10501-DQB10201	178	HERDEMGHALLHWAA	EMGHALLHW	4	0.2294	4180.0	55.00	0.49	tr_D0A254_
HLA-DQA10501-DQB10201	179	YAKHRNHPEVQYVLE	HPEVQYVLE	6	0.2289	4202.8	55.00	0.44	tr_D0A254_
HLA-DQA10501-DQB10201	180	LSPIVDMKSVGTDDG	MKSVGTDDG	6	0.2255	4359.7	55.00	0.32	tr_D0A254_
HLA-DQA10501-DQB10201	181	RDEMGHALLHWAAYN	EMGHALLHW	2	0.2254	4363.6	55.00	0.46	tr_D0A254_
HLA-DQA10501-DQB10201	182	HMRISPSNLEEACAS	ISPSNLEEA	3	0.2246	4399.3	55.00	0.23	tr_D0A254_
HLA-DQA10501-DQB10201	183	LLLLAEHRPIDIHER	LAEHRPIDI	3	0.2216	4548.6	60.00	0.39	tr_D0A254_
HLA-DQA10501-DQB10201	184	EEACASGSLGYVIMF	GSLGYVIMF	6	0.2213	4560.5	60.00	0.38	tr_D0A254_
HLA-DQA10501-DQB10201	185	HRPIDIHERDEMGHA	IDIHERDEM	3	0.2211	4568.8	60.00	0.36	tr_D0A254_
HLA-DQA10501-DQB10201	186	PTKKNHQGYSAYCVA	QGYSAYCVA	6	0.2200	4624.7	60.00	0.54	tr_D0A254_
HLA-DQA10501-DQB10201	187	QYVLENYPLAQRGAT	VLENYPLAQ	2	0.2171	4775.9	60.00	0.40	tr_D0A254_
HLA-DQA10501-DQB10201	188	IMFWAQGQNIKGHES	MFWAQGQNI	1	0.2167	4791.8	60.00	0.74	tr_D0A254_
HLA-DQA10501-DQB10201	189	GHALLHWAAYNNSVA	WAAYNNSVA	6	0.2166	4798.0	60.00	0.51	tr_D0A254_
HLA-DQA10501-DQB10201	190	PIDIHERDEMGHALL	IDIHERDEM	1	0.2144	4914.9	60.00	0.31	tr_D0A254_
HLA-DQA10501-DQB10201	191	AYKGHIHVVRFLVDA	HVVRFLVDA	6	0.2123	5025.4	60.00	0.28	tr_D0A254_
HLA-DQA10501-DQB10201	192	RPIDIHERDEMGHAL	IDIHERDEM	2	0.2118	5055.5	60.00	0.35	tr_D0A254_
HLA-DQA10501-DQB10201	193	YLPGSDAFYFKRQLH	LPGSDAFYF	1	0.2102	5140.7	60.00	0.35	tr_D0A254_
HLA-DQA10501-DQB10201	194	DEMGHALLHWAAYNN	EMGHALLHW	1	0.2099	5162.8	60.00	0.44	tr_D0A254_
HLA-DQA10501-DQB10201	195	VDTDGYTALNYAKHR	TDGYTALNY	2	0.2097	5170.4	60.00	0.44	tr_D0A254_
HLA-DQA10501-DQB10201	196	KTSVHMRISPSNLEE	HMRISPSNL	4	0.2052	5427.0	65.00	0.31	tr_D0A254_
HLA-DQA10501-DQB10201	197	MLSPIVDMKSVGTDD	DMKSVGTDD	6	0.2046	5466.0	65.00	0.33	tr_D0A254_
HLA-DQA10501-DQB10201	198	AAYKGHIHVVRFLVD	IHVVRFLVD	6	0.2026	5583.3	65.00	0.28	tr_D0A254_
HLA-DQA10501-DQB10201	199	VRFLLDHGADPTKKN	FLLDHGADP	2	0.1984	5845.6	65.00	0.27	tr_D0A254_
HLA-DQA10501-DQB10201	200	MRISPSNLEEACASG	ISPSNLEEA	2	0.1980	5869.8	65.00	0.28	tr_D0A254_
HLA-DQA10501-DQB10201	201	VDAGADINATNSAGF	INATNSAGF	6	0.1937	6151.6	65.00	0.40	tr_D0A254_
HLA-DQA10501-DQB10201	202	YLVEVCKMDINVVGK	LVEVCKMDI	1	0.1926	6220.3	65.00	0.34	tr_D0A254_
HLA-DQA10501-DQB10201	203	PCAFPVEWLGHQGKM	PCAFPVEWL	0	0.1905	6364.4	65.00	0.44	tr_D0A254_
HLA-DQA10501-DQB10201	204	NIKGHESVVGGGDTL	SVVGGGDTL	6	0.1894	6438.4	70.00	0.57	tr_D0A254_
HLA-DQA10501-DQB10201	205	DTDGYTALNYAKHRN	TDGYTALNY	1	0.1884	6513.6	70.00	0.44	tr_D0A254_
HLA-DQA10501-DQB10201	206	HKTSVHMRISPSNLE	HMRISPSNL	5	0.1847	6778.9	70.00	0.40	tr_D0A254_
HLA-DQA10501-DQB10201	207	IDIHERDEMGHALLH	RDEMGHALL	5	0.1845	6795.4	70.00	0.29	tr_D0A254_
HLA-DQA10501-DQB10201	208	LVDAGADINATNSAG	LVDAGADIN	0	0.1830	6901.6	70.00	0.31	tr_D0A254_
HLA-DQA10501-DQB10201	209	EMGHALLHWAAYNNS	EMGHALLHW	0	0.1828	6917.0	70.00	0.32	tr_D0A254_
HLA-DQA10501-DQB10201	210	LVEVCKMDINVVGKD	LVEVCKMDI	0	0.1823	6958.1	70.00	0.23	tr_D0A254_
HLA-DQA10501-DQB10201	211	DAGADINATNSAGFS	INATNSAGF	5	0.1819	6989.6	70.00	0.40	tr_D0A254_
HLA-DQA10501-DQB10201	212	MFWAQGQNIKGHESV	MFWAQGQNI	0	0.1802	7114.4	70.00	0.65	tr_D0A254_
HLA-DQA10501-DQB10201	213	MTAAYKGHIHVVRFL	KGHIHVVRF	5	0.1794	7174.1	70.00	0.42	tr_D0A254_
HLA-DQA10501-DQB10201	214	TAAYKGHIHVVRFLV	KGHIHVVRF	4	0.1788	7224.4	70.00	0.36	tr_D0A254_
HLA-DQA10501-DQB10201	215	LEEACASGSLGYVIM	SGSLGYVIM	6	0.1746	7558.2	70.00	0.34	tr_D0A254_

HLA-DQA10501-DQB10201	216	AFYFKRQLHYQPYTP	QLHYQPYTP	6	0.1737	7631.6	70.00	0.16	tr_D0A254_
HLA-DQA10501-DQB10201	217	YVLENYPLAQRGATG	VLENYPLAQ	1	0.1737	7636.9	70.00	0.46	tr_D0A254_
HLA-DQA10501-DQB10201	218	DAFYFKRQLHYQPYT	FYFKRQLHY	2	0.1726	7729.4	70.00	0.27	tr_D0A254_
HLA-DQA10501-DQB10201	219	LMTAAYKGHIHVVRF	KGHIHVVRF	6	0.1725	7735.7	70.00	0.50	tr_D0A254_
HLA-DQA10501-DQB10201	220	SDAFYFKRQLHYQPY	FYFKRQLHY	3	0.1714	7825.6	75.00	0.28	tr_D0A254_
HLA-DQA10501-DQB10201	221	ENYPLAQRGATGMAA	QRGATGMAA	6	0.1707	7884.0	75.00	0.75	tr_D0A254_
HLA-DQA10501-DQB10201	222	GADINATNSAGFSAL	INATNSAGF	3	0.1686	8068.4	75.00	0.44	tr_D0A254_
HLA-DQA10501-DQB10201	223	ADINATNSAGFSALH	INATNSAGF	2	0.1682	8104.4	75.00	0.43	tr_D0A254_
HLA-DQA10501-DQB10201	224	RISPSNLEEACASGS	ISPSNLEEA	1	0.1671	8198.1	75.00	0.29	tr_D0A254_
HLA-DQA10501-DQB10201	225	AGADINATNSAGFSA	INATNSAGF	4	0.1669	8215.3	75.00	0.43	tr_D0A254_
HLA-DQA10501-DQB10201	226	VGGGDTLLMTAAYKG	DTLLMTAAY	4	0.1667	8232.6	75.00	0.26	tr_D0A254_
HLA-DQA10501-DQB10201	227	LPGSDAFYFKRQLHY	FYFKRQLHY	6	0.1665	8256.9	75.00	0.28	tr_D0A254_
HLA-DQA10501-DQB10201	228	YPLAQRGATGMAAGD	QRGATGMAA	4	0.1650	8391.8	75.00	0.69	tr_D0A254_
HLA-DQA10501-DQB10201	229	PLAQRGATGMAAGDG	QRGATGMAA	3	0.1645	8431.0	75.00	0.54	tr_D0A254_
HLA-DQA10501-DQB10201	230	MHKTSVHMRISPSNL	HMRISPSNL	6	0.1606	8796.7	75.00	0.51	tr_D0A254_
HLA-DQA10501-DQB10201	231	MGHALLHWAAYNNSV	HWAAYNNSV	6	0.1604	8820.3	75.00	0.29	tr_D0A254_
HLA-DQA10501-DQB10201	232	FYFKRQLHYQPYTPC	QLHYQPYTP	5	0.1583	9018.2	75.00	0.20	tr_D0A254_
HLA-DQA10501-DQB10201	233	RFLLDHGADPTKKNH	FLLDHGADP	1	0.1575	9099.9	75.00	0.37	tr_D0A254_
HLA-DQA10501-DQB10201	234	GSDAFYFKRQLHYQP	FYFKRQLHY	4	0.1553	9315.5	75.00	0.32	tr_D0A254_
HLA-DQA10501-DQB10201	235	NYAKHRNHPEVQYVL	RNHPEVQYV	5	0.1532	9525.0	80.00	0.38	tr_D0A254_
HLA-DQA10501-DQB10201	236	ISPSNLEEACASGSL	ISPSNLEEA	0	0.1520	9658.0	80.00	0.21	tr_D0A254_
HLA-DQA10501-DQB10201	237	NYPLAQRGATGMAAG	QRGATGMAA	5	0.1515	9710.2	80.00	0.69	tr_D0A254_
HLA-DQA10501-DQB10201	238	VEVCKMDINVVGKDG	MDINVVGKD	5	0.1504	9826.2	80.00	0.21	tr_D0A254_
HLA-DQA10501-DQB10201	239	DPTKKNHQGYSAYCV	HQGYSAYCV	6	0.1503	9829.1	80.00	0.62	tr_D0A254_
HLA-DQA10501-DQB10201	240	TDGYTALNYAKHRNH	DGYTALNYA	1	0.1491	9957.9	80.00	0.34	tr_D0A254_
HLA-DQA10501-DQB10201	241	INVVGKDGRTALVWA	DGRTALVWA	6	0.1462	10280.8	80.00	0.41	tr_D0A254_
HLA-DQA10501-DQB10201	242	NLEEACASGSLGYVI	ASGSLGYVI	6	0.1430	10643.7	80.00	0.52	tr_D0A254_
HLA-DQA10501-DQB10201	243	NVVGKDGRTALVWAA	KDGRTALVW	4	0.1390	11113.4	80.00	0.35	tr_D0A254_
HLA-DQA10501-DQB10201	244	PGSDAFYFKRQLHYQ	FYFKRQLHY	5	0.1383	11195.4	80.00	0.36	tr_D0A254_
HLA-DQA10501-DQB10201	245	GGGDTLLMTAAYKGH	DTLLMTAAY	3	0.1352	11572.8	80.00	0.32	tr_D0A254_
HLA-DQA10501-DQB10201	246	YFKRQLHYQPYTPCA	QLHYQPYTP	4	0.1334	11808.3	85.00	0.23	tr_D0A254_
HLA-DQA10501-DQB10201	247	EVCKMDINVVGKDGR	MDINVVGKD	4	0.1325	11922.3	85.00	0.25	tr_D0A254_
HLA-DQA10501-DQB10201	248	GGDTLLMTAAYKGHI	TLLMTAAYK	3	0.1321	11975.2	85.00	0.28	tr_D0A254_
HLA-DQA10501-DQB10201	249	VVGKDGRTALVWAAR	KDGRTALVW	3	0.1309	12132.9	85.00	0.31	tr_D0A254_
HLA-DQA10501-DQB10201	250	LNYAKHRNHPEVQYV	RNHPEVQYV	6	0.1295	12321.9	85.00	0.46	tr_D0A254_
HLA-DQA10501-DQB10201	251	SPSNLEEACASGSLG	SNLEEACAS	2	0.1256	12840.6	85.00	0.22	tr_D0A254_
HLA-DQA10501-DQB10201	252	DINVVGKDGRTALVW	KDGRTALVW	6	0.1255	12859.6	85.00	0.50	tr_D0A254_
HLA-DQA10501-DQB10201	253	VCKMDINVVGKDGRT	MDINVVGKD	3	0.1240	13075.9	85.00	0.27	tr_D0A254_
HLA-DQA10501-DQB10201	254	CAFPVEWLGHQGKMH	VEWLGHQGK	4	0.1232	13184.0	85.00	0.34	tr_D0A254_
HLA-DQA10501-DQB10201	255	FLLDHGADPTKKNHQ	LLDHGADPT	1	0.1231	13198.7	85.00	0.46	tr_D0A254_
HLA-DQA10501-DQB10201	256	VLENYPLAQRGATGM	VLENYPLAQ	0	0.1225	13278.2	85.00	0.41	tr_D0A254_
HLA-DQA10501-DQB10201	257	GDTLLMTAAYKGHIH	TLLMTAAYK	2	0.1223	13311.3	85.00	0.29	tr_D0A254_
HLA-DQA10501-DQB10201	258	PSNLEEACASGSLGY	EACASGSLG	5	0.1152	14379.5	85.00	0.22	tr_D0A254_
HLA-DQA10501-DQB10201	259	SNLEEACASGSLGYV	CASGSLGYV	6	0.1146	14468.4	85.00	0.19	tr_D0A254_
HLA-DQA10501-DQB10201	260	LLMTAAYKGHIHVVR	YKGHIHVVR	6	0.1141	14552.4	85.00	0.27	tr_D0A254_
HLA-DQA10501-DQB10201	261	CKMDINVVGKDGRTA	MDINVVGKD	2	0.1136	14624.2	90.00	0.25	tr_D0A254_
HLA-DQA10501-DQB10201	262	DTLLMTAAYKGHIHV	TLLMTAAYK	1	0.1135	14638.2	90.00	0.29	tr_D0A254_
HLA-DQA10501-DQB10201	263	KMHKTSVHMRISPSN	SVHMRISPS	5	0.1112	15014.6	90.00	0.30	tr_D0A254_
HLA-DQA10501-DQB10201	264	QNIKGHESVVGGGDT	ESVVGGGDT	6	0.1103	15167.0	90.00	0.29	tr_D0A254_
HLA-DQA10501-DQB10201	265	AFPVEWLGHQGKMHK	VEWLGHQGK	3	0.1101	15190.3	90.00	0.32	tr_D0A254_

HLA-DQA10501-DQB10201	266	KMDINVVGKDGRTAL	MDINVVGKD	1	0.1086	15434.2	90.00	0.24	tr_D0A254_
HLA-DQA10501-DQB10201	267	TLLMTAAYKGHIHVV	AYKGHIHVV	6	0.1074	15635.9	90.00	0.22	tr_D0A254_
HLA-DQA10501-DQB10201	268	GKMHKTSVHMRISPS	SVHMRISPS	6	0.1035	16324.1	90.00	0.37	tr_D0A254_
HLA-DQA10501-DQB10201	269	WAQGQNIKGHESVVG	IKGHESVVG	6	0.1006	16828.2	90.00	0.38	tr_D0A254_
HLA-DQA10501-DQB10201	270	DGYTALNYAKHRNHP	DGYTALNYA	0	0.1002	16913.4	90.00	0.40	tr_D0A254_
HLA-DQA10501-DQB10201	271	GQNIKGHESVVGGGD	HESVVGGGD	6	0.0999	16966.6	90.00	0.28	tr_D0A254_
HLA-DQA10501-DQB10201	272	FPVEWLGHQGKMHKT	VEWLGHQGK	2	0.0990	17126.9	90.00	0.34	tr_D0A254_
HLA-DQA10501-DQB10201	273	AQGQNIKGHESVVGG	IKGHESVVG	5	0.0968	17543.2	90.00	0.31	tr_D0A254_
HLA-DQA10501-DQB10201	274	MDINVVGKDGRTALV	DINVVGKDG	1	0.0965	17599.3	90.00	0.31	tr_D0A254_
HLA-DQA10501-DQB10201	275	ALNYAKHRNHPEVQY	HRNHPEVQY	6	0.0933	18215.2	90.00	0.43	tr_D0A254_
HLA-DQA10501-DQB10201	276	FWAQGQNIKGHESVV	QNIKGHESV	5	0.0916	18554.4	90.00	0.25	tr_D0A254_
HLA-DQA10501-DQB10201	277	QGQNIKGHESVVGGG	IKGHESVVG	4	0.0899	18909.2	95.00	0.31	tr_D0A254_
HLA-DQA10501-DQB10201	278	LENYPLAQRGATGMA	LAQRGATGM	5	0.0850	19922.0	95.00	0.35	tr_D0A254_
HLA-DQA10501-DQB10201	279	GHQGKMHKTSVHMRI	HKTSVHMRI	6	0.0831	20349.4	95.00	0.56	tr_D0A254_
HLA-DQA10501-DQB10201	280	QGKMHKTSVHMRISP	HKTSVHMRI	4	0.0817	20661.1	95.00	0.47	tr_D0A254_
HLA-DQA10501-DQB10201	281	PVEWLGHQGKMHKTS	VEWLGHQGK	1	0.0771	21700.4	95.00	0.35	tr_D0A254_
HLA-DQA10501-DQB10201	282	HQGKMHKTSVHMRIS	HKTSVHMRI	5	0.0737	22532.8	95.00	0.50	tr_D0A254_
HLA-DQA10501-DQB10201	283	ADPTKKNHQGYSAYC	NHQGYSAYC	6	0.0705	23328.9	95.00	0.54	tr_D0A254_
HLA-DQA10501-DQB10201	284	LLDHGADPTKKNHQG	LLDHGADPT	0	0.0673	24133.6	95.00	0.66	tr_D0A254_
HLA-DQA10501-DQB10201	285	TALNYAKHRNHPEVQ	KHRNHPEVQ	6	0.0633	25211.6	100.00	0.46	tr_D0A254_
HLA-DQA10501-DQB10201	286	GYTALNYAKHRNHPE	YTALNYAKH	1	0.0608	25906.8	100.00	0.37	tr_D0A254_
HLA-DQA10501-DQB10201	287	YTALNYAKHRNHPEV	AKHRNHPEV	6	0.0530	28186.2	100.00	0.24	tr_D0A254_
HLA-DQA10501-DQB10201	288	VEWLGHQGKMHKTSV	WLGHQGKMH	2	0.0520	28470.4	100.00	0.34	tr_D0A254_
HLA-DQA10501-DQB10201	289	LGHQGKMHKTSVHMR	KMHKTSVHM	5	0.0430	31404.0	100.00	0.50	tr_D0A254_
HLA-DQA10501-DQB10201	290	WLGHQGKMHKTSVHM	KMHKTSVHM	6	0.0415	31920.6	100.00	0.63	tr_D0A254_
HLA-DQA10501-DQB10201	291	GADPTKKNHQGYSAY	KKNHQGYSA	5	0.0353	34119.1	100.00	0.37	tr_D0A254_
HLA-DQA10501-DQB10201	292	EWLGHQGKMHKTSVH	WLGHQGKMH	1	0.0318	35426.8	100.00	0.51	tr_D0A254_
HLA-DQA10501-DQB10201	293	LDHGADPTKKNHQGY	DHGADPTKK	1	0.0283	36829.1	100.00	0.40	tr_D0A254_
HLA-DQA10501-DQB10201	294	HGADPTKKNHQGYSA	KKNHQGYSA	6	0.0262	37651.4	100.00	0.57	tr_D0A254_
HLA-DQA10501-DQB10201	295	DHGADPTKKNHQGYS	DHGADPTKK	0	0.0189	40742.4	100.00	0.41	tr_D0A254_

Allele: HLA-DQA10501-DQB10201. Number of high binders 0. Number of weak binders 24. Number of peptides 295

Allele	pos	peptide	core	Of	1-log50k(aff)	affinity(nM)	%Rank	Relia	Identity	Bind Level
HLA-DQA10501-DQB10201	1	MGFEPFLPFLSTRNS	GFEPFLPFL	1	0.2186	4694.4	60.00	0.35	tr_D0A254_	
HLA-DQA10501-DQB10201	2	GFEPFLPFLSTRNSV	PFLSTRNSV	6	0.2129	4993.4	60.00	0.19	tr_D0A254_	
HLA-DQA10501-DQB10201	3	FEPFLPFLSTRNSVV	FLSTRNSVV	6	0.2031	5556.0	65.00	0.28	tr_D0A254_	
HLA-DQA10501-DQB10201	4	EPFLPFLSTRNSVVK	FLSTRNSVV	5	0.1674	8169.5	75.00	0.34	tr_D0A254_	
HLA-DQA10501-DQB10201	5	PFLPFLSTRNSVVKL	FLSTRNSVV	4	0.1640	8481.3	75.00	0.37	tr_D0A254_	
HLA-DQA10501-DQB10201	6	FLPFLSTRNSVVKLN	FLSTRNSVV	3	0.1562	9221.9	75.00	0.38	tr_D0A254_	
HLA-DQA10501-DQB10201	7	VVKLNTSLNTTDKDL	TSLNTTDKD	5	0.1420	10762.7	80.00	0.38	tr_D0A254_	
HLA-DQA10501-DQB10201	8	LPFLSTRNSVVKLNT	FLSTRNSVV	2	0.1392	11083.7	80.00	0.32	tr_D0A254_	
HLA-DQA10501-DQB10201	9	KLNTSLNTTDKDLHD	TSLNTTDKD	3	0.1383	11202.6	80.00	0.38	tr_D0A254_	
HLA-DQA10501-DQB10201	10	SVVKLNTSLNTTDKD	TSLNTTDKD	6	0.1345	11661.8	80.00	0.39	tr_D0A254_	
HLA-DQA10501-DQB10201	11	VKLNTSLNTTDKDLH	TSLNTTDKD	4	0.1305	12183.3	85.00	0.35	tr_D0A254_	
HLA-DQA10501-DQB10201	12	LNTSLNTTDKDLHDG	TSLNTTDKD	2	0.1270	12654.4	85.00	0.40	tr_D0A254_	

HLA-DQA10501-DQB10201	13	PFLSTRNSVVKLNTS	FLSTRNSVV	1	0.1233	13173.7	85.00	0.34	tr_D0A254_	
HLA-DQA10501-DQB10201	14	RNSVVKLNTSLNTTD	VKLNTSLNT	4	0.1153	14367.0	85.00	0.28	tr_D0A254_	
HLA-DQA10501-DQB10201	15	STRNSVVKLNTSLNT	VKLNTSLNT	6	0.1122	14849.5	90.00	0.34	tr_D0A254_	
HLA-DQA10501-DQB10201	16	NTSLNTTDKDLHDGG	TSLNTTDKD	1	0.1115	14965.8	90.00	0.40	tr_D0A254_	
HLA-DQA10501-DQB10201	17	TRNSVVKLNTSLNTT	VKLNTSLNT	5	0.1080	15539.7	90.00	0.28	tr_D0A254_	
HLA-DQA10501-DQB10201	18	NSVVKLNTSLNTTDK	VKLNTSLNT	3	0.1056	15944.2	90.00	0.28	tr_D0A254_	
HLA-DQA10501-DQB10201	19	FLSTRNSVVKLNTSL	FLSTRNSVV	0	0.0990	17132.6	90.00	0.23	tr_D0A254_	
HLA-DQA10501-DQB10201	20	LSTRNSVVKLNTSLN	VVKLNTSLN	6	0.0957	17754.6	90.00	0.27	tr_D0A254_	
HLA-DQA10501-DQB10201	21	TSLNTTDKDLHDGGS	TSLNTTDKD	0	0.0924	18400.8	90.00	0.33	tr_D0A254_	
HLA-DQA10501-DQB10201	22	SLNTTDKDLHDGGSR	SLNTTDKDL	0	0.0632	25243.0	100.00	0.29	tr_D0A254_	

Allele: HLA-DQA10501-DQB10201. Number of high binders 0. Number of weak binders 0. Number of peptides 22

Allele	pos	peptide	core	Of	1-log50k(aff)	affinity(nM)	%Rank	Relia	Identity	Bind Level
HLA-DQA10501-DQB10201	1	RYDGYSAVMDCPVAA	YSAVMDCPV	4	0.4493	387.1	9.50	0.31	tr_D0A254_	WB
HLA-DQA10501-DQB10201	2	YDGYSAVMDCPVAAS	YSAVMDCPV	3	0.4146	563.5	14.00	0.36	tr_D0A254_	
HLA-DQA10501-DQB10201	3	SSAWNLLVHALPCRD	WNLLVHALP	3	0.4083	603.4	15.00	0.42	tr_D0A254_	
HLA-DQA10501-DQB10201	4	SAWNLLVHALPCRDP	LVHALPCRD	5	0.4069	612.3	15.00	0.34	tr_D0A254_	
HLA-DQA10501-DQB10201	5	YFWVFMFRYIIPTTT	FRYIIPTTT	6	0.4049	625.5	15.00	0.44	tr_D0A254_	
HLA-DQA10501-DQB10201	6	AWNLLVHALPCRDPM	LVHALPCRD	4	0.3902	733.5	17.00	0.36	tr_D0A254_	
HLA-DQA10501-DQB10201	7	FWVFMFRYIIPTTTN	FRYIIPTTT	5	0.3814	807.1	19.00	0.48	tr_D0A254_	
HLA-DQA10501-DQB10201	8	ISSSAWNLLVHALPC	WNLLVHALP	5	0.3737	877.1	20.00	0.55	tr_D0A254_	
HLA-DQA10501-DQB10201	9	YISSSAWNLLVHALP	WNLLVHALP	6	0.3724	889.0	20.00	0.51	tr_D0A254_	
HLA-DQA10501-DQB10201	10	DGYSAVMDCPVAASN	YSAVMDCPV	2	0.3624	990.8	22.00	0.43	tr_D0A254_	
HLA-DQA10501-DQB10201	11	SYFWVFMFRYIIPTT	MFRYIIPTT	6	0.3547	1077.6	23.00	0.31	tr_D0A254_	
HLA-DQA10501-DQB10201	12	SSSAWNLLVHALPCR	WNLLVHALP	4	0.3522	1106.3	24.00	0.54	tr_D0A254_	
HLA-DQA10501-DQB10201	13	WVFMFRYIIPTTTNR	FRYIIPTTT	4	0.3505	1127.0	24.00	0.50	tr_D0A254_	
HLA-DQA10501-DQB10201	14	WNLLVHALPCRDPMT	LVHALPCRD	3	0.3456	1189.1	25.00	0.45	tr_D0A254_	
HLA-DQA10501-DQB10201	15	ASNHAWFFIAVFCFM	HAWFFIAVF	3	0.3402	1259.6	26.00	0.31	tr_D0A254_	
HLA-DQA10501-DQB10201	16	VFMFRYIIPTTTNRL	FRYIIPTTT	3	0.3391	1275.2	26.00	0.54	tr_D0A254_	
HLA-DQA10501-DQB10201	17	PVAASNHAWFFIAVF	HAWFFIAVF	6	0.3365	1311.9	27.00	0.55	tr_D0A254_	
HLA-DQA10501-DQB10201	18	KSYFWVFMFRYIIPT	FMFRYIIPT	6	0.3330	1362.7	28.00	0.26	tr_D0A254_	
HLA-DQA10501-DQB10201	19	AASNHAWFFIAVFCF	HAWFFIAVF	4	0.3329	1363.3	28.00	0.38	tr_D0A254_	
HLA-DQA10501-DQB10201	20	SNHAWFFIAVFCFMV	HAWFFIAVF	2	0.3308	1395.6	28.00	0.29	tr_D0A254_	
HLA-DQA10501-DQB10201	21	WFFIAVFCFMVLQWL	VFCFMVLQW	5	0.3285	1430.8	29.00	0.23	tr_D0A254_	
HLA-DQA10501-DQB10201	22	AWFFIAVFCFMVLQW	VFCFMVLQW	6	0.3276	1444.7	29.00	0.28	tr_D0A254_	
HLA-DQA10501-DQB10201	23	NHAWFFIAVFCFMVL	HAWFFIAVF	1	0.3253	1479.8	29.00	0.23	tr_D0A254_	
HLA-DQA10501-DQB10201	24	VAASNHAWFFIAVFC	HAWFFIAVF	5	0.3146	1661.6	32.00	0.47	tr_D0A254_	
HLA-DQA10501-DQB10201	25	FMFRYIIPTTTNRLG	FRYIIPTTT	2	0.3128	1694.4	32.00	0.58	tr_D0A254_	
HLA-DQA10501-DQB10201	26	HAWFFIAVFCFMVLQ	WFFIAVFCF	2	0.3127	1696.6	32.00	0.20	tr_D0A254_	
HLA-DQA10501-DQB10201	27	GYSAVMDCPVAASNH	YSAVMDCPV	1	0.3109	1730.1	33.00	0.47	tr_D0A254_	
HLA-DQA10501-DQB10201	28	GKSYFWVFMFRYIIP	YFWVFMFRY	3	0.3086	1772.9	33.00	0.25	tr_D0A254_	
HLA-DQA10501-DQB10201	29	DSLGEYISSSAWNLL	YISSSAWNL	5	0.3037	1869.6	34.00	0.23	tr_D0A254_	
HLA-DQA10501-DQB10201	30	SLGEYISSSAWNLLV	YISSSAWNL	4	0.2999	1949.1	35.00	0.26	tr_D0A254_	
HLA-DQA10501-DQB10201	31	FFIAVFCFMVLQWLI	VFCFMVLQW	4	0.2982	1984.0	36.00	0.30	tr_D0A254_	
HLA-DQA10501-DQB10201	32	SDSLGEYISSSAWNL	YISSSAWNL	6	0.2970	2010.1	36.00	0.25	tr_D0A254_	

HLA-DQA10501-DQB10201	33	FIAVFCFMVLQWLIF	VFCFMVLQW	3	0.2933	2092.0	37.00	0.27	tr_D0A254_
HLA-DQA10501-DQB10201	34	DVTGKSYFWVFMFRY	YFWVFMFRY	6	0.2931	2096.6	37.00	0.35	tr_D0A254_
HLA-DQA10501-DQB10201	35	RSDSLGEYISSSAWN	GEYISSSAW	5	0.2868	2245.3	38.00	0.29	tr_D0A254_
HLA-DQA10501-DQB10201	36	IAVFCFMVLQWLIFI	VFCFMVLQW	2	0.2867	2247.2	38.00	0.26	tr_D0A254_
HLA-DQA10501-DQB10201	37	PRSDSLGEYISSSAW	GEYISSSAW	6	0.2843	2307.8	39.00	0.30	tr_D0A254_
HLA-DQA10501-DQB10201	38	NLLVHALPCRDPMTI	LVHALPCRD	2	0.2791	2441.1	40.00	0.63	tr_D0A254_
HLA-DQA10501-DQB10201	39	TGKSYFWVFMFRYII	YFWVFMFRY	4	0.2778	2474.0	41.00	0.31	tr_D0A254_
HLA-DQA10501-DQB10201	40	LGEYISSSAWNLLVH	YISSSAWNL	3	0.2767	2504.1	41.00	0.25	tr_D0A254_
HLA-DQA10501-DQB10201	41	VTGKSYFWVFMFRYI	YFWVFMFRY	5	0.2763	2516.6	41.00	0.32	tr_D0A254_
HLA-DQA10501-DQB10201	42	EYISSSAWNLLVHAL	AWNLLVHAL	6	0.2681	2748.1	43.00	0.31	tr_D0A254_
HLA-DQA10501-DQB10201	43	AVFCFMVLQWLIFIS	VFCFMVLQW	1	0.2673	2773.0	43.00	0.25	tr_D0A254_
HLA-DQA10501-DQB10201	44	FCFMVLQWLIFISGW	QWLIFISGW	6	0.2655	2827.5	44.00	0.30	tr_D0A254_
HLA-DQA10501-DQB10201	45	GEYISSSAWNLLVHA	YISSSAWNL	2	0.2594	3020.9	46.00	0.22	tr_D0A254_
HLA-DQA10501-DQB10201	46	LLVHALPCRDPMTID	LVHALPCRD	1	0.2584	3054.2	46.00	0.65	tr_D0A254_
HLA-DQA10501-DQB10201	47	VFCFMVLQWLIFISG	FCFMVLQWL	1	0.2573	3088.4	46.00	0.25	tr_D0A254_
HLA-DQA10501-DQB10201	48	TIDVTGKSYFWVFMF	KSYFWVFMF	6	0.2567	3111.2	46.00	0.29	tr_D0A254_
HLA-DQA10501-DQB10201	49	VMDCPVAASNHAWFF	VAASNHAWF	5	0.2539	3205.4	47.00	0.46	tr_D0A254_
HLA-DQA10501-DQB10201	50	MDCPVAASNHAWFFI	VAASNHAWF	4	0.2530	3236.2	47.00	0.42	tr_D0A254_
HLA-DQA10501-DQB10201	51	YSAVMDCPVAASNHA	YSAVMDCPV	0	0.2486	3395.3	49.00	0.45	tr_D0A254_
HLA-DQA10501-DQB10201	52	CPVAASNHAWFFIAV	VAASNHAWF	2	0.2466	3470.7	49.00	0.32	tr_D0A254_
HLA-DQA10501-DQB10201	53	DCPVAASNHAWFFIA	VAASNHAWF	3	0.2450	3531.3	50.00	0.38	tr_D0A254_
HLA-DQA10501-DQB10201	54	CPRSDSLGEYISSSA	RSDSLGEYI	2	0.2438	3574.8	50.00	0.38	tr_D0A254_
HLA-DQA10501-DQB10201	55	IDVTGKSYFWVFMFR	KSYFWVFMF	5	0.2425	3624.7	50.00	0.22	tr_D0A254_
HLA-DQA10501-DQB10201	56	VMLKCPRSDSLGEYI	RSDSLGEYI	6	0.2425	3625.7	50.00	0.67	tr_D0A254_
HLA-DQA10501-DQB10201	57	FMVLQWLIFISGWRQ	QWLIFISGW	4	0.2373	3838.3	55.00	0.25	tr_D0A254_
HLA-DQA10501-DQB10201	58	CFMVLQWLIFISGWR	QWLIFISGW	5	0.2367	3862.7	55.00	0.29	tr_D0A254_
HLA-DQA10501-DQB10201	59	AVMDCPVAASNHAWF	VAASNHAWF	6	0.2357	3904.4	55.00	0.52	tr_D0A254_
HLA-DQA10501-DQB10201	60	QWLIFISGWRQARVM	ISGWRQARV	5	0.2308	4116.0	55.00	0.25	tr_D0A254_
HLA-DQA10501-DQB10201	61	MLKCPRSDSLGEYIS	RSDSLGEYI	5	0.2293	4181.3	55.00	0.67	tr_D0A254_
HLA-DQA10501-DQB10201	62	WLIFISGWRQARVML	ISGWRQARV	4	0.2214	4554.4	60.00	0.27	tr_D0A254_
HLA-DQA10501-DQB10201	63	LQWLIFISGWRQARV	ISGWRQARV	6	0.2203	4608.5	60.00	0.22	tr_D0A254_
HLA-DQA10501-DQB10201	64	LKCPRSDSLGEYISS	RSDSLGEYI	4	0.2189	4681.5	60.00	0.62	tr_D0A254_
HLA-DQA10501-DQB10201	65	LVHALPCRDPMTIDV	LVHALPCRD	0	0.2176	4745.5	60.00	0.65	tr_D0A254_
HLA-DQA10501-DQB10201	66	MVLQWLIFISGWRQA	QWLIFISGW	3	0.2161	4823.9	60.00	0.26	tr_D0A254_
HLA-DQA10501-DQB10201	67	MTIDVTGKSYFWVFM	GKSYFWVFM	6	0.2157	4846.1	60.00	0.35	tr_D0A254_
HLA-DQA10501-DQB10201	68	LIFISGWRQARVMLK	WRQARVMLK	6	0.2145	4910.3	60.00	0.37	tr_D0A254_
HLA-DQA10501-DQB10201	69	KCPRSDSLGEYISSS	RSDSLGEYI	3	0.2144	4916.5	60.00	0.55	tr_D0A254_
HLA-DQA10501-DQB10201	70	VLQWLIFISGWRQAR	QWLIFISGW	2	0.2057	5401.1	65.00	0.28	tr_D0A254_
HLA-DQA10501-DQB10201	71	IFISGWRQARVMLKC	WRQARVMLK	5	0.2027	5577.5	65.00	0.40	tr_D0A254_
HLA-DQA10501-DQB10201	72	FISGWRQARVMLKCP	WRQARVMLK	4	0.2013	5666.0	65.00	0.44	tr_D0A254_
HLA-DQA10501-DQB10201	73	PMTIDVTGKSYFWVF	DVTGKSYFW	4	0.1952	6048.5	65.00	0.21	tr_D0A254_
HLA-DQA10501-DQB10201	74	SAVMDCPVAASNHAW	AVMDCPVAA	1	0.1838	6846.1	70.00	0.31	tr_D0A254_
HLA-DQA10501-DQB10201	75	DPMTIDVTGKSYFWV	DVTGKSYFW	5	0.1811	7044.0	70.00	0.19	tr_D0A254_
HLA-DQA10501-DQB10201	76	RDPMTIDVTGKSYFW	DVTGKSYFW	6	0.1794	7180.6	70.00	0.21	tr_D0A254_
HLA-DQA10501-DQB10201	77	ISGWRQARVMLKCPR	WRQARVMLK	3	0.1743	7583.7	70.00	0.47	tr_D0A254_
HLA-DQA10501-DQB10201	78	CRDPMTIDVTGKSYF	TIDVTGKSY	5	0.1629	8581.6	75.00	0.25	tr_D0A254_
HLA-DQA10501-DQB10201	79	HALPCRDPMTIDVTG	RDPMTIDVT	5	0.1621	8653.1	75.00	0.44	tr_D0A254_
HLA-DQA10501-DQB10201	80	VHALPCRDPMTIDVT	RDPMTIDVT	6	0.1581	9034.2	75.00	0.48	tr_D0A254_
HLA-DQA10501-DQB10201	81	SGWRQARVMLKCPRS	WRQARVMLK	2	0.1561	9240.3	75.00	0.47	tr_D0A254_
HLA-DQA10501-DQB10201	82	PCRDPMTIDVTGKSY	TIDVTGKSY	6	0.1557	9273.2	75.00	0.25	tr_D0A254_

HLA-DQA10501-DQB10201	83	ALPCRDPMTIDVTGK	RDPMTIDVT	4	0.1517	9688.8	80.00	0.33	tr_D0A254_
HLA-DQA10501-DQB10201	84	LPCRDPMTIDVTGKS	RDPMTIDVT	3	0.1420	10754.8	80.00	0.33	tr_D0A254_
HLA-DQA10501-DQB10201	85	GWRQARVMLKCPRSD	WRQARVMLK	1	0.1418	10782.7	80.00	0.45	tr_D0A254_
HLA-DQA10501-DQB10201	86	RVMLKCPRSDSLGEY	PRSDSLGEY	6	0.1309	12131.4	85.00	0.53	tr_D0A254_
HLA-DQA10501-DQB10201	87	WRQARVMLKCPRSDS	WRQARVMLK	0	0.1179	13969.7	85.00	0.38	tr_D0A254_
HLA-DQA10501-DQB10201	88	RQARVMLKCPRSDSL	VMLKCPRSD	4	0.0905	18779.5	95.00	0.20	tr_D0A254_
HLA-DQA10501-DQB10201	89	QARVMLKCPRSDSLG	RVMLKCPRS	2	0.0821	20569.9	95.00	0.31	tr_D0A254_
HLA-DQA10501-DQB10201	90	ARVMLKCPRSDSLGE	VMLKCPRSD	2	0.0776	21585.2	95.00	0.21	tr_D0A254_

Allele: HLA-DQA10501-DQB10201. Number of high binders 0. Number of weak binders 1. Number of peptides 90

Allele	pos	peptide	core	Of	1-log50k(aff)	affinity(nM)	%Rank	Relia	Identity	Bind Level
HLA-DQA10501-DQB10201	1	YRAVVSFVAQDDIVM	VSFVAQDDI	4	0.6864	29.7	0.25	0.34	tr_C9ZXW1_	SB
HLA-DQA10501-DQB10201	2	RAVVSFVAQDDIVMA	VSFVAQDDI	3	0.6736	34.2	0.30	0.32	tr_C9ZXW1_	SB
HLA-DQA10501-DQB10201	3	RYRAVVSFVAQDDIV	VSFVAQDDI	5	0.6686	36.1	0.40	0.40	tr_C9ZXW1_	SB
HLA-DQA10501-DQB10201	4	AVVSFVAQDDIVMAM	VSFVAQDDI	2	0.6618	38.8	0.40	0.33	tr_C9ZXW1_	SB
HLA-DQA10501-DQB10201	5	DRYRAVVSFVAQDDI	VVSFVAQDD	5	0.6451	46.5	0.60	0.42	tr_C9ZXW1_	WB
HLA-DQA10501-DQB10201	6	VVSFVAQDDIVMAMD	VSFVAQDDI	1	0.6353	51.7	0.70	0.29	tr_C9ZXW1_	WB
HLA-DQA10501-DQB10201	7	SPSSELVETFDDLLL	LVETFDDLL	5	0.6154	64.2	1.00	0.40	tr_C9ZXW1_	WB
HLA-DQA10501-DQB10201	8	HSPSSELVETFDDLL	LVETFDDLL	6	0.6110	67.3	1.10	0.41	tr_C9ZXW1_	WB
HLA-DQA10501-DQB10201	9	PSSELVETFDDLLLL	LVETFDDLL	4	0.6051	71.7	1.20	0.41	tr_C9ZXW1_	WB
HLA-DQA10501-DQB10201	10	AQDDIVMAMDTPYEA	VMAMDTPYE	5	0.5890	85.4	1.50	0.35	tr_C9ZXW1_	WB
HLA-DQA10501-DQB10201	11	QDDIVMAMDTPYEAT	VMAMDTPYE	4	0.5826	91.5	1.70	0.38	tr_C9ZXW1_	WB
HLA-DQA10501-DQB10201	12	DDIVMAMDTPYEATY	VMAMDTPYE	3	0.5805	93.6	1.80	0.40	tr_C9ZXW1_	WB
HLA-DQA10501-DQB10201	13	SSELVETFDDLLLLS	LVETFDDLL	3	0.5754	98.9	1.90	0.45	tr_C9ZXW1_	WB
HLA-DQA10501-DQB10201	14	DIVMAMDTPYEATYF	VMAMDTPYE	2	0.5730	101.5	2.00	0.38	tr_C9ZXW1_	WB
HLA-DQA10501-DQB10201	15	VAQDDIVMAMDTPYE	VMAMDTPYE	6	0.5696	105.3	2.50	0.34	tr_C9ZXW1_	WB
HLA-DQA10501-DQB10201	16	VSFVAQDDIVMAMDT	FVAQDDIVM	2	0.5621	114.3	2.50	0.32	tr_C9ZXW1_	WB
HLA-DQA10501-DQB10201	17	TDRYRAVVSFVAQDD	VVSFVAQDD	6	0.5543	124.3	3.00	0.65	tr_C9ZXW1_	WB
HLA-DQA10501-DQB10201	18	FVAQDDIVMAMDTPY	IVMAMDTPY	6	0.5525	126.7	3.00	0.38	tr_C9ZXW1_	WB
HLA-DQA10501-DQB10201	19	SELVETFDDLLLLSE	LVETFDDLL	2	0.5501	130.0	3.00	0.47	tr_C9ZXW1_	WB
HLA-DQA10501-DQB10201	20	VHSPSSELVETFDDL	ELVETFDDL	6	0.5305	160.7	4.00	0.34	tr_C9ZXW1_	WB
HLA-DQA10501-DQB10201	21	RVVNYTWENLAFQVP	WENLAFQVP	6	0.5272	166.7	4.00	0.37	tr_C9ZXW1_	WB
HLA-DQA10501-DQB10201	22	IVMAMDTPYEATYFA	VMAMDTPYE	1	0.5260	168.7	4.00	0.40	tr_C9ZXW1_	WB
HLA-DQA10501-DQB10201	23	SFVAQDDIVMAMDTP	FVAQDDIVM	1	0.5234	173.6	4.00	0.32	tr_C9ZXW1_	WB
HLA-DQA10501-DQB10201	24	VVNYTWENLAFQVPA	WENLAFQVP	5	0.5101	200.5	5.00	0.36	tr_C9ZXW1_	WB
HLA-DQA10501-DQB10201	25	ELVETFDDLLLLSEG	LVETFDDLL	1	0.5070	207.2	5.00	0.48	tr_C9ZXW1_	WB
HLA-DQA10501-DQB10201	26	RTNPCEYFMELLQLP	EYFMELLQL	5	0.4768	287.4	7.50	0.35	tr_C9ZXW1_	WB
HLA-DQA10501-DQB10201	27	ERVVNYTWENLAFQV	VVNYTWENL	2	0.4732	299.0	7.50	0.25	tr_C9ZXW1_	WB
HLA-DQA10501-DQB10201	28	SVNALRVGQMLQDLA	VGQMLQDLA	6	0.4664	321.6	8.00	0.43	tr_C9ZXW1_	WB
HLA-DQA10501-DQB10201	29	RAPINLEGELCMERV	EGELCMERV	6	0.4652	325.8	8.00	0.31	tr_C9ZXW1_	WB
HLA-DQA10501-DQB10201	30	TVHSPSSELVETFDD	PSSELVETF	4	0.4636	331.7	8.50	0.48	tr_C9ZXW1_	WB
HLA-DQA10501-DQB10201	31	KKKGSSWIVQLVELT	WIVQLVELT	6	0.4500	383.9	9.50	0.43	tr_C9ZXW1_	WB
HLA-DQA10501-DQB10201	32	VMAMDTPYEATYFAC	MAMDTPYEA	1	0.4469	397.2	10.00	0.32	tr_C9ZXW1_	
HLA-DQA10501-DQB10201	33	NPCEYFMELLQLPKE	EYFMELLQL	3	0.4467	398.3	10.00	0.27	tr_C9ZXW1_	
HLA-DQA10501-DQB10201	34	PRTNPCEYFMELLQL	EYFMELLQL	6	0.4454	403.6	10.00	0.46	tr_C9ZXW1_	

HLA-DQA10501-DQB10201	35	MERVVNYTWENLAFQ	VVNYTWENL	3	0.4444	408.1	10.00	0.32	tr_C9ZXW1_
HLA-DQA10501-DQB10201	36	VNYTWENLAFQVPAK	WENLAFQVP	4	0.4431	413.7	11.00	0.41	tr_C9ZXW1_
HLA-DQA10501-DQB10201	37	TNPCEYFMELLQLPK	EYFMELLQL	4	0.4426	415.9	11.00	0.31	tr_C9ZXW1_
HLA-DQA10501-DQB10201	38	ATVHSPSSELVETFD	PSSELVETF	5	0.4424	416.8	11.00	0.51	tr_C9ZXW1_
HLA-DQA10501-DQB10201	39	NYTWENLAFQVPAKD	WENLAFQVP	3	0.4412	422.5	11.00	0.43	tr_C9ZXW1_
HLA-DQA10501-DQB10201	40	CMERVVNYTWENLAF	VVNYTWENL	4	0.4406	425.0	11.00	0.37	tr_C9ZXW1_
HLA-DQA10501-DQB10201	41	TGRAPINLEGELCME	PINLEGELC	4	0.4381	436.7	11.00	0.35	tr_C9ZXW1_
HLA-DQA10501-DQB10201	42	APINLEGELCMERVV	EGELCMERV	5	0.4374	440.4	11.00	0.29	tr_C9ZXW1_
HLA-DQA10501-DQB10201	43	CEYFMELLQLPKEEL	EYFMELLQL	1	0.4315	469.2	12.00	0.22	tr_C9ZXW1_
HLA-DQA10501-DQB10201	44	PCEYFMELLQLPKEE	EYFMELLQL	2	0.4308	472.6	12.00	0.25	tr_C9ZXW1_
HLA-DQA10501-DQB10201	45	LVETFDDLLLLSEGH	LVETFDDLL	0	0.4252	502.4	13.00	0.44	tr_C9ZXW1_
HLA-DQA10501-DQB10201	46	LCMERVVNYTWENLA	VVNYTWENL	5	0.4241	508.2	13.00	0.40	tr_C9ZXW1_
HLA-DQA10501-DQB10201	47	ETGRAPINLEGELCM	PINLEGELC	5	0.4240	508.9	13.00	0.43	tr_C9ZXW1_
HLA-DQA10501-DQB10201	48	YTWENLAFQVPAKDS	WENLAFQVP	2	0.4218	521.0	13.00	0.45	tr_C9ZXW1_
HLA-DQA10501-DQB10201	49	VNALRVGQMLQDLAK	VGQMLQDLA	5	0.4216	522.5	13.00	0.42	tr_C9ZXW1_
HLA-DQA10501-DQB10201	50	FTDRYRAVVSFVAQD	RYRAVVSFV	3	0.4198	532.5	13.00	0.34	tr_C9ZXW1_
HLA-DQA10501-DQB10201	51	EATAYFASIGHQVPP	YFASIGHQV	4	0.4187	539.1	13.00	0.38	tr_C9ZXW1_
HLA-DQA10501-DQB10201	52	GRAPINLEGELCMER	PINLEGELC	3	0.4175	546.1	14.00	0.31	tr_C9ZXW1_
HLA-DQA10501-DQB10201	53	EYFMELLQLPKEELE	LLQLPKEEL	5	0.4142	565.9	14.00	0.22	tr_C9ZXW1_
HLA-DQA10501-DQB10201	54	KKGSSWIVQLVELTK	WIVQLVELT	5	0.4131	572.3	14.00	0.41	tr_C9ZXW1_
HLA-DQA10501-DQB10201	55	MELLQLPKEELEVLW	PKEELEVLW	6	0.4056	621.1	15.00	0.39	tr_C9ZXW1_
HLA-DQA10501-DQB10201	56	AMDTPYEATYFACRV	EATYFACRV	6	0.4051	624.2	15.00	0.58	tr_C9ZXW1_
HLA-DQA10501-DQB10201	57	AIFTDRYRAVVSFVA	RYRAVVSFV	5	0.4021	644.7	16.00	0.46	tr_C9ZXW1_
HLA-DQA10501-DQB10201	58	LKKKGSSWIVQLVEL	SSWIVQLVE	5	0.4017	647.8	16.00	0.41	tr_C9ZXW1_
HLA-DQA10501-DQB10201	59	DSVNALRVGQMLQDL	RVGQMLQDL	6	0.3980	674.0	16.00	0.46	tr_C9ZXW1_
HLA-DQA10501-DQB10201	60	EEATAYFASIGHQVP	YFASIGHQV	5	0.3959	689.5	17.00	0.34	tr_C9ZXW1_
HLA-DQA10501-DQB10201	61	PINLEGELCMERVVN	EGELCMERV	4	0.3943	701.6	17.00	0.29	tr_C9ZXW1_
HLA-DQA10501-DQB10201	62	KGSSWIVQLVELTKR	WIVQLVELT	4	0.3918	720.6	17.00	0.41	tr_C9ZXW1_
HLA-DQA10501-DQB10201	63	ATAYFASIGHQVPPR	YFASIGHQV	3	0.3916	722.4	17.00	0.38	tr_C9ZXW1_
HLA-DQA10501-DQB10201	64	GLGPRESEVLVNEVI	ESEVLVNEV	5	0.3897	737.6	17.00	0.47	tr_C9ZXW1_
HLA-DQA10501-DQB10201	65	IFTDRYRAVVSFVAQ	RYRAVVSFV	4	0.3889	744.2	18.00	0.44	tr_C9ZXW1_
HLA-DQA10501-DQB10201	66	TALVANPCVIVLDEP	PCVIVLDEP	6	0.3844	780.9	18.00	0.41	tr_C9ZXW1_
HLA-DQA10501-DQB10201	67	LGPRESEVLVNEVIN	ESEVLVNEV	4	0.3842	782.9	18.00	0.41	tr_C9ZXW1_
HLA-DQA10501-DQB10201	68	TWENLAFQVPAKDSD	WENLAFQVP	1	0.3834	789.9	18.00	0.38	tr_C9ZXW1_
HLA-DQA10501-DQB10201	69	NEVINRLHLVECQDT	RLHLVECQD	5	0.3826	796.3	19.00	0.38	tr_C9ZXW1_
HLA-DQA10501-DQB10201	70	PRESEVLVNEVINRL	ESEVLVNEV	2	0.3820	801.4	19.00	0.31	tr_C9ZXW1_
HLA-DQA10501-DQB10201	71	IATVHSPSSELVETF	PSSELVETF	6	0.3819	802.3	19.00	0.62	tr_C9ZXW1_
HLA-DQA10501-DQB10201	72	TAYFASIGHQVPPRT	YFASIGHQV	2	0.3804	815.8	19.00	0.35	tr_C9ZXW1_
HLA-DQA10501-DQB10201	73	VINRLHLVECQDTVL	RLHLVECQD	3	0.3798	820.7	19.00	0.38	tr_C9ZXW1_
HLA-DQA10501-DQB10201	74	EELEVLWRAWELYLT	WRAWELYLT	6	0.3772	844.5	19.00	0.36	tr_C9ZXW1_
HLA-DQA10501-DQB10201	75	YFMELLQLPKEELEV	LLQLPKEEL	4	0.3761	854.7	20.00	0.30	tr_C9ZXW1_
HLA-DQA10501-DQB10201	76	NAIFTDRYRAVVSFV	RYRAVVSFV	6	0.3746	868.4	20.00	0.71	tr_C9ZXW1_
HLA-DQA10501-DQB10201	77	GSSWIVQLVELTKRS	WIVQLVELT	3	0.3741	873.0	20.00	0.43	tr_C9ZXW1_
HLA-DQA10501-DQB10201	78	LEVLWRAWELYLTTP	WRAWELYLT	4	0.3736	878.2	20.00	0.37	tr_C9ZXW1_
HLA-DQA10501-DQB10201	79	ELLQLPKEELEVLWR	PKEELEVLW	5	0.3734	879.8	20.00	0.34	tr_C9ZXW1_
HLA-DQA10501-DQB10201	80	NALRVGQMLQDLAKK	VGQMLQDLA	4	0.3730	883.7	20.00	0.43	tr_C9ZXW1_
HLA-DQA10501-DQB10201	81	EVINRLHLVECQDTV	RLHLVECQD	4	0.3726	887.1	20.00	0.41	tr_C9ZXW1_
HLA-DQA10501-DQB10201	82	VNEVINRLHLVECQD	RLHLVECQD	6	0.3722	891.5	20.00	0.44	tr_C9ZXW1_
HLA-DQA10501-DQB10201	83	ALVANPCVIVLDEPT	PCVIVLDEP	5	0.3706	906.4	21.00	0.37	tr_C9ZXW1_
HLA-DQA10501-DQB10201	84	EGCCFLNNAIFTDRY	NNAIFTDRY	6	0.3663	950.5	21.00	0.54	tr_C9ZXW1_

HLA-DQA10501-DQB10201	85	EVLWRAWELYLTTPA	WRAWELYLT	3	0.3658	954.8	21.00	0.32	tr_C9ZXW1_
HLA-DQA10501-DQB10201	86	DEPTTGLDSVNALRV	LDSVNALRV	6	0.3625	990.2	22.00	0.44	tr_C9ZXW1_
HLA-DQA10501-DQB10201	87	ELEVLWRAWELYLTT	WRAWELYLT	5	0.3624	990.9	22.00	0.34	tr_C9ZXW1_
HLA-DQA10501-DQB10201	88	LVANPCVIVLDEPTT	PCVIVLDEP	4	0.3610	1006.0	22.00	0.34	tr_C9ZXW1_
HLA-DQA10501-DQB10201	89	GPRESEVLVNEVINR	ESEVLVNEV	3	0.3607	1009.3	22.00	0.39	tr_C9ZXW1_
HLA-DQA10501-DQB10201	90	PPRTNPCEYFMELLQ	PCEYFMELL	5	0.3605	1011.9	22.00	0.49	tr_C9ZXW1_
HLA-DQA10501-DQB10201	91	VLWRAWELYLTTPAA	WRAWELYLT	2	0.3597	1020.5	22.00	0.28	tr_C9ZXW1_
HLA-DQA10501-DQB10201	92	AYFASIGHQVPPRTN	YFASIGHQV	1	0.3591	1026.9	23.00	0.42	tr_C9ZXW1_
HLA-DQA10501-DQB10201	93	VPPRTNPCEYFMELL	PCEYFMELL	6	0.3574	1046.0	23.00	0.58	tr_C9ZXW1_
HLA-DQA10501-DQB10201	94	MDTPYEATYFACRVR	EATYFACRV	5	0.3567	1053.9	23.00	0.57	tr_C9ZXW1_
HLA-DQA10501-DQB10201	95	RESEVLVNEVINRLH	ESEVLVNEV	1	0.3565	1056.7	23.00	0.31	tr_C9ZXW1_
HLA-DQA10501-DQB10201	96	FMELLQLPKEELEVL	LLQLPKEEL	3	0.3562	1059.7	23.00	0.29	tr_C9ZXW1_
HLA-DQA10501-DQB10201	97	ELCMERVVNYTWENL	VVNYTWENL	6	0.3559	1063.2	23.00	0.54	tr_C9ZXW1_
HLA-DQA10501-DQB10201	98	PETGRAPINLEGELC	PINLEGELC	6	0.3552	1071.5	23.00	0.51	tr_C9ZXW1_
HLA-DQA10501-DQB10201	99	DTPYEATYFACRVRL	EATYFACRV	4	0.3548	1076.2	23.00	0.53	tr_C9ZXW1_
HLA-DQA10501-DQB10201	100	LPKEELEVLWRAWEL	EVLWRAWEL	6	0.3548	1076.5	23.00	0.34	tr_C9ZXW1_
HLA-DQA10501-DQB10201	101	INRLHLVECQDTVLG	RLHLVECQD	2	0.3543	1081.4	23.00	0.38	tr_C9ZXW1_
HLA-DQA10501-DQB10201	102	YHGPREEATAYFASI	PREEATAYF	3	0.3543	1081.6	23.00	0.38	tr_C9ZXW1_
HLA-DQA10501-DQB10201	103	ALRVGQMLQDLAKKE	VGQMLQDLA	3	0.3509	1122.9	24.00	0.51	tr_C9ZXW1_
HLA-DQA10501-DQB10201	104	VANPCVIVLDEPTTG	PCVIVLDEP	3	0.3499	1134.6	24.00	0.31	tr_C9ZXW1_
HLA-DQA10501-DQB10201	105	HVVYHGPREEATAYF	PREEATAYF	6	0.3493	1142.3	24.00	0.51	tr_C9ZXW1_
HLA-DQA10501-DQB10201	106	TGLDSVNALRVGQML	VNALRVGQM	5	0.3481	1157.2	25.00	0.33	tr_C9ZXW1_
HLA-DQA10501-DQB10201	107	LGLGPRESEVLVNEV	ESEVLVNEV	6	0.3468	1173.2	25.00	0.68	tr_C9ZXW1_
HLA-DQA10501-DQB10201	108	VVYHGPREEATAYFA	PREEATAYF	5	0.3453	1192.4	25.00	0.46	tr_C9ZXW1_
HLA-DQA10501-DQB10201	109	LLQLPKEELEVLWRA	PKEELEVLW	4	0.3445	1203.0	25.00	0.38	tr_C9ZXW1_
HLA-DQA10501-DQB10201	110	TTGLDSVNALRVGQM	VNALRVGQM	6	0.3445	1203.3	25.00	0.34	tr_C9ZXW1_
HLA-DQA10501-DQB10201	111	INLEGELCMERVVNY	EGELCMERV	3	0.3443	1204.7	25.00	0.45	tr_C9ZXW1_
HLA-DQA10501-DQB10201	112	SSWIVQLVELTKRSY	WIVQLVELT	2	0.3410	1249.3	26.00	0.46	tr_C9ZXW1_
HLA-DQA10501-DQB10201	113	KEELEVLWRAWELYL	EVLWRAWEL	4	0.3400	1263.3	26.00	0.31	tr_C9ZXW1_
HLA-DQA10501-DQB10201	114	ANPCVIVLDEPTTGL	PCVIVLDEP	2	0.3394	1271.1	26.00	0.29	tr_C9ZXW1_
HLA-DQA10501-DQB10201	115	KRANVATALVANPCV	RANVATALV	1	0.3383	1285.9	27.00	0.22	tr_C9ZXW1_
HLA-DQA10501-DQB10201	116	HLKKKGSSWIVQLVE	SSWIVQLVE	6	0.3378	1292.9	27.00	0.68	tr_C9ZXW1_
HLA-DQA10501-DQB10201	117	PKEELEVLWRAWELY	EVLWRAWEL	5	0.3347	1337.6	27.00	0.32	tr_C9ZXW1_
HLA-DQA10501-DQB10201	118	NRLHLVECQDTVLGI	RLHLVECQD	1	0.3345	1339.6	27.00	0.32	tr_C9ZXW1_
HLA-DQA10501-DQB10201	119	REEATAYFASIGHQV	YFASIGHQV	6	0.3345	1340.8	27.00	0.43	tr_C9ZXW1_
HLA-DQA10501-DQB10201	120	LWRAWELYLTTPAAS	WRAWELYLT	1	0.3331	1360.3	28.00	0.25	tr_C9ZXW1_
HLA-DQA10501-DQB10201	121	MAMDTPYEATYFACR	MAMDTPYEA	0	0.3306	1397.8	28.00	0.38	tr_C9ZXW1_
HLA-DQA10501-DQB10201	122	LQLPKEELEVLWRAW	PKEELEVLW	3	0.3305	1399.2	28.00	0.41	tr_C9ZXW1_
HLA-DQA10501-DQB10201	123	RKRANVATALVANPC	RANVATALV	2	0.3300	1407.1	28.00	0.26	tr_C9ZXW1_
HLA-DQA10501-DQB10201	124	RANVATALVANPCVI	ATALVANPC	4	0.3299	1408.0	28.00	0.22	tr_C9ZXW1_
HLA-DQA10501-DQB10201	125	EPTTGLDSVNALRVG	LDSVNALRV	5	0.3297	1412.3	28.00	0.43	tr_C9ZXW1_
HLA-DQA10501-DQB10201	126	HGPREEATAYFASIG	PREEATAYF	2	0.3289	1423.6	29.00	0.31	tr_C9ZXW1_
HLA-DQA10501-DQB10201	127	GCCFLNNAIFTDRYR	NNAIFTDRY	5	0.3283	1432.6	29.00	0.53	tr_C9ZXW1_
HLA-DQA10501-DQB10201	128	ESEVLVNEVINRLHL	LVNEVINRL	4	0.3277	1441.7	29.00	0.22	tr_C9ZXW1_
HLA-DQA10501-DQB10201	129	LDSVNALRVGQMLQD	VNALRVGQM	3	0.3266	1460.6	29.00	0.35	tr_C9ZXW1_
HLA-DQA10501-DQB10201	130	YFASIGHQVPPRTNP	FASIGHQVP	1	0.3258	1472.0	29.00	0.40	tr_C9ZXW1_
HLA-DQA10501-DQB10201	131	GPREEATAYFASIGH	PREEATAYF	1	0.3248	1488.9	29.00	0.27	tr_C9ZXW1_
HLA-DQA10501-DQB10201	132	NPCVIVLDEPTTGLD	PCVIVLDEP	1	0.3228	1521.1	30.00	0.27	tr_C9ZXW1_
HLA-DQA10501-DQB10201	133	LRVGQMLQDLAKKEK	VGQMLQDLA	2	0.3201	1566.3	30.00	0.55	tr_C9ZXW1_
HLA-DQA10501-DQB10201	134	ATALVANPCVIVLDE	NPCVIVLDE	6	0.3169	1620.6	31.00	0.40	tr_C9ZXW1_

HLA-DQA10501-DQB10201	135	QLPKEELEVLWRAWE	PKEELEVLW	2	0.3164	1630.1	31.00	0.41	tr_C9ZXW1_
HLA-DQA10501-DQB10201	136	GLDSVNALRVGQMLQ	VNALRVGQM	4	0.3156	1644.9	31.00	0.35	tr_C9ZXW1_
HLA-DQA10501-DQB10201	137	VETFDDLLLLSEGHV	ETFDDLLLL	1	0.3139	1675.0	32.00	0.26	tr_C9ZXW1_
HLA-DQA10501-DQB10201	138	VYHGPREEATAYFAS	PREEATAYF	4	0.3131	1689.1	32.00	0.47	tr_C9ZXW1_
HLA-DQA10501-DQB10201	139	WENLAFQVPAKDSDG	WENLAFQVP	0	0.3118	1714.0	32.00	0.35	tr_C9ZXW1_
HLA-DQA10501-DQB10201	140	CCFLNNAIFTDRYRA	NNAIFTDRY	4	0.3101	1744.2	33.00	0.52	tr_C9ZXW1_
HLA-DQA10501-DQB10201	141	RLHLVECQDTVLGIP	LVECQDTVL	3	0.3101	1745.0	33.00	0.29	tr_C9ZXW1_
HLA-DQA10501-DQB10201	142	PTTGLDSVNALRVGQ	LDSVNALRV	4	0.3099	1748.1	33.00	0.43	tr_C9ZXW1_
HLA-DQA10501-DQB10201	143	TPYEATYFACRVRLG	EATYFACRV	3	0.3096	1754.7	33.00	0.60	tr_C9ZXW1_
HLA-DQA10501-DQB10201	144	ANVATALVANPCVIV	ATALVANPC	3	0.3095	1755.7	33.00	0.25	tr_C9ZXW1_
HLA-DQA10501-DQB10201	145	GGERKRANVATALVA	RANVATALV	5	0.3091	1763.2	33.00	0.43	tr_C9ZXW1_
HLA-DQA10501-DQB10201	146	ERKRANVATALVANP	RANVATALV	3	0.3070	1804.6	33.00	0.37	tr_C9ZXW1_
HLA-DQA10501-DQB10201	147	VLDEPTTGLDSVNAL	TGLDSVNAL	6	0.3037	1870.2	34.00	0.51	tr_C9ZXW1_
HLA-DQA10501-DQB10201	148	PREEATAYFASIGHQ	PREEATAYF	0	0.3026	1892.2	34.00	0.17	tr_C9ZXW1_
HLA-DQA10501-DQB10201	149	WRAWELYLTTPAAST	ELYLTTPAA	4	0.2994	1958.7	35.00	0.26	tr_C9ZXW1_
HLA-DQA10501-DQB10201	150	CFLNNAIFTDRYRAV	NNAIFTDRY	3	0.2984	1981.3	36.00	0.50	tr_C9ZXW1_
HLA-DQA10501-DQB10201	151	KVSGPITERDPYLED	PITERDPYL	4	0.2968	2015.6	36.00	0.44	tr_C9ZXW1_
HLA-DQA10501-DQB10201	152	PYEATYFACRVRLGL	EATYFACRV	2	0.2967	2017.6	36.00	0.56	tr_C9ZXW1_
HLA-DQA10501-DQB10201	153	LVECQDTVLGIPGLV	TVLGIPGLV	6	0.2963	2025.1	36.00	0.38	tr_C9ZXW1_
HLA-DQA10501-DQB10201	154	NVATALVANPCVIVL	ATALVANPC	2	0.2956	2040.6	36.00	0.26	tr_C9ZXW1_
HLA-DQA10501-DQB10201	155	LVNEVINRLHLVECQ	NRLHLVECQ	6	0.2950	2054.5	36.00	0.34	tr_C9ZXW1_
HLA-DQA10501-DQB10201	156	NNAIFTDRYRAVVSF	DRYRAVVSF	6	0.2938	2080.7	37.00	0.47	tr_C9ZXW1_
HLA-DQA10501-DQB10201	157	SWIVQLVELTKRSYR	WIVQLVELT	1	0.2924	2112.8	37.00	0.56	tr_C9ZXW1_
HLA-DQA10501-DQB10201	158	SGGERKRANVATALV	RANVATALV	6	0.2924	2113.3	37.00	0.64	tr_C9ZXW1_
HLA-DQA10501-DQB10201	159	SGPITERDPYLEDHL	PITERDPYL	2	0.2896	2179.2	38.00	0.34	tr_C9ZXW1_
HLA-DQA10501-DQB10201	160	TALGGRVLAIMGPSG	VLAIMGPSG	6	0.2894	2183.1	38.00	0.39	tr_C9ZXW1_
HLA-DQA10501-DQB10201	161	VATALVANPCVIVLD	TALVANPCV	2	0.2889	2194.8	38.00	0.23	tr_C9ZXW1_
HLA-DQA10501-DQB10201	162	LDEPTTGLDSVNALR	TGLDSVNAL	5	0.2886	2202.6	38.00	0.50	tr_C9ZXW1_
HLA-DQA10501-DQB10201	163	NLEGELCMERVVNYT	EGELCMERV	2	0.2878	2221.4	38.00	0.55	tr_C9ZXW1_
HLA-DQA10501-DQB10201	164	SEVLVNEVINRLHLV	LVNEVINRL	3	0.2857	2273.1	39.00	0.31	tr_C9ZXW1_
HLA-DQA10501-DQB10201	165	GERKRANVATALVAN	RANVATALV	4	0.2853	2281.1	39.00	0.43	tr_C9ZXW1_
HLA-DQA10501-DQB10201	166	EVLVNEVINRLHLVE	LVNEVINRL	2	0.2848	2294.3	39.00	0.31	tr_C9ZXW1_
HLA-DQA10501-DQB10201	167	FLNNAIFTDRYRAVV	NNAIFTDRY	2	0.2798	2421.2	40.00	0.52	tr_C9ZXW1_
HLA-DQA10501-DQB10201	168	LKVSGPITERDPYLE	PITERDPYL	5	0.2794	2431.9	40.00	0.44	tr_C9ZXW1_
HLA-DQA10501-DQB10201	169	PCVIVLDEPTTGLDS	VIVLDEPTT	2	0.2793	2434.4	40.00	0.28	tr_C9ZXW1_
HLA-DQA10501-DQB10201	170	ALGGRVLAIMGPSGA	VLAIMGPSG	5	0.2786	2454.3	40.00	0.43	tr_C9ZXW1_
HLA-DQA10501-DQB10201	171	VSGPITERDPYLEDH	PITERDPYL	3	0.2784	2460.2	41.00	0.44	tr_C9ZXW1_
HLA-DQA10501-DQB10201	172	VLVNEVINRLHLVEC	VINRLHLVE	5	0.2764	2512.8	41.00	0.28	tr_C9ZXW1_
HLA-DQA10501-DQB10201	173	VECQDTVLGIPGLVK	TVLGIPGLV	5	0.2746	2562.4	42.00	0.38	tr_C9ZXW1_
HLA-DQA10501-DQB10201	174	EGELCMERVVNYTWE	ERVVNYTWE	6	0.2744	2567.1	42.00	0.34	tr_C9ZXW1_
HLA-DQA10501-DQB10201	175	LEGELCMERVVNYTW	EGELCMERV	1	0.2744	2568.5	42.00	0.52	tr_C9ZXW1_
HLA-DQA10501-DQB10201	176	YEATYFACRVRLGLG	EATYFACRV	1	0.2728	2612.9	42.00	0.60	tr_C9ZXW1_
HLA-DQA10501-DQB10201	177	ETFDDLLLLSEGHVV	DLLLLSEGH	4	0.2720	2636.5	42.00	0.19	tr_C9ZXW1_
HLA-DQA10501-DQB10201	178	LLKVSGPITERDPYL	PITERDPYL	6	0.2716	2647.0	42.00	0.36	tr_C9ZXW1_
HLA-DQA10501-DQB10201	179	SEGHVVYHGPREEAT	VVYHGPREE	4	0.2698	2698.3	43.00	0.36	tr_C9ZXW1_
HLA-DQA10501-DQB10201	180	RVGQMLQDLAKKEKR	VGQMLQDLA	1	0.2697	2702.0	43.00	0.62	tr_C9ZXW1_
HLA-DQA10501-DQB10201	181	LSEGHVVYHGPREEA	VVYHGPREE	5	0.2690	2722.8	43.00	0.42	tr_C9ZXW1_
HLA-DQA10501-DQB10201	182	GPITERDPYLEDHLK	PITERDPYL	1	0.2649	2845.2	44.00	0.37	tr_C9ZXW1_
HLA-DQA10501-DQB10201	183	RTVIATVHSPSSELV	VHSPSSELV	6	0.2636	2885.5	44.00	0.40	tr_C9ZXW1_
HLA-DQA10501-DQB10201	184	GTALGGRVLAIMGPS	RVLAIMGPS	6	0.2628	2911.5	45.00	0.31	tr_C9ZXW1_

HLA-DQA10501-DQB10201	185	HLVECQDTVLGIPGL	LVECQDTVL	1	0.2620	2937.8	45.00	0.35	tr_C9ZXW1_
HLA-DQA10501-DQB10201	186	CVIVLDEPTTGLDSV	VIVLDEPTT	1	0.2605	2984.8	45.00	0.29	tr_C9ZXW1_
HLA-DQA10501-DQB10201	187	EGHVVYHGPREEATA	VVYHGPREE	3	0.2601	2997.2	45.00	0.36	tr_C9ZXW1_
HLA-DQA10501-DQB10201	188	RAWELYLTTPAASTN	ELYLTTPAA	3	0.2584	3054.1	46.00	0.29	tr_C9ZXW1_
HLA-DQA10501-DQB10201	189	AWELYLTTPAASTNP	ELYLTTPAA	2	0.2576	3079.3	46.00	0.31	tr_C9ZXW1_
HLA-DQA10501-DQB10201	190	ECQDTVLGIPGLVKG	TVLGIPGLV	4	0.2575	3081.9	46.00	0.40	tr_C9ZXW1_
HLA-DQA10501-DQB10201	191	TVIATVHSPSSELVE	VHSPSSELV	5	0.2574	3086.0	46.00	0.44	tr_C9ZXW1_
HLA-DQA10501-DQB10201	192	LGGRVLAIMGPSGAG	VLAIMGPSG	4	0.2569	3104.1	46.00	0.46	tr_C9ZXW1_
HLA-DQA10501-DQB10201	193	LHLVECQDTVLGIPG	LVECQDTVL	2	0.2548	3175.3	47.00	0.40	tr_C9ZXW1_
HLA-DQA10501-DQB10201	194	GHVVYHGPREEATAY	VVYHGPREE	2	0.2546	3180.2	47.00	0.34	tr_C9ZXW1_
HLA-DQA10501-DQB10201	195	CQDTVLGIPGLVKGV	TVLGIPGLV	3	0.2511	3302.5	48.00	0.41	tr_C9ZXW1_
HLA-DQA10501-DQB10201	196	VIATVHSPSSELVET	VHSPSSELV	4	0.2485	3397.5	49.00	0.41	tr_C9ZXW1_
HLA-DQA10501-DQB10201	197	SPETGRAPINLEGEL	RAPINLEGE	5	0.2480	3418.3	49.00	0.44	tr_C9ZXW1_
HLA-DQA10501-DQB10201	198	QDTVLGIPGLVKGVS	TVLGIPGLV	2	0.2476	3431.6	49.00	0.43	tr_C9ZXW1_
HLA-DQA10501-DQB10201	199	TFDDLLLLSEGHVVY	DLLLLSEGH	3	0.2442	3558.6	50.00	0.25	tr_C9ZXW1_
HLA-DQA10501-DQB10201	200	PCLLKVSGPITERDP	VSGPITERD	5	0.2432	3600.4	50.00	0.30	tr_C9ZXW1_
HLA-DQA10501-DQB10201	201	CLLKVSGPITERDPY	VSGPITERD	4	0.2413	3674.8	55.00	0.32	tr_C9ZXW1_
HLA-DQA10501-DQB10201	202	GELCMERVVNYTWEN	ERVVNYTWE	5	0.2380	3806.9	55.00	0.42	tr_C9ZXW1_
HLA-DQA10501-DQB10201	203	SGTALGGRVLAIMGP	GRVLAIMGP	6	0.2380	3809.3	55.00	0.26	tr_C9ZXW1_
HLA-DQA10501-DQB10201	204	LLSEGHVVYHGPREE	VVYHGPREE	6	0.2370	3847.1	55.00	0.43	tr_C9ZXW1_
HLA-DQA10501-DQB10201	205	LNNAIFTDRYRAVVS	NNAIFTDRY	1	0.2367	3861.1	55.00	0.50	tr_C9ZXW1_
HLA-DQA10501-DQB10201	206	WELYLTTPAASTNPC	ELYLTTPAA	1	0.2351	3926.9	55.00	0.31	tr_C9ZXW1_
HLA-DQA10501-DQB10201	207	LYLTTPAASTNPCLL	AASTNPCLL	6	0.2344	3958.2	55.00	0.51	tr_C9ZXW1_
HLA-DQA10501-DQB10201	208	NPCLLKVSGPITERD	LLKVSGPIT	3	0.2319	4067.4	55.00	0.34	tr_C9ZXW1_
HLA-DQA10501-DQB10201	209	PITERDPYLEDHLKK	PITERDPYL	0	0.2304	4134.6	55.00	0.35	tr_C9ZXW1_
HLA-DQA10501-DQB10201	210	QVPPRTNPCEYFMEL	PRTNPCEYF	3	0.2298	4159.3	55.00	0.41	tr_C9ZXW1_
HLA-DQA10501-DQB10201	211	DTVLGIPGLVKGVSG	TVLGIPGLV	1	0.2283	4227.6	55.00	0.44	tr_C9ZXW1_
HLA-DQA10501-DQB10201	212	GGRVLAIMGPSGAGK	VLAIMGPSG	3	0.2256	4354.9	55.00	0.47	tr_C9ZXW1_
HLA-DQA10501-DQB10201	213	FASIGHQVPPRTNPC	FASIGHQVP	0	0.2252	4372.6	55.00	0.44	tr_C9ZXW1_
HLA-DQA10501-DQB10201	214	FDDLLLLSEGHVVYH	DLLLLSEGH	2	0.2234	4458.5	60.00	0.33	tr_C9ZXW1_
HLA-DQA10501-DQB10201	215	WIVQLVELTKRSYRM	WIVQLVELT	0	0.2228	4487.7	60.00	0.63	tr_C9ZXW1_
HLA-DQA10501-DQB10201	216	LEGCCFLNNAIFTDR	FLNNAIFTD	5	0.2215	4552.9	60.00	0.50	tr_C9ZXW1_
HLA-DQA10501-DQB10201	217	VIVLDEPTTGLDSVN	IVLDEPTTG	1	0.2205	4598.5	60.00	0.34	tr_C9ZXW1_
HLA-DQA10501-DQB10201	218	ENLAFQVPAKDSDGR	LAFQVPAKD	2	0.2179	4732.9	60.00	0.52	tr_C9ZXW1_
HLA-DQA10501-DQB10201	219	EATYFACRVRLGLGP	EATYFACRV	0	0.2176	4749.9	60.00	0.50	tr_C9ZXW1_
HLA-DQA10501-DQB10201	220	KRTVIATVHSPSSEL	TVHSPSSEL	6	0.2166	4801.3	60.00	0.24	tr_C9ZXW1_
HLA-DQA10501-DQB10201	221	IVLDEPTTGLDSVNA	TTGLDSVNA	6	0.2161	4826.5	60.00	0.41	tr_C9ZXW1_
HLA-DQA10501-DQB10201	222	NLEGCCFLNNAIFTD	FLNNAIFTD	6	0.2145	4911.3	60.00	0.57	tr_C9ZXW1_
HLA-DQA10501-DQB10201	223	KKEKRTVIATVHSPS	VIATVHSPS	6	0.2129	4997.4	60.00	0.27	tr_C9ZXW1_
HLA-DQA10501-DQB10201	224	KEKRTVIATVHSPSS	VIATVHSPS	5	0.2104	5131.3	60.00	0.24	tr_C9ZXW1_
HLA-DQA10501-DQB10201	225	RVRLGLGPRESEVLV	VRLGLGPRE	1	0.2095	5184.5	60.00	0.35	tr_C9ZXW1_
HLA-DQA10501-DQB10201	226	RLGLGPRESEVLVNE	RESEVLVNE	6	0.2089	5217.3	60.00	0.38	tr_C9ZXW1_
HLA-DQA10501-DQB10201	227	GRVLAIMGPSGAGKT	VLAIMGPSG	2	0.2077	5284.4	60.00	0.51	tr_C9ZXW1_
HLA-DQA10501-DQB10201	228	ELYLTTPAASTNPCL	LYLTTPAAS	1	0.2054	5415.1	65.00	0.25	tr_C9ZXW1_
HLA-DQA10501-DQB10201	229	EKRTVIATVHSPSSE	VIATVHSPS	4	0.2046	5463.7	65.00	0.25	tr_C9ZXW1_
HLA-DQA10501-DQB10201	230	VRLGLGPRESEVLVN	PRESEVLVN	6	0.2035	5529.2	65.00	0.24	tr_C9ZXW1_
HLA-DQA10501-DQB10201	231	KMSGTALGGRVLAIM	LGGRVLAIM	6	0.2028	5573.6	65.00	0.40	tr_C9ZXW1_
HLA-DQA10501-DQB10201	232	HQVPPRTNPCEYFME	PRTNPCEYF	4	0.2028	5574.2	65.00	0.50	tr_C9ZXW1_
HLA-DQA10501-DQB10201	233	GHQVPPRTNPCEYFM	PRTNPCEYF	5	0.2010	5683.0	65.00	0.54	tr_C9ZXW1_
HLA-DQA10501-DQB10201	234	IGHQVPPRTNPCEYF	PRTNPCEYF	6	0.2007	5698.4	65.00	0.55	tr_C9ZXW1_

HLA-DQA10501-DQB10201	235	VSGGERKRANVATAL	KRANVATAL	6	0.2003	5727.6	65.00	0.81	tr_C9ZXW1_
HLA-DQA10501-DQB10201	236	CRVRLGLGPRESEVL	VRLGLGPRE	2	0.1995	5774.3	65.00	0.41	tr_C9ZXW1_
HLA-DQA10501-DQB10201	237	DSPETGRAPINLEGE	RAPINLEGE	6	0.1993	5786.8	65.00	0.52	tr_C9ZXW1_
HLA-DQA10501-DQB10201	238	AKKEKRTVIATVHSP	KRTVIATVH	4	0.1990	5807.6	65.00	0.36	tr_C9ZXW1_
HLA-DQA10501-DQB10201	239	MSGTALGGRVLAIMG	LGGRVLAIM	5	0.1966	5961.0	65.00	0.29	tr_C9ZXW1_
HLA-DQA10501-DQB10201	240	KLHGTEKNLEGCCFL	EKNLEGCCF	5	0.1897	6418.3	65.00	0.52	tr_C9ZXW1_
HLA-DQA10501-DQB10201	241	STNPCLLKVSGPITE	LLKVSGPIT	5	0.1893	6446.2	70.00	0.54	tr_C9ZXW1_
HLA-DQA10501-DQB10201	242	YFACRVRLGLGPRES	VRLGLGPRE	5	0.1892	6458.6	70.00	0.47	tr_C9ZXW1_
HLA-DQA10501-DQB10201	243	YLTTPAASTNPCLLK	AASTNPCLL	5	0.1888	6484.5	70.00	0.63	tr_C9ZXW1_
HLA-DQA10501-DQB10201	244	NLAFQVPAKDSDGRK	LAFQVPAKD	1	0.1877	6560.8	70.00	0.56	tr_C9ZXW1_
HLA-DQA10501-DQB10201	245	DDLLLLSEGHVVYHG	DLLLLSEGH	1	0.1869	6619.4	70.00	0.35	tr_C9ZXW1_
HLA-DQA10501-DQB10201	246	TYFACRVRLGLGPRE	VRLGLGPRE	6	0.1861	6673.9	70.00	0.47	tr_C9ZXW1_
HLA-DQA10501-DQB10201	247	LLLSEGHVVYHGPRE	HVVYHGPRE	6	0.1856	6712.1	70.00	0.38	tr_C9ZXW1_
HLA-DQA10501-DQB10201	248	FACRVRLGLGPRESE	VRLGLGPRE	4	0.1847	6779.1	70.00	0.50	tr_C9ZXW1_
HLA-DQA10501-DQB10201	249	GAGKTTLMSAITGKL	LMSAITGKL	6	0.1843	6807.1	70.00	0.55	tr_C9ZXW1_
HLA-DQA10501-DQB10201	250	TNPCLLKVSGPITER	LLKVSGPIT	4	0.1838	6840.9	70.00	0.49	tr_C9ZXW1_
HLA-DQA10501-DQB10201	251	DLLLLSEGHVVYHGP	DLLLLSEGH	0	0.1823	6957.4	70.00	0.23	tr_C9ZXW1_
HLA-DQA10501-DQB10201	252	ACRVRLGLGPRESEV	VRLGLGPRE	3	0.1822	6965.8	70.00	0.52	tr_C9ZXW1_
HLA-DQA10501-DQB10201	253	DHLKKKGSSWIVQLV	GSSWIVQLV	6	0.1805	7089.6	70.00	0.65	tr_C9ZXW1_
HLA-DQA10501-DQB10201	254	RVLAIMGPSGAGKTT	VLAIMGPSG	1	0.1782	7270.2	70.00	0.58	tr_C9ZXW1_
HLA-DQA10501-DQB10201	255	LTTPAASTNPCLLKV	AASTNPCLL	4	0.1780	7290.1	70.00	0.67	tr_C9ZXW1_
HLA-DQA10501-DQB10201	256	LAKKEKRTVIATVHS	KRTVIATVH	5	0.1766	7400.7	70.00	0.41	tr_C9ZXW1_
HLA-DQA10501-DQB10201	257	AGKTTLMSAITGKLH	LMSAITGKL	5	0.1741	7604.4	70.00	0.44	tr_C9ZXW1_
HLA-DQA10501-DQB10201	258	LHGTEKNLEGCCFLN	EKNLEGCCF	4	0.1733	7667.7	70.00	0.51	tr_C9ZXW1_
HLA-DQA10501-DQB10201	259	TVLGIPGLVKGVSGG	TVLGIPGLV	0	0.1707	7889.5	75.00	0.47	tr_C9ZXW1_
HLA-DQA10501-DQB10201	260	VGQMLQDLAKKEKRT	VGQMLQDLA	0	0.1701	7938.2	75.00	0.76	tr_C9ZXW1_
HLA-DQA10501-DQB10201	261	TTPAASTNPCLLKVS	AASTNPCLL	3	0.1662	8277.0	75.00	0.69	tr_C9ZXW1_
HLA-DQA10501-DQB10201	262	DLAKKEKRTVIATVH	KRTVIATVH	6	0.1657	8320.8	75.00	0.61	tr_C9ZXW1_
HLA-DQA10501-DQB10201	263	HGTEKNLEGCCFLNN	EKNLEGCCF	3	0.1648	8406.3	75.00	0.48	tr_C9ZXW1_
HLA-DQA10501-DQB10201	264	GKLHGTEKNLEGCCF	EKNLEGCCF	6	0.1642	8459.9	75.00	0.63	tr_C9ZXW1_
HLA-DQA10501-DQB10201	265	ASTNPCLLKVSGPIT	LLKVSGPIT	6	0.1627	8597.5	75.00	0.62	tr_C9ZXW1_
HLA-DQA10501-DQB10201	266	GTEKNLEGCCFLNNA	EKNLEGCCF	2	0.1586	8987.8	75.00	0.45	tr_C9ZXW1_
HLA-DQA10501-DQB10201	267	ITERDPYLEDHLKKK	ITERDPYLE	0	0.1573	9112.2	75.00	0.25	tr_C9ZXW1_
HLA-DQA10501-DQB10201	268	GKTTLMSAITGKLHG	LMSAITGKL	4	0.1566	9190.7	75.00	0.47	tr_C9ZXW1_
HLA-DQA10501-DQB10201	269	HKMSGTALGGRVLAI	ALGGRVLAI	6	0.1554	9305.0	75.00	0.52	tr_C9ZXW1_
HLA-DQA10501-DQB10201	270	PSGAGKTTLMSAITG	TTLMSAITG	6	0.1551	9339.4	75.00	0.38	tr_C9ZXW1_
HLA-DQA10501-DQB10201	271	TPAASTNPCLLKVSG	AASTNPCLL	2	0.1547	9371.7	75.00	0.70	tr_C9ZXW1_
HLA-DQA10501-DQB10201	272	TEKNLEGCCFLNNAI	EKNLEGCCF	1	0.1530	9546.7	80.00	0.41	tr_C9ZXW1_
HLA-DQA10501-DQB10201	273	KNLEGCCFLNNAIFT	CFLNNAIFT	6	0.1479	10092.1	80.00	0.44	tr_C9ZXW1_
HLA-DQA10501-DQB10201	274	EKNLEGCCFLNNAIF	EKNLEGCCF	0	0.1466	10235.6	80.00	0.28	tr_C9ZXW1_
HLA-DQA10501-DQB10201	275	KIKKTLIHKMSGTAL	IHKMSGTAL	6	0.1466	10239.1	80.00	0.83	tr_C9ZXW1_
HLA-DQA10501-DQB10201	276	LLLLSEGHVVYHGPR	LLSEGHVVY	2	0.1437	10561.4	80.00	0.28	tr_C9ZXW1_
HLA-DQA10501-DQB10201	277	KTTLMSAITGKLHGT	LMSAITGKL	3	0.1426	10688.9	80.00	0.51	tr_C9ZXW1_
HLA-DQA10501-DQB10201	278	PAASTNPCLLKVSGP	AASTNPCLL	1	0.1399	11005.1	80.00	0.67	tr_C9ZXW1_
HLA-DQA10501-DQB10201	279	SIGHQVPPRTNPCEY	PPRTNPCEY	6	0.1397	11031.7	80.00	0.33	tr_C9ZXW1_
HLA-DQA10501-DQB10201	280	SGAGKTTLMSAITGK	KTTLMSAIT	4	0.1374	11309.5	80.00	0.37	tr_C9ZXW1_
HLA-DQA10501-DQB10201	281	LAFQVPAKDSDGRKI	LAFQVPAKD	0	0.1343	11691.2	85.00	0.58	tr_C9ZXW1_
HLA-DQA10501-DQB10201	282	ATYFACRVRLGLGPR	TYFACRVRL	1	0.1343	11698.3	85.00	0.26	tr_C9ZXW1_
HLA-DQA10501-DQB10201	283	TTLMSAITGKLHGTE	LMSAITGKL	2	0.1330	11858.1	85.00	0.54	tr_C9ZXW1_
HLA-DQA10501-DQB10201	284	VLAIMGPSGAGKTTL	VLAIMGPSG	0	0.1327	11891.8	85.00	0.61	tr_C9ZXW1_

HLA-DQA10501-DQB10201	285	AASTNPCLLKVSGPI	AASTNPCLL	0	0.1326	11911.5	85.00	0.50	tr_C9ZXW1_
HLA-DQA10501-DQB10201	286	LGIPGLVKGVSGGER	VKGVSGGER	6	0.1307	12159.2	85.00	0.44	tr_C9ZXW1_
HLA-DQA10501-DQB10201	287	GPSGAGKTTLMSAIT	KTTLMSAIT	6	0.1305	12181.8	85.00	0.57	tr_C9ZXW1_
HLA-DQA10501-DQB10201	288	IKKTLIHKMSGTALG	IHKMSGTAL	5	0.1290	12388.0	85.00	0.80	tr_C9ZXW1_
HLA-DQA10501-DQB10201	289	KKTLIHKMSGTALGG	IHKMSGTAL	4	0.1254	12877.2	85.00	0.67	tr_C9ZXW1_
HLA-DQA10501-DQB10201	290	ASIGHQVPPRTNPCE	IGHQVPPRT	2	0.1250	12930.8	85.00	0.57	tr_C9ZXW1_
HLA-DQA10501-DQB10201	291	MPDSPETGRAPINLE	TGRAPINLE	6	0.1240	13064.4	85.00	0.38	tr_C9ZXW1_
HLA-DQA10501-DQB10201	292	PDSPETGRAPINLEG	TGRAPINLE	5	0.1219	13364.5	85.00	0.34	tr_C9ZXW1_
HLA-DQA10501-DQB10201	293	LIHKMSGTALGGRVL	IHKMSGTAL	1	0.1218	13381.0	85.00	0.39	tr_C9ZXW1_
HLA-DQA10501-DQB10201	294	IHKMSGTALGGRVLA	TALGGRVLA	6	0.1210	13503.8	85.00	0.23	tr_C9ZXW1_
HLA-DQA10501-DQB10201	295	GIPGLVKGVSGGERK	VKGVSGGER	5	0.1200	13649.1	85.00	0.38	tr_C9ZXW1_
HLA-DQA10501-DQB10201	296	QDLAKKEKRTVIATV	EKRTVIATV	6	0.1192	13760.4	85.00	0.84	tr_C9ZXW1_
HLA-DQA10501-DQB10201	297	TLIHKMSGTALGGRV	IHKMSGTAL	2	0.1180	13944.8	85.00	0.52	tr_C9ZXW1_
HLA-DQA10501-DQB10201	298	VLGIPGLVKGVSGGE	LVKGVSGGE	6	0.1167	14140.2	85.00	0.31	tr_C9ZXW1_
HLA-DQA10501-DQB10201	299	TLMSAITGKLHGTEK	LMSAITGKL	1	0.1144	14497.1	85.00	0.56	tr_C9ZXW1_
HLA-DQA10501-DQB10201	300	KTLIHKMSGTALGGR	IHKMSGTAL	3	0.1131	14707.5	90.00	0.62	tr_C9ZXW1_
HLA-DQA10501-DQB10201	301	IPGLVKGVSGGERKR	VKGVSGGER	4	0.1065	15801.9	90.00	0.38	tr_C9ZXW1_
HLA-DQA10501-DQB10201	302	TERDPYLEDHLKKKG	RDPYLEDHL	2	0.1037	16278.0	90.00	0.44	tr_C9ZXW1_
HLA-DQA10501-DQB10201	303	EDHLKKKGSSWIVQL	KGSSWIVQL	6	0.1008	16803.1	90.00	0.76	tr_C9ZXW1_
HLA-DQA10501-DQB10201	304	PGLVKGVSGGERKRA	VKGVSGGER	3	0.0979	17334.0	90.00	0.42	tr_C9ZXW1_
HLA-DQA10501-DQB10201	305	IVQLVELTKRSYRMY	IVQLVELTK	0	0.0952	17845.5	90.00	0.38	tr_C9ZXW1_
HLA-DQA10501-DQB10201	306	GLVKGVSGGERKRAN	VKGVSGGER	2	0.0911	18661.9	95.00	0.50	tr_C9ZXW1_
HLA-DQA10501-DQB10201	307	LMSAITGKLHGTEKN	LMSAITGKL	0	0.0880	19304.1	95.00	0.47	tr_C9ZXW1_
HLA-DQA10501-DQB10201	308	MGPSGAGKTTLMSAI	AGKTTLMSA	5	0.0826	20458.4	95.00	0.36	tr_C9ZXW1_
HLA-DQA10501-DQB10201	309	SAITGKLHGTEKNLE	LHGTEKNLE	6	0.0819	20621.3	95.00	0.59	tr_C9ZXW1_
HLA-DQA10501-DQB10201	310	LVKGVSGGERKRANV	VKGVSGGER	1	0.0808	20866.4	95.00	0.51	tr_C9ZXW1_
HLA-DQA10501-DQB10201	311	ERDPYLEDHLKKKGS	RDPYLEDHL	1	0.0789	21303.5	95.00	0.50	tr_C9ZXW1_
HLA-DQA10501-DQB10201	312	AITGKLHGTEKNLEG	LHGTEKNLE	5	0.0779	21526.7	95.00	0.53	tr_C9ZXW1_
HLA-DQA10501-DQB10201	313	TGKLHGTEKNLEGCC	LHGTEKNLE	3	0.0767	21815.3	95.00	0.53	tr_C9ZXW1_
HLA-DQA10501-DQB10201	314	ITGKLHGTEKNLEGC	LHGTEKNLE	4	0.0762	21919.2	95.00	0.54	tr_C9ZXW1_
HLA-DQA10501-DQB10201	315	IMGPSGAGKTTLMSA	AGKTTLMSA	6	0.0727	22760.9	95.00	0.51	tr_C9ZXW1_
HLA-DQA10501-DQB10201	316	LAIMGPSGAGKTTLM	LAIMGPSGA	0	0.0723	22860.9	95.00	0.32	tr_C9ZXW1_
HLA-DQA10501-DQB10201	317	VQLVELTKRSYRMYP	VQLVELTKR	0	0.0631	25273.3	100.00	0.33	tr_C9ZXW1_
HLA-DQA10501-DQB10201	318	GVSGGERKRANVATA	RKRANVATA	6	0.0631	25274.4	100.00	0.61	tr_C9ZXW1_
HLA-DQA10501-DQB10201	319	AFQVPAKDSDGRKIK	FQVPAKDSD	1	0.0609	25863.1	100.00	0.35	tr_C9ZXW1_
HLA-DQA10501-DQB10201	320	MSAITGKLHGTEKNL	MSAITGKLH	0	0.0591	26376.9	100.00	0.32	tr_C9ZXW1_
HLA-DQA10501-DQB10201	321	VELTKRSYRMYPRHP	SYRMYPRHP	6	0.0574	26882.8	100.00	0.29	tr_C9ZXW1_
HLA-DQA10501-DQB10201	322	AIMGPSGAGKTTLMS	SGAGKTTLM	5	0.0568	27036.5	100.00	0.34	tr_C9ZXW1_
HLA-DQA10501-DQB10201	323	ELTKRSYRMYPRHPS	SYRMYPRHP	5	0.0563	27196.4	100.00	0.28	tr_C9ZXW1_
HLA-DQA10501-DQB10201	324	LTKRSYRMYPRHPSA	SYRMYPRHP	4	0.0557	27372.4	100.00	0.23	tr_C9ZXW1_
HLA-DQA10501-DQB10201	325	GQMLQDLAKKEKRTV	MLQDLAKKE	2	0.0544	27749.0	100.00	0.55	tr_C9ZXW1_
HLA-DQA10501-DQB10201	326	LVELTKRSYRMYPRH	RSYRMYPRH	6	0.0543	27776.0	100.00	0.25	tr_C9ZXW1_
HLA-DQA10501-DQB10201	327	VKGVSGGERKRANVA	VKGVSGGER	0	0.0539	27892.9	100.00	0.51	tr_C9ZXW1_
HLA-DQA10501-DQB10201	328	QLVELTKRSYRMYPR	LVELTKRSY	1	0.0529	28213.7	100.00	0.21	tr_C9ZXW1_
HLA-DQA10501-DQB10201	329	RDPYLEDHLKKKGSS	RDPYLEDHL	0	0.0527	28265.7	100.00	0.50	tr_C9ZXW1_
HLA-DQA10501-DQB10201	330	DSDGRKIKKTLIHKM	IKKTLIHKM	6	0.0463	30293.3	100.00	0.65	tr_C9ZXW1_
HLA-DQA10501-DQB10201	331	FQVPAKDSDGRKIKK	FQVPAKDSD	0	0.0443	30948.6	100.00	0.31	tr_C9ZXW1_
HLA-DQA10501-DQB10201	332	RKIKKTLIHKMSGTA	IKKTLIHKM	2	0.0441	31042.8	100.00	0.38	tr_C9ZXW1_
HLA-DQA10501-DQB10201	333	SDGRKIKKTLIHKMS	IKKTLIHKM	5	0.0438	31142.8	100.00	0.56	tr_C9ZXW1_
HLA-DQA10501-DQB10201	334	QMLQDLAKKEKRTVI	MLQDLAKKE	1	0.0418	31823.3	100.00	0.63	tr_C9ZXW1_

HLA-DQA10501-DQB10201	335	GRKIKKTLIHKMSGT	IKKTLIHKM	3	0.0408	32165.3	100.00	0.44	tr_C9ZXW1_
HLA-DQA10501-DQB10201	336	DGRKIKKTLIHKMSG	IKKTLIHKM	4	0.0403	32336.0	100.00	0.50	tr_C9ZXW1_
HLA-DQA10501-DQB10201	337	KGVSGGERKRANVAT	ERKRANVAT	6	0.0394	32634.7	100.00	0.29	tr_C9ZXW1_
HLA-DQA10501-DQB10201	338	MLQDLAKKEKRTVIA	MLQDLAKKE	0	0.0359	33908.2	100.00	0.52	tr_C9ZXW1_
HLA-DQA10501-DQB10201	339	LEDHLKKKGSSWIVQ	KKGSSWIVQ	6	0.0291	36475.8	100.00	0.35	tr_C9ZXW1_
HLA-DQA10501-DQB10201	340	PAKDSDGRKIKKTLI	GRKIKKTLI	6	0.0278	37005.3	100.00	0.58	tr_C9ZXW1_
HLA-DQA10501-DQB10201	341	LQDLAKKEKRTVIAT	KEKRTVIAT	6	0.0276	37097.1	100.00	0.42	tr_C9ZXW1_
HLA-DQA10501-DQB10201	342	QVPAKDSDGRKIKKT	VPAKDSDGR	1	0.0260	37747.3	100.00	0.31	tr_C9ZXW1_
HLA-DQA10501-DQB10201	343	DPYLEDHLKKKGSSW	DPYLEDHLK	0	0.0255	37952.5	100.00	0.34	tr_C9ZXW1_
HLA-DQA10501-DQB10201	344	YLEDHLKKKGSSWIV	LKKKGSSWI	5	0.0253	38026.1	100.00	0.40	tr_C9ZXW1_
HLA-DQA10501-DQB10201	345	AKDSDGRKIKKTLIH	GRKIKKTLI	5	0.0234	38822.6	100.00	0.55	tr_C9ZXW1_
HLA-DQA10501-DQB10201	346	PYLEDHLKKKGSSWI	LKKKGSSWI	6	0.0232	38908.0	100.00	0.44	tr_C9ZXW1_
HLA-DQA10501-DQB10201	347	KDSDGRKIKKTLIHK	GRKIKKTLI	4	0.0218	39472.8	100.00	0.48	tr_C9ZXW1_
HLA-DQA10501-DQB10201	348	VPAKDSDGRKIKKTL	VPAKDSDGR	0	0.0203	40139.9	100.00	0.30	tr_C9ZXW1_

Allele: HLA-DQA10501-DQB10201. Number of high binders 4. Number of weak binders 27. Number of peptides 348

Allele	pos	peptide	core	Of	1-log50k(aff)	affinity(nM)	%Rank	Relia	Identity	Bind Level
HLA-DQA10501-DQB10201	1	PDGQSVIEFFNFDSW	IEFFNFDSW	6	0.4696	310.5	8.00	0.39	tr_C9ZXW1_	WB
HLA-DQA10501-DQB10201	2	LHCRYPDGQSVIEFF	DGQSVIEFF	6	0.4475	394.5	10.00	0.60	tr_C9ZXW1_	
HLA-DQA10501-DQB10201	3	QSVIEFFNFDSWSWW	IEFFNFDSW	3	0.4467	398.0	10.00	0.40	tr_C9ZXW1_	
HLA-DQA10501-DQB10201	4	DGQSVIEFFNFDSWS	IEFFNFDSW	5	0.4444	408.3	11.00	0.38	tr_C9ZXW1_	
HLA-DQA10501-DQB10201	5	RYPDGQSVIEFFNFD	DGQSVIEFF	3	0.4390	432.4	11.00	0.46	tr_C9ZXW1_	
HLA-DQA10501-DQB10201	6	GQSVIEFFNFDSWSW	IEFFNFDSW	4	0.4341	456.4	12.00	0.40	tr_C9ZXW1_	
HLA-DQA10501-DQB10201	7	CRYPDGQSVIEFFNF	DGQSVIEFF	4	0.4320	466.4	12.00	0.50	tr_C9ZXW1_	
HLA-DQA10501-DQB10201	8	HCRYPDGQSVIEFFN	DGQSVIEFF	5	0.4308	472.7	12.00	0.51	tr_C9ZXW1_	
HLA-DQA10501-DQB10201	9	YPDGQSVIEFFNFDS	DGQSVIEFF	2	0.4241	508.6	13.00	0.47	tr_C9ZXW1_	
HLA-DQA10501-DQB10201	10	SVIEFFNFDSWSWWK	IEFFNFDSW	2	0.4188	538.5	13.00	0.43	tr_C9ZXW1_	
HLA-DQA10501-DQB10201	11	VIEFFNFDSWSWWKS	IEFFNFDSW	1	0.3893	741.1	18.00	0.41	tr_C9ZXW1_	
HLA-DQA10501-DQB10201	12	DLHCRYPDGQSVIEF	PDGQSVIEF	6	0.2877	2224.2	38.00	0.63	tr_C9ZXW1_	
HLA-DQA10501-DQB10201	13	NEFSRLEKICDPPDL	SRLEKICDP	3	0.2230	4478.8	60.00	0.22	tr_C9ZXW1_	
HLA-DQA10501-DQB10201	14	EFSRLEKICDPPDLH	SRLEKICDP	2	0.2047	5461.3	65.00	0.23	tr_C9ZXW1_	
HLA-DQA10501-DQB10201	15	FSRLEKICDPPDLHC	LEKICDPPD	3	0.1837	6852.6	70.00	0.19	tr_C9ZXW1_	
HLA-DQA10501-DQB10201	16	SRLEKICDPPDLHCR	EKICDPPDL	3	0.1543	9421.3	75.00	0.22	tr_C9ZXW1_	
HLA-DQA10501-DQB10201	17	RLEKICDPPDLHCRY	EKICDPPDL	2	0.1393	11070.5	80.00	0.26	tr_C9ZXW1_	
HLA-DQA10501-DQB10201	18	PDLHCRYPDGQSVIE	YPDGQSVIE	6	0.1347	11640.5	80.00	0.39	tr_C9ZXW1_	
HLA-DQA10501-DQB10201	19	LEKICDPPDLHCRYP	EKICDPPDL	1	0.1233	13170.2	85.00	0.31	tr_C9ZXW1_	
HLA-DQA10501-DQB10201	20	EKICDPPDLHCRYPD	KICDPPDLH	1	0.1150	14401.4	85.00	0.29	tr_C9ZXW1_	
HLA-DQA10501-DQB10201	21	PPDLHCRYPDGQSVI	RYPDGQSVI	6	0.1005	16847.7	90.00	0.37	tr_C9ZXW1_	
HLA-DQA10501-DQB10201	22	KICDPPDLHCRYPDG	KICDPPDLH	0	0.0892	19044.5	95.00	0.28	tr_C9ZXW1_	
HLA-DQA10501-DQB10201	23	ICDPPDLHCRYPDGQ	LHCRYPDGQ	6	0.0850	19923.5	95.00	0.27	tr_C9ZXW1_	
HLA-DQA10501-DQB10201	24	DPPDLHCRYPDGQSV	LHCRYPDGQ	4	0.0847	19999.7	95.00	0.26	tr_C9ZXW1_	
HLA-DQA10501-DQB10201	25	CDPPDLHCRYPDGQS	LHCRYPDGQ	5	0.0807	20885.6	95.00	0.29	tr_C9ZXW1_	

Allele: HLA-DQA10501-DQB10201. Number of high binders 0. Number of weak binders 1. Number of peptides 25

Allele	pos	peptide	core	Of	1-log50k(aff)	affinity(nM)	%Rank	Relia	Identity	Bind Level
HLA-DQA10501-DQB10201	1	VKPLTAVVSDIFPLF	LTAVVSDIF	3	0.5957	79.4	1.40	0.48	tr_D0A423_	WB
HLA-DQA10501-DQB10201	2	KPLTAVVSDIFPLFG	LTAVVSDIF	2	0.5928	81.9	1.50	0.40	tr_D0A423_	WB
HLA-DQA10501-DQB10201	3	PLTAVVSDIFPLFGY	LTAVVSDIF	1	0.5804	93.7	1.80	0.40	tr_D0A423_	WB
HLA-DQA10501-DQB10201	4	SVKPLTAVVSDIFPL	LTAVVSDIF	4	0.5704	104.4	2.50	0.56	tr_D0A423_	WB
HLA-DQA10501-DQB10201	5	WSVKPLTAVVSDIFP	LTAVVSDIF	5	0.5698	105.1	2.50	0.61	tr_D0A423_	WB
HLA-DQA10501-DQB10201	6	LTAVVSDIFPLFGYS	LTAVVSDIF	0	0.5434	139.7	3.50	0.34	tr_D0A423_	WB
HLA-DQA10501-DQB10201	7	GWSVKPLTAVVSDIF	LTAVVSDIF	6	0.5269	167.1	4.00	0.76	tr_D0A423_	WB
HLA-DQA10501-DQB10201	8	RRLPMFGTAAEGYGP	MFGTAAEGY	4	0.4779	283.9	7.00	0.75	tr_D0A423_	WB
HLA-DQA10501-DQB10201	9	CLRRLPMFGTAAEGY	MFGTAAEGY	6	0.4585	350.2	9.00	0.80	tr_D0A423_	WB
HLA-DQA10501-DQB10201	10	LRRLPMFGTAAEGYG	MFGTAAEGY	5	0.4541	367.2	9.00	0.76	tr_D0A423_	WB
HLA-DQA10501-DQB10201	11	TAVVSDIFPLFGYSK	VVSDIFPLF	2	0.4462	400.1	10.00	0.33	tr_D0A423_	
HLA-DQA10501-DQB10201	12	RLPMFGTAAEGYGPK	MFGTAAEGY	3	0.4430	414.3	11.00	0.75	tr_D0A423_	
HLA-DQA10501-DQB10201	13	LPMFGTAAEGYGPKA	MFGTAAEGY	2	0.4344	454.5	12.00	0.75	tr_D0A423_	
HLA-DQA10501-DQB10201	14	LSDYLVRDSLLAMFT	RDSLLAMFT	6	0.4329	462.4	12.00	0.25	tr_D0A423_	
HLA-DQA10501-DQB10201	15	GLSDYLVRDSLLAMF	DYLVRDSLL	3	0.4223	518.6	13.00	0.31	tr_D0A423_	
HLA-DQA10501-DQB10201	16	SDYLVRDSLLAMFTQ	DYLVRDSLL	1	0.4154	558.3	14.00	0.25	tr_D0A423_	
HLA-DQA10501-DQB10201	17	PMFGTAAEGYGPKAV	MFGTAAEGY	1	0.4027	641.1	16.00	0.73	tr_D0A423_	
HLA-DQA10501-DQB10201	18	KGLSDYLVRDSLLAM	DYLVRDSLL	4	0.3981	673.5	16.00	0.40	tr_D0A423_	
HLA-DQA10501-DQB10201	19	AVVSDIFPLFGYSKR	VVSDIFPLF	1	0.3906	730.0	17.00	0.39	tr_D0A423_	
HLA-DQA10501-DQB10201	20	CKGLSDYLVRDSLLA	DYLVRDSLL	5	0.3866	762.5	18.00	0.44	tr_D0A423_	
HLA-DQA10501-DQB10201	21	YLVRDSLLAMFTQRF	LLAMFTQRF	6	0.3747	867.4	20.00	0.38	tr_D0A423_	
HLA-DQA10501-DQB10201	22	DYLVRDSLLAMFTQR	RDSLLAMFT	4	0.3676	936.6	21.00	0.26	tr_D0A423_	
HLA-DQA10501-DQB10201	23	LAMFTQRFGVDVSVY	RFGVDVSVY	6	0.3570	1050.7	23.00	0.43	tr_D0A423_	
HLA-DQA10501-DQB10201	24	AMFTQRFGVDVSVYQ	RFGVDVSVY	5	0.3558	1064.0	23.00	0.36	tr_D0A423_	
HLA-DQA10501-DQB10201	25	MFGTAAEGYGPKAVM	MFGTAAEGY	0	0.3552	1071.8	23.00	0.65	tr_D0A423_	
HLA-DQA10501-DQB10201	26	LCKGLSDYLVRDSLL	DYLVRDSLL	6	0.3523	1105.6	24.00	0.53	tr_D0A423_	
HLA-DQA10501-DQB10201	27	FTQRFGVDVSVYQRL	RFGVDVSVY	3	0.3325	1369.5	28.00	0.31	tr_D0A423_	
HLA-DQA10501-DQB10201	28	MFTQRFGVDVSVYQR	RFGVDVSVY	4	0.3322	1374.6	28.00	0.34	tr_D0A423_	
HLA-DQA10501-DQB10201	29	LVRDSLLAMFTQRFG	LLAMFTQRF	5	0.3299	1408.2	28.00	0.38	tr_D0A423_	
HLA-DQA10501-DQB10201	30	MGWSVKPLTAVVSDI	PLTAVVSDI	6	0.3281	1435.9	29.00	0.50	tr_D0A423_	
HLA-DQA10501-DQB10201	31	GYGPKAVMSISLSYF	VMSISLSYF	6	0.3225	1525.9	30.00	0.49	tr_D0A423_	
HLA-DQA10501-DQB10201	32	YGPKAVMSISLSYFL	VMSISLSYF	5	0.3160	1638.0	31.00	0.42	tr_D0A423_	
HLA-DQA10501-DQB10201	33	VRDSLLAMFTQRFGV	LLAMFTQRF	4	0.3046	1852.8	34.00	0.40	tr_D0A423_	
HLA-DQA10501-DQB10201	34	GPKAVMSISLSYFLC	VMSISLSYF	4	0.3016	1912.5	35.00	0.41	tr_D0A423_	
HLA-DQA10501-DQB10201	35	LLAMFTQRFGVDVSV	QRFGVDVSV	6	0.2954	2045.4	36.00	0.34	tr_D0A423_	
HLA-DQA10501-DQB10201	36	TQRFGVDVSVYQRLS	RFGVDVSVY	2	0.2952	2050.0	36.00	0.34	tr_D0A423_	
HLA-DQA10501-DQB10201	37	NEVNEPLPTLQSEES	LPTLQSEES	6	0.2818	2370.8	40.00	0.50	tr_D0A423_	
HLA-DQA10501-DQB10201	38	PKAVMSISLSYFLCK	VMSISLSYF	3	0.2811	2387.2	40.00	0.36	tr_D0A423_	
HLA-DQA10501-DQB10201	39	QSEESGEATYVHPDA	EATYVHPDA	6	0.2808	2397.1	40.00	0.41	tr_D0A423_	
HLA-DQA10501-DQB10201	40	TRSLPNEVNEPLPTL	LPNEVNEPL	3	0.2785	2456.9	41.00	0.29	tr_D0A423_	
HLA-DQA10501-DQB10201	41	SLLAMFTQRFGVDVS	LLAMFTQRF	1	0.2773	2489.0	41.00	0.32	tr_D0A423_	
HLA-DQA10501-DQB10201	42	RDSLLAMFTQRFGVD	LLAMFTQRF	3	0.2723	2625.9	42.00	0.52	tr_D0A423_	
HLA-DQA10501-DQB10201	43	RSLPNEVNEPLPTLQ	LPNEVNEPL	2	0.2715	2649.0	42.00	0.27	tr_D0A423_	
HLA-DQA10501-DQB10201	44	EVNEPLPTLQSEESG	LPTLQSEES	5	0.2706	2676.0	43.00	0.50	tr_D0A423_	
HLA-DQA10501-DQB10201	45	QRFGVDVSVYQRLSN	RFGVDVSVY	1	0.2703	2685.6	43.00	0.37	tr_D0A423_	

HLA-DQA10501-DQB10201	46	VSVYQRLSNVANMGW	LSNVANMGW	6	0.2666	2793.6	44.00	0.31	tr_D0A423_
HLA-DQA10501-DQB10201	47	DSLLAMFTQRFGVDV	LLAMFTQRF	2	0.2666	2794.0	44.00	0.49	tr_D0A423_
HLA-DQA10501-DQB10201	48	KAVMSISLSYFLCKG	VMSISLSYF	2	0.2663	2804.5	44.00	0.40	tr_D0A423_
HLA-DQA10501-DQB10201	49	SEESGEATYVHPDAR	EATYVHPDA	5	0.2643	2864.6	44.00	0.38	tr_D0A423_
HLA-DQA10501-DQB10201	50	EESGEATYVHPDARA	EATYVHPDA	4	0.2594	3019.7	46.00	0.34	tr_D0A423_
HLA-DQA10501-DQB10201	51	FLCKGLSDYLVRDSL	SDYLVRDSL	6	0.2581	3061.7	46.00	0.42	tr_D0A423_
HLA-DQA10501-DQB10201	52	SLPNEVNEPLPTLQS	LPNEVNEPL	1	0.2571	3097.2	46.00	0.26	tr_D0A423_
HLA-DQA10501-DQB10201	53	SLSYFLCKGLSDYLV	YFLCKGLSD	3	0.2568	3105.8	46.00	0.31	tr_D0A423_
HLA-DQA10501-DQB10201	54	SGEATYVHPDARALF	EATYVHPDA	2	0.2553	3157.7	47.00	0.32	tr_D0A423_
HLA-DQA10501-DQB10201	55	NMGWSVKPLTAVVSD	KPLTAVVSD	6	0.2530	3235.5	47.00	0.25	tr_D0A423_
HLA-DQA10501-DQB10201	56	VNEPLPTLQSEESGE	LPTLQSEES	4	0.2525	3254.8	48.00	0.57	tr_D0A423_
HLA-DQA10501-DQB10201	57	EGYGPKAVMSISLSY	AVMSISLSY	6	0.2516	3286.7	48.00	0.40	tr_D0A423_
HLA-DQA10501-DQB10201	58	GEATYVHPDARALFN	EATYVHPDA	1	0.2505	3325.1	48.00	0.28	tr_D0A423_
HLA-DQA10501-DQB10201	59	AVMSISLSYFLCKGL	VMSISLSYF	1	0.2502	3335.3	48.00	0.41	tr_D0A423_
HLA-DQA10501-DQB10201	60	SVYQRLSNVANMGWS	LSNVANMGW	5	0.2492	3371.6	48.00	0.31	tr_D0A423_
HLA-DQA10501-DQB10201	61	APTRSLPNEVNEPLP	LPNEVNEPL	5	0.2478	3422.7	49.00	0.38	tr_D0A423_
HLA-DQA10501-DQB10201	62	SYFLCKGLSDYLVRD	YFLCKGLSD	1	0.2460	3492.4	49.00	0.24	tr_D0A423_
HLA-DQA10501-DQB10201	63	ESGEATYVHPDARAL	EATYVHPDA	3	0.2453	3519.0	49.00	0.39	tr_D0A423_
HLA-DQA10501-DQB10201	64	RFGVDVSVYQRLSNV	RFGVDVSVY	0	0.2452	3523.0	50.00	0.28	tr_D0A423_
HLA-DQA10501-DQB10201	65	NEPLPTLQSEESGEA	LPTLQSEES	3	0.2431	3604.1	50.00	0.50	tr_D0A423_
HLA-DQA10501-DQB10201	66	LSYFLCKGLSDYLVR	YFLCKGLSD	2	0.2429	3611.9	50.00	0.27	tr_D0A423_
HLA-DQA10501-DQB10201	67	FAPTRSLPNEVNEPL	LPNEVNEPL	6	0.2413	3672.5	55.00	0.32	tr_D0A423_
HLA-DQA10501-DQB10201	68	PTRSLPNEVNEPLPT	LPNEVNEPL	4	0.2390	3764.7	55.00	0.38	tr_D0A423_
HLA-DQA10501-DQB10201	69	LPNEVNEPLPTLQSE	EVNEPLPTL	3	0.2388	3772.7	55.00	0.34	tr_D0A423_
HLA-DQA10501-DQB10201	70	VYQRLSNVANMGWSV	LSNVANMGW	4	0.2374	3831.5	55.00	0.34	tr_D0A423_
HLA-DQA10501-DQB10201	71	EPLPTLQSEESGEAT	LPTLQSEES	2	0.2370	3849.6	55.00	0.47	tr_D0A423_
HLA-DQA10501-DQB10201	72	VDVSVYQRLSNVANM	QRLSNVANM	6	0.2364	3875.1	55.00	0.34	tr_D0A423_
HLA-DQA10501-DQB10201	73	ISLSYFLCKGLSDYL	YFLCKGLSD	4	0.2359	3894.1	55.00	0.37	tr_D0A423_
HLA-DQA10501-DQB10201	74	LPTLQSEESGEATYV	EESGEATYV	6	0.2358	3900.7	55.00	0.37	tr_D0A423_
HLA-DQA10501-DQB10201	75	FGVDVSVYQRLSNVA	VYQRLSNVA	6	0.2312	4099.9	55.00	0.35	tr_D0A423_
HLA-DQA10501-DQB10201	76	DVSVYQRLSNVANMG	VYQRLSNVA	3	0.2310	4106.7	55.00	0.28	tr_D0A423_
HLA-DQA10501-DQB10201	77	ANMGWSVKPLTAVVS	VKPLTAVVS	6	0.2309	4110.0	55.00	0.27	tr_D0A423_
HLA-DQA10501-DQB10201	78	LQSEESGEATYVHPD	EESGEATYV	3	0.2297	4164.1	55.00	0.42	tr_D0A423_
HLA-DQA10501-DQB10201	79	YFLCKGLSDYLVRDS	LCKGLSDYL	2	0.2288	4205.1	55.00	0.23	tr_D0A423_
HLA-DQA10501-DQB10201	80	PNEVNEPLPTLQSEE	EVNEPLPTL	2	0.2275	4263.4	55.00	0.27	tr_D0A423_
HLA-DQA10501-DQB10201	81	EATYVHPDARALFNK	EATYVHPDA	0	0.2238	4441.5	60.00	0.25	tr_D0A423_
HLA-DQA10501-DQB10201	82	TLQSEESGEATYVHP	EESGEATYV	4	0.2223	4510.4	60.00	0.40	tr_D0A423_
HLA-DQA10501-DQB10201	83	PLPTLQSEESGEATY	LPTLQSEES	1	0.2177	4743.1	60.00	0.39	tr_D0A423_
HLA-DQA10501-DQB10201	84	VANMGWSVKPLTAVV	MGWSVKPLT	3	0.2173	4764.5	60.00	0.25	tr_D0A423_
HLA-DQA10501-DQB10201	85	HPDARALFNKVPCLR	ALFNKVPCL	5	0.2163	4814.4	60.00	0.49	tr_D0A423_
HLA-DQA10501-DQB10201	86	PCLRRLPMFGTAAEG	PMFGTAAEG	6	0.2162	4819.1	60.00	0.51	tr_D0A423_
HLA-DQA10501-DQB10201	87	NVANMGWSVKPLTAV	MGWSVKPLT	4	0.2159	4834.7	60.00	0.31	tr_D0A423_
HLA-DQA10501-DQB10201	88	VHPDARALFNKVPCL	ALFNKVPCL	6	0.2145	4910.6	60.00	0.55	tr_D0A423_
HLA-DQA10501-DQB10201	89	AAEGYGPKAVMSISL	PKAVMSISL	6	0.2137	4951.0	60.00	0.60	tr_D0A423_
HLA-DQA10501-DQB10201	90	SNVANMGWSVKPLTA	MGWSVKPLT	5	0.2102	5141.8	60.00	0.34	tr_D0A423_
HLA-DQA10501-DQB10201	91	YQRLSNVANMGWSVK	LSNVANMGW	3	0.2078	5280.4	60.00	0.35	tr_D0A423_
HLA-DQA10501-DQB10201	92	LSNVANMGWSVKPLT	MGWSVKPLT	6	0.2068	5339.0	65.00	0.34	tr_D0A423_
HLA-DQA10501-DQB10201	93	PTLQSEESGEATYVH	EESGEATYV	5	0.2028	5570.4	65.00	0.40	tr_D0A423_
HLA-DQA10501-DQB10201	94	SISLSYFLCKGLSDY	YFLCKGLSD	5	0.2015	5651.0	65.00	0.47	tr_D0A423_
HLA-DQA10501-DQB10201	95	AEGYGPKAVMSISLS	PKAVMSISL	5	0.2014	5656.5	65.00	0.48	tr_D0A423_

HLA-DQA10501-DQB10201	96	SFAPTRSLPNEVNEP	PTRSLPNEV	3	0.2004	5720.2	65.00	0.57	tr_D0A423_
HLA-DQA10501-DQB10201	97	PDARALFNKVPCLRR	ALFNKVPCL	4	0.1982	5855.9	65.00	0.46	tr_D0A423_
HLA-DQA10501-DQB10201	98	DARALFNKVPCLRRL	ALFNKVPCL	3	0.1978	5878.9	65.00	0.46	tr_D0A423_
HLA-DQA10501-DQB10201	99	GVDVSVYQRLSNVAN	VYQRLSNVA	5	0.1965	5963.5	65.00	0.44	tr_D0A423_
HLA-DQA10501-DQB10201	100	RLSNVANMGWSVKPL	NMGWSVKPL	6	0.1935	6165.1	65.00	0.31	tr_D0A423_
HLA-DQA10501-DQB10201	101	MSFAPTRSLPNEVNE	PTRSLPNEV	4	0.1930	6198.6	65.00	0.60	tr_D0A423_
HLA-DQA10501-DQB10201	102	VMSISLSYFLCKGLS	VMSISLSYF	0	0.1911	6321.5	65.00	0.41	tr_D0A423_
HLA-DQA10501-DQB10201	103	MSISLSYFLCKGLSD	YFLCKGLSD	6	0.1911	6322.1	65.00	0.46	tr_D0A423_
HLA-DQA10501-DQB10201	104	ARALFNKVPCLRRLP	ALFNKVPCL	2	0.1893	6449.1	70.00	0.45	tr_D0A423_
HLA-DQA10501-DQB10201	105	RALFNKVPCLRRLPM	ALFNKVPCL	1	0.1862	6669.1	70.00	0.42	tr_D0A423_
HLA-DQA10501-DQB10201	106	QRLSNVANMGWSVKP	LSNVANMGW	2	0.1783	7263.9	70.00	0.39	tr_D0A423_
HLA-DQA10501-DQB10201	107	ALFNKVPCLRRLPMF	ALFNKVPCL	0	0.1778	7306.5	70.00	0.31	tr_D0A423_
HLA-DQA10501-DQB10201	108	ATYVHPDARALFNKV	VHPDARALF	3	0.1713	7835.8	75.00	0.24	tr_D0A423_
HLA-DQA10501-DQB10201	109	TYVHPDARALFNKVP	VHPDARALF	2	0.1570	9144.7	75.00	0.23	tr_D0A423_
HLA-DQA10501-DQB10201	110	VPCLRRLPMFGTAAE	LPMFGTAAE	6	0.1562	9229.6	75.00	0.38	tr_D0A423_
HLA-DQA10501-DQB10201	111	FGTAAEGYGPKAVMS	FGTAAEGYG	0	0.1517	9682.1	80.00	0.25	tr_D0A423_
HLA-DQA10501-DQB10201	112	GTAAEGYGPKAVMSI	YGPKAVMSI	6	0.1432	10613.8	80.00	0.31	tr_D0A423_
HLA-DQA10501-DQB10201	113	YVHPDARALFNKVPC	VHPDARALF	1	0.1394	11061.6	80.00	0.25	tr_D0A423_
HLA-DQA10501-DQB10201	114	TAAEGYGPKAVMSIS	YGPKAVMSI	5	0.1333	11820.9	85.00	0.28	tr_D0A423_
HLA-DQA10501-DQB10201	115	LFNKVPCLRRLPMFG	LFNKVPCLR	0	0.1330	11858.6	85.00	0.23	tr_D0A423_
HLA-DQA10501-DQB10201	116	KVPCLRRLPMFGTAA	RLPMFGTAA	6	0.1254	12876.7	85.00	0.35	tr_D0A423_
HLA-DQA10501-DQB10201	117	FNKVPCLRRLPMFGT	PCLRRLPMF	4	0.1140	14560.8	85.00	0.26	tr_D0A423_
HLA-DQA10501-DQB10201	118	NKVPCLRRLPMFGTA	PCLRRLPMF	3	0.1047	16103.0	90.00	0.26	tr_D0A423_

Allele: HLA-DQA10501-DQB10201. Number of high binders 0. Number of weak binders 10. Number of peptides 118

Allele	pos	peptide	core	Of	1-log50k(aff)	affinity(nM)	%Rank	Relia	Identity	Bind Level
HLA-DQA10501-DQB10201	1	LPNREERYGDALLLY	RYGDALLLY	6	0.4786	282.0	7.00	0.48	tr_D0A423_	WB
HLA-DQA10501-DQB10201	2	PNREERYGDALLLYK	RYGDALLLY	5	0.4361	446.2	11.00	0.45	tr_D0A423_	
HLA-DQA10501-DQB10201	3	NREERYGDALLLYKK	RYGDALLLY	4	0.3953	694.5	17.00	0.41	tr_D0A423_	
HLA-DQA10501-DQB10201	4	ELPNREERYGDALLL	ERYGDALLL	6	0.3810	810.5	19.00	0.62	tr_D0A423_	
HLA-DQA10501-DQB10201	5	STEGPDAAGLVPYEG	DAAGLVPYE	5	0.3699	913.3	21.00	0.35	tr_D0A423_	
HLA-DQA10501-DQB10201	6	REERYGDALLLYKKK	RYGDALLLY	3	0.3636	978.0	22.00	0.40	tr_D0A423_	
HLA-DQA10501-DQB10201	7	LSTEGPDAAGLVPYE	DAAGLVPYE	6	0.3522	1106.5	24.00	0.41	tr_D0A423_	
HLA-DQA10501-DQB10201	8	TEGPDAAGLVPYEGG	DAAGLVPYE	4	0.3472	1168.6	25.00	0.35	tr_D0A423_	
HLA-DQA10501-DQB10201	9	EERYGDALLLYKKKR	RYGDALLLY	2	0.3363	1313.9	27.00	0.44	tr_D0A423_	
HLA-DQA10501-DQB10201	10	EGPDAAGLVPYEGGA	DAAGLVPYE	3	0.3305	1400.1	28.00	0.33	tr_D0A423_	
HLA-DQA10501-DQB10201	11	GPDAAGLVPYEGGAP	AAGLVPYEG	3	0.3154	1648.0	32.00	0.42	tr_D0A423_	
HLA-DQA10501-DQB10201	12	CCCGVFEYNKEVVER	FEYNKEVVE	5	0.3144	1666.0	32.00	0.43	tr_D0A423_	
HLA-DQA10501-DQB10201	13	CCGVFEYNKEVVERN	FEYNKEVVE	4	0.3140	1673.6	32.00	0.34	tr_D0A423_	
HLA-DQA10501-DQB10201	14	SCCCGVFEYNKEVVE	FEYNKEVVE	6	0.3062	1821.1	34.00	0.48	tr_D0A423_	
HLA-DQA10501-DQB10201	15	PLSTEGPDAAGLVPY	PDAAGLVPY	6	0.3002	1941.4	35.00	0.32	tr_D0A423_	
HLA-DQA10501-DQB10201	16	CGVFEYNKEVVERNG	FEYNKEVVE	3	0.2961	2030.2	36.00	0.33	tr_D0A423_	
HLA-DQA10501-DQB10201	17	PDAAGLVPYEGGAPT	AAGLVPYEG	2	0.2937	2085.1	37.00	0.43	tr_D0A423_	
HLA-DQA10501-DQB10201	18	ERYGDALLLYKKKRE	RYGDALLLY	1	0.2828	2345.1	39.00	0.51	tr_D0A423_	
HLA-DQA10501-DQB10201	19	DAAGLVPYEGGAPTG	AAGLVPYEG	1	0.2654	2829.2	44.00	0.46	tr_D0A423_	

HLA-DQA10501-DQB10201	20	NPLSTEGPDAAGLVP	PLSTEGPDA	1	0.2607	2978.2	45.00	0.29	tr_D0A423_
HLA-DQA10501-DQB10201	21	SNPLSTEGPDAAGLV	PLSTEGPDA	2	0.2412	3678.4	55.00	0.31	tr_D0A423_
HLA-DQA10501-DQB10201	22	DGDLSSFHFQEPGSC	LSSFHFQEP	3	0.2302	4143.6	55.00	0.29	tr_D0A423_
HLA-DQA10501-DQB10201	23	GSCCCGVFEYNKEVV	VFEYNKEVV	6	0.2224	4505.8	60.00	0.71	tr_D0A423_
HLA-DQA10501-DQB10201	24	ESNPLSTEGPDAAGL	PLSTEGPDA	3	0.2219	4531.8	60.00	0.43	tr_D0A423_
HLA-DQA10501-DQB10201	25	PTGDGDLSSFHFQEP	LSSFHFQEP	6	0.2216	4545.0	60.00	0.43	tr_D0A423_
HLA-DQA10501-DQB10201	26	TGDGDLSSFHFQEPG	LSSFHFQEP	5	0.2166	4801.4	60.00	0.38	tr_D0A423_
HLA-DQA10501-DQB10201	27	GDLSSFHFQEPGSCC	LSSFHFQEP	2	0.2157	4845.3	60.00	0.31	tr_D0A423_
HLA-DQA10501-DQB10201	28	GDGDLSSFHFQEPGS	LSSFHFQEP	4	0.2120	5041.9	60.00	0.34	tr_D0A423_
HLA-DQA10501-DQB10201	29	NSESNPLSTEGPDAA	PLSTEGPDA	5	0.2104	5132.5	60.00	0.43	tr_D0A423_
HLA-DQA10501-DQB10201	30	SESNPLSTEGPDAAG	PLSTEGPDA	4	0.2093	5191.8	60.00	0.44	tr_D0A423_
HLA-DQA10501-DQB10201	31	DLSSFHFQEPGSCCC	LSSFHFQEP	1	0.2051	5435.0	65.00	0.29	tr_D0A423_
HLA-DQA10501-DQB10201	32	KCDPFSDSLTQPDRA	DSLTQPDRA	6	0.2046	5462.0	65.00	0.41	tr_D0A423_
HLA-DQA10501-DQB10201	33	CDPFSDSLTQPDRAI	DSLTQPDRA	5	0.2014	5658.2	65.00	0.44	tr_D0A423_
HLA-DQA10501-DQB10201	34	TQPDRAINSESNPLS	AINSESNPL	5	0.1944	6101.0	65.00	0.44	tr_D0A423_
HLA-DQA10501-DQB10201	35	DPFSDSLTQPDRAIN	DSLTQPDRA	4	0.1907	6349.2	65.00	0.44	tr_D0A423_
HLA-DQA10501-DQB10201	36	LTQPDRAINSESNPL	AINSESNPL	6	0.1907	6349.9	65.00	0.46	tr_D0A423_
HLA-DQA10501-DQB10201	37	QPDRAINSESNPLST	AINSESNPL	4	0.1886	6499.3	70.00	0.41	tr_D0A423_
HLA-DQA10501-DQB10201	38	AAGLVPYEGGAPTGD	AAGLVPYEG	0	0.1872	6593.3	70.00	0.32	tr_D0A423_
HLA-DQA10501-DQB10201	39	PDRAINSESNPLSTE	AINSESNPL	3	0.1800	7131.5	70.00	0.42	tr_D0A423_
HLA-DQA10501-DQB10201	40	RYGDALLLYKKKREE	RYGDALLLY	0	0.1743	7584.6	70.00	0.68	tr_D0A423_
HLA-DQA10501-DQB10201	41	APTGDGDLSSFHFQE	DLSSFHFQE	6	0.1731	7684.4	70.00	0.32	tr_D0A423_
HLA-DQA10501-DQB10201	42	GGAPTGDGDLSSFHF	DGDLSSFHF	6	0.1723	7749.1	70.00	0.35	tr_D0A423_
HLA-DQA10501-DQB10201	43	PFSDSLTQPDRAINS	DSLTQPDRA	3	0.1697	7973.5	75.00	0.51	tr_D0A423_
HLA-DQA10501-DQB10201	44	DRAINSESNPLSTEG	AINSESNPL	2	0.1689	8040.4	75.00	0.45	tr_D0A423_
HLA-DQA10501-DQB10201	45	LSSFHFQEPGSCCCG	FHFQEPGSC	3	0.1663	8267.1	75.00	0.34	tr_D0A423_
HLA-DQA10501-DQB10201	46	INSESNPLSTEGPDA	PLSTEGPDA	6	0.1644	8438.7	75.00	0.55	tr_D0A423_
HLA-DQA10501-DQB10201	47	EKKCDPFSDSLTQPD	PFSDSLTQP	5	0.1629	8580.0	75.00	0.44	tr_D0A423_
HLA-DQA10501-DQB10201	48	FSDSLTQPDRAINSE	DSLTQPDRA	2	0.1620	8667.5	75.00	0.55	tr_D0A423_
HLA-DQA10501-DQB10201	49	KKCDPFSDSLTQPDR	PFSDSLTQP	4	0.1590	8950.2	75.00	0.37	tr_D0A423_
HLA-DQA10501-DQB10201	50	YEGGAPTGDGDLSSF	TGDGDLSSF	6	0.1567	9177.9	75.00	0.20	tr_D0A423_
HLA-DQA10501-DQB10201	51	GAPTGDGDLSSFHFQ	DGDLSSFHF	5	0.1532	9525.5	80.00	0.29	tr_D0A423_
HLA-DQA10501-DQB10201	52	RAINSESNPLSTEGP	AINSESNPL	1	0.1499	9871.9	80.00	0.43	tr_D0A423_
HLA-DQA10501-DQB10201	53	PYEGGAPTGDGDLSS	YEGGAPTGD	1	0.1443	10498.8	80.00	0.25	tr_D0A423_
HLA-DQA10501-DQB10201	54	GLVPYEGGAPTGDGD	YEGGAPTGD	4	0.1405	10936.5	80.00	0.32	tr_D0A423_
HLA-DQA10501-DQB10201	55	VPYEGGAPTGDGDLS	YEGGAPTGD	2	0.1401	10979.0	80.00	0.31	tr_D0A423_
HLA-DQA10501-DQB10201	56	AGLVPYEGGAPTGDG	YEGGAPTGD	5	0.1394	11068.1	80.00	0.30	tr_D0A423_
HLA-DQA10501-DQB10201	57	LVPYEGGAPTGDGDL	YEGGAPTGD	3	0.1391	11099.8	80.00	0.34	tr_D0A423_
HLA-DQA10501-DQB10201	58	EGGAPTGDGDLSSFH	TGDGDLSSF	5	0.1352	11584.8	80.00	0.21	tr_D0A423_
HLA-DQA10501-DQB10201	59	SSFHFQEPGSCCCGV	FHFQEPGSC	2	0.1345	11660.9	80.00	0.41	tr_D0A423_
HLA-DQA10501-DQB10201	60	DSLTQPDRAINSESN	DRAINSESN	6	0.1335	11794.0	85.00	0.39	tr_D0A423_
HLA-DQA10501-DQB10201	61	FHFQEPGSCCCGVFE	GSCCCGVFE	6	0.1332	11830.3	85.00	0.35	tr_D0A423_
HLA-DQA10501-DQB10201	62	SDSLTQPDRAINSES	DSLTQPDRA	1	0.1320	11982.5	85.00	0.56	tr_D0A423_
HLA-DQA10501-DQB10201	63	SLTQPDRAINSESNP	DRAINSESN	5	0.1311	12103.6	85.00	0.46	tr_D0A423_
HLA-DQA10501-DQB10201	64	AINSESNPLSTEGPD	AINSESNPL	0	0.1306	12166.4	85.00	0.34	tr_D0A423_
HLA-DQA10501-DQB10201	65	SFHFQEPGSCCCGVF	FHFQEPGSC	1	0.1296	12306.7	85.00	0.33	tr_D0A423_
HLA-DQA10501-DQB10201	66	HFQEPGSCCCGVFEY	GSCCCGVFE	5	0.1271	12643.2	85.00	0.37	tr_D0A423_
HLA-DQA10501-DQB10201	67	REKKCDPFSDSLTQP	PFSDSLTQP	6	0.1269	12670.2	85.00	0.57	tr_D0A423_
HLA-DQA10501-DQB10201	68	FQEPGSCCCGVFEYN	GSCCCGVFE	4	0.1211	13481.3	85.00	0.34	tr_D0A423_
HLA-DQA10501-DQB10201	69	EPGSCCCGVFEYNKE	GSCCCGVFE	2	0.1124	14815.0	90.00	0.34	tr_D0A423_

HLA-DQA10501-DQB10201	70	PGSCCCGVFEYNKEV	GSCCCGVFE	1	0.1094	15301.2	90.00	0.28	tr_D0A423_
HLA-DQA10501-DQB10201	71	QEPGSCCCGVFEYNK	GSCCCGVFE	3	0.1033	16346.9	90.00	0.41	tr_D0A423_
HLA-DQA10501-DQB10201	72	EREKKCDPFSDSLTQ	DPFSDSLTQ	6	0.0759	21987.8	95.00	0.31	tr_D0A423_
HLA-DQA10501-DQB10201	73	EEREKKCDPFSDSLT	CDPFSDSLT	6	0.0676	24051.4	95.00	0.35	tr_D0A423_
HLA-DQA10501-DQB10201	74	REEREKKCDPFSDSL	KCDPFSDSL	6	0.0579	26712.9	100.00	0.28	tr_D0A423_
HLA-DQA10501-DQB10201	75	YGDALLLYKKKREER	YGDALLLYK	0	0.0554	27448.9	100.00	0.62	tr_D0A423_
HLA-DQA10501-DQB10201	76	KKREEREKKCDPFSD	EREKKCDPF	4	0.0514	28663.9	100.00	0.35	tr_D0A423_
HLA-DQA10501-DQB10201	77	KREEREKKCDPFSDS	EREKKCDPF	3	0.0463	30306.4	100.00	0.34	tr_D0A423_
HLA-DQA10501-DQB10201	78	GDALLLYKKKREERE	YKKKREERE	6	0.0452	30671.9	100.00	0.56	tr_D0A423_
HLA-DQA10501-DQB10201	79	YKKKREEREKKCDPF	EREKKCDPF	6	0.0447	30810.6	100.00	0.51	tr_D0A423_
HLA-DQA10501-DQB10201	80	KKKREEREKKCDPFS	EREKKCDPF	5	0.0402	32350.0	100.00	0.43	tr_D0A423_
HLA-DQA10501-DQB10201	81	DALLLYKKKREEREK	YKKKREERE	5	0.0393	32670.8	100.00	0.51	tr_D0A423_
HLA-DQA10501-DQB10201	82	ALLLYKKKREEREKK	YKKKREERE	4	0.0357	33993.1	100.00	0.50	tr_D0A423_
HLA-DQA10501-DQB10201	83	LLLYKKKREEREKKC	YKKKREERE	3	0.0353	34108.4	100.00	0.48	tr_D0A423_
HLA-DQA10501-DQB10201	84	LLYKKKREEREKKCD	YKKKREERE	2	0.0337	34725.4	100.00	0.47	tr_D0A423_
HLA-DQA10501-DQB10201	85	LYKKKREEREKKCDP	YKKKREERE	1	0.0320	35354.0	100.00	0.40	tr_D0A423_

Allele: HLA-DQA10501-DQB10201. Number of high binders 0. Number of weak binders 1. Number of peptides 85

Allele	pos	peptide	core	Of	1-log50k(aff)	affinity(nM)	%Rank	Relia	Identity	Bind Level
HLA-DQA10501-DQB10201	1	ALSNFFIAEADCLAD	FFIAEADCL	4	0.7453	15.7	0.05	0.50	tr_D0A423_	SB
HLA-DQA10501-DQB10201	2	SNFFIAEADCLADGP	FFIAEADCL	2	0.7329	18.0	0.07	0.44	tr_D0A423_	SB
HLA-DQA10501-DQB10201	3	LSNFFIAEADCLADG	FFIAEADCL	3	0.7328	18.0	0.08	0.49	tr_D0A423_	SB
HLA-DQA10501-DQB10201	4	GALSNFFIAEADCLA	FFIAEADCL	5	0.7230	20.0	0.09	0.53	tr_D0A423_	SB
HLA-DQA10501-DQB10201	5	NFFIAEADCLADGPH	FFIAEADCL	1	0.7168	21.4	0.12	0.45	tr_D0A423_	SB
HLA-DQA10501-DQB10201	6	FFIAEADCLADGPHF	FFIAEADCL	0	0.6919	28.0	0.20	0.43	tr_D0A423_	SB
HLA-DQA10501-DQB10201	7	PGALSNFFIAEADCL	FFIAEADCL	6	0.6751	33.6	0.30	0.62	tr_D0A423_	SB
HLA-DQA10501-DQB10201	8	SFPGALSNFFIAEAD	SNFFIAEAD	6	0.5186	182.9	4.50	0.44	tr_D0A423_	WB
HLA-DQA10501-DQB10201	9	FPGALSNFFIAEADC	SNFFIAEAD	5	0.5111	198.3	5.00	0.41	tr_D0A423_	WB
HLA-DQA10501-DQB10201	10	FIAEADCLADGPHFS	FIAEADCLA	0	0.5011	221.0	5.50	0.54	tr_D0A423_	WB
HLA-DQA10501-DQB10201	11	VSFPGALSNFFIAEA	FPGALSNFF	2	0.4417	420.2	11.00	0.43	tr_D0A423_	
HLA-DQA10501-DQB10201	12	ATSVSFPGALSNFFI	FPGALSNFF	5	0.4338	457.6	12.00	0.61	tr_D0A423_	
HLA-DQA10501-DQB10201	13	TSVSFPGALSNFFIA	FPGALSNFF	4	0.4114	583.2	14.00	0.62	tr_D0A423_	
HLA-DQA10501-DQB10201	14	SVSFPGALSNFFIAE	FPGALSNFF	3	0.4094	596.1	15.00	0.59	tr_D0A423_	
HLA-DQA10501-DQB10201	15	RATSVSFPGALSNFF	FPGALSNFF	6	0.4050	625.3	15.00	0.70	tr_D0A423_	
HLA-DQA10501-DQB10201	16	NLFGYLQRATSVSFP	LQRATSVSF	5	0.3126	1699.4	32.00	0.47	tr_D0A423_	
HLA-DQA10501-DQB10201	17	LFGYLQRATSVSFPG	LQRATSVSF	4	0.2919	2124.5	37.00	0.46	tr_D0A423_	
HLA-DQA10501-DQB10201	18	ANLFGYLQRATSVSF	LQRATSVSF	6	0.2915	2134.6	37.00	0.53	tr_D0A423_	
HLA-DQA10501-DQB10201	19	DCLADGPHFSFAFYQ	PHFSFAFYQ	6	0.2844	2305.7	39.00	0.43	tr_D0A423_	
HLA-DQA10501-DQB10201	20	FGYLQRATSVSFPGA	LQRATSVSF	3	0.2743	2571.3	42.00	0.44	tr_D0A423_	
HLA-DQA10501-DQB10201	21	GYLQRATSVSFPGAL	LQRATSVSF	2	0.2639	2878.0	44.00	0.43	tr_D0A423_	
HLA-DQA10501-DQB10201	22	YLQRATSVSFPGALS	LQRATSVSF	1	0.2538	3207.8	47.00	0.36	tr_D0A423_	
HLA-DQA10501-DQB10201	23	IAEADCLADGPHFSF	IAEADCLAD	0	0.2467	3464.3	49.00	0.24	tr_D0A423_	
HLA-DQA10501-DQB10201	24	QRATSVSFPGALSNF	SFPGALSNF	6	0.2269	4295.3	55.00	0.25	tr_D0A423_	
HLA-DQA10501-DQB10201	25	LQRATSVSFPGALSN	LQRATSVSF	0	0.2258	4343.6	55.00	0.25	tr_D0A423_	
HLA-DQA10501-DQB10201	26	ADCLADGPHFSFAFY	GPHFSFAFY	6	0.2135	4962.1	60.00	0.45	tr_D0A423_	

HLA-DQA10501-DQB10201	27	KANLFGYLQRATSVS	GYLQRATSV	5	0.2024	5596.5	65.00	0.31	tr_D0A423_	
HLA-DQA10501-DQB10201	28	AEADCLADGPHFSFA	EADCLADGP	1	0.1784	7256.3	70.00	0.30	tr_D0A423_	
HLA-DQA10501-DQB10201	29	EADCLADGPHFSFAF	DCLADGPHF	2	0.1692	8019.3	75.00	0.24	tr_D0A423_	

Allele: HLA-DQA10501-DQB10201. Number of high binders 7. Number of weak binders 3. Number of peptides 29

Allele	pos	peptide	core	Of	1-log50k(aff)	affinity(nM)	%Rank	Relia	Identity	Bind Level
HLA-DQA10501-DQB10201	1	IADNIIRSSLFPMFT	RSSLFPMFT	6	0.4195	534.5	13.00	0.45	tr_C9ZVF1_	
HLA-DQA10501-DQB10201	2	ADNIIRSSLFPMFTY	RSSLFPMFT	5	0.4145	564.1	14.00	0.45	tr_C9ZVF1_	
HLA-DQA10501-DQB10201	3	DNIIRSSLFPMFTYT	RSSLFPMFT	4	0.3912	726.0	17.00	0.46	tr_C9ZVF1_	
HLA-DQA10501-DQB10201	4	FPMFTYTFGADAKLY	YTFGADAKL	5	0.3907	729.5	17.00	0.37	tr_C9ZVF1_	
HLA-DQA10501-DQB10201	5	NIIRSSLFPMFTYTF	RSSLFPMFT	3	0.3765	850.8	19.00	0.49	tr_C9ZVF1_	
HLA-DQA10501-DQB10201	6	PMFTYTFGADAKLYQ	YTFGADAKL	4	0.3676	936.5	21.00	0.39	tr_C9ZVF1_	
HLA-DQA10501-DQB10201	7	LFPMFTYTFGADAKL	YTFGADAKL	6	0.3659	954.3	21.00	0.41	tr_C9ZVF1_	
HLA-DQA10501-DQB10201	8	IIRSSLFPMFTYTFG	RSSLFPMFT	2	0.3577	1042.3	23.00	0.51	tr_C9ZVF1_	
HLA-DQA10501-DQB10201	9	GIADNIIRSSLFPMF	IRSSLFPMF	6	0.3558	1064.2	23.00	0.41	tr_C9ZVF1_	
HLA-DQA10501-DQB10201	10	MFTYTFGADAKLYQR	YTFGADAKL	3	0.3422	1233.1	26.00	0.39	tr_C9ZVF1_	
HLA-DQA10501-DQB10201	11	IRSSLFPMFTYTFGA	RSSLFPMFT	1	0.3235	1509.3	30.00	0.51	tr_C9ZVF1_	
HLA-DQA10501-DQB10201	12	FTYTFGADAKLYQRM	YTFGADAKL	2	0.3151	1653.0	32.00	0.42	tr_C9ZVF1_	
HLA-DQA10501-DQB10201	13	SSLFPMFTYTFGADA	FTYTFGADA	6	0.3062	1820.4	34.00	0.50	tr_C9ZVF1_	
HLA-DQA10501-DQB10201	14	GFCELFGKGIADNII	FGKGIADNI	5	0.3048	1848.4	34.00	0.50	tr_C9ZVF1_	
HLA-DQA10501-DQB10201	15	SLFPMFTYTFGADAK	FTYTFGADA	5	0.2997	1953.0	35.00	0.41	tr_C9ZVF1_	
HLA-DQA10501-DQB10201	16	FCELFGKGIADNIIR	FGKGIADNI	4	0.2846	2300.5	39.00	0.49	tr_C9ZVF1_	
HLA-DQA10501-DQB10201	17	RSSLFPMFTYTFGAD	RSSLFPMFT	0	0.2807	2399.3	40.00	0.40	tr_C9ZVF1_	
HLA-DQA10501-DQB10201	18	KGIADNIIRSSLFPM	IIRSSLFPM	6	0.2793	2435.1	40.00	0.33	tr_C9ZVF1_	
HLA-DQA10501-DQB10201	19	CELFGKGIADNIIRS	FGKGIADNI	3	0.2643	2863.4	44.00	0.49	tr_C9ZVF1_	
HLA-DQA10501-DQB10201	20	TYTFGADAKLYQRMS	YTFGADAKL	1	0.2537	3212.1	47.00	0.45	tr_C9ZVF1_	
HLA-DQA10501-DQB10201	21	ELFGKGIADNIIRSS	FGKGIADNI	2	0.2536	3217.1	47.00	0.50	tr_C9ZVF1_	
HLA-DQA10501-DQB10201	22	GKGIADNIIRSSLFP	DNIIRSSLF	5	0.2392	3756.9	55.00	0.41	tr_C9ZVF1_	
HLA-DQA10501-DQB10201	23	FGKGIADNIIRSSLF	DNIIRSSLF	6	0.2308	4116.7	55.00	0.40	tr_C9ZVF1_	
HLA-DQA10501-DQB10201	24	LFGKGIADNIIRSSL	FGKGIADNI	1	0.2238	4438.0	60.00	0.50	tr_C9ZVF1_	
HLA-DQA10501-DQB10201	25	YTFGADAKLYQRMSS	YTFGADAKL	0	0.2003	5726.2	65.00	0.41	tr_C9ZVF1_	

Allele: HLA-DQA10501-DQB10201. Number of high binders 0. Number of weak binders 0. Number of peptides 25

Allele	pos	peptide	core	Of	1-log50k(aff)	affinity(nM)	%Rank	Relia	Identity	Bind Level
HLA-DQA10501-DQB10201	1	MVSWSRPEFAEAFFG	VSWSRPEFA	1	0.5545	124.0	3.00	0.33	tr_C9ZRE0_	WB
HLA-DQA10501-DQB10201	2	VSWSRPEFAEAFFGK	RPEFAEAFF	4	0.4716	304.1	7.50	0.31	tr_C9ZRE0_	WB
HLA-DQA10501-DQB10201	3	SWSRPEFAEAFFGKL	RPEFAEAFF	3	0.4451	405.2	10.00	0.38	tr_C9ZRE0_	
HLA-DQA10501-DQB10201	4	WSRPEFAEAFFGKLV	RPEFAEAFF	2	0.4410	423.3	11.00	0.34	tr_C9ZRE0_	
HLA-DQA10501-DQB10201	5	SRPEFAEAFFGKLVR	RPEFAEAFF	1	0.3915	723.0	17.00	0.33	tr_C9ZRE0_	
HLA-DQA10501-DQB10201	6	RPEFAEAFFGKLVRR	RPEFAEAFF	0	0.3294	1416.7	28.00	0.25	tr_C9ZRE0_	

HLA-DQA10501-DQB10201	7	CIYATKAALDSSPFV	AALDSSPFV	6	0.3266	1459.3	29.00	0.41	tr_C9ZRE0_
HLA-DQA10501-DQB10201	8	IYATKAALDSSPFVR	AALDSSPFV	5	0.2946	2064.8	37.00	0.38	tr_C9ZRE0_
HLA-DQA10501-DQB10201	9	RCIYATKAALDSSPF	IYATKAALD	2	0.2723	2627.3	42.00	0.34	tr_C9ZRE0_
HLA-DQA10501-DQB10201	10	VRRRCIYATKAALDS	IYATKAALD	5	0.2612	2961.2	45.00	0.38	tr_C9ZRE0_
HLA-DQA10501-DQB10201	11	RRCIYATKAALDSSP	IYATKAALD	3	0.2574	3086.9	46.00	0.41	tr_C9ZRE0_
HLA-DQA10501-DQB10201	12	PEFAEAFFGKLVRRR	FAEAFFGKL	2	0.2556	3146.5	47.00	0.33	tr_C9ZRE0_
HLA-DQA10501-DQB10201	13	RRRCIYATKAALDSS	IYATKAALD	4	0.2544	3188.8	47.00	0.38	tr_C9ZRE0_
HLA-DQA10501-DQB10201	14	LVRRRCIYATKAALD	IYATKAALD	6	0.2457	3502.3	49.00	0.49	tr_C9ZRE0_
HLA-DQA10501-DQB10201	15	EFAEAFFGKLVRRRC	FAEAFFGKL	1	0.2145	4909.0	60.00	0.37	tr_C9ZRE0_
HLA-DQA10501-DQB10201	16	FAEAFFGKLVRRRCI	AEAFFGKLV	1	0.1813	7031.6	70.00	0.34	tr_C9ZRE0_
HLA-DQA10501-DQB10201	17	KLVRRRCIYATKAAL	RRCIYATKA	4	0.1750	7529.3	70.00	0.49	tr_C9ZRE0_
HLA-DQA10501-DQB10201	18	FGKLVRRRCIYATKA	RRCIYATKA	6	0.1652	8366.7	75.00	0.55	tr_C9ZRE0_
HLA-DQA10501-DQB10201	19	GKLVRRRCIYATKAA	RRCIYATKA	5	0.1572	9121.7	75.00	0.58	tr_C9ZRE0_
HLA-DQA10501-DQB10201	20	AEAFFGKLVRRRCIY	AEAFFGKLV	0	0.1108	15077.3	90.00	0.34	tr_C9ZRE0_
HLA-DQA10501-DQB10201	21	EAFFGKLVRRRCIYA	LVRRRCIYA	6	0.0870	19512.8	95.00	0.21	tr_C9ZRE0_
HLA-DQA10501-DQB10201	22	AFFGKLVRRRCIYAT	LVRRRCIYA	5	0.0856	19795.6	95.00	0.25	tr_C9ZRE0_
HLA-DQA10501-DQB10201	23	FFGKLVRRRCIYATK	LVRRRCIYA	4	0.0813	20752.3	95.00	0.23	tr_C9ZRE0_

Allele: HLA-DQA10501-DQB10201. Number of high binders 0. Number of weak binders 2. Number of peptides 23

Allele	pos	peptide	core	Of	1-log50k(aff)	affinity(nM)	%Rank	Relia	Identity	Bind Level
HLA-DQA10501-DQB10201	1	CAVSVSWSAYVQAFL	WSAYVQAFL	6	0.5687	106.3	2.50	0.69	tr_C9ZRE0_	WB
HLA-DQA10501-DQB10201	2	AVSVSWSAYVQAFLK	WSAYVQAFL	5	0.5365	150.6	3.50	0.64	tr_C9ZRE0_	WB
HLA-DQA10501-DQB10201	3	QAFLKRFSFVLPQPL	FSFVLPQPL	6	0.5166	186.9	4.50	0.62	tr_C9ZRE0_	WB
HLA-DQA10501-DQB10201	4	SVSWSAYVQAFLKRF	WSAYVQAFL	3	0.5049	212.1	5.00	0.64	tr_C9ZRE0_	WB
HLA-DQA10501-DQB10201	5	VSVSWSAYVQAFLKR	WSAYVQAFL	4	0.5034	215.5	5.50	0.65	tr_C9ZRE0_	WB
HLA-DQA10501-DQB10201	6	AFLKRFSFVLPQPLR	FSFVLPQPL	5	0.4863	259.4	6.50	0.62	tr_C9ZRE0_	WB
HLA-DQA10501-DQB10201	7	VSWSAYVQAFLKRFS	WSAYVQAFL	2	0.4806	276.0	7.00	0.69	tr_C9ZRE0_	WB
HLA-DQA10501-DQB10201	8	FLKRFSFVLPQPLRK	FSFVLPQPL	4	0.4506	381.6	9.50	0.59	tr_C9ZRE0_	WB
HLA-DQA10501-DQB10201	9	SWSAYVQAFLKRFSF	WSAYVQAFL	1	0.4417	420.3	11.00	0.69	tr_C9ZRE0_	
HLA-DQA10501-DQB10201	10	LKRFSFVLPQPLRKS	FSFVLPQPL	3	0.4222	518.8	13.00	0.63	tr_C9ZRE0_	
HLA-DQA10501-DQB10201	11	AYTHTSVALGEFASW	TSVALGEFA	4	0.4220	519.9	13.00	0.49	tr_C9ZRE0_	
HLA-DQA10501-DQB10201	12	GSAYTHTSVALGEFA	TSVALGEFA	6	0.4186	539.2	13.00	0.51	tr_C9ZRE0_	
HLA-DQA10501-DQB10201	13	YTHTSVALGEFASWT	TSVALGEFA	3	0.4182	541.6	13.00	0.46	tr_C9ZRE0_	
HLA-DQA10501-DQB10201	14	KRFSFVLPQPLRKSP	FSFVLPQPL	2	0.4034	636.0	15.00	0.64	tr_C9ZRE0_	
HLA-DQA10501-DQB10201	15	THTSVALGEFASWTV	TSVALGEFA	2	0.4028	640.2	16.00	0.46	tr_C9ZRE0_	
HLA-DQA10501-DQB10201	16	SAYTHTSVALGEFAS	TSVALGEFA	5	0.4009	653.5	16.00	0.51	tr_C9ZRE0_	
HLA-DQA10501-DQB10201	17	VALGEFASWTVAVCM	ASWTVAVCM	6	0.3856	770.8	18.00	0.37	tr_C9ZRE0_	
HLA-DQA10501-DQB10201	18	RFSFVLPQPLRKSPI	FSFVLPQPL	1	0.3746	868.2	20.00	0.68	tr_C9ZRE0_	
HLA-DQA10501-DQB10201	19	HTSVALGEFASWTVA	TSVALGEFA	1	0.3724	889.4	20.00	0.47	tr_C9ZRE0_	
HLA-DQA10501-DQB10201	20	VQAFLKRFSFVLPQP	RFSFVLPQP	6	0.3704	908.6	21.00	0.56	tr_C9ZRE0_	
HLA-DQA10501-DQB10201	21	TSVALGEFASWTVAV	TSVALGEFA	0	0.3687	925.5	21.00	0.31	tr_C9ZRE0_	
HLA-DQA10501-DQB10201	22	GCAVSVSWSAYVQAF	SWSAYVQAF	6	0.3638	975.8	22.00	0.50	tr_C9ZRE0_	
HLA-DQA10501-DQB10201	23	FASWTVAVCMTLECL	AVCMTLECL	6	0.3621	994.6	22.00	0.38	tr_C9ZRE0_	
HLA-DQA10501-DQB10201	24	WSAYVQAFLKRFSFV	WSAYVQAFL	0	0.3581	1038.3	23.00	0.64	tr_C9ZRE0_	
HLA-DQA10501-DQB10201	25	LGEFASWTVAVCMTL	ASWTVAVCM	4	0.3494	1140.1	24.00	0.29	tr_C9ZRE0_	

HLA-DQA10501-DQB10201	26	ALGEFASWTVAVCMT	ASWTVAVCM	5	0.3493	1142.5	24.00	0.32	tr_C9ZRE0_	
HLA-DQA10501-DQB10201	27	ASWTVAVCMTLECLV	AVCMTLECL	5	0.3446	1200.9	25.00	0.38	tr_C9ZRE0_	
HLA-DQA10501-DQB10201	28	AGSAYTHTSVALGEF	HTSVALGEF	6	0.3395	1269.4	26.00	0.47	tr_C9ZRE0_	
HLA-DQA10501-DQB10201	29	GEFASWTVAVCMTLE	ASWTVAVCM	3	0.3387	1280.9	26.00	0.29	tr_C9ZRE0_	
HLA-DQA10501-DQB10201	30	SVALGEFASWTVAVC	EFASWTVAV	5	0.3383	1286.7	27.00	0.25	tr_C9ZRE0_	
HLA-DQA10501-DQB10201	31	SWTVAVCMTLECLVS	AVCMTLECL	4	0.3252	1482.1	29.00	0.41	tr_C9ZRE0_	
HLA-DQA10501-DQB10201	32	EFASWTVAVCMTLEC	ASWTVAVCM	2	0.3208	1554.6	30.00	0.31	tr_C9ZRE0_	
HLA-DQA10501-DQB10201	33	WTVAVCMTLECLVSG	AVCMTLECL	3	0.3101	1745.8	33.00	0.42	tr_C9ZRE0_	
HLA-DQA10501-DQB10201	34	TVAVCMTLECLVSGC	AVCMTLECL	2	0.2884	2207.5	38.00	0.44	tr_C9ZRE0_	
HLA-DQA10501-DQB10201	35	FSFVLPQPLRKSPID	FSFVLPQPL	0	0.2871	2239.2	38.00	0.74	tr_C9ZRE0_	
HLA-DQA10501-DQB10201	36	VAVCMTLECLVSGCA	AVCMTLECL	1	0.2792	2436.8	40.00	0.38	tr_C9ZRE0_	
HLA-DQA10501-DQB10201	37	SGCAVSVSWSAYVQA	VSWSAYVQA	6	0.2695	2707.4	43.00	0.52	tr_C9ZRE0_	
HLA-DQA10501-DQB10201	38	AVCMTLECLVSGCAV	AVCMTLECL	0	0.2648	2849.8	44.00	0.29	tr_C9ZRE0_	
HLA-DQA10501-DQB10201	39	YVQAFLKRFSFVLPQ	KRFSFVLPQ	6	0.2628	2912.4	45.00	0.41	tr_C9ZRE0_	
HLA-DQA10501-DQB10201	40	TLECLVSGCAVSVSW	LVSGCAVSV	4	0.2581	3062.4	46.00	0.31	tr_C9ZRE0_	
HLA-DQA10501-DQB10201	41	VAGSAYTHTSVALGE	THTSVALGE	6	0.2545	3186.3	47.00	0.56	tr_C9ZRE0_	
HLA-DQA10501-DQB10201	42	LVSGCAVSVSWSAYV	VSVSWSAYV	6	0.2536	3214.8	47.00	0.34	tr_C9ZRE0_	
HLA-DQA10501-DQB10201	43	LECLVSGCAVSVSWS	LVSGCAVSV	3	0.2384	3789.7	55.00	0.32	tr_C9ZRE0_	
HLA-DQA10501-DQB10201	44	ECLVSGCAVSVSWSA	LVSGCAVSV	2	0.2304	4132.2	55.00	0.36	tr_C9ZRE0_	
HLA-DQA10501-DQB10201	45	MTLECLVSGCAVSVS	LVSGCAVSV	5	0.2279	4246.7	55.00	0.40	tr_C9ZRE0_	
HLA-DQA10501-DQB10201	46	CMTLECLVSGCAVSV	LVSGCAVSV	6	0.2255	4360.4	55.00	0.44	tr_C9ZRE0_	
HLA-DQA10501-DQB10201	47	CLVSGCAVSVSWSAY	LVSGCAVSV	1	0.2213	4561.4	60.00	0.34	tr_C9ZRE0_	
HLA-DQA10501-DQB10201	48	VSGCAVSVSWSAYVQ	VSVSWSAYV	5	0.2213	4563.3	60.00	0.37	tr_C9ZRE0_	
HLA-DQA10501-DQB10201	49	AYVQAFLKRFSFVLP	LKRFSFVLP	6	0.2207	4592.1	60.00	0.34	tr_C9ZRE0_	
HLA-DQA10501-DQB10201	50	VCMTLECLVSGCAVS	LECLVSGCA	4	0.1970	5932.7	65.00	0.29	tr_C9ZRE0_	
HLA-DQA10501-DQB10201	51	SAYVQAFLKRFSFVL	YVQAFLKRF	2	0.1965	5964.6	65.00	0.41	tr_C9ZRE0_	
HLA-DQA10501-DQB10201	52	LPVAGSAYTHTSVAL	AYTHTSVAL	6	0.1861	6678.0	70.00	0.58	tr_C9ZRE0_	
HLA-DQA10501-DQB10201	53	PVAGSAYTHTSVALG	AYTHTSVAL	5	0.1795	7172.2	70.00	0.49	tr_C9ZRE0_	
HLA-DQA10501-DQB10201	54	FVLPQPLRKSPIDVV	LRKSPIDVV	6	0.1603	8821.2	75.00	0.58	tr_C9ZRE0_	
HLA-DQA10501-DQB10201	55	AMLPVAGSAYTHTSV	MLPVAGSAY	1	0.1512	9734.0	80.00	0.23	tr_C9ZRE0_	
HLA-DQA10501-DQB10201	56	VLPQPLRKSPIDVVG	LRKSPIDVV	5	0.1457	10339.7	80.00	0.60	tr_C9ZRE0_	
HLA-DQA10501-DQB10201	57	LPQPLRKSPIDVVGG	LRKSPIDVV	4	0.1378	11262.1	80.00	0.53	tr_C9ZRE0_	
HLA-DQA10501-DQB10201	58	MLPVAGSAYTHTSVA	VAGSAYTHT	3	0.1337	11771.1	85.00	0.23	tr_C9ZRE0_	
HLA-DQA10501-DQB10201	59	PQPLRKSPIDVVGGR	LRKSPIDVV	3	0.1291	12366.9	85.00	0.51	tr_C9ZRE0_	
HLA-DQA10501-DQB10201	60	SFVLPQPLRKSPIDV	FVLPQPLRK	1	0.1159	14268.3	85.00	0.44	tr_C9ZRE0_	

Allele: HLA-DQA10501-DQB10201. Number of high binders 0. Number of weak binders 8. Number of peptides 60

Allele	pos	peptide	core	Of	1-log50k(aff)	affinity(nM)	%Rank	Relia	Identity	Bind Level
HLA-DQA10501-DQB10201	1	WCFFRYASSYAREFS	YASSYAREF	5	0.3987	668.9	16.00	0.41	tr_C9ZVS2_	
HLA-DQA10501-DQB10201	2	TWCFFRYASSYAREF	YASSYAREF	6	0.3986	669.9	16.00	0.43	tr_C9ZVS2_	
HLA-DQA10501-DQB10201	3	CFFRYASSYAREFSG	YASSYAREF	4	0.3706	906.4	21.00	0.38	tr_C9ZVS2_	
HLA-DQA10501-DQB10201	4	FFRYASSYAREFSGF	YASSYAREF	3	0.3686	927.0	21.00	0.41	tr_C9ZVS2_	
HLA-DQA10501-DQB10201	5	FRYASSYAREFSGFL	YASSYAREF	2	0.3586	1032.5	23.00	0.34	tr_C9ZVS2_	
HLA-DQA10501-DQB10201	6	RYASSYAREFSGFLS	YAREFSGFL	5	0.3360	1319.1	27.00	0.31	tr_C9ZVS2_	
HLA-DQA10501-DQB10201	7	ITWCFFRYASSYARE	FFRYASSYA	4	0.3175	1611.5	31.00	0.41	tr_C9ZVS2_	

HLA-DQA10501-DQB10201	8	YASSYAREFSGFLSG	YAREFSGFL	4	0.3028	1889.3	34.00	0.37	tr_C9ZVS2_
HLA-DQA10501-DQB10201	9	QITWCFFRYASSYAR	FFRYASSYA	5	0.2851	2286.9	39.00	0.56	tr_C9ZVS2_
HLA-DQA10501-DQB10201	10	LQITWCFFRYASSYA	FFRYASSYA	6	0.2818	2370.2	40.00	0.67	tr_C9ZVS2_
HLA-DQA10501-DQB10201	11	SSYAREFSGFLSGHA	YAREFSGFL	2	0.2636	2886.1	44.00	0.35	tr_C9ZVS2_
HLA-DQA10501-DQB10201	12	ASSYAREFSGFLSGH	YAREFSGFL	3	0.2609	2972.9	45.00	0.45	tr_C9ZVS2_
HLA-DQA10501-DQB10201	13	SYAREFSGFLSGHAR	YAREFSGFL	1	0.2405	3706.3	55.00	0.34	tr_C9ZVS2_
HLA-DQA10501-DQB10201	14	YAREFSGFLSGHART	FSGFLSGHA	4	0.2110	5099.0	60.00	0.29	tr_C9ZVS2_
HLA-DQA10501-DQB10201	15	PLQITWCFFRYASSY	CFFRYASSY	6	0.1824	6949.2	70.00	0.37	tr_C9ZVS2_
HLA-DQA10501-DQB10201	16	AREFSGFLSGHARTL	FSGFLSGHA	3	0.1820	6976.8	70.00	0.40	tr_C9ZVS2_
HLA-DQA10501-DQB10201	17	MPLQITWCFFRYASS	PLQITWCFF	1	0.1480	10084.1	80.00	0.34	tr_C9ZVS2_

Allele: HLA-DQA10501-DQB10201. Number of high binders 0. Number of weak binders 0. Number of peptides 17

Allele	pos	peptide	core	Of	1-log50k(aff)	affinity(nM)	%Rank	Relia	Identity	Bind Level
HLA-DQA10501-DQB10201	1	YGCKWSGTTCKFENP	WSGTTCKFE	4	0.1238	13092.2	85.00	0.63	tr_D0A3U1_	
HLA-DQA10501-DQB10201	2	KIYGCKWSGTTCKFE	WSGTTCKFE	6	0.1129	14744.8	90.00	0.59	tr_D0A3U1_	
HLA-DQA10501-DQB10201	3	IYGCKWSGTTCKFEN	WSGTTCKFE	5	0.1124	14824.5	90.00	0.65	tr_D0A3U1_	
HLA-DQA10501-DQB10201	4	GCKWSGTTCKFENPK	WSGTTCKFE	3	0.1079	15558.2	90.00	0.61	tr_D0A3U1_	
HLA-DQA10501-DQB10201	5	CKWSGTTCKFENPKC	WSGTTCKFE	2	0.1032	16364.7	90.00	0.59	tr_D0A3U1_	
HLA-DQA10501-DQB10201	6	ENPKCSEGSDPSDSC	EGSDPSDSC	6	0.1002	16903.7	90.00	0.31	tr_D0A3U1_	
HLA-DQA10501-DQB10201	7	ATECKFTTEGACGGA	KFTTEGACG	4	0.0983	17267.0	90.00	0.34	tr_D0A3U1_	
HLA-DQA10501-DQB10201	8	TEGACGGAKIYGCKW	GAKIYGCKW	6	0.0970	17503.4	90.00	0.65	tr_D0A3U1_	
HLA-DQA10501-DQB10201	9	ECKFTTEGACGGAKI	KFTTEGACG	2	0.0952	17844.8	90.00	0.26	tr_D0A3U1_	
HLA-DQA10501-DQB10201	10	KWSGTTCKFENPKCS	WSGTTCKFE	1	0.0946	17966.4	90.00	0.62	tr_D0A3U1_	
HLA-DQA10501-DQB10201	11	EGACGGAKIYGCKWS	GAKIYGCKW	5	0.0882	19243.6	95.00	0.67	tr_D0A3U1_	
HLA-DQA10501-DQB10201	12	TECKFTTEGACGGAK	KFTTEGACG	3	0.0864	19635.2	95.00	0.32	tr_D0A3U1_	
HLA-DQA10501-DQB10201	13	NPKCSEGSDPSDSCK	EGSDPSDSC	5	0.0859	19747.3	95.00	0.29	tr_D0A3U1_	
HLA-DQA10501-DQB10201	14	CKFTTEGACGGAKIY	KFTTEGACG	1	0.0855	19823.3	95.00	0.23	tr_D0A3U1_	
HLA-DQA10501-DQB10201	15	CSEGSDPSDSCKNEV	EGSDPSDSC	2	0.0806	20911.4	95.00	0.26	tr_D0A3U1_	
HLA-DQA10501-DQB10201	16	PKCSEGSDPSDSCKN	EGSDPSDSC	4	0.0795	21153.5	95.00	0.30	tr_D0A3U1_	
HLA-DQA10501-DQB10201	17	ACGGAKIYGCKWSGT	GAKIYGCKW	3	0.0789	21299.6	95.00	0.62	tr_D0A3U1_	
HLA-DQA10501-DQB10201	18	GACGGAKIYGCKWSG	GAKIYGCKW	4	0.0777	21567.5	95.00	0.70	tr_D0A3U1_	
HLA-DQA10501-DQB10201	19	WSGTTCKFENPKCSE	WSGTTCKFE	0	0.0771	21706.8	95.00	0.52	tr_D0A3U1_	
HLA-DQA10501-DQB10201	20	FENPKCSEGSDPSDS	SEGSDPSDS	6	0.0761	21947.6	95.00	0.33	tr_D0A3U1_	
HLA-DQA10501-DQB10201	21	KCSEGSDPSDSCKNE	EGSDPSDSC	3	0.0743	22377.3	95.00	0.31	tr_D0A3U1_	
HLA-DQA10501-DQB10201	22	CGGAKIYGCKWSGTT	GAKIYGCKW	2	0.0733	22610.5	95.00	0.60	tr_D0A3U1_	
HLA-DQA10501-DQB10201	23	KFTTEGACGGAKIYG	FTTEGACGG	1	0.0730	22683.8	95.00	0.21	tr_D0A3U1_	
HLA-DQA10501-DQB10201	24	SEGSDPSDSCKNEVA	EGSDPSDSC	1	0.0727	22780.4	95.00	0.28	tr_D0A3U1_	
HLA-DQA10501-DQB10201	25	GGAKIYGCKWSGTTC	GAKIYGCKW	1	0.0705	23328.2	95.00	0.58	tr_D0A3U1_	
HLA-DQA10501-DQB10201	26	KFENPKCSEGSDPSD	CSEGSDPSD	6	0.0703	23361.0	95.00	0.31	tr_D0A3U1_	
HLA-DQA10501-DQB10201	27	AKIYGCKWSGTTCKF	KWSGTTCKF	6	0.0604	26013.5	100.00	0.56	tr_D0A3U1_	
HLA-DQA10501-DQB10201	28	FTTEGACGGAKIYGC	EGACGGAKI	3	0.0591	26381.7	100.00	0.31	tr_D0A3U1_	
HLA-DQA10501-DQB10201	29	GAKIYGCKWSGTTCK	GAKIYGCKW	0	0.0558	27335.1	100.00	0.54	tr_D0A3U1_	
HLA-DQA10501-DQB10201	30	CKFENPKCSEGSDPS	PKCSEGSDP	5	0.0532	28106.8	100.00	0.29	tr_D0A3U1_	
HLA-DQA10501-DQB10201	31	TCKFENPKCSEGSDP	PKCSEGSDP	6	0.0514	28682.5	100.00	0.37	tr_D0A3U1_	
HLA-DQA10501-DQB10201	32	TTEGACGGAKIYGCK	EGACGGAKI	2	0.0429	31435.3	100.00	0.40	tr_D0A3U1_	

HLA-DQA10501-DQB10201	33	TTCKFENPKCSEGSD	KFENPKCSE	3	0.0404	32287.0	100.00	0.38	tr_D0A3U1_	
HLA-DQA10501-DQB10201	34	SGTTCKFENPKCSEG	KFENPKCSE	5	0.0394	32634.0	100.00	0.46	tr_D0A3U1_	
HLA-DQA10501-DQB10201	35	GTTCKFENPKCSEGS	KFENPKCSE	4	0.0376	33294.9	100.00	0.44	tr_D0A3U1_	

Allele: HLA-DQA10501-DQB10201. Number of high binders 0. Number of weak binders 0. Number of peptides 35

Allele	pos	peptide	core	Of	1-log50k(aff)	affinity(nM)	%Rank	Relia	Identity	Bind Level
HLA-DQA10501-DQB10201	1	EKSVGGFADVSLMIF	VGGFADVSL	3	0.4582	351.4	9.00	0.32	tr_D0A0N1_	WB
HLA-DQA10501-DQB10201	2	KSVGGFADVSLMIFL	VGGFADVSL	2	0.4323	465.2	12.00	0.31	tr_D0A0N1_	
HLA-DQA10501-DQB10201	3	SVGGFADVSLMIFLP	FADVSLMIF	4	0.4287	483.4	12.00	0.31	tr_D0A0N1_	
HLA-DQA10501-DQB10201	4	VGGFADVSLMIFLPS	FADVSLMIF	3	0.3891	742.0	18.00	0.35	tr_D0A0N1_	
HLA-DQA10501-DQB10201	5	GGFADVSLMIFLPSL	FADVSLMIF	2	0.3760	855.6	20.00	0.35	tr_D0A0N1_	
HLA-DQA10501-DQB10201	6	GFADVSLMIFLPSLT	FADVSLMIF	1	0.3513	1117.7	24.00	0.32	tr_D0A0N1_	
HLA-DQA10501-DQB10201	7	FADVSLMIFLPSLTF	FADVSLMIF	0	0.3432	1219.4	26.00	0.24	tr_D0A0N1_	
HLA-DQA10501-DQB10201	8	ADVSLMIFLPSLTFV	SLMIFLPSL	3	0.3010	1924.8	35.00	0.33	tr_D0A0N1_	
HLA-DQA10501-DQB10201	9	DVSLMIFLPSLTFVS	SLMIFLPSL	2	0.2860	2266.1	39.00	0.34	tr_D0A0N1_	
HLA-DQA10501-DQB10201	10	SLMIFLPSLTFVSIT	PSLTFVSIT	6	0.2809	2394.4	40.00	0.22	tr_D0A0N1_	
HLA-DQA10501-DQB10201	11	VSLMIFLPSLTFVSI	SLMIFLPSL	1	0.2805	2404.1	40.00	0.28	tr_D0A0N1_	
HLA-DQA10501-DQB10201	12	MIFLPSLTFVSITKF	LTFVSITKF	6	0.2464	3474.8	49.00	0.29	tr_D0A0N1_	
HLA-DQA10501-DQB10201	13	IFLPSLTFVSITKFE	LTFVSITKF	5	0.2463	3479.4	49.00	0.28	tr_D0A0N1_	
HLA-DQA10501-DQB10201	14	FLPSLTFVSITKFEN	LTFVSITKF	4	0.2443	3556.8	50.00	0.27	tr_D0A0N1_	
HLA-DQA10501-DQB10201	15	LMIFLPSLTFVSITK	PSLTFVSIT	5	0.2328	4025.6	55.00	0.23	tr_D0A0N1_	
HLA-DQA10501-DQB10201	16	LPSLTFVSITKFENA	FVSITKFEN	5	0.2328	4026.7	55.00	0.23	tr_D0A0N1_	
HLA-DQA10501-DQB10201	17	PSLTFVSITKFENAE	FVSITKFEN	4	0.2196	4647.6	60.00	0.29	tr_D0A0N1_	
HLA-DQA10501-DQB10201	18	SLTFVSITKFENAER	FVSITKFEN	3	0.2098	5168.0	60.00	0.22	tr_D0A0N1_	

Allele: HLA-DQA10501-DQB10201. Number of high binders 0. Number of weak binders 1. Number of peptides 18

Allele	pos	peptide	core	Of	1-log50k(aff)	affinity(nM)	%Rank	Relia	Identity	Bind Level
HLA-DQA10501-DQB10201	1	WKVHATTASERADAA	HATTASERA	3	0.3096	1754.7	33.00	0.27	tr_D0A0N1_	
HLA-DQA10501-DQB10201	2	TWKVHATTASERADA	HATTASERA	4	0.2878	2222.4	38.00	0.37	tr_D0A0N1_	
HLA-DQA10501-DQB10201	3	KVHATTASERADAAQ	TASERADAA	5	0.2844	2304.0	39.00	0.31	tr_D0A0N1_	
HLA-DQA10501-DQB10201	4	RTWKVHATTASERAD	HATTASERA	5	0.2824	2353.9	40.00	0.38	tr_D0A0N1_	
HLA-DQA10501-DQB10201	5	PLHFLLLGYEAGRTW	LLGYEAGRT	5	0.2731	2603.8	42.00	0.25	tr_D0A0N1_	
HLA-DQA10501-DQB10201	6	GRTWKVHATTASERA	HATTASERA	6	0.2659	2815.4	44.00	0.37	tr_D0A0N1_	
HLA-DQA10501-DQB10201	7	HATTASERADAAQGD	TASERADAA	3	0.2638	2878.6	44.00	0.32	tr_D0A0N1_	
HLA-DQA10501-DQB10201	8	VHATTASERADAAQG	TASERADAA	4	0.2597	3010.2	46.00	0.31	tr_D0A0N1_	
HLA-DQA10501-DQB10201	9	LHFLLLGYEAGRTWK	FLLLGYEAG	2	0.2475	3433.8	49.00	0.25	tr_D0A0N1_	
HLA-DQA10501-DQB10201	10	ATTASERADAAQGDS	TASERADAA	2	0.2461	3486.0	49.00	0.37	tr_D0A0N1_	
HLA-DQA10501-DQB10201	11	TTASERADAAQGDSR	TASERADAA	1	0.2367	3861.5	55.00	0.31	tr_D0A0N1_	
HLA-DQA10501-DQB10201	12	SRDSHGEVSGWETSA	SHGEVSGWE	3	0.2349	3937.1	55.00	0.35	tr_D0A0N1_	
HLA-DQA10501-DQB10201	13	GDSRDSHGEVSGWET	SHGEVSGWE	5	0.2343	3963.9	55.00	0.42	tr_D0A0N1_	

HLA-DQA10501-DQB10201	14	TASERADAAQGDSRD	ERADAAQGD	3	0.2326	4035.2	55.00	0.30	tr_D0A0N1_
HLA-DQA10501-DQB10201	15	DSRDSHGEVSGWETS	SHGEVSGWE	4	0.2283	4229.0	55.00	0.41	tr_D0A0N1_
HLA-DQA10501-DQB10201	16	HFLLLGYEAGRTWKV	LLGYEAGRT	3	0.2270	4290.6	55.00	0.28	tr_D0A0N1_
HLA-DQA10501-DQB10201	17	RDSHGEVSGWETSAG	SHGEVSGWE	2	0.2252	4372.9	55.00	0.34	tr_D0A0N1_
HLA-DQA10501-DQB10201	18	AGRTWKVHATTASER	VHATTASER	6	0.2141	4931.3	60.00	0.50	tr_D0A0N1_
HLA-DQA10501-DQB10201	19	DSHGEVSGWETSAGR	SHGEVSGWE	1	0.2081	5263.7	60.00	0.34	tr_D0A0N1_
HLA-DQA10501-DQB10201	20	ASERADAAQGDSRDS	ERADAAQGD	2	0.2007	5701.6	65.00	0.37	tr_D0A0N1_
HLA-DQA10501-DQB10201	21	FLLLGYEAGRTWKVH	LLGYEAGRT	2	0.1948	6076.8	65.00	0.38	tr_D0A0N1_
HLA-DQA10501-DQB10201	22	QGDSRDSHGEVSGWE	SHGEVSGWE	6	0.1914	6303.2	65.00	0.56	tr_D0A0N1_
HLA-DQA10501-DQB10201	23	SERADAAQGDSRDSH	ERADAAQGD	1	0.1829	6913.6	70.00	0.35	tr_D0A0N1_
HLA-DQA10501-DQB10201	24	SHGEVSGWETSAGRR	HGEVSGWET	1	0.1662	8275.1	75.00	0.26	tr_D0A0N1_
HLA-DQA10501-DQB10201	25	EAGRTWKVHATTASE	KVHATTASE	6	0.1652	8370.7	75.00	0.49	tr_D0A0N1_
HLA-DQA10501-DQB10201	26	ERADAAQGDSRDSHG	ADAAQGDSR	2	0.1566	9187.1	75.00	0.34	tr_D0A0N1_
HLA-DQA10501-DQB10201	27	HGEVSGWETSAGRRD	WETSAGRRD	6	0.1465	10245.8	80.00	0.38	tr_D0A0N1_
HLA-DQA10501-DQB10201	28	WETSAGRRDSDDTQI	RRDSDDTQI	6	0.1419	10767.9	80.00	0.32	tr_D0A0N1_
HLA-DQA10501-DQB10201	29	LLLGYEAGRTWKVHA	LLGYEAGRT	1	0.1405	10930.6	80.00	0.46	tr_D0A0N1_
HLA-DQA10501-DQB10201	30	SGWETSAGRRDSDDT	WETSAGRRD	2	0.1395	11050.2	80.00	0.41	tr_D0A0N1_
HLA-DQA10501-DQB10201	31	VSGWETSAGRRDSDD	WETSAGRRD	3	0.1390	11106.9	80.00	0.41	tr_D0A0N1_
HLA-DQA10501-DQB10201	32	GWETSAGRRDSDDTQ	WETSAGRRD	1	0.1369	11371.5	80.00	0.32	tr_D0A0N1_
HLA-DQA10501-DQB10201	33	EVSGWETSAGRRDSD	WETSAGRRD	4	0.1344	11678.0	80.00	0.49	tr_D0A0N1_
HLA-DQA10501-DQB10201	34	GEVSGWETSAGRRDS	WETSAGRRD	5	0.1269	12664.2	85.00	0.47	tr_D0A0N1_
HLA-DQA10501-DQB10201	35	YEAGRTWKVHATTAS	TWKVHATTA	5	0.1178	13977.0	85.00	0.41	tr_D0A0N1_
HLA-DQA10501-DQB10201	36	RADAAQGDSRDSHGE	ADAAQGDSR	1	0.1170	14092.8	85.00	0.41	tr_D0A0N1_
HLA-DQA10501-DQB10201	37	ETSAGRRDSDDTQIK	RRDSDDTQI	5	0.1116	14951.3	90.00	0.35	tr_D0A0N1_
HLA-DQA10501-DQB10201	38	GRRDSDDTQIKTFVL	DTQIKTFVL	6	0.1114	14978.0	90.00	0.32	tr_D0A0N1_
HLA-DQA10501-DQB10201	39	LLGYEAGRTWKVHAT	LLGYEAGRT	0	0.1105	15130.4	90.00	0.42	tr_D0A0N1_
HLA-DQA10501-DQB10201	40	AQGDSRDSHGEVSGW	DSHGEVSGW	6	0.1091	15355.2	90.00	0.40	tr_D0A0N1_
HLA-DQA10501-DQB10201	41	SAGRRDSDDTQIKTF	RRDSDDTQI	3	0.1085	15449.2	90.00	0.29	tr_D0A0N1_
HLA-DQA10501-DQB10201	42	AGRRDSDDTQIKTFV	RRDSDDTQI	2	0.1070	15711.5	90.00	0.25	tr_D0A0N1_
HLA-DQA10501-DQB10201	43	TSAGRRDSDDTQIKT	RRDSDDTQI	4	0.1050	16060.6	90.00	0.35	tr_D0A0N1_
HLA-DQA10501-DQB10201	44	ADAAQGDSRDSHGEV	ADAAQGDSR	0	0.1041	16218.4	90.00	0.31	tr_D0A0N1_
HLA-DQA10501-DQB10201	45	GYEAGRTWKVHATTA	TWKVHATTA	6	0.0877	19350.7	95.00	0.53	tr_D0A0N1_
HLA-DQA10501-DQB10201	46	DAAQGDSRDSHGEVS	SRDSHGEVS	6	0.0849	19950.9	95.00	0.38	tr_D0A0N1_
HLA-DQA10501-DQB10201	47	LGYEAGRTWKVHATT	LGYEAGRTW	0	0.0761	21943.6	95.00	0.31	tr_D0A0N1_
HLA-DQA10501-DQB10201	48	AAQGDSRDSHGEVSG	SRDSHGEVS	5	0.0694	23587.3	95.00	0.43	tr_D0A0N1_

Allele: HLA-DQA10501-DQB10201. Number of high binders 0. Number of weak binders 0. Number of peptides 48

Allele	pos	peptide	core	Of	1-log50k(aff)	affinity(nM)	%Rank	Relia	Identity	Bind Level
HLA-DQA10501-DQB10201	1	NYKDLINRTVFSLIM	NRTVFSLIM	6	0.2732	2602.7	42.00	0.69	tr_C9ZRP4_	
HLA-DQA10501-DQB10201	2	SSKQVLLPLQGNISP	LLPLQGNIS	5	0.2091	5202.9	60.00	0.34	tr_C9ZRP4_	
HLA-DQA10501-DQB10201	3	KSSKQVLLPLQGNIS	LLPLQGNIS	6	0.1964	5972.9	65.00	0.28	tr_C9ZRP4_	
HLA-DQA10501-DQB10201	4	SKQVLLPLQGNISPS	LLPLQGNIS	4	0.1943	6106.2	65.00	0.34	tr_C9ZRP4_	
HLA-DQA10501-DQB10201	5	QVLLPLQGNISPSAE	LLPLQGNIS	2	0.1838	6844.3	70.00	0.33	tr_C9ZRP4_	
HLA-DQA10501-DQB10201	6	MKNKSSKQVLLPLQG	SKQVLLPLQ	5	0.1821	6971.3	70.00	0.37	tr_C9ZRP4_	
HLA-DQA10501-DQB10201	7	NKSSKQVLLPLQGNI	SKQVLLPLQ	3	0.1798	7147.5	70.00	0.31	tr_C9ZRP4_	

HLA-DQA10501-DQB10201	8	KQVLLPLQGNISPSA	LLPLQGNIS	3	0.1789	7213.1	70.00	0.36	tr_C9ZRP4_
HLA-DQA10501-DQB10201	9	KNKSSKQVLLPLQGN	SKQVLLPLQ	4	0.1764	7416.5	70.00	0.35	tr_C9ZRP4_
HLA-DQA10501-DQB10201	10	VLLPLQGNISPSAES	LLPLQGNIS	1	0.1675	8162.9	75.00	0.29	tr_C9ZRP4_
HLA-DQA10501-DQB10201	11	RNYKDLINRTVFSLI	INRTVFSLI	6	0.1664	8261.6	75.00	0.46	tr_C9ZRP4_
HLA-DQA10501-DQB10201	12	LLPLQGNISPSAESP	LQGNISPSA	3	0.1660	8301.4	75.00	0.22	tr_C9ZRP4_
HLA-DQA10501-DQB10201	13	LPLQGNISPSAESPR	LQGNISPSA	2	0.1388	11135.9	80.00	0.31	tr_C9ZRP4_
HLA-DQA10501-DQB10201	14	PLQGNISPSAESPRP	LQGNISPSA	1	0.1320	11987.5	85.00	0.27	tr_C9ZRP4_
HLA-DQA10501-DQB10201	15	TRNYKDLINRTVFSL	LINRTVFSL	6	0.1314	12058.4	85.00	0.38	tr_C9ZRP4_
HLA-DQA10501-DQB10201	16	LQGNISPSAESPRPS	QGNISPSAE	1	0.1209	13522.9	85.00	0.30	tr_C9ZRP4_
HLA-DQA10501-DQB10201	17	AESPRPSSLMTTEGR	PSSLMTTEG	5	0.1114	14983.5	90.00	0.29	tr_C9ZRP4_
HLA-DQA10501-DQB10201	18	RTRNYKDLINRTVFS	DLINRTVFS	6	0.1111	15026.5	90.00	0.29	tr_C9ZRP4_
HLA-DQA10501-DQB10201	19	ESPRPSSLMTTEGRT	PSSLMTTEG	4	0.1102	15169.3	90.00	0.25	tr_C9ZRP4_
HLA-DQA10501-DQB10201	20	GRTRNYKDLINRTVF	NYKDLINRT	4	0.1071	15694.9	90.00	0.29	tr_C9ZRP4_
HLA-DQA10501-DQB10201	21	SAESPRPSSLMTTEG	PSSLMTTEG	6	0.1068	15743.7	90.00	0.43	tr_C9ZRP4_
HLA-DQA10501-DQB10201	22	EGRTRNYKDLINRTV	NYKDLINRT	5	0.1039	16239.7	90.00	0.32	tr_C9ZRP4_
HLA-DQA10501-DQB10201	23	QGNISPSAESPRPSS	NISPSAESP	2	0.0973	17449.9	90.00	0.32	tr_C9ZRP4_
HLA-DQA10501-DQB10201	24	TEGRTRNYKDLINRT	NYKDLINRT	6	0.0960	17694.8	90.00	0.35	tr_C9ZRP4_
HLA-DQA10501-DQB10201	25	SPRPSSLMTTEGRTR	PSSLMTTEG	3	0.0958	17737.3	90.00	0.22	tr_C9ZRP4_
HLA-DQA10501-DQB10201	26	MTTEGRTRNYKDLIN	RTRNYKDLI	5	0.0891	19061.8	95.00	0.49	tr_C9ZRP4_
HLA-DQA10501-DQB10201	27	PRPSSLMTTEGRTRN	PSSLMTTEG	2	0.0885	19191.2	95.00	0.20	tr_C9ZRP4_
HLA-DQA10501-DQB10201	28	PSAESPRPSSLMTTE	RPSSLMTTE	6	0.0868	19545.1	95.00	0.41	tr_C9ZRP4_
HLA-DQA10501-DQB10201	29	GNISPSAESPRPSSL	NISPSAESP	1	0.0841	20130.4	95.00	0.34	tr_C9ZRP4_
HLA-DQA10501-DQB10201	30	RPSSLMTTEGRTRNY	SSLMTTEGR	2	0.0828	20415.1	95.00	0.26	tr_C9ZRP4_
HLA-DQA10501-DQB10201	31	TTEGRTRNYKDLINR	RTRNYKDLI	4	0.0808	20850.0	95.00	0.47	tr_C9ZRP4_
HLA-DQA10501-DQB10201	32	NISPSAESPRPSSLM	NISPSAESP	0	0.0755	22097.5	95.00	0.24	tr_C9ZRP4_
HLA-DQA10501-DQB10201	33	SPSAESPRPSSLMTT	PRPSSLMTT	6	0.0753	22135.8	95.00	0.40	tr_C9ZRP4_
HLA-DQA10501-DQB10201	34	LMTTEGRTRNYKDLI	RTRNYKDLI	6	0.0745	22333.3	95.00	0.67	tr_C9ZRP4_
HLA-DQA10501-DQB10201	35	PSSLMTTEGRTRNYK	SSLMTTEGR	1	0.0687	23766.9	95.00	0.37	tr_C9ZRP4_
HLA-DQA10501-DQB10201	36	ISPSAESPRPSSLMT	SPSAESPRP	1	0.0624	25445.4	100.00	0.27	tr_C9ZRP4_
HLA-DQA10501-DQB10201	37	SSLMTTEGRTRNYKD	SLMTTEGRT	1	0.0555	27416.3	100.00	0.36	tr_C9ZRP4_
HLA-DQA10501-DQB10201	38	SLMTTEGRTRNYKDL	SLMTTEGRT	0	0.0391	32735.5	100.00	0.32	tr_C9ZRP4_

Allele: HLA-DQA10501-DQB10201. Number of high binders 0. Number of weak binders 0. Number of peptides 38

Allele	pos	peptide	core	Of	1-log50k(aff)	affinity(nM)	%Rank	Relia	Identity	Bind Level
HLA-DQA10501-DQB10201	1	IMGFFTWVYLQSYVY	FTWVYLQSY	4	0.4709	306.4	7.50	0.41	tr_C9ZRP4_	WB
HLA-DQA10501-DQB10201	2	MGFFTWVYLQSYVYR	FTWVYLQSY	3	0.4440	410.0	11.00	0.39	tr_C9ZRP4_	
HLA-DQA10501-DQB10201	3	QGIMGFFTWVYLQSY	FTWVYLQSY	6	0.4427	415.8	11.00	0.57	tr_C9ZRP4_	
HLA-DQA10501-DQB10201	4	GFFTWVYLQSYVYRD	FTWVYLQSY	2	0.4393	431.1	11.00	0.36	tr_C9ZRP4_	
HLA-DQA10501-DQB10201	5	FFTWVYLQSYVYRDE	WVYLQSYVY	3	0.4338	457.4	12.00	0.28	tr_C9ZRP4_	
HLA-DQA10501-DQB10201	6	GIMGFFTWVYLQSYV	FTWVYLQSY	5	0.4285	484.6	12.00	0.56	tr_C9ZRP4_	
HLA-DQA10501-DQB10201	7	SWHTISSCALQLPEE	WHTISSCAL	1	0.4140	566.9	14.00	0.42	tr_C9ZRP4_	
HLA-DQA10501-DQB10201	8	WHTISSCALQLPEEQ	WHTISSCAL	0	0.4112	584.5	14.00	0.23	tr_C9ZRP4_	
HLA-DQA10501-DQB10201	9	PSWHTISSCALQLPE	WHTISSCAL	2	0.4039	632.6	15.00	0.53	tr_C9ZRP4_	
HLA-DQA10501-DQB10201	10	CPSWHTISSCALQLP	WHTISSCAL	3	0.4022	644.5	16.00	0.55	tr_C9ZRP4_	
HLA-DQA10501-DQB10201	11	FTWVYLQSYVYRDEN	WVYLQSYVY	2	0.3845	780.1	18.00	0.31	tr_C9ZRP4_	

HLA-DQA10501-DQB10201	12	NCPSWHTISSCALQL	WHTISSCAL	4	0.3805	814.6	19.00	0.57	tr_C9ZRP4_
HLA-DQA10501-DQB10201	13	DENCPSWHTISSCAL	WHTISSCAL	6	0.3578	1041.9	23.00	0.83	tr_C9ZRP4_
HLA-DQA10501-DQB10201	14	HTISSCALQLPEEQR	CALQLPEEQ	5	0.3548	1075.7	23.00	0.34	tr_C9ZRP4_
HLA-DQA10501-DQB10201	15	TWVYLQSYVYRDENC	WVYLQSYVY	1	0.3532	1094.5	24.00	0.34	tr_C9ZRP4_
HLA-DQA10501-DQB10201	16	WVYLQSYVYRDENCP	WVYLQSYVY	0	0.3443	1204.9	25.00	0.23	tr_C9ZRP4_
HLA-DQA10501-DQB10201	17	ENCPSWHTISSCALQ	WHTISSCAL	5	0.3394	1270.4	26.00	0.83	tr_C9ZRP4_
HLA-DQA10501-DQB10201	18	RAFKMKDFGDLIPGH	KMKDFGDLI	3	0.3319	1378.5	28.00	0.47	tr_C9ZRP4_
HLA-DQA10501-DQB10201	19	AFKMKDFGDLIPGHG	KMKDFGDLI	2	0.3286	1427.9	29.00	0.43	tr_C9ZRP4_
HLA-DQA10501-DQB10201	20	TISSCALQLPEEQRR	CALQLPEEQ	4	0.3276	1444.8	29.00	0.34	tr_C9ZRP4_
HLA-DQA10501-DQB10201	21	KRAFKMKDFGDLIPG	KMKDFGDLI	4	0.3269	1455.4	29.00	0.51	tr_C9ZRP4_
HLA-DQA10501-DQB10201	22	CQGIMGFFTWVYLQS	FFTWVYLQS	6	0.3131	1690.0	32.00	0.41	tr_C9ZRP4_
HLA-DQA10501-DQB10201	23	FKMKDFGDLIPGHGG	KMKDFGDLI	1	0.3074	1796.5	33.00	0.38	tr_C9ZRP4_
HLA-DQA10501-DQB10201	24	VYLQSYVYRDENCPS	SYVYRDENC	4	0.3007	1932.6	35.00	0.28	tr_C9ZRP4_
HLA-DQA10501-DQB10201	25	RMDCQGIMGFFTWVY	IMGFFTWVY	6	0.2991	1965.2	35.00	0.37	tr_C9ZRP4_
HLA-DQA10501-DQB10201	26	MDCQGIMGFFTWVYL	IMGFFTWVY	5	0.2975	2000.4	36.00	0.29	tr_C9ZRP4_
HLA-DQA10501-DQB10201	27	YLQSYVYRDENCPSW	SYVYRDENC	3	0.2928	2105.3	37.00	0.29	tr_C9ZRP4_
HLA-DQA10501-DQB10201	28	DCQGIMGFFTWVYLQ	IMGFFTWVY	4	0.2820	2366.4	40.00	0.25	tr_C9ZRP4_
HLA-DQA10501-DQB10201	29	ISSCALQLPEEQRRS	CALQLPEEQ	3	0.2809	2392.5	40.00	0.43	tr_C9ZRP4_
HLA-DQA10501-DQB10201	30	ITDRMDCQGIMGFFT	CQGIMGFFT	6	0.2738	2584.5	42.00	0.37	tr_C9ZRP4_
HLA-DQA10501-DQB10201	31	TDRMDCQGIMGFFTW	CQGIMGFFT	5	0.2700	2691.9	43.00	0.34	tr_C9ZRP4_
HLA-DQA10501-DQB10201	32	DRMDCQGIMGFFTWV	CQGIMGFFT	4	0.2677	2759.6	43.00	0.35	tr_C9ZRP4_
HLA-DQA10501-DQB10201	33	KMKDFGDLIPGHGGI	KMKDFGDLI	0	0.2610	2968.4	45.00	0.29	tr_C9ZRP4_
HLA-DQA10501-DQB10201	34	SSCALQLPEEQRRSL	CALQLPEEQ	2	0.2545	3186.2	47.00	0.51	tr_C9ZRP4_
HLA-DQA10501-DQB10201	35	LQSYVYRDENCPSWH	SYVYRDENC	2	0.2481	3414.5	49.00	0.32	tr_C9ZRP4_
HLA-DQA10501-DQB10201	36	GITDRMDCQGIMGFF	RMDCQGIMG	4	0.2348	3940.3	55.00	0.25	tr_C9ZRP4_
HLA-DQA10501-DQB10201	37	SCALQLPEEQRRSLL	CALQLPEEQ	1	0.2251	4379.1	55.00	0.53	tr_C9ZRP4_
HLA-DQA10501-DQB10201	38	GGITDRMDCQGIMGF	RMDCQGIMG	5	0.2204	4603.8	60.00	0.27	tr_C9ZRP4_
HLA-DQA10501-DQB10201	39	QSYVYRDENCPSWHT	SYVYRDENC	1	0.2164	4811.1	60.00	0.38	tr_C9ZRP4_
HLA-DQA10501-DQB10201	40	MKDFGDLIPGHGGIT	FGDLIPGHG	3	0.2109	5103.3	60.00	0.27	tr_C9ZRP4_
HLA-DQA10501-DQB10201	41	GHGGITDRMDCQGIM	DRMDCQGIM	6	0.2062	5368.9	65.00	0.24	tr_C9ZRP4_
HLA-DQA10501-DQB10201	42	HGGITDRMDCQGIMG	RMDCQGIMG	6	0.2055	5409.3	65.00	0.29	tr_C9ZRP4_
HLA-DQA10501-DQB10201	43	LIPGHGGITDRMDCQ	GHGGITDRM	3	0.2037	5520.9	65.00	0.38	tr_C9ZRP4_
HLA-DQA10501-DQB10201	44	IPGHGGITDRMDCQG	GHGGITDRM	2	0.1975	5902.1	65.00	0.32	tr_C9ZRP4_
HLA-DQA10501-DQB10201	45	GDLIPGHGGITDRMD	GHGGITDRM	5	0.1946	6091.7	65.00	0.54	tr_C9ZRP4_
HLA-DQA10501-DQB10201	46	PGHGGITDRMDCQGI	GHGGITDRM	1	0.1929	6204.6	65.00	0.25	tr_C9ZRP4_
HLA-DQA10501-DQB10201	47	DLIPGHGGITDRMDC	GHGGITDRM	4	0.1922	6252.2	65.00	0.47	tr_C9ZRP4_
HLA-DQA10501-DQB10201	48	SYVYRDENCPSWHTI	SYVYRDENC	0	0.1835	6867.5	70.00	0.33	tr_C9ZRP4_
HLA-DQA10501-DQB10201	49	KDFGDLIPGHGGITD	FGDLIPGHG	2	0.1694	7994.1	75.00	0.40	tr_C9ZRP4_
HLA-DQA10501-DQB10201	50	FGDLIPGHGGITDRM	GHGGITDRM	6	0.1625	8616.3	75.00	0.58	tr_C9ZRP4_
HLA-DQA10501-DQB10201	51	QLPEEQRRSLLSTLN	RRSLLSTLN	6	0.1594	8907.4	75.00	0.53	tr_C9ZRP4_
HLA-DQA10501-DQB10201	52	CALQLPEEQRRSLLS	CALQLPEEQ	0	0.1585	9000.8	75.00	0.52	tr_C9ZRP4_
HLA-DQA10501-DQB10201	53	LPEEQRRSLLSTLNR	RRSLLSTLN	5	0.1560	9247.5	75.00	0.41	tr_C9ZRP4_
HLA-DQA10501-DQB10201	54	PEEQRRSLLSTLNRS	RRSLLSTLN	4	0.1476	10126.4	80.00	0.38	tr_C9ZRP4_
HLA-DQA10501-DQB10201	55	DFGDLIPGHGGITDR	FGDLIPGHG	1	0.1475	10141.4	80.00	0.43	tr_C9ZRP4_
HLA-DQA10501-DQB10201	56	EEQRRSLLSTLNRSL	RRSLLSTLN	3	0.1471	10181.6	80.00	0.37	tr_C9ZRP4_
HLA-DQA10501-DQB10201	57	EQRRSLLSTLNRSLT	RRSLLSTLN	2	0.1368	11381.2	80.00	0.34	tr_C9ZRP4_
HLA-DQA10501-DQB10201	58	QRRSLLSTLNRSLTE	RRSLLSTLN	1	0.1339	11746.4	85.00	0.20	tr_C9ZRP4_
HLA-DQA10501-DQB10201	59	YVYRDENCPSWHTIS	VYRDENCPS	1	0.1278	12542.9	85.00	0.34	tr_C9ZRP4_
HLA-DQA10501-DQB10201	60	LQLPEEQRRSLLSTL	QRRSLLSTL	6	0.1158	14277.5	85.00	0.62	tr_C9ZRP4_
HLA-DQA10501-DQB10201	61	RDENCPSWHTISSCA	SWHTISSCA	6	0.1049	16069.8	90.00	0.55	tr_C9ZRP4_

HLA-DQA10501-DQB10201	62	ALQLPEEQRRSLLST	ALQLPEEQR	0	0.0873	19450.4	95.00	0.32	tr_C9ZRP4_	
HLA-DQA10501-DQB10201	63	VYRDENCPSWHTISS	YRDENCPSW	1	0.0800	21051.5	95.00	0.40	tr_C9ZRP4_	
HLA-DQA10501-DQB10201	64	YRDENCPSWHTISSC	PSWHTISSC	6	0.0783	21439.7	95.00	0.37	tr_C9ZRP4_	

Allele: HLA-DQA10501-DQB10201. Number of high binders 0. Number of weak binders 1. Number of peptides 64

Allele	pos	peptide	core	Of	1-log50k(aff)	affinity(nM)	%Rank	Relia	Identity	Bind Level
HLA-DQA10501-DQB10201	1	HHDDHTHGVVDDGID	THGVVDDGI	5	0.4610	341.0	8.50	0.76	tr_D0A888_	WB
HLA-DQA10501-DQB10201	2	DDHTHGVVDDGIDGD	THGVVDDGI	3	0.4575	354.1	9.00	0.69	tr_D0A888_	WB
HLA-DQA10501-DQB10201	3	HDDHTHGVVDDGIDG	THGVVDDGI	4	0.4399	428.4	11.00	0.74	tr_D0A888_	
HLA-DQA10501-DQB10201	4	HHHDDHTHGVVDDGI	THGVVDDGI	6	0.4373	440.6	11.00	0.81	tr_D0A888_	
HLA-DQA10501-DQB10201	5	DHTHGVVDDGIDGDT	THGVVDDGI	2	0.4347	453.1	12.00	0.67	tr_D0A888_	
HLA-DQA10501-DQB10201	6	HTHGVVDDGIDGDTH	THGVVDDGI	1	0.3966	684.7	16.00	0.67	tr_D0A888_	
HLA-DQA10501-DQB10201	7	THGVVDDGIDGDTHG	THGVVDDGI	0	0.3332	1359.1	28.00	0.57	tr_D0A888_	
HLA-DQA10501-DQB10201	8	HGVVDDGIDGDTHGG	VVDDGIDGD	2	0.2120	5045.6	60.00	0.49	tr_D0A888_	
HLA-DQA10501-DQB10201	9	GVVDDGIDGDTHGGC	VVDDGIDGD	1	0.1844	6799.4	70.00	0.58	tr_D0A888_	
HLA-DQA10501-DQB10201	10	HHHHDDHTHGVVDDG	HTHGVVDDG	6	0.1660	8299.8	75.00	0.59	tr_D0A888_	
HLA-DQA10501-DQB10201	11	VVDDGIDGDTHGGCE	VVDDGIDGD	0	0.1570	9144.7	75.00	0.47	tr_D0A888_	
HLA-DQA10501-DQB10201	12	HHHHHDDHTHGVVDD	DHTHGVVDD	6	0.1060	15880.4	90.00	0.65	tr_D0A888_	
HLA-DQA10501-DQB10201	13	VDDGIDGDTHGGCES	DDGIDGDTH	1	0.0908	18711.2	95.00	0.39	tr_D0A888_	
HLA-DQA10501-DQB10201	14	DDGIDGDTHGGCESG	DDGIDGDTH	0	0.0746	22295.9	95.00	0.33	tr_D0A888_	
HLA-DQA10501-DQB10201	15	SHHHHHDDHTHGVVD	DDHTHGVVD	6	0.0590	26400.0	100.00	0.34	tr_D0A888_	
HLA-DQA10501-DQB10201	16	DGIDGDTHGGCESGH	GDTHGGCES	4	0.0555	27425.5	100.00	0.19	tr_D0A888_	
HLA-DQA10501-DQB10201	17	MTRPPSHHHHHDDHT	HHHHHDDHT	6	0.0555	27441.2	100.00	0.47	tr_D0A888_	
HLA-DQA10501-DQB10201	18	TRPPSHHHHHDDHTH	HHHHHDDHT	5	0.0530	28185.9	100.00	0.44	tr_D0A888_	
HLA-DQA10501-DQB10201	19	PSHHHHHDDHTHGVV	HHHHHDDHT	2	0.0528	28248.9	100.00	0.27	tr_D0A888_	
HLA-DQA10501-DQB10201	20	PPSHHHHHDDHTHGV	HHHHHDDHT	3	0.0503	29026.2	100.00	0.33	tr_D0A888_	
HLA-DQA10501-DQB10201	21	RPPSHHHHHDDHTHG	HHHHHDDHT	4	0.0490	29432.9	100.00	0.34	tr_D0A888_	
HLA-DQA10501-DQB10201	22	GIDGDTHGGCESGHG	THGGCESGH	5	0.0472	30005.6	100.00	0.19	tr_D0A888_	
HLA-DQA10501-DQB10201	23	IDGDTHGGCESGHGT	THGGCESGH	4	0.0436	31181.5	100.00	0.20	tr_D0A888_	
HLA-DQA10501-DQB10201	24	HHHTHDMTRPPSHHH	HHTHDMTRP	1	0.0431	31350.7	100.00	0.32	tr_D0A888_	
HLA-DQA10501-DQB10201	25	DMTRPPSHHHHHDDH	SHHHHHDDH	6	0.0431	31372.1	100.00	0.60	tr_D0A888_	
HLA-DQA10501-DQB10201	26	DGDTHGGCESGHGTY	THGGCESGH	3	0.0396	32568.8	100.00	0.23	tr_D0A888_	
HLA-DQA10501-DQB10201	27	GDTHGGCESGHGTYS	THGGCESGH	2	0.0335	34799.9	100.00	0.25	tr_D0A888_	
HLA-DQA10501-DQB10201	28	HHTHDMTRPPSHHHH	THDMTRPPS	2	0.0330	34974.3	100.00	0.35	tr_D0A888_	
HLA-DQA10501-DQB10201	29	HDMTRPPSHHHHHDD	PSHHHHHDD	6	0.0271	37277.7	100.00	0.50	tr_D0A888_	
HLA-DQA10501-DQB10201	30	HTHDMTRPPSHHHHH	THDMTRPPS	1	0.0245	38345.4	100.00	0.41	tr_D0A888_	
HLA-DQA10501-DQB10201	31	THDMTRPPSHHHHHD	THDMTRPPS	0	0.0220	39387.9	100.00	0.34	tr_D0A888_	

Allele: HLA-DQA10501-DQB10201. Number of high binders 0. Number of weak binders 2. Number of peptides 31

Allele	pos	peptide	core	Of	1-log50k(aff)	affinity(nM)	%Rank	Relia	Identity	Bind Level

HLA-DQA10501-DQB10201	1	RVILLHVEEMSPDTL	VEEMSPDTL	6	0.6277	56.2	0.80	0.43	tr_D0A0U2_	WB
HLA-DQA10501-DQB10201	2	VIVLFAEFSFLYDLF	EFSFLYDLF	6	0.6272	56.5	0.80	0.53	tr_D0A0U2_	WB
HLA-DQA10501-DQB10201	3	VILLHVEEMSPDTLV	VEEMSPDTL	5	0.6155	64.1	1.00	0.44	tr_D0A0U2_	WB
HLA-DQA10501-DQB10201	4	IVLFAEFSFLYDLFA	EFSFLYDLF	5	0.6046	72.1	1.20	0.44	tr_D0A0U2_	WB
HLA-DQA10501-DQB10201	5	VLFAEFSFLYDLFAT	EFSFLYDLF	4	0.5775	96.7	1.80	0.44	tr_D0A0U2_	WB
HLA-DQA10501-DQB10201	6	LMRVILLHVEEMSPD	VILLHVEEM	3	0.5663	109.1	2.50	0.50	tr_D0A0U2_	WB
HLA-DQA10501-DQB10201	7	FAEFSFLYDLFATAL	EFSFLYDLF	2	0.5624	113.8	2.50	0.34	tr_D0A0U2_	WB
HLA-DQA10501-DQB10201	8	MRVILLHVEEMSPDT	VILLHVEEM	2	0.5579	119.5	2.50	0.49	tr_D0A0U2_	WB
HLA-DQA10501-DQB10201	9	LFAEFSFLYDLFATA	EFSFLYDLF	3	0.5567	121.0	2.50	0.41	tr_D0A0U2_	WB
HLA-DQA10501-DQB10201	10	RLMRVILLHVEEMSP	VILLHVEEM	4	0.5501	130.0	3.00	0.53	tr_D0A0U2_	WB
HLA-DQA10501-DQB10201	11	ILLHVEEMSPDTLVN	VEEMSPDTL	4	0.5430	140.5	3.50	0.60	tr_D0A0U2_	WB
HLA-DQA10501-DQB10201	12	VNFFYADSSIISDDM	FFYADSSII	2	0.5285	164.2	4.00	0.30	tr_D0A0U2_	WB
HLA-DQA10501-DQB10201	13	LVNFFYADSSIISDD	FFYADSSII	3	0.5285	164.3	4.00	0.34	tr_D0A0U2_	WB
HLA-DQA10501-DQB10201	14	TLVNFFYADSSIISD	FFYADSSII	4	0.5283	164.6	4.00	0.34	tr_D0A0U2_	WB
HLA-DQA10501-DQB10201	15	RVIVLFAEFSFLYDL	FAEFSFLYD	5	0.5203	179.5	4.50	0.37	tr_D0A0U2_	WB
HLA-DQA10501-DQB10201	16	LLHVEEMSPDTLVNF	VEEMSPDTL	3	0.5190	182.1	4.50	0.70	tr_D0A0U2_	WB
HLA-DQA10501-DQB10201	17	GRLMRVILLHVEEMS	VILLHVEEM	5	0.5168	186.4	4.50	0.56	tr_D0A0U2_	WB
HLA-DQA10501-DQB10201	18	ISGTVFVIGLSVPDV	VIGLSVPDV	6	0.5150	190.0	4.50	0.73	tr_D0A0U2_	WB
HLA-DQA10501-DQB10201	19	DTLVNFFYADSSIIS	FFYADSSII	5	0.5142	191.7	4.50	0.34	tr_D0A0U2_	WB
HLA-DQA10501-DQB10201	20	PDTLVNFFYADSSII	FFYADSSII	6	0.5138	192.7	4.50	0.37	tr_D0A0U2_	WB
HLA-DQA10501-DQB10201	21	LHVEEMSPDTLVNFF	VEEMSPDTL	2	0.5070	207.3	5.00	0.65	tr_D0A0U2_	WB
HLA-DQA10501-DQB10201	22	AEFSFLYDLFATALN	EFSFLYDLF	1	0.5050	211.8	5.00	0.34	tr_D0A0U2_	WB
HLA-DQA10501-DQB10201	23	LRVIVLFAEFSFLYD	FAEFSFLYD	6	0.5049	212.1	5.00	0.41	tr_D0A0U2_	WB
HLA-DQA10501-DQB10201	24	GTVFVIGLSVPDVKE	VIGLSVPDV	4	0.5043	213.5	5.50	0.62	tr_D0A0U2_	WB
HLA-DQA10501-DQB10201	25	TVFVIGLSVPDVKEI	VIGLSVPDV	3	0.5031	216.2	5.50	0.62	tr_D0A0U2_	WB
HLA-DQA10501-DQB10201	26	SGTVFVIGLSVPDVK	VIGLSVPDV	5	0.5022	218.3	5.50	0.64	tr_D0A0U2_	WB
HLA-DQA10501-DQB10201	27	HVEEMSPDTLVNFFY	VEEMSPDTL	1	0.4938	239.2	6.00	0.62	tr_D0A0U2_	WB
HLA-DQA10501-DQB10201	28	TGRLMRVILLHVEEM	VILLHVEEM	6	0.4922	243.3	6.00	0.73	tr_D0A0U2_	WB
HLA-DQA10501-DQB10201	29	VFVIGLSVPDVKEIA	VIGLSVPDV	2	0.4910	246.4	6.00	0.62	tr_D0A0U2_	WB
HLA-DQA10501-DQB10201	30	NFFYADSSIISDDMR	FFYADSSII	1	0.4904	248.1	6.00	0.30	tr_D0A0U2_	WB
HLA-DQA10501-DQB10201	31	FFYADSSIISDDMRY	DSSIISDDM	4	0.4692	312.1	8.00	0.25	tr_D0A0U2_	WB
HLA-DQA10501-DQB10201	32	EFSFLYDLFATALNN	LYDLFATAL	4	0.4681	315.9	8.00	0.38	tr_D0A0U2_	WB
HLA-DQA10501-DQB10201	33	FVIGLSVPDVKEIAR	VIGLSVPDV	1	0.4629	334.2	8.50	0.63	tr_D0A0U2_	WB
HLA-DQA10501-DQB10201	34	SPDTLVNFFYADSSI	VNFFYADSS	5	0.4516	377.3	9.50	0.49	tr_D0A0U2_	WB
HLA-DQA10501-DQB10201	35	DLRVIVLFAEFSFLY	VIVLFAEFS	3	0.4253	501.8	13.00	0.36	tr_D0A0U2_	
HLA-DQA10501-DQB10201	36	VEEMSPDTLVNFFYA	VEEMSPDTL	0	0.4247	504.8	13.00	0.56	tr_D0A0U2_	
HLA-DQA10501-DQB10201	37	FSFLYDLFATALNNT	LYDLFATAL	3	0.4153	559.2	14.00	0.46	tr_D0A0U2_	
HLA-DQA10501-DQB10201	38	MSPDTLVNFFYADSS	VNFFYADSS	6	0.4072	610.6	15.00	0.47	tr_D0A0U2_	
HLA-DQA10501-DQB10201	39	SDDMRYGVFDDTKCD	MRYGVFDDT	3	0.4051	624.3	15.00	0.48	tr_D0A0U2_	
HLA-DQA10501-DQB10201	40	SIISDDMRYGVFDDT	MRYGVFDDT	6	0.4045	628.5	15.00	0.64	tr_D0A0U2_	
HLA-DQA10501-DQB10201	41	ISDDMRYGVFDDTKC	MRYGVFDDT	4	0.3993	664.8	16.00	0.47	tr_D0A0U2_	
HLA-DQA10501-DQB10201	42	NDLRVIVLFAEFSFL	VIVLFAEFS	4	0.3970	681.4	16.00	0.43	tr_D0A0U2_	
HLA-DQA10501-DQB10201	43	IISDDMRYGVFDDTK	MRYGVFDDT	5	0.3960	688.7	17.00	0.50	tr_D0A0U2_	
HLA-DQA10501-DQB10201	44	FYADSSIISDDMRYG	DSSIISDDM	3	0.3934	708.7	17.00	0.38	tr_D0A0U2_	
HLA-DQA10501-DQB10201	45	RNDLRVIVLFAEFSF	VIVLFAEFS	5	0.3926	714.5	17.00	0.42	tr_D0A0U2_	
HLA-DQA10501-DQB10201	46	DDMRYGVFDDTKCDT	MRYGVFDDT	2	0.3917	722.1	17.00	0.46	tr_D0A0U2_	
HLA-DQA10501-DQB10201	47	SFLYDLFATALNNTA	LYDLFATAL	2	0.3818	803.8	19.00	0.43	tr_D0A0U2_	
HLA-DQA10501-DQB10201	48	AGAARLVFATSLPHW	ARLVFATSL	3	0.3815	805.9	19.00	0.36	tr_D0A0U2_	
HLA-DQA10501-DQB10201	49	VIGLSVPDVKEIARK	VIGLSVPDV	0	0.3806	814.1	19.00	0.61	tr_D0A0U2_	
HLA-DQA10501-DQB10201	50	GGASVAFDSNTPQLI	FDSNTPQLI	6	0.3791	827.5	19.00	0.55	tr_D0A0U2_	

HLA-DQA10501-DQB10201	51	ERNDLRVIVLFAEFS	VIVLFAEFS	6	0.3711	901.9	20.00	0.48	tr_D0A0U2_
HLA-DQA10501-DQB10201	52	GAARLVFATSLPHWG	ARLVFATSL	2	0.3603	1013.5	22.00	0.37	tr_D0A0U2_
HLA-DQA10501-DQB10201	53	GASVAFDSNTPQLIR	FDSNTPQLI	5	0.3594	1023.4	22.00	0.56	tr_D0A0U2_
HLA-DQA10501-DQB10201	54	DMRYGVFDDTKCDTA	MRYGVFDDT	1	0.3572	1048.6	23.00	0.45	tr_D0A0U2_
HLA-DQA10501-DQB10201	55	FLYDLFATALNNTAG	LYDLFATAL	1	0.3560	1061.8	23.00	0.41	tr_D0A0U2_
HLA-DQA10501-DQB10201	56	AARLVFATSLPHWGD	VFATSLPHW	4	0.3554	1069.4	23.00	0.28	tr_D0A0U2_
HLA-DQA10501-DQB10201	57	TSSKTAQLFHDVEKD	TAQLFHDVE	4	0.3518	1111.8	24.00	0.60	tr_D0A0U2_
HLA-DQA10501-DQB10201	58	NTAGAARLVFATSLP	ARLVFATSL	5	0.3514	1116.2	24.00	0.51	tr_D0A0U2_
HLA-DQA10501-DQB10201	59	SSKTAQLFHDVEKDS	TAQLFHDVE	3	0.3457	1187.1	25.00	0.56	tr_D0A0U2_
HLA-DQA10501-DQB10201	60	TAGAARLVFATSLPH	ARLVFATSL	4	0.3425	1229.7	26.00	0.45	tr_D0A0U2_
HLA-DQA10501-DQB10201	61	YADSSIISDDMRYGV	DSSIISDDM	2	0.3387	1280.0	26.00	0.44	tr_D0A0U2_
HLA-DQA10501-DQB10201	62	PARLIGGASVAFDSN	IGGASVAFD	4	0.3378	1293.6	27.00	0.48	tr_D0A0U2_
HLA-DQA10501-DQB10201	63	SKTAQLFHDVEKDSR	TAQLFHDVE	2	0.3349	1333.8	27.00	0.54	tr_D0A0U2_
HLA-DQA10501-DQB10201	64	ARLIGGASVAFDSNT	IGGASVAFD	3	0.3271	1451.4	29.00	0.43	tr_D0A0U2_
HLA-DQA10501-DQB10201	65	ARLVFATSLPHWGDT	VFATSLPHW	3	0.3258	1472.6	29.00	0.34	tr_D0A0U2_
HLA-DQA10501-DQB10201	66	KTAQLFHDVEKDSRL	TAQLFHDVE	1	0.3254	1478.5	29.00	0.50	tr_D0A0U2_
HLA-DQA10501-DQB10201	67	TETSSKTAQLFHDVE	TAQLFHDVE	6	0.3254	1479.0	29.00	0.75	tr_D0A0U2_
HLA-DQA10501-DQB10201	68	ASVAFDSNTPQLIRK	FDSNTPQLI	4	0.3244	1494.5	30.00	0.56	tr_D0A0U2_
HLA-DQA10501-DQB10201	69	EERNDLRVIVLFAEF	RVIVLFAEF	6	0.3220	1533.9	30.00	0.34	tr_D0A0U2_
HLA-DQA10501-DQB10201	70	EMSPDTLVNFFYADS	LVNFFYADS	6	0.3168	1622.3	31.00	0.26	tr_D0A0U2_
HLA-DQA10501-DQB10201	71	RLIGGASVAFDSNTP	IGGASVAFD	2	0.3151	1654.1	32.00	0.41	tr_D0A0U2_
HLA-DQA10501-DQB10201	72	NNTAGAARLVFATSL	ARLVFATSL	6	0.3146	1661.6	32.00	0.57	tr_D0A0U2_
HLA-DQA10501-DQB10201	73	LYDLFATALNNTAGA	LYDLFATAL	0	0.3050	1844.8	34.00	0.34	tr_D0A0U2_
HLA-DQA10501-DQB10201	74	EEMSPDTLVNFFYAD	SPDTLVNFF	3	0.3030	1885.3	34.00	0.35	tr_D0A0U2_
HLA-DQA10501-DQB10201	75	ADSSIISDDMRYGVF	DSSIISDDM	1	0.3017	1910.2	35.00	0.39	tr_D0A0U2_
HLA-DQA10501-DQB10201	76	ATGRLMRVILLHVEE	RVILLHVEE	6	0.3005	1935.8	35.00	0.56	tr_D0A0U2_
HLA-DQA10501-DQB10201	77	SVAFDSNTPQLIRKG	FDSNTPQLI	3	0.3005	1936.5	35.00	0.57	tr_D0A0U2_
HLA-DQA10501-DQB10201	78	EKDSRLWTPLSLLAF	WTPLSLLAF	6	0.3004	1938.2	35.00	0.36	tr_D0A0U2_
HLA-DQA10501-DQB10201	79	ETSSKTAQLFHDVEK	TAQLFHDVE	5	0.3001	1944.3	35.00	0.73	tr_D0A0U2_
HLA-DQA10501-DQB10201	80	LIGGASVAFDSNTPQ	IGGASVAFD	1	0.2992	1962.9	35.00	0.37	tr_D0A0U2_
HLA-DQA10501-DQB10201	81	TAQLFHDVEKDSRLW	TAQLFHDVE	0	0.2987	1974.5	35.00	0.35	tr_D0A0U2_
HLA-DQA10501-DQB10201	82	PISGTVFVIGLSVPD	FVIGLSVPD	6	0.2976	1997.3	36.00	0.51	tr_D0A0U2_
HLA-DQA10501-DQB10201	83	MRYGVFDDTKCDTAE	MRYGVFDDT	0	0.2969	2012.8	36.00	0.38	tr_D0A0U2_
HLA-DQA10501-DQB10201	84	KDSRLWTPLSLLAFA	WTPLSLLAF	5	0.2922	2118.9	37.00	0.34	tr_D0A0U2_
HLA-DQA10501-DQB10201	85	IGGASVAFDSNTPQL	AFDSNTPQL	6	0.2903	2163.1	38.00	0.28	tr_D0A0U2_
HLA-DQA10501-DQB10201	86	RLVFATSLPHWGDTE	VFATSLPHW	2	0.2844	2303.9	39.00	0.41	tr_D0A0U2_
HLA-DQA10501-DQB10201	87	DSSIISDDMRYGVFD	SIISDDMRY	2	0.2840	2315.7	39.00	0.28	tr_D0A0U2_
HLA-DQA10501-DQB10201	88	DSRLWTPLSLLAFAT	WTPLSLLAF	4	0.2823	2357.0	40.00	0.29	tr_D0A0U2_
HLA-DQA10501-DQB10201	89	LEERNDLRVIVLFAE	RNDLRVIVL	3	0.2821	2361.5	40.00	0.34	tr_D0A0U2_
HLA-DQA10501-DQB10201	90	KLEERNDLRVIVLFA	RNDLRVIVL	4	0.2751	2548.9	41.00	0.43	tr_D0A0U2_
HLA-DQA10501-DQB10201	91	VAFDSNTPQLIRKGG	FDSNTPQLI	2	0.2740	2580.1	42.00	0.65	tr_D0A0U2_
HLA-DQA10501-DQB10201	92	SSIISDDMRYGVFDD	DMRYGVFDD	6	0.2727	2615.6	42.00	0.31	tr_D0A0U2_
HLA-DQA10501-DQB10201	93	RKLEERNDLRVIVLF	RNDLRVIVL	5	0.2611	2966.0	45.00	0.49	tr_D0A0U2_
HLA-DQA10501-DQB10201	94	LLAFATGRLMRVILL	FATGRLMRV	3	0.2593	3024.2	46.00	0.28	tr_D0A0U2_
HLA-DQA10501-DQB10201	95	SLLAFATGRLMRVIL	FATGRLMRV	4	0.2582	3061.0	46.00	0.29	tr_D0A0U2_
HLA-DQA10501-DQB10201	96	ARKLEERNDLRVIVL	RNDLRVIVL	6	0.2554	3155.5	47.00	0.56	tr_D0A0U2_
HLA-DQA10501-DQB10201	97	LWTPLSLLAFATGRL	LLAFATGRL	6	0.2552	3159.6	47.00	0.44	tr_D0A0U2_
HLA-DQA10501-DQB10201	98	WTPLSLLAFATGRLM	LLAFATGRL	5	0.2521	3268.9	48.00	0.36	tr_D0A0U2_
HLA-DQA10501-DQB10201	99	LVFATSLPHWGDTET	VFATSLPHW	1	0.2496	3356.8	48.00	0.49	tr_D0A0U2_
HLA-DQA10501-DQB10201	100	SRLWTPLSLLAFATG	WTPLSLLAF	3	0.2482	3411.2	49.00	0.32	tr_D0A0U2_

HLA-DQA10501-DQB10201	101	GKYLPISGTVFVIGL	SGTVFVIGL	6	0.2450	3528.1	50.00	0.31	tr_D0A0U2_
HLA-DQA10501-DQB10201	102	AQLFHDVEKDSRLWT	QLFHDVEKD	1	0.2413	3673.9	55.00	0.35	tr_D0A0U2_
HLA-DQA10501-DQB10201	103	PLSLLAFATGRLMRV	FATGRLMRV	6	0.2412	3679.7	55.00	0.34	tr_D0A0U2_
HLA-DQA10501-DQB10201	104	KGGAVTTIREDGEGV	VTTIREDGE	4	0.2388	3772.5	55.00	0.41	tr_D0A0U2_
HLA-DQA10501-DQB10201	105	VEKDSRLWTPLSLLA	SRLWTPLSL	4	0.2383	3794.5	55.00	0.47	tr_D0A0U2_
HLA-DQA10501-DQB10201	106	LSLLAFATGRLMRVI	FATGRLMRV	5	0.2380	3806.0	55.00	0.32	tr_D0A0U2_
HLA-DQA10501-DQB10201	107	YDLFATALNNTAGAA	FATALNNTA	3	0.2379	3810.4	55.00	0.33	tr_D0A0U2_
HLA-DQA10501-DQB10201	108	TALNNTAGAARLVFA	AGAARLVFA	6	0.2374	3830.8	55.00	0.39	tr_D0A0U2_
HLA-DQA10501-DQB10201	109	LAFATGRLMRVILLH	FATGRLMRV	2	0.2335	3995.9	55.00	0.34	tr_D0A0U2_
HLA-DQA10501-DQB10201	110	DVEKDSRLWTPLSLL	SRLWTPLSL	5	0.2329	4024.0	55.00	0.52	tr_D0A0U2_
HLA-DQA10501-DQB10201	111	AFDSNTPQLIRKGGA	FDSNTPQLI	1	0.2323	4049.1	55.00	0.73	tr_D0A0U2_
HLA-DQA10501-DQB10201	112	GGAVTTIREDGEGVG	VTTIREDGE	3	0.2317	4074.2	55.00	0.38	tr_D0A0U2_
HLA-DQA10501-DQB10201	113	VGKYLPISGTVFVIG	YLPISGTVF	3	0.2307	4122.4	55.00	0.29	tr_D0A0U2_
HLA-DQA10501-DQB10201	114	FATGRLMRVILLHVE	MRVILLHVE	6	0.2297	4165.9	55.00	0.25	tr_D0A0U2_
HLA-DQA10501-DQB10201	115	AFATGRLMRVILLHV	FATGRLMRV	1	0.2286	4213.1	55.00	0.28	tr_D0A0U2_
HLA-DQA10501-DQB10201	116	KYLPISGTVFVIGLS	SGTVFVIGL	5	0.2257	4347.6	55.00	0.27	tr_D0A0U2_
HLA-DQA10501-DQB10201	117	RLWTPLSLLAFATGR	WTPLSLLAF	2	0.2240	4432.0	60.00	0.35	tr_D0A0U2_
HLA-DQA10501-DQB10201	118	YLPISGTVFVIGLSV	SGTVFVIGL	4	0.2239	4435.4	60.00	0.22	tr_D0A0U2_
HLA-DQA10501-DQB10201	119	VFATSLPHWGDTETS	VFATSLPHW	0	0.2220	4527.1	60.00	0.39	tr_D0A0U2_
HLA-DQA10501-DQB10201	120	ALNNTAGAARLVFAT	AGAARLVFA	5	0.2216	4548.5	60.00	0.38	tr_D0A0U2_
HLA-DQA10501-DQB10201	121	GVGKYLPISGTVFVI	YLPISGTVF	4	0.2209	4580.2	60.00	0.36	tr_D0A0U2_
HLA-DQA10501-DQB10201	122	IRKGGAVTTIREDGE	VTTIREDGE	6	0.2180	4726.3	60.00	0.41	tr_D0A0U2_
HLA-DQA10501-DQB10201	123	LNNTAGAARLVFATS	AGAARLVFA	4	0.2162	4822.1	60.00	0.34	tr_D0A0U2_
HLA-DQA10501-DQB10201	124	GEGVGKYLPISGTVF	YLPISGTVF	6	0.2156	4851.6	60.00	0.49	tr_D0A0U2_
HLA-DQA10501-DQB10201	125	TPLSLLAFATGRLMR	LLAFATGRL	4	0.2153	4865.8	60.00	0.38	tr_D0A0U2_
HLA-DQA10501-DQB10201	126	QLFHDVEKDSRLWTP	QLFHDVEKD	0	0.2141	4933.2	60.00	0.29	tr_D0A0U2_
HLA-DQA10501-DQB10201	127	LPISGTVFVIGLSVP	SGTVFVIGL	3	0.2132	4980.9	60.00	0.26	tr_D0A0U2_
HLA-DQA10501-DQB10201	128	HDVEKDSRLWTPLSL	SRLWTPLSL	6	0.2126	5010.2	60.00	0.66	tr_D0A0U2_
HLA-DQA10501-DQB10201	129	GAVTTIREDGEGVGK	VTTIREDGE	2	0.2110	5100.8	60.00	0.41	tr_D0A0U2_
HLA-DQA10501-DQB10201	130	EGVGKYLPISGTVFV	YLPISGTVF	5	0.2087	5228.1	60.00	0.41	tr_D0A0U2_
HLA-DQA10501-DQB10201	131	ATALNNTAGAARLVF	TAGAARLVF	6	0.2078	5279.7	60.00	0.52	tr_D0A0U2_
HLA-DQA10501-DQB10201	132	RKGGAVTTIREDGEG	VTTIREDGE	5	0.2072	5314.7	60.00	0.45	tr_D0A0U2_
HLA-DQA10501-DQB10201	133	RYGVFDDTKCDTAEK	RYGVFDDTK	0	0.2054	5415.9	65.00	0.40	tr_D0A0U2_
HLA-DQA10501-DQB10201	134	AVTTIREDGEGVGKY	VTTIREDGE	1	0.1998	5757.6	65.00	0.40	tr_D0A0U2_
HLA-DQA10501-DQB10201	135	DLFATALNNTAGAAR	FATALNNTA	2	0.1963	5977.6	65.00	0.43	tr_D0A0U2_
HLA-DQA10501-DQB10201	136	YGVFDDTKCDTAEKL	TKCDTAEKL	6	0.1927	6213.0	65.00	0.41	tr_D0A0U2_
HLA-DQA10501-DQB10201	137	IGLSVPDVKEIARKL	IGLSVPDVK	0	0.1876	6564.9	70.00	0.54	tr_D0A0U2_
HLA-DQA10501-DQB10201	138	QLIRKGGAVTTIRED	GGAVTTIRE	5	0.1874	6579.7	70.00	0.48	tr_D0A0U2_
HLA-DQA10501-DQB10201	139	VTTIREDGEGVGKYL	TIREDGEGV	2	0.1854	6726.3	70.00	0.35	tr_D0A0U2_
HLA-DQA10501-DQB10201	140	LIRKGGAVTTIREDG	GGAVTTIRE	4	0.1799	7134.9	70.00	0.41	tr_D0A0U2_
HLA-DQA10501-DQB10201	141	GVFDDTKCDTAEKLP	TKCDTAEKL	5	0.1793	7188.8	70.00	0.54	tr_D0A0U2_
HLA-DQA10501-DQB10201	142	LPKDDCASNYGATQV	ASNYGATQV	6	0.1791	7201.2	70.00	0.78	tr_D0A0U2_
HLA-DQA10501-DQB10201	143	PKDDCASNYGATQVS	ASNYGATQV	5	0.1770	7363.5	70.00	0.68	tr_D0A0U2_
HLA-DQA10501-DQB10201	144	LFATALNNTAGAARL	FATALNNTA	1	0.1765	7407.0	70.00	0.34	tr_D0A0U2_
HLA-DQA10501-DQB10201	145	PQLIRKGGAVTTIRE	GGAVTTIRE	6	0.1731	7687.9	70.00	0.54	tr_D0A0U2_
HLA-DQA10501-DQB10201	146	LFHDVEKDSRLWTPL	FHDVEKDSR	1	0.1639	8491.6	75.00	0.29	tr_D0A0U2_
HLA-DQA10501-DQB10201	147	IARKLEERNDLRVIV	ERNDLRVIV	6	0.1611	8751.7	75.00	0.37	tr_D0A0U2_
HLA-DQA10501-DQB10201	148	FDSNTPQLIRKGGAV	FDSNTPQLI	0	0.1564	9210.0	75.00	0.82	tr_D0A0U2_
HLA-DQA10501-DQB10201	149	TTIREDGEGVGKYLP	TIREDGEGV	1	0.1561	9235.4	75.00	0.41	tr_D0A0U2_
HLA-DQA10501-DQB10201	150	VFDDTKCDTAEKLPK	TKCDTAEKL	4	0.1516	9692.8	80.00	0.56	tr_D0A0U2_

HLA-DQA10501-DQB10201	151	FATALNNTAGAARLV	NNTAGAARL	5	0.1510	9757.3	80.00	0.28	tr_D0A0U2_	
HLA-DQA10501-DQB10201	152	FATSLPHWGDTETSS	PHWGDTETS	5	0.1508	9783.3	80.00	0.29	tr_D0A0U2_	
HLA-DQA10501-DQB10201	153	TIREDGEGVGKYLPI	TIREDGEGV	0	0.1498	9888.9	80.00	0.30	tr_D0A0U2_	
HLA-DQA10501-DQB10201	154	FHDVEKDSRLWTPLS	FHDVEKDSR	0	0.1452	10386.0	80.00	0.23	tr_D0A0U2_	
HLA-DQA10501-DQB10201	155	FDDTKCDTAEKLPKD	TKCDTAEKL	3	0.1448	10435.8	80.00	0.71	tr_D0A0U2_	
HLA-DQA10501-DQB10201	156	GDTETSSKTAQLFHD	TSSKTAQLF	4	0.1442	10505.5	80.00	0.56	tr_D0A0U2_	
HLA-DQA10501-DQB10201	157	DTETSSKTAQLFHDV	TSSKTAQLF	3	0.1438	10552.3	80.00	0.46	tr_D0A0U2_	
HLA-DQA10501-DQB10201	158	DGEGVGKYLPISGTV	KYLPISGTV	6	0.1428	10669.3	80.00	0.30	tr_D0A0U2_	
HLA-DQA10501-DQB10201	159	HWGDTETSSKTAQLF	TSSKTAQLF	6	0.1415	10820.3	80.00	0.73	tr_D0A0U2_	
HLA-DQA10501-DQB10201	160	DVKEIARKLEERNDL	EIARKLEER	3	0.1372	11329.6	80.00	0.34	tr_D0A0U2_	
HLA-DQA10501-DQB10201	161	KEIARKLEERNDLRV	LEERNDLRV	6	0.1340	11726.8	85.00	0.31	tr_D0A0U2_	
HLA-DQA10501-DQB10201	162	EIARKLEERNDLRVI	LEERNDLRV	5	0.1318	12007.6	85.00	0.29	tr_D0A0U2_	
HLA-DQA10501-DQB10201	163	SVPDVKEIARKLEER	EIARKLEER	6	0.1283	12481.9	85.00	0.39	tr_D0A0U2_	
HLA-DQA10501-DQB10201	164	WGDTETSSKTAQLFH	TSSKTAQLF	5	0.1262	12765.8	85.00	0.68	tr_D0A0U2_	
HLA-DQA10501-DQB10201	165	DDTKCDTAEKLPKDD	TKCDTAEKL	2	0.1262	12767.6	85.00	0.68	tr_D0A0U2_	
HLA-DQA10501-DQB10201	166	REDGEGVGKYLPISG	VGKYLPISG	6	0.1254	12874.3	85.00	0.37	tr_D0A0U2_	
HLA-DQA10501-DQB10201	167	PDVKEIARKLEERND	EIARKLEER	4	0.1248	12957.3	85.00	0.41	tr_D0A0U2_	
HLA-DQA10501-DQB10201	168	VKEIARKLEERNDLR	EIARKLEER	2	0.1235	13138.4	85.00	0.31	tr_D0A0U2_	
HLA-DQA10501-DQB10201	169	ATSLPHWGDTETSSK	PHWGDTETS	4	0.1219	13372.9	85.00	0.25	tr_D0A0U2_	
HLA-DQA10501-DQB10201	170	EDGEGVGKYLPISGT	VGKYLPISG	5	0.1216	13411.8	85.00	0.34	tr_D0A0U2_	
HLA-DQA10501-DQB10201	171	VPDVKEIARKLEERN	EIARKLEER	5	0.1205	13581.3	85.00	0.40	tr_D0A0U2_	
HLA-DQA10501-DQB10201	172	NTPQLIRKGGAVTTI	RKGGAVTTI	6	0.1181	13933.2	85.00	0.55	tr_D0A0U2_	
HLA-DQA10501-DQB10201	173	TPQLIRKGGAVTTIR	RKGGAVTTI	5	0.1157	14301.1	85.00	0.40	tr_D0A0U2_	
HLA-DQA10501-DQB10201	174	LSVPDVKEIARKLEE	KEIARKLEE	6	0.1145	14479.4	85.00	0.31	tr_D0A0U2_	
HLA-DQA10501-DQB10201	175	TSLPHWGDTETSSKT	PHWGDTETS	3	0.1125	14807.5	90.00	0.26	tr_D0A0U2_	
HLA-DQA10501-DQB10201	176	IREDGEGVGKYLPIS	DGEGVGKYL	3	0.1115	14971.5	90.00	0.24	tr_D0A0U2_	
HLA-DQA10501-DQB10201	177	DTKCDTAEKLPKDDC	TKCDTAEKL	1	0.1089	15382.5	90.00	0.62	tr_D0A0U2_	
HLA-DQA10501-DQB10201	178	GLSVPDVKEIARKLE	VKEIARKLE	6	0.1089	15392.8	90.00	0.16	tr_D0A0U2_	
HLA-DQA10501-DQB10201	179	SLPHWGDTETSSKTA	PHWGDTETS	2	0.1009	16781.5	90.00	0.28	tr_D0A0U2_	
HLA-DQA10501-DQB10201	180	TKCDTAEKLPKDDCA	TKCDTAEKL	0	0.0841	20135.4	95.00	0.47	tr_D0A0U2_	
HLA-DQA10501-DQB10201	181	LPHWGDTETSSKTAQ	PHWGDTETS	1	0.0784	21411.7	95.00	0.34	tr_D0A0U2_	
HLA-DQA10501-DQB10201	182	PHWGDTETSSKTAQL	ETSSKTAQL	6	0.0766	21828.0	95.00	0.45	tr_D0A0U2_	
HLA-DQA10501-DQB10201	183	DTAEKLPKDDCASNY	PKDDCASNY	6	0.0711	23172.0	95.00	0.38	tr_D0A0U2_	
HLA-DQA10501-DQB10201	184	SNTPQLIRKGGAVTT	IRKGGAVTT	6	0.0694	23603.1	95.00	0.48	tr_D0A0U2_	
HLA-DQA10501-DQB10201	185	KLPKDDCASNYGATQ	CASNYGATQ	6	0.0646	24849.5	95.00	0.37	tr_D0A0U2_	
HLA-DQA10501-DQB10201	186	TAEKLPKDDCASNYG	PKDDCASNY	5	0.0638	25078.8	95.00	0.38	tr_D0A0U2_	
HLA-DQA10501-DQB10201	187	AEKLPKDDCASNYGA	PKDDCASNY	4	0.0610	25844.9	100.00	0.41	tr_D0A0U2_	
HLA-DQA10501-DQB10201	188	EKLPKDDCASNYGAT	PKDDCASNY	3	0.0548	27648.6	100.00	0.46	tr_D0A0U2_	
HLA-DQA10501-DQB10201	189	KCDTAEKLPKDDCAS	EKLPKDDCA	5	0.0535	28013.0	100.00	0.26	tr_D0A0U2_	
HLA-DQA10501-DQB10201	190	CDTAEKLPKDDCASN	EKLPKDDCA	4	0.0487	29510.7	100.00	0.27	tr_D0A0U2_	
HLA-DQA10501-DQB10201	191	DSNTPQLIRKGGAVT	LIRKGGAVT	6	0.0458	30477.1	100.00	0.49	tr_D0A0U2_	

Allele: HLA-DQA10501-DQB10201. Number of high binders 0. Number of weak binders 34. Number of peptides 191

Allele	pos	peptide	core	Of	1-log50k(aff)	affinity(nM)	%Rank	Relia	Identity	Bind Level
HLA-DQA10501-DQB10201	1	HSQCCGFVCALPVPR	FVCALPVPR	6	0.3022	1901.5	35.00	0.37	tr_C9ZWK4_	

HLA-DQA10501-DQB10201	2	SQCCGFVCALPVPRN	FVCALPVPR	5	0.2962	2028.2	36.00	0.38	tr_C9ZWK4_
HLA-DQA10501-DQB10201	3	QCCGFVCALPVPRNS	FVCALPVPR	4	0.2834	2329.3	39.00	0.43	tr_C9ZWK4_
HLA-DQA10501-DQB10201	4	CCGFVCALPVPRNSP	FVCALPVPR	3	0.2728	2613.3	42.00	0.50	tr_C9ZWK4_
HLA-DQA10501-DQB10201	5	AHSQCCGFVCALPVP	CGFVCALPV	5	0.2648	2850.5	44.00	0.41	tr_C9ZWK4_
HLA-DQA10501-DQB10201	6	CGFVCALPVPRNSPL	FVCALPVPR	2	0.2601	2997.3	45.00	0.55	tr_C9ZWK4_
HLA-DQA10501-DQB10201	7	GFVCALPVPRNSPLF	FVCALPVPR	1	0.2401	3723.8	55.00	0.56	tr_C9ZWK4_
HLA-DQA10501-DQB10201	8	GAHSQCCGFVCALPV	CGFVCALPV	6	0.2326	4038.3	55.00	0.49	tr_C9ZWK4_
HLA-DQA10501-DQB10201	9	FVCALPVPRNSPLFQ	FVCALPVPR	0	0.2232	4470.8	60.00	0.44	tr_C9ZWK4_
HLA-DQA10501-DQB10201	10	MNGIVPVCSGMSEKI	MNGIVPVCS	0	0.2104	5134.7	60.00	0.48	tr_C9ZWK4_
HLA-DQA10501-DQB10201	11	LPVPRNSPLFQHLSF	SPLFQHLSF	6	0.1859	6693.4	70.00	0.31	tr_C9ZWK4_
HLA-DQA10501-DQB10201	12	EKINKKRMNATDVHS	RMNATDVHS	6	0.1768	7378.6	70.00	0.46	tr_C9ZWK4_
HLA-DQA10501-DQB10201	13	KKRMNATDVHSHSLF	RMNATDVHS	2	0.1696	7984.5	75.00	0.34	tr_C9ZWK4_
HLA-DQA10501-DQB10201	14	PVPRNSPLFQHLSFS	SPLFQHLSF	5	0.1693	8008.9	75.00	0.25	tr_C9ZWK4_
HLA-DQA10501-DQB10201	15	KINKKRMNATDVHSH	RMNATDVHS	5	0.1671	8202.9	75.00	0.45	tr_C9ZWK4_
HLA-DQA10501-DQB10201	16	PRNSPLFQHLSFSST	FQHLSFSST	6	0.1650	8387.1	75.00	0.44	tr_C9ZWK4_
HLA-DQA10501-DQB10201	17	CALPVPRNSPLFQHL	VPRNSPLFQ	4	0.1636	8511.5	75.00	0.46	tr_C9ZWK4_
HLA-DQA10501-DQB10201	18	INKKRMNATDVHSHS	RMNATDVHS	4	0.1594	8912.3	75.00	0.43	tr_C9ZWK4_
HLA-DQA10501-DQB10201	19	NKKRMNATDVHSHSL	RMNATDVHS	3	0.1591	8939.9	75.00	0.41	tr_C9ZWK4_
HLA-DQA10501-DQB10201	20	ALPVPRNSPLFQHLS	VPRNSPLFQ	3	0.1590	8947.3	75.00	0.46	tr_C9ZWK4_
HLA-DQA10501-DQB10201	21	VCALPVPRNSPLFQH	VPRNSPLFQ	5	0.1570	9148.8	75.00	0.47	tr_C9ZWK4_
HLA-DQA10501-DQB10201	22	KGAHSQCCGFVCALP	QCCGFVCAL	5	0.1550	9347.6	75.00	0.32	tr_C9ZWK4_
HLA-DQA10501-DQB10201	23	KRMNATDVHSHSLFK	RMNATDVHS	1	0.1475	10140.8	80.00	0.32	tr_C9ZWK4_
HLA-DQA10501-DQB10201	24	VPRNSPLFQHLSFSS	SPLFQHLSF	4	0.1419	10768.2	80.00	0.28	tr_C9ZWK4_
HLA-DQA10501-DQB10201	25	NGIVPVCSGMSEKIN	VPVCSGMSE	3	0.1384	11181.8	80.00	0.35	tr_C9ZWK4_
HLA-DQA10501-DQB10201	26	FKGAHSQCCGFVCAL	QCCGFVCAL	6	0.1373	11322.7	80.00	0.33	tr_C9ZWK4_
HLA-DQA10501-DQB10201	27	SEKINKKRMNATDVH	KRMNATDVH	6	0.1348	11628.7	80.00	0.63	tr_C9ZWK4_
HLA-DQA10501-DQB10201	28	HSLFKGAHSQCCGFV	GAHSQCCGF	5	0.1328	11888.2	85.00	0.32	tr_C9ZWK4_
HLA-DQA10501-DQB10201	29	SLFKGAHSQCCGFVC	GAHSQCCGF	4	0.1255	12857.6	85.00	0.33	tr_C9ZWK4_
HLA-DQA10501-DQB10201	30	SHSLFKGAHSQCCGF	GAHSQCCGF	6	0.1232	13180.6	85.00	0.41	tr_C9ZWK4_
HLA-DQA10501-DQB10201	31	GIVPVCSGMSEKINK	VPVCSGMSE	2	0.1231	13200.6	85.00	0.41	tr_C9ZWK4_
HLA-DQA10501-DQB10201	32	RMNATDVHSHSLFKG	RMNATDVHS	0	0.1230	13218.9	85.00	0.28	tr_C9ZWK4_
HLA-DQA10501-DQB10201	33	LFKGAHSQCCGFVCA	GAHSQCCGF	3	0.1214	13436.6	85.00	0.28	tr_C9ZWK4_
HLA-DQA10501-DQB10201	34	IVPVCSGMSEKINKK	VPVCSGMSE	1	0.1094	15300.8	90.00	0.41	tr_C9ZWK4_
HLA-DQA10501-DQB10201	35	VHSHSLFKGAHSQCC	FKGAHSQCC	6	0.0999	16969.5	90.00	0.33	tr_C9ZWK4_
HLA-DQA10501-DQB10201	36	MNATDVHSHSLFKGA	TDVHSHSLF	3	0.0945	17977.5	90.00	0.28	tr_C9ZWK4_
HLA-DQA10501-DQB10201	37	DVHSHSLFKGAHSQC	LFKGAHSQC	6	0.0911	18652.6	95.00	0.32	tr_C9ZWK4_
HLA-DQA10501-DQB10201	38	HSHSLFKGAHSQCCG	FKGAHSQCC	5	0.0892	19055.0	95.00	0.30	tr_C9ZWK4_
HLA-DQA10501-DQB10201	39	ATDVHSHSLFKGAHS	HSLFKGAHS	6	0.0845	20036.7	95.00	0.21	tr_C9ZWK4_
HLA-DQA10501-DQB10201	40	VPVCSGMSEKINKKR	VPVCSGMSE	0	0.0845	20041.5	95.00	0.41	tr_C9ZWK4_
HLA-DQA10501-DQB10201	41	NATDVHSHSLFKGAH	TDVHSHSLF	2	0.0828	20423.3	95.00	0.29	tr_C9ZWK4_
HLA-DQA10501-DQB10201	42	TDVHSHSLFKGAHSQ	HSLFKGAHS	5	0.0753	22141.3	95.00	0.25	tr_C9ZWK4_
HLA-DQA10501-DQB10201	43	MSEKINKKRMNATDV	KKRMNATDV	6	0.0737	22523.3	95.00	0.75	tr_C9ZWK4_
HLA-DQA10501-DQB10201	44	PVCSGMSEKINKKRM	VCSGMSEKI	1	0.0420	31752.5	100.00	0.50	tr_C9ZWK4_
HLA-DQA10501-DQB10201	45	VCSGMSEKINKKRMN	VCSGMSEKI	0	0.0286	36679.6	100.00	0.51	tr_C9ZWK4_
HLA-DQA10501-DQB10201	46	GMSEKINKKRMNATD	NKKRMNATD	6	0.0204	40102.6	100.00	0.31	tr_C9ZWK4_
HLA-DQA10501-DQB10201	47	CSGMSEKINKKRMNA	KINKKRMNA	6	0.0159	42093.4	100.00	0.23	tr_C9ZWK4_
HLA-DQA10501-DQB10201	48	SGMSEKINKKRMNAT	INKKRMNAT	6	0.0154	42315.8	100.00	0.31	tr_C9ZWK4_

Allele: HLA-DQA10501-DQB10201. Number of high binders 0. Number of weak binders 0. Number of peptides 48

Allele	pos	peptide	core	Of	1-log50k(aff)	affinity(nM)	%Rank	Relia	Identity	Bind Level
HLA-DQA10501-DQB10201	1	GWGPYAPVLVELSDI	WGPYAPVLV	1	0.4913	245.6	6.00	0.31	tr_D0A5M0_	WB
HLA-DQA10501-DQB10201	2	TGWGPYAPVLVELSD	WGPYAPVLV	2	0.4719	303.0	7.50	0.40	tr_D0A5M0_	WB
HLA-DQA10501-DQB10201	3	WGPYAPVLVELSDIR	WGPYAPVLV	0	0.4623	336.2	8.50	0.23	tr_D0A5M0_	WB
HLA-DQA10501-DQB10201	4	GPYAPVLVELSDIRY	PVLVELSDI	4	0.4558	360.7	9.00	0.22	tr_D0A5M0_	WB
HLA-DQA10501-DQB10201	5	STGWGPYAPVLVELS	WGPYAPVLV	3	0.4552	363.2	9.00	0.47	tr_D0A5M0_	WB
HLA-DQA10501-DQB10201	6	VSTGWGPYAPVLVEL	WGPYAPVLV	4	0.4458	402.0	10.00	0.58	tr_D0A5M0_	
HLA-DQA10501-DQB10201	7	PYAPVLVELSDIRYL	PVLVELSDI	3	0.4416	420.8	11.00	0.24	tr_D0A5M0_	
HLA-DQA10501-DQB10201	8	YAPVLVELSDIRYLV	PVLVELSDI	2	0.4279	487.6	12.00	0.26	tr_D0A5M0_	
HLA-DQA10501-DQB10201	9	APVLVELSDIRYLVW	LVELSDIRY	3	0.4137	569.0	14.00	0.20	tr_D0A5M0_	
HLA-DQA10501-DQB10201	10	VVSTGWGPYAPVLVE	WGPYAPVLV	5	0.3988	668.3	16.00	0.70	tr_D0A5M0_	
HLA-DQA10501-DQB10201	11	CVVSTGWGPYAPVLV	WGPYAPVLV	6	0.3952	695.0	17.00	0.73	tr_D0A5M0_	
HLA-DQA10501-DQB10201	12	PVLVELSDIRYLVWK	LVELSDIRY	2	0.3796	822.8	19.00	0.23	tr_D0A5M0_	
HLA-DQA10501-DQB10201	13	VLVELSDIRYLVWKG	LVELSDIRY	1	0.3231	1516.5	30.00	0.31	tr_D0A5M0_	
HLA-DQA10501-DQB10201	14	LVELSDIRYLVWKGE	LVELSDIRY	0	0.2787	2450.6	40.00	0.29	tr_D0A5M0_	
HLA-DQA10501-DQB10201	15	VELSDIRYLVWKGEG	LSDIRYLVW	2	0.2280	4243.4	55.00	0.34	tr_D0A5M0_	
HLA-DQA10501-DQB10201	16	ELSDIRYLVWKGEGN	LSDIRYLVW	1	0.1909	6336.3	65.00	0.36	tr_D0A5M0_	
HLA-DQA10501-DQB10201	17	SCVVSTGWGPYAPVL	GWGPYAPVL	6	0.1836	6859.2	70.00	0.26	tr_D0A5M0_	
HLA-DQA10501-DQB10201	18	LSDIRYLVWKGEGNT	LSDIRYLVW	0	0.1558	9264.1	75.00	0.28	tr_D0A5M0_	
HLA-DQA10501-DQB10201	19	VWKGEGNTCPAVPAF	EGNTCPAVP	4	0.1306	12171.9	85.00	0.26	tr_D0A5M0_	
HLA-DQA10501-DQB10201	20	DIRYLVWKGEGNTCP	YLVWKGEGN	3	0.1300	12246.3	85.00	0.26	tr_D0A5M0_	
HLA-DQA10501-DQB10201	21	SDIRYLVWKGEGNTC	IRYLVWKGE	2	0.1236	13130.8	85.00	0.26	tr_D0A5M0_	
HLA-DQA10501-DQB10201	22	YLVWKGEGNTCPAVP	EGNTCPAVP	6	0.1219	13375.8	85.00	0.25	tr_D0A5M0_	
HLA-DQA10501-DQB10201	23	IRYLVWKGEGNTCPA	YLVWKGEGN	2	0.1167	14149.7	85.00	0.29	tr_D0A5M0_	
HLA-DQA10501-DQB10201	24	LVWKGEGNTCPAVPA	EGNTCPAVP	5	0.1149	14427.8	85.00	0.28	tr_D0A5M0_	
HLA-DQA10501-DQB10201	25	RYLVWKGEGNTCPAV	YLVWKGEGN	1	0.1104	15140.3	90.00	0.30	tr_D0A5M0_	

Allele: HLA-DQA10501-DQB10201. Number of high binders 0. Number of weak binders 5. Number of peptides 25

Allele	pos	peptide	core	Of	1-log50k(aff)	affinity(nM)	%Rank	Relia	Identity	Bind Level
HLA-DQA10501-DQB10201	1	GHVAVVVGVTGDRMF	VVGVTGDRM	5	0.5172	185.7	4.50	0.77	tr_C9ZJ95_	WB
HLA-DQA10501-DQB10201	2	HVAVVVGVTGDRMFV	VVGVTGDRM	4	0.5164	187.2	4.50	0.75	tr_C9ZJ95_	WB
HLA-DQA10501-DQB10201	3	VAVVVGVTGDRMFVA	VVGVTGDRM	3	0.5071	207.1	5.00	0.73	tr_C9ZJ95_	WB
HLA-DQA10501-DQB10201	4	AVVVGVTGDRMFVAE	VVGVTGDRM	2	0.4968	231.4	6.00	0.72	tr_C9ZJ95_	WB
HLA-DQA10501-DQB10201	5	ATFGSVDGAADIWDL	SVDGAADIW	4	0.4919	244.2	6.00	0.47	tr_C9ZJ95_	WB
HLA-DQA10501-DQB10201	6	PATFGSVDGAADIWD	SVDGAADIW	5	0.4913	245.7	6.00	0.47	tr_C9ZJ95_	WB
HLA-DQA10501-DQB10201	7	CGHVAVVVGVTGDRM	VVGVTGDRM	6	0.4894	250.9	6.50	0.76	tr_C9ZJ95_	WB
HLA-DQA10501-DQB10201	8	VVVGVTGDRMFVAEQ	VVGVTGDRM	1	0.4685	314.6	8.00	0.73	tr_C9ZJ95_	WB
HLA-DQA10501-DQB10201	9	TFGSVDGAADIWDLK	SVDGAADIW	3	0.4510	379.8	9.50	0.47	tr_C9ZJ95_	WB
HLA-DQA10501-DQB10201	10	GVTGDRMFVAEQNWE	RMFVAEQNW	5	0.4480	392.7	10.00	0.44	tr_C9ZJ95_	
HLA-DQA10501-DQB10201	11	FGSVDGAADIWDLKD	SVDGAADIW	2	0.4411	423.1	11.00	0.49	tr_C9ZJ95_	
HLA-DQA10501-DQB10201	12	VGVTGDRMFVAEQNW	RMFVAEQNW	6	0.4400	428.2	11.00	0.50	tr_C9ZJ95_	

HLA-DQA10501-DQB10201	13	VVGVTGDRMFVAEQN	VVGVTGDRM	0	0.4377	438.7	11.00	0.54	tr_C9ZJ95_
HLA-DQA10501-DQB10201	14	VTGDRMFVAEQNWEN	RMFVAEQNW	4	0.4326	463.7	12.00	0.42	tr_C9ZJ95_
HLA-DQA10501-DQB10201	15	QPATFGSVDGAADIW	SVDGAADIW	6	0.4264	495.7	13.00	0.55	tr_C9ZJ95_
HLA-DQA10501-DQB10201	16	TGDRMFVAEQNWENA	RMFVAEQNW	3	0.4195	534.4	13.00	0.41	tr_C9ZJ95_
HLA-DQA10501-DQB10201	17	GDRMFVAEQNWENAA	RMFVAEQNW	2	0.4023	643.4	16.00	0.44	tr_C9ZJ95_
HLA-DQA10501-DQB10201	18	GSVDGAADIWDLKDI	SVDGAADIW	1	0.3971	680.7	16.00	0.47	tr_C9ZJ95_
HLA-DQA10501-DQB10201	19	DRMFVAEQNWENAAW	RMFVAEQNW	1	0.3927	713.8	17.00	0.41	tr_C9ZJ95_
HLA-DQA10501-DQB10201	20	SVDGAADIWDLKDIQ	SVDGAADIW	0	0.3695	917.4	21.00	0.35	tr_C9ZJ95_
HLA-DQA10501-DQB10201	21	DGAADIWDLKDIQLL	IWDLKDIQL	5	0.3624	991.4	22.00	0.38	tr_C9ZJ95_
HLA-DQA10501-DQB10201	22	RMFVAEQNWENAAWP	MFVAEQNWE	1	0.3580	1039.4	23.00	0.25	tr_C9ZJ95_
HLA-DQA10501-DQB10201	23	GAADIWDLKDIQLLN	IWDLKDIQL	4	0.3564	1058.0	23.00	0.38	tr_C9ZJ95_
HLA-DQA10501-DQB10201	24	AADIWDLKDIQLLNG	IWDLKDIQL	3	0.3446	1201.3	25.00	0.35	tr_C9ZJ95_
HLA-DQA10501-DQB10201	25	VDGAADIWDLKDIQL	IWDLKDIQL	6	0.3429	1223.2	26.00	0.44	tr_C9ZJ95_
HLA-DQA10501-DQB10201	26	LNPVDAILGAHEGVF	VDAILGAHE	3	0.3389	1278.3	26.00	0.29	tr_C9ZJ95_
HLA-DQA10501-DQB10201	27	NPVDAILGAHEGVFS	VDAILGAHE	2	0.3319	1378.7	28.00	0.25	tr_C9ZJ95_
HLA-DQA10501-DQB10201	28	PVDAILGAHEGVFSY	AILGAHEGV	3	0.3312	1388.5	28.00	0.26	tr_C9ZJ95_
HLA-DQA10501-DQB10201	29	ADIWDLKDIQLLNGQ	IWDLKDIQL	2	0.3215	1543.1	30.00	0.37	tr_C9ZJ95_
HLA-DQA10501-DQB10201	30	VDAILGAHEGVFSYS	AILGAHEGV	2	0.3123	1704.4	32.00	0.29	tr_C9ZJ95_
HLA-DQA10501-DQB10201	31	DIWDLKDIQLLNGQK	WDLKDIQLL	2	0.2948	2058.9	36.00	0.34	tr_C9ZJ95_
HLA-DQA10501-DQB10201	32	CLNPVDAILGAHEGV	VDAILGAHE	4	0.2934	2090.7	37.00	0.40	tr_C9ZJ95_
HLA-DQA10501-DQB10201	33	MFVAEQNWENAAWPG	MFVAEQNWE	0	0.2900	2168.9	38.00	0.29	tr_C9ZJ95_
HLA-DQA10501-DQB10201	34	PQPATFGSVDGAADI	FGSVDGAAD	5	0.2898	2172.6	38.00	0.31	tr_C9ZJ95_
HLA-DQA10501-DQB10201	35	TSYQSGLKWQCVEYA	LKWQCVEYA	6	0.2829	2342.8	39.00	0.51	tr_C9ZJ95_
HLA-DQA10501-DQB10201	36	RRYWMLRGTPQPATF	WMLRGTPQP	3	0.2808	2395.0	40.00	0.28	tr_C9ZJ95_
HLA-DQA10501-DQB10201	37	YARRYWMLRGTPQPA	WMLRGTPQP	5	0.2762	2517.6	41.00	0.29	tr_C9ZJ95_
HLA-DQA10501-DQB10201	38	DAILGAHEGVFSYSN	AILGAHEGV	1	0.2757	2530.7	41.00	0.32	tr_C9ZJ95_
HLA-DQA10501-DQB10201	39	ARRYWMLRGTPQPAT	WMLRGTPQP	4	0.2733	2599.2	42.00	0.30	tr_C9ZJ95_
HLA-DQA10501-DQB10201	40	TPQPATFGSVDGAAD	TFGSVDGAA	5	0.2707	2673.8	43.00	0.32	tr_C9ZJ95_
HLA-DQA10501-DQB10201	41	KHCLNPVDAILGAHE	VDAILGAHE	6	0.2677	2762.2	43.00	0.52	tr_C9ZJ95_
HLA-DQA10501-DQB10201	42	VAEQNWENAAWPGPY	QNWENAAWP	3	0.2659	2814.9	44.00	0.28	tr_C9ZJ95_
HLA-DQA10501-DQB10201	43	RYWMLRGTPQPATFG	WMLRGTPQP	2	0.2649	2846.1	44.00	0.32	tr_C9ZJ95_
HLA-DQA10501-DQB10201	44	YQSGLKWQCVEYARR	LKWQCVEYA	4	0.2623	2928.6	45.00	0.48	tr_C9ZJ95_
HLA-DQA10501-DQB10201	45	SGLKWQCVEYARRYW	LKWQCVEYA	2	0.2604	2989.1	45.00	0.45	tr_C9ZJ95_
HLA-DQA10501-DQB10201	46	SYQSGLKWQCVEYAR	LKWQCVEYA	5	0.2596	3013.1	46.00	0.53	tr_C9ZJ95_
HLA-DQA10501-DQB10201	47	QSGLKWQCVEYARRY	LKWQCVEYA	3	0.2590	3033.9	46.00	0.47	tr_C9ZJ95_
HLA-DQA10501-DQB10201	48	FVAEQNWENAAWPGP	QNWENAAWP	4	0.2541	3199.9	47.00	0.27	tr_C9ZJ95_
HLA-DQA10501-DQB10201	49	GLKWQCVEYARRYWM	LKWQCVEYA	1	0.2533	3228.1	47.00	0.34	tr_C9ZJ95_
HLA-DQA10501-DQB10201	50	HCLNPVDAILGAHEG	VDAILGAHE	5	0.2515	3289.6	48.00	0.50	tr_C9ZJ95_
HLA-DQA10501-DQB10201	51	EYARRYWMLRGTPQP	YWMLRGTPQ	5	0.2501	3338.5	48.00	0.40	tr_C9ZJ95_
HLA-DQA10501-DQB10201	52	AILGAHEGVFSYSNC	AILGAHEGV	0	0.2496	3359.4	48.00	0.26	tr_C9ZJ95_
HLA-DQA10501-DQB10201	53	IWDLKDIQLLNGQKR	WDLKDIQLL	1	0.2480	3417.1	49.00	0.37	tr_C9ZJ95_
HLA-DQA10501-DQB10201	54	YWMLRGTPQPATFGS	WMLRGTPQP	1	0.2466	3467.1	49.00	0.32	tr_C9ZJ95_
HLA-DQA10501-DQB10201	55	AEQNWENAAWPGPYH	QNWENAAWP	2	0.2425	3626.6	50.00	0.27	tr_C9ZJ95_
HLA-DQA10501-DQB10201	56	PYHNYSRVLNLSCNP	RVLNLSCNP	6	0.2410	3687.2	55.00	0.35	tr_C9ZJ95_
HLA-DQA10501-DQB10201	57	GKVEKHCLNPVDAIL	HCLNPVDAI	5	0.2332	4011.8	55.00	0.27	tr_C9ZJ95_
HLA-DQA10501-DQB10201	58	ANSKPRVGDLLIYPR	RVGDLLIYP	5	0.2330	4016.8	55.00	0.34	tr_C9ZJ95_
HLA-DQA10501-DQB10201	59	KVEKHCLNPVDAILG	HCLNPVDAI	4	0.2329	4022.1	55.00	0.22	tr_C9ZJ95_
HLA-DQA10501-DQB10201	60	SANSKPRVGDLLIYP	RVGDLLIYP	6	0.2314	4090.0	55.00	0.44	tr_C9ZJ95_
HLA-DQA10501-DQB10201	61	PCGHVAVVVGVTGDR	VVVGVTGDR	6	0.2312	4096.1	55.00	0.28	tr_C9ZJ95_
HLA-DQA10501-DQB10201	62	SKPRVGDLLIYPRQP	RVGDLLIYP	3	0.2301	4148.3	55.00	0.34	tr_C9ZJ95_

HLA-DQA10501-DQB10201	63	AWPGPYHNYSRVLNL	HNYSRVLNL	6	0.2294	4179.6	55.00	0.40	tr_C9ZJ95_
HLA-DQA10501-DQB10201	64	LKWQCVEYARRYWML	WQCVEYARR	2	0.2281	4238.1	55.00	0.25	tr_C9ZJ95_
HLA-DQA10501-DQB10201	65	VEKHCLNPVDAILGA	HCLNPVDAI	3	0.2252	4374.6	55.00	0.19	tr_C9ZJ95_
HLA-DQA10501-DQB10201	66	YHNYSRVLNLSCNPN	RVLNLSCNP	5	0.2246	4403.2	55.00	0.34	tr_C9ZJ95_
HLA-DQA10501-DQB10201	67	GTPQPATFGSVDGAA	TFGSVDGAA	6	0.2231	4471.0	60.00	0.40	tr_C9ZJ95_
HLA-DQA10501-DQB10201	68	WPGPYHNYSRVLNLS	PYHNYSRVL	3	0.2223	4511.3	60.00	0.31	tr_C9ZJ95_
HLA-DQA10501-DQB10201	69	EQNWENAAWPGPYHN	QNWENAAWP	1	0.2221	4522.0	60.00	0.26	tr_C9ZJ95_
HLA-DQA10501-DQB10201	70	PGPYHNYSRVLNLSC	PYHNYSRVL	2	0.2210	4573.9	60.00	0.25	tr_C9ZJ95_
HLA-DQA10501-DQB10201	71	NSKPRVGDLLIYPRQ	RVGDLLIYP	4	0.2208	4586.9	60.00	0.34	tr_C9ZJ95_
HLA-DQA10501-DQB10201	72	GPYHNYSRVLNLSCN	PYHNYSRVL	1	0.2147	4899.4	60.00	0.25	tr_C9ZJ95_
HLA-DQA10501-DQB10201	73	KPRVGDLLIYPRQPN	RVGDLLIYP	2	0.2118	5052.7	60.00	0.35	tr_C9ZJ95_
HLA-DQA10501-DQB10201	74	FPCGHVAVVVGVTGD	AVVVGVTGD	6	0.2110	5099.9	60.00	0.22	tr_C9ZJ95_
HLA-DQA10501-DQB10201	75	GTSYQSGLKWQCVEY	GLKWQCVEY	6	0.2085	5238.2	60.00	0.36	tr_C9ZJ95_
HLA-DQA10501-DQB10201	76	EKHCLNPVDAILGAH	HCLNPVDAI	2	0.2083	5250.7	60.00	0.22	tr_C9ZJ95_
HLA-DQA10501-DQB10201	77	NTTTYNNVTVAGTSY	NVTVAGTSY	6	0.2061	5379.4	65.00	0.35	tr_C9ZJ95_
HLA-DQA10501-DQB10201	78	SGKVEKHCLNPVDAI	HCLNPVDAI	6	0.2057	5400.2	65.00	0.32	tr_C9ZJ95_
HLA-DQA10501-DQB10201	79	TTTYNNVTVAGTSYQ	NVTVAGTSY	5	0.2037	5518.6	65.00	0.28	tr_C9ZJ95_
HLA-DQA10501-DQB10201	80	HNYSRVLNLSCNPNG	RVLNLSCNP	4	0.1996	5766.7	65.00	0.39	tr_C9ZJ95_
HLA-DQA10501-DQB10201	81	TTYNNVTVAGTSYQS	NVTVAGTSY	4	0.1991	5801.1	65.00	0.28	tr_C9ZJ95_
HLA-DQA10501-DQB10201	82	RVGDLLIYPRQPNGF	IYPRQPNGF	6	0.1962	5985.1	65.00	0.31	tr_C9ZJ95_
HLA-DQA10501-DQB10201	83	PRVGDLLIYPRQPNG	RVGDLLIYP	1	0.1959	6000.8	65.00	0.39	tr_C9ZJ95_
HLA-DQA10501-DQB10201	84	CTVREKDNVTVQGWV	KDNVTVQGW	5	0.1956	6025.7	65.00	0.47	tr_C9ZJ95_
HLA-DQA10501-DQB10201	85	WMLRGTPQPATFGSV	MLRGTPQPA	1	0.1954	6034.6	65.00	0.42	tr_C9ZJ95_
HLA-DQA10501-DQB10201	86	GFPCGHVAVVVGVTG	VAVVVGVTG	6	0.1937	6148.0	65.00	0.29	tr_C9ZJ95_
HLA-DQA10501-DQB10201	87	VEYARRYWMLRGTPQ	YWMLRGTPQ	6	0.1936	6158.3	65.00	0.43	tr_C9ZJ95_
HLA-DQA10501-DQB10201	88	HEGVFSYSNCGATEN	VFSYSNCGA	3	0.1922	6251.6	65.00	0.38	tr_C9ZJ95_
HLA-DQA10501-DQB10201	89	AHEGVFSYSNCGATE	VFSYSNCGA	4	0.1918	6273.6	65.00	0.40	tr_C9ZJ95_
HLA-DQA10501-DQB10201	90	REKDNVTVQGWVRYE	KDNVTVQGW	2	0.1917	6284.2	65.00	0.31	tr_C9ZJ95_
HLA-DQA10501-DQB10201	91	VGDLLIYPRQPNGFP	IYPRQPNGF	5	0.1909	6337.2	65.00	0.44	tr_C9ZJ95_
HLA-DQA10501-DQB10201	92	VREKDNVTVQGWVRY	KDNVTVQGW	3	0.1893	6448.4	70.00	0.34	tr_C9ZJ95_
HLA-DQA10501-DQB10201	93	KWQCVEYARRYWMLR	WQCVEYARR	1	0.1869	6614.5	70.00	0.29	tr_C9ZJ95_
HLA-DQA10501-DQB10201	94	TYNNVTVAGTSYQSG	NVTVAGTSY	3	0.1862	6671.2	70.00	0.26	tr_C9ZJ95_
HLA-DQA10501-DQB10201	95	TENTTTYNNVTVAGT	YNNVTVAGT	6	0.1854	6723.0	70.00	0.47	tr_C9ZJ95_
HLA-DQA10501-DQB10201	96	NVTVAGTSYQSGLKW	TSYQSGLKW	6	0.1851	6746.5	70.00	0.37	tr_C9ZJ95_
HLA-DQA10501-DQB10201	97	NGFPCGHVAVVVGVT	GHVAVVVGV	5	0.1841	6819.3	70.00	0.32	tr_C9ZJ95_
HLA-DQA10501-DQB10201	98	QNWENAAWPGPYHNY	ENAAWPGPY	3	0.1840	6829.5	70.00	0.28	tr_C9ZJ95_
HLA-DQA10501-DQB10201	99	EGVFSYSNCGATENT	VFSYSNCGA	2	0.1831	6893.5	70.00	0.40	tr_C9ZJ95_
HLA-DQA10501-DQB10201	100	LGAHEGVFSYSNCGA	VFSYSNCGA	6	0.1831	6896.1	70.00	0.47	tr_C9ZJ95_
HLA-DQA10501-DQB10201	101	WDLKDIQLLNGQKRK	WDLKDIQLL	0	0.1825	6938.3	70.00	0.41	tr_C9ZJ95_
HLA-DQA10501-DQB10201	102	ILGAHEGVFSYSNCG	LGAHEGVFS	1	0.1820	6975.9	70.00	0.25	tr_C9ZJ95_
HLA-DQA10501-DQB10201	103	RGTPQPATFGSVDGA	ATFGSVDGA	6	0.1820	6977.4	70.00	0.52	tr_C9ZJ95_
HLA-DQA10501-DQB10201	104	NYSRVLNLSCNPNGT	RVLNLSCNP	3	0.1812	7038.7	70.00	0.46	tr_C9ZJ95_
HLA-DQA10501-DQB10201	105	AAWPGPYHNYSRVLN	PYHNYSRVL	5	0.1808	7070.6	70.00	0.52	tr_C9ZJ95_
HLA-DQA10501-DQB10201	106	GDLLIYPRQPNGFPC	IYPRQPNGF	4	0.1801	7120.1	70.00	0.51	tr_C9ZJ95_
HLA-DQA10501-DQB10201	107	PNGFPCGHVAVVVGV	GHVAVVVGV	6	0.1796	7161.0	70.00	0.44	tr_C9ZJ95_
HLA-DQA10501-DQB10201	108	TVREKDNVTVQGWVR	KDNVTVQGW	4	0.1790	7206.6	70.00	0.38	tr_C9ZJ95_
HLA-DQA10501-DQB10201	109	TSANSKPRVGDLLIY	PRVGDLLIY	6	0.1785	7248.2	70.00	0.49	tr_C9ZJ95_
HLA-DQA10501-DQB10201	110	ENTTTYNNVTVAGTS	YNNVTVAGT	5	0.1779	7294.8	70.00	0.43	tr_C9ZJ95_
HLA-DQA10501-DQB10201	111	YNNVTVAGTSYQSGL	NVTVAGTSY	2	0.1776	7321.9	70.00	0.28	tr_C9ZJ95_
HLA-DQA10501-DQB10201	112	WQCVEYARRYWMLRG	VEYARRYWM	3	0.1731	7686.2	70.00	0.25	tr_C9ZJ95_

HLA-DQA10501-DQB10201	113	GAHEGVFSYSNCGAT	VFSYSNCGA	5	0.1677	8142.2	75.00	0.53	tr_C9ZJ95_
HLA-DQA10501-DQB10201	114	NAAWPGPYHNYSRVL	PYHNYSRVL	6	0.1653	8362.0	75.00	0.62	tr_C9ZJ95_
HLA-DQA10501-DQB10201	115	GVFSYSNCGATENTT	VFSYSNCGA	1	0.1640	8482.2	75.00	0.35	tr_C9ZJ95_
HLA-DQA10501-DQB10201	116	YSRVLNLSCNPNGTA	RVLNLSCNP	2	0.1639	8483.5	75.00	0.54	tr_C9ZJ95_
HLA-DQA10501-DQB10201	117	DLLIYPRQPNGFPCG	IYPRQPNGF	3	0.1633	8540.4	75.00	0.50	tr_C9ZJ95_
HLA-DQA10501-DQB10201	118	CVEYARRYWMLRGTP	RYWMLRGTP	6	0.1602	8839.5	75.00	0.22	tr_C9ZJ95_
HLA-DQA10501-DQB10201	119	GSGKVEKHCLNPVDA	KHCLNPVDA	6	0.1588	8970.8	75.00	0.40	tr_C9ZJ95_
HLA-DQA10501-DQB10201	120	RQPNGFPCGHVAVVV	PCGHVAVVV	6	0.1574	9109.0	75.00	0.59	tr_C9ZJ95_
HLA-DQA10501-DQB10201	121	VTVAGTSYQSGLKWQ	TSYQSGLKW	5	0.1571	9138.5	75.00	0.41	tr_C9ZJ95_
HLA-DQA10501-DQB10201	122	AGTSYQSGLKWQCVE	TSYQSGLKW	2	0.1564	9204.5	75.00	0.34	tr_C9ZJ95_
HLA-DQA10501-DQB10201	123	ATENTTTYNNVTVAG	TTYNNVTVA	5	0.1562	9229.7	75.00	0.45	tr_C9ZJ95_
HLA-DQA10501-DQB10201	124	NWENAAWPGPYHNYS	ENAAWPGPY	2	0.1551	9339.0	75.00	0.34	tr_C9ZJ95_
HLA-DQA10501-DQB10201	125	QCVEYARRYWMLRGT	VEYARRYWM	2	0.1526	9587.4	80.00	0.28	tr_C9ZJ95_
HLA-DQA10501-DQB10201	126	ACTVREKDNVTVQGW	KDNVTVQGW	6	0.1522	9632.7	80.00	0.65	tr_C9ZJ95_
HLA-DQA10501-DQB10201	127	VAGTSYQSGLKWQCV	TSYQSGLKW	3	0.1513	9731.6	80.00	0.39	tr_C9ZJ95_
HLA-DQA10501-DQB10201	128	QPNGFPCGHVAVVVG	PCGHVAVVV	5	0.1493	9936.4	80.00	0.51	tr_C9ZJ95_
HLA-DQA10501-DQB10201	129	NNVTVAGTSYQSGLK	NVTVAGTSY	1	0.1488	9996.4	80.00	0.31	tr_C9ZJ95_
HLA-DQA10501-DQB10201	130	TVAGTSYQSGLKWQC	TSYQSGLKW	4	0.1430	10641.4	80.00	0.43	tr_C9ZJ95_
HLA-DQA10501-DQB10201	131	VFSYSNCGATENTTT	SYSNCGATE	2	0.1413	10834.4	80.00	0.37	tr_C9ZJ95_
HLA-DQA10501-DQB10201	132	MLRGTPQPATFGSVD	MLRGTPQPA	0	0.1393	11074.4	80.00	0.31	tr_C9ZJ95_
HLA-DQA10501-DQB10201	133	LLIYPRQPNGFPCGH	IYPRQPNGF	2	0.1381	11222.9	80.00	0.55	tr_C9ZJ95_
HLA-DQA10501-DQB10201	134	GATENTTTYNNVTVA	TTYNNVTVA	6	0.1365	11419.5	80.00	0.66	tr_C9ZJ95_
HLA-DQA10501-DQB10201	135	SRVLNLSCNPNGTAC	RVLNLSCNP	1	0.1365	11422.3	80.00	0.59	tr_C9ZJ95_
HLA-DQA10501-DQB10201	136	WENAAWPGPYHNYSR	ENAAWPGPY	1	0.1348	11631.7	80.00	0.35	tr_C9ZJ95_
HLA-DQA10501-DQB10201	137	LIYPRQPNGFPCGHV	IYPRQPNGF	1	0.1331	11844.9	85.00	0.50	tr_C9ZJ95_
HLA-DQA10501-DQB10201	138	LRGTPQPATFGSVDG	PATFGSVDG	6	0.1326	11912.5	85.00	0.43	tr_C9ZJ95_
HLA-DQA10501-DQB10201	139	ATSANSKPRVGDLLI	KPRVGDLLI	6	0.1249	12938.4	85.00	0.51	tr_C9ZJ95_
HLA-DQA10501-DQB10201	140	EGSGKVEKHCLNPVD	VEKHCLNPV	5	0.1229	13227.3	85.00	0.49	tr_C9ZJ95_
HLA-DQA10501-DQB10201	141	FSYSNCGATENTTTY	SYSNCGATE	1	0.1140	14559.0	85.00	0.46	tr_C9ZJ95_
HLA-DQA10501-DQB10201	142	DLKDIQLLNGQKRKP	IQLLNGQKR	4	0.1131	14713.4	90.00	0.31	tr_C9ZJ95_
HLA-DQA10501-DQB10201	143	IYPRQPNGFPCGHVA	IYPRQPNGF	0	0.1089	15386.6	90.00	0.41	tr_C9ZJ95_
HLA-DQA10501-DQB10201	144	RVLNLSCNPNGTACT	RVLNLSCNP	0	0.1075	15620.7	90.00	0.58	tr_C9ZJ95_
HLA-DQA10501-DQB10201	145	PEGSGKVEKHCLNPV	VEKHCLNPV	6	0.1028	16447.8	90.00	0.56	tr_C9ZJ95_
HLA-DQA10501-DQB10201	146	ENAAWPGPYHNYSRV	ENAAWPGPY	0	0.0962	17663.4	90.00	0.33	tr_C9ZJ95_
HLA-DQA10501-DQB10201	147	LKDIQLLNGQKRKPL	IQLLNGQKR	3	0.0957	17746.4	90.00	0.39	tr_C9ZJ95_
HLA-DQA10501-DQB10201	148	SYSNCGATENTTTYN	SYSNCGATE	0	0.0895	18986.9	95.00	0.32	tr_C9ZJ95_
HLA-DQA10501-DQB10201	149	NATSANSKPRVGDLL	SKPRVGDLL	6	0.0894	19010.6	95.00	0.69	tr_C9ZJ95_
HLA-DQA10501-DQB10201	150	SCNPNGTACTVREKD	TACTVREKD	6	0.0816	20680.6	95.00	0.37	tr_C9ZJ95_
HLA-DQA10501-DQB10201	151	NPNGTACTVREKDNV	TACTVREKD	4	0.0796	21140.0	95.00	0.37	tr_C9ZJ95_
HLA-DQA10501-DQB10201	152	KDIQLLNGQKRKPLL	IQLLNGQKR	2	0.0795	21163.4	95.00	0.53	tr_C9ZJ95_
HLA-DQA10501-DQB10201	153	PRQPNGFPCGHVAVV	FPCGHVAVV	6	0.0792	21213.4	95.00	0.44	tr_C9ZJ95_
HLA-DQA10501-DQB10201	154	TACTVREKDNVTVQG	EKDNVTVQG	6	0.0791	21247.8	95.00	0.29	tr_C9ZJ95_
HLA-DQA10501-DQB10201	155	CNPNGTACTVREKDN	TACTVREKD	5	0.0779	21524.6	95.00	0.41	tr_C9ZJ95_
HLA-DQA10501-DQB10201	156	NLSCNPNGTACTVRE	NGTACTVRE	6	0.0766	21835.6	95.00	0.56	tr_C9ZJ95_
HLA-DQA10501-DQB10201	157	PNGTACTVREKDNVT	TACTVREKD	3	0.0759	21987.6	95.00	0.31	tr_C9ZJ95_
HLA-DQA10501-DQB10201	158	NGTACTVREKDNVTV	TACTVREKD	2	0.0749	22242.4	95.00	0.31	tr_C9ZJ95_
HLA-DQA10501-DQB10201	159	RKPLLKYHNGNATSA	YHNGNATSA	6	0.0743	22376.6	95.00	0.63	tr_C9ZJ95_
HLA-DQA10501-DQB10201	160	GTACTVREKDNVTVQ	TACTVREKD	1	0.0740	22455.2	95.00	0.28	tr_C9ZJ95_
HLA-DQA10501-DQB10201	161	KPLLKYHNGNATSAN	YHNGNATSA	5	0.0733	22629.8	95.00	0.60	tr_C9ZJ95_
HLA-DQA10501-DQB10201	162	PLLKYHNGNATSANS	YHNGNATSA	4	0.0727	22774.0	95.00	0.51	tr_C9ZJ95_

HLA-DQA10501-DQB10201	163	CGATENTTTYNNVTV	TTTYNNVTV	6	0.0727	22779.9	95.00	0.44	tr_C9ZJ95_	
HLA-DQA10501-DQB10201	164	YPRQPNGFPCGHVAV	PNGFPCGHV	4	0.0689	23724.0	95.00	0.27	tr_C9ZJ95_	
HLA-DQA10501-DQB10201	165	VLNLSCNPNGTACTV	NPNGTACTV	6	0.0682	23908.5	95.00	0.34	tr_C9ZJ95_	
HLA-DQA10501-DQB10201	166	LKYHNGNATSANSKP	YHNGNATSA	2	0.0681	23930.0	95.00	0.39	tr_C9ZJ95_	
HLA-DQA10501-DQB10201	167	DIQLLNGQKRKPLLK	IQLLNGQKR	1	0.0659	24516.8	95.00	0.58	tr_C9ZJ95_	
HLA-DQA10501-DQB10201	168	LLKYHNGNATSANSK	YHNGNATSA	3	0.0650	24760.1	95.00	0.45	tr_C9ZJ95_	
HLA-DQA10501-DQB10201	169	LSCNPNGTACTVREK	NGTACTVRE	5	0.0641	24999.7	95.00	0.47	tr_C9ZJ95_	
HLA-DQA10501-DQB10201	170	YHNGNATSANSKPRV	YHNGNATSA	0	0.0604	26012.1	100.00	0.25	tr_C9ZJ95_	
HLA-DQA10501-DQB10201	171	KYHNGNATSANSKPR	YHNGNATSA	1	0.0600	26123.5	100.00	0.41	tr_C9ZJ95_	
HLA-DQA10501-DQB10201	172	SNCGATENTTTYNNV	ENTTTYNNV	6	0.0590	26417.4	100.00	0.38	tr_C9ZJ95_	
HLA-DQA10501-DQB10201	173	SGIRYPEGSGKVEKH	YPEGSGKVE	4	0.0585	26558.4	100.00	0.25	tr_C9ZJ95_	
HLA-DQA10501-DQB10201	174	LNLSCNPNGTACTVR	NPNGTACTV	5	0.0578	26739.8	100.00	0.36	tr_C9ZJ95_	
HLA-DQA10501-DQB10201	175	NCGATENTTTYNNVT	ENTTTYNNV	5	0.0573	26892.7	100.00	0.34	tr_C9ZJ95_	
HLA-DQA10501-DQB10201	176	YSNCGATENTTTYNN	YSNCGATEN	0	0.0562	27217.3	100.00	0.23	tr_C9ZJ95_	
HLA-DQA10501-DQB10201	177	IRYPEGSGKVEKHCL	YPEGSGKVE	2	0.0550	27582.3	100.00	0.29	tr_C9ZJ95_	
HLA-DQA10501-DQB10201	178	GIRYPEGSGKVEKHC	YPEGSGKVE	3	0.0529	28218.0	100.00	0.32	tr_C9ZJ95_	
HLA-DQA10501-DQB10201	179	RYPEGSGKVEKHCLN	YPEGSGKVE	1	0.0513	28709.2	100.00	0.26	tr_C9ZJ95_	
HLA-DQA10501-DQB10201	180	YPEGSGKVEKHCLNP	EGSGKVEKH	2	0.0484	29612.8	100.00	0.17	tr_C9ZJ95_	
HLA-DQA10501-DQB10201	181	HNGNATSANSKPRVG	TSANSKPRV	5	0.0470	30078.4	100.00	0.28	tr_C9ZJ95_	
HLA-DQA10501-DQB10201	182	NGNATSANSKPRVGD	TSANSKPRV	4	0.0448	30781.3	100.00	0.36	tr_C9ZJ95_	
HLA-DQA10501-DQB10201	183	GNATSANSKPRVGDL	TSANSKPRV	3	0.0421	31691.7	100.00	0.38	tr_C9ZJ95_	
HLA-DQA10501-DQB10201	184	IQLLNGQKRKPLLKY	IQLLNGQKR	0	0.0399	32474.5	100.00	0.55	tr_C9ZJ95_	
HLA-DQA10501-DQB10201	185	KRKPLLKYHNGNATS	LKYHNGNAT	5	0.0386	32915.6	100.00	0.45	tr_C9ZJ95_	
HLA-DQA10501-DQB10201	186	QKRKPLLKYHNGNAT	LKYHNGNAT	6	0.0301	36116.8	100.00	0.55	tr_C9ZJ95_	
HLA-DQA10501-DQB10201	187	QLLNGQKRKPLLKYH	QKRKPLLKY	5	0.0184	40990.0	100.00	0.23	tr_C9ZJ95_	
HLA-DQA10501-DQB10201	188	GQKRKPLLKYHNGNA	LLKYHNGNA	6	0.0174	41412.2	100.00	0.34	tr_C9ZJ95_	
HLA-DQA10501-DQB10201	189	LLNGQKRKPLLKYHN	NGQKRKPLL	2	0.0155	42278.3	100.00	0.14	tr_C9ZJ95_	
HLA-DQA10501-DQB10201	190	NGQKRKPLLKYHNGN	PLLKYHNGN	6	0.0151	42459.8	100.00	0.36	tr_C9ZJ95_	
HLA-DQA10501-DQB10201	191	LNGQKRKPLLKYHNG	NGQKRKPLL	1	0.0134	43262.0	100.00	0.16	tr_C9ZJ95_	

Allele: HLA-DQA10501-DQB10201. Number of high binders 0. Number of weak binders 9. Number of peptides 191

Allele	pos	peptide	core	Of	1-log50k(aff)	affinity(nM)	%Rank	Relia	Identity	Bind Level
HLA-DQA10501-DQB10201	1	EKGTAWVFAMYPRDS	WVFAMYPRD	5	0.4150	561.0	14.00	0.43	tr_C9ZZZ3_	
HLA-DQA10501-DQB10201	2	GEKGTAWVFAMYPRD	WVFAMYPRD	6	0.4147	562.5	14.00	0.42	tr_C9ZZZ3_	
HLA-DQA10501-DQB10201	3	KGTAWVFAMYPRDSN	WVFAMYPRD	4	0.3993	664.6	16.00	0.44	tr_C9ZZZ3_	
HLA-DQA10501-DQB10201	4	GTAWVFAMYPRDSNG	WVFAMYPRD	3	0.3783	834.3	19.00	0.46	tr_C9ZZZ3_	
HLA-DQA10501-DQB10201	5	AFGEKGTAWVFAMYP	TAWVFAMYP	6	0.3505	1126.9	24.00	0.57	tr_C9ZZZ3_	
HLA-DQA10501-DQB10201	6	FGEKGTAWVFAMYPR	TAWVFAMYP	5	0.3205	1558.5	30.00	0.50	tr_C9ZZZ3_	
HLA-DQA10501-DQB10201	7	TAFGEKGTAWVFAMY	GTAWVFAMY	6	0.2418	3652.3	50.00	0.30	tr_C9ZZZ3_	
HLA-DQA10501-DQB10201	8	LTAFGEKGTAWVFAM	KGTAWVFAM	6	0.2098	5165.7	60.00	0.29	tr_C9ZZZ3_	
HLA-DQA10501-DQB10201	9	ELTAFGEKGTAWVFA	EKGTAWVFA	6	0.1967	5951.6	65.00	0.34	tr_C9ZZZ3_	
HLA-DQA10501-DQB10201	10	GGELTAFGEKGTAWV	ELTAFGEKG	2	0.1830	6906.8	70.00	0.31	tr_C9ZZZ3_	
HLA-DQA10501-DQB10201	11	GELTAFGEKGTAWVF	AFGEKGTAW	4	0.1807	7074.4	70.00	0.28	tr_C9ZZZ3_	
HLA-DQA10501-DQB10201	12	LLKAHGGGELTAFGE	GGELTAFGE	6	0.1753	7500.9	70.00	0.32	tr_C9ZZZ3_	
HLA-DQA10501-DQB10201	13	KAHGGGELTAFGEKG	ELTAFGEKG	6	0.1708	7877.8	75.00	0.40	tr_C9ZZZ3_	

```
 HLA-DQA10501-DQB10201   14      GGGELTAFGEKGTAW  ELTAFGEKG  3        0.1685       8072.8    75.00  0.37   tr_C9ZZZ3_
 HLA-DQA10501-DQB10201   15      AHGGGELTAFGEKGT  ELTAFGEKG  5        0.1610       8754.8    75.00  0.40   tr_C9ZZZ3_
 HLA-DQA10501-DQB10201   16      LKAHGGGELTAFGEK  GGELTAFGE  5        0.1549       9359.4    75.00  0.28   tr_C9ZZZ3_
 HLA-DQA10501-DQB10201   17      HGGGELTAFGEKGTA  ELTAFGEKG  4        0.1490       9971.9    80.00  0.46   tr_C9ZZZ3_
 HLA-DQA10501-DQB10201   18      DLLKAHGGGELTAFG  HGGGELTAF  5        0.1419      10771.9    80.00  0.25   tr_C9ZZZ3_
-----------------------------------------------------------------------------------------------

Allele: HLA-DQA10501-DQB10201. Number of high binders 0. Number of weak binders 0. Number of peptides 18

-----------------------------------------------------------------------------------------------
-----------------------------------------------------------------------------------------------
                Allele  pos              peptide       core Of 1-log50k(aff) affinity(nM)    %Rank Relia     Identity Bind Level
-----------------------------------------------------------------------------------------------
 HLA-DQA10501-DQB10201    1      THSVWHGSTVAARDV  HGSTVAARD  5        0.2493       3369.0    48.00  0.33   tr_D0A148_
 HLA-DQA10501-DQB10201    2      YTHSVWHGSTVAARD  WHGSTVAAR  5        0.2307       4118.8    55.00  0.34   tr_D0A148_
 HLA-DQA10501-DQB10201    3      HSVWHGSTVAARDVK  HGSTVAARD  4        0.2188       4686.3    60.00  0.30   tr_D0A148_
 HLA-DQA10501-DQB10201    4      SVWHGSTVAARDVKR  WHGSTVAAR  2        0.2014       5656.9    65.00  0.28   tr_D0A148_
 HLA-DQA10501-DQB10201    5      VWHGSTVAARDVKRS  HGSTVAARD  2        0.1809       7062.1    70.00  0.32   tr_D0A148_
 HLA-DQA10501-DQB10201    6      WHGSTVAARDVKRST  HGSTVAARD  1        0.1474      10144.0    80.00  0.35   tr_D0A148_
 HLA-DQA10501-DQB10201    7      VAARDVKRSTSHQFC  VKRSTSHQF  5        0.1130      14727.6    90.00  0.54   tr_D0A148_
 HLA-DQA10501-DQB10201    8      HGSTVAARDVKRSTS  HGSTVAARD  0        0.1010      16763.1    90.00  0.33   tr_D0A148_
 HLA-DQA10501-DQB10201    9      AARDVKRSTSHQFCK  VKRSTSHQF  4        0.0935      18190.2    90.00  0.53   tr_D0A148_
 HLA-DQA10501-DQB10201   10      TVAARDVKRSTSHQF  VKRSTSHQF  6        0.0931      18262.8    90.00  0.71   tr_D0A148_
 HLA-DQA10501-DQB10201   11      ARDVKRSTSHQFCKS  VKRSTSHQF  3        0.0886      19176.6    95.00  0.49   tr_D0A148_
 HLA-DQA10501-DQB10201   12      RDVKRSTSHQFCKSG  VKRSTSHQF  2        0.0793      21203.5    95.00  0.53   tr_D0A148_
 HLA-DQA10501-DQB10201   13      DVKRSTSHQFCKSGV  VKRSTSHQF  1        0.0785      21391.1    95.00  0.40   tr_D0A148_
 HLA-DQA10501-DQB10201   14      KRSTSHQFCKSGVYS  QFCKSGVYS  6        0.0775      21618.2    95.00  0.39   tr_D0A148_
 HLA-DQA10501-DQB10201   15      RSTSHQFCKSGVYSS  QFCKSGVYS  5        0.0717      23015.0    95.00  0.41   tr_D0A148_
 HLA-DQA10501-DQB10201   16      VKRSTSHQFCKSGVY  KRSTSHQFC  1        0.0692      23657.1    95.00  0.27   tr_D0A148_
 HLA-DQA10501-DQB10201   17      GSTVAARDVKRSTSH  TVAARDVKR  2        0.0668      24274.2    95.00  0.40   tr_D0A148_
 HLA-DQA10501-DQB10201   18      STVAARDVKRSTSHQ  TVAARDVKR  1        0.0510      28796.0   100.00  0.50   tr_D0A148_
-----------------------------------------------------------------------------------------------

Allele: HLA-DQA10501-DQB10201. Number of high binders 0. Number of weak binders 0. Number of peptides 18

-----------------------------------------------------------------------------------------------
-----------------------------------------------------------------------------------------------
                Allele  pos              peptide       core Of 1-log50k(aff) affinity(nM)    %Rank Relia     Identity Bind Level
-----------------------------------------------------------------------------------------------
 HLA-DQA10501-DQB10201    1      DKAMAEAESVEAEVM  AESVEAEVM  6        0.7131         22.3     0.12  0.41   tr_D0A6D7_          SB
 HLA-DQA10501-DQB10201    2      KAMAEAESVEAEVMK  AESVEAEVM  5        0.6696         35.7     0.40  0.39   tr_D0A6D7_          SB
 HLA-DQA10501-DQB10201    3      VDKAMAEAESVEAEV  MAEAESVEA  4        0.6619         38.8     0.40  0.26   tr_D0A6D7_          SB
 HLA-DQA10501-DQB10201    4      AMAEAESVEAEVMKK  AESVEAEVM  4        0.6247         58.0     0.80  0.41   tr_D0A6D7_          WB
 HLA-DQA10501-DQB10201    5      GVDKAMAEAESVEAE  MAEAESVEA  5        0.6153         64.2     1.00  0.36   tr_D0A6D7_          WB
 HLA-DQA10501-DQB10201    6      MAEAESVEAEVMKKP  AESVEAEVM  3        0.5916         83.0     1.50  0.51   tr_D0A6D7_          WB
 HLA-DQA10501-DQB10201    7      GGVDKAMAEAESVEA  KAMAEAESV  4        0.5857         88.4     1.60  0.31   tr_D0A6D7_          WB
 HLA-DQA10501-DQB10201    8      AEAESVEAEVMKKPS  AESVEAEVM  2        0.5314        159.1     4.00  0.65   tr_D0A6D7_          WB
 HLA-DQA10501-DQB10201    9      AHVLMFSACGDDEAA  MFSACGDDE  4        0.5244        171.6     4.00  0.60   tr_D0A6D7_          WB
 HLA-DQA10501-DQB10201   10      HVLMFSACGDDEAAA  MFSACGDDE  3        0.5164        187.2     4.50  0.61   tr_D0A6D7_          WB
 HLA-DQA10501-DQB10201   11      NAHVLMFSACGDDEA  MFSACGDDE  5        0.5154        189.4     4.50  0.61   tr_D0A6D7_          WB
```

HLA-DQA10501-DQB10201	12	VLMFSACGDDEAAAD	MFSACGDDE	2	0.5125	195.3	5.00	0.61	tr_D0A6D7_	WB
HLA-DQA10501-DQB10201	13	KGGVDKAMAEAESVE	KAMAEAESV	5	0.5065	208.5	5.00	0.57	tr_D0A6D7_	WB
HLA-DQA10501-DQB10201	14	LMFSACGDDEAAADL	MFSACGDDE	1	0.4877	255.5	6.50	0.61	tr_D0A6D7_	WB
HLA-DQA10501-DQB10201	15	KFTLTELFSVAEPCP	ELFSVAEPC	5	0.4810	274.7	7.00	0.38	tr_D0A6D7_	WB
HLA-DQA10501-DQB10201	16	EAESVEAEVMKKPSK	AESVEAEVM	1	0.4726	300.7	7.50	0.74	tr_D0A6D7_	WB
HLA-DQA10501-DQB10201	17	FTLTELFSVAEPCPN	ELFSVAEPC	4	0.4726	300.7	7.50	0.35	tr_D0A6D7_	WB
HLA-DQA10501-DQB10201	18	VNAHVLMFSACGDDE	MFSACGDDE	6	0.4577	353.3	9.00	0.69	tr_D0A6D7_	WB
HLA-DQA10501-DQB10201	19	TLTELFSVAEPCPNI	ELFSVAEPC	3	0.4573	354.8	9.00	0.37	tr_D0A6D7_	WB
HLA-DQA10501-DQB10201	20	PKGGVDKAMAEAESV	KAMAEAESV	6	0.4559	360.5	9.00	0.72	tr_D0A6D7_	WB
HLA-DQA10501-DQB10201	21	EKFTLTELFSVAEPC	ELFSVAEPC	6	0.4536	369.5	9.50	0.42	tr_D0A6D7_	WB
HLA-DQA10501-DQB10201	22	LTELFSVAEPCPNIL	ELFSVAEPC	2	0.4526	373.5	9.50	0.39	tr_D0A6D7_	WB
HLA-DQA10501-DQB10201	23	MFSACGDDEAAADLP	MFSACGDDE	0	0.4523	374.5	9.50	0.47	tr_D0A6D7_	WB
HLA-DQA10501-DQB10201	24	KQQRSFRALFIGIDY	RALFIGIDY	6	0.4376	439.2	11.00	0.25	tr_D0A6D7_	
HLA-DQA10501-DQB10201	25	TELFSVAEPCPNILT	ELFSVAEPC	1	0.4259	498.3	13.00	0.38	tr_D0A6D7_	
HLA-DQA10501-DQB10201	26	TIAMELLQVAVPYVL	LLQVAVPYV	5	0.4157	556.9	14.00	0.28	tr_D0A6D7_	
HLA-DQA10501-DQB10201	27	QQRSFRALFIGIDYK	QRSFRALFI	1	0.4150	560.6	14.00	0.30	tr_D0A6D7_	
HLA-DQA10501-DQB10201	28	YGAQVRAEEDKEEEF	AEEDKEEEF	6	0.4131	572.6	14.00	0.26	tr_D0A6D7_	
HLA-DQA10501-DQB10201	29	SKQQRSFRALFIGID	QRSFRALFI	3	0.4063	616.3	15.00	0.44	tr_D0A6D7_	
HLA-DQA10501-DQB10201	30	ITERCVLMDTDDPRF	ERCVLMDTD	2	0.4058	619.6	15.00	0.30	tr_D0A6D7_	
HLA-DQA10501-DQB10201	31	TEKFTLTELFSVAEP	TELFSVAEP	6	0.4032	637.1	15.00	0.40	tr_D0A6D7_	
HLA-DQA10501-DQB10201	32	GAQVRAEEDKEEEFD	AEEDKEEEF	5	0.4006	655.3	16.00	0.36	tr_D0A6D7_	
HLA-DQA10501-DQB10201	33	VFDCSHAGTLLDLPF	AGTLLDLPF	6	0.3974	678.7	16.00	0.32	tr_D0A6D7_	
HLA-DQA10501-DQB10201	34	IAMELLQVAVPYVLV	LLQVAVPYV	4	0.3965	685.0	16.00	0.29	tr_D0A6D7_	
HLA-DQA10501-DQB10201	35	LHYSGYGAQVRAEED	GAQVRAEED	6	0.3930	711.6	17.00	0.52	tr_D0A6D7_	
HLA-DQA10501-DQB10201	36	GTPAELRGCQADAVM	LRGCQADAV	5	0.3929	712.6	17.00	0.50	tr_D0A6D7_	
HLA-DQA10501-DQB10201	37	TPAELRGCQADAVMM	LRGCQADAV	4	0.3926	714.8	17.00	0.46	tr_D0A6D7_	
HLA-DQA10501-DQB10201	38	TERCVLMDTDDPRFN	LMDTDDPRF	5	0.3898	736.8	17.00	0.34	tr_D0A6D7_	
HLA-DQA10501-DQB10201	39	PAELRGCQADAVMMA	LRGCQADAV	3	0.3836	787.6	18.00	0.44	tr_D0A6D7_	
HLA-DQA10501-DQB10201	40	YSGYGAQVRAEEDKE	GAQVRAEED	4	0.3826	796.2	19.00	0.41	tr_D0A6D7_	
HLA-DQA10501-DQB10201	41	SGYGAQVRAEEDKEE	GAQVRAEED	3	0.3817	804.3	19.00	0.38	tr_D0A6D7_	
HLA-DQA10501-DQB10201	42	GYGAQVRAEEDKEEE	GAQVRAEED	2	0.3781	836.5	19.00	0.37	tr_D0A6D7_	
HLA-DQA10501-DQB10201	43	FDCSHAGTLLDLPFS	HAGTLLDLP	4	0.3772	844.8	19.00	0.33	tr_D0A6D7_	
HLA-DQA10501-DQB10201	44	QRSFRALFIGIDYKG	RALFIGIDY	4	0.3763	852.5	20.00	0.22	tr_D0A6D7_	
HLA-DQA10501-DQB10201	45	DCSHAGTLLDLPFSL	HAGTLLDLP	3	0.3762	854.0	20.00	0.32	tr_D0A6D7_	
HLA-DQA10501-DQB10201	46	ELFSVAEPCPNILTG	LFSVAEPCP	1	0.3760	855.4	20.00	0.38	tr_D0A6D7_	
HLA-DQA10501-DQB10201	47	CILDNELHEIISTLP	LHEIISTLP	6	0.3743	871.4	20.00	0.31	tr_D0A6D7_	
HLA-DQA10501-DQB10201	48	AQVRAEEDKEEEFDQ	AEEDKEEEF	4	0.3731	883.1	20.00	0.38	tr_D0A6D7_	
HLA-DQA10501-DQB10201	49	AMELLQVAVPYVLVT	LLQVAVPYV	3	0.3697	915.7	21.00	0.31	tr_D0A6D7_	
HLA-DQA10501-DQB10201	50	IPITERCVLMDTDDP	ERCVLMDTD	4	0.3683	929.6	21.00	0.47	tr_D0A6D7_	
HLA-DQA10501-DQB10201	51	DKEEEFDQCIVPCDY	DQCIVPCDY	6	0.3670	942.5	21.00	0.42	tr_D0A6D7_	
HLA-DQA10501-DQB10201	52	QVRAEEDKEEEFDQC	AEEDKEEEF	3	0.3657	956.2	21.00	0.40	tr_D0A6D7_	
HLA-DQA10501-DQB10201	53	HYSGYGAQVRAEEDK	GAQVRAEED	5	0.3653	960.3	21.00	0.51	tr_D0A6D7_	
HLA-DQA10501-DQB10201	54	AELRGCQADAVMMAG	LRGCQADAV	2	0.3623	991.6	22.00	0.47	tr_D0A6D7_	
HLA-DQA10501-DQB10201	55	PITERCVLMDTDDPR	ERCVLMDTD	3	0.3614	1001.6	22.00	0.43	tr_D0A6D7_	
HLA-DQA10501-DQB10201	56	ERCVLMDTDDPRFNA	LMDTDDPRF	4	0.3610	1005.8	22.00	0.37	tr_D0A6D7_	
HLA-DQA10501-DQB10201	57	CQADAVMMAGTMEKI	MMAGTMEKI	6	0.3605	1011.8	22.00	0.47	tr_D0A6D7_	
HLA-DQA10501-DQB10201	58	TLPRGVRLTAVFDCS	GVRLTAVFD	4	0.3596	1021.8	22.00	0.28	tr_D0A6D7_	
HLA-DQA10501-DQB10201	59	EEEFDQCIVPCDYEE	DQCIVPCDY	4	0.3579	1040.4	23.00	0.40	tr_D0A6D7_	
HLA-DQA10501-DQB10201	60	CSHAGTLLDLPFSLI	HAGTLLDLP	2	0.3574	1046.0	23.00	0.30	tr_D0A6D7_	
HLA-DQA10501-DQB10201	61	KEEEFDQCIVPCDYE	DQCIVPCDY	5	0.3555	1067.9	23.00	0.41	tr_D0A6D7_	

HLA-DQA10501-DQB10201	62	KKPSKQQRSFRALFI	QRSFRALFI	6	0.3551	1072.8	23.00	0.75	tr_D0A6D7_
HLA-DQA10501-DQB10201	63	LSLTEKFTLTELFSV	EKFTLTELF	4	0.3545	1079.2	23.00	0.40	tr_D0A6D7_
HLA-DQA10501-DQB10201	64	CLSLTEKFTLTELFS	EKFTLTELF	5	0.3541	1083.7	23.00	0.49	tr_D0A6D7_
HLA-DQA10501-DQB10201	65	ILDNELHEIISTLPR	LHEIISTLP	5	0.3535	1091.5	24.00	0.29	tr_D0A6D7_
HLA-DQA10501-DQB10201	66	PCDYEENGCILDNEL	NGCILDNEL	6	0.3528	1099.0	24.00	0.44	tr_D0A6D7_
HLA-DQA10501-DQB10201	67	LPRGVRLTAVFDCSH	GVRLTAVFD	3	0.3515	1114.7	24.00	0.25	tr_D0A6D7_
HLA-DQA10501-DQB10201	68	AESVEAEVMKKPSKQ	AESVEAEVM	0	0.3507	1124.6	24.00	0.77	tr_D0A6D7_
HLA-DQA10501-DQB10201	69	EEFDQCIVPCDYEEN	DQCIVPCDY	3	0.3504	1129.0	24.00	0.37	tr_D0A6D7_
HLA-DQA10501-DQB10201	70	KGTPAELRGCQADAV	LRGCQADAV	6	0.3484	1152.9	25.00	0.56	tr_D0A6D7_
HLA-DQA10501-DQB10201	71	RSFRALFIGIDYKGT	RALFIGIDY	3	0.3457	1187.3	25.00	0.26	tr_D0A6D7_
HLA-DQA10501-DQB10201	72	VRAEEDKEEEFDQCI	AEEDKEEEF	2	0.3450	1196.2	25.00	0.39	tr_D0A6D7_
HLA-DQA10501-DQB10201	73	RCLSLTEKFTLTELF	EKFTLTELF	6	0.3449	1197.9	25.00	0.63	tr_D0A6D7_
HLA-DQA10501-DQB10201	74	MELLQVAVPYVLVTL	LLQVAVPYV	2	0.3448	1198.8	25.00	0.33	tr_D0A6D7_
HLA-DQA10501-DQB10201	75	EENGCILDNELHEII	ILDNELHEI	5	0.3437	1212.9	25.00	0.27	tr_D0A6D7_
HLA-DQA10501-DQB10201	76	STLPRGVRLTAVFDC	GVRLTAVFD	5	0.3434	1216.7	26.00	0.34	tr_D0A6D7_
HLA-DQA10501-DQB10201	77	YEENGCILDNELHEI	NGCILDNEL	3	0.3431	1220.9	26.00	0.35	tr_D0A6D7_
HLA-DQA10501-DQB10201	78	PSKQQRSFRALFIGI	QRSFRALFI	4	0.3423	1231.5	26.00	0.62	tr_D0A6D7_
HLA-DQA10501-DQB10201	79	AVFDCSHAGTLLDLP	SHAGTLLDL	5	0.3415	1242.3	26.00	0.47	tr_D0A6D7_
HLA-DQA10501-DQB10201	80	LTEKFTLTELFSVAE	EKFTLTELF	2	0.3383	1286.9	27.00	0.35	tr_D0A6D7_
HLA-DQA10501-DQB10201	81	SHAGTLLDLPFSLIC	HAGTLLDLP	1	0.3364	1312.9	27.00	0.31	tr_D0A6D7_
HLA-DQA10501-DQB10201	82	SFRALFIGIDYKGTP	RALFIGIDY	2	0.3362	1316.1	27.00	0.28	tr_D0A6D7_
HLA-DQA10501-DQB10201	83	SLTEKFTLTELFSVA	EKFTLTELF	3	0.3356	1324.0	27.00	0.36	tr_D0A6D7_
HLA-DQA10501-DQB10201	84	ISTLPRGVRLTAVFD	GVRLTAVFD	6	0.3329	1364.3	28.00	0.38	tr_D0A6D7_
HLA-DQA10501-DQB10201	85	GIPITERCVLMDTDD	ERCVLMDTD	5	0.3322	1374.3	28.00	0.52	tr_D0A6D7_
HLA-DQA10501-DQB10201	86	CDYEENGCILDNELH	NGCILDNEL	5	0.3319	1377.8	28.00	0.41	tr_D0A6D7_
HLA-DQA10501-DQB10201	87	GCILDNELHEIISTL	ELHEIISTL	6	0.3301	1404.9	28.00	0.38	tr_D0A6D7_
HLA-DQA10501-DQB10201	88	PRGVRLTAVFDCSHA	GVRLTAVFD	2	0.3300	1406.9	28.00	0.23	tr_D0A6D7_
HLA-DQA10501-DQB10201	89	QADAVMMAGTMEKIG	MMAGTMEKI	5	0.3296	1413.6	28.00	0.47	tr_D0A6D7_
HLA-DQA10501-DQB10201	90	RAEEDKEEEFDQCIV	AEEDKEEEF	1	0.3279	1439.2	29.00	0.34	tr_D0A6D7_
HLA-DQA10501-DQB10201	91	ELRGCQADAVMMAGT	LRGCQADAV	1	0.3250	1485.7	29.00	0.47	tr_D0A6D7_
HLA-DQA10501-DQB10201	92	KPSKQQRSFRALFIG	QRSFRALFI	5	0.3231	1515.5	30.00	0.74	tr_D0A6D7_
HLA-DQA10501-DQB10201	93	RCVLMDTDDPRFNAI	LMDTDDPRF	3	0.3231	1516.1	30.00	0.47	tr_D0A6D7_
HLA-DQA10501-DQB10201	94	EFDQCIVPCDYEENG	DQCIVPCDY	2	0.3231	1516.9	30.00	0.41	tr_D0A6D7_
HLA-DQA10501-DQB10201	95	DYEENGCILDNELHE	NGCILDNEL	4	0.3228	1521.8	30.00	0.40	tr_D0A6D7_
HLA-DQA10501-DQB10201	96	IGIPITERCVLMDTD	ERCVLMDTD	6	0.3217	1539.5	30.00	0.52	tr_D0A6D7_
HLA-DQA10501-DQB10201	97	LDNELHEIISTLPRG	LHEIISTLP	4	0.3217	1539.8	30.00	0.32	tr_D0A6D7_
HLA-DQA10501-DQB10201	98	ADAVMMAGTMEKIGI	MMAGTMEKI	4	0.3194	1578.5	31.00	0.47	tr_D0A6D7_
HLA-DQA10501-DQB10201	99	ENGCILDNELHEIIS	ILDNELHEI	4	0.3189	1585.8	31.00	0.32	tr_D0A6D7_
HLA-DQA10501-DQB10201	100	FSACGDDEAAADLPN	FSACGDDEA	0	0.3175	1610.3	31.00	0.35	tr_D0A6D7_
HLA-DQA10501-DQB10201	101	DAVMMAGTMEKIGIP	MMAGTMEKI	3	0.3170	1620.4	31.00	0.53	tr_D0A6D7_
HLA-DQA10501-DQB10201	102	DDEAAADLPNAGDFV	DLPNAGDFV	6	0.3146	1662.0	32.00	0.35	tr_D0A6D7_
HLA-DQA10501-DQB10201	103	KTPGTIYSLLSTTRD	YSLLSTTRD	6	0.3134	1684.7	32.00	0.40	tr_D0A6D7_
HLA-DQA10501-DQB10201	104	ELLQVAVPYVLVTLG	LLQVAVPYV	1	0.3131	1689.9	32.00	0.34	tr_D0A6D7_
HLA-DQA10501-DQB10201	105	DNELHEIISTLPRGV	LHEIISTLP	3	0.3106	1734.8	33.00	0.33	tr_D0A6D7_
HLA-DQA10501-DQB10201	106	DEAAADLPNAGDFVE	DLPNAGDFV	5	0.3087	1771.9	33.00	0.40	tr_D0A6D7_
HLA-DQA10501-DQB10201	107	FRALFIGIDYKGTPA	RALFIGIDY	1	0.3049	1845.6	34.00	0.31	tr_D0A6D7_
HLA-DQA10501-DQB10201	108	CVLMDTDDPRFNAIK	LMDTDDPRF	2	0.3031	1883.2	34.00	0.54	tr_D0A6D7_
HLA-DQA10501-DQB10201	109	AADLPNAGDFVEGAS	DLPNAGDFV	2	0.3020	1905.8	35.00	0.31	tr_D0A6D7_
HLA-DQA10501-DQB10201	110	GCQADAVMMAGTMEK	VMMAGTMEK	6	0.3014	1917.2	35.00	0.36	tr_D0A6D7_
HLA-DQA10501-DQB10201	111	FLHYSGYGAQVRAEE	YGAQVRAEE	6	0.2997	1953.5	35.00	0.28	tr_D0A6D7_

HLA-DQA10501-DQB10201	112	AAADLPNAGDFVEGA	DLPNAGDFV	3	0.2985	1979.2	35.00	0.34	tr_D0A6D7_
HLA-DQA10501-DQB10201	113	RGVRLTAVFDCSHAG	GVRLTAVFD	1	0.2964	2023.0	36.00	0.25	tr_D0A6D7_
HLA-DQA10501-DQB10201	114	EDKEEEFDQCIVPCD	EEEFDQCIV	3	0.2964	2024.8	36.00	0.22	tr_D0A6D7_
HLA-DQA10501-DQB10201	115	NGCILDNELHEIIST	ILDNELHEI	3	0.2956	2042.2	36.00	0.28	tr_D0A6D7_
HLA-DQA10501-DQB10201	116	AEEDKEEEFDQCIVP	AEEDKEEEF	0	0.2942	2071.9	37.00	0.22	tr_D0A6D7_
HLA-DQA10501-DQB10201	117	DVNAHVLMFSACGDD	VLMFSACGD	5	0.2942	2072.7	37.00	0.44	tr_D0A6D7_
HLA-DQA10501-DQB10201	118	RGCQADAVMMAGTME	AVMMAGTME	6	0.2922	2117.2	37.00	0.49	tr_D0A6D7_
HLA-DQA10501-DQB10201	119	LLQVAVPYVLVTLGL	LQVAVPYVL	1	0.2920	2123.3	37.00	0.31	tr_D0A6D7_
HLA-DQA10501-DQB10201	120	EAAADLPNAGDFVEG	DLPNAGDFV	4	0.2903	2161.6	38.00	0.39	tr_D0A6D7_
HLA-DQA10501-DQB10201	121	IDYKGTPAELRGCQA	PAELRGCQA	6	0.2897	2175.3	38.00	0.35	tr_D0A6D7_
HLA-DQA10501-DQB10201	122	LFIGIDYKGTPAELR	DYKGTPAEL	5	0.2877	2223.7	38.00	0.43	tr_D0A6D7_
HLA-DQA10501-DQB10201	123	SACGDDEAAADLPNA	DDEAAADLP	4	0.2876	2225.3	38.00	0.34	tr_D0A6D7_
HLA-DQA10501-DQB10201	124	GSGGAATQCFISMLL	TQCFISMLL	6	0.2871	2238.3	38.00	0.49	tr_D0A6D7_
HLA-DQA10501-DQB10201	125	FDQCIVPCDYEENGC	DQCIVPCDY	1	0.2870	2240.2	38.00	0.38	tr_D0A6D7_
HLA-DQA10501-DQB10201	126	AVMMAGTMEKIGIPI	MMAGTMEKI	2	0.2870	2241.0	38.00	0.51	tr_D0A6D7_
HLA-DQA10501-DQB10201	127	PGTIYSLLSTTRDKL	YSLLSTTRD	4	0.2868	2246.3	38.00	0.41	tr_D0A6D7_
HLA-DQA10501-DQB10201	128	ALFIGIDYKGTPAEL	DYKGTPAEL	6	0.2863	2258.0	39.00	0.47	tr_D0A6D7_
HLA-DQA10501-DQB10201	129	DPRFNAIKPTRANIL	FNAIKPTRA	3	0.2858	2271.0	39.00	0.61	tr_D0A6D7_
HLA-DQA10501-DQB10201	130	HAGTLLDLPFSLICS	AGTLLDLPF	1	0.2857	2273.4	39.00	0.28	tr_D0A6D7_
HLA-DQA10501-DQB10201	131	NELHEIISTLPRGVR	LHEIISTLP	2	0.2854	2280.0	39.00	0.35	tr_D0A6D7_
HLA-DQA10501-DQB10201	132	EEDKEEEFDQCIVPC	EEEFDQCIV	4	0.2853	2282.4	39.00	0.23	tr_D0A6D7_
HLA-DQA10501-DQB10201	133	LRGCQADAVMMAGTM	LRGCQADAV	0	0.2850	2290.8	39.00	0.31	tr_D0A6D7_
HLA-DQA10501-DQB10201	134	TPGTIYSLLSTTRDK	YSLLSTTRD	5	0.2836	2324.4	39.00	0.40	tr_D0A6D7_
HLA-DQA10501-DQB10201	135	TAVFDCSHAGTLLDL	SHAGTLLDL	6	0.2821	2361.7	40.00	0.60	tr_D0A6D7_
HLA-DQA10501-DQB10201	136	ADLPNAGDFVEGASG	DLPNAGDFV	1	0.2799	2419.0	40.00	0.31	tr_D0A6D7_
HLA-DQA10501-DQB10201	137	GDDEAAADLPNAGDF	EAAADLPNA	3	0.2795	2430.8	40.00	0.35	tr_D0A6D7_
HLA-DQA10501-DQB10201	138	GDVNAHVLMFSACGD	VLMFSACGD	6	0.2788	2447.7	40.00	0.45	tr_D0A6D7_
HLA-DQA10501-DQB10201	139	IRTGGDVNAHVLMFS	DVNAHVLMF	5	0.2763	2515.1	41.00	0.36	tr_D0A6D7_
HLA-DQA10501-DQB10201	140	DDPRFNAIKPTRANI	FNAIKPTRA	4	0.2736	2591.6	42.00	0.68	tr_D0A6D7_
HLA-DQA10501-DQB10201	141	SGGAATQCFISMLLN	TQCFISMLL	5	0.2731	2605.7	42.00	0.41	tr_D0A6D7_
HLA-DQA10501-DQB10201	142	FIGIDYKGTPAELRG	DYKGTPAEL	4	0.2730	2608.0	42.00	0.42	tr_D0A6D7_
HLA-DQA10501-DQB10201	143	IISTLPRGVRLTAVF	PRGVRLTAV	5	0.2723	2627.8	42.00	0.49	tr_D0A6D7_
HLA-DQA10501-DQB10201	144	TDDPRFNAIKPTRAN	FNAIKPTRA	5	0.2708	2669.9	43.00	0.68	tr_D0A6D7_
HLA-DQA10501-DQB10201	145	DYKGTPAELRGCQAD	PAELRGCQA	5	0.2700	2691.7	43.00	0.44	tr_D0A6D7_
HLA-DQA10501-DQB10201	146	VPCDYEENGCILDNE	ENGCILDNE	6	0.2698	2697.5	43.00	0.38	tr_D0A6D7_
HLA-DQA10501-DQB10201	147	DTDDPRFNAIKPTRA	FNAIKPTRA	6	0.2690	2721.8	43.00	0.64	tr_D0A6D7_
HLA-DQA10501-DQB10201	148	CGDDEAAADLPNAGD	EAAADLPNA	4	0.2688	2727.8	43.00	0.34	tr_D0A6D7_
HLA-DQA10501-DQB10201	149	AGTMEKIGIPITERC	KIGIPITER	5	0.2681	2748.9	43.00	0.28	tr_D0A6D7_
HLA-DQA10501-DQB10201	150	VMMAGTMEKIGIPIT	MMAGTMEKI	1	0.2669	2785.3	44.00	0.46	tr_D0A6D7_
HLA-DQA10501-DQB10201	151	MAGTMEKIGIPITER	KIGIPITER	6	0.2668	2787.6	44.00	0.30	tr_D0A6D7_
HLA-DQA10501-DQB10201	152	ACGDDEAAADLPNAG	DDEAAADLP	3	0.2665	2797.8	44.00	0.35	tr_D0A6D7_
HLA-DQA10501-DQB10201	153	GVRLTAVFDCSHAGT	RLTAVFDCS	2	0.2646	2855.3	44.00	0.30	tr_D0A6D7_
HLA-DQA10501-DQB10201	154	PRFNAIKPTRANILQ	FNAIKPTRA	2	0.2643	2863.9	44.00	0.58	tr_D0A6D7_
HLA-DQA10501-DQB10201	155	MMAGTMEKIGIPITE	EKIGIPITE	6	0.2641	2869.5	44.00	0.29	tr_D0A6D7_
HLA-DQA10501-DQB10201	156	ELHEIISTLPRGVRL	LHEIISTLP	1	0.2635	2888.6	44.00	0.35	tr_D0A6D7_
HLA-DQA10501-DQB10201	157	FNAIKPTRANILQHM	TRANILQHM	6	0.2622	2930.8	45.00	0.44	tr_D0A6D7_
HLA-DQA10501-DQB10201	158	GTMEKIGIPITERCV	KIGIPITER	4	0.2608	2976.0	45.00	0.28	tr_D0A6D7_
HLA-DQA10501-DQB10201	159	KPTRANILQHMAWLV	TRANILQHM	2	0.2606	2980.9	45.00	0.26	tr_D0A6D7_
HLA-DQA10501-DQB10201	160	LFSVAEPCPNILTGE	LFSVAEPCP	0	0.2602	2993.9	45.00	0.38	tr_D0A6D7_
HLA-DQA10501-DQB10201	161	TMEKIGIPITERCVL	KIGIPITER	3	0.2595	3018.6	46.00	0.29	tr_D0A6D7_

HLA-DQA10501-DQB10201	162	RIRTGGDVNAHVLMF	DVNAHVLMF	6	0.2589	3038.1	46.00	0.44	tr_D0A6D7_
HLA-DQA10501-DQB10201	163	RTGGDVNAHVLMFSA	DVNAHVLMF	4	0.2583	3055.4	46.00	0.35	tr_D0A6D7_
HLA-DQA10501-DQB10201	164	IKPTRANILQHMAWL	TRANILQHM	3	0.2578	3072.1	46.00	0.32	tr_D0A6D7_
HLA-DQA10501-DQB10201	165	GTIYSLLSTTRDKLR	YSLLSTTRD	3	0.2573	3091.4	46.00	0.44	tr_D0A6D7_
HLA-DQA10501-DQB10201	166	IGIDYKGTPAELRGC	DYKGTPAEL	3	0.2571	3097.5	46.00	0.45	tr_D0A6D7_
HLA-DQA10501-DQB10201	167	PPKGGVDKAMAEAES	VDKAMAEAE	5	0.2554	3153.1	47.00	0.50	tr_D0A6D7_
HLA-DQA10501-DQB10201	168	LFLHYSGYGAQVRAE	YSGYGAQVR	4	0.2535	3220.7	47.00	0.34	tr_D0A6D7_
HLA-DQA10501-DQB10201	169	VLMDTDDPRFNAIKP	LMDTDDPRF	1	0.2530	3236.2	47.00	0.60	tr_D0A6D7_
HLA-DQA10501-DQB10201	170	DLPNAGDFVEGASGS	NAGDFVEGA	3	0.2493	3369.4	48.00	0.28	tr_D0A6D7_
HLA-DQA10501-DQB10201	171	RFNAIKPTRANILQH	FNAIKPTRA	1	0.2484	3402.6	49.00	0.57	tr_D0A6D7_
HLA-DQA10501-DQB10201	172	MEKIGIPITERCVLM	KIGIPITER	2	0.2479	3420.3	49.00	0.29	tr_D0A6D7_
HLA-DQA10501-DQB10201	173	GAATQCFISMLLNKT	FISMLLNKT	6	0.2457	3502.3	49.00	0.36	tr_D0A6D7_
HLA-DQA10501-DQB10201	174	YKGTPAELRGCQADA	PAELRGCQA	4	0.2444	3553.6	50.00	0.49	tr_D0A6D7_
HLA-DQA10501-DQB10201	175	DQCIVPCDYEENGCI	DQCIVPCDY	0	0.2438	3577.3	50.00	0.31	tr_D0A6D7_
HLA-DQA10501-DQB10201	176	AATQCFISMLLNKTP	FISMLLNKT	5	0.2430	3604.8	50.00	0.43	tr_D0A6D7_
HLA-DQA10501-DQB10201	177	TGGDVNAHVLMFSAC	DVNAHVLMF	3	0.2424	3629.7	50.00	0.35	tr_D0A6D7_
HLA-DQA10501-DQB10201	178	CIVPCDYEENGCILD	DYEENGCIL	5	0.2421	3643.7	50.00	0.43	tr_D0A6D7_
HLA-DQA10501-DQB10201	179	NILQHMAWLVKDAKP	MAWLVKDAK	5	0.2420	3644.4	50.00	0.37	tr_D0A6D7_
HLA-DQA10501-DQB10201	180	TIYSLLSTTRDKLRE	YSLLSTTRD	2	0.2412	3676.4	55.00	0.49	tr_D0A6D7_
HLA-DQA10501-DQB10201	181	QCIVPCDYEENGCIL	DYEENGCIL	6	0.2409	3688.2	55.00	0.44	tr_D0A6D7_
HLA-DQA10501-DQB10201	182	GIDYKGTPAELRGCQ	DYKGTPAEL	2	0.2406	3702.1	55.00	0.44	tr_D0A6D7_
HLA-DQA10501-DQB10201	183	TRANILQHMAWLVKD	QHMAWLVKD	6	0.2404	3708.7	55.00	0.20	tr_D0A6D7_
HLA-DQA10501-DQB10201	184	NKTPGTIYSLLSTTR	IYSLLSTTR	6	0.2403	3712.6	55.00	0.34	tr_D0A6D7_
HLA-DQA10501-DQB10201	185	TLLDLPFSLICSSND	FSLICSSND	6	0.2389	3771.4	55.00	0.43	tr_D0A6D7_
HLA-DQA10501-DQB10201	186	AIKPTRANILQHMAW	TRANILQHM	4	0.2385	3785.3	55.00	0.41	tr_D0A6D7_
HLA-DQA10501-DQB10201	187	AGTLLDLPFSLICSS	TLLDLPFSL	2	0.2379	3811.2	55.00	0.37	tr_D0A6D7_
HLA-DQA10501-DQB10201	188	DALFLHYSGYGAQVR	YSGYGAQVR	6	0.2378	3814.9	55.00	0.43	tr_D0A6D7_
HLA-DQA10501-DQB10201	189	LQVAVPYVLVTLGLQ	LQVAVPYVL	0	0.2366	3866.7	55.00	0.27	tr_D0A6D7_
HLA-DQA10501-DQB10201	190	RALFIGIDYKGTPAE	ALFIGIDYK	1	0.2365	3870.9	55.00	0.27	tr_D0A6D7_
HLA-DQA10501-DQB10201	191	PTRANILQHMAWLVK	TRANILQHM	1	0.2362	3880.1	55.00	0.28	tr_D0A6D7_
HLA-DQA10501-DQB10201	192	EKIGIPITERCVLMD	KIGIPITER	1	0.2358	3898.0	55.00	0.25	tr_D0A6D7_
HLA-DQA10501-DQB10201	193	ALFLHYSGYGAQVRA	YSGYGAQVR	5	0.2350	3933.0	55.00	0.41	tr_D0A6D7_
HLA-DQA10501-DQB10201	194	ANILQHMAWLVKDAK	MAWLVKDAK	6	0.2349	3936.9	55.00	0.30	tr_D0A6D7_
HLA-DQA10501-DQB10201	195	LLNKTPGTIYSLLST	PGTIYSLLS	5	0.2347	3945.3	55.00	0.51	tr_D0A6D7_
HLA-DQA10501-DQB10201	196	GGAATQCFISMLLNK	TQCFISMLL	4	0.2298	4160.2	55.00	0.43	tr_D0A6D7_
HLA-DQA10501-DQB10201	197	EIISTLPRGVRLTAV	PRGVRLTAV	6	0.2295	4173.9	55.00	0.56	tr_D0A6D7_
HLA-DQA10501-DQB10201	198	MLLNKTPGTIYSLLS	PGTIYSLLS	6	0.2294	4180.0	55.00	0.62	tr_D0A6D7_
HLA-DQA10501-DQB10201	199	RANILQHMAWLVKDA	HMAWLVKDA	6	0.2293	4183.8	55.00	0.24	tr_D0A6D7_
HLA-DQA10501-DQB10201	200	EGASGSGGAATQCFI	GGAATQCFI	6	0.2290	4194.5	55.00	0.44	tr_D0A6D7_
HLA-DQA10501-DQB10201	201	LNKTPGTIYSLLSTT	PGTIYSLLS	4	0.2288	4206.4	55.00	0.44	tr_D0A6D7_
HLA-DQA10501-DQB10201	202	LLDLPFSLICSSNDC	FSLICSSND	5	0.2286	4215.2	55.00	0.52	tr_D0A6D7_
HLA-DQA10501-DQB10201	203	IVPCDYEENGCILDN	DYEENGCIL	4	0.2258	4346.6	55.00	0.43	tr_D0A6D7_
HLA-DQA10501-DQB10201	204	LDLPFSLICSSNDCS	FSLICSSND	4	0.2255	4360.4	55.00	0.47	tr_D0A6D7_
HLA-DQA10501-DQB10201	205	GGDVNAHVLMFSACG	DVNAHVLMF	2	0.2251	4377.6	55.00	0.37	tr_D0A6D7_
HLA-DQA10501-DQB10201	206	SRCLSLTEKFTLTEL	TEKFTLTEL	6	0.2249	4385.3	55.00	0.69	tr_D0A6D7_
HLA-DQA10501-DQB10201	207	DLPFSLICSSNDCSA	FSLICSSND	3	0.2239	4433.1	60.00	0.45	tr_D0A6D7_
HLA-DQA10501-DQB10201	208	SGSGGAATQCFISML	GGAATQCFI	3	0.2234	4460.3	60.00	0.31	tr_D0A6D7_
HLA-DQA10501-DQB10201	209	VAEPCPNILTGEPRW	NILTGEPRW	6	0.2225	4500.7	60.00	0.41	tr_D0A6D7_
HLA-DQA10501-DQB10201	210	ATQCFISMLLNKTPG	FISMLLNKT	4	0.2216	4545.5	60.00	0.43	tr_D0A6D7_
HLA-DQA10501-DQB10201	211	VRLTAVFDCSHAGTL	RLTAVFDCS	1	0.2215	4551.9	60.00	0.37	tr_D0A6D7_

HLA-DQA10501-DQB10201	212	KIGIPITERCVLMDT	TERCVLMDT	6	0.2194	4658.5	60.00	0.21	tr_D0A6D7_
HLA-DQA10501-DQB10201	213	NAIKPTRANILQHMA	TRANILQHM	5	0.2177	4743.8	60.00	0.47	tr_D0A6D7_
HLA-DQA10501-DQB10201	214	ASGSGGAATQCFISM	GGAATQCFI	4	0.2150	4882.7	60.00	0.36	tr_D0A6D7_
HLA-DQA10501-DQB10201	215	RPPKGGVDKAMAEAE	VDKAMAEAE	6	0.2139	4940.2	60.00	0.68	tr_D0A6D7_
HLA-DQA10501-DQB10201	216	GASGSGGAATQCFIS	GGAATQCFI	5	0.2100	5154.2	60.00	0.43	tr_D0A6D7_
HLA-DQA10501-DQB10201	217	LQHMAWLVKDAKPGD	MAWLVKDAK	3	0.2095	5179.9	60.00	0.41	tr_D0A6D7_
HLA-DQA10501-DQB10201	218	ILQHMAWLVKDAKPG	MAWLVKDAK	4	0.2090	5208.5	60.00	0.38	tr_D0A6D7_
HLA-DQA10501-DQB10201	219	LPFSLICSSNDCSAV	FSLICSSND	2	0.2069	5333.0	65.00	0.45	tr_D0A6D7_
HLA-DQA10501-DQB10201	220	LHEIISTLPRGVRLT	LHEIISTLP	0	0.2067	5340.2	65.00	0.32	tr_D0A6D7_
HLA-DQA10501-DQB10201	221	IYSLLSTTRDKLREK	YSLLSTTRD	1	0.2053	5422.9	65.00	0.56	tr_D0A6D7_
HLA-DQA10501-DQB10201	222	GTLLDLPFSLICSSN	TLLDLPFSL	1	0.2044	5475.3	65.00	0.44	tr_D0A6D7_
HLA-DQA10501-DQB10201	223	QHMAWLVKDAKPGDA	MAWLVKDAK	2	0.2027	5577.6	65.00	0.42	tr_D0A6D7_
HLA-DQA10501-DQB10201	224	GDALFLHYSGYGAQV	HYSGYGAQV	6	0.1994	5781.5	65.00	0.44	tr_D0A6D7_
HLA-DQA10501-DQB10201	225	AEPCPNILTGEPRWK	NILTGEPRW	5	0.1984	5842.9	65.00	0.41	tr_D0A6D7_
HLA-DQA10501-DQB10201	226	TQCFISMLLNKTPGT	FISMLLNKT	3	0.1973	5911.8	65.00	0.50	tr_D0A6D7_
HLA-DQA10501-DQB10201	227	LMDTDDPRFNAIKPT	LMDTDDPRF	0	0.1969	5940.0	65.00	0.57	tr_D0A6D7_
HLA-DQA10501-DQB10201	228	QVAVPYVLVTLGLQR	PYVLVTLGL	4	0.1960	5995.2	65.00	0.24	tr_D0A6D7_
HLA-DQA10501-DQB10201	229	EPCPNILTGEPRWKT	NILTGEPRW	4	0.1907	6350.7	65.00	0.43	tr_D0A6D7_
HLA-DQA10501-DQB10201	230	LPNAGDFVEGASGSG	NAGDFVEGA	2	0.1898	6410.3	65.00	0.43	tr_D0A6D7_
HLA-DQA10501-DQB10201	231	LVKDAKPGDALFLHY	PGDALFLHY	6	0.1897	6421.2	65.00	0.44	tr_D0A6D7_
HLA-DQA10501-DQB10201	232	RLTAVFDCSHAGTLL	RLTAVFDCS	0	0.1893	6448.7	70.00	0.31	tr_D0A6D7_
HLA-DQA10501-DQB10201	233	KRIRTGGDVNAHVLM	GGDVNAHVL	5	0.1878	6553.6	70.00	0.20	tr_D0A6D7_
HLA-DQA10501-DQB10201	234	PFSLICSSNDCSAVG	FSLICSSND	1	0.1862	6670.2	70.00	0.46	tr_D0A6D7_
HLA-DQA10501-DQB10201	235	PQMSASRCLSLTEKF	RCLSLTEKF	6	0.1843	6810.3	70.00	0.43	tr_D0A6D7_
HLA-DQA10501-DQB10201	236	VEGASGSGGAATQCF	SGGAATQCF	6	0.1842	6811.8	70.00	0.70	tr_D0A6D7_
HLA-DQA10501-DQB10201	237	HMAWLVKDAKPGDAL	MAWLVKDAK	1	0.1821	6973.4	70.00	0.41	tr_D0A6D7_
HLA-DQA10501-DQB10201	238	AVPYVLVTLGLQRRP	YVLVTLGLQ	3	0.1816	7008.8	70.00	0.35	tr_D0A6D7_
HLA-DQA10501-DQB10201	239	VAVPYVLVTLGLQRR	PYVLVTLGL	3	0.1798	7143.2	70.00	0.25	tr_D0A6D7_
HLA-DQA10501-DQB10201	240	PCPNILTGEPRWKTT	NILTGEPRW	3	0.1795	7168.1	70.00	0.44	tr_D0A6D7_
HLA-DQA10501-DQB10201	241	VPYVLVTLGLQRRPP	YVLVTLGLQ	2	0.1790	7210.9	70.00	0.34	tr_D0A6D7_
HLA-DQA10501-DQB10201	242	MKRIRTGGDVNAHVL	GGDVNAHVL	6	0.1759	7454.3	70.00	0.25	tr_D0A6D7_
HLA-DQA10501-DQB10201	243	FSVAEPCPNILTGEP	VAEPCPNIL	2	0.1736	7640.2	70.00	0.38	tr_D0A6D7_
HLA-DQA10501-DQB10201	244	KPGDALFLHYSGYGA	FLHYSGYGA	6	0.1728	7710.7	70.00	0.35	tr_D0A6D7_
HLA-DQA10501-DQB10201	245	CPNILTGEPRWKTTP	NILTGEPRW	2	0.1726	7725.1	70.00	0.44	tr_D0A6D7_
HLA-DQA10501-DQB10201	246	QCFISMLLNKTPGTI	FISMLLNKT	2	0.1723	7747.5	70.00	0.58	tr_D0A6D7_
HLA-DQA10501-DQB10201	247	VKDAKPGDALFLHYS	PGDALFLHY	5	0.1704	7911.5	75.00	0.42	tr_D0A6D7_
HLA-DQA10501-DQB10201	248	PNAGDFVEGASGSGG	NAGDFVEGA	1	0.1690	8028.6	75.00	0.42	tr_D0A6D7_
HLA-DQA10501-DQB10201	249	SVAEPCPNILTGEPR	PNILTGEPR	6	0.1687	8056.6	75.00	0.50	tr_D0A6D7_
HLA-DQA10501-DQB10201	250	AWLVKDAKPGDALFL	AKPGDALFL	6	0.1673	8184.1	75.00	0.30	tr_D0A6D7_
HLA-DQA10501-DQB10201	251	QMSASRCLSLTEKFT	RCLSLTEKF	5	0.1664	8259.5	75.00	0.38	tr_D0A6D7_
HLA-DQA10501-DQB10201	252	MSASRCLSLTEKFTL	RCLSLTEKF	4	0.1641	8471.6	75.00	0.37	tr_D0A6D7_
HLA-DQA10501-DQB10201	253	FSLICSSNDCSAVGE	FSLICSSND	0	0.1640	8476.2	75.00	0.37	tr_D0A6D7_
HLA-DQA10501-DQB10201	254	LTAVFDCSHAGTLLD	CSHAGTLLD	6	0.1639	8490.3	75.00	0.23	tr_D0A6D7_
HLA-DQA10501-DQB10201	255	PYVLVTLGLQRRPPK	VLVTLGLQR	2	0.1632	8554.4	75.00	0.28	tr_D0A6D7_
HLA-DQA10501-DQB10201	256	MAWLVKDAKPGDALF	MAWLVKDAK	0	0.1630	8574.6	75.00	0.22	tr_D0A6D7_
HLA-DQA10501-DQB10201	257	AKPGDALFLHYSGYG	PGDALFLHY	2	0.1605	8806.6	75.00	0.35	tr_D0A6D7_
HLA-DQA10501-DQB10201	258	PNILTGEPRWKTTPL	NILTGEPRW	1	0.1601	8841.0	75.00	0.47	tr_D0A6D7_
HLA-DQA10501-DQB10201	259	DAKPGDALFLHYSGY	PGDALFLHY	3	0.1591	8941.6	75.00	0.38	tr_D0A6D7_
HLA-DQA10501-DQB10201	260	KDAKPGDALFLHYSG	PGDALFLHY	4	0.1588	8973.4	75.00	0.38	tr_D0A6D7_
HLA-DQA10501-DQB10201	261	PGDALFLHYSGYGAQ	FLHYSGYGA	5	0.1580	9050.1	75.00	0.38	tr_D0A6D7_

HLA-DQA10501-DQB10201	262	EMKRIRTGGDVNAHV	TGGDVNAHV	6	0.1559	9253.0	75.00	0.32	tr_D0A6D7_
HLA-DQA10501-DQB10201	263	HEIISTLPRGVRLTA	IISTLPRGV	2	0.1558	9261.6	75.00	0.38	tr_D0A6D7_
HLA-DQA10501-DQB10201	264	CFISMLLNKTPGTIY	FISMLLNKT	1	0.1548	9371.3	75.00	0.56	tr_D0A6D7_
HLA-DQA10501-DQB10201	265	SASRCLSLTEKFTLT	RCLSLTEKF	3	0.1500	9861.2	80.00	0.38	tr_D0A6D7_
HLA-DQA10501-DQB10201	266	NAGDFVEGASGSGGA	NAGDFVEGA	0	0.1487	10006.7	80.00	0.34	tr_D0A6D7_
HLA-DQA10501-DQB10201	267	YSLLSTTRDKLREKG	YSLLSTTRD	0	0.1453	10383.4	80.00	0.56	tr_D0A6D7_
HLA-DQA10501-DQB10201	268	ASRCLSLTEKFTLTE	RCLSLTEKF	2	0.1443	10490.9	80.00	0.34	tr_D0A6D7_
HLA-DQA10501-DQB10201	269	WLVKDAKPGDALFLH	AKPGDALFL	5	0.1441	10511.4	80.00	0.34	tr_D0A6D7_
HLA-DQA10501-DQB10201	270	YVLVTLGLQRRPPKG	VLVTLGLQR	1	0.1439	10536.8	80.00	0.38	tr_D0A6D7_
HLA-DQA10501-DQB10201	271	MDTDDPRFNAIKPTR	PRFNAIKPT	5	0.1428	10670.0	80.00	0.43	tr_D0A6D7_
HLA-DQA10501-DQB10201	272	QSPQMSASRCLSLTE	MSASRCLSL	4	0.1402	10975.0	80.00	0.26	tr_D0A6D7_
HLA-DQA10501-DQB10201	273	SMLLNKTPGTIYSLL	TPGTIYSLL	6	0.1358	11509.4	80.00	0.56	tr_D0A6D7_
HLA-DQA10501-DQB10201	274	SPQMSASRCLSLTEK	SRCLSLTEK	6	0.1355	11546.2	80.00	0.22	tr_D0A6D7_
HLA-DQA10501-DQB10201	275	VGEMKRIRTGGDVNA	RIRTGGDVN	5	0.1353	11568.2	80.00	0.42	tr_D0A6D7_
HLA-DQA10501-DQB10201	276	FKQSPQMSASRCLSL	MSASRCLSL	6	0.1337	11764.6	85.00	0.36	tr_D0A6D7_
HLA-DQA10501-DQB10201	277	FISMLLNKTPGTIYS	FISMLLNKT	0	0.1314	12070.7	85.00	0.51	tr_D0A6D7_
HLA-DQA10501-DQB10201	278	GEMKRIRTGGDVNAH	RIRTGGDVN	4	0.1297	12289.4	85.00	0.41	tr_D0A6D7_
HLA-DQA10501-DQB10201	279	KQSPQMSASRCLSLT	MSASRCLSL	5	0.1248	12955.8	85.00	0.34	tr_D0A6D7_
HLA-DQA10501-DQB10201	280	SLICSSNDCSAVGEM	NDCSAVGEM	6	0.1240	13066.4	85.00	0.29	tr_D0A6D7_
HLA-DQA10501-DQB10201	281	MKKPSKQQRSFRALF	QQRSFRALF	6	0.1213	13462.2	85.00	0.73	tr_D0A6D7_
HLA-DQA10501-DQB10201	282	NILTGEPRWKTTPLG	NILTGEPRW	0	0.1203	13607.5	85.00	0.47	tr_D0A6D7_
HLA-DQA10501-DQB10201	283	RRPPKGGVDKAMAEA	GVDKAMAEA	6	0.1171	14083.5	85.00	0.38	tr_D0A6D7_
HLA-DQA10501-DQB10201	284	LICSSNDCSAVGEMK	NDCSAVGEM	5	0.1095	15283.9	90.00	0.28	tr_D0A6D7_
HLA-DQA10501-DQB10201	285	ESVEAEVMKKPSKQQ	SVEAEVMKK	1	0.1083	15492.9	90.00	0.47	tr_D0A6D7_
HLA-DQA10501-DQB10201	286	AVGEMKRIRTGGDVN	RIRTGGDVN	6	0.1069	15731.8	90.00	0.57	tr_D0A6D7_
HLA-DQA10501-DQB10201	287	GFKQSPQMSASRCLS	PQMSASRCL	5	0.1061	15858.0	90.00	0.44	tr_D0A6D7_
HLA-DQA10501-DQB10201	288	KGFKQSPQMSASRCL	PQMSASRCL	6	0.1038	16265.5	90.00	0.47	tr_D0A6D7_
HLA-DQA10501-DQB10201	289	AGDFVEGASGSGGAA	FVEGASGSG	3	0.1012	16719.8	90.00	0.26	tr_D0A6D7_
HLA-DQA10501-DQB10201	290	VLVTLGLQRRPPKGG	VLVTLGLQR	0	0.0968	17552.0	90.00	0.49	tr_D0A6D7_
HLA-DQA10501-DQB10201	291	CSSNDCSAVGEMKRI	DCSAVGEMK	4	0.0943	18016.6	90.00	0.25	tr_D0A6D7_
HLA-DQA10501-DQB10201	292	ICSSNDCSAVGEMKR	DCSAVGEMK	5	0.0940	18078.3	90.00	0.25	tr_D0A6D7_
HLA-DQA10501-DQB10201	293	QRRPPKGGVDKAMAE	GGVDKAMAE	6	0.0915	18582.9	90.00	0.37	tr_D0A6D7_
HLA-DQA10501-DQB10201	294	GDFVEGASGSGGAAT	FVEGASGSG	2	0.0869	19525.7	95.00	0.33	tr_D0A6D7_
HLA-DQA10501-DQB10201	295	SSNDCSAVGEMKRIR	NDCSAVGEM	2	0.0849	19951.9	95.00	0.27	tr_D0A6D7_
HLA-DQA10501-DQB10201	296	KLREKGFKQSPQMSA	KGFKQSPQM	4	0.0846	20023.3	95.00	0.38	tr_D0A6D7_
HLA-DQA10501-DQB10201	297	ISMLLNKTPGTIYSL	LNKTPGTIY	4	0.0827	20437.9	95.00	0.23	tr_D0A6D7_
HLA-DQA10501-DQB10201	298	SNDCSAVGEMKRIRT	DCSAVGEMK	2	0.0809	20828.5	95.00	0.20	tr_D0A6D7_
HLA-DQA10501-DQB10201	299	EKGFKQSPQMSASRC	KGFKQSPQM	1	0.0794	21170.7	95.00	0.26	tr_D0A6D7_
HLA-DQA10501-DQB10201	300	LREKGFKQSPQMSAS	KGFKQSPQM	3	0.0787	21336.5	95.00	0.38	tr_D0A6D7_
HLA-DQA10501-DQB10201	301	FVEGASGSGGAATQC	GSGGAATQC	6	0.0774	21642.0	95.00	0.31	tr_D0A6D7_
HLA-DQA10501-DQB10201	302	DFVEGASGSGGAATQ	FVEGASGSG	1	0.0747	22286.7	95.00	0.34	tr_D0A6D7_
HLA-DQA10501-DQB10201	303	DKLREKGFKQSPQMS	KGFKQSPQM	5	0.0732	22658.5	95.00	0.51	tr_D0A6D7_
HLA-DQA10501-DQB10201	304	REKGFKQSPQMSASR	KGFKQSPQM	2	0.0716	23046.7	95.00	0.37	tr_D0A6D7_
HLA-DQA10501-DQB10201	305	ILTGEPRWKTTPLGG	RWKTTPLGG	6	0.0707	23275.5	95.00	0.29	tr_D0A6D7_
HLA-DQA10501-DQB10201	306	RDKLREKGFKQSPQM	KGFKQSPQM	6	0.0697	23513.7	95.00	0.59	tr_D0A6D7_
HLA-DQA10501-DQB10201	307	SAVGEMKRIRTGGDV	KRIRTGGDV	6	0.0687	23767.7	95.00	0.37	tr_D0A6D7_
HLA-DQA10501-DQB10201	308	SLLSTTRDKLREKGF	LLSTTRDKL	1	0.0680	23969.1	95.00	0.58	tr_D0A6D7_
HLA-DQA10501-DQB10201	309	LQRRPPKGGVDKAMA	PKGGVDKAM	5	0.0678	24012.7	95.00	0.41	tr_D0A6D7_
HLA-DQA10501-DQB10201	310	NDCSAVGEMKRIRTG	DCSAVGEMK	1	0.0653	24657.5	95.00	0.28	tr_D0A6D7_
HLA-DQA10501-DQB10201	311	SVEAEVMKKPSKQQR	SVEAEVMKK	0	0.0640	25025.4	95.00	0.52	tr_D0A6D7_

HLA-DQA10501-DQB10201	312	GLQRRPPKGGVDKAM	PKGGVDKAM	6	0.0571	26945.1	100.00	0.58	tr_D0A6D7_
HLA-DQA10501-DQB10201	313	LTGEPRWKTTPLGGK	RWKTTPLGG	5	0.0539	27914.9	100.00	0.43	tr_D0A6D7_
HLA-DQA10501-DQB10201	314	LVTLGLQRRPPKGGV	LVTLGLQRR	0	0.0523	28383.9	100.00	0.52	tr_D0A6D7_
HLA-DQA10501-DQB10201	315	DCSAVGEMKRIRTGG	DCSAVGEMK	0	0.0518	28547.5	100.00	0.25	tr_D0A6D7_
HLA-DQA10501-DQB10201	316	LLSTTRDKLREKGFK	LLSTTRDKL	0	0.0499	29144.9	100.00	0.52	tr_D0A6D7_
HLA-DQA10501-DQB10201	317	CSAVGEMKRIRTGGD	VGEMKRIRT	3	0.0482	29668.9	100.00	0.24	tr_D0A6D7_
HLA-DQA10501-DQB10201	318	TRDKLREKGFKQSPQ	EKGFKQSPQ	6	0.0403	32331.4	100.00	0.44	tr_D0A6D7_
HLA-DQA10501-DQB10201	319	VMKKPSKQQRSFRAL	KQQRSFRAL	6	0.0340	34605.0	100.00	0.29	tr_D0A6D7_
HLA-DQA10501-DQB10201	320	LGLQRRPPKGGVDKA	PPKGGVDKA	6	0.0317	35493.1	100.00	0.63	tr_D0A6D7_
HLA-DQA10501-DQB10201	321	VTLGLQRRPPKGGVD	TLGLQRRPP	1	0.0286	36692.7	100.00	0.29	tr_D0A6D7_
HLA-DQA10501-DQB10201	322	VEAEVMKKPSKQQRS	VEAEVMKKP	0	0.0284	36754.7	100.00	0.35	tr_D0A6D7_
HLA-DQA10501-DQB10201	323	LSTTRDKLREKGFKQ	RDKLREKGF	4	0.0284	36776.6	100.00	0.32	tr_D0A6D7_
HLA-DQA10501-DQB10201	324	TTRDKLREKGFKQSP	RDKLREKGF	2	0.0270	37317.7	100.00	0.28	tr_D0A6D7_
HLA-DQA10501-DQB10201	325	STTRDKLREKGFKQS	RDKLREKGF	3	0.0255	37957.0	100.00	0.35	tr_D0A6D7_
HLA-DQA10501-DQB10201	326	EVMKKPSKQQRSFRA	SKQQRSFRA	6	0.0244	38406.1	100.00	0.42	tr_D0A6D7_
HLA-DQA10501-DQB10201	327	TLGLQRRPPKGGVDK	TLGLQRRPP	0	0.0208	39920.7	100.00	0.31	tr_D0A6D7_
HLA-DQA10501-DQB10201	328	EAEVMKKPSKQQRSF	EVMKKPSKQ	2	0.0192	40641.1	100.00	0.37	tr_D0A6D7_
HLA-DQA10501-DQB10201	329	AEVMKKPSKQQRSFR	PSKQQRSFR	6	0.0187	40845.2	100.00	0.24	tr_D0A6D7_

Allele: HLA-DQA10501-DQB10201. Number of high binders 3. Number of weak binders 20. Number of peptides 329

Allele	pos	peptide	core	Of	1-log50k(aff)	affinity(nM)	%Rank	Relia	Identity	Bind Level
HLA-DQA10501-DQB10201	1	HHHDDHTHGADDHEG	HTHGADDHE	5	0.1577	9072.8	75.00	0.43	tr_D0A886_	
HLA-DQA10501-DQB10201	2	HHDDHTHGADDHEGH	HTHGADDHE	4	0.1529	9564.5	80.00	0.38	tr_D0A886_	
HLA-DQA10501-DQB10201	3	HDDHTHGADDHEGHG	HTHGADDHE	3	0.1411	10860.4	80.00	0.38	tr_D0A886_	
HLA-DQA10501-DQB10201	4	HHHHDDHTHGADDHE	HTHGADDHE	6	0.1384	11186.8	80.00	0.62	tr_D0A886_	
HLA-DQA10501-DQB10201	5	DDHTHGADDHEGHGH	HTHGADDHE	2	0.1312	12093.0	85.00	0.40	tr_D0A886_	
HLA-DQA10501-DQB10201	6	DHTHGADDHEGHGHS	HTHGADDHE	1	0.1152	14369.5	85.00	0.41	tr_D0A886_	
HLA-DQA10501-DQB10201	7	HTHGADDHEGHGHSH	THGADDHEG	1	0.0891	19077.3	95.00	0.38	tr_D0A886_	
HLA-DQA10501-DQB10201	8	HHHHHDDHTHGADDH	DHTHGADDH	6	0.0831	20348.7	95.00	0.58	tr_D0A886_	
HLA-DQA10501-DQB10201	9	THGADDHEGHGHSHG	THGADDHEG	0	0.0591	26378.0	100.00	0.46	tr_D0A886_	
HLA-DQA10501-DQB10201	10	HHHHHHDDHTHGADD	DDHTHGADD	6	0.0531	28149.1	100.00	0.30	tr_D0A886_	
HLA-DQA10501-DQB10201	11	DHGHHHHHHHHDDHT	HHHHHDDHT	6	0.0503	29007.4	100.00	0.52	tr_D0A886_	
HLA-DQA10501-DQB10201	12	HGHHHHHHHHDDHTH	HHHHHDDHT	5	0.0480	29740.9	100.00	0.47	tr_D0A886_	
HLA-DQA10501-DQB10201	13	DDHEGHGHSHGGCES	GHSHGGCES	6	0.0479	29792.7	100.00	0.57	tr_D0A886_	
HLA-DQA10501-DQB10201	14	HHHHHHHDDHTHGAD	HHHHHDDHT	2	0.0475	29892.5	100.00	0.28	tr_D0A886_	
HLA-DQA10501-DQB10201	15	DHEGHGHSHGGCESG	GHSHGGCES	5	0.0464	30268.4	100.00	0.50	tr_D0A886_	
HLA-DQA10501-DQB10201	16	HEGHGHSHGGCESGH	GHSHGGCES	4	0.0439	31078.8	100.00	0.44	tr_D0A886_	
HLA-DQA10501-DQB10201	17	GHHHHHHHHDDHTHG	HHHHHDDHT	4	0.0438	31140.1	100.00	0.38	tr_D0A886_	
HLA-DQA10501-DQB10201	18	HHHHHHHHDDHTHGA	HHHHHDDHT	3	0.0422	31676.0	100.00	0.35	tr_D0A886_	
HLA-DQA10501-DQB10201	19	EGHGHSHGGCESGHG	GHSHGGCES	3	0.0417	31838.8	100.00	0.38	tr_D0A886_	
HLA-DQA10501-DQB10201	20	GHGHSHGGCESGHGT	GHSHGGCES	2	0.0402	32371.0	100.00	0.34	tr_D0A886_	
HLA-DQA10501-DQB10201	21	HGHSHGGCESGHGTY	GHSHGGCES	1	0.0384	33003.0	100.00	0.31	tr_D0A886_	
HLA-DQA10501-DQB10201	22	HDHGHHHHHHHHDDH	HHHHHHDDH	6	0.0369	33529.5	100.00	0.61	tr_D0A886_	
HLA-DQA10501-DQB10201	23	HGADDHEGHGHSHGG	HGADDHEGH	0	0.0342	34534.0	100.00	0.42	tr_D0A886_	
HLA-DQA10501-DQB10201	24	GHSHGGCESGHGTYS	GHSHGGCES	0	0.0333	34867.4	100.00	0.22	tr_D0A886_	

HLA-DQA10501-DQB10201	25	ADDHEGHGHSHGGCE	HGHSHGGCE	6	0.0281	36890.1	100.00	0.34	tr_D0A886_	
HLA-DQA10501-DQB10201	26	GADDHEGHGHSHGGC	DHEGHGHSH	3	0.0253	38015.0	100.00	0.20	tr_D0A886_	
HLA-DQA10501-DQB10201	27	THDHGHHHHHHHHDD	HHHHHHHDD	6	0.0235	38779.8	100.00	0.48	tr_D0A886_	
HLA-DQA10501-DQB10201	28	HTHDHGHHHHHHHHD	HHHHHHHHD	6	0.0174	41436.8	100.00	0.47	tr_D0A886_	
HLA-DQA10501-DQB10201	29	HHHTHDHGHHHHHHH	DHGHHHHHH	5	0.0167	41733.3	100.00	0.24	tr_D0A886_	
HLA-DQA10501-DQB10201	30	HHTHDHGHHHHHHHH	DHGHHHHHH	4	0.0143	42833.1	100.00	0.23	tr_D0A886_	

Allele: HLA-DQA10501-DQB10201. Number of high binders 0. Number of weak binders 0. Number of peptides 30

Allele	pos	peptide	core	Of	1-log50k(aff)	affinity(nM)	%Rank	Relia	Identity	Bind Level
HLA-DQA10501-DQB10201	1	HHDDHTHGVVDDGID	THGVVDDGI	5	0.4610	341.0	8.50	0.76	tr_D0A889_	WB
HLA-DQA10501-DQB10201	2	DDHTHGVVDDGIDGD	THGVVDDGI	3	0.4575	354.1	9.00	0.69	tr_D0A889_	WB
HLA-DQA10501-DQB10201	3	HDDHTHGVVDDGIDG	THGVVDDGI	4	0.4399	428.4	11.00	0.74	tr_D0A889_	
HLA-DQA10501-DQB10201	4	HHHDDHTHGVVDDGI	THGVVDDGI	6	0.4373	440.6	11.00	0.81	tr_D0A889_	
HLA-DQA10501-DQB10201	5	DHTHGVVDDGIDGDT	THGVVDDGI	2	0.4347	453.1	12.00	0.67	tr_D0A889_	
HLA-DQA10501-DQB10201	6	HTHGVVDDGIDGDTH	THGVVDDGI	1	0.3966	684.7	16.00	0.67	tr_D0A889_	
HLA-DQA10501-DQB10201	7	THGVVDDGIDGDTHG	THGVVDDGI	0	0.3332	1359.1	28.00	0.57	tr_D0A889_	
HLA-DQA10501-DQB10201	8	HGVVDDGIDGDTHGG	VVDDGIDGD	2	0.2120	5045.6	60.00	0.49	tr_D0A889_	
HLA-DQA10501-DQB10201	9	GVVDDGIDGDTHGGC	VVDDGIDGD	1	0.1844	6799.4	70.00	0.58	tr_D0A889_	
HLA-DQA10501-DQB10201	10	HHHHDDHTHGVVDDG	HTHGVVDDG	6	0.1660	8299.8	75.00	0.59	tr_D0A889_	
HLA-DQA10501-DQB10201	11	VVDDGIDGDTHGGCE	VVDDGIDGD	0	0.1570	9144.7	75.00	0.47	tr_D0A889_	
HLA-DQA10501-DQB10201	12	HHHHHDDHTHGVVDD	DHTHGVVDD	6	0.1060	15880.4	90.00	0.65	tr_D0A889_	
HLA-DQA10501-DQB10201	13	VDDGIDGDTHGGCES	DDGIDGDTH	1	0.0908	18711.2	95.00	0.39	tr_D0A889_	
HLA-DQA10501-DQB10201	14	DDGIDGDTHGGCESG	DDGIDGDTH	0	0.0746	22295.9	95.00	0.33	tr_D0A889_	
HLA-DQA10501-DQB10201	15	DGIDGDTHGGCESGH	GDTHGGCES	4	0.0555	27425.5	100.00	0.19	tr_D0A889_	
HLA-DQA10501-DQB10201	16	GIDGDTHGGCESGHG	THGGCESGH	5	0.0472	30005.6	100.00	0.19	tr_D0A889_	
HLA-DQA10501-DQB10201	17	IDGDTHGGCESGHGT	THGGCESGH	4	0.0436	31181.5	100.00	0.20	tr_D0A889_	
HLA-DQA10501-DQB10201	18	DGDTHGGCESGHGTY	THGGCESGH	3	0.0396	32568.8	100.00	0.23	tr_D0A889_	
HLA-DQA10501-DQB10201	19	GDTHGGCESGHGTYS	THGGCESGH	2	0.0335	34799.9	100.00	0.25	tr_D0A889_	

Allele: HLA-DQA10501-DQB10201. Number of high binders 0. Number of weak binders 2. Number of peptides 19

Allele	pos	peptide	core	Of	1-log50k(aff)	affinity(nM)	%Rank	Relia	Identity	Bind Level
HLA-DQA10501-DQB10201	1	IPSLSQGNVLELPPD	LSQGNVLEL	3	0.3901	734.3	17.00	0.28	tr_D0AAF8_	
HLA-DQA10501-DQB10201	2	GGMASNVFNLESATL	VFNLESATL	6	0.3784	833.1	19.00	0.57	tr_D0AAF8_	
HLA-DQA10501-DQB10201	3	PSLSQGNVLELPPDS	LSQGNVLEL	2	0.3759	856.3	20.00	0.26	tr_D0AAF8_	
HLA-DQA10501-DQB10201	4	SIPSLSQGNVLELPP	LSQGNVLEL	4	0.3738	876.0	20.00	0.31	tr_D0AAF8_	
HLA-DQA10501-DQB10201	5	SLSQGNVLELPPDSD	SQGNVLELP	2	0.3685	927.3	21.00	0.29	tr_D0AAF8_	
HLA-DQA10501-DQB10201	6	GMASNVFNLESATLG	VFNLESATL	5	0.3554	1069.4	23.00	0.52	tr_D0AAF8_	
HLA-DQA10501-DQB10201	7	TSIPSLSQGNVLELP	LSQGNVLEL	5	0.3550	1073.2	23.00	0.38	tr_D0AAF8_	
HLA-DQA10501-DQB10201	8	MASNVFNLESATLGA	VFNLESATL	4	0.3347	1336.9	27.00	0.54	tr_D0AAF8_	
HLA-DQA10501-DQB10201	9	DGEHRGCLNTVFDPI	CLNTVFDPI	6	0.3328	1365.1	28.00	0.52	tr_D0AAF8_	

HLA-DQA10501-DQB10201	10	LSQGNVLELPPDSDK	SQGNVLELP	1	0.3283	1433.4	29.00	0.29	tr_D0AAF8_
HLA-DQA10501-DQB10201	11	PSGGMASNVFNLESA	MASNVFNLE	4	0.3213	1545.8	30.00	0.34	tr_D0AAF8_
HLA-DQA10501-DQB10201	12	VVPSGGMASNVFNLE	MASNVFNLE	6	0.3192	1581.6	31.00	0.40	tr_D0AAF8_
HLA-DQA10501-DQB10201	13	SPRATEPIINEDEHP	PIINEDEHP	6	0.3174	1612.8	31.00	0.26	tr_D0AAF8_
HLA-DQA10501-DQB10201	14	VPSGGMASNVFNLES	MASNVFNLE	5	0.3070	1804.1	33.00	0.40	tr_D0AAF8_
HLA-DQA10501-DQB10201	15	PRATEPIINEDEHPT	PIINEDEHP	5	0.3029	1887.5	34.00	0.32	tr_D0AAF8_
HLA-DQA10501-DQB10201	16	PTSIPSLSQGNVLEL	LSQGNVLEL	6	0.3020	1904.4	35.00	0.50	tr_D0AAF8_
HLA-DQA10501-DQB10201	17	ASNVFNLESATLGAG	VFNLESATL	3	0.3005	1935.9	35.00	0.59	tr_D0AAF8_
HLA-DQA10501-DQB10201	18	GEHRGCLNTVFDPIK	CLNTVFDPI	5	0.2938	2081.4	37.00	0.43	tr_D0AAF8_
HLA-DQA10501-DQB10201	19	SGGMASNVFNLESAT	MASNVFNLE	3	0.2913	2138.2	37.00	0.31	tr_D0AAF8_
HLA-DQA10501-DQB10201	20	SNVFNLESATLGAGI	VFNLESATL	2	0.2907	2152.8	37.00	0.55	tr_D0AAF8_
HLA-DQA10501-DQB10201	21	RATEPIINEDEHPTS	PIINEDEHP	4	0.2888	2196.9	38.00	0.34	tr_D0AAF8_
HLA-DQA10501-DQB10201	22	LESATLGAGIVMLPS	GAGIVMLPS	6	0.2838	2318.7	39.00	0.29	tr_D0AAF8_
HLA-DQA10501-DQB10201	23	ATLGAGIVMLPSGFL	IVMLPSGFL	6	0.2774	2485.5	41.00	0.26	tr_D0AAF8_
HLA-DQA10501-DQB10201	24	EHRGCLNTVFDPIKG	CLNTVFDPI	4	0.2764	2513.2	41.00	0.41	tr_D0AAF8_
HLA-DQA10501-DQB10201	25	ATEPIINEDEHPTSI	PIINEDEHP	3	0.2721	2632.1	42.00	0.36	tr_D0AAF8_
HLA-DQA10501-DQB10201	26	MSPRATEPIINEDEH	TEPIINEDE	5	0.2705	2677.7	43.00	0.34	tr_D0AAF8_
HLA-DQA10501-DQB10201	27	NVFNLESATLGAGIV	VFNLESATL	1	0.2683	2743.6	43.00	0.52	tr_D0AAF8_
HLA-DQA10501-DQB10201	28	SQGNVLELPPDSDKS	VLELPPDSD	4	0.2670	2781.3	44.00	0.27	tr_D0AAF8_
HLA-DQA10501-DQB10201	29	GVVPSGGMASNVFNL	SGGMASNVF	4	0.2658	2819.4	44.00	0.58	tr_D0AAF8_
HLA-DQA10501-DQB10201	30	NLESATLGAGIVMLP	TLGAGIVML	5	0.2628	2910.8	45.00	0.28	tr_D0AAF8_
HLA-DQA10501-DQB10201	31	SATLGAGIVMLPSGF	GAGIVMLPS	4	0.2627	2915.9	45.00	0.26	tr_D0AAF8_
HLA-DQA10501-DQB10201	32	TLGAGIVMLPSGFLN	IVMLPSGFL	5	0.2615	2953.9	45.00	0.25	tr_D0AAF8_
HLA-DQA10501-DQB10201	33	KGVVPSGGMASNVFN	SGGMASNVF	5	0.2583	3058.1	46.00	0.62	tr_D0AAF8_
HLA-DQA10501-DQB10201	34	HRGCLNTVFDPIKGV	CLNTVFDPI	3	0.2569	3103.1	46.00	0.43	tr_D0AAF8_
HLA-DQA10501-DQB10201	35	ESATLGAGIVMLPSG	GAGIVMLPS	5	0.2562	3125.1	46.00	0.25	tr_D0AAF8_
HLA-DQA10501-DQB10201	36	IKGVVPSGGMASNVF	SGGMASNVF	6	0.2548	3174.1	47.00	0.71	tr_D0AAF8_
HLA-DQA10501-DQB10201	37	VFNLESATLGAGIVM	VFNLESATL	0	0.2504	3330.4	48.00	0.31	tr_D0AAF8_
HLA-DQA10501-DQB10201	38	TEPIINEDEHPTSIP	PIINEDEHP	2	0.2436	3583.9	50.00	0.44	tr_D0AAF8_
HLA-DQA10501-DQB10201	39	FNLESATLGAGIVML	TLGAGIVML	6	0.2366	3867.3	55.00	0.35	tr_D0AAF8_
HLA-DQA10501-DQB10201	40	LGAGIVMLPSGFLNS	IVMLPSGFL	4	0.2358	3900.7	55.00	0.29	tr_D0AAF8_
HLA-DQA10501-DQB10201	41	RGCLNTVFDPIKGVV	CLNTVFDPI	2	0.2344	3959.8	55.00	0.43	tr_D0AAF8_
HLA-DQA10501-DQB10201	42	ADGEHRGCLNTVFDP	RGCLNTVFD	5	0.2341	3971.9	55.00	0.46	tr_D0AAF8_
HLA-DQA10501-DQB10201	43	GCLNTVFDPIKGVVP	CLNTVFDPI	1	0.2297	4166.2	55.00	0.38	tr_D0AAF8_
HLA-DQA10501-DQB10201	44	QGNVLELPPDSDKST	VLELPPDSD	3	0.2169	4785.5	60.00	0.43	tr_D0AAF8_
HLA-DQA10501-DQB10201	45	AADGEHRGCLNTVFD	RGCLNTVFD	6	0.2107	5117.7	60.00	0.50	tr_D0AAF8_
HLA-DQA10501-DQB10201	46	NTVFDPIKGVVPSGG	PIKGVVPSG	5	0.2070	5326.1	60.00	0.29	tr_D0AAF8_
HLA-DQA10501-DQB10201	47	TVFDPIKGVVPSGGM	PIKGVVPSG	4	0.2049	5445.8	65.00	0.29	tr_D0AAF8_
HLA-DQA10501-DQB10201	48	HPTSIPSLSQGNVLE	PSLSQGNVL	5	0.2022	5607.1	65.00	0.57	tr_D0AAF8_
HLA-DQA10501-DQB10201	49	GAGIVMLPSGFLNSG	IVMLPSGFL	3	0.2018	5634.0	65.00	0.32	tr_D0AAF8_
HLA-DQA10501-DQB10201	50	PDSDKSTAAADGEHR	KSTAAADGE	4	0.2015	5650.1	65.00	0.44	tr_D0AAF8_
HLA-DQA10501-DQB10201	51	EPIINEDEHPTSIPS	PIINEDEHP	1	0.2011	5676.5	65.00	0.54	tr_D0AAF8_
HLA-DQA10501-DQB10201	52	CLNTVFDPIKGVVPS	CLNTVFDPI	0	0.2010	5683.3	65.00	0.31	tr_D0AAF8_
HLA-DQA10501-DQB10201	53	PIINEDEHPTSIPSL	PIINEDEHP	0	0.1985	5838.9	65.00	0.37	tr_D0AAF8_
HLA-DQA10501-DQB10201	54	LNTVFDPIKGVVPSG	PIKGVVPSG	6	0.1983	5849.7	65.00	0.32	tr_D0AAF8_
HLA-DQA10501-DQB10201	55	DSDKSTAAADGEHRG	KSTAAADGE	3	0.1940	6126.4	65.00	0.40	tr_D0AAF8_
HLA-DQA10501-DQB10201	56	VFDPIKGVVPSGGMA	IKGVVPSGG	4	0.1935	6162.6	65.00	0.38	tr_D0AAF8_
HLA-DQA10501-DQB10201	57	PPDSDKSTAAADGEH	KSTAAADGE	5	0.1887	6490.9	70.00	0.64	tr_D0AAF8_
HLA-DQA10501-DQB10201	58	SDKSTAAADGEHRGC	KSTAAADGE	2	0.1886	6500.8	70.00	0.37	tr_D0AAF8_
HLA-DQA10501-DQB10201	59	AGIVMLPSGFLNSGI	IVMLPSGFL	2	0.1879	6549.8	70.00	0.34	tr_D0AAF8_

HLA-DQA10501-DQB10201	60	EHPTSIPSLSQGNVL	PSLSQGNVL	6	0.1858	6698.3	70.00	0.54	tr_D0AAF8_
HLA-DQA10501-DQB10201	61	DKSTAAADGEHRGCL	TAAADGEHR	3	0.1821	6972.9	70.00	0.30	tr_D0AAF8_
HLA-DQA10501-DQB10201	62	GNVLELPPDSDKSTA	VLELPPDSD	2	0.1816	7010.7	70.00	0.56	tr_D0AAF8_
HLA-DQA10501-DQB10201	63	FDPIKGVVPSGGMAS	IKGVVPSGG	3	0.1789	7212.6	70.00	0.41	tr_D0AAF8_
HLA-DQA10501-DQB10201	64	LPPDSDKSTAAADGE	KSTAAADGE	6	0.1669	8217.3	75.00	0.84	tr_D0AAF8_
HLA-DQA10501-DQB10201	65	KSTAAADGEHRGCLN	TAAADGEHR	2	0.1588	8965.9	75.00	0.35	tr_D0AAF8_
HLA-DQA10501-DQB10201	66	IINEDEHPTSIPSLS	EHPTSIPSL	5	0.1571	9141.0	75.00	0.43	tr_D0AAF8_
HLA-DQA10501-DQB10201	67	INEDEHPTSIPSLSQ	EHPTSIPSL	4	0.1540	9447.3	75.00	0.40	tr_D0AAF8_
HLA-DQA10501-DQB10201	68	NVLELPPDSDKSTAA	VLELPPDSD	1	0.1514	9718.3	80.00	0.66	tr_D0AAF8_
HLA-DQA10501-DQB10201	69	DPIKGVVPSGGMASN	IKGVVPSGG	2	0.1495	9916.1	80.00	0.44	tr_D0AAF8_
HLA-DQA10501-DQB10201	70	PIKGVVPSGGMASNV	IKGVVPSGG	1	0.1403	10956.9	80.00	0.35	tr_D0AAF8_
HLA-DQA10501-DQB10201	71	NEDEHPTSIPSLSQG	EHPTSIPSL	3	0.1385	11176.1	80.00	0.43	tr_D0AAF8_
HLA-DQA10501-DQB10201	72	AAADGEHRGCLNTVF	HRGCLNTVF	6	0.1372	11336.4	80.00	0.44	tr_D0AAF8_
HLA-DQA10501-DQB10201	73	EDEHPTSIPSLSQGN	EHPTSIPSL	2	0.1313	12083.4	85.00	0.40	tr_D0AAF8_
HLA-DQA10501-DQB10201	74	STAAADGEHRGCLNT	TAAADGEHR	1	0.1287	12424.9	85.00	0.50	tr_D0AAF8_
HLA-DQA10501-DQB10201	75	DEHPTSIPSLSQGNV	EHPTSIPSL	1	0.1269	12661.7	85.00	0.35	tr_D0AAF8_
HLA-DQA10501-DQB10201	76	TAAADGEHRGCLNTV	EHRGCLNTV	6	0.1264	12731.9	85.00	0.33	tr_D0AAF8_
HLA-DQA10501-DQB10201	77	VLELPPDSDKSTAAA	VLELPPDSD	0	0.1034	16338.9	90.00	0.81	tr_D0AAF8_
HLA-DQA10501-DQB10201	78	ELPPDSDKSTAAADG	DKSTAAADG	6	0.0539	27919.2	100.00	0.54	tr_D0AAF8_
HLA-DQA10501-DQB10201	79	LELPPDSDKSTAAAD	SDKSTAAAD	6	0.0393	32689.5	100.00	0.40	tr_D0AAF8_

Allele: HLA-DQA10501-DQB10201. Number of high binders 0. Number of weak binders 0. Number of peptides 79

Allele	pos	peptide	core	Of	1-log50k(aff)	affinity(nM)	%Rank	Relia	Identity	Bind Level
HLA-DQA10501-DQB10201	1	FQHAGSMGEVSVGVP	MGEVSVGVP	6	0.2708	2671.3	43.00	0.31	tr_C9ZL05_	
HLA-DQA10501-DQB10201	2	QHAGSMGEVSVGVPR	MGEVSVGVP	5	0.2451	3524.5	50.00	0.34	tr_C9ZL05_	
HLA-DQA10501-DQB10201	3	HAGSMGEVSVGVPRR	MGEVSVGVP	4	0.2388	3776.4	55.00	0.29	tr_C9ZL05_	
HLA-DQA10501-DQB10201	4	GESFQHAGSMGEVSV	HAGSMGEVS	5	0.2357	3904.4	55.00	0.31	tr_C9ZL05_	
HLA-DQA10501-DQB10201	5	SFQHAGSMGEVSVGV	SMGEVSVGV	6	0.2322	4052.6	55.00	0.28	tr_C9ZL05_	
HLA-DQA10501-DQB10201	6	AGSMGEVSVGVPRRS	MGEVSVGVP	3	0.2177	4740.0	60.00	0.32	tr_C9ZL05_	
HLA-DQA10501-DQB10201	7	ESFQHAGSMGEVSVG	HAGSMGEVS	4	0.2157	4848.7	60.00	0.31	tr_C9ZL05_	
HLA-DQA10501-DQB10201	8	PGESFQHAGSMGEVS	HAGSMGEVS	6	0.2040	5497.5	65.00	0.39	tr_C9ZL05_	
HLA-DQA10501-DQB10201	9	GSMGEVSVGVPRRST	MGEVSVGVP	2	0.1994	5779.3	65.00	0.34	tr_C9ZL05_	
HLA-DQA10501-DQB10201	10	PPGESFQHAGSMGEV	FQHAGSMGE	5	0.1789	7219.3	70.00	0.44	tr_C9ZL05_	
HLA-DQA10501-DQB10201	11	SMGEVSVGVPRRSTR	MGEVSVGVP	1	0.1734	7659.0	70.00	0.35	tr_C9ZL05_	
HLA-DQA10501-DQB10201	12	HPPGESFQHAGSMGE	FQHAGSMGE	6	0.1484	10041.8	80.00	0.62	tr_C9ZL05_	
HLA-DQA10501-DQB10201	13	RRSTRNSRMDTDRSV	TRNSRMDTD	3	0.1343	11688.5	85.00	0.33	tr_C9ZL05_	
HLA-DQA10501-DQB10201	14	MGEVSVGVPRRSTRN	EVSVGVPRR	2	0.1314	12065.2	85.00	0.37	tr_C9ZL05_	
HLA-DQA10501-DQB10201	15	RSTRNSRMDTDRSVR	TRNSRMDTD	2	0.1295	12317.0	85.00	0.35	tr_C9ZL05_	
HLA-DQA10501-DQB10201	16	TRNSRMDTDRSVRNW	SRMDTDRSV	3	0.1291	12366.9	85.00	0.26	tr_C9ZL05_	
HLA-DQA10501-DQB10201	17	STRNSRMDTDRSVRN	TRNSRMDTD	1	0.1280	12522.3	85.00	0.28	tr_C9ZL05_	
HLA-DQA10501-DQB10201	18	PRRSTRNSRMDTDRS	TRNSRMDTD	4	0.1154	14341.7	85.00	0.46	tr_C9ZL05_	
HLA-DQA10501-DQB10201	19	GVPRRSTRNSRMDTD	TRNSRMDTD	6	0.1135	14635.6	90.00	0.64	tr_C9ZL05_	
HLA-DQA10501-DQB10201	20	VPRRSTRNSRMDTDR	TRNSRMDTD	5	0.1092	15340.4	90.00	0.60	tr_C9ZL05_	
HLA-DQA10501-DQB10201	21	RNSRMDTDRSVRNWG	SRMDTDRSV	2	0.1066	15772.8	90.00	0.32	tr_C9ZL05_	
HLA-DQA10501-DQB10201	22	NSRMDTDRSVRNWGA	SRMDTDRSV	1	0.0964	17627.9	90.00	0.30	tr_C9ZL05_	

HLA-DQA10501-DQB10201	23	GEVSVGVPRRSTRNS	EVSVGVPRR	1	0.0945	17984.1	90.00	0.59	tr_C9ZL05_	
HLA-DQA10501-DQB10201	24	DHPPGESFQHAGSMG	ESFQHAGSM	5	0.0835	20261.3	95.00	0.29	tr_C9ZL05_	
HLA-DQA10501-DQB10201	25	ARANDHPPGESFQHA	PPGESFQHA	6	0.0834	20271.8	95.00	0.38	tr_C9ZL05_	
HLA-DQA10501-DQB10201	26	NDHPPGESFQHAGSM	ESFQHAGSM	6	0.0826	20463.5	95.00	0.40	tr_C9ZL05_	
HLA-DQA10501-DQB10201	27	RANDHPPGESFQHAG	PPGESFQHA	5	0.0760	21974.2	95.00	0.34	tr_C9ZL05_	
HLA-DQA10501-DQB10201	28	SRMDTDRSVRNWGAG	SRMDTDRSV	0	0.0760	21980.4	95.00	0.26	tr_C9ZL05_	
HLA-DQA10501-DQB10201	29	EVSVGVPRRSTRNSR	EVSVGVPRR	0	0.0753	22134.9	95.00	0.56	tr_C9ZL05_	
HLA-DQA10501-DQB10201	30	EARANDHPPGESFQH	RANDHPPGE	2	0.0719	22979.2	95.00	0.30	tr_C9ZL05_	
HLA-DQA10501-DQB10201	31	ANDHPPGESFQHAGS	PPGESFQHA	4	0.0680	23961.6	95.00	0.34	tr_C9ZL05_	
HLA-DQA10501-DQB10201	32	MDTDRSVRNWGAGHG	VRNWGAGHG	6	0.0675	24090.8	95.00	0.29	tr_C9ZL05_	
HLA-DQA10501-DQB10201	33	RMDTDRSVRNWGAGH	SVRNWGAGH	6	0.0673	24147.1	95.00	0.18	tr_C9ZL05_	
HLA-DQA10501-DQB10201	34	VGVPRRSTRNSRMDT	RRSTRNSRM	4	0.0521	28460.2	100.00	0.49	tr_C9ZL05_	
HLA-DQA10501-DQB10201	35	SVGVPRRSTRNSRMD	RRSTRNSRM	5	0.0491	29386.1	100.00	0.54	tr_C9ZL05_	
HLA-DQA10501-DQB10201	36	VSVGVPRRSTRNSRM	RRSTRNSRM	6	0.0469	30117.5	100.00	0.46	tr_C9ZL05_	

Allele: HLA-DQA10501-DQB10201. Number of high binders 0. Number of weak binders 0. Number of peptides 36

Allele	pos	peptide	core	Of	1-log50k(aff)	affinity(nM)	%Rank	Relia	Identity	Bind Level
HLA-DQA10501-DQB10201	1	VGRDVALSYDTPWCL	VALSYDTPW	4	0.4918	244.4	6.00	0.39	tr_C9ZL05_	WB
HLA-DQA10501-DQB10201	2	GRDVALSYDTPWCLY	VALSYDTPW	3	0.4859	260.6	6.50	0.41	tr_C9ZL05_	WB
HLA-DQA10501-DQB10201	3	RVGRDVALSYDTPWC	VALSYDTPW	5	0.4783	282.8	7.00	0.40	tr_C9ZL05_	WB
HLA-DQA10501-DQB10201	4	PRVGRDVALSYDTPW	VALSYDTPW	6	0.4715	304.2	7.50	0.40	tr_C9ZL05_	WB
HLA-DQA10501-DQB10201	5	RDVALSYDTPWCLYE	VALSYDTPW	2	0.4697	310.4	8.00	0.43	tr_C9ZL05_	WB
HLA-DQA10501-DQB10201	6	DVALSYDTPWCLYEV	VALSYDTPW	1	0.4433	412.9	11.00	0.43	tr_C9ZL05_	
HLA-DQA10501-DQB10201	7	VALSYDTPWCLYEVM	VALSYDTPW	0	0.4031	638.1	16.00	0.32	tr_C9ZL05_	
HLA-DQA10501-DQB10201	8	PPRVGRDVALSYDTP	DVALSYDTP	6	0.3413	1245.1	26.00	0.34	tr_C9ZL05_	
HLA-DQA10501-DQB10201	9	VRPRMLRLGDSYLTE	RMLRLGDSY	3	0.3344	1342.1	27.00	0.41	tr_C9ZL05_	
HLA-DQA10501-DQB10201	10	LYVRPRMLRLGDSYL	RMLRLGDSY	5	0.3255	1477.0	29.00	0.52	tr_C9ZL05_	
HLA-DQA10501-DQB10201	11	YVRPRMLRLGDSYLT	RMLRLGDSY	4	0.3194	1577.5	31.00	0.47	tr_C9ZL05_	
HLA-DQA10501-DQB10201	12	RPRMLRLGDSYLTEK	RMLRLGDSY	2	0.3095	1757.4	33.00	0.43	tr_C9ZL05_	
HLA-DQA10501-DQB10201	13	TENVRDLLYVRPRML	LLYVRPRML	6	0.3069	1807.0	33.00	0.27	tr_C9ZL05_	
HLA-DQA10501-DQB10201	14	PRMLRLGDSYLTEKP	RMLRLGDSY	1	0.3004	1937.9	35.00	0.40	tr_C9ZL05_	
HLA-DQA10501-DQB10201	15	ALSYDTPWCLYEVMK	LSYDTPWCL	1	0.3001	1943.7	35.00	0.29	tr_C9ZL05_	
HLA-DQA10501-DQB10201	16	EKPPRVGRDVALSYD	VGRDVALSY	5	0.2965	2020.9	36.00	0.43	tr_C9ZL05_	
HLA-DQA10501-DQB10201	17	DTPWCLYEVMKTTFF	YEVMKTTFF	6	0.2940	2077.2	37.00	0.35	tr_C9ZL05_	
HLA-DQA10501-DQB10201	18	KPPRVGRDVALSYDT	VGRDVALSY	4	0.2848	2294.5	39.00	0.41	tr_C9ZL05_	
HLA-DQA10501-DQB10201	19	RMLRLGDSYLTEKPP	MLRLGDSYL	1	0.2798	2421.0	40.00	0.31	tr_C9ZL05_	
HLA-DQA10501-DQB10201	20	TPWCLYEVMKTTFFA	YEVMKTTFF	5	0.2748	2555.6	41.00	0.34	tr_C9ZL05_	
HLA-DQA10501-DQB10201	21	LSYDTPWCLYEVMKT	TPWCLYEVM	4	0.2735	2593.6	42.00	0.27	tr_C9ZL05_	
HLA-DQA10501-DQB10201	22	LLYVRPRMLRLGDSY	RMLRLGDSY	6	0.2720	2636.0	42.00	0.63	tr_C9ZL05_	
HLA-DQA10501-DQB10201	23	ENVRDLLYVRPRMLR	LLYVRPRML	5	0.2695	2706.8	43.00	0.37	tr_C9ZL05_	
HLA-DQA10501-DQB10201	24	NVRDLLYVRPRMLRL	LLYVRPRML	4	0.2686	2734.4	43.00	0.33	tr_C9ZL05_	
HLA-DQA10501-DQB10201	25	PWCLYEVMKTTFFAV	YEVMKTTFF	4	0.2524	3256.3	48.00	0.37	tr_C9ZL05_	
HLA-DQA10501-DQB10201	26	YDTPWCLYEVMKTTF	LYEVMKTTF	6	0.2496	3356.6	48.00	0.31	tr_C9ZL05_	
HLA-DQA10501-DQB10201	27	TEKPPRVGRDVALSY	VGRDVALSY	6	0.2488	3388.4	49.00	0.61	tr_C9ZL05_	
HLA-DQA10501-DQB10201	28	WCLYEVMKTTFFAVT	YEVMKTTFF	3	0.2463	3480.1	49.00	0.33	tr_C9ZL05_	

HLA-DQA10501-DQB10201	29	VRDLLYVRPRMLRLG	LLYVRPRML	3	0.2400	3726.3	55.00	0.37	tr_C9ZL05_
HLA-DQA10501-DQB10201	30	SYDTPWCLYEVMKTT	TPWCLYEVM	3	0.2254	4364.2	55.00	0.32	tr_C9ZL05_
HLA-DQA10501-DQB10201	31	CLYEVMKTTFFAVTG	YEVMKTTFF	2	0.2216	4548.6	60.00	0.32	tr_C9ZL05_
HLA-DQA10501-DQB10201	32	MLRLGDSYLTEKPPR	RLGDSYLTE	2	0.2162	4822.0	60.00	0.36	tr_C9ZL05_
HLA-DQA10501-DQB10201	33	RDLLYVRPRMLRLGD	LLYVRPRML	2	0.2135	4961.8	60.00	0.41	tr_C9ZL05_
HLA-DQA10501-DQB10201	34	DLLYVRPRMLRLGDS	LLYVRPRML	1	0.2011	5672.6	65.00	0.40	tr_C9ZL05_
HLA-DQA10501-DQB10201	35	LRLGDSYLTEKPPRV	YLTEKPPRV	6	0.1999	5750.5	65.00	0.34	tr_C9ZL05_
HLA-DQA10501-DQB10201	36	RLGDSYLTEKPPRVG	YLTEKPPRV	5	0.1673	8178.7	75.00	0.43	tr_C9ZL05_
HLA-DQA10501-DQB10201	37	LTEKPPRVGRDVALS	PRVGRDVAL	5	0.1462	10283.7	80.00	0.46	tr_C9ZL05_
HLA-DQA10501-DQB10201	38	YLTEKPPRVGRDVAL	PRVGRDVAL	6	0.1401	10984.6	80.00	0.45	tr_C9ZL05_
HLA-DQA10501-DQB10201	39	LGDSYLTEKPPRVGR	YLTEKPPRV	4	0.1365	11420.8	80.00	0.63	tr_C9ZL05_
HLA-DQA10501-DQB10201	40	GDSYLTEKPPRVGRD	YLTEKPPRV	3	0.1364	11428.0	80.00	0.71	tr_C9ZL05_
HLA-DQA10501-DQB10201	41	DSYLTEKPPRVGRDV	YLTEKPPRV	2	0.1293	12344.2	85.00	0.74	tr_C9ZL05_
HLA-DQA10501-DQB10201	42	SYLTEKPPRVGRDVA	YLTEKPPRV	1	0.1197	13693.9	85.00	0.62	tr_C9ZL05_

Allele: HLA-DQA10501-DQB10201. Number of high binders 0. Number of weak binders 5. Number of peptides 42

Allele	pos	peptide	core	Of	1-log50k(aff)	affinity(nM)	%Rank	Relia	Identity	Bind Level
HLA-DQA10501-DQB10201	1	ASAPHSNADSEGSET	APHSNADSE	2	0.1541	9440.7	75.00	0.31	tr_D0A070_	
HLA-DQA10501-DQB10201	2	EASAPHSNADSEGSE	APHSNADSE	3	0.1530	9546.1	80.00	0.34	tr_D0A070_	
HLA-DQA10501-DQB10201	3	SAPHSNADSEGSETG	APHSNADSE	1	0.1444	10481.5	80.00	0.27	tr_D0A070_	
HLA-DQA10501-DQB10201	4	REASAPHSNADSEGS	APHSNADSE	4	0.1410	10879.5	80.00	0.38	tr_D0A070_	
HLA-DQA10501-DQB10201	5	APHSNADSEGSETGG	PHSNADSEG	1	0.1271	12639.8	85.00	0.22	tr_D0A070_	
HLA-DQA10501-DQB10201	6	PHSNADSEGSETGGN	NADSEGSET	3	0.0976	17400.3	90.00	0.33	tr_D0A070_	
HLA-DQA10501-DQB10201	7	SETGGNKKTEEAEKA	KKTEEAEKA	6	0.0825	20485.2	95.00	0.58	tr_D0A070_	
HLA-DQA10501-DQB10201	8	HSNADSEGSETGGNK	NADSEGSET	2	0.0817	20655.1	95.00	0.43	tr_D0A070_	
HLA-DQA10501-DQB10201	9	ETGGNKKTEEAEKAN	KKTEEAEKA	5	0.0767	21795.7	95.00	0.57	tr_D0A070_	
HLA-DQA10501-DQB10201	10	TGGNKKTEEAEKANS	KKTEEAEKA	4	0.0717	23005.8	95.00	0.54	tr_D0A070_	
HLA-DQA10501-DQB10201	11	SNADSEGSETGGNKK	NADSEGSET	1	0.0683	23868.2	95.00	0.50	tr_D0A070_	
HLA-DQA10501-DQB10201	12	EGSETGGNKKTEEAE	GNKKTEEAE	6	0.0520	28497.8	100.00	0.35	tr_D0A070_	
HLA-DQA10501-DQB10201	13	GSETGGNKKTEEAEK	GNKKTEEAE	5	0.0490	29412.9	100.00	0.29	tr_D0A070_	
HLA-DQA10501-DQB10201	14	NADSEGSETGGNKKT	NADSEGSET	0	0.0452	30647.4	100.00	0.53	tr_D0A070_	
HLA-DQA10501-DQB10201	15	SEGSETGGNKKTEEA	GGNKKTEEA	6	0.0405	32256.3	100.00	0.46	tr_D0A070_	
HLA-DQA10501-DQB10201	16	DSEGSETGGNKKTEE	TGGNKKTEE	6	0.0309	35795.5	100.00	0.52	tr_D0A070_	
HLA-DQA10501-DQB10201	17	ADSEGSETGGNKKTE	ADSEGSETG	0	0.0254	37999.7	100.00	0.32	tr_D0A070_	

Allele: HLA-DQA10501-DQB10201. Number of high binders 0. Number of weak binders 0. Number of peptides 17

Allele	pos	peptide	core	Of	1-log50k(aff)	affinity(nM)	%Rank	Relia	Identity	Bind Level
HLA-DQA10501-DQB10201	1	STDWLLRYDVIPQAY	RYDVIPQAY	6	0.5611	115.4	2.50	0.45	tr_D0A3E0_	WB
HLA-DQA10501-DQB10201	2	YGQLYAFELLYDHEV	AFELLYDHE	5	0.5602	116.6	2.50	0.31	tr_D0A3E0_	WB
HLA-DQA10501-DQB10201	3	PYGQLYAFELLYDHE	AFELLYDHE	6	0.5472	134.1	3.00	0.34	tr_D0A3E0_	WB

HLA-DQA10501-DQB10201	4	TDWLLRYDVIPQAYA	RYDVIPQAY	5	0.5435	139.6	3.00	0.45	tr_D0A3E0_	WB
HLA-DQA10501-DQB10201	5	DWLLRYDVIPQAYAF	RYDVIPQAY	4	0.5430	140.5	3.50	0.46	tr_D0A3E0_	WB
HLA-DQA10501-DQB10201	6	TPPYGQLYAFELLYD	QLYAFELLY	5	0.5329	156.6	3.50	0.28	tr_D0A3E0_	WB
HLA-DQA10501-DQB10201	7	ITPPYGQLYAFELLY	QLYAFELLY	6	0.5229	174.5	4.00	0.33	tr_D0A3E0_	WB
HLA-DQA10501-DQB10201	8	LRGMFPNASEFFPAI	MFPNASEFF	3	0.5191	182.0	4.50	0.57	tr_D0A3E0_	WB
HLA-DQA10501-DQB10201	9	GQLYAFELLYDHEVK	AFELLYDHE	4	0.5174	185.3	4.50	0.33	tr_D0A3E0_	WB
HLA-DQA10501-DQB10201	10	CLLRGMFPNASEFFP	MFPNASEFF	5	0.5149	190.4	4.50	0.61	tr_D0A3E0_	WB
HLA-DQA10501-DQB10201	11	LLRGMFPNASEFFPA	MFPNASEFF	4	0.5123	195.8	5.00	0.58	tr_D0A3E0_	WB
HLA-DQA10501-DQB10201	12	FFPAIHTADVSTDWL	TADVSTDWL	6	0.5086	203.7	5.00	0.59	tr_D0A3E0_	WB
HLA-DQA10501-DQB10201	13	RGMFPNASEFFPAIH	MFPNASEFF	2	0.5081	204.8	5.00	0.56	tr_D0A3E0_	WB
HLA-DQA10501-DQB10201	14	PPYGQLYAFELLYDH	QLYAFELLY	4	0.5056	210.4	5.00	0.23	tr_D0A3E0_	WB
HLA-DQA10501-DQB10201	15	FPAIHTADVSTDWLL	TADVSTDWL	5	0.5055	210.6	5.00	0.62	tr_D0A3E0_	WB
HLA-DQA10501-DQB10201	16	WLLRYDVIPQAYAFS	RYDVIPQAY	3	0.5038	214.5	5.50	0.52	tr_D0A3E0_	WB
HLA-DQA10501-DQB10201	17	IPQAYAFSHLDEHWW	AYAFSHLDE	3	0.5001	223.5	5.50	0.29	tr_D0A3E0_	WB
HLA-DQA10501-DQB10201	18	GMFPNASEFFPAIHT	MFPNASEFF	1	0.4983	227.7	5.50	0.54	tr_D0A3E0_	WB
HLA-DQA10501-DQB10201	19	VSTDWLLRYDVIPQA	LRYDVIPQA	6	0.4857	261.2	6.50	0.36	tr_D0A3E0_	WB
HLA-DQA10501-DQB10201	20	AIHTADVSTDWLLRY	TADVSTDWL	3	0.4834	267.6	6.50	0.60	tr_D0A3E0_	WB
HLA-DQA10501-DQB10201	21	QLYAFELLYDHEVKG	AFELLYDHE	3	0.4785	282.3	7.00	0.35	tr_D0A3E0_	WB
HLA-DQA10501-DQB10201	22	GCLLRGMFPNASEFF	MFPNASEFF	6	0.4780	283.8	7.00	0.65	tr_D0A3E0_	WB
HLA-DQA10501-DQB10201	23	PQAYAFSHLDEHWWR	AYAFSHLDE	2	0.4731	299.2	7.50	0.30	tr_D0A3E0_	WB
HLA-DQA10501-DQB10201	24	PAIHTADVSTDWLLR	TADVSTDWL	4	0.4725	301.2	7.50	0.64	tr_D0A3E0_	WB
HLA-DQA10501-DQB10201	25	IHTADVSTDWLLRYD	TADVSTDWL	2	0.4699	309.8	8.00	0.61	tr_D0A3E0_	WB
HLA-DQA10501-DQB10201	26	VIPQAYAFSHLDEHW	AYAFSHLDE	4	0.4696	310.8	8.00	0.35	tr_D0A3E0_	WB
HLA-DQA10501-DQB10201	27	CEEGYTLNSVTLECV	LNSVTLECV	6	0.4667	320.5	8.00	0.49	tr_D0A3E0_	WB
HLA-DQA10501-DQB10201	28	GITPPYGQLYAFELL	YGQLYAFEL	5	0.4620	337.2	8.50	0.32	tr_D0A3E0_	WB
HLA-DQA10501-DQB10201	29	MFPNASEFFPAIHTA	MFPNASEFF	0	0.4605	342.8	8.50	0.47	tr_D0A3E0_	WB
HLA-DQA10501-DQB10201	30	NAAGKMMLINAGEFL	MLINAGEFL	6	0.4604	343.3	8.50	0.46	tr_D0A3E0_	WB
HLA-DQA10501-DQB10201	31	EEGYTLNSVTLECVC	LNSVTLECV	5	0.4558	360.7	9.00	0.47	tr_D0A3E0_	WB
HLA-DQA10501-DQB10201	32	QAYAFSHLDEHWWRN	AYAFSHLDE	1	0.4526	373.4	9.50	0.28	tr_D0A3E0_	WB
HLA-DQA10501-DQB10201	33	LLRYDVIPQAYAFSH	RYDVIPQAY	2	0.4509	380.5	9.50	0.59	tr_D0A3E0_	WB
HLA-DQA10501-DQB10201	34	TADVSTDWLLRYDVI	TADVSTDWL	0	0.4465	399.0	10.00	0.38	tr_D0A3E0_	
HLA-DQA10501-DQB10201	35	HTADVSTDWLLRYDV	TADVSTDWL	1	0.4456	402.7	10.00	0.57	tr_D0A3E0_	
HLA-DQA10501-DQB10201	36	LYAFELLYDHEVKGY	AFELLYDHE	2	0.4452	404.6	10.00	0.37	tr_D0A3E0_	
HLA-DQA10501-DQB10201	37	EGYTLNSVTLECVCS	LNSVTLECV	4	0.4342	455.7	12.00	0.46	tr_D0A3E0_	
HLA-DQA10501-DQB10201	38	DVSTDWLLRYDVIPQ	DWLLRYDVI	4	0.4334	459.6	12.00	0.35	tr_D0A3E0_	
HLA-DQA10501-DQB10201	39	ADVSTDWLLRYDVIP	DWLLRYDVI	5	0.4295	479.2	12.00	0.37	tr_D0A3E0_	
HLA-DQA10501-DQB10201	40	AAGKMMLINAGEFLR	MLINAGEFL	5	0.4288	483.2	12.00	0.45	tr_D0A3E0_	
HLA-DQA10501-DQB10201	41	AGKMMLINAGEFLRN	MLINAGEFL	4	0.4145	564.2	14.00	0.45	tr_D0A3E0_	
HLA-DQA10501-DQB10201	42	AYAFSHLDEHWWRNV	AFSHLDEHW	2	0.4136	569.3	14.00	0.37	tr_D0A3E0_	
HLA-DQA10501-DQB10201	43	GYTLNSVTLECVCSS	LNSVTLECV	3	0.4130	573.5	14.00	0.44	tr_D0A3E0_	
HLA-DQA10501-DQB10201	44	KPEEGYAFSWGEFQM	YAFSWGEFQ	5	0.4121	578.6	14.00	0.49	tr_D0A3E0_	
HLA-DQA10501-DQB10201	45	TGITPPYGQLYAFEL	YGQLYAFEL	6	0.4096	594.5	15.00	0.50	tr_D0A3E0_	
HLA-DQA10501-DQB10201	46	YAFELLYDHEVKGYI	AFELLYDHE	1	0.4086	600.8	15.00	0.40	tr_D0A3E0_	
HLA-DQA10501-DQB10201	47	VSREVFSEGFCADPQ	SREVFSEGF	1	0.4074	608.8	15.00	0.40	tr_D0A3E0_	
HLA-DQA10501-DQB10201	48	LRYDVIPQAYAFSHL	RYDVIPQAY	1	0.4054	622.5	15.00	0.64	tr_D0A3E0_	
HLA-DQA10501-DQB10201	49	NTLLSVSREVFSEGF	SREVFSEGF	6	0.4032	637.1	15.00	0.63	tr_D0A3E0_	
HLA-DQA10501-DQB10201	50	EFFPAIHTADVSTDW	PAIHTADVS	3	0.4009	653.2	16.00	0.41	tr_D0A3E0_	
HLA-DQA10501-DQB10201	51	GKMMLINAGEFLRNH	MLINAGEFL	3	0.3953	694.4	17.00	0.45	tr_D0A3E0_	
HLA-DQA10501-DQB10201	52	LSVSREVFSEGFCAD	SREVFSEGF	3	0.3940	704.2	17.00	0.62	tr_D0A3E0_	
HLA-DQA10501-DQB10201	53	EGYAFSWGEFQMKCM	YAFSWGEFQ	2	0.3910	727.6	17.00	0.38	tr_D0A3E0_	

HLA-DQA10501-DQB10201	54	SVSREVFSEGFCADP	SREVFSEGF	2	0.3900	735.0	17.00	0.56	tr_D0A3E0_
HLA-DQA10501-DQB10201	55	NHYNSNASEPFFPEE	YNSNASEPF	2	0.3894	739.9	17.00	0.50	tr_D0A3E0_
HLA-DQA10501-DQB10201	56	RNHYNSNASEPFFPE	YNSNASEPF	3	0.3893	740.6	18.00	0.53	tr_D0A3E0_
HLA-DQA10501-DQB10201	57	DVIPQAYAFSHLDEH	AYAFSHLDE	5	0.3891	742.5	18.00	0.49	tr_D0A3E0_
HLA-DQA10501-DQB10201	58	YTLNSVTLECVCSSR	LNSVTLECV	2	0.3890	743.3	18.00	0.47	tr_D0A3E0_
HLA-DQA10501-DQB10201	59	LRNHYNSNASEPFFP	YNSNASEPF	4	0.3886	746.4	18.00	0.56	tr_D0A3E0_
HLA-DQA10501-DQB10201	60	EDSHRFVYNASDRTH	FVYNASDRT	5	0.3879	752.3	18.00	0.74	tr_D0A3E0_
HLA-DQA10501-DQB10201	61	TLLSVSREVFSEGFC	SREVFSEGF	5	0.3875	755.1	18.00	0.64	tr_D0A3E0_
HLA-DQA10501-DQB10201	62	PEEGYAFSWGEFQMK	YAFSWGEFQ	4	0.3875	755.4	18.00	0.44	tr_D0A3E0_
HLA-DQA10501-DQB10201	63	YDVIPQAYAFSHLDE	AYAFSHLDE	6	0.3853	773.9	18.00	0.60	tr_D0A3E0_
HLA-DQA10501-DQB10201	64	FEDSHRFVYNASDRT	FVYNASDRT	6	0.3851	774.8	18.00	0.74	tr_D0A3E0_
HLA-DQA10501-DQB10201	65	KMMLINAGEFLRNHY	MLINAGEFL	2	0.3847	778.7	18.00	0.44	tr_D0A3E0_
HLA-DQA10501-DQB10201	66	DSHRFVYNASDRTHA	FVYNASDRT	4	0.3835	788.6	18.00	0.75	tr_D0A3E0_
HLA-DQA10501-DQB10201	67	LNAAGKMMLINAGEF	MMLINAGEF	6	0.3825	796.9	19.00	0.75	tr_D0A3E0_
HLA-DQA10501-DQB10201	68	EEGYAFSWGEFQMKC	YAFSWGEFQ	3	0.3790	827.9	19.00	0.49	tr_D0A3E0_
HLA-DQA10501-DQB10201	69	NASEFFPAIHTADVS	PAIHTADVS	6	0.3790	828.3	19.00	0.35	tr_D0A3E0_
HLA-DQA10501-DQB10201	70	LLSVSREVFSEGFCA	SREVFSEGF	4	0.3785	832.2	19.00	0.62	tr_D0A3E0_
HLA-DQA10501-DQB10201	71	SREVFSEGFCADPQN	SEGFCADPQ	5	0.3771	845.3	19.00	0.33	tr_D0A3E0_
HLA-DQA10501-DQB10201	72	QKPEEGYAFSWGEFQ	YAFSWGEFQ	6	0.3768	847.8	19.00	0.50	tr_D0A3E0_
HLA-DQA10501-DQB10201	73	SEFFPAIHTADVSTD	PAIHTADVS	4	0.3757	858.0	20.00	0.42	tr_D0A3E0_
HLA-DQA10501-DQB10201	74	HYNSNASEPFFPEES	YNSNASEPF	1	0.3746	868.8	20.00	0.44	tr_D0A3E0_
HLA-DQA10501-DQB10201	75	ASEFFPAIHTADVST	PAIHTADVS	5	0.3721	892.2	20.00	0.37	tr_D0A3E0_
HLA-DQA10501-DQB10201	76	FLRNHYNSNASEPFF	YNSNASEPF	5	0.3709	903.5	20.00	0.60	tr_D0A3E0_
HLA-DQA10501-DQB10201	77	SCEEGYTLNSVTLEC	YTLNSVTLE	5	0.3701	911.7	21.00	0.45	tr_D0A3E0_
HLA-DQA10501-DQB10201	78	GYAFSWGEFQMKCMN	YAFSWGEFQ	1	0.3654	959.3	21.00	0.37	tr_D0A3E0_
HLA-DQA10501-DQB10201	79	LLSCLGAQAVQTLHL	AQAVQTLHL	6	0.3612	1003.6	22.00	0.33	tr_D0A3E0_
HLA-DQA10501-DQB10201	80	AFELLYDHEVKGYII	FELLYDHEV	1	0.3581	1037.8	23.00	0.39	tr_D0A3E0_
HLA-DQA10501-DQB10201	81	CAATLFDIGVAMQSD	DIGVAMQSD	6	0.3531	1096.3	24.00	0.55	tr_D0A3E0_
HLA-DQA10501-DQB10201	82	YNSNASEPFFPEESY	YNSNASEPF	0	0.3527	1100.5	24.00	0.29	tr_D0A3E0_
HLA-DQA10501-DQB10201	83	SHRFVYNASDRTHAK	FVYNASDRT	3	0.3526	1102.0	24.00	0.73	tr_D0A3E0_
HLA-DQA10501-DQB10201	84	MMLINAGEFLRNHYN	MLINAGEFL	1	0.3501	1131.8	24.00	0.51	tr_D0A3E0_
HLA-DQA10501-DQB10201	85	YAFSWGEFQMKCMNE	YAFSWGEFQ	0	0.3499	1134.5	24.00	0.29	tr_D0A3E0_
HLA-DQA10501-DQB10201	86	RYDVIPQAYAFSHLD	RYDVIPQAY	0	0.3482	1155.8	25.00	0.54	tr_D0A3E0_
HLA-DQA10501-DQB10201	87	ISLLSCLGAQAVQTL	CLGAQAVQT	5	0.3481	1156.5	25.00	0.37	tr_D0A3E0_
HLA-DQA10501-DQB10201	88	MSVFYPPLIPSEHYF	MSVFYPPLI	0	0.3478	1160.2	25.00	0.34	tr_D0A3E0_
HLA-DQA10501-DQB10201	89	TLFDIGVAMQSDGRI	DIGVAMQSD	3	0.3456	1188.5	25.00	0.38	tr_D0A3E0_
HLA-DQA10501-DQB10201	90	LFDIGVAMQSDGRID	DIGVAMQSD	2	0.3456	1189.1	25.00	0.33	tr_D0A3E0_
HLA-DQA10501-DQB10201	91	KLYHYSAHDTTIAPL	YHYSAHDTT	2	0.3441	1207.7	25.00	0.34	tr_D0A3E0_
HLA-DQA10501-DQB10201	92	PNASEFFPAIHTADV	FPAIHTADV	6	0.3418	1237.9	26.00	0.34	tr_D0A3E0_
HLA-DQA10501-DQB10201	93	YAFSHLDEHWWRNVC	AFSHLDEHW	1	0.3393	1272.2	26.00	0.48	tr_D0A3E0_
HLA-DQA10501-DQB10201	94	HRFVYNASDRTHAKM	FVYNASDRT	2	0.3386	1281.7	27.00	0.75	tr_D0A3E0_
HLA-DQA10501-DQB10201	95	AATLFDIGVAMQSDG	DIGVAMQSD	5	0.3364	1313.5	27.00	0.49	tr_D0A3E0_
HLA-DQA10501-DQB10201	96	LYHYSAHDTTIAPLA	YHYSAHDTT	1	0.3362	1315.2	27.00	0.32	tr_D0A3E0_
HLA-DQA10501-DQB10201	97	FPNASEFFPAIHTAD	ASEFFPAIH	3	0.3342	1344.7	27.00	0.23	tr_D0A3E0_
HLA-DQA10501-DQB10201	98	IKDKTGAEAMPYACL	KTGAEAMPY	3	0.3317	1381.8	28.00	0.42	tr_D0A3E0_
HLA-DQA10501-DQB10201	99	LSCLGAQAVQTLHLV	AQAVQTLHL	5	0.3295	1414.1	28.00	0.32	tr_D0A3E0_
HLA-DQA10501-DQB10201	100	TLNSVTLECVCSSRK	LNSVTLECV	1	0.3287	1427.2	29.00	0.54	tr_D0A3E0_
HLA-DQA10501-DQB10201	101	ETIKDKTGAEAMPYA	KTGAEAMPY	5	0.3273	1449.5	29.00	0.50	tr_D0A3E0_
HLA-DQA10501-DQB10201	102	TIKDKTGAEAMPYAC	KTGAEAMPY	4	0.3250	1484.9	29.00	0.47	tr_D0A3E0_
HLA-DQA10501-DQB10201	103	AKMGSLGQHLAQEIL	GQHLAQEIL	6	0.3239	1503.1	30.00	0.50	tr_D0A3E0_

HLA-DQA10501-DQB10201	104	WSCEEGYTLNSVTLE	YTLNSVTLE	6	0.3217	1539.6	30.00	0.49	tr_D0A3E0_
HLA-DQA10501-DQB10201	105	SLLSCLGAQAVQTLH	CLGAQAVQT	4	0.3211	1549.0	30.00	0.35	tr_D0A3E0_
HLA-DQA10501-DQB10201	106	KCMNENHTVYTVEDN	HTVYTVEDN	6	0.3203	1563.3	30.00	0.39	tr_D0A3E0_
HLA-DQA10501-DQB10201	107	TTGITPPYGQLYAFE	PYGQLYAFE	6	0.3202	1563.7	30.00	0.64	tr_D0A3E0_
HLA-DQA10501-DQB10201	108	EFLRNHYNSNASEPF	YNSNASEPF	6	0.3182	1598.9	31.00	0.73	tr_D0A3E0_
HLA-DQA10501-DQB10201	109	ATLFDIGVAMQSDGR	DIGVAMQSD	4	0.3171	1618.7	31.00	0.47	tr_D0A3E0_
HLA-DQA10501-DQB10201	110	LISLLSCLGAQAVQT	CLGAQAVQT	6	0.3157	1643.1	31.00	0.40	tr_D0A3E0_
HLA-DQA10501-DQB10201	111	KDKTGAEAMPYACLH	KTGAEAMPY	2	0.3155	1646.6	32.00	0.41	tr_D0A3E0_
HLA-DQA10501-DQB10201	112	NENHTVYTVEDNKCP	HTVYTVEDN	3	0.3152	1651.9	32.00	0.28	tr_D0A3E0_
HLA-DQA10501-DQB10201	113	YHYSAHDTTIAPLAA	AHDTTIAPL	4	0.3146	1662.7	32.00	0.28	tr_D0A3E0_
HLA-DQA10501-DQB10201	114	FDIGVAMQSDGRIDK	DIGVAMQSD	1	0.3133	1685.2	32.00	0.37	tr_D0A3E0_
HLA-DQA10501-DQB10201	115	RFVYNASDRTHAKMG	FVYNASDRT	1	0.3114	1720.3	32.00	0.77	tr_D0A3E0_
HLA-DQA10501-DQB10201	116	ENHTVYTVEDNKCPY	HTVYTVEDN	2	0.3112	1724.1	33.00	0.27	tr_D0A3E0_
HLA-DQA10501-DQB10201	117	PLIPSEHYFQETIKD	SEHYFQETI	4	0.3112	1724.5	33.00	0.31	tr_D0A3E0_
HLA-DQA10501-DQB10201	118	LIDTNTLLSVSREVF	LLSVSREVF	6	0.3106	1735.3	33.00	0.32	tr_D0A3E0_
HLA-DQA10501-DQB10201	119	REVFSEGFCADPQNR	SEGFCADPQ	4	0.3086	1774.2	33.00	0.44	tr_D0A3E0_
HLA-DQA10501-DQB10201	120	YKLYHYSAHDTTIAP	YHYSAHDTT	3	0.3065	1814.2	34.00	0.51	tr_D0A3E0_
HLA-DQA10501-DQB10201	121	LGAQAVQTLHLVQLV	QTLHLVQLV	6	0.3054	1835.2	34.00	0.28	tr_D0A3E0_
HLA-DQA10501-DQB10201	122	MNENHTVYTVEDNKC	HTVYTVEDN	4	0.3049	1847.0	34.00	0.28	tr_D0A3E0_
HLA-DQA10501-DQB10201	123	SCLGAQAVQTLHLVQ	AQAVQTLHL	4	0.3026	1892.8	34.00	0.34	tr_D0A3E0_
HLA-DQA10501-DQB10201	124	CMNENHTVYTVEDNK	HTVYTVEDN	5	0.2974	2001.9	36.00	0.31	tr_D0A3E0_
HLA-DQA10501-DQB10201	125	TSYKLYHYSAHDTTI	YHYSAHDTT	5	0.2973	2003.9	36.00	0.56	tr_D0A3E0_
HLA-DQA10501-DQB10201	126	SYKLYHYSAHDTTIA	YHYSAHDTT	4	0.2972	2007.5	36.00	0.54	tr_D0A3E0_
HLA-DQA10501-DQB10201	127	AGCLLRGMFPNASEF	LRGMFPNAS	4	0.2967	2016.8	36.00	0.44	tr_D0A3E0_
HLA-DQA10501-DQB10201	128	CLGAQAVQTLHLVQL	AQAVQTLHL	3	0.2967	2016.9	36.00	0.29	tr_D0A3E0_
HLA-DQA10501-DQB10201	129	IDTNTLLSVSREVFS	LLSVSREVF	5	0.2947	2061.5	36.00	0.29	tr_D0A3E0_
HLA-DQA10501-DQB10201	130	EVFSEGFCADPQNRC	SEGFCADPQ	3	0.2933	2091.9	37.00	0.47	tr_D0A3E0_
HLA-DQA10501-DQB10201	131	DKTGAEAMPYACLHG	KTGAEAMPY	1	0.2933	2093.1	37.00	0.41	tr_D0A3E0_
HLA-DQA10501-DQB10201	132	VFRRVCPAWSCEEGY	PAWSCEEGY	6	0.2933	2093.7	37.00	0.40	tr_D0A3E0_
HLA-DQA10501-DQB10201	133	YPPLIPSEHYFQETI	SEHYFQETI	6	0.2894	2184.2	38.00	0.47	tr_D0A3E0_
HLA-DQA10501-DQB10201	134	QETIKDKTGAEAMPY	KTGAEAMPY	6	0.2891	2190.6	38.00	0.66	tr_D0A3E0_
HLA-DQA10501-DQB10201	135	KMGSLGQHLAQEILK	GQHLAQEIL	5	0.2887	2199.1	38.00	0.44	tr_D0A3E0_
HLA-DQA10501-DQB10201	136	SAGCLLRGMFPNASE	LRGMFPNAS	5	0.2860	2264.0	39.00	0.45	tr_D0A3E0_
HLA-DQA10501-DQB10201	137	CLLISLLSCLGAQAV	LSCLGAQAV	6	0.2854	2280.2	39.00	0.37	tr_D0A3E0_
HLA-DQA10501-DQB10201	138	DTNTLLSVSREVFSE	LLSVSREVF	4	0.2852	2284.6	39.00	0.29	tr_D0A3E0_
HLA-DQA10501-DQB10201	139	LIPSEHYFQETIKDK	SEHYFQETI	3	0.2848	2295.0	39.00	0.29	tr_D0A3E0_
HLA-DQA10501-DQB10201	140	LTSYKLYHYSAHDTT	YHYSAHDTT	6	0.2841	2311.4	39.00	0.60	tr_D0A3E0_
HLA-DQA10501-DQB10201	141	KCMESRDLSISTDTL	DLSISTDTL	6	0.2834	2328.9	39.00	0.71	tr_D0A3E0_
HLA-DQA10501-DQB10201	142	DTTIAPLAATLGDST	LAATLGDST	6	0.2816	2375.9	40.00	0.46	tr_D0A3E0_
HLA-DQA10501-DQB10201	143	LLISLLSCLGAQAVQ	LSCLGAQAV	5	0.2815	2378.9	40.00	0.37	tr_D0A3E0_
HLA-DQA10501-DQB10201	144	GAQAVQTLHLVQLVH	AQAVQTLHL	1	0.2794	2432.2	40.00	0.28	tr_D0A3E0_
HLA-DQA10501-DQB10201	145	TLIDTNTLLSVSREV	NTLLSVSRE	5	0.2787	2452.0	40.00	0.32	tr_D0A3E0_
HLA-DQA10501-DQB10201	146	GQKPEEGYAFSWGEF	GYAFSWGEF	6	0.2784	2458.4	41.00	0.34	tr_D0A3E0_
HLA-DQA10501-DQB10201	147	DTLIDTNTLLSVSRE	NTLLSVSRE	6	0.2782	2465.6	41.00	0.37	tr_D0A3E0_
HLA-DQA10501-DQB10201	148	AFSWGEFQMKCMNEN	WGEFQMKCM	3	0.2776	2480.6	41.00	0.44	tr_D0A3E0_
HLA-DQA10501-DQB10201	149	PPLIPSEHYFQETIK	SEHYFQETI	5	0.2775	2482.3	41.00	0.35	tr_D0A3E0_
HLA-DQA10501-DQB10201	150	NSNASEPFFPEESYN	EPFFPEESY	5	0.2764	2513.7	41.00	0.32	tr_D0A3E0_
HLA-DQA10501-DQB10201	151	YSAHDTTIAPLAATL	AHDTTIAPL	2	0.2760	2523.8	41.00	0.28	tr_D0A3E0_
HLA-DQA10501-DQB10201	152	HNQSQICGEVPCGYL	QSQICGEVP	2	0.2755	2537.8	41.00	0.39	tr_D0A3E0_
HLA-DQA10501-DQB10201	153	MLINAGEFLRNHYNS	MLINAGEFL	0	0.2753	2542.8	41.00	0.68	tr_D0A3E0_

HLA-DQA10501-DQB10201	154	KHNQSQICGEVPCGY	QSQICGEVP	3	0.2750	2551.4	41.00	0.40	tr_D0A3E0_
HLA-DQA10501-DQB10201	155	NHTVYTVEDNKCPYH	HTVYTVEDN	1	0.2737	2586.8	42.00	0.28	tr_D0A3E0_
HLA-DQA10501-DQB10201	156	TTIAPLAATLGDSTT	LAATLGDST	5	0.2728	2611.4	42.00	0.53	tr_D0A3E0_
HLA-DQA10501-DQB10201	157	MGSLGQHLAQEILKN	GQHLAQEIL	4	0.2722	2630.6	42.00	0.47	tr_D0A3E0_
HLA-DQA10501-DQB10201	158	PKLDTLIDTNTLLSV	IDTNTLLSV	6	0.2715	2651.0	42.00	0.25	tr_D0A3E0_
HLA-DQA10501-DQB10201	159	MKCMNENHTVYTVED	NHTVYTVED	6	0.2707	2672.1	43.00	0.60	tr_D0A3E0_
HLA-DQA10501-DQB10201	160	HYSAHDTTIAPLAAT	AHDTTIAPL	3	0.2696	2705.7	43.00	0.34	tr_D0A3E0_
HLA-DQA10501-DQB10201	161	AFSHLDEHWWRNVCN	AFSHLDEHW	0	0.2692	2717.0	43.00	0.46	tr_D0A3E0_
HLA-DQA10501-DQB10201	162	QSAGCLLRGMFPNAS	LRGMFPNAS	6	0.2688	2729.2	43.00	0.46	tr_D0A3E0_
HLA-DQA10501-DQB10201	163	AHDTTIAPLAATLGD	IAPLAATLG	5	0.2676	2764.1	43.00	0.26	tr_D0A3E0_
HLA-DQA10501-DQB10201	164	SAHDTTIAPLAATLG	DTTIAPLAA	3	0.2672	2775.2	43.00	0.22	tr_D0A3E0_
HLA-DQA10501-DQB10201	165	IPSEHYFQETIKDKT	SEHYFQETI	2	0.2671	2777.7	44.00	0.30	tr_D0A3E0_
HLA-DQA10501-DQB10201	166	CMESRDLSISTDTLG	DLSISTDTL	5	0.2645	2858.5	44.00	0.67	tr_D0A3E0_
HLA-DQA10501-DQB10201	167	KQCKVFRRVCPAWSC	FRRVCPAWS	5	0.2641	2872.0	44.00	0.46	tr_D0A3E0_
HLA-DQA10501-DQB10201	168	NKQCKVFRRVCPAWS	FRRVCPAWS	6	0.2639	2878.3	44.00	0.43	tr_D0A3E0_
HLA-DQA10501-DQB10201	169	CKVFRRVCPAWSCEE	FRRVCPAWS	3	0.2636	2886.5	44.00	0.41	tr_D0A3E0_
HLA-DQA10501-DQB10201	170	LNSVTLECVCSSRKC	LNSVTLECV	0	0.2635	2889.9	44.00	0.51	tr_D0A3E0_
HLA-DQA10501-DQB10201	171	TIAPLAATLGDSTTT	LAATLGDST	4	0.2628	2911.7	45.00	0.56	tr_D0A3E0_
HLA-DQA10501-DQB10201	172	TNTLLSVSREVFSEG	LLSVSREVF	3	0.2622	2930.7	45.00	0.28	tr_D0A3E0_
HLA-DQA10501-DQB10201	173	QCKVFRRVCPAWSCE	FRRVCPAWS	4	0.2611	2964.7	45.00	0.46	tr_D0A3E0_
HLA-DQA10501-DQB10201	174	SVFYPPLIPSEHYFQ	FYPPLIPSE	2	0.2611	2965.4	45.00	0.22	tr_D0A3E0_
HLA-DQA10501-DQB10201	175	DIGVAMQSDGRIDKH	VAMQSDGRI	3	0.2602	2994.3	45.00	0.35	tr_D0A3E0_
HLA-DQA10501-DQB10201	176	VFSEGFCADPQNRCH	SEGFCADPQ	2	0.2597	3009.6	46.00	0.55	tr_D0A3E0_
HLA-DQA10501-DQB10201	177	RLRDIKFFEDSHRFV	FFEDSHRFV	6	0.2597	3010.5	46.00	0.38	tr_D0A3E0_
HLA-DQA10501-DQB10201	178	CPAWSCEEGYTLNSV	EEGYTLNSV	6	0.2595	3015.9	46.00	0.41	tr_D0A3E0_
HLA-DQA10501-DQB10201	179	NPKLDTLIDTNTLLS	TLIDTNTLL	5	0.2556	3146.5	47.00	0.24	tr_D0A3E0_
HLA-DQA10501-DQB10201	180	FELLYDHEVKGYIIR	FELLYDHEV	0	0.2553	3156.7	47.00	0.41	tr_D0A3E0_
HLA-DQA10501-DQB10201	181	HAKMGSLGQHLAQEI	LGQHLAQEI	6	0.2553	3157.3	47.00	0.62	tr_D0A3E0_
HLA-DQA10501-DQB10201	182	NQSQICGEVPCGYLN	QSQICGEVP	1	0.2552	3160.5	47.00	0.38	tr_D0A3E0_
HLA-DQA10501-DQB10201	183	GSLGQHLAQEILKNA	GQHLAQEIL	3	0.2548	3173.9	47.00	0.47	tr_D0A3E0_
HLA-DQA10501-DQB10201	184	KVFRRVCPAWSCEEG	FRRVCPAWS	2	0.2546	3181.7	47.00	0.41	tr_D0A3E0_
HLA-DQA10501-DQB10201	185	HDTTIAPLAATLGDS	IAPLAATLG	4	0.2539	3206.4	47.00	0.26	tr_D0A3E0_
HLA-DQA10501-DQB10201	186	LRDIKFFEDSHRFVY	FFEDSHRFV	5	0.2533	3227.9	47.00	0.41	tr_D0A3E0_
HLA-DQA10501-DQB10201	187	KLDTLIDTNTLLSVS	DTLIDTNTL	2	0.2524	3257.8	48.00	0.19	tr_D0A3E0_
HLA-DQA10501-DQB10201	188	AQAVQTLHLVQLVHR	QTLHLVQLV	4	0.2510	3306.0	48.00	0.23	tr_D0A3E0_
HLA-DQA10501-DQB10201	189	SYNSCVTYSRSTDVP	VTYSRSTDV	5	0.2481	3413.6	49.00	0.57	tr_D0A3E0_
HLA-DQA10501-DQB10201	190	PAWSCEEGYTLNSVT	EEGYTLNSV	5	0.2462	3485.9	49.00	0.38	tr_D0A3E0_
HLA-DQA10501-DQB10201	191	FVYNASDRTHAKMGS	FVYNASDRT	0	0.2454	3516.2	49.00	0.82	tr_D0A3E0_
HLA-DQA10501-DQB10201	192	CNPKLDTLIDTNTLL	TLIDTNTLL	6	0.2451	3526.1	50.00	0.28	tr_D0A3E0_
HLA-DQA10501-DQB10201	193	AWSCEEGYTLNSVTL	EEGYTLNSV	4	0.2444	3552.5	50.00	0.38	tr_D0A3E0_
HLA-DQA10501-DQB10201	194	SLGQHLAQEILKNAE	GQHLAQEIL	2	0.2440	3567.8	50.00	0.44	tr_D0A3E0_
HLA-DQA10501-DQB10201	195	SNASEPFFPEESYNS	EPFFPEESY	4	0.2436	3584.1	50.00	0.34	tr_D0A3E0_
HLA-DQA10501-DQB10201	196	MESRDLSISTDTLGR	DLSISTDTL	4	0.2428	3614.9	50.00	0.63	tr_D0A3E0_
HLA-DQA10501-DQB10201	197	LDEHWWRNVCNPKLD	WRNVCNPKL	5	0.2419	3650.0	50.00	0.62	tr_D0A3E0_
HLA-DQA10501-DQB10201	198	HCAATLFDIGVAMQS	TLFDIGVAM	4	0.2416	3663.3	55.00	0.18	tr_D0A3E0_
HLA-DQA10501-DQB10201	199	MPYACLHGGSLCLLI	LHGGSLCLL	5	0.2405	3703.7	55.00	0.49	tr_D0A3E0_
HLA-DQA10501-DQB10201	200	FRRVCPAWSCEEGYT	PAWSCEEGY	5	0.2403	3715.2	55.00	0.49	tr_D0A3E0_
HLA-DQA10501-DQB10201	201	FYPPLIPSEHYFQET	PSEHYFQET	6	0.2403	3715.4	55.00	0.22	tr_D0A3E0_
HLA-DQA10501-DQB10201	202	IAPLAATLGDSTTTG	LAATLGDST	3	0.2396	3743.7	55.00	0.61	tr_D0A3E0_
HLA-DQA10501-DQB10201	203	RCHCAATLFDIGVAM	TLFDIGVAM	6	0.2390	3764.5	55.00	0.35	tr_D0A3E0_

HLA-DQA10501-DQB10201	204	VKHNQSQICGEVPCG	QSQICGEVP	4	0.2383	3796.4	55.00	0.50	tr_D0A3E0_
HLA-DQA10501-DQB10201	205	KTGAEAMPYACLHGG	KTGAEAMPY	0	0.2380	3807.9	55.00	0.27	tr_D0A3E0_
HLA-DQA10501-DQB10201	206	FSWGEFQMKCMNENH	WGEFQMKCM	2	0.2376	3824.8	55.00	0.49	tr_D0A3E0_
HLA-DQA10501-DQB10201	207	RDIKFFEDSHRFVYN	FFEDSHRFV	4	0.2371	3844.9	55.00	0.46	tr_D0A3E0_
HLA-DQA10501-DQB10201	208	CHCAATLFDIGVAMQ	TLFDIGVAM	5	0.2370	3847.3	55.00	0.23	tr_D0A3E0_
HLA-DQA10501-DQB10201	209	HTVYTVEDNKCPYHD	TVYTVEDNK	1	0.2369	3853.8	55.00	0.28	tr_D0A3E0_
HLA-DQA10501-DQB10201	210	PSEHYFQETIKDKTG	SEHYFQETI	1	0.2363	3879.9	55.00	0.28	tr_D0A3E0_
HLA-DQA10501-DQB10201	211	LDTLIDTNTLLSVSR	TLIDTNTLL	2	0.2357	3905.0	55.00	0.25	tr_D0A3E0_
HLA-DQA10501-DQB10201	212	RRVCPAWSCEEGYTL	PAWSCEEGY	4	0.2349	3937.4	55.00	0.51	tr_D0A3E0_
HLA-DQA10501-DQB10201	213	YACLHGGSLCLLISL	LHGGSLCLL	3	0.2336	3993.4	55.00	0.40	tr_D0A3E0_
HLA-DQA10501-DQB10201	214	KVKHNQSQICGEVPC	QSQICGEVP	5	0.2328	4028.9	55.00	0.53	tr_D0A3E0_
HLA-DQA10501-DQB10201	215	DEHWWRNVCNPKLDT	WRNVCNPKL	4	0.2321	4056.4	55.00	0.61	tr_D0A3E0_
HLA-DQA10501-DQB10201	216	LCLLISLLSCLGAQA	LLSCLGAQA	6	0.2310	4107.5	55.00	0.40	tr_D0A3E0_
HLA-DQA10501-DQB10201	217	ESRDLSISTDTLGRN	DLSISTDTL	3	0.2307	4120.2	55.00	0.63	tr_D0A3E0_
HLA-DQA10501-DQB10201	218	PKVKHNQSQICGEVP	QSQICGEVP	6	0.2302	4142.8	55.00	0.54	tr_D0A3E0_
HLA-DQA10501-DQB10201	219	VFYPPLIPSEHYFQE	PPLIPSEHY	3	0.2292	4187.7	55.00	0.27	tr_D0A3E0_
HLA-DQA10501-DQB10201	220	HLDEHWWRNVCNPKL	WRNVCNPKL	6	0.2287	4212.1	55.00	0.61	tr_D0A3E0_
HLA-DQA10501-DQB10201	221	NRCHCAATLFDIGVA	ATLFDIGVA	6	0.2280	4240.1	55.00	0.26	tr_D0A3E0_
HLA-DQA10501-DQB10201	222	PYACLHGGSLCLLIS	LHGGSLCLL	4	0.2279	4245.9	55.00	0.45	tr_D0A3E0_
HLA-DQA10501-DQB10201	223	YNSCVTYSRSTDVPR	VTYSRSTDV	4	0.2264	4318.7	55.00	0.54	tr_D0A3E0_
HLA-DQA10501-DQB10201	224	SCVTYSRSTDVPRTL	VTYSRSTDV	2	0.2263	4322.1	55.00	0.41	tr_D0A3E0_
HLA-DQA10501-DQB10201	225	FSEGFCADPQNRCHC	SEGFCADPQ	1	0.2262	4327.7	55.00	0.58	tr_D0A3E0_
HLA-DQA10501-DQB10201	226	DIKFFEDSHRFVYNA	FFEDSHRFV	3	0.2258	4343.5	55.00	0.46	tr_D0A3E0_
HLA-DQA10501-DQB10201	227	NASEPFFPEESYNSC	EPFFPEESY	3	0.2256	4356.0	55.00	0.32	tr_D0A3E0_
HLA-DQA10501-DQB10201	228	QSQICGEVPCGYLNA	ICGEVPCGY	3	0.2253	4366.1	55.00	0.45	tr_D0A3E0_
HLA-DQA10501-DQB10201	229	EHWWRNVCNPKLDTL	WRNVCNPKL	3	0.2250	4381.4	55.00	0.63	tr_D0A3E0_
HLA-DQA10501-DQB10201	230	RVCPAWSCEEGYTLN	PAWSCEEGY	3	0.2248	4390.0	55.00	0.50	tr_D0A3E0_
HLA-DQA10501-DQB10201	231	AMPYACLHGGSLCLL	LHGGSLCLL	6	0.2237	4446.1	60.00	0.61	tr_D0A3E0_
HLA-DQA10501-DQB10201	232	RENLGRLRDIKFFED	LRDIKFFED	6	0.2233	4462.1	60.00	0.47	tr_D0A3E0_
HLA-DQA10501-DQB10201	233	ACLHGGSLCLLISLL	LHGGSLCLL	2	0.2211	4573.3	60.00	0.34	tr_D0A3E0_
HLA-DQA10501-DQB10201	234	GRLRDIKFFEDSHRF	LRDIKFFED	2	0.2210	4573.7	60.00	0.26	tr_D0A3E0_
HLA-DQA10501-DQB10201	235	ELLYDHEVKGYIIRI	EVKGYIIRI	6	0.2208	4585.8	60.00	0.44	tr_D0A3E0_
HLA-DQA10501-DQB10201	236	NLGRLRDIKFFEDSH	LRDIKFFED	4	0.2202	4617.4	60.00	0.40	tr_D0A3E0_
HLA-DQA10501-DQB10201	237	QNRCHCAATLFDIGV	AATLFDIGV	6	0.2201	4622.7	60.00	0.34	tr_D0A3E0_
HLA-DQA10501-DQB10201	238	PGQKPEEGYAFSWGE	PEEGYAFSW	4	0.2195	4652.5	60.00	0.40	tr_D0A3E0_
HLA-DQA10501-DQB10201	239	FFEDSHRFVYNASDR	RFVYNASDR	6	0.2183	4710.2	60.00	0.30	tr_D0A3E0_
HLA-DQA10501-DQB10201	240	NSCVTYSRSTDVPRT	VTYSRSTDV	3	0.2180	4727.6	60.00	0.50	tr_D0A3E0_
HLA-DQA10501-DQB10201	241	APLAATLGDSTTTGI	LAATLGDST	2	0.2177	4740.4	60.00	0.63	tr_D0A3E0_
HLA-DQA10501-DQB10201	242	QAVQTLHLVQLVHRH	QTLHLVQLV	3	0.2175	4750.6	60.00	0.29	tr_D0A3E0_
HLA-DQA10501-DQB10201	243	VCPAWSCEEGYTLNS	PAWSCEEGY	2	0.2143	4922.7	60.00	0.52	tr_D0A3E0_
HLA-DQA10501-DQB10201	244	KFFEDSHRFVYNASD	FFEDSHRFV	1	0.2128	4998.7	60.00	0.36	tr_D0A3E0_
HLA-DQA10501-DQB10201	245	VCNPKLDTLIDTNTL	DTLIDTNTL	6	0.2119	5049.0	60.00	0.36	tr_D0A3E0_
HLA-DQA10501-DQB10201	246	LGQHLAQEILKNAEN	GQHLAQEIL	1	0.2109	5104.7	60.00	0.47	tr_D0A3E0_
HLA-DQA10501-DQB10201	247	ENLGRLRDIKFFEDS	LRDIKFFED	5	0.2103	5138.3	60.00	0.43	tr_D0A3E0_
HLA-DQA10501-DQB10201	248	ASEPFFPEESYNSCV	EPFFPEESY	2	0.2101	5147.0	60.00	0.31	tr_D0A3E0_
HLA-DQA10501-DQB10201	249	STDVPRTLQSAGCLL	TLQSAGCLL	6	0.2101	5150.0	60.00	0.55	tr_D0A3E0_
HLA-DQA10501-DQB10201	250	CLHGGSLCLLISLLS	LHGGSLCLL	1	0.2092	5202.0	60.00	0.32	tr_D0A3E0_
HLA-DQA10501-DQB10201	251	GSLCLLISLLSCLGA	LISLLSCLG	5	0.2079	5271.9	60.00	0.38	tr_D0A3E0_
HLA-DQA10501-DQB10201	252	HWWRNVCNPKLDTLI	WRNVCNPKL	2	0.2068	5334.2	65.00	0.65	tr_D0A3E0_
HLA-DQA10501-DQB10201	253	LGRLRDIKFFEDSHR	LRDIKFFED	3	0.2066	5345.8	65.00	0.37	tr_D0A3E0_

HLA-DQA10501-DQB10201	254	LQSAGCLLRGMFPNA	LLRGMFPNA	6	0.2058	5394.5	65.00	0.33	tr_D0A3E0_
HLA-DQA10501-DQB10201	255	SWGEFQMKCMNENHT	WGEFQMKCM	1	0.2055	5413.1	65.00	0.46	tr_D0A3E0_
HLA-DQA10501-DQB10201	256	IKFFEDSHRFVYNAS	FFEDSHRFV	2	0.2054	5420.1	65.00	0.46	tr_D0A3E0_
HLA-DQA10501-DQB10201	257	APGQKPEEGYAFSWG	PEEGYAFSW	5	0.2048	5454.4	65.00	0.45	tr_D0A3E0_
HLA-DQA10501-DQB10201	258	CVTYSRSTDVPRTLQ	VTYSRSTDV	1	0.2035	5530.6	65.00	0.38	tr_D0A3E0_
HLA-DQA10501-DQB10201	259	RTLQSAGCLLRGMFP	TLQSAGCLL	1	0.2030	5562.4	65.00	0.28	tr_D0A3E0_
HLA-DQA10501-DQB10201	260	GGSLCLLISLLSCLG	LISLLSCLG	6	0.2026	5581.8	65.00	0.47	tr_D0A3E0_
HLA-DQA10501-DQB10201	261	TLGDSTTTGITPPYG	TTGITPPYG	6	0.2018	5631.1	65.00	0.49	tr_D0A3E0_
HLA-DQA10501-DQB10201	262	YLNAAGKMMLINAGE	KMMLINAGE	6	0.2011	5677.3	65.00	0.49	tr_D0A3E0_
HLA-DQA10501-DQB10201	263	ESYNSCVTYSRSTDV	VTYSRSTDV	6	0.2009	5689.4	65.00	0.64	tr_D0A3E0_
HLA-DQA10501-DQB10201	264	TTTGITPPYGQLYAF	TTGITPPYG	1	0.1994	5780.3	65.00	0.37	tr_D0A3E0_
HLA-DQA10501-DQB10201	265	DSTTTGITPPYGQLY	TTGITPPYG	3	0.1975	5902.8	65.00	0.51	tr_D0A3E0_
HLA-DQA10501-DQB10201	266	PNKQCKVFRRVCPAW	VFRRVCPAW	6	0.1963	5978.3	65.00	0.59	tr_D0A3E0_
HLA-DQA10501-DQB10201	267	LLYDHEVKGYIIRIR	EVKGYIIRI	5	0.1962	5985.8	65.00	0.42	tr_D0A3E0_
HLA-DQA10501-DQB10201	268	SEHYFQETIKDKTGA	EHYFQETIK	1	0.1955	6027.4	65.00	0.34	tr_D0A3E0_
HLA-DQA10501-DQB10201	269	AVQTLHLVQLVHRHG	QTLHLVQLV	2	0.1948	6078.6	65.00	0.31	tr_D0A3E0_
HLA-DQA10501-DQB10201	270	IGVAMQSDGRIDKHP	VAMQSDGRI	2	0.1944	6104.7	65.00	0.47	tr_D0A3E0_
HLA-DQA10501-DQB10201	271	SLCLLISLLSCLGAQ	LISLLSCLG	4	0.1942	6115.9	65.00	0.40	tr_D0A3E0_
HLA-DQA10501-DQB10201	272	TDVPRTLQSAGCLLR	TLQSAGCLL	5	0.1941	6120.6	65.00	0.47	tr_D0A3E0_
HLA-DQA10501-DQB10201	273	EPFFPEESYNSCVTY	ESYNSCVTY	6	0.1931	6189.2	65.00	0.28	tr_D0A3E0_
HLA-DQA10501-DQB10201	274	DPQNRCHCAATLFDI	CHCAATLFD	5	0.1922	6248.2	65.00	0.31	tr_D0A3E0_
HLA-DQA10501-DQB10201	275	LHGGSLCLLISLLSC	LHGGSLCLL	0	0.1918	6273.2	65.00	0.28	tr_D0A3E0_
HLA-DQA10501-DQB10201	276	GDSTTTGITPPYGQL	TTGITPPYG	4	0.1915	6294.7	65.00	0.56	tr_D0A3E0_
HLA-DQA10501-DQB10201	277	WWRNVCNPKLDTLID	WRNVCNPKL	1	0.1909	6335.0	65.00	0.60	tr_D0A3E0_
HLA-DQA10501-DQB10201	278	LGDSTTTGITPPYGQ	TTGITPPYG	5	0.1908	6342.6	65.00	0.55	tr_D0A3E0_
HLA-DQA10501-DQB10201	279	SEPFFPEESYNSCVT	EPFFPEESY	1	0.1902	6384.0	65.00	0.25	tr_D0A3E0_
HLA-DQA10501-DQB10201	280	PQNRCHCAATLFDIG	CHCAATLFD	4	0.1901	6391.4	65.00	0.28	tr_D0A3E0_
HLA-DQA10501-DQB10201	281	KNAENHMNGLTSYKL	MNGLTSYKL	6	0.1878	6557.2	70.00	0.62	tr_D0A3E0_
HLA-DQA10501-DQB10201	282	WRNVCNPKLDTLIDT	PKLDTLIDT	6	0.1864	6651.8	70.00	0.38	tr_D0A3E0_
HLA-DQA10501-DQB10201	283	FSHLDEHWWRNVCNP	FSHLDEHWW	0	0.1856	6709.9	70.00	0.53	tr_D0A3E0_
HLA-DQA10501-DQB10201	284	VPCGYLNAAGKMMLI	YLNAAGKMM	4	0.1847	6779.6	70.00	0.33	tr_D0A3E0_
HLA-DQA10501-DQB10201	285	PLAATLGDSTTTGIT	LAATLGDST	1	0.1846	6781.9	70.00	0.68	tr_D0A3E0_
HLA-DQA10501-DQB10201	286	GAPGQKPEEGYAFSW	PEEGYAFSW	6	0.1845	6792.0	70.00	0.58	tr_D0A3E0_
HLA-DQA10501-DQB10201	287	NAENHMNGLTSYKLY	MNGLTSYKL	5	0.1842	6814.1	70.00	0.61	tr_D0A3E0_
HLA-DQA10501-DQB10201	288	STTTGITPPYGQLYA	TTGITPPYG	2	0.1833	6877.8	70.00	0.50	tr_D0A3E0_
HLA-DQA10501-DQB10201	289	LYDHEVKGYIIRIRR	EVKGYIIRI	4	0.1828	6921.1	70.00	0.38	tr_D0A3E0_
HLA-DQA10501-DQB10201	290	SQICGEVPCGYLNAA	ICGEVPCGY	2	0.1821	6969.5	70.00	0.51	tr_D0A3E0_
HLA-DQA10501-DQB10201	291	TVYTVEDNKCPYHDF	VYTVEDNKC	1	0.1812	7042.4	70.00	0.38	tr_D0A3E0_
HLA-DQA10501-DQB10201	292	TGAEAMPYACLHGGS	TGAEAMPYA	0	0.1808	7068.0	70.00	0.26	tr_D0A3E0_
HLA-DQA10501-DQB10201	293	DVPRTLQSAGCLLRG	TLQSAGCLL	4	0.1800	7131.7	70.00	0.42	tr_D0A3E0_
HLA-DQA10501-DQB10201	294	WGEFQMKCMNENHTV	WGEFQMKCM	0	0.1799	7140.8	70.00	0.34	tr_D0A3E0_
HLA-DQA10501-DQB10201	295	TLQSAGCLLRGMFPN	TLQSAGCLL	0	0.1775	7324.4	70.00	0.25	tr_D0A3E0_
HLA-DQA10501-DQB10201	296	ADPQNRCHCAATLFD	CHCAATLFD	6	0.1767	7393.7	70.00	0.37	tr_D0A3E0_
HLA-DQA10501-DQB10201	297	NVCNPKLDTLIDTNT	PKLDTLIDT	4	0.1747	7549.7	70.00	0.31	tr_D0A3E0_
HLA-DQA10501-DQB10201	298	PRTLQSAGCLLRGMF	TLQSAGCLL	2	0.1746	7561.5	70.00	0.35	tr_D0A3E0_
HLA-DQA10501-DQB10201	299	QHLAQEILKNAENHM	ILKNAENHM	6	0.1740	7607.9	70.00	0.44	tr_D0A3E0_
HLA-DQA10501-DQB10201	300	RNVCNPKLDTLIDTN	PKLDTLIDT	5	0.1720	7779.8	75.00	0.36	tr_D0A3E0_
HLA-DQA10501-DQB10201	301	VPRTLQSAGCLLRGM	TLQSAGCLL	3	0.1716	7813.1	75.00	0.44	tr_D0A3E0_
HLA-DQA10501-DQB10201	302	GLTSYKLYHYSAHDT	LYHYSAHDT	6	0.1715	7816.5	75.00	0.62	tr_D0A3E0_
HLA-DQA10501-DQB10201	303	EVPCGYLNAAGKMML	YLNAAGKMM	5	0.1712	7842.2	75.00	0.39	tr_D0A3E0_

HLA-DQA10501-DQB10201	304	PCGYLNAAGKMMLIN	YLNAAGKMM	3	0.1712	7847.5	75.00	0.34	tr_D0A3E0_
HLA-DQA10501-DQB10201	305	VTYSRSTDVPRTLQS	VTYSRSTDV	0	0.1708	7875.2	75.00	0.29	tr_D0A3E0_
HLA-DQA10501-DQB10201	306	PFFPEESYNSCVTYS	EESYNSCVT	4	0.1696	7980.7	75.00	0.27	tr_D0A3E0_
HLA-DQA10501-DQB10201	307	CGYLNAAGKMMLINA	YLNAAGKMM	2	0.1688	8046.7	75.00	0.31	tr_D0A3E0_
HLA-DQA10501-DQB10201	308	QMKCMNENHTVYTVE	ENHTVYTVE	6	0.1676	8151.2	75.00	0.60	tr_D0A3E0_
HLA-DQA10501-DQB10201	309	GQHLAQEILKNAENH	GQHLAQEIL	0	0.1672	8190.4	75.00	0.33	tr_D0A3E0_
HLA-DQA10501-DQB10201	310	GVAMQSDGRIDKHPL	VAMQSDGRI	1	0.1670	8208.1	75.00	0.47	tr_D0A3E0_
HLA-DQA10501-DQB10201	311	HLAQEILKNAENHMN	ILKNAENHM	5	0.1658	8314.3	75.00	0.51	tr_D0A3E0_
HLA-DQA10501-DQB10201	312	HGGSLCLLISLLSCL	LLISLLSCL	6	0.1656	8336.3	75.00	0.31	tr_D0A3E0_
HLA-DQA10501-DQB10201	313	VQTLHLVQLVHRHGA	QTLHLVQLV	1	0.1652	8373.8	75.00	0.34	tr_D0A3E0_
HLA-DQA10501-DQB10201	314	SEGFCADPQNRCHCA	SEGFCADPQ	0	0.1651	8380.8	75.00	0.55	tr_D0A3E0_
HLA-DQA10501-DQB10201	315	YDHEVKGYIIRIRRG	EVKGYIIRI	3	0.1640	8475.7	75.00	0.40	tr_D0A3E0_
HLA-DQA10501-DQB10201	316	AENHMNGLTSYKLYH	MNGLTSYKL	4	0.1640	8479.8	75.00	0.62	tr_D0A3E0_
HLA-DQA10501-DQB10201	317	NSVTLECVCSSRKCM	VTLECVCSS	2	0.1632	8552.0	75.00	0.33	tr_D0A3E0_
HLA-DQA10501-DQB10201	318	ENHMNGLTSYKLYHY	MNGLTSYKL	3	0.1632	8552.8	75.00	0.62	tr_D0A3E0_
HLA-DQA10501-DQB10201	319	THAKMGSLGQHLAQE	KMGSLGQHL	3	0.1621	8658.7	75.00	0.35	tr_D0A3E0_
HLA-DQA10501-DQB10201	320	QICGEVPCGYLNAAG	ICGEVPCGY	1	0.1617	8696.0	75.00	0.50	tr_D0A3E0_
HLA-DQA10501-DQB10201	321	GEVPCGYLNAAGKMM	YLNAAGKMM	6	0.1587	8981.3	75.00	0.50	tr_D0A3E0_
HLA-DQA10501-DQB10201	322	LRENLGRLRDIKFFE	LGRLRDIKF	4	0.1574	9104.8	75.00	0.35	tr_D0A3E0_
HLA-DQA10501-DQB10201	323	GYLNAAGKMMLINAG	YLNAAGKMM	1	0.1538	9468.7	75.00	0.31	tr_D0A3E0_
HLA-DQA10501-DQB10201	324	DRTHAKMGSLGQHLA	KMGSLGQHL	5	0.1534	9508.5	80.00	0.53	tr_D0A3E0_
HLA-DQA10501-DQB10201	325	FFPEESYNSCVTYSR	EESYNSCVT	3	0.1525	9602.1	80.00	0.25	tr_D0A3E0_
HLA-DQA10501-DQB10201	326	LAQEILKNAENHMNG	ILKNAENHM	4	0.1520	9651.9	80.00	0.54	tr_D0A3E0_
HLA-DQA10501-DQB10201	327	RTHAKMGSLGQHLAQ	KMGSLGQHL	4	0.1512	9742.2	80.00	0.46	tr_D0A3E0_
HLA-DQA10501-DQB10201	328	ATLGDSTTTGITPPY	TTTGITPPY	6	0.1504	9818.9	80.00	0.49	tr_D0A3E0_
HLA-DQA10501-DQB10201	329	CYLNRRYRELFHCPG	YRELFHCPG	6	0.1503	9834.4	80.00	0.25	tr_D0A3E0_
HLA-DQA10501-DQB10201	330	RKCMESRDLSISTDT	SRDLSISTD	5	0.1498	9891.8	80.00	0.34	tr_D0A3E0_
HLA-DQA10501-DQB10201	331	AQEILKNAENHMNGL	ILKNAENHM	3	0.1497	9899.4	80.00	0.55	tr_D0A3E0_
HLA-DQA10501-DQB10201	332	EHYFQETIKDKTGAE	HYFQETIKD	1	0.1464	10258.6	80.00	0.34	tr_D0A3E0_
HLA-DQA10501-DQB10201	333	SRKCMESRDLSISTD	SRDLSISTD	6	0.1463	10266.8	80.00	0.40	tr_D0A3E0_
HLA-DQA10501-DQB10201	334	NHMNGLTSYKLYHYS	MNGLTSYKL	2	0.1462	10275.8	80.00	0.62	tr_D0A3E0_
HLA-DQA10501-DQB10201	335	GAEAMPYACLHGGSL	EAMPYACLH	2	0.1461	10290.7	80.00	0.23	tr_D0A3E0_
HLA-DQA10501-DQB10201	336	DHEVKGYIIRIRRGA	EVKGYIIRI	2	0.1455	10356.7	80.00	0.40	tr_D0A3E0_
HLA-DQA10501-DQB10201	337	RSTDVPRTLQSAGCL	RTLQSAGCL	6	0.1449	10429.3	80.00	0.49	tr_D0A3E0_
HLA-DQA10501-DQB10201	338	LAATLGDSTTTGITP	LAATLGDST	0	0.1432	10615.9	80.00	0.56	tr_D0A3E0_
HLA-DQA10501-DQB10201	339	FQETIKDKTGAEAMP	KDKTGAEAM	5	0.1425	10696.8	80.00	0.46	tr_D0A3E0_
HLA-DQA10501-DQB10201	340	LLRENLGRLRDIKFF	LGRLRDIKF	5	0.1421	10748.9	80.00	0.44	tr_D0A3E0_
HLA-DQA10501-DQB10201	341	YLNRRYRELFHCPGN	YRELFHCPG	5	0.1395	11052.0	80.00	0.25	tr_D0A3E0_
HLA-DQA10501-DQB10201	342	SVTLECVCSSRKCME	VTLECVCSS	1	0.1391	11098.4	80.00	0.39	tr_D0A3E0_
HLA-DQA10501-DQB10201	343	PLLRENLGRLRDIKF	LGRLRDIKF	6	0.1382	11212.9	80.00	0.49	tr_D0A3E0_
HLA-DQA10501-DQB10201	344	EAMPYACLHGGSLCL	CLHGGSLCL	6	0.1378	11256.9	80.00	0.28	tr_D0A3E0_
HLA-DQA10501-DQB10201	345	LNRRYRELFHCPGNV	YRELFHCPG	4	0.1375	11300.1	80.00	0.25	tr_D0A3E0_
HLA-DQA10501-DQB10201	346	GEFLRNHYNSNASEP	NHYNSNASE	5	0.1372	11336.1	80.00	0.41	tr_D0A3E0_
HLA-DQA10501-DQB10201	347	QEILKNAENHMNGLT	ILKNAENHM	2	0.1355	11538.4	80.00	0.56	tr_D0A3E0_
HLA-DQA10501-DQB10201	348	FPEESYNSCVTYSRS	EESYNSCVT	2	0.1354	11554.8	80.00	0.25	tr_D0A3E0_
HLA-DQA10501-DQB10201	349	HEVKGYIIRIRRGAP	EVKGYIIRI	1	0.1353	11562.5	80.00	0.38	tr_D0A3E0_
HLA-DQA10501-DQB10201	350	LCYLNRRYRELFHCP	LNRRYRELF	3	0.1351	11589.0	80.00	0.26	tr_D0A3E0_
HLA-DQA10501-DQB10201	351	HMNGLTSYKLYHYSA	MNGLTSYKL	1	0.1347	11647.4	80.00	0.60	tr_D0A3E0_
HLA-DQA10501-DQB10201	352	CADPQNRCHCAATLF	RCHCAATLF	6	0.1346	11650.2	80.00	0.56	tr_D0A3E0_
HLA-DQA10501-DQB10201	353	GEFQMKCMNENHTVY	EFQMKCMNE	1	0.1339	11744.4	85.00	0.35	tr_D0A3E0_

HLA-DQA10501-DQB10201	354	SDRTHAKMGSLGQHL	KMGSLGQHL	6	0.1337	11768.9	85.00	0.71	tr_D0A3E0_
HLA-DQA10501-DQB10201	355	SPKVKHNQSQICGEV	HNQSQICGE	5	0.1317	12021.6	85.00	0.34	tr_D0A3E0_
HLA-DQA10501-DQB10201	356	CPYHDFRRFVKSTKP	RRFVKSTKP	6	0.1301	12231.3	85.00	0.43	tr_D0A3E0_
HLA-DQA10501-DQB10201	357	VAMQSDGRIDKHPLL	VAMQSDGRI	0	0.1295	12314.9	85.00	0.41	tr_D0A3E0_
HLA-DQA10501-DQB10201	358	QTLHLVQLVHRHGAR	QTLHLVQLV	0	0.1286	12438.9	85.00	0.32	tr_D0A3E0_
HLA-DQA10501-DQB10201	359	TYSRSTDVPRTLQSA	RSTDVPRTL	3	0.1274	12600.2	85.00	0.34	tr_D0A3E0_
HLA-DQA10501-DQB10201	360	ILKNAENHMNGLTSY	NHMNGLTSY	6	0.1273	12609.3	85.00	0.41	tr_D0A3E0_
HLA-DQA10501-DQB10201	361	VYTVEDNKCPYHDFR	VYTVEDNKC	0	0.1267	12688.3	85.00	0.40	tr_D0A3E0_
HLA-DQA10501-DQB10201	362	AEAMPYACLHGGSLC	YACLHGGSL	5	0.1267	12688.4	85.00	0.28	tr_D0A3E0_
HLA-DQA10501-DQB10201	363	EILKNAENHMNGLTS	ILKNAENHM	1	0.1261	12779.1	85.00	0.47	tr_D0A3E0_
HLA-DQA10501-DQB10201	364	NRRYRELFHCPGNVG	YRELFHCPG	3	0.1249	12937.6	85.00	0.23	tr_D0A3E0_
HLA-DQA10501-DQB10201	365	EVKGYIIRIRRGAPG	EVKGYIIRI	0	0.1242	13047.2	85.00	0.30	tr_D0A3E0_
HLA-DQA10501-DQB10201	366	HDFRRFVKSTKPHDP	RRFVKSTKP	3	0.1238	13093.7	85.00	0.44	tr_D0A3E0_
HLA-DQA10501-DQB10201	367	EFQMKCMNENHTVYT	EFQMKCMNE	0	0.1222	13327.0	85.00	0.24	tr_D0A3E0_
HLA-DQA10501-DQB10201	368	PAGLCYLNRRYRELF	LNRRYRELF	6	0.1220	13350.1	85.00	0.48	tr_D0A3E0_
HLA-DQA10501-DQB10201	369	SHLDEHWWRNVCNPK	HWWRNVCNP	5	0.1215	13428.2	85.00	0.27	tr_D0A3E0_
HLA-DQA10501-DQB10201	370	VCSSRKCMESRDLSI	CMESRDLSI	6	0.1213	13464.7	85.00	0.50	tr_D0A3E0_
HLA-DQA10501-DQB10201	371	DFRRFVKSTKPHDPA	RRFVKSTKP	2	0.1209	13516.5	85.00	0.43	tr_D0A3E0_
HLA-DQA10501-DQB10201	372	GLCYLNRRYRELFHC	LNRRYRELF	4	0.1206	13555.9	85.00	0.34	tr_D0A3E0_
HLA-DQA10501-DQB10201	373	LINAGEFLRNHYNSN	AGEFLRNHY	3	0.1198	13674.5	85.00	0.33	tr_D0A3E0_
HLA-DQA10501-DQB10201	374	STKPHDPAGLCYLNR	PAGLCYLNR	6	0.1190	13802.3	85.00	0.26	tr_D0A3E0_
HLA-DQA10501-DQB10201	375	TKPHDPAGLCYLNRR	PAGLCYLNR	5	0.1186	13861.3	85.00	0.27	tr_D0A3E0_
HLA-DQA10501-DQB10201	376	KPHDPAGLCYLNRRY	PAGLCYLNR	4	0.1178	13981.3	85.00	0.26	tr_D0A3E0_
HLA-DQA10501-DQB10201	377	CGEVPCGYLNAAGKM	GYLNAAGKM	6	0.1170	14093.6	85.00	0.60	tr_D0A3E0_
HLA-DQA10501-DQB10201	378	AGLCYLNRRYRELFH	LNRRYRELF	5	0.1167	14142.2	85.00	0.40	tr_D0A3E0_
HLA-DQA10501-DQB10201	379	YHDFRRFVKSTKPHD	RRFVKSTKP	4	0.1161	14242.7	85.00	0.47	tr_D0A3E0_
HLA-DQA10501-DQB10201	380	RSPKVKHNQSQICGE	HNQSQICGE	6	0.1159	14274.1	85.00	0.42	tr_D0A3E0_
HLA-DQA10501-DQB10201	381	PYHDFRRFVKSTKPH	RRFVKSTKP	5	0.1155	14331.5	85.00	0.45	tr_D0A3E0_
HLA-DQA10501-DQB10201	382	ICGEVPCGYLNAAGK	ICGEVPCGY	0	0.1153	14358.8	85.00	0.47	tr_D0A3E0_
HLA-DQA10501-DQB10201	383	VTLECVCSSRKCMES	VTLECVCSS	0	0.1145	14483.3	85.00	0.38	tr_D0A3E0_
HLA-DQA10501-DQB10201	384	SSRKCMESRDLSIST	CMESRDLSI	4	0.1141	14554.0	85.00	0.41	tr_D0A3E0_
HLA-DQA10501-DQB10201	385	PEESYNSCVTYSRST	SYNSCVTYS	3	0.1132	14698.8	90.00	0.29	tr_D0A3E0_
HLA-DQA10501-DQB10201	386	AGEFLRNHYNSNASE	NHYNSNASE	6	0.1128	14754.6	90.00	0.47	tr_D0A3E0_
HLA-DQA10501-DQB10201	387	CSSRKCMESRDLSIS	CMESRDLSI	5	0.1125	14808.6	90.00	0.49	tr_D0A3E0_
HLA-DQA10501-DQB10201	388	SDGRIDKHPLLRENL	KHPLLRENL	6	0.1124	14812.9	90.00	0.44	tr_D0A3E0_
HLA-DQA10501-DQB10201	389	RRYRELFHCPGNVGK	YRELFHCPG	2	0.1109	15055.0	90.00	0.29	tr_D0A3E0_
HLA-DQA10501-DQB10201	390	HYFQETIKDKTGAEA	HYFQETIKD	0	0.1098	15234.1	90.00	0.31	tr_D0A3E0_
HLA-DQA10501-DQB10201	391	KSTKPHDPAGLCYLN	HDPAGLCYL	5	0.1089	15384.7	90.00	0.35	tr_D0A3E0_
HLA-DQA10501-DQB10201	392	YFQETIKDKTGAEAM	KDKTGAEAM	6	0.1087	15425.5	90.00	0.46	tr_D0A3E0_
HLA-DQA10501-DQB10201	393	VKSTKPHDPAGLCYL	HDPAGLCYL	6	0.1086	15445.9	90.00	0.41	tr_D0A3E0_
HLA-DQA10501-DQB10201	394	EESYNSCVTYSRSTD	SYNSCVTYS	2	0.1071	15700.5	90.00	0.28	tr_D0A3E0_
HLA-DQA10501-DQB10201	395	RYRELFHCPGNVGKP	ELFHCPGNV	3	0.1060	15885.7	90.00	0.31	tr_D0A3E0_
HLA-DQA10501-DQB10201	396	DGRIDKHPLLRENLG	KHPLLRENL	5	0.1058	15915.8	90.00	0.34	tr_D0A3E0_
HLA-DQA10501-DQB10201	397	MNGLTSYKLYHYSAH	MNGLTSYKL	0	0.1049	16066.5	90.00	0.44	tr_D0A3E0_
HLA-DQA10501-DQB10201	398	FRRFVKSTKPHDPAG	RRFVKSTKP	1	0.1049	16074.0	90.00	0.41	tr_D0A3E0_
HLA-DQA10501-DQB10201	399	PHDPAGLCYLNRRYR	PAGLCYLNR	3	0.1038	16263.4	90.00	0.28	tr_D0A3E0_
HLA-DQA10501-DQB10201	400	VEDNKCPYHDFRRFV	PYHDFRRFV	6	0.1031	16387.6	90.00	0.63	tr_D0A3E0_
HLA-DQA10501-DQB10201	401	IRIRRGAPGQKPEEG	APGQKPEEG	6	0.1028	16434.1	90.00	0.35	tr_D0A3E0_
HLA-DQA10501-DQB10201	402	VKGYIIRIRRGAPGQ	RIRRGAPGQ	6	0.1022	16541.3	90.00	0.20	tr_D0A3E0_
HLA-DQA10501-DQB10201	403	FQMKCMNENHTVYTV	NENHTVYTV	6	0.1015	16671.2	90.00	0.17	tr_D0A3E0_

HLA-DQA10501-DQB10201	404	LKNAENHMNGLTSYK	NHMNGLTSY	5	0.1011	16748.6	90.00	0.40	tr_D0A3E0_
HLA-DQA10501-DQB10201	405	RIRRGAPGQKPEEGY	APGQKPEEG	5	0.1009	16790.2	90.00	0.38	tr_D0A3E0_
HLA-DQA10501-DQB10201	406	YSRSTDVPRTLQSAG	RSTDVPRTL	2	0.1005	16861.5	90.00	0.41	tr_D0A3E0_
HLA-DQA10501-DQB10201	407	SRSTDVPRTLQSAGC	RSTDVPRTL	1	0.1002	16913.2	90.00	0.33	tr_D0A3E0_
HLA-DQA10501-DQB10201	408	INAGEFLRNHYNSNA	AGEFLRNHY	2	0.0998	16986.1	90.00	0.43	tr_D0A3E0_
HLA-DQA10501-DQB10201	409	GYIIRIRRGAPGQKP	RRGAPGQKP	6	0.0997	17006.3	90.00	0.26	tr_D0A3E0_
HLA-DQA10501-DQB10201	410	NGLTSYKLYHYSAHD	KLYHYSAHD	6	0.0996	17028.9	90.00	0.43	tr_D0A3E0_
HLA-DQA10501-DQB10201	411	AATLGDSTTTGITPP	LGDSTTTGI	3	0.0995	17043.3	90.00	0.29	tr_D0A3E0_
HLA-DQA10501-DQB10201	412	KHPLLRENLGRLRDI	ENLGRLRDI	6	0.0988	17166.6	90.00	0.26	tr_D0A3E0_
HLA-DQA10501-DQB10201	413	YRELFHCPGNVGKPP	ELFHCPGNV	2	0.0972	17473.7	90.00	0.36	tr_D0A3E0_
HLA-DQA10501-DQB10201	414	HDPAGLCYLNRRYRE	PAGLCYLNR	2	0.0955	17788.7	90.00	0.28	tr_D0A3E0_
HLA-DQA10501-DQB10201	415	FVKSTKPHDPAGLCY	PHDPAGLCY	6	0.0952	17851.1	90.00	0.43	tr_D0A3E0_
HLA-DQA10501-DQB10201	416	YIIRIRRGAPGQKPE	RRGAPGQKP	5	0.0939	18105.6	90.00	0.25	tr_D0A3E0_
HLA-DQA10501-DQB10201	417	GRIDKHPLLRENLGR	KHPLLRENL	4	0.0937	18145.6	90.00	0.32	tr_D0A3E0_
HLA-DQA10501-DQB10201	418	IIRIRRGAPGQKPEE	RRGAPGQKP	4	0.0935	18177.4	90.00	0.25	tr_D0A3E0_
HLA-DQA10501-DQB10201	419	RRGAPGQKPEEGYAF	APGQKPEEG	3	0.0924	18391.7	90.00	0.40	tr_D0A3E0_
HLA-DQA10501-DQB10201	420	EDNKCPYHDFRRFVK	PYHDFRRFV	5	0.0917	18539.7	90.00	0.57	tr_D0A3E0_
HLA-DQA10501-DQB10201	421	MQSDGRIDKHPLLRE	IDKHPLLRE	6	0.0913	18613.3	90.00	0.28	tr_D0A3E0_
HLA-DQA10501-DQB10201	422	NAGEFLRNHYNSNAS	AGEFLRNHY	1	0.0910	18679.4	95.00	0.39	tr_D0A3E0_
HLA-DQA10501-DQB10201	423	RIDKHPLLRENLGRL	KHPLLRENL	3	0.0902	18833.7	95.00	0.29	tr_D0A3E0_
HLA-DQA10501-DQB10201	424	DPAGLCYLNRRYREL	YLNRRYREL	6	0.0902	18845.7	95.00	0.32	tr_D0A3E0_
HLA-DQA10501-DQB10201	425	KCPYHDFRRFVKSTK	PYHDFRRFV	2	0.0896	18968.2	95.00	0.46	tr_D0A3E0_
HLA-DQA10501-DQB10201	426	RGAPGQKPEEGYAFS	APGQKPEEG	2	0.0887	19141.8	95.00	0.40	tr_D0A3E0_
HLA-DQA10501-DQB10201	427	NKCPYHDFRRFVKST	PYHDFRRFV	3	0.0876	19381.1	95.00	0.49	tr_D0A3E0_
HLA-DQA10501-DQB10201	428	DNKCPYHDFRRFVKS	PYHDFRRFV	4	0.0873	19436.7	95.00	0.55	tr_D0A3E0_
HLA-DQA10501-DQB10201	429	KGYIIRIRRGAPGQK	RIRRGAPGQ	5	0.0870	19513.4	95.00	0.21	tr_D0A3E0_
HLA-DQA10501-DQB10201	430	DKHPLLRENLGRLRD	KHPLLRENL	1	0.0869	19523.4	95.00	0.19	tr_D0A3E0_
HLA-DQA10501-DQB10201	431	TLHLVQLVHRHGARS	LHLVQLVHR	1	0.0867	19561.4	95.00	0.38	tr_D0A3E0_
HLA-DQA10501-DQB10201	432	IRRGAPGQKPEEGYA	APGQKPEEG	4	0.0865	19601.9	95.00	0.41	tr_D0A3E0_
HLA-DQA10501-DQB10201	433	GARSPKVKHNQSQIC	VKHNQSQIC	6	0.0855	19823.3	95.00	0.68	tr_D0A3E0_
HLA-DQA10501-DQB10201	434	AMQSDGRIDKHPLLR	GRIDKHPLL	5	0.0847	20007.7	95.00	0.38	tr_D0A3E0_
HLA-DQA10501-DQB10201	435	EGFCADPQNRCHCAA	GFCADPQNR	1	0.0846	20026.8	95.00	0.33	tr_D0A3E0_
HLA-DQA10501-DQB10201	436	RRFVKSTKPHDPAGL	FVKSTKPHD	2	0.0844	20055.8	95.00	0.37	tr_D0A3E0_
HLA-DQA10501-DQB10201	437	PPNKQCKVFRRVCPA	KVFRRVCPA	6	0.0837	20208.1	95.00	0.73	tr_D0A3E0_
HLA-DQA10501-DQB10201	438	IDKHPLLRENLGRLR	KHPLLRENL	2	0.0833	20309.1	95.00	0.28	tr_D0A3E0_
HLA-DQA10501-DQB10201	439	QSDGRIDKHPLLREN	GRIDKHPLL	3	0.0825	20473.1	95.00	0.28	tr_D0A3E0_
HLA-DQA10501-DQB10201	440	YTVEDNKCPYHDFRR	TVEDNKCPY	1	0.0818	20640.5	95.00	0.43	tr_D0A3E0_
HLA-DQA10501-DQB10201	441	ARSPKVKHNQSQICG	VKHNQSQIC	5	0.0816	20683.0	95.00	0.58	tr_D0A3E0_
HLA-DQA10501-DQB10201	442	ECVCSSRKCMESRDL	RKCMESRDL	6	0.0813	20754.5	95.00	0.52	tr_D0A3E0_
HLA-DQA10501-DQB10201	443	HPLLRENLGRLRDIK	ENLGRLRDI	5	0.0805	20936.3	95.00	0.28	tr_D0A3E0_
HLA-DQA10501-DQB10201	444	CVCSSRKCMESRDLS	RKCMESRDL	5	0.0755	22082.0	95.00	0.49	tr_D0A3E0_
HLA-DQA10501-DQB10201	445	RELFHCPGNVGKPPN	ELFHCPGNV	1	0.0750	22213.1	95.00	0.38	tr_D0A3E0_
HLA-DQA10501-DQB10201	446	TLECVCSSRKCMESR	LECVCSSRK	1	0.0714	23103.8	95.00	0.41	tr_D0A3E0_
HLA-DQA10501-DQB10201	447	NASDRTHAKMGSLGQ	RTHAKMGSL	4	0.0705	23328.4	95.00	0.37	tr_D0A3E0_
HLA-DQA10501-DQB10201	448	RFVKSTKPHDPAGLC	FVKSTKPHD	1	0.0697	23527.4	95.00	0.41	tr_D0A3E0_
HLA-DQA10501-DQB10201	449	VYNASDRTHAKMGSL	RTHAKMGSL	6	0.0695	23560.0	95.00	0.47	tr_D0A3E0_
HLA-DQA10501-DQB10201	450	FCADPQNRCHCAATL	NRCHCAATL	6	0.0695	23565.4	95.00	0.57	tr_D0A3E0_
HLA-DQA10501-DQB10201	451	LVQLVHRHGARSPKV	RHGARSPKV	6	0.0682	23917.8	95.00	0.52	tr_D0A3E0_
HLA-DQA10501-DQB10201	452	YNASDRTHAKMGSLG	RTHAKMGSL	5	0.0665	24355.5	95.00	0.44	tr_D0A3E0_
HLA-DQA10501-DQB10201	453	LHLVQLVHRHGARSP	LHLVQLVHR	0	0.0662	24437.0	95.00	0.31	tr_D0A3E0_

HLA-DQA10501-DQB10201	454	ASDRTHAKMGSLGQH	RTHAKMGSL	3	0.0654	24638.0	95.00	0.31	tr_D0A3E0_
HLA-DQA10501-DQB10201	455	ELFHCPGNVGKPPNK	LFHCPGNVG	1	0.0648	24791.5	95.00	0.35	tr_D0A3E0_
HLA-DQA10501-DQB10201	456	TVEDNKCPYHDFRRF	TVEDNKCPY	0	0.0619	25580.9	100.00	0.32	tr_D0A3E0_
HLA-DQA10501-DQB10201	457	LECVCSSRKCMESRD	SRKCMESRD	6	0.0599	26157.7	100.00	0.29	tr_D0A3E0_
HLA-DQA10501-DQB10201	458	VQLVHRHGARSPKVK	RHGARSPKV	5	0.0551	27551.2	100.00	0.52	tr_D0A3E0_
HLA-DQA10501-DQB10201	459	GFCADPQNRCHCAAT	GFCADPQNR	0	0.0547	27665.7	100.00	0.35	tr_D0A3E0_
HLA-DQA10501-DQB10201	460	QLVHRHGARSPKVKH	RHGARSPKV	4	0.0481	29710.0	100.00	0.53	tr_D0A3E0_
HLA-DQA10501-DQB10201	461	HLVQLVHRHGARSPK	VHRHGARSP	5	0.0446	30844.3	100.00	0.21	tr_D0A3E0_
HLA-DQA10501-DQB10201	462	LVHRHGARSPKVKHN	RHGARSPKV	3	0.0434	31255.2	100.00	0.56	tr_D0A3E0_
HLA-DQA10501-DQB10201	463	LFHCPGNVGKPPNKQ	LFHCPGNVG	0	0.0417	31852.6	100.00	0.44	tr_D0A3E0_
HLA-DQA10501-DQB10201	464	VHRHGARSPKVKHNQ	RHGARSPKV	2	0.0371	33463.6	100.00	0.62	tr_D0A3E0_
HLA-DQA10501-DQB10201	465	HGARSPKVKHNQSQI	KVKHNQSQI	6	0.0352	34150.1	100.00	0.56	tr_D0A3E0_
HLA-DQA10501-DQB10201	466	HRHGARSPKVKHNQS	RHGARSPKV	1	0.0318	35461.3	100.00	0.62	tr_D0A3E0_
HLA-DQA10501-DQB10201	467	KPPNKQCKVFRRVCP	CKVFRRVCP	6	0.0271	37277.7	100.00	0.37	tr_D0A3E0_
HLA-DQA10501-DQB10201	468	RHGARSPKVKHNQSQ	RHGARSPKV	0	0.0258	37833.2	100.00	0.55	tr_D0A3E0_
HLA-DQA10501-DQB10201	469	FHCPGNVGKPPNKQC	NVGKPPNKQ	5	0.0231	38932.0	100.00	0.26	tr_D0A3E0_
HLA-DQA10501-DQB10201	470	GNVGKPPNKQCKVFR	PNKQCKVFR	6	0.0209	39862.0	100.00	0.30	tr_D0A3E0_
HLA-DQA10501-DQB10201	471	PGNVGKPPNKQCKVF	PPNKQCKVF	6	0.0202	40186.4	100.00	0.29	tr_D0A3E0_
HLA-DQA10501-DQB10201	472	VGKPPNKQCKVFRRV	PNKQCKVFR	4	0.0202	40193.3	100.00	0.31	tr_D0A3E0_
HLA-DQA10501-DQB10201	473	NVGKPPNKQCKVFRR	PNKQCKVFR	5	0.0200	40271.3	100.00	0.28	tr_D0A3E0_
HLA-DQA10501-DQB10201	474	GKPPNKQCKVFRRVC	PNKQCKVFR	3	0.0196	40443.3	100.00	0.28	tr_D0A3E0_
HLA-DQA10501-DQB10201	475	HCPGNVGKPPNKQCK	NVGKPPNKQ	4	0.0182	41054.8	100.00	0.32	tr_D0A3E0_
HLA-DQA10501-DQB10201	476	CPGNVGKPPNKQCKV	NVGKPPNKQ	3	0.0179	41208.4	100.00	0.33	tr_D0A3E0_

Allele: HLA-DQA10501-DQB10201. Number of high binders 0. Number of weak binders 33. Number of peptides 476

Allele	pos	peptide	core	Of	1-log50k(aff)	affinity(nM)	%Rank	Relia	Identity	Bind Level
HLA-DQA10501-DQB10201	1	EACKAVGSYGCEWKD	AVGSYGCEW	4	0.3645	968.8	22.00	0.75	tr_D0A3U3_	
HLA-DQA10501-DQB10201	2	ACKAVGSYGCEWKDT	AVGSYGCEW	3	0.3550	1074.1	23.00	0.74	tr_D0A3U3_	
HLA-DQA10501-DQB10201	3	CKAVGSYGCEWKDTE	AVGSYGCEW	2	0.3442	1207.0	25.00	0.71	tr_D0A3U3_	
HLA-DQA10501-DQB10201	4	TEACKAVGSYGCEWK	AVGSYGCEW	5	0.3426	1227.1	26.00	0.74	tr_D0A3U3_	
HLA-DQA10501-DQB10201	5	TTEACKAVGSYGCEW	AVGSYGCEW	6	0.3377	1294.5	27.00	0.84	tr_D0A3U3_	
HLA-DQA10501-DQB10201	6	KAVGSYGCEWKDTEV	AVGSYGCEW	1	0.3346	1338.3	27.00	0.69	tr_D0A3U3_	
HLA-DQA10501-DQB10201	7	AVGSYGCEWKDTEVC	AVGSYGCEW	0	0.2908	2150.9	37.00	0.63	tr_D0A3U3_	
HLA-DQA10501-DQB10201	8	GSYGCEWKDTEVCSW	WKDTEVCSW	6	0.2541	3198.1	47.00	0.69	tr_D0A3U3_	
HLA-DQA10501-DQB10201	9	SYGCEWKDTEVCSWK	WKDTEVCSW	5	0.2282	4233.2	55.00	0.69	tr_D0A3U3_	
HLA-DQA10501-DQB10201	10	YGCEWKDTEVCSWKK	WKDTEVCSW	4	0.2014	5657.6	65.00	0.70	tr_D0A3U3_	
HLA-DQA10501-DQB10201	11	GCEWKDTEVCSWKKE	WKDTEVCSW	3	0.1905	6366.8	65.00	0.75	tr_D0A3U3_	
HLA-DQA10501-DQB10201	12	ECDSDSDGVNPCESL	SDGVNPCES	5	0.1866	6638.0	70.00	0.44	tr_D0A3U3_	
HLA-DQA10501-DQB10201	13	CDSDSDGVNPCESLI	SDGVNPCES	4	0.1809	7060.1	70.00	0.44	tr_D0A3U3_	
HLA-DQA10501-DQB10201	14	CEWKDTEVCSWKKEC	WKDTEVCSW	2	0.1808	7072.5	70.00	0.79	tr_D0A3U3_	
HLA-DQA10501-DQB10201	15	EWKDTEVCSWKKECD	WKDTEVCSW	1	0.1745	7568.2	70.00	0.77	tr_D0A3U3_	
HLA-DQA10501-DQB10201	16	DSDSDGVNPCESLIG	SDGVNPCES	3	0.1722	7761.4	70.00	0.38	tr_D0A3U3_	
HLA-DQA10501-DQB10201	17	KECDSDSDGVNPCES	SDGVNPCES	6	0.1687	8061.0	75.00	0.47	tr_D0A3U3_	
HLA-DQA10501-DQB10201	18	ATNCSLFKTTEACKA	FKTTEACKA	6	0.1558	9268.4	75.00	0.64	tr_D0A3U3_	
HLA-DQA10501-DQB10201	19	TNCSLFKTTEACKAV	FKTTEACKA	5	0.1531	9541.3	80.00	0.67	tr_D0A3U3_	

HLA-DQA10501-DQB10201	20	NCSLFKTTEACKAVG	FKTTEACKA	4	0.1405	10937.9	80.00	0.67	tr_D0A3U3_
HLA-DQA10501-DQB10201	21	WKDTEVCSWKKECDS	WKDTEVCSW	0	0.1368	11378.1	80.00	0.69	tr_D0A3U3_
HLA-DQA10501-DQB10201	22	CSLFKTTEACKAVGS	FKTTEACKA	3	0.1358	11510.1	80.00	0.62	tr_D0A3U3_
HLA-DQA10501-DQB10201	23	SLFKTTEACKAVGSY	FKTTEACKA	2	0.1355	11540.2	80.00	0.58	tr_D0A3U3_
HLA-DQA10501-DQB10201	24	VGSYGCEWKDTEVCS	VGSYGCEWK	0	0.1348	11631.9	80.00	0.25	tr_D0A3U3_
HLA-DQA10501-DQB10201	25	EVATNCSLFKTTEAC	EVATNCSLF	0	0.1246	12991.1	85.00	0.28	tr_D0A3U3_
HLA-DQA10501-DQB10201	26	KKECDSDSDGVNPCE	DSDGVNPCE	6	0.1231	13201.0	85.00	0.22	tr_D0A3U3_
HLA-DQA10501-DQB10201	27	SWKKECDSDSDGVNP	ECDSDSDGV	4	0.1179	13968.2	85.00	0.28	tr_D0A3U3_
HLA-DQA10501-DQB10201	28	LFKTTEACKAVGSYG	FKTTEACKA	1	0.1174	14033.5	85.00	0.59	tr_D0A3U3_
HLA-DQA10501-DQB10201	29	WKKECDSDSDGVNPC	ECDSDSDGV	3	0.1155	14337.4	85.00	0.28	tr_D0A3U3_
HLA-DQA10501-DQB10201	30	VCSWKKECDSDSDGV	ECDSDSDGV	6	0.1112	15005.6	90.00	0.31	tr_D0A3U3_
HLA-DQA10501-DQB10201	31	CSWKKECDSDSDGVN	ECDSDSDGV	5	0.1067	15759.3	90.00	0.30	tr_D0A3U3_
HLA-DQA10501-DQB10201	32	TEVCSWKKECDSDSD	KKECDSDSD	6	0.0968	17538.1	90.00	0.31	tr_D0A3U3_
HLA-DQA10501-DQB10201	33	VATNCSLFKTTEACK	CSLFKTTEA	4	0.0945	17985.7	90.00	0.27	tr_D0A3U3_
HLA-DQA10501-DQB10201	34	EVCSWKKECDSDSDG	WKKECDSDS	4	0.0943	18016.3	90.00	0.26	tr_D0A3U3_
HLA-DQA10501-DQB10201	35	FKTTEACKAVGSYGC	FKTTEACKA	0	0.0943	18030.5	90.00	0.50	tr_D0A3U3_
HLA-DQA10501-DQB10201	36	DTEVCSWKKECDSDS	WKKECDSDS	6	0.0747	22286.2	95.00	0.34	tr_D0A3U3_
HLA-DQA10501-DQB10201	37	KTTEACKAVGSYGCE	KAVGSYGCE	6	0.0746	22298.5	95.00	0.31	tr_D0A3U3_
HLA-DQA10501-DQB10201	38	KDTEVCSWKKECDSD	SWKKECDSD	6	0.0610	25844.6	100.00	0.29	tr_D0A3U3_

Allele: HLA-DQA10501-DQB10201. Number of high binders 0. Number of weak binders 0. Number of peptides 38

Allele	pos	peptide	core	Of	1-log50k(aff)	affinity(nM)	%Rank	Relia	Identity	Bind Level
HLA-DQA10501-DQB10201	1	VELREQDYEAVASVL	DYEAVASVL	6	0.4416	420.6	11.00	0.69	tr_C9ZPP9_	
HLA-DQA10501-DQB10201	2	ELREQDYEAVASVLH	DYEAVASVL	5	0.4302	475.7	12.00	0.57	tr_C9ZPP9_	
HLA-DQA10501-DQB10201	3	LREQDYEAVASVLHG	DYEAVASVL	4	0.4069	612.0	15.00	0.56	tr_C9ZPP9_	
HLA-DQA10501-DQB10201	4	REQDYEAVASVLHGA	DYEAVASVL	3	0.3875	755.6	18.00	0.56	tr_C9ZPP9_	
HLA-DQA10501-DQB10201	5	EQDYEAVASVLHGAG	DYEAVASVL	2	0.3694	918.5	21.00	0.60	tr_C9ZPP9_	
HLA-DQA10501-DQB10201	6	KTQAVELREQDYEAV	AVELREQDY	3	0.3487	1149.9	25.00	0.31	tr_C9ZPP9_	
HLA-DQA10501-DQB10201	7	QDYEAVASVLHGAGL	DYEAVASVL	1	0.3430	1222.7	26.00	0.56	tr_C9ZPP9_	
HLA-DQA10501-DQB10201	8	TQAVELREQDYEAVA	AVELREQDY	2	0.3429	1223.4	26.00	0.32	tr_C9ZPP9_	
HLA-DQA10501-DQB10201	9	GKTQAVELREQDYEA	AVELREQDY	4	0.3428	1225.7	26.00	0.36	tr_C9ZPP9_	
HLA-DQA10501-DQB10201	10	RGKTQAVELREQDYE	AVELREQDY	5	0.3383	1286.5	27.00	0.40	tr_C9ZPP9_	
HLA-DQA10501-DQB10201	11	AVELREQDYEAVASV	QDYEAVASV	6	0.3298	1409.6	28.00	0.25	tr_C9ZPP9_	
HLA-DQA10501-DQB10201	12	QAVELREQDYEAVAS	AVELREQDY	1	0.3259	1470.6	29.00	0.30	tr_C9ZPP9_	
HLA-DQA10501-DQB10201	13	SRGKTQAVELREQDY	AVELREQDY	6	0.3252	1481.3	29.00	0.41	tr_C9ZPP9_	
HLA-DQA10501-DQB10201	14	DYEAVASVLHGAGLH	DYEAVASVL	0	0.2977	1995.1	36.00	0.52	tr_C9ZPP9_	
HLA-DQA10501-DQB10201	15	GGTVRRIEQFLKDTF	IEQFLKDTF	6	0.2860	2266.2	39.00	0.48	tr_C9ZPP9_	
HLA-DQA10501-DQB10201	16	GTVRRIEQFLKDTFS	IEQFLKDTF	5	0.2727	2616.7	42.00	0.43	tr_C9ZPP9_	
HLA-DQA10501-DQB10201	17	RIEQFLKDTFSLNLE	KDTFSLNLE	6	0.2613	2960.3	45.00	0.32	tr_C9ZPP9_	
HLA-DQA10501-DQB10201	18	TVRRIEQFLKDTFSL	IEQFLKDTF	4	0.2580	3066.6	46.00	0.42	tr_C9ZPP9_	
HLA-DQA10501-DQB10201	19	GSRGKTQAVELREQD	RGKTQAVEL	2	0.2577	3075.2	46.00	0.25	tr_C9ZPP9_	
HLA-DQA10501-DQB10201	20	EQFLKDTFSLNLEGS	TFSLNLEGS	6	0.2519	3276.1	48.00	0.36	tr_C9ZPP9_	
HLA-DQA10501-DQB10201	21	IEQFLKDTFSLNLEG	KDTFSLNLE	5	0.2399	3730.1	55.00	0.31	tr_C9ZPP9_	
HLA-DQA10501-DQB10201	22	RRIEQFLKDTFSLNL	IEQFLKDTF	2	0.2394	3748.7	55.00	0.37	tr_C9ZPP9_	
HLA-DQA10501-DQB10201	23	EGSRGKTQAVELREQ	RGKTQAVEL	3	0.2393	3753.8	55.00	0.37	tr_C9ZPP9_	

HLA-DQA10501-DQB10201	24	VRRIEQFLKDTFSLN	IEQFLKDTF	3	0.2346	3950.9	55.00	0.47	tr_C9ZPP9_
HLA-DQA10501-DQB10201	25	LEGSRGKTQAVELRE	RGKTQAVEL	4	0.2334	4000.5	55.00	0.51	tr_C9ZPP9_
HLA-DQA10501-DQB10201	26	QFLKDTFSLNLEGSR	TFSLNLEGS	5	0.2329	4023.0	55.00	0.35	tr_C9ZPP9_
HLA-DQA10501-DQB10201	27	LNLEGSRGKTQAVEL	RGKTQAVEL	6	0.2270	4289.6	55.00	0.85	tr_C9ZPP9_
HLA-DQA10501-DQB10201	28	QRGGTVRRIEQFLKD	TVRRIEQFL	4	0.2191	4671.4	60.00	0.44	tr_C9ZPP9_
HLA-DQA10501-DQB10201	29	FLKDTFSLNLEGSRG	TFSLNLEGS	4	0.2187	4691.1	60.00	0.34	tr_C9ZPP9_
HLA-DQA10501-DQB10201	30	SQQRGGTVRRIEQFL	TVRRIEQFL	6	0.2165	4805.9	60.00	0.62	tr_C9ZPP9_
HLA-DQA10501-DQB10201	31	RGGTVRRIEQFLKDT	TVRRIEQFL	3	0.2133	4974.6	60.00	0.40	tr_C9ZPP9_
HLA-DQA10501-DQB10201	32	NLEGSRGKTQAVELR	RGKTQAVEL	5	0.2064	5357.4	65.00	0.79	tr_C9ZPP9_
HLA-DQA10501-DQB10201	33	YEAVASVLHGAGLHN	YEAVASVLH	0	0.1973	5913.8	65.00	0.27	tr_C9ZPP9_
HLA-DQA10501-DQB10201	34	QQRGGTVRRIEQFLK	TVRRIEQFL	5	0.1893	6448.4	70.00	0.55	tr_C9ZPP9_
HLA-DQA10501-DQB10201	35	LKDTFSLNLEGSRGK	TFSLNLEGS	3	0.1856	6709.6	70.00	0.34	tr_C9ZPP9_
HLA-DQA10501-DQB10201	36	KDTFSLNLEGSRGKT	TFSLNLEGS	2	0.1664	8265.8	75.00	0.39	tr_C9ZPP9_
HLA-DQA10501-DQB10201	37	RSTLKNIISLCHHNE	IISLCHHNE	6	0.1530	9554.8	80.00	0.31	tr_C9ZPP9_
HLA-DQA10501-DQB10201	38	PTRSTLKNIISLCHH	LKNIISLCH	5	0.1501	9851.2	80.00	0.35	tr_C9ZPP9_
HLA-DQA10501-DQB10201	39	STLKNIISLCHHNEH	IISLCHHNE	5	0.1491	9965.2	80.00	0.31	tr_C9ZPP9_
HLA-DQA10501-DQB10201	40	HPTRSTLKNIISLCH	LKNIISLCH	6	0.1490	9969.5	80.00	0.43	tr_C9ZPP9_
HLA-DQA10501-DQB10201	41	TLKNIISLCHHNEHP	IISLCHHNE	4	0.1489	9982.8	80.00	0.36	tr_C9ZPP9_
HLA-DQA10501-DQB10201	42	DTFSLNLEGSRGKTQ	TFSLNLEGS	1	0.1444	10476.4	80.00	0.45	tr_C9ZPP9_
HLA-DQA10501-DQB10201	43	TRSTLKNIISLCHHN	LKNIISLCH	4	0.1364	11432.9	80.00	0.36	tr_C9ZPP9_
HLA-DQA10501-DQB10201	44	KNIISLCHHNEHPNL	IISLCHHNE	2	0.1345	11669.4	80.00	0.41	tr_C9ZPP9_
HLA-DQA10501-DQB10201	45	HNEHPNLKGIGDTSQ	LKGIGDTSQ	6	0.1337	11763.1	85.00	0.49	tr_C9ZPP9_
HLA-DQA10501-DQB10201	46	NEHPNLKGIGDTSQQ	LKGIGDTSQ	5	0.1313	12078.7	85.00	0.47	tr_C9ZPP9_
HLA-DQA10501-DQB10201	47	LKNIISLCHHNEHPN	IISLCHHNE	3	0.1300	12244.5	85.00	0.41	tr_C9ZPP9_
HLA-DQA10501-DQB10201	48	EHPNLKGIGDTSQQR	LKGIGDTSQ	4	0.1298	12273.6	85.00	0.41	tr_C9ZPP9_
HLA-DQA10501-DQB10201	49	HPNLKGIGDTSQQRG	LKGIGDTSQ	3	0.1280	12516.0	85.00	0.34	tr_C9ZPP9_
HLA-DQA10501-DQB10201	50	TSQQRGGTVRRIEQF	GTVRRIEQF	6	0.1261	12779.8	85.00	0.55	tr_C9ZPP9_
HLA-DQA10501-DQB10201	51	NIISLCHHNEHPNLK	IISLCHHNE	1	0.1204	13590.9	85.00	0.42	tr_C9ZPP9_
HLA-DQA10501-DQB10201	52	PNLKGIGDTSQQRGG	LKGIGDTSQ	2	0.1157	14303.0	85.00	0.38	tr_C9ZPP9_
HLA-DQA10501-DQB10201	53	MHPTRSTLKNIISLC	TLKNIISLC	6	0.1151	14396.7	85.00	0.34	tr_C9ZPP9_
HLA-DQA10501-DQB10201	54	ARPYLQMHPTRSTLK	YLQMHPTRS	3	0.1132	14695.6	90.00	0.33	tr_C9ZPP9_
HLA-DQA10501-DQB10201	55	TFSLNLEGSRGKTQA	TFSLNLEGS	0	0.1098	15240.3	90.00	0.43	tr_C9ZPP9_
HLA-DQA10501-DQB10201	56	NLKGIGDTSQQRGGT	LKGIGDTSQ	1	0.1030	16397.5	90.00	0.37	tr_C9ZPP9_
HLA-DQA10501-DQB10201	57	QMHPTRSTLKNIISL	TRSTLKNII	4	0.0991	17113.0	90.00	0.36	tr_C9ZPP9_
HLA-DQA10501-DQB10201	58	RPYLQMHPTRSTLKN	YLQMHPTRS	2	0.0974	17421.4	90.00	0.40	tr_C9ZPP9_
HLA-DQA10501-DQB10201	59	IISLCHHNEHPNLKG	IISLCHHNE	0	0.0965	17593.2	90.00	0.38	tr_C9ZPP9_
HLA-DQA10501-DQB10201	60	YLQMHPTRSTLKNII	TRSTLKNII	6	0.0875	19390.9	95.00	0.42	tr_C9ZPP9_
HLA-DQA10501-DQB10201	61	HHNEHPNLKGIGDTS	NLKGIGDTS	6	0.0863	19644.8	95.00	0.49	tr_C9ZPP9_
HLA-DQA10501-DQB10201	62	LQMHPTRSTLKNIIS	TRSTLKNII	5	0.0841	20122.3	95.00	0.41	tr_C9ZPP9_
HLA-DQA10501-DQB10201	63	PYLQMHPTRSTLKNI	YLQMHPTRS	1	0.0830	20366.8	95.00	0.40	tr_C9ZPP9_
HLA-DQA10501-DQB10201	64	LKGIGDTSQQRGGTV	LKGIGDTSQ	0	0.0794	21178.5	95.00	0.28	tr_C9ZPP9_
HLA-DQA10501-DQB10201	65	DTSQQRGGTVRRIEQ	QRGGTVRRI	4	0.0668	24257.4	95.00	0.41	tr_C9ZPP9_
HLA-DQA10501-DQB10201	66	FSLNLEGSRGKTQAV	SLNLEGSRG	1	0.0651	24708.0	95.00	0.43	tr_C9ZPP9_
HLA-DQA10501-DQB10201	67	GDTSQQRGGTVRRIE	QRGGTVRRI	5	0.0622	25508.8	100.00	0.49	tr_C9ZPP9_
HLA-DQA10501-DQB10201	68	CHHNEHPNLKGIGDT	HPNLKGIGD	5	0.0619	25582.6	100.00	0.31	tr_C9ZPP9_
HLA-DQA10501-DQB10201	69	SLNLEGSRGKTQAVE	SRGKTQAVE	6	0.0610	25844.4	100.00	0.39	tr_C9ZPP9_
HLA-DQA10501-DQB10201	70	ISLCHHNEHPNLKGI	CHHNEHPNL	3	0.0607	25917.4	100.00	0.41	tr_C9ZPP9_
HLA-DQA10501-DQB10201	71	LCHHNEHPNLKGIGD	HPNLKGIGD	6	0.0601	26086.0	100.00	0.38	tr_C9ZPP9_
HLA-DQA10501-DQB10201	72	KGIGDTSQQRGGTVR	IGDTSQQRG	2	0.0587	26481.0	100.00	0.41	tr_C9ZPP9_
HLA-DQA10501-DQB10201	73	IGDTSQQRGGTVRRI	QRGGTVRRI	6	0.0586	26527.7	100.00	0.52	tr_C9ZPP9_

HLA-DQA10501-DQB10201	74	GIGDTSQQRGGTVRR	IGDTSQQRG	1	0.0507	28901.5	100.00	0.42	tr_C9ZPP9_
HLA-DQA10501-DQB10201	75	SLCHHNEHPNLKGIG	CHHNEHPNL	2	0.0506	28922.8	100.00	0.44	tr_C9ZPP9_

Allele: HLA-DQA10501-DQB10201. Number of high binders 0. Number of weak binders 0. Number of peptides 75

Allele	pos	peptide	core	Of	1-log50k(aff)	affinity(nM)	%Rank	Relia	Identity	Bind Level
HLA-DQA10501-DQB10201	1	TDEGEGYGAMSAAEE	YGAMSAAEE	6	0.5347	153.6	3.50	0.83	tr_C9ZVS3_	WB
HLA-DQA10501-DQB10201	2	DEGEGYGAMSAAEEK	YGAMSAAEE	5	0.5089	203.0	5.00	0.81	tr_C9ZVS3_	WB
HLA-DQA10501-DQB10201	3	VYSCVWQSLLPLYEL	WQSLLPLYE	5	0.4997	224.4	5.50	0.51	tr_C9ZVS3_	WB
HLA-DQA10501-DQB10201	4	EGEGYGAMSAAEEKS	YGAMSAAEE	4	0.4974	230.0	5.50	0.79	tr_C9ZVS3_	WB
HLA-DQA10501-DQB10201	5	LVYSCVWQSLLPLYE	WQSLLPLYE	6	0.4882	254.2	6.50	0.50	tr_C9ZVS3_	WB
HLA-DQA10501-DQB10201	6	YSCVWQSLLPLYELA	WQSLLPLYE	4	0.4782	283.0	7.00	0.53	tr_C9ZVS3_	WB
HLA-DQA10501-DQB10201	7	GEGYGAMSAAEEKSH	YGAMSAAEE	3	0.4715	304.4	7.50	0.78	tr_C9ZVS3_	WB
HLA-DQA10501-DQB10201	8	EGYGAMSAAEEKSHH	YGAMSAAEE	2	0.4578	353.2	9.00	0.81	tr_C9ZVS3_	WB
HLA-DQA10501-DQB10201	9	VWQSLLPLYELARFF	WQSLLPLYE	1	0.4403	426.5	11.00	0.49	tr_C9ZVS3_	
HLA-DQA10501-DQB10201	10	SCVWQSLLPLYELAR	WQSLLPLYE	3	0.4401	427.4	11.00	0.62	tr_C9ZVS3_	
HLA-DQA10501-DQB10201	11	CVWQSLLPLYELARF	WQSLLPLYE	2	0.4381	436.6	11.00	0.56	tr_C9ZVS3_	
HLA-DQA10501-DQB10201	12	GYGAMSAAEEKSHHK	YGAMSAAEE	1	0.4177	544.8	14.00	0.80	tr_C9ZVS3_	
HLA-DQA10501-DQB10201	13	GKAHTEVVTDEGEGY	TEVVTDEGE	4	0.4107	587.8	14.00	0.34	tr_C9ZVS3_	
HLA-DQA10501-DQB10201	14	VLVYSCVWQSLLPLY	VWQSLLPLY	6	0.3965	685.4	16.00	0.26	tr_C9ZVS3_	
HLA-DQA10501-DQB10201	15	KAHTEVVTDEGEGYG	TEVVTDEGE	3	0.3940	703.8	17.00	0.34	tr_C9ZVS3_	
HLA-DQA10501-DQB10201	16	GSGKAHTEVVTDEGE	TEVVTDEGE	6	0.3927	713.8	17.00	0.37	tr_C9ZVS3_	
HLA-DQA10501-DQB10201	17	SGKAHTEVVTDEGEG	TEVVTDEGE	5	0.3820	801.9	19.00	0.41	tr_C9ZVS3_	
HLA-DQA10501-DQB10201	18	AHTEVVTDEGEGYGA	TEVVTDEGE	2	0.3766	849.4	19.00	0.35	tr_C9ZVS3_	
HLA-DQA10501-DQB10201	19	QQTHLLNMLSPTEPL	LLNMLSPTE	4	0.3754	861.4	20.00	0.41	tr_C9ZVS3_	
HLA-DQA10501-DQB10201	20	LVLVYSCVWQSLLPL	VYSCVWQSL	3	0.3708	904.6	20.00	0.28	tr_C9ZVS3_	
HLA-DQA10501-DQB10201	21	WQSLLPLYELARFFS	WQSLLPLYE	0	0.3627	987.9	22.00	0.41	tr_C9ZVS3_	
HLA-DQA10501-DQB10201	22	MQVAMPVSLVDSCLG	PVSLVDSCL	5	0.3608	1008.2	22.00	0.29	tr_C9ZVS3_	
HLA-DQA10501-DQB10201	23	LPLYELARFFSFPFQ	ARFFSFPFQ	6	0.3593	1024.9	22.00	0.38	tr_C9ZVS3_	
HLA-DQA10501-DQB10201	24	LLVLVYSCVWQSLLP	VYSCVWQSL	4	0.3560	1062.4	23.00	0.31	tr_C9ZVS3_	
HLA-DQA10501-DQB10201	25	PLYELARFFSFPFQS	ARFFSFPFQ	5	0.3555	1067.3	23.00	0.31	tr_C9ZVS3_	
HLA-DQA10501-DQB10201	26	VSLVDSCLGMQELLV	CLGMQELLV	6	0.3550	1073.9	23.00	0.38	tr_C9ZVS3_	
HLA-DQA10501-DQB10201	27	QTHLLNMLSPTEPLG	LLNMLSPTE	3	0.3546	1078.7	23.00	0.41	tr_C9ZVS3_	
HLA-DQA10501-DQB10201	28	SLVDSCLGMQELLVL	CLGMQELLV	5	0.3516	1113.7	24.00	0.37	tr_C9ZVS3_	
HLA-DQA10501-DQB10201	29	LYELARFFSFPFQSS	ARFFSFPFQ	4	0.3475	1164.2	25.00	0.29	tr_C9ZVS3_	
HLA-DQA10501-DQB10201	30	TTDSKFMQCINAIIP	KFMQCINAI	4	0.3453	1192.7	25.00	0.34	tr_C9ZVS3_	
HLA-DQA10501-DQB10201	31	DSKFMQCINAIIPHG	KFMQCINAI	2	0.3437	1213.8	26.00	0.29	tr_C9ZVS3_	
HLA-DQA10501-DQB10201	32	TQQTHLLNMLSPTEP	LLNMLSPTE	5	0.3431	1221.1	26.00	0.48	tr_C9ZVS3_	
HLA-DQA10501-DQB10201	33	HTEVVTDEGEGYGAM	VVTDEGEGY	3	0.3423	1231.6	26.00	0.37	tr_C9ZVS3_	
HLA-DQA10501-DQB10201	34	VDSCLGMQELLVLVY	CLGMQELLV	3	0.3394	1271.5	26.00	0.33	tr_C9ZVS3_	
HLA-DQA10501-DQB10201	35	THLLNMLSPTEPLGS	LLNMLSPTE	2	0.3391	1275.8	26.00	0.43	tr_C9ZVS3_	
HLA-DQA10501-DQB10201	36	ELLVLVYSCVWQSLL	VYSCVWQSL	5	0.3383	1286.9	27.00	0.32	tr_C9ZVS3_	
HLA-DQA10501-DQB10201	37	LVDSCLGMQELLVLV	CLGMQELLV	4	0.3366	1310.3	27.00	0.37	tr_C9ZVS3_	
HLA-DQA10501-DQB10201	38	TDSKFMQCINAIIPH	KFMQCINAI	3	0.3353	1328.9	27.00	0.31	tr_C9ZVS3_	
HLA-DQA10501-DQB10201	39	LGSGKAHTEVVTDEG	AHTEVVTDE	5	0.3348	1336.2	27.00	0.40	tr_C9ZVS3_	
HLA-DQA10501-DQB10201	40	YGAMSAAEEKSHHKN	YGAMSAAEE	0	0.3314	1386.5	28.00	0.71	tr_C9ZVS3_	

HLA-DQA10501-DQB10201	41	LLPLYELARFFSFPF	LARFFSFPF	6	0.3284	1431.7	29.00	0.31	tr_C9ZVS3_
HLA-DQA10501-DQB10201	42	QVAMPVSLVDSCLGM	PVSLVDSCL	4	0.3267	1457.6	29.00	0.35	tr_C9ZVS3_
HLA-DQA10501-DQB10201	43	YELARFFSFPFQSSS	ARFFSFPFQ	3	0.3263	1464.8	29.00	0.29	tr_C9ZVS3_
HLA-DQA10501-DQB10201	44	TLGAGVISLAIAFQM	ISLAIAFQM	6	0.3260	1469.4	29.00	0.38	tr_C9ZVS3_
HLA-DQA10501-DQB10201	45	SKFMQCINAIIPHGG	MQCINAIIP	3	0.3232	1513.8	30.00	0.30	tr_C9ZVS3_
HLA-DQA10501-DQB10201	46	ELARFFSFPFQSSSY	ARFFSFPFQ	2	0.3221	1532.9	30.00	0.26	tr_C9ZVS3_
HLA-DQA10501-DQB10201	47	HLLNMLSPTEPLGSG	LLNMLSPTE	1	0.3171	1617.2	31.00	0.44	tr_C9ZVS3_
HLA-DQA10501-DQB10201	48	QELLVLVYSCVWQSL	VYSCVWQSL	6	0.3142	1669.6	32.00	0.36	tr_C9ZVS3_
HLA-DQA10501-DQB10201	49	PVSLVDSCLGMQELL	DSCLGMQEL	5	0.3108	1731.9	33.00	0.28	tr_C9ZVS3_
HLA-DQA10501-DQB10201	50	DSCLGMQELLVLVYS	CLGMQELLV	2	0.3082	1781.2	33.00	0.34	tr_C9ZVS3_
HLA-DQA10501-DQB10201	51	MPVSLVDSCLGMQEL	DSCLGMQEL	6	0.3072	1801.2	33.00	0.32	tr_C9ZVS3_
HLA-DQA10501-DQB10201	52	SLLPLYELARFFSFP	PLYELARFF	3	0.3008	1929.9	35.00	0.35	tr_C9ZVS3_
HLA-DQA10501-DQB10201	53	TEVVTDEGEGYGAMS	VVTDEGEGY	2	0.2996	1955.5	35.00	0.46	tr_C9ZVS3_
HLA-DQA10501-DQB10201	54	LARFFSFPFQSSSYQ	ARFFSFPFQ	1	0.2991	1965.5	35.00	0.25	tr_C9ZVS3_
HLA-DQA10501-DQB10201	55	KFMQCINAIIPHGGA	MQCINAIIP	2	0.2983	1982.0	36.00	0.31	tr_C9ZVS3_
HLA-DQA10501-DQB10201	56	VAMPVSLVDSCLGMQ	PVSLVDSCL	3	0.2947	2061.4	36.00	0.36	tr_C9ZVS3_
HLA-DQA10501-DQB10201	57	LGMQELLVLVYSCVW	LVLVYSCVW	6	0.2923	2114.7	37.00	0.46	tr_C9ZVS3_
HLA-DQA10501-DQB10201	58	HTQQTHLLNMLSPTE	LLNMLSPTE	6	0.2911	2143.8	37.00	0.50	tr_C9ZVS3_
HLA-DQA10501-DQB10201	59	ATLGAGVISLAIAFQ	VISLAIAFQ	6	0.2901	2166.0	38.00	0.37	tr_C9ZVS3_
HLA-DQA10501-DQB10201	60	PTTDSKFMQCINAII	KFMQCINAI	5	0.2853	2281.5	39.00	0.36	tr_C9ZVS3_
HLA-DQA10501-DQB10201	61	QSLLPLYELARFFSF	PLYELARFF	4	0.2851	2288.0	39.00	0.37	tr_C9ZVS3_
HLA-DQA10501-DQB10201	62	PLGSGKAHTEVVTDE	AHTEVVTDE	6	0.2821	2362.6	40.00	0.64	tr_C9ZVS3_
HLA-DQA10501-DQB10201	63	GMQELLVLVYSCVWQ	LVLVYSCVW	5	0.2796	2427.8	40.00	0.41	tr_C9ZVS3_
HLA-DQA10501-DQB10201	64	AMPVSLVDSCLGMQE	PVSLVDSCL	2	0.2795	2429.7	40.00	0.35	tr_C9ZVS3_
HLA-DQA10501-DQB10201	65	LLNMLSPTEPLGSGK	LLNMLSPTE	0	0.2791	2440.2	40.00	0.35	tr_C9ZVS3_
HLA-DQA10501-DQB10201	66	LGSATLGAGVISLAI	GAGVISLAI	6	0.2776	2480.2	41.00	0.41	tr_C9ZVS3_
HLA-DQA10501-DQB10201	67	SCLGMQELLVLVYSC	CLGMQELLV	1	0.2776	2480.5	41.00	0.34	tr_C9ZVS3_
HLA-DQA10501-DQB10201	68	ARFFSFPFQSSSYQA	RFFSFPFQS	1	0.2771	2493.7	41.00	0.25	tr_C9ZVS3_
HLA-DQA10501-DQB10201	69	MQELLVLVYSCVWQS	LVLVYSCVW	4	0.2717	2644.8	42.00	0.41	tr_C9ZVS3_
HLA-DQA10501-DQB10201	70	SATLGAGVISLAIAF	GAGVISLAI	4	0.2664	2800.3	44.00	0.29	tr_C9ZVS3_
HLA-DQA10501-DQB10201	71	EVVTDEGEGYGAMSA	VVTDEGEGY	1	0.2654	2830.9	44.00	0.52	tr_C9ZVS3_
HLA-DQA10501-DQB10201	72	GSATLGAGVISLAIA	GAGVISLAI	5	0.2622	2930.3	45.00	0.32	tr_C9ZVS3_
HLA-DQA10501-DQB10201	73	VTDEGEGYGAMSAAE	GYGAMSAAE	6	0.2566	3111.6	46.00	0.44	tr_C9ZVS3_
HLA-DQA10501-DQB10201	74	RFFSFPFQSSSYQAA	FQSSSYQAA	6	0.2561	3129.3	47.00	0.19	tr_C9ZVS3_
HLA-DQA10501-DQB10201	75	FMQCINAIIPHGGAL	MQCINAIIP	1	0.2509	3311.7	48.00	0.34	tr_C9ZVS3_
HLA-DQA10501-DQB10201	76	VVTDEGEGYGAMSAA	VVTDEGEGY	0	0.2483	3404.3	49.00	0.33	tr_C9ZVS3_
HLA-DQA10501-DQB10201	77	CLGMQELLVLVYSCV	MQELLVLVY	3	0.2440	3567.0	50.00	0.22	tr_C9ZVS3_
HLA-DQA10501-DQB10201	78	NLGSATLGAGVISLA	TLGAGVISL	5	0.2340	3975.0	55.00	0.44	tr_C9ZVS3_
HLA-DQA10501-DQB10201	79	TPTTDSKFMQCINAI	KFMQCINAI	6	0.2300	4150.3	55.00	0.53	tr_C9ZVS3_
HLA-DQA10501-DQB10201	80	FNLGSATLGAGVISL	TLGAGVISL	6	0.2300	4153.6	55.00	0.53	tr_C9ZVS3_
HLA-DQA10501-DQB10201	81	HVHTQQTHLLNMLSP	THLLNMLSP	6	0.2298	4162.1	55.00	0.35	tr_C9ZVS3_
HLA-DQA10501-DQB10201	82	FFSFPFQSSSYQAAH	FQSSSYQAA	5	0.2219	4530.9	60.00	0.28	tr_C9ZVS3_
HLA-DQA10501-DQB10201	83	VHTQQTHLLNMLSPT	THLLNMLSP	5	0.2212	4563.8	60.00	0.36	tr_C9ZVS3_
HLA-DQA10501-DQB10201	84	FSFPFQSSSYQAAHT	FQSSSYQAA	4	0.2096	5174.5	60.00	0.26	tr_C9ZVS3_
HLA-DQA10501-DQB10201	85	MQCINAIIPHGGALS	CINAIIPHG	2	0.1961	5987.9	65.00	0.34	tr_C9ZVS3_
HLA-DQA10501-DQB10201	86	HTHVHTQQTHLLNML	VHTQQTHLL	3	0.1955	6028.9	65.00	0.33	tr_C9ZVS3_
HLA-DQA10501-DQB10201	87	LNMLSPTEPLGSGKA	LNMLSPTEP	0	0.1859	6692.0	70.00	0.47	tr_C9ZVS3_
HLA-DQA10501-DQB10201	88	THVHTQQTHLLNMLS	VHTQQTHLL	2	0.1856	6710.5	70.00	0.31	tr_C9ZVS3_
HLA-DQA10501-DQB10201	89	SFPFQSSSYQAAHTH	FQSSSYQAA	3	0.1820	6977.1	70.00	0.31	tr_C9ZVS3_
HLA-DQA10501-DQB10201	90	AIIPHGGALSTTFNL	HGGALSTTF	4	0.1815	7019.3	70.00	0.31	tr_C9ZVS3_

HLA-DQA10501-DQB10201	91	GGALSTTFNLGSATL	TFNLGSATL	6	0.1799	7140.9	70.00	0.69	tr_C9ZVS3_
HLA-DQA10501-DQB10201	92	GALSTTFNLGSATLG	TFNLGSATL	5	0.1794	7176.1	70.00	0.56	tr_C9ZVS3_
HLA-DQA10501-DQB10201	93	ALSTTFNLGSATLGA	TFNLGSATL	4	0.1778	7301.9	70.00	0.50	tr_C9ZVS3_
HLA-DQA10501-DQB10201	94	NAIIPHGGALSTTFN	HGGALSTTF	5	0.1775	7330.4	70.00	0.38	tr_C9ZVS3_
HLA-DQA10501-DQB10201	95	INAIIPHGGALSTTF	HGGALSTTF	6	0.1772	7350.1	70.00	0.42	tr_C9ZVS3_
HLA-DQA10501-DQB10201	96	THTHTHVHTQQTHLL	VHTQQTHLL	6	0.1740	7611.4	70.00	0.60	tr_C9ZVS3_
HLA-DQA10501-DQB10201	97	TTFNLGSATLGAGVI	TFNLGSATL	1	0.1738	7626.2	70.00	0.29	tr_C9ZVS3_
HLA-DQA10501-DQB10201	98	THTHVHTQQTHLLNM	VHTQQTHLL	4	0.1732	7678.5	70.00	0.38	tr_C9ZVS3_
HLA-DQA10501-DQB10201	99	TFNLGSATLGAGVIS	SATLGAGVI	5	0.1722	7759.2	70.00	0.29	tr_C9ZVS3_
HLA-DQA10501-DQB10201	100	EPLGSGKAHTEVVTD	KAHTEVVTD	6	0.1686	8063.7	75.00	0.58	tr_C9ZVS3_
HLA-DQA10501-DQB10201	101	FPFQSSSYQAAHTHT	FQSSSYQAA	2	0.1646	8427.3	75.00	0.27	tr_C9ZVS3_
HLA-DQA10501-DQB10201	102	HTHTHVHTQQTHLLN	VHTQQTHLL	5	0.1640	8474.3	75.00	0.49	tr_C9ZVS3_
HLA-DQA10501-DQB10201	103	IIPHGGALSTTFNLG	HGGALSTTF	3	0.1633	8540.7	75.00	0.32	tr_C9ZVS3_
HLA-DQA10501-DQB10201	104	STTFNLGSATLGAGV	TFNLGSATL	2	0.1630	8572.8	75.00	0.43	tr_C9ZVS3_
HLA-DQA10501-DQB10201	105	LSTTFNLGSATLGAG	TFNLGSATL	3	0.1627	8596.8	75.00	0.50	tr_C9ZVS3_
HLA-DQA10501-DQB10201	106	QCINAIIPHGGALST	CINAIIPHG	1	0.1571	9135.4	75.00	0.33	tr_C9ZVS3_
HLA-DQA10501-DQB10201	107	CINAIIPHGGALSTT	INAIIPHGG	1	0.1486	10011.0	80.00	0.26	tr_C9ZVS3_
HLA-DQA10501-DQB10201	108	IPHGGALSTTFNLGS	HGGALSTTF	2	0.1448	10438.9	80.00	0.34	tr_C9ZVS3_
HLA-DQA10501-DQB10201	109	PFQSSSYQAAHTHTH	SSSYQAAHT	3	0.1326	11915.1	85.00	0.25	tr_C9ZVS3_
HLA-DQA10501-DQB10201	110	DTPTTDSKFMQCINA	SKFMQCINA	6	0.1302	12220.8	85.00	0.37	tr_C9ZVS3_
HLA-DQA10501-DQB10201	111	PHGGALSTTFNLGSA	HGGALSTTF	1	0.1269	12661.6	85.00	0.29	tr_C9ZVS3_
HLA-DQA10501-DQB10201	112	GAMSAAEEKSHHKNG	AMSAAEEKS	1	0.1207	13552.4	85.00	0.50	tr_C9ZVS3_
HLA-DQA10501-DQB10201	113	FQSSSYQAAHTHTHT	SSSYQAAHT	2	0.1115	14969.1	90.00	0.34	tr_C9ZVS3_
HLA-DQA10501-DQB10201	114	HGGALSTTFNLGSAT	HGGALSTTF	0	0.1109	15060.2	90.00	0.23	tr_C9ZVS3_
HLA-DQA10501-DQB10201	115	NMLSPTEPLGSGKAH	NMLSPTEPL	0	0.1084	15469.3	90.00	0.54	tr_C9ZVS3_
HLA-DQA10501-DQB10201	116	HTHTHTHVHTQQTHL	HVHTQQTHL	6	0.1051	16042.4	90.00	0.43	tr_C9ZVS3_
HLA-DQA10501-DQB10201	117	GDTPTTDSKFMQCIN	DSKFMQCIN	6	0.1050	16060.1	90.00	0.25	tr_C9ZVS3_
HLA-DQA10501-DQB10201	118	NGDTPTTDSKFMQCI	TDSKFMQCI	6	0.1013	16701.6	90.00	0.32	tr_C9ZVS3_
HLA-DQA10501-DQB10201	119	TEPLGSGKAHTEVVT	SGKAHTEVV	5	0.0995	17038.3	90.00	0.39	tr_C9ZVS3_
HLA-DQA10501-DQB10201	120	KNGDTPTTDSKFMQC	TTDSKFMQC	6	0.0919	18488.6	90.00	0.31	tr_C9ZVS3_
HLA-DQA10501-DQB10201	121	QSSSYQAAHTHTHTH	SSSYQAAHT	1	0.0860	19710.6	95.00	0.37	tr_C9ZVS3_
HLA-DQA10501-DQB10201	122	PTEPLGSGKAHTEVV	SGKAHTEVV	6	0.0856	19810.4	95.00	0.55	tr_C9ZVS3_
HLA-DQA10501-DQB10201	123	SSSYQAAHTHTHTHT	SYQAAHTHT	2	0.0737	22518.7	95.00	0.38	tr_C9ZVS3_
HLA-DQA10501-DQB10201	124	HKNGDTPTTDSKFMQ	TPTTDSKFM	5	0.0704	23331.2	95.00	0.29	tr_C9ZVS3_
HLA-DQA10501-DQB10201	125	HTHTHTHTHVHTQQT	THTHVHTQQ	5	0.0700	23434.2	95.00	0.44	tr_C9ZVS3_
HLA-DQA10501-DQB10201	126	THTHTHTHVHTQQTH	THTHVHTQQ	4	0.0690	23711.4	95.00	0.34	tr_C9ZVS3_
HLA-DQA10501-DQB10201	127	HHKNGDTPTTDSKFM	TPTTDSKFM	6	0.0675	24094.7	95.00	0.31	tr_C9ZVS3_
HLA-DQA10501-DQB10201	128	AMSAAEEKSHHKNGD	AMSAAEEKS	0	0.0643	24943.3	95.00	0.76	tr_C9ZVS3_
HLA-DQA10501-DQB10201	129	SHHKNGDTPTTDSKF	KNGDTPTTD	3	0.0603	26041.1	100.00	0.30	tr_C9ZVS3_
HLA-DQA10501-DQB10201	130	AHTHTHTHTHVHTQQ	THTHVHTQQ	6	0.0596	26224.6	100.00	0.67	tr_C9ZVS3_
HLA-DQA10501-DQB10201	131	EKSHHKNGDTPTTDS	KNGDTPTTD	5	0.0576	26807.3	100.00	0.44	tr_C9ZVS3_
HLA-DQA10501-DQB10201	132	EEKSHHKNGDTPTTD	KNGDTPTTD	6	0.0575	26838.9	100.00	0.48	tr_C9ZVS3_
HLA-DQA10501-DQB10201	133	SSYQAAHTHTHTHTH	SYQAAHTHT	1	0.0530	28170.1	100.00	0.51	tr_C9ZVS3_
HLA-DQA10501-DQB10201	134	SPTEPLGSGKAHTEV	GSGKAHTEV	6	0.0530	28172.8	100.00	0.37	tr_C9ZVS3_
HLA-DQA10501-DQB10201	135	MLSPTEPLGSGKAHT	MLSPTEPLG	0	0.0488	29496.0	100.00	0.33	tr_C9ZVS3_
HLA-DQA10501-DQB10201	136	KSHHKNGDTPTTDSK	KNGDTPTTD	4	0.0487	29524.1	100.00	0.44	tr_C9ZVS3_
HLA-DQA10501-DQB10201	137	SYQAAHTHTHTHTHV	THTHTHTHV	6	0.0483	29646.4	100.00	0.35	tr_C9ZVS3_
HLA-DQA10501-DQB10201	138	LSPTEPLGSGKAHTE	LGSGKAHTE	6	0.0431	31355.4	100.00	0.38	tr_C9ZVS3_
HLA-DQA10501-DQB10201	139	QAAHTHTHTHTHVHT	THTHTHVHT	6	0.0359	33913.4	100.00	0.38	tr_C9ZVS3_
HLA-DQA10501-DQB10201	140	AAHTHTHTHTHVHTQ	THTHTHVHT	5	0.0345	34427.6	100.00	0.32	tr_C9ZVS3_

HLA-DQA10501-DQB10201	141	AEEKSHHKNGDTPTT	SHHKNGDTP	4	0.0339	34642.1	100.00	0.26	tr_C9ZVS3_	
HLA-DQA10501-DQB10201	142	YQAAHTHTHTHTHVH	THTHTHTHV	5	0.0339	34651.1	100.00	0.49	tr_C9ZVS3_	
HLA-DQA10501-DQB10201	143	AAEEKSHHKNGDTPT	SHHKNGDTP	5	0.0328	35080.8	100.00	0.35	tr_C9ZVS3_	
HLA-DQA10501-DQB10201	144	SAAEEKSHHKNGDTP	SHHKNGDTP	6	0.0297	36246.8	100.00	0.44	tr_C9ZVS3_	
HLA-DQA10501-DQB10201	145	MSAAEEKSHHKNGDT	MSAAEEKSH	0	0.0220	39423.7	100.00	0.31	tr_C9ZVS3_	

Allele: HLA-DQA10501-DQB10201. Number of high binders 0. Number of weak binders 8. Number of peptides 145

Allele	pos	peptide	core	Of	1-log50k(aff)	affinity(nM)	%Rank	Relia	Identity	Bind Level
HLA-DQA10501-DQB10201	1	NFICTEFGLLLDDLF	EFGLLLDDL	5	0.7144	22.0	0.12	0.45	tr_D0A1S7_	SB
HLA-DQA10501-DQB10201	2	FICTEFGLLLDDLFA	EFGLLLDDL	4	0.7098	23.1	0.15	0.41	tr_D0A1S7_	SB
HLA-DQA10501-DQB10201	3	ICTEFGLLLDDLFAF	EFGLLLDDL	3	0.7088	23.4	0.15	0.41	tr_D0A1S7_	SB
HLA-DQA10501-DQB10201	4	CTEFGLLLDDLFAFS	EFGLLLDDL	2	0.6902	28.5	0.25	0.40	tr_D0A1S7_	SB
HLA-DQA10501-DQB10201	5	PLWEVLIDVESELHY	EVLIDVESE	3	0.6805	31.7	0.30	0.48	tr_D0A1S7_	SB
HLA-DQA10501-DQB10201	6	LYNIAFYAAQDIYYL	IAFYAAQDI	3	0.6805	31.7	0.30	0.35	tr_D0A1S7_	SB
HLA-DQA10501-DQB10201	7	TEFGLLLDDLFAFSC	FGLLLDDLF	2	0.6793	32.1	0.30	0.45	tr_D0A1S7_	SB
HLA-DQA10501-DQB10201	8	RLYNIAFYAAQDIYY	IAFYAAQDI	4	0.6722	34.7	0.40	0.38	tr_D0A1S7_	SB
HLA-DQA10501-DQB10201	9	YNIAFYAAQDIYYLI	IAFYAAQDI	2	0.6708	35.2	0.40	0.32	tr_D0A1S7_	SB
HLA-DQA10501-DQB10201	10	LWEVLIDVESELHYS	EVLIDVESE	2	0.6700	35.5	0.40	0.50	tr_D0A1S7_	SB
HLA-DQA10501-DQB10201	11	WEVLIDVESELHYSI	EVLIDVESE	1	0.6528	42.8	0.50	0.50	tr_D0A1S7_	WB
HLA-DQA10501-DQB10201	12	EFGLLLDDLFAFSCR	FGLLLDDLF	1	0.6438	47.2	0.60	0.46	tr_D0A1S7_	WB
HLA-DQA10501-DQB10201	13	FRLYNIAFYAAQDIY	IAFYAAQDI	5	0.6412	48.5	0.60	0.52	tr_D0A1S7_	WB
HLA-DQA10501-DQB10201	14	NIAFYAAQDIYYLIQ	IAFYAAQDI	1	0.6371	50.7	0.70	0.31	tr_D0A1S7_	WB
HLA-DQA10501-DQB10201	15	SGRILCWADAFEFFL	WADAFEFFL	6	0.6335	52.7	0.70	0.33	tr_D0A1S7_	WB
HLA-DQA10501-DQB10201	16	LNFICTEFGLLLDDL	EFGLLLDDL	6	0.6249	57.9	0.80	0.75	tr_D0A1S7_	WB
HLA-DQA10501-DQB10201	17	GRILCWADAFEFFLQ	WADAFEFFL	5	0.6138	65.3	1.00	0.31	tr_D0A1S7_	WB
HLA-DQA10501-DQB10201	18	EVLIDVESELHYSIL	EVLIDVESE	0	0.6016	74.5	1.30	0.44	tr_D0A1S7_	WB
HLA-DQA10501-DQB10201	19	RILCWADAFEFFLQL	WADAFEFFL	4	0.5991	76.5	1.30	0.34	tr_D0A1S7_	WB
HLA-DQA10501-DQB10201	20	IAFYAAQDIYYLIQA	FYAAQDIYY	2	0.5961	79.1	1.40	0.31	tr_D0A1S7_	WB
HLA-DQA10501-DQB10201	21	FFRLYNIAFYAAQDI	IAFYAAQDI	6	0.5910	83.5	1.50	0.71	tr_D0A1S7_	WB
HLA-DQA10501-DQB10201	22	QSGRILCWADAFEFF	RILCWADAF	3	0.5862	88.0	1.60	0.31	tr_D0A1S7_	WB
HLA-DQA10501-DQB10201	23	AFYAAQDIYYLIQAF	FYAAQDIYY	1	0.5537	125.1	3.00	0.30	tr_D0A1S7_	WB
HLA-DQA10501-DQB10201	24	ILCWADAFEFFLQLA	WADAFEFFL	3	0.5528	126.3	3.00	0.38	tr_D0A1S7_	WB
HLA-DQA10501-DQB10201	25	YQSGRILCWADAFEF	RILCWADAF	4	0.5397	145.5	3.50	0.41	tr_D0A1S7_	WB
HLA-DQA10501-DQB10201	26	GEPPRAISSEEMTLL	PRAISSEEM	3	0.5248	171.0	4.00	0.67	tr_D0A1S7_	WB
HLA-DQA10501-DQB10201	27	RYELVDRYGEELAQL	RYGEELAQL	6	0.5228	174.6	4.00	0.41	tr_D0A1S7_	WB
HLA-DQA10501-DQB10201	28	TGEPPRAISSEEMTL	PRAISSEEM	4	0.5177	184.7	4.50	0.73	tr_D0A1S7_	WB
HLA-DQA10501-DQB10201	29	EVTGEPPRAISSEEM	PRAISSEEM	6	0.5167	186.7	4.50	0.86	tr_D0A1S7_	WB
HLA-DQA10501-DQB10201	30	FGLLLDDLFAFSCRV	FGLLLDDLF	0	0.5130	194.3	5.00	0.59	tr_D0A1S7_	WB
HLA-DQA10501-DQB10201	31	QQRRAYINFDLVPTV	RAYINFDLV	3	0.5113	197.9	5.00	0.34	tr_D0A1S7_	WB
HLA-DQA10501-DQB10201	32	VTGEPPRAISSEEMT	PRAISSEEM	5	0.5083	204.4	5.00	0.81	tr_D0A1S7_	WB
HLA-DQA10501-DQB10201	33	TLTMGVFTVYDTLFI	VFTVYDTLF	5	0.5068	207.7	5.00	0.43	tr_D0A1S7_	WB
HLA-DQA10501-DQB10201	34	LCWADAFEFFLQLAF	WADAFEFFL	2	0.5059	209.7	5.00	0.41	tr_D0A1S7_	WB
HLA-DQA10501-DQB10201	35	YELVDRYGEELAQLR	RYGEELAQL	5	0.5042	213.7	5.50	0.35	tr_D0A1S7_	WB
HLA-DQA10501-DQB10201	36	QRRAYINFDLVPTVN	RAYINFDLV	2	0.5016	219.9	5.50	0.31	tr_D0A1S7_	WB
HLA-DQA10501-DQB10201	37	EPPRAISSEEMTLLK	PRAISSEEM	2	0.4976	229.5	5.50	0.71	tr_D0A1S7_	WB

HLA-DQA10501-DQB10201	38	AQQRRAYINFDLVPT	RAYINFDLV	4	0.4914	245.5	6.00	0.44	tr_D0A1S7_	WB
HLA-DQA10501-DQB10201	39	LTMGVFTVYDTLFIS	VFTVYDTLF	4	0.4882	254.0	6.50	0.41	tr_D0A1S7_	WB
HLA-DQA10501-DQB10201	40	FYAAQDIYYLIQAFN	FYAAQDIYY	0	0.4879	254.9	6.50	0.29	tr_D0A1S7_	WB
HLA-DQA10501-DQB10201	41	MYQSGRILCWADAFE	RILCWADAF	5	0.4849	263.2	6.50	0.49	tr_D0A1S7_	WB
HLA-DQA10501-DQB10201	42	RTLTMGVFTVYDTLF	VFTVYDTLF	6	0.4845	264.4	6.50	0.52	tr_D0A1S7_	WB
HLA-DQA10501-DQB10201	43	CWADAFEFFLQLAFG	WADAFEFFL	1	0.4824	270.5	7.00	0.38	tr_D0A1S7_	WB
HLA-DQA10501-DQB10201	44	RRAYINFDLVPTVNS	RAYINFDLV	1	0.4780	283.7	7.00	0.30	tr_D0A1S7_	WB
HLA-DQA10501-DQB10201	45	SERYELVDRYGEELA	LVDRYGEEL	5	0.4767	287.6	7.50	0.42	tr_D0A1S7_	WB
HLA-DQA10501-DQB10201	46	DNEAVNWFDVVSTRF	WFDVVSTRF	6	0.4762	289.3	7.50	0.50	tr_D0A1S7_	WB
HLA-DQA10501-DQB10201	47	PSKVDVLFRVRDEFA	LFRVRDEFA	6	0.4760	289.9	7.50	0.49	tr_D0A1S7_	WB
HLA-DQA10501-DQB10201	48	CAQQRRAYINFDLVP	RAYINFDLV	5	0.4739	296.4	7.50	0.56	tr_D0A1S7_	WB
HLA-DQA10501-DQB10201	49	YVLSHLYHMTPNLLD	LYHMTPNLL	5	0.4733	298.6	7.50	0.70	tr_D0A1S7_	WB
HLA-DQA10501-DQB10201	50	ELVDRYGEELAQLRS	RYGEELAQL	4	0.4722	302.1	7.50	0.35	tr_D0A1S7_	WB
HLA-DQA10501-DQB10201	51	VDVLFRVRDEFANRM	LFRVRDEFA	3	0.4722	302.1	7.50	0.43	tr_D0A1S7_	WB
HLA-DQA10501-DQB10201	52	TMGVFTVYDTLFISG	VFTVYDTLF	3	0.4710	305.9	7.50	0.40	tr_D0A1S7_	WB
HLA-DQA10501-DQB10201	53	SHLYHMTPNLLDNIF	LYHMTPNLL	2	0.4666	320.9	8.00	0.57	tr_D0A1S7_	WB
HLA-DQA10501-DQB10201	54	LYREAMFFAGGPLIL	FFAGGPLIL	6	0.4665	321.3	8.00	0.61	tr_D0A1S7_	WB
HLA-DQA10501-DQB10201	55	SKVDVLFRVRDEFAN	LFRVRDEFA	5	0.4654	325.0	8.00	0.54	tr_D0A1S7_	WB
HLA-DQA10501-DQB10201	56	VIVVHTVFLCELLWF	VHTVFLCEL	3	0.4612	340.2	8.50	0.51	tr_D0A1S7_	WB
HLA-DQA10501-DQB10201	57	TVIVVHTVFLCELLW	VHTVFLCEL	4	0.4609	341.4	8.50	0.54	tr_D0A1S7_	WB
HLA-DQA10501-DQB10201	58	LSHLYHMTPNLLDNI	LYHMTPNLL	3	0.4604	343.3	8.50	0.66	tr_D0A1S7_	WB
HLA-DQA10501-DQB10201	59	MGVFTVYDTLFISGF	VFTVYDTLF	2	0.4599	345.1	8.50	0.37	tr_D0A1S7_	WB
HLA-DQA10501-DQB10201	60	IVVHTVFLCELLWFP	VHTVFLCEL	2	0.4590	348.4	9.00	0.47	tr_D0A1S7_	WB
HLA-DQA10501-DQB10201	61	WADAFEFFLQLAFGV	WADAFEFFL	0	0.4578	352.9	9.00	0.28	tr_D0A1S7_	WB
HLA-DQA10501-DQB10201	62	DVLFRVRDEFANRML	LFRVRDEFA	2	0.4577	353.2	9.00	0.41	tr_D0A1S7_	WB
HLA-DQA10501-DQB10201	63	PPRAISSEEMTLLKG	PRAISSEEM	1	0.4575	354.1	9.00	0.71	tr_D0A1S7_	WB
HLA-DQA10501-DQB10201	64	VLSHLYHMTPNLLDN	LYHMTPNLL	4	0.4573	354.8	9.00	0.69	tr_D0A1S7_	WB
HLA-DQA10501-DQB10201	65	ERYELVDRYGEELAQ	LVDRYGEEL	4	0.4551	363.6	9.00	0.44	tr_D0A1S7_	WB
HLA-DQA10501-DQB10201	66	HLYHMTPNLLDNIFM	LYHMTPNLL	1	0.4537	368.9	9.50	0.52	tr_D0A1S7_	WB
HLA-DQA10501-DQB10201	67	LVDRYGEELAQLRSL	RYGEELAQL	3	0.4536	369.4	9.50	0.37	tr_D0A1S7_	WB
HLA-DQA10501-DQB10201	68	NEAVNWFDVVSTRFA	WFDVVSTRF	5	0.4531	371.4	9.50	0.52	tr_D0A1S7_	WB
HLA-DQA10501-DQB10201	69	VKRSYVDLYREAMFF	DLYREAMFF	6	0.4527	373.1	9.50	0.40	tr_D0A1S7_	WB
HLA-DQA10501-DQB10201	70	TRRLSYLNFICTEFG	YLNFICTEF	5	0.4493	387.0	9.50	0.25	tr_D0A1S7_	WB
HLA-DQA10501-DQB10201	71	PTRRLSYLNFICTEF	YLNFICTEF	6	0.4466	398.6	10.00	0.34	tr_D0A1S7_	
HLA-DQA10501-DQB10201	72	RLTVIVVHTVFLCEL	VHTVFLCEL	6	0.4452	404.6	10.00	0.74	tr_D0A1S7_	
HLA-DQA10501-DQB10201	73	KVDVLFRVRDEFANR	LFRVRDEFA	4	0.4446	407.1	10.00	0.54	tr_D0A1S7_	
HLA-DQA10501-DQB10201	74	RAYINFDLVPTVNSQ	RAYINFDLV	0	0.4444	408.1	10.00	0.23	tr_D0A1S7_	
HLA-DQA10501-DQB10201	75	YREAMFFAGGPLILH	FFAGGPLIL	5	0.4441	409.6	11.00	0.59	tr_D0A1S7_	
HLA-DQA10501-DQB10201	76	IYVLSHLYHMTPNLL	LYHMTPNLL	6	0.4440	409.8	11.00	0.74	tr_D0A1S7_	
HLA-DQA10501-DQB10201	77	VVHTVFLCELLWFPW	VHTVFLCEL	1	0.4438	410.9	11.00	0.41	tr_D0A1S7_	
HLA-DQA10501-DQB10201	78	RRLSYLNFICTEFGL	YLNFICTEF	4	0.4417	420.0	11.00	0.25	tr_D0A1S7_	
HLA-DQA10501-DQB10201	79	GSERYELVDRYGEEL	LVDRYGEEL	6	0.4399	428.6	11.00	0.49	tr_D0A1S7_	
HLA-DQA10501-DQB10201	80	RLSYLNFICTEFGLL	YLNFICTEF	3	0.4397	429.2	11.00	0.25	tr_D0A1S7_	
HLA-DQA10501-DQB10201	81	LSYLNFICTEFGLLL	LNFICTEFG	3	0.4395	430.4	11.00	0.28	tr_D0A1S7_	
HLA-DQA10501-DQB10201	82	LTVIVVHTVFLCELL	VHTVFLCEL	5	0.4383	435.7	11.00	0.64	tr_D0A1S7_	
HLA-DQA10501-DQB10201	83	SYLNFICTEFGLLLD	LNFICTEFG	2	0.4359	447.5	11.00	0.24	tr_D0A1S7_	
HLA-DQA10501-DQB10201	84	TDSEVKVSFDLPMLS	EVKVSFDLP	3	0.4351	451.1	12.00	0.31	tr_D0A1S7_	
HLA-DQA10501-DQB10201	85	YLNFICTEFGLLLDD	TEFGLLLDD	6	0.4336	458.9	12.00	0.25	tr_D0A1S7_	
HLA-DQA10501-DQB10201	86	REAMFFAGGPLILHC	FFAGGPLIL	4	0.4330	461.8	12.00	0.61	tr_D0A1S7_	
HLA-DQA10501-DQB10201	87	FDLVPTVNSQEVAFV	VNSQEVAFV	6	0.4319	467.2	12.00	0.34	tr_D0A1S7_	

HLA-DQA10501-DQB10201	88	EAMFFAGGPLILHCL	FFAGGPLIL	3	0.4288	483.0	12.00	0.59	tr_D0A1S7_
HLA-DQA10501-DQB10201	89	GVFTVYDTLFISGFR	VFTVYDTLF	1	0.4288	483.1	12.00	0.38	tr_D0A1S7_
HLA-DQA10501-DQB10201	90	KRSYVDLYREAMFFA	DLYREAMFF	5	0.4266	494.7	12.00	0.34	tr_D0A1S7_
HLA-DQA10501-DQB10201	91	GVDNEAVNWFDVVST	VNWFDVVST	6	0.4255	500.8	13.00	0.31	tr_D0A1S7_
HLA-DQA10501-DQB10201	92	VLIDVESELHYSILG	LIDVESELH	1	0.4231	513.6	13.00	0.37	tr_D0A1S7_
HLA-DQA10501-DQB10201	93	CTDSEVKVSFDLPML	EVKVSFDLP	4	0.4231	513.8	13.00	0.38	tr_D0A1S7_
HLA-DQA10501-DQB10201	94	DSEVKVSFDLPMLSA	EVKVSFDLP	2	0.4223	518.3	13.00	0.31	tr_D0A1S7_
HLA-DQA10501-DQB10201	95	TVNSQEVAFVAVTRE	VAFVAVTRE	6	0.4201	530.8	13.00	0.47	tr_D0A1S7_
HLA-DQA10501-DQB10201	96	EAVNWFDVVSTRFAK	WFDVVSTRF	4	0.4191	536.8	13.00	0.50	tr_D0A1S7_
HLA-DQA10501-DQB10201	97	VLFRVRDEFANRMLT	LFRVRDEFA	1	0.4189	537.9	13.00	0.41	tr_D0A1S7_
HLA-DQA10501-DQB10201	98	VFFRLYNIAFYAAQD	NIAFYAAQD	6	0.4184	540.6	13.00	0.39	tr_D0A1S7_
HLA-DQA10501-DQB10201	99	NSQEVAFVAVTREHP	VAFVAVTRE	4	0.4166	551.3	14.00	0.43	tr_D0A1S7_
HLA-DQA10501-DQB10201	100	QMYQSGRILCWADAF	RILCWADAF	6	0.4128	574.4	14.00	0.56	tr_D0A1S7_
HLA-DQA10501-DQB10201	101	LCELLWFPWEPPILS	WFPWEPPIL	5	0.4124	577.0	14.00	0.43	tr_D0A1S7_
HLA-DQA10501-DQB10201	102	CELLWFPWEPPILSV	WFPWEPPIL	4	0.4117	581.2	14.00	0.46	tr_D0A1S7_
HLA-DQA10501-DQB10201	103	ELLWFPWEPPILSVP	WFPWEPPIL	3	0.4112	584.3	14.00	0.53	tr_D0A1S7_
HLA-DQA10501-DQB10201	104	RCAQQRRAYINFDLV	RAYINFDLV	6	0.4103	590.3	14.00	0.69	tr_D0A1S7_
HLA-DQA10501-DQB10201	105	AMFFAGGPLILHCLQ	FFAGGPLIL	2	0.4094	595.6	15.00	0.59	tr_D0A1S7_
HLA-DQA10501-DQB10201	106	VHTVFLCELLWFPWE	VHTVFLCEL	0	0.4092	597.2	15.00	0.31	tr_D0A1S7_
HLA-DQA10501-DQB10201	107	LKGVDNEAVNWFDVV	EAVNWFDVV	6	0.4071	611.1	15.00	0.37	tr_D0A1S7_
HLA-DQA10501-DQB10201	108	NFDLVPTVNSQEVAF	VPTVNSQEV	4	0.4060	618.5	15.00	0.41	tr_D0A1S7_
HLA-DQA10501-DQB10201	109	MVKRSYVDLYREAMF	YVDLYREAM	5	0.4016	648.2	16.00	0.31	tr_D0A1S7_
HLA-DQA10501-DQB10201	110	RCTDSEVKVSFDLPM	EVKVSFDLP	5	0.3998	661.4	16.00	0.50	tr_D0A1S7_
HLA-DQA10501-DQB10201	111	SQEVAFVAVTREHPR	VAFVAVTRE	3	0.3996	662.3	16.00	0.41	tr_D0A1S7_
HLA-DQA10501-DQB10201	112	FLCELLWFPWEPPIL	WFPWEPPIL	6	0.3993	665.1	16.00	0.46	tr_D0A1S7_
HLA-DQA10501-DQB10201	113	DLVPTVNSQEVAFVA	VNSQEVAFV	5	0.3971	680.6	16.00	0.31	tr_D0A1S7_
HLA-DQA10501-DQB10201	114	SEVKVSFDLPMLSAT	EVKVSFDLP	1	0.3961	687.9	16.00	0.30	tr_D0A1S7_
HLA-DQA10501-DQB10201	115	LPTRRLSYLNFICTE	SYLNFICTE	6	0.3950	696.4	17.00	0.39	tr_D0A1S7_
HLA-DQA10501-DQB10201	116	GSKEFSWFLQVMCRT	WFLQVMCRT	6	0.3945	699.9	17.00	0.34	tr_D0A1S7_
HLA-DQA10501-DQB10201	117	VDRYGEELAQLRSLV	RYGEELAQL	2	0.3944	700.9	17.00	0.41	tr_D0A1S7_
HLA-DQA10501-DQB10201	118	LLWFPWEPPILSVPD	WFPWEPPIL	2	0.3942	702.5	17.00	0.54	tr_D0A1S7_
HLA-DQA10501-DQB10201	119	RSYVDLYREAMFFAG	DLYREAMFF	4	0.3936	706.7	17.00	0.32	tr_D0A1S7_
HLA-DQA10501-DQB10201	120	VDNEAVNWFDVVSTR	VNWFDVVST	5	0.3929	712.2	17.00	0.30	tr_D0A1S7_
HLA-DQA10501-DQB10201	121	SKEFSWFLQVMCRTL	WFLQVMCRT	5	0.3912	725.3	17.00	0.34	tr_D0A1S7_
HLA-DQA10501-DQB10201	122	PRAISSEEMTLLKGV	PRAISSEEM	0	0.3911	726.4	17.00	0.60	tr_D0A1S7_
HLA-DQA10501-DQB10201	123	KGVDNEAVNWFDVVS	EAVNWFDVV	5	0.3897	737.7	17.00	0.35	tr_D0A1S7_
HLA-DQA10501-DQB10201	124	AYINFDLVPTVNSQE	INFDLVPTV	2	0.3887	745.2	18.00	0.31	tr_D0A1S7_
HLA-DQA10501-DQB10201	125	ADAFEFFLQLAFGVI	EFFLQLAFG	4	0.3886	746.3	18.00	0.28	tr_D0A1S7_
HLA-DQA10501-DQB10201	126	YAAQDIYYLIQAFNG	DIYYLIQAF	4	0.3884	747.7	18.00	0.34	tr_D0A1S7_
HLA-DQA10501-DQB10201	127	LYHMTPNLLDNIFMK	LYHMTPNLL	0	0.3878	752.8	18.00	0.46	tr_D0A1S7_
HLA-DQA10501-DQB10201	128	LVPTVNSQEVAFVAV	VNSQEVAFV	4	0.3870	759.0	18.00	0.31	tr_D0A1S7_
HLA-DQA10501-DQB10201	129	APSKVDVLFRVRDEF	VLFRVRDEF	6	0.3865	763.2	18.00	0.66	tr_D0A1S7_
HLA-DQA10501-DQB10201	130	QEVAFVAVTREHPRA	VAFVAVTRE	2	0.3863	765.1	18.00	0.43	tr_D0A1S7_
HLA-DQA10501-DQB10201	131	VFTVYDTLFISGFRV	VFTVYDTLF	0	0.3853	773.5	18.00	0.38	tr_D0A1S7_
HLA-DQA10501-DQB10201	132	DRYGEELAQLRSLVF	RYGEELAQL	1	0.3846	779.5	18.00	0.34	tr_D0A1S7_
HLA-DQA10501-DQB10201	133	DAFEFFLQLAFGVIY	EFFLQLAFG	3	0.3828	794.3	18.00	0.26	tr_D0A1S7_
HLA-DQA10501-DQB10201	134	YINFDLVPTVNSQEV	VPTVNSQEV	6	0.3818	803.3	19.00	0.43	tr_D0A1S7_
HLA-DQA10501-DQB10201	135	AVNWFDVVSTRFAKQ	WFDVVSTRF	3	0.3814	807.1	19.00	0.55	tr_D0A1S7_
HLA-DQA10501-DQB10201	136	MFFAGGPLILHCLQM	FFAGGPLIL	1	0.3810	810.0	19.00	0.61	tr_D0A1S7_
HLA-DQA10501-DQB10201	137	VNSQEVAFVAVTREH	VAFVAVTRE	5	0.3795	824.0	19.00	0.47	tr_D0A1S7_

HLA-DQA10501-DQB10201	138	AAQDIYYLIQAFNGD	DIYYLIQAF	3	0.3774	842.6	19.00	0.34	tr_D0A1S7_
HLA-DQA10501-DQB10201	139	KEFSWFLQVMCRTLT	WFLQVMCRT	4	0.3734	880.0	20.00	0.34	tr_D0A1S7_
HLA-DQA10501-DQB10201	140	LLAKGSERYELVDRY	ERYELVDRY	6	0.3732	881.6	20.00	0.67	tr_D0A1S7_
HLA-DQA10501-DQB10201	141	INFDLVPTVNSQEVA	VPTVNSQEV	5	0.3720	893.2	20.00	0.46	tr_D0A1S7_
HLA-DQA10501-DQB10201	142	EVKVSFDLPMLSATV	VSFDLPMLS	3	0.3716	896.7	20.00	0.27	tr_D0A1S7_
HLA-DQA10501-DQB10201	143	CRTLTMGVFTVYDTL	GVFTVYDTL	6	0.3696	916.6	21.00	0.43	tr_D0A1S7_
HLA-DQA10501-DQB10201	144	AFEFFLQLAFGVIYV	EFFLQLAFG	2	0.3692	921.1	21.00	0.26	tr_D0A1S7_
HLA-DQA10501-DQB10201	145	GMVKRSYVDLYREAM	YVDLYREAM	6	0.3689	923.2	21.00	0.31	tr_D0A1S7_
HLA-DQA10501-DQB10201	146	TLLKGVDNEAVNWFD	VDNEAVNWF	5	0.3670	943.3	21.00	0.31	tr_D0A1S7_
HLA-DQA10501-DQB10201	147	EVAFVAVTREHPRAP	VAFVAVTRE	1	0.3668	945.0	21.00	0.44	tr_D0A1S7_
HLA-DQA10501-DQB10201	148	LWFPWEPPILSVPDR	WFPWEPPIL	1	0.3644	969.6	22.00	0.57	tr_D0A1S7_
HLA-DQA10501-DQB10201	149	VYDTLFISGFRVIVL	ISGFRVIVL	6	0.3642	972.0	22.00	0.51	tr_D0A1S7_
HLA-DQA10501-DQB10201	150	LLKGVDNEAVNWFDV	VDNEAVNWF	4	0.3641	973.3	22.00	0.28	tr_D0A1S7_
HLA-DQA10501-DQB10201	151	SLVFFRLYNIAFYAA	FRLYNIAFY	4	0.3617	999.0	22.00	0.40	tr_D0A1S7_
HLA-DQA10501-DQB10201	152	RPPLEHILGVRLTDA	ILGVRLTDA	6	0.3614	1002.2	22.00	0.58	tr_D0A1S7_
HLA-DQA10501-DQB10201	153	AQDIYYLIQAFNGDA	DIYYLIQAF	2	0.3609	1007.3	22.00	0.37	tr_D0A1S7_
HLA-DQA10501-DQB10201	154	FEFFLQLAFGVIYVL	FFLQLAFGV	2	0.3593	1024.6	22.00	0.25	tr_D0A1S7_
HLA-DQA10501-DQB10201	155	PTVNSQEVAFVAVTR	EVAFVAVTR	6	0.3591	1027.0	23.00	0.37	tr_D0A1S7_
HLA-DQA10501-DQB10201	156	RSLVFFRLYNIAFYA	FRLYNIAFY	5	0.3586	1033.0	23.00	0.42	tr_D0A1S7_
HLA-DQA10501-DQB10201	157	EFSWFLQVMCRTLTM	WFLQVMCRT	3	0.3583	1035.9	23.00	0.33	tr_D0A1S7_
HLA-DQA10501-DQB10201	158	SYVDLYREAMFFAGG	DLYREAMFF	3	0.3575	1045.1	23.00	0.34	tr_D0A1S7_
HLA-DQA10501-DQB10201	159	LFRVRDEFANRMLTT	LFRVRDEFA	0	0.3551	1072.1	23.00	0.34	tr_D0A1S7_
HLA-DQA10501-DQB10201	160	LVFFRLYNIAFYAAQ	FRLYNIAFY	3	0.3540	1085.5	23.00	0.35	tr_D0A1S7_
HLA-DQA10501-DQB10201	161	WFPWEPPILSVPDRK	WFPWEPPIL	0	0.3534	1092.7	24.00	0.40	tr_D0A1S7_
HLA-DQA10501-DQB10201	162	VPTVNSQEVAFVAVT	VNSQEVAFV	3	0.3532	1094.8	24.00	0.36	tr_D0A1S7_
HLA-DQA10501-DQB10201	163	VNWFDVVSTRFAKQM	WFDVVSTRF	2	0.3524	1104.2	24.00	0.57	tr_D0A1S7_
HLA-DQA10501-DQB10201	164	PPLEHILGVRLTDAT	ILGVRLTDA	5	0.3507	1125.1	24.00	0.60	tr_D0A1S7_
HLA-DQA10501-DQB10201	165	RYGEELAQLRSLVFF	RYGEELAQL	0	0.3501	1132.5	24.00	0.21	tr_D0A1S7_
HLA-DQA10501-DQB10201	166	DLYREAMFFAGGPLI	MFFAGGPLI	6	0.3497	1136.9	24.00	0.41	tr_D0A1S7_
HLA-DQA10501-DQB10201	167	QDIYYLIQAFNGDAH	DIYYLIQAF	1	0.3492	1142.5	24.00	0.33	tr_D0A1S7_
HLA-DQA10501-DQB10201	168	MTLLKGVDNEAVNWF	VDNEAVNWF	6	0.3484	1152.5	25.00	0.31	tr_D0A1S7_
HLA-DQA10501-DQB10201	169	EFFLQLAFGVIYVLS	AFGVIYVLS	6	0.3484	1153.5	25.00	0.23	tr_D0A1S7_
HLA-DQA10501-DQB10201	170	NGSKEFSWFLQVMCR	EFSWFLQVM	4	0.3475	1163.9	25.00	0.49	tr_D0A1S7_
HLA-DQA10501-DQB10201	171	YDTLFISGFRVIVLL	ISGFRVIVL	5	0.3473	1166.3	25.00	0.50	tr_D0A1S7_
HLA-DQA10501-DQB10201	172	LAKGSERYELVDRYG	ERYELVDRY	5	0.3466	1175.8	25.00	0.63	tr_D0A1S7_
HLA-DQA10501-DQB10201	173	LRSLVFFRLYNIAFY	FRLYNIAFY	6	0.3461	1181.7	25.00	0.55	tr_D0A1S7_
HLA-DQA10501-DQB10201	174	IIGMVKRSYVDLYRE	RSYVDLYRE	6	0.3459	1184.8	25.00	0.38	tr_D0A1S7_
HLA-DQA10501-DQB10201	175	HTVFLCELLWFPWEP	TVFLCELLW	1	0.3437	1213.6	26.00	0.22	tr_D0A1S7_
HLA-DQA10501-DQB10201	176	TVFLCELLWFPWEPP	ELLWFPWEP	5	0.3427	1226.5	26.00	0.19	tr_D0A1S7_
HLA-DQA10501-DQB10201	177	AKGSERYELVDRYGE	ERYELVDRY	4	0.3393	1272.3	26.00	0.60	tr_D0A1S7_
HLA-DQA10501-DQB10201	178	DIYYLIQAFNGDAHI	DIYYLIQAF	0	0.3386	1282.5	27.00	0.24	tr_D0A1S7_
HLA-DQA10501-DQB10201	179	YVDLYREAMFFAGGP	DLYREAMFF	2	0.3382	1287.1	27.00	0.32	tr_D0A1S7_
HLA-DQA10501-DQB10201	180	TLFISGFRVIVLLQV	ISGFRVIVL	3	0.3380	1290.5	27.00	0.42	tr_D0A1S7_
HLA-DQA10501-DQB10201	181	PLEHILGVRLTDATH	ILGVRLTDA	4	0.3346	1338.8	27.00	0.57	tr_D0A1S7_
HLA-DQA10501-DQB10201	182	LFISGFRVIVLLQVL	ISGFRVIVL	2	0.3345	1340.1	27.00	0.38	tr_D0A1S7_
HLA-DQA10501-DQB10201	183	INGSKEFSWFLQVMC	EFSWFLQVM	5	0.3316	1382.3	28.00	0.57	tr_D0A1S7_
HLA-DQA10501-DQB10201	184	VKVSFDLPMLSATVI	VSFDLPMLS	2	0.3310	1392.2	28.00	0.33	tr_D0A1S7_
HLA-DQA10501-DQB10201	185	AKSSAEEWEIIGMVK	EWEIIGMVK	6	0.3309	1393.8	28.00	0.40	tr_D0A1S7_
HLA-DQA10501-DQB10201	186	SCAKSSAEEWEIIGM	AKSSAEEWE	2	0.3302	1403.4	28.00	0.32	tr_D0A1S7_
HLA-DQA10501-DQB10201	187	IGMVKRSYVDLYREA	RSYVDLYRE	5	0.3293	1417.0	28.00	0.34	tr_D0A1S7_

HLA-DQA10501-DQB10201	188	KGSERYELVDRYGEE	ERYELVDRY	3	0.3280	1438.5	29.00	0.60	tr_D0A1S7_
HLA-DQA10501-DQB10201	189	LIDVESELHYSILGI	LIDVESELH	0	0.3273	1448.1	29.00	0.40	tr_D0A1S7_
HLA-DQA10501-DQB10201	190	LEHILGVRLTDATHS	ILGVRLTDA	3	0.3263	1465.1	29.00	0.54	tr_D0A1S7_
HLA-DQA10501-DQB10201	191	CVSCAKSSAEEWEII	AKSSAEEWE	4	0.3261	1468.0	29.00	0.40	tr_D0A1S7_
HLA-DQA10501-DQB10201	192	VFLCELLWFPWEPPI	ELLWFPWEP	4	0.3253	1479.9	29.00	0.19	tr_D0A1S7_
HLA-DQA10501-DQB10201	193	DTLFISGFRVIVLLQ	ISGFRVIVL	4	0.3239	1503.8	30.00	0.47	tr_D0A1S7_
HLA-DQA10501-DQB10201	194	CAKSSAEEWEIIGMV	AKSSAEEWE	1	0.3209	1552.6	30.00	0.28	tr_D0A1S7_
HLA-DQA10501-DQB10201	195	KLPTRRLSYLNFICT	RRLSYLNFI	4	0.3207	1555.9	30.00	0.52	tr_D0A1S7_
HLA-DQA10501-DQB10201	196	HINGSKEFSWFLQVM	EFSWFLQVM	6	0.3201	1566.8	30.00	0.69	tr_D0A1S7_
HLA-DQA10501-DQB10201	197	EFANRMLTTLRPPLE	LTTLRPPLE	6	0.3193	1580.0	31.00	0.33	tr_D0A1S7_
HLA-DQA10501-DQB10201	198	FISGFRVIVLLQVLF	ISGFRVIVL	1	0.3190	1584.6	31.00	0.34	tr_D0A1S7_
HLA-DQA10501-DQB10201	199	LDDLFAFSCRVPSVY	DLFAFSCRV	2	0.3168	1622.9	31.00	0.26	tr_D0A1S7_
HLA-DQA10501-DQB10201	200	RRCTDSEVKVSFDLP	EVKVSFDLP	6	0.3146	1661.8	32.00	0.64	tr_D0A1S7_
HLA-DQA10501-DQB10201	201	YCVSCAKSSAEEWEI	AKSSAEEWE	5	0.3145	1664.5	32.00	0.42	tr_D0A1S7_
HLA-DQA10501-DQB10201	202	VAFVAVTREHPRAPS	FVAVTREHP	2	0.3140	1672.7	32.00	0.43	tr_D0A1S7_
HLA-DQA10501-DQB10201	203	VHMMKIQHAHDVLLA	IQHAHDVLL	5	0.3136	1680.4	32.00	0.30	tr_D0A1S7_
HLA-DQA10501-DQB10201	204	EHILGVRLTDATHSE	ILGVRLTDA	2	0.3125	1699.9	32.00	0.56	tr_D0A1S7_
HLA-DQA10501-DQB10201	205	FFLQLAFGVIYVLSH	AFGVIYVLS	5	0.3120	1708.9	32.00	0.23	tr_D0A1S7_
HLA-DQA10501-DQB10201	206	VSCAKSSAEEWEIIG	CAKSSAEEW	2	0.3116	1716.3	32.00	0.39	tr_D0A1S7_
HLA-DQA10501-DQB10201	207	GLLLDDLFAFSCRVP	DLFAFSCRV	5	0.3115	1719.1	32.00	0.43	tr_D0A1S7_
HLA-DQA10501-DQB10201	208	HILGVRLTDATHSEI	ILGVRLTDA	1	0.3095	1755.8	33.00	0.49	tr_D0A1S7_
HLA-DQA10501-DQB10201	209	FFAGGPLILHCLQMY	FFAGGPLIL	0	0.3093	1761.2	33.00	0.61	tr_D0A1S7_
HLA-DQA10501-DQB10201	210	EMTLLKGVDNEAVNW	LLKGVDNEA	3	0.3089	1767.9	33.00	0.29	tr_D0A1S7_
HLA-DQA10501-DQB10201	211	KVSFDLPMLSATVIS	VSFDLPMLS	1	0.3069	1806.6	33.00	0.29	tr_D0A1S7_
HLA-DQA10501-DQB10201	212	IKLPTRRLSYLNFIC	RRLSYLNFI	5	0.3057	1830.3	34.00	0.63	tr_D0A1S7_
HLA-DQA10501-DQB10201	213	NWFDVVSTRFAKQMR	WFDVVSTRF	1	0.3056	1831.4	34.00	0.65	tr_D0A1S7_
HLA-DQA10501-DQB10201	214	FRVRDEFANRMLTTL	VRDEFANRM	2	0.3051	1842.9	34.00	0.41	tr_D0A1S7_
HLA-DQA10501-DQB10201	215	RVHMMKIQHAHDVLL	IQHAHDVLL	6	0.3029	1885.7	34.00	0.40	tr_D0A1S7_
HLA-DQA10501-DQB10201	216	VMCRTLTMGVFTVYD	TMGVFTVYD	6	0.3019	1907.0	35.00	0.60	tr_D0A1S7_
HLA-DQA10501-DQB10201	217	KSSAEEWEIIGMVKR	EWEIIGMVK	5	0.3013	1920.4	35.00	0.39	tr_D0A1S7_
HLA-DQA10501-DQB10201	218	MCRTLTMGVFTVYDT	TMGVFTVYD	5	0.3003	1941.2	35.00	0.47	tr_D0A1S7_
HLA-DQA10501-DQB10201	219	DDLFAFSCRVPSVYR	DLFAFSCRV	1	0.2998	1950.5	35.00	0.26	tr_D0A1S7_
HLA-DQA10501-DQB10201	220	DEFANRMLTTLRPPL	NRMLTTLRP	4	0.2996	1955.6	35.00	0.29	tr_D0A1S7_
HLA-DQA10501-DQB10201	221	VDLYREAMFFAGGPL	DLYREAMFF	1	0.2991	1965.4	35.00	0.28	tr_D0A1S7_
HLA-DQA10501-DQB10201	222	WEIIGMVKRSYVDLY	VKRSYVDLY	6	0.2987	1974.6	35.00	0.64	tr_D0A1S7_
HLA-DQA10501-DQB10201	223	FSWFLQVMCRTLTMG	WFLQVMCRT	2	0.2986	1976.6	35.00	0.43	tr_D0A1S7_
HLA-DQA10501-DQB10201	224	YIKLPTRRLSYLNFI	RRLSYLNFI	6	0.2978	1992.6	36.00	0.65	tr_D0A1S7_
HLA-DQA10501-DQB10201	225	FANRMLTTLRPPLEH	LTTLRPPLE	5	0.2966	2020.5	36.00	0.38	tr_D0A1S7_
HLA-DQA10501-DQB10201	226	FYCVSCAKSSAEEWE	CAKSSAEEW	5	0.2963	2026.1	36.00	0.46	tr_D0A1S7_
HLA-DQA10501-DQB10201	227	VSFDLPMLSATVIST	MLSATVIST	6	0.2955	2044.6	36.00	0.31	tr_D0A1S7_
HLA-DQA10501-DQB10201	228	YGEELAQLRSLVFFR	EELAQLRSL	2	0.2954	2044.9	36.00	0.28	tr_D0A1S7_
HLA-DQA10501-DQB10201	229	ISGFRVIVLLQVLFY	FRVIVLLQV	3	0.2950	2054.1	36.00	0.19	tr_D0A1S7_
HLA-DQA10501-DQB10201	230	SEEMTLLKGVDNEAV	LLKGVDNEA	5	0.2948	2060.2	36.00	0.31	tr_D0A1S7_
HLA-DQA10501-DQB10201	231	LLDDLFAFSCRVPSV	DLFAFSCRV	3	0.2943	2071.1	37.00	0.41	tr_D0A1S7_
HLA-DQA10501-DQB10201	232	EIIGMVKRSYVDLYR	VKRSYVDLY	5	0.2910	2145.7	37.00	0.55	tr_D0A1S7_
HLA-DQA10501-DQB10201	233	FDLPMLSATVISTIV	SATVISTIV	6	0.2909	2146.9	37.00	0.26	tr_D0A1S7_
HLA-DQA10501-DQB10201	234	DLFAFSCRVPSVYRL	FSCRVPSVY	4	0.2873	2233.0	38.00	0.27	tr_D0A1S7_
HLA-DQA10501-DQB10201	235	FLQLAFGVIYVLSHL	AFGVIYVLS	4	0.2872	2236.9	38.00	0.32	tr_D0A1S7_
HLA-DQA10501-DQB10201	236	LQLAFGVIYVLSHLY	AFGVIYVLS	3	0.2859	2267.9	39.00	0.26	tr_D0A1S7_
HLA-DQA10501-DQB10201	237	GEELAQLRSLVFFRL	LRSLVFFRL	6	0.2834	2329.5	39.00	0.25	tr_D0A1S7_

HLA-DQA10501-DQB10201	238	IYYLIQAFNGDAHIN	IQAFNGDAH	4	0.2832	2335.1	39.00	0.28	tr_D0A1S7_
HLA-DQA10501-DQB10201	239	SSAEEWEIIGMVKRS	EWEIIGMVK	4	0.2827	2346.9	39.00	0.39	tr_D0A1S7_
HLA-DQA10501-DQB10201	240	VRDEFANRMLTTLRP	NRMLTTLRP	6	0.2824	2354.3	40.00	0.30	tr_D0A1S7_
HLA-DQA10501-DQB10201	241	EEMTLLKGVDNEAVN	LLKGVDNEA	4	0.2822	2360.0	40.00	0.32	tr_D0A1S7_
HLA-DQA10501-DQB10201	242	SFDLPMLSATVISTI	MLSATVIST	5	0.2819	2368.7	40.00	0.34	tr_D0A1S7_
HLA-DQA10501-DQB10201	243	DLPMLSATVISTIVL	SATVISTIV	5	0.2817	2373.4	40.00	0.21	tr_D0A1S7_
HLA-DQA10501-DQB10201	244	DVESELHYSILGIPQ	HYSILGIPQ	6	0.2812	2385.0	40.00	0.35	tr_D0A1S7_
HLA-DQA10501-DQB10201	245	FTVYDTLFISGFRVI	FTVYDTLFI	0	0.2793	2435.3	40.00	0.32	tr_D0A1S7_
HLA-DQA10501-DQB10201	246	ANRMLTTLRPPLEHI	LTTLRPPLE	4	0.2783	2462.7	41.00	0.43	tr_D0A1S7_
HLA-DQA10501-DQB10201	247	PMLSATVISTIVLLY	SATVISTIV	3	0.2782	2464.9	41.00	0.19	tr_D0A1S7_
HLA-DQA10501-DQB10201	248	SAEEWEIIGMVKRSY	EWEIIGMVK	3	0.2776	2480.1	41.00	0.39	tr_D0A1S7_
HLA-DQA10501-DQB10201	249	LLLDDLFAFSCRVPS	DLFAFSCRV	4	0.2772	2492.1	41.00	0.44	tr_D0A1S7_
HLA-DQA10501-DQB10201	250	SELHYSILGIPQGSD	HYSILGIPQ	3	0.2763	2515.5	41.00	0.28	tr_D0A1S7_
HLA-DQA10501-DQB10201	251	TVYDTLFISGFRVIV	FISGFRVIV	6	0.2748	2556.0	41.00	0.31	tr_D0A1S7_
HLA-DQA10501-DQB10201	252	HMMKIQHAHDVLLAK	IQHAHDVLL	4	0.2739	2582.0	42.00	0.30	tr_D0A1S7_
HLA-DQA10501-DQB10201	253	EELAQLRSLVFFRLY	LAQLRSLVF	2	0.2734	2595.2	42.00	0.25	tr_D0A1S7_
HLA-DQA10501-DQB10201	254	ILGVRLTDATHSEIV	ILGVRLTDA	0	0.2726	2619.4	42.00	0.32	tr_D0A1S7_
HLA-DQA10501-DQB10201	255	SSEEMTLLKGVDNEA	LLKGVDNEA	6	0.2723	2626.1	42.00	0.29	tr_D0A1S7_
HLA-DQA10501-DQB10201	256	LPMLSATVISTIVLL	SATVISTIV	4	0.2720	2636.8	42.00	0.22	tr_D0A1S7_
HLA-DQA10501-DQB10201	257	VESELHYSILGIPQG	HYSILGIPQ	5	0.2719	2637.6	42.00	0.34	tr_D0A1S7_
HLA-DQA10501-DQB10201	258	CLQMYQSGRILCWAD	YQSGRILCW	4	0.2705	2678.1	43.00	0.32	tr_D0A1S7_
HLA-DQA10501-DQB10201	259	RDEFANRMLTTLRPP	NRMLTTLRP	5	0.2689	2724.0	43.00	0.33	tr_D0A1S7_
HLA-DQA10501-DQB10201	260	YHMTPNLLDNIFMKK	TPNLLDNIF	3	0.2681	2750.2	43.00	0.28	tr_D0A1S7_
HLA-DQA10501-DQB10201	261	FPWEPPILSVPDRKD	PILSVPDRK	5	0.2674	2770.8	43.00	0.56	tr_D0A1S7_
HLA-DQA10501-DQB10201	262	NRMLTTLRPPLEHIL	LTTLRPPLE	3	0.2669	2785.7	44.00	0.52	tr_D0A1S7_
HLA-DQA10501-DQB10201	263	ESELHYSILGIPQGS	HYSILGIPQ	4	0.2668	2787.3	44.00	0.28	tr_D0A1S7_
HLA-DQA10501-DQB10201	264	LQMYQSGRILCWADA	YQSGRILCW	3	0.2651	2840.8	44.00	0.29	tr_D0A1S7_
HLA-DQA10501-DQB10201	265	HCLQMYQSGRILCWA	YQSGRILCW	5	0.2640	2873.5	44.00	0.32	tr_D0A1S7_
HLA-DQA10501-DQB10201	266	MLSATVISTIVLLYH	SATVISTIV	2	0.2638	2879.2	44.00	0.26	tr_D0A1S7_
HLA-DQA10501-DQB10201	267	RVRDEFANRMLTTLR	VRDEFANRM	1	0.2638	2880.7	44.00	0.38	tr_D0A1S7_
HLA-DQA10501-DQB10201	268	WFDVVSTRFAKQMRS	WFDVVSTRF	0	0.2629	2909.6	45.00	0.64	tr_D0A1S7_
HLA-DQA10501-DQB10201	269	ELHYSILGIPQGSDA	HYSILGIPQ	2	0.2610	2968.6	45.00	0.26	tr_D0A1S7_
HLA-DQA10501-DQB10201	270	AEEWEIIGMVKRSYV	EWEIIGMVK	2	0.2607	2977.1	45.00	0.37	tr_D0A1S7_
HLA-DQA10501-DQB10201	271	IDVESELHYSILGIP	ELHYSILGI	5	0.2598	3006.0	46.00	0.41	tr_D0A1S7_
HLA-DQA10501-DQB10201	272	QLAFGVIYVLSHLYH	AFGVIYVLS	2	0.2587	3042.2	46.00	0.28	tr_D0A1S7_
HLA-DQA10501-DQB10201	273	MMKIQHAHDVLLAKG	KIQHAHDVL	2	0.2586	3046.4	46.00	0.30	tr_D0A1S7_
HLA-DQA10501-DQB10201	274	QLRSLVFFRLYNIAF	FFRLYNIAF	6	0.2579	3071.1	46.00	0.55	tr_D0A1S7_
HLA-DQA10501-DQB10201	275	YYLIQAFNGDAHING	IQAFNGDAH	3	0.2567	3110.6	46.00	0.28	tr_D0A1S7_
HLA-DQA10501-DQB10201	276	RAISSEEMTLLKGVD	AISSEEMTL	1	0.2550	3167.3	47.00	0.27	tr_D0A1S7_
HLA-DQA10501-DQB10201	277	SWFLQVMCRTLTMGV	WFLQVMCRT	1	0.2522	3264.6	48.00	0.47	tr_D0A1S7_
HLA-DQA10501-DQB10201	278	SGFRVIVLLQVLFYC	FRVIVLLQV	2	0.2520	3271.9	48.00	0.28	tr_D0A1S7_
HLA-DQA10501-DQB10201	279	RMLTTLRPPLEHILG	LTTLRPPLE	2	0.2511	3304.8	48.00	0.54	tr_D0A1S7_
HLA-DQA10501-DQB10201	280	LAFGVIYVLSHLYHM	AFGVIYVLS	1	0.2509	3312.4	48.00	0.27	tr_D0A1S7_
HLA-DQA10501-DQB10201	281	CRVHMMKIQHAHDVL	KIQHAHDVL	6	0.2480	3418.5	49.00	0.47	tr_D0A1S7_
HLA-DQA10501-DQB10201	282	EHPRAPSKVDVLFRV	RAPSKVDVL	3	0.2470	3453.8	49.00	0.32	tr_D0A1S7_
HLA-DQA10501-DQB10201	283	WFLQVMCRTLTMGVF	WFLQVMCRT	0	0.2466	3467.9	49.00	0.31	tr_D0A1S7_
HLA-DQA10501-DQB10201	284	RAPSKVDVLFRVRDE	DVLFRVRDE	6	0.2450	3528.2	50.00	0.34	tr_D0A1S7_
HLA-DQA10501-DQB10201	285	HRCAQQRRAYINFDL	RRAYINFDL	6	0.2446	3545.2	50.00	0.54	tr_D0A1S7_
HLA-DQA10501-DQB10201	286	LFAFSCRVPSVYRLT	FSCRVPSVY	3	0.2434	3592.4	50.00	0.37	tr_D0A1S7_
HLA-DQA10501-DQB10201	287	YRLTVIVVHTVFLCE	VVHTVFLCE	6	0.2425	3628.2	50.00	0.37	tr_D0A1S7_

HLA-DQA10501-DQB10201	288	ISSEEMTLLKGVDNE	TLLKGVDNE	6	0.2420	3645.3	50.00	0.29	tr_D0A1S7_
HLA-DQA10501-DQB10201	289	LSATVISTIVLLYHF	SATVISTIV	1	0.2399	3730.9	55.00	0.26	tr_D0A1S7_
HLA-DQA10501-DQB10201	290	EEWEIIGMVKRSYVD	EWEIIGMVK	1	0.2378	3815.0	55.00	0.38	tr_D0A1S7_
HLA-DQA10501-DQB10201	291	GFRVIVLLQVLFYCV	FRVIVLLQV	1	0.2367	3862.6	55.00	0.28	tr_D0A1S7_
HLA-DQA10501-DQB10201	292	AISSEEMTLLKGVDN	ISSEEMTLL	1	0.2365	3868.3	55.00	0.19	tr_D0A1S7_
HLA-DQA10501-DQB10201	293	MKIQHAHDVLLAKGS	KIQHAHDVL	1	0.2352	3924.3	55.00	0.32	tr_D0A1S7_
HLA-DQA10501-DQB10201	294	REHPRAPSKVDVLFR	RAPSKVDVL	4	0.2333	4006.4	55.00	0.38	tr_D0A1S7_
HLA-DQA10501-DQB10201	295	AFVAVTREHPRAPSK	FVAVTREHP	1	0.2328	4028.7	55.00	0.75	tr_D0A1S7_
HLA-DQA10501-DQB10201	296	AFSCRVPSVYRLTVI	PSVYRLTVI	6	0.2327	4033.6	55.00	0.32	tr_D0A1S7_
HLA-DQA10501-DQB10201	297	MAPHREVTGEPPRAI	HREVTGEPP	3	0.2322	4055.0	55.00	0.35	tr_D0A1S7_
HLA-DQA10501-DQB10201	298	LHCLQMYQSGRILCW	YQSGRILCW	6	0.2318	4071.2	55.00	0.38	tr_D0A1S7_
HLA-DQA10501-DQB10201	299	ELAQLRSLVFFRLYN	LAQLRSLVF	1	0.2318	4071.9	55.00	0.25	tr_D0A1S7_
HLA-DQA10501-DQB10201	300	LFYCVSCAKSSAEEW	CAKSSAEEW	6	0.2312	4099.0	55.00	0.60	tr_D0A1S7_
HLA-DQA10501-DQB10201	301	LRPPLEHILGVRLTD	LEHILGVRL	4	0.2311	4104.0	55.00	0.40	tr_D0A1S7_
HLA-DQA10501-DQB10201	302	FAFSCRVPSVYRLTV	FSCRVPSVY	2	0.2283	4228.6	55.00	0.38	tr_D0A1S7_
HLA-DQA10501-DQB10201	303	SVYRLTVIVVHTVFL	RLTVIVVHT	3	0.2282	4231.7	55.00	0.24	tr_D0A1S7_
HLA-DQA10501-DQB10201	304	RVPSVYRLTVIVVHT	RLTVIVVHT	6	0.2282	4234.3	55.00	0.35	tr_D0A1S7_
HLA-DQA10501-DQB10201	305	TREHPRAPSKVDVLF	RAPSKVDVL	5	0.2276	4261.1	55.00	0.49	tr_D0A1S7_
HLA-DQA10501-DQB10201	306	PSVYRLTVIVVHTVF	RLTVIVVHT	4	0.2268	4296.0	55.00	0.27	tr_D0A1S7_
HLA-DQA10501-DQB10201	307	APHREVTGEPPRAIS	HREVTGEPP	2	0.2262	4324.3	55.00	0.35	tr_D0A1S7_
HLA-DQA10501-DQB10201	308	PRAPSKVDVLFRVRD	RAPSKVDVL	1	0.2260	4336.5	55.00	0.30	tr_D0A1S7_
HLA-DQA10501-DQB10201	309	AFGVIYVLSHLYHMT	AFGVIYVLS	0	0.2252	4372.2	55.00	0.17	tr_D0A1S7_
HLA-DQA10501-DQB10201	310	LHYSILGIPQGSDAR	HYSILGIPQ	1	0.2251	4375.4	55.00	0.28	tr_D0A1S7_
HLA-DQA10501-DQB10201	311	VPSVYRLTVIVVHTV	RLTVIVVHT	5	0.2251	4378.8	55.00	0.31	tr_D0A1S7_
HLA-DQA10501-DQB10201	312	HPRAPSKVDVLFRVR	RAPSKVDVL	2	0.2249	4388.3	55.00	0.33	tr_D0A1S7_
HLA-DQA10501-DQB10201	313	DVLLAKGSERYELVD	KGSERYELV	5	0.2247	4394.5	55.00	0.32	tr_D0A1S7_
HLA-DQA10501-DQB10201	314	MLTTLRPPLEHILGV	LTTLRPPLE	1	0.2239	4436.4	60.00	0.55	tr_D0A1S7_
HLA-DQA10501-DQB10201	315	HMTPNLLDNIFMKKC	TPNLLDNIF	2	0.2225	4502.4	60.00	0.34	tr_D0A1S7_
HLA-DQA10501-DQB10201	316	PWEPPILSVPDRKDK	PILSVPDRK	4	0.2215	4551.5	60.00	0.66	tr_D0A1S7_
HLA-DQA10501-DQB10201	317	YLIQAFNGDAHINGS	IQAFNGDAH	2	0.2207	4592.0	60.00	0.31	tr_D0A1S7_
HLA-DQA10501-DQB10201	318	VLLAKGSERYELVDR	KGSERYELV	4	0.2204	4607.0	60.00	0.31	tr_D0A1S7_
HLA-DQA10501-DQB10201	319	EWEIIGMVKRSYVDL	EWEIIGMVK	0	0.2203	4611.6	60.00	0.22	tr_D0A1S7_
HLA-DQA10501-DQB10201	320	LGVRLTDATHSEIVS	LTDATHSEI	4	0.2193	4658.6	60.00	0.32	tr_D0A1S7_
HLA-DQA10501-DQB10201	321	HDVLLAKGSERYELV	KGSERYELV	6	0.2177	4741.3	60.00	0.31	tr_D0A1S7_
HLA-DQA10501-DQB10201	322	FLQVMCRTLTMGVFT	RTLTMGVFT	6	0.2171	4772.3	60.00	0.38	tr_D0A1S7_
HLA-DQA10501-DQB10201	323	SCRVPSVYRLTVIVV	VYRLTVIVV	6	0.2170	4778.9	60.00	0.34	tr_D0A1S7_
HLA-DQA10501-DQB10201	324	FRVIVLLQVLFYCVS	IVLLQVLFY	3	0.2163	4813.0	60.00	0.25	tr_D0A1S7_
HLA-DQA10501-DQB10201	325	VIYVLSHLYHMTPNL	HLYHMTPNL	6	0.2145	4908.5	60.00	0.32	tr_D0A1S7_
HLA-DQA10501-DQB10201	326	VYRLTVIVVHTVFLC	RLTVIVVHT	2	0.2129	4994.5	60.00	0.23	tr_D0A1S7_
HLA-DQA10501-DQB10201	327	KIQHAHDVLLAKGSE	IQHAHDVLL	1	0.2120	5044.8	60.00	0.28	tr_D0A1S7_
HLA-DQA10501-DQB10201	328	PHREVTGEPPRAISS	HREVTGEPP	1	0.2119	5049.9	60.00	0.33	tr_D0A1S7_
HLA-DQA10501-DQB10201	329	SATVISTIVLLYHFL	VISTIVLLY	3	0.2114	5078.4	60.00	0.28	tr_D0A1S7_
HLA-DQA10501-DQB10201	330	DMAPHREVTGEPPRA	HREVTGEPP	4	0.2104	5133.2	60.00	0.44	tr_D0A1S7_
HLA-DQA10501-DQB10201	331	IVLLQVLFYCVSCAK	LFYCVSCAK	6	0.2103	5135.6	60.00	0.35	tr_D0A1S7_
HLA-DQA10501-DQB10201	332	FGVIYVLSHLYHMTP	YVLSHLYHM	4	0.2087	5229.0	60.00	0.25	tr_D0A1S7_
HLA-DQA10501-DQB10201	333	HYSILGIPQGSDARA	YSILGIPQG	1	0.2074	5301.4	60.00	0.25	tr_D0A1S7_
HLA-DQA10501-DQB10201	334	QVMCRTLTMGVFTVY	RTLTMGVFT	4	0.2072	5315.4	60.00	0.32	tr_D0A1S7_
HLA-DQA10501-DQB10201	335	FSCRVPSVYRLTVIV	PSVYRLTVI	5	0.2064	5357.0	65.00	0.35	tr_D0A1S7_
HLA-DQA10501-DQB10201	336	LAQLRSLVFFRLYNI	LRSLVFFRL	3	0.2061	5375.2	65.00	0.27	tr_D0A1S7_
HLA-DQA10501-DQB10201	337	CRVPSVYRLTVIVVH	VYRLTVIVV	5	0.2058	5395.2	65.00	0.31	tr_D0A1S7_

HLA-DQA10501-DQB10201	338	REVTGEPPRAISSEE	PPRAISSEE	6	0.2056	5404.4	65.00	0.37	tr_D0A1S7_
HLA-DQA10501-DQB10201	339	WEPPILSVPDRKDKG	PILSVPDRK	3	0.2052	5430.8	65.00	0.67	tr_D0A1S7_
HLA-DQA10501-DQB10201	340	LQVMCRTLTMGVFTV	RTLTMGVFT	5	0.2036	5526.6	65.00	0.35	tr_D0A1S7_
HLA-DQA10501-DQB10201	341	VLLQVLFYCVSCAKS	LFYCVSCAK	5	0.2031	5555.1	65.00	0.36	tr_D0A1S7_
HLA-DQA10501-DQB10201	342	MTPNLLDNIFMKKCS	TPNLLDNIF	1	0.2015	5651.3	65.00	0.39	tr_D0A1S7_
HLA-DQA10501-DQB10201	343	GVRLTDATHSEIVSR	LTDATHSEI	3	0.2005	5709.9	65.00	0.26	tr_D0A1S7_
HLA-DQA10501-DQB10201	344	HREVTGEPPRAISSE	EVTGEPPRA	2	0.1985	5839.4	65.00	0.39	tr_D0A1S7_
HLA-DQA10501-DQB10201	345	AQLRSLVFFRLYNIA	LRSLVFFRL	2	0.1969	5939.9	65.00	0.31	tr_D0A1S7_
HLA-DQA10501-DQB10201	346	VIVLLQVLFYCVSCA	VLFYCVSCA	6	0.1967	5952.4	65.00	0.34	tr_D0A1S7_
HLA-DQA10501-DQB10201	347	HTDMAPHREVTGEPP	HREVTGEPP	6	0.1942	6118.6	65.00	0.53	tr_D0A1S7_
HLA-DQA10501-DQB10201	348	LLQVLFYCVSCAKSS	LFYCVSCAK	4	0.1935	6159.2	65.00	0.36	tr_D0A1S7_
HLA-DQA10501-DQB10201	349	HSEIVSRHARLWEEN	SRHARLWEE	5	0.1898	6411.8	65.00	0.20	tr_D0A1S7_
HLA-DQA10501-DQB10201	350	RVIVLLQVLFYCVSC	IVLLQVLFY	2	0.1877	6564.2	70.00	0.28	tr_D0A1S7_
HLA-DQA10501-DQB10201	351	DNIFMKKCSIPLAYI	FMKKCSIPL	3	0.1872	6595.0	70.00	0.32	tr_D0A1S7_
HLA-DQA10501-DQB10201	352	GSDARAIKGAYREAV	IKGAYREAV	6	0.1861	6674.0	70.00	0.44	tr_D0A1S7_
HLA-DQA10501-DQB10201	353	SCRVHMMKIQHAHDV	VHMMKIQHA	3	0.1859	6687.3	70.00	0.34	tr_D0A1S7_
HLA-DQA10501-DQB10201	354	FAGGPLILHCLQMYQ	LILHCLQMY	5	0.1856	6712.0	70.00	0.28	tr_D0A1S7_
HLA-DQA10501-DQB10201	355	LQVLFYCVSCAKSSA	LFYCVSCAK	3	0.1854	6728.9	70.00	0.36	tr_D0A1S7_
HLA-DQA10501-DQB10201	356	THSEIVSRHARLWEE	SRHARLWEE	6	0.1848	6769.8	70.00	0.26	tr_D0A1S7_
HLA-DQA10501-DQB10201	357	TLRPPLEHILGVRLT	LEHILGVRL	5	0.1847	6779.9	70.00	0.51	tr_D0A1S7_
HLA-DQA10501-DQB10201	358	VRLTDATHSEIVSRH	LTDATHSEI	2	0.1834	6872.0	70.00	0.28	tr_D0A1S7_
HLA-DQA10501-DQB10201	359	AHDVLLAKGSERYEL	LLAKGSERY	4	0.1821	6969.8	70.00	0.33	tr_D0A1S7_
HLA-DQA10501-DQB10201	360	LDNIFMKKCSIPLAY	FMKKCSIPL	4	0.1820	6982.2	70.00	0.35	tr_D0A1S7_
HLA-DQA10501-DQB10201	361	EPPILSVPDRKDKGK	PILSVPDRK	2	0.1815	7015.0	70.00	0.70	tr_D0A1S7_
HLA-DQA10501-DQB10201	362	SDARAIKGAYREAVR	IKGAYREAV	5	0.1810	7053.7	70.00	0.40	tr_D0A1S7_
HLA-DQA10501-DQB10201	363	QVLFYCVSCAKSSAE	LFYCVSCAK	2	0.1801	7119.7	70.00	0.36	tr_D0A1S7_
HLA-DQA10501-DQB10201	364	TTLRPPLEHILGVRL	LEHILGVRL	6	0.1794	7176.6	70.00	0.62	tr_D0A1S7_
HLA-DQA10501-DQB10201	365	GVIYVLSHLYHMTPN	YVLSHLYHM	3	0.1790	7206.7	70.00	0.29	tr_D0A1S7_
HLA-DQA10501-DQB10201	366	LILHCLQMYQSGRIL	LQMYQSGRI	5	0.1785	7244.2	70.00	0.32	tr_D0A1S7_
HLA-DQA10501-DQB10201	367	ARAIKGAYREAVRRW	IKGAYREAV	3	0.1785	7251.5	70.00	0.34	tr_D0A1S7_
HLA-DQA10501-DQB10201	368	WRRRCTDSEVKVSFD	DSEVKVSFD	6	0.1777	7307.8	70.00	0.46	tr_D0A1S7_
HLA-DQA10501-DQB10201	369	AGGPLILHCLQMYQS	LILHCLQMY	4	0.1768	7381.1	70.00	0.24	tr_D0A1S7_
HLA-DQA10501-DQB10201	370	TDMAPHREVTGEPPR	HREVTGEPP	5	0.1764	7412.4	70.00	0.62	tr_D0A1S7_
HLA-DQA10501-DQB10201	371	FVAVTREHPRAPSKV	FVAVTREHP	0	0.1761	7434.9	70.00	0.74	tr_D0A1S7_
HLA-DQA10501-DQB10201	372	DSCRVHMMKIQHAHD	VHMMKIQHA	4	0.1758	7462.5	70.00	0.41	tr_D0A1S7_
HLA-DQA10501-DQB10201	373	ILHCLQMYQSGRILC	QMYQSGRIL	5	0.1757	7468.0	70.00	0.30	tr_D0A1S7_
HLA-DQA10501-DQB10201	374	LIQAFNGDAHINGSK	IQAFNGDAH	1	0.1752	7513.0	70.00	0.38	tr_D0A1S7_
HLA-DQA10501-DQB10201	375	GDAHINGSKEFSWFL	INGSKEFSW	4	0.1733	7663.2	70.00	0.29	tr_D0A1S7_
HLA-DQA10501-DQB10201	376	YSILGIPQGSDARAI	SILGIPQGS	1	0.1730	7691.3	70.00	0.25	tr_D0A1S7_
HLA-DQA10501-DQB10201	377	SEIVSRHARLWEENK	SRHARLWEE	4	0.1724	7746.4	70.00	0.20	tr_D0A1S7_
HLA-DQA10501-DQB10201	378	RRRCTDSEVKVSFDL	DSEVKVSFD	5	0.1706	7897.7	75.00	0.50	tr_D0A1S7_
HLA-DQA10501-DQB10201	379	IQHAHDVLLAKGSER	QHAHDVLLA	1	0.1701	7936.2	75.00	0.25	tr_D0A1S7_
HLA-DQA10501-DQB10201	380	NGDAHINGSKEFSWF	INGSKEFSW	5	0.1701	7938.7	75.00	0.32	tr_D0A1S7_
HLA-DQA10501-DQB10201	381	RLTDATHSEIVSRHA	LTDATHSEI	1	0.1686	8069.1	75.00	0.23	tr_D0A1S7_
HLA-DQA10501-DQB10201	382	NIFMKKCSIPLAYIK	FMKKCSIPL	2	0.1664	8261.0	75.00	0.34	tr_D0A1S7_
HLA-DQA10501-DQB10201	383	LTTLRPPLEHILGVR	LTTLRPPLE	0	0.1661	8285.6	75.00	0.51	tr_D0A1S7_
HLA-DQA10501-DQB10201	384	IHRCAQQRRAYINFD	QRRAYINFD	6	0.1660	8295.1	75.00	0.68	tr_D0A1S7_
HLA-DQA10501-DQB10201	385	DARAIKGAYREAVRR	IKGAYREAV	4	0.1654	8348.4	75.00	0.37	tr_D0A1S7_
HLA-DQA10501-DQB10201	386	VLFYCVSCAKSSAEE	LFYCVSCAK	1	0.1645	8433.9	75.00	0.37	tr_D0A1S7_
HLA-DQA10501-DQB10201	387	DAHINGSKEFSWFLQ	INGSKEFSW	3	0.1641	8474.0	75.00	0.28	tr_D0A1S7_

HLA-DQA10501-DQB10201	388	IFMKKCSIPLAYIKL	FMKKCSIPL	1	0.1627	8599.3	75.00	0.30	tr_D0A1S7_
HLA-DQA10501-DQB10201	389	FNGDAHINGSKEFSW	INGSKEFSW	6	0.1611	8748.7	75.00	0.31	tr_D0A1S7_
HLA-DQA10501-DQB10201	390	GGPLILHCLQMYQSG	LILHCLQMY	3	0.1609	8767.8	75.00	0.25	tr_D0A1S7_
HLA-DQA10501-DQB10201	391	AHINGSKEFSWFLQV	KEFSWFLQV	6	0.1606	8796.9	75.00	0.25	tr_D0A1S7_
HLA-DQA10501-DQB10201	392	TPNLLDNIFMKKCSI	PNLLDNIFM	1	0.1604	8813.5	75.00	0.41	tr_D0A1S7_
HLA-DQA10501-DQB10201	393	LLDNIFMKKCSIPLA	FMKKCSIPL	5	0.1596	8896.2	75.00	0.46	tr_D0A1S7_
HLA-DQA10501-DQB10201	394	QHAHDVLLAKGSERY	LLAKGSERY	6	0.1591	8936.5	75.00	0.28	tr_D0A1S7_
HLA-DQA10501-DQB10201	395	PPILSVPDRKDKGKN	PILSVPDRK	1	0.1591	8939.7	75.00	0.75	tr_D0A1S7_
HLA-DQA10501-DQB10201	396	ATHSEIVSRHARLWE	EIVSRHARL	4	0.1589	8958.0	75.00	0.33	tr_D0A1S7_
HLA-DQA10501-DQB10201	397	EIVSRHARLWEENKR	SRHARLWEE	3	0.1581	9042.4	75.00	0.19	tr_D0A1S7_
HLA-DQA10501-DQB10201	398	PLILHCLQMYQSGRI	LQMYQSGRI	6	0.1577	9074.8	75.00	0.43	tr_D0A1S7_
HLA-DQA10501-DQB10201	399	NCDSCRVHMMKIQHA	VHMMKIQHA	6	0.1565	9199.2	75.00	0.65	tr_D0A1S7_
HLA-DQA10501-DQB10201	400	RAIKGAYREAVRRWH	IKGAYREAV	2	0.1563	9212.2	75.00	0.39	tr_D0A1S7_
HLA-DQA10501-DQB10201	401	FMKKCSIPLAYIKLP	KKCSIPLAY	2	0.1557	9273.9	75.00	0.22	tr_D0A1S7_
HLA-DQA10501-DQB10201	402	HAHDVLLAKGSERYE	LLAKGSERY	5	0.1554	9309.2	75.00	0.35	tr_D0A1S7_
HLA-DQA10501-DQB10201	403	NPSDTWRRRCTDSEV	WRRRCTDSE	5	0.1551	9339.9	75.00	0.60	tr_D0A1S7_
HLA-DQA10501-DQB10201	404	IQAFNGDAHINGSKE	QAFNGDAHI	1	0.1519	9663.9	80.00	0.29	tr_D0A1S7_
HLA-DQA10501-DQB10201	405	AYIKLPTRRLSYLNF	TRRLSYLNF	6	0.1517	9682.5	80.00	0.62	tr_D0A1S7_
HLA-DQA10501-DQB10201	406	EEEGKHTDMAPHREV	HTDMAPHRE	5	0.1505	9817.5	80.00	0.53	tr_D0A1S7_
HLA-DQA10501-DQB10201	407	NLLDNIFMKKCSIPL	FMKKCSIPL	6	0.1497	9900.4	80.00	0.60	tr_D0A1S7_
HLA-DQA10501-DQB10201	408	TWRRRCTDSEVKVSF	WRRRCTDSE	1	0.1468	10214.3	80.00	0.38	tr_D0A1S7_
HLA-DQA10501-DQB10201	409	REEEGKHTDMAPHRE	HTDMAPHRE	6	0.1460	10303.2	80.00	0.57	tr_D0A1S7_
HLA-DQA10501-DQB10201	410	SDTWRRRCTDSEVKV	WRRRCTDSE	3	0.1447	10445.2	80.00	0.48	tr_D0A1S7_
HLA-DQA10501-DQB10201	411	AIKGAYREAVRRWHP	IKGAYREAV	1	0.1444	10480.8	80.00	0.36	tr_D0A1S7_
HLA-DQA10501-DQB10201	412	GPLILHCLQMYQSGR	LILHCLQMY	2	0.1442	10507.2	80.00	0.27	tr_D0A1S7_
HLA-DQA10501-DQB10201	413	DATHSEIVSRHARLW	EIVSRHARL	5	0.1438	10550.1	80.00	0.34	tr_D0A1S7_
HLA-DQA10501-DQB10201	414	TDATHSEIVSRHARL	EIVSRHARL	6	0.1430	10641.6	80.00	0.32	tr_D0A1S7_
HLA-DQA10501-DQB10201	415	PSDTWRRRCTDSEVK	WRRRCTDSE	4	0.1426	10693.7	80.00	0.51	tr_D0A1S7_
HLA-DQA10501-DQB10201	416	VTREHPRAPSKVDVL	RAPSKVDVL	6	0.1424	10712.0	80.00	0.74	tr_D0A1S7_
HLA-DQA10501-DQB10201	417	LTDATHSEIVSRHAR	TDATHSEIV	1	0.1411	10860.9	80.00	0.26	tr_D0A1S7_
HLA-DQA10501-DQB10201	418	EEGKHTDMAPHREVT	HTDMAPHRE	4	0.1408	10901.2	80.00	0.54	tr_D0A1S7_
HLA-DQA10501-DQB10201	419	CDSCRVHMMKIQHAH	VHMMKIQHA	5	0.1394	11064.5	80.00	0.61	tr_D0A1S7_
HLA-DQA10501-DQB10201	420	QAFNGDAHINGSKEF	AHINGSKEF	6	0.1382	11211.2	80.00	0.32	tr_D0A1S7_
HLA-DQA10501-DQB10201	421	DTWRRRCTDSEVKVS	WRRRCTDSE	2	0.1380	11238.3	80.00	0.50	tr_D0A1S7_
HLA-DQA10501-DQB10201	422	KHTDMAPHREVTGEP	HTDMAPHRE	1	0.1357	11514.9	80.00	0.41	tr_D0A1S7_
HLA-DQA10501-DQB10201	423	QGSDARAIKGAYREA	ARAIKGAYR	4	0.1342	11709.9	85.00	0.41	tr_D0A1S7_
HLA-DQA10501-DQB10201	424	KKCSIPLAYIKLPTR	LAYIKLPTR	6	0.1326	11909.8	85.00	0.36	tr_D0A1S7_
HLA-DQA10501-DQB10201	425	MKKCSIPLAYIKLPT	PLAYIKLPT	6	0.1326	11912.8	85.00	0.28	tr_D0A1S7_
HLA-DQA10501-DQB10201	426	PQGSDARAIKGAYRE	ARAIKGAYR	5	0.1294	12323.4	85.00	0.47	tr_D0A1S7_
HLA-DQA10501-DQB10201	427	IKGAYREAVRRWHPD	AYREAVRRW	3	0.1267	12696.0	85.00	0.35	tr_D0A1S7_
HLA-DQA10501-DQB10201	428	EGKHTDMAPHREVTG	HTDMAPHRE	3	0.1263	12750.4	85.00	0.55	tr_D0A1S7_
HLA-DQA10501-DQB10201	429	SILGIPQGSDARAIK	SILGIPQGS	0	0.1252	12897.3	85.00	0.26	tr_D0A1S7_
HLA-DQA10501-DQB10201	430	PILSVPDRKDKGKNV	PILSVPDRK	0	0.1252	12901.1	85.00	0.76	tr_D0A1S7_
HLA-DQA10501-DQB10201	431	CSIPLAYIKLPTRRL	LAYIKLPTR	4	0.1251	12922.5	85.00	0.36	tr_D0A1S7_
HLA-DQA10501-DQB10201	432	IVSRHARLWEENKRK	SRHARLWEE	2	0.1238	13092.7	85.00	0.29	tr_D0A1S7_
HLA-DQA10501-DQB10201	433	KNPSDTWRRRCTDSE	WRRRCTDSE	6	0.1222	13325.6	85.00	0.81	tr_D0A1S7_
HLA-DQA10501-DQB10201	434	FDVVSTRFAKQMRSA	FDVVSTRFA	0	0.1216	13409.7	85.00	0.34	tr_D0A1S7_
HLA-DQA10501-DQB10201	435	GKHTDMAPHREVTGE	HTDMAPHRE	2	0.1203	13597.3	85.00	0.52	tr_D0A1S7_
HLA-DQA10501-DQB10201	436	KCSIPLAYIKLPTRR	LAYIKLPTR	5	0.1203	13608.4	85.00	0.37	tr_D0A1S7_
HLA-DQA10501-DQB10201	437	AFNGDAHINGSKEFS	AHINGSKEF	5	0.1202	13620.4	85.00	0.34	tr_D0A1S7_

HLA-DQA10501-DQB10201	438	SIPLAYIKLPTRRLS	LAYIKLPTR	3	0.1174	14037.3	85.00	0.34	tr_D0A1S7_
HLA-DQA10501-DQB10201	439	PNLLDNIFMKKCSIP	PNLLDNIFM	0	0.1152	14377.8	85.00	0.37	tr_D0A1S7_
HLA-DQA10501-DQB10201	440	TRFAKQMRSAIHRCA	MRSAIHRCA	6	0.1107	15094.5	90.00	0.37	tr_D0A1S7_
HLA-DQA10501-DQB10201	441	IPLAYIKLPTRRLSY	LAYIKLPTR	2	0.1076	15606.3	90.00	0.37	tr_D0A1S7_
HLA-DQA10501-DQB10201	442	RFAKQMRSAIHRCAQ	MRSAIHRCA	5	0.1057	15926.5	90.00	0.35	tr_D0A1S7_
HLA-DQA10501-DQB10201	443	GAYREAVRRWHPDRN	AYREAVRRW	1	0.1051	16035.8	90.00	0.37	tr_D0A1S7_
HLA-DQA10501-DQB10201	444	IPQGSDARAIKGAYR	ARAIKGAYR	6	0.1045	16141.8	90.00	0.53	tr_D0A1S7_
HLA-DQA10501-DQB10201	445	DVVSTRFAKQMRSAI	TRFAKQMRS	4	0.1041	16205.3	90.00	0.29	tr_D0A1S7_
HLA-DQA10501-DQB10201	446	KGAYREAVRRWHPDR	AYREAVRRW	2	0.1023	16536.0	90.00	0.45	tr_D0A1S7_
HLA-DQA10501-DQB10201	447	PLAYIKLPTRRLSYL	LAYIKLPTR	1	0.1001	16931.2	90.00	0.37	tr_D0A1S7_
HLA-DQA10501-DQB10201	448	DRKDKGKNVHREEEG	KNVHREEEG	6	0.0998	16985.7	90.00	0.42	tr_D0A1S7_
HLA-DQA10501-DQB10201	449	AYREAVRRWHPDRNP	VRRWHPDRN	5	0.0993	17078.9	90.00	0.28	tr_D0A1S7_
HLA-DQA10501-DQB10201	450	VSRHARLWEENKRKN	SRHARLWEE	1	0.0982	17285.9	90.00	0.34	tr_D0A1S7_
HLA-DQA10501-DQB10201	451	ILGIPQGSDARAIKG	IPQGSDARA	3	0.0968	17543.6	90.00	0.25	tr_D0A1S7_
HLA-DQA10501-DQB10201	452	VVSTRFAKQMRSAIH	TRFAKQMRS	3	0.0947	17941.9	90.00	0.28	tr_D0A1S7_
HLA-DQA10501-DQB10201	453	FAKQMRSAIHRCAQQ	MRSAIHRCA	4	0.0945	17991.9	90.00	0.38	tr_D0A1S7_
HLA-DQA10501-DQB10201	454	KQMRSAIHRCAQQRR	MRSAIHRCA	2	0.0934	18202.4	90.00	0.32	tr_D0A1S7_
HLA-DQA10501-DQB10201	455	AKQMRSAIHRCAQQR	MRSAIHRCA	3	0.0920	18476.2	90.00	0.35	tr_D0A1S7_
HLA-DQA10501-DQB10201	456	LAYIKLPTRRLSYLN	YIKLPTRRL	2	0.0911	18650.3	95.00	0.25	tr_D0A1S7_
HLA-DQA10501-DQB10201	457	RKDKGKNVHREEEGK	GKNVHREEE	4	0.0911	18652.0	95.00	0.28	tr_D0A1S7_
HLA-DQA10501-DQB10201	458	QMRSAIHRCAQQRRA	MRSAIHRCA	1	0.0900	18883.0	95.00	0.25	tr_D0A1S7_
HLA-DQA10501-DQB10201	459	EAVRRWHPDRNPNCD	VRRWHPDRN	2	0.0866	19586.2	95.00	0.37	tr_D0A1S7_
HLA-DQA10501-DQB10201	460	STRFAKQMRSAIHRC	QMRSAIHRC	6	0.0865	19621.8	95.00	0.24	tr_D0A1S7_
HLA-DQA10501-DQB10201	461	KDKGKNVHREEEGKH	GKNVHREEE	3	0.0860	19722.9	95.00	0.28	tr_D0A1S7_
HLA-DQA10501-DQB10201	462	AIHRCAQQRRAYINF	QQRRAYINF	6	0.0853	19860.2	95.00	0.36	tr_D0A1S7_
HLA-DQA10501-DQB10201	463	GIPQGSDARAIKGAY	DARAIKGAY	6	0.0847	20002.3	95.00	0.25	tr_D0A1S7_
HLA-DQA10501-DQB10201	464	VSTRFAKQMRSAIHR	TRFAKQMRS	2	0.0836	20246.6	95.00	0.28	tr_D0A1S7_
HLA-DQA10501-DQB10201	465	NVHREEEGKHTDMAP	EGKHTDMAP	6	0.0833	20293.1	95.00	0.32	tr_D0A1S7_
HLA-DQA10501-DQB10201	466	DKGKNVHREEEGKHT	GKNVHREEE	2	0.0826	20450.3	95.00	0.28	tr_D0A1S7_
HLA-DQA10501-DQB10201	467	REAVRRWHPDRNPNC	VRRWHPDRN	3	0.0812	20771.4	95.00	0.41	tr_D0A1S7_
HLA-DQA10501-DQB10201	468	LGIPQGSDARAIKGA	IPQGSDARA	2	0.0809	20833.5	95.00	0.28	tr_D0A1S7_
HLA-DQA10501-DQB10201	469	YREAVRRWHPDRNPN	VRRWHPDRN	4	0.0807	20873.4	95.00	0.38	tr_D0A1S7_
HLA-DQA10501-DQB10201	470	HREEEGKHTDMAPHR	EGKHTDMAP	4	0.0802	20999.4	95.00	0.26	tr_D0A1S7_
HLA-DQA10501-DQB10201	471	PNCDSCRVHMMKIQH	RVHMMKIQH	6	0.0798	21092.8	95.00	0.40	tr_D0A1S7_
HLA-DQA10501-DQB10201	472	AVRRWHPDRNPNCDS	VRRWHPDRN	1	0.0793	21199.6	95.00	0.33	tr_D0A1S7_
HLA-DQA10501-DQB10201	473	MRSAIHRCAQQRRAY	AIHRCAQQR	3	0.0774	21637.8	95.00	0.28	tr_D0A1S7_
HLA-DQA10501-DQB10201	474	GKNVHREEEGKHTDM	EEEGKHTDM	6	0.0771	21720.8	95.00	0.21	tr_D0A1S7_
HLA-DQA10501-DQB10201	475	VHREEEGKHTDMAPH	EGKHTDMAP	5	0.0766	21822.6	95.00	0.32	tr_D0A1S7_
HLA-DQA10501-DQB10201	476	KGKNVHREEEGKHTD	GKNVHREEE	1	0.0762	21933.9	95.00	0.25	tr_D0A1S7_
HLA-DQA10501-DQB10201	477	SRHARLWEENKRKNP	SRHARLWEE	0	0.0744	22356.8	95.00	0.29	tr_D0A1S7_
HLA-DQA10501-DQB10201	478	PDRKDKGKNVHREEE	GKNVHREEE	6	0.0742	22401.8	95.00	0.58	tr_D0A1S7_
HLA-DQA10501-DQB10201	479	RSAIHRCAQQRRAYI	AIHRCAQQR	2	0.0722	22898.8	95.00	0.30	tr_D0A1S7_
HLA-DQA10501-DQB10201	480	SAIHRCAQQRRAYIN	AIHRCAQQR	1	0.0686	23793.4	95.00	0.31	tr_D0A1S7_
HLA-DQA10501-DQB10201	481	KNVHREEEGKHTDMA	EEGKHTDMA	6	0.0675	24083.0	95.00	0.23	tr_D0A1S7_
HLA-DQA10501-DQB10201	482	VRRWHPDRNPNCDSC	WHPDRNPNC	3	0.0674	24123.4	95.00	0.27	tr_D0A1S7_
HLA-DQA10501-DQB10201	483	NPNCDSCRVHMMKIQ	CRVHMMKIQ	6	0.0659	24499.0	95.00	0.23	tr_D0A1S7_
HLA-DQA10501-DQB10201	484	RNPNCDSCRVHMMKI	CDSCRVHMM	4	0.0592	26355.2	100.00	0.31	tr_D0A1S7_
HLA-DQA10501-DQB10201	485	RWHPDRNPNCDSCRV	NPNCDSCRV	6	0.0564	27158.8	100.00	0.34	tr_D0A1S7_
HLA-DQA10501-DQB10201	486	DRNPNCDSCRVHMMK	CDSCRVHMM	5	0.0547	27661.2	100.00	0.35	tr_D0A1S7_
HLA-DQA10501-DQB10201	487	VAVTREHPRAPSKVD	AVTREHPRA	1	0.0546	27698.3	100.00	0.37	tr_D0A1S7_

HLA-DQA10501-DQB10201	488	PDRNPNCDSCRVHMM	CDSCRVHMM	6	0.0541	27852.5	100.00	0.38	tr_D0A1S7_	
HLA-DQA10501-DQB10201	489	AVTREHPRAPSKVDV	AVTREHPRA	0	0.0532	28127.5	100.00	0.20	tr_D0A1S7_	
HLA-DQA10501-DQB10201	490	RRWHPDRNPNCDSCR	WHPDRNPNC	2	0.0524	28367.1	100.00	0.33	tr_D0A1S7_	
HLA-DQA10501-DQB10201	491	WHPDRNPNCDSCRVH	NPNCDSCRV	5	0.0510	28792.9	100.00	0.31	tr_D0A1S7_	
HLA-DQA10501-DQB10201	492	RHARLWEENKRKNPS	ARLWEENKR	2	0.0502	29060.8	100.00	0.38	tr_D0A1S7_	
HLA-DQA10501-DQB10201	493	RLWEENKRKNPSDTW	KRKNPSDTW	6	0.0466	30187.3	100.00	0.70	tr_D0A1S7_	
HLA-DQA10501-DQB10201	494	LWEENKRKNPSDTWR	KRKNPSDTW	5	0.0443	30949.9	100.00	0.64	tr_D0A1S7_	
HLA-DQA10501-DQB10201	495	HPDRNPNCDSCRVHM	NPNCDSCRV	4	0.0433	31313.7	100.00	0.37	tr_D0A1S7_	
HLA-DQA10501-DQB10201	496	WEENKRKNPSDTWRR	KRKNPSDTW	4	0.0404	32282.8	100.00	0.65	tr_D0A1S7_	
HLA-DQA10501-DQB10201	497	HARLWEENKRKNPSD	ARLWEENKR	1	0.0380	33130.0	100.00	0.46	tr_D0A1S7_	
HLA-DQA10501-DQB10201	498	VPDRKDKGKNVHREE	KGKNVHREE	6	0.0374	33356.6	100.00	0.62	tr_D0A1S7_	
HLA-DQA10501-DQB10201	499	EENKRKNPSDTWRRR	KRKNPSDTW	3	0.0371	33453.5	100.00	0.63	tr_D0A1S7_	
HLA-DQA10501-DQB10201	500	ENKRKNPSDTWRRRC	KRKNPSDTW	2	0.0364	33732.2	100.00	0.54	tr_D0A1S7_	
HLA-DQA10501-DQB10201	501	KRKNPSDTWRRRCTD	KRKNPSDTW	0	0.0334	34834.9	100.00	0.34	tr_D0A1S7_	
HLA-DQA10501-DQB10201	502	NKRKNPSDTWRRRCT	KRKNPSDTW	1	0.0322	35278.3	100.00	0.52	tr_D0A1S7_	
HLA-DQA10501-DQB10201	503	ILSVPDRKDKGKNVH	ILSVPDRKD	0	0.0299	36170.0	100.00	0.58	tr_D0A1S7_	
HLA-DQA10501-DQB10201	504	RKNPSDTWRRRCTDS	TWRRRCTDS	6	0.0288	36609.8	100.00	0.28	tr_D0A1S7_	
HLA-DQA10501-DQB10201	505	ARLWEENKRKNPSDT	ARLWEENKR	0	0.0287	36649.0	100.00	0.38	tr_D0A1S7_	
HLA-DQA10501-DQB10201	506	SVPDRKDKGKNVHRE	DKGKNVHRE	6	0.0176	41316.9	100.00	0.61	tr_D0A1S7_	
HLA-DQA10501-DQB10201	507	LSVPDRKDKGKNVHR	SVPDRKDKG	1	0.0099	44897.3	100.00	0.28	tr_D0A1S7_	

Allele: HLA-DQA10501-DQB10201. Number of high binders 10. Number of weak binders 60. Number of peptides 507

Allele	pos	peptide	core	Of	1-log50k(aff)	affinity(nM)	%Rank	Relia	Identity	Bind Level
HLA-DQA10501-DQB10201	1	DEHMPSQEDEELSEI	MPSQEDEEL	3	0.3638	975.9	22.00	0.46	tr_C9ZKG2_	
HLA-DQA10501-DQB10201	2	GLCDEHMPSQEDEEL	MPSQEDEEL	6	0.3519	1110.7	24.00	0.59	tr_C9ZKG2_	
HLA-DQA10501-DQB10201	3	CDEHMPSQEDEELSE	MPSQEDEEL	4	0.3499	1133.9	24.00	0.52	tr_C9ZKG2_	
HLA-DQA10501-DQB10201	4	LCDEHMPSQEDEELS	MPSQEDEEL	5	0.3488	1148.1	24.00	0.54	tr_C9ZKG2_	
HLA-DQA10501-DQB10201	5	EHMPSQEDEELSEIG	MPSQEDEEL	2	0.3303	1401.9	28.00	0.49	tr_C9ZKG2_	
HLA-DQA10501-DQB10201	6	HMPSQEDEELSEIGA	MPSQEDEEL	1	0.2993	1961.2	35.00	0.46	tr_C9ZKG2_	
HLA-DQA10501-DQB10201	7	SGYCFDKGLCDEHMP	DKGLCDEHM	5	0.2850	2290.3	39.00	0.59	tr_C9ZKG2_	
HLA-DQA10501-DQB10201	8	VSGYCFDKGLCDEHM	DKGLCDEHM	6	0.2741	2575.3	42.00	0.51	tr_C9ZKG2_	
HLA-DQA10501-DQB10201	9	GYCFDKGLCDEHMPS	DKGLCDEHM	4	0.2673	2771.7	43.00	0.61	tr_C9ZKG2_	
HLA-DQA10501-DQB10201	10	MHLCGDEREDDCSAY	MHLCGDERE	0	0.2531	3231.9	47.00	0.50	tr_C9ZKG2_	
HLA-DQA10501-DQB10201	11	MPSQEDEELSEIGAP	MPSQEDEEL	0	0.2483	3405.4	49.00	0.40	tr_C9ZKG2_	
HLA-DQA10501-DQB10201	12	YCFDKGLCDEHMPSQ	DKGLCDEHM	3	0.2452	3523.3	50.00	0.64	tr_C9ZKG2_	
HLA-DQA10501-DQB10201	13	KGLCDEHMPSQEDEE	HMPSQEDEE	6	0.2302	4141.8	55.00	0.56	tr_C9ZKG2_	
HLA-DQA10501-DQB10201	14	SQEDEELSEIGAPSS	LSEIGAPSS	6	0.2211	4569.3	60.00	0.35	tr_C9ZKG2_	
HLA-DQA10501-DQB10201	15	FDKGLCDEHMPSQED	DKGLCDEHM	1	0.2144	4915.2	60.00	0.54	tr_C9ZKG2_	
HLA-DQA10501-DQB10201	16	CFDKGLCDEHMPSQE	DKGLCDEHM	2	0.2126	5010.8	60.00	0.66	tr_C9ZKG2_	
HLA-DQA10501-DQB10201	17	GPVSGYCFDKGLCDE	VSGYCFDKG	2	0.2021	5616.5	65.00	0.31	tr_C9ZKG2_	
HLA-DQA10501-DQB10201	18	PSQEDEELSEIGAPS	QEDEELSEI	2	0.2004	5716.7	65.00	0.23	tr_C9ZKG2_	
HLA-DQA10501-DQB10201	19	PVSGYCFDKGLCDEH	VSGYCFDKG	1	0.1988	5821.7	65.00	0.29	tr_C9ZKG2_	
HLA-DQA10501-DQB10201	20	QEDEELSEIGAPSSG	LSEIGAPSS	5	0.1956	6022.0	65.00	0.38	tr_C9ZKG2_	
HLA-DQA10501-DQB10201	21	EDEELSEIGAPSSGV	LSEIGAPSS	4	0.1909	6338.4	65.00	0.38	tr_C9ZKG2_	
HLA-DQA10501-DQB10201	22	NGPVSGYCFDKGLCD	VSGYCFDKG	3	0.1907	6353.0	65.00	0.37	tr_C9ZKG2_	

```
 HLA-DQA10501-DQB10201   23      DKGLCDEHMPSQEDE  DKGLCDEHM  0         0.1892       6456.0    70.00  0.34    tr_C9ZKG2_
 HLA-DQA10501-DQB10201   24      DEELSEIGAPSSGVN  LSEIGAPSS  3         0.1776       7320.8    70.00  0.41    tr_C9ZKG2_
 HLA-DQA10501-DQB10201   25      EELSEIGAPSSGVNP  LSEIGAPSS  2         0.1628       8586.0    75.00  0.37    tr_C9ZKG2_
 HLA-DQA10501-DQB10201   26      YTGNGPVSGYCFDKG  VSGYCFDKG  6         0.1608       8772.9    75.00  0.44    tr_C9ZKG2_
 HLA-DQA10501-DQB10201   27      TGNGPVSGYCFDKGL  VSGYCFDKG  5         0.1602       8831.5    75.00  0.47    tr_C9ZKG2_
 HLA-DQA10501-DQB10201   28      HLCGDEREDDCSAYT  LCGDEREDD  1         0.1582       9031.8    75.00  0.23    tr_C9ZKG2_
 HLA-DQA10501-DQB10201   29      GNGPVSGYCFDKGLC  VSGYCFDKG  4         0.1572       9125.2    75.00  0.45    tr_C9ZKG2_
 HLA-DQA10501-DQB10201   30      SAYTGNGPVSGYCFD  YTGNGPVSG  2         0.1472      10173.4    80.00  0.28    tr_C9ZKG2_
 HLA-DQA10501-DQB10201   31      CSAYTGNGPVSGYCF  YTGNGPVSG  3         0.1384      11188.3    80.00  0.32    tr_C9ZKG2_
 HLA-DQA10501-DQB10201   32      ELSEIGAPSSGVNPA  LSEIGAPSS  1         0.1379      11244.5    80.00  0.35    tr_C9ZKG2_
 HLA-DQA10501-DQB10201   33      LCGDEREDDCSAYTG  CGDEREDDC  1         0.1344      11685.7    85.00  0.25    tr_C9ZKG2_
 HLA-DQA10501-DQB10201   34      AYTGNGPVSGYCFDK  YTGNGPVSG  1         0.1297      12289.6    85.00  0.28    tr_C9ZKG2_
 HLA-DQA10501-DQB10201   35      DDCSAYTGNGPVSGY  YTGNGPVSG  5         0.1219      13373.8    85.00  0.40    tr_C9ZKG2_
 HLA-DQA10501-DQB10201   36      DCSAYTGNGPVSGYC  YTGNGPVSG  4         0.1216      13418.4    85.00  0.41    tr_C9ZKG2_
 HLA-DQA10501-DQB10201   37      LSEIGAPSSGVNPAA  EIGAPSSGV  2         0.1176      14009.8    85.00  0.34    tr_C9ZKG2_
 HLA-DQA10501-DQB10201   38      EDDCSAYTGNGPVSG  YTGNGPVSG  6         0.1156      14321.1    85.00  0.39    tr_C9ZKG2_
 HLA-DQA10501-DQB10201   39      PSSGVNPAATSLPQR  PAATSLPQR  6         0.1154      14350.7    85.00  0.25    tr_C9ZKG2_
 HLA-DQA10501-DQB10201   40      SEIGAPSSGVNPAAT  SSGVNPAAT  6         0.1144      14498.0    85.00  0.34    tr_C9ZKG2_
 HLA-DQA10501-DQB10201   41      GAPSSGVNPAATSLP  SSGVNPAAT  3         0.1116      14955.0    90.00  0.33    tr_C9ZKG2_
 HLA-DQA10501-DQB10201   42      APSSGVNPAATSLPQ  SSGVNPAAT  2         0.1112      15007.0    90.00  0.31    tr_C9ZKG2_
 HLA-DQA10501-DQB10201   43      EIGAPSSGVNPAATS  SSGVNPAAT  5         0.1026      16481.8    90.00  0.35    tr_C9ZKG2_
 HLA-DQA10501-DQB10201   44      SSGVNPAATSLPQRK  PAATSLPQR  5         0.1023      16523.1    90.00  0.21    tr_C9ZKG2_
 HLA-DQA10501-DQB10201   45      CGDEREDDCSAYTGN  REDDCSAYT  4         0.1017      16634.1    90.00  0.25    tr_C9ZKG2_
 HLA-DQA10501-DQB10201   46      IGAPSSGVNPAATSL  SSGVNPAAT  4         0.0990      17130.0    90.00  0.37    tr_C9ZKG2_
 HLA-DQA10501-DQB10201   47      REDDCSAYTGNGPVS  AYTGNGPVS  6         0.0925      18379.1    90.00  0.46    tr_C9ZKG2_
 HLA-DQA10501-DQB10201   48      SGVNPAATSLPQRKK  PAATSLPQR  4         0.0852      19881.5    95.00  0.24    tr_C9ZKG2_
 HLA-DQA10501-DQB10201   49      GDEREDDCSAYTGNG  REDDCSAYT  3         0.0798      21081.3    95.00  0.34    tr_C9ZKG2_
 HLA-DQA10501-DQB10201   50      DEREDDCSAYTGNGP  REDDCSAYT  2         0.0777      21571.7    95.00  0.31    tr_C9ZKG2_
 HLA-DQA10501-DQB10201   51      GVNPAATSLPQRKKN  AATSLPQRK  4         0.0770      21723.9    95.00  0.25    tr_C9ZKG2_
 HLA-DQA10501-DQB10201   52      EREDDCSAYTGNGPV  SAYTGNGPV  6         0.0756      22058.8    95.00  0.25    tr_C9ZKG2_
 HLA-DQA10501-DQB10201   53      VNPAATSLPQRKKNL  AATSLPQRK  3         0.0687      23764.3    95.00  0.32    tr_C9ZKG2_
 HLA-DQA10501-DQB10201   54      NPAATSLPQRKKNLV  AATSLPQRK  2         0.0571      26950.7   100.00  0.45    tr_C9ZKG2_
-----------------------------------------------------------------------------------------------------------------------

Allele: HLA-DQA10501-DQB10201. Number of high binders 0. Number of weak binders 0. Number of peptides 54

-----------------------------------------------------------------------------------------------------------------------
-----------------------------------------------------------------------------------------------------------------------
                Allele  pos                peptide       core Of 1-log50k(aff) affinity(nM)    %Rank Relia     Identity Bind Level
-----------------------------------------------------------------------------------------------------------------------
 HLA-DQA10501-DQB10201    1      WTKFWLVTHRACIYL  LVTHRACIY  5         0.2566       3114.9    46.00  0.37    tr_C9ZKG2_
 HLA-DQA10501-DQB10201    2      GWTKFWLVTHRACIY  LVTHRACIY  6         0.2476       3432.0    49.00  0.36    tr_C9ZKG2_
 HLA-DQA10501-DQB10201    3      FWLVTHRACIYLRLP  LVTHRACIY  2         0.2416       3660.2    50.00  0.26    tr_C9ZKG2_
 HLA-DQA10501-DQB10201    4      KFWLVTHRACIYLRL  LVTHRACIY  3         0.2378       3815.9    55.00  0.29    tr_C9ZKG2_
 HLA-DQA10501-DQB10201    5      TKFWLVTHRACIYLR  LVTHRACIY  4         0.2351       3930.3    55.00  0.35    tr_C9ZKG2_
 HLA-DQA10501-DQB10201    6      LVTHRACIYLRLPRW  CIYLRLPRW  6         0.2293       4184.6    55.00  0.32    tr_C9ZKG2_
 HLA-DQA10501-DQB10201    7      VTHRACIYLRLPRWV  CIYLRLPRW  5         0.2272       4278.3    55.00  0.32    tr_C9ZKG2_
 HLA-DQA10501-DQB10201    8      WLVTHRACIYLRLPR  LVTHRACIY  1         0.2037       5520.4    65.00  0.28    tr_C9ZKG2_
 HLA-DQA10501-DQB10201    9      RRSPTRSDDNCDEPW  SDDNCDEPW  6         0.2032       5549.8    65.00  0.38    tr_C9ZKG2_
 HLA-DQA10501-DQB10201   10      THRACIYLRLPRWVR  CIYLRLPRW  4         0.2029       5568.0    65.00  0.31    tr_C9ZKG2_
```

HLA-DQA10501-DQB10201	11	FGWTKFWLVTHRACI	KFWLVTHRA	4	0.1989	5813.7	65.00	0.29	tr_C9ZKG2_
HLA-DQA10501-DQB10201	12	SKFGWTKFWLVTHRA	KFWLVTHRA	6	0.1951	6054.4	65.00	0.40	tr_C9ZKG2_
HLA-DQA10501-DQB10201	13	RSPTRSDDNCDEPWN	SDDNCDEPW	5	0.1947	6084.9	65.00	0.38	tr_C9ZKG2_
HLA-DQA10501-DQB10201	14	KFGWTKFWLVTHRAC	KFWLVTHRA	5	0.1863	6657.7	70.00	0.37	tr_C9ZKG2_
HLA-DQA10501-DQB10201	15	SPTRSDDNCDEPWNR	SDDNCDEPW	4	0.1822	6965.3	70.00	0.38	tr_C9ZKG2_
HLA-DQA10501-DQB10201	16	HRACIYLRLPRWVRR	CIYLRLPRW	3	0.1784	7254.7	70.00	0.35	tr_C9ZKG2_
HLA-DQA10501-DQB10201	17	GSKFGWTKFWLVTHR	FGWTKFWLV	3	0.1609	8763.6	75.00	0.28	tr_C9ZKG2_
HLA-DQA10501-DQB10201	18	LLRGSKFGWTKFWLV	FGWTKFWLV	6	0.1588	8965.5	75.00	0.48	tr_C9ZKG2_
HLA-DQA10501-DQB10201	19	RACIYLRLPRWVRRS	CIYLRLPRW	2	0.1584	9011.9	75.00	0.43	tr_C9ZKG2_
HLA-DQA10501-DQB10201	20	RGSKFGWTKFWLVTH	FGWTKFWLV	4	0.1583	9016.1	75.00	0.37	tr_C9ZKG2_
HLA-DQA10501-DQB10201	21	AYRLLQLLLRGSKFG	AYRLLQLLL	0	0.1508	9785.5	80.00	0.31	tr_C9ZKG2_
HLA-DQA10501-DQB10201	22	VRRSPTRSDDNCDEP	TRSDDNCDE	5	0.1491	9963.3	80.00	0.42	tr_C9ZKG2_
HLA-DQA10501-DQB10201	23	LRGSKFGWTKFWLVT	FGWTKFWLV	5	0.1483	10043.8	80.00	0.47	tr_C9ZKG2_
HLA-DQA10501-DQB10201	24	ACIYLRLPRWVRRSP	CIYLRLPRW	1	0.1454	10372.9	80.00	0.47	tr_C9ZKG2_
HLA-DQA10501-DQB10201	25	WVRRSPTRSDDNCDE	TRSDDNCDE	6	0.1321	11975.1	85.00	0.43	tr_C9ZKG2_
HLA-DQA10501-DQB10201	26	CIYLRLPRWVRRSPT	CIYLRLPRW	0	0.1306	12170.9	85.00	0.38	tr_C9ZKG2_
HLA-DQA10501-DQB10201	27	YRLLQLLLRGSKFGW	QLLLRGSKF	4	0.1214	13439.9	85.00	0.38	tr_C9ZKG2_
HLA-DQA10501-DQB10201	28	LLLRGSKFGWTKFWL	KFGWTKFWL	6	0.1191	13780.2	85.00	0.47	tr_C9ZKG2_
HLA-DQA10501-DQB10201	29	RLLQLLLRGSKFGWT	QLLLRGSKF	3	0.1088	15409.0	90.00	0.44	tr_C9ZKG2_
HLA-DQA10501-DQB10201	30	RWVRRSPTRSDDNCD	WVRRSPTRS	1	0.1020	16580.2	90.00	0.33	tr_C9ZKG2_
HLA-DQA10501-DQB10201	31	LLQLLLRGSKFGWTK	QLLLRGSKF	2	0.0984	17242.1	90.00	0.54	tr_C9ZKG2_
HLA-DQA10501-DQB10201	32	LQLLLRGSKFGWTKF	QLLLRGSKF	1	0.0974	17439.0	90.00	0.45	tr_C9ZKG2_
HLA-DQA10501-DQB10201	33	QLLLRGSKFGWTKFW	QLLLRGSKF	0	0.0955	17786.0	90.00	0.34	tr_C9ZKG2_
HLA-DQA10501-DQB10201	34	LPRWVRRSPTRSDDN	WVRRSPTRS	3	0.0914	18591.9	90.00	0.51	tr_C9ZKG2_
HLA-DQA10501-DQB10201	35	PRWVRRSPTRSDDNC	WVRRSPTRS	2	0.0903	18815.8	95.00	0.50	tr_C9ZKG2_
HLA-DQA10501-DQB10201	36	RLPRWVRRSPTRSDD	WVRRSPTRS	4	0.0879	19326.8	95.00	0.58	tr_C9ZKG2_
HLA-DQA10501-DQB10201	37	IYLRLPRWVRRSPTR	IYLRLPRWV	0	0.0858	19757.7	95.00	0.44	tr_C9ZKG2_
HLA-DQA10501-DQB10201	38	LRLPRWVRRSPTRSD	WVRRSPTRS	5	0.0829	20397.7	95.00	0.56	tr_C9ZKG2_
HLA-DQA10501-DQB10201	39	YLRLPRWVRRSPTRS	WVRRSPTRS	6	0.0750	22199.6	95.00	0.52	tr_C9ZKG2_

Allele: HLA-DQA10501-DQB10201. Number of high binders 0. Number of weak binders 0. Number of peptides 39

Allele	pos	peptide	core	Of	1-log50k(aff)	affinity(nM)	%Rank	Relia	Identity	Bind Level
HLA-DQA10501-DQB10201	1	FVDSLLHQVVSEDLL	LHQVVSEDL	5	0.5775	96.7	1.80	0.57	tr_C9ZY45_	WB
HLA-DQA10501-DQB10201	2	CFVDSLLHQVVSEDL	LHQVVSEDL	6	0.5628	113.3	2.50	0.74	tr_C9ZY45_	WB
HLA-DQA10501-DQB10201	3	DSLLHQVVSEDLLHF	LHQVVSEDL	3	0.5492	131.4	3.00	0.58	tr_C9ZY45_	WB
HLA-DQA10501-DQB10201	4	VDSLLHQVVSEDLLH	LHQVVSEDL	4	0.5443	138.5	3.00	0.58	tr_C9ZY45_	WB
HLA-DQA10501-DQB10201	5	SLLHQVVSEDLLHFV	LHQVVSEDL	2	0.5315	159.1	4.00	0.53	tr_C9ZY45_	WB
HLA-DQA10501-DQB10201	6	LLHQVVSEDLLHFVC	LHQVVSEDL	1	0.5044	213.3	5.50	0.51	tr_C9ZY45_	WB
HLA-DQA10501-DQB10201	7	DVNQTDVIGLTALQL	VIGLTALQL	6	0.4908	247.1	6.00	0.68	tr_C9ZY45_	WB
HLA-DQA10501-DQB10201	8	TFHESLGGDWLFPSV	HESLGGDWL	2	0.4843	265.0	6.50	0.41	tr_C9ZY45_	WB
HLA-DQA10501-DQB10201	9	NLLSFEGALVFADGL	GALVFADGL	6	0.4824	270.5	7.00	0.47	tr_C9ZY45_	WB
HLA-DQA10501-DQB10201	10	SFPLAHYLQHEEQND	AHYLQHEEQ	4	0.4812	274.1	7.00	0.69	tr_C9ZY45_	WB
HLA-DQA10501-DQB10201	11	FPLAHYLQHEEQNDV	AHYLQHEEQ	3	0.4773	285.7	7.00	0.69	tr_C9ZY45_	WB
HLA-DQA10501-DQB10201	12	VNQTDVIGLTALQLA	VIGLTALQL	5	0.4727	300.3	7.50	0.61	tr_C9ZY45_	WB
HLA-DQA10501-DQB10201	13	LLSFEGALVFADGLI	GALVFADGL	5	0.4702	308.6	8.00	0.49	tr_C9ZY45_	WB

HLA-DQA10501-DQB10201	14	FHESLGGDWLFPSVV	HESLGGDWL	1	0.4678	316.9	8.00	0.34	tr_C9ZY45_	WB
HLA-DQA10501-DQB10201	15	GRTFHESLGGDWLFP	HESLGGDWL	4	0.4653	325.5	8.00	0.49	tr_C9ZY45_	WB
HLA-DQA10501-DQB10201	16	GQSFPLAHYLQHEEQ	AHYLQHEEQ	6	0.4653	325.6	8.00	0.75	tr_C9ZY45_	WB
HLA-DQA10501-DQB10201	17	QSFPLAHYLQHEEQN	AHYLQHEEQ	5	0.4630	333.7	8.50	0.71	tr_C9ZY45_	WB
HLA-DQA10501-DQB10201	18	RTFHESLGGDWLFPS	HESLGGDWL	3	0.4628	334.6	8.50	0.46	tr_C9ZY45_	WB
HLA-DQA10501-DQB10201	19	LSFEGALVFADGLIV	GALVFADGL	4	0.4560	359.8	9.00	0.50	tr_C9ZY45_	WB
HLA-DQA10501-DQB10201	20	LHQVVSEDLLHFVCQ	LHQVVSEDL	0	0.4472	396.1	10.00	0.44	tr_C9ZY45_	
HLA-DQA10501-DQB10201	21	NQTDVIGLTALQLAA	VIGLTALQL	4	0.4471	396.4	10.00	0.62	tr_C9ZY45_	
HLA-DQA10501-DQB10201	22	PLAHYLQHEEQNDVP	AHYLQHEEQ	2	0.4426	416.1	11.00	0.64	tr_C9ZY45_	
HLA-DQA10501-DQB10201	23	CGRTFHESLGGDWLF	HESLGGDWL	5	0.4421	418.5	11.00	0.52	tr_C9ZY45_	
HLA-DQA10501-DQB10201	24	SFEGALVFADGLIVV	GALVFADGL	3	0.4416	420.7	11.00	0.50	tr_C9ZY45_	
HLA-DQA10501-DQB10201	25	LGREPFLGECSNEMI	FLGECSNEM	5	0.4338	457.8	12.00	0.62	tr_C9ZY45_	
HLA-DQA10501-DQB10201	26	GREPFLGECSNEMII	FLGECSNEM	4	0.4329	462.1	12.00	0.62	tr_C9ZY45_	
HLA-DQA10501-DQB10201	27	SQNLLSFEGALVFAD	LLSFEGALV	3	0.4283	485.5	12.00	0.40	tr_C9ZY45_	
HLA-DQA10501-DQB10201	28	PFSLQLLQCFVDSLL	LLQCFVDSL	5	0.4254	501.4	13.00	0.39	tr_C9ZY45_	
HLA-DQA10501-DQB10201	29	QTDVIGLTALQLAAN	VIGLTALQL	3	0.4250	503.1	13.00	0.61	tr_C9ZY45_	
HLA-DQA10501-DQB10201	30	SPSQNLLSFEGALVF	LLSFEGALV	5	0.4247	505.0	13.00	0.48	tr_C9ZY45_	
HLA-DQA10501-DQB10201	31	LAHYLQHEEQNDVPL	AHYLQHEEQ	1	0.4217	521.9	13.00	0.61	tr_C9ZY45_	
HLA-DQA10501-DQB10201	32	PSQNLLSFEGALVFA	LLSFEGALV	4	0.4190	537.2	13.00	0.40	tr_C9ZY45_	
HLA-DQA10501-DQB10201	33	FVILFLVEQLGMDVN	LVEQLGMDV	5	0.4177	544.6	14.00	0.28	tr_C9ZY45_	
HLA-DQA10501-DQB10201	34	FEGALVFADGLIVVH	GALVFADGL	2	0.4168	550.0	14.00	0.51	tr_C9ZY45_	
HLA-DQA10501-DQB10201	35	QNLLSFEGALVFADG	LLSFEGALV	2	0.4133	571.6	14.00	0.37	tr_C9ZY45_	
HLA-DQA10501-DQB10201	36	NFVILFLVEQLGMDV	LVEQLGMDV	6	0.4055	621.4	15.00	0.28	tr_C9ZY45_	
HLA-DQA10501-DQB10201	37	REPFLGECSNEMIIR	FLGECSNEM	3	0.4052	623.7	15.00	0.63	tr_C9ZY45_	
HLA-DQA10501-DQB10201	38	EPFLGECSNEMIIRF	FLGECSNEM	2	0.4034	635.6	15.00	0.61	tr_C9ZY45_	
HLA-DQA10501-DQB10201	39	QLLQCFVDSLLHQVV	FVDSLLHQV	5	0.4033	636.8	15.00	0.22	tr_C9ZY45_	
HLA-DQA10501-DQB10201	40	TDVIGLTALQLAANN	VIGLTALQL	2	0.4018	646.7	16.00	0.66	tr_C9ZY45_	
HLA-DQA10501-DQB10201	41	FSLQLLQCFVDSLLH	LLQCFVDSL	4	0.4010	653.0	16.00	0.41	tr_C9ZY45_	
HLA-DQA10501-DQB10201	42	EGALVFADGLIVVHA	GALVFADGL	1	0.3978	676.0	16.00	0.46	tr_C9ZY45_	
HLA-DQA10501-DQB10201	43	SPFSLQLLQCFVDSL	LLQCFVDSL	6	0.3970	681.7	16.00	0.44	tr_C9ZY45_	
HLA-DQA10501-DQB10201	44	VILFLVEQLGMDVNA	LVEQLGMDV	4	0.3969	682.5	16.00	0.32	tr_C9ZY45_	
HLA-DQA10501-DQB10201	45	HESLGGDWLFPSVVT	HESLGGDWL	0	0.3952	695.0	17.00	0.31	tr_C9ZY45_	
HLA-DQA10501-DQB10201	46	LCGRTFHESLGGDWL	HESLGGDWL	6	0.3938	705.3	17.00	0.57	tr_C9ZY45_	
HLA-DQA10501-DQB10201	47	NSPSQNLLSFEGALV	LLSFEGALV	6	0.3909	727.7	17.00	0.56	tr_C9ZY45_	
HLA-DQA10501-DQB10201	48	EMIIRFLIDNGADIN	FLIDNGADI	5	0.3894	739.6	17.00	0.34	tr_C9ZY45_	
HLA-DQA10501-DQB10201	49	NEMIIRFLIDNGADI	FLIDNGADI	6	0.3882	749.8	18.00	0.36	tr_C9ZY45_	
HLA-DQA10501-DQB10201	50	LQLLQCFVDSLLHQV	LLQCFVDSL	2	0.3876	754.2	18.00	0.35	tr_C9ZY45_	
HLA-DQA10501-DQB10201	51	PLHRAVLGGDVALVC	RAVLGGDVA	3	0.3870	759.7	18.00	0.40	tr_C9ZY45_	
HLA-DQA10501-DQB10201	52	LHRAVLGGDVALVCA	RAVLGGDVA	2	0.3855	771.5	18.00	0.37	tr_C9ZY45_	
HLA-DQA10501-DQB10201	53	MIIRFLIDNGADINA	FLIDNGADI	4	0.3803	816.9	19.00	0.34	tr_C9ZY45_	
HLA-DQA10501-DQB10201	54	QCFVDSLLHQVVSED	LLHQVVSED	6	0.3794	824.1	19.00	0.36	tr_C9ZY45_	
HLA-DQA10501-DQB10201	55	PFLGECSNEMIIRFL	FLGECSNEM	1	0.3790	828.1	19.00	0.55	tr_C9ZY45_	
HLA-DQA10501-DQB10201	56	GALVFADGLIVVHAL	GALVFADGL	0	0.3783	834.6	19.00	0.31	tr_C9ZY45_	
HLA-DQA10501-DQB10201	57	KPEETSVMSAVAVRS	VMSAVAVRS	6	0.3780	837.3	19.00	0.49	tr_C9ZY45_	
HLA-DQA10501-DQB10201	58	HRAVLGGDVALVCAL	RAVLGGDVA	1	0.3777	839.4	19.00	0.31	tr_C9ZY45_	
HLA-DQA10501-DQB10201	59	DVIGLTALQLAANNA	VIGLTALQL	1	0.3753	862.3	20.00	0.63	tr_C9ZY45_	
HLA-DQA10501-DQB10201	60	RAVLGGDVALVCALL	RAVLGGDVA	0	0.3744	870.1	20.00	0.19	tr_C9ZY45_	
HLA-DQA10501-DQB10201	61	TPLHRAVLGGDVALV	RAVLGGDVA	4	0.3708	905.2	20.00	0.46	tr_C9ZY45_	
HLA-DQA10501-DQB10201	62	TLGREPFLGECSNEM	FLGECSNEM	6	0.3686	926.5	21.00	0.72	tr_C9ZY45_	
HLA-DQA10501-DQB10201	63	PEETSVMSAVAVRSS	VMSAVAVRS	5	0.3685	928.0	21.00	0.47	tr_C9ZY45_	

HLA-DQA10501-DQB10201	64	ANFVILFLVEQLGMD	VILFLVEQL	3	0.3673	940.3	21.00	0.30	tr_C9ZY45_
HLA-DQA10501-DQB10201	65	LFLVEQLGMDVNARE	LVEQLGMDV	2	0.3661	952.4	21.00	0.29	tr_C9ZY45_
HLA-DQA10501-DQB10201	66	AHYLQHEEQNDVPLP	AHYLQHEEQ	0	0.3661	952.5	21.00	0.44	tr_C9ZY45_
HLA-DQA10501-DQB10201	67	LLQCFVDSLLHQVVS	FVDSLLHQV	4	0.3655	958.2	21.00	0.29	tr_C9ZY45_
HLA-DQA10501-DQB10201	68	SLQLLQCFVDSLLHQ	LLQCFVDSL	3	0.3632	982.9	22.00	0.46	tr_C9ZY45_
HLA-DQA10501-DQB10201	69	ESLGGDWLFPSVVTF	GDWLFPSVV	4	0.3622	992.9	22.00	0.26	tr_C9ZY45_
HLA-DQA10501-DQB10201	70	EETSVMSAVAVRSSY	VMSAVAVRS	4	0.3617	998.6	22.00	0.49	tr_C9ZY45_
HLA-DQA10501-DQB10201	71	ILFLVEQLGMDVNAR	LVEQLGMDV	3	0.3608	1008.0	22.00	0.30	tr_C9ZY45_
HLA-DQA10501-DQB10201	72	LGGDWLFPSVVTFLL	FPSVVTFLL	6	0.3594	1024.0	22.00	0.24	tr_C9ZY45_
HLA-DQA10501-DQB10201	73	GKGANFVILFLVEQL	VILFLVEQL	6	0.3579	1040.7	23.00	0.47	tr_C9ZY45_
HLA-DQA10501-DQB10201	74	SLGGDWLFPSVVTFL	GDWLFPSVV	3	0.3577	1042.3	23.00	0.25	tr_C9ZY45_
HLA-DQA10501-DQB10201	75	IIRFLIDNGADINAR	FLIDNGADI	3	0.3552	1071.5	23.00	0.35	tr_C9ZY45_
HLA-DQA10501-DQB10201	76	GANFVILFLVEQLGM	VILFLVEQL	4	0.3535	1091.1	24.00	0.32	tr_C9ZY45_
HLA-DQA10501-DQB10201	77	KGANFVILFLVEQLG	VILFLVEQL	5	0.3467	1174.3	25.00	0.36	tr_C9ZY45_
HLA-DQA10501-DQB10201	78	LQCFVDSLLHQVVSE	FVDSLLHQV	3	0.3447	1199.9	25.00	0.28	tr_C9ZY45_
HLA-DQA10501-DQB10201	79	AREGECVKASTGEEL	VKASTGEEL	6	0.3446	1201.6	25.00	0.88	tr_C9ZY45_
HLA-DQA10501-DQB10201	80	ETSVMSAVAVRSSYT	VMSAVAVRS	3	0.3415	1242.1	26.00	0.48	tr_C9ZY45_
HLA-DQA10501-DQB10201	81	AVLGGDVALVCALLS	VLGGDVALV	1	0.3403	1259.3	26.00	0.18	tr_C9ZY45_
HLA-DQA10501-DQB10201	82	YTPLHRAVLGGDVAL	RAVLGGDVA	5	0.3381	1288.9	27.00	0.55	tr_C9ZY45_
HLA-DQA10501-DQB10201	83	ECVKASTGEELKPEE	VKASTGEEL	2	0.3360	1318.4	27.00	0.77	tr_C9ZY45_
HLA-DQA10501-DQB10201	84	ADVNQTDVIGLTALQ	QTDVIGLTA	4	0.3358	1321.8	27.00	0.27	tr_C9ZY45_
HLA-DQA10501-DQB10201	85	LLLERGAVITQRDDA	RGAVITQRD	4	0.3357	1323.2	27.00	0.41	tr_C9ZY45_
HLA-DQA10501-DQB10201	86	TSVMSAVAVRSSYTP	VMSAVAVRS	2	0.3352	1330.4	27.00	0.46	tr_C9ZY45_
HLA-DQA10501-DQB10201	87	FLVEQLGMDVNAREG	LVEQLGMDV	1	0.3336	1353.1	28.00	0.34	tr_C9ZY45_
HLA-DQA10501-DQB10201	88	SNEMIIRFLIDNGAD	IRFLIDNGA	5	0.3324	1370.3	28.00	0.40	tr_C9ZY45_
HLA-DQA10501-DQB10201	89	EGECVKASTGEELKP	VKASTGEEL	4	0.3320	1376.3	28.00	0.88	tr_C9ZY45_
HLA-DQA10501-DQB10201	90	GECVKASTGEELKPE	VKASTGEEL	3	0.3299	1408.5	28.00	0.83	tr_C9ZY45_
HLA-DQA10501-DQB10201	91	HQVVSEDLLHFVCQK	HQVVSEDLL	0	0.3298	1409.8	28.00	0.38	tr_C9ZY45_
HLA-DQA10501-DQB10201	92	PSVVTFLLGGADVNA	FLLGGADVN	5	0.3269	1455.5	29.00	0.29	tr_C9ZY45_
HLA-DQA10501-DQB10201	93	ELKPEETSVMSAVAV	TSVMSAVAV	6	0.3253	1480.1	29.00	0.40	tr_C9ZY45_
HLA-DQA10501-DQB10201	94	GGDWLFPSVVTFLLG	GDWLFPSVV	1	0.3237	1506.6	30.00	0.17	tr_C9ZY45_
HLA-DQA10501-DQB10201	95	GADVNQTDVIGLTAL	QTDVIGLTA	5	0.3235	1509.4	30.00	0.28	tr_C9ZY45_
HLA-DQA10501-DQB10201	96	CVKASTGEELKPEET	VKASTGEEL	1	0.3233	1513.0	30.00	0.61	tr_C9ZY45_
HLA-DQA10501-DQB10201	97	CGADVNQTDVIGLTA	QTDVIGLTA	6	0.3229	1519.2	30.00	0.31	tr_C9ZY45_
HLA-DQA10501-DQB10201	98	SRSPFSLQLLQCFVD	FSLQLLQCF	4	0.3227	1522.7	30.00	0.31	tr_C9ZY45_
HLA-DQA10501-DQB10201	99	VVTFLLGGADVNARD	FLLGGADVN	3	0.3226	1524.6	30.00	0.30	tr_C9ZY45_
HLA-DQA10501-DQB10201	100	SVMSAVAVRSSYTPL	VMSAVAVRS	1	0.3218	1538.1	30.00	0.45	tr_C9ZY45_
HLA-DQA10501-DQB10201	101	AAAKGNITAMRLLLE	KGNITAMRL	3	0.3164	1629.9	31.00	0.44	tr_C9ZY45_
HLA-DQA10501-DQB10201	102	LLERGAVITQRDDAG	RGAVITQRD	3	0.3162	1633.5	31.00	0.42	tr_C9ZY45_
HLA-DQA10501-DQB10201	103	ASRSPFSLQLLQCFV	FSLQLLQCF	5	0.3148	1658.1	32.00	0.32	tr_C9ZY45_
HLA-DQA10501-DQB10201	104	REGECVKASTGEELK	VKASTGEEL	5	0.3144	1665.6	32.00	0.88	tr_C9ZY45_
HLA-DQA10501-DQB10201	105	SWLQRFVNIRSLWCP	QRFVNIRSL	3	0.3140	1673.5	32.00	0.31	tr_C9ZY45_
HLA-DQA10501-DQB10201	106	RLLLERGAVITQRDD	RGAVITQRD	5	0.3132	1686.9	32.00	0.47	tr_C9ZY45_
HLA-DQA10501-DQB10201	107	IRFLIDNGADINARN	FLIDNGADI	2	0.3129	1692.5	32.00	0.46	tr_C9ZY45_
HLA-DQA10501-DQB10201	108	FLGECSNEMIIRFLI	FLGECSNEM	0	0.3126	1698.8	32.00	0.50	tr_C9ZY45_
HLA-DQA10501-DQB10201	109	ALVFADGLIVVHALM	ADGLIVVHA	4	0.3113	1721.7	32.00	0.28	tr_C9ZY45_
HLA-DQA10501-DQB10201	110	VLGGDVALVCALLSC	DVALVCALL	4	0.3104	1738.9	33.00	0.25	tr_C9ZY45_
HLA-DQA10501-DQB10201	111	VAVTQGNDIATVCTL	NDIATVCTL	6	0.3104	1739.6	33.00	0.30	tr_C9ZY45_
HLA-DQA10501-DQB10201	112	SVVTFLLGGADVNAR	FLLGGADVN	4	0.3091	1763.5	33.00	0.29	tr_C9ZY45_
HLA-DQA10501-DQB10201	113	CQKGGKGANFVILFL	GANFVILFL	6	0.3086	1774.3	33.00	0.45	tr_C9ZY45_

HLA-DQA10501-DQB10201	114	LERGAVITQRDDAGY	RGAVITQRD	2	0.3085	1776.2	33.00	0.41	tr_C9ZY45_
HLA-DQA10501-DQB10201	115	RSPFSLQLLQCFVDS	FSLQLLQCF	3	0.3076	1793.4	33.00	0.29	tr_C9ZY45_
HLA-DQA10501-DQB10201	116	LSCGADVNQTDVIGL	VNQTDVIGL	6	0.3074	1797.2	33.00	0.34	tr_C9ZY45_
HLA-DQA10501-DQB10201	117	MRLLLERGAVITQRD	RGAVITQRD	6	0.3073	1799.0	33.00	0.44	tr_C9ZY45_
HLA-DQA10501-DQB10201	118	WLQRFVNIRSLWCPT	QRFVNIRSL	2	0.3065	1815.3	34.00	0.29	tr_C9ZY45_
HLA-DQA10501-DQB10201	119	LKPEETSVMSAVAVR	ETSVMSAVA	4	0.3059	1826.8	34.00	0.37	tr_C9ZY45_
HLA-DQA10501-DQB10201	120	LMVAAAKGNITAMRL	KGNITAMRL	6	0.3040	1864.1	34.00	0.73	tr_C9ZY45_
HLA-DQA10501-DQB10201	121	NAGVSAVGGSWLQRF	VSAVGGSWL	3	0.3038	1868.3	34.00	0.43	tr_C9ZY45_
HLA-DQA10501-DQB10201	122	VAAAKGNITAMRLLL	KGNITAMRL	4	0.3038	1868.7	34.00	0.58	tr_C9ZY45_
HLA-DQA10501-DQB10201	123	CSNEMIIRFLIDNGA	IRFLIDNGA	6	0.3016	1913.5	35.00	0.38	tr_C9ZY45_
HLA-DQA10501-DQB10201	124	VIGLTALQLAANNAG	VIGLTALQL	0	0.2998	1950.0	35.00	0.57	tr_C9ZY45_
HLA-DQA10501-DQB10201	125	GGKGANFVILFLVEQ	FVILFLVEQ	6	0.2974	2003.1	36.00	0.28	tr_C9ZY45_
HLA-DQA10501-DQB10201	126	AAKGNITAMRLLLER	KGNITAMRL	2	0.2966	2019.1	36.00	0.42	tr_C9ZY45_
HLA-DQA10501-DQB10201	127	HASRSPFSLQLLQCF	FSLQLLQCF	6	0.2965	2021.0	36.00	0.46	tr_C9ZY45_
HLA-DQA10501-DQB10201	128	FPSVVTFLLGGADVN	FLLGGADVN	6	0.2965	2022.0	36.00	0.32	tr_C9ZY45_
HLA-DQA10501-DQB10201	129	LVEQLGMDVNAREGE	LGMDVNARE	4	0.2954	2044.9	36.00	0.25	tr_C9ZY45_
HLA-DQA10501-DQB10201	130	VTFLLGGADVNARDK	FLLGGADVN	2	0.2945	2066.6	37.00	0.33	tr_C9ZY45_
HLA-DQA10501-DQB10201	131	LQRFVNIRSLWCPTA	FVNIRSLWC	3	0.2943	2070.0	37.00	0.26	tr_C9ZY45_
HLA-DQA10501-DQB10201	132	MVAAAKGNITAMRLL	KGNITAMRL	5	0.2933	2093.9	37.00	0.65	tr_C9ZY45_
HLA-DQA10501-DQB10201	133	SYTPLHRAVLGGDVA	RAVLGGDVA	6	0.2926	2108.5	37.00	0.64	tr_C9ZY45_
HLA-DQA10501-DQB10201	134	AGVSAVGGSWLQRFV	VSAVGGSWL	2	0.2923	2116.0	37.00	0.41	tr_C9ZY45_
HLA-DQA10501-DQB10201	135	SCGADVNQTDVIGLT	VNQTDVIGL	5	0.2922	2118.5	37.00	0.32	tr_C9ZY45_
HLA-DQA10501-DQB10201	136	GSWLQRFVNIRSLWC	QRFVNIRSL	4	0.2910	2144.6	37.00	0.36	tr_C9ZY45_
HLA-DQA10501-DQB10201	137	ADGLIVVHALMVLCA	VHALMVLCA	6	0.2883	2210.3	38.00	0.48	tr_C9ZY45_
HLA-DQA10501-DQB10201	138	VMSAVAVRSSYTPLH	VMSAVAVRS	0	0.2879	2218.8	38.00	0.34	tr_C9ZY45_
HLA-DQA10501-DQB10201	139	GATALHVAVTQGNDI	VAVTQGNDI	6	0.2870	2241.7	38.00	0.52	tr_C9ZY45_
HLA-DQA10501-DQB10201	140	LHVAVTQGNDIATVC	VAVTQGNDI	2	0.2865	2253.2	38.00	0.44	tr_C9ZY45_
HLA-DQA10501-DQB10201	141	ATALHVAVTQGNDIA	VAVTQGNDI	5	0.2843	2306.5	39.00	0.52	tr_C9ZY45_
HLA-DQA10501-DQB10201	142	QKGGKGANFVILFLV	GANFVILFL	5	0.2841	2310.8	39.00	0.38	tr_C9ZY45_
HLA-DQA10501-DQB10201	143	VKASTGEELKPEETS	VKASTGEEL	0	0.2833	2332.5	39.00	0.50	tr_C9ZY45_
HLA-DQA10501-DQB10201	144	LVFADGLIVVHALMV	ADGLIVVHA	3	0.2830	2338.9	39.00	0.30	tr_C9ZY45_
HLA-DQA10501-DQB10201	145	ALHVAVTQGNDIATV	VAVTQGNDI	3	0.2829	2342.7	39.00	0.50	tr_C9ZY45_
HLA-DQA10501-DQB10201	146	HVAVTQGNDIATVCT	VAVTQGNDI	1	0.2825	2351.7	39.00	0.39	tr_C9ZY45_
HLA-DQA10501-DQB10201	147	ERGAVITQRDDAGYT	RGAVITQRD	1	0.2811	2389.5	40.00	0.39	tr_C9ZY45_
HLA-DQA10501-DQB10201	148	KGGKGANFVILFLVE	GANFVILFL	4	0.2808	2397.3	40.00	0.31	tr_C9ZY45_
HLA-DQA10501-DQB10201	149	DINARNASGETPLMV	ASGETPLMV	6	0.2806	2400.9	40.00	0.47	tr_C9ZY45_
HLA-DQA10501-DQB10201	150	AANNAGVSAVGGSWL	VSAVGGSWL	6	0.2799	2419.9	40.00	0.57	tr_C9ZY45_
HLA-DQA10501-DQB10201	151	LFPSVVTFLLGGADV	TFLLGGADV	6	0.2752	2545.9	41.00	0.47	tr_C9ZY45_
HLA-DQA10501-DQB10201	152	DGLIVVHALMVLCAT	VHALMVLCA	5	0.2741	2577.2	42.00	0.37	tr_C9ZY45_
HLA-DQA10501-DQB10201	153	TALHVAVTQGNDIAT	VAVTQGNDI	4	0.2739	2582.0	42.00	0.55	tr_C9ZY45_
HLA-DQA10501-DQB10201	154	ALLSCGADVNQTDVI	ADVNQTDVI	6	0.2734	2595.3	42.00	0.44	tr_C9ZY45_
HLA-DQA10501-DQB10201	155	QRFVNIRSLWCPTAA	FVNIRSLWC	2	0.2732	2600.4	42.00	0.25	tr_C9ZY45_
HLA-DQA10501-DQB10201	156	GVSAVGGSWLQRFVN	VSAVGGSWL	1	0.2729	2609.7	42.00	0.40	tr_C9ZY45_
HLA-DQA10501-DQB10201	157	INARNASGETPLMVA	ASGETPLMV	5	0.2728	2612.2	42.00	0.47	tr_C9ZY45_
HLA-DQA10501-DQB10201	158	AKGNITAMRLLLERG	KGNITAMRL	1	0.2727	2615.0	42.00	0.40	tr_C9ZY45_
HLA-DQA10501-DQB10201	159	AVTQGNDIATVCTLG	NDIATVCTL	5	0.2725	2620.5	42.00	0.29	tr_C9ZY45_
HLA-DQA10501-DQB10201	160	HYLQHEEQNDVPLPE	EQNDVPLPE	6	0.2725	2622.4	42.00	0.20	tr_C9ZY45_
HLA-DQA10501-DQB10201	161	RFLIDNGADINARNA	FLIDNGADI	1	0.2725	2622.5	42.00	0.56	tr_C9ZY45_
HLA-DQA10501-DQB10201	162	TFLLGGADVNARDKD	FLLGGADVN	1	0.2711	2660.5	42.00	0.34	tr_C9ZY45_
HLA-DQA10501-DQB10201	163	GNSPSQNLLSFEGAL	QNLLSFEGA	5	0.2705	2678.1	43.00	0.37	tr_C9ZY45_

HLA-DQA10501-DQB10201	164	EQLGMDVNAREGECV	VNAREGECV	6	0.2699	2696.5	43.00	0.30	tr_C9ZY45_
HLA-DQA10501-DQB10201	165	LGGDVALVCALLSCG	DVALVCALL	3	0.2682	2745.7	43.00	0.31	tr_C9ZY45_
HLA-DQA10501-DQB10201	166	EELKPEETSVMSAVA	ETSVMSAVA	6	0.2678	2757.0	43.00	0.58	tr_C9ZY45_
HLA-DQA10501-DQB10201	167	GDWLFPSVVTFLLGG	WLFPSVVTF	2	0.2675	2767.8	43.00	0.20	tr_C9ZY45_
HLA-DQA10501-DQB10201	168	VFADGLIVVHALMVL	ADGLIVVHA	2	0.2662	2805.8	44.00	0.32	tr_C9ZY45_
HLA-DQA10501-DQB10201	169	YLQHEEQNDVPLPEP	EQNDVPLPE	5	0.2662	2807.0	44.00	0.22	tr_C9ZY45_
HLA-DQA10501-DQB10201	170	ANNAGVSAVGGSWLQ	VSAVGGSWL	5	0.2651	2840.4	44.00	0.56	tr_C9ZY45_
HLA-DQA10501-DQB10201	171	GGSWLQRFVNIRSLW	QRFVNIRSL	5	0.2648	2849.6	44.00	0.43	tr_C9ZY45_
HLA-DQA10501-DQB10201	172	LLSCGADVNQTDVIG	ADVNQTDVI	5	0.2644	2861.5	44.00	0.44	tr_C9ZY45_
HLA-DQA10501-DQB10201	173	KETSLSGREALLQRF	GREALLQRF	6	0.2639	2877.1	44.00	0.50	tr_C9ZY45_
HLA-DQA10501-DQB10201	174	ECSNEMIIRFLIDNG	IIRFLIDNG	6	0.2613	2959.7	45.00	0.41	tr_C9ZY45_
HLA-DQA10501-DQB10201	175	LLCGRTFHESLGGDW	FHESLGGDW	6	0.2583	3057.5	46.00	0.70	tr_C9ZY45_
HLA-DQA10501-DQB10201	176	VTQGNDIATVCTLGR	NDIATVCTL	4	0.2580	3067.9	46.00	0.25	tr_C9ZY45_
HLA-DQA10501-DQB10201	177	NARNASGETPLMVAA	ASGETPLMV	4	0.2570	3099.1	46.00	0.47	tr_C9ZY45_
HLA-DQA10501-DQB10201	178	VVHALMVLCATLTNE	VHALMVLCA	1	0.2559	3138.4	47.00	0.24	tr_C9ZY45_
HLA-DQA10501-DQB10201	179	LIVVHALMVLCATLT	VHALMVLCA	3	0.2549	3172.3	47.00	0.31	tr_C9ZY45_
HLA-DQA10501-DQB10201	180	GLIVVHALMVLCATL	VHALMVLCA	4	0.2548	3173.1	47.00	0.38	tr_C9ZY45_
HLA-DQA10501-DQB10201	181	GGQSFPLAHYLQHEE	LAHYLQHEE	6	0.2541	3197.5	47.00	0.46	tr_C9ZY45_
HLA-DQA10501-DQB10201	182	FADGLIVVHALMVLC	DGLIVVHAL	2	0.2521	3269.5	48.00	0.25	tr_C9ZY45_
HLA-DQA10501-DQB10201	183	ETSLSGREALLQRFA	GREALLQRF	5	0.2515	3289.6	48.00	0.41	tr_C9ZY45_
HLA-DQA10501-DQB10201	184	NDIATVCTLGREPFL	VCTLGREPF	5	0.2512	3299.5	48.00	0.17	tr_C9ZY45_
HLA-DQA10501-DQB10201	185	NNAGVSAVGGSWLQR	VSAVGGSWL	4	0.2500	3343.2	48.00	0.56	tr_C9ZY45_
HLA-DQA10501-DQB10201	186	ARNASGETPLMVAAA	ASGETPLMV	3	0.2500	3344.5	48.00	0.47	tr_C9ZY45_
HLA-DQA10501-DQB10201	187	VSAVGGSWLQRFVNI	VGGSWLQRF	3	0.2495	3362.3	48.00	0.37	tr_C9ZY45_
HLA-DQA10501-DQB10201	188	IVVHALMVLCATLTN	VHALMVLCA	2	0.2469	3456.1	49.00	0.31	tr_C9ZY45_
HLA-DQA10501-DQB10201	189	TQGNDIATVCTLGRE	NDIATVCTL	3	0.2466	3470.1	49.00	0.28	tr_C9ZY45_
HLA-DQA10501-DQB10201	190	GNDIATVCTLGREPF	NDIATVCTL	1	0.2460	3491.3	49.00	0.20	tr_C9ZY45_
HLA-DQA10501-DQB10201	191	DWLFPSVVTFLLGGA	LFPSVVTFL	2	0.2459	3497.1	49.00	0.25	tr_C9ZY45_
HLA-DQA10501-DQB10201	192	VEQLGMDVNAREGEC	LGMDVNARE	3	0.2455	3509.1	49.00	0.31	tr_C9ZY45_
HLA-DQA10501-DQB10201	193	GGDVALVCALLSCGA	DVALVCALL	2	0.2453	3518.2	49.00	0.34	tr_C9ZY45_
HLA-DQA10501-DQB10201	194	VCQKGGKGANFVILF	KGANFVILF	6	0.2438	3575.5	50.00	0.67	tr_C9ZY45_
HLA-DQA10501-DQB10201	195	QVVSEDLLHFVCQKG	VVSEDLLHF	1	0.2436	3582.7	50.00	0.33	tr_C9ZY45_
HLA-DQA10501-DQB10201	196	RGAVITQRDDAGYTV	AVITQRDDA	2	0.2397	3738.5	55.00	0.32	tr_C9ZY45_
HLA-DQA10501-DQB10201	197	QGNDIATVCTLGREP	NDIATVCTL	2	0.2394	3750.2	55.00	0.26	tr_C9ZY45_
HLA-DQA10501-DQB10201	198	KGNITAMRLLLERGA	KGNITAMRL	0	0.2388	3772.8	55.00	0.24	tr_C9ZY45_
HLA-DQA10501-DQB10201	199	QLGMDVNAREGECVK	VNAREGECV	5	0.2387	3780.6	55.00	0.32	tr_C9ZY45_
HLA-DQA10501-DQB10201	200	RFVNIRSLWCPTAAE	FVNIRSLWC	1	0.2358	3897.2	55.00	0.30	tr_C9ZY45_
HLA-DQA10501-DQB10201	201	LGECSNEMIIRFLID	EMIIRFLID	6	0.2348	3941.6	55.00	0.28	tr_C9ZY45_
HLA-DQA10501-DQB10201	202	WLFPSVVTFLLGGAD	VTFLLGGAD	6	0.2342	3968.7	55.00	0.22	tr_C9ZY45_
HLA-DQA10501-DQB10201	203	MSAVAVRSSYTPLHR	AVAVRSSYT	2	0.2335	3995.6	55.00	0.29	tr_C9ZY45_
HLA-DQA10501-DQB10201	204	TSLSGREALLQRFAC	GREALLQRF	4	0.2325	4041.7	55.00	0.43	tr_C9ZY45_
HLA-DQA10501-DQB10201	205	RDKDGATALHVAVTQ	TALHVAVTQ	6	0.2317	4076.4	55.00	0.46	tr_C9ZY45_
HLA-DQA10501-DQB10201	206	FLLGGADVNARDKDG	FLLGGADVN	0	0.2296	4169.0	55.00	0.38	tr_C9ZY45_
HLA-DQA10501-DQB10201	207	GDVALVCALLSCGAD	DVALVCALL	1	0.2294	4177.2	55.00	0.33	tr_C9ZY45_
HLA-DQA10501-DQB10201	208	RNASGETPLMVAAAK	ASGETPLMV	2	0.2277	4255.8	55.00	0.51	tr_C9ZY45_
HLA-DQA10501-DQB10201	209	HHASRSPFSLQLLQC	PFSLQLLQC	6	0.2274	4271.8	55.00	0.32	tr_C9ZY45_
HLA-DQA10501-DQB10201	210	FLIDNGADINARNAS	FLIDNGADI	0	0.2258	4344.2	55.00	0.50	tr_C9ZY45_
HLA-DQA10501-DQB10201	211	VGGSWLQRFVNIRSL	QRFVNIRSL	6	0.2258	4344.6	55.00	0.41	tr_C9ZY45_
HLA-DQA10501-DQB10201	212	GECSNEMIIRFLIDN	EMIIRFLID	5	0.2248	4393.7	55.00	0.31	tr_C9ZY45_
HLA-DQA10501-DQB10201	213	DKDGATALHVAVTQG	TALHVAVTQ	5	0.2244	4410.5	55.00	0.32	tr_C9ZY45_

HLA-DQA10501-DQB10201	214	CGNSPSQNLLSFEGA	QNLLSFEGA	6	0.2240	4430.9	60.00	0.54	tr_C9ZY45_
HLA-DQA10501-DQB10201	215	DVALVCALLSCGADV	ALLSCGADV	6	0.2228	4488.7	60.00	0.24	tr_C9ZY45_
HLA-DQA10501-DQB10201	216	AMRLLLERGAVITQR	ERGAVITQR	6	0.2224	4509.0	60.00	0.41	tr_C9ZY45_
HLA-DQA10501-DQB10201	217	DIATVCTLGREPFLG	CTLGREPFL	5	0.2210	4574.2	60.00	0.20	tr_C9ZY45_
HLA-DQA10501-DQB10201	218	LQHEEQNDVPLPEPH	EQNDVPLPE	4	0.2203	4609.1	60.00	0.30	tr_C9ZY45_
HLA-DQA10501-DQB10201	219	NITAMRLLLERGAVI	LLLERGAVI	6	0.2182	4714.9	60.00	0.25	tr_C9ZY45_
HLA-DQA10501-DQB10201	220	MWVFRDRMMERGGQS	WVFRDRMME	1	0.2174	4756.5	60.00	0.32	tr_C9ZY45_
HLA-DQA10501-DQB10201	221	GNITAMRLLLERGAV	ITAMRLLLE	2	0.2160	4828.6	60.00	0.30	tr_C9ZY45_
HLA-DQA10501-DQB10201	222	RGGQSFPLAHYLQHE	PLAHYLQHE	6	0.2146	4905.5	60.00	0.53	tr_C9ZY45_
HLA-DQA10501-DQB10201	223	DGATALHVAVTQGND	TALHVAVTQ	3	0.2145	4908.2	60.00	0.31	tr_C9ZY45_
HLA-DQA10501-DQB10201	224	VLHHASRSPFSLQLL	RSPFSLQLL	6	0.2143	4922.3	60.00	0.58	tr_C9ZY45_
HLA-DQA10501-DQB10201	225	LGMDVNAREGECVKA	VNAREGECV	4	0.2134	4969.1	60.00	0.35	tr_C9ZY45_
HLA-DQA10501-DQB10201	226	RDRMMERGGQSFPLA	MMERGGQSF	3	0.2126	5012.0	60.00	0.34	tr_C9ZY45_
HLA-DQA10501-DQB10201	227	GAVITQRDDAGYTVL	AVITQRDDA	1	0.2120	5045.0	60.00	0.33	tr_C9ZY45_
HLA-DQA10501-DQB10201	228	QHEEQNDVPLPEPHM	EQNDVPLPE	3	0.2100	5154.6	60.00	0.33	tr_C9ZY45_
HLA-DQA10501-DQB10201	229	RVRSKEVYNLLKAYC	VYNLLKAYC	6	0.2092	5199.3	60.00	0.31	tr_C9ZY45_
HLA-DQA10501-DQB10201	230	NASGETPLMVAAAKG	ASGETPLMV	1	0.2091	5206.1	60.00	0.44	tr_C9ZY45_
HLA-DQA10501-DQB10201	231	SAVAVRSSYTPLHRA	AVAVRSSYT	1	0.2088	5221.4	60.00	0.30	tr_C9ZY45_
HLA-DQA10501-DQB10201	232	CALLSCGADVNQTDV	LLSCGADVN	2	0.2083	5249.2	60.00	0.32	tr_C9ZY45_
HLA-DQA10501-DQB10201	233	VALVCALLSCGADVN	LLSCGADVN	6	0.2079	5273.0	60.00	0.28	tr_C9ZY45_
HLA-DQA10501-DQB10201	234	KDGATALHVAVTQGN	TALHVAVTQ	4	0.2069	5328.7	65.00	0.30	tr_C9ZY45_
HLA-DQA10501-DQB10201	235	YCKETSLSGREALLQ	LSGREALLQ	6	0.2066	5347.5	65.00	0.28	tr_C9ZY45_
HLA-DQA10501-DQB10201	236	SLSGREALLQRFACG	GREALLQRF	3	0.2066	5349.6	65.00	0.44	tr_C9ZY45_
HLA-DQA10501-DQB10201	237	WVFRDRMMERGGQSF	MMERGGQSF	6	0.2064	5360.3	65.00	0.29	tr_C9ZY45_
HLA-DQA10501-DQB10201	238	TGEELKPEETSVMSA	PEETSVMSA	6	0.2060	5382.1	65.00	0.43	tr_C9ZY45_
HLA-DQA10501-DQB10201	239	VCALLSCGADVNQTD	LLSCGADVN	3	0.2050	5443.5	65.00	0.32	tr_C9ZY45_
HLA-DQA10501-DQB10201	240	IATVCTLGREPFLGE	CTLGREPFL	4	0.2047	5457.4	65.00	0.25	tr_C9ZY45_
HLA-DQA10501-DQB10201	241	SAVGGSWLQRFVNIR	VGGSWLQRF	2	0.2043	5483.1	65.00	0.46	tr_C9ZY45_
HLA-DQA10501-DQB10201	242	FVNIRSLWCPTAAER	FVNIRSLWC	0	0.2038	5514.2	65.00	0.31	tr_C9ZY45_
HLA-DQA10501-DQB10201	243	ADINARNASGETPLM	NASGETPLM	6	0.2033	5540.5	65.00	0.32	tr_C9ZY45_
HLA-DQA10501-DQB10201	244	KASTGEELKPEETSV	GEELKPEET	4	0.2011	5676.6	65.00	0.47	tr_C9ZY45_
HLA-DQA10501-DQB10201	245	ITAMRLLLERGAVIT	LLLERGAVI	5	0.2007	5698.5	65.00	0.23	tr_C9ZY45_
HLA-DQA10501-DQB10201	246	GEELKPEETSVMSAV	PEETSVMSA	5	0.2000	5744.1	65.00	0.36	tr_C9ZY45_
HLA-DQA10501-DQB10201	247	HEEQNDVPLPEPHML	EQNDVPLPE	2	0.1998	5753.7	65.00	0.29	tr_C9ZY45_
HLA-DQA10501-DQB10201	248	ALVCALLSCGADVNQ	LLSCGADVN	5	0.1986	5832.4	65.00	0.31	tr_C9ZY45_
HLA-DQA10501-DQB10201	249	DRMMERGGQSFPLAH	MMERGGQSF	2	0.1984	5844.9	65.00	0.35	tr_C9ZY45_
HLA-DQA10501-DQB10201	250	LHHASRSPFSLQLLQ	RSPFSLQLL	5	0.1966	5961.5	65.00	0.49	tr_C9ZY45_
HLA-DQA10501-DQB10201	251	ASTGEELKPEETSVM	GEELKPEET	3	0.1961	5991.4	65.00	0.50	tr_C9ZY45_
HLA-DQA10501-DQB10201	252	IGLTALQLAANNAGV	ALQLAANNA	4	0.1959	6005.1	65.00	0.31	tr_C9ZY45_
HLA-DQA10501-DQB10201	253	RRVRSKEVYNLLKAY	EVYNLLKAY	6	0.1955	6030.4	65.00	0.38	tr_C9ZY45_
HLA-DQA10501-DQB10201	254	EEQNDVPLPEPHMLL	EQNDVPLPE	1	0.1953	6041.7	65.00	0.28	tr_C9ZY45_
HLA-DQA10501-DQB10201	255	FRDRMMERGGQSFPL	MMERGGQSF	4	0.1951	6058.7	65.00	0.46	tr_C9ZY45_
HLA-DQA10501-DQB10201	256	TVCTLGREPFLGECS	REPFLGECS	6	0.1938	6141.2	65.00	0.25	tr_C9ZY45_
HLA-DQA10501-DQB10201	257	RMMERGGQSFPLAHY	MMERGGQSF	1	0.1932	6181.3	65.00	0.31	tr_C9ZY45_
HLA-DQA10501-DQB10201	258	VVSEDLLHFVCQKGG	VSEDLLHFV	1	0.1917	6279.8	65.00	0.26	tr_C9ZY45_
HLA-DQA10501-DQB10201	259	LVCALLSCGADVNQT	LLSCGADVN	4	0.1917	6281.6	65.00	0.32	tr_C9ZY45_
HLA-DQA10501-DQB10201	260	VRSKEVYNLLKAYCK	VYNLLKAYC	5	0.1912	6318.6	65.00	0.28	tr_C9ZY45_
HLA-DQA10501-DQB10201	261	ARDKDGATALHVAVT	KDGATALHV	3	0.1904	6372.9	65.00	0.30	tr_C9ZY45_
HLA-DQA10501-DQB10201	262	CTLGREPFLGECSNE	PFLGECSNE	6	0.1900	6402.5	65.00	0.28	tr_C9ZY45_
HLA-DQA10501-DQB10201	263	AVITQRDDAGYTVLH	AVITQRDDA	0	0.1888	6486.7	70.00	0.27	tr_C9ZY45_

HLA-DQA10501-DQB10201	264	ATVCTLGREPFLGEC	CTLGREPFL	3	0.1879	6549.0	70.00	0.23	tr_C9ZY45_
HLA-DQA10501-DQB10201	265	AVGGSWLQRFVNIRS	VGGSWLQRF	1	0.1866	6642.3	70.00	0.46	tr_C9ZY45_
HLA-DQA10501-DQB10201	266	CKETSLSGREALLQR	LSGREALLQ	5	0.1865	6649.9	70.00	0.28	tr_C9ZY45_
HLA-DQA10501-DQB10201	267	MMERGGQSFPLAHYL	RGGQSFPLA	3	0.1858	6698.0	70.00	0.25	tr_C9ZY45_
HLA-DQA10501-DQB10201	268	TAMRLLLERGAVITQ	LLLERGAVI	4	0.1846	6784.7	70.00	0.29	tr_C9ZY45_
HLA-DQA10501-DQB10201	269	LSGREALLQRFACGN	GREALLQRF	2	0.1846	6786.9	70.00	0.48	tr_C9ZY45_
HLA-DQA10501-DQB10201	270	AYCKETSLSGREALL	SLSGREALL	6	0.1841	6818.8	70.00	0.34	tr_C9ZY45_
HLA-DQA10501-DQB10201	271	LAANNAGVSAVGGSW	GVSAVGGSW	6	0.1839	6834.7	70.00	0.37	tr_C9ZY45_
HLA-DQA10501-DQB10201	272	AVAVRSSYTPLHRAV	AVAVRSSYT	0	0.1834	6869.9	70.00	0.25	tr_C9ZY45_
HLA-DQA10501-DQB10201	273	NARDKDGATALHVAV	KDGATALHV	4	0.1821	6970.4	70.00	0.38	tr_C9ZY45_
HLA-DQA10501-DQB10201	274	RSKEVYNLLKAYCKE	VYNLLKAYC	4	0.1820	6979.0	70.00	0.31	tr_C9ZY45_
HLA-DQA10501-DQB10201	275	STGEELKPEETSVMS	GEELKPEET	2	0.1816	7008.5	70.00	0.50	tr_C9ZY45_
HLA-DQA10501-DQB10201	276	VNIRSLWCPTAAERR	IRSLWCPTA	2	0.1804	7102.0	70.00	0.28	tr_C9ZY45_
HLA-DQA10501-DQB10201	277	VCTLGREPFLGECSN	REPFLGECS	5	0.1803	7104.2	70.00	0.28	tr_C9ZY45_
HLA-DQA10501-DQB10201	278	VFRDRMMERGGQSFP	MMERGGQSF	5	0.1776	7315.2	70.00	0.50	tr_C9ZY45_
HLA-DQA10501-DQB10201	279	EQNDVPLPEPHMLLL	EQNDVPLPE	0	0.1774	7335.3	70.00	0.22	tr_C9ZY45_
HLA-DQA10501-DQB10201	280	AAERRVRSKEVYNLL	RSKEVYNLL	6	0.1753	7503.2	70.00	0.37	tr_C9ZY45_
HLA-DQA10501-DQB10201	281	GMDVNAREGECVKAS	VNAREGECV	3	0.1747	7554.8	70.00	0.43	tr_C9ZY45_
HLA-DQA10501-DQB10201	282	TALQLAANNAGVSAV	AANNAGVSA	5	0.1726	7722.1	70.00	0.29	tr_C9ZY45_
HLA-DQA10501-DQB10201	283	SSYTPLHRAVLGGDV	HRAVLGGDV	6	0.1710	7863.5	75.00	0.51	tr_C9ZY45_
HLA-DQA10501-DQB10201	284	SKEVYNLLKAYCKET	VYNLLKAYC	3	0.1705	7907.3	75.00	0.28	tr_C9ZY45_
HLA-DQA10501-DQB10201	285	LTALQLAANNAGVSA	ALQLAANNA	2	0.1699	7957.8	75.00	0.29	tr_C9ZY45_
HLA-DQA10501-DQB10201	286	VITQRDDAGYTVLHH	DAGYTVLHH	6	0.1685	8079.4	75.00	0.41	tr_C9ZY45_
HLA-DQA10501-DQB10201	287	VNARDKDGATALHVA	KDGATALHV	5	0.1678	8138.6	75.00	0.52	tr_C9ZY45_
HLA-DQA10501-DQB10201	288	LQLAANNAGVSAVGG	NAGVSAVGG	6	0.1670	8205.2	75.00	0.30	tr_C9ZY45_
HLA-DQA10501-DQB10201	289	ALQLAANNAGVSAVG	AANNAGVSA	4	0.1669	8219.3	75.00	0.29	tr_C9ZY45_
HLA-DQA10501-DQB10201	290	ERRVRSKEVYNLLKA	RSKEVYNLL	4	0.1649	8399.0	75.00	0.30	tr_C9ZY45_
HLA-DQA10501-DQB10201	291	NIRSLWCPTAAERRV	WCPTAAERR	5	0.1642	8464.1	75.00	0.26	tr_C9ZY45_
HLA-DQA10501-DQB10201	292	MERGGQSFPLAHYLQ	RGGQSFPLA	2	0.1636	8518.6	75.00	0.23	tr_C9ZY45_
HLA-DQA10501-DQB10201	293	MDVNAREGECVKAST	VNAREGECV	2	0.1633	8545.4	75.00	0.47	tr_C9ZY45_
HLA-DQA10501-DQB10201	294	GADINARNASGETPL	ARNASGETP	5	0.1625	8619.3	75.00	0.49	tr_C9ZY45_
HLA-DQA10501-DQB10201	295	KEVYNLLKAYCKETS	VYNLLKAYC	2	0.1609	8765.0	75.00	0.28	tr_C9ZY45_
HLA-DQA10501-DQB10201	296	EVYNLLKAYCKETSL	VYNLLKAYC	1	0.1585	8998.4	75.00	0.27	tr_C9ZY45_
HLA-DQA10501-DQB10201	297	ITQRDDAGYTVLHHA	DAGYTVLHH	5	0.1578	9067.6	75.00	0.32	tr_C9ZY45_
HLA-DQA10501-DQB10201	298	ASGETPLMVAAAKGN	PLMVAAAKG	5	0.1575	9096.5	75.00	0.34	tr_C9ZY45_
HLA-DQA10501-DQB10201	299	QNDVPLPEPHMLLLC	VPLPEPHML	3	0.1567	9178.9	75.00	0.23	tr_C9ZY45_
HLA-DQA10501-DQB10201	300	SGREALLQRFACGNS	GREALLQRF	1	0.1561	9239.3	75.00	0.50	tr_C9ZY45_
HLA-DQA10501-DQB10201	301	ACGNSPSQNLLSFEG	PSQNLLSFE	5	0.1550	9346.9	75.00	0.53	tr_C9ZY45_
HLA-DQA10501-DQB10201	302	AERRVRSKEVYNLLK	RSKEVYNLL	5	0.1549	9359.7	75.00	0.35	tr_C9ZY45_
HLA-DQA10501-DQB10201	303	TPLMVAAAKGNITAM	VAAAKGNIT	4	0.1538	9470.0	75.00	0.34	tr_C9ZY45_
HLA-DQA10501-DQB10201	304	ERGGQSFPLAHYLQH	RGGQSFPLA	1	0.1532	9527.7	80.00	0.22	tr_C9ZY45_
HLA-DQA10501-DQB10201	305	FACGNSPSQNLLSFE	PSQNLLSFE	6	0.1527	9576.7	80.00	0.68	tr_C9ZY45_
HLA-DQA10501-DQB10201	306	QLAANNAGVSAVGGS	NAGVSAVGG	5	0.1526	9590.1	80.00	0.30	tr_C9ZY45_
HLA-DQA10501-DQB10201	307	GETPLMVAAAKGNIT	PLMVAAAKG	3	0.1515	9707.2	80.00	0.32	tr_C9ZY45_
HLA-DQA10501-DQB10201	308	GLTALQLAANNAGVS	ALQLAANNA	3	0.1515	9708.5	80.00	0.40	tr_C9ZY45_
HLA-DQA10501-DQB10201	309	PLMVAAAKGNITAMR	VAAAKGNIT	3	0.1503	9830.0	80.00	0.34	tr_C9ZY45_
HLA-DQA10501-DQB10201	310	DVNARDKDGATALHV	KDGATALHV	6	0.1503	9834.4	80.00	0.69	tr_C9ZY45_
HLA-DQA10501-DQB10201	311	VAVRSSYTPLHRAVL	VRSSYTPLH	2	0.1496	9912.3	80.00	0.26	tr_C9ZY45_
HLA-DQA10501-DQB10201	312	KAYCKETSLSGREAL	ETSLSGREA	5	0.1490	9974.9	80.00	0.50	tr_C9ZY45_
HLA-DQA10501-DQB10201	313	ETPLMVAAAKGNITA	PLMVAAAKG	2	0.1478	10098.9	80.00	0.34	tr_C9ZY45_

HLA-DQA10501-DQB10201	314	DVPLPEPHMLLLCGR	PHMLLLCGR	6	0.1476	10130.2	80.00	0.34	tr_C9ZY45_
HLA-DQA10501-DQB10201	315	VSEDLLHFVCQKGGK	VSEDLLHFV	0	0.1439	10534.9	80.00	0.31	tr_C9ZY45_
HLA-DQA10501-DQB10201	316	NDVPLPEPHMLLLCG	VPLPEPHML	2	0.1432	10619.9	80.00	0.23	tr_C9ZY45_
HLA-DQA10501-DQB10201	317	IRSLWCPTAAERRVR	WCPTAAERR	4	0.1428	10659.1	80.00	0.31	tr_C9ZY45_
HLA-DQA10501-DQB10201	318	TQRDDAGYTVLHHAS	DAGYTVLHH	4	0.1419	10773.6	80.00	0.34	tr_C9ZY45_
HLA-DQA10501-DQB10201	319	TVLHHASRSPFSLQL	SRSPFSLQL	6	0.1405	10936.6	80.00	0.35	tr_C9ZY45_
HLA-DQA10501-DQB10201	320	LLGGADVNARDKDGA	LLGGADVNA	0	0.1401	10983.2	80.00	0.37	tr_C9ZY45_
HLA-DQA10501-DQB10201	321	SGETPLMVAAAKGNI	PLMVAAAKG	4	0.1390	11118.1	80.00	0.45	tr_C9ZY45_
HLA-DQA10501-DQB10201	322	GREALLQRFACGNSP	GREALLQRF	0	0.1373	11322.7	80.00	0.34	tr_C9ZY45_
HLA-DQA10501-DQB10201	323	DVNAREGECVKASTG	VNAREGECV	1	0.1366	11404.9	80.00	0.55	tr_C9ZY45_
HLA-DQA10501-DQB10201	324	GYTVLHHASRSPFSL	LHHASRSPF	4	0.1358	11509.6	80.00	0.28	tr_C9ZY45_
HLA-DQA10501-DQB10201	325	DAGYTVLHHASRSPF	LHHASRSPF	6	0.1352	11583.8	80.00	0.31	tr_C9ZY45_
HLA-DQA10501-DQB10201	326	AGYTVLHHASRSPFS	LHHASRSPF	5	0.1317	12027.0	85.00	0.31	tr_C9ZY45_
HLA-DQA10501-DQB10201	327	TAAERRVRSKEVYNL	RRVRSKEVY	4	0.1310	12111.5	85.00	0.32	tr_C9ZY45_
HLA-DQA10501-DQB10201	328	NGADINARNASGETP	ARNASGETP	6	0.1293	12336.6	85.00	0.57	tr_C9ZY45_
HLA-DQA10501-DQB10201	329	QRDDAGYTVLHHASR	DAGYTVLHH	3	0.1274	12593.9	85.00	0.30	tr_C9ZY45_
HLA-DQA10501-DQB10201	330	VYNLLKAYCKETSLS	VYNLLKAYC	0	0.1266	12705.1	85.00	0.23	tr_C9ZY45_
HLA-DQA10501-DQB10201	331	PLPEPHMLLLCGRTF	PHMLLLCGR	4	0.1264	12729.7	85.00	0.32	tr_C9ZY45_
HLA-DQA10501-DQB10201	332	AVRSSYTPLHRAVLG	VRSSYTPLH	1	0.1259	12809.6	85.00	0.26	tr_C9ZY45_
HLA-DQA10501-DQB10201	333	PHMLLLCGRTFHESL	CGRTFHESL	6	0.1256	12846.6	85.00	0.37	tr_C9ZY45_
HLA-DQA10501-DQB10201	334	RSLWCPTAAERRVRS	WCPTAAERR	3	0.1250	12924.1	85.00	0.36	tr_C9ZY45_
HLA-DQA10501-DQB10201	335	RDDAGYTVLHHASRS	DAGYTVLHH	2	0.1245	13001.7	85.00	0.32	tr_C9ZY45_
HLA-DQA10501-DQB10201	336	LIDNGADINARNASG	LIDNGADIN	0	0.1243	13028.7	85.00	0.36	tr_C9ZY45_
HLA-DQA10501-DQB10201	337	YTVLHHASRSPFSLQ	LHHASRSPF	3	0.1233	13167.0	85.00	0.30	tr_C9ZY45_
HLA-DQA10501-DQB10201	338	DDAGYTVLHHASRSP	DAGYTVLHH	1	0.1230	13220.0	85.00	0.30	tr_C9ZY45_
HLA-DQA10501-DQB10201	339	VPLPEPHMLLLCGRT	PHMLLLCGR	5	0.1209	13510.1	85.00	0.31	tr_C9ZY45_
HLA-DQA10501-DQB10201	340	LLLCGRTFHESLGGD	CGRTFHESL	3	0.1185	13877.2	85.00	0.33	tr_C9ZY45_
HLA-DQA10501-DQB10201	341	RSSYTPLHRAVLGGD	LHRAVLGGD	6	0.1150	14413.7	85.00	0.32	tr_C9ZY45_
HLA-DQA10501-DQB10201	342	CPTAAERRVRSKEVY	RRVRSKEVY	6	0.1126	14789.9	90.00	0.47	tr_C9ZY45_
HLA-DQA10501-DQB10201	343	LKAYCKETSLSGREA	ETSLSGREA	6	0.1121	14870.9	90.00	0.63	tr_C9ZY45_
HLA-DQA10501-DQB10201	344	VRSSYTPLHRAVLGG	PLHRAVLGG	6	0.1120	14882.2	90.00	0.23	tr_C9ZY45_
HLA-DQA10501-DQB10201	345	SEDLLHFVCQKGGKG	DLLHFVCQK	2	0.1114	14986.6	90.00	0.36	tr_C9ZY45_
HLA-DQA10501-DQB10201	346	LPEPHMLLLCGRTFH	PHMLLLCGR	3	0.1113	14990.5	90.00	0.31	tr_C9ZY45_
HLA-DQA10501-DQB10201	347	MLLLCGRTFHESLGG	CGRTFHESL	4	0.1093	15324.5	90.00	0.34	tr_C9ZY45_
HLA-DQA10501-DQB10201	348	HMLLLCGRTFHESLG	CGRTFHESL	5	0.1089	15388.8	90.00	0.35	tr_C9ZY45_
HLA-DQA10501-DQB10201	349	SLWCPTAAERRVRSK	WCPTAAERR	2	0.1084	15467.8	90.00	0.44	tr_C9ZY45_
HLA-DQA10501-DQB10201	350	PTAAERRVRSKEVYN	RRVRSKEVY	5	0.1077	15593.5	90.00	0.40	tr_C9ZY45_
HLA-DQA10501-DQB10201	351	PEPHMLLLCGRTFHE	PHMLLLCGR	2	0.1075	15629.8	90.00	0.31	tr_C9ZY45_
HLA-DQA10501-DQB10201	352	VNAREGECVKASTGE	VNAREGECV	0	0.1058	15923.5	90.00	0.44	tr_C9ZY45_
HLA-DQA10501-DQB10201	353	FVCQKGGKGANFVIL	GKGANFVIL	6	0.1050	16049.3	90.00	0.54	tr_C9ZY45_
HLA-DQA10501-DQB10201	354	EALLQRFACGNSPSQ	QRFACGNSP	4	0.1029	16422.9	90.00	0.38	tr_C9ZY45_
HLA-DQA10501-DQB10201	355	EPHMLLLCGRTFHES	PHMLLLCGR	1	0.1017	16634.7	90.00	0.31	tr_C9ZY45_
HLA-DQA10501-DQB10201	356	LLQRFACGNSPSQNL	QRFACGNSP	2	0.1010	16766.9	90.00	0.34	tr_C9ZY45_
HLA-DQA10501-DQB10201	357	REALLQRFACGNSPS	QRFACGNSP	5	0.1007	16814.7	90.00	0.38	tr_C9ZY45_
HLA-DQA10501-DQB10201	358	LQRFACGNSPSQNLL	FACGNSPSQ	3	0.0986	17206.4	90.00	0.30	tr_C9ZY45_
HLA-DQA10501-DQB10201	359	YNLLKAYCKETSLSG	LKAYCKETS	3	0.0985	17231.5	90.00	0.21	tr_C9ZY45_
HLA-DQA10501-DQB10201	360	ALLQRFACGNSPSQN	QRFACGNSP	3	0.0975	17403.3	90.00	0.38	tr_C9ZY45_
HLA-DQA10501-DQB10201	361	NAREGECVKASTGEE	CVKASTGEE	6	0.0967	17563.9	90.00	0.49	tr_C9ZY45_
HLA-DQA10501-DQB10201	362	LGGADVNARDKDGAT	GGADVNARD	1	0.0961	17676.8	90.00	0.26	tr_C9ZY45_
HLA-DQA10501-DQB10201	363	EDLLHFVCQKGGKGA	DLLHFVCQK	1	0.0927	18345.3	90.00	0.40	tr_C9ZY45_

HLA-DQA10501-DQB10201	364	RFACGNSPSQNLLSF	FACGNSPSQ	1	0.0906	18751.7	95.00	0.28	tr_C9ZY45_	
HLA-DQA10501-DQB10201	365	QRFACGNSPSQNLLS	FACGNSPSQ	2	0.0871	19494.2	95.00	0.32	tr_C9ZY45_	
HLA-DQA10501-DQB10201	366	IDNGADINARNASGE	GADINARNA	3	0.0858	19770.1	95.00	0.19	tr_C9ZY45_	
HLA-DQA10501-DQB10201	367	WCPTAAERRVRSKEV	ERRVRSKEV	6	0.0845	20034.6	95.00	0.50	tr_C9ZY45_	
HLA-DQA10501-DQB10201	368	LWCPTAAERRVRSKE	WCPTAAERR	1	0.0836	20237.2	95.00	0.51	tr_C9ZY45_	
HLA-DQA10501-DQB10201	369	DNGADINARNASGET	NARNASGET	6	0.0828	20416.7	95.00	0.23	tr_C9ZY45_	
HLA-DQA10501-DQB10201	370	GGADVNARDKDGATA	GGADVNARD	0	0.0790	21261.4	95.00	0.20	tr_C9ZY45_	
HLA-DQA10501-DQB10201	371	GADVNARDKDGATAL	RDKDGATAL	6	0.0781	21486.9	95.00	0.31	tr_C9ZY45_	
HLA-DQA10501-DQB10201	372	NLLKAYCKETSLSGR	LKAYCKETS	2	0.0765	21852.4	95.00	0.25	tr_C9ZY45_	
HLA-DQA10501-DQB10201	373	ADVNARDKDGATALH	RDKDGATAL	5	0.0761	21946.0	95.00	0.29	tr_C9ZY45_	
HLA-DQA10501-DQB10201	374	LLKAYCKETSLSGRE	KETSLSGRE	6	0.0713	23121.1	95.00	0.25	tr_C9ZY45_	
HLA-DQA10501-DQB10201	375	DLLHFVCQKGGKGAN	DLLHFVCQK	0	0.0679	23973.0	95.00	0.42	tr_C9ZY45_	
HLA-DQA10501-DQB10201	376	HFVCQKGGKGANFVI	KGGKGANFV	5	0.0653	24676.4	95.00	0.43	tr_C9ZY45_	
HLA-DQA10501-DQB10201	377	LHFVCQKGGKGANFV	KGGKGANFV	6	0.0568	27035.1	100.00	0.50	tr_C9ZY45_	
HLA-DQA10501-DQB10201	378	LLHFVCQKGGKGANF	QKGGKGANF	6	0.0522	28414.1	100.00	0.36	tr_C9ZY45_	

Allele: HLA-DQA10501-DQB10201. Number of high binders 0. Number of weak binders 19. Number of peptides 378

Allele	pos	peptide	core	Of	1-log50k(aff)	affinity(nM)	%Rank	Relia	Identity	Bind Level
HLA-DQA10501-DQB10201	1	PSKGLTSAEAEELLL	LTSAEAEEL	4	0.6790	32.2	0.30	0.50	tr_D0A564_	SB
HLA-DQA10501-DQB10201	2	PPSKGLTSAEAEELL	LTSAEAEEL	5	0.6591	40.0	0.50	0.61	tr_D0A564_	WB
HLA-DQA10501-DQB10201	3	SKGLTSAEAEELLLK	LTSAEAEEL	3	0.6399	49.2	0.60	0.50	tr_D0A564_	WB
HLA-DQA10501-DQB10201	4	KGLTSAEAEELLLKY	LTSAEAEEL	2	0.6228	59.2	0.90	0.50	tr_D0A564_	WB
HLA-DQA10501-DQB10201	5	LPPSKGLTSAEAEEL	LTSAEAEEL	6	0.5974	77.9	1.40	0.70	tr_D0A564_	WB
HLA-DQA10501-DQB10201	6	GLTSAEAEELLLKYG	LTSAEAEEL	1	0.5940	80.9	1.40	0.55	tr_D0A564_	WB
HLA-DQA10501-DQB10201	7	CTINEGVIDVDEAAL	EGVIDVDEA	4	0.5552	123.1	3.00	0.46	tr_D0A564_	WB
HLA-DQA10501-DQB10201	8	TINEGVIDVDEAALT	EGVIDVDEA	3	0.5503	129.7	3.00	0.41	tr_D0A564_	WB
HLA-DQA10501-DQB10201	9	LTSAEAEELLLKYGR	LTSAEAEEL	0	0.5306	160.6	4.00	0.44	tr_D0A564_	WB
HLA-DQA10501-DQB10201	10	INEGVIDVDEAALTG	EGVIDVDEA	2	0.5280	165.1	4.00	0.44	tr_D0A564_	WB
HLA-DQA10501-DQB10201	11	DCTINEGVIDVDEAA	EGVIDVDEA	5	0.5238	172.9	4.00	0.56	tr_D0A564_	WB
HLA-DQA10501-DQB10201	12	RDGTWQQIDAALLVP	WQQIDAALL	4	0.5169	186.2	4.50	0.56	tr_D0A564_	WB
HLA-DQA10501-DQB10201	13	ADCTINEGVIDVDEA	EGVIDVDEA	6	0.5161	187.8	4.50	0.63	tr_D0A564_	WB
HLA-DQA10501-DQB10201	14	VIDVDEAALTGESLP	EAALTGESL	5	0.5006	222.2	5.50	0.70	tr_D0A564_	WB
HLA-DQA10501-DQB10201	15	DGTWQQIDAALLVPG	WQQIDAALL	3	0.5001	223.4	5.50	0.54	tr_D0A564_	WB
HLA-DQA10501-DQB10201	16	NEGVIDVDEAALTGE	EGVIDVDEA	1	0.4999	223.9	5.50	0.41	tr_D0A564_	WB
HLA-DQA10501-DQB10201	17	GVIDVDEAALTGESL	EAALTGESL	6	0.4996	224.5	5.50	0.62	tr_D0A564_	WB
HLA-DQA10501-DQB10201	18	AYRDGTWQQIDAALL	WQQIDAALL	6	0.4987	226.7	5.50	0.65	tr_D0A564_	WB
HLA-DQA10501-DQB10201	19	YRDGTWQQIDAALLV	WQQIDAALL	5	0.4958	234.1	6.00	0.56	tr_D0A564_	WB
HLA-DQA10501-DQB10201	20	TWQQIDAALLVPGDL	WQQIDAALL	1	0.4883	253.8	6.50	0.44	tr_D0A564_	WB
HLA-DQA10501-DQB10201	21	GTWQQIDAALLVPGD	WQQIDAALL	2	0.4812	273.9	7.00	0.51	tr_D0A564_	WB
HLA-DQA10501-DQB10201	22	IDVDEAALTGESLPV	EAALTGESL	4	0.4780	283.6	7.00	0.70	tr_D0A564_	WB
HLA-DQA10501-DQB10201	23	GKTATLLQSVEADIG	LQSVEADIG	6	0.4728	300.0	7.50	0.32	tr_D0A564_	WB
HLA-DQA10501-DQB10201	24	WQQIDAALLVPGDLV	WQQIDAALL	0	0.4685	314.4	8.00	0.32	tr_D0A564_	WB
HLA-DQA10501-DQB10201	25	TATLLQSVEADIGSI	LQSVEADIG	4	0.4665	321.3	8.00	0.35	tr_D0A564_	WB
HLA-DQA10501-DQB10201	26	DVDEAALTGESLPVT	EAALTGESL	3	0.4647	327.7	8.50	0.72	tr_D0A564_	WB
HLA-DQA10501-DQB10201	27	KTATLLQSVEADIGS	LQSVEADIG	5	0.4640	330.0	8.50	0.34	tr_D0A564_	WB

HLA-DQA10501-DQB10201	28	FGKTATLLQSVEADI	LLQSVEADI	6	0.4508	380.9	9.50	0.38	tr_D0A564_	WB
HLA-DQA10501-DQB10201	29	VDEAALTGESLPVTM	EAALTGESL	2	0.4506	381.8	9.50	0.65	tr_D0A564_	WB
HLA-DQA10501-DQB10201	30	EGVIDVDEAALTGES	EGVIDVDEA	0	0.4494	386.7	9.50	0.34	tr_D0A564_	WB
HLA-DQA10501-DQB10201	31	ATLLQSVEADIGSIR	LQSVEADIG	3	0.4442	409.1	11.00	0.35	tr_D0A564_	
HLA-DQA10501-DQB10201	32	TLLQSVEADIGSIRI	LQSVEADIG	2	0.4267	494.4	12.00	0.34	tr_D0A564_	
HLA-DQA10501-DQB10201	33	SNVVRGEVDATVQYT	VRGEVDATV	3	0.4262	496.9	13.00	0.32	tr_D0A564_	
HLA-DQA10501-DQB10201	34	GSNVVRGEVDATVQY	VRGEVDATV	4	0.4174	546.7	14.00	0.37	tr_D0A564_	
HLA-DQA10501-DQB10201	35	DEAALTGESLPVTMG	EAALTGESL	1	0.4136	569.4	14.00	0.65	tr_D0A564_	
HLA-DQA10501-DQB10201	36	MGSNVVRGEVDATVQ	VRGEVDATV	5	0.4117	581.2	14.00	0.37	tr_D0A564_	
HLA-DQA10501-DQB10201	37	KMGSNVVRGEVDATV	VRGEVDATV	6	0.4101	591.1	14.00	0.40	tr_D0A564_	
HLA-DQA10501-DQB10201	38	FFGKTATLLQSVEAD	TLLQSVEAD	6	0.3972	680.4	16.00	0.37	tr_D0A564_	
HLA-DQA10501-DQB10201	39	NVVRGEVDATVQYTG	VRGEVDATV	2	0.3940	703.9	17.00	0.36	tr_D0A564_	
HLA-DQA10501-DQB10201	40	LLGIQLANALIGWYE	ANALIGWYE	6	0.3831	792.0	18.00	0.22	tr_D0A564_	
HLA-DQA10501-DQB10201	41	IFLRNLWGPMPIVLW	LWGPMPIVL	5	0.3818	803.0	19.00	0.21	tr_D0A564_	
HLA-DQA10501-DQB10201	42	PADCTINEGVIDVDE	INEGVIDVD	5	0.3779	838.3	19.00	0.34	tr_D0A564_	
HLA-DQA10501-DQB10201	43	LLQSVEADIGSIRII	LQSVEADIG	1	0.3735	879.2	20.00	0.39	tr_D0A564_	
HLA-DQA10501-DQB10201	44	QQIDAALLVPGDLVK	AALLVPGDL	4	0.3720	893.0	20.00	0.33	tr_D0A564_	
HLA-DQA10501-DQB10201	45	IQFALQHFADGAVLL	HFADGAVLL	6	0.3674	938.7	21.00	0.43	tr_D0A564_	
HLA-DQA10501-DQB10201	46	VLLGIQLANALIGWY	LANALIGWY	6	0.3653	960.9	21.00	0.17	tr_D0A564_	
HLA-DQA10501-DQB10201	47	LGIQLANALIGWYET	ANALIGWYE	5	0.3644	969.5	22.00	0.28	tr_D0A564_	
HLA-DQA10501-DQB10201	48	VVRGEVDATVQYTGQ	VRGEVDATV	1	0.3622	993.5	22.00	0.34	tr_D0A564_	
HLA-DQA10501-DQB10201	49	GIQLANALIGWYETI	ANALIGWYE	4	0.3621	994.2	22.00	0.31	tr_D0A564_	
HLA-DQA10501-DQB10201	50	FLRNLWGPMPIVLWI	LWGPMPIVL	4	0.3600	1017.4	22.00	0.23	tr_D0A564_	
HLA-DQA10501-DQB10201	51	TSAEAEELLLKYGRN	TSAEAEELL	0	0.3586	1032.5	23.00	0.46	tr_D0A564_	
HLA-DQA10501-DQB10201	52	AVLLGIQLANALIGW	LLGIQLANA	2	0.3553	1070.1	23.00	0.23	tr_D0A564_	
HLA-DQA10501-DQB10201	53	EVDATVQYTGQSTFF	QYTGQSTFF	6	0.3513	1117.2	24.00	0.44	tr_D0A564_	
HLA-DQA10501-DQB10201	54	FALQHFADGAVLLGI	HFADGAVLL	4	0.3501	1132.4	24.00	0.31	tr_D0A564_	
HLA-DQA10501-DQB10201	55	QIDAALLVPGDLVKL	AALLVPGDL	3	0.3494	1140.6	24.00	0.38	tr_D0A564_	
HLA-DQA10501-DQB10201	56	LIFLRNLWGPMPIVL	LWGPMPIVL	6	0.3458	1185.7	25.00	0.28	tr_D0A564_	
HLA-DQA10501-DQB10201	57	LLPPSKGLTSAEAEE	KGLTSAEAE	5	0.3434	1216.8	26.00	0.49	tr_D0A564_	
HLA-DQA10501-DQB10201	58	EAALTGESLPVTMGT	EAALTGESL	0	0.3432	1220.2	26.00	0.55	tr_D0A564_	
HLA-DQA10501-DQB10201	59	QFALQHFADGAVLLG	HFADGAVLL	5	0.3392	1273.4	26.00	0.38	tr_D0A564_	
HLA-DQA10501-DQB10201	60	TAYRDGTWQQIDAAL	TWQQIDAAL	6	0.3358	1321.0	27.00	0.47	tr_D0A564_	
HLA-DQA10501-DQB10201	61	TFFGKTATLLQSVEA	ATLLQSVEA	6	0.3353	1328.3	27.00	0.49	tr_D0A564_	
HLA-DQA10501-DQB10201	62	WLIFLRNLWGPMPIV	RNLWGPMPI	5	0.3338	1350.2	27.00	0.26	tr_D0A564_	
HLA-DQA10501-DQB10201	63	LQSVEADIGSIRIIL	LQSVEADIG	0	0.3321	1374.8	28.00	0.30	tr_D0A564_	
HLA-DQA10501-DQB10201	64	ALQHFADGAVLLGIQ	HFADGAVLL	3	0.3321	1375.7	28.00	0.28	tr_D0A564_	
HLA-DQA10501-DQB10201	65	SWLIFLRNLWGPMPI	RNLWGPMPI	6	0.3313	1387.7	28.00	0.28	tr_D0A564_	
HLA-DQA10501-DQB10201	66	LQHFADGAVLLGIQL	HFADGAVLL	2	0.3301	1405.4	28.00	0.22	tr_D0A564_	
HLA-DQA10501-DQB10201	67	DGAVLLGIQLANALI	LLGIQLANA	4	0.3279	1440.1	29.00	0.42	tr_D0A564_	
HLA-DQA10501-DQB10201	68	VPADCTINEGVIDVD	INEGVIDVD	6	0.3278	1440.9	29.00	0.55	tr_D0A564_	
HLA-DQA10501-DQB10201	69	LRNLWGPMPIVLWIV	LWGPMPIVL	3	0.3273	1448.8	29.00	0.27	tr_D0A564_	
HLA-DQA10501-DQB10201	70	GAVLLGIQLANALIG	LLGIQLANA	3	0.3241	1500.5	30.00	0.34	tr_D0A564_	
HLA-DQA10501-DQB10201	71	QSVEADIGSIRIILM	IGSIRIILM	6	0.3224	1528.4	30.00	0.38	tr_D0A564_	
HLA-DQA10501-DQB10201	72	IQLANALIGWYETIK	ANALIGWYE	3	0.3222	1531.0	30.00	0.29	tr_D0A564_	
HLA-DQA10501-DQB10201	73	VDATVQYTGQSTFFG	QYTGQSTFF	5	0.3217	1539.6	30.00	0.47	tr_D0A564_	
HLA-DQA10501-DQB10201	74	WIVIIIQFALQHFAD	IIQFALQHF	4	0.3202	1564.8	30.00	0.29	tr_D0A564_	
HLA-DQA10501-DQB10201	75	IIQFALQHFADGAVL	QHFADGAVL	6	0.3187	1590.4	31.00	0.38	tr_D0A564_	
HLA-DQA10501-DQB10201	76	QLANALIGWYETIKA	IGWYETIKA	6	0.3174	1612.1	31.00	0.22	tr_D0A564_	
HLA-DQA10501-DQB10201	77	GWYETIKAGDAVAAL	KAGDAVAAL	6	0.3174	1612.9	31.00	0.34	tr_D0A564_	

HLA-DQA10501-DQB10201	78	ADGAVLLGIQLANAL	LLGIQLANA	5	0.3167	1625.3	31.00	0.41	tr_D0A564_
HLA-DQA10501-DQB10201	79	PKMGSNVVRGEVDAT	SNVVRGEVD	4	0.3146	1662.5	32.00	0.44	tr_D0A564_
HLA-DQA10501-DQB10201	80	VLWIVIIIQFALQHF	IIQFALQHF	6	0.3131	1689.6	32.00	0.49	tr_D0A564_
HLA-DQA10501-DQB10201	81	QHFADGAVLLGIQLA	HFADGAVLL	1	0.3100	1747.4	33.00	0.22	tr_D0A564_
HLA-DQA10501-DQB10201	82	IDAALLVPGDLVKLG	AALLVPGDL	2	0.3079	1786.5	33.00	0.47	tr_D0A564_
HLA-DQA10501-DQB10201	83	FADGAVLLGIQLANA	LLGIQLANA	6	0.3072	1799.7	33.00	0.45	tr_D0A564_
HLA-DQA10501-DQB10201	84	RNLWGPMPIVLWIVI	LWGPMPIVL	2	0.3017	1910.4	35.00	0.32	tr_D0A564_
HLA-DQA10501-DQB10201	85	LWIVIIIQFALQHFA	IIQFALQHF	5	0.3005	1936.0	35.00	0.38	tr_D0A564_
HLA-DQA10501-DQB10201	86	WYETIKAGDAVAALK	KAGDAVAAL	5	0.2991	1964.8	35.00	0.28	tr_D0A564_
HLA-DQA10501-DQB10201	87	VRGEVDATVQYTGQS	EVDATVQYT	3	0.2979	1990.5	36.00	0.40	tr_D0A564_
HLA-DQA10501-DQB10201	88	PLLPPSKGLTSAEAE	KGLTSAEAE	6	0.2939	2079.8	37.00	0.60	tr_D0A564_
HLA-DQA10501-DQB10201	89	PSWLIFLRNLWGPMP	WLIFLRNLW	2	0.2927	2106.5	37.00	0.31	tr_D0A564_
HLA-DQA10501-DQB10201	90	SVEADIGSIRIILMR	IGSIRIILM	5	0.2923	2114.9	37.00	0.34	tr_D0A564_
HLA-DQA10501-DQB10201	91	IVIIIQFALQHFADG	IIQFALQHF	3	0.2916	2131.2	37.00	0.31	tr_D0A564_
HLA-DQA10501-DQB10201	92	VIIIQFALQHFADGA	IIQFALQHF	2	0.2900	2169.3	38.00	0.26	tr_D0A564_
HLA-DQA10501-DQB10201	93	IIIQFALQHFADGAV	IIQFALQHF	1	0.2884	2207.2	38.00	0.21	tr_D0A564_
HLA-DQA10501-DQB10201	94	IGWYETIKAGDAVAA	YETIKAGDA	3	0.2881	2214.9	38.00	0.23	tr_D0A564_
HLA-DQA10501-DQB10201	95	ALIGWYETIKAGDAV	YETIKAGDA	5	0.2857	2273.1	39.00	0.29	tr_D0A564_
HLA-DQA10501-DQB10201	96	HFADGAVLLGIQLAN	ADGAVLLGI	2	0.2852	2283.7	39.00	0.18	tr_D0A564_
HLA-DQA10501-DQB10201	97	LIGWYETIKAGDAVA	YETIKAGDA	4	0.2852	2283.9	39.00	0.26	tr_D0A564_
HLA-DQA10501-DQB10201	98	DATVQYTGQSTFFGK	QYTGQSTFF	4	0.2849	2292.8	39.00	0.46	tr_D0A564_
HLA-DQA10501-DQB10201	99	STFFGKTATLLQSVE	FFGKTATLL	2	0.2833	2331.5	39.00	0.38	tr_D0A564_
HLA-DQA10501-DQB10201	100	TPSWLIFLRNLWGPM	WLIFLRNLW	3	0.2820	2364.0	40.00	0.34	tr_D0A564_
HLA-DQA10501-DQB10201	101	DAALLVPGDLVKLGS	AALLVPGDL	1	0.2816	2376.4	40.00	0.47	tr_D0A564_
HLA-DQA10501-DQB10201	102	TGESLPVTMGTEHMP	PVTMGTEHM	5	0.2764	2512.0	41.00	0.55	tr_D0A564_
HLA-DQA10501-DQB10201	103	GEVDATVQYTGQSTF	EVDATVQYT	1	0.2761	2520.8	41.00	0.35	tr_D0A564_
HLA-DQA10501-DQB10201	104	LANALIGWYETIKAG	ANALIGWYE	1	0.2748	2557.7	41.00	0.26	tr_D0A564_
HLA-DQA10501-DQB10201	105	TGQSTFFGKTATLLQ	FFGKTATLL	5	0.2714	2651.9	42.00	0.63	tr_D0A564_
HLA-DQA10501-DQB10201	106	NLWGPMPIVLWIVII	LWGPMPIVL	1	0.2712	2657.3	42.00	0.29	tr_D0A564_
HLA-DQA10501-DQB10201	107	QSTFFGKTATLLQSV	FFGKTATLL	3	0.2700	2693.5	43.00	0.51	tr_D0A564_
HLA-DQA10501-DQB10201	108	YETIKAGDAVAALKN	KAGDAVAAL	4	0.2694	2710.0	43.00	0.30	tr_D0A564_
HLA-DQA10501-DQB10201	109	MPKMGSNVVRGEVDA	SNVVRGEVD	5	0.2686	2732.8	43.00	0.60	tr_D0A564_
HLA-DQA10501-DQB10201	110	YTGQSTFFGKTATLL	FFGKTATLL	6	0.2686	2735.6	43.00	0.79	tr_D0A564_
HLA-DQA10501-DQB10201	111	ATVQYTGQSTFFGKT	QYTGQSTFF	3	0.2660	2811.5	44.00	0.49	tr_D0A564_
HLA-DQA10501-DQB10201	112	NALIGWYETIKAGDA	YETIKAGDA	6	0.2603	2992.0	45.00	0.34	tr_D0A564_
HLA-DQA10501-DQB10201	113	GQSTFFGKTATLLQS	FFGKTATLL	4	0.2601	2997.8	45.00	0.60	tr_D0A564_
HLA-DQA10501-DQB10201	114	HMPKMGSNVVRGEVD	SNVVRGEVD	6	0.2595	3016.9	46.00	0.64	tr_D0A564_
HLA-DQA10501-DQB10201	115	ATAYRDGTWQQIDAA	GTWQQIDAA	6	0.2579	3069.1	46.00	0.41	tr_D0A564_
HLA-DQA10501-DQB10201	116	ANALIGWYETIKAGD	IGWYETIKA	4	0.2541	3197.7	47.00	0.28	tr_D0A564_
HLA-DQA10501-DQB10201	117	EKKTPSWLIFLRNLW	WLIFLRNLW	6	0.2520	3273.1	48.00	0.44	tr_D0A564_
HLA-DQA10501-DQB10201	118	KTPSWLIFLRNLWGP	WLIFLRNLW	4	0.2489	3382.4	48.00	0.44	tr_D0A564_
HLA-DQA10501-DQB10201	119	LTGESLPVTMGTEHM	PVTMGTEHM	6	0.2479	3421.6	49.00	0.55	tr_D0A564_
HLA-DQA10501-DQB10201	120	IVLWIVIIIQFALQH	IIIQFALQH	6	0.2452	3522.8	50.00	0.37	tr_D0A564_
HLA-DQA10501-DQB10201	121	VKLGSGSAVPADCTI	GSAVPADCT	5	0.2449	3534.6	50.00	0.34	tr_D0A564_
HLA-DQA10501-DQB10201	122	RGEVDATVQYTGQST	EVDATVQYT	2	0.2445	3548.4	50.00	0.50	tr_D0A564_
HLA-DQA10501-DQB10201	123	TVQYTGQSTFFGKTA	QYTGQSTFF	2	0.2436	3585.1	50.00	0.51	tr_D0A564_
HLA-DQA10501-DQB10201	124	GESLPVTMGTEHMPK	PVTMGTEHM	4	0.2426	3624.1	50.00	0.55	tr_D0A564_
HLA-DQA10501-DQB10201	125	ESLPVTMGTEHMPKM	PVTMGTEHM	3	0.2412	3678.2	55.00	0.52	tr_D0A564_
HLA-DQA10501-DQB10201	126	LGSGSAVPADCTINE	GSAVPADCT	3	0.2388	3775.2	55.00	0.34	tr_D0A564_
HLA-DQA10501-DQB10201	127	ETIKAGDAVAALKNS	KAGDAVAAL	3	0.2375	3829.8	55.00	0.35	tr_D0A564_

HLA-DQA10501-DQB10201	128	KKTPSWLIFLRNLWG	WLIFLRNLW	5	0.2362	3881.5	55.00	0.43	tr_D0A564_
HLA-DQA10501-DQB10201	129	KLGSGSAVPADCTIN	GSAVPADCT	4	0.2349	3937.7	55.00	0.34	tr_D0A564_
HLA-DQA10501-DQB10201	130	GSGSAVPADCTINEG	GSAVPADCT	2	0.2333	4004.2	55.00	0.32	tr_D0A564_
HLA-DQA10501-DQB10201	131	AALLVPGDLVKLGSG	ALLVPGDLV	1	0.2315	4086.3	55.00	0.40	tr_D0A564_
HLA-DQA10501-DQB10201	132	LVKLGSGSAVPADCT	GSAVPADCT	6	0.2296	4168.8	55.00	0.35	tr_D0A564_
HLA-DQA10501-DQB10201	133	TIKAGDAVAALKNSL	KAGDAVAAL	2	0.2273	4273.8	55.00	0.34	tr_D0A564_
HLA-DQA10501-DQB10201	134	PMPIVLWIVIIIQFA	WIVIIIQFA	6	0.2270	4287.0	55.00	0.56	tr_D0A564_
HLA-DQA10501-DQB10201	135	AALTGESLPVTMGTE	TGESLPVTM	3	0.2239	4434.6	60.00	0.34	tr_D0A564_
HLA-DQA10501-DQB10201	136	AVPADCTINEGVIDV	TINEGVIDV	6	0.2227	4491.1	60.00	0.46	tr_D0A564_
HLA-DQA10501-DQB10201	137	SGSAVPADCTINEGV	GSAVPADCT	1	0.2216	4548.1	60.00	0.30	tr_D0A564_
HLA-DQA10501-DQB10201	138	SLPVTMGTEHMPKMG	PVTMGTEHM	2	0.2215	4550.4	60.00	0.51	tr_D0A564_
HLA-DQA10501-DQB10201	139	IATAYRDGTWQQIDA	YRDGTWQQI	4	0.2193	4659.6	60.00	0.28	tr_D0A564_
HLA-DQA10501-DQB10201	140	PIVLWIVIIIQFALQ	WIVIIIQFA	4	0.2187	4689.8	60.00	0.44	tr_D0A564_
HLA-DQA10501-DQB10201	141	MPIVLWIVIIIQFAL	WIVIIIQFA	5	0.2170	4778.7	60.00	0.54	tr_D0A564_
HLA-DQA10501-DQB10201	142	LWGPMPIVLWIVIII	WGPMPIVLW	1	0.2165	4803.6	60.00	0.38	tr_D0A564_
HLA-DQA10501-DQB10201	143	PIATAYRDGTWQQID	YRDGTWQQI	5	0.2156	4852.8	60.00	0.31	tr_D0A564_
HLA-DQA10501-DQB10201	144	EELLLKYGRNELPEK	LKYGRNELP	4	0.2117	5061.6	60.00	0.34	tr_D0A564_
HLA-DQA10501-DQB10201	145	AEELLLKYGRNELPE	LLKYGRNEL	4	0.2087	5225.4	60.00	0.38	tr_D0A564_
HLA-DQA10501-DQB10201	146	KPIATAYRDGTWQQI	YRDGTWQQI	6	0.2061	5374.3	65.00	0.29	tr_D0A564_
HLA-DQA10501-DQB10201	147	VQYTGQSTFFGKTAT	QYTGQSTFF	1	0.2056	5404.5	65.00	0.58	tr_D0A564_
HLA-DQA10501-DQB10201	148	EHREGDDGSVPLLPP	DDGSVPLLP	5	0.2042	5488.6	65.00	0.29	tr_D0A564_
HLA-DQA10501-DQB10201	149	LPVTMGTEHMPKMGS	PVTMGTEHM	1	0.2032	5548.8	65.00	0.52	tr_D0A564_
HLA-DQA10501-DQB10201	150	DLVKLGSGSAVPADC	SGSAVPADC	6	0.2017	5641.3	65.00	0.34	tr_D0A564_
HLA-DQA10501-DQB10201	151	IKAGDAVAALKNSLK	KAGDAVAAL	1	0.1981	5863.2	65.00	0.37	tr_D0A564_
HLA-DQA10501-DQB10201	152	EAEELLLKYGRNELP	LLKYGRNEL	5	0.1964	5971.5	65.00	0.38	tr_D0A564_
HLA-DQA10501-DQB10201	153	SAEAEELLLKYGRNE	SAEAEELLL	0	0.1926	6221.8	65.00	0.61	tr_D0A564_
HLA-DQA10501-DQB10201	154	WGPMPIVLWIVIIIQ	WGPMPIVLW	0	0.1911	6325.6	65.00	0.32	tr_D0A564_
HLA-DQA10501-DQB10201	155	ALTGESLPVTMGTEH	TGESLPVTM	2	0.1905	6365.8	65.00	0.37	tr_D0A564_
HLA-DQA10501-DQB10201	156	HREGDDGSVPLLPPS	DDGSVPLLP	4	0.1902	6386.8	65.00	0.31	tr_D0A564_
HLA-DQA10501-DQB10201	157	ELLLKYGRNELPEKK	LLKYGRNEL	2	0.1900	6402.9	65.00	0.31	tr_D0A564_
HLA-DQA10501-DQB10201	158	SAVPADCTINEGVID	CTINEGVID	6	0.1874	6581.0	70.00	0.25	tr_D0A564_
HLA-DQA10501-DQB10201	159	SEHREGDDGSVPLLP	DDGSVPLLP	6	0.1870	6614.2	70.00	0.31	tr_D0A564_
HLA-DQA10501-DQB10201	160	GSAVPADCTINEGVI	GSAVPADCT	0	0.1832	6888.3	70.00	0.22	tr_D0A564_
HLA-DQA10501-DQB10201	161	SLKPIATAYRDGTWQ	TAYRDGTWQ	6	0.1757	7473.1	70.00	0.25	tr_D0A564_
HLA-DQA10501-DQB10201	162	QYTGQSTFFGKTATL	QYTGQSTFF	0	0.1748	7544.9	70.00	0.45	tr_D0A564_
HLA-DQA10501-DQB10201	163	LLLKYGRNELPEKKT	LKYGRNELP	2	0.1737	7637.9	70.00	0.34	tr_D0A564_
HLA-DQA10501-DQB10201	164	REGDDGSVPLLPPSK	GDDGSVPLL	2	0.1736	7643.3	70.00	0.25	tr_D0A564_
HLA-DQA10501-DQB10201	165	KAGDAVAALKNSLKP	AGDAVAALK	1	0.1718	7794.6	75.00	0.28	tr_D0A564_
HLA-DQA10501-DQB10201	166	PEKKTPSWLIFLRNL	PSWLIFLRN	5	0.1687	8055.4	75.00	0.41	tr_D0A564_
HLA-DQA10501-DQB10201	167	PVTMGTEHMPKMGSN	PVTMGTEHM	0	0.1663	8272.9	75.00	0.49	tr_D0A564_
HLA-DQA10501-DQB10201	168	NSLKPIATAYRDGTW	ATAYRDGTW	6	0.1645	8432.9	75.00	0.31	tr_D0A564_
HLA-DQA10501-DQB10201	169	LKPIATAYRDGTWQQ	TAYRDGTWQ	5	0.1643	8446.9	75.00	0.27	tr_D0A564_
HLA-DQA10501-DQB10201	170	ISEHREGDDGSVPLL	GDDGSVPLL	6	0.1640	8478.2	75.00	0.32	tr_D0A564_
HLA-DQA10501-DQB10201	171	VPLLPPSKGLTSAEA	SKGLTSAEA	6	0.1638	8499.3	75.00	0.82	tr_D0A564_
HLA-DQA10501-DQB10201	172	GPMPIVLWIVIIIQF	VLWIVIIIQ	5	0.1630	8567.0	75.00	0.30	tr_D0A564_
HLA-DQA10501-DQB10201	173	GDLVKLGSGSAVPAD	LGSGSAVPA	5	0.1619	8671.5	75.00	0.45	tr_D0A564_
HLA-DQA10501-DQB10201	174	EGDDGSVPLLPPSKG	GDDGSVPLL	1	0.1609	8771.2	75.00	0.27	tr_D0A564_
HLA-DQA10501-DQB10201	175	ALLVPGDLVKLGSGS	ALLVPGDLV	0	0.1589	8955.9	75.00	0.53	tr_D0A564_
HLA-DQA10501-DQB10201	176	LPEKKTPSWLIFLRN	PSWLIFLRN	6	0.1541	9435.0	75.00	0.47	tr_D0A564_
HLA-DQA10501-DQB10201	177	LLKYGRNELPEKKTP	YGRNELPEK	3	0.1538	9469.0	75.00	0.25	tr_D0A564_

HLA-DQA10501-DQB10201	178	PGDLVKLGSGSAVPA	LGSGSAVPA	6	0.1449	10420.6	80.00	0.53	tr_D0A564_
HLA-DQA10501-DQB10201	179	SVLSKAISEHREGDD	SKAISEHRE	3	0.1447	10448.1	80.00	0.32	tr_D0A564_
HLA-DQA10501-DQB10201	180	AEAEELLLKYGRNEL	LLKYGRNEL	6	0.1440	10525.4	80.00	0.44	tr_D0A564_
HLA-DQA10501-DQB10201	181	AGDAVAALKNSLKPI	AVAALKNSL	3	0.1403	10953.9	80.00	0.33	tr_D0A564_
HLA-DQA10501-DQB10201	182	RRQSVLSKAISEHRE	SKAISEHRE	6	0.1399	11004.7	80.00	0.30	tr_D0A564_
HLA-DQA10501-DQB10201	183	VLSKAISEHREGDDG	SKAISEHRE	2	0.1394	11061.6	80.00	0.34	tr_D0A564_
HLA-DQA10501-DQB10201	184	QSVLSKAISEHREGD	SKAISEHRE	4	0.1382	11214.1	80.00	0.31	tr_D0A564_
HLA-DQA10501-DQB10201	185	GDDGSVPLLPPSKGL	DDGSVPLLP	1	0.1340	11729.8	85.00	0.35	tr_D0A564_
HLA-DQA10501-DQB10201	186	AISEHREGDDGSVPL	EGDDGSVPL	6	0.1318	12017.5	85.00	0.30	tr_D0A564_
HLA-DQA10501-DQB10201	187	RQSVLSKAISEHREG	SKAISEHRE	5	0.1307	12157.3	85.00	0.28	tr_D0A564_
HLA-DQA10501-DQB10201	188	KNSLKPIATAYRDGT	IATAYRDGT	6	0.1299	12262.0	85.00	0.27	tr_D0A564_
HLA-DQA10501-DQB10201	189	EHMPKMGSNVVRGEV	MGSNVVRGE	5	0.1295	12311.7	85.00	0.31	tr_D0A564_
HLA-DQA10501-DQB10201	190	LSKAISEHREGDDGS	SKAISEHRE	1	0.1277	12554.7	85.00	0.29	tr_D0A564_
HLA-DQA10501-DQB10201	191	ALKNSLKPIATAYRD	KPIATAYRD	6	0.1259	12810.5	85.00	0.36	tr_D0A564_
HLA-DQA10501-DQB10201	192	KPQRRQSVLSKAISE	SVLSKAISE	6	0.1227	13262.8	85.00	0.56	tr_D0A564_
HLA-DQA10501-DQB10201	193	LKNSLKPIATAYRDG	KPIATAYRD	5	0.1216	13410.6	85.00	0.33	tr_D0A564_
HLA-DQA10501-DQB10201	194	PQRRQSVLSKAISEH	SVLSKAISE	5	0.1172	14076.2	85.00	0.45	tr_D0A564_
HLA-DQA10501-DQB10201	195	SKAISEHREGDDGSV	SKAISEHRE	0	0.1160	14246.7	85.00	0.19	tr_D0A564_
HLA-DQA10501-DQB10201	196	LKYGRNELPEKKTPS	YGRNELPEK	2	0.1153	14364.4	85.00	0.38	tr_D0A564_
HLA-DQA10501-DQB10201	197	QRRQSVLSKAISEHR	SVLSKAISE	4	0.1150	14413.3	85.00	0.35	tr_D0A564_
HLA-DQA10501-DQB10201	198	GPKGVPGTNDAGEVH	PGTNDAGEV	5	0.1147	14447.3	85.00	0.34	tr_D0A564_
HLA-DQA10501-DQB10201	199	TGPKGVPGTNDAGEV	PGTNDAGEV	6	0.1141	14550.2	85.00	0.43	tr_D0A564_
HLA-DQA10501-DQB10201	200	DAVAALKNSLKPIAT	AVAALKNSL	1	0.1131	14708.3	90.00	0.35	tr_D0A564_
HLA-DQA10501-DQB10201	201	KAISEHREGDDGSVP	EHREGDDGS	4	0.1130	14717.9	90.00	0.20	tr_D0A564_
HLA-DQA10501-DQB10201	202	GDAVAALKNSLKPIA	AVAALKNSL	2	0.1119	14904.6	90.00	0.39	tr_D0A564_
HLA-DQA10501-DQB10201	203	TEHMPKMGSNVVRGE	MGSNVVRGE	6	0.1070	15710.7	90.00	0.35	tr_D0A564_
HLA-DQA10501-DQB10201	204	NELPEKKTPSWLIFL	KTPSWLIFL	6	0.1060	15886.4	90.00	0.49	tr_D0A564_
HLA-DQA10501-DQB10201	205	ELPEKKTPSWLIFLR	KTPSWLIFL	5	0.1007	16819.5	90.00	0.39	tr_D0A564_
HLA-DQA10501-DQB10201	206	AALKNSLKPIATAYR	LKPIATAYR	6	0.1003	16898.3	90.00	0.31	tr_D0A564_
HLA-DQA10501-DQB10201	207	PKGVPGTNDAGEVHK	PGTNDAGEV	4	0.0992	17102.2	90.00	0.29	tr_D0A564_
HLA-DQA10501-DQB10201	208	VPGDLVKLGSGSAVP	VKLGSGSAV	5	0.0991	17106.3	90.00	0.41	tr_D0A564_
HLA-DQA10501-DQB10201	209	KGVPGTNDAGEVHKP	GTNDAGEVH	4	0.0987	17187.2	90.00	0.28	tr_D0A564_
HLA-DQA10501-DQB10201	210	AVAALKNSLKPIATA	AVAALKNSL	0	0.0978	17345.2	90.00	0.27	tr_D0A564_
HLA-DQA10501-DQB10201	211	VAALKNSLKPIATAY	SLKPIATAY	6	0.0964	17615.5	90.00	0.29	tr_D0A564_
HLA-DQA10501-DQB10201	212	LVPGDLVKLGSGSAV	VKLGSGSAV	6	0.0938	18115.4	90.00	0.38	tr_D0A564_
HLA-DQA10501-DQB10201	213	GVPGTNDAGEVHKPQ	PGTNDAGEV	2	0.0937	18134.6	90.00	0.31	tr_D0A564_
HLA-DQA10501-DQB10201	214	DDGSVPLLPPSKGLT	DDGSVPLLP	0	0.0916	18558.8	90.00	0.31	tr_D0A564_
HLA-DQA10501-DQB10201	215	DTGPKGVPGTNDAGE	VPGTNDAGE	6	0.0905	18781.6	95.00	0.45	tr_D0A564_
HLA-DQA10501-DQB10201	216	LLVPGDLVKLGSGSA	LVPGDLVKL	1	0.0883	19226.5	95.00	0.29	tr_D0A564_
HLA-DQA10501-DQB10201	217	VTMGTEHMPKMGSNV	VTMGTEHMP	0	0.0872	19455.4	95.00	0.40	tr_D0A564_
HLA-DQA10501-DQB10201	218	PQKPQRRQSVLSKAI	RQSVLSKAI	6	0.0864	19640.7	95.00	0.41	tr_D0A564_
HLA-DQA10501-DQB10201	219	KYGRNELPEKKTPSW	YGRNELPEK	1	0.0836	20228.7	95.00	0.46	tr_D0A564_
HLA-DQA10501-DQB10201	220	VPGTNDAGEVHKPQK	PGTNDAGEV	1	0.0833	20307.2	95.00	0.29	tr_D0A564_
HLA-DQA10501-DQB10201	221	GTEHMPKMGSNVVRG	KMGSNVVRG	6	0.0826	20447.4	95.00	0.43	tr_D0A564_
HLA-DQA10501-DQB10201	222	QKPQRRQSVLSKAIS	RQSVLSKAI	5	0.0818	20629.4	95.00	0.35	tr_D0A564_
HLA-DQA10501-DQB10201	223	YGRNELPEKKTPSWL	YGRNELPEK	0	0.0815	20705.6	95.00	0.31	tr_D0A564_
HLA-DQA10501-DQB10201	224	TMGTEHMPKMGSNVV	MPKMGSNVV	6	0.0712	23136.6	95.00	0.59	tr_D0A564_
HLA-DQA10501-DQB10201	225	PGTNDAGEVHKPQKP	GTNDAGEVH	1	0.0712	23148.6	95.00	0.32	tr_D0A564_
HLA-DQA10501-DQB10201	226	MGTEHMPKMGSNVVR	MPKMGSNVV	5	0.0701	23420.7	95.00	0.56	tr_D0A564_
HLA-DQA10501-DQB10201	227	MGDTGPKGVPGTNDA	PKGVPGTND	5	0.0697	23516.7	95.00	0.50	tr_D0A564_

HLA-DQA10501-DQB10201	228	KPQKPQRRQSVLSKA	RRQSVLSKA	6	0.0691	23669.4	95.00	0.57	tr_D0A564_
HLA-DQA10501-DQB10201	229	GDTGPKGVPGTNDAG	PKGVPGTND	4	0.0654	24629.7	95.00	0.50	tr_D0A564_
HLA-DQA10501-DQB10201	230	DGSVPLLPPSKGLTS	SVPLLPPSK	2	0.0640	25017.3	95.00	0.31	tr_D0A564_
HLA-DQA10501-DQB10201	231	RNELPEKKTPSWLIF	PEKKTPSWL	4	0.0579	26736.6	100.00	0.55	tr_D0A564_
HLA-DQA10501-DQB10201	232	GRNELPEKKTPSWLI	PEKKTPSWL	5	0.0564	27157.0	100.00	0.47	tr_D0A564_
HLA-DQA10501-DQB10201	233	SVPLLPPSKGLTSAE	PSKGLTSAE	6	0.0547	27676.1	100.00	0.28	tr_D0A564_
HLA-DQA10501-DQB10201	234	GTNDAGEVHKPQKPQ	GTNDAGEVH	0	0.0542	27816.9	100.00	0.35	tr_D0A564_
HLA-DQA10501-DQB10201	235	GSVPLLPPSKGLTSA	SVPLLPPSK	1	0.0490	29434.2	100.00	0.36	tr_D0A564_
HLA-DQA10501-DQB10201	236	HKPQKPQRRQSVLSK	PQRRQSVLS	5	0.0411	32066.3	100.00	0.42	tr_D0A564_
HLA-DQA10501-DQB10201	237	VHKPQKPQRRQSVLS	PQRRQSVLS	6	0.0391	32763.5	100.00	0.64	tr_D0A564_
HLA-DQA10501-DQB10201	238	TNDAGEVHKPQKPQR	DAGEVHKPQ	2	0.0381	33095.6	100.00	0.29	tr_D0A564_
HLA-DQA10501-DQB10201	239	NDAGEVHKPQKPQRR	DAGEVHKPQ	1	0.0338	34682.2	100.00	0.33	tr_D0A564_
HLA-DQA10501-DQB10201	240	DAGEVHKPQKPQRRQ	GEVHKPQKP	2	0.0277	37038.1	100.00	0.25	tr_D0A564_
HLA-DQA10501-DQB10201	241	AGEVHKPQKPQRRQS	GEVHKPQKP	1	0.0203	40146.8	100.00	0.31	tr_D0A564_
HLA-DQA10501-DQB10201	242	GEVHKPQKPQRRQSV	QKPQRRQSV	6	0.0168	41699.9	100.00	0.25	tr_D0A564_
HLA-DQA10501-DQB10201	243	EVHKPQKPQRRQSVL	KPQRRQSVL	6	0.0152	42407.5	100.00	0.31	tr_D0A564_

Allele: HLA-DQA10501-DQB10201. Number of high binders 1. Number of weak binders 29. Number of peptides 243

Allele	pos	peptide	core	Of	1-log50k(aff)	affinity(nM)	%Rank	Relia	Identity	Bind Level
HLA-DQA10501-DQB10201	1	LDTMVLGAADLDECD	MVLGAADLD	3	0.5980	77.5	1.30	0.38	tr_D0A564_	WB
HLA-DQA10501-DQB10201	2	DTMVLGAADLDECDN	MVLGAADLD	2	0.5871	87.1	1.60	0.40	tr_D0A564_	WB
HLA-DQA10501-DQB10201	3	ALDTMVLGAADLDEC	MVLGAADLD	4	0.5822	91.9	1.70	0.38	tr_D0A564_	WB
HLA-DQA10501-DQB10201	4	DALDTMVLGAADLDE	MVLGAADLD	5	0.5692	105.8	2.50	0.40	tr_D0A564_	WB
HLA-DQA10501-DQB10201	5	TMVLGAADLDECDNY	MVLGAADLD	1	0.5668	108.5	2.50	0.36	tr_D0A564_	WB
HLA-DQA10501-DQB10201	6	MVLGAADLDECDNYE	VLGAADLDE	1	0.5341	154.6	3.50	0.38	tr_D0A564_	WB
HLA-DQA10501-DQB10201	7	KHKIIVTRLTAIETM	TRLTAIETM	6	0.5298	161.9	4.00	0.62	tr_D0A564_	WB
HLA-DQA10501-DQB10201	8	DMMLSVGGFAQVFPE	SVGGFAQVF	4	0.5076	205.9	5.00	0.40	tr_D0A564_	WB
HLA-DQA10501-DQB10201	9	HKIIVTRLTAIETMS	TRLTAIETM	5	0.5033	215.7	5.50	0.58	tr_D0A564_	WB
HLA-DQA10501-DQB10201	10	ECDNYEQLEFVPFDP	DNYEQLEFV	2	0.4983	227.9	5.50	0.41	tr_D0A564_	WB
HLA-DQA10501-DQB10201	11	RDALDTMVLGAADLD	MVLGAADLD	6	0.4980	228.6	5.50	0.55	tr_D0A564_	WB
HLA-DQA10501-DQB10201	12	DECDNYEQLEFVPFD	DNYEQLEFV	3	0.4970	231.1	6.00	0.48	tr_D0A564_	WB
HLA-DQA10501-DQB10201	13	CDNYEQLEFVPFDPT	DNYEQLEFV	1	0.4966	232.1	6.00	0.32	tr_D0A564_	WB
HLA-DQA10501-DQB10201	14	MMLSVGGFAQVFPEH	SVGGFAQVF	3	0.4951	235.7	6.00	0.39	tr_D0A564_	WB
HLA-DQA10501-DQB10201	15	GDMMLSVGGFAQVFP	SVGGFAQVF	5	0.4859	260.5	6.50	0.49	tr_D0A564_	WB
HLA-DQA10501-DQB10201	16	LDECDNYEQLEFVPF	DNYEQLEFV	4	0.4809	274.8	7.00	0.55	tr_D0A564_	WB
HLA-DQA10501-DQB10201	17	AKEMCRMLDLDPNIL	MLDLDPNIL	6	0.4771	286.4	7.00	0.38	tr_D0A564_	WB
HLA-DQA10501-DQB10201	18	KIIVTRLTAIETMSG	TRLTAIETM	4	0.4767	287.7	7.50	0.53	tr_D0A564_	WB
HLA-DQA10501-DQB10201	19	MLSVGGFAQVFPEHK	SVGGFAQVF	2	0.4714	304.8	7.50	0.34	tr_D0A564_	WB
HLA-DQA10501-DQB10201	20	DNYEQLEFVPFDPTT	DNYEQLEFV	0	0.4680	316.3	8.00	0.25	tr_D0A564_	WB
HLA-DQA10501-DQB10201	21	QDEINDSVVDIIDKL	INDSVVDII	3	0.4669	319.8	8.00	0.29	tr_D0A564_	WB
HLA-DQA10501-DQB10201	22	RMLDLDPNILTAEKL	PNILTAEKL	6	0.4661	322.7	8.00	0.52	tr_D0A564_	WB
HLA-DQA10501-DQB10201	23	IIVTRLTAIETMSGV	TRLTAIETM	3	0.4641	329.7	8.50	0.52	tr_D0A564_	WB
HLA-DQA10501-DQB10201	24	KEMCRMLDLDPNILT	MLDLDPNIL	5	0.4635	332.1	8.50	0.39	tr_D0A564_	WB
HLA-DQA10501-DQB10201	25	LSVGGFAQVFPEHKF	SVGGFAQVF	1	0.4609	341.2	8.50	0.31	tr_D0A564_	WB
HLA-DQA10501-DQB10201	26	DLDECDNYEQLEFVP	DNYEQLEFV	5	0.4576	353.9	9.00	0.62	tr_D0A564_	WB

HLA-DQA10501-DQB10201	27	HNQDEINDSVVDIID	INDSVVDII	5	0.4535	369.7	9.50	0.44	tr_D0A564_	WB
HLA-DQA10501-DQB10201	28	DEINDSVVDIIDKLA	INDSVVDII	2	0.4493	387.2	9.50	0.28	tr_D0A564_	WB
HLA-DQA10501-DQB10201	29	EMCRMLDLDPNILTA	MLDLDPNIL	4	0.4402	427.0	11.00	0.44	tr_D0A564_	
HLA-DQA10501-DQB10201	30	IVTRLTAIETMSGVN	TRLTAIETM	2	0.4361	446.2	11.00	0.54	tr_D0A564_	
HLA-DQA10501-DQB10201	31	ADLDECDNYEQLEFV	DNYEQLEFV	6	0.4350	451.8	12.00	0.63	tr_D0A564_	
HLA-DQA10501-DQB10201	32	VHNQDEINDSVVDII	INDSVVDII	6	0.4338	457.8	12.00	0.56	tr_D0A564_	
HLA-DQA10501-DQB10201	33	VLGAADLDECDNYEQ	VLGAADLDE	0	0.4313	470.0	12.00	0.45	tr_D0A564_	
HLA-DQA10501-DQB10201	34	SVGGFAQVFPEHKFL	FAQVFPEHK	4	0.4249	503.8	13.00	0.22	tr_D0A564_	
HLA-DQA10501-DQB10201	35	MLDLDPNILTAEKLP	PNILTAEKL	5	0.4206	527.7	13.00	0.59	tr_D0A564_	
HLA-DQA10501-DQB10201	36	VTRLTAIETMSGVNM	TRLTAIETM	1	0.4198	532.8	13.00	0.50	tr_D0A564_	
HLA-DQA10501-DQB10201	37	NQDEINDSVVDIIDK	INDSVVDII	4	0.4191	536.5	13.00	0.43	tr_D0A564_	
HLA-DQA10501-DQB10201	38	VFPEHKFLIVEALRQ	FLIVEALRQ	6	0.4184	540.5	13.00	0.38	tr_D0A564_	
HLA-DQA10501-DQB10201	39	IAKEMCRMLDLDPNI	RMLDLDPNI	6	0.4167	550.5	14.00	0.35	tr_D0A564_	
HLA-DQA10501-DQB10201	40	EINDSVVDIIDKLAS	SVVDIIDKL	4	0.4123	577.2	14.00	0.29	tr_D0A564_	
HLA-DQA10501-DQB10201	41	MCRMLDLDPNILTAE	MLDLDPNIL	3	0.4096	594.9	15.00	0.52	tr_D0A564_	
HLA-DQA10501-DQB10201	42	GTLTLNKMEIQEQCF	KMEIQEQCF	6	0.4032	637.0	15.00	0.52	tr_D0A564_	
HLA-DQA10501-DQB10201	43	APHVIIEMVHNQDEI	IIEMVHNQD	4	0.4008	654.2	16.00	0.32	tr_D0A564_	
HLA-DQA10501-DQB10201	44	HVIIEMVHNQDEIND	IIEMVHNQD	2	0.4006	655.2	16.00	0.31	tr_D0A564_	
HLA-DQA10501-DQB10201	45	YGDMMLSVGGFAQVF	SVGGFAQVF	6	0.3971	680.5	16.00	0.60	tr_D0A564_	
HLA-DQA10501-DQB10201	46	PHVIIEMVHNQDEIN	IIEMVHNQD	3	0.3964	685.9	16.00	0.30	tr_D0A564_	
HLA-DQA10501-DQB10201	47	PRDALDTMVLGAADL	TMVLGAADL	6	0.3946	699.4	17.00	0.60	tr_D0A564_	
HLA-DQA10501-DQB10201	48	NYEQLEFVPFDPTTK	EQLEFVPFD	2	0.3926	714.7	17.00	0.22	tr_D0A564_	
HLA-DQA10501-DQB10201	49	AQVFPEHKFLIVEAL	HKFLIVEAL	6	0.3893	740.7	18.00	0.56	tr_D0A564_	
HLA-DQA10501-DQB10201	50	LTLNKMEIQEQCFTF	KMEIQEQCF	4	0.3884	748.1	18.00	0.49	tr_D0A564_	
HLA-DQA10501-DQB10201	51	TLTLNKMEIQEQCFT	KMEIQEQCF	5	0.3879	752.2	18.00	0.50	tr_D0A564_	
HLA-DQA10501-DQB10201	52	QVFPEHKFLIVEALR	HKFLIVEAL	5	0.3869	760.2	18.00	0.44	tr_D0A564_	
HLA-DQA10501-DQB10201	53	FPEHKFLIVEALRQR	FLIVEALRQ	5	0.3863	765.5	18.00	0.40	tr_D0A564_	
HLA-DQA10501-DQB10201	54	TRLTAIETMSGVNML	TRLTAIETM	0	0.3831	792.1	18.00	0.34	tr_D0A564_	
HLA-DQA10501-DQB10201	55	VIIEMVHNQDEINDS	EMVHNQDEI	3	0.3821	800.6	19.00	0.28	tr_D0A564_	
HLA-DQA10501-DQB10201	56	CRMLDLDPNILTAEK	MLDLDPNIL	2	0.3814	806.8	19.00	0.57	tr_D0A564_	
HLA-DQA10501-DQB10201	57	TLNKMEIQEQCFTFE	KMEIQEQCF	3	0.3813	807.3	19.00	0.47	tr_D0A564_	
HLA-DQA10501-DQB10201	58	YEQLEFVPFDPTTKR	EQLEFVPFD	1	0.3661	952.0	21.00	0.23	tr_D0A564_	
HLA-DQA10501-DQB10201	59	IETMSGVNMLCSDKT	VNMLCSDKT	6	0.3593	1025.1	23.00	0.36	tr_D0A564_	
HLA-DQA10501-DQB10201	60	LIAKEMCRMLDLDPN	EMCRMLDLD	4	0.3572	1048.2	23.00	0.40	tr_D0A564_	
HLA-DQA10501-DQB10201	61	GRWHLCGILTFLDPP	WHLCGILTF	2	0.3566	1054.7	23.00	0.25	tr_D0A564_	
HLA-DQA10501-DQB10201	62	PEHKFLIVEALRQRG	FLIVEALRQ	4	0.3562	1059.6	23.00	0.42	tr_D0A564_	
HLA-DQA10501-DQB10201	63	SKHKIIVTRLTAIET	VTRLTAIET	6	0.3532	1094.1	24.00	0.62	tr_D0A564_	
HLA-DQA10501-DQB10201	64	INDSVVDIIDKLASR	SVVDIIDKL	3	0.3512	1118.5	24.00	0.37	tr_D0A564_	
HLA-DQA10501-DQB10201	65	DLRSLLVLSALAAKW	VLSALAAKW	6	0.3503	1130.2	24.00	0.47	tr_D0A564_	
HLA-DQA10501-DQB10201	66	VGGFAQVFPEHKFLI	FAQVFPEHK	3	0.3496	1137.6	24.00	0.37	tr_D0A564_	
HLA-DQA10501-DQB10201	67	IIEMVHNQDEINDSV	EMVHNQDEI	2	0.3488	1147.7	24.00	0.30	tr_D0A564_	
HLA-DQA10501-DQB10201	68	GAPHVIIEMVHNQDE	IIEMVHNQD	5	0.3488	1147.8	24.00	0.43	tr_D0A564_	
HLA-DQA10501-DQB10201	69	VLIAKEMCRMLDLDP	EMCRMLDLD	5	0.3487	1149.4	25.00	0.46	tr_D0A564_	
HLA-DQA10501-DQB10201	70	RWHLCGILTFLDPPR	WHLCGILTF	1	0.3474	1165.6	25.00	0.26	tr_D0A564_	
HLA-DQA10501-DQB10201	71	WHLCGILTFLDPPRP	GILTFLDPP	4	0.3470	1170.0	25.00	0.22	tr_D0A564_	
HLA-DQA10501-DQB10201	72	LNKMEIQEQCFTFEK	KMEIQEQCF	2	0.3461	1182.0	25.00	0.50	tr_D0A564_	
HLA-DQA10501-DQB10201	73	TAIETMSGVNMLCSD	MSGVNMLCS	5	0.3461	1182.4	25.00	0.26	tr_D0A564_	
HLA-DQA10501-DQB10201	74	EHKFLIVEALRQRGY	FLIVEALRQ	3	0.3454	1191.2	25.00	0.47	tr_D0A564_	
HLA-DQA10501-DQB10201	75	KLPKVDVNDMPSDLG	DVNDMPSDL	5	0.3398	1265.3	26.00	0.26	tr_D0A564_	
HLA-DQA10501-DQB10201	76	LDLDPNILTAEKLPK	PNILTAEKL	4	0.3378	1293.8	27.00	0.76	tr_D0A564_	

HLA-DQA10501-DQB10201	77	LTAIETMSGVNMLCS	IETMSGVNM	3	0.3367	1308.1	27.00	0.23	tr_D0A564_
HLA-DQA10501-DQB10201	78	MVHNQDEINDSVVDI	EINDSVVDI	6	0.3352	1330.6	27.00	0.60	tr_D0A564_
HLA-DQA10501-DQB10201	79	ETMSGVNMLCSDKTG	VNMLCSDKT	5	0.3332	1359.3	28.00	0.38	tr_D0A564_
HLA-DQA10501-DQB10201	80	GVDVKMITGDHVLIA	VKMITGDHV	3	0.3304	1400.9	28.00	0.50	tr_D0A564_
HLA-DQA10501-DQB10201	81	RLTAIETMSGVNMLC	IETMSGVNM	4	0.3301	1405.5	28.00	0.34	tr_D0A564_
HLA-DQA10501-DQB10201	82	LPKVDVNDMPSDLGE	DVNDMPSDL	4	0.3301	1406.3	28.00	0.29	tr_D0A564_
HLA-DQA10501-DQB10201	83	EKLPKVDVNDMPSDL	DVNDMPSDL	6	0.3285	1430.3	29.00	0.28	tr_D0A564_
HLA-DQA10501-DQB10201	84	AGRWHLCGILTFLDP	WHLCGILTF	3	0.3280	1437.8	29.00	0.40	tr_D0A564_
HLA-DQA10501-DQB10201	85	TCAMTGDGVNDAPAL	MTGDGVNDA	3	0.3276	1444.1	29.00	0.41	tr_D0A564_
HLA-DQA10501-DQB10201	86	DLDPNILTAEKLPKV	PNILTAEKL	3	0.3271	1452.6	29.00	0.75	tr_D0A564_
HLA-DQA10501-DQB10201	87	YGVDVKMITGDHVLI	VKMITGDHV	4	0.3262	1466.9	29.00	0.59	tr_D0A564_
HLA-DQA10501-DQB10201	88	LGAADLDECDNYEQL	LGAADLDEC	0	0.3246	1492.0	29.00	0.25	tr_D0A564_
HLA-DQA10501-DQB10201	89	EKGHDLRSLLVLSAL	RSLLVLSAL	6	0.3232	1514.1	30.00	0.37	tr_D0A564_
HLA-DQA10501-DQB10201	90	LRSLLVLSALAAKWR	VLSALAAKW	5	0.3227	1523.3	30.00	0.49	tr_D0A564_
HLA-DQA10501-DQB10201	91	HLCGILTFLDPPRPD	GILTFLDPP	3	0.3214	1545.0	30.00	0.28	tr_D0A564_
HLA-DQA10501-DQB10201	92	KGAPHVIIEMVHNQD	IIEMVHNQD	6	0.3213	1546.2	30.00	0.50	tr_D0A564_
HLA-DQA10501-DQB10201	93	EQLEFVPFDPTTKRT	QLEFVPFDP	1	0.3205	1559.2	30.00	0.29	tr_D0A564_
HLA-DQA10501-DQB10201	94	QYGVDVKMITGDHVL	VKMITGDHV	5	0.3199	1569.2	30.00	0.65	tr_D0A564_
HLA-DQA10501-DQB10201	95	RQYGVDVKMITGDHV	VKMITGDHV	6	0.3199	1569.7	30.00	0.61	tr_D0A564_
HLA-DQA10501-DQB10201	96	KGHDLRSLLVLSALA	LRSLLVLSA	4	0.3176	1608.3	31.00	0.31	tr_D0A564_
HLA-DQA10501-DQB10201	97	HVLIAKEMCRMLDLD	EMCRMLDLD	6	0.3164	1630.2	31.00	0.46	tr_D0A564_
HLA-DQA10501-DQB10201	98	AIETMSGVNMLCSDK	MSGVNMLCS	4	0.3158	1641.4	31.00	0.22	tr_D0A564_
HLA-DQA10501-DQB10201	99	GHDLRSLLVLSALAA	LRSLLVLSA	3	0.3145	1664.1	32.00	0.24	tr_D0A564_
HLA-DQA10501-DQB10201	100	PSDLGEKYGDMMLSV	KYGDMMLSV	6	0.3135	1682.3	32.00	0.54	tr_D0A564_
HLA-DQA10501-DQB10201	101	GYTCAMTGDGVNDAP	MTGDGVNDA	5	0.3133	1685.9	32.00	0.43	tr_D0A564_
HLA-DQA10501-DQB10201	102	KWREPPRDALDTMVL	PRDALDTMV	5	0.3118	1714.1	32.00	0.44	tr_D0A564_
HLA-DQA10501-DQB10201	103	FAQVFPEHKFLIVEA	EHKFLIVEA	6	0.3109	1729.8	33.00	0.52	tr_D0A564_
HLA-DQA10501-DQB10201	104	YTCAMTGDGVNDAPA	MTGDGVNDA	4	0.3104	1739.0	33.00	0.42	tr_D0A564_
HLA-DQA10501-DQB10201	105	SAGRWHLCGILTFLD	WHLCGILTF	4	0.3098	1750.0	33.00	0.42	tr_D0A564_
HLA-DQA10501-DQB10201	106	NKMEIQEQCFTFEKG	KMEIQEQCF	1	0.3086	1772.8	33.00	0.52	tr_D0A564_
HLA-DQA10501-DQB10201	107	GEKYGDMMLSVGGFA	MMLSVGGFA	6	0.3069	1806.7	33.00	0.36	tr_D0A564_
HLA-DQA10501-DQB10201	108	VDVKMITGDHVLIAK	VKMITGDHV	2	0.3069	1807.0	33.00	0.54	tr_D0A564_
HLA-DQA10501-DQB10201	109	HKFLIVEALRQRGYT	FLIVEALRQ	2	0.3060	1824.2	34.00	0.52	tr_D0A564_
HLA-DQA10501-DQB10201	110	RSLLVLSALAAKWRE	VLSALAAKW	4	0.3058	1828.7	34.00	0.53	tr_D0A564_
HLA-DQA10501-DQB10201	111	LDPNILTAEKLPKVD	PNILTAEKL	2	0.3049	1846.3	34.00	0.75	tr_D0A564_
HLA-DQA10501-DQB10201	112	NDSVVDIIDKLASRG	SVVDIIDKL	2	0.3034	1876.7	34.00	0.47	tr_D0A564_
HLA-DQA10501-DQB10201	113	SDLGEKYGDMMLSVG	KYGDMMLSV	5	0.3009	1927.5	35.00	0.41	tr_D0A564_
HLA-DQA10501-DQB10201	114	SLLVLSALAAKWREP	VLSALAAKW	3	0.2982	1984.9	36.00	0.63	tr_D0A564_
HLA-DQA10501-DQB10201	115	IEMVHNQDEINDSVV	EMVHNQDEI	1	0.2973	2003.7	36.00	0.38	tr_D0A564_
HLA-DQA10501-DQB10201	116	CAMTGDGVNDAPALK	MTGDGVNDA	2	0.2973	2004.6	36.00	0.43	tr_D0A564_
HLA-DQA10501-DQB10201	117	AADLDECDNYEQLEF	CDNYEQLEF	6	0.2969	2012.0	36.00	0.43	tr_D0A564_
HLA-DQA10501-DQB10201	118	LCGILTFLDPPRPDT	GILTFLDPP	2	0.2968	2015.7	36.00	0.31	tr_D0A564_
HLA-DQA10501-DQB10201	119	EKYGDMMLSVGGFAQ	MMLSVGGFA	5	0.2957	2039.4	36.00	0.34	tr_D0A564_
HLA-DQA10501-DQB10201	120	WREPPRDALDTMVLG	PRDALDTMV	4	0.2943	2071.0	37.00	0.39	tr_D0A564_
HLA-DQA10501-DQB10201	121	PPRDALDTMVLGAAD	DTMVLGAAD	6	0.2920	2121.6	37.00	0.26	tr_D0A564_
HLA-DQA10501-DQB10201	122	DSAGRWHLCGILTFL	WHLCGILTF	5	0.2901	2166.9	38.00	0.44	tr_D0A564_
HLA-DQA10501-DQB10201	123	GGFAQVFPEHKFLIV	FAQVFPEHK	2	0.2896	2178.1	38.00	0.48	tr_D0A564_
HLA-DQA10501-DQB10201	124	FEKGHDLRSLLVLSA	LRSLLVLSA	6	0.2880	2216.0	38.00	0.41	tr_D0A564_
HLA-DQA10501-DQB10201	125	REPPRDALDTMVLGA	PRDALDTMV	3	0.2879	2218.6	38.00	0.34	tr_D0A564_
HLA-DQA10501-DQB10201	126	LGEKYGDMMLSVGGF	KYGDMMLSV	3	0.2872	2236.9	38.00	0.29	tr_D0A564_

HLA-DQA10501-DQB10201	127	TMSGVNMLCSDKTGT	VNMLCSDKT	4	0.2870	2241.1	38.00	0.44	tr_D0A564_
HLA-DQA10501-DQB10201	128	VKMITGDHVLIAKEM	DHVLIAKEM	6	0.2862	2261.2	39.00	0.37	tr_D0A564_
HLA-DQA10501-DQB10201	129	DVKMITGDHVLIAKE	VKMITGDHV	1	0.2860	2264.9	39.00	0.51	tr_D0A564_
HLA-DQA10501-DQB10201	130	AEKLPKVDVNDMPSD	LPKVDVNDM	3	0.2827	2347.9	39.00	0.30	tr_D0A564_
HLA-DQA10501-DQB10201	131	PKVDVNDMPSDLGEK	DVNDMPSDL	3	0.2825	2352.1	39.00	0.29	tr_D0A564_
HLA-DQA10501-DQB10201	132	TDSAGRWHLCGILTF	WHLCGILTF	6	0.2803	2408.5	40.00	0.56	tr_D0A564_
HLA-DQA10501-DQB10201	133	HDLRSLLVLSALAAK	LRSLLVLSA	2	0.2802	2412.1	40.00	0.29	tr_D0A564_
HLA-DQA10501-DQB10201	134	LLVLSALAAKWREPP	VLSALAAKW	2	0.2801	2414.1	40.00	0.67	tr_D0A564_
HLA-DQA10501-DQB10201	135	AKWREPPRDALDTMV	PRDALDTMV	6	0.2798	2422.8	40.00	0.62	tr_D0A564_
HLA-DQA10501-DQB10201	136	DPNILTAEKLPKVDV	PNILTAEKL	1	0.2795	2429.9	40.00	0.73	tr_D0A564_
HLA-DQA10501-DQB10201	137	KYGDMMLSVGGFAQV	MMLSVGGFA	4	0.2777	2478.9	41.00	0.38	tr_D0A564_
HLA-DQA10501-DQB10201	138	DSVVDIIDKLASRGI	SVVDIIDKL	1	0.2763	2515.0	41.00	0.46	tr_D0A564_
HLA-DQA10501-DQB10201	139	EPPRDALDTMVLGAA	PRDALDTMV	2	0.2757	2531.6	41.00	0.33	tr_D0A564_
HLA-DQA10501-DQB10201	140	AMTGDGVNDAPALKR	MTGDGVNDA	1	0.2748	2556.3	41.00	0.43	tr_D0A564_
HLA-DQA10501-DQB10201	141	KVDVNDMPSDLGEKY	DVNDMPSDL	2	0.2737	2587.0	42.00	0.28	tr_D0A564_
HLA-DQA10501-DQB10201	142	TGTLTLNKMEIQEQC	LNKMEIQEQ	5	0.2737	2588.2	42.00	0.37	tr_D0A564_
HLA-DQA10501-DQB10201	143	DLGEKYGDMMLSVGG	KYGDMMLSV	4	0.2717	2644.5	42.00	0.39	tr_D0A564_
HLA-DQA10501-DQB10201	144	GAADLDECDNYEQLE	DLDECDNYE	3	0.2699	2697.3	43.00	0.17	tr_D0A564_
HLA-DQA10501-DQB10201	145	RGYTCAMTGDGVNDA	MTGDGVNDA	6	0.2684	2740.0	43.00	0.38	tr_D0A564_
HLA-DQA10501-DQB10201	146	CGILTFLDPPRPDTK	GILTFLDPP	1	0.2659	2815.3	44.00	0.31	tr_D0A564_
HLA-DQA10501-DQB10201	147	MSGVNMLCSDKTGTL	VNMLCSDKT	3	0.2649	2846.2	44.00	0.51	tr_D0A564_
HLA-DQA10501-DQB10201	148	SVTKGAPHVIIEMVH	PHVIIEMVH	6	0.2646	2855.3	44.00	0.44	tr_D0A564_
HLA-DQA10501-DQB10201	149	KTGTLTLNKMEIQEQ	LNKMEIQEQ	6	0.2625	2920.9	45.00	0.44	tr_D0A564_
HLA-DQA10501-DQB10201	150	KFLIVEALRQRGYTC	FLIVEALRQ	1	0.2570	3100.9	46.00	0.61	tr_D0A564_
HLA-DQA10501-DQB10201	151	GFAQVFPEHKFLIVE	FAQVFPEHK	1	0.2566	3114.5	46.00	0.50	tr_D0A564_
HLA-DQA10501-DQB10201	152	EMVHNQDEINDSVVD	MVHNQDEIN	1	0.2561	3131.0	47.00	0.34	tr_D0A564_
HLA-DQA10501-DQB10201	153	VDVNDMPSDLGEKYG	DVNDMPSDL	1	0.2555	3152.2	47.00	0.29	tr_D0A564_
HLA-DQA10501-DQB10201	154	LVLSALAAKWREPPR	VLSALAAKW	1	0.2553	3158.3	47.00	0.71	tr_D0A564_
HLA-DQA10501-DQB10201	155	KMITGDHVLIAKEMC	DHVLIAKEM	5	0.2541	3197.9	47.00	0.40	tr_D0A564_
HLA-DQA10501-DQB10201	156	QLEFVPFDPTTKRTA	LEFVPFDPT	1	0.2537	3213.9	47.00	0.38	tr_D0A564_
HLA-DQA10501-DQB10201	157	IRRSRQYGVDVKMIT	QYGVDVKMI	5	0.2476	3431.9	49.00	0.26	tr_D0A564_
HLA-DQA10501-DQB10201	158	TAEKLPKVDVNDMPS	LPKVDVNDM	4	0.2474	3439.6	49.00	0.38	tr_D0A564_
HLA-DQA10501-DQB10201	159	KMEIQEQCFTFEKGH	KMEIQEQCF	0	0.2474	3440.6	49.00	0.47	tr_D0A564_
HLA-DQA10501-DQB10201	160	CFTFEKGHDLRSLLV	GHDLRSLLV	6	0.2469	3459.6	49.00	0.55	tr_D0A564_
HLA-DQA10501-DQB10201	161	VTKGAPHVIIEMVHN	PHVIIEMVH	5	0.2463	3479.4	49.00	0.39	tr_D0A564_
HLA-DQA10501-DQB10201	162	GILTFLDPPRPDTKE	LTFLDPPRP	2	0.2419	3650.1	50.00	0.32	tr_D0A564_
HLA-DQA10501-DQB10201	163	FTFEKGHDLRSLLVL	GHDLRSLLV	5	0.2418	3653.3	50.00	0.47	tr_D0A564_
HLA-DQA10501-DQB10201	164	DHVLIAKEMCRMLDL	KEMCRMLDL	6	0.2406	3703.3	55.00	0.31	tr_D0A564_
HLA-DQA10501-DQB10201	165	TFEKGHDLRSLLVLS	GHDLRSLLV	4	0.2396	3742.9	55.00	0.46	tr_D0A564_
HLA-DQA10501-DQB10201	166	SGVNMLCSDKTGTLT	VNMLCSDKT	2	0.2371	3844.9	55.00	0.60	tr_D0A564_
HLA-DQA10501-DQB10201	167	TKGAPHVIIEMVHNQ	PHVIIEMVH	4	0.2367	3862.6	55.00	0.34	tr_D0A564_
HLA-DQA10501-DQB10201	168	TIRRSRQYGVDVKMI	QYGVDVKMI	6	0.2357	3903.9	55.00	0.33	tr_D0A564_
HLA-DQA10501-DQB10201	169	LTAEKLPKVDVNDMP	LPKVDVNDM	5	0.2320	4064.4	55.00	0.50	tr_D0A564_
HLA-DQA10501-DQB10201	170	RSRQYGVDVKMITGD	QYGVDVKMI	3	0.2281	4236.3	55.00	0.31	tr_D0A564_
HLA-DQA10501-DQB10201	171	FSVTKGAPHVIIEMV	APHVIIEMV	6	0.2258	4344.0	55.00	0.57	tr_D0A564_
HLA-DQA10501-DQB10201	172	SVVDIIDKLASRGIR	SVVDIIDKL	0	0.2252	4370.8	55.00	0.42	tr_D0A564_
HLA-DQA10501-DQB10201	173	RRSRQYGVDVKMITG	QYGVDVKMI	4	0.2251	4375.4	55.00	0.29	tr_D0A564_
HLA-DQA10501-DQB10201	174	MITGDHVLIAKEMCR	DHVLIAKEM	4	0.2247	4397.3	55.00	0.38	tr_D0A564_
HLA-DQA10501-DQB10201	175	DVNDMPSDLGEKYGD	MPSDLGEKY	4	0.2240	4428.9	60.00	0.31	tr_D0A564_
HLA-DQA10501-DQB10201	176	LSKHKIIVTRLTAIE	IVTRLTAIE	6	0.2239	4435.6	60.00	0.50	tr_D0A564_

HLA-DQA10501-DQB10201	177	SRQYGVDVKMITGDH	QYGVDVKMI	2	0.2184	4704.6	60.00	0.31	tr_D0A564_
HLA-DQA10501-DQB10201	178	MPSDLGEKYGDMMLS	LGEKYGDMM	4	0.2181	4720.0	60.00	0.28	tr_D0A564_
HLA-DQA10501-DQB10201	179	VLSALAAKWREPPRD	VLSALAAKW	0	0.2175	4752.1	60.00	0.63	tr_D0A564_
HLA-DQA10501-DQB10201	180	ITGDHVLIAKEMCRM	DHVLIAKEM	3	0.2173	4761.3	60.00	0.40	tr_D0A564_
HLA-DQA10501-DQB10201	181	PNILTAEKLPKVDVN	PNILTAEKL	0	0.2160	4833.0	60.00	0.65	tr_D0A564_
HLA-DQA10501-DQB10201	182	DMPSDLGEKYGDMML	LGEKYGDMM	5	0.2126	5009.1	60.00	0.33	tr_D0A564_
HLA-DQA10501-DQB10201	183	QRGYTCAMTGDGVND	TCAMTGDGV	4	0.2117	5062.9	60.00	0.31	tr_D0A564_
HLA-DQA10501-DQB10201	184	GDHVLIAKEMCRMLD	DHVLIAKEM	1	0.2115	5069.6	60.00	0.29	tr_D0A564_
HLA-DQA10501-DQB10201	185	DKTGTLTLNKMEIQE	LTLNKMEIQ	5	0.2107	5113.2	60.00	0.29	tr_D0A564_
HLA-DQA10501-DQB10201	186	FLIVEALRQRGYTCA	FLIVEALRQ	0	0.2040	5498.6	65.00	0.65	tr_D0A564_
HLA-DQA10501-DQB10201	187	TGDHVLIAKEMCRML	DHVLIAKEM	2	0.2037	5521.0	65.00	0.38	tr_D0A564_
HLA-DQA10501-DQB10201	188	ETIRRSRQYGVDVKM	RQYGVDVKM	6	0.1985	5840.2	65.00	0.51	tr_D0A564_
HLA-DQA10501-DQB10201	189	GVNMLCSDKTGTLTL	VNMLCSDKT	1	0.1975	5902.8	65.00	0.68	tr_D0A564_
HLA-DQA10501-DQB10201	190	SDKTGTLTLNKMEIQ	LTLNKMEIQ	6	0.1940	6127.8	65.00	0.34	tr_D0A564_
HLA-DQA10501-DQB10201	191	NDMPSDLGEKYGDMM	LGEKYGDMM	6	0.1907	6354.4	65.00	0.40	tr_D0A564_
HLA-DQA10501-DQB10201	192	PFDPTTKRTAATLVD	KRTAATLVD	6	0.1885	6506.2	70.00	0.52	tr_D0A564_
HLA-DQA10501-DQB10201	193	FDPTTKRTAATLVDK	KRTAATLVD	5	0.1864	6656.3	70.00	0.40	tr_D0A564_
HLA-DQA10501-DQB10201	194	LRQRGYTCAMTGDGV	TCAMTGDGV	6	0.1854	6722.9	70.00	0.43	tr_D0A564_
HLA-DQA10501-DQB10201	195	RQRGYTCAMTGDGVN	TCAMTGDGV	5	0.1830	6902.1	70.00	0.40	tr_D0A564_
HLA-DQA10501-DQB10201	196	ILTFLDPPRPDTKET	LTFLDPPRP	1	0.1818	6997.3	70.00	0.47	tr_D0A564_
HLA-DQA10501-DQB10201	197	LASRGIRCLSVAKTD	IRCLSVAKT	5	0.1813	7033.1	70.00	0.37	tr_D0A564_
HLA-DQA10501-DQB10201	198	DPTTKRTAATLVDKR	KRTAATLVD	4	0.1802	7116.8	70.00	0.37	tr_D0A564_
HLA-DQA10501-DQB10201	199	EIQEQCFTFEKGHDL	FTFEKGHDL	6	0.1800	7127.9	70.00	0.47	tr_D0A564_
HLA-DQA10501-DQB10201	200	IIDKLASRGIRCLSV	SRGIRCLSV	6	0.1780	7287.2	70.00	0.51	tr_D0A564_
HLA-DQA10501-DQB10201	201	CSDKTGTLTLNKMEI	TLTLNKMEI	6	0.1777	7310.8	70.00	0.49	tr_D0A564_
HLA-DQA10501-DQB10201	202	SRGIRCLSVAKTDSA	IRCLSVAKT	3	0.1775	7324.5	70.00	0.33	tr_D0A564_
HLA-DQA10501-DQB10201	203	VNDMPSDLGEKYGDM	MPSDLGEKY	3	0.1762	7430.4	70.00	0.46	tr_D0A564_
HLA-DQA10501-DQB10201	204	KLASRGIRCLSVAKT	IRCLSVAKT	6	0.1757	7467.1	70.00	0.35	tr_D0A564_
HLA-DQA10501-DQB10201	205	IDKLASRGIRCLSVA	SRGIRCLSV	5	0.1747	7552.2	70.00	0.43	tr_D0A564_
HLA-DQA10501-DQB10201	206	KTDSAGRWHLCGILT	RWHLCGILT	6	0.1715	7820.6	75.00	0.61	tr_D0A564_
HLA-DQA10501-DQB10201	207	ASRGIRCLSVAKTDS	IRCLSVAKT	4	0.1698	7959.6	75.00	0.40	tr_D0A564_
HLA-DQA10501-DQB10201	208	LEFVPFDPTTKRTAA	LEFVPFDPT	0	0.1683	8096.2	75.00	0.46	tr_D0A564_
HLA-DQA10501-DQB10201	209	AAKWREPPRDALDTM	PPRDALDTM	6	0.1667	8234.5	75.00	0.56	tr_D0A564_
HLA-DQA10501-DQB10201	210	PTTKRTAATLVDKRS	KRTAATLVD	3	0.1653	8364.1	75.00	0.35	tr_D0A564_
HLA-DQA10501-DQB10201	211	RGIRCLSVAKTDSAG	IRCLSVAKT	2	0.1615	8714.2	75.00	0.37	tr_D0A564_
HLA-DQA10501-DQB10201	212	ILTAEKLPKVDVNDM	LPKVDVNDM	6	0.1602	8834.6	75.00	0.68	tr_D0A564_
HLA-DQA10501-DQB10201	213	MEIQEQCFTFEKGHD	IQEQCFTFE	2	0.1601	8848.5	75.00	0.34	tr_D0A564_
HLA-DQA10501-DQB10201	214	IQEQCFTFEKGHDLR	FTFEKGHDL	5	0.1586	8992.6	75.00	0.51	tr_D0A564_
HLA-DQA10501-DQB10201	215	EALRQRGYTCAMTGD	RGYTCAMTG	5	0.1581	9034.0	75.00	0.34	tr_D0A564_
HLA-DQA10501-DQB10201	216	DKLASRGIRCLSVAK	SRGIRCLSV	4	0.1547	9375.9	75.00	0.39	tr_D0A564_
HLA-DQA10501-DQB10201	217	TTKRTAATLVDKRSG	KRTAATLVD	2	0.1533	9519.7	80.00	0.38	tr_D0A564_
HLA-DQA10501-DQB10201	218	KFSVTKGAPHVIIEM	GAPHVIIEM	6	0.1518	9673.9	80.00	0.31	tr_D0A564_
HLA-DQA10501-DQB10201	219	QCFTFEKGHDLRSLL	FTFEKGHDL	2	0.1506	9804.1	80.00	0.48	tr_D0A564_
HLA-DQA10501-DQB10201	220	VVDIIDKLASRGIRC	VVDIIDKLA	0	0.1494	9924.6	80.00	0.46	tr_D0A564_
HLA-DQA10501-DQB10201	221	ALRQRGYTCAMTGDG	RGYTCAMTG	4	0.1491	9967.3	80.00	0.32	tr_D0A564_
HLA-DQA10501-DQB10201	222	VEALRQRGYTCAMTG	RGYTCAMTG	6	0.1490	9971.3	80.00	0.35	tr_D0A564_
HLA-DQA10501-DQB10201	223	IVEALRQRGYTCAMT	QRGYTCAMT	6	0.1478	10100.3	80.00	0.41	tr_D0A564_
HLA-DQA10501-DQB10201	224	KLSKHKIIVTRLTAI	KHKIIVTRL	3	0.1446	10459.4	80.00	0.39	tr_D0A564_
HLA-DQA10501-DQB10201	225	VNMLCSDKTGTLTLN	VNMLCSDKT	0	0.1445	10468.8	80.00	0.64	tr_D0A564_
HLA-DQA10501-DQB10201	226	GIRCLSVAKTDSAGR	IRCLSVAKT	1	0.1420	10758.1	80.00	0.37	tr_D0A564_

HLA-DQA10501-DQB10201	227	EQCFTFEKGHDLRSL	FTFEKGHDL	3	0.1418	10786.1	80.00	0.57	tr_D0A564_
HLA-DQA10501-DQB10201	228	QEQCFTFEKGHDLRS	FTFEKGHDL	4	0.1404	10940.7	80.00	0.60	tr_D0A564_
HLA-DQA10501-DQB10201	229	IRCLSVAKTDSAGRW	LSVAKTDSA	3	0.1402	10965.0	80.00	0.37	tr_D0A564_
HLA-DQA10501-DQB10201	230	KETIRRSRQYGVDVK	SRQYGVDVK	6	0.1397	11027.5	80.00	0.44	tr_D0A564_
HLA-DQA10501-DQB10201	231	TKRTAATLVDKRSGE	KRTAATLVD	1	0.1380	11237.7	80.00	0.35	tr_D0A564_
HLA-DQA10501-DQB10201	232	GEKFSVTKGAPHVII	VTKGAPHVI	5	0.1349	11620.4	80.00	0.41	tr_D0A564_
HLA-DQA10501-DQB10201	233	EKFSVTKGAPHVIIE	VTKGAPHVI	4	0.1315	12045.9	85.00	0.41	tr_D0A564_
HLA-DQA10501-DQB10201	234	VPFDPTTKRTAATLV	TKRTAATLV	6	0.1311	12105.1	85.00	0.80	tr_D0A564_
HLA-DQA10501-DQB10201	235	SKKLSKHKIIVTRLT	KHKIIVTRL	5	0.1308	12146.2	85.00	0.70	tr_D0A564_
HLA-DQA10501-DQB10201	236	KKLSKHKIIVTRLTA	KHKIIVTRL	4	0.1296	12298.1	85.00	0.58	tr_D0A564_
HLA-DQA10501-DQB10201	237	LTFLDPPRPDTKETI	LTFLDPPRP	0	0.1268	12674.7	85.00	0.55	tr_D0A564_
HLA-DQA10501-DQB10201	238	MLCSDKTGTLTLNKM	TGTLTLNKM	6	0.1230	13219.7	85.00	0.76	tr_D0A564_
HLA-DQA10501-DQB10201	239	LIVEALRQRGYTCAM	RQRGYTCAM	6	0.1223	13320.4	85.00	0.37	tr_D0A564_
HLA-DQA10501-DQB10201	240	SGEKFSVTKGAPHVI	VTKGAPHVI	6	0.1218	13384.9	85.00	0.50	tr_D0A564_
HLA-DQA10501-DQB10201	241	LCSDKTGTLTLNKME	TGTLTLNKM	5	0.1212	13471.0	85.00	0.69	tr_D0A564_
HLA-DQA10501-DQB10201	242	RCLSVAKTDSAGRWH	LSVAKTDSA	2	0.1207	13548.3	85.00	0.44	tr_D0A564_
HLA-DQA10501-DQB10201	243	TKETIRRSRQYGVDV	RSRQYGVDV	6	0.1194	13744.5	85.00	0.50	tr_D0A564_
HLA-DQA10501-DQB10201	244	DIIDKLASRGIRCLS	KLASRGIRC	4	0.1162	14215.2	85.00	0.27	tr_D0A564_
HLA-DQA10501-DQB10201	245	CLSVAKTDSAGRWHL	LSVAKTDSA	1	0.1140	14569.4	90.00	0.43	tr_D0A564_
HLA-DQA10501-DQB10201	246	VDIIDKLASRGIRCL	KLASRGIRC	5	0.1121	14870.9	90.00	0.31	tr_D0A564_
HLA-DQA10501-DQB10201	247	KRTAATLVDKRSGEK	TAATLVDKR	2	0.1085	15452.9	90.00	0.33	tr_D0A564_
HLA-DQA10501-DQB10201	248	AKTDSAGRWHLCGIL	GRWHLCGIL	6	0.1044	16153.7	90.00	0.37	tr_D0A564_
HLA-DQA10501-DQB10201	249	RTAATLVDKRSGEKF	VDKRSGEKF	6	0.1037	16282.6	90.00	0.35	tr_D0A564_
HLA-DQA10501-DQB10201	250	LSALAAKWREPPRDA	LSALAAKWR	0	0.1035	16317.0	90.00	0.28	tr_D0A564_
HLA-DQA10501-DQB10201	251	NILTAEKLPKVDVND	NILTAEKLP	0	0.1031	16393.6	90.00	0.21	tr_D0A564_
HLA-DQA10501-DQB10201	252	LSVAKTDSAGRWHLC	AKTDSAGRW	3	0.1003	16888.2	90.00	0.34	tr_D0A564_
HLA-DQA10501-DQB10201	253	ALAAKWREPPRDALD	WREPPRDAL	5	0.0980	17313.0	90.00	0.35	tr_D0A564_
HLA-DQA10501-DQB10201	254	SALAAKWREPPRDAL	AKWREPPRD	4	0.0974	17439.1	90.00	0.27	tr_D0A564_
HLA-DQA10501-DQB10201	255	LAAKWREPPRDALDT	WREPPRDAL	4	0.0966	17578.4	90.00	0.33	tr_D0A564_
HLA-DQA10501-DQB10201	256	VAKTDSAGRWHLCGI	AGRWHLCGI	6	0.0861	19688.2	95.00	0.35	tr_D0A564_
HLA-DQA10501-DQB10201	257	EFVPFDPTTKRTAAT	EFVPFDPTT	0	0.0826	20450.7	95.00	0.36	tr_D0A564_
HLA-DQA10501-DQB10201	258	TAATLVDKRSGEKFS	VDKRSGEKF	5	0.0818	20639.0	95.00	0.46	tr_D0A564_
HLA-DQA10501-DQB10201	259	RSGEKFSVTKGAPHV	SVTKGAPHV	6	0.0797	21108.5	95.00	0.32	tr_D0A564_
HLA-DQA10501-DQB10201	260	SVAKTDSAGRWHLCG	AKTDSAGRW	2	0.0762	21928.2	95.00	0.44	tr_D0A564_
HLA-DQA10501-DQB10201	261	DTKETIRRSRQYGVD	IRRSRQYGV	5	0.0743	22369.6	95.00	0.39	tr_D0A564_
HLA-DQA10501-DQB10201	262	ATLVDKRSGEKFSVT	VDKRSGEKF	3	0.0696	23543.7	95.00	0.55	tr_D0A564_
HLA-DQA10501-DQB10201	263	AATLVDKRSGEKFSV	VDKRSGEKF	4	0.0694	23591.4	95.00	0.61	tr_D0A564_
HLA-DQA10501-DQB10201	264	TFLDPPRPDTKETIR	PRPDTKETI	5	0.0652	24689.0	95.00	0.32	tr_D0A564_
HLA-DQA10501-DQB10201	265	FVPFDPTTKRTAATL	TTKRTAATL	6	0.0637	25084.8	95.00	0.52	tr_D0A564_
HLA-DQA10501-DQB10201	266	TLVDKRSGEKFSVTK	VDKRSGEKF	2	0.0633	25218.4	100.00	0.57	tr_D0A564_
HLA-DQA10501-DQB10201	267	KRSGEKFSVTKGAPH	FSVTKGAPH	6	0.0623	25473.2	100.00	0.38	tr_D0A564_
HLA-DQA10501-DQB10201	268	LVDKRSGEKFSVTKG	VDKRSGEKF	1	0.0570	26989.5	100.00	0.54	tr_D0A564_
HLA-DQA10501-DQB10201	269	PDTKETIRRSRQYGV	IRRSRQYGV	6	0.0548	27637.2	100.00	0.50	tr_D0A564_
HLA-DQA10501-DQB10201	270	VDKRSGEKFSVTKGA	VDKRSGEKF	0	0.0510	28781.7	100.00	0.34	tr_D0A564_
HLA-DQA10501-DQB10201	271	DKRSGEKFSVTKGAP	KFSVTKGAP	6	0.0493	29323.6	100.00	0.34	tr_D0A564_
HLA-DQA10501-DQB10201	272	FLDPPRPDTKETIRR	PRPDTKETI	4	0.0465	30242.5	100.00	0.44	tr_D0A564_
HLA-DQA10501-DQB10201	273	NMLCSDKTGTLTLNK	DKTGTLTLN	5	0.0416	31893.0	100.00	0.19	tr_D0A564_
HLA-DQA10501-DQB10201	274	PRPDTKETIRRSRQY	ETIRRSRQY	6	0.0404	32281.1	100.00	0.33	tr_D0A564_
HLA-DQA10501-DQB10201	275	LDPPRPDTKETIRRS	PRPDTKETI	3	0.0352	34164.2	100.00	0.51	tr_D0A564_
HLA-DQA10501-DQB10201	276	PPRPDTKETIRRSRQ	PRPDTKETI	1	0.0329	35039.4	100.00	0.38	tr_D0A564_

HLA-DQA10501-DQB10201	277	RPDTKETIRRSRQYG	ETIRRSRQY	5	0.0323	35261.2	100.00	0.36	tr_D0A564_
HLA-DQA10501-DQB10201	278	DPPRPDTKETIRRSR	PRPDTKETI	2	0.0316	35524.6	100.00	0.51	tr_D0A564_

Allele: HLA-DQA10501-DQB10201. Number of high binders 0. Number of weak binders 28. Number of peptides 278

Allele	pos	peptide	core	Of	1-log50k(aff)	affinity(nM)	%Rank	Relia	Identity	Bind Level
HLA-DQA10501-DQB10201	1	RRKMERYESVDTFEP	ERYESVDTF	4	0.5541	124.5	3.00	0.50	tr_D0A4W6_	WB
HLA-DQA10501-DQB10201	2	RKMERYESVDTFEPA	ERYESVDTF	3	0.5441	138.7	3.00	0.49	tr_D0A4W6_	WB
HLA-DQA10501-DQB10201	3	KMERYESVDTFEPAT	ERYESVDTF	2	0.5298	161.9	4.00	0.50	tr_D0A4W6_	WB
HLA-DQA10501-DQB10201	4	ERRKMERYESVDTFE	ERYESVDTF	5	0.5211	178.0	4.50	0.56	tr_D0A4W6_	WB
HLA-DQA10501-DQB10201	5	MERYESVDTFEPATT	ERYESVDTF	1	0.5134	193.4	4.50	0.48	tr_D0A4W6_	WB
HLA-DQA10501-DQB10201	6	ERYESVDTFEPATTV	RYESVDTFE	1	0.4589	349.0	9.00	0.34	tr_D0A4W6_	WB
HLA-DQA10501-DQB10201	7	EERRKMERYESVDTF	ERYESVDTF	6	0.4556	361.4	9.00	0.78	tr_D0A4W6_	WB
HLA-DQA10501-DQB10201	8	RCTNRFSTNMPLLEE	FSTNMPLLE	5	0.3782	835.4	19.00	0.47	tr_D0A4W6_	
HLA-DQA10501-DQB10201	9	CTNRFSTNMPLLEER	FSTNMPLLE	4	0.3515	1115.1	24.00	0.44	tr_D0A4W6_	
HLA-DQA10501-DQB10201	10	RYESVDTFEPATTVY	RYESVDTFE	0	0.3493	1141.8	24.00	0.38	tr_D0A4W6_	
HLA-DQA10501-DQB10201	11	RRCTNRFSTNMPLLE	FSTNMPLLE	6	0.3421	1234.3	26.00	0.55	tr_D0A4W6_	
HLA-DQA10501-DQB10201	12	TNRFSTNMPLLEERH	FSTNMPLLE	3	0.3394	1271.4	26.00	0.39	tr_D0A4W6_	
HLA-DQA10501-DQB10201	13	NRFSTNMPLLEERHD	FSTNMPLLE	2	0.3289	1424.2	29.00	0.37	tr_D0A4W6_	
HLA-DQA10501-DQB10201	14	RFSTNMPLLEERHDL	FSTNMPLLE	1	0.3164	1630.5	31.00	0.32	tr_D0A4W6_	
HLA-DQA10501-DQB10201	15	FSTNMPLLEERHDLS	FSTNMPLLE	0	0.2777	2479.1	41.00	0.27	tr_D0A4W6_	
HLA-DQA10501-DQB10201	16	CRVPLLGAGSSDDCD	LGAGSSDDC	5	0.2562	3126.0	46.00	0.25	tr_D0A4W6_	
HLA-DQA10501-DQB10201	17	VPLLGAGSSDDCDGD	GAGSSDDCD	4	0.2482	3408.9	49.00	0.29	tr_D0A4W6_	
HLA-DQA10501-DQB10201	18	RVPLLGAGSSDDCDG	GAGSSDDCD	5	0.2461	3486.5	49.00	0.26	tr_D0A4W6_	
HLA-DQA10501-DQB10201	19	IPTRHPRFYSSEERR	PRFYSSEER	5	0.2458	3500.7	49.00	0.67	tr_D0A4W6_	
HLA-DQA10501-DQB10201	20	YESVDTFEPATTVYK	YESVDTFEP	0	0.2453	3516.8	49.00	0.23	tr_D0A4W6_	
HLA-DQA10501-DQB10201	21	TIPTRHPRFYSSEER	PRFYSSEER	6	0.2409	3688.7	55.00	0.81	tr_D0A4W6_	
HLA-DQA10501-DQB10201	22	VRRCTNRFSTNMPLL	RFSTNMPLL	6	0.2369	3854.2	55.00	0.56	tr_D0A4W6_	
HLA-DQA10501-DQB10201	23	PLLGAGSSDDCDGDN	GAGSSDDCD	3	0.2334	3999.7	55.00	0.29	tr_D0A4W6_	
HLA-DQA10501-DQB10201	24	STNMPLLEERHDLSL	LLEERHDLS	5	0.2205	4600.6	60.00	0.23	tr_D0A4W6_	
HLA-DQA10501-DQB10201	25	PTRHPRFYSSEERRK	PRFYSSEER	4	0.2174	4758.8	60.00	0.62	tr_D0A4W6_	
HLA-DQA10501-DQB10201	26	TRHPRFYSSEERRKM	PRFYSSEER	3	0.2142	4925.2	60.00	0.60	tr_D0A4W6_	
HLA-DQA10501-DQB10201	27	LLGAGSSDDCDGDNN	GAGSSDDCD	2	0.2109	5106.6	60.00	0.33	tr_D0A4W6_	
HLA-DQA10501-DQB10201	28	QCRVPLLGAGSSDDC	LLGAGSSDD	5	0.2089	5216.5	60.00	0.34	tr_D0A4W6_	
HLA-DQA10501-DQB10201	29	RHPRFYSSEERRKME	PRFYSSEER	2	0.2062	5371.7	65.00	0.61	tr_D0A4W6_	
HLA-DQA10501-DQB10201	30	SEERRKMERYESVDT	MERYESVDT	6	0.1993	5787.6	65.00	0.46	tr_D0A4W6_	
HLA-DQA10501-DQB10201	31	HPRFYSSEERRKMER	PRFYSSEER	1	0.1943	6112.1	65.00	0.58	tr_D0A4W6_	
HLA-DQA10501-DQB10201	32	ESVDTFEPATTVYKD	VDTFEPATT	2	0.1925	6228.1	65.00	0.22	tr_D0A4W6_	
HLA-DQA10501-DQB10201	33	TNMPLLEERHDLSLR	LLEERHDLS	4	0.1890	6467.7	70.00	0.28	tr_D0A4W6_	
HLA-DQA10501-DQB10201	34	NMPLLEERHDLSLRQ	LLEERHDLS	3	0.1869	6619.5	70.00	0.29	tr_D0A4W6_	
HLA-DQA10501-DQB10201	35	VDTFEPATTVYKDHL	ATTVYKDHL	6	0.1842	6815.9	70.00	0.50	tr_D0A4W6_	
HLA-DQA10501-DQB10201	36	LGAGSSDDCDGDNNA	GAGSSDDCD	1	0.1752	7510.6	70.00	0.41	tr_D0A4W6_	
HLA-DQA10501-DQB10201	37	DTFEPATTVYKDHLA	ATTVYKDHL	5	0.1715	7819.1	75.00	0.50	tr_D0A4W6_	
HLA-DQA10501-DQB10201	38	SQCRVPLLGAGSSDD	LLGAGSSDD	6	0.1659	8306.5	75.00	0.38	tr_D0A4W6_	
HLA-DQA10501-DQB10201	39	PRFYSSEERRKMERY	PRFYSSEER	0	0.1655	8340.6	75.00	0.49	tr_D0A4W6_	
HLA-DQA10501-DQB10201	40	SLRQKLQATIPTRHP	LQATIPTRH	5	0.1654	8349.8	75.00	0.37	tr_D0A4W6_	

HLA-DQA10501-DQB10201	41	MPLLEERHDLSLRQK	LLEERHDLS	2	0.1642	8456.7	75.00	0.31	tr_D0A4W6_
HLA-DQA10501-DQB10201	42	SVDTFEPATTVYKDH	VDTFEPATT	1	0.1586	8993.7	75.00	0.28	tr_D0A4W6_
HLA-DQA10501-DQB10201	43	RQKLQATIPTRHPRF	LQATIPTRH	3	0.1572	9128.6	75.00	0.34	tr_D0A4W6_
HLA-DQA10501-DQB10201	44	LSLRQKLQATIPTRH	LQATIPTRH	6	0.1550	9345.3	75.00	0.35	tr_D0A4W6_
HLA-DQA10501-DQB10201	45	QKLQATIPTRHPRFY	LQATIPTRH	2	0.1523	9620.5	80.00	0.34	tr_D0A4W6_
HLA-DQA10501-DQB10201	46	TFEPATTVYKDHLAG	ATTVYKDHL	4	0.1511	9745.6	80.00	0.55	tr_D0A4W6_
HLA-DQA10501-DQB10201	47	LRQKLQATIPTRHPR	LQATIPTRH	4	0.1494	9926.5	80.00	0.38	tr_D0A4W6_
HLA-DQA10501-DQB10201	48	PLLEERHDLSLRQKL	LLEERHDLS	1	0.1463	10263.7	80.00	0.28	tr_D0A4W6_
HLA-DQA10501-DQB10201	49	TVYKDHLAGRSQEPR	HLAGRSQEP	5	0.1430	10638.3	80.00	0.39	tr_D0A4W6_
HLA-DQA10501-DQB10201	50	SSEERRKMERYESVD	RKMERYESV	5	0.1430	10638.4	80.00	0.56	tr_D0A4W6_
HLA-DQA10501-DQB10201	51	TTVYKDHLAGRSQEP	HLAGRSQEP	6	0.1401	10980.8	80.00	0.41	tr_D0A4W6_
HLA-DQA10501-DQB10201	52	AVRRCTNRFSTNMPL	NRFSTNMPL	6	0.1381	11224.2	80.00	0.63	tr_D0A4W6_
HLA-DQA10501-DQB10201	53	KLQATIPTRHPRFYS	LQATIPTRH	1	0.1362	11458.8	80.00	0.34	tr_D0A4W6_
HLA-DQA10501-DQB10201	54	FEPATTVYKDHLAGR	ATTVYKDHL	3	0.1351	11589.0	80.00	0.52	tr_D0A4W6_
HLA-DQA10501-DQB10201	55	DCDGDNNANEPVAAV	NNANEPVAA	5	0.1336	11785.0	85.00	0.35	tr_D0A4W6_
HLA-DQA10501-DQB10201	56	DGDNNANEPVAAVRR	NEPVAAVRR	6	0.1308	12147.5	85.00	0.23	tr_D0A4W6_
HLA-DQA10501-DQB10201	57	YSSEERRKMERYESV	RKMERYESV	6	0.1307	12157.6	85.00	0.61	tr_D0A4W6_
HLA-DQA10501-DQB10201	58	GAGSSDDCDGDNNAN	GAGSSDDCD	0	0.1286	12433.2	85.00	0.42	tr_D0A4W6_
HLA-DQA10501-DQB10201	59	SSQCRVPLLGAGSSD	PLLGAGSSD	6	0.1284	12464.7	85.00	0.43	tr_D0A4W6_
HLA-DQA10501-DQB10201	60	DLSLRQKLQATIPTR	RQKLQATIP	4	0.1260	12784.4	85.00	0.36	tr_D0A4W6_
HLA-DQA10501-DQB10201	61	CDGDNNANEPVAAVR	NNANEPVAA	4	0.1242	13038.9	85.00	0.31	tr_D0A4W6_
HLA-DQA10501-DQB10201	62	EPATTVYKDHLAGRS	ATTVYKDHL	2	0.1239	13082.1	85.00	0.47	tr_D0A4W6_
HLA-DQA10501-DQB10201	63	GDNNANEPVAAVRRC	NEPVAAVRR	5	0.1233	13172.7	85.00	0.23	tr_D0A4W6_
HLA-DQA10501-DQB10201	64	DDCDGDNNANEPVAA	NNANEPVAA	6	0.1233	13172.9	85.00	0.38	tr_D0A4W6_
HLA-DQA10501-DQB10201	65	MKGQSSQCRVPLLGA	SSQCRVPLL	4	0.1197	13686.8	85.00	0.37	tr_D0A4W6_
HLA-DQA10501-DQB10201	66	DNNANEPVAAVRRCT	NEPVAAVRR	4	0.1157	14299.2	85.00	0.23	tr_D0A4W6_
HLA-DQA10501-DQB10201	67	HDLSLRQKLQATIPT	RQKLQATIP	5	0.1148	14441.8	85.00	0.44	tr_D0A4W6_
HLA-DQA10501-DQB10201	68	SSDDCDGDNNANEPV	DGDNNANEP	5	0.1124	14823.8	90.00	0.34	tr_D0A4W6_
HLA-DQA10501-DQB10201	69	GQSSQCRVPLLGAGS	SSQCRVPLL	2	0.1123	14837.3	90.00	0.34	tr_D0A4W6_
HLA-DQA10501-DQB10201	70	QSSQCRVPLLGAGSS	SSQCRVPLL	1	0.1118	14909.1	90.00	0.28	tr_D0A4W6_
HLA-DQA10501-DQB10201	71	SDDCDGDNNANEPVA	DGDNNANEP	4	0.1110	15046.2	90.00	0.30	tr_D0A4W6_
HLA-DQA10501-DQB10201	72	RHDLSLRQKLQATIP	RQKLQATIP	6	0.1103	15165.3	90.00	0.56	tr_D0A4W6_
HLA-DQA10501-DQB10201	73	LLEERHDLSLRQKLQ	ERHDLSLRQ	3	0.1089	15397.5	90.00	0.31	tr_D0A4W6_
HLA-DQA10501-DQB10201	74	PATTVYKDHLAGRSQ	ATTVYKDHL	1	0.1080	15538.4	90.00	0.43	tr_D0A4W6_
HLA-DQA10501-DQB10201	75	ATTVYKDHLAGRSQE	ATTVYKDHL	0	0.1075	15623.2	90.00	0.25	tr_D0A4W6_
HLA-DQA10501-DQB10201	76	LQATIPTRHPRFYSS	TIPTRHPRF	3	0.1073	15661.1	90.00	0.38	tr_D0A4W6_
HLA-DQA10501-DQB10201	77	NNANEPVAAVRRCTN	NEPVAAVRR	3	0.1069	15718.8	90.00	0.22	tr_D0A4W6_
HLA-DQA10501-DQB10201	78	KGQSSQCRVPLLGAG	SSQCRVPLL	3	0.1068	15750.1	90.00	0.40	tr_D0A4W6_
HLA-DQA10501-DQB10201	79	GSSDDCDGDNNANEP	DGDNNANEP	6	0.1051	16040.8	90.00	0.39	tr_D0A4W6_
HLA-DQA10501-DQB10201	80	ATIPTRHPRFYSSEE	HPRFYSSEE	6	0.0991	17116.7	90.00	0.40	tr_D0A4W6_
HLA-DQA10501-DQB10201	81	RFYSSEERRKMERYE	RFYSSEERR	0	0.0990	17126.3	90.00	0.50	tr_D0A4W6_
HLA-DQA10501-DQB10201	82	ANEPVAAVRRCTNRF	AVRRCTNRF	6	0.0928	18320.0	90.00	0.16	tr_D0A4W6_
HLA-DQA10501-DQB10201	83	NANEPVAAVRRCTNR	NEPVAAVRR	2	0.0926	18363.8	90.00	0.25	tr_D0A4W6_
HLA-DQA10501-DQB10201	84	AGSSDDCDGDNNANE	AGSSDDCDG	0	0.0913	18613.9	90.00	0.17	tr_D0A4W6_
HLA-DQA10501-DQB10201	85	LEERHDLSLRQKLQA	ERHDLSLRQ	2	0.0891	19064.5	95.00	0.37	tr_D0A4W6_
HLA-DQA10501-DQB10201	86	QATIPTRHPRFYSSE	TIPTRHPRF	2	0.0857	19774.4	95.00	0.47	tr_D0A4W6_
HLA-DQA10501-DQB10201	87	NEPVAAVRRCTNRFS	AVRRCTNRF	5	0.0813	20739.0	95.00	0.22	tr_D0A4W6_
HLA-DQA10501-DQB10201	88	EERHDLSLRQKLQAT	ERHDLSLRQ	1	0.0806	20904.0	95.00	0.34	tr_D0A4W6_
HLA-DQA10501-DQB10201	89	ERHDLSLRQKLQATI	LRQKLQATI	6	0.0795	21149.0	95.00	0.25	tr_D0A4W6_
HLA-DQA10501-DQB10201	90	AAVRRCTNRFSTNMP	TNRFSTNMP	6	0.0727	22780.2	95.00	0.49	tr_D0A4W6_

HLA-DQA10501-DQB10201	91	EPVAAVRRCTNRFST	AVRRCTNRF	4	0.0710	23191.3	95.00	0.28	tr_D0A4W6_	
HLA-DQA10501-DQB10201	92	FYSSEERRKMERYES	EERRKMERY	4	0.0701	23409.6	95.00	0.35	tr_D0A4W6_	
HLA-DQA10501-DQB10201	93	PVAAVRRCTNRFSTN	AVRRCTNRF	3	0.0595	26279.4	100.00	0.34	tr_D0A4W6_	
HLA-DQA10501-DQB10201	94	VAAVRRCTNRFSTNM	AVRRCTNRF	2	0.0579	26731.7	100.00	0.34	tr_D0A4W6_	

Allele: HLA-DQA10501-DQB10201. Number of high binders 0. Number of weak binders 7. Number of peptides 94

Allele	pos	peptide	core	Of	1-log50k(aff)	affinity(nM)	%Rank	Relia	Identity	Bind Level
HLA-DQA10501-DQB10201	1	HSYASLAFANADSAL	LAFANADSA	5	0.4969	231.1	6.00	0.46	tr_D0A4W6_	WB
HLA-DQA10501-DQB10201	2	SYASLAFANADSALQ	LAFANADSA	4	0.4881	254.4	6.50	0.44	tr_D0A4W6_	WB
HLA-DQA10501-DQB10201	3	YASLAFANADSALQT	LAFANADSA	3	0.4767	287.8	7.50	0.44	tr_D0A4W6_	WB
HLA-DQA10501-DQB10201	4	ASLAFANADSALQTL	LAFANADSA	2	0.4665	321.4	8.00	0.44	tr_D0A4W6_	WB
HLA-DQA10501-DQB10201	5	SLAFANADSALQTLL	LAFANADSA	1	0.4555	362.0	9.00	0.38	tr_D0A4W6_	WB
HLA-DQA10501-DQB10201	6	LHSYASLAFANADSA	LAFANADSA	6	0.4324	464.9	12.00	0.46	tr_D0A4W6_	
HLA-DQA10501-DQB10201	7	DVSVISFYGANGFFC	ISFYGANGF	4	0.4186	539.7	13.00	0.37	tr_D0A4W6_	
HLA-DQA10501-DQB10201	8	VSVISFYGANGFFCA	ISFYGANGF	3	0.4151	560.3	14.00	0.34	tr_D0A4W6_	
HLA-DQA10501-DQB10201	9	LAFANADSALQTLLS	LAFANADSA	0	0.4122	578.1	14.00	0.27	tr_D0A4W6_	
HLA-DQA10501-DQB10201	10	SVISFYGANGFFCAA	ISFYGANGF	2	0.4064	615.9	15.00	0.30	tr_D0A4W6_	
HLA-DQA10501-DQB10201	11	VISFYGANGFFCAAP	FYGANGFFC	3	0.4014	650.0	16.00	0.32	tr_D0A4W6_	
HLA-DQA10501-DQB10201	12	IVLHSYASLAFANAD	YASLAFANA	5	0.3768	848.1	19.00	0.30	tr_D0A4W6_	
HLA-DQA10501-DQB10201	13	RDVSVISFYGANGFF	ISFYGANGF	5	0.3736	877.6	20.00	0.46	tr_D0A4W6_	
HLA-DQA10501-DQB10201	14	ISFYGANGFFCAAPN	FYGANGFFC	2	0.3705	907.8	21.00	0.35	tr_D0A4W6_	
HLA-DQA10501-DQB10201	15	VLHSYASLAFANADS	YASLAFANA	4	0.3645	969.0	22.00	0.26	tr_D0A4W6_	
HLA-DQA10501-DQB10201	16	VIPVTATVHEQQVWI	VTATVHEQQ	3	0.3605	1011.8	22.00	0.41	tr_D0A4W6_	
HLA-DQA10501-DQB10201	17	AFANADSALQTLLSL	FANADSALQ	1	0.3590	1028.2	23.00	0.17	tr_D0A4W6_	
HLA-DQA10501-DQB10201	18	QVWIVLHSYASLAFA	LHSYASLAF	5	0.3522	1106.4	24.00	0.38	tr_D0A4W6_	
HLA-DQA10501-DQB10201	19	IPVTATVHEQQVWIV	VTATVHEQQ	2	0.3516	1113.6	24.00	0.36	tr_D0A4W6_	
HLA-DQA10501-DQB10201	20	QQVWIVLHSYASLAF	LHSYASLAF	6	0.3512	1118.9	24.00	0.50	tr_D0A4W6_	
HLA-DQA10501-DQB10201	21	WIVLHSYASLAFANA	YASLAFANA	6	0.3475	1164.2	25.00	0.29	tr_D0A4W6_	
HLA-DQA10501-DQB10201	22	TVIPVTATVHEQQVW	VTATVHEQQ	4	0.3391	1274.4	26.00	0.49	tr_D0A4W6_	
HLA-DQA10501-DQB10201	23	DSALQTLLSLRTETM	LLSLRTETM	6	0.3390	1276.8	26.00	0.56	tr_D0A4W6_	
HLA-DQA10501-DQB10201	24	VWIVLHSYASLAFAN	LHSYASLAF	4	0.3340	1347.8	27.00	0.34	tr_D0A4W6_	
HLA-DQA10501-DQB10201	25	PVTATVHEQQVWIVL	VTATVHEQQ	1	0.3303	1401.8	28.00	0.35	tr_D0A4W6_	
HLA-DQA10501-DQB10201	26	SFYGANGFFCAAPNN	FYGANGFFC	1	0.3242	1497.7	30.00	0.39	tr_D0A4W6_	
HLA-DQA10501-DQB10201	27	NTVIPVTATVHEQQV	VTATVHEQQ	5	0.3181	1600.8	31.00	0.52	tr_D0A4W6_	
HLA-DQA10501-DQB10201	28	SRDVSVISFYGANGF	ISFYGANGF	6	0.3139	1675.1	32.00	0.53	tr_D0A4W6_	
HLA-DQA10501-DQB10201	29	VTATVHEQQVWIVLH	VTATVHEQQ	0	0.3038	1868.7	34.00	0.25	tr_D0A4W6_	
HLA-DQA10501-DQB10201	30	NNTVIPVTATVHEQQ	VTATVHEQQ	6	0.2971	2008.0	36.00	0.57	tr_D0A4W6_	
HLA-DQA10501-DQB10201	31	LRWGNSSAVSPHFVD	SSAVSPHFV	5	0.2899	2170.7	38.00	0.47	tr_D0A4W6_	
HLA-DQA10501-DQB10201	32	FYGANGFFCAAPNNT	FFCAAPNNT	6	0.2897	2175.1	38.00	0.28	tr_D0A4W6_	
HLA-DQA10501-DQB10201	33	FANADSALQTLLSLR	NADSALQTL	2	0.2886	2201.4	38.00	0.24	tr_D0A4W6_	
HLA-DQA10501-DQB10201	34	WGNSSAVSPHFVDAQ	SSAVSPHFV	3	0.2812	2386.4	40.00	0.41	tr_D0A4W6_	
HLA-DQA10501-DQB10201	35	TLRWGNSSAVSPHFV	SSAVSPHFV	6	0.2807	2398.5	40.00	0.43	tr_D0A4W6_	
HLA-DQA10501-DQB10201	36	NSSAVSPHFVDAQSG	PHFVDAQSG	6	0.2759	2526.3	41.00	0.38	tr_D0A4W6_	
HLA-DQA10501-DQB10201	37	RWGNSSAVSPHFVDA	SSAVSPHFV	4	0.2743	2570.6	42.00	0.49	tr_D0A4W6_	
HLA-DQA10501-DQB10201	38	QTLSDSRDVSVISFY	SRDVSVISF	5	0.2738	2584.6	42.00	0.53	tr_D0A4W6_	

HLA-DQA10501-DQB10201	39	EQQVWIVLHSYASLA	VLHSYASLA	6	0.2634	2893.9	45.00	0.47	tr_D0A4W6_
HLA-DQA10501-DQB10201	40	SAVSPHFVDAQSGPC	PHFVDAQSG	4	0.2596	3012.4	46.00	0.44	tr_D0A4W6_
HLA-DQA10501-DQB10201	41	SSAVSPHFVDAQSGP	PHFVDAQSG	5	0.2591	3028.9	46.00	0.46	tr_D0A4W6_
HLA-DQA10501-DQB10201	42	AVSPHFVDAQSGPCV	PHFVDAQSG	3	0.2589	3036.2	46.00	0.38	tr_D0A4W6_
HLA-DQA10501-DQB10201	43	TATVHEQQVWIVLHS	TVHEQQVWI	2	0.2584	3054.4	46.00	0.34	tr_D0A4W6_
HLA-DQA10501-DQB10201	44	PQTLSDSRDVSVISF	SRDVSVISF	6	0.2582	3059.9	46.00	0.70	tr_D0A4W6_
HLA-DQA10501-DQB10201	45	GNSSAVSPHFVDAQS	SSAVSPHFV	2	0.2570	3099.7	46.00	0.43	tr_D0A4W6_
HLA-DQA10501-DQB10201	46	ATVHEQQVWIVLHSY	QVWIVLHSY	6	0.2546	3181.7	47.00	0.20	tr_D0A4W6_
HLA-DQA10501-DQB10201	47	TLSDSRDVSVISFYG	SRDVSVISF	4	0.2541	3198.2	47.00	0.50	tr_D0A4W6_
HLA-DQA10501-DQB10201	48	ANADSALQTLLSLRT	LQTLLSLRT	6	0.2516	3286.5	48.00	0.25	tr_D0A4W6_
HLA-DQA10501-DQB10201	49	FFCAAPNNTVIPVTA	NNTVIPVTA	6	0.2487	3390.6	49.00	0.33	tr_D0A4W6_
HLA-DQA10501-DQB10201	50	VSPHFVDAQSGPCVA	PHFVDAQSG	2	0.2482	3410.0	49.00	0.36	tr_D0A4W6_
HLA-DQA10501-DQB10201	51	NADSALQTLLSLRTE	LQTLLSLRT	5	0.2482	3410.1	49.00	0.25	tr_D0A4W6_
HLA-DQA10501-DQB10201	52	LSDSRDVSVISFYGA	SRDVSVISF	3	0.2471	3448.8	49.00	0.47	tr_D0A4W6_
HLA-DQA10501-DQB10201	53	YGANGFFCAAPNNTV	FFCAAPNNT	5	0.2467	3467.1	49.00	0.47	tr_D0A4W6_
HLA-DQA10501-DQB10201	54	ADSALQTLLSLRTET	LQTLLSLRT	4	0.2461	3488.1	49.00	0.26	tr_D0A4W6_
HLA-DQA10501-DQB10201	55	GFFCAAPNNTVIPVT	FFCAAPNNT	1	0.2437	3578.1	50.00	0.47	tr_D0A4W6_
HLA-DQA10501-DQB10201	56	DSRDVSVISFYGANG	SRDVSVISF	1	0.2390	3765.9	55.00	0.33	tr_D0A4W6_
HLA-DQA10501-DQB10201	57	PNNTVIPVTATVHEQ	PVTATVHEQ	6	0.2368	3856.9	55.00	0.33	tr_D0A4W6_
HLA-DQA10501-DQB10201	58	ANGFFCAAPNNTVIP	FFCAAPNNT	3	0.2368	3857.2	55.00	0.63	tr_D0A4W6_
HLA-DQA10501-DQB10201	59	SDSRDVSVISFYGAN	SRDVSVISF	2	0.2367	3860.9	55.00	0.46	tr_D0A4W6_
HLA-DQA10501-DQB10201	60	PTDNTTAGGLTTLRW	AGGLTTLRW	6	0.2348	3943.5	55.00	0.64	tr_D0A4W6_
HLA-DQA10501-DQB10201	61	NGFFCAAPNNTVIPV	FFCAAPNNT	2	0.2330	4020.8	55.00	0.61	tr_D0A4W6_
HLA-DQA10501-DQB10201	62	GANGFFCAAPNNTVI	FFCAAPNNT	4	0.2311	4103.8	55.00	0.55	tr_D0A4W6_
HLA-DQA10501-DQB10201	63	SPHFVDAQSGPCVAV	PHFVDAQSG	1	0.2286	4216.5	55.00	0.33	tr_D0A4W6_
HLA-DQA10501-DQB10201	64	TVHEQQVWIVLHSYA	QVWIVLHSY	5	0.2279	4245.9	55.00	0.22	tr_D0A4W6_
HLA-DQA10501-DQB10201	65	TTLRWGNSSAVSPHF	NSSAVSPHF	6	0.2273	4276.0	55.00	0.40	tr_D0A4W6_
HLA-DQA10501-DQB10201	66	AAPNNTVIPVTATVH	NNTVIPVTA	3	0.2226	4498.2	60.00	0.32	tr_D0A4W6_
HLA-DQA10501-DQB10201	67	CAAPNNTVIPVTATV	NNTVIPVTA	4	0.2224	4507.3	60.00	0.41	tr_D0A4W6_
HLA-DQA10501-DQB10201	68	APNNTVIPVTATVHE	NNTVIPVTA	2	0.2220	4526.1	60.00	0.27	tr_D0A4W6_
HLA-DQA10501-DQB10201	69	HEQQVWIVLHSYASL	IVLHSYASL	6	0.2155	4856.8	60.00	0.32	tr_D0A4W6_
HLA-DQA10501-DQB10201	70	FCAAPNNTVIPVTAT	NNTVIPVTA	5	0.2150	4882.3	60.00	0.44	tr_D0A4W6_
HLA-DQA10501-DQB10201	71	PHFVDAQSGPCVAVP	PHFVDAQSG	0	0.2087	5226.6	60.00	0.22	tr_D0A4W6_
HLA-DQA10501-DQB10201	72	TDNTTAGGLTTLRWG	AGGLTTLRW	5	0.2012	5671.2	65.00	0.61	tr_D0A4W6_
HLA-DQA10501-DQB10201	73	VHEQQVWIVLHSYAS	QVWIVLHSY	4	0.1961	5993.7	65.00	0.26	tr_D0A4W6_
HLA-DQA10501-DQB10201	74	VDAQSGPCVAVPQTL	PCVAVPQTL	6	0.1940	6128.0	65.00	0.46	tr_D0A4W6_
HLA-DQA10501-DQB10201	75	AQSGPCVAVPQTLSD	PCVAVPQTL	4	0.1872	6596.9	70.00	0.50	tr_D0A4W6_
HLA-DQA10501-DQB10201	76	DNTTAGGLTTLRWGN	AGGLTTLRW	4	0.1855	6719.5	70.00	0.62	tr_D0A4W6_
HLA-DQA10501-DQB10201	77	HFVDAQSGPCVAVPQ	VDAQSGPCV	2	0.1807	7080.6	70.00	0.28	tr_D0A4W6_
HLA-DQA10501-DQB10201	78	GLTTLRWGNSSAVSP	WGNSSAVSP	6	0.1797	7151.6	70.00	0.49	tr_D0A4W6_
HLA-DQA10501-DQB10201	79	GPCVAVPQTLSDSRD	PCVAVPQTL	1	0.1789	7216.1	70.00	0.37	tr_D0A4W6_
HLA-DQA10501-DQB10201	80	QSGPCVAVPQTLSDS	PCVAVPQTL	3	0.1773	7340.6	70.00	0.49	tr_D0A4W6_
HLA-DQA10501-DQB10201	81	DAQSGPCVAVPQTLS	PCVAVPQTL	5	0.1773	7342.3	70.00	0.48	tr_D0A4W6_
HLA-DQA10501-DQB10201	82	FVDAQSGPCVAVPQT	VDAQSGPCV	1	0.1747	7550.5	70.00	0.26	tr_D0A4W6_
HLA-DQA10501-DQB10201	83	SGPCVAVPQTLSDSR	PCVAVPQTL	2	0.1728	7706.1	70.00	0.47	tr_D0A4W6_
HLA-DQA10501-DQB10201	84	LTTLRWGNSSAVSPH	WGNSSAVSP	5	0.1716	7812.8	75.00	0.50	tr_D0A4W6_
HLA-DQA10501-DQB10201	85	NTTAGGLTTLRWGNS	AGGLTTLRW	3	0.1686	8063.9	75.00	0.65	tr_D0A4W6_
HLA-DQA10501-DQB10201	86	PCVAVPQTLSDSRDV	PCVAVPQTL	0	0.1651	8374.6	75.00	0.24	tr_D0A4W6_
HLA-DQA10501-DQB10201	87	TTAGGLTTLRWGNSS	AGGLTTLRW	2	0.1558	9269.1	75.00	0.66	tr_D0A4W6_
HLA-DQA10501-DQB10201	88	VAVPQTLSDSRDVSV	LSDSRDVSV	6	0.1468	10214.0	80.00	0.26	tr_D0A4W6_

HLA-DQA10501-DQB10201	89	CVAVPQTLSDSRDVS	PQTLSDSRD	4	0.1368	11377.2	80.00	0.29	tr_D0A4W6_	
HLA-DQA10501-DQB10201	90	AVPQTLSDSRDVSVI	LSDSRDVSV	5	0.1365	11419.7	80.00	0.30	tr_D0A4W6_	
HLA-DQA10501-DQB10201	91	VPQTLSDSRDVSVIS	LSDSRDVSV	4	0.1331	11849.2	85.00	0.29	tr_D0A4W6_	
HLA-DQA10501-DQB10201	92	TAGGLTTLRWGNSSA	AGGLTTLRW	1	0.1292	12357.6	85.00	0.72	tr_D0A4W6_	
HLA-DQA10501-DQB10201	93	AGGLTTLRWGNSSAV	LRWGNSSAV	6	0.1233	13170.7	85.00	0.42	tr_D0A4W6_	
HLA-DQA10501-DQB10201	94	NPTDNTTAGGLTTLR	TTAGGLTTL	5	0.1170	14103.5	85.00	0.35	tr_D0A4W6_	
HLA-DQA10501-DQB10201	95	GGLTTLRWGNSSAVS	LRWGNSSAV	5	0.1131	14712.9	90.00	0.44	tr_D0A4W6_	
HLA-DQA10501-DQB10201	96	LNPTDNTTAGGLTTL	TTAGGLTTL	6	0.1107	15099.5	90.00	0.47	tr_D0A4W6_	
HLA-DQA10501-DQB10201	97	GWSPPSSALNPTDNT	PSSALNPTD	4	0.1061	15862.0	90.00	0.34	tr_D0A4W6_	
HLA-DQA10501-DQB10201	98	AGWSPPSSALNPTDN	PSSALNPTD	5	0.1033	16354.3	90.00	0.40	tr_D0A4W6_	
HLA-DQA10501-DQB10201	99	SAGWSPPSSALNPTD	PSSALNPTD	6	0.1007	16820.7	90.00	0.45	tr_D0A4W6_	
HLA-DQA10501-DQB10201	100	WSPPSSALNPTDNTT	PSSALNPTD	3	0.0999	16970.3	90.00	0.34	tr_D0A4W6_	
HLA-DQA10501-DQB10201	101	SPPSSALNPTDNTTA	PSSALNPTD	2	0.0891	19062.7	95.00	0.33	tr_D0A4W6_	
HLA-DQA10501-DQB10201	102	AFGSAGWSPPSSALN	SAGWSPPSS	3	0.0856	19794.3	95.00	0.28	tr_D0A4W6_	
HLA-DQA10501-DQB10201	103	FGSAGWSPPSSALNP	SAGWSPPSS	2	0.0810	20810.3	95.00	0.28	tr_D0A4W6_	
HLA-DQA10501-DQB10201	104	PPSSALNPTDNTTAG	PSSALNPTD	1	0.0785	21392.7	95.00	0.31	tr_D0A4W6_	
HLA-DQA10501-DQB10201	105	GSAGWSPPSSALNPT	SAGWSPPSS	1	0.0752	22157.4	95.00	0.20	tr_D0A4W6_	
HLA-DQA10501-DQB10201	106	ALNPTDNTTAGGLTT	NTTAGGLTT	6	0.0714	23101.6	95.00	0.37	tr_D0A4W6_	
HLA-DQA10501-DQB10201	107	PSSALNPTDNTTAGG	PSSALNPTD	0	0.0671	24185.6	95.00	0.23	tr_D0A4W6_	
HLA-DQA10501-DQB10201	108	SALNPTDNTTAGGLT	DNTTAGGLT	6	0.0653	24663.3	95.00	0.33	tr_D0A4W6_	
HLA-DQA10501-DQB10201	109	SSALNPTDNTTAGGL	TDNTTAGGL	6	0.0613	25769.8	100.00	0.37	tr_D0A4W6_	

Allele: HLA-DQA10501-DQB10201. Number of high binders 0. Number of weak binders 5. Number of peptides 109

Allele	pos	peptide	core	Of	1-log50k(aff)	affinity(nM)	%Rank	Relia	Identity	Bind Level
HLA-DQA10501-DQB10201	1	SWHDLTTYTACSDMT	TYTACSDMT	6	0.3170	1619.5	31.00	0.43	tr_C9ZPL1_	
HLA-DQA10501-DQB10201	2	WHDLTTYTACSDMTN	TYTACSDMT	5	0.3034	1877.1	34.00	0.48	tr_C9ZPL1_	
HLA-DQA10501-DQB10201	3	FSWHDLTTYTACSDM	LTTYTACSD	5	0.2770	2497.4	41.00	0.35	tr_C9ZPL1_	
HLA-DQA10501-DQB10201	4	HDLTTYTACSDMTNG	TYTACSDMT	4	0.2727	2614.7	42.00	0.52	tr_C9ZPL1_	
HLA-DQA10501-DQB10201	5	GFSWHDLTTYTACSD	LTTYTACSD	6	0.2666	2793.1	44.00	0.40	tr_C9ZPL1_	
HLA-DQA10501-DQB10201	6	LRHGFSWHDLTTYTA	WHDLTTYTA	6	0.2564	3118.3	46.00	0.40	tr_C9ZPL1_	
HLA-DQA10501-DQB10201	7	DLTTYTACSDMTNGT	TYTACSDMT	3	0.2557	3145.2	47.00	0.56	tr_C9ZPL1_	
HLA-DQA10501-DQB10201	8	WFLRHGFSWHDLTTY	FSWHDLTTY	6	0.2431	3603.3	50.00	0.29	tr_C9ZPL1_	
HLA-DQA10501-DQB10201	9	RHGFSWHDLTTYTAC	WHDLTTYTA	5	0.2430	3607.5	50.00	0.41	tr_C9ZPL1_	
HLA-DQA10501-DQB10201	10	LTTYTACSDMTNGTC	TYTACSDMT	2	0.2264	4317.5	55.00	0.65	tr_C9ZPL1_	
HLA-DQA10501-DQB10201	11	FLRHGFSWHDLTTYT	FSWHDLTTY	5	0.2226	4498.3	60.00	0.32	tr_C9ZPL1_	
HLA-DQA10501-DQB10201	12	TACSDMTNGTCLLLA	TNGTCLLLA	6	0.2221	4523.1	60.00	0.43	tr_C9ZPL1_	
HLA-DQA10501-DQB10201	13	HGFSWHDLTTYTACS	WHDLTTYTA	4	0.2199	4632.4	60.00	0.46	tr_C9ZPL1_	
HLA-DQA10501-DQB10201	14	TYTACSDMTNGTCLL	DMTNGTCLL	6	0.2110	5098.7	60.00	0.35	tr_C9ZPL1_	
HLA-DQA10501-DQB10201	15	SDMTNGTCLLLANPQ	TNGTCLLLA	3	0.2067	5342.3	65.00	0.34	tr_C9ZPL1_	
HLA-DQA10501-DQB10201	16	CSDMTNGTCLLLANP	TNGTCLLLA	4	0.2049	5446.9	65.00	0.44	tr_C9ZPL1_	
HLA-DQA10501-DQB10201	17	ACSDMTNGTCLLLAN	TNGTCLLLA	5	0.2042	5489.8	65.00	0.41	tr_C9ZPL1_	
HLA-DQA10501-DQB10201	18	TTYTACSDMTNGTCL	TYTACSDMT	1	0.2034	5538.7	65.00	0.64	tr_C9ZPL1_	
HLA-DQA10501-DQB10201	19	YTACSDMTNGTCLLL	DMTNGTCLL	5	0.1858	6694.7	70.00	0.42	tr_C9ZPL1_	

Allele: HLA-DQA10501-DQB10201. Number of high binders 0. Number of weak binders 0. Number of peptides 19

Allele	pos	peptide	core	Of	1-log50k(aff)	affinity(nM)	%Rank	Relia	Identity	Bind Level
HLA-DQA10501-DQB10201	1	AQPWSILTPTNFDDW	WSILTPTNF	3	0.4231	513.9	13.00	0.73	tr_C9ZPL1_	
HLA-DQA10501-DQB10201	2	FAKLFNIVPLGVDPH	LFNIVPLGV	3	0.4174	546.8	14.00	0.41	tr_C9ZPL1_	
HLA-DQA10501-DQB10201	3	AKLFNIVPLGVDPHV	LFNIVPLGV	2	0.4124	577.1	14.00	0.32	tr_C9ZPL1_	
HLA-DQA10501-DQB10201	4	QPWSILTPTNFDDWK	WSILTPTNF	2	0.4047	627.3	15.00	0.73	tr_C9ZPL1_	
HLA-DQA10501-DQB10201	5	KLFNIVPLGVDPHVV	LFNIVPLGV	1	0.4045	628.6	15.00	0.26	tr_C9ZPL1_	
HLA-DQA10501-DQB10201	6	QAQPWSILTPTNFDD	WSILTPTNF	4	0.4042	630.5	15.00	0.76	tr_C9ZPL1_	
HLA-DQA10501-DQB10201	7	QQAQPWSILTPTNFD	WSILTPTNF	5	0.3888	745.0	18.00	0.76	tr_C9ZPL1_	
HLA-DQA10501-DQB10201	8	LFNIVPLGVDPHVVQ	LFNIVPLGV	0	0.3793	825.2	19.00	0.25	tr_C9ZPL1_	
HLA-DQA10501-DQB10201	9	VQQAQPWSILTPTNF	WSILTPTNF	6	0.3619	996.0	22.00	0.77	tr_C9ZPL1_	
HLA-DQA10501-DQB10201	10	FNIVPLGVDPHVVQQ	IVPLGVDPH	2	0.3201	1566.5	30.00	0.31	tr_C9ZPL1_	
HLA-DQA10501-DQB10201	11	GVDPHVVQQAQPWSI	HVVQQAQPW	4	0.2941	2076.0	37.00	0.43	tr_C9ZPL1_	
HLA-DQA10501-DQB10201	12	PLGVDPHVVQQAQPW	HVVQQAQPW	6	0.2931	2098.2	37.00	0.50	tr_C9ZPL1_	
HLA-DQA10501-DQB10201	13	VDPHVVQQAQPWSIL	HVVQQAQPW	3	0.2905	2158.4	37.00	0.44	tr_C9ZPL1_	
HLA-DQA10501-DQB10201	14	LGVDPHVVQQAQPWS	HVVQQAQPW	5	0.2830	2340.9	39.00	0.51	tr_C9ZPL1_	
HLA-DQA10501-DQB10201	15	DPHVVQQAQPWSILT	HVVQQAQPW	2	0.2716	2646.1	42.00	0.44	tr_C9ZPL1_	
HLA-DQA10501-DQB10201	16	NIVPLGVDPHVVQQA	IVPLGVDPH	1	0.2673	2773.9	43.00	0.31	tr_C9ZPL1_	
HLA-DQA10501-DQB10201	17	PHVVQQAQPWSILTP	HVVQQAQPW	1	0.2523	3260.1	48.00	0.41	tr_C9ZPL1_	
HLA-DQA10501-DQB10201	18	IVPLGVDPHVVQQAQ	PLGVDPHVV	2	0.2352	3925.7	55.00	0.26	tr_C9ZPL1_	
HLA-DQA10501-DQB10201	19	VPLGVDPHVVQQAQP	PHVVQQAQP	6	0.2319	4066.6	55.00	0.37	tr_C9ZPL1_	
HLA-DQA10501-DQB10201	20	HVVQQAQPWSILTPT	HVVQQAQPW	0	0.2179	4733.2	60.00	0.35	tr_C9ZPL1_	
HLA-DQA10501-DQB10201	21	VVQQAQPWSILTPTN	PWSILTPTN	6	0.1904	6369.3	65.00	0.26	tr_C9ZPL1_	

Allele: HLA-DQA10501-DQB10201. Number of high binders 0. Number of weak binders 0. Number of peptides 21

Allele	pos	peptide	core	Of	1-log50k(aff)	affinity(nM)	%Rank	Relia	Identity	Bind Level
HLA-DQA10501-DQB10201	1	QESILSLPDMVENFF	LPDMVENFF	6	0.5433	139.9	3.50	0.39	tr_C9ZXD7_	WB
HLA-DQA10501-DQB10201	2	LNWRVPVMRLAEEVA	VMRLAEEVA	6	0.5350	153.1	3.50	0.54	tr_C9ZXD7_	WB
HLA-DQA10501-DQB10201	3	NWRVPVMRLAEEVAQ	VMRLAEEVA	5	0.5250	170.6	4.00	0.51	tr_C9ZXD7_	WB
HLA-DQA10501-DQB10201	4	ESILSLPDMVENFFT	LPDMVENFF	5	0.5189	182.3	4.50	0.37	tr_C9ZXD7_	WB
HLA-DQA10501-DQB10201	5	VAQRMRCAYVEFPFM	MRCAYVEFP	4	0.4970	231.1	6.00	0.38	tr_C9ZXD7_	WB
HLA-DQA10501-DQB10201	6	WRVPVMRLAEEVAQR	VMRLAEEVA	4	0.4943	238.0	6.00	0.51	tr_C9ZXD7_	WB
HLA-DQA10501-DQB10201	7	RVPVMRLAEEVAQRM	VMRLAEEVA	3	0.4941	238.4	6.00	0.46	tr_C9ZXD7_	WB
HLA-DQA10501-DQB10201	8	AQRMRCAYVEFPFMS	MRCAYVEFP	3	0.4819	272.0	7.00	0.39	tr_C9ZXD7_	WB
HLA-DQA10501-DQB10201	9	SILSLPDMVENFFTS	LPDMVENFF	4	0.4765	288.3	7.50	0.42	tr_C9ZXD7_	WB
HLA-DQA10501-DQB10201	10	QRMRCAYVEFPFMSV	MRCAYVEFP	2	0.4725	301.0	7.50	0.35	tr_C9ZXD7_	WB
HLA-DQA10501-DQB10201	11	VPVMRLAEEVAQRMR	VMRLAEEVA	2	0.4719	303.1	7.50	0.50	tr_C9ZXD7_	WB
HLA-DQA10501-DQB10201	12	VQESILSLPDMVENF	SILSLPDMV	3	0.4661	322.7	8.00	0.36	tr_C9ZXD7_	WB
HLA-DQA10501-DQB10201	13	QYVQESILSLPDMVE	SILSLPDMV	5	0.4649	326.9	8.00	0.32	tr_C9ZXD7_	WB
HLA-DQA10501-DQB10201	14	ILSLPDMVENFFTSL	LPDMVENFF	3	0.4598	345.3	8.50	0.41	tr_C9ZXD7_	WB
HLA-DQA10501-DQB10201	15	EVAQRMRCAYVEFPF	MRCAYVEFP	5	0.4523	374.5	9.50	0.51	tr_C9ZXD7_	WB

HLA-DQA10501-DQB10201	16	RQYVQESILSLPDMV	SILSLPDMV	6	0.4498	384.8	9.50	0.32	tr_C9ZXD7_	WB
HLA-DQA10501-DQB10201	17	YVQESILSLPDMVEN	SILSLPDMV	4	0.4486	389.8	10.00	0.35	tr_C9ZXD7_	
HLA-DQA10501-DQB10201	18	RMRCAYVEFPFMSVL	MRCAYVEFP	1	0.4472	395.8	10.00	0.32	tr_C9ZXD7_	
HLA-DQA10501-DQB10201	19	LPDMVENFFTSLERV	NFFTSLERV	6	0.4443	408.4	11.00	0.31	tr_C9ZXD7_	
HLA-DQA10501-DQB10201	20	MRCAYVEFPFMSVLL	CAYVEFPFM	2	0.4410	423.3	11.00	0.32	tr_C9ZXD7_	
HLA-DQA10501-DQB10201	21	LSLPDMVENFFTSLE	LPDMVENFF	2	0.4368	443.3	11.00	0.38	tr_C9ZXD7_	
HLA-DQA10501-DQB10201	22	DRQYVQESILSLPDM	ESILSLPDM	6	0.4361	446.4	11.00	0.31	tr_C9ZXD7_	
HLA-DQA10501-DQB10201	23	PVMRLAEEVAQRMRC	VMRLAEEVA	1	0.4352	450.8	12.00	0.50	tr_C9ZXD7_	
HLA-DQA10501-DQB10201	24	SLPDMVENFFTSLER	LPDMVENFF	1	0.4345	454.0	12.00	0.30	tr_C9ZXD7_	
HLA-DQA10501-DQB10201	25	PDMVENFFTSLERVY	NFFTSLERV	5	0.4342	455.7	12.00	0.29	tr_C9ZXD7_	
HLA-DQA10501-DQB10201	26	EEVAQRMRCAYVEFP	MRCAYVEFP	6	0.4281	486.9	12.00	0.52	tr_C9ZXD7_	
HLA-DQA10501-DQB10201	27	SLNWRVPVMRLAEEV	PVMRLAEEV	6	0.4250	503.4	13.00	0.64	tr_C9ZXD7_	
HLA-DQA10501-DQB10201	28	MVENFFTSLERVYTD	NFFTSLERV	3	0.4209	526.3	13.00	0.28	tr_C9ZXD7_	
HLA-DQA10501-DQB10201	29	DMVENFFTSLERVYT	NFFTSLERV	4	0.4204	529.0	13.00	0.27	tr_C9ZXD7_	
HLA-DQA10501-DQB10201	30	DDRQYVQESILSLPD	RQYVQESIL	2	0.4047	627.3	15.00	0.32	tr_C9ZXD7_	
HLA-DQA10501-DQB10201	31	DDDRQYVQESILSLP	RQYVQESIL	3	0.4031	637.9	16.00	0.41	tr_C9ZXD7_	
HLA-DQA10501-DQB10201	32	VENFFTSLERVYTDG	NFFTSLERV	2	0.3963	686.6	16.00	0.34	tr_C9ZXD7_	
HLA-DQA10501-DQB10201	33	AYVEFPFMSVLLREA	FMSVLLREA	6	0.3961	687.9	16.00	0.44	tr_C9ZXD7_	
HLA-DQA10501-DQB10201	34	CAYVEFPFMSVLLRE	PFMSVLLRE	6	0.3941	702.9	17.00	0.26	tr_C9ZXD7_	
HLA-DQA10501-DQB10201	35	SDDDRQYVQESILSL	RQYVQESIL	4	0.3936	706.8	17.00	0.40	tr_C9ZXD7_	
HLA-DQA10501-DQB10201	36	RCAYVEFPFMSVLLR	CAYVEFPFM	1	0.3891	742.7	18.00	0.35	tr_C9ZXD7_	
HLA-DQA10501-DQB10201	37	YVEFPFMSVLLREAV	FMSVLLREA	5	0.3791	827.3	19.00	0.38	tr_C9ZXD7_	
HLA-DQA10501-DQB10201	38	NDEKRSIDWEIYERI	IDWEIYERI	6	0.3779	838.1	19.00	0.40	tr_C9ZXD7_	
HLA-DQA10501-DQB10201	39	LASDDDRQYVQESIL	RQYVQESIL	6	0.3769	847.0	19.00	0.62	tr_C9ZXD7_	
HLA-DQA10501-DQB10201	40	RMGHLFLGDIVVVGP	GHLFLGDIV	2	0.3767	849.1	19.00	0.28	tr_C9ZXD7_	
HLA-DQA10501-DQB10201	41	HLFLGDIVVVGPQSL	IVVVGPQSL	6	0.3675	937.7	21.00	0.37	tr_C9ZXD7_	
HLA-DQA10501-DQB10201	42	ASDDDRQYVQESILS	RQYVQESIL	5	0.3649	964.4	22.00	0.55	tr_C9ZXD7_	
HLA-DQA10501-DQB10201	43	MGHLFLGDIVVVGPQ	GHLFLGDIV	1	0.3610	1005.6	22.00	0.27	tr_C9ZXD7_	
HLA-DQA10501-DQB10201	44	RIKRMGHLFLGDIVV	GHLFLGDIV	5	0.3607	1009.5	22.00	0.45	tr_C9ZXD7_	
HLA-DQA10501-DQB10201	45	IKRMGHLFLGDIVVV	GHLFLGDIV	4	0.3596	1021.0	22.00	0.41	tr_C9ZXD7_	
HLA-DQA10501-DQB10201	46	ERIKRMGHLFLGDIV	GHLFLGDIV	6	0.3586	1032.0	23.00	0.54	tr_C9ZXD7_	
HLA-DQA10501-DQB10201	47	VMRLAEEVAQRMRCA	VMRLAEEVA	0	0.3573	1047.0	23.00	0.40	tr_C9ZXD7_	
HLA-DQA10501-DQB10201	48	LFLGDIVVVGPQSLN	IVVVGPQSL	5	0.3556	1066.9	23.00	0.38	tr_C9ZXD7_	
HLA-DQA10501-DQB10201	49	GHLFLGDIVVVGPQS	GHLFLGDIV	0	0.3553	1070.1	23.00	0.14	tr_C9ZXD7_	
HLA-DQA10501-DQB10201	50	ENFFTSLERVYTDGK	NFFTSLERV	1	0.3551	1072.3	23.00	0.34	tr_C9ZXD7_	
HLA-DQA10501-DQB10201	51	EFPFMSVLLREAVSY	FMSVLLREA	3	0.3548	1075.8	23.00	0.40	tr_C9ZXD7_	
HLA-DQA10501-DQB10201	52	KRMGHLFLGDIVVVG	GHLFLGDIV	3	0.3533	1093.2	24.00	0.36	tr_C9ZXD7_	
HLA-DQA10501-DQB10201	53	VEFPFMSVLLREAVS	FMSVLLREA	4	0.3532	1095.1	24.00	0.39	tr_C9ZXD7_	
HLA-DQA10501-DQB10201	54	FLGDIVVVGPQSLNW	IVVVGPQSL	4	0.3525	1103.5	24.00	0.41	tr_C9ZXD7_	
HLA-DQA10501-DQB10201	55	KQAFATYHSIQTIVS	YHSIQTIVS	6	0.3474	1165.9	25.00	0.40	tr_C9ZXD7_	
HLA-DQA10501-DQB10201	56	FATYHSIQTIVSVLC	YHSIQTIVS	3	0.3454	1191.0	25.00	0.29	tr_C9ZXD7_	
HLA-DQA10501-DQB10201	57	QAFATYHSIQTIVSV	YHSIQTIVS	5	0.3421	1234.2	26.00	0.37	tr_C9ZXD7_	
HLA-DQA10501-DQB10201	58	DEKRSIDWEIYERIK	IDWEIYERI	5	0.3393	1272.6	26.00	0.40	tr_C9ZXD7_	
HLA-DQA10501-DQB10201	59	AFATYHSIQTIVSVL	YHSIQTIVS	4	0.3338	1350.7	28.00	0.39	tr_C9ZXD7_	
HLA-DQA10501-DQB10201	60	FPFMSVLLREAVSYS	FMSVLLREA	2	0.3222	1531.6	30.00	0.43	tr_C9ZXD7_	
HLA-DQA10501-DQB10201	61	MLPHPILASDDDRQY	PILASDDDR	4	0.3204	1561.1	30.00	0.28	tr_C9ZXD7_	
HLA-DQA10501-DQB10201	62	NFFTSLERVYTDGKC	NFFTSLERV	0	0.3197	1573.1	31.00	0.30	tr_C9ZXD7_	
HLA-DQA10501-DQB10201	63	ATYHSIQTIVSVLCR	YHSIQTIVS	2	0.3190	1585.6	31.00	0.28	tr_C9ZXD7_	
HLA-DQA10501-DQB10201	64	LPHPILASDDDRQYV	PILASDDDR	3	0.3165	1627.6	31.00	0.30	tr_C9ZXD7_	
HLA-DQA10501-DQB10201	65	LGDIVVVGPQSLNWR	IVVVGPQSL	3	0.3094	1758.5	33.00	0.50	tr_C9ZXD7_	

HLA-DQA10501-DQB10201	66	EKRSIDWEIYERIKR	IDWEIYERI	4	0.3079	1786.7	33.00	0.41	tr_C9ZXD7_
HLA-DQA10501-DQB10201	67	AKMLPHPILASDDDR	PILASDDDR	6	0.3076	1793.5	33.00	0.32	tr_C9ZXD7_
HLA-DQA10501-DQB10201	68	KMLPHPILASDDDRQ	PILASDDDR	5	0.3069	1807.2	33.00	0.30	tr_C9ZXD7_
HLA-DQA10501-DQB10201	69	TYHSIQTIVSVLCRV	YHSIQTIVS	1	0.3055	1834.5	34.00	0.22	tr_C9ZXD7_
HLA-DQA10501-DQB10201	70	AEEVAQRMRCAYVEF	RMRCAYVEF	6	0.3050	1843.5	34.00	0.63	tr_C9ZXD7_
HLA-DQA10501-DQB10201	71	AKQAFATYHSIQTIV	TYHSIQTIV	6	0.3022	1901.7	35.00	0.44	tr_C9ZXD7_
HLA-DQA10501-DQB10201	72	PHPILASDDDRQYVQ	PILASDDDR	2	0.2963	2026.2	36.00	0.33	tr_C9ZXD7_
HLA-DQA10501-DQB10201	73	TNDEKRSIDWEIYER	SIDWEIYER	6	0.2950	2054.1	36.00	0.27	tr_C9ZXD7_
HLA-DQA10501-DQB10201	74	KRSIDWEIYERIKRM	IDWEIYERI	3	0.2933	2093.6	37.00	0.41	tr_C9ZXD7_
HLA-DQA10501-DQB10201	75	ILASDDDRQYVQESI	DRQYVQESI	6	0.2920	2123.0	37.00	0.51	tr_C9ZXD7_
HLA-DQA10501-DQB10201	76	IAKMLPHPILASDDD	HPILASDDD	6	0.2906	2154.9	37.00	0.43	tr_C9ZXD7_
HLA-DQA10501-DQB10201	77	YHSIQTIVSVLCRVP	TIVSVLCRV	5	0.2853	2281.3	39.00	0.23	tr_C9ZXD7_
HLA-DQA10501-DQB10201	78	QSLNWRVPVMRLAEE	VPVMRLAEE	6	0.2837	2321.0	39.00	0.31	tr_C9ZXD7_
HLA-DQA10501-DQB10201	79	GDIVVVGPQSLNWRV	IVVVGPQSL	2	0.2832	2335.7	39.00	0.53	tr_C9ZXD7_
HLA-DQA10501-DQB10201	80	HPILASDDDRQYVQE	PILASDDDR	1	0.2810	2390.7	40.00	0.32	tr_C9ZXD7_
HLA-DQA10501-DQB10201	81	RTNDEKRSIDWEIYE	RSIDWEIYE	6	0.2778	2474.0	41.00	0.37	tr_C9ZXD7_
HLA-DQA10501-DQB10201	82	DIVVVGPQSLNWRVP	IVVVGPQSL	1	0.2708	2669.8	43.00	0.54	tr_C9ZXD7_
HLA-DQA10501-DQB10201	83	RSIDWEIYERIKRMG	IDWEIYERI	2	0.2646	2856.2	44.00	0.43	tr_C9ZXD7_
HLA-DQA10501-DQB10201	84	HSIQTIVSVLCRVPA	TIVSVLCRV	4	0.2631	2901.9	45.00	0.25	tr_C9ZXD7_
HLA-DQA10501-DQB10201	85	MRLAEEVAQRMRCAY	LAEEVAQRM	2	0.2616	2949.6	45.00	0.56	tr_C9ZXD7_
HLA-DQA10501-DQB10201	86	YERIKRMGHLFLGDI	MGHLFLGDI	6	0.2586	3045.6	46.00	0.48	tr_C9ZXD7_
HLA-DQA10501-DQB10201	87	PQSLNWRVPVMRLAE	NWRVPVMRL	4	0.2576	3081.1	46.00	0.22	tr_C9ZXD7_
HLA-DQA10501-DQB10201	88	IQTIVSVLCRVPASA	VLCRVPASA	6	0.2527	3247.7	47.00	0.22	tr_C9ZXD7_
HLA-DQA10501-DQB10201	89	SIQTIVSVLCRVPAS	TIVSVLCRV	3	0.2522	3265.0	48.00	0.26	tr_C9ZXD7_
HLA-DQA10501-DQB10201	90	GPQSLNWRVPVMRLA	NWRVPVMRL	5	0.2521	3267.1	48.00	0.25	tr_C9ZXD7_
HLA-DQA10501-DQB10201	91	EAKQAFATYHSIQTI	ATYHSIQTI	6	0.2505	3324.4	48.00	0.48	tr_C9ZXD7_
HLA-DQA10501-DQB10201	92	FFTSLERVYTDGKCE	FFTSLERVY	0	0.2488	3389.0	49.00	0.25	tr_C9ZXD7_
HLA-DQA10501-DQB10201	93	PILASDDDRQYVQES	ILASDDDRQ	1	0.2453	3517.5	49.00	0.41	tr_C9ZXD7_
HLA-DQA10501-DQB10201	94	QTIVSVLCRVPASAY	VLCRVPASA	5	0.2405	3704.1	55.00	0.31	tr_C9ZXD7_
HLA-DQA10501-DQB10201	95	RLAEEVAQRMRCAYV	LAEEVAQRM	1	0.2378	3816.9	55.00	0.57	tr_C9ZXD7_
HLA-DQA10501-DQB10201	96	VGPQSLNWRVPVMRL	NWRVPVMRL	6	0.2362	3881.4	55.00	0.33	tr_C9ZXD7_
HLA-DQA10501-DQB10201	97	RIAKMLPHPILASDD	PHPILASDD	6	0.2338	3985.7	55.00	0.40	tr_C9ZXD7_
HLA-DQA10501-DQB10201	98	SIDWEIYERIKRMGH	IDWEIYERI	1	0.2259	4338.7	55.00	0.49	tr_C9ZXD7_
HLA-DQA10501-DQB10201	99	YRTNDEKRSIDWEIY	KRSIDWEIY	6	0.2252	4372.7	55.00	0.39	tr_C9ZXD7_
HLA-DQA10501-DQB10201	100	FTSLERVYTDGKCEE	VYTDGKCEE	6	0.2249	4387.8	55.00	0.34	tr_C9ZXD7_
HLA-DQA10501-DQB10201	101	TSLERVYTDGKCEEA	VYTDGKCEE	5	0.2194	4658.2	60.00	0.35	tr_C9ZXD7_
HLA-DQA10501-DQB10201	102	LAEEVAQRMRCAYVE	LAEEVAQRM	0	0.2166	4797.3	60.00	0.38	tr_C9ZXD7_
HLA-DQA10501-DQB10201	103	TIVSVLCRVPASAYR	VLCRVPASA	4	0.2149	4887.1	60.00	0.30	tr_C9ZXD7_
HLA-DQA10501-DQB10201	104	IVVVGPQSLNWRVPV	IVVVGPQSL	0	0.2116	5066.8	60.00	0.52	tr_C9ZXD7_
HLA-DQA10501-DQB10201	105	PTCDRIAKMLPHPIL	IAKMLPHPI	5	0.2075	5297.4	60.00	0.59	tr_C9ZXD7_
HLA-DQA10501-DQB10201	106	IYERIKRMGHLFLGD	IKRMGHLFL	4	0.2024	5599.1	65.00	0.39	tr_C9ZXD7_
HLA-DQA10501-DQB10201	107	TCDRIAKMLPHPILA	IAKMLPHPI	4	0.2012	5667.1	65.00	0.56	tr_C9ZXD7_
HLA-DQA10501-DQB10201	108	GPTCDRIAKMLPHPI	IAKMLPHPI	6	0.2005	5712.7	65.00	0.65	tr_C9ZXD7_
HLA-DQA10501-DQB10201	109	DRIAKMLPHPILASD	IAKMLPHPI	2	0.1943	6112.1	65.00	0.52	tr_C9ZXD7_
HLA-DQA10501-DQB10201	110	VVGPQSLNWRVPVMR	LNWRVPVMR	6	0.1929	6205.4	65.00	0.34	tr_C9ZXD7_
HLA-DQA10501-DQB10201	111	WEIYERIKRMGHLFL	IKRMGHLFL	6	0.1928	6206.7	65.00	0.51	tr_C9ZXD7_
HLA-DQA10501-DQB10201	112	IVSVLCRVPASAYRT	VLCRVPASA	3	0.1922	6249.0	65.00	0.36	tr_C9ZXD7_
HLA-DQA10501-DQB10201	113	CDRIAKMLPHPILAS	IAKMLPHPI	3	0.1918	6279.2	65.00	0.55	tr_C9ZXD7_
HLA-DQA10501-DQB10201	114	SLERVYTDGKCEEAK	VYTDGKCEE	4	0.1912	6314.6	65.00	0.37	tr_C9ZXD7_
HLA-DQA10501-DQB10201	115	EEAKQAFATYHSIQT	FATYHSIQT	6	0.1854	6725.0	70.00	0.38	tr_C9ZXD7_

```
 HLA-DQA10501-DQB10201  116      LERVYTDGKCEEAKQ  VYTDGKCEE  3        0.1758       7460.0    70.00  0.37    tr_C9ZXD7_
 HLA-DQA10501-DQB10201  117      AYRTNDEKRSIDWEI  EKRSIDWEI  6        0.1709       7866.4    75.00  0.45    tr_C9ZXD7_
 HLA-DQA10501-DQB10201  118      VSVLCRVPASAYRTN  VLCRVPASA  2        0.1705       7899.6    75.00  0.41    tr_C9ZXD7_
 HLA-DQA10501-DQB10201  119      VVVGPQSLNWRVPVM  SLNWRVPVM  6        0.1702       7931.4    75.00  0.44    tr_C9ZXD7_
 HLA-DQA10501-DQB10201  120      EIYERIKRMGHLFLG  IKRMGHLFL  5        0.1686       8064.5    75.00  0.48    tr_C9ZXD7_
 HLA-DQA10501-DQB10201  121      IDWEIYERIKRMGHL  IDWEIYERI  0        0.1625       8614.6    75.00  0.41    tr_C9ZXD7_
 HLA-DQA10501-DQB10201  122      ERVYTDGKCEEAKQA  VYTDGKCEE  2        0.1606       8799.9    75.00  0.39    tr_C9ZXD7_
 HLA-DQA10501-DQB10201  123      VLCRVPASAYRTNDE  ASAYRTNDE  6        0.1568       9167.7    75.00  0.43    tr_C9ZXD7_
 HLA-DQA10501-DQB10201  124      CEEAKQAFATYHSIQ  AFATYHSIQ  6        0.1566       9187.9    75.00  0.35    tr_C9ZXD7_
 HLA-DQA10501-DQB10201  125      SVLCRVPASAYRTND  VLCRVPASA  1        0.1532       9531.7    80.00  0.41    tr_C9ZXD7_
 HLA-DQA10501-DQB10201  126      RVYTDGKCEEAKQAF  VYTDGKCEE  1        0.1395      11051.5    80.00  0.40    tr_C9ZXD7_
 HLA-DQA10501-DQB10201  127      KCEEAKQAFATYHSI  QAFATYHSI  6        0.1345      11667.2    80.00  0.43    tr_C9ZXD7_
 HLA-DQA10501-DQB10201  128      SAYRTNDEKRSIDWE  DEKRSIDWE  6        0.1325      11923.2    85.00  0.51    tr_C9ZXD7_
 HLA-DQA10501-DQB10201  129      DWEIYERIKRMGHLF  RIKRMGHLF  6        0.1314      12063.8    85.00  0.35    tr_C9ZXD7_
 HLA-DQA10501-DQB10201  130      LCRVPASAYRTNDEK  ASAYRTNDE  5        0.1284      12465.6    85.00  0.44    tr_C9ZXD7_
 HLA-DQA10501-DQB10201  131      VYTDGKCEEAKQAFA  VYTDGKCEE  0        0.1238      13103.9    85.00  0.31    tr_C9ZXD7_
 HLA-DQA10501-DQB10201  132      TDGKCEEAKQAFATY  EAKQAFATY  6        0.1235      13136.6    85.00  0.46    tr_C9ZXD7_
 HLA-DQA10501-DQB10201  133      CRVPASAYRTNDEKR  ASAYRTNDE  4        0.1173      14046.5    85.00  0.44    tr_C9ZXD7_
 HLA-DQA10501-DQB10201  134      DGKCEEAKQAFATYH  EAKQAFATY  5        0.1165      14173.1    85.00  0.34    tr_C9ZXD7_
 HLA-DQA10501-DQB10201  135      MGPTCDRIAKMLPHP  RIAKMLPHP  6        0.1156      14307.9    85.00  0.31    tr_C9ZXD7_
 HLA-DQA10501-DQB10201  136      GKCEEAKQAFATYHS  EAKQAFATY  4        0.1132      14684.5    90.00  0.31    tr_C9ZXD7_
 HLA-DQA10501-DQB10201  137      RVPASAYRTNDEKRS  ASAYRTNDE  3        0.1109      15058.6    90.00  0.40    tr_C9ZXD7_
 HLA-DQA10501-DQB10201  138      VPASAYRTNDEKRSI  ASAYRTNDE  2        0.1039      16239.7    90.00  0.43    tr_C9ZXD7_
 HLA-DQA10501-DQB10201  139      PASAYRTNDEKRSID  ASAYRTNDE  1        0.0980      17318.2    90.00  0.39    tr_C9ZXD7_
 HLA-DQA10501-DQB10201  140      YTDGKCEEAKQAFAT  CEEAKQAFA  5        0.0916      18553.9    90.00  0.32    tr_C9ZXD7_
 HLA-DQA10501-DQB10201  141      ASAYRTNDEKRSIDW  AYRTNDEKR  2        0.0912      18642.7    95.00  0.28    tr_C9ZXD7_
------------------------------------------------------------------------------------------------
```

Allele: HLA-DQA10501-DQB10201. Number of high binders 0. Number of weak binders 16. Number of peptides 141

```
------------------------------------------------------------------------------------------------
------------------------------------------------------------------------------------------------
                Allele  pos                  peptide       core Of 1-log50k(aff) affinity(nM)    %Rank Relia     Identity Bind Level
------------------------------------------------------------------------------------------------
 HLA-DQA10501-DQB10201    1      AVKMFYFTEDLEESI  MFYFTEDLE  3        0.7148         21.9     0.12  0.34    tr_C9ZXD7_         SB
 HLA-DQA10501-DQB10201    2      MAVKMFYFTEDLEES  MFYFTEDLE  4        0.7041         24.6     0.15  0.40    tr_C9ZXD7_         SB
 HLA-DQA10501-DQB10201    3      VKMFYFTEDLEESIN  MFYFTEDLE  2        0.7031         24.8     0.15  0.34    tr_C9ZXD7_         SB
 HLA-DQA10501-DQB10201    4      KMAVKMFYFTEDLEE  MFYFTEDLE  5        0.6999         25.7     0.17  0.48    tr_C9ZXD7_         SB
 HLA-DQA10501-DQB10201    5      KMFYFTEDLEESINA  MFYFTEDLE  1        0.6848         30.3     0.25  0.34    tr_C9ZXD7_         SB
 HLA-DQA10501-DQB10201    6      EDVQGTLRYMAPEVY  LRYMAPEVY  6        0.6640         37.9     0.40  0.67    tr_C9ZXD7_         SB
 HLA-DQA10501-DQB10201    7      MFYFTEDLEESINAL  FYFTEDLEE  1        0.6535         42.5     0.50  0.37    tr_C9ZXD7_         WB
 HLA-DQA10501-DQB10201    8      DVQGTLRYMAPEVYR  LRYMAPEVY  5        0.6419         48.2     0.60  0.69    tr_C9ZXD7_         WB
 HLA-DQA10501-DQB10201    9      GKMAVKMFYFTEDLE  MFYFTEDLE  6        0.6342         52.3     0.70  0.69    tr_C9ZXD7_         WB
 HLA-DQA10501-DQB10201   10      VQGTLRYMAPEVYRG  LRYMAPEVY  4        0.6178         62.5     1.00  0.69    tr_C9ZXD7_         WB
 HLA-DQA10501-DQB10201   11      GMLAEWEWTSSEQLL  WEWTSSEQL  5        0.6170         63.0     1.00  0.70    tr_C9ZXD7_         WB
 HLA-DQA10501-DQB10201   12      QVEELARGMLAEWEW  ARGMLAEWE  5        0.6166         63.3     1.00  0.61    tr_C9ZXD7_         WB
 HLA-DQA10501-DQB10201   13      FPCQITAALEDIDDA  QITAALEDI  3        0.6066         70.6     1.20  0.33    tr_C9ZXD7_         WB
 HLA-DQA10501-DQB10201   14      QGTLRYMAPEVYRGE  LRYMAPEVY  3        0.6055         71.4     1.20  0.69    tr_C9ZXD7_         WB
 HLA-DQA10501-DQB10201   15      VEELARGMLAEWEWT  ARGMLAEWE  4        0.5989         76.7     1.30  0.62    tr_C9ZXD7_         WB
 HLA-DQA10501-DQB10201   16      CQITAALEDIDDATF  QITAALEDI  1        0.5982         77.2     1.30  0.25    tr_C9ZXD7_         WB
```

HLA-DQA10501-DQB10201	17	MLAEWEWTSSEQLLG	WEWTSSEQL	4	0.5972	78.1	1.40	0.70	tr_C9ZXD7_	WB
HLA-DQA10501-DQB10201	18	SSSCSAEVESLPDIF	EVESLPDIF	6	0.5935	81.3	1.40	0.71	tr_C9ZXD7_	WB
HLA-DQA10501-DQB10201	19	PCQITAALEDIDDAT	QITAALEDI	2	0.5924	82.3	1.50	0.27	tr_C9ZXD7_	WB
HLA-DQA10501-DQB10201	20	GTLRYMAPEVYRGED	LRYMAPEVY	2	0.5905	83.9	1.50	0.68	tr_C9ZXD7_	WB
HLA-DQA10501-DQB10201	21	KYRITRWNMAAEVVT	RWNMAAEVV	5	0.5902	84.3	1.50	0.53	tr_C9ZXD7_	WB
HLA-DQA10501-DQB10201	22	RFPCQITAALEDIDD	QITAALEDI	4	0.5891	85.2	1.50	0.47	tr_C9ZXD7_	WB
HLA-DQA10501-DQB10201	23	SSCSAEVESLPDIFS	EVESLPDIF	5	0.5873	87.0	1.60	0.72	tr_C9ZXD7_	WB
HLA-DQA10501-DQB10201	24	KQVEELARGMLAEWE	ARGMLAEWE	6	0.5836	90.5	1.70	0.68	tr_C9ZXD7_	WB
HLA-DQA10501-DQB10201	25	LAEWEWTSSEQLLGQ	WEWTSSEQL	3	0.5803	93.8	1.80	0.69	tr_C9ZXD7_	WB
HLA-DQA10501-DQB10201	26	RGMLAEWEWTSSEQL	WEWTSSEQL	6	0.5777	96.5	1.80	0.75	tr_C9ZXD7_	WB
HLA-DQA10501-DQB10201	27	EELARGMLAEWEWTS	ARGMLAEWE	3	0.5775	96.7	1.80	0.62	tr_C9ZXD7_	WB
HLA-DQA10501-DQB10201	28	ITRWNMAAEVVTGFL	RWNMAAEVV	2	0.5758	98.5	1.90	0.42	tr_C9ZXD7_	WB
HLA-DQA10501-DQB10201	29	MLADMTDVDYYVDGL	DVDYYVDGL	6	0.5727	101.9	2.00	0.41	tr_C9ZXD7_	WB
HLA-DQA10501-DQB10201	30	CDIWSVGCVAAELFD	VGCVAAELF	5	0.5719	102.8	2.00	0.50	tr_C9ZXD7_	WB
HLA-DQA10501-DQB10201	31	RITRWNMAAEVVTGF	RWNMAAEVV	3	0.5710	103.7	2.00	0.46	tr_C9ZXD7_	WB
HLA-DQA10501-DQB10201	32	QITAALEDIDDATFR	ITAALEDID	1	0.5700	104.8	2.50	0.35	tr_C9ZXD7_	WB
HLA-DQA10501-DQB10201	33	PCDIWSVGCVAAELF	VGCVAAELF	6	0.5673	108.0	2.50	0.50	tr_C9ZXD7_	WB
HLA-DQA10501-DQB10201	34	YRITRWNMAAEVVTG	RWNMAAEVV	4	0.5657	109.8	2.50	0.47	tr_C9ZXD7_	WB
HLA-DQA10501-DQB10201	35	TLRYMAPEVYRGEDH	LRYMAPEVY	1	0.5651	110.6	2.50	0.71	tr_C9ZXD7_	WB
HLA-DQA10501-DQB10201	36	FYFTEDLEESINALL	FYFTEDLEE	0	0.5641	111.8	2.50	0.37	tr_C9ZXD7_	WB
HLA-DQA10501-DQB10201	37	AEWEWTSSEQLLGQM	WEWTSSEQL	2	0.5635	112.4	2.50	0.69	tr_C9ZXD7_	WB
HLA-DQA10501-DQB10201	38	TRWNMAAEVVTGFLE	RWNMAAEVV	1	0.5635	112.5	2.50	0.35	tr_C9ZXD7_	WB
HLA-DQA10501-DQB10201	39	DIWSVGCVAAELFDC	VGCVAAELF	4	0.5591	118.0	2.50	0.49	tr_C9ZXD7_	WB
HLA-DQA10501-DQB10201	40	SCSAEVESLPDIFSR	EVESLPDIF	4	0.5591	118.0	2.50	0.70	tr_C9ZXD7_	WB
HLA-DQA10501-DQB10201	41	ETPHMLADMTDVDYY	MLADMTDVD	4	0.5590	118.2	2.50	0.28	tr_C9ZXD7_	WB
HLA-DQA10501-DQB10201	42	IWSVGCVAAELFDCP	VGCVAAELF	3	0.5582	119.2	2.50	0.50	tr_C9ZXD7_	WB
HLA-DQA10501-DQB10201	43	FRFPCQITAALEDID	QITAALEDI	5	0.5563	121.5	3.00	0.56	tr_C9ZXD7_	WB
HLA-DQA10501-DQB10201	44	DKYRITRWNMAAEVV	RWNMAAEVV	6	0.5545	124.0	3.00	0.75	tr_C9ZXD7_	WB
HLA-DQA10501-DQB10201	45	TPHMLADMTDVDYYV	MLADMTDVD	3	0.5539	124.8	3.00	0.29	tr_C9ZXD7_	WB
HLA-DQA10501-DQB10201	46	ELARGMLAEWEWTSS	ARGMLAEWE	2	0.5498	130.5	3.00	0.63	tr_C9ZXD7_	WB
HLA-DQA10501-DQB10201	47	LADMTDVDYYVDGLT	DVDYYVDGL	5	0.5497	130.6	3.00	0.44	tr_C9ZXD7_	WB
HLA-DQA10501-DQB10201	48	PHMLADMTDVDYYVD	MLADMTDVD	2	0.5486	132.2	3.00	0.28	tr_C9ZXD7_	WB
HLA-DQA10501-DQB10201	49	CSAEVESLPDIFSRE	EVESLPDIF	3	0.5429	140.5	3.50	0.75	tr_C9ZXD7_	WB
HLA-DQA10501-DQB10201	50	EWEWTSSEQLLGQML	WEWTSSEQL	1	0.5425	141.2	3.50	0.65	tr_C9ZXD7_	WB
HLA-DQA10501-DQB10201	51	METPHMLADMTDVDY	MLADMTDVD	5	0.5408	143.8	3.50	0.31	tr_C9ZXD7_	WB
HLA-DQA10501-DQB10201	52	WSVGCVAAELFDCPP	VGCVAAELF	2	0.5388	146.9	3.50	0.51	tr_C9ZXD7_	WB
HLA-DQA10501-DQB10201	53	ADMTDVDYYVDGLTS	DVDYYVDGL	4	0.5318	158.5	4.00	0.45	tr_C9ZXD7_	WB
HLA-DQA10501-DQB10201	54	RWNMAAEVVTGFLES	WNMAAEVVT	1	0.5294	162.6	4.00	0.33	tr_C9ZXD7_	WB
HLA-DQA10501-DQB10201	55	HMLADMTDVDYYVDG	MLADMTDVD	1	0.5290	163.3	4.00	0.30	tr_C9ZXD7_	WB
HLA-DQA10501-DQB10201	56	SAEVESLPDIFSREK	EVESLPDIF	2	0.5283	164.6	4.00	0.81	tr_C9ZXD7_	WB
HLA-DQA10501-DQB10201	57	RGSFGAVYEATSDLT	VYEATSDLT	6	0.5129	194.5	5.00	0.54	tr_C9ZXD7_	WB
HLA-DQA10501-DQB10201	58	LARGMLAEWEWTSSE	ARGMLAEWE	1	0.5103	200.1	5.00	0.63	tr_C9ZXD7_	WB
HLA-DQA10501-DQB10201	59	VGCVAAELFDCPPLF	ELFDCPPLF	6	0.5075	206.1	5.00	0.34	tr_C9ZXD7_	WB
HLA-DQA10501-DQB10201	60	FMETPHMLADMTDVD	MLADMTDVD	6	0.5042	213.8	5.50	0.32	tr_C9ZXD7_	WB
HLA-DQA10501-DQB10201	61	DMTDVDYYVDGLTSN	DVDYYVDGL	3	0.4982	227.9	5.50	0.49	tr_C9ZXD7_	WB
HLA-DQA10501-DQB10201	62	GSFGAVYEATSDLTG	VYEATSDLT	5	0.4968	231.5	6.00	0.55	tr_C9ZXD7_	WB
HLA-DQA10501-DQB10201	63	TDVDYYVDGLTSNPV	DVDYYVDGL	1	0.4965	232.3	6.00	0.38	tr_C9ZXD7_	WB
HLA-DQA10501-DQB10201	64	GCVAAELFDCPPLFM	ELFDCPPLF	5	0.4952	235.6	6.00	0.42	tr_C9ZXD7_	WB
HLA-DQA10501-DQB10201	65	IVCDRGWLNSVQDLV	WLNSVQDLV	6	0.4934	240.1	6.00	0.69	tr_C9ZXD7_	WB
HLA-DQA10501-DQB10201	66	SVGCVAAELFDCPPL	VGCVAAELF	1	0.4911	246.3	6.00	0.49	tr_C9ZXD7_	WB

HLA-DQA10501-DQB10201	67	VCDRGWLNSVQDLVH	WLNSVQDLV	5	0.4903	248.3	6.00	0.70	tr_C9ZXD7_	WB
HLA-DQA10501-DQB10201	68	AEVESLPDIFSREKQ	EVESLPDIF	1	0.4900	249.2	6.00	0.84	tr_C9ZXD7_	WB
HLA-DQA10501-DQB10201	69	ITAALEDIDDATFRP	AALEDIDDA	2	0.4888	252.5	6.50	0.33	tr_C9ZXD7_	WB
HLA-DQA10501-DQB10201	70	CVAAELFDCPPLFME	ELFDCPPLF	4	0.4839	266.3	6.50	0.43	tr_C9ZXD7_	WB
HLA-DQA10501-DQB10201	71	VFRFPCQITAALEDI	QITAALEDI	6	0.4837	266.8	6.50	0.66	tr_C9ZXD7_	WB
HLA-DQA10501-DQB10201	72	MTDVDYYVDGLTSNP	DVDYYVDGL	2	0.4832	268.1	6.50	0.44	tr_C9ZXD7_	WB
HLA-DQA10501-DQB10201	73	CDRGWLNSVQDLVHT	WLNSVQDLV	4	0.4813	273.8	7.00	0.71	tr_C9ZXD7_	WB
HLA-DQA10501-DQB10201	74	DRGWLNSVQDLVHTL	WLNSVQDLV	3	0.4800	277.6	7.00	0.72	tr_C9ZXD7_	WB
HLA-DQA10501-DQB10201	75	CLQPEGRATAADLLL	GRATAADLL	5	0.4798	278.1	7.00	0.54	tr_C9ZXD7_	WB
HLA-DQA10501-DQB10201	76	DVDYYVDGLTSNPVL	VDGLTSNPV	5	0.4797	278.5	7.00	0.22	tr_C9ZXD7_	WB
HLA-DQA10501-DQB10201	77	YRTYIRDYVVSEVNL	IRDYVVSEV	4	0.4727	300.4	7.50	0.35	tr_C9ZXD7_	WB
HLA-DQA10501-DQB10201	78	GEYRTYIRDYVVSEV	IRDYVVSEV	6	0.4687	313.6	8.00	0.58	tr_C9ZXD7_	WB
HLA-DQA10501-DQB10201	79	EYRTYIRDYVVSEVN	IRDYVVSEV	5	0.4674	318.3	8.00	0.41	tr_C9ZXD7_	WB
HLA-DQA10501-DQB10201	80	SFGAVYEATSDLTGG	VYEATSDLT	4	0.4658	323.7	8.00	0.56	tr_C9ZXD7_	WB
HLA-DQA10501-DQB10201	81	LFMETPHMLADMTDV	PHMLADMTD	5	0.4606	342.6	8.50	0.28	tr_C9ZXD7_	WB
HLA-DQA10501-DQB10201	82	YVVSEVNLSLSEILE	VNLSLSEIL	5	0.4604	343.3	8.50	0.37	tr_C9ZXD7_	WB
HLA-DQA10501-DQB10201	83	RGWLNSVQDLVHTLS	WLNSVQDLV	2	0.4603	343.7	8.50	0.67	tr_C9ZXD7_	WB
HLA-DQA10501-DQB10201	84	SPCDIWSVGCVAAEL	SVGCVAAEL	6	0.4594	346.9	9.00	0.55	tr_C9ZXD7_	WB
HLA-DQA10501-DQB10201	85	VAAELFDCPPLFMET	ELFDCPPLF	3	0.4591	348.2	9.00	0.44	tr_C9ZXD7_	WB
HLA-DQA10501-DQB10201	86	PTGDIERDLAPLQVF	ERDLAPLQV	5	0.4556	361.4	9.00	0.45	tr_C9ZXD7_	WB
HLA-DQA10501-DQB10201	87	VVSEVNLSLSEILEG	VNLSLSEIL	4	0.4551	363.5	9.00	0.31	tr_C9ZXD7_	WB
HLA-DQA10501-DQB10201	88	WEWTSSEQLLGQMLR	WEWTSSEQL	0	0.4537	368.9	9.50	0.61	tr_C9ZXD7_	WB
HLA-DQA10501-DQB10201	89	SFLEYGYGKQVEELA	YGKQVEELA	6	0.4521	375.4	9.50	0.40	tr_C9ZXD7_	WB
HLA-DQA10501-DQB10201	90	ALKVLHTWILEERMY	HTWILEERM	5	0.4514	378.2	9.50	0.47	tr_C9ZXD7_	WB
HLA-DQA10501-DQB10201	91	KALKVLHTWILEERM	HTWILEERM	6	0.4493	387.0	9.50	0.45	tr_C9ZXD7_	WB
HLA-DQA10501-DQB10201	92	DYVVSEVNLSLSEIL	VNLSLSEIL	6	0.4493	387.2	9.50	0.44	tr_C9ZXD7_	WB
HLA-DQA10501-DQB10201	93	GGKMAVKMFYFTEDL	KMFYFTEDL	6	0.4487	389.4	10.00	0.75	tr_C9ZXD7_	
HLA-DQA10501-DQB10201	94	WNMAAEVVTGFLESG	WNMAAEVVT	0	0.4478	393.2	10.00	0.28	tr_C9ZXD7_	
HLA-DQA10501-DQB10201	95	LQPEGRATAADLLLH	GRATAADLL	4	0.4466	398.5	10.00	0.52	tr_C9ZXD7_	
HLA-DQA10501-DQB10201	96	LRYMAPEVYRGEDHC	LRYMAPEVY	0	0.4461	400.5	10.00	0.76	tr_C9ZXD7_	
HLA-DQA10501-DQB10201	97	RTYIRDYVVSEVNLS	IRDYVVSEV	3	0.4442	409.2	11.00	0.35	tr_C9ZXD7_	
HLA-DQA10501-DQB10201	98	ARGMLAEWEWTSSEQ	ARGMLAEWE	0	0.4436	411.5	11.00	0.56	tr_C9ZXD7_	
HLA-DQA10501-DQB10201	99	PLFMETPHMLADMTD	PHMLADMTD	6	0.4433	412.8	11.00	0.37	tr_C9ZXD7_	
HLA-DQA10501-DQB10201	100	TYIRDYVVSEVNLSL	IRDYVVSEV	2	0.4432	413.6	11.00	0.33	tr_C9ZXD7_	
HLA-DQA10501-DQB10201	101	LDHKTSVDSLFPGTL	VDSLFPGTL	6	0.4423	417.4	11.00	0.22	tr_C9ZXD7_	
HLA-DQA10501-DQB10201	102	LKVLHTWILEERMYT	HTWILEERM	4	0.4413	421.9	11.00	0.43	tr_C9ZXD7_	
HLA-DQA10501-DQB10201	103	TPTGDIERDLAPLQV	ERDLAPLQV	6	0.4412	422.4	11.00	0.50	tr_C9ZXD7_	
HLA-DQA10501-DQB10201	104	ERDLAPLQVFSGEVL	LQVFSGEVL	6	0.4350	451.9	12.00	0.56	tr_C9ZXD7_	
HLA-DQA10501-DQB10201	105	AAELFDCPPLFMETP	ELFDCPPLF	2	0.4349	452.3	12.00	0.44	tr_C9ZXD7_	
HLA-DQA10501-DQB10201	106	DLVHTLSYDIIVVPI	VHTLSYDII	2	0.4344	454.9	12.00	0.38	tr_C9ZXD7_	
HLA-DQA10501-DQB10201	107	GWLNSVQDLVHTLSY	WLNSVQDLV	1	0.4320	466.5	12.00	0.69	tr_C9ZXD7_	
HLA-DQA10501-DQB10201	108	TEDLEESINALLNEI	SINALLNEI	6	0.4310	472.0	12.00	0.42	tr_C9ZXD7_	
HLA-DQA10501-DQB10201	109	FCLQPEGRATAADLL	GRATAADLL	6	0.4295	479.5	12.00	0.80	tr_C9ZXD7_	
HLA-DQA10501-DQB10201	110	KLKLRAFATVPVTLF	AFATVPVTL	5	0.4293	480.4	12.00	0.40	tr_C9ZXD7_	
HLA-DQA10501-DQB10201	111	YFTEDLEESINALLN	FTEDLEESI	1	0.4290	482.3	12.00	0.31	tr_C9ZXD7_	
HLA-DQA10501-DQB10201	112	FLEYGYGKQVEELAR	YGKQVEELA	5	0.4274	490.6	12.00	0.41	tr_C9ZXD7_	
HLA-DQA10501-DQB10201	113	KVLHTWILEERMYTV	HTWILEERM	3	0.4269	493.0	12.00	0.44	tr_C9ZXD7_	
HLA-DQA10501-DQB10201	114	HVDAQVVMIGFDDKY	VMIGFDDKY	6	0.4258	499.1	13.00	0.47	tr_C9ZXD7_	
HLA-DQA10501-DQB10201	115	DHKTSVDSLFPGTLC	VDSLFPGTL	5	0.4245	506.3	13.00	0.26	tr_C9ZXD7_	
HLA-DQA10501-DQB10201	116	GRGSFGAVYEATSDL	SFGAVYEAT	3	0.4234	512.4	13.00	0.29	tr_C9ZXD7_	

HLA-DQA10501-DQB10201	117	YVDGLTSNPVLCDFL	SNPVLCDFL	6	0.4199	532.1	13.00	0.25	tr_C9ZXD7_
HLA-DQA10501-DQB10201	118	TGDIERDLAPLQVFS	ERDLAPLQV	4	0.4191	536.4	13.00	0.49	tr_C9ZXD7_
HLA-DQA10501-DQB10201	119	VSEVNLSLSEILEGK	VNLSLSEIL	3	0.4155	557.8	14.00	0.33	tr_C9ZXD7_
HLA-DQA10501-DQB10201	120	LKLKLRAFATVPVTL	AFATVPVTL	6	0.4146	563.2	14.00	0.50	tr_C9ZXD7_
HLA-DQA10501-DQB10201	121	QPEGRATAADLLLHR	GRATAADLL	3	0.4143	565.4	14.00	0.50	tr_C9ZXD7_
HLA-DQA10501-DQB10201	122	YIRDYVVSEVNLSLS	IRDYVVSEV	1	0.4132	571.7	14.00	0.32	tr_C9ZXD7_
HLA-DQA10501-DQB10201	123	FGAVYEATSDLTGGK	VYEATSDLT	3	0.4120	579.6	14.00	0.62	tr_C9ZXD7_
HLA-DQA10501-DQB10201	124	EVESLPDIFSREKQY	EVESLPDIF	0	0.4112	584.4	14.00	0.80	tr_C9ZXD7_
HLA-DQA10501-DQB10201	125	VLHTWILEERMYTVL	HTWILEERM	2	0.4105	588.6	14.00	0.45	tr_C9ZXD7_
HLA-DQA10501-DQB10201	126	LKLRAFATVPVTLFT	AFATVPVTL	4	0.4080	605.2	15.00	0.41	tr_C9ZXD7_
HLA-DQA10501-DQB10201	127	AELFDCPPLFMETPH	ELFDCPPLF	1	0.4077	606.9	15.00	0.46	tr_C9ZXD7_
HLA-DQA10501-DQB10201	128	QDLVHTLSYDIIVVP	VHTLSYDII	3	0.4067	613.8	15.00	0.50	tr_C9ZXD7_
HLA-DQA10501-DQB10201	129	DLAPLQVFSGEVLKL	LQVFSGEVL	4	0.4049	625.6	15.00	0.50	tr_C9ZXD7_
HLA-DQA10501-DQB10201	130	YYVDGLTSNPVLCDF	VDGLTSNPV	2	0.4045	628.4	15.00	0.31	tr_C9ZXD7_
HLA-DQA10501-DQB10201	131	PLDHKTSVDSLFPGT	KTSVDSLFP	4	0.4034	635.6	15.00	0.28	tr_C9ZXD7_
HLA-DQA10501-DQB10201	132	VDYYVDGLTSNPVLC	VDGLTSNPV	4	0.4033	636.8	15.00	0.33	tr_C9ZXD7_
HLA-DQA10501-DQB10201	133	PEGRATAADLLLHRL	GRATAADLL	2	0.4029	639.3	16.00	0.45	tr_C9ZXD7_
HLA-DQA10501-DQB10201	134	VDAQVVMIGFDDKYR	VMIGFDDKY	5	0.4022	644.1	16.00	0.49	tr_C9ZXD7_
HLA-DQA10501-DQB10201	135	EDLEESINALLNEIK	SINALLNEI	5	0.4018	647.2	16.00	0.37	tr_C9ZXD7_
HLA-DQA10501-DQB10201	136	HTLSYDIIVVPISEP	DIIVVPISE	5	0.4008	654.0	16.00	0.32	tr_C9ZXD7_
HLA-DQA10501-DQB10201	137	LVHTLSYDIIVVPIS	VHTLSYDII	1	0.4006	655.5	16.00	0.33	tr_C9ZXD7_
HLA-DQA10501-DQB10201	138	SEDVQGTLRYMAPEV	TLRYMAPEV	6	0.3997	662.0	16.00	0.63	tr_C9ZXD7_
HLA-DQA10501-DQB10201	139	LGRGSFGAVYEATSD	SFGAVYEAT	4	0.3997	662.1	16.00	0.32	tr_C9ZXD7_
HLA-DQA10501-DQB10201	140	TAALEDIDDATFRPA	AALEDIDDA	1	0.3985	670.6	16.00	0.37	tr_C9ZXD7_
HLA-DQA10501-DQB10201	141	LEYGYGKQVEELARG	YGKQVEELA	4	0.3979	674.9	16.00	0.41	tr_C9ZXD7_
HLA-DQA10501-DQB10201	142	SVQDLVHTLSYDIIV	VHTLSYDII	5	0.3974	678.7	16.00	0.56	tr_C9ZXD7_
HLA-DQA10501-DQB10201	143	VHTLSYDIIVVPISE	DIIVVPISE	6	0.3973	679.4	16.00	0.29	tr_C9ZXD7_
HLA-DQA10501-DQB10201	144	DAQVVMIGFDDKYRI	VMIGFDDKY	4	0.3968	683.0	16.00	0.51	tr_C9ZXD7_
HLA-DQA10501-DQB10201	145	NSVQDLVHTLSYDII	VHTLSYDII	6	0.3951	695.5	17.00	0.63	tr_C9ZXD7_
HLA-DQA10501-DQB10201	146	FSSSCSAEVESLPDI	SCSAEVESL	3	0.3951	695.6	17.00	0.23	tr_C9ZXD7_
HLA-DQA10501-DQB10201	147	DYYVDGLTSNPVLCD	VDGLTSNPV	3	0.3942	702.3	17.00	0.38	tr_C9ZXD7_
HLA-DQA10501-DQB10201	148	HKTSVDSLFPGTLCL	VDSLFPGTL	4	0.3933	709.0	17.00	0.31	tr_C9ZXD7_
HLA-DQA10501-DQB10201	149	SEVNLSLSEILEGKA	VNLSLSEIL	2	0.3925	715.3	17.00	0.34	tr_C9ZXD7_
HLA-DQA10501-DQB10201	150	VQDLVHTLSYDIIVV	VHTLSYDII	4	0.3901	734.2	17.00	0.56	tr_C9ZXD7_
HLA-DQA10501-DQB10201	151	KLRAFATVPVTLFTV	AFATVPVTL	3	0.3895	739.1	17.00	0.41	tr_C9ZXD7_
HLA-DQA10501-DQB10201	152	DLEESINALLNEIKI	SINALLNEI	4	0.3890	742.8	18.00	0.38	tr_C9ZXD7_
HLA-DQA10501-DQB10201	153	LFSSSCSAEVESLPD	SCSAEVESL	4	0.3890	742.9	18.00	0.27	tr_C9ZXD7_
HLA-DQA10501-DQB10201	154	RDYVVSEVNLSLSEI	EVNLSLSEI	6	0.3872	758.0	18.00	0.49	tr_C9ZXD7_
HLA-DQA10501-DQB10201	155	NTFALQIVATPVAQA	QIVATPVAQ	5	0.3866	762.4	18.00	0.25	tr_C9ZXD7_
HLA-DQA10501-DQB10201	156	TLSYDIIVVPISEPG	DIIVVPISE	4	0.3860	767.4	18.00	0.29	tr_C9ZXD7_
HLA-DQA10501-DQB10201	157	WLNSVQDLVHTLSYD	WLNSVQDLV	0	0.3847	778.8	18.00	0.55	tr_C9ZXD7_
HLA-DQA10501-DQB10201	158	LHTWILEERMYTVLL	HTWILEERM	1	0.3846	779.6	18.00	0.34	tr_C9ZXD7_
HLA-DQA10501-DQB10201	159	LSYDIIVVPISEPGV	DIIVVPISE	3	0.3842	783.1	18.00	0.30	tr_C9ZXD7_
HLA-DQA10501-DQB10201	160	ELFDCPPLFMETPHM	LFDCPPLFM	1	0.3834	789.7	18.00	0.37	tr_C9ZXD7_
HLA-DQA10501-DQB10201	161	SSRNTFALQIVATPV	ALQIVATPV	6	0.3831	792.2	18.00	0.38	tr_C9ZXD7_
HLA-DQA10501-DQB10201	162	EGRATAADLLLHRLF	GRATAADLL	1	0.3817	804.6	19.00	0.44	tr_C9ZXD7_
HLA-DQA10501-DQB10201	163	RDLAPLQVFSGEVLK	LQVFSGEVL	5	0.3816	805.5	19.00	0.60	tr_C9ZXD7_
HLA-DQA10501-DQB10201	164	GDIERDLAPLQVFSG	ERDLAPLQV	3	0.3815	806.2	19.00	0.52	tr_C9ZXD7_
HLA-DQA10501-DQB10201	165	RNTFALQIVATPVAQ	ALQIVATPV	4	0.3814	807.2	19.00	0.27	tr_C9ZXD7_
HLA-DQA10501-DQB10201	166	FTEDLEESINALLNE	ESINALLNE	6	0.3799	819.6	19.00	0.29	tr_C9ZXD7_

HLA-DQA10501-DQB10201	167	LGEYRTYIRDYVVSE	YIRDYVVSE	6	0.3788	830.3	19.00	0.39	tr_C9ZXD7_
HLA-DQA10501-DQB10201	168	EYGYGKQVEELARGM	YGKQVEELA	3	0.3783	834.5	19.00	0.44	tr_C9ZXD7_
HLA-DQA10501-DQB10201	169	TFALQIVATPVAQAA	QIVATPVAQ	4	0.3769	847.0	19.00	0.25	tr_C9ZXD7_
HLA-DQA10501-DQB10201	170	AQVVMIGFDDKYRIT	VMIGFDDKY	3	0.3766	850.1	19.00	0.54	tr_C9ZXD7_
HLA-DQA10501-DQB10201	171	EQLLGQMLRLSPLDH	MLRLSPLDH	6	0.3764	851.6	20.00	0.38	tr_C9ZXD7_
HLA-DQA10501-DQB10201	172	PPLFMETPHMLADMT	TPHMLADMT	6	0.3752	862.7	20.00	0.25	tr_C9ZXD7_
HLA-DQA10501-DQB10201	173	SRNTFALQIVATPVA	ALQIVATPV	5	0.3745	869.5	20.00	0.34	tr_C9ZXD7_
HLA-DQA10501-DQB10201	174	LLGRGSFGAVYEATS	SFGAVYEAT	5	0.3737	877.4	20.00	0.34	tr_C9ZXD7_
HLA-DQA10501-DQB10201	175	DDATFRPADALNERY	PADALNERY	6	0.3733	880.4	20.00	0.47	tr_C9ZXD7_
HLA-DQA10501-DQB10201	176	NMAAEVVTGFLESGC	AAEVVTGFL	2	0.3732	881.6	20.00	0.30	tr_C9ZXD7_
HLA-DQA10501-DQB10201	177	ISEPGVRLALEGFQY	RLALEGFQY	6	0.3729	884.2	20.00	0.31	tr_C9ZXD7_
HLA-DQA10501-DQB10201	178	FALQIVATPVAQAAP	QIVATPVAQ	3	0.3727	886.1	20.00	0.29	tr_C9ZXD7_
HLA-DQA10501-DQB10201	179	LEESINALLNEIKIM	SINALLNEI	3	0.3714	899.1	20.00	0.41	tr_C9ZXD7_
HLA-DQA10501-DQB10201	180	SPLDHKTSVDSLFPG	KTSVDSLFP	5	0.3712	900.5	20.00	0.30	tr_C9ZXD7_
HLA-DQA10501-DQB10201	181	TPRGLSVIGPLQSFL	GLSVIGPLQ	3	0.3704	908.8	21.00	0.23	tr_C9ZXD7_
HLA-DQA10501-DQB10201	182	LRAFATVPVTLFTVA	AFATVPVTL	2	0.3699	913.4	21.00	0.44	tr_C9ZXD7_
HLA-DQA10501-DQB10201	183	GYYTIPYGNALGYIE	PYGNALGYI	5	0.3674	939.0	21.00	0.41	tr_C9ZXD7_
HLA-DQA10501-DQB10201	184	LAPLQVFSGEVLKLK	LQVFSGEVL	3	0.3669	943.9	21.00	0.52	tr_C9ZXD7_
HLA-DQA10501-DQB10201	185	VTPRGLSVIGPLQSF	GLSVIGPLQ	4	0.3669	944.0	21.00	0.28	tr_C9ZXD7_
HLA-DQA10501-DQB10201	186	GKQVEELARGMLAEW	LARGMLAEW	6	0.3665	947.9	21.00	0.43	tr_C9ZXD7_
HLA-DQA10501-DQB10201	187	PRGLSVIGPLQSFLE	GLSVIGPLQ	2	0.3660	953.6	21.00	0.24	tr_C9ZXD7_
HLA-DQA10501-DQB10201	188	VDGLTSNPVLCDFLR	SNPVLCDFL	5	0.3654	959.1	21.00	0.26	tr_C9ZXD7_
HLA-DQA10501-DQB10201	189	SEPGVRLALEGFQYV	VRLALEGFQ	4	0.3651	962.0	22.00	0.29	tr_C9ZXD7_
HLA-DQA10501-DQB10201	190	EPGVRLALEGFQYVI	VRLALEGFQ	3	0.3650	963.9	22.00	0.29	tr_C9ZXD7_
HLA-DQA10501-DQB10201	191	DIERDLAPLQVFSGE	ERDLAPLQV	2	0.3634	980.2	22.00	0.55	tr_C9ZXD7_
HLA-DQA10501-DQB10201	192	ILLRTMFVSLTSVRE	FVSLTSVRE	6	0.3620	994.9	22.00	0.67	tr_C9ZXD7_
HLA-DQA10501-DQB10201	193	QSFLEYGYGKQVEEL	GYGKQVEEL	6	0.3611	1004.8	22.00	0.52	tr_C9ZXD7_
HLA-DQA10501-DQB10201	194	SYDIIVVPISEPGVR	DIIVVPISE	2	0.3610	1006.1	22.00	0.34	tr_C9ZXD7_
HLA-DQA10501-DQB10201	195	EVNLSLSEILEGKAV	VNLSLSEIL	1	0.3609	1006.9	22.00	0.34	tr_C9ZXD7_
HLA-DQA10501-DQB10201	196	TVNVLTLITNLVDQH	LITNLVDQH	6	0.3604	1012.6	22.00	0.37	tr_C9ZXD7_
HLA-DQA10501-DQB10201	197	IERDLAPLQVFSGEV	ERDLAPLQV	1	0.3601	1016.1	22.00	0.42	tr_C9ZXD7_
HLA-DQA10501-DQB10201	198	GAVYEATSDLTGGKM	VYEATSDLT	2	0.3595	1022.7	22.00	0.70	tr_C9ZXD7_
HLA-DQA10501-DQB10201	199	VLLGRGSFGAVYEAT	SFGAVYEAT	6	0.3594	1023.5	22.00	0.41	tr_C9ZXD7_
HLA-DQA10501-DQB10201	200	RLFSSSCSAEVESLP	SCSAEVESL	5	0.3593	1024.8	22.00	0.29	tr_C9ZXD7_
HLA-DQA10501-DQB10201	201	DATFRPADALNERYV	PADALNERY	5	0.3591	1027.3	23.00	0.50	tr_C9ZXD7_
HLA-DQA10501-DQB10201	202	PGVRLALEGFQYVIN	VRLALEGFQ	2	0.3562	1059.2	23.00	0.25	tr_C9ZXD7_
HLA-DQA10501-DQB10201	203	IRDYVVSEVNLSLSE	IRDYVVSEV	0	0.3562	1060.3	23.00	0.26	tr_C9ZXD7_
HLA-DQA10501-DQB10201	204	APLQVFSGEVLKLKL	LQVFSGEVL	2	0.3560	1062.1	23.00	0.52	tr_C9ZXD7_
HLA-DQA10501-DQB10201	205	MAAEVVTGFLESGCV	AAEVVTGFL	1	0.3559	1062.9	23.00	0.29	tr_C9ZXD7_
HLA-DQA10501-DQB10201	206	RGLSVIGPLQSFLEY	LSVIGPLQS	2	0.3551	1072.6	23.00	0.26	tr_C9ZXD7_
HLA-DQA10501-DQB10201	207	LQSSPSDIDVIVCDR	DIDVIVCDR	6	0.3549	1074.9	23.00	0.38	tr_C9ZXD7_
HLA-DQA10501-DQB10201	208	SSPSDIDVIVCDRGW	DIDVIVCDR	4	0.3544	1080.0	23.00	0.38	tr_C9ZXD7_
HLA-DQA10501-DQB10201	209	EESINALLNEIKIMC	SINALLNEI	2	0.3540	1085.2	23.00	0.42	tr_C9ZXD7_
HLA-DQA10501-DQB10201	210	YYTIPYGNALGYIEG	PYGNALGYI	4	0.3532	1095.0	24.00	0.41	tr_C9ZXD7_
HLA-DQA10501-DQB10201	211	CPPLFMETPHMLADM	FMETPHMLA	4	0.3523	1104.9	24.00	0.32	tr_C9ZXD7_
HLA-DQA10501-DQB10201	212	VITPTGDIERDLAPL	TGDIERDLA	4	0.3523	1105.9	24.00	0.40	tr_C9ZXD7_
HLA-DQA10501-DQB10201	213	VNVLTLITNLVDQHN	LITNLVDQH	5	0.3518	1110.9	24.00	0.37	tr_C9ZXD7_
HLA-DQA10501-DQB10201	214	DDKYRITRWNMAAEV	TRWNMAAEV	6	0.3508	1123.9	24.00	0.80	tr_C9ZXD7_
HLA-DQA10501-DQB10201	215	SPSDIDVIVCDRGWL	DIDVIVCDR	3	0.3497	1137.4	24.00	0.37	tr_C9ZXD7_
HLA-DQA10501-DQB10201	216	LLRTMFVSLTSVRER	FVSLTSVRE	5	0.3490	1145.6	24.00	0.62	tr_C9ZXD7_

HLA-DQA10501-DQB10201	217	ITPTGDIERDLAPLQ	TGDIERDLA	3	0.3486	1150.4	25.00	0.37	tr_C9ZXD7_
HLA-DQA10501-DQB10201	218	QVVMIGFDDKYRITR	VMIGFDDKY	2	0.3486	1150.7	25.00	0.58	tr_C9ZXD7_
HLA-DQA10501-DQB10201	219	RAFATVPVTLFTVAV	AFATVPVTL	1	0.3482	1155.5	25.00	0.41	tr_C9ZXD7_
HLA-DQA10501-DQB10201	220	YGYGKQVEELARGML	YGKQVEELA	2	0.3482	1155.8	25.00	0.47	tr_C9ZXD7_
HLA-DQA10501-DQB10201	221	TVPVTLFTVAVPILN	LFTVAVPIL	5	0.3473	1167.1	25.00	0.30	tr_C9ZXD7_
HLA-DQA10501-DQB10201	222	QSSPSDIDVIVCDRG	DIDVIVCDR	5	0.3453	1192.6	25.00	0.35	tr_C9ZXD7_
HLA-DQA10501-DQB10201	223	HTWILEERMYTVLLS	ERMYTVLLS	6	0.3452	1193.6	25.00	0.28	tr_C9ZXD7_
HLA-DQA10501-DQB10201	224	PVTLFTVAVPILNQE	FTVAVPILN	4	0.3440	1208.8	25.00	0.28	tr_C9ZXD7_
HLA-DQA10501-DQB10201	225	DGLTSNPVLCDFLRM	SNPVLCDFL	4	0.3440	1209.3	25.00	0.27	tr_C9ZXD7_
HLA-DQA10501-DQB10201	226	QLLGQMLRLSPLDHK	MLRLSPLDH	5	0.3439	1210.5	25.00	0.38	tr_C9ZXD7_
HLA-DQA10501-DQB10201	227	NVLTLITNLVDQHNI	LITNLVDQH	4	0.3437	1212.6	25.00	0.37	tr_C9ZXD7_
HLA-DQA10501-DQB10201	228	LVITPTGDIERDLAP	TGDIERDLA	5	0.3430	1222.2	26.00	0.38	tr_C9ZXD7_
HLA-DQA10501-DQB10201	229	KTSVDSLFPGTLCLH	VDSLFPGTL	3	0.3424	1230.7	26.00	0.36	tr_C9ZXD7_
HLA-DQA10501-DQB10201	230	DNLGEYRTYIRDYVV	YRTYIRDYV	5	0.3420	1235.4	26.00	0.47	tr_C9ZXD7_
HLA-DQA10501-DQB10201	231	LSPLDHKTSVDSLFP	KTSVDSLFP	6	0.3417	1239.1	26.00	0.31	tr_C9ZXD7_
HLA-DQA10501-DQB10201	232	LRTMFVSLTSVRERC	FVSLTSVRE	4	0.3417	1240.3	26.00	0.62	tr_C9ZXD7_
HLA-DQA10501-DQB10201	233	RLALEGFQYVINTPI	FQYVINTPI	6	0.3416	1241.0	26.00	0.36	tr_C9ZXD7_
HLA-DQA10501-DQB10201	234	ATFRPADALNERYVI	PADALNERY	4	0.3409	1251.1	26.00	0.53	tr_C9ZXD7_
HLA-DQA10501-DQB10201	235	HRLFSSSCSAEVESL	FSSSCSAEV	3	0.3407	1253.3	26.00	0.35	tr_C9ZXD7_
HLA-DQA10501-DQB10201	236	TMFVSLTSVRERCEL	FVSLTSVRE	2	0.3402	1259.6	26.00	0.62	tr_C9ZXD7_
HLA-DQA10501-DQB10201	237	GVRLALEGFQYVINT	RLALEGFQY	2	0.3402	1260.4	26.00	0.23	tr_C9ZXD7_
HLA-DQA10501-DQB10201	238	AQAAPSTAQISELRE	TAQISELRE	6	0.3397	1266.6	26.00	0.46	tr_C9ZXD7_
HLA-DQA10501-DQB10201	239	DLVITPTGDIERDLA	TGDIERDLA	6	0.3393	1272.3	26.00	0.33	tr_C9ZXD7_
HLA-DQA10501-DQB10201	240	VPVTLFTVAVPILNQ	FTVAVPILN	5	0.3388	1279.7	26.00	0.29	tr_C9ZXD7_
HLA-DQA10501-DQB10201	241	NSSRNTFALQIVATP	RNTFALQIV	3	0.3385	1283.7	27.00	0.33	tr_C9ZXD7_
HLA-DQA10501-DQB10201	242	AALEDIDDATFRPAD	AALEDIDDA	0	0.3381	1289.4	27.00	0.31	tr_C9ZXD7_
HLA-DQA10501-DQB10201	243	YDIIVVPISEPGVRL	DIIVVPISE	1	0.3380	1290.7	27.00	0.37	tr_C9ZXD7_
HLA-DQA10501-DQB10201	244	CSPCDIWSVGCVAAE	WSVGCVAAE	6	0.3379	1291.4	27.00	0.40	tr_C9ZXD7_
HLA-DQA10501-DQB10201	245	DCPPLFMETPHMLAD	FMETPHMLA	5	0.3370	1304.3	27.00	0.34	tr_C9ZXD7_
HLA-DQA10501-DQB10201	246	ALQIVATPVAQAAPS	QIVATPVAQ	2	0.3366	1309.6	27.00	0.28	tr_C9ZXD7_
HLA-DQA10501-DQB10201	247	AVTPRGLSVIGPLQS	GLSVIGPLQ	5	0.3364	1312.9	27.00	0.32	tr_C9ZXD7_
HLA-DQA10501-DQB10201	248	PSDIDVIVCDRGWLN	DIDVIVCDR	2	0.3361	1317.3	27.00	0.39	tr_C9ZXD7_
HLA-DQA10501-DQB10201	249	ESINALLNEIKIMCS	SINALLNEI	1	0.3359	1319.8	27.00	0.42	tr_C9ZXD7_
HLA-DQA10501-DQB10201	250	TFRPADALNERYVID	PADALNERY	3	0.3351	1330.9	27.00	0.54	tr_C9ZXD7_
HLA-DQA10501-DQB10201	251	AKALKVLHTWILEER	LHTWILEER	6	0.3341	1345.5	27.00	0.41	tr_C9ZXD7_
HLA-DQA10501-DQB10201	252	VSLRKNAATAQEERE	AATAQEERE	6	0.3339	1348.9	27.00	0.47	tr_C9ZXD7_
HLA-DQA10501-DQB10201	253	RTMFVSLTSVRERCE	FVSLTSVRE	3	0.3338	1350.6	28.00	0.65	tr_C9ZXD7_
HLA-DQA10501-DQB10201	254	DFGTARQGVGSEDVQ	RQGVGSEDV	5	0.3326	1368.2	28.00	0.57	tr_C9ZXD7_
HLA-DQA10501-DQB10201	255	GYIEGKHVDAQVVMI	HVDAQVVMI	6	0.3326	1368.4	28.00	0.28	tr_C9ZXD7_
HLA-DQA10501-DQB10201	256	SEQLLGQMLRLSPLD	QMLRLSPLD	6	0.3324	1371.3	28.00	0.33	tr_C9ZXD7_
HLA-DQA10501-DQB10201	257	GLSVIGPLQSFLEYG	LSVIGPLQS	1	0.3311	1390.6	28.00	0.27	tr_C9ZXD7_
HLA-DQA10501-DQB10201	258	NGYYTIPYGNALGYI	PYGNALGYI	6	0.3303	1402.7	28.00	0.45	tr_C9ZXD7_
HLA-DQA10501-DQB10201	259	ATVPVTLFTVAVPIL	LFTVAVPIL	6	0.3296	1413.6	28.00	0.40	tr_C9ZXD7_
HLA-DQA10501-DQB10201	260	VVFRFPCQITAALED	PCQITAALE	5	0.3295	1415.3	28.00	0.28	tr_C9ZXD7_
HLA-DQA10501-DQB10201	261	VLKLKLRAFATVPVT	RAFATVPVT	6	0.3283	1433.9	29.00	0.45	tr_C9ZXD7_
HLA-DQA10501-DQB10201	262	GYGKQVEELARGMLA	YGKQVEELA	1	0.3277	1442.7	29.00	0.35	tr_C9ZXD7_
HLA-DQA10501-DQB10201	263	PISEPGVRLALEGFQ	GVRLALEGF	5	0.3269	1454.4	29.00	0.35	tr_C9ZXD7_
HLA-DQA10501-DQB10201	264	LLGQMLRLSPLDHKT	MLRLSPLDH	4	0.3264	1463.4	29.00	0.38	tr_C9ZXD7_
HLA-DQA10501-DQB10201	265	QILNSSRNTFALQIV	RNTFALQIV	6	0.3261	1467.4	29.00	0.49	tr_C9ZXD7_
HLA-DQA10501-DQB10201	266	YTIPYGNALGYIEGK	PYGNALGYI	3	0.3253	1481.1	29.00	0.40	tr_C9ZXD7_

HLA-DQA10501-DQB10201	267	ALEGFQYVINTPISS	FQYVINTPI	4	0.3248	1489.3	29.00	0.29	tr_C9ZXD7_
HLA-DQA10501-DQB10201	268	STTGPSAGAAVPQVT	SAGAAVPQV	5	0.3247	1489.6	29.00	0.54	tr_C9ZXD7_
HLA-DQA10501-DQB10201	269	LFDCPPLFMETPHML	PLFMETPHM	5	0.3245	1493.5	29.00	0.27	tr_C9ZXD7_
HLA-DQA10501-DQB10201	270	VRLALEGFQYVINTP	RLALEGFQY	1	0.3243	1496.4	30.00	0.21	tr_C9ZXD7_
HLA-DQA10501-DQB10201	271	PLQVFSGEVLKLKLR	LQVFSGEVL	1	0.3241	1500.6	30.00	0.56	tr_C9ZXD7_
HLA-DQA10501-DQB10201	272	AVYEATSDLTGGKMA	VYEATSDLT	1	0.3234	1510.6	30.00	0.75	tr_C9ZXD7_
HLA-DQA10501-DQB10201	273	LQIVATPVAQAAPST	QIVATPVAQ	1	0.3234	1511.4	30.00	0.29	tr_C9ZXD7_
HLA-DQA10501-DQB10201	274	RPADALNERYVIDQT	NERYVIDQT	6	0.3230	1518.5	30.00	0.40	tr_C9ZXD7_
HLA-DQA10501-DQB10201	275	KHVDAQVVMIGFDDK	VVMIGFDDK	6	0.3229	1518.8	30.00	0.28	tr_C9ZXD7_
HLA-DQA10501-DQB10201	276	VTLFTVAVPILNQEG	LFTVAVPIL	2	0.3226	1525.1	30.00	0.26	tr_C9ZXD7_
HLA-DQA10501-DQB10201	277	NLGEYRTYIRDYVVS	YRTYIRDYV	4	0.3223	1529.6	30.00	0.47	tr_C9ZXD7_
HLA-DQA10501-DQB10201	278	AFATVPVTLFTVAVP	AFATVPVTL	0	0.3218	1537.4	30.00	0.29	tr_C9ZXD7_
HLA-DQA10501-DQB10201	279	FDCPPLFMETPHMLA	FMETPHMLA	6	0.3208	1554.3	30.00	0.32	tr_C9ZXD7_
HLA-DQA10501-DQB10201	280	LALEGFQYVINTPIS	FQYVINTPI	5	0.3204	1561.9	30.00	0.35	tr_C9ZXD7_
HLA-DQA10501-DQB10201	281	GKHVDAQVVMIGFDD	QVVMIGFDD	6	0.3202	1564.1	30.00	0.29	tr_C9ZXD7_
HLA-DQA10501-DQB10201	282	VLTLITNLVDQHNIS	LITNLVDQH	3	0.3197	1572.4	31.00	0.43	tr_C9ZXD7_
HLA-DQA10501-DQB10201	283	MFVSLTSVRERCELE	FVSLTSVRE	1	0.3196	1575.4	31.00	0.62	tr_C9ZXD7_
HLA-DQA10501-DQB10201	284	GRATAADLLLHRLFS	RATAADLLL	1	0.3188	1587.8	31.00	0.43	tr_C9ZXD7_
HLA-DQA10501-DQB10201	285	KDNLGEYRTYIRDYV	YRTYIRDYV	6	0.3184	1595.3	31.00	0.62	tr_C9ZXD7_
HLA-DQA10501-DQB10201	286	EVRKVLMAIGTAVSL	MAIGTAVSL	6	0.3178	1604.9	31.00	0.41	tr_C9ZXD7_
HLA-DQA10501-DQB10201	287	LGYIEGKHVDAQVVM	KHVDAQVVM	6	0.3178	1605.4	31.00	0.34	tr_C9ZXD7_
HLA-DQA10501-DQB10201	288	FGTARQGVGSEDVQG	RQGVGSEDV	4	0.3171	1618.6	31.00	0.52	tr_C9ZXD7_
HLA-DQA10501-DQB10201	289	LFTVAVPILNQEGSL	FTVAVPILN	1	0.3155	1646.4	32.00	0.26	tr_C9ZXD7_
HLA-DQA10501-DQB10201	290	LNSSRNTFALQIVAT	RNTFALQIV	4	0.3153	1649.9	32.00	0.37	tr_C9ZXD7_
HLA-DQA10501-DQB10201	291	GVLLGRGSFGAVYEA	RGSFGAVYE	5	0.3146	1662.3	32.00	0.50	tr_C9ZXD7_
HLA-DQA10501-DQB10201	292	TDFGTARQGVGSEDV	RQGVGSEDV	6	0.3142	1670.2	32.00	0.65	tr_C9ZXD7_
HLA-DQA10501-DQB10201	293	FRPADALNERYVIDQ	PADALNERY	2	0.3139	1675.1	32.00	0.57	tr_C9ZXD7_
HLA-DQA10501-DQB10201	294	SDIDVIVCDRGWLNS	DIDVIVCDR	1	0.3136	1680.4	32.00	0.43	tr_C9ZXD7_
HLA-DQA10501-DQB10201	295	ENSVNLFMELCCCSL	FMELCCCSL	6	0.3131	1688.8	32.00	0.47	tr_C9ZXD7_
HLA-DQA10501-DQB10201	296	REDDGTVVFRFPCQI	VVFRFPCQI	6	0.3130	1691.9	32.00	0.43	tr_C9ZXD7_
HLA-DQA10501-DQB10201	297	CTVNVLTLITNLVDQ	VLTLITNLV	4	0.3128	1694.0	32.00	0.29	tr_C9ZXD7_
HLA-DQA10501-DQB10201	298	VVMIGFDDKYRITRW	VMIGFDDKY	1	0.3121	1708.6	32.00	0.59	tr_C9ZXD7_
HLA-DQA10501-DQB10201	299	ALNERYVIDQTRAIV	RYVIDQTRA	4	0.3088	1768.9	33.00	0.27	tr_C9ZXD7_
HLA-DQA10501-DQB10201	300	SLRKNAATAQEERER	AATAQEERE	5	0.3083	1778.6	33.00	0.50	tr_C9ZXD7_
HLA-DQA10501-DQB10201	301	GTARQGVGSEDVQGT	RQGVGSEDV	3	0.3071	1801.9	33.00	0.50	tr_C9ZXD7_
HLA-DQA10501-DQB10201	302	FATVPVTLFTVAVPI	TLFTVAVPI	6	0.3070	1805.0	33.00	0.38	tr_C9ZXD7_
HLA-DQA10501-DQB10201	303	LGQMLRLSPLDHKTS	MLRLSPLDH	3	0.3068	1807.6	33.00	0.40	tr_C9ZXD7_
HLA-DQA10501-DQB10201	304	TVVFRFPCQITAALE	PCQITAALE	6	0.3066	1812.2	34.00	0.29	tr_C9ZXD7_
HLA-DQA10501-DQB10201	305	LEGFQYVINTPISST	FQYVINTPI	3	0.3065	1814.0	34.00	0.31	tr_C9ZXD7_
HLA-DQA10501-DQB10201	306	KGVLLGRGSFGAVYE	RGSFGAVYE	6	0.3065	1815.4	34.00	0.63	tr_C9ZXD7_
HLA-DQA10501-DQB10201	307	NCTVNVLTLITNLVD	VLTLITNLV	5	0.3064	1816.1	34.00	0.37	tr_C9ZXD7_
HLA-DQA10501-DQB10201	308	LSVIGPLQSFLEYGY	LQSFLEYGY	6	0.3058	1827.4	34.00	0.19	tr_C9ZXD7_
HLA-DQA10501-DQB10201	309	VIVCDRGWLNSVQDL	RGWLNSVQD	5	0.3055	1833.3	34.00	0.41	tr_C9ZXD7_
HLA-DQA10501-DQB10201	310	RLQSSPSDIDVIVCD	SDIDVIVCD	6	0.3052	1840.9	34.00	0.26	tr_C9ZXD7_
HLA-DQA10501-DQB10201	311	YIEGKHVDAQVVMIG	HVDAQVVMI	5	0.3050	1844.7	34.00	0.25	tr_C9ZXD7_
HLA-DQA10501-DQB10201	312	AAEVVTGFLESGCVG	AAEVVTGFL	0	0.3043	1857.4	34.00	0.29	tr_C9ZXD7_
HLA-DQA10501-DQB10201	313	ILNSSRNTFALQIVA	RNTFALQIV	5	0.3035	1874.1	34.00	0.45	tr_C9ZXD7_
HLA-DQA10501-DQB10201	314	GLTSNPVLCDFLRMC	SNPVLCDFL	3	0.3034	1876.9	34.00	0.35	tr_C9ZXD7_
HLA-DQA10501-DQB10201	315	DALNERYVIDQTRAI	RYVIDQTRA	5	0.3032	1879.4	34.00	0.28	tr_C9ZXD7_
HLA-DQA10501-DQB10201	316	KAVTPRGLSVIGPLQ	RGLSVIGPL	5	0.3030	1885.2	34.00	0.39	tr_C9ZXD7_

HLA-DQA10501-DQB10201	317	LRKNAATAQEERERI	AATAQEERE	4	0.3028	1888.1	34.00	0.51	tr_C9ZXD7_
HLA-DQA10501-DQB10201	318	APSTAQISELREKLL	TAQISELRE	3	0.3026	1893.1	34.00	0.37	tr_C9ZXD7_
HLA-DQA10501-DQB10201	319	TARQGVGSEDVQGTL	RQGVGSEDV	2	0.3021	1903.8	35.00	0.49	tr_C9ZXD7_
HLA-DQA10501-DQB10201	320	TLFTVAVPILNQEGS	FTVAVPILN	2	0.3020	1904.3	35.00	0.34	tr_C9ZXD7_
HLA-DQA10501-DQB10201	321	TTGPSAGAAVPQVTR	SAGAAVPQV	4	0.3014	1917.3	35.00	0.51	tr_C9ZXD7_
HLA-DQA10501-DQB10201	322	EDDGTVVFRFPCQIT	VVFRFPCQI	5	0.3012	1921.6	35.00	0.43	tr_C9ZXD7_
HLA-DQA10501-DQB10201	323	PADALNERYVIDQTR	NERYVIDQT	5	0.3011	1922.7	35.00	0.34	tr_C9ZXD7_
HLA-DQA10501-DQB10201	324	LLHRLFSSSCSAEVE	FSSSCSAEV	5	0.3010	1926.1	35.00	0.50	tr_C9ZXD7_
HLA-DQA10501-DQB10201	325	AAPSTAQISELREKL	TAQISELRE	4	0.3006	1933.2	35.00	0.42	tr_C9ZXD7_
HLA-DQA10501-DQB10201	326	QAAPSTAQISELREK	TAQISELRE	5	0.3005	1935.6	35.00	0.44	tr_C9ZXD7_
HLA-DQA10501-DQB10201	327	EGKHVDAQVVMIGFD	HVDAQVVMI	3	0.3000	1945.7	35.00	0.26	tr_C9ZXD7_
HLA-DQA10501-DQB10201	328	SDLVITPTGDIERDL	VITPTGDIE	3	0.2998	1951.7	35.00	0.41	tr_C9ZXD7_
HLA-DQA10501-DQB10201	329	LNERYVIDQTRAIVN	RYVIDQTRA	3	0.2996	1954.2	35.00	0.26	tr_C9ZXD7_
HLA-DQA10501-DQB10201	330	VRKVLMAIGTAVSLR	MAIGTAVSL	5	0.2993	1961.5	35.00	0.41	tr_C9ZXD7_
HLA-DQA10501-DQB10201	331	IEGKHVDAQVVMIGF	HVDAQVVMI	4	0.2989	1970.6	35.00	0.26	tr_C9ZXD7_
HLA-DQA10501-DQB10201	332	QIVATPVAQAAPSTA	IVATPVAQA	1	0.2988	1972.3	35.00	0.23	tr_C9ZXD7_
HLA-DQA10501-DQB10201	333	SINALLNEIKIMCSL	SINALLNEI	0	0.2985	1978.1	35.00	0.31	tr_C9ZXD7_
HLA-DQA10501-DQB10201	334	ADALNERYVIDQTRA	RYVIDQTRA	6	0.2984	1981.6	36.00	0.28	tr_C9ZXD7_
HLA-DQA10501-DQB10201	335	TIPYGNALGYIEGKH	PYGNALGYI	2	0.2975	1999.7	36.00	0.39	tr_C9ZXD7_
HLA-DQA10501-DQB10201	336	TSVDSLFPGTLCLHP	VDSLFPGTL	2	0.2974	2001.9	36.00	0.41	tr_C9ZXD7_
HLA-DQA10501-DQB10201	337	FTVAVPILNQEGSLI	ILNQEGSLI	6	0.2971	2008.6	36.00	0.25	tr_C9ZXD7_
HLA-DQA10501-DQB10201	338	IPYGNALGYIEGKHV	PYGNALGYI	1	0.2953	2047.5	36.00	0.31	tr_C9ZXD7_
HLA-DQA10501-DQB10201	339	VNLSLSEILEGKAVT	VNLSLSEIL	0	0.2948	2059.1	36.00	0.32	tr_C9ZXD7_
HLA-DQA10501-DQB10201	340	CERKENSVNLFMELC	SVNLFMELC	6	0.2947	2060.9	36.00	0.41	tr_C9ZXD7_
HLA-DQA10501-DQB10201	341	DIIVVPISEPGVRLA	DIIVVPISE	0	0.2944	2067.9	37.00	0.34	tr_C9ZXD7_
HLA-DQA10501-DQB10201	342	VPISEPGVRLALEGF	GVRLALEGF	6	0.2928	2104.4	37.00	0.52	tr_C9ZXD7_
HLA-DQA10501-DQB10201	343	TWILEERMYTVLLSS	ERMYTVLLS	5	0.2928	2105.4	37.00	0.24	tr_C9ZXD7_
HLA-DQA10501-DQB10201	344	WILEERMYTVLLSSA	MYTVLLSSA	6	0.2927	2107.6	37.00	0.28	tr_C9ZXD7_
HLA-DQA10501-DQB10201	345	RKNAATAQEERERIL	AATAQEERE	3	0.2924	2112.7	37.00	0.58	tr_C9ZXD7_
HLA-DQA10501-DQB10201	346	EGFQYVINTPISSTE	FQYVINTPI	2	0.2919	2125.8	37.00	0.31	tr_C9ZXD7_
HLA-DQA10501-DQB10201	347	LHRLFSSSCSAEVES	FSSSCSAEV	4	0.2915	2134.2	37.00	0.45	tr_C9ZXD7_
HLA-DQA10501-DQB10201	348	TVIKVLRQLLTALTY	RQLLTALTY	6	0.2915	2134.9	37.00	0.55	tr_C9ZXD7_
HLA-DQA10501-DQB10201	349	DIDDATFRPADALNE	FRPADALNE	6	0.2908	2149.4	37.00	0.36	tr_C9ZXD7_
HLA-DQA10501-DQB10201	350	LTLITNLVDQHNISM	LITNLVDQH	2	0.2898	2174.2	38.00	0.47	tr_C9ZXD7_
HLA-DQA10501-DQB10201	351	YGKQVEELARGMLAE	EELARGMLA	5	0.2895	2180.5	38.00	0.36	tr_C9ZXD7_
HLA-DQA10501-DQB10201	352	PSTAQISELREKLLP	TAQISELRE	2	0.2876	2226.9	38.00	0.36	tr_C9ZXD7_
HLA-DQA10501-DQB10201	353	ERKENSVNLFMELCC	SVNLFMELC	5	0.2872	2235.5	38.00	0.35	tr_C9ZXD7_
HLA-DQA10501-DQB10201	354	VLCDFLRMCFCLQPE	RMCFCLQPE	6	0.2869	2243.8	38.00	0.57	tr_C9ZXD7_
HLA-DQA10501-DQB10201	355	LTSNPVLCDFLRMCF	SNPVLCDFL	2	0.2850	2290.3	39.00	0.34	tr_C9ZXD7_
HLA-DQA10501-DQB10201	356	LSDLVITPTGDIERD	VITPTGDIE	4	0.2844	2304.0	39.00	0.42	tr_C9ZXD7_
HLA-DQA10501-DQB10201	357	ARQGVGSEDVQGTLR	RQGVGSEDV	1	0.2841	2311.7	39.00	0.43	tr_C9ZXD7_
HLA-DQA10501-DQB10201	358	GSIREDDGTVVFRFP	DDGTVVFRF	5	0.2838	2319.3	39.00	0.34	tr_C9ZXD7_
HLA-DQA10501-DQB10201	359	NERYVIDQTRAIVNC	RYVIDQTRA	2	0.2832	2334.0	39.00	0.27	tr_C9ZXD7_
HLA-DQA10501-DQB10201	360	EWTSSEQLLGQMLRL	SEQLLGQML	4	0.2825	2351.4	39.00	0.25	tr_C9ZXD7_
HLA-DQA10501-DQB10201	361	VIKVLRQLLTALTYL	RQLLTALTY	5	0.2825	2351.6	39.00	0.46	tr_C9ZXD7_
HLA-DQA10501-DQB10201	362	RKENSVNLFMELCCC	SVNLFMELC	4	0.2812	2386.2	40.00	0.31	tr_C9ZXD7_
HLA-DQA10501-DQB10201	363	GFQYVINTPISSTEV	YVINTPISS	3	0.2809	2393.2	40.00	0.28	tr_C9ZXD7_
HLA-DQA10501-DQB10201	364	TGPSAGAAVPQVTRG	SAGAAVPQV	3	0.2806	2402.5	40.00	0.47	tr_C9ZXD7_
HLA-DQA10501-DQB10201	365	PVAQAAPSTAQISEL	PSTAQISEL	6	0.2803	2409.2	40.00	0.54	tr_C9ZXD7_
HLA-DQA10501-DQB10201	366	GQMLRLSPLDHKTSV	MLRLSPLDH	2	0.2801	2413.3	40.00	0.44	tr_C9ZXD7_

HLA-DQA10501-DQB10201	367	VNCTVNVLTLITNLV	VLTLITNLV	6	0.2800	2417.8	40.00	0.47	tr_C9ZXD7_
HLA-DQA10501-DQB10201	368	ALGYIEGKHVDAQVV	GKHVDAQVV	6	0.2789	2445.8	40.00	0.38	tr_C9ZXD7_
HLA-DQA10501-DQB10201	369	DDGTVVFRFPCQITA	VVFRFPCQI	4	0.2787	2450.6	40.00	0.46	tr_C9ZXD7_
HLA-DQA10501-DQB10201	370	SAKALKVLHTWILEE	VLHTWILEE	6	0.2782	2464.3	41.00	0.61	tr_C9ZXD7_
HLA-DQA10501-DQB10201	371	GKAVTPRGLSVIGPL	RGLSVIGPL	6	0.2779	2471.2	41.00	0.54	tr_C9ZXD7_
HLA-DQA10501-DQB10201	372	EVLKLKLRAFATVPV	LRAFATVPV	6	0.2775	2483.2	41.00	0.69	tr_C9ZXD7_
HLA-DQA10501-DQB10201	373	RKVLMAIGTAVSLRK	MAIGTAVSL	4	0.2772	2490.5	41.00	0.35	tr_C9ZXD7_
HLA-DQA10501-DQB10201	374	IDDATFRPADALNER	FRPADALNE	5	0.2772	2491.3	41.00	0.34	tr_C9ZXD7_
HLA-DQA10501-DQB10201	375	GTVVFRFPCQITAAL	VVFRFPCQI	2	0.2770	2496.2	41.00	0.38	tr_C9ZXD7_
HLA-DQA10501-DQB10201	376	ERERILTLRQDSPWT	ILTLRQDSP	4	0.2766	2506.7	41.00	0.29	tr_C9ZXD7_
HLA-DQA10501-DQB10201	377	RYVIDQTRAIVNCTV	TRAIVNCTV	6	0.2763	2515.4	41.00	0.38	tr_C9ZXD7_
HLA-DQA10501-DQB10201	378	EERERILTLRQDSPW	ILTLRQDSP	5	0.2761	2520.8	41.00	0.32	tr_C9ZXD7_
HLA-DQA10501-DQB10201	379	EERMYTVLLSSASSE	VLLSSASSE	6	0.2747	2560.4	42.00	0.41	tr_C9ZXD7_
HLA-DQA10501-DQB10201	380	CLHPCIPEAVATLLN	IPEAVATLL	5	0.2739	2582.0	42.00	0.32	tr_C9ZXD7_
HLA-DQA10501-DQB10201	381	DIDVIVCDRGWLNSV	DIDVIVCDR	0	0.2735	2592.4	42.00	0.37	tr_C9ZXD7_
HLA-DQA10501-DQB10201	382	LQVFSGEVLKLKLRA	LQVFSGEVL	0	0.2735	2593.4	42.00	0.52	tr_C9ZXD7_
HLA-DQA10501-DQB10201	383	HPCIPEAVATLLNSL	AVATLLNSL	6	0.2731	2603.8	42.00	0.34	tr_C9ZXD7_
HLA-DQA10501-DQB10201	384	MYTVLLSSASSEGDL	VLLSSASSE	3	0.2726	2617.1	42.00	0.41	tr_C9ZXD7_
HLA-DQA10501-DQB10201	385	FVSLTSVRERCELEK	FVSLTSVRE	0	0.2726	2619.5	42.00	0.55	tr_C9ZXD7_
HLA-DQA10501-DQB10201	386	KNAATAQEERERILT	AATAQEERE	2	0.2722	2629.9	42.00	0.65	tr_C9ZXD7_
HLA-DQA10501-DQB10201	387	DGTVVFRFPCQITAA	VVFRFPCQI	3	0.2705	2679.5	43.00	0.46	tr_C9ZXD7_
HLA-DQA10501-DQB10201	388	KVLRQLLTALTYLHA	RQLLTALTY	3	0.2704	2681.3	43.00	0.33	tr_C9ZXD7_
HLA-DQA10501-DQB10201	389	HCSPCDIWSVGCVAA	DIWSVGCVA	5	0.2697	2701.0	43.00	0.53	tr_C9ZXD7_
HLA-DQA10501-DQB10201	390	SIREDDGTVVFRFPC	DDGTVVFRF	4	0.2697	2701.4	43.00	0.33	tr_C9ZXD7_
HLA-DQA10501-DQB10201	391	LNSVQDLVHTLSYDI	VQDLVHTLS	3	0.2695	2706.5	43.00	0.28	tr_C9ZXD7_
HLA-DQA10501-DQB10201	392	KPLSDLVITPTGDIE	VITPTGDIE	6	0.2695	2707.9	43.00	0.37	tr_C9ZXD7_
HLA-DQA10501-DQB10201	393	LSTTGPSAGAAVPQV	SAGAAVPQV	6	0.2693	2714.0	43.00	0.79	tr_C9ZXD7_
HLA-DQA10501-DQB10201	394	PLSDLVITPTGDIER	VITPTGDIE	5	0.2688	2729.4	43.00	0.39	tr_C9ZXD7_
HLA-DQA10501-DQB10201	395	NSVNLFMELCCCSLG	FMELCCCSL	5	0.2685	2737.4	43.00	0.46	tr_C9ZXD7_
HLA-DQA10501-DQB10201	396	RMYTVLLSSASSEGD	VLLSSASSE	4	0.2675	2765.6	43.00	0.44	tr_C9ZXD7_
HLA-DQA10501-DQB10201	397	LCLHPCIPEAVATLL	IPEAVATLL	6	0.2672	2775.7	43.00	0.45	tr_C9ZXD7_
HLA-DQA10501-DQB10201	398	LCDFLRMCFCLQPEG	RMCFCLQPE	5	0.2671	2778.3	44.00	0.52	tr_C9ZXD7_
HLA-DQA10501-DQB10201	399	ITNLVDQHNISMILL	QHNISMILL	6	0.2664	2799.4	44.00	0.60	tr_C9ZXD7_
HLA-DQA10501-DQB10201	400	YTVLLSSASSEGDLS	VLLSSASSE	2	0.2654	2832.0	44.00	0.41	tr_C9ZXD7_
HLA-DQA10501-DQB10201	401	IREDDGTVVFRFPCQ	DDGTVVFRF	3	0.2640	2872.9	44.00	0.32	tr_C9ZXD7_
HLA-DQA10501-DQB10201	402	KVLMAIGTAVSLRKN	MAIGTAVSL	3	0.2639	2876.0	44.00	0.37	tr_C9ZXD7_
HLA-DQA10501-DQB10201	403	SSEQLLGQMLRLSPL	GQMLRLSPL	6	0.2638	2879.9	44.00	0.28	tr_C9ZXD7_
HLA-DQA10501-DQB10201	404	CIPEAVATLLNSLRV	AVATLLNSL	4	0.2633	2894.0	45.00	0.22	tr_C9ZXD7_
HLA-DQA10501-DQB10201	405	KRLQSSPSDIDVIVC	PSDIDVIVC	6	0.2631	2902.2	45.00	0.26	tr_C9ZXD7_
HLA-DQA10501-DQB10201	406	PYGNALGYIEGKHVD	YGNALGYIE	1	0.2630	2906.1	45.00	0.28	tr_C9ZXD7_
HLA-DQA10501-DQB10201	407	GPSAGAAVPQVTRGH	SAGAAVPQV	2	0.2618	2944.4	45.00	0.47	tr_C9ZXD7_
HLA-DQA10501-DQB10201	408	KENSVNLFMELCCCS	SVNLFMELC	3	0.2617	2944.6	45.00	0.32	tr_C9ZXD7_
HLA-DQA10501-DQB10201	409	ERYVIDQTRAIVNCT	RYVIDQTRA	1	0.2612	2961.0	45.00	0.29	tr_C9ZXD7_
HLA-DQA10501-DQB10201	410	ERMYTVLLSSASSEG	VLLSSASSE	5	0.2611	2964.5	45.00	0.41	tr_C9ZXD7_
HLA-DQA10501-DQB10201	411	VAQAAPSTAQISELR	PSTAQISEL	5	0.2611	2965.5	45.00	0.52	tr_C9ZXD7_
HLA-DQA10501-DQB10201	412	SVIGPLQSFLEYGYG	LQSFLEYGY	5	0.2610	2968.9	45.00	0.22	tr_C9ZXD7_
HLA-DQA10501-DQB10201	413	SVDSLFPGTLCLHPC	VDSLFPGTL	1	0.2606	2981.6	45.00	0.41	tr_C9ZXD7_
HLA-DQA10501-DQB10201	414	ILEERMYTVLLSSAS	MYTVLLSSA	5	0.2604	2988.3	45.00	0.31	tr_C9ZXD7_
HLA-DQA10501-DQB10201	415	LQILNSSRNTFALQI	SRNTFALQI	6	0.2601	2998.2	45.00	0.73	tr_C9ZXD7_
HLA-DQA10501-DQB10201	416	SMILLRTMFVSLTSV	TMFVSLTSV	6	0.2599	3003.3	45.00	0.35	tr_C9ZXD7_

HLA-DQA10501-DQB10201	417	TVAVPILNQEGSLIG	ILNQEGSLI	5	0.2595	3015.5	46.00	0.34	tr_C9ZXD7_
HLA-DQA10501-DQB10201	418	IPEAVATLLNSLRVP	AVATLLNSL	3	0.2595	3016.7	46.00	0.28	tr_C9ZXD7_
HLA-DQA10501-DQB10201	419	LLLHRLFSSSCSAEV	FSSSCSAEV	6	0.2593	3023.5	46.00	0.62	tr_C9ZXD7_
HLA-DQA10501-DQB10201	420	IKVLRQLLTALTYLH	RQLLTALTY	4	0.2586	3047.5	46.00	0.44	tr_C9ZXD7_
HLA-DQA10501-DQB10201	421	LHPCIPEAVATLLNS	IPEAVATLL	4	0.2582	3060.6	46.00	0.31	tr_C9ZXD7_
HLA-DQA10501-DQB10201	422	SVNLFMELCCCSLGD	FMELCCCSL	4	0.2573	3090.2	46.00	0.52	tr_C9ZXD7_
HLA-DQA10501-DQB10201	423	AVSLRKNAATAQEER	KNAATAQEE	5	0.2568	3105.6	46.00	0.63	tr_C9ZXD7_
HLA-DQA10501-DQB10201	424	LEDIDDATFRPADAL	ATFRPADAL	6	0.2565	3116.8	46.00	0.41	tr_C9ZXD7_
HLA-DQA10501-DQB10201	425	CGSIREDDGTVVFRF	DDGTVVFRF	6	0.2556	3146.0	47.00	0.35	tr_C9ZXD7_
HLA-DQA10501-DQB10201	426	HCERKENSVNLFMEL	NSVNLFMEL	6	0.2551	3163.1	47.00	0.32	tr_C9ZXD7_
HLA-DQA10501-DQB10201	427	NAATAQEERERILTL	AATAQEERE	1	0.2551	3164.6	47.00	0.64	tr_C9ZXD7_
HLA-DQA10501-DQB10201	428	PCIPEAVATLLNSLR	AVATLLNSL	5	0.2550	3167.8	47.00	0.28	tr_C9ZXD7_
HLA-DQA10501-DQB10201	429	VLRQLLTALTYLHAR	RQLLTALTY	2	0.2548	3173.9	47.00	0.37	tr_C9ZXD7_
HLA-DQA10501-DQB10201	430	IVATPVAQAAPSTAQ	VAQAAPSTA	5	0.2540	3201.2	47.00	0.31	tr_C9ZXD7_
HLA-DQA10501-DQB10201	431	CDFLRMCFCLQPEGR	RMCFCLQPE	4	0.2526	3250.1	47.00	0.48	tr_C9ZXD7_
HLA-DQA10501-DQB10201	432	FQYVINTPISSTEVR	YVINTPISS	2	0.2522	3264.4	48.00	0.37	tr_C9ZXD7_
HLA-DQA10501-DQB10201	433	RERILTLRQDSPWTK	ILTLRQDSP	3	0.2519	3276.7	48.00	0.29	tr_C9ZXD7_
HLA-DQA10501-DQB10201	434	RQGVGSEDVQGTLRY	RQGVGSEDV	0	0.2517	3283.0	48.00	0.28	tr_C9ZXD7_
HLA-DQA10501-DQB10201	435	TLITNLVDQHNISMI	LITNLVDQH	1	0.2514	3293.9	48.00	0.50	tr_C9ZXD7_
HLA-DQA10501-DQB10201	436	NALGYIEGKHVDAQV	YIEGKHVDA	4	0.2501	3340.3	48.00	0.26	tr_C9ZXD7_
HLA-DQA10501-DQB10201	437	VLMAIGTAVSLRKNA	MAIGTAVSL	2	0.2501	3341.8	48.00	0.41	tr_C9ZXD7_
HLA-DQA10501-DQB10201	438	TAVSLRKNAATAQEE	KNAATAQEE	6	0.2491	3377.5	48.00	0.79	tr_C9ZXD7_
HLA-DQA10501-DQB10201	439	PSAGAAVPQVTRGHN	SAGAAVPQV	1	0.2481	3412.5	49.00	0.46	tr_C9ZXD7_
HLA-DQA10501-DQB10201	440	LQSFLEYGYGKQVEE	EYGYGKQVE	5	0.2481	3413.9	49.00	0.34	tr_C9ZXD7_
HLA-DQA10501-DQB10201	441	PEAVATLLNSLRVPC	AVATLLNSL	2	0.2471	3450.3	49.00	0.29	tr_C9ZXD7_
HLA-DQA10501-DQB10201	442	DVIVCDRGWLNSVQD	RGWLNSVQD	6	0.2467	3464.6	49.00	0.55	tr_C9ZXD7_
HLA-DQA10501-DQB10201	443	IVNCTVNVLTLITNL	NVLTLITNL	6	0.2466	3467.5	49.00	0.28	tr_C9ZXD7_
HLA-DQA10501-DQB10201	444	LEERMYTVLLSSASS	MYTVLLSSA	4	0.2465	3471.6	49.00	0.28	tr_C9ZXD7_
HLA-DQA10501-DQB10201	445	GCVGKPLSDLVITPT	LSDLVITPT	6	0.2459	3494.8	49.00	0.50	tr_C9ZXD7_
HLA-DQA10501-DQB10201	446	EDIDDATFRPADALN	ATFRPADAL	5	0.2455	3509.8	49.00	0.46	tr_C9ZXD7_
HLA-DQA10501-DQB10201	447	RLSPLDHKTSVDSLF	HKTSVDSLF	6	0.2443	3558.1	50.00	0.49	tr_C9ZXD7_
HLA-DQA10501-DQB10201	448	TVLLSSASSEGDLST	VLLSSASSE	1	0.2439	3573.1	50.00	0.37	tr_C9ZXD7_
HLA-DQA10501-DQB10201	449	AEVVTGFLESGCVGK	VVTGFLESG	2	0.2430	3607.6	50.00	0.26	tr_C9ZXD7_
HLA-DQA10501-DQB10201	450	STAQISELREKLLPH	TAQISELRE	1	0.2424	3631.0	50.00	0.38	tr_C9ZXD7_
HLA-DQA10501-DQB10201	451	EKRLQSSPSDIDVIV	SPSDIDVIV	6	0.2423	3632.7	50.00	0.32	tr_C9ZXD7_
HLA-DQA10501-DQB10201	452	ALEDIDDATFRPADA	LEDIDDATF	1	0.2423	3634.2	50.00	0.28	tr_C9ZXD7_
HLA-DQA10501-DQB10201	453	YGNALGYIEGKHVDA	YIEGKHVDA	6	0.2423	3634.4	50.00	0.26	tr_C9ZXD7_
HLA-DQA10501-DQB10201	454	TSNPVLCDFLRMCFC	SNPVLCDFL	1	0.2420	3644.8	50.00	0.31	tr_C9ZXD7_
HLA-DQA10501-DQB10201	455	QEERERILTLRQDSP	ILTLRQDSP	6	0.2417	3658.6	50.00	0.29	tr_C9ZXD7_
HLA-DQA10501-DQB10201	456	DQTRAIVNCTVNVLT	IVNCTVNVL	5	0.2416	3660.5	50.00	0.34	tr_C9ZXD7_
HLA-DQA10501-DQB10201	457	MILLRTMFVSLTSVR	TMFVSLTSV	5	0.2412	3678.4	55.00	0.29	tr_C9ZXD7_
HLA-DQA10501-DQB10201	458	YVIDQTRAIVNCTVN	TRAIVNCTV	5	0.2410	3684.6	55.00	0.39	tr_C9ZXD7_
HLA-DQA10501-DQB10201	459	DFLRMCFCLQPEGRA	RMCFCLQPE	3	0.2406	3703.6	55.00	0.47	tr_C9ZXD7_
HLA-DQA10501-DQB10201	460	RAIVNCTVNVLTLIT	TVNVLTLIT	6	0.2401	3721.4	55.00	0.29	tr_C9ZXD7_
HLA-DQA10501-DQB10201	461	QMLRLSPLDHKTSVD	MLRLSPLDH	1	0.2399	3730.2	55.00	0.50	tr_C9ZXD7_
HLA-DQA10501-DQB10201	462	TEVRKVLMAIGTAVS	LMAIGTAVS	6	0.2394	3750.3	55.00	0.36	tr_C9ZXD7_
HLA-DQA10501-DQB10201	463	INALLNEIKIMCSLN	INALLNEIK	0	0.2392	3758.5	55.00	0.17	tr_C9ZXD7_
HLA-DQA10501-DQB10201	464	DHCSPCDIWSVGCVA	DIWSVGCVA	6	0.2391	3761.5	55.00	0.71	tr_C9ZXD7_
HLA-DQA10501-DQB10201	465	LITNLVDQHNISMIL	LITNLVDQH	0	0.2373	3834.7	55.00	0.31	tr_C9ZXD7_
HLA-DQA10501-DQB10201	466	FPGTLCLHPCIPEAV	LHPCIPEAV	6	0.2372	3840.4	55.00	0.50	tr_C9ZXD7_

HLA-DQA10501-DQB10201	467	TGGKMAVKMFYFTED	VKMFYFTED	6	0.2362	3880.9	55.00	0.53	tr_C9ZXD7_
HLA-DQA10501-DQB10201	468	REKQYVSSLTITTQP	VSSLTITTQ	5	0.2361	3885.4	55.00	0.24	tr_C9ZXD7_
HLA-DQA10501-DQB10201	469	VNLFMELCCCSLGDI	FMELCCCSL	3	0.2358	3900.2	55.00	0.57	tr_C9ZXD7_
HLA-DQA10501-DQB10201	470	QNILIKGDVVKITDF	NILIKGDVV	1	0.2357	3902.6	55.00	0.25	tr_C9ZXD7_
HLA-DQA10501-DQB10201	471	NLSLSEILEGKAVTP	LSLSEILEG	1	0.2355	3911.2	55.00	0.31	tr_C9ZXD7_
HLA-DQA10501-DQB10201	472	DIFSREKQYVSSLTI	KQYVSSLTI	6	0.2354	3915.6	55.00	0.58	tr_C9ZXD7_
HLA-DQA10501-DQB10201	473	EAVATLLNSLRVPCH	AVATLLNSL	1	0.2351	3929.7	55.00	0.28	tr_C9ZXD7_
HLA-DQA10501-DQB10201	474	VYEATSDLTGGKMAV	VYEATSDLT	0	0.2349	3935.1	55.00	0.82	tr_C9ZXD7_
HLA-DQA10501-DQB10201	475	VPILNQEGSLIGRIL	EGSLIGRIL	6	0.2349	3937.1	55.00	0.40	tr_C9ZXD7_
HLA-DQA10501-DQB10201	476	QTRAIVNCTVNVLTL	IVNCTVNVL	4	0.2345	3954.2	55.00	0.34	tr_C9ZXD7_
HLA-DQA10501-DQB10201	477	TRAIVNCTVNVLTLI	IVNCTVNVL	3	0.2345	3956.1	55.00	0.31	tr_C9ZXD7_
HLA-DQA10501-DQB10201	478	ISMILLRTMFVSLTS	LRTMFVSLT	5	0.2341	3971.0	55.00	0.33	tr_C9ZXD7_
HLA-DQA10501-DQB10201	479	VMIGFDDKYRITRWN	VMIGFDDKY	0	0.2340	3977.7	55.00	0.61	tr_C9ZXD7_
HLA-DQA10501-DQB10201	480	AIVNCTVNVLTLITN	TVNVLTLIT	5	0.2323	4048.8	55.00	0.24	tr_C9ZXD7_
HLA-DQA10501-DQB10201	481	VATPVAQAAPSTAQI	VAQAAPSTA	4	0.2321	4058.5	55.00	0.47	tr_C9ZXD7_
HLA-DQA10501-DQB10201	482	KNGYYTIPYGNALGY	IPYGNALGY	6	0.2314	4089.5	55.00	0.38	tr_C9ZXD7_
HLA-DQA10501-DQB10201	483	SREKQYVSSLTITTQ	KQYVSSLTI	3	0.2313	4091.6	55.00	0.28	tr_C9ZXD7_
HLA-DQA10501-DQB10201	484	ERILTLRQDSPWTKG	ILTLRQDSP	2	0.2312	4097.9	55.00	0.31	tr_C9ZXD7_
HLA-DQA10501-DQB10201	485	NLFMELCCCSLGDII	FMELCCCSL	2	0.2310	4106.6	55.00	0.53	tr_C9ZXD7_
HLA-DQA10501-DQB10201	486	LRQLLTALTYLHARG	RQLLTALTY	1	0.2306	4125.3	55.00	0.37	tr_C9ZXD7_
HLA-DQA10501-DQB10201	487	FSREKQYVSSLTITT	KQYVSSLTI	4	0.2300	4149.7	55.00	0.36	tr_C9ZXD7_
HLA-DQA10501-DQB10201	488	IDQTRAIVNCTVNVL	IVNCTVNVL	6	0.2300	4153.1	55.00	0.35	tr_C9ZXD7_
HLA-DQA10501-DQB10201	489	GKPLSDLVITPTGDI	LSDLVITPT	3	0.2298	4160.7	55.00	0.38	tr_C9ZXD7_
HLA-DQA10501-DQB10201	490	AVATLLNSLRVPCHL	NSLRVPCHL	6	0.2290	4195.6	55.00	0.21	tr_C9ZXD7_
HLA-DQA10501-DQB10201	491	NILIKGDVVKITDFG	NILIKGDVV	0	0.2281	4236.4	55.00	0.22	tr_C9ZXD7_
HLA-DQA10501-DQB10201	492	TSSEQLLGQMLRLSP	QLLGQMLRL	4	0.2281	4239.3	55.00	0.36	tr_C9ZXD7_
HLA-DQA10501-DQB10201	493	RATAADLLLHRLFSS	RATAADLLL	0	0.2274	4268.1	55.00	0.49	tr_C9ZXD7_
HLA-DQA10501-DQB10201	494	VGKPLSDLVITPTGD	LSDLVITPT	4	0.2268	4297.5	55.00	0.39	tr_C9ZXD7_
HLA-DQA10501-DQB10201	495	VAVPILNQEGSLIGR	ILNQEGSLI	4	0.2265	4311.0	55.00	0.37	tr_C9ZXD7_
HLA-DQA10501-DQB10201	496	PLQSFLEYGYGKQVE	EYGYGKQVE	6	0.2260	4337.5	55.00	0.40	tr_C9ZXD7_
HLA-DQA10501-DQB10201	497	FHCERKENSVNLFME	ENSVNLFME	6	0.2255	4359.5	55.00	0.34	tr_C9ZXD7_
HLA-DQA10501-DQB10201	498	VIGPLQSFLEYGYGK	PLQSFLEYG	3	0.2240	4428.3	60.00	0.23	tr_C9ZXD7_
HLA-DQA10501-DQB10201	499	NISMILLRTMFVSLT	LRTMFVSLT	6	0.2239	4433.8	60.00	0.44	tr_C9ZXD7_
HLA-DQA10501-DQB10201	500	IFSREKQYVSSLTIT	KQYVSSLTI	5	0.2226	4499.1	60.00	0.49	tr_C9ZXD7_
HLA-DQA10501-DQB10201	501	IVVPISEPGVRLALE	EPGVRLALE	6	0.2217	4540.3	60.00	0.44	tr_C9ZXD7_
HLA-DQA10501-DQB10201	502	NLVDQHNISMILLRT	QHNISMILL	4	0.2214	4554.9	60.00	0.44	tr_C9ZXD7_
HLA-DQA10501-DQB10201	503	LMAIGTAVSLRKNAA	MAIGTAVSL	1	0.2196	4643.6	60.00	0.45	tr_C9ZXD7_
HLA-DQA10501-DQB10201	504	TNLVDQHNISMILLR	QHNISMILL	5	0.2193	4660.3	60.00	0.53	tr_C9ZXD7_
HLA-DQA10501-DQB10201	505	VLLSSASSEGDLSTT	VLLSSASSE	0	0.2188	4684.4	60.00	0.28	tr_C9ZXD7_
HLA-DQA10501-DQB10201	506	EKQYVSSLTITTQPH	VSSLTITTQ	4	0.2184	4704.4	60.00	0.22	tr_C9ZXD7_
HLA-DQA10501-DQB10201	507	TAQISELREKLLPHL	TAQISELRE	0	0.2178	4739.1	60.00	0.28	tr_C9ZXD7_
HLA-DQA10501-DQB10201	508	LFMELCCCSLGDIIY	FMELCCCSL	1	0.2173	4764.8	60.00	0.52	tr_C9ZXD7_
HLA-DQA10501-DQB10201	509	ATPVAQAAPSTAQIS	VAQAAPSTA	3	0.2172	4766.6	60.00	0.52	tr_C9ZXD7_
HLA-DQA10501-DQB10201	510	WTSSEQLLGQMLRLS	SEQLLGQML	3	0.2172	4768.3	60.00	0.30	tr_C9ZXD7_
HLA-DQA10501-DQB10201	511	STCGSIREDDGTVVF	IREDDGTVV	5	0.2170	4779.0	60.00	0.40	tr_C9ZXD7_
HLA-DQA10501-DQB10201	512	CVGKPLSDLVITPTG	LSDLVITPT	5	0.2169	4786.2	60.00	0.44	tr_C9ZXD7_
HLA-DQA10501-DQB10201	513	SAGAAVPQVTRGHNT	AGAAVPQVT	1	0.2163	4817.1	60.00	0.50	tr_C9ZXD7_
HLA-DQA10501-DQB10201	514	GSEDVQGTLRYMAPE	QGTLRYMAP	5	0.2152	4873.0	60.00	0.22	tr_C9ZXD7_
HLA-DQA10501-DQB10201	515	PILNQEGSLIGRILI	EGSLIGRIL	5	0.2148	4892.0	60.00	0.40	tr_C9ZXD7_
HLA-DQA10501-DQB10201	516	PGTLCLHPCIPEAVA	LHPCIPEAV	5	0.2147	4898.9	60.00	0.57	tr_C9ZXD7_

HLA-DQA10501-DQB10201	517	IIVVPISEPGVRLAL	IIVVPISEP	0	0.2146	4903.1	60.00	0.25	tr_C9ZXD7_
HLA-DQA10501-DQB10201	518	LVDQHNISMILLRTM	QHNISMILL	3	0.2146	4903.4	60.00	0.39	tr_C9ZXD7_
HLA-DQA10501-DQB10201	519	FLRMCFCLQPEGRAT	RMCFCLQPE	2	0.2141	4931.0	60.00	0.51	tr_C9ZXD7_
HLA-DQA10501-DQB10201	520	ALLNEIKIMCSLNHP	EIKIMCSLN	4	0.2140	4936.6	60.00	0.23	tr_C9ZXD7_
HLA-DQA10501-DQB10201	521	STEVRKVLMAIGTAV	VLMAIGTAV	6	0.2136	4959.0	60.00	0.38	tr_C9ZXD7_
HLA-DQA10501-DQB10201	522	PQNILIKGDVVKITD	NILIKGDVV	2	0.2134	4969.8	60.00	0.35	tr_C9ZXD7_
HLA-DQA10501-DQB10201	523	YMAPEVYRGEDHCSP	PEVYRGEDH	3	0.2132	4978.8	60.00	0.38	tr_C9ZXD7_
HLA-DQA10501-DQB10201	524	QGVGSEDVQGTLRYM	SEDVQGTLR	4	0.2130	4992.6	60.00	0.25	tr_C9ZXD7_
HLA-DQA10501-DQB10201	525	SLNHPNIVHYFHCER	IVHYFHCER	6	0.2128	4999.8	60.00	0.43	tr_C9ZXD7_
HLA-DQA10501-DQB10201	526	AKDNLGEYRTYIRDY	EYRTYIRDY	6	0.2123	5026.9	60.00	0.47	tr_C9ZXD7_
HLA-DQA10501-DQB10201	527	VVPISEPGVRLALEG	EPGVRLALE	5	0.2122	5032.3	60.00	0.43	tr_C9ZXD7_
HLA-DQA10501-DQB10201	528	VDQHNISMILLRTMF	QHNISMILL	2	0.2110	5096.7	60.00	0.35	tr_C9ZXD7_
HLA-DQA10501-DQB10201	529	VIDQTRAIVNCTVNV	TRAIVNCTV	4	0.2109	5103.8	60.00	0.46	tr_C9ZXD7_
HLA-DQA10501-DQB10201	530	NALLNEIKIMCSLNH	LNEIKIMCS	3	0.2102	5143.6	60.00	0.26	tr_C9ZXD7_
HLA-DQA10501-DQB10201	531	AVPILNQEGSLIGRI	ILNQEGSLI	3	0.2102	5145.0	60.00	0.39	tr_C9ZXD7_
HLA-DQA10501-DQB10201	532	VGSEDVQGTLRYMAP	QGTLRYMAP	6	0.2099	5161.1	60.00	0.19	tr_C9ZXD7_
HLA-DQA10501-DQB10201	533	GNALGYIEGKHVDAQ	YIEGKHVDA	5	0.2097	5170.7	60.00	0.29	tr_C9ZXD7_
HLA-DQA10501-DQB10201	534	TLCLHPCIPEAVATL	LHPCIPEAV	3	0.2093	5191.9	60.00	0.51	tr_C9ZXD7_
HLA-DQA10501-DQB10201	535	AATAQEERERILTLR	AATAQEERE	0	0.2064	5361.6	65.00	0.50	tr_C9ZXD7_
HLA-DQA10501-DQB10201	536	LEKRLQSSPSDIDVI	RLQSSPSDI	3	0.2062	5372.8	65.00	0.34	tr_C9ZXD7_
HLA-DQA10501-DQB10201	537	TPVAQAAPSTAQISE	VAQAAPSTA	2	0.2050	5444.0	65.00	0.50	tr_C9ZXD7_
HLA-DQA10501-DQB10201	538	AQEERERILTLRQDS	ERILTLRQD	5	0.2045	5469.1	65.00	0.38	tr_C9ZXD7_
HLA-DQA10501-DQB10201	539	GTLCLHPCIPEAVAT	LHPCIPEAV	4	0.2040	5501.8	65.00	0.58	tr_C9ZXD7_
HLA-DQA10501-DQB10201	540	TCGSIREDDGTVVFR	IREDDGTVV	4	0.2038	5511.4	65.00	0.35	tr_C9ZXD7_
HLA-DQA10501-DQB10201	541	RQLLTALTYLHARGV	RQLLTALTY	0	0.2032	5546.1	65.00	0.26	tr_C9ZXD7_
HLA-DQA10501-DQB10201	542	DQHNISMILLRTMFV	QHNISMILL	1	0.2026	5585.3	65.00	0.29	tr_C9ZXD7_
HLA-DQA10501-DQB10201	543	LTVIKVLRQLLTALT	LRQLLTALT	6	0.2022	5607.2	65.00	0.40	tr_C9ZXD7_
HLA-DQA10501-DQB10201	544	RYMAPEVYRGEDHCS	PEVYRGEDH	4	0.2020	5618.2	65.00	0.40	tr_C9ZXD7_
HLA-DQA10501-DQB10201	545	VKPQNILIKGDVVKI	NILIKGDVV	4	0.2020	5621.1	65.00	0.53	tr_C9ZXD7_
HLA-DQA10501-DQB10201	546	KPQNILIKGDVVKIT	NILIKGDVV	3	0.2018	5630.6	65.00	0.45	tr_C9ZXD7_
HLA-DQA10501-DQB10201	547	EVVTGFLESGCVGKP	VVTGFLESG	1	0.2012	5667.1	65.00	0.29	tr_C9ZXD7_
HLA-DQA10501-DQB10201	548	CFCLQPEGRATAADL	EGRATAADL	6	0.2010	5681.6	65.00	0.71	tr_C9ZXD7_
HLA-DQA10501-DQB10201	549	ILIKGDVVKITDFGT	LIKGDVVKI	1	0.2007	5699.0	65.00	0.20	tr_C9ZXD7_
HLA-DQA10501-DQB10201	550	TAQEERERILTLRQD	ERILTLRQD	6	0.2004	5721.8	65.00	0.44	tr_C9ZXD7_
HLA-DQA10501-DQB10201	551	QYVINTPISSTEVRK	YVINTPISS	1	0.1996	5771.3	65.00	0.47	tr_C9ZXD7_
HLA-DQA10501-DQB10201	552	PVLCDFLRMCFCLQP	FLRMCFCLQ	5	0.1987	5824.5	65.00	0.35	tr_C9ZXD7_
HLA-DQA10501-DQB10201	553	MAPEVYRGEDHCSPC	PEVYRGEDH	2	0.1983	5851.4	65.00	0.36	tr_C9ZXD7_
HLA-DQA10501-DQB10201	554	VDSLFPGTLCLHPCI	VDSLFPGTL	0	0.1970	5935.5	65.00	0.28	tr_C9ZXD7_
HLA-DQA10501-DQB10201	555	LNHPNIVHYFHCERK	IVHYFHCER	5	0.1965	5967.6	65.00	0.35	tr_C9ZXD7_
HLA-DQA10501-DQB10201	556	IGPLQSFLEYGYGKQ	PLQSFLEYG	2	0.1954	6037.0	65.00	0.25	tr_C9ZXD7_
HLA-DQA10501-DQB10201	557	SNPVLCDFLRMCFCL	SNPVLCDFL	0	0.1950	6065.7	65.00	0.28	tr_C9ZXD7_
HLA-DQA10501-DQB10201	558	NQEGSLIGRILICAN	EGSLIGRIL	2	0.1950	6065.9	65.00	0.31	tr_C9ZXD7_
HLA-DQA10501-DQB10201	559	KQYVSSLTITTQPHS	VSSLTITTQ	3	0.1946	6089.3	65.00	0.25	tr_C9ZXD7_
HLA-DQA10501-DQB10201	560	GVGSEDVQGTLRYMA	SEDVQGTLR	3	0.1940	6132.0	65.00	0.23	tr_C9ZXD7_
HLA-DQA10501-DQB10201	561	LNQEGSLIGRILICA	EGSLIGRIL	3	0.1932	6184.7	65.00	0.40	tr_C9ZXD7_
HLA-DQA10501-DQB10201	562	RILTLRQDSPWTKGV	ILTLRQDSP	1	0.1930	6196.7	65.00	0.34	tr_C9ZXD7_
HLA-DQA10501-DQB10201	563	LSLSEILEGKAVTPR	ILEGKAVTP	5	0.1927	6216.2	65.00	0.22	tr_C9ZXD7_
HLA-DQA10501-DQB10201	564	NHPNIVHYFHCERKE	IVHYFHCER	4	0.1926	6223.6	65.00	0.35	tr_C9ZXD7_
HLA-DQA10501-DQB10201	565	APEVYRGEDHCSPCD	PEVYRGEDH	1	0.1923	6243.2	65.00	0.31	tr_C9ZXD7_
HLA-DQA10501-DQB10201	566	NPVLCDFLRMCFCLQ	FLRMCFCLQ	6	0.1898	6412.3	65.00	0.37	tr_C9ZXD7_

HLA-DQA10501-DQB10201	567	FMELCCCSLGDIIYG	FMELCCCSL	0	0.1888	6481.2	70.00	0.46	tr_C9ZXD7_
HLA-DQA10501-DQB10201	568	PEVYRGEDHCSPCDI	EVYRGEDHC	1	0.1887	6487.5	70.00	0.23	tr_C9ZXD7_
HLA-DQA10501-DQB10201	569	RDVKPQNILIKGDVV	NILIKGDVV	6	0.1886	6495.1	70.00	0.71	tr_C9ZXD7_
HLA-DQA10501-DQB10201	570	QLLTALTYLHARGVA	LTALTYLHA	2	0.1885	6503.0	70.00	0.31	tr_C9ZXD7_
HLA-DQA10501-DQB10201	571	ILNQEGSLIGRILIC	EGSLIGRIL	4	0.1880	6541.1	70.00	0.45	tr_C9ZXD7_
HLA-DQA10501-DQB10201	572	LLNEIKIMCSLNHPN	EIKIMCSLN	3	0.1877	6562.2	70.00	0.26	tr_C9ZXD7_
HLA-DQA10501-DQB10201	573	YFHCERKENSVNLFM	RKENSVNLF	5	0.1872	6598.9	70.00	0.44	tr_C9ZXD7_
HLA-DQA10501-DQB10201	574	DSLFPGTLCLHPCIP	FPGTLCLHP	3	0.1866	6640.0	70.00	0.22	tr_C9ZXD7_
HLA-DQA10501-DQB10201	575	LFPGTLCLHPCIPEA	FPGTLCLHP	1	0.1859	6692.2	70.00	0.20	tr_C9ZXD7_
HLA-DQA10501-DQB10201	576	SSTEVRKVLMAIGTA	KVLMAIGTA	6	0.1843	6805.4	70.00	0.28	tr_C9ZXD7_
HLA-DQA10501-DQB10201	577	VATLLNSLRVPCHLR	NSLRVPCHL	5	0.1834	6872.2	70.00	0.28	tr_C9ZXD7_
HLA-DQA10501-DQB10201	578	NTKNGYYTIPYGNAL	YTIPYGNAL	6	0.1833	6878.8	70.00	0.28	tr_C9ZXD7_
HLA-DQA10501-DQB10201	579	GPLQSFLEYGYGKQV	LQSFLEYGY	2	0.1832	6886.1	70.00	0.20	tr_C9ZXD7_
HLA-DQA10501-DQB10201	580	HNISMILLRTMFVSL	LLRTMFVSL	6	0.1819	6984.3	70.00	0.30	tr_C9ZXD7_
HLA-DQA10501-DQB10201	581	PPDLTVIKVLRQLLT	VIKVLRQLL	5	0.1818	6996.6	70.00	0.25	tr_C9ZXD7_
HLA-DQA10501-DQB10201	582	ALTYLHARGVAHRDV	ARGVAHRDV	6	0.1816	7006.3	70.00	0.38	tr_C9ZXD7_
HLA-DQA10501-DQB10201	583	TKNGYYTIPYGNALG	YTIPYGNAL	5	0.1812	7038.9	70.00	0.27	tr_C9ZXD7_
HLA-DQA10501-DQB10201	584	IDVIVCDRGWLNSVQ	VIVCDRGWL	2	0.1802	7113.5	70.00	0.25	tr_C9ZXD7_
HLA-DQA10501-DQB10201	585	PDLTVIKVLRQLLTA	VIKVLRQLL	4	0.1801	7125.1	70.00	0.22	tr_C9ZXD7_
HLA-DQA10501-DQB10201	586	DVKPQNILIKGDVVK	NILIKGDVV	5	0.1800	7128.1	70.00	0.67	tr_C9ZXD7_
HLA-DQA10501-DQB10201	587	ELEKRLQSSPSDIDV	RLQSSPSDI	4	0.1800	7133.8	70.00	0.47	tr_C9ZXD7_
HLA-DQA10501-DQB10201	588	EVYRGEDHCSPCDIW	EDHCSPCDI	5	0.1797	7154.3	70.00	0.18	tr_C9ZXD7_
HLA-DQA10501-DQB10201	589	TKGVLLGRGSFGAVY	GRGSFGAVY	6	0.1793	7185.8	70.00	0.62	tr_C9ZXD7_
HLA-DQA10501-DQB10201	590	DLTVIKVLRQLLTAL	VLRQLLTAL	6	0.1793	7187.2	70.00	0.22	tr_C9ZXD7_
HLA-DQA10501-DQB10201	591	SGCVGKPLSDLVITP	VGKPLSDLV	3	0.1792	7190.9	70.00	0.32	tr_C9ZXD7_
HLA-DQA10501-DQB10201	592	ISSTEVRKVLMAIGT	RKVLMAIGT	6	0.1791	7204.7	70.00	0.31	tr_C9ZXD7_
HLA-DQA10501-DQB10201	593	LIKGDVVKITDFGTA	DVVKITDFG	4	0.1789	7216.4	70.00	0.19	tr_C9ZXD7_
HLA-DQA10501-DQB10201	594	RSTCGSIREDDGTVV	IREDDGTVV	6	0.1784	7254.5	70.00	0.54	tr_C9ZXD7_
HLA-DQA10501-DQB10201	595	SLFPGTLCLHPCIPE	FPGTLCLHP	2	0.1781	7277.5	70.00	0.23	tr_C9ZXD7_
HLA-DQA10501-DQB10201	596	LIGRILICANAKDNL	RILICANAK	3	0.1781	7282.7	70.00	0.26	tr_C9ZXD7_
HLA-DQA10501-DQB10201	597	HPNIVHYFHCERKEN	IVHYFHCER	3	0.1776	7315.4	70.00	0.38	tr_C9ZXD7_
HLA-DQA10501-DQB10201	598	QEGSLIGRILICANA	EGSLIGRIL	1	0.1776	7321.3	70.00	0.30	tr_C9ZXD7_
HLA-DQA10501-DQB10201	599	LRMCFCLQPEGRATA	RMCFCLQPE	1	0.1768	7385.7	70.00	0.54	tr_C9ZXD7_
HLA-DQA10501-DQB10201	600	KPPDLTVIKVLRQLL	VIKVLRQLL	6	0.1764	7415.9	70.00	0.31	tr_C9ZXD7_
HLA-DQA10501-DQB10201	601	QHNISMILLRTMFVS	QHNISMILL	0	0.1762	7431.8	70.00	0.22	tr_C9ZXD7_
HLA-DQA10501-DQB10201	602	MAIGTAVSLRKNAAT	MAIGTAVSL	0	0.1755	7488.7	70.00	0.44	tr_C9ZXD7_
HLA-DQA10501-DQB10201	603	ATLLNSLRVPCHLRL	NSLRVPCHL	4	0.1753	7506.0	70.00	0.32	tr_C9ZXD7_
HLA-DQA10501-DQB10201	604	VVTGFLESGCVGKPL	TGFLESGCV	2	0.1727	7718.8	70.00	0.27	tr_C9ZXD7_
HLA-DQA10501-DQB10201	605	LNEIKIMCSLNHPNI	EIKIMCSLN	2	0.1727	7720.5	70.00	0.25	tr_C9ZXD7_
HLA-DQA10501-DQB10201	606	CSLNHPNIVHYFHCE	NHPNIVHYF	3	0.1726	7726.8	70.00	0.34	tr_C9ZXD7_
HLA-DQA10501-DQB10201	607	LLTALTYLHARGVAH	LTALTYLHA	1	0.1707	7886.7	75.00	0.25	tr_C9ZXD7_
HLA-DQA10501-DQB10201	608	EGSLIGRILICANAK	RILICANAK	6	0.1703	7918.6	75.00	0.26	tr_C9ZXD7_
HLA-DQA10501-DQB10201	609	GSLIGRILICANAKD	RILICANAK	5	0.1701	7941.3	75.00	0.31	tr_C9ZXD7_
HLA-DQA10501-DQB10201	610	QVFSGEVLKLKLRAF	VFSGEVLKL	1	0.1700	7947.3	75.00	0.45	tr_C9ZXD7_
HLA-DQA10501-DQB10201	611	CELEKRLQSSPSDID	RLQSSPSDI	5	0.1700	7949.7	75.00	0.50	tr_C9ZXD7_
HLA-DQA10501-DQB10201	612	LLSSASSEGDLSTTG	SASSEGDLS	3	0.1689	8039.9	75.00	0.35	tr_C9ZXD7_
HLA-DQA10501-DQB10201	613	ITDFGTARQGVGSED	ARQGVGSED	6	0.1689	8044.8	75.00	0.40	tr_C9ZXD7_
HLA-DQA10501-DQB10201	614	SLIGRILICANAKDN	RILICANAK	4	0.1688	8047.7	75.00	0.29	tr_C9ZXD7_
HLA-DQA10501-DQB10201	615	YVINTPISSTEVRKV	YVINTPISS	0	0.1684	8084.5	75.00	0.44	tr_C9ZXD7_
HLA-DQA10501-DQB10201	616	IMCSLNHPNIVHYFH	NHPNIVHYF	5	0.1683	8095.9	75.00	0.36	tr_C9ZXD7_

HLA-DQA10501-DQB10201	617	AQISELREKLLPHLR	ISELREKLL	2	0.1674	8168.2	75.00	0.40	tr_C9ZXD7_
HLA-DQA10501-DQB10201	618	KGDVVKITDFGTARQ	ITDFGTARQ	6	0.1673	8181.8	75.00	0.35	tr_C9ZXD7_
HLA-DQA10501-DQB10201	619	SLTSVRERCELEKRL	SVRERCELE	3	0.1669	8215.8	75.00	0.29	tr_C9ZXD7_
HLA-DQA10501-DQB10201	620	RGHNTKNGYYTIPYG	NGYYTIPYG	6	0.1662	8277.4	75.00	0.43	tr_C9ZXD7_
HLA-DQA10501-DQB10201	621	IGRILICANAKDNLG	RILICANAK	2	0.1654	8347.8	75.00	0.31	tr_C9ZXD7_
HLA-DQA10501-DQB10201	622	DVVKITDFGTARQGV	ITDFGTARQ	4	0.1650	8383.7	75.00	0.30	tr_C9ZXD7_
HLA-DQA10501-DQB10201	623	PNIVHYFHCERKENS	IVHYFHCER	2	0.1650	8386.1	75.00	0.41	tr_C9ZXD7_
HLA-DQA10501-DQB10201	624	NEIKIMCSLNHPNIV	EIKIMCSLN	1	0.1636	8515.6	75.00	0.23	tr_C9ZXD7_
HLA-DQA10501-DQB10201	625	EGKAVTPRGLSVIGP	PRGLSVIGP	6	0.1634	8537.7	75.00	0.59	tr_C9ZXD7_
HLA-DQA10501-DQB10201	626	VSLTSVRERCELEKR	SVRERCELE	4	0.1633	8539.6	75.00	0.28	tr_C9ZXD7_
HLA-DQA10501-DQB10201	627	LESGCVGKPLSDLVI	VGKPLSDLV	5	0.1632	8552.7	75.00	0.42	tr_C9ZXD7_
HLA-DQA10501-DQB10201	628	LTYLHARGVAHRDVK	ARGVAHRDV	5	0.1629	8584.2	75.00	0.33	tr_C9ZXD7_
HLA-DQA10501-DQB10201	629	HNTKNGYYTIPYGNA	NGYYTIPYG	4	0.1627	8596.4	75.00	0.30	tr_C9ZXD7_
HLA-DQA10501-DQB10201	630	SLGDIIYGRSQKPPD	IIYGRSQKP	4	0.1626	8605.2	75.00	0.43	tr_C9ZXD7_
HLA-DQA10501-DQB10201	631	PISSTEVRKVLMAIG	VRKVLMAIG	6	0.1626	8606.6	75.00	0.34	tr_C9ZXD7_
HLA-DQA10501-DQB10201	632	PDIFSREKQYVSSLT	EKQYVSSLT	6	0.1620	8661.3	75.00	0.45	tr_C9ZXD7_
HLA-DQA10501-DQB10201	633	CSLGDIIYGRSQKPP	IIYGRSQKP	5	0.1615	8710.2	75.00	0.41	tr_C9ZXD7_
HLA-DQA10501-DQB10201	634	ILTLRQDSPWTKGVL	ILTLRQDSP	0	0.1597	8880.3	75.00	0.31	tr_C9ZXD7_
HLA-DQA10501-DQB10201	635	ESGCVGKPLSDLVIT	VGKPLSDLV	4	0.1585	8996.6	75.00	0.41	tr_C9ZXD7_
HLA-DQA10501-DQB10201	636	SLSEILEGKAVTPRG	ILEGKAVTP	4	0.1581	9036.8	75.00	0.30	tr_C9ZXD7_
HLA-DQA10501-DQB10201	637	MCSLNHPNIVHYFHC	NHPNIVHYF	4	0.1576	9086.8	75.00	0.37	tr_C9ZXD7_
HLA-DQA10501-DQB10201	638	TYLHARGVAHRDVKP	ARGVAHRDV	4	0.1576	9089.7	75.00	0.37	tr_C9ZXD7_
HLA-DQA10501-DQB10201	639	LSEILEGKAVTPRGL	ILEGKAVTP	3	0.1575	9093.5	75.00	0.31	tr_C9ZXD7_
HLA-DQA10501-DQB10201	640	DLLLHRLFSSSCSAE	RLFSSSCSA	5	0.1574	9109.8	75.00	0.49	tr_C9ZXD7_
HLA-DQA10501-DQB10201	641	KIMCSLNHPNIVHYF	NHPNIVHYF	6	0.1571	9139.3	75.00	0.37	tr_C9ZXD7_
HLA-DQA10501-DQB10201	642	LSSASSEGDLSTTGP	SASSEGDLS	2	0.1569	9156.8	75.00	0.40	tr_C9ZXD7_
HLA-DQA10501-DQB10201	643	TSAKALKVLHTWILE	KVLHTWILE	6	0.1565	9196.7	75.00	0.26	tr_C9ZXD7_
HLA-DQA10501-DQB10201	644	GHNTKNGYYTIPYGN	NGYYTIPYG	5	0.1563	9217.2	75.00	0.41	tr_C9ZXD7_
HLA-DQA10501-DQB10201	645	QKPPDLTVIKVLRQL	LTVIKVLRQ	5	0.1561	9235.3	75.00	0.31	tr_C9ZXD7_
HLA-DQA10501-DQB10201	646	FLESGCVGKPLSDLV	VGKPLSDLV	6	0.1561	9239.9	75.00	0.50	tr_C9ZXD7_
HLA-DQA10501-DQB10201	647	LGDIIYGRSQKPPDL	IIYGRSQKP	3	0.1555	9294.3	75.00	0.46	tr_C9ZXD7_
HLA-DQA10501-DQB10201	648	SQKPPDLTVIKVLRQ	LTVIKVLRQ	6	0.1554	9307.9	75.00	0.35	tr_C9ZXD7_
HLA-DQA10501-DQB10201	649	LRLSPLDHKTSVDSL	DHKTSVDSL	6	0.1550	9350.5	75.00	0.71	tr_C9ZXD7_
HLA-DQA10501-DQB10201	650	ELCCCSLGDIIYGRS	LGDIIYGRS	6	0.1546	9386.4	75.00	0.34	tr_C9ZXD7_
HLA-DQA10501-DQB10201	651	ADLLLHRLFSSSCSA	RLFSSSCSA	6	0.1541	9437.5	75.00	0.50	tr_C9ZXD7_
HLA-DQA10501-DQB10201	652	NIVHYFHCERKENSV	IVHYFHCER	1	0.1538	9467.2	75.00	0.40	tr_C9ZXD7_
HLA-DQA10501-DQB10201	653	TLLNSLRVPCHLRLQ	NSLRVPCHL	3	0.1538	9473.2	80.00	0.37	tr_C9ZXD7_
HLA-DQA10501-DQB10201	654	CCSLGDIIYGRSQKP	IIYGRSQKP	6	0.1536	9491.1	80.00	0.32	tr_C9ZXD7_
HLA-DQA10501-DQB10201	655	TPISSTEVRKVLMAI	EVRKVLMAI	6	0.1527	9577.0	80.00	0.44	tr_C9ZXD7_
HLA-DQA10501-DQB10201	656	SDLTGGKMAVKMFYF	KMAVKMFYF	6	0.1525	9606.6	80.00	0.41	tr_C9ZXD7_
HLA-DQA10501-DQB10201	657	HYFHCERKENSVNLF	RKENSVNLF	6	0.1524	9612.3	80.00	0.69	tr_C9ZXD7_
HLA-DQA10501-DQB10201	658	GDVVKITDFGTARQG	ITDFGTARQ	5	0.1524	9615.3	80.00	0.30	tr_C9ZXD7_
HLA-DQA10501-DQB10201	659	RILICANAKDNLGEY	RILICANAK	0	0.1522	9629.7	80.00	0.22	tr_C9ZXD7_
HLA-DQA10501-DQB10201	660	GRILICANAKDNLGE	RILICANAK	1	0.1513	9725.7	80.00	0.30	tr_C9ZXD7_
HLA-DQA10501-DQB10201	661	IKGDVVKITDFGTAR	GDVVKITDF	2	0.1510	9758.9	80.00	0.24	tr_C9ZXD7_
HLA-DQA10501-DQB10201	662	LTSVRERCELEKRLQ	SVRERCELE	2	0.1509	9772.9	80.00	0.33	tr_C9ZXD7_
HLA-DQA10501-DQB10201	663	TALTYLHARGVAHRD	YLHARGVAH	4	0.1504	9817.9	80.00	0.31	tr_C9ZXD7_
HLA-DQA10501-DQB10201	664	LTGGKMAVKMFYFTE	KMAVKMFYF	4	0.1501	9851.2	80.00	0.31	tr_C9ZXD7_
HLA-DQA10501-DQB10201	665	MLRLSPLDHKTSVDS	MLRLSPLDH	0	0.1500	9865.7	80.00	0.62	tr_C9ZXD7_
HLA-DQA10501-DQB10201	666	VYRGEDHCSPCDIWS	EDHCSPCDI	4	0.1496	9904.9	80.00	0.23	tr_C9ZXD7_

HLA-DQA10501-DQB10201	667	QISELREKLLPHLRS	ISELREKLL	1	0.1481	10071.8	80.00	0.40	tr_C9ZXD7_
HLA-DQA10501-DQB10201	668	GEVLKLKLRAFATVP	KLRAFATVP	6	0.1480	10077.8	80.00	0.57	tr_C9ZXD7_
HLA-DQA10501-DQB10201	669	LNSLRVPCHLRLQIL	PCHLRLQIL	6	0.1477	10110.2	80.00	0.30	tr_C9ZXD7_
HLA-DQA10501-DQB10201	670	VESLPDIFSREKQYV	IFSREKQYV	6	0.1469	10197.0	80.00	0.28	tr_C9ZXD7_
HLA-DQA10501-DQB10201	671	ATAQEERERILTLRQ	RERILTLRQ	6	0.1469	10197.5	80.00	0.29	tr_C9ZXD7_
HLA-DQA10501-DQB10201	672	NAKDNLGEYRTYIRD	AKDNLGEYR	1	0.1463	10272.7	80.00	0.22	tr_C9ZXD7_
HLA-DQA10501-DQB10201	673	DLTGGKMAVKMFYFT	KMAVKMFYF	5	0.1454	10364.2	80.00	0.38	tr_C9ZXD7_
HLA-DQA10501-DQB10201	674	RMCFCLQPEGRATAA	RMCFCLQPE	0	0.1441	10517.1	80.00	0.49	tr_C9ZXD7_
HLA-DQA10501-DQB10201	675	LTALTYLHARGVAHR	YLHARGVAH	5	0.1439	10533.6	80.00	0.28	tr_C9ZXD7_
HLA-DQA10501-DQB10201	676	VTGFLESGCVGKPLS	TGFLESGCV	1	0.1437	10561.9	80.00	0.32	tr_C9ZXD7_
HLA-DQA10501-DQB10201	677	PHLRSTCGSIREDDG	CGSIREDDG	6	0.1435	10579.3	80.00	0.28	tr_C9ZXD7_
HLA-DQA10501-DQB10201	678	SEILEGKAVTPRGLS	ILEGKAVTP	2	0.1428	10659.5	80.00	0.34	tr_C9ZXD7_
HLA-DQA10501-DQB10201	679	LCCCSLGDIIYGRSQ	LGDIIYGRS	5	0.1425	10705.3	80.00	0.38	tr_C9ZXD7_
HLA-DQA10501-DQB10201	680	ILICANAKDNLGEYR	AKDNLGEYR	6	0.1424	10708.7	80.00	0.28	tr_C9ZXD7_
HLA-DQA10501-DQB10201	681	LRVPCHLRLQILNSS	LRLQILNSS	6	0.1423	10725.9	80.00	0.28	tr_C9ZXD7_
HLA-DQA10501-DQB10201	682	HARGVAHRDVKPQNI	HRDVKPQNI	6	0.1419	10772.9	80.00	0.38	tr_C9ZXD7_
HLA-DQA10501-DQB10201	683	LLNSLRVPCHLRLQI	NSLRVPCHL	2	0.1417	10792.4	80.00	0.37	tr_C9ZXD7_
HLA-DQA10501-DQB10201	684	GDIIYGRSQKPPDLT	IIYGRSQKP	2	0.1413	10839.4	80.00	0.51	tr_C9ZXD7_
HLA-DQA10501-DQB10201	685	VVKITDFGTARQGVG	ITDFGTARQ	3	0.1409	10892.2	80.00	0.34	tr_C9ZXD7_
HLA-DQA10501-DQB10201	686	LTLRQDSPWTKGVLL	SPWTKGVLL	6	0.1402	10973.6	80.00	0.38	tr_C9ZXD7_
HLA-DQA10501-DQB10201	687	EIKIMCSLNHPNIVH	EIKIMCSLN	0	0.1400	10993.0	80.00	0.19	tr_C9ZXD7_
HLA-DQA10501-DQB10201	688	VFSGEVLKLKLRAFA	VFSGEVLKL	0	0.1396	11045.5	80.00	0.40	tr_C9ZXD7_
HLA-DQA10501-DQB10201	689	ATAADLLLHRLFSSS	AADLLLHRL	2	0.1392	11092.5	80.00	0.39	tr_C9ZXD7_
HLA-DQA10501-DQB10201	690	RSQKPPDLTVIKVLR	DLTVIKVLR	6	0.1380	11231.7	80.00	0.37	tr_C9ZXD7_
HLA-DQA10501-DQB10201	691	HLRSTCGSIREDDGT	CGSIREDDG	5	0.1377	11263.8	80.00	0.28	tr_C9ZXD7_
HLA-DQA10501-DQB10201	692	YLHARGVAHRDVKPQ	ARGVAHRDV	3	0.1369	11367.6	80.00	0.42	tr_C9ZXD7_
HLA-DQA10501-DQB10201	693	FDDKYRITRWNMAAE	ITRWNMAAE	6	0.1361	11470.3	80.00	0.35	tr_C9ZXD7_
HLA-DQA10501-DQB10201	694	LRSTCGSIREDDGTV	CGSIREDDG	4	0.1358	11499.3	80.00	0.27	tr_C9ZXD7_
HLA-DQA10501-DQB10201	695	SASSEGDLSTTGPSA	DLSTTGPSA	6	0.1355	11543.5	80.00	0.29	tr_C9ZXD7_
HLA-DQA10501-DQB10201	696	HLRLQILNSSRNTFA	LNSSRNTFA	6	0.1352	11575.1	80.00	0.27	tr_C9ZXD7_
HLA-DQA10501-DQB10201	697	SLRVPCHLRLQILNS	HLRLQILNS	6	0.1351	11587.6	80.00	0.41	tr_C9ZXD7_
HLA-DQA10501-DQB10201	698	RVPCHLRLQILNSSR	LRLQILNSS	5	0.1342	11709.1	85.00	0.31	tr_C9ZXD7_
HLA-DQA10501-DQB10201	699	SSASSEGDLSTTGPS	SASSEGDLS	1	0.1335	11797.5	85.00	0.44	tr_C9ZXD7_
HLA-DQA10501-DQB10201	700	TSVRERCELEKRLQS	SVRERCELE	1	0.1332	11832.4	85.00	0.35	tr_C9ZXD7_
HLA-DQA10501-DQB10201	701	MELCCCSLGDIIYGR	CCCSLGDII	3	0.1326	11913.3	85.00	0.22	tr_C9ZXD7_
HLA-DQA10501-DQB10201	702	YRGEDHCSPCDIWSV	EDHCSPCDI	3	0.1315	12057.1	85.00	0.29	tr_C9ZXD7_
HLA-DQA10501-DQB10201	703	ARGVAHRDVKPQNIL	HRDVKPQNI	5	0.1313	12079.8	85.00	0.44	tr_C9ZXD7_
HLA-DQA10501-DQB10201	704	RCELEKRLQSSPSDI	RLQSSPSDI	6	0.1311	12102.2	85.00	0.62	tr_C9ZXD7_
HLA-DQA10501-DQB10201	705	LRLQILNSSRNTFAL	LNSSRNTFA	5	0.1306	12166.0	85.00	0.34	tr_C9ZXD7_
HLA-DQA10501-DQB10201	706	VKITDFGTARQGVGS	DFGTARQGV	4	0.1301	12233.9	85.00	0.33	tr_C9ZXD7_
HLA-DQA10501-DQB10201	707	RQDSPWTKGVLLGRG	WTKGVLLGR	5	0.1296	12295.6	85.00	0.34	tr_C9ZXD7_
HLA-DQA10501-DQB10201	708	TAADLLLHRLFSSSC	AADLLLHRL	1	0.1295	12316.3	85.00	0.38	tr_C9ZXD7_
HLA-DQA10501-DQB10201	709	NSLRVPCHLRLQILN	PCHLRLQIL	5	0.1294	12331.8	85.00	0.33	tr_C9ZXD7_
HLA-DQA10501-DQB10201	710	ANAKDNLGEYRTYIR	AKDNLGEYR	2	0.1294	12334.5	85.00	0.32	tr_C9ZXD7_
HLA-DQA10501-DQB10201	711	VPCHLRLQILNSSRN	LRLQILNSS	4	0.1293	12343.6	85.00	0.28	tr_C9ZXD7_
HLA-DQA10501-DQB10201	712	RGVAHRDVKPQNILI	HRDVKPQNI	4	0.1286	12442.6	85.00	0.47	tr_C9ZXD7_
HLA-DQA10501-DQB10201	713	DIIYGRSQKPPDLTV	IIYGRSQKP	1	0.1283	12473.2	85.00	0.50	tr_C9ZXD7_
HLA-DQA10501-DQB10201	714	EILEGKAVTPRGLSV	ILEGKAVTP	1	0.1282	12495.3	85.00	0.33	tr_C9ZXD7_
HLA-DQA10501-DQB10201	715	TRGHNTKNGYYTIPY	KNGYYTIPY	6	0.1279	12533.3	85.00	0.67	tr_C9ZXD7_
HLA-DQA10501-DQB10201	716	CCCSLGDIIYGRSQK	LGDIIYGRS	4	0.1279	12536.6	85.00	0.36	tr_C9ZXD7_

HLA-DQA10501-DQB10201	717	KITDFGTARQGVGSE	DFGTARQGV	3	0.1274	12596.9	85.00	0.32	tr_C9ZXD7_
HLA-DQA10501-DQB10201	718	DSPWTKGVLLGRGSF	WTKGVLLGR	3	0.1262	12759.5	85.00	0.31	tr_C9ZXD7_
HLA-DQA10501-DQB10201	719	LICANAKDNLGEYRT	AKDNLGEYR	5	0.1251	12912.5	85.00	0.34	tr_C9ZXD7_
HLA-DQA10501-DQB10201	720	CANAKDNLGEYRTYI	AKDNLGEYR	3	0.1250	12931.4	85.00	0.36	tr_C9ZXD7_
HLA-DQA10501-DQB10201	721	LPHLRSTCGSIREDD	TCGSIREDD	6	0.1249	12938.0	85.00	0.28	tr_C9ZXD7_
HLA-DQA10501-DQB10201	722	DLSTTGPSAGAAVPQ	PSAGAAVPQ	6	0.1239	13079.9	85.00	0.41	tr_C9ZXD7_
HLA-DQA10501-DQB10201	723	GRSQKPPDLTVIKVL	PDLTVIKVL	6	0.1235	13139.6	85.00	0.49	tr_C9ZXD7_
HLA-DQA10501-DQB10201	724	AADLLLHRLFSSSCS	HRLFSSSCS	6	0.1227	13259.7	85.00	0.23	tr_C9ZXD7_
HLA-DQA10501-DQB10201	725	IVHYFHCERKENSVN	IVHYFHCER	0	0.1226	13275.9	85.00	0.35	tr_C9ZXD7_
HLA-DQA10501-DQB10201	726	QDSPWTKGVLLGRGS	WTKGVLLGR	4	0.1225	13289.4	85.00	0.35	tr_C9ZXD7_
HLA-DQA10501-DQB10201	727	RLQILNSSRNTFALQ	LNSSRNTFA	4	0.1223	13314.5	85.00	0.38	tr_C9ZXD7_
HLA-DQA10501-DQB10201	728	CHLRLQILNSSRNTF	LRLQILNSS	2	0.1219	13364.5	85.00	0.26	tr_C9ZXD7_
HLA-DQA10501-DQB10201	729	TGFLESGCVGKPLSD	GFLESGCVG	1	0.1217	13403.8	85.00	0.25	tr_C9ZXD7_
HLA-DQA10501-DQB10201	730	LRQDSPWTKGVLLGR	WTKGVLLGR	6	0.1209	13521.6	85.00	0.45	tr_C9ZXD7_
HLA-DQA10501-DQB10201	731	NTPISSTEVRKVLMA	STEVRKVLM	5	0.1202	13622.2	85.00	0.31	tr_C9ZXD7_
HLA-DQA10501-DQB10201	732	IKIMCSLNHPNIVHY	CSLNHPNIV	4	0.1200	13652.0	85.00	0.28	tr_C9ZXD7_
HLA-DQA10501-DQB10201	733	ICANAKDNLGEYRTY	AKDNLGEYR	4	0.1195	13721.0	85.00	0.38	tr_C9ZXD7_
HLA-DQA10501-DQB10201	734	TSDLTGGKMAVKMFY	GKMAVKMFY	6	0.1190	13790.1	85.00	0.37	tr_C9ZXD7_
HLA-DQA10501-DQB10201	735	SPWTKGVLLGRGSFG	WTKGVLLGR	2	0.1190	13797.6	85.00	0.28	tr_C9ZXD7_
HLA-DQA10501-DQB10201	736	PCHLRLQILNSSRNT	LRLQILNSS	3	0.1190	13799.2	85.00	0.31	tr_C9ZXD7_
HLA-DQA10501-DQB10201	737	INTPISSTEVRKVLM	STEVRKVLM	6	0.1187	13837.2	85.00	0.33	tr_C9ZXD7_
HLA-DQA10501-DQB10201	738	LPDIFSREKQYVSSL	REKQYVSSL	6	0.1176	14003.7	85.00	0.33	tr_C9ZXD7_
HLA-DQA10501-DQB10201	739	AGAAVPQVTRGHNTK	AGAAVPQVT	0	0.1176	14006.6	85.00	0.75	tr_C9ZXD7_
HLA-DQA10501-DQB10201	740	TLRQDSPWTKGVLLG	SPWTKGVLL	5	0.1168	14129.5	85.00	0.42	tr_C9ZXD7_
HLA-DQA10501-DQB10201	741	LHARGVAHRDVKPQN	ARGVAHRDV	2	0.1164	14187.2	85.00	0.41	tr_C9ZXD7_
HLA-DQA10501-DQB10201	742	ASSEGDLSTTGPSAG	DLSTTGPSA	5	0.1157	14292.6	85.00	0.44	tr_C9ZXD7_
HLA-DQA10501-DQB10201	743	EDHCSPCDIWSVGCV	CDIWSVGCV	6	0.1155	14323.3	85.00	0.28	tr_C9ZXD7_
HLA-DQA10501-DQB10201	744	ISELREKLLPHLRST	LREKLLPHL	3	0.1154	14343.7	85.00	0.28	tr_C9ZXD7_
HLA-DQA10501-DQB10201	745	ESLPDIFSREKQYVS	IFSREKQYV	5	0.1150	14410.9	85.00	0.38	tr_C9ZXD7_
HLA-DQA10501-DQB10201	746	GTAVSLRKNAATAQE	RKNAATAQE	6	0.1136	14620.1	90.00	0.62	tr_C9ZXD7_
HLA-DQA10501-DQB10201	747	GFLESGCVGKPLSDL	CVGKPLSDL	6	0.1124	14821.1	90.00	0.26	tr_C9ZXD7_
HLA-DQA10501-DQB10201	748	VINTPISSTEVRKVL	NTPISSTEV	2	0.1105	15121.3	90.00	0.26	tr_C9ZXD7_
HLA-DQA10501-DQB10201	749	SSEGDLSTTGPSAGA	DLSTTGPSA	4	0.1100	15213.5	90.00	0.46	tr_C9ZXD7_
HLA-DQA10501-DQB10201	750	RGEDHCSPCDIWSVG	EDHCSPCDI	2	0.1098	15236.2	90.00	0.36	tr_C9ZXD7_
HLA-DQA10501-DQB10201	751	GFDDKYRITRWNMAA	YRITRWNMA	5	0.1092	15334.1	90.00	0.40	tr_C9ZXD7_
HLA-DQA10501-DQB10201	752	GEDHCSPCDIWSVGC	DHCSPCDIW	2	0.1083	15483.9	90.00	0.36	tr_C9ZXD7_
HLA-DQA10501-DQB10201	753	GVAHRDVKPQNILIK	HRDVKPQNI	3	0.1083	15494.6	90.00	0.47	tr_C9ZXD7_
HLA-DQA10501-DQB10201	754	IGFDDKYRITRWNMA	YRITRWNMA	6	0.1082	15506.6	90.00	0.41	tr_C9ZXD7_
HLA-DQA10501-DQB10201	755	MIGFDDKYRITRWNM	MIGFDDKYR	0	0.1078	15580.0	90.00	0.23	tr_C9ZXD7_
HLA-DQA10501-DQB10201	756	ILEGKAVTPRGLSVI	ILEGKAVTP	0	0.1059	15900.0	90.00	0.23	tr_C9ZXD7_
HLA-DQA10501-DQB10201	757	PWTKGVLLGRGSFGA	WTKGVLLGR	1	0.1054	15985.7	90.00	0.26	tr_C9ZXD7_
HLA-DQA10501-DQB10201	758	SVRERCELEKRLQSS	SVRERCELE	0	0.1049	16078.8	90.00	0.30	tr_C9ZXD7_
HLA-DQA10501-DQB10201	759	SLPDIFSREKQYVSS	IFSREKQYV	4	0.1044	16151.2	90.00	0.40	tr_C9ZXD7_
HLA-DQA10501-DQB10201	760	SEGDLSTTGPSAGAA	DLSTTGPSA	3	0.1044	16159.2	90.00	0.48	tr_C9ZXD7_
HLA-DQA10501-DQB10201	761	LLPHLRSTCGSIRED	LRSTCGSIR	4	0.1039	16241.6	90.00	0.28	tr_C9ZXD7_
HLA-DQA10501-DQB10201	762	GDLSTTGPSAGAAVP	DLSTTGPSA	1	0.1033	16343.9	90.00	0.31	tr_C9ZXD7_
HLA-DQA10501-DQB10201	763	EGDLSTTGPSAGAAV	DLSTTGPSA	2	0.1022	16553.2	90.00	0.38	tr_C9ZXD7_
HLA-DQA10501-DQB10201	764	MCFCLQPEGRATAAD	CFCLQPEGR	1	0.1016	16653.2	90.00	0.34	tr_C9ZXD7_
HLA-DQA10501-DQB10201	765	WTKGVLLGRGSFGAV	VLLGRGSFG	4	0.1009	16781.1	90.00	0.25	tr_C9ZXD7_
HLA-DQA10501-DQB10201	766	VAHRDVKPQNILIKG	HRDVKPQNI	2	0.1004	16875.8	90.00	0.49	tr_C9ZXD7_

HLA-DQA10501-DQB10201	767	AHRDVKPQNILIKGD	HRDVKPQNI	1	0.0996	17016.0	90.00	0.40	tr_C9ZXD7_	
HLA-DQA10501-DQB10201	768	SGEVLKLKLRAFATV	LKLRAFATV	6	0.0992	17092.6	90.00	0.30	tr_C9ZXD7_	
HLA-DQA10501-DQB10201	769	FSGEVLKLKLRAFAT	KLKLRAFAT	6	0.0980	17323.9	90.00	0.28	tr_C9ZXD7_	
HLA-DQA10501-DQB10201	770	AIGTAVSLRKNAATA	IGTAVSLRK	1	0.0975	17402.6	90.00	0.43	tr_C9ZXD7_	
HLA-DQA10501-DQB10201	771	ATSDLTGGKMAVKMF	GGKMAVKMF	6	0.0971	17490.7	90.00	0.34	tr_C9ZXD7_	
HLA-DQA10501-DQB10201	772	IIYGRSQKPPDLTVI	IIYGRSQKP	0	0.0964	17614.0	90.00	0.41	tr_C9ZXD7_	
HLA-DQA10501-DQB10201	773	HRDVKPQNILIKGDV	QNILIKGDV	6	0.0937	18140.7	90.00	0.25	tr_C9ZXD7_	
HLA-DQA10501-DQB10201	774	LEGKAVTPRGLSVIG	GKAVTPRGL	2	0.0933	18224.3	90.00	0.29	tr_C9ZXD7_	
HLA-DQA10501-DQB10201	775	KLLPHLRSTCGSIRE	LRSTCGSIR	5	0.0898	18918.2	95.00	0.33	tr_C9ZXD7_	
HLA-DQA10501-DQB10201	776	SELREKLLPHLRSTC	LREKLLPHL	2	0.0878	19329.1	95.00	0.37	tr_C9ZXD7_	
HLA-DQA10501-DQB10201	777	VHYFHCERKENSVNL	HYFHCERKE	1	0.0875	19410.2	95.00	0.28	tr_C9ZXD7_	
HLA-DQA10501-DQB10201	778	YGRSQKPPDLTVIKV	PPDLTVIKV	6	0.0851	19900.6	95.00	0.60	tr_C9ZXD7_	
HLA-DQA10501-DQB10201	779	ELREKLLPHLRSTCG	LREKLLPHL	1	0.0827	20444.1	95.00	0.36	tr_C9ZXD7_	
HLA-DQA10501-DQB10201	780	IGTAVSLRKNAATAQ	LRKNAATAQ	6	0.0806	20910.5	95.00	0.41	tr_C9ZXD7_	
HLA-DQA10501-DQB10201	781	YEATSDLTGGKMAVK	TSDLTGGKM	3	0.0779	21518.3	95.00	0.31	tr_C9ZXD7_	
HLA-DQA10501-DQB10201	782	LREKLLPHLRSTCGS	LREKLLPHL	0	0.0776	21588.9	95.00	0.22	tr_C9ZXD7_	
HLA-DQA10501-DQB10201	783	EATSDLTGGKMAVKM	TGGKMAVKM	6	0.0776	21594.3	95.00	0.41	tr_C9ZXD7_	
HLA-DQA10501-DQB10201	784	ERCELEKRLQSSPSD	KRLQSSPSD	6	0.0775	21615.6	95.00	0.52	tr_C9ZXD7_	
HLA-DQA10501-DQB10201	785	EKLLPHLRSTCGSIR	LRSTCGSIR	6	0.0761	21939.8	95.00	0.34	tr_C9ZXD7_	
HLA-DQA10501-DQB10201	786	VRERCELEKRLQSSP	ERCELEKRL	2	0.0747	22276.6	95.00	0.30	tr_C9ZXD7_	
HLA-DQA10501-DQB10201	787	REKLLPHLRSTCGSI	HLRSTCGSI	6	0.0726	22791.0	95.00	0.19	tr_C9ZXD7_	
HLA-DQA10501-DQB10201	788	RERCELEKRLQSSPS	ERCELEKRL	1	0.0618	25607.5	100.00	0.31	tr_C9ZXD7_	
HLA-DQA10501-DQB10201	789	IYGRSQKPPDLTVIK	GRSQKPPDL	2	0.0566	27101.3	100.00	0.41	tr_C9ZXD7_	
HLA-DQA10501-DQB10201	790	VTRGHNTKNGYYTIP	TKNGYYTIP	6	0.0479	29784.3	100.00	0.34	tr_C9ZXD7_	
HLA-DQA10501-DQB10201	791	QVTRGHNTKNGYYTI	RGHNTKNGY	3	0.0358	33947.5	100.00	0.29	tr_C9ZXD7_	
HLA-DQA10501-DQB10201	792	GAAVPQVTRGHNTKN	AAVPQVTRG	1	0.0342	34544.4	100.00	0.26	tr_C9ZXD7_	
HLA-DQA10501-DQB10201	793	PQVTRGHNTKNGYYT	RGHNTKNGY	4	0.0332	34905.5	100.00	0.36	tr_C9ZXD7_	
HLA-DQA10501-DQB10201	794	VPQVTRGHNTKNGYY	RGHNTKNGY	5	0.0331	34954.2	100.00	0.38	tr_C9ZXD7_	
HLA-DQA10501-DQB10201	795	AVPQVTRGHNTKNGY	RGHNTKNGY	6	0.0319	35401.8	100.00	0.38	tr_C9ZXD7_	
HLA-DQA10501-DQB10201	796	AAVPQVTRGHNTKNG	PQVTRGHNT	3	0.0259	37773.9	100.00	0.28	tr_C9ZXD7_	

Allele: HLA-DQA10501-DQB10201. Number of high binders 6. Number of weak binders 86. Number of peptides 796

Allele	pos	peptide	core	Of	1-log50k(aff)	affinity(nM)	%Rank	Relia	Identity	Bind Level
HLA-DQA10501-DQB10201	1	SPMINCFDGVAEFFF	FDGVAEFFF	6	0.5996	76.1	1.30	0.48	tr_C9ZR23_	WB
HLA-DQA10501-DQB10201	2	PMINCFDGVAEFFFS	FDGVAEFFF	5	0.5750	99.3	1.90	0.48	tr_C9ZR23_	WB
HLA-DQA10501-DQB10201	3	MINCFDGVAEFFFSR	FDGVAEFFF	4	0.5433	140.0	3.50	0.46	tr_C9ZR23_	WB
HLA-DQA10501-DQB10201	4	INCFDGVAEFFFSRS	FDGVAEFFF	3	0.5239	172.6	4.00	0.46	tr_C9ZR23_	WB
HLA-DQA10501-DQB10201	5	PIVYIRTDNLDGELD	IVYIRTDNL	1	0.5084	204.2	5.00	0.31	tr_C9ZR23_	WB
HLA-DQA10501-DQB10201	6	NPIVYIRTDNLDGEL	IVYIRTDNL	2	0.5056	210.4	5.00	0.37	tr_C9ZR23_	WB
HLA-DQA10501-DQB10201	7	IVYIRTDNLDGELDL	IVYIRTDNL	0	0.5023	218.1	5.50	0.25	tr_C9ZR23_	WB
HLA-DQA10501-DQB10201	8	NCFDGVAEFFFSRSP	FDGVAEFFF	2	0.4987	226.8	5.50	0.47	tr_C9ZR23_	WB
HLA-DQA10501-DQB10201	9	PSPMINCFDGVAEFF	CFDGVAEFF	6	0.4884	253.5	6.50	0.68	tr_C9ZR23_	WB
HLA-DQA10501-DQB10201	10	TNPIVYIRTDNLDGE	IVYIRTDNL	3	0.4855	261.6	6.50	0.44	tr_C9ZR23_	WB
HLA-DQA10501-DQB10201	11	AATNPIVYIRTDNLD	IVYIRTDNL	5	0.4594	346.8	9.00	0.61	tr_C9ZR23_	WB
HLA-DQA10501-DQB10201	12	ATNPIVYIRTDNLDG	IVYIRTDNL	4	0.4589	348.8	9.00	0.54	tr_C9ZR23_	WB

HLA-DQA10501-DQB10201	13	CFDGVAEFFFSRSPA	FDGVAEFFF	1	0.4573	354.9	9.00	0.50	tr_C9ZR23_	WB
HLA-DQA10501-DQB10201	14	DVILLREGEDIPCDV	ILLREGEDI	2	0.4405	425.5	11.00	0.44	tr_C9ZR23_	
HLA-DQA10501-DQB10201	15	NAIAHQLLSVDDSCA	QLLSVDDSC	5	0.4210	525.4	13.00	0.30	tr_C9ZR23_	
HLA-DQA10501-DQB10201	16	VYIRTDNLDGELDLK	TDNLDGELD	4	0.4182	541.9	13.00	0.23	tr_C9ZR23_	
HLA-DQA10501-DQB10201	17	PNAIAHQLLSVDDSC	QLLSVDDSC	6	0.4170	548.9	14.00	0.27	tr_C9ZR23_	
HLA-DQA10501-DQB10201	18	RVGDVILLREGEDIP	ILLREGEDI	5	0.4170	549.2	14.00	0.51	tr_C9ZR23_	
HLA-DQA10501-DQB10201	19	PCDVVVLAATNPIVY	VVLAATNPI	4	0.4160	555.1	14.00	0.38	tr_C9ZR23_	
HLA-DQA10501-DQB10201	20	CDVVVLAATNPIVYI	VVLAATNPI	3	0.4150	561.2	14.00	0.37	tr_C9ZR23_	
HLA-DQA10501-DQB10201	21	GDVILLREGEDIPCD	ILLREGEDI	3	0.4139	567.8	14.00	0.55	tr_C9ZR23_	
HLA-DQA10501-DQB10201	22	VGDVILLREGEDIPC	ILLREGEDI	4	0.4099	592.5	15.00	0.51	tr_C9ZR23_	
HLA-DQA10501-DQB10201	23	LAATNPIVYIRTDNL	IVYIRTDNL	6	0.4095	595.2	15.00	0.70	tr_C9ZR23_	
HLA-DQA10501-DQB10201	24	VILLREGEDIPCDVV	ILLREGEDI	1	0.4095	595.4	15.00	0.41	tr_C9ZR23_	
HLA-DQA10501-DQB10201	25	AIAHQLLSVDDSCAS	QLLSVDDSC	4	0.4086	601.1	15.00	0.30	tr_C9ZR23_	
HLA-DQA10501-DQB10201	26	IRVGDVILLREGEDI	ILLREGEDI	6	0.4043	629.9	15.00	0.41	tr_C9ZR23_	
HLA-DQA10501-DQB10201	27	GELDLKPRDVVAPQL	PRDVVAPQL	6	0.4038	633.0	15.00	0.87	tr_C9ZR23_	
HLA-DQA10501-DQB10201	28	DVVVLAATNPIVYIR	VVLAATNPI	2	0.3980	674.1	16.00	0.39	tr_C9ZR23_	
HLA-DQA10501-DQB10201	29	IAHQLLSVDDSCASI	QLLSVDDSC	3	0.3925	715.3	17.00	0.34	tr_C9ZR23_	
HLA-DQA10501-DQB10201	30	ELDLKPRDVVAPQLS	PRDVVAPQL	5	0.3924	716.0	17.00	0.75	tr_C9ZR23_	
HLA-DQA10501-DQB10201	31	VPNAIAHQLLSVDDS	HQLLSVDDS	6	0.3913	724.7	17.00	0.28	tr_C9ZR23_	
HLA-DQA10501-DQB10201	32	TNHSIRVGDVILLRE	VGDVILLRE	6	0.3861	766.7	18.00	0.37	tr_C9ZR23_	
HLA-DQA10501-DQB10201	33	TGGDDVPNAIAHQLL	PNAIAHQLL	6	0.3851	775.3	18.00	0.58	tr_C9ZR23_	
HLA-DQA10501-DQB10201	34	DLKPRDVVAPQLSSD	PRDVVAPQL	3	0.3824	798.0	19.00	0.70	tr_C9ZR23_	
HLA-DQA10501-DQB10201	35	DVPNAIAHQLLSVDD	PNAIAHQLL	2	0.3797	821.9	19.00	0.34	tr_C9ZR23_	
HLA-DQA10501-DQB10201	36	YIRTDNLDGELDLKP	TDNLDGELD	3	0.3795	823.3	19.00	0.31	tr_C9ZR23_	
HLA-DQA10501-DQB10201	37	IPCDVVVLAATNPIV	VVLAATNPI	5	0.3776	841.2	19.00	0.46	tr_C9ZR23_	
HLA-DQA10501-DQB10201	38	ILLREGEDIPCDVVV	ILLREGEDI	0	0.3760	855.8	20.00	0.34	tr_C9ZR23_	
HLA-DQA10501-DQB10201	39	VVVLAATNPIVYIRT	VVLAATNPI	1	0.3737	876.8	20.00	0.38	tr_C9ZR23_	
HLA-DQA10501-DQB10201	40	LDLKPRDVVAPQLSS	PRDVVAPQL	4	0.3728	885.3	20.00	0.74	tr_C9ZR23_	
HLA-DQA10501-DQB10201	41	FDGVAEFFFSRSPAA	FDGVAEFFF	0	0.3663	950.3	21.00	0.49	tr_C9ZR23_	
HLA-DQA10501-DQB10201	42	AHQLLSVDDSCASIV	QLLSVDDSC	2	0.3657	955.9	21.00	0.34	tr_C9ZR23_	
HLA-DQA10501-DQB10201	43	DDVPNAIAHQLLSVD	PNAIAHQLL	3	0.3626	989.0	22.00	0.44	tr_C9ZR23_	
HLA-DQA10501-DQB10201	44	LKPRDVVAPQLSSDH	PRDVVAPQL	2	0.3591	1026.4	23.00	0.68	tr_C9ZR23_	
HLA-DQA10501-DQB10201	45	GDDVPNAIAHQLLSV	PNAIAHQLL	4	0.3585	1033.6	23.00	0.47	tr_C9ZR23_	
HLA-DQA10501-DQB10201	46	DIPCDVVVLAATNPI	VVLAATNPI	6	0.3570	1050.8	23.00	0.52	tr_C9ZR23_	
HLA-DQA10501-DQB10201	47	SIRVGDVILLREGED	VGDVILLRE	3	0.3544	1080.5	23.00	0.29	tr_C9ZR23_	
HLA-DQA10501-DQB10201	48	NHSIRVGDVILLREG	VGDVILLRE	5	0.3535	1091.0	24.00	0.34	tr_C9ZR23_	
HLA-DQA10501-DQB10201	49	GGDDVPNAIAHQLLS	PNAIAHQLL	5	0.3534	1092.9	24.00	0.55	tr_C9ZR23_	
HLA-DQA10501-DQB10201	50	HSIRVGDVILLREGE	VGDVILLRE	4	0.3502	1131.4	24.00	0.32	tr_C9ZR23_	
HLA-DQA10501-DQB10201	51	RNPKVKWAQIDRDIS	VKWAQIDRD	4	0.3438	1211.5	25.00	0.54	tr_C9ZR23_	
HLA-DQA10501-DQB10201	52	KPRDVVAPQLSSDHT	PRDVVAPQL	1	0.3430	1222.8	26.00	0.61	tr_C9ZR23_	
HLA-DQA10501-DQB10201	53	RTNHSIRVGDVILLR	RVGDVILLR	6	0.3375	1297.0	27.00	0.28	tr_C9ZR23_	
HLA-DQA10501-DQB10201	54	VVLAATNPIVYIRTD	VVLAATNPI	0	0.3356	1323.9	27.00	0.32	tr_C9ZR23_	
HLA-DQA10501-DQB10201	55	TRTNHSIRVGDVILL	IRVGDVILL	6	0.3334	1356.0	28.00	0.32	tr_C9ZR23_	
HLA-DQA10501-DQB10201	56	KRNPKVKWAQIDRDI	VKWAQIDRD	5	0.3297	1412.0	28.00	0.67	tr_C9ZR23_	
HLA-DQA10501-DQB10201	57	LTFSLTAIKAARDDI	LTAIKAARD	4	0.3266	1459.6	29.00	0.34	tr_C9ZR23_	
HLA-DQA10501-DQB10201	58	HQLLSVDDSCASIVG	QLLSVDDSC	1	0.3243	1497.2	30.00	0.41	tr_C9ZR23_	
HLA-DQA10501-DQB10201	59	IRTDNLDGELDLKPR	TDNLDGELD	2	0.3228	1521.1	30.00	0.38	tr_C9ZR23_	
HLA-DQA10501-DQB10201	60	DPSPMINCFDGVAEF	NCFDGVAEF	6	0.3187	1589.7	31.00	0.28	tr_C9ZR23_	
HLA-DQA10501-DQB10201	61	NPKVKWAQIDRDISK	VKWAQIDRD	3	0.3179	1604.7	31.00	0.50	tr_C9ZR23_	
HLA-DQA10501-DQB10201	62	LLREGEDIPCDVVVL	EGEDIPCDV	3	0.3174	1611.8	31.00	0.38	tr_C9ZR23_	

HLA-DQA10501-DQB10201	63	TKTAICLAVYTGEDT	LAVYTGEDT	6	0.3131	1688.8	32.00	0.57	tr_C9ZR23_
HLA-DQA10501-DQB10201	64	NKRNPKVKWAQIDRD	VKWAQIDRD	6	0.3115	1718.0	32.00	0.77	tr_C9ZR23_
HLA-DQA10501-DQB10201	65	VAEFFFSRSPAAETV	FFFSRSPAA	3	0.3018	1909.0	35.00	0.34	tr_C9ZR23_
HLA-DQA10501-DQB10201	66	LREGEDIPCDVVVLA	EGEDIPCDV	2	0.3013	1920.1	35.00	0.38	tr_C9ZR23_
HLA-DQA10501-DQB10201	67	PRDVVAPQLSSDHTG	PRDVVAPQL	0	0.3007	1932.0	35.00	0.51	tr_C9ZR23_
HLA-DQA10501-DQB10201	68	TFSLTAIKAARDDIK	LTAIKAARD	3	0.2989	1969.4	35.00	0.34	tr_C9ZR23_
HLA-DQA10501-DQB10201	69	ALTFSLTAIKAARDD	LTAIKAARD	5	0.2978	1993.0	36.00	0.36	tr_C9ZR23_
HLA-DQA10501-DQB10201	70	TAICLAVYTGEDTKC	LAVYTGEDT	4	0.2971	2009.0	36.00	0.62	tr_C9ZR23_
HLA-DQA10501-DQB10201	71	GVAEFFFSRSPAAET	FFFSRSPAA	4	0.2963	2026.0	36.00	0.38	tr_C9ZR23_
HLA-DQA10501-DQB10201	72	KTAICLAVYTGEDTK	LAVYTGEDT	5	0.2961	2029.5	36.00	0.60	tr_C9ZR23_
HLA-DQA10501-DQB10201	73	AEFFFSRSPAAETVA	FFFSRSPAA	2	0.2938	2081.9	37.00	0.32	tr_C9ZR23_
HLA-DQA10501-DQB10201	74	REGEDIPCDVVVLAA	EGEDIPCDV	1	0.2914	2136.5	37.00	0.34	tr_C9ZR23_
HLA-DQA10501-DQB10201	75	AICLAVYTGEDTKCC	LAVYTGEDT	3	0.2874	2230.1	38.00	0.63	tr_C9ZR23_
HLA-DQA10501-DQB10201	76	LALTFSLTAIKAARD	TFSLTAIKA	3	0.2872	2235.1	38.00	0.41	tr_C9ZR23_
HLA-DQA10501-DQB10201	77	ETRTNHSIRVGDVIL	HSIRVGDVI	5	0.2850	2288.9	39.00	0.37	tr_C9ZR23_
HLA-DQA10501-DQB10201	78	DGVAEFFFSRSPAAE	FFFSRSPAA	5	0.2813	2382.7	40.00	0.45	tr_C9ZR23_
HLA-DQA10501-DQB10201	79	ICLAVYTGEDTKCCL	LAVYTGEDT	2	0.2769	2498.7	41.00	0.63	tr_C9ZR23_
HLA-DQA10501-DQB10201	80	EFFFSRSPAAETVAA	FFFSRSPAA	1	0.2764	2513.7	41.00	0.30	tr_C9ZR23_
HLA-DQA10501-DQB10201	81	RTDNLDGELDLKPRD	TDNLDGELD	1	0.2735	2591.9	42.00	0.44	tr_C9ZR23_
HLA-DQA10501-DQB10201	82	MRVSLSENNILPQSC	VSLSENNIL	2	0.2727	2615.5	42.00	0.29	tr_C9ZR23_
HLA-DQA10501-DQB10201	83	QLLSVDDSCASIVGK	QLLSVDDSC	0	0.2708	2670.6	43.00	0.43	tr_C9ZR23_
HLA-DQA10501-DQB10201	84	SDPSPMINCFDGVAE	PMINCFDGV	4	0.2698	2697.9	43.00	0.35	tr_C9ZR23_
HLA-DQA10501-DQB10201	85	AAETVAANNSAPMRV	ANNSAPMRV	6	0.2693	2714.0	43.00	0.29	tr_C9ZR23_
HLA-DQA10501-DQB10201	86	FSLTAIKAARDDIKR	LTAIKAARD	2	0.2691	2718.7	43.00	0.38	tr_C9ZR23_
HLA-DQA10501-DQB10201	87	VLAATNPIVYIRTDN	LAATNPIVY	1	0.2686	2734.3	43.00	0.36	tr_C9ZR23_
HLA-DQA10501-DQB10201	88	APMRVSLSENNILPQ	VSLSENNIL	4	0.2655	2827.1	44.00	0.31	tr_C9ZR23_
HLA-DQA10501-DQB10201	89	LLPLALTFSLTAIKA	TFSLTAIKA	6	0.2607	2979.6	45.00	0.70	tr_C9ZR23_
HLA-DQA10501-DQB10201	90	LGQMRVTCSDPSPMI	MRVTCSDPS	3	0.2602	2995.4	45.00	0.32	tr_C9ZR23_
HLA-DQA10501-DQB10201	91	SAPMRVSLSENNILP	VSLSENNIL	5	0.2578	3073.8	46.00	0.35	tr_C9ZR23_
HLA-DQA10501-DQB10201	92	LPLALTFSLTAIKAA	TFSLTAIKA	5	0.2574	3088.1	46.00	0.65	tr_C9ZR23_
HLA-DQA10501-DQB10201	93	PMRVSLSENNILPQS	VSLSENNIL	3	0.2562	3126.3	46.00	0.31	tr_C9ZR23_
HLA-DQA10501-DQB10201	94	KLGQMRVTCSDPSPM	MRVTCSDPS	4	0.2553	3156.9	47.00	0.35	tr_C9ZR23_
HLA-DQA10501-DQB10201	95	EGEDIPCDVVVLAAT	EGEDIPCDV	0	0.2553	3158.2	47.00	0.26	tr_C9ZR23_
HLA-DQA10501-DQB10201	96	RVSLSENNILPQSCV	VSLSENNIL	1	0.2545	3185.8	47.00	0.28	tr_C9ZR23_
HLA-DQA10501-DQB10201	97	HTGGDDVPNAIAHQL	VPNAIAHQL	6	0.2540	3200.6	47.00	0.62	tr_C9ZR23_
HLA-DQA10501-DQB10201	98	FFFSRSPAAETVAAN	FFSRSPAAE	1	0.2503	3334.3	48.00	0.23	tr_C9ZR23_
HLA-DQA10501-DQB10201	99	EDIPCDVVVLAATNP	VVVLAATNP	6	0.2487	3392.8	49.00	0.36	tr_C9ZR23_
HLA-DQA10501-DQB10201	100	SPAAETVAANNSAPM	VAANNSAPM	6	0.2477	3427.2	49.00	0.46	tr_C9ZR23_
HLA-DQA10501-DQB10201	101	AETVAANNSAPMRVS	VAANNSAPM	3	0.2451	3526.5	50.00	0.34	tr_C9ZR23_
HLA-DQA10501-DQB10201	102	GQMRVTCSDPSPMIN	MRVTCSDPS	2	0.2439	3571.8	50.00	0.33	tr_C9ZR23_
HLA-DQA10501-DQB10201	103	CLAVYTGEDTKCCLN	LAVYTGEDT	1	0.2437	3578.2	50.00	0.67	tr_C9ZR23_
HLA-DQA10501-DQB10201	104	NSAPMRVSLSENNIL	VSLSENNIL	6	0.2436	3582.4	50.00	0.38	tr_C9ZR23_
HLA-DQA10501-DQB10201	105	TDNLDGELDLKPRDV	TDNLDGELD	0	0.2432	3598.0	50.00	0.31	tr_C9ZR23_
HLA-DQA10501-DQB10201	106	PLALTFSLTAIKAAR	TFSLTAIKA	4	0.2426	3621.8	50.00	0.62	tr_C9ZR23_
HLA-DQA10501-DQB10201	107	KKERKVLNREAMTWE	VLNREAMTW	5	0.2408	3694.2	55.00	0.50	tr_C9ZR23_
HLA-DQA10501-DQB10201	108	VSLSENNILPQSCVL	ENNILPQSC	4	0.2395	3747.1	55.00	0.27	tr_C9ZR23_
HLA-DQA10501-DQB10201	109	CSDPSPMINCFDGVA	PMINCFDGV	5	0.2374	3831.8	55.00	0.41	tr_C9ZR23_
HLA-DQA10501-DQB10201	110	WETRTNHSIRVGDVI	HSIRVGDVI	6	0.2353	3919.2	55.00	0.55	tr_C9ZR23_
HLA-DQA10501-DQB10201	111	PAAETVAANNSAPMR	VAANNSAPM	5	0.2352	3924.4	55.00	0.41	tr_C9ZR23_
HLA-DQA10501-DQB10201	112	KERKVLNREAMTWET	VLNREAMTW	4	0.2340	3974.4	55.00	0.46	tr_C9ZR23_

HLA-DQA10501-DQB10201	113	TCSDPSPMINCFDGV	PMINCFDGV	6	0.2310	4104.8	55.00	0.44	tr_C9ZR23_
HLA-DQA10501-DQB10201	114	ERKVLNREAMTWETR	VLNREAMTW	3	0.2301	4147.8	55.00	0.42	tr_C9ZR23_
HLA-DQA10501-DQB10201	115	RKVLNREAMTWETRT	VLNREAMTW	2	0.2290	4197.0	55.00	0.36	tr_C9ZR23_
HLA-DQA10501-DQB10201	116	ETVAANNSAPMRVSL	ANNSAPMRV	4	0.2286	4213.0	55.00	0.34	tr_C9ZR23_
HLA-DQA10501-DQB10201	117	GKLGQMRVTCSDPSP	MRVTCSDPS	5	0.2285	4218.9	55.00	0.43	tr_C9ZR23_
HLA-DQA10501-DQB10201	118	SLTAIKAARDDIKRH	LTAIKAARD	1	0.2266	4308.9	55.00	0.38	tr_C9ZR23_
HLA-DQA10501-DQB10201	119	NTKTAICLAVYTGED	CLAVYTGED	6	0.2251	4375.7	55.00	0.28	tr_C9ZR23_
HLA-DQA10501-DQB10201	120	LSSDHTGGDDVPNAI	TGGDDVPNA	5	0.2249	4386.6	55.00	0.21	tr_C9ZR23_
HLA-DQA10501-DQB10201	121	NKKERKVLNREAMTW	VLNREAMTW	6	0.2219	4533.1	60.00	0.57	tr_C9ZR23_
HLA-DQA10501-DQB10201	122	QMRVTCSDPSPMINC	MRVTCSDPS	1	0.2194	4654.4	60.00	0.37	tr_C9ZR23_
HLA-DQA10501-DQB10201	123	GEDIPCDVVVLAATN	DVVVLAATN	6	0.2130	4990.0	60.00	0.17	tr_C9ZR23_
HLA-DQA10501-DQB10201	124	SSDHTGGDDVPNAIA	GGDDVPNAI	5	0.2126	5011.8	60.00	0.22	tr_C9ZR23_
HLA-DQA10501-DQB10201	125	VAANNSAPMRVSLSE	ANNSAPMRV	2	0.2105	5128.7	60.00	0.35	tr_C9ZR23_
HLA-DQA10501-DQB10201	126	NNSAPMRVSLSENNI	RVSLSENNI	6	0.2086	5231.4	60.00	0.40	tr_C9ZR23_
HLA-DQA10501-DQB10201	127	RVTCSDPSPMINCFD	PSPMINCFD	6	0.2061	5375.6	65.00	0.46	tr_C9ZR23_
HLA-DQA10501-DQB10201	128	KVLNREAMTWETRTN	VLNREAMTW	1	0.2057	5400.7	65.00	0.38	tr_C9ZR23_
HLA-DQA10501-DQB10201	129	VGKLGQMRVTCSDPS	MRVTCSDPS	6	0.2046	5466.3	65.00	0.42	tr_C9ZR23_
HLA-DQA10501-DQB10201	130	QLSSDHTGGDDVPNA	TGGDDVPNA	6	0.2039	5507.1	65.00	0.28	tr_C9ZR23_
HLA-DQA10501-DQB10201	131	TVAANNSAPMRVSLS	ANNSAPMRV	3	0.2025	5591.5	65.00	0.38	tr_C9ZR23_
HLA-DQA10501-DQB10201	132	AANNSAPMRVSLSEN	PMRVSLSEN	6	0.2004	5719.4	65.00	0.28	tr_C9ZR23_
HLA-DQA10501-DQB10201	133	LAVYTGEDTKCCLNK	LAVYTGEDT	0	0.1971	5926.6	65.00	0.65	tr_C9ZR23_
HLA-DQA10501-DQB10201	134	FFSRSPAAETVAANN	FFSRSPAAE	0	0.1935	6161.0	65.00	0.20	tr_C9ZR23_
HLA-DQA10501-DQB10201	135	SDHTGGDDVPNAIAH	GGDDVPNAI	4	0.1930	6194.1	65.00	0.24	tr_C9ZR23_
HLA-DQA10501-DQB10201	136	LTAIKAARDDIKRHK	AIKAARDDI	2	0.1927	6217.6	65.00	0.40	tr_C9ZR23_
HLA-DQA10501-DQB10201	137	SLSENNILPQSCVLK	ENNILPQSC	3	0.1894	6443.8	70.00	0.32	tr_C9ZR23_
HLA-DQA10501-DQB10201	138	RSPAAETVAANNSAP	AETVAANNS	4	0.1879	6545.2	70.00	0.34	tr_C9ZR23_
HLA-DQA10501-DQB10201	139	ANNSAPMRVSLSENN	PMRVSLSEN	5	0.1876	6568.3	70.00	0.31	tr_C9ZR23_
HLA-DQA10501-DQB10201	140	VLKNTKTAICLAVYT	KTAICLAVY	5	0.1875	6572.7	70.00	0.41	tr_C9ZR23_
HLA-DQA10501-DQB10201	141	STLLPLALTFSLTAI	ALTFSLTAI	6	0.1872	6597.7	70.00	0.32	tr_C9ZR23_
HLA-DQA10501-DQB10201	142	KNTKTAICLAVYTGE	KTAICLAVY	3	0.1864	6657.5	70.00	0.34	tr_C9ZR23_
HLA-DQA10501-DQB10201	143	VTCSDPSPMINCFDG	PSPMINCFD	5	0.1803	7111.7	70.00	0.46	tr_C9ZR23_
HLA-DQA10501-DQB10201	144	DHTGGDDVPNAIAHQ	TGGDDVPNA	2	0.1797	7154.3	70.00	0.25	tr_C9ZR23_
HLA-DQA10501-DQB10201	145	MRVTCSDPSPMINCF	VTCSDPSPM	2	0.1785	7249.3	70.00	0.34	tr_C9ZR23_
HLA-DQA10501-DQB10201	146	DNLDGELDLKPRDVV	NLDGELDLK	1	0.1778	7300.8	70.00	0.23	tr_C9ZR23_
HLA-DQA10501-DQB10201	147	FSRSPAAETVAANNS	AETVAANNS	6	0.1777	7310.3	70.00	0.28	tr_C9ZR23_
HLA-DQA10501-DQB10201	148	PQLSSDHTGGDDVPN	SDHTGGDDV	4	0.1777	7313.6	70.00	0.43	tr_C9ZR23_
HLA-DQA10501-DQB10201	149	SRSPAAETVAANNSA	AETVAANNS	5	0.1769	7378.1	70.00	0.30	tr_C9ZR23_
HLA-DQA10501-DQB10201	150	VLNREAMTWETRTNH	VLNREAMTW	0	0.1766	7401.0	70.00	0.32	tr_C9ZR23_
HLA-DQA10501-DQB10201	151	CVLKNTKTAICLAVY	KTAICLAVY	6	0.1765	7410.0	70.00	0.55	tr_C9ZR23_
HLA-DQA10501-DQB10201	152	APQLSSDHTGGDDVP	SDHTGGDDV	5	0.1754	7495.3	70.00	0.44	tr_C9ZR23_
HLA-DQA10501-DQB10201	153	LKNTKTAICLAVYTG	KTAICLAVY	4	0.1751	7517.5	70.00	0.40	tr_C9ZR23_
HLA-DQA10501-DQB10201	154	RDVVAPQLSSDHTGG	RDVVAPQLS	0	0.1684	8085.7	75.00	0.43	tr_C9ZR23_
HLA-DQA10501-DQB10201	155	IVGKLGQMRVTCSDP	QMRVTCSDP	6	0.1647	8410.4	75.00	0.29	tr_C9ZR23_
HLA-DQA10501-DQB10201	156	LSENNILPQSCVLKN	ENNILPQSC	2	0.1639	8487.0	75.00	0.36	tr_C9ZR23_
HLA-DQA10501-DQB10201	157	TLLPLALTFSLTAIK	ALTFSLTAI	5	0.1609	8769.5	75.00	0.24	tr_C9ZR23_
HLA-DQA10501-DQB10201	158	LLSVDDSCASIVGKL	LLSVDDSCA	0	0.1572	9131.2	75.00	0.40	tr_C9ZR23_
HLA-DQA10501-DQB10201	159	DSCASIVGKLGQMRV	VGKLGQMRV	6	0.1534	9510.4	80.00	0.46	tr_C9ZR23_
HLA-DQA10501-DQB10201	160	SIVGKLGQMRVTCSD	VGKLGQMRV	2	0.1489	9980.4	80.00	0.28	tr_C9ZR23_
HLA-DQA10501-DQB10201	161	TAIKAARDDIKRHKQ	AIKAARDDI	1	0.1485	10028.5	80.00	0.51	tr_C9ZR23_
HLA-DQA10501-DQB10201	162	TWETRTNHSIRVGDV	NHSIRVGDV	6	0.1466	10235.5	80.00	0.55	tr_C9ZR23_

HLA-DQA10501-DQB10201	163	ASIVGKLGQMRVTCS	VGKLGQMRV	3	0.1417	10788.0	80.00	0.34	tr_C9ZR23_
HLA-DQA10501-DQB10201	164	SCASIVGKLGQMRVT	VGKLGQMRV	5	0.1408	10897.6	80.00	0.44	tr_C9ZR23_
HLA-DQA10501-DQB10201	165	NLDGELDLKPRDVVA	ELDLKPRDV	4	0.1408	10898.7	80.00	0.37	tr_C9ZR23_
HLA-DQA10501-DQB10201	166	CASIVGKLGQMRVTC	VGKLGQMRV	4	0.1375	11296.3	80.00	0.43	tr_C9ZR23_
HLA-DQA10501-DQB10201	167	SENNILPQSCVLKNT	ENNILPQSC	1	0.1342	11703.5	85.00	0.41	tr_C9ZR23_
HLA-DQA10501-DQB10201	168	VAPQLSSDHTGGDDV	SDHTGGDDV	6	0.1317	12029.8	85.00	0.43	tr_C9ZR23_
HLA-DQA10501-DQB10201	169	LNREAMTWETRTNHS	EAMTWETRT	3	0.1298	12276.8	85.00	0.31	tr_C9ZR23_
HLA-DQA10501-DQB10201	170	YNKKERKVLNREAMT	RKVLNREAM	5	0.1237	13114.7	85.00	0.38	tr_C9ZR23_
HLA-DQA10501-DQB10201	171	DVVAPQLSSDHTGGD	APQLSSDHT	3	0.1218	13385.1	85.00	0.40	tr_C9ZR23_
HLA-DQA10501-DQB10201	172	NREAMTWETRTNHSI	EAMTWETRT	2	0.1208	13525.4	85.00	0.29	tr_C9ZR23_
HLA-DQA10501-DQB10201	173	AIKAARDDIKRHKQD	AIKAARDDI	0	0.1207	13542.3	85.00	0.46	tr_C9ZR23_
HLA-DQA10501-DQB10201	174	DGELDLKPRDVVAPQ	ELDLKPRDV	2	0.1183	13903.6	85.00	0.49	tr_C9ZR23_
HLA-DQA10501-DQB10201	175	SCVLKNTKTAICLAV	TKTAICLAV	6	0.1173	14057.6	85.00	0.39	tr_C9ZR23_
HLA-DQA10501-DQB10201	176	LDGELDLKPRDVVAP	ELDLKPRDV	3	0.1165	14174.6	85.00	0.50	tr_C9ZR23_
HLA-DQA10501-DQB10201	177	VVAPQLSSDHTGGDD	APQLSSDHT	2	0.1145	14493.3	85.00	0.39	tr_C9ZR23_
HLA-DQA10501-DQB10201	178	EAMTWETRTNHSIRV	TRTNHSIRV	6	0.1082	15503.8	90.00	0.31	tr_C9ZR23_
HLA-DQA10501-DQB10201	179	ENNILPQSCVLKNTK	NILPQSCVL	2	0.1075	15629.4	90.00	0.38	tr_C9ZR23_
HLA-DQA10501-DQB10201	180	LSVDDSCASIVGKLG	VDDSCASIV	2	0.1060	15885.0	90.00	0.34	tr_C9ZR23_
HLA-DQA10501-DQB10201	181	LNKRNPKVKWAQIDR	PKVKWAQID	5	0.1054	15983.8	90.00	0.47	tr_C9ZR23_
HLA-DQA10501-DQB10201	182	REAMTWETRTNHSIR	WETRTNHSI	5	0.1044	16156.1	90.00	0.34	tr_C9ZR23_
HLA-DQA10501-DQB10201	183	DDSCASIVGKLGQMR	IVGKLGQMR	6	0.1042	16200.6	90.00	0.29	tr_C9ZR23_
HLA-DQA10501-DQB10201	184	QSCVLKNTKTAICLA	NTKTAICLA	6	0.1006	16843.5	90.00	0.56	tr_C9ZR23_
HLA-DQA10501-DQB10201	185	IYNKKERKVLNREAM	RKVLNREAM	6	0.0996	17012.2	90.00	0.60	tr_C9ZR23_
HLA-DQA10501-DQB10201	186	VDDSCASIVGKLGQM	SIVGKLGQM	6	0.0982	17276.3	90.00	0.31	tr_C9ZR23_
HLA-DQA10501-DQB10201	187	SVDDSCASIVGKLGQ	VDDSCASIV	1	0.0950	17895.4	90.00	0.34	tr_C9ZR23_
HLA-DQA10501-DQB10201	188	MTWETRTNHSIRVGD	TRTNHSIRV	4	0.0929	18299.0	90.00	0.32	tr_C9ZR23_
HLA-DQA10501-DQB10201	189	AVYTGEDTKCCLNKR	VYTGEDTKC	1	0.0910	18674.6	95.00	0.38	tr_C9ZR23_
HLA-DQA10501-DQB10201	190	AMTWETRTNHSIRVG	TRTNHSIRV	5	0.0886	19169.0	95.00	0.32	tr_C9ZR23_
HLA-DQA10501-DQB10201	191	ARDDIKRHKQDAIYN	KRHKQDAIY	5	0.0876	19383.8	95.00	0.35	tr_C9ZR23_
HLA-DQA10501-DQB10201	192	CLNKRNPKVKWAQID	PKVKWAQID	6	0.0836	20236.3	95.00	0.62	tr_C9ZR23_
HLA-DQA10501-DQB10201	193	AARDDIKRHKQDAIY	KRHKQDAIY	6	0.0769	21747.9	95.00	0.48	tr_C9ZR23_
HLA-DQA10501-DQB10201	194	NNILPQSCVLKNTKT	NILPQSCVL	1	0.0747	22276.1	95.00	0.53	tr_C9ZR23_
HLA-DQA10501-DQB10201	195	RDDIKRHKQDAIYNK	KRHKQDAIY	4	0.0745	22330.7	95.00	0.31	tr_C9ZR23_
HLA-DQA10501-DQB10201	196	PQSCVLKNTKTAICL	KNTKTAICL	6	0.0667	24286.5	95.00	0.34	tr_C9ZR23_
HLA-DQA10501-DQB10201	197	DDIKRHKQDAIYNKK	KRHKQDAIY	3	0.0658	24521.8	95.00	0.28	tr_C9ZR23_
HLA-DQA10501-DQB10201	198	DIKRHKQDAIYNKKE	RHKQDAIYN	3	0.0631	25273.9	100.00	0.23	tr_C9ZR23_
HLA-DQA10501-DQB10201	199	NILPQSCVLKNTKTA	NILPQSCVL	0	0.0628	25341.2	100.00	0.50	tr_C9ZR23_
HLA-DQA10501-DQB10201	200	VYTGEDTKCCLNKRN	VYTGEDTKC	0	0.0565	27124.1	100.00	0.39	tr_C9ZR23_
HLA-DQA10501-DQB10201	201	IKAARDDIKRHKQDA	IKAARDDIK	0	0.0562	27225.0	100.00	0.56	tr_C9ZR23_
HLA-DQA10501-DQB10201	202	AIYNKKERKVLNREA	ERKVLNREA	6	0.0556	27389.9	100.00	0.65	tr_C9ZR23_
HLA-DQA10501-DQB10201	203	IKRHKQDAIYNKKER	RHKQDAIYN	2	0.0536	27985.1	100.00	0.23	tr_C9ZR23_
HLA-DQA10501-DQB10201	204	KAARDDIKRHKQDAI	IKRHKQDAI	6	0.0536	27998.1	100.00	0.57	tr_C9ZR23_
HLA-DQA10501-DQB10201	205	LPQSCVLKNTKTAIC	VLKNTKTAI	5	0.0506	28929.3	100.00	0.38	tr_C9ZR23_
HLA-DQA10501-DQB10201	206	KRHKQDAIYNKKERK	RHKQDAIYN	1	0.0445	30896.1	100.00	0.26	tr_C9ZR23_
HLA-DQA10501-DQB10201	207	ILPQSCVLKNTKTAI	VLKNTKTAI	6	0.0438	31124.9	100.00	0.47	tr_C9ZR23_
HLA-DQA10501-DQB10201	208	RHKQDAIYNKKERKV	RHKQDAIYN	0	0.0351	34183.4	100.00	0.20	tr_C9ZR23_
HLA-DQA10501-DQB10201	209	YTGEDTKCCLNKRNP	TGEDTKCCL	1	0.0335	34814.2	100.00	0.48	tr_C9ZR23_
HLA-DQA10501-DQB10201	210	DAIYNKKERKVLNRE	KERKVLNRE	6	0.0332	34893.0	100.00	0.55	tr_C9ZR23_
HLA-DQA10501-DQB10201	211	HKQDAIYNKKERKVL	AIYNKKERK	4	0.0302	36061.4	100.00	0.27	tr_C9ZR23_
HLA-DQA10501-DQB10201	212	KQDAIYNKKERKVLN	AIYNKKERK	3	0.0270	37322.1	100.00	0.30	tr_C9ZR23_

HLA-DQA10501-DQB10201	213	CCLNKRNPKVKWAQI	NPKVKWAQI	6	0.0246	38301.5	100.00	0.50	tr_C9ZR23_
HLA-DQA10501-DQB10201	214	QDAIYNKKERKVLNR	AIYNKKERK	2	0.0246	38321.8	100.00	0.32	tr_C9ZR23_
HLA-DQA10501-DQB10201	215	TGEDTKCCLNKRNPK	TGEDTKCCL	0	0.0227	39127.5	100.00	0.46	tr_C9ZR23_
HLA-DQA10501-DQB10201	216	TKCCLNKRNPKVKWA	KRNPKVKWA	6	0.0134	43244.3	100.00	0.31	tr_C9ZR23_
HLA-DQA10501-DQB10201	217	KCCLNKRNPKVKWAQ	KRNPKVKWA	5	0.0127	43572.6	100.00	0.32	tr_C9ZR23_
HLA-DQA10501-DQB10201	218	GEDTKCCLNKRNPKV	GEDTKCCLN	0	0.0126	43608.9	100.00	0.19	tr_C9ZR23_
HLA-DQA10501-DQB10201	219	DTKCCLNKRNPKVKW	NKRNPKVKW	6	0.0111	44354.1	100.00	0.29	tr_C9ZR23_
HLA-DQA10501-DQB10201	220	EDTKCCLNKRNPKVK	DTKCCLNKR	1	0.0101	44814.3	100.00	0.22	tr_C9ZR23_

Allele: HLA-DQA10501-DQB10201. Number of high binders 0. Number of weak binders 13. Number of peptides 220

Allele	pos	peptide	core	Of	1-log50k(aff)	affinity(nM)	%Rank	Relia	Identity	Bind Level
HLA-DQA10501-DQB10201	1	DMSKYYFALVIENDV	YFALVIEND	5	0.6509	43.7	0.50	0.41	tr_C9ZR23_	WB
HLA-DQA10501-DQB10201	2	MSKYYFALVIENDVA	YFALVIEND	4	0.6473	45.4	0.60	0.35	tr_C9ZR23_	WB
HLA-DQA10501-DQB10201	3	SKYYFALVIENDVAM	YFALVIEND	3	0.6429	47.7	0.60	0.37	tr_C9ZR23_	WB
HLA-DQA10501-DQB10201	4	KYYFALVIENDVAMH	YFALVIEND	2	0.6259	57.3	0.80	0.37	tr_C9ZR23_	WB
HLA-DQA10501-DQB10201	5	CNTVEVVYDDVSQEM	VYDDVSQEM	6	0.6253	57.7	0.80	0.40	tr_C9ZR23_	WB
HLA-DQA10501-DQB10201	6	NTVEVVYDDVSQEMT	VYDDVSQEM	5	0.6164	63.5	1.00	0.37	tr_C9ZR23_	WB
HLA-DQA10501-DQB10201	7	YYFALVIENDVAMHH	YFALVIEND	1	0.6095	68.4	1.10	0.37	tr_C9ZR23_	WB
HLA-DQA10501-DQB10201	8	EWFVHYVFAFASEFK	YVFAFASEF	5	0.6072	70.1	1.20	0.45	tr_C9ZR23_	WB
HLA-DQA10501-DQB10201	9	TTAAVEVNGIKEEWF	VNGIKEEWF	6	0.5990	76.6	1.30	0.39	tr_C9ZR23_	WB
HLA-DQA10501-DQB10201	10	GERRCLAPVEAEEVQ	LAPVEAEEV	5	0.5978	77.6	1.30	0.55	tr_C9ZR23_	WB
HLA-DQA10501-DQB10201	11	NGERRCLAPVEAEEV	LAPVEAEEV	6	0.5934	81.4	1.40	0.56	tr_C9ZR23_	WB
HLA-DQA10501-DQB10201	12	EEWFVHYVFAFASEF	YVFAFASEF	6	0.5930	81.8	1.50	0.60	tr_C9ZR23_	WB
HLA-DQA10501-DQB10201	13	WFVHYVFAFASEFKT	YVFAFASEF	4	0.5927	82.0	1.50	0.42	tr_C9ZR23_	WB
HLA-DQA10501-DQB10201	14	TAAVEVNGIKEEWFV	VNGIKEEWF	5	0.5918	82.8	1.50	0.40	tr_C9ZR23_	WB
HLA-DQA10501-DQB10201	15	TVEVVYDDVSQEMTQ	VYDDVSQEM	4	0.5871	87.2	1.60	0.38	tr_C9ZR23_	WB
HLA-DQA10501-DQB10201	16	TDMSKYYFALVIEND	YFALVIEND	6	0.5870	87.2	1.60	0.62	tr_C9ZR23_	WB
HLA-DQA10501-DQB10201	17	ERRCLAPVEAEEVQG	LAPVEAEEV	4	0.5804	93.7	1.80	0.54	tr_C9ZR23_	WB
HLA-DQA10501-DQB10201	18	FVHYVFAFASEFKTM	YVFAFASEF	3	0.5726	101.9	2.00	0.44	tr_C9ZR23_	WB
HLA-DQA10501-DQB10201	19	RMSSKAAILAEVEHY	KAAILAEVE	4	0.5714	103.2	2.00	0.61	tr_C9ZR23_	WB
HLA-DQA10501-DQB10201	20	MENSVTILGVTAIED	ILGVTAIED	6	0.5656	110.0	2.50	0.70	tr_C9ZR23_	WB
HLA-DQA10501-DQB10201	21	AFESWEGYMESLLQF	WEGYMESLL	4	0.5648	110.9	2.50	0.50	tr_C9ZR23_	WB
HLA-DQA10501-DQB10201	22	MSSKAAILAEVEHYA	KAAILAEVE	3	0.5636	112.4	2.50	0.56	tr_C9ZR23_	WB
HLA-DQA10501-DQB10201	23	YFALVIENDVAMHHD	FALVIENDV	1	0.5634	112.6	2.50	0.40	tr_C9ZR23_	WB
HLA-DQA10501-DQB10201	24	AAVEVNGIKEEWFVH	VNGIKEEWF	4	0.5625	113.8	2.50	0.41	tr_C9ZR23_	WB
HLA-DQA10501-DQB10201	25	DAFESWEGYMESLLQ	WEGYMESLL	5	0.5618	114.6	2.50	0.50	tr_C9ZR23_	WB
HLA-DQA10501-DQB10201	26	LCNTVEVVYDDVSQE	TVEVVYDDV	3	0.5544	124.2	3.00	0.46	tr_C9ZR23_	WB
HLA-DQA10501-DQB10201	27	LDAFESWEGYMESLL	WEGYMESLL	6	0.5521	127.3	3.00	0.55	tr_C9ZR23_	WB
HLA-DQA10501-DQB10201	28	SSKAAILAEVEHYAV	KAAILAEVE	2	0.5518	127.6	3.00	0.56	tr_C9ZR23_	WB
HLA-DQA10501-DQB10201	29	RRCLAPVEAEEVQGS	LAPVEAEEV	3	0.5513	128.3	3.00	0.56	tr_C9ZR23_	WB
HLA-DQA10501-DQB10201	30	FESWEGYMESLLQFS	WEGYMESLL	3	0.5462	135.6	3.00	0.50	tr_C9ZR23_	WB
HLA-DQA10501-DQB10201	31	GERMSSKAAILAEVE	KAAILAEVE	6	0.5427	140.9	3.50	0.80	tr_C9ZR23_	WB
HLA-DQA10501-DQB10201	32	GTMRYQAASPDEVAL	YQAASPDEV	4	0.5426	141.0	3.50	0.63	tr_C9ZR23_	WB
HLA-DQA10501-DQB10201	33	NSVTILGVTAIEDKL	ILGVTAIED	4	0.5410	143.4	3.50	0.64	tr_C9ZR23_	WB
HLA-DQA10501-DQB10201	34	VLSLCNTVEVVYDDV	TVEVVYDDV	6	0.5401	145.0	3.50	0.67	tr_C9ZR23_	WB

HLA-DQA10501-DQB10201	35	LSLCNTVEVVYDDVS	TVEVVYDDV	5	0.5400	145.0	3.50	0.56	tr_C9ZR23_	WB
HLA-DQA10501-DQB10201	36	AVEVNGIKEEWFVHY	VNGIKEEWF	3	0.5386	147.3	3.50	0.47	tr_C9ZR23_	WB
HLA-DQA10501-DQB10201	37	SKAAILAEVEHYAVF	KAAILAEVE	1	0.5379	148.3	3.50	0.50	tr_C9ZR23_	WB
HLA-DQA10501-DQB10201	38	VGTMRYQAASPDEVA	YQAASPDEV	5	0.5370	149.8	3.50	0.62	tr_C9ZR23_	WB
HLA-DQA10501-DQB10201	39	VEVVYDDVSQEMTQS	VYDDVSQEM	3	0.5368	150.1	3.50	0.49	tr_C9ZR23_	WB
HLA-DQA10501-DQB10201	40	VHYVFAFASEFKTMG	YVFAFASEF	2	0.5365	150.6	3.50	0.44	tr_C9ZR23_	WB
HLA-DQA10501-DQB10201	41	ERMSSKAAILAEVEH	KAAILAEVE	5	0.5365	150.7	3.50	0.74	tr_C9ZR23_	WB
HLA-DQA10501-DQB10201	42	TMRYQAASPDEVALC	YQAASPDEV	3	0.5353	152.6	3.50	0.64	tr_C9ZR23_	WB
HLA-DQA10501-DQB10201	43	ENSVTILGVTAIEDK	ILGVTAIED	5	0.5350	153.1	3.50	0.68	tr_C9ZR23_	WB
HLA-DQA10501-DQB10201	44	SLCNTVEVVYDDVSQ	TVEVVYDDV	4	0.5345	153.9	3.50	0.50	tr_C9ZR23_	WB
HLA-DQA10501-DQB10201	45	KGADDRILEMALDEN	RILEMALDE	5	0.5336	155.5	3.50	0.44	tr_C9ZR23_	WB
HLA-DQA10501-DQB10201	46	RCLAPVEAEEVQGSC	LAPVEAEEV	2	0.5303	161.1	4.00	0.60	tr_C9ZR23_	WB
HLA-DQA10501-DQB10201	47	AVGTMRYQAASPDEV	YQAASPDEV	6	0.5262	168.4	4.00	0.61	tr_C9ZR23_	WB
HLA-DQA10501-DQB10201	48	SVTILGVTAIEDKLQ	ILGVTAIED	3	0.5241	172.3	4.00	0.65	tr_C9ZR23_	WB
HLA-DQA10501-DQB10201	49	GADDRILEMALDENS	RILEMALDE	4	0.5199	180.3	4.50	0.44	tr_C9ZR23_	WB
HLA-DQA10501-DQB10201	50	MRYQAASPDEVALCN	YQAASPDEV	2	0.5158	188.5	4.50	0.67	tr_C9ZR23_	WB
HLA-DQA10501-DQB10201	51	VTILGVTAIEDKLQD	ILGVTAIED	2	0.5115	197.5	5.00	0.65	tr_C9ZR23_	WB
HLA-DQA10501-DQB10201	52	HYVFAFASEFKTMGV	YVFAFASEF	1	0.5112	198.1	5.00	0.46	tr_C9ZR23_	WB
HLA-DQA10501-DQB10201	53	ASEFKTMGVIVEEKS	TMGVIVEEK	5	0.5108	198.9	5.00	0.60	tr_C9ZR23_	WB
HLA-DQA10501-DQB10201	54	FASEFKTMGVIVEEK	TMGVIVEEK	6	0.5093	202.1	5.00	0.70	tr_C9ZR23_	WB
HLA-DQA10501-DQB10201	55	ADDRILEMALDENSS	RILEMALDE	3	0.5055	210.8	5.00	0.43	tr_C9ZR23_	WB
HLA-DQA10501-DQB10201	56	ESWEGYMESLLQFSK	WEGYMESLL	2	0.5034	215.5	5.50	0.50	tr_C9ZR23_	WB
HLA-DQA10501-DQB10201	57	AEKRLTRNELDEFLE	TRNELDEFL	5	0.5007	221.9	5.50	0.56	tr_C9ZR23_	WB
HLA-DQA10501-DQB10201	58	EVVYDDVSQEMTQSG	VYDDVSQEM	2	0.5001	223.3	5.50	0.52	tr_C9ZR23_	WB
HLA-DQA10501-DQB10201	59	VEVNGIKEEWFVHYV	VNGIKEEWF	2	0.5001	223.3	5.50	0.52	tr_C9ZR23_	WB
HLA-DQA10501-DQB10201	60	KAAILAEVEHYAVFG	KAAILAEVE	0	0.4970	231.1	6.00	0.35	tr_C9ZR23_	WB
HLA-DQA10501-DQB10201	61	VAEKRLTRNELDEFL	TRNELDEFL	6	0.4947	236.7	6.00	0.54	tr_C9ZR23_	WB
HLA-DQA10501-DQB10201	62	SEFKTMGVIVEEKST	TMGVIVEEK	4	0.4936	239.6	6.00	0.57	tr_C9ZR23_	WB
HLA-DQA10501-DQB10201	63	CLAPVEAEEVQGSCG	LAPVEAEEV	1	0.4916	244.9	6.00	0.61	tr_C9ZR23_	WB
HLA-DQA10501-DQB10201	64	DDRILEMALDENSST	RILEMALDE	2	0.4890	251.8	6.50	0.42	tr_C9ZR23_	WB
HLA-DQA10501-DQB10201	65	ATTAAVEVNGIKEEW	EVNGIKEEW	6	0.4843	265.0	6.50	0.37	tr_C9ZR23_	WB
HLA-DQA10501-DQB10201	66	EVNGIKEEWFVHYVF	VNGIKEEWF	1	0.4826	269.8	7.00	0.43	tr_C9ZR23_	WB
HLA-DQA10501-DQB10201	67	EFKTMGVIVEEKSTN	TMGVIVEEK	3	0.4761	289.5	7.50	0.61	tr_C9ZR23_	WB
HLA-DQA10501-DQB10201	68	TILGVTAIEDKLQDH	ILGVTAIED	1	0.4754	291.8	7.50	0.66	tr_C9ZR23_	WB
HLA-DQA10501-DQB10201	69	VKGADDRILEMALDE	RILEMALDE	6	0.4732	298.9	7.50	0.73	tr_C9ZR23_	WB
HLA-DQA10501-DQB10201	70	NSSIVEDLGQVDYVL	EDLGQVDYV	5	0.4708	306.7	8.00	0.29	tr_C9ZR23_	WB
HLA-DQA10501-DQB10201	71	RYQAASPDEVALCNG	YQAASPDEV	1	0.4704	308.1	8.00	0.74	tr_C9ZR23_	WB
HLA-DQA10501-DQB10201	72	SIVEDLGQVDYVLSD	EDLGQVDYV	3	0.4699	309.8	8.00	0.28	tr_C9ZR23_	WB
HLA-DQA10501-DQB10201	73	YVFAFASEFKTMGVI	VFAFASEFK	1	0.4695	310.9	8.00	0.49	tr_C9ZR23_	WB
HLA-DQA10501-DQB10201	74	SSIVEDLGQVDYVLS	EDLGQVDYV	4	0.4667	320.5	8.00	0.28	tr_C9ZR23_	WB
HLA-DQA10501-DQB10201	75	KRLTRNELDEFLEKV	TRNELDEFL	3	0.4637	331.2	8.50	0.49	tr_C9ZR23_	WB
HLA-DQA10501-DQB10201	76	EKRLTRNELDEFLEK	TRNELDEFL	4	0.4632	333.0	8.50	0.54	tr_C9ZR23_	WB
HLA-DQA10501-DQB10201	77	SWEGYMESLLQFSKG	WEGYMESLL	1	0.4606	342.3	8.50	0.49	tr_C9ZR23_	WB
HLA-DQA10501-DQB10201	78	RNSSIVEDLGQVDYV	EDLGQVDYV	6	0.4581	351.9	9.00	0.34	tr_C9ZR23_	WB
HLA-DQA10501-DQB10201	79	DRILEMALDENSSTG	ILEMALDEN	2	0.4545	365.7	9.00	0.40	tr_C9ZR23_	WB
HLA-DQA10501-DQB10201	80	KEEWFVHYVFAFASE	HYVFAFASE	6	0.4545	365.9	9.00	0.31	tr_C9ZR23_	WB
HLA-DQA10501-DQB10201	81	VMADVQYGDAAFPTG	VQYGDAAFP	4	0.4521	375.3	9.50	0.27	tr_C9ZR23_	WB
HLA-DQA10501-DQB10201	82	FKTMGVIVEEKSTNA	TMGVIVEEK	2	0.4520	375.8	9.50	0.61	tr_C9ZR23_	WB
HLA-DQA10501-DQB10201	83	GVMADVQYGDAAFPT	VQYGDAAFP	5	0.4496	385.6	9.50	0.29	tr_C9ZR23_	WB
HLA-DQA10501-DQB10201	84	DKTGTLTQNVMEFLF	LTQNVMEFL	5	0.4470	396.8	10.00	0.39	tr_C9ZR23_	

HLA-DQA10501-DQB10201	85	VVYDDVSQEMTQSGL	VYDDVSQEM	1	0.4455	403.1	10.00	0.57	tr_C9ZR23_
HLA-DQA10501-DQB10201	86	SLLQFSKGVMADVQY	SKGVMADVQ	5	0.4438	410.5	11.00	0.48	tr_C9ZR23_
HLA-DQA10501-DQB10201	87	RLTRNELDEFLEKVR	TRNELDEFL	2	0.4414	421.5	11.00	0.54	tr_C9ZR23_
HLA-DQA10501-DQB10201	88	AAILAEVEHYAVFGL	AILAEVEHY	1	0.4414	421.6	11.00	0.41	tr_C9ZR23_
HLA-DQA10501-DQB10201	89	INGERRCLAPVEAEE	RRCLAPVEA	4	0.4402	427.1	11.00	0.43	tr_C9ZR23_
HLA-DQA10501-DQB10201	90	FALVIENDVAMHHDG	FALVIENDV	0	0.4392	431.8	11.00	0.44	tr_C9ZR23_
HLA-DQA10501-DQB10201	91	IVEDLGQVDYVLSDK	EDLGQVDYV	2	0.4390	432.8	11.00	0.28	tr_C9ZR23_
HLA-DQA10501-DQB10201	92	KTGTLTQNVMEFLFA	LTQNVMEFL	4	0.4366	444.2	11.00	0.34	tr_C9ZR23_
HLA-DQA10501-DQB10201	93	RKVPRRVLEQTPALY	VLEQTPALY	6	0.4365	444.3	11.00	0.46	tr_C9ZR23_
HLA-DQA10501-DQB10201	94	LAPVEAEEVQGSCGE	LAPVEAEEV	0	0.4329	462.3	12.00	0.54	tr_C9ZR23_
HLA-DQA10501-DQB10201	95	VEDLGQVDYVLSDKT	VDYVLSDKT	6	0.4327	463.3	12.00	0.25	tr_C9ZR23_
HLA-DQA10501-DQB10201	96	VTDMSKYYFALVIEN	KYYFALVIE	5	0.4319	467.2	12.00	0.38	tr_C9ZR23_
HLA-DQA10501-DQB10201	97	YSGGSFWDGFGLTLW	WDGFGLTLW	6	0.4317	468.1	12.00	0.57	tr_C9ZR23_
HLA-DQA10501-DQB10201	98	NAIYYFVKGADDRIL	FVKGADDRI	5	0.4308	472.6	12.00	0.49	tr_C9ZR23_
HLA-DQA10501-DQB10201	99	MADVQYGDAAFPTGS	VQYGDAAFP	3	0.4289	482.5	12.00	0.28	tr_C9ZR23_
HLA-DQA10501-DQB10201	100	IYYFVKGADDRILEM	FVKGADDRI	3	0.4278	488.6	12.00	0.44	tr_C9ZR23_
HLA-DQA10501-DQB10201	101	VRNSSIVEDLGQVDY	IVEDLGQVD	5	0.4274	490.6	12.00	0.26	tr_C9ZR23_
HLA-DQA10501-DQB10201	102	AIYYFVKGADDRILE	FVKGADDRI	4	0.4271	492.3	12.00	0.49	tr_C9ZR23_
HLA-DQA10501-DQB10201	103	LQFSKGVMADVQYGD	SKGVMADVQ	3	0.4249	503.7	13.00	0.48	tr_C9ZR23_
HLA-DQA10501-DQB10201	104	VLRVLADKLDAFESW	VLRVLADKL	0	0.4240	508.9	13.00	0.23	tr_C9ZR23_
HLA-DQA10501-DQB10201	105	IKEEWFVHYVFAFAS	FVHYVFAFA	5	0.4226	516.6	13.00	0.23	tr_C9ZR23_
HLA-DQA10501-DQB10201	106	QFSKGVMADVQYGDA	SKGVMADVQ	2	0.4225	517.4	13.00	0.38	tr_C9ZR23_
HLA-DQA10501-DQB10201	107	DCVLRVLADKLDAFE	VLRVLADKL	2	0.4212	524.6	13.00	0.44	tr_C9ZR23_
HLA-DQA10501-DQB10201	108	KGVMADVQYGDAAFP	VQYGDAAFP	6	0.4204	529.1	13.00	0.30	tr_C9ZR23_
HLA-DQA10501-DQB10201	109	GDCVLRVLADKLDAF	VLRVLADKL	3	0.4200	531.4	13.00	0.46	tr_C9ZR23_
HLA-DQA10501-DQB10201	110	GIKEEWFVHYVFAFA	FVHYVFAFA	6	0.4157	556.6	14.00	0.26	tr_C9ZR23_
HLA-DQA10501-DQB10201	111	TGTLTQNVMEFLFAT	LTQNVMEFL	3	0.4154	558.7	14.00	0.31	tr_C9ZR23_
HLA-DQA10501-DQB10201	112	SGGSFWDGFGLTLWN	WDGFGLTLW	5	0.4152	559.7	14.00	0.54	tr_C9ZR23_
HLA-DQA10501-DQB10201	113	LLQFSKGVMADVQYG	SKGVMADVQ	4	0.4141	566.6	14.00	0.47	tr_C9ZR23_
HLA-DQA10501-DQB10201	114	TNAIYYFVKGADDRI	FVKGADDRI	6	0.4111	584.9	14.00	0.44	tr_C9ZR23_
HLA-DQA10501-DQB10201	115	LTRNELDEFLEKVRE	TRNELDEFL	1	0.4098	593.2	15.00	0.53	tr_C9ZR23_
HLA-DQA10501-DQB10201	116	TINGERRCLAPVEAE	RRCLAPVEA	5	0.4088	600.0	15.00	0.53	tr_C9ZR23_
HLA-DQA10501-DQB10201	117	FVTDMSKYYFALVIE	KYYFALVIE	6	0.4064	615.9	15.00	0.50	tr_C9ZR23_
HLA-DQA10501-DQB10201	118	GTLTQNVMEFLFATI	LTQNVMEFL	2	0.4045	628.6	15.00	0.29	tr_C9ZR23_
HLA-DQA10501-DQB10201	119	CVLRVLADKLDAFES	VLRVLADKL	1	0.4024	642.7	16.00	0.38	tr_C9ZR23_
HLA-DQA10501-DQB10201	120	SDKTGTLTQNVMEFL	LTQNVMEFL	6	0.4011	651.8	16.00	0.55	tr_C9ZR23_
HLA-DQA10501-DQB10201	121	ADVQYGDAAFPTGSS	VQYGDAAFP	2	0.3995	663.6	16.00	0.32	tr_C9ZR23_
HLA-DQA10501-DQB10201	122	NVRNSSIVEDLGQVD	IVEDLGQVD	6	0.3991	666.2	16.00	0.25	tr_C9ZR23_
HLA-DQA10501-DQB10201	123	GSFWDGFGLTLWNGL	WDGFGLTLW	3	0.3980	674.5	16.00	0.50	tr_C9ZR23_
HLA-DQA10501-DQB10201	124	ESLLQFSKGVMADVQ	SKGVMADVQ	6	0.3975	677.8	16.00	0.56	tr_C9ZR23_
HLA-DQA10501-DQB10201	125	GEWCNVRNSSIVEDL	RNSSIVEDL	6	0.3972	680.2	16.00	0.51	tr_C9ZR23_
HLA-DQA10501-DQB10201	126	AILAEVEHYAVFGLR	AILAEVEHY	0	0.3957	691.0	17.00	0.35	tr_C9ZR23_
HLA-DQA10501-DQB10201	127	KTMGVIVEEKSTNAI	TMGVIVEEK	1	0.3948	698.2	17.00	0.60	tr_C9ZR23_
HLA-DQA10501-DQB10201	128	LRVLADKLDAFESWE	VLADKLDAF	2	0.3945	700.5	17.00	0.25	tr_C9ZR23_
HLA-DQA10501-DQB10201	129	KVPRRVLEQTPALYK	VLEQTPALY	5	0.3943	701.6	17.00	0.46	tr_C9ZR23_
HLA-DQA10501-DQB10201	130	FSKGVMADVQYGDAA	SKGVMADVQ	1	0.3940	703.9	17.00	0.34	tr_C9ZR23_
HLA-DQA10501-DQB10201	131	YYFVKGADDRILEMA	FVKGADDRI	2	0.3938	705.7	17.00	0.47	tr_C9ZR23_
HLA-DQA10501-DQB10201	132	NGIKEEWFVHYVFAF	WFVHYVFAF	6	0.3931	710.9	17.00	0.38	tr_C9ZR23_
HLA-DQA10501-DQB10201	133	EDLGQVDYVLSDKTG	VDYVLSDKT	5	0.3919	720.0	17.00	0.29	tr_C9ZR23_
HLA-DQA10501-DQB10201	134	RILEMALDENSSTGG	ILEMALDEN	1	0.3896	738.2	17.00	0.46	tr_C9ZR23_

HLA-DQA10501-DQB10201	135	GGSFWDGFGLTLWNG	WDGFGLTLW	4	0.3892	741.2	18.00	0.55	tr_C9ZR23_
HLA-DQA10501-DQB10201	136	VNGIKEEWFVHYVFA	VNGIKEEWF	0	0.3887	745.5	18.00	0.38	tr_C9ZR23_
HLA-DQA10501-DQB10201	137	RVLSLCNTVEVVYDD	SLCNTVEVV	3	0.3885	747.2	18.00	0.23	tr_C9ZR23_
HLA-DQA10501-DQB10201	138	GDGGNDVAMIQEAQV	VAMIQEAQV	6	0.3880	751.5	18.00	0.65	tr_C9ZR23_
HLA-DQA10501-DQB10201	139	VPRRVLEQTPALYKV	VLEQTPALY	4	0.3866	763.0	18.00	0.48	tr_C9ZR23_
HLA-DQA10501-DQB10201	140	ILGVTAIEDKLQDHV	ILGVTAIED	0	0.3859	768.5	18.00	0.64	tr_C9ZR23_
HLA-DQA10501-DQB10201	141	EWCNVRNSSIVEDLG	RNSSIVEDL	5	0.3857	770.0	18.00	0.37	tr_C9ZR23_
HLA-DQA10501-DQB10201	142	SFWDGFGLTLWNGLY	WDGFGLTLW	2	0.3853	773.6	18.00	0.50	tr_C9ZR23_
HLA-DQA10501-DQB10201	143	MEFLFATINGERRCL	FATINGERR	4	0.3852	774.6	18.00	0.44	tr_C9ZR23_
HLA-DQA10501-DQB10201	144	AFASEFKTMGVIVEE	KTMGVIVEE	6	0.3834	789.3	18.00	0.58	tr_C9ZR23_
HLA-DQA10501-DQB10201	145	KLDAFESWEGYMESL	SWEGYMESL	6	0.3826	796.1	19.00	0.38	tr_C9ZR23_
HLA-DQA10501-DQB10201	146	VMEFLFATINGERRC	FATINGERR	5	0.3817	803.8	19.00	0.41	tr_C9ZR23_
HLA-DQA10501-DQB10201	147	EFLFATINGERRCLA	FATINGERR	3	0.3799	820.4	19.00	0.40	tr_C9ZR23_
HLA-DQA10501-DQB10201	148	HFGRVLSLCNTVEVV	SLCNTVEVV	6	0.3799	820.4	19.00	0.29	tr_C9ZR23_
HLA-DQA10501-DQB10201	149	SKGVMADVQYGDAAF	DVQYGDAAF	6	0.3796	823.0	19.00	0.22	tr_C9ZR23_
HLA-DQA10501-DQB10201	150	SYVLVIEGGQVLERI	EGGQVLERI	6	0.3786	831.9	19.00	0.34	tr_C9ZR23_
HLA-DQA10501-DQB10201	151	TLTQNVMEFLFATIN	LTQNVMEFL	1	0.3785	832.7	19.00	0.29	tr_C9ZR23_
HLA-DQA10501-DQB10201	152	DATTAAVEVNGIKEE	TAAVEVNGI	3	0.3759	855.9	20.00	0.34	tr_C9ZR23_
HLA-DQA10501-DQB10201	153	IYKLRLEMENSVTIL	EMENSVTIL	6	0.3750	864.3	20.00	0.32	tr_C9ZR23_
HLA-DQA10501-DQB10201	154	WEGYMESLLQFSKGV	WEGYMESLL	0	0.3749	865.2	20.00	0.47	tr_C9ZR23_
HLA-DQA10501-DQB10201	155	PGDCVLRVLADKLDA	VLRVLADKL	4	0.3749	865.2	20.00	0.63	tr_C9ZR23_
HLA-DQA10501-DQB10201	156	LVAEKRLTRNELDEF	LTRNELDEF	6	0.3746	868.7	20.00	0.70	tr_C9ZR23_
HLA-DQA10501-DQB10201	157	NVMEFLFATINGERR	FLFATINGE	4	0.3738	876.4	20.00	0.34	tr_C9ZR23_
HLA-DQA10501-DQB10201	158	FGRVLSLCNTVEVVY	SLCNTVEVV	5	0.3732	881.6	20.00	0.28	tr_C9ZR23_
HLA-DQA10501-DQB10201	159	SPGDCVLRVLADKLD	VLRVLADKL	5	0.3728	885.2	20.00	0.72	tr_C9ZR23_
HLA-DQA10501-DQB10201	160	GRVLSLCNTVEVVYD	SLCNTVEVV	4	0.3720	893.0	20.00	0.30	tr_C9ZR23_
HLA-DQA10501-DQB10201	161	YQAASPDEVALCNGC	YQAASPDEV	0	0.3714	898.9	20.00	0.81	tr_C9ZR23_
HLA-DQA10501-DQB10201	162	WCNVRNSSIVEDLGQ	RNSSIVEDL	4	0.3682	931.1	21.00	0.37	tr_C9ZR23_
HLA-DQA10501-DQB10201	163	DGGNDVAMIQEAQVG	VAMIQEAQV	5	0.3677	936.0	21.00	0.58	tr_C9ZR23_
HLA-DQA10501-DQB10201	164	YVLVIEGGQVLERIL	EGGQVLERI	5	0.3672	940.5	21.00	0.35	tr_C9ZR23_
HLA-DQA10501-DQB10201	165	PRRVLEQTPALYKVT	VLEQTPALY	3	0.3670	942.6	21.00	0.50	tr_C9ZR23_
HLA-DQA10501-DQB10201	166	CSPGDCVLRVLADKL	VLRVLADKL	6	0.3639	975.0	22.00	0.78	tr_C9ZR23_
HLA-DQA10501-DQB10201	167	FWDGFGLTLWNGLYS	WDGFGLTLW	1	0.3638	976.5	22.00	0.47	tr_C9ZR23_
HLA-DQA10501-DQB10201	168	RVLADKLDAFESWEG	VLADKLDAF	1	0.3631	983.8	22.00	0.28	tr_C9ZR23_
HLA-DQA10501-DQB10201	169	GAVGTMRYQAASPDE	RYQAASPDE	6	0.3618	997.0	22.00	0.35	tr_C9ZR23_
HLA-DQA10501-DQB10201	170	GGNDVAMIQEAQVGV	VAMIQEAQV	4	0.3596	1021.8	22.00	0.55	tr_C9ZR23_
HLA-DQA10501-DQB10201	171	DVQYGDAAFPTGSSS	VQYGDAAFP	1	0.3594	1023.4	22.00	0.34	tr_C9ZR23_
HLA-DQA10501-DQB10201	172	CNVRNSSIVEDLGQV	RNSSIVEDL	3	0.3587	1031.6	23.00	0.34	tr_C9ZR23_
HLA-DQA10501-DQB10201	173	LHFGRVLSLCNTVEV	VLSLCNTVE	5	0.3576	1044.3	23.00	0.29	tr_C9ZR23_
HLA-DQA10501-DQB10201	174	EVEHYAVFGLRTLLV	VFGLRTLLV	6	0.3560	1061.9	23.00	0.38	tr_C9ZR23_
HLA-DQA10501-DQB10201	175	TQNVMEFLFATINGE	FLFATINGE	6	0.3560	1062.1	23.00	0.44	tr_C9ZR23_
HLA-DQA10501-DQB10201	176	RDATTAAVEVNGIKE	TAAVEVNGI	4	0.3557	1065.4	23.00	0.38	tr_C9ZR23_
HLA-DQA10501-DQB10201	177	FLFATINGERRCLAP	FATINGERR	2	0.3550	1073.6	23.00	0.41	tr_C9ZR23_
HLA-DQA10501-DQB10201	178	ATINGERRCLAPVEA	RRCLAPVEA	6	0.3547	1076.9	23.00	0.58	tr_C9ZR23_
HLA-DQA10501-DQB10201	179	RRVLEQTPALYKVTR	VLEQTPALY	2	0.3517	1113.0	24.00	0.50	tr_C9ZR23_
HLA-DQA10501-DQB10201	180	EMENSVTILGVTAIE	TILGVTAIE	6	0.3498	1135.9	24.00	0.38	tr_C9ZR23_
HLA-DQA10501-DQB10201	181	VFAFASEFKTMGVIV	VFAFASEFK	0	0.3479	1159.7	25.00	0.44	tr_C9ZR23_
HLA-DQA10501-DQB10201	182	VARDATTAAVEVNGI	TAAVEVNGI	6	0.3473	1166.9	25.00	0.50	tr_C9ZR23_
HLA-DQA10501-DQB10201	183	VIKYSFYKSVLIGII	FYKSVLIGI	5	0.3462	1181.0	25.00	0.38	tr_C9ZR23_
HLA-DQA10501-DQB10201	184	VYDDVSQEMTQSGLS	VYDDVSQEM	0	0.3459	1184.5	25.00	0.62	tr_C9ZR23_

HLA-DQA10501-DQB10201	185	QNVMEFLFATINGER	FLFATINGE	5	0.3424	1230.5	26.00	0.47	tr_C9ZR23_
HLA-DQA10501-DQB10201	186	VLADKLDAFESWEGY	DKLDAFESW	3	0.3420	1235.8	26.00	0.20	tr_C9ZR23_
HLA-DQA10501-DQB10201	187	VLHFGRVLSLCNTVE	VLSLCNTVE	6	0.3403	1258.8	26.00	0.32	tr_C9ZR23_
HLA-DQA10501-DQB10201	188	YKLRLEMENSVTILG	EMENSVTIL	5	0.3402	1259.6	26.00	0.31	tr_C9ZR23_
HLA-DQA10501-DQB10201	189	KLRLEMENSVTILGV	ENSVTILGV	6	0.3399	1263.8	26.00	0.27	tr_C9ZR23_
HLA-DQA10501-DQB10201	190	ILAEVEHYAVFGLRT	HYAVFGLRT	6	0.3398	1265.0	26.00	0.31	tr_C9ZR23_
HLA-DQA10501-DQB10201	191	IKYSFYKSVLIGIIQ	FYKSVLIGI	4	0.3396	1268.7	26.00	0.32	tr_C9ZR23_
HLA-DQA10501-DQB10201	192	DFSISRFSDLRSLVF	FSDLRSLVF	6	0.3396	1268.8	26.00	0.50	tr_C9ZR23_
HLA-DQA10501-DQB10201	193	FVKGADDRILEMALD	DRILEMALD	6	0.3395	1269.8	26.00	0.43	tr_C9ZR23_
HLA-DQA10501-DQB10201	194	YFVKGADDRILEMAL	FVKGADDRI	1	0.3370	1303.9	27.00	0.51	tr_C9ZR23_
HLA-DQA10501-DQB10201	195	LTQNVMEFLFATING	LTQNVMEFL	0	0.3367	1308.8	27.00	0.25	tr_C9ZR23_
HLA-DQA10501-DQB10201	196	VLVIEGGQVLERILT	EGGQVLERI	4	0.3367	1308.9	27.00	0.37	tr_C9ZR23_
HLA-DQA10501-DQB10201	197	KYSFYKSVLIGIIQL	FYKSVLIGI	3	0.3361	1317.5	27.00	0.32	tr_C9ZR23_
HLA-DQA10501-DQB10201	198	TMGVIVEEKSTNAIY	TMGVIVEEK	0	0.3359	1320.5	27.00	0.56	tr_C9ZR23_
HLA-DQA10501-DQB10201	199	GNDVAMIQEAQVGVG	VAMIQEAQV	3	0.3335	1355.4	28.00	0.54	tr_C9ZR23_
HLA-DQA10501-DQB10201	200	EIYKLRLEMENSVTI	LRLEMENSV	4	0.3330	1362.6	28.00	0.31	tr_C9ZR23_
HLA-DQA10501-DQB10201	201	SSYVLVIEGGQVLER	IEGGQVLER	6	0.3326	1367.6	28.00	0.20	tr_C9ZR23_
HLA-DQA10501-DQB10201	202	ADKLDAFESWEGYME	AFESWEGYM	5	0.3305	1398.8	28.00	0.23	tr_C9ZR23_
HLA-DQA10501-DQB10201	203	VEHYAVFGLRTLLVA	VFGLRTLLV	5	0.3301	1404.8	28.00	0.34	tr_C9ZR23_
HLA-DQA10501-DQB10201	204	YSFYKSVLIGIIQLV	FYKSVLIGI	2	0.3296	1413.4	28.00	0.30	tr_C9ZR23_
HLA-DQA10501-DQB10201	205	GKQAARAADFSISRF	AADFSISRF	6	0.3295	1415.3	28.00	0.37	tr_C9ZR23_
HLA-DQA10501-DQB10201	206	DLGQVDYVLSDKTGT	VDYVLSDKT	4	0.3282	1434.0	29.00	0.44	tr_C9ZR23_
HLA-DQA10501-DQB10201	207	LADKLDAFESWEGYM	DKLDAFESW	2	0.3281	1435.6	29.00	0.22	tr_C9ZR23_
HLA-DQA10501-DQB10201	208	STNAIYYFVKGADDR	YYFVKGADD	5	0.3271	1451.1	29.00	0.46	tr_C9ZR23_
HLA-DQA10501-DQB10201	209	KFVTDMSKYYFALVI	SKYYFALVI	6	0.3270	1453.5	29.00	0.50	tr_C9ZR23_
HLA-DQA10501-DQB10201	210	FVIKYSFYKSVLIGI	FYKSVLIGI	6	0.3269	1454.3	29.00	0.53	tr_C9ZR23_
HLA-DQA10501-DQB10201	211	DKLDAFESWEGYMES	FESWEGYME	5	0.3255	1477.0	29.00	0.24	tr_C9ZR23_
HLA-DQA10501-DQB10201	212	GGQVLERILTTPSLL	RILTTPSLL	6	0.3252	1482.3	29.00	0.47	tr_C9ZR23_
HLA-DQA10501-DQB10201	213	EEIYKLRLEMENSVT	LRLEMENSV	5	0.3249	1486.6	29.00	0.32	tr_C9ZR23_
HLA-DQA10501-DQB10201	214	GSSYVLVIEGGQVLE	VLVIEGGQV	4	0.3247	1490.0	29.00	0.19	tr_C9ZR23_
HLA-DQA10501-DQB10201	215	KEEIYKLRLEMENSV	IYKLRLEME	3	0.3240	1500.7	30.00	0.28	tr_C9ZR23_
HLA-DQA10501-DQB10201	216	ELDEFLEKVREAELS	LEKVREAEL	5	0.3236	1507.8	30.00	0.47	tr_C9ZR23_
HLA-DQA10501-DQB10201	217	AEVEHYAVFGLRTLL	HYAVFGLRT	4	0.3224	1527.5	30.00	0.32	tr_C9ZR23_
HLA-DQA10501-DQB10201	218	NELDEFLEKVREAEL	LEKVREAEL	6	0.3224	1527.9	30.00	0.50	tr_C9ZR23_
HLA-DQA10501-DQB10201	219	NDVAMIQEAQVGVGI	VAMIQEAQV	2	0.3216	1540.1	30.00	0.51	tr_C9ZR23_
HLA-DQA10501-DQB10201	220	ARDATTAAVEVNGIK	TAAVEVNGI	5	0.3216	1541.1	30.00	0.47	tr_C9ZR23_
HLA-DQA10501-DQB10201	221	LVIEGGQVLERILTT	EGGQVLERI	3	0.3216	1541.2	30.00	0.35	tr_C9ZR23_
HLA-DQA10501-DQB10201	222	LAEVEHYAVFGLRTL	HYAVFGLRT	5	0.3206	1557.5	30.00	0.33	tr_C9ZR23_
HLA-DQA10501-DQB10201	223	TRNELDEFLEKVREA	TRNELDEFL	0	0.3203	1562.1	30.00	0.51	tr_C9ZR23_
HLA-DQA10501-DQB10201	224	FSISRFSDLRSLVFV	FSDLRSLVF	5	0.3186	1591.7	31.00	0.49	tr_C9ZR23_
HLA-DQA10501-DQB10201	225	GREGKQAARAADFSI	QAARAADFS	5	0.3183	1596.5	31.00	0.45	tr_C9ZR23_
HLA-DQA10501-DQB10201	226	TGSSYVLVIEGGQVL	VLVIEGGQV	5	0.3179	1603.6	31.00	0.22	tr_C9ZR23_
HLA-DQA10501-DQB10201	227	DRKVPRRVLEQTPAL	RVLEQTPAL	6	0.3172	1616.8	31.00	0.55	tr_C9ZR23_
HLA-DQA10501-DQB10201	228	LDEFLEKVREAELSM	LEKVREAEL	4	0.3162	1633.8	31.00	0.43	tr_C9ZR23_
HLA-DQA10501-DQB10201	229	STGSSYVLVIEGGQV	VLVIEGGQV	6	0.3158	1640.2	31.00	0.25	tr_C9ZR23_
HLA-DQA10501-DQB10201	230	RVLEQTPALYKVTRS	VLEQTPALY	1	0.3150	1655.2	32.00	0.51	tr_C9ZR23_
HLA-DQA10501-DQB10201	231	SFYKSVLIGIIQLVH	FYKSVLIGI	1	0.3141	1670.9	32.00	0.30	tr_C9ZR23_
HLA-DQA10501-DQB10201	232	KSTNAIYYFVKGADD	YYFVKGADD	6	0.3129	1692.4	32.00	0.56	tr_C9ZR23_
HLA-DQA10501-DQB10201	233	EHYAVFGLRTLLVAE	VFGLRTLLV	4	0.3121	1708.5	32.00	0.35	tr_C9ZR23_
HLA-DQA10501-DQB10201	234	VIENDVAMHHDGEWC	VAMHHDGEW	5	0.3118	1712.8	32.00	0.29	tr_C9ZR23_

HLA-DQA10501-DQB10201	235	WDGFGLTLWNGLYSL	WDGFGLTLW	0	0.3111	1725.9	33.00	0.35	tr_C9ZR23_
HLA-DQA10501-DQB10201	236	YAVFGLRTLLVAEKR	RTLLVAEKR	6	0.3110	1728.6	33.00	0.34	tr_C9ZR23_
HLA-DQA10501-DQB10201	237	LVIENDVAMHHDGEW	VAMHHDGEW	6	0.3097	1753.1	33.00	0.29	tr_C9ZR23_
HLA-DQA10501-DQB10201	238	NPLSTGSSYVLVIEG	GSSYVLVIE	5	0.3096	1753.7	33.00	0.28	tr_C9ZR23_
HLA-DQA10501-DQB10201	239	LTLWNGLYSLPQTML	GLYSLPQTM	5	0.3088	1770.7	33.00	0.27	tr_C9ZR23_
HLA-DQA10501-DQB10201	240	VNPLSTGSSYVLVIE	GSSYVLVIE	6	0.3078	1789.8	33.00	0.38	tr_C9ZR23_
HLA-DQA10501-DQB10201	241	DKVQTAEQIALTCSL	EQIALTCSL	6	0.3073	1797.9	33.00	0.34	tr_C9ZR23_
HLA-DQA10501-DQB10201	242	TLWNGLYSLPQTMLY	GLYSLPQTM	4	0.3071	1802.9	33.00	0.25	tr_C9ZR23_
HLA-DQA10501-DQB10201	243	AVFGLRTLLVAEKRL	RTLLVAEKR	5	0.3065	1813.5	34.00	0.34	tr_C9ZR23_
HLA-DQA10501-DQB10201	244	VRLVARDATTAAVEV	DATTAAVEV	6	0.3049	1845.9	34.00	0.57	tr_C9ZR23_
HLA-DQA10501-DQB10201	245	PLSTGSSYVLVIEGG	SYVLVIEGG	6	0.3048	1848.5	34.00	0.28	tr_C9ZR23_
HLA-DQA10501-DQB10201	246	REGKQAARAADFSIS	QAARAADFS	4	0.3036	1872.5	34.00	0.45	tr_C9ZR23_
HLA-DQA10501-DQB10201	247	LSTGSSYVLVIEGGQ	SYVLVIEGG	5	0.3029	1886.6	34.00	0.17	tr_C9ZR23_
HLA-DQA10501-DQB10201	248	ALVIENDVAMHHDGE	ALVIENDVA	0	0.3023	1899.3	35.00	0.22	tr_C9ZR23_
HLA-DQA10501-DQB10201	249	DVAMIQEAQVGVGIT	VAMIQEAQV	1	0.2991	1965.9	35.00	0.52	tr_C9ZR23_
HLA-DQA10501-DQB10201	250	VIEGGQVLERILTTP	EGGQVLERI	2	0.2989	1969.2	35.00	0.37	tr_C9ZR23_
HLA-DQA10501-DQB10201	251	LRLEMENSVTILGVT	EMENSVTIL	3	0.2986	1976.6	35.00	0.30	tr_C9ZR23_
HLA-DQA10501-DQB10201	252	HYAVFGLRTLLVAEK	VFGLRTLLV	3	0.2985	1977.5	35.00	0.29	tr_C9ZR23_
HLA-DQA10501-DQB10201	253	LQAGIKVWMLTGDKV	VWMLTGDKV	6	0.2978	1992.6	36.00	0.80	tr_C9ZR23_
HLA-DQA10501-DQB10201	254	LSDKTGTLTQNVMEF	TLTQNVMEF	6	0.2977	1995.5	36.00	0.57	tr_C9ZR23_
HLA-DQA10501-DQB10201	255	HVLHFGRVLSLCNTV	LHFGRVLSL	2	0.2977	1996.4	36.00	0.25	tr_C9ZR23_
HLA-DQA10501-DQB10201	256	DEFLEKVREAELSMN	LEKVREAEL	3	0.2976	1997.1	36.00	0.41	tr_C9ZR23_
HLA-DQA10501-DQB10201	257	FVHGQLAYNRTAFVI	LAYNRTAFV	5	0.2976	1998.3	36.00	0.54	tr_C9ZR23_
HLA-DQA10501-DQB10201	258	FGLTLWNGLYSLPQT	WNGLYSLPQ	5	0.2966	2019.1	36.00	0.31	tr_C9ZR23_
HLA-DQA10501-DQB10201	259	GFGLTLWNGLYSLPQ	WNGLYSLPQ	6	0.2951	2051.7	36.00	0.37	tr_C9ZR23_
HLA-DQA10501-DQB10201	260	GQVLERILTTPSLLK	RILTTPSLL	5	0.2946	2063.0	36.00	0.45	tr_C9ZR23_
HLA-DQA10501-DQB10201	261	DGEWCNVRNSSIVED	VRNSSIVED	6	0.2944	2068.0	37.00	0.69	tr_C9ZR23_
HLA-DQA10501-DQB10201	262	GLTLWNGLYSLPQTM	GLYSLPQTM	6	0.2939	2080.4	37.00	0.31	tr_C9ZR23_
HLA-DQA10501-DQB10201	263	IENDVAMHHDGEWCN	VAMHHDGEW	4	0.2937	2083.0	37.00	0.31	tr_C9ZR23_
HLA-DQA10501-DQB10201	264	TGDKVQTAEQIALTC	VQTAEQIAL	4	0.2931	2098.5	37.00	0.27	tr_C9ZR23_
HLA-DQA10501-DQB10201	265	QAGIKVWMLTGDKVQ	VWMLTGDKV	5	0.2930	2100.3	37.00	0.71	tr_C9ZR23_
HLA-DQA10501-DQB10201	266	RLEMENSVTILGVTA	EMENSVTIL	2	0.2926	2108.7	37.00	0.30	tr_C9ZR23_
HLA-DQA10501-DQB10201	267	LEMENSVTILGVTAI	EMENSVTIL	1	0.2925	2111.9	37.00	0.24	tr_C9ZR23_
HLA-DQA10501-DQB10201	268	TGREGKQAARAADFS	QAARAADFS	6	0.2919	2124.2	37.00	0.48	tr_C9ZR23_
HLA-DQA10501-DQB10201	269	QVLERILTTPSLLKL	RILTTPSLL	4	0.2919	2126.0	37.00	0.46	tr_C9ZR23_
HLA-DQA10501-DQB10201	270	RLVARDATTAAVEVN	DATTAAVEV	5	0.2912	2141.1	37.00	0.48	tr_C9ZR23_
HLA-DQA10501-DQB10201	271	KQAARAADFSISRFS	AADFSISRF	5	0.2908	2150.1	37.00	0.37	tr_C9ZR23_
HLA-DQA10501-DQB10201	272	FYKSVLIGIIQLVHN	YKSVLIGII	1	0.2895	2181.8	38.00	0.19	tr_C9ZR23_
HLA-DQA10501-DQB10201	273	LWNGLYSLPQTMLYC	GLYSLPQTM	3	0.2893	2186.5	38.00	0.28	tr_C9ZR23_
HLA-DQA10501-DQB10201	274	KGERMSSKAAILAEV	SKAAILAEV	6	0.2890	2193.4	38.00	0.47	tr_C9ZR23_
HLA-DQA10501-DQB10201	275	LGQVDYVLSDKTGTL	VDYVLSDKT	3	0.2890	2193.6	38.00	0.54	tr_C9ZR23_
HLA-DQA10501-DQB10201	276	TAIEDKLQDHVPETI	LQDHVPETI	6	0.2888	2196.5	38.00	0.78	tr_C9ZR23_
HLA-DQA10501-DQB10201	277	CGAVGTMRYQAASPD	MRYQAASPD	6	0.2883	2208.2	38.00	0.47	tr_C9ZR23_
HLA-DQA10501-DQB10201	278	SISRFSDLRSLVFVH	FSDLRSLVF	4	0.2878	2221.6	38.00	0.48	tr_C9ZR23_
HLA-DQA10501-DQB10201	279	AGIKVWMLTGDKVQT	VWMLTGDKV	4	0.2877	2222.9	38.00	0.69	tr_C9ZR23_
HLA-DQA10501-DQB10201	280	GIKVWMLTGDKVQTA	VWMLTGDKV	3	0.2875	2227.4	38.00	0.62	tr_C9ZR23_
HLA-DQA10501-DQB10201	281	VQYGDAAFPTGSSSD	VQYGDAAFP	0	0.2874	2229.7	38.00	0.38	tr_C9ZR23_
HLA-DQA10501-DQB10201	282	MESLLQFSKGVMADV	FSKGVMADV	6	0.2863	2258.6	39.00	0.58	tr_C9ZR23_
HLA-DQA10501-DQB10201	283	EHVLHFGRVLSLCNT	LHFGRVLSL	3	0.2853	2282.0	39.00	0.29	tr_C9ZR23_
HLA-DQA10501-DQB10201	284	VFVHGQLAYNRTAFV	LAYNRTAFV	6	0.2838	2319.0	39.00	0.55	tr_C9ZR23_

HLA-DQA10501-DQB10201	285	FATINGERRCLAPVE	FATINGERR	0	0.2837	2323.1	39.00	0.34	tr_C9ZR23_
HLA-DQA10501-DQB10201	286	EGKQAARAADFSISR	QAARAADFS	3	0.2833	2332.2	39.00	0.41	tr_C9ZR23_
HLA-DQA10501-DQB10201	287	LFATINGERRCLAPV	FATINGERR	1	0.2831	2337.8	39.00	0.51	tr_C9ZR23_
HLA-DQA10501-DQB10201	288	ENDVAMHHDGEWCNV	VAMHHDGEW	3	0.2830	2339.4	39.00	0.32	tr_C9ZR23_
HLA-DQA10501-DQB10201	289	VFGLRTLLVAEKRLT	RTLLVAEKR	4	0.2830	2340.5	39.00	0.37	tr_C9ZR23_
HLA-DQA10501-DQB10201	290	KVQTAEQIALTCSLC	EQIALTCSL	5	0.2820	2365.0	40.00	0.30	tr_C9ZR23_
HLA-DQA10501-DQB10201	291	IKVWMLTGDKVQTAE	VWMLTGDKV	2	0.2818	2370.1	40.00	0.58	tr_C9ZR23_
HLA-DQA10501-DQB10201	292	HYSGGSFWDGFGLTL	FWDGFGLTL	6	0.2815	2377.1	40.00	0.47	tr_C9ZR23_
HLA-DQA10501-DQB10201	293	VLERILTTPSLLKLL	RILTTPSLL	3	0.2810	2390.9	40.00	0.47	tr_C9ZR23_
HLA-DQA10501-DQB10201	294	RKEEIYKLRLEMENS	IYKLRLEME	4	0.2808	2395.5	40.00	0.44	tr_C9ZR23_
HLA-DQA10501-DQB10201	295	VGDGGNDVAMIQEAQ	DVAMIQEAQ	6	0.2807	2399.1	40.00	0.37	tr_C9ZR23_
HLA-DQA10501-DQB10201	296	GDKVQTAEQIALTCS	VQTAEQIAL	3	0.2805	2405.0	40.00	0.22	tr_C9ZR23_
HLA-DQA10501-DQB10201	297	GEHVLHFGRVLSLCN	LHFGRVLSL	4	0.2789	2444.8	40.00	0.34	tr_C9ZR23_
HLA-DQA10501-DQB10201	298	NGLYSLPQTMLYCLD	PQTMLYCLD	6	0.2786	2454.4	40.00	0.35	tr_C9ZR23_
HLA-DQA10501-DQB10201	299	HGQLAYNRTAFVIKY	LAYNRTAFV	3	0.2776	2481.2	41.00	0.44	tr_C9ZR23_
HLA-DQA10501-DQB10201	300	NNRKEEIYKLRLEME	IYKLRLEME	6	0.2771	2493.0	41.00	0.58	tr_C9ZR23_
HLA-DQA10501-DQB10201	301	EGGQVLERILTTPSL	ERILTTPSL	6	0.2762	2519.5	41.00	0.43	tr_C9ZR23_
HLA-DQA10501-DQB10201	302	VQTAEQIALTCSLCS	EQIALTCSL	4	0.2750	2550.5	41.00	0.30	tr_C9ZR23_
HLA-DQA10501-DQB10201	303	NRKEEIYKLRLEMEN	IYKLRLEME	5	0.2743	2571.0	42.00	0.51	tr_C9ZR23_
HLA-DQA10501-DQB10201	304	KSVLIGIIQLVHNIF	IIQLVHNIF	6	0.2737	2587.8	42.00	0.42	tr_C9ZR23_
HLA-DQA10501-DQB10201	305	APVEAEEVQGSCGEH	PVEAEEVQG	1	0.2730	2605.7	42.00	0.36	tr_C9ZR23_
HLA-DQA10501-DQB10201	306	FAFASEFKTMGVIVE	EFKTMGVIV	5	0.2729	2608.7	42.00	0.41	tr_C9ZR23_
HLA-DQA10501-DQB10201	307	PVEAEEVQGSCGEHV	VQGSCGEHV	6	0.2726	2618.4	42.00	0.43	tr_C9ZR23_
HLA-DQA10501-DQB10201	308	ILEMALDENSSTGGP	ILEMALDEN	0	0.2723	2626.3	42.00	0.62	tr_C9ZR23_
HLA-DQA10501-DQB10201	309	VRSRGFITLAVGDGG	FITLAVGDG	5	0.2720	2635.7	42.00	0.26	tr_C9ZR23_
HLA-DQA10501-DQB10201	310	LTGDKVQTAEQIALT	VQTAEQIAL	5	0.2717	2645.1	42.00	0.33	tr_C9ZR23_
HLA-DQA10501-DQB10201	311	TTETNESADVNPLST	SADVNPLST	6	0.2709	2666.7	43.00	0.28	tr_C9ZR23_
HLA-DQA10501-DQB10201	312	IFHTHYSGGSFWDGF	THYSGGSFW	3	0.2707	2671.5	43.00	0.43	tr_C9ZR23_
HLA-DQA10501-DQB10201	313	DKLQDHVPETIRSFL	LQDHVPETI	2	0.2700	2693.1	43.00	0.59	tr_C9ZR23_
HLA-DQA10501-DQB10201	314	CGEHVLHFGRVLSLC	LHFGRVLSL	5	0.2696	2704.8	43.00	0.44	tr_C9ZR23_
HLA-DQA10501-DQB10201	315	WNGLYSLPQTMLYCL	GLYSLPQTM	2	0.2693	2713.3	43.00	0.29	tr_C9ZR23_
HLA-DQA10501-DQB10201	316	ISRFSDLRSLVFVHG	FSDLRSLVF	3	0.2684	2741.4	43.00	0.46	tr_C9ZR23_
HLA-DQA10501-DQB10201	317	EFLEKVREAELSMNN	LEKVREAEL	2	0.2680	2753.2	43.00	0.38	tr_C9ZR23_
HLA-DQA10501-DQB10201	318	PSLLKLLTELSENCV	LTELSENCV	6	0.2679	2753.5	43.00	0.35	tr_C9ZR23_
HLA-DQA10501-DQB10201	319	VWMLTGDKVQTAEQI	VWMLTGDKV	0	0.2679	2754.9	43.00	0.32	tr_C9ZR23_
HLA-DQA10501-DQB10201	320	LVRSRGFITLAVGDG	FITLAVGDG	6	0.2677	2759.5	43.00	0.31	tr_C9ZR23_
HLA-DQA10501-DQB10201	321	LAVGDGGNDVAMIQE	GNDVAMIQE	6	0.2659	2814.5	44.00	0.46	tr_C9ZR23_
HLA-DQA10501-DQB10201	322	LERILTTPSLLKLLT	RILTTPSLL	2	0.2657	2819.9	44.00	0.48	tr_C9ZR23_
HLA-DQA10501-DQB10201	323	LVARDATTAAVEVNG	DATTAAVEV	4	0.2642	2867.4	44.00	0.43	tr_C9ZR23_
HLA-DQA10501-DQB10201	324	QTAEQIALTCSLCSP	EQIALTCSL	3	0.2639	2875.4	44.00	0.35	tr_C9ZR23_
HLA-DQA10501-DQB10201	325	MLTGDKVQTAEQIAL	VQTAEQIAL	6	0.2635	2889.0	44.00	0.40	tr_C9ZR23_
HLA-DQA10501-DQB10201	326	GQVDYVLSDKTGTLT	VDYVLSDKT	2	0.2634	2892.3	45.00	0.59	tr_C9ZR23_
HLA-DQA10501-DQB10201	327	QAARAADFSISRFSD	AADFSISRF	4	0.2632	2897.8	45.00	0.44	tr_C9ZR23_
HLA-DQA10501-DQB10201	328	AIEDKLQDHVPETIR	LQDHVPETI	5	0.2626	2918.3	45.00	0.79	tr_C9ZR23_
HLA-DQA10501-DQB10201	329	GQLAYNRTAFVIKYS	LAYNRTAFV	2	0.2623	2926.8	45.00	0.41	tr_C9ZR23_
HLA-DQA10501-DQB10201	330	KLQDHVPETIRSFLQ	LQDHVPETI	1	0.2621	2932.7	45.00	0.49	tr_C9ZR23_
HLA-DQA10501-DQB10201	331	AVGDGGNDVAMIQEA	GNDVAMIQE	5	0.2618	2942.4	45.00	0.36	tr_C9ZR23_
HLA-DQA10501-DQB10201	332	KVWMLTGDKVQTAEQ	VWMLTGDKV	1	0.2613	2958.3	45.00	0.54	tr_C9ZR23_
HLA-DQA10501-DQB10201	333	RAADFSISRFSDLRS	AADFSISRF	1	0.2609	2972.8	45.00	0.31	tr_C9ZR23_
HLA-DQA10501-DQB10201	334	IEGGQVLERILTTPS	EGGQVLERI	1	0.2608	2973.9	45.00	0.38	tr_C9ZR23_

HLA-DQA10501-DQB10201	335	SRGFITLAVGDGGND	FITLAVGDG	3	0.2600	2999.7	45.00	0.35	tr_C9ZR23_
HLA-DQA10501-DQB10201	336	RLVRSRGFITLAVGD	SRGFITLAV	4	0.2598	3008.8	46.00	0.41	tr_C9ZR23_
HLA-DQA10501-DQB10201	337	RSRGFITLAVGDGGN	FITLAVGDG	4	0.2592	3026.5	46.00	0.29	tr_C9ZR23_
HLA-DQA10501-DQB10201	338	KVTRSGVDLGVCQFF	GVDLGVCQF	5	0.2589	3035.3	46.00	0.37	tr_C9ZR23_
HLA-DQA10501-DQB10201	339	AADFSISRFSDLRSL	FSISRFSDL	3	0.2589	3038.0	46.00	0.20	tr_C9ZR23_
HLA-DQA10501-DQB10201	340	RGFITLAVGDGGNDV	FITLAVGDG	2	0.2587	3041.7	46.00	0.34	tr_C9ZR23_
HLA-DQA10501-DQB10201	341	DVNPLSTGSSYVLVI	TGSSYVLVI	6	0.2587	3044.0	46.00	0.56	tr_C9ZR23_
HLA-DQA10501-DQB10201	342	GFITLAVGDGGNDVA	ITLAVGDGG	2	0.2586	3047.6	46.00	0.32	tr_C9ZR23_
HLA-DQA10501-DQB10201	343	SLLKLLTELSENCVS	LTELSENCV	5	0.2578	3072.8	46.00	0.32	tr_C9ZR23_
HLA-DQA10501-DQB10201	344	VHGQLAYNRTAFVIK	LAYNRTAFV	4	0.2577	3075.5	46.00	0.53	tr_C9ZR23_
HLA-DQA10501-DQB10201	345	IGIIQLVHNIFHTHY	VHNIFHTHY	6	0.2576	3079.2	46.00	0.46	tr_C9ZR23_
HLA-DQA10501-DQB10201	346	DGFGLTLWNGLYSLP	FGLTLWNGL	2	0.2576	3079.6	46.00	0.25	tr_C9ZR23_
HLA-DQA10501-DQB10201	347	VTRLVRSRGFITLAV	SRGFITLAV	6	0.2571	3096.7	46.00	0.68	tr_C9ZR23_
HLA-DQA10501-DQB10201	348	VTTETNESADVNPLS	ETNESADVN	3	0.2571	3096.8	46.00	0.22	tr_C9ZR23_
HLA-DQA10501-DQB10201	349	NDVAMHHDGEWCNVR	VAMHHDGEW	2	0.2569	3103.1	46.00	0.37	tr_C9ZR23_
HLA-DQA10501-DQB10201	350	FHTHYSGGSFWDGFG	THYSGGSFW	2	0.2564	3119.6	46.00	0.41	tr_C9ZR23_
HLA-DQA10501-DQB10201	351	VEAEEVQGSCGEHVL	VQGSCGEHV	5	0.2564	3120.8	46.00	0.51	tr_C9ZR23_
HLA-DQA10501-DQB10201	352	SRFSDLRSLVFVHGQ	FSDLRSLVF	2	0.2562	3127.0	46.00	0.46	tr_C9ZR23_
HLA-DQA10501-DQB10201	353	QLAYNRTAFVIKYSF	LAYNRTAFV	1	0.2559	3138.4	47.00	0.34	tr_C9ZR23_
HLA-DQA10501-DQB10201	354	HTHYSGGSFWDGFGL	THYSGGSFW	1	0.2554	3154.3	47.00	0.31	tr_C9ZR23_
HLA-DQA10501-DQB10201	355	SVTTETNESADVNPL	ETNESADVN	4	0.2547	3176.2	47.00	0.27	tr_C9ZR23_
HLA-DQA10501-DQB10201	356	EDKLQDHVPETIRSF	LQDHVPETI	3	0.2547	3176.5	47.00	0.78	tr_C9ZR23_
HLA-DQA10501-DQB10201	357	AARAADFSISRFSDL	AADFSISRF	3	0.2546	3183.0	47.00	0.46	tr_C9ZR23_
HLA-DQA10501-DQB10201	358	VIVEEKSTNAIYYFV	STNAIYYFV	6	0.2542	3195.1	47.00	0.34	tr_C9ZR23_
HLA-DQA10501-DQB10201	359	RNELDEFLEKVREAE	ELDEFLEKV	2	0.2539	3204.1	47.00	0.30	tr_C9ZR23_
HLA-DQA10501-DQB10201	360	SCGEHVLHFGRVLSL	LHFGRVLSL	6	0.2538	3207.9	47.00	0.50	tr_C9ZR23_
HLA-DQA10501-DQB10201	361	GLYSLPQTMLYCLDR	PQTMLYCLD	5	0.2535	3218.4	47.00	0.31	tr_C9ZR23_
HLA-DQA10501-DQB10201	362	FGLRTLLVAEKRLTR	RTLLVAEKR	3	0.2533	3226.5	47.00	0.44	tr_C9ZR23_
HLA-DQA10501-DQB10201	363	RFSDLRSLVFVHGQL	FSDLRSLVF	1	0.2526	3250.3	47.00	0.37	tr_C9ZR23_
HLA-DQA10501-DQB10201	364	FSDLRSLVFVHGQLA	LVFVHGQLA	6	0.2519	3275.0	48.00	0.28	tr_C9ZR23_
HLA-DQA10501-DQB10201	365	ERILTTPSLLKLLTE	RILTTPSLL	1	0.2512	3299.3	48.00	0.47	tr_C9ZR23_
HLA-DQA10501-DQB10201	366	ADFSISRFSDLRSLV	FSISRFSDL	2	0.2512	3302.1	48.00	0.17	tr_C9ZR23_
HLA-DQA10501-DQB10201	367	YKVTRSGVDLGVCQF	GVDLGVCQF	6	0.2505	3325.8	48.00	0.42	tr_C9ZR23_
HLA-DQA10501-DQB10201	368	ARAADFSISRFSDLR	AADFSISRF	2	0.2498	3352.3	48.00	0.44	tr_C9ZR23_
HLA-DQA10501-DQB10201	369	TAFVIKYSFYKSVLI	YSFYKSVLI	6	0.2495	3360.1	48.00	0.57	tr_C9ZR23_
HLA-DQA10501-DQB10201	370	TETNESADVNPLSTG	SADVNPLST	5	0.2484	3403.3	49.00	0.29	tr_C9ZR23_
HLA-DQA10501-DQB10201	371	LLTELSENCVSVICA	ENCVSVICA	6	0.2477	3429.7	49.00	0.38	tr_C9ZR23_
HLA-DQA10501-DQB10201	372	IEDKLQDHVPETIRS	LQDHVPETI	4	0.2472	3447.9	49.00	0.80	tr_C9ZR23_
HLA-DQA10501-DQB10201	373	HNIFHTHYSGGSFWD	THYSGGSFW	5	0.2469	3456.8	49.00	0.60	tr_C9ZR23_
HLA-DQA10501-DQB10201	374	SVLIGIIQLVHNIFH	IIQLVHNIF	5	0.2467	3466.1	49.00	0.41	tr_C9ZR23_
HLA-DQA10501-DQB10201	375	TRLVRSRGFITLAVG	SRGFITLAV	5	0.2465	3472.0	49.00	0.49	tr_C9ZR23_
HLA-DQA10501-DQB10201	376	DLRSLVFVHGQLAYN	LVFVHGQLA	4	0.2454	3512.9	49.00	0.28	tr_C9ZR23_
HLA-DQA10501-DQB10201	377	LLKLLTELSENCVSV	LTELSENCV	4	0.2433	3596.3	50.00	0.35	tr_C9ZR23_
HLA-DQA10501-DQB10201	378	VTRSGVDLGVCQFFG	GVDLGVCQF	4	0.2431	3601.2	50.00	0.34	tr_C9ZR23_
HLA-DQA10501-DQB10201	379	SDLRSLVFVHGQLAY	LVFVHGQLA	5	0.2413	3674.5	55.00	0.34	tr_C9ZR23_
HLA-DQA10501-DQB10201	380	DVAMHHDGEWCNVRN	VAMHHDGEW	1	0.2398	3734.3	55.00	0.34	tr_C9ZR23_
HLA-DQA10501-DQB10201	381	EGYMESLLQFSKGVM	YMESLLQFS	2	0.2388	3775.6	55.00	0.31	tr_C9ZR23_
HLA-DQA10501-DQB10201	382	GIIQLVHNIFHTHYS	VHNIFHTHY	5	0.2380	3807.3	55.00	0.44	tr_C9ZR23_
HLA-DQA10501-DQB10201	383	DHVPETIRSFLQAGI	IRSFLQAGI	6	0.2380	3808.6	55.00	0.43	tr_C9ZR23_
HLA-DQA10501-DQB10201	384	FITLAVGDGGNDVAM	ITLAVGDGG	1	0.2379	3811.5	55.00	0.35	tr_C9ZR23_

HLA-DQA10501-DQB10201	385	AFVIKYSFYKSVLIG	YSFYKSVLI	5	0.2368	3855.7	55.00	0.46	tr_C9ZR23_
HLA-DQA10501-DQB10201	386	THYSGGSFWDGFGLT	THYSGGSFW	0	0.2368	3858.1	55.00	0.25	tr_C9ZR23_
HLA-DQA10501-DQB10201	387	VAMIQEAQVGVGITG	VAMIQEAQV	0	0.2367	3860.0	55.00	0.47	tr_C9ZR23_
HLA-DQA10501-DQB10201	388	YKSVLIGIIQLVHNI	LIGIIQLVH	4	0.2367	3861.3	55.00	0.21	tr_C9ZR23_
HLA-DQA10501-DQB10201	389	LQDHVPETIRSFLQA	LQDHVPETI	0	0.2362	3881.2	55.00	0.34	tr_C9ZR23_
HLA-DQA10501-DQB10201	390	NVRLVARDATTAAVE	RDATTAAVE	6	0.2355	3913.2	55.00	0.31	tr_C9ZR23_
HLA-DQA10501-DQB10201	391	NIFHTHYSGGSFWDG	THYSGGSFW	4	0.2350	3932.4	55.00	0.56	tr_C9ZR23_
HLA-DQA10501-DQB10201	392	LYSLPQTMLYCLDRK	PQTMLYCLD	4	0.2349	3937.9	55.00	0.26	tr_C9ZR23_
HLA-DQA10501-DQB10201	393	EAEEVQGSCGEHVLH	VQGSCGEHV	4	0.2346	3948.9	55.00	0.55	tr_C9ZR23_
HLA-DQA10501-DQB10201	394	RSLVFVHGQLAYNRT	LVFVHGQLA	2	0.2341	3970.7	55.00	0.28	tr_C9ZR23_
HLA-DQA10501-DQB10201	395	LAYNRTAFVIKYSFY	LAYNRTAFV	0	0.2340	3973.8	55.00	0.22	tr_C9ZR23_
HLA-DQA10501-DQB10201	396	FLEKVREAELSMNNR	LEKVREAEL	1	0.2336	3994.2	55.00	0.36	tr_C9ZR23_
HLA-DQA10501-DQB10201	397	LRSLVFVHGQLAYNR	LVFVHGQLA	3	0.2329	4025.2	55.00	0.28	tr_C9ZR23_
HLA-DQA10501-DQB10201	398	AEEVQGSCGEHVLHF	VQGSCGEHV	3	0.2328	4027.6	55.00	0.56	tr_C9ZR23_
HLA-DQA10501-DQB10201	399	QVDYVLSDKTGTLTQ	VDYVLSDKT	1	0.2326	4037.7	55.00	0.60	tr_C9ZR23_
HLA-DQA10501-DQB10201	400	TAEQIALTCSLCSPG	EQIALTCSL	2	0.2321	4059.7	55.00	0.38	tr_C9ZR23_
HLA-DQA10501-DQB10201	401	VLIGIIQLVHNIFHT	IIQLVHNIF	4	0.2315	4084.7	55.00	0.41	tr_C9ZR23_
HLA-DQA10501-DQB10201	402	TPSLLKLLTELSENC	LKLLTELSE	4	0.2306	4124.7	55.00	0.29	tr_C9ZR23_
HLA-DQA10501-DQB10201	403	LKLLTELSENCVSVI	LTELSENCV	3	0.2300	4152.6	55.00	0.38	tr_C9ZR23_
HLA-DQA10501-DQB10201	404	WMLTGDKVQTAEQIA	DKVQTAEQI	5	0.2289	4203.2	55.00	0.34	tr_C9ZR23_
HLA-DQA10501-DQB10201	405	GSVTTETNESADVNP	ETNESADVN	5	0.2262	4326.4	55.00	0.29	tr_C9ZR23_
HLA-DQA10501-DQB10201	406	TIRSFLQAGIKVWML	IRSFLQAGI	1	0.2250	4384.3	55.00	0.23	tr_C9ZR23_
HLA-DQA10501-DQB10201	407	LTTPSLLKLLTELSE	LKLLTELSE	6	0.2247	4398.3	55.00	0.39	tr_C9ZR23_
HLA-DQA10501-DQB10201	408	ETNESADVNPLSTGS	SADVNPLST	4	0.2240	4431.3	60.00	0.31	tr_C9ZR23_
HLA-DQA10501-DQB10201	409	GKGERMSSKAAILAE	RMSSKAAIL	4	0.2239	4433.6	60.00	0.32	tr_C9ZR23_
HLA-DQA10501-DQB10201	410	GVIVEEKSTNAIYYF	KSTNAIYYF	6	0.2238	4439.8	60.00	0.44	tr_C9ZR23_
HLA-DQA10501-DQB10201	411	IVEEKSTNAIYYFVK	STNAIYYFV	5	0.2237	4445.5	60.00	0.29	tr_C9ZR23_
HLA-DQA10501-DQB10201	412	ITLAVGDGGNDVAMI	DGGNDVAMI	6	0.2230	4475.8	60.00	0.22	tr_C9ZR23_
HLA-DQA10501-DQB10201	413	TRSGVDLGVCQFFGS	GVDLGVCQF	3	0.2223	4510.7	60.00	0.37	tr_C9ZR23_
HLA-DQA10501-DQB10201	414	TTPSLLKLLTELSEN	LKLLTELSE	5	0.2217	4541.6	60.00	0.34	tr_C9ZR23_
HLA-DQA10501-DQB10201	415	RSGVDLGVCQFFGSF	GVDLGVCQF	2	0.2214	4556.1	60.00	0.33	tr_C9ZR23_
HLA-DQA10501-DQB10201	416	LGVTAIEDKLQDHVP	GVTAIEDKL	1	0.2211	4571.5	60.00	0.37	tr_C9ZR23_
HLA-DQA10501-DQB10201	417	SLVFVHGQLAYNRTA	LVFVHGQLA	1	0.2192	4663.6	60.00	0.29	tr_C9ZR23_
HLA-DQA10501-DQB10201	418	EKSTNAIYYFVKGAD	IYYFVKGAD	6	0.2191	4672.6	60.00	0.29	tr_C9ZR23_
HLA-DQA10501-DQB10201	419	LNVRLVARDATTAAV	VRLVARDAT	2	0.2174	4757.0	60.00	0.25	tr_C9ZR23_
HLA-DQA10501-DQB10201	420	KLLTELSENCVSVIC	LTELSENCV	2	0.2167	4794.1	60.00	0.40	tr_C9ZR23_
HLA-DQA10501-DQB10201	421	GLRTLLVAEKRLTRN	RTLLVAEKR	2	0.2161	4825.5	60.00	0.44	tr_C9ZR23_
HLA-DQA10501-DQB10201	422	HVPETIRSFLQAGIK	IRSFLQAGI	5	0.2158	4838.6	60.00	0.35	tr_C9ZR23_
HLA-DQA10501-DQB10201	423	EEVQGSCGEHVLHFG	VQGSCGEHV	2	0.2152	4873.9	60.00	0.59	tr_C9ZR23_
HLA-DQA10501-DQB10201	424	LVFVHGQLAYNRTAF	FVHGQLAYN	2	0.2140	4934.2	60.00	0.17	tr_C9ZR23_
HLA-DQA10501-DQB10201	425	LTELSENCVSVICAR	ENCVSVICA	5	0.2140	4934.3	60.00	0.39	tr_C9ZR23_
HLA-DQA10501-DQB10201	426	ETIRSFLQAGIKVWM	IRSFLQAGI	2	0.2138	4945.2	60.00	0.31	tr_C9ZR23_
HLA-DQA10501-DQB10201	427	PETIRSFLQAGIKVW	IRSFLQAGI	3	0.2136	4957.9	60.00	0.40	tr_C9ZR23_
HLA-DQA10501-DQB10201	428	RILTTPSLLKLLTEL	RILTTPSLL	0	0.2134	4966.4	60.00	0.41	tr_C9ZR23_
HLA-DQA10501-DQB10201	429	YSLPQTMLYCLDRKV	PQTMLYCLD	3	0.2129	4997.8	60.00	0.29	tr_C9ZR23_
HLA-DQA10501-DQB10201	430	AEQIALTCSLCSPGD	EQIALTCSL	1	0.2128	4998.1	60.00	0.40	tr_C9ZR23_
HLA-DQA10501-DQB10201	431	VHNIFHTHYSGGSFW	THYSGGSFW	6	0.2127	5007.5	60.00	0.61	tr_C9ZR23_
HLA-DQA10501-DQB10201	432	KLNVRLVARDATTAA	VRLVARDAT	3	0.2115	5072.0	60.00	0.32	tr_C9ZR23_
HLA-DQA10501-DQB10201	433	DPAVGEKACAMGVMQ	AVGEKACAM	2	0.2108	5109.9	60.00	0.30	tr_C9ZR23_
HLA-DQA10501-DQB10201	434	EKLNVRLVARDATTA	VRLVARDAT	4	0.2099	5161.0	60.00	0.38	tr_C9ZR23_

HLA-DQA10501-DQB10201	435	NGSVTTETNESADVN	VTTETNESA	3	0.2093	5192.6	60.00	0.31	tr_C9ZR23_
HLA-DQA10501-DQB10201	436	ITGREGKQAARAADF	KQAARAADF	6	0.2091	5202.9	60.00	0.74	tr_C9ZR23_
HLA-DQA10501-DQB10201	437	IIQLVHNIFHTHYSG	VHNIFHTHY	4	0.2081	5259.9	60.00	0.46	tr_C9ZR23_
HLA-DQA10501-DQB10201	438	VPETIRSFLQAGIKV	IRSFLQAGI	4	0.2080	5269.5	60.00	0.38	tr_C9ZR23_
HLA-DQA10501-DQB10201	439	TLAVGDGGNDVAMIQ	DGGNDVAMI	5	0.2077	5283.5	60.00	0.22	tr_C9ZR23_
HLA-DQA10501-DQB10201	440	TELSENCVSVICART	ENCVSVICA	4	0.2074	5301.6	60.00	0.42	tr_C9ZR23_
HLA-DQA10501-DQB10201	441	VEEKSTNAIYYFVKG	STNAIYYFV	4	0.2058	5395.0	65.00	0.29	tr_C9ZR23_
HLA-DQA10501-DQB10201	442	SLPQTMLYCLDRKVP	TMLYCLDRK	4	0.2057	5397.9	65.00	0.38	tr_C9ZR23_
HLA-DQA10501-DQB10201	443	CEKLNVRLVARDATT	VRLVARDAT	5	0.2046	5466.8	65.00	0.45	tr_C9ZR23_
HLA-DQA10501-DQB10201	444	LIGIIQLVHNIFHTH	IIQLVHNIF	3	0.2038	5511.1	65.00	0.44	tr_C9ZR23_
HLA-DQA10501-DQB10201	445	ELSENCVSVICARTT	ENCVSVICA	3	0.2028	5574.5	65.00	0.34	tr_C9ZR23_
HLA-DQA10501-DQB10201	446	PALYKVTRSGVDLGV	TRSGVDLGV	6	0.2023	5600.8	65.00	0.29	tr_C9ZR23_
HLA-DQA10501-DQB10201	447	IRSFLQAGIKVWMLT	QAGIKVWML	5	0.2023	5601.5	65.00	0.22	tr_C9ZR23_
HLA-DQA10501-DQB10201	448	ALYKVTRSGVDLGVC	TRSGVDLGV	5	0.2012	5668.1	65.00	0.23	tr_C9ZR23_
HLA-DQA10501-DQB10201	449	RNGSVTTETNESADV	VTTETNESA	4	0.2003	5722.5	65.00	0.42	tr_C9ZR23_
HLA-DQA10501-DQB10201	450	LSENCVSVICARTTP	ENCVSVICA	2	0.1989	5814.0	65.00	0.33	tr_C9ZR23_
HLA-DQA10501-DQB10201	451	GCEKLNVRLVARDAT	VRLVARDAT	6	0.1984	5842.3	65.00	0.47	tr_C9ZR23_
HLA-DQA10501-DQB10201	452	MGVIVEEKSTNAIYY	MGVIVEEKS	0	0.1972	5920.2	65.00	0.34	tr_C9ZR23_
HLA-DQA10501-DQB10201	453	LYKVTRSGVDLGVCQ	TRSGVDLGV	4	0.1967	5950.5	65.00	0.21	tr_C9ZR23_
HLA-DQA10501-DQB10201	454	TNESADVNPLSTGSS	SADVNPLST	3	0.1952	6050.4	65.00	0.38	tr_C9ZR23_
HLA-DQA10501-DQB10201	455	GCGAVGTMRYQAASP	TMRYQAASP	6	0.1950	6059.9	65.00	0.49	tr_C9ZR23_
HLA-DQA10501-DQB10201	456	YGDAAFPTGSSSDPA	PTGSSSDPA	6	0.1950	6065.7	65.00	0.45	tr_C9ZR23_
HLA-DQA10501-DQB10201	457	VLSDKTGTLTQNVME	TGTLTQNVM	5	0.1945	6094.5	65.00	0.56	tr_C9ZR23_
HLA-DQA10501-DQB10201	458	GYMESLLQFSKGVMA	YMESLLQFS	1	0.1944	6100.5	65.00	0.37	tr_C9ZR23_
HLA-DQA10501-DQB10201	459	EEKSTNAIYYFVKGA	STNAIYYFV	3	0.1941	6121.7	65.00	0.25	tr_C9ZR23_
HLA-DQA10501-DQB10201	460	VLEQTPALYKVTRSG	VLEQTPALY	0	0.1940	6128.3	65.00	0.67	tr_C9ZR23_
HLA-DQA10501-DQB10201	461	ILTTPSLLKLLTELS	LLKLLTELS	6	0.1939	6135.1	65.00	0.50	tr_C9ZR23_
HLA-DQA10501-DQB10201	462	MNNRKEEIYKLRLEM	EIYKLRLEM	6	0.1938	6144.1	65.00	0.38	tr_C9ZR23_
HLA-DQA10501-DQB10201	463	SDPAVGEKACAMGVM	AVGEKACAM	3	0.1937	6145.7	65.00	0.37	tr_C9ZR23_
HLA-DQA10501-DQB10201	464	ASPDEVALCNGCEKL	VALCNGCEK	5	0.1933	6172.0	65.00	0.35	tr_C9ZR23_
HLA-DQA10501-DQB10201	465	WGKGERMSSKAAILA	RMSSKAAIL	5	0.1933	6173.8	65.00	0.47	tr_C9ZR23_
HLA-DQA10501-DQB10201	466	YDDVSQEMTQSGLST	YDDVSQEMT	0	0.1933	6174.9	65.00	0.37	tr_C9ZR23_
HLA-DQA10501-DQB10201	467	SLCSPGDCVLRVLAD	DCVLRVLAD	6	0.1907	6349.9	65.00	0.23	tr_C9ZR23_
HLA-DQA10501-DQB10201	468	LRTLLVAEKRLTRNE	RTLLVAEKR	1	0.1896	6428.9	70.00	0.52	tr_C9ZR23_
HLA-DQA10501-DQB10201	469	VAMHHDGEWCNVRNS	AMHHDGEWC	1	0.1894	6441.3	70.00	0.42	tr_C9ZR23_
HLA-DQA10501-DQB10201	470	AYNRTAFVIKYSFYK	NRTAFVIKY	2	0.1875	6577.7	70.00	0.32	tr_C9ZR23_
HLA-DQA10501-DQB10201	471	QDHVPETIRSFLQAG	PETIRSFLQ	4	0.1869	6619.0	70.00	0.28	tr_C9ZR23_
HLA-DQA10501-DQB10201	472	PAVGEKACAMGVMQK	AVGEKACAM	1	0.1866	6638.7	70.00	0.28	tr_C9ZR23_
HLA-DQA10501-DQB10201	473	LPQTMLYCLDRKVPR	TMLYCLDRK	3	0.1863	6664.1	70.00	0.38	tr_C9ZR23_
HLA-DQA10501-DQB10201	474	LCSPGDCVLRVLADK	CVLRVLADK	6	0.1862	6669.3	70.00	0.20	tr_C9ZR23_
HLA-DQA10501-DQB10201	475	EQIALTCSLCSPGDC	IALTCSLCS	2	0.1860	6679.4	70.00	0.32	tr_C9ZR23_
HLA-DQA10501-DQB10201	476	LDRKVPRRVLEQTPA	RRVLEQTPA	6	0.1853	6730.5	70.00	0.32	tr_C9ZR23_
HLA-DQA10501-DQB10201	477	QYGDAAFPTGSSSDP	YGDAAFPTG	1	0.1833	6878.4	70.00	0.33	tr_C9ZR23_
HLA-DQA10501-DQB10201	478	TPALYKVTRSGVDLG	VTRSGVDLG	6	0.1833	6879.5	70.00	0.30	tr_C9ZR23_
HLA-DQA10501-DQB10201	479	LLVAEKRLTRNELDE	KRLTRNELD	5	0.1830	6902.3	70.00	0.47	tr_C9ZR23_
HLA-DQA10501-DQB10201	480	AASPDEVALCNGCEK	EVALCNGCE	5	0.1822	6959.6	70.00	0.34	tr_C9ZR23_
HLA-DQA10501-DQB10201	481	GDAAFPTGSSSDPAV	PTGSSSDPA	5	0.1808	7070.2	70.00	0.44	tr_C9ZR23_
HLA-DQA10501-DQB10201	482	YMESLLQFSKGVMAD	LQFSKGVMA	5	0.1804	7098.4	70.00	0.28	tr_C9ZR23_
HLA-DQA10501-DQB10201	483	GVTAIEDKLQDHVPE	GVTAIEDKL	0	0.1801	7119.7	70.00	0.27	tr_C9ZR23_
HLA-DQA10501-DQB10201	484	EVQGSCGEHVLHFGR	VQGSCGEHV	1	0.1800	7128.4	70.00	0.65	tr_C9ZR23_

HLA-DQA10501-DQB10201	485	SPDEVALCNGCEKLN	VALCNGCEK	4	0.1769	7374.6	70.00	0.34	tr_C9ZR23_
HLA-DQA10501-DQB10201	486	NESADVNPLSTGSSY	SADVNPLST	2	0.1766	7400.9	70.00	0.42	tr_C9ZR23_
HLA-DQA10501-DQB10201	487	SENCVSVICARTTPK	ENCVSVICA	1	0.1760	7448.8	70.00	0.32	tr_C9ZR23_
HLA-DQA10501-DQB10201	488	RSFLQAGIKVWMLTG	QAGIKVWML	4	0.1733	7667.1	70.00	0.25	tr_C9ZR23_
HLA-DQA10501-DQB10201	489	AMIQEAQVGVGITGR	MIQEAQVGV	1	0.1730	7694.3	70.00	0.24	tr_C9ZR23_
HLA-DQA10501-DQB10201	490	QAASPDEVALCNGCE	EVALCNGCE	6	0.1729	7698.7	70.00	0.34	tr_C9ZR23_
HLA-DQA10501-DQB10201	491	RTAFVIKYSFYKSVL	KYSFYKSVL	6	0.1727	7721.3	70.00	0.43	tr_C9ZR23_
HLA-DQA10501-DQB10201	492	QTPALYKVTRSGVDL	KVTRSGVDL	6	0.1721	7771.5	70.00	0.39	tr_C9ZR23_
HLA-DQA10501-DQB10201	493	YVLSDKTGTLTQNVM	TGTLTQNVM	6	0.1710	7858.2	75.00	0.70	tr_C9ZR23_
HLA-DQA10501-DQB10201	494	DAAFPTGSSSDPAVG	PTGSSSDPA	4	0.1702	7928.2	75.00	0.41	tr_C9ZR23_
HLA-DQA10501-DQB10201	495	AVGEKACAMGVMQKR	KACAMGVMQ	4	0.1700	7948.0	75.00	0.29	tr_C9ZR23_
HLA-DQA10501-DQB10201	496	KRNGSVTTETNESAD	VTTETNESA	5	0.1698	7967.0	75.00	0.50	tr_C9ZR23_
HLA-DQA10501-DQB10201	497	PDEVALCNGCEKLNV	VALCNGCEK	3	0.1692	8014.9	75.00	0.36	tr_C9ZR23_
HLA-DQA10501-DQB10201	498	SFLQAGIKVWMLTGD	QAGIKVWML	3	0.1691	8019.6	75.00	0.28	tr_C9ZR23_
HLA-DQA10501-DQB10201	499	IQLVHNIFHTHYSGG	VHNIFHTHY	3	0.1683	8096.5	75.00	0.60	tr_C9ZR23_
HLA-DQA10501-DQB10201	500	AAFPTGSSSDPAVGE	PTGSSSDPA	3	0.1675	8164.7	75.00	0.41	tr_C9ZR23_
HLA-DQA10501-DQB10201	501	ADVNPLSTGSSYVLV	STGSSYVLV	6	0.1671	8196.1	75.00	0.34	tr_C9ZR23_
HLA-DQA10501-DQB10201	502	SMNNRKEEIYKLRLE	EEIYKLRLE	6	0.1650	8387.3	75.00	0.39	tr_C9ZR23_
HLA-DQA10501-DQB10201	503	LEKVREAELSMNNRK	EKVREAELS	1	0.1649	8392.3	75.00	0.25	tr_C9ZR23_
HLA-DQA10501-DQB10201	504	MIQEAQVGVGITGRE	VGVGITGRE	6	0.1644	8444.9	75.00	0.20	tr_C9ZR23_
HLA-DQA10501-DQB10201	505	TCSLCSPGDCVLRVL	PGDCVLRVL	6	0.1635	8523.6	75.00	0.36	tr_C9ZR23_
HLA-DQA10501-DQB10201	506	CSLCSPGDCVLRVLA	PGDCVLRVL	5	0.1635	8526.1	75.00	0.31	tr_C9ZR23_
HLA-DQA10501-DQB10201	507	PQTMLYCLDRKVPRR	TMLYCLDRK	2	0.1622	8642.3	75.00	0.41	tr_C9ZR23_
HLA-DQA10501-DQB10201	508	TLLVAEKRLTRNELD	KRLTRNELD	6	0.1622	8646.2	75.00	0.50	tr_C9ZR23_
HLA-DQA10501-DQB10201	509	YNRTAFVIKYSFYKS	NRTAFVIKY	1	0.1619	8673.7	75.00	0.31	tr_C9ZR23_
HLA-DQA10501-DQB10201	510	QWGKGERMSSKAAIL	RMSSKAAIL	6	0.1604	8817.4	75.00	0.73	tr_C9ZR23_
HLA-DQA10501-DQB10201	511	ESADVNPLSTGSSYV	SADVNPLST	1	0.1598	8872.0	75.00	0.41	tr_C9ZR23_
HLA-DQA10501-DQB10201	512	CLDRKVPRRVLEQTP	PRRVLEQTP	6	0.1579	9055.8	75.00	0.46	tr_C9ZR23_
HLA-DQA10501-DQB10201	513	RTLLVAEKRLTRNEL	RTLLVAEKR	0	0.1577	9075.3	75.00	0.44	tr_C9ZR23_
HLA-DQA10501-DQB10201	514	DEVALCNGCEKLNVR	VALCNGCEK	2	0.1577	9076.1	75.00	0.40	tr_C9ZR23_
HLA-DQA10501-DQB10201	515	GSSSDPAVGEKACAM	AVGEKACAM	6	0.1561	9238.1	75.00	0.62	tr_C9ZR23_
HLA-DQA10501-DQB10201	516	LVHNIFHTHYSGGSF	VHNIFHTHY	1	0.1558	9267.6	75.00	0.52	tr_C9ZR23_
HLA-DQA10501-DQB10201	517	QKRNGSVTTETNESA	VTTETNESA	6	0.1551	9332.0	75.00	0.47	tr_C9ZR23_
HLA-DQA10501-DQB10201	518	SSDPAVGEKACAMGV	AVGEKACAM	4	0.1547	9378.3	75.00	0.56	tr_C9ZR23_
HLA-DQA10501-DQB10201	519	QIALTCSLCSPGDCV	IALTCSLCS	1	0.1539	9453.2	75.00	0.38	tr_C9ZR23_
HLA-DQA10501-DQB10201	520	QLVHNIFHTHYSGGS	VHNIFHTHY	2	0.1531	9544.1	80.00	0.60	tr_C9ZR23_
HLA-DQA10501-DQB10201	521	NGCEKLNVRLVARDA	NVRLVARDA	6	0.1530	9545.8	80.00	0.42	tr_C9ZR23_
HLA-DQA10501-DQB10201	522	SADVNPLSTGSSYVL	PLSTGSSYV	5	0.1523	9620.7	80.00	0.33	tr_C9ZR23_
HLA-DQA10501-DQB10201	523	FLQAGIKVWMLTGDK	QAGIKVWML	2	0.1507	9793.5	80.00	0.35	tr_C9ZR23_
HLA-DQA10501-DQB10201	524	ENCVSVICARTTPKQ	ENCVSVICA	0	0.1502	9845.5	80.00	0.23	tr_C9ZR23_
HLA-DQA10501-DQB10201	525	AFPTGSSSDPAVGEK	PTGSSSDPA	2	0.1502	9848.6	80.00	0.41	tr_C9ZR23_
HLA-DQA10501-DQB10201	526	VGEKACAMGVMQKRN	KACAMGVMQ	3	0.1502	9849.6	80.00	0.34	tr_C9ZR23_
HLA-DQA10501-DQB10201	527	IQEAQVGVGITGREG	VGVGITGRE	5	0.1501	9852.9	80.00	0.23	tr_C9ZR23_
HLA-DQA10501-DQB10201	528	VDYVLSDKTGTLTQN	VDYVLSDKT	0	0.1491	9963.0	80.00	0.60	tr_C9ZR23_
HLA-DQA10501-DQB10201	529	GSCGEHVLHFGRVLS	VLHFGRVLS	6	0.1489	9982.6	80.00	0.39	tr_C9ZR23_
HLA-DQA10501-DQB10201	530	AVTRLVRSRGFITLA	RSRGFITLA	6	0.1482	10060.8	80.00	0.34	tr_C9ZR23_
HLA-DQA10501-DQB10201	531	STGCGAVGTMRYQAA	VGTMRYQAA	6	0.1480	10083.9	80.00	0.37	tr_C9ZR23_
HLA-DQA10501-DQB10201	532	VTAIEDKLQDHVPET	IEDKLQDHV	3	0.1476	10121.0	80.00	0.18	tr_C9ZR23_
HLA-DQA10501-DQB10201	533	SSSDPAVGEKACAMG	AVGEKACAM	5	0.1471	10179.8	80.00	0.62	tr_C9ZR23_
HLA-DQA10501-DQB10201	534	NRTAFVIKYSFYKSV	IKYSFYKSV	6	0.1458	10319.6	80.00	0.19	tr_C9ZR23_

HLA-DQA10501-DQB10201	535	VQGSCGEHVLHFGRV	VQGSCGEHV	0	0.1440	10523.9	80.00	0.52	tr_C9ZR23_
HLA-DQA10501-DQB10201	536	REAELSMNNRKEEIY	SMNNRKEEI	5	0.1402	10967.2	80.00	0.40	tr_C9ZR23_
HLA-DQA10501-DQB10201	537	TGCGAVGTMRYQAAS	VGTMRYQAA	5	0.1394	11064.9	80.00	0.38	tr_C9ZR23_
HLA-DQA10501-DQB10201	538	QTMLYCLDRKVPRRV	TMLYCLDRK	1	0.1386	11161.3	80.00	0.47	tr_C9ZR23_
HLA-DQA10501-DQB10201	539	FPTGSSSDPAVGEKA	PTGSSSDPA	1	0.1384	11181.9	80.00	0.39	tr_C9ZR23_
HLA-DQA10501-DQB10201	540	DDVSQEMTQSGLSTG	EMTQSGLST	5	0.1375	11295.9	80.00	0.36	tr_C9ZR23_
HLA-DQA10501-DQB10201	541	IALTCSLCSPGDCVL	IALTCSLCS	0	0.1336	11786.2	85.00	0.28	tr_C9ZR23_
HLA-DQA10501-DQB10201	542	GEKACAMGVMQKRNG	KACAMGVMQ	2	0.1332	11832.1	85.00	0.36	tr_C9ZR23_
HLA-DQA10501-DQB10201	543	AAVTRLVRSRGFITL	LVRSRGFIT	5	0.1321	11971.2	85.00	0.24	tr_C9ZR23_
HLA-DQA10501-DQB10201	544	HDGEWCNVRNSSIVE	NVRNSSIVE	6	0.1314	12066.5	85.00	0.59	tr_C9ZR23_
HLA-DQA10501-DQB10201	545	LSMNNRKEEIYKLRL	KEEIYKLRL	6	0.1306	12168.9	85.00	0.40	tr_C9ZR23_
HLA-DQA10501-DQB10201	546	QEAQVGVGITGREGK	VGVGITGRE	4	0.1297	12282.3	85.00	0.25	tr_C9ZR23_
HLA-DQA10501-DQB10201	547	EVALCNGCEKLNVRL	VALCNGCEK	1	0.1294	12326.7	85.00	0.43	tr_C9ZR23_
HLA-DQA10501-DQB10201	548	LEMALDENSSTGGPQ	LEMALDENS	0	0.1293	12340.9	85.00	0.43	tr_C9ZR23_
HLA-DQA10501-DQB10201	549	EKVREAELSMNNRKE	KVREAELSM	1	0.1291	12369.1	85.00	0.32	tr_C9ZR23_
HLA-DQA10501-DQB10201	550	LTCSLCSPGDCVLRV	SPGDCVLRV	6	0.1283	12476.8	85.00	0.40	tr_C9ZR23_
HLA-DQA10501-DQB10201	551	DVSQEMTQSGLSTGC	EMTQSGLST	4	0.1274	12597.1	85.00	0.37	tr_C9ZR23_
HLA-DQA10501-DQB10201	552	EQTPALYKVTRSGVD	PALYKVTRS	3	0.1261	12777.4	85.00	0.27	tr_C9ZR23_
HLA-DQA10501-DQB10201	553	SGLSTGCGAVGTMRY	CGAVGTMRY	6	0.1237	13115.6	85.00	0.47	tr_C9ZR23_
HLA-DQA10501-DQB10201	554	AELSMNNRKEEIYKL	SMNNRKEEI	3	0.1229	13222.2	85.00	0.38	tr_C9ZR23_
HLA-DQA10501-DQB10201	555	AMHHDGEWCNVRNSS	AMHHDGEWC	0	0.1223	13312.3	85.00	0.44	tr_C9ZR23_
HLA-DQA10501-DQB10201	556	EAELSMNNRKEEIYK	SMNNRKEEI	4	0.1221	13342.3	85.00	0.38	tr_C9ZR23_
HLA-DQA10501-DQB10201	557	QGSCGEHVLHFGRVL	HVLHFGRVL	6	0.1221	13347.1	85.00	0.41	tr_C9ZR23_
HLA-DQA10501-DQB10201	558	VREAELSMNNRKEEI	SMNNRKEEI	6	0.1200	13646.9	85.00	0.44	tr_C9ZR23_
HLA-DQA10501-DQB10201	559	LSTGCGAVGTMRYQA	CGAVGTMRY	4	0.1181	13937.5	85.00	0.38	tr_C9ZR23_
HLA-DQA10501-DQB10201	560	KAAVTRLVRSRGFIT	LVRSRGFIT	6	0.1170	14095.3	85.00	0.28	tr_C9ZR23_
HLA-DQA10501-DQB10201	561	EAQVGVGITGREGKQ	VGVGITGRE	3	0.1168	14131.0	85.00	0.27	tr_C9ZR23_
HLA-DQA10501-DQB10201	562	MQKRNGSVTTETNES	SVTTETNES	6	0.1159	14267.4	85.00	0.25	tr_C9ZR23_
HLA-DQA10501-DQB10201	563	CNGCEKLNVRLVARD	LNVRLVARD	6	0.1155	14329.1	85.00	0.30	tr_C9ZR23_
HLA-DQA10501-DQB10201	564	NCVSVICARTTPKQK	VSVICARTT	2	0.1149	14423.9	85.00	0.42	tr_C9ZR23_
HLA-DQA10501-DQB10201	565	EKACAMGVMQKRNGS	KACAMGVMQ	1	0.1136	14624.9	90.00	0.41	tr_C9ZR23_
HLA-DQA10501-DQB10201	566	TTPKQKAAVTRLVRS	QKAAVTRLV	4	0.1136	14634.4	90.00	0.31	tr_C9ZR23_
HLA-DQA10501-DQB10201	567	SQEMTQSGLSTGCGA	EMTQSGLST	2	0.1133	14674.6	90.00	0.29	tr_C9ZR23_
HLA-DQA10501-DQB10201	568	NSSTGGPQWGKGERM	PQWGKGERM	6	0.1129	14742.0	90.00	0.72	tr_C9ZR23_
HLA-DQA10501-DQB10201	569	ARTTPKQKAAVTRLV	QKAAVTRLV	6	0.1125	14810.4	90.00	0.56	tr_C9ZR23_
HLA-DQA10501-DQB10201	570	QKAAVTRLVRSRGFI	TRLVRSRGF	5	0.1117	14923.5	90.00	0.34	tr_C9ZR23_
HLA-DQA10501-DQB10201	571	KQKAAVTRLVRSRGF	TRLVRSRGF	6	0.1106	15114.2	90.00	0.38	tr_C9ZR23_
HLA-DQA10501-DQB10201	572	VSQEMTQSGLSTGCG	EMTQSGLST	3	0.1104	15139.9	90.00	0.37	tr_C9ZR23_
HLA-DQA10501-DQB10201	573	GLSTGCGAVGTMRYQ	CGAVGTMRY	5	0.1095	15293.9	90.00	0.50	tr_C9ZR23_
HLA-DQA10501-DQB10201	574	PTGSSSDPAVGEKAC	PTGSSSDPA	0	0.1092	15333.3	90.00	0.32	tr_C9ZR23_
HLA-DQA10501-DQB10201	575	ELSMNNRKEEIYKLR	SMNNRKEEI	2	0.1089	15387.7	90.00	0.40	tr_C9ZR23_
HLA-DQA10501-DQB10201	576	VMQKRNGSVTTETNE	RNGSVTTET	4	0.1081	15519.4	90.00	0.38	tr_C9ZR23_
HLA-DQA10501-DQB10201	577	YCLDRKVPRRVLEQT	VPRRVLEQT	6	0.1065	15796.7	90.00	0.61	tr_C9ZR23_
HLA-DQA10501-DQB10201	578	LEQTPALYKVTRSGV	PALYKVTRS	4	0.1064	15819.0	90.00	0.43	tr_C9ZR23_
HLA-DQA10501-DQB10201	579	RTTPKQKAAVTRLVR	QKAAVTRLV	5	0.1057	15929.1	90.00	0.47	tr_C9ZR23_
HLA-DQA10501-DQB10201	580	TMLYCLDRKVPRRVL	TMLYCLDRK	0	0.1054	15992.3	90.00	0.49	tr_C9ZR23_
HLA-DQA10501-DQB10201	581	GVMQKRNGSVTTETN	RNGSVTTET	5	0.1044	16163.4	90.00	0.43	tr_C9ZR23_
HLA-DQA10501-DQB10201	582	STGGPQWGKGERMSS	PQWGKGERM	4	0.1021	16572.3	90.00	0.71	tr_C9ZR23_
HLA-DQA10501-DQB10201	583	ALTCSLCSPGDCVLR	SLCSPGDCV	4	0.1020	16582.6	90.00	0.31	tr_C9ZR23_
HLA-DQA10501-DQB10201	584	AQVGVGITGREGKQA	VGVGITGRE	2	0.1016	16660.3	90.00	0.34	tr_C9ZR23_

HLA-DQA10501-DQB10201	585	TPKQKAAVTRLVRSR	QKAAVTRLV	3	0.1011	16743.7	90.00	0.34	tr_C9ZR23_
HLA-DQA10501-DQB10201	586	ALCNGCEKLNVRLVA	EKLNVRLVA	6	0.1009	16787.6	90.00	0.54	tr_C9ZR23_
HLA-DQA10501-DQB10201	587	QEMTQSGLSTGCGAV	EMTQSGLST	1	0.1005	16863.7	90.00	0.29	tr_C9ZR23_
HLA-DQA10501-DQB10201	588	KVREAELSMNNRKEE	VREAELSMN	1	0.1004	16873.4	90.00	0.22	tr_C9ZR23_
HLA-DQA10501-DQB10201	589	SSTGGPQWGKGERMS	PQWGKGERM	5	0.1002	16901.9	90.00	0.77	tr_C9ZR23_
HLA-DQA10501-DQB10201	590	EMALDENSSTGGPQW	NSSTGGPQW	6	0.0976	17390.5	90.00	0.35	tr_C9ZR23_
HLA-DQA10501-DQB10201	591	CVSVICARTTPKQKA	VSVICARTT	1	0.0957	17758.3	90.00	0.41	tr_C9ZR23_
HLA-DQA10501-DQB10201	592	PKQKAAVTRLVRSRG	QKAAVTRLV	2	0.0949	17916.7	90.00	0.32	tr_C9ZR23_
HLA-DQA10501-DQB10201	593	PQWGKGERMSSKAAI	ERMSSKAAI	6	0.0933	18213.2	90.00	0.34	tr_C9ZR23_
HLA-DQA10501-DQB10201	594	MGVMQKRNGSVTTET	RNGSVTTET	6	0.0929	18289.6	90.00	0.64	tr_C9ZR23_
HLA-DQA10501-DQB10201	595	LCNGCEKLNVRLVAR	EKLNVRLVA	5	0.0921	18462.6	90.00	0.43	tr_C9ZR23_
HLA-DQA10501-DQB10201	596	VALCNGCEKLNVRLV	VALCNGCEK	0	0.0916	18559.8	90.00	0.39	tr_C9ZR23_
HLA-DQA10501-DQB10201	597	TGSSSDPAVGEKACA	SDPAVGEKA	4	0.0915	18586.1	90.00	0.28	tr_C9ZR23_
HLA-DQA10501-DQB10201	598	QVGVGITGREGKQAA	VGVGITGRE	1	0.0896	18970.9	95.00	0.35	tr_C9ZR23_
HLA-DQA10501-DQB10201	599	MHHDGEWCNVRNSSI	WCNVRNSSI	6	0.0889	19104.6	95.00	0.38	tr_C9ZR23_
HLA-DQA10501-DQB10201	600	TGGPQWGKGERMSSK	PQWGKGERM	3	0.0885	19194.1	95.00	0.70	tr_C9ZR23_
HLA-DQA10501-DQB10201	601	TQSGLSTGCGAVGTM	TGCGAVGTM	6	0.0878	19329.6	95.00	0.26	tr_C9ZR23_
HLA-DQA10501-DQB10201	602	EMTQSGLSTGCGAVG	SGLSTGCGA	4	0.0869	19535.2	95.00	0.32	tr_C9ZR23_
HLA-DQA10501-DQB10201	603	GGPQWGKGERMSSKA	PQWGKGERM	2	0.0847	20001.2	95.00	0.63	tr_C9ZR23_
HLA-DQA10501-DQB10201	604	DYVLSDKTGTLTQNV	KTGTLTQNV	6	0.0841	20130.4	95.00	0.37	tr_C9ZR23_
HLA-DQA10501-DQB10201	605	HHDGEWCNVRNSSIV	WCNVRNSSI	5	0.0826	20463.5	95.00	0.41	tr_C9ZR23_
HLA-DQA10501-DQB10201	606	MALDENSSTGGPQWG	NSSTGGPQW	5	0.0823	20518.5	95.00	0.44	tr_C9ZR23_
HLA-DQA10501-DQB10201	607	QSGLSTGCGAVGTMR	TGCGAVGTM	5	0.0810	20819.3	95.00	0.25	tr_C9ZR23_
HLA-DQA10501-DQB10201	608	GPQWGKGERMSSKAA	PQWGKGERM	1	0.0787	21328.7	95.00	0.61	tr_C9ZR23_
HLA-DQA10501-DQB10201	609	MTQSGLSTGCGAVGT	SGLSTGCGA	3	0.0780	21509.0	95.00	0.34	tr_C9ZR23_
HLA-DQA10501-DQB10201	610	KACAMGVMQKRNGSV	KACAMGVMQ	0	0.0756	22063.8	95.00	0.39	tr_C9ZR23_
HLA-DQA10501-DQB10201	611	CARTTPKQKAAVTRL	KQKAAVTRL	6	0.0744	22352.4	95.00	0.88	tr_C9ZR23_
HLA-DQA10501-DQB10201	612	GITGREGKQAARAAD	REGKQAARA	4	0.0710	23195.5	95.00	0.50	tr_C9ZR23_
HLA-DQA10501-DQB10201	613	GVGITGREGKQAARA	REGKQAARA	6	0.0682	23895.8	95.00	0.50	tr_C9ZR23_
HLA-DQA10501-DQB10201	614	VGVGITGREGKQAAR	GVGITGREG	1	0.0674	24105.6	95.00	0.38	tr_C9ZR23_
HLA-DQA10501-DQB10201	615	ALDENSSTGGPQWGK	NSSTGGPQW	4	0.0671	24184.3	95.00	0.51	tr_C9ZR23_
HLA-DQA10501-DQB10201	616	VSVICARTTPKQKAA	VSVICARTT	0	0.0663	24411.4	95.00	0.42	tr_C9ZR23_
HLA-DQA10501-DQB10201	617	VGITGREGKQAARAA	REGKQAARA	5	0.0657	24573.3	95.00	0.52	tr_C9ZR23_
HLA-DQA10501-DQB10201	618	LDENSSTGGPQWGKG	NSSTGGPQW	3	0.0611	25818.9	100.00	0.53	tr_C9ZR23_
HLA-DQA10501-DQB10201	619	AMGVMQKRNGSVTTE	KRNGSVTTE	6	0.0591	26380.9	100.00	0.67	tr_C9ZR23_
HLA-DQA10501-DQB10201	620	DENSSTGGPQWGKGE	NSSTGGPQW	2	0.0581	26664.7	100.00	0.50	tr_C9ZR23_
HLA-DQA10501-DQB10201	621	LYCLDRKVPRRVLEQ	KVPRRVLEQ	6	0.0578	26758.9	100.00	0.41	tr_C9ZR23_
HLA-DQA10501-DQB10201	622	MLYCLDRKVPRRVLE	MLYCLDRKV	0	0.0553	27486.3	100.00	0.31	tr_C9ZR23_
HLA-DQA10501-DQB10201	623	ENSSTGGPQWGKGER	NSSTGGPQW	1	0.0523	28380.0	100.00	0.46	tr_C9ZR23_
HLA-DQA10501-DQB10201	624	ACAMGVMQKRNGSVT	CAMGVMQKR	1	0.0497	29193.8	100.00	0.26	tr_C9ZR23_
HLA-DQA10501-DQB10201	625	CAMGVMQKRNGSVTT	CAMGVMQKR	0	0.0395	32595.6	100.00	0.21	tr_C9ZR23_
HLA-DQA10501-DQB10201	626	SVICARTTPKQKAAV	VICARTTPK	1	0.0356	34027.3	100.00	0.44	tr_C9ZR23_
HLA-DQA10501-DQB10201	627	VICARTTPKQKAAVT	VICARTTPK	0	0.0264	37594.1	100.00	0.34	tr_C9ZR23_
HLA-DQA10501-DQB10201	628	ICARTTPKQKAAVTR	PKQKAAVTR	6	0.0186	40882.4	100.00	0.32	tr_C9ZR23_

Allele: HLA-DQA10501-DQB10201. Number of high binders 0. Number of weak binders 83. Number of peptides 628

Allele	pos	peptide	core	Of	1-log50k(aff)	affinity(nM)	%Rank	Relia	Identity	Bind Level
HLA-DQA10501-DQB10201	1	STYVTVVLADDELTI	TVVLADDEL	4	0.7159	21.6	0.12	0.46	tr_C9ZR23_	SB
HLA-DQA10501-DQB10201	2	PSTYVTVVLADDELT	TVVLADDEL	5	0.7117	22.6	0.12	0.50	tr_C9ZR23_	SB
HLA-DQA10501-DQB10201	3	EPSTYVTVVLADDEL	TVVLADDEL	6	0.7066	23.9	0.15	0.51	tr_C9ZR23_	SB
HLA-DQA10501-DQB10201	4	TYVTVVLADDELTIR	TVVLADDEL	3	0.6962	26.8	0.17	0.46	tr_C9ZR23_	SB
HLA-DQA10501-DQB10201	5	YVTVVLADDELTIRN	TVVLADDEL	2	0.6770	33.0	0.30	0.47	tr_C9ZR23_	SB
HLA-DQA10501-DQB10201	6	VTVVLADDELTIRNA	TVVLADDEL	1	0.6221	59.7	0.90	0.53	tr_C9ZR23_	WB
HLA-DQA10501-DQB10201	7	EEPSTYVTVVLADDE	VTVVLADDE	6	0.6037	72.8	1.20	0.64	tr_C9ZR23_	WB
HLA-DQA10501-DQB10201	8	TVVLADDELTIRNAN	TVVLADDEL	0	0.5349	153.2	3.50	0.62	tr_C9ZR23_	WB
HLA-DQA10501-DQB10201	9	PEEPSTYVTVVLADD	YVTVVLADD	6	0.4481	391.9	10.00	0.62	tr_C9ZR23_	
HLA-DQA10501-DQB10201	10	VVLADDELTIRNANS	VVLADDELT	0	0.3384	1284.2	27.00	0.68	tr_C9ZR23_	
HLA-DQA10501-DQB10201	11	LSRCLWCVPEEPSTY	CLWCVPEEP	3	0.3179	1603.4	31.00	0.48	tr_C9ZR23_	
HLA-DQA10501-DQB10201	12	VSRLSRCLWCVPEEP	CLWCVPEEP	6	0.3168	1622.4	31.00	0.51	tr_C9ZR23_	
HLA-DQA10501-DQB10201	13	SRCLWCVPEEPSTYV	CLWCVPEEP	2	0.3162	1632.9	31.00	0.43	tr_C9ZR23_	
HLA-DQA10501-DQB10201	14	SRLSRCLWCVPEEPS	CLWCVPEEP	5	0.3131	1689.2	32.00	0.50	tr_C9ZR23_	
HLA-DQA10501-DQB10201	15	RLSRCLWCVPEEPST	CLWCVPEEP	4	0.3083	1779.3	33.00	0.50	tr_C9ZR23_	
HLA-DQA10501-DQB10201	16	VPEEPSTYVTVVLAD	TYVTVVLAD	6	0.3042	1860.7	34.00	0.36	tr_C9ZR23_	
HLA-DQA10501-DQB10201	17	RCLWCVPEEPSTYVT	CLWCVPEEP	1	0.2905	2157.9	37.00	0.44	tr_C9ZR23_	
HLA-DQA10501-DQB10201	18	CVPEEPSTYVTVVLA	STYVTVVLA	6	0.2624	2923.1	45.00	0.34	tr_C9ZR23_	
HLA-DQA10501-DQB10201	19	WCVPEEPSTYVTVVL	PSTYVTVVL	6	0.2536	3216.1	47.00	0.54	tr_C9ZR23_	
HLA-DQA10501-DQB10201	20	WRPDPRDVMRSAELR	RDVMRSAEL	5	0.2445	3548.7	50.00	0.44	tr_C9ZR23_	
HLA-DQA10501-DQB10201	21	RPDPRDVMRSAELRR	DVMRSAELR	5	0.2410	3683.9	55.00	0.41	tr_C9ZR23_	
HLA-DQA10501-DQB10201	22	PDPRDVMRSAELRRQ	DVMRSAELR	4	0.2360	3889.5	55.00	0.35	tr_C9ZR23_	
HLA-DQA10501-DQB10201	23	DPRDVMRSAELRRQA	DVMRSAELR	3	0.2280	4243.4	55.00	0.37	tr_C9ZR23_	
HLA-DQA10501-DQB10201	24	CLWCVPEEPSTYVTV	CLWCVPEEP	0	0.2262	4325.4	55.00	0.43	tr_C9ZR23_	
HLA-DQA10501-DQB10201	25	SAELRRQANDPLAVE	LRRQANDPL	3	0.2227	4494.8	60.00	0.32	tr_C9ZR23_	
HLA-DQA10501-DQB10201	26	IWRPDPRDVMRSAEL	RDVMRSAEL	6	0.2207	4590.2	60.00	0.67	tr_C9ZR23_	
HLA-DQA10501-DQB10201	27	SVSRLSRCLWCVPEE	RCLWCVPEE	6	0.2192	4667.5	60.00	0.43	tr_C9ZR23_	
HLA-DQA10501-DQB10201	28	PRDVMRSAELRRQAN	DVMRSAELR	2	0.2088	5219.0	60.00	0.35	tr_C9ZR23_	
HLA-DQA10501-DQB10201	29	RSAELRRQANDPLAV	LRRQANDPL	4	0.2058	5396.0	65.00	0.38	tr_C9ZR23_	
HLA-DQA10501-DQB10201	30	AELRRQANDPLAVEK	LRRQANDPL	2	0.2041	5495.0	65.00	0.33	tr_C9ZR23_	
HLA-DQA10501-DQB10201	31	RDVMRSAELRRQAND	DVMRSAELR	1	0.1933	6176.1	65.00	0.37	tr_C9ZR23_	
HLA-DQA10501-DQB10201	32	LWCVPEEPSTYVTVV	EPSTYVTVV	6	0.1913	6309.7	65.00	0.43	tr_C9ZR23_	
HLA-DQA10501-DQB10201	33	ELRRQANDPLAVEKR	LRRQANDPL	1	0.1875	6577.4	70.00	0.28	tr_C9ZR23_	
HLA-DQA10501-DQB10201	34	VMRSAELRRQANDPL	LRRQANDPL	6	0.1723	7752.6	70.00	0.58	tr_C9ZR23_	
HLA-DQA10501-DQB10201	35	MRSAELRRQANDPLA	LRRQANDPL	5	0.1707	7887.3	75.00	0.53	tr_C9ZR23_	
HLA-DQA10501-DQB10201	36	DVMRSAELRRQANDP	DVMRSAELR	0	0.1648	8404.1	75.00	0.32	tr_C9ZR23_	
HLA-DQA10501-DQB10201	37	LRRQANDPLAVEKRA	RQANDPLAV	2	0.1641	8472.8	75.00	0.28	tr_C9ZR23_	
HLA-DQA10501-DQB10201	38	VLADDELTIRNANSV	VLADDELTI	0	0.1554	9310.7	75.00	0.50	tr_C9ZR23_	
HLA-DQA10501-DQB10201	39	ASVSRLSRCLWCVPE	VSRLSRCLW	2	0.1549	9359.1	75.00	0.41	tr_C9ZR23_	
HLA-DQA10501-DQB10201	40	VEKRASVSRLSRCLW	VSRLSRCLW	6	0.1524	9614.2	80.00	0.51	tr_C9ZR23_	
HLA-DQA10501-DQB10201	41	EKRASVSRLSRCLWC	VSRLSRCLW	5	0.1509	9770.9	80.00	0.46	tr_C9ZR23_	
HLA-DQA10501-DQB10201	42	RASVSRLSRCLWCVP	VSRLSRCLW	3	0.1470	10194.9	80.00	0.51	tr_C9ZR23_	
HLA-DQA10501-DQB10201	43	KRASVSRLSRCLWCV	VSRLSRCLW	4	0.1439	10541.3	80.00	0.49	tr_C9ZR23_	
HLA-DQA10501-DQB10201	44	LIKIWRPDPRDVMRS	WRPDPRDVM	4	0.1343	11687.4	85.00	0.34	tr_C9ZR23_	
HLA-DQA10501-DQB10201	45	RRQANDPLAVEKRAS	RQANDPLAV	1	0.1325	11924.0	85.00	0.34	tr_C9ZR23_	
HLA-DQA10501-DQB10201	46	KIWRPDPRDVMRSAE	PRDVMRSAE	6	0.1199	13660.3	85.00	0.34	tr_C9ZR23_	
HLA-DQA10501-DQB10201	47	IKIWRPDPRDVMRSA	WRPDPRDVM	3	0.1178	13975.3	85.00	0.38	tr_C9ZR23_	
HLA-DQA10501-DQB10201	48	RQANDPLAVEKRASV	QANDPLAVE	1	0.1121	14859.0	90.00	0.31	tr_C9ZR23_	

HLA-DQA10501-DQB10201	49	DPLAVEKRASVSRLS	EKRASVSRL	5	0.1017	16628.9	90.00	0.46	tr_C9ZR23_	
HLA-DQA10501-DQB10201	50	LAVEKRASVSRLSRC	EKRASVSRL	3	0.0987	17179.0	90.00	0.38	tr_C9ZR23_	
HLA-DQA10501-DQB10201	51	PLAVEKRASVSRLSR	EKRASVSRL	4	0.0979	17332.1	90.00	0.42	tr_C9ZR23_	
HLA-DQA10501-DQB10201	52	AVEKRASVSRLSRCL	EKRASVSRL	2	0.0976	17389.0	90.00	0.32	tr_C9ZR23_	
HLA-DQA10501-DQB10201	53	NDPLAVEKRASVSRL	EKRASVSRL	6	0.0938	18125.7	90.00	0.56	tr_C9ZR23_	
HLA-DQA10501-DQB10201	54	QANDPLAVEKRASVS	QANDPLAVE	0	0.0850	19934.3	95.00	0.29	tr_C9ZR23_	
HLA-DQA10501-DQB10201	55	ANDPLAVEKRASVSR	DPLAVEKRA	2	0.0606	25963.2	100.00	0.24	tr_C9ZR23_	

Allele: HLA-DQA10501-DQB10201. Number of high binders 5. Number of weak binders 3. Number of peptides 55

Allele	pos	peptide	core	Of	1-log50k(aff)	affinity(nM)	%Rank	Relia	Identity	Bind Level
HLA-DQA10501-DQB10201	1	RSVTAVFGVVDDAML	VFGVVDDAM	5	0.7096	23.2	0.15	0.53	tr_C9ZN43_	SB
HLA-DQA10501-DQB10201	2	SVTAVFGVVDDAMLS	VFGVVDDAM	4	0.6967	26.6	0.17	0.54	tr_C9ZN43_	SB
HLA-DQA10501-DQB10201	3	TRSVTAVFGVVDDAM	VFGVVDDAM	6	0.6939	27.4	0.20	0.63	tr_C9ZN43_	SB
HLA-DQA10501-DQB10201	4	VTAVFGVVDDAMLSI	VFGVVDDAM	3	0.6872	29.5	0.25	0.56	tr_C9ZN43_	SB
HLA-DQA10501-DQB10201	5	DGELMVYGWIAGEVL	YGWIAGEVL	6	0.6814	31.4	0.25	0.61	tr_C9ZN43_	SB
HLA-DQA10501-DQB10201	6	LIPFFDVALMYDELV	VALMYDELV	6	0.6722	34.7	0.40	0.50	tr_C9ZN43_	SB
HLA-DQA10501-DQB10201	7	ELMVYGWIAGEVLSQ	YGWIAGEVL	4	0.6678	36.4	0.40	0.56	tr_C9ZN43_	SB
HLA-DQA10501-DQB10201	8	TWREALEADGVEFFL	LEADGVEFF	5	0.6677	36.4	0.40	0.25	tr_C9ZN43_	SB
HLA-DQA10501-DQB10201	9	TAVFGVVDDAMLSIA	VFGVVDDAM	2	0.6669	36.8	0.40	0.58	tr_C9ZN43_	SB
HLA-DQA10501-DQB10201	10	DTWREALEADGVEFF	LEADGVEFF	6	0.6658	37.2	0.40	0.27	tr_C9ZN43_	SB
HLA-DQA10501-DQB10201	11	IPFFDVALMYDELVS	VALMYDELV	5	0.6648	37.6	0.40	0.47	tr_C9ZN43_	SB
HLA-DQA10501-DQB10201	12	GELMVYGWIAGEVLS	YGWIAGEVL	5	0.6646	37.7	0.40	0.61	tr_C9ZN43_	SB
HLA-DQA10501-DQB10201	13	LMVYGWIAGEVLSQA	YGWIAGEVL	3	0.6611	39.1	0.40	0.52	tr_C9ZN43_	SB
HLA-DQA10501-DQB10201	14	PFFDVALMYDELVSA	VALMYDELV	4	0.6570	40.9	0.50	0.44	tr_C9ZN43_	WB
HLA-DQA10501-DQB10201	15	FFDVALMYDELVSAF	VALMYDELV	3	0.6549	41.8	0.50	0.45	tr_C9ZN43_	WB
HLA-DQA10501-DQB10201	16	MVYGWIAGEVLSQAL	YGWIAGEVL	2	0.6488	44.7	0.50	0.50	tr_C9ZN43_	WB
HLA-DQA10501-DQB10201	17	WREALEADGVEFFLG	LEADGVEFF	4	0.6450	46.6	0.60	0.26	tr_C9ZN43_	WB
HLA-DQA10501-DQB10201	18	AVFGVVDDAMLSIAE	VFGVVDDAM	1	0.6366	51.0	0.70	0.57	tr_C9ZN43_	WB
HLA-DQA10501-DQB10201	19	SPSNATYLALQSELD	YLALQSELD	6	0.6311	54.1	0.70	0.51	tr_C9ZN43_	WB
HLA-DQA10501-DQB10201	20	PSNATYLALQSELDT	YLALQSELD	5	0.6285	55.7	0.80	0.51	tr_C9ZN43_	WB
HLA-DQA10501-DQB10201	21	MLSIAEVAFVDPVML	EVAFVDPVM	5	0.6262	57.1	0.80	0.29	tr_C9ZN43_	WB
HLA-DQA10501-DQB10201	22	LDTIYMQSIITADEM	QSIITADEM	6	0.6230	59.1	0.90	0.74	tr_C9ZN43_	WB
HLA-DQA10501-DQB10201	23	FDVALMYDELVSAFN	VALMYDELV	2	0.6219	59.8	0.90	0.47	tr_C9ZN43_	WB
HLA-DQA10501-DQB10201	24	AMLSIAEVAFVDPVM	EVAFVDPVM	6	0.6179	62.5	1.00	0.31	tr_C9ZN43_	WB
HLA-DQA10501-DQB10201	25	LDTWREALEADGVEF	ALEADGVEF	6	0.6157	64.0	1.00	0.34	tr_C9ZN43_	WB
HLA-DQA10501-DQB10201	26	VYGWIAGEVLSQALS	YGWIAGEVL	1	0.6118	66.7	1.10	0.50	tr_C9ZN43_	WB
HLA-DQA10501-DQB10201	27	TIYMQSIITADEMRY	QSIITADEM	4	0.6109	67.3	1.10	0.63	tr_C9ZN43_	WB
HLA-DQA10501-DQB10201	28	REALEADGVEFFLGQ	LEADGVEFF	3	0.6093	68.6	1.10	0.30	tr_C9ZN43_	WB
HLA-DQA10501-DQB10201	29	LSIAEVAFVDPVMLT	EVAFVDPVM	4	0.6050	71.8	1.20	0.31	tr_C9ZN43_	WB
HLA-DQA10501-DQB10201	30	SNATYLALQSELDTR	YLALQSELD	4	0.6036	72.9	1.20	0.51	tr_C9ZN43_	WB
HLA-DQA10501-DQB10201	31	MYDVAYYDPAYVPPA	VAYYDPAYV	3	0.5984	77.1	1.30	0.47	tr_C9ZN43_	WB
HLA-DQA10501-DQB10201	32	DTIYMQSIITADEMR	QSIITADEM	5	0.5965	78.7	1.40	0.68	tr_C9ZN43_	WB
HLA-DQA10501-DQB10201	33	SIAEVAFVDPVMLTP	EVAFVDPVM	3	0.5945	80.4	1.40	0.35	tr_C9ZN43_	WB
HLA-DQA10501-DQB10201	34	DAMLSIAEVAFVDPV	AEVAFVDPV	6	0.5918	82.8	1.50	0.28	tr_C9ZN43_	WB
HLA-DQA10501-DQB10201	35	IYMQSIITADEMRYG	QSIITADEM	3	0.5877	86.5	1.60	0.62	tr_C9ZN43_	WB

HLA-DQA10501-DQB10201	36	VLIPFFDVALMYDEL	DVALMYDEL	6	0.5859	88.3	1.60	0.55	tr_C9ZN43_	WB
HLA-DQA10501-DQB10201	37	NATYLALQSELDTRS	YLALQSELD	3	0.5833	90.8	1.70	0.54	tr_C9ZN43_	WB
HLA-DQA10501-DQB10201	38	YDVAYYDPAYVPPAL	VAYYDPAYV	2	0.5823	91.8	1.70	0.51	tr_C9ZN43_	WB
HLA-DQA10501-DQB10201	39	VFGVVDDAMLSIAEV	VFGVVDDAM	0	0.5804	93.7	1.80	0.49	tr_C9ZN43_	WB
HLA-DQA10501-DQB10201	40	EALEADGVEFFLGQL	LEADGVEFF	2	0.5802	93.9	1.80	0.34	tr_C9ZN43_	WB
HLA-DQA10501-DQB10201	41	YMQSIITADEMRYGP	QSIITADEM	2	0.5744	100.0	1.90	0.60	tr_C9ZN43_	WB
HLA-DQA10501-DQB10201	42	DVALMYDELVSAFNG	VALMYDELV	1	0.5699	105.0	2.50	0.50	tr_C9ZN43_	WB
HLA-DQA10501-DQB10201	43	DDAMLSIAEVAFVDP	AMLSIAEVA	2	0.5692	105.8	2.50	0.22	tr_C9ZN43_	WB
HLA-DQA10501-DQB10201	44	SLYNQRRYVIDDLVI	RRYVIDDLV	5	0.5659	109.6	2.50	0.54	tr_C9ZN43_	WB
HLA-DQA10501-DQB10201	45	ESLYNQRRYVIDDLV	RRYVIDDLV	6	0.5580	119.4	2.50	0.63	tr_C9ZN43_	WB
HLA-DQA10501-DQB10201	46	IAEVAFVDPVMLTPR	EVAFVDPVM	2	0.5579	119.5	2.50	0.41	tr_C9ZN43_	WB
HLA-DQA10501-DQB10201	47	ATYLALQSELDTRSV	YLALQSELD	2	0.5573	120.3	2.50	0.56	tr_C9ZN43_	WB
HLA-DQA10501-DQB10201	48	DTRSVTAVFGVVDDA	AVFGVVDDA	6	0.5522	127.1	3.00	0.49	tr_C9ZN43_	WB
HLA-DQA10501-DQB10201	49	YGWIAGEVLSQALSS	YGWIAGEVL	0	0.5432	140.1	3.50	0.46	tr_C9ZN43_	WB
HLA-DQA10501-DQB10201	50	DVAYYDPAYVPPALS	VAYYDPAYV	1	0.5380	148.2	3.50	0.51	tr_C9ZN43_	WB
HLA-DQA10501-DQB10201	51	YNQRRYVIDDLVIGD	RRYVIDDLV	3	0.5375	149.0	3.50	0.55	tr_C9ZN43_	WB
HLA-DQA10501-DQB10201	52	NQRRYVIDDLVIGDF	RRYVIDDLV	2	0.5364	150.8	3.50	0.49	tr_C9ZN43_	WB
HLA-DQA10501-DQB10201	53	LYNQRRYVIDDLVIG	RRYVIDDLV	4	0.5360	151.4	3.50	0.56	tr_C9ZN43_	WB
HLA-DQA10501-DQB10201	54	LDRELAQVSSEALYY	LAQVSSEAL	4	0.5325	157.3	3.50	0.42	tr_C9ZN43_	WB
HLA-DQA10501-DQB10201	55	MQSIITADEMRYGPF	QSIITADEM	1	0.5308	160.1	4.00	0.57	tr_C9ZN43_	WB
HLA-DQA10501-DQB10201	56	ALDTWREALEADGVE	REALEADGV	5	0.5301	161.5	4.00	0.51	tr_C9ZN43_	WB
HLA-DQA10501-DQB10201	57	VDDAMLSIAEVAFVD	AMLSIAEVA	3	0.5290	163.4	4.00	0.33	tr_C9ZN43_	WB
HLA-DQA10501-DQB10201	58	ALEADGVEFFLGQLM	LEADGVEFF	1	0.5283	164.6	4.00	0.32	tr_C9ZN43_	WB
HLA-DQA10501-DQB10201	59	TADEMRYGPFEEEEE	MRYGPFEEE	4	0.5264	168.0	4.00	0.34	tr_C9ZN43_	WB
HLA-DQA10501-DQB10201	60	GYAAARFAQAVLPRM	FAQAVLPRM	6	0.5264	168.0	4.00	0.50	tr_C9ZN43_	WB
HLA-DQA10501-DQB10201	61	GNVLYINVVGNDERF	YINVVGNDE	4	0.5215	177.2	4.50	0.62	tr_C9ZN43_	WB
HLA-DQA10501-DQB10201	62	PSPSNATYLALQSEL	TYLALQSEL	6	0.5193	181.6	4.50	0.49	tr_C9ZN43_	WB
HLA-DQA10501-DQB10201	63	YAAARFAQAVLPRME	FAQAVLPRM	5	0.5154	189.3	4.50	0.51	tr_C9ZN43_	WB
HLA-DQA10501-DQB10201	64	VVDDAMLSIAEVAFV	AMLSIAEVA	4	0.5146	191.0	4.50	0.34	tr_C9ZN43_	WB
HLA-DQA10501-DQB10201	65	YLLSSSSLEFALDTW	SLEFALDTW	6	0.5111	198.2	5.00	0.49	tr_C9ZN43_	WB
HLA-DQA10501-DQB10201	66	QRRYVIDDLVIGDFG	RRYVIDDLV	1	0.5078	205.5	5.00	0.49	tr_C9ZN43_	WB
HLA-DQA10501-DQB10201	67	AEVAFVDPVMLTPRL	EVAFVDPVM	1	0.5043	213.4	5.50	0.46	tr_C9ZN43_	WB
HLA-DQA10501-DQB10201	68	FGVVDDAMLSIAEVA	AMLSIAEVA	6	0.5035	215.3	5.50	0.41	tr_C9ZN43_	WB
HLA-DQA10501-DQB10201	69	NVLYINVVGNDERFH	YINVVGNDE	3	0.5024	217.9	5.50	0.63	tr_C9ZN43_	WB
HLA-DQA10501-DQB10201	70	TYLALQSELDTRSVT	YLALQSELD	1	0.5020	218.7	5.50	0.65	tr_C9ZN43_	WB
HLA-DQA10501-DQB10201	71	DRELAQVSSEALYYG	LAQVSSEAL	3	0.5017	219.6	5.50	0.41	tr_C9ZN43_	WB
HLA-DQA10501-DQB10201	72	GVVDDAMLSIAEVAF	AMLSIAEVA	5	0.5007	222.0	5.50	0.41	tr_C9ZN43_	WB
HLA-DQA10501-DQB10201	73	AAARFAQAVLPRMEY	FAQAVLPRM	4	0.5006	222.2	5.50	0.53	tr_C9ZN43_	WB
HLA-DQA10501-DQB10201	74	ILDRELAQVSSEALY	LAQVSSEAL	5	0.4972	230.4	5.50	0.54	tr_C9ZN43_	WB
HLA-DQA10501-DQB10201	75	VLYINVVGNDERFHT	YINVVGNDE	2	0.4934	240.0	6.00	0.64	tr_C9ZN43_	WB
HLA-DQA10501-DQB10201	76	ADEMRYGPFEEEEEK	YGPFEEEEE	5	0.4931	240.9	6.00	0.32	tr_C9ZN43_	WB
HLA-DQA10501-DQB10201	77	VDGELMVYGWIAGEV	VYGWIAGEV	6	0.4910	246.6	6.00	0.74	tr_C9ZN43_	WB
HLA-DQA10501-DQB10201	78	VALMYDELVSAFNGN	VALMYDELV	0	0.4885	253.1	6.50	0.48	tr_C9ZN43_	WB
HLA-DQA10501-DQB10201	79	GGNVLYINVVGNDER	YINVVGNDE	5	0.4879	254.9	6.50	0.68	tr_C9ZN43_	WB
HLA-DQA10501-DQB10201	80	DEMRYGPFEEEEEKE	YGPFEEEEE	4	0.4866	258.6	6.50	0.32	tr_C9ZN43_	WB
HLA-DQA10501-DQB10201	81	AARFAQAVLPRMEYV	FAQAVLPRM	3	0.4819	271.9	7.00	0.56	tr_C9ZN43_	WB
HLA-DQA10501-DQB10201	82	DAYLLSSSSLEFALD	LLSSSSLEF	3	0.4819	272.1	7.00	0.47	tr_C9ZN43_	WB
HLA-DQA10501-DQB10201	83	QGGNVLYINVVGNDE	YINVVGNDE	6	0.4811	274.4	7.00	0.70	tr_C9ZN43_	WB
HLA-DQA10501-DQB10201	84	FALDTWREALEADGV	REALEADGV	6	0.4785	282.2	7.00	0.54	tr_C9ZN43_	WB
HLA-DQA10501-DQB10201	85	LILDRELAQVSSEAL	LAQVSSEAL	6	0.4756	291.2	7.50	0.61	tr_C9ZN43_	WB

HLA-DQA10501-DQB10201	86	DVRVLIPFFDVALMY	PFFDVALMY	6	0.4725	301.0	7.50	0.25	tr_C9ZN43_	WB
HLA-DQA10501-DQB10201	87	LYINVVGNDERFHTV	YINVVGNDE	1	0.4716	304.0	7.50	0.62	tr_C9ZN43_	WB
HLA-DQA10501-DQB10201	88	RELAQVSSEALYYGA	LAQVSSEAL	2	0.4716	304.0	7.50	0.44	tr_C9ZN43_	WB
HLA-DQA10501-DQB10201	89	VRVLIPFFDVALMYD	FFDVALMYD	6	0.4694	311.2	8.00	0.32	tr_C9ZN43_	WB
HLA-DQA10501-DQB10201	90	TVRVYSLLYNPLIPD	VYSLLYNPL	3	0.4675	317.8	8.00	0.35	tr_C9ZN43_	WB
HLA-DQA10501-DQB10201	91	THDAYLLSSSSLEFA	LLSSSSLEF	5	0.4669	319.8	8.00	0.54	tr_C9ZN43_	WB
HLA-DQA10501-DQB10201	92	ITADEMRYGPFEEEE	MRYGPFEEE	5	0.4667	320.8	8.00	0.49	tr_C9ZN43_	WB
HLA-DQA10501-DQB10201	93	QSIITADEMRYGPFE	QSIITADEM	0	0.4662	322.5	8.00	0.45	tr_C9ZN43_	WB
HLA-DQA10501-DQB10201	94	HDAYLLSSSSLEFAL	LLSSSSLEF	4	0.4649	326.8	8.00	0.53	tr_C9ZN43_	WB
HLA-DQA10501-DQB10201	95	RVYSLLYNPLIPDNL	YNPLIPDNL	6	0.4624	335.9	8.50	0.26	tr_C9ZN43_	WB
HLA-DQA10501-DQB10201	96	RVLIPFFDVALMYDE	FFDVALMYD	5	0.4622	336.7	8.50	0.33	tr_C9ZN43_	WB
HLA-DQA10501-DQB10201	97	LLGYAAARFAQAVLP	ARFAQAVLP	6	0.4603	343.6	8.50	0.38	tr_C9ZN43_	WB
HLA-DQA10501-DQB10201	98	AYLLSSSSLEFALDT	LLSSSSLEF	2	0.4598	345.3	8.50	0.45	tr_C9ZN43_	WB
HLA-DQA10501-DQB10201	99	EMRYGPFEEEEEKEC	YGPFEEEEE	3	0.4588	349.2	9.00	0.34	tr_C9ZN43_	WB
HLA-DQA10501-DQB10201	100	DNFDHDDVDGELMVY	HDDVDGELM	4	0.4585	350.4	9.00	0.26	tr_C9ZN43_	WB
HLA-DQA10501-DQB10201	101	MTVRVYSLLYNPLIP	VYSLLYNPL	4	0.4577	353.3	9.00	0.35	tr_C9ZN43_	WB
HLA-DQA10501-DQB10201	102	PDVRVLIPFFDVALM	DVRVLIPFF	1	0.4572	355.2	9.00	0.35	tr_C9ZN43_	WB
HLA-DQA10501-DQB10201	103	VAYYDPAYVPPALSE	VAYYDPAYV	0	0.4547	365.0	9.00	0.44	tr_C9ZN43_	WB
HLA-DQA10501-DQB10201	104	PLNTLMFLILDRELA	LMFLILDRE	4	0.4541	367.3	9.50	0.45	tr_C9ZN43_	WB
HLA-DQA10501-DQB10201	105	ARFAQAVLPRMEYVT	FAQAVLPRM	2	0.4503	382.7	9.50	0.58	tr_C9ZN43_	WB
HLA-DQA10501-DQB10201	106	LEADGVEFFLGQLML	LEADGVEFF	0	0.4503	382.8	9.50	0.29	tr_C9ZN43_	WB
HLA-DQA10501-DQB10201	107	LLSSSSLEFALDTWR	SLEFALDTW	5	0.4502	383.1	9.50	0.49	tr_C9ZN43_	WB
HLA-DQA10501-DQB10201	108	RTHDAYLLSSSSLEF	LLSSSSLEF	6	0.4494	386.7	9.50	0.66	tr_C9ZN43_	WB
HLA-DQA10501-DQB10201	109	LDTRSVTAVFGVVDD	TAVFGVVDD	6	0.4487	389.4	10.00	0.34	tr_C9ZN43_	
HLA-DQA10501-DQB10201	110	LDRNPDVRVLIPFFD	DVRVLIPFF	5	0.4483	391.2	10.00	0.51	tr_C9ZN43_	
HLA-DQA10501-DQB10201	111	EVAFVDPVMLTPRLH	EVAFVDPVM	0	0.4454	403.5	10.00	0.44	tr_C9ZN43_	
HLA-DQA10501-DQB10201	112	SPLNTLMFLILDREL	LMFLILDRE	5	0.4453	404.3	10.00	0.50	tr_C9ZN43_	
HLA-DQA10501-DQB10201	113	VRVYSLLYNPLIPDN	VYSLLYNPL	2	0.4444	408.1	10.00	0.34	tr_C9ZN43_	
HLA-DQA10501-DQB10201	114	HGGTLQTVAVLDDNA	LQTVAVLDD	4	0.4431	413.7	11.00	0.32	tr_C9ZN43_	
HLA-DQA10501-DQB10201	115	NPDVRVLIPFFDVAL	DVRVLIPFF	2	0.4428	415.4	11.00	0.41	tr_C9ZN43_	
HLA-DQA10501-DQB10201	116	NTLMFLILDRELAQV	LMFLILDRE	2	0.4427	415.7	11.00	0.37	tr_C9ZN43_	
HLA-DQA10501-DQB10201	117	LNTLMFLILDRELAQ	LMFLILDRE	3	0.4424	416.8	11.00	0.41	tr_C9ZN43_	
HLA-DQA10501-DQB10201	118	VYSLLYNPLIPDNLC	YNPLIPDNL	5	0.4421	418.5	11.00	0.28	tr_C9ZN43_	
HLA-DQA10501-DQB10201	119	RRYVIDDLVIGDFGG	RRYVIDDLV	0	0.4417	420.1	11.00	0.42	tr_C9ZN43_	
HLA-DQA10501-DQB10201	120	GTDNFDHDDVDGELM	HDDVDGELM	6	0.4410	423.5	11.00	0.30	tr_C9ZN43_	
HLA-DQA10501-DQB10201	121	IITADEMRYGPFEEE	MRYGPFEEE	6	0.4397	429.4	11.00	0.51	tr_C9ZN43_	
HLA-DQA10501-DQB10201	122	ELAQVSSEALYYGAN	LAQVSSEAL	1	0.4396	430.0	11.00	0.44	tr_C9ZN43_	
HLA-DQA10501-DQB10201	123	PVGDGQALASLPLLE	ALASLPLLE	6	0.4394	430.8	11.00	0.41	tr_C9ZN43_	
HLA-DQA10501-DQB10201	124	DRNPDVRVLIPFFDV	DVRVLIPFF	4	0.4375	439.8	11.00	0.51	tr_C9ZN43_	
HLA-DQA10501-DQB10201	125	TDNFDHDDVDGELMV	HDDVDGELM	5	0.4370	441.9	11.00	0.28	tr_C9ZN43_	
HLA-DQA10501-DQB10201	126	VMTVRVYSLLYNPLI	VYSLLYNPL	5	0.4353	450.4	11.00	0.41	tr_C9ZN43_	
HLA-DQA10501-DQB10201	127	RNPDVRVLIPFFDVA	DVRVLIPFF	3	0.4340	456.6	12.00	0.47	tr_C9ZN43_	
HLA-DQA10501-DQB10201	128	WMHGGTLQTVAVLDD	LQTVAVLDD	6	0.4329	462.3	12.00	0.47	tr_C9ZN43_	
HLA-DQA10501-DQB10201	129	GGTLQTVAVLDDNAT	TVAVLDDNA	5	0.4309	472.1	12.00	0.32	tr_C9ZN43_	
HLA-DQA10501-DQB10201	130	GTLQTVAVLDDNATL	TVAVLDDNA	4	0.4280	487.2	12.00	0.31	tr_C9ZN43_	
HLA-DQA10501-DQB10201	131	MHGGTLQTVAVLDDN	LQTVAVLDD	5	0.4260	498.2	13.00	0.43	tr_C9ZN43_	
HLA-DQA10501-DQB10201	132	NFDHDDVDGELMVYG	HDDVDGELM	3	0.4257	499.5	13.00	0.28	tr_C9ZN43_	
HLA-DQA10501-DQB10201	133	GDGQALASLPLLERE	ALASLPLLE	4	0.4251	502.7	13.00	0.38	tr_C9ZN43_	
HLA-DQA10501-DQB10201	134	MESLYNQRRYVIDDL	QRRYVIDDL	6	0.4245	506.1	13.00	0.89	tr_C9ZN43_	
HLA-DQA10501-DQB10201	135	LSSSSLEFALDTWRE	SLEFALDTW	4	0.4242	507.7	13.00	0.51	tr_C9ZN43_	

HLA-DQA10501-DQB10201	136	LGYAAARFAQAVLPR	ARFAQAVLP	5	0.4240	508.8	13.00	0.38	tr_C9ZN43_
HLA-DQA10501-DQB10201	137	DDFNTAWEQFAETRP	FNTAWEQFA	2	0.4238	509.9	13.00	0.22	tr_C9ZN43_
HLA-DQA10501-DQB10201	138	RFAQAVLPRMEYVTP	FAQAVLPRM	1	0.4238	510.1	13.00	0.56	tr_C9ZN43_
HLA-DQA10501-DQB10201	139	MRYGPFEEEEEKECF	YGPFEEEEE	2	0.4235	511.6	13.00	0.38	tr_C9ZN43_
HLA-DQA10501-DQB10201	140	TLMFLILDRELAQVS	LMFLILDRE	1	0.4234	512.1	13.00	0.37	tr_C9ZN43_
HLA-DQA10501-DQB10201	141	VGDGQALASLPLLER	ALASLPLLE	5	0.4228	515.5	13.00	0.40	tr_C9ZN43_
HLA-DQA10501-DQB10201	142	FDHDDVDGELMVYGW	HDDVDGELM	2	0.4204	529.3	13.00	0.27	tr_C9ZN43_
HLA-DQA10501-DQB10201	143	DVMTVRVYSLLYNPL	VYSLLYNPL	6	0.4194	535.0	13.00	0.50	tr_C9ZN43_
HLA-DQA10501-DQB10201	144	SHVYFLTASPNAELL	YFLTASPNA	3	0.4156	557.2	14.00	0.31	tr_C9ZN43_
HLA-DQA10501-DQB10201	145	YINVVGNDERFHTVQ	YINVVGNDE	0	0.4149	561.7	14.00	0.60	tr_C9ZN43_
HLA-DQA10501-DQB10201	146	LLDTIYMQSIITADE	MQSIITADE	6	0.4144	564.3	14.00	0.45	tr_C9ZN43_
HLA-DQA10501-DQB10201	147	NDDDFNTAWEQFAET	FNTAWEQFA	4	0.4141	566.7	14.00	0.35	tr_C9ZN43_
HLA-DQA10501-DQB10201	148	TLQTVAVLDDNATLV	TVAVLDDNA	3	0.4128	574.5	14.00	0.30	tr_C9ZN43_
HLA-DQA10501-DQB10201	149	PLLGYAAARFAQAVL	LLGYAAARF	1	0.4123	577.4	14.00	0.32	tr_C9ZN43_
HLA-DQA10501-DQB10201	150	YSPLNTLMFLILDRE	LMFLILDRE	6	0.4102	591.0	14.00	0.74	tr_C9ZN43_
HLA-DQA10501-DQB10201	151	GQALASLPLLERENL	ALASLPLLE	2	0.4100	591.8	15.00	0.38	tr_C9ZN43_
HLA-DQA10501-DQB10201	152	NRGNAFVIGLAPGDP	FVIGLAPGD	5	0.4089	599.0	15.00	0.38	tr_C9ZN43_
HLA-DQA10501-DQB10201	153	DGQALASLPLLEREN	ALASLPLLE	3	0.4081	604.6	15.00	0.40	tr_C9ZN43_
HLA-DQA10501-DQB10201	154	DDDFNTAWEQFAETR	FNTAWEQFA	3	0.4061	617.3	15.00	0.32	tr_C9ZN43_
HLA-DQA10501-DQB10201	155	YSLLYNPLIPDNLCD	YNPLIPDNL	4	0.4060	618.5	15.00	0.41	tr_C9ZN43_
HLA-DQA10501-DQB10201	156	GFMYLRGISFGDTEY	GISFGDTEY	6	0.4058	619.3	15.00	0.31	tr_C9ZN43_
HLA-DQA10501-DQB10201	157	MSAIVRGADATIIEV	VRGADATII	4	0.4055	621.5	15.00	0.44	tr_C9ZN43_
HLA-DQA10501-DQB10201	158	RGNAFVIGLAPGDPS	FVIGLAPGD	4	0.4055	621.7	15.00	0.36	tr_C9ZN43_
HLA-DQA10501-DQB10201	159	VNDDDFNTAWEQFAE	FNTAWEQFA	5	0.4054	622.6	15.00	0.45	tr_C9ZN43_
HLA-DQA10501-DQB10201	160	RVNDDDFNTAWEQFA	FNTAWEQFA	6	0.4050	624.7	15.00	0.44	tr_C9ZN43_
HLA-DQA10501-DQB10201	161	LLTLVRYAVSQLRLL	RYAVSQLRL	5	0.4047	626.8	15.00	0.38	tr_C9ZN43_
HLA-DQA10501-DQB10201	162	IPSPSNATYLALQSE	ATYLALQSE	6	0.4047	626.9	15.00	0.62	tr_C9ZN43_
HLA-DQA10501-DQB10201	163	LAQVSSEALYYGANV	LAQVSSEAL	0	0.4041	631.3	15.00	0.29	tr_C9ZN43_
HLA-DQA10501-DQB10201	164	NGTDNFDHDDVDGEL	FDHDDVDGE	5	0.4037	633.9	15.00	0.34	tr_C9ZN43_
HLA-DQA10501-DQB10201	165	NSHVYFLTASPNAEL	YFLTASPNA	4	0.4030	638.7	16.00	0.35	tr_C9ZN43_
HLA-DQA10501-DQB10201	166	GNAFVIGLAPGDPSL	FVIGLAPGD	3	0.4030	638.8	16.00	0.38	tr_C9ZN43_
HLA-DQA10501-DQB10201	167	GRELCGVFTVDGLMR	CGVFTVDGL	4	0.4029	639.3	16.00	0.31	tr_C9ZN43_
HLA-DQA10501-DQB10201	168	RHKGEFFVVLGPVGD	FFVVLGPVG	5	0.4028	639.8	16.00	0.28	tr_C9ZN43_
HLA-DQA10501-DQB10201	169	SSSSLEFALDTWREA	SLEFALDTW	3	0.4028	639.9	16.00	0.51	tr_C9ZN43_
HLA-DQA10501-DQB10201	170	HVYFLTASPNAELLT	YFLTASPNA	2	0.4024	643.0	16.00	0.34	tr_C9ZN43_
HLA-DQA10501-DQB10201	171	YDPAYVPPALSEDVM	PPALSEDVM	6	0.4021	644.8	16.00	0.36	tr_C9ZN43_
HLA-DQA10501-DQB10201	172	QALASLPLLERENLV	ALASLPLLE	1	0.4020	645.7	16.00	0.31	tr_C9ZN43_
HLA-DQA10501-DQB10201	173	SAIVRGADATIIEVA	VRGADATII	3	0.4020	645.9	16.00	0.38	tr_C9ZN43_
HLA-DQA10501-DQB10201	174	GWIAGEVLSQALSSR	WIAGEVLSQ	1	0.4008	654.1	16.00	0.37	tr_C9ZN43_
HLA-DQA10501-DQB10201	175	ELLTLVRYAVSQLRL	RYAVSQLRL	6	0.4008	654.3	16.00	0.46	tr_C9ZN43_
HLA-DQA10501-DQB10201	176	AIVRGADATIIEVAL	VRGADATII	2	0.4004	657.0	16.00	0.36	tr_C9ZN43_
HLA-DQA10501-DQB10201	177	SSSLEFALDTWREAL	SLEFALDTW	2	0.4000	660.1	16.00	0.49	tr_C9ZN43_
HLA-DQA10501-DQB10201	178	HHRGRNVIQLSPTLE	VIQLSPTLE	6	0.3978	675.6	16.00	0.52	tr_C9ZN43_
HLA-DQA10501-DQB10201	179	DPAYVPPALSEDVMT	PPALSEDVM	5	0.3976	676.8	16.00	0.33	tr_C9ZN43_
HLA-DQA10501-DQB10201	180	WKPLPLLGYAAARFA	LLGYAAARF	5	0.3966	684.6	16.00	0.67	tr_C9ZN43_
HLA-DQA10501-DQB10201	181	HRGRNVIQLSPTLEQ	VIQLSPTLE	5	0.3964	685.8	16.00	0.47	tr_C9ZN43_
HLA-DQA10501-DQB10201	182	NAFVIGLAPGDPSLL	FVIGLAPGD	2	0.3964	686.3	16.00	0.38	tr_C9ZN43_
HLA-DQA10501-DQB10201	183	VGRELCGVFTVDGLM	CGVFTVDGL	5	0.3963	687.0	16.00	0.35	tr_C9ZN43_
HLA-DQA10501-DQB10201	184	VTVFNGTDNFDHDDV	TVFNGTDNF	1	0.3962	687.7	16.00	0.38	tr_C9ZN43_
HLA-DQA10501-DQB10201	185	AWKPLPLLGYAAARF	LLGYAAARF	6	0.3957	691.1	17.00	0.83	tr_C9ZN43_

HLA-DQA10501-DQB10201	186	HKGEFFVVLGPVGDG	FVVLGPVGD	5	0.3957	691.5	17.00	0.28	tr_C9ZN43_
HLA-DQA10501-DQB10201	187	YFLTASPNAELLTLV	FLTASPNAE	1	0.3942	702.3	17.00	0.29	tr_C9ZN43_
HLA-DQA10501-DQB10201	188	VYFLTASPNAELLTL	FLTASPNAE	2	0.3941	703.3	17.00	0.32	tr_C9ZN43_
HLA-DQA10501-DQB10201	189	YLALQSELDTRSVTA	YLALQSELD	0	0.3933	709.5	17.00	0.72	tr_C9ZN43_
HLA-DQA10501-DQB10201	190	KGEFFVVLGPVGDGQ	FVVLGPVGD	4	0.3932	710.4	17.00	0.27	tr_C9ZN43_
HLA-DQA10501-DQB10201	191	DFNTAWEQFAETRPQ	WEQFAETRP	5	0.3912	725.4	17.00	0.23	tr_C9ZN43_
HLA-DQA10501-DQB10201	192	RELCGVFTVDGLMRG	CGVFTVDGL	3	0.3875	755.6	18.00	0.32	tr_C9ZN43_
HLA-DQA10501-DQB10201	193	GEFFVVLGPVGDGQA	FVVLGPVGD	3	0.3870	759.0	18.00	0.27	tr_C9ZN43_
HLA-DQA10501-DQB10201	194	ELDTRSVTAVFGVVD	VTAVFGVVD	6	0.3859	768.1	18.00	0.37	tr_C9ZN43_
HLA-DQA10501-DQB10201	195	LMFLILDRELAQVSS	LMFLILDRE	0	0.3857	770.3	18.00	0.31	tr_C9ZN43_
HLA-DQA10501-DQB10201	196	LPLLGYAAARFAQAV	LLGYAAARF	2	0.3838	786.0	18.00	0.46	tr_C9ZN43_
HLA-DQA10501-DQB10201	197	PMSAIVRGADATIIE	VRGADATII	5	0.3833	790.0	18.00	0.51	tr_C9ZN43_
HLA-DQA10501-DQB10201	198	SIITADEMRYGPFEE	IITADEMRY	1	0.3833	790.7	18.00	0.41	tr_C9ZN43_
HLA-DQA10501-DQB10201	199	YKIKPVEVLQVALER	EVLQVALER	6	0.3829	794.1	18.00	0.43	tr_C9ZN43_
HLA-DQA10501-DQB10201	200	AQVSSEALYYGANVL	ALYYGANVL	6	0.3825	797.1	19.00	0.37	tr_C9ZN43_
HLA-DQA10501-DQB10201	201	EADGVEFFLGQLMLT	VEFFLGQLM	4	0.3819	802.5	19.00	0.35	tr_C9ZN43_
HLA-DQA10501-DQB10201	202	GVTVFNGTDNFDHDD	TVFNGTDNF	2	0.3807	812.6	19.00	0.47	tr_C9ZN43_
HLA-DQA10501-DQB10201	203	WNSHVYFLTASPNAE	YFLTASPNA	5	0.3807	812.7	19.00	0.39	tr_C9ZN43_
HLA-DQA10501-DQB10201	204	FMYLRGISFGDTEYK	GISFGDTEY	5	0.3805	815.1	19.00	0.32	tr_C9ZN43_
HLA-DQA10501-DQB10201	205	TVFNGTDNFDHDDVD	VFNGTDNFD	1	0.3796	822.4	19.00	0.30	tr_C9ZN43_
HLA-DQA10501-DQB10201	206	HLDRNPDVRVLIPFF	DVRVLIPFF	6	0.3787	830.6	19.00	0.66	tr_C9ZN43_
HLA-DQA10501-DQB10201	207	SSLEFALDTWREALE	SLEFALDTW	1	0.3782	835.1	19.00	0.47	tr_C9ZN43_
HLA-DQA10501-DQB10201	208	WSLARALNASVPPLT	ALNASVPPL	5	0.3781	836.6	19.00	0.33	tr_C9ZN43_
HLA-DQA10501-DQB10201	209	LTLVRYAVSQLRLLR	RYAVSQLRL	4	0.3766	850.1	19.00	0.39	tr_C9ZN43_
HLA-DQA10501-DQB10201	210	SVGRELCGVFTVDGL	CGVFTVDGL	6	0.3762	853.6	20.00	0.49	tr_C9ZN43_
HLA-DQA10501-DQB10201	211	RGRNVIQLSPTLEQQ	VIQLSPTLE	4	0.3762	853.9	20.00	0.47	tr_C9ZN43_
HLA-DQA10501-DQB10201	212	SEIVREFHTALPDSS	FHTALPDSS	6	0.3758	857.2	20.00	0.32	tr_C9ZN43_
HLA-DQA10501-DQB10201	213	GQSDRLFISMIPSPS	FISMIPSPS	6	0.3757	858.2	20.00	0.31	tr_C9ZN43_
HLA-DQA10501-DQB10201	214	TLVRYAVSQLRLLRI	RYAVSQLRL	3	0.3754	861.1	20.00	0.34	tr_C9ZN43_
HLA-DQA10501-DQB10201	215	PAYVPPALSEDVMTV	PPALSEDVM	4	0.3752	862.8	20.00	0.37	tr_C9ZN43_
HLA-DQA10501-DQB10201	216	PGVTVFNGTDNFDHD	TVFNGTDNF	3	0.3751	864.0	20.00	0.52	tr_C9ZN43_
HLA-DQA10501-DQB10201	217	EIVREFHTALPDSSA	FHTALPDSS	5	0.3741	873.4	20.00	0.37	tr_C9ZN43_
HLA-DQA10501-DQB10201	218	IVREFHTALPDSSAW	FHTALPDSS	4	0.3740	874.2	20.00	0.41	tr_C9ZN43_
HLA-DQA10501-DQB10201	219	GRNVIQLSPTLEQQL	VIQLSPTLE	3	0.3732	881.3	20.00	0.49	tr_C9ZN43_
HLA-DQA10501-DQB10201	220	RNVIQLSPTLEQQLF	VIQLSPTLE	2	0.3730	883.4	20.00	0.47	tr_C9ZN43_
HLA-DQA10501-DQB10201	221	PLPLLGYAAARFAQA	LLGYAAARF	3	0.3729	884.9	20.00	0.54	tr_C9ZN43_
HLA-DQA10501-DQB10201	222	KPLPLLGYAAARFAQ	LLGYAAARF	4	0.3720	893.2	20.00	0.67	tr_C9ZN43_
HLA-DQA10501-DQB10201	223	FAQAVLPRMEYVTPK	FAQAVLPRM	0	0.3715	898.2	20.00	0.51	tr_C9ZN43_
HLA-DQA10501-DQB10201	224	LQTVAVLDDNATLVG	TVAVLDDNA	2	0.3715	898.4	20.00	0.41	tr_C9ZN43_
HLA-DQA10501-DQB10201	225	ANPGVTVFNGTDNFD	TVFNGTDNF	5	0.3714	899.2	20.00	0.59	tr_C9ZN43_
HLA-DQA10501-DQB10201	226	MWSLARALNASVPPL	ALNASVPPL	6	0.3713	899.9	20.00	0.35	tr_C9ZN43_
HLA-DQA10501-DQB10201	227	PNRGNAFVIGLAPGD	FVIGLAPGD	6	0.3712	900.8	20.00	0.35	tr_C9ZN43_
HLA-DQA10501-DQB10201	228	NVIQLSPTLEQQLFV	VIQLSPTLE	1	0.3710	903.3	20.00	0.40	tr_C9ZN43_
HLA-DQA10501-DQB10201	229	GPVGDGQALASLPLL	QALASLPLL	6	0.3701	912.2	21.00	0.54	tr_C9ZN43_
HLA-DQA10501-DQB10201	230	IVRGADATIIEVALR	VRGADATII	1	0.3700	913.1	21.00	0.33	tr_C9ZN43_
HLA-DQA10501-DQB10201	231	FLILDRELAQVSSEA	ELAQVSSEA	6	0.3699	913.7	21.00	0.32	tr_C9ZN43_
HLA-DQA10501-DQB10201	232	AFVIGLAPGDPSLLA	FVIGLAPGD	1	0.3694	918.5	21.00	0.39	tr_C9ZN43_
HLA-DQA10501-DQB10201	233	EFFVVLGPVGDGQAL	FVVLGPVGD	2	0.3692	920.7	21.00	0.29	tr_C9ZN43_
HLA-DQA10501-DQB10201	234	REWLTSRKAFMESLY	RKAFMESLY	6	0.3689	923.7	21.00	0.42	tr_C9ZN43_
HLA-DQA10501-DQB10201	235	KIKPVEVLQVALERH	EVLQVALER	5	0.3683	930.2	21.00	0.34	tr_C9ZN43_

HLA-DQA10501-DQB10201	236	RYVIDDLVIGDFGGD	IDDLVIGDF	3	0.3674	938.8	21.00	0.28	tr_C9ZN43_
HLA-DQA10501-DQB10201	237	YYDPAYVPPALSEDV	PAYVPPALS	3	0.3672	941.1	21.00	0.32	tr_C9ZN43_
HLA-DQA10501-DQB10201	238	ERHKGEFFVVLGPVG	FFVVLGPVG	6	0.3669	944.1	21.00	0.32	tr_C9ZN43_
HLA-DQA10501-DQB10201	239	FGQSDRLFISMIPSP	LFISMIPSP	6	0.3652	961.7	21.00	0.38	tr_C9ZN43_
HLA-DQA10501-DQB10201	240	SLEFALDTWREALEA	SLEFALDTW	0	0.3642	971.7	22.00	0.31	tr_C9ZN43_
HLA-DQA10501-DQB10201	241	AYYDPAYVPPALSED	PAYVPPALS	4	0.3642	971.8	22.00	0.31	tr_C9ZN43_
HLA-DQA10501-DQB10201	242	QSDRLFISMIPSPSN	FISMIPSPS	5	0.3635	979.6	22.00	0.35	tr_C9ZN43_
HLA-DQA10501-DQB10201	243	EWLTSRKAFMESLYN	RKAFMESLY	5	0.3632	982.7	22.00	0.38	tr_C9ZN43_
HLA-DQA10501-DQB10201	244	GWNSHVYFLTASPNA	YFLTASPNA	6	0.3616	999.3	22.00	0.44	tr_C9ZN43_
HLA-DQA10501-DQB10201	245	ELCGVFTVDGLMRGR	CGVFTVDGL	2	0.3616	1000.0	22.00	0.37	tr_C9ZN43_
HLA-DQA10501-DQB10201	246	DHDDVDGELMVYGWI	DVDGELMVY	3	0.3614	1001.4	22.00	0.26	tr_C9ZN43_
HLA-DQA10501-DQB10201	247	SLLYNPLIPDNLCDA	YNPLIPDNL	3	0.3613	1002.5	22.00	0.48	tr_C9ZN43_
HLA-DQA10501-DQB10201	248	WIAGEVLSQALSSRE	WIAGEVLSQ	0	0.3608	1008.2	22.00	0.30	tr_C9ZN43_
HLA-DQA10501-DQB10201	249	MYLRGISFGDTEYKM	GISFGDTEY	4	0.3607	1008.9	22.00	0.29	tr_C9ZN43_
HLA-DQA10501-DQB10201	250	RYGPFEEEEEKECFT	YGPFEEEEE	1	0.3603	1014.1	22.00	0.41	tr_C9ZN43_
HLA-DQA10501-DQB10201	251	APMSAIVRGADATII	VRGADATII	6	0.3590	1027.7	23.00	0.54	tr_C9ZN43_
HLA-DQA10501-DQB10201	252	LERHKGEFFVVLGPV	EFFVVLGPV	6	0.3586	1032.6	23.00	0.43	tr_C9ZN43_
HLA-DQA10501-DQB10201	253	NPGVTVFNGTDNFDH	TVFNGTDNF	4	0.3582	1036.9	23.00	0.54	tr_C9ZN43_
HLA-DQA10501-DQB10201	254	EFALDTWREALEADG	TWREALEAD	5	0.3582	1037.3	23.00	0.25	tr_C9ZN43_
HLA-DQA10501-DQB10201	255	MFLILDRELAQVSSE	RELAQVSSE	6	0.3577	1042.6	23.00	0.34	tr_C9ZN43_
HLA-DQA10501-DQB10201	256	LARALNASVPPLTSP	ALNASVPPL	3	0.3568	1052.5	23.00	0.38	tr_C9ZN43_
HLA-DQA10501-DQB10201	257	LEFALDTWREALEAD	DTWREALEA	5	0.3564	1056.9	23.00	0.25	tr_C9ZN43_
HLA-DQA10501-DQB10201	258	QVSSEALYYGANVLT	ALYYGANVL	5	0.3548	1075.6	23.00	0.37	tr_C9ZN43_
HLA-DQA10501-DQB10201	259	IGFMYLRGISFGDTE	RGISFGDTE	6	0.3546	1078.1	23.00	0.31	tr_C9ZN43_
HLA-DQA10501-DQB10201	260	RMEYVTPKTLLDTIY	PKTLLDTIY	6	0.3545	1079.0	23.00	0.40	tr_C9ZN43_
HLA-DQA10501-DQB10201	261	KWTVAPDVKVELIPP	APDVKVELI	4	0.3531	1096.0	24.00	0.31	tr_C9ZN43_
HLA-DQA10501-DQB10201	262	SYKIKPVEVLQVALE	VEVLQVALE	6	0.3521	1107.5	24.00	0.39	tr_C9ZN43_
HLA-DQA10501-DQB10201	263	WLTSRKAFMESLYNQ	RKAFMESLY	4	0.3503	1130.2	24.00	0.34	tr_C9ZN43_
HLA-DQA10501-DQB10201	264	YVIDDLVIGDFGGDC	IDDLVIGDF	2	0.3497	1137.0	24.00	0.34	tr_C9ZN43_
HLA-DQA10501-DQB10201	265	TLLDTIYMQSIITAD	YMQSIITAD	6	0.3494	1140.4	24.00	0.30	tr_C9ZN43_
HLA-DQA10501-DQB10201	266	ALASLPLLERENLVG	ALASLPLLE	0	0.3482	1155.9	25.00	0.22	tr_C9ZN43_
HLA-DQA10501-DQB10201	267	SDRLFISMIPSPSNA	FISMIPSPS	4	0.3481	1156.2	25.00	0.38	tr_C9ZN43_
HLA-DQA10501-DQB10201	268	GRFGQSDRLFISMIP	DRLFISMIP	6	0.3474	1165.8	25.00	0.32	tr_C9ZN43_
HLA-DQA10501-DQB10201	269	SSEIVREFHTALPDS	EFHTALPDS	6	0.3473	1167.1	25.00	0.37	tr_C9ZN43_
HLA-DQA10501-DQB10201	270	SLARALNASVPPLTS	ALNASVPPL	4	0.3468	1173.4	25.00	0.38	tr_C9ZN43_
HLA-DQA10501-DQB10201	271	SPVTPLIRYADPNAI	IRYADPNAI	6	0.3461	1181.8	25.00	0.57	tr_C9ZN43_
HLA-DQA10501-DQB10201	272	LVRYAVSQLRLLRIG	RYAVSQLRL	2	0.3452	1194.2	25.00	0.38	tr_C9ZN43_
HLA-DQA10501-DQB10201	273	RKWTVAPDVKVELIP	APDVKVELI	5	0.3450	1196.8	25.00	0.32	tr_C9ZN43_
HLA-DQA10501-DQB10201	274	AERVQFATSLPHWAD	FATSLPHWA	5	0.3446	1201.9	25.00	0.28	tr_C9ZN43_
HLA-DQA10501-DQB10201	275	YLRGISFGDTEYKMT	GISFGDTEY	3	0.3431	1220.5	26.00	0.31	tr_C9ZN43_
HLA-DQA10501-DQB10201	276	LTSRKAFMESLYNQR	RKAFMESLY	3	0.3430	1222.2	26.00	0.34	tr_C9ZN43_
HLA-DQA10501-DQB10201	277	NAERVQFATSLPHWA	FATSLPHWA	6	0.3426	1227.5	26.00	0.23	tr_C9ZN43_
HLA-DQA10501-DQB10201	278	LCGVFTVDGLMRGRV	CGVFTVDGL	1	0.3425	1229.3	26.00	0.31	tr_C9ZN43_
HLA-DQA10501-DQB10201	279	FFVVLGPVGDGQALA	FVVLGPVGD	1	0.3422	1233.7	26.00	0.28	tr_C9ZN43_
HLA-DQA10501-DQB10201	280	VREFHTALPDSSAWK	FHTALPDSS	3	0.3420	1235.6	26.00	0.42	tr_C9ZN43_
HLA-DQA10501-DQB10201	281	SLPLLERENLVGFAP	ERENLVGFA	5	0.3415	1242.9	26.00	0.19	tr_C9ZN43_
HLA-DQA10501-DQB10201	282	ADGVEFFLGQLMLTR	VEFFLGQLM	3	0.3410	1249.6	26.00	0.35	tr_C9ZN43_
HLA-DQA10501-DQB10201	283	ARALNASVPPLTSPV	ALNASVPPL	2	0.3399	1264.8	26.00	0.41	tr_C9ZN43_
HLA-DQA10501-DQB10201	284	VWMHGGTLQTVAVLD	TLQTVAVLD	6	0.3398	1266.1	26.00	0.44	tr_C9ZN43_
HLA-DQA10501-DQB10201	285	SRKWTVAPDVKVELI	APDVKVELI	6	0.3397	1266.5	26.00	0.29	tr_C9ZN43_

HLA-DQA10501-DQB10201	286	TSYKIKPVEVLQVAL	SYKIKPVEV	1	0.3391	1274.6	26.00	0.28	tr_C9ZN43_
HLA-DQA10501-DQB10201	287	ASLPLLERENLVGFA	ERENLVGFA	6	0.3387	1280.0	26.00	0.18	tr_C9ZN43_
HLA-DQA10501-DQB10201	288	FNGTDNFDHDDVDGE	FDHDDVDGE	6	0.3374	1299.4	27.00	0.37	tr_C9ZN43_
HLA-DQA10501-DQB10201	289	WTVAPDVKVELIPPT	APDVKVELI	3	0.3371	1302.4	27.00	0.32	tr_C9ZN43_
HLA-DQA10501-DQB10201	290	VTPLIRYADPNAIKL	IRYADPNAI	4	0.3370	1304.3	27.00	0.55	tr_C9ZN43_
HLA-DQA10501-DQB10201	291	FVIGLAPGDPSLLAA	FVIGLAPGD	0	0.3366	1309.4	27.00	0.38	tr_C9ZN43_
HLA-DQA10501-DQB10201	292	EDVMTVRVYSLLYNP	TVRVYSLLY	4	0.3364	1313.0	27.00	0.46	tr_C9ZN43_
HLA-DQA10501-DQB10201	293	PNAERVQFATSLPHW	QFATSLPHW	6	0.3362	1315.2	27.00	0.24	tr_C9ZN43_
HLA-DQA10501-DQB10201	294	AYVPPALSEDVMTVR	PPALSEDVM	3	0.3358	1321.6	27.00	0.36	tr_C9ZN43_
HLA-DQA10501-DQB10201	295	ALMYDELVSAFNGNP	LMYDELVSA	1	0.3349	1334.5	27.00	0.28	tr_C9ZN43_
HLA-DQA10501-DQB10201	296	RFGQSDRLFISMIPS	DRLFISMIP	5	0.3339	1348.3	27.00	0.25	tr_C9ZN43_
HLA-DQA10501-DQB10201	297	QSELDTRSVTAVFGV	TRSVTAVFG	5	0.3335	1355.1	28.00	0.48	tr_C9ZN43_
HLA-DQA10501-DQB10201	298	VSSEALYYGANVLTG	ALYYGANVL	4	0.3334	1356.0	28.00	0.34	tr_C9ZN43_
HLA-DQA10501-DQB10201	299	LLYNPLIPDNLCDAI	YNPLIPDNL	2	0.3326	1367.4	28.00	0.50	tr_C9ZN43_
HLA-DQA10501-DQB10201	300	LASLPLLERENLVGF	PLLERENLV	4	0.3324	1371.7	28.00	0.32	tr_C9ZN43_
HLA-DQA10501-DQB10201	301	PPTSYKIKPVEVLQV	SYKIKPVEV	3	0.3317	1381.9	28.00	0.37	tr_C9ZN43_
HLA-DQA10501-DQB10201	302	NTAWEQFAETRPQGV	FAETRPQGV	6	0.3314	1385.4	28.00	0.32	tr_C9ZN43_
HLA-DQA10501-DQB10201	303	FNTAWEQFAETRPQG	WEQFAETRP	4	0.3303	1403.0	28.00	0.31	tr_C9ZN43_
HLA-DQA10501-DQB10201	304	LTGNGRFGQSDRLFI	GRFGQSDRL	4	0.3303	1403.1	28.00	0.31	tr_C9ZN43_
HLA-DQA10501-DQB10201	305	YVPPALSEDVMTVRV	PPALSEDVM	2	0.3296	1413.1	28.00	0.29	tr_C9ZN43_
HLA-DQA10501-DQB10201	306	PTSYKIKPVEVLQVA	SYKIKPVEV	2	0.3295	1414.5	28.00	0.38	tr_C9ZN43_
HLA-DQA10501-DQB10201	307	IKPVEVLQVALERHK	EVLQVALER	4	0.3295	1414.9	28.00	0.31	tr_C9ZN43_
HLA-DQA10501-DQB10201	308	LSEDVMTVRVYSLLY	TVRVYSLLY	6	0.3283	1433.9	29.00	0.50	tr_C9ZN43_
HLA-DQA10501-DQB10201	309	SELDTRSVTAVFGVV	TRSVTAVFG	4	0.3280	1437.5	29.00	0.47	tr_C9ZN43_
HLA-DQA10501-DQB10201	310	VIDDLVIGDFGGDCK	IDDLVIGDF	1	0.3273	1448.6	29.00	0.32	tr_C9ZN43_
HLA-DQA10501-DQB10201	311	DGVEFFLGQLMLTRT	VEFFLGQLM	2	0.3262	1466.2	29.00	0.34	tr_C9ZN43_
HLA-DQA10501-DQB10201	312	LQSELDTRSVTAVFG	TRSVTAVFG	6	0.3257	1473.4	29.00	0.58	tr_C9ZN43_
HLA-DQA10501-DQB10201	313	PVTPLIRYADPNAIK	IRYADPNAI	5	0.3257	1474.8	29.00	0.57	tr_C9ZN43_
HLA-DQA10501-DQB10201	314	ERVQFATSLPHWADA	FATSLPHWA	4	0.3253	1480.2	29.00	0.32	tr_C9ZN43_
HLA-DQA10501-DQB10201	315	TAWEQFAETRPQGVI	FAETRPQGV	5	0.3246	1491.3	29.00	0.39	tr_C9ZN43_
HLA-DQA10501-DQB10201	316	FAETRPQGVIAFAPP	PQGVIAFAP	5	0.3242	1498.2	30.00	0.32	tr_C9ZN43_
HLA-DQA10501-DQB10201	317	DRLFISMIPSPSNAT	FISMIPSPS	3	0.3242	1498.6	30.00	0.41	tr_C9ZN43_
HLA-DQA10501-DQB10201	318	HDDVDGELMVYGWIA	DVDGELMVY	2	0.3240	1501.1	30.00	0.29	tr_C9ZN43_
HLA-DQA10501-DQB10201	319	QFAETRPQGVIAFAP	PQGVIAFAP	6	0.3234	1511.3	30.00	0.29	tr_C9ZN43_
HLA-DQA10501-DQB10201	320	GRVNDDDFNTAWEQF	DFNTAWEQF	6	0.3226	1523.8	30.00	0.58	tr_C9ZN43_
HLA-DQA10501-DQB10201	321	SEDVMTVRVYSLLYN	TVRVYSLLY	5	0.3224	1528.1	30.00	0.44	tr_C9ZN43_
HLA-DQA10501-DQB10201	322	VRGADATIIEVALRK	VRGADATII	0	0.3220	1535.1	30.00	0.25	tr_C9ZN43_
HLA-DQA10501-DQB10201	323	SSEALYYGANVLTGN	ALYYGANVL	3	0.3217	1539.8	30.00	0.34	tr_C9ZN43_
HLA-DQA10501-DQB10201	324	SMWSLARALNASVPP	WSLARALNA	2	0.3214	1543.5	30.00	0.38	tr_C9ZN43_
HLA-DQA10501-DQB10201	325	MEYVTPKTLLDTIYM	PKTLLDTIY	5	0.3202	1564.5	30.00	0.38	tr_C9ZN43_
HLA-DQA10501-DQB10201	326	VLPRMEYVTPKTLLD	MEYVTPKTL	4	0.3201	1566.9	30.00	0.55	tr_C9ZN43_
HLA-DQA10501-DQB10201	327	PKTLLDTIYMQSIIT	TIYMQSIIT	6	0.3197	1572.3	31.00	0.25	tr_C9ZN43_
HLA-DQA10501-DQB10201	328	TSRKAFMESLYNQRR	RKAFMESLY	2	0.3196	1573.9	31.00	0.34	tr_C9ZN43_
HLA-DQA10501-DQB10201	329	GNGRFGQSDRLFISM	RFGQSDRLF	3	0.3191	1582.5	31.00	0.31	tr_C9ZN43_
HLA-DQA10501-DQB10201	330	TPLIRYADPNAIKLS	IRYADPNAI	3	0.3191	1583.9	31.00	0.58	tr_C9ZN43_
HLA-DQA10501-DQB10201	331	LPLLERENLVGFAPS	ERENLVGFA	4	0.3181	1601.2	31.00	0.18	tr_C9ZN43_
HLA-DQA10501-DQB10201	332	VTPKTLLDTIYMQSI	LDTIYMQSI	6	0.3173	1614.1	31.00	0.38	tr_C9ZN43_
HLA-DQA10501-DQB10201	333	TGNGRFGQSDRLFIS	GRFGQSDRL	3	0.3169	1621.8	31.00	0.31	tr_C9ZN43_
HLA-DQA10501-DQB10201	334	RALNASVPPLTSPVT	ALNASVPPL	1	0.3168	1623.7	31.00	0.41	tr_C9ZN43_
HLA-DQA10501-DQB10201	335	TVAPDVKVELIPPTS	APDVKVELI	2	0.3168	1623.8	31.00	0.30	tr_C9ZN43_

HLA-DQA10501-DQB10201	336	VAPDVKVELIPPTSY	APDVKVELI	1	0.3166	1626.0	31.00	0.22	tr_C9ZN43_
HLA-DQA10501-DQB10201	337	TPKTLLDTIYMQSII	LDTIYMQSI	5	0.3165	1628.7	31.00	0.35	tr_C9ZN43_
HLA-DQA10501-DQB10201	338	KTLLDTIYMQSIITA	TIYMQSIIT	5	0.3162	1633.3	31.00	0.23	tr_C9ZN43_
HLA-DQA10501-DQB10201	339	VIQLSPTLEQQLFVV	VIQLSPTLE	0	0.3160	1636.6	31.00	0.34	tr_C9ZN43_
HLA-DQA10501-DQB10201	340	QTVAVLDDNATLVGR	TVAVLDDNA	1	0.3153	1649.9	32.00	0.51	tr_C9ZN43_
HLA-DQA10501-DQB10201	341	AVLPRMEYVTPKTLL	MEYVTPKTL	5	0.3136	1680.6	32.00	0.54	tr_C9ZN43_
HLA-DQA10501-DQB10201	342	FLTASPNAELLTLVR	FLTASPNAE	0	0.3130	1692.0	32.00	0.28	tr_C9ZN43_
HLA-DQA10501-DQB10201	343	ASRKWTVAPDVKVEL	WTVAPDVKV	4	0.3129	1692.7	32.00	0.31	tr_C9ZN43_
HLA-DQA10501-DQB10201	344	NGRFGQSDRLFISMI	FGQSDRLFI	3	0.3125	1699.8	32.00	0.26	tr_C9ZN43_
HLA-DQA10501-DQB10201	345	AETRPQGVIAFAPPA	PQGVIAFAP	4	0.3125	1700.1	32.00	0.33	tr_C9ZN43_
HLA-DQA10501-DQB10201	346	ISMWSLARALNASVP	WSLARALNA	3	0.3125	1700.3	32.00	0.47	tr_C9ZN43_
HLA-DQA10501-DQB10201	347	RYAVSQLRLLRIGFM	LRLLRIGFM	6	0.3124	1703.0	32.00	0.39	tr_C9ZN43_
HLA-DQA10501-DQB10201	348	VPPALSEDVMTVRVY	PPALSEDVM	1	0.3121	1708.3	32.00	0.25	tr_C9ZN43_
HLA-DQA10501-DQB10201	349	QAVLPRMEYVTPKTL	MEYVTPKTL	6	0.3115	1719.3	32.00	0.53	tr_C9ZN43_
HLA-DQA10501-DQB10201	350	VRYAVSQLRLLRIGF	RYAVSQLRL	1	0.3113	1721.8	32.00	0.38	tr_C9ZN43_
HLA-DQA10501-DQB10201	351	ALERHKGEFFVVLGP	LERHKGEFF	1	0.3111	1726.4	33.00	0.28	tr_C9ZN43_
HLA-DQA10501-DQB10201	352	GLAPGDPSLLAAHLD	PSLLAAHLD	6	0.3109	1730.2	33.00	0.62	tr_C9ZN43_
HLA-DQA10501-DQB10201	353	REFHTALPDSSAWKP	FHTALPDSS	2	0.3107	1734.7	33.00	0.46	tr_C9ZN43_
HLA-DQA10501-DQB10201	354	VFNGTDNFDHDDVDG	TDNFDHDDV	4	0.3101	1744.5	33.00	0.34	tr_C9ZN43_
HLA-DQA10501-DQB10201	355	CGVFTVDGLMRGRVN	VFTVDGLMR	2	0.3099	1749.6	33.00	0.28	tr_C9ZN43_
HLA-DQA10501-DQB10201	356	TRPQGVIAFAPPAKD	VIAFAPPAK	5	0.3093	1760.5	33.00	0.22	tr_C9ZN43_
HLA-DQA10501-DQB10201	357	DVDGELMVYGWIAGE	MVYGWIAGE	6	0.3076	1793.3	33.00	0.38	tr_C9ZN43_
HLA-DQA10501-DQB10201	358	PGDPSLLAAHLDRNP	PSLLAAHLD	3	0.3054	1836.3	34.00	0.50	tr_C9ZN43_
HLA-DQA10501-DQB10201	359	GDPSLLAAHLDRNPD	PSLLAAHLD	2	0.3050	1843.9	34.00	0.49	tr_C9ZN43_
HLA-DQA10501-DQB10201	360	AELLTLVRYAVSQLR	VRYAVSQLR	6	0.3050	1844.2	34.00	0.56	tr_C9ZN43_
HLA-DQA10501-DQB10201	361	LRGISFGDTEYKMTT	GISFGDTEY	2	0.3045	1854.4	34.00	0.34	tr_C9ZN43_
HLA-DQA10501-DQB10201	362	RIGFMYLRGISFGDT	FMYLRGISF	3	0.3045	1855.0	34.00	0.35	tr_C9ZN43_
HLA-DQA10501-DQB10201	363	NPNAERVQFATSLPH	ERVQFATSL	4	0.3042	1860.5	34.00	0.31	tr_C9ZN43_
HLA-DQA10501-DQB10201	364	RLPNRGNAFVIGLAP	NAFVIGLAP	6	0.3041	1862.7	34.00	0.37	tr_C9ZN43_
HLA-DQA10501-DQB10201	365	AWEQFAETRPQGVIA	FAETRPQGV	4	0.3039	1866.5	34.00	0.46	tr_C9ZN43_
HLA-DQA10501-DQB10201	366	LAPGDPSLLAAHLDR	PSLLAAHLD	5	0.3036	1872.4	34.00	0.55	tr_C9ZN43_
HLA-DQA10501-DQB10201	367	YGPFEEEEEKECFTA	YGPFEEEEE	0	0.3036	1873.1	34.00	0.31	tr_C9ZN43_
HLA-DQA10501-DQB10201	368	EEKECFTANDPVPEQ	FTANDPVPE	5	0.3034	1876.4	34.00	0.35	tr_C9ZN43_
HLA-DQA10501-DQB10201	369	VAFVDPVMLTPRLHH	VAFVDPVML	0	0.3032	1881.1	34.00	0.51	tr_C9ZN43_
HLA-DQA10501-DQB10201	370	ATRISMWSLARALNA	WSLARALNA	6	0.3024	1896.3	35.00	0.50	tr_C9ZN43_
HLA-DQA10501-DQB10201	371	LYNPLIPDNLCDAIN	YNPLIPDNL	1	0.3020	1905.4	35.00	0.44	tr_C9ZN43_
HLA-DQA10501-DQB10201	372	SRKAFMESLYNQRRY	RKAFMESLY	1	0.3014	1916.5	35.00	0.32	tr_C9ZN43_
HLA-DQA10501-DQB10201	373	NQGGNVLYINVVGND	LYINVVGND	6	0.3006	1933.4	35.00	0.25	tr_C9ZN43_
HLA-DQA10501-DQB10201	374	LPRMEYVTPKTLLDT	MEYVTPKTL	3	0.3006	1933.5	35.00	0.55	tr_C9ZN43_
HLA-DQA10501-DQB10201	375	TSSEIVREFHTALPD	REFHTALPD	6	0.3000	1946.9	35.00	0.31	tr_C9ZN43_
HLA-DQA10501-DQB10201	376	PLIRYADPNAIKLSS	IRYADPNAI	2	0.3000	1947.4	35.00	0.61	tr_C9ZN43_
HLA-DQA10501-DQB10201	377	SEALYYGANVLTGNG	ALYYGANVL	2	0.2997	1953.7	35.00	0.34	tr_C9ZN43_
HLA-DQA10501-DQB10201	378	PRMEYVTPKTLLDTI	MEYVTPKTL	2	0.2991	1966.3	35.00	0.50	tr_C9ZN43_
HLA-DQA10501-DQB10201	379	RISMWSLARALNASV	WSLARALNA	4	0.2991	1966.7	35.00	0.47	tr_C9ZN43_
HLA-DQA10501-DQB10201	380	SRTHDAYLLSSSSLE	AYLLSSSSL	5	0.2990	1967.3	35.00	0.44	tr_C9ZN43_
HLA-DQA10501-DQB10201	381	ETRPQGVIAFAPPAK	PQGVIAFAP	3	0.2987	1974.4	35.00	0.27	tr_C9ZN43_
HLA-DQA10501-DQB10201	382	RPQGVIAFAPPAKDL	VIAFAPPAK	4	0.2979	1991.9	36.00	0.25	tr_C9ZN43_
HLA-DQA10501-DQB10201	383	WEQFAETRPQGVIAF	FAETRPQGV	3	0.2974	2001.6	36.00	0.50	tr_C9ZN43_
HLA-DQA10501-DQB10201	384	APGDPSLLAAHLDRN	PSLLAAHLD	4	0.2974	2003.0	36.00	0.50	tr_C9ZN43_
HLA-DQA10501-DQB10201	385	LIPPTSYKIKPVEVL	SYKIKPVEV	5	0.2967	2016.5	36.00	0.62	tr_C9ZN43_

HLA-DQA10501-DQB10201	386	IVWMHGGTLQTVAVL	MHGGTLQTV	3	0.2957	2040.4	36.00	0.23	tr_C9ZN43_
HLA-DQA10501-DQB10201	387	TRISMWSLARALNAS	WSLARALNA	5	0.2953	2048.9	36.00	0.50	tr_C9ZN43_
HLA-DQA10501-DQB10201	388	GNPNAERVQFATSLP	ERVQFATSL	5	0.2952	2050.5	36.00	0.36	tr_C9ZN43_
HLA-DQA10501-DQB10201	389	HWADANTSSEIVREF	TSSEIVREF	6	0.2950	2054.4	36.00	0.37	tr_C9ZN43_
HLA-DQA10501-DQB10201	390	LASRKWTVAPDVKVE	WTVAPDVKV	5	0.2948	2058.3	36.00	0.40	tr_C9ZN43_
HLA-DQA10501-DQB10201	391	KPVEVLQVALERHKG	EVLQVALER	3	0.2938	2080.6	37.00	0.35	tr_C9ZN43_
HLA-DQA10501-DQB10201	392	LPNRGNAFVIGLAPG	NAFVIGLAP	5	0.2938	2081.3	37.00	0.34	tr_C9ZN43_
HLA-DQA10501-DQB10201	393	SLASRKWTVAPDVKV	RKWTVAPDV	4	0.2937	2084.3	37.00	0.28	tr_C9ZN43_
HLA-DQA10501-DQB10201	394	EKECFTANDPVPEQG	FTANDPVPE	4	0.2934	2090.6	37.00	0.34	tr_C9ZN43_
HLA-DQA10501-DQB10201	395	MSSVGRELCGVFTVD	GRELCGVFT	4	0.2933	2091.9	37.00	0.27	tr_C9ZN43_
HLA-DQA10501-DQB10201	396	RGWNSHVYFLTASPN	VYFLTASPN	6	0.2929	2102.7	37.00	0.35	tr_C9ZN43_
HLA-DQA10501-DQB10201	397	IPPTSYKIKPVEVLQ	SYKIKPVEV	4	0.2920	2122.1	37.00	0.59	tr_C9ZN43_
HLA-DQA10501-DQB10201	398	IAGEVLSQALSSREW	EVLSQALSS	3	0.2915	2133.2	37.00	0.33	tr_C9ZN43_
HLA-DQA10501-DQB10201	399	GVEFFLGQLMLTRTG	VEFFLGQLM	1	0.2915	2133.3	37.00	0.34	tr_C9ZN43_
HLA-DQA10501-DQB10201	400	ERFHTVQGGTTVFEP	VQGGTTVFE	5	0.2912	2141.7	37.00	0.45	tr_C9ZN43_
HLA-DQA10501-DQB10201	401	LGPVGDGQALASLPL	GQALASLPL	6	0.2906	2153.9	37.00	0.47	tr_C9ZN43_
HLA-DQA10501-DQB10201	402	LRIGFMYLRGISFGD	FMYLRGISF	4	0.2906	2154.1	37.00	0.47	tr_C9ZN43_
HLA-DQA10501-DQB10201	403	WADANTSSEIVREFH	TSSEIVREF	5	0.2906	2155.4	37.00	0.31	tr_C9ZN43_
HLA-DQA10501-DQB10201	404	RVQFATSLPHWADAN	FATSLPHWA	3	0.2906	2155.6	37.00	0.38	tr_C9ZN43_
HLA-DQA10501-DQB10201	405	IDDLVIGDFGGDCKG	DDLVIGDFG	1	0.2901	2166.5	38.00	0.32	tr_C9ZN43_
HLA-DQA10501-DQB10201	406	SANPGVTVFNGTDNF	TVFNGTDNF	6	0.2898	2172.7	38.00	0.71	tr_C9ZN43_
HLA-DQA10501-DQB10201	407	AGEVLSQALSSREWL	QALSSREWL	6	0.2897	2175.3	38.00	0.29	tr_C9ZN43_
HLA-DQA10501-DQB10201	408	RLFISMIPSPSNATY	FISMIPSPS	2	0.2893	2185.1	38.00	0.51	tr_C9ZN43_
HLA-DQA10501-DQB10201	409	EQFAETRPQGVIAFA	FAETRPQGV	2	0.2893	2186.1	38.00	0.50	tr_C9ZN43_
HLA-DQA10501-DQB10201	410	YAVSQLRLLRIGFMY	LRLLRIGFM	5	0.2888	2198.2	38.00	0.38	tr_C9ZN43_
HLA-DQA10501-DQB10201	411	DPSLLAAHLDRNPDV	PSLLAAHLD	1	0.2887	2200.9	38.00	0.47	tr_C9ZN43_
HLA-DQA10501-DQB10201	412	RLLRIGFMYLRGISF	FMYLRGISF	6	0.2884	2206.8	38.00	0.57	tr_C9ZN43_
HLA-DQA10501-DQB10201	413	LHHRGRNVIQLSPTL	NVIQLSPTL	6	0.2879	2219.9	38.00	0.41	tr_C9ZN43_
HLA-DQA10501-DQB10201	414	QQLFVVVGYVTNTSA	VVGYVTNTS	5	0.2875	2228.9	38.00	0.35	tr_C9ZN43_
HLA-DQA10501-DQB10201	415	SREWLTSRKAFMESL	SRKAFMESL	6	0.2870	2239.8	38.00	0.60	tr_C9ZN43_
HLA-DQA10501-DQB10201	416	TVDGLMRGRVNDDDF	RGRVNDDDF	6	0.2863	2258.4	39.00	0.57	tr_C9ZN43_
HLA-DQA10501-DQB10201	417	VDGLMRGRVNDDDFN	RGRVNDDDF	5	0.2856	2274.1	39.00	0.54	tr_C9ZN43_
HLA-DQA10501-DQB10201	418	EYVTPKTLLDTIYMQ	PKTLLDTIY	4	0.2844	2305.6	39.00	0.40	tr_C9ZN43_
HLA-DQA10501-DQB10201	419	TVAVLDDNATLVGRL	TVAVLDDNA	0	0.2838	2320.7	39.00	0.34	tr_C9ZN43_
HLA-DQA10501-DQB10201	420	FVVLGPVGDGQALAS	FVVLGPVGD	0	0.2833	2331.8	39.00	0.26	tr_C9ZN43_
HLA-DQA10501-DQB10201	421	DDVDGELMVYGWIAG	DVDGELMVY	1	0.2833	2333.3	39.00	0.36	tr_C9ZN43_
HLA-DQA10501-DQB10201	422	YVTPKTLLDTIYMQS	PKTLLDTIY	3	0.2831	2335.9	39.00	0.35	tr_C9ZN43_
HLA-DQA10501-DQB10201	423	SLMSSVGRELCGVFT	LMSSVGREL	1	0.2830	2340.9	39.00	0.29	tr_C9ZN43_
HLA-DQA10501-DQB10201	424	EEEKECFTANDPVPE	FTANDPVPE	6	0.2829	2343.1	39.00	0.48	tr_C9ZN43_
HLA-DQA10501-DQB10201	425	KIVWMHGGTLQTVAV	MHGGTLQTV	4	0.2825	2352.3	39.00	0.31	tr_C9ZN43_
HLA-DQA10501-DQB10201	426	ALNASVPPLTSPVTP	LNASVPPLT	1	0.2825	2353.6	40.00	0.47	tr_C9ZN43_
HLA-DQA10501-DQB10201	427	FVVVGYVTNTSASAP	YVTNTSASA	5	0.2821	2363.1	40.00	0.31	tr_C9ZN43_
HLA-DQA10501-DQB10201	428	LMYDELVSAFNGNPN	MYDELVSAF	1	0.2819	2367.7	40.00	0.36	tr_C9ZN43_
HLA-DQA10501-DQB10201	429	EVLQVALERHKGEFF	LERHKGEFF	6	0.2816	2376.2	40.00	0.44	tr_C9ZN43_
HLA-DQA10501-DQB10201	430	PQGVIAFAPPAKDLI	VIAFAPPAK	3	0.2812	2386.3	40.00	0.28	tr_C9ZN43_
HLA-DQA10501-DQB10201	431	SSVGRELCGVFTVDG	GRELCGVFT	3	0.2809	2393.0	40.00	0.26	tr_C9ZN43_
HLA-DQA10501-DQB10201	432	RFHTVQGGTTVFEPS	VQGGTTVFE	4	0.2799	2419.5	40.00	0.44	tr_C9ZN43_
HLA-DQA10501-DQB10201	433	PVEVLQVALERHKGE	EVLQVALER	2	0.2797	2424.5	40.00	0.41	tr_C9ZN43_
HLA-DQA10501-DQB10201	434	KECFTANDPVPEQGE	FTANDPVPE	3	0.2792	2438.7	40.00	0.37	tr_C9ZN43_
HLA-DQA10501-DQB10201	435	DGLMRGRVNDDDFNT	RGRVNDDDF	4	0.2786	2453.4	40.00	0.51	tr_C9ZN43_

HLA-DQA10501-DQB10201	436	LIRYADPNAIKLSSA	IRYADPNAI	1	0.2783	2460.8	41.00	0.62	tr_C9ZN43_
HLA-DQA10501-DQB10201	437	LMSSVGRELCGVFTV	GRELCGVFT	5	0.2777	2479.1	41.00	0.27	tr_C9ZN43_
HLA-DQA10501-DQB10201	438	QVALERHKGEFFVVL	LERHKGEFF	3	0.2774	2486.9	41.00	0.49	tr_C9ZN43_
HLA-DQA10501-DQB10201	439	DERFHTVQGGTTVFE	VQGGTTVFE	6	0.2772	2490.2	41.00	0.47	tr_C9ZN43_
HLA-DQA10501-DQB10201	440	NDPVPEQGEVCVVNY	QGEVCVVNY	6	0.2771	2493.2	41.00	0.27	tr_C9ZN43_
HLA-DQA10501-DQB10201	441	LTASPNAELLTLVRY	PNAELLTLV	4	0.2767	2504.3	41.00	0.29	tr_C9ZN43_
HLA-DQA10501-DQB10201	442	LFVVVGYVTNTSASA	VVGYVTNTS	3	0.2765	2510.8	41.00	0.32	tr_C9ZN43_
HLA-DQA10501-DQB10201	443	PLLERENLVGFAPST	ERENLVGFA	3	0.2760	2522.9	41.00	0.22	tr_C9ZN43_
HLA-DQA10501-DQB10201	444	VALERHKGEFFVVLG	LERHKGEFF	2	0.2759	2526.1	41.00	0.47	tr_C9ZN43_
HLA-DQA10501-DQB10201	445	EQQLFVVVGYVTNTS	VVGYVTNTS	6	0.2752	2544.5	41.00	0.44	tr_C9ZN43_
HLA-DQA10501-DQB10201	446	LRKIVWMHGGTLQTV	MHGGTLQTV	6	0.2748	2556.5	41.00	0.44	tr_C9ZN43_
HLA-DQA10501-DQB10201	447	APDVKVELIPPTSYK	DVKVELIPP	2	0.2744	2566.9	42.00	0.20	tr_C9ZN43_
HLA-DQA10501-DQB10201	448	ESVRLYSPLNTLMFL	RLYSPLNTL	3	0.2739	2583.1	42.00	0.22	tr_C9ZN43_
HLA-DQA10501-DQB10201	449	AESVRLYSPLNTLMF	VRLYSPLNT	3	0.2730	2605.8	42.00	0.21	tr_C9ZN43_
HLA-DQA10501-DQB10201	450	YADPNAIKLSSAQLA	IKLSSAQLA	6	0.2730	2606.4	42.00	0.52	tr_C9ZN43_
HLA-DQA10501-DQB10201	451	AVSQLRLLRIGFMYL	LRLLRIGFM	4	0.2729	2609.0	42.00	0.44	tr_C9ZN43_
HLA-DQA10501-DQB10201	452	EALYYGANVLTGNGR	ALYYGANVL	1	0.2728	2613.1	42.00	0.34	tr_C9ZN43_
HLA-DQA10501-DQB10201	453	RGADATIIEVALRKI	ADATIIEVA	2	0.2713	2656.7	42.00	0.25	tr_C9ZN43_
HLA-DQA10501-DQB10201	454	RKIVWMHGGTLQTVA	MHGGTLQTV	5	0.2707	2673.6	43.00	0.40	tr_C9ZN43_
HLA-DQA10501-DQB10201	455	RGISFGDTEYKMTTS	GISFGDTEY	1	0.2705	2677.7	43.00	0.39	tr_C9ZN43_
HLA-DQA10501-DQB10201	456	ADANTSSEIVREFHT	TSSEIVREF	4	0.2705	2677.8	43.00	0.31	tr_C9ZN43_
HLA-DQA10501-DQB10201	457	VLTGNGRFGQSDRLF	GRFGQSDRL	5	0.2692	2715.9	43.00	0.44	tr_C9ZN43_
HLA-DQA10501-DQB10201	458	GLMRGRVNDDDFNTA	RGRVNDDDF	3	0.2687	2729.9	43.00	0.51	tr_C9ZN43_
HLA-DQA10501-DQB10201	459	QLFVVVGYVTNTSAS	VVGYVTNTS	4	0.2686	2734.5	43.00	0.40	tr_C9ZN43_
HLA-DQA10501-DQB10201	460	PPALSEDVMTVRVYS	PALSEDVMT	1	0.2683	2742.8	43.00	0.25	tr_C9ZN43_
HLA-DQA10501-DQB10201	461	MRGWNSHVYFLTASP	HVYFLTASP	6	0.2658	2817.0	44.00	0.29	tr_C9ZN43_
HLA-DQA10501-DQB10201	462	DSRTHDAYLLSSSSL	AYLLSSSSL	6	0.2657	2821.2	44.00	0.63	tr_C9ZN43_
HLA-DQA10501-DQB10201	463	LLRIGFMYLRGISFG	FMYLRGISF	5	0.2653	2833.4	44.00	0.53	tr_C9ZN43_
HLA-DQA10501-DQB10201	464	VQFATSLPHWADANT	FATSLPHWA	2	0.2648	2850.2	44.00	0.50	tr_C9ZN43_
HLA-DQA10501-DQB10201	465	ECFTANDPVPEQGEV	FTANDPVPE	2	0.2644	2860.1	44.00	0.41	tr_C9ZN43_
HLA-DQA10501-DQB10201	466	TSLMSSVGRELCGVF	LMSSVGREL	2	0.2641	2870.0	44.00	0.46	tr_C9ZN43_
HLA-DQA10501-DQB10201	467	MRGRVNDDDFNTAWE	RGRVNDDDF	1	0.2640	2874.8	44.00	0.43	tr_C9ZN43_
HLA-DQA10501-DQB10201	468	LLERENLVGFAPSTG	ERENLVGFA	2	0.2639	2875.3	44.00	0.23	tr_C9ZN43_
HLA-DQA10501-DQB10201	469	GEVLSQALSSREWLT	QALSSREWL	5	0.2637	2882.3	44.00	0.31	tr_C9ZN43_
HLA-DQA10501-DQB10201	470	NGNPNAERVQFATSL	ERVQFATSL	6	0.2629	2909.0	45.00	0.43	tr_C9ZN43_
HLA-DQA10501-DQB10201	471	LMRGRVNDDDFNTAW	RGRVNDDDF	2	0.2628	2912.3	45.00	0.49	tr_C9ZN43_
HLA-DQA10501-DQB10201	472	SAPMSAIVRGADATI	IVRGADATI	6	0.2623	2925.5	45.00	0.46	tr_C9ZN43_
HLA-DQA10501-DQB10201	473	SVRLYSPLNTLMFLI	RLYSPLNTL	2	0.2619	2939.3	45.00	0.20	tr_C9ZN43_
HLA-DQA10501-DQB10201	474	CNQGGNVLYINVVGN	GNVLYINVV	4	0.2619	2941.1	45.00	0.38	tr_C9ZN43_
HLA-DQA10501-DQB10201	475	VVVGYVTNTSASAPM	YVTNTSASA	4	0.2618	2941.5	45.00	0.31	tr_C9ZN43_
HLA-DQA10501-DQB10201	476	PALSEDVMTVRVYSL	VMTVRVYSL	6	0.2618	2943.2	45.00	0.30	tr_C9ZN43_
HLA-DQA10501-DQB10201	477	TANDPVPEQGEVCVV	PEQGEVCVV	6	0.2606	2981.3	45.00	0.38	tr_C9ZN43_
HLA-DQA10501-DQB10201	478	EFHTALPDSSAWKPL	FHTALPDSS	1	0.2594	3020.3	46.00	0.56	tr_C9ZN43_
HLA-DQA10501-DQB10201	479	YNPLIPDNLCDAINA	YNPLIPDNL	0	0.2594	3021.5	46.00	0.35	tr_C9ZN43_
HLA-DQA10501-DQB10201	480	QLRLLRIGFMYLRGI	LRLLRIGFM	1	0.2591	3031.0	46.00	0.34	tr_C9ZN43_
HLA-DQA10501-DQB10201	481	NAELLTLVRYAVSQL	LVRYAVSQL	6	0.2583	3055.4	46.00	0.62	tr_C9ZN43_
HLA-DQA10501-DQB10201	482	VEFFLGQLMLTRTGP	VEFFLGQLM	0	0.2573	3089.1	46.00	0.26	tr_C9ZN43_
HLA-DQA10501-DQB10201	483	FHTVQGGTTVFEPSR	VQGGTTVFE	3	0.2570	3099.3	46.00	0.47	tr_C9ZN43_
HLA-DQA10501-DQB10201	484	EVCVVNYGATRISMW	VVNYGATRI	3	0.2568	3106.5	46.00	0.43	tr_C9ZN43_
HLA-DQA10501-DQB10201	485	TTSLMSSVGRELCGV	LMSSVGREL	3	0.2565	3116.2	46.00	0.51	tr_C9ZN43_

HLA-DQA10501-DQB10201	486	VEVLQVALERHKGEF	EVLQVALER	1	0.2562	3125.4	46.00	0.46	tr_C9ZN43_
HLA-DQA10501-DQB10201	487	ADPNAIKLSSAQLAG	IKLSSAQLA	5	0.2555	3150.5	47.00	0.54	tr_C9ZN43_
HLA-DQA10501-DQB10201	488	LYSPLNTLMFLILDR	TLMFLILDR	6	0.2548	3173.4	47.00	0.29	tr_C9ZN43_
HLA-DQA10501-DQB10201	489	TASPNAELLTLVRYA	PNAELLTLV	3	0.2547	3177.7	47.00	0.34	tr_C9ZN43_
HLA-DQA10501-DQB10201	490	LFISMIPSPSNATYL	FISMIPSPS	1	0.2544	3188.9	47.00	0.55	tr_C9ZN43_
HLA-DQA10501-DQB10201	491	ELIPPTSYKIKPVEV	SYKIKPVEV	6	0.2542	3194.9	47.00	0.80	tr_C9ZN43_
HLA-DQA10501-DQB10201	492	CFTANDPVPEQGEVC	FTANDPVPE	1	0.2541	3198.9	47.00	0.37	tr_C9ZN43_
HLA-DQA10501-DQB10201	493	RKAFMESLYNQRRYV	FMESLYNQR	3	0.2526	3251.7	47.00	0.28	tr_C9ZN43_
HLA-DQA10501-DQB10201	494	LAESVRLYSPLNTLM	VRLYSPLNT	4	0.2525	3252.8	48.00	0.30	tr_C9ZN43_
HLA-DQA10501-DQB10201	495	DANTSSEIVREFHTA	TSSEIVREF	3	0.2525	3253.3	48.00	0.30	tr_C9ZN43_
HLA-DQA10501-DQB10201	496	KMTTSLMSSVGRELC	LMSSVGREL	5	0.2524	3259.0	48.00	0.58	tr_C9ZN43_
HLA-DQA10501-DQB10201	497	LQVALERHKGEFFVV	LERHKGEFF	4	0.2523	3261.7	48.00	0.58	tr_C9ZN43_
HLA-DQA10501-DQB10201	498	SQLRLLRIGFMYLRG	LRLLRIGFM	2	0.2519	3276.8	48.00	0.44	tr_C9ZN43_
HLA-DQA10501-DQB10201	499	QGVIAFAPPAKDLIK	VIAFAPPAK	2	0.2519	3276.9	48.00	0.32	tr_C9ZN43_
HLA-DQA10501-DQB10201	500	NTSSEIVREFHTALP	IVREFHTAL	5	0.2513	3298.1	48.00	0.23	tr_C9ZN43_
HLA-DQA10501-DQB10201	501	FTANDPVPEQGEVCV	FTANDPVPE	0	0.2510	3308.0	48.00	0.23	tr_C9ZN43_
HLA-DQA10501-DQB10201	502	MIPSPSNATYLALQS	NATYLALQS	6	0.2493	3368.6	48.00	0.50	tr_C9ZN43_
HLA-DQA10501-DQB10201	503	FFLGQLMLTRTGPLA	MLTRTGPLA	6	0.2487	3392.6	49.00	0.30	tr_C9ZN43_
HLA-DQA10501-DQB10201	504	DPVPEQGEVCVVNYG	QGEVCVVNY	5	0.2486	3396.0	49.00	0.25	tr_C9ZN43_
HLA-DQA10501-DQB10201	505	GVFTVDGLMRGRVND	VFTVDGLMR	1	0.2485	3397.9	49.00	0.35	tr_C9ZN43_
HLA-DQA10501-DQB10201	506	VSQLRLLRIGFMYLR	LRLLRIGFM	3	0.2484	3402.9	49.00	0.47	tr_C9ZN43_
HLA-DQA10501-DQB10201	507	LSPTLEQQLFVVVGY	QQLFVVVGY	6	0.2483	3407.0	49.00	0.31	tr_C9ZN43_
HLA-DQA10501-DQB10201	508	ISFGDTEYKMTTSLM	EYKMTTSLM	6	0.2482	3410.0	49.00	0.62	tr_C9ZN43_
HLA-DQA10501-DQB10201	509	VRLYSPLNTLMFLIL	RLYSPLNTL	1	0.2477	3426.5	49.00	0.19	tr_C9ZN43_
HLA-DQA10501-DQB10201	510	SLPHWADANTSSEIV	DANTSSEIV	6	0.2477	3426.9	49.00	0.42	tr_C9ZN43_
HLA-DQA10501-DQB10201	511	YKMTTSLMSSVGREL	LMSSVGREL	6	0.2473	3444.2	49.00	0.67	tr_C9ZN43_
HLA-DQA10501-DQB10201	512	CLAESVRLYSPLNTL	RLYSPLNTL	6	0.2466	3467.9	49.00	0.34	tr_C9ZN43_
HLA-DQA10501-DQB10201	513	VCVVNYGATRISMWS	VVNYGATRI	2	0.2466	3468.7	49.00	0.41	tr_C9ZN43_
HLA-DQA10501-DQB10201	514	ANTSSEIVREFHTAL	TSSEIVREF	2	0.2456	3506.8	49.00	0.23	tr_C9ZN43_
HLA-DQA10501-DQB10201	515	EVLSQALSSREWLTS	QALSSREWL	4	0.2450	3529.9	50.00	0.35	tr_C9ZN43_
HLA-DQA10501-DQB10201	516	EFFLGQLMLTRTGPL	LMLTRTGPL	6	0.2449	3533.5	50.00	0.31	tr_C9ZN43_
HLA-DQA10501-DQB10201	517	IQLSPTLEQQLFVVV	PTLEQQLFV	4	0.2447	3541.1	50.00	0.34	tr_C9ZN43_
HLA-DQA10501-DQB10201	518	RLYSPLNTLMFLILD	NTLMFLILD	6	0.2443	3557.4	50.00	0.23	tr_C9ZN43_
HLA-DQA10501-DQB10201	519	CVVNYGATRISMWSL	VVNYGATRI	1	0.2434	3590.4	50.00	0.31	tr_C9ZN43_
HLA-DQA10501-DQB10201	520	PHWADANTSSEIVRE	DANTSSEIV	4	0.2433	3595.3	50.00	0.34	tr_C9ZN43_
HLA-DQA10501-DQB10201	521	ASPNAELLTLVRYAV	PNAELLTLV	2	0.2429	3609.2	50.00	0.35	tr_C9ZN43_
HLA-DQA10501-DQB10201	522	NCNQGGNVLYINVVG	GNVLYINVV	5	0.2426	3622.8	50.00	0.47	tr_C9ZN43_
HLA-DQA10501-DQB10201	523	ANDPVPEQGEVCVVN	PEQGEVCVV	5	0.2424	3628.7	50.00	0.37	tr_C9ZN43_
HLA-DQA10501-DQB10201	524	LERENLVGFAPSTGS	LVGFAPSTG	5	0.2421	3640.8	50.00	0.29	tr_C9ZN43_
HLA-DQA10501-DQB10201	525	ALSEDVMTVRVYSLL	VMTVRVYSL	5	0.2419	3650.2	50.00	0.31	tr_C9ZN43_
HLA-DQA10501-DQB10201	526	QFATSLPHWADANTS	FATSLPHWA	1	0.2414	3671.4	55.00	0.45	tr_C9ZN43_
HLA-DQA10501-DQB10201	527	ALQSELDTRSVTAVF	DTRSVTAVF	6	0.2412	3677.7	55.00	0.50	tr_C9ZN43_
HLA-DQA10501-DQB10201	528	AQAVLPRMEYVTPKT	RMEYVTPKT	6	0.2406	3703.2	55.00	0.23	tr_C9ZN43_
HLA-DQA10501-DQB10201	529	GADATIIEVALRKIV	ADATIIEVA	1	0.2401	3721.1	55.00	0.28	tr_C9ZN43_
HLA-DQA10501-DQB10201	530	NAMRGWNSHVYFLTA	WNSHVYFLT	5	0.2391	3762.0	55.00	0.31	tr_C9ZN43_
HLA-DQA10501-DQB10201	531	VLGPVGDGQALASLP	PVGDGQALA	3	0.2385	3788.2	55.00	0.31	tr_C9ZN43_
HLA-DQA10501-DQB10201	532	LRLLRIGFMYLRGIS	LRLLRIGFM	0	0.2385	3788.3	55.00	0.31	tr_C9ZN43_
HLA-DQA10501-DQB10201	533	ASLASRKWTVAPDVK	RKWTVAPDV	5	0.2384	3790.8	55.00	0.50	tr_C9ZN43_
HLA-DQA10501-DQB10201	534	PDVKVELIPPTSYKI	VELIPPTSY	4	0.2383	3796.4	55.00	0.25	tr_C9ZN43_
HLA-DQA10501-DQB10201	535	CNCNQGGNVLYINVV	GNVLYINVV	6	0.2382	3799.9	55.00	0.55	tr_C9ZN43_

HLA-DQA10501-DQB10201	536	SPTLEQQLFVVVGYV	QQLFVVVGY	5	0.2378	3816.6	55.00	0.23	tr_C9ZN43_
HLA-DQA10501-DQB10201	537	HTVQGGTTVFEPSRC	VQGGTTVFE	2	0.2365	3870.3	55.00	0.40	tr_C9ZN43_
HLA-DQA10501-DQB10201	538	SAFNGNPNAERVQFA	PNAERVQFA	6	0.2365	3870.8	55.00	0.52	tr_C9ZN43_
HLA-DQA10501-DQB10201	539	VLQVALERHKGEFFV	LERHKGEFF	5	0.2364	3873.4	55.00	0.63	tr_C9ZN43_
HLA-DQA10501-DQB10201	540	VVLGPVGDGQALASL	PVGDGQALA	4	0.2353	3921.8	55.00	0.31	tr_C9ZN43_
HLA-DQA10501-DQB10201	541	SNAMRGWNSHVYFLT	WNSHVYFLT	6	0.2352	3924.6	55.00	0.34	tr_C9ZN43_
HLA-DQA10501-DQB10201	542	MTTSLMSSVGRELCG	LMSSVGREL	4	0.2348	3940.8	55.00	0.60	tr_C9ZN43_
HLA-DQA10501-DQB10201	543	GRLPNRGNAFVIGLA	RGNAFVIGL	5	0.2339	3981.2	55.00	0.44	tr_C9ZN43_
HLA-DQA10501-DQB10201	544	DDLVIGDFGGDCKGG	DDLVIGDFG	0	0.2332	4008.4	55.00	0.33	tr_C9ZN43_
HLA-DQA10501-DQB10201	545	PSLLAAHLDRNPDVR	PSLLAAHLD	0	0.2332	4009.2	55.00	0.43	tr_C9ZN43_
HLA-DQA10501-DQB10201	546	QLSPTLEQQLFVVVG	PTLEQQLFV	3	0.2320	4061.3	55.00	0.32	tr_C9ZN43_
HLA-DQA10501-DQB10201	547	RGRVNDDDFNTAWEQ	RGRVNDDDF	0	0.2314	4088.1	55.00	0.33	tr_C9ZN43_
HLA-DQA10501-DQB10201	548	EQGEVCVVNYGATRI	VVNYGATRI	6	0.2310	4106.3	55.00	0.63	tr_C9ZN43_
HLA-DQA10501-DQB10201	549	TVFEPSRCLAESVRL	PSRCLAESV	4	0.2308	4115.6	55.00	0.38	tr_C9ZN43_
HLA-DQA10501-DQB10201	550	VVGYVTNTSASAPMS	YVTNTSASA	3	0.2299	4154.1	55.00	0.37	tr_C9ZN43_
HLA-DQA10501-DQB10201	551	DHMRSYLSANPGVTV	SYLSANPGV	4	0.2295	4171.8	55.00	0.35	tr_C9ZN43_
HLA-DQA10501-DQB10201	552	HMRSYLSANPGVTVF	SYLSANPGV	3	0.2295	4172.7	55.00	0.34	tr_C9ZN43_
HLA-DQA10501-DQB10201	553	GVIAFAPPAKDLIKF	VIAFAPPAK	1	0.2294	4180.2	55.00	0.36	tr_C9ZN43_
HLA-DQA10501-DQB10201	554	GEVCVVNYGATRISM	VVNYGATRI	4	0.2290	4198.5	55.00	0.54	tr_C9ZN43_
HLA-DQA10501-DQB10201	555	VVNYGATRISMWSLA	YGATRISMW	3	0.2287	4210.6	55.00	0.28	tr_C9ZN43_
HLA-DQA10501-DQB10201	556	AMRGWNSHVYFLTAS	WNSHVYFLT	4	0.2284	4224.9	55.00	0.26	tr_C9ZN43_
HLA-DQA10501-DQB10201	557	MYDELVSAFNGNPNA	MYDELVSAF	0	0.2283	4228.1	55.00	0.32	tr_C9ZN43_
HLA-DQA10501-DQB10201	558	NASLASRKWTVAPDV	RKWTVAPDV	6	0.2282	4232.7	55.00	0.78	tr_C9ZN43_
HLA-DQA10501-DQB10201	559	PVPEQGEVCVVNYGA	QGEVCVVNY	4	0.2281	4236.6	55.00	0.22	tr_C9ZN43_
HLA-DQA10501-DQB10201	560	VFEPSRCLAESVRLY	PSRCLAESV	3	0.2281	4237.8	55.00	0.38	tr_C9ZN43_
HLA-DQA10501-DQB10201	561	SFGDTEYKMTTSLMS	EYKMTTSLM	5	0.2279	4245.0	55.00	0.58	tr_C9ZN43_
HLA-DQA10501-DQB10201	562	ALYYGANVLTGNGRF	YYGANVLTG	2	0.2271	4283.3	55.00	0.37	tr_C9ZN43_
HLA-DQA10501-DQB10201	563	LEQQLFVVVGYVTNT	VVVGYVTNT	6	0.2263	4322.4	55.00	0.31	tr_C9ZN43_
HLA-DQA10501-DQB10201	564	FQDHMRSYLSANPGV	SYLSANPGV	6	0.2260	4334.8	55.00	0.39	tr_C9ZN43_
HLA-DQA10501-DQB10201	565	VGYVTNTSASAPMSA	YVTNTSASA	2	0.2253	4368.9	55.00	0.34	tr_C9ZN43_
HLA-DQA10501-DQB10201	566	INVVGNDERFHTVQG	NVVGNDERF	1	0.2250	4384.4	55.00	0.49	tr_C9ZN43_
HLA-DQA10501-DQB10201	567	ADATIIEVALRKIVW	DATIIEVAL	1	0.2248	4392.4	55.00	0.18	tr_C9ZN43_
HLA-DQA10501-DQB10201	568	QDHMRSYLSANPGVT	SYLSANPGV	5	0.2246	4399.4	55.00	0.37	tr_C9ZN43_
HLA-DQA10501-DQB10201	569	GPFEEEEEKECFTAN	PFEEEEEKE	1	0.2245	4405.1	55.00	0.51	tr_C9ZN43_
HLA-DQA10501-DQB10201	570	VGRLPNRGNAFVIGL	RGNAFVIGL	6	0.2245	4405.8	55.00	0.63	tr_C9ZN43_
HLA-DQA10501-DQB10201	571	SPNAELLTLVRYAVS	PNAELLTLV	1	0.2241	4423.5	55.00	0.33	tr_C9ZN43_
HLA-DQA10501-DQB10201	572	VAVLDDNATLVGRLP	VLDDNATLV	2	0.2240	4427.9	60.00	0.38	tr_C9ZN43_
HLA-DQA10501-DQB10201	573	LPHWADANTSSEIVR	DANTSSEIV	5	0.2235	4453.2	60.00	0.40	tr_C9ZN43_
HLA-DQA10501-DQB10201	574	GDTEYKMTTSLMSSV	EYKMTTSLM	3	0.2231	4472.4	60.00	0.45	tr_C9ZN43_
HLA-DQA10501-DQB10201	575	PTLEQQLFVVVGYVT	QQLFVVVGY	4	0.2219	4531.2	60.00	0.25	tr_C9ZN43_
HLA-DQA10501-DQB10201	576	LCDAINAGLNASLAS	NAGLNASLA	5	0.2217	4540.9	60.00	0.49	tr_C9ZN43_
HLA-DQA10501-DQB10201	577	DLIKFVTRLLSDSRT	VTRLLSDSR	5	0.2210	4577.4	60.00	0.28	tr_C9ZN43_
HLA-DQA10501-DQB10201	578	TVQGGTTVFEPSRCL	VQGGTTVFE	1	0.2209	4580.6	60.00	0.39	tr_C9ZN43_
HLA-DQA10501-DQB10201	579	GTTVFEPSRCLAESV	PSRCLAESV	6	0.2207	4592.1	60.00	0.57	tr_C9ZN43_
HLA-DQA10501-DQB10201	580	YGATRISMWSLARAL	ISMWSLARA	5	0.2192	4664.5	60.00	0.32	tr_C9ZN43_
HLA-DQA10501-DQB10201	581	NLCDAINAGLNASLA	NAGLNASLA	6	0.2190	4675.6	60.00	0.50	tr_C9ZN43_
HLA-DQA10501-DQB10201	582	NYGATRISMWSLARA	ISMWSLARA	6	0.2185	4701.9	60.00	0.34	tr_C9ZN43_
HLA-DQA10501-DQB10201	583	MRSYLSANPGVTVFN	SYLSANPGV	2	0.2180	4727.2	60.00	0.36	tr_C9ZN43_
HLA-DQA10501-DQB10201	584	TSPVTPLIRYADPNA	LIRYADPNA	6	0.2179	4729.9	60.00	0.41	tr_C9ZN43_
HLA-DQA10501-DQB10201	585	AFNGNPNAERVQFAT	PNAERVQFA	5	0.2175	4751.5	60.00	0.53	tr_C9ZN43_

HLA-DQA10501-DQB10201	586	FGDTEYKMTTSLMSS	EYKMTTSLM	4	0.2172	4767.2	60.00	0.52	tr_C9ZN43_
HLA-DQA10501-DQB10201	587	RLHHRGRNVIQLSPT	RNVIQLSPT	6	0.2170	4778.4	60.00	0.35	tr_C9ZN43_
HLA-DQA10501-DQB10201	588	FEPSRCLAESVRLYS	PSRCLAESV	2	0.2169	4783.6	60.00	0.37	tr_C9ZN43_
HLA-DQA10501-DQB10201	589	LGQLMLTRTGPLARD	MLTRTGPLA	4	0.2156	4852.5	60.00	0.41	tr_C9ZN43_
HLA-DQA10501-DQB10201	590	LNASVPPLTSPVTPL	LNASVPPLT	0	0.2154	4863.5	60.00	0.35	tr_C9ZN43_
HLA-DQA10501-DQB10201	591	ERENLVGFAPSTGSN	LVGFAPSTG	4	0.2150	4881.8	60.00	0.34	tr_C9ZN43_
HLA-DQA10501-DQB10201	592	FLGQLMLTRTGPLAR	MLTRTGPLA	5	0.2137	4954.5	60.00	0.38	tr_C9ZN43_
HLA-DQA10501-DQB10201	593	RYADPNAIKLSSAQL	AIKLSSAQL	6	0.2132	4979.7	60.00	0.72	tr_C9ZN43_
HLA-DQA10501-DQB10201	594	RCLAESVRLYSPLNT	VRLYSPLNT	6	0.2127	5007.1	60.00	0.38	tr_C9ZN43_
HLA-DQA10501-DQB10201	595	QGEVCVVNYGATRIS	VVNYGATRI	5	0.2110	5100.5	60.00	0.65	tr_C9ZN43_
HLA-DQA10501-DQB10201	596	ALRKIVWMHGGTLQT	WMHGGTLQT	6	0.2105	5126.5	60.00	0.49	tr_C9ZN43_
HLA-DQA10501-DQB10201	597	KDLIKFVTRLLSDSR	VTRLLSDSR	6	0.2105	5126.8	60.00	0.31	tr_C9ZN43_
HLA-DQA10501-DQB10201	598	EPSRCLAESVRLYSP	RCLAESVRL	3	0.2103	5135.5	60.00	0.30	tr_C9ZN43_
HLA-DQA10501-DQB10201	599	GATRISMWSLARALN	ISMWSLARA	4	0.2090	5212.3	60.00	0.29	tr_C9ZN43_
HLA-DQA10501-DQB10201	600	EEEEKECFTANDPVP	ECFTANDPV	5	0.2086	5232.1	60.00	0.44	tr_C9ZN43_
HLA-DQA10501-DQB10201	601	DTEYKMTTSLMSSVG	EYKMTTSLM	2	0.2086	5233.5	60.00	0.47	tr_C9ZN43_
HLA-DQA10501-DQB10201	602	GQLMLTRTGPLARDT	MLTRTGPLA	3	0.2074	5302.6	60.00	0.44	tr_C9ZN43_
HLA-DQA10501-DQB10201	603	VLSQALSSREWLTSR	QALSSREWL	3	0.2071	5319.7	60.00	0.44	tr_C9ZN43_
HLA-DQA10501-DQB10201	604	ASAPMSAIVRGADAT	SAIVRGADA	5	0.2070	5325.0	60.00	0.37	tr_C9ZN43_
HLA-DQA10501-DQB10201	605	LLSDSRTHDAYLLSS	THDAYLLSS	6	0.2070	5327.1	60.00	0.40	tr_C9ZN43_
HLA-DQA10501-DQB10201	606	GYVTNTSASAPMSAI	YVTNTSASA	1	0.2063	5365.1	65.00	0.28	tr_C9ZN43_
HLA-DQA10501-DQB10201	607	VIGLAPGDPSLLAAH	VIGLAPGDP	0	0.2058	5391.7	65.00	0.41	tr_C9ZN43_
HLA-DQA10501-DQB10201	608	AHLDRNPDVRVLIPF	PDVRVLIPF	6	0.2040	5501.0	65.00	0.29	tr_C9ZN43_
HLA-DQA10501-DQB10201	609	TLEQQLFVVVGYVTN	QQLFVVVGY	3	0.2034	5534.4	65.00	0.28	tr_C9ZN43_
HLA-DQA10501-DQB10201	610	PNAELLTLVRYAVSQ	PNAELLTLV	0	0.2034	5536.0	65.00	0.20	tr_C9ZN43_
HLA-DQA10501-DQB10201	611	DVKVELIPPTSYKIK	KVELIPPTS	2	0.2033	5539.7	65.00	0.32	tr_C9ZN43_
HLA-DQA10501-DQB10201	612	GISFGDTEYKMTTSL	GISFGDTEY	0	0.2031	5551.8	65.00	0.37	tr_C9ZN43_
HLA-DQA10501-DQB10201	613	KAFMESLYNQRRYVI	FMESLYNQR	2	0.2028	5571.6	65.00	0.42	tr_C9ZN43_
HLA-DQA10501-DQB10201	614	YVTNTSASAPMSAIV	TSASAPMSA	4	0.2028	5574.8	65.00	0.34	tr_C9ZN43_
HLA-DQA10501-DQB10201	615	VIAFAPPAKDLIKFV	VIAFAPPAK	0	0.2027	5579.8	65.00	0.27	tr_C9ZN43_
HLA-DQA10501-DQB10201	616	TTVFEPSRCLAESVR	PSRCLAESV	5	0.2025	5587.4	65.00	0.51	tr_C9ZN43_
HLA-DQA10501-DQB10201	617	IRYADPNAIKLSSAQ	IRYADPNAI	0	0.2020	5620.7	65.00	0.62	tr_C9ZN43_
HLA-DQA10501-DQB10201	618	LIKFVTRLLSDSRTH	VTRLLSDSR	4	0.2019	5623.6	65.00	0.29	tr_C9ZN43_
HLA-DQA10501-DQB10201	619	FNGNPNAERVQFATS	PNAERVQFA	4	0.2015	5649.1	65.00	0.55	tr_C9ZN43_
HLA-DQA10501-DQB10201	620	AKDLIKFVTRLLSDS	FVTRLLSDS	6	0.2005	5711.0	65.00	0.34	tr_C9ZN43_
HLA-DQA10501-DQB10201	621	DATIIEVALRKIVWM	EVALRKIVW	5	0.2001	5737.4	65.00	0.21	tr_C9ZN43_
HLA-DQA10501-DQB10201	622	QLMLTRTGPLARDTN	MLTRTGPLA	2	0.1997	5762.3	65.00	0.45	tr_C9ZN43_
HLA-DQA10501-DQB10201	623	CDAINAGLNASLASR	NAGLNASLA	4	0.1994	5780.5	65.00	0.50	tr_C9ZN43_
HLA-DQA10501-DQB10201	624	IKFVTRLLSDSRTHD	VTRLLSDSR	3	0.1993	5789.6	65.00	0.32	tr_C9ZN43_
HLA-DQA10501-DQB10201	625	SASAPMSAIVRGADA	SAIVRGADA	6	0.1979	5872.7	65.00	0.42	tr_C9ZN43_
HLA-DQA10501-DQB10201	626	AVLDDNATLVGRLPN	VLDDNATLV	1	0.1978	5883.9	65.00	0.38	tr_C9ZN43_
HLA-DQA10501-DQB10201	627	PSRCLAESVRLYSPL	RCLAESVRL	2	0.1976	5892.1	65.00	0.32	tr_C9ZN43_
HLA-DQA10501-DQB10201	628	NDERFHTVQGGTTVF	FHTVQGGTT	4	0.1972	5920.3	65.00	0.46	tr_C9ZN43_
HLA-DQA10501-DQB10201	629	VNYGATRISMWSLAR	YGATRISMW	2	0.1971	5925.8	65.00	0.37	tr_C9ZN43_
HLA-DQA10501-DQB10201	630	GSNAMRGWNSHVYFL	RGWNSHVYF	5	0.1963	5977.7	65.00	0.47	tr_C9ZN43_
HLA-DQA10501-DQB10201	631	VPEQGEVCVVNYGAT	QGEVCVVNY	3	0.1961	5991.3	65.00	0.28	tr_C9ZN43_
HLA-DQA10501-DQB10201	632	PFEEEEEKECFTAND	PFEEEEEKE	0	0.1961	5994.0	65.00	0.41	tr_C9ZN43_
HLA-DQA10501-DQB10201	633	NASVPPLTSPVTPLI	LTSPVTPLI	6	0.1953	6045.1	65.00	0.31	tr_C9ZN43_
HLA-DQA10501-DQB10201	634	TSLPHWADANTSSEI	WADANTSSE	5	0.1951	6053.2	65.00	0.40	tr_C9ZN43_
HLA-DQA10501-DQB10201	635	VQGGTTVFEPSRCLA	VQGGTTVFE	0	0.1941	6120.1	65.00	0.28	tr_C9ZN43_

HLA-DQA10501-DQB10201	636	PLIPDNLCDAINAGL	LIPDNLCDA	1	0.1939	6136.0	65.00	0.32	tr_C9ZN43_
HLA-DQA10501-DQB10201	637	NPLIPDNLCDAINAG	LIPDNLCDA	2	0.1934	6166.7	65.00	0.34	tr_C9ZN43_
HLA-DQA10501-DQB10201	638	IGLAPGDPSLLAAHL	DPSLLAAHL	6	0.1926	6223.7	65.00	0.44	tr_C9ZN43_
HLA-DQA10501-DQB10201	639	RSYLSANPGVTVFNG	SYLSANPGV	1	0.1925	6231.3	65.00	0.38	tr_C9ZN43_
HLA-DQA10501-DQB10201	640	VFTVDGLMRGRVNDD	VFTVDGLMR	0	0.1914	6305.3	65.00	0.32	tr_C9ZN43_
HLA-DQA10501-DQB10201	641	AFMESLYNQRRYVID	FMESLYNQR	1	0.1909	6339.3	65.00	0.36	tr_C9ZN43_
HLA-DQA10501-DQB10201	642	TEYKMTTSLMSSVGR	EYKMTTSLM	1	0.1897	6423.4	70.00	0.44	tr_C9ZN43_
HLA-DQA10501-DQB10201	643	LSDSRTHDAYLLSSS	THDAYLLSS	5	0.1881	6532.9	70.00	0.35	tr_C9ZN43_
HLA-DQA10501-DQB10201	644	TGSNAMRGWNSHVYF	RGWNSHVYF	6	0.1876	6567.9	70.00	0.61	tr_C9ZN43_
HLA-DQA10501-DQB10201	645	NVLTGNGRFGQSDRL	GRFGQSDRL	6	0.1874	6583.7	70.00	0.69	tr_C9ZN43_
HLA-DQA10501-DQB10201	646	SRCLAESVRLYSPLN	RCLAESVRL	1	0.1873	6588.9	70.00	0.34	tr_C9ZN43_
HLA-DQA10501-DQB10201	647	ASVPPLTSPVTPLIR	LTSPVTPLI	5	0.1870	6611.7	70.00	0.31	tr_C9ZN43_
HLA-DQA10501-DQB10201	648	RFQDHMRSYLSANPG	RSYLSANPG	6	0.1860	6682.4	70.00	0.38	tr_C9ZN43_
HLA-DQA10501-DQB10201	649	LSQALSSREWLTSRK	QALSSREWL	2	0.1860	6683.6	70.00	0.50	tr_C9ZN43_
HLA-DQA10501-DQB10201	650	LPDSSAWKPLPLLGY	WKPLPLLGY	6	0.1856	6714.4	70.00	0.28	tr_C9ZN43_
HLA-DQA10501-DQB10201	651	SMIPSPSNATYLALQ	SNATYLALQ	6	0.1854	6724.6	70.00	0.42	tr_C9ZN43_
HLA-DQA10501-DQB10201	652	KFVTRLLSDSRTHDA	VTRLLSDSR	2	0.1830	6900.6	70.00	0.35	tr_C9ZN43_
HLA-DQA10501-DQB10201	653	RENLVGFAPSTGSNA	LVGFAPSTG	3	0.1830	6904.1	70.00	0.41	tr_C9ZN43_
HLA-DQA10501-DQB10201	654	YDELVSAFNGNPNAE	YDELVSAFN	0	0.1829	6911.2	70.00	0.22	tr_C9ZN43_
HLA-DQA10501-DQB10201	655	DLVIGDFGGDCKGGA	VIGDFGGDC	2	0.1825	6944.2	70.00	0.32	tr_C9ZN43_
HLA-DQA10501-DQB10201	656	SVPPLTSPVTPLIRY	LTSPVTPLI	4	0.1823	6953.4	70.00	0.30	tr_C9ZN43_
HLA-DQA10501-DQB10201	657	LMLTRTGPLARDTNY	MLTRTGPLA	1	0.1821	6967.7	70.00	0.41	tr_C9ZN43_
HLA-DQA10501-DQB10201	658	FATSLPHWADANTSS	FATSLPHWA	0	0.1818	6992.7	70.00	0.44	tr_C9ZN43_
HLA-DQA10501-DQB10201	659	EEEEEKECFTANDPV	ECFTANDPV	6	0.1813	7029.0	70.00	0.38	tr_C9ZN43_
HLA-DQA10501-DQB10201	660	LYYGANVLTGNGRFG	YYGANVLTG	1	0.1806	7088.0	70.00	0.44	tr_C9ZN43_
HLA-DQA10501-DQB10201	661	FISMIPSPSNATYLA	FISMIPSPS	0	0.1804	7103.1	70.00	0.62	tr_C9ZN43_
HLA-DQA10501-DQB10201	662	EYKMTTSLMSSVGRE	EYKMTTSLM	0	0.1792	7194.2	70.00	0.28	tr_C9ZN43_
HLA-DQA10501-DQB10201	663	FTVDGLMRGRVNDDD	MRGRVNDDD	6	0.1786	7237.5	70.00	0.36	tr_C9ZN43_
HLA-DQA10501-DQB10201	664	DAINAGLNASLASRK	NAGLNASLA	3	0.1786	7243.1	70.00	0.45	tr_C9ZN43_
HLA-DQA10501-DQB10201	665	SDSRTHDAYLLSSSS	THDAYLLSS	4	0.1775	7323.3	70.00	0.31	tr_C9ZN43_
HLA-DQA10501-DQB10201	666	AFVDPVMLTPRLHHR	FVDPVMLTP	1	0.1769	7372.1	70.00	0.28	tr_C9ZN43_
HLA-DQA10501-DQB10201	667	PLTSPVTPLIRYADP	TPLIRYADP	6	0.1767	7393.9	70.00	0.28	tr_C9ZN43_
HLA-DQA10501-DQB10201	668	DSSAWKPLPLLGYAA	WKPLPLLGY	4	0.1766	7399.2	70.00	0.22	tr_C9ZN43_
HLA-DQA10501-DQB10201	669	LTSPVTPLIRYADPN	TPLIRYADP	5	0.1765	7408.3	70.00	0.27	tr_C9ZN43_
HLA-DQA10501-DQB10201	670	SAWKPLPLLGYAAAR	PLLGYAAAR	6	0.1760	7444.7	70.00	0.26	tr_C9ZN43_
HLA-DQA10501-DQB10201	671	NVVGNDERFHTVQGG	NVVGNDERF	0	0.1758	7462.3	70.00	0.46	tr_C9ZN43_
HLA-DQA10501-DQB10201	672	LIPDNLCDAINAGLN	LIPDNLCDA	0	0.1757	7472.5	70.00	0.23	tr_C9ZN43_
HLA-DQA10501-DQB10201	673	PDSSAWKPLPLLGYA	WKPLPLLGY	5	0.1751	7516.3	70.00	0.26	tr_C9ZN43_
HLA-DQA10501-DQB10201	674	PEQGEVCVVNYGATR	QGEVCVVNY	2	0.1749	7537.3	70.00	0.31	tr_C9ZN43_
HLA-DQA10501-DQB10201	675	PAKDLIKFVTRLLSD	KFVTRLLSD	6	0.1746	7561.8	70.00	0.43	tr_C9ZN43_
HLA-DQA10501-DQB10201	676	SSREWLTSRKAFMES	WLTSRKAFM	4	0.1739	7614.1	70.00	0.54	tr_C9ZN43_
HLA-DQA10501-DQB10201	677	PRLHHRGRNVIQLSP	RGRNVIQLS	5	0.1721	7767.7	70.00	0.41	tr_C9ZN43_
HLA-DQA10501-DQB10201	678	VTNTSASAPMSAIVR	TSASAPMSA	3	0.1712	7842.2	75.00	0.40	tr_C9ZN43_
HLA-DQA10501-DQB10201	679	LALQSELDTRSVTAV	ELDTRSVTA	5	0.1708	7873.8	75.00	0.29	tr_C9ZN43_
HLA-DQA10501-DQB10201	680	VPPLTSPVTPLIRYA	LTSPVTPLI	3	0.1706	7894.1	75.00	0.30	tr_C9ZN43_
HLA-DQA10501-DQB10201	681	ATSLPHWADANTSSE	WADANTSSE	6	0.1699	7953.3	75.00	0.45	tr_C9ZN43_
HLA-DQA10501-DQB10201	682	AAHLDRNPDVRVLIP	NPDVRVLIP	6	0.1695	7986.7	75.00	0.35	tr_C9ZN43_
HLA-DQA10501-DQB10201	683	ATIIEVALRKIVWMH	EVALRKIVW	4	0.1693	8009.5	75.00	0.25	tr_C9ZN43_
HLA-DQA10501-DQB10201	684	FVTRLLSDSRTHDAY	VTRLLSDSR	1	0.1689	8045.0	75.00	0.37	tr_C9ZN43_
HLA-DQA10501-DQB10201	685	RLLSDSRTHDAYLLS	RTHDAYLLS	6	0.1688	8051.3	75.00	0.38	tr_C9ZN43_

HLA-DQA10501-DQB10201	686	SYLSANPGVTVFNGT	YLSANPGVT	1	0.1688	8052.4	75.00	0.31	tr_C9ZN43_
HLA-DQA10501-DQB10201	687	LSSREWLTSRKAFME	WLTSRKAFM	5	0.1677	8149.2	75.00	0.56	tr_C9ZN43_
HLA-DQA10501-DQB10201	688	SSAWKPLPLLGYAAA	WKPLPLLGY	3	0.1675	8165.1	75.00	0.23	tr_C9ZN43_
HLA-DQA10501-DQB10201	689	DNLCDAINAGLNASL	INAGLNASL	6	0.1666	8242.7	75.00	0.34	tr_C9ZN43_
HLA-DQA10501-DQB10201	690	TSASAPMSAIVRGAD	PMSAIVRGA	5	0.1662	8275.5	75.00	0.30	tr_C9ZN43_
HLA-DQA10501-DQB10201	691	SLLAAHLDRNPDVRV	LLAAHLDRN	1	0.1660	8297.3	75.00	0.31	tr_C9ZN43_
HLA-DQA10501-DQB10201	692	SQALSSREWLTSRKA	QALSSREWL	1	0.1659	8308.8	75.00	0.52	tr_C9ZN43_
HLA-DQA10501-DQB10201	693	IPDNLCDAINAGLNA	DAINAGLNA	6	0.1639	8488.6	75.00	0.25	tr_C9ZN43_
HLA-DQA10501-DQB10201	694	FHTALPDSSAWKPLP	FHTALPDSS	0	0.1635	8523.1	75.00	0.64	tr_C9ZN43_
HLA-DQA10501-DQB10201	695	GNDERFHTVQGGTTV	FHTVQGGTT	5	0.1632	8553.5	75.00	0.58	tr_C9ZN43_
HLA-DQA10501-DQB10201	696	VALRKIVWMHGGTLQ	IVWMHGGTL	5	0.1628	8589.5	75.00	0.39	tr_C9ZN43_
HLA-DQA10501-DQB10201	697	ENLVGFAPSTGSNAM	LVGFAPSTG	2	0.1623	8639.6	75.00	0.49	tr_C9ZN43_
HLA-DQA10501-DQB10201	698	FMESLYNQRRYVIDD	FMESLYNQR	0	0.1622	8643.1	75.00	0.27	tr_C9ZN43_
HLA-DQA10501-DQB10201	699	NTSASAPMSAIVRGA	TSASAPMSA	1	0.1621	8655.5	75.00	0.31	tr_C9ZN43_
HLA-DQA10501-DQB10201	700	FEEEEEKECFTANDP	KECFTANDP	6	0.1609	8766.2	75.00	0.32	tr_C9ZN43_
HLA-DQA10501-DQB10201	701	AINAGLNASLASRKW	NAGLNASLA	2	0.1598	8871.6	75.00	0.42	tr_C9ZN43_
HLA-DQA10501-DQB10201	702	RDTNYRAIRRFQDHM	YRAIRRFQD	4	0.1594	8906.8	75.00	0.35	tr_C9ZN43_
HLA-DQA10501-DQB10201	703	ISMIPSPSNATYLAL	PSNATYLAL	6	0.1587	8979.2	75.00	0.59	tr_C9ZN43_
HLA-DQA10501-DQB10201	704	PPLTSPVTPLIRYAD	LTSPVTPLI	2	0.1585	8997.3	75.00	0.30	tr_C9ZN43_
HLA-DQA10501-DQB10201	705	VLDDNATLVGRLPNR	VLDDNATLV	0	0.1572	9128.1	75.00	0.28	tr_C9ZN43_
HLA-DQA10501-DQB10201	706	ALPDSSAWKPLPLLG	SAWKPLPLL	5	0.1572	9129.2	75.00	0.35	tr_C9ZN43_
HLA-DQA10501-DQB10201	707	RRFQDHMRSYLSANP	MRSYLSANP	6	0.1572	9130.7	75.00	0.36	tr_C9ZN43_
HLA-DQA10501-DQB10201	708	VKVELIPPTSYKIKP	KVELIPPTS	1	0.1566	9184.2	75.00	0.43	tr_C9ZN43_
HLA-DQA10501-DQB10201	709	VGNDERFHTVQGGTT	FHTVQGGTT	6	0.1558	9261.6	75.00	0.58	tr_C9ZN43_
HLA-DQA10501-DQB10201	710	IAFAPPAKDLIKFVT	PAKDLIKFV	5	0.1557	9273.6	75.00	0.31	tr_C9ZN43_
HLA-DQA10501-DQB10201	711	TALPDSSAWKPLPLL	SAWKPLPLL	6	0.1554	9305.6	75.00	0.40	tr_C9ZN43_
HLA-DQA10501-DQB10201	712	LARDTNYRAIRRFQD	YRAIRRFQD	6	0.1554	9309.5	75.00	0.38	tr_C9ZN43_
HLA-DQA10501-DQB10201	713	TNTSASAPMSAIVRG	TSASAPMSA	2	0.1547	9372.1	75.00	0.42	tr_C9ZN43_
HLA-DQA10501-DQB10201	714	EVALRKIVWMHGGTL	IVWMHGGTL	6	0.1540	9444.0	75.00	0.42	tr_C9ZN43_
HLA-DQA10501-DQB10201	715	ALSSREWLTSRKAFM	WLTSRKAFM	6	0.1533	9522.6	80.00	0.56	tr_C9ZN43_
HLA-DQA10501-DQB10201	716	ACNCNQGGNVLYINV	GGNVLYINV	6	0.1532	9533.4	80.00	0.36	tr_C9ZN43_
HLA-DQA10501-DQB10201	717	QGGTTVFEPSRCLAE	TTVFEPSRC	3	0.1531	9539.7	80.00	0.25	tr_C9ZN43_
HLA-DQA10501-DQB10201	718	TIIEVALRKIVWMHG	EVALRKIVW	3	0.1527	9583.1	80.00	0.28	tr_C9ZN43_
HLA-DQA10501-DQB10201	719	LVIGDFGGDCKGGAG	IGDFGGDCK	2	0.1498	9887.4	80.00	0.45	tr_C9ZN43_
HLA-DQA10501-DQB10201	720	DTNYRAIRRFQDHMR	YRAIRRFQD	3	0.1480	10081.4	80.00	0.28	tr_C9ZN43_
HLA-DQA10501-DQB10201	721	TRLLSDSRTHDAYLL	SRTHDAYLL	6	0.1478	10099.2	80.00	0.42	tr_C9ZN43_
HLA-DQA10501-DQB10201	722	PDNLCDAINAGLNAS	DAINAGLNA	5	0.1453	10379.6	80.00	0.28	tr_C9ZN43_
HLA-DQA10501-DQB10201	723	ARDTNYRAIRRFQDH	YRAIRRFQD	5	0.1450	10414.2	80.00	0.38	tr_C9ZN43_
HLA-DQA10501-DQB10201	724	TNYRAIRRFQDHMRS	YRAIRRFQD	2	0.1448	10436.8	80.00	0.25	tr_C9ZN43_
HLA-DQA10501-DQB10201	725	DELVSAFNGNPNAER	AFNGNPNAE	5	0.1440	10524.1	80.00	0.22	tr_C9ZN43_
HLA-DQA10501-DQB10201	726	GGTTVFEPSRCLAES	TTVFEPSRC	2	0.1422	10730.5	80.00	0.25	tr_C9ZN43_
HLA-DQA10501-DQB10201	727	TPRLHHRGRNVIQLS	RGRNVIQLS	6	0.1414	10827.8	80.00	0.50	tr_C9ZN43_
HLA-DQA10501-DQB10201	728	AIRRFQDHMRSYLSA	DHMRSYLSA	6	0.1390	11112.7	80.00	0.23	tr_C9ZN43_
HLA-DQA10501-DQB10201	729	PPAKDLIKFVTRLLS	IKFVTRLLS	6	0.1383	11195.8	80.00	0.28	tr_C9ZN43_
HLA-DQA10501-DQB10201	730	YRAIRRFQDHMRSYL	FQDHMRSYL	6	0.1380	11235.6	80.00	0.25	tr_C9ZN43_
HLA-DQA10501-DQB10201	731	VTRLLSDSRTHDAYL	TRLLSDSRT	1	0.1378	11256.1	80.00	0.28	tr_C9ZN43_
HLA-DQA10501-DQB10201	732	INAGLNASLASRKWT	NAGLNASLA	1	0.1376	11277.3	80.00	0.46	tr_C9ZN43_
HLA-DQA10501-DQB10201	733	QALSSREWLTSRKAF	QALSSREWL	0	0.1362	11452.9	80.00	0.43	tr_C9ZN43_
HLA-DQA10501-DQB10201	734	APPAKDLIKFVTRLL	LIKFVTRLL	6	0.1362	11453.2	80.00	0.38	tr_C9ZN43_
HLA-DQA10501-DQB10201	735	NLVGFAPSTGSNAMR	LVGFAPSTG	1	0.1358	11503.7	80.00	0.55	tr_C9ZN43_

HLA-DQA10501-DQB10201	736	MLTRTGPLARDTNYR	MLTRTGPLA	0	0.1355	11536.1	80.00	0.44	tr_C9ZN43_
HLA-DQA10501-DQB10201	737	FVDPVMLTPRLHHRG	PVMLTPRLH	3	0.1340	11729.4	85.00	0.37	tr_C9ZN43_
HLA-DQA10501-DQB10201	738	TLVGRLPNRGNAFVI	PNRGNAFVI	6	0.1336	11786.1	85.00	0.27	tr_C9ZN43_
HLA-DQA10501-DQB10201	739	YYGANVLTGNGRFGQ	YYGANVLTG	0	0.1332	11828.0	85.00	0.35	tr_C9ZN43_
HLA-DQA10501-DQB10201	740	LAAHLDRNPDVRVLI	RNPDVRVLI	6	0.1325	11924.9	85.00	0.28	tr_C9ZN43_
HLA-DQA10501-DQB10201	741	VSAFNGNPNAERVQF	NPNAERVQF	6	0.1315	12053.7	85.00	0.25	tr_C9ZN43_
HLA-DQA10501-DQB10201	742	LVGRLPNRGNAFVIG	PNRGNAFVI	5	0.1315	12057.5	85.00	0.25	tr_C9ZN43_
HLA-DQA10501-DQB10201	743	LDDNATLVGRLPNRG	NATLVGRLP	3	0.1312	12097.0	85.00	0.36	tr_C9ZN43_
HLA-DQA10501-DQB10201	744	ELVSAFNGNPNAERV	AFNGNPNAE	4	0.1299	12260.4	85.00	0.23	tr_C9ZN43_
HLA-DQA10501-DQB10201	745	LLAAHLDRNPDVRVL	LLAAHLDRN	0	0.1299	12261.3	85.00	0.26	tr_C9ZN43_
HLA-DQA10501-DQB10201	746	IIEVALRKIVWMHGG	EVALRKIVW	2	0.1290	12379.9	85.00	0.34	tr_C9ZN43_
HLA-DQA10501-DQB10201	747	NYRAIRRFQDHMRSY	AIRRFQDHM	3	0.1286	12431.1	85.00	0.25	tr_C9ZN43_
HLA-DQA10501-DQB10201	748	KVELIPPTSYKIKPV	VELIPPTSY	1	0.1275	12583.2	85.00	0.47	tr_C9ZN43_
HLA-DQA10501-DQB10201	749	RAIRRFQDHMRSYLS	FQDHMRSYL	5	0.1274	12603.3	85.00	0.25	tr_C9ZN43_
HLA-DQA10501-DQB10201	750	HTALPDSSAWKPLPL	SSAWKPLPL	6	0.1262	12761.3	85.00	0.53	tr_C9ZN43_
HLA-DQA10501-DQB10201	751	AACNCNQGGNVLYIN	NQGGNVLYI	5	0.1253	12885.9	85.00	0.49	tr_C9ZN43_
HLA-DQA10501-DQB10201	752	IRRFQDHMRSYLSAN	DHMRSYLSA	5	0.1243	13031.0	85.00	0.23	tr_C9ZN43_
HLA-DQA10501-DQB10201	753	GPLARDTNYRAIRRF	TNYRAIRRF	6	0.1242	13035.6	85.00	0.47	tr_C9ZN43_
HLA-DQA10501-DQB10201	754	FAPPAKDLIKFVTRL	PAKDLIKFV	3	0.1234	13154.1	85.00	0.35	tr_C9ZN43_
HLA-DQA10501-DQB10201	755	AFAPPAKDLIKFVTR	PAKDLIKFV	4	0.1205	13571.3	85.00	0.40	tr_C9ZN43_
HLA-DQA10501-DQB10201	756	DDNATLVGRLPNRGN	NATLVGRLP	2	0.1194	13744.7	85.00	0.40	tr_C9ZN43_
HLA-DQA10501-DQB10201	757	LSANPGVTVFNGTDN	VTVFNGTDN	6	0.1189	13805.2	85.00	0.34	tr_C9ZN43_
HLA-DQA10501-DQB10201	758	VIGDFGGDCKGGAGE	IGDFGGDCK	1	0.1188	13823.4	85.00	0.54	tr_C9ZN43_
HLA-DQA10501-DQB10201	759	IEVALRKIVWMHGGT	EVALRKIVW	1	0.1187	13842.3	85.00	0.30	tr_C9ZN43_
HLA-DQA10501-DQB10201	760	YLSANPGVTVFNGTD	YLSANPGVT	0	0.1182	13910.1	85.00	0.27	tr_C9ZN43_
HLA-DQA10501-DQB10201	761	PLARDTNYRAIRRFQ	TNYRAIRRF	5	0.1161	14231.5	85.00	0.38	tr_C9ZN43_
HLA-DQA10501-DQB10201	762	LVSAFNGNPNAERVQ	AFNGNPNAE	3	0.1151	14398.6	85.00	0.26	tr_C9ZN43_
HLA-DQA10501-DQB10201	763	ATLVGRLPNRGNAFV	RLPNRGNAF	5	0.1145	14479.7	85.00	0.31	tr_C9ZN43_
HLA-DQA10501-DQB10201	764	VVGNDERFHTVQGGT	ERFHTVQGG	5	0.1132	14685.4	90.00	0.28	tr_C9ZN43_
HLA-DQA10501-DQB10201	765	GAACNCNQGGNVLYI	NQGGNVLYI	6	0.1124	14815.5	90.00	0.65	tr_C9ZN43_
HLA-DQA10501-DQB10201	766	LVGFAPSTGSNAMRG	LVGFAPSTG	0	0.1076	15603.1	90.00	0.44	tr_C9ZN43_
HLA-DQA10501-DQB10201	767	NAGLNASLASRKWTV	NAGLNASLA	0	0.1047	16108.8	90.00	0.34	tr_C9ZN43_
HLA-DQA10501-DQB10201	768	STGSNAMRGWNSHVY	MRGWNSHVY	6	0.1045	16147.2	90.00	0.56	tr_C9ZN43_
HLA-DQA10501-DQB10201	769	DNATLVGRLPNRGNA	NATLVGRLP	1	0.1013	16715.9	90.00	0.36	tr_C9ZN43_
HLA-DQA10501-DQB10201	770	NATLVGRLPNRGNAF	RLPNRGNAF	6	0.1011	16747.2	90.00	0.26	tr_C9ZN43_
HLA-DQA10501-DQB10201	771	LTPRLHHRGRNVIQL	HRGRNVIQL	6	0.0988	17176.6	90.00	0.63	tr_C9ZN43_
HLA-DQA10501-DQB10201	772	VELIPPTSYKIKPVE	VELIPPTSY	0	0.0982	17287.4	90.00	0.49	tr_C9ZN43_
HLA-DQA10501-DQB10201	773	VGFAPSTGSNAMRGW	STGSNAMRG	5	0.0948	17924.1	90.00	0.32	tr_C9ZN43_
HLA-DQA10501-DQB10201	774	VDPVMLTPRLHHRGR	PVMLTPRLH	2	0.0946	17957.9	90.00	0.51	tr_C9ZN43_
HLA-DQA10501-DQB10201	775	LTRTGPLARDTNYRA	LARDTNYRA	6	0.0945	17987.0	90.00	0.23	tr_C9ZN43_
HLA-DQA10501-DQB10201	776	ANVLTGNGRFGQSDR	NVLTGNGRF	1	0.0937	18135.4	90.00	0.28	tr_C9ZN43_
HLA-DQA10501-DQB10201	777	GANVLTGNGRFGQSD	NVLTGNGRF	2	0.0929	18297.2	90.00	0.33	tr_C9ZN43_
HLA-DQA10501-DQB10201	778	YGANVLTGNGRFGQS	NVLTGNGRF	3	0.0922	18444.9	90.00	0.32	tr_C9ZN43_
HLA-DQA10501-DQB10201	779	GFAPSTGSNAMRGWN	STGSNAMRG	4	0.0894	19011.8	95.00	0.34	tr_C9ZN43_
HLA-DQA10501-DQB10201	780	FAPSTGSNAMRGWNS	STGSNAMRG	3	0.0860	19719.3	95.00	0.31	tr_C9ZN43_
HLA-DQA10501-DQB10201	781	LNASLASRKWTVAPD	SRKWTVAPD	6	0.0850	19940.1	95.00	0.49	tr_C9ZN43_
HLA-DQA10501-DQB10201	782	TRTGPLARDTNYRAI	LARDTNYRA	5	0.0845	20038.7	95.00	0.29	tr_C9ZN43_
HLA-DQA10501-DQB10201	783	TGPLARDTNYRAIRR	DTNYRAIRR	6	0.0789	21295.7	95.00	0.28	tr_C9ZN43_
HLA-DQA10501-DQB10201	784	DPVMLTPRLHHRGRN	PVMLTPRLH	1	0.0778	21540.9	95.00	0.55	tr_C9ZN43_
HLA-DQA10501-DQB10201	785	AGLNASLASRKWTVA	NASLASRKW	3	0.0764	21868.0	95.00	0.31	tr_C9ZN43_

HLA-DQA10501-DQB10201	786	KGGAGERGAACNCNQ	RGAACNCNQ	6	0.0744	22363.0	95.00	0.35	tr_C9ZN43_	
HLA-DQA10501-DQB10201	787	IGDFGGDCKGGAGER	IGDFGGDCK	0	0.0728	22752.8	95.00	0.56	tr_C9ZN43_	
HLA-DQA10501-DQB10201	788	RTGPLARDTNYRAIR	LARDTNYRA	4	0.0713	23118.3	95.00	0.34	tr_C9ZN43_	
HLA-DQA10501-DQB10201	789	APSTGSNAMRGWNSH	STGSNAMRG	2	0.0701	23430.4	95.00	0.33	tr_C9ZN43_	
HLA-DQA10501-DQB10201	790	PSTGSNAMRGWNSHV	STGSNAMRG	1	0.0685	23824.8	95.00	0.25	tr_C9ZN43_	
HLA-DQA10501-DQB10201	791	GGAGERGAACNCNQG	RGAACNCNQ	5	0.0678	24003.6	95.00	0.37	tr_C9ZN43_	
HLA-DQA10501-DQB10201	792	CKGGAGERGAACNCN	ERGAACNCN	6	0.0651	24729.9	95.00	0.30	tr_C9ZN43_	
HLA-DQA10501-DQB10201	793	GAGERGAACNCNQGG	RGAACNCNQ	4	0.0647	24829.6	95.00	0.34	tr_C9ZN43_	
HLA-DQA10501-DQB10201	794	DCKGGAGERGAACNC	GERGAACNC	6	0.0639	25051.2	95.00	0.22	tr_C9ZN43_	
HLA-DQA10501-DQB10201	795	GLNASLASRKWTVAP	NASLASRKW	2	0.0592	26354.3	100.00	0.38	tr_C9ZN43_	
HLA-DQA10501-DQB10201	796	RGAACNCNQGGNVLY	CNQGGNVLY	6	0.0589	26422.0	100.00	0.38	tr_C9ZN43_	
HLA-DQA10501-DQB10201	797	AGERGAACNCNQGGN	RGAACNCNQ	3	0.0574	26859.3	100.00	0.36	tr_C9ZN43_	
HLA-DQA10501-DQB10201	798	GDCKGGAGERGAACN	CKGGAGERG	2	0.0560	27291.6	100.00	0.31	tr_C9ZN43_	
HLA-DQA10501-DQB10201	799	PVMLTPRLHHRGRNV	PVMLTPRLH	0	0.0547	27679.7	100.00	0.55	tr_C9ZN43_	
HLA-DQA10501-DQB10201	800	DFGGDCKGGAGERGA	CKGGAGERG	5	0.0541	27840.1	100.00	0.41	tr_C9ZN43_	
HLA-DQA10501-DQB10201	801	GDFGGDCKGGAGERG	CKGGAGERG	6	0.0529	28221.4	100.00	0.41	tr_C9ZN43_	
HLA-DQA10501-DQB10201	802	GERGAACNCNQGGNV	RGAACNCNQ	2	0.0529	28224.1	100.00	0.34	tr_C9ZN43_	
HLA-DQA10501-DQB10201	803	GGDCKGGAGERGAAC	CKGGAGERG	3	0.0528	28234.2	100.00	0.32	tr_C9ZN43_	
HLA-DQA10501-DQB10201	804	ERGAACNCNQGGNVL	RGAACNCNQ	1	0.0522	28426.1	100.00	0.31	tr_C9ZN43_	
HLA-DQA10501-DQB10201	805	FGGDCKGGAGERGAA	CKGGAGERG	4	0.0514	28672.9	100.00	0.41	tr_C9ZN43_	
HLA-DQA10501-DQB10201	806	MLTPRLHHRGRNVIQ	HHRGRNVIQ	6	0.0461	30369.4	100.00	0.32	tr_C9ZN43_	
HLA-DQA10501-DQB10201	807	VMLTPRLHHRGRNVI	LHHRGRNVI	6	0.0377	33262.5	100.00	0.33	tr_C9ZN43_	

Allele: HLA-DQA10501-DQB10201. Number of high binders 13. Number of weak binders 95. Number of peptides 807

Allele	pos	peptide	core	Of	1-log50k(aff)	affinity(nM)	%Rank	Relia	Identity	Bind Level
HLA-DQA10501-DQB10201	1	FRSLVINLASPDDWE	INLASPDDW	5	0.5620	114.3	2.50	0.49	tr_C9ZL71_	WB
HLA-DQA10501-DQB10201	2	RFRSLVINLASPDDW	INLASPDDW	6	0.5598	117.1	2.50	0.43	tr_C9ZL71_	WB
HLA-DQA10501-DQB10201	3	SLVINLASPDDWEVF	INLASPDDW	3	0.5549	123.5	3.00	0.58	tr_C9ZL71_	WB
HLA-DQA10501-DQB10201	4	RSLVINLASPDDWEV	INLASPDDW	4	0.5468	134.8	3.00	0.56	tr_C9ZL71_	WB
HLA-DQA10501-DQB10201	5	LVINLASPDDWEVFH	INLASPDDW	2	0.5343	154.3	3.50	0.58	tr_C9ZL71_	WB
HLA-DQA10501-DQB10201	6	VINLASPDDWEVFHP	INLASPDDW	1	0.4922	243.4	6.00	0.62	tr_C9ZL71_	WB
HLA-DQA10501-DQB10201	7	LRFRSLVINLASPDD	FRSLVINLA	2	0.4772	286.1	7.00	0.41	tr_C9ZL71_	WB
HLA-DQA10501-DQB10201	8	ERRFTLRVAIPFMEE	RVAIPFMEE	6	0.4671	319.2	8.00	0.46	tr_C9ZL71_	WB
HLA-DQA10501-DQB10201	9	RRFTLRVAIPFMEEN	RVAIPFMEE	5	0.4656	324.5	8.00	0.42	tr_C9ZL71_	WB
HLA-DQA10501-DQB10201	10	GRPYSVDFGDPLLRF	YSVDFGDPL	3	0.4525	373.7	9.50	0.41	tr_C9ZL71_	WB
HLA-DQA10501-DQB10201	11	RFTLRVAIPFMEENS	RVAIPFMEE	4	0.4514	378.5	9.50	0.43	tr_C9ZL71_	WB
HLA-DQA10501-DQB10201	12	LLRFRSLVINLASPD	FRSLVINLA	3	0.4476	394.3	10.00	0.52	tr_C9ZL71_	
HLA-DQA10501-DQB10201	13	WNGRPYSVDFGDPLL	YSVDFGDPL	5	0.4377	439.0	11.00	0.54	tr_C9ZL71_	
HLA-DQA10501-DQB10201	14	RPYSVDFGDPLLRFR	YSVDFGDPL	2	0.4320	466.7	12.00	0.44	tr_C9ZL71_	
HLA-DQA10501-DQB10201	15	FTLRVAIPFMEENSN	RVAIPFMEE	3	0.4304	474.7	12.00	0.43	tr_C9ZL71_	
HLA-DQA10501-DQB10201	16	PLLRFRSLVINLASP	FRSLVINLA	4	0.4259	498.6	13.00	0.57	tr_C9ZL71_	
HLA-DQA10501-DQB10201	17	GDPLLRFRSLVINLA	FRSLVINLA	6	0.4193	535.2	13.00	0.59	tr_C9ZL71_	
HLA-DQA10501-DQB10201	18	NGRPYSVDFGDPLLR	YSVDFGDPL	4	0.4156	557.5	14.00	0.51	tr_C9ZL71_	
HLA-DQA10501-DQB10201	19	PYSVDFGDPLLRFRS	YSVDFGDPL	1	0.4098	593.1	15.00	0.44	tr_C9ZL71_	
HLA-DQA10501-DQB10201	20	TLRVAIPFMEENSNW	RVAIPFMEE	2	0.4060	618.1	15.00	0.37	tr_C9ZL71_	

HLA-DQA10501-DQB10201	21	DPLLRFRSLVINLAS	FRSLVINLA	5	0.4056	621.0	15.00	0.57	tr_C9ZL71_
HLA-DQA10501-DQB10201	22	QWNGRPYSVDFGDPL	YSVDFGDPL	6	0.4028	640.3	16.00	0.57	tr_C9ZL71_
HLA-DQA10501-DQB10201	23	INLASPDDWEVFHPD	INLASPDDW	0	0.3983	671.9	16.00	0.62	tr_C9ZL71_
HLA-DQA10501-DQB10201	24	GTWNSNCATIELEDR	SNCATIELE	4	0.3716	897.1	20.00	0.38	tr_C9ZL71_
HLA-DQA10501-DQB10201	25	YGTWNSNCATIELED	SNCATIELE	5	0.3714	899.2	20.00	0.45	tr_C9ZL71_
HLA-DQA10501-DQB10201	26	LRVAIPFMEENSNWS	RVAIPFMEE	1	0.3707	905.6	21.00	0.43	tr_C9ZL71_
HLA-DQA10501-DQB10201	27	DERRFTLRVAIPFME	TLRVAIPFM	5	0.3675	937.7	21.00	0.37	tr_C9ZL71_
HLA-DQA10501-DQB10201	28	YSVDFGDPLLRFRSL	YSVDFGDPL	0	0.3663	949.7	21.00	0.34	tr_C9ZL71_
HLA-DQA10501-DQB10201	29	TWNSNCATIELEDRH	SNCATIELE	3	0.3587	1031.3	23.00	0.34	tr_C9ZL71_
HLA-DQA10501-DQB10201	30	KYGTWNSNCATIELE	SNCATIELE	6	0.3380	1290.5	27.00	0.52	tr_C9ZL71_
HLA-DQA10501-DQB10201	31	PDERRFTLRVAIPFM	TLRVAIPFM	6	0.3380	1290.7	27.00	0.46	tr_C9ZL71_
HLA-DQA10501-DQB10201	32	WNSNCATIELEDRHT	SNCATIELE	2	0.3371	1302.4	27.00	0.34	tr_C9ZL71_
HLA-DQA10501-DQB10201	33	SITWRVLDICGRDAP	VLDICGRDA	5	0.3370	1303.8	27.00	0.26	tr_C9ZL71_
HLA-DQA10501-DQB10201	34	WRVLDICGRDAPDMW	CGRDAPDMW	6	0.3355	1325.9	27.00	0.25	tr_C9ZL71_
HLA-DQA10501-DQB10201	35	PSITWRVLDICGRDA	VLDICGRDA	6	0.3338	1350.3	27.00	0.28	tr_C9ZL71_
HLA-DQA10501-DQB10201	36	SPDDWEVFHPDERRF	WEVFHPDER	4	0.3319	1377.7	28.00	0.41	tr_C9ZL71_
HLA-DQA10501-DQB10201	37	LASPDDWEVFHPDER	WEVFHPDER	6	0.3318	1379.2	28.00	0.38	tr_C9ZL71_
HLA-DQA10501-DQB10201	38	ITWRVLDICGRDAPD	VLDICGRDA	4	0.3270	1452.8	29.00	0.35	tr_C9ZL71_
HLA-DQA10501-DQB10201	39	ASPDDWEVFHPDERR	WEVFHPDER	5	0.3257	1474.1	29.00	0.38	tr_C9ZL71_
HLA-DQA10501-DQB10201	40	PDDWEVFHPDERRFT	WEVFHPDER	3	0.3209	1553.3	30.00	0.43	tr_C9ZL71_
HLA-DQA10501-DQB10201	41	DTGGGRVSLSLSDVP	VSLSLSDVP	6	0.3187	1590.5	31.00	0.48	tr_C9ZL71_
HLA-DQA10501-DQB10201	42	RVAIPFMEENSNWST	RVAIPFMEE	0	0.3174	1613.2	31.00	0.38	tr_C9ZL71_
HLA-DQA10501-DQB10201	43	GRVSLSLSDVPGSIA	VSLSLSDVP	2	0.3148	1657.7	32.00	0.38	tr_C9ZL71_
HLA-DQA10501-DQB10201	44	GGRVSLSLSDVPGSI	VSLSLSDVP	3	0.3134	1684.1	32.00	0.41	tr_C9ZL71_
HLA-DQA10501-DQB10201	45	TGGGRVSLSLSDVPG	VSLSLSDVP	5	0.3131	1688.7	32.00	0.49	tr_C9ZL71_
HLA-DQA10501-DQB10201	46	DDWEVFHPDERRFTL	WEVFHPDER	2	0.3130	1691.3	32.00	0.46	tr_C9ZL71_
HLA-DQA10501-DQB10201	47	TWRVLDICGRDAPDM	VLDICGRDA	3	0.3115	1719.7	32.00	0.35	tr_C9ZL71_
HLA-DQA10501-DQB10201	48	EENSNWSTAPLPIVC	WSTAPLPIV	5	0.3109	1730.1	33.00	0.47	tr_C9ZL71_
HLA-DQA10501-DQB10201	49	GGGRVSLSLSDVPGS	VSLSLSDVP	4	0.3066	1812.4	34.00	0.47	tr_C9ZL71_
HLA-DQA10501-DQB10201	50	NLASPDDWEVFHPDE	DWEVFHPDE	6	0.3006	1934.5	35.00	0.40	tr_C9ZL71_
HLA-DQA10501-DQB10201	51	MEENSNWSTAPLPIV	WSTAPLPIV	6	0.2997	1952.1	35.00	0.49	tr_C9ZL71_
HLA-DQA10501-DQB10201	52	NSNCATIELEDRHTD	SNCATIELE	1	0.2968	2015.1	36.00	0.34	tr_C9ZL71_
HLA-DQA10501-DQB10201	53	ENSNWSTAPLPIVCR	WSTAPLPIV	4	0.2908	2151.4	37.00	0.47	tr_C9ZL71_
HLA-DQA10501-DQB10201	54	RVSLSLSDVPGSIAR	VSLSLSDVP	1	0.2907	2152.2	37.00	0.41	tr_C9ZL71_
HLA-DQA10501-DQB10201	55	RVLDICGRDAPDMWK	CGRDAPDMW	5	0.2903	2162.7	38.00	0.32	tr_C9ZL71_
HLA-DQA10501-DQB10201	56	GDDRCVYINLPPGAV	VYINLPPGA	5	0.2876	2226.6	38.00	0.37	tr_C9ZL71_
HLA-DQA10501-DQB10201	57	DDRCVYINLPPGAVL	VYINLPPGA	4	0.2855	2277.7	39.00	0.40	tr_C9ZL71_
HLA-DQA10501-DQB10201	58	FGDPLLRFRSLVINL	RFRSLVINL	6	0.2852	2284.7	39.00	0.56	tr_C9ZL71_
HLA-DQA10501-DQB10201	59	HPDERRFTLRVAIPF	FTLRVAIPF	6	0.2834	2330.8	39.00	0.50	tr_C9ZL71_
HLA-DQA10501-DQB10201	60	SVDFGDPLLRFRSLV	DFGDPLLRF	2	0.2756	2533.8	41.00	0.37	tr_C9ZL71_
HLA-DQA10501-DQB10201	61	RGDDRCVYINLPPGA	VYINLPPGA	6	0.2750	2551.3	41.00	0.38	tr_C9ZL71_
HLA-DQA10501-DQB10201	62	DWEVFHPDERRFTLR	WEVFHPDER	1	0.2731	2605.5	42.00	0.49	tr_C9ZL71_
HLA-DQA10501-DQB10201	63	NSNWSTAPLPIVCRR	WSTAPLPIV	3	0.2693	2712.3	43.00	0.47	tr_C9ZL71_
HLA-DQA10501-DQB10201	64	AVGILFQGRAYALTV	QGRAYALTV	6	0.2680	2752.1	43.00	0.44	tr_C9ZL71_
HLA-DQA10501-DQB10201	65	VLDICGRDAPDMWKY	CGRDAPDMW	4	0.2680	2752.4	43.00	0.46	tr_C9ZL71_
HLA-DQA10501-DQB10201	66	SNCATIELEDRHTDT	CATIELEDR	2	0.2669	2786.3	44.00	0.25	tr_C9ZL71_
HLA-DQA10501-DQB10201	67	VDFGDPLLRFRSLVI	LLRFRSLVI	6	0.2649	2845.9	44.00	0.38	tr_C9ZL71_
HLA-DQA10501-DQB10201	68	VQWNGRPYSVDFGDP	GRPYSVDFG	4	0.2623	2926.0	45.00	0.38	tr_C9ZL71_
HLA-DQA10501-DQB10201	69	DRCVYINLPPGAVLN	VYINLPPGA	3	0.2602	2994.0	45.00	0.42	tr_C9ZL71_
HLA-DQA10501-DQB10201	70	IARNINNSAVGILFQ	NNSAVGILF	5	0.2554	3153.3	47.00	0.40	tr_C9ZL71_

HLA-DQA10501-DQB10201	71	SIARNINNSAVGILF	NNSAVGILF	6	0.2522	3265.5	48.00	0.50	tr_C9ZL71_
HLA-DQA10501-DQB10201	72	SNWSTAPLPIVCRRG	WSTAPLPIV	2	0.2511	3304.3	48.00	0.50	tr_C9ZL71_
HLA-DQA10501-DQB10201	73	WKYGTWNSNCATIEL	WNSNCATIE	5	0.2410	3685.9	55.00	0.62	tr_C9ZL71_
HLA-DQA10501-DQB10201	74	FMEENSNWSTAPLPI	NWSTAPLPI	6	0.2409	3691.1	55.00	0.44	tr_C9ZL71_
HLA-DQA10501-DQB10201	75	VSLSLSDVPGSIARN	VSLSLSDVP	0	0.2397	3736.8	55.00	0.37	tr_C9ZL71_
HLA-DQA10501-DQB10201	76	RCVYINLPPGAVLND	VYINLPPGA	2	0.2346	3951.7	55.00	0.44	tr_C9ZL71_
HLA-DQA10501-DQB10201	77	CVYINLPPGAVLNDT	VYINLPPGA	1	0.2320	4062.6	55.00	0.34	tr_C9ZL71_
HLA-DQA10501-DQB10201	78	NDTGGGRVSLSLSDV	RVSLSLSDV	6	0.2315	4082.6	55.00	0.67	tr_C9ZL71_
HLA-DQA10501-DQB10201	79	RRGDDRCVYINLPPG	DRCVYINLP	4	0.2302	4144.4	55.00	0.31	tr_C9ZL71_
HLA-DQA10501-DQB10201	80	ARNINNSAVGILFQG	NNSAVGILF	4	0.2301	4145.8	55.00	0.33	tr_C9ZL71_
HLA-DQA10501-DQB10201	81	VAIPFMEENSNWSTA	PFMEENSNW	3	0.2255	4359.5	55.00	0.26	tr_C9ZL71_
HLA-DQA10501-DQB10201	82	DFGDPLLRFRSLVIN	LLRFRSLVI	5	0.2229	4485.0	60.00	0.43	tr_C9ZL71_
HLA-DQA10501-DQB10201	83	VYINLPPGAVLNDTG	VYINLPPGA	0	0.2227	4493.8	60.00	0.27	tr_C9ZL71_
HLA-DQA10501-DQB10201	84	LDICGRDAPDMWKYG	CGRDAPDMW	3	0.2212	4566.4	60.00	0.57	tr_C9ZL71_
HLA-DQA10501-DQB10201	85	RVQWNGRPYSVDFGD	GRPYSVDFG	5	0.2196	4644.2	60.00	0.46	tr_C9ZL71_
HLA-DQA10501-DQB10201	86	NWSTAPLPIVCRRGD	WSTAPLPIV	1	0.2181	4720.9	60.00	0.57	tr_C9ZL71_
HLA-DQA10501-DQB10201	87	NCATIELEDRHTDTY	CATIELEDR	1	0.2179	4732.6	60.00	0.30	tr_C9ZL71_
HLA-DQA10501-DQB10201	88	SAVGILFQGRAYALT	FQGRAYALT	6	0.2175	4751.8	60.00	0.25	tr_C9ZL71_
HLA-DQA10501-DQB10201	89	NINNSAVGILFQGRA	NNSAVGILF	2	0.2169	4784.3	60.00	0.23	tr_C9ZL71_
HLA-DQA10501-DQB10201	90	RNINNSAVGILFQGR	NNSAVGILF	3	0.2148	4896.0	60.00	0.31	tr_C9ZL71_
HLA-DQA10501-DQB10201	91	CRRGDDRCVYINLPP	DRCVYINLP	5	0.2141	4928.4	60.00	0.44	tr_C9ZL71_
HLA-DQA10501-DQB10201	92	MWKYGTWNSNCATIE	WNSNCATIE	6	0.2111	5094.1	60.00	0.65	tr_C9ZL71_
HLA-DQA10501-DQB10201	93	INNSAVGILFQGRAY	NNSAVGILF	1	0.2055	5411.2	65.00	0.20	tr_C9ZL71_
HLA-DQA10501-DQB10201	94	DICGRDAPDMWKYGT	CGRDAPDMW	2	0.2035	5528.3	65.00	0.60	tr_C9ZL71_
HLA-DQA10501-DQB10201	95	PFMEENSNWSTAPLP	SNWSTAPLP	6	0.2030	5560.3	65.00	0.35	tr_C9ZL71_
HLA-DQA10501-DQB10201	96	NSAVGILFQGRAYAL	LFQGRAYAL	6	0.2028	5572.2	65.00	0.25	tr_C9ZL71_
HLA-DQA10501-DQB10201	97	AIPFMEENSNWSTAP	FMEENSNWS	3	0.1957	6014.2	65.00	0.40	tr_C9ZL71_
HLA-DQA10501-DQB10201	98	FHPDERRFTLRVAIP	RFTLRVAIP	6	0.1953	6043.6	65.00	0.58	tr_C9ZL71_
HLA-DQA10501-DQB10201	99	NNSAVGILFQGRAYA	NSAVGILFQ	1	0.1899	6403.8	65.00	0.16	tr_C9ZL71_
HLA-DQA10501-DQB10201	100	IPFMEENSNWSTAPL	FMEENSNWS	2	0.1883	6521.2	70.00	0.40	tr_C9ZL71_
HLA-DQA10501-DQB10201	101	YRVQWNGRPYSVDFG	GRPYSVDFG	6	0.1848	6772.1	70.00	0.47	tr_C9ZL71_
HLA-DQA10501-DQB10201	102	CATIELEDRHTDTYR	CATIELEDR	0	0.1826	6935.9	70.00	0.28	tr_C9ZL71_
HLA-DQA10501-DQB10201	103	SLSLSDVPGSIARNI	LSDVPGSIA	3	0.1795	7170.7	70.00	0.27	tr_C9ZL71_
HLA-DQA10501-DQB10201	104	GSIARNINNSAVGIL	INNSAVGIL	6	0.1750	7525.1	70.00	0.43	tr_C9ZL71_
HLA-DQA10501-DQB10201	105	WSTAPLPIVCRRGDD	WSTAPLPIV	0	0.1737	7632.6	70.00	0.56	tr_C9ZL71_
HLA-DQA10501-DQB10201	106	YINLPPGAVLNDTGG	PGAVLNDTG	5	0.1734	7659.1	70.00	0.28	tr_C9ZL71_
HLA-DQA10501-DQB10201	107	ICGRDAPDMWKYGTW	CGRDAPDMW	1	0.1734	7661.4	70.00	0.60	tr_C9ZL71_
HLA-DQA10501-DQB10201	108	WEVFHPDERRFTLRV	WEVFHPDER	0	0.1728	7708.2	70.00	0.55	tr_C9ZL71_
HLA-DQA10501-DQB10201	109	VCRRGDDRCVYINLP	DRCVYINLP	6	0.1727	7716.1	70.00	0.47	tr_C9ZL71_
HLA-DQA10501-DQB10201	110	LSLSDVPGSIARNIN	LSDVPGSIA	2	0.1598	8869.3	75.00	0.31	tr_C9ZL71_
HLA-DQA10501-DQB10201	111	INLPPGAVLNDTGGG	PGAVLNDTG	4	0.1436	10571.8	80.00	0.39	tr_C9ZL71_
HLA-DQA10501-DQB10201	112	TYRVQWNGRPYSVDF	NGRPYSVDF	6	0.1436	10575.7	80.00	0.22	tr_C9ZL71_
HLA-DQA10501-DQB10201	113	PGSIARNINNSAVGI	NINNSAVGI	6	0.1414	10830.0	80.00	0.51	tr_C9ZL71_
HLA-DQA10501-DQB10201	114	ELEDRHTDTYRVQWN	TDTYRVQWN	6	0.1369	11362.9	80.00	0.39	tr_C9ZL71_
HLA-DQA10501-DQB10201	115	SLSDVPGSIARNINN	LSDVPGSIA	1	0.1348	11629.0	80.00	0.32	tr_C9ZL71_
HLA-DQA10501-DQB10201	116	LPIVCRRGDDRCVYI	RGDDRCVYI	6	0.1333	11815.0	85.00	0.34	tr_C9ZL71_
HLA-DQA10501-DQB10201	117	CGRDAPDMWKYGTWN	CGRDAPDMW	0	0.1321	11974.2	85.00	0.57	tr_C9ZL71_
HLA-DQA10501-DQB10201	118	ATIELEDRHTDTYRV	TIELEDRHT	1	0.1317	12023.5	85.00	0.30	tr_C9ZL71_
HLA-DQA10501-DQB10201	119	DTYRVQWNGRPYSVD	WNGRPYSVD	6	0.1316	12036.3	85.00	0.22	tr_C9ZL71_
HLA-DQA10501-DQB10201	120	RHTDTYRVQWNGRPY	TDTYRVQWN	2	0.1314	12059.2	85.00	0.22	tr_C9ZL71_

HLA-DQA10501-DQB10201	121	DRHTDTYRVQWNGRP	TDTYRVQWN	3	0.1291	12364.9	85.00	0.31	tr_C9ZL71_
HLA-DQA10501-DQB10201	122	HTDTYRVQWNGRPYS	TDTYRVQWN	1	0.1291	12367.2	85.00	0.17	tr_C9ZL71_
HLA-DQA10501-DQB10201	123	LPPGAVLNDTGGGRV	PGAVLNDTG	2	0.1280	12517.2	85.00	0.32	tr_C9ZL71_
HLA-DQA10501-DQB10201	124	NLPPGAVLNDTGGGR	PGAVLNDTG	3	0.1280	12520.0	85.00	0.40	tr_C9ZL71_
HLA-DQA10501-DQB10201	125	LNDTGGGRVSLSLSD	GRVSLSLSD	6	0.1273	12615.2	85.00	0.38	tr_C9ZL71_
HLA-DQA10501-DQB10201	126	PIVCRRGDDRCVYIN	RGDDRCVYI	5	0.1213	13453.5	85.00	0.31	tr_C9ZL71_
HLA-DQA10501-DQB10201	127	TDTYRVQWNGRPYSV	RVQWNGRPY	4	0.1203	13600.0	85.00	0.18	tr_C9ZL71_
HLA-DQA10501-DQB10201	128	IVCRRGDDRCVYINL	RGDDRCVYI	4	0.1201	13630.0	85.00	0.31	tr_C9ZL71_
HLA-DQA10501-DQB10201	129	LEDRHTDTYRVQWNG	TDTYRVQWN	5	0.1179	13962.1	85.00	0.36	tr_C9ZL71_
HLA-DQA10501-DQB10201	130	EDRHTDTYRVQWNGR	TDTYRVQWN	4	0.1149	14415.4	85.00	0.32	tr_C9ZL71_
HLA-DQA10501-DQB10201	131	IELEDRHTDTYRVQW	HTDTYRVQW	6	0.1127	14772.1	90.00	0.40	tr_C9ZL71_
HLA-DQA10501-DQB10201	132	EVFHPDERRFTLRVA	ERRFTLRVA	6	0.1126	14787.3	90.00	0.48	tr_C9ZL71_
HLA-DQA10501-DQB10201	133	VFHPDERRFTLRVAI	RRFTLRVAI	6	0.1120	14890.6	90.00	0.45	tr_C9ZL71_
HLA-DQA10501-DQB10201	134	DVPGSIARNINNSAV	IARNINNSA	5	0.1102	15183.7	90.00	0.35	tr_C9ZL71_
HLA-DQA10501-DQB10201	135	DMWKYGTWNSNCATI	YGTWNSNCA	4	0.1100	15215.8	90.00	0.34	tr_C9ZL71_
HLA-DQA10501-DQB10201	136	AVLNDTGGGRVSLSL	GGGRVSLSL	6	0.1095	15297.8	90.00	0.44	tr_C9ZL71_
HLA-DQA10501-DQB10201	137	PLPIVCRRGDDRCVY	RRGDDRCVY	6	0.1076	15603.3	90.00	0.40	tr_C9ZL71_
HLA-DQA10501-DQB10201	138	LSDVPGSIARNINNS	VPGSIARNI	3	0.1063	15826.8	90.00	0.39	tr_C9ZL71_
HLA-DQA10501-DQB10201	139	VLNDTGGGRVSLSLS	GGGRVSLSL	5	0.1054	15990.4	90.00	0.34	tr_C9ZL71_
HLA-DQA10501-DQB10201	140	SDVPGSIARNINNSA	IARNINNSA	6	0.1050	16049.8	90.00	0.38	tr_C9ZL71_
HLA-DQA10501-DQB10201	141	PPGAVLNDTGGGRVS	PGAVLNDTG	1	0.1043	16168.5	90.00	0.37	tr_C9ZL71_
HLA-DQA10501-DQB10201	142	PDMWKYGTWNSNCAT	YGTWNSNCA	5	0.1007	16819.6	90.00	0.38	tr_C9ZL71_
HLA-DQA10501-DQB10201	143	APDMWKYGTWNSNCA	YGTWNSNCA	6	0.0998	16980.0	90.00	0.41	tr_C9ZL71_
HLA-DQA10501-DQB10201	144	TIELEDRHTDTYRVQ	TIELEDRHT	0	0.0949	17908.8	90.00	0.28	tr_C9ZL71_
HLA-DQA10501-DQB10201	145	VPGSIARNINNSAVG	IARNINNSA	4	0.0933	18224.9	90.00	0.38	tr_C9ZL71_
HLA-DQA10501-DQB10201	146	STAPLPIVCRRGDDR	IVCRRGDDR	6	0.0922	18440.7	90.00	0.32	tr_C9ZL71_
HLA-DQA10501-DQB10201	147	TAPLPIVCRRGDDRC	IVCRRGDDR	5	0.0839	20171.6	95.00	0.37	tr_C9ZL71_
HLA-DQA10501-DQB10201	148	APLPIVCRRGDDRCV	IVCRRGDDR	4	0.0805	20922.1	95.00	0.41	tr_C9ZL71_
HLA-DQA10501-DQB10201	149	PGAVLNDTGGGRVSL	PGAVLNDTG	0	0.0801	21027.3	95.00	0.31	tr_C9ZL71_
HLA-DQA10501-DQB10201	150	DAPDMWKYGTWNSNC	DMWKYGTWN	3	0.0786	21351.5	95.00	0.31	tr_C9ZL71_
HLA-DQA10501-DQB10201	151	RDAPDMWKYGTWNSN	DMWKYGTWN	4	0.0735	22572.1	95.00	0.34	tr_C9ZL71_
HLA-DQA10501-DQB10201	152	GRDAPDMWKYGTWNS	DMWKYGTWN	5	0.0728	22753.1	95.00	0.44	tr_C9ZL71_
HLA-DQA10501-DQB10201	153	GAVLNDTGGGRVSLS	TGGGRVSLS	6	0.0705	23320.1	95.00	0.31	tr_C9ZL71_

Allele: HLA-DQA10501-DQB10201. Number of high binders 0. Number of weak binders 11. Number of peptides 153

Allele	pos	peptide	core	Of	1-log50k(aff)	affinity(nM)	%Rank	Relia	Identity	Bind Level
HLA-DQA10501-DQB10201	1	RRMLREIEYPLDEVT	EIEYPLDEV	5	0.5695	105.4	2.50	0.44	tr_C9ZL71_	WB
HLA-DQA10501-DQB10201	2	YKPIAVFEALDVAAV	IAVFEALDV	3	0.5682	106.9	2.50	0.22	tr_C9ZL71_	WB
HLA-DQA10501-DQB10201	3	KPIAVFEALDVAAVC	FEALDVAAV	5	0.5668	108.5	2.50	0.16	tr_C9ZL71_	WB
HLA-DQA10501-DQB10201	4	VRRMLREIEYPLDEV	EIEYPLDEV	6	0.5667	108.6	2.50	0.51	tr_C9ZL71_	WB
HLA-DQA10501-DQB10201	5	PIAVFEALDVAAVCA	FEALDVAAV	4	0.5559	122.1	3.00	0.14	tr_C9ZL71_	WB
HLA-DQA10501-DQB10201	6	RMLREIEYPLDEVTV	EIEYPLDEV	4	0.5553	122.9	3.00	0.46	tr_C9ZL71_	WB
HLA-DQA10501-DQB10201	7	GYKPIAVFEALDVAA	IAVFEALDV	4	0.5546	123.8	3.00	0.29	tr_C9ZL71_	WB
HLA-DQA10501-DQB10201	8	QYGYKPIAVFEALDV	IAVFEALDV	6	0.5525	126.7	3.00	0.42	tr_C9ZL71_	WB
HLA-DQA10501-DQB10201	9	YGYKPIAVFEALDVA	IAVFEALDV	5	0.5491	131.5	3.00	0.35	tr_C9ZL71_	WB

HLA-DQA10501-DQB10201	10	RTFRVVNESDEIATF	RVVNESDEI	3	0.5466	135.0	3.00	0.49	tr_C9ZL71_	WB
HLA-DQA10501-DQB10201	11	MLREIEYPLDEVTVY	EIEYPLDEV	3	0.5423	141.4	3.50	0.50	tr_C9ZL71_	WB
HLA-DQA10501-DQB10201	12	LYLPFFNLETAIDCF	NLETAIDCF	6	0.5418	142.2	3.50	0.43	tr_C9ZL71_	WB
HLA-DQA10501-DQB10201	13	LDVAAVCAVMDTEFL	AVCAVMDTE	4	0.5417	142.4	3.50	0.25	tr_C9ZL71_	WB
HLA-DQA10501-DQB10201	14	IPRTFRVVNESDEIA	RVVNESDEI	5	0.5400	145.1	3.50	0.50	tr_C9ZL71_	WB
HLA-DQA10501-DQB10201	15	PRTFRVVNESDEIAT	RVVNESDEI	4	0.5352	152.8	3.50	0.46	tr_C9ZL71_	WB
HLA-DQA10501-DQB10201	16	YLPFFNLETAIDCFN	NLETAIDCF	5	0.5344	154.0	3.50	0.39	tr_C9ZL71_	WB
HLA-DQA10501-DQB10201	17	IAVFEALDVAAVCAV	FEALDVAAV	3	0.5271	166.8	4.00	0.17	tr_C9ZL71_	WB
HLA-DQA10501-DQB10201	18	TFRVVNESDEIATFR	RVVNESDEI	2	0.5263	168.3	4.00	0.51	tr_C9ZL71_	WB
HLA-DQA10501-DQB10201	19	ALDVAAVCAVMDTEF	AVCAVMDTE	5	0.5247	171.2	4.00	0.28	tr_C9ZL71_	WB
HLA-DQA10501-DQB10201	20	LREIEYPLDEVTVYT	EIEYPLDEV	2	0.5196	181.0	4.50	0.56	tr_C9ZL71_	WB
HLA-DQA10501-DQB10201	21	AIPRTFRVVNESDEI	RVVNESDEI	6	0.5183	183.4	4.50	0.58	tr_C9ZL71_	WB
HLA-DQA10501-DQB10201	22	LPFFNLETAIDCFNI	NLETAIDCF	4	0.5138	192.5	4.50	0.40	tr_C9ZL71_	WB
HLA-DQA10501-DQB10201	23	DVAAVCAVMDTEFLH	AVCAVMDTE	3	0.5113	197.9	5.00	0.26	tr_C9ZL71_	WB
HLA-DQA10501-DQB10201	24	VFEALDVAAVCAVMD	ALDVAAVCA	3	0.5051	211.7	5.00	0.22	tr_C9ZL71_	WB
HLA-DQA10501-DQB10201	25	AVFEALDVAAVCAVM	FEALDVAAV	2	0.5030	216.4	5.50	0.20	tr_C9ZL71_	WB
HLA-DQA10501-DQB10201	26	AHAFERAETYLLDAV	AETYLLDAV	6	0.5007	221.8	5.50	0.30	tr_C9ZL71_	WB
HLA-DQA10501-DQB10201	27	KSYARSLNALALDTS	LNALALDTS	6	0.4968	231.6	6.00	0.28	tr_C9ZL71_	WB
HLA-DQA10501-DQB10201	28	EALDVAAVCAVMDTE	AVCAVMDTE	6	0.4964	232.4	6.00	0.32	tr_C9ZL71_	WB
HLA-DQA10501-DQB10201	29	AFERAETYLLDAVQQ	TYLLDAVQQ	6	0.4958	234.1	6.00	0.37	tr_C9ZL71_	WB
HLA-DQA10501-DQB10201	30	FRVVNESDEIATFRL	RVVNESDEI	1	0.4913	245.7	6.00	0.53	tr_C9ZL71_	WB
HLA-DQA10501-DQB10201	31	KQYGYKPIAVFEALD	PIAVFEALD	6	0.4902	248.5	6.00	0.54	tr_C9ZL71_	WB
HLA-DQA10501-DQB10201	32	REIEYPLDEVTVYTF	EIEYPLDEV	1	0.4902	248.5	6.00	0.55	tr_C9ZL71_	WB
HLA-DQA10501-DQB10201	33	SYARSLNALALDTSC	LNALALDTS	5	0.4884	253.6	6.50	0.28	tr_C9ZL71_	WB
HLA-DQA10501-DQB10201	34	FEALDVAAVCAVMDT	ALDVAAVCA	2	0.4870	257.4	6.50	0.22	tr_C9ZL71_	WB
HLA-DQA10501-DQB10201	35	PFFNLETAIDCFNIA	NLETAIDCF	3	0.4869	257.5	6.50	0.41	tr_C9ZL71_	WB
HLA-DQA10501-DQB10201	36	PAHAFERAETYLLDA	FERAETYLL	4	0.4835	267.2	6.50	0.21	tr_C9ZL71_	WB
HLA-DQA10501-DQB10201	37	VAAVCAVMDTEFLHH	AVCAVMDTE	2	0.4825	270.1	7.00	0.31	tr_C9ZL71_	WB
HLA-DQA10501-DQB10201	38	HAFERAETYLLDAVQ	AETYLLDAV	5	0.4814	273.6	7.00	0.28	tr_C9ZL71_	WB
HLA-DQA10501-DQB10201	39	WEPTVHSGFLEIWEA	VHSGFLEIW	4	0.4759	290.1	7.50	0.49	tr_C9ZL71_	WB
HLA-DQA10501-DQB10201	40	CWEPTVHSGFLEIWE	VHSGFLEIW	5	0.4751	292.7	7.50	0.53	tr_C9ZL71_	WB
HLA-DQA10501-DQB10201	41	RPAHAFERAETYLLD	FERAETYLL	5	0.4746	294.3	7.50	0.26	tr_C9ZL71_	WB
HLA-DQA10501-DQB10201	42	FERAETYLLDAVQQR	TYLLDAVQQ	5	0.4692	311.9	8.00	0.35	tr_C9ZL71_	WB
HLA-DQA10501-DQB10201	43	ERAETYLLDAVQQRM	TYLLDAVQQ	4	0.4620	337.5	8.50	0.35	tr_C9ZL71_	WB
HLA-DQA10501-DQB10201	44	VKSYARSLNALALDT	SLNALALDT	6	0.4612	340.1	8.50	0.31	tr_C9ZL71_	WB
HLA-DQA10501-DQB10201	45	FFNLETAIDCFNIAF	NLETAIDCF	2	0.4607	342.0	8.50	0.43	tr_C9ZL71_	WB
HLA-DQA10501-DQB10201	46	PLYLPFFNLETAIDC	FFNLETAID	5	0.4490	388.4	10.00	0.40	tr_C9ZL71_	
HLA-DQA10501-DQB10201	47	YARSLNALALDTSCK	LNALALDTS	4	0.4485	390.4	10.00	0.28	tr_C9ZL71_	
HLA-DQA10501-DQB10201	48	EPTVHSGFLEIWEAH	VHSGFLEIW	3	0.4472	396.0	10.00	0.47	tr_C9ZL71_	
HLA-DQA10501-DQB10201	49	SVRRMLREIEYPLDE	RMLREIEYP	3	0.4467	398.2	10.00	0.23	tr_C9ZL71_	
HLA-DQA10501-DQB10201	50	AAVCAVMDTEFLHHR	AVCAVMDTE	1	0.4425	416.4	11.00	0.34	tr_C9ZL71_	
HLA-DQA10501-DQB10201	51	EIEYPLDEVTVYTFG	EIEYPLDEV	0	0.4372	441.0	11.00	0.49	tr_C9ZL71_	
HLA-DQA10501-DQB10201	52	YSVRRMLREIEYPLD	RMLREIEYP	4	0.4342	455.7	12.00	0.28	tr_C9ZL71_	
HLA-DQA10501-DQB10201	53	CCWEPTVHSGFLEIW	VHSGFLEIW	6	0.4331	461.3	12.00	0.65	tr_C9ZL71_	
HLA-DQA10501-DQB10201	54	ERPAHAFERAETYLL	FERAETYLL	6	0.4317	468.3	12.00	0.34	tr_C9ZL71_	
HLA-DQA10501-DQB10201	55	RAETYLLDAVQQRMH	TYLLDAVQQ	3	0.4306	474.0	12.00	0.37	tr_C9ZL71_	
HLA-DQA10501-DQB10201	56	QVKSYARSLNALALD	RSLNALALD	6	0.4284	485.0	12.00	0.33	tr_C9ZL71_	
HLA-DQA10501-DQB10201	57	PTVHSGFLEIWEAHQ	VHSGFLEIW	2	0.4271	491.9	12.00	0.44	tr_C9ZL71_	
HLA-DQA10501-DQB10201	58	FNLETAIDCFNIAFE	NLETAIDCF	1	0.4169	549.7	14.00	0.41	tr_C9ZL71_	
HLA-DQA10501-DQB10201	59	EPLYLPFFNLETAID	FFNLETAID	6	0.4165	551.6	14.00	0.45	tr_C9ZL71_	

HLA-DQA10501-DQB10201	60	AVQQRMHEPLYLPFF	QRMHEPLYL	3	0.4164	552.3	14.00	0.42	tr_C9ZL71_
HLA-DQA10501-DQB10201	61	ARSLNALALDTSCKI	LNALALDTS	3	0.4154	558.7	14.00	0.29	tr_C9ZL71_
HLA-DQA10501-DQB10201	62	AETYLLDAVQQRMHE	TYLLDAVQQ	2	0.4140	567.0	14.00	0.38	tr_C9ZL71_
HLA-DQA10501-DQB10201	63	VQQRMHEPLYLPFFN	QRMHEPLYL	2	0.4138	568.0	14.00	0.38	tr_C9ZL71_
HLA-DQA10501-DQB10201	64	RVVNESDEIATFRLY	RVVNESDEI	0	0.4101	591.3	14.00	0.44	tr_C9ZL71_
HLA-DQA10501-DQB10201	65	TVHSGFLEIWEAHQT	VHSGFLEIW	1	0.4065	615.1	15.00	0.43	tr_C9ZL71_
HLA-DQA10501-DQB10201	66	AVCAVMDTEFLHHRG	CAVMDTEFL	2	0.4030	638.4	16.00	0.28	tr_C9ZL71_
HLA-DQA10501-DQB10201	67	RDTRWKESVWPDDWY	KESVWPDDW	5	0.4014	649.9	16.00	0.40	tr_C9ZL71_
HLA-DQA10501-DQB10201	68	GMLAWQPLIQLPALL	PLIQLPALL	6	0.3974	678.6	16.00	0.32	tr_C9ZL71_
HLA-DQA10501-DQB10201	69	AYSVRRMLREIEYPL	RMLREIEYP	5	0.3935	707.9	17.00	0.32	tr_C9ZL71_
HLA-DQA10501-DQB10201	70	QQRMHEPLYLPFFNL	QRMHEPLYL	1	0.3902	733.5	17.00	0.34	tr_C9ZL71_
HLA-DQA10501-DQB10201	71	FRLYGTQVGTEVDIN	YGTQVGTEV	3	0.3878	752.7	18.00	0.35	tr_C9ZL71_
HLA-DQA10501-DQB10201	72	ETYLLDAVQQRMHEP	TYLLDAVQQ	1	0.3852	774.7	18.00	0.40	tr_C9ZL71_
HLA-DQA10501-DQB10201	73	FERPAHAFERAETYL	HAFERAETY	5	0.3825	797.4	19.00	0.35	tr_C9ZL71_
HLA-DQA10501-DQB10201	74	TFRLYGTQVGTEVDI	YGTQVGTEV	4	0.3785	832.6	19.00	0.39	tr_C9ZL71_
HLA-DQA10501-DQB10201	75	ICNRGAAEIVNMDMT	AAEIVNMDM	5	0.3782	835.0	19.00	0.31	tr_C9ZL71_
HLA-DQA10501-DQB10201	76	AAEIVNMDMTSTCFL	MDMTSTCFL	6	0.3768	848.2	19.00	0.28	tr_C9ZL71_
HLA-DQA10501-DQB10201	77	KAIPRTFRVVNESDE	TFRVVNESD	5	0.3766	849.7	19.00	0.50	tr_C9ZL71_
HLA-DQA10501-DQB10201	78	KSVWQALRGVISASV	LRGVISASV	6	0.3756	858.9	20.00	0.55	tr_C9ZL71_
HLA-DQA10501-DQB10201	79	DTRWKESVWPDDWYR	KESVWPDDW	4	0.3753	862.0	20.00	0.41	tr_C9ZL71_
HLA-DQA10501-DQB10201	80	CAYSVRRMLREIEYP	RRMLREIEY	5	0.3752	862.4	20.00	0.37	tr_C9ZL71_
HLA-DQA10501-DQB10201	81	MLAWQPLIQLPALLV	PLIQLPALL	5	0.3747	867.9	20.00	0.33	tr_C9ZL71_
HLA-DQA10501-DQB10201	82	CNRGAAEIVNMDMTS	AAEIVNMDM	4	0.3741	872.8	20.00	0.29	tr_C9ZL71_
HLA-DQA10501-DQB10201	83	GSMYIFHTEKEEASF	IFHTEKEEA	4	0.3740	873.8	20.00	0.44	tr_C9ZL71_
HLA-DQA10501-DQB10201	84	SICNRGAAEIVNMDM	AAEIVNMDM	6	0.3719	894.3	20.00	0.46	tr_C9ZL71_
HLA-DQA10501-DQB10201	85	ATFRLYGTQVGTEVD	YGTQVGTEV	5	0.3718	895.4	20.00	0.44	tr_C9ZL71_
HLA-DQA10501-DQB10201	86	RSLNALALDTSCKIR	LNALALDTS	2	0.3703	910.1	21.00	0.41	tr_C9ZL71_
HLA-DQA10501-DQB10201	87	VHSGFLEIWEAHQTS	VHSGFLEIW	0	0.3680	932.9	21.00	0.35	tr_C9ZL71_
HLA-DQA10501-DQB10201	88	TRWKESVWPDDWYRW	KESVWPDDW	3	0.3679	933.9	21.00	0.44	tr_C9ZL71_
HLA-DQA10501-DQB10201	89	PRDTRWKESVWPDDW	KESVWPDDW	6	0.3668	944.9	21.00	0.47	tr_C9ZL71_
HLA-DQA10501-DQB10201	90	GAAEIVNMDMTSTCF	NMDMTSTCF	6	0.3667	945.6	21.00	0.26	tr_C9ZL71_
HLA-DQA10501-DQB10201	91	HGGSMYIFHTEKEEA	IFHTEKEEA	6	0.3667	946.2	21.00	0.41	tr_C9ZL71_
HLA-DQA10501-DQB10201	92	LETAIDCFNIAFESY	CFNIAFESY	6	0.3661	952.3	21.00	0.47	tr_C9ZL71_
HLA-DQA10501-DQB10201	93	AWQPLIQLPALLVWP	PLIQLPALL	3	0.3645	968.3	22.00	0.41	tr_C9ZL71_
HLA-DQA10501-DQB10201	94	QRMHEPLYLPFFNLE	HEPLYLPFF	3	0.3636	978.6	22.00	0.34	tr_C9ZL71_
HLA-DQA10501-DQB10201	95	LAWQPLIQLPALLVW	PLIQLPALL	4	0.3628	986.2	22.00	0.37	tr_C9ZL71_
HLA-DQA10501-DQB10201	96	NRGAAEIVNMDMTST	AAEIVNMDM	3	0.3624	991.2	22.00	0.24	tr_C9ZL71_
HLA-DQA10501-DQB10201	97	GGSMYIFHTEKEEAS	IFHTEKEEA	5	0.3623	992.5	22.00	0.43	tr_C9ZL71_
HLA-DQA10501-DQB10201	98	PGMLAWQPLIQLPAL	MLAWQPLIQ	2	0.3617	998.3	22.00	0.32	tr_C9ZL71_
HLA-DQA10501-DQB10201	99	DFRIPGMLAWQPLIQ	MLAWQPLIQ	6	0.3616	999.2	22.00	0.30	tr_C9ZL71_
HLA-DQA10501-DQB10201	100	VNMDMTSTCFLPYVE	MDMTSTCFL	2	0.3611	1005.0	22.00	0.26	tr_C9ZL71_
HLA-DQA10501-DQB10201	101	NLETAIDCFNIAFES	NLETAIDCF	0	0.3609	1007.3	22.00	0.32	tr_C9ZL71_
HLA-DQA10501-DQB10201	102	HEPLYLPFFNLETAI	PFFNLETAI	6	0.3605	1011.5	22.00	0.37	tr_C9ZL71_
HLA-DQA10501-DQB10201	103	SVWQALRGVISASVR	LRGVISASV	5	0.3601	1015.7	22.00	0.52	tr_C9ZL71_
HLA-DQA10501-DQB10201	104	TAIDCFNIAFESYNW	CFNIAFESY	4	0.3599	1018.4	22.00	0.38	tr_C9ZL71_
HLA-DQA10501-DQB10201	105	SMYIFHTEKEEASFN	IFHTEKEEA	3	0.3588	1030.0	23.00	0.44	tr_C9ZL71_
HLA-DQA10501-DQB10201	106	AEIVNMDMTSTCFLP	MDMTSTCFL	5	0.3582	1036.6	23.00	0.31	tr_C9ZL71_
HLA-DQA10501-DQB10201	107	EIVNMDMTSTCFLPY	MDMTSTCFL	4	0.3580	1039.4	23.00	0.28	tr_C9ZL71_
HLA-DQA10501-DQB10201	108	IVNMDMTSTCFLPYV	MDMTSTCFL	3	0.3579	1039.9	23.00	0.28	tr_C9ZL71_
HLA-DQA10501-DQB10201	109	VLDFRIPGMLAWQPL	PGMLAWQPL	6	0.3579	1040.8	23.00	0.24	tr_C9ZL71_

HLA-DQA10501-DQB10201	110	FRIPGMLAWQPLIQL	MLAWQPLIQ	5	0.3578	1041.2	23.00	0.28	tr_C9ZL71_
HLA-DQA10501-DQB10201	111	IATFRLYGTQVGTEV	YGTQVGTEV	6	0.3556	1066.3	23.00	0.44	tr_C9ZL71_
HLA-DQA10501-DQB10201	112	NMDMTSTCFLPYVEA	MDMTSTCFL	1	0.3552	1070.8	23.00	0.24	tr_C9ZL71_
HLA-DQA10501-DQB10201	113	DAVQQRMHEPLYLPF	QRMHEPLYL	4	0.3539	1086.9	24.00	0.52	tr_C9ZL71_
HLA-DQA10501-DQB10201	114	RLYGTQVGTEVDINR	YGTQVGTEV	2	0.3528	1099.2	24.00	0.31	tr_C9ZL71_
HLA-DQA10501-DQB10201	115	RIPGMLAWQPLIQLP	MLAWQPLIQ	4	0.3518	1111.8	24.00	0.37	tr_C9ZL71_
HLA-DQA10501-DQB10201	116	RWKESVWPDDWYRWL	KESVWPDDW	2	0.3505	1126.8	24.00	0.44	tr_C9ZL71_
HLA-DQA10501-DQB10201	117	VKQYGYKPIAVFEAL	KPIAVFEAL	6	0.3467	1174.3	25.00	0.34	tr_C9ZL71_
HLA-DQA10501-DQB10201	118	LDFRIPGMLAWQPLI	PGMLAWQPL	5	0.3466	1175.9	25.00	0.22	tr_C9ZL71_
HLA-DQA10501-DQB10201	119	HQVKSYARSLNALAL	ARSLNALAL	6	0.3462	1180.8	25.00	0.52	tr_C9ZL71_
HLA-DQA10501-DQB10201	120	RGAAEIVNMDMTSTC	AAEIVNMDM	2	0.3445	1202.4	25.00	0.25	tr_C9ZL71_
HLA-DQA10501-DQB10201	121	ETAIDCFNIAFESYN	CFNIAFESY	5	0.3445	1203.0	25.00	0.44	tr_C9ZL71_
HLA-DQA10501-DQB10201	122	MYIFHTEKEEASFNW	IFHTEKEEA	2	0.3441	1208.6	25.00	0.44	tr_C9ZL71_
HLA-DQA10501-DQB10201	123	IPGMLAWQPLIQLPA	MLAWQPLIQ	3	0.3430	1222.7	26.00	0.34	tr_C9ZL71_
HLA-DQA10501-DQB10201	124	YVLDFRIPGMLAWQP	VLDFRIPGM	1	0.3426	1227.7	26.00	0.34	tr_C9ZL71_
HLA-DQA10501-DQB10201	125	WQPLIQLPALLVWPV	PLIQLPALL	2	0.3419	1237.2	26.00	0.40	tr_C9ZL71_
HLA-DQA10501-DQB10201	126	LFERPAHAFERAETY	HAFERAETY	6	0.3411	1248.0	26.00	0.38	tr_C9ZL71_
HLA-DQA10501-DQB10201	127	TYLLDAVQQRMHEPL	LLDAVQQRM	2	0.3370	1304.3	27.00	0.31	tr_C9ZL71_
HLA-DQA10501-DQB10201	128	LLDAVQQRMHEPLYL	QRMHEPLYL	6	0.3345	1339.7	27.00	0.56	tr_C9ZL71_
HLA-DQA10501-DQB10201	129	VWQALRGVISASVRG	LRGVISASV	4	0.3344	1342.3	27.00	0.54	tr_C9ZL71_
HLA-DQA10501-DQB10201	130	LDAVQQRMHEPLYLP	QRMHEPLYL	5	0.3323	1372.2	28.00	0.57	tr_C9ZL71_
HLA-DQA10501-DQB10201	131	QPLIQLPALLVWPVL	PLIQLPALL	1	0.3305	1399.2	28.00	0.31	tr_C9ZL71_
HLA-DQA10501-DQB10201	132	MHEPLYLPFFNLETA	HEPLYLPFF	1	0.3291	1420.2	29.00	0.28	tr_C9ZL71_
HLA-DQA10501-DQB10201	133	MDMTSTCFLPYVEAG	TSTCFLPYV	3	0.3283	1433.9	29.00	0.22	tr_C9ZL71_
HLA-DQA10501-DQB10201	134	DKAIPRTFRVVNESD	TFRVVNESD	6	0.3279	1438.7	29.00	0.70	tr_C9ZL71_
HLA-DQA10501-DQB10201	135	AIDCFNIAFESYNWK	CFNIAFESY	3	0.3276	1444.7	29.00	0.36	tr_C9ZL71_
HLA-DQA10501-DQB10201	136	RASGDPEASYVAEPG	EASYVAEPG	6	0.3261	1468.1	29.00	0.57	tr_C9ZL71_
HLA-DQA10501-DQB10201	137	WKESVWPDDWYRWLA	KESVWPDDW	1	0.3260	1469.1	29.00	0.46	tr_C9ZL71_
HLA-DQA10501-DQB10201	138	VCAVMDTEFLHHRGK	CAVMDTEFL	1	0.3253	1480.6	29.00	0.47	tr_C9ZL71_
HLA-DQA10501-DQB10201	139	LYGTQVGTEVDINRH	YGTQVGTEV	1	0.3253	1480.8	29.00	0.33	tr_C9ZL71_
HLA-DQA10501-DQB10201	140	TCFLPYVEAGRENDL	LPYVEAGRE	3	0.3238	1505.1	30.00	0.31	tr_C9ZL71_
HLA-DQA10501-DQB10201	141	PLIQLPALLVWPVLV	ALLVWPVLV	6	0.3198	1571.1	31.00	0.25	tr_C9ZL71_
HLA-DQA10501-DQB10201	142	GFLEIWEAHQTSIEE	EAHQTSIEE	6	0.3193	1580.2	31.00	0.31	tr_C9ZL71_
HLA-DQA10501-DQB10201	143	FCTGHSMGGAVACLC	MGGAVACLC	6	0.3187	1589.8	31.00	0.50	tr_C9ZL71_
HLA-DQA10501-DQB10201	144	GDPEASYVAEPGREP	EASYVAEPG	3	0.3182	1599.2	31.00	0.46	tr_C9ZL71_
HLA-DQA10501-DQB10201	145	ESICNRGAAEIVNMD	RGAAEIVNM	5	0.3171	1618.0	31.00	0.44	tr_C9ZL71_
HLA-DQA10501-DQB10201	146	GQPPMGNAAFQTAYD	NAAFQTAYD	6	0.3146	1661.6	32.00	0.32	tr_C9ZL71_
HLA-DQA10501-DQB10201	147	DPEASYVAEPGREPD	EASYVAEPG	2	0.3141	1671.9	32.00	0.44	tr_C9ZL71_
HLA-DQA10501-DQB10201	148	RMHEPLYLPFFNLET	HEPLYLPFF	2	0.3138	1676.2	32.00	0.38	tr_C9ZL71_
HLA-DQA10501-DQB10201	149	LEIWEAHQTSIEEKL	EAHQTSIEE	4	0.3135	1681.7	32.00	0.22	tr_C9ZL71_
HLA-DQA10501-DQB10201	150	TGHSMGGAVACLCAY	MGGAVACLC	4	0.3129	1692.8	32.00	0.43	tr_C9ZL71_
HLA-DQA10501-DQB10201	151	LCAYSVRRMLREIEY	RRMLREIEY	6	0.3098	1750.8	33.00	0.54	tr_C9ZL71_
HLA-DQA10501-DQB10201	152	HSMGGAVACLCAYSV	MGGAVACLC	2	0.3088	1769.6	33.00	0.33	tr_C9ZL71_
HLA-DQA10501-DQB10201	153	CTGHSMGGAVACLCA	MGGAVACLC	5	0.3081	1783.4	33.00	0.47	tr_C9ZL71_
HLA-DQA10501-DQB10201	154	SGDPEASYVAEPGRE	EASYVAEPG	4	0.3078	1789.3	33.00	0.51	tr_C9ZL71_
HLA-DQA10501-DQB10201	155	WQALRGVISASVRGA	LRGVISASV	3	0.3068	1808.9	33.00	0.57	tr_C9ZL71_
HLA-DQA10501-DQB10201	156	SLNALALDTSCKIRA	LNALALDTS	1	0.3067	1809.7	33.00	0.46	tr_C9ZL71_
HLA-DQA10501-DQB10201	157	CFLPYVEAGRENDLG	LPYVEAGRE	2	0.3065	1814.5	34.00	0.33	tr_C9ZL71_
HLA-DQA10501-DQB10201	158	FLPYVEAGRENDLGP	LPYVEAGRE	1	0.3055	1834.6	34.00	0.30	tr_C9ZL71_
HLA-DQA10501-DQB10201	159	STCFLPYVEAGREND	LPYVEAGRE	4	0.3036	1871.5	34.00	0.40	tr_C9ZL71_

HLA-DQA10501-DQB10201	160	DVKQYGYKPIAVFEA	YKPIAVFEA	6	0.3027	1890.2	34.00	0.40	tr_C9ZL71_
HLA-DQA10501-DQB10201	161	MTSTCFLPYVEAGRE	LPYVEAGRE	6	0.3026	1891.7	34.00	0.30	tr_C9ZL71_
HLA-DQA10501-DQB10201	162	DMTSTCFLPYVEAGR	TSTCFLPYV	2	0.3024	1897.6	35.00	0.28	tr_C9ZL71_
HLA-DQA10501-DQB10201	163	ASGDPEASYVAEPGR	EASYVAEPG	5	0.3021	1902.1	35.00	0.56	tr_C9ZL71_
HLA-DQA10501-DQB10201	164	ENDGGALNGSVALRL	LNGSVALRL	6	0.3010	1924.7	35.00	0.67	tr_C9ZL71_
HLA-DQA10501-DQB10201	165	RHGGSMYIFHTEKEE	YIFHTEKEE	6	0.3005	1936.7	35.00	0.35	tr_C9ZL71_
HLA-DQA10501-DQB10201	166	FLEIWEAHQTSIEEK	EAHQTSIEE	5	0.2998	1951.0	35.00	0.25	tr_C9ZL71_
HLA-DQA10501-DQB10201	167	GHSMGGAVACLCAYS	MGGAVACLC	3	0.2983	1983.1	36.00	0.43	tr_C9ZL71_
HLA-DQA10501-DQB10201	168	PLDEVTVYTFGQPPM	VYTFGQPPM	6	0.2979	1990.7	36.00	0.42	tr_C9ZL71_
HLA-DQA10501-DQB10201	169	SGFLEIWEAHQTSIE	WEAHQTSIE	6	0.2977	1994.6	36.00	0.22	tr_C9ZL71_
HLA-DQA10501-DQB10201	170	RESICNRGAAEIVNM	RGAAEIVNM	6	0.2976	1997.2	36.00	0.51	tr_C9ZL71_
HLA-DQA10501-DQB10201	171	IDCFNIAFESYNWKG	CFNIAFESY	2	0.2968	2015.8	36.00	0.40	tr_C9ZL71_
HLA-DQA10501-DQB10201	172	GPSDPSINYTIMCSV	INYTIMCSV	6	0.2940	2076.3	37.00	0.58	tr_C9ZL71_
HLA-DQA10501-DQB10201	173	LPALLVWPVLVNVMT	VWPVLVNVM	5	0.2919	2124.4	37.00	0.25	tr_C9ZL71_
HLA-DQA10501-DQB10201	174	PALLVWPVLVNVMTL	VWPVLVNVM	4	0.2910	2146.5	37.00	0.24	tr_C9ZL71_
HLA-DQA10501-DQB10201	175	EIATFRLYGTQVGTE	TFRLYGTQV	3	0.2899	2170.8	38.00	0.35	tr_C9ZL71_
HLA-DQA10501-DQB10201	176	QPPMGNAAFQTAYDK	NAAFQTAYD	5	0.2877	2222.7	38.00	0.28	tr_C9ZL71_
HLA-DQA10501-DQB10201	177	LDEVTVYTFGQPPMG	VYTFGQPPM	5	0.2875	2227.6	38.00	0.38	tr_C9ZL71_
HLA-DQA10501-DQB10201	178	PPMGNAAFQTAYDKA	NAAFQTAYD	4	0.2875	2229.7	38.00	0.25	tr_C9ZL71_
HLA-DQA10501-DQB10201	179	HSGFLEIWEAHQTSI	FLEIWEAHQ	3	0.2872	2235.4	38.00	0.19	tr_C9ZL71_
HLA-DQA10501-DQB10201	180	TSCKIRASGDPEASY	IRASGDPEA	4	0.2867	2246.9	38.00	0.41	tr_C9ZL71_
HLA-DQA10501-DQB10201	181	YIFHTEKEEASFNWK	IFHTEKEEA	1	0.2866	2249.8	38.00	0.50	tr_C9ZL71_
HLA-DQA10501-DQB10201	182	IMCSVHSRPQDIEFW	SRPQDIEFW	6	0.2862	2260.1	39.00	0.41	tr_C9ZL71_
HLA-DQA10501-DQB10201	183	TSTCFLPYVEAGREN	LPYVEAGRE	5	0.2858	2268.9	39.00	0.38	tr_C9ZL71_
HLA-DQA10501-DQB10201	184	SCKIRASGDPEASYV	IRASGDPEA	3	0.2857	2273.4	39.00	0.41	tr_C9ZL71_
HLA-DQA10501-DQB10201	185	VVNESDEIATFRLYG	VVNESDEIA	0	0.2852	2284.8	39.00	0.40	tr_C9ZL71_
HLA-DQA10501-DQB10201	186	NPKSVWQALRGVISA	WQALRGVIS	5	0.2845	2300.9	39.00	0.58	tr_C9ZL71_
HLA-DQA10501-DQB10201	187	PEASYVAEPGREPDA	EASYVAEPG	1	0.2841	2311.3	39.00	0.41	tr_C9ZL71_
HLA-DQA10501-DQB10201	188	LLVWPVLVNVMTLIY	LVNVMTLIY	6	0.2840	2315.6	39.00	0.26	tr_C9ZL71_
HLA-DQA10501-DQB10201	189	PMGNAAFQTAYDKAI	NAAFQTAYD	3	0.2831	2336.9	39.00	0.25	tr_C9ZL71_
HLA-DQA10501-DQB10201	190	PQDIEFWKGLRESIC	FWKGLRESI	5	0.2826	2349.2	39.00	0.34	tr_C9ZL71_
HLA-DQA10501-DQB10201	191	QLPALLVWPVLVNVM	VWPVLVNVM	6	0.2814	2379.9	40.00	0.30	tr_C9ZL71_
HLA-DQA10501-DQB10201	192	YGTQVGTEVDINRHG	QVGTEVDIN	3	0.2810	2391.4	40.00	0.34	tr_C9ZL71_
HLA-DQA10501-DQB10201	193	EIWEAHQTSIEEKLG	WEAHQTSIE	2	0.2802	2411.2	40.00	0.20	tr_C9ZL71_
HLA-DQA10501-DQB10201	194	IEYPLDEVTVYTFGQ	YPLDEVTVY	2	0.2801	2413.3	40.00	0.22	tr_C9ZL71_
HLA-DQA10501-DQB10201	195	NNPKSVWQALRGVIS	WQALRGVIS	6	0.2801	2415.1	40.00	0.60	tr_C9ZL71_
HLA-DQA10501-DQB10201	196	ALLVWPVLVNVMTLI	VWPVLVNVM	3	0.2798	2423.4	40.00	0.26	tr_C9ZL71_
HLA-DQA10501-DQB10201	197	MGNAAFQTAYDKAIP	AFQTAYDKA	4	0.2790	2444.3	40.00	0.26	tr_C9ZL71_
HLA-DQA10501-DQB10201	198	PKSVWQALRGVISAS	WQALRGVIS	4	0.2777	2477.6	41.00	0.57	tr_C9ZL71_
HLA-DQA10501-DQB10201	199	DEVTVYTFGQPPMGN	VYTFGQPPM	4	0.2772	2491.2	41.00	0.36	tr_C9ZL71_
HLA-DQA10501-DQB10201	200	NLFERPAHAFERAET	AHAFERAET	6	0.2770	2496.3	41.00	0.28	tr_C9ZL71_
HLA-DQA10501-DQB10201	201	LVWPVLVNVMTLIYT	LVNVMTLIY	5	0.2764	2511.6	41.00	0.22	tr_C9ZL71_
HLA-DQA10501-DQB10201	202	YLLDAVQQRMHEPLY	LLDAVQQRM	1	0.2762	2519.5	41.00	0.38	tr_C9ZL71_
HLA-DQA10501-DQB10201	203	KIRASGDPEASYVAE	IRASGDPEA	1	0.2760	2523.7	41.00	0.40	tr_C9ZL71_
HLA-DQA10501-DQB10201	204	LIQLPALLVWPVLVN	ALLVWPVLV	5	0.2758	2529.0	41.00	0.32	tr_C9ZL71_
HLA-DQA10501-DQB10201	205	SMGGAVACLCAYSVR	MGGAVACLC	1	0.2758	2529.3	41.00	0.34	tr_C9ZL71_
HLA-DQA10501-DQB10201	206	NDGGALNGSVALRLS	LNGSVALRL	5	0.2736	2591.2	42.00	0.63	tr_C9ZL71_
HLA-DQA10501-DQB10201	207	QDIEFWKGLRESICN	FWKGLRESI	4	0.2735	2591.8	42.00	0.33	tr_C9ZL71_
HLA-DQA10501-DQB10201	208	EASYVAEPGREPDAM	SYVAEPGRE	2	0.2719	2638.2	42.00	0.29	tr_C9ZL71_
HLA-DQA10501-DQB10201	209	SDEIATFRLYGTQVG	TFRLYGTQV	5	0.2717	2642.6	42.00	0.52	tr_C9ZL71_

HLA-DQA10501-DQB10201	210	CKIRASGDPEASYVA	IRASGDPEA	2	0.2711	2661.8	42.00	0.41	tr_C9ZL71_
HLA-DQA10501-DQB10201	211	PSDPSINYTIMCSVH	INYTIMCSV	5	0.2705	2679.1	43.00	0.50	tr_C9ZL71_
HLA-DQA10501-DQB10201	212	DTSCKIRASGDPEAS	IRASGDPEA	5	0.2652	2837.0	44.00	0.46	tr_C9ZL71_
HLA-DQA10501-DQB10201	213	IQLPALLVWPVLVNV	ALLVWPVLV	4	0.2651	2840.7	44.00	0.31	tr_C9ZL71_
HLA-DQA10501-DQB10201	214	DCFNIAFESYNWKGQ	CFNIAFESY	1	0.2647	2850.9	44.00	0.44	tr_C9ZL71_
HLA-DQA10501-DQB10201	215	SCCWEPTVHSGFLEI	TVHSGFLEI	6	0.2629	2909.1	45.00	0.41	tr_C9ZL71_
HLA-DQA10501-DQB10201	216	DEIATFRLYGTQVGT	TFRLYGTQV	4	0.2626	2916.1	45.00	0.47	tr_C9ZL71_
HLA-DQA10501-DQB10201	217	ARHGGSMYIFHTEKE	MYIFHTEKE	6	0.2624	2925.2	45.00	0.46	tr_C9ZL71_
HLA-DQA10501-DQB10201	218	FGQPPMGNAAFQTAY	MGNAAFQTA	5	0.2613	2960.5	45.00	0.39	tr_C9ZL71_
HLA-DQA10501-DQB10201	219	EVTVYTFGQPPMGNA	VYTFGQPPM	3	0.2609	2971.0	45.00	0.39	tr_C9ZL71_
HLA-DQA10501-DQB10201	220	SDPSINYTIMCSVHS	INYTIMCSV	4	0.2609	2971.0	45.00	0.43	tr_C9ZL71_
HLA-DQA10501-DQB10201	221	IRASGDPEASYVAEP	PEASYVAEP	6	0.2599	3002.6	45.00	0.34	tr_C9ZL71_
HLA-DQA10501-DQB10201	222	KESVWPDDWYRWLAR	KESVWPDDW	0	0.2593	3024.8	46.00	0.37	tr_C9ZL71_
HLA-DQA10501-DQB10201	223	VHNLSLLLQSIPNDS	LLLQSIPND	5	0.2592	3027.0	46.00	0.31	tr_C9ZL71_
HLA-DQA10501-DQB10201	224	LPYVEAGRENDLGPE	YVEAGREND	2	0.2592	3028.1	46.00	0.28	tr_C9ZL71_
HLA-DQA10501-DQB10201	225	CAVMDTEFLHHRGKA	CAVMDTEFL	0	0.2580	3067.3	46.00	0.49	tr_C9ZL71_
HLA-DQA10501-DQB10201	226	ESDEIATFRLYGTQV	TFRLYGTQV	6	0.2566	3112.5	46.00	0.63	tr_C9ZL71_
HLA-DQA10501-DQB10201	227	IDVKQYGYKPIAVFE	QYGYKPIAV	4	0.2562	3128.0	46.00	0.44	tr_C9ZL71_
HLA-DQA10501-DQB10201	228	GVHNLSLLLQSIPND	LLLQSIPND	6	0.2560	3134.4	47.00	0.34	tr_C9ZL71_
HLA-DQA10501-DQB10201	229	AFRGTANMSNVREDI	NMSNVREDI	6	0.2555	3150.5	47.00	0.38	tr_C9ZL71_
HLA-DQA10501-DQB10201	230	YVEAGRENDLGPERP	ENDLGPERP	6	0.2552	3159.9	47.00	0.32	tr_C9ZL71_
HLA-DQA10501-DQB10201	231	PSINYTIMCSVHSRP	INYTIMCSV	2	0.2551	3165.6	47.00	0.35	tr_C9ZL71_
HLA-DQA10501-DQB10201	232	QALRGVISASVRGAG	LRGVISASV	2	0.2547	3179.0	47.00	0.69	tr_C9ZL71_
HLA-DQA10501-DQB10201	233	GNAAFQTAYDKAIPR	AFQTAYDKA	3	0.2542	3194.5	47.00	0.29	tr_C9ZL71_
HLA-DQA10501-DQB10201	234	YPLDEVTVYTFGQPP	TVYTFGQPP	6	0.2539	3205.6	47.00	0.29	tr_C9ZL71_
HLA-DQA10501-DQB10201	235	DGGALNGSVALRLSS	LNGSVALRL	4	0.2536	3216.1	47.00	0.62	tr_C9ZL71_
HLA-DQA10501-DQB10201	236	VWPVLVNVMTLIYTT	LVNVMTLIY	4	0.2533	3226.9	47.00	0.23	tr_C9ZL71_
HLA-DQA10501-DQB10201	237	LDTSCKIRASGDPEA	IRASGDPEA	6	0.2527	3246.0	47.00	0.51	tr_C9ZL71_
HLA-DQA10501-DQB10201	238	DIEFWKGLRESICNR	FWKGLRESI	3	0.2518	3277.9	48.00	0.34	tr_C9ZL71_
HLA-DQA10501-DQB10201	239	IWEAHQTSIEEKLGG	EAHQTSIEE	2	0.2509	3311.7	48.00	0.26	tr_C9ZL71_
HLA-DQA10501-DQB10201	240	TFGQPPMGNAAFQTA	MGNAAFQTA	6	0.2508	3316.5	48.00	0.44	tr_C9ZL71_
HLA-DQA10501-DQB10201	241	MCSVHSRPQDIEFWK	SRPQDIEFW	5	0.2507	3319.3	48.00	0.41	tr_C9ZL71_
HLA-DQA10501-DQB10201	242	EYPLDEVTVYTFGQP	YPLDEVTVY	1	0.2482	3408.6	49.00	0.22	tr_C9ZL71_
HLA-DQA10501-DQB10201	243	HNLSLLLQSIPNDSS	LLLQSIPND	4	0.2480	3415.9	49.00	0.31	tr_C9ZL71_
HLA-DQA10501-DQB10201	244	RNLFERPAHAFERAE	RPAHAFERA	5	0.2469	3459.5	49.00	0.32	tr_C9ZL71_
HLA-DQA10501-DQB10201	245	NAAFQTAYDKAIPRT	AFQTAYDKA	2	0.2437	3578.5	50.00	0.26	tr_C9ZL71_
HLA-DQA10501-DQB10201	246	DPSINYTIMCSVHSR	INYTIMCSV	3	0.2428	3615.5	50.00	0.41	tr_C9ZL71_
HLA-DQA10501-DQB10201	247	LKRRGGVHNLSLLLQ	VHNLSLLLQ	6	0.2422	3639.5	50.00	0.50	tr_C9ZL71_
HLA-DQA10501-DQB10201	248	SINYTIMCSVHSRPQ	INYTIMCSV	1	0.2417	3657.0	50.00	0.36	tr_C9ZL71_
HLA-DQA10501-DQB10201	249	VFCTGHSMGGAVACL	SMGGAVACL	6	0.2404	3708.6	55.00	0.62	tr_C9ZL71_
HLA-DQA10501-DQB10201	250	FRGTANMSNVREDIK	NMSNVREDI	5	0.2401	3721.1	55.00	0.32	tr_C9ZL71_
HLA-DQA10501-DQB10201	251	GGALNGSVALRLSST	LNGSVALRL	3	0.2382	3800.7	55.00	0.63	tr_C9ZL71_
HLA-DQA10501-DQB10201	252	WPVLVNVMTLIYTTR	LVNVMTLIY	3	0.2375	3829.3	55.00	0.19	tr_C9ZL71_
HLA-DQA10501-DQB10201	253	VTVYTFGQPPMGNAA	VYTFGQPPM	2	0.2372	3838.5	55.00	0.43	tr_C9ZL71_
HLA-DQA10501-DQB10201	254	HPRDTRWKESVWPDD	WKESVWPDD	6	0.2372	3840.4	55.00	0.44	tr_C9ZL71_
HLA-DQA10501-DQB10201	255	TIDVKQYGYKPIAVF	QYGYKPIAV	5	0.2367	3860.5	55.00	0.57	tr_C9ZL71_
HLA-DQA10501-DQB10201	256	CSVHSRPQDIEFWKG	SRPQDIEFW	4	0.2356	3908.8	55.00	0.38	tr_C9ZL71_
HLA-DQA10501-DQB10201	257	RPQDIEFWKGLRESI	FWKGLRESI	6	0.2354	3914.4	55.00	0.40	tr_C9ZL71_
HLA-DQA10501-DQB10201	258	NLSLLLQSIPNDSSS	LLLQSIPND	3	0.2350	3934.3	55.00	0.34	tr_C9ZL71_
HLA-DQA10501-DQB10201	259	AAFQTAYDKAIPRTF	AFQTAYDKA	1	0.2344	3958.5	55.00	0.26	tr_C9ZL71_

HLA-DQA10501-DQB10201	260	NPDACTTSQGDVEMR	TTSQGDVEM	5	0.2335	3997.6	55.00	0.47	tr_C9ZL71_
HLA-DQA10501-DQB10201	261	RGGVHNLSLLLQSIP	VHNLSLLLQ	3	0.2314	4088.8	55.00	0.40	tr_C9ZL71_
HLA-DQA10501-DQB10201	262	GGVHNLSLLLQSIPN	VHNLSLLLQ	2	0.2297	4167.0	55.00	0.35	tr_C9ZL71_
HLA-DQA10501-DQB10201	263	RGTANMSNVREDIKM	NMSNVREDI	4	0.2296	4170.2	55.00	0.31	tr_C9ZL71_
HLA-DQA10501-DQB10201	264	LSLLLQSIPNDSSSP	LLQSIPNDS	3	0.2295	4175.7	55.00	0.38	tr_C9ZL71_
HLA-DQA10501-DQB10201	265	GALNGSVALRLSSTT	LNGSVALRL	2	0.2291	4189.9	55.00	0.63	tr_C9ZL71_
HLA-DQA10501-DQB10201	266	PYVEAGRENDLGPER	VEAGRENDL	2	0.2287	4208.5	55.00	0.34	tr_C9ZL71_
HLA-DQA10501-DQB10201	267	TNPDACTTSQGDVEM	TTSQGDVEM	6	0.2282	4231.6	55.00	0.57	tr_C9ZL71_
HLA-DQA10501-DQB10201	268	MGGAVACLCAYSVRR	MGGAVACLC	0	0.2281	4238.8	55.00	0.28	tr_C9ZL71_
HLA-DQA10501-DQB10201	269	RRGGVHNLSLLLQSI	VHNLSLLLQ	4	0.2278	4249.3	55.00	0.41	tr_C9ZL71_
HLA-DQA10501-DQB10201	270	PSTEQQSVAATPDHN	QQSVAATPD	4	0.2277	4254.4	55.00	0.37	tr_C9ZL71_
HLA-DQA10501-DQB10201	271	ALRGVISASVRGAGR	LRGVISASV	1	0.2277	4257.3	55.00	0.69	tr_C9ZL71_
HLA-DQA10501-DQB10201	272	WEAHQTSIEEKLGGF	HQTSIEEKL	3	0.2275	4263.5	55.00	0.32	tr_C9ZL71_
HLA-DQA10501-DQB10201	273	GHQVKSYARSLNALA	YARSLNALA	6	0.2268	4297.1	55.00	0.40	tr_C9ZL71_
HLA-DQA10501-DQB10201	274	FCPSTEQQSVAATPD	QQSVAATPD	6	0.2257	4348.1	55.00	0.52	tr_C9ZL71_
HLA-DQA10501-DQB10201	275	CFNIAFESYNWKGQS	CFNIAFESY	0	0.2255	4358.5	55.00	0.38	tr_C9ZL71_
HLA-DQA10501-DQB10201	276	WYRWLARHGGSMYIF	RHGGSMYIF	6	0.2255	4359.2	55.00	0.49	tr_C9ZL71_
HLA-DQA10501-DQB10201	277	DACTTSQGDVEMRKF	TTSQGDVEM	3	0.2234	4459.8	60.00	0.34	tr_C9ZL71_
HLA-DQA10501-DQB10201	278	CLCAYSVRRMLREIE	VRRMLREIE	6	0.2233	4462.6	60.00	0.61	tr_C9ZL71_
HLA-DQA10501-DQB10201	279	IEFWKGLRESICNRG	FWKGLRESI	2	0.2232	4468.3	60.00	0.35	tr_C9ZL71_
HLA-DQA10501-DQB10201	280	HPMKNKAFGIEGHQV	AFGIEGHQV	6	0.2226	4498.7	60.00	0.71	tr_C9ZL71_
HLA-DQA10501-DQB10201	281	SVHSRPQDIEFWKGL	SRPQDIEFW	3	0.2220	4528.1	60.00	0.38	tr_C9ZL71_
HLA-DQA10501-DQB10201	282	PVLVNVMTLIYTTRH	VMTLIYTTR	5	0.2219	4532.1	60.00	0.20	tr_C9ZL71_
HLA-DQA10501-DQB10201	283	INYTIMCSVHSRPQD	IMCSVHSRP	4	0.2207	4590.5	60.00	0.26	tr_C9ZL71_
HLA-DQA10501-DQB10201	284	KRRGGVHNLSLLLQS	VHNLSLLLQ	5	0.2204	4603.5	60.00	0.47	tr_C9ZL71_
HLA-DQA10501-DQB10201	285	VNESDEIATFRLYGT	NESDEIATF	1	0.2201	4622.8	60.00	0.29	tr_C9ZL71_
HLA-DQA10501-DQB10201	286	GRNLFERPAHAFERA	RPAHAFERA	6	0.2198	4636.5	60.00	0.38	tr_C9ZL71_
HLA-DQA10501-DQB10201	287	CPSTEQQSVAATPDH	QQSVAATPD	5	0.2193	4659.3	60.00	0.47	tr_C9ZL71_
HLA-DQA10501-DQB10201	288	GTQVGTEVDINRHGN	QVGTEVDIN	2	0.2192	4666.8	60.00	0.46	tr_C9ZL71_
HLA-DQA10501-DQB10201	289	TIMCSVHSRPQDIEF	SVHSRPQDI	4	0.2174	4755.6	60.00	0.37	tr_C9ZL71_
HLA-DQA10501-DQB10201	290	DRDNASLNSSCCWEP	ASLNSSCCW	4	0.2169	4782.7	60.00	0.49	tr_C9ZL71_
HLA-DQA10501-DQB10201	291	HSRPQDIEFWKGLRE	SRPQDIEFW	1	0.2168	4788.1	60.00	0.36	tr_C9ZL71_
HLA-DQA10501-DQB10201	292	STEQQSVAATPDHNG	QQSVAATPD	3	0.2161	4827.1	60.00	0.34	tr_C9ZL71_
HLA-DQA10501-DQB10201	293	SLLLQSIPNDSSSPP	LLQSIPNDS	2	0.2155	4856.0	60.00	0.41	tr_C9ZL71_
HLA-DQA10501-DQB10201	294	YRWLARHGGSMYIFH	RHGGSMYIF	5	0.2141	4928.5	60.00	0.44	tr_C9ZL71_
HLA-DQA10501-DQB10201	295	LNALALDTSCKIRAS	LNALALDTS	0	0.2137	4952.3	60.00	0.44	tr_C9ZL71_
HLA-DQA10501-DQB10201	296	WLARHGGSMYIFHTE	RHGGSMYIF	3	0.2107	5117.4	60.00	0.41	tr_C9ZL71_
HLA-DQA10501-DQB10201	297	RDNASLNSSCCWEPT	ASLNSSCCW	3	0.2102	5144.9	60.00	0.47	tr_C9ZL71_
HLA-DQA10501-DQB10201	298	DNASLNSSCCWEPTV	ASLNSSCCW	2	0.2093	5195.5	60.00	0.43	tr_C9ZL71_
HLA-DQA10501-DQB10201	299	PDACTTSQGDVEMRK	TTSQGDVEM	4	0.2088	5220.4	60.00	0.38	tr_C9ZL71_
HLA-DQA10501-DQB10201	300	GTANMSNVREDIKMR	NMSNVREDI	3	0.2085	5240.6	60.00	0.29	tr_C9ZL71_
HLA-DQA10501-DQB10201	301	TVYTFGQPPMGNAAF	VYTFGQPPM	1	0.2078	5275.9	60.00	0.45	tr_C9ZL71_
HLA-DQA10501-DQB10201	302	YTIMCSVHSRPQDIE	SVHSRPQDI	5	0.2063	5364.2	65.00	0.34	tr_C9ZL71_
HLA-DQA10501-DQB10201	303	KNKAFGIEGHQVKSY	AFGIEGHQV	3	0.2054	5420.3	65.00	0.61	tr_C9ZL71_
HLA-DQA10501-DQB10201	304	NYTIMCSVHSRPQDI	IMCSVHSRP	3	0.2053	5423.1	65.00	0.31	tr_C9ZL71_
HLA-DQA10501-DQB10201	305	YTFGQPPMGNAAFQT	PMGNAAFQT	6	0.2043	5483.5	65.00	0.52	tr_C9ZL71_
HLA-DQA10501-DQB10201	306	ALNGSVALRLSSTTG	LNGSVALRL	1	0.2042	5487.6	65.00	0.62	tr_C9ZL71_
HLA-DQA10501-DQB10201	307	RWLARHGGSMYIFHT	RHGGSMYIF	4	0.2040	5497.8	65.00	0.42	tr_C9ZL71_
HLA-DQA10501-DQB10201	308	ACTTSQGDVEMRKFC	TTSQGDVEM	2	0.2038	5513.9	65.00	0.31	tr_C9ZL71_
HLA-DQA10501-DQB10201	309	LARHGGSMYIFHTEK	RHGGSMYIF	2	0.2037	5518.5	65.00	0.32	tr_C9ZL71_

HLA-DQA10501-DQB10201	310	EAHQTSIEEKLGGFL	IEEKLGGFL	6	0.2036	5521.9	65.00	0.31	tr_C9ZL71_
HLA-DQA10501-DQB10201	311	TEQQSVAATPDHNGT	QQSVAATPD	2	0.2034	5537.4	65.00	0.35	tr_C9ZL71_
HLA-DQA10501-DQB10201	312	VEAGRENDLGPERPS	ENDLGPERP	5	0.2034	5538.7	65.00	0.43	tr_C9ZL71_
HLA-DQA10501-DQB10201	313	IAFRGTANMSNVRED	TANMSNVRE	5	0.2032	5546.7	65.00	0.40	tr_C9ZL71_
HLA-DQA10501-DQB10201	314	SSCCWEPTVHSGFLE	PTVHSGFLE	6	0.2029	5563.7	65.00	0.41	tr_C9ZL71_
HLA-DQA10501-DQB10201	315	PMKNKAFGIEGHQVK	AFGIEGHQV	5	0.2024	5595.2	65.00	0.66	tr_C9ZL71_
HLA-DQA10501-DQB10201	316	SRPQDIEFWKGLRES	IEFWKGLRE	5	0.2007	5698.5	65.00	0.41	tr_C9ZL71_
HLA-DQA10501-DQB10201	317	NKAFGIEGHQVKSYA	AFGIEGHQV	2	0.1996	5768.9	65.00	0.60	tr_C9ZL71_
HLA-DQA10501-DQB10201	318	AFQTAYDKAIPRTFR	YDKAIPRTF	5	0.1995	5772.4	65.00	0.20	tr_C9ZL71_
HLA-DQA10501-DQB10201	319	TEKEEASFNWKQLEY	SFNWKQLEY	6	0.1985	5839.0	65.00	0.47	tr_C9ZL71_
HLA-DQA10501-DQB10201	320	PGDGERRLGSVDDHS	RRLGSVDDH	5	0.1982	5855.5	65.00	0.37	tr_C9ZL71_
HLA-DQA10501-DQB10201	321	EKEEASFNWKQLEYR	SFNWKQLEY	5	0.1979	5874.8	65.00	0.41	tr_C9ZL71_
HLA-DQA10501-DQB10201	322	KEEASFNWKQLEYRV	SFNWKQLEY	4	0.1976	5893.5	65.00	0.38	tr_C9ZL71_
HLA-DQA10501-DQB10201	323	KAPRIVIAFRGTANM	VIAFRGTAN	5	0.1975	5899.6	65.00	0.26	tr_C9ZL71_
HLA-DQA10501-DQB10201	324	IFHTEKEEASFNWKQ	IFHTEKEEA	0	0.1961	5990.1	65.00	0.51	tr_C9ZL71_
HLA-DQA10501-DQB10201	325	NDRDNASLNSSCCWE	ASLNSSCCW	5	0.1959	6004.2	65.00	0.50	tr_C9ZL71_
HLA-DQA10501-DQB10201	326	APRIVIAFRGTANMS	VIAFRGTAN	4	0.1953	6044.1	65.00	0.28	tr_C9ZL71_
HLA-DQA10501-DQB10201	327	VIAFRGTANMSNVRE	TANMSNVRE	6	0.1944	6104.9	65.00	0.34	tr_C9ZL71_
HLA-DQA10501-DQB10201	328	LRESICNRGAAEIVN	CNRGAAEIV	5	0.1941	6121.6	65.00	0.46	tr_C9ZL71_
HLA-DQA10501-DQB10201	329	MKNKAFGIEGHQVKS	AFGIEGHQV	4	0.1930	6197.2	65.00	0.67	tr_C9ZL71_
HLA-DQA10501-DQB10201	330	LLLQSIPNDSSSPPV	LLQSIPNDS	1	0.1925	6232.4	65.00	0.40	tr_C9ZL71_
HLA-DQA10501-DQB10201	331	KMRRRAWDEMKNDRD	WDEMKNDRD	6	0.1923	6240.3	65.00	0.36	tr_C9ZL71_
HLA-DQA10501-DQB10201	332	PTIDVKQYGYKPIAV	QYGYKPIAV	6	0.1921	6254.8	65.00	0.75	tr_C9ZL71_
HLA-DQA10501-DQB10201	333	VHSRPQDIEFWKGLR	SRPQDIEFW	2	0.1918	6276.2	65.00	0.50	tr_C9ZL71_
HLA-DQA10501-DQB10201	334	GGAVACLCAYSVRRM	VACLCAYSV	3	0.1917	6280.4	65.00	0.32	tr_C9ZL71_
HLA-DQA10501-DQB10201	335	VLVNVMTLIYTTRHR	LVNVMTLIY	1	0.1915	6298.2	65.00	0.24	tr_C9ZL71_
HLA-DQA10501-DQB10201	336	EGPSDPSINYTIMCS	SINYTIMCS	6	0.1913	6310.5	65.00	0.56	tr_C9ZL71_
HLA-DQA10501-DQB10201	337	RIVIAFRGTANMSNV	VIAFRGTAN	2	0.1907	6351.1	65.00	0.25	tr_C9ZL71_
HLA-DQA10501-DQB10201	338	EFWKGLRESICNRGA	FWKGLRESI	1	0.1906	6356.9	65.00	0.38	tr_C9ZL71_
HLA-DQA10501-DQB10201	339	EQQSVAATPDHNGTP	SVAATPDHN	3	0.1900	6396.5	65.00	0.35	tr_C9ZL71_
HLA-DQA10501-DQB10201	340	TQVGTEVDINRHGNY	QVGTEVDIN	1	0.1887	6493.0	70.00	0.53	tr_C9ZL71_
HLA-DQA10501-DQB10201	341	NASLNSSCCWEPTVH	ASLNSSCCW	1	0.1883	6521.3	70.00	0.43	tr_C9ZL71_
HLA-DQA10501-DQB10201	342	NESDEIATFRLYGTQ	ESDEIATFR	1	0.1882	6522.8	70.00	0.22	tr_C9ZL71_
HLA-DQA10501-DQB10201	343	GDGERRLGSVDDHSG	RRLGSVDDH	4	0.1881	6535.9	70.00	0.35	tr_C9ZL71_
HLA-DQA10501-DQB10201	344	EGHQVKSYARSLNAL	SYARSLNAL	6	0.1875	6578.7	70.00	0.49	tr_C9ZL71_
HLA-DQA10501-DQB10201	345	PRIVIAFRGTANMSN	VIAFRGTAN	3	0.1874	6581.5	70.00	0.25	tr_C9ZL71_
HLA-DQA10501-DQB10201	346	CTTSQGDVEMRKFCP	TTSQGDVEM	1	0.1873	6589.4	70.00	0.26	tr_C9ZL71_
HLA-DQA10501-DQB10201	347	TANMSNVREDIKMRR	NMSNVREDI	2	0.1867	6630.0	70.00	0.33	tr_C9ZL71_
HLA-DQA10501-DQB10201	348	GKAPRIVIAFRGTAN	VIAFRGTAN	6	0.1865	6643.6	70.00	0.29	tr_C9ZL71_
HLA-DQA10501-DQB10201	349	EEASFNWKQLEYRVR	SFNWKQLEY	3	0.1859	6692.9	70.00	0.32	tr_C9ZL71_
HLA-DQA10501-DQB10201	350	TNNPKSVWQALRGVI	VWQALRGVI	6	0.1849	6760.5	70.00	0.32	tr_C9ZL71_
HLA-DQA10501-DQB10201	351	GAVACLCAYSVRRML	VACLCAYSV	2	0.1836	6859.5	70.00	0.38	tr_C9ZL71_
HLA-DQA10501-DQB10201	352	KAFGIEGHQVKSYAR	AFGIEGHQV	1	0.1827	6923.9	70.00	0.61	tr_C9ZL71_
HLA-DQA10501-DQB10201	353	GLRESICNRGAAEIV	CNRGAAEIV	6	0.1819	6983.8	70.00	0.59	tr_C9ZL71_
HLA-DQA10501-DQB10201	354	VYTFGQPPMGNAAFQ	VYTFGQPPM	0	0.1817	6998.5	70.00	0.37	tr_C9ZL71_
HLA-DQA10501-DQB10201	355	NSSTVYRVFCTGHSM	RVFCTGHSM	6	0.1810	7054.7	70.00	0.28	tr_C9ZL71_
HLA-DQA10501-DQB10201	356	RLKRRGGVHNLSLLL	GVHNLSLLL	6	0.1803	7111.0	70.00	0.51	tr_C9ZL71_
HLA-DQA10501-DQB10201	357	GAGRNLFERPAHAFE	FERPAHAFE	6	0.1800	7134.2	70.00	0.40	tr_C9ZL71_
HLA-DQA10501-DQB10201	358	MRRRAWDEMKNDRDN	WDEMKNDRD	5	0.1787	7229.0	70.00	0.44	tr_C9ZL71_
HLA-DQA10501-DQB10201	359	GREGGGSRAPLLEND	GGGSRAPLL	3	0.1786	7239.2	70.00	0.37	tr_C9ZL71_

HLA-DQA10501-DQB10201	360	YRSNARESAAYPRET	RESAAYPRE	5	0.1785	7247.1	70.00	0.35	tr_C9ZL71_
HLA-DQA10501-DQB10201	361	NYRSNARESAAYPRE	RESAAYPRE	6	0.1783	7259.9	70.00	0.43	tr_C9ZL71_
HLA-DQA10501-DQB10201	362	YDKAIPRTFRVVNES	PRTFRVVNE	5	0.1783	7266.7	70.00	0.37	tr_C9ZL71_
HLA-DQA10501-DQB10201	363	AYDKAIPRTFRVVNE	PRTFRVVNE	6	0.1779	7295.2	70.00	0.49	tr_C9ZL71_
HLA-DQA10501-DQB10201	364	DGERRLGSVDDHSGG	RRLGSVDDH	3	0.1777	7309.0	70.00	0.32	tr_C9ZL71_
HLA-DQA10501-DQB10201	365	AGRNLFERPAHAFER	FERPAHAFE	5	0.1770	7368.0	70.00	0.37	tr_C9ZL71_
HLA-DQA10501-DQB10201	366	EAGRENDLGPERPSG	ENDLGPERP	4	0.1766	7400.7	70.00	0.52	tr_C9ZL71_
HLA-DQA10501-DQB10201	367	LRGVISASVRGAGRN	LRGVISASV	0	0.1759	7458.4	70.00	0.60	tr_C9ZL71_
HLA-DQA10501-DQB10201	368	LVNVMTLIYTTRHRP	VMTLIYTTR	3	0.1758	7460.3	70.00	0.26	tr_C9ZL71_
HLA-DQA10501-DQB10201	369	PPGDGERRLGSVDDH	RRLGSVDDH	6	0.1756	7480.2	70.00	0.51	tr_C9ZL71_
HLA-DQA10501-DQB10201	370	SASVRGAGRNLFERP	VRGAGRNLF	3	0.1738	7627.5	70.00	0.44	tr_C9ZL71_
HLA-DQA10501-DQB10201	371	HHRGKAPRIVIAFRG	PRIVIAFRG	6	0.1737	7634.0	70.00	0.47	tr_C9ZL71_
HLA-DQA10501-DQB10201	372	ESVWPDDWYRWLARH	ESVWPDDWY	0	0.1733	7671.3	70.00	0.47	tr_C9ZL71_
HLA-DQA10501-DQB10201	373	SNARESAAYPRETNP	RESAAYPRE	3	0.1729	7697.0	70.00	0.44	tr_C9ZL71_
HLA-DQA10501-DQB10201	374	IVIAFRGTANMSNVR	VIAFRGTAN	1	0.1717	7802.9	75.00	0.26	tr_C9ZL71_
HLA-DQA10501-DQB10201	375	RRAWDEMKNDRDNAS	WDEMKNDRD	3	0.1709	7866.6	75.00	0.42	tr_C9ZL71_
HLA-DQA10501-DQB10201	376	REGGGSRAPLLENDG	GGGSRAPLL	2	0.1703	7917.3	75.00	0.36	tr_C9ZL71_
HLA-DQA10501-DQB10201	377	SVALRLSSTTGDAPP	LSSTTGDAP	5	0.1699	7957.5	75.00	0.37	tr_C9ZL71_
HLA-DQA10501-DQB10201	378	RRRAWDEMKNDRDNA	WDEMKNDRD	4	0.1699	7958.4	75.00	0.47	tr_C9ZL71_
HLA-DQA10501-DQB10201	379	FQTAYDKAIPRTFRV	YDKAIPRTF	4	0.1698	7959.4	75.00	0.29	tr_C9ZL71_
HLA-DQA10501-DQB10201	380	FLKENSSTVYRVFCT	STVYRVFCT	6	0.1691	8025.9	75.00	0.28	tr_C9ZL71_
HLA-DQA10501-DQB10201	381	NARESAAYPRETNPD	RESAAYPRE	2	0.1690	8033.4	75.00	0.41	tr_C9ZL71_
HLA-DQA10501-DQB10201	382	VISASVRGAGRNLFE	VRGAGRNLF	5	0.1687	8057.4	75.00	0.47	tr_C9ZL71_
HLA-DQA10501-DQB10201	383	RGKAPRIVIAFRGTA	PRIVIAFRG	4	0.1685	8079.8	75.00	0.34	tr_C9ZL71_
HLA-DQA10501-DQB10201	384	INRHGNYICKPTYME	NYICKPTYM	5	0.1674	8177.0	75.00	0.39	tr_C9ZL71_
HLA-DQA10501-DQB10201	385	SSTVYRVFCTGHSMG	RVFCTGHSM	5	0.1670	8207.5	75.00	0.29	tr_C9ZL71_
HLA-DQA10501-DQB10201	386	ALDTSCKIRASGDPE	KIRASGDPE	6	0.1669	8214.2	75.00	0.81	tr_C9ZL71_
HLA-DQA10501-DQB10201	387	EASFNWKQLEYRVRR	SFNWKQLEY	2	0.1668	8228.9	75.00	0.34	tr_C9ZL71_
HLA-DQA10501-DQB10201	388	RAWDEMKNDRDNASL	WDEMKNDRD	2	0.1663	8274.6	75.00	0.43	tr_C9ZL71_
HLA-DQA10501-DQB10201	389	LNGSVALRLSSTTGD	LNGSVALRL	0	0.1660	8299.6	75.00	0.45	tr_C9ZL71_
HLA-DQA10501-DQB10201	390	ASVRGAGRNLFERPA	VRGAGRNLF	2	0.1651	8380.6	75.00	0.41	tr_C9ZL71_
HLA-DQA10501-DQB10201	391	GERRLGSVDDHSGGN	RLGSVDDHS	3	0.1649	8393.2	75.00	0.38	tr_C9ZL71_
HLA-DQA10501-DQB10201	392	IYTTRHRPETVPIHP	RPETVPIHP	6	0.1648	8409.3	75.00	0.28	tr_C9ZL71_
HLA-DQA10501-DQB10201	393	HRGKAPRIVIAFRGT	PRIVIAFRG	5	0.1647	8414.2	75.00	0.40	tr_C9ZL71_
HLA-DQA10501-DQB10201	394	ENSSTVYRVFCTGHS	VYRVFCTGH	5	0.1646	8427.0	75.00	0.21	tr_C9ZL71_
HLA-DQA10501-DQB10201	395	ARESAAYPRETNPDA	RESAAYPRE	1	0.1644	8441.3	75.00	0.39	tr_C9ZL71_
HLA-DQA10501-DQB10201	396	AGRENDLGPERPSGA	ENDLGPERP	3	0.1642	8456.9	75.00	0.54	tr_C9ZL71_
HLA-DQA10501-DQB10201	397	ALRLSSTTGDAPPGD	LSSTTGDAP	3	0.1642	8457.5	75.00	0.39	tr_C9ZL71_
HLA-DQA10501-DQB10201	398	RSNARESAAYPRETN	RESAAYPRE	4	0.1642	8464.1	75.00	0.42	tr_C9ZL71_
HLA-DQA10501-DQB10201	399	EGGGSRAPLLENDGG	GGGSRAPLL	1	0.1640	8483.0	75.00	0.34	tr_C9ZL71_
HLA-DQA10501-DQB10201	400	FNIAFESYNWKGQSH	NIAFESYNW	1	0.1633	8540.2	75.00	0.47	tr_C9ZL71_
HLA-DQA10501-DQB10201	401	AVACLCAYSVRRMLR	VACLCAYSV	1	0.1633	8544.6	75.00	0.44	tr_C9ZL71_
HLA-DQA10501-DQB10201	402	AHQTSIEEKLGGFLK	IEEKLGGFL	5	0.1631	8557.4	75.00	0.35	tr_C9ZL71_
HLA-DQA10501-DQB10201	403	DIKMRRRAWDEMKND	MRRRAWDEM	3	0.1631	8559.0	75.00	0.44	tr_C9ZL71_
HLA-DQA10501-DQB10201	404	ASLNSSCCWEPTVHS	ASLNSSCCW	0	0.1625	8614.3	75.00	0.41	tr_C9ZL71_
HLA-DQA10501-DQB10201	405	KNDRDNASLNSSCCW	ASLNSSCCW	6	0.1624	8623.0	75.00	0.64	tr_C9ZL71_
HLA-DQA10501-DQB10201	406	ASFNWKQLEYRVRRY	SFNWKQLEY	1	0.1616	8701.1	75.00	0.26	tr_C9ZL71_
HLA-DQA10501-DQB10201	407	NRHGNYICKPTYMEQ	NYICKPTYM	4	0.1615	8710.0	75.00	0.39	tr_C9ZL71_
HLA-DQA10501-DQB10201	408	VACLCAYSVRRMLRE	YSVRRMLRE	6	0.1608	8772.9	75.00	0.21	tr_C9ZL71_
HLA-DQA10501-DQB10201	409	NVGREGGGSRAPLLE	GGGSRAPLL	5	0.1605	8803.3	75.00	0.52	tr_C9ZL71_

HLA-DQA10501-DQB10201	410	KENSSTVYRVFCTGH	VYRVFCTGH	6	0.1603	8826.4	75.00	0.22	tr_C9ZL71_
HLA-DQA10501-DQB10201	411	WKQLEYRVRRYLVRL	RVRRYLVRL	6	0.1601	8842.8	75.00	0.44	tr_C9ZL71_
HLA-DQA10501-DQB10201	412	DINRHGNYICKPTYM	NYICKPTYM	6	0.1591	8937.9	75.00	0.38	tr_C9ZL71_
HLA-DQA10501-DQB10201	413	TTRHRPETVPIHPRD	RPETVPIHP	4	0.1586	8984.2	75.00	0.33	tr_C9ZL71_
HLA-DQA10501-DQB10201	414	HGNYICKPTYMEQRC	NYICKPTYM	2	0.1577	9074.1	75.00	0.37	tr_C9ZL71_
HLA-DQA10501-DQB10201	415	RESAAYPRETNPDAC	ESAAYPRET	1	0.1577	9081.3	75.00	0.25	tr_C9ZL71_
HLA-DQA10501-DQB10201	416	HQTSIEEKLGGFLKE	IEEKLGGFL	4	0.1574	9105.5	75.00	0.37	tr_C9ZL71_
HLA-DQA10501-DQB10201	417	YTTRHRPETVPIHPR	RPETVPIHP	5	0.1570	9142.6	75.00	0.31	tr_C9ZL71_
HLA-DQA10501-DQB10201	418	ANMSNVREDIKMRRR	NMSNVREDI	1	0.1568	9163.2	75.00	0.35	tr_C9ZL71_
HLA-DQA10501-DQB10201	419	DWYRWLARHGGSMYI	ARHGGSMYI	6	0.1564	9205.8	75.00	0.37	tr_C9ZL71_
HLA-DQA10501-DQB10201	420	GSVALRLSSTTGDAP	LSSTTGDAP	6	0.1558	9261.0	75.00	0.28	tr_C9ZL71_
HLA-DQA10501-DQB10201	421	AFGIEGHQVKSYARS	AFGIEGHQV	0	0.1558	9263.1	75.00	0.54	tr_C9ZL71_
HLA-DQA10501-DQB10201	422	VNVMTLIYTTRHRPE	VMTLIYTTR	2	0.1553	9310.9	75.00	0.32	tr_C9ZL71_
HLA-DQA10501-DQB10201	423	IKMRRRAWDEMKNDR	MRRRAWDEM	2	0.1552	9330.8	75.00	0.44	tr_C9ZL71_
HLA-DQA10501-DQB10201	424	QQSVAATPDHNGTPT	SVAATPDHN	2	0.1549	9354.3	75.00	0.47	tr_C9ZL71_
HLA-DQA10501-DQB10201	425	LKENSSTVYRVFCTG	STVYRVFCT	5	0.1548	9361.5	75.00	0.25	tr_C9ZL71_
HLA-DQA10501-DQB10201	426	FWKGLRESICNRGAA	WKGLRESIC	1	0.1547	9379.4	75.00	0.46	tr_C9ZL71_
HLA-DQA10501-DQB10201	427	RHGNYICKPTYMEQR	NYICKPTYM	3	0.1541	9440.3	75.00	0.38	tr_C9ZL71_
HLA-DQA10501-DQB10201	428	VGREGGGSRAPLLEN	GGGSRAPLL	4	0.1540	9447.2	75.00	0.51	tr_C9ZL71_
HLA-DQA10501-DQB10201	429	VALRLSSTTGDAPPG	LSSTTGDAP	4	0.1540	9452.9	75.00	0.37	tr_C9ZL71_
HLA-DQA10501-DQB10201	430	SVRGAGRNLFERPAH	VRGAGRNLF	1	0.1538	9467.5	75.00	0.40	tr_C9ZL71_
HLA-DQA10501-DQB10201	431	IHPRDTRWKESVWPD	TRWKESVWP	5	0.1538	9473.3	80.00	0.47	tr_C9ZL71_
HLA-DQA10501-DQB10201	432	AWDEMKNDRDNASLN	WDEMKNDRD	1	0.1536	9485.4	80.00	0.43	tr_C9ZL71_
HLA-DQA10501-DQB10201	433	GRENDLGPERPSGAS	ENDLGPERP	2	0.1534	9505.1	80.00	0.56	tr_C9ZL71_
HLA-DQA10501-DQB10201	434	LLENDGGALNGSVAL	GALNGSVAL	6	0.1525	9600.3	80.00	0.44	tr_C9ZL71_
HLA-DQA10501-DQB10201	435	TTSQGDVEMRKFCPS	TSQGDVEMR	1	0.1525	9603.1	80.00	0.28	tr_C9ZL71_
HLA-DQA10501-DQB10201	436	LRLSSTTGDAPPGDG	LSSTTGDAP	2	0.1521	9647.8	80.00	0.42	tr_C9ZL71_
HLA-DQA10501-DQB10201	437	VREDIKMRRRAWDEM	MRRRAWDEM	6	0.1512	9739.8	80.00	0.70	tr_C9ZL71_
HLA-DQA10501-DQB10201	438	SYNWKGQSHARAQER	QSHARAQER	6	0.1510	9755.6	80.00	0.38	tr_C9ZL71_
HLA-DQA10501-DQB10201	439	VMTLIYTTRHRPETV	TTRHRPETV	6	0.1509	9768.6	80.00	0.34	tr_C9ZL71_
HLA-DQA10501-DQB10201	440	ERRLGSVDDHSGGNG	RLGSVDDHS	2	0.1508	9780.1	80.00	0.44	tr_C9ZL71_
HLA-DQA10501-DQB10201	441	QVGTEVDINRHGNYI	QVGTEVDIN	0	0.1506	9803.4	80.00	0.47	tr_C9ZL71_
HLA-DQA10501-DQB10201	442	ISASVRGAGRNLFER	VRGAGRNLF	4	0.1504	9820.7	80.00	0.49	tr_C9ZL71_
HLA-DQA10501-DQB10201	443	SQGDVEMRKFCPSTE	MRKFCPSTE	6	0.1500	9867.1	80.00	0.49	tr_C9ZL71_
HLA-DQA10501-DQB10201	444	KFCPSTEQQSVAATP	EQQSVAATP	6	0.1499	9879.7	80.00	0.37	tr_C9ZL71_
HLA-DQA10501-DQB10201	445	GNYICKPTYMEQRCH	NYICKPTYM	1	0.1491	9957.6	80.00	0.34	tr_C9ZL71_
HLA-DQA10501-DQB10201	446	STVYRVFCTGHSMGG	RVFCTGHSM	4	0.1491	9960.0	80.00	0.31	tr_C9ZL71_
HLA-DQA10501-DQB10201	447	STNNPKSVWQALRGV	KSVWQALRG	5	0.1488	9992.2	80.00	0.28	tr_C9ZL71_
HLA-DQA10501-DQB10201	448	EDIKMRRRAWDEMKN	MRRRAWDEM	4	0.1488	9993.6	80.00	0.50	tr_C9ZL71_
HLA-DQA10501-DQB10201	449	GVISASVRGAGRNLF	VRGAGRNLF	6	0.1483	10048.0	80.00	0.52	tr_C9ZL71_
HLA-DQA10501-DQB10201	450	YRVFCTGHSMGGAVA	GHSMGGAVA	6	0.1482	10058.3	80.00	0.46	tr_C9ZL71_
HLA-DQA10501-DQB10201	451	LENDGGALNGSVALR	GALNGSVAL	5	0.1478	10101.9	80.00	0.37	tr_C9ZL71_
HLA-DQA10501-DQB10201	452	GFLKENSSTVYRVFC	SSTVYRVFC	6	0.1477	10117.0	80.00	0.25	tr_C9ZL71_
HLA-DQA10501-DQB10201	453	TRHRPETVPIHPRDT	RPETVPIHP	3	0.1475	10136.5	80.00	0.35	tr_C9ZL71_
HLA-DQA10501-DQB10201	454	TSIEEKLGGFLKENS	IEEKLGGFL	2	0.1471	10174.8	80.00	0.34	tr_C9ZL71_
HLA-DQA10501-DQB10201	455	ACLCAYSVRRMLREI	YSVRRMLRE	5	0.1470	10185.9	80.00	0.28	tr_C9ZL71_
HLA-DQA10501-DQB10201	456	GGGSRAPLLENDGGA	GGGSRAPLL	0	0.1466	10239.0	80.00	0.20	tr_C9ZL71_
HLA-DQA10501-DQB10201	457	SSNYRSNARESAAYP	YRSNARESA	3	0.1459	10310.0	80.00	0.44	tr_C9ZL71_
HLA-DQA10501-DQB10201	458	FNWKQLEYRVRRYLV	EYRVRRYLV	6	0.1444	10481.1	80.00	0.38	tr_C9ZL71_
HLA-DQA10501-DQB10201	459	NYICKPTYMEQRCHP	NYICKPTYM	0	0.1441	10518.3	80.00	0.23	tr_C9ZL71_

HLA-DQA10501-DQB10201	460	RVFCTGHSMGGAVAC	GHSMGGAVA	5	0.1434	10590.7	80.00	0.48	tr_C9ZL71_
HLA-DQA10501-DQB10201	461	REDIKMRRRAWDEMK	MRRRAWDEM	5	0.1433	10605.0	80.00	0.57	tr_C9ZL71_
HLA-DQA10501-DQB10201	462	QGDVEMRKFCPSTEQ	MRKFCPSTE	5	0.1429	10652.2	80.00	0.51	tr_C9ZL71_
HLA-DQA10501-DQB10201	463	HRPETVPIHPRDTRW	RPETVPIHP	1	0.1429	10654.8	80.00	0.31	tr_C9ZL71_
HLA-DQA10501-DQB10201	464	AVMDTEFLHHRGKAP	AVMDTEFLH	0	0.1423	10726.2	80.00	0.34	tr_C9ZL71_
HLA-DQA10501-DQB10201	465	SNYRSNARESAAYPR	YRSNARESA	2	0.1417	10797.2	80.00	0.38	tr_C9ZL71_
HLA-DQA10501-DQB10201	466	ETNPDACTTSQGDVE	CTTSQGDVE	6	0.1416	10808.8	80.00	0.62	tr_C9ZL71_
HLA-DQA10501-DQB10201	467	NALALDTSCKIRASG	LALDTSCKI	2	0.1415	10810.4	80.00	0.62	tr_C9ZL71_
HLA-DQA10501-DQB10201	468	NVMTLIYTTRHRPET	VMTLIYTTR	1	0.1413	10837.2	80.00	0.28	tr_C9ZL71_
HLA-DQA10501-DQB10201	469	QTSIEEKLGGFLKEN	IEEKLGGFL	3	0.1408	10899.1	80.00	0.43	tr_C9ZL71_
HLA-DQA10501-DQB10201	470	NSSCCWEPTVHSGFL	WEPTVHSGF	5	0.1402	10965.9	80.00	0.43	tr_C9ZL71_
HLA-DQA10501-DQB10201	471	HTEKEEASFNWKQLE	ASFNWKQLE	6	0.1398	11012.6	80.00	0.38	tr_C9ZL71_
HLA-DQA10501-DQB10201	472	QTAYDKAIPRTFRVV	YDKAIPRTF	3	0.1397	11029.5	80.00	0.40	tr_C9ZL71_
HLA-DQA10501-DQB10201	473	LLQSIPNDSSSPPVE	LLQSIPNDS	0	0.1391	11102.9	80.00	0.37	tr_C9ZL71_
HLA-DQA10501-DQB10201	474	YNWKGQSHARAQERK	WKGQSHARA	2	0.1386	11160.7	80.00	0.36	tr_C9ZL71_
HLA-DQA10501-DQB10201	475	VRGAGRNLFERPAHA	VRGAGRNLF	0	0.1382	11208.7	80.00	0.25	tr_C9ZL71_
HLA-DQA10501-DQB10201	476	GSTNNPKSVWQALRG	KSVWQALRG	6	0.1379	11246.3	80.00	0.37	tr_C9ZL71_
HLA-DQA10501-DQB10201	477	GDVEMRKFCPSTEQQ	MRKFCPSTE	4	0.1378	11255.4	80.00	0.49	tr_C9ZL71_
HLA-DQA10501-DQB10201	478	MTLIYTTRHRPETVP	TTRHRPETV	5	0.1378	11256.7	80.00	0.47	tr_C9ZL71_
HLA-DQA10501-DQB10201	479	RLSSTTGDAPPGDGE	LSSTTGDAP	1	0.1374	11303.3	80.00	0.41	tr_C9ZL71_
HLA-DQA10501-DQB10201	480	GGSRAPLLENDGGAL	LLENDGGAL	6	0.1374	11304.3	80.00	0.28	tr_C9ZL71_
HLA-DQA10501-DQB10201	481	SFNWKQLEYRVRRYL	FNWKQLEYR	1	0.1373	11320.2	80.00	0.25	tr_C9ZL71_
HLA-DQA10501-DQB10201	482	RHRPETVPIHPRDTR	RPETVPIHP	2	0.1369	11363.7	80.00	0.35	tr_C9ZL71_
HLA-DQA10501-DQB10201	483	TLIYTTRHRPETVPI	TTRHRPETV	4	0.1369	11366.4	80.00	0.47	tr_C9ZL71_
HLA-DQA10501-DQB10201	484	RENDLGPERPSGASQ	ENDLGPERP	1	0.1359	11492.2	80.00	0.60	tr_C9ZL71_
HLA-DQA10501-DQB10201	485	DVEMRKFCPSTEQQS	MRKFCPSTE	3	0.1351	11586.0	80.00	0.47	tr_C9ZL71_
HLA-DQA10501-DQB10201	486	RGAGRNLFERPAHAF	LFERPAHAF	6	0.1351	11586.2	80.00	0.25	tr_C9ZL71_
HLA-DQA10501-DQB10201	487	TVYRVFCTGHSMGGA	RVFCTGHSM	3	0.1351	11594.9	80.00	0.34	tr_C9ZL71_
HLA-DQA10501-DQB10201	488	SIEEKLGGFLKENSS	IEEKLGGFL	1	0.1350	11598.3	80.00	0.32	tr_C9ZL71_
HLA-DQA10501-DQB10201	489	KQLEYRVRRYLVRLK	RVRRYLVRL	5	0.1349	11613.3	80.00	0.38	tr_C9ZL71_
HLA-DQA10501-DQB10201	490	QLEYRVRRYLVRLKR	RVRRYLVRL	4	0.1339	11743.7	85.00	0.33	tr_C9ZL71_
HLA-DQA10501-DQB10201	491	NWKGQSHARAQERKV	QSHARAQER	4	0.1324	11934.5	85.00	0.35	tr_C9ZL71_
HLA-DQA10501-DQB10201	492	SVWPDDWYRWLARHG	WYRWLARHG	6	0.1314	12061.7	85.00	0.31	tr_C9ZL71_
HLA-DQA10501-DQB10201	493	GGFLKENSSTVYRVF	ENSSTVYRV	5	0.1314	12065.9	85.00	0.31	tr_C9ZL71_
HLA-DQA10501-DQB10201	494	GSSNYRSNARESAAY	YRSNARESA	4	0.1311	12108.8	85.00	0.51	tr_C9ZL71_
HLA-DQA10501-DQB10201	495	YICKPTYMEQRCHPM	YMEQRCHPM	6	0.1309	12124.5	85.00	0.24	tr_C9ZL71_
HLA-DQA10501-DQB10201	496	ESAAYPRETNPDACT	PRETNPDAC	5	0.1301	12229.8	85.00	0.32	tr_C9ZL71_
HLA-DQA10501-DQB10201	497	WDEMKNDRDNASLNS	WDEMKNDRD	0	0.1300	12245.3	85.00	0.33	tr_C9ZL71_
HLA-DQA10501-DQB10201	498	LIYTTRHRPETVPIH	TTRHRPETV	3	0.1294	12327.3	85.00	0.45	tr_C9ZL71_
HLA-DQA10501-DQB10201	499	VYRVFCTGHSMGGAV	RVFCTGHSM	2	0.1289	12393.2	85.00	0.32	tr_C9ZL71_
HLA-DQA10501-DQB10201	500	DDWYRWLARHGGSMY	WLARHGGSM	5	0.1287	12427.3	85.00	0.28	tr_C9ZL71_
HLA-DQA10501-DQB10201	501	TAYDKAIPRTFRVVN	YDKAIPRTF	2	0.1285	12455.4	85.00	0.44	tr_C9ZL71_
HLA-DQA10501-DQB10201	502	LHHRGKAPRIVIAFR	APRIVIAFR	6	0.1282	12484.0	85.00	0.45	tr_C9ZL71_
HLA-DQA10501-DQB10201	503	EKLGGFLKENSSTVY	LKENSSTVY	6	0.1280	12516.4	85.00	0.38	tr_C9ZL71_
HLA-DQA10501-DQB10201	504	QSVAATPDHNGTPTI	SVAATPDHN	1	0.1277	12561.0	85.00	0.63	tr_C9ZL71_
HLA-DQA10501-DQB10201	505	EMRKFCPSTEQQSVA	MRKFCPSTE	1	0.1276	12567.5	85.00	0.35	tr_C9ZL71_
HLA-DQA10501-DQB10201	506	GSRAPLLENDGGALN	LLENDGGAL	5	0.1276	12574.6	85.00	0.29	tr_C9ZL71_
HLA-DQA10501-DQB10201	507	VEMRKFCPSTEQQSV	MRKFCPSTE	2	0.1276	12577.5	85.00	0.46	tr_C9ZL71_
HLA-DQA10501-DQB10201	508	VRLKRRGGVHNLSLL	RGGVHNLSL	5	0.1273	12618.6	85.00	0.37	tr_C9ZL71_
HLA-DQA10501-DQB10201	509	TSQGDVEMRKFCPST	TSQGDVEMR	0	0.1270	12650.8	85.00	0.22	tr_C9ZL71_

HLA-DQA10501-DQB10201	510	NGSVALRLSSTTGDA	LRLSSTTGD	5	0.1262	12757.4	85.00	0.25	tr_C9ZL71_
HLA-DQA10501-DQB10201	511	FHTEKEEASFNWKQL	EKEEASFNW	3	0.1259	12806.5	85.00	0.20	tr_C9ZL71_
HLA-DQA10501-DQB10201	512	QNVGREGGGSRAPLL	GGGSRAPLL	6	0.1258	12812.9	85.00	0.70	tr_C9ZL71_
HLA-DQA10501-DQB10201	513	LGPERPSGASQVMRQ	SGASQVMRQ	6	0.1257	12830.2	85.00	0.46	tr_C9ZL71_
HLA-DQA10501-DQB10201	514	NWKQLEYRVRRYLVR	EYRVRRYLV	5	0.1252	12899.7	85.00	0.40	tr_C9ZL71_
HLA-DQA10501-DQB10201	515	VDINRHGNYICKPTY	RHGNYICKP	4	0.1243	13024.4	85.00	0.32	tr_C9ZL71_
HLA-DQA10501-DQB10201	516	TEVDINRHGNYICKP	RHGNYICKP	6	0.1239	13090.1	85.00	0.42	tr_C9ZL71_
HLA-DQA10501-DQB10201	517	RGSSNYRSNARESAA	YRSNARESA	5	0.1231	13205.6	85.00	0.56	tr_C9ZL71_
HLA-DQA10501-DQB10201	518	NMSNVREDIKMRRRA	MSNVREDIK	1	0.1229	13225.7	85.00	0.42	tr_C9ZL71_
HLA-DQA10501-DQB10201	519	PIHPRDTRWKESVWP	TRWKESVWP	6	0.1225	13281.7	85.00	0.54	tr_C9ZL71_
HLA-DQA10501-DQB10201	520	APPGDGERRLGSVDD	ERRLGSVDD	6	0.1223	13311.0	85.00	0.70	tr_C9ZL71_
HLA-DQA10501-DQB10201	521	ESYNWKGQSHARAQE	WKGQSHARA	4	0.1223	13311.6	85.00	0.56	tr_C9ZL71_
HLA-DQA10501-DQB10201	522	LGGFLKENSSTVYRV	ENSSTVYRV	6	0.1220	13352.3	85.00	0.34	tr_C9ZL71_
HLA-DQA10501-DQB10201	523	DSSSPPVEGPSDPSI	VEGPSDPSI	6	0.1219	13368.7	85.00	0.32	tr_C9ZL71_
HLA-DQA10501-DQB10201	524	SRAPLLENDGGALNG	LLENDGGAL	4	0.1218	13390.3	85.00	0.29	tr_C9ZL71_
HLA-DQA10501-DQB10201	525	VWPDDWYRWLARHGG	WYRWLARHG	5	0.1214	13437.3	85.00	0.31	tr_C9ZL71_
HLA-DQA10501-DQB10201	526	VEGPSDPSINYTIMC	PSINYTIMC	6	0.1210	13501.2	85.00	0.46	tr_C9ZL71_
HLA-DQA10501-DQB10201	527	LEYRVRRYLVRLKRR	RVRRYLVRL	3	0.1205	13578.4	85.00	0.32	tr_C9ZL71_
HLA-DQA10501-DQB10201	528	MRKFCPSTEQQSVAA	STEQQSVAA	6	0.1203	13599.2	85.00	0.31	tr_C9ZL71_
HLA-DQA10501-DQB10201	529	AFESYNWKGQSHARA	WKGQSHARA	6	0.1202	13621.3	85.00	0.60	tr_C9ZL71_
HLA-DQA10501-DQB10201	530	RRLGSVDDHSGGNGD	RLGSVDDHS	1	0.1202	13624.0	85.00	0.50	tr_C9ZL71_
HLA-DQA10501-DQB10201	531	PLLENDGGALNGSVA	GGALNGSVA	6	0.1196	13708.1	85.00	0.35	tr_C9ZL71_
HLA-DQA10501-DQB10201	532	PERPSGASQVMRQNV	SGASQVMRQ	4	0.1191	13789.5	85.00	0.35	tr_C9ZL71_
HLA-DQA10501-DQB10201	533	SVAATPDHNGTPTID	SVAATPDHN	0	0.1186	13855.3	85.00	0.42	tr_C9ZL71_
HLA-DQA10501-DQB10201	534	PDDWYRWLARHGGSM	WLARHGGSM	6	0.1186	13856.2	85.00	0.29	tr_C9ZL71_
HLA-DQA10501-DQB10201	535	EVDINRHGNYICKPT	RHGNYICKP	5	0.1180	13944.3	85.00	0.40	tr_C9ZL71_
HLA-DQA10501-DQB10201	536	RPETVPIHPRDTRWK	RPETVPIHP	0	0.1180	13949.0	85.00	0.29	tr_C9ZL71_
HLA-DQA10501-DQB10201	537	CRGSSNYRSNARESA	YRSNARESA	6	0.1177	13999.7	85.00	0.64	tr_C9ZL71_
HLA-DQA10501-DQB10201	538	VAATPDHNGTPTIDV	HNGTPTIDV	6	0.1158	14283.6	85.00	0.34	tr_C9ZL71_
HLA-DQA10501-DQB10201	539	IEEKLGGFLKENSST	LGGFLKENS	4	0.1150	14402.7	85.00	0.33	tr_C9ZL71_
HLA-DQA10501-DQB10201	540	ALALDTSCKIRASGD	LALDTSCKI	1	0.1146	14473.3	85.00	0.73	tr_C9ZL71_
HLA-DQA10501-DQB10201	541	IEGHQVKSYARSLNA	KSYARSLNA	6	0.1145	14493.5	85.00	0.42	tr_C9ZL71_
HLA-DQA10501-DQB10201	542	FESYNWKGQSHARAQ	WKGQSHARA	5	0.1144	14501.3	85.00	0.60	tr_C9ZL71_
HLA-DQA10501-DQB10201	543	NIAFESYNWKGQSHA	NIAFESYNW	0	0.1138	14596.2	90.00	0.47	tr_C9ZL71_
HLA-DQA10501-DQB10201	544	GPERPSGASQVMRQN	SGASQVMRQ	5	0.1136	14625.3	90.00	0.46	tr_C9ZL71_
HLA-DQA10501-DQB10201	545	NDSSSPPVEGPSDPS	SPPVEGPSD	4	0.1134	14656.1	90.00	0.26	tr_C9ZL71_
HLA-DQA10501-DQB10201	546	PNDSSSPPVEGPSDP	SPPVEGPSD	5	0.1128	14752.6	90.00	0.32	tr_C9ZL71_
HLA-DQA10501-DQB10201	547	GGSTNNPKSVWQALR	PKSVWQALR	6	0.1126	14779.8	90.00	0.47	tr_C9ZL71_
HLA-DQA10501-DQB10201	548	SAAYPRETNPDACTT	PRETNPDAC	4	0.1123	14833.2	90.00	0.37	tr_C9ZL71_
HLA-DQA10501-DQB10201	549	RAPLLENDGGALNGS	LLENDGGAL	3	0.1121	14864.3	90.00	0.31	tr_C9ZL71_
HLA-DQA10501-DQB10201	550	TPDHNGTPTIDVKQY	TPTIDVKQY	6	0.1116	14955.3	90.00	0.32	tr_C9ZL71_
HLA-DQA10501-DQB10201	551	LVRLKRRGGVHNLSL	RGGVHNLSL	6	0.1111	15021.0	90.00	0.47	tr_C9ZL71_
HLA-DQA10501-DQB10201	552	LSSTTGDAPPGDGER	LSSTTGDAP	0	0.1106	15106.4	90.00	0.40	tr_C9ZL71_
HLA-DQA10501-DQB10201	553	LNSSCCWEPTVHSGF	WEPTVHSGF	6	0.1104	15146.3	90.00	0.56	tr_C9ZL71_
HLA-DQA10501-DQB10201	554	WKGQSHARAQERKVG	QSHARAQER	3	0.1101	15192.4	90.00	0.38	tr_C9ZL71_
HLA-DQA10501-DQB10201	555	WPDDWYRWLARHGGS	WYRWLARHG	4	0.1092	15340.4	90.00	0.34	tr_C9ZL71_
HLA-DQA10501-DQB10201	556	APLLENDGGALNGSV	LLENDGGAL	2	0.1091	15358.2	90.00	0.33	tr_C9ZL71_
HLA-DQA10501-DQB10201	557	ERPSGASQVMRQNVG	SGASQVMRQ	3	0.1088	15404.3	90.00	0.31	tr_C9ZL71_
HLA-DQA10501-DQB10201	558	SSSPPVEGPSDPSIN	VEGPSDPSI	5	0.1086	15434.5	90.00	0.31	tr_C9ZL71_
HLA-DQA10501-DQB10201	559	KLGGFLKENSSTVYR	LKENSSTVY	5	0.1073	15652.8	90.00	0.39	tr_C9ZL71_

HLA-DQA10501-DQB10201	560	EEKLGGFLKENSSTV	FLKENSSTV	6	0.1072	15682.1	90.00	0.30	tr_C9ZL71_
HLA-DQA10501-DQB10201	561	ENDLGPERPSGASQV	ENDLGPERP	0	0.1066	15777.1	90.00	0.51	tr_C9ZL71_
HLA-DQA10501-DQB10201	562	IPNDSSSPPVEGPSD	SPPVEGPSD	6	0.1054	15981.4	90.00	0.29	tr_C9ZL71_
HLA-DQA10501-DQB10201	563	EYRVRRYLVRLKRRG	RVRRYLVRL	2	0.1052	16026.4	90.00	0.37	tr_C9ZL71_
HLA-DQA10501-DQB10201	564	RKFCPSTEQQSVAAT	STEQQSVAA	5	0.1050	16055.5	90.00	0.26	tr_C9ZL71_
HLA-DQA10501-DQB10201	565	PDHNGTPTIDVKQYG	TPTIDVKQY	5	0.1046	16126.1	90.00	0.29	tr_C9ZL71_
HLA-DQA10501-DQB10201	566	SSPPVEGPSDPSINY	VEGPSDPSI	4	0.1045	16136.0	90.00	0.32	tr_C9ZL71_
HLA-DQA10501-DQB10201	567	VGTEVDINRHGNYIC	DINRHGNYI	5	0.1044	16165.4	90.00	0.34	tr_C9ZL71_
HLA-DQA10501-DQB10201	568	ICKPTYMEQRCHPMK	YMEQRCHPM	5	0.1042	16200.7	90.00	0.25	tr_C9ZL71_
HLA-DQA10501-DQB10201	569	RGVISASVRGAGRNL	VISASVRGA	2	0.1032	16368.5	90.00	0.32	tr_C9ZL71_
HLA-DQA10501-DQB10201	570	AATPDHNGTPTIDVK	HNGTPTIDV	5	0.1022	16543.0	90.00	0.31	tr_C9ZL71_
HLA-DQA10501-DQB10201	571	DHNGTPTIDVKQYGY	TPTIDVKQY	4	0.1016	16653.9	90.00	0.28	tr_C9ZL71_
HLA-DQA10501-DQB10201	572	SPPVEGPSDPSINYT	VEGPSDPSI	3	0.1001	16936.7	90.00	0.29	tr_C9ZL71_
HLA-DQA10501-DQB10201	573	KGLRESICNRGAAEI	ICNRGAAEI	6	0.0997	16994.7	90.00	0.43	tr_C9ZL71_
HLA-DQA10501-DQB10201	574	AAYPRETNPDACTTS	PRETNPDAC	3	0.0993	17077.6	90.00	0.40	tr_C9ZL71_
HLA-DQA10501-DQB10201	575	SLNSSCCWEPTVHSG	SLNSSCCWE	0	0.0992	17092.4	90.00	0.41	tr_C9ZL71_
HLA-DQA10501-DQB10201	576	LQSIPNDSSSPPVEG	LQSIPNDSS	0	0.0987	17190.9	90.00	0.28	tr_C9ZL71_
HLA-DQA10501-DQB10201	577	PVEGPSDPSINYTIM	DPSINYTIM	6	0.0974	17435.9	90.00	0.34	tr_C9ZL71_
HLA-DQA10501-DQB10201	578	PSGASQVMRQNVGRE	VMRQNVGRE	6	0.0973	17457.1	90.00	0.40	tr_C9ZL71_
HLA-DQA10501-DQB10201	579	SGASQVMRQNVGREG	VMRQNVGRE	5	0.0971	17493.0	90.00	0.32	tr_C9ZL71_
HLA-DQA10501-DQB10201	580	FLHHRGKAPRIVIAF	KAPRIVIAF	6	0.0968	17538.3	90.00	0.47	tr_C9ZL71_
HLA-DQA10501-DQB10201	581	ATPDHNGTPTIDVKQ	DHNGTPTID	3	0.0966	17574.2	90.00	0.25	tr_C9ZL71_
HLA-DQA10501-DQB10201	582	NDLGPERPSGASQVM	RPSGASQVM	6	0.0962	17653.9	90.00	0.53	tr_C9ZL71_
HLA-DQA10501-DQB10201	583	QSIPNDSSSPPVEGP	NDSSSPPVE	4	0.0959	17723.9	90.00	0.23	tr_C9ZL71_
HLA-DQA10501-DQB10201	584	FGIEGHQVKSYARSL	QVKSYARSL	6	0.0954	17812.9	90.00	0.37	tr_C9ZL71_
HLA-DQA10501-DQB10201	585	DLGPERPSGASQVMR	RPSGASQVM	5	0.0951	17873.9	90.00	0.43	tr_C9ZL71_
HLA-DQA10501-DQB10201	586	RPSGASQVMRQNVGR	SGASQVMRQ	2	0.0946	17965.4	90.00	0.35	tr_C9ZL71_
HLA-DQA10501-DQB10201	587	GSVDDHSGGNGDDGG	SGGNGDDGG	6	0.0937	18136.5	90.00	0.41	tr_C9ZL71_
HLA-DQA10501-DQB10201	588	WKGLRESICNRGAAE	WKGLRESIC	0	0.0928	18311.6	90.00	0.34	tr_C9ZL71_
HLA-DQA10501-DQB10201	589	AYPRETNPDACTTSQ	PRETNPDAC	2	0.0926	18352.3	90.00	0.47	tr_C9ZL71_
HLA-DQA10501-DQB10201	590	DEMKNDRDNASLNSS	EMKNDRDNA	1	0.0924	18393.8	90.00	0.31	tr_C9ZL71_
HLA-DQA10501-DQB10201	591	CKPTYMEQRCHPMKN	YMEQRCHPM	4	0.0915	18586.1	90.00	0.28	tr_C9ZL71_
HLA-DQA10501-DQB10201	592	STTGDAPPGDGERRL	TTGDAPPGD	1	0.0912	18639.5	95.00	0.34	tr_C9ZL71_
HLA-DQA10501-DQB10201	593	RCHPMKNKAFGIEGH	NKAFGIEGH	6	0.0908	18714.6	95.00	0.47	tr_C9ZL71_
HLA-DQA10501-DQB10201	594	CHPMKNKAFGIEGHQ	NKAFGIEGH	5	0.0908	18725.2	95.00	0.34	tr_C9ZL71_
HLA-DQA10501-DQB10201	595	SIPNDSSSPPVEGPS	NDSSSPPVE	3	0.0907	18743.0	95.00	0.25	tr_C9ZL71_
HLA-DQA10501-DQB10201	596	LALDTSCKIRASGDP	LALDTSCKI	0	0.0883	19227.6	95.00	0.64	tr_C9ZL71_
HLA-DQA10501-DQB10201	597	SVDDHSGGNGDDGGS	SGGNGDDGG	5	0.0877	19368.3	95.00	0.42	tr_C9ZL71_
HLA-DQA10501-DQB10201	598	RLGSVDDHSGGNGDD	RLGSVDDHS	0	0.0874	19427.5	95.00	0.50	tr_C9ZL71_
HLA-DQA10501-DQB10201	599	YRVRRYLVRLKRRGG	RVRRYLVRL	1	0.0870	19512.8	95.00	0.41	tr_C9ZL71_
HLA-DQA10501-DQB10201	600	GTEVDINRHGNYICK	DINRHGNYI	4	0.0865	19605.3	95.00	0.38	tr_C9ZL71_
HLA-DQA10501-DQB10201	601	GASQVMRQNVGREGG	VMRQNVGRE	4	0.0863	19651.6	95.00	0.32	tr_C9ZL71_
HLA-DQA10501-DQB10201	602	GIEGHQVKSYARSLN	QVKSYARSL	5	0.0852	19886.4	95.00	0.35	tr_C9ZL71_
HLA-DQA10501-DQB10201	603	SSTTGDAPPGDGERR	TTGDAPPGD	2	0.0851	19917.2	95.00	0.48	tr_C9ZL71_
HLA-DQA10501-DQB10201	604	PPVEGPSDPSINYTI	VEGPSDPSI	2	0.0847	19998.2	95.00	0.35	tr_C9ZL71_
HLA-DQA10501-DQB10201	605	KGQSHARAQERKVGT	QSHARAQER	2	0.0833	20312.6	95.00	0.44	tr_C9ZL71_
HLA-DQA10501-DQB10201	606	EMKNDRDNASLNSSC	EMKNDRDNA	0	0.0832	20332.0	95.00	0.23	tr_C9ZL71_
HLA-DQA10501-DQB10201	607	VDDHSGGNGDDGGST	SGGNGDDGG	4	0.0827	20442.5	95.00	0.40	tr_C9ZL71_
HLA-DQA10501-DQB10201	608	MSNVREDIKMRRRAW	MSNVREDIK	0	0.0825	20478.6	95.00	0.38	tr_C9ZL71_
HLA-DQA10501-DQB10201	609	PETVPIHPRDTRWKE	PIHPRDTRW	4	0.0813	20754.1	95.00	0.32	tr_C9ZL71_

HLA-DQA10501-DQB10201	610	GTPTIDVKQYGYKPI	VKQYGYKPI	6	0.0808	20862.6	95.00	0.23	tr_C9ZL71_
HLA-DQA10501-DQB10201	611	TTGDAPPGDGERRLG	TTGDAPPGD	0	0.0799	21057.4	95.00	0.28	tr_C9ZL71_
HLA-DQA10501-DQB10201	612	TPTIDVKQYGYKPIA	VKQYGYKPI	5	0.0793	21209.7	95.00	0.19	tr_C9ZL71_
HLA-DQA10501-DQB10201	613	DDHSGGNGDDGGSTN	SGGNGDDGG	3	0.0785	21375.1	95.00	0.38	tr_C9ZL71_
HLA-DQA10501-DQB10201	614	YPRETNPDACTTSQG	PRETNPDAC	1	0.0779	21525.0	95.00	0.46	tr_C9ZL71_
HLA-DQA10501-DQB10201	615	ASQVMRQNVGREGGG	VMRQNVGRE	3	0.0772	21686.8	95.00	0.32	tr_C9ZL71_
HLA-DQA10501-DQB10201	616	LGSVDDHSGGNGDDG	HSGGNGDDG	6	0.0766	21822.1	95.00	0.49	tr_C9ZL71_
HLA-DQA10501-DQB10201	617	HNGTPTIDVKQYGYK	TPTIDVKQY	3	0.0756	22067.6	95.00	0.31	tr_C9ZL71_
HLA-DQA10501-DQB10201	618	GQSHARAQERKVGTS	QSHARAQER	1	0.0749	22227.5	95.00	0.48	tr_C9ZL71_
HLA-DQA10501-DQB10201	619	VMDTEFLHHRGKAPR	VMDTEFLHH	0	0.0747	22281.4	95.00	0.41	tr_C9ZL71_
HLA-DQA10501-DQB10201	620	NGTPTIDVKQYGYKP	TPTIDVKQY	2	0.0747	22284.3	95.00	0.30	tr_C9ZL71_
HLA-DQA10501-DQB10201	621	KPTYMEQRCHPMKNK	YMEQRCHPM	3	0.0740	22461.3	95.00	0.31	tr_C9ZL71_
HLA-DQA10501-DQB10201	622	YLVRLKRRGGVHNLS	RRGGVHNLS	6	0.0731	22670.5	95.00	0.62	tr_C9ZL71_
HLA-DQA10501-DQB10201	623	IAFESYNWKGQSHAR	IAFESYNWK	0	0.0710	23186.5	95.00	0.31	tr_C9ZL71_
HLA-DQA10501-DQB10201	624	DHSGGNGDDGGSTNN	SGGNGDDGG	2	0.0702	23405.3	95.00	0.41	tr_C9ZL71_
HLA-DQA10501-DQB10201	625	PRETNPDACTTSQGD	DACTTSQGD	6	0.0696	23534.8	95.00	0.34	tr_C9ZL71_
HLA-DQA10501-DQB10201	626	RVRRYLVRLKRRGGV	RRYLVRLKR	2	0.0692	23638.1	95.00	0.28	tr_C9ZL71_
HLA-DQA10501-DQB10201	627	CCRGSSNYRSNARES	SNYRSNARE	5	0.0685	23815.3	95.00	0.41	tr_C9ZL71_
HLA-DQA10501-DQB10201	628	SQVMRQNVGREGGGS	VMRQNVGRE	2	0.0685	23830.0	95.00	0.34	tr_C9ZL71_
HLA-DQA10501-DQB10201	629	ETVPIHPRDTRWKES	PIHPRDTRW	3	0.0682	23909.8	95.00	0.35	tr_C9ZL71_
HLA-DQA10501-DQB10201	630	EFLHHRGKAPRIVIA	HRGKAPRIV	4	0.0677	24037.1	95.00	0.25	tr_C9ZL71_
HLA-DQA10501-DQB10201	631	TEFLHHRGKAPRIVI	HRGKAPRIV	5	0.0665	24352.6	95.00	0.28	tr_C9ZL71_
HLA-DQA10501-DQB10201	632	QRCHPMKNKAFGIEG	KNKAFGIEG	6	0.0659	24496.6	95.00	0.44	tr_C9ZL71_
HLA-DQA10501-DQB10201	633	MKNDRDNASLNSSCC	RDNASLNSS	4	0.0658	24543.0	95.00	0.25	tr_C9ZL71_
HLA-DQA10501-DQB10201	634	SNVREDIKMRRRAWD	VREDIKMRR	2	0.0650	24740.0	95.00	0.33	tr_C9ZL71_
HLA-DQA10501-DQB10201	635	DGGSTNNPKSVWQAL	NPKSVWQAL	6	0.0644	24916.5	95.00	0.40	tr_C9ZL71_
HLA-DQA10501-DQB10201	636	RETNPDACTTSQGDV	DACTTSQGD	5	0.0639	25051.4	95.00	0.41	tr_C9ZL71_
HLA-DQA10501-DQB10201	637	GCCRGSSNYRSNARE	SNYRSNARE	6	0.0632	25226.1	100.00	0.53	tr_C9ZL71_
HLA-DQA10501-DQB10201	638	NVREDIKMRRRAWDE	VREDIKMRR	1	0.0629	25329.2	100.00	0.22	tr_C9ZL71_
HLA-DQA10501-DQB10201	639	VPIHPRDTRWKESVW	DTRWKESVW	6	0.0628	25331.9	100.00	0.34	tr_C9ZL71_
HLA-DQA10501-DQB10201	640	PTYMEQRCHPMKNKA	YMEQRCHPM	2	0.0625	25425.3	100.00	0.40	tr_C9ZL71_
HLA-DQA10501-DQB10201	641	DTEFLHHRGKAPRIV	HRGKAPRIV	6	0.0622	25506.8	100.00	0.29	tr_C9ZL71_
HLA-DQA10501-DQB10201	642	HSGGNGDDGGSTNNP	SGGNGDDGG	1	0.0618	25622.2	100.00	0.41	tr_C9ZL71_
HLA-DQA10501-DQB10201	643	DAPPGDGERRLGSVD	PPGDGERRL	2	0.0615	25712.7	100.00	0.31	tr_C9ZL71_
HLA-DQA10501-DQB10201	644	TGDAPPGDGERRLGS	PPGDGERRL	4	0.0614	25730.5	100.00	0.38	tr_C9ZL71_
HLA-DQA10501-DQB10201	645	GDAPPGDGERRLGSV	PPGDGERRL	3	0.0600	26119.0	100.00	0.39	tr_C9ZL71_
HLA-DQA10501-DQB10201	646	QVMRQNVGREGGGSR	VMRQNVGRE	1	0.0598	26181.0	100.00	0.34	tr_C9ZL71_
HLA-DQA10501-DQB10201	647	MDTEFLHHRGKAPRI	HHRGKAPRI	6	0.0588	26478.7	100.00	0.34	tr_C9ZL71_
HLA-DQA10501-DQB10201	648	TVPIHPRDTRWKESV	PIHPRDTRW	2	0.0578	26749.9	100.00	0.41	tr_C9ZL71_
HLA-DQA10501-DQB10201	649	EQRCHPMKNKAFGIE	PMKNKAFGI	5	0.0553	27476.2	100.00	0.50	tr_C9ZL71_
HLA-DQA10501-DQB10201	650	QSHARAQERKVGTSG	QSHARAQER	0	0.0549	27595.4	100.00	0.40	tr_C9ZL71_
HLA-DQA10501-DQB10201	651	RAQERKVGTSGCCRG	KVGTSGCCR	5	0.0540	27872.1	100.00	0.37	tr_C9ZL71_
HLA-DQA10501-DQB10201	652	RQNVGREGGGSRAPL	EGGGSRAPL	6	0.0536	28011.4	100.00	0.37	tr_C9ZL71_
HLA-DQA10501-DQB10201	653	TYMEQRCHPMKNKAF	YMEQRCHPM	1	0.0522	28427.0	100.00	0.44	tr_C9ZL71_
HLA-DQA10501-DQB10201	654	VMRQNVGREGGGSRA	MRQNVGREG	1	0.0519	28521.9	100.00	0.37	tr_C9ZL71_
HLA-DQA10501-DQB10201	655	ARAQERKVGTSGCCR	KVGTSGCCR	6	0.0518	28550.3	100.00	0.52	tr_C9ZL71_
HLA-DQA10501-DQB10201	656	AQERKVGTSGCCRGS	KVGTSGCCR	4	0.0506	28914.0	100.00	0.36	tr_C9ZL71_
HLA-DQA10501-DQB10201	657	VRRYLVRLKRRGGVH	RRYLVRLKR	1	0.0506	28932.1	100.00	0.34	tr_C9ZL71_
HLA-DQA10501-DQB10201	658	QERKVGTSGCCRGSS	KVGTSGCCR	3	0.0477	29826.6	100.00	0.38	tr_C9ZL71_
HLA-DQA10501-DQB10201	659	SGGNGDDGGSTNNPK	SGGNGDDGG	0	0.0477	29853.1	100.00	0.38	tr_C9ZL71_

```
 HLA-DQA10501-DQB10201  660      MRQNVGREGGGSRAP  MRQNVGREG  0       0.0465      30224.6   100.00  0.29   tr_C9ZL71_
 HLA-DQA10501-DQB10201  661      ERKVGTSGCCRGSSN  KVGTSGCCR  2       0.0445      30895.1   100.00  0.39   tr_C9ZL71_
 HLA-DQA10501-DQB10201  662      RYLVRLKRRGGVHNL  KRRGGVHNL  6       0.0443      30967.0   100.00  0.43   tr_C9ZL71_
 HLA-DQA10501-DQB10201  663      MEQRCHPMKNKAFGI  PMKNKAFGI  6       0.0428      31475.1   100.00  0.65   tr_C9ZL71_
 HLA-DQA10501-DQB10201  664      RRYLVRLKRRGGVHN  RYLVRLKRR  1       0.0386      32934.5   100.00  0.38   tr_C9ZL71_
 HLA-DQA10501-DQB10201  665      RKVGTSGCCRGSSNY  KVGTSGCCR  1       0.0381      33103.8   100.00  0.41   tr_C9ZL71_
 HLA-DQA10501-DQB10201  666      HARAQERKVGTSGCC  RKVGTSGCC  6       0.0379      33197.4   100.00  0.24   tr_C9ZL71_
 HLA-DQA10501-DQB10201  667      SHARAQERKVGTSGC  HARAQERKV  1       0.0360      33867.5   100.00  0.26   tr_C9ZL71_
 HLA-DQA10501-DQB10201  668      GDDGGSTNNPKSVWQ  STNNPKSVW  5       0.0341      34570.6   100.00  0.23   tr_C9ZL71_
 HLA-DQA10501-DQB10201  669      YMEQRCHPMKNKAFG  YMEQRCHPM  0       0.0339      34641.0   100.00  0.51   tr_C9ZL71_
 HLA-DQA10501-DQB10201  670      NGDDGGSTNNPKSVW  STNNPKSVW  6       0.0331      34965.2   100.00  0.31   tr_C9ZL71_
 HLA-DQA10501-DQB10201  671      DDGGSTNNPKSVWQA  STNNPKSVW  4       0.0322      35281.4   100.00  0.24   tr_C9ZL71_
 HLA-DQA10501-DQB10201  672      SGCCRGSSNYRSNAR  SSNYRSNAR  6       0.0318      35432.1   100.00  0.50   tr_C9ZL71_
 HLA-DQA10501-DQB10201  673      KVGTSGCCRGSSNYR  KVGTSGCCR  0       0.0309      35776.9   100.00  0.37   tr_C9ZL71_
 HLA-DQA10501-DQB10201  674      GGNGDDGGSTNNPKS  GNGDDGGST  1       0.0298      36222.5   100.00  0.32   tr_C9ZL71_
 HLA-DQA10501-DQB10201  675      GNGDDGGSTNNPKSV  GSTNNPKSV  6       0.0276      37075.4   100.00  0.25   tr_C9ZL71_
 HLA-DQA10501-DQB10201  676      VGTSGCCRGSSNYRS  CRGSSNYRS  6       0.0242      38472.2   100.00  0.37   tr_C9ZL71_
 HLA-DQA10501-DQB10201  677      TSGCCRGSSNYRSNA  CRGSSNYRS  4       0.0233      38857.5   100.00  0.34   tr_C9ZL71_
 HLA-DQA10501-DQB10201  678      GTSGCCRGSSNYRSN  CRGSSNYRS  5       0.0199      40320.5   100.00  0.39   tr_C9ZL71_
-----------------------------------------------------------------------------------------------

Allele: HLA-DQA10501-DQB10201. Number of high binders 0. Number of weak binders 45. Number of peptides 678

-----------------------------------------------------------------------------------------------
-----------------------------------------------------------------------------------------------
                Allele  pos                  peptide       core Of 1-log50k(aff) affinity(nM)    %Rank Relia     Identity Bind Level
-----------------------------------------------------------------------------------------------
 HLA-DQA10501-DQB10201    1      YLHNVFFGVEEYDVA  NVFFGVEEY  3       0.6441         47.0     0.60  0.34   tr_D0A0U3_          WB
 HLA-DQA10501-DQB10201    2      KITTAVLGVVDEAML  VLGVVDEAM  5       0.6426         47.8     0.60  0.48   tr_D0A0U3_          WB
 HLA-DQA10501-DQB10201    3      MYLHNVFFGVEEYDV  NVFFGVEEY  4       0.6394         49.5     0.60  0.40   tr_D0A0U3_          WB
 HLA-DQA10501-DQB10201    4      TKITTAVLGVVDEAM  VLGVVDEAM  6       0.6355         51.6     0.70  0.53   tr_D0A0U3_          WB
 HLA-DQA10501-DQB10201    5      DVYVIGLTAADPVVI  IGLTAADPV  4       0.6293         55.2     0.80  0.50   tr_D0A0U3_          WB
 HLA-DQA10501-DQB10201    6      DGQLLVYGWITGEVL  YGWITGEVL  6       0.6251         57.8     0.80  0.70   tr_D0A0U3_          WB
 HLA-DQA10501-DQB10201    7      SGDVYVIGLTAADPV  IGLTAADPV  6       0.6231         59.0     0.90  0.56   tr_D0A0U3_          WB
 HLA-DQA10501-DQB10201    8      GDVYVIGLTAADPVV  IGLTAADPV  5       0.6226         59.3     0.90  0.55   tr_D0A0U3_          WB
 HLA-DQA10501-DQB10201    9      LHNVFFGVEEYDVAQ  NVFFGVEEY  2       0.6199         61.1     0.90  0.34   tr_D0A0U3_          WB
 HLA-DQA10501-DQB10201   10      LVYGWITGEVLAQAL  YGWITGEVL  2       0.6168         63.2     1.00  0.48   tr_D0A0U3_          WB
 HLA-DQA10501-DQB10201   11      VYVIGLTAADPVVIA  IGLTAADPV  3       0.6140         65.2     1.00  0.51   tr_D0A0U3_          WB
 HLA-DQA10501-DQB10201   12      GQLLVYGWITGEVLA  YGWITGEVL  5       0.6122         66.4     1.10  0.70   tr_D0A0U3_          WB
 HLA-DQA10501-DQB10201   13      QLLVYGWITGEVLAQ  YGWITGEVL  4       0.6110         67.3     1.10  0.65   tr_D0A0U3_          WB
 HLA-DQA10501-DQB10201   14      FMYLHNVFFGVEEYD  NVFFGVEEY  5       0.6102         67.9     1.10  0.46   tr_D0A0U3_          WB
 HLA-DQA10501-DQB10201   15      LLVYGWITGEVLAQA  YGWITGEVL  3       0.6092         68.6     1.10  0.56   tr_D0A0U3_          WB
 HLA-DQA10501-DQB10201   16      ITTAVLGVVDEAMLK  VLGVVDEAM  4       0.6060         71.0     1.20  0.50   tr_D0A0U3_          WB
 HLA-DQA10501-DQB10201   17      FVPFFDVVLLHEEFF  VVLLHEEFF  6       0.6053         71.6     1.20  0.41   tr_D0A0U3_          WB
 HLA-DQA10501-DQB10201   18      HNVFFGVEEYDVAQR  NVFFGVEEY  1       0.5994         76.3     1.30  0.34   tr_D0A0U3_          WB
 HLA-DQA10501-DQB10201   19      NVFFGVEEYDVAQRI  FFGVEEYDV  2       0.5979         77.5     1.30  0.25   tr_D0A0U3_          WB
 HLA-DQA10501-DQB10201   20      YVIGLTAADPVVIAA  IGLTAADPV  2       0.5897         84.7     1.50  0.51   tr_D0A0U3_          WB
 HLA-DQA10501-DQB10201   21      VPFFDVVLLHEEFFN  VVLLHEEFF  5       0.5854         88.7     1.60  0.42   tr_D0A0U3_          WB
 HLA-DQA10501-DQB10201   22      VYGWITGEVLAQALS  YGWITGEVL  1       0.5850         89.1     1.70  0.46   tr_D0A0U3_          WB
 HLA-DQA10501-DQB10201   23      TTAVLGVVDEAMLKT  VLGVVDEAM  3       0.5828         91.3     1.70  0.51   tr_D0A0U3_          WB
```

HLA-DQA10501-DQB10201	24	DTIVSPFSGAAEELQ	PFSGAAEEL	5	0.5805	93.6	1.80	0.59	tr_D0A0U3_	WB
HLA-DQA10501-DQB10201	25	SLYDQRRYVIDDLVI	RRYVIDDLV	5	0.5783	95.8	1.80	0.54	tr_D0A0U3_	WB
HLA-DQA10501-DQB10201	26	IVSPFSGAAEELQRE	PFSGAAEEL	3	0.5721	102.5	2.00	0.50	tr_D0A0U3_	WB
HLA-DQA10501-DQB10201	27	VSPFSGAAEELQREW	PFSGAAEEL	2	0.5715	103.2	2.00	0.45	tr_D0A0U3_	WB
HLA-DQA10501-DQB10201	28	QSLYDQRRYVIDDLV	RRYVIDDLV	6	0.5705	104.3	2.50	0.62	tr_D0A0U3_	WB
HLA-DQA10501-DQB10201	29	TIVSPFSGAAEELQR	PFSGAAEEL	4	0.5693	105.6	2.50	0.54	tr_D0A0U3_	WB
HLA-DQA10501-DQB10201	30	IVIRSTDAAAIEDVL	DAAAIEDVL	6	0.5658	109.7	2.50	0.45	tr_D0A0U3_	WB
HLA-DQA10501-DQB10201	31	PFFDVVLLHEEFFNA	VVLLHEEFF	4	0.5655	110.1	2.50	0.44	tr_D0A0U3_	WB
HLA-DQA10501-DQB10201	32	GFMYLHNVFFGVEEY	NVFFGVEEY	6	0.5633	112.8	2.50	0.66	tr_D0A0U3_	WB
HLA-DQA10501-DQB10201	33	FFDVVLLHEEFFNAF	VVLLHEEFF	3	0.5625	113.8	2.50	0.44	tr_D0A0U3_	WB
HLA-DQA10501-DQB10201	34	TAVLGVVDEAMLKTP	VLGVVDEAM	2	0.5580	119.3	2.50	0.57	tr_D0A0U3_	WB
HLA-DQA10501-DQB10201	35	YDQRRYVIDDLVIGD	RRYVIDDLV	3	0.5494	131.1	3.00	0.56	tr_D0A0U3_	WB
HLA-DQA10501-DQB10201	36	LYDQRRYVIDDLVIG	RRYVIDDLV	4	0.5486	132.2	3.00	0.56	tr_D0A0U3_	WB
HLA-DQA10501-DQB10201	37	YGWITGEVLAQALSV	YGWITGEVL	0	0.5483	132.5	3.00	0.37	tr_D0A0U3_	WB
HLA-DQA10501-DQB10201	38	SPFSGAAEELQREWS	FSGAAEELQ	2	0.5452	137.2	3.00	0.42	tr_D0A0U3_	WB
HLA-DQA10501-DQB10201	39	VFFGVEEYDVAQRIL	FFGVEEYDV	1	0.5425	141.1	3.50	0.32	tr_D0A0U3_	WB
HLA-DQA10501-DQB10201	40	STKITTAVLGVVDEA	AVLGVVDEA	6	0.5387	147.1	3.50	0.58	tr_D0A0U3_	WB
HLA-DQA10501-DQB10201	41	SSLITLLAEQDVVAF	LITLLAEQD	2	0.5373	149.3	3.50	0.29	tr_D0A0U3_	WB
HLA-DQA10501-DQB10201	42	VSSLITLLAEQDVVA	LITLLAEQD	3	0.5364	150.9	3.50	0.35	tr_D0A0U3_	WB
HLA-DQA10501-DQB10201	43	DQRRYVIDDLVIGDY	RRYVIDDLV	2	0.5350	153.1	3.50	0.50	tr_D0A0U3_	WB
HLA-DQA10501-DQB10201	44	TVSSLITLLAEQDVV	LITLLAEQD	4	0.5349	153.3	3.50	0.41	tr_D0A0U3_	WB
HLA-DQA10501-DQB10201	45	VIGLTAADPVVIAAH	IGLTAADPV	1	0.5312	159.6	4.00	0.55	tr_D0A0U3_	WB
HLA-DQA10501-DQB10201	46	DRPTALHAAVEMEEG	LHAAVEMEE	5	0.5301	161.4	4.00	0.32	tr_D0A0U3_	WB
HLA-DQA10501-DQB10201	47	VFVPFFDVVLLHEEF	DVVLLHEEF	6	0.5292	163.0	4.00	0.47	tr_D0A0U3_	WB
HLA-DQA10501-DQB10201	48	VIRSTDAAAIEDVLK	DAAAIEDVL	5	0.5283	164.7	4.00	0.43	tr_D0A0U3_	WB
HLA-DQA10501-DQB10201	49	PDRPTALHAAVEMEE	LHAAVEMEE	6	0.5265	167.9	4.00	0.50	tr_D0A0U3_	WB
HLA-DQA10501-DQB10201	50	FDVVLLHEEFFNAFE	VVLLHEEFF	2	0.5249	170.8	4.00	0.47	tr_D0A0U3_	WB
HLA-DQA10501-DQB10201	51	VVLLHEEFFNAFEGV	EFFNAFEGV	6	0.5245	171.6	4.00	0.34	tr_D0A0U3_	WB
HLA-DQA10501-DQB10201	52	MWREALAFAASPFVD	LAFAASPFV	5	0.5225	175.3	4.50	0.47	tr_D0A0U3_	WB
HLA-DQA10501-DQB10201	53	PMKAMNATALVDSIF	ATALVDSIF	6	0.5210	178.1	4.50	0.38	tr_D0A0U3_	WB
HLA-DQA10501-DQB10201	54	SLITLLAEQDVVAFA	LITLLAEQD	1	0.5197	180.7	4.50	0.28	tr_D0A0U3_	WB
HLA-DQA10501-DQB10201	55	HDSARVFVPFFDVVL	SARVFVPFF	2	0.5190	182.0	4.50	0.43	tr_D0A0U3_	WB
HLA-DQA10501-DQB10201	56	DSARVFVPFFDVVLL	SARVFVPFF	1	0.5184	183.3	4.50	0.41	tr_D0A0U3_	WB
HLA-DQA10501-DQB10201	57	LNSHDSARVFVPFFD	SARVFVPFF	5	0.5157	188.8	4.50	0.60	tr_D0A0U3_	WB
HLA-DQA10501-DQB10201	58	TGEVLAQALSVPERL	QALSVPERL	6	0.5153	189.5	4.50	0.54	tr_D0A0U3_	WB
HLA-DQA10501-DQB10201	59	AVLGVVDEAMLKTPG	VLGVVDEAM	1	0.5146	190.9	4.50	0.61	tr_D0A0U3_	WB
HLA-DQA10501-DQB10201	60	GTVSSLITLLAEQDV	LITLLAEQD	5	0.5141	191.9	4.50	0.48	tr_D0A0U3_	WB
HLA-DQA10501-DQB10201	61	MKAMNATALVDSIFA	ATALVDSIF	5	0.5130	194.3	5.00	0.34	tr_D0A0U3_	WB
HLA-DQA10501-DQB10201	62	SHDSARVFVPFFDVV	SARVFVPFF	3	0.5116	197.2	5.00	0.52	tr_D0A0U3_	WB
HLA-DQA10501-DQB10201	63	RPTALHAAVEMEEGA	LHAAVEMEE	4	0.5105	199.5	5.00	0.33	tr_D0A0U3_	WB
HLA-DQA10501-DQB10201	64	FDTIVSPFSGAAEEL	PFSGAAEEL	6	0.5098	201.2	5.00	0.80	tr_D0A0U3_	WB
HLA-DQA10501-DQB10201	65	NMWREALAFAASPFV	LAFAASPFV	6	0.5088	203.4	5.00	0.47	tr_D0A0U3_	WB
HLA-DQA10501-DQB10201	66	QDSRLFFDTIVSPFS	FFDTIVSPF	5	0.5050	211.9	5.00	0.43	tr_D0A0U3_	WB
HLA-DQA10501-DQB10201	67	DVVLLHEEFFNAFEG	VVLLHEEFF	1	0.5050	211.9	5.00	0.46	tr_D0A0U3_	WB
HLA-DQA10501-DQB10201	68	LITLLAEQDVVAFAP	LITLLAEQD	0	0.5042	213.7	5.50	0.17	tr_D0A0U3_	WB
HLA-DQA10501-DQB10201	69	NQDSRLFFDTIVSPF	FFDTIVSPF	6	0.5036	215.1	5.50	0.48	tr_D0A0U3_	WB
HLA-DQA10501-DQB10201	70	KTPGVAFIDPVPLAP	VAFIDPVPL	4	0.5009	221.3	5.50	0.42	tr_D0A0U3_	WB
HLA-DQA10501-DQB10201	71	NSHDSARVFVPFFDV	SARVFVPFF	4	0.5007	221.9	5.50	0.58	tr_D0A0U3_	WB
HLA-DQA10501-DQB10201	72	WREALAFAASPFVDG	LAFAASPFV	4	0.4982	228.0	5.50	0.47	tr_D0A0U3_	WB
HLA-DQA10501-DQB10201	73	MLKTPGVAFIDPVPL	VAFIDPVPL	6	0.4980	228.4	5.50	0.45	tr_D0A0U3_	WB

HLA-DQA10501-DQB10201	74	EVLAQALSVPERLTD	QALSVPERL	4	0.4969	231.3	6.00	0.58	tr_D0A0U3_	WB
HLA-DQA10501-DQB10201	75	GEVLAQALSVPERLT	QALSVPERL	5	0.4962	233.1	6.00	0.54	tr_D0A0U3_	WB
HLA-DQA10501-DQB10201	76	PFSGAAEELQREWST	FSGAAEELQ	1	0.4954	234.9	6.00	0.46	tr_D0A0U3_	WB
HLA-DQA10501-DQB10201	77	QRRYVIDDLVIGDYG	RRYVIDDLV	1	0.4939	238.8	6.00	0.51	tr_D0A0U3_	WB
HLA-DQA10501-DQB10201	78	LKTPGVAFIDPVPLA	VAFIDPVPL	5	0.4919	244.1	6.00	0.42	tr_D0A0U3_	WB
HLA-DQA10501-DQB10201	79	PTALHAAVEMEEGAS	LHAAVEMEE	3	0.4914	245.4	6.00	0.34	tr_D0A0U3_	WB
HLA-DQA10501-DQB10201	80	TALHAAVEMEEGASL	LHAAVEMEE	2	0.4887	252.7	6.50	0.32	tr_D0A0U3_	WB
HLA-DQA10501-DQB10201	81	HIVIRSTDAAAIEDV	TDAAAIEDV	6	0.4868	257.8	6.50	0.47	tr_D0A0U3_	WB
HLA-DQA10501-DQB10201	82	KAMNATALVDSIFAQ	ATALVDSIF	4	0.4851	262.8	6.50	0.34	tr_D0A0U3_	WB
HLA-DQA10501-DQB10201	83	IRSTDAAAIEDVLKK	DAAAIEDVL	4	0.4845	264.5	6.50	0.43	tr_D0A0U3_	WB
HLA-DQA10501-DQB10201	84	FGTVSSLITLLAEQD	LITLLAEQD	6	0.4825	270.1	7.00	0.56	tr_D0A0U3_	WB
HLA-DQA10501-DQB10201	85	SARVFVPFFDVVLLH	SARVFVPFF	0	0.4793	279.8	7.00	0.31	tr_D0A0U3_	WB
HLA-DQA10501-DQB10201	86	DSRLFFDTIVSPFSG	FFDTIVSPF	4	0.4770	286.7	7.00	0.42	tr_D0A0U3_	WB
HLA-DQA10501-DQB10201	87	HEEFFNAFEGVSSAD	EFFNAFEGV	2	0.4758	290.5	7.50	0.38	tr_D0A0U3_	WB
HLA-DQA10501-DQB10201	88	VLLHEEFFNAFEGVS	EFFNAFEGV	5	0.4755	291.5	7.50	0.47	tr_D0A0U3_	WB
HLA-DQA10501-DQB10201	89	EEFFNAFEGVSSADR	EFFNAFEGV	1	0.4747	294.1	7.50	0.34	tr_D0A0U3_	WB
HLA-DQA10501-DQB10201	90	TPGVAFIDPVPLAPR	VAFIDPVPL	3	0.4741	295.9	7.50	0.43	tr_D0A0U3_	WB
HLA-DQA10501-DQB10201	91	ENMWREALAFAASPF	ALAFAASPF	6	0.4695	311.1	8.00	0.57	tr_D0A0U3_	WB
HLA-DQA10501-DQB10201	92	ALHAAVEMEEGASLL	LHAAVEMEE	1	0.4693	311.7	8.00	0.29	tr_D0A0U3_	WB
HLA-DQA10501-DQB10201	93	FFGVEEYDVAQRILR	FFGVEEYDV	0	0.4688	313.6	8.00	0.31	tr_D0A0U3_	WB
HLA-DQA10501-DQB10201	94	LLHEEFFNAFEGVSS	EFFNAFEGV	4	0.4684	314.8	8.00	0.43	tr_D0A0U3_	WB
HLA-DQA10501-DQB10201	95	EFFNAFEGVSSADRV	EFFNAFEGV	0	0.4681	315.8	8.00	0.21	tr_D0A0U3_	WB
HLA-DQA10501-DQB10201	96	REALAFAASPFVDGQ	LAFAASPFV	3	0.4676	317.6	8.00	0.49	tr_D0A0U3_	WB
HLA-DQA10501-DQB10201	97	GWITGEVLAQALSVP	EVLAQALSV	5	0.4668	320.3	8.00	0.31	tr_D0A0U3_	WB
HLA-DQA10501-DQB10201	98	ITLLAEQDVVAFAPL	LLAEQDVVA	2	0.4661	322.8	8.00	0.28	tr_D0A0U3_	WB
HLA-DQA10501-DQB10201	99	MNATALVDSIFAQSV	VDSIFAQSV	6	0.4648	327.1	8.00	0.35	tr_D0A0U3_	WB
HLA-DQA10501-DQB10201	100	DSGDVYVIGLTAADP	VIGLTAADP	6	0.4644	328.8	8.50	0.66	tr_D0A0U3_	WB
HLA-DQA10501-DQB10201	101	AMNATALVDSIFAQS	ATALVDSIF	3	0.4618	338.0	8.50	0.34	tr_D0A0U3_	WB
HLA-DQA10501-DQB10201	102	VLVPSMLQFAIENMW	MLQFAIENM	5	0.4601	344.4	8.50	0.41	tr_D0A0U3_	WB
HLA-DQA10501-DQB10201	103	QAPFNGVSVLIPDRP	FNGVSVLIP	3	0.4585	350.3	9.00	0.34	tr_D0A0U3_	WB
HLA-DQA10501-DQB10201	104	LKKTLATFSVEPQSV	LATFSVEPQ	4	0.4584	350.6	9.00	0.43	tr_D0A0U3_	WB
HLA-DQA10501-DQB10201	105	ARVFVPFFDVVLLHE	FFDVVLLHE	6	0.4568	356.8	9.00	0.38	tr_D0A0U3_	WB
HLA-DQA10501-DQB10201	106	VPSMLQFAIENMWRE	MLQFAIENM	3	0.4556	361.7	9.00	0.33	tr_D0A0U3_	WB
HLA-DQA10501-DQB10201	107	RSTDAAAIEDVLKKT	DAAAIEDVL	3	0.4553	362.7	9.00	0.45	tr_D0A0U3_	WB
HLA-DQA10501-DQB10201	108	WITGEVLAQALSVPE	EVLAQALSV	4	0.4552	363.1	9.00	0.32	tr_D0A0U3_	WB
HLA-DQA10501-DQB10201	109	LVPSMLQFAIENMWR	MLQFAIENM	4	0.4538	368.6	9.50	0.35	tr_D0A0U3_	WB
HLA-DQA10501-DQB10201	110	KKTLATFSVEPQSVV	LATFSVEPQ	3	0.4528	372.5	9.50	0.41	tr_D0A0U3_	WB
HLA-DQA10501-DQB10201	111	VLAQALSVPERLTDR	QALSVPERL	3	0.4518	376.5	9.50	0.66	tr_D0A0U3_	WB
HLA-DQA10501-DQB10201	112	TLLAEQDVVAFAPLT	LLAEQDVVA	1	0.4518	376.8	9.50	0.25	tr_D0A0U3_	WB
HLA-DQA10501-DQB10201	113	LHEEFFNAFEGVSSA	EFFNAFEGV	3	0.4511	379.4	9.50	0.44	tr_D0A0U3_	WB
HLA-DQA10501-DQB10201	114	EALAFAASPFVDGQV	LAFAASPFV	2	0.4511	379.8	9.50	0.48	tr_D0A0U3_	WB
HLA-DQA10501-DQB10201	115	IPDRPTALHAAVEME	TALHAAVEM	5	0.4508	380.7	9.50	0.54	tr_D0A0U3_	WB
HLA-DQA10501-DQB10201	116	APFNGVSVLIPDRPT	VSVLIPDRP	5	0.4506	381.6	9.50	0.38	tr_D0A0U3_	WB
HLA-DQA10501-DQB10201	117	SPMKAMNATALVDSI	NATALVDSI	6	0.4496	385.7	9.50	0.34	tr_D0A0U3_	WB
HLA-DQA10501-DQB10201	118	HAAVEMEEGASLLEV	EEGASLLEV	6	0.4485	390.5	10.00	0.48	tr_D0A0U3_	
HLA-DQA10501-DQB10201	119	SRLFFDTIVSPFSGA	FFDTIVSPF	3	0.4468	397.4	10.00	0.42	tr_D0A0U3_	
HLA-DQA10501-DQB10201	120	PGVAFIDPVPLAPRL	VAFIDPVPL	2	0.4463	399.9	10.00	0.48	tr_D0A0U3_	
HLA-DQA10501-DQB10201	121	HLNSHDSARVFVPFF	SARVFVPFF	6	0.4457	402.6	10.00	0.75	tr_D0A0U3_	
HLA-DQA10501-DQB10201	122	KTLATFSVEPQSVVV	LATFSVEPQ	2	0.4457	402.6	10.00	0.43	tr_D0A0U3_	
HLA-DQA10501-DQB10201	123	IGLTAADPVVIAAHL	IGLTAADPV	0	0.4421	418.4	11.00	0.50	tr_D0A0U3_	

HLA-DQA10501-DQB10201	124	DTARFIRNLVADSRT	IRNLVADSR	5	0.4408	424.2	11.00	0.47	tr_D0A0U3_
HLA-DQA10501-DQB10201	125	PSMLQFAIENMWREA	MLQFAIENM	2	0.4375	439.5	11.00	0.32	tr_D0A0U3_
HLA-DQA10501-DQB10201	126	NATALVDSIFAQSVV	VDSIFAQSV	5	0.4373	440.6	11.00	0.38	tr_D0A0U3_
HLA-DQA10501-DQB10201	127	GDTARFIRNLVADSR	IRNLVADSR	6	0.4357	448.2	11.00	0.50	tr_D0A0U3_
HLA-DQA10501-DQB10201	128	MQSLYDQRRYVIDDL	QRRYVIDDL	6	0.4349	452.1	12.00	0.90	tr_D0A0U3_
HLA-DQA10501-DQB10201	129	PFNGVSVLIPDRPTA	VSVLIPDRP	4	0.4331	461.4	12.00	0.40	tr_D0A0U3_
HLA-DQA10501-DQB10201	130	SHCYPSEVEMQAPFN	EVEMQAPFN	6	0.4325	464.0	12.00	0.40	tr_D0A0U3_
HLA-DQA10501-DQB10201	131	RVFVPFFDVVLLHEE	FFDVVLLHE	5	0.4321	466.3	12.00	0.34	tr_D0A0U3_
HLA-DQA10501-DQB10201	132	PLSLLGFATAYFMRV	LLGFATAYF	3	0.4320	466.5	12.00	0.41	tr_D0A0U3_
HLA-DQA10501-DQB10201	133	TLATFSVEPQSVVVL	TFSVEPQSV	3	0.4314	469.8	12.00	0.32	tr_D0A0U3_
HLA-DQA10501-DQB10201	134	GFATAYFMRVIISPM	FMRVIISPM	6	0.4311	471.1	12.00	0.50	tr_D0A0U3_
HLA-DQA10501-DQB10201	135	SMLQFAIENMWREAL	MLQFAIENM	1	0.4302	475.6	12.00	0.30	tr_D0A0U3_
HLA-DQA10501-DQB10201	136	HWADEITASEVVQKF	TASEVVQKF	6	0.4299	477.2	12.00	0.25	tr_D0A0U3_
HLA-DQA10501-DQB10201	137	STDAAAIEDVLKKTL	DAAAIEDVL	2	0.4299	477.3	12.00	0.51	tr_D0A0U3_
HLA-DQA10501-DQB10201	138	LSLLGFATAYFMRVI	LLGFATAYF	2	0.4295	479.7	12.00	0.37	tr_D0A0U3_
HLA-DQA10501-DQB10201	139	GDFGTVSSLITLLAE	FGTVSSLIT	2	0.4289	482.4	12.00	0.25	tr_D0A0U3_
HLA-DQA10501-DQB10201	140	LPHWADEITASEVVQ	ADEITASEV	4	0.4287	483.5	12.00	0.28	tr_D0A0U3_
HLA-DQA10501-DQB10201	141	LHAAVEMEEGASLLE	HAAVEMEEG	1	0.4282	486.1	12.00	0.25	tr_D0A0U3_
HLA-DQA10501-DQB10201	142	WADEITASEVVQKFY	EITASEVVQ	3	0.4271	492.1	12.00	0.26	tr_D0A0U3_
HLA-DQA10501-DQB10201	143	NLPHWADEITASEVV	ADEITASEV	5	0.4256	500.1	13.00	0.34	tr_D0A0U3_
HLA-DQA10501-DQB10201	144	LGDFGTVSSLITLLA	FGTVSSLIT	3	0.4248	504.5	13.00	0.31	tr_D0A0U3_
HLA-DQA10501-DQB10201	145	RTPLSLLGFATAYFM	LLGFATAYF	5	0.4242	507.8	13.00	0.55	tr_D0A0U3_
HLA-DQA10501-DQB10201	146	RRYVIDDLVIGDYGG	RRYVIDDLV	0	0.4234	512.0	13.00	0.49	tr_D0A0U3_
HLA-DQA10501-DQB10201	147	KIEKRFVEAVNAGFN	FVEAVNAGF	5	0.4231	513.9	13.00	0.43	tr_D0A0U3_
HLA-DQA10501-DQB10201	148	YVLVPSMLQFAIENM	MLQFAIENM	6	0.4231	513.9	13.00	0.59	tr_D0A0U3_
HLA-DQA10501-DQB10201	149	DVLKKTLATFSVEPQ	LATFSVEPQ	6	0.4224	517.8	13.00	0.55	tr_D0A0U3_
HLA-DQA10501-DQB10201	150	IEKRFVEAVNAGFNA	FVEAVNAGF	4	0.4204	529.0	13.00	0.41	tr_D0A0U3_
HLA-DQA10501-DQB10201	151	LAQALSVPERLTDRK	QALSVPERL	2	0.4198	532.3	13.00	0.73	tr_D0A0U3_
HLA-DQA10501-DQB10201	152	FFNAFEGVSSADRVL	FEGVSSADR	4	0.4190	536.9	13.00	0.28	tr_D0A0U3_
HLA-DQA10501-DQB10201	153	LLAEQDVVAFAPLTG	DVVAFAPLT	5	0.4188	538.5	13.00	0.28	tr_D0A0U3_
HLA-DQA10501-DQB10201	154	SLLGFATAYFMRVII	LLGFATAYF	1	0.4187	538.7	13.00	0.31	tr_D0A0U3_
HLA-DQA10501-DQB10201	155	VLKKTLATFSVEPQS	LATFSVEPQ	5	0.4185	540.0	13.00	0.51	tr_D0A0U3_
HLA-DQA10501-DQB10201	156	RRTPLSLLGFATAYF	LLGFATAYF	6	0.4184	540.8	13.00	0.71	tr_D0A0U3_
HLA-DQA10501-DQB10201	157	QKIEKRFVEAVNAGF	FVEAVNAGF	6	0.4182	541.9	13.00	0.49	tr_D0A0U3_
HLA-DQA10501-DQB10201	158	RLFFDTIVSPFSGAA	FFDTIVSPF	2	0.4177	544.9	14.00	0.45	tr_D0A0U3_
HLA-DQA10501-DQB10201	159	ALAFAASPFVDGQVV	LAFAASPFV	1	0.4171	548.2	14.00	0.47	tr_D0A0U3_
HLA-DQA10501-DQB10201	160	ITGEVLAQALSVPER	EVLAQALSV	3	0.4162	553.8	14.00	0.32	tr_D0A0U3_
HLA-DQA10501-DQB10201	161	HCYPSEVEMQAPFNG	EVEMQAPFN	5	0.4137	568.8	14.00	0.44	tr_D0A0U3_
HLA-DQA10501-DQB10201	162	LIPDRPTALHAAVEM	TALHAAVEM	6	0.4117	581.5	14.00	0.75	tr_D0A0U3_
HLA-DQA10501-DQB10201	163	TARFIRNLVADSRTR	IRNLVADSR	4	0.4116	582.0	14.00	0.46	tr_D0A0U3_
HLA-DQA10501-DQB10201	164	FNAFEGVSSADRVLF	FEGVSSADR	3	0.4108	587.1	14.00	0.34	tr_D0A0U3_
HLA-DQA10501-DQB10201	165	GVAFIDPVPLAPRLN	VAFIDPVPL	1	0.4097	594.0	15.00	0.57	tr_D0A0U3_
HLA-DQA10501-DQB10201	166	ISPMKAMNATALVDS	AMNATALVD	5	0.4096	594.6	15.00	0.41	tr_D0A0U3_
HLA-DQA10501-DQB10201	167	FNGVSVLIPDRPTAL	VSVLIPDRP	3	0.4095	595.5	15.00	0.43	tr_D0A0U3_
HLA-DQA10501-DQB10201	168	EMQAPFNGVSVLIPD	FNGVSVLIP	5	0.4093	596.9	15.00	0.50	tr_D0A0U3_
HLA-DQA10501-DQB10201	169	GYRLLDAPDGIMMFD	RLLDAPDGI	2	0.4088	600.0	15.00	0.50	tr_D0A0U3_
HLA-DQA10501-DQB10201	170	WSTKITTAVLGVVDE	TAVLGVVDE	6	0.4085	602.1	15.00	0.50	tr_D0A0U3_
HLA-DQA10501-DQB10201	171	GEMRYGPFADDGCFL	PFADDGCFL	6	0.4080	605.3	15.00	0.49	tr_D0A0U3_
HLA-DQA10501-DQB10201	172	PLGDFGTVSSLITLL	FGTVSSLIT	4	0.4074	608.8	15.00	0.44	tr_D0A0U3_
HLA-DQA10501-DQB10201	173	ATALVDSIFAQSVVN	VDSIFAQSV	4	0.4065	615.0	15.00	0.41	tr_D0A0U3_

HLA-DQA10501-DQB10201	174	ATAYFMRVIISPMKA	FMRVIISPM	4	0.4058	619.8	15.00	0.41	tr_D0A0U3_
HLA-DQA10501-DQB10201	175	AAVEMEEGASLLEVR	EEGASLLEV	5	0.4051	624.4	15.00	0.44	tr_D0A0U3_
HLA-DQA10501-DQB10201	176	AMLKTPGVAFIDPVP	PGVAFIDPV	5	0.4049	625.6	15.00	0.52	tr_D0A0U3_
HLA-DQA10501-DQB10201	177	EKRFVEAVNAGFNAS	FVEAVNAGF	3	0.4041	631.2	15.00	0.41	tr_D0A0U3_
HLA-DQA10501-DQB10201	178	IISPMKAMNATALVD	AMNATALVD	6	0.4039	632.8	15.00	0.44	tr_D0A0U3_
HLA-DQA10501-DQB10201	179	MLQFAIENMWREALA	MLQFAIENM	0	0.4036	634.7	15.00	0.23	tr_D0A0U3_
HLA-DQA10501-DQB10201	180	CYPSEVEMQAPFNGV	EVEMQAPFN	4	0.4030	638.9	16.00	0.46	tr_D0A0U3_
HLA-DQA10501-DQB10201	181	FATAYFMRVIISPMK	FMRVIISPM	5	0.4017	647.8	16.00	0.47	tr_D0A0U3_
HLA-DQA10501-DQB10201	182	GPLGDFGTVSSLITL	FGTVSSLIT	5	0.4015	649.4	16.00	0.47	tr_D0A0U3_
HLA-DQA10501-DQB10201	183	RGYRLLDAPDGIMMF	RLLDAPDGI	3	0.4005	656.3	16.00	0.52	tr_D0A0U3_
HLA-DQA10501-DQB10201	184	DFGTVSSLITLLAEQ	FGTVSSLIT	1	0.3994	664.3	16.00	0.27	tr_D0A0U3_
HLA-DQA10501-DQB10201	185	LHIVIRSTDAAAIED	STDAAAIED	6	0.3990	667.2	16.00	0.31	tr_D0A0U3_
HLA-DQA10501-DQB10201	186	SIFAQSVVNVGEMRY	VVNVGEMRY	6	0.3984	671.3	16.00	0.43	tr_D0A0U3_
HLA-DQA10501-DQB10201	187	LGFMYLHNVFFGVEE	HNVFFGVEE	6	0.3984	671.6	16.00	0.34	tr_D0A0U3_
HLA-DQA10501-DQB10201	188	LATFSVEPQSVVVLD	TFSVEPQSV	2	0.3978	676.0	16.00	0.40	tr_D0A0U3_
HLA-DQA10501-DQB10201	189	VLGVVDEAMLKTPGV	VLGVVDEAM	0	0.3978	676.0	16.00	0.69	tr_D0A0U3_
HLA-DQA10501-DQB10201	190	VEMQAPFNGVSVLIP	FNGVSVLIP	6	0.3971	680.5	16.00	0.56	tr_D0A0U3_
HLA-DQA10501-DQB10201	191	IGRGYRLLDAPDGIM	RLLDAPDGI	5	0.3969	682.1	16.00	0.50	tr_D0A0U3_
HLA-DQA10501-DQB10201	192	TNLPHWADEITASEV	ADEITASEV	6	0.3967	683.8	16.00	0.42	tr_D0A0U3_
HLA-DQA10501-DQB10201	193	VGPLGDFGTVSSLIT	FGTVSSLIT	6	0.3958	690.6	17.00	0.51	tr_D0A0U3_
HLA-DQA10501-DQB10201	194	AVEMEEGASLLEVRE	EEGASLLEV	4	0.3952	694.9	17.00	0.40	tr_D0A0U3_
HLA-DQA10501-DQB10201	195	MQAPFNGVSVLIPDR	FNGVSVLIP	4	0.3939	704.8	17.00	0.50	tr_D0A0U3_
HLA-DQA10501-DQB10201	196	PHWADEITASEVVQK	EITASEVVQ	5	0.3937	706.0	17.00	0.28	tr_D0A0U3_
HLA-DQA10501-DQB10201	197	TAYFMRVIISPMKAM	FMRVIISPM	3	0.3934	708.2	17.00	0.44	tr_D0A0U3_
HLA-DQA10501-DQB10201	198	HNQDSRLFFDTIVSP	LFFDTIVSP	6	0.3925	715.2	17.00	0.31	tr_D0A0U3_
HLA-DQA10501-DQB10201	199	ARFIRNLVADSRTRS	IRNLVADSR	3	0.3913	724.6	17.00	0.49	tr_D0A0U3_
HLA-DQA10501-DQB10201	200	LFFDTIVSPFSGAAE	FFDTIVSPF	1	0.3911	726.7	17.00	0.49	tr_D0A0U3_
HLA-DQA10501-DQB10201	201	YRLLDAPDGIMMFDS	RLLDAPDGI	1	0.3906	730.1	17.00	0.49	tr_D0A0U3_
HLA-DQA10501-DQB10201	202	TPLSLLGFATAYFMR	LLGFATAYF	4	0.3902	733.9	17.00	0.56	tr_D0A0U3_
HLA-DQA10501-DQB10201	203	GRGYRLLDAPDGIMM	RLLDAPDGI	4	0.3901	734.1	17.00	0.51	tr_D0A0U3_
HLA-DQA10501-DQB10201	204	NAFEGVSSADRVLFA	EGVSSADRV	3	0.3891	742.0	18.00	0.28	tr_D0A0U3_
HLA-DQA10501-DQB10201	205	TALVDSIFAQSVVNV	VDSIFAQSV	3	0.3884	747.8	18.00	0.37	tr_D0A0U3_
HLA-DQA10501-DQB10201	206	VIGRGYRLLDAPDGI	RLLDAPDGI	6	0.3868	761.2	18.00	0.49	tr_D0A0U3_
HLA-DQA10501-DQB10201	207	ADEITASEVVQKFYT	TASEVVQKF	4	0.3860	768.0	18.00	0.27	tr_D0A0U3_
HLA-DQA10501-DQB10201	208	KMERNVIHLSPTLEQ	VIHLSPTLE	5	0.3858	769.6	18.00	0.46	tr_D0A0U3_
HLA-DQA10501-DQB10201	209	WSMARALNASIPPLT	ALNASIPPL	5	0.3856	771.2	18.00	0.35	tr_D0A0U3_
HLA-DQA10501-DQB10201	210	KRFVEAVNAGFNASM	FVEAVNAGF	2	0.3845	780.2	18.00	0.39	tr_D0A0U3_
HLA-DQA10501-DQB10201	211	DSRTRSAYVLVPSML	SAYVLVPSM	5	0.3843	782.2	18.00	0.37	tr_D0A0U3_
HLA-DQA10501-DQB10201	212	LAEQDVVAFAPLTGS	DVVAFAPLT	4	0.3821	800.7	19.00	0.33	tr_D0A0U3_
HLA-DQA10501-DQB10201	213	VWSMARALNASIPPL	ALNASIPPL	6	0.3816	805.4	19.00	0.35	tr_D0A0U3_
HLA-DQA10501-DQB10201	214	IDGQLLVYGWITGEV	VYGWITGEV	6	0.3810	809.9	19.00	0.72	tr_D0A0U3_
HLA-DQA10501-DQB10201	215	KADIEIEVFSLLHHQ	KADIEIEVF	0	0.3804	815.3	19.00	0.54	tr_D0A0U3_
HLA-DQA10501-DQB10201	216	EAMLKTPGVAFIDPV	PGVAFIDPV	6	0.3787	830.8	19.00	0.57	tr_D0A0U3_
HLA-DQA10501-DQB10201	217	NKMERNVIHLSPTLE	VIHLSPTLE	6	0.3785	832.9	19.00	0.55	tr_D0A0U3_
HLA-DQA10501-DQB10201	218	AYFMRVIISPMKAMN	FMRVIISPM	2	0.3780	837.3	19.00	0.44	tr_D0A0U3_
HLA-DQA10501-DQB10201	219	LQFAIENMWREALAF	NMWREALAF	6	0.3772	844.4	19.00	0.32	tr_D0A0U3_
HLA-DQA10501-DQB10201	220	RGKLFVVVGPLGDFG	FVVVGPLGD	4	0.3767	848.9	19.00	0.28	tr_D0A0U3_
HLA-DQA10501-DQB10201	221	TDAAAIEDVLKKTLA	DAAAIEDVL	1	0.3765	851.2	20.00	0.56	tr_D0A0U3_
HLA-DQA10501-DQB10201	222	ERNVIHLSPTLEQQF	VIHLSPTLE	3	0.3761	854.9	20.00	0.49	tr_D0A0U3_
HLA-DQA10501-DQB10201	223	LLGFATAYFMRVIIS	FATAYFMRV	3	0.3750	865.0	20.00	0.28	tr_D0A0U3_

HLA-DQA10501-DQB10201	224	AQALSVPERLTDRKT	QALSVPERL	1	0.3742	871.8	20.00	0.80	tr_D0A0U3_
HLA-DQA10501-DQB10201	225	GKLFVVVGPLGDFGT	FVVVGPLGD	3	0.3734	879.9	20.00	0.26	tr_D0A0U3_
HLA-DQA10501-DQB10201	226	YPSEVEMQAPFNGVS	EVEMQAPFN	3	0.3731	882.2	20.00	0.50	tr_D0A0U3_
HLA-DQA10501-DQB10201	227	EMRYGPFADDGCFLK	PFADDGCFL	5	0.3724	889.0	20.00	0.48	tr_D0A0U3_
HLA-DQA10501-DQB10201	228	IFAQSVVNVGEMRYG	VVNVGEMRY	5	0.3723	890.3	20.00	0.41	tr_D0A0U3_
HLA-DQA10501-DQB10201	229	FAQSVVNVGEMRYGP	VVNVGEMRY	4	0.3721	892.0	20.00	0.44	tr_D0A0U3_
HLA-DQA10501-DQB10201	230	NRGKLFVVVGPLGDF	FVVVGPLGD	5	0.3712	900.7	20.00	0.34	tr_D0A0U3_
HLA-DQA10501-DQB10201	231	SNFDHDDIDGQLLVY	DIDGQLLVY	6	0.3712	900.9	20.00	0.26	tr_D0A0U3_
HLA-DQA10501-DQB10201	232	VGDTARFIRNLVADS	FIRNLVADS	6	0.3705	907.7	21.00	0.48	tr_D0A0U3_
HLA-DQA10501-DQB10201	233	LAFAASPFVDGQVVV	LAFAASPFV	0	0.3693	919.7	21.00	0.38	tr_D0A0U3_
HLA-DQA10501-DQB10201	234	FGVEEYDVAQRILRR	EEYDVAQRI	3	0.3693	920.0	21.00	0.43	tr_D0A0U3_
HLA-DQA10501-DQB10201	235	SRTRSAYVLVPSMLQ	SAYVLVPSM	4	0.3687	925.4	21.00	0.34	tr_D0A0U3_
HLA-DQA10501-DQB10201	236	VEMEEGASLLEVREH	EEGASLLEV	3	0.3686	926.8	21.00	0.40	tr_D0A0U3_
HLA-DQA10501-DQB10201	237	MERNVIHLSPTLEQQ	VIHLSPTLE	4	0.3682	930.7	21.00	0.46	tr_D0A0U3_
HLA-DQA10501-DQB10201	238	SSHCYPSEVEMQAPF	YPSEVEMQA	4	0.3675	937.5	21.00	0.24	tr_D0A0U3_
HLA-DQA10501-DQB10201	239	VTVFNASSNFDHDDI	VFNASSNFD	2	0.3673	939.6	21.00	0.41	tr_D0A0U3_
HLA-DQA10501-DQB10201	240	DSAWNTFVESRPQAV	FVESRPQAV	6	0.3670	942.7	21.00	0.34	tr_D0A0U3_
HLA-DQA10501-DQB10201	241	RNVIHLSPTLEQQFY	VIHLSPTLE	2	0.3660	952.9	21.00	0.47	tr_D0A0U3_
HLA-DQA10501-DQB10201	242	YFTRASPSAELLALI	PSAELLALI	6	0.3652	961.0	21.00	0.28	tr_D0A0U3_
HLA-DQA10501-DQB10201	243	RTRSAYVLVPSMLQF	SAYVLVPSM	3	0.3644	970.3	22.00	0.36	tr_D0A0U3_
HLA-DQA10501-DQB10201	244	RFIRNLVADSRTRSA	IRNLVADSR	2	0.3631	983.2	22.00	0.50	tr_D0A0U3_
HLA-DQA10501-DQB10201	245	TVFNASSNFDHDDID	VFNASSNFD	1	0.3628	987.1	22.00	0.35	tr_D0A0U3_
HLA-DQA10501-DQB10201	246	KLFVVVGPLGDFGTV	FVVVGPLGD	2	0.3626	988.8	22.00	0.31	tr_D0A0U3_
HLA-DQA10501-DQB10201	247	ADSRTRSAYVLVPSM	SAYVLVPSM	6	0.3624	991.4	22.00	0.54	tr_D0A0U3_
HLA-DQA10501-DQB10201	248	SAWNTFVESRPQAVI	FVESRPQAV	5	0.3614	1002.2	22.00	0.44	tr_D0A0U3_
HLA-DQA10501-DQB10201	249	LGFATAYFMRVIISP	FATAYFMRV	2	0.3606	1010.0	22.00	0.27	tr_D0A0U3_
HLA-DQA10501-DQB10201	250	AFEGVSSADRVLFAT	EGVSSADRV	2	0.3601	1016.2	22.00	0.29	tr_D0A0U3_
HLA-DQA10501-DQB10201	251	MARALNASIPPLTNP	ALNASIPPL	3	0.3599	1018.6	22.00	0.42	tr_D0A0U3_
HLA-DQA10501-DQB10201	252	FSGAAEELQREWSTK	FSGAAEELQ	0	0.3598	1018.8	22.00	0.55	tr_D0A0U3_
HLA-DQA10501-DQB10201	253	LTDRKTFMQSLYDQR	FMQSLYDQR	6	0.3595	1022.1	22.00	0.34	tr_D0A0U3_
HLA-DQA10501-DQB10201	254	SAYVLVPSMLQFAIE	PSMLQFAIE	6	0.3591	1027.3	23.00	0.31	tr_D0A0U3_
HLA-DQA10501-DQB10201	255	FFDTIVSPFSGAAEE	FFDTIVSPF	0	0.3588	1029.8	23.00	0.40	tr_D0A0U3_
HLA-DQA10501-DQB10201	256	VIDDLVIGDYGGECA	IGDYGGECA	6	0.3583	1036.4	23.00	0.41	tr_D0A0U3_
HLA-DQA10501-DQB10201	257	RLGFMYLHNVFFGVE	LHNVFFGVE	6	0.3581	1037.9	23.00	0.31	tr_D0A0U3_
HLA-DQA10501-DQB10201	258	AEQDVVAFAPLTGSS	DVVAFAPLT	3	0.3578	1041.5	23.00	0.38	tr_D0A0U3_
HLA-DQA10501-DQB10201	259	YFMRVIISPMKAMNA	FMRVIISPM	1	0.3577	1043.0	23.00	0.44	tr_D0A0U3_
HLA-DQA10501-DQB10201	260	LFVVVGPLGDFGTVS	FVVVGPLGD	1	0.3567	1054.2	23.00	0.30	tr_D0A0U3_
HLA-DQA10501-DQB10201	261	ASSNFDHDDIDGQLL	HDDIDGQLL	6	0.3563	1058.5	23.00	0.23	tr_D0A0U3_
HLA-DQA10501-DQB10201	262	VAFIDPVPLAPRLNK	VAFIDPVPL	0	0.3559	1063.3	23.00	0.49	tr_D0A0U3_
HLA-DQA10501-DQB10201	263	RTIRLGFMYLHNVFF	FMYLHNVFF	6	0.3556	1066.3	23.00	0.45	tr_D0A0U3_
HLA-DQA10501-DQB10201	264	ALVDSIFAQSVVNVG	VDSIFAQSV	2	0.3554	1069.1	23.00	0.43	tr_D0A0U3_
HLA-DQA10501-DQB10201	265	AQSVVNVGEMRYGPF	VVNVGEMRY	3	0.3554	1069.4	23.00	0.44	tr_D0A0U3_
HLA-DQA10501-DQB10201	266	SRPQAVIVFAPPVGD	AVIVFAPPV	4	0.3551	1072.8	23.00	0.32	tr_D0A0U3_
HLA-DQA10501-DQB10201	267	VDSIFAQSVVNVGEM	QSVVNVGEM	6	0.3544	1080.4	23.00	0.29	tr_D0A0U3_
HLA-DQA10501-DQB10201	268	NVIHLSPTLEQQFYV	VIHLSPTLE	1	0.3542	1083.0	23.00	0.44	tr_D0A0U3_
HLA-DQA10501-DQB10201	269	DLVIGDYGGECAGWA	IGDYGGECA	3	0.3526	1101.9	24.00	0.41	tr_D0A0U3_
HLA-DQA10501-DQB10201	270	DDLVIGDYGGECAGW	IGDYGGECA	4	0.3523	1105.4	24.00	0.46	tr_D0A0U3_
HLA-DQA10501-DQB10201	271	IENMWREALAFAASP	EALAFAASP	6	0.3514	1115.8	24.00	0.31	tr_D0A0U3_
HLA-DQA10501-DQB10201	272	DEITASEVVQKFYTA	TASEVVQKF	3	0.3512	1118.3	24.00	0.29	tr_D0A0U3_
HLA-DQA10501-DQB10201	273	NGVSVLIPDRPTALH	VSVLIPDRP	2	0.3509	1122.6	24.00	0.55	tr_D0A0U3_

HLA-DQA10501-DQB10201	274	SMARALNASIPPLTN	ALNASIPPL	4	0.3508	1124.1	24.00	0.41	tr_D0A0U3_
HLA-DQA10501-DQB10201	275	TRSAYVLVPSMLQFA	SAYVLVPSM	2	0.3502	1130.5	24.00	0.35	tr_D0A0U3_
HLA-DQA10501-DQB10201	276	NFDHDDIDGQLLVYG	DIDGQLLVY	5	0.3494	1141.2	24.00	0.23	tr_D0A0U3_
HLA-DQA10501-DQB10201	277	LVDSIFAQSVVNVGE	VDSIFAQSV	1	0.3493	1142.0	24.00	0.34	tr_D0A0U3_
HLA-DQA10501-DQB10201	278	AWNTFVESRPQAVIV	FVESRPQAV	4	0.3491	1143.7	24.00	0.46	tr_D0A0U3_
HLA-DQA10501-DQB10201	279	GVTVFNASSNFDHDD	VFNASSNFD	3	0.3479	1159.6	25.00	0.55	tr_D0A0U3_
HLA-DQA10501-DQB10201	280	SSNFDHDDIDGQLLV	HDDIDGQLL	5	0.3466	1176.2	25.00	0.23	tr_D0A0U3_
HLA-DQA10501-DQB10201	281	RFVEAVNAGFNASMT	FVEAVNAGF	1	0.3463	1179.1	25.00	0.47	tr_D0A0U3_
HLA-DQA10501-DQB10201	282	ATFSVEPQSVVVLDG	TFSVEPQSV	1	0.3460	1182.9	25.00	0.45	tr_D0A0U3_
HLA-DQA10501-DQB10201	283	ADRVLFATNLPHWAD	FATNLPHWA	5	0.3454	1191.5	25.00	0.28	tr_D0A0U3_
HLA-DQA10501-DQB10201	284	RPQAVIVFAPPVGDT	AVIVFAPPV	3	0.3452	1193.5	25.00	0.32	tr_D0A0U3_
HLA-DQA10501-DQB10201	285	RLHIVIRSTDAAAIE	IRSTDAAAI	5	0.3447	1199.8	25.00	0.28	tr_D0A0U3_
HLA-DQA10501-DQB10201	286	LYFTRASPSAELLAL	RASPSAELL	4	0.3444	1204.6	25.00	0.30	tr_D0A0U3_
HLA-DQA10501-DQB10201	287	SADRVLFATNLPHWA	FATNLPHWA	6	0.3434	1217.2	26.00	0.26	tr_D0A0U3_
HLA-DQA10501-DQB10201	288	FDHDDIDGQLLVYGW	DIDGQLLVY	4	0.3432	1219.3	26.00	0.23	tr_D0A0U3_
HLA-DQA10501-DQB10201	289	PNLYFTRASPSAELL	RASPSAELL	6	0.3428	1224.8	26.00	0.32	tr_D0A0U3_
HLA-DQA10501-DQB10201	290	RHFDSAWNTFVESRP	WNTFVESRP	6	0.3422	1232.6	26.00	0.40	tr_D0A0U3_
HLA-DQA10501-DQB10201	291	ARALNASIPPLTNPM	ALNASIPPL	2	0.3416	1241.2	26.00	0.45	tr_D0A0U3_
HLA-DQA10501-DQB10201	292	PQAVIVFAPPVGDTA	AVIVFAPPV	2	0.3397	1266.9	26.00	0.30	tr_D0A0U3_
HLA-DQA10501-DQB10201	293	TDRKTFMQSLYDQRR	FMQSLYDQR	5	0.3397	1267.2	26.00	0.34	tr_D0A0U3_
HLA-DQA10501-DQB10201	294	IRLGFMYLHNVFFGV	FMYLHNVFF	4	0.3393	1272.5	26.00	0.34	tr_D0A0U3_
HLA-DQA10501-DQB10201	295	ILRRMGYGFCGVFTL	GYGFCGVFT	5	0.3389	1277.7	26.00	0.49	tr_D0A0U3_
HLA-DQA10501-DQB10201	296	FTRASPSAELLALIR	PSAELLALI	5	0.3384	1284.7	27.00	0.28	tr_D0A0U3_
HLA-DQA10501-DQB10201	297	NLYFTRASPSAELLA	RASPSAELL	5	0.3381	1289.2	27.00	0.31	tr_D0A0U3_
HLA-DQA10501-DQB10201	298	MRYGPFADDGCFLKG	PFADDGCFL	4	0.3381	1289.6	27.00	0.54	tr_D0A0U3_
HLA-DQA10501-DQB10201	299	EMEEGASLLEVREHN	EEGASLLEV	2	0.3375	1296.7	27.00	0.44	tr_D0A0U3_
HLA-DQA10501-DQB10201	300	RSAYVLVPSMLQFAI	SAYVLVPSM	1	0.3375	1296.7	27.00	0.37	tr_D0A0U3_
HLA-DQA10501-DQB10201	301	NASSNFDHDDIDGQL	DHDDIDGQL	6	0.3372	1301.1	27.00	0.26	tr_D0A0U3_
HLA-DQA10501-DQB10201	302	HFDSAWNTFVESRPQ	WNTFVESRP	5	0.3362	1315.4	27.00	0.41	tr_D0A0U3_
HLA-DQA10501-DQB10201	303	TIRLGFMYLHNVFFG	FMYLHNVFF	5	0.3362	1315.6	27.00	0.37	tr_D0A0U3_
HLA-DQA10501-DQB10201	304	DRKTFMQSLYDQRRY	FMQSLYDQR	4	0.3352	1330.4	27.00	0.36	tr_D0A0U3_
HLA-DQA10501-DQB10201	305	LVIGDYGGECAGWAA	IGDYGGECA	2	0.3350	1332.6	27.00	0.42	tr_D0A0U3_
HLA-DQA10501-DQB10201	306	SVWSMARALNASIPP	WSMARALNA	2	0.3340	1347.6	27.00	0.40	tr_D0A0U3_
HLA-DQA10501-DQB10201	307	RVLFATNLPHWADEI	FATNLPHWA	3	0.3337	1352.1	28.00	0.37	tr_D0A0U3_
HLA-DQA10501-DQB10201	308	WNTFVESRPQAVIVF	FVESRPQAV	3	0.3336	1352.7	28.00	0.55	tr_D0A0U3_
HLA-DQA10501-DQB10201	309	RLLDAPDGIMMFDSS	RLLDAPDGI	0	0.3333	1357.1	28.00	0.41	tr_D0A0U3_
HLA-DQA10501-DQB10201	310	DSIFAQSVVNVGEMR	QSVVNVGEM	5	0.3333	1358.2	28.00	0.28	tr_D0A0U3_
HLA-DQA10501-DQB10201	311	EQDVVAFAPLTGSSG	DVVAFAPLT	2	0.3331	1361.0	28.00	0.43	tr_D0A0U3_
HLA-DQA10501-DQB10201	312	VSVWSMARALNASIP	WSMARALNA	3	0.3329	1363.2	28.00	0.49	tr_D0A0U3_
HLA-DQA10501-DQB10201	313	TGCAVNYGATQVSVW	YGATQVSVW	6	0.3324	1370.4	28.00	0.32	tr_D0A0U3_
HLA-DQA10501-DQB10201	314	DSSHCYPSEVEMQAP	YPSEVEMQA	5	0.3323	1372.2	28.00	0.29	tr_D0A0U3_
HLA-DQA10501-DQB10201	315	IDDLVIGDYGGECAG	IGDYGGECA	5	0.3322	1374.1	28.00	0.44	tr_D0A0U3_
HLA-DQA10501-DQB10201	316	FAIENMWREALAFAA	WREALAFAA	6	0.3321	1375.7	28.00	0.35	tr_D0A0U3_
HLA-DQA10501-DQB10201	317	FDSAWNTFVESRPQA	WNTFVESRP	4	0.3313	1386.7	28.00	0.41	tr_D0A0U3_
HLA-DQA10501-DQB10201	318	DRVLFATNLPHWADE	FATNLPHWA	4	0.3310	1391.7	28.00	0.33	tr_D0A0U3_
HLA-DQA10501-DQB10201	319	EVEMQAPFNGVSVLI	PFNGVSVLI	6	0.3309	1393.4	28.00	0.41	tr_D0A0U3_
HLA-DQA10501-DQB10201	320	PGVTVFNASSNFDHD	VFNASSNFD	4	0.3304	1400.5	28.00	0.56	tr_D0A0U3_
HLA-DQA10501-DQB10201	321	HHQKIEKRFVEAVNA	KRFVEAVNA	6	0.3303	1401.8	28.00	0.60	tr_D0A0U3_
HLA-DQA10501-DQB10201	322	LFATNLPHWADEITA	FATNLPHWA	1	0.3303	1401.9	28.00	0.25	tr_D0A0U3_
HLA-DQA10501-DQB10201	323	PSEVEMQAPFNGVSV	EVEMQAPFN	2	0.3297	1411.4	28.00	0.55	tr_D0A0U3_

HLA-DQA10501-DQB10201	324	QFAIENMWREALAFA	NMWREALAF	5	0.3295	1413.9	28.00	0.34	tr_D0A0U3_
HLA-DQA10501-DQB10201	325	TRASPSAELLALIRY	PSAELLALI	4	0.3284	1432.0	29.00	0.28	tr_D0A0U3_
HLA-DQA10501-DQB10201	326	VVGPLGDFGTVSSLI	DFGTVSSLI	6	0.3279	1439.0	29.00	0.51	tr_D0A0U3_
HLA-DQA10501-DQB10201	327	ESRPQAVIVFAPPVG	AVIVFAPPV	5	0.3275	1446.4	29.00	0.38	tr_D0A0U3_
HLA-DQA10501-DQB10201	328	FVVVGPLGDFGTVSS	VVGPLGDFG	2	0.3271	1451.6	29.00	0.28	tr_D0A0U3_
HLA-DQA10501-DQB10201	329	QSVVNVGEMRYGPFA	VVNVGEMRY	2	0.3267	1457.9	29.00	0.46	tr_D0A0U3_
HLA-DQA10501-DQB10201	330	HQKIEKRFVEAVNAG	KRFVEAVNA	5	0.3265	1461.3	29.00	0.54	tr_D0A0U3_
HLA-DQA10501-DQB10201	331	FIRNLVADSRTRSAY	IRNLVADSR	1	0.3255	1477.4	29.00	0.52	tr_D0A0U3_
HLA-DQA10501-DQB10201	332	AYVLVPSMLQFAIEN	PSMLQFAIE	5	0.3252	1481.3	29.00	0.33	tr_D0A0U3_
HLA-DQA10501-DQB10201	333	EHNQDSRLFFDTIVS	SRLFFDTIV	5	0.3252	1481.9	29.00	0.41	tr_D0A0U3_
HLA-DQA10501-DQB10201	334	QAVIVFAPPVGDTAR	AVIVFAPPV	1	0.3245	1492.8	29.00	0.31	tr_D0A0U3_
HLA-DQA10501-DQB10201	335	RNRGKLFVVVGPLGD	FVVVGPLGD	6	0.3243	1497.2	30.00	0.38	tr_D0A0U3_
HLA-DQA10501-DQB10201	336	GVSVLIPDRPTALHA	VSVLIPDRP	1	0.3235	1510.4	30.00	0.55	tr_D0A0U3_
HLA-DQA10501-DQB10201	337	LRRMGYGFCGVFTLS	GYGFCGVFT	4	0.3230	1518.3	30.00	0.46	tr_D0A0U3_
HLA-DQA10501-DQB10201	338	RYVIDDLVIGDYGGE	IDDLVIGDY	3	0.3225	1526.3	30.00	0.22	tr_D0A0U3_
HLA-DQA10501-DQB10201	339	GCAVNYGATQVSVWS	YGATQVSVW	5	0.3224	1528.4	30.00	0.32	tr_D0A0U3_
HLA-DQA10501-DQB10201	340	ATNLPHWADEITASE	WADEITASE	6	0.3213	1545.2	30.00	0.34	tr_D0A0U3_
HLA-DQA10501-DQB10201	341	VLFATNLPHWADEIT	FATNLPHWA	2	0.3212	1548.2	30.00	0.34	tr_D0A0U3_
HLA-DQA10501-DQB10201	342	QVSVWSMARALNASI	WSMARALNA	4	0.3205	1558.6	30.00	0.51	tr_D0A0U3_
HLA-DQA10501-DQB10201	343	SSADRVLFATNLPHW	RVLFATNLP	4	0.3199	1570.0	30.00	0.31	tr_D0A0U3_
HLA-DQA10501-DQB10201	344	VESRPQAVIVFAPPV	AVIVFAPPV	6	0.3181	1600.1	31.00	0.45	tr_D0A0U3_
HLA-DQA10501-DQB10201	345	CAVNYGATQVSVWSM	YGATQVSVW	4	0.3181	1600.9	31.00	0.32	tr_D0A0U3_
HLA-DQA10501-DQB10201	346	TFSVEPQSVVVLDGN	TFSVEPQSV	0	0.3169	1621.8	31.00	0.29	tr_D0A0U3_
HLA-DQA10501-DQB10201	347	RALNASIPPLTNPMT	ALNASIPPL	1	0.3168	1622.7	31.00	0.45	tr_D0A0U3_
HLA-DQA10501-DQB10201	348	ATQVSVWSMARALNA	WSMARALNA	6	0.3160	1636.9	31.00	0.53	tr_D0A0U3_
HLA-DQA10501-DQB10201	349	RLTDRKTFMQSLYDQ	KTFMQSLYD	5	0.3159	1638.3	31.00	0.39	tr_D0A0U3_
HLA-DQA10501-DQB10201	350	SNPGVTVFNASSNFD	VFNASSNFD	6	0.3153	1648.7	32.00	0.57	tr_D0A0U3_
HLA-DQA10501-DQB10201	351	VFNASSNFDHDDIDG	VFNASSNFD	0	0.3153	1650.1	32.00	0.29	tr_D0A0U3_
HLA-DQA10501-DQB10201	352	EWSTKITTAVLGVVD	TTAVLGVVD	6	0.3149	1656.2	32.00	0.52	tr_D0A0U3_
HLA-DQA10501-DQB10201	353	SEVEMQAPFNGVSVL	EVEMQAPFN	1	0.3140	1672.4	32.00	0.55	tr_D0A0U3_
HLA-DQA10501-DQB10201	354	NTFVESRPQAVIVFA	FVESRPQAV	2	0.3140	1672.6	32.00	0.55	tr_D0A0U3_
HLA-DQA10501-DQB10201	355	AVIVFAPPVGDTARF	VIVFAPPVG	1	0.3134	1684.8	32.00	0.28	tr_D0A0U3_
HLA-DQA10501-DQB10201	356	VGEMRYGPFADDGCF	YGPFADDGC	5	0.3132	1688.3	32.00	0.29	tr_D0A0U3_
HLA-DQA10501-DQB10201	357	FVEAVNAGFNASMTS	FVEAVNAGF	0	0.3130	1691.4	32.00	0.44	tr_D0A0U3_
HLA-DQA10501-DQB10201	358	TFVESRPQAVIVFAP	FVESRPQAV	1	0.3129	1692.9	32.00	0.44	tr_D0A0U3_
HLA-DQA10501-DQB10201	359	KRLHIVIRSTDAAAI	IRSTDAAAI	6	0.3127	1697.3	32.00	0.31	tr_D0A0U3_
HLA-DQA10501-DQB10201	360	RYGPFADDGCFLKGV	PFADDGCFL	3	0.3124	1702.3	32.00	0.60	tr_D0A0U3_
HLA-DQA10501-DQB10201	361	RILRRMGYGFCGVFT	GYGFCGVFT	6	0.3122	1706.8	32.00	0.56	tr_D0A0U3_
HLA-DQA10501-DQB10201	362	GVEEYDVAQRILRRM	EEYDVAQRI	2	0.3117	1714.5	32.00	0.50	tr_D0A0U3_
HLA-DQA10501-DQB10201	363	RRMGYGFCGVFTLSS	GYGFCGVFT	3	0.3112	1723.7	32.00	0.44	tr_D0A0U3_
HLA-DQA10501-DQB10201	364	TQVSVWSMARALNAS	WSMARALNA	5	0.3111	1726.1	33.00	0.55	tr_D0A0U3_
HLA-DQA10501-DQB10201	365	VIVFAPPVGDTARFI	PVGDTARFI	6	0.3104	1738.7	33.00	0.32	tr_D0A0U3_
HLA-DQA10501-DQB10201	366	GVSSADRVLFATNLP	RVLFATNLP	6	0.3099	1748.4	33.00	0.40	tr_D0A0U3_
HLA-DQA10501-DQB10201	367	RKTFMQSLYDQRRYV	FMQSLYDQR	3	0.3095	1757.4	33.00	0.41	tr_D0A0U3_
HLA-DQA10501-DQB10201	368	AFAASPFVDGQVVVT	FVDGQVVVT	6	0.3093	1760.2	33.00	0.26	tr_D0A0U3_
HLA-DQA10501-DQB10201	369	AIENMWREALAFAAS	WREALAFAA	5	0.3086	1773.9	33.00	0.41	tr_D0A0U3_
HLA-DQA10501-DQB10201	370	VIHLSPTLEQQFYVC	VIHLSPTLE	0	0.3083	1779.6	33.00	0.34	tr_D0A0U3_
HLA-DQA10501-DQB10201	371	FMRVIISPMKAMNAT	FMRVIISPM	0	0.3080	1786.1	33.00	0.41	tr_D0A0U3_
HLA-DQA10501-DQB10201	372	NPGVTVFNASSNFDH	VFNASSNFD	5	0.3075	1795.5	33.00	0.59	tr_D0A0U3_
HLA-DQA10501-DQB10201	373	FEGVSSADRVLFATN	EGVSSADRV	1	0.3073	1798.1	33.00	0.35	tr_D0A0U3_

HLA-DQA10501-DQB10201	374	YVIDDLVIGDYGGEC	IDDLVIGDY	2	0.3042	1859.7	34.00	0.25	tr_D0A0U3_
HLA-DQA10501-DQB10201	375	SVVNVGEMRYGPFAD	VVNVGEMRY	1	0.3041	1861.3	34.00	0.43	tr_D0A0U3_
HLA-DQA10501-DQB10201	376	REHNQDSRLFFDTIV	SRLFFDTIV	6	0.3040	1863.7	34.00	0.49	tr_D0A0U3_
HLA-DQA10501-DQB10201	377	LRTIRLGFMYLHNVF	GFMYLHNVF	6	0.3031	1882.6	34.00	0.41	tr_D0A0U3_
HLA-DQA10501-DQB10201	378	NPMTPSMRYLDPDAG	MRYLDPDAG	6	0.3031	1883.3	34.00	0.43	tr_D0A0U3_
HLA-DQA10501-DQB10201	379	GLTAADPVVIAAHLN	LTAADPVVI	1	0.3028	1887.5	34.00	0.50	tr_D0A0U3_
HLA-DQA10501-DQB10201	380	VIGDYGGECAGWAAG	IGDYGGECA	1	0.3027	1889.6	34.00	0.43	tr_D0A0U3_
HLA-DQA10501-DQB10201	381	RASPSAELLALIRYA	PSAELLALI	3	0.3025	1894.4	35.00	0.30	tr_D0A0U3_
HLA-DQA10501-DQB10201	382	NVGEMRYGPFADDGC	YGPFADDGC	6	0.3025	1894.5	35.00	0.26	tr_D0A0U3_
HLA-DQA10501-DQB10201	383	NPNLYFTRASPSAEL	YFTRASPSA	4	0.3023	1899.5	35.00	0.31	tr_D0A0U3_
HLA-DQA10501-DQB10201	384	ERLTDRKTFMQSLYD	KTFMQSLYD	6	0.3007	1931.9	35.00	0.55	tr_D0A0U3_
HLA-DQA10501-DQB10201	385	QDVVAFAPLTGSSGG	DVVAFAPLT	1	0.3003	1941.3	35.00	0.47	tr_D0A0U3_
HLA-DQA10501-DQB10201	386	AVNYGATQVSVWSMA	YGATQVSVW	3	0.2997	1953.5	35.00	0.34	tr_D0A0U3_
HLA-DQA10501-DQB10201	387	RMGYGFCGVFTLSSS	GYGFCGVFT	2	0.2995	1957.3	35.00	0.47	tr_D0A0U3_
HLA-DQA10501-DQB10201	388	FNASSNFDHDDIDGQ	SSNFDHDDI	3	0.2988	1972.1	35.00	0.28	tr_D0A0U3_
HLA-DQA10501-DQB10201	389	ECAGWAAGQGAMCWC	WAAGQGAMC	4	0.2985	1978.1	35.00	0.34	tr_D0A0U3_
HLA-DQA10501-DQB10201	390	VSSADRVLFATNLPH	RVLFATNLP	5	0.2980	1990.2	36.00	0.32	tr_D0A0U3_
HLA-DQA10501-DQB10201	391	FVESRPQAVIVFAPP	FVESRPQAV	0	0.2977	1995.5	36.00	0.28	tr_D0A0U3_
HLA-DQA10501-DQB10201	392	RVIGRGYRLLDAPDG	YRLLDAPDG	6	0.2964	2024.7	36.00	0.31	tr_D0A0U3_
HLA-DQA10501-DQB10201	393	EGVSSADRVLFATNL	DRVLFATNL	6	0.2963	2026.7	36.00	0.34	tr_D0A0U3_
HLA-DQA10501-DQB10201	394	VIISPMKAMNATALV	MKAMNATAL	5	0.2951	2051.7	36.00	0.46	tr_D0A0U3_
HLA-DQA10501-DQB10201	395	FVDGQVVVTGTNPLA	VVVTGTNPL	5	0.2949	2056.0	36.00	0.34	tr_D0A0U3_
HLA-DQA10501-DQB10201	396	FATNLPHWADEITAS	PHWADEITA	5	0.2943	2069.6	37.00	0.25	tr_D0A0U3_
HLA-DQA10501-DQB10201	397	MEEGASLLEVREHNQ	EEGASLLEV	1	0.2939	2078.4	37.00	0.47	tr_D0A0U3_
HLA-DQA10501-DQB10201	398	DHDDIDGQLLVYGWI	DIDGQLLVY	3	0.2939	2078.5	37.00	0.32	tr_D0A0U3_
HLA-DQA10501-DQB10201	399	GECAGWAAGQGAMCW	WAAGQGAMC	5	0.2922	2118.9	37.00	0.37	tr_D0A0U3_
HLA-DQA10501-DQB10201	400	FDSSHCYPSEVEMQA	YPSEVEMQA	6	0.2917	2128.4	37.00	0.35	tr_D0A0U3_
HLA-DQA10501-DQB10201	401	GYGFCGVFTLSSSLT	VFTLSSSLT	6	0.2906	2154.4	37.00	0.34	tr_D0A0U3_
HLA-DQA10501-DQB10201	402	ASPSAELLALIRYAL	PSAELLALI	2	0.2901	2166.4	38.00	0.34	tr_D0A0U3_
HLA-DQA10501-DQB10201	403	LTGCAVNYGATQVSV	AVNYGATQV	4	0.2894	2183.1	38.00	0.40	tr_D0A0U3_
HLA-DQA10501-DQB10201	404	MGYGFCGVFTLSSSL	GYGFCGVFT	1	0.2887	2200.3	38.00	0.43	tr_D0A0U3_
HLA-DQA10501-DQB10201	405	PVGDTARFIRNLVAD	ARFIRNLVA	5	0.2877	2224.4	38.00	0.27	tr_D0A0U3_
HLA-DQA10501-DQB10201	406	PFVDGQVVVTGTNPL	VVVTGTNPL	6	0.2867	2247.5	38.00	0.34	tr_D0A0U3_
HLA-DQA10501-DQB10201	407	PTLEQQFYVCTSYLS	QFYVCTSYL	5	0.2862	2260.6	39.00	0.41	tr_D0A0U3_
HLA-DQA10501-DQB10201	408	EITASEVVQKFYTAE	TASEVVQKF	2	0.2856	2275.5	39.00	0.40	tr_D0A0U3_
HLA-DQA10501-DQB10201	409	KTFMQSLYDQRRYVI	FMQSLYDQR	2	0.2854	2279.6	39.00	0.48	tr_D0A0U3_
HLA-DQA10501-DQB10201	410	PDSGDVYVIGLTAAD	YVIGLTAAD	6	0.2845	2302.3	39.00	0.38	tr_D0A0U3_
HLA-DQA10501-DQB10201	411	RYALGRLRTIRLGFM	LRTIRLGFM	6	0.2842	2309.6	39.00	0.56	tr_D0A0U3_
HLA-DQA10501-DQB10201	412	YALGRLRTIRLGFMY	LRTIRLGFM	5	0.2833	2331.7	39.00	0.54	tr_D0A0U3_
HLA-DQA10501-DQB10201	413	TLEQQFYVCTSYLSR	QFYVCTSYL	4	0.2832	2333.4	39.00	0.41	tr_D0A0U3_
HLA-DQA10501-DQB10201	414	MTPSMRYLDPDAGKL	MRYLDPDAG	4	0.2830	2340.3	39.00	0.49	tr_D0A0U3_
HLA-DQA10501-DQB10201	415	ALGRLRTIRLGFMYL	LRTIRLGFM	4	0.2827	2346.6	39.00	0.53	tr_D0A0U3_
HLA-DQA10501-DQB10201	416	VVNVGEMRYGPFADD	VVNVGEMRY	0	0.2823	2357.0	40.00	0.31	tr_D0A0U3_
HLA-DQA10501-DQB10201	417	YGPFADDGCFLKGVP	PFADDGCFL	2	0.2821	2362.1	40.00	0.69	tr_D0A0U3_
HLA-DQA10501-DQB10201	418	PSASPISGFQQAVER	ISGFQQAVE	5	0.2819	2366.5	40.00	0.43	tr_D0A0U3_
HLA-DQA10501-DQB10201	419	DGQVVVTGTNPLARD	VVVTGTNPL	3	0.2815	2378.3	40.00	0.37	tr_D0A0U3_
HLA-DQA10501-DQB10201	420	ALNASIPPLTNPMTP	LNASIPPLT	1	0.2814	2380.1	40.00	0.44	tr_D0A0U3_
HLA-DQA10501-DQB10201	421	CAGWAAGQGAMCWCN	WAAGQGAMC	3	0.2812	2386.8	40.00	0.35	tr_D0A0U3_
HLA-DQA10501-DQB10201	422	TPSASPISGFQQAVE	ISGFQQAVE	6	0.2801	2415.4	40.00	0.51	tr_D0A0U3_
HLA-DQA10501-DQB10201	423	PMTPSMRYLDPDAGK	MRYLDPDAG	5	0.2794	2432.8	40.00	0.46	tr_D0A0U3_

HLA-DQA10501-DQB10201	424	VEEYDVAQRILRRMG	EEYDVAQRI	1	0.2792	2438.4	40.00	0.50	tr_D0A0U3_
HLA-DQA10501-DQB10201	425	EDVLKKTLATFSVEP	TLATFSVEP	6	0.2787	2450.9	40.00	0.63	tr_D0A0U3_
HLA-DQA10501-DQB10201	426	FAASPFVDGQVVVTG	FVDGQVVVT	5	0.2785	2455.5	41.00	0.27	tr_D0A0U3_
HLA-DQA10501-DQB10201	427	APDGIMMFDSSHCYP	MMFDSSHCY	5	0.2754	2539.7	41.00	0.28	tr_D0A0U3_
HLA-DQA10501-DQB10201	428	VVVGPLGDFGTVSSL	VVGPLGDFG	1	0.2750	2550.0	41.00	0.32	tr_D0A0U3_
HLA-DQA10501-DQB10201	429	SPTLEQQFYVCTSYL	QFYVCTSYL	6	0.2750	2551.4	41.00	0.50	tr_D0A0U3_
HLA-DQA10501-DQB10201	430	QALSVPERLTDRKTF	QALSVPERL	0	0.2746	2563.6	42.00	0.83	tr_D0A0U3_
HLA-DQA10501-DQB10201	431	AGWAAGQGAMCWCNQ	WAAGQGAMC	2	0.2743	2572.0	42.00	0.41	tr_D0A0U3_
HLA-DQA10501-DQB10201	432	SASPISGFQQAVERN	ISGFQQAVE	4	0.2740	2577.8	42.00	0.44	tr_D0A0U3_
HLA-DQA10501-DQB10201	433	DAAAIEDVLKKTLAT	DAAAIEDVL	0	0.2721	2631.5	42.00	0.62	tr_D0A0U3_
HLA-DQA10501-DQB10201	434	VRVIGRGYRLLDAPD	GRGYRLLDA	4	0.2712	2657.0	42.00	0.38	tr_D0A0U3_
HLA-DQA10501-DQB10201	435	DAPDGIMMFDSSHCY	MMFDSSHCY	6	0.2703	2684.8	43.00	0.26	tr_D0A0U3_
HLA-DQA10501-DQB10201	436	VDGQVVVTGTNPLAR	VVVTGTNPL	4	0.2702	2688.5	43.00	0.37	tr_D0A0U3_
HLA-DQA10501-DQB10201	437	LEQQFYVCTSYLSRN	QFYVCTSYL	3	0.2699	2695.1	43.00	0.41	tr_D0A0U3_
HLA-DQA10501-DQB10201	438	VADSRTRSAYVLVPS	TRSAYVLVP	5	0.2690	2721.2	43.00	0.34	tr_D0A0U3_
HLA-DQA10501-DQB10201	439	RVIISPMKAMNATAL	MKAMNATAL	6	0.2688	2729.0	43.00	0.53	tr_D0A0U3_
HLA-DQA10501-DQB10201	440	TPSMRYLDPDAGKLS	MRYLDPDAG	3	0.2680	2753.4	43.00	0.50	tr_D0A0U3_
HLA-DQA10501-DQB10201	441	PPVGDTARFIRNLVA	ARFIRNLVA	6	0.2679	2755.9	43.00	0.31	tr_D0A0U3_
HLA-DQA10501-DQB10201	442	EQQFYVCTSYLSRND	QFYVCTSYL	2	0.2672	2774.5	43.00	0.40	tr_D0A0U3_
HLA-DQA10501-DQB10201	443	IMMFDSSHCYPSEVE	SHCYPSEVE	6	0.2669	2783.8	44.00	0.29	tr_D0A0U3_
HLA-DQA10501-DQB10201	444	SPSAELLALIRYALG	PSAELLALI	1	0.2665	2797.3	44.00	0.31	tr_D0A0U3_
HLA-DQA10501-DQB10201	445	TRHFDSAWNTFVESR	AWNTFVESR	6	0.2656	2825.1	44.00	0.27	tr_D0A0U3_
HLA-DQA10501-DQB10201	446	DVVAFAPLTGSSGGR	VVAFAPLTG	1	0.2644	2860.3	44.00	0.46	tr_D0A0U3_
HLA-DQA10501-DQB10201	447	VNYGATQVSVWSMAR	YGATQVSVW	2	0.2641	2869.7	44.00	0.43	tr_D0A0U3_
HLA-DQA10501-DQB10201	448	HDDIDGQLLVYGWIT	DIDGQLLVY	2	0.2635	2889.6	44.00	0.36	tr_D0A0U3_
HLA-DQA10501-DQB10201	449	LNKMERNVIHLSPTL	NVIHLSPTL	6	0.2631	2901.4	45.00	0.43	tr_D0A0U3_
HLA-DQA10501-DQB10201	450	LGRLRTIRLGFMYLH	LRTIRLGFM	3	0.2629	2908.5	45.00	0.50	tr_D0A0U3_
HLA-DQA10501-DQB10201	451	GQVVVTGTNPLARDT	VVVTGTNPL	2	0.2627	2914.9	45.00	0.36	tr_D0A0U3_
HLA-DQA10501-DQB10201	452	ERNRGKLFVVVGPLG	LFVVVGPLG	6	0.2626	2918.4	45.00	0.41	tr_D0A0U3_
HLA-DQA10501-DQB10201	453	ADIEIEVFSLLHHQK	EIEVFSLLH	3	0.2623	2926.7	45.00	0.37	tr_D0A0U3_
HLA-DQA10501-DQB10201	454	EGHLPDSGDVYVIGL	SGDVYVIGL	6	0.2607	2979.4	45.00	0.44	tr_D0A0U3_
HLA-DQA10501-DQB10201	455	KKRLHIVIRSTDAAA	VIRSTDAAA	6	0.2605	2984.4	45.00	0.36	tr_D0A0U3_
HLA-DQA10501-DQB10201	456	VNVGEMRYGPFADDG	RYGPFADDG	6	0.2593	3024.0	46.00	0.22	tr_D0A0U3_
HLA-DQA10501-DQB10201	457	AASPFVDGQVVVTGT	FVDGQVVVT	4	0.2588	3039.3	46.00	0.29	tr_D0A0U3_
HLA-DQA10501-DQB10201	458	FSVEPQSVVVLDGNA	PQSVVVLDG	4	0.2584	3054.3	46.00	0.32	tr_D0A0U3_
HLA-DQA10501-DQB10201	459	DIEIEVFSLLHHQKI	EIEVFSLLH	2	0.2575	3084.2	46.00	0.31	tr_D0A0U3_
HLA-DQA10501-DQB10201	460	WNPNLYFTRASPSAE	YFTRASPSA	5	0.2565	3116.2	46.00	0.41	tr_D0A0U3_
HLA-DQA10501-DQB10201	461	HLPDSGDVYVIGLTA	DVYVIGLTA	6	0.2563	3123.1	46.00	0.35	tr_D0A0U3_
HLA-DQA10501-DQB10201	462	IVFAPPVGDTARFIR	PVGDTARFI	5	0.2555	3151.4	47.00	0.41	tr_D0A0U3_
HLA-DQA10501-DQB10201	463	ATVEGHLPDSGDVYV	HLPDSGDVY	5	0.2546	3181.2	47.00	0.26	tr_D0A0U3_
HLA-DQA10501-DQB10201	464	IGDYGGECAGWAAGQ	IGDYGGECA	0	0.2538	3208.8	47.00	0.40	tr_D0A0U3_
HLA-DQA10501-DQB10201	465	PDGIMMFDSSHCYPS	MMFDSSHCY	4	0.2537	3212.8	47.00	0.31	tr_D0A0U3_
HLA-DQA10501-DQB10201	466	GRLRTIRLGFMYLHN	LRTIRLGFM	2	0.2536	3217.0	47.00	0.47	tr_D0A0U3_
HLA-DQA10501-DQB10201	467	LVADSRTRSAYVLVP	TRSAYVLVP	6	0.2535	3218.8	47.00	0.51	tr_D0A0U3_
HLA-DQA10501-DQB10201	468	TFMQSLYDQRRYVID	FMQSLYDQR	1	0.2533	3224.9	47.00	0.46	tr_D0A0U3_
HLA-DQA10501-DQB10201	469	ASPISGFQQAVERNR	ISGFQQAVE	3	0.2527	3246.3	47.00	0.42	tr_D0A0U3_
HLA-DQA10501-DQB10201	470	RLTGCAVNYGATQVS	AVNYGATQV	5	0.2525	3253.9	48.00	0.49	tr_D0A0U3_
HLA-DQA10501-DQB10201	471	MFDSSHCYPSEVEMQ	SHCYPSEVE	4	0.2521	3268.9	48.00	0.29	tr_D0A0U3_
HLA-DQA10501-DQB10201	472	HVRVIGRGYRLLDAP	GRGYRLLDA	5	0.2516	3287.9	48.00	0.44	tr_D0A0U3_
HLA-DQA10501-DQB10201	473	NYGATQVSVWSMARA	YGATQVSVW	1	0.2515	3291.2	48.00	0.39	tr_D0A0U3_

HLA-DQA10501-DQB10201	474	TVEGHLPDSGDVYVI	HLPDSGDVY	4	0.2514	3294.8	48.00	0.28	tr_D0A0U3_
HLA-DQA10501-DQB10201	475	LDAPDGIMMFDSSHC	IMMFDSSHC	6	0.2511	3302.7	48.00	0.18	tr_D0A0U3_
HLA-DQA10501-DQB10201	476	STRHFDSAWNTFVES	SAWNTFVES	6	0.2511	3303.6	48.00	0.31	tr_D0A0U3_
HLA-DQA10501-DQB10201	477	MMFDSSHCYPSEVEM	SHCYPSEVE	5	0.2510	3306.3	48.00	0.26	tr_D0A0U3_
HLA-DQA10501-DQB10201	478	TNPMTPSMRYLDPDA	SMRYLDPDA	6	0.2506	3322.5	48.00	0.40	tr_D0A0U3_
HLA-DQA10501-DQB10201	479	RLRTIRLGFMYLHNV	LRTIRLGFM	1	0.2504	3329.3	48.00	0.43	tr_D0A0U3_
HLA-DQA10501-DQB10201	480	DGIMMFDSSHCYPSE	MMFDSSHCY	3	0.2503	3331.4	48.00	0.34	tr_D0A0U3_
HLA-DQA10501-DQB10201	481	QVVVTGTNPLARDTQ	VVVTGTNPL	1	0.2497	3353.4	48.00	0.35	tr_D0A0U3_
HLA-DQA10501-DQB10201	482	DEAMLKTPGVAFIDP	TPGVAFIDP	6	0.2496	3356.8	48.00	0.43	tr_D0A0U3_
HLA-DQA10501-DQB10201	483	EPQSVVVLDGNATVE	VVVLDGNAT	4	0.2495	3363.0	48.00	0.25	tr_D0A0U3_
HLA-DQA10501-DQB10201	484	GWAAGQGAMCWCNQG	WAAGQGAMC	1	0.2490	3380.7	48.00	0.44	tr_D0A0U3_
HLA-DQA10501-DQB10201	485	DIDGQLLVYGWITGE	LVYGWITGE	6	0.2489	3382.2	48.00	0.31	tr_D0A0U3_
HLA-DQA10501-DQB10201	486	SPFVDGQVVVTGTNP	FVDGQVVVT	2	0.2489	3382.6	48.00	0.22	tr_D0A0U3_
HLA-DQA10501-DQB10201	487	PSMRYLDPDAGKLSR	MRYLDPDAG	2	0.2489	3385.2	49.00	0.53	tr_D0A0U3_
HLA-DQA10501-DQB10201	488	VEPQSVVVLDGNATV	PQSVVVLDG	2	0.2484	3402.2	49.00	0.25	tr_D0A0U3_
HLA-DQA10501-DQB10201	489	LLDAPDGIMMFDSSH	DAPDGIMMF	2	0.2474	3437.7	49.00	0.20	tr_D0A0U3_
HLA-DQA10501-DQB10201	490	EEGASLLEVREHNQD	EEGASLLEV	0	0.2467	3465.1	49.00	0.35	tr_D0A0U3_
HLA-DQA10501-DQB10201	491	SVEPQSVVVLDGNAT	PQSVVVLDG	3	0.2467	3465.3	49.00	0.29	tr_D0A0U3_
HLA-DQA10501-DQB10201	492	GIMMFDSSHCYPSEV	MMFDSSHCY	2	0.2464	3475.6	49.00	0.27	tr_D0A0U3_
HLA-DQA10501-DQB10201	493	YGFCGVFTLSSSLTG	VFTLSSSLT	5	0.2453	3516.9	49.00	0.46	tr_D0A0U3_
HLA-DQA10501-DQB10201	494	ASPFVDGQVVVTGTN	FVDGQVVVT	3	0.2420	3645.0	50.00	0.32	tr_D0A0U3_
HLA-DQA10501-DQB10201	495	QQFYVCTSYLSRNDK	QFYVCTSYL	1	0.2416	3661.8	55.00	0.41	tr_D0A0U3_
HLA-DQA10501-DQB10201	496	APPVGDTARFIRNLV	PVGDTARFI	2	0.2413	3672.0	55.00	0.41	tr_D0A0U3_
HLA-DQA10501-DQB10201	497	PRLTGCAVNYGATQV	AVNYGATQV	6	0.2412	3676.5	55.00	0.63	tr_D0A0U3_
HLA-DQA10501-DQB10201	498	LTAADPVVIAAHLNS	LTAADPVVI	0	0.2412	3677.6	55.00	0.43	tr_D0A0U3_
HLA-DQA10501-DQB10201	499	VREHNQDSRLFFDTI	DSRLFFDTI	6	0.2398	3734.0	55.00	0.48	tr_D0A0U3_
HLA-DQA10501-DQB10201	500	AFIDPVPLAPRLNKM	FIDPVPLAP	1	0.2396	3741.0	55.00	0.43	tr_D0A0U3_
HLA-DQA10501-DQB10201	501	GHLPDSGDVYVIGLT	SGDVYVIGL	5	0.2392	3758.4	55.00	0.35	tr_D0A0U3_
HLA-DQA10501-DQB10201	502	TASEVVQKFYTAERN	QKFYTAERN	6	0.2382	3798.8	55.00	0.45	tr_D0A0U3_
HLA-DQA10501-DQB10201	503	VSVLIPDRPTALHAA	VSVLIPDRP	0	0.2378	3816.9	55.00	0.55	tr_D0A0U3_
HLA-DQA10501-DQB10201	504	DDIDGQLLVYGWITG	DIDGQLLVY	1	0.2378	3817.1	55.00	0.38	tr_D0A0U3_
HLA-DQA10501-DQB10201	505	GPFADDGCFLKGVPR	PFADDGCFL	1	0.2375	3827.1	55.00	0.74	tr_D0A0U3_
HLA-DQA10501-DQB10201	506	GFCGVFTLSSSLTGV	VFTLSSSLT	4	0.2375	3828.9	55.00	0.47	tr_D0A0U3_
HLA-DQA10501-DQB10201	507	LPDSGDVYVIGLTAA	DVYVIGLTA	5	0.2350	3933.1	55.00	0.27	tr_D0A0U3_
HLA-DQA10501-DQB10201	508	LDGNATVEGHLPDSG	VEGHLPDSG	6	0.2341	3969.7	55.00	0.30	tr_D0A0U3_
HLA-DQA10501-DQB10201	509	VERNRGKLFVVVGPL	KLFVVVGPL	6	0.2338	3983.1	55.00	0.54	tr_D0A0U3_
HLA-DQA10501-DQB10201	510	PQSVVVLDGNATVEG	VLDGNATVE	5	0.2323	4049.4	55.00	0.19	tr_D0A0U3_
HLA-DQA10501-DQB10201	511	DGNATVEGHLPDSGD	VEGHLPDSG	5	0.2322	4054.6	55.00	0.34	tr_D0A0U3_
HLA-DQA10501-DQB10201	512	SPISGFQQAVERNRG	ISGFQQAVE	2	0.2322	4054.9	55.00	0.44	tr_D0A0U3_
HLA-DQA10501-DQB10201	513	PSAELLALIRYALGR	SAELLALIR	1	0.2321	4057.4	55.00	0.33	tr_D0A0U3_
HLA-DQA10501-DQB10201	514	IEIEVFSLLHHQKIE	EIEVFSLLH	1	0.2307	4119.9	55.00	0.31	tr_D0A0U3_
HLA-DQA10501-DQB10201	515	GWNPNLYFTRASPSA	YFTRASPSA	6	0.2304	4132.3	55.00	0.46	tr_D0A0U3_
HLA-DQA10501-DQB10201	516	FCGVFTLSSSLTGVA	VFTLSSSLT	3	0.2289	4199.4	55.00	0.43	tr_D0A0U3_
HLA-DQA10501-DQB10201	517	NATVEGHLPDSGDVY	VEGHLPDSG	3	0.2283	4227.7	55.00	0.34	tr_D0A0U3_
HLA-DQA10501-DQB10201	518	VVLDGNATVEGHLPD	NATVEGHLP	5	0.2280	4244.2	55.00	0.20	tr_D0A0U3_
HLA-DQA10501-DQB10201	519	IHLSPTLEQQFYVCT	LSPTLEQQF	2	0.2273	4272.7	55.00	0.21	tr_D0A0U3_
HLA-DQA10501-DQB10201	520	YGATQVSVWSMARAL	YGATQVSVW	0	0.2270	4289.5	55.00	0.27	tr_D0A0U3_
HLA-DQA10501-DQB10201	521	ITASEVVQKFYTAER	TASEVVQKF	1	0.2267	4302.4	55.00	0.46	tr_D0A0U3_
HLA-DQA10501-DQB10201	522	VLDGNATVEGHLPDS	NATVEGHLP	4	0.2266	4306.1	55.00	0.23	tr_D0A0U3_
HLA-DQA10501-DQB10201	523	VLIPDRPTALHAAVE	PTALHAAVE	6	0.2262	4323.5	55.00	0.59	tr_D0A0U3_

HLA-DQA10501-DQB10201	524	ASTRHFDSAWNTFVE	FDSAWNTFV	5	0.2260	4333.5	55.00	0.38	tr_D0A0U3_
HLA-DQA10501-DQB10201	525	VVVTGTNPLARDTQY	VVVTGTNPLA	1	0.2249	4387.8	55.00	0.31	tr_D0A0U3_
HLA-DQA10501-DQB10201	526	VEGHLPDSGDVYVIG	HLPDSGDVY	3	0.2224	4508.6	60.00	0.31	tr_D0A0U3_
HLA-DQA10501-DQB10201	527	SRRTPLSLLGFATAY	SLLGFATAY	6	0.2216	4546.4	60.00	0.52	tr_D0A0U3_
HLA-DQA10501-DQB10201	528	VASTRHFDSAWNTFV	FDSAWNTFV	6	0.2209	4579.7	60.00	0.41	tr_D0A0U3_
HLA-DQA10501-DQB10201	529	VVVLDGNATVEGHLP	VLDGNATVE	2	0.2209	4581.1	60.00	0.22	tr_D0A0U3_
HLA-DQA10501-DQB10201	530	SMRYLDPDAGKLSRG	MRYLDPDAG	1	0.2203	4611.7	60.00	0.64	tr_D0A0U3_
HLA-DQA10501-DQB10201	531	SVVVLDGNATVEGHL	VLDGNATVE	3	0.2198	4634.5	60.00	0.20	tr_D0A0U3_
HLA-DQA10501-DQB10201	532	QSVVVLDGNATVEGH	VVVLDGNAT	2	0.2194	4655.1	60.00	0.28	tr_D0A0U3_
HLA-DQA10501-DQB10201	533	DKKRLHIVIRSTDAA	HIVIRSTDA	5	0.2190	4677.0	60.00	0.38	tr_D0A0U3_
HLA-DQA10501-DQB10201	534	PFADDGCFLKGVPRL	PFADDGCFL	0	0.2185	4700.4	60.00	0.57	tr_D0A0U3_
HLA-DQA10501-DQB10201	535	QRILRRMGYGFCGVF	RMGYGFCGV	5	0.2177	4742.6	60.00	0.41	tr_D0A0U3_
HLA-DQA10501-DQB10201	536	MRVIISPMKAMNATA	RVIISPMKA	1	0.2174	4757.7	60.00	0.43	tr_D0A0U3_
HLA-DQA10501-DQB10201	537	HLSPTLEQQFYVCTS	SPTLEQQFY	2	0.2172	4765.9	60.00	0.16	tr_D0A0U3_
HLA-DQA10501-DQB10201	538	VFAPPVGDTARFIRN	PVGDTARFI	4	0.2160	4829.2	60.00	0.54	tr_D0A0U3_
HLA-DQA10501-DQB10201	539	EEYDVAQRILRRMGY	EEYDVAQRI	0	0.2158	4838.4	60.00	0.41	tr_D0A0U3_
HLA-DQA10501-DQB10201	540	WAAGQGAMCWCNQGG	AAGQGAMCW	1	0.2157	4844.8	60.00	0.41	tr_D0A0U3_
HLA-DQA10501-DQB10201	541	LSPTLEQQFYVCTSY	QQFYVCTSY	6	0.2149	4889.0	60.00	0.25	tr_D0A0U3_
HLA-DQA10501-DQB10201	542	QFYVCTSYLSRNDKK	QFYVCTSYL	0	0.2129	4994.6	60.00	0.37	tr_D0A0U3_
HLA-DQA10501-DQB10201	543	GNATVEGHLPDSGDV	VEGHLPDSG	4	0.2113	5081.8	60.00	0.38	tr_D0A0U3_
HLA-DQA10501-DQB10201	544	VHVRVIGRGYRLLDA	GRGYRLLDA	6	0.2112	5087.4	60.00	0.49	tr_D0A0U3_
HLA-DQA10501-DQB10201	545	SAELLALIRYALGRL	LIRYALGRL	6	0.2108	5107.8	60.00	0.34	tr_D0A0U3_
HLA-DQA10501-DQB10201	546	REWSTKITTAVLGVV	ITTAVLGVV	6	0.2106	5119.6	60.00	0.34	tr_D0A0U3_
HLA-DQA10501-DQB10201	547	ASEVVQKFYTAERNA	QKFYTAERN	5	0.2102	5145.6	60.00	0.58	tr_D0A0U3_
HLA-DQA10501-DQB10201	548	CGVFTLSSSLTGVAS	VFTLSSSLT	2	0.2102	5145.6	60.00	0.37	tr_D0A0U3_
HLA-DQA10501-DQB10201	549	FAPPVGDTARFIRNL	PVGDTARFI	3	0.2101	5151.7	60.00	0.60	tr_D0A0U3_
HLA-DQA10501-DQB10201	550	AHLNSHDSARVFVPF	DSARVFVPF	6	0.2082	5256.7	60.00	0.28	tr_D0A0U3_
HLA-DQA10501-DQB10201	551	GATQVSVWSMARALN	VWSMARALN	6	0.2079	5271.1	60.00	0.25	tr_D0A0U3_
HLA-DQA10501-DQB10201	552	FMQSLYDQRRYVIDD	FMQSLYDQR	0	0.2073	5304.9	60.00	0.40	tr_D0A0U3_
HLA-DQA10501-DQB10201	553	TAPSVHVKVMHPFTP	HVKVMHPFT	5	0.2060	5384.6	65.00	0.35	tr_D0A0U3_
HLA-DQA10501-DQB10201	554	SEVVQKFYTAERNAS	QKFYTAERN	4	0.2051	5434.3	65.00	0.58	tr_D0A0U3_
HLA-DQA10501-DQB10201	555	GGECAGWAAGQGAMC	WAAGQGAMC	6	0.2039	5503.7	65.00	0.52	tr_D0A0U3_
HLA-DQA10501-DQB10201	556	LHHQKIEKRFVEAVN	IEKRFVEAV	5	0.2034	5535.3	65.00	0.46	tr_D0A0U3_
HLA-DQA10501-DQB10201	557	PERLTDRKTFMQSLY	RKTFMQSLY	6	0.2033	5542.4	65.00	0.46	tr_D0A0U3_
HLA-DQA10501-DQB10201	558	LGVVDEAMLKTPGVA	LGVVDEAML	0	0.2027	5578.1	65.00	0.42	tr_D0A0U3_
HLA-DQA10501-DQB10201	559	PISGFQQAVERNRGK	ISGFQQAVE	1	0.2016	5646.4	65.00	0.46	tr_D0A0U3_
HLA-DQA10501-DQB10201	560	GVFTLSSSLTGVAST	VFTLSSSLT	1	0.2007	5700.6	65.00	0.32	tr_D0A0U3_
HLA-DQA10501-DQB10201	561	IRNLVADSRTRSAYV	IRNLVADSR	0	0.1989	5810.1	65.00	0.63	tr_D0A0U3_
HLA-DQA10501-DQB10201	562	KSNPGVTVFNASSNF	TVFNASSNF	6	0.1987	5825.9	65.00	0.65	tr_D0A0U3_
HLA-DQA10501-DQB10201	563	LTNPMTPSMRYLDPD	PSMRYLDPD	6	0.1985	5840.5	65.00	0.44	tr_D0A0U3_
HLA-DQA10501-DQB10201	564	APSVHVKVMHPFTPS	HVKVMHPFT	4	0.1982	5856.3	65.00	0.34	tr_D0A0U3_
HLA-DQA10501-DQB10201	565	ALIRYALGRLRTIRL	LGRLRTIRL	6	0.1967	5953.4	65.00	0.37	tr_D0A0U3_
HLA-DQA10501-DQB10201	566	KTAPSVHVKVMHPFT	HVKVMHPFT	6	0.1964	5974.3	65.00	0.35	tr_D0A0U3_
HLA-DQA10501-DQB10201	567	EIEVFSLLHHQKIEK	EIEVFSLLH	0	0.1958	6011.0	65.00	0.31	tr_D0A0U3_
HLA-DQA10501-DQB10201	568	DYGGECAGWAAGQGA	YGGECAGWA	1	0.1943	6110.7	65.00	0.34	tr_D0A0U3_
HLA-DQA10501-DQB10201	569	PSVHVKVMHPFTPSA	HVKVMHPFT	3	0.1942	6112.8	65.00	0.31	tr_D0A0U3_
HLA-DQA10501-DQB10201	570	FLKGVPRLTGCAVNY	RLTGCAVNY	6	0.1941	6123.5	65.00	0.36	tr_D0A0U3_
HLA-DQA10501-DQB10201	571	QREWSTKITTAVLGV	KITTAVLGV	6	0.1910	6333.7	65.00	0.44	tr_D0A0U3_
HLA-DQA10501-DQB10201	572	SGAAEELQREWSTKI	GAAEELQRE	1	0.1903	6378.9	65.00	0.38	tr_D0A0U3_
HLA-DQA10501-DQB10201	573	KGVPRLTGCAVNYGA	RLTGCAVNY	4	0.1895	6436.3	70.00	0.25	tr_D0A0U3_

HLA-DQA10501-DQB10201	574	IRYALGRLRTIRLGF	RLRTIRLGF	6	0.1893	6448.2	70.00	0.29	tr_D0A0U3_
HLA-DQA10501-DQB10201	575	EVVQKFYTAERNASR	QKFYTAERN	3	0.1881	6530.0	70.00	0.55	tr_D0A0U3_
HLA-DQA10501-DQB10201	576	LKGVPRLTGCAVNYG	RLTGCAVNY	5	0.1881	6532.9	70.00	0.30	tr_D0A0U3_
HLA-DQA10501-DQB10201	577	YHAIRRFQRDVRSYL	FQRDVRSYL	6	0.1872	6594.6	70.00	0.37	tr_D0A0U3_
HLA-DQA10501-DQB10201	578	LEVREHNQDSRLFFD	REHNQDSRL	3	0.1870	6610.2	70.00	0.28	tr_D0A0U3_
HLA-DQA10501-DQB10201	579	GCFLKGVPRLTGCAV	VPRLTGCAV	6	0.1870	6613.7	70.00	0.49	tr_D0A0U3_
HLA-DQA10501-DQB10201	580	LSSSLTGVASTRHFD	LTGVASTRH	4	0.1868	6625.9	70.00	0.51	tr_D0A0U3_
HLA-DQA10501-DQB10201	581	SVHVKVMHPFTPSAS	HVKVMHPFT	2	0.1863	6658.5	70.00	0.34	tr_D0A0U3_
HLA-DQA10501-DQB10201	582	GDYGGECAGWAAGQG	YGGECAGWA	2	0.1855	6719.4	70.00	0.49	tr_D0A0U3_
HLA-DQA10501-DQB10201	583	AQRILRRMGYGFCGV	RMGYGFCGV	6	0.1850	6756.7	70.00	0.57	tr_D0A0U3_
HLA-DQA10501-DQB10201	584	FTPSASPISGFQQAV	PISGFQQAV	6	0.1847	6778.2	70.00	0.35	tr_D0A0U3_
HLA-DQA10501-DQB10201	585	AAHLNSHDSARVFVP	SHDSARVFV	5	0.1845	6788.6	70.00	0.38	tr_D0A0U3_
HLA-DQA10501-DQB10201	586	EGASLLEVREHNQDS	GASLLEVRE	1	0.1844	6796.1	70.00	0.34	tr_D0A0U3_
HLA-DQA10501-DQB10201	587	VEAVNAGFNASMTSR	VEAVNAGFN	0	0.1832	6889.7	70.00	0.26	tr_D0A0U3_
HLA-DQA10501-DQB10201	588	TLSSSLTGVASTRHF	LTGVASTRH	5	0.1815	7016.4	70.00	0.44	tr_D0A0U3_
HLA-DQA10501-DQB10201	589	SSSLTGVASTRHFDS	LTGVASTRH	3	0.1797	7152.7	70.00	0.47	tr_D0A0U3_
HLA-DQA10501-DQB10201	590	IAAHLNSHDSARVFV	SHDSARVFV	6	0.1796	7162.6	70.00	0.35	tr_D0A0U3_
HLA-DQA10501-DQB10201	591	EVREHNQDSRLFFDT	REHNQDSRL	2	0.1791	7198.7	70.00	0.28	tr_D0A0U3_
HLA-DQA10501-DQB10201	592	RLNKMERNVIHLSPT	ERNVIHLSP	5	0.1790	7207.9	70.00	0.45	tr_D0A0U3_
HLA-DQA10501-DQB10201	593	GVPRLTGCAVNYGAT	RLTGCAVNY	3	0.1786	7243.8	70.00	0.26	tr_D0A0U3_
HLA-DQA10501-DQB10201	594	GVVDEAMLKTPGVAF	MLKTPGVAF	6	0.1782	7270.1	70.00	0.31	tr_D0A0U3_
HLA-DQA10501-DQB10201	595	FTLSSSLTGVASTRH	LTGVASTRH	6	0.1780	7286.8	70.00	0.34	tr_D0A0U3_
HLA-DQA10501-DQB10201	596	VDEAMLKTPGVAFID	MLKTPGVAF	4	0.1776	7315.1	70.00	0.34	tr_D0A0U3_
HLA-DQA10501-DQB10201	597	WKTAPSVHVKVMHPF	VHVKVMHPF	6	0.1769	7370.6	70.00	0.42	tr_D0A0U3_
HLA-DQA10501-DQB10201	598	GVASTRHFDSAWNTF	HFDSAWNTF	6	0.1767	7392.1	70.00	0.29	tr_D0A0U3_
HLA-DQA10501-DQB10201	599	VFTLSSSLTGVASTR	VFTLSSSLT	0	0.1763	7421.3	70.00	0.25	tr_D0A0U3_
HLA-DQA10501-DQB10201	600	NDKKRLHIVIRSTDA	HIVIRSTDA	6	0.1758	7459.1	70.00	0.54	tr_D0A0U3_
HLA-DQA10501-DQB10201	601	LLALIRYALGRLRTI	LIRYALGRL	3	0.1758	7463.9	70.00	0.27	tr_D0A0U3_
HLA-DQA10501-DQB10201	602	NLVADSRTRSAYVLV	RTRSAYVLV	6	0.1753	7501.0	70.00	0.57	tr_D0A0U3_
HLA-DQA10501-DQB10201	603	DPVVIAAHLNSHDSA	AHLNSHDSA	6	0.1748	7543.4	70.00	0.24	tr_D0A0U3_
HLA-DQA10501-DQB10201	604	SLTGVASTRHFDSAW	LTGVASTRH	1	0.1744	7576.7	70.00	0.39	tr_D0A0U3_
HLA-DQA10501-DQB10201	605	LLEVREHNQDSRLFF	REHNQDSRL	4	0.1743	7584.1	70.00	0.29	tr_D0A0U3_
HLA-DQA10501-DQB10201	606	PRLNKMERNVIHLSP	ERNVIHLSP	6	0.1738	7622.6	70.00	0.54	tr_D0A0U3_
HLA-DQA10501-DQB10201	607	AELLALIRYALGRLR	LIRYALGRL	5	0.1738	7624.0	70.00	0.33	tr_D0A0U3_
HLA-DQA10501-DQB10201	608	CFLKGVPRLTGCAVN	VPRLTGCAV	5	0.1735	7653.7	70.00	0.46	tr_D0A0U3_
HLA-DQA10501-DQB10201	609	ELLALIRYALGRLRT	LIRYALGRL	4	0.1734	7657.4	70.00	0.34	tr_D0A0U3_
HLA-DQA10501-DQB10201	610	VVQKFYTAERNASRR	QKFYTAERN	2	0.1726	7725.9	70.00	0.54	tr_D0A0U3_
HLA-DQA10501-DQB10201	611	VPRLTGCAVNYGATQ	RLTGCAVNY	2	0.1723	7751.4	70.00	0.27	tr_D0A0U3_
HLA-DQA10501-DQB10201	612	SSLTGVASTRHFDSA	LTGVASTRH	2	0.1720	7777.0	70.00	0.45	tr_D0A0U3_
HLA-DQA10501-DQB10201	613	VVDEAMLKTPGVAFI	MLKTPGVAF	5	0.1719	7782.7	75.00	0.28	tr_D0A0U3_
HLA-DQA10501-DQB10201	614	LIRYALGRLRTIRLG	LGRLRTIRL	5	0.1708	7877.6	75.00	0.35	tr_D0A0U3_
HLA-DQA10501-DQB10201	615	RGWNPNLYFTRASPS	LYFTRASPS	6	0.1705	7900.3	75.00	0.38	tr_D0A0U3_
HLA-DQA10501-DQB10201	616	TAADPVVIAAHLNSH	DPVVIAAHL	3	0.1693	8010.6	75.00	0.33	tr_D0A0U3_
HLA-DQA10501-DQB10201	617	LLHHQKIEKRFVEAV	IEKRFVEAV	6	0.1689	8044.1	75.00	0.62	tr_D0A0U3_
HLA-DQA10501-DQB10201	618	AVERNRGKLFVVVGP	GKLFVVVGP	6	0.1685	8076.6	75.00	0.31	tr_D0A0U3_
HLA-DQA10501-DQB10201	619	LNASIPPLTNPMTPS	LNASIPPLT	0	0.1684	8085.2	75.00	0.58	tr_D0A0U3_
HLA-DQA10501-DQB10201	620	HAIRRFQRDVRSYLK	FQRDVRSYL	5	0.1679	8130.9	75.00	0.34	tr_D0A0U3_
HLA-DQA10501-DQB10201	621	VHVKVMHPFTPSASP	HVKVMHPFT	1	0.1677	8144.4	75.00	0.37	tr_D0A0U3_
HLA-DQA10501-DQB10201	622	AADPVVIAAHLNSHD	DPVVIAAHL	2	0.1670	8207.9	75.00	0.29	tr_D0A0U3_
HLA-DQA10501-DQB10201	623	ADPVVIAAHLNSHDS	DPVVIAAHL	1	0.1652	8371.9	75.00	0.25	tr_D0A0U3_

HLA-DQA10501-DQB10201	624	LTGVASTRHFDSAWN	TRHFDSAWN	6	0.1647	8417.5	75.00	0.25	tr_D0A0U3_
HLA-DQA10501-DQB10201	625	VVTGTNPLARDTQYH	VVTGTNPLA	0	0.1624	8629.8	75.00	0.34	tr_D0A0U3_
HLA-DQA10501-DQB10201	626	NASRRTPLSLLGFAT	PLSLLGFAT	6	0.1619	8670.3	75.00	0.25	tr_D0A0U3_
HLA-DQA10501-DQB10201	627	SVLIPDRPTALHAAV	LIPDRPTAL	2	0.1615	8709.4	75.00	0.41	tr_D0A0U3_
HLA-DQA10501-DQB10201	628	IEDVLKKTLATFSVE	KTLATFSVE	6	0.1607	8782.4	75.00	0.47	tr_D0A0U3_
HLA-DQA10501-DQB10201	629	TGSSGGRGWNPNLYF	RGWNPNLYF	6	0.1603	8824.7	75.00	0.47	tr_D0A0U3_
HLA-DQA10501-DQB10201	630	LALIRYALGRLRTIR	LIRYALGRL	2	0.1599	8859.7	75.00	0.28	tr_D0A0U3_
HLA-DQA10501-DQB10201	631	TQYHAIRRFQRDVRS	RRFQRDVRS	6	0.1595	8897.8	75.00	0.22	tr_D0A0U3_
HLA-DQA10501-DQB10201	632	ASRRTPLSLLGFATA	PLSLLGFAT	5	0.1581	9042.2	75.00	0.25	tr_D0A0U3_
HLA-DQA10501-DQB10201	633	FADDGCFLKGVPRLT	FLKGVPRLT	6	0.1577	9079.4	75.00	0.44	tr_D0A0U3_
HLA-DQA10501-DQB10201	634	VMHPFTPSASPISGF	PSASPISGF	6	0.1570	9150.5	75.00	0.33	tr_D0A0U3_
HLA-DQA10501-DQB10201	635	AIRRFQRDVRSYLKS	FQRDVRSYL	4	0.1561	9239.5	75.00	0.35	tr_D0A0U3_
HLA-DQA10501-DQB10201	636	ERNASRRTPLSLLGF	RTPLSLLGF	6	0.1552	9321.1	75.00	0.46	tr_D0A0U3_
HLA-DQA10501-DQB10201	637	VVAFAPLTGSSGGRG	VVAFAPLTG	0	0.1539	9453.8	75.00	0.61	tr_D0A0U3_
HLA-DQA10501-DQB10201	638	TGVASTRHFDSAWNT	TRHFDSAWN	5	0.1539	9455.2	75.00	0.26	tr_D0A0U3_
HLA-DQA10501-DQB10201	639	YGGECAGWAAGQGAM	AGWAAGQGA	5	0.1531	9540.0	80.00	0.45	tr_D0A0U3_
HLA-DQA10501-DQB10201	640	QYHAIRRFQRDVRSY	IRRFQRDVR	4	0.1529	9557.2	80.00	0.29	tr_D0A0U3_
HLA-DQA10501-DQB10201	641	GRGWNPNLYFTRASP	NLYFTRASP	6	0.1528	9568.2	80.00	0.25	tr_D0A0U3_
HLA-DQA10501-DQB10201	642	SLLEVREHNQDSRLF	REHNQDSRL	5	0.1527	9586.0	80.00	0.34	tr_D0A0U3_
HLA-DQA10501-DQB10201	643	GAAEELQREWSTKIT	GAAEELQRE	0	0.1525	9598.2	80.00	0.29	tr_D0A0U3_
HLA-DQA10501-DQB10201	644	RNASRRTPLSLLGFA	RTPLSLLGF	5	0.1519	9665.2	80.00	0.41	tr_D0A0U3_
HLA-DQA10501-DQB10201	645	FIDPVPLAPRLNKME	FIDPVPLAP	0	0.1517	9686.8	80.00	0.53	tr_D0A0U3_
HLA-DQA10501-DQB10201	646	LQREWSTKITTAVLG	TKITTAVLG	6	0.1515	9702.0	80.00	0.32	tr_D0A0U3_
HLA-DQA10501-DQB10201	647	VQKFYTAERNASRRT	QKFYTAERN	1	0.1515	9706.8	80.00	0.55	tr_D0A0U3_
HLA-DQA10501-DQB10201	648	IEVFSLLHHQKIEKR	FSLLHHQKI	3	0.1515	9709.0	80.00	0.38	tr_D0A0U3_
HLA-DQA10501-DQB10201	649	NPLARDTQYHAIRRF	TQYHAIRRF	6	0.1513	9728.7	80.00	0.52	tr_D0A0U3_
HLA-DQA10501-DQB10201	650	VIAAHLNSHDSARVF	AHLNSHDSA	3	0.1507	9787.1	80.00	0.34	tr_D0A0U3_
HLA-DQA10501-DQB10201	651	DTQYHAIRRFQRDVR	IRRFQRDVR	6	0.1505	9808.9	80.00	0.24	tr_D0A0U3_
HLA-DQA10501-DQB10201	652	ISGFQQAVERNRGKL	ISGFQQAVE	0	0.1489	9987.4	80.00	0.40	tr_D0A0U3_
HLA-DQA10501-DQB10201	653	GSSGGRGWNPNLYFT	GRGWNPNLY	4	0.1489	9988.1	80.00	0.46	tr_D0A0U3_
HLA-DQA10501-DQB10201	654	HVKVMHPFTPSASPI	HVKVMHPFT	0	0.1478	10098.0	80.00	0.27	tr_D0A0U3_
HLA-DQA10501-DQB10201	655	ADDGCFLKGVPRLTG	FLKGVPRLT	5	0.1471	10182.2	80.00	0.47	tr_D0A0U3_
HLA-DQA10501-DQB10201	656	ASLLEVREHNQDSRL	REHNQDSRL	6	0.1464	10257.3	80.00	0.35	tr_D0A0U3_
HLA-DQA10501-DQB10201	657	EAVNAGFNASMTSRR	NAGFNASMT	3	0.1454	10369.0	80.00	0.26	tr_D0A0U3_
HLA-DQA10501-DQB10201	658	HPFTPSASPISGFQQ	PSASPISGF	4	0.1448	10437.5	80.00	0.28	tr_D0A0U3_
HLA-DQA10501-DQB10201	659	ELQREWSTKITTAVL	STKITTAVL	6	0.1447	10452.5	80.00	0.42	tr_D0A0U3_
HLA-DQA10501-DQB10201	660	DDGCFLKGVPRLTGC	FLKGVPRLT	4	0.1446	10456.3	80.00	0.49	tr_D0A0U3_
HLA-DQA10501-DQB10201	661	ARDTQYHAIRRFQRD	TQYHAIRRF	3	0.1438	10551.0	80.00	0.38	tr_D0A0U3_
HLA-DQA10501-DQB10201	662	MHPFTPSASPISGFQ	PSASPISGF	5	0.1435	10579.1	80.00	0.32	tr_D0A0U3_
HLA-DQA10501-DQB10201	663	GASLLEVREHNQDSR	GASLLEVRE	0	0.1434	10593.4	80.00	0.23	tr_D0A0U3_
HLA-DQA10501-DQB10201	664	SMTSRRWKTAPSVHV	WKTAPSVHV	6	0.1431	10632.5	80.00	0.60	tr_D0A0U3_
HLA-DQA10501-DQB10201	665	RDTQYHAIRRFQRDV	TQYHAIRRF	2	0.1427	10675.8	80.00	0.31	tr_D0A0U3_
HLA-DQA10501-DQB10201	666	SGGRGWNPNLYFTRA	GRGWNPNLY	2	0.1426	10686.6	80.00	0.36	tr_D0A0U3_
HLA-DQA10501-DQB10201	667	PVVIAAHLNSHDSAR	AHLNSHDSA	5	0.1421	10742.5	80.00	0.32	tr_D0A0U3_
HLA-DQA10501-DQB10201	668	IRRFQRDVRSYLKSN	FQRDVRSYL	3	0.1413	10840.8	80.00	0.38	tr_D0A0U3_
HLA-DQA10501-DQB10201	669	AVNAGFNASMTSRRW	NAGFNASMT	2	0.1404	10950.1	80.00	0.21	tr_D0A0U3_
HLA-DQA10501-DQB10201	670	PFTPSASPISGFQQA	PSASPISGF	3	0.1402	10973.9	80.00	0.31	tr_D0A0U3_
HLA-DQA10501-DQB10201	671	RWKTAPSVHVKVMHP	SVHVKVMHP	6	0.1394	11058.8	80.00	0.41	tr_D0A0U3_
HLA-DQA10501-DQB10201	672	TVHVRVIGRGYRLLD	RVIGRGYRL	4	0.1386	11158.7	80.00	0.35	tr_D0A0U3_
HLA-DQA10501-DQB10201	673	GGRGWNPNLYFTRAS	RGWNPNLYF	2	0.1386	11159.5	80.00	0.25	tr_D0A0U3_

HLA-DQA10501-DQB10201	674	EVFSLLHHQKIEKRF	FSLLHHQKI	2	0.1380	11239.4	80.00	0.40	tr_D0A0U3_
HLA-DQA10501-DQB10201	675	FYVCTSYLSRNDKKR	FYVCTSYLS	0	0.1379	11250.7	80.00	0.47	tr_D0A0U3_
HLA-DQA10501-DQB10201	676	DGCFLKGVPRLTGCA	FLKGVPRLT	3	0.1369	11370.1	80.00	0.51	tr_D0A0U3_
HLA-DQA10501-DQB10201	677	MRYLDPDAGKLSRGQ	MRYLDPDAG	0	0.1362	11453.7	80.00	0.73	tr_D0A0U3_
HLA-DQA10501-DQB10201	678	PLARDTQYHAIRRFQ	TQYHAIRRF	5	0.1359	11490.1	80.00	0.47	tr_D0A0U3_
HLA-DQA10501-DQB10201	679	SSGGRGWNPNLYFTR	GRGWNPNLY	3	0.1355	11545.3	80.00	0.44	tr_D0A0U3_
HLA-DQA10501-DQB10201	680	VVIAAHLNSHDSARV	AHLNSHDSA	4	0.1348	11631.8	80.00	0.35	tr_D0A0U3_
HLA-DQA10501-DQB10201	681	VPERLTDRKTFMQSL	DRKTFMQSL	6	0.1344	11683.3	80.00	0.55	tr_D0A0U3_
HLA-DQA10501-DQB10201	682	VKVMHPFTPSASPIS	FTPSASPIS	6	0.1323	11942.2	85.00	0.27	tr_D0A0U3_
HLA-DQA10501-DQB10201	683	EYDVAQRILRRMGYG	EYDVAQRIL	0	0.1323	11953.7	85.00	0.44	tr_D0A0U3_
HLA-DQA10501-DQB10201	684	TSRRWKTAPSVHVKV	WKTAPSVHV	4	0.1309	12125.8	85.00	0.54	tr_D0A0U3_
HLA-DQA10501-DQB10201	685	QAVERNRGKLFVVVG	RGKLFVVVG	6	0.1291	12375.2	85.00	0.37	tr_D0A0U3_
HLA-DQA10501-DQB10201	686	SRRWKTAPSVHVKVM	WKTAPSVHV	3	0.1289	12392.4	85.00	0.51	tr_D0A0U3_
HLA-DQA10501-DQB10201	687	PLTNPMTPSMRYLDP	TPSMRYLDP	6	0.1287	12427.2	85.00	0.36	tr_D0A0U3_
HLA-DQA10501-DQB10201	688	NTVHVRVIGRGYRLL	RVIGRGYRL	5	0.1284	12463.3	85.00	0.34	tr_D0A0U3_
HLA-DQA10501-DQB10201	689	KVMHPFTPSASPISG	FTPSASPIS	5	0.1276	12573.6	85.00	0.23	tr_D0A0U3_
HLA-DQA10501-DQB10201	690	RRFQRDVRSYLKSNP	FQRDVRSYL	2	0.1270	12648.4	85.00	0.40	tr_D0A0U3_
HLA-DQA10501-DQB10201	691	LARDTQYHAIRRFQR	TQYHAIRRF	4	0.1269	12673.1	85.00	0.41	tr_D0A0U3_
HLA-DQA10501-DQB10201	692	MTSRRWKTAPSVHVK	WKTAPSVHV	5	0.1261	12777.4	85.00	0.58	tr_D0A0U3_
HLA-DQA10501-DQB10201	693	AAGQGAMCWCNQGGN	AAGQGAMCW	0	0.1239	13085.2	85.00	0.50	tr_D0A0U3_
HLA-DQA10501-DQB10201	694	VAQRILRRMGYGFCG	ILRRMGYGF	4	0.1237	13107.2	85.00	0.30	tr_D0A0U3_
HLA-DQA10501-DQB10201	695	QQAVERNRGKLFVVV	NRGKLFVVV	6	0.1229	13230.9	85.00	0.37	tr_D0A0U3_
HLA-DQA10501-DQB10201	696	TGTNPLARDTQYHAI	NPLARDTQY	3	0.1211	13487.0	85.00	0.23	tr_D0A0U3_
HLA-DQA10501-DQB10201	697	AAAIEDVLKKTLATF	AAAIEDVLK	0	0.1208	13527.8	85.00	0.46	tr_D0A0U3_
HLA-DQA10501-DQB10201	698	RRWKTAPSVHVKVMH	WKTAPSVHV	2	0.1206	13561.3	85.00	0.50	tr_D0A0U3_
HLA-DQA10501-DQB10201	699	GNTVHVRVIGRGYRL	RVIGRGYRL	6	0.1201	13627.5	85.00	0.36	tr_D0A0U3_
HLA-DQA10501-DQB10201	700	RFQRDVRSYLKSNPG	FQRDVRSYL	1	0.1195	13729.3	85.00	0.38	tr_D0A0U3_
HLA-DQA10501-DQB10201	701	DVAQRILRRMGYGFC	ILRRMGYGF	5	0.1193	13755.5	85.00	0.35	tr_D0A0U3_
HLA-DQA10501-DQB10201	702	VTGTNPLARDTQYHA	NPLARDTQY	4	0.1190	13793.5	85.00	0.23	tr_D0A0U3_
HLA-DQA10501-DQB10201	703	RNLVADSRTRSAYVL	SRTRSAYVL	6	0.1174	14045.5	85.00	0.37	tr_D0A0U3_
HLA-DQA10501-DQB10201	704	QKFYTAERNASRRTP	QKFYTAERN	0	0.1163	14204.4	85.00	0.47	tr_D0A0U3_
HLA-DQA10501-DQB10201	705	VFSLLHHQKIEKRFV	FSLLHHQKI	1	0.1157	14296.5	85.00	0.41	tr_D0A0U3_
HLA-DQA10501-DQB10201	706	RNDKKRLHIVIRSTD	RLHIVIRST	5	0.1153	14365.3	85.00	0.42	tr_D0A0U3_
HLA-DQA10501-DQB10201	707	SGFQQAVERNRGKLF	VERNRGKLF	6	0.1150	14413.3	85.00	0.31	tr_D0A0U3_
HLA-DQA10501-DQB10201	708	FQRDVRSYLKSNPGV	FQRDVRSYL	0	0.1148	14435.7	85.00	0.25	tr_D0A0U3_
HLA-DQA10501-DQB10201	709	RSYLKSNPGVTVFNA	NPGVTVFNA	6	0.1136	14620.3	90.00	0.30	tr_D0A0U3_
HLA-DQA10501-DQB10201	710	VNAGFNASMTSRRWK	AGFNASMTS	2	0.1133	14673.5	90.00	0.25	tr_D0A0U3_
HLA-DQA10501-DQB10201	711	EELQREWSTKITTAV	WSTKITTAV	6	0.1130	14716.8	90.00	0.50	tr_D0A0U3_
HLA-DQA10501-DQB10201	712	SYLKSNPGVTVFNAS	NPGVTVFNA	5	0.1123	14828.7	90.00	0.25	tr_D0A0U3_
HLA-DQA10501-DQB10201	713	LTGSSGGRGWNPNLY	GRGWNPNLY	6	0.1100	15207.4	90.00	0.70	tr_D0A0U3_
HLA-DQA10501-DQB10201	714	AIEDVLKKTLATFSV	KKTLATFSV	6	0.1099	15226.8	90.00	0.34	tr_D0A0U3_
HLA-DQA10501-DQB10201	715	YDVAQRILRRMGYGF	ILRRMGYGF	6	0.1086	15439.7	90.00	0.41	tr_D0A0U3_
HLA-DQA10501-DQB10201	716	DVRSYLKSNPGVTVF	LKSNPGVTV	5	0.1082	15503.5	90.00	0.28	tr_D0A0U3_
HLA-DQA10501-DQB10201	717	GTNPLARDTQYHAIR	NPLARDTQY	2	0.1068	15750.8	90.00	0.23	tr_D0A0U3_
HLA-DQA10501-DQB10201	718	APRLNKMERNVIHLS	MERNVIHLS	6	0.1064	15806.5	90.00	0.30	tr_D0A0U3_
HLA-DQA10501-DQB10201	719	AAEELQREWSTKITT	AEELQREWS	1	0.1063	15830.8	90.00	0.44	tr_D0A0U3_
HLA-DQA10501-DQB10201	720	AERNASRRTPLSLLG	ASRRTPLSL	4	0.1058	15912.0	90.00	0.34	tr_D0A0U3_
HLA-DQA10501-DQB10201	721	TAERNASRRTPLSLL	ASRRTPLSL	5	0.1044	16153.7	90.00	0.44	tr_D0A0U3_
HLA-DQA10501-DQB10201	722	QGGNTVHVRVIGRGY	HVRVIGRGY	6	0.1044	16166.9	90.00	0.25	tr_D0A0U3_
HLA-DQA10501-DQB10201	723	RDVRSYLKSNPGVTV	LKSNPGVTV	6	0.1041	16214.8	90.00	0.31	tr_D0A0U3_

HLA-DQA10501-DQB10201	724	LAPRLNKMERNVIHL	KMERNVIHL	6	0.1040	16226.5	90.00	0.29	tr_D0A0U3_
HLA-DQA10501-DQB10201	725	VRSYLKSNPGVTVFN	LKSNPGVTV	4	0.1040	16232.8	90.00	0.25	tr_D0A0U3_
HLA-DQA10501-DQB10201	726	FQQAVERNRGKLFVV	VERNRGKLF	4	0.1037	16288.4	90.00	0.32	tr_D0A0U3_
HLA-DQA10501-DQB10201	727	DPVPLAPRLNKMERN	PRLNKMERN	6	0.1032	16365.1	90.00	0.34	tr_D0A0U3_
HLA-DQA10501-DQB10201	728	SRNDKKRLHIVIRST	RLHIVIRST	6	0.1030	16411.2	90.00	0.53	tr_D0A0U3_
HLA-DQA10501-DQB10201	729	TNPLARDTQYHAIRR	NPLARDTQY	1	0.1027	16451.7	90.00	0.22	tr_D0A0U3_
HLA-DQA10501-DQB10201	730	CWCNQGGNTVHVRVI	GGNTVHVRV	5	0.1026	16478.5	90.00	0.41	tr_D0A0U3_
HLA-DQA10501-DQB10201	731	YLKSNPGVTVFNASS	NPGVTVFNA	4	0.1025	16500.6	90.00	0.26	tr_D0A0U3_
HLA-DQA10501-DQB10201	732	LKSNPGVTVFNASSN	VTVFNASSN	6	0.1019	16601.2	90.00	0.28	tr_D0A0U3_
HLA-DQA10501-DQB10201	733	CNQGGNTVHVRVIGR	TVHVRVIGR	6	0.0998	16979.8	90.00	0.30	tr_D0A0U3_
HLA-DQA10501-DQB10201	734	VPLAPRLNKMERNVI	PRLNKMERN	4	0.0998	16989.4	90.00	0.33	tr_D0A0U3_
HLA-DQA10501-DQB10201	735	GFQQAVERNRGKLFV	VERNRGKLF	5	0.0993	17082.1	90.00	0.39	tr_D0A0U3_
HLA-DQA10501-DQB10201	736	NASIPPLTNPMTPSM	IPPLTNPMT	3	0.0983	17264.9	90.00	0.25	tr_D0A0U3_
HLA-DQA10501-DQB10201	737	WCNQGGNTVHVRVIG	GGNTVHVRV	4	0.0967	17559.8	90.00	0.32	tr_D0A0U3_
HLA-DQA10501-DQB10201	738	NQGGNTVHVRVIGRG	TVHVRVIGR	5	0.0953	17835.7	90.00	0.28	tr_D0A0U3_
HLA-DQA10501-DQB10201	739	MCWCNQGGNTVHVRV	GGNTVHVRV	6	0.0942	18050.0	90.00	0.48	tr_D0A0U3_
HLA-DQA10501-DQB10201	740	SLLHHQKIEKRFVEA	KIEKRFVEA	6	0.0942	18052.9	90.00	0.47	tr_D0A0U3_
HLA-DQA10501-DQB10201	741	QRDVRSYLKSNPGVT	SYLKSNPGV	5	0.0931	18253.3	90.00	0.28	tr_D0A0U3_
HLA-DQA10501-DQB10201	742	PVPLAPRLNKMERNV	PRLNKMERN	5	0.0930	18286.7	90.00	0.40	tr_D0A0U3_
HLA-DQA10501-DQB10201	743	AEELQREWSTKITTA	EWSTKITTA	6	0.0922	18429.7	90.00	0.40	tr_D0A0U3_
HLA-DQA10501-DQB10201	744	FSLLHHQKIEKRFVE	FSLLHHQKI	0	0.0922	18434.9	90.00	0.29	tr_D0A0U3_
HLA-DQA10501-DQB10201	745	GGNTVHVRVIGRGYR	HVRVIGRGY	5	0.0922	18445.5	90.00	0.26	tr_D0A0U3_
HLA-DQA10501-DQB10201	746	ALSVPERLTDRKTFM	RLTDRKTFM	6	0.0909	18695.2	95.00	0.47	tr_D0A0U3_
HLA-DQA10501-DQB10201	747	IPPLTNPMTPSMRYL	PMTPSMRYL	6	0.0909	18701.1	95.00	0.20	tr_D0A0U3_
HLA-DQA10501-DQB10201	748	NAGFNASMTSRRWKT	AGFNASMTS	1	0.0902	18845.7	95.00	0.29	tr_D0A0U3_
HLA-DQA10501-DQB10201	749	AAIEDVLKKTLATFS	LKKTLATFS	6	0.0899	18908.6	95.00	0.44	tr_D0A0U3_
HLA-DQA10501-DQB10201	750	SIPPLTNPMTPSMRY	NPMTPSMRY	6	0.0897	18946.3	95.00	0.19	tr_D0A0U3_
HLA-DQA10501-DQB10201	751	PLAPRLNKMERNVIH	PRLNKMERN	3	0.0891	19073.8	95.00	0.31	tr_D0A0U3_
HLA-DQA10501-DQB10201	752	PPLTNPMTPSMRYLD	PMTPSMRYL	5	0.0885	19188.7	95.00	0.23	tr_D0A0U3_
HLA-DQA10501-DQB10201	753	ASIPPLTNPMTPSMR	IPPLTNPMT	2	0.0884	19209.9	95.00	0.28	tr_D0A0U3_
HLA-DQA10501-DQB10201	754	YTAERNASRRTPLSL	ASRRTPLSL	6	0.0858	19765.2	95.00	0.58	tr_D0A0U3_
HLA-DQA10501-DQB10201	755	NASMTSRRWKTAPSV	RRWKTAPSV	6	0.0831	20335.7	95.00	0.74	tr_D0A0U3_
HLA-DQA10501-DQB10201	756	ASMTSRRWKTAPSVH	RRWKTAPSV	5	0.0819	20611.3	95.00	0.64	tr_D0A0U3_
HLA-DQA10501-DQB10201	757	LSVPERLTDRKTFMQ	RLTDRKTFM	5	0.0814	20731.9	95.00	0.52	tr_D0A0U3_
HLA-DQA10501-DQB10201	758	IDPVPLAPRLNKMER	PVPLAPRLN	2	0.0799	21058.3	95.00	0.29	tr_D0A0U3_
HLA-DQA10501-DQB10201	759	SVPERLTDRKTFMQS	RLTDRKTFM	4	0.0787	21341.8	95.00	0.51	tr_D0A0U3_
HLA-DQA10501-DQB10201	760	KFYTAERNASRRTPL	FYTAERNAS	1	0.0758	22018.0	95.00	0.30	tr_D0A0U3_
HLA-DQA10501-DQB10201	761	AGFNASMTSRRWKTA	NASMTSRRW	3	0.0726	22799.6	95.00	0.31	tr_D0A0U3_
HLA-DQA10501-DQB10201	762	LSRNDKKRLHIVIRS	KRLHIVIRS	6	0.0723	22870.1	95.00	0.64	tr_D0A0U3_
HLA-DQA10501-DQB10201	763	AGQGAMCWCNQGGNT	AGQGAMCWC	0	0.0673	24131.2	95.00	0.31	tr_D0A0U3_
HLA-DQA10501-DQB10201	764	YLDPDAGKLSRGQLA	GKLSRGQLA	6	0.0670	24226.2	95.00	0.53	tr_D0A0U3_
HLA-DQA10501-DQB10201	765	GQGAMCWCNQGGNTV	WCNQGGNTV	6	0.0661	24466.1	95.00	0.45	tr_D0A0U3_
HLA-DQA10501-DQB10201	766	VAFAPLTGSSGGRGW	FAPLTGSSG	2	0.0658	24538.8	95.00	0.33	tr_D0A0U3_
HLA-DQA10501-DQB10201	767	FYTAERNASRRTPLS	FYTAERNAS	0	0.0634	25187.9	100.00	0.30	tr_D0A0U3_
HLA-DQA10501-DQB10201	768	YVCTSYLSRNDKKRL	YVCTSYLSR	0	0.0623	25480.3	100.00	0.39	tr_D0A0U3_
HLA-DQA10501-DQB10201	769	AMCWCNQGGNTVHVR	WCNQGGNTV	3	0.0601	26106.3	100.00	0.31	tr_D0A0U3_
HLA-DQA10501-DQB10201	770	QGAMCWCNQGGNTVH	WCNQGGNTV	5	0.0577	26782.9	100.00	0.47	tr_D0A0U3_
HLA-DQA10501-DQB10201	771	GAMCWCNQGGNTVHV	WCNQGGNTV	4	0.0576	26803.5	100.00	0.41	tr_D0A0U3_
HLA-DQA10501-DQB10201	772	GFNASMTSRRWKTAP	NASMTSRRW	2	0.0570	26989.5	100.00	0.44	tr_D0A0U3_
HLA-DQA10501-DQB10201	773	RYLDPDAGKLSRGQL	YLDPDAGKL	1	0.0522	28413.8	100.00	0.25	tr_D0A0U3_

HLA-DQA10501-DQB10201	774	AFAPLTGSSGGRGWN	FAPLTGSSG	1	0.0512	28721.3	100.00	0.40	tr_D0A0U3_
HLA-DQA10501-DQB10201	775	FAPLTGSSGGRGWNP	LTGSSGGRG	3	0.0493	29339.7	100.00	0.31	tr_D0A0U3_
HLA-DQA10501-DQB10201	776	FNASMTSRRWKTAPS	NASMTSRRW	1	0.0468	30136.1	100.00	0.51	tr_D0A0U3_
HLA-DQA10501-DQB10201	777	PLTGSSGGRGWNPNL	GGRGWNPNL	6	0.0455	30567.9	100.00	0.25	tr_D0A0U3_
HLA-DQA10501-DQB10201	778	APLTGSSGGRGWNPN	LTGSSGGRG	2	0.0403	32322.7	100.00	0.34	tr_D0A0U3_
HLA-DQA10501-DQB10201	779	SYLSRNDKKRLHIVI	DKKRLHIVI	6	0.0389	32817.1	100.00	0.47	tr_D0A0U3_
HLA-DQA10501-DQB10201	780	YLSRNDKKRLHIVIR	DKKRLHIVI	5	0.0387	32897.8	100.00	0.35	tr_D0A0U3_
HLA-DQA10501-DQB10201	781	VCTSYLSRNDKKRLH	TSYLSRNDK	2	0.0349	34291.9	100.00	0.29	tr_D0A0U3_
HLA-DQA10501-DQB10201	782	CTSYLSRNDKKRLHI	TSYLSRNDK	1	0.0311	35694.9	100.00	0.28	tr_D0A0U3_
HLA-DQA10501-DQB10201	783	TSYLSRNDKKRLHIV	SYLSRNDKK	1	0.0275	37149.3	100.00	0.28	tr_D0A0U3_

Allele: HLA-DQA10501-DQB10201. Number of high binders 0. Number of weak binders 117. Number of peptides 783

Allele	pos	peptide	core	Of	1-log50k(aff)	affinity(nM)	%Rank	Relia	Identity	Bind Level
HLA-DQA10501-DQB10201	1	HAATVMRADEEFDSL	TVMRADEEF	3	0.7077	23.6	0.15	0.47	tr_C9ZWY7_	SB
HLA-DQA10501-DQB10201	2	AATVMRADEEFDSLW	TVMRADEEF	2	0.7076	23.7	0.15	0.42	tr_C9ZWY7_	SB
HLA-DQA10501-DQB10201	3	KMHAATVMRADEEFD	TVMRADEEF	5	0.7001	25.7	0.17	0.50	tr_C9ZWY7_	SB
HLA-DQA10501-DQB10201	4	MHAATVMRADEEFDS	TVMRADEEF	4	0.6992	25.9	0.17	0.49	tr_C9ZWY7_	SB
HLA-DQA10501-DQB10201	5	ATVMRADEEFDSLWE	TVMRADEEF	1	0.6882	29.2	0.25	0.44	tr_C9ZWY7_	SB
HLA-DQA10501-DQB10201	6	GIFSQSVIVADDMWY	QSVIVADDM	4	0.6676	36.5	0.40	0.50	tr_C9ZWY7_	SB
HLA-DQA10501-DQB10201	7	TKMHAATVMRADEEF	TVMRADEEF	6	0.6581	40.4	0.50	0.54	tr_C9ZWY7_	WB
HLA-DQA10501-DQB10201	8	TVMRADEEFDSLWEE	TVMRADEEF	0	0.6457	46.2	0.60	0.38	tr_C9ZWY7_	WB
HLA-DQA10501-DQB10201	9	IFSQSVIVADDMWYG	QSVIVADDM	3	0.6442	47.0	0.60	0.47	tr_C9ZWY7_	WB
HLA-DQA10501-DQB10201	10	NGIFSQSVIVADDMW	QSVIVADDM	5	0.6434	47.4	0.60	0.61	tr_C9ZWY7_	WB
HLA-DQA10501-DQB10201	11	FSQSVIVADDMWYGP	QSVIVADDM	2	0.6384	50.0	0.70	0.46	tr_C9ZWY7_	WB
HLA-DQA10501-DQB10201	12	MRADEEFDSLWEEFV	FDSLWEEFV	6	0.6342	52.4	0.70	0.34	tr_C9ZWY7_	WB
HLA-DQA10501-DQB10201	13	RADEEFDSLWEEFVM	FDSLWEEFV	5	0.6236	58.7	0.90	0.35	tr_C9ZWY7_	WB
HLA-DQA10501-DQB10201	14	VMRADEEFDSLWEEF	EFDSLWEEF	6	0.6184	62.1	1.00	0.33	tr_C9ZWY7_	WB
HLA-DQA10501-DQB10201	15	FVSFFDVALLYSEFV	VALLYSEFV	6	0.6169	63.1	1.00	0.60	tr_C9ZWY7_	WB
HLA-DQA10501-DQB10201	16	SLYNQRRYVVDDLVF	RRYVVDDLV	5	0.6077	69.7	1.10	0.56	tr_C9ZWY7_	WB
HLA-DQA10501-DQB10201	17	ADEEFDSLWEEFVMT	FDSLWEEFV	4	0.6024	73.9	1.20	0.35	tr_C9ZWY7_	WB
HLA-DQA10501-DQB10201	18	SQSVIVADDMWYGPF	QSVIVADDM	1	0.5998	75.9	1.30	0.39	tr_C9ZWY7_	WB
HLA-DQA10501-DQB10201	19	GVTAFNASDDFDHDD	TAFNASDDF	2	0.5952	79.9	1.40	0.54	tr_C9ZWY7_	WB
HLA-DQA10501-DQB10201	20	PGVTAFNASDDFDHD	TAFNASDDF	3	0.5920	82.6	1.50	0.55	tr_C9ZWY7_	WB
HLA-DQA10501-DQB10201	21	INGIFSQSVIVADDM	QSVIVADDM	6	0.5894	85.0	1.50	0.76	tr_C9ZWY7_	WB
HLA-DQA10501-DQB10201	22	YHPGVTAFNASDDFD	TAFNASDDF	5	0.5875	86.8	1.60	0.58	tr_C9ZWY7_	WB
HLA-DQA10501-DQB10201	23	VTAFNASDDFDHDDA	TAFNASDDF	1	0.5802	93.9	1.80	0.47	tr_C9ZWY7_	WB
HLA-DQA10501-DQB10201	24	LYNQRRYVVDDLVFG	RRYVVDDLV	4	0.5784	95.7	1.80	0.56	tr_C9ZWY7_	WB
HLA-DQA10501-DQB10201	25	HPGVTAFNASDDFDH	TAFNASDDF	4	0.5766	97.6	1.90	0.55	tr_C9ZWY7_	WB
HLA-DQA10501-DQB10201	26	HGDTLVFGLTEADIT	VFGLTEADI	5	0.5730	101.6	2.00	0.49	tr_C9ZWY7_	WB
HLA-DQA10501-DQB10201	27	KSLYNQRRYVVDDLV	RRYVVDDLV	6	0.5728	101.7	2.00	0.64	tr_C9ZWY7_	WB
HLA-DQA10501-DQB10201	28	DEEFDSLWEEFVMTH	FDSLWEEFV	3	0.5685	106.5	2.50	0.34	tr_C9ZWY7_	WB
HLA-DQA10501-DQB10201	29	GDTLVFGLTEADITT	VFGLTEADI	4	0.5661	109.4	2.50	0.48	tr_C9ZWY7_	WB
HLA-DQA10501-DQB10201	30	VSFFDVALLYSEFVK	VALLYSEFV	5	0.5636	112.3	2.50	0.61	tr_C9ZWY7_	WB
HLA-DQA10501-DQB10201	31	DTLVFGLTEADITTV	VFGLTEADI	3	0.5630	113.2	2.50	0.45	tr_C9ZWY7_	WB
HLA-DQA10501-DQB10201	32	YNQRRYVVDDLVFGN	RRYVVDDLV	3	0.5599	117.0	2.50	0.56	tr_C9ZWY7_	WB

HLA-DQA10501-DQB10201	33	QSVIVADDMWYGPFE	SVIVADDMW	1	0.5529	126.2	3.00	0.38	tr_C9ZWY7_	WB
HLA-DQA10501-DQB10201	34	ATWRTAVEEAAVPYI	WRTAVEEAA	2	0.5525	126.6	3.00	0.46	tr_C9ZWY7_	WB
HLA-DQA10501-DQB10201	35	TLVFGLTEADITTVA	VFGLTEADI	2	0.5429	140.6	3.50	0.41	tr_C9ZWY7_	WB
HLA-DQA10501-DQB10201	36	SFFDVALLYSEFVKV	VALLYSEFV	4	0.5395	145.8	3.50	0.66	tr_C9ZWY7_	WB
HLA-DQA10501-DQB10201	37	PHGDTLVFGLTEADI	VFGLTEADI	6	0.5390	146.6	3.50	0.70	tr_C9ZWY7_	WB
HLA-DQA10501-DQB10201	38	GATWRTAVEEAAVPY	WRTAVEEAA	3	0.5376	148.9	3.50	0.56	tr_C9ZWY7_	WB
HLA-DQA10501-DQB10201	39	FFDVALLYSEFVKVF	VALLYSEFV	3	0.5353	152.6	3.50	0.64	tr_C9ZWY7_	WB
HLA-DQA10501-DQB10201	40	EEFDSLWEEFVMTHP	FDSLWEEFV	2	0.5340	154.8	3.50	0.34	tr_C9ZWY7_	WB
HLA-DQA10501-DQB10201	41	NQRRYVVDDLVFGNF	RRYVVDDLV	2	0.5332	156.1	3.50	0.53	tr_C9ZWY7_	WB
HLA-DQA10501-DQB10201	42	TWRTAVEEAAVPYIP	WRTAVEEAA	1	0.5327	156.9	3.50	0.37	tr_C9ZWY7_	WB
HLA-DQA10501-DQB10201	43	LVFGLTEADITTVAE	FGLTEADIT	2	0.5284	164.5	4.00	0.42	tr_C9ZWY7_	WB
HLA-DQA10501-DQB10201	44	TAFNASDDFDHDDAD	AFNASDDFD	1	0.5279	165.4	4.00	0.47	tr_C9ZWY7_	WB
HLA-DQA10501-DQB10201	45	IGATWRTAVEEAAVP	WRTAVEEAA	4	0.5240	172.6	4.00	0.56	tr_C9ZWY7_	WB
HLA-DQA10501-DQB10201	46	SVIVADDMWYGPFED	SVIVADDMW	0	0.5235	173.5	4.00	0.32	tr_C9ZWY7_	WB
HLA-DQA10501-DQB10201	47	TRSVTAVFGVVDLAM	VFGVVDLAM	6	0.5141	192.0	4.50	0.41	tr_C9ZWY7_	WB
HLA-DQA10501-DQB10201	48	RYHPGVTAFNASDDF	TAFNASDDF	6	0.5138	192.7	4.50	0.75	tr_C9ZWY7_	WB
HLA-DQA10501-DQB10201	49	GVRVFVSFFDVALLY	VSFFDVALL	5	0.5091	202.7	5.00	0.26	tr_C9ZWY7_	WB
HLA-DQA10501-DQB10201	50	RSVTAVFGVVDLAML	VFGVVDLAM	5	0.5044	213.2	5.50	0.37	tr_C9ZWY7_	WB
HLA-DQA10501-DQB10201	51	WATAENVTVEIIPPP	AENVTVEII	3	0.5019	219.0	5.50	0.40	tr_C9ZWY7_	WB
HLA-DQA10501-DQB10201	52	VFVSFFDVALLYSEF	DVALLYSEF	6	0.5003	222.8	5.50	0.25	tr_C9ZWY7_	WB
HLA-DQA10501-DQB10201	53	WRTAVEEAAVPYIPD	VEEAAVPYI	4	0.4990	226.0	5.50	0.31	tr_C9ZWY7_	WB
HLA-DQA10501-DQB10201	54	VVLVVDDSAAVSAFF	DSAAVSAFF	6	0.4985	227.2	5.50	0.51	tr_C9ZWY7_	WB
HLA-DQA10501-DQB10201	55	QRRYVVDDLVFGNFG	RRYVVDDLV	1	0.4981	228.1	5.50	0.57	tr_C9ZWY7_	WB
HLA-DQA10501-DQB10201	56	VRVFVSFFDVALLYS	VSFFDVALL	4	0.4979	228.7	5.50	0.25	tr_C9ZWY7_	WB
HLA-DQA10501-DQB10201	57	VIGATWRTAVEEAAV	WRTAVEEAA	5	0.4975	229.7	5.50	0.56	tr_C9ZWY7_	WB
HLA-DQA10501-DQB10201	58	ATAENVTVEIIPPPP	AENVTVEII	2	0.4965	232.3	6.00	0.34	tr_C9ZWY7_	WB
HLA-DQA10501-DQB10201	59	MVSGWVIGEVLSQAL	SGWVIGEVL	2	0.4958	234.1	6.00	0.26	tr_C9ZWY7_	WB
HLA-DQA10501-DQB10201	60	FDVALLYSEFVKVFK	VALLYSEFV	2	0.4948	236.5	6.00	0.68	tr_C9ZWY7_	WB
HLA-DQA10501-DQB10201	61	HWATAENVTVEIIPP	AENVTVEII	4	0.4930	241.3	6.00	0.43	tr_C9ZWY7_	WB
HLA-DQA10501-DQB10201	62	DGALMVSGWVIGEVL	SGWVIGEVL	6	0.4916	244.8	6.00	0.59	tr_C9ZWY7_	WB
HLA-DQA10501-DQB10201	63	MLSVGRVAFIEPITL	VAFIEPITL	6	0.4885	253.3	6.50	0.37	tr_C9ZWY7_	WB
HLA-DQA10501-DQB10201	64	LMVSGWVIGEVLSQA	SGWVIGEVL	3	0.4881	254.3	6.50	0.37	tr_C9ZWY7_	WB
HLA-DQA10501-DQB10201	65	FVIGATWRTAVEEAA	WRTAVEEAA	6	0.4862	259.7	6.50	0.47	tr_C9ZWY7_	WB
HLA-DQA10501-DQB10201	66	RGVRVFVSFFDVALL	VSFFDVALL	6	0.4861	260.0	6.50	0.34	tr_C9ZWY7_	WB
HLA-DQA10501-DQB10201	67	FTKMHAATVMRADEE	ATVMRADEE	6	0.4822	271.2	7.00	0.56	tr_C9ZWY7_	WB
HLA-DQA10501-DQB10201	68	SVTAVFGVVDLAMLS	VFGVVDLAM	4	0.4793	279.8	7.00	0.34	tr_C9ZWY7_	WB
HLA-DQA10501-DQB10201	69	RHWATAENVTVEIIP	AENVTVEII	5	0.4771	286.4	7.00	0.43	tr_C9ZWY7_	WB
HLA-DQA10501-DQB10201	70	ARHWATAENVTVEII	AENVTVEII	6	0.4759	290.4	7.50	0.47	tr_C9ZWY7_	WB
HLA-DQA10501-DQB10201	71	SVVSRMGRVNSEELI	MGRVNSEEL	5	0.4750	293.1	7.50	0.72	tr_C9ZWY7_	WB
HLA-DQA10501-DQB10201	72	ALMVSGWVIGEVLSQ	SGWVIGEVL	4	0.4750	293.2	7.50	0.47	tr_C9ZWY7_	WB
HLA-DQA10501-DQB10201	73	VFGLTEADITTVAEH	FGLTEADIT	1	0.4740	296.3	7.50	0.49	tr_C9ZWY7_	WB
HLA-DQA10501-DQB10201	74	RVFVSFFDVALLYSE	VSFFDVALL	3	0.4720	302.8	7.50	0.27	tr_C9ZWY7_	WB
HLA-DQA10501-DQB10201	75	EFDSLWEEFVMTHPQ	FDSLWEEFV	1	0.4711	305.7	7.50	0.36	tr_C9ZWY7_	WB
HLA-DQA10501-DQB10201	76	TPLIVVVVLVVDDSA	VVLVVDDSA	6	0.4687	313.8	8.00	0.50	tr_C9ZWY7_	WB
HLA-DQA10501-DQB10201	77	LIVVVVLVVDDSAAV	VVLVVDDSA	4	0.4675	317.7	8.00	0.46	tr_C9ZWY7_	WB
HLA-DQA10501-DQB10201	78	VDDSAAVSAFFSLVV	DSAAVSAFF	2	0.4673	318.4	8.00	0.38	tr_C9ZWY7_	WB
HLA-DQA10501-DQB10201	79	TAENVTVEIIPPPPR	AENVTVEII	1	0.4672	318.7	8.00	0.35	tr_C9ZWY7_	WB
HLA-DQA10501-DQB10201	80	PLIVVVVLVVDDSAA	VVLVVDDSA	5	0.4656	324.3	8.00	0.49	tr_C9ZWY7_	WB
HLA-DQA10501-DQB10201	81	VSGWVIGEVLSQALS	SGWVIGEVL	1	0.4634	332.2	8.50	0.26	tr_C9ZWY7_	WB
HLA-DQA10501-DQB10201	82	VVSRMGRVNSEELIN	MGRVNSEEL	4	0.4632	333.0	8.50	0.69	tr_C9ZWY7_	WB

HLA-DQA10501-DQB10201	83	VLVVDDSAAVSAFFS	DSAAVSAFF	5	0.4616	338.9	8.50	0.51	tr_C9ZWY7_	WB
HLA-DQA10501-DQB10201	84	GALMVSGWVIGEVLS	SGWVIGEVL	5	0.4613	339.7	8.50	0.56	tr_C9ZWY7_	WB
HLA-DQA10501-DQB10201	85	DTRSVTAVFGVVDLA	AVFGVVDLA	6	0.4612	340.4	8.50	0.35	tr_C9ZWY7_	WB
HLA-DQA10501-DQB10201	86	IVVVVLVVDDSAAVS	VVLVVDDSA	3	0.4608	341.9	8.50	0.41	tr_C9ZWY7_	WB
HLA-DQA10501-DQB10201	87	LSVGRVAFIEPITLS	VAFIEPITL	5	0.4586	350.1	9.00	0.38	tr_C9ZWY7_	WB
HLA-DQA10501-DQB10201	88	VTAVFGVVDLAMLSV	VFGVVDLAM	3	0.4581	351.8	9.00	0.34	tr_C9ZWY7_	WB
HLA-DQA10501-DQB10201	89	SVGRVAFIEPITLSP	VAFIEPITL	4	0.4564	358.4	9.00	0.40	tr_C9ZWY7_	WB
HLA-DQA10501-DQB10201	90	LAVLPLLRQEDLVAF	PLLRQEDLV	4	0.4518	376.7	9.50	0.38	tr_C9ZWY7_	WB
HLA-DQA10501-DQB10201	91	LVVDDSAAVSAFFSL	DSAAVSAFF	4	0.4493	386.9	9.50	0.51	tr_C9ZWY7_	WB
HLA-DQA10501-DQB10201	92	TAVEEAAVPYIPDRV	VEEAAVPYI	2	0.4467	398.1	10.00	0.29	tr_C9ZWY7_	
HLA-DQA10501-DQB10201	93	VVVVLVVDDSAAVSA	VVLVVDDSA	2	0.4450	405.3	10.00	0.41	tr_C9ZWY7_	
HLA-DQA10501-DQB10201	94	HRGVRVFVSFFDVAL	FVSFFDVAL	6	0.4440	410.0	11.00	0.37	tr_C9ZWY7_	
HLA-DQA10501-DQB10201	95	VVDDSAAVSAFFSLV	DSAAVSAFF	3	0.4418	419.9	11.00	0.49	tr_C9ZWY7_	
HLA-DQA10501-DQB10201	96	AVLPLLRQEDLVAFA	PLLRQEDLV	3	0.4416	420.6	11.00	0.35	tr_C9ZWY7_	
HLA-DQA10501-DQB10201	97	DVALLYSEFVKVFKK	VALLYSEFV	1	0.4408	424.4	11.00	0.69	tr_C9ZWY7_	
HLA-DQA10501-DQB10201	98	YIPDRVIVAAPNPLA	VIVAAPNPL	5	0.4400	427.9	11.00	0.52	tr_C9ZWY7_	
HLA-DQA10501-DQB10201	99	VLPLLRQEDLVAFAP	PLLRQEDLV	2	0.4387	433.8	11.00	0.34	tr_C9ZWY7_	
HLA-DQA10501-DQB10201	100	VSRMGRVNSEELING	MGRVNSEEL	3	0.4381	436.7	11.00	0.67	tr_C9ZWY7_	
HLA-DQA10501-DQB10201	101	AMLSVGRVAFIEPIT	GRVAFIEPI	5	0.4381	437.1	11.00	0.34	tr_C9ZWY7_	
HLA-DQA10501-DQB10201	102	LAMLSVGRVAFIEPI	GRVAFIEPI	6	0.4373	440.6	11.00	0.47	tr_C9ZWY7_	
HLA-DQA10501-DQB10201	103	VIVADDMWYGPFEDS	VIVADDMWY	0	0.4355	449.3	11.00	0.34	tr_C9ZWY7_	
HLA-DQA10501-DQB10201	104	DDSAAVSAFFSLVVG	DSAAVSAFF	1	0.4349	452.4	12.00	0.40	tr_C9ZWY7_	
HLA-DQA10501-DQB10201	105	AFFSLVVGITNADAI	VVGITNADA	5	0.4338	457.7	12.00	0.41	tr_C9ZWY7_	
HLA-DQA10501-DQB10201	106	GPMGDTRTLAVLPLL	RTLAVLPLL	6	0.4335	459.0	12.00	0.46	tr_C9ZWY7_	
HLA-DQA10501-DQB10201	107	SGWVIGEVLSQALSS	IGEVLSQAL	4	0.4322	465.8	12.00	0.19	tr_C9ZWY7_	
HLA-DQA10501-DQB10201	108	AARHWATAENVTVEI	RHWATAENV	2	0.4319	467.3	12.00	0.32	tr_C9ZWY7_	
HLA-DQA10501-DQB10201	109	AENVTVEIIPPPPRS	AENVTVEII	0	0.4304	475.1	12.00	0.29	tr_C9ZWY7_	
HLA-DQA10501-DQB10201	110	IPDRVIVAAPNPLAS	VIVAAPNPL	4	0.4299	477.6	12.00	0.51	tr_C9ZWY7_	
HLA-DQA10501-DQB10201	111	VVVLVVDDSAAVSAF	VVLVVDDSA	1	0.4297	478.3	12.00	0.38	tr_C9ZWY7_	
HLA-DQA10501-DQB10201	112	RTAVEEAAVPYIPDR	VEEAAVPYI	3	0.4286	483.9	12.00	0.38	tr_C9ZWY7_	
HLA-DQA10501-DQB10201	113	LPLLRQEDLVAFAPL	PLLRQEDLV	1	0.4284	485.2	12.00	0.28	tr_C9ZWY7_	
HLA-DQA10501-DQB10201	114	ESVVSRMGRVNSEEL	MGRVNSEEL	6	0.4270	492.5	12.00	0.82	tr_C9ZWY7_	
HLA-DQA10501-DQB10201	115	LDTRSVTAVFGVVDL	TAVFGVVDL	6	0.4264	495.9	13.00	0.31	tr_C9ZWY7_	
HLA-DQA10501-DQB10201	116	FGLTEADITTVAEHL	DITTVAEHL	6	0.4258	499.0	13.00	0.38	tr_C9ZWY7_	
HLA-DQA10501-DQB10201	117	PYIPDRVIVAAPNPL	VIVAAPNPL	6	0.4258	499.0	13.00	0.57	tr_C9ZWY7_	
HLA-DQA10501-DQB10201	118	GLTEADITTVAEHLD	DITTVAEHL	5	0.4253	501.7	13.00	0.44	tr_C9ZWY7_	
HLA-DQA10501-DQB10201	119	IKSLYNQRRYVVDDL	QRRYVVDDL	6	0.4253	502.0	13.00	0.90	tr_C9ZWY7_	
HLA-DQA10501-DQB10201	120	PMGDTRTLAVLPLLR	RTLAVLPLL	5	0.4240	508.8	13.00	0.35	tr_C9ZWY7_	
HLA-DQA10501-DQB10201	121	RTLAVLPLLRQEDLV	PLLRQEDLV	6	0.4238	509.9	13.00	0.30	tr_C9ZWY7_	
HLA-DQA10501-DQB10201	122	PDRVIVAAPNPLASE	VIVAAPNPL	3	0.4227	516.3	13.00	0.52	tr_C9ZWY7_	
HLA-DQA10501-DQB10201	123	TAVFGVVDLAMLSVG	VFGVVDLAM	2	0.4226	516.4	13.00	0.40	tr_C9ZWY7_	
HLA-DQA10501-DQB10201	124	TLAVLPLLRQEDLVA	PLLRQEDLV	5	0.4220	520.2	13.00	0.40	tr_C9ZWY7_	
HLA-DQA10501-DQB10201	125	RRYVVDDLVFGNFGG	RRYVVDDLV	0	0.4218	520.9	13.00	0.51	tr_C9ZWY7_	
HLA-DQA10501-DQB10201	126	AVEEAAVPYIPDRVI	VEEAAVPYI	1	0.4214	523.3	13.00	0.31	tr_C9ZWY7_	
HLA-DQA10501-DQB10201	127	LTEADITTVAEHLDN	DITTVAEHL	4	0.4212	524.4	13.00	0.41	tr_C9ZWY7_	
HLA-DQA10501-DQB10201	128	SRMGRVNSEELINGI	MGRVNSEEL	2	0.4206	527.7	13.00	0.64	tr_C9ZWY7_	
HLA-DQA10501-DQB10201	129	VGRVAFIEPITLSPK	VAFIEPITL	3	0.4196	533.5	13.00	0.41	tr_C9ZWY7_	
HLA-DQA10501-DQB10201	130	ALLGYATARAMESVV	YATARAMES	4	0.4179	543.4	14.00	0.35	tr_C9ZWY7_	
HLA-DQA10501-DQB10201	131	DRVIVAAPNPLASET	VIVAAPNPL	2	0.4156	557.1	14.00	0.54	tr_C9ZWY7_	
HLA-DQA10501-DQB10201	132	QNKGEFFVVVGPMGD	EFFVVVGPM	4	0.4132	571.7	14.00	0.35	tr_C9ZWY7_	

HLA-DQA10501-DQB10201	133	PLLRQEDLVAFAPLT	DLVAFAPLT	6	0.4122	578.1	14.00	0.28	tr_C9ZWY7_
HLA-DQA10501-DQB10201	134	RMGRVNSEELINGIF	MGRVNSEEL	1	0.4119	580.0	14.00	0.56	tr_C9ZWY7_
HLA-DQA10501-DQB10201	135	FFSLVVGITNADAIR	VGITNADAI	5	0.4110	585.8	14.00	0.37	tr_C9ZWY7_
HLA-DQA10501-DQB10201	136	VNSEELINGIFSQSV	INGIFSQSV	6	0.4109	586.4	14.00	0.61	tr_C9ZWY7_
HLA-DQA10501-DQB10201	137	TSRNTYGRMQGELDT	YGRMQGELD	5	0.4108	587.0	14.00	0.73	tr_C9ZWY7_
HLA-DQA10501-DQB10201	138	ADDMWYGPFEDSCVP	DMWYGPFED	2	0.4106	588.3	14.00	0.31	tr_C9ZWY7_
HLA-DQA10501-DQB10201	139	LLGYATARAMESVVS	YATARAMES	3	0.4093	596.5	15.00	0.34	tr_C9ZWY7_
HLA-DQA10501-DQB10201	140	NKGEFFVVVGPMGDT	EFFVVVGPM	3	0.4089	599.1	15.00	0.31	tr_C9ZWY7_
HLA-DQA10501-DQB10201	141	ATSRNTYGRMQGELD	YGRMQGELD	6	0.4083	603.1	15.00	0.72	tr_C9ZWY7_
HLA-DQA10501-DQB10201	142	TRTLAVLPLLRQEDL	LPLLRQEDL	6	0.4078	606.3	15.00	0.34	tr_C9ZWY7_
HLA-DQA10501-DQB10201	143	QFVIGATWRTAVEEA	TWRTAVEEA	6	0.4069	612.3	15.00	0.56	tr_C9ZWY7_
HLA-DQA10501-DQB10201	144	FAARHWATAENVTVE	RHWATAENV	3	0.4066	614.2	15.00	0.54	tr_C9ZWY7_
HLA-DQA10501-DQB10201	145	SFAARHWATAENVTV	RHWATAENV	4	0.4066	614.2	15.00	0.62	tr_C9ZWY7_
HLA-DQA10501-DQB10201	146	FSLVVGITNADAIRS	VGITNADAI	4	0.4060	618.1	15.00	0.39	tr_C9ZWY7_
HLA-DQA10501-DQB10201	147	VADDMWYGPFEDSCV	DMWYGPFED	3	0.4058	619.9	15.00	0.34	tr_C9ZWY7_
HLA-DQA10501-DQB10201	148	LALLGYATARAMESV	YATARAMES	5	0.4017	647.8	16.00	0.41	tr_C9ZWY7_
HLA-DQA10501-DQB10201	149	IVADDMWYGPFEDSC	DMWYGPFED	4	0.4010	652.7	16.00	0.34	tr_C9ZWY7_
HLA-DQA10501-DQB10201	150	NSEELINGIFSQSVI	INGIFSQSV	5	0.4002	658.0	16.00	0.56	tr_C9ZWY7_
HLA-DQA10501-DQB10201	151	TEADITTVAEHLDNH	DITTVAEHL	3	0.3993	664.6	16.00	0.41	tr_C9ZWY7_
HLA-DQA10501-DQB10201	152	MGDTRTLAVLPLLRQ	RTLAVLPLL	4	0.3987	669.1	16.00	0.35	tr_C9ZWY7_
HLA-DQA10501-DQB10201	153	NVIHFSPTIEQQLFL	VIHFSPTIE	1	0.3982	672.5	16.00	0.23	tr_C9ZWY7_
HLA-DQA10501-DQB10201	154	DSAAVSAFFSLVVGI	VSAFFSLVV	4	0.3980	674.4	16.00	0.28	tr_C9ZWY7_
HLA-DQA10501-DQB10201	155	NHRGVRVFVSFFDVA	VFVSFFDVA	6	0.3979	675.1	16.00	0.43	tr_C9ZWY7_
HLA-DQA10501-DQB10201	156	LLRQEDLVAFAPLTG	LVAFAPLTG	6	0.3960	688.9	17.00	0.29	tr_C9ZWY7_
HLA-DQA10501-DQB10201	157	QGGNVVYMNSLGDDH	YMNSLGDDH	6	0.3942	702.8	17.00	0.28	tr_C9ZWY7_
HLA-DQA10501-DQB10201	158	SRNTYGRMQGELDTR	YGRMQGELD	4	0.3925	715.4	17.00	0.69	tr_C9ZWY7_
HLA-DQA10501-DQB10201	159	AFNASDDFDHDDADG	AFNASDDFD	0	0.3909	728.0	17.00	0.56	tr_C9ZWY7_
HLA-DQA10501-DQB10201	160	SLVVGITNADAIRSI	VGITNADAI	3	0.3908	729.2	17.00	0.42	tr_C9ZWY7_
HLA-DQA10501-DQB10201	161	KGEFFVVVGPMGDTR	EFFVVVGPM	2	0.3902	733.2	17.00	0.32	tr_C9ZWY7_
HLA-DQA10501-DQB10201	162	DDMWYGPFEDSCVPT	DMWYGPFED	1	0.3897	737.2	17.00	0.28	tr_C9ZWY7_
HLA-DQA10501-DQB10201	163	AVFGVVDLAMLSVGR	VFGVVDLAM	1	0.3883	748.4	18.00	0.41	tr_C9ZWY7_
HLA-DQA10501-DQB10201	164	SEELINGIFSQSVIV	INGIFSQSV	4	0.3880	751.6	18.00	0.57	tr_C9ZWY7_
HLA-DQA10501-DQB10201	165	SLGGSMNGIVVPDND	MNGIVVPDN	5	0.3874	756.1	18.00	0.43	tr_C9ZWY7_
HLA-DQA10501-DQB10201	166	NASFAARHWATAENV	RHWATAENV	6	0.3868	761.2	18.00	0.84	tr_C9ZWY7_
HLA-DQA10501-DQB10201	167	ADQNKGEFFVVVGPM	EFFVVVGPM	6	0.3864	764.2	18.00	0.62	tr_C9ZWY7_
HLA-DQA10501-DQB10201	168	GGNVVYMNSLGDDHR	YMNSLGDDH	5	0.3864	764.5	18.00	0.34	tr_C9ZWY7_
HLA-DQA10501-DQB10201	169	GDTRTLAVLPLLRQE	RTLAVLPLL	3	0.3863	765.0	18.00	0.37	tr_C9ZWY7_
HLA-DQA10501-DQB10201	170	RLLRVGFMYLQGVHF	FMYLQGVHF	6	0.3862	765.8	18.00	0.57	tr_C9ZWY7_
HLA-DQA10501-DQB10201	171	ELDTRSVTAVFGVVD	VTAVFGVVD	6	0.3859	768.1	18.00	0.37	tr_C9ZWY7_
HLA-DQA10501-DQB10201	172	PTPLIVVVVLVVDDS	VVVLVVDDS	6	0.3858	769.4	18.00	0.75	tr_C9ZWY7_
HLA-DQA10501-DQB10201	173	GRVAFIEPITLSPKL	VAFIEPITL	2	0.3855	771.6	18.00	0.47	tr_C9ZWY7_
HLA-DQA10501-DQB10201	174	RNVIHFSPTIEQQLF	VIHFSPTIE	2	0.3847	778.7	18.00	0.29	tr_C9ZWY7_
HLA-DQA10501-DQB10201	175	RNTYGRMQGELDTRS	YGRMQGELD	3	0.3845	779.9	18.00	0.67	tr_C9ZWY7_
HLA-DQA10501-DQB10201	176	MGRVNSEELINGIFS	MGRVNSEEL	0	0.3842	782.6	18.00	0.34	tr_C9ZWY7_
HLA-DQA10501-DQB10201	177	RTRGVYVLAPSAMQF	VYVLAPSAM	4	0.3838	786.0	18.00	0.42	tr_C9ZWY7_
HLA-DQA10501-DQB10201	178	VEEAAVPYIPDRVIV	AVPYIPDRV	4	0.3832	791.3	18.00	0.40	tr_C9ZWY7_
HLA-DQA10501-DQB10201	179	GEFFVVVGPMGDTRT	EFFVVVGPM	1	0.3825	797.5	19.00	0.32	tr_C9ZWY7_
HLA-DQA10501-DQB10201	180	VGQSAGFGGFAGVIT	FGGFAGVIT	6	0.3822	800.0	19.00	0.69	tr_C9ZWY7_
HLA-DQA10501-DQB10201	181	GWVIGEVLSQALSSR	VIGEVLSQA	2	0.3807	813.2	19.00	0.30	tr_C9ZWY7_
HLA-DQA10501-DQB10201	182	LINGIFSQSVIVADD	SQSVIVADD	6	0.3804	815.2	19.00	0.31	tr_C9ZWY7_

HLA-DQA10501-DQB10201	183	DTRTLAVLPLLRQED	RTLAVLPLL	2	0.3799	820.2	19.00	0.35	tr_C9ZWY7_
HLA-DQA10501-DQB10201	184	GNVVYMNSLGDDHRM	YMNSLGDDH	4	0.3799	820.3	19.00	0.34	tr_C9ZWY7_
HLA-DQA10501-DQB10201	185	RVIVAAPNPLASETK	VIVAAPNPL	1	0.3793	825.0	19.00	0.57	tr_C9ZWY7_
HLA-DQA10501-DQB10201	186	PLALLGYATARAMES	YATARAMES	6	0.3782	835.1	19.00	0.46	tr_C9ZWY7_
HLA-DQA10501-DQB10201	187	DQNKGEFFVVVGPMG	EFFVVVGPM	5	0.3778	839.3	19.00	0.45	tr_C9ZWY7_
HLA-DQA10501-DQB10201	188	TRGVYVLAPSAMQFV	VYVLAPSAM	3	0.3773	843.6	19.00	0.34	tr_C9ZWY7_
HLA-DQA10501-DQB10201	189	VIHFSPTIEQQLFLL	FSPTIEQQL	3	0.3769	846.7	19.00	0.22	tr_C9ZWY7_
HLA-DQA10501-DQB10201	190	LGYATARAMESVVSR	YATARAMES	2	0.3764	851.6	20.00	0.34	tr_C9ZWY7_
HLA-DQA10501-DQB10201	191	AAERLLFATSLPHWA	ERLLFATSL	2	0.3764	852.1	20.00	0.38	tr_C9ZWY7_
HLA-DQA10501-DQB10201	192	EELINGIFSQSVIVA	INGIFSQSV	3	0.3762	853.1	20.00	0.51	tr_C9ZWY7_
HLA-DQA10501-DQB10201	193	NTYGRMQGELDTRSV	YGRMQGELD	2	0.3756	859.0	20.00	0.66	tr_C9ZWY7_
HLA-DQA10501-DQB10201	194	LGGSMNGIVVPDNDT	MNGIVVPDN	4	0.3751	864.0	20.00	0.42	tr_C9ZWY7_
HLA-DQA10501-DQB10201	195	AERLLFATSLPHWAD	ERLLFATSL	1	0.3740	874.4	20.00	0.28	tr_C9ZWY7_
HLA-DQA10501-DQB10201	196	QAAERLLFATSLPHW	ERLLFATSL	3	0.3739	875.5	20.00	0.42	tr_C9ZWY7_
HLA-DQA10501-DQB10201	197	ASFAARHWATAENVT	RHWATAENV	5	0.3730	883.8	20.00	0.79	tr_C9ZWY7_
HLA-DQA10501-DQB10201	198	DARTRGVYVLAPSAM	VYVLAPSAM	6	0.3713	899.8	20.00	0.51	tr_C9ZWY7_
HLA-DQA10501-DQB10201	199	LVVGITNADAIRSIT	VGITNADAI	2	0.3696	916.2	21.00	0.43	tr_C9ZWY7_
HLA-DQA10501-DQB10201	200	SAFFSLVVGITNADA	VVGITNADA	6	0.3694	918.4	21.00	0.50	tr_C9ZWY7_
HLA-DQA10501-DQB10201	201	GRNVIHFSPTIEQQL	VIHFSPTIE	3	0.3689	924.1	21.00	0.34	tr_C9ZWY7_
HLA-DQA10501-DQB10201	202	VFTKMHAATVMRADE	MHAATVMRA	4	0.3688	924.9	21.00	0.54	tr_C9ZWY7_
HLA-DQA10501-DQB10201	203	EADITTVAEHLDNHR	DITTVAEHL	2	0.3687	925.2	21.00	0.46	tr_C9ZWY7_
HLA-DQA10501-DQB10201	204	NVVYMNSLGDDHRMI	YMNSLGDDH	3	0.3664	948.8	21.00	0.38	tr_C9ZWY7_
HLA-DQA10501-DQB10201	205	ARTRGVYVLAPSAMQ	VYVLAPSAM	5	0.3661	952.1	21.00	0.47	tr_C9ZWY7_
HLA-DQA10501-DQB10201	206	LRQEDLVAFAPLTGS	LVAFAPLTG	5	0.3651	962.2	22.00	0.34	tr_C9ZWY7_
HLA-DQA10501-DQB10201	207	HPQAAERLLFATSLP	ERLLFATSL	5	0.3639	975.4	22.00	0.48	tr_C9ZWY7_
HLA-DQA10501-DQB10201	208	VGPMGDTRTLAVLPL	TRTLAVLPL	6	0.3634	980.8	22.00	0.65	tr_C9ZWY7_
HLA-DQA10501-DQB10201	209	VALLYSEFVKVFKKH	VALLYSEFV	0	0.3629	985.8	22.00	0.69	tr_C9ZWY7_
HLA-DQA10501-DQB10201	210	ELINGIFSQSVIVAD	INGIFSQSV	2	0.3621	994.5	22.00	0.43	tr_C9ZWY7_
HLA-DQA10501-DQB10201	211	GQSAGFGGFAGVITG	FGGFAGVIT	5	0.3610	1006.2	22.00	0.65	tr_C9ZWY7_
HLA-DQA10501-DQB10201	212	RGVYVLAPSAMQFVI	VYVLAPSAM	2	0.3610	1006.6	22.00	0.40	tr_C9ZWY7_
HLA-DQA10501-DQB10201	213	EFFVVVGPMGDTRTL	FVVVGPMGD	2	0.3605	1011.4	22.00	0.34	tr_C9ZWY7_
HLA-DQA10501-DQB10201	214	NQGGNVVYMNSLGDD	VVYMNSLGD	5	0.3604	1012.7	22.00	0.34	tr_C9ZWY7_
HLA-DQA10501-DQB10201	215	FDSLWEEFVMTHPQG	FDSLWEEFV	0	0.3597	1019.8	22.00	0.36	tr_C9ZWY7_
HLA-DQA10501-DQB10201	216	GSSAVRGWDPHFYFL	VRGWDPHFY	4	0.3596	1020.9	22.00	0.54	tr_C9ZWY7_
HLA-DQA10501-DQB10201	217	LRVGFMYLQGVHFGD	FMYLQGVHF	4	0.3589	1029.8	23.00	0.51	tr_C9ZWY7_
HLA-DQA10501-DQB10201	218	ALSSRTWLESREAFI	WLESREAFI	6	0.3586	1032.6	23.00	0.56	tr_C9ZWY7_
HLA-DQA10501-DQB10201	219	GGSMNGIVVPDNDTT	MNGIVVPDN	3	0.3575	1045.5	23.00	0.41	tr_C9ZWY7_
HLA-DQA10501-DQB10201	220	GYATARAMESVVSRM	YATARAMES	1	0.3573	1046.7	23.00	0.33	tr_C9ZWY7_
HLA-DQA10501-DQB10201	221	DLAMLSVGRVAFIEP	VGRVAFIEP	6	0.3569	1051.8	23.00	0.36	tr_C9ZWY7_
HLA-DQA10501-DQB10201	222	LLRVGFMYLQGVHFG	FMYLQGVHF	5	0.3568	1052.7	23.00	0.51	tr_C9ZWY7_
HLA-DQA10501-DQB10201	223	GWDPHFYFLTPAPNA	FYFLTPAPN	5	0.3565	1056.8	23.00	0.32	tr_C9ZWY7_
HLA-DQA10501-DQB10201	224	WDPHFYFLTPAPNAE	FYFLTPAPN	4	0.3564	1057.0	23.00	0.32	tr_C9ZWY7_
HLA-DQA10501-DQB10201	225	AVRGWDPHFYFLTPA	VRGWDPHFY	1	0.3550	1074.1	23.00	0.38	tr_C9ZWY7_
HLA-DQA10501-DQB10201	226	TGSSAVRGWDPHFYF	VRGWDPHFY	5	0.3538	1087.1	24.00	0.54	tr_C9ZWY7_
HLA-DQA10501-DQB10201	227	DPHFYFLTPAPNAEL	YFLTPAPNA	4	0.3532	1095.2	24.00	0.25	tr_C9ZWY7_
HLA-DQA10501-DQB10201	228	SAAVSAFFSLVVGIT	VSAFFSLVV	3	0.3530	1097.4	24.00	0.33	tr_C9ZWY7_
HLA-DQA10501-DQB10201	229	PQAAERLLFATSLPH	ERLLFATSL	4	0.3508	1123.2	24.00	0.44	tr_C9ZWY7_
HLA-DQA10501-DQB10201	230	ENVTVEIIPPPPRSV	NVTVEIIPP	1	0.3502	1130.8	24.00	0.28	tr_C9ZWY7_
HLA-DQA10501-DQB10201	231	SSAVRGWDPHFYFLT	VRGWDPHFY	3	0.3494	1141.1	24.00	0.52	tr_C9ZWY7_
HLA-DQA10501-DQB10201	232	SAVRGWDPHFYFLTP	VRGWDPHFY	2	0.3489	1147.2	24.00	0.47	tr_C9ZWY7_

HLA-DQA10501-DQB10201	233	ESLGGSMNGIVVPDN	MNGIVVPDN	6	0.3483	1154.7	25.00	0.51	tr_C9ZWY7_
HLA-DQA10501-DQB10201	234	YFLTPAPNAELIALL	PNAELIALL	6	0.3482	1155.2	25.00	0.41	tr_C9ZWY7_
HLA-DQA10501-DQB10201	235	ERLLFATSLPHWADN	LFATSLPHW	3	0.3482	1155.3	25.00	0.26	tr_C9ZWY7_
HLA-DQA10501-DQB10201	236	GSMNGIVVPDNDTTL	MNGIVVPDN	2	0.3478	1161.0	25.00	0.41	tr_C9ZWY7_
HLA-DQA10501-DQB10201	237	GVFTKMHAATVMRAD	MHAATVMRA	5	0.3477	1161.9	25.00	0.64	tr_C9ZWY7_
HLA-DQA10501-DQB10201	238	DNHRGVRVFVSFFDV	VRVFVSFFD	5	0.3475	1163.9	25.00	0.25	tr_C9ZWY7_
HLA-DQA10501-DQB10201	239	TYGRMQGELDTRSVT	YGRMQGELD	1	0.3465	1176.4	25.00	0.69	tr_C9ZWY7_
HLA-DQA10501-DQB10201	240	WVIGEVLSQALSSRT	IGEVLSQAL	2	0.3464	1177.8	25.00	0.31	tr_C9ZWY7_
HLA-DQA10501-DQB10201	241	LDNHRGVRVFVSFFD	VRVFVSFFD	6	0.3464	1178.2	25.00	0.31	tr_C9ZWY7_
HLA-DQA10501-DQB10201	242	ADGALMVSGWVIGEV	VSGWVIGEV	6	0.3457	1186.8	25.00	0.63	tr_C9ZWY7_
HLA-DQA10501-DQB10201	243	VFGVVDLAMLSVGRV	VFGVVDLAM	0	0.3446	1201.8	25.00	0.30	tr_C9ZWY7_
HLA-DQA10501-DQB10201	244	VVYMNSLGDDHRMIP	YMNSLGDDH	2	0.3437	1213.4	25.00	0.41	tr_C9ZWY7_
HLA-DQA10501-DQB10201	245	RVAFIEPITLSPKLS	VAFIEPITL	1	0.3433	1218.5	26.00	0.53	tr_C9ZWY7_
HLA-DQA10501-DQB10201	246	QSAGFGGFAGVITGR	FGGFAGVIT	4	0.3430	1222.1	26.00	0.62	tr_C9ZWY7_
HLA-DQA10501-DQB10201	247	VGFMYLQGVHFGDRE	FMYLQGVHF	2	0.3425	1229.1	26.00	0.47	tr_C9ZWY7_
HLA-DQA10501-DQB10201	248	GVYVLAPSAMQFVIG	VYVLAPSAM	1	0.3422	1232.8	26.00	0.43	tr_C9ZWY7_
HLA-DQA10501-DQB10201	249	SAGFGGFAGVITGRH	FGGFAGVIT	3	0.3419	1236.6	26.00	0.50	tr_C9ZWY7_
HLA-DQA10501-DQB10201	250	RQEDLVAFAPLTGSS	LVAFAPLTG	4	0.3413	1245.1	26.00	0.36	tr_C9ZWY7_
HLA-DQA10501-DQB10201	251	PHFYFLTPAPNAELI	YFLTPAPNA	3	0.3411	1247.5	26.00	0.25	tr_C9ZWY7_
HLA-DQA10501-DQB10201	252	EALKFVRRVAMDART	FVRRVAMDA	4	0.3411	1248.1	26.00	0.43	tr_C9ZWY7_
HLA-DQA10501-DQB10201	253	DMWYGPFEDSCVPTT	WYGPFEDSC	2	0.3409	1250.8	26.00	0.31	tr_C9ZWY7_
HLA-DQA10501-DQB10201	254	CGVFTKMHAATVMRA	MHAATVMRA	6	0.3403	1258.1	26.00	0.65	tr_C9ZWY7_
HLA-DQA10501-DQB10201	255	VRGWDPHFYFLTPAP	HFYFLTPAP	6	0.3400	1263.2	26.00	0.31	tr_C9ZWY7_
HLA-DQA10501-DQB10201	256	SPHGDTLVFGLTEAD	LVFGLTEAD	6	0.3378	1293.1	27.00	0.46	tr_C9ZWY7_
HLA-DQA10501-DQB10201	257	GFMYLQGVHFGDREY	FMYLQGVHF	1	0.3366	1309.7	27.00	0.41	tr_C9ZWY7_
HLA-DQA10501-DQB10201	258	HPGRNVIHFSPTIEQ	VIHFSPTIE	5	0.3360	1318.7	27.00	0.48	tr_C9ZWY7_
HLA-DQA10501-DQB10201	259	IHFSPTIEQQLFLLV	PTIEQQLFL	4	0.3356	1324.6	27.00	0.25	tr_C9ZWY7_
HLA-DQA10501-DQB10201	260	HFYFLTPAPNAELIA	YFLTPAPNA	2	0.3348	1335.2	27.00	0.25	tr_C9ZWY7_
HLA-DQA10501-DQB10201	261	LSSRTWLESREAFIK	WLESREAFI	5	0.3344	1341.6	27.00	0.58	tr_C9ZWY7_
HLA-DQA10501-DQB10201	262	RLRLLRVGFMYLQGV	VGFMYLQGV	6	0.3340	1347.0	27.00	0.39	tr_C9ZWY7_
HLA-DQA10501-DQB10201	263	RVGFMYLQGVHFGDR	FMYLQGVHF	3	0.3337	1351.5	28.00	0.50	tr_C9ZWY7_
HLA-DQA10501-DQB10201	264	NPDRVAFHSVVATSR	FHSVVATSR	6	0.3329	1363.9	28.00	0.44	tr_C9ZWY7_
HLA-DQA10501-DQB10201	265	REALKFVRRVAMDAR	FVRRVAMDA	5	0.3327	1366.8	28.00	0.52	tr_C9ZWY7_
HLA-DQA10501-DQB10201	266	AGFGGFAGVITGRHI	FGGFAGVIT	2	0.3326	1367.5	28.00	0.50	tr_C9ZWY7_
HLA-DQA10501-DQB10201	267	AAVSAFFSLVVGITN	VSAFFSLVV	2	0.3323	1371.8	28.00	0.31	tr_C9ZWY7_
HLA-DQA10501-DQB10201	268	QGELDTRSVTAVFGV	TRSVTAVFG	5	0.3315	1384.5	28.00	0.49	tr_C9ZWY7_
HLA-DQA10501-DQB10201	269	KHPQAAERLLFATSL	ERLLFATSL	6	0.3312	1389.4	28.00	0.56	tr_C9ZWY7_
HLA-DQA10501-DQB10201	270	ADITTVAEHLDNHRG	DITTVAEHL	1	0.3298	1409.4	28.00	0.51	tr_C9ZWY7_
HLA-DQA10501-DQB10201	271	PGRNVIHFSPTIEQQ	VIHFSPTIE	4	0.3291	1421.0	29.00	0.46	tr_C9ZWY7_
HLA-DQA10501-DQB10201	272	QEDLVAFAPLTGSSA	LVAFAPLTG	3	0.3289	1423.2	29.00	0.38	tr_C9ZWY7_
HLA-DQA10501-DQB10201	273	RGWDPHFYFLTPAPN	FYFLTPAPN	6	0.3289	1423.5	29.00	0.30	tr_C9ZWY7_
HLA-DQA10501-DQB10201	274	VVGITNADAIRSITN	VGITNADAI	1	0.3271	1452.6	29.00	0.50	tr_C9ZWY7_
HLA-DQA10501-DQB10201	275	GELDTRSVTAVFGVV	TRSVTAVFG	4	0.3265	1461.8	29.00	0.47	tr_C9ZWY7_
HLA-DQA10501-DQB10201	276	RTWLESREAFIKSLY	WLESREAFI	2	0.3262	1466.9	29.00	0.50	tr_C9ZWY7_
HLA-DQA10501-DQB10201	277	EEAAVPYIPDRVIVA	AVPYIPDRV	3	0.3241	1499.0	30.00	0.56	tr_C9ZWY7_
HLA-DQA10501-DQB10201	278	LTPAPNAELIALLRY	PNAELIALL	4	0.3241	1500.6	30.00	0.34	tr_C9ZWY7_
HLA-DQA10501-DQB10201	279	MQGELDTRSVTAVFG	TRSVTAVFG	6	0.3238	1504.0	30.00	0.60	tr_C9ZWY7_
HLA-DQA10501-DQB10201	280	RRPPAITFFLIPLLL	TFFLIPLLL	6	0.3236	1508.1	30.00	0.44	tr_C9ZWY7_
HLA-DQA10501-DQB10201	281	MQFVIGATWRTAVEE	ATWRTAVEE	6	0.3221	1532.9	30.00	0.55	tr_C9ZWY7_
HLA-DQA10501-DQB10201	282	SSRTWLESREAFIKS	WLESREAFI	4	0.3220	1534.4	30.00	0.61	tr_C9ZWY7_

HLA-DQA10501-DQB10201	283	FYFLTPAPNAELIAL	YFLTPAPNA	1	0.3208	1554.2	30.00	0.25	tr_C9ZWY7_
HLA-DQA10501-DQB10201	284	FLTPAPNAELIALLR	PNAELIALL	5	0.3207	1555.7	30.00	0.36	tr_C9ZWY7_
HLA-DQA10501-DQB10201	285	PDRVAFHSVVATSRN	FHSVVATSR	5	0.3200	1568.5	30.00	0.46	tr_C9ZWY7_
HLA-DQA10501-DQB10201	286	LTGSSAVRGWDPHFY	VRGWDPHFY	6	0.3192	1580.8	31.00	0.57	tr_C9ZWY7_
HLA-DQA10501-DQB10201	287	VPTTGFAAEGCAVNY	TTGFAAEGC	2	0.3189	1585.8	31.00	0.32	tr_C9ZWY7_
HLA-DQA10501-DQB10201	288	SRTWLESREAFIKSL	WLESREAFI	3	0.3189	1586.0	31.00	0.62	tr_C9ZWY7_
HLA-DQA10501-DQB10201	289	ALKFVRRVAMDARTR	FVRRVAMDA	3	0.3188	1587.7	31.00	0.46	tr_C9ZWY7_
HLA-DQA10501-DQB10201	290	TWLESREAFIKSLYN	WLESREAFI	1	0.3187	1589.7	31.00	0.46	tr_C9ZWY7_
HLA-DQA10501-DQB10201	291	VIVAAPNPLASETKY	VIVAAPNPL	0	0.3175	1610.6	31.00	0.65	tr_C9ZWY7_
HLA-DQA10501-DQB10201	292	DSLWEEFVMTHPQGV	FVMTHPQGV	6	0.3165	1627.6	31.00	0.33	tr_C9ZWY7_
HLA-DQA10501-DQB10201	293	SMNGIVVPDNDTTLN	MNGIVVPDN	1	0.3160	1636.3	31.00	0.41	tr_C9ZWY7_
HLA-DQA10501-DQB10201	294	SHPGRNVIHFSPTIE	VIHFSPTIE	6	0.3152	1651.9	32.00	0.57	tr_C9ZWY7_
HLA-DQA10501-DQB10201	295	TPLALLGYATARAME	LGYATARAM	5	0.3132	1687.3	32.00	0.31	tr_C9ZWY7_
HLA-DQA10501-DQB10201	296	FMYLQGVHFGDREYQ	FMYLQGVHF	0	0.3112	1723.5	32.00	0.32	tr_C9ZWY7_
HLA-DQA10501-DQB10201	297	AVSAFFSLVVGITNA	VSAFFSLVV	1	0.3097	1751.8	33.00	0.30	tr_C9ZWY7_
HLA-DQA10501-DQB10201	298	SVSTLKLLETAADQN	LLETAADQN	6	0.3072	1800.2	33.00	0.48	tr_C9ZWY7_
HLA-DQA10501-DQB10201	299	YATARAMESVVSRMG	YATARAMES	0	0.3072	1800.4	33.00	0.23	tr_C9ZWY7_
HLA-DQA10501-DQB10201	300	NASDDFDHDDADGAL	FDHDDADGA	5	0.3067	1810.1	33.00	0.32	tr_C9ZWY7_
HLA-DQA10501-DQB10201	301	SCVPTTGFAAEGCAV	TTGFAAEGC	4	0.3063	1817.7	34.00	0.37	tr_C9ZWY7_
HLA-DQA10501-DQB10201	302	GFGGFAGVITGRHIT	FGGFAGVIT	1	0.3056	1832.3	34.00	0.48	tr_C9ZWY7_
HLA-DQA10501-DQB10201	303	SLWEEFVMTHPQGVI	FVMTHPQGV	5	0.3056	1832.6	34.00	0.38	tr_C9ZWY7_
HLA-DQA10501-DQB10201	304	VYMNSLGDDHRMIPL	YMNSLGDDH	1	0.3049	1847.1	34.00	0.45	tr_C9ZWY7_
HLA-DQA10501-DQB10201	305	IREALKFVRRVAMDA	FVRRVAMDA	6	0.3045	1853.2	34.00	0.68	tr_C9ZWY7_
HLA-DQA10501-DQB10201	306	VYVLAPSAMQFVIGA	VYVLAPSAM	0	0.3044	1855.8	34.00	0.32	tr_C9ZWY7_
HLA-DQA10501-DQB10201	307	AMDARTRGVYVLAPS	TRGVYVLAP	5	0.3044	1856.9	34.00	0.47	tr_C9ZWY7_
HLA-DQA10501-DQB10201	308	WEEFVMTHPQGVIVF	FVMTHPQGV	3	0.3043	1857.2	34.00	0.41	tr_C9ZWY7_
HLA-DQA10501-DQB10201	309	EDLVAFAPLTGSSAV	LVAFAPLTG	2	0.3034	1876.0	34.00	0.38	tr_C9ZWY7_
HLA-DQA10501-DQB10201	310	YAINRLRLLRVGFMY	LRLLRVGFM	5	0.3030	1885.1	34.00	0.44	tr_C9ZWY7_
HLA-DQA10501-DQB10201	311	CNQGGNVVYMNSLGD	VVYMNSLGD	6	0.3027	1890.0	34.00	0.41	tr_C9ZWY7_
HLA-DQA10501-DQB10201	312	TPAPNAELIALLRYA	PNAELIALL	3	0.3027	1890.6	34.00	0.37	tr_C9ZWY7_
HLA-DQA10501-DQB10201	313	RPPAITFFLIPLLLV	TFFLIPLLL	5	0.3027	1890.7	34.00	0.41	tr_C9ZWY7_
HLA-DQA10501-DQB10201	314	VPYIPDRVIVAAPNP	RVIVAAPNP	6	0.3024	1896.9	35.00	0.38	tr_C9ZWY7_
HLA-DQA10501-DQB10201	315	VSAFFSLVVGITNAD	FFSLVVGIT	3	0.3023	1898.7	35.00	0.24	tr_C9ZWY7_
HLA-DQA10501-DQB10201	316	LKFVRRVAMDARTRG	FVRRVAMDA	2	0.3022	1900.8	35.00	0.46	tr_C9ZWY7_
HLA-DQA10501-DQB10201	317	RLLFATSLPHWADNN	LFATSLPHW	2	0.3022	1900.9	35.00	0.36	tr_C9ZWY7_
HLA-DQA10501-DQB10201	318	RYAINRLRLLRVGFM	LRLLRVGFM	6	0.3022	1901.7	35.00	0.47	tr_C9ZWY7_
HLA-DQA10501-DQB10201	319	MDARTRGVYVLAPSA	TRGVYVLAP	4	0.3018	1909.4	35.00	0.40	tr_C9ZWY7_
HLA-DQA10501-DQB10201	320	VAMDARTRGVYVLAP	TRGVYVLAP	6	0.3015	1915.0	35.00	0.55	tr_C9ZWY7_
HLA-DQA10501-DQB10201	321	LRLLRVGFMYLQGVH	VGFMYLQGV	5	0.3008	1928.9	35.00	0.37	tr_C9ZWY7_
HLA-DQA10501-DQB10201	322	FNASDDFDHDDADGA	FDHDDADGA	6	0.3006	1933.2	35.00	0.28	tr_C9ZWY7_
HLA-DQA10501-DQB10201	323	DRVAFHSVVATSRNT	FHSVVATSR	4	0.3001	1944.5	35.00	0.46	tr_C9ZWY7_
HLA-DQA10501-DQB10201	324	HFSPTIEQQLFLLVG	PTIEQQLFL	3	0.2996	1955.8	35.00	0.25	tr_C9ZWY7_
HLA-DQA10501-DQB10201	325	LWEEFVMTHPQGVIV	FVMTHPQGV	4	0.2985	1978.5	35.00	0.41	tr_C9ZWY7_
HLA-DQA10501-DQB10201	326	PTTGFAAEGCAVNYG	GFAAEGCAV	3	0.2980	1989.5	36.00	0.31	tr_C9ZWY7_
HLA-DQA10501-DQB10201	327	FFVVVGPMGDTRTLA	FVVVGPMGD	1	0.2978	1993.6	36.00	0.41	tr_C9ZWY7_
HLA-DQA10501-DQB10201	328	GRVNSEELINGIFSQ	EELINGIFS	5	0.2977	1995.1	36.00	0.36	tr_C9ZWY7_
HLA-DQA10501-DQB10201	329	CVPTTGFAAEGCAVN	TTGFAAEGC	3	0.2970	2010.0	36.00	0.34	tr_C9ZWY7_
HLA-DQA10501-DQB10201	330	EAAVPYIPDRVIVAA	AVPYIPDRV	2	0.2960	2032.8	36.00	0.59	tr_C9ZWY7_
HLA-DQA10501-DQB10201	331	SDDFDHDDADGALMV	FDHDDADGA	3	0.2959	2035.9	36.00	0.31	tr_C9ZWY7_
HLA-DQA10501-DQB10201	332	PPAITFFLIPLLLVL	TFFLIPLLL	4	0.2947	2060.7	36.00	0.40	tr_C9ZWY7_

HLA-DQA10501-DQB10201	333	ASDDFDHDDADGALM	FDHDDADGA	4	0.2946	2064.1	37.00	0.35	tr_C9ZWY7_
HLA-DQA10501-DQB10201	334	PAITFFLIPLLLVLP	TFFLIPLLL	3	0.2944	2068.2	37.00	0.38	tr_C9ZWY7_
HLA-DQA10501-DQB10201	335	THPQGVIVFTPPIRE	IVFTPPIRE	6	0.2921	2119.2	37.00	0.30	tr_C9ZWY7_
HLA-DQA10501-DQB10201	336	VDLAMLSVGRVAFIE	LAMLSVGRV	2	0.2921	2120.5	37.00	0.25	tr_C9ZWY7_
HLA-DQA10501-DQB10201	337	RYVVDDLVFGNFGGE	RYVVDDLVF	0	0.2917	2129.7	37.00	0.44	tr_C9ZWY7_
HLA-DQA10501-DQB10201	338	WTPLALLGYATARAM	LLGYATARA	5	0.2916	2131.9	37.00	0.45	tr_C9ZWY7_
HLA-DQA10501-DQB10201	339	VVDDLVFGNFGGECH	VFGNFGGEC	5	0.2916	2132.1	37.00	0.35	tr_C9ZWY7_
HLA-DQA10501-DQB10201	340	PAPNAELIALLRYAI	PNAELIALL	2	0.2912	2140.8	37.00	0.36	tr_C9ZWY7_
HLA-DQA10501-DQB10201	341	TNPDRVAFHSVVATS	RVAFHSVVA	4	0.2909	2148.5	37.00	0.26	tr_C9ZWY7_
HLA-DQA10501-DQB10201	342	AINRLRLLRVGFMYL	LRLLRVGFM	4	0.2907	2153.6	37.00	0.46	tr_C9ZWY7_
HLA-DQA10501-DQB10201	343	NVTVEIIPPPPRSVS	TVEIIPPPP	2	0.2896	2177.4	38.00	0.32	tr_C9ZWY7_
HLA-DQA10501-DQB10201	344	IPLRDGITVFNIDRC	ITVFNIDRC	6	0.2895	2180.4	38.00	0.35	tr_C9ZWY7_
HLA-DQA10501-DQB10201	345	YLQGVHFGDREYQET	QGVHFGDRE	2	0.2895	2180.5	38.00	0.31	tr_C9ZWY7_
HLA-DQA10501-DQB10201	346	HISVWSMARVLNPSV	WSMARVLNP	4	0.2893	2184.5	38.00	0.31	tr_C9ZWY7_
HLA-DQA10501-DQB10201	347	ISVWSMARVLNPSVP	WSMARVLNP	3	0.2873	2232.9	38.00	0.33	tr_C9ZWY7_
HLA-DQA10501-DQB10201	348	SIETMGVGLNASFAA	GVGLNASFA	5	0.2869	2242.2	38.00	0.35	tr_C9ZWY7_
HLA-DQA10501-DQB10201	349	YGRMQGELDTRSVTA	YGRMQGELD	0	0.2863	2256.8	39.00	0.58	tr_C9ZWY7_
HLA-DQA10501-DQB10201	350	KFVRRVAMDARTRGV	FVRRVAMDA	1	0.2860	2264.2	39.00	0.46	tr_C9ZWY7_
HLA-DQA10501-DQB10201	351	DITTVAEHLDNHRGV	DITTVAEHL	0	0.2850	2289.4	39.00	0.44	tr_C9ZWY7_
HLA-DQA10501-DQB10201	352	RVNSEELINGIFSQS	EELINGIFS	4	0.2842	2308.5	39.00	0.34	tr_C9ZWY7_
HLA-DQA10501-DQB10201	353	SVWSMARVLNPSVPP	MARVLNPSV	4	0.2839	2317.5	39.00	0.32	tr_C9ZWY7_
HLA-DQA10501-DQB10201	354	ITNPDRVAFHSVVAT	VAFHSVVAT	6	0.2825	2352.5	39.00	0.37	tr_C9ZWY7_
HLA-DQA10501-DQB10201	355	FGVVDLAMLSVGRVA	LAMLSVGRV	5	0.2814	2380.0	40.00	0.34	tr_C9ZWY7_
HLA-DQA10501-DQB10201	356	ATHISVWSMARVLNP	WSMARVLNP	6	0.2812	2384.5	40.00	0.35	tr_C9ZWY7_
HLA-DQA10501-DQB10201	357	AADQNKGEFFVVVGP	GEFFVVVGP	6	0.2811	2388.3	40.00	0.27	tr_C9ZWY7_
HLA-DQA10501-DQB10201	358	HLDNHRGVRVFVSFF	GVRVFVSFF	6	0.2808	2397.2	40.00	0.47	tr_C9ZWY7_
HLA-DQA10501-DQB10201	359	LQGVHFGDREYQETL	FGDREYQET	5	0.2807	2398.9	40.00	0.19	tr_C9ZWY7_
HLA-DQA10501-DQB10201	360	RVAFHSVVATSRNTY	FHSVVATSR	3	0.2805	2404.1	40.00	0.56	tr_C9ZWY7_
HLA-DQA10501-DQB10201	361	NRLRLLRVGFMYLQG	LRLLRVGFM	2	0.2803	2410.2	40.00	0.41	tr_C9ZWY7_
HLA-DQA10501-DQB10201	362	VIGEVLSQALSSRTW	IGEVLSQAL	1	0.2802	2412.9	40.00	0.34	tr_C9ZWY7_
HLA-DQA10501-DQB10201	363	AVPYIPDRVIVAAPN	AVPYIPDRV	0	0.2799	2418.3	40.00	0.31	tr_C9ZWY7_
HLA-DQA10501-DQB10201	364	VHFGDREYQETLKLM	REYQETLKL	5	0.2796	2427.2	40.00	0.31	tr_C9ZWY7_
HLA-DQA10501-DQB10201	365	AITFFLIPLLLVLPH	TFFLIPLLL	2	0.2796	2428.4	40.00	0.43	tr_C9ZWY7_
HLA-DQA10501-DQB10201	366	AAVPYIPDRVIVAAP	AVPYIPDRV	1	0.2793	2435.6	40.00	0.53	tr_C9ZWY7_
HLA-DQA10501-DQB10201	367	VSTLKLLETAADQNK	LLETAADQN	5	0.2793	2436.4	40.00	0.54	tr_C9ZWY7_
HLA-DQA10501-DQB10201	368	EFVMTHPQGVIVFTP	FVMTHPQGV	1	0.2792	2436.7	40.00	0.35	tr_C9ZWY7_
HLA-DQA10501-DQB10201	369	HPQGVIVFTPPIREA	IVFTPPIRE	5	0.2791	2439.8	40.00	0.33	tr_C9ZWY7_
HLA-DQA10501-DQB10201	370	VWSMARVLNPSVPPV	MARVLNPSV	3	0.2781	2466.3	41.00	0.34	tr_C9ZWY7_
HLA-DQA10501-DQB10201	371	GVHFGDREYQETLKL	REYQETLKL	6	0.2779	2472.1	41.00	0.38	tr_C9ZWY7_
HLA-DQA10501-DQB10201	372	FVMTHPQGVIVFTPP	PQGVIVFTP	5	0.2777	2479.0	41.00	0.25	tr_C9ZWY7_
HLA-DQA10501-DQB10201	373	VVDLAMLSVGRVAFI	LAMLSVGRV	3	0.2775	2483.5	41.00	0.31	tr_C9ZWY7_
HLA-DQA10501-DQB10201	374	IKVKVYNLLFTNRAP	YNLLFTNRA	5	0.2771	2493.1	41.00	0.47	tr_C9ZWY7_
HLA-DQA10501-DQB10201	375	MYLQGVHFGDREYQE	QGVHFGDRE	3	0.2765	2509.3	41.00	0.34	tr_C9ZWY7_
HLA-DQA10501-DQB10201	376	APNAELIALLRYAIN	PNAELIALL	1	0.2765	2510.9	41.00	0.37	tr_C9ZWY7_
HLA-DQA10501-DQB10201	377	TTGFAAEGCAVNYGA	GFAAEGCAV	2	0.2762	2517.3	41.00	0.31	tr_C9ZWY7_
HLA-DQA10501-DQB10201	378	YVVDDLVFGNFGGEC	VFGNFGGEC	6	0.2761	2521.4	41.00	0.41	tr_C9ZWY7_
HLA-DQA10501-DQB10201	379	PLRDGITVFNIDRCS	ITVFNIDRC	5	0.2749	2552.9	41.00	0.34	tr_C9ZWY7_
HLA-DQA10501-DQB10201	380	INRLRLLRVGFMYLQ	LRLLRVGFM	3	0.2744	2567.6	42.00	0.47	tr_C9ZWY7_
HLA-DQA10501-DQB10201	381	EEFVMTHPQGVIVFT	FVMTHPQGV	2	0.2738	2584.3	42.00	0.43	tr_C9ZWY7_
HLA-DQA10501-DQB10201	382	VAFIEPITLSPKLSH	VAFIEPITL	0	0.2738	2584.9	42.00	0.55	tr_C9ZWY7_

HLA-DQA10501-DQB10201	383	DDFDHDDADGALMVS	FDHDDADGA	2	0.2731	2603.7	42.00	0.33	tr_C9ZWY7_
HLA-DQA10501-DQB10201	384	VDDLVFGNFGGECHG	VFGNFGGEC	4	0.2729	2608.6	42.00	0.37	tr_C9ZWY7_
HLA-DQA10501-DQB10201	385	PQGVIVFTPPIREAL	IVFTPPIRE	4	0.2724	2625.4	42.00	0.34	tr_C9ZWY7_
HLA-DQA10501-DQB10201	386	ITFFLIPLLLVLPHI	TFFLIPLLL	1	0.2722	2630.1	42.00	0.40	tr_C9ZWY7_
HLA-DQA10501-DQB10201	387	FSPTIEQQLFLLVGY	PTIEQQLFL	2	0.2714	2652.3	42.00	0.25	tr_C9ZWY7_
HLA-DQA10501-DQB10201	388	KLLETAADQNKGEFF	LLETAADQN	1	0.2704	2682.5	43.00	0.40	tr_C9ZWY7_
HLA-DQA10501-DQB10201	389	IESLGGSMNGIVVPD	SMNGIVVPD	6	0.2701	2691.5	43.00	0.44	tr_C9ZWY7_
HLA-DQA10501-DQB10201	390	THISVWSMARVLNPS	WSMARVLNP	5	0.2699	2695.4	43.00	0.35	tr_C9ZWY7_
HLA-DQA10501-DQB10201	391	RSIETMGVGLNASFA	GVGLNASFA	6	0.2693	2713.0	43.00	0.37	tr_C9ZWY7_
HLA-DQA10501-DQB10201	392	LLFATSLPHWADNNT	FATSLPHWA	2	0.2692	2717.7	43.00	0.47	tr_C9ZWY7_
HLA-DQA10501-DQB10201	393	MWYGPFEDSCVPTTG	WYGPFEDSC	1	0.2684	2739.0	43.00	0.37	tr_C9ZWY7_
HLA-DQA10501-DQB10201	394	SIKVKVYNLLFTNRA	YNLLFTNRA	6	0.2684	2740.5	43.00	0.42	tr_C9ZWY7_
HLA-DQA10501-DQB10201	395	RRRPPAITFFLIPLL	ITFFLIPLL	6	0.2678	2757.7	43.00	0.47	tr_C9ZWY7_
HLA-DQA10501-DQB10201	396	WLESREAFIKSLYNQ	WLESREAFI	0	0.2676	2763.7	43.00	0.37	tr_C9ZWY7_
HLA-DQA10501-DQB10201	397	VFKKHPQAAERLLFA	QAAERLLFA	6	0.2676	2763.8	43.00	0.29	tr_C9ZWY7_
HLA-DQA10501-DQB10201	398	LRDGITVFNIDRCST	ITVFNIDRC	4	0.2667	2791.7	44.00	0.35	tr_C9ZWY7_
HLA-DQA10501-DQB10201	399	VLAPSAMQFVIGATW	MQFVIGATW	6	0.2641	2870.1	44.00	0.38	tr_C9ZWY7_
HLA-DQA10501-DQB10201	400	VGITNADAIRSITNP	VGITNADAI	0	0.2640	2872.4	44.00	0.41	tr_C9ZWY7_
HLA-DQA10501-DQB10201	401	DDLVFGNFGGECHGM	VFGNFGGEC	3	0.2640	2873.2	44.00	0.38	tr_C9ZWY7_
HLA-DQA10501-DQB10201	402	DLVAFAPLTGSSAVR	LVAFAPLTG	1	0.2631	2901.1	45.00	0.41	tr_C9ZWY7_
HLA-DQA10501-DQB10201	403	LSPHGDTLVFGLTEA	DTLVFGLTE	5	0.2624	2922.7	45.00	0.32	tr_C9ZWY7_
HLA-DQA10501-DQB10201	404	DLVFGNFGGECHGMA	VFGNFGGEC	2	0.2618	2943.7	45.00	0.32	tr_C9ZWY7_
HLA-DQA10501-DQB10201	405	FVRRVAMDARTRGVY	FVRRVAMDA	0	0.2606	2981.2	45.00	0.37	tr_C9ZWY7_
HLA-DQA10501-DQB10201	406	IETMGVGLNASFAAR	GVGLNASFA	4	0.2605	2983.4	45.00	0.37	tr_C9ZWY7_
HLA-DQA10501-DQB10201	407	MNGIVVPDNDTTLNE	NGIVVPDND	1	0.2600	3001.3	45.00	0.35	tr_C9ZWY7_
HLA-DQA10501-DQB10201	408	STLKLLETAADQNKG	LLETAADQN	4	0.2595	3016.1	46.00	0.60	tr_C9ZWY7_
HLA-DQA10501-DQB10201	409	GVVDLAMLSVGRVAF	LAMLSVGRV	4	0.2591	3030.9	46.00	0.42	tr_C9ZWY7_
HLA-DQA10501-DQB10201	410	RSVSTLKLLETAADQ	KLLETAADQ	6	0.2584	3052.7	46.00	0.34	tr_C9ZWY7_
HLA-DQA10501-DQB10201	411	FGGFAGVITGRHITT	FGGFAGVIT	0	0.2573	3089.5	46.00	0.43	tr_C9ZWY7_
HLA-DQA10501-DQB10201	412	WSMARVLNPSVPPVT	MARVLNPSV	2	0.2570	3100.6	46.00	0.34	tr_C9ZWY7_
HLA-DQA10501-DQB10201	413	LAPSAMQFVIGATWR	MQFVIGATW	5	0.2569	3104.2	46.00	0.31	tr_C9ZWY7_
HLA-DQA10501-DQB10201	414	DSCVPTTGFAAEGCA	TTGFAAEGC	5	0.2566	3113.6	46.00	0.56	tr_C9ZWY7_
HLA-DQA10501-DQB10201	415	VAFHSVVATSRNTYG	FHSVVATSR	2	0.2564	3121.0	46.00	0.64	tr_C9ZWY7_
HLA-DQA10501-DQB10201	416	MTHPQGVIVFTPPIR	VIVFTPPIR	6	0.2552	3160.5	47.00	0.26	tr_C9ZWY7_
HLA-DQA10501-DQB10201	417	FKKHPQAAERLLFAT	QAAERLLFA	5	0.2550	3166.9	47.00	0.28	tr_C9ZWY7_
HLA-DQA10501-DQB10201	418	VMTHPQGVIVFTPPI	PQGVIVFTP	4	0.2543	3192.2	47.00	0.29	tr_C9ZWY7_
HLA-DQA10501-DQB10201	419	RDGITVFNIDRCSTI	ITVFNIDRC	3	0.2537	3213.8	47.00	0.32	tr_C9ZWY7_
HLA-DQA10501-DQB10201	420	QGVHFGDREYQETLK	FGDREYQET	4	0.2536	3215.7	47.00	0.20	tr_C9ZWY7_
HLA-DQA10501-DQB10201	421	HFGDREYQETLKLMT	REYQETLKL	4	0.2536	3216.8	47.00	0.38	tr_C9ZWY7_
HLA-DQA10501-DQB10201	422	TLKLLETAADQNKGE	LLETAADQN	3	0.2536	3217.7	47.00	0.64	tr_C9ZWY7_
HLA-DQA10501-DQB10201	423	PLLLVLPHIVRPLAA	PHIVRPLAA	6	0.2534	3222.1	47.00	0.40	tr_C9ZWY7_
HLA-DQA10501-DQB10201	424	RMIPLRDGITVFNID	RDGITVFNI	5	0.2524	3257.6	48.00	0.31	tr_C9ZWY7_
HLA-DQA10501-DQB10201	425	KVFKKHPQAAERLLF	PQAAERLLF	6	0.2521	3268.7	48.00	0.38	tr_C9ZWY7_
HLA-DQA10501-DQB10201	426	DLSPHGDTLVFGLTE	DTLVFGLTE	6	0.2521	3269.3	48.00	0.49	tr_C9ZWY7_
HLA-DQA10501-DQB10201	427	KWTPLALLGYATARA	LLGYATARA	6	0.2516	3286.5	48.00	0.56	tr_C9ZWY7_
HLA-DQA10501-DQB10201	428	ALLRYAINRLRLLRV	INRLRLLRV	6	0.2509	3310.4	48.00	0.34	tr_C9ZWY7_
HLA-DQA10501-DQB10201	429	VVPDNDTTLNEDLSP	TTLNEDLSP	6	0.2504	3328.9	48.00	0.29	tr_C9ZWY7_
HLA-DQA10501-DQB10201	430	LRYHPGVTAFNASDD	VTAFNASDD	6	0.2495	3361.5	48.00	0.46	tr_C9ZWY7_
HLA-DQA10501-DQB10201	431	EDSCVPTTGFAAEGC	TTGFAAEGC	6	0.2495	3363.5	48.00	0.64	tr_C9ZWY7_
HLA-DQA10501-DQB10201	432	KVKVYNLLFTNRAPR	YNLLFTNRA	4	0.2493	3370.3	48.00	0.47	tr_C9ZWY7_

HLA-DQA10501-DQB10201	433	VPTPLIVVVVLVVDD	VVVVLVVDD	6	0.2485	3398.8	49.00	0.73	tr_C9ZWY7_
HLA-DQA10501-DQB10201	434	CAVNYGATHISVWSM	YGATHISVW	4	0.2484	3401.4	49.00	0.19	tr_C9ZWY7_
HLA-DQA10501-DQB10201	435	APSAMQFVIGATWRT	MQFVIGATW	4	0.2476	3430.2	49.00	0.30	tr_C9ZWY7_
HLA-DQA10501-DQB10201	436	VTRGGDVPGIGSVFQ	VPGIGSVFQ	6	0.2473	3443.5	49.00	0.48	tr_C9ZWY7_
HLA-DQA10501-DQB10201	437	QGVIVFTPPIREALK	IVFTPPIRE	3	0.2473	3443.8	49.00	0.34	tr_C9ZWY7_
HLA-DQA10501-DQB10201	438	TMGVGLNASFAARHW	GVGLNASFA	2	0.2454	3514.0	49.00	0.31	tr_C9ZWY7_
HLA-DQA10501-DQB10201	439	FDHDDADGALMVSGW	DGALMVSGW	6	0.2451	3525.1	50.00	0.25	tr_C9ZWY7_
HLA-DQA10501-DQB10201	440	LKLLETAADQNKGEF	LLETAADQN	2	0.2451	3525.5	50.00	0.65	tr_C9ZWY7_
HLA-DQA10501-DQB10201	441	YMNSLGDDHRMIPLR	YMNSLGDDH	0	0.2446	3545.5	50.00	0.44	tr_C9ZWY7_
HLA-DQA10501-DQB10201	442	YVLAPSAMQFVIGAT	LAPSAMQFV	2	0.2443	3558.3	50.00	0.27	tr_C9ZWY7_
HLA-DQA10501-DQB10201	443	MIPLRDGITVFNIDR	RDGITVFNI	4	0.2432	3600.9	50.00	0.25	tr_C9ZWY7_
HLA-DQA10501-DQB10201	444	ETMGVGLNASFAARH	GVGLNASFA	3	0.2431	3601.1	50.00	0.35	tr_C9ZWY7_
HLA-DQA10501-DQB10201	445	DFDHDDADGALMVSG	FDHDDADGA	1	0.2430	3608.7	50.00	0.29	tr_C9ZWY7_
HLA-DQA10501-DQB10201	446	TFFLIPLLLVLPHIV	TFFLIPLLL	0	0.2416	3662.5	55.00	0.29	tr_C9ZWY7_
HLA-DQA10501-DQB10201	447	GVIVFTPPIREALKF	IVFTPPIRE	2	0.2416	3663.2	55.00	0.35	tr_C9ZWY7_
HLA-DQA10501-DQB10201	448	FVVVGPMGDTRTLAV	FVVVGPMGD	0	0.2412	3679.5	55.00	0.36	tr_C9ZWY7_
HLA-DQA10501-DQB10201	449	NAELIALLRYAINRL	LLRYAINRL	6	0.2410	3686.6	55.00	0.48	tr_C9ZWY7_
HLA-DQA10501-DQB10201	450	DHDDADGALMVSGWV	GALMVSGWV	6	0.2406	3701.6	55.00	0.31	tr_C9ZWY7_
HLA-DQA10501-DQB10201	451	EQQLFLLVGYIARDK	LVGYIARDK	6	0.2401	3723.8	55.00	0.34	tr_C9ZWY7_
HLA-DQA10501-DQB10201	452	VPGIGSVFQKTIESL	VFQKTIESL	6	0.2398	3734.3	55.00	0.55	tr_C9ZWY7_
HLA-DQA10501-DQB10201	453	LVFGNFGGECHGMAG	FGNFGGECH	2	0.2395	3747.4	55.00	0.35	tr_C9ZWY7_
HLA-DQA10501-DQB10201	454	LIALLRYAINRLRLL	LLRYAINRL	3	0.2394	3748.6	55.00	0.28	tr_C9ZWY7_
HLA-DQA10501-DQB10201	455	RMQGELDTRSVTAVF	DTRSVTAVF	6	0.2384	3789.1	55.00	0.50	tr_C9ZWY7_
HLA-DQA10501-DQB10201	456	TNADAIRSITNPDRV	RSITNPDRV	6	0.2383	3794.2	55.00	0.40	tr_C9ZWY7_
HLA-DQA10501-DQB10201	457	PNAELIALLRYAINR	NAELIALLR	1	0.2379	3810.3	55.00	0.31	tr_C9ZWY7_
HLA-DQA10501-DQB10201	458	EGCAVNYGATHISVW	NYGATHISV	5	0.2377	3819.2	55.00	0.29	tr_C9ZWY7_
HLA-DQA10501-DQB10201	459	RRSIETMGVGLNASF	ETMGVGLNA	4	0.2375	3826.7	55.00	0.22	tr_C9ZWY7_
HLA-DQA10501-DQB10201	460	YHAVRRFQEDARSYL	FQEDARSYL	6	0.2371	3844.2	55.00	0.28	tr_C9ZWY7_
HLA-DQA10501-DQB10201	461	IGEVLSQALSSRTWL	EVLSQALSS	2	0.2371	3845.8	55.00	0.43	tr_C9ZWY7_
HLA-DQA10501-DQB10201	462	APRRSIETMGVGLNA	RRSIETMGV	2	0.2366	3865.9	55.00	0.29	tr_C9ZWY7_
HLA-DQA10501-DQB10201	463	PSAMQFVIGATWRTA	MQFVIGATW	3	0.2362	3882.0	55.00	0.28	tr_C9ZWY7_
HLA-DQA10501-DQB10201	464	SAMQFVIGATWRTAV	MQFVIGATW	2	0.2345	3954.7	55.00	0.29	tr_C9ZWY7_
HLA-DQA10501-DQB10201	465	AVNYGATHISVWSMA	YGATHISVW	3	0.2340	3977.6	55.00	0.19	tr_C9ZWY7_
HLA-DQA10501-DQB10201	466	SITNPDRVAFHSVVA	RVAFHSVVA	6	0.2336	3993.8	55.00	0.54	tr_C9ZWY7_
HLA-DQA10501-DQB10201	467	PRSVSTLKLLETAAD	VSTLKLLET	3	0.2336	3994.0	55.00	0.34	tr_C9ZWY7_
HLA-DQA10501-DQB10201	468	QALSSRTWLESREAF	TWLESREAF	6	0.2336	3994.9	55.00	0.34	tr_C9ZWY7_
HLA-DQA10501-DQB10201	469	KKHPQAAERLLFATS	QAAERLLFA	4	0.2331	4012.6	55.00	0.26	tr_C9ZWY7_
HLA-DQA10501-DQB10201	470	TAADQNKGEFFVVVG	ADQNKGEFF	2	0.2331	4013.8	55.00	0.31	tr_C9ZWY7_
HLA-DQA10501-DQB10201	471	FTNRAPRRSIETMGV	RRSIETMGV	6	0.2328	4028.0	55.00	0.58	tr_C9ZWY7_
HLA-DQA10501-DQB10201	472	ATARAMESVVSRMGR	AMESVVSRM	4	0.2323	4048.5	55.00	0.25	tr_C9ZWY7_
HLA-DQA10501-DQB10201	473	SPTIEQQLFLLVGYI	PTIEQQLFL	1	0.2323	4049.7	55.00	0.26	tr_C9ZWY7_
HLA-DQA10501-DQB10201	474	LRYAINRLRLLRVGF	INRLRLLRV	4	0.2323	4051.3	55.00	0.32	tr_C9ZWY7_
HLA-DQA10501-DQB10201	475	HDDADGALMVSGWVI	GALMVSGWV	5	0.2322	4052.2	55.00	0.26	tr_C9ZWY7_
HLA-DQA10501-DQB10201	476	GCAVNYGATHISVWS	NYGATHISV	4	0.2321	4058.1	55.00	0.26	tr_C9ZWY7_
HLA-DQA10501-DQB10201	477	AMQFVIGATWRTAVE	MQFVIGATW	1	0.2320	4064.6	55.00	0.28	tr_C9ZWY7_
HLA-DQA10501-DQB10201	478	NRAPRRSIETMGVGL	RRSIETMGV	4	0.2319	4067.9	55.00	0.44	tr_C9ZWY7_
HLA-DQA10501-DQB10201	479	PPPPRSVSTLKLLET	VSTLKLLET	6	0.2317	4075.8	55.00	0.47	tr_C9ZWY7_
HLA-DQA10501-DQB10201	480	TGFAAEGCAVNYGAT	GFAAEGCAV	1	0.2315	4082.9	55.00	0.38	tr_C9ZWY7_
HLA-DQA10501-DQB10201	481	VKVYNLLFTNRAPRR	YNLLFTNRA	3	0.2313	4093.6	55.00	0.45	tr_C9ZWY7_
HLA-DQA10501-DQB10201	482	DGITVFNIDRCSTIG	ITVFNIDRC	2	0.2311	4102.0	55.00	0.38	tr_C9ZWY7_

HLA-DQA10501-DQB10201	483	LFATSLPHWADNNTT	FATSLPHWA	1	0.2310	4107.4	55.00	0.48	tr_C9ZWY7_
HLA-DQA10501-DQB10201	484	IEQQLFLLVGYIARD	LLVGYIARD	6	0.2302	4143.6	55.00	0.41	tr_C9ZWY7_
HLA-DQA10501-DQB10201	485	ETVKRFHDEMKDDES	FHDEMKDDE	5	0.2301	4146.0	55.00	0.30	tr_C9ZWY7_
HLA-DQA10501-DQB10201	486	ADAIRSITNPDRVAF	RSITNPDRV	4	0.2298	4160.3	55.00	0.47	tr_C9ZWY7_
HLA-DQA10501-DQB10201	487	VPDNDTTLNEDLSPH	TTLNEDLSP	5	0.2286	4215.8	55.00	0.28	tr_C9ZWY7_
HLA-DQA10501-DQB10201	488	CLCNQGGNVVYMNSL	GNVVYMNSL	6	0.2285	4218.1	55.00	0.50	tr_C9ZWY7_
HLA-DQA10501-DQB10201	489	DADGALMVSGWVIGE	MVSGWVIGE	6	0.2285	4220.0	55.00	0.31	tr_C9ZWY7_
HLA-DQA10501-DQB10201	490	LLETAADQNKGEFFV	ADQNKGEFF	5	0.2284	4224.8	55.00	0.45	tr_C9ZWY7_
HLA-DQA10501-DQB10201	491	PRRSIETMGVGLNAS	RRSIETMGV	1	0.2278	4249.7	55.00	0.25	tr_C9ZWY7_
HLA-DQA10501-DQB10201	492	RAPRRSIETMGVGLN	RRSIETMGV	3	0.2276	4261.1	55.00	0.36	tr_C9ZWY7_
HLA-DQA10501-DQB10201	493	ETAADQNKGEFFVVV	ADQNKGEFF	3	0.2267	4300.4	55.00	0.40	tr_C9ZWY7_
HLA-DQA10501-DQB10201	494	MGVGLNASFAARHWA	GVGLNASFA	1	0.2264	4316.8	55.00	0.31	tr_C9ZWY7_
HLA-DQA10501-DQB10201	495	NADAIRSITNPDRVA	RSITNPDRV	5	0.2257	4348.4	55.00	0.42	tr_C9ZWY7_
HLA-DQA10501-DQB10201	496	HRMIPLRDGITVFNI	RDGITVFNI	6	0.2255	4356.8	55.00	0.36	tr_C9ZWY7_
HLA-DQA10501-DQB10201	497	VTVEIIPPPPRSVST	TVEIIPPPP	1	0.2253	4368.0	55.00	0.51	tr_C9ZWY7_
HLA-DQA10501-DQB10201	498	SETVKRFHDEMKDDE	FHDEMKDDE	6	0.2251	4376.8	55.00	0.32	tr_C9ZWY7_
HLA-DQA10501-DQB10201	499	EHLDNHRGVRVFVSF	HRGVRVFVS	5	0.2248	4392.0	55.00	0.34	tr_C9ZWY7_
HLA-DQA10501-DQB10201	500	PPRSVSTLKLLETAA	VSTLKLLET	4	0.2247	4397.1	55.00	0.38	tr_C9ZWY7_
HLA-DQA10501-DQB10201	501	YGATHISVWSMARVL	ISVWSMARV	5	0.2246	4402.0	55.00	0.29	tr_C9ZWY7_
HLA-DQA10501-DQB10201	502	KDDESKWTPLALLGY	WTPLALLGY	6	0.2240	4429.6	60.00	0.50	tr_C9ZWY7_
HLA-DQA10501-DQB10201	503	QQLFLLVGYIARDKK	LVGYIARDK	5	0.2239	4436.1	60.00	0.27	tr_C9ZWY7_
HLA-DQA10501-DQB10201	504	VIVFTPPIREALKFV	IVFTPPIRE	1	0.2235	4455.3	60.00	0.33	tr_C9ZWY7_
HLA-DQA10501-DQB10201	505	PPPRSVSTLKLLETA	VSTLKLLET	5	0.2232	4466.4	60.00	0.43	tr_C9ZWY7_
HLA-DQA10501-DQB10201	506	AFHSVVATSRNTYGR	FHSVVATSR	1	0.2229	4484.4	60.00	0.70	tr_C9ZWY7_
HLA-DQA10501-DQB10201	507	GITNADAIRSITNPD	ITNADAIRS	1	0.2229	4485.4	60.00	0.31	tr_C9ZWY7_
HLA-DQA10501-DQB10201	508	PTIEQQLFLLVGYIA	PTIEQQLFL	0	0.2227	4491.0	60.00	0.20	tr_C9ZWY7_
HLA-DQA10501-DQB10201	509	IALLRYAINRLRLLR	LLRYAINRL	2	0.2221	4524.0	60.00	0.34	tr_C9ZWY7_
HLA-DQA10501-DQB10201	510	GGDVPGIGSVFQKTI	VPGIGSVFQ	3	0.2218	4539.1	60.00	0.38	tr_C9ZWY7_
HLA-DQA10501-DQB10201	511	DDHRMIPLRDGITVF	PLRDGITVF	6	0.2218	4539.1	60.00	0.31	tr_C9ZWY7_
HLA-DQA10501-DQB10201	512	LLRYAINRLRLLRVG	INRLRLLRV	5	0.2217	4539.8	60.00	0.31	tr_C9ZWY7_
HLA-DQA10501-DQB10201	513	WYGPFEDSCVPTTGF	WYGPFEDSC	0	0.2216	4544.3	60.00	0.30	tr_C9ZWY7_
HLA-DQA10501-DQB10201	514	NYGATHISVWSMARV	ISVWSMARV	6	0.2212	4568.3	60.00	0.33	tr_C9ZWY7_
HLA-DQA10501-DQB10201	515	NGIVVPDNDTTLNED	NGIVVPDND	0	0.2199	4631.1	60.00	0.29	tr_C9ZWY7_
HLA-DQA10501-DQB10201	516	ELIALLRYAINRLRL	LLRYAINRL	4	0.2195	4650.6	60.00	0.38	tr_C9ZWY7_
HLA-DQA10501-DQB10201	517	TRGGDVPGIGSVFQK	VPGIGSVFQ	5	0.2187	4692.4	60.00	0.47	tr_C9ZWY7_
HLA-DQA10501-DQB10201	518	FGDREYQETLKLMTR	REYQETLKL	3	0.2178	4735.2	60.00	0.40	tr_C9ZWY7_
HLA-DQA10501-DQB10201	519	MNSLGDDHRMIPLRD	DHRMIPLRD	6	0.2174	4759.6	60.00	0.50	tr_C9ZWY7_
HLA-DQA10501-DQB10201	520	IPLLLVLPHIVRPLA	LPHIVRPLA	6	0.2164	4809.2	60.00	0.34	tr_C9ZWY7_
HLA-DQA10501-DQB10201	521	LLLVLPHIVRPLAAK	PHIVRPLAA	5	0.2157	4846.6	60.00	0.41	tr_C9ZWY7_
HLA-DQA10501-DQB10201	522	LCNQGGNVVYMNSLG	GNVVYMNSL	5	0.2153	4865.8	60.00	0.43	tr_C9ZWY7_
HLA-DQA10501-DQB10201	523	GATHISVWSMARVLN	ISVWSMARV	4	0.2149	4887.3	60.00	0.28	tr_C9ZWY7_
HLA-DQA10501-DQB10201	524	DAIRSITNPDRVAFH	RSITNPDRV	3	0.2145	4910.2	60.00	0.48	tr_C9ZWY7_
HLA-DQA10501-DQB10201	525	LESREAFIKSLYNQR	REAFIKSLY	3	0.2144	4916.8	60.00	0.29	tr_C9ZWY7_
HLA-DQA10501-DQB10201	526	GDVPGIGSVFQKTIE	VPGIGSVFQ	2	0.2136	4959.9	60.00	0.35	tr_C9ZWY7_
HLA-DQA10501-DQB10201	527	PGIGSVFQKTIESLG	VFQKTIESL	5	0.2129	4996.4	60.00	0.51	tr_C9ZWY7_
HLA-DQA10501-DQB10201	528	MRRRPPAITFFLIPL	AITFFLIPL	6	0.2128	4998.5	60.00	0.41	tr_C9ZWY7_
HLA-DQA10501-DQB10201	529	AEGCAVNYGATHISV	NYGATHISV	6	0.2128	4999.3	60.00	0.36	tr_C9ZWY7_
HLA-DQA10501-DQB10201	530	SMARVLNPSVPPVTK	MARVLNPSV	1	0.2127	5007.2	60.00	0.37	tr_C9ZWY7_
HLA-DQA10501-DQB10201	531	VFGNFGGECHGMAGE	FGNFGGECH	1	0.2126	5012.4	60.00	0.38	tr_C9ZWY7_
HLA-DQA10501-DQB10201	532	IVVPDNDTTLNEDLS	DTTLNEDLS	6	0.2125	5017.5	60.00	0.28	tr_C9ZWY7_

HLA-DQA10501-DQB10201	533	RLCGVFTKMHAATVM	TKMHAATVM	6	0.2121	5037.9	60.00	0.62	tr_C9ZWY7_
HLA-DQA10501-DQB10201	534	TIGVKVPTPLIVVVV	PTPLIVVVV	6	0.2116	5063.7	60.00	0.22	tr_C9ZWY7_
HLA-DQA10501-DQB10201	535	VVGPMGDTRTLAVLP	DTRTLAVLP	6	0.2108	5112.5	60.00	0.28	tr_C9ZWY7_
HLA-DQA10501-DQB10201	536	KYHAVRRFQEDARSY	VRRFQEDAR	4	0.2108	5112.7	60.00	0.29	tr_C9ZWY7_
HLA-DQA10501-DQB10201	537	DDADGALMVSGWVIG	GALMVSGWV	4	0.2105	5124.6	60.00	0.28	tr_C9ZWY7_
HLA-DQA10501-DQB10201	538	KSIKVKVYNLLFTNR	VYNLLFTNR	6	0.2105	5127.4	60.00	0.27	tr_C9ZWY7_
HLA-DQA10501-DQB10201	539	QLFLLVGYIARDKKT	LVGYIARDK	4	0.2105	5129.3	60.00	0.26	tr_C9ZWY7_
HLA-DQA10501-DQB10201	540	VNYGATHISVWSMAR	YGATHISVW	2	0.2102	5145.5	60.00	0.23	tr_C9ZWY7_
HLA-DQA10501-DQB10201	541	TIESLGGSMNGIVVP	GGSMNGIVV	5	0.2099	5157.8	60.00	0.22	tr_C9ZWY7_
HLA-DQA10501-DQB10201	542	RCSTIGVKVPTPLIV	IGVKVPTPL	4	0.2099	5160.6	60.00	0.28	tr_C9ZWY7_
HLA-DQA10501-DQB10201	543	DDESKWTPLALLGYA	WTPLALLGY	5	0.2097	5172.1	60.00	0.44	tr_C9ZWY7_
HLA-DQA10501-DQB10201	544	HAVRRFQEDARSYLR	FQEDARSYL	5	0.2096	5178.3	60.00	0.28	tr_C9ZWY7_
HLA-DQA10501-DQB10201	545	PDNDTTLNEDLSPHG	TTLNEDLSP	4	0.2095	5181.7	60.00	0.26	tr_C9ZWY7_
HLA-DQA10501-DQB10201	546	TKYHAVRRFQEDARS	VRRFQEDAR	5	0.2095	5184.7	60.00	0.28	tr_C9ZWY7_
HLA-DQA10501-DQB10201	547	KVYNLLFTNRAPRRS	YNLLFTNRA	2	0.2091	5207.6	60.00	0.47	tr_C9ZWY7_
HLA-DQA10501-DQB10201	548	PLTGSSAVRGWDPHF	AVRGWDPHF	6	0.2083	5248.0	60.00	0.34	tr_C9ZWY7_
HLA-DQA10501-DQB10201	549	DHRMIPLRDGITVFN	PLRDGITVF	5	0.2083	5250.5	60.00	0.28	tr_C9ZWY7_
HLA-DQA10501-DQB10201	550	TNRAPRRSIETMGVG	RRSIETMGV	5	0.2078	5277.8	60.00	0.55	tr_C9ZWY7_
HLA-DQA10501-DQB10201	551	GFAAEGCAVNYGATH	GFAAEGCAV	0	0.2077	5284.3	60.00	0.34	tr_C9ZWY7_
HLA-DQA10501-DQB10201	552	ETKYHAVRRFQEDAR	VRRFQEDAR	6	0.2076	5289.7	60.00	0.33	tr_C9ZWY7_
HLA-DQA10501-DQB10201	553	RGGDVPGIGSVFQKT	VPGIGSVFQ	4	0.2072	5311.8	60.00	0.45	tr_C9ZWY7_
HLA-DQA10501-DQB10201	554	CSTIGVKVPTPLIVV	IGVKVPTPL	3	0.2071	5316.8	60.00	0.25	tr_C9ZWY7_
HLA-DQA10501-DQB10201	555	AKKSIKVKVYNLLFT	KVKVYNLLF	5	0.2070	5325.1	60.00	0.50	tr_C9ZWY7_
HLA-DQA10501-DQB10201	556	AVRRFQEDARSYLRY	FQEDARSYL	4	0.2069	5328.0	65.00	0.29	tr_C9ZWY7_
HLA-DQA10501-DQB10201	557	DESKWTPLALLGYAT	WTPLALLGY	4	0.2066	5346.9	65.00	0.41	tr_C9ZWY7_
HLA-DQA10501-DQB10201	558	STIGVKVPTPLIVVV	IGVKVPTPL	2	0.2063	5362.4	65.00	0.25	tr_C9ZWY7_
HLA-DQA10501-DQB10201	559	ITNADAIRSITNPDR	AIRSITNPD	5	0.2063	5367.6	65.00	0.25	tr_C9ZWY7_
HLA-DQA10501-DQB10201	560	SVFQKTIESLGGSMN	TIESLGGSM	5	0.2051	5434.5	65.00	0.30	tr_C9ZWY7_
HLA-DQA10501-DQB10201	561	NEDLSPHGDTLVFGL	PHGDTLVFG	5	0.2049	5444.3	65.00	0.41	tr_C9ZWY7_
HLA-DQA10501-DQB10201	562	GITVFNIDRCSTIGV	ITVFNIDRC	1	0.2038	5513.9	65.00	0.34	tr_C9ZWY7_
HLA-DQA10501-DQB10201	563	LCGVFTKMHAATVMR	TKMHAATVM	5	0.2029	5564.9	65.00	0.50	tr_C9ZWY7_
HLA-DQA10501-DQB10201	564	DNDTTLNEDLSPHGD	TTLNEDLSP	3	0.2022	5610.7	65.00	0.29	tr_C9ZWY7_
HLA-DQA10501-DQB10201	565	TARAMESVVSRMGRV	AMESVVSRM	3	0.2011	5674.3	65.00	0.31	tr_C9ZWY7_
HLA-DQA10501-DQB10201	566	ESKWTPLALLGYATA	WTPLALLGY	3	0.2011	5677.4	65.00	0.38	tr_C9ZWY7_
HLA-DQA10501-DQB10201	567	LVAFAPLTGSSAVRG	LVAFAPLTG	0	0.2008	5692.6	65.00	0.37	tr_C9ZWY7_
HLA-DQA10501-DQB10201	568	GSVFQKTIESLGGSM	VFQKTIESL	2	0.2003	5727.6	65.00	0.34	tr_C9ZWY7_
HLA-DQA10501-DQB10201	569	TVKRFHDEMKDDESK	FHDEMKDDE	4	0.2002	5728.0	65.00	0.33	tr_C9ZWY7_
HLA-DQA10501-DQB10201	570	GEVLSQALSSRTWLE	EVLSQALSS	1	0.1998	5756.0	65.00	0.50	tr_C9ZWY7_
HLA-DQA10501-DQB10201	571	FAAEGCAVNYGATHI	AVNYGATHI	6	0.1991	5801.3	65.00	0.41	tr_C9ZWY7_
HLA-DQA10501-DQB10201	572	DVPGIGSVFQKTIES	VPGIGSVFQ	1	0.1989	5809.1	65.00	0.34	tr_C9ZWY7_
HLA-DQA10501-DQB10201	573	SVGQSAGFGGFAGVI	GFGGFAGVI	6	0.1981	5863.9	65.00	0.51	tr_C9ZWY7_
HLA-DQA10501-DQB10201	574	GIVVPDNDTTLNEDL	NDTTLNEDL	6	0.1974	5907.5	65.00	0.28	tr_C9ZWY7_
HLA-DQA10501-DQB10201	575	ESREAFIKSLYNQRR	REAFIKSLY	2	0.1972	5917.0	65.00	0.29	tr_C9ZWY7_
HLA-DQA10501-DQB10201	576	VKRFHDEMKDDESKW	FHDEMKDDE	3	0.1970	5932.4	65.00	0.38	tr_C9ZWY7_
HLA-DQA10501-DQB10201	577	GIGSVFQKTIESLGG	VFQKTIESL	4	0.1969	5938.1	65.00	0.47	tr_C9ZWY7_
HLA-DQA10501-DQB10201	578	GVGLNASFAARHWAT	NASFAARHW	4	0.1963	5979.9	65.00	0.24	tr_C9ZWY7_
HLA-DQA10501-DQB10201	579	LGDDHRMIPLRDGIT	DHRMIPLRD	3	0.1960	5995.7	65.00	0.46	tr_C9ZWY7_
HLA-DQA10501-DQB10201	580	DRCSTIGVKVPTPLI	IGVKVPTPL	5	0.1958	6012.9	65.00	0.38	tr_C9ZWY7_
HLA-DQA10501-DQB10201	581	SETKYHAVRRFQEDA	YHAVRRFQE	4	0.1956	6023.5	65.00	0.37	tr_C9ZWY7_
HLA-DQA10501-DQB10201	582	SLGDDHRMIPLRDGI	DHRMIPLRD	4	0.1955	6029.3	65.00	0.51	tr_C9ZWY7_

HLA-DQA10501-DQB10201	583	RVAMDARTRGVYVLA	RTRGVYVLA	6	0.1950	6059.7	65.00	0.42	tr_C9ZWY7_
HLA-DQA10501-DQB10201	584	RSYLRYHPGVTAFNA	HPGVTAFNA	6	0.1948	6077.7	65.00	0.25	tr_C9ZWY7_
HLA-DQA10501-DQB10201	585	LFLLVGYIARDKKTT	LVGYIARDK	3	0.1948	6078.7	65.00	0.28	tr_C9ZWY7_
HLA-DQA10501-DQB10201	586	TSLHMVTRGGDVPGI	MVTRGGDVP	4	0.1943	6109.7	65.00	0.35	tr_C9ZWY7_
HLA-DQA10501-DQB10201	587	SKWTPLALLGYATAR	WTPLALLGY	2	0.1941	6120.3	65.00	0.37	tr_C9ZWY7_
HLA-DQA10501-DQB10201	588	KKSIKVKVYNLLFTN	KVKVYNLLF	4	0.1940	6128.5	65.00	0.44	tr_C9ZWY7_
HLA-DQA10501-DQB10201	589	LETAADQNKGEFFVV	ADQNKGEFF	4	0.1935	6159.6	65.00	0.56	tr_C9ZWY7_
HLA-DQA10501-DQB10201	590	FFLIPLLLVLPHIVR	PLLLVLPHI	4	0.1934	6169.9	65.00	0.34	tr_C9ZWY7_
HLA-DQA10501-DQB10201	591	EDLSPHGDTLVFGLT	PHGDTLVFG	4	0.1930	6193.5	65.00	0.40	tr_C9ZWY7_
HLA-DQA10501-DQB10201	592	GNFGGECHGMAGERG	CHGMAGERG	6	0.1930	6194.9	65.00	0.50	tr_C9ZWY7_
HLA-DQA10501-DQB10201	593	GDDHRMIPLRDGITV	DHRMIPLRD	2	0.1929	6202.0	65.00	0.41	tr_C9ZWY7_
HLA-DQA10501-DQB10201	594	LIPLLLVLPHIVRPL	PLLLVLPHI	2	0.1925	6230.6	65.00	0.29	tr_C9ZWY7_
HLA-DQA10501-DQB10201	595	AEHLDNHRGVRVFVS	HRGVRVFVS	6	0.1924	6236.1	65.00	0.41	tr_C9ZWY7_
HLA-DQA10501-DQB10201	596	KTIESLGGSMNGIVV	GGSMNGIVV	6	0.1921	6253.5	65.00	0.23	tr_C9ZWY7_
HLA-DQA10501-DQB10201	597	FLIPLLLVLPHIVRP	PLLLVLPHI	3	0.1915	6298.7	65.00	0.40	tr_C9ZWY7_
HLA-DQA10501-DQB10201	598	VYNLLFTNRAPRRSI	YNLLFTNRA	1	0.1910	6330.4	65.00	0.47	tr_C9ZWY7_
HLA-DQA10501-DQB10201	599	AIRSITNPDRVAFHS	RSITNPDRV	2	0.1898	6413.4	65.00	0.49	tr_C9ZWY7_
HLA-DQA10501-DQB10201	600	LNEDLSPHGDTLVFG	PHGDTLVFG	6	0.1891	6462.1	70.00	0.51	tr_C9ZWY7_
HLA-DQA10501-DQB10201	601	SLHMVTRGGDVPGIG	MVTRGGDVP	3	0.1886	6497.3	70.00	0.31	tr_C9ZWY7_
HLA-DQA10501-DQB10201	602	RSITNPDRVAFHSVV	DRVAFHSVV	6	0.1884	6508.8	70.00	0.45	tr_C9ZWY7_
HLA-DQA10501-DQB10201	603	IDRCSTIGVKVPTPL	IGVKVPTPL	6	0.1883	6518.1	70.00	0.41	tr_C9ZWY7_
HLA-DQA10501-DQB10201	604	KLMTRMGRRLCGVFT	GRRLCGVFT	6	0.1881	6531.0	70.00	0.47	tr_C9ZWY7_
HLA-DQA10501-DQB10201	605	AELIALLRYAINRLR	LLRYAINRL	5	0.1880	6539.5	70.00	0.50	tr_C9ZWY7_
HLA-DQA10501-DQB10201	606	TSETVKRFHDEMKDD	TVKRFHDEM	3	0.1879	6545.5	70.00	0.47	tr_C9ZWY7_
HLA-DQA10501-DQB10201	607	MARVLNPSVPPVTKA	MARVLNPSV	0	0.1879	6545.9	70.00	0.31	tr_C9ZWY7_
HLA-DQA10501-DQB10201	608	LLVLPHIVRPLAAKK	PHIVRPLAA	4	0.1878	6551.0	70.00	0.42	tr_C9ZWY7_
HLA-DQA10501-DQB10201	609	TIEQQLFLLVGYIAR	QLFLLVGYI	4	0.1876	6570.2	70.00	0.20	tr_C9ZWY7_
HLA-DQA10501-DQB10201	610	VVVGPMGDTRTLAVL	MGDTRTLAV	5	0.1874	6583.5	70.00	0.34	tr_C9ZWY7_
HLA-DQA10501-DQB10201	611	VFQKTIESLGGSMNG	TIESLGGSM	4	0.1873	6588.7	70.00	0.31	tr_C9ZWY7_
HLA-DQA10501-DQB10201	612	ARSYLRYHPGVTAFN	RYHPGVTAF	5	0.1873	6592.8	70.00	0.28	tr_C9ZWY7_
HLA-DQA10501-DQB10201	613	VATSRNTYGRMQGEL	TYGRMQGEL	6	0.1866	6639.2	70.00	0.58	tr_C9ZWY7_
HLA-DQA10501-DQB10201	614	SREAFIKSLYNQRRY	FIKSLYNQR	4	0.1861	6674.0	70.00	0.28	tr_C9ZWY7_
HLA-DQA10501-DQB10201	615	YLRYHPGVTAFNASD	HPGVTAFNA	4	0.1859	6692.6	70.00	0.25	tr_C9ZWY7_
HLA-DQA10501-DQB10201	616	VKVFKKHPQAAERLL	KHPQAAERL	5	0.1858	6698.1	70.00	0.63	tr_C9ZWY7_
HLA-DQA10501-DQB10201	617	GDREYQETLKLMTRM	REYQETLKL	2	0.1857	6707.9	70.00	0.37	tr_C9ZWY7_
HLA-DQA10501-DQB10201	618	SYLRYHPGVTAFNAS	RYHPGVTAF	3	0.1853	6734.9	70.00	0.22	tr_C9ZWY7_
HLA-DQA10501-DQB10201	619	IGVKVPTPLIVVVVL	PTPLIVVVV	5	0.1848	6771.7	70.00	0.23	tr_C9ZWY7_
HLA-DQA10501-DQB10201	620	MVTRGGDVPGIGSVF	DVPGIGSVF	6	0.1847	6779.9	70.00	0.31	tr_C9ZWY7_
HLA-DQA10501-DQB10201	621	VRRVAMDARTRGVYV	DARTRGVYV	6	0.1838	6844.0	70.00	0.34	tr_C9ZWY7_
HLA-DQA10501-DQB10201	622	LSHPGRNVIHFSPTI	NVIHFSPTI	6	0.1837	6850.2	70.00	0.44	tr_C9ZWY7_
HLA-DQA10501-DQB10201	623	KRFHDEMKDDESKWT	FHDEMKDDE	2	0.1832	6890.9	70.00	0.35	tr_C9ZWY7_
HLA-DQA10501-DQB10201	624	DARSYLRYHPGVTAF	RYHPGVTAF	6	0.1830	6906.4	70.00	0.29	tr_C9ZWY7_
HLA-DQA10501-DQB10201	625	NSLGDDHRMIPLRDG	DHRMIPLRD	5	0.1822	6963.6	70.00	0.56	tr_C9ZWY7_
HLA-DQA10501-DQB10201	626	LHMVTRGGDVPGIGS	MVTRGGDVP	2	0.1816	7006.6	70.00	0.35	tr_C9ZWY7_
HLA-DQA10501-DQB10201	627	IVFTPPIREALKFVR	IVFTPPIRE	0	0.1815	7019.6	70.00	0.36	tr_C9ZWY7_
HLA-DQA10501-DQB10201	628	EVLSQALSSRTWLES	EVLSQALSS	0	0.1812	7042.3	70.00	0.38	tr_C9ZWY7_
HLA-DQA10501-DQB10201	629	FAPLTGSSAVRGWDP	LTGSSAVRG	3	0.1809	7060.9	70.00	0.46	tr_C9ZWY7_
HLA-DQA10501-DQB10201	630	SQALSSRTWLESREA	RTWLESREA	6	0.1809	7062.4	70.00	0.47	tr_C9ZWY7_
HLA-DQA10501-DQB10201	631	GGFAGVITGRHITTY	FAGVITGRH	2	0.1801	7120.2	70.00	0.44	tr_C9ZWY7_
HLA-DQA10501-DQB10201	632	TMRRRPPAITFFLIP	PAITFFLIP	6	0.1800	7127.4	70.00	0.31	tr_C9ZWY7_

HLA-DQA10501-DQB10201	633	IGSVFQKTIESLGGS	VFQKTIESL	3	0.1798	7146.3	70.00	0.50	tr_C9ZWY7_
HLA-DQA10501-DQB10201	634	NFGGECHGMAGERGA	CHGMAGERG	5	0.1788	7224.4	70.00	0.55	tr_C9ZWY7_
HLA-DQA10501-DQB10201	635	VRRFQEDARSYLRYH	FQEDARSYL	3	0.1782	7270.5	70.00	0.33	tr_C9ZWY7_
HLA-DQA10501-DQB10201	636	ASETKYHAVRRFQED	YHAVRRFQE	5	0.1770	7365.3	70.00	0.39	tr_C9ZWY7_
HLA-DQA10501-DQB10201	637	AAEGCAVNYGATHIS	AVNYGATHI	5	0.1766	7397.3	70.00	0.43	tr_C9ZWY7_
HLA-DQA10501-DQB10201	638	MTRMGRRLCGVFTKM	GRRLCGVFT	4	0.1761	7434.9	70.00	0.38	tr_C9ZWY7_
HLA-DQA10501-DQB10201	639	LVLPHIVRPLAAKKS	PHIVRPLAA	3	0.1761	7441.8	70.00	0.44	tr_C9ZWY7_
HLA-DQA10501-DQB10201	640	QKTIESLGGSMNGIV	TIESLGGSM	2	0.1758	7464.9	70.00	0.28	tr_C9ZWY7_
HLA-DQA10501-DQB10201	641	FLLVGYIARDKKTTS	LVGYIARDK	2	0.1756	7481.0	70.00	0.34	tr_C9ZWY7_
HLA-DQA10501-DQB10201	642	AAKKSIKVKVYNLLF	KVKVYNLLF	6	0.1754	7492.8	70.00	0.65	tr_C9ZWY7_
HLA-DQA10501-DQB10201	643	FHSVVATSRNTYGRM	FHSVVATSR	0	0.1746	7563.7	70.00	0.65	tr_C9ZWY7_
HLA-DQA10501-DQB10201	644	AFIEPITLSPKLSHP	FIEPITLSP	1	0.1737	7631.0	70.00	0.36	tr_C9ZWY7_
HLA-DQA10501-DQB10201	645	RFHDEMKDDESKWTP	FHDEMKDDE	1	0.1737	7631.6	70.00	0.36	tr_C9ZWY7_
HLA-DQA10501-DQB10201	646	ITTVAEHLDNHRGVR	ITTVAEHLD	0	0.1733	7667.0	70.00	0.41	tr_C9ZWY7_
HLA-DQA10501-DQB10201	647	FQKTIESLGGSMNGI	TIESLGGSM	3	0.1729	7698.2	70.00	0.38	tr_C9ZWY7_
HLA-DQA10501-DQB10201	648	AFAPLTGSSAVRGWD	LTGSSAVRG	4	0.1723	7750.1	70.00	0.46	tr_C9ZWY7_
HLA-DQA10501-DQB10201	649	IRSITNPDRVAFHSV	RSITNPDRV	1	0.1717	7799.9	75.00	0.46	tr_C9ZWY7_
HLA-DQA10501-DQB10201	650	HMVTRGGDVPGIGSV	MVTRGGDVP	1	0.1705	7904.4	75.00	0.34	tr_C9ZWY7_
HLA-DQA10501-DQB10201	651	NDTTLNEDLSPHGDT	TTLNEDLSP	2	0.1705	7905.6	75.00	0.34	tr_C9ZWY7_
HLA-DQA10501-DQB10201	652	ARAMESVVSRMGRVN	AMESVVSRM	2	0.1699	7956.3	75.00	0.44	tr_C9ZWY7_
HLA-DQA10501-DQB10201	653	ALLYSEFVKVFKKHP	LYSEFVKVF	2	0.1698	7963.9	75.00	0.34	tr_C9ZWY7_
HLA-DQA10501-DQB10201	654	IPPPPRSVSTLKLLE	PRSVSTLKL	4	0.1697	7967.5	75.00	0.44	tr_C9ZWY7_
HLA-DQA10501-DQB10201	655	FVKVFKKHPQAAERL	KHPQAAERL	6	0.1691	8026.6	75.00	0.75	tr_C9ZWY7_
HLA-DQA10501-DQB10201	656	LMTRMGRRLCGVFTK	GRRLCGVFT	5	0.1687	8055.4	75.00	0.44	tr_C9ZWY7_
HLA-DQA10501-DQB10201	657	VAFAPLTGSSAVRGW	LTGSSAVRG	5	0.1677	8148.6	75.00	0.44	tr_C9ZWY7_
HLA-DQA10501-DQB10201	658	ITVFNIDRCSTIGVK	ITVFNIDRC	0	0.1667	8233.1	75.00	0.28	tr_C9ZWY7_
HLA-DQA10501-DQB10201	659	REAFIKSLYNQRRYV	FIKSLYNQR	3	0.1662	8276.3	75.00	0.30	tr_C9ZWY7_
HLA-DQA10501-DQB10201	660	EMKDDESKWTPLALL	SKWTPLALL	6	0.1649	8392.9	75.00	0.45	tr_C9ZWY7_
HLA-DQA10501-DQB10201	661	FGNFGGECHGMAGER	FGNFGGECH	0	0.1643	8448.5	75.00	0.37	tr_C9ZWY7_
HLA-DQA10501-DQB10201	662	GFAGVITGRHITTYS	FAGVITGRH	1	0.1638	8501.1	75.00	0.42	tr_C9ZWY7_
HLA-DQA10501-DQB10201	663	GRMQGELDTRSVTAV	ELDTRSVTA	5	0.1631	8557.2	75.00	0.36	tr_C9ZWY7_
HLA-DQA10501-DQB10201	664	VLPHIVRPLAAKKSI	PHIVRPLAA	2	0.1628	8590.1	75.00	0.48	tr_C9ZWY7_
HLA-DQA10501-DQB10201	665	TTSETVKRFHDEMKD	TVKRFHDEM	4	0.1621	8659.4	75.00	0.57	tr_C9ZWY7_
HLA-DQA10501-DQB10201	666	TRMGRRLCGVFTKMH	GRRLCGVFT	3	0.1610	8754.0	75.00	0.37	tr_C9ZWY7_
HLA-DQA10501-DQB10201	667	RRFQEDARSYLRYHP	FQEDARSYL	2	0.1608	8778.6	75.00	0.38	tr_C9ZWY7_
HLA-DQA10501-DQB10201	668	VGLNASFAARHWATA	NASFAARHW	3	0.1608	8780.8	75.00	0.34	tr_C9ZWY7_
HLA-DQA10501-DQB10201	669	FGGECHGMAGERGAS	CHGMAGERG	4	0.1606	8792.2	75.00	0.58	tr_C9ZWY7_
HLA-DQA10501-DQB10201	670	DREYQETLKLMTRMG	REYQETLKL	1	0.1606	8797.6	75.00	0.37	tr_C9ZWY7_
HLA-DQA10501-DQB10201	671	NNTTSETVKRFHDEM	TVKRFHDEM	6	0.1605	8806.0	75.00	0.71	tr_C9ZWY7_
HLA-DQA10501-DQB10201	672	GVKVPTPLIVVVVLV	PTPLIVVVV	4	0.1602	8835.2	75.00	0.28	tr_C9ZWY7_
HLA-DQA10501-DQB10201	673	ECHGMAGERGASCLC	CHGMAGERG	1	0.1595	8900.0	75.00	0.47	tr_C9ZWY7_
HLA-DQA10501-DQB10201	674	GECHGMAGERGASCL	CHGMAGERG	2	0.1594	8913.9	75.00	0.52	tr_C9ZWY7_
HLA-DQA10501-DQB10201	675	MESVVSRMGRVNSEE	RMGRVNSEE	6	0.1593	8924.2	75.00	0.47	tr_C9ZWY7_
HLA-DQA10501-DQB10201	676	FQEDARSYLRYHPGV	SYLRYHPGV	6	0.1584	9010.6	75.00	0.34	tr_C9ZWY7_
HLA-DQA10501-DQB10201	677	IVAAPNPLASETKYH	IVAAPNPLA	0	0.1582	9031.3	75.00	0.48	tr_C9ZWY7_
HLA-DQA10501-DQB10201	678	RRVAMDARTRGVYVL	DARTRGVYV	5	0.1581	9034.4	75.00	0.44	tr_C9ZWY7_
HLA-DQA10501-DQB10201	679	YGPFEDSCVPTTGFA	FEDSCVPTT	3	0.1576	9088.4	75.00	0.37	tr_C9ZWY7_
HLA-DQA10501-DQB10201	680	VAEHLDNHRGVRVFV	NHRGVRVFV	6	0.1570	9141.4	75.00	0.44	tr_C9ZWY7_
HLA-DQA10501-DQB10201	681	PIREALKFVRRVAMD	KFVRRVAMD	6	0.1568	9165.9	75.00	0.26	tr_C9ZWY7_
HLA-DQA10501-DQB10201	682	LFTNRAPRRSIETMG	PRRSIETMG	6	0.1557	9270.7	75.00	0.46	tr_C9ZWY7_

HLA-DQA10501-DQB10201	683	GGECHGMAGERGASC	CHGMAGERG	3	0.1556	9282.0	75.00	0.57	tr_C9ZWY7_
HLA-DQA10501-DQB10201	684	TVEIIPPPPRSVSTL	TVEIIPPPP	0	0.1552	9330.8	75.00	0.62	tr_C9ZWY7_
HLA-DQA10501-DQB10201	685	APLTGSSAVRGWDPH	LTGSSAVRG	2	0.1549	9355.7	75.00	0.46	tr_C9ZWY7_
HLA-DQA10501-DQB10201	686	MKDDESKWTPLALLG	SKWTPLALL	5	0.1548	9368.0	75.00	0.40	tr_C9ZWY7_
HLA-DQA10501-DQB10201	687	SLPHWADNNTTSETV	WADNNTTSE	4	0.1545	9400.4	75.00	0.41	tr_C9ZWY7_
HLA-DQA10501-DQB10201	688	GTMRRRPPAITFFLI	PPAITFFLI	6	0.1530	9551.6	80.00	0.30	tr_C9ZWY7_
HLA-DQA10501-DQB10201	689	FATSLPHWADNNTTS	FATSLPHWA	0	0.1526	9587.8	80.00	0.62	tr_C9ZWY7_
HLA-DQA10501-DQB10201	690	IIPPPPRSVSTLKLL	PRSVSTLKL	5	0.1525	9604.3	80.00	0.58	tr_C9ZWY7_
HLA-DQA10501-DQB10201	691	LSQALSSRTWLESRE	SRTWLESRE	6	0.1494	9927.2	80.00	0.32	tr_C9ZWY7_
HLA-DQA10501-DQB10201	692	LNASFAARHWATAEN	ARHWATAEN	6	0.1494	9927.4	80.00	0.29	tr_C9ZWY7_
HLA-DQA10501-DQB10201	693	EDARSYLRYHPGVTA	SYLRYHPGV	4	0.1490	9969.0	80.00	0.31	tr_C9ZWY7_
HLA-DQA10501-DQB10201	694	GLNASFAARHWATAE	NASFAARHW	2	0.1480	10077.9	80.00	0.36	tr_C9ZWY7_
HLA-DQA10501-DQB10201	695	RMGRRLCGVFTKMHA	GRRLCGVFT	2	0.1478	10100.3	80.00	0.37	tr_C9ZWY7_
HLA-DQA10501-DQB10201	696	TTSLHMVTRGGDVPG	MVTRGGDVP	5	0.1476	10130.0	80.00	0.49	tr_C9ZWY7_
HLA-DQA10501-DQB10201	697	SCLCNQGGNVVYMNS	NQGGNVVYM	4	0.1473	10157.0	80.00	0.37	tr_C9ZWY7_
HLA-DQA10501-DQB10201	698	FHDEMKDDESKWTPL	FHDEMKDDE	0	0.1472	10171.2	80.00	0.32	tr_C9ZWY7_
HLA-DQA10501-DQB10201	699	SVPPVTKAATPSMRY	VTKAATPSM	4	0.1457	10332.5	80.00	0.49	tr_C9ZWY7_
HLA-DQA10501-DQB10201	700	LASETKYHAVRRFQE	YHAVRRFQE	6	0.1453	10383.0	80.00	0.49	tr_C9ZWY7_
HLA-DQA10501-DQB10201	701	RFQEDARSYLRYHPG	FQEDARSYL	1	0.1450	10409.5	80.00	0.38	tr_C9ZWY7_
HLA-DQA10501-DQB10201	702	LLYSEFVKVFKKHPQ	LYSEFVKVF	1	0.1443	10495.5	80.00	0.38	tr_C9ZWY7_
HLA-DQA10501-DQB10201	703	FEDSCVPTTGFAAEG	PTTGFAAEG	6	0.1441	10520.7	80.00	0.39	tr_C9ZWY7_
HLA-DQA10501-DQB10201	704	DTTLNEDLSPHGDTL	TTLNEDLSP	1	0.1439	10535.9	80.00	0.37	tr_C9ZWY7_
HLA-DQA10501-DQB10201	705	GGTMRRRPPAITFFL	RPPAITFFL	6	0.1430	10642.9	80.00	0.35	tr_C9ZWY7_
HLA-DQA10501-DQB10201	706	EAFIKSLYNQRRYVV	FIKSLYNQR	2	0.1429	10651.0	80.00	0.38	tr_C9ZWY7_
HLA-DQA10501-DQB10201	707	RAMESVVSRMGRVNS	AMESVVSRM	1	0.1429	10656.8	80.00	0.50	tr_C9ZWY7_
HLA-DQA10501-DQB10201	708	QEDARSYLRYHPGVT	SYLRYHPGV	5	0.1428	10667.0	80.00	0.36	tr_C9ZWY7_
HLA-DQA10501-DQB10201	709	YNLLFTNRAPRRSIE	YNLLFTNRA	0	0.1421	10743.1	80.00	0.43	tr_C9ZWY7_
HLA-DQA10501-DQB10201	710	KTTSLHMVTRGGDVP	MVTRGGDVP	6	0.1418	10776.8	80.00	0.49	tr_C9ZWY7_
HLA-DQA10501-DQB10201	711	NIDRCSTIGVKVPTP	TIGVKVPTP	6	0.1403	10957.1	80.00	0.51	tr_C9ZWY7_
HLA-DQA10501-DQB10201	712	CHGMAGERGASCLCN	CHGMAGERG	0	0.1393	11079.3	80.00	0.31	tr_C9ZWY7_
HLA-DQA10501-DQB10201	713	FIEPITLSPKLSHPG	FIEPITLSP	0	0.1392	11093.8	80.00	0.30	tr_C9ZWY7_
HLA-DQA10501-DQB10201	714	KVPTPLIVVVVLVVD	IVVVVLVVD	6	0.1391	11103.4	80.00	0.35	tr_C9ZWY7_
HLA-DQA10501-DQB10201	715	LLVGYIARDKKTTSL	LVGYIARDK	1	0.1390	11109.6	80.00	0.37	tr_C9ZWY7_
HLA-DQA10501-DQB10201	716	NTTSETVKRFHDEMK	TVKRFHDEM	5	0.1390	11118.3	80.00	0.67	tr_C9ZWY7_
HLA-DQA10501-DQB10201	717	ASVGQSAGFGGFAGV	QSAGFGGFA	4	0.1388	11138.3	80.00	0.29	tr_C9ZWY7_
HLA-DQA10501-DQB10201	718	VPPVTKAATPSMRYA	VTKAATPSM	3	0.1375	11290.1	80.00	0.51	tr_C9ZWY7_
HLA-DQA10501-DQB10201	719	PPIREALKFVRRVAM	LKFVRRVAM	6	0.1374	11300.6	80.00	0.38	tr_C9ZWY7_
HLA-DQA10501-DQB10201	720	FAGVITGRHITTYST	FAGVITGRH	0	0.1374	11304.6	80.00	0.37	tr_C9ZWY7_
HLA-DQA10501-DQB10201	721	MASVGQSAGFGGFAG	QSAGFGGFA	5	0.1372	11332.9	80.00	0.27	tr_C9ZWY7_
HLA-DQA10501-DQB10201	722	DEMKDDESKWTPLAL	ESKWTPLAL	6	0.1370	11353.9	80.00	0.54	tr_C9ZWY7_
HLA-DQA10501-DQB10201	723	ARVLNPSVPPVTKAA	VLNPSVPPV	2	0.1365	11414.9	80.00	0.31	tr_C9ZWY7_
HLA-DQA10501-DQB10201	724	AFIKSLYNQRRYVVD	FIKSLYNQR	1	0.1365	11421.9	80.00	0.31	tr_C9ZWY7_
HLA-DQA10501-DQB10201	725	RRLCGVFTKMHAATV	FTKMHAATV	6	0.1363	11441.2	80.00	0.61	tr_C9ZWY7_
HLA-DQA10501-DQB10201	726	FIKSLYNQRRYVVDD	YNQRRYVVD	5	0.1363	11444.3	80.00	0.25	tr_C9ZWY7_
HLA-DQA10501-DQB10201	727	EIIPPPPRSVSTLKL	PRSVSTLKL	6	0.1351	11591.0	80.00	0.76	tr_C9ZWY7_
HLA-DQA10501-DQB10201	728	AMESVVSRMGRVNSE	AMESVVSRM	0	0.1345	11664.3	80.00	0.35	tr_C9ZWY7_
HLA-DQA10501-DQB10201	729	ASCLCNQGGNVVYMN	NQGGNVVYM	5	0.1336	11785.4	85.00	0.44	tr_C9ZWY7_
HLA-DQA10501-DQB10201	730	LKLMTRMGRRLCGVF	RMGRRLCGV	5	0.1331	11844.4	85.00	0.54	tr_C9ZWY7_
HLA-DQA10501-DQB10201	731	VKVPTPLIVVVVLVV	PTPLIVVVV	3	0.1322	11956.3	85.00	0.31	tr_C9ZWY7_
HLA-DQA10501-DQB10201	732	VFTPPIREALKFVRR	PIREALKFV	4	0.1305	12177.5	85.00	0.35	tr_C9ZWY7_

HLA-DQA10501-DQB10201	733	TLNEDLSPHGDTLVF	SPHGDTLVF	6	0.1305	12182.7	85.00	0.27	tr_C9ZWY7_
HLA-DQA10501-DQB10201	734	TVFNIDRCSTIGVKV	VFNIDRCST	1	0.1303	12213.4	85.00	0.28	tr_C9ZWY7_
HLA-DQA10501-DQB10201	735	LPHIVRPLAAKKSIK	PHIVRPLAA	1	0.1296	12302.9	85.00	0.55	tr_C9ZWY7_
HLA-DQA10501-DQB10201	736	NPSVPPVTKAATPSM	VTKAATPSM	6	0.1291	12363.6	85.00	0.80	tr_C9ZWY7_
HLA-DQA10501-DQB10201	737	VLSQALSSRTWLESR	QALSSRTWL	3	0.1289	12401.8	85.00	0.38	tr_C9ZWY7_
HLA-DQA10501-DQB10201	738	PPVTKAATPSMRYAN	VTKAATPSM	2	0.1280	12516.4	85.00	0.51	tr_C9ZWY7_
HLA-DQA10501-DQB10201	739	LPHWADNNTTSETVK	WADNNTTSE	3	0.1279	12537.2	85.00	0.41	tr_C9ZWY7_
HLA-DQA10501-DQB10201	740	FTPPIREALKFVRRV	PIREALKFV	3	0.1271	12644.7	85.00	0.34	tr_C9ZWY7_
HLA-DQA10501-DQB10201	741	TSLPHWADNNTTSET	WADNNTTSE	5	0.1260	12787.4	85.00	0.52	tr_C9ZWY7_
HLA-DQA10501-DQB10201	742	MGRRLCGVFTKMHAA	GRRLCGVFT	1	0.1258	12822.0	85.00	0.37	tr_C9ZWY7_
HLA-DQA10501-DQB10201	743	ATSLPHWADNNTTSE	WADNNTTSE	6	0.1246	12982.9	85.00	0.53	tr_C9ZWY7_
HLA-DQA10501-DQB10201	744	TTLNEDLSPHGDTLV	TTLNEDLSP	0	0.1246	12984.4	85.00	0.22	tr_C9ZWY7_
HLA-DQA10501-DQB10201	745	HGMAGERGASCLCNQ	RGASCLCNQ	6	0.1244	13016.5	85.00	0.29	tr_C9ZWY7_
HLA-DQA10501-DQB10201	746	REYQETLKLMTRMGR	EYQETLKLM	1	0.1241	13055.4	85.00	0.29	tr_C9ZWY7_
HLA-DQA10501-DQB10201	747	LLFTNRAPRRSIETM	APRRSIETM	6	0.1240	13072.8	85.00	0.36	tr_C9ZWY7_
HLA-DQA10501-DQB10201	748	GPFEDSCVPTTGFAA	FEDSCVPTT	2	0.1237	13112.0	85.00	0.44	tr_C9ZWY7_
HLA-DQA10501-DQB10201	749	PFEDSCVPTTGFAAE	FEDSCVPTT	1	0.1214	13438.5	85.00	0.39	tr_C9ZWY7_
HLA-DQA10501-DQB10201	750	PVTKAATPSMRYANR	VTKAATPSM	1	0.1203	13608.7	85.00	0.50	tr_C9ZWY7_
HLA-DQA10501-DQB10201	751	HWADNNTTSETVKRF	WADNNTTSE	1	0.1187	13834.6	85.00	0.38	tr_C9ZWY7_
HLA-DQA10501-DQB10201	752	TGGTMRRRPPAITFF	RRPPAITFF	6	0.1184	13882.3	85.00	0.41	tr_C9ZWY7_
HLA-DQA10501-DQB10201	753	PSVPPVTKAATPSMR	VTKAATPSM	5	0.1183	13904.2	85.00	0.71	tr_C9ZWY7_
HLA-DQA10501-DQB10201	754	TPPIREALKFVRRVA	PIREALKFV	2	0.1176	14014.8	85.00	0.34	tr_C9ZWY7_
HLA-DQA10501-DQB10201	755	LYSEFVKVFKKHPQA	LYSEFVKVF	0	0.1174	14031.0	85.00	0.36	tr_C9ZWY7_
HLA-DQA10501-DQB10201	756	VFNIDRCSTIGVKVP	IDRCSTIGV	3	0.1163	14208.4	85.00	0.25	tr_C9ZWY7_
HLA-DQA10501-DQB10201	757	GASCLCNQGGNVVYM	NQGGNVVYM	6	0.1146	14472.2	85.00	0.61	tr_C9ZWY7_
HLA-DQA10501-DQB10201	758	PHWADNNTTSETVKR	WADNNTTSE	2	0.1143	14513.9	85.00	0.47	tr_C9ZWY7_
HLA-DQA10501-DQB10201	759	TVAEHLDNHRGVRVF	LDNHRGVRV	5	0.1140	14570.4	90.00	0.34	tr_C9ZWY7_
HLA-DQA10501-DQB10201	760	RVLNPSVPPVTKAAT	VLNPSVPPV	1	0.1126	14779.6	90.00	0.38	tr_C9ZWY7_
HLA-DQA10501-DQB10201	761	NPLASETKYHAVRRF	TKYHAVRRF	6	0.1113	14991.4	90.00	0.57	tr_C9ZWY7_
HLA-DQA10501-DQB10201	762	GMAGERGASCLCNQG	RGASCLCNQ	5	0.1110	15052.4	90.00	0.28	tr_C9ZWY7_
HLA-DQA10501-DQB10201	763	TTVAEHLDNHRGVRV	LDNHRGVRV	6	0.1107	15086.1	90.00	0.26	tr_C9ZWY7_
HLA-DQA10501-DQB10201	764	KLSHPGRNVIHFSPT	PGRNVIHFS	4	0.1103	15164.8	90.00	0.38	tr_C9ZWY7_
HLA-DQA10501-DQB10201	765	VTKAATPSMRYANRN	VTKAATPSM	0	0.1094	15309.9	90.00	0.37	tr_C9ZWY7_
HLA-DQA10501-DQB10201	766	GRRLCGVFTKMHAAT	GRRLCGVFT	0	0.1090	15374.2	90.00	0.26	tr_C9ZWY7_
HLA-DQA10501-DQB10201	767	TLKLMTRMGRRLCGV	RMGRRLCGV	6	0.1090	15375.0	90.00	0.58	tr_C9ZWY7_
HLA-DQA10501-DQB10201	768	PKLSHPGRNVIHFSP	PGRNVIHFS	5	0.1058	15918.4	90.00	0.47	tr_C9ZWY7_
HLA-DQA10501-DQB10201	769	PLASETKYHAVRRFQ	TKYHAVRRF	5	0.1024	16507.5	90.00	0.48	tr_C9ZWY7_
HLA-DQA10501-DQB10201	770	WADNNTTSETVKRFH	DNNTTSETV	2	0.1002	16910.7	90.00	0.34	tr_C9ZWY7_
HLA-DQA10501-DQB10201	771	HDEMKDDESKWTPLA	EMKDDESKW	2	0.0988	17175.1	90.00	0.36	tr_C9ZWY7_
HLA-DQA10501-DQB10201	772	MAGERGASCLCNQGG	RGASCLCNQ	4	0.0987	17188.7	90.00	0.26	tr_C9ZWY7_
HLA-DQA10501-DQB10201	773	FNIDRCSTIGVKVPT	IDRCSTIGV	2	0.0969	17528.1	90.00	0.27	tr_C9ZWY7_
HLA-DQA10501-DQB10201	774	KKTTSLHMVTRGGDV	HMVTRGGDV	6	0.0950	17897.2	90.00	0.47	tr_C9ZWY7_
HLA-DQA10501-DQB10201	775	NLLFTNRAPRRSIET	LFTNRAPRR	2	0.0940	18092.2	90.00	0.38	tr_C9ZWY7_
HLA-DQA10501-DQB10201	776	PHIVRPLAAKKSIKV	PHIVRPLAA	0	0.0914	18589.1	90.00	0.57	tr_C9ZWY7_
HLA-DQA10501-DQB10201	777	TGRHITTYSTGGTMR	TTYSTGGTM	5	0.0904	18796.2	95.00	0.40	tr_C9ZWY7_
HLA-DQA10501-DQB10201	778	EYQETLKLMTRMGRR	EYQETLKLM	0	0.0891	19067.8	95.00	0.32	tr_C9ZWY7_
HLA-DQA10501-DQB10201	779	GRHITTYSTGGTMRR	TTYSTGGTM	4	0.0886	19169.2	95.00	0.38	tr_C9ZWY7_
HLA-DQA10501-DQB10201	780	LVGYIARDKKTTSLH	LVGYIARDK	0	0.0886	19172.5	95.00	0.50	tr_C9ZWY7_
HLA-DQA10501-DQB10201	781	VAAPNPLASETKYHA	PNPLASETK	3	0.0878	19327.1	95.00	0.23	tr_C9ZWY7_
HLA-DQA10501-DQB10201	782	ITGRHITTYSTGGTM	TTYSTGGTM	6	0.0875	19401.4	95.00	0.50	tr_C9ZWY7_

HLA-DQA10501-DQB10201	783	VVATSRNTYGRMQGE	NTYGRMQGE	6	0.0873	19443.7	95.00	0.43	tr_C9ZWY7_
HLA-DQA10501-DQB10201	784	VLNPSVPPVTKAATP	LNPSVPPVT	1	0.0871	19483.9	95.00	0.41	tr_C9ZWY7_
HLA-DQA10501-DQB10201	785	IEPITLSPKLSHPGR	EPITLSPKL	1	0.0863	19657.9	95.00	0.41	tr_C9ZWY7_
HLA-DQA10501-DQB10201	786	LAAKKSIKVKVYNLL	IKVKVYNLL	6	0.0851	19901.3	95.00	0.52	tr_C9ZWY7_
HLA-DQA10501-DQB10201	787	AGERGASCLCNQGGN	RGASCLCNQ	3	0.0841	20131.3	95.00	0.31	tr_C9ZWY7_
HLA-DQA10501-DQB10201	788	AGVITGRHITTYSTG	GRHITTYST	5	0.0813	20741.7	95.00	0.19	tr_C9ZWY7_
HLA-DQA10501-DQB10201	789	SPKLSHPGRNVIHFS	PGRNVIHFS	6	0.0801	21009.8	95.00	0.58	tr_C9ZWY7_
HLA-DQA10501-DQB10201	790	HITTYSTGGTMRRRP	TTYSTGGTM	2	0.0792	21223.0	95.00	0.35	tr_C9ZWY7_
HLA-DQA10501-DQB10201	791	ADNNTTSETVKRFHD	DNNTTSETV	1	0.0789	21292.0	95.00	0.39	tr_C9ZWY7_
HLA-DQA10501-DQB10201	792	RYANRNRGSLSGKQL	RGSLSGKQL	6	0.0786	21362.6	95.00	0.73	tr_C9ZWY7_
HLA-DQA10501-DQB10201	793	RHITTYSTGGTMRRR	TTYSTGGTM	3	0.0785	21396.2	95.00	0.38	tr_C9ZWY7_
HLA-DQA10501-DQB10201	794	STGGTMRRRPPAITF	RRRPPAITF	6	0.0770	21733.1	95.00	0.32	tr_C9ZWY7_
HLA-DQA10501-DQB10201	795	HSVVATSRNTYGRMQ	VVATSRNTY	2	0.0766	21831.6	95.00	0.34	tr_C9ZWY7_
HLA-DQA10501-DQB10201	796	YSEFVKVFKKHPQAA	YSEFVKVFK	0	0.0764	21867.3	95.00	0.32	tr_C9ZWY7_
HLA-DQA10501-DQB10201	797	TKAATPSMRYANRNR	KAATPSMRY	1	0.0758	22011.8	95.00	0.36	tr_C9ZWY7_
HLA-DQA10501-DQB10201	798	GERGASCLCNQGGNV	RGASCLCNQ	2	0.0752	22150.2	95.00	0.27	tr_C9ZWY7_
HLA-DQA10501-DQB10201	799	YQETLKLMTRMGRRL	LMTRMGRRL	6	0.0751	22189.3	95.00	0.29	tr_C9ZWY7_
HLA-DQA10501-DQB10201	800	AAPNPLASETKYHAV	PNPLASETK	2	0.0749	22228.4	95.00	0.25	tr_C9ZWY7_
HLA-DQA10501-DQB10201	801	ITTYSTGGTMRRRPP	TYSTGGTMR	2	0.0747	22283.1	95.00	0.29	tr_C9ZWY7_
HLA-DQA10501-DQB10201	802	YANRNRGSLSGKQLA	RGSLSGKQL	5	0.0744	22348.5	95.00	0.65	tr_C9ZWY7_
HLA-DQA10501-DQB10201	803	ARDKKTTSLHMVTRG	KTTSLHMVT	4	0.0741	22433.3	95.00	0.32	tr_C9ZWY7_
HLA-DQA10501-DQB10201	804	SVVATSRNTYGRMQG	VVATSRNTY	1	0.0717	23008.8	95.00	0.33	tr_C9ZWY7_
HLA-DQA10501-DQB10201	805	DNNTTSETVKRFHDE	TTSETVKRF	3	0.0713	23116.4	95.00	0.38	tr_C9ZWY7_
HLA-DQA10501-DQB10201	806	YIARDKKTTSLHMVT	KTTSLHMVT	6	0.0708	23245.5	95.00	0.51	tr_C9ZWY7_
HLA-DQA10501-DQB10201	807	DKKTTSLHMVTRGGD	KTTSLHMVT	2	0.0707	23271.9	95.00	0.25	tr_C9ZWY7_
HLA-DQA10501-DQB10201	808	GVITGRHITTYSTGG	GRHITTYST	4	0.0699	23478.1	95.00	0.22	tr_C9ZWY7_
HLA-DQA10501-DQB10201	809	ERGASCLCNQGGNVV	RGASCLCNQ	1	0.0682	23910.3	95.00	0.30	tr_C9ZWY7_
HLA-DQA10501-DQB10201	810	IARDKKTTSLHMVTR	KTTSLHMVT	5	0.0676	24069.7	95.00	0.41	tr_C9ZWY7_
HLA-DQA10501-DQB10201	811	RGASCLCNQGGNVVY	CNQGGNVVY	6	0.0671	24186.6	95.00	0.23	tr_C9ZWY7_
HLA-DQA10501-DQB10201	812	EPITLSPKLSHPGRN	PITLSPKLS	1	0.0671	24199.2	95.00	0.42	tr_C9ZWY7_
HLA-DQA10501-DQB10201	813	RDKKTTSLHMVTRGG	KTTSLHMVT	3	0.0662	24419.1	95.00	0.29	tr_C9ZWY7_
HLA-DQA10501-DQB10201	814	VITGRHITTYSTGGT	GRHITTYST	3	0.0652	24696.7	95.00	0.20	tr_C9ZWY7_
HLA-DQA10501-DQB10201	815	ANRNRGSLSGKQLAG	RGSLSGKQL	4	0.0650	24740.9	95.00	0.67	tr_C9ZWY7_
HLA-DQA10501-DQB10201	816	KAATPSMRYANRNRG	KAATPSMRY	0	0.0647	24832.6	95.00	0.26	tr_C9ZWY7_
HLA-DQA10501-DQB10201	817	QETLKLMTRMGRRLC	LMTRMGRRL	5	0.0626	25396.4	100.00	0.39	tr_C9ZWY7_
HLA-DQA10501-DQB10201	818	PNPLASETKYHAVRR	ETKYHAVRR	6	0.0624	25444.8	100.00	0.25	tr_C9ZWY7_
HLA-DQA10501-DQB10201	819	VEIIPPPPRSVSTLK	VEIIPPPPR	0	0.0622	25519.5	100.00	0.40	tr_C9ZWY7_
HLA-DQA10501-DQB10201	820	TTYSTGGTMRRRPPA	TYSTGGTMR	1	0.0615	25694.1	100.00	0.29	tr_C9ZWY7_
HLA-DQA10501-DQB10201	821	TYSTGGTMRRRPPAI	TMRRRPPAI	6	0.0610	25854.7	100.00	0.42	tr_C9ZWY7_
HLA-DQA10501-DQB10201	822	YSTGGTMRRRPPAIT	TMRRRPPAI	5	0.0600	26119.0	100.00	0.44	tr_C9ZWY7_
HLA-DQA10501-DQB10201	823	LNPSVPPVTKAATPS	LNPSVPPVT	0	0.0594	26291.1	100.00	0.32	tr_C9ZWY7_
HLA-DQA10501-DQB10201	824	APNPLASETKYHAVR	PNPLASETK	1	0.0592	26350.3	100.00	0.26	tr_C9ZWY7_
HLA-DQA10501-DQB10201	825	ETLKLMTRMGRRLCG	LMTRMGRRL	4	0.0585	26540.6	100.00	0.41	tr_C9ZWY7_
HLA-DQA10501-DQB10201	826	ATPSMRYANRNRGSL	MRYANRNRG	4	0.0529	28195.4	100.00	0.26	tr_C9ZWY7_
HLA-DQA10501-DQB10201	827	SEFVKVFKKHPQAAE	VFKKHPQAA	5	0.0528	28238.1	100.00	0.22	tr_C9ZWY7_
HLA-DQA10501-DQB10201	828	VGYIARDKKTTSLHM	VGYIARDKK	0	0.0515	28626.1	100.00	0.43	tr_C9ZWY7_
HLA-DQA10501-DQB10201	829	TPSMRYANRNRGSLS	MRYANRNRG	3	0.0513	28706.7	100.00	0.22	tr_C9ZWY7_
HLA-DQA10501-DQB10201	830	AATPSMRYANRNRGS	PSMRYANRN	3	0.0512	28731.3	100.00	0.31	tr_C9ZWY7_
HLA-DQA10501-DQB10201	831	PLAAKKSIKVKVYNL	SIKVKVYNL	6	0.0509	28836.5	100.00	0.47	tr_C9ZWY7_
HLA-DQA10501-DQB10201	832	EFVKVFKKHPQAAER	VFKKHPQAA	4	0.0497	29197.3	100.00	0.24	tr_C9ZWY7_

HLA-DQA10501-DQB10201	833	PSMRYANRNRGSLSG	MRYANRNRG	2	0.0470	30069.3	100.00	0.21	tr_C9ZWY7_
HLA-DQA10501-DQB10201	834	GYIARDKKTTSLHMV	KKTTSLHMV	6	0.0463	30308.1	100.00	0.56	tr_C9ZWY7_
HLA-DQA10501-DQB10201	835	LSPKLSHPGRNVIHF	HPGRNVIHF	6	0.0462	30343.8	100.00	0.45	tr_C9ZWY7_
HLA-DQA10501-DQB10201	836	HIVRPLAAKKSIKVK	VRPLAAKKS	2	0.0454	30578.5	100.00	0.29	tr_C9ZWY7_
HLA-DQA10501-DQB10201	837	VRPLAAKKSIKVKVY	KKSIKVKVY	6	0.0436	31208.2	100.00	0.37	tr_C9ZWY7_
HLA-DQA10501-DQB10201	838	PITLSPKLSHPGRNV	PITLSPKLS	0	0.0431	31374.4	100.00	0.49	tr_C9ZWY7_
HLA-DQA10501-DQB10201	839	IVRPLAAKKSIKVKV	VRPLAAKKS	1	0.0398	32497.7	100.00	0.25	tr_C9ZWY7_
HLA-DQA10501-DQB10201	840	RPLAAKKSIKVKVYN	KKSIKVKVY	5	0.0377	33241.6	100.00	0.41	tr_C9ZWY7_
HLA-DQA10501-DQB10201	841	SMRYANRNRGSLSGK	MRYANRNRG	1	0.0364	33721.3	100.00	0.22	tr_C9ZWY7_
HLA-DQA10501-DQB10201	842	MRYANRNRGSLSGKQ	NRGSLSGKQ	6	0.0355	34039.1	100.00	0.31	tr_C9ZWY7_
HLA-DQA10501-DQB10201	843	ITLSPKLSHPGRNVI	KLSHPGRNV	5	0.0312	35689.1	100.00	0.25	tr_C9ZWY7_
HLA-DQA10501-DQB10201	844	TLSPKLSHPGRNVIH	KLSHPGRNV	4	0.0293	36417.8	100.00	0.22	tr_C9ZWY7_

Allele: HLA-DQA10501-DQB10201. Number of high binders 6. Number of weak binders 85. Number of peptides 844

Allele	pos	peptide	core	Of	1-log50k(aff)	affinity(nM)	%Rank	Relia	Identity	Bind Level
HLA-DQA10501-DQB10201	1	GNVVYVREVEDDFSM	YVREVEDDF	4	0.6815	31.4	0.25	0.51	tr_C9ZZQ4_	SB
HLA-DQA10501-DQB10201	2	GGNVVYVREVEDDFS	YVREVEDDF	5	0.6782	32.5	0.30	0.52	tr_C9ZZQ4_	SB
HLA-DQA10501-DQB10201	3	QGGNVVYVREVEDDF	YVREVEDDF	6	0.6713	35.0	0.40	0.55	tr_C9ZZQ4_	SB
HLA-DQA10501-DQB10201	4	NVVYVREVEDDFSMQ	YVREVEDDF	3	0.6652	37.4	0.40	0.49	tr_C9ZZQ4_	SB
HLA-DQA10501-DQB10201	5	VVYVREVEDDFSMQT	YVREVEDDF	2	0.6459	46.1	0.60	0.52	tr_C9ZZQ4_	WB
HLA-DQA10501-DQB10201	6	AVFVFGISASDVDVI	VFGISASDV	3	0.6453	46.4	0.60	0.54	tr_C9ZZQ4_	WB
HLA-DQA10501-DQB10201	7	VFVFGISASDVDVIE	VFGISASDV	2	0.6425	47.9	0.60	0.50	tr_C9ZZQ4_	WB
HLA-DQA10501-DQB10201	8	GAVFVFGISASDVDV	VFGISASDV	4	0.6321	53.6	0.70	0.61	tr_C9ZZQ4_	WB
HLA-DQA10501-DQB10201	9	NQRRYVIADFVIGDF	RRYVIADFV	2	0.6254	57.6	0.80	0.51	tr_C9ZZQ4_	WB
HLA-DQA10501-DQB10201	10	SGAVFVFGISASDVD	VFGISASDV	5	0.6245	58.1	0.80	0.63	tr_C9ZZQ4_	WB
HLA-DQA10501-DQB10201	11	FVFGISASDVDVIER	VFGISASDV	1	0.6213	60.2	0.90	0.48	tr_C9ZZQ4_	WB
HLA-DQA10501-DQB10201	12	DGEMMVYGWAAGEVL	YGWAAGEVL	6	0.6207	60.6	0.90	0.62	tr_C9ZZQ4_	WB
HLA-DQA10501-DQB10201	13	VYVREVEDDFSMQTP	YVREVEDDF	1	0.6154	64.1	1.00	0.53	tr_C9ZZQ4_	WB
HLA-DQA10501-DQB10201	14	DSLYNQRRYVIADFV	RRYVIADFV	6	0.6139	65.2	1.00	0.79	tr_C9ZZQ4_	WB
HLA-DQA10501-DQB10201	15	SLYNQRRYVIADFVI	RRYVIADFV	5	0.6098	68.2	1.10	0.79	tr_C9ZZQ4_	WB
HLA-DQA10501-DQB10201	16	EMMVYGWAAGEVLSQ	YGWAAGEVL	4	0.6054	71.5	1.20	0.56	tr_C9ZZQ4_	WB
HLA-DQA10501-DQB10201	17	GEMMVYGWAAGEVLS	YGWAAGEVL	5	0.6053	71.6	1.20	0.62	tr_C9ZZQ4_	WB
HLA-DQA10501-DQB10201	18	QRRYVIADFVIGDFG	RRYVIADFV	1	0.5975	77.9	1.40	0.50	tr_C9ZZQ4_	WB
HLA-DQA10501-DQB10201	19	MMVYGWAAGEVLSQA	YGWAAGEVL	3	0.5944	80.5	1.40	0.52	tr_C9ZZQ4_	WB
HLA-DQA10501-DQB10201	20	YNQRRYVIADFVIGD	RRYVIADFV	3	0.5911	83.4	1.50	0.73	tr_C9ZZQ4_	WB
HLA-DQA10501-DQB10201	21	MVYGWAAGEVLSQAL	YGWAAGEVL	2	0.5829	91.1	1.70	0.50	tr_C9ZZQ4_	WB
HLA-DQA10501-DQB10201	22	LYNQRRYVIADFVIG	RRYVIADFV	4	0.5814	92.7	1.70	0.76	tr_C9ZZQ4_	WB
HLA-DQA10501-DQB10201	23	GEKEYEVALRVMEEM	VALRVMEEM	6	0.5727	101.8	2.00	0.50	tr_C9ZZQ4_	WB
HLA-DQA10501-DQB10201	24	VFGISASDVDVIERH	VFGISASDV	0	0.5569	120.8	2.50	0.34	tr_C9ZZQ4_	WB
HLA-DQA10501-DQB10201	25	AGEVLSQALSVPEWL	QALSVPEWL	6	0.5501	130.0	3.00	0.60	tr_C9ZZQ4_	WB
HLA-DQA10501-DQB10201	26	TSGAVFVFGISASDV	VFGISASDV	6	0.5464	135.4	3.00	0.76	tr_C9ZZQ4_	WB
HLA-DQA10501-DQB10201	27	VYGWAAGEVLSQALS	YGWAAGEVL	1	0.5438	139.1	3.00	0.53	tr_C9ZZQ4_	WB
HLA-DQA10501-DQB10201	28	RRYVIADFVIGDFGG	RRYVIADFV	0	0.5416	142.6	3.50	0.43	tr_C9ZZQ4_	WB
HLA-DQA10501-DQB10201	29	NQGGNVVYVREVEDD	VYVREVEDD	6	0.5377	148.7	3.50	0.34	tr_C9ZZQ4_	WB
HLA-DQA10501-DQB10201	30	EKEYEVALRVMEEMG	VALRVMEEM	5	0.5354	152.5	3.50	0.43	tr_C9ZZQ4_	WB

HLA-DQA10501-DQB10201	31	KEYEVALRVMEEMGY	VALRVMEEM	4	0.5275	166.0	4.00	0.43	tr_C9ZZQ4_	WB
HLA-DQA10501-DQB10201	32	YVREVEDDFSMQTPK	YVREVEDDF	0	0.5273	166.4	4.00	0.51	tr_C9ZZQ4_	WB
HLA-DQA10501-DQB10201	33	PAPDKEFNAVFERFA	EFNAVFERF	5	0.5208	178.6	4.50	0.49	tr_C9ZZQ4_	WB
HLA-DQA10501-DQB10201	34	EVLSQALSVPEWLKD	QALSVPEWL	4	0.5131	194.0	5.00	0.62	tr_C9ZZQ4_	WB
HLA-DQA10501-DQB10201	35	GEVLSQALSVPEWLK	QALSVPEWL	5	0.5104	199.8	5.00	0.60	tr_C9ZZQ4_	WB
HLA-DQA10501-DQB10201	36	APDKEFNAVFERFAA	EFNAVFERF	4	0.5032	215.9	5.50	0.49	tr_C9ZZQ4_	WB
HLA-DQA10501-DQB10201	37	YGWAAGEVLSQALSV	YGWAAGEVL	0	0.4949	236.2	6.00	0.38	tr_C9ZZQ4_	WB
HLA-DQA10501-DQB10201	38	EYEVALRVMEEMGYK	EVALRVMEE	2	0.4947	236.8	6.00	0.46	tr_C9ZZQ4_	WB
HLA-DQA10501-DQB10201	39	KPAPDKEFNAVFERF	EFNAVFERF	6	0.4892	251.2	6.50	0.67	tr_C9ZZQ4_	WB
HLA-DQA10501-DQB10201	40	VIADFVIGDFGGDCE	IADFVIGDF	1	0.4857	261.0	6.50	0.28	tr_C9ZZQ4_	WB
HLA-DQA10501-DQB10201	41	PDKEFNAVFERFAAT	EFNAVFERF	3	0.4806	275.8	7.00	0.47	tr_C9ZZQ4_	WB
HLA-DQA10501-DQB10201	42	YGEKEYEVALRVMEE	EVALRVMEE	6	0.4802	277.2	7.00	0.70	tr_C9ZZQ4_	WB
HLA-DQA10501-DQB10201	43	VLSQALSVPEWLKDR	QALSVPEWL	3	0.4771	286.6	7.00	0.68	tr_C9ZZQ4_	WB
HLA-DQA10501-DQB10201	44	FGISASDVDVIERHL	FGISASDVD	0	0.4770	286.6	7.00	0.31	tr_C9ZZQ4_	WB
HLA-DQA10501-DQB10201	45	RYVIADFVIGDFGGD	IADFVIGDF	3	0.4713	305.1	7.50	0.47	tr_C9ZZQ4_	WB
HLA-DQA10501-DQB10201	46	CNQGGNVVYVREVED	VVYVREVED	6	0.4671	319.2	8.00	0.56	tr_C9ZZQ4_	WB
HLA-DQA10501-DQB10201	47	YEVALRVMEEMGYKL	EVALRVMEE	1	0.4633	332.6	8.50	0.41	tr_C9ZZQ4_	WB
HLA-DQA10501-DQB10201	48	LRVLVLFSEVALLYE	FSEVALLYE	6	0.4627	334.8	8.50	0.44	tr_C9ZZQ4_	WB
HLA-DQA10501-DQB10201	49	YVIADFVIGDFGGDC	IADFVIGDF	2	0.4595	346.6	9.00	0.45	tr_C9ZZQ4_	WB
HLA-DQA10501-DQB10201	50	TDGEMMVYGWAAGEV	VYGWAAGEV	6	0.4543	366.5	9.00	0.74	tr_C9ZZQ4_	WB
HLA-DQA10501-DQB10201	51	IADFVIGDFGGDCEG	IGDFGGDCE	5	0.4539	368.4	9.50	0.37	tr_C9ZZQ4_	WB
HLA-DQA10501-DQB10201	52	LSQALSVPEWLKDRT	QALSVPEWL	2	0.4519	376.3	9.50	0.70	tr_C9ZZQ4_	WB
HLA-DQA10501-DQB10201	53	VLVLFSEVALLYEKF	EVALLYEKF	6	0.4505	382.1	9.50	0.30	tr_C9ZZQ4_	WB
HLA-DQA10501-DQB10201	54	VLAPSGAQVFLELMW	GAQVFLELM	5	0.4467	398.0	10.00	0.41	tr_C9ZZQ4_	
HLA-DQA10501-DQB10201	55	DKEFNAVFERFAATK	EFNAVFERF	2	0.4442	408.8	11.00	0.54	tr_C9ZZQ4_	
HLA-DQA10501-DQB10201	56	LVLFSEVALLYEKFV	FSEVALLYE	3	0.4413	422.2	11.00	0.32	tr_C9ZZQ4_	
HLA-DQA10501-DQB10201	57	YVAIKRFQEVMREYL	FQEVMREYL	6	0.4306	474.0	12.00	0.51	tr_C9ZZQ4_	
HLA-DQA10501-DQB10201	58	YVLAPSGAQVFLELM	GAQVFLELM	6	0.4292	481.1	12.00	0.44	tr_C9ZZQ4_	
HLA-DQA10501-DQB10201	59	ITIVFGPVGDDTTLD	VFGPVGDDT	3	0.4270	492.9	12.00	0.40	tr_C9ZZQ4_	
HLA-DQA10501-DQB10201	60	KALMKMTNTLFIDPI	TNTLFIDPI	6	0.4232	513.2	13.00	0.61	tr_C9ZZQ4_	
HLA-DQA10501-DQB10201	61	AAGEVLSQALSVPEW	SQALSVPEW	6	0.4198	532.3	13.00	0.40	tr_C9ZZQ4_	
HLA-DQA10501-DQB10201	62	DFVIGDFGGDCEGEA	IGDFGGDCE	3	0.4184	540.4	13.00	0.44	tr_C9ZZQ4_	
HLA-DQA10501-DQB10201	63	ADFVIGDFGGDCEGE	IGDFGGDCE	4	0.4171	548.5	14.00	0.44	tr_C9ZZQ4_	
HLA-DQA10501-DQB10201	64	VLFSEVALLYEKFVT	FSEVALLYE	2	0.4169	549.3	14.00	0.34	tr_C9ZZQ4_	
HLA-DQA10501-DQB10201	65	RVLVLFSEVALLYEK	FSEVALLYE	5	0.4159	555.8	14.00	0.41	tr_C9ZZQ4_	
HLA-DQA10501-DQB10201	66	MTNTLFIDPITIEPQ	TNTLFIDPI	1	0.4154	558.3	14.00	0.28	tr_C9ZZQ4_	
HLA-DQA10501-DQB10201	67	SQALSVPEWLKDRTT	QALSVPEWL	1	0.4150	560.7	14.00	0.74	tr_C9ZZQ4_	
HLA-DQA10501-DQB10201	68	TIVFGPVGDDTTLDS	VFGPVGDDT	2	0.4095	595.2	15.00	0.39	tr_C9ZZQ4_	
HLA-DQA10501-DQB10201	69	FVIGDFGGDCEGEAA	IGDFGGDCE	2	0.4088	600.0	15.00	0.38	tr_C9ZZQ4_	
HLA-DQA10501-DQB10201	70	GITIVFGPVGDDTTL	VFGPVGDDT	4	0.4079	605.4	15.00	0.41	tr_C9ZZQ4_	
HLA-DQA10501-DQB10201	71	KMTNTLFIDPITIEP	TNTLFIDPI	2	0.4076	607.7	15.00	0.40	tr_C9ZZQ4_	
HLA-DQA10501-DQB10201	72	LMKMTNTLFIDPITI	TNTLFIDPI	4	0.4064	615.6	15.00	0.49	tr_C9ZZQ4_	
HLA-DQA10501-DQB10201	73	GWAAGEVLSQALSVP	EVLSQALSV	5	0.4061	617.3	15.00	0.32	tr_C9ZZQ4_	
HLA-DQA10501-DQB10201	74	LAPSGAQVFLELMWK	GAQVFLELM	4	0.4056	620.8	15.00	0.40	tr_C9ZZQ4_	
HLA-DQA10501-DQB10201	75	PVGDDTTLDSISELQ	TTLDSISEL	5	0.4037	634.0	15.00	0.46	tr_C9ZZQ4_	
HLA-DQA10501-DQB10201	76	TNTLFIDPITIEPQL	IDPITIEPQ	5	0.4033	636.9	15.00	0.22	tr_C9ZZQ4_	
HLA-DQA10501-DQB10201	77	EVALRVMEEMGYKLC	VALRVMEEM	1	0.4006	655.3	16.00	0.47	tr_C9ZZQ4_	
HLA-DQA10501-DQB10201	78	ALMKMTNTLFIDPIT	TNTLFIDPI	5	0.3995	663.0	16.00	0.53	tr_C9ZZQ4_	
HLA-DQA10501-DQB10201	79	SGITIVFGPVGDDTT	VFGPVGDDT	5	0.3993	664.6	16.00	0.41	tr_C9ZZQ4_	
HLA-DQA10501-DQB10201	80	NTLFIDPITIEPQLN	PITIEPQLN	6	0.3991	666.0	16.00	0.25	tr_C9ZZQ4_	

HLA-DQA10501-DQB10201	81	KEFNAVFERFAATKP	EFNAVFERF	1	0.3991	666.4	16.00	0.56	tr_C9ZZQ4_
HLA-DQA10501-DQB10201	82	APRLVFATNLPHWYD	FATNLPHWY	5	0.3975	677.6	16.00	0.37	tr_C9ZZQ4_
HLA-DQA10501-DQB10201	83	IVFGPVGDDTTLDSI	VFGPVGDDT	1	0.3962	687.6	16.00	0.37	tr_C9ZZQ4_
HLA-DQA10501-DQB10201	84	ELRVLVLFSEVALLY	LFSEVALLY	6	0.3954	693.3	17.00	0.28	tr_C9ZZQ4_
HLA-DQA10501-DQB10201	85	AAPRLVFATNLPHWY	FATNLPHWY	6	0.3951	696.0	17.00	0.32	tr_C9ZZQ4_
HLA-DQA10501-DQB10201	86	MKMTNTLFIDPITIE	TNTLFIDPI	3	0.3937	706.7	17.00	0.47	tr_C9ZZQ4_
HLA-DQA10501-DQB10201	87	VAIKRFQEVMREYLK	FQEVMREYL	5	0.3811	809.2	19.00	0.50	tr_C9ZZQ4_
HLA-DQA10501-DQB10201	88	WAAGEVLSQALSVPE	EVLSQALSV	4	0.3804	816.0	19.00	0.34	tr_C9ZZQ4_
HLA-DQA10501-DQB10201	89	NSGKEANAIVCAAEA	NAIVCAAEA	6	0.3803	816.5	19.00	0.46	tr_C9ZZQ4_
HLA-DQA10501-DQB10201	90	LFSEVALLYEKFVTA	FSEVALLYE	1	0.3796	822.4	19.00	0.34	tr_C9ZZQ4_
HLA-DQA10501-DQB10201	91	GISASDVDVIERHLA	ISASDVDVI	1	0.3784	833.2	19.00	0.35	tr_C9ZZQ4_
HLA-DQA10501-DQB10201	92	SGKEANAIVCAAEAH	NAIVCAAEA	5	0.3764	851.9	20.00	0.37	tr_C9ZZQ4_
HLA-DQA10501-DQB10201	93	PRLVFATNLPHWYDN	FATNLPHWY	4	0.3762	853.4	20.00	0.41	tr_C9ZZQ4_
HLA-DQA10501-DQB10201	94	GPVGDDTTLDSISEL	TTLDSISEL	6	0.3757	858.2	20.00	0.62	tr_C9ZZQ4_
HLA-DQA10501-DQB10201	95	VGDDTTLDSISELQK	TTLDSISEL	4	0.3754	861.0	20.00	0.43	tr_C9ZZQ4_
HLA-DQA10501-DQB10201	96	RHDTGSLEYSGQLFL	SLEYSGQLF	5	0.3740	874.1	20.00	0.55	tr_C9ZZQ4_
HLA-DQA10501-DQB10201	97	MWKRVLLDSQVSPFP	WKRVLLDSQ	1	0.3735	878.5	20.00	0.32	tr_C9ZZQ4_
HLA-DQA10501-DQB10201	98	LMWKRVLLDSQVSPF	WKRVLLDSQ	2	0.3732	882.0	20.00	0.37	tr_C9ZZQ4_
HLA-DQA10501-DQB10201	99	LNGLVIFFGDNTIAV	FFGDNTIAV	6	0.3712	900.9	20.00	0.38	tr_C9ZZQ4_
HLA-DQA10501-DQB10201	100	KSGITIVFGPVGDDT	VFGPVGDDT	6	0.3708	904.9	20.00	0.44	tr_C9ZZQ4_
HLA-DQA10501-DQB10201	101	APSGAQVFLELMWKR	GAQVFLELM	3	0.3689	923.8	21.00	0.42	tr_C9ZZQ4_
HLA-DQA10501-DQB10201	102	TLFIDPITIEPQLNK	PITIEPQLN	5	0.3648	965.2	22.00	0.26	tr_C9ZZQ4_
HLA-DQA10501-DQB10201	103	PEWLKDRTTFMDSLY	RTTFMDSLY	6	0.3630	984.4	22.00	0.35	tr_C9ZZQ4_
HLA-DQA10501-DQB10201	104	EWLKDRTTFMDSLYN	RTTFMDSLY	5	0.3629	985.4	22.00	0.31	tr_C9ZZQ4_
HLA-DQA10501-DQB10201	105	AIKRFQEVMREYLKT	FQEVMREYL	4	0.3599	1018.4	22.00	0.49	tr_C9ZZQ4_
HLA-DQA10501-DQB10201	106	SELRVLVLFSEVALL	VLVLFSEVA	4	0.3592	1025.4	23.00	0.41	tr_C9ZZQ4_
HLA-DQA10501-DQB10201	107	GEVRRWVRELYFLRP	RRWVRELYF	3	0.3592	1026.2	23.00	0.42	tr_C9ZZQ4_
HLA-DQA10501-DQB10201	108	VIGDFGGDCEGEAAK	IGDFGGDCE	1	0.3584	1035.3	23.00	0.37	tr_C9ZZQ4_
HLA-DQA10501-DQB10201	109	EFNAVFERFAATKPQ	EFNAVFERF	0	0.3572	1047.9	23.00	0.46	tr_C9ZZQ4_
HLA-DQA10501-DQB10201	110	RLVFATNLPHWYDNE	FATNLPHWY	3	0.3570	1050.1	23.00	0.46	tr_C9ZZQ4_
HLA-DQA10501-DQB10201	111	ELMWKRVLLDSQVSP	WKRVLLDSQ	3	0.3569	1051.8	23.00	0.52	tr_C9ZZQ4_
HLA-DQA10501-DQB10201	112	GKEANAIVCAAEAHG	NAIVCAAEA	4	0.3555	1067.3	23.00	0.34	tr_C9ZZQ4_
HLA-DQA10501-DQB10201	113	GSGEVRRWVRELYFL	RRWVRELYF	5	0.3549	1075.1	23.00	0.52	tr_C9ZZQ4_
HLA-DQA10501-DQB10201	114	TGSLEYSGQLFLHAF	SLEYSGQLF	2	0.3543	1081.8	23.00	0.34	tr_C9ZZQ4_
HLA-DQA10501-DQB10201	115	GDDTTLDSISELQKH	TTLDSISEL	3	0.3515	1114.8	24.00	0.44	tr_C9ZZQ4_
HLA-DQA10501-DQB10201	116	FSEVALLYEKFVTAF	EVALLYEKF	2	0.3514	1116.3	24.00	0.29	tr_C9ZZQ4_
HLA-DQA10501-DQB10201	117	WLKDRTTFMDSLYNQ	RTTFMDSLY	4	0.3499	1134.4	24.00	0.31	tr_C9ZZQ4_
HLA-DQA10501-DQB10201	118	AYVLAPSGAQVFLEL	SGAQVFLEL	6	0.3498	1135.5	24.00	0.31	tr_C9ZZQ4_
HLA-DQA10501-DQB10201	119	KEANAIVCAAEAHGI	NAIVCAAEA	3	0.3485	1152.3	25.00	0.32	tr_C9ZZQ4_
HLA-DQA10501-DQB10201	120	NGLVIFFGDNTIAVK	FFGDNTIAV	5	0.3482	1155.6	25.00	0.34	tr_C9ZZQ4_
HLA-DQA10501-DQB10201	121	TGSGEVRRWVRELYF	RRWVRELYF	6	0.3473	1167.3	25.00	0.58	tr_C9ZZQ4_
HLA-DQA10501-DQB10201	122	LKDRTTFMDSLYNQR	RTTFMDSLY	3	0.3472	1167.6	25.00	0.26	tr_C9ZZQ4_
HLA-DQA10501-DQB10201	123	EANAIVCAAEAHGIM	NAIVCAAEA	2	0.3466	1176.2	25.00	0.34	tr_C9ZZQ4_
HLA-DQA10501-DQB10201	124	HDTGSLEYSGQLFLH	SLEYSGQLF	4	0.3462	1180.4	25.00	0.53	tr_C9ZZQ4_
HLA-DQA10501-DQB10201	125	HHYGEKEYEVALRVM	KEYEVALRV	5	0.3462	1180.6	25.00	0.37	tr_C9ZZQ4_
HLA-DQA10501-DQB10201	126	LFIDPITIEPQLNKF	PITIEPQLN	4	0.3454	1191.5	25.00	0.31	tr_C9ZZQ4_
HLA-DQA10501-DQB10201	127	KSHKTPEEFIKDELS	PEEFIKDEL	5	0.3449	1197.5	25.00	0.58	tr_C9ZZQ4_
HLA-DQA10501-DQB10201	128	PSGAQVFLELMWKRV	GAQVFLELM	2	0.3431	1220.5	26.00	0.42	tr_C9ZZQ4_
HLA-DQA10501-DQB10201	129	DTGSLEYSGQLFLHA	SLEYSGQLF	3	0.3421	1233.8	26.00	0.49	tr_C9ZZQ4_
HLA-DQA10501-DQB10201	130	WKRVLLDSQVSPFPG	WKRVLLDSQ	0	0.3420	1235.6	26.00	0.25	tr_C9ZZQ4_

HLA-DQA10501-DQB10201	131	EVRRWVRELYFLRPS	RRWVRELYF	2	0.3419	1237.0	26.00	0.40	tr_C9ZZQ4_
HLA-DQA10501-DQB10201	132	VREVEDDFSMQTPKD	VREVEDDFS	0	0.3415	1242.9	26.00	0.47	tr_C9ZZQ4_
HLA-DQA10501-DQB10201	133	FLELMWKRVLLDSQV	WKRVLLDSQ	5	0.3411	1247.3	26.00	0.56	tr_C9ZZQ4_
HLA-DQA10501-DQB10201	134	ISASDVDVIERHLAA	SASDVDVIE	1	0.3400	1263.4	26.00	0.29	tr_C9ZZQ4_
HLA-DQA10501-DQB10201	135	AAAPRLVFATNLPHW	PRLVFATNL	3	0.3397	1266.4	26.00	0.40	tr_C9ZZQ4_
HLA-DQA10501-DQB10201	136	VFGPVGDDTTLDSIS	VFGPVGDDT	0	0.3396	1267.6	26.00	0.32	tr_C9ZZQ4_
HLA-DQA10501-DQB10201	137	DDTTLDSISELQKHE	TLDSISELQ	3	0.3394	1271.5	26.00	0.39	tr_C9ZZQ4_
HLA-DQA10501-DQB10201	138	KFRRHVIHLSPTVEQ	VIHLSPTVE	5	0.3390	1276.6	26.00	0.49	tr_C9ZZQ4_
HLA-DQA10501-DQB10201	139	QYVAIKRFQEVMREY	KRFQEVMRE	5	0.3377	1294.5	27.00	0.41	tr_C9ZZQ4_
HLA-DQA10501-DQB10201	140	GLVIFFGDNTIAVKA	FFGDNTIAV	4	0.3368	1307.3	27.00	0.34	tr_C9ZZQ4_
HLA-DQA10501-DQB10201	141	LVFATNLPHWYDNET	FATNLPHWY	2	0.3366	1309.6	27.00	0.50	tr_C9ZZQ4_
HLA-DQA10501-DQB10201	142	GVFVVLNVNDKPAPD	VVLNVNDKP	3	0.3361	1317.7	27.00	0.54	tr_C9ZZQ4_
HLA-DQA10501-DQB10201	143	LELMWKRVLLDSQVS	WKRVLLDSQ	4	0.3352	1330.3	27.00	0.56	tr_C9ZZQ4_
HLA-DQA10501-DQB10201	144	ERHLAAHSELRVLVL	HSELRVLVL	6	0.3329	1363.0	28.00	0.23	tr_C9ZZQ4_
HLA-DQA10501-DQB10201	145	LSTFSRTGMLYVMYV	TGMLYVMYV	6	0.3324	1370.7	28.00	0.34	tr_C9ZZQ4_
HLA-DQA10501-DQB10201	146	VFLELMWKRVLLDSQ	WKRVLLDSQ	6	0.3317	1381.2	28.00	0.60	tr_C9ZZQ4_
HLA-DQA10501-DQB10201	147	PLNGLVIFFGDNTIA	LVIFFGDNT	4	0.3315	1384.0	28.00	0.26	tr_C9ZZQ4_
HLA-DQA10501-DQB10201	148	GSLEYSGQLFLHAFG	SLEYSGQLF	1	0.3313	1387.6	28.00	0.35	tr_C9ZZQ4_
HLA-DQA10501-DQB10201	149	SGEVRRWVRELYFLR	RRWVRELYF	4	0.3311	1391.0	28.00	0.50	tr_C9ZZQ4_
HLA-DQA10501-DQB10201	150	CGVFVVLNVNDKPAP	VVLNVNDKP	4	0.3305	1399.0	28.00	0.50	tr_C9ZZQ4_
HLA-DQA10501-DQB10201	151	NKFRRHVIHLSPTVE	VIHLSPTVE	6	0.3302	1403.5	28.00	0.52	tr_C9ZZQ4_
HLA-DQA10501-DQB10201	152	GHHYGEKEYEVALRV	KEYEVALRV	6	0.3301	1405.7	28.00	0.47	tr_C9ZZQ4_
HLA-DQA10501-DQB10201	153	HSELRVLVLFSEVAL	VLVLFSEVA	5	0.3295	1415.1	28.00	0.48	tr_C9ZZQ4_
HLA-DQA10501-DQB10201	154	LRHDTGSLEYSGQLF	SLEYSGQLF	6	0.3292	1419.0	28.00	0.74	tr_C9ZZQ4_
HLA-DQA10501-DQB10201	155	IERHLAAHSELRVLV	RHLAAHSEL	2	0.3288	1425.8	29.00	0.23	tr_C9ZZQ4_
HLA-DQA10501-DQB10201	156	VRRWVRELYFLRPSP	RRWVRELYF	1	0.3287	1426.6	29.00	0.38	tr_C9ZZQ4_
HLA-DQA10501-DQB10201	157	AHSELRVLVLFSEVA	VLVLFSEVA	6	0.3287	1426.8	29.00	0.52	tr_C9ZZQ4_
HLA-DQA10501-DQB10201	158	IKRFQEVMREYLKTH	FQEVMREYL	3	0.3287	1426.9	29.00	0.47	tr_C9ZZQ4_
HLA-DQA10501-DQB10201	159	MDSLYNQRRYVIADF	QRRYVIADF	6	0.3280	1438.0	29.00	0.81	tr_C9ZZQ4_
HLA-DQA10501-DQB10201	160	NASMESRNWTIADRV	RNWTIADRV	6	0.3273	1449.5	29.00	0.77	tr_C9ZZQ4_
HLA-DQA10501-DQB10201	161	HYGEKEYEVALRVME	KEYEVALRV	4	0.3269	1455.1	29.00	0.38	tr_C9ZZQ4_
HLA-DQA10501-DQB10201	162	SPLNGLVIFFGDNTI	LVIFFGDNT	5	0.3265	1461.3	29.00	0.30	tr_C9ZZQ4_
HLA-DQA10501-DQB10201	163	SPTIRKFHATVESRE	FHATVESRE	6	0.3261	1467.5	29.00	0.51	tr_C9ZZQ4_
HLA-DQA10501-DQB10201	164	YCNQGGNVVYVREVE	NVVYVREVE	6	0.3257	1474.8	29.00	0.52	tr_C9ZZQ4_
HLA-DQA10501-DQB10201	165	FERFAATKPQAVLLF	FAATKPQAV	3	0.3255	1477.8	29.00	0.55	tr_C9ZZQ4_
HLA-DQA10501-DQB10201	166	VIERHLAAHSELRVL	RHLAAHSEL	3	0.3253	1481.2	29.00	0.32	tr_C9ZZQ4_
HLA-DQA10501-DQB10201	167	SKSHKTPEEFIKDEL	PEEFIKDEL	6	0.3251	1482.9	29.00	0.76	tr_C9ZZQ4_
HLA-DQA10501-DQB10201	168	FRRHVIHLSPTVEQQ	VIHLSPTVE	4	0.3251	1484.4	29.00	0.48	tr_C9ZZQ4_
HLA-DQA10501-DQB10201	169	KLCGVFVVLNVNDKP	VVLNVNDKP	6	0.3232	1514.2	30.00	0.47	tr_C9ZZQ4_
HLA-DQA10501-DQB10201	170	LVIFFGDNTIAVKAT	FFGDNTIAV	3	0.3230	1517.7	30.00	0.36	tr_C9ZZQ4_
HLA-DQA10501-DQB10201	171	DTTLDSISELQKHEV	TLDSISELQ	2	0.3230	1518.5	30.00	0.37	tr_C9ZZQ4_
HLA-DQA10501-DQB10201	172	RRHVIHLSPTVEQQI	VIHLSPTVE	3	0.3226	1524.8	30.00	0.43	tr_C9ZZQ4_
HLA-DQA10501-DQB10201	173	VFVVLNVNDKPAPDK	VVLNVNDKP	2	0.3223	1528.8	30.00	0.57	tr_C9ZZQ4_
HLA-DQA10501-DQB10201	174	VFERFAATKPQAVLL	FAATKPQAV	4	0.3217	1539.9	30.00	0.50	tr_C9ZZQ4_
HLA-DQA10501-DQB10201	175	LCGVFVVLNVNDKPA	VVLNVNDKP	5	0.3204	1561.5	30.00	0.45	tr_C9ZZQ4_
HLA-DQA10501-DQB10201	176	NNSGKEANAIVCAAE	EANAIVCAA	5	0.3202	1563.6	30.00	0.56	tr_C9ZZQ4_
HLA-DQA10501-DQB10201	177	KDRTTFMDSLYNQRR	RTTFMDSLY	2	0.3193	1580.5	31.00	0.28	tr_C9ZZQ4_
HLA-DQA10501-DQB10201	178	RHVIHLSPTVEQQIY	VIHLSPTVE	2	0.3192	1582.2	31.00	0.41	tr_C9ZZQ4_
HLA-DQA10501-DQB10201	179	NYFLKHDTDGEMMVY	KHDTDGEMM	4	0.3191	1583.1	31.00	0.27	tr_C9ZZQ4_
HLA-DQA10501-DQB10201	180	GSAAAPRLVFATNLP	PRLVFATNL	5	0.3171	1617.4	31.00	0.55	tr_C9ZZQ4_

HLA-DQA10501-DQB10201	181	DVIERHLAAHSELRV	RHLAAHSEL	4	0.3170	1619.6	31.00	0.37	tr_C9ZZQ4_
HLA-DQA10501-DQB10201	182	HVIHLSPTVEQQIYV	VIHLSPTVE	1	0.3167	1624.3	31.00	0.41	tr_C9ZZQ4_
HLA-DQA10501-DQB10201	183	QALSVPEWLKDRTTF	QALSVPEWL	0	0.3163	1631.4	31.00	0.81	tr_C9ZZQ4_
HLA-DQA10501-DQB10201	184	ELQKHEVVAFGPMTG	EVVAFGPMT	5	0.3160	1638.0	31.00	0.38	tr_C9ZZQ4_
HLA-DQA10501-DQB10201	185	AVFERFAATKPQAVL	FAATKPQAV	5	0.3155	1646.1	32.00	0.50	tr_C9ZZQ4_
HLA-DQA10501-DQB10201	186	STFSRTGMLYVMYVL	TGMLYVMYV	5	0.3153	1650.3	32.00	0.25	tr_C9ZZQ4_
HLA-DQA10501-DQB10201	187	VPEWLKDRTTFMDSL	DRTTFMDSL	6	0.3147	1659.6	32.00	0.54	tr_C9ZZQ4_
HLA-DQA10501-DQB10201	188	PTSGAVFVFGISASD	FVFGISASD	6	0.3139	1675.1	32.00	0.58	tr_C9ZZQ4_
HLA-DQA10501-DQB10201	189	VFATNLPHWYDNETS	FATNLPHWY	1	0.3129	1692.4	32.00	0.46	tr_C9ZZQ4_
HLA-DQA10501-DQB10201	190	IGDFGGDCEGEAAKQ	IGDFGGDCE	0	0.3126	1698.9	32.00	0.34	tr_C9ZZQ4_
HLA-DQA10501-DQB10201	191	DVDVIERHLAAHSEL	RHLAAHSEL	6	0.3118	1713.8	32.00	0.60	tr_C9ZZQ4_
HLA-DQA10501-DQB10201	192	DKSGITIVFGPVGDD	TIVFGPVGD	5	0.3110	1728.4	33.00	0.28	tr_C9ZZQ4_
HLA-DQA10501-DQB10201	193	PNYFLKHDTDGEMMV	KHDTDGEMM	5	0.3109	1730.3	33.00	0.29	tr_C9ZZQ4_
HLA-DQA10501-DQB10201	194	FIDPITIEPQLNKFR	PITIEPQLN	3	0.3082	1781.7	33.00	0.32	tr_C9ZZQ4_
HLA-DQA10501-DQB10201	195	HKTPEEFIKDELSKE	PEEFIKDEL	3	0.3065	1813.6	34.00	0.45	tr_C9ZZQ4_
HLA-DQA10501-DQB10201	196	RHLAAHSELRVLVLF	HSELRVLVL	5	0.3063	1817.9	34.00	0.25	tr_C9ZZQ4_
HLA-DQA10501-DQB10201	197	RTVTAVFGIVTKALM	FGIVTKALM	6	0.3062	1821.2	34.00	0.32	tr_C9ZZQ4_
HLA-DQA10501-DQB10201	198	DPNYFLKHDTDGEMM	KHDTDGEMM	6	0.3061	1823.1	34.00	0.29	tr_C9ZZQ4_
HLA-DQA10501-DQB10201	199	SAAAPRLVFATNLPH	PRLVFATNL	4	0.3043	1858.0	34.00	0.47	tr_C9ZZQ4_
HLA-DQA10501-DQB10201	200	DRTTFMDSLYNQRRY	RTTFMDSLY	1	0.3041	1861.5	34.00	0.32	tr_C9ZZQ4_
HLA-DQA10501-DQB10201	201	PTIRKFHATVESREK	FHATVESRE	5	0.3040	1863.7	34.00	0.52	tr_C9ZZQ4_
HLA-DQA10501-DQB10201	202	SMESRNWTIADRVKV	RNWTIADRV	4	0.3040	1865.2	34.00	0.61	tr_C9ZZQ4_
HLA-DQA10501-DQB10201	203	ALSTFSRTGMLYVMY	RTGMLYVMY	6	0.3036	1872.5	34.00	0.38	tr_C9ZZQ4_
HLA-DQA10501-DQB10201	204	GAYVLAPSGAQVFLE	PSGAQVFLE	6	0.3030	1883.4	34.00	0.38	tr_C9ZZQ4_
HLA-DQA10501-DQB10201	205	SGAQVFLELMWKRVL	GAQVFLELM	1	0.3030	1884.3	34.00	0.47	tr_C9ZZQ4_
HLA-DQA10501-DQB10201	206	SHKTPEEFIKDELSK	PEEFIKDEL	4	0.3018	1909.3	35.00	0.56	tr_C9ZZQ4_
HLA-DQA10501-DQB10201	207	ANAIVCAAEAHGIMK	NAIVCAAEA	1	0.3017	1911.3	35.00	0.34	tr_C9ZZQ4_
HLA-DQA10501-DQB10201	208	RWVRELYFLRPSPTI	YFLRPSPTI	6	0.3016	1912.7	35.00	0.26	tr_C9ZZQ4_
HLA-DQA10501-DQB10201	209	SLEYSGQLFLHAFGT	SLEYSGQLF	0	0.3013	1918.4	35.00	0.28	tr_C9ZZQ4_
HLA-DQA10501-DQB10201	210	VIHLSPTVEQQIYVL	VIHLSPTVE	0	0.3011	1924.5	35.00	0.28	tr_C9ZZQ4_
HLA-DQA10501-DQB10201	211	NAVFERFAATKPQAV	FAATKPQAV	6	0.3006	1934.0	35.00	0.44	tr_C9ZZQ4_
HLA-DQA10501-DQB10201	212	LQKHEVVAFGPMTGS	EVVAFGPMT	4	0.3003	1939.9	35.00	0.38	tr_C9ZZQ4_
HLA-DQA10501-DQB10201	213	SNNSGKEANAIVCAA	EANAIVCAA	6	0.2987	1973.4	35.00	0.68	tr_C9ZZQ4_
HLA-DQA10501-DQB10201	214	TFSRTGMLYVMYVLL	TGMLYVMYV	4	0.2972	2005.9	36.00	0.26	tr_C9ZZQ4_
HLA-DQA10501-DQB10201	215	EKKKRTVTAVFGIVT	VTAVFGIVT	6	0.2968	2015.4	36.00	0.43	tr_C9ZZQ4_
HLA-DQA10501-DQB10201	216	VALRVMEEMGYKLCG	VALRVMEEM	0	0.2964	2023.1	36.00	0.53	tr_C9ZZQ4_
HLA-DQA10501-DQB10201	217	WVRELYFLRPSPTIE	YFLRPSPTI	5	0.2954	2047.0	36.00	0.28	tr_C9ZZQ4_
HLA-DQA10501-DQB10201	218	EQYVAIKRFQEVMRE	KRFQEVMRE	6	0.2951	2053.2	36.00	0.55	tr_C9ZZQ4_
HLA-DQA10501-DQB10201	219	RRWVRELYFLRPSPT	RRWVRELYF	0	0.2948	2058.7	36.00	0.22	tr_C9ZZQ4_
HLA-DQA10501-DQB10201	220	SELQKHEVVAFGPMT	EVVAFGPMT	6	0.2947	2061.1	36.00	0.43	tr_C9ZZQ4_
HLA-DQA10501-DQB10201	221	SEVALLYEKFVTAFN	EVALLYEKF	1	0.2943	2069.5	37.00	0.32	tr_C9ZZQ4_
HLA-DQA10501-DQB10201	222	KTPEEFIKDELSKET	PEEFIKDEL	2	0.2941	2073.9	37.00	0.44	tr_C9ZZQ4_
HLA-DQA10501-DQB10201	223	HSPLNGLVIFFGDNT	LVIFFGDNT	6	0.2931	2096.9	37.00	0.40	tr_C9ZZQ4_
HLA-DQA10501-DQB10201	224	HLAAHSELRVLVLFS	HSELRVLVL	4	0.2915	2133.3	37.00	0.19	tr_C9ZZQ4_
HLA-DQA10501-DQB10201	225	DKPAPDKEFNAVFER	DKEFNAVFE	5	0.2898	2173.2	38.00	0.44	tr_C9ZZQ4_
HLA-DQA10501-DQB10201	226	KRFQEVMREYLKTHT	FQEVMREYL	2	0.2897	2176.2	38.00	0.56	tr_C9ZZQ4_
HLA-DQA10501-DQB10201	227	YFLKHDTDGEMMVYG	KHDTDGEMM	3	0.2889	2196.1	38.00	0.28	tr_C9ZZQ4_
HLA-DQA10501-DQB10201	228	MESRNWTIADRVKVQ	RNWTIADRV	3	0.2883	2209.2	38.00	0.60	tr_C9ZZQ4_
HLA-DQA10501-DQB10201	229	NGSAAAPRLVFATNL	PRLVFATNL	6	0.2876	2225.1	38.00	0.67	tr_C9ZZQ4_
HLA-DQA10501-DQB10201	230	TPEEFIKDELSKETD	PEEFIKDEL	1	0.2870	2240.0	38.00	0.39	tr_C9ZZQ4_

HLA-DQA10501-DQB10201	231	ERFAATKPQAVLLFG	FAATKPQAV	2	0.2869	2242.1	38.00	0.57	tr_C9ZZQ4_
HLA-DQA10501-DQB10201	232	KRTVTAVFGIVTKAL	VFGIVTKAL	6	0.2867	2248.4	38.00	0.37	tr_C9ZZQ4_
HLA-DQA10501-DQB10201	233	ASMESRNWTIADRVK	RNWTIADRV	5	0.2861	2262.9	39.00	0.75	tr_C9ZZQ4_
HLA-DQA10501-DQB10201	234	QLFLHAFGTTTSDSA	FGTTTSDSA	6	0.2857	2272.9	39.00	0.42	tr_C9ZZQ4_
HLA-DQA10501-DQB10201	235	VDVIERHLAAHSELR	RHLAAHSEL	5	0.2854	2278.8	39.00	0.50	tr_C9ZZQ4_
HLA-DQA10501-DQB10201	236	ELYFLRPSPTIETMV	YFLRPSPTI	2	0.2853	2282.3	39.00	0.25	tr_C9ZZQ4_
HLA-DQA10501-DQB10201	237	PFPGKLLIAGTNPLA	LLIAGTNPL	5	0.2842	2309.0	39.00	0.40	tr_C9ZZQ4_
HLA-DQA10501-DQB10201	238	NDKPAPDKEFNAVFE	DKEFNAVFE	6	0.2833	2332.1	39.00	0.57	tr_C9ZZQ4_
HLA-DQA10501-DQB10201	239	TTLDSISELQKHEVV	TLDSISELQ	1	0.2830	2338.7	39.00	0.36	tr_C9ZZQ4_
HLA-DQA10501-DQB10201	240	ESRNWTIADRVKVQV	RNWTIADRV	2	0.2830	2339.0	39.00	0.56	tr_C9ZZQ4_
HLA-DQA10501-DQB10201	241	FSRTGMLYVMYVLLL	TGMLYVMYV	3	0.2825	2352.2	39.00	0.28	tr_C9ZZQ4_
HLA-DQA10501-DQB10201	242	FVVLNVNDKPAPDKE	VVLNVNDKP	1	0.2821	2361.5	40.00	0.62	tr_C9ZZQ4_
HLA-DQA10501-DQB10201	243	FLKHDTDGEMMVYGW	KHDTDGEMM	2	0.2815	2376.8	40.00	0.22	tr_C9ZZQ4_
HLA-DQA10501-DQB10201	244	AAHSELRVLVLFSEV	RVLVLFSEV	6	0.2814	2381.0	40.00	0.25	tr_C9ZZQ4_
HLA-DQA10501-DQB10201	245	QKHEVVAFGPMTGSG	EVVAFGPMT	3	0.2809	2393.7	40.00	0.41	tr_C9ZZQ4_
HLA-DQA10501-DQB10201	246	TLHSPLNGLVIFFGD	PLNGLVIFF	4	0.2808	2394.9	40.00	0.34	tr_C9ZZQ4_
HLA-DQA10501-DQB10201	247	LAAHSELRVLVLFSE	LRVLVLFSE	6	0.2804	2405.4	40.00	0.21	tr_C9ZZQ4_
HLA-DQA10501-DQB10201	248	VRELYFLRPSPTIET	YFLRPSPTI	4	0.2798	2421.3	40.00	0.31	tr_C9ZZQ4_
HLA-DQA10501-DQB10201	249	TKALMKMTNTLFIDP	MTNTLFIDP	6	0.2789	2445.7	40.00	0.29	tr_C9ZZQ4_
HLA-DQA10501-DQB10201	250	VIFFGDNTIAVKATA	FFGDNTIAV	2	0.2775	2484.2	41.00	0.46	tr_C9ZZQ4_
HLA-DQA10501-DQB10201	251	TDKSGITIVFGPVGD	TIVFGPVGD	6	0.2774	2486.3	41.00	0.28	tr_C9ZZQ4_
HLA-DQA10501-DQB10201	252	NWTIADRVKVQVVQS	RVKVQVVQS	6	0.2773	2489.0	41.00	0.53	tr_C9ZZQ4_
HLA-DQA10501-DQB10201	253	RELYFLRPSPTIETM	YFLRPSPTI	3	0.2772	2490.6	41.00	0.31	tr_C9ZZQ4_
HLA-DQA10501-DQB10201	254	NAIVCAAEAHGIMKV	AIVCAAEAH	1	0.2769	2499.5	41.00	0.25	tr_C9ZZQ4_
HLA-DQA10501-DQB10201	255	SRNWTIADRVKVQVV	RNWTIADRV	1	0.2759	2527.2	41.00	0.44	tr_C9ZZQ4_
HLA-DQA10501-DQB10201	256	GVLRLGFMYLQGHHY	FMYLQGHHY	6	0.2758	2529.9	41.00	0.56	tr_C9ZZQ4_
HLA-DQA10501-DQB10201	257	KFVTAFNGSAAAPRL	NGSAAAPRL	6	0.2748	2557.7	41.00	0.29	tr_C9ZZQ4_
HLA-DQA10501-DQB10201	258	EVALLYEKFVTAFNG	VALLYEKFV	1	0.2738	2584.4	42.00	0.22	tr_C9ZZQ4_
HLA-DQA10501-DQB10201	259	THTSETGITDPNYFL	TGITDPNYF	5	0.2734	2595.3	42.00	0.44	tr_C9ZZQ4_
HLA-DQA10501-DQB10201	260	FGPVGDDTTLDSISE	PVGDDTTLD	2	0.2728	2613.6	42.00	0.24	tr_C9ZZQ4_
HLA-DQA10501-DQB10201	261	KHEVVAFGPMTGSGE	EVVAFGPMT	2	0.2701	2689.5	43.00	0.41	tr_C9ZZQ4_
HLA-DQA10501-DQB10201	262	KRVLLDSQVSPFPGK	LLDSQVSPF	3	0.2696	2704.2	43.00	0.31	tr_C9ZZQ4_
HLA-DQA10501-DQB10201	263	VTLHSPLNGLVIFFG	PLNGLVIFF	5	0.2692	2716.7	43.00	0.36	tr_C9ZZQ4_
HLA-DQA10501-DQB10201	264	TVTAVFGIVTKALMK	FGIVTKALM	5	0.2689	2724.8	43.00	0.31	tr_C9ZZQ4_
HLA-DQA10501-DQB10201	265	LYFLRPSPTIETMVL	YFLRPSPTI	1	0.2685	2737.8	43.00	0.23	tr_C9ZZQ4_
HLA-DQA10501-DQB10201	266	DTDGEMMVYGWAAGE	MVYGWAAGE	6	0.2684	2739.2	43.00	0.43	tr_C9ZZQ4_
HLA-DQA10501-DQB10201	267	VMEEMGYKLCGVFVV	EMGYKLCGV	3	0.2678	2756.5	43.00	0.34	tr_C9ZZQ4_
HLA-DQA10501-DQB10201	268	KKKRTVTAVFGIVTK	VTAVFGIVT	5	0.2668	2787.7	44.00	0.40	tr_C9ZZQ4_
HLA-DQA10501-DQB10201	269	FVTAFNGSAAAPRLV	NGSAAAPRL	5	0.2666	2793.7	44.00	0.26	tr_C9ZZQ4_
HLA-DQA10501-DQB10201	270	LFLHAFGTTTSDSAK	FGTTTSDSA	5	0.2664	2801.2	44.00	0.46	tr_C9ZZQ4_
HLA-DQA10501-DQB10201	271	IAVKATASWLVGALR	ASWLVGALR	6	0.2661	2809.5	44.00	0.39	tr_C9ZZQ4_
HLA-DQA10501-DQB10201	272	MEEMGYKLCGVFVVL	GYKLCGVFV	4	0.2655	2828.6	44.00	0.33	tr_C9ZZQ4_
HLA-DQA10501-DQB10201	273	MSCLRSISLSVPPHA	ISLSVPPHA	6	0.2650	2842.2	44.00	0.32	tr_C9ZZQ4_
HLA-DQA10501-DQB10201	274	SCLRSISLSVPPHAL	ISLSVPPHA	5	0.2639	2878.3	44.00	0.35	tr_C9ZZQ4_
HLA-DQA10501-DQB10201	275	KTHTSETGITDPNYF	TGITDPNYF	6	0.2637	2882.9	44.00	0.44	tr_C9ZZQ4_
HLA-DQA10501-DQB10201	276	FPGKLLIAGTNPLAK	LIAGTNPLA	5	0.2635	2888.7	44.00	0.28	tr_C9ZZQ4_
HLA-DQA10501-DQB10201	277	TDPNYFLKHDTDGEM	NYFLKHDTD	3	0.2631	2901.3	45.00	0.28	tr_C9ZZQ4_
HLA-DQA10501-DQB10201	278	KKRTVTAVFGIVTKA	VTAVFGIVT	4	0.2630	2905.2	45.00	0.34	tr_C9ZZQ4_
HLA-DQA10501-DQB10201	279	RVMEEMGYKLCGVFV	GYKLCGVFV	6	0.2630	2905.5	45.00	0.37	tr_C9ZZQ4_
HLA-DQA10501-DQB10201	280	VADRRTSGAYVLAPS	TSGAYVLAP	5	0.2627	2913.0	45.00	0.23	tr_C9ZZQ4_

HLA-DQA10501-DQB10201	281	WLVGALRHDTGSLEY	RHDTGSLEY	6	0.2625	2921.1	45.00	0.43	tr_C9ZZQ4_
HLA-DQA10501-DQB10201	282	FTTAVNAGFNASMES	AGFNASMES	6	0.2623	2925.6	45.00	0.33	tr_C9ZZQ4_
HLA-DQA10501-DQB10201	283	ATASWLVGALRHDTG	ASWLVGALR	2	0.2623	2928.6	45.00	0.28	tr_C9ZZQ4_
HLA-DQA10501-DQB10201	284	WTIADRVKVQVVQSS	RVKVQVVQS	5	0.2622	2930.9	45.00	0.49	tr_C9ZZQ4_
HLA-DQA10501-DQB10201	285	LHSPLNGLVIFFGDN	PLNGLVIFF	3	0.2619	2940.9	45.00	0.39	tr_C9ZZQ4_
HLA-DQA10501-DQB10201	286	RFAATKPQAVLLFGA	FAATKPQAV	1	0.2618	2943.9	45.00	0.52	tr_C9ZZQ4_
HLA-DQA10501-DQB10201	287	EEMGYKLCGVFVVLN	GYKLCGVFV	3	0.2614	2954.4	45.00	0.28	tr_C9ZZQ4_
HLA-DQA10501-DQB10201	288	SASDVDVIERHLAAH	ASDVDVIER	1	0.2604	2987.6	45.00	0.37	tr_C9ZZQ4_
HLA-DQA10501-DQB10201	289	FNAVFERFAATKPQA	FNAVFERFA	0	0.2604	2988.9	45.00	0.26	tr_C9ZZQ4_
HLA-DQA10501-DQB10201	290	VLRLGFMYLQGHHYG	FMYLQGHHY	5	0.2601	2998.8	45.00	0.54	tr_C9ZZQ4_
HLA-DQA10501-DQB10201	291	VKATASWLVGALRHD	ASWLVGALR	4	0.2598	3007.8	46.00	0.32	tr_C9ZZQ4_
HLA-DQA10501-DQB10201	292	RVLLDSQVSPFPGKL	LLDSQVSPF	2	0.2593	3024.5	46.00	0.34	tr_C9ZZQ4_
HLA-DQA10501-DQB10201	293	VLIRHALGHLGVLRL	LGHLGVLRL	6	0.2590	3034.8	46.00	0.40	tr_C9ZZQ4_
HLA-DQA10501-DQB10201	294	RHALGHLGVLRLGFM	LGVLRLGFM	6	0.2578	3074.7	46.00	0.34	tr_C9ZZQ4_
HLA-DQA10501-DQB10201	295	AVKATASWLVGALRH	ASWLVGALR	5	0.2575	3084.1	46.00	0.29	tr_C9ZZQ4_
HLA-DQA10501-DQB10201	296	EMGYKLCGVFVVLNV	GYKLCGVFV	2	0.2565	3117.5	46.00	0.26	tr_C9ZZQ4_
HLA-DQA10501-DQB10201	297	IFTTAVNAGFNASME	NAGFNASME	6	0.2557	3142.9	47.00	0.38	tr_C9ZZQ4_
HLA-DQA10501-DQB10201	298	VTAFNGSAAAPRLVF	NGSAAAPRL	4	0.2557	3145.3	47.00	0.35	tr_C9ZZQ4_
HLA-DQA10501-DQB10201	299	IHLSPTVEQQIYVLT	TVEQQIYVL	5	0.2553	3159.0	47.00	0.26	tr_C9ZZQ4_
HLA-DQA10501-DQB10201	300	KATASWLVGALRHDT	ASWLVGALR	3	0.2550	3167.4	47.00	0.34	tr_C9ZZQ4_
HLA-DQA10501-DQB10201	301	SVTLHSPLNGLVIFF	PLNGLVIFF	6	0.2545	3184.0	47.00	0.54	tr_C9ZZQ4_
HLA-DQA10501-DQB10201	302	QGHHYGEKEYEVALR	YGEKEYEVA	4	0.2542	3194.4	47.00	0.26	tr_C9ZZQ4_
HLA-DQA10501-DQB10201	303	YFLRPSPTIETMVLI	PTIETMVLI	6	0.2538	3208.1	47.00	0.20	tr_C9ZZQ4_
HLA-DQA10501-DQB10201	304	HEVVAFGPMTGSGEV	EVVAFGPMT	1	0.2536	3216.3	47.00	0.42	tr_C9ZZQ4_
HLA-DQA10501-DQB10201	305	VMYVLLLLMPYPLQV	LLMPYPLQV	6	0.2535	3219.0	47.00	0.32	tr_C9ZZQ4_
HLA-DQA10501-DQB10201	306	RFQEVMREYLKTHTS	FQEVMREYL	1	0.2533	3228.1	47.00	0.69	tr_C9ZZQ4_
HLA-DQA10501-DQB10201	307	TRVPKIFTTAVNAGF	FTTAVNAGF	6	0.2517	3284.2	48.00	0.53	tr_C9ZZQ4_
HLA-DQA10501-DQB10201	308	VPKIFTTAVNAGFNA	FTTAVNAGF	4	0.2512	3301.7	48.00	0.46	tr_C9ZZQ4_
HLA-DQA10501-DQB10201	309	LQGHHYGEKEYEVAL	YGEKEYEVA	5	0.2510	3308.5	48.00	0.34	tr_C9ZZQ4_
HLA-DQA10501-DQB10201	310	SRTGMLYVMYVLLLL	TGMLYVMYV	2	0.2509	3310.3	48.00	0.29	tr_C9ZZQ4_
HLA-DQA10501-DQB10201	311	FLHAFGTTTSDSAKA	FGTTTSDSA	4	0.2507	3317.8	48.00	0.51	tr_C9ZZQ4_
HLA-DQA10501-DQB10201	312	LRLGFMYLQGHHYGE	FMYLQGHHY	4	0.2505	3325.7	48.00	0.57	tr_C9ZZQ4_
HLA-DQA10501-DQB10201	313	LVADRRTSGAYVLAP	TSGAYVLAP	6	0.2503	3332.4	48.00	0.31	tr_C9ZZQ4_
HLA-DQA10501-DQB10201	314	GAQVFLELMWKRVLL	GAQVFLELM	0	0.2500	3345.5	48.00	0.45	tr_C9ZZQ4_
HLA-DQA10501-DQB10201	315	SGAYVLAPSGAQVFL	LAPSGAQVF	5	0.2498	3350.2	48.00	0.34	tr_C9ZZQ4_
HLA-DQA10501-DQB10201	316	GDNTIAVKATASWLV	AVKATASWL	5	0.2497	3355.3	48.00	0.34	tr_C9ZZQ4_
HLA-DQA10501-DQB10201	317	PGKLLIAGTNPLAKN	LIAGTNPLA	4	0.2495	3360.4	48.00	0.32	tr_C9ZZQ4_
HLA-DQA10501-DQB10201	318	TGHLPTSGAVFVFGI	TSGAVFVFG	5	0.2493	3368.6	48.00	0.27	tr_C9ZZQ4_
HLA-DQA10501-DQB10201	319	AIVCAAEAHGIMKVI	AIVCAAEAH	0	0.2482	3410.9	49.00	0.25	tr_C9ZZQ4_
HLA-DQA10501-DQB10201	320	SDTGRFLRKLVADRR	LRKLVADRR	6	0.2481	3412.5	49.00	0.56	tr_C9ZZQ4_
HLA-DQA10501-DQB10201	321	RVPKIFTTAVNAGFN	FTTAVNAGF	5	0.2477	3429.8	49.00	0.45	tr_C9ZZQ4_
HLA-DQA10501-DQB10201	322	CLRSISLSVPPHALS	ISLSVPPHA	4	0.2470	3452.9	49.00	0.38	tr_C9ZZQ4_
HLA-DQA10501-DQB10201	323	YEKFVTAFNGSAAAP	VTAFNGSAA	4	0.2465	3471.3	49.00	0.25	tr_C9ZZQ4_
HLA-DQA10501-DQB10201	324	LSPTVEQQIYVLTRY	QQIYVLTRY	6	0.2465	3472.8	49.00	0.26	tr_C9ZZQ4_
HLA-DQA10501-DQB10201	325	YLQGHHYGEKEYEVA	YGEKEYEVA	6	0.2464	3475.5	49.00	0.31	tr_C9ZZQ4_
HLA-DQA10501-DQB10201	326	DTGRFLRKLVADRRT	LRKLVADRR	5	0.2464	3477.0	49.00	0.51	tr_C9ZZQ4_
HLA-DQA10501-DQB10201	327	LKHDTDGEMMVYGWA	KHDTDGEMM	1	0.2458	3498.8	49.00	0.22	tr_C9ZZQ4_
HLA-DQA10501-DQB10201	328	HALSTFSRTGMLYVM	FSRTGMLYV	5	0.2453	3517.9	49.00	0.47	tr_C9ZZQ4_
HLA-DQA10501-DQB10201	329	KHDTDGEMMVYGWAA	EMMVYGWAA	6	0.2451	3527.6	50.00	0.26	tr_C9ZZQ4_
HLA-DQA10501-DQB10201	330	PEEFIKDELSKETDK	EEFIKDELS	1	0.2444	3553.6	50.00	0.44	tr_C9ZZQ4_

HLA-DQA10501-DQB10201	331	RTTFMDSLYNQRRYV	FMDSLYNQR	3	0.2437	3580.0	50.00	0.28	tr_C9ZZQ4_
HLA-DQA10501-DQB10201	332	VTAVFGIVTKALMKM	FGIVTKALM	4	0.2436	3583.8	50.00	0.38	tr_C9ZZQ4_
HLA-DQA10501-DQB10201	333	IFFGDNTIAVKATAS	FFGDNTIAV	1	0.2432	3600.1	50.00	0.44	tr_C9ZZQ4_
HLA-DQA10501-DQB10201	334	ADRRTSGAYVLAPSG	RRTSGAYVL	2	0.2429	3609.1	50.00	0.22	tr_C9ZZQ4_
HLA-DQA10501-DQB10201	335	GKLLIAGTNPLAKNE	LIAGTNPLA	3	0.2429	3611.1	50.00	0.34	tr_C9ZZQ4_
HLA-DQA10501-DQB10201	336	SPFPGKLLIAGTNPL	LLIAGTNPL	6	0.2427	3617.0	50.00	0.51	tr_C9ZZQ4_
HLA-DQA10501-DQB10201	337	TASWLVGALRHDTGS	ASWLVGALR	1	0.2425	3625.7	50.00	0.26	tr_C9ZZQ4_
HLA-DQA10501-DQB10201	338	ALRVMEEMGYKLCGV	EMGYKLCGV	6	0.2420	3646.7	50.00	0.42	tr_C9ZZQ4_
HLA-DQA10501-DQB10201	339	TSGAYVLAPSGAQVF	LAPSGAQVF	6	0.2416	3662.5	55.00	0.33	tr_C9ZZQ4_
HLA-DQA10501-DQB10201	340	PHALSTFSRTGMLYV	FSRTGMLYV	6	0.2413	3674.7	55.00	0.54	tr_C9ZZQ4_
HLA-DQA10501-DQB10201	341	EKFVTAFNGSAAAPR	AFNGSAAAP	5	0.2412	3677.0	55.00	0.27	tr_C9ZZQ4_
HLA-DQA10501-DQB10201	342	HTSETGITDPNYFLK	TGITDPNYF	4	0.2411	3680.6	55.00	0.45	tr_C9ZZQ4_
HLA-DQA10501-DQB10201	343	ISELQKHEVVAFGPM	KHEVVAFGP	5	0.2409	3687.8	55.00	0.32	tr_C9ZZQ4_
HLA-DQA10501-DQB10201	344	IDPITIEPQLNKFRR	PITIEPQLN	2	0.2409	3687.8	55.00	0.44	tr_C9ZZQ4_
HLA-DQA10501-DQB10201	345	ETDKSGITIVFGPVG	KSGITIVFG	3	0.2409	3690.2	55.00	0.32	tr_C9ZZQ4_
HLA-DQA10501-DQB10201	346	YKLCGVFVVLNVNDK	FVVLNVNDK	6	0.2404	3708.2	55.00	0.33	tr_C9ZZQ4_
HLA-DQA10501-DQB10201	347	KIFTTAVNAGFNASM	FTTAVNAGF	2	0.2401	3722.8	55.00	0.38	tr_C9ZZQ4_
HLA-DQA10501-DQB10201	348	MYVLLLLMPYPLQVH	LLMPYPLQV	5	0.2398	3735.6	55.00	0.31	tr_C9ZZQ4_
HLA-DQA10501-DQB10201	349	HALGHLGVLRLGFMY	LGVLRLGFM	5	0.2396	3741.3	55.00	0.36	tr_C9ZZQ4_
HLA-DQA10501-DQB10201	350	LVGALRHDTGSLEYS	RHDTGSLEY	5	0.2396	3741.4	55.00	0.41	tr_C9ZZQ4_
HLA-DQA10501-DQB10201	351	GDFGGDCEGEAAKQG	FGGDCEGEA	2	0.2394	3748.2	55.00	0.43	tr_C9ZZQ4_
HLA-DQA10501-DQB10201	352	TAFNGSAAAPRLVFA	NGSAAAPRL	3	0.2390	3765.4	55.00	0.33	tr_C9ZZQ4_
HLA-DQA10501-DQB10201	353	PKIFTTAVNAGFNAS	FTTAVNAGF	3	0.2389	3769.9	55.00	0.44	tr_C9ZZQ4_
HLA-DQA10501-DQB10201	354	FATNLPHWYDNETSS	FATNLPHWY	0	0.2367	3861.1	55.00	0.46	tr_C9ZZQ4_
HLA-DQA10501-DQB10201	355	LEYSGQLFLHAFGTT	LEYSGQLFL	0	0.2365	3868.5	55.00	0.32	tr_C9ZZQ4_
HLA-DQA10501-DQB10201	356	SPTVEQQIYVLTRYL	TVEQQIYVL	2	0.2364	3873.6	55.00	0.22	tr_C9ZZQ4_
HLA-DQA10501-DQB10201	357	DRRTSGAYVLAPSGA	AYVLAPSGA	6	0.2359	3894.7	55.00	0.18	tr_C9ZZQ4_
HLA-DQA10501-DQB10201	358	HLSPTVEQQIYVLTR	TVEQQIYVL	4	0.2354	3915.0	55.00	0.25	tr_C9ZZQ4_
HLA-DQA10501-DQB10201	359	TIAVKATASWLVGAL	AVKATASWL	2	0.2354	3916.5	55.00	0.28	tr_C9ZZQ4_
HLA-DQA10501-DQB10201	360	HLPTSGAVFVFGISA	TSGAVFVFG	3	0.2353	3919.1	55.00	0.21	tr_C9ZZQ4_
HLA-DQA10501-DQB10201	361	EEKKKRTVTAVFGIV	KRTVTAVFG	4	0.2352	3923.5	55.00	0.45	tr_C9ZZQ4_
HLA-DQA10501-DQB10201	362	GYKLCGVFVVLNVND	VFVVLNVND	6	0.2348	3943.4	55.00	0.26	tr_C9ZZQ4_
HLA-DQA10501-DQB10201	363	GHLGVLRLGFMYLQG	RLGFMYLQG	6	0.2345	3953.6	55.00	0.27	tr_C9ZZQ4_
HLA-DQA10501-DQB10201	364	LNKFRRHVIHLSPTV	HVIHLSPTV	6	0.2340	3973.7	55.00	0.37	tr_C9ZZQ4_
HLA-DQA10501-DQB10201	365	GHLPTSGAVFVFGIS	TSGAVFVFG	4	0.2340	3974.6	55.00	0.25	tr_C9ZZQ4_
HLA-DQA10501-DQB10201	366	LHAFGTTTSDSAKAL	FGTTTSDSA	3	0.2339	3978.2	55.00	0.68	tr_C9ZZQ4_
HLA-DQA10501-DQB10201	367	RNWTIADRVKVQVVQ	RNWTIADRV	0	0.2334	3999.6	55.00	0.35	tr_C9ZZQ4_
HLA-DQA10501-DQB10201	368	IRHALGHLGVLRLGF	LGHLGVLRL	4	0.2330	4018.7	55.00	0.38	tr_C9ZZQ4_
HLA-DQA10501-DQB10201	369	DNTIAVKATASWLVG	AVKATASWL	4	0.2325	4039.4	55.00	0.35	tr_C9ZZQ4_
HLA-DQA10501-DQB10201	370	EVVAFGPMTGSGEVR	EVVAFGPMT	0	0.2324	4046.6	55.00	0.34	tr_C9ZZQ4_
HLA-DQA10501-DQB10201	371	FGDNTIAVKATASWL	AVKATASWL	6	0.2321	4060.4	55.00	0.37	tr_C9ZZQ4_
HLA-DQA10501-DQB10201	372	TGRFLRKLVADRRTS	LRKLVADRR	4	0.2320	4061.6	55.00	0.49	tr_C9ZZQ4_
HLA-DQA10501-DQB10201	373	REVEDDFSMQTPKDG	VEDDFSMQT	2	0.2319	4065.2	55.00	0.32	tr_C9ZZQ4_
HLA-DQA10501-DQB10201	374	LLYEKFVTAFNGSAA	VTAFNGSAA	6	0.2316	4079.5	55.00	0.23	tr_C9ZZQ4_
HLA-DQA10501-DQB10201	375	ALGHLGVLRLGFMYL	LGVLRLGFM	4	0.2315	4086.7	55.00	0.39	tr_C9ZZQ4_
HLA-DQA10501-DQB10201	376	QTPKDGTVGLAALRC	TVGLAALRC	6	0.2312	4097.8	55.00	0.57	tr_C9ZZQ4_
HLA-DQA10501-DQB10201	377	GQLFLHAFGTTTSDS	FLHAFGTTT	3	0.2311	4102.4	55.00	0.38	tr_C9ZZQ4_
HLA-DQA10501-DQB10201	378	TTAVNAGFNASMESR	AGFNASMES	5	0.2310	4106.0	55.00	0.31	tr_C9ZZQ4_
HLA-DQA10501-DQB10201	379	KEEKKKRTVTAVFGI	KRTVTAVFG	5	0.2310	4108.8	55.00	0.50	tr_C9ZZQ4_
HLA-DQA10501-DQB10201	380	HLGVLRLGFMYLQGH	RLGFMYLQG	5	0.2307	4120.7	55.00	0.22	tr_C9ZZQ4_

HLA-DQA10501-DQB10201	381	EYSGQLFLHAFGTTT	FLHAFGTTT	6	0.2301	4145.1	55.00	0.44	tr_C9ZZQ4_
HLA-DQA10501-DQB10201	382	LRVMEEMGYKLCGVF	EMGYKLCGV	5	0.2300	4149.7	55.00	0.46	tr_C9ZZQ4_
HLA-DQA10501-DQB10201	383	KLLIAGTNPLAKNEQ	LIAGTNPLA	2	0.2297	4163.3	55.00	0.34	tr_C9ZZQ4_
HLA-DQA10501-DQB10201	384	LYVMYVLLLLMPYPL	LLLLMPYPL	6	0.2297	4166.3	55.00	0.33	tr_C9ZZQ4_
HLA-DQA10501-DQB10201	385	CYCNQGGNVVYVREV	GGNVVYVRE	5	0.2296	4171.7	55.00	0.41	tr_C9ZZQ4_
HLA-DQA10501-DQB10201	386	LTGHLPTSGAVFVFG	TSGAVFVFG	6	0.2288	4206.8	55.00	0.37	tr_C9ZZQ4_
HLA-DQA10501-DQB10201	387	VALLYEKFVTAFNGS	LYEKFVTAF	3	0.2282	4233.9	55.00	0.31	tr_C9ZZQ4_
HLA-DQA10501-DQB10201	388	SGQLFLHAFGTTTSD	FLHAFGTTT	4	0.2280	4242.7	55.00	0.44	tr_C9ZZQ4_
HLA-DQA10501-DQB10201	389	AVNAGFNASMESRNW	AGFNASMES	3	0.2273	4274.4	55.00	0.29	tr_C9ZZQ4_
HLA-DQA10501-DQB10201	390	KETDKSGITIVFGPV	KSGITIVFG	4	0.2272	4281.5	55.00	0.51	tr_C9ZZQ4_
HLA-DQA10501-DQB10201	391	SISELQKHEVVAFGP	KHEVVAFGP	6	0.2266	4309.4	55.00	0.35	tr_C9ZZQ4_
HLA-DQA10501-DQB10201	392	LRSISLSVPPHALST	ISLSVPPHA	3	0.2265	4311.5	55.00	0.45	tr_C9ZZQ4_
HLA-DQA10501-DQB10201	393	TAVNAGFNASMESRN	AGFNASMES	4	0.2261	4330.6	55.00	0.26	tr_C9ZZQ4_
HLA-DQA10501-DQB10201	394	LIRHALGHLGVLRLG	LGHLGVLRL	5	0.2257	4347.6	55.00	0.37	tr_C9ZZQ4_
HLA-DQA10501-DQB10201	395	GTVGLAALRCNADSV	TVGLAALRC	1	0.2256	4351.7	55.00	0.36	tr_C9ZZQ4_
HLA-DQA10501-DQB10201	396	LYEKFVTAFNGSAAA	VTAFNGSAA	5	0.2255	4357.7	55.00	0.25	tr_C9ZZQ4_
HLA-DQA10501-DQB10201	397	YSGQLFLHAFGTTTS	FLHAFGTTT	5	0.2244	4411.3	55.00	0.41	tr_C9ZZQ4_
HLA-DQA10501-DQB10201	398	NTIAVKATASWLVGA	AVKATASWL	3	0.2234	4459.3	60.00	0.35	tr_C9ZZQ4_
HLA-DQA10501-DQB10201	399	TSETGITDPNYFLKH	TGITDPNYF	3	0.2230	4479.5	60.00	0.45	tr_C9ZZQ4_
HLA-DQA10501-DQB10201	400	YVLLLLMPYPLQVHG	LLMPYPLQV	4	0.2227	4493.8	60.00	0.29	tr_C9ZZQ4_
HLA-DQA10501-DQB10201	401	VLLDSQVSPFPGKLL	LLDSQVSPF	1	0.2219	4530.2	60.00	0.41	tr_C9ZZQ4_
HLA-DQA10501-DQB10201	402	AQVFLELMWKRVLLD	LMWKRVLLD	6	0.2218	4538.2	60.00	0.31	tr_C9ZZQ4_
HLA-DQA10501-DQB10201	403	FMYLQGHHYGEKEYE	FMYLQGHHY	0	0.2214	4556.8	60.00	0.32	tr_C9ZZQ4_
HLA-DQA10501-DQB10201	404	VTKALMKMTNTLFID	LMKMTNTLF	4	0.2213	4558.9	60.00	0.38	tr_C9ZZQ4_
HLA-DQA10501-DQB10201	405	YVMYVLLLLMPYPLQ	LLLLMPYPL	5	0.2212	4566.2	60.00	0.30	tr_C9ZZQ4_
HLA-DQA10501-DQB10201	406	ALLYEKFVTAFNGSA	LYEKFVTAF	2	0.2209	4580.5	60.00	0.25	tr_C9ZZQ4_
HLA-DQA10501-DQB10201	407	LPTSGAVFVFGISAS	TSGAVFVFG	2	0.2206	4598.4	60.00	0.22	tr_C9ZZQ4_
HLA-DQA10501-DQB10201	408	SKETDKSGITIVFGP	KSGITIVFG	5	0.2203	4608.7	60.00	0.56	tr_C9ZZQ4_
HLA-DQA10501-DQB10201	409	TPKDGTVGLAALRCN	TVGLAALRC	5	0.2200	4628.3	60.00	0.52	tr_C9ZZQ4_
HLA-DQA10501-DQB10201	410	FFGDNTIAVKATASW	FFGDNTIAV	0	0.2198	4633.9	60.00	0.25	tr_C9ZZQ4_
HLA-DQA10501-DQB10201	411	LGHLGVLRLGFMYLQ	LGVLRLGFM	3	0.2197	4642.6	60.00	0.41	tr_C9ZZQ4_
HLA-DQA10501-DQB10201	412	TIADRVKVQVVQSSK	RVKVQVVQS	4	0.2195	4649.0	60.00	0.55	tr_C9ZZQ4_
HLA-DQA10501-DQB10201	413	FAATKPQAVLLFGAP	FAATKPQAV	0	0.2189	4680.8	60.00	0.40	tr_C9ZZQ4_
HLA-DQA10501-DQB10201	414	TLDSISELQKHEVVA	TLDSISELQ	0	0.2182	4718.2	60.00	0.42	tr_C9ZZQ4_
HLA-DQA10501-DQB10201	415	LGFMYLQGHHYGEKE	FMYLQGHHY	2	0.2179	4731.0	60.00	0.60	tr_C9ZZQ4_
HLA-DQA10501-DQB10201	416	MGYKLCGVFVVLNVN	GYKLCGVFV	1	0.2177	4743.8	60.00	0.25	tr_C9ZZQ4_
HLA-DQA10501-DQB10201	417	RLGFMYLQGHHYGEK	FMYLQGHHY	3	0.2174	4756.0	60.00	0.60	tr_C9ZZQ4_
HLA-DQA10501-DQB10201	418	MPYPLQVHGAPANIN	QVHGAPANI	5	0.2174	4758.0	60.00	0.37	tr_C9ZZQ4_
HLA-DQA10501-DQB10201	419	MYLQGHHYGEKEYEV	HHYGEKEYE	5	0.2172	4765.9	60.00	0.34	tr_C9ZZQ4_
HLA-DQA10501-DQB10201	420	RRTSGAYVLAPSGAQ	AYVLAPSGA	5	0.2172	4769.3	60.00	0.19	tr_C9ZZQ4_
HLA-DQA10501-DQB10201	421	KDGTVGLAALRCNAD	TVGLAALRC	3	0.2164	4811.5	60.00	0.47	tr_C9ZZQ4_
HLA-DQA10501-DQB10201	422	GFMYLQGHHYGEKEY	FMYLQGHHY	1	0.2161	4823.9	60.00	0.51	tr_C9ZZQ4_
HLA-DQA10501-DQB10201	423	RTGMLYVMYVLLLLM	TGMLYVMYV	1	0.2160	4831.7	60.00	0.31	tr_C9ZZQ4_
HLA-DQA10501-DQB10201	424	ITDPNYFLKHDTDGE	NYFLKHDTD	4	0.2159	4835.4	60.00	0.39	tr_C9ZZQ4_
HLA-DQA10501-DQB10201	425	TVGLAALRCNADSVT	ALRCNADSV	5	0.2155	4855.7	60.00	0.29	tr_C9ZZQ4_
HLA-DQA10501-DQB10201	426	HAFGTTTSDSAKALK	FGTTTSDSA	2	0.2155	4856.5	60.00	0.72	tr_C9ZZQ4_
HLA-DQA10501-DQB10201	427	DFGGDCEGEAAKQGA	FGGDCEGEA	1	0.2154	4862.2	60.00	0.41	tr_C9ZZQ4_
HLA-DQA10501-DQB10201	428	HDTDGEMMVYGWAAG	EMMVYGWAA	5	0.2153	4865.3	60.00	0.26	tr_C9ZZQ4_
HLA-DQA10501-DQB10201	429	PTVEQQIYVLTRYLS	TVEQQIYVL	1	0.2153	4865.5	60.00	0.19	tr_C9ZZQ4_
HLA-DQA10501-DQB10201	430	TAVFGIVTKALMKMT	FGIVTKALM	3	0.2151	4876.5	60.00	0.48	tr_C9ZZQ4_

HLA-DQA10501-DQB10201	431	AFNGSAAAPRLVFAT	NGSAAAPRL	2	0.2151	4880.3	60.00	0.34	tr_C9ZZQ4_
HLA-DQA10501-DQB10201	432	VGALRHDTGSLEYSG	RHDTGSLEY	4	0.2148	4894.5	60.00	0.44	tr_C9ZZQ4_
HLA-DQA10501-DQB10201	433	GRFLRKLVADRRTSG	LRKLVADRR	3	0.2147	4899.0	60.00	0.50	tr_C9ZZQ4_
HLA-DQA10501-DQB10201	434	SWLVGALRHDTGSLE	LRHDTGSLE	6	0.2145	4909.4	60.00	0.33	tr_C9ZZQ4_
HLA-DQA10501-DQB10201	435	PYPLQVHGAPANINV	QVHGAPANI	4	0.2144	4915.9	60.00	0.37	tr_C9ZZQ4_
HLA-DQA10501-DQB10201	436	RTSGAYVLAPSGAQV	AYVLAPSGA	4	0.2137	4952.1	60.00	0.21	tr_C9ZZQ4_
HLA-DQA10501-DQB10201	437	SSPTIRKFHATVESR	KFHATVESR	6	0.2129	4996.8	60.00	0.52	tr_C9ZZQ4_
HLA-DQA10501-DQB10201	438	KNEQYVAIKRFQEVM	AIKRFQEVM	6	0.2126	5011.5	60.00	0.32	tr_C9ZZQ4_
HLA-DQA10501-DQB10201	439	SDVDVIERHLAAHSE	ERHLAAHSE	6	0.2124	5021.3	60.00	0.57	tr_C9ZZQ4_
HLA-DQA10501-DQB10201	440	ASWLVGALRHDTGSL	ASWLVGALR	0	0.2124	5021.4	60.00	0.17	tr_C9ZZQ4_
HLA-DQA10501-DQB10201	441	MLYVMYVLLLLMPYP	VLLLLMPYP	6	0.2116	5068.2	60.00	0.29	tr_C9ZZQ4_
HLA-DQA10501-DQB10201	442	QVFLELMWKRVLLDS	LMWKRVLLD	5	0.2107	5117.9	60.00	0.31	tr_C9ZZQ4_
HLA-DQA10501-DQB10201	443	DGTVGLAALRCNADS	TVGLAALRC	2	0.2100	5152.7	60.00	0.46	tr_C9ZZQ4_
HLA-DQA10501-DQB10201	444	PKDGTVGLAALRCNA	TVGLAALRC	4	0.2098	5163.3	60.00	0.47	tr_C9ZZQ4_
HLA-DQA10501-DQB10201	445	VNAGFNASMESRNWT	AGFNASMES	2	0.2098	5163.5	60.00	0.32	tr_C9ZZQ4_
HLA-DQA10501-DQB10201	446	IVCAAEAHGIMKVIR	EAHGIMKVI	5	0.2098	5164.2	60.00	0.26	tr_C9ZZQ4_
HLA-DQA10501-DQB10201	447	SETGITDPNYFLKHD	TGITDPNYF	2	0.2096	5178.7	60.00	0.49	tr_C9ZZQ4_
HLA-DQA10501-DQB10201	448	KVLLCTWNTRVPKIF	WNTRVPKIF	6	0.2092	5199.8	60.00	0.53	tr_C9ZZQ4_
HLA-DQA10501-DQB10201	449	LLIAGTNPLAKNEQY	LIAGTNPLA	1	0.2089	5215.6	60.00	0.38	tr_C9ZZQ4_
HLA-DQA10501-DQB10201	450	LSVPEWLKDRTTFMD	WLKDRTTFM	5	0.2088	5220.2	60.00	0.67	tr_C9ZZQ4_
HLA-DQA10501-DQB10201	451	LLLLMPYPLQVHGAP	LLMPYPLQV	2	0.2088	5223.6	60.00	0.30	tr_C9ZZQ4_
HLA-DQA10501-DQB10201	452	VLLLLMPYPLQVHGA	LLMPYPLQV	3	0.2080	5269.0	60.00	0.29	tr_C9ZZQ4_
HLA-DQA10501-DQB10201	453	LSKETDKSGITIVFG	KSGITIVFG	6	0.2076	5292.6	60.00	0.64	tr_C9ZZQ4_
HLA-DQA10501-DQB10201	454	FLRPSPTIETMVLIR	PTIETMVLI	5	0.2072	5315.7	60.00	0.24	tr_C9ZZQ4_
HLA-DQA10501-DQB10201	455	VLLCTWNTRVPKIFT	WNTRVPKIF	5	0.2065	5353.7	65.00	0.56	tr_C9ZZQ4_
HLA-DQA10501-DQB10201	456	VCYCNQGGNVVYVRE	GGNVVYVRE	6	0.2065	5354.5	65.00	0.54	tr_C9ZZQ4_
HLA-DQA10501-DQB10201	457	MTGSGEVRRWVRELY	VRRWVRELY	6	0.2063	5363.5	65.00	0.65	tr_C9ZZQ4_
HLA-DQA10501-DQB10201	458	SVPEWLKDRTTFMDS	WLKDRTTFM	4	0.2061	5375.3	65.00	0.64	tr_C9ZZQ4_
HLA-DQA10501-DQB10201	459	FQEVMREYLKTHTSE	FQEVMREYL	0	0.2061	5378.5	65.00	0.61	tr_C9ZZQ4_
HLA-DQA10501-DQB10201	460	LKEEKKKRTVTAVFG	KRTVTAVFG	6	0.2057	5402.1	65.00	0.69	tr_C9ZZQ4_
HLA-DQA10501-DQB10201	461	IADRVKVQVVQSSKS	RVKVQVVQS	3	0.2054	5420.3	65.00	0.56	tr_C9ZZQ4_
HLA-DQA10501-DQB10201	462	LGVLRLGFMYLQGHH	RLGFMYLQG	4	0.2046	5461.7	65.00	0.26	tr_C9ZZQ4_
HLA-DQA10501-DQB10201	463	RSISLSVPPHALSTF	ISLSVPPHA	2	0.2046	5466.9	65.00	0.51	tr_C9ZZQ4_
HLA-DQA10501-DQB10201	464	TGITDPNYFLKHDTD	NYFLKHDTD	6	0.2045	5468.9	65.00	0.47	tr_C9ZZQ4_
HLA-DQA10501-DQB10201	465	NPLAKNEQYVAIKRF	EQYVAIKRF	6	0.2045	5472.1	65.00	0.58	tr_C9ZZQ4_
HLA-DQA10501-DQB10201	466	LMPYPLQVHGAPANI	QVHGAPANI	6	0.2039	5503.7	65.00	0.41	tr_C9ZZQ4_
HLA-DQA10501-DQB10201	467	IVTKALMKMTNTLFI	LMKMTNTLF	5	0.2031	5552.9	65.00	0.46	tr_C9ZZQ4_
HLA-DQA10501-DQB10201	468	KLVADRRTSGAYVLA	RRTSGAYVL	5	0.2029	5567.6	65.00	0.41	tr_C9ZZQ4_
HLA-DQA10501-DQB10201	469	AVFGIVTKALMKMTN	FGIVTKALM	2	0.2028	5571.1	65.00	0.50	tr_C9ZZQ4_
HLA-DQA10501-DQB10201	470	LLLMPYPLQVHGAPA	LLMPYPLQV	1	0.2024	5598.7	65.00	0.28	tr_C9ZZQ4_
HLA-DQA10501-DQB10201	471	ALTGHLPTSGAVFVF	PTSGAVFVF	6	0.2023	5599.7	65.00	0.37	tr_C9ZZQ4_
HLA-DQA10501-DQB10201	472	VGLAALRCNADSVTL	ALRCNADSV	4	0.2022	5609.3	65.00	0.36	tr_C9ZZQ4_
HLA-DQA10501-DQB10201	473	LKTHTSETGITDPNY	ETGITDPNY	6	0.2018	5634.2	65.00	0.62	tr_C9ZZQ4_
HLA-DQA10501-DQB10201	474	ATNLPHWYDNETSSP	WYDNETSSP	6	0.2013	5665.4	65.00	0.31	tr_C9ZZQ4_
HLA-DQA10501-DQB10201	475	EVEDDFSMQTPKDGT	VEDDFSMQT	1	0.2011	5674.4	65.00	0.36	tr_C9ZZQ4_
HLA-DQA10501-DQB10201	476	LLCTWNTRVPKIFTT	WNTRVPKIF	4	0.2003	5727.9	65.00	0.58	tr_C9ZZQ4_
HLA-DQA10501-DQB10201	477	RFLRKLVADRRTSGA	LRKLVADRR	2	0.2001	5735.3	65.00	0.55	tr_C9ZZQ4_
HLA-DQA10501-DQB10201	478	LDSISELQKHEVVAF	LQKHEVVAF	6	0.1997	5760.7	65.00	0.35	tr_C9ZZQ4_
HLA-DQA10501-DQB10201	479	NEQYVAIKRFQEVMR	AIKRFQEVM	5	0.1997	5763.7	65.00	0.26	tr_C9ZZQ4_
HLA-DQA10501-DQB10201	480	TTFMDSLYNQRRYVI	FMDSLYNQR	2	0.1989	5809.6	65.00	0.44	tr_C9ZZQ4_

HLA-DQA10501-DQB10201	481	GLAALRCNADSVTLH	ALRCNADSV	3	0.1981	5861.0	65.00	0.37	tr_C9ZZQ4_
HLA-DQA10501-DQB10201	482	TVEQQIYVLTRYLSN	QQIYVLTRY	3	0.1975	5898.7	65.00	0.25	tr_C9ZZQ4_
HLA-DQA10501-DQB10201	483	GALRHDTGSLEYSGQ	RHDTGSLEY	3	0.1975	5899.4	65.00	0.42	tr_C9ZZQ4_
HLA-DQA10501-DQB10201	484	WNTRVPKIFTTAVNA	KIFTTAVNA	6	0.1967	5953.2	65.00	0.47	tr_C9ZZQ4_
HLA-DQA10501-DQB10201	485	ALRHDTGSLEYSGQL	RHDTGSLEY	2	0.1967	5953.4	65.00	0.40	tr_C9ZZQ4_
HLA-DQA10501-DQB10201	486	KSLEKWGGSLNISLV	WGGSLNISL	5	0.1948	6078.1	65.00	0.56	tr_C9ZZQ4_
HLA-DQA10501-DQB10201	487	SISLSVPPHALSTFS	ISLSVPPHA	1	0.1946	6090.8	65.00	0.44	tr_C9ZZQ4_
HLA-DQA10501-DQB10201	488	AFGTTTSDSAKALKE	FGTTTSDSA	1	0.1934	6170.4	65.00	0.71	tr_C9ZZQ4_
HLA-DQA10501-DQB10201	489	TNLPHWYDNETSSPT	WYDNETSSP	5	0.1923	6241.5	65.00	0.33	tr_C9ZZQ4_
HLA-DQA10501-DQB10201	490	GAPANINVKVLLCTW	NVKVLLCTW	6	0.1923	6243.0	65.00	0.40	tr_C9ZZQ4_
HLA-DQA10501-DQB10201	491	TGMLYVMYVLLLLMP	TGMLYVMYV	0	0.1922	6248.5	65.00	0.28	tr_C9ZZQ4_
HLA-DQA10501-DQB10201	492	MVLIRHALGHLGVLR	ALGHLGVLR	6	0.1921	6258.4	65.00	0.30	tr_C9ZZQ4_
HLA-DQA10501-DQB10201	493	LRPSPTIETMVLIRH	PTIETMVLI	4	0.1917	6280.6	65.00	0.19	tr_C9ZZQ4_
HLA-DQA10501-DQB10201	494	NLPHWYDNETSSPTI	WYDNETSSP	4	0.1916	6288.0	65.00	0.35	tr_C9ZZQ4_
HLA-DQA10501-DQB10201	495	VVQSSKSHKTPEEFI	KSHKTPEEF	5	0.1916	6290.0	65.00	0.56	tr_C9ZZQ4_
HLA-DQA10501-DQB10201	496	CTWNTRVPKIFTTAV	WNTRVPKIF	2	0.1908	6345.3	65.00	0.52	tr_C9ZZQ4_
HLA-DQA10501-DQB10201	497	ETGITDPNYFLKHDT	TGITDPNYF	1	0.1907	6351.4	65.00	0.50	tr_C9ZZQ4_
HLA-DQA10501-DQB10201	498	FNGSAAAPRLVFATN	NGSAAAPRL	1	0.1897	6419.1	65.00	0.34	tr_C9ZZQ4_
HLA-DQA10501-DQB10201	499	ASDVDVIERHLAAHS	ASDVDVIER	0	0.1882	6522.2	70.00	0.34	tr_C9ZZQ4_
HLA-DQA10501-DQB10201	500	GMLYVMYVLLLLMPY	YVLLLLMPY	6	0.1882	6523.0	70.00	0.39	tr_C9ZZQ4_
HLA-DQA10501-DQB10201	501	ALSVPEWLKDRTTFM	WLKDRTTFM	6	0.1880	6543.2	70.00	0.65	tr_C9ZZQ4_
HLA-DQA10501-DQB10201	502	AKNEQYVAIKRFQEV	EQYVAIKRF	3	0.1879	6545.9	70.00	0.39	tr_C9ZZQ4_
HLA-DQA10501-DQB10201	503	ADRVKVQVVQSSKSH	RVKVQVVQS	2	0.1876	6565.7	70.00	0.60	tr_C9ZZQ4_
HLA-DQA10501-DQB10201	504	LCTWNTRVPKIFTTA	WNTRVPKIF	3	0.1875	6573.1	70.00	0.58	tr_C9ZZQ4_
HLA-DQA10501-DQB10201	505	LAKNEQYVAIKRFQE	EQYVAIKRF	4	0.1860	6685.2	70.00	0.43	tr_C9ZZQ4_
HLA-DQA10501-DQB10201	506	DSVTLHSPLNGLVIF	TLHSPLNGL	3	0.1858	6697.4	70.00	0.23	tr_C9ZZQ4_
HLA-DQA10501-DQB10201	507	LAALRCNADSVTLHS	ALRCNADSV	2	0.1855	6715.6	70.00	0.40	tr_C9ZZQ4_
HLA-DQA10501-DQB10201	508	SLEKWGGSLNISLVR	WGGSLNISL	4	0.1847	6776.4	70.00	0.49	tr_C9ZZQ4_
HLA-DQA10501-DQB10201	509	NAGFNASMESRNWTI	AGFNASMES	1	0.1845	6795.1	70.00	0.35	tr_C9ZZQ4_
HLA-DQA10501-DQB10201	510	RKSLEKWGGSLNISL	WGGSLNISL	6	0.1844	6799.4	70.00	0.74	tr_C9ZZQ4_
HLA-DQA10501-DQB10201	511	GITDPNYFLKHDTDG	NYFLKHDTD	5	0.1841	6818.7	70.00	0.56	tr_C9ZZQ4_
HLA-DQA10501-DQB10201	512	LLMPYPLQVHGAPAN	LLMPYPLQV	0	0.1841	6820.2	70.00	0.22	tr_C9ZZQ4_
HLA-DQA10501-DQB10201	513	KSDTGRFLRKLVADR	FLRKLVADR	6	0.1837	6851.8	70.00	0.40	tr_C9ZZQ4_
HLA-DQA10501-DQB10201	514	YPLQVHGAPANINVK	QVHGAPANI	3	0.1835	6862.9	70.00	0.38	tr_C9ZZQ4_
HLA-DQA10501-DQB10201	515	VVLNVNDKPAPDKEF	VVLNVNDKP	0	0.1833	6880.2	70.00	0.69	tr_C9ZZQ4_
HLA-DQA10501-DQB10201	516	DSISELQKHEVVAFG	LQKHEVVAF	5	0.1831	6895.0	70.00	0.30	tr_C9ZZQ4_
HLA-DQA10501-DQB10201	517	QLNKFRRHVIHLSPT	RRHVIHLSP	5	0.1818	6992.1	70.00	0.39	tr_C9ZZQ4_
HLA-DQA10501-DQB10201	518	DPITIEPQLNKFRRH	PITIEPQLN	1	0.1817	6998.0	70.00	0.61	tr_C9ZZQ4_
HLA-DQA10501-DQB10201	519	TWNTRVPKIFTTAVN	WNTRVPKIF	1	0.1815	7017.3	70.00	0.44	tr_C9ZZQ4_
HLA-DQA10501-DQB10201	520	LEKWGGSLNISLVRR	WGGSLNISL	3	0.1807	7078.3	70.00	0.44	tr_C9ZZQ4_
HLA-DQA10501-DQB10201	521	RPSPTIETMVLIRHA	PTIETMVLI	3	0.1804	7098.4	70.00	0.19	tr_C9ZZQ4_
HLA-DQA10501-DQB10201	522	GIVTKALMKMTNTLF	LMKMTNTLF	6	0.1801	7122.4	70.00	0.61	tr_C9ZZQ4_
HLA-DQA10501-DQB10201	523	TMVLIRHALGHLGVL	RHALGHLGV	5	0.1792	7195.5	70.00	0.31	tr_C9ZZQ4_
HLA-DQA10501-DQB10201	524	AALRCNADSVTLHSP	ALRCNADSV	1	0.1784	7255.7	70.00	0.35	tr_C9ZZQ4_
HLA-DQA10501-DQB10201	525	APANINVKVLLCTWN	NVKVLLCTW	5	0.1782	7272.2	70.00	0.32	tr_C9ZZQ4_
HLA-DQA10501-DQB10201	526	LSNNSGKEANAIVCA	KEANAIVCA	6	0.1780	7287.2	70.00	0.53	tr_C9ZZQ4_
HLA-DQA10501-DQB10201	527	PLAKNEQYVAIKRFQ	EQYVAIKRF	5	0.1779	7292.8	70.00	0.53	tr_C9ZZQ4_
HLA-DQA10501-DQB10201	528	VFGIVTKALMKMTNT	FGIVTKALM	1	0.1779	7297.9	70.00	0.48	tr_C9ZZQ4_
HLA-DQA10501-DQB10201	529	EEFIKDELSKETDKS	EEFIKDELS	0	0.1775	7326.4	70.00	0.47	tr_C9ZZQ4_
HLA-DQA10501-DQB10201	530	PPHALSTFSRTGMLY	TFSRTGMLY	6	0.1770	7364.3	70.00	0.62	tr_C9ZZQ4_

HLA-DQA10501-DQB10201	531	LLDSQVSPFPGKLLI	LLDSQVSPF	0	0.1757	7468.7	70.00	0.44	tr_C9ZZQ4_
HLA-DQA10501-DQB10201	532	VCAAEAHGIMKVIRK	EAHGIMKVI	4	0.1756	7476.0	70.00	0.28	tr_C9ZZQ4_
HLA-DQA10501-DQB10201	533	TFMDSLYNQRRYVIA	FMDSLYNQR	1	0.1749	7536.0	70.00	0.41	tr_C9ZZQ4_
HLA-DQA10501-DQB10201	534	PSPTIETMVLIRHAL	TIETMVLIR	3	0.1743	7584.6	70.00	0.19	tr_C9ZZQ4_
HLA-DQA10501-DQB10201	535	FLRKLVADRRTSGAY	LRKLVADRR	1	0.1736	7638.9	70.00	0.62	tr_C9ZZQ4_
HLA-DQA10501-DQB10201	536	PQLNKFRRHVIHLSP	RRHVIHLSP	6	0.1705	7904.1	75.00	0.48	tr_C9ZZQ4_
HLA-DQA10501-DQB10201	537	NTRVPKIFTTAVNAG	KIFTTAVNA	5	0.1702	7926.0	75.00	0.40	tr_C9ZZQ4_
HLA-DQA10501-DQB10201	538	QSSKSHKTPEEFIKD	KSHKTPEEF	3	0.1698	7962.5	75.00	0.55	tr_C9ZZQ4_
HLA-DQA10501-DQB10201	539	QVHGAPANINVKVLL	ANINVKVLL	6	0.1696	7979.0	75.00	0.29	tr_C9ZZQ4_
HLA-DQA10501-DQB10201	540	ADSVTLHSPLNGLVI	TLHSPLNGL	4	0.1695	7990.3	75.00	0.28	tr_C9ZZQ4_
HLA-DQA10501-DQB10201	541	VVAFGPMTGSGEVRR	VVAFGPMTG	0	0.1694	7994.1	75.00	0.32	tr_C9ZZQ4_
HLA-DQA10501-DQB10201	542	LPHWYDNETSSPTIR	WYDNETSSP	3	0.1688	8051.8	75.00	0.35	tr_C9ZZQ4_
HLA-DQA10501-DQB10201	543	ETMVLIRHALGHLGV	RHALGHLGV	6	0.1684	8082.2	75.00	0.37	tr_C9ZZQ4_
HLA-DQA10501-DQB10201	544	LQVHGAPANINVKVL	QVHGAPANI	1	0.1681	8110.3	75.00	0.38	tr_C9ZZQ4_
HLA-DQA10501-DQB10201	545	VEQQIYVLTRYLSNN	QIYVLTRYL	3	0.1674	8174.2	75.00	0.25	tr_C9ZZQ4_
HLA-DQA10501-DQB10201	546	ALRCNADSVTLHSPL	ALRCNADSV	0	0.1660	8299.5	75.00	0.25	tr_C9ZZQ4_
HLA-DQA10501-DQB10201	547	PANINVKVLLCTWNT	NVKVLLCTW	4	0.1657	8327.3	75.00	0.32	tr_C9ZZQ4_
HLA-DQA10501-DQB10201	548	DRVKVQVVQSSKSHK	RVKVQVVQS	1	0.1654	8349.8	75.00	0.63	tr_C9ZZQ4_
HLA-DQA10501-DQB10201	549	SSKSHKTPEEFIKDE	KSHKTPEEF	2	0.1649	8398.1	75.00	0.47	tr_C9ZZQ4_
HLA-DQA10501-DQB10201	550	RKLVADRRTSGAYVL	RRTSGAYVL	6	0.1640	8481.5	75.00	0.55	tr_C9ZZQ4_
HLA-DQA10501-DQB10201	551	TKPQAVLLFGAPKSD	VLLFGAPKS	5	0.1638	8496.6	75.00	0.28	tr_C9ZZQ4_
HLA-DQA10501-DQB10201	552	EKWGGSLNISLVRRR	WGGSLNISL	2	0.1637	8506.8	75.00	0.44	tr_C9ZZQ4_
HLA-DQA10501-DQB10201	553	VQSSKSHKTPEEFIK	KSHKTPEEF	4	0.1633	8545.8	75.00	0.55	tr_C9ZZQ4_
HLA-DQA10501-DQB10201	554	AALTGHLPTSGAVFV	LPTSGAVFV	6	0.1629	8577.5	75.00	0.55	tr_C9ZZQ4_
HLA-DQA10501-DQB10201	555	PLQVHGAPANINVKV	QVHGAPANI	2	0.1621	8650.6	75.00	0.41	tr_C9ZZQ4_
HLA-DQA10501-DQB10201	556	ATKPQAVLLFGAPKS	VLLFGAPKS	6	0.1613	8732.6	75.00	0.28	tr_C9ZZQ4_
HLA-DQA10501-DQB10201	557	VHGAPANINVKVLLC	NINVKVLLC	6	0.1611	8752.0	75.00	0.28	tr_C9ZZQ4_
HLA-DQA10501-DQB10201	558	VEDDFSMQTPKDGTV	DFSMQTPKD	3	0.1605	8807.5	75.00	0.34	tr_C9ZZQ4_
HLA-DQA10501-DQB10201	559	NADSVTLHSPLNGLV	TLHSPLNGL	5	0.1596	8890.9	75.00	0.28	tr_C9ZZQ4_
HLA-DQA10501-DQB10201	560	SMQTPKDGTVGLAAL	DGTVGLAAL	6	0.1589	8964.6	75.00	0.56	tr_C9ZZQ4_
HLA-DQA10501-DQB10201	561	VSPFPGKLLIAGTNP	KLLIAGTNP	6	0.1588	8966.5	75.00	0.54	tr_C9ZZQ4_
HLA-DQA10501-DQB10201	562	SPTIETMVLIRHALG	TIETMVLIR	2	0.1588	8967.8	75.00	0.25	tr_C9ZZQ4_
HLA-DQA10501-DQB10201	563	VNDKPAPDKEFNAVF	PDKEFNAVF	6	0.1586	8986.9	75.00	0.54	tr_C9ZZQ4_
HLA-DQA10501-DQB10201	564	AATKPQAVLLFGAPK	PQAVLLFGA	4	0.1583	9019.1	75.00	0.26	tr_C9ZZQ4_
HLA-DQA10501-DQB10201	565	KPQAVLLFGAPKSDT	VLLFGAPKS	4	0.1582	9029.6	75.00	0.28	tr_C9ZZQ4_
HLA-DQA10501-DQB10201	566	LVRRRGAALTGHLPT	GAALTGHLP	5	0.1582	9031.7	75.00	0.26	tr_C9ZZQ4_
HLA-DQA10501-DQB10201	567	ISLSVPPHALSTFSR	ISLSVPPHA	0	0.1560	9243.5	75.00	0.38	tr_C9ZZQ4_
HLA-DQA10501-DQB10201	568	CAAEAHGIMKVIRKS	EAHGIMKVI	3	0.1538	9464.0	75.00	0.32	tr_C9ZZQ4_
HLA-DQA10501-DQB10201	569	CNADSVTLHSPLNGL	TLHSPLNGL	6	0.1538	9470.4	75.00	0.29	tr_C9ZZQ4_
HLA-DQA10501-DQB10201	570	ANINVKVLLCTWNTR	NVKVLLCTW	3	0.1532	9525.3	80.00	0.32	tr_C9ZZQ4_
HLA-DQA10501-DQB10201	571	LIAGTNPLAKNEQYV	LIAGTNPLA	0	0.1527	9582.6	80.00	0.38	tr_C9ZZQ4_
HLA-DQA10501-DQB10201	572	AGFNASMESRNWTIA	GFNASMESR	1	0.1516	9691.5	80.00	0.25	tr_C9ZZQ4_
HLA-DQA10501-DQB10201	573	MQTPKDGTVGLAALR	DGTVGLAAL	5	0.1512	9734.4	80.00	0.43	tr_C9ZZQ4_
HLA-DQA10501-DQB10201	574	FGGDCEGEAAKQGAV	FGGDCEGEA	0	0.1509	9764.9	80.00	0.37	tr_C9ZZQ4_
HLA-DQA10501-DQB10201	575	VRRRGAALTGHLPTS	GAALTGHLP	4	0.1500	9861.2	80.00	0.28	tr_C9ZZQ4_
HLA-DQA10501-DQB10201	576	VAFGPMTGSGEVRRW	PMTGSGEVR	4	0.1498	9886.7	80.00	0.35	tr_C9ZZQ4_
HLA-DQA10501-DQB10201	577	AFGPMTGSGEVRRWV	PMTGSGEVR	3	0.1490	9974.3	80.00	0.37	tr_C9ZZQ4_
HLA-DQA10501-DQB10201	578	HGAPANINVKVLLCT	NINVKVLLC	5	0.1486	10011.0	80.00	0.25	tr_C9ZZQ4_
HLA-DQA10501-DQB10201	579	FMDSLYNQRRYVIAD	FMDSLYNQR	0	0.1485	10027.0	80.00	0.30	tr_C9ZZQ4_
HLA-DQA10501-DQB10201	580	PQAVLLFGAPKSDTG	VLLFGAPKS	3	0.1481	10065.9	80.00	0.29	tr_C9ZZQ4_

HLA-DQA10501-DQB10201	581	SLVRRRGAALTGHLP	RGAALTGHL	5	0.1481	10071.0	80.00	0.28	tr_C9ZZQ4_
HLA-DQA10501-DQB10201	582	DCEGEAAKQGAVCYC	AAKQGAVCY	5	0.1473	10157.2	80.00	0.32	tr_C9ZZQ4_
HLA-DQA10501-DQB10201	583	EGEAAKQGAVCYCNQ	QGAVCYCNQ	6	0.1473	10161.9	80.00	0.28	tr_C9ZZQ4_
HLA-DQA10501-DQB10201	584	AAEAHGIMKVIRKSL	EAHGIMKVI	2	0.1463	10265.9	80.00	0.35	tr_C9ZZQ4_
HLA-DQA10501-DQB10201	585	NINVKVLLCTWNTRV	NVKVLLCTW	2	0.1461	10291.7	80.00	0.31	tr_C9ZZQ4_
HLA-DQA10501-DQB10201	586	KWGGSLNISLVRRRG	WGGSLNISL	1	0.1455	10358.5	80.00	0.44	tr_C9ZZQ4_
HLA-DQA10501-DQB10201	587	QVVQSSKSHKTPEEF	KSHKTPEEF	6	0.1451	10404.6	80.00	0.83	tr_C9ZZQ4_
HLA-DQA10501-DQB10201	588	INVKVLLCTWNTRVP	NVKVLLCTW	1	0.1449	10426.8	80.00	0.35	tr_C9ZZQ4_
HLA-DQA10501-DQB10201	589	EQQIYVLTRYLSNNS	QIYVLTRYL	2	0.1446	10455.6	80.00	0.29	tr_C9ZZQ4_
HLA-DQA10501-DQB10201	590	PHWYDNETSSPTIRK	WYDNETSSP	2	0.1440	10525.6	80.00	0.49	tr_C9ZZQ4_
HLA-DQA10501-DQB10201	591	FGTTTSDSAKALKEE	FGTTTSDSA	0	0.1433	10612.3	80.00	0.62	tr_C9ZZQ4_
HLA-DQA10501-DQB10201	592	RRRGAALTGHLPTSG	GAALTGHLP	3	0.1411	10857.5	80.00	0.28	tr_C9ZZQ4_
HLA-DQA10501-DQB10201	593	GDCEGEAAKQGAVCY	AAKQGAVCY	6	0.1404	10946.3	80.00	0.38	tr_C9ZZQ4_
HLA-DQA10501-DQB10201	594	PTIETMVLIRHALGH	TIETMVLIR	1	0.1395	11055.2	80.00	0.26	tr_C9ZZQ4_
HLA-DQA10501-DQB10201	595	FGIVTKALMKMTNTL	FGIVTKALM	0	0.1386	11157.0	80.00	0.42	tr_C9ZZQ4_
HLA-DQA10501-DQB10201	596	PKSDTGRFLRKLVAD	RFLRKLVAD	6	0.1386	11161.7	80.00	0.42	tr_C9ZZQ4_
HLA-DQA10501-DQB10201	597	LRKLVADRRTSGAYV	DRRTSGAYV	6	0.1386	11165.6	80.00	0.42	tr_C9ZZQ4_
HLA-DQA10501-DQB10201	598	IETMVLIRHALGHLG	IRHALGHLG	6	0.1385	11175.0	80.00	0.39	tr_C9ZZQ4_
HLA-DQA10501-DQB10201	599	TIETMVLIRHALGHL	LIRHALGHL	6	0.1381	11225.0	80.00	0.25	tr_C9ZZQ4_
HLA-DQA10501-DQB10201	600	FGPMTGSGEVRRWVR	PMTGSGEVR	2	0.1361	11468.6	80.00	0.43	tr_C9ZZQ4_
HLA-DQA10501-DQB10201	601	AEAHGIMKVIRKSLE	EAHGIMKVI	1	0.1357	11511.5	80.00	0.30	tr_C9ZZQ4_
HLA-DQA10501-DQB10201	602	CEGEAAKQGAVCYCN	AAKQGAVCY	4	0.1356	11529.6	80.00	0.34	tr_C9ZZQ4_
HLA-DQA10501-DQB10201	603	QAVLLFGAPKSDTGR	VLLFGAPKS	2	0.1352	11579.1	80.00	0.35	tr_C9ZZQ4_
HLA-DQA10501-DQB10201	604	TSSPTIRKFHATVES	IRKFHATVE	5	0.1351	11596.6	80.00	0.47	tr_C9ZZQ4_
HLA-DQA10501-DQB10201	605	RCNADSVTLHSPLNG	VTLHSPLNG	6	0.1348	11629.2	80.00	0.28	tr_C9ZZQ4_
HLA-DQA10501-DQB10201	606	AVCYCNQGGNVVYVR	NQGGNVVYV	5	0.1346	11658.3	80.00	0.41	tr_C9ZZQ4_
HLA-DQA10501-DQB10201	607	GEAAKQGAVCYCNQG	QGAVCYCNQ	5	0.1326	11910.2	85.00	0.28	tr_C9ZZQ4_
HLA-DQA10501-DQB10201	608	RRGAALTGHLPTSGA	GAALTGHLP	2	0.1320	11981.0	85.00	0.29	tr_C9ZZQ4_
HLA-DQA10501-DQB10201	609	LRCNADSVTLHSPLN	DSVTLHSPL	5	0.1307	12162.2	85.00	0.14	tr_C9ZZQ4_
HLA-DQA10501-DQB10201	610	RVKVQVVQSSKSHKT	RVKVQVVQS	0	0.1298	12280.0	85.00	0.54	tr_C9ZZQ4_
HLA-DQA10501-DQB10201	611	VKVLLCTWNTRVPKI	TWNTRVPKI	6	0.1295	12311.3	85.00	0.38	tr_C9ZZQ4_
HLA-DQA10501-DQB10201	612	QQIYVLTRYLSNNSG	QIYVLTRYL	1	0.1277	12564.0	85.00	0.28	tr_C9ZZQ4_
HLA-DQA10501-DQB10201	613	NVKVLLCTWNTRVPK	NVKVLLCTW	0	0.1273	12614.7	85.00	0.31	tr_C9ZZQ4_
HLA-DQA10501-DQB10201	614	WGGSLNISLVRRRGA	WGGSLNISL	0	0.1270	12655.0	85.00	0.32	tr_C9ZZQ4_
HLA-DQA10501-DQB10201	615	GAVCYCNQGGNVVYV	NQGGNVVYV	6	0.1262	12766.5	85.00	0.52	tr_C9ZZQ4_
HLA-DQA10501-DQB10201	616	ELSKETDKSGITIVF	DKSGITIVF	6	0.1250	12928.9	85.00	0.60	tr_C9ZZQ4_
HLA-DQA10501-DQB10201	617	AVLLFGAPKSDTGRF	VLLFGAPKS	1	0.1243	13030.4	85.00	0.34	tr_C9ZZQ4_
HLA-DQA10501-DQB10201	618	ISLVRRRGAALTGHL	RGAALTGHL	6	0.1243	13033.8	85.00	0.31	tr_C9ZZQ4_
HLA-DQA10501-DQB10201	619	VLLFGAPKSDTGRFL	PKSDTGRFL	6	0.1219	13368.2	85.00	0.23	tr_C9ZZQ4_
HLA-DQA10501-DQB10201	620	FNASMESRNWTIADR	SRNWTIADR	6	0.1217	13399.6	85.00	0.34	tr_C9ZZQ4_
HLA-DQA10501-DQB10201	621	GAALTGHLPTSGAVF	HLPTSGAVF	6	0.1216	13415.8	85.00	0.31	tr_C9ZZQ4_
HLA-DQA10501-DQB10201	622	EDDFSMQTPKDGTVG	DFSMQTPKD	2	0.1211	13485.0	85.00	0.51	tr_C9ZZQ4_
HLA-DQA10501-DQB10201	623	PITIEPQLNKFRRHV	PITIEPQLN	0	0.1211	13490.5	85.00	0.71	tr_C9ZZQ4_
HLA-DQA10501-DQB10201	624	EAAKQGAVCYCNQGG	QGAVCYCNQ	4	0.1206	13554.0	85.00	0.25	tr_C9ZZQ4_
HLA-DQA10501-DQB10201	625	ALKEEKKKRTVTAVF	KKRTVTAVF	6	0.1206	13566.3	85.00	0.87	tr_C9ZZQ4_
HLA-DQA10501-DQB10201	626	HWYDNETSSPTIRKF	WYDNETSSP	1	0.1202	13620.7	85.00	0.62	tr_C9ZZQ4_
HLA-DQA10501-DQB10201	627	EYLKTHTSETGITDP	TSETGITDP	6	0.1199	13669.5	85.00	0.28	tr_C9ZZQ4_
HLA-DQA10501-DQB10201	628	PMTGSGEVRRWVREL	EVRRWVREL	6	0.1198	13682.0	85.00	0.30	tr_C9ZZQ4_
HLA-DQA10501-DQB10201	629	GFNASMESRNWTIAD	FNASMESRN	1	0.1196	13709.5	85.00	0.25	tr_C9ZZQ4_
HLA-DQA10501-DQB10201	630	QEVMREYLKTHTSET	EYLKTHTSE	5	0.1192	13760.0	85.00	0.38	tr_C9ZZQ4_

HLA-DQA10501-DQB10201	631	YLSNNSGKEANAIVC	GKEANAIVC	6	0.1183	13902.7	85.00	0.35	tr_C9ZZQ4_
HLA-DQA10501-DQB10201	632	VMREYLKTHTSETGI	EYLKTHTSE	3	0.1168	14132.7	85.00	0.31	tr_C9ZZQ4_
HLA-DQA10501-DQB10201	633	AGTNPLAKNEQYVAI	PLAKNEQYV	4	0.1164	14196.4	85.00	0.33	tr_C9ZZQ4_
HLA-DQA10501-DQB10201	634	LDSQVSPFPGKLLIA	LDSQVSPFP	0	0.1161	14242.7	85.00	0.29	tr_C9ZZQ4_
HLA-DQA10501-DQB10201	635	RGAALTGHLPTSGAV	GAALTGHLP	1	0.1152	14375.6	85.00	0.29	tr_C9ZZQ4_
HLA-DQA10501-DQB10201	636	EVMREYLKTHTSETG	EYLKTHTSE	4	0.1144	14509.0	85.00	0.33	tr_C9ZZQ4_
HLA-DQA10501-DQB10201	637	GPMTGSGEVRRWVRE	PMTGSGEVR	1	0.1126	14791.3	90.00	0.40	tr_C9ZZQ4_
HLA-DQA10501-DQB10201	638	REYLKTHTSETGITD	EYLKTHTSE	1	0.1116	14954.5	90.00	0.28	tr_C9ZZQ4_
HLA-DQA10501-DQB10201	639	ETSSPTIRKFHATVE	IRKFHATVE	6	0.1111	15022.9	90.00	0.76	tr_C9ZZQ4_
HLA-DQA10501-DQB10201	640	EAHGIMKVIRKSLEK	AHGIMKVIR	1	0.1110	15052.9	90.00	0.25	tr_C9ZZQ4_
HLA-DQA10501-DQB10201	641	EFIKDELSKETDKSG	FIKDELSKE	1	0.1106	15106.9	90.00	0.60	tr_C9ZZQ4_
HLA-DQA10501-DQB10201	642	MREYLKTHTSETGIT	EYLKTHTSE	2	0.1087	15421.8	90.00	0.32	tr_C9ZZQ4_
HLA-DQA10501-DQB10201	643	EPQLNKFRRHVIHLS	FRRHVIHLS	6	0.1084	15473.0	90.00	0.56	tr_C9ZZQ4_
HLA-DQA10501-DQB10201	644	TNPLAKNEQYVAIKR	NEQYVAIKR	6	0.1076	15616.1	90.00	0.29	tr_C9ZZQ4_
HLA-DQA10501-DQB10201	645	GGDCEGEAAKQGAVC	EAAKQGAVC	6	0.1072	15668.7	90.00	0.29	tr_C9ZZQ4_
HLA-DQA10501-DQB10201	646	AHGIMKVIRKSLEKW	VIRKSLEKW	6	0.1069	15728.9	90.00	0.40	tr_C9ZZQ4_
HLA-DQA10501-DQB10201	647	YLKTHTSETGITDPN	TSETGITDP	5	0.1064	15815.7	90.00	0.29	tr_C9ZZQ4_
HLA-DQA10501-DQB10201	648	QIYVLTRYLSNNSGK	QIYVLTRYL	0	0.1056	15943.0	90.00	0.29	tr_C9ZZQ4_
HLA-DQA10501-DQB10201	649	SLSVPPHALSTFSRT	PPHALSTFS	4	0.1055	15974.6	90.00	0.31	tr_C9ZZQ4_
HLA-DQA10501-DQB10201	650	IAGTNPLAKNEQYVA	PLAKNEQYV	5	0.1033	16355.2	90.00	0.37	tr_C9ZZQ4_
HLA-DQA10501-DQB10201	651	APKSDTGRFLRKLVA	GRFLRKLVA	6	0.1011	16738.3	90.00	0.42	tr_C9ZZQ4_
HLA-DQA10501-DQB10201	652	NISLVRRRGAALTGH	VRRRGAALT	4	0.1002	16908.5	90.00	0.28	tr_C9ZZQ4_
HLA-DQA10501-DQB10201	653	GTNPLAKNEQYVAIK	PLAKNEQYV	3	0.0995	17030.4	90.00	0.31	tr_C9ZZQ4_
HLA-DQA10501-DQB10201	654	VPPHALSTFSRTGML	STFSRTGML	6	0.0994	17054.6	90.00	0.29	tr_C9ZZQ4_
HLA-DQA10501-DQB10201	655	FSMQTPKDGTVGLAA	KDGTVGLAA	6	0.0992	17086.1	90.00	0.58	tr_C9ZZQ4_
HLA-DQA10501-DQB10201	656	LNISLVRRRGAALTG	VRRRGAALT	5	0.0986	17199.3	90.00	0.35	tr_C9ZZQ4_
HLA-DQA10501-DQB10201	657	SQVSPFPGKLLIAGT	PGKLLIAGT	6	0.0981	17307.0	90.00	0.38	tr_C9ZZQ4_
HLA-DQA10501-DQB10201	658	DDFSMQTPKDGTVGL	DFSMQTPKD	1	0.0973	17453.9	90.00	0.55	tr_C9ZZQ4_
HLA-DQA10501-DQB10201	659	AAKQGAVCYCNQGGN	QGAVCYCNQ	3	0.0967	17567.4	90.00	0.31	tr_C9ZZQ4_
HLA-DQA10501-DQB10201	660	QVSPFPGKLLIAGTN	PGKLLIAGT	5	0.0948	17928.7	90.00	0.36	tr_C9ZZQ4_
HLA-DQA10501-DQB10201	661	WYDNETSSPTIRKFH	WYDNETSSP	0	0.0941	18054.3	90.00	0.58	tr_C9ZZQ4_
HLA-DQA10501-DQB10201	662	SLNISLVRRRGAALT	VRRRGAALT	6	0.0930	18282.1	90.00	0.44	tr_C9ZZQ4_
HLA-DQA10501-DQB10201	663	AKQGAVCYCNQGGNV	QGAVCYCNQ	2	0.0918	18510.6	90.00	0.31	tr_C9ZZQ4_
HLA-DQA10501-DQB10201	664	LLFGAPKSDTGRFLR	PKSDTGRFL	5	0.0911	18652.6	95.00	0.35	tr_C9ZZQ4_
HLA-DQA10501-DQB10201	665	HGIMKVIRKSLEKWG	VIRKSLEKW	5	0.0909	18705.9	95.00	0.41	tr_C9ZZQ4_
HLA-DQA10501-DQB10201	666	RYLSNNSGKEANAIV	SGKEANAIV	6	0.0904	18794.6	95.00	0.31	tr_C9ZZQ4_
HLA-DQA10501-DQB10201	667	SVPPHALSTFSRTGM	PPHALSTFS	2	0.0888	19131.9	95.00	0.26	tr_C9ZZQ4_
HLA-DQA10501-DQB10201	668	IYVLTRYLSNNSGKE	YVLTRYLSN	1	0.0888	19139.3	95.00	0.26	tr_C9ZZQ4_
HLA-DQA10501-DQB10201	669	KQGAVCYCNQGGNVV	QGAVCYCNQ	1	0.0874	19412.8	95.00	0.30	tr_C9ZZQ4_
HLA-DQA10501-DQB10201	670	QGAVCYCNQGGNVVY	YCNQGGNVV	5	0.0871	19480.1	95.00	0.25	tr_C9ZZQ4_
HLA-DQA10501-DQB10201	671	LSVPPHALSTFSRTG	PPHALSTFS	3	0.0863	19654.3	95.00	0.38	tr_C9ZZQ4_
HLA-DQA10501-DQB10201	672	DSQVSPFPGKLLIAG	VSPFPGKLL	3	0.0860	19707.8	95.00	0.27	tr_C9ZZQ4_
HLA-DQA10501-DQB10201	673	DFSMQTPKDGTVGLA	DFSMQTPKD	0	0.0852	19879.1	95.00	0.35	tr_C9ZZQ4_
HLA-DQA10501-DQB10201	674	FIKDELSKETDKSGI	FIKDELSKE	0	0.0849	19961.7	95.00	0.50	tr_C9ZZQ4_
HLA-DQA10501-DQB10201	675	GSLNISLVRRRGAAL	LVRRRGAAL	6	0.0839	20168.8	95.00	0.38	tr_C9ZZQ4_
HLA-DQA10501-DQB10201	676	GGSLNISLVRRRGAA	SLNISLVRR	2	0.0836	20227.3	95.00	0.38	tr_C9ZZQ4_
HLA-DQA10501-DQB10201	677	VIRKSLEKWGGSLNI	EKWGGSLNI	6	0.0834	20286.7	95.00	0.43	tr_C9ZZQ4_
HLA-DQA10501-DQB10201	678	GIMKVIRKSLEKWGG	VIRKSLEKW	4	0.0832	20315.3	95.00	0.40	tr_C9ZZQ4_
HLA-DQA10501-DQB10201	679	YVLTRYLSNNSGKEA	TRYLSNNSG	3	0.0830	20360.6	95.00	0.22	tr_C9ZZQ4_
HLA-DQA10501-DQB10201	680	TTTSDSAKALKEEKK	SAKALKEEK	5	0.0823	20521.8	95.00	0.27	tr_C9ZZQ4_

HLA-DQA10501-DQB10201	681	GTTTSDSAKALKEEK	SAKALKEEK	6	0.0820	20592.1	95.00	0.31	tr_C9ZZQ4_
HLA-DQA10501-DQB10201	682	IRKSLEKWGGSLNIS	EKWGGSLNI	5	0.0802	21001.4	95.00	0.43	tr_C9ZZQ4_
HLA-DQA10501-DQB10201	683	GAPKSDTGRFLRKLV	TGRFLRKLV	6	0.0786	21359.6	95.00	0.34	tr_C9ZZQ4_
HLA-DQA10501-DQB10201	684	IMKVIRKSLEKWGGS	VIRKSLEKW	3	0.0753	22141.1	95.00	0.43	tr_C9ZZQ4_
HLA-DQA10501-DQB10201	685	TRYLSNNSGKEANAI	NSGKEANAI	6	0.0741	22426.8	95.00	0.30	tr_C9ZZQ4_
HLA-DQA10501-DQB10201	686	VLNVNDKPAPDKEFN	VLNVNDKPA	0	0.0723	22865.9	95.00	0.25	tr_C9ZZQ4_
HLA-DQA10501-DQB10201	687	VLTRYLSNNSGKEAN	YLSNNSGKE	4	0.0713	23129.4	95.00	0.26	tr_C9ZZQ4_
HLA-DQA10501-DQB10201	688	VKVQVVQSSKSHKTP	VQVVQSSKS	2	0.0690	23705.5	95.00	0.41	tr_C9ZZQ4_
HLA-DQA10501-DQB10201	689	LTRYLSNNSGKEANA	YLSNNSGKE	3	0.0679	23990.9	95.00	0.25	tr_C9ZZQ4_
HLA-DQA10501-DQB10201	690	MKVIRKSLEKWGGSL	VIRKSLEKW	2	0.0672	24159.9	95.00	0.50	tr_C9ZZQ4_
HLA-DQA10501-DQB10201	691	LFGAPKSDTGRFLRK	PKSDTGRFL	4	0.0672	24162.8	95.00	0.49	tr_C9ZZQ4_
HLA-DQA10501-DQB10201	692	TTSDSAKALKEEKKK	SAKALKEEK	4	0.0670	24213.3	95.00	0.28	tr_C9ZZQ4_
HLA-DQA10501-DQB10201	693	NVNDKPAPDKEFNAV	APDKEFNAV	6	0.0653	24681.0	95.00	0.19	tr_C9ZZQ4_
HLA-DQA10501-DQB10201	694	KVIRKSLEKWGGSLN	VIRKSLEKW	1	0.0643	24947.8	95.00	0.44	tr_C9ZZQ4_
HLA-DQA10501-DQB10201	695	FGAPKSDTGRFLRKL	PKSDTGRFL	3	0.0640	25021.1	95.00	0.52	tr_C9ZZQ4_
HLA-DQA10501-DQB10201	696	LNVNDKPAPDKEFNA	NVNDKPAPD	1	0.0609	25879.9	100.00	0.25	tr_C9ZZQ4_
HLA-DQA10501-DQB10201	697	DELSKETDKSGITIV	TDKSGITIV	6	0.0602	26066.5	100.00	0.44	tr_C9ZZQ4_
HLA-DQA10501-DQB10201	698	IEPQLNKFRRHVIHL	KFRRHVIHL	6	0.0586	26527.7	100.00	0.50	tr_C9ZZQ4_
HLA-DQA10501-DQB10201	699	TSDSAKALKEEKKKR	SAKALKEEK	3	0.0562	27228.5	100.00	0.28	tr_C9ZZQ4_
HLA-DQA10501-DQB10201	700	ITIEPQLNKFRRHVI	TIEPQLNKF	1	0.0526	28297.2	100.00	0.42	tr_C9ZZQ4_
HLA-DQA10501-DQB10201	701	SDSAKALKEEKKKRT	SAKALKEEK	2	0.0496	29250.4	100.00	0.32	tr_C9ZZQ4_
HLA-DQA10501-DQB10201	702	VQVVQSSKSHKTPEE	SKSHKTPEE	6	0.0471	30044.2	100.00	0.36	tr_C9ZZQ4_
HLA-DQA10501-DQB10201	703	KVQVVQSSKSHKTPE	VQVVQSSKS	1	0.0469	30101.5	100.00	0.56	tr_C9ZZQ4_
HLA-DQA10501-DQB10201	704	KDELSKETDKSGITI	ETDKSGITI	6	0.0440	31054.6	100.00	0.35	tr_C9ZZQ4_
HLA-DQA10501-DQB10201	705	NETSSPTIRKFHATV	TIRKFHATV	6	0.0439	31096.0	100.00	0.55	tr_C9ZZQ4_
HLA-DQA10501-DQB10201	706	YDNETSSPTIRKFHA	DNETSSPTI	1	0.0432	31345.6	100.00	0.36	tr_C9ZZQ4_
HLA-DQA10501-DQB10201	707	TIEPQLNKFRRHVIH	TIEPQLNKF	0	0.0430	31407.0	100.00	0.35	tr_C9ZZQ4_
HLA-DQA10501-DQB10201	708	DSAKALKEEKKKRTV	SAKALKEEK	1	0.0412	32031.6	100.00	0.35	tr_C9ZZQ4_
HLA-DQA10501-DQB10201	709	IKDELSKETDKSGIT	ELSKETDKS	3	0.0396	32568.8	100.00	0.31	tr_C9ZZQ4_
HLA-DQA10501-DQB10201	710	DNETSSPTIRKFHAT	DNETSSPTI	0	0.0337	34725.0	100.00	0.28	tr_C9ZZQ4_
HLA-DQA10501-DQB10201	711	SAKALKEEKKKRTVT	AKALKEEKK	1	0.0283	36824.7	100.00	0.57	tr_C9ZZQ4_
HLA-DQA10501-DQB10201	712	KALKEEKKKRTVTAV	KKKRTVTAV	6	0.0204	40086.5	100.00	0.65	tr_C9ZZQ4_
HLA-DQA10501-DQB10201	713	AKALKEEKKKRTVTA	AKALKEEKK	0	0.0144	42804.8	100.00	0.51	tr_C9ZZQ4_

Allele: HLA-DQA10501-DQB10201. Number of high binders 4. Number of weak binders 49. Number of peptides 713

Allele	pos	peptide	core	Of	1-log50k(aff)	affinity(nM)	%Rank	Relia	Identity	Bind Level
HLA-DQA10501-DQB10201	1	VKVGHIVDVAAVERV	IVDVAAVER	5	0.5483	132.6	3.00	0.41	tr_C9ZZQ4_	WB
HLA-DQA10501-DQB10201	2	ATYFSLSTAEREQLD	FSLSTAERE	3	0.5398	145.4	3.50	0.57	tr_C9ZZQ4_	WB
HLA-DQA10501-DQB10201	3	RATYFSLSTAEREQL	FSLSTAERE	4	0.5274	166.2	4.00	0.57	tr_C9ZZQ4_	WB
HLA-DQA10501-DQB10201	4	GVPEPVEIYQLDAVP	VEIYQLDAV	5	0.5250	170.7	4.00	0.50	tr_C9ZZQ4_	WB
HLA-DQA10501-DQB10201	5	TYFSLSTAEREQLDV	FSLSTAERE	2	0.5220	176.2	4.50	0.56	tr_C9ZZQ4_	WB
HLA-DQA10501-DQB10201	6	VPEPVEIYQLDAVPG	VEIYQLDAV	4	0.5180	184.0	4.50	0.44	tr_C9ZZQ4_	WB
HLA-DQA10501-DQB10201	7	RLDHEAYVADESSDV	HEAYVADES	3	0.5177	184.7	4.50	0.31	tr_C9ZZQ4_	WB
HLA-DQA10501-DQB10201	8	KVGHIVDVAAVERVG	IVDVAAVER	4	0.5168	186.3	4.50	0.43	tr_C9ZZQ4_	WB
HLA-DQA10501-DQB10201	9	AVKVGHIVDVAAVER	IVDVAAVER	6	0.5158	188.4	4.50	0.46	tr_C9ZZQ4_	WB

HLA-DQA10501-DQB10201	10	LTRATYFSLSTAERE	FSLSTAERE	6	0.5111	198.3	5.00	0.61	tr_C9ZZQ4_	WB
HLA-DQA10501-DQB10201	11	LDHEAYVADESSDVS	HEAYVADES	2	0.5084	204.2	5.00	0.28	tr_C9ZZQ4_	WB
HLA-DQA10501-DQB10201	12	TRATYFSLSTAEREQ	FSLSTAERE	5	0.5079	205.4	5.00	0.61	tr_C9ZZQ4_	WB
HLA-DQA10501-DQB10201	13	LRLDHEAYVADESSD	HEAYVADES	4	0.5062	209.1	5.00	0.39	tr_C9ZZQ4_	WB
HLA-DQA10501-DQB10201	14	PLRLDHEAYVADESS	HEAYVADES	5	0.4937	239.4	6.00	0.38	tr_C9ZZQ4_	WB
HLA-DQA10501-DQB10201	15	PEPVEIYQLDAVPGR	VEIYQLDAV	3	0.4917	244.7	6.00	0.43	tr_C9ZZQ4_	WB
HLA-DQA10501-DQB10201	16	VGHIVDVAAVERVGR	IVDVAAVER	3	0.4873	256.4	6.50	0.46	tr_C9ZZQ4_	WB
HLA-DQA10501-DQB10201	17	YFSLSTAEREQLDVT	FSLSTAERE	1	0.4846	264.1	6.50	0.60	tr_C9ZZQ4_	WB
HLA-DQA10501-DQB10201	18	DHEAYVADESSDVSH	EAYVADESS	2	0.4820	271.6	7.00	0.34	tr_C9ZZQ4_	WB
HLA-DQA10501-DQB10201	19	EPVEIYQLDAVPGRT	VEIYQLDAV	2	0.4797	278.7	7.00	0.44	tr_C9ZZQ4_	WB
HLA-DQA10501-DQB10201	20	GHIVDVAAVERVGRP	IVDVAAVER	2	0.4762	289.4	7.50	0.43	tr_C9ZZQ4_	WB
HLA-DQA10501-DQB10201	21	RGVPEPVEIYQLDAV	VEIYQLDAV	6	0.4759	290.4	7.50	0.56	tr_C9ZZQ4_	WB
HLA-DQA10501-DQB10201	22	FLNYRWGTDSIDNFY	WGTDSIDNF	5	0.4749	293.3	7.50	0.46	tr_C9ZZQ4_	WB
HLA-DQA10501-DQB10201	23	AVDQSTEDLEGDAFE	TEDLEGDAF	5	0.4732	299.0	7.50	0.50	tr_C9ZZQ4_	WB
HLA-DQA10501-DQB10201	24	VDQSTEDLEGDAFEC	TEDLEGDAF	4	0.4666	320.9	8.00	0.54	tr_C9ZZQ4_	WB
HLA-DQA10501-DQB10201	25	QSTEDLEGDAFECGW	TEDLEGDAF	2	0.4657	324.2	8.00	0.36	tr_C9ZZQ4_	WB
HLA-DQA10501-DQB10201	26	APLRLDHEAYVADES	HEAYVADES	6	0.4596	346.2	8.50	0.52	tr_C9ZZQ4_	WB
HLA-DQA10501-DQB10201	27	PVEIYQLDAVPGRTF	VEIYQLDAV	1	0.4576	353.7	9.00	0.41	tr_C9ZZQ4_	WB
HLA-DQA10501-DQB10201	28	PAVDQSTEDLEGDAF	TEDLEGDAF	6	0.4574	354.5	9.00	0.54	tr_C9ZZQ4_	WB
HLA-DQA10501-DQB10201	29	STEDLEGDAFECGWE	TEDLEGDAF	1	0.4546	365.3	9.00	0.34	tr_C9ZZQ4_	WB
HLA-DQA10501-DQB10201	30	HIVDVAAVERVGRPT	IVDVAAVER	1	0.4511	379.6	9.50	0.43	tr_C9ZZQ4_	WB
HLA-DQA10501-DQB10201	31	LNYRWGTDSIDNFYR	WGTDSIDNF	4	0.4445	407.5	10.00	0.49	tr_C9ZZQ4_	
HLA-DQA10501-DQB10201	32	DQSTEDLEGDAFECG	TEDLEGDAF	3	0.4425	416.5	11.00	0.48	tr_C9ZZQ4_	
HLA-DQA10501-DQB10201	33	LAVKVGHIVDVAAVE	HIVDVAAVE	6	0.4423	417.4	11.00	0.32	tr_C9ZZQ4_	
HLA-DQA10501-DQB10201	34	CFLNYRWGTDSIDNF	WGTDSIDNF	6	0.4403	426.6	11.00	0.53	tr_C9ZZQ4_	
HLA-DQA10501-DQB10201	35	TEDLEGDAFECGWEE	TEDLEGDAF	0	0.4315	469.0	12.00	0.26	tr_C9ZZQ4_	
HLA-DQA10501-DQB10201	36	HEAYVADESSDVSHT	EAYVADESS	1	0.4302	475.8	12.00	0.34	tr_C9ZZQ4_	
HLA-DQA10501-DQB10201	37	QWASHPNVMADAVAA	PNVMADAVA	5	0.4288	483.3	12.00	0.34	tr_C9ZZQ4_	
HLA-DQA10501-DQB10201	38	NYRWGTDSIDNFYRA	WGTDSIDNF	3	0.4266	494.8	12.00	0.50	tr_C9ZZQ4_	
HLA-DQA10501-DQB10201	39	WKEAVWNDEYYQEAI	EAVWNDEYY	2	0.4259	498.5	13.00	0.28	tr_C9ZZQ4_	
HLA-DQA10501-DQB10201	40	NYDCYEVKTIGDSFM	EVKTIGDSF	5	0.4240	509.0	13.00	0.57	tr_C9ZZQ4_	
HLA-DQA10501-DQB10201	41	SLIRTLIGNYDCYEV	LIGNYDCYE	5	0.4234	512.3	13.00	0.34	tr_C9ZZQ4_	
HLA-DQA10501-DQB10201	42	YDCYEVKTIGDSFMI	EVKTIGDSF	4	0.4232	513.5	13.00	0.55	tr_C9ZZQ4_	
HLA-DQA10501-DQB10201	43	PWKEAVWNDEYYQEA	EAVWNDEYY	3	0.4195	534.1	13.00	0.26	tr_C9ZZQ4_	
HLA-DQA10501-DQB10201	44	VEIYQLDAVPGRTFA	VEIYQLDAV	0	0.4174	546.7	14.00	0.38	tr_C9ZZQ4_	
HLA-DQA10501-DQB10201	45	FSLSTAEREQLDVTA	FSLSTAERE	0	0.4168	549.8	14.00	0.48	tr_C9ZZQ4_	
HLA-DQA10501-DQB10201	46	DCYEVKTIGDSFMIA	EVKTIGDSF	3	0.4129	573.6	14.00	0.52	tr_C9ZZQ4_	
HLA-DQA10501-DQB10201	47	TDPVTLIFTDIENST	VTLIFTDIE	3	0.4107	587.8	14.00	0.46	tr_C9ZZQ4_	
HLA-DQA10501-DQB10201	48	HSLIRTLIGNYDCYE	IRTLIGNYD	3	0.4104	589.8	14.00	0.41	tr_C9ZZQ4_	
HLA-DQA10501-DQB10201	49	WASHPNVMADAVAAH	PNVMADAVA	4	0.4098	593.4	15.00	0.28	tr_C9ZZQ4_	
HLA-DQA10501-DQB10201	50	SQWASHPNVMADAVA	PNVMADAVA	6	0.4046	627.4	15.00	0.41	tr_C9ZZQ4_	
HLA-DQA10501-DQB10201	51	PTDPVTLIFTDIENS	VTLIFTDIE	4	0.4023	643.7	16.00	0.51	tr_C9ZZQ4_	
HLA-DQA10501-DQB10201	52	YRWGTDSIDNFYRAE	WGTDSIDNF	2	0.4008	654.2	16.00	0.53	tr_C9ZZQ4_	
HLA-DQA10501-DQB10201	53	RLAVKVGHIVDVAAV	KVGHIVDVA	4	0.4005	656.3	16.00	0.44	tr_C9ZZQ4_	
HLA-DQA10501-DQB10201	54	DPVTLIFTDIENSTS	VTLIFTDIE	2	0.3992	665.6	16.00	0.44	tr_C9ZZQ4_	
HLA-DQA10501-DQB10201	55	KEAVWNDEYYQEAIH	EAVWNDEYY	1	0.3968	683.1	16.00	0.22	tr_C9ZZQ4_	
HLA-DQA10501-DQB10201	56	CYEVKTIGDSFMIAS	EVKTIGDSF	2	0.3951	695.4	17.00	0.50	tr_C9ZZQ4_	
HLA-DQA10501-DQB10201	57	PLPPWKEAVWNDEYY	EAVWNDEYY	6	0.3950	696.5	17.00	0.47	tr_C9ZZQ4_	
HLA-DQA10501-DQB10201	58	FAPLRLDHEAYVADE	DHEAYVADE	6	0.3949	696.9	17.00	0.26	tr_C9ZZQ4_	
HLA-DQA10501-DQB10201	59	TKGYDYYGQTANMAA	YYGQTANMA	5	0.3935	707.6	17.00	0.57	tr_C9ZZQ4_	

HLA-DQA10501-DQB10201	60	PPWKEAVWNDEYYQE	EAVWNDEYY	4	0.3934	708.3	17.00	0.37	tr_C9ZZQ4_
HLA-DQA10501-DQB10201	61	LIRTLIGNYDCYEVK	LIGNYDCYE	4	0.3908	729.1	17.00	0.35	tr_C9ZZQ4_
HLA-DQA10501-DQB10201	62	DPTDPVTLIFTDIEN	VTLIFTDIE	5	0.3860	768.0	18.00	0.66	tr_C9ZZQ4_
HLA-DQA10501-DQB10201	63	LPPWKEAVWNDEYYQ	EAVWNDEYY	5	0.3857	770.0	18.00	0.40	tr_C9ZZQ4_
HLA-DQA10501-DQB10201	64	LRGVPEPVEIYQLDA	PVEIYQLDA	6	0.3857	770.6	18.00	0.38	tr_C9ZZQ4_
HLA-DQA10501-DQB10201	65	AVWNDEYYQEAIHRL	YYQEAIHRL	6	0.3856	770.8	18.00	0.31	tr_C9ZZQ4_
HLA-DQA10501-DQB10201	66	GSVPLRGVPEPVEIY	PLRGVPEPV	3	0.3846	779.6	18.00	0.43	tr_C9ZZQ4_
HLA-DQA10501-DQB10201	67	MDPTDPVTLIFTDIE	VTLIFTDIE	6	0.3835	788.7	18.00	0.75	tr_C9ZZQ4_
HLA-DQA10501-DQB10201	68	YEVKTIGDSFMIASK	EVKTIGDSF	1	0.3832	791.5	18.00	0.50	tr_C9ZZQ4_
HLA-DQA10501-DQB10201	69	HRLAVKVGHIVDVAA	KVGHIVDVA	5	0.3811	809.4	19.00	0.50	tr_C9ZZQ4_
HLA-DQA10501-DQB10201	70	PLRGVPEPVEIYQLD	PLRGVPEPV	0	0.3808	812.0	19.00	0.22	tr_C9ZZQ4_
HLA-DQA10501-DQB10201	71	EAVWNDEYYQEAIHR	WNDEYYQEA	3	0.3802	817.3	19.00	0.22	tr_C9ZZQ4_
HLA-DQA10501-DQB10201	72	SVPLRGVPEPVEIYQ	PLRGVPEPV	2	0.3794	824.3	19.00	0.43	tr_C9ZZQ4_
HLA-DQA10501-DQB10201	73	VTKGYDYYGQTANMA	YYGQTANMA	6	0.3781	836.4	19.00	0.61	tr_C9ZZQ4_
HLA-DQA10501-DQB10201	74	ASHPNVMADAVAAHH	PNVMADAVA	3	0.3777	840.1	19.00	0.28	tr_C9ZZQ4_
HLA-DQA10501-DQB10201	75	VPLRGVPEPVEIYQL	PLRGVPEPV	1	0.3766	849.7	19.00	0.32	tr_C9ZZQ4_
HLA-DQA10501-DQB10201	76	LGSVPLRGVPEPVEI	PLRGVPEPV	4	0.3763	852.5	20.00	0.44	tr_C9ZZQ4_
HLA-DQA10501-DQB10201	77	VTLIFTDIENSTSQW	VTLIFTDIE	0	0.3748	866.5	20.00	0.25	tr_C9ZZQ4_
HLA-DQA10501-DQB10201	78	IVDVAAVERVGRPTE	IVDVAAVER	0	0.3733	881.2	20.00	0.44	tr_C9ZZQ4_
HLA-DQA10501-DQB10201	79	PVTLIFTDIENSTSQ	VTLIFTDIE	1	0.3730	883.4	20.00	0.43	tr_C9ZZQ4_
HLA-DQA10501-DQB10201	80	EREQLDVTALGSVPL	VTALGSVPL	6	0.3722	891.6	20.00	0.47	tr_C9ZZQ4_
HLA-DQA10501-DQB10201	81	TFAPLRLDHEAYVAD	FAPLRLDHE	1	0.3719	894.4	20.00	0.26	tr_C9ZZQ4_
HLA-DQA10501-DQB10201	82	GRTFAPLRLDHEAYV	FAPLRLDHE	3	0.3710	902.9	20.00	0.32	tr_C9ZZQ4_
HLA-DQA10501-DQB10201	83	EAYVADESSDVSHTT	YVADESSDV	2	0.3686	926.2	21.00	0.42	tr_C9ZZQ4_
HLA-DQA10501-DQB10201	84	LLTRATYFSLSTAER	YFSLSTAER	6	0.3686	926.7	21.00	0.44	tr_C9ZZQ4_
HLA-DQA10501-DQB10201	85	EDLEGDAFECGWEEK	EGDAFECGW	3	0.3680	933.0	21.00	0.24	tr_C9ZZQ4_
HLA-DQA10501-DQB10201	86	KGYDYYGQTANMAAR	YYGQTANMA	4	0.3660	953.3	21.00	0.56	tr_C9ZZQ4_
HLA-DQA10501-DQB10201	87	PGRTFAPLRLDHEAY	FAPLRLDHE	4	0.3658	954.7	21.00	0.35	tr_C9ZZQ4_
HLA-DQA10501-DQB10201	88	LISNPAVDQSTEDLE	AVDQSTEDL	5	0.3638	976.0	22.00	0.45	tr_C9ZZQ4_
HLA-DQA10501-DQB10201	89	SKAEPPSAQLDPEVY	SAQLDPEVY	6	0.3635	979.4	22.00	0.57	tr_C9ZZQ4_
HLA-DQA10501-DQB10201	90	SNPAVDQSTEDLEGD	AVDQSTEDL	3	0.3632	982.8	22.00	0.44	tr_C9ZZQ4_
HLA-DQA10501-DQB10201	91	ESVSNVLHALLGTFT	LHALLGTFT	6	0.3631	983.3	22.00	0.49	tr_C9ZZQ4_
HLA-DQA10501-DQB10201	92	VWNDEYYQEAIHRLA	YYQEAIHRL	5	0.3611	1004.8	22.00	0.31	tr_C9ZZQ4_
HLA-DQA10501-DQB10201	93	GYDYYGQTANMAART	YYGQTANMA	3	0.3596	1021.8	22.00	0.50	tr_C9ZZQ4_
HLA-DQA10501-DQB10201	94	AVPGRTFAPLRLDHE	FAPLRLDHE	6	0.3592	1025.4	23.00	0.41	tr_C9ZZQ4_
HLA-DQA10501-DQB10201	95	RTFAPLRLDHEAYVA	FAPLRLDHE	2	0.3583	1036.0	23.00	0.34	tr_C9ZZQ4_
HLA-DQA10501-DQB10201	96	SHPNVMADAVAAHHS	PNVMADAVA	2	0.3563	1059.1	23.00	0.29	tr_C9ZZQ4_
HLA-DQA10501-DQB10201	97	YDYYGQTANMAARTE	YYGQTANMA	2	0.3559	1062.7	23.00	0.46	tr_C9ZZQ4_
HLA-DQA10501-DQB10201	98	ALGSVPLRGVPEPVE	PLRGVPEPV	5	0.3555	1067.4	23.00	0.46	tr_C9ZZQ4_
HLA-DQA10501-DQB10201	99	SVSNVLHALLGTFTI	LHALLGTFT	5	0.3553	1069.7	23.00	0.49	tr_C9ZZQ4_
HLA-DQA10501-DQB10201	100	IRTLIGNYDCYEVKT	LIGNYDCYE	3	0.3526	1101.6	24.00	0.40	tr_C9ZZQ4_
HLA-DQA10501-DQB10201	101	NPAVDQSTEDLEGDA	AVDQSTEDL	2	0.3520	1108.8	24.00	0.41	tr_C9ZZQ4_
HLA-DQA10501-DQB10201	102	VPGRTFAPLRLDHEA	FAPLRLDHE	5	0.3518	1111.5	24.00	0.40	tr_C9ZZQ4_
HLA-DQA10501-DQB10201	103	HHSLIRTLIGNYDCY	IRTLIGNYD	4	0.3513	1117.2	24.00	0.61	tr_C9ZZQ4_
HLA-DQA10501-DQB10201	104	ILISNPAVDQSTEDL	AVDQSTEDL	6	0.3510	1121.2	24.00	0.46	tr_C9ZZQ4_
HLA-DQA10501-DQB10201	105	TLIFTDIENSTSQWA	LIFTDIENS	1	0.3504	1128.5	24.00	0.25	tr_C9ZZQ4_
HLA-DQA10501-DQB10201	106	IHRLAVKVGHIVDVA	KVGHIVDVA	6	0.3500	1133.2	24.00	0.56	tr_C9ZZQ4_
HLA-DQA10501-DQB10201	107	REQLDVTALGSVPLR	VTALGSVPL	5	0.3493	1141.7	24.00	0.43	tr_C9ZZQ4_
HLA-DQA10501-DQB10201	108	GNYDCYEVKTIGDSF	EVKTIGDSF	6	0.3483	1154.2	25.00	0.86	tr_C9ZZQ4_
HLA-DQA10501-DQB10201	109	KAEPPSAQLDPEVYR	SAQLDPEVY	5	0.3449	1196.9	25.00	0.60	tr_C9ZZQ4_

HLA-DQA10501-DQB10201	110	EQLDVTALGSVPLRG	VTALGSVPL	4	0.3442	1206.2	25.00	0.33	tr_C9ZZQ4_
HLA-DQA10501-DQB10201	111	ISNPAVDQSTEDLEG	AVDQSTEDL	4	0.3430	1222.7	26.00	0.46	tr_C9ZZQ4_
HLA-DQA10501-DQB10201	112	RWGTDSIDNFYRAEE	WGTDSIDNF	1	0.3409	1250.2	26.00	0.56	tr_C9ZZQ4_
HLA-DQA10501-DQB10201	113	AHHSLIRTLIGNYDC	IRTLIGNYD	5	0.3405	1256.5	26.00	0.62	tr_C9ZZQ4_
HLA-DQA10501-DQB10201	114	VSNVLHALLGTFTIV	LHALLGTFT	4	0.3354	1327.0	27.00	0.53	tr_C9ZZQ4_
HLA-DQA10501-DQB10201	115	TSQWASHPNVMADAV	HPNVMADAV	6	0.3353	1328.7	27.00	0.53	tr_C9ZZQ4_
HLA-DQA10501-DQB10201	116	HPNVMADAVAAHHSL	NVMADAVAA	2	0.3347	1337.8	27.00	0.29	tr_C9ZZQ4_
HLA-DQA10501-DQB10201	117	AAHHSLIRTLIGNYD	IRTLIGNYD	6	0.3339	1348.6	27.00	0.64	tr_C9ZZQ4_
HLA-DQA10501-DQB10201	118	WNDEYYQEAIHRLAV	YYQEAIHRL	4	0.3307	1396.5	28.00	0.35	tr_C9ZZQ4_
HLA-DQA10501-DQB10201	119	TALGSVPLRGVPEPV	PLRGVPEPV	6	0.3273	1449.3	29.00	0.49	tr_C9ZZQ4_
HLA-DQA10501-DQB10201	120	WGTDSIDNFYRAEEK	WGTDSIDNF	0	0.3260	1468.9	29.00	0.31	tr_C9ZZQ4_
HLA-DQA10501-DQB10201	121	AGIHTGLCDIRYDEV	LCDIRYDEV	6	0.3257	1474.5	29.00	0.35	tr_C9ZZQ4_
HLA-DQA10501-DQB10201	122	QLDVTALGSVPLRGV	VTALGSVPL	3	0.3253	1480.2	29.00	0.33	tr_C9ZZQ4_
HLA-DQA10501-DQB10201	123	EVKTIGDSFMIASKS	EVKTIGDSF	0	0.3235	1509.2	30.00	0.41	tr_C9ZZQ4_
HLA-DQA10501-DQB10201	124	GIHTGLCDIRYDEVT	LCDIRYDEV	5	0.3228	1520.5	30.00	0.37	tr_C9ZZQ4_
HLA-DQA10501-DQB10201	125	LIFTDIENSTSQWAS	IENSTSQWA	5	0.3197	1572.4	31.00	0.28	tr_C9ZZQ4_
HLA-DQA10501-DQB10201	126	SLSTAEREQLDVTAL	REQLDVTAL	6	0.3191	1582.5	31.00	0.27	tr_C9ZZQ4_
HLA-DQA10501-DQB10201	127	EIYQLDAVPGRTFAP	IYQLDAVPG	1	0.3164	1630.1	31.00	0.35	tr_C9ZZQ4_
HLA-DQA10501-DQB10201	128	VLLTRATYFSLSTAE	TYFSLSTAE	6	0.3152	1650.8	32.00	0.50	tr_C9ZZQ4_
HLA-DQA10501-DQB10201	129	SNVLHALLGTFTIVQ	LHALLGTFT	3	0.3143	1668.3	32.00	0.52	tr_C9ZZQ4_
HLA-DQA10501-DQB10201	130	DYYGQTANMAARTES	YYGQTANMA	1	0.3129	1693.7	32.00	0.47	tr_C9ZZQ4_
HLA-DQA10501-DQB10201	131	NVLHALLGTFTIVQR	LHALLGTFT	2	0.3127	1697.1	32.00	0.47	tr_C9ZZQ4_
HLA-DQA10501-DQB10201	132	AEPPSAQLDPEVYRK	SAQLDPEVY	4	0.3118	1714.1	32.00	0.61	tr_C9ZZQ4_
HLA-DQA10501-DQB10201	133	LDAVPGRTFAPLRLD	GRTFAPLRL	5	0.3102	1743.8	33.00	0.63	tr_C9ZZQ4_
HLA-DQA10501-DQB10201	134	IDNFYRAEEKQVAEL	FYRAEEKQV	3	0.3100	1746.2	33.00	0.37	tr_C9ZZQ4_
HLA-DQA10501-DQB10201	135	LDVTALGSVPLRGVP	VTALGSVPL	2	0.3096	1754.6	33.00	0.34	tr_C9ZZQ4_
HLA-DQA10501-DQB10201	136	RTLIGNYDCYEVKTI	LIGNYDCYE	2	0.3086	1773.0	33.00	0.50	tr_C9ZZQ4_
HLA-DQA10501-DQB10201	137	EPPSAQLDPEVYRKL	SAQLDPEVY	3	0.3072	1799.8	33.00	0.62	tr_C9ZZQ4_
HLA-DQA10501-DQB10201	138	LSTAEREQLDVTALG	REQLDVTAL	5	0.3051	1841.2	34.00	0.25	tr_C9ZZQ4_
HLA-DQA10501-DQB10201	139	VPLPPWKEAVWNDEY	KEAVWNDEY	6	0.3044	1856.2	34.00	0.43	tr_C9ZZQ4_
HLA-DQA10501-DQB10201	140	RCFLNYRWGTDSIDN	YRWGTDSID	5	0.3036	1872.6	34.00	0.50	tr_C9ZZQ4_
HLA-DQA10501-DQB10201	141	DNFYRAEEKQVAELN	FYRAEEKQV	2	0.3027	1890.6	34.00	0.37	tr_C9ZZQ4_
HLA-DQA10501-DQB10201	142	PNVMADAVAAHHSLI	NVMADAVAA	1	0.3026	1893.0	34.00	0.31	tr_C9ZZQ4_
HLA-DQA10501-DQB10201	143	STAEREQLDVTALGS	QLDVTALGS	6	0.3026	1893.0	34.00	0.24	tr_C9ZZQ4_
HLA-DQA10501-DQB10201	144	DLEGDAFECGWEEKG	EGDAFECGW	2	0.3003	1940.0	35.00	0.28	tr_C9ZZQ4_
HLA-DQA10501-DQB10201	145	AYVADESSDVSHTTF	YVADESSDV	1	0.3003	1940.4	35.00	0.53	tr_C9ZZQ4_
HLA-DQA10501-DQB10201	146	IHTGLCDIRYDEVTK	LCDIRYDEV	4	0.2993	1961.7	35.00	0.35	tr_C9ZZQ4_
HLA-DQA10501-DQB10201	147	YYGQTANMAARTESI	NMAARTESI	6	0.2976	1998.4	36.00	0.38	tr_C9ZZQ4_
HLA-DQA10501-DQB10201	148	IRAGIHTGLCDIRYD	HTGLCDIRY	5	0.2966	2020.0	36.00	0.33	tr_C9ZZQ4_
HLA-DQA10501-DQB10201	149	DAVPGRTFAPLRLDH	GRTFAPLRL	4	0.2961	2030.8	36.00	0.59	tr_C9ZZQ4_
HLA-DQA10501-DQB10201	150	DVTALGSVPLRGVPE	ALGSVPLRG	3	0.2949	2056.8	36.00	0.28	tr_C9ZZQ4_
HLA-DQA10501-DQB10201	151	SSSVILISNPAVDQS	LISNPAVDQ	5	0.2946	2064.7	37.00	0.35	tr_C9ZZQ4_
HLA-DQA10501-DQB10201	152	VILISNPAVDQSTED	LISNPAVDQ	2	0.2945	2066.1	37.00	0.29	tr_C9ZZQ4_
HLA-DQA10501-DQB10201	153	DSIDNFYRAEEKQVA	FYRAEEKQV	5	0.2945	2066.4	37.00	0.39	tr_C9ZZQ4_
HLA-DQA10501-DQB10201	154	IVQRQKELTAICERW	ELTAICERW	6	0.2943	2069.9	37.00	0.50	tr_C9ZZQ4_
HLA-DQA10501-DQB10201	155	TAAVRLARDLQRCFL	ARDLQRCFL	6	0.2936	2085.7	37.00	0.32	tr_C9ZZQ4_
HLA-DQA10501-DQB10201	156	AEREQLDVTALGSVP	QLDVTALGS	4	0.2929	2101.6	37.00	0.28	tr_C9ZZQ4_
HLA-DQA10501-DQB10201	157	VLHALLGTFTIVQRQ	LHALLGTFT	1	0.2927	2107.6	37.00	0.47	tr_C9ZZQ4_
HLA-DQA10501-DQB10201	158	GTGGESVSNVLHALL	VSNVLHALL	6	0.2913	2139.1	37.00	0.61	tr_C9ZZQ4_
HLA-DQA10501-DQB10201	159	HTGLCDIRYDEVTKG	LCDIRYDEV	3	0.2908	2150.4	37.00	0.34	tr_C9ZZQ4_

HLA-DQA10501-DQB10201	160	RIRAGIHTGLCDIRY	HTGLCDIRY	6	0.2908	2151.3	37.00	0.38	tr_C9ZZQ4_
HLA-DQA10501-DQB10201	161	PPSAQLDPEVYRKLW	SAQLDPEVY	2	0.2900	2170.1	38.00	0.64	tr_C9ZZQ4_
HLA-DQA10501-DQB10201	162	TDSIDNFYRAEEKQV	FYRAEEKQV	6	0.2895	2180.7	38.00	0.39	tr_C9ZZQ4_
HLA-DQA10501-DQB10201	163	DSSSVILISNPAVDQ	LISNPAVDQ	6	0.2892	2187.5	38.00	0.35	tr_C9ZZQ4_
HLA-DQA10501-DQB10201	164	QRCFLNYRWGTDSID	YRWGTDSID	6	0.2892	2188.7	38.00	0.53	tr_C9ZZQ4_
HLA-DQA10501-DQB10201	165	QLDAVPGRTFAPLRL	GRTFAPLRL	6	0.2891	2191.1	38.00	0.64	tr_C9ZZQ4_
HLA-DQA10501-DQB10201	166	TLIGNYDCYEVKTIG	LIGNYDCYE	1	0.2884	2207.5	38.00	0.50	tr_C9ZZQ4_
HLA-DQA10501-DQB10201	167	SSVILISNPAVDQST	LISNPAVDQ	4	0.2877	2223.0	38.00	0.35	tr_C9ZZQ4_
HLA-DQA10501-DQB10201	168	TAEREQLDVTALGSV	QLDVTALGS	5	0.2857	2271.9	39.00	0.28	tr_C9ZZQ4_
HLA-DQA10501-DQB10201	169	SIDNFYRAEEKQVAE	FYRAEEKQV	4	0.2851	2287.0	39.00	0.40	tr_C9ZZQ4_
HLA-DQA10501-DQB10201	170	RAGIHTGLCDIRYDE	HTGLCDIRY	4	0.2806	2400.6	40.00	0.34	tr_C9ZZQ4_
HLA-DQA10501-DQB10201	171	NDEYYQEAIHRLAVK	YYQEAIHRL	3	0.2806	2402.3	40.00	0.37	tr_C9ZZQ4_
HLA-DQA10501-DQB10201	172	SVILISNPAVDQSTE	LISNPAVDQ	3	0.2801	2415.3	40.00	0.38	tr_C9ZZQ4_
HLA-DQA10501-DQB10201	173	VTALGSVPLRGVPEP	ALGSVPLRG	2	0.2795	2429.2	40.00	0.32	tr_C9ZZQ4_
HLA-DQA10501-DQB10201	174	GESVSNVLHALLGTF	VSNVLHALL	3	0.2781	2467.8	41.00	0.44	tr_C9ZZQ4_
HLA-DQA10501-DQB10201	175	TGLCDIRYDEVTKGY	LCDIRYDEV	2	0.2752	2546.7	41.00	0.36	tr_C9ZZQ4_
HLA-DQA10501-DQB10201	176	TGGESVSNVLHALLG	VSNVLHALL	5	0.2723	2627.1	42.00	0.54	tr_C9ZZQ4_
HLA-DQA10501-DQB10201	177	NFYRAEEKQVAELNS	FYRAEEKQV	1	0.2718	2639.8	42.00	0.31	tr_C9ZZQ4_
HLA-DQA10501-DQB10201	178	VQRQKELTAICERWR	ELTAICERW	5	0.2702	2686.4	43.00	0.49	tr_C9ZZQ4_
HLA-DQA10501-DQB10201	179	GLCDIRYDEVTKGYD	LCDIRYDEV	1	0.2700	2694.0	43.00	0.31	tr_C9ZZQ4_
HLA-DQA10501-DQB10201	180	YGQTANMAARTESIA	NMAARTESI	5	0.2654	2829.6	44.00	0.49	tr_C9ZZQ4_
HLA-DQA10501-DQB10201	181	SKSATAAVRLARDLQ	TAAVRLARD	4	0.2650	2842.9	44.00	0.34	tr_C9ZZQ4_
HLA-DQA10501-DQB10201	182	RQKELTAICERWRVP	ELTAICERW	3	0.2626	2917.9	45.00	0.53	tr_C9ZZQ4_
HLA-DQA10501-DQB10201	183	PSAQLDPEVYRKLWN	SAQLDPEVY	1	0.2611	2964.3	45.00	0.70	tr_C9ZZQ4_
HLA-DQA10501-DQB10201	184	QRQKELTAICERWRV	ELTAICERW	4	0.2610	2969.7	45.00	0.49	tr_C9ZZQ4_
HLA-DQA10501-DQB10201	185	ATAAVRLARDLQRCF	AVRLARDLQ	3	0.2607	2979.2	45.00	0.32	tr_C9ZZQ4_
HLA-DQA10501-DQB10201	186	GGESVSNVLHALLGT	VSNVLHALL	4	0.2604	2988.3	45.00	0.51	tr_C9ZZQ4_
HLA-DQA10501-DQB10201	187	KSATAAVRLARDLQR	TAAVRLARD	3	0.2584	3052.5	46.00	0.27	tr_C9ZZQ4_
HLA-DQA10501-DQB10201	188	DARDNVSAPMDPTDP	RDNVSAPMD	2	0.2560	3134.2	47.00	0.28	tr_C9ZZQ4_
HLA-DQA10501-DQB10201	189	DEYYQEAIHRLAVKV	YYQEAIHRL	2	0.2553	3158.5	47.00	0.41	tr_C9ZZQ4_
HLA-DQA10501-DQB10201	190	NVMADAVAAHHSLIR	NVMADAVAA	0	0.2542	3193.8	47.00	0.28	tr_C9ZZQ4_
HLA-DQA10501-DQB10201	191	SATAAVRLARDLQRC	TAAVRLARD	2	0.2538	3209.2	47.00	0.28	tr_C9ZZQ4_
HLA-DQA10501-DQB10201	192	RDARDNVSAPMDPTD	RDNVSAPMD	3	0.2537	3211.9	47.00	0.31	tr_C9ZZQ4_
HLA-DQA10501-DQB10201	193	QVLLTRATYFSLSTA	ATYFSLSTA	6	0.2533	3227.0	47.00	0.31	tr_C9ZZQ4_
HLA-DQA10501-DQB10201	194	LCDIRYDEVTKGYDY	IRYDEVTKG	3	0.2527	3249.1	47.00	0.29	tr_C9ZZQ4_
HLA-DQA10501-DQB10201	195	IFTDIENSTSQWASH	IENSTSQWA	4	0.2526	3252.4	47.00	0.41	tr_C9ZZQ4_
HLA-DQA10501-DQB10201	196	AAVRLARDLQRCFLN	ARDLQRCFL	5	0.2518	3279.3	48.00	0.34	tr_C9ZZQ4_
HLA-DQA10501-DQB10201	197	GTDSIDNFYRAEEKQ	DNFYRAEEK	5	0.2511	3305.6	48.00	0.42	tr_C9ZZQ4_
HLA-DQA10501-DQB10201	198	GQTANMAARTESIAN	NMAARTESI	4	0.2503	3331.7	48.00	0.49	tr_C9ZZQ4_
HLA-DQA10501-DQB10201	199	QKELTAICERWRVPL	ELTAICERW	2	0.2484	3402.8	49.00	0.56	tr_C9ZZQ4_
HLA-DQA10501-DQB10201	200	YVADESSDVSHTTFN	YVADESSDV	0	0.2476	3432.3	49.00	0.51	tr_C9ZZQ4_
HLA-DQA10501-DQB10201	201	LRDARDNVSAPMDPT	RDNVSAPMD	4	0.2474	3440.2	49.00	0.30	tr_C9ZZQ4_
HLA-DQA10501-DQB10201	202	SDSSSVILISNPAVD	ILISNPAVD	6	0.2472	3447.6	49.00	0.35	tr_C9ZZQ4_
HLA-DQA10501-DQB10201	203	NSKAEPPSAQLDPEV	PSAQLDPEV	6	0.2470	3454.4	49.00	0.55	tr_C9ZZQ4_
HLA-DQA10501-DQB10201	204	TLRDARDNVSAPMDP	RDNVSAPMD	5	0.2464	3475.7	49.00	0.33	tr_C9ZZQ4_
HLA-DQA10501-DQB10201	205	LEGDAFECGWEEKGK	EGDAFECGW	1	0.2446	3545.4	50.00	0.35	tr_C9ZZQ4_
HLA-DQA10501-DQB10201	206	EVTKGYDYYGQTANM	DYYGQTANM	6	0.2445	3547.1	50.00	0.48	tr_C9ZZQ4_
HLA-DQA10501-DQB10201	207	LIGNYDCYEVKTIGD	LIGNYDCYE	0	0.2429	3610.1	50.00	0.47	tr_C9ZZQ4_
HLA-DQA10501-DQB10201	208	STSQWASHPNVMADA	SHPNVMADA	6	0.2424	3630.6	50.00	0.29	tr_C9ZZQ4_
HLA-DQA10501-DQB10201	209	ARDNVSAPMDPTDPV	NVSAPMDPT	3	0.2402	3716.1	55.00	0.29	tr_C9ZZQ4_

HLA-DQA10501-DQB10201	210	ASKSATAAVRLARDL	TAAVRLARD	5	0.2398	3734.9	55.00	0.40	tr_C9ZZQ4_
HLA-DQA10501-DQB10201	211	CDIRYDEVTKGYDYY	IRYDEVTKG	2	0.2394	3751.9	55.00	0.26	tr_C9ZZQ4_
HLA-DQA10501-DQB10201	212	ITLRDARDNVSAPMD	RDNVSAPMD	6	0.2377	3821.2	55.00	0.38	tr_C9ZZQ4_
HLA-DQA10501-DQB10201	213	GQVLLTRATYFSLST	VLLTRATYF	2	0.2373	3837.1	55.00	0.25	tr_C9ZZQ4_
HLA-DQA10501-DQB10201	214	LHALLGTFTIVQRQK	LHALLGTFT	0	0.2346	3949.3	55.00	0.42	tr_C9ZZQ4_
HLA-DQA10501-DQB10201	215	VDVAAVERVGRPTES	VDVAAVERV	0	0.2339	3981.3	55.00	0.56	tr_C9ZZQ4_
HLA-DQA10501-DQB10201	216	FTDIENSTSQWASHP	IENSTSQWA	3	0.2328	4025.9	55.00	0.54	tr_C9ZZQ4_
HLA-DQA10501-DQB10201	217	AVRLARDLQRCFLNY	ARDLQRCFL	4	0.2326	4037.3	55.00	0.40	tr_C9ZZQ4_
HLA-DQA10501-DQB10201	218	QTANMAARTESIANG	NMAARTESI	3	0.2325	4042.5	55.00	0.46	tr_C9ZZQ4_
HLA-DQA10501-DQB10201	219	IYQLDAVPGRTFAPL	IYQLDAVPG	0	0.2322	4052.5	55.00	0.30	tr_C9ZZQ4_
HLA-DQA10501-DQB10201	220	IASKSATAAVRLARD	TAAVRLARD	6	0.2307	4122.4	55.00	0.40	tr_C9ZZQ4_
HLA-DQA10501-DQB10201	221	AIHRLAVKVGHIVDV	VKVGHIVDV	6	0.2247	4396.0	55.00	0.32	tr_C9ZZQ4_
HLA-DQA10501-DQB10201	222	TAICERWRVPLPPWK	RWRVPLPPW	5	0.2239	4436.9	60.00	0.39	tr_C9ZZQ4_
HLA-DQA10501-DQB10201	223	PMDPTDPVTLIFTDI	PVTLIFTDI	6	0.2235	4454.9	60.00	0.47	tr_C9ZZQ4_
HLA-DQA10501-DQB10201	224	LRIRAGIHTGLCDIR	AGIHTGLCD	4	0.2227	4490.7	60.00	0.25	tr_C9ZZQ4_
HLA-DQA10501-DQB10201	225	KELTAICERWRVPLP	ELTAICERW	1	0.2225	4500.7	60.00	0.56	tr_C9ZZQ4_
HLA-DQA10501-DQB10201	226	FYRAEEKQVAELNSK	FYRAEEKQV	0	0.2217	4542.7	60.00	0.25	tr_C9ZZQ4_
HLA-DQA10501-DQB10201	227	AICERWRVPLPPWKE	RWRVPLPPW	4	0.2216	4545.9	60.00	0.38	tr_C9ZZQ4_
HLA-DQA10501-DQB10201	228	GGQVLLTRATYFSLS	VLLTRATYF	3	0.2214	4555.2	60.00	0.31	tr_C9ZZQ4_
HLA-DQA10501-DQB10201	229	ARTESIANGGQVLLT	ANGGQVLLT	6	0.2210	4578.5	60.00	0.31	tr_C9ZZQ4_
HLA-DQA10501-DQB10201	230	SFMIASKSATAAVRL	KSATAAVRL	6	0.2202	4613.9	60.00	0.57	tr_C9ZZQ4_
HLA-DQA10501-DQB10201	231	LTAICERWRVPLPPW	RWRVPLPPW	6	0.2193	4660.3	60.00	0.44	tr_C9ZZQ4_
HLA-DQA10501-DQB10201	232	SSDVSHTTFNDCMSI	SHTTFNDCM	4	0.2183	4713.8	60.00	0.41	tr_C9ZZQ4_
HLA-DQA10501-DQB10201	233	DIRYDEVTKGYDYYG	IRYDEVTKG	1	0.2170	4779.5	60.00	0.28	tr_C9ZZQ4_
HLA-DQA10501-DQB10201	234	ADAVAAHHSLIRTLI	HHSLIRTLI	6	0.2165	4802.4	60.00	0.33	tr_C9ZZQ4_
HLA-DQA10501-DQB10201	235	EYYQEAIHRLAVKVG	YYQEAIHRL	1	0.2159	4837.2	60.00	0.38	tr_C9ZZQ4_
HLA-DQA10501-DQB10201	236	RVPLPPWKEAVWNDE	WKEAVWNDE	6	0.2150	4881.2	60.00	0.62	tr_C9ZZQ4_
HLA-DQA10501-DQB10201	237	RDNVSAPMDPTDPVT	NVSAPMDPT	2	0.2136	4958.2	60.00	0.32	tr_C9ZZQ4_
HLA-DQA10501-DQB10201	238	GLRIRAGIHTGLCDI	AGIHTGLCD	5	0.2128	5002.1	60.00	0.28	tr_C9ZZQ4_
HLA-DQA10501-DQB10201	239	ICERWRVPLPPWKEA	RWRVPLPPW	3	0.2126	5012.5	60.00	0.38	tr_C9ZZQ4_
HLA-DQA10501-DQB10201	240	ELTAICERWRVPLPP	ELTAICERW	0	0.2119	5049.2	60.00	0.39	tr_C9ZZQ4_
HLA-DQA10501-DQB10201	241	VKTIGDSFMIASKSA	VKTIGDSFM	0	0.2117	5058.9	60.00	0.43	tr_C9ZZQ4_
HLA-DQA10501-DQB10201	242	NGGQVLLTRATYFSL	VLLTRATYF	4	0.2092	5200.3	60.00	0.41	tr_C9ZZQ4_
HLA-DQA10501-DQB10201	243	IANGGQVLLTRATYF	VLLTRATYF	6	0.2088	5221.5	60.00	0.44	tr_C9ZZQ4_
HLA-DQA10501-DQB10201	244	FTIVQRQKELTAICE	QKELTAICE	6	0.2085	5237.4	60.00	0.48	tr_C9ZZQ4_
HLA-DQA10501-DQB10201	245	VAAHHSLIRTLIGNY	LIRTLIGNY	6	0.2082	5256.4	60.00	0.29	tr_C9ZZQ4_
HLA-DQA10501-DQB10201	246	TANMAARTESIANGG	NMAARTESI	2	0.2077	5284.9	60.00	0.47	tr_C9ZZQ4_
HLA-DQA10501-DQB10201	247	VMADAVAAHHSLIRT	MADAVAAHH	1	0.2073	5308.7	60.00	0.33	tr_C9ZZQ4_
HLA-DQA10501-DQB10201	248	TIVQRQKELTAICER	QKELTAICE	5	0.2064	5359.1	65.00	0.38	tr_C9ZZQ4_
HLA-DQA10501-DQB10201	249	ESSDVSHTTFNDCMS	SHTTFNDCM	5	0.2061	5376.7	65.00	0.44	tr_C9ZZQ4_
HLA-DQA10501-DQB10201	250	SSQLGTGGESVSNVL	QLGTGGESV	2	0.2060	5384.9	65.00	0.40	tr_C9ZZQ4_
HLA-DQA10501-DQB10201	251	AARTESIANGGQVLL	SIANGGQVL	5	0.2058	5396.6	65.00	0.41	tr_C9ZZQ4_
HLA-DQA10501-DQB10201	252	NSTSQWASHPNVMAD	WASHPNVMA	5	0.2048	5454.4	65.00	0.31	tr_C9ZZQ4_
HLA-DQA10501-DQB10201	253	SDVSHTTFNDCMSIS	SHTTFNDCM	3	0.2034	5535.7	65.00	0.40	tr_C9ZZQ4_
HLA-DQA10501-DQB10201	254	DESSDVSHTTFNDCM	SHTTFNDCM	6	0.2021	5614.2	65.00	0.50	tr_C9ZZQ4_
HLA-DQA10501-DQB10201	255	RTESIANGGQVLLTR	SIANGGQVL	3	0.2018	5633.8	65.00	0.29	tr_C9ZZQ4_
HLA-DQA10501-DQB10201	256	SSDSSSVILISNPAV	VILISNPAV	6	0.2014	5654.5	65.00	0.38	tr_C9ZZQ4_
HLA-DQA10501-DQB10201	257	CERWRVPLPPWKEAV	RWRVPLPPW	2	0.2010	5680.4	65.00	0.38	tr_C9ZZQ4_
HLA-DQA10501-DQB10201	258	SAQLDPEVYRKLWNG	SAQLDPEVY	0	0.2007	5701.8	65.00	0.73	tr_C9ZZQ4_
HLA-DQA10501-DQB10201	259	FMIASKSATAAVRLA	KSATAAVRL	5	0.2002	5733.5	65.00	0.54	tr_C9ZZQ4_

HLA-DQA10501-DQB10201	260	DNVSAPMDPTDPVTL	NVSAPMDPT	1	0.1992	5791.6	65.00	0.31	tr_C9ZZQ4_
HLA-DQA10501-DQB10201	261	DAVAAHHSLIRTLIG	HHSLIRTLI	5	0.1982	5856.9	65.00	0.28	tr_C9ZZQ4_
HLA-DQA10501-DQB10201	262	ALLGTFTIVQRQKEL	FTIVQRQKE	5	0.1975	5900.5	65.00	0.36	tr_C9ZZQ4_
HLA-DQA10501-DQB10201	263	TESIANGGQVLLTRA	SIANGGQVL	2	0.1975	5900.8	65.00	0.27	tr_C9ZZQ4_
HLA-DQA10501-DQB10201	264	TDIENSTSQWASHPN	IENSTSQWA	2	0.1975	5903.9	65.00	0.59	tr_C9ZZQ4_
HLA-DQA10501-DQB10201	265	RLARDLQRCFLNYRW	ARDLQRCFL	2	0.1970	5930.3	65.00	0.40	tr_C9ZZQ4_
HLA-DQA10501-DQB10201	266	MADAVAAHHSLIRTL	AHHSLIRTL	6	0.1970	5933.1	65.00	0.23	tr_C9ZZQ4_
HLA-DQA10501-DQB10201	267	NGLRIRAGIHTGLCD	AGIHTGLCD	6	0.1953	6041.2	65.00	0.31	tr_C9ZZQ4_
HLA-DQA10501-DQB10201	268	EGDAFECGWEEKGKQ	EGDAFECGW	0	0.1945	6092.4	65.00	0.35	tr_C9ZZQ4_
HLA-DQA10501-DQB10201	269	SQLGTGGESVSNVLH	QLGTGGESV	1	0.1944	6099.2	65.00	0.37	tr_C9ZZQ4_
HLA-DQA10501-DQB10201	270	ERWRVPLPPWKEAVW	RWRVPLPPW	1	0.1940	6126.5	65.00	0.35	tr_C9ZZQ4_
HLA-DQA10501-DQB10201	271	QLGTGGESVSNVLHA	GGESVSNVL	4	0.1934	6170.3	65.00	0.30	tr_C9ZZQ4_
HLA-DQA10501-DQB10201	272	ANGGQVLLTRATYFS	VLLTRATYF	5	0.1928	6212.0	65.00	0.50	tr_C9ZZQ4_
HLA-DQA10501-DQB10201	273	YDEVTKGYDYYGQTA	GYDYYGQTA	6	0.1927	6214.3	65.00	0.51	tr_C9ZZQ4_
HLA-DQA10501-DQB10201	274	DVSHTTFNDCMSISS	SHTTFNDCM	2	0.1921	6256.1	65.00	0.37	tr_C9ZZQ4_
HLA-DQA10501-DQB10201	275	LLGTFTIVQRQKELT	FTIVQRQKE	4	0.1913	6308.6	65.00	0.32	tr_C9ZZQ4_
HLA-DQA10501-DQB10201	276	HALLGTFTIVQRQKE	FTIVQRQKE	6	0.1912	6316.4	65.00	0.29	tr_C9ZZQ4_
HLA-DQA10501-DQB10201	277	LGTGGESVSNVLHAL	SVSNVLHAL	6	0.1894	6440.7	70.00	0.27	tr_C9ZZQ4_
HLA-DQA10501-DQB10201	278	YQLDAVPGRTFAPLR	QLDAVPGRT	1	0.1888	6483.8	70.00	0.35	tr_C9ZZQ4_
HLA-DQA10501-DQB10201	279	VRLARDLQRCFLNYR	ARDLQRCFL	3	0.1878	6554.0	70.00	0.50	tr_C9ZZQ4_
HLA-DQA10501-DQB10201	280	LQRCFLNYRWGTDSI	NYRWGTDSI	6	0.1842	6813.2	70.00	0.54	tr_C9ZZQ4_
HLA-DQA10501-DQB10201	281	DEVTKGYDYYGQTAN	GYDYYGQTA	5	0.1840	6832.4	70.00	0.52	tr_C9ZZQ4_
HLA-DQA10501-DQB10201	282	ESIANGGQVLLTRAT	SIANGGQVL	1	0.1836	6860.8	70.00	0.26	tr_C9ZZQ4_
HLA-DQA10501-DQB10201	283	LGTFTIVQRQKELTA	FTIVQRQKE	3	0.1827	6924.3	70.00	0.31	tr_C9ZZQ4_
HLA-DQA10501-DQB10201	284	DIENSTSQWASHPNV	IENSTSQWA	1	0.1797	7152.2	70.00	0.53	tr_C9ZZQ4_
HLA-DQA10501-DQB10201	285	LARDLQRCFLNYRWG	ARDLQRCFL	1	0.1780	7289.4	70.00	0.43	tr_C9ZZQ4_
HLA-DQA10501-DQB10201	286	ANMAARTESIANGGQ	NMAARTESI	1	0.1773	7341.1	70.00	0.47	tr_C9ZZQ4_
HLA-DQA10501-DQB10201	287	EAIHRLAVKVGHIVD	RLAVKVGHI	4	0.1767	7389.2	70.00	0.34	tr_C9ZZQ4_
HLA-DQA10501-DQB10201	288	IRYDEVTKGYDYYGQ	EVTKGYDYY	4	0.1767	7392.6	70.00	0.32	tr_C9ZZQ4_
HLA-DQA10501-DQB10201	289	AVAAHHSLIRTLIGN	HHSLIRTLI	4	0.1756	7475.2	70.00	0.30	tr_C9ZZQ4_
HLA-DQA10501-DQB10201	290	MIASKSATAAVRLAR	KSATAAVRL	4	0.1754	7491.4	70.00	0.58	tr_C9ZZQ4_
HLA-DQA10501-DQB10201	291	SIANGGQVLLTRATY	ANGGQVLLT	2	0.1751	7516.3	70.00	0.24	tr_C9ZZQ4_
HLA-DQA10501-DQB10201	292	MAARTESIANGGQVL	SIANGGQVL	6	0.1743	7586.6	70.00	0.54	tr_C9ZZQ4_
HLA-DQA10501-DQB10201	293	ESSDSSSVILISNPA	SVILISNPA	6	0.1740	7607.2	70.00	0.38	tr_C9ZZQ4_
HLA-DQA10501-DQB10201	294	SHTTFNDCMSISSQL	DCMSISSQL	6	0.1725	7732.4	70.00	0.30	tr_C9ZZQ4_
HLA-DQA10501-DQB10201	295	VSHTTFNDCMSISSQ	SHTTFNDCM	1	0.1719	7782.7	75.00	0.34	tr_C9ZZQ4_
HLA-DQA10501-DQB10201	296	APMDPTDPVTLIFTD	TDPVTLIFT	5	0.1717	7797.7	75.00	0.25	tr_C9ZZQ4_
HLA-DQA10501-DQB10201	297	RWRVPLPPWKEAVWN	WRVPLPPWK	1	0.1690	8035.8	75.00	0.35	tr_C9ZZQ4_
HLA-DQA10501-DQB10201	298	GTFTIVQRQKELTAI	FTIVQRQKE	2	0.1689	8037.1	75.00	0.35	tr_C9ZZQ4_
HLA-DQA10501-DQB10201	299	YYQEAIHRLAVKVGH	YYQEAIHRL	0	0.1688	8052.5	75.00	0.37	tr_C9ZZQ4_
HLA-DQA10501-DQB10201	300	ISSQLGTGGESVSNV	QLGTGGESV	3	0.1680	8119.1	75.00	0.61	tr_C9ZZQ4_
HLA-DQA10501-DQB10201	301	NVSAPMDPTDPVTLI	NVSAPMDPT	0	0.1674	8173.9	75.00	0.22	tr_C9ZZQ4_
HLA-DQA10501-DQB10201	302	TFTIVQRQKELTAIC	FTIVQRQKE	1	0.1668	8228.5	75.00	0.34	tr_C9ZZQ4_
HLA-DQA10501-DQB10201	303	HTTFNDCMSISSQLG	CMSISSQLG	6	0.1661	8291.1	75.00	0.40	tr_C9ZZQ4_
HLA-DQA10501-DQB10201	304	YRAEEKQVAELNSKA	EEKQVAELN	3	0.1644	8444.6	75.00	0.38	tr_C9ZZQ4_
HLA-DQA10501-DQB10201	305	CMSISSQLGTGGESV	QLGTGGESV	6	0.1643	8449.9	75.00	0.75	tr_C9ZZQ4_
HLA-DQA10501-DQB10201	306	TTFNDCMSISSQLGT	CMSISSQLG	5	0.1634	8536.9	75.00	0.41	tr_C9ZZQ4_
HLA-DQA10501-DQB10201	307	VSAPMDPTDPVTLIF	PTDPVTLIF	6	0.1633	8545.2	75.00	0.26	tr_C9ZZQ4_
HLA-DQA10501-DQB10201	308	SAPMDPTDPVTLIFT	TDPVTLIFT	6	0.1622	8643.2	75.00	0.23	tr_C9ZZQ4_
HLA-DQA10501-DQB10201	309	ENSTSQWASHPNVMA	QWASHPNVM	5	0.1619	8670.5	75.00	0.38	tr_C9ZZQ4_

HLA-DQA10501-DQB10201	310	MSISSQLGTGGESVS	QLGTGGESV	5	0.1598	8874.4	75.00	0.77	tr_C9ZZQ4_
HLA-DQA10501-DQB10201	311	NMAARTESIANGGQV	NMAARTESI	0	0.1588	8972.2	75.00	0.32	tr_C9ZZQ4_
HLA-DQA10501-DQB10201	312	LNSKAEPPSAQLDPE	PPSAQLDPE	6	0.1582	9029.6	75.00	0.64	tr_C9ZZQ4_
HLA-DQA10501-DQB10201	313	SISSQLGTGGESVSN	QLGTGGESV	4	0.1546	9381.7	75.00	0.76	tr_C9ZZQ4_
HLA-DQA10501-DQB10201	314	RYDEVTKGYDYYGQT	EVTKGYDYY	3	0.1509	9771.4	80.00	0.35	tr_C9ZZQ4_
HLA-DQA10501-DQB10201	315	TIGDSFMIASKSATA	MIASKSATA	6	0.1507	9795.3	80.00	0.31	tr_C9ZZQ4_
HLA-DQA10501-DQB10201	316	TESSDSSSVILISNP	SDSSSVILI	3	0.1494	9929.7	80.00	0.23	tr_C9ZZQ4_
HLA-DQA10501-DQB10201	317	YQEAIHRLAVKVGHI	RLAVKVGHI	6	0.1485	10029.9	80.00	0.39	tr_C9ZZQ4_
HLA-DQA10501-DQB10201	318	GDSFMIASKSATAAV	MIASKSATA	4	0.1483	10049.2	80.00	0.37	tr_C9ZZQ4_
HLA-DQA10501-DQB10201	319	ARDLQRCFLNYRWGT	ARDLQRCFL	0	0.1473	10155.0	80.00	0.38	tr_C9ZZQ4_
HLA-DQA10501-DQB10201	320	QEAIHRLAVKVGHIV	RLAVKVGHI	5	0.1472	10167.1	80.00	0.38	tr_C9ZZQ4_
HLA-DQA10501-DQB10201	321	IENSTSQWASHPNVM	QWASHPNVM	6	0.1467	10221.4	80.00	0.34	tr_C9ZZQ4_
HLA-DQA10501-DQB10201	322	RAEEKQVAELNSKAE	EEKQVAELN	2	0.1463	10273.1	80.00	0.37	tr_C9ZZQ4_
HLA-DQA10501-DQB10201	323	IGDSFMIASKSATAA	MIASKSATA	5	0.1450	10410.3	80.00	0.38	tr_C9ZZQ4_
HLA-DQA10501-DQB10201	324	TFNDCMSISSQLGTG	CMSISSQLG	4	0.1444	10476.5	80.00	0.43	tr_C9ZZQ4_
HLA-DQA10501-DQB10201	325	PTESSDSSSVILISN	SSSVILISN	6	0.1441	10517.4	80.00	0.31	tr_C9ZZQ4_
HLA-DQA10501-DQB10201	326	IGNYDCYEVKTIGDS	IGNYDCYEV	0	0.1434	10601.4	80.00	0.22	tr_C9ZZQ4_
HLA-DQA10501-DQB10201	327	RPTESSDSSSVILIS	DSSSVILIS	6	0.1433	10602.4	80.00	0.32	tr_C9ZZQ4_
HLA-DQA10501-DQB10201	328	VADESSDVSHTTFND	VADESSDVS	0	0.1430	10641.4	80.00	0.38	tr_C9ZZQ4_
HLA-DQA10501-DQB10201	329	LWNGLRIRAGIHTGL	IRAGIHTGL	6	0.1419	10765.0	80.00	0.45	tr_C9ZZQ4_
HLA-DQA10501-DQB10201	330	KTIGDSFMIASKSAT	TIGDSFMIA	1	0.1417	10797.0	80.00	0.30	tr_C9ZZQ4_
HLA-DQA10501-DQB10201	331	WNGLRIRAGIHTGLC	IRAGIHTGL	5	0.1408	10897.9	80.00	0.43	tr_C9ZZQ4_
HLA-DQA10501-DQB10201	332	DSFMIASKSATAAVR	MIASKSATA	3	0.1405	10933.9	80.00	0.36	tr_C9ZZQ4_
HLA-DQA10501-DQB10201	333	EEKQVAELNSKAEPP	VAELNSKAE	4	0.1402	10969.2	80.00	0.23	tr_C9ZZQ4_
HLA-DQA10501-DQB10201	334	AEEKQVAELNSKAEP	EEKQVAELN	1	0.1391	11101.4	80.00	0.30	tr_C9ZZQ4_
HLA-DQA10501-DQB10201	335	GRPTESSDSSSVILI	SDSSSVILI	6	0.1380	11232.7	80.00	0.44	tr_C9ZZQ4_
HLA-DQA10501-DQB10201	336	WRVPLPPWKEAVWND	WRVPLPPWK	0	0.1350	11606.6	80.00	0.25	tr_C9ZZQ4_
HLA-DQA10501-DQB10201	337	DVAAVERVGRPTESS	VAAVERVGR	1	0.1327	11891.4	85.00	0.17	tr_C9ZZQ4_
HLA-DQA10501-DQB10201	338	LDPEVYRKLWNGLRI	RKLWNGLRI	6	0.1326	11908.8	85.00	0.40	tr_C9ZZQ4_
HLA-DQA10501-DQB10201	339	FNDCMSISSQLGTGG	CMSISSQLG	3	0.1303	12206.6	85.00	0.44	tr_C9ZZQ4_
HLA-DQA10501-DQB10201	340	ADESSDVSHTTFNDC	SSDVSHTTF	3	0.1281	12501.9	85.00	0.35	tr_C9ZZQ4_
HLA-DQA10501-DQB10201	341	RDLQRCFLNYRWGTD	QRCFLNYRW	3	0.1259	12803.2	85.00	0.37	tr_C9ZZQ4_
HLA-DQA10501-DQB10201	342	VYRKLWNGLRIRAGI	WNGLRIRAG	5	0.1247	12976.7	85.00	0.26	tr_C9ZZQ4_
HLA-DQA10501-DQB10201	343	EKQVAELNSKAEPPS	ELNSKAEPP	5	0.1231	13193.3	85.00	0.32	tr_C9ZZQ4_
HLA-DQA10501-DQB10201	344	DLQRCFLNYRWGTDS	QRCFLNYRW	2	0.1196	13702.9	85.00	0.35	tr_C9ZZQ4_
HLA-DQA10501-DQB10201	345	EVYRKLWNGLRIRAG	RKLWNGLRI	3	0.1195	13724.2	85.00	0.22	tr_C9ZZQ4_
HLA-DQA10501-DQB10201	346	KQVAELNSKAEPPSA	ELNSKAEPP	4	0.1186	13856.8	85.00	0.33	tr_C9ZZQ4_
HLA-DQA10501-DQB10201	347	DPEVYRKLWNGLRIR	RKLWNGLRI	5	0.1182	13910.4	85.00	0.34	tr_C9ZZQ4_
HLA-DQA10501-DQB10201	348	VAAVERVGRPTESSD	VERVGRPTE	3	0.1171	14078.9	85.00	0.20	tr_C9ZZQ4_
HLA-DQA10501-DQB10201	349	YRKLWNGLRIRAGIH	WNGLRIRAG	4	0.1158	14282.9	85.00	0.27	tr_C9ZZQ4_
HLA-DQA10501-DQB10201	350	PEVYRKLWNGLRIRA	RKLWNGLRI	4	0.1148	14436.2	85.00	0.31	tr_C9ZZQ4_
HLA-DQA10501-DQB10201	351	VGRPTESSDSSSVIL	SSDSSSVIL	6	0.1135	14643.1	90.00	0.48	tr_C9ZZQ4_
HLA-DQA10501-DQB10201	352	RKLWNGLRIRAGIHT	LRIRAGIHT	6	0.1118	14915.1	90.00	0.19	tr_C9ZZQ4_
HLA-DQA10501-DQB10201	353	NDCMSISSQLGTGGE	CMSISSQLG	2	0.1107	15091.0	90.00	0.43	tr_C9ZZQ4_
HLA-DQA10501-DQB10201	354	QVAELNSKAEPPSAQ	ELNSKAEPP	3	0.1089	15396.5	90.00	0.33	tr_C9ZZQ4_
HLA-DQA10501-DQB10201	355	AQLDPEVYRKLWNGL	VYRKLWNGL	6	0.1078	15566.8	90.00	0.33	tr_C9ZZQ4_
HLA-DQA10501-DQB10201	356	KLWNGLRIRAGIHTG	WNGLRIRAG	2	0.1021	16557.5	90.00	0.25	tr_C9ZZQ4_
HLA-DQA10501-DQB10201	357	AAVERVGRPTESSDS	VERVGRPTE	2	0.1012	16733.4	90.00	0.26	tr_C9ZZQ4_
HLA-DQA10501-DQB10201	358	DCMSISSQLGTGGES	CMSISSQLG	1	0.0999	16967.0	90.00	0.40	tr_C9ZZQ4_
HLA-DQA10501-DQB10201	359	QLDPEVYRKLWNGLR	VYRKLWNGL	5	0.0982	17286.2	90.00	0.34	tr_C9ZZQ4_

HLA-DQA10501-DQB10201	360	AVERVGRPTESSDSS	ERVGRPTES	2	0.0965	17596.5	90.00	0.20	tr_C9ZZQ4_	
HLA-DQA10501-DQB10201	361	VAELNSKAEPPSAQL	ELNSKAEPP	2	0.0963	17644.1	90.00	0.38	tr_C9ZZQ4_	
HLA-DQA10501-DQB10201	362	AELNSKAEPPSAQLD	ELNSKAEPP	1	0.0879	19326.8	95.00	0.38	tr_C9ZZQ4_	
HLA-DQA10501-DQB10201	363	VERVGRPTESSDSSS	ERVGRPTES	1	0.0866	19594.5	95.00	0.19	tr_C9ZZQ4_	
HLA-DQA10501-DQB10201	364	RVGRPTESSDSSSVI	ESSDSSSVI	6	0.0864	19625.4	95.00	0.38	tr_C9ZZQ4_	
HLA-DQA10501-DQB10201	365	ELNSKAEPPSAQLDP	ELNSKAEPP	0	0.0842	20103.6	95.00	0.25	tr_C9ZZQ4_	
HLA-DQA10501-DQB10201	366	ERVGRPTESSDSSSV	RVGRPTESS	1	0.0746	22308.7	95.00	0.19	tr_C9ZZQ4_	

Allele: HLA-DQA10501-DQB10201. Number of high binders 0. Number of weak binders 30. Number of peptides 366

Allele	pos	peptide	core	Of	1-log50k(aff)	affinity(nM)	%Rank	Relia	Identity	Bind Level
HLA-DQA10501-DQB10201	1	YIAIKRFQGVMSEYL	FQGVMSEYL	6	0.6599	39.6	0.40	0.77	tr_D0A0W7_	SB
HLA-DQA10501-DQB10201	2	RVILLHVEEMSPETL	VEEMSPETL	6	0.6410	48.6	0.60	0.48	tr_D0A0W7_	WB
HLA-DQA10501-DQB10201	3	VILLHVEEMSPETLV	VEEMSPETL	5	0.6246	58.1	0.80	0.46	tr_D0A0W7_	WB
HLA-DQA10501-DQB10201	4	IAIKRFQGVMSEYLK	FQGVMSEYL	5	0.6237	58.7	0.90	0.77	tr_D0A0W7_	WB
HLA-DQA10501-DQB10201	5	AIKRFQGVMSEYLKA	FQGVMSEYL	4	0.6064	70.7	1.20	0.77	tr_D0A0W7_	WB
HLA-DQA10501-DQB10201	6	DTAKFLMMMVAEERI	LMMMVAEER	5	0.5989	76.7	1.30	0.43	tr_D0A0W7_	WB
HLA-DQA10501-DQB10201	7	MLDTPNITFIDPLEL	ITFIDPLEL	6	0.5880	86.3	1.60	0.50	tr_D0A0W7_	WB
HLA-DQA10501-DQB10201	8	TAKFLMMMVAEERIA	LMMMVAEER	4	0.5846	89.6	1.70	0.43	tr_D0A0W7_	WB
HLA-DQA10501-DQB10201	9	IKRFQGVMSEYLKAH	FQGVMSEYL	3	0.5805	93.5	1.80	0.75	tr_D0A0W7_	WB
HLA-DQA10501-DQB10201	10	VIVLFGEFSLLYDLF	EFSLLYDLF	6	0.5803	93.8	1.80	0.50	tr_D0A0W7_	WB
HLA-DQA10501-DQB10201	11	EVFERFAAALPQAII	FAAALPQAI	5	0.5764	97.8	1.90	0.52	tr_D0A0W7_	WB
HLA-DQA10501-DQB10201	12	REVFERFAAALPQAI	FAAALPQAI	6	0.5757	98.6	1.90	0.49	tr_D0A0W7_	WB
HLA-DQA10501-DQB10201	13	VFERFAAALPQAIIV	FAAALPQAI	4	0.5677	107.5	2.50	0.55	tr_D0A0W7_	WB
HLA-DQA10501-DQB10201	14	ILLHVEEMSPETLVN	VEEMSPETL	4	0.5654	110.2	2.50	0.63	tr_D0A0W7_	WB
HLA-DQA10501-DQB10201	15	EGELMVYGWICGEVL	YGWICGEVL	6	0.5598	117.1	2.50	0.62	tr_D0A0W7_	WB
HLA-DQA10501-DQB10201	16	FERFAAALPQAIIVF	FAAALPQAI	3	0.5597	117.2	2.50	0.58	tr_D0A0W7_	WB
HLA-DQA10501-DQB10201	17	AKFLMMMVAEERIAR	LMMMVAEER	3	0.5585	118.7	2.50	0.42	tr_D0A0W7_	WB
HLA-DQA10501-DQB10201	18	DTPNITFIDPLELKP	ITFIDPLEL	4	0.5558	122.3	3.00	0.52	tr_D0A0W7_	WB
HLA-DQA10501-DQB10201	19	LMRVILLHVEEMSPE	VILLHVEEM	3	0.5554	122.7	3.00	0.48	tr_D0A0W7_	WB
HLA-DQA10501-DQB10201	20	KRFQGVMSEYLKAHV	FQGVMSEYL	2	0.5554	122.8	3.00	0.76	tr_D0A0W7_	WB
HLA-DQA10501-DQB10201	21	LDTPNITFIDPLELK	ITFIDPLEL	5	0.5523	126.9	3.00	0.49	tr_D0A0W7_	WB
HLA-DQA10501-DQB10201	22	IVLFGEFSLLYDLFT	EFSLLYDLF	5	0.5510	128.8	3.00	0.40	tr_D0A0W7_	WB
HLA-DQA10501-DQB10201	23	MRVILLHVEEMSPET	VILLHVEEM	2	0.5508	129.1	3.00	0.46	tr_D0A0W7_	WB
HLA-DQA10501-DQB10201	24	RLMRVILLHVEEMSP	VILLHVEEM	4	0.5501	130.0	3.00	0.53	tr_D0A0W7_	WB
HLA-DQA10501-DQB10201	25	PYLYFLRADPAAETL	LYFLRADPA	2	0.5481	132.9	3.00	0.35	tr_D0A0W7_	WB
HLA-DQA10501-DQB10201	26	LLHVEEMSPETLVNF	VEEMSPETL	3	0.5472	134.2	3.00	0.71	tr_D0A0W7_	WB
HLA-DQA10501-DQB10201	27	AHVSETNITEADYFL	TNITEADYF	5	0.5437	139.4	3.00	0.60	tr_D0A0W7_	WB
HLA-DQA10501-DQB10201	28	NPYLYFLRADPAAET	LYFLRADPA	3	0.5435	139.6	3.00	0.40	tr_D0A0W7_	WB
HLA-DQA10501-DQB10201	29	WNPYLYFLRADPAAE	LYFLRADPA	4	0.5434	139.8	3.50	0.44	tr_D0A0W7_	WB
HLA-DQA10501-DQB10201	30	KFLMMMVAEERIARS	LMMMVAEER	2	0.5422	141.7	3.50	0.43	tr_D0A0W7_	WB
HLA-DQA10501-DQB10201	31	GELMVYGWICGEVLS	YGWICGEVL	5	0.5393	146.2	3.50	0.62	tr_D0A0W7_	WB
HLA-DQA10501-DQB10201	32	PLNGLAVFMEDDDTA	AVFMEDDDT	5	0.5385	147.5	3.50	0.25	tr_D0A0W7_	WB
HLA-DQA10501-DQB10201	33	ELMVYGWICGEVLSQ	YGWICGEVL	4	0.5373	149.4	3.50	0.57	tr_D0A0W7_	WB
HLA-DQA10501-DQB10201	34	GPLNGLAVFMEDDDT	AVFMEDDDT	6	0.5370	149.9	3.50	0.31	tr_D0A0W7_	WB
HLA-DQA10501-DQB10201	35	KAHVSETNITEADYF	TNITEADYF	6	0.5369	150.0	3.50	0.65	tr_D0A0W7_	WB

HLA-DQA10501-DQB10201	36	HVSETNITEADYFLT	TNITEADYF	4	0.5367	150.3	3.50	0.56	tr_D0A0W7_	WB
HLA-DQA10501-DQB10201	37	YLYFLRADPAAETLA	LYFLRADPA	1	0.5300	161.7	4.00	0.36	tr_D0A0W7_	WB
HLA-DQA10501-DQB10201	38	FLMMMVAEERIARSY	MMMVAEERI	2	0.5295	162.4	4.00	0.43	tr_D0A0W7_	WB
HLA-DQA10501-DQB10201	39	LHVEEMSPETLVNFF	VEEMSPETL	2	0.5285	164.2	4.00	0.70	tr_D0A0W7_	WB
HLA-DQA10501-DQB10201	40	VNFFYADSSIISDDM	FFYADSSII	2	0.5285	164.2	4.00	0.30	tr_D0A0W7_	WB
HLA-DQA10501-DQB10201	41	LVNFFYADSSIISDD	FFYADSSII	3	0.5285	164.3	4.00	0.34	tr_D0A0W7_	WB
HLA-DQA10501-DQB10201	42	TLVNFFYADSSIISD	FFYADSSII	4	0.5283	164.6	4.00	0.34	tr_D0A0W7_	WB
HLA-DQA10501-DQB10201	43	LMVYGWICGEVLSQA	YGWICGEVL	3	0.5277	165.7	4.00	0.54	tr_D0A0W7_	WB
HLA-DQA10501-DQB10201	44	TPNITFIDPLELKPR	ITFIDPLEL	3	0.5258	169.1	4.00	0.55	tr_D0A0W7_	WB
HLA-DQA10501-DQB10201	45	QWNPYLYFLRADPAA	LYFLRADPA	5	0.5254	169.8	4.00	0.52	tr_D0A0W7_	WB
HLA-DQA10501-DQB10201	46	RFQGVMSEYLKAHVS	FQGVMSEYL	1	0.5244	171.8	4.00	0.79	tr_D0A0W7_	WB
HLA-DQA10501-DQB10201	47	VLFGEFSLLYDLFTT	EFSLLYDLF	4	0.5230	174.3	4.00	0.42	tr_D0A0W7_	WB
HLA-DQA10501-DQB10201	48	MVYGWICGEVLSQAL	YGWICGEVL	2	0.5201	179.9	4.50	0.54	tr_D0A0W7_	WB
HLA-DQA10501-DQB10201	49	KDTAKFLMMMVAEER	LMMMVAEER	6	0.5199	180.4	4.50	0.71	tr_D0A0W7_	WB
HLA-DQA10501-DQB10201	50	LNGLAVFMEDDDTAS	AVFMEDDDT	4	0.5197	180.7	4.50	0.26	tr_D0A0W7_	WB
HLA-DQA10501-DQB10201	51	GRLMRVILLHVEEMS	VILLHVEEM	5	0.5168	186.4	4.50	0.56	tr_D0A0W7_	WB
HLA-DQA10501-DQB10201	52	MYLQGVHYGDEEYAL	GVHYGDEEY	4	0.5145	191.2	4.50	0.38	tr_D0A0W7_	WB
HLA-DQA10501-DQB10201	53	ETLVNFFYADSSIIS	FFYADSSII	5	0.5123	195.8	5.00	0.34	tr_D0A0W7_	WB
HLA-DQA10501-DQB10201	54	PETLVNFFYADSSII	FFYADSSII	6	0.5114	197.6	5.00	0.35	tr_D0A0W7_	WB
HLA-DQA10501-DQB10201	55	ERFAAALPQAIIVFG	FAAALPQAI	2	0.5112	198.0	5.00	0.61	tr_D0A0W7_	WB
HLA-DQA10501-DQB10201	56	AVICSNEADGIADFL	EADGIADFL	6	0.5106	199.4	5.00	0.73	tr_D0A0W7_	WB
HLA-DQA10501-DQB10201	57	VSETNITEADYFLTH	TNITEADYF	3	0.5102	200.2	5.00	0.56	tr_D0A0W7_	WB
HLA-DQA10501-DQB10201	58	FREVFERFAAALPQA	RFAAALPQA	6	0.5085	204.0	5.00	0.46	tr_D0A0W7_	WB
HLA-DQA10501-DQB10201	59	YLQGVHYGDEEYALT	GVHYGDEEY	3	0.5069	207.5	5.00	0.38	tr_D0A0W7_	WB
HLA-DQA10501-DQB10201	60	QGPLNGLAVFMEDDD	LAVFMEDDD	6	0.5037	214.9	5.50	0.28	tr_D0A0W7_	WB
HLA-DQA10501-DQB10201	61	FMYLQGVHYGDEEYA	GVHYGDEEY	5	0.5036	215.1	5.50	0.40	tr_D0A0W7_	WB
HLA-DQA10501-DQB10201	62	GAVCRVAMDTLPVIL	AMDTLPVIL	6	0.5035	215.2	5.50	0.32	tr_D0A0W7_	WB
HLA-DQA10501-DQB10201	63	HVEEMSPETLVNFFY	VEEMSPETL	1	0.5018	219.3	5.50	0.67	tr_D0A0W7_	WB
HLA-DQA10501-DQB10201	64	PNITFIDPLELKPRL	ITFIDPLEL	2	0.4984	227.6	5.50	0.57	tr_D0A0W7_	WB
HLA-DQA10501-DQB10201	65	VLSATWNSYMPQEYV	WNSYMPQEY	5	0.4978	229.0	5.50	0.62	tr_D0A0W7_	WB
HLA-DQA10501-DQB10201	66	LRVIVLFGEFSLLYD	FGEFSLLYD	6	0.4961	233.4	6.00	0.38	tr_D0A0W7_	WB
HLA-DQA10501-DQB10201	67	RQWNPYLYFLRADPA	LYFLRADPA	6	0.4946	237.1	6.00	0.58	tr_D0A0W7_	WB
HLA-DQA10501-DQB10201	68	IVTAVFGPVTEAMLD	VFGPVTEAM	4	0.4925	242.5	6.00	0.24	tr_D0A0W7_	WB
HLA-DQA10501-DQB10201	69	TGRLMRVILLHVEEM	VILLHVEEM	6	0.4922	243.3	6.00	0.73	tr_D0A0W7_	WB
HLA-DQA10501-DQB10201	70	NFFYADSSIISDDMR	FFYADSSII	1	0.4904	248.1	6.00	0.30	tr_D0A0W7_	WB
HLA-DQA10501-DQB10201	71	RIVTAVFGPVTEAML	VTAVFGPVT	2	0.4895	250.6	6.50	0.21	tr_D0A0W7_	WB
HLA-DQA10501-DQB10201	72	LFGEFSLLYDLFTTA	EFSLLYDLF	3	0.4893	251.0	6.50	0.46	tr_D0A0W7_	WB
HLA-DQA10501-DQB10201	73	LQGVHYGDEEYALTV	GVHYGDEEY	2	0.4876	255.7	6.50	0.35	tr_D0A0W7_	WB
HLA-DQA10501-DQB10201	74	RVIVLFGEFSLLYDL	FGEFSLLYD	5	0.4851	262.8	6.50	0.34	tr_D0A0W7_	WB
HLA-DQA10501-DQB10201	75	SETNITEADYFLTHG	TNITEADYF	2	0.4840	265.8	6.50	0.58	tr_D0A0W7_	WB
HLA-DQA10501-DQB10201	76	VVYPRNLDLMPQDFI	NLDLMPQDF	5	0.4840	265.9	6.50	0.51	tr_D0A0W7_	WB
HLA-DQA10501-DQB10201	77	AFNTTIAEAANDLRY	TIAEAANDL	4	0.4831	268.4	6.50	0.41	tr_D0A0W7_	WB
HLA-DQA10501-DQB10201	78	VYGWICGEVLSQALS	YGWICGEVL	1	0.4830	268.9	7.00	0.53	tr_D0A0W7_	WB
HLA-DQA10501-DQB10201	79	LHAFNTTIAEAANDL	TIAEAANDL	6	0.4824	270.5	7.00	0.41	tr_D0A0W7_	WB
HLA-DQA10501-DQB10201	80	LSATWNSYMPQEYVN	WNSYMPQEY	4	0.4816	273.0	7.00	0.62	tr_D0A0W7_	WB
HLA-DQA10501-DQB10201	81	VICSNEADGIADFLR	EADGIADFL	5	0.4802	277.0	7.00	0.71	tr_D0A0W7_	WB
HLA-DQA10501-DQB10201	82	PLGDIVTYLALPILM	TYLALPILM	6	0.4802	277.1	7.00	0.31	tr_D0A0W7_	WB
HLA-DQA10501-DQB10201	83	KVLSATWNSYMPQEY	WNSYMPQEY	6	0.4797	278.6	7.00	0.67	tr_D0A0W7_	WB
HLA-DQA10501-DQB10201	84	NGLAVFMEDDDTASK	AVFMEDDDT	3	0.4796	278.9	7.00	0.25	tr_D0A0W7_	WB
HLA-DQA10501-DQB10201	85	GVFTVMSSDGEPAPD	FTVMSSDGE	2	0.4790	280.6	7.00	0.47	tr_D0A0W7_	WB

HLA-DQA10501-DQB10201	86	LYFLRADPAAETLAL	LYFLRADPA	0	0.4782	283.0	7.00	0.31	tr_D0A0W7_	WB
HLA-DQA10501-DQB10201	87	HGVFTVMSSDGEPAP	FTVMSSDGE	3	0.4761	289.5	7.50	0.50	tr_D0A0W7_	WB
HLA-DQA10501-DQB10201	88	ADYFLTHGTEGELMV	THGTEGELM	5	0.4757	290.8	7.50	0.61	tr_D0A0W7_	WB
HLA-DQA10501-DQB10201	89	EVVYPRNLDLMPQDF	NLDLMPQDF	6	0.4745	294.6	7.50	0.55	tr_D0A0W7_	WB
HLA-DQA10501-DQB10201	90	DYFLTHGTEGELMVY	THGTEGELM	4	0.4734	298.3	7.50	0.61	tr_D0A0W7_	WB
HLA-DQA10501-DQB10201	91	HAFNTTIAEAANDLR	TIAEAANDL	5	0.4732	298.8	7.50	0.41	tr_D0A0W7_	WB
HLA-DQA10501-DQB10201	92	VFTVMSSDGEPAPDD	FTVMSSDGE	1	0.4719	303.2	7.50	0.45	tr_D0A0W7_	WB
HLA-DQA10501-DQB10201	93	EADYFLTHGTEGELM	THGTEGELM	6	0.4693	311.8	8.00	0.63	tr_D0A0W7_	WB
HLA-DQA10501-DQB10201	94	FFYADSSIISDDMRY	DSSIISDDM	4	0.4692	312.1	8.00	0.25	tr_D0A0W7_	WB
HLA-DQA10501-DQB10201	95	EFREVFERFAAALPQ	ERFAAALPQ	6	0.4686	314.1	8.00	0.41	tr_D0A0W7_	WB
HLA-DQA10501-DQB10201	96	VHYGDEEYALTVNVM	EYALTVNVM	6	0.4678	317.0	8.00	0.57	tr_D0A0W7_	WB
HLA-DQA10501-DQB10201	97	AMLDTPNITFIDPLE	PNITFIDPL	5	0.4677	317.1	8.00	0.43	tr_D0A0W7_	WB
HLA-DQA10501-DQB10201	98	FGEFSLLYDLFTTAL	EFSLLYDLF	2	0.4670	319.4	8.00	0.46	tr_D0A0W7_	WB
HLA-DQA10501-DQB10201	99	AVCRVAMDTLPVILR	VAMDTLPVI	4	0.4667	320.7	8.00	0.35	tr_D0A0W7_	WB
HLA-DQA10501-DQB10201	100	LQGPLNGLAVFMEDD	GLAVFMEDD	6	0.4659	323.3	8.00	0.28	tr_D0A0W7_	WB
HLA-DQA10501-DQB10201	101	FNTTIAEAANDLRYE	TIAEAANDL	3	0.4654	325.2	8.00	0.49	tr_D0A0W7_	WB
HLA-DQA10501-DQB10201	102	VTAVFGPVTEAMLDT	VFGPVTEAM	3	0.4653	325.4	8.00	0.25	tr_D0A0W7_	WB
HLA-DQA10501-DQB10201	103	SATWNSYMPQEYVNA	WNSYMPQEY	3	0.4652	325.9	8.00	0.62	tr_D0A0W7_	WB
HLA-DQA10501-DQB10201	104	MPQDFIKEQLELETD	FIKEQLELE	4	0.4643	329.0	8.50	0.47	tr_D0A0W7_	WB
HLA-DQA10501-DQB10201	105	GPLGDIVTYLALPIL	VTYLALPIL	6	0.4639	330.3	8.50	0.31	tr_D0A0W7_	WB
HLA-DQA10501-DQB10201	106	TAVFGPVTEAMLDTP	FGPVTEAML	3	0.4639	330.5	8.50	0.32	tr_D0A0W7_	WB
HLA-DQA10501-DQB10201	107	ERIVTAVFGPVTEAM	VTAVFGPVT	3	0.4619	337.7	8.50	0.36	tr_D0A0W7_	WB
HLA-DQA10501-DQB10201	108	QGVHYGDEEYALTVN	VHYGDEEYA	2	0.4611	340.6	8.50	0.34	tr_D0A0W7_	WB
HLA-DQA10501-DQB10201	109	RFAAALPQAIIVFGA	FAAALPQAI	1	0.4588	349.2	9.00	0.68	tr_D0A0W7_	WB
HLA-DQA10501-DQB10201	110	IVIVYGPLGDIVTYL	VYGPLGDIV	3	0.4587	349.7	9.00	0.35	tr_D0A0W7_	WB
HLA-DQA10501-DQB10201	111	NITFIDPLELKPRLN	ITFIDPLEL	1	0.4560	359.9	9.00	0.68	tr_D0A0W7_	WB
HLA-DQA10501-DQB10201	112	GFMYLQGVHYGDEEY	GVHYGDEEY	6	0.4535	369.7	9.50	0.50	tr_D0A0W7_	WB
HLA-DQA10501-DQB10201	113	LMMMVAEERIARSYI	MMMVAEERI	1	0.4528	372.6	9.50	0.48	tr_D0A0W7_	WB
HLA-DQA10501-DQB10201	114	LHGVFTVMSSDGEPA	FTVMSSDGE	4	0.4525	373.9	9.50	0.50	tr_D0A0W7_	WB
HLA-DQA10501-DQB10201	115	GGAVCRVAMDTLPVI	VAMDTLPVI	6	0.4524	374.1	9.50	0.41	tr_D0A0W7_	WB
HLA-DQA10501-DQB10201	116	CGEVLSQALSSLEWL	SQALSSLEW	5	0.4519	376.1	9.50	0.44	tr_D0A0W7_	WB
HLA-DQA10501-DQB10201	117	VIVYGPLGDIVTYLA	VYGPLGDIV	2	0.4518	376.7	9.50	0.33	tr_D0A0W7_	WB
HLA-DQA10501-DQB10201	118	SPETLVNFFYADSSI	VNFFYADSS	5	0.4501	383.5	9.50	0.47	tr_D0A0W7_	WB
HLA-DQA10501-DQB10201	119	ATWNSYMPQEYVNAI	WNSYMPQEY	2	0.4480	392.5	10.00	0.62	tr_D0A0W7_	
HLA-DQA10501-DQB10201	120	ETNITEADYFLTHGT	TNITEADYF	1	0.4472	395.9	10.00	0.56	tr_D0A0W7_	
HLA-DQA10501-DQB10201	121	EAMLDTPNITFIDPL	PNITFIDPL	6	0.4465	398.8	10.00	0.56	tr_D0A0W7_	
HLA-DQA10501-DQB10201	122	ICSNEADGIADFLRR	EADGIADFL	4	0.4451	404.8	10.00	0.70	tr_D0A0W7_	
HLA-DQA10501-DQB10201	123	SQRRYVINDIVIGDY	INDIVIGDY	6	0.4449	405.8	10.00	0.38	tr_D0A0W7_	
HLA-DQA10501-DQB10201	124	PQDFIKEQLELETDQ	FIKEQLELE	3	0.4441	409.5	11.00	0.47	tr_D0A0W7_	
HLA-DQA10501-DQB10201	125	QDFIKEQLELETDQN	FIKEQLELE	2	0.4438	410.5	11.00	0.46	tr_D0A0W7_	
HLA-DQA10501-DQB10201	126	VYPRNLDLMPQDFIK	NLDLMPQDF	4	0.4431	413.8	11.00	0.50	tr_D0A0W7_	
HLA-DQA10501-DQB10201	127	YFLTHGTEGELMVYG	THGTEGELM	3	0.4426	416.0	11.00	0.62	tr_D0A0W7_	
HLA-DQA10501-DQB10201	128	DLMPQDFIKEQLELE	FIKEQLELE	6	0.4421	418.4	11.00	0.50	tr_D0A0W7_	
HLA-DQA10501-DQB10201	129	VCRVAMDTLPVILRA	VAMDTLPVI	3	0.4417	420.1	11.00	0.38	tr_D0A0W7_	
HLA-DQA10501-DQB10201	130	ELHGVFTVMSSDGEP	FTVMSSDGE	5	0.4417	420.3	11.00	0.54	tr_D0A0W7_	
HLA-DQA10501-DQB10201	131	LMPQDFIKEQLELET	FIKEQLELE	5	0.4409	424.0	11.00	0.50	tr_D0A0W7_	
HLA-DQA10501-DQB10201	132	RFFLHAFNTTIAEAA	FNTTIAEAA	6	0.4387	434.1	11.00	0.32	tr_D0A0W7_	
HLA-DQA10501-DQB10201	133	GLAVFMEDDDTASKA	AVFMEDDDT	2	0.4387	434.2	11.00	0.32	tr_D0A0W7_	
HLA-DQA10501-DQB10201	134	AVFGPVTEAMLDTPN	FGPVTEAML	2	0.4343	455.3	12.00	0.34	tr_D0A0W7_	
HLA-DQA10501-DQB10201	135	YGPLGDIVTYLALPI	IVTYLALPI	6	0.4337	458.2	12.00	0.28	tr_D0A0W7_	

HLA-DQA10501-DQB10201	136	DEFREVFERFAAALP	FERFAAALP	6	0.4316	468.5	12.00	0.34	tr_D0A0W7_
HLA-DQA10501-DQB10201	137	LGPSSVQASLAQMWL	VQASLAQMW	5	0.4309	472.4	12.00	0.37	tr_D0A0W7_
HLA-DQA10501-DQB10201	138	NTTIAEAANDLRYEQ	TIAEAANDL	2	0.4308	472.8	12.00	0.50	tr_D0A0W7_
HLA-DQA10501-DQB10201	139	YGWICGEVLSQALSS	YGWICGEVL	0	0.4307	473.3	12.00	0.46	tr_D0A0W7_
HLA-DQA10501-DQB10201	140	YFLRADPAAETLALI	FLRADPAAE	1	0.4307	473.3	12.00	0.29	tr_D0A0W7_
HLA-DQA10501-DQB10201	141	PAPDDEFREVFERFA	EFREVFERF	5	0.4305	474.6	12.00	0.44	tr_D0A0W7_
HLA-DQA10501-DQB10201	142	VYGPLGDIVTYLALP	PLGDIVTYL	3	0.4290	482.0	12.00	0.33	tr_D0A0W7_
HLA-DQA10501-DQB10201	143	GERIVTAVFGPVTEA	VTAVFGPVT	4	0.4289	482.6	12.00	0.40	tr_D0A0W7_
HLA-DQA10501-DQB10201	144	FQGVMSEYLKAHVSE	FQGVMSEYL	0	0.4288	483.2	12.00	0.73	tr_D0A0W7_
HLA-DQA10501-DQB10201	145	IVYGPLGDIVTYLAL	VYGPLGDIV	1	0.4285	484.8	12.00	0.29	tr_D0A0W7_
HLA-DQA10501-DQB10201	146	VEEMSPETLVNFFYA	VEEMSPETL	0	0.4268	493.6	12.00	0.61	tr_D0A0W7_
HLA-DQA10501-DQB10201	147	QGERIVTAVFGPVTE	VTAVFGPVT	5	0.4266	494.7	12.00	0.46	tr_D0A0W7_
HLA-DQA10501-DQB10201	148	LGDIVTYLALPILMK	VTYLALPIL	4	0.4261	497.3	13.00	0.28	tr_D0A0W7_
HLA-DQA10501-DQB10201	149	FLHAFNTTIAEAAND	FNTTIAEAA	4	0.4258	498.9	13.00	0.41	tr_D0A0W7_
HLA-DQA10501-DQB10201	150	FTVMSSDGEPAPDDE	VMSSDGEPA	2	0.4245	505.9	13.00	0.39	tr_D0A0W7_
HLA-DQA10501-DQB10201	151	DLRVIVLFGEFSLLY	LFGEFSLLY	6	0.4244	506.9	13.00	0.35	tr_D0A0W7_
HLA-DQA10501-DQB10201	152	GVHYGDEEYALTVNV	VHYGDEEYA	1	0.4232	513.5	13.00	0.37	tr_D0A0W7_
HLA-DQA10501-DQB10201	153	DRFFLHAFNTTIAEA	AFNTTIAEA	6	0.4229	515.1	13.00	0.43	tr_D0A0W7_
HLA-DQA10501-DQB10201	154	VFGPVTEAMLDTPNI	FGPVTEAML	1	0.4214	523.4	13.00	0.31	tr_D0A0W7_
HLA-DQA10501-DQB10201	155	FLTHGTEGELMVYGW	THGTEGELM	2	0.4211	525.2	13.00	0.57	tr_D0A0W7_
HLA-DQA10501-DQB10201	156	YPRNLDLMPQDFIKE	NLDLMPQDF	3	0.4210	525.8	13.00	0.50	tr_D0A0W7_
HLA-DQA10501-DQB10201	157	CSNEADGIADFLRRS	EADGIADFL	3	0.4202	530.0	13.00	0.75	tr_D0A0W7_
HLA-DQA10501-DQB10201	158	TTIAEAANDLRYEQG	TIAEAANDL	1	0.4200	531.5	13.00	0.48	tr_D0A0W7_
HLA-DQA10501-DQB10201	159	FFLHAFNTTIAEAAN	FNTTIAEAA	5	0.4193	535.2	13.00	0.32	tr_D0A0W7_
HLA-DQA10501-DQB10201	160	CRVAMDTLPVILRAP	AMDTLPVIL	3	0.4185	539.8	13.00	0.38	tr_D0A0W7_
HLA-DQA10501-DQB10201	161	TWNSYMPQEYVNAIN	WNSYMPQEY	1	0.4153	559.3	14.00	0.61	tr_D0A0W7_
HLA-DQA10501-DQB10201	162	WSMARALNASIPPLA	LNASIPPLA	6	0.4140	567.2	14.00	0.37	tr_D0A0W7_
HLA-DQA10501-DQB10201	163	SNEADGIADFLRRSL	EADGIADFL	2	0.4135	570.0	14.00	0.75	tr_D0A0W7_
HLA-DQA10501-DQB10201	164	QRRYVINDIVIGDYG	INDIVIGDY	5	0.4126	575.8	14.00	0.35	tr_D0A0W7_
HLA-DQA10501-DQB10201	165	KIVIVYGPLGDIVTY	VYGPLGDIV	4	0.4124	576.7	14.00	0.49	tr_D0A0W7_
HLA-DQA10501-DQB10201	166	DFIKEQLELETDQNK	FIKEQLELE	1	0.4121	578.8	14.00	0.49	tr_D0A0W7_
HLA-DQA10501-DQB10201	167	APDDEFREVFERFAA	EFREVFERF	4	0.4118	580.9	14.00	0.44	tr_D0A0W7_
HLA-DQA10501-DQB10201	168	QWTVAGSVKVEVVYP	AGSVKVEVV	4	0.4108	586.9	14.00	0.41	tr_D0A0W7_
HLA-DQA10501-DQB10201	169	DDEFREVFERFAAAL	EFREVFERF	2	0.4101	591.2	14.00	0.33	tr_D0A0W7_
HLA-DQA10501-DQB10201	170	ELQGPLNGLAVFMED	NGLAVFMED	6	0.4095	595.1	15.00	0.35	tr_D0A0W7_
HLA-DQA10501-DQB10201	171	EPAPDDEFREVFERF	EFREVFERF	6	0.4092	597.3	15.00	0.61	tr_D0A0W7_
HLA-DQA10501-DQB10201	172	YELHGVFTVMSSDGE	FTVMSSDGE	6	0.4092	597.6	15.00	0.48	tr_D0A0W7_
HLA-DQA10501-DQB10201	173	NKIVIVYGPLGDIVT	VYGPLGDIV	5	0.4072	610.5	15.00	0.47	tr_D0A0W7_
HLA-DQA10501-DQB10201	174	EQGERIVTAVFGPVT	VTAVFGPVT	6	0.4059	619.0	15.00	0.49	tr_D0A0W7_
HLA-DQA10501-DQB10201	175	SDDMRYGVFDDTKCD	MRYGVFDDT	3	0.4051	624.3	15.00	0.48	tr_D0A0W7_
HLA-DQA10501-DQB10201	176	SIISDDMRYGVFDDT	MRYGVFDDT	6	0.4045	628.5	15.00	0.64	tr_D0A0W7_
HLA-DQA10501-DQB10201	177	GEFSLLYDLFTTALN	EFSLLYDLF	1	0.4030	638.7	16.00	0.48	tr_D0A0W7_
HLA-DQA10501-DQB10201	178	EVLSQALSSLEWLKD	SQALSSLEW	3	0.4025	642.3	16.00	0.40	tr_D0A0W7_
HLA-DQA10501-DQB10201	179	ICGEVLSQALSSLEW	SQALSSLEW	6	0.4010	652.6	16.00	0.62	tr_D0A0W7_
HLA-DQA10501-DQB10201	180	GEVLSQALSSLEWLK	SQALSSLEW	4	0.4008	654.3	16.00	0.41	tr_D0A0W7_
HLA-DQA10501-DQB10201	181	MSPETLVNFFYADSS	VNFFYADSS	6	0.4004	656.6	16.00	0.50	tr_D0A0W7_
HLA-DQA10501-DQB10201	182	QNKIVIVYGPLGDIV	VYGPLGDIV	6	0.4002	658.1	16.00	0.49	tr_D0A0W7_
HLA-DQA10501-DQB10201	183	ISDDMRYGVFDDTKC	MRYGVFDDT	4	0.3993	664.8	16.00	0.47	tr_D0A0W7_
HLA-DQA10501-DQB10201	184	RVAMDTLPVILRAPV	AMDTLPVIL	2	0.3993	664.9	16.00	0.41	tr_D0A0W7_
HLA-DQA10501-DQB10201	185	SLYSQRRYVINDIVI	RRYVINDIV	5	0.3990	666.7	16.00	0.64	tr_D0A0W7_

HLA-DQA10501-DQB10201	186	HYGDEEYALTVNVMS	EYALTVNVM	5	0.3990	667.2	16.00	0.62	tr_D0A0W7_
HLA-DQA10501-DQB10201	187	YSQRRYVINDIVIGD	RRYVINDIV	3	0.3983	672.4	16.00	0.53	tr_D0A0W7_
HLA-DQA10501-DQB10201	188	RNLDLMPQDFIKEQL	NLDLMPQDF	1	0.3974	678.4	16.00	0.42	tr_D0A0W7_
HLA-DQA10501-DQB10201	189	IISDDMRYGVFDDTK	MRYGVFDDT	5	0.3960	688.7	17.00	0.50	tr_D0A0W7_
HLA-DQA10501-DQB10201	190	ILGPSSVQASLAQMW	VQASLAQMW	6	0.3950	696.1	17.00	0.49	tr_D0A0W7_
HLA-DQA10501-DQB10201	191	GDIVTYLALPILMKH	VTYLALPIL	3	0.3949	697.0	17.00	0.29	tr_D0A0W7_
HLA-DQA10501-DQB10201	192	MARALNASIPPLANP	LNASIPPLA	4	0.3938	705.3	17.00	0.47	tr_D0A0W7_
HLA-DQA10501-DQB10201	193	GPSSVQASLAQMWLH	VQASLAQMW	4	0.3936	707.3	17.00	0.37	tr_D0A0W7_
HLA-DQA10501-DQB10201	194	FYADSSIISDDMRYG	DSSIISDDM	3	0.3934	708.7	17.00	0.38	tr_D0A0W7_
HLA-DQA10501-DQB10201	195	NLDLMPQDFIKEQLE	PQDFIKEQL	5	0.3933	709.2	17.00	0.28	tr_D0A0W7_
HLA-DQA10501-DQB10201	196	WTVAGSVKVEVVYPR	AGSVKVEVV	3	0.3921	718.6	17.00	0.38	tr_D0A0W7_
HLA-DQA10501-DQB10201	197	RSLYSQRRYVINDIV	RRYVINDIV	6	0.3921	718.9	17.00	0.75	tr_D0A0W7_
HLA-DQA10501-DQB10201	198	DDMRYGVFDDTKCDT	MRYGVFDDT	2	0.3917	722.1	17.00	0.46	tr_D0A0W7_
HLA-DQA10501-DQB10201	199	LAVFMEDDDTASKAA	AVFMEDDDT	1	0.3910	727.2	17.00	0.38	tr_D0A0W7_
HLA-DQA10501-DQB10201	200	PDDEFREVFERFAAA	EFREVFERF	3	0.3909	728.3	17.00	0.43	tr_D0A0W7_
HLA-DQA10501-DQB10201	201	SRQWTVAGSVKVEVV	AGSVKVEVV	6	0.3900	734.8	17.00	0.46	tr_D0A0W7_
HLA-DQA10501-DQB10201	202	AEAANDLRYEQGERI	LRYEQGERI	6	0.3894	739.6	17.00	0.68	tr_D0A0W7_
HLA-DQA10501-DQB10201	203	WNSYMPQEYVNAINA	WNSYMPQEY	0	0.3889	744.0	18.00	0.44	tr_D0A0W7_
HLA-DQA10501-DQB10201	204	EAANDLRYEQGERIV	LRYEQGERI	5	0.3882	749.9	18.00	0.69	tr_D0A0W7_
HLA-DQA10501-DQB10201	205	TNITEADYFLTHGTE	TNITEADYF	0	0.3877	753.4	18.00	0.45	tr_D0A0W7_
HLA-DQA10501-DQB10201	206	RQWTVAGSVKVEVVY	AGSVKVEVV	5	0.3871	758.5	18.00	0.44	tr_D0A0W7_
HLA-DQA10501-DQB10201	207	QYIAIKRFQGVMSEY	RFQGVMSEY	6	0.3842	782.6	18.00	0.57	tr_D0A0W7_
HLA-DQA10501-DQB10201	208	SMARALNASIPPLAN	LNASIPPLA	5	0.3838	786.3	18.00	0.44	tr_D0A0W7_
HLA-DQA10501-DQB10201	209	TGGAVCRVAMDTLPV	RVAMDTLPV	6	0.3826	796.3	19.00	0.32	tr_D0A0W7_
HLA-DQA10501-DQB10201	210	VWSMARALNASIPPL	ALNASIPPL	6	0.3816	805.4	19.00	0.35	tr_D0A0W7_
HLA-DQA10501-DQB10201	211	AGAARLVFATSLPHW	ARLVFATSL	3	0.3815	805.9	19.00	0.36	tr_D0A0W7_
HLA-DQA10501-DQB10201	212	NEADGIADFLRRSLT	EADGIADFL	1	0.3784	833.2	19.00	0.77	tr_D0A0W7_
HLA-DQA10501-DQB10201	213	KFRRHVIHLSPTLEQ	VIHLSPTLE	5	0.3783	834.0	19.00	0.49	tr_D0A0W7_
HLA-DQA10501-DQB10201	214	LPQAIIVFGAPEKDT	IVFGAPEKD	5	0.3778	839.2	19.00	0.51	tr_D0A0W7_
HLA-DQA10501-DQB10201	215	ARALNASIPPLANPM	LNASIPPLA	3	0.3773	843.8	19.00	0.50	tr_D0A0W7_
HLA-DQA10501-DQB10201	216	LTHGTEGELMVYGWI	THGTEGELM	1	0.3766	849.9	19.00	0.52	tr_D0A0W7_
HLA-DQA10501-DQB10201	217	TIAEAANDLRYEQGE	TIAEAANDL	0	0.3754	860.7	20.00	0.43	tr_D0A0W7_
HLA-DQA10501-DQB10201	218	LYSQRRYVINDIVIG	RRYVINDIV	4	0.3752	862.6	20.00	0.61	tr_D0A0W7_
HLA-DQA10501-DQB10201	219	YGDEEYALTVNVMSQ	EYALTVNVM	4	0.3746	868.6	20.00	0.62	tr_D0A0W7_
HLA-DQA10501-DQB10201	220	AANDLRYEQGERIVT	LRYEQGERI	4	0.3744	870.2	20.00	0.70	tr_D0A0W7_
HLA-DQA10501-DQB10201	221	PRNLDLMPQDFIKEQ	NLDLMPQDF	2	0.3736	878.3	20.00	0.56	tr_D0A0W7_
HLA-DQA10501-DQB10201	222	ALPQAIIVFGAPEKD	IVFGAPEKD	6	0.3731	883.0	20.00	0.48	tr_D0A0W7_
HLA-DQA10501-DQB10201	223	YLAGDGVGTVDAVIC	GVGTVDAVI	5	0.3730	883.6	20.00	0.34	tr_D0A0W7_
HLA-DQA10501-DQB10201	224	TEGELMVYGWICGEV	VYGWICGEV	6	0.3726	887.1	20.00	0.44	tr_D0A0W7_
HLA-DQA10501-DQB10201	225	PSSVQASLAQMWLHA	VQASLAQMW	3	0.3718	895.6	20.00	0.38	tr_D0A0W7_
HLA-DQA10501-DQB10201	226	GDEEYALTVNVMSQM	EYALTVNVM	3	0.3717	896.1	20.00	0.54	tr_D0A0W7_
HLA-DQA10501-DQB10201	227	SDRFFLHAFNTTIAE	LHAFNTTIA	5	0.3716	896.7	20.00	0.26	tr_D0A0W7_
HLA-DQA10501-DQB10201	228	YMPQEYVNAINAGFN	YVNAINAGF	5	0.3715	897.6	20.00	0.30	tr_D0A0W7_
HLA-DQA10501-DQB10201	229	PQAIIVFGAPEKDTA	IVFGAPEKD	4	0.3715	897.8	20.00	0.52	tr_D0A0W7_
HLA-DQA10501-DQB10201	230	VAMDTLPVILRAPVA	AMDTLPVIL	1	0.3711	901.9	20.00	0.41	tr_D0A0W7_
HLA-DQA10501-DQB10201	231	NKFRRHVIHLSPTLE	VIHLSPTLE	6	0.3709	903.7	20.00	0.54	tr_D0A0W7_
HLA-DQA10501-DQB10201	232	FGPVTEAMLDTPNIT	VTEAMLDTP	3	0.3708	905.0	20.00	0.28	tr_D0A0W7_
HLA-DQA10501-DQB10201	233	ISGTVFVIGLSVPNV	VIGLSVPNV	6	0.3707	905.6	21.00	0.63	tr_D0A0W7_
HLA-DQA10501-DQB10201	234	DIVTYLALPILMKHN	VTYLALPIL	2	0.3704	908.5	21.00	0.33	tr_D0A0W7_
HLA-DQA10501-DQB10201	235	RVLRLGFMYLQGVHY	FMYLQGVHY	6	0.3701	911.3	21.00	0.50	tr_D0A0W7_

HLA-DQA10501-DQB10201	236	VLSQALSSLEWLKDR	SQALSSLEW	2	0.3700	912.3	21.00	0.43	tr_D0A0W7_
HLA-DQA10501-DQB10201	237	RRYVINDIVIGDYGG	INDIVIGDY	4	0.3698	914.3	21.00	0.37	tr_D0A0W7_
HLA-DQA10501-DQB10201	238	LKAHVSETNITEADY	ETNITEADY	6	0.3692	920.9	21.00	0.47	tr_D0A0W7_
HLA-DQA10501-DQB10201	239	EKDTAKFLMMMVAEE	FLMMMVAEE	6	0.3681	931.4	21.00	0.70	tr_D0A0W7_
HLA-DQA10501-DQB10201	240	DLRYEQGERIVTAVF	LRYEQGERI	1	0.3679	933.6	21.00	0.48	tr_D0A0W7_
HLA-DQA10501-DQB10201	241	TVAGSVKVEVVYPRN	AGSVKVEVV	2	0.3661	952.4	21.00	0.37	tr_D0A0W7_
HLA-DQA10501-DQB10201	242	FIKEQLELETDQNKI	FIKEQLELE	0	0.3659	954.2	21.00	0.41	tr_D0A0W7_
HLA-DQA10501-DQB10201	243	SQMGYELHGVFTVMS	LHGVFTVMS	6	0.3650	963.3	22.00	0.26	tr_D0A0W7_
HLA-DQA10501-DQB10201	244	LGFMYLQGVHYGDEE	FMYLQGVHY	2	0.3649	964.7	22.00	0.45	tr_D0A0W7_
HLA-DQA10501-DQB10201	245	FLRADPAAETLALIR	PAAETLALI	5	0.3645	969.1	22.00	0.25	tr_D0A0W7_
HLA-DQA10501-DQB10201	246	GTVFVIGLSVPNVRE	VIGLSVPNV	4	0.3637	977.1	22.00	0.56	tr_D0A0W7_
HLA-DQA10501-DQB10201	247	RNDLRVIVLFGEFSL	VIVLFGEFS	5	0.3637	977.3	22.00	0.49	tr_D0A0W7_
HLA-DQA10501-DQB10201	248	SSVQASLAQMWLHAL	VQASLAQMW	2	0.3628	986.5	22.00	0.39	tr_D0A0W7_
HLA-DQA10501-DQB10201	249	NDLRVIVLFGEFSLL	VIVLFGEFS	4	0.3628	986.8	22.00	0.46	tr_D0A0W7_
HLA-DQA10501-DQB10201	250	SYMPQEYVNAINAGF	YVNAINAGF	6	0.3623	991.8	22.00	0.35	tr_D0A0W7_
HLA-DQA10501-DQB10201	251	VEVVYPRNLDLMPQD	RNLDLMPQD	6	0.3621	994.3	22.00	0.25	tr_D0A0W7_
HLA-DQA10501-DQB10201	252	TVFVIGLSVPNVREI	VIGLSVPNV	3	0.3620	995.3	22.00	0.55	tr_D0A0W7_
HLA-DQA10501-DQB10201	253	EFSLLYDLFTTALNN	EFSLLYDLF	0	0.3618	997.4	22.00	0.37	tr_D0A0W7_
HLA-DQA10501-DQB10201	254	FRRHVIHLSPTLEQQ	VIHLSPTLE	4	0.3609	1006.7	22.00	0.50	tr_D0A0W7_
HLA-DQA10501-DQB10201	255	SGTVFVIGLSVPNVR	VIGLSVPNV	5	0.3607	1009.8	22.00	0.58	tr_D0A0W7_
HLA-DQA10501-DQB10201	256	GAARLVFATSLPHWG	ARLVFATSL	2	0.3603	1013.5	22.00	0.37	tr_D0A0W7_
HLA-DQA10501-DQB10201	257	YALTVNVMSQMGYEL	VMSQMGYEL	6	0.3601	1016.0	22.00	0.55	tr_D0A0W7_
HLA-DQA10501-DQB10201	258	ERNDLRVIVLFGEFS	VIVLFGEFS	6	0.3575	1044.5	23.00	0.44	tr_D0A0W7_
HLA-DQA10501-DQB10201	259	LDLMPQDFIKEQLEL	PQDFIKEQL	4	0.3575	1045.5	23.00	0.35	tr_D0A0W7_
HLA-DQA10501-DQB10201	260	MPQEYVNAINAGFNA	YVNAINAGF	4	0.3572	1048.5	23.00	0.33	tr_D0A0W7_
HLA-DQA10501-DQB10201	261	DMRYGVFDDTKCDTA	MRYGVFDDT	1	0.3572	1048.6	23.00	0.45	tr_D0A0W7_
HLA-DQA10501-DQB10201	262	DEEYALTVNVMSQMG	EYALTVNVM	2	0.3565	1056.4	23.00	0.55	tr_D0A0W7_
HLA-DQA10501-DQB10201	263	ITFIDPLELKPRLNK	ITFIDPLEL	0	0.3562	1059.3	23.00	0.69	tr_D0A0W7_
HLA-DQA10501-DQB10201	264	FAAALPQAIIVFGAP	FAAALPQAI	0	0.3562	1059.9	23.00	0.71	tr_D0A0W7_
HLA-DQA10501-DQB10201	265	ANDLRYEQGERIVTA	LRYEQGERI	3	0.3560	1061.9	23.00	0.73	tr_D0A0W7_
HLA-DQA10501-DQB10201	266	LAGDGVGTVDAVICS	GVGTVDAVI	4	0.3560	1062.2	23.00	0.34	tr_D0A0W7_
HLA-DQA10501-DQB10201	267	RALNASIPPLANPMT	LNASIPPLA	2	0.3555	1067.8	23.00	0.51	tr_D0A0W7_
HLA-DQA10501-DQB10201	268	AARLVFATSLPHWGD	VFATSLPHW	4	0.3554	1069.4	23.00	0.28	tr_D0A0W7_
HLA-DQA10501-DQB10201	269	MSQMGYELHGVFTVM	ELHGVFTVM	6	0.3548	1075.7	23.00	0.29	tr_D0A0W7_
HLA-DQA10501-DQB10201	270	NVMSQMGYELHGVFT	VMSQMGYEL	1	0.3544	1080.7	23.00	0.26	tr_D0A0W7_
HLA-DQA10501-DQB10201	271	AGDGVGTVDAVICSN	GVGTVDAVI	3	0.3542	1082.9	23.00	0.30	tr_D0A0W7_
HLA-DQA10501-DQB10201	272	RRHVIHLSPTLEQQL	VIHLSPTLE	3	0.3539	1086.3	23.00	0.50	tr_D0A0W7_
HLA-DQA10501-DQB10201	273	VAGSVKVEVVYPRNL	AGSVKVEVV	1	0.3523	1105.8	24.00	0.28	tr_D0A0W7_
HLA-DQA10501-DQB10201	274	TSSKTAQLFHDVEKD	TAQLFHDVE	4	0.3518	1111.8	24.00	0.60	tr_D0A0W7_
HLA-DQA10501-DQB10201	275	VNVMSQMGYELHGVF	VMSQMGYEL	2	0.3515	1114.5	24.00	0.34	tr_D0A0W7_
HLA-DQA10501-DQB10201	276	NDLRYEQGERIVTAV	LRYEQGERI	2	0.3515	1114.5	24.00	0.65	tr_D0A0W7_
HLA-DQA10501-DQB10201	277	NTAGAARLVFATSLP	ARLVFATSL	5	0.3514	1116.2	24.00	0.51	tr_D0A0W7_
HLA-DQA10501-DQB10201	278	GYELHGVFTVMSSDG	VFTVMSSDG	6	0.3514	1116.5	24.00	0.28	tr_D0A0W7_
HLA-DQA10501-DQB10201	279	VFVIGLSVPNVREIA	VIGLSVPNV	2	0.3512	1118.2	24.00	0.56	tr_D0A0W7_
HLA-DQA10501-DQB10201	280	QMGYELHGVFTVMSS	LHGVFTVMS	5	0.3505	1127.7	24.00	0.20	tr_D0A0W7_
HLA-DQA10501-DQB10201	281	SYLAGDGVGTVDAVI	GVGTVDAVI	6	0.3500	1133.6	24.00	0.36	tr_D0A0W7_
HLA-DQA10501-DQB10201	282	HVIHLSPTLEQQLYV	VIHLSPTLE	1	0.3490	1145.9	24.00	0.44	tr_D0A0W7_
HLA-DQA10501-DQB10201	283	LRLGFMYLQGVHYGD	FMYLQGVHY	4	0.3484	1153.2	25.00	0.52	tr_D0A0W7_
HLA-DQA10501-DQB10201	284	MGYELHGVFTVMSSD	LHGVFTVMS	4	0.3482	1155.7	25.00	0.25	tr_D0A0W7_
HLA-DQA10501-DQB10201	285	RHVIHLSPTLEQQLY	VIHLSPTLE	2	0.3481	1156.7	25.00	0.50	tr_D0A0W7_

HLA-DQA10501-DQB10201	286	QAIIVFGAPEKDTAK	IVFGAPEKD	3	0.3480	1158.3	25.00	0.50	tr_D0A0W7_
HLA-DQA10501-DQB10201	287	ALTVNVMSQMGYELH	VMSQMGYEL	5	0.3463	1179.4	25.00	0.46	tr_D0A0W7_
HLA-DQA10501-DQB10201	288	SSKTAQLFHDVEKDS	TAQLFHDVE	3	0.3457	1187.1	25.00	0.56	tr_D0A0W7_
HLA-DQA10501-DQB10201	289	RLGFMYLQGVHYGDE	FMYLQGVHY	3	0.3455	1189.9	25.00	0.51	tr_D0A0W7_
HLA-DQA10501-DQB10201	290	LSQALSSLEWLKDRT	QALSSLEWL	2	0.3451	1195.3	25.00	0.47	tr_D0A0W7_
HLA-DQA10501-DQB10201	291	VLRLGFMYLQGVHYG	FMYLQGVHY	5	0.3436	1214.0	26.00	0.50	tr_D0A0W7_
HLA-DQA10501-DQB10201	292	IRQWNPYLYFLRADP	YLYFLRADP	6	0.3435	1215.9	26.00	0.32	tr_D0A0W7_
HLA-DQA10501-DQB10201	293	DGVGTVDAVICSNEA	DAVICSNEA	6	0.3427	1226.5	26.00	0.23	tr_D0A0W7_
HLA-DQA10501-DQB10201	294	TAGAARLVFATSLPH	ARLVFATSL	4	0.3425	1229.7	26.00	0.45	tr_D0A0W7_
HLA-DQA10501-DQB10201	295	EEYALTVNVMSQMGY	EYALTVNVM	1	0.3410	1249.6	26.00	0.47	tr_D0A0W7_
HLA-DQA10501-DQB10201	296	HSDRFFLHAFNTTIA	LHAFNTTIA	6	0.3402	1260.1	26.00	0.31	tr_D0A0W7_
HLA-DQA10501-DQB10201	297	PQEYVNAINAGFNAS	YVNAINAGF	3	0.3401	1261.2	26.00	0.33	tr_D0A0W7_
HLA-DQA10501-DQB10201	298	YADSSIISDDMRYGV	DSSIISDDM	2	0.3387	1280.0	26.00	0.44	tr_D0A0W7_
HLA-DQA10501-DQB10201	299	SVQASLAQMWLHALA	VQASLAQMW	1	0.3385	1284.0	27.00	0.34	tr_D0A0W7_
HLA-DQA10501-DQB10201	300	YEQGERIVTAVFGPV	ERIVTAVFG	4	0.3373	1299.7	27.00	0.32	tr_D0A0W7_
HLA-DQA10501-DQB10201	301	LRYEQGERIVTAVFG	ERIVTAVFG	6	0.3369	1305.3	27.00	0.37	tr_D0A0W7_
HLA-DQA10501-DQB10201	302	GVGTVDAVICSNEAD	DAVICSNEA	5	0.3364	1313.3	27.00	0.28	tr_D0A0W7_
HLA-DQA10501-DQB10201	303	SELQGPLNGLAVFME	PLNGLAVFM	5	0.3360	1318.0	27.00	0.46	tr_D0A0W7_
HLA-DQA10501-DQB10201	304	GDGVGTVDAVICSNE	GVGTVDAVI	2	0.3354	1327.7	27.00	0.30	tr_D0A0W7_
HLA-DQA10501-DQB10201	305	KVEVVYPRNLDLMPQ	PRNLDLMPQ	6	0.3351	1331.6	27.00	0.26	tr_D0A0W7_
HLA-DQA10501-DQB10201	306	TVMSSDGEPAPDDEF	VMSSDGEPA	1	0.3349	1333.7	27.00	0.50	tr_D0A0W7_
HLA-DQA10501-DQB10201	307	SKTAQLFHDVEKDSR	TAQLFHDVE	2	0.3349	1333.8	27.00	0.54	tr_D0A0W7_
HLA-DQA10501-DQB10201	308	VMSQMGYELHGVFTV	GYELHGVFT	5	0.3348	1336.5	27.00	0.25	tr_D0A0W7_
HLA-DQA10501-DQB10201	309	MMMVAEERIARSYIL	MMMVAEERI	0	0.3347	1337.5	27.00	0.58	tr_D0A0W7_
HLA-DQA10501-DQB10201	310	TLALIRYALCQLRVL	RYALCQLRV	5	0.3343	1343.3	27.00	0.40	tr_D0A0W7_
HLA-DQA10501-DQB10201	311	SVWSMARALNASIPP	WSMARALNA	2	0.3340	1347.6	27.00	0.40	tr_D0A0W7_
HLA-DQA10501-DQB10201	312	ESRQWTVAGSVKVEV	VAGSVKVEV	6	0.3334	1356.7	28.00	0.44	tr_D0A0W7_
HLA-DQA10501-DQB10201	313	TVNVMSQMGYELHGV	VMSQMGYEL	3	0.3334	1356.8	28.00	0.42	tr_D0A0W7_
HLA-DQA10501-DQB10201	314	VSVWSMARALNASIP	WSMARALNA	3	0.3329	1363.2	28.00	0.49	tr_D0A0W7_
HLA-DQA10501-DQB10201	315	AIIVFGAPEKDTAKF	IVFGAPEKD	2	0.3327	1366.1	28.00	0.52	tr_D0A0W7_
HLA-DQA10501-DQB10201	316	PTGGAVCRVAMDTLP	VCRVAMDTL	5	0.3321	1375.0	28.00	0.40	tr_D0A0W7_
HLA-DQA10501-DQB10201	317	EYVNAINAGFNASLE	NAGFNASLE	6	0.3320	1376.7	28.00	0.29	tr_D0A0W7_
HLA-DQA10501-DQB10201	318	QEYVNAINAGFNASL	YVNAINAGF	2	0.3320	1376.9	28.00	0.34	tr_D0A0W7_
HLA-DQA10501-DQB10201	319	VQASLAQMWLHALAT	QMWLHALAT	6	0.3299	1408.6	28.00	0.29	tr_D0A0W7_
HLA-DQA10501-DQB10201	320	THGTEGELMVYGWIC	THGTEGELM	0	0.3285	1429.4	29.00	0.42	tr_D0A0W7_
HLA-DQA10501-DQB10201	321	RYVINDIVIGDYGGT	INDIVIGDY	3	0.3278	1441.7	29.00	0.44	tr_D0A0W7_
HLA-DQA10501-DQB10201	322	SSYLAGDGVGTVDAV	DGVGTVDAV	6	0.3274	1447.1	29.00	0.30	tr_D0A0W7_
HLA-DQA10501-DQB10201	323	AGSVKVEVVYPRNLD	AGSVKVEVV	0	0.3262	1466.5	29.00	0.21	tr_D0A0W7_
HLA-DQA10501-DQB10201	324	FVIGLSVPNVREIAR	VIGLSVPNV	1	0.3261	1468.4	29.00	0.56	tr_D0A0W7_
HLA-DQA10501-DQB10201	325	ARLVFATSLPHWGDT	VFATSLPHW	3	0.3258	1472.6	29.00	0.34	tr_D0A0W7_
HLA-DQA10501-DQB10201	326	QQLYVLSSYLAGDGV	SSYLAGDGV	6	0.3256	1475.3	29.00	0.51	tr_D0A0W7_
HLA-DQA10501-DQB10201	327	KTAQLFHDVEKDSRL	TAQLFHDVE	1	0.3254	1478.5	29.00	0.50	tr_D0A0W7_
HLA-DQA10501-DQB10201	328	TETSSKTAQLFHDVE	TAQLFHDVE	6	0.3254	1479.0	29.00	0.75	tr_D0A0W7_
HLA-DQA10501-DQB10201	329	LRADPAAETLALIRY	PAAETLALI	4	0.3251	1483.5	29.00	0.29	tr_D0A0W7_
HLA-DQA10501-DQB10201	330	EYALTVNVMSQMGYE	EYALTVNVM	0	0.3249	1486.5	29.00	0.32	tr_D0A0W7_
HLA-DQA10501-DQB10201	331	ALNASIPPLANPMTP	LNASIPPLA	1	0.3248	1488.2	29.00	0.49	tr_D0A0W7_
HLA-DQA10501-DQB10201	332	TEADYFLTHGTEGEL	FLTHGTEGE	5	0.3247	1489.6	29.00	0.39	tr_D0A0W7_
HLA-DQA10501-DQB10201	333	QVSVWSMARALNASI	WSMARALNA	4	0.3205	1558.6	30.00	0.51	tr_D0A0W7_
HLA-DQA10501-DQB10201	334	LTVNVMSQMGYELHG	VMSQMGYEL	4	0.3201	1565.8	30.00	0.46	tr_D0A0W7_
HLA-DQA10501-DQB10201	335	TLPVILRAPVALLLL	VILRAPVAL	3	0.3190	1585.3	31.00	0.25	tr_D0A0W7_

HLA-DQA10501-DQB10201	336	ETLALIRYALCQLRV	RYALCQLRV	6	0.3185	1593.7	31.00	0.47	tr_D0A0W7_
HLA-DQA10501-DQB10201	337	DAVICSNEADGIADF	NEADGIADF	6	0.3171	1618.6	31.00	0.31	tr_D0A0W7_
HLA-DQA10501-DQB10201	338	IVTYLALPILMKHNV	VTYLALPIL	1	0.3169	1620.8	31.00	0.35	tr_D0A0W7_
HLA-DQA10501-DQB10201	339	VKVEVVYPRNLDLMP	VYPRNLDLM	5	0.3167	1624.3	31.00	0.26	tr_D0A0W7_
HLA-DQA10501-DQB10201	340	ATQVSVWSMARALNA	WSMARALNA	6	0.3160	1636.9	31.00	0.53	tr_D0A0W7_
HLA-DQA10501-DQB10201	341	NSYMPQEYVNAINAG	QEYVNAINA	5	0.3155	1645.7	31.00	0.45	tr_D0A0W7_
HLA-DQA10501-DQB10201	342	NNTAGAARLVFATSL	ARLVFATSL	6	0.3146	1661.6	32.00	0.57	tr_D0A0W7_
HLA-DQA10501-DQB10201	343	QLYVLSSYLAGDGVG	SSYLAGDGV	5	0.3141	1670.9	32.00	0.56	tr_D0A0W7_
HLA-DQA10501-DQB10201	344	AVFMEDDDTASKAAA	VFMEDDDTA	1	0.3139	1675.2	32.00	0.45	tr_D0A0W7_
HLA-DQA10501-DQB10201	345	RYEQGERIVTAVFGP	ERIVTAVFG	5	0.3133	1685.4	32.00	0.40	tr_D0A0W7_
HLA-DQA10501-DQB10201	346	YVLSSYLAGDGVGTV	SSYLAGDGV	3	0.3130	1691.7	32.00	0.48	tr_D0A0W7_
HLA-DQA10501-DQB10201	347	DTLPVILRAPVALLL	VILRAPVAL	4	0.3126	1698.6	32.00	0.26	tr_D0A0W7_
HLA-DQA10501-DQB10201	348	FIRQWNPYLYFLRAD	PYLYFLRAD	6	0.3124	1702.9	32.00	0.33	tr_D0A0W7_
HLA-DQA10501-DQB10201	349	CNQGSKAVYVKEMLE	AVYVKEMLE	6	0.3120	1710.2	32.00	0.46	tr_D0A0W7_
HLA-DQA10501-DQB10201	350	QLRVLRLGFMYLQGV	LGFMYLQGV	6	0.3117	1714.8	32.00	0.37	tr_D0A0W7_
HLA-DQA10501-DQB10201	351	GWICGEVLSQALSSL	EVLSQALSS	5	0.3114	1721.4	32.00	0.27	tr_D0A0W7_
HLA-DQA10501-DQB10201	352	TQVSVWSMARALNAS	WSMARALNA	5	0.3111	1726.1	33.00	0.55	tr_D0A0W7_
HLA-DQA10501-DQB10201	353	VLSSYLAGDGVGTVD	SSYLAGDGV	2	0.3109	1729.7	33.00	0.40	tr_D0A0W7_
HLA-DQA10501-DQB10201	354	EMSPETLVNFFYADS	LVNFFYADS	6	0.3105	1738.1	33.00	0.26	tr_D0A0W7_
HLA-DQA10501-DQB10201	355	YVINDIVIGDYGGTC	INDIVIGDY	2	0.3104	1738.6	33.00	0.43	tr_D0A0W7_
HLA-DQA10501-DQB10201	356	LYVLSSYLAGDGVGT	SSYLAGDGV	4	0.3098	1751.5	33.00	0.50	tr_D0A0W7_
HLA-DQA10501-DQB10201	357	AMDTLPVILRAPVAL	AMDTLPVIL	0	0.3094	1757.7	33.00	0.28	tr_D0A0W7_
HLA-DQA10501-DQB10201	358	EADGIADFLRRSLTE	EADGIADFL	0	0.3093	1759.8	33.00	0.71	tr_D0A0W7_
HLA-DQA10501-DQB10201	359	GPVTEAMLDTPNITF	VTEAMLDTP	2	0.3085	1775.3	33.00	0.31	tr_D0A0W7_
HLA-DQA10501-DQB10201	360	LALIRYALCQLRVLR	RYALCQLRV	4	0.3085	1775.6	33.00	0.35	tr_D0A0W7_
HLA-DQA10501-DQB10201	361	YVNAINAGFNASLES	NAGFNASLE	5	0.3083	1780.2	33.00	0.32	tr_D0A0W7_
HLA-DQA10501-DQB10201	362	VLPQLSVGAEANATV	PQLSVGAEA	2	0.3073	1798.3	33.00	0.27	tr_D0A0W7_
HLA-DQA10501-DQB10201	363	GTEGELMVYGWICGE	MVYGWICGE	6	0.3068	1808.7	33.00	0.44	tr_D0A0W7_
HLA-DQA10501-DQB10201	364	ILMKHNVVAFSPMTG	VVAFSPMTG	6	0.3066	1811.6	34.00	0.40	tr_D0A0W7_
HLA-DQA10501-DQB10201	365	LPVILRAPVALLLLL	VILRAPVAL	2	0.3061	1823.0	34.00	0.28	tr_D0A0W7_
HLA-DQA10501-DQB10201	366	NQGSKAVYVKEMLEN	AVYVKEMLE	5	0.3060	1825.0	34.00	0.43	tr_D0A0W7_
HLA-DQA10501-DQB10201	367	TEAMLDTPNITFIDP	TPNITFIDP	6	0.3055	1833.9	34.00	0.31	tr_D0A0W7_
HLA-DQA10501-DQB10201	368	GHSDRFFLHAFNTTI	FLHAFNTTI	6	0.3051	1841.5	34.00	0.44	tr_D0A0W7_
HLA-DQA10501-DQB10201	369	DQNKIVIVYGPLGDI	VIVYGPLGD	5	0.3049	1847.0	34.00	0.28	tr_D0A0W7_
HLA-DQA10501-DQB10201	370	EERNDLRVIVLFGEF	RVIVLFGEF	6	0.3045	1854.4	34.00	0.30	tr_D0A0W7_
HLA-DQA10501-DQB10201	371	ALIRYALCQLRVLRL	RYALCQLRV	3	0.3030	1883.8	34.00	0.34	tr_D0A0W7_
HLA-DQA10501-DQB10201	372	LSSYLAGDGVGTVDA	SSYLAGDGV	1	0.3030	1884.9	34.00	0.34	tr_D0A0W7_
HLA-DQA10501-DQB10201	373	QGSKAVYVKEMLENG	AVYVKEMLE	4	0.3023	1899.5	35.00	0.41	tr_D0A0W7_
HLA-DQA10501-DQB10201	374	VIHLSPTLEQQLYVL	VIHLSPTLE	0	0.3022	1901.1	35.00	0.33	tr_D0A0W7_
HLA-DQA10501-DQB10201	375	DDCASNYGATQVSVW	YGATQVSVW	6	0.3022	1901.5	35.00	0.41	tr_D0A0W7_
HLA-DQA10501-DQB10201	376	ADSSIISDDMRYGVF	DSSIISDDM	1	0.3017	1910.2	35.00	0.39	tr_D0A0W7_
HLA-DQA10501-DQB10201	377	SQALSSLEWLKDRTT	QALSSLEWL	1	0.3016	1913.4	35.00	0.52	tr_D0A0W7_
HLA-DQA10501-DQB10201	378	GTVDAVICSNEADGI	DAVICSNEA	3	0.3014	1917.2	35.00	0.32	tr_D0A0W7_
HLA-DQA10501-DQB10201	379	GSVKVEVVYPRNLDL	VEVVYPRNL	4	0.3007	1931.3	35.00	0.23	tr_D0A0W7_
HLA-DQA10501-DQB10201	380	ATGRLMRVILLHVEE	RVILLHVEE	6	0.3005	1935.8	35.00	0.56	tr_D0A0W7_
HLA-DQA10501-DQB10201	381	EKDSRLWTPLSLLAF	WTPLSLLAF	6	0.3004	1938.2	35.00	0.36	tr_D0A0W7_
HLA-DQA10501-DQB10201	382	ETSSKTAQLFHDVEK	TAQLFHDVE	5	0.3001	1944.3	35.00	0.73	tr_D0A0W7_
HLA-DQA10501-DQB10201	383	YILGPSSVQASLAQM	PSSVQASLA	4	0.2999	1949.3	35.00	0.46	tr_D0A0W7_
HLA-DQA10501-DQB10201	384	PVTEAMLDTPNITFI	VTEAMLDTP	1	0.2988	1972.9	35.00	0.28	tr_D0A0W7_
HLA-DQA10501-DQB10201	385	TAQLFHDVEKDSRLW	TAQLFHDVE	0	0.2987	1974.5	35.00	0.35	tr_D0A0W7_

HLA-DQA10501-DQB10201	386	DGEPAPDDEFREVFE	PDDEFREVF	5	0.2986	1976.7	35.00	0.34	tr_D0A0W7_
HLA-DQA10501-DQB10201	387	IIVFGAPEKDTAKFL	IVFGAPEKD	1	0.2986	1977.4	35.00	0.57	tr_D0A0W7_
HLA-DQA10501-DQB10201	388	LPTGGAVCRVAMDTL	VCRVAMDTL	6	0.2985	1978.9	35.00	0.43	tr_D0A0W7_
HLA-DQA10501-DQB10201	389	AQMWLHALATAGTSF	ALATAGTSF	6	0.2979	1992.3	36.00	0.29	tr_D0A0W7_
HLA-DQA10501-DQB10201	390	FSLLYDLFTTALNNT	LYDLFTTAL	3	0.2976	1997.4	36.00	0.38	tr_D0A0W7_
HLA-DQA10501-DQB10201	391	MRYGVFDDTKCDTAE	MRYGVFDDT	0	0.2969	2012.8	36.00	0.38	tr_D0A0W7_
HLA-DQA10501-DQB10201	392	SVKVEVVYPRNLDLM	VEVVYPRNL	3	0.2956	2042.1	36.00	0.22	tr_D0A0W7_
HLA-DQA10501-DQB10201	393	RADPAAETLALIRYA	PAAETLALI	3	0.2954	2045.5	36.00	0.37	tr_D0A0W7_
HLA-DQA10501-DQB10201	394	VGTVDAVICSNEADG	DAVICSNEA	4	0.2945	2066.1	37.00	0.32	tr_D0A0W7_
HLA-DQA10501-DQB10201	395	RSYILGPSSVQASLA	PSSVQASLA	6	0.2934	2090.6	37.00	0.57	tr_D0A0W7_
HLA-DQA10501-DQB10201	396	VINDIVIGDYGGTCE	INDIVIGDY	1	0.2927	2107.5	37.00	0.41	tr_D0A0W7_
HLA-DQA10501-DQB10201	397	KDSRLWTPLSLLAFA	WTPLSLLAF	5	0.2922	2118.9	37.00	0.34	tr_D0A0W7_
HLA-DQA10501-DQB10201	398	PVILRAPVALLLLLI	VILRAPVAL	1	0.2920	2121.8	37.00	0.25	tr_D0A0W7_
HLA-DQA10501-DQB10201	399	WICGEVLSQALSSLE	EVLSQALSS	4	0.2915	2133.3	37.00	0.31	tr_D0A0W7_
HLA-DQA10501-DQB10201	400	SLAQMWLHALATAGT	WLHALATAG	5	0.2909	2148.3	37.00	0.31	tr_D0A0W7_
HLA-DQA10501-DQB10201	401	TVDAVICSNEADGIA	DAVICSNEA	2	0.2903	2161.6	38.00	0.34	tr_D0A0W7_
HLA-DQA10501-DQB10201	402	DCASNYGATQVSVWS	YGATQVSVW	5	0.2901	2167.6	38.00	0.43	tr_D0A0W7_
HLA-DQA10501-DQB10201	403	ITEADYFLTHGTEGE	FLTHGTEGE	6	0.2898	2174.4	38.00	0.35	tr_D0A0W7_
HLA-DQA10501-DQB10201	404	EEMSPETLVNFFYAD	SPETLVNFF	3	0.2897	2176.9	38.00	0.33	tr_D0A0W7_
HLA-DQA10501-DQB10201	405	MDTLPVILRAPVALL	VILRAPVAL	5	0.2892	2187.2	38.00	0.32	tr_D0A0W7_
HLA-DQA10501-DQB10201	406	QASLAQMWLHALATA	QMWLHALAT	5	0.2889	2193.8	38.00	0.34	tr_D0A0W7_
HLA-DQA10501-DQB10201	407	CASNYGATQVSVWSM	YGATQVSVW	4	0.2888	2198.2	38.00	0.44	tr_D0A0W7_
HLA-DQA10501-DQB10201	408	TEFGVSLRSAVIRED	LRSAVIRED	6	0.2874	2230.7	38.00	0.41	tr_D0A0W7_
HLA-DQA10501-DQB10201	409	LMKHNVVAFSPMTGS	VVAFSPMTG	5	0.2871	2239.3	38.00	0.38	tr_D0A0W7_
HLA-DQA10501-DQB10201	410	GSKAVYVKEMLENGQ	AVYVKEMLE	3	0.2861	2263.0	39.00	0.37	tr_D0A0W7_
HLA-DQA10501-DQB10201	411	ADPAAETLALIRYAL	PAAETLALI	2	0.2857	2272.7	39.00	0.37	tr_D0A0W7_
HLA-DQA10501-DQB10201	412	LRVLRLGFMYLQGVH	LGFMYLQGV	5	0.2855	2277.2	39.00	0.36	tr_D0A0W7_
HLA-DQA10501-DQB10201	413	SSELQGPLNGLAVFM	PLNGLAVFM	6	0.2849	2293.1	39.00	0.63	tr_D0A0W7_
HLA-DQA10501-DQB10201	414	ASLAQMWLHALATAG	WLHALATAG	6	0.2848	2295.0	39.00	0.31	tr_D0A0W7_
HLA-DQA10501-DQB10201	415	IVLPQLSVGAEANAT	PQLSVGAEA	3	0.2845	2303.0	39.00	0.37	tr_D0A0W7_
HLA-DQA10501-DQB10201	416	RLVFATSLPHWGDTE	VFATSLPHW	2	0.2844	2303.9	39.00	0.41	tr_D0A0W7_
HLA-DQA10501-DQB10201	417	NITEADYFLTHGTEG	DYFLTHGTE	5	0.2842	2309.6	39.00	0.23	tr_D0A0W7_
HLA-DQA10501-DQB10201	418	DSSIISDDMRYGVFD	SIISDDMRY	2	0.2840	2315.7	39.00	0.28	tr_D0A0W7_
HLA-DQA10501-DQB10201	419	QMWLHALATAGTSFA	ALATAGTSF	5	0.2839	2316.4	39.00	0.25	tr_D0A0W7_
HLA-DQA10501-DQB10201	420	MWLHALATAGTSFAP	ALATAGTSF	4	0.2830	2338.6	39.00	0.30	tr_D0A0W7_
HLA-DQA10501-DQB10201	421	VMSSDGEPAPDDEFR	VMSSDGEPA	0	0.2827	2346.3	39.00	0.44	tr_D0A0W7_
HLA-DQA10501-DQB10201	422	DSRLWTPLSLLAFAT	WTPLSLLAF	4	0.2823	2357.0	40.00	0.29	tr_D0A0W7_
HLA-DQA10501-DQB10201	423	SYILGPSSVQASLAQ	PSSVQASLA	5	0.2821	2361.6	40.00	0.55	tr_D0A0W7_
HLA-DQA10501-DQB10201	424	GSTFIRQWNPYLYFL	FIRQWNPYL	3	0.2808	2395.1	40.00	0.33	tr_D0A0W7_
HLA-DQA10501-DQB10201	425	LLIVLPQLSVGAEAN	PQLSVGAEA	5	0.2803	2409.2	40.00	0.43	tr_D0A0W7_
HLA-DQA10501-DQB10201	426	RYALCQLRVLRLGFM	LRVLRLGFM	6	0.2800	2417.9	40.00	0.40	tr_D0A0W7_
HLA-DQA10501-DQB10201	427	LPQLSVGAEANATVK	PQLSVGAEA	1	0.2796	2427.6	40.00	0.27	tr_D0A0W7_
HLA-DQA10501-DQB10201	428	VDAVICSNEADGIAD	DAVICSNEA	1	0.2782	2463.7	41.00	0.32	tr_D0A0W7_
HLA-DQA10501-DQB10201	429	TGSTFIRQWNPYLYF	FIRQWNPYL	4	0.2781	2467.8	41.00	0.34	tr_D0A0W7_
HLA-DQA10501-DQB10201	430	KHNVVAFSPMTGSTF	VVAFSPMTG	3	0.2778	2475.3	41.00	0.38	tr_D0A0W7_
HLA-DQA10501-DQB10201	431	VSLRSAVIREDGEGV	VIREDGEGV	6	0.2771	2495.2	41.00	0.27	tr_D0A0W7_
HLA-DQA10501-DQB10201	432	FGVSLRSAVIREDGE	LRSAVIRED	4	0.2770	2497.6	41.00	0.31	tr_D0A0W7_
HLA-DQA10501-DQB10201	433	VTEAMLDTPNITFID	EAMLDTPNI	2	0.2767	2504.0	41.00	0.32	tr_D0A0W7_
HLA-DQA10501-DQB10201	434	GEPAPDDEFREVFER	PDDEFREVF	4	0.2765	2511.2	41.00	0.31	tr_D0A0W7_
HLA-DQA10501-DQB10201	435	PILMKHNVVAFSPMT	KHNVVAFSP	4	0.2746	2561.6	42.00	0.27	tr_D0A0W7_

HLA-DQA10501-DQB10201	436	ASNYGATQVSVWSMA	YGATQVSVW	3	0.2746	2562.6	42.00	0.44	tr_D0A0W7_
HLA-DQA10501-DQB10201	437	LAQMWLHALATAGTS	WLHALATAG	4	0.2745	2565.0	42.00	0.29	tr_D0A0W7_
HLA-DQA10501-DQB10201	438	LIVLPQLSVGAEANA	PQLSVGAEA	4	0.2744	2568.1	42.00	0.42	tr_D0A0W7_
HLA-DQA10501-DQB10201	439	EFGVSLRSAVIREDG	LRSAVIRED	5	0.2741	2575.1	42.00	0.38	tr_D0A0W7_
HLA-DQA10501-DQB10201	440	LIRYALCQLRVLRLG	RYALCQLRV	2	0.2736	2591.6	42.00	0.39	tr_D0A0W7_
HLA-DQA10501-DQB10201	441	SSIISDDMRYGVFDD	DMRYGVFDD	6	0.2727	2615.6	42.00	0.31	tr_D0A0W7_
HLA-DQA10501-DQB10201	442	IAEAANDLRYEQGER	IAEAANDLR	0	0.2726	2618.9	42.00	0.25	tr_D0A0W7_
HLA-DQA10501-DQB10201	443	STFIRQWNPYLYFLR	FIRQWNPYL	2	0.2725	2621.7	42.00	0.34	tr_D0A0W7_
HLA-DQA10501-DQB10201	444	TFIRQWNPYLYFLRA	FIRQWNPYL	1	0.2724	2624.4	42.00	0.29	tr_D0A0W7_
HLA-DQA10501-DQB10201	445	SKAVYVKEMLENGQT	AVYVKEMLE	2	0.2718	2640.2	42.00	0.40	tr_D0A0W7_
HLA-DQA10501-DQB10201	446	TDQNKIVIVYGPLGD	VIVYGPLGD	6	0.2713	2654.5	42.00	0.31	tr_D0A0W7_
HLA-DQA10501-DQB10201	447	VKVLSATWNSYMPQE	TWNSYMPQE	6	0.2708	2669.1	43.00	0.34	tr_D0A0W7_
HLA-DQA10501-DQB10201	448	MKHNVVAFSPMTGST	VVAFSPMTG	4	0.2703	2683.1	43.00	0.39	tr_D0A0W7_
HLA-DQA10501-DQB10201	449	AAETLALIRYALCQL	LIRYALCQL	6	0.2703	2683.7	43.00	0.53	tr_D0A0W7_
HLA-DQA10501-DQB10201	450	LGHSDRFFLHAFNTT	FFLHAFNTT	6	0.2695	2706.9	43.00	0.41	tr_D0A0W7_
HLA-DQA10501-DQB10201	451	ADGIADFLRRSLTEF	FLRRSLTEF	6	0.2689	2724.8	43.00	0.69	tr_D0A0W7_
HLA-DQA10501-DQB10201	452	YALCQLRVLRLGFMY	LRVLRLGFM	5	0.2683	2742.3	43.00	0.44	tr_D0A0W7_
HLA-DQA10501-DQB10201	453	IVIGDYGGTCEGEAA	YGGTCEGEA	5	0.2683	2742.7	43.00	0.37	tr_D0A0W7_
HLA-DQA10501-DQB10201	454	SDGEPAPDDEFREVF	PDDEFREVF	6	0.2682	2746.1	43.00	0.44	tr_D0A0W7_
HLA-DQA10501-DQB10201	455	EYLKAHVSETNITEA	VSETNITEA	6	0.2680	2750.5	43.00	0.50	tr_D0A0W7_
HLA-DQA10501-DQB10201	456	SLLYDLFTTALNNTA	LYDLFTTAL	2	0.2678	2757.4	43.00	0.41	tr_D0A0W7_
HLA-DQA10501-DQB10201	457	YLKAHVSETNITEAD	VSETNITEA	5	0.2678	2758.7	43.00	0.52	tr_D0A0W7_
HLA-DQA10501-DQB10201	458	HNVVAFSPMTGSTFI	VVAFSPMTG	2	0.2676	2764.3	43.00	0.38	tr_D0A0W7_
HLA-DQA10501-DQB10201	459	VIGLSVPNVREIARK	VIGLSVPNV	0	0.2675	2767.1	43.00	0.49	tr_D0A0W7_
HLA-DQA10501-DQB10201	460	DPAAETLALIRYALC	PAAETLALI	1	0.2675	2767.2	43.00	0.36	tr_D0A0W7_
HLA-DQA10501-DQB10201	461	LEERNDLRVIVLFGE	RNDLRVIVL	3	0.2672	2775.5	43.00	0.36	tr_D0A0W7_
HLA-DQA10501-DQB10201	462	TSQCYTESSELQGPL	CYTESSELQ	3	0.2659	2816.1	44.00	0.38	tr_D0A0W7_
HLA-DQA10501-DQB10201	463	LLLIVLPQLSVGAEA	PQLSVGAEA	6	0.2654	2829.8	44.00	0.45	tr_D0A0W7_
HLA-DQA10501-DQB10201	464	AALPQAIIVFGAPEK	IIVFGAPEK	6	0.2650	2843.9	44.00	0.35	tr_D0A0W7_
HLA-DQA10501-DQB10201	465	GIADFLRRSLTEFGV	FLRRSLTEF	4	0.2645	2857.4	44.00	0.55	tr_D0A0W7_
HLA-DQA10501-DQB10201	466	KTSQCYTESSELQGP	CYTESSELQ	4	0.2644	2860.4	44.00	0.41	tr_D0A0W7_
HLA-DQA10501-DQB10201	467	SQCYTESSELQGPLN	CYTESSELQ	2	0.2643	2863.5	44.00	0.35	tr_D0A0W7_
HLA-DQA10501-DQB10201	468	SLRSAVIREDGEGVG	VIREDGEGV	5	0.2629	2908.5	45.00	0.31	tr_D0A0W7_
HLA-DQA10501-DQB10201	469	CQLRVLRLGFMYLQG	LRVLRLGFM	2	0.2626	2918.2	45.00	0.38	tr_D0A0W7_
HLA-DQA10501-DQB10201	470	SPTLEQQLYVLSSYL	QLYVLSSYL	6	0.2620	2936.4	45.00	0.38	tr_D0A0W7_
HLA-DQA10501-DQB10201	471	AAALPQAIIVFGAPE	AIIVFGAPE	6	0.2617	2944.9	45.00	0.47	tr_D0A0W7_
HLA-DQA10501-DQB10201	472	RKLEERNDLRVIVLF	RNDLRVIVL	5	0.2611	2966.0	45.00	0.49	tr_D0A0W7_
HLA-DQA10501-DQB10201	473	PQLSVGAEANATVKV	QLSVGAEAN	1	0.2607	2977.8	45.00	0.28	tr_D0A0W7_
HLA-DQA10501-DQB10201	474	LNKFRRHVIHLSPTL	HVIHLSPTL	6	0.2606	2981.3	45.00	0.42	tr_D0A0W7_
HLA-DQA10501-DQB10201	475	VTYLALPILMKHNVV	TYLALPILM	1	0.2604	2987.1	45.00	0.49	tr_D0A0W7_
HLA-DQA10501-DQB10201	476	LPILMKHNVVAFSPM	KHNVVAFSP	5	0.2601	2997.6	45.00	0.32	tr_D0A0W7_
HLA-DQA10501-DQB10201	477	IKEQLELETDQNKIV	QLELETDQN	3	0.2597	3011.1	46.00	0.43	tr_D0A0W7_
HLA-DQA10501-DQB10201	478	LLAFATGRLMRVILL	FATGRLMRV	3	0.2593	3024.2	46.00	0.28	tr_D0A0W7_
HLA-DQA10501-DQB10201	479	ATAGTSFAPGQLLFT	TSFAPGQLL	4	0.2592	3026.5	46.00	0.34	tr_D0A0W7_
HLA-DQA10501-DQB10201	480	PTLEQQLYVLSSYLA	QLYVLSSYL	5	0.2582	3060.2	46.00	0.32	tr_D0A0W7_
HLA-DQA10501-DQB10201	481	SLLAFATGRLMRVIL	FATGRLMRV	4	0.2582	3061.0	46.00	0.29	tr_D0A0W7_
HLA-DQA10501-DQB10201	482	ALCQLRVLRLGFMYL	LRVLRLGFM	4	0.2579	3071.2	46.00	0.52	tr_D0A0W7_
HLA-DQA10501-DQB10201	483	LEQQLYVLSSYLAGD	QLYVLSSYL	3	0.2571	3095.0	46.00	0.31	tr_D0A0W7_
HLA-DQA10501-DQB10201	484	DIVIGDYGGTCEGEA	YGGTCEGEA	6	0.2571	3096.7	46.00	0.34	tr_D0A0W7_
HLA-DQA10501-DQB10201	485	DGIADFLRRSLTEFG	FLRRSLTEF	5	0.2560	3133.0	47.00	0.58	tr_D0A0W7_

HLA-DQA10501-DQB10201	486	WLHALATAGTSFAPG	ALATAGTSF	3	0.2555	3149.5	47.00	0.32	tr_D0A0W7_
HLA-DQA10501-DQB10201	487	SNYGATQVSVWSMAR	YGATQVSVW	2	0.2555	3152.2	47.00	0.46	tr_D0A0W7_
HLA-DQA10501-DQB10201	488	ARKLEERNDLRVIVL	RNDLRVIVL	6	0.2554	3155.5	47.00	0.56	tr_D0A0W7_
HLA-DQA10501-DQB10201	489	LWTPLSLLAFATGRL	LLAFATGRL	6	0.2552	3159.6	47.00	0.44	tr_D0A0W7_
HLA-DQA10501-DQB10201	490	ADFLRRSLTEFGVSL	FLRRSLTEF	2	0.2546	3181.1	47.00	0.43	tr_D0A0W7_
HLA-DQA10501-DQB10201	491	IADFLRRSLTEFGVS	FLRRSLTEF	3	0.2544	3188.8	47.00	0.52	tr_D0A0W7_
HLA-DQA10501-DQB10201	492	IRYALCQLRVLRLGF	RYALCQLRV	1	0.2540	3201.9	47.00	0.33	tr_D0A0W7_
HLA-DQA10501-DQB10201	493	FAPGQLLFTGTNPLA	LLFTGTNPL	5	0.2537	3211.9	47.00	0.42	tr_D0A0W7_
HLA-DQA10501-DQB10201	494	KLEERNDLRVIVLFG	RNDLRVIVL	4	0.2536	3215.2	47.00	0.44	tr_D0A0W7_
HLA-DQA10501-DQB10201	495	MTGSTFIRQWNPYLY	FIRQWNPYL	5	0.2536	3217.2	47.00	0.34	tr_D0A0W7_
HLA-DQA10501-DQB10201	496	VRSLYSQRRYVINDI	QRRYVINDI	6	0.2531	3234.4	47.00	0.79	tr_D0A0W7_
HLA-DQA10501-DQB10201	497	LESRQWTVAGSVKVE	WTVAGSVKV	5	0.2525	3254.4	48.00	0.55	tr_D0A0W7_
HLA-DQA10501-DQB10201	498	VILRAPVALLLLLIV	LRAPVALLL	2	0.2523	3261.0	48.00	0.29	tr_D0A0W7_
HLA-DQA10501-DQB10201	499	WTPLSLLAFATGRLM	LLAFATGRL	5	0.2521	3268.9	48.00	0.36	tr_D0A0W7_
HLA-DQA10501-DQB10201	500	GVSLRSAVIREDGEG	LRSAVIRED	3	0.2517	3283.1	48.00	0.31	tr_D0A0W7_
HLA-DQA10501-DQB10201	501	NYGATQVSVWSMARA	YGATQVSVW	1	0.2515	3291.2	48.00	0.39	tr_D0A0W7_
HLA-DQA10501-DQB10201	502	VNAINAGFNASLESR	NAGFNASLE	4	0.2514	3294.4	48.00	0.41	tr_D0A0W7_
HLA-DQA10501-DQB10201	503	QLSVGAEANATVKVL	EANATVKVL	6	0.2510	3306.7	48.00	0.23	tr_D0A0W7_
HLA-DQA10501-DQB10201	504	DLGHSDRFFLHAFNT	DRFFLHAFN	5	0.2507	3319.6	48.00	0.31	tr_D0A0W7_
HLA-DQA10501-DQB10201	505	PVALLLLLIVLPQLS	LLLIVLPQL	5	0.2497	3352.9	48.00	0.46	tr_D0A0W7_
HLA-DQA10501-DQB10201	506	LLYDLFTTALNNTAG	LYDLFTTAL	1	0.2497	3356.2	48.00	0.38	tr_D0A0W7_
HLA-DQA10501-DQB10201	507	LVFATSLPHWGDTET	VFATSLPHW	1	0.2496	3356.8	48.00	0.49	tr_D0A0W7_
HLA-DQA10501-DQB10201	508	LCQLRVLRLGFMYLQ	LRVLRLGFM	3	0.2496	3358.3	48.00	0.48	tr_D0A0W7_
HLA-DQA10501-DQB10201	509	INDIVIGDYGGTCEG	INDIVIGDY	0	0.2491	3377.6	48.00	0.35	tr_D0A0W7_
HLA-DQA10501-DQB10201	510	GDLGHSDRFFLHAFN	DRFFLHAFN	6	0.2486	3395.4	49.00	0.34	tr_D0A0W7_
HLA-DQA10501-DQB10201	511	SRLWTPLSLLAFATG	WTPLSLLAF	3	0.2482	3411.2	49.00	0.32	tr_D0A0W7_
HLA-DQA10501-DQB10201	512	AINAGFNASLESRQW	NAGFNASLE	2	0.2479	3421.6	49.00	0.26	tr_D0A0W7_
HLA-DQA10501-DQB10201	513	AETLALIRYALCQLR	LIRYALCQL	5	0.2472	3447.1	49.00	0.44	tr_D0A0W7_
HLA-DQA10501-DQB10201	514	KAVYVKEMLENGQTT	AVYVKEMLE	1	0.2468	3460.1	49.00	0.35	tr_D0A0W7_
HLA-DQA10501-DQB10201	515	ALATAGTSFAPGQLL	TSFAPGQLL	6	0.2464	3476.2	49.00	0.33	tr_D0A0W7_
HLA-DQA10501-DQB10201	516	EQQLYVLSSYLAGDG	QLYVLSSYL	2	0.2462	3482.5	49.00	0.33	tr_D0A0W7_
HLA-DQA10501-DQB10201	517	GKYLPISGTVFVIGL	SGTVFVIGL	6	0.2450	3528.1	50.00	0.31	tr_D0A0W7_
HLA-DQA10501-DQB10201	518	SLESRQWTVAGSVKV	WTVAGSVKV	6	0.2441	3562.9	50.00	0.58	tr_D0A0W7_
HLA-DQA10501-DQB10201	519	TVKVLSATWNSYMPQ	TVKVLSATW	0	0.2439	3572.7	50.00	0.27	tr_D0A0W7_
HLA-DQA10501-DQB10201	520	VALLLLLIVLPQLSV	LLLIVLPQL	4	0.2439	3573.1	50.00	0.44	tr_D0A0W7_
HLA-DQA10501-DQB10201	521	LATAGTSFAPGQLLF	TSFAPGQLL	5	0.2438	3574.9	50.00	0.39	tr_D0A0W7_
HLA-DQA10501-DQB10201	522	ATVKVLSATWNSYMP	TVKVLSATW	1	0.2433	3593.7	50.00	0.35	tr_D0A0W7_
HLA-DQA10501-DQB10201	523	NVVAFSPMTGSTFIR	VVAFSPMTG	1	0.2432	3597.5	50.00	0.43	tr_D0A0W7_
HLA-DQA10501-DQB10201	524	PAAETLALIRYALCQ	PAAETLALI	0	0.2430	3605.5	50.00	0.25	tr_D0A0W7_
HLA-DQA10501-DQB10201	525	AEANATVKVLSATWN	TVKVLSATW	5	0.2416	3662.5	55.00	0.41	tr_D0A0W7_
HLA-DQA10501-DQB10201	526	NAINAGFNASLESRQ	NAGFNASLE	3	0.2416	3663.5	55.00	0.35	tr_D0A0W7_
HLA-DQA10501-DQB10201	527	QCYTESSELQGPLNG	CYTESSELQ	1	0.2414	3667.9	55.00	0.34	tr_D0A0W7_
HLA-DQA10501-DQB10201	528	VKTSQCYTESSELQG	CYTESSELQ	5	0.2414	3669.6	55.00	0.41	tr_D0A0W7_
HLA-DQA10501-DQB10201	529	GAEANATVKVLSATW	TVKVLSATW	6	0.2413	3673.8	55.00	0.47	tr_D0A0W7_
HLA-DQA10501-DQB10201	530	AQLFHDVEKDSRLWT	QLFHDVEKD	1	0.2413	3673.9	55.00	0.35	tr_D0A0W7_
HLA-DQA10501-DQB10201	531	PLSLLAFATGRLMRV	FATGRLMRV	6	0.2412	3679.7	55.00	0.34	tr_D0A0W7_
HLA-DQA10501-DQB10201	532	ECNQGSKAVYVKEML	KAVYVKEML	6	0.2404	3710.1	55.00	0.61	tr_D0A0W7_
HLA-DQA10501-DQB10201	533	TLEQQLYVLSSYLAG	QLYVLSSYL	4	0.2397	3737.1	55.00	0.34	tr_D0A0W7_
HLA-DQA10501-DQB10201	534	PISGTVFVIGLSVPN	FVIGLSVPN	6	0.2396	3740.4	55.00	0.28	tr_D0A0W7_
HLA-DQA10501-DQB10201	535	VIGDYGGTCEGEAAK	YGGTCEGEA	4	0.2395	3746.2	55.00	0.38	tr_D0A0W7_

HLA-DQA10501-DQB10201	536	KDDCASNYGATQVSV	NYGATQVSV	6	0.2393	3755.6	55.00	0.44	tr_D0A0W7_
HLA-DQA10501-DQB10201	537	LSPTLEQQLYVLSSY	QQLYVLSSY	6	0.2392	3758.4	55.00	0.34	tr_D0A0W7_
HLA-DQA10501-DQB10201	538	VEKDSRLWTPLSLLA	SRLWTPLSL	4	0.2383	3794.5	55.00	0.47	tr_D0A0W7_
HLA-DQA10501-DQB10201	539	LSLLAFATGRLMRVI	FATGRLMRV	5	0.2380	3806.0	55.00	0.32	tr_D0A0W7_
HLA-DQA10501-DQB10201	540	PGQLLFTGTNPLAKD	LLFTGTNPL	3	0.2380	3807.7	55.00	0.40	tr_D0A0W7_
HLA-DQA10501-DQB10201	541	VMSEYLKAHVSETNI	YLKAHVSET	4	0.2377	3818.3	55.00	0.25	tr_D0A0W7_
HLA-DQA10501-DQB10201	542	TALNNTAGAARLVFA	AGAARLVFA	6	0.2374	3830.8	55.00	0.39	tr_D0A0W7_
HLA-DQA10501-DQB10201	543	HGTEGELMVYGWICG	EGELMVYGW	3	0.2374	3832.1	55.00	0.28	tr_D0A0W7_
HLA-DQA10501-DQB10201	544	APVALLLLLIVLPQL	LLLIVLPQL	6	0.2364	3875.7	55.00	0.61	tr_D0A0W7_
HLA-DQA10501-DQB10201	545	TAGTSFAPGQLLFTG	TSFAPGQLL	3	0.2361	3888.2	55.00	0.36	tr_D0A0W7_
HLA-DQA10501-DQB10201	546	DFLRRSLTEFGVSLR	FLRRSLTEF	1	0.2358	3899.3	55.00	0.42	tr_D0A0W7_
HLA-DQA10501-DQB10201	547	LAFATGRLMRVILLH	FATGRLMRV	2	0.2335	3995.9	55.00	0.34	tr_D0A0W7_
HLA-DQA10501-DQB10201	548	SQYIAIKRFQGVMSE	KRFQGVMSE	6	0.2335	3998.5	55.00	0.49	tr_D0A0W7_
HLA-DQA10501-DQB10201	549	DVEKDSRLWTPLSLL	SRLWTPLSL	5	0.2329	4024.0	55.00	0.52	tr_D0A0W7_
HLA-DQA10501-DQB10201	550	ALPILMKHNVVAFSP	KHNVVAFSP	6	0.2327	4031.6	55.00	0.44	tr_D0A0W7_
HLA-DQA10501-DQB10201	551	NATVKVLSATWNSYM	TVKVLSATW	2	0.2322	4052.5	55.00	0.41	tr_D0A0W7_
HLA-DQA10501-DQB10201	552	SLPTGGAVCRVAMDT	AVCRVAMDT	6	0.2307	4118.5	55.00	0.39	tr_D0A0W7_
HLA-DQA10501-DQB10201	553	VGKYLPISGTVFVIG	YLPISGTVF	3	0.2307	4122.4	55.00	0.29	tr_D0A0W7_
HLA-DQA10501-DQB10201	554	KEQLELETDQNKIVI	QLELETDQN	2	0.2306	4126.8	55.00	0.50	tr_D0A0W7_
HLA-DQA10501-DQB10201	555	AGTSFAPGQLLFTGT	TSFAPGQLL	2	0.2304	4131.7	55.00	0.36	tr_D0A0W7_
HLA-DQA10501-DQB10201	556	NDIVIGDYGGTCEGE	IGDYGGTCE	4	0.2301	4145.5	55.00	0.31	tr_D0A0W7_
HLA-DQA10501-DQB10201	557	DRTTFVRSLYSQRRY	VRSLYSQRR	5	0.2300	4152.0	55.00	0.22	tr_D0A0W7_
HLA-DQA10501-DQB10201	558	INAGFNASLESRQWT	NAGFNASLE	1	0.2297	4162.8	55.00	0.23	tr_D0A0W7_
HLA-DQA10501-DQB10201	559	FATGRLMRVILLHVE	MRVILLHVE	6	0.2297	4165.9	55.00	0.25	tr_D0A0W7_
HLA-DQA10501-DQB10201	560	ALLLLLIVLPQLSVG	LLLIVLPQL	3	0.2297	4166.7	55.00	0.43	tr_D0A0W7_
HLA-DQA10501-DQB10201	561	LTEFGVSLRSAVIRE	SLRSAVIRE	6	0.2287	4208.5	55.00	0.26	tr_D0A0W7_
HLA-DQA10501-DQB10201	562	AFATGRLMRVILLHV	FATGRLMRV	1	0.2286	4213.1	55.00	0.28	tr_D0A0W7_
HLA-DQA10501-DQB10201	563	QALSSLEWLKDRTTF	QALSSLEWL	0	0.2285	4218.8	55.00	0.56	tr_D0A0W7_
HLA-DQA10501-DQB10201	564	VVKTSQCYTESSELQ	CYTESSELQ	6	0.2284	4224.9	55.00	0.45	tr_D0A0W7_
HLA-DQA10501-DQB10201	565	SFAPGQLLFTGTNPL	LLFTGTNPL	6	0.2284	4225.9	55.00	0.46	tr_D0A0W7_
HLA-DQA10501-DQB10201	566	GQLLFTGTNPLAKDS	LLFTGTNPL	2	0.2278	4251.0	55.00	0.42	tr_D0A0W7_
HLA-DQA10501-DQB10201	567	QGVMSEYLKAHVSET	EYLKAHVSE	5	0.2274	4269.5	55.00	0.36	tr_D0A0W7_
HLA-DQA10501-DQB10201	568	YGATQVSVWSMARAL	YGATQVSVW	0	0.2270	4289.5	55.00	0.27	tr_D0A0W7_
HLA-DQA10501-DQB10201	569	LSVGAEANATVKVLS	EANATVKVL	5	0.2268	4297.8	55.00	0.26	tr_D0A0W7_
HLA-DQA10501-DQB10201	570	APGQLLFTGTNPLAK	LLFTGTNPL	4	0.2267	4302.1	55.00	0.40	tr_D0A0W7_
HLA-DQA10501-DQB10201	571	LYDLFTTALNNTAGA	LYDLFTTAL	0	0.2265	4311.2	55.00	0.31	tr_D0A0W7_
HLA-DQA10501-DQB10201	572	GVMSEYLKAHVSETN	EYLKAHVSE	4	0.2263	4320.0	55.00	0.31	tr_D0A0W7_
HLA-DQA10501-DQB10201	573	KYLPISGTVFVIGLS	SGTVFVIGL	5	0.2257	4347.6	55.00	0.27	tr_D0A0W7_
HLA-DQA10501-DQB10201	574	LLLLLIVLPQLSVGA	LLLIVLPQL	2	0.2256	4354.1	55.00	0.41	tr_D0A0W7_
HLA-DQA10501-DQB10201	575	RSLTEFGVSLRSAVI	EFGVSLRSA	4	0.2250	4380.4	55.00	0.31	tr_D0A0W7_
HLA-DQA10501-DQB10201	576	EWLKDRTTFVRSLYS	WLKDRTTFV	1	0.2250	4381.0	55.00	0.25	tr_D0A0W7_
HLA-DQA10501-DQB10201	577	ETDQNKIVIVYGPLG	IVIVYGPLG	6	0.2248	4389.7	55.00	0.46	tr_D0A0W7_
HLA-DQA10501-DQB10201	578	LRSAVIREDGEGVGK	VIREDGEGV	4	0.2243	4414.2	55.00	0.38	tr_D0A0W7_
HLA-DQA10501-DQB10201	579	RLWTPLSLLAFATGR	WTPLSLLAF	2	0.2240	4432.0	60.00	0.35	tr_D0A0W7_
HLA-DQA10501-DQB10201	580	YLPISGTVFVIGLSV	SGTVFVIGL	4	0.2239	4435.4	60.00	0.22	tr_D0A0W7_
HLA-DQA10501-DQB10201	581	IVFGAPEKDTAKFLM	IVFGAPEKD	0	0.2237	4442.3	60.00	0.68	tr_D0A0W7_
HLA-DQA10501-DQB10201	582	MSEYLKAHVSETNIT	YLKAHVSET	3	0.2237	4442.5	60.00	0.26	tr_D0A0W7_
HLA-DQA10501-DQB10201	583	RIARSYILGPSSVQA	ILGPSSVQA	6	0.2235	4451.7	60.00	0.34	tr_D0A0W7_
HLA-DQA10501-DQB10201	584	SVGAEANATVKVLSA	NATVKVLSA	6	0.2229	4482.0	60.00	0.28	tr_D0A0W7_
HLA-DQA10501-DQB10201	585	KGDLGHSDRFFLHAF	DLGHSDRFF	2	0.2221	4524.3	60.00	0.31	tr_D0A0W7_

HLA-DQA10501-DQB10201	586	VFATSLPHWGDTETS	VFATSLPHW	0	0.2220	4527.1	60.00	0.39	tr_D0A0W7_
HLA-DQA10501-DQB10201	587	ALNNTAGAARLVFAT	AGAARLVFA	5	0.2216	4548.5	60.00	0.38	tr_D0A0W7_
HLA-DQA10501-DQB10201	588	EANATVKVLSATWNS	TVKVLSATW	4	0.2214	4558.1	60.00	0.45	tr_D0A0W7_
HLA-DQA10501-DQB10201	589	GVGKYLPISGTVFVI	YLPISGTVF	4	0.2209	4580.2	60.00	0.36	tr_D0A0W7_
HLA-DQA10501-DQB10201	590	CYTESSELQGPLNGL	CYTESSELQ	0	0.2206	4596.0	60.00	0.28	tr_D0A0W7_
HLA-DQA10501-DQB10201	591	IHLSPTLEQQLYVLS	PTLEQQLYV	4	0.2206	4597.3	60.00	0.29	tr_D0A0W7_
HLA-DQA10501-DQB10201	592	ANATVKVLSATWNSY	TVKVLSATW	3	0.2204	4605.9	60.00	0.46	tr_D0A0W7_
HLA-DQA10501-DQB10201	593	KDRTTFVRSLYSQRR	VRSLYSQRR	6	0.2202	4617.7	60.00	0.26	tr_D0A0W7_
HLA-DQA10501-DQB10201	594	LLLLIVLPQLSVGAE	LLLIVLPQL	1	0.2187	4692.6	60.00	0.38	tr_D0A0W7_
HLA-DQA10501-DQB10201	595	QLLFTGTNPLAKDSQ	LLFTGTNPL	1	0.2180	4726.4	60.00	0.40	tr_D0A0W7_
HLA-DQA10501-DQB10201	596	LNASIPPLANPMTPS	LNASIPPLA	0	0.2172	4767.7	60.00	0.55	tr_D0A0W7_
HLA-DQA10501-DQB10201	597	VFMEDDDTASKAAAL	VFMEDDDTA	0	0.2164	4808.4	60.00	0.58	tr_D0A0W7_
HLA-DQA10501-DQB10201	598	LNNTAGAARLVFATS	AGAARLVFA	4	0.2162	4822.1	60.00	0.34	tr_D0A0W7_
HLA-DQA10501-DQB10201	599	IGDYGGTCEGEAAKH	YGGTCEGEA	3	0.2158	4839.7	60.00	0.41	tr_D0A0W7_
HLA-DQA10501-DQB10201	600	GEGVGKYLPISGTVF	YLPISGTVF	6	0.2156	4851.6	60.00	0.49	tr_D0A0W7_
HLA-DQA10501-DQB10201	601	EQLELETDQNKIVIV	QLELETDQN	1	0.2156	4852.6	60.00	0.50	tr_D0A0W7_
HLA-DQA10501-DQB10201	602	TPLSLLAFATGRLMR	LLAFATGRL	4	0.2153	4865.8	60.00	0.38	tr_D0A0W7_
HLA-DQA10501-DQB10201	603	IARSYILGPSSVQAS	ILGPSSVQA	5	0.2148	4892.8	60.00	0.35	tr_D0A0W7_
HLA-DQA10501-DQB10201	604	PEKDTAKFLMMMVAE	KFLMMMVAE	6	0.2148	4894.7	60.00	0.43	tr_D0A0W7_
HLA-DQA10501-DQB10201	605	ARSYILGPSSVQASL	ILGPSSVQA	4	0.2147	4898.7	60.00	0.35	tr_D0A0W7_
HLA-DQA10501-DQB10201	606	QLFHDVEKDSRLWTP	QLFHDVEKD	0	0.2141	4933.2	60.00	0.29	tr_D0A0W7_
HLA-DQA10501-DQB10201	607	HLSPTLEQQLYVLSS	PTLEQQLYV	3	0.2136	4956.7	60.00	0.29	tr_D0A0W7_
HLA-DQA10501-DQB10201	608	HALATAGTSFAPGQL	ALATAGTSF	1	0.2134	4967.0	60.00	0.30	tr_D0A0W7_
HLA-DQA10501-DQB10201	609	LPISGTVFVIGLSVP	SGTVFVIGL	3	0.2132	4980.9	60.00	0.26	tr_D0A0W7_
HLA-DQA10501-DQB10201	610	LKDRTTFVRSLYSQR	FVRSLYSQR	6	0.2129	4993.7	60.00	0.25	tr_D0A0W7_
HLA-DQA10501-DQB10201	611	HDVEKDSRLWTPLSL	SRLWTPLSL	6	0.2126	5010.2	60.00	0.66	tr_D0A0W7_
HLA-DQA10501-DQB10201	612	LEWLKDRTTFVRSLY	WLKDRTTFV	2	0.2120	5043.2	60.00	0.40	tr_D0A0W7_
HLA-DQA10501-DQB10201	613	RTTFVRSLYSQRRYV	VRSLYSQRR	4	0.2113	5082.3	60.00	0.27	tr_D0A0W7_
HLA-DQA10501-DQB10201	614	GTSFAPGQLLFTGTN	TSFAPGQLL	1	0.2109	5106.0	60.00	0.32	tr_D0A0W7_
HLA-DQA10501-DQB10201	615	SEYLKAHVSETNITE	YLKAHVSET	2	0.2107	5112.8	60.00	0.24	tr_D0A0W7_
HLA-DQA10501-DQB10201	616	LHALATAGTSFAPGQ	ALATAGTSF	2	0.2100	5157.0	60.00	0.37	tr_D0A0W7_
HLA-DQA10501-DQB10201	617	EERIARSYILGPSSV	SYILGPSSV	6	0.2094	5190.2	60.00	0.24	tr_D0A0W7_
HLA-DQA10501-DQB10201	618	SLTEFGVSLRSAVIR	EFGVSLRSA	3	0.2091	5206.8	60.00	0.26	tr_D0A0W7_
HLA-DQA10501-DQB10201	619	EGVGKYLPISGTVFV	YLPISGTVF	5	0.2087	5228.1	60.00	0.41	tr_D0A0W7_
HLA-DQA10501-DQB10201	620	GATQVSVWSMARALN	VWSMARALN	6	0.2079	5271.1	60.00	0.25	tr_D0A0W7_
HLA-DQA10501-DQB10201	621	FLRRSLTEFGVSLRS	FLRRSLTEF	0	0.2079	5273.6	60.00	0.28	tr_D0A0W7_
HLA-DQA10501-DQB10201	622	SSDGEPAPDDEFREV	GEPAPDDEF	3	0.2079	5273.8	60.00	0.37	tr_D0A0W7_
HLA-DQA10501-DQB10201	623	TTALNNTAGAARLVF	TAGAARLVF	6	0.2077	5281.7	60.00	0.52	tr_D0A0W7_
HLA-DQA10501-DQB10201	624	MVAEERIARSYILGP	ERIARSYIL	4	0.2065	5355.9	65.00	0.32	tr_D0A0W7_
HLA-DQA10501-DQB10201	625	MSSDGEPAPDDEFRE	GEPAPDDEF	4	0.2061	5377.9	65.00	0.35	tr_D0A0W7_
HLA-DQA10501-DQB10201	626	ALSSLEWLKDRTTFV	WLKDRTTFV	6	0.2058	5396.4	65.00	0.54	tr_D0A0W7_
HLA-DQA10501-DQB10201	627	RYGVFDDTKCDTAEK	RYGVFDDTK	0	0.2054	5415.9	65.00	0.40	tr_D0A0W7_
HLA-DQA10501-DQB10201	628	ILRAPVALLLLLIVL	LRAPVALLL	1	0.2046	5462.6	65.00	0.34	tr_D0A0W7_
HLA-DQA10501-DQB10201	629	NAGFNASLESRQWTV	FNASLESRQ	3	0.2042	5487.1	65.00	0.25	tr_D0A0W7_
HLA-DQA10501-DQB10201	630	PMTGSTFIRQWNPYL	FIRQWNPYL	6	0.2039	5503.4	65.00	0.44	tr_D0A0W7_
HLA-DQA10501-DQB10201	631	RRSLTEFGVSLRSAV	EFGVSLRSA	5	0.2039	5505.3	65.00	0.39	tr_D0A0W7_
HLA-DQA10501-DQB10201	632	RSAVIREDGEGVGKY	VIREDGEGV	3	0.2039	5506.1	65.00	0.51	tr_D0A0W7_
HLA-DQA10501-DQB10201	633	WLKDRTTFVRSLYSQ	TTFVRSLYS	5	0.2037	5516.5	65.00	0.31	tr_D0A0W7_
HLA-DQA10501-DQB10201	634	AEERIARSYILGPSS	ERIARSYIL	2	0.2035	5532.6	65.00	0.25	tr_D0A0W7_
HLA-DQA10501-DQB10201	635	TSFAPGQLLFTGTNP	QLLFTGTNP	6	0.2018	5629.9	65.00	0.28	tr_D0A0W7_

HLA-DQA10501-DQB10201	636	ERIARSYILGPSSVQ	SYILGPSSV	5	0.2012	5669.4	65.00	0.28	tr_D0A0W7_
HLA-DQA10501-DQB10201	637	SAVIREDGEGVGKYL	VIREDGEGV	2	0.2012	5671.9	65.00	0.50	tr_D0A0W7_
HLA-DQA10501-DQB10201	638	AVYVKEMLENGQTTT	AVYVKEMLE	0	0.2009	5688.1	65.00	0.34	tr_D0A0W7_
HLA-DQA10501-DQB10201	639	LVGKGDLGHSDRFFL	DLGHSDRFF	5	0.2005	5713.7	65.00	0.50	tr_D0A0W7_
HLA-DQA10501-DQB10201	640	VAEERIARSYILGPS	ERIARSYIL	3	0.1977	5887.5	65.00	0.30	tr_D0A0W7_
HLA-DQA10501-DQB10201	641	VVAFSPMTGSTFIRQ	VVAFSPMTG	0	0.1970	5935.3	65.00	0.32	tr_D0A0W7_
HLA-DQA10501-DQB10201	642	MEDDDTASKAAALWQ	TASKAAALW	5	0.1969	5936.8	65.00	0.57	tr_D0A0W7_
HLA-DQA10501-DQB10201	643	NPLAKDSQYIAIKRF	SQYIAIKRF	6	0.1968	5946.5	65.00	0.52	tr_D0A0W7_
HLA-DQA10501-DQB10201	644	TTFVRSLYSQRRYVI	VRSLYSQRR	3	0.1957	6016.9	65.00	0.32	tr_D0A0W7_
HLA-DQA10501-DQB10201	645	FMEDDDTASKAAALW	TASKAAALW	6	0.1952	6050.1	65.00	0.67	tr_D0A0W7_
HLA-DQA10501-DQB10201	646	MMVAEERIARSYILG	ERIARSYIL	5	0.1949	6066.3	65.00	0.31	tr_D0A0W7_
HLA-DQA10501-DQB10201	647	LRRSLTEFGVSLRSA	EFGVSLRSA	6	0.1948	6073.0	65.00	0.37	tr_D0A0W7_
HLA-DQA10501-DQB10201	648	SLEWLKDRTTFVRSL	WLKDRTTFV	3	0.1934	6165.4	65.00	0.51	tr_D0A0W7_
HLA-DQA10501-DQB10201	649	GKGDLGHSDRFFLHA	DLGHSDRFF	3	0.1934	6171.8	65.00	0.44	tr_D0A0W7_
HLA-DQA10501-DQB10201	650	YGVFDDTKCDTAEKL	TKCDTAEKL	6	0.1927	6213.0	65.00	0.41	tr_D0A0W7_
HLA-DQA10501-DQB10201	651	VGKGDLGHSDRFFLH	DLGHSDRFF	4	0.1915	6294.4	65.00	0.49	tr_D0A0W7_
HLA-DQA10501-DQB10201	652	LLFTGTNPLAKDSQY	LFTGTNPLA	1	0.1914	6301.8	65.00	0.33	tr_D0A0W7_
HLA-DQA10501-DQB10201	653	GDYGGTCEGEAAKHG	YGGTCEGEA	2	0.1902	6383.0	65.00	0.47	tr_D0A0W7_
HLA-DQA10501-DQB10201	654	LSSLEWLKDRTTFVR	WLKDRTTFV	5	0.1902	6388.9	65.00	0.57	tr_D0A0W7_
HLA-DQA10501-DQB10201	655	GQTTTMESGFTVVKT	ESGFTVVKT	6	0.1901	6392.5	65.00	0.41	tr_D0A0W7_
HLA-DQA10501-DQB10201	656	ESSELQGPLNGLAVF	GPLNGLAVF	6	0.1883	6516.8	70.00	0.25	tr_D0A0W7_
HLA-DQA10501-DQB10201	657	HSLPTGGAVCRVAMD	PTGGAVCRV	3	0.1861	6673.7	70.00	0.47	tr_D0A0W7_
HLA-DQA10501-DQB10201	658	YDLFTTALNNTAGAA	FTTALNNTA	3	0.1847	6778.2	70.00	0.34	tr_D0A0W7_
HLA-DQA10501-DQB10201	659	LETDQNKIVIVYGPL	KIVIVYGPL	6	0.1833	6879.2	70.00	0.41	tr_D0A0W7_
HLA-DQA10501-DQB10201	660	ENGQTTTMESGFTVV	TMESGFTVV	6	0.1824	6946.3	70.00	0.22	tr_D0A0W7_
HLA-DQA10501-DQB10201	661	VGAEANATVKVLSAT	EANATVKVL	3	0.1819	6983.7	70.00	0.25	tr_D0A0W7_
HLA-DQA10501-DQB10201	662	AVIREDGEGVGKYLP	VIREDGEGV	1	0.1818	6997.3	70.00	0.49	tr_D0A0W7_
HLA-DQA10501-DQB10201	663	RLNKFRRHVIHLSPT	RRHVIHLSP	5	0.1816	7005.1	70.00	0.39	tr_D0A0W7_
HLA-DQA10501-DQB10201	664	TVVKTSQCYTESSEL	QCYTESSEL	6	0.1807	7073.5	70.00	0.41	tr_D0A0W7_
HLA-DQA10501-DQB10201	665	QLELETDQNKIVIVY	QLELETDQN	0	0.1804	7104.0	70.00	0.36	tr_D0A0W7_
HLA-DQA10501-DQB10201	666	TYLALPILMKHNVVA	TYLALPILM	0	0.1803	7105.5	70.00	0.56	tr_D0A0W7_
HLA-DQA10501-DQB10201	667	SSLEWLKDRTTFVRS	WLKDRTTFV	4	0.1786	7237.7	70.00	0.62	tr_D0A0W7_
HLA-DQA10501-DQB10201	668	LSKDDCASNYGATQV	ASNYGATQV	6	0.1781	7278.9	70.00	0.81	tr_D0A0W7_
HLA-DQA10501-DQB10201	669	RAPVALLLLLIVLPQ	LLLLIVLPQ	6	0.1780	7287.2	70.00	0.41	tr_D0A0W7_
HLA-DQA10501-DQB10201	670	AGFNASLESRQWTVA	FNASLESRQ	2	0.1774	7338.3	70.00	0.32	tr_D0A0W7_
HLA-DQA10501-DQB10201	671	GAPEKDTAKFLMMMV	TAKFLMMMV	6	0.1770	7362.7	70.00	0.47	tr_D0A0W7_
HLA-DQA10501-DQB10201	672	YTESSELQGPLNGLA	SSELQGPLN	3	0.1768	7381.2	70.00	0.27	tr_D0A0W7_
HLA-DQA10501-DQB10201	673	SKDDCASNYGATQVS	ASNYGATQV	5	0.1767	7388.8	70.00	0.68	tr_D0A0W7_
HLA-DQA10501-DQB10201	674	TFVRSLYSQRRYVIN	VRSLYSQRR	2	0.1759	7454.1	70.00	0.38	tr_D0A0W7_
HLA-DQA10501-DQB10201	675	APEKDTAKFLMMMVA	TAKFLMMMV	5	0.1753	7504.3	70.00	0.31	tr_D0A0W7_
HLA-DQA10501-DQB10201	676	PLAKDSQYIAIKRFQ	SQYIAIKRF	5	0.1751	7515.6	70.00	0.46	tr_D0A0W7_
HLA-DQA10501-DQB10201	677	VAFSPMTGSTFIRQW	FSPMTGSTF	2	0.1745	7567.4	70.00	0.31	tr_D0A0W7_
HLA-DQA10501-DQB10201	678	LRAPVALLLLLIVLP	RAPVALLLL	1	0.1737	7635.6	70.00	0.31	tr_D0A0W7_
HLA-DQA10501-DQB10201	679	LENGQTTTMESGFTV	TTTMESGFT	5	0.1708	7881.4	75.00	0.32	tr_D0A0W7_
HLA-DQA10501-DQB10201	680	DYGGTCEGEAAKHGA	YGGTCEGEA	1	0.1705	7906.1	75.00	0.47	tr_D0A0W7_
HLA-DQA10501-DQB10201	681	MESGFTVVKTSQCYT	VVKTSQCYT	6	0.1702	7924.6	75.00	0.24	tr_D0A0W7_
HLA-DQA10501-DQB10201	682	PRLNKFRRHVIHLSP	RRHVIHLSP	6	0.1700	7947.3	75.00	0.49	tr_D0A0W7_
HLA-DQA10501-DQB10201	683	DSQYIAIKRFQGVMS	IKRFQGVMS	6	0.1689	8037.2	75.00	0.41	tr_D0A0W7_
HLA-DQA10501-DQB10201	684	TESSELQGPLNGLAV	ELQGPLNGL	4	0.1686	8070.4	75.00	0.26	tr_D0A0W7_
HLA-DQA10501-DQB10201	685	EHSLPTGGAVCRVAM	PTGGAVCRV	4	0.1682	8101.6	75.00	0.50	tr_D0A0W7_

HLA-DQA10501-DQB10201	686	AFSPMTGSTFIRQWN	FSPMTGSTF	1	0.1655	8345.8	75.00	0.29	tr_D0A0W7_
HLA-DQA10501-DQB10201	687	EDDDTASKAAALWQK	TASKAAALW	4	0.1654	8347.7	75.00	0.52	tr_D0A0W7_
HLA-DQA10501-DQB10201	688	NASLESRQWTVAGSV	RQWTVAGSV	6	0.1653	8362.5	75.00	0.68	tr_D0A0W7_
HLA-DQA10501-DQB10201	689	HLVGKGDLGHSDRFF	DLGHSDRFF	6	0.1650	8391.6	75.00	0.54	tr_D0A0W7_
HLA-DQA10501-DQB10201	690	VIREDGEGVGKYLPI	VIREDGEGV	0	0.1647	8413.0	75.00	0.37	tr_D0A0W7_
HLA-DQA10501-DQB10201	691	ESGFTVVKTSQCYTE	VVKTSQCYT	5	0.1641	8469.0	75.00	0.26	tr_D0A0W7_
HLA-DQA10501-DQB10201	692	LFHDVEKDSRLWTPL	FHDVEKDSR	1	0.1639	8491.6	75.00	0.29	tr_D0A0W7_
HLA-DQA10501-DQB10201	693	MLENGQTTTMESGFT	TTTMESGFT	6	0.1638	8499.8	75.00	0.34	tr_D0A0W7_
HLA-DQA10501-DQB10201	694	QTTTMESGFTVVKTS	ESGFTVVKT	5	0.1636	8517.8	75.00	0.40	tr_D0A0W7_
HLA-DQA10501-DQB10201	695	FSPMTGSTFIRQWNP	MTGSTFIRQ	3	0.1636	8519.1	75.00	0.32	tr_D0A0W7_
HLA-DQA10501-DQB10201	696	TMESGFTVVKTSQCY	ESGFTVVKT	2	0.1629	8581.5	75.00	0.23	tr_D0A0W7_
HLA-DQA10501-DQB10201	697	IARKLEERNDLRVIV	ERNDLRVIV	6	0.1611	8751.7	75.00	0.37	tr_D0A0W7_
HLA-DQA10501-DQB10201	698	LALPILMKHNVVAFS	MKHNVVAFS	6	0.1609	8764.7	75.00	0.37	tr_D0A0W7_
HLA-DQA10501-DQB10201	699	GVFDDTKCDTAEKLS	TKCDTAEKL	5	0.1608	8782.3	75.00	0.50	tr_D0A0W7_
HLA-DQA10501-DQB10201	700	GEHSLPTGGAVCRVA	PTGGAVCRV	5	0.1605	8803.3	75.00	0.59	tr_D0A0W7_
HLA-DQA10501-DQB10201	701	NGQTTTMESGFTVVK	TMESGFTVV	5	0.1599	8859.5	75.00	0.23	tr_D0A0W7_
HLA-DQA10501-DQB10201	702	GGEHSLPTGGAVCRV	PTGGAVCRV	6	0.1586	8993.0	75.00	0.69	tr_D0A0W7_
HLA-DQA10501-DQB10201	703	LFTGTNPLAKDSQYI	PLAKDSQYI	6	0.1571	9138.5	75.00	0.28	tr_D0A0W7_
HLA-DQA10501-DQB10201	704	ASLESRQWTVAGSVK	RQWTVAGSV	5	0.1568	9163.0	75.00	0.55	tr_D0A0W7_
HLA-DQA10501-DQB10201	705	CECNQGSKAVYVKEM	SKAVYVKEM	6	0.1562	9222.3	75.00	0.63	tr_D0A0W7_
HLA-DQA10501-DQB10201	706	EMLENGQTTTMESGF	QTTTMESGF	6	0.1553	9313.5	75.00	0.34	tr_D0A0W7_
HLA-DQA10501-DQB10201	707	VYVKEMLENGQTTTM	YVKEMLENG	1	0.1546	9389.3	75.00	0.24	tr_D0A0W7_
HLA-DQA10501-DQB10201	708	FVRSLYSQRRYVIND	VRSLYSQRR	1	0.1539	9457.5	75.00	0.38	tr_D0A0W7_
HLA-DQA10501-DQB10201	709	YVKEMLENGQTTTME	ENGQTTTME	6	0.1537	9474.5	80.00	0.25	tr_D0A0W7_
HLA-DQA10501-DQB10201	710	LAKDSQYIAIKRFQG	SQYIAIKRF	4	0.1537	9481.0	80.00	0.43	tr_D0A0W7_
HLA-DQA10501-DQB10201	711	DLFTTALNNTAGAAR	FTTALNNTA	2	0.1535	9495.4	80.00	0.41	tr_D0A0W7_
HLA-DQA10501-DQB10201	712	SPMTGSTFIRQWNPY	TGSTFIRQW	3	0.1524	9616.0	80.00	0.25	tr_D0A0W7_
HLA-DQA10501-DQB10201	713	LFTTALNNTAGAARL	NNTAGAARL	6	0.1522	9629.4	80.00	0.30	tr_D0A0W7_
HLA-DQA10501-DQB10201	714	GFTVVKTSQCYTESS	VVKTSQCYT	3	0.1513	9728.9	80.00	0.28	tr_D0A0W7_
HLA-DQA10501-DQB10201	715	FATSLPHWGDTETSS	PHWGDTETS	5	0.1508	9783.3	80.00	0.29	tr_D0A0W7_
HLA-DQA10501-DQB10201	716	SGFTVVKTSQCYTES	VVKTSQCYT	4	0.1503	9834.7	80.00	0.30	tr_D0A0W7_
HLA-DQA10501-DQB10201	717	FTVVKTSQCYTESSE	VVKTSQCYT	2	0.1499	9873.0	80.00	0.29	tr_D0A0W7_
HLA-DQA10501-DQB10201	718	DDDTASKAAALWQKG	TASKAAALW	3	0.1495	9921.2	80.00	0.54	tr_D0A0W7_
HLA-DQA10501-DQB10201	719	ELETDQNKIVIVYGP	QNKIVIVYG	5	0.1493	9936.3	80.00	0.30	tr_D0A0W7_
HLA-DQA10501-DQB10201	720	YLALPILMKHNVVAF	LMKHNVVAF	6	0.1490	9977.4	80.00	0.46	tr_D0A0W7_
HLA-DQA10501-DQB10201	721	IGLSVPNVREIARKL	IGLSVPNVR	0	0.1481	10071.3	80.00	0.29	tr_D0A0W7_
HLA-DQA10501-DQB10201	722	TTMESGFTVVKTSQC	ESGFTVVKT	3	0.1478	10098.6	80.00	0.34	tr_D0A0W7_
HLA-DQA10501-DQB10201	723	NVREIARKLEERNDL	EIARKLEER	3	0.1468	10212.9	80.00	0.29	tr_D0A0W7_
HLA-DQA10501-DQB10201	724	FGAPEKDTAKFLMMM	DTAKFLMMM	6	0.1457	10340.9	80.00	0.40	tr_D0A0W7_
HLA-DQA10501-DQB10201	725	TFIDPLELKPRLNKF	FIDPLELKP	1	0.1453	10383.7	80.00	0.38	tr_D0A0W7_
HLA-DQA10501-DQB10201	726	FHDVEKDSRLWTPLS	FHDVEKDSR	0	0.1452	10386.0	80.00	0.23	tr_D0A0W7_
HLA-DQA10501-DQB10201	727	SVPNVREIARKLEER	EIARKLEER	6	0.1448	10432.5	80.00	0.28	tr_D0A0W7_
HLA-DQA10501-DQB10201	728	GFNASLESRQWTVAG	FNASLESRQ	1	0.1442	10502.1	80.00	0.35	tr_D0A0W7_
HLA-DQA10501-DQB10201	729	TTTMESGFTVVKTSQ	ESGFTVVKT	4	0.1442	10502.1	80.00	0.37	tr_D0A0W7_
HLA-DQA10501-DQB10201	730	GDTETSSKTAQLFHD	TSSKTAQLF	4	0.1442	10505.5	80.00	0.56	tr_D0A0W7_
HLA-DQA10501-DQB10201	731	AKDSQYIAIKRFQGV	SQYIAIKRF	3	0.1441	10518.6	80.00	0.43	tr_D0A0W7_
HLA-DQA10501-DQB10201	732	LELETDQNKIVIVYG	QNKIVIVYG	6	0.1439	10536.0	80.00	0.33	tr_D0A0W7_
HLA-DQA10501-DQB10201	733	DTETSSKTAQLFHDV	TSSKTAQLF	3	0.1438	10552.3	80.00	0.46	tr_D0A0W7_
HLA-DQA10501-DQB10201	734	TGTNPLAKDSQYIAI	PLAKDSQYI	4	0.1433	10608.1	80.00	0.38	tr_D0A0W7_
HLA-DQA10501-DQB10201	735	DGEGVGKYLPISGTV	KYLPISGTV	6	0.1428	10669.3	80.00	0.30	tr_D0A0W7_

HLA-DQA10501-DQB10201	736	FTTALNNTAGAARLV	NNTAGAARL	5	0.1428	10669.9	80.00	0.33	tr_D0A0W7_
HLA-DQA10501-DQB10201	737	LSVPNVREIARKLEE	REIARKLEE	6	0.1422	10737.9	80.00	0.31	tr_D0A0W7_
HLA-DQA10501-DQB10201	738	VKEMLENGQTTTMES	ENGQTTTME	5	0.1416	10805.3	80.00	0.25	tr_D0A0W7_
HLA-DQA10501-DQB10201	739	HWGDTETSSKTAQLF	TSSKTAQLF	6	0.1415	10820.3	80.00	0.73	tr_D0A0W7_
HLA-DQA10501-DQB10201	740	KDSQYIAIKRFQGVM	SQYIAIKRF	2	0.1414	10832.0	80.00	0.38	tr_D0A0W7_
HLA-DQA10501-DQB10201	741	ANPMTPSMSFRDPSE	SMSFRDPSE	6	0.1399	11009.0	80.00	0.38	tr_D0A0W7_
HLA-DQA10501-DQB10201	742	PNVREIARKLEERND	EIARKLEER	4	0.1382	11208.4	80.00	0.35	tr_D0A0W7_
HLA-DQA10501-DQB10201	743	DDTASKAAALWQKGA	TASKAAALW	2	0.1380	11235.1	80.00	0.51	tr_D0A0W7_
HLA-DQA10501-DQB10201	744	REIARKLEERNDLRV	LEERNDLRV	6	0.1378	11263.2	80.00	0.29	tr_D0A0W7_
HLA-DQA10501-DQB10201	745	VPNVREIARKLEERN	EIARKLEER	5	0.1346	11651.6	80.00	0.34	tr_D0A0W7_
HLA-DQA10501-DQB10201	746	VFDDTKCDTAEKLSK	TKCDTAEKL	4	0.1338	11754.0	85.00	0.52	tr_D0A0W7_
HLA-DQA10501-DQB10201	747	VREIARKLEERNDLR	EIARKLEER	2	0.1332	11834.0	85.00	0.29	tr_D0A0W7_
HLA-DQA10501-DQB10201	748	SIPPLANPMTPSMSF	PPLANPMTP	2	0.1320	11993.0	85.00	0.22	tr_D0A0W7_
HLA-DQA10501-DQB10201	749	EIARKLEERNDLRVI	LEERNDLRV	5	0.1318	12007.6	85.00	0.29	tr_D0A0W7_
HLA-DQA10501-DQB10201	750	NASIPPLANPMTPSM	PPLANPMTP	4	0.1318	12015.2	85.00	0.24	tr_D0A0W7_
HLA-DQA10501-DQB10201	751	NPMTPSMSFRDPSEG	SMSFRDPSE	5	0.1290	12378.2	85.00	0.38	tr_D0A0W7_
HLA-DQA10501-DQB10201	752	KAAALWQKGASHLVG	WQKGASHLV	5	0.1290	12380.6	85.00	0.58	tr_D0A0W7_
HLA-DQA10501-DQB10201	753	FTGTNPLAKDSQYIA	PLAKDSQYI	5	0.1288	12404.9	85.00	0.40	tr_D0A0W7_
HLA-DQA10501-DQB10201	754	KEMLENGQTTTMESG	ENGQTTTME	4	0.1287	12421.8	85.00	0.23	tr_D0A0W7_
HLA-DQA10501-DQB10201	755	SKAAALWQKGASHLV	WQKGASHLV	6	0.1281	12499.0	85.00	0.68	tr_D0A0W7_
HLA-DQA10501-DQB10201	756	TNPLAKDSQYIAIKR	PLAKDSQYI	2	0.1262	12762.2	85.00	0.31	tr_D0A0W7_
HLA-DQA10501-DQB10201	757	WGDTETSSKTAQLFH	TSSKTAQLF	5	0.1262	12765.8	85.00	0.68	tr_D0A0W7_
HLA-DQA10501-DQB10201	758	REDGEGVGKYLPISG	VGKYLPISG	6	0.1254	12874.3	85.00	0.37	tr_D0A0W7_
HLA-DQA10501-DQB10201	759	GLSVPNVREIARKLE	VREIARKLE	6	0.1245	13003.0	85.00	0.29	tr_D0A0W7_
HLA-DQA10501-DQB10201	760	VFGAPEKDTAKFLMM	PEKDTAKFL	4	0.1238	13101.7	85.00	0.31	tr_D0A0W7_
HLA-DQA10501-DQB10201	761	ASIPPLANPMTPSMS	PPLANPMTP	3	0.1232	13189.6	85.00	0.27	tr_D0A0W7_
HLA-DQA10501-DQB10201	762	FDDTKCDTAEKLSKD	TKCDTAEKL	3	0.1230	13210.9	85.00	0.73	tr_D0A0W7_
HLA-DQA10501-DQB10201	763	GTNPLAKDSQYIAIK	PLAKDSQYI	3	0.1224	13294.7	85.00	0.37	tr_D0A0W7_
HLA-DQA10501-DQB10201	764	ATSLPHWGDTETSSK	PHWGDTETS	4	0.1219	13372.9	85.00	0.25	tr_D0A0W7_
HLA-DQA10501-DQB10201	765	EDGEGVGKYLPISGT	VGKYLPISG	5	0.1216	13411.8	85.00	0.34	tr_D0A0W7_
HLA-DQA10501-DQB10201	766	MSMLHLSDRNASLAP	SMLHLSDRN	1	0.1200	13648.9	85.00	0.30	tr_D0A0W7_
HLA-DQA10501-DQB10201	767	DTASKAAALWQKGAS	TASKAAALW	1	0.1193	13751.9	85.00	0.53	tr_D0A0W7_
HLA-DQA10501-DQB10201	768	IPPLANPMTPSMSFR	PPLANPMTP	1	0.1162	14220.4	85.00	0.22	tr_D0A0W7_
HLA-DQA10501-DQB10201	769	MTPSMSFRDPSEGKL	SMSFRDPSE	3	0.1155	14335.5	85.00	0.37	tr_D0A0W7_
HLA-DQA10501-DQB10201	770	SMLHLSDRNASLAPS	DRNASLAPS	6	0.1147	14458.4	85.00	0.28	tr_D0A0W7_
HLA-DQA10501-DQB10201	771	FNASLESRQWTVAGS	NASLESRQW	1	0.1144	14494.7	85.00	0.28	tr_D0A0W7_
HLA-DQA10501-DQB10201	772	LANPMTPSMSFRDPS	PSMSFRDPS	6	0.1141	14543.0	85.00	0.25	tr_D0A0W7_
HLA-DQA10501-DQB10201	773	YGGTCEGEAAKHGAT	YGGTCEGEA	0	0.1130	14719.8	90.00	0.44	tr_D0A0W7_
HLA-DQA10501-DQB10201	774	TSLPHWGDTETSSKT	PHWGDTETS	3	0.1125	14807.5	90.00	0.26	tr_D0A0W7_
HLA-DQA10501-DQB10201	775	AAALWQKGASHLVGK	WQKGASHLV	4	0.1118	14913.3	90.00	0.61	tr_D0A0W7_
HLA-DQA10501-DQB10201	776	EGEAAKHGATCECNQ	KHGATCECN	5	0.1117	14935.7	90.00	0.23	tr_D0A0W7_
HLA-DQA10501-DQB10201	777	IREDGEGVGKYLPIS	DGEGVGKYL	3	0.1115	14971.5	90.00	0.24	tr_D0A0W7_
HLA-DQA10501-DQB10201	778	PMTPSMSFRDPSEGK	SMSFRDPSE	4	0.1107	15086.6	90.00	0.38	tr_D0A0W7_
HLA-DQA10501-DQB10201	779	PPLANPMTPSMSFRD	NPMTPSMSF	4	0.1092	15342.1	90.00	0.19	tr_D0A0W7_
HLA-DQA10501-DQB10201	780	KPRLNKFRRHVIHLS	FRRHVIHLS	6	0.1076	15608.7	90.00	0.58	tr_D0A0W7_
HLA-DQA10501-DQB10201	781	PLANPMTPSMSFRDP	NPMTPSMSF	3	0.1075	15627.6	90.00	0.22	tr_D0A0W7_
HLA-DQA10501-DQB10201	782	CEGEAAKHGATCECN	KHGATCECN	6	0.1059	15894.3	90.00	0.29	tr_D0A0W7_
HLA-DQA10501-DQB10201	783	RDPSEGKLSGASLVG	EGKLSGASL	4	0.1053	15999.9	90.00	0.46	tr_D0A0W7_
HLA-DQA10501-DQB10201	784	TPSMSFRDPSEGKLS	SMSFRDPSE	2	0.1051	16038.9	90.00	0.41	tr_D0A0W7_
HLA-DQA10501-DQB10201	785	DDTKCDTAEKLSKDD	TKCDTAEKL	2	0.1042	16189.2	90.00	0.69	tr_D0A0W7_

HLA-DQA10501-DQB10201	786	LWQKGASHLVGKGDL	WQKGASHLV	1	0.1023	16529.5	90.00	0.48	tr_D0A0W7_
HLA-DQA10501-DQB10201	787	AALWQKGASHLVGKG	WQKGASHLV	3	0.1023	16533.8	90.00	0.63	tr_D0A0W7_
HLA-DQA10501-DQB10201	788	GEAAKHGATCECNQG	KHGATCECN	4	0.1018	16616.5	90.00	0.23	tr_D0A0W7_
HLA-DQA10501-DQB10201	789	FRDPSEGKLSGASLV	EGKLSGASL	5	0.1017	16637.9	90.00	0.57	tr_D0A0W7_
HLA-DQA10501-DQB10201	790	SLPHWGDTETSSKTA	PHWGDTETS	2	0.1009	16781.5	90.00	0.28	tr_D0A0W7_
HLA-DQA10501-DQB10201	791	ALWQKGASHLVGKGD	WQKGASHLV	2	0.0995	17034.3	90.00	0.59	tr_D0A0W7_
HLA-DQA10501-DQB10201	792	MLHLSDRNASLAPSG	RNASLAPSG	6	0.0991	17108.2	90.00	0.21	tr_D0A0W7_
HLA-DQA10501-DQB10201	793	SHLVGKGDLGHSDRF	GDLGHSDRF	6	0.0990	17124.8	90.00	0.32	tr_D0A0W7_
HLA-DQA10501-DQB10201	794	SFRDPSEGKLSGASL	EGKLSGASL	6	0.0977	17377.2	90.00	0.73	tr_D0A0W7_
HLA-DQA10501-DQB10201	795	TCEGEAAKHGATCEC	AKHGATCEC	6	0.0965	17606.7	90.00	0.38	tr_D0A0W7_
HLA-DQA10501-DQB10201	796	EAAKHGATCECNQGS	KHGATCECN	3	0.0962	17662.1	90.00	0.25	tr_D0A0W7_
HLA-DQA10501-DQB10201	797	DTKCDTAEKLSKDDC	TKCDTAEKL	1	0.0956	17772.3	90.00	0.55	tr_D0A0W7_
HLA-DQA10501-DQB10201	798	WQKGASHLVGKGDLG	WQKGASHLV	0	0.0938	18125.5	90.00	0.31	tr_D0A0W7_
HLA-DQA10501-DQB10201	799	TKCDTAEKLSKDDCA	EKLSKDDCA	6	0.0925	18379.1	90.00	0.35	tr_D0A0W7_
HLA-DQA10501-DQB10201	800	PSMSFRDPSEGKLSG	SMSFRDPSE	1	0.0923	18418.9	90.00	0.42	tr_D0A0W7_
HLA-DQA10501-DQB10201	801	GATCECNQGSKAVYV	NQGSKAVYV	6	0.0918	18510.6	90.00	0.75	tr_D0A0W7_
HLA-DQA10501-DQB10201	802	TASKAAALWQKGASH	TASKAAALW	0	0.0911	18664.1	95.00	0.40	tr_D0A0W7_
HLA-DQA10501-DQB10201	803	FIDPLELKPRLNKFR	FIDPLELKP	0	0.0903	18821.2	95.00	0.41	tr_D0A0W7_
HLA-DQA10501-DQB10201	804	TCECNQGSKAVYVKE	NQGSKAVYV	4	0.0894	19011.6	95.00	0.53	tr_D0A0W7_
HLA-DQA10501-DQB10201	805	LHLSDRNASLAPSGG	RNASLAPSG	5	0.0874	19419.3	95.00	0.22	tr_D0A0W7_
HLA-DQA10501-DQB10201	806	NASLAPSGGEHSLPT	LAPSGGEHS	3	0.0846	20024.8	95.00	0.34	tr_D0A0W7_
HLA-DQA10501-DQB10201	807	HLSDRNASLAPSGGE	RNASLAPSG	4	0.0841	20134.5	95.00	0.23	tr_D0A0W7_
HLA-DQA10501-DQB10201	808	RNASLAPSGGEHSLP	LAPSGGEHS	4	0.0824	20496.1	95.00	0.37	tr_D0A0W7_
HLA-DQA10501-DQB10201	809	ASLAPSGGEHSLPTG	LAPSGGEHS	2	0.0814	20728.1	95.00	0.34	tr_D0A0W7_
HLA-DQA10501-DQB10201	810	AAKHGATCECNQGSK	KHGATCECN	2	0.0801	21016.4	95.00	0.28	tr_D0A0W7_
HLA-DQA10501-DQB10201	811	DTAEKLSKDDCASNY	EKLSKDDCA	3	0.0785	21392.9	95.00	0.36	tr_D0A0W7_
HLA-DQA10501-DQB10201	812	LPHWGDTETSSKTAQ	PHWGDTETS	1	0.0784	21411.7	95.00	0.34	tr_D0A0W7_
HLA-DQA10501-DQB10201	813	ATCECNQGSKAVYVK	NQGSKAVYV	5	0.0772	21681.9	95.00	0.72	tr_D0A0W7_
HLA-DQA10501-DQB10201	814	ASHLVGKGDLGHSDR	HLVGKGDLG	2	0.0771	21700.6	95.00	0.34	tr_D0A0W7_
HLA-DQA10501-DQB10201	815	PHWGDTETSSKTAQL	ETSSKTAQL	6	0.0766	21828.0	95.00	0.45	tr_D0A0W7_
HLA-DQA10501-DQB10201	816	SMSFRDPSEGKLSGA	SMSFRDPSE	0	0.0752	22163.4	95.00	0.36	tr_D0A0W7_
HLA-DQA10501-DQB10201	817	GASHLVGKGDLGHSD	HLVGKGDLG	3	0.0745	22324.1	95.00	0.34	tr_D0A0W7_
HLA-DQA10501-DQB10201	818	LSDRNASLAPSGGEH	DRNASLAPS	2	0.0745	22332.1	95.00	0.25	tr_D0A0W7_
HLA-DQA10501-DQB10201	819	KCDTAEKLSKDDCAS	EKLSKDDCA	5	0.0740	22439.6	95.00	0.46	tr_D0A0W7_
HLA-DQA10501-DQB10201	820	SDRNASLAPSGGEHS	LAPSGGEHS	6	0.0739	22466.4	95.00	0.29	tr_D0A0W7_
HLA-DQA10501-DQB10201	821	QKGASHLVGKGDLGH	HLVGKGDLG	5	0.0736	22547.5	95.00	0.34	tr_D0A0W7_
HLA-DQA10501-DQB10201	822	DRNASLAPSGGEHSL	LAPSGGEHS	5	0.0730	22692.8	95.00	0.34	tr_D0A0W7_
HLA-DQA10501-DQB10201	823	TAEKLSKDDCASNYG	EKLSKDDCA	2	0.0718	22985.7	95.00	0.34	tr_D0A0W7_
HLA-DQA10501-DQB10201	824	GTCEGEAAKHGATCE	AAKHGATCE	6	0.0716	23035.5	95.00	0.52	tr_D0A0W7_
HLA-DQA10501-DQB10201	825	SLAPSGGEHSLPTGG	LAPSGGEHS	1	0.0710	23191.8	95.00	0.34	tr_D0A0W7_
HLA-DQA10501-DQB10201	826	CDTAEKLSKDDCASN	EKLSKDDCA	4	0.0701	23417.9	95.00	0.46	tr_D0A0W7_
HLA-DQA10501-DQB10201	827	ASKAAALWQKGASHL	ASKAAALWQ	0	0.0700	23437.7	95.00	0.37	tr_D0A0W7_
HLA-DQA10501-DQB10201	828	KGASHLVGKGDLGHS	HLVGKGDLG	4	0.0690	23707.0	95.00	0.38	tr_D0A0W7_
HLA-DQA10501-DQB10201	829	AKHGATCECNQGSKA	KHGATCECN	1	0.0685	23822.7	95.00	0.30	tr_D0A0W7_
HLA-DQA10501-DQB10201	830	AEKLSKDDCASNYGA	EKLSKDDCA	1	0.0665	24338.9	95.00	0.32	tr_D0A0W7_
HLA-DQA10501-DQB10201	831	LAPSGGEHSLPTGGA	PSGGEHSLP	2	0.0651	24717.3	95.00	0.21	tr_D0A0W7_
HLA-DQA10501-DQB10201	832	GGTCEGEAAKHGATC	GGTCEGEAA	0	0.0618	25608.0	100.00	0.36	tr_D0A0W7_
HLA-DQA10501-DQB10201	833	SGGEHSLPTGGAVCR	LPTGGAVCR	6	0.0604	26000.3	100.00	0.26	tr_D0A0W7_
HLA-DQA10501-DQB10201	834	KLSKDDCASNYGATQ	CASNYGATQ	6	0.0598	26171.9	100.00	0.42	tr_D0A0W7_
HLA-DQA10501-DQB10201	835	PSGGEHSLPTGGAVC	SLPTGGAVC	6	0.0580	26706.3	100.00	0.25	tr_D0A0W7_

HLA-DQA10501-DQB10201	836	APSGGEHSLPTGGAV	PSGGEHSLP	1	0.0566	27112.7	100.00	0.24	tr_D0A0W7_
HLA-DQA10501-DQB10201	837	LKPRLNKFRRHVIHL	KFRRHVIHL	6	0.0564	27168.5	100.00	0.51	tr_D0A0W7_
HLA-DQA10501-DQB10201	838	EKLSKDDCASNYGAT	SKDDCASNY	3	0.0560	27288.4	100.00	0.31	tr_D0A0W7_
HLA-DQA10501-DQB10201	839	MSFRDPSEGKLSGAS	FRDPSEGKL	2	0.0520	28479.9	100.00	0.34	tr_D0A0W7_
HLA-DQA10501-DQB10201	840	KHGATCECNQGSKAV	HGATCECNQ	1	0.0517	28580.6	100.00	0.34	tr_D0A0W7_
HLA-DQA10501-DQB10201	841	IDPLELKPRLNKFRR	PLELKPRLN	2	0.0509	28815.6	100.00	0.38	tr_D0A0W7_
HLA-DQA10501-DQB10201	842	HGATCECNQGSKAVY	CNQGSKAVY	6	0.0422	31687.3	100.00	0.22	tr_D0A0W7_
HLA-DQA10501-DQB10201	843	DPLELKPRLNKFRRH	PLELKPRLN	1	0.0418	31813.7	100.00	0.38	tr_D0A0W7_
HLA-DQA10501-DQB10201	844	LELKPRLNKFRRHVI	LNKFRRHVI	6	0.0330	34995.5	100.00	0.50	tr_D0A0W7_
HLA-DQA10501-DQB10201	845	ELKPRLNKFRRHVIH	LNKFRRHVI	5	0.0310	35740.5	100.00	0.40	tr_D0A0W7_
HLA-DQA10501-DQB10201	846	PLELKPRLNKFRRHV	PLELKPRLN	0	0.0285	36718.1	100.00	0.34	tr_D0A0W7_

Allele: HLA-DQA10501-DQB10201. Number of high binders 1. Number of weak binders 117. Number of peptides 846

Allele	pos	peptide	core	Of	1-log50k(aff)	affinity(nM)	%Rank	Relia	Identity	Bind Level
HLA-DQA10501-DQB10201	1	ACIQIITAVVPPELP	ITAVVPPEL	5	0.6762	33.2	0.30	0.64	tr_C9ZUI4_	SB
HLA-DQA10501-DQB10201	2	CIQIITAVVPPELPM	ITAVVPPEL	4	0.6708	35.2	0.40	0.67	tr_C9ZUI4_	SB
HLA-DQA10501-DQB10201	3	IQIITAVVPPELPME	ITAVVPPEL	3	0.6667	36.8	0.40	0.62	tr_C9ZUI4_	SB
HLA-DQA10501-DQB10201	4	LACIQIITAVVPPEL	ITAVVPPEL	6	0.6586	40.2	0.50	0.63	tr_C9ZUI4_	WB
HLA-DQA10501-DQB10201	5	QIITAVVPPELPMEL	ITAVVPPEL	2	0.6508	43.7	0.50	0.62	tr_C9ZUI4_	WB
HLA-DQA10501-DQB10201	6	IITAVVPPELPMELT	ITAVVPPEL	1	0.6186	62.0	0.90	0.64	tr_C9ZUI4_	WB
HLA-DQA10501-DQB10201	7	CFDKTGTLTTDEMLF	TGTLTTDEM	4	0.5465	135.2	3.00	0.58	tr_C9ZUI4_	WB
HLA-DQA10501-DQB10201	8	DFLMESIFNADDADM	IFNADDADM	6	0.5412	143.1	3.50	0.44	tr_C9ZUI4_	WB
HLA-DQA10501-DQB10201	9	FLMESIFNADDADMG	IFNADDADM	5	0.5408	143.8	3.50	0.44	tr_C9ZUI4_	WB
HLA-DQA10501-DQB10201	10	VETVRLVEEADAEGV	RLVEEADAE	4	0.5366	150.5	3.50	0.32	tr_C9ZUI4_	WB
HLA-DQA10501-DQB10201	11	FDKTGTLTTDEMLFS	TGTLTTDEM	3	0.5287	164.0	4.00	0.58	tr_C9ZUI4_	WB
HLA-DQA10501-DQB10201	12	ETVRLVEEADAEGVA	RLVEEADAE	3	0.5261	168.5	4.00	0.34	tr_C9ZUI4_	WB
HLA-DQA10501-DQB10201	13	SMVETVRLVEEADAE	RLVEEADAE	6	0.5184	183.2	4.50	0.35	tr_C9ZUI4_	WB
HLA-DQA10501-DQB10201	14	CCFDKTGTLTTDEML	TGTLTTDEM	5	0.5175	185.0	4.50	0.68	tr_C9ZUI4_	WB
HLA-DQA10501-DQB10201	15	MVETVRLVEEADAEG	RLVEEADAE	5	0.5151	190.0	4.50	0.35	tr_C9ZUI4_	WB
HLA-DQA10501-DQB10201	16	LMESIFNADDADMGG	IFNADDADM	4	0.5121	196.2	5.00	0.47	tr_C9ZUI4_	WB
HLA-DQA10501-DQB10201	17	ITAVVPPELPMELTL	ITAVVPPEL	0	0.5084	204.1	5.00	0.77	tr_C9ZUI4_	WB
HLA-DQA10501-DQB10201	18	LTSAYSMSVLQMDGV	MSVLQMDGV	6	0.5061	209.3	5.00	0.47	tr_C9ZUI4_	WB
HLA-DQA10501-DQB10201	19	TVRLVEEADAEGVAT	RLVEEADAE	2	0.5041	213.9	5.50	0.35	tr_C9ZUI4_	WB
HLA-DQA10501-DQB10201	20	MESIFNADDADMGGA	IFNADDADM	3	0.4956	234.4	6.00	0.47	tr_C9ZUI4_	WB
HLA-DQA10501-DQB10201	21	FLACIQIITAVVPPE	IITAVVPPE	6	0.4923	243.1	6.00	0.70	tr_C9ZUI4_	WB
HLA-DQA10501-DQB10201	22	ALGYRVNIDDTVVYD	YRVNIDDTV	3	0.4872	256.7	6.50	0.47	tr_C9ZUI4_	WB
HLA-DQA10501-DQB10201	23	DKTGTLTTDEMLFSG	TGTLTTDEM	2	0.4838	266.3	6.50	0.57	tr_C9ZUI4_	WB
HLA-DQA10501-DQB10201	24	LGYRVNIDDTVVYDP	YRVNIDDTV	2	0.4791	280.3	7.00	0.40	tr_C9ZUI4_	WB
HLA-DQA10501-DQB10201	25	PEMNAPASDFLMESI	ASDFLMESI	6	0.4782	283.0	7.00	0.41	tr_C9ZUI4_	WB
HLA-DQA10501-DQB10201	26	GALGYRVNIDDTVVY	YRVNIDDTV	4	0.4779	284.0	7.00	0.46	tr_C9ZUI4_	WB
HLA-DQA10501-DQB10201	27	ESIFNADDADMGGAP	IFNADDADM	2	0.4768	287.5	7.50	0.47	tr_C9ZUI4_	WB
HLA-DQA10501-DQB10201	28	YSMVETVRLVEEADA	VRLVEEADA	6	0.4754	291.7	7.50	0.28	tr_C9ZUI4_	WB
HLA-DQA10501-DQB10201	29	EMNAPASDFLMESIF	ASDFLMESI	5	0.4752	292.4	7.50	0.37	tr_C9ZUI4_	WB
HLA-DQA10501-DQB10201	30	TSAYSMSVLQMDGVK	MSVLQMDGV	5	0.4742	295.6	7.50	0.45	tr_C9ZUI4_	WB
HLA-DQA10501-DQB10201	31	VRLVEEADAEGVATM	VEEADAEGV	3	0.4735	298.0	7.50	0.35	tr_C9ZUI4_	WB

HLA-DQA10501-DQB10201	32	SDFLMESIFNADDAD	ESIFNADDA	5	0.4718	303.5	7.50	0.35	tr_C9ZUI4_	WB
HLA-DQA10501-DQB10201	33	LGALGYRVNIDDTVV	YRVNIDDTV	5	0.4656	324.3	8.00	0.50	tr_C9ZUI4_	WB
HLA-DQA10501-DQB10201	34	GYRVNIDDTVVYDPP	YRVNIDDTV	1	0.4629	334.1	8.50	0.36	tr_C9ZUI4_	WB
HLA-DQA10501-DQB10201	35	TCCFDKTGTLTTDEM	TGTLTTDEM	6	0.4622	336.7	8.50	0.90	tr_C9ZUI4_	WB
HLA-DQA10501-DQB10201	36	AHAGIAVLNATSMDA	VLNATSMDA	6	0.4590	348.3	9.00	0.44	tr_C9ZUI4_	WB
HLA-DQA10501-DQB10201	37	LEGTDTVAGDAMEKA	VAGDAMEKA	6	0.4584	350.7	9.00	0.37	tr_C9ZUI4_	WB
HLA-DQA10501-DQB10201	38	LYSMVETVRLVEEAD	VETVRLVEE	4	0.4547	365.1	9.00	0.39	tr_C9ZUI4_	WB
HLA-DQA10501-DQB10201	39	SAYSMSVLQMDGVKH	MSVLQMDGV	4	0.4545	365.8	9.00	0.44	tr_C9ZUI4_	WB
HLA-DQA10501-DQB10201	40	SLGALGYRVNIDDTV	YRVNIDDTV	6	0.4502	383.3	9.50	0.53	tr_C9ZUI4_	WB
HLA-DQA10501-DQB10201	41	KTGTLTTDEMLFSGV	TGTLTTDEM	1	0.4499	384.4	9.50	0.50	tr_C9ZUI4_	WB
HLA-DQA10501-DQB10201	42	HAGIAVLNATSMDAS	VLNATSMDA	5	0.4489	388.8	10.00	0.46	tr_C9ZUI4_	
HLA-DQA10501-DQB10201	43	PEAIAQLCESIPPDF	LCESIPPDF	6	0.4481	392.1	10.00	0.27	tr_C9ZUI4_	
HLA-DQA10501-DQB10201	44	EGTDTVAGDAMEKAS	VAGDAMEKA	5	0.4470	396.6	10.00	0.40	tr_C9ZUI4_	
HLA-DQA10501-DQB10201	45	GTDTVAGDAMEKASL	VAGDAMEKA	4	0.4400	427.8	11.00	0.43	tr_C9ZUI4_	
HLA-DQA10501-DQB10201	46	LHLYSMVETVRLVEE	VETVRLVEE	6	0.4360	447.1	11.00	0.41	tr_C9ZUI4_	
HLA-DQA10501-DQB10201	47	RLVEEADAEGVATMR	VEEADAEGV	2	0.4316	468.5	12.00	0.41	tr_C9ZUI4_	
HLA-DQA10501-DQB10201	48	EAIAQLCESIPPDFH	LCESIPPDF	5	0.4316	468.6	12.00	0.29	tr_C9ZUI4_	
HLA-DQA10501-DQB10201	49	AGIAVLNATSMDASQ	VLNATSMDA	4	0.4314	469.8	12.00	0.46	tr_C9ZUI4_	
HLA-DQA10501-DQB10201	50	ASDFLMESIFNADDA	ESIFNADDA	6	0.4313	470.4	12.00	0.34	tr_C9ZUI4_	
HLA-DQA10501-DQB10201	51	QFGLHLYSMVETVRL	YSMVETVRL	6	0.4301	476.3	12.00	0.47	tr_C9ZUI4_	
HLA-DQA10501-DQB10201	52	TTDEMLFSGVDMADG	EMLFSGVDM	3	0.4294	480.2	12.00	0.31	tr_C9ZUI4_	
HLA-DQA10501-DQB10201	53	HLYSMVETVRLVEEA	VETVRLVEE	5	0.4292	480.9	12.00	0.40	tr_C9ZUI4_	
HLA-DQA10501-DQB10201	54	MNAPASDFLMESIFN	ASDFLMESI	4	0.4257	499.7	13.00	0.39	tr_C9ZUI4_	
HLA-DQA10501-DQB10201	55	SIFNADDADMGGAPQ	IFNADDADM	1	0.4251	502.8	13.00	0.52	tr_C9ZUI4_	
HLA-DQA10501-DQB10201	56	AYSMSVLQMDGVKHG	MSVLQMDGV	3	0.4245	506.1	13.00	0.48	tr_C9ZUI4_	
HLA-DQA10501-DQB10201	57	LTTDEMLFSGVDMAD	EMLFSGVDM	4	0.4218	521.4	13.00	0.41	tr_C9ZUI4_	
HLA-DQA10501-DQB10201	58	GSPEAIAQLCESIPP	EAIAQLCES	3	0.4206	528.0	13.00	0.34	tr_C9ZUI4_	
HLA-DQA10501-DQB10201	59	SPEAIAQLCESIPPD	EAIAQLCES	2	0.4202	530.5	13.00	0.30	tr_C9ZUI4_	
HLA-DQA10501-DQB10201	60	TGTLTTDEMLFSGVD	TGTLTTDEM	0	0.4195	534.3	13.00	0.37	tr_C9ZUI4_	
HLA-DQA10501-DQB10201	61	NCLTSAYSMSVLQMD	AYSMSVLQM	5	0.4158	556.2	14.00	0.41	tr_C9ZUI4_	
HLA-DQA10501-DQB10201	62	GIAVLNATSMDASQN	VLNATSMDA	3	0.4156	557.1	14.00	0.46	tr_C9ZUI4_	
HLA-DQA10501-DQB10201	63	TDTVAGDAMEKASLG	VAGDAMEKA	3	0.4118	580.6	14.00	0.44	tr_C9ZUI4_	
HLA-DQA10501-DQB10201	64	QPEMNAPASDFLMES	MNAPASDFL	3	0.4109	586.3	14.00	0.41	tr_C9ZUI4_	
HLA-DQA10501-DQB10201	65	APASDFLMESIFNAD	ASDFLMESI	2	0.4068	612.9	15.00	0.38	tr_C9ZUI4_	
HLA-DQA10501-DQB10201	66	GTLTTDEMLFSGVDM	EMLFSGVDM	6	0.4067	613.4	15.00	0.51	tr_C9ZUI4_	
HLA-DQA10501-DQB10201	67	KGSPEAIAQLCESIP	EAIAQLCES	4	0.4063	616.6	15.00	0.35	tr_C9ZUI4_	
HLA-DQA10501-DQB10201	68	IAVLNATSMDASQNV	VLNATSMDA	2	0.4044	628.8	15.00	0.45	tr_C9ZUI4_	
HLA-DQA10501-DQB10201	69	YRVNIDDTVVYDPPA	YRVNIDDTV	0	0.4024	642.7	16.00	0.29	tr_C9ZUI4_	
HLA-DQA10501-DQB10201	70	TTTLATLIAQYSEHI	LIAQYSEHI	6	0.4020	645.4	16.00	0.46	tr_C9ZUI4_	
HLA-DQA10501-DQB10201	71	TDEMLFSGVDMADGK	EMLFSGVDM	2	0.4020	645.5	16.00	0.34	tr_C9ZUI4_	
HLA-DQA10501-DQB10201	72	FGLHLYSMVETVRLV	YSMVETVRL	5	0.4011	651.6	16.00	0.43	tr_C9ZUI4_	
HLA-DQA10501-DQB10201	73	SQPEMNAPASDFLME	MNAPASDFL	4	0.4006	655.3	16.00	0.50	tr_C9ZUI4_	
HLA-DQA10501-DQB10201	74	TTLATLIAQYSEHIA	LIAQYSEHI	5	0.4006	655.5	16.00	0.38	tr_C9ZUI4_	
HLA-DQA10501-DQB10201	75	CLTSAYSMSVLQMDG	AYSMSVLQM	4	0.4001	658.8	16.00	0.35	tr_C9ZUI4_	
HLA-DQA10501-DQB10201	76	QLEGTDTVAGDAMEK	TDTVAGDAM	4	0.3979	675.2	16.00	0.47	tr_C9ZUI4_	
HLA-DQA10501-DQB10201	77	VSQPEMNAPASDFLM	MNAPASDFL	5	0.3978	675.6	16.00	0.53	tr_C9ZUI4_	
HLA-DQA10501-DQB10201	78	VVSQPEMNAPASDFL	MNAPASDFL	6	0.3977	676.5	16.00	0.56	tr_C9ZUI4_	
HLA-DQA10501-DQB10201	79	NAPASDFLMESIFNA	ASDFLMESI	3	0.3969	682.1	16.00	0.41	tr_C9ZUI4_	
HLA-DQA10501-DQB10201	80	PASDFLMESIFNADD	ASDFLMESI	1	0.3961	688.1	16.00	0.28	tr_C9ZUI4_	
HLA-DQA10501-DQB10201	81	LATLIAQYSEHIAVW	LIAQYSEHI	3	0.3931	711.0	17.00	0.41	tr_C9ZUI4_	

HLA-DQA10501-DQB10201	82	TLTTDEMLFSGVDMA	EMLFSGVDM	5	0.3906	730.4	17.00	0.44	tr_C9ZUI4_
HLA-DQA10501-DQB10201	83	LQLEGTDTVAGDAME	TDTVAGDAM	5	0.3904	731.8	17.00	0.57	tr_C9ZUI4_
HLA-DQA10501-DQB10201	84	TLATLIAQYSEHIAV	LIAQYSEHI	4	0.3903	732.5	17.00	0.39	tr_C9ZUI4_
HLA-DQA10501-DQB10201	85	LVEEADAEGVATMRQ	VEEADAEGV	1	0.3897	737.4	17.00	0.47	tr_C9ZUI4_
HLA-DQA10501-DQB10201	86	GLHLYSMVETVRLVE	YSMVETVRL	4	0.3889	744.2	18.00	0.40	tr_C9ZUI4_
HLA-DQA10501-DQB10201	87	ATLIAQYSEHIAVWA	LIAQYSEHI	2	0.3886	746.4	18.00	0.41	tr_C9ZUI4_
HLA-DQA10501-DQB10201	88	FMQFGLHLYSMVETV	HLYSMVETV	6	0.3855	772.1	18.00	0.44	tr_C9ZUI4_
HLA-DQA10501-DQB10201	89	QAHAGIAVLNATSMD	IAVLNATSM	5	0.3846	779.7	18.00	0.44	tr_C9ZUI4_
HLA-DQA10501-DQB10201	90	YSMSVLQMDGVKHGE	MSVLQMDGV	2	0.3839	785.6	18.00	0.54	tr_C9ZUI4_
HLA-DQA10501-DQB10201	91	AIAQLCESIPPDFHS	LCESIPPDF	4	0.3828	795.0	19.00	0.37	tr_C9ZUI4_
HLA-DQA10501-DQB10201	92	AKGSPEAIAQLCESI	EAIAQLCES	5	0.3801	817.9	19.00	0.34	tr_C9ZUI4_
HLA-DQA10501-DQB10201	93	DEMLFSGVDMADGKG	EMLFSGVDM	1	0.3780	836.8	19.00	0.32	tr_C9ZUI4_
HLA-DQA10501-DQB10201	94	LLQLEGTDTVAGDAM	TDTVAGDAM	6	0.3737	876.9	20.00	0.55	tr_C9ZUI4_
HLA-DQA10501-DQB10201	95	VAKGSPEAIAQLCES	EAIAQLCES	6	0.3734	880.0	20.00	0.41	tr_C9ZUI4_
HLA-DQA10501-DQB10201	96	KNLIFAGLAVFQCPL	IFAGLAVFQ	3	0.3704	908.4	21.00	0.25	tr_C9ZUI4_
HLA-DQA10501-DQB10201	97	PQVKLGDASIAAPFT	DASIAAPFT	6	0.3693	920.1	21.00	0.63	tr_C9ZUI4_
HLA-DQA10501-DQB10201	98	TTLQMYKILALNCLT	YKILALNCL	5	0.3673	939.7	21.00	0.52	tr_C9ZUI4_
HLA-DQA10501-DQB10201	99	LNCLTSAYSMSVLQM	AYSMSVLQM	6	0.3669	943.8	21.00	0.56	tr_C9ZUI4_
HLA-DQA10501-DQB10201	100	SDDEWDLCVNAESIP	DLCVNAESI	5	0.3668	944.6	21.00	0.50	tr_C9ZUI4_
HLA-DQA10501-DQB10201	101	QVKLGDASIAAPFTC	DASIAAPFT	5	0.3661	951.9	21.00	0.62	tr_C9ZUI4_
HLA-DQA10501-DQB10201	102	DTVAGDAMEKASLGA	VAGDAMEKA	2	0.3648	965.5	22.00	0.54	tr_C9ZUI4_
HLA-DQA10501-DQB10201	103	AIKGYRVIALAYRPL	GYRVIALAY	3	0.3631	983.1	22.00	0.20	tr_C9ZUI4_
HLA-DQA10501-DQB10201	104	VTTLQMYKILALNCL	YKILALNCL	6	0.3621	993.7	22.00	0.60	tr_C9ZUI4_
HLA-DQA10501-DQB10201	105	TLQMYKILALNCLTS	YKILALNCL	4	0.3618	997.8	22.00	0.53	tr_C9ZUI4_
HLA-DQA10501-DQB10201	106	MQFGLHLYSMVETVR	HLYSMVETV	5	0.3618	997.9	22.00	0.38	tr_C9ZUI4_
HLA-DQA10501-DQB10201	107	DDEWDLCVNAESIPT	DLCVNAESI	4	0.3613	1003.0	22.00	0.49	tr_C9ZUI4_
HLA-DQA10501-DQB10201	108	FTCRSRALTSVCDIV	RALTSVCDI	5	0.3583	1036.3	23.00	0.56	tr_C9ZUI4_
HLA-DQA10501-DQB10201	109	KQAHAGIAVLNATSM	IAVLNATSM	6	0.3582	1036.8	23.00	0.44	tr_C9ZUI4_
HLA-DQA10501-DQB10201	110	TCHSLLQLEGTDTVA	LLQLEGTDT	4	0.3570	1050.1	23.00	0.28	tr_C9ZUI4_
HLA-DQA10501-DQB10201	111	VPPAPSADAPFMEQV	APSADAPFM	3	0.3570	1050.5	23.00	0.31	tr_C9ZUI4_
HLA-DQA10501-DQB10201	112	TLIAQYSEHIAVWAR	LIAQYSEHI	1	0.3542	1083.2	23.00	0.43	tr_C9ZUI4_
HLA-DQA10501-DQB10201	113	HRCVIITGDSVQTAI	ITGDSVQTA	5	0.3540	1084.7	23.00	0.33	tr_C9ZUI4_
HLA-DQA10501-DQB10201	114	VVPPAPSADAPFMEQ	APSADAPFM	4	0.3539	1086.6	23.00	0.38	tr_C9ZUI4_
HLA-DQA10501-DQB10201	115	LQMYKILALNCLTSA	YKILALNCL	3	0.3525	1103.0	24.00	0.49	tr_C9ZUI4_
HLA-DQA10501-DQB10201	116	QMYKILALNCLTSAY	YKILALNCL	2	0.3522	1106.3	24.00	0.49	tr_C9ZUI4_
HLA-DQA10501-DQB10201	117	EKNLIFAGLAVFQCP	IFAGLAVFQ	4	0.3512	1118.8	24.00	0.31	tr_C9ZUI4_
HLA-DQA10501-DQB10201	118	CRSRALTSVCDIVRL	RALTSVCDI	3	0.3508	1123.6	24.00	0.44	tr_C9ZUI4_
HLA-DQA10501-DQB10201	119	IPTTTLATLIAQYSE	LATLIAQYS	5	0.3503	1129.7	24.00	0.42	tr_C9ZUI4_
HLA-DQA10501-DQB10201	120	VTCHSLLQLEGTDTV	LLQLEGTDT	5	0.3502	1130.5	24.00	0.31	tr_C9ZUI4_
HLA-DQA10501-DQB10201	121	DEWDLCVNAESIPTT	DLCVNAESI	3	0.3494	1140.4	24.00	0.46	tr_C9ZUI4_
HLA-DQA10501-DQB10201	122	PFTCRSRALTSVCDI	RALTSVCDI	6	0.3492	1142.6	24.00	0.70	tr_C9ZUI4_
HLA-DQA10501-DQB10201	123	LLGLVKQNVFCTEPF	QNVFCTEPF	6	0.3474	1166.1	25.00	0.58	tr_C9ZUI4_
HLA-DQA10501-DQB10201	124	AVLNATSMDASQNVG	VLNATSMDA	1	0.3466	1176.1	25.00	0.58	tr_C9ZUI4_
HLA-DQA10501-DQB10201	125	HAIKGYRVIALAYRP	GYRVIALAY	4	0.3462	1180.5	25.00	0.23	tr_C9ZUI4_
HLA-DQA10501-DQB10201	126	IAQLCESIPPDFHSV	LCESIPPDF	3	0.3450	1196.8	25.00	0.47	tr_C9ZUI4_
HLA-DQA10501-DQB10201	127	PSDDEWDLCVNAESI	DLCVNAESI	6	0.3449	1197.8	25.00	0.47	tr_C9ZUI4_
HLA-DQA10501-DQB10201	128	IFNADDADMGGAPQV	IFNADDADM	0	0.3428	1224.5	26.00	0.47	tr_C9ZUI4_
HLA-DQA10501-DQB10201	129	TVVPPAPSADAPFME	APSADAPFM	5	0.3414	1244.1	26.00	0.45	tr_C9ZUI4_
HLA-DQA10501-DQB10201	130	SHRCVIITGDSVQTA	ITGDSVQTA	6	0.3403	1258.9	26.00	0.32	tr_C9ZUI4_
HLA-DQA10501-DQB10201	131	EMLFSGVDMADGKGL	EMLFSGVDM	0	0.3395	1269.3	26.00	0.26	tr_C9ZUI4_

HLA-DQA10501-DQB10201	132	VATMRQAGAEGEFKP	MRQAGAEGE	3	0.3385	1283.5	27.00	0.38	tr_C9ZUI4_
HLA-DQA10501-DQB10201	133	NLIFAGLAVFQCPLK	IFAGLAVFQ	2	0.3384	1284.4	27.00	0.30	tr_C9ZUI4_
HLA-DQA10501-DQB10201	134	CHSLLQLEGTDTVAG	LLQLEGTDT	3	0.3375	1297.6	27.00	0.26	tr_C9ZUI4_
HLA-DQA10501-DQB10201	135	RCVIITGDSVQTAIS	ITGDSVQTA	4	0.3374	1298.1	27.00	0.32	tr_C9ZUI4_
HLA-DQA10501-DQB10201	136	LFLACIQIITAVVPP	QIITAVVPP	6	0.3372	1302.2	27.00	0.31	tr_C9ZUI4_
HLA-DQA10501-DQB10201	137	VKLGDASIAAPFTCR	DASIAAPFT	4	0.3370	1304.8	27.00	0.62	tr_C9ZUI4_
HLA-DQA10501-DQB10201	138	CVIITGDSVQTAISV	ITGDSVQTA	3	0.3366	1310.7	27.00	0.31	tr_C9ZUI4_
HLA-DQA10501-DQB10201	139	PPAPSADAPFMEQVR	APSADAPFM	2	0.3360	1318.2	27.00	0.31	tr_C9ZUI4_
HLA-DQA10501-DQB10201	140	MYKILALNCLTSAYS	YKILALNCL	1	0.3360	1319.0	27.00	0.44	tr_C9ZUI4_
HLA-DQA10501-DQB10201	141	HSLLQLEGTDTVAGD	LLQLEGTDT	2	0.3351	1331.3	27.00	0.27	tr_C9ZUI4_
HLA-DQA10501-DQB10201	142	RVNIDDTVVYDPPAQ	NIDDTVVYD	2	0.3348	1335.1	27.00	0.24	tr_C9ZUI4_
HLA-DQA10501-DQB10201	143	TCRSRALTSVCDIVR	RALTSVCDI	4	0.3348	1335.8	27.00	0.52	tr_C9ZUI4_
HLA-DQA10501-DQB10201	144	LVKQNVFCTEPFRIP	QNVFCTEPF	3	0.3340	1347.4	27.00	0.44	tr_C9ZUI4_
HLA-DQA10501-DQB10201	145	SIPTTTLATLIAQYS	LATLIAQYS	6	0.3336	1352.7	28.00	0.44	tr_C9ZUI4_
HLA-DQA10501-DQB10201	146	VVAKGSPEAIAQLCE	PEAIAQLCE	6	0.3336	1353.3	28.00	0.40	tr_C9ZUI4_
HLA-DQA10501-DQB10201	147	VEEADAEGVATMRQA	VEEADAEGV	0	0.3331	1361.3	28.00	0.38	tr_C9ZUI4_
HLA-DQA10501-DQB10201	148	IKGYRVIALAYRPLK	GYRVIALAY	2	0.3321	1375.6	28.00	0.21	tr_C9ZUI4_
HLA-DQA10501-DQB10201	149	RSRALTSVCDIVRLG	RALTSVCDI	2	0.3308	1395.4	28.00	0.47	tr_C9ZUI4_
HLA-DQA10501-DQB10201	150	CEKNLIFAGLAVFQC	IFAGLAVFQ	5	0.3304	1400.2	28.00	0.31	tr_C9ZUI4_
HLA-DQA10501-DQB10201	151	PTTTLATLIAQYSEH	LATLIAQYS	4	0.3296	1413.3	28.00	0.41	tr_C9ZUI4_
HLA-DQA10501-DQB10201	152	GALKQAHAGIAVLNA	HAGIAVLNA	6	0.3295	1414.9	28.00	0.47	tr_C9ZUI4_
HLA-DQA10501-DQB10201	153	IFMQFGLHLYSMVET	LHLYSMVET	6	0.3288	1425.2	29.00	0.38	tr_C9ZUI4_
HLA-DQA10501-DQB10201	154	GLVKQNVFCTEPFRI	QNVFCTEPF	4	0.3283	1432.5	29.00	0.44	tr_C9ZUI4_
HLA-DQA10501-DQB10201	155	KGYRVIALAYRPLKE	GYRVIALAY	1	0.3277	1442.9	29.00	0.19	tr_C9ZUI4_
HLA-DQA10501-DQB10201	156	KGNGEDEVDDCIVWT	EVDDCIVWT	6	0.3262	1465.6	29.00	0.25	tr_C9ZUI4_
HLA-DQA10501-DQB10201	157	EGVATMRQAGAEGEF	MRQAGAEGE	5	0.3256	1476.3	29.00	0.50	tr_C9ZUI4_
HLA-DQA10501-DQB10201	158	KKGNGEDEVDDCIVW	EDEVDDCIV	5	0.3221	1532.7	30.00	0.28	tr_C9ZUI4_
HLA-DQA10501-DQB10201	159	VKQNVFCTEPFRIPY	QNVFCTEPF	2	0.3217	1539.7	30.00	0.44	tr_C9ZUI4_
HLA-DQA10501-DQB10201	160	KLGDASIAAPFTCRS	DASIAAPFT	3	0.3206	1556.9	30.00	0.63	tr_C9ZUI4_
HLA-DQA10501-DQB10201	161	EWDLCVNAESIPTTT	DLCVNAESI	2	0.3202	1565.2	30.00	0.43	tr_C9ZUI4_
HLA-DQA10501-DQB10201	162	KIPPGFKLTVVPPAP	FKLTVVPPA	5	0.3190	1584.5	31.00	0.56	tr_C9ZUI4_
HLA-DQA10501-DQB10201	163	GVATMRQAGAEGEFK	MRQAGAEGE	4	0.3181	1600.9	31.00	0.40	tr_C9ZUI4_
HLA-DQA10501-DQB10201	164	LGLVKQNVFCTEPFR	QNVFCTEPF	5	0.3179	1603.4	31.00	0.52	tr_C9ZUI4_
HLA-DQA10501-DQB10201	165	AHAIKGYRVIALAYR	GYRVIALAY	5	0.3179	1603.9	31.00	0.29	tr_C9ZUI4_
HLA-DQA10501-DQB10201	166	KKKGNGEDEVDDCIV	EDEVDDCIV	6	0.3177	1607.3	31.00	0.28	tr_C9ZUI4_
HLA-DQA10501-DQB10201	167	LIAQYSEHIAVWARC	LIAQYSEHI	0	0.3164	1630.7	31.00	0.33	tr_C9ZUI4_
HLA-DQA10501-DQB10201	168	GYRVIALAYRPLKEE	ALAYRPLKE	5	0.3161	1634.8	31.00	0.21	tr_C9ZUI4_
HLA-DQA10501-DQB10201	169	GNGEDEVDDCIVWTD	EVDDCIVWT	5	0.3156	1644.4	31.00	0.25	tr_C9ZUI4_
HLA-DQA10501-DQB10201	170	PAPSADAPFMEQVRH	APSADAPFM	1	0.3149	1656.3	32.00	0.32	tr_C9ZUI4_
HLA-DQA10501-DQB10201	171	ASLGALGYRVNIDDT	GYRVNIDDT	6	0.3146	1661.3	32.00	0.35	tr_C9ZUI4_
HLA-DQA10501-DQB10201	172	LIFAGLAVFQCPLKK	IFAGLAVFQ	1	0.3143	1668.5	32.00	0.35	tr_C9ZUI4_
HLA-DQA10501-DQB10201	173	SMSVLQMDGVKHGES	MSVLQMDGV	1	0.3142	1668.7	32.00	0.60	tr_C9ZUI4_
HLA-DQA10501-DQB10201	174	LVTCHSLLQLEGTDT	HSLLQLEGT	4	0.3141	1670.6	32.00	0.34	tr_C9ZUI4_
HLA-DQA10501-DQB10201	175	REHMVLMAGDGTNDV	MVLMAGDGT	3	0.3140	1673.1	32.00	0.43	tr_C9ZUI4_
HLA-DQA10501-DQB10201	176	ALKQAHAGIAVLNAT	HAGIAVLNA	5	0.3129	1693.0	32.00	0.40	tr_C9ZUI4_
HLA-DQA10501-DQB10201	177	IPPGFKLTVVPPAPS	FKLTVVPPA	4	0.3119	1712.0	32.00	0.55	tr_C9ZUI4_
HLA-DQA10501-DQB10201	178	NAHAIKGYRVIALAY	GYRVIALAY	6	0.3105	1738.1	33.00	0.43	tr_C9ZUI4_
HLA-DQA10501-DQB10201	179	LGDASIAAPFTCRSR	DASIAAPFT	2	0.3078	1788.2	33.00	0.66	tr_C9ZUI4_
HLA-DQA10501-DQB10201	180	VIITGDSVQTAISVG	ITGDSVQTA	2	0.3074	1796.1	33.00	0.40	tr_C9ZUI4_
HLA-DQA10501-DQB10201	181	SRALTSVCDIVRLGR	RALTSVCDI	1	0.3066	1812.4	34.00	0.51	tr_C9ZUI4_

HLA-DQA10501-DQB10201	182	YRVIALAYRPLKEEE	ALAYRPLKE	4	0.3045	1853.1	34.00	0.25	tr_C9ZUI4_
HLA-DQA10501-DQB10201	183	TVAGDAMEKASLGAL	VAGDAMEKA	1	0.3031	1883.3	34.00	0.56	tr_C9ZUI4_
HLA-DQA10501-DQB10201	184	VNIDDTVVYDPPAQK	NIDDTVVYD	1	0.3029	1886.0	34.00	0.26	tr_C9ZUI4_
HLA-DQA10501-DQB10201	185	DCEKNLIFAGLAVFQ	IFAGLAVFQ	6	0.3029	1886.5	34.00	0.46	tr_C9ZUI4_
HLA-DQA10501-DQB10201	186	KQNVFCTEPFRIPYA	QNVFCTEPF	1	0.3028	1887.7	34.00	0.39	tr_C9ZUI4_
HLA-DQA10501-DQB10201	187	SLLQLEGTDTVAGDA	LQLEGTDTV	2	0.3028	1889.4	34.00	0.38	tr_C9ZUI4_
HLA-DQA10501-DQB10201	188	EHMVLMAGDGTNDVG	MVLMAGDGT	2	0.3016	1913.2	35.00	0.43	tr_C9ZUI4_
HLA-DQA10501-DQB10201	189	PPGFKLTVVPPAPSA	FKLTVVPPA	3	0.3013	1920.5	35.00	0.54	tr_C9ZUI4_
HLA-DQA10501-DQB10201	190	NGEDEVDDCIVWTDA	EVDDCIVWT	4	0.2998	1950.7	35.00	0.27	tr_C9ZUI4_
HLA-DQA10501-DQB10201	191	DRWKLFLACIQIITA	WKLFLACIQ	2	0.2994	1959.0	35.00	0.34	tr_C9ZUI4_
HLA-DQA10501-DQB10201	192	AQARRKAEIVQIARW	AEIVQIARW	6	0.2990	1967.8	35.00	0.28	tr_C9ZUI4_
HLA-DQA10501-DQB10201	193	AQLCESIPPDFHSVA	LCESIPPDF	2	0.2988	1971.6	35.00	0.55	tr_C9ZUI4_
HLA-DQA10501-DQB10201	194	PGFKLTVVPPAPSAD	FKLTVVPPA	2	0.2987	1973.9	35.00	0.55	tr_C9ZUI4_
HLA-DQA10501-DQB10201	195	RVIALAYRPLKEEER	ALAYRPLKE	3	0.2978	1993.4	36.00	0.26	tr_C9ZUI4_
HLA-DQA10501-DQB10201	196	TAVVPPELPMELTLA	TAVVPPELP	0	0.2977	1995.4	36.00	0.27	tr_C9ZUI4_
HLA-DQA10501-DQB10201	197	KLFLACIQIITAVVP	IQIITAVVP	6	0.2967	2017.2	36.00	0.31	tr_C9ZUI4_
HLA-DQA10501-DQB10201	198	YKILALNCLTSAYSM	YKILALNCL	0	0.2964	2024.3	36.00	0.38	tr_C9ZUI4_
HLA-DQA10501-DQB10201	199	QREHMVLMAGDGTND	MVLMAGDGT	4	0.2943	2069.9	37.00	0.50	tr_C9ZUI4_
HLA-DQA10501-DQB10201	200	LKQAHAGIAVLNATS	HAGIAVLNA	4	0.2938	2081.7	37.00	0.42	tr_C9ZUI4_
HLA-DQA10501-DQB10201	201	GSHRCVIITGDSVQT	IITGDSVQT	6	0.2909	2148.7	37.00	0.37	tr_C9ZUI4_
HLA-DQA10501-DQB10201	202	RDRWKLFLACIQIIT	WKLFLACIQ	3	0.2904	2160.7	38.00	0.40	tr_C9ZUI4_
HLA-DQA10501-DQB10201	203	HKIPPGFKLTVVPPA	FKLTVVPPA	6	0.2903	2162.7	38.00	0.58	tr_C9ZUI4_
HLA-DQA10501-DQB10201	204	ESIPTTTLATLIAQY	TLATLIAQY	6	0.2899	2171.5	38.00	0.46	tr_C9ZUI4_
HLA-DQA10501-DQB10201	205	IFAGLAVFQCPLKKD	FAGLAVFQC	1	0.2897	2175.8	38.00	0.26	tr_C9ZUI4_
HLA-DQA10501-DQB10201	206	LTVVPPAPSADAPFM	APSADAPFM	6	0.2894	2182.5	38.00	0.52	tr_C9ZUI4_
HLA-DQA10501-DQB10201	207	WDLCVNAESIPTTTL	DLCVNAESI	1	0.2886	2201.6	38.00	0.45	tr_C9ZUI4_
HLA-DQA10501-DQB10201	208	QYKILVRFPFLANLR	RFPFLANLR	6	0.2869	2243.7	38.00	0.31	tr_C9ZUI4_
HLA-DQA10501-DQB10201	209	LKQREHMVLMAGDGT	MVLMAGDGT	6	0.2866	2250.0	38.00	0.45	tr_C9ZUI4_
HLA-DQA10501-DQB10201	210	GFKLTVVPPAPSADA	FKLTVVPPA	1	0.2856	2275.2	39.00	0.54	tr_C9ZUI4_
HLA-DQA10501-DQB10201	211	MKKKGNGEDEVDDCI	GEDEVDDCI	6	0.2844	2303.3	39.00	0.31	tr_C9ZUI4_
HLA-DQA10501-DQB10201	212	YMMCTIFMQFGLHLY	FMQFGLHLY	6	0.2839	2317.9	39.00	0.34	tr_C9ZUI4_
HLA-DQA10501-DQB10201	213	RWKLFLACIQIITAV	WKLFLACIQ	1	0.2835	2327.5	39.00	0.31	tr_C9ZUI4_
HLA-DQA10501-DQB10201	214	YKILVRFPFLANLRR	RFPFLANLR	5	0.2834	2328.5	39.00	0.25	tr_C9ZUI4_
HLA-DQA10501-DQB10201	215	ELPMELTLAVNTALL	TLAVNTALL	6	0.2832	2334.6	39.00	0.40	tr_C9ZUI4_
HLA-DQA10501-DQB10201	216	YVVAKGSPEAIAQLC	VAKGSPEAI	2	0.2825	2351.7	39.00	0.38	tr_C9ZUI4_
HLA-DQA10501-DQB10201	217	QARRKAEIVQIARWN	AEIVQIARW	5	0.2824	2356.1	40.00	0.23	tr_C9ZUI4_
HLA-DQA10501-DQB10201	218	KQREHMVLMAGDGTN	MVLMAGDGT	5	0.2818	2371.3	40.00	0.48	tr_C9ZUI4_
HLA-DQA10501-DQB10201	219	MELTLAVNTALLGLV	VNTALLGLV	6	0.2816	2374.2	40.00	0.23	tr_C9ZUI4_
HLA-DQA10501-DQB10201	220	NRDRWKLFLACIQII	WKLFLACIQ	4	0.2814	2380.1	40.00	0.51	tr_C9ZUI4_
HLA-DQA10501-DQB10201	221	SIPPDFHSVANAHAI	FHSVANAHA	5	0.2813	2382.5	40.00	0.58	tr_C9ZUI4_
HLA-DQA10501-DQB10201	222	WKLFLACIQIITAVV	WKLFLACIQ	0	0.2812	2384.6	40.00	0.24	tr_C9ZUI4_
HLA-DQA10501-DQB10201	223	MLFSGVDMADGKGLL	FSGVDMADG	2	0.2807	2399.5	40.00	0.32	tr_C9ZUI4_
HLA-DQA10501-DQB10201	224	PMELTLAVNTALLGL	TLAVNTALL	4	0.2801	2413.1	40.00	0.27	tr_C9ZUI4_
HLA-DQA10501-DQB10201	225	TIFMQFGLHLYSMVE	GLHLYSMVE	6	0.2798	2422.6	40.00	0.20	tr_C9ZUI4_
HLA-DQA10501-DQB10201	226	KILVRFPFLANLRRM	RFPFLANLR	4	0.2785	2455.1	40.00	0.25	tr_C9ZUI4_
HLA-DQA10501-DQB10201	227	RSKEAIHNMDREDCE	EAIHNMDRE	3	0.2784	2458.4	41.00	0.38	tr_C9ZUI4_
HLA-DQA10501-DQB10201	228	ITKVFHPYMMCTIFM	PYMMCTIFM	6	0.2779	2471.7	41.00	0.34	tr_C9ZUI4_
HLA-DQA10501-DQB10201	229	GDASIAAPFTCRSRA	DASIAAPFT	1	0.2770	2496.3	41.00	0.69	tr_C9ZUI4_
HLA-DQA10501-DQB10201	230	LALNCLTSAYSMSVL	TSAYSMSVL	6	0.2769	2499.6	41.00	0.38	tr_C9ZUI4_
HLA-DQA10501-DQB10201	231	ALNCLTSAYSMSVLQ	TSAYSMSVL	5	0.2759	2526.7	41.00	0.31	tr_C9ZUI4_

HLA-DQA10501-DQB10201	232	HMVLMAGDGTNDVGA	MVLMAGDGT	1	0.2753	2542.7	41.00	0.46	tr_C9ZUI4_
HLA-DQA10501-DQB10201	233	VLNATSMDASQNVGG	VLNATSMDA	0	0.2747	2558.5	42.00	0.51	tr_C9ZUI4_
HLA-DQA10501-DQB10201	234	VIALAYRPLKEEERS	ALAYRPLKE	2	0.2745	2564.2	42.00	0.28	tr_C9ZUI4_
HLA-DQA10501-DQB10201	235	VLVTCHSLLQLEGTD	HSLLQLEGT	5	0.2726	2619.2	42.00	0.47	tr_C9ZUI4_
HLA-DQA10501-DQB10201	236	GEDEVDDCIVWTDAA	EVDDCIVWT	3	0.2720	2634.7	42.00	0.29	tr_C9ZUI4_
HLA-DQA10501-DQB10201	237	KQYKILVRFPFLANL	KILVRFPFL	3	0.2709	2667.6	43.00	0.29	tr_C9ZUI4_
HLA-DQA10501-DQB10201	238	QLCESIPPDFHSVAN	LCESIPPDF	1	0.2699	2694.7	43.00	0.51	tr_C9ZUI4_
HLA-DQA10501-DQB10201	239	SKEAIHNMDREDCEK	EAIHNMDRE	2	0.2696	2704.3	43.00	0.38	tr_C9ZUI4_
HLA-DQA10501-DQB10201	240	LPMELTLAVNTALLG	TLAVNTALL	5	0.2692	2717.4	43.00	0.28	tr_C9ZUI4_
HLA-DQA10501-DQB10201	241	KMAQARRKAEIVQIA	RRKAEIVQI	5	0.2691	2720.7	43.00	0.51	tr_C9ZUI4_
HLA-DQA10501-DQB10201	242	ESIPPDFHSVANAHA	FHSVANAHA	6	0.2688	2729.0	43.00	0.62	tr_C9ZUI4_
HLA-DQA10501-DQB10201	243	KVFHPYMMCTIFMQF	PYMMCTIFM	4	0.2687	2730.7	43.00	0.27	tr_C9ZUI4_
HLA-DQA10501-DQB10201	244	KVPSDDEWDLCVNAE	EWDLCVNAE	6	0.2684	2738.8	43.00	0.52	tr_C9ZUI4_
HLA-DQA10501-DQB10201	245	DPNRDRWKLFLACIQ	WKLFLACIQ	6	0.2683	2743.6	43.00	0.61	tr_C9ZUI4_
HLA-DQA10501-DQB10201	246	CTIFMQFGLHLYSMV	IFMQFGLHL	2	0.2668	2788.5	44.00	0.28	tr_C9ZUI4_
HLA-DQA10501-DQB10201	247	NIDDTVVYDPPAQKE	TVVYDPPAQ	4	0.2655	2826.5	44.00	0.23	tr_C9ZUI4_
HLA-DQA10501-DQB10201	248	RALTSVCDIVRLGRS	RALTSVCDI	0	0.2654	2831.6	44.00	0.46	tr_C9ZUI4_
HLA-DQA10501-DQB10201	249	VGALKQAHAGIAVLN	QAHAGIAVL	5	0.2652	2836.4	44.00	0.44	tr_C9ZUI4_
HLA-DQA10501-DQB10201	250	APSADAPFMEQVRHK	APSADAPFM	0	0.2650	2843.0	44.00	0.34	tr_C9ZUI4_
HLA-DQA10501-DQB10201	251	KVVSQPEMNAPASDF	EMNAPASDF	6	0.2647	2850.6	44.00	0.52	tr_C9ZUI4_
HLA-DQA10501-DQB10201	252	PNRDRWKLFLACIQI	WKLFLACIQ	5	0.2647	2850.8	44.00	0.57	tr_C9ZUI4_
HLA-DQA10501-DQB10201	253	RRHKVPSDDEWDLCV	KVPSDDEWD	3	0.2645	2859.7	44.00	0.31	tr_C9ZUI4_
HLA-DQA10501-DQB10201	254	QNVFCTEPFRIPYAG	QNVFCTEPF	0	0.2643	2864.6	44.00	0.34	tr_C9ZUI4_
HLA-DQA10501-DQB10201	255	VPSDDEWDLCVNAES	EWDLCVNAE	5	0.2643	2865.2	44.00	0.54	tr_C9ZUI4_
HLA-DQA10501-DQB10201	256	ILVRFPFLANLRRMP	RFPFLANLR	3	0.2636	2885.6	44.00	0.32	tr_C9ZUI4_
HLA-DQA10501-DQB10201	257	PYMMCTIFMQFGLHL	IFMQFGLHL	6	0.2626	2917.7	45.00	0.40	tr_C9ZUI4_
HLA-DQA10501-DQB10201	258	ERSKEAIHNMDREDC	EAIHNMDRE	4	0.2614	2955.7	45.00	0.44	tr_C9ZUI4_
HLA-DQA10501-DQB10201	259	SVQTAISVGRDVTIL	SVGRDVTIL	6	0.2612	2961.8	45.00	0.26	tr_C9ZUI4_
HLA-DQA10501-DQB10201	260	MMCTIFMQFGLHLYS	IFMQFGLHL	4	0.2610	2967.2	45.00	0.32	tr_C9ZUI4_
HLA-DQA10501-DQB10201	261	TRRHKVPSDDEWDLC	KVPSDDEWD	4	0.2606	2980.6	45.00	0.31	tr_C9ZUI4_
HLA-DQA10501-DQB10201	262	MCTIFMQFGLHLYSM	IFMQFGLHL	3	0.2599	3003.3	45.00	0.31	tr_C9ZUI4_
HLA-DQA10501-DQB10201	263	MSVLQMDGVKHGESQ	MSVLQMDGV	0	0.2593	3022.7	46.00	0.56	tr_C9ZUI4_
HLA-DQA10501-DQB10201	264	PLKKDAKDTIEMLQS	DAKDTIEML	4	0.2590	3033.3	46.00	0.45	tr_C9ZUI4_
HLA-DQA10501-DQB10201	265	KASLGALGYRVNIDD	ALGYRVNID	5	0.2589	3037.1	46.00	0.47	tr_C9ZUI4_
HLA-DQA10501-DQB10201	266	DGKYVVAKGSPEAIA	VAKGSPEAI	5	0.2582	3060.6	46.00	0.50	tr_C9ZUI4_
HLA-DQA10501-DQB10201	267	MVLMAGDGTNDVGAL	MVLMAGDGT	0	0.2577	3076.7	46.00	0.39	tr_C9ZUI4_
HLA-DQA10501-DQB10201	268	RPITKVFHPYMMCTI	ITKVFHPYM	2	0.2574	3087.9	46.00	0.32	tr_C9ZUI4_
HLA-DQA10501-DQB10201	269	FEKQYKILVRFPFLA	KILVRFPFL	5	0.2568	3104.9	46.00	0.34	tr_C9ZUI4_
HLA-DQA10501-DQB10201	270	PDGKYVVAKGSPEAI	VAKGSPEAI	6	0.2567	3109.6	46.00	0.56	tr_C9ZUI4_
HLA-DQA10501-DQB10201	271	IPPDFHSVANAHAIK	FHSVANAHA	4	0.2557	3145.0	47.00	0.56	tr_C9ZUI4_
HLA-DQA10501-DQB10201	272	DEVDDCIVWTDAATG	IVWTDAATG	6	0.2549	3170.9	47.00	0.35	tr_C9ZUI4_
HLA-DQA10501-DQB10201	273	VFHPYMMCTIFMQFG	PYMMCTIFM	3	0.2549	3171.1	47.00	0.25	tr_C9ZUI4_
HLA-DQA10501-DQB10201	274	IITGDSVQTAISVGR	ITGDSVQTA	1	0.2546	3180.3	47.00	0.43	tr_C9ZUI4_
HLA-DQA10501-DQB10201	275	ANAHAIKGYRVIALA	IKGYRVIAL	5	0.2542	3196.2	47.00	0.48	tr_C9ZUI4_
HLA-DQA10501-DQB10201	276	ELTLAVNTALLGLVK	TLAVNTALL	2	0.2540	3201.9	47.00	0.23	tr_C9ZUI4_
HLA-DQA10501-DQB10201	277	ARRKAEIVQIARWNK	AEIVQIARW	4	0.2533	3226.1	47.00	0.25	tr_C9ZUI4_
HLA-DQA10501-DQB10201	278	LVLVTCHSLLQLEGT	HSLLQLEGT	6	0.2528	3244.2	47.00	0.50	tr_C9ZUI4_
HLA-DQA10501-DQB10201	279	TDLKQREHMVLMAGD	EHMVLMAGD	6	0.2523	3261.6	48.00	0.60	tr_C9ZUI4_
HLA-DQA10501-DQB10201	280	APQVKLGDASIAAPF	GDASIAAPF	6	0.2523	3263.1	48.00	0.38	tr_C9ZUI4_
HLA-DQA10501-DQB10201	281	MAQARRKAEIVQIAR	RRKAEIVQI	4	0.2520	3271.5	48.00	0.44	tr_C9ZUI4_

HLA-DQA10501-DQB10201	282	PITKVFHPYMMCTIF	ITKVFHPYM	1	0.2515	3288.8	48.00	0.30	tr_C9ZUI4_
HLA-DQA10501-DQB10201	283	KEAIHNMDREDCEKN	EAIHNMDRE	1	0.2515	3291.1	48.00	0.37	tr_C9ZUI4_
HLA-DQA10501-DQB10201	284	TKVFHPYMMCTIFMQ	PYMMCTIFM	5	0.2501	3338.5	48.00	0.29	tr_C9ZUI4_
HLA-DQA10501-DQB10201	285	RHKVPSDDEWDLCVN	KVPSDDEWD	2	0.2500	3343.6	48.00	0.32	tr_C9ZUI4_
HLA-DQA10501-DQB10201	286	EDCEKNLIFAGLAVF	KNLIFAGLA	4	0.2498	3350.8	48.00	0.41	tr_C9ZUI4_
HLA-DQA10501-DQB10201	287	EKQYKILVRFPFLAN	KILVRFPFL	4	0.2488	3385.8	49.00	0.35	tr_C9ZUI4_
HLA-DQA10501-DQB10201	288	QTRRHKVPSDDEWDL	KVPSDDEWD	5	0.2484	3402.1	49.00	0.33	tr_C9ZUI4_
HLA-DQA10501-DQB10201	289	KYVVAKGSPEAIAQL	VAKGSPEAI	3	0.2479	3421.4	49.00	0.47	tr_C9ZUI4_
HLA-DQA10501-DQB10201	290	IAQYSEHIAVWARCA	YSEHIAVWA	3	0.2463	3481.4	49.00	0.22	tr_C9ZUI4_
HLA-DQA10501-DQB10201	291	FHPYMMCTIFMQFGL	PYMMCTIFM	2	0.2458	3500.0	49.00	0.24	tr_C9ZUI4_
HLA-DQA10501-DQB10201	292	SMKKKGNGEDEVDDC	NGEDEVDDC	6	0.2457	3502.8	49.00	0.29	tr_C9ZUI4_
HLA-DQA10501-DQB10201	293	GKYVVAKGSPEAIAQ	VAKGSPEAI	4	0.2454	3514.4	49.00	0.50	tr_C9ZUI4_
HLA-DQA10501-DQB10201	294	AEGVATMRQAGAEGE	MRQAGAEGE	6	0.2450	3531.0	50.00	0.62	tr_C9ZUI4_
HLA-DQA10501-DQB10201	295	AVVPPELPMELTLAV	VPPELPMEL	2	0.2448	3539.0	50.00	0.31	tr_C9ZUI4_
HLA-DQA10501-DQB10201	296	FKLTVVPPAPSADAP	FKLTVVPPA	0	0.2442	3562.0	50.00	0.47	tr_C9ZUI4_
HLA-DQA10501-DQB10201	297	SGSHRCVIITGDSVQ	CVIITGDSV	5	0.2438	3575.1	50.00	0.44	tr_C9ZUI4_
HLA-DQA10501-DQB10201	298	PELPMELTLAVNTAL	ELTLAVNTA	5	0.2432	3597.2	50.00	0.35	tr_C9ZUI4_
HLA-DQA10501-DQB10201	299	PPDFHSVANAHAIKG	FHSVANAHA	3	0.2432	3598.2	50.00	0.51	tr_C9ZUI4_
HLA-DQA10501-DQB10201	300	EDEVDDCIVWTDAAT	EVDDCIVWT	2	0.2425	3627.2	50.00	0.34	tr_C9ZUI4_
HLA-DQA10501-DQB10201	301	LVRFPFLANLRRMPC	RFPFLANLR	2	0.2423	3633.0	50.00	0.33	tr_C9ZUI4_
HLA-DQA10501-DQB10201	302	VVPPELPMELTLAVN	VPPELPMEL	1	0.2420	3646.9	50.00	0.26	tr_C9ZUI4_
HLA-DQA10501-DQB10201	303	HKMAQARRKAEIVQI	RRKAEIVQI	6	0.2414	3667.9	55.00	0.75	tr_C9ZUI4_
HLA-DQA10501-DQB10201	304	CPLKKDAKDTIEMLQ	DAKDTIEML	5	0.2412	3676.1	55.00	0.58	tr_C9ZUI4_
HLA-DQA10501-DQB10201	305	DLCVNAESIPTTTLA	DLCVNAESI	0	0.2412	3678.8	55.00	0.37	tr_C9ZUI4_
HLA-DQA10501-DQB10201	306	EEADAEGVATMRQAG	AEGVATMRQ	4	0.2411	3681.3	55.00	0.41	tr_C9ZUI4_
HLA-DQA10501-DQB10201	307	ESQMILSGIILTVCF	LSGIILTVC	5	0.2401	3721.2	55.00	0.22	tr_C9ZUI4_
HLA-DQA10501-DQB10201	308	DSVQTAISVGRDVTI	QTAISVGRD	3	0.2399	3729.7	55.00	0.29	tr_C9ZUI4_
HLA-DQA10501-DQB10201	309	AQYSEHIAVWARCAP	YSEHIAVWA	2	0.2399	3731.5	55.00	0.20	tr_C9ZUI4_
HLA-DQA10501-DQB10201	310	EERSKEAIHNMDRED	EAIHNMDRE	5	0.2397	3736.7	55.00	0.51	tr_C9ZUI4_
HLA-DQA10501-DQB10201	311	DLKQREHMVLMAGDG	EHMVLMAGD	5	0.2391	3761.1	55.00	0.52	tr_C9ZUI4_
HLA-DQA10501-DQB10201	312	DREDCEKNLIFAGLA	KNLIFAGLA	6	0.2380	3808.3	55.00	0.54	tr_C9ZUI4_
HLA-DQA10501-DQB10201	313	PDFHSVANAHAIKGY	FHSVANAHA	2	0.2376	3825.5	55.00	0.48	tr_C9ZUI4_
HLA-DQA10501-DQB10201	314	PPELPMELTLAVNTA	ELTLAVNTA	6	0.2374	3832.0	55.00	0.38	tr_C9ZUI4_
HLA-DQA10501-DQB10201	315	REDCEKNLIFAGLAV	KNLIFAGLA	5	0.2373	3837.6	55.00	0.47	tr_C9ZUI4_
HLA-DQA10501-DQB10201	316	IDDTVVYDPPAQKED	TVVYDPPAQ	3	0.2367	3862.4	55.00	0.35	tr_C9ZUI4_
HLA-DQA10501-DQB10201	317	LKKDAKDTIEMLQSG	DAKDTIEML	3	0.2365	3871.1	55.00	0.43	tr_C9ZUI4_
HLA-DQA10501-DQB10201	318	SQMILSGIILTVCFL	LSGIILTVC	4	0.2364	3873.9	55.00	0.20	tr_C9ZUI4_
HLA-DQA10501-DQB10201	319	VQTAISVGRDVTILK	SVGRDVTIL	5	0.2358	3899.0	55.00	0.25	tr_C9ZUI4_
HLA-DQA10501-DQB10201	320	GESQMILSGIILTVC	LSGIILTVC	6	0.2353	3919.4	55.00	0.25	tr_C9ZUI4_
HLA-DQA10501-DQB10201	321	ILALNCLTSAYSMSV	LTSAYSMSV	6	0.2351	3928.3	55.00	0.47	tr_C9ZUI4_
HLA-DQA10501-DQB10201	322	LTLAVNTALLGLVKQ	TLAVNTALL	1	0.2348	3939.3	55.00	0.22	tr_C9ZUI4_
HLA-DQA10501-DQB10201	323	AEAGKVVSQPEMNAP	KVVSQPEMN	4	0.2347	3946.5	55.00	0.29	tr_C9ZUI4_
HLA-DQA10501-DQB10201	324	QYSEHIAVWARCAPT	YSEHIAVWA	1	0.2328	4026.1	55.00	0.18	tr_C9ZUI4_
HLA-DQA10501-DQB10201	325	LCESIPPDFHSVANA	LCESIPPDF	0	0.2318	4073.1	55.00	0.43	tr_C9ZUI4_
HLA-DQA10501-DQB10201	326	VRFPFLANLRRMPCI	RFPFLANLR	1	0.2317	4074.1	55.00	0.30	tr_C9ZUI4_
HLA-DQA10501-DQB10201	327	VQTRRHKVPSDDEWD	KVPSDDEWD	6	0.2317	4075.9	55.00	0.34	tr_C9ZUI4_
HLA-DQA10501-DQB10201	328	VAGDAMEKASLGALG	VAGDAMEKA	0	0.2314	4089.0	55.00	0.47	tr_C9ZUI4_
HLA-DQA10501-DQB10201	329	EAIHNMDREDCEKNL	EAIHNMDRE	0	0.2313	4095.0	55.00	0.31	tr_C9ZUI4_
HLA-DQA10501-DQB10201	330	VANAHAIKGYRVIAL	IKGYRVIAL	6	0.2295	4176.1	55.00	0.56	tr_C9ZUI4_
HLA-DQA10501-DQB10201	331	ITGDSVQTAISVGRD	QTAISVGRD	6	0.2292	4185.5	55.00	0.31	tr_C9ZUI4_

HLA-DQA10501-DQB10201	332	VNAESIPTTTLATLI	PTTTLATLI	6	0.2290	4194.8	55.00	0.61	tr_C9ZUI4_
HLA-DQA10501-DQB10201	333	EKASLGALGYRVNID	ALGYRVNID	6	0.2289	4199.0	55.00	0.46	tr_C9ZUI4_
HLA-DQA10501-DQB10201	334	DVGALKQAHAGIAVL	QAHAGIAVL	6	0.2289	4203.0	55.00	0.61	tr_C9ZUI4_
HLA-DQA10501-DQB10201	335	KLTVVPPAPSADAPF	PAPSADAPF	6	0.2283	4228.4	55.00	0.43	tr_C9ZUI4_
HLA-DQA10501-DQB10201	336	VPPELPMELTLAVNT	PMELTLAVN	5	0.2280	4240.4	55.00	0.19	tr_C9ZUI4_
HLA-DQA10501-DQB10201	337	GDSVQTAISVGRDVT	QTAISVGRD	4	0.2277	4253.9	55.00	0.37	tr_C9ZUI4_
HLA-DQA10501-DQB10201	338	VKHGESQMILSGIIL	QMILSGIIL	6	0.2270	4288.7	55.00	0.41	tr_C9ZUI4_
HLA-DQA10501-DQB10201	339	NAESIPTTTLATLIA	PTTTLATLI	5	0.2268	4298.0	55.00	0.47	tr_C9ZUI4_
HLA-DQA10501-DQB10201	340	TAEAGKVVSQPEMNA	AGKVVSQPE	3	0.2266	4305.5	55.00	0.26	tr_C9ZUI4_
HLA-DQA10501-DQB10201	341	IVRLGRSTLVTTLQM	STLVTTLQM	6	0.2258	4346.2	55.00	0.45	tr_C9ZUI4_
HLA-DQA10501-DQB10201	342	DASIAAPFTCRSRAL	DASIAAPFT	0	0.2256	4356.2	55.00	0.65	tr_C9ZUI4_
HLA-DQA10501-DQB10201	343	IALAYRPLKEEERSK	ALAYRPLKE	1	0.2255	4359.2	55.00	0.32	tr_C9ZUI4_
HLA-DQA10501-DQB10201	344	QTAISVGRDVTILKC	SVGRDVTIL	4	0.2253	4366.6	55.00	0.25	tr_C9ZUI4_
HLA-DQA10501-DQB10201	345	TGDSVQTAISVGRDV	QTAISVGRD	5	0.2253	4368.8	55.00	0.38	tr_C9ZUI4_
HLA-DQA10501-DQB10201	346	AESIPTTTLATLIAQ	PTTTLATLI	4	0.2251	4377.3	55.00	0.35	tr_C9ZUI4_
HLA-DQA10501-DQB10201	347	AVNTALLGLVKQNVF	LLGLVKQNV	5	0.2248	4393.8	55.00	0.25	tr_C9ZUI4_
HLA-DQA10501-DQB10201	348	GFEKQYKILVRFPFL	KILVRFPFL	6	0.2247	4398.0	55.00	0.41	tr_C9ZUI4_
HLA-DQA10501-DQB10201	349	LFSGVDMADGKGLLN	FSGVDMADG	1	0.2239	4433.2	60.00	0.38	tr_C9ZUI4_
HLA-DQA10501-DQB10201	350	ETAEAGKVVSQPEMN	AGKVVSQPE	4	0.2239	4434.0	60.00	0.33	tr_C9ZUI4_
HLA-DQA10501-DQB10201	351	FRIPYAGKVDTCCFD	AGKVDTCCF	5	0.2239	4434.1	60.00	0.28	tr_C9ZUI4_
HLA-DQA10501-DQB10201	352	RFPFLANLRRMPCIV	NLRRMPCIV	6	0.2236	4447.3	60.00	0.18	tr_C9ZUI4_
HLA-DQA10501-DQB10201	353	HGESQMILSGIILTV	ILSGIILTV	6	0.2235	4455.1	60.00	0.31	tr_C9ZUI4_
HLA-DQA10501-DQB10201	354	RRKAEIVQIARWNKQ	AEIVQIARW	3	0.2230	4478.4	60.00	0.28	tr_C9ZUI4_
HLA-DQA10501-DQB10201	355	KKDAKDTIEMLQSGS	DAKDTIEML	2	0.2229	4481.6	60.00	0.42	tr_C9ZUI4_
HLA-DQA10501-DQB10201	356	HKVPSDDEWDLCVNA	KVPSDDEWD	1	0.2228	4489.2	60.00	0.36	tr_C9ZUI4_
HLA-DQA10501-DQB10201	357	FAGLAVFQCPLKKDA	GLAVFQCPL	2	0.2223	4511.1	60.00	0.32	tr_C9ZUI4_
HLA-DQA10501-DQB10201	358	LVTTLQMYKILALNC	MYKILALNC	6	0.2221	4523.3	60.00	0.32	tr_C9ZUI4_
HLA-DQA10501-DQB10201	359	DDTVVYDPPAQKEDI	TVVYDPPAQ	2	0.2217	4539.6	60.00	0.34	tr_C9ZUI4_
HLA-DQA10501-DQB10201	360	SKSQPMPTLCPQRPI	MPTLCPQRP	5	0.2217	4541.6	60.00	0.55	tr_C9ZUI4_
HLA-DQA10501-DQB10201	361	HPYMMCTIFMQFGLH	MMCTIFMQF	3	0.2216	4545.9	60.00	0.23	tr_C9ZUI4_
HLA-DQA10501-DQB10201	362	EAGKVVSQPEMNAPA	KVVSQPEMN	3	0.2213	4560.7	60.00	0.31	tr_C9ZUI4_
HLA-DQA10501-DQB10201	363	EEERSKEAIHNMDRE	EAIHNMDRE	6	0.2211	4569.2	60.00	0.57	tr_C9ZUI4_
HLA-DQA10501-DQB10201	364	QRPITKVFHPYMMCT	ITKVFHPYM	3	0.2207	4592.8	60.00	0.48	tr_C9ZUI4_
HLA-DQA10501-DQB10201	365	EVDDCIVWTDAATGK	IVWTDAATG	5	0.2204	4604.1	60.00	0.37	tr_C9ZUI4_
HLA-DQA10501-DQB10201	366	VRLGRSTLVTTLQMY	STLVTTLQM	5	0.2188	4687.2	60.00	0.40	tr_C9ZUI4_
HLA-DQA10501-DQB10201	367	PFRIPYAGKVDTCCF	AGKVDTCCF	6	0.2187	4692.9	60.00	0.29	tr_C9ZUI4_
HLA-DQA10501-DQB10201	368	QSGSHRCVIITGDSV	CVIITGDSV	6	0.2182	4716.9	60.00	0.56	tr_C9ZUI4_
HLA-DQA10501-DQB10201	369	DDCIVWTDAATGKEV	IVWTDAATG	3	0.2182	4717.4	60.00	0.36	tr_C9ZUI4_
HLA-DQA10501-DQB10201	370	VDDCIVWTDAATGKE	IVWTDAATG	4	0.2182	4718.5	60.00	0.38	tr_C9ZUI4_
HLA-DQA10501-DQB10201	371	QCPLKKDAKDTIEML	DAKDTIEML	6	0.2165	4805.1	60.00	0.81	tr_C9ZUI4_
HLA-DQA10501-DQB10201	372	QMILSGIILTVCFLC	LSGIILTVC	3	0.2154	4862.8	60.00	0.25	tr_C9ZUI4_
HLA-DQA10501-DQB10201	373	ALLGLVKQNVFCTEP	VKQNVFCTE	5	0.2151	4879.9	60.00	0.30	tr_C9ZUI4_
HLA-DQA10501-DQB10201	374	KSQPMPTLCPQRPIT	MPTLCPQRP	4	0.2148	4891.5	60.00	0.56	tr_C9ZUI4_
HLA-DQA10501-DQB10201	375	DFHSVANAHAIKGYR	FHSVANAHA	1	0.2134	4968.6	60.00	0.52	tr_C9ZUI4_
HLA-DQA10501-DQB10201	376	KDAKDTIEMLQSGSH	DAKDTIEML	1	0.2129	4994.9	60.00	0.41	tr_C9ZUI4_
HLA-DQA10501-DQB10201	377	MSKSQPMPTLCPQRP	MPTLCPQRP	6	0.2128	5000.1	60.00	0.49	tr_C9ZUI4_
HLA-DQA10501-DQB10201	378	MILSGIILTVCFLCM	LSGIILTVC	2	0.2127	5008.0	60.00	0.26	tr_C9ZUI4_
HLA-DQA10501-DQB10201	379	PQRPITKVFHPYMMC	ITKVFHPYM	4	0.2118	5052.7	60.00	0.57	tr_C9ZUI4_
HLA-DQA10501-DQB10201	380	CAPTHKEDIVTDLKQ	KEDIVTDLK	5	0.2118	5057.0	60.00	0.38	tr_C9ZUI4_
HLA-DQA10501-DQB10201	381	EADAEGVATMRQAGA	AEGVATMRQ	3	0.2116	5067.2	60.00	0.44	tr_C9ZUI4_

HLA-DQA10501-DQB10201	382	SSMKKKGNGEDEVDD	KKGNGEDEV	4	0.2110	5099.6	60.00	0.35	tr_C9ZUI4_
HLA-DQA10501-DQB10201	383	TLAVNTALLGLVKQN	VNTALLGLV	3	0.2103	5138.2	60.00	0.25	tr_C9ZUI4_
HLA-DQA10501-DQB10201	384	KHGESQMILSGIILT	QMILSGIIL	5	0.2091	5206.8	60.00	0.35	tr_C9ZUI4_
HLA-DQA10501-DQB10201	385	CPQRPITKVFHPYMM	ITKVFHPYM	5	0.2089	5214.6	60.00	0.62	tr_C9ZUI4_
HLA-DQA10501-DQB10201	386	LANLRRMPCIVSAPD	MPCIVSAPD	6	0.2087	5225.6	60.00	0.39	tr_C9ZUI4_
HLA-DQA10501-DQB10201	387	ANLRRMPCIVSAPDG	MPCIVSAPD	5	0.2080	5267.9	60.00	0.41	tr_C9ZUI4_
HLA-DQA10501-DQB10201	388	LAVNTALLGLVKQNV	LLGLVKQNV	6	0.2077	5285.2	60.00	0.29	tr_C9ZUI4_
HLA-DQA10501-DQB10201	389	KILALNCLTSAYSMS	LNCLTSAYS	4	0.2073	5307.0	60.00	0.23	tr_C9ZUI4_
HLA-DQA10501-DQB10201	390	FNADDADMGGAPQVK	FNADDADMG	0	0.2063	5364.9	65.00	0.38	tr_C9ZUI4_
HLA-DQA10501-DQB10201	391	RETAEAGKVVSQPEM	AGKVVSQPE	5	0.2060	5384.6	65.00	0.41	tr_C9ZUI4_
HLA-DQA10501-DQB10201	392	YSEHIAVWARCAPTH	HIAVWARCA	3	0.2055	5410.4	65.00	0.22	tr_C9ZUI4_
HLA-DQA10501-DQB10201	393	GVKHGESQMILSGII	SQMILSGII	6	0.2051	5432.3	65.00	0.34	tr_C9ZUI4_
HLA-DQA10501-DQB10201	394	ILSGIILTVCFLCMS	LSGIILTVC	1	0.2051	5436.4	65.00	0.23	tr_C9ZUI4_
HLA-DQA10501-DQB10201	395	TALLGLVKQNVFCTE	VKQNVFCTE	6	0.2048	5450.9	65.00	0.28	tr_C9ZUI4_
HLA-DQA10501-DQB10201	396	VNTALLGLVKQNVFC	LLGLVKQNV	4	0.2044	5475.2	65.00	0.30	tr_C9ZUI4_
HLA-DQA10501-DQB10201	397	VTILKCRQQLVASSM	RQQLVASSM	6	0.2037	5516.6	65.00	0.78	tr_C9ZUI4_
HLA-DQA10501-DQB10201	398	AGDAMEKASLGALGY	KASLGALGY	6	0.2029	5565.4	65.00	0.44	tr_C9ZUI4_
HLA-DQA10501-DQB10201	399	DCIVWTDAATGKEVN	IVWTDAATG	2	0.2013	5662.7	65.00	0.37	tr_C9ZUI4_
HLA-DQA10501-DQB10201	400	PSADAPFMEQVRHKM	PSADAPFME	0	0.2006	5706.8	65.00	0.26	tr_C9ZUI4_
HLA-DQA10501-DQB10201	401	LQMDGVKHGESQMIL	VKHGESQMI	5	0.2001	5738.0	65.00	0.42	tr_C9ZUI4_
HLA-DQA10501-DQB10201	402	GKVVSQPEMNAPASD	PEMNAPASD	6	0.1987	5825.0	65.00	0.29	tr_C9ZUI4_
HLA-DQA10501-DQB10201	403	DIVRLGRSTLVTTLQ	RSTLVTTLQ	6	0.1986	5830.3	65.00	0.41	tr_C9ZUI4_
HLA-DQA10501-DQB10201	404	LMAGDGTNDVGALKQ	MAGDGTNDV	1	0.1985	5835.4	65.00	0.35	tr_C9ZUI4_
HLA-DQA10501-DQB10201	405	AELVLVTCHSLLQLE	VTCHSLLQL	5	0.1976	5895.6	65.00	0.29	tr_C9ZUI4_
HLA-DQA10501-DQB10201	406	RCAPTHKEDIVTDLK	KEDIVTDLK	6	0.1973	5913.3	65.00	0.46	tr_C9ZUI4_
HLA-DQA10501-DQB10201	407	AGKVVSQPEMNAPAS	KVVSQPEMN	2	0.1973	5914.5	65.00	0.34	tr_C9ZUI4_
HLA-DQA10501-DQB10201	408	QMDGVKHGESQMILS	VKHGESQMI	4	0.1963	5978.2	65.00	0.35	tr_C9ZUI4_
HLA-DQA10501-DQB10201	409	NVFCTEPFRIPYAGK	VFCTEPFRI	1	0.1962	5985.1	65.00	0.49	tr_C9ZUI4_
HLA-DQA10501-DQB10201	410	SQPMPTLCPQRPITK	MPTLCPQRP	3	0.1959	6006.9	65.00	0.56	tr_C9ZUI4_
HLA-DQA10501-DQB10201	411	ADAEGVATMRQAGAE	AEGVATMRQ	2	0.1954	6039.7	65.00	0.44	tr_C9ZUI4_
HLA-DQA10501-DQB10201	412	ALAYRPLKEEERSKE	YRPLKEEER	3	0.1945	6097.0	65.00	0.31	tr_C9ZUI4_
HLA-DQA10501-DQB10201	413	AEIVQIARWNKQLEE	ARWNKQLEE	6	0.1942	6113.6	65.00	0.38	tr_C9ZUI4_
HLA-DQA10501-DQB10201	414	DGVKHGESQMILSGI	ESQMILSGI	6	0.1938	6143.3	65.00	0.34	tr_C9ZUI4_
HLA-DQA10501-DQB10201	415	DTVVYDPPAQKEDIK	TVVYDPPAQ	1	0.1934	6167.4	65.00	0.38	tr_C9ZUI4_
HLA-DQA10501-DQB10201	416	EVNLDRRSILAKTFV	RRSILAKTF	5	0.1930	6198.5	65.00	0.50	tr_C9ZUI4_
HLA-DQA10501-DQB10201	417	STLVTTLQMYKILAL	LQMYKILAL	6	0.1927	6216.5	65.00	0.31	tr_C9ZUI4_
HLA-DQA10501-DQB10201	418	VLMAGDGTNDVGALK	MAGDGTNDV	2	0.1926	6219.2	65.00	0.53	tr_C9ZUI4_
HLA-DQA10501-DQB10201	419	LCPQRPITKVFHPYM	ITKVFHPYM	6	0.1926	6224.9	65.00	0.75	tr_C9ZUI4_
HLA-DQA10501-DQB10201	420	NADDADMGGAPQVKL	MGGAPQVKL	6	0.1925	6227.0	65.00	0.34	tr_C9ZUI4_
HLA-DQA10501-DQB10201	421	FPFLANLRRMPCIVS	NLRRMPCIV	5	0.1924	6234.9	65.00	0.21	tr_C9ZUI4_
HLA-DQA10501-DQB10201	422	TAISVGRDVTILKCR	SVGRDVTIL	3	0.1924	6237.9	65.00	0.29	tr_C9ZUI4_
HLA-DQA10501-DQB10201	423	ELVLVTCHSLLQLEG	VTCHSLLQL	4	0.1924	6238.0	65.00	0.25	tr_C9ZUI4_
HLA-DQA10501-DQB10201	424	KAELVLVTCHSLLQL	VTCHSLLQL	6	0.1922	6250.5	65.00	0.32	tr_C9ZUI4_
HLA-DQA10501-DQB10201	425	DAMEKASLGALGYRV	KASLGALGY	4	0.1907	6348.1	65.00	0.28	tr_C9ZUI4_
HLA-DQA10501-DQB10201	426	RIPYAGKVDTCCFDK	AGKVDTCCF	4	0.1904	6372.2	65.00	0.29	tr_C9ZUI4_
HLA-DQA10501-DQB10201	427	LGRSTLVTTLQMYKI	STLVTTLQM	3	0.1901	6390.4	65.00	0.23	tr_C9ZUI4_
HLA-DQA10501-DQB10201	428	APFTCRSRALTSVCD	SRALTSVCD	6	0.1901	6396.0	65.00	0.64	tr_C9ZUI4_
HLA-DQA10501-DQB10201	429	DAEGVATMRQAGAEG	AEGVATMRQ	1	0.1900	6399.4	65.00	0.40	tr_C9ZUI4_
HLA-DQA10501-DQB10201	430	TEPFRIPYAGKVDTC	PYAGKVDTC	6	0.1884	6512.7	70.00	0.23	tr_C9ZUI4_
HLA-DQA10501-DQB10201	431	APTHKEDIVTDLKQR	KEDIVTDLK	4	0.1879	6545.2	70.00	0.35	tr_C9ZUI4_

HLA-DQA10501-DQB10201	432	ASSMKKKGNGEDEVD	KKGNGEDEV	5	0.1876	6571.6	70.00	0.44	tr_C9ZUI4_
HLA-DQA10501-DQB10201	433	ALTSVCDIVRLGRST	TSVCDIVRL	2	0.1875	6577.4	70.00	0.32	tr_C9ZUI4_
HLA-DQA10501-DQB10201	434	CIVWTDAATGKEVNL	WTDAATGKE	3	0.1873	6589.4	70.00	0.32	tr_C9ZUI4_
HLA-DQA10501-DQB10201	435	IVWTDAATGKEVNLD	WTDAATGKE	2	0.1869	6614.7	70.00	0.33	tr_C9ZUI4_
HLA-DQA10501-DQB10201	436	RKAEIVQIARWNKQL	AEIVQIARW	2	0.1868	6624.1	70.00	0.41	tr_C9ZUI4_
HLA-DQA10501-DQB10201	437	EPFRIPYAGKVDTCC	PYAGKVDTC	5	0.1867	6630.0	70.00	0.28	tr_C9ZUI4_
HLA-DQA10501-DQB10201	438	RLGRSTLVTTLQMYK	STLVTTLQM	4	0.1867	6630.2	70.00	0.34	tr_C9ZUI4_
HLA-DQA10501-DQB10201	439	NLRRMPCIVSAPDGK	MPCIVSAPD	4	0.1867	6632.6	70.00	0.39	tr_C9ZUI4_
HLA-DQA10501-DQB10201	440	FHSVANAHAIKGYRV	AHAIKGYRV	6	0.1867	6635.5	70.00	0.31	tr_C9ZUI4_
HLA-DQA10501-DQB10201	441	DAKDTIEMLQSGSHR	DAKDTIEML	0	0.1865	6648.8	70.00	0.33	tr_C9ZUI4_
HLA-DQA10501-DQB10201	442	PTHKEDIVTDLKQRE	KEDIVTDLK	3	0.1860	6679.6	70.00	0.30	tr_C9ZUI4_
HLA-DQA10501-DQB10201	443	LSGIILTVCFLCMSK	GIILTVCFL	2	0.1853	6730.7	70.00	0.19	tr_C9ZUI4_
HLA-DQA10501-DQB10201	444	IPYAGKVDTCCFDKT	AGKVDTCCF	3	0.1852	6740.7	70.00	0.28	tr_C9ZUI4_
HLA-DQA10501-DQB10201	445	NTALLGLVKQNVFCT	LLGLVKQNV	3	0.1846	6783.9	70.00	0.33	tr_C9ZUI4_
HLA-DQA10501-DQB10201	446	LRRMPCIVSAPDGKY	MPCIVSAPD	3	0.1845	6794.8	70.00	0.38	tr_C9ZUI4_
HLA-DQA10501-DQB10201	447	EIVQIARWNKQLEES	ARWNKQLEE	5	0.1838	6843.9	70.00	0.41	tr_C9ZUI4_
HLA-DQA10501-DQB10201	448	QPMPTLCPQRPITKV	MPTLCPQRP	2	0.1831	6899.4	70.00	0.57	tr_C9ZUI4_
HLA-DQA10501-DQB10201	449	FVQTRRHKVPSDDEW	HKVPSDDEW	6	0.1830	6905.3	70.00	0.41	tr_C9ZUI4_
HLA-DQA10501-DQB10201	450	CESIPPDFHSVANAH	DFHSVANAH	6	0.1830	6906.4	70.00	0.43	tr_C9ZUI4_
HLA-DQA10501-DQB10201	451	TLVTTLQMYKILALN	LQMYKILAL	5	0.1829	6908.3	70.00	0.29	tr_C9ZUI4_
HLA-DQA10501-DQB10201	452	GDAMEKASLGALGYR	KASLGALGY	5	0.1816	7007.5	70.00	0.37	tr_C9ZUI4_
HLA-DQA10501-DQB10201	453	AISVGRDVTILKCRQ	SVGRDVTIL	2	0.1806	7087.8	70.00	0.28	tr_C9ZUI4_
HLA-DQA10501-DQB10201	454	PPKAELVLVTCHSLL	ELVLVTCHS	4	0.1799	7142.4	70.00	0.22	tr_C9ZUI4_
HLA-DQA10501-DQB10201	455	KEVNLDRRSILAKTF	RRSILAKTF	6	0.1798	7148.7	70.00	0.68	tr_C9ZUI4_
HLA-DQA10501-DQB10201	456	TVPPKAELVLVTCHS	ELVLVTCHS	6	0.1782	7271.1	70.00	0.27	tr_C9ZUI4_
HLA-DQA10501-DQB10201	457	AIHNMDREDCEKNLI	HNMDREDCE	2	0.1782	7274.8	70.00	0.32	tr_C9ZUI4_
HLA-DQA10501-DQB10201	458	MDGVKHGESQMILSG	VKHGESQMI	3	0.1780	7285.5	70.00	0.34	tr_C9ZUI4_
HLA-DQA10501-DQB10201	459	PFLANLRRMPCIVSA	NLRRMPCIV	4	0.1771	7358.0	70.00	0.26	tr_C9ZUI4_
HLA-DQA10501-DQB10201	460	GRSTLVTTLQMYKIL	STLVTTLQM	2	0.1769	7371.7	70.00	0.26	tr_C9ZUI4_
HLA-DQA10501-DQB10201	461	LKTVPPKAELVLVTC	PKAELVLVT	5	0.1768	7384.5	70.00	0.34	tr_C9ZUI4_
HLA-DQA10501-DQB10201	462	DHKIPPGFKLTVVPP	GFKLTVVPP	6	0.1765	7408.2	70.00	0.46	tr_C9ZUI4_
HLA-DQA10501-DQB10201	463	VPPKAELVLVTCHSL	ELVLVTCHS	5	0.1761	7441.4	70.00	0.25	tr_C9ZUI4_
HLA-DQA10501-DQB10201	464	RRMPCIVSAPDGKYV	MPCIVSAPD	2	0.1756	7475.7	70.00	0.38	tr_C9ZUI4_
HLA-DQA10501-DQB10201	465	APDGKYVVAKGSPEA	YVVAKGSPE	5	0.1750	7530.1	70.00	0.51	tr_C9ZUI4_
HLA-DQA10501-DQB10201	466	CDIVRLGRSTLVTTL	GRSTLVTTL	6	0.1750	7530.7	70.00	0.38	tr_C9ZUI4_
HLA-DQA10501-DQB10201	467	AMEKASLGALGYRVN	KASLGALGY	3	0.1750	7530.7	70.00	0.33	tr_C9ZUI4_
HLA-DQA10501-DQB10201	468	GAPQVKLGDASIAAP	LGDASIAAP	6	0.1747	7551.6	70.00	0.29	tr_C9ZUI4_
HLA-DQA10501-DQB10201	469	THKEDIVTDLKQREH	KEDIVTDLK	2	0.1746	7560.1	70.00	0.30	tr_C9ZUI4_
HLA-DQA10501-DQB10201	470	KAEIVQIARWNKQLE	AEIVQIARW	1	0.1741	7599.3	70.00	0.47	tr_C9ZUI4_
HLA-DQA10501-DQB10201	471	ARCAPTHKEDIVTDL	HKEDIVTDL	6	0.1739	7621.1	70.00	0.57	tr_C9ZUI4_
HLA-DQA10501-DQB10201	472	EDPNRDRWKLFLACI	RWKLFLACI	6	0.1738	7623.4	70.00	0.67	tr_C9ZUI4_
HLA-DQA10501-DQB10201	473	SEHIAVWARCAPTHK	HIAVWARCA	2	0.1734	7662.4	70.00	0.23	tr_C9ZUI4_
HLA-DQA10501-DQB10201	474	MEKASLGALGYRVNI	SLGALGYRV	4	0.1733	7668.1	70.00	0.24	tr_C9ZUI4_
HLA-DQA10501-DQB10201	475	TILKCRQQLVASSMK	RQQLVASSM	5	0.1733	7670.7	70.00	0.64	tr_C9ZUI4_
HLA-DQA10501-DQB10201	476	VFCTEPFRIPYAGKV	VFCTEPFRI	0	0.1730	7694.4	70.00	0.38	tr_C9ZUI4_
HLA-DQA10501-DQB10201	477	SRETAEAGKVVSQPE	AGKVVSQPE	6	0.1722	7760.5	70.00	0.58	tr_C9ZUI4_
HLA-DQA10501-DQB10201	478	VNLDRRSILAKTFVQ	RRSILAKTF	4	0.1720	7776.9	70.00	0.49	tr_C9ZUI4_
HLA-DQA10501-DQB10201	479	TLKTVPPKAELVLVT	PKAELVLVT	6	0.1717	7800.3	75.00	0.44	tr_C9ZUI4_
HLA-DQA10501-DQB10201	480	LCVNAESIPTTTLAT	VNAESIPTT	2	0.1716	7809.4	75.00	0.28	tr_C9ZUI4_
HLA-DQA10501-DQB10201	481	KTVPPKAELVLVTCH	PKAELVLVT	4	0.1714	7828.3	75.00	0.28	tr_C9ZUI4_

HLA-DQA10501-DQB10201	482	ADDADMGGAPQVKLG	MGGAPQVKL	5	0.1711	7848.2	75.00	0.31	tr_C9ZUI4_
HLA-DQA10501-DQB10201	483	SGIILTVCFLCMSKS	GIILTVCFL	1	0.1709	7868.4	75.00	0.22	tr_C9ZUI4_
HLA-DQA10501-DQB10201	484	PKAELVLVTCHSLLQ	ELVLVTCHS	3	0.1708	7875.9	75.00	0.23	tr_C9ZUI4_
HLA-DQA10501-DQB10201	485	FLANLRRMPCIVSAP	NLRRMPCIV	3	0.1696	7983.3	75.00	0.34	tr_C9ZUI4_
HLA-DQA10501-DQB10201	486	TSVCDIVRLGRSTLV	VRLGRSTLV	6	0.1695	7988.5	75.00	0.42	tr_C9ZUI4_
HLA-DQA10501-DQB10201	487	DDADMGGAPQVKLGD	MGGAPQVKL	4	0.1685	8072.5	75.00	0.32	tr_C9ZUI4_
HLA-DQA10501-DQB10201	488	RSTLVTTLQMYKILA	STLVTTLQM	1	0.1684	8082.1	75.00	0.28	tr_C9ZUI4_
HLA-DQA10501-DQB10201	489	SGFEKQYKILVRFPF	QYKILVRFP	5	0.1679	8130.7	75.00	0.37	tr_C9ZUI4_
HLA-DQA10501-DQB10201	490	MDREDCEKNLIFAGL	EKNLIFAGL	6	0.1663	8268.1	75.00	0.62	tr_C9ZUI4_
HLA-DQA10501-DQB10201	491	FSGVDMADGKGLLNT	FSGVDMADG	0	0.1659	8302.9	75.00	0.37	tr_C9ZUI4_
HLA-DQA10501-DQB10201	492	CTEPFRIPYAGKVDT	IPYAGKVDT	6	0.1642	8458.8	75.00	0.28	tr_C9ZUI4_
HLA-DQA10501-DQB10201	493	PMPTLCPQRPITKVF	MPTLCPQRP	1	0.1642	8459.0	75.00	0.57	tr_C9ZUI4_
HLA-DQA10501-DQB10201	494	VLQMDGVKHGESQMI	VKHGESQMI	6	0.1639	8489.1	75.00	0.53	tr_C9ZUI4_
HLA-DQA10501-DQB10201	495	EHIAVWARCAPTHKE	HIAVWARCA	1	0.1628	8588.1	75.00	0.22	tr_C9ZUI4_
HLA-DQA10501-DQB10201	496	ILTVCFLCMSKSQPM	FLCMSKSQP	5	0.1626	8612.0	75.00	0.19	tr_C9ZUI4_
HLA-DQA10501-DQB10201	497	NLDRRSILAKTFVQT	RRSILAKTF	3	0.1624	8625.1	75.00	0.44	tr_C9ZUI4_
HLA-DQA10501-DQB10201	498	TVVYDPPAQKEDIKG	VVYDPPAQK	1	0.1624	8626.7	75.00	0.41	tr_C9ZUI4_
HLA-DQA10501-DQB10201	499	MAGDGTNDVGALKQA	TNDVGALKQ	5	0.1613	8728.5	75.00	0.34	tr_C9ZUI4_
HLA-DQA10501-DQB10201	500	VWTDAATGKEVNLDR	WTDAATGKE	1	0.1608	8778.5	75.00	0.32	tr_C9ZUI4_
HLA-DQA10501-DQB10201	501	TVCFLCMSKSQPMPT	MSKSQPMPT	6	0.1607	8788.2	75.00	0.26	tr_C9ZUI4_
HLA-DQA10501-DQB10201	502	RMPCIVSAPDGKYVV	MPCIVSAPD	1	0.1606	8794.0	75.00	0.39	tr_C9ZUI4_
HLA-DQA10501-DQB10201	503	PYAGKVDTCCFDKTG	AGKVDTCCF	2	0.1603	8824.8	75.00	0.29	tr_C9ZUI4_
HLA-DQA10501-DQB10201	504	CVNAESIPTTTLATL	IPTTTLATL	6	0.1602	8839.2	75.00	0.33	tr_C9ZUI4_
HLA-DQA10501-DQB10201	505	VTDLKQREHMVLMAG	REHMVLMAG	6	0.1598	8873.9	75.00	0.34	tr_C9ZUI4_
HLA-DQA10501-DQB10201	506	GIILTVCFLCMSKSQ	GIILTVCFL	0	0.1596	8894.6	75.00	0.18	tr_C9ZUI4_
HLA-DQA10501-DQB10201	507	AGLAVFQCPLKKDAK	GLAVFQCPL	1	0.1594	8913.7	75.00	0.40	tr_C9ZUI4_
HLA-DQA10501-DQB10201	508	HKEDIVTDLKQREHM	KEDIVTDLK	1	0.1592	8929.5	75.00	0.29	tr_C9ZUI4_
HLA-DQA10501-DQB10201	509	IHNMDREDCEKNLIF	HNMDREDCE	1	0.1582	9031.5	75.00	0.29	tr_C9ZUI4_
HLA-DQA10501-DQB10201	510	LAYRPLKEEERSKEA	YRPLKEEER	2	0.1580	9049.1	75.00	0.41	tr_C9ZUI4_
HLA-DQA10501-DQB10201	511	IVQIARWNKQLEESK	ARWNKQLEE	4	0.1572	9129.3	75.00	0.34	tr_C9ZUI4_
HLA-DQA10501-DQB10201	512	LTSVCDIVRLGRSTL	TSVCDIVRL	1	0.1570	9147.6	75.00	0.34	tr_C9ZUI4_
HLA-DQA10501-DQB10201	513	IILTVCFLCMSKSQP	ILTVCFLCM	1	0.1565	9197.2	75.00	0.25	tr_C9ZUI4_
HLA-DQA10501-DQB10201	514	LTVCFLCMSKSQPMP	FLCMSKSQP	4	0.1561	9233.7	75.00	0.25	tr_C9ZUI4_
HLA-DQA10501-DQB10201	515	IVTDLKQREHMVLMA	QREHMVLMA	6	0.1544	9410.1	75.00	0.43	tr_C9ZUI4_
HLA-DQA10501-DQB10201	516	FCTEPFRIPYAGKVD	TEPFRIPYA	2	0.1537	9482.7	80.00	0.25	tr_C9ZUI4_
HLA-DQA10501-DQB10201	517	SADAPFMEQVRHKMA	FMEQVRHKM	5	0.1536	9493.1	80.00	0.24	tr_C9ZUI4_
HLA-DQA10501-DQB10201	518	LDRRSILAKTFVQTR	RRSILAKTF	2	0.1527	9581.0	80.00	0.41	tr_C9ZUI4_
HLA-DQA10501-DQB10201	519	ISVGRDVTILKCRQQ	SVGRDVTIL	1	0.1522	9634.6	80.00	0.31	tr_C9ZUI4_
HLA-DQA10501-DQB10201	520	SVCDIVRLGRSTLVT	VRLGRSTLV	5	0.1516	9700.1	80.00	0.44	tr_C9ZUI4_
HLA-DQA10501-DQB10201	521	NSGFEKQYKILVRFP	QYKILVRFP	6	0.1512	9734.4	80.00	0.46	tr_C9ZUI4_
HLA-DQA10501-DQB10201	522	NTLKTVPPKAELVLV	PPKAELVLV	6	0.1509	9767.5	80.00	0.34	tr_C9ZUI4_
HLA-DQA10501-DQB10201	523	LNATSMDASQNVGGS	TSMDASQNV	3	0.1507	9790.7	80.00	0.32	tr_C9ZUI4_
HLA-DQA10501-DQB10201	524	DADMGGAPQVKLGDA	MGGAPQVKL	3	0.1504	9821.6	80.00	0.37	tr_C9ZUI4_
HLA-DQA10501-DQB10201	525	SVANAHAIKGYRVIA	AHAIKGYRV	4	0.1489	9981.6	80.00	0.47	tr_C9ZUI4_
HLA-DQA10501-DQB10201	526	MLQSGSHRCVIITGD	HRCVIITGD	6	0.1485	10031.8	80.00	0.60	tr_C9ZUI4_
HLA-DQA10501-DQB10201	527	VCDIVRLGRSTLVTT	VRLGRSTLV	4	0.1473	10161.6	80.00	0.41	tr_C9ZUI4_
HLA-DQA10501-DQB10201	528	VCFLCMSKSQPMPTL	MSKSQPMPT	5	0.1467	10220.0	80.00	0.25	tr_C9ZUI4_
HLA-DQA10501-DQB10201	529	HIAVWARCAPTHKED	VWARCAPTH	3	0.1452	10393.6	80.00	0.18	tr_C9ZUI4_
HLA-DQA10501-DQB10201	530	WTDAATGKEVNLDRR	WTDAATGKE	0	0.1451	10401.8	80.00	0.23	tr_C9ZUI4_
HLA-DQA10501-DQB10201	531	ILKCRQQLVASSMKK	RQQLVASSM	4	0.1447	10442.8	80.00	0.62	tr_C9ZUI4_

HLA-DQA10501-DQB10201	532	LQSGSHRCVIITGDS	HRCVIITGD	5	0.1419	10764.3	80.00	0.58	tr_C9ZUI4_
HLA-DQA10501-DQB10201	533	HSVANAHAIKGYRVI	AHAIKGYRV	5	0.1412	10851.1	80.00	0.49	tr_C9ZUI4_
HLA-DQA10501-DQB10201	534	DRRSILAKTFVQTRR	RRSILAKTF	1	0.1411	10867.7	80.00	0.36	tr_C9ZUI4_
HLA-DQA10501-DQB10201	535	GDGTNDVGALKQAHA	TNDVGALKQ	3	0.1402	10971.8	80.00	0.31	tr_C9ZUI4_
HLA-DQA10501-DQB10201	536	MPCIVSAPDGKYVVA	MPCIVSAPD	0	0.1386	11154.8	80.00	0.29	tr_C9ZUI4_
HLA-DQA10501-DQB10201	537	GKGLLNTLKTVPPKA	TLKTVPPKA	6	0.1386	11160.1	80.00	0.42	tr_C9ZUI4_
HLA-DQA10501-DQB10201	538	CFLCMSKSQPMPTLC	MSKSQPMPT	4	0.1383	11198.2	80.00	0.24	tr_C9ZUI4_
HLA-DQA10501-DQB10201	539	GGAPQVKLGDASIAA	VKLGDASIA	5	0.1382	11205.3	80.00	0.34	tr_C9ZUI4_
HLA-DQA10501-DQB10201	540	SVGRDVTILKCRQQL	SVGRDVTIL	0	0.1381	11225.5	80.00	0.23	tr_C9ZUI4_
HLA-DQA10501-DQB10201	541	SAPDGKYVVAKGSPE	YVVAKGSPE	6	0.1371	11342.4	80.00	0.69	tr_C9ZUI4_
HLA-DQA10501-DQB10201	542	VGRDVTILKCRQQLV	ILKCRQQLV	6	0.1371	11343.5	80.00	0.34	tr_C9ZUI4_
HLA-DQA10501-DQB10201	543	VQIARWNKQLEESKK	ARWNKQLEE	3	0.1367	11392.7	80.00	0.33	tr_C9ZUI4_
HLA-DQA10501-DQB10201	544	KEDIVTDLKQREHMV	IVTDLKQRE	3	0.1364	11423.9	80.00	0.31	tr_C9ZUI4_
HLA-DQA10501-DQB10201	545	SGVDMADGKGLLNTL	DGKGLLNTL	6	0.1352	11576.6	80.00	0.22	tr_C9ZUI4_
HLA-DQA10501-DQB10201	546	EDIVTDLKQREHMVL	IVTDLKQRE	2	0.1346	11648.3	80.00	0.29	tr_C9ZUI4_
HLA-DQA10501-DQB10201	547	KGLLNTLKTVPPKAE	TLKTVPPKA	5	0.1342	11705.3	85.00	0.44	tr_C9ZUI4_
HLA-DQA10501-DQB10201	548	LLNTLKTVPPKAELV	TLKTVPPKA	3	0.1327	11902.9	85.00	0.47	tr_C9ZUI4_
HLA-DQA10501-DQB10201	549	ADGKGLLNTLKTVPP	LNTLKTVPP	6	0.1326	11909.1	85.00	0.23	tr_C9ZUI4_
HLA-DQA10501-DQB10201	550	GLLNTLKTVPPKAEL	TLKTVPPKA	4	0.1322	11966.0	85.00	0.47	tr_C9ZUI4_
HLA-DQA10501-DQB10201	551	AYRPLKEEERSKEAI	YRPLKEEER	1	0.1321	11979.9	85.00	0.41	tr_C9ZUI4_
HLA-DQA10501-DQB10201	552	FLCMSKSQPMPTLCP	MSKSQPMPT	3	0.1318	12016.5	85.00	0.32	tr_C9ZUI4_
HLA-DQA10501-DQB10201	553	DGTNDVGALKQAHAG	TNDVGALKQ	2	0.1302	12229.2	85.00	0.31	tr_C9ZUI4_
HLA-DQA10501-DQB10201	554	SVLQMDGVKHGESQM	VLQMDGVKH	1	0.1301	12240.3	85.00	0.32	tr_C9ZUI4_
HLA-DQA10501-DQB10201	555	ADMGGAPQVKLGDAS	MGGAPQVKL	2	0.1294	12333.9	85.00	0.38	tr_C9ZUI4_
HLA-DQA10501-DQB10201	556	LNTLKTVPPKAELVL	TLKTVPPKA	2	0.1288	12414.5	85.00	0.45	tr_C9ZUI4_
HLA-DQA10501-DQB10201	557	GTNDVGALKQAHAGI	VGALKQAHA	4	0.1283	12479.0	85.00	0.29	tr_C9ZUI4_
HLA-DQA10501-DQB10201	558	GRDVTILKCRQQLVA	ILKCRQQLV	5	0.1281	12503.8	85.00	0.35	tr_C9ZUI4_
HLA-DQA10501-DQB10201	559	YAGKVDTCCFDKTGT	AGKVDTCCF	1	0.1279	12534.1	85.00	0.37	tr_C9ZUI4_
HLA-DQA10501-DQB10201	560	MGGAPQVKLGDASIA	VKLGDASIA	6	0.1273	12606.5	85.00	0.41	tr_C9ZUI4_
HLA-DQA10501-DQB10201	561	DIVTDLKQREHMVLM	IVTDLKQRE	1	0.1267	12693.8	85.00	0.28	tr_C9ZUI4_
HLA-DQA10501-DQB10201	562	AGDGTNDVGALKQAH	TNDVGALKQ	4	0.1263	12743.1	85.00	0.47	tr_C9ZUI4_
HLA-DQA10501-DQB10201	563	HNMDREDCEKNLIFA	NMDREDCEK	1	0.1255	12857.2	85.00	0.25	tr_C9ZUI4_
HLA-DQA10501-DQB10201	564	QIARWNKQLEESKKS	ARWNKQLEE	2	0.1244	13007.5	85.00	0.34	tr_C9ZUI4_
HLA-DQA10501-DQB10201	565	TFVQTRRHKVPSDDE	RHKVPSDDE	6	0.1244	13011.5	85.00	0.64	tr_C9ZUI4_
HLA-DQA10501-DQB10201	566	AKDTIEMLQSGSHRC	KDTIEMLQS	1	0.1232	13186.3	85.00	0.23	tr_C9ZUI4_
HLA-DQA10501-DQB10201	567	NDVGALKQAHAGIAV	KQAHAGIAV	6	0.1231	13200.3	85.00	0.28	tr_C9ZUI4_
HLA-DQA10501-DQB10201	568	CMSKSQPMPTLCPQR	PMPTLCPQR	6	0.1230	13218.6	85.00	0.22	tr_C9ZUI4_
HLA-DQA10501-DQB10201	569	IAVWARCAPTHKEDI	WARCAPTHK	3	0.1229	13223.9	85.00	0.25	tr_C9ZUI4_
HLA-DQA10501-DQB10201	570	VDMADGKGLLNTLKT	KGLLNTLKT	6	0.1223	13314.3	85.00	0.25	tr_C9ZUI4_
HLA-DQA10501-DQB10201	571	RRSILAKTFVQTRRH	RRSILAKTF	0	0.1213	13462.2	85.00	0.22	tr_C9ZUI4_
HLA-DQA10501-DQB10201	572	KEEERSKEAIHNMDR	SKEAIHNMD	5	0.1204	13583.9	85.00	0.41	tr_C9ZUI4_
HLA-DQA10501-DQB10201	573	NATSMDASQNVGGSG	TSMDASQNV	2	0.1203	13611.3	85.00	0.47	tr_C9ZUI4_
HLA-DQA10501-DQB10201	574	DMGGAPQVKLGDASI	QVKLGDASI	6	0.1201	13629.9	85.00	0.32	tr_C9ZUI4_
HLA-DQA10501-DQB10201	575	GVDMADGKGLLNTLK	DMADGKGLL	2	0.1192	13760.6	85.00	0.21	tr_C9ZUI4_
HLA-DQA10501-DQB10201	576	LKCRQQLVASSMKKK	RQQLVASSM	3	0.1184	13883.5	85.00	0.63	tr_C9ZUI4_
HLA-DQA10501-DQB10201	577	TNDVGALKQAHAGIA	VGALKQAHA	3	0.1181	13931.7	85.00	0.33	tr_C9ZUI4_
HLA-DQA10501-DQB10201	578	ADAPFMEQVRHKMAQ	FMEQVRHKM	4	0.1179	13969.2	85.00	0.34	tr_C9ZUI4_
HLA-DQA10501-DQB10201	579	LKEEERSKEAIHNMD	SKEAIHNMD	6	0.1178	13977.1	85.00	0.47	tr_C9ZUI4_
HLA-DQA10501-DQB10201	580	VASSMKKKGNGEDEV	KKGNGEDEV	6	0.1177	13990.0	85.00	0.73	tr_C9ZUI4_
HLA-DQA10501-DQB10201	581	GLAVFQCPLKKDAKD	GLAVFQCPL	0	0.1172	14070.9	85.00	0.41	tr_C9ZUI4_

HLA-DQA10501-DQB10201	582	DGKGLLNTLKTVPPK	LNTLKTVPP	5	0.1171	14091.3	85.00	0.23	tr_C9ZUI4_
HLA-DQA10501-DQB10201	583	RDVTILKCRQQLVAS	ILKCRQQLV	4	0.1162	14214.9	85.00	0.38	tr_C9ZUI4_
HLA-DQA10501-DQB10201	584	AATGKEVNLDRRSIL	VNLDRRSIL	6	0.1157	14292.1	85.00	0.23	tr_C9ZUI4_
HLA-DQA10501-DQB10201	585	ATGKEVNLDRRSILA	VNLDRRSIL	5	0.1156	14317.4	85.00	0.20	tr_C9ZUI4_
HLA-DQA10501-DQB10201	586	KDHKIPPGFKLTVVP	PPGFKLTVV	5	0.1155	14322.6	85.00	0.47	tr_C9ZUI4_
HLA-DQA10501-DQB10201	587	MPTLCPQRPITKVFH	MPTLCPQRP	0	0.1153	14361.1	85.00	0.44	tr_C9ZUI4_
HLA-DQA10501-DQB10201	588	MADGKGLLNTLKTVP	KGLLNTLKT	4	0.1145	14490.0	85.00	0.25	tr_C9ZUI4_
HLA-DQA10501-DQB10201	589	TDAATGKEVNLDRRS	ATGKEVNLD	3	0.1142	14525.2	85.00	0.29	tr_C9ZUI4_
HLA-DQA10501-DQB10201	590	DMADGKGLLNTLKTV	KGLLNTLKT	5	0.1139	14584.9	90.00	0.25	tr_C9ZUI4_
HLA-DQA10501-DQB10201	591	NNSGFEKQYKILVRF	KQYKILVRF	6	0.1131	14714.2	90.00	0.43	tr_C9ZUI4_
HLA-DQA10501-DQB10201	592	DTCCFDKTGTLTTDE	KTGTLTTDE	6	0.1125	14804.9	90.00	0.54	tr_C9ZUI4_
HLA-DQA10501-DQB10201	593	DAATGKEVNLDRRSI	ATGKEVNLD	2	0.1122	14852.3	90.00	0.29	tr_C9ZUI4_
HLA-DQA10501-DQB10201	594	KSRETAEAGKVVSQP	EAGKVVSQP	6	0.1118	14921.5	90.00	0.28	tr_C9ZUI4_
HLA-DQA10501-DQB10201	595	DVTILKCRQQLVASS	ILKCRQQLV	3	0.1111	15032.7	90.00	0.35	tr_C9ZUI4_
HLA-DQA10501-DQB10201	596	LCMSKSQPMPTLCPQ	MSKSQPMPT	2	0.1111	15033.8	90.00	0.37	tr_C9ZUI4_
HLA-DQA10501-DQB10201	597	WARCAPTHKEDIVTD	THKEDIVTD	6	0.1088	15412.8	90.00	0.35	tr_C9ZUI4_
HLA-DQA10501-DQB10201	598	DAPFMEQVRHKMAQA	FMEQVRHKM	3	0.1084	15479.8	90.00	0.38	tr_C9ZUI4_
HLA-DQA10501-DQB10201	599	KCRQQLVASSMKKKG	RQQLVASSM	2	0.1079	15556.1	90.00	0.65	tr_C9ZUI4_
HLA-DQA10501-DQB10201	600	GEHNSNEPHNEPDVP	EPHNEPDVP	6	0.1077	15592.3	90.00	0.44	tr_C9ZUI4_
HLA-DQA10501-DQB10201	601	IARWNKQLEESKKSR	ARWNKQLEE	1	0.1071	15696.4	90.00	0.33	tr_C9ZUI4_
HLA-DQA10501-DQB10201	602	ATSMDASQNVGGSGE	TSMDASQNV	1	0.1046	16120.7	90.00	0.50	tr_C9ZUI4_
HLA-DQA10501-DQB10201	603	PCIVSAPDGKYVVAK	IVSAPDGKY	2	0.1025	16496.3	90.00	0.39	tr_C9ZUI4_
HLA-DQA10501-DQB10201	604	RHKMAQARRKAEIVQ	QARRKAEIV	5	0.1023	16521.9	90.00	0.60	tr_C9ZUI4_
HLA-DQA10501-DQB10201	605	AVWARCAPTHKEDIV	WARCAPTHK	2	0.1017	16630.4	90.00	0.31	tr_C9ZUI4_
HLA-DQA10501-DQB10201	606	CRQQLVASSMKKKGN	RQQLVASSM	1	0.1005	16848.4	90.00	0.64	tr_C9ZUI4_
HLA-DQA10501-DQB10201	607	VRHKMAQARRKAEIV	QARRKAEIV	6	0.0993	17081.0	90.00	0.67	tr_C9ZUI4_
HLA-DQA10501-DQB10201	608	AGKVDTCCFDKTGTL	AGKVDTCCF	0	0.0990	17133.4	90.00	0.31	tr_C9ZUI4_
HLA-DQA10501-DQB10201	609	HNSNEPHNEPDVPKD	EPHNEPDVP	4	0.0983	17257.6	90.00	0.43	tr_C9ZUI4_
HLA-DQA10501-DQB10201	610	ASIAAPFTCRSRALT	IAAPFTCRS	2	0.0974	17426.3	90.00	0.28	tr_C9ZUI4_
HLA-DQA10501-DQB10201	611	KDTIEMLQSGSHRCV	IEMLQSGSH	3	0.0971	17482.8	90.00	0.29	tr_C9ZUI4_
HLA-DQA10501-DQB10201	612	TGKEVNLDRRSILAK	VNLDRRSIL	4	0.0965	17596.1	90.00	0.22	tr_C9ZUI4_
HLA-DQA10501-DQB10201	613	EHNSNEPHNEPDVPK	EPHNEPDVP	5	0.0964	17626.6	90.00	0.38	tr_C9ZUI4_
HLA-DQA10501-DQB10201	614	ESKKSRETAEAGKVV	ETAEAGKVV	6	0.0962	17649.1	90.00	0.30	tr_C9ZUI4_
HLA-DQA10501-DQB10201	615	GKEVNLDRRSILAKT	DRRSILAKT	6	0.0958	17743.5	90.00	0.24	tr_C9ZUI4_
HLA-DQA10501-DQB10201	616	VVYDPPAQKEDIKGK	VVYDPPAQK	0	0.0954	17811.0	90.00	0.47	tr_C9ZUI4_
HLA-DQA10501-DQB10201	617	APFMEQVRHKMAQAR	FMEQVRHKM	2	0.0951	17864.8	90.00	0.36	tr_C9ZUI4_
HLA-DQA10501-DQB10201	618	KKSRETAEAGKVVSQ	ETAEAGKVV	4	0.0950	17884.4	90.00	0.25	tr_C9ZUI4_
HLA-DQA10501-DQB10201	619	AAPFTCRSRALTSVC	RSRALTSVC	6	0.0946	17968.0	90.00	0.34	tr_C9ZUI4_
HLA-DQA10501-DQB10201	620	NMDREDCEKNLIFAG	NMDREDCEK	0	0.0941	18057.8	90.00	0.20	tr_C9ZUI4_
HLA-DQA10501-DQB10201	621	SKKSRETAEAGKVVS	ETAEAGKVV	5	0.0941	18070.5	90.00	0.25	tr_C9ZUI4_
HLA-DQA10501-DQB10201	622	CIVSAPDGKYVVAKG	IVSAPDGKY	1	0.0936	18151.6	90.00	0.37	tr_C9ZUI4_
HLA-DQA10501-DQB10201	623	VWARCAPTHKEDIVT	WARCAPTHK	1	0.0925	18380.5	90.00	0.28	tr_C9ZUI4_
HLA-DQA10501-DQB10201	624	KRGLEDPNRDRWKLF	RGLEDPNRD	1	0.0922	18446.3	90.00	0.36	tr_C9ZUI4_
HLA-DQA10501-DQB10201	625	PFMEQVRHKMAQARR	FMEQVRHKM	1	0.0920	18485.0	90.00	0.31	tr_C9ZUI4_
HLA-DQA10501-DQB10201	626	NSNEPHNEPDVPKDH	EPHNEPDVP	3	0.0906	18761.5	95.00	0.46	tr_C9ZUI4_
HLA-DQA10501-DQB10201	627	DTIEMLQSGSHRCVI	IEMLQSGSH	2	0.0904	18805.0	95.00	0.28	tr_C9ZUI4_
HLA-DQA10501-DQB10201	628	RSILAKTFVQTRRHK	RSILAKTFV	0	0.0900	18883.5	95.00	0.25	tr_C9ZUI4_
HLA-DQA10501-DQB10201	629	SQNVGGSGEHNSNEP	NVGGSGEHN	2	0.0882	19253.6	95.00	0.31	tr_C9ZUI4_
HLA-DQA10501-DQB10201	630	QNVGGSGEHNSNEPH	NVGGSGEHN	1	0.0870	19498.4	95.00	0.29	tr_C9ZUI4_
HLA-DQA10501-DQB10201	631	LEDPNRDRWKLFLAC	DRWKLFLAC	6	0.0868	19537.7	95.00	0.29	tr_C9ZUI4_

HLA-DQA10501-DQB10201	632	RGLEDPNRDRWKLFL	NRDRWKLFL	6	0.0866	19591.5	95.00	0.31	tr_C9ZUI4_
HLA-DQA10501-DQB10201	633	PKDHKIPPGFKLTVV	PPGFKLTVV	6	0.0865	19612.1	95.00	0.62	tr_C9ZUI4_
HLA-DQA10501-DQB10201	634	TIEMLQSGSHRCVII	IEMLQSGSH	1	0.0853	19870.1	95.00	0.23	tr_C9ZUI4_
HLA-DQA10501-DQB10201	635	IAAPFTCRSRALTSV	CRSRALTSV	6	0.0852	19882.3	95.00	0.31	tr_C9ZUI4_
HLA-DQA10501-DQB10201	636	VDTCCFDKTGTLTTD	FDKTGTLTT	5	0.0844	20055.0	95.00	0.27	tr_C9ZUI4_
HLA-DQA10501-DQB10201	637	RKNNSGFEKQYKILV	FEKQYKILV	6	0.0839	20172.5	95.00	0.47	tr_C9ZUI4_
HLA-DQA10501-DQB10201	638	KNNSGFEKQYKILVR	FEKQYKILV	5	0.0838	20192.8	95.00	0.33	tr_C9ZUI4_
HLA-DQA10501-DQB10201	639	TSMDASQNVGGSGEH	TSMDASQNV	0	0.0838	20200.0	95.00	0.38	tr_C9ZUI4_
HLA-DQA10501-DQB10201	640	YRPLKEEERSKEAIH	YRPLKEEER	0	0.0831	20343.2	95.00	0.36	tr_C9ZUI4_
HLA-DQA10501-DQB10201	641	RQQLVASSMKKKGNG	RQQLVASSM	0	0.0830	20369.4	95.00	0.56	tr_C9ZUI4_
HLA-DQA10501-DQB10201	642	SNEPHNEPDVPKDHK	EPHNEPDVP	2	0.0826	20454.0	95.00	0.49	tr_C9ZUI4_
HLA-DQA10501-DQB10201	643	NVGGSGEHNSNEPHN	SGEHNSNEP	4	0.0817	20647.2	95.00	0.23	tr_C9ZUI4_
HLA-DQA10501-DQB10201	644	SIAAPFTCRSRALTS	IAAPFTCRS	1	0.0811	20784.4	95.00	0.28	tr_C9ZUI4_
HLA-DQA10501-DQB10201	645	ARWNKQLEESKKSRE	RWNKQLEES	1	0.0809	20831.9	95.00	0.34	tr_C9ZUI4_
HLA-DQA10501-DQB10201	646	MDASQNVGGSGEHNS	NVGGSGEHN	5	0.0806	20905.8	95.00	0.44	tr_C9ZUI4_
HLA-DQA10501-DQB10201	647	EMLQSGSHRCVIITG	SHRCVIITG	6	0.0806	20909.4	95.00	0.32	tr_C9ZUI4_
HLA-DQA10501-DQB10201	648	GKVDTCCFDKTGTLT	VDTCCFDKT	2	0.0805	20937.5	95.00	0.33	tr_C9ZUI4_
HLA-DQA10501-DQB10201	649	GLEDPNRDRWKLFLA	RDRWKLFLA	6	0.0804	20948.8	95.00	0.31	tr_C9ZUI4_
HLA-DQA10501-DQB10201	650	GSGEHNSNEPHNEPD	HNSNEPHNE	4	0.0801	21020.8	95.00	0.29	tr_C9ZUI4_
HLA-DQA10501-DQB10201	651	DASQNVGGSGEHNSN	NVGGSGEHN	4	0.0800	21040.3	95.00	0.42	tr_C9ZUI4_
HLA-DQA10501-DQB10201	652	SGEHNSNEPHNEPDV	HNSNEPHNE	3	0.0796	21121.5	95.00	0.25	tr_C9ZUI4_
HLA-DQA10501-DQB10201	653	TLCPQRPITKVFHPY	PITKVFHPY	6	0.0796	21128.4	95.00	0.37	tr_C9ZUI4_
HLA-DQA10501-DQB10201	654	GGSGEHNSNEPHNEP	HNSNEPHNE	5	0.0795	21164.3	95.00	0.28	tr_C9ZUI4_
HLA-DQA10501-DQB10201	655	ASQNVGGSGEHNSNE	NVGGSGEHN	3	0.0783	21440.0	95.00	0.40	tr_C9ZUI4_
HLA-DQA10501-DQB10201	656	EESKKSRETAEAGKV	RETAEAGKV	6	0.0780	21500.6	95.00	0.56	tr_C9ZUI4_
HLA-DQA10501-DQB10201	657	KVDTCCFDKTGTLTT	FDKTGTLTT	6	0.0779	21533.4	95.00	0.25	tr_C9ZUI4_
HLA-DQA10501-DQB10201	658	SMDASQNVGGSGEHN	NVGGSGEHN	6	0.0777	21578.0	95.00	0.44	tr_C9ZUI4_
HLA-DQA10501-DQB10201	659	LAVFQCPLKKDAKDT	LAVFQCPLK	0	0.0771	21717.3	95.00	0.45	tr_C9ZUI4_
HLA-DQA10501-DQB10201	660	NEPHNEPDVPKDHKI	EPHNEPDVP	1	0.0766	21821.4	95.00	0.50	tr_C9ZUI4_
HLA-DQA10501-DQB10201	661	VGGSGEHNSNEPHNE	HNSNEPHNE	6	0.0764	21887.2	95.00	0.30	tr_C9ZUI4_
HLA-DQA10501-DQB10201	662	FMEQVRHKMAQARRK	FMEQVRHKM	0	0.0761	21942.7	95.00	0.25	tr_C9ZUI4_
HLA-DQA10501-DQB10201	663	PLKEEERSKEAIHNM	RSKEAIHNM	6	0.0760	21979.7	95.00	0.44	tr_C9ZUI4_
HLA-DQA10501-DQB10201	664	IVSAPDGKYVVAKGS	IVSAPDGKY	0	0.0749	22223.6	95.00	0.34	tr_C9ZUI4_
HLA-DQA10501-DQB10201	665	VSAPDGKYVVAKGSP	KYVVAKGSP	6	0.0749	22226.5	95.00	0.29	tr_C9ZUI4_
HLA-DQA10501-DQB10201	666	SILAKTFVQTRRHKV	AKTFVQTRR	3	0.0744	22347.3	95.00	0.23	tr_C9ZUI4_
HLA-DQA10501-DQB10201	667	IEMLQSGSHRCVIIT	SGSHRCVII	5	0.0735	22563.3	95.00	0.17	tr_C9ZUI4_
HLA-DQA10501-DQB10201	668	ILAKTFVQTRRHKVP	AKTFVQTRR	2	0.0670	24225.6	95.00	0.22	tr_C9ZUI4_
HLA-DQA10501-DQB10201	669	EPHNEPDVPKDHKIP	EPHNEPDVP	0	0.0629	25308.1	100.00	0.44	tr_C9ZUI4_
HLA-DQA10501-DQB10201	670	PTLCPQRPITKVFHP	QRPITKVFH	5	0.0619	25578.7	100.00	0.44	tr_C9ZUI4_
HLA-DQA10501-DQB10201	671	FQCPLKKDAKDTIEM	KDAKDTIEM	6	0.0608	25886.1	100.00	0.37	tr_C9ZUI4_
HLA-DQA10501-DQB10201	672	KTFVQTRRHKVPSDD	RRHKVPSDD	6	0.0606	25957.6	100.00	0.31	tr_C9ZUI4_
HLA-DQA10501-DQB10201	673	NRKNNSGFEKQYKIL	NNSGFEKQY	3	0.0603	26051.8	100.00	0.23	tr_C9ZUI4_
HLA-DQA10501-DQB10201	674	KSQNRKNNSGFEKQY	NNSGFEKQY	6	0.0594	26305.6	100.00	0.37	tr_C9ZUI4_
HLA-DQA10501-DQB10201	675	AKTFVQTRRHKVPSD	TRRHKVPSD	6	0.0569	27022.8	100.00	0.26	tr_C9ZUI4_
HLA-DQA10501-DQB10201	676	SQNRKNNSGFEKQYK	NNSGFEKQY	5	0.0564	27157.0	100.00	0.31	tr_C9ZUI4_
HLA-DQA10501-DQB10201	677	MEQVRHKMAQARRKA	RHKMAQARR	4	0.0561	27235.9	100.00	0.25	tr_C9ZUI4_
HLA-DQA10501-DQB10201	678	QNRKNNSGFEKQYKI	NNSGFEKQY	4	0.0558	27333.0	100.00	0.23	tr_C9ZUI4_
HLA-DQA10501-DQB10201	679	RPLKEEERSKEAIHN	EEERSKEAI	4	0.0551	27551.2	100.00	0.28	tr_C9ZUI4_
HLA-DQA10501-DQB10201	680	LAKTFVQTRRHKVPS	AKTFVQTRR	1	0.0546	27685.7	100.00	0.24	tr_C9ZUI4_
HLA-DQA10501-DQB10201	681	RWNKQLEESKKSRET	WNKQLEESK	1	0.0544	27743.0	100.00	0.46	tr_C9ZUI4_

HLA-DQA10501-DQB10201	682	VYDPPAQKEDIKGKS	VYDPPAQKE	0	0.0529	28207.6	100.00	0.40	tr_C9ZUI4_
HLA-DQA10501-DQB10201	683	AVFQCPLKKDAKDTI	LKKDAKDTI	6	0.0522	28423.0	100.00	0.33	tr_C9ZUI4_
HLA-DQA10501-DQB10201	684	NEPDVPKDHKIPPGF	KDHKIPPGF	6	0.0519	28514.8	100.00	0.44	tr_C9ZUI4_
HLA-DQA10501-DQB10201	685	EQVRHKMAQARRKAE	RHKMAQARR	3	0.0513	28699.9	100.00	0.28	tr_C9ZUI4_
HLA-DQA10501-DQB10201	686	QVRHKMAQARRKAEI	AQARRKAEI	6	0.0509	28814.7	100.00	0.27	tr_C9ZUI4_
HLA-DQA10501-DQB10201	687	VFQCPLKKDAKDTIE	LKKDAKDTI	5	0.0507	28888.0	100.00	0.35	tr_C9ZUI4_
HLA-DQA10501-DQB10201	688	EPDVPKDHKIPPGFK	KDHKIPPGF	5	0.0490	29437.7	100.00	0.38	tr_C9ZUI4_
HLA-DQA10501-DQB10201	689	VPKDHKIPPGFKLTV	KDHKIPPGF	2	0.0483	29640.3	100.00	0.28	tr_C9ZUI4_
HLA-DQA10501-DQB10201	690	LEESKKSRETAEAGK	KSRETAEAG	5	0.0479	29785.0	100.00	0.41	tr_C9ZUI4_
HLA-DQA10501-DQB10201	691	QLEESKKSRETAEAG	KSRETAEAG	6	0.0478	29824.7	100.00	0.50	tr_C9ZUI4_
HLA-DQA10501-DQB10201	692	PDVPKDHKIPPGFKL	KDHKIPPGF	4	0.0467	30170.0	100.00	0.40	tr_C9ZUI4_
HLA-DQA10501-DQB10201	693	DVPKDHKIPPGFKLT	KDHKIPPGF	3	0.0449	30768.3	100.00	0.38	tr_C9ZUI4_
HLA-DQA10501-DQB10201	694	SKSQNRKNNSGFEKQ	KNNSGFEKQ	6	0.0448	30803.3	100.00	0.51	tr_C9ZUI4_
HLA-DQA10501-DQB10201	695	QQLVASSMKKKGNGE	QQLVASSMK	0	0.0426	31546.0	100.00	0.41	tr_C9ZUI4_
HLA-DQA10501-DQB10201	696	YDPPAQKEDIKGKST	DPPAQKEDI	1	0.0399	32458.0	100.00	0.28	tr_C9ZUI4_
HLA-DQA10501-DQB10201	697	KQLEESKKSRETAEA	KKSRETAEA	6	0.0388	32849.1	100.00	0.56	tr_C9ZUI4_
HLA-DQA10501-DQB10201	698	LVASSMKKKGNGEDE	KKKGNGEDE	6	0.0379	33176.3	100.00	0.74	tr_C9ZUI4_
HLA-DQA10501-DQB10201	699	IKGKSTTGSSKNETS	TTGSSKNET	5	0.0377	33255.0	100.00	0.50	tr_C9ZUI4_
HLA-DQA10501-DQB10201	700	PHNEPDVPKDHKIPP	EPDVPKDHK	3	0.0374	33374.6	100.00	0.22	tr_C9ZUI4_
HLA-DQA10501-DQB10201	701	HNEPDVPKDHKIPPG	PKDHKIPPG	6	0.0373	33394.1	100.00	0.17	tr_C9ZUI4_
HLA-DQA10501-DQB10201	702	DIKGKSTTGSSKNET	TTGSSKNET	6	0.0359	33910.8	100.00	0.67	tr_C9ZUI4_
HLA-DQA10501-DQB10201	703	KGKSTTGSSKNETSG	TTGSSKNET	4	0.0344	34466.8	100.00	0.51	tr_C9ZUI4_
HLA-DQA10501-DQB10201	704	WNKQLEESKKSRETA	WNKQLEESK	0	0.0328	35071.3	100.00	0.42	tr_C9ZUI4_
HLA-DQA10501-DQB10201	705	DPPAQKEDIKGKSTT	DPPAQKEDI	0	0.0322	35307.0	100.00	0.23	tr_C9ZUI4_
HLA-DQA10501-DQB10201	706	GKSTTGSSKNETSGS	TTGSSKNET	3	0.0321	35333.3	100.00	0.50	tr_C9ZUI4_
HLA-DQA10501-DQB10201	707	KSTTGSSKNETSGSS	TTGSSKNET	2	0.0313	35640.1	100.00	0.50	tr_C9ZUI4_
HLA-DQA10501-DQB10201	708	SSKSQNRKNNSGFEK	RKNNSGFEK	6	0.0309	35795.1	100.00	0.49	tr_C9ZUI4_
HLA-DQA10501-DQB10201	709	STTGSSKNETSGSSK	TTGSSKNET	1	0.0292	36473.8	100.00	0.52	tr_C9ZUI4_
HLA-DQA10501-DQB10201	710	GSSKSQNRKNNSGFE	NRKNNSGFE	6	0.0264	37563.6	100.00	0.74	tr_C9ZUI4_
HLA-DQA10501-DQB10201	711	NKQLEESKKSRETAE	SKKSRETAE	6	0.0249	38191.0	100.00	0.36	tr_C9ZUI4_
HLA-DQA10501-DQB10201	712	TTGSSKNETSGSSKS	TTGSSKNET	0	0.0235	38775.2	100.00	0.38	tr_C9ZUI4_
HLA-DQA10501-DQB10201	713	PPAQKEDIKGKSTTG	KEDIKGKST	4	0.0231	38955.1	100.00	0.32	tr_C9ZUI4_
HLA-DQA10501-DQB10201	714	QLVASSMKKKGNGED	QLVASSMKK	0	0.0229	39016.3	100.00	0.54	tr_C9ZUI4_
HLA-DQA10501-DQB10201	715	PAQKEDIKGKSTTGS	KEDIKGKST	3	0.0222	39325.3	100.00	0.34	tr_C9ZUI4_
HLA-DQA10501-DQB10201	716	SSKNETSGSSKSQNR	SGSSKSQNR	6	0.0208	39920.3	100.00	0.29	tr_C9ZUI4_
HLA-DQA10501-DQB10201	717	AQKEDIKGKSTTGSS	KEDIKGKST	2	0.0206	40021.9	100.00	0.37	tr_C9ZUI4_
HLA-DQA10501-DQB10201	718	TGSSKNETSGSSKSQ	ETSGSSKSQ	6	0.0202	40188.6	100.00	0.34	tr_C9ZUI4_
HLA-DQA10501-DQB10201	719	GSSKNETSGSSKSQN	ETSGSSKSQ	5	0.0192	40603.8	100.00	0.30	tr_C9ZUI4_
HLA-DQA10501-DQB10201	720	EDIKGKSTTGSSKNE	STTGSSKNE	6	0.0184	40988.2	100.00	0.34	tr_C9ZUI4_
HLA-DQA10501-DQB10201	721	QKEDIKGKSTTGSSK	KEDIKGKST	1	0.0181	41116.2	100.00	0.34	tr_C9ZUI4_
HLA-DQA10501-DQB10201	722	SKNETSGSSKSQNRK	SGSSKSQNR	5	0.0178	41232.9	100.00	0.26	tr_C9ZUI4_
HLA-DQA10501-DQB10201	723	KEDIKGKSTTGSSKN	IKGKSTTGS	3	0.0160	42051.1	100.00	0.18	tr_C9ZUI4_
HLA-DQA10501-DQB10201	724	KNETSGSSKSQNRKN	SGSSKSQNR	4	0.0157	42196.0	100.00	0.29	tr_C9ZUI4_
HLA-DQA10501-DQB10201	725	NETSGSSKSQNRKNN	SGSSKSQNR	3	0.0144	42794.2	100.00	0.29	tr_C9ZUI4_
HLA-DQA10501-DQB10201	726	ETSGSSKSQNRKNNS	SGSSKSQNR	2	0.0133	43304.2	100.00	0.29	tr_C9ZUI4_
HLA-DQA10501-DQB10201	727	SGSSKSQNRKNNSGF	QNRKNNSGF	6	0.0125	43681.1	100.00	0.36	tr_C9ZUI4_
HLA-DQA10501-DQB10201	728	TSGSSKSQNRKNNSG	SGSSKSQNR	1	0.0111	44335.4	100.00	0.31	tr_C9ZUI4_

Allele: HLA-DQA10501-DQB10201. Number of high binders 3. Number of weak binders 38. Number of peptides 728

Allele	pos	peptide	core	Of	1-log50k(aff)	affinity(nM)	%Rank	Relia	Identity	Bind Level
HLA-DQA10501-DQB10201	1	FIPFFDVALFYDDFI	VALFYDDFI	6	0.7019	25.2	0.15	0.51	tr_C9ZNH3_	SB
HLA-DQA10501-DQB10201	2	GRMIYMMILGDDYLP	YMMILGDDY	4	0.6856	30.0	0.25	0.49	tr_C9ZNH3_	SB
HLA-DQA10501-DQB10201	3	IPFFDVALFYDDFIR	VALFYDDFI	5	0.6783	32.5	0.30	0.50	tr_C9ZNH3_	SB
HLA-DQA10501-DQB10201	4	MIYMMILGDDYLPRP	YMMILGDDY	2	0.6735	34.2	0.30	0.43	tr_C9ZNH3_	SB
HLA-DQA10501-DQB10201	5	RMIYMMILGDDYLPR	YMMILGDDY	3	0.6700	35.5	0.40	0.47	tr_C9ZNH3_	SB
HLA-DQA10501-DQB10201	6	FFDVALFYDDFIRAF	VALFYDDFI	3	0.6692	35.8	0.40	0.49	tr_C9ZNH3_	SB
HLA-DQA10501-DQB10201	7	PFFDVALFYDDFIRA	VALFYDDFI	4	0.6622	38.7	0.40	0.50	tr_C9ZNH3_	SB
HLA-DQA10501-DQB10201	8	IYMMILGDDYLPRPA	YMMILGDDY	1	0.6572	40.8	0.50	0.41	tr_C9ZNH3_	WB
HLA-DQA10501-DQB10201	9	VHFGDSEYELAVELM	EYELAVELM	6	0.6494	44.4	0.50	0.58	tr_C9ZNH3_	WB
HLA-DQA10501-DQB10201	10	GGRMIYMMILGDDYL	YMMILGDDY	5	0.6478	45.2	0.60	0.55	tr_C9ZNH3_	WB
HLA-DQA10501-DQB10201	11	YGGQVMFGWIVGEVL	FGWIVGEVL	6	0.6376	50.4	0.70	0.68	tr_C9ZNH3_	WB
HLA-DQA10501-DQB10201	12	SVDDMQYGAFADEDC	QYGAFADED	5	0.6356	51.6	0.70	0.53	tr_C9ZNH3_	WB
HLA-DQA10501-DQB10201	13	FDVALFYDDFIRAFR	VALFYDDFI	2	0.6337	52.7	0.70	0.50	tr_C9ZNH3_	WB
HLA-DQA10501-DQB10201	14	VDDMQYGAFADEDCS	QYGAFADED	4	0.6285	55.7	0.80	0.53	tr_C9ZNH3_	WB
HLA-DQA10501-DQB10201	15	GGQVMFGWIVGEVLA	FGWIVGEVL	5	0.6177	62.5	1.00	0.65	tr_C9ZNH3_	WB
HLA-DQA10501-DQB10201	16	TIYTHSVISVDDMQY	HSVISVDDM	4	0.6140	65.1	1.00	0.35	tr_C9ZNH3_	WB
HLA-DQA10501-DQB10201	17	YMMILGDDYLPRPAA	MMILGDDYL	1	0.6127	66.0	1.10	0.46	tr_C9ZNH3_	WB
HLA-DQA10501-DQB10201	18	VFIPFFDVALFYDDF	DVALFYDDF	6	0.6127	66.1	1.10	0.47	tr_C9ZNH3_	WB
HLA-DQA10501-DQB10201	19	GQVMFGWIVGEVLAR	FGWIVGEVL	4	0.6055	71.4	1.20	0.62	tr_C9ZNH3_	WB
HLA-DQA10501-DQB10201	20	DDMQYGAFADEDCSR	QYGAFADED	3	0.6037	72.8	1.20	0.54	tr_C9ZNH3_	WB
HLA-DQA10501-DQB10201	21	HFGDSEYELAVELMS	EYELAVELM	5	0.6032	73.2	1.20	0.55	tr_C9ZNH3_	WB
HLA-DQA10501-DQB10201	22	QVMFGWIVGEVLARA	FGWIVGEVL	3	0.5973	78.0	1.40	0.60	tr_C9ZNH3_	WB
HLA-DQA10501-DQB10201	23	IYTHSVISVDDMQYG	HSVISVDDM	3	0.5937	81.1	1.40	0.34	tr_C9ZNH3_	WB
HLA-DQA10501-DQB10201	24	VMFGWIVGEVLARAL	FGWIVGEVL	2	0.5867	87.5	1.60	0.56	tr_C9ZNH3_	WB
HLA-DQA10501-DQB10201	25	DVALFYDDFIRAFRG	VALFYDDFI	1	0.5842	89.9	1.70	0.55	tr_C9ZNH3_	WB
HLA-DQA10501-DQB10201	26	DMQYGAFADEDCSRI	QYGAFADED	2	0.5829	91.2	1.70	0.52	tr_C9ZNH3_	WB
HLA-DQA10501-DQB10201	27	KVYSLLYDPFLPDAY	VYSLLYDPF	1	0.5792	94.9	1.80	0.38	tr_C9ZNH3_	WB
HLA-DQA10501-DQB10201	28	ALSGTGAVEFDPLAF	LSGTGAVEF	1	0.5754	98.9	1.90	0.31	tr_C9ZNH3_	WB
HLA-DQA10501-DQB10201	29	FGDSEYELAVELMSQ	EYELAVELM	4	0.5749	99.4	1.90	0.50	tr_C9ZNH3_	WB
HLA-DQA10501-DQB10201	30	YTHSVISVDDMQYGA	HSVISVDDM	2	0.5748	99.5	1.90	0.34	tr_C9ZNH3_	WB
HLA-DQA10501-DQB10201	31	RAVTSVLGVVDDATL	VLGVVDDAT	5	0.5740	100.4	2.00	0.43	tr_C9ZNH3_	WB
HLA-DQA10501-DQB10201	32	EGGRMIYMMILGDDY	YMMILGDDY	6	0.5729	101.6	2.00	0.79	tr_C9ZNH3_	WB
HLA-DQA10501-DQB10201	33	AYALSGTGAVEFDPL	LSGTGAVEF	3	0.5716	103.1	2.00	0.46	tr_C9ZNH3_	WB
HLA-DQA10501-DQB10201	34	ISVDDMQYGAFADED	QYGAFADED	6	0.5701	104.8	2.50	0.67	tr_C9ZNH3_	WB
HLA-DQA10501-DQB10201	35	TRAVTSVLGVVDDAT	VLGVVDDAT	6	0.5694	105.5	2.50	0.38	tr_C9ZNH3_	WB
HLA-DQA10501-DQB10201	36	TTIYTHSVISVDDMQ	HSVISVDDM	5	0.5673	108.0	2.50	0.50	tr_C9ZNH3_	WB
HLA-DQA10501-DQB10201	37	LSGTGAVEFDPLAFD	AVEFDPLAF	5	0.5650	110.7	2.50	0.32	tr_C9ZNH3_	WB
HLA-DQA10501-DQB10201	38	DTLSVLPSLEEEDLV	LPSLEEEDL	5	0.5600	116.9	2.50	0.24	tr_C9ZNH3_	WB
HLA-DQA10501-DQB10201	39	MFGWIVGEVLARALE	FGWIVGEVL	1	0.5567	121.0	2.50	0.57	tr_C9ZNH3_	WB
HLA-DQA10501-DQB10201	40	AVTSVLGVVDDATLS	VLGVVDDAT	4	0.5558	122.3	3.00	0.47	tr_C9ZNH3_	WB
HLA-DQA10501-DQB10201	41	YALSGTGAVEFDPLA	LSGTGAVEF	2	0.5555	122.7	3.00	0.43	tr_C9ZNH3_	WB
HLA-DQA10501-DQB10201	42	THSVISVDDMQYGAF	VISVDDMQY	3	0.5479	133.2	3.00	0.36	tr_C9ZNH3_	WB
HLA-DQA10501-DQB10201	43	MQYGAFADEDCSRIV	QYGAFADED	1	0.5474	133.8	3.00	0.52	tr_C9ZNH3_	WB
HLA-DQA10501-DQB10201	44	SDTLSVLPSLEEEDL	LPSLEEEDL	6	0.5450	137.5	3.00	0.28	tr_C9ZNH3_	WB
HLA-DQA10501-DQB10201	45	LTTIYTHSVISVDDM	HSVISVDDM	6	0.5444	138.3	3.00	0.63	tr_C9ZNH3_	WB

HLA-DQA10501-DQB10201	46	VYSLLYDPFLPDAYN	YSLLYDPFL	1	0.5433	140.0	3.50	0.35	tr_C9ZNH3_	WB
HLA-DQA10501-DQB10201	47	GDSEYELAVELMSQM	EYELAVELM	3	0.5422	141.6	3.50	0.56	tr_C9ZNH3_	WB
HLA-DQA10501-DQB10201	48	LSVLPSLEEEDLVAF	LPSLEEEDL	3	0.5404	144.4	3.50	0.29	tr_C9ZNH3_	WB
HLA-DQA10501-DQB10201	49	TLSVLPSLEEEDLVA	LPSLEEEDL	4	0.5397	145.6	3.50	0.28	tr_C9ZNH3_	WB
HLA-DQA10501-DQB10201	50	NVHFGDSEYELAVEL	SEYELAVEL	6	0.5356	152.1	3.50	0.29	tr_C9ZNH3_	WB
HLA-DQA10501-DQB10201	51	KAYALSGTGAVEFDP	LSGTGAVEF	4	0.5356	152.2	3.50	0.61	tr_C9ZNH3_	WB
HLA-DQA10501-DQB10201	52	VTSVLGVVDDATLST	VLGVVDDAT	3	0.5347	153.7	3.50	0.50	tr_C9ZNH3_	WB
HLA-DQA10501-DQB10201	53	NVRVFIPFFDVALFY	PFFDVALFY	6	0.5317	158.7	4.00	0.38	tr_C9ZNH3_	WB
HLA-DQA10501-DQB10201	54	SLYNQRRYVIDDIVI	RRYVIDDIV	5	0.5274	166.2	4.00	0.58	tr_C9ZNH3_	WB
HLA-DQA10501-DQB10201	55	SGTGAVEFDPLAFDG	AVEFDPLAF	4	0.5241	172.2	4.00	0.34	tr_C9ZNH3_	WB
HLA-DQA10501-DQB10201	56	IKAYALSGTGAVEFD	LSGTGAVEF	5	0.5225	175.2	4.00	0.60	tr_C9ZNH3_	WB
HLA-DQA10501-DQB10201	57	DTRAVTSVLGVVDDA	SVLGVVDDA	6	0.5224	175.4	4.50	0.50	tr_C9ZNH3_	WB
HLA-DQA10501-DQB10201	58	GTGAVEFDPLAFDGD	AVEFDPLAF	3	0.5194	181.2	4.50	0.34	tr_C9ZNH3_	WB
HLA-DQA10501-DQB10201	59	DSEYELAVELMSQMG	EYELAVELM	2	0.5192	181.7	4.50	0.60	tr_C9ZNH3_	WB
HLA-DQA10501-DQB10201	60	SVLPSLEEEDLVAFA	LPSLEEEDL	2	0.5180	184.1	4.50	0.26	tr_C9ZNH3_	WB
HLA-DQA10501-DQB10201	61	GFTSASFLDAVAQHI	FLDAVAQHI	6	0.5158	188.4	4.50	0.46	tr_C9ZNH3_	WB
HLA-DQA10501-DQB10201	62	DSLYNQRRYVIDDIV	RRYVIDDIV	6	0.5153	189.5	4.50	0.64	tr_C9ZNH3_	WB
HLA-DQA10501-DQB10201	63	HSVISVDDMQYGAFA	VISVDDMQY	2	0.5133	193.7	5.00	0.43	tr_C9ZNH3_	WB
HLA-DQA10501-DQB10201	64	LALLGFTSASFLDAV	FTSASFLDA	5	0.5130	194.3	5.00	0.33	tr_C9ZNH3_	WB
HLA-DQA10501-DQB10201	65	NQRRYVIDDIVIGDF	RRYVIDDIV	2	0.5127	194.8	5.00	0.46	tr_C9ZNH3_	WB
HLA-DQA10501-DQB10201	66	DSDTLSVLPSLEEED	TLSVLPSLE	3	0.5083	204.3	5.00	0.34	tr_C9ZNH3_	WB
HLA-DQA10501-DQB10201	67	TSVLGVVDDATLSTP	VLGVVDDAT	2	0.5078	205.4	5.00	0.52	tr_C9ZNH3_	WB
HLA-DQA10501-DQB10201	68	VRVFIPFFDVALFYD	PFFDVALFY	5	0.5073	206.5	5.00	0.34	tr_C9ZNH3_	WB
HLA-DQA10501-DQB10201	69	FGWIVGEVLARALEC	FGWIVGEVL	0	0.5069	207.5	5.00	0.47	tr_C9ZNH3_	WB
HLA-DQA10501-DQB10201	70	YNQRRYVIDDIVIGD	RRYVIDDIV	3	0.5056	210.5	5.00	0.56	tr_C9ZNH3_	WB
HLA-DQA10501-DQB10201	71	PLYTLLFSSVNDPFA	LFSSVNDPF	5	0.5025	217.6	5.50	0.49	tr_C9ZNH3_	WB
HLA-DQA10501-DQB10201	72	QNVHFGDSEYELAVE	FGDSEYELA	4	0.5025	217.8	5.50	0.28	tr_C9ZNH3_	WB
HLA-DQA10501-DQB10201	73	VALFYDDFIRAFRGL	VALFYDDFI	0	0.5020	218.8	5.50	0.56	tr_C9ZNH3_	WB
HLA-DQA10501-DQB10201	74	RVFIPFFDVALFYDD	PFFDVALFY	4	0.5020	218.9	5.50	0.37	tr_C9ZNH3_	WB
HLA-DQA10501-DQB10201	75	ALLGFTSASFLDAVA	FTSASFLDA	4	0.5011	220.9	5.50	0.29	tr_C9ZNH3_	WB
HLA-DQA10501-DQB10201	76	LYNQRRYVIDDIVIG	RRYVIDDIV	4	0.4998	224.1	5.50	0.57	tr_C9ZNH3_	WB
HLA-DQA10501-DQB10201	77	VGEVLARALECSEFL	RALECSEFL	6	0.4987	226.8	5.50	0.44	tr_C9ZNH3_	WB
HLA-DQA10501-DQB10201	78	LESSFDGEADDAEFT	SFDGEADDA	3	0.4970	231.0	6.00	0.28	tr_C9ZNH3_	WB
HLA-DQA10501-DQB10201	79	PAADGLITFDACDES	LITFDACDE	5	0.4956	234.5	6.00	0.50	tr_C9ZNH3_	WB
HLA-DQA10501-DQB10201	80	TLESSFDGEADDAEF	SFDGEADDA	4	0.4949	236.2	6.00	0.31	tr_C9ZNH3_	WB
HLA-DQA10501-DQB10201	81	DATLSTPDVVFIDPI	PDVVFIDPI	6	0.4940	238.7	6.00	0.43	tr_C9ZNH3_	WB
HLA-DQA10501-DQB10201	82	TGAVEFDPLAFDGDA	AVEFDPLAF	2	0.4927	241.9	6.00	0.40	tr_C9ZNH3_	WB
HLA-DQA10501-DQB10201	83	VLPSLEEEDLVAFAP	LPSLEEEDL	1	0.4916	244.8	6.00	0.24	tr_C9ZNH3_	WB
HLA-DQA10501-DQB10201	84	LLGFTSASFLDAVAQ	FTSASFLDA	3	0.4903	248.3	6.00	0.29	tr_C9ZNH3_	WB
HLA-DQA10501-DQB10201	85	ESSFDGEADDAEFTA	SFDGEADDA	2	0.4892	251.3	6.50	0.28	tr_C9ZNH3_	WB
HLA-DQA10501-DQB10201	86	FTSASFLDAVAQHIV	FLDAVAQHI	5	0.4891	251.5	6.50	0.52	tr_C9ZNH3_	WB
HLA-DQA10501-DQB10201	87	AADGLITFDACDESG	LITFDACDE	4	0.4891	251.6	6.50	0.47	tr_C9ZNH3_	WB
HLA-DQA10501-DQB10201	88	YLQNVHFGDSEYELA	FGDSEYELA	6	0.4887	252.6	6.50	0.31	tr_C9ZNH3_	WB
HLA-DQA10501-DQB10201	89	AVEFDPLAFDGDAAY	PLAFDGDAA	5	0.4878	255.1	6.50	0.34	tr_C9ZNH3_	WB
HLA-DQA10501-DQB10201	90	LQNVHFGDSEYELAV	FGDSEYELA	5	0.4877	255.4	6.50	0.31	tr_C9ZNH3_	WB
HLA-DQA10501-DQB10201	91	QRRYVIDDIVIGDFG	RRYVIDDIV	1	0.4854	261.9	6.50	0.49	tr_C9ZNH3_	WB
HLA-DQA10501-DQB10201	92	APLYTLLFSSVNDPF	LFSSVNDPF	6	0.4853	262.1	6.50	0.61	tr_C9ZNH3_	WB
HLA-DQA10501-DQB10201	93	LYTLLFSSVNDPFAR	LFSSVNDPF	4	0.4843	265.0	6.50	0.49	tr_C9ZNH3_	WB
HLA-DQA10501-DQB10201	94	PNAYFLHVSPAAELL	FLHVSPAAE	4	0.4838	266.4	6.50	0.31	tr_C9ZNH3_	WB
HLA-DQA10501-DQB10201	95	QYGAFADEDCSRIVG	QYGAFADED	0	0.4829	269.0	7.00	0.48	tr_C9ZNH3_	WB

HLA-DQA10501-DQB10201	96	KNEFFVVVGPLSDSD	FFVVVGPLS	3	0.4813	273.7	7.00	0.34	tr_C9ZNH3_	WB
HLA-DQA10501-DQB10201	97	SDSDTLSVLPSLEEE	TLSVLPSLE	4	0.4796	278.9	7.00	0.41	tr_C9ZNH3_	WB
HLA-DQA10501-DQB10201	98	PLALLGFTSASFLDA	FTSASFLDA	6	0.4792	279.9	7.00	0.44	tr_C9ZNH3_	WB
HLA-DQA10501-DQB10201	99	GAVEFDPLAFDGDAA	AVEFDPLAF	1	0.4786	281.9	7.00	0.35	tr_C9ZNH3_	WB
HLA-DQA10501-DQB10201	100	RPAADGLITFDACDE	LITFDACDE	6	0.4779	284.1	7.00	0.54	tr_C9ZNH3_	WB
HLA-DQA10501-DQB10201	101	AYFLHVSPAAELLAL	FLHVSPAAE	2	0.4772	286.3	7.00	0.28	tr_C9ZNH3_	WB
HLA-DQA10501-DQB10201	102	RNVRVFIPFFDVALF	NVRVFIPFF	1	0.4766	287.9	7.50	0.29	tr_C9ZNH3_	WB
HLA-DQA10501-DQB10201	103	NAYFLHVSPAAELLA	FLHVSPAAE	3	0.4766	288.0	7.50	0.30	tr_C9ZNH3_	WB
HLA-DQA10501-DQB10201	104	EADDAEFTATWELFA	DAEFTATWE	3	0.4761	289.6	7.50	0.42	tr_C9ZNH3_	WB
HLA-DQA10501-DQB10201	105	ADGLITFDACDESGV	LITFDACDE	3	0.4760	290.0	7.50	0.51	tr_C9ZNH3_	WB
HLA-DQA10501-DQB10201	106	NEFFVVVGPLSDSDT	FFVVVGPLS	2	0.4753	292.0	7.50	0.35	tr_C9ZNH3_	WB
HLA-DQA10501-DQB10201	107	YFLHVSPAAELLALL	FLHVSPAAE	1	0.4744	294.8	7.50	0.20	tr_C9ZNH3_	WB
HLA-DQA10501-DQB10201	108	ENKNEFFVVVGPLSD	FFVVVGPLS	5	0.4744	294.9	7.50	0.32	tr_C9ZNH3_	WB
HLA-DQA10501-DQB10201	109	TPSMHYHNLEEDDHP	MHYHNLEED	3	0.4737	297.3	7.50	0.41	tr_C9ZNH3_	WB
HLA-DQA10501-DQB10201	110	MMILGDDYLPRPAAD	MMILGDDYL	0	0.4730	299.6	7.50	0.60	tr_C9ZNH3_	WB
HLA-DQA10501-DQB10201	111	TLSTPDVVFIDPITL	PDVVFIDPI	4	0.4728	300.2	7.50	0.41	tr_C9ZNH3_	WB
HLA-DQA10501-DQB10201	112	DIVIGDFGGECEPLA	IGDFGGECE	3	0.4727	300.4	7.50	0.38	tr_C9ZNH3_	WB
HLA-DQA10501-DQB10201	113	SPNAYFLHVSPAAEL	FLHVSPAAE	5	0.4717	303.8	7.50	0.34	tr_C9ZNH3_	WB
HLA-DQA10501-DQB10201	114	DIKAYALSGTGAVEF	LSGTGAVEF	6	0.4706	307.4	8.00	0.70	tr_C9ZNH3_	WB
HLA-DQA10501-DQB10201	115	GEADDAEFTATWELF	DAEFTATWE	4	0.4692	311.9	8.00	0.44	tr_C9ZNH3_	WB
HLA-DQA10501-DQB10201	116	NKNEFFVVVGPLSDS	FFVVVGPLS	4	0.4681	315.6	8.00	0.33	tr_C9ZNH3_	WB
HLA-DQA10501-DQB10201	117	YTLLFSSVNDPFARS	LFSSVNDPF	3	0.4680	316.3	8.00	0.50	tr_C9ZNH3_	WB
HLA-DQA10501-DQB10201	118	SEYELAVELMSQMGR	EYELAVELM	1	0.4677	317.0	8.00	0.61	tr_C9ZNH3_	WB
HLA-DQA10501-DQB10201	119	SSFDGEADDAEFTAT	SFDGEADDA	1	0.4659	323.4	8.00	0.27	tr_C9ZNH3_	WB
HLA-DQA10501-DQB10201	120	SVLGVVDDATLSTPD	VLGVVDDAT	1	0.4652	325.7	8.00	0.56	tr_C9ZNH3_	WB
HLA-DQA10501-DQB10201	121	VIHPLSYEVPPPELL	SYEVPPPEL	5	0.4648	327.1	8.00	0.34	tr_C9ZNH3_	WB
HLA-DQA10501-DQB10201	122	NRNVRVFIPFFDVAL	NVRVFIPFF	2	0.4636	331.6	8.50	0.34	tr_C9ZNH3_	WB
HLA-DQA10501-DQB10201	123	ATLSTPDVVFIDPIT	PDVVFIDPI	5	0.4624	335.7	8.50	0.41	tr_C9ZNH3_	WB
HLA-DQA10501-DQB10201	124	EFFVVVGPLSDSDTL	FVVVGPLSD	2	0.4623	336.3	8.50	0.31	tr_C9ZNH3_	WB
HLA-DQA10501-DQB10201	125	SVISVDDMQYGAFAD	VISVDDMQY	1	0.4623	336.4	8.50	0.48	tr_C9ZNH3_	WB
HLA-DQA10501-DQB10201	126	LPSLEEEDLVAFAPF	PSLEEEDLV	1	0.4604	343.3	8.50	0.31	tr_C9ZNH3_	WB
HLA-DQA10501-DQB10201	127	IVIGDFGGECEPLAA	IGDFGGECE	2	0.4601	344.3	8.50	0.35	tr_C9ZNH3_	WB
HLA-DQA10501-DQB10201	128	YSLLYDPFLPDAYNN	YSLLYDPFL	0	0.4593	347.2	9.00	0.35	tr_C9ZNH3_	WB
HLA-DQA10501-DQB10201	129	FDGEADDAEFTATWE	DAEFTATWE	6	0.4588	349.3	9.00	0.41	tr_C9ZNH3_	WB
HLA-DQA10501-DQB10201	130	DGLITFDACDESGVQ	LITFDACDE	2	0.4585	350.5	9.00	0.54	tr_C9ZNH3_	WB
HLA-DQA10501-DQB10201	131	AVAQHIVGVDPQKVL	IVGVDPQKV	5	0.4578	353.0	9.00	0.40	tr_C9ZNH3_	WB
HLA-DQA10501-DQB10201	132	QQLFVIAGYLASENA	AGYLASENA	6	0.4573	354.8	9.00	0.38	tr_C9ZNH3_	WB
HLA-DQA10501-DQB10201	133	LGFTSASFLDAVAQH	FTSASFLDA	2	0.4569	356.6	9.00	0.31	tr_C9ZNH3_	WB
HLA-DQA10501-DQB10201	134	WPLARALNAAVPPLT	ALNAAVPPL	5	0.4562	359.3	9.00	0.33	tr_C9ZNH3_	WB
HLA-DQA10501-DQB10201	135	ETPSMHYHNLEEDDH	MHYHNLEED	4	0.4561	359.6	9.00	0.41	tr_C9ZNH3_	WB
HLA-DQA10501-DQB10201	136	DAVAQHIVGVDPQKV	IVGVDPQKV	6	0.4558	360.8	9.00	0.37	tr_C9ZNH3_	WB
HLA-DQA10501-DQB10201	137	GEVLARALECSEFLK	RALECSEFL	5	0.4557	361.1	9.00	0.41	tr_C9ZNH3_	WB
HLA-DQA10501-DQB10201	138	DDIVIGDFGGECEPL	IGDFGGECE	4	0.4547	365.0	9.00	0.44	tr_C9ZNH3_	WB
HLA-DQA10501-DQB10201	139	ADDAEFTATWELFAG	DAEFTATWE	2	0.4538	368.7	9.50	0.40	tr_C9ZNH3_	WB
HLA-DQA10501-DQB10201	140	PGQVVLSGVLPLVSD	LSGVLPLVS	5	0.4530	371.8	9.50	0.38	tr_C9ZNH3_	WB
HLA-DQA10501-DQB10201	141	FPGQVVLSGVLPLVS	LSGVLPLVS	6	0.4529	372.1	9.50	0.37	tr_C9ZNH3_	WB
HLA-DQA10501-DQB10201	142	PETPSMHYHNLEEDD	MHYHNLEED	5	0.4526	373.4	9.50	0.52	tr_C9ZNH3_	WB
HLA-DQA10501-DQB10201	143	RSLNSVVSLDRDAPL	NSVVSLDRD	3	0.4525	373.8	9.50	0.28	tr_C9ZNH3_	WB
HLA-DQA10501-DQB10201	144	LSANRNVRVFIPFFD	NVRVFIPFF	5	0.4525	374.0	9.50	0.56	tr_C9ZNH3_	WB
HLA-DQA10501-DQB10201	145	LSDSDTLSVLPSLEE	TLSVLPSLE	5	0.4517	376.9	9.50	0.50	tr_C9ZNH3_	WB

HLA-DQA10501-DQB10201	146	LSTPDVVFIDPITLN	PDVVFIDPI	3	0.4499	384.5	9.50	0.40	tr_C9ZNH3_	WB
HLA-DQA10501-DQB10201	147	FTLESSFDGEADDAE	SFDGEADDA	5	0.4489	388.7	10.00	0.36	tr_C9ZNH3_	
HLA-DQA10501-DQB10201	148	STPDVVFIDPITLNP	PDVVFIDPI	2	0.4485	390.2	10.00	0.36	tr_C9ZNH3_	
HLA-DQA10501-DQB10201	149	EVIHPLSYEVPPPEL	SYEVPPPEL	6	0.4480	392.5	10.00	0.31	tr_C9ZNH3_	
HLA-DQA10501-DQB10201	150	ANRNVRVFIPFFDVA	NVRVFIPFF	3	0.4469	397.4	10.00	0.48	tr_C9ZNH3_	
HLA-DQA10501-DQB10201	151	FLDAVAQHIVGVDPQ	FLDAVAQHI	0	0.4466	398.7	10.00	0.26	tr_C9ZNH3_	
HLA-DQA10501-DQB10201	152	TSASFLDAVAQHIVG	FLDAVAQHI	4	0.4454	403.6	10.00	0.58	tr_C9ZNH3_	
HLA-DQA10501-DQB10201	153	PSMHYHNLEEDDHPK	MHYHNLEED	2	0.4447	406.7	10.00	0.45	tr_C9ZNH3_	
HLA-DQA10501-DQB10201	154	MYLQNVHFGDSEYEL	VHFGDSEYE	5	0.4446	407.3	10.00	0.42	tr_C9ZNH3_	
HLA-DQA10501-DQB10201	155	WSTASNVNVEVIHPL	ASNVNVEVI	3	0.4439	410.2	11.00	0.26	tr_C9ZNH3_	
HLA-DQA10501-DQB10201	156	SANRNVRVFIPFFDV	NVRVFIPFF	4	0.4434	412.4	11.00	0.55	tr_C9ZNH3_	
HLA-DQA10501-DQB10201	157	EFLKSRMTFMDSLYN	SRMTFMDSL	4	0.4430	414.5	11.00	0.40	tr_C9ZNH3_	
HLA-DQA10501-DQB10201	158	SFDGEADDAEFTATW	FDGEADDAE	1	0.4425	416.7	11.00	0.25	tr_C9ZNH3_	
HLA-DQA10501-DQB10201	159	VEFDPLAFDGDAAYG	PLAFDGDAA	4	0.4411	423.1	11.00	0.43	tr_C9ZNH3_	
HLA-DQA10501-DQB10201	160	VAQHIVGVDPQKVLT	IVGVDPQKV	4	0.4398	428.9	11.00	0.42	tr_C9ZNH3_	
HLA-DQA10501-DQB10201	161	QLFVIAGYLASENAS	AGYLASENA	5	0.4393	431.2	11.00	0.39	tr_C9ZNH3_	
HLA-DQA10501-DQB10201	162	SEFLKSRMTFMDSLY	SRMTFMDSL	5	0.4391	432.1	11.00	0.38	tr_C9ZNH3_	
HLA-DQA10501-DQB10201	163	TLLFSSVNDPFARSV	LFSSVNDPF	2	0.4383	435.8	11.00	0.51	tr_C9ZNH3_	
HLA-DQA10501-DQB10201	164	IDDIVIGDFGGECEP	IGDFGGECE	5	0.4365	444.4	11.00	0.46	tr_C9ZNH3_	
HLA-DQA10501-DQB10201	165	EVLARALECSEFLKS	RALECSEFL	4	0.4364	444.8	11.00	0.43	tr_C9ZNH3_	
HLA-DQA10501-DQB10201	166	GQVVLSGVLPLVSDN	LSGVLPLVS	4	0.4359	447.4	11.00	0.37	tr_C9ZNH3_	
HLA-DQA10501-DQB10201	167	HPGRNVIYLSPTLEQ	VIYLSPTLE	5	0.4358	447.8	11.00	0.53	tr_C9ZNH3_	
HLA-DQA10501-DQB10201	168	IHPLSYEVPPPELLR	SYEVPPPEL	4	0.4346	453.6	12.00	0.35	tr_C9ZNH3_	
HLA-DQA10501-DQB10201	169	VIGDFGGECEPLAAA	IGDFGGECE	1	0.4345	454.1	12.00	0.35	tr_C9ZNH3_	
HLA-DQA10501-DQB10201	170	DGSLLLIGLTASDVG	LIGLTASDV	5	0.4345	454.3	12.00	0.68	tr_C9ZNH3_	
HLA-DQA10501-DQB10201	171	DGEADDAEFTATWEL	DAEFTATWE	5	0.4315	469.5	12.00	0.49	tr_C9ZNH3_	
HLA-DQA10501-DQB10201	172	RNVIYLSPTLEQQLF	VIYLSPTLE	2	0.4297	478.5	12.00	0.44	tr_C9ZNH3_	
HLA-DQA10501-DQB10201	173	DDAEFTATWELFAGT	DAEFTATWE	1	0.4295	479.4	12.00	0.40	tr_C9ZNH3_	
HLA-DQA10501-DQB10201	174	NVIYLSPTLEQQLFV	VIYLSPTLE	1	0.4283	485.9	12.00	0.41	tr_C9ZNH3_	
HLA-DQA10501-DQB10201	175	SASFLDAVAQHIVGV	FLDAVAQHI	3	0.4283	486.0	12.00	0.64	tr_C9ZNH3_	
HLA-DQA10501-DQB10201	176	LWPLARALNAAVPPL	ALNAAVPPL	6	0.4279	487.8	12.00	0.46	tr_C9ZNH3_	
HLA-DQA10501-DQB10201	177	VISVDDMQYGAFADE	MQYGAFADE	6	0.4279	487.8	12.00	0.37	tr_C9ZNH3_	
HLA-DQA10501-DQB10201	178	PLSDSDTLSVLPSLE	TLSVLPSLE	6	0.4277	489.0	12.00	0.50	tr_C9ZNH3_	
HLA-DQA10501-DQB10201	179	SFNRSLNSVVSLDRD	NSVVSLDRD	6	0.4268	493.6	12.00	0.28	tr_C9ZNH3_	
HLA-DQA10501-DQB10201	180	VIDDIVIGDFGGECE	IGDFGGECE	6	0.4262	496.9	13.00	0.44	tr_C9ZNH3_	
HLA-DQA10501-DQB10201	181	PGRNVIYLSPTLEQQ	VIYLSPTLE	4	0.4259	498.5	13.00	0.50	tr_C9ZNH3_	
HLA-DQA10501-DQB10201	182	EENKNEFFVVVGPLS	FFVVVGPLS	6	0.4257	499.5	13.00	0.40	tr_C9ZNH3_	
HLA-DQA10501-DQB10201	183	SLNSVVSLDRDAPLN	NSVVSLDRD	2	0.4255	500.9	13.00	0.29	tr_C9ZNH3_	
HLA-DQA10501-DQB10201	184	IVGEVLARALECSEF	ARALECSEF	6	0.4253	502.0	13.00	0.34	tr_C9ZNH3_	
HLA-DQA10501-DQB10201	185	FLKSRMTFMDSLYNQ	SRMTFMDSL	3	0.4252	502.2	13.00	0.33	tr_C9ZNH3_	
HLA-DQA10501-DQB10201	186	LARALNAAVPPLTKP	LNAAVPPLT	4	0.4252	502.3	13.00	0.43	tr_C9ZNH3_	
HLA-DQA10501-DQB10201	187	GRNVIYLSPTLEQQL	VIYLSPTLE	3	0.4252	502.5	13.00	0.47	tr_C9ZNH3_	
HLA-DQA10501-DQB10201	188	GLITFDACDESGVQM	LITFDACDE	1	0.4246	505.8	13.00	0.54	tr_C9ZNH3_	
HLA-DQA10501-DQB10201	189	RRYVIDDIVIGDFGG	RRYVIDDIV	0	0.4240	509.1	13.00	0.37	tr_C9ZNH3_	
HLA-DQA10501-DQB10201	190	FLHVSPAAELLALLR	PAAELLALL	5	0.4239	509.4	13.00	0.20	tr_C9ZNH3_	
HLA-DQA10501-DQB10201	191	TDGSLLLIGLTASDV	LIGLTASDV	6	0.4238	510.0	13.00	0.81	tr_C9ZNH3_	
HLA-DQA10501-DQB10201	192	PSLEEEDLVAFAPFT	DLVAFAPFT	6	0.4236	511.1	13.00	0.22	tr_C9ZNH3_	
HLA-DQA10501-DQB10201	193	LFVIAGYLASENAST	AGYLASENA	4	0.4236	511.1	13.00	0.38	tr_C9ZNH3_	
HLA-DQA10501-DQB10201	194	TPLALLGFTSASFLD	LGFTSASFL	5	0.4223	518.1	13.00	0.49	tr_C9ZNH3_	
HLA-DQA10501-DQB10201	195	SFLDAVAQHIVGVDP	FLDAVAQHI	1	0.4222	518.9	13.00	0.46	tr_C9ZNH3_	

HLA-DQA10501-DQB10201	196	ASFLDAVAQHIVGVD	FLDAVAQHI	2	0.4222	519.1	13.00	0.61	tr_C9ZNH3_
HLA-DQA10501-DQB10201	197	LDTRAVTSVLGVVDD	VTSVLGVVD	5	0.4219	520.4	13.00	0.33	tr_C9ZNH3_
HLA-DQA10501-DQB10201	198	VFTLESSFDGEADDA	SFDGEADDA	6	0.4218	520.8	13.00	0.30	tr_C9ZNH3_
HLA-DQA10501-DQB10201	199	SMHYHNLEEDDHPKS	MHYHNLEED	1	0.4211	524.9	13.00	0.47	tr_C9ZNH3_
HLA-DQA10501-DQB10201	200	RHPGRNVIYLSPTLE	VIYLSPTLE	6	0.4210	525.5	13.00	0.60	tr_C9ZNH3_
HLA-DQA10501-DQB10201	201	STASNVNVEVIHPLS	ASNVNVEVI	2	0.4202	530.1	13.00	0.25	tr_C9ZNH3_
HLA-DQA10501-DQB10201	202	TASNVNVEVIHPLSY	ASNVNVEVI	1	0.4184	540.5	13.00	0.21	tr_C9ZNH3_
HLA-DQA10501-DQB10201	203	WSPNAYFLHVSPAAE	FLHVSPAAE	6	0.4165	551.8	14.00	0.40	tr_C9ZNH3_
HLA-DQA10501-DQB10201	204	ESGVQMLAPLYTLLF	GVQMLAPLY	2	0.4164	552.3	14.00	0.31	tr_C9ZNH3_
HLA-DQA10501-DQB10201	205	LKSRMTFMDSLYNQR	RMTFMDSLY	3	0.4162	553.8	14.00	0.31	tr_C9ZNH3_
HLA-DQA10501-DQB10201	206	PLARALNAAVPPLTK	LNAAVPPLT	5	0.4161	554.2	14.00	0.37	tr_C9ZNH3_
HLA-DQA10501-DQB10201	207	GVMIFAPQVSDAMRF	FAPQVSDAM	4	0.4157	556.6	14.00	0.26	tr_C9ZNH3_
HLA-DQA10501-DQB10201	208	TPDVVFIDPITLNPR	VVFIDPITL	3	0.4156	557.1	14.00	0.27	tr_C9ZNH3_
HLA-DQA10501-DQB10201	209	FVIAGYLASENASTL	AGYLASENA	3	0.4156	557.3	14.00	0.41	tr_C9ZNH3_
HLA-DQA10501-DQB10201	210	AQHIVGVDPQKVLTT	IVGVDPQKV	3	0.4151	560.6	14.00	0.49	tr_C9ZNH3_
HLA-DQA10501-DQB10201	211	EFDPLAFDGDAAYGG	PLAFDGDAA	3	0.4146	563.5	14.00	0.44	tr_C9ZNH3_
HLA-DQA10501-DQB10201	212	FFVVVGPLSDSDTLS	FVVVGPLSD	1	0.4142	565.6	14.00	0.38	tr_C9ZNH3_
HLA-DQA10501-DQB10201	213	QVVLSGVLPLVSDNG	LSGVLPLVS	3	0.4140	566.9	14.00	0.36	tr_C9ZNH3_
HLA-DQA10501-DQB10201	214	AYGGQVMFGWIVGEV	MFGWIVGEV	6	0.4126	575.5	14.00	0.62	tr_C9ZNH3_
HLA-DQA10501-DQB10201	215	NRSLNSVVSLDRDAP	NSVVSLDRD	4	0.4118	580.5	14.00	0.31	tr_C9ZNH3_
HLA-DQA10501-DQB10201	216	FNRSLNSVVSLDRDA	NSVVSLDRD	5	0.4113	583.9	14.00	0.28	tr_C9ZNH3_
HLA-DQA10501-DQB10201	217	GSLLLIGLTASDVGG	LIGLTASDV	4	0.4107	587.8	14.00	0.68	tr_C9ZNH3_
HLA-DQA10501-DQB10201	218	DDATLSTPDVVFIDP	ATLSTPDVV	2	0.4104	589.6	14.00	0.42	tr_C9ZNH3_
HLA-DQA10501-DQB10201	219	GVFTLESSFDGEADD	VFTLESSFD	1	0.4075	608.1	15.00	0.31	tr_C9ZNH3_
HLA-DQA10501-DQB10201	220	CGVFTLESSFDGEAD	VFTLESSFD	2	0.4066	614.0	15.00	0.37	tr_C9ZNH3_
HLA-DQA10501-DQB10201	221	ACDESGVQMLAPLYT	GVQMLAPLY	5	0.4066	614.3	15.00	0.44	tr_C9ZNH3_
HLA-DQA10501-DQB10201	222	RTPLALLGFTSASFL	LLGFTSASF	5	0.4065	614.7	15.00	0.43	tr_C9ZNH3_
HLA-DQA10501-DQB10201	223	SLLLIGLTASDVGGI	LIGLTASDV	3	0.4060	618.2	15.00	0.69	tr_C9ZNH3_
HLA-DQA10501-DQB10201	224	PQGVMIFAPQVSDAM	FAPQVSDAM	6	0.4056	621.1	15.00	0.32	tr_C9ZNH3_
HLA-DQA10501-DQB10201	225	CDESGVQMLAPLYTL	GVQMLAPLY	4	0.4055	621.4	15.00	0.40	tr_C9ZNH3_
HLA-DQA10501-DQB10201	226	EYELAVELMSQMGRS	EYELAVELM	0	0.4041	631.0	15.00	0.56	tr_C9ZNH3_
HLA-DQA10501-DQB10201	227	ARALNAAVPPLTKPE	LNAAVPPLT	3	0.4041	631.2	15.00	0.46	tr_C9ZNH3_
HLA-DQA10501-DQB10201	228	ASNVNVEVIHPLSYE	VNVEVIHPL	3	0.4039	632.7	15.00	0.23	tr_C9ZNH3_
HLA-DQA10501-DQB10201	229	DESGVQMLAPLYTLL	GVQMLAPLY	3	0.4035	635.0	15.00	0.41	tr_C9ZNH3_
HLA-DQA10501-DQB10201	230	HPLSYEVPPPELLRS	SYEVPPPEL	3	0.4035	635.5	15.00	0.41	tr_C9ZNH3_
HLA-DQA10501-DQB10201	231	MFPGQVVLSGVLPLV	VLSGVLPLV	6	0.4011	651.9	16.00	0.32	tr_C9ZNH3_
HLA-DQA10501-DQB10201	232	LLFSSVNDPFARSVN	LFSSVNDPF	1	0.4001	658.8	16.00	0.54	tr_C9ZNH3_
HLA-DQA10501-DQB10201	233	VEVIHPLSYEVPPPE	IHPLSYEVP	3	0.3999	660.2	16.00	0.33	tr_C9ZNH3_
HLA-DQA10501-DQB10201	234	VVLSGVLPLVSDNGF	LSGVLPLVS	2	0.3996	662.3	16.00	0.37	tr_C9ZNH3_
HLA-DQA10501-DQB10201	235	LNSVVSLDRDAPLNA	NSVVSLDRD	1	0.3986	669.6	16.00	0.30	tr_C9ZNH3_
HLA-DQA10501-DQB10201	236	QWSTASNVNVEVIHP	ASNVNVEVI	4	0.3970	681.3	16.00	0.41	tr_C9ZNH3_
HLA-DQA10501-DQB10201	237	EQQLFVIAGYLASEN	IAGYLASEN	6	0.3964	686.0	16.00	0.46	tr_C9ZNH3_
HLA-DQA10501-DQB10201	238	NVNVEVIHPLSYEVP	IHPLSYEVP	6	0.3962	687.7	16.00	0.22	tr_C9ZNH3_
HLA-DQA10501-DQB10201	239	IGDFGGECEPLAAAY	FGGECEPLA	3	0.3945	700.6	17.00	0.34	tr_C9ZNH3_
HLA-DQA10501-DQB10201	240	VMIFAPQVSDAMRFL	FAPQVSDAM	3	0.3942	702.2	17.00	0.32	tr_C9ZNH3_
HLA-DQA10501-DQB10201	241	FMYLQNVHFGDSEYE	VHFGDSEYE	6	0.3937	706.6	17.00	0.43	tr_C9ZNH3_
HLA-DQA10501-DQB10201	242	LLLIGLTASDVGGIA	LIGLTASDV	2	0.3930	711.7	17.00	0.68	tr_C9ZNH3_
HLA-DQA10501-DQB10201	243	FDPLAFDGDAAYGGQ	PLAFDGDAA	2	0.3926	715.1	17.00	0.47	tr_C9ZNH3_
HLA-DQA10501-DQB10201	244	SLCGVFTLESSFDGE	VFTLESSFD	4	0.3922	718.0	17.00	0.48	tr_C9ZNH3_
HLA-DQA10501-DQB10201	245	VLTTIYTHSVISVDD	THSVISVDD	6	0.3919	720.3	17.00	0.53	tr_C9ZNH3_

HLA-DQA10501-DQB10201	246	LDAVAQHIVGVDPQK	QHIVGVDPQ	5	0.3917	721.4	17.00	0.39	tr_C9ZNH3_
HLA-DQA10501-DQB10201	247	IEENKNEFFVVVGPL	EFFVVVGPL	6	0.3917	722.0	17.00	0.53	tr_C9ZNH3_
HLA-DQA10501-DQB10201	248	VLARALECSEFLKSR	RALECSEFL	3	0.3914	723.7	17.00	0.48	tr_C9ZNH3_
HLA-DQA10501-DQB10201	249	GWIVGEVLARALECS	EVLARALEC	5	0.3908	729.1	17.00	0.26	tr_C9ZNH3_
HLA-DQA10501-DQB10201	250	LCGVFTLESSFDGEA	VFTLESSFD	3	0.3907	729.6	17.00	0.43	tr_C9ZNH3_
HLA-DQA10501-DQB10201	251	QGVMIFAPQVSDAMR	FAPQVSDAM	5	0.3894	740.2	17.00	0.30	tr_C9ZNH3_
HLA-DQA10501-DQB10201	252	MDSLYNQRRYVIDDI	QRRYVIDDI	6	0.3884	748.0	18.00	0.89	tr_C9ZNH3_
HLA-DQA10501-DQB10201	253	PLSYEVPPPELLRSI	SYEVPPPEL	2	0.3874	755.9	18.00	0.45	tr_C9ZNH3_
HLA-DQA10501-DQB10201	254	SGVQMLAPLYTLLFS	VQMLAPLYT	2	0.3873	756.6	18.00	0.35	tr_C9ZNH3_
HLA-DQA10501-DQB10201	255	KPETPSMHYHNLEED	MHYHNLEED	6	0.3872	758.1	18.00	0.63	tr_C9ZNH3_
HLA-DQA10501-DQB10201	256	KSRMTFMDSLYNQRR	RMTFMDSLY	2	0.3870	759.8	18.00	0.33	tr_C9ZNH3_
HLA-DQA10501-DQB10201	257	WIVGEVLARALECSE	EVLARALEC	4	0.3848	777.5	18.00	0.24	tr_C9ZNH3_
HLA-DQA10501-DQB10201	258	LITFDACDESGVQML	LITFDACDE	0	0.3841	783.4	18.00	0.43	tr_C9ZNH3_
HLA-DQA10501-DQB10201	259	NVEVIHPLSYEVPPP	IHPLSYEVP	4	0.3818	803.2	19.00	0.35	tr_C9ZNH3_
HLA-DQA10501-DQB10201	260	SLEEEDLVAFAPFTG	DLVAFAPFT	5	0.3816	805.3	19.00	0.24	tr_C9ZNH3_
HLA-DQA10501-DQB10201	261	VNVEVIHPLSYEVPP	IHPLSYEVP	5	0.3802	817.8	19.00	0.32	tr_C9ZNH3_
HLA-DQA10501-DQB10201	262	DAEFTATWELFAGTH	EFTATWELF	2	0.3794	824.2	19.00	0.26	tr_C9ZNH3_
HLA-DQA10501-DQB10201	263	QHIVGVDPQKVLTTI	IVGVDPQKV	2	0.3787	830.4	19.00	0.55	tr_C9ZNH3_
HLA-DQA10501-DQB10201	264	GRSLCGVFTLESSFD	VFTLESSFD	6	0.3779	837.9	19.00	0.52	tr_C9ZNH3_
HLA-DQA10501-DQB10201	265	LSFNRSLNSVVSLDR	RSLNSVVSL	4	0.3777	840.0	19.00	0.38	tr_C9ZNH3_
HLA-DQA10501-DQB10201	266	LHVSPAAELLALLRY	PAAELLALL	4	0.3769	847.5	19.00	0.27	tr_C9ZNH3_
HLA-DQA10501-DQB10201	267	SNVNVEVIHPLSYEV	VNVEVIHPL	2	0.3752	863.2	20.00	0.25	tr_C9ZNH3_
HLA-DQA10501-DQB10201	268	CSEFLKSRMTFMDSL	SRMTFMDSL	6	0.3748	866.2	20.00	0.64	tr_C9ZNH3_
HLA-DQA10501-DQB10201	269	HLSANRNVRVFIPFF	NVRVFIPFF	6	0.3744	870.0	20.00	0.68	tr_C9ZNH3_
HLA-DQA10501-DQB10201	270	LLIGLTASDVGGIAA	LIGLTASDV	1	0.3739	875.3	20.00	0.60	tr_C9ZNH3_
HLA-DQA10501-DQB10201	271	PDVVFIDPITLNPRL	VVFIDPITL	2	0.3734	880.1	20.00	0.34	tr_C9ZNH3_
HLA-DQA10501-DQB10201	272	VRQWSTASNVNVEVI	ASNVNVEVI	6	0.3732	882.1	20.00	0.57	tr_C9ZNH3_
HLA-DQA10501-DQB10201	273	VIYLSPTLEQQLFVI	VIYLSPTLE	0	0.3726	887.6	20.00	0.35	tr_C9ZNH3_
HLA-DQA10501-DQB10201	274	RALNAAVPPLTKPET	LNAAVPPLT	2	0.3703	909.4	21.00	0.50	tr_C9ZNH3_
HLA-DQA10501-DQB10201	275	MIFAPQVSDAMRFLM	FAPQVSDAM	2	0.3697	915.6	21.00	0.34	tr_C9ZNH3_
HLA-DQA10501-DQB10201	276	VDDATLSTPDVVFID	ATLSTPDVV	3	0.3693	919.5	21.00	0.55	tr_C9ZNH3_
HLA-DQA10501-DQB10201	277	RSLCGVFTLESSFDG	VFTLESSFD	5	0.3669	944.2	21.00	0.51	tr_C9ZNH3_
HLA-DQA10501-DQB10201	278	MHYHNLEEDDHPKSV	MHYHNLEED	0	0.3663	949.7	21.00	0.36	tr_C9ZNH3_
HLA-DQA10501-DQB10201	279	MLDTRAVTSVLGVVD	VTSVLGVVD	6	0.3663	949.9	21.00	0.44	tr_C9ZNH3_
HLA-DQA10501-DQB10201	280	LLSFNRSLNSVVSLD	RSLNSVVSL	5	0.3658	955.5	21.00	0.51	tr_C9ZNH3_
HLA-DQA10501-DQB10201	281	DACDESGVQMLAPLY	GVQMLAPLY	6	0.3652	961.8	22.00	0.55	tr_C9ZNH3_
HLA-DQA10501-DQB10201	282	PRPAADGLITFDACD	DGLITFDAC	5	0.3647	966.3	22.00	0.25	tr_C9ZNH3_
HLA-DQA10501-DQB10201	283	VIAGYLASENASTLH	AGYLASENA	2	0.3643	970.8	22.00	0.44	tr_C9ZNH3_
HLA-DQA10501-DQB10201	284	LSYEVPPPELLRSII	SYEVPPPEL	1	0.3643	971.1	22.00	0.44	tr_C9ZNH3_
HLA-DQA10501-DQB10201	285	LLALLRYAVSQLHLL	RYAVSQLHL	5	0.3641	972.8	22.00	0.35	tr_C9ZNH3_
HLA-DQA10501-DQB10201	286	FDGDAAYGGQVMFGW	DAAYGGQVM	3	0.3639	974.8	22.00	0.30	tr_C9ZNH3_
HLA-DQA10501-DQB10201	287	HLLRIGFMYLQNVHF	FMYLQNVHF	6	0.3639	975.0	22.00	0.48	tr_C9ZNH3_
HLA-DQA10501-DQB10201	288	VVDDATLSTPDVVFI	ATLSTPDVV	4	0.3618	997.2	22.00	0.56	tr_C9ZNH3_
HLA-DQA10501-DQB10201	289	SRMTFMDSLYNQRRY	RMTFMDSLY	1	0.3615	1001.0	22.00	0.33	tr_C9ZNH3_
HLA-DQA10501-DQB10201	290	GWSPNAYFLHVSPAA	YFLHVSPAA	6	0.3611	1004.6	22.00	0.51	tr_C9ZNH3_
HLA-DQA10501-DQB10201	291	ELLRSIIEENKNEFF	IEENKNEFF	6	0.3604	1013.1	22.00	0.35	tr_C9ZNH3_
HLA-DQA10501-DQB10201	292	RYVIDDIVIGDFGGE	IDDIVIGDF	3	0.3593	1025.3	23.00	0.31	tr_C9ZNH3_
HLA-DQA10501-DQB10201	293	YVIDDIVIGDFGGEC	IDDIVIGDF	2	0.3586	1032.0	23.00	0.30	tr_C9ZNH3_
HLA-DQA10501-DQB10201	294	HPQGVMIFAPQVSDA	VMIFAPQVS	4	0.3581	1037.8	23.00	0.32	tr_C9ZNH3_
HLA-DQA10501-DQB10201	295	GLTASDVGGIAAHLS	VGGIAAHLS	6	0.3579	1040.7	23.00	0.57	tr_C9ZNH3_

HLA-DQA10501-DQB10201	296	NSVVSLDRDAPLNAA	VSLDRDAPL	3	0.3572	1048.3	23.00	0.36	tr_C9ZNH3_
HLA-DQA10501-DQB10201	297	SAERLIFATNLPHWA	ERLIFATNL	2	0.3567	1054.6	23.00	0.28	tr_C9ZNH3_
HLA-DQA10501-DQB10201	298	KMFPGQVVLSGVLPL	VVLSGVLPL	6	0.3565	1056.6	23.00	0.34	tr_C9ZNH3_
HLA-DQA10501-DQB10201	299	VLSGVLPLVSDNGFR	LSGVLPLVS	1	0.3564	1057.1	23.00	0.41	tr_C9ZNH3_
HLA-DQA10501-DQB10201	300	AERLIFATNLPHWAD	IFATNLPHW	4	0.3562	1060.0	23.00	0.25	tr_C9ZNH3_
HLA-DQA10501-DQB10201	301	LPRPAADGLITFDAC	DGLITFDAC	6	0.3558	1064.8	23.00	0.23	tr_C9ZNH3_
HLA-DQA10501-DQB10201	302	VLGVVDDATLSTPDV	VLGVVDDAT	0	0.3556	1066.3	23.00	0.55	tr_C9ZNH3_
HLA-DQA10501-DQB10201	303	RQWSTASNVNVEVIH	ASNVNVEVI	5	0.3556	1066.6	23.00	0.49	tr_C9ZNH3_
HLA-DQA10501-DQB10201	304	ASTLHAVMRDDATRL	LHAVMRDDA	3	0.3554	1069.3	23.00	0.34	tr_C9ZNH3_
HLA-DQA10501-DQB10201	305	PLAFDGDAAYGGQVM	DAAYGGQVM	6	0.3536	1089.9	24.00	0.34	tr_C9ZNH3_
HLA-DQA10501-DQB10201	306	DPLAFDGDAAYGGQV	PLAFDGDAA	1	0.3535	1091.3	24.00	0.50	tr_C9ZNH3_
HLA-DQA10501-DQB10201	307	GVVDDATLSTPDVVF	ATLSTPDVV	5	0.3534	1092.8	24.00	0.60	tr_C9ZNH3_
HLA-DQA10501-DQB10201	308	SPTPHVTNIEIAWTL	VTNIEIAWT	5	0.3530	1096.8	24.00	0.34	tr_C9ZNH3_
HLA-DQA10501-DQB10201	309	EVPPPELLRSIIEEN	LLRSIIEEN	6	0.3506	1126.3	24.00	0.44	tr_C9ZNH3_
HLA-DQA10501-DQB10201	310	GVQMLAPLYTLLFSS	VQMLAPLYT	1	0.3505	1127.1	24.00	0.38	tr_C9ZNH3_
HLA-DQA10501-DQB10201	311	SENASTLHAVMRDDA	LHAVMRDDA	6	0.3495	1139.3	24.00	0.49	tr_C9ZNH3_
HLA-DQA10501-DQB10201	312	ENASTLHAVMRDDAT	LHAVMRDDA	5	0.3495	1139.6	24.00	0.44	tr_C9ZNH3_
HLA-DQA10501-DQB10201	313	LTASDVGGIAAHLSA	VGGIAAHLS	5	0.3488	1147.6	24.00	0.55	tr_C9ZNH3_
HLA-DQA10501-DQB10201	314	LARALECSEFLKSRM	RALECSEFL	2	0.3488	1148.0	24.00	0.56	tr_C9ZNH3_
HLA-DQA10501-DQB10201	315	DGDAAYGGQVMFGWI	AYGGQVMFG	4	0.3485	1151.8	25.00	0.29	tr_C9ZNH3_
HLA-DQA10501-DQB10201	316	GPLSDSDTLSVLPSL	DTLSVLPSL	6	0.3473	1166.8	25.00	0.49	tr_C9ZNH3_
HLA-DQA10501-DQB10201	317	LEEEDLVAFAPFTGS	DLVAFAPFT	4	0.3465	1177.2	25.00	0.26	tr_C9ZNH3_
HLA-DQA10501-DQB10201	318	CLVNYGAMHISLWPL	YGAMHISLW	4	0.3457	1187.7	25.00	0.25	tr_C9ZNH3_
HLA-DQA10501-DQB10201	319	SLLYDPFLPDAYNNG	YDPFLPDAY	3	0.3451	1194.7	25.00	0.45	tr_C9ZNH3_
HLA-DQA10501-DQB10201	320	PTPHVTNIEIAWTLA	VTNIEIAWT	4	0.3438	1212.0	25.00	0.30	tr_C9ZNH3_
HLA-DQA10501-DQB10201	321	STLHAVMRDDATRLI	VMRDDATRL	5	0.3425	1229.5	26.00	0.30	tr_C9ZNH3_
HLA-DQA10501-DQB10201	322	HVSPAAELLALLRYA	PAAELLALL	3	0.3416	1240.7	26.00	0.31	tr_C9ZNH3_
HLA-DQA10501-DQB10201	323	AEFTATWELFAGTHP	WELFAGTHP	6	0.3411	1247.5	26.00	0.32	tr_C9ZNH3_
HLA-DQA10501-DQB10201	324	LSPTPHVTNIEIAWT	VTNIEIAWT	6	0.3407	1253.5	26.00	0.40	tr_C9ZNH3_
HLA-DQA10501-DQB10201	325	THPQGVMIFAPQVSD	VMIFAPQVS	5	0.3404	1257.2	26.00	0.37	tr_C9ZNH3_
HLA-DQA10501-DQB10201	326	ELLALLRYAVSQLHL	RYAVSQLHL	6	0.3384	1285.4	27.00	0.43	tr_C9ZNH3_
HLA-DQA10501-DQB10201	327	GFMYLQNVHFGDSEY	NVHFGDSEY	6	0.3378	1293.8	27.00	0.35	tr_C9ZNH3_
HLA-DQA10501-DQB10201	328	LRIGFMYLQNVHFGD	FMYLQNVHF	4	0.3369	1306.5	27.00	0.44	tr_C9ZNH3_
HLA-DQA10501-DQB10201	329	LALLRYAVSQLHLLR	RYAVSQLHL	4	0.3355	1325.2	27.00	0.29	tr_C9ZNH3_
HLA-DQA10501-DQB10201	330	SLWPLARALNAAVPP	RALNAAVPP	6	0.3355	1325.4	27.00	0.31	tr_C9ZNH3_
HLA-DQA10501-DQB10201	331	TLLSFNRSLNSVVSL	RSLNSVVSL	6	0.3355	1325.9	27.00	0.63	tr_C9ZNH3_
HLA-DQA10501-DQB10201	332	AFDGDAAYGGQVMFG	DAAYGGQVM	4	0.3353	1329.0	27.00	0.38	tr_C9ZNH3_
HLA-DQA10501-DQB10201	333	KSAERLIFATNLPHW	ERLIFATNL	3	0.3348	1336.4	27.00	0.32	tr_C9ZNH3_
HLA-DQA10501-DQB10201	334	SYEVPPPELLRSIIE	SYEVPPPEL	0	0.3344	1341.8	27.00	0.37	tr_C9ZNH3_
HLA-DQA10501-DQB10201	335	LFSSVNDPFARSVNG	LFSSVNDPF	0	0.3326	1368.0	28.00	0.45	tr_C9ZNH3_
HLA-DQA10501-DQB10201	336	LEQQLFVIAGYLASE	VIAGYLASE	6	0.3325	1369.8	28.00	0.49	tr_C9ZNH3_
HLA-DQA10501-DQB10201	337	LLRIGFMYLQNVHFG	FMYLQNVHF	5	0.3323	1371.9	28.00	0.43	tr_C9ZNH3_
HLA-DQA10501-DQB10201	338	LVNYGAMHISLWPLA	YGAMHISLW	3	0.3322	1373.7	28.00	0.26	tr_C9ZNH3_
HLA-DQA10501-DQB10201	339	VPPPELLRSIIEENK	LLRSIIEEN	5	0.3318	1379.2	28.00	0.38	tr_C9ZNH3_
HLA-DQA10501-DQB10201	340	GCLVNYGAMHISLWP	YGAMHISLW	5	0.3317	1381.2	28.00	0.29	tr_C9ZNH3_
HLA-DQA10501-DQB10201	341	GDAAYGGQVMFGWIV	AYGGQVMFG	3	0.3312	1388.4	28.00	0.28	tr_C9ZNH3_
HLA-DQA10501-DQB10201	342	TPHVTNIEIAWTLAG	VTNIEIAWT	3	0.3312	1389.3	28.00	0.28	tr_C9ZNH3_
HLA-DQA10501-DQB10201	343	LAFDGDAAYGGQVMF	DAAYGGQVM	5	0.3305	1398.9	28.00	0.46	tr_C9ZNH3_
HLA-DQA10501-DQB10201	344	ALLRYAVSQLHLLRI	RYAVSQLHL	3	0.3304	1400.3	28.00	0.27	tr_C9ZNH3_
HLA-DQA10501-DQB10201	345	YLPRPAADGLITFDA	PAADGLITF	4	0.3304	1400.3	28.00	0.26	tr_C9ZNH3_

HLA-DQA10501-DQB10201	346	DRTPLALLGFTSASF	LLGFTSASF	6	0.3299	1408.6	28.00	0.76	tr_C9ZNH3_
HLA-DQA10501-DQB10201	347	EEEDLVAFAPFTGSD	DLVAFAPFT	3	0.3298	1409.9	28.00	0.37	tr_C9ZNH3_
HLA-DQA10501-DQB10201	348	TASDVGGIAAHLSAN	VGGIAAHLS	4	0.3298	1410.2	28.00	0.56	tr_C9ZNH3_
HLA-DQA10501-DQB10201	349	ERLIFATNLPHWADH	FATNLPHWA	4	0.3289	1424.1	29.00	0.34	tr_C9ZNH3_
HLA-DQA10501-DQB10201	350	NEGGRMIYMMILGDD	IYMMILGDD	6	0.3289	1424.2	29.00	0.50	tr_C9ZNH3_
HLA-DQA10501-DQB10201	351	IIEENKNEFFVVVGP	IEENKNEFF	1	0.3267	1457.6	29.00	0.35	tr_C9ZNH3_
HLA-DQA10501-DQB10201	352	HIVGVDPQKVLTTIY	IVGVDPQKV	1	0.3260	1468.7	29.00	0.62	tr_C9ZNH3_
HLA-DQA10501-DQB10201	353	LFYDDFIRAFRGLKS	FYDDFIRAF	1	0.3260	1469.0	29.00	0.34	tr_C9ZNH3_
HLA-DQA10501-DQB10201	354	ALNAAVPPLTKPETP	LNAAVPPLT	1	0.3259	1471.0	29.00	0.56	tr_C9ZNH3_
HLA-DQA10501-DQB10201	355	LGVVDDATLSTPDVV	ATLSTPDVV	6	0.3258	1472.8	29.00	0.55	tr_C9ZNH3_
HLA-DQA10501-DQB10201	356	IFAPQVSDAMRFLMK	FAPQVSDAM	1	0.3250	1485.5	29.00	0.37	tr_C9ZNH3_
HLA-DQA10501-DQB10201	357	DYLPRPAADGLITFD	PAADGLITF	5	0.3246	1492.1	29.00	0.31	tr_C9ZNH3_
HLA-DQA10501-DQB10201	358	LLRSIIEENKNEFFV	IEENKNEFF	5	0.3237	1505.7	30.00	0.47	tr_C9ZNH3_
HLA-DQA10501-DQB10201	359	EFTATWELFAGTHPQ	WELFAGTHP	5	0.3234	1511.3	30.00	0.34	tr_C9ZNH3_
HLA-DQA10501-DQB10201	360	FVVVGPLSDSDTLSV	FVVVGPLSD	0	0.3232	1515.2	30.00	0.41	tr_C9ZNH3_
HLA-DQA10501-DQB10201	361	PHVTNIEIAWTLAGT	VTNIEIAWT	2	0.3228	1521.4	30.00	0.28	tr_C9ZNH3_
HLA-DQA10501-DQB10201	362	YEVPPPELLRSIIEE	ELLRSIIEE	6	0.3224	1527.9	30.00	0.48	tr_C9ZNH3_
HLA-DQA10501-DQB10201	363	LIGLTASDVGGIAAH	LIGLTASDV	0	0.3222	1530.3	30.00	0.52	tr_C9ZNH3_
HLA-DQA10501-DQB10201	364	NASTLHAVMRDDATR	LHAVMRDDA	4	0.3219	1536.3	30.00	0.41	tr_C9ZNH3_
HLA-DQA10501-DQB10201	365	ALFYDDFIRAFRGLK	FYDDFIRAF	2	0.3196	1574.2	31.00	0.38	tr_C9ZNH3_
HLA-DQA10501-DQB10201	366	PPPELLRSIIEENKN	LLRSIIEEN	4	0.3196	1575.1	31.00	0.38	tr_C9ZNH3_
HLA-DQA10501-DQB10201	367	TLHAVMRDDATRLIE	VMRDDATRL	4	0.3180	1601.9	31.00	0.33	tr_C9ZNH3_
HLA-DQA10501-DQB10201	368	EEDLVAFAPFTGSDA	DLVAFAPFT	2	0.3178	1604.8	31.00	0.31	tr_C9ZNH3_
HLA-DQA10501-DQB10201	369	DGCLVNYGAMHISLW	YGAMHISLW	6	0.3173	1614.6	31.00	0.31	tr_C9ZNH3_
HLA-DQA10501-DQB10201	370	YGAFADEDCSRIVGN	YGAFADEDC	0	0.3159	1638.4	31.00	0.57	tr_C9ZNH3_
HLA-DQA10501-DQB10201	371	VSPAAELLALLRYAV	PAAELLALL	2	0.3158	1641.1	31.00	0.35	tr_C9ZNH3_
HLA-DQA10501-DQB10201	372	QLHLLRIGFMYLQNV	IGFMYLQNV	6	0.3157	1642.6	31.00	0.40	tr_C9ZNH3_
HLA-DQA10501-DQB10201	373	PELLRSIIEENKNEF	LLRSIIEEN	2	0.3153	1648.8	32.00	0.34	tr_C9ZNH3_
HLA-DQA10501-DQB10201	374	GDFGGECEPLAAAYG	FGGECEPLA	2	0.3152	1650.8	32.00	0.46	tr_C9ZNH3_
HLA-DQA10501-DQB10201	375	GTHPQGVMIFAPQVS	VMIFAPQVS	6	0.3150	1655.0	32.00	0.43	tr_C9ZNH3_
HLA-DQA10501-DQB10201	376	LAPLYTLLFSSVNDP	LLFSSVNDP	6	0.3147	1660.9	32.00	0.41	tr_C9ZNH3_
HLA-DQA10501-DQB10201	377	AVMRDDATRLIETVV	ATRLIETVV	6	0.3146	1662.6	32.00	0.42	tr_C9ZNH3_
HLA-DQA10501-DQB10201	378	DVVFIDPITLNPRLR	VVFIDPITL	1	0.3140	1672.2	32.00	0.44	tr_C9ZNH3_
HLA-DQA10501-DQB10201	379	RIGFMYLQNVHFGDS	FMYLQNVHF	3	0.3129	1692.1	32.00	0.46	tr_C9ZNH3_
HLA-DQA10501-DQB10201	380	GLKSAERLIFATNLP	ERLIFATNL	5	0.3113	1723.3	32.00	0.49	tr_C9ZNH3_
HLA-DQA10501-DQB10201	381	PPELLRSIIEENKNE	LLRSIIEEN	3	0.3104	1740.0	33.00	0.38	tr_C9ZNH3_
HLA-DQA10501-DQB10201	382	LRSIIEENKNEFFVV	IEENKNEFF	4	0.3104	1740.3	33.00	0.51	tr_C9ZNH3_
HLA-DQA10501-DQB10201	383	RSIIEENKNEFFVVV	IEENKNEFF	3	0.3102	1743.2	33.00	0.51	tr_C9ZNH3_
HLA-DQA10501-DQB10201	384	HVTNIEIAWTLAGTT	VTNIEIAWT	1	0.3085	1775.5	33.00	0.28	tr_C9ZNH3_
HLA-DQA10501-DQB10201	385	RIVGNHTDAVDGCLV	TDAVDGCLV	6	0.3082	1781.8	33.00	0.38	tr_C9ZNH3_
HLA-DQA10501-DQB10201	386	EDLVAFAPFTGSDAV	DLVAFAPFT	1	0.3079	1787.5	33.00	0.28	tr_C9ZNH3_
HLA-DQA10501-DQB10201	387	SVVSLDRDAPLNAAL	VSLDRDAPL	2	0.3077	1791.0	33.00	0.44	tr_C9ZNH3_
HLA-DQA10501-DQB10201	388	ASDVGGIAAHLSANR	VGGIAAHLS	3	0.3062	1820.6	34.00	0.55	tr_C9ZNH3_
HLA-DQA10501-DQB10201	389	IGFMYLQNVHFGDSE	FMYLQNVHF	2	0.3061	1822.7	34.00	0.41	tr_C9ZNH3_
HLA-DQA10501-DQB10201	390	LLRYAVSQLHLLRIG	RYAVSQLHL	2	0.3045	1853.7	34.00	0.28	tr_C9ZNH3_
HLA-DQA10501-DQB10201	391	AAYGGQVMFGWIVGE	VMFGWIVGE	6	0.3041	1861.1	34.00	0.32	tr_C9ZNH3_
HLA-DQA10501-DQB10201	392	SIIEENKNEFFVVVG	IEENKNEFF	2	0.3034	1877.3	34.00	0.47	tr_C9ZNH3_
HLA-DQA10501-DQB10201	393	DAAYGGQVMFGWIVG	AYGGQVMFG	2	0.3028	1888.4	34.00	0.29	tr_C9ZNH3_
HLA-DQA10501-DQB10201	394	ARALECSEFLKSRMT	RALECSEFL	1	0.3021	1903.0	35.00	0.62	tr_C9ZNH3_
HLA-DQA10501-DQB10201	395	IAGYLASENASTLHA	AGYLASENA	1	0.3020	1905.1	35.00	0.50	tr_C9ZNH3_

HLA-DQA10501-DQB10201	396	ILSPTPHVTNIEIAW	PHVTNIEIA	5	0.3018	1909.5	35.00	0.48	tr_C9ZNH3_
HLA-DQA10501-DQB10201	397	LKSAERLIFATNLPH	ERLIFATNL	4	0.3009	1928.2	35.00	0.43	tr_C9ZNH3_
HLA-DQA10501-DQB10201	398	DFIRAFRGLKSAERL	RGLKSAERL	6	0.3005	1936.7	35.00	0.39	tr_C9ZNH3_
HLA-DQA10501-DQB10201	399	IRAFRGLKSAERLIF	RGLKSAERL	4	0.2998	1951.8	35.00	0.41	tr_C9ZNH3_
HLA-DQA10501-DQB10201	400	LHLLRIGFMYLQNVH	IGFMYLQNV	5	0.2990	1967.2	35.00	0.31	tr_C9ZNH3_
HLA-DQA10501-DQB10201	401	VNYGAMHISLWPLAR	YGAMHISLW	2	0.2989	1970.5	35.00	0.31	tr_C9ZNH3_
HLA-DQA10501-DQB10201	402	WELFAGTHPQGVMIF	FAGTHPQGV	3	0.2987	1975.2	35.00	0.35	tr_C9ZNH3_
HLA-DQA10501-DQB10201	403	GVNAGLHASFAVRQW	LHASFAVRQ	5	0.2982	1985.8	36.00	0.31	tr_C9ZNH3_
HLA-DQA10501-DQB10201	404	LLYDPFLPDAYNNGV	YDPFLPDAY	2	0.2981	1986.0	36.00	0.50	tr_C9ZNH3_
HLA-DQA10501-DQB10201	405	VKMFPGQVVLSGVLP	QVVLSGVLP	6	0.2981	1986.4	36.00	0.44	tr_C9ZNH3_
HLA-DQA10501-DQB10201	406	ATWELFAGTHPQGVM	WELFAGTHP	2	0.2978	1993.5	36.00	0.34	tr_C9ZNH3_
HLA-DQA10501-DQB10201	407	TATWELFAGTHPQGV	WELFAGTHP	3	0.2971	2008.2	36.00	0.34	tr_C9ZNH3_
HLA-DQA10501-DQB10201	408	LSGVLPLVSDNGFRA	LSGVLPLVS	0	0.2969	2013.2	36.00	0.35	tr_C9ZNH3_
HLA-DQA10501-DQB10201	409	MGRSLCGVFTLESSF	GVFTLESSF	6	0.2949	2056.4	36.00	0.27	tr_C9ZNH3_
HLA-DQA10501-DQB10201	410	RGLKSAERLIFATNL	ERLIFATNL	6	0.2932	2094.8	37.00	0.56	tr_C9ZNH3_
HLA-DQA10501-DQB10201	411	TWELFAGTHPQGVMI	FAGTHPQGV	4	0.2931	2096.9	37.00	0.36	tr_C9ZNH3_
HLA-DQA10501-DQB10201	412	FIRAFRGLKSAERLI	RGLKSAERL	5	0.2927	2106.8	37.00	0.40	tr_C9ZNH3_
HLA-DQA10501-DQB10201	413	SDVGGIAAHLSANRN	VGGIAAHLS	2	0.2922	2118.8	37.00	0.54	tr_C9ZNH3_
HLA-DQA10501-DQB10201	414	EPLAAAYGATCYCNE	AAAYGATCY	3	0.2921	2119.9	37.00	0.35	tr_C9ZNH3_
HLA-DQA10501-DQB10201	415	VMRDDATRLIETVVN	ATRLIETVV	5	0.2918	2128.2	37.00	0.36	tr_C9ZNH3_
HLA-DQA10501-DQB10201	416	RKMLDTRAVTSVLGV	RAVTSVLGV	6	0.2916	2131.2	37.00	0.41	tr_C9ZNH3_
HLA-DQA10501-DQB10201	417	RMTFMDSLYNQRRYV	RMTFMDSLY	0	0.2913	2138.3	37.00	0.32	tr_C9ZNH3_
HLA-DQA10501-DQB10201	418	RYAVSQLHLLRIGFM	LHLLRIGFM	6	0.2909	2148.1	37.00	0.34	tr_C9ZNH3_
HLA-DQA10501-DQB10201	419	ECEPLAAAYGATCYC	AAAYGATCY	5	0.2907	2152.7	37.00	0.45	tr_C9ZNH3_
HLA-DQA10501-DQB10201	420	SPAAELLALLRYAVS	PAAELLALL	1	0.2891	2189.7	38.00	0.35	tr_C9ZNH3_
HLA-DQA10501-DQB10201	421	KMLDTRAVTSVLGVV	RAVTSVLGV	5	0.2890	2191.5	38.00	0.34	tr_C9ZNH3_
HLA-DQA10501-DQB10201	422	IGLTASDVGGIAAHL	DVGGIAAHL	6	0.2871	2239.3	38.00	0.44	tr_C9ZNH3_
HLA-DQA10501-DQB10201	423	RAFRGLKSAERLIFA	RGLKSAERL	3	0.2864	2254.9	38.00	0.38	tr_C9ZNH3_
HLA-DQA10501-DQB10201	424	FAPQVSDAMRFLMKL	FAPQVSDAM	0	0.2859	2267.1	39.00	0.29	tr_C9ZNH3_
HLA-DQA10501-DQB10201	425	FYDDFIRAFRGLKSA	FYDDFIRAF	0	0.2854	2278.6	39.00	0.29	tr_C9ZNH3_
HLA-DQA10501-DQB10201	426	HSVPSTSGNVMSDLR	TSGNVMSDL	5	0.2849	2292.1	39.00	0.41	tr_C9ZNH3_
HLA-DQA10501-DQB10201	427	LRHPGRNVIYLSPTL	NVIYLSPTL	6	0.2839	2317.2	39.00	0.45	tr_C9ZNH3_
HLA-DQA10501-DQB10201	428	VRGWSPNAYFLHVSP	NAYFLHVSP	6	0.2837	2321.4	39.00	0.31	tr_C9ZNH3_
HLA-DQA10501-DQB10201	429	RLIFATNLPHWADHR	IFATNLPHW	2	0.2833	2332.2	39.00	0.37	tr_C9ZNH3_
HLA-DQA10501-DQB10201	430	DFGGECEPLAAAYGA	FGGECEPLA	1	0.2830	2340.0	39.00	0.46	tr_C9ZNH3_
HLA-DQA10501-DQB10201	431	AVRQWSTASNVNVEV	TASNVNVEV	6	0.2828	2345.0	39.00	0.45	tr_C9ZNH3_
HLA-DQA10501-DQB10201	432	FTATWELFAGTHPQG	WELFAGTHP	4	0.2818	2370.6	40.00	0.41	tr_C9ZNH3_
HLA-DQA10501-DQB10201	433	HAVMRDDATRLIETV	VMRDDATRL	2	0.2817	2373.3	40.00	0.41	tr_C9ZNH3_
HLA-DQA10501-DQB10201	434	IYLSPTLEQQLFVIA	PTLEQQLFV	4	0.2813	2382.8	40.00	0.28	tr_C9ZNH3_
HLA-DQA10501-DQB10201	435	ISLWPLARALNAAVP	ARALNAAVP	6	0.2808	2395.5	40.00	0.34	tr_C9ZNH3_
HLA-DQA10501-DQB10201	436	VDGCLVNYGAMHISL	LVNYGAMHI	4	0.2806	2400.0	40.00	0.41	tr_C9ZNH3_
HLA-DQA10501-DQB10201	437	RGWSPNAYFLHVSPA	NAYFLHVSP	5	0.2806	2402.6	40.00	0.23	tr_C9ZNH3_
HLA-DQA10501-DQB10201	438	LHAVMRDDATRLIET	VMRDDATRL	3	0.2804	2407.1	40.00	0.40	tr_C9ZNH3_
HLA-DQA10501-DQB10201	439	VNAGLHASFAVRQWS	LHASFAVRQ	4	0.2793	2435.1	40.00	0.34	tr_C9ZNH3_
HLA-DQA10501-DQB10201	440	VVSLDRDAPLNAALP	VSLDRDAPL	1	0.2786	2452.6	40.00	0.41	tr_C9ZNH3_
HLA-DQA10501-DQB10201	441	VTNIEIAWTLAGTTS	IAWTLAGTT	5	0.2783	2461.3	41.00	0.25	tr_C9ZNH3_
HLA-DQA10501-DQB10201	442	IVGNHTDAVDGCLVN	TDAVDGCLV	5	0.2783	2461.7	41.00	0.41	tr_C9ZNH3_
HLA-DQA10501-DQB10201	443	VQMLAPLYTLLFSSV	VQMLAPLYT	0	0.2782	2464.0	41.00	0.32	tr_C9ZNH3_
HLA-DQA10501-DQB10201	444	DDYLPRPAADGLITF	PAADGLITF	6	0.2777	2476.5	41.00	0.38	tr_C9ZNH3_
HLA-DQA10501-DQB10201	445	DLVAFAPFTGSDAVR	DLVAFAPFT	0	0.2772	2491.8	41.00	0.25	tr_C9ZNH3_

HLA-DQA10501-DQB10201	446	SRIVGNHTDAVDGCL	IVGNHTDAV	2	0.2765	2510.7	41.00	0.37	tr_C9ZNH3_
HLA-DQA10501-DQB10201	447	LRYAVSQLHLLRIGF	RYAVSQLHL	1	0.2758	2530.5	41.00	0.31	tr_C9ZNH3_
HLA-DQA10501-DQB10201	448	CEPLAAAYGATCYCN	AAAYGATCY	4	0.2754	2539.6	41.00	0.41	tr_C9ZNH3_
HLA-DQA10501-DQB10201	449	NYGAMHISLWPLARA	YGAMHISLW	1	0.2750	2552.3	41.00	0.29	tr_C9ZNH3_
HLA-DQA10501-DQB10201	450	QMGRSLCGVFTLESS	GRSLCGVFT	2	0.2731	2603.6	42.00	0.24	tr_C9ZNH3_
HLA-DQA10501-DQB10201	451	DAMRFLMKLVTDNRT	LMKLVTDNR	5	0.2722	2630.4	42.00	0.31	tr_C9ZNH3_
HLA-DQA10501-DQB10201	452	ASENASTLHAVMRDD	STLHAVMRD	5	0.2717	2643.2	42.00	0.38	tr_C9ZNH3_
HLA-DQA10501-DQB10201	453	GECEPLAAAYGATCY	AAAYGATCY	6	0.2714	2653.9	42.00	0.54	tr_C9ZNH3_
HLA-DQA10501-DQB10201	454	NGVNAGLHASFAVRQ	LHASFAVRQ	6	0.2713	2656.6	42.00	0.35	tr_C9ZNH3_
HLA-DQA10501-DQB10201	455	GKSERLFIHSVPSTS	FIHSVPSTS	6	0.2703	2683.0	43.00	0.32	tr_C9ZNH3_
HLA-DQA10501-DQB10201	456	AELLALLRYAVSQLH	LRYAVSQLH	6	0.2701	2688.8	43.00	0.46	tr_C9ZNH3_
HLA-DQA10501-DQB10201	457	MSQMGRSLCGVFTLE	GRSLCGVFT	4	0.2692	2716.7	43.00	0.34	tr_C9ZNH3_
HLA-DQA10501-DQB10201	458	AFRGLKSAERLIFAT	RGLKSAERL	2	0.2691	2719.5	43.00	0.38	tr_C9ZNH3_
HLA-DQA10501-DQB10201	459	SQMGRSLCGVFTLES	GRSLCGVFT	3	0.2689	2724.5	43.00	0.27	tr_C9ZNH3_
HLA-DQA10501-DQB10201	460	SPTLEQQLFVIAGYL	QLFVIAGYL	6	0.2688	2727.6	43.00	0.39	tr_C9ZNH3_
HLA-DQA10501-DQB10201	461	EDCSRIVGNHTDAVD	IVGNHTDAV	5	0.2683	2741.8	43.00	0.47	tr_C9ZNH3_
HLA-DQA10501-DQB10201	462	AMRFLMKLVTDNRTS	LMKLVTDNR	4	0.2672	2776.9	43.00	0.34	tr_C9ZNH3_
HLA-DQA10501-DQB10201	463	YAVSQLHLLRIGFMY	LHLLRIGFM	5	0.2668	2788.3	44.00	0.32	tr_C9ZNH3_
HLA-DQA10501-DQB10201	464	KVLTTIYTHSVISVD	YTHSVISVD	6	0.2663	2803.3	44.00	0.37	tr_C9ZNH3_
HLA-DQA10501-DQB10201	465	IHSVPSTSGNVMSDL	TSGNVMSDL	6	0.2663	2803.3	44.00	0.58	tr_C9ZNH3_
HLA-DQA10501-DQB10201	466	YELAVELMSQMGRSL	ELAVELMSQ	1	0.2663	2803.9	44.00	0.43	tr_C9ZNH3_
HLA-DQA10501-DQB10201	467	PLAAAYGATCYCNEG	AAAYGATCY	2	0.2655	2828.5	44.00	0.34	tr_C9ZNH3_
HLA-DQA10501-DQB10201	468	SQLHLLRIGFMYLQN	RIGFMYLQN	6	0.2651	2841.1	44.00	0.31	tr_C9ZNH3_
HLA-DQA10501-DQB10201	469	YILSPTPHVTNIEIA	PHVTNIEIA	6	0.2650	2842.1	44.00	0.56	tr_C9ZNH3_
HLA-DQA10501-DQB10201	470	AAELLALLRYAVSQL	LLRYAVSQL	6	0.2648	2848.2	44.00	0.46	tr_C9ZNH3_
HLA-DQA10501-DQB10201	471	CSRIVGNHTDAVDGC	IVGNHTDAV	3	0.2635	2887.8	44.00	0.43	tr_C9ZNH3_
HLA-DQA10501-DQB10201	472	VGNHTDAVDGCLVNY	TDAVDGCLV	4	0.2632	2898.0	45.00	0.40	tr_C9ZNH3_
HLA-DQA10501-DQB10201	473	DVGGIAAHLSANRNV	VGGIAAHLS	1	0.2616	2950.4	45.00	0.54	tr_C9ZNH3_
HLA-DQA10501-DQB10201	474	AVRGWSPNAYFLHVS	VRGWSPNAY	1	0.2604	2989.3	45.00	0.29	tr_C9ZNH3_
HLA-DQA10501-DQB10201	475	TFDACDESGVQMLAP	ESGVQMLAP	6	0.2599	3003.9	45.00	0.38	tr_C9ZNH3_
HLA-DQA10501-DQB10201	476	LMSQMGRSLCGVFTL	GRSLCGVFT	5	0.2595	3017.6	46.00	0.40	tr_C9ZNH3_
HLA-DQA10501-DQB10201	477	ITFDACDESGVQMLA	TFDACDESG	1	0.2593	3024.6	46.00	0.29	tr_C9ZNH3_
HLA-DQA10501-DQB10201	478	DAVDGCLVNYGAMHI	LVNYGAMHI	6	0.2592	3025.8	46.00	0.53	tr_C9ZNH3_
HLA-DQA10501-DQB10201	479	LIFATNLPHWADHRP	FATNLPHWA	2	0.2589	3037.0	46.00	0.47	tr_C9ZNH3_
HLA-DQA10501-DQB10201	480	KSERLFIHSVPSTSG	FIHSVPSTS	5	0.2584	3052.6	46.00	0.32	tr_C9ZNH3_
HLA-DQA10501-DQB10201	481	YNNGVNAGLHASFAV	NAGLHASFA	5	0.2573	3090.8	46.00	0.40	tr_C9ZNH3_
HLA-DQA10501-DQB10201	482	RDDATRLIETVVNRT	ATRLIETVV	3	0.2572	3092.3	46.00	0.26	tr_C9ZNH3_
HLA-DQA10501-DQB10201	483	GSDAVRGWSPNAYFL	VRGWSPNAY	4	0.2570	3099.0	46.00	0.56	tr_C9ZNH3_
HLA-DQA10501-DQB10201	484	FDACDESGVQMLAPL	ESGVQMLAP	5	0.2565	3117.6	46.00	0.41	tr_C9ZNH3_
HLA-DQA10501-DQB10201	485	MRFLMKLVTDNRTSS	LMKLVTDNR	3	0.2564	3118.5	46.00	0.34	tr_C9ZNH3_
HLA-DQA10501-DQB10201	486	VAFAPFTGSDAVRGW	FTGSDAVRG	5	0.2563	3122.7	46.00	0.32	tr_C9ZNH3_
HLA-DQA10501-DQB10201	487	MLAPLYTLLFSSVND	TLLFSSVND	6	0.2558	3141.7	47.00	0.38	tr_C9ZNH3_
HLA-DQA10501-DQB10201	488	PAAELLALLRYAVSQ	AAELLALLR	1	0.2556	3148.5	47.00	0.34	tr_C9ZNH3_
HLA-DQA10501-DQB10201	489	SVPSTSGNVMSDLRK	TSGNVMSDL	4	0.2549	3172.8	47.00	0.40	tr_C9ZNH3_
HLA-DQA10501-DQB10201	490	AGTHPQGVMIFAPQV	GVMIFAPQV	6	0.2542	3194.7	47.00	0.50	tr_C9ZNH3_
HLA-DQA10501-DQB10201	491	IVGVDPQKVLTTIYT	IVGVDPQKV	0	0.2533	3226.6	47.00	0.55	tr_C9ZNH3_
HLA-DQA10501-DQB10201	492	MRDDATRLIETVVNR	ATRLIETVV	4	0.2530	3235.8	47.00	0.38	tr_C9ZNH3_
HLA-DQA10501-DQB10201	493	CNEGGRMIYMMILGD	MIYMMILGD	6	0.2527	3248.1	47.00	0.37	tr_C9ZNH3_
HLA-DQA10501-DQB10201	494	DDATRLIETVVNRTL	ATRLIETVV	2	0.2525	3252.8	48.00	0.25	tr_C9ZNH3_
HLA-DQA10501-DQB10201	495	LGKSERLFIHSVPST	LFIHSVPST	6	0.2521	3270.1	48.00	0.36	tr_C9ZNH3_

HLA-DQA10501-DQB10201	496	DCSRIVGNHTDAVDG	IVGNHTDAV	4	0.2520	3271.4	48.00	0.47	tr_C9ZNH3_
HLA-DQA10501-DQB10201	497	TGSDAVRGWSPNAYF	VRGWSPNAY	5	0.2520	3272.8	48.00	0.60	tr_C9ZNH3_
HLA-DQA10501-DQB10201	498	NRTSSAYILSPTPHV	YILSPTPHV	6	0.2518	3279.5	48.00	0.28	tr_C9ZNH3_
HLA-DQA10501-DQB10201	499	VGPLSDSDTLSVLPS	SDTLSVLPS	6	0.2516	3287.6	48.00	0.22	tr_C9ZNH3_
HLA-DQA10501-DQB10201	500	PTLEQQLFVIAGYLA	QLFVIAGYL	5	0.2514	3295.0	48.00	0.38	tr_C9ZNH3_
HLA-DQA10501-DQB10201	501	MILGDDYLPRPAADG	MILGDDYLP	0	0.2512	3299.7	48.00	0.44	tr_C9ZNH3_
HLA-DQA10501-DQB10201	502	LSPTLEQQLFVIAGY	QQLFVIAGY	6	0.2498	3352.0	48.00	0.34	tr_C9ZNH3_
HLA-DQA10501-DQB10201	503	LVAFAPFTGSDAVRG	FTGSDAVRG	6	0.2497	3353.6	48.00	0.28	tr_C9ZNH3_
HLA-DQA10501-DQB10201	504	DAVRGWSPNAYFLHV	VRGWSPNAY	2	0.2497	3356.1	48.00	0.46	tr_C9ZNH3_
HLA-DQA10501-DQB10201	505	ELFAGTHPQGVMIFA	FAGTHPQGV	2	0.2478	3425.3	49.00	0.42	tr_C9ZNH3_
HLA-DQA10501-DQB10201	506	NAGLHASFAVRQWST	LHASFAVRQ	3	0.2477	3426.7	49.00	0.35	tr_C9ZNH3_
HLA-DQA10501-DQB10201	507	VPSTSGNVMSDLRKM	TSGNVMSDL	3	0.2477	3427.6	49.00	0.38	tr_C9ZNH3_
HLA-DQA10501-DQB10201	508	SLDRDAPLNAALPTD	PLNAALPTD	6	0.2474	3440.0	49.00	0.44	tr_C9ZNH3_
HLA-DQA10501-DQB10201	509	LYDPFLPDAYNNGVN	YDPFLPDAY	1	0.2463	3481.8	49.00	0.54	tr_C9ZNH3_
HLA-DQA10501-DQB10201	510	SERLFIHSVPSTSGN	FIHSVPSTS	4	0.2455	3511.9	49.00	0.34	tr_C9ZNH3_
HLA-DQA10501-DQB10201	511	LAAAYGATCYCNEGG	AAAYGATCY	1	0.2452	3522.4	49.00	0.34	tr_C9ZNH3_
HLA-DQA10501-DQB10201	512	GNHTDAVDGCLVNYG	TDAVDGCLV	3	0.2443	3556.2	50.00	0.34	tr_C9ZNH3_
HLA-DQA10501-DQB10201	513	AVDGCLVNYGAMHIS	LVNYGAMHI	5	0.2436	3583.1	50.00	0.53	tr_C9ZNH3_
HLA-DQA10501-DQB10201	514	YLSPTLEQQLFVIAG	PTLEQQLFV	3	0.2435	3587.7	50.00	0.32	tr_C9ZNH3_
HLA-DQA10501-DQB10201	515	SDAMRFLMKLVTDNR	LMKLVTDNR	6	0.2432	3597.7	50.00	0.35	tr_C9ZNH3_
HLA-DQA10501-DQB10201	516	AFAPFTGSDAVRGWS	FTGSDAVRG	4	0.2425	3624.9	50.00	0.33	tr_C9ZNH3_
HLA-DQA10501-DQB10201	517	SDAVRGWSPNAYFLH	VRGWSPNAY	3	0.2424	3628.9	50.00	0.53	tr_C9ZNH3_
HLA-DQA10501-DQB10201	518	VVFIDPITLNPRLRH	VVFIDPITL	0	0.2422	3636.3	50.00	0.48	tr_C9ZNH3_
HLA-DQA10501-DQB10201	519	TNIEIAWTLAGTTSG	IAWTLAGTT	4	0.2418	3655.6	50.00	0.29	tr_C9ZNH3_
HLA-DQA10501-DQB10201	520	LRKMLDTRAVTSVLG	TRAVTSVLG	6	0.2417	3659.7	50.00	0.45	tr_C9ZNH3_
HLA-DQA10501-DQB10201	521	YGAMHISLWPLARAL	YGAMHISLW	0	0.2410	3686.6	55.00	0.25	tr_C9ZNH3_
HLA-DQA10501-DQB10201	522	LFAGTHPQGVMIFAP	FAGTHPQGV	1	0.2408	3692.1	55.00	0.31	tr_C9ZNH3_
HLA-DQA10501-DQB10201	523	RTSSAYILSPTPHVT	YILSPTPHV	5	0.2408	3693.0	55.00	0.30	tr_C9ZNH3_
HLA-DQA10501-DQB10201	524	FTGSDAVRGWSPNAY	VRGWSPNAY	6	0.2408	3694.7	55.00	0.55	tr_C9ZNH3_
HLA-DQA10501-DQB10201	525	STSGNVMSDLRKMLD	SGNVMSDLR	2	0.2404	3708.5	55.00	0.35	tr_C9ZNH3_
HLA-DQA10501-DQB10201	526	RFLMKLVTDNRTSSA	LMKLVTDNR	2	0.2402	3718.2	55.00	0.35	tr_C9ZNH3_
HLA-DQA10501-DQB10201	527	LASENASTLHAVMRD	STLHAVMRD	6	0.2399	3731.6	55.00	0.49	tr_C9ZNH3_
HLA-DQA10501-DQB10201	528	AGYLASENASTLHAV	AGYLASENA	0	0.2398	3733.1	55.00	0.41	tr_C9ZNH3_
HLA-DQA10501-DQB10201	529	FRGLKSAERLIFATN	RGLKSAERL	1	0.2394	3750.8	55.00	0.37	tr_C9ZNH3_
HLA-DQA10501-DQB10201	530	TLEQQLFVIAGYLAS	QLFVIAGYL	4	0.2392	3757.6	55.00	0.32	tr_C9ZNH3_
HLA-DQA10501-DQB10201	531	VVGPLSDSDTLSVLP	PLSDSDTLS	3	0.2389	3772.3	55.00	0.25	tr_C9ZNH3_
HLA-DQA10501-DQB10201	532	AVSQLHLLRIGFMYL	LHLLRIGFM	4	0.2385	3786.7	55.00	0.46	tr_C9ZNH3_
HLA-DQA10501-DQB10201	533	PSTSGNVMSDLRKML	SGNVMSDLR	3	0.2384	3790.8	55.00	0.40	tr_C9ZNH3_
HLA-DQA10501-DQB10201	534	HISLWPLARALNAAV	WPLARALNA	4	0.2382	3800.3	55.00	0.34	tr_C9ZNH3_
HLA-DQA10501-DQB10201	535	NHTDAVDGCLVNYGA	TDAVDGCLV	2	0.2377	3821.3	55.00	0.38	tr_C9ZNH3_
HLA-DQA10501-DQB10201	536	FAPFTGSDAVRGWSP	FTGSDAVRG	3	0.2375	3828.3	55.00	0.40	tr_C9ZNH3_
HLA-DQA10501-DQB10201	537	LDRDAPLNAALPTDG	PLNAALPTD	5	0.2372	3841.1	55.00	0.41	tr_C9ZNH3_
HLA-DQA10501-DQB10201	538	ADIKAYALSGTGAVE	AYALSGTGA	4	0.2365	3870.4	55.00	0.44	tr_C9ZNH3_
HLA-DQA10501-DQB10201	539	DDFIRAFRGLKSAER	FRGLKSAER	6	0.2351	3930.3	55.00	0.29	tr_C9ZNH3_
HLA-DQA10501-DQB10201	540	VSLDRDAPLNAALPT	APLNAALPT	6	0.2347	3944.3	55.00	0.26	tr_C9ZNH3_
HLA-DQA10501-DQB10201	541	NNGVNAGLHASFAVR	NAGLHASFA	4	0.2325	4038.9	55.00	0.38	tr_C9ZNH3_
HLA-DQA10501-DQB10201	542	YDDFIRAFRGLKSAE	IRAFRGLKS	4	0.2322	4055.0	55.00	0.37	tr_C9ZNH3_
HLA-DQA10501-DQB10201	543	ELMSQMGRSLCGVFT	GRSLCGVFT	6	0.2320	4062.9	55.00	0.50	tr_C9ZNH3_
HLA-DQA10501-DQB10201	544	DATRLIETVVNRTLL	LIETVVNRT	4	0.2320	4064.3	55.00	0.26	tr_C9ZNH3_
HLA-DQA10501-DQB10201	545	VVVGPLSDSDTLSVL	VVGPLSDSD	1	0.2319	4065.6	55.00	0.19	tr_C9ZNH3_

HLA-DQA10501-DQB10201	546	NIEIAWTLAGTTSGV	IAWTLAGTT	3	0.2308	4115.7	55.00	0.29	tr_C9ZNH3_
HLA-DQA10501-DQB10201	547	RATRRLRADIKAYAL	RADIKAYAL	6	0.2301	4146.7	55.00	0.36	tr_C9ZNH3_
HLA-DQA10501-DQB10201	548	RALECSEFLKSRMTF	RALECSEFL	0	0.2300	4150.4	55.00	0.62	tr_C9ZNH3_
HLA-DQA10501-DQB10201	549	VSQLHLLRIGFMYLQ	LHLLRIGFM	3	0.2283	4229.1	55.00	0.44	tr_C9ZNH3_
HLA-DQA10501-DQB10201	550	DAPLNAALPTDGSLL	PLNAALPTD	2	0.2280	4242.5	55.00	0.34	tr_C9ZNH3_
HLA-DQA10501-DQB10201	551	TSSAYILSPTPHVTN	YILSPTPHV	4	0.2278	4250.7	55.00	0.32	tr_C9ZNH3_
HLA-DQA10501-DQB10201	552	DRDAPLNAALPTDGS	PLNAALPTD	4	0.2278	4252.0	55.00	0.39	tr_C9ZNH3_
HLA-DQA10501-DQB10201	553	CYCNEGGRMIYMMIL	GRMIYMMIL	6	0.2266	4307.7	55.00	0.40	tr_C9ZNH3_
HLA-DQA10501-DQB10201	554	RDAPLNAALPTDGSL	PLNAALPTD	3	0.2260	4336.6	55.00	0.38	tr_C9ZNH3_
HLA-DQA10501-DQB10201	555	TDNRTSSAYILSPTP	SAYILSPTP	6	0.2251	4377.3	55.00	0.28	tr_C9ZNH3_
HLA-DQA10501-DQB10201	556	APQVSDAMRFLMKLV	PQVSDAMRF	1	0.2245	4407.1	55.00	0.30	tr_C9ZNH3_
HLA-DQA10501-DQB10201	557	DNRTSSAYILSPTPH	SAYILSPTP	5	0.2243	4414.1	55.00	0.25	tr_C9ZNH3_
HLA-DQA10501-DQB10201	558	SGVLPLVSDNGFRAT	LPLVSDNGF	3	0.2238	4437.7	60.00	0.33	tr_C9ZNH3_
HLA-DQA10501-DQB10201	559	MHISLWPLARALNAA	WPLARALNA	5	0.2237	4446.4	60.00	0.44	tr_C9ZNH3_
HLA-DQA10501-DQB10201	560	AYNNGVNAGLHASFA	NAGLHASFA	6	0.2236	4449.9	60.00	0.46	tr_C9ZNH3_
HLA-DQA10501-DQB10201	561	ERLFIHSVPSTSGNV	FIHSVPSTS	3	0.2222	4519.1	60.00	0.38	tr_C9ZNH3_
HLA-DQA10501-DQB10201	562	AGLHASFAVRQWSTA	LHASFAVRQ	2	0.2213	4563.0	60.00	0.41	tr_C9ZNH3_
HLA-DQA10501-DQB10201	563	VSDAMRFLMKLVTDN	FLMKLVTDN	6	0.2212	4568.3	60.00	0.35	tr_C9ZNH3_
HLA-DQA10501-DQB10201	564	APLNAALPTDGSLLL	PLNAALPTD	1	0.2206	4594.0	60.00	0.28	tr_C9ZNH3_
HLA-DQA10501-DQB10201	565	QKVLTTIYTHSVISV	IYTHSVISV	6	0.2205	4601.8	60.00	0.48	tr_C9ZNH3_
HLA-DQA10501-DQB10201	566	IFATNLPHWADHRPS	FATNLPHWA	1	0.2197	4641.7	60.00	0.47	tr_C9ZNH3_
HLA-DQA10501-DQB10201	567	QMLAPLYTLLFSSVN	LAPLYTLLF	2	0.2196	4645.2	60.00	0.31	tr_C9ZNH3_
HLA-DQA10501-DQB10201	568	AMHISLWPLARALNA	WPLARALNA	6	0.2188	4684.5	60.00	0.47	tr_C9ZNH3_
HLA-DQA10501-DQB10201	569	ELAVELMSQMGRSLC	ELAVELMSQ	0	0.2187	4690.9	60.00	0.32	tr_C9ZNH3_
HLA-DQA10501-DQB10201	570	PLNAALPTDGSLLLI	PTDGSLLLI	6	0.2185	4700.5	60.00	0.25	tr_C9ZNH3_
HLA-DQA10501-DQB10201	571	HTDAVDGCLVNYGAM	TDAVDGCLV	1	0.2185	4702.3	60.00	0.38	tr_C9ZNH3_
HLA-DQA10501-DQB10201	572	TRLIETVVNRTLLSF	VVNRTLLSF	6	0.2171	4775.5	60.00	0.34	tr_C9ZNH3_
HLA-DQA10501-DQB10201	573	FAGTHPQGVMIFAPQ	FAGTHPQGV	0	0.2165	4804.0	60.00	0.20	tr_C9ZNH3_
HLA-DQA10501-DQB10201	574	GVKMFPGQVVLSGVL	PGQVVLSGV	5	0.2165	4804.3	60.00	0.31	tr_C9ZNH3_
HLA-DQA10501-DQB10201	575	HLGKSERLFIHSVPS	ERLFIHSVP	5	0.2159	4834.7	60.00	0.42	tr_C9ZNH3_
HLA-DQA10501-DQB10201	576	RADIKAYALSGTGAV	AYALSGTGA	5	0.2154	4860.2	60.00	0.50	tr_C9ZNH3_
HLA-DQA10501-DQB10201	577	FLMKLVTDNRTSSAY	LMKLVTDNR	1	0.2146	4901.6	60.00	0.40	tr_C9ZNH3_
HLA-DQA10501-DQB10201	578	ATRRLRADIKAYALS	RADIKAYAL	5	0.2140	4937.2	60.00	0.32	tr_C9ZNH3_
HLA-DQA10501-DQB10201	579	TRRLRADIKAYALSG	RADIKAYAL	4	0.2139	4944.1	60.00	0.29	tr_C9ZNH3_
HLA-DQA10501-DQB10201	580	MTFMDSLYNQRRYVI	FMDSLYNQR	2	0.2134	4969.6	60.00	0.38	tr_C9ZNH3_
HLA-DQA10501-DQB10201	581	TKPETPSMHYHNLEE	SMHYHNLEE	6	0.2132	4977.5	60.00	0.50	tr_C9ZNH3_
HLA-DQA10501-DQB10201	582	GHLGKSERLFIHSVP	ERLFIHSVP	6	0.2131	4985.5	60.00	0.48	tr_C9ZNH3_
HLA-DQA10501-DQB10201	583	QVSDAMRFLMKLVTD	RFLMKLVTD	6	0.2127	5004.8	60.00	0.33	tr_C9ZNH3_
HLA-DQA10501-DQB10201	584	SSAYILSPTPHVTNI	YILSPTPHV	3	0.2125	5017.8	60.00	0.41	tr_C9ZNH3_
HLA-DQA10501-DQB10201	585	YCNEGGRMIYMMILG	GRMIYMMIL	5	0.2121	5036.8	60.00	0.30	tr_C9ZNH3_
HLA-DQA10501-DQB10201	586	FAVRQWSTASNVNVE	WSTASNVNV	5	0.2119	5051.5	60.00	0.54	tr_C9ZNH3_
HLA-DQA10501-DQB10201	587	TSGNVMSDLRKMLDT	SGNVMSDLR	1	0.2115	5072.7	60.00	0.34	tr_C9ZNH3_
HLA-DQA10501-DQB10201	588	IEIAWTLAGTTSGVK	IAWTLAGTT	2	0.2114	5078.2	60.00	0.36	tr_C9ZNH3_
HLA-DQA10501-DQB10201	589	ECSEFLKSRMTFMDS	FLKSRMTFM	4	0.2114	5079.0	60.00	0.50	tr_C9ZNH3_
HLA-DQA10501-DQB10201	590	AYILSPTPHVTNIEI	YILSPTPHV	1	0.2105	5128.3	60.00	0.31	tr_C9ZNH3_
HLA-DQA10501-DQB10201	591	GYLASENASTLHAVM	NASTLHAVM	6	0.2099	5160.9	60.00	0.38	tr_C9ZNH3_
HLA-DQA10501-DQB10201	592	DEDCSRIVGNHTDAV	IVGNHTDAV	6	0.2090	5210.7	60.00	0.55	tr_C9ZNH3_
HLA-DQA10501-DQB10201	593	RLFIHSVPSTSGNVM	FIHSVPSTS	2	0.2088	5219.9	60.00	0.34	tr_C9ZNH3_
HLA-DQA10501-DQB10201	594	LRADIKAYALSGTGA	AYALSGTGA	6	0.2085	5237.6	60.00	0.46	tr_C9ZNH3_
HLA-DQA10501-DQB10201	595	RLRHPGRNVIYLSPT	RNVIYLSPT	6	0.2069	5330.1	65.00	0.37	tr_C9ZNH3_

HLA-DQA10501-DQB10201	596	SGVKMFPGQVVLSGV	PGQVVLSGV	6	0.2061	5376.1	65.00	0.37	tr_C9ZNH3_
HLA-DQA10501-DQB10201	597	RRLRADIKAYALSGT	RADIKAYAL	3	0.2057	5398.0	65.00	0.31	tr_C9ZNH3_
HLA-DQA10501-DQB10201	598	FGGECEPLAAAYGAT	FGGECEPLA	0	0.2050	5438.6	65.00	0.38	tr_C9ZNH3_
HLA-DQA10501-DQB10201	599	GVLPLVSDNGFRATR	LPLVSDNGF	2	0.2043	5480.0	65.00	0.37	tr_C9ZNH3_
HLA-DQA10501-DQB10201	600	DLRKMLDTRAVTSVL	MLDTRAVTS	4	0.2031	5556.0	65.00	0.38	tr_C9ZNH3_
HLA-DQA10501-DQB10201	601	TDAVDGCLVNYGAMH	TDAVDGCLV	0	0.2023	5603.5	65.00	0.26	tr_C9ZNH3_
HLA-DQA10501-DQB10201	602	AAAYGATCYCNEGGR	AAAYGATCY	0	0.2013	5664.9	65.00	0.31	tr_C9ZNH3_
HLA-DQA10501-DQB10201	603	GDDYLPRPAADGLIT	PRPAADGLI	5	0.2009	5685.3	65.00	0.38	tr_C9ZNH3_
HLA-DQA10501-DQB10201	604	ATRLIETVVNRTLLS	LIETVVNRT	3	0.2007	5699.1	65.00	0.31	tr_C9ZNH3_
HLA-DQA10501-DQB10201	605	PQVSDAMRFLMKLVT	QVSDAMRFL	1	0.2003	5723.8	65.00	0.23	tr_C9ZNH3_
HLA-DQA10501-DQB10201	606	SAYILSPTPHVTNIE	YILSPTPHV	2	0.2003	5727.7	65.00	0.40	tr_C9ZNH3_
HLA-DQA10501-DQB10201	607	FSSVNDPFARSVNGA	FSSVNDPFA	0	0.2001	5736.1	65.00	0.66	tr_C9ZNH3_
HLA-DQA10501-DQB10201	608	SFAVRQWSTASNVNV	WSTASNVNV	6	0.1995	5773.6	65.00	0.58	tr_C9ZNH3_
HLA-DQA10501-DQB10201	609	LECSEFLKSRMTFMD	FLKSRMTFM	5	0.1982	5855.8	65.00	0.57	tr_C9ZNH3_
HLA-DQA10501-DQB10201	610	VTDNRTSSAYILSPT	TSSAYILSP	5	0.1974	5910.5	65.00	0.30	tr_C9ZNH3_
HLA-DQA10501-DQB10201	611	RLIETVVNRTLLSFN	VVNRTLLSF	5	0.1971	5928.9	65.00	0.26	tr_C9ZNH3_
HLA-DQA10501-DQB10201	612	FRATRRLRADIKAYA	TRRLRADIK	3	0.1970	5935.0	65.00	0.33	tr_C9ZNH3_
HLA-DQA10501-DQB10201	613	LVTDNRTSSAYILSP	TSSAYILSP	6	0.1962	5986.0	65.00	0.38	tr_C9ZNH3_
HLA-DQA10501-DQB10201	614	APFTGSDAVRGWSPN	FTGSDAVRG	2	0.1957	6013.9	65.00	0.45	tr_C9ZNH3_
HLA-DQA10501-DQB10201	615	VLPLVSDNGFRATRR	LPLVSDNGF	1	0.1957	6014.3	65.00	0.37	tr_C9ZNH3_
HLA-DQA10501-DQB10201	616	GLHASFAVRQWSTAS	LHASFAVRQ	1	0.1952	6050.4	65.00	0.43	tr_C9ZNH3_
HLA-DQA10501-DQB10201	617	PTDGSLLLIGLTASD	LLIGLTASD	6	0.1947	6084.6	65.00	0.38	tr_C9ZNH3_
HLA-DQA10501-DQB10201	618	EIAWTLAGTTSGVKM	IAWTLAGTT	1	0.1935	6160.4	65.00	0.29	tr_C9ZNH3_
HLA-DQA10501-DQB10201	619	LNAAVPPLTKPETPS	LNAAVPPLT	0	0.1934	6169.6	65.00	0.77	tr_C9ZNH3_
HLA-DQA10501-DQB10201	620	LFIHSVPSTSGNVMS	FIHSVPSTS	1	0.1894	6443.6	70.00	0.37	tr_C9ZNH3_
HLA-DQA10501-DQB10201	621	LGDDYLPRPAADGLI	PRPAADGLI	6	0.1886	6497.9	70.00	0.40	tr_C9ZNH3_
HLA-DQA10501-DQB10201	622	GFRATRRLRADIKAY	TRRLRADIK	4	0.1867	6634.5	70.00	0.38	tr_C9ZNH3_
HLA-DQA10501-DQB10201	623	TCYCNEGGRMIYMMI	EGGRMIYMM	5	0.1866	6636.8	70.00	0.28	tr_C9ZNH3_
HLA-DQA10501-DQB10201	624	ALECSEFLKSRMTFM	FLKSRMTFM	6	0.1856	6709.9	70.00	0.56	tr_C9ZNH3_
HLA-DQA10501-DQB10201	625	AHLSANRNVRVFIPF	RNVRVFIPF	6	0.1852	6739.2	70.00	0.25	tr_C9ZNH3_
HLA-DQA10501-DQB10201	626	RLRADIKAYALSGTG	RADIKAYAL	2	0.1849	6761.7	70.00	0.32	tr_C9ZNH3_
HLA-DQA10501-DQB10201	627	GAMHISLWPLARALN	AMHISLWPL	1	0.1838	6842.5	70.00	0.20	tr_C9ZNH3_
HLA-DQA10501-DQB10201	628	YDPFLPDAYNNGVNA	YDPFLPDAY	0	0.1836	6857.2	70.00	0.49	tr_C9ZNH3_
HLA-DQA10501-DQB10201	629	YLASENASTLHAVMR	NASTLHAVM	5	0.1827	6927.9	70.00	0.34	tr_C9ZNH3_
HLA-DQA10501-DQB10201	630	DAYNNGVNAGLHASF	VNAGLHASF	6	0.1803	7105.7	70.00	0.42	tr_C9ZNH3_
HLA-DQA10501-DQB10201	631	GGECEPLAAAYGATC	LAAAYGATC	6	0.1798	7145.1	70.00	0.42	tr_C9ZNH3_
HLA-DQA10501-DQB10201	632	RPNASDRTPLALLGF	RTPLALLGF	6	0.1794	7177.5	70.00	0.44	tr_C9ZNH3_
HLA-DQA10501-DQB10201	633	NAALPTDGSLLLIGL	DGSLLLIGL	6	0.1790	7205.0	70.00	0.29	tr_C9ZNH3_
HLA-DQA10501-DQB10201	634	TFMDSLYNQRRYVID	FMDSLYNQR	1	0.1790	7211.7	70.00	0.36	tr_C9ZNH3_
HLA-DQA10501-DQB10201	635	SDLRKMLDTRAVTSV	MLDTRAVTS	5	0.1788	7225.3	70.00	0.44	tr_C9ZNH3_
HLA-DQA10501-DQB10201	636	NGFRATRRLRADIKA	TRRLRADIK	5	0.1782	7268.6	70.00	0.41	tr_C9ZNH3_
HLA-DQA10501-DQB10201	637	TTSGVKMFPGQVVLS	VKMFPGQVV	4	0.1778	7304.9	70.00	0.31	tr_C9ZNH3_
HLA-DQA10501-DQB10201	638	MSDLRKMLDTRAVTS	MLDTRAVTS	6	0.1770	7367.9	70.00	0.44	tr_C9ZNH3_
HLA-DQA10501-DQB10201	639	TSGVKMFPGQVVLSG	FPGQVVLSG	6	0.1764	7417.3	70.00	0.26	tr_C9ZNH3_
HLA-DQA10501-DQB10201	640	VGGIAAHLSANRNVR	VGGIAAHLS	0	0.1761	7434.8	70.00	0.47	tr_C9ZNH3_
HLA-DQA10501-DQB10201	641	RTLLSFNRSLNSVVS	FNRSLNSVV	5	0.1756	7481.9	70.00	0.31	tr_C9ZNH3_
HLA-DQA10501-DQB10201	642	ARSVNGAIHRGALFV	AIHRGALFV	6	0.1748	7540.3	70.00	0.49	tr_C9ZNH3_
HLA-DQA10501-DQB10201	643	LIETVVNRTLLSFNR	VVNRTLLSF	4	0.1748	7544.2	70.00	0.29	tr_C9ZNH3_
HLA-DQA10501-DQB10201	644	ALPTDGSLLLIGLTA	SLLLIGLTA	6	0.1743	7581.0	70.00	0.31	tr_C9ZNH3_
HLA-DQA10501-DQB10201	645	LNAALPTDGSLLLIG	PTDGSLLLI	5	0.1735	7646.7	70.00	0.22	tr_C9ZNH3_

HLA-DQA10501-DQB10201	646	VELMSQMGRSLCGVF	QMGRSLCGV	5	0.1730	7696.0	70.00	0.35	tr_C9ZNH3_
HLA-DQA10501-DQB10201	647	PFTGSDAVRGWSPNA	FTGSDAVRG	1	0.1729	7701.9	70.00	0.46	tr_C9ZNH3_
HLA-DQA10501-DQB10201	648	DNGFRATRRLRADIK	TRRLRADIK	6	0.1725	7737.3	70.00	0.42	tr_C9ZNH3_
HLA-DQA10501-DQB10201	649	AGTTSGVKMFPGQVV	VKMFPGQVV	6	0.1720	7778.7	70.00	0.41	tr_C9ZNH3_
HLA-DQA10501-DQB10201	650	NRTLLSFNRSLNSVV	FNRSLNSVV	6	0.1719	7781.8	75.00	0.38	tr_C9ZNH3_
HLA-DQA10501-DQB10201	651	LMKLVTDNRTSSAYI	MKLVTDNRT	1	0.1715	7821.3	75.00	0.39	tr_C9ZNH3_
HLA-DQA10501-DQB10201	652	PNASDRTPLALLGFT	RTPLALLGF	5	0.1710	7861.9	75.00	0.35	tr_C9ZNH3_
HLA-DQA10501-DQB10201	653	AALPTDGSLLLIGLT	DGSLLLIGL	5	0.1706	7892.0	75.00	0.20	tr_C9ZNH3_
HLA-DQA10501-DQB10201	654	DPQKVLTTIYTHSVI	LTTIYTHSV	5	0.1705	7907.2	75.00	0.25	tr_C9ZNH3_
HLA-DQA10501-DQB10201	655	GTTSGVKMFPGQVVL	VKMFPGQVV	5	0.1694	8001.4	75.00	0.43	tr_C9ZNH3_
HLA-DQA10501-DQB10201	656	ATCYCNEGGRMIYMM	EGGRMIYMM	6	0.1691	8024.3	75.00	0.41	tr_C9ZNH3_
HLA-DQA10501-DQB10201	657	LPLVSDNGFRATRRL	LPLVSDNGF	0	0.1686	8065.8	75.00	0.32	tr_C9ZNH3_
HLA-DQA10501-DQB10201	658	RSVNGAIHRGALFVS	AIHRGALFV	5	0.1684	8088.0	75.00	0.39	tr_C9ZNH3_
HLA-DQA10501-DQB10201	659	FIHSVPSTSGNVMSD	VPSTSGNVM	4	0.1682	8100.6	75.00	0.36	tr_C9ZNH3_
HLA-DQA10501-DQB10201	660	AAHLSANRNVRVFIP	ANRNVRVFI	5	0.1681	8110.4	75.00	0.38	tr_C9ZNH3_
HLA-DQA10501-DQB10201	661	SVNGAIHRGALFVSG	AIHRGALFV	4	0.1667	8234.7	75.00	0.36	tr_C9ZNH3_
HLA-DQA10501-DQB10201	662	VDPQKVLTTIYTHSV	PQKVLTTIY	2	0.1662	8283.2	75.00	0.25	tr_C9ZNH3_
HLA-DQA10501-DQB10201	663	LHASFAVRQWSTASN	HASFAVRQW	1	0.1648	8408.9	75.00	0.34	tr_C9ZNH3_
HLA-DQA10501-DQB10201	664	NASDRTPLALLGFTS	RTPLALLGF	4	0.1642	8456.9	75.00	0.32	tr_C9ZNH3_
HLA-DQA10501-DQB10201	665	PQKVLTTIYTHSVIS	LTTIYTHSV	4	0.1632	8553.9	75.00	0.25	tr_C9ZNH3_
HLA-DQA10501-DQB10201	666	PRLRHPGRNVIYLSP	GRNVIYLSP	6	0.1606	8794.7	75.00	0.44	tr_C9ZNH3_
HLA-DQA10501-DQB10201	667	AVELMSQMGRSLCGV	QMGRSLCGV	6	0.1603	8820.9	75.00	0.34	tr_C9ZNH3_
HLA-DQA10501-DQB10201	668	SGNVMSDLRKMLDTR	SGNVMSDLR	0	0.1598	8873.0	75.00	0.34	tr_C9ZNH3_
HLA-DQA10501-DQB10201	669	ASDRTPLALLGFTSA	RTPLALLGF	3	0.1592	8931.3	75.00	0.30	tr_C9ZNH3_
HLA-DQA10501-DQB10201	670	VFIDPITLNPRLRHP	FIDPITLNP	1	0.1588	8971.0	75.00	0.34	tr_C9ZNH3_
HLA-DQA10501-DQB10201	671	AAYGATCYCNEGGRM	YGATCYCNE	2	0.1582	9029.8	75.00	0.39	tr_C9ZNH3_
HLA-DQA10501-DQB10201	672	IETVVNRTLLSFNRS	VVNRTLLSF	3	0.1582	9031.6	75.00	0.31	tr_C9ZNH3_
HLA-DQA10501-DQB10201	673	LAVELMSQMGRSLCG	AVELMSQMG	1	0.1581	9038.7	75.00	0.23	tr_C9ZNH3_
HLA-DQA10501-DQB10201	674	ETVVNRTLLSFNRSL	VVNRTLLSF	2	0.1570	9146.1	75.00	0.25	tr_C9ZNH3_
HLA-DQA10501-DQB10201	675	ILGDDYLPRPAADGL	LPRPAADGL	6	0.1569	9151.5	75.00	0.31	tr_C9ZNH3_
HLA-DQA10501-DQB10201	676	VNGAIHRGALFVSGN	AIHRGALFV	3	0.1569	9156.1	75.00	0.35	tr_C9ZNH3_
HLA-DQA10501-DQB10201	677	LPTDGSLLLIGLTAS	SLLLIGLTA	5	0.1562	9222.4	75.00	0.23	tr_C9ZNH3_
HLA-DQA10501-DQB10201	678	FMDSLYNQRRYVIDD	FMDSLYNQR	0	0.1562	9225.8	75.00	0.25	tr_C9ZNH3_
HLA-DQA10501-DQB10201	679	VGVDPQKVLTTIYTH	PQKVLTTIY	4	0.1555	9295.2	75.00	0.41	tr_C9ZNH3_
HLA-DQA10501-DQB10201	680	SVNDPFARSVNGAIH	FARSVNGAI	5	0.1545	9398.0	75.00	0.42	tr_C9ZNH3_
HLA-DQA10501-DQB10201	681	GVDPQKVLTTIYTHS	PQKVLTTIY	3	0.1527	9578.8	80.00	0.34	tr_C9ZNH3_
HLA-DQA10501-DQB10201	682	IAWTLAGTTSGVKMF	AGTTSGVKM	5	0.1520	9656.9	80.00	0.17	tr_C9ZNH3_
HLA-DQA10501-DQB10201	683	MKLVTDNRTSSAYIL	NRTSSAYIL	6	0.1517	9685.8	80.00	0.44	tr_C9ZNH3_
HLA-DQA10501-DQB10201	684	VNRTLLSFNRSLNSV	SFNRSLNSV	6	0.1506	9801.9	80.00	0.27	tr_C9ZNH3_
HLA-DQA10501-DQB10201	685	SDNGFRATRRLRADI	FRATRRLRA	4	0.1501	9850.9	80.00	0.42	tr_C9ZNH3_
HLA-DQA10501-DQB10201	686	LPDAYNNGVNAGLHA	NGVNAGLHA	6	0.1493	9937.8	80.00	0.34	tr_C9ZNH3_
HLA-DQA10501-DQB10201	687	LTKPETPSMHYHNLE	PSMHYHNLE	6	0.1484	10037.1	80.00	0.67	tr_C9ZNH3_
HLA-DQA10501-DQB10201	688	IAAHLSANRNVRVFI	ANRNVRVFI	6	0.1481	10072.2	80.00	0.37	tr_C9ZNH3_
HLA-DQA10501-DQB10201	689	HASFAVRQWSTASNV	RQWSTASNV	6	0.1479	10094.4	80.00	0.44	tr_C9ZNH3_
HLA-DQA10501-DQB10201	690	SDRTPLALLGFTSAS	RTPLALLGF	2	0.1478	10104.6	80.00	0.29	tr_C9ZNH3_
HLA-DQA10501-DQB10201	691	VNDPFARSVNGAIHR	FARSVNGAI	4	0.1475	10132.6	80.00	0.37	tr_C9ZNH3_
HLA-DQA10501-DQB10201	692	AWTLAGTTSGVKMFP	AGTTSGVKM	4	0.1475	10135.1	80.00	0.26	tr_C9ZNH3_
HLA-DQA10501-DQB10201	693	SSVNDPFARSVNGAI	FARSVNGAI	6	0.1469	10199.3	80.00	0.52	tr_C9ZNH3_
HLA-DQA10501-DQB10201	694	NGAIHRGALFVSGNG	AIHRGALFV	2	0.1462	10279.4	80.00	0.34	tr_C9ZNH3_
HLA-DQA10501-DQB10201	695	KLVTDNRTSSAYILS	NRTSSAYIL	5	0.1456	10346.7	80.00	0.41	tr_C9ZNH3_

HLA-DQA10501-DQB10201	696	GAFADEDCSRIVGNH	AFADEDCSR	1	0.1453	10376.6	80.00	0.37	tr_C9ZNH3_
HLA-DQA10501-DQB10201	697	FARSVNGAIHRGALF	GAIHRGALF	6	0.1443	10491.9	80.00	0.45	tr_C9ZNH3_
HLA-DQA10501-DQB10201	698	FATNLPHWADHRPSS	FATNLPHWA	0	0.1442	10509.0	80.00	0.60	tr_C9ZNH3_
HLA-DQA10501-DQB10201	699	WTLAGTTSGVKMFPG	AGTTSGVKM	3	0.1441	10513.7	80.00	0.23	tr_C9ZNH3_
HLA-DQA10501-DQB10201	700	VMSDLRKMLDTRAVT	LRKMLDTRA	4	0.1438	10545.5	80.00	0.29	tr_C9ZNH3_
HLA-DQA10501-DQB10201	701	NVMSDLRKMLDTRAV	LRKMLDTRA	5	0.1417	10791.7	80.00	0.32	tr_C9ZNH3_
HLA-DQA10501-DQB10201	702	PFLPDAYNNGVNAGL	YNNGVNAGL	6	0.1415	10819.0	80.00	0.31	tr_C9ZNH3_
HLA-DQA10501-DQB10201	703	TVVNRTLLSFNRSLN	VVNRTLLSF	1	0.1412	10849.3	80.00	0.25	tr_C9ZNH3_
HLA-DQA10501-DQB10201	704	VSGNGHLGKSERLFI	LGKSERLFI	6	0.1403	10962.7	80.00	0.41	tr_C9ZNH3_
HLA-DQA10501-DQB10201	705	ETIRRFHANRPNASD	FHANRPNAS	5	0.1395	11051.5	80.00	0.39	tr_C9ZNH3_
HLA-DQA10501-DQB10201	706	GNVMSDLRKMLDTRA	LRKMLDTRA	6	0.1377	11268.5	80.00	0.32	tr_C9ZNH3_
HLA-DQA10501-DQB10201	707	NDPFARSVNGAIHRG	FARSVNGAI	3	0.1371	11339.9	80.00	0.34	tr_C9ZNH3_
HLA-DQA10501-DQB10201	708	PDAYNNGVNAGLHAS	NGVNAGLHA	5	0.1351	11594.4	80.00	0.30	tr_C9ZNH3_
HLA-DQA10501-DQB10201	709	GAIHRGALFVSGNGH	AIHRGALFV	1	0.1348	11626.0	80.00	0.31	tr_C9ZNH3_
HLA-DQA10501-DQB10201	710	ANRPNASDRTPLALL	ASDRTPLAL	5	0.1347	11638.1	80.00	0.43	tr_C9ZNH3_
HLA-DQA10501-DQB10201	711	NGHLGKSERLFIHSV	SERLFIHSV	6	0.1345	11668.1	80.00	0.34	tr_C9ZNH3_
HLA-DQA10501-DQB10201	712	FLPDAYNNGVNAGLH	YNNGVNAGL	5	0.1343	11696.1	85.00	0.28	tr_C9ZNH3_
HLA-DQA10501-DQB10201	713	DPFARSVNGAIHRGA	FARSVNGAI	2	0.1339	11741.8	85.00	0.32	tr_C9ZNH3_
HLA-DQA10501-DQB10201	714	LAGTTSGVKMFPGQV	GVKMFPGQV	6	0.1338	11754.2	85.00	0.26	tr_C9ZNH3_
HLA-DQA10501-DQB10201	715	VVNRTLLSFNRSLNS	RTLLSFNRS	3	0.1328	11878.2	85.00	0.17	tr_C9ZNH3_
HLA-DQA10501-DQB10201	716	NRPNASDRTPLALLG	ASDRTPLAL	4	0.1323	11951.0	85.00	0.31	tr_C9ZNH3_
HLA-DQA10501-DQB10201	717	LVSDNGFRATRRLRA	FRATRRLRA	6	0.1319	11994.7	85.00	0.38	tr_C9ZNH3_
HLA-DQA10501-DQB10201	718	VSDNGFRATRRLRAD	FRATRRLRA	5	0.1305	12182.9	85.00	0.49	tr_C9ZNH3_
HLA-DQA10501-DQB10201	719	AIHRGALFVSGNGHL	AIHRGALFV	0	0.1299	12261.8	85.00	0.25	tr_C9ZNH3_
HLA-DQA10501-DQB10201	720	ATNLPHWADHRPSSE	WADHRPSSE	6	0.1287	12416.0	85.00	0.56	tr_C9ZNH3_
HLA-DQA10501-DQB10201	721	TIRRFHANRPNASDR	FHANRPNAS	4	0.1276	12565.8	85.00	0.38	tr_C9ZNH3_
HLA-DQA10501-DQB10201	722	HANRPNASDRTPLAL	ASDRTPLAL	6	0.1276	12573.4	85.00	0.44	tr_C9ZNH3_
HLA-DQA10501-DQB10201	723	ASFAVRQWSTASNVN	RQWSTASNV	5	0.1275	12589.8	85.00	0.45	tr_C9ZNH3_
HLA-DQA10501-DQB10201	724	TLAGTTSGVKMFPGQ	TTSGVKMFP	4	0.1272	12625.9	85.00	0.23	tr_C9ZNH3_
HLA-DQA10501-DQB10201	725	IRRFHANRPNASDRT	FHANRPNAS	3	0.1264	12734.1	85.00	0.35	tr_C9ZNH3_
HLA-DQA10501-DQB10201	726	NLPHWADHRPSSETI	WADHRPSSE	4	0.1260	12787.7	85.00	0.55	tr_C9ZNH3_
HLA-DQA10501-DQB10201	727	SGNGHLGKSERLFIH	LGKSERLFI	5	0.1259	12801.4	85.00	0.39	tr_C9ZNH3_
HLA-DQA10501-DQB10201	728	GGIAAHLSANRNVRV	AAHLSANRN	3	0.1258	12825.1	85.00	0.28	tr_C9ZNH3_
HLA-DQA10501-DQB10201	729	ADEDCSRIVGNHTDA	RIVGNHTDA	6	0.1246	12990.9	85.00	0.43	tr_C9ZNH3_
HLA-DQA10501-DQB10201	730	SETIRRFHANRPNAS	FHANRPNAS	6	0.1244	13019.2	85.00	0.34	tr_C9ZNH3_
HLA-DQA10501-DQB10201	731	TNLPHWADHRPSSET	WADHRPSSE	5	0.1243	13033.2	85.00	0.58	tr_C9ZNH3_
HLA-DQA10501-DQB10201	732	AYGATCYCNEGGRMI	YGATCYCNE	1	0.1242	13040.0	85.00	0.46	tr_C9ZNH3_
HLA-DQA10501-DQB10201	733	FIDPITLNPRLRHPG	FIDPITLNP	0	0.1216	13413.9	85.00	0.34	tr_C9ZNH3_
HLA-DQA10501-DQB10201	734	HWADHRPSSETIRRF	WADHRPSSE	1	0.1210	13494.7	85.00	0.44	tr_C9ZNH3_
HLA-DQA10501-DQB10201	735	RRFHANRPNASDRTP	FHANRPNAS	2	0.1200	13647.2	85.00	0.34	tr_C9ZNH3_
HLA-DQA10501-DQB10201	736	GNGHLGKSERLFIHS	LGKSERLFI	4	0.1190	13799.0	85.00	0.35	tr_C9ZNH3_
HLA-DQA10501-DQB10201	737	PFARSVNGAIHRGAL	FARSVNGAI	1	0.1188	13830.0	85.00	0.28	tr_C9ZNH3_
HLA-DQA10501-DQB10201	738	PLVSDNGFRATRRLR	LVSDNGFRA	1	0.1187	13845.9	85.00	0.31	tr_C9ZNH3_
HLA-DQA10501-DQB10201	739	GIAAHLSANRNVRVF	AAHLSANRN	2	0.1186	13863.7	85.00	0.28	tr_C9ZNH3_
HLA-DQA10501-DQB10201	740	AFADEDCSRIVGNHT	AFADEDCSR	0	0.1171	14085.5	85.00	0.28	tr_C9ZNH3_
HLA-DQA10501-DQB10201	741	DPFLPDAYNNGVNAG	DAYNNGVNA	5	0.1164	14195.5	85.00	0.29	tr_C9ZNH3_
HLA-DQA10501-DQB10201	742	RFHANRPNASDRTPL	FHANRPNAS	1	0.1153	14355.1	85.00	0.30	tr_C9ZNH3_
HLA-DQA10501-DQB10201	743	LPHWADHRPSSETIR	WADHRPSSE	3	0.1130	14724.4	90.00	0.57	tr_C9ZNH3_
HLA-DQA10501-DQB10201	744	GATCYCNEGGRMIYM	NEGGRMIYM	6	0.1114	14985.3	90.00	0.47	tr_C9ZNH3_
HLA-DQA10501-DQB10201	745	IHRGALFVSGNGHLG	LFVSGNGHL	5	0.1068	15742.3	90.00	0.29	tr_C9ZNH3_

HLA-DQA10501-DQB10201	746	SSETIRRFHANRPNA	IRRFHANRP	4	0.1052	16017.0	90.00	0.44	tr_C9ZNH3_
HLA-DQA10501-DQB10201	747	PHWADHRPSSETIRR	WADHRPSSE	2	0.1049	16068.6	90.00	0.59	tr_C9ZNH3_
HLA-DQA10501-DQB10201	748	YGATCYCNEGGRMIY	YGATCYCNE	0	0.1028	16441.6	90.00	0.34	tr_C9ZNH3_
HLA-DQA10501-DQB10201	749	NPRLRHPGRNVIYLS	PGRNVIYLS	6	0.1023	16529.0	90.00	0.49	tr_C9ZNH3_
HLA-DQA10501-DQB10201	750	FVSGNGHLGKSERLF	GHLGKSERL	5	0.1005	16853.0	90.00	0.47	tr_C9ZNH3_
HLA-DQA10501-DQB10201	751	WADHRPSSETIRRFH	WADHRPSSE	0	0.1005	16853.3	90.00	0.38	tr_C9ZNH3_
HLA-DQA10501-DQB10201	752	FHANRPNASDRTPLA	NRPNASDRT	3	0.0998	16987.3	90.00	0.24	tr_C9ZNH3_
HLA-DQA10501-DQB10201	753	FADEDCSRIVGNHTD	CSRIVGNHT	5	0.0990	17128.0	90.00	0.40	tr_C9ZNH3_
HLA-DQA10501-DQB10201	754	RPSSETIRRFHANRP	IRRFHANRP	6	0.0971	17485.4	90.00	0.49	tr_C9ZNH3_
HLA-DQA10501-DQB10201	755	PSSETIRRFHANRPN	IRRFHANRP	5	0.0923	18423.7	90.00	0.49	tr_C9ZNH3_
HLA-DQA10501-DQB10201	756	HRGALFVSGNGHLGK	LFVSGNGHL	4	0.0902	18845.3	95.00	0.30	tr_C9ZNH3_
HLA-DQA10501-DQB10201	757	LFVSGNGHLGKSERL	GHLGKSERL	6	0.0881	19283.0	95.00	0.53	tr_C9ZNH3_
HLA-DQA10501-DQB10201	758	RGALFVSGNGHLGKS	LFVSGNGHL	3	0.0809	20837.1	95.00	0.31	tr_C9ZNH3_
HLA-DQA10501-DQB10201	759	GALFVSGNGHLGKSE	LFVSGNGHL	2	0.0786	21359.2	95.00	0.31	tr_C9ZNH3_
HLA-DQA10501-DQB10201	760	ALFVSGNGHLGKSER	LFVSGNGHL	1	0.0714	23095.1	95.00	0.31	tr_C9ZNH3_
HLA-DQA10501-DQB10201	761	NAAVPPLTKPETPSM	PPLTKPETP	4	0.0710	23181.5	95.00	0.34	tr_C9ZNH3_
HLA-DQA10501-DQB10201	762	IDPITLNPRLRHPGR	DPITLNPRL	1	0.0702	23384.5	95.00	0.35	tr_C9ZNH3_
HLA-DQA10501-DQB10201	763	ADHRPSSETIRRFHA	PSSETIRRF	4	0.0701	23425.3	95.00	0.51	tr_C9ZNH3_
HLA-DQA10501-DQB10201	764	LNPRLRHPGRNVIYL	HPGRNVIYL	6	0.0682	23894.3	95.00	0.38	tr_C9ZNH3_
HLA-DQA10501-DQB10201	765	DHRPSSETIRRFHAN	PSSETIRRF	3	0.0638	25060.7	95.00	0.53	tr_C9ZNH3_
HLA-DQA10501-DQB10201	766	HRPSSETIRRFHANR	PSSETIRRF	2	0.0632	25241.9	100.00	0.48	tr_C9ZNH3_
HLA-DQA10501-DQB10201	767	AVPPLTKPETPSMHY	PPLTKPETP	2	0.0621	25530.6	100.00	0.33	tr_C9ZNH3_
HLA-DQA10501-DQB10201	768	AAVPPLTKPETPSMH	PPLTKPETP	3	0.0602	26054.6	100.00	0.39	tr_C9ZNH3_
HLA-DQA10501-DQB10201	769	VPPLTKPETPSMHYH	PPLTKPETP	1	0.0542	27808.5	100.00	0.29	tr_C9ZNH3_
HLA-DQA10501-DQB10201	770	DPITLNPRLRHPGRN	DPITLNPRL	0	0.0526	28290.7	100.00	0.28	tr_C9ZNH3_
HLA-DQA10501-DQB10201	771	TLNPRLRHPGRNVIY	RHPGRNVIY	6	0.0493	29329.3	100.00	0.47	tr_C9ZNH3_
HLA-DQA10501-DQB10201	772	PPLTKPETPSMHYHN	KPETPSMHY	4	0.0427	31507.1	100.00	0.27	tr_C9ZNH3_
HLA-DQA10501-DQB10201	773	PLTKPETPSMHYHNL	KPETPSMHY	3	0.0420	31746.3	100.00	0.28	tr_C9ZNH3_
HLA-DQA10501-DQB10201	774	PITLNPRLRHPGRNV	PITLNPRLR	0	0.0361	33847.7	100.00	0.31	tr_C9ZNH3_
HLA-DQA10501-DQB10201	775	ITLNPRLRHPGRNVI	RLRHPGRNV	5	0.0314	35584.6	100.00	0.25	tr_C9ZNH3_

Allele: HLA-DQA10501-DQB10201. Number of high binders 7. Number of weak binders 139. Number of peptides 775

Allele	pos	peptide	core	Of	1-log50k(aff)	affinity(nM)	%Rank	Relia	Identity	Bind Level
HLA-DQA10501-DQB10201	1	VNLRDSVVAASPLDY	VVAASPLDY	6	0.5606	116.1	2.50	0.61	tr_C9ZZ34_	WB
HLA-DQA10501-DQB10201	2	PSSSVSWLCDETLSE	VSWLCDETL	4	0.5605	116.2	2.50	0.63	tr_C9ZZ34_	WB
HLA-DQA10501-DQB10201	3	LRPSSSVSWLCDETL	VSWLCDETL	6	0.5595	117.5	2.50	0.68	tr_C9ZZ34_	WB
HLA-DQA10501-DQB10201	4	NLRDSVVAASPLDYS	VVAASPLDY	5	0.5589	118.2	2.50	0.57	tr_C9ZZ34_	WB
HLA-DQA10501-DQB10201	5	LRDSVVAASPLDYSE	VVAASPLDY	4	0.5532	125.8	3.00	0.58	tr_C9ZZ34_	WB
HLA-DQA10501-DQB10201	6	RPSSSVSWLCDETLS	VSWLCDETL	5	0.5482	132.8	3.00	0.70	tr_C9ZZ34_	WB
HLA-DQA10501-DQB10201	7	SSSVSWLCDETLSEG	VSWLCDETL	3	0.5431	140.3	3.50	0.60	tr_C9ZZ34_	WB
HLA-DQA10501-DQB10201	8	RDSVVAASPLDYSEE	VVAASPLDY	3	0.5429	140.5	3.50	0.59	tr_C9ZZ34_	WB
HLA-DQA10501-DQB10201	9	SSVSWLCDETLSEGL	VSWLCDETL	2	0.5292	163.1	4.00	0.59	tr_C9ZZ34_	WB
HLA-DQA10501-DQB10201	10	DSVVAASPLDYSEEH	VVAASPLDY	2	0.5277	165.8	4.00	0.61	tr_C9ZZ34_	WB
HLA-DQA10501-DQB10201	11	PLGWEGLEGQSLPFF	WEGLEGQSL	3	0.5092	202.5	5.00	0.62	tr_C9ZZ34_	WB
HLA-DQA10501-DQB10201	12	LGWEGLEGQSLPFFG	WEGLEGQSL	2	0.4930	241.3	6.00	0.62	tr_C9ZZ34_	WB

HLA-DQA10501-DQB10201	13	SVSWLCDETLSEGLA	VSWLCDETL	1	0.4928	241.8	6.00	0.61	tr_C9ZZ34_	WB
HLA-DQA10501-DQB10201	14	SVVAASPLDYSEEHS	VVAASPLDY	1	0.4804	276.5	7.00	0.63	tr_C9ZZ34_	WB
HLA-DQA10501-DQB10201	15	NPLGWEGLEGQSLPF	WEGLEGQSL	4	0.4768	287.5	7.50	0.69	tr_C9ZZ34_	WB
HLA-DQA10501-DQB10201	16	GNPLGWEGLEGQSLP	WEGLEGQSL	5	0.4626	335.2	8.50	0.71	tr_C9ZZ34_	WB
HLA-DQA10501-DQB10201	17	GWEGLEGQSLPFFGM	WEGLEGQSL	1	0.4613	339.9	8.50	0.60	tr_C9ZZ34_	WB
HLA-DQA10501-DQB10201	18	SGNPLGWEGLEGQSL	WEGLEGQSL	6	0.4349	452.2	12.00	0.71	tr_C9ZZ34_	
HLA-DQA10501-DQB10201	19	VSWLCDETLSEGLAG	VSWLCDETL	0	0.4049	626.0	15.00	0.54	tr_C9ZZ34_	
HLA-DQA10501-DQB10201	20	VVAASPLDYSEEHSP	VVAASPLDY	0	0.4020	645.9	16.00	0.61	tr_C9ZZ34_	
HLA-DQA10501-DQB10201	21	WEGLEGQSLPFFGMR	WEGLEGQSL	0	0.3994	663.9	16.00	0.49	tr_C9ZZ34_	
HLA-DQA10501-DQB10201	22	FVNLRDSVVAASPLD	SVVAASPLD	6	0.3805	814.5	19.00	0.37	tr_C9ZZ34_	
HLA-DQA10501-DQB10201	23	RFVNLRDSVVAASPL	DSVVAASPL	6	0.3360	1318.8	27.00	0.41	tr_C9ZZ34_	
HLA-DQA10501-DQB10201	24	LGKQLQGALANPTLW	LQGALANPT	4	0.3240	1501.0	30.00	0.38	tr_C9ZZ34_	
HLA-DQA10501-DQB10201	25	PRVGYSGNCLESPDL	YSGNCLESP	4	0.3203	1563.0	30.00	0.31	tr_C9ZZ34_	
HLA-DQA10501-DQB10201	26	GKQLQGALANPTLWC	LQGALANPT	3	0.3195	1576.7	31.00	0.36	tr_C9ZZ34_	
HLA-DQA10501-DQB10201	27	VASPRFVNLRDSVVA	FVNLRDSVV	5	0.3169	1622.0	31.00	0.35	tr_C9ZZ34_	
HLA-DQA10501-DQB10201	28	SPRVGYSGNCLESPD	YSGNCLESP	5	0.3163	1631.5	31.00	0.31	tr_C9ZZ34_	
HLA-DQA10501-DQB10201	29	ASPRFVNLRDSVVAA	FVNLRDSVV	4	0.3101	1745.1	33.00	0.35	tr_C9ZZ34_	
HLA-DQA10501-DQB10201	30	NLRPSSSVSWLCDET	SVSWLCDET	6	0.3085	1774.9	33.00	0.36	tr_C9ZZ34_	
HLA-DQA10501-DQB10201	31	LVASPRFVNLRDSVV	RFVNLRDSV	5	0.3076	1792.9	33.00	0.37	tr_C9ZZ34_	
HLA-DQA10501-DQB10201	32	HSPRVGYSGNCLESP	RVGYSGNCL	3	0.3061	1822.9	34.00	0.30	tr_C9ZZ34_	
HLA-DQA10501-DQB10201	33	MCALRDIASTSPLGD	DIASTSPLG	5	0.3057	1829.6	34.00	0.28	tr_C9ZZ34_	
HLA-DQA10501-DQB10201	34	KQLQGALANPTLWCS	LQGALANPT	2	0.3008	1929.8	35.00	0.38	tr_C9ZZ34_	
HLA-DQA10501-DQB10201	35	CALRDIASTSPLGDA	DIASTSPLG	4	0.2990	1968.5	35.00	0.28	tr_C9ZZ34_	
HLA-DQA10501-DQB10201	36	ALGKQLQGALANPTL	LQGALANPT	5	0.2979	1990.7	36.00	0.47	tr_C9ZZ34_	
HLA-DQA10501-DQB10201	37	SPRFVNLRDSVVAAS	FVNLRDSVV	3	0.2956	2041.8	36.00	0.34	tr_C9ZZ34_	
HLA-DQA10501-DQB10201	38	ALRDIASTSPLGDAI	DIASTSPLG	3	0.2931	2098.2	37.00	0.29	tr_C9ZZ34_	
HLA-DQA10501-DQB10201	39	RVGYSGNCLESPDLK	YSGNCLESP	3	0.2925	2112.1	37.00	0.28	tr_C9ZZ34_	
HLA-DQA10501-DQB10201	40	SPKSSFSPLVASPRF	SPLVASPRF	6	0.2915	2134.4	37.00	0.32	tr_C9ZZ34_	
HLA-DQA10501-DQB10201	41	PKSSFSPLVASPRFV	SPLVASPRF	5	0.2825	2352.9	39.00	0.29	tr_C9ZZ34_	
HLA-DQA10501-DQB10201	42	PRFVNLRDSVVAASP	FVNLRDSVV	2	0.2822	2360.8	40.00	0.31	tr_C9ZZ34_	
HLA-DQA10501-DQB10201	43	EGLEGQSLPFFGMRS	LEGQSLPFF	2	0.2736	2589.0	42.00	0.54	tr_C9ZZ34_	
HLA-DQA10501-DQB10201	44	QLQGALANPTLWCSK	LQGALANPT	1	0.2729	2610.1	42.00	0.40	tr_C9ZZ34_	
HLA-DQA10501-DQB10201	45	PLVASPRFVNLRDSV	RFVNLRDSV	6	0.2668	2786.5	44.00	0.49	tr_C9ZZ34_	
HLA-DQA10501-DQB10201	46	LRDIASTSPLGDAIR	DIASTSPLG	2	0.2650	2843.3	44.00	0.32	tr_C9ZZ34_	
HLA-DQA10501-DQB10201	47	PHSPRVGYSGNCLES	RVGYSGNCL	4	0.2616	2948.5	45.00	0.46	tr_C9ZZ34_	
HLA-DQA10501-DQB10201	48	SPHSPRVGYSGNCLE	RVGYSGNCL	5	0.2592	3025.6	46.00	0.50	tr_C9ZZ34_	
HLA-DQA10501-DQB10201	49	VGYSGNCLESPDLKV	YSGNCLESP	2	0.2508	3314.5	48.00	0.39	tr_C9ZZ34_	
HLA-DQA10501-DQB10201	50	AGSPKSSFSPLVASP	KSSFSPLVA	4	0.2504	3328.0	48.00	0.41	tr_C9ZZ34_	
HLA-DQA10501-DQB10201	51	IALGKQLQGALANPT	LQGALANPT	6	0.2501	3339.0	48.00	0.50	tr_C9ZZ34_	
HLA-DQA10501-DQB10201	52	SWLCDETLSEGLAGS	WLCDETLSE	1	0.2474	3439.0	49.00	0.54	tr_C9ZZ34_	
HLA-DQA10501-DQB10201	53	KSSFSPLVASPRFVN	SPLVASPRF	4	0.2453	3519.5	49.00	0.34	tr_C9ZZ34_	
HLA-DQA10501-DQB10201	54	GSPKSSFSPLVASPR	KSSFSPLVA	3	0.2430	3607.4	50.00	0.38	tr_C9ZZ34_	
HLA-DQA10501-DQB10201	55	GLAGSPKSSFSPLVA	KSSFSPLVA	6	0.2412	3676.9	55.00	0.49	tr_C9ZZ34_	
HLA-DQA10501-DQB10201	56	RNLRPSSSVSWLCDE	SSVSWLCDE	6	0.2378	3816.4	55.00	0.34	tr_C9ZZ34_	
HLA-DQA10501-DQB10201	57	GLEGQSLPFFGMRSR	LEGQSLPFF	1	0.2366	3864.5	55.00	0.55	tr_C9ZZ34_	
HLA-DQA10501-DQB10201	58	VAASPLDYSEEHSPR	PLDYSEEHS	4	0.2310	4104.7	55.00	0.35	tr_C9ZZ34_	
HLA-DQA10501-DQB10201	59	LAGSPKSSFSPLVAS	KSSFSPLVA	5	0.2310	4107.7	55.00	0.44	tr_C9ZZ34_	
HLA-DQA10501-DQB10201	60	SSFSPLVASPRFVNL	SPLVASPRF	3	0.2309	4113.3	55.00	0.38	tr_C9ZZ34_	
HLA-DQA10501-DQB10201	61	RDIASTSPLGDAIRD	DIASTSPLG	1	0.2305	4129.2	55.00	0.33	tr_C9ZZ34_	
HLA-DQA10501-DQB10201	62	RSGNPLGWEGLEGQS	PLGWEGLEG	4	0.2296	4169.7	55.00	0.42	tr_C9ZZ34_	

HLA-DQA10501-DQB10201	63	RGGSFCSQFSDNIAL	FCSQFSDNI	4	0.2292	4187.2	55.00	0.29	tr_C9ZZ34_
HLA-DQA10501-DQB10201	64	PRSGNPLGWEGLEGQ	PLGWEGLEG	5	0.2274	4267.7	55.00	0.45	tr_C9ZZ34_
HLA-DQA10501-DQB10201	65	LQGALANPTLWCSKE	GALANPTLW	2	0.2267	4304.1	55.00	0.30	tr_C9ZZ34_
HLA-DQA10501-DQB10201	66	AASPLDYSEEHSPRF	PLDYSEEHS	3	0.2257	4349.2	55.00	0.44	tr_C9ZZ34_
HLA-DQA10501-DQB10201	67	SERGGSFCSQFSDNI	FCSQFSDNI	6	0.2243	4414.1	55.00	0.31	tr_C9ZZ34_
HLA-DQA10501-DQB10201	68	ERGGSFCSQFSDNIA	FCSQFSDNI	5	0.2231	4474.0	60.00	0.30	tr_C9ZZ34_
HLA-DQA10501-DQB10201	69	HSYDQPHVCPISMER	QPHVCPISM	4	0.2201	4620.0	60.00	0.34	tr_C9ZZ34_
HLA-DQA10501-DQB10201	70	FSPLVASPRFVNLRD	SPLVASPRF	1	0.2185	4701.5	60.00	0.37	tr_C9ZZ34_
HLA-DQA10501-DQB10201	71	DRVKGGIIVTTGEGA	IIVTTGEGA	6	0.2166	4801.1	60.00	0.55	tr_C9ZZ34_
HLA-DQA10501-DQB10201	72	SHSYDQPHVCPISME	QPHVCPISM	5	0.2146	4902.5	60.00	0.35	tr_C9ZZ34_
HLA-DQA10501-DQB10201	73	SPRSGNPLGWEGLEG	PLGWEGLEG	6	0.2133	4972.6	60.00	0.57	tr_C9ZZ34_
HLA-DQA10501-DQB10201	74	SFSPLVASPRFVNLR	SPLVASPRF	2	0.2132	4977.0	60.00	0.41	tr_C9ZZ34_
HLA-DQA10501-DQB10201	75	ASPLDYSEEHSPRFA	PLDYSEEHS	2	0.2117	5058.0	60.00	0.41	tr_C9ZZ34_
HLA-DQA10501-DQB10201	76	SYDQPHVCPISMERN	QPHVCPISM	3	0.2114	5074.6	60.00	0.29	tr_C9ZZ34_
HLA-DQA10501-DQB10201	77	GGSFCSQFSDNIALG	FCSQFSDNI	3	0.2097	5173.7	60.00	0.25	tr_C9ZZ34_
HLA-DQA10501-DQB10201	78	IASTSPLGDAIRDRV	LGDAIRDRV	6	0.2066	5349.3	65.00	0.47	tr_C9ZZ34_
HLA-DQA10501-DQB10201	79	RVKGGIIVTTGEGAG	IIVTTGEGA	5	0.2063	5364.3	65.00	0.58	tr_C9ZZ34_
HLA-DQA10501-DQB10201	80	SPLVASPRFVNLRDS	PRFVNLRDS	6	0.2062	5369.6	65.00	0.25	tr_C9ZZ34_
HLA-DQA10501-DQB10201	81	DIASTSPLGDAIRDR	IASTSPLGD	1	0.2049	5449.9	65.00	0.34	tr_C9ZZ34_
HLA-DQA10501-DQB10201	82	WLCDETLSEGLAGSP	WLCDETLSE	0	0.2048	5451.9	65.00	0.43	tr_C9ZZ34_
HLA-DQA10501-DQB10201	83	YDQPHVCPISMERNL	QPHVCPISM	2	0.2036	5526.8	65.00	0.28	tr_C9ZZ34_
HLA-DQA10501-DQB10201	84	QPHVCPISMERNLRP	ISMERNLRP	6	0.2006	5706.8	65.00	0.20	tr_C9ZZ34_
HLA-DQA10501-DQB10201	85	LEGQSLPFFGMRSRD	LEGQSLPFF	0	0.2003	5726.7	65.00	0.47	tr_C9ZZ34_
HLA-DQA10501-DQB10201	86	GPSERGGSFCSQFSD	GGSFCSQFS	5	0.1959	6002.5	65.00	0.41	tr_C9ZZ34_
HLA-DQA10501-DQB10201	87	VKGGIIVTTGEGAGS	IIVTTGEGA	4	0.1959	6005.5	65.00	0.61	tr_C9ZZ34_
HLA-DQA10501-DQB10201	88	HSHSYDQPHVCPISM	QPHVCPISM	6	0.1938	6141.8	65.00	0.46	tr_C9ZZ34_
HLA-DQA10501-DQB10201	89	ERNLRPSSSVSWLCD	SSSVSWLCD	6	0.1930	6197.3	65.00	0.28	tr_C9ZZ34_
HLA-DQA10501-DQB10201	90	GSSPASTINSSPLGM	TINSSPLGM	6	0.1923	6245.7	65.00	0.56	tr_C9ZZ34_
HLA-DQA10501-DQB10201	91	SPLDYSEEHSPRFAS	PLDYSEEHS	1	0.1905	6367.1	65.00	0.47	tr_C9ZZ34_
HLA-DQA10501-DQB10201	92	GYSGNCLESPDLKVA	YSGNCLESP	1	0.1888	6484.2	70.00	0.50	tr_C9ZZ34_
HLA-DQA10501-DQB10201	93	CIHSHSYDQPHVCPI	IHSHSYDQP	1	0.1877	6557.6	70.00	0.30	tr_C9ZZ34_
HLA-DQA10501-DQB10201	94	PSERGGSFCSQFSDN	GGSFCSQFS	4	0.1875	6571.9	70.00	0.40	tr_C9ZZ34_
HLA-DQA10501-DQB10201	95	GSFCSQFSDNIALGK	FCSQFSDNI	2	0.1868	6621.7	70.00	0.30	tr_C9ZZ34_
HLA-DQA10501-DQB10201	96	TCIHSHSYDQPHVCP	IHSHSYDQP	2	0.1867	6633.1	70.00	0.31	tr_C9ZZ34_
HLA-DQA10501-DQB10201	97	GGIIVTTGEGAGSSP	IIVTTGEGA	2	0.1857	6707.0	70.00	0.68	tr_C9ZZ34_
HLA-DQA10501-DQB10201	98	DQPHVCPISMERNLR	PHVCPISME	2	0.1851	6747.8	70.00	0.25	tr_C9ZZ34_
HLA-DQA10501-DQB10201	99	EEHSPRFASGPSERG	PRFASGPSE	4	0.1848	6772.9	70.00	0.55	tr_C9ZZ34_
HLA-DQA10501-DQB10201	100	KGGIIVTTGEGAGSS	IIVTTGEGA	3	0.1845	6790.6	70.00	0.67	tr_C9ZZ34_
HLA-DQA10501-DQB10201	101	SEEHSPRFASGPSER	PRFASGPSE	5	0.1839	6839.7	70.00	0.62	tr_C9ZZ34_
HLA-DQA10501-DQB10201	102	PSPHSPRVGYSGNCL	RVGYSGNCL	6	0.1833	6880.0	70.00	0.83	tr_C9ZZ34_
HLA-DQA10501-DQB10201	103	SSPASTINSSPLGMG	TINSSPLGM	5	0.1788	7221.1	70.00	0.58	tr_C9ZZ34_
HLA-DQA10501-DQB10201	104	DTCIHSHSYDQPHVC	IHSHSYDQP	3	0.1785	7247.7	70.00	0.35	tr_C9ZZ34_
HLA-DQA10501-DQB10201	105	PHVCPISMERNLRPS	ISMERNLRP	5	0.1784	7255.4	70.00	0.23	tr_C9ZZ34_
HLA-DQA10501-DQB10201	106	QGALANPTLWCSKEE	GALANPTLW	1	0.1783	7260.6	70.00	0.43	tr_C9ZZ34_
HLA-DQA10501-DQB10201	107	NIALGKQLQGALANP	GKQLQGALA	4	0.1771	7358.5	70.00	0.40	tr_C9ZZ34_
HLA-DQA10501-DQB10201	108	YSEEHSPRFASGPSE	PRFASGPSE	6	0.1759	7451.2	70.00	0.75	tr_C9ZZ34_
HLA-DQA10501-DQB10201	109	IRDRVKGGIIVTTGE	GGIIVTTGE	6	0.1755	7485.3	70.00	0.35	tr_C9ZZ34_
HLA-DQA10501-DQB10201	110	SGPSERGGSFCSQFS	GGSFCSQFS	6	0.1755	7487.4	70.00	0.40	tr_C9ZZ34_
HLA-DQA10501-DQB10201	111	GIIVTTGEGAGSSPA	IIVTTGEGA	1	0.1745	7566.6	70.00	0.68	tr_C9ZZ34_
HLA-DQA10501-DQB10201	112	EGLAGSPKSSFSPLV	PKSSFSPLV	6	0.1737	7635.4	70.00	0.73	tr_C9ZZ34_

HLA-DQA10501-DQB10201	113	EHSPRFASGPSERGG	PRFASGPSE	3	0.1734	7654.8	70.00	0.53	tr_C9ZZ34_
HLA-DQA10501-DQB10201	114	SFCSQFSDNIALGKQ	FCSQFSDNI	1	0.1706	7897.2	75.00	0.29	tr_C9ZZ34_
HLA-DQA10501-DQB10201	115	IHSHSYDQPHVCPIS	HSYDQPHVC	3	0.1701	7940.2	75.00	0.29	tr_C9ZZ34_
HLA-DQA10501-DQB10201	116	ESPDLKVASASQPGP	DLKVASASQ	3	0.1652	8366.3	75.00	0.36	tr_C9ZZ34_
HLA-DQA10501-DQB10201	117	HSPRFASGPSERGGS	PRFASGPSE	2	0.1652	8368.9	75.00	0.52	tr_C9ZZ34_
HLA-DQA10501-DQB10201	118	SPASTINSSPLGMGR	TINSSPLGM	4	0.1633	8543.8	75.00	0.56	tr_C9ZZ34_
HLA-DQA10501-DQB10201	119	ASTSPLGDAIRDRVK	LGDAIRDRV	5	0.1626	8606.1	75.00	0.55	tr_C9ZZ34_
HLA-DQA10501-DQB10201	120	SPDLKVASASQPGPA	DLKVASASQ	2	0.1623	8637.4	75.00	0.31	tr_C9ZZ34_
HLA-DQA10501-DQB10201	121	RDRVKGGIIVTTGEG	GGIIVTTGE	5	0.1615	8710.9	75.00	0.32	tr_C9ZZ34_
HLA-DQA10501-DQB10201	122	SPRFASGPSERGGSF	PRFASGPSE	1	0.1596	8889.8	75.00	0.47	tr_C9ZZ34_
HLA-DQA10501-DQB10201	123	IIVTTGEGAGSSPAS	IIVTTGEGA	0	0.1587	8975.0	75.00	0.60	tr_C9ZZ34_
HLA-DQA10501-DQB10201	124	MERNLRPSSSVSWLC	LRPSSSVSW	4	0.1578	9070.0	75.00	0.34	tr_C9ZZ34_
HLA-DQA10501-DQB10201	125	TDTCIHSHSYDQPHV	IHSHSYDQP	4	0.1573	9117.7	75.00	0.44	tr_C9ZZ34_
HLA-DQA10501-DQB10201	126	FCSQFSDNIALGKQL	QFSDNIALG	3	0.1544	9404.7	75.00	0.17	tr_C9ZZ34_
HLA-DQA10501-DQB10201	127	CLESPDLKVASASQP	DLKVASASQ	5	0.1540	9446.0	75.00	0.44	tr_C9ZZ34_
HLA-DQA10501-DQB10201	128	PLDYSEEHSPRFASG	PLDYSEEHS	0	0.1539	9459.7	75.00	0.43	tr_C9ZZ34_
HLA-DQA10501-DQB10201	129	AIRDRVKGGIIVTTG	VKGGIIVTT	5	0.1537	9476.7	80.00	0.31	tr_C9ZZ34_
HLA-DQA10501-DQB10201	130	PDLKVASASQPGPAA	LKVASASQP	2	0.1528	9570.9	80.00	0.32	tr_C9ZZ34_
HLA-DQA10501-DQB10201	131	HDPWKKVLRCLTITT	WKKVLRCLT	3	0.1509	9771.3	80.00	0.35	tr_C9ZZ34_
HLA-DQA10501-DQB10201	132	YSGNCLESPDLKVAS	YSGNCLESP	0	0.1498	9890.8	80.00	0.41	tr_C9ZZ34_
HLA-DQA10501-DQB10201	133	PRFASGPSERGGSFC	PRFASGPSE	0	0.1493	9936.6	80.00	0.34	tr_C9ZZ34_
HLA-DQA10501-DQB10201	134	DAIRDRVKGGIIVTT	VKGGIIVTT	6	0.1489	9982.4	80.00	0.51	tr_C9ZZ34_
HLA-DQA10501-DQB10201	135	LESPDLKVASASQPG	DLKVASASQ	4	0.1488	9998.6	80.00	0.38	tr_C9ZZ34_
HLA-DQA10501-DQB10201	136	DGTDTCIHSHSYDQP	IHSHSYDQP	6	0.1481	10066.4	80.00	0.63	tr_C9ZZ34_
HLA-DQA10501-DQB10201	137	PASTINSSPLGMGRG	TINSSPLGM	3	0.1477	10117.8	80.00	0.55	tr_C9ZZ34_
HLA-DQA10501-DQB10201	138	HVCPISMERNLRPSS	ISMERNLRP	4	0.1471	10183.2	80.00	0.28	tr_C9ZZ34_
HLA-DQA10501-DQB10201	139	STSPLGDAIRDRVKG	LGDAIRDRV	4	0.1445	10471.7	80.00	0.55	tr_C9ZZ34_
HLA-DQA10501-DQB10201	140	GTDTCIHSHSYDQPH	IHSHSYDQP	5	0.1442	10508.2	80.00	0.58	tr_C9ZZ34_
HLA-DQA10501-DQB10201	141	DLKVASASQPGPAAP	LKVASASQP	1	0.1431	10633.7	80.00	0.31	tr_C9ZZ34_
HLA-DQA10501-DQB10201	142	EGQSLPFFGMRSRDG	FFGMRSRDG	6	0.1421	10748.0	80.00	0.35	tr_C9ZZ34_
HLA-DQA10501-DQB10201	143	GALANPTLWCSKEES	GALANPTLW	0	0.1419	10765.2	80.00	0.31	tr_C9ZZ34_
HLA-DQA10501-DQB10201	144	DNIALGKQLQGALAN	GKQLQGALA	5	0.1411	10863.7	80.00	0.47	tr_C9ZZ34_
HLA-DQA10501-DQB10201	145	SMERNLRPSSSVSWL	LRPSSSVSW	5	0.1397	11029.0	80.00	0.40	tr_C9ZZ34_
HLA-DQA10501-DQB10201	146	QSLPFFGMRSRDGTD	FFGMRSRDG	4	0.1393	11071.7	80.00	0.41	tr_C9ZZ34_
HLA-DQA10501-DQB10201	147	SDNIALGKQLQGALA	GKQLQGALA	6	0.1388	11136.2	80.00	0.48	tr_C9ZZ34_
HLA-DQA10501-DQB10201	148	PSHDPWKKVLRCLTI	WKKVLRCLT	5	0.1385	11174.9	80.00	0.52	tr_C9ZZ34_
HLA-DQA10501-DQB10201	149	GQSLPFFGMRSRDGT	FFGMRSRDG	5	0.1377	11268.1	80.00	0.40	tr_C9ZZ34_
HLA-DQA10501-DQB10201	150	SHDPWKKVLRCLTIT	WKKVLRCLT	4	0.1364	11426.4	80.00	0.48	tr_C9ZZ34_
HLA-DQA10501-DQB10201	151	ASTINSSPLGMGRGS	TINSSPLGM	2	0.1362	11453.2	80.00	0.64	tr_C9ZZ34_
HLA-DQA10501-DQB10201	152	PFFGMRSRDGTDTCI	FFGMRSRDG	1	0.1340	11732.6	85.00	0.31	tr_C9ZZ34_
HLA-DQA10501-DQB10201	153	SLPFFGMRSRDGTDT	FFGMRSRDG	3	0.1337	11769.2	85.00	0.39	tr_C9ZZ34_
HLA-DQA10501-DQB10201	154	ASGPSERGGSFCSQF	RGGSFCSQF	6	0.1329	11866.4	85.00	0.50	tr_C9ZZ34_
HLA-DQA10501-DQB10201	155	HSPRSGNPLGWEGLE	PRSGNPLGW	2	0.1322	11960.2	85.00	0.28	tr_C9ZZ34_
HLA-DQA10501-DQB10201	156	ALANPTLWCSKEESP	PTLWCSKEE	4	0.1310	12114.4	85.00	0.32	tr_C9ZZ34_
HLA-DQA10501-DQB10201	157	LPFFGMRSRDGTDTC	FFGMRSRDG	2	0.1310	12118.6	85.00	0.39	tr_C9ZZ34_
HLA-DQA10501-DQB10201	158	LCDETLSEGLAGSPK	DETLSEGLA	2	0.1301	12234.8	85.00	0.28	tr_C9ZZ34_
HLA-DQA10501-DQB10201	159	TSPLGDAIRDRVKGG	LGDAIRDRV	3	0.1289	12391.1	85.00	0.60	tr_C9ZZ34_
HLA-DQA10501-DQB10201	160	ISMERNLRPSSSVSW	LRPSSSVSW	6	0.1278	12548.5	85.00	0.49	tr_C9ZZ34_
HLA-DQA10501-DQB10201	161	APSHDPWKKVLRCLT	WKKVLRCLT	6	0.1276	12569.4	85.00	0.67	tr_C9ZZ34_
HLA-DQA10501-DQB10201	162	VCPISMERNLRPSSS	ISMERNLRP	3	0.1259	12799.7	85.00	0.30	tr_C9ZZ34_

HLA-DQA10501-DQB10201	163	NSSPLGMGRGSDDSA	MGRGSDDSA	6	0.1220	13351.1	85.00	0.58	tr_C9ZZ34_
HLA-DQA10501-DQB10201	164	CSQFSDNIALGKQLQ	NIALGKQLQ	6	0.1219	13365.1	85.00	0.20	tr_C9ZZ34_
HLA-DQA10501-DQB10201	165	STINSSPLGMGRGSD	TINSSPLGM	1	0.1210	13506.9	85.00	0.67	tr_C9ZZ34_
HLA-DQA10501-DQB10201	166	CDETLSEGLAGSPKS	ETLSEGLAG	2	0.1206	13555.3	85.00	0.26	tr_C9ZZ34_
HLA-DQA10501-DQB10201	167	SSPLGMGRGSDDSAH	MGRGSDDSA	5	0.1175	14015.9	85.00	0.55	tr_C9ZZ34_
HLA-DQA10501-DQB10201	168	SPLGDAIRDRVKGGI	LGDAIRDRV	2	0.1173	14051.1	85.00	0.60	tr_C9ZZ34_
HLA-DQA10501-DQB10201	169	AHSPRSGNPLGWEGL	PRSGNPLGW	3	0.1169	14118.6	85.00	0.35	tr_C9ZZ34_
HLA-DQA10501-DQB10201	170	PLGMGRGSDDSAHSP	MGRGSDDSA	3	0.1169	14120.1	85.00	0.55	tr_C9ZZ34_
HLA-DQA10501-DQB10201	171	PLGDAIRDRVKGGII	LGDAIRDRV	1	0.1167	14145.2	85.00	0.47	tr_C9ZZ34_
HLA-DQA10501-DQB10201	172	LANPTLWCSKEESPR	TLWCSKEES	4	0.1166	14156.9	85.00	0.32	tr_C9ZZ34_
HLA-DQA10501-DQB10201	173	AGSSPASTINSSPLG	ASTINSSPL	5	0.1159	14261.2	85.00	0.47	tr_C9ZZ34_
HLA-DQA10501-DQB10201	174	FFGMRSRDGTDTCIH	FFGMRSRDG	0	0.1156	14307.8	85.00	0.25	tr_C9ZZ34_
HLA-DQA10501-DQB10201	175	NCLESPDLKVASASQ	DLKVASASQ	6	0.1145	14492.4	85.00	0.56	tr_C9ZZ34_
HLA-DQA10501-DQB10201	176	CPISMERNLRPSSSV	ISMERNLRP	2	0.1131	14701.7	90.00	0.32	tr_C9ZZ34_
HLA-DQA10501-DQB10201	177	GAGSSPASTINSSPL	ASTINSSPL	6	0.1130	14719.5	90.00	0.54	tr_C9ZZ34_
HLA-DQA10501-DQB10201	178	ANPTLWCSKEESPRS	TLWCSKEES	3	0.1123	14838.1	90.00	0.31	tr_C9ZZ34_
HLA-DQA10501-DQB10201	179	SPLGMGRGSDDSAHS	MGRGSDDSA	4	0.1121	14874.0	90.00	0.52	tr_C9ZZ34_
HLA-DQA10501-DQB10201	180	LGMGRGSDDSAHSPR	MGRGSDDSA	2	0.1114	14976.7	90.00	0.58	tr_C9ZZ34_
HLA-DQA10501-DQB10201	181	SQFSDNIALGKQLQG	NIALGKQLQ	5	0.1110	15049.1	90.00	0.19	tr_C9ZZ34_
HLA-DQA10501-DQB10201	182	QFSDNIALGKQLQGA	NIALGKQLQ	4	0.1083	15483.2	90.00	0.19	tr_C9ZZ34_
HLA-DQA10501-DQB10201	183	DSAHSPRSGNPLGWE	PRSGNPLGW	5	0.1066	15775.9	90.00	0.45	tr_C9ZZ34_
HLA-DQA10501-DQB10201	184	FSDNIALGKQLQGAL	ALGKQLQGA	5	0.1060	15875.0	90.00	0.29	tr_C9ZZ34_
HLA-DQA10501-DQB10201	185	LKVASASQPGPAAPS	VASASQPGP	2	0.1058	15918.7	90.00	0.25	tr_C9ZZ34_
HLA-DQA10501-DQB10201	186	LDYSEEHSPRFASGP	YSEEHSPRF	2	0.1056	15952.5	90.00	0.47	tr_C9ZZ34_
HLA-DQA10501-DQB10201	187	TINSSPLGMGRGSDD	TINSSPLGM	0	0.1053	16008.4	90.00	0.50	tr_C9ZZ34_
HLA-DQA10501-DQB10201	188	NPTLWCSKEESPRST	TLWCSKEES	2	0.1047	16103.0	90.00	0.31	tr_C9ZZ34_
HLA-DQA10501-DQB10201	189	GDAIRDRVKGGIIVT	RVKGGIIVT	6	0.1044	16153.8	90.00	0.45	tr_C9ZZ34_
HLA-DQA10501-DQB10201	190	PISMERNLRPSSSVS	ISMERNLRP	1	0.1027	16453.2	90.00	0.32	tr_C9ZZ34_
HLA-DQA10501-DQB10201	191	LGDAIRDRVKGGIIV	LGDAIRDRV	0	0.1007	16809.4	90.00	0.35	tr_C9ZZ34_
HLA-DQA10501-DQB10201	192	VTTGEGAGSSPASTI	GAGSSPAST	5	0.1002	16905.0	90.00	0.22	tr_C9ZZ34_
HLA-DQA10501-DQB10201	193	SAHSPRSGNPLGWEG	PRSGNPLGW	4	0.0998	16975.6	90.00	0.46	tr_C9ZZ34_
HLA-DQA10501-DQB10201	194	RFASGPSERGGSFCS	FASGPSERG	1	0.0997	17007.8	90.00	0.36	tr_C9ZZ34_
HLA-DQA10501-DQB10201	195	IVTTGEGAGSSPAST	GAGSSPAST	6	0.0993	17069.9	90.00	0.22	tr_C9ZZ34_
HLA-DQA10501-DQB10201	196	GMGRGSDDSAHSPRS	MGRGSDDSA	1	0.0992	17090.4	90.00	0.58	tr_C9ZZ34_
HLA-DQA10501-DQB10201	197	SGNCLESPDLKVASA	GNCLESPDL	1	0.0988	17174.9	90.00	0.36	tr_C9ZZ34_
HLA-DQA10501-DQB10201	198	DETLSEGLAGSPKSS	ETLSEGLAG	1	0.0978	17350.1	90.00	0.32	tr_C9ZZ34_
HLA-DQA10501-DQB10201	199	FASGPSERGGSFCSQ	ERGGSFCSQ	6	0.0965	17594.4	90.00	0.38	tr_C9ZZ34_
HLA-DQA10501-DQB10201	200	SASQPGPAAPSHDPW	PAAPSHDPW	6	0.0957	17753.3	90.00	0.45	tr_C9ZZ34_
HLA-DQA10501-DQB10201	201	PTLWCSKEESPRSTA	TLWCSKEES	1	0.0930	18287.5	90.00	0.34	tr_C9ZZ34_
HLA-DQA10501-DQB10201	202	FGMRSRDGTDTCIHS	SRDGTDTCI	4	0.0894	19007.9	95.00	0.25	tr_C9ZZ34_
HLA-DQA10501-DQB10201	203	TTGEGAGSSPASTIN	GAGSSPAST	4	0.0891	19061.0	95.00	0.26	tr_C9ZZ34_
HLA-DQA10501-DQB10201	204	GNCLESPDLKVASAS	GNCLESPDL	0	0.0889	19098.8	95.00	0.26	tr_C9ZZ34_
HLA-DQA10501-DQB10201	205	ETLSEGLAGSPKSSF	ETLSEGLAG	0	0.0884	19218.2	95.00	0.19	tr_C9ZZ34_
HLA-DQA10501-DQB10201	206	DYSEEHSPRFASGPS	YSEEHSPRF	1	0.0874	19421.8	95.00	0.50	tr_C9ZZ34_
HLA-DQA10501-DQB10201	207	DDSAHSPRSGNPLGW	PRSGNPLGW	6	0.0837	20207.2	95.00	0.67	tr_C9ZZ34_
HLA-DQA10501-DQB10201	208	KVASASQPGPAAPSH	VASASQPGP	1	0.0823	20524.5	95.00	0.34	tr_C9ZZ34_
HLA-DQA10501-DQB10201	209	ASQPGPAAPSHDPWK	PAAPSHDPW	5	0.0821	20571.6	95.00	0.47	tr_C9ZZ34_
HLA-DQA10501-DQB10201	210	TGEGAGSSPASTINS	GAGSSPAST	3	0.0794	21188.1	95.00	0.28	tr_C9ZZ34_
HLA-DQA10501-DQB10201	211	INSSPLGMGRGSDDS	GMGRGSDDS	6	0.0775	21627.3	95.00	0.33	tr_C9ZZ34_
HLA-DQA10501-DQB10201	212	VASASQPGPAAPSHD	ASASQPGPA	1	0.0774	21646.0	95.00	0.32	tr_C9ZZ34_

HLA-DQA10501-DQB10201	213	EGAGSSPASTINSSP	PASTINSSP	6	0.0763	21891.4	95.00	0.30	tr_C9ZZ34_
HLA-DQA10501-DQB10201	214	MGRGSDDSAHSPRSG	MGRGSDDSA	0	0.0757	22048.3	95.00	0.56	tr_C9ZZ34_
HLA-DQA10501-DQB10201	215	TLSEGLAGSPKSSFS	LAGSPKSSF	5	0.0729	22719.1	95.00	0.25	tr_C9ZZ34_
HLA-DQA10501-DQB10201	216	GMRSRDGTDTCIHSH	SRDGTDTCI	3	0.0720	22937.0	95.00	0.28	tr_C9ZZ34_
HLA-DQA10501-DQB10201	217	SQPGPAAPSHDPWKK	PAAPSHDPW	4	0.0717	23014.0	95.00	0.47	tr_C9ZZ34_
HLA-DQA10501-DQB10201	218	TLWCSKEESPRSTAP	TLWCSKEES	0	0.0705	23330.2	95.00	0.37	tr_C9ZZ34_
HLA-DQA10501-DQB10201	219	GEGAGSSPASTINSS	GAGSSPAST	2	0.0699	23462.6	95.00	0.33	tr_C9ZZ34_
HLA-DQA10501-DQB10201	220	QPGPAAPSHDPWKKV	PAAPSHDPW	3	0.0697	23532.2	95.00	0.47	tr_C9ZZ34_
HLA-DQA10501-DQB10201	221	LSEGLAGSPKSSFSP	LAGSPKSSF	4	0.0681	23920.6	95.00	0.31	tr_C9ZZ34_
HLA-DQA10501-DQB10201	222	MRSRDGTDTCIHSHS	SRDGTDTCI	2	0.0669	24253.7	95.00	0.28	tr_C9ZZ34_
HLA-DQA10501-DQB10201	223	RSRDGTDTCIHSHSY	SRDGTDTCI	1	0.0656	24574.9	95.00	0.23	tr_C9ZZ34_
HLA-DQA10501-DQB10201	224	SEGLAGSPKSSFSPL	LAGSPKSSF	3	0.0653	24676.7	95.00	0.36	tr_C9ZZ34_
HLA-DQA10501-DQB10201	225	SRDGTDTCIHSHSYD	DTCIHSHSY	5	0.0643	24934.6	95.00	0.22	tr_C9ZZ34_
HLA-DQA10501-DQB10201	226	PGPAAPSHDPWKKVL	PAAPSHDPW	2	0.0639	25056.6	95.00	0.47	tr_C9ZZ34_
HLA-DQA10501-DQB10201	227	RDGTDTCIHSHSYDQ	CIHSHSYDQ	6	0.0623	25485.3	100.00	0.29	tr_C9ZZ34_
HLA-DQA10501-DQB10201	228	ASASQPGPAAPSHDP	PGPAAPSHD	5	0.0622	25522.8	100.00	0.34	tr_C9ZZ34_
HLA-DQA10501-DQB10201	229	KEESPRSTAPSPHSP	PRSTAPSPH	4	0.0586	26526.0	100.00	0.42	tr_C9ZZ34_
HLA-DQA10501-DQB10201	230	AAPSHDPWKKVLRCL	PWKKVLRCL	6	0.0562	27209.1	100.00	0.54	tr_C9ZZ34_
HLA-DQA10501-DQB10201	231	GPAAPSHDPWKKVLR	PAAPSHDPW	1	0.0554	27457.5	100.00	0.58	tr_C9ZZ34_
HLA-DQA10501-DQB10201	232	EESPRSTAPSPHSPR	PRSTAPSPH	3	0.0534	28057.8	100.00	0.41	tr_C9ZZ34_
HLA-DQA10501-DQB10201	233	CSKEESPRSTAPSPH	PRSTAPSPH	6	0.0532	28130.8	100.00	0.53	tr_C9ZZ34_
HLA-DQA10501-DQB10201	234	SKEESPRSTAPSPHS	PRSTAPSPH	5	0.0528	28230.2	100.00	0.44	tr_C9ZZ34_
HLA-DQA10501-DQB10201	235	ESPRSTAPSPHSPRV	PRSTAPSPH	2	0.0498	29171.0	100.00	0.40	tr_C9ZZ34_
HLA-DQA10501-DQB10201	236	PAAPSHDPWKKVLRC	PAAPSHDPW	0	0.0477	29825.9	100.00	0.49	tr_C9ZZ34_
HLA-DQA10501-DQB10201	237	LWCSKEESPRSTAPS	LWCSKEESP	0	0.0439	31077.8	100.00	0.34	tr_C9ZZ34_
HLA-DQA10501-DQB10201	238	SPRSTAPSPHSPRVG	PRSTAPSPH	1	0.0422	31683.5	100.00	0.38	tr_C9ZZ34_
HLA-DQA10501-DQB10201	239	APSPHSPRVGYSGNC	PRVGYSGNC	6	0.0413	31988.4	100.00	0.43	tr_C9ZZ34_
HLA-DQA10501-DQB10201	240	WCSKEESPRSTAPSP	SPRSTAPSP	6	0.0397	32543.4	100.00	0.34	tr_C9ZZ34_
HLA-DQA10501-DQB10201	241	GRGSDDSAHSPRSGN	GSDDSAHSP	2	0.0375	33325.9	100.00	0.25	tr_C9ZZ34_
HLA-DQA10501-DQB10201	242	PRSTAPSPHSPRVGY	PRSTAPSPH	0	0.0348	34315.3	100.00	0.31	tr_C9ZZ34_
HLA-DQA10501-DQB10201	243	GSDDSAHSPRSGNPL	HSPRSGNPL	6	0.0342	34519.0	100.00	0.29	tr_C9ZZ34_
HLA-DQA10501-DQB10201	244	SDDSAHSPRSGNPLG	HSPRSGNPL	5	0.0333	34891.1	100.00	0.29	tr_C9ZZ34_
HLA-DQA10501-DQB10201	245	RGSDDSAHSPRSGNP	GSDDSAHSP	1	0.0330	34986.4	100.00	0.31	tr_C9ZZ34_
HLA-DQA10501-DQB10201	246	RSTAPSPHSPRVGYS	PHSPRVGYS	6	0.0286	36679.2	100.00	0.24	tr_C9ZZ34_
HLA-DQA10501-DQB10201	247	STAPSPHSPRVGYSG	PHSPRVGYS	5	0.0245	38349.2	100.00	0.23	tr_C9ZZ34_
HLA-DQA10501-DQB10201	248	TAPSPHSPRVGYSGN	PHSPRVGYS	4	0.0233	38851.6	100.00	0.27	tr_C9ZZ34_

Allele: HLA-DQA10501-DQB10201. Number of high binders 0. Number of weak binders 17. Number of peptides 248

Allele	pos	peptide	core	Of	1-log50k(aff)	affinity(nM)	%Rank	Relia	Identity	Bind Level
HLA-DQA10501-DQB10201	1	TRYENFHELAGPDFA	FHELAGPDF	5	0.5395	145.8	3.50	0.57	tr_C9ZZ34_	WB
HLA-DQA10501-DQB10201	2	NTRYENFHELAGPDF	FHELAGPDF	6	0.5350	153.1	3.50	0.67	tr_C9ZZ34_	WB
HLA-DQA10501-DQB10201	3	CRLFLPALDLEMEGV	ALDLEMEGV	6	0.5262	168.3	4.00	0.33	tr_C9ZZ34_	WB
HLA-DQA10501-DQB10201	4	EPPFDLMDVHFADFV	MDVHFADFV	6	0.5228	174.7	4.00	0.40	tr_C9ZZ34_	WB
HLA-DQA10501-DQB10201	5	RLFLPALDLEMEGVV	ALDLEMEGV	5	0.5212	177.8	4.50	0.31	tr_C9ZZ34_	WB
HLA-DQA10501-DQB10201	6	LFLPALDLEMEGVVT	ALDLEMEGV	4	0.5127	194.9	5.00	0.31	tr_C9ZZ34_	WB

HLA-DQA10501-DQB10201	7	LSDFGGAVECAMQPP	FGGAVECAM	3	0.5122	195.9	5.00	0.50	tr_C9ZZ34_	WB
HLA-DQA10501-DQB10201	8	RYENFHELAGPDFAG	FHELAGPDF	4	0.5092	202.4	5.00	0.61	tr_C9ZZ34_	WB
HLA-DQA10501-DQB10201	9	KLSDFGGAVECAMQP	FGGAVECAM	4	0.5077	205.8	5.00	0.48	tr_C9ZZ34_	WB
HLA-DQA10501-DQB10201	10	SFQPNEIQSEYALVT	PNEIQSEYA	3	0.5014	220.2	5.50	0.51	tr_C9ZZ34_	WB
HLA-DQA10501-DQB10201	11	LVCRLFLPALDLEME	FLPALDLEM	5	0.4992	225.5	5.50	0.38	tr_C9ZZ34_	WB
HLA-DQA10501-DQB10201	12	GSFQPNEIQSEYALV	PNEIQSEYA	4	0.4992	225.5	5.50	0.58	tr_C9ZZ34_	WB
HLA-DQA10501-DQB10201	13	FQPNEIQSEYALVTL	PNEIQSEYA	2	0.4959	233.8	6.00	0.44	tr_C9ZZ34_	WB
HLA-DQA10501-DQB10201	14	DVFRVCVTAVPVDVI	VTAVPVDVI	6	0.4958	234.0	6.00	0.40	tr_C9ZZ34_	WB
HLA-DQA10501-DQB10201	15	VCRLFLPALDLEMEG	FLPALDLEM	4	0.4952	235.4	6.00	0.31	tr_C9ZZ34_	WB
HLA-DQA10501-DQB10201	16	VCKLSDFGGAVECAM	FGGAVECAM	6	0.4944	237.5	6.00	0.50	tr_C9ZZ34_	WB
HLA-DQA10501-DQB10201	17	PPFDLMDVHFADFVR	MDVHFADFV	5	0.4900	249.1	6.00	0.40	tr_C9ZZ34_	WB
HLA-DQA10501-DQB10201	18	SDFGGAVECAMQPPT	FGGAVECAM	2	0.4880	254.5	6.50	0.50	tr_C9ZZ34_	WB
HLA-DQA10501-DQB10201	19	CKLSDFGGAVECAMQ	FGGAVECAM	5	0.4852	262.5	6.50	0.52	tr_C9ZZ34_	WB
HLA-DQA10501-DQB10201	20	PFDLMDVHFADFVRQ	MDVHFADFV	4	0.4808	275.1	7.00	0.38	tr_C9ZZ34_	WB
HLA-DQA10501-DQB10201	21	AALVETFHGLPCDII	FHGLPCDII	6	0.4781	283.2	7.00	0.49	tr_C9ZZ34_	WB
HLA-DQA10501-DQB10201	22	QQFTEVVLRVAREEF	VLRVAREEF	6	0.4777	284.7	7.00	0.58	tr_C9ZZ34_	WB
HLA-DQA10501-DQB10201	23	FLVCRLFLPALDLEM	FLPALDLEM	6	0.4774	285.4	7.00	0.41	tr_C9ZZ34_	WB
HLA-DQA10501-DQB10201	24	FLPALDLEMEGVVTT	ALDLEMEGV	3	0.4768	287.3	7.50	0.34	tr_C9ZZ34_	WB
HLA-DQA10501-DQB10201	25	YENFHELAGPDFAGG	FHELAGPDF	3	0.4755	291.6	7.50	0.64	tr_C9ZZ34_	WB
HLA-DQA10501-DQB10201	26	EFVSGGSLAALVETF	SLAALVETF	6	0.4711	305.8	7.50	0.52	tr_C9ZZ34_	WB
HLA-DQA10501-DQB10201	27	VFRVCVTAVPVDVIH	VTAVPVDVI	5	0.4704	308.1	8.00	0.41	tr_C9ZZ34_	WB
HLA-DQA10501-DQB10201	28	FDLMDVHFADFVRQC	MDVHFADFV	3	0.4648	327.2	8.00	0.40	tr_C9ZZ34_	WB
HLA-DQA10501-DQB10201	29	PGSFQPNEIQSEYAL	PNEIQSEYA	5	0.4645	328.2	8.50	0.65	tr_C9ZZ34_	WB
HLA-DQA10501-DQB10201	30	DLMDVHFADFVRQCL	MDVHFADFV	2	0.4619	337.7	8.50	0.35	tr_C9ZZ34_	WB
HLA-DQA10501-DQB10201	31	QFTEVVLRVAREEFG	VLRVAREEF	5	0.4608	341.6	8.50	0.51	tr_C9ZZ34_	WB
HLA-DQA10501-DQB10201	32	APGSFQPNEIQSEYA	PNEIQSEYA	6	0.4592	347.7	9.00	0.67	tr_C9ZZ34_	WB
HLA-DQA10501-DQB10201	33	GVPFDIRLDSVVVTF	RLDSVVVTF	6	0.4588	349.4	9.00	0.25	tr_C9ZZ34_	WB
HLA-DQA10501-DQB10201	34	EVVLRVAREEFGVPF	VLRVAREEF	2	0.4587	349.7	9.00	0.51	tr_C9ZZ34_	WB
HLA-DQA10501-DQB10201	35	FVSGGSLAALVETFH	SLAALVETF	5	0.4547	365.2	9.00	0.40	tr_C9ZZ34_	WB
HLA-DQA10501-DQB10201	36	VVLRVAREEFGVPFD	VLRVAREEF	1	0.4544	366.4	9.00	0.44	tr_C9ZZ34_	WB
HLA-DQA10501-DQB10201	37	FTEVVLRVAREEFGV	VLRVAREEF	4	0.4533	370.4	9.50	0.50	tr_C9ZZ34_	WB
HLA-DQA10501-DQB10201	38	ALVETFHGLPCDIIR	FHGLPCDII	5	0.4533	370.6	9.50	0.50	tr_C9ZZ34_	WB
HLA-DQA10501-DQB10201	39	TEVVLRVAREEFGVP	VLRVAREEF	3	0.4526	373.6	9.50	0.57	tr_C9ZZ34_	WB
HLA-DQA10501-DQB10201	40	TKRLRAVQAMSQQFT	VQAMSQQFT	6	0.4512	379.3	9.50	0.32	tr_C9ZZ34_	WB
HLA-DQA10501-DQB10201	41	LMCEKRFSQASEQLE	RFSQASEQL	5	0.4479	392.8	10.00	0.60	tr_C9ZZ34_	
HLA-DQA10501-DQB10201	42	ENFHELAGPDFAGGR	FHELAGPDF	2	0.4477	393.6	10.00	0.65	tr_C9ZZ34_	
HLA-DQA10501-DQB10201	43	DFGGAVECAMQPPTS	FGGAVECAM	1	0.4452	404.6	10.00	0.52	tr_C9ZZ34_	
HLA-DQA10501-DQB10201	44	REEFGVPFDIRLDSV	PFDIRLDSV	6	0.4420	418.6	11.00	0.47	tr_C9ZZ34_	
HLA-DQA10501-DQB10201	45	KRLRAVQAMSQQFTE	VQAMSQQFT	5	0.4407	425.0	11.00	0.34	tr_C9ZZ34_	
HLA-DQA10501-DQB10201	46	FRVCVTAVPVDVIHR	VTAVPVDVI	4	0.4405	425.6	11.00	0.42	tr_C9ZZ34_	
HLA-DQA10501-DQB10201	47	QPNEIQSEYALVTLR	PNEIQSEYA	1	0.4394	430.9	11.00	0.43	tr_C9ZZ34_	
HLA-DQA10501-DQB10201	48	FGVPFDIRLDSVVVT	PFDIRLDSV	3	0.4379	437.8	11.00	0.44	tr_C9ZZ34_	
HLA-DQA10501-DQB10201	49	LRAVQAMSQQFTEVV	VQAMSQQFT	3	0.4359	447.6	11.00	0.32	tr_C9ZZ34_	
HLA-DQA10501-DQB10201	50	EKDAIVYQLQEATSF	AIVYQLQEA	3	0.4355	449.5	11.00	0.32	tr_C9ZZ34_	
HLA-DQA10501-DQB10201	51	EFGVPFDIRLDSVVV	PFDIRLDSV	4	0.4335	459.3	12.00	0.49	tr_C9ZZ34_	
HLA-DQA10501-DQB10201	52	PEPPFDLMDVHFADF	LMDVHFADF	6	0.4329	462.4	12.00	0.28	tr_C9ZZ34_	
HLA-DQA10501-DQB10201	53	VLRVAREEFGVPFDI	VLRVAREEF	0	0.4299	477.2	12.00	0.26	tr_C9ZZ34_	
HLA-DQA10501-DQB10201	54	RLRAVQAMSQQFTEV	VQAMSQQFT	4	0.4296	478.7	12.00	0.35	tr_C9ZZ34_	
HLA-DQA10501-DQB10201	55	YDVFRVCVTAVPVDV	CVTAVPVDV	6	0.4288	483.2	12.00	0.31	tr_C9ZZ34_	
HLA-DQA10501-DQB10201	56	TRKRTMVLTTESDYC	MVLTTESDY	5	0.4287	483.5	12.00	0.38	tr_C9ZZ34_	

HLA-DQA10501-DQB10201	57	VPFDIRLDSVVVTFH	PFDIRLDSV	1	0.4284	485.2	12.00	0.31	tr_C9ZZ34_
HLA-DQA10501-DQB10201	58	MLSNSSFLAQQVTFL	FLAQQVTFL	6	0.4275	489.7	12.00	0.56	tr_C9ZZ34_
HLA-DQA10501-DQB10201	59	RAVQAMSQQFTEVVL	VQAMSQQFT	2	0.4267	494.2	12.00	0.25	tr_C9ZZ34_
HLA-DQA10501-DQB10201	60	LVETFHGLPCDIIRR	FHGLPCDII	4	0.4263	496.2	13.00	0.50	tr_C9ZZ34_
HLA-DQA10501-DQB10201	61	TTKRLRAVQAMSQQF	LRAVQAMSQ	4	0.4262	497.1	13.00	0.38	tr_C9ZZ34_
HLA-DQA10501-DQB10201	62	RKRTMVLTTESDYCS	MVLTTESDY	4	0.4257	499.7	13.00	0.36	tr_C9ZZ34_
HLA-DQA10501-DQB10201	63	GRTSSLYGEEVRLAY	TSSLYGEEV	2	0.4257	499.9	13.00	0.50	tr_C9ZZ34_
HLA-DQA10501-DQB10201	64	PSGRTSSLYGEEVRL	TSSLYGEEV	4	0.4228	515.5	13.00	0.65	tr_C9ZZ34_
HLA-DQA10501-DQB10201	65	EEFGVPFDIRLDSVV	PFDIRLDSV	5	0.4217	521.8	13.00	0.49	tr_C9ZZ34_
HLA-DQA10501-DQB10201	66	NFHELAGPDFAGGRV	FHELAGPDF	1	0.4217	521.8	13.00	0.63	tr_C9ZZ34_
HLA-DQA10501-DQB10201	67	PFVWGIAMHLSQLLV	IAMHLSQLL	5	0.4205	528.3	13.00	0.26	tr_C9ZZ34_
HLA-DQA10501-DQB10201	68	CEKRFSQASEQLEKV	RFSQASEQL	3	0.4202	530.3	13.00	0.49	tr_C9ZZ34_
HLA-DQA10501-DQB10201	69	LPALDLEMEGVVTTK	ALDLEMEGV	2	0.4197	533.3	13.00	0.43	tr_C9ZZ34_
HLA-DQA10501-DQB10201	70	SGRTSSLYGEEVRLA	TSSLYGEEV	3	0.4193	535.6	13.00	0.58	tr_C9ZZ34_
HLA-DQA10501-DQB10201	71	EKRFSQASEQLEKVL	RFSQASEQL	2	0.4185	539.9	13.00	0.51	tr_C9ZZ34_
HLA-DQA10501-DQB10201	72	KRTMVLTTESDYCST	MVLTTESDY	3	0.4182	541.7	13.00	0.35	tr_C9ZZ34_
HLA-DQA10501-DQB10201	73	EEVRLAYRVTELCRI	RLAYRVTEL	3	0.4173	547.3	14.00	0.39	tr_C9ZZ34_
HLA-DQA10501-DQB10201	74	LSNSSFLAQQVTFLV	FLAQQVTFL	5	0.4158	556.1	14.00	0.49	tr_C9ZZ34_
HLA-DQA10501-DQB10201	75	MCEKRFSQASEQLEK	RFSQASEQL	4	0.4147	562.5	14.00	0.56	tr_C9ZZ34_
HLA-DQA10501-DQB10201	76	LPFVWGIAMHLSQLL	IAMHLSQLL	6	0.4147	562.8	14.00	0.35	tr_C9ZZ34_
HLA-DQA10501-DQB10201	77	VETFHGLPCDIIRRY	FHGLPCDII	3	0.4144	564.5	14.00	0.55	tr_C9ZZ34_
HLA-DQA10501-DQB10201	78	SVSQYPFNDVVEPGC	PFNDVVEPG	5	0.4142	565.7	14.00	0.29	tr_C9ZZ34_
HLA-DQA10501-DQB10201	79	PNEIQSEYALVTLRQ	EYALVTLRQ	6	0.4135	570.3	14.00	0.23	tr_C9ZZ34_
HLA-DQA10501-DQB10201	80	VSGGSLAALVETFHG	SLAALVETF	4	0.4134	570.8	14.00	0.43	tr_C9ZZ34_
HLA-DQA10501-DQB10201	81	KDAIVYQLQEATSFS	AIVYQLQEA	2	0.4111	585.1	14.00	0.34	tr_C9ZZ34_
HLA-DQA10501-DQB10201	82	KDRPSASELLFHDFM	SELLFHDFM	6	0.4086	600.8	15.00	0.51	tr_C9ZZ34_
HLA-DQA10501-DQB10201	83	NKAERLEFDIYGPVG	EFDIYGPVG	6	0.4085	601.7	15.00	0.28	tr_C9ZZ34_
HLA-DQA10501-DQB10201	84	RTPSGRTSSLYGEEV	TSSLYGEEV	6	0.4079	605.7	15.00	0.85	tr_C9ZZ34_
HLA-DQA10501-DQB10201	85	YGEEVRLAYRVTELC	RLAYRVTEL	5	0.4074	608.9	15.00	0.63	tr_C9ZZ34_
HLA-DQA10501-DQB10201	86	LMDVHFADFVRQCLK	MDVHFADFV	1	0.4061	617.5	15.00	0.34	tr_C9ZZ34_
HLA-DQA10501-DQB10201	87	LYGEEVRLAYRVTEL	RLAYRVTEL	6	0.4056	621.1	15.00	0.70	tr_C9ZZ34_
HLA-DQA10501-DQB10201	88	SPNNVLVSIDGVCKL	NVLVSIDGV	3	0.4047	627.0	15.00	0.38	tr_C9ZZ34_
HLA-DQA10501-DQB10201	89	LSVSQYPFNDVVEPG	PFNDVVEPG	6	0.4038	632.9	15.00	0.33	tr_C9ZZ34_
HLA-DQA10501-DQB10201	90	LQQEKDAIVYQLQEA	AIVYQLQEA	6	0.4029	639.4	16.00	0.55	tr_C9ZZ34_
HLA-DQA10501-DQB10201	91	EVRLAYRVTELCRIL	RLAYRVTEL	2	0.4018	646.9	16.00	0.34	tr_C9ZZ34_
HLA-DQA10501-DQB10201	92	QQEKDAIVYQLQEAT	AIVYQLQEA	5	0.4014	650.1	16.00	0.44	tr_C9ZZ34_
HLA-DQA10501-DQB10201	93	RTMVLTTESDYCSTG	MVLTTESDY	2	0.4009	653.3	16.00	0.37	tr_C9ZZ34_
HLA-DQA10501-DQB10201	94	KAERLEFDIYGPVGA	EFDIYGPVG	5	0.4003	657.8	16.00	0.31	tr_C9ZZ34_
HLA-DQA10501-DQB10201	95	VRLAYRVTELCRILD	YRVTELCRI	4	0.4001	658.8	16.00	0.38	tr_C9ZZ34_
HLA-DQA10501-DQB10201	96	TPSGRTSSLYGEEVR	TSSLYGEEV	5	0.3995	663.5	16.00	0.79	tr_C9ZZ34_
HLA-DQA10501-DQB10201	97	DISPNNVLVSIDGVC	NVLVSIDGV	5	0.3989	667.5	16.00	0.47	tr_C9ZZ34_
HLA-DQA10501-DQB10201	98	GDCYNKAERLEFDIY	AERLEFDIY	6	0.3979	674.8	16.00	0.47	tr_C9ZZ34_
HLA-DQA10501-DQB10201	99	SNSSFLAQQVTFLVC	FLAQQVTFL	4	0.3976	676.9	16.00	0.51	tr_C9ZZ34_
HLA-DQA10501-DQB10201	100	TFLVCRLFLPALDLE	RLFLPALDL	5	0.3974	678.3	16.00	0.35	tr_C9ZZ34_
HLA-DQA10501-DQB10201	101	VSQYPFNDVVEPGCA	PFNDVVEPG	4	0.3973	679.0	16.00	0.30	tr_C9ZZ34_
HLA-DQA10501-DQB10201	102	GLMCEKRFSQASEQL	RFSQASEQL	6	0.3969	682.3	16.00	0.83	tr_C9ZZ34_
HLA-DQA10501-DQB10201	103	CYNKAERLEFDIYGP	AERLEFDIY	4	0.3961	688.5	17.00	0.37	tr_C9ZZ34_
HLA-DQA10501-DQB10201	104	PRDCAFRLVSELQKL	AFRLVSELQ	4	0.3958	690.3	17.00	0.50	tr_C9ZZ34_
HLA-DQA10501-DQB10201	105	RVCVTAVPVDVIHRR	VTAVPVDVI	3	0.3954	693.3	17.00	0.46	tr_C9ZZ34_
HLA-DQA10501-DQB10201	106	LESQWARTSSLPFVW	WARTSSLPF	4	0.3952	694.8	17.00	0.49	tr_C9ZZ34_

HLA-DQA10501-DQB10201	107	QEKDAIVYQLQEATS	AIVYQLQEA	4	0.3948	698.3	17.00	0.37	tr_C9ZZ34_
HLA-DQA10501-DQB10201	108	PFDIRLDSVVVTFHT	RLDSVVVTF	4	0.3945	700.2	17.00	0.26	tr_C9ZZ34_
HLA-DQA10501-DQB10201	109	YNKAERLEFDIYGPV	AERLEFDIY	3	0.3944	700.8	17.00	0.38	tr_C9ZZ34_
HLA-DQA10501-DQB10201	110	RTSSLYGEEVRLAYR	TSSLYGEEV	1	0.3942	702.8	17.00	0.52	tr_C9ZZ34_
HLA-DQA10501-DQB10201	111	FVWGIAMHLSQLLVG	IAMHLSQLL	4	0.3926	714.5	17.00	0.24	tr_C9ZZ34_
HLA-DQA10501-DQB10201	112	VRPRDCAFRLVSELQ	AFRLVSELQ	6	0.3915	723.5	17.00	0.77	tr_C9ZZ34_
HLA-DQA10501-DQB10201	113	RVAREEFGVPFDIRL	EEFGVPFDI	4	0.3907	729.4	17.00	0.23	tr_C9ZZ34_
HLA-DQA10501-DQB10201	114	SGGSLAALVETFHGL	SLAALVETF	3	0.3905	731.1	17.00	0.47	tr_C9ZZ34_
HLA-DQA10501-DQB10201	115	VAREEFGVPFDIRLD	EEFGVPFDI	3	0.3901	734.2	17.00	0.22	tr_C9ZZ34_
HLA-DQA10501-DQB10201	116	ETFHGLPCDIIRRYT	FHGLPCDII	2	0.3892	741.9	18.00	0.57	tr_C9ZZ34_
HLA-DQA10501-DQB10201	117	DRPSASELLFHDFMV	SELLFHDFM	5	0.3887	745.3	18.00	0.50	tr_C9ZZ34_
HLA-DQA10501-DQB10201	118	KRFSQASEQLEKVLE	RFSQASEQL	1	0.3887	745.8	18.00	0.50	tr_C9ZZ34_
HLA-DQA10501-DQB10201	119	NNVLVSIDGVCKLSD	NVLVSIDGV	1	0.3885	747.4	18.00	0.37	tr_C9ZZ34_
HLA-DQA10501-DQB10201	120	NYKSDRTIAVVAPPH	TIAVVAPPH	6	0.3880	751.6	18.00	0.55	tr_C9ZZ34_
HLA-DQA10501-DQB10201	121	PNNVLVSIDGVCKLS	NVLVSIDGV	2	0.3873	757.2	18.00	0.38	tr_C9ZZ34_
HLA-DQA10501-DQB10201	122	PALDLEMEGVVTTKR	ALDLEMEGV	1	0.3868	761.2	18.00	0.41	tr_C9ZZ34_
HLA-DQA10501-DQB10201	123	APLMAAFGLMCEKRF	MAAFGLMCE	3	0.3867	761.6	18.00	0.25	tr_C9ZZ34_
HLA-DQA10501-DQB10201	124	DAIVYQLQEATSFSR	AIVYQLQEA	1	0.3865	763.5	18.00	0.34	tr_C9ZZ34_
HLA-DQA10501-DQB10201	125	PTRKRTMVLTTESDY	MVLTTESDY	6	0.3864	764.3	18.00	0.44	tr_C9ZZ34_
HLA-DQA10501-DQB10201	126	RDCAFRLVSELQKLE	AFRLVSELQ	3	0.3851	775.5	18.00	0.50	tr_C9ZZ34_
HLA-DQA10501-DQB10201	127	AVQAMSQQFTEVVLR	VQAMSQQFT	1	0.3850	776.1	18.00	0.23	tr_C9ZZ34_
HLA-DQA10501-DQB10201	128	DCYNKAERLEFDIYG	AERLEFDIY	5	0.3847	778.4	18.00	0.39	tr_C9ZZ34_
HLA-DQA10501-DQB10201	129	LAALVETFHGLPCDI	TFHGLPCDI	6	0.3844	780.9	18.00	0.53	tr_C9ZZ34_
HLA-DQA10501-DQB10201	130	TEFVSGGSLAALVET	GGSLAALVE	5	0.3843	782.0	18.00	0.29	tr_C9ZZ34_
HLA-DQA10501-DQB10201	131	ITEFVSGGSLAALVE	GGSLAALVE	6	0.3841	783.9	18.00	0.40	tr_C9ZZ34_
HLA-DQA10501-DQB10201	132	RPRDCAFRLVSELQK	AFRLVSELQ	5	0.3833	790.5	18.00	0.60	tr_C9ZZ34_
HLA-DQA10501-DQB10201	133	EPGCAQEISEEYRLL	CAQEISEEY	3	0.3822	799.7	19.00	0.48	tr_C9ZZ34_
HLA-DQA10501-DQB10201	134	RLAYRVTELCRILDC	YRVTELCRI	3	0.3817	804.6	19.00	0.40	tr_C9ZZ34_
HLA-DQA10501-DQB10201	135	CYDVFRVCVTAVPVD	RVCVTAVPV	5	0.3810	810.7	19.00	0.47	tr_C9ZZ34_
HLA-DQA10501-DQB10201	136	YKSDRTIAVVAPPHG	TIAVVAPPH	5	0.3803	816.1	19.00	0.49	tr_C9ZZ34_
HLA-DQA10501-DQB10201	137	ISPNNVLVSIDGVCK	NVLVSIDGV	4	0.3803	816.8	19.00	0.46	tr_C9ZZ34_
HLA-DQA10501-DQB10201	138	SPEPPFDLMDVHFAD	PFDLMDVHF	4	0.3787	830.5	19.00	0.44	tr_C9ZZ34_
HLA-DQA10501-DQB10201	139	PQYYAAPGSFQPNEI	QYYAAPGSF	1	0.3786	831.8	19.00	0.34	tr_C9ZZ34_
HLA-DQA10501-DQB10201	140	GEEVRLAYRVTELCR	RLAYRVTEL	4	0.3781	836.1	19.00	0.58	tr_C9ZZ34_
HLA-DQA10501-DQB10201	141	VEPGCAQEISEEYRL	CAQEISEEY	4	0.3779	837.8	19.00	0.50	tr_C9ZZ34_
HLA-DQA10501-DQB10201	142	NVLVSIDGVCKLSDF	NVLVSIDGV	0	0.3770	846.1	19.00	0.31	tr_C9ZZ34_
HLA-DQA10501-DQB10201	143	TMVLTTESDYCSTGS	MVLTTESDY	1	0.3758	857.1	20.00	0.39	tr_C9ZZ34_
HLA-DQA10501-DQB10201	144	FHELAGPDFAGGRVP	FHELAGPDF	0	0.3757	857.9	20.00	0.58	tr_C9ZZ34_
HLA-DQA10501-DQB10201	145	RFSQASEQLEKVLEL	FSQASEQLE	1	0.3757	858.3	20.00	0.35	tr_C9ZZ34_
HLA-DQA10501-DQB10201	146	SQYPFNDVVEPGCAQ	PFNDVVEPG	3	0.3756	858.7	20.00	0.31	tr_C9ZZ34_
HLA-DQA10501-DQB10201	147	DCAFRLVSELQKLES	AFRLVSELQ	2	0.3746	868.7	20.00	0.50	tr_C9ZZ34_
HLA-DQA10501-DQB10201	148	VTFLVCRLFLPALDL	RLFLPALDL	6	0.3742	872.2	20.00	0.50	tr_C9ZZ34_
[truncated: 673,924 more chars]
